# Identification of tandem repeat families from long-read sequences of *Humulus lupulus*

**DOI:** 10.1371/journal.pone.0233971

**Published:** 2020-06-05

**Authors:** Katherine A. Easterling, Nicholi J. Pitra, Taylan B. Morcol, Jenna R. Aquino, Lauren G. Lopes, Kristin C. Bussey, Paul D. Matthews, Hank W. Bass

**Affiliations:** 1 Department of Biological Science, Florida State University, Tallahassee, FL, United States America; 2 Hopsteiner, S.S. Steiner, Inc., New York, New York, United States America; 3 Department of Biological Sciences, Lehman College, City University of New York, Bronx, New York, United States America; 4 The Graduate Center, City University of New York, New York, New York, United States America; Leibniz-Institute of Plant Genetics and Crop Plant Research (IPK), GERMANY

## Abstract

Hop (*Humulus lupulus* L.) is known for its use as a bittering agent in beer and has a rich history of cultivation, beginning in Europe and now spanning the globe. There are five wild varieties worldwide, which may have been introgressed with cultivated varieties. As a dioecious species, its obligate outcrossing, non-Mendelian inheritance, and genomic structural variability have confounded directed breeding efforts. Consequently, understanding the hop genome represents a considerable challenge, requiring additional resources. In order to facilitate investigations into the transmission genetics of hop, we report here a tandem repeat discovery pipeline developed using k-mer filtering and dot plot analysis of PacBio long-read sequences from the hop cultivar Apollo. From this we identified 17 new and distinct tandem repeat sequence families, which represent candidates for FISH probe development. For two of these candidates, HuluTR120 and HuluTR225, we produced oligonucleotide FISH probes from conserved regions of and demonstrated their utility by staining meiotic chromosomes from wild hop, var. *neomexicanus* to address, for example, questions about hop transmission genetics. Collectively, these tandem repeat sequence families represent new resources suitable for development of additional cytogenomic tools for hop research.

## Introduction

*Humulus lupulus* (hop) is a dioecious twining bine in the Cannabaceae family of flowering plants with a long history of cultivation [1,2] for various uses including medicine (as reviewed by [3,4] and animal fodder [5], but is most commonly known as a flavoring agent in the brewing industry. The quest for complex taste and aromas in the rapidly expanding craft brewing industry has placed increasing demands on breeders to produce new varieties of plants with specific desirable traits including disease resistance [6–8]. However, hop presents multiple challenges to the production of new varieties due to its extended juvenile phase of two years to first flowers and its non-Mendelian inheritance patterns [9].

Cytogenetic analysis of male meiosis in hop has revealed a tendency for unusual meiotic configurations such as multivalent chromosomal complexes [9–12]. Recent 3D molecular cytology has shown that pervasive whole chromosome or segmental aneuploidy exists in hop and is exacerbated by passage through meiosis, particularly in cultivated hop [7]. Inheritance patterns of hop wild varieties remains largely unexplored. To date, there are limited cytological tools for assessing segregation patterns and establishing hop karyotypes (9 autosomes, XY). These tools have included telomere, 5S rDNA, HSR1 (Humulus subtelomeric repeat 1) [13,14], and more recently HSR0 (Humulus subtelomeric repeat 0) [7]. Despite these advances, most genomes of model hop varieties remain to be sequenced, assembled, and fully annotated, except for partial assemblies of Shinshu Wase, *H lupulus* var. *cordifolius* [15] and Teamaker [16]. Given the importance of cytogenetics in guiding studies of chromosomal structural genomics and the challenge presented by hop transmission genetics, more cytogenetic tools are needed. Among the more valuable FISH probes are those corresponding to tandemly repeated sequences [7,17–22]. Here, we set out to identify new tandem repeat sequences that could serve as candidates for future FISH probe development in hop.

Tandem repeats are among the fastest evolving components in genomes [23–25] and are typically found in heterochromatic, noncoding DNA at centromeric, pericentromeric, or subtelomeric regions. Plants, particularly angiosperms, are characteristically rich in repetitive DNA, which can account for the vast majority of plant nuclear genomes [26]. Hop has been previously reported to contain around 34% repetitive elements in the assembled portions of the genome [15], but that value will likely increase as more complete genome assemblies are produced.

Here we use long-read genomic sequences to find and characterize new families of hop tandem repeats. We describe our discovery pipeline using k-mer filtering and dot plot analysis of single molecule long read sequence data from cultivar Apollo, resulting in the identification of 17 new tandem repeat families. We also include evidence that aberrant meiosis, previously observed in cultivated hop, extends to two wild-collected neomexicanus hop accessions. As proof of concept, we developed and used FISH probes from two of the tandem repeat families, HuluTR120 and HuluTR225, to show their utility in marking meiotic chromosomes from non-cultivated wild hops.

## Methods

### Plant materials, collection, and fixation

Forest Products Free Use Permit for collections of botanical specimens to be used for

scientific purposes was obtained from the USDA (Permit Number: RO-289). Male panicles were collected before pollen shedding and fixed in Farmer's fluid as previously described [7,9]. Developing male flowers from wild hops, *H*. *lupulus* var. *neomexicanus* were collected from the Coronado National Forest in Arizona (U.S.A.). The hop variety named Apollo is a patented cultivar from Hopsteiner, Inc. Flowers from plant SH2 were collected from plants growing wild on Mt. Lemmon. Flowers from plant TM2-82C were collected on Mt. Bigelow.

### Identification of tandem repeats in long-read PacBio sequences

Tandemly repeated sequences were discovered essentially using the approach previously described for the tandem repeat HSR0 [7]. Previously unreported details, parameters, and procedures are further described. DNA sequence input was hop (Apollo) genomic DNA from long-read PacBio DNA Single Molecule, Real-Time (SMRT) cells (libraries submitted Dec 2014, University of Washington PacBio Sequencing Services, Center https://pacbio.gs.washington.edu/) using single molecule sequencing without circular consensus error correction. The sequences from 32 SMRT cells had a library size range 3–20 kb, an average RQ (read quality) range of 81.5–82.55, and an Average Polymerase Mean Read Length (bp) ranged of 4,093–5,048. For repeat detection, PacBio single molecule FASTA sequences greater than 5 kb (n = 1,037,871) were subjected to k-mer analysis in which all 12mers were counted and sorted by abundance for each read. Sequences were filtered for retention if meeting the criterion where the fifth most repeated 12mer occurred at least eight times within a single read using "ksift" (https://github.com/dvera/ksift) as previously reported [7]. These settings were derived by trial and error to optimize TR detection and simple repeat avoidance. This filter reduced the total list to 1,121 sequences (S2 file, FASTA sequences), reflecting ~1000-fold enrichment. These k-mer filtered sequences were then used to produce a document, referred to as the "HuluTR PDF book".

### Characterization of tandem repeat families

**U**sing the online YASS dot-plot genome server (https://bioinfo.lifl.fr/yass/yass.php), reads with TRs were grouped into families if their pairwise dot-plots between two different reads displayed parallel diagonals indicating repeating units of similar sequences between the two. For this, we used the default parameters from the YASS genome server which included Scoring matrix [match = +5, transversion = -4, transition = -3, other = -4 (composition bias correction)]; Gap costs [opening = -16, extension = -4]; [E-value threshold = 10]; [X-drop threshold = 30], and display DNA strain [fwd&rc] [27]. To facilitate this process, we concatenated sequences representing each TR family into a single customized file here named "polySeq" (S2 Fig) and used it in each pairwise alignment with unclassified reads. New families (those not matching any of the repeats in the polySeq file) were added to the end of the polySeq file as they were discovered and included in blocks of sequence at 1kb intervals for ease of positional recognition in the output dot plots. The S2 Fig contains the full "polySeq34_v7" FASTA sequence with embedded locators, a table of synonyms to guide location to 1Kbp blocks, and individual dot plots of the polySeq vs. each HuluTR consensus sequence. We also used FlexiDot [28], flexidot_v1.06.py from https://github.com/molbio-dresden/flexidot/, to produce self-alignment PNG plots with the following command line settings (python flexidot_v1.06.py -i filename.fas -k 10 -c 0 -p 0 -B green -C purple -M 1 -f 0 -s 1 -E 12).

For each TR family grouped by sequence similarity, we established an average consensus unit length based on results from the Tandem Repeats Finder server at https://tandem.bu.edu/trf/trf.html [29]. Parameter settings used for TRF were default and as follows: alignment parameters (match = 2, mismatch = 2, indels = 7), minimum alignment score to report repeat = 50, Maximum period size = 1000, Maximum TR array size (bp, millions) = 2. Because of minor variation in the exact repeat lengths as determined by TRF, we rounded to the nearest 5bp and designated each HuluTR family accordingly (S3 Fig). The nomenclature used here is illustrated the example "HuluTR120-r479", which refers to *Humulus* *lupulus* Tandem Repeat of ~120 bp PacBio read number 479 from the k-mer filtered set of 1,121 reads (S1 and S2 Datas).

For analysis of monomer divergence within and between reads of HuluTR120, we extracted 11 monomers from an internal contiguous cluster for each of 10 reads. These were analyzed using a multiple sequence alignment tool, Clustal Omega (Clustal 2.1, https://www.ebi.ac.uk/Tools/msa/clustalo/). The resulting Percent Identity Matrix was imported into MS Excel and the sequence identity values were visualized for the individual monomers or their read-to-read averages (Fig 3) using the Conditional Formatting tool with 2-Color Scale set from 40 (black) to 70 (yellow).

### FISH and 3D cytology

Male meiocytes from hop plants were prepared, analyzed, and imaged using 3D deconvolution microscopy as previously described [7]. Prehybridization, hybridization, post-hybridization washes, DAPI counterstaining, and slide mounting were done as described [20] using denaturation temperature of 92°C. Nucleoli were measured using the Measure Distances program in the DeltaVision Software. Their diameter measurements were taken from central optical sections of each nucleolus, which are primarily spherical. Seventeen nucleoli were measured for cells with only one nucleolus (n = 17 cells) and sixteen were measured for cells with two nucleoli (n = 8 cells). Average diameters were converted to volume in cubic microns.

Tandem repeat oligo names, sequences, and associated dyes utilized and reported in FISH experiments are as follows: “TR120Y” is 5’ -[ATTO647N]-GAGCACGAGATATTGATAAAAA, “TR225Y” is 5’-[ATTO647N]-TTAGTGCAATGTTATCTAGT. Additional resources for synthetic consensus sequences were designed in order to provide new information as additional tools for hop cytogenetics. The synthetic consensus sequences were made (GenScript Biotech Corp.) and inserted into plasmids to enable their use as templates to make FISH probes via conventional labeling techniques. These plasmids (pHTR120syn, pHTR225syn, pHTR600syn, pHTR390syn, an pHTR060syn) and their descriptions are available from AddGene (addgene.org).

## Results

In this study, we set out to develop new FISH probe candidates that can be used for cytogenetic tracking of individual chromosomes in the *Humulus lupulus* species. To date, there exist only a few such probes including those for rDNA repeats and other tandemly repeated clusters. These have served to establish basic hop karyotypes, but more cytogenomic information is necessary in order to further delineate individual chromosomes, integrate physical and linkage maps, and to explore questions about transmission genetics in both cultivated and wild varieties for this group of plants.

### Finding tandem repeats with K-mer and dot plot analyses of PacBio long-read sequence data

**Fig 1:**
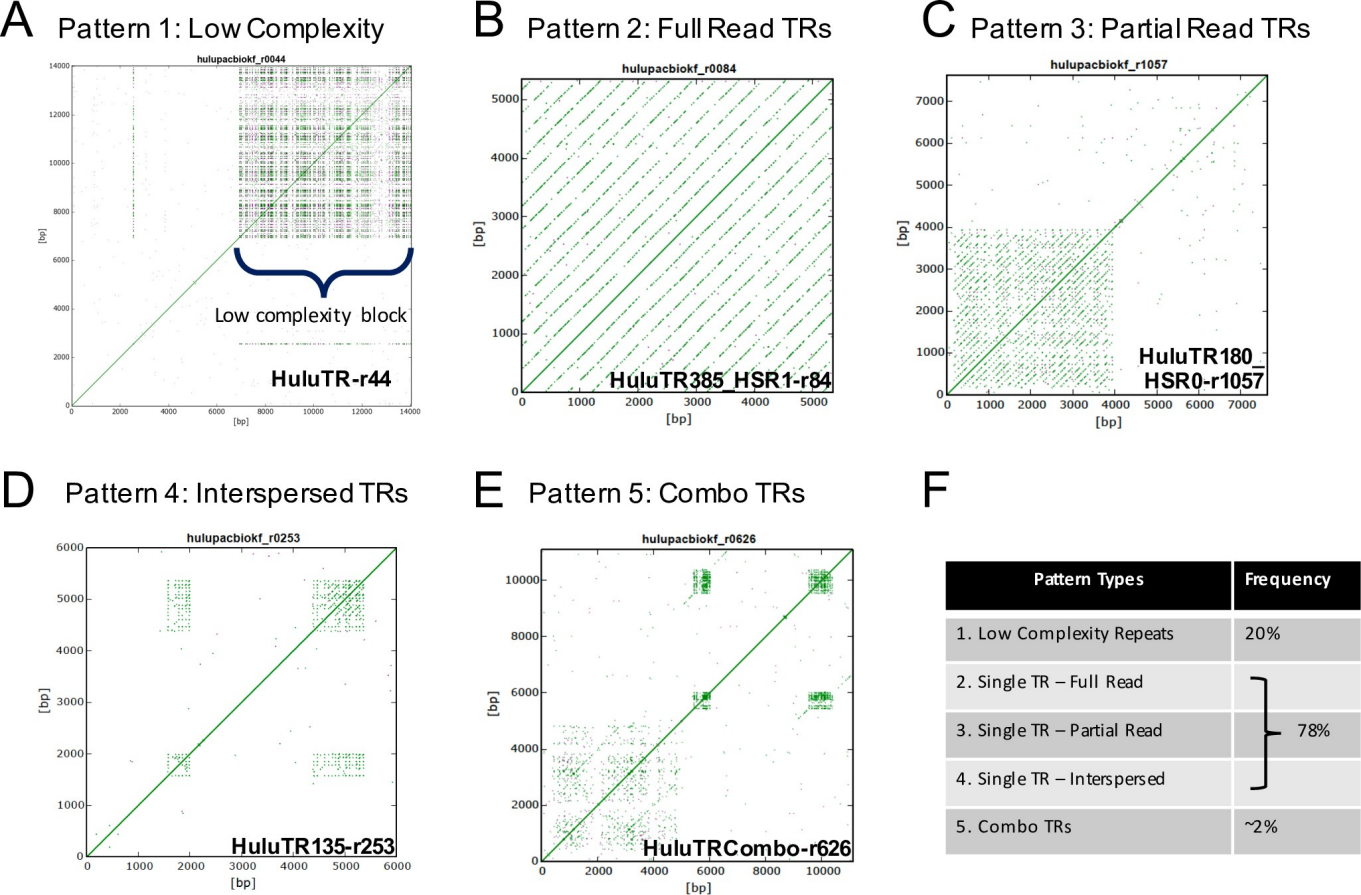
Dot plot outputs of k-mer analysis, showing different pattern types. PacBio Single Molecule, Real-Time (SMRT) DNA sequences were screened for tandem repeats. For each read, a self-aligned dot-plot is shown. The parallel diagonals represent internal tandem repeats. (A) Pattern 1: Example showing the HuluTR-r44 read showing no conspicuous parallel diagonals, indicating lack of long or regular tandem repeats. The low complexity simple repeats often present as blocks in these dot plots (Low complexity block) which are distinct from uniform tandem arrays. (B) Pattern 2: Example showing the HuluTR385_HSR1-r84 read in which the tandem repeats occupy an entire read. (C) Pattern 3: Example showing the HuluTR180_HSR0-r1057 read in which the tandem repeats occupy part of the read. (D) Pattern 4: Example showing the HuluTR135-r253 read in which the tandem repeats occupy multiple but interspersed regions of the read. (E) Pattern 5: Example showing the HuluTRCombo-r626 read in which more than one tandem repeat family is present in the same read. (F) Percentages of each of the five HuluTR pattern types from the 1,121 k-mer-filtered reads.

We and others have successfully mined sequence data to identify tandem repeats that have been developed into FISH probes [7,26,30–32]. Here we carried out a thorough analysis of PacBio Single Molecule, Real-Time (SMRT) reads (n = 1,037,871 reads), each consisting of sequences greater than 5,000 bp long. These reads, from 2014, produced single molecule DNA sequence, not circular consensus corrected, and are expected to contain an estimated error rate of ~10% based on alignments with a telomeric test case (S4 Fig). A k-mer computational filter designed to detect repetitive sequences resulted in a list of 1,121 reads which were visualized as self-aligned Dot Plots using the YASS program [27] and FlexiDot [28] as summarized in Fig 1. Self-aligned dot plots using the same sequence on the X and Y axis produce a single main diagonal line of identity and for tandem repeats, a series of parallel diagonals whose frequency and spacing reflect their abundance and unit lengths.

Several types of repeat sequence patterns were observed among the 1,121 reads that passed the k-mer screen. The dot-plot pattern types can be grouped as those with low complexity and no obvious tandem repeats (Fig 1A, no conspicuous parallel diagonals) or those with more clearly revealed tandem repeats, which fall into several subgroups (Fig 1B–1E). The spacing between the diagonals resulting from tandem repeats is proportional to the repeat unit length, and these plots provide easy to interpret summary diagrams. Low complexity reads (e.g. Fig 1A) comprised ~20% of the k-mer filtered reads and included homopolymeric runs of single or simple sequence repeats or microsatellites, but were not further analyzed. In contrast, desirable reads of larger tandem repeats showed more conspicuous dot-plot diagonals. These could be further subdivided into groups where the tandem repeats fill an entire read (Full Read TRs, Fig 1B), a single portion of a read (Partial Read TRs, Fig 1C), multiple but separate patches of the same repeat in a read (Interspersed TRs, Fig 1D), or separate patches of dissimilar repeats in a read (Combo TRs, Fig 1E). The reads with the Combo TRs account for ~2% of the full k-mer set (Fig 1F) and often include repetitive sequence clusters with relatively short repeat lengths of ~30–50 bp, but were not prioritized for further analysis. By mining long-read sequence data, our pipeline identified nearly 900 PacBio SMRT reads with tandem repeats. Among these were reads housing known tandem repeat families (5S rDNA, HSR1, HSR0) and those housing new uncharacterized tandem repeat families.

### Defining HuluTRs: The tandem repeat families of hop

**Fig 2:**
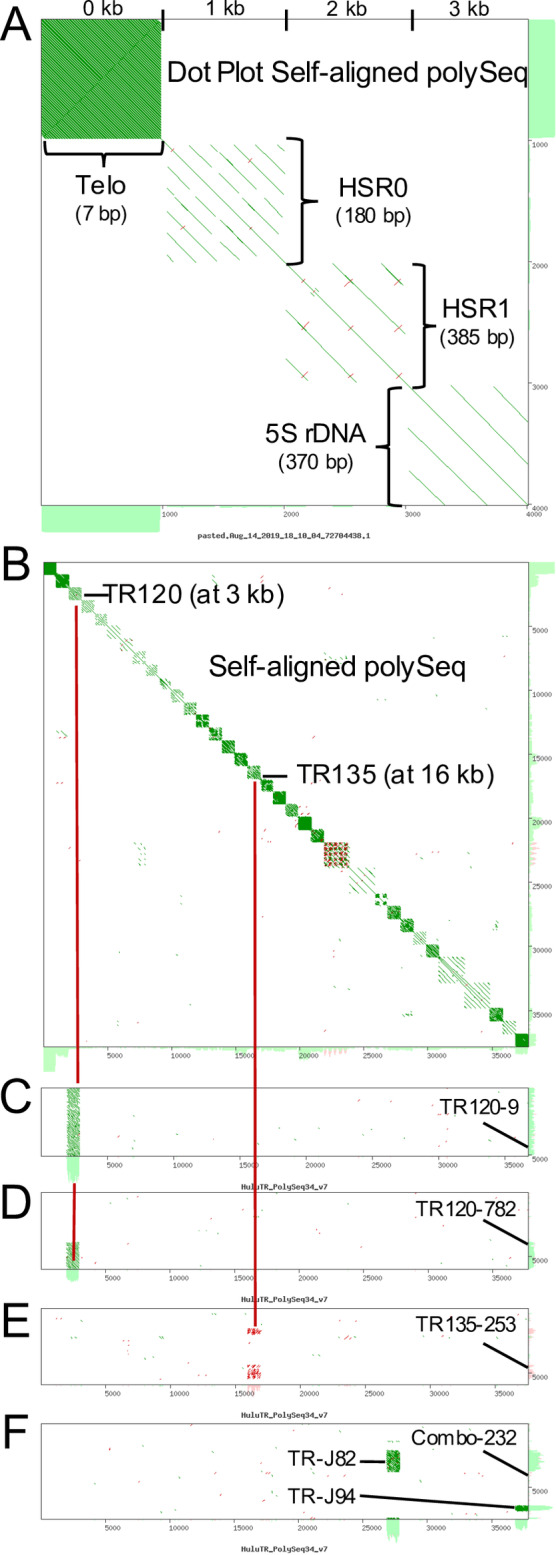
Using polySeq to define HuluTR families. A concatenation of consensus sequences for each known repeat family was made for use in the YASS dot-plot analysis to group tandem repeats into existing or new families, one read at a time. (A) Previously known repeats, Telo, HSR0, HSR1 and 5S rDNA showing 1 kb blocks in a dot plot of self-aligned polySeq. (B) Dot plot output of all 34 polySeq repeat sequences (FASTA sequence in S3). Examples of read matching are indicated by the dark red bars denoting alignment to polySeq regions at 3 kb, corresponding to TR120, and at 16 kb, corresponding to TR 135. (C) HuluTR120-r9 with a Full read TR pattern, matching with the polySeq at 3 kb. (D) HuluTR120-r782 with a Partial read TR pattern, matching with the polySeq at 3 kb. (E) HuluTR135-r253 with an Interspersed TR pattern, matching with the polySeq at 16 kb. (F) HuluTRCombo-r232 with a Combo TR pattern, matching with the polySeq at two locations, 27 and 37 kb.

To consolidate and sort out the newly discovered TR families, we grouped them by sequence similarity into families using dot plot analysis as summarized in Fig 2. The process is illustrated for four previously known TRs (Fig 2A): telomere, HSR0, HSR1, and 5S rDNA. For each TR, a 1kb block representing a TR family was made by a concatenation of a single repeating unit or consensus sequence repeat. These 1-kb TR family-specific sequence blocks provide convenient visual delineations on the dot plot and were concatenated to produce a customized file called "polySeq". The resulting 4-TR polySeq (shown as self-aligned in Fig 2A) was used as one of the two inputs to screen new reads by dot plotting, one at a time. For each new, uncharacterized read (those not matching sequences in the existing polySeq), we gave them a name (based on unit repeat length or discovery number) and appended them to the polySeq file as a 1kb block of repeats, or 2kb blocks for large repeats. This process was repeated for each read, eventually producing a polySeq set of 34 distinct TR families (S2 Fig), shown as a self-aligned dot plot (Fig 2B). The TR family assignment procedure is illustrated for four different reads in panels C-F (Fig 2). For these examples, the dot plot shows the result with the polySeq on the X-axis and the query read on the Y-axis. The TR patterns shown include examples designated full read TRs (Fig 2C), partial read TRs (Fig 2D), interspersed TRs (Fig 2E), or combo TRs (Fig 2F). The fact that the self-aligned polySeq-34 dot plot (Fig 2, panel B) as well as the pairwise queries (Fig 2C–2F) show sequence similarity diagonals within but not between the different TR families demonstrates the strength and specificity of this approach, even when using error-prone long reads.

Using this approach to define families of TRs by dot plot-guided sequence similarity grouping, we selected a subset omitting smaller repeats (<50 bp), and avoiding those that tended to occur in combination with other TRs in the same read (e.g. Fig 2F). This resulted in a total of 17 new HuluTR families, listed in Table 1, sorted sequentially by relative abundance then by repeat length. The six most abundant TR families found in the library range from 34 to 232 TR-containing reads per million, and included previously known sequences HSR1, HSR0, and 5S rDNA, and newly discovered sequences, HuluTR120, HuluTR225, and HuluTR060. Their relative abundance makes them good candidates for FISH probes. The other families were found to occur at a lesser frequency, including six that were found in only one read of the k-mer-filtered set.

**Table 1:** Hop tandem repeats.

HuluTR Family(A)	Repeat Size (bp)(B)	TR-containing reads per million(C)	% A+T	Representative PacBio Read(D)	GenBank Accession
HuluTR385 (HSR1)	385	232	61	r55	GU831574
HuluTR180 (HSR0)	180	163	62	r120	MH188533.1
*HuluTR120	120	69	67	r782	MN537570
HuluTR335 (5SrDNA)	335	63	55	r243	MN537579(E)
*HuluTR225	225	46	64	r397	MN537574
*HuluTR060	60	34	59	r91	MN537567
*HuluTR450	450	8	77	r873	MN537581
*HuluTR135	135	5	42	r253	MN537571
*HuluTR600	600	4	69	r823	MN537582
*HuluTR390	390	2	79	r15	MN537580
*HuluTR360	360	2	66	r642	MN537578
*HuluTR240	240	2	71	r1001	MN537575
*HuluTR185	185	2	49	r424	MN537573
*HuluTR100	100	2	62	r983	MN537569
*HuluTR350	350	1	67	r625	MN537577
*HuluTR280	280	1	69	r934	MN537576
*HuluTR150	150	1	60	r390	MN537572
*HuluTR070	70	1	73	r541	MN537568
*HuluTR055	55	1	64	r292	MN537566
*HuluTR050	50	1	57	r33	MN537565

A. * Indicates Tandem Repeat Families newly described in this study.

B. Repeat size rounded to nearest 5 or 10 bp using the most abundant consensus sequence reported by Tandem Repeats Finder for the Representative PacBio Read listed.

C. Rounded number of reads harboring the corresponding TR family per million original reads before k-mer filtering and polySeq family assignments.

D. PacBio Read Number assigned the 1,121 seqeunces after k-mer filtering (e.g. the associated fasta title for r55 is >hulupacbiokf_r0055)

E. The hop 5S rDNA repeat in this clone matches E00464 for 5S rDNA from the 5sRNAdb [33]

Several TR clusters feature a high %A+T (AT content), as is often observed for tandemly-repeated macrosatellite sequences [34]. The average AT content ranged from an unusually low value of 42% for HuluTR135 to a high value of 79% for HuluTR390. The AT content for these TR sequence families is higher than global library average, as is generally expected for satellite DNA. This TR discovery strategy greatly expands the number of published hop TR sequence families while illustrating an approach that could be applied to other plant species for which long-read sequence datasets are available.

### Development of new tandem repeat FISH probes: Selection of representative sequences for TR FISH probe production

**Fig 3:**
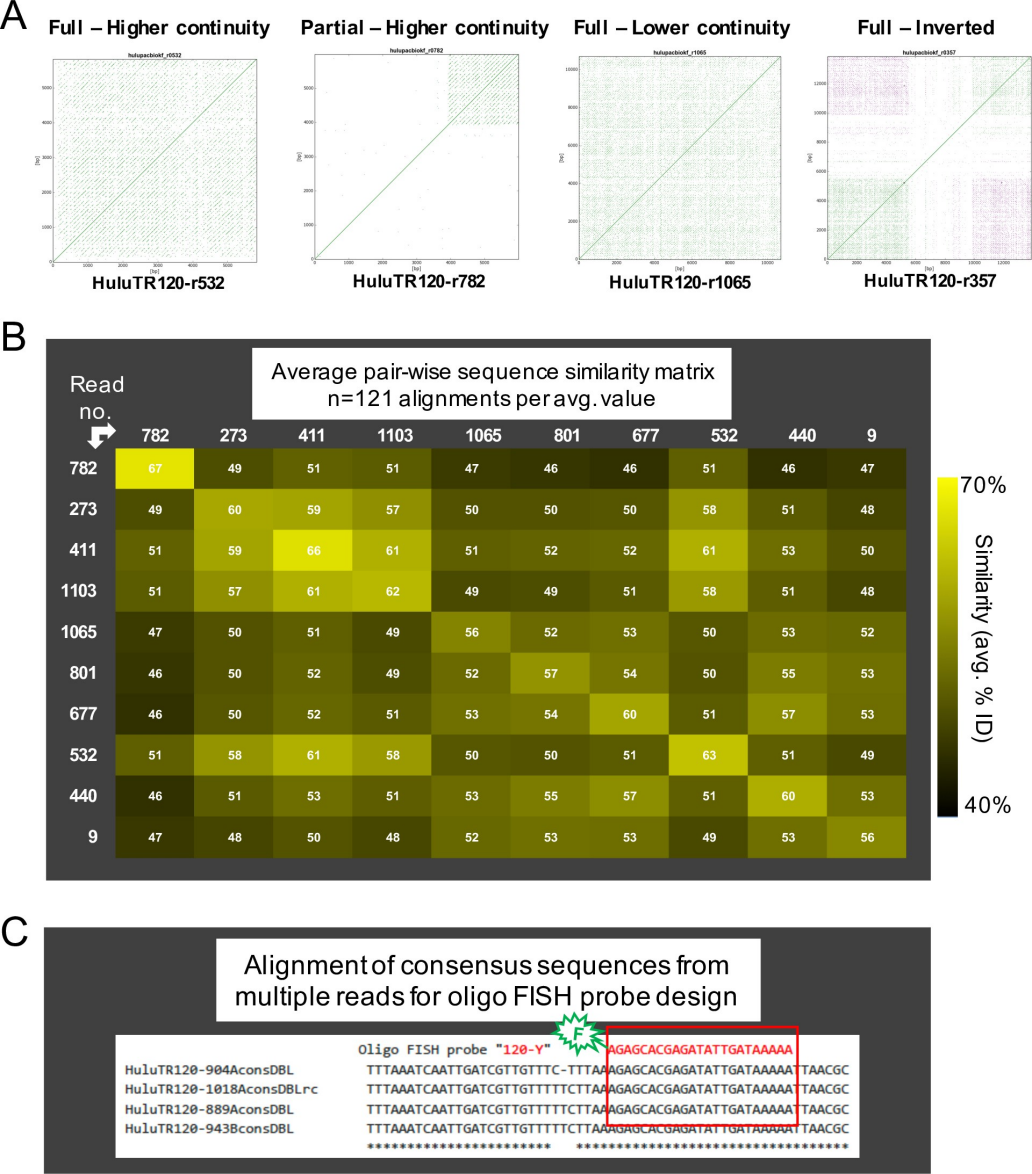
Sequence variation in the HuluTR120 family. (A) Differences observed in diagonal patterns from reads that match the HuluTR120 family. Dot plot patterns are designated according to the aspects of the parallel diagonal morphology and designated as "Higher continuity" or "Lower continuity" in reads with Full or Partial TR occupancy. (B) Pair-wise heat map matrix of average sequence similarity (% identity)between any two reads. The diagonal boxes report within-read average. The numbers on the left and across the top identify the PacBio source read. The color scheme is shown to the right. (D) CLUSTAL Omega sequence alignment example for identification of short sequence OLIGO for fluorescent labeling and subsequent FISH probe design.

Once the tandemly repeated DNA sequences were categorized by family, we aimed to produce representative oligonucleotide FISH probes for cytogenetic detection of the corresponding chromosomal loci. Oligo FISH probes are advantageous because of their small size, uniformity of labeling, and consistency across experiments. The goal of identifying the best region of a tandem repeat family to use as a FISH probe is complicated by considerable sequence variation that is commonly observed in tandem repeat sequence families [35]. For instance, as summarized in Fig 3 for sequences of HuluTR120 family, we observed variation from one read to another in the dot plot patterns. We consider the more continuous, parallel diagonals to reflect tandem repeats with a high degree of similarity (Fig 3A, 1st two plots). Such sequences were given high priority for probe development. However, some reads exhibited a less continuous appearance of diagonals (Fig 3, 3rd plot), which we interpret as having undergone sequence divergence, and were excluded from use in probe development.

To illustrate the range of sequence similarity variation both between and within reads, we selected 10 reads assigned to the HuluTR120 family (Fig 3B). For each read, we extracted an internal, contiguous 11-repeat block of HuluTR120 monomers and separated them to quantify all possible monomer-to-monomer pairwise sequence similarities. This resulted in 122 pairwise similarity values for each read-to-read comparison. The average value for these 122 are shown in the cells of the grid (Fig 3B). The highest within-read average was surprisingly low at 67% (for 782 x 782), even if adjusted for the known long read error rates. In contrast, the between-read averages were 46% (for 782 x 801, 677, or 440).

Given that the sequences of the monomeric repeating units tended to vary within and between individual reads, we decided to use consensus sequence data to guide oligo FISH design (Fig 3C). For high priority reads, those with higher continuity parallel diagonals, we used the Tandem Repeats Finder program [29] to define read-specific consensus sequences. We next carried out multiple sequence alignments of these consensus sequences to identify the most highly conserved sequence regions which were considered ideal for design and production of fluorescent oligonucleotide probes (Fig 3C). A list of new and previously published tandem repeats and FISH probes for hop are summarized in Table 2. Collectively, these represent the beginning of a new toolkit for hop cytogenomics, suitable for future investigations for structural genomics, segregation patterns, and chromosome evolution in hop. Their utility is demonstrated below using two of these new reagents, the oligo FISH probes for HuluTR120 and HuluTR225, in wild collected var. *neomexicanus* hop.

**Table 2:** HuluTR family-specific oligo FISH probes.

Probe name(A)	Sequence(B)	Fluorophores(C)	Reference(D)
MTLF	5'-F-CCCTAAACCCTAAACCCTAAACCCTAAA	F	1
5SBob1	5'-F-GCACCGGATCCCATCAGAACTCC	F	2
5SBob2	5'-F-AGTTAAGCGTGCTTGGGCGAGAG	F	2
5SBob3	5'-F-GTGACCTCCTGGGAAGTCCTCGTG	F, R, Y	2
HSR1	5'-F-GGTACCCCTCTGGTGAATTGGA	F	2
HSR0/ZERO	5'-F-AGAAATATGAGTGAATTACGAAATCGC	R, Y	2
TR120	5'-F-AGAGCACGAGATATTGATAAAAA	F, R, Y	
TR225	5'-F-TTAGTGCAATGTTATCTAGT	F, Y	

A. Probes 5SBob1-3, HSR1, HSR0, and TELO were previously published; others are newly described in this study

B. Oligonucleotides synthesized with 5' fluorophores (5'-F-)

C. Co-synthetically attached fluorophore designations are F, FITC channel, Alexa448; R, TRITC channel, Alexa546; Y, Cy5 channel, ATTO646N

D. 1 = Bass et al. (1997) [17], 2 = Easterling et al. (2018)[7].

### Aberrant meiosis and HuluTR FISH in wild hop

**Fig 4:**
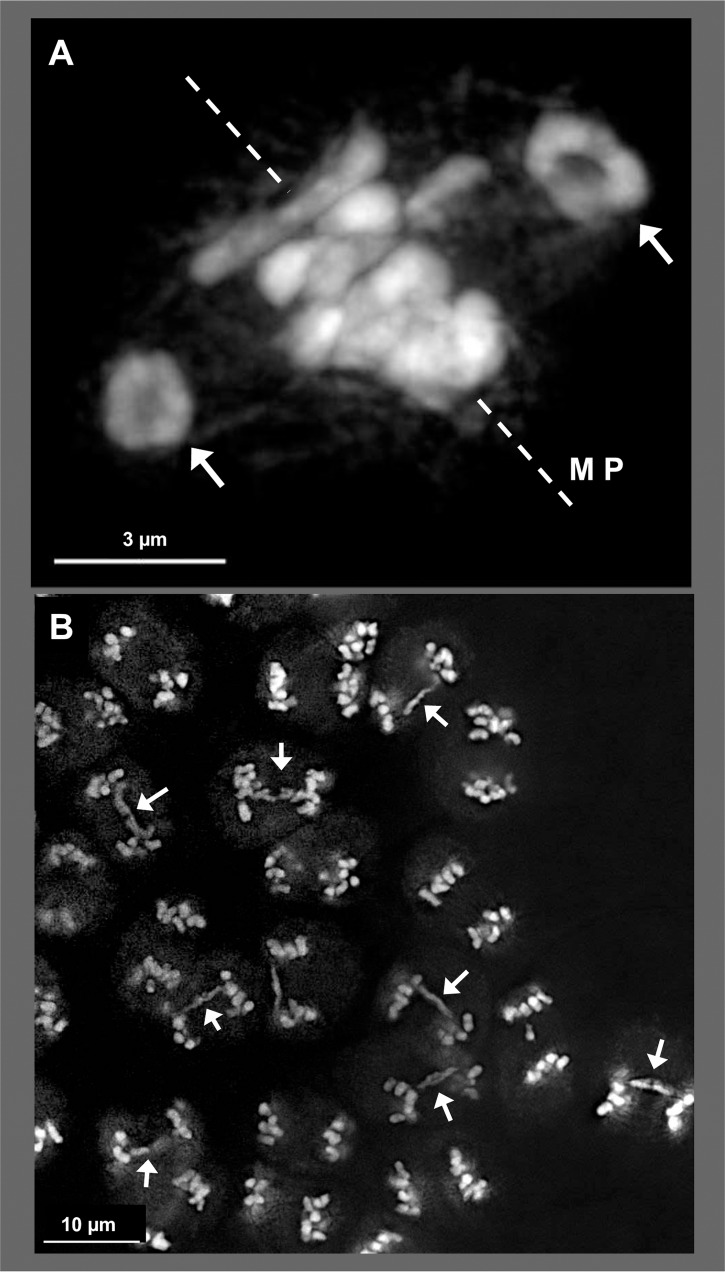
Meiotic abnormalities in Arizona Sky Island wild *neomexicaus* hop, plant TM2-82C. DAPI stained through-focus projections of (A) metaphase I, with bivalents (arrows) outside of the metaphase plate (MP), indicated by dashed line; (B) group of meiotic cells at anaphase I where half of the dividing nuclei exhibit anaphase bridges (arrows). The length of the scale bars are indicated in micrometers. More than 30 nuclei from plant TM2-82C were imaged and analyzed over multiple slides (n = 6) during metaphase I. More than 30 nuclei from plant TM2-82C were imaged and analyzed over multiple slides (n = 6) during anaphase I.

**Fig 5:**
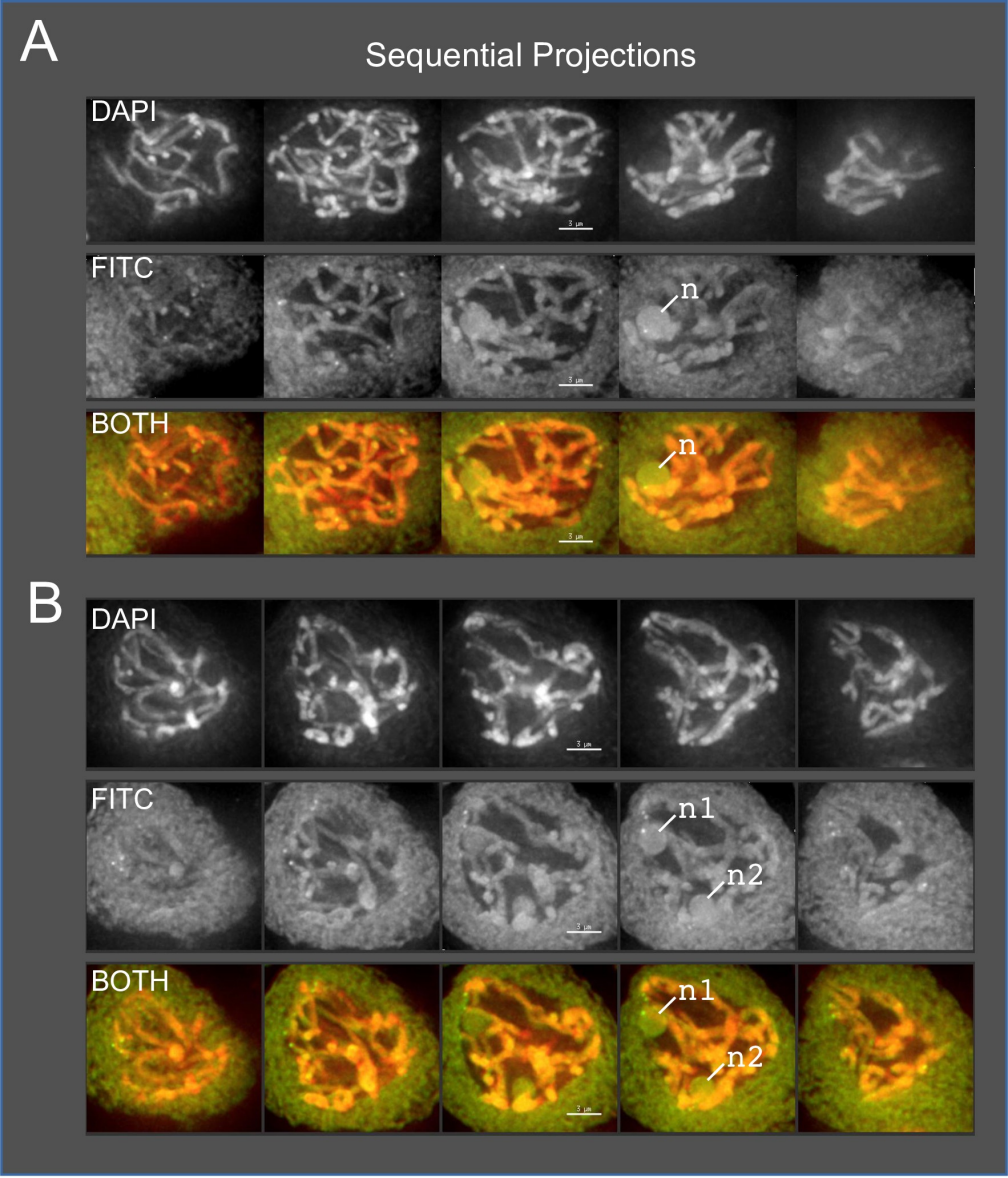
Single and double nucleoli during pachytene in Arizona Sky Island wild *neomexicanus* hop, plant SH2. Male flower buds were harvested and fixed in Farmer’s Fluid, then exchanged into Buffer A and formaldehyde fixed before microdissecting pollen mother cells from anthers for 3D acrylamide telomere FISH. The background fluorescence in the FISH channel reveals the location and number of nucleoli. Through-focus maximum-intensity sequential projections through two individual nuclei are shown in gray-scale for individual wavelengths or in color for overlay images, as labeled on the left. (A) Hop nucleus at mid-prophase showing a single nucleolus (‘n’ in FITC and BOTH). (B) Hop nucleus at mid-prophase showing two separate nucleoli (‘n1’, ‘n2’ in FITC and BOTH). The lengths of the scale bars (3 microns) are indicated. More than 70 nuclei from plant SH2 were imaged and analyzed over multiple slides (n = 14) during pachytene.

**Fig 6:**
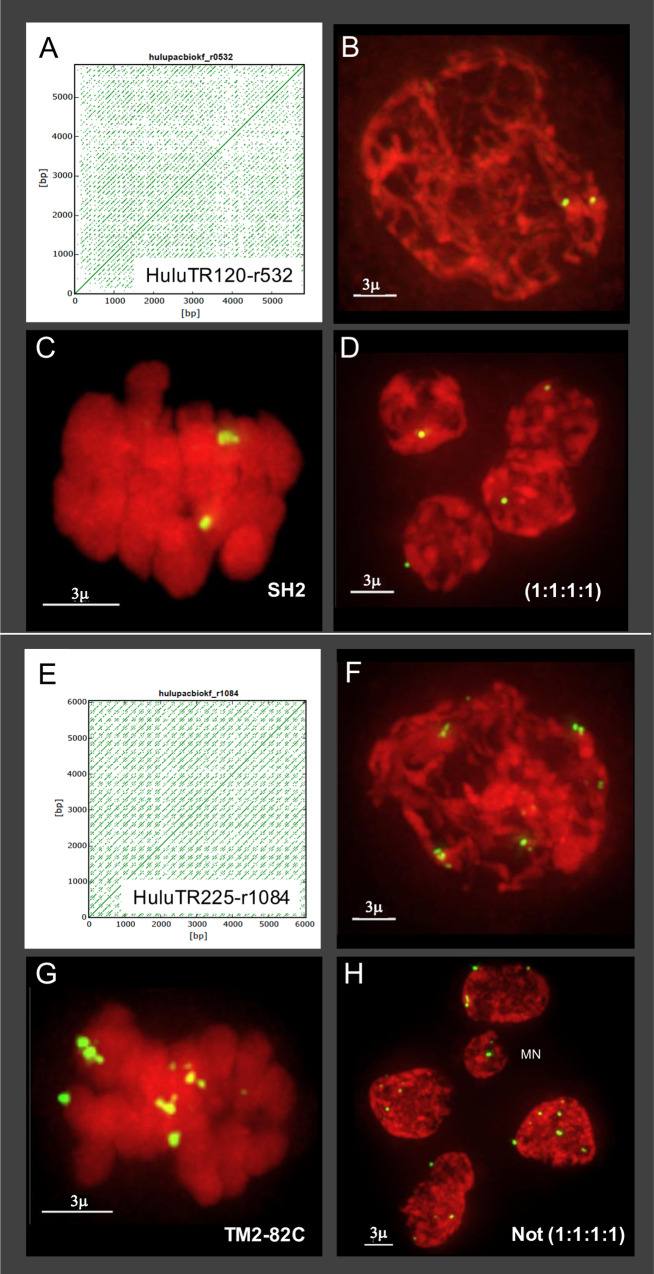
HuluTR120, 225 dot plots and FISH signals during meiosis in plants SH2 and TM2-82C. (A) Dot plot for HuluTR120. (B-D) Hop nucleus from plant SH2 hybridized with oligo FISH probe (TR120-Y) for HuluTR120 at (B) mid-prophase, (C) metaphase I and (D) tetrad stage. Meiotic prophase nuclei show two signals and an equal distribution of 1:1:1:1 after meiosis II at tetrad stage. (E) Dot plot for HuluTR225. (F-H) Hop nucleus from plant TM2-82C hybridized with oligo FISH probe (TR225-Y) for HuluTR225 at (F) mid-prophase, (G) metaphase I and (H) tetrad stage. Meiotic prophase nuclei show approximately 10–12 FISH signals per nucleus with variable size and brightness per signal spot. The tetrad-stage cell shows highly variable signals at the second meiotic division of approximately (5:4:2:4:7) and a micronucleus, labeled MN. The lengths of the scale bars are indicated in micrometers. More than 80 nuclei from plant SH2 were imaged and analyzed over multiple slides (n = 5) stained with HuluTR120 during various meiotic stages. More than 80 nuclei from plant TM2-82C were imaged and analyzed over multiple slides (n = 4) stained with HuluTR225 during various meiotic stages.

An important question in hop genome evolution is whether or not aberrant meiosis is a natural, intrinsic feature of hop or whether it can be explained entirely as a result of breeding and cultivation with structurally diverse genomes. To begin to address this issue and to demonstrate a possible application of these new FISH probes, wild hop was collected from what are thought to be isolated populations [36] in the Arizona Sky Islands and male meiosis analyzed cytologically as shown in Figs 4–6.

We found evidence of aberrant chromosomal behavior at metaphase I (Fig 4A) and anaphase I (Fig 4B) using 3D imaging of DAPI-stained meiocytes. At metaphase, chromosomes typically congress on the metaphase plate, but in the example shown, two presumed ring bivalents (arrows, Fig 4A) are seen to be excluded from the metaphase plate, indicative of a chromosomal positioning problem. The frequency of irregularities was conspicuous and occasionally extreme as seen in the low magnification image of 22 anaphase-stage cells from a single plant (TM2-82C from Mt. Bigelow), 11 of which exhibited chromosome bridges (arrows, Fig 4B). Another way to track chromosome bivalency is through observation of nucleoli, which we were able to observe via autofluorescence. When homologous rDNA regions with NORs pair and synapse, their associated nucleoli fuse into a single nucleolus. Given that reported hop karyotypes have a single NOR locus [14,37], we would expect that normal pairing would result in fusion of the homologous NORs to give one large nucleolar region by mid-prophase. However, tracking nucleoli number in mid-late meiotic prophase, we found that the meiocytes from wild hop (plant SH2 from Mt. Lemmon) could show either of two different patterns, single nucleoli (‴n" in Fig 5A) or double (“n1”, “n2” in Fig 5B). Interestingly, the double nuclei occurred at an unusually high frequency, observed in 11 of the 22 nuclei imaged in 3D. We interpret the presence of double nucleoli as deviation from normal disomic, homologous pairing at the NOR regions. Consistent with this interpretation, we observed that the average nucleolar volumes were 14 μ^3^ for single-nucleolus cells (n = 17 nucleoli) and 5 μ^3^ for double-nucleolus cells (n = 16 nucleoli in 8 cells), a 2.8 fold difference.

In order to test our new FISH probes on wild and non-Apollo hops, we applied two of them, HuluTR120 and HuluTR225, to meiocytes of two var. *neomexicanus* plants, SH2 and TM2-82C, as shown in Fig 6. We show that both of these HuluTR probes, designed from Apollo sequence data, successfully hybridized as discrete foci on the chromosomes of wild hop. In one case, the HuluTR120 probe gave two bright signals in plant SH2 as seen at mid-prophase (Fig 6B) and metaphase I (Fig 6C), a pattern indicative of paired homologous loci. At the tetrad stage, the HuluTR120 signals were distributed equally (Fig 6D, 1:1:1:1). In another case, the HuluTR225 probe gave more complex patterns in plant TM2-82C, with variable brightness and size. The 10–12 FISH signals are seen at mid-prophase (Fig 6F) and at metaphase I (Fig 6G). The FISH signals appear to be distributed in an irregular pattern at both metaphase I and the post-meiotic tetrad-like stage (Fig 6H). The examples represent multiple occurrences of meiotic abnormalities from a single plant (Figs 4B and 6E–6H). Therefore, TR probes designed from one genotype can be used in others, and wild hops show both balanced (D) and unbalanced FISH signal distribution (H), similar to recent observations with 5S rDNA FISH [7]. his approach to develop new cytogenomic tools enabled the discovery and characterization of a class of tandem repeats with demonstrated utility for investigating the mysterious mechanisms of hop genome transmission and chromosomal evolution.

## Discussion

Interest in tandem repeats has prompted investigators to develop new software programs to find or characterize tandem repeats using DNA sequencing data [24,26,38]. Among the programs used are Tandem Repeat Finder [29], which uses string matching algorithms, and those utilizing graph-based clustering, such as RepeatExplorer [39] and TAREAN [32]. These programs allow for the mining of existing and public repositories of genomic data to identify tandem repeats for various studies related to phylogenetics, genome evolution, and cytogenetics [26,40,41]. More recently, long-read sequence data has been used to support FISH probe development in plants, with the aid of RepeatExplorer and TAREAN [42,43].

Here, we describe an approach using long-read sequences that allows for TR discovery aided by direct visual inspection of single self-aligned read dot plots. Even with these error-prone early generation single-molecule reads, we were able to uniquely and unambiguously find and group tandemly repeated sequence families and build consensus sequences. The DNA sequences from these reads were screened by k-mer analysis using criteria that yielded ~1000X enrichment for reads with the desired sequence features. The k-mer filtered dot plots provide highly informative way to visualize the data, making it easy to quickly interpret tandem repeat patterns within their genomic context one read at a time without any requirement for assembly. Compared to other methods, the approach described here has several notable advantages including (1) intuitive visualization of the genomic structure of the repeats, (2) highly sensitive ability to detect tandem repeats, as illustrated by the discovery of reads with HuluTR families present once per million reads (e.g. HuluTR050, HuluTR055, HuluTR070, HuluTR150, HuluTR280, and HuluTR350), (3) the retention of adjacent flanking genomic sequence, possibly useful for guiding genome assembly efforts, and (4) the retention of the individuality of TR clusters, which may come from multiple different loci. This last advantage may be helpful for future consideration of homologous alleles, homeologous alleles from hybrids, or multi-chromosomal loci on different paths of divergence. In contrast, the approach reported here has disadvantages such as the requirement for long-read sequences as the input data and the fact that the larger repeats, the less likely they will meet our k-mer threshold for 5–10 kbp reads. On the whole, we consider this a robust and versatile approach as evidenced by our ability to find both known (5S rDNA, HSR1, HSR0, and telomere repeats) and many new TRs (Table 1).

Repetitive sequences pose the greatest challenge for assembling complete genomes. The 1C genome size estimates for hop range from 2.5–3.0 Gb according to flow cytometric methods [15,44,45] but only 2.1 Gb according to a recent from genome assembly [15]. Therefore, sequence assemblies currently account for only 80% of the known genome size, indicating that a large fraction of the genome is not represented in contemporary assemblies. Tandem repeat sequences are often mis-assembled and under-represented, being particularly prone to the repeat collapse problem in genome assembly. These discrepancies contribute to the genome size under-estimations while exacerbating problems associated with accurate contig assembly. For instance, markers flanking a TR cluster may be separated by only a few Kbp of TR, but reside on different contigs if only short read sequences guide the assemblies. Accurate incorporation of TR clusters is especially important in hop given its high degree of structural variability and segregation distortion [7,9].

A primary goal of this study was to produce new molecular cytology tools for hop chromosome research. To that end, we have described 17 new tandem repeat families (Table 1) and shown FISH results with probes for HuluTR120 and HuluTR225. To date, most of the hop chromosomes are numbered and distinguished by their relative size and in some cases their centromere locations as inferred from the primary constriction on mitotic chromosomes [12]. The most current hop karyotype includes HSR1, 5S rDNA, NOR, and telomere signals, which together uniquely tag 4 of the 10 chromosomes [13,14,37]. Notably, centromere-specific sequences have yet to be identified in hop. It is possible that among our HuluTR families are one or more that reside at centromeres. Alternatively, hop centromere repeats may not be organized as tandem repeats or their size and copy number may have resulted in their exclusion from our k-mer filtered subset of 1,121 reads. Indeed, a recent study in wheat found that centromeric tandem repeats enriched at CENH3 ChIP seq peaks can exceed 500 bp in repeat unit length [25].

FISH probes are also invaluable for tracking meiotic chromosome interactions and post-meiotic transmission of discrete genetic loci. For instance, hop 5S rDNA FISH probes were previously used to document abnormal chromosomal interactions during pairing at late prophase and cytological segregation distortion in tetrads [7]. Here we present two new FISH probes that hybridize to a small number of discrete foci in wild hop plants. HuluTR120 FISH signals showed equal distribution of signals at the tetrad stage (1:1:1:1) in meiocytes from one wild plant, SH2. HuluTR225 FISH signals showed clear irregularities in meiocytes from a different wild plant, TM2-82C. An emerging picture is that there is considerable variation in FISH patterns even when using the same probe on cells from the one plant, siblings, or different varieties. This highlights the magnitude of the challenge of sorting out the hop genome and the importance of developing new markers of all types. With advances in hop genomics, and as the connections between physical chromosomes and linkage groups are elucidated, a cytological toolkit of TR FISH probes will accelerate an integrated view of the hop genome.

Wild hop populations occur naturally across the US in three varieties and are morphologically distinct but are not necessarily reproductively isolated [36]. They have been described as monophyletic [46] and are known to exhibit high levels of genetic diversity, particularly var. *neomexicanus* [47]. It is worth noting that cultivated, escaped hop plants, also referred to as ferals, can be mistaken for wild varieties, especially near areas where hop is cultivated or bred. In this study, we intentionally wanted wild *neomexicanus* hops and collected, therefore, from remote southwest US regions in the Arizona Sky Islands where the hop plants are morphologically distinct var. *neomexicanus*. Our cytological data in these wild plants (Figs 4–6), together with previously reported meiotic segregation irregularities [7,9] establish that such meiotic abnormalities are clearly not limited to cultivated hop and can also occur in the wild. These findings, while limited in scope, highlight the recurrent observations of genomic instability in some members of the species. Similar phenomena have been observed *Oenothera* sp. and *Clarkia* sp., members of the Onagraceae family [48,49]. Interestingly, some of these have stabilized structural variation though specialized meiotic behavior possibly contributing directly to speciation events [50]. It remains to be determined whether the evolutionary dynamics of hop has contributed to speciation or divergence in the wild, questions that can be addressed using chromosome-marking FISH probes.

Here we considerably increased the number of known tandem repeat sequence families in hop using an innovative bioinformatic pipeline for de novo identification, visualization, and classification of TRs from long-read sequence data. This approach and the resulting cytogenetic resources should prove useful for further investigations into evolutionary, cytogenetic, or structural genomic research in hop.

## Supporting information

S1 Data (PDF)

S2 Data (PDF)

S1 File (FAS)

S1 Fig (PDF)

S2 Fig (PDF)

S3 Fig (PDF)

S4 Fig (PDF)
