# Supplementary material for: Identification of tandem repeat families from long-read sequences of Humulus lupulus
Source: PLoS One. 2020 Jun 5;15(6):e0233971. doi: 10.1371/journal.pone.0233971 (PMC7274563; doi:10.1371/journal.pone.0233971)
Supplement: S1 Fig — (PDF) [file pone.0233971.s004.pdf]

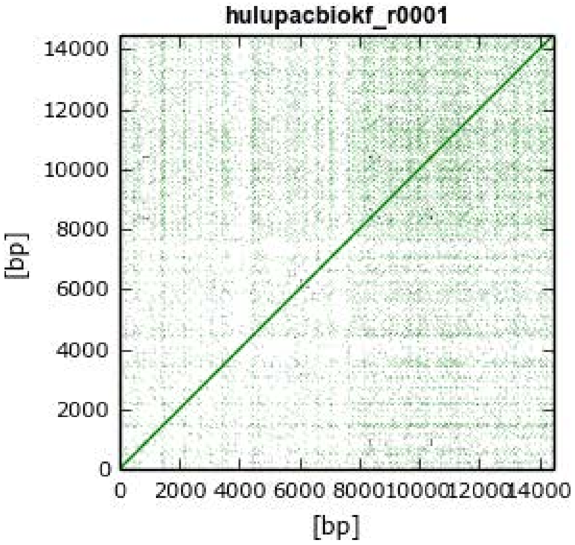
hulupacbiokf_r0001
14000
12000
10000
8000
6000
4000
2000
0
[bp]
0
2000
4000
6000
8000
10000
12000
14000
[bp]

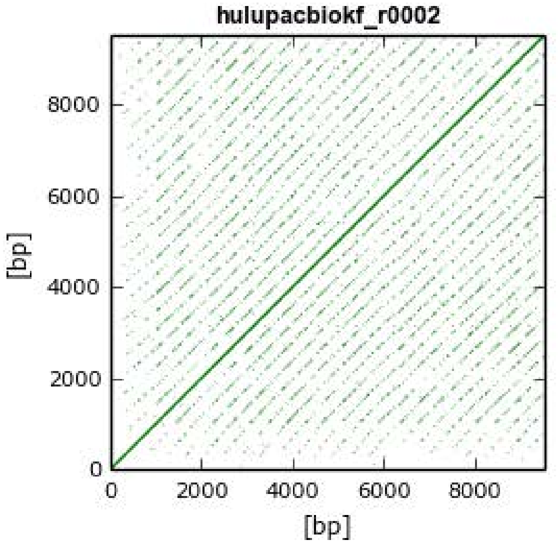
hulupacbiokf_r0002
8000
6000
4000
2000
0
[bp]
0
2000
4000
6000
8000
[bp]

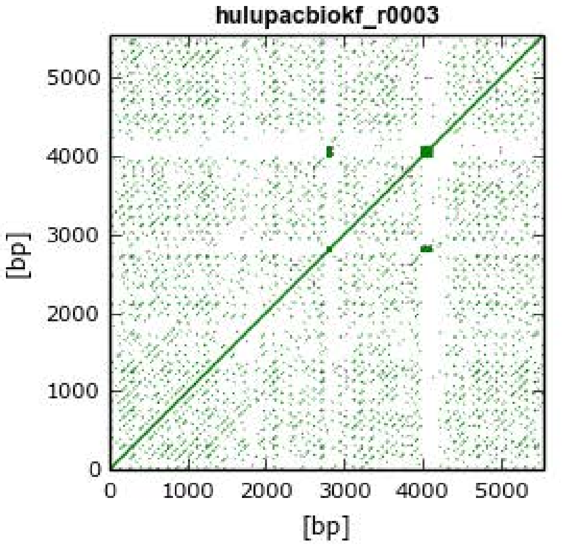
hulupacbiokf_r0003
5000
4000
3000
2000
1000
0
[bp]
0
1000
2000
3000
4000
5000
[bp]

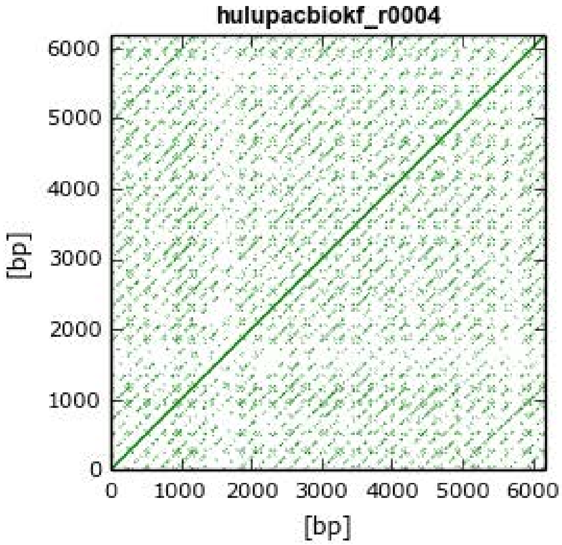
hulupacbiokf_r0004
6000
5000
4000
3000
2000
1000
0
[bp]
0
1000
2000
3000
4000
5000
6000
[bp]

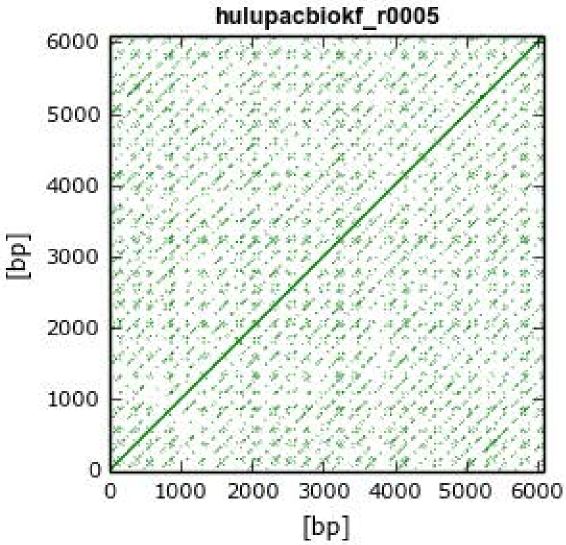
hulupacbiokf_r0005
6000
5000
4000
3000
2000
1000
0
[bp]
0
1000
2000
3000
4000
5000
6000
[bp]

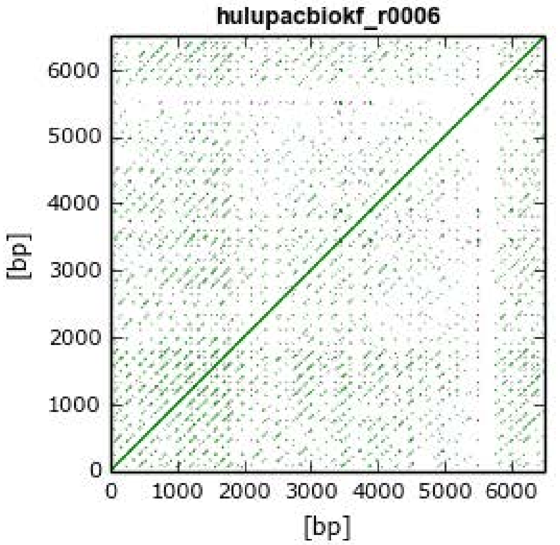
hulupacbiokf_r0006
6000
5000
4000
3000
2000
1000
0
[bp]
0
1000
2000
3000
4000
5000
6000
[bp]

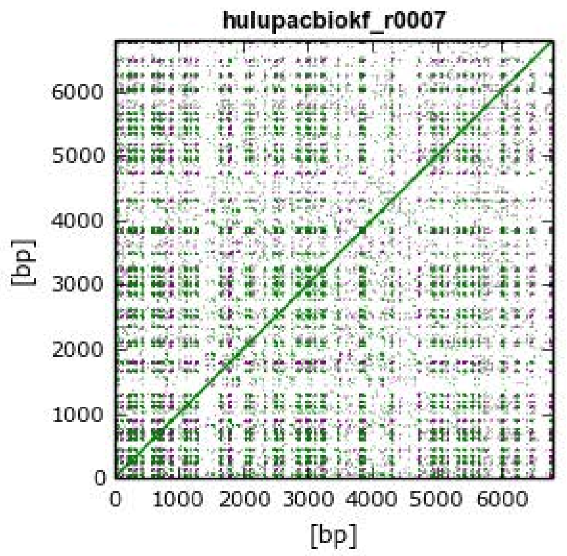
hulupacbiokf_r0007
[bp]
[bp]

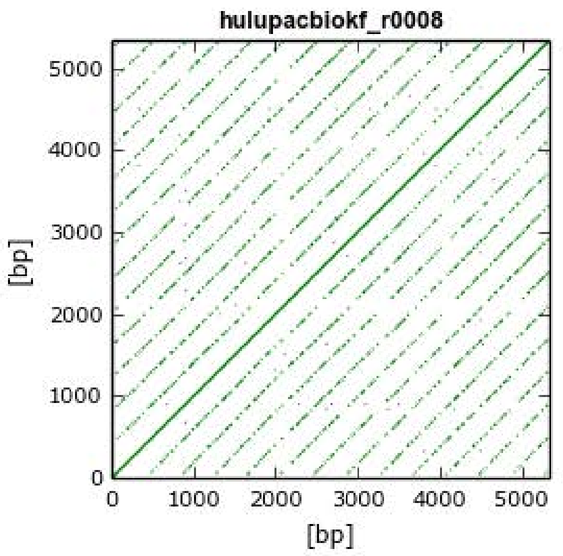
hulupacbiokf_r0008
[bp]
[bp]

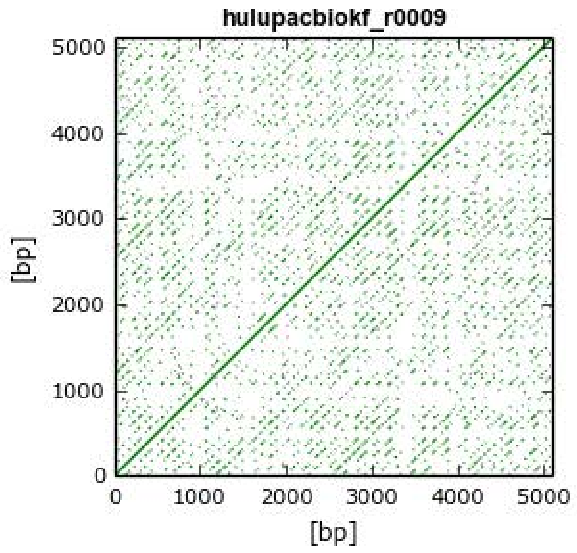
hulupacbiokf_r0009
[bp]
[bp]

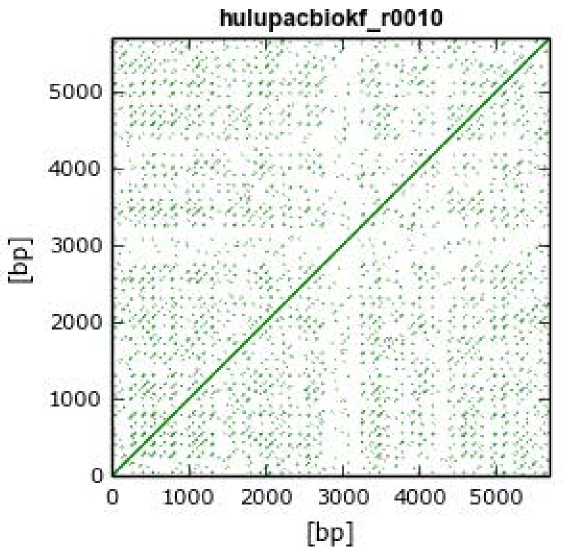
hulupacbiokf_r0010
[bp]
[bp]

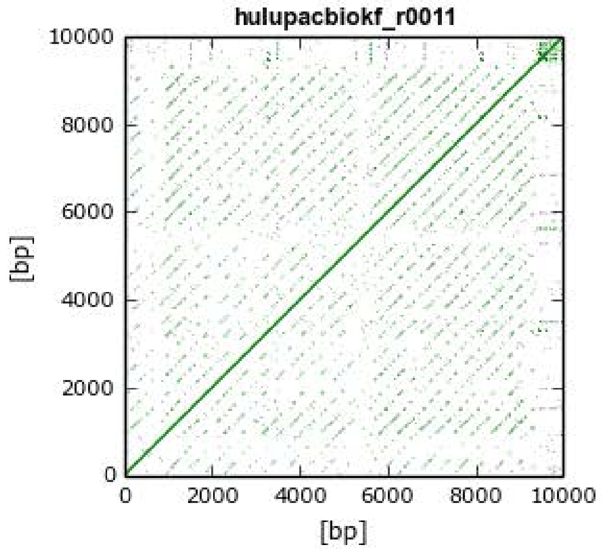
hulupacbiokf_r0011
[bp]
[bp]

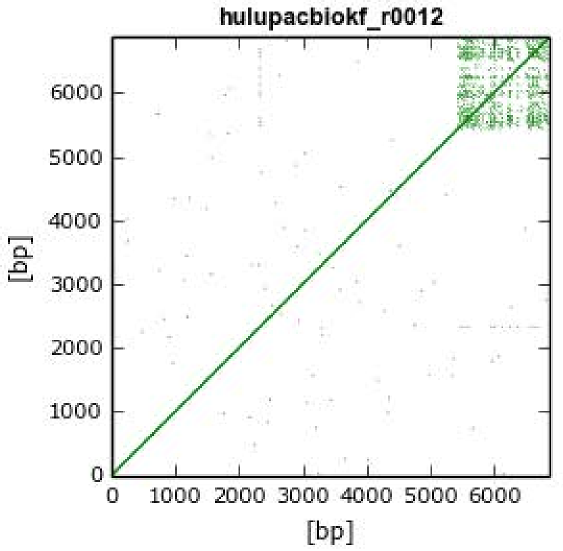
hulupacbiokf_r0012
[bp]
[bp]

hulupacbiokf_r0013

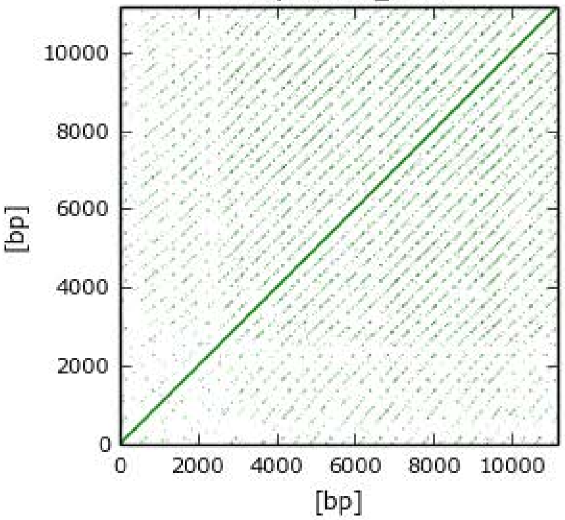

hulupacbiokf_r0014

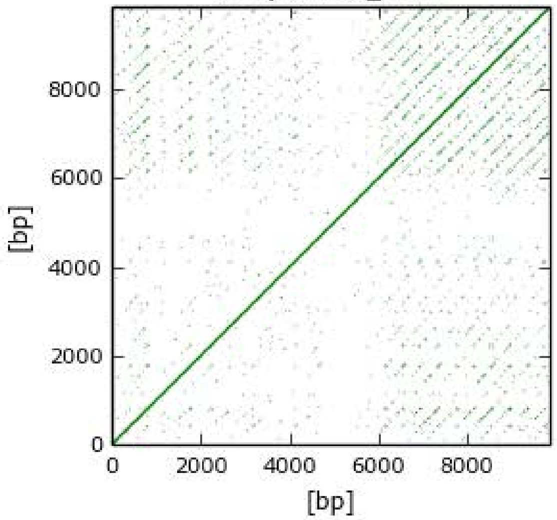

HuluTR390 from read r0015
is in GenBank Acc. MN537580

hulupacbiokf_r0015

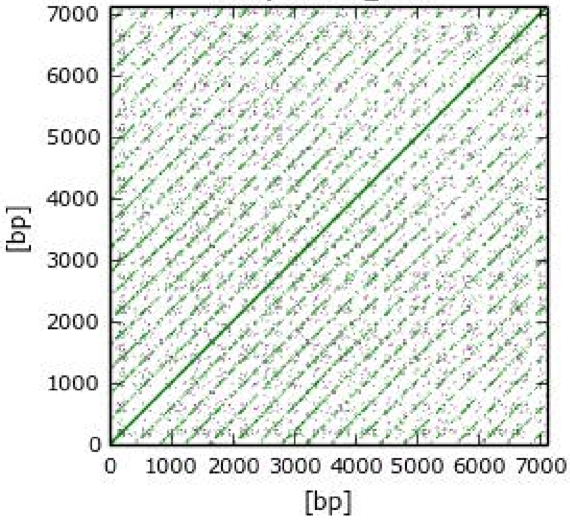

hulupacbiokf_r0016

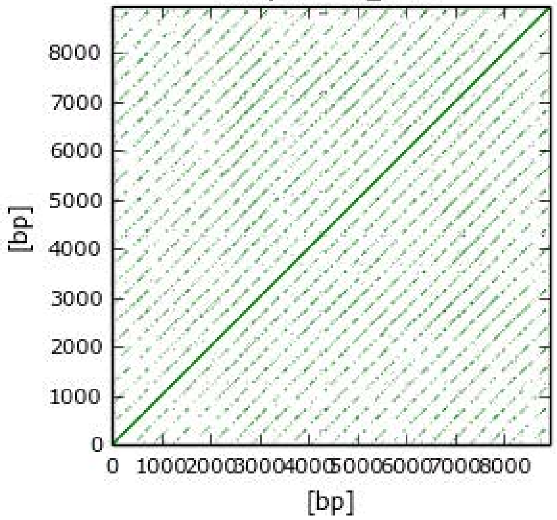

hulupacbiokf_r0017

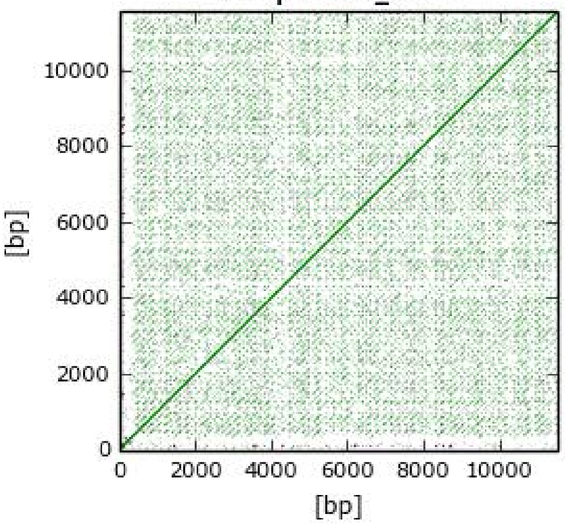

hulupacbiokf_r0018

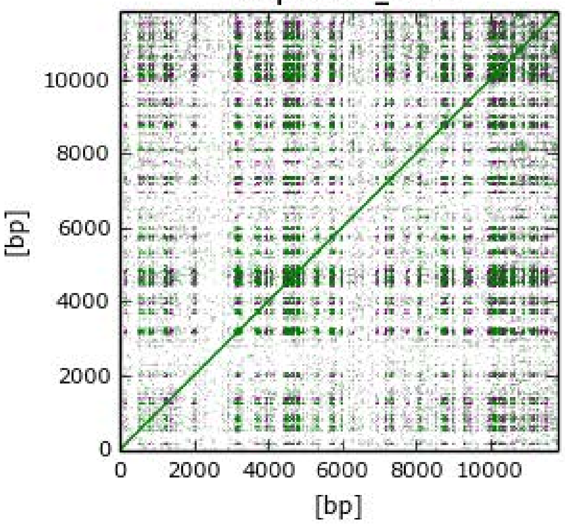

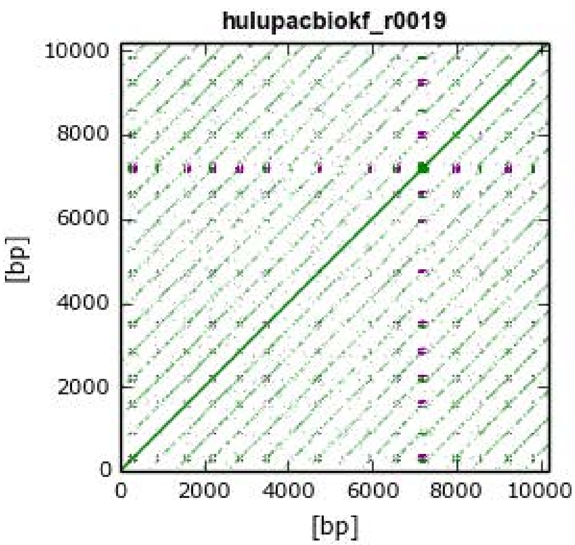
hulupacbiokf_r0019
10000
8000
6000
4000
2000
0
[bp]
0 2000 4000 6000 8000 10000
[bp]

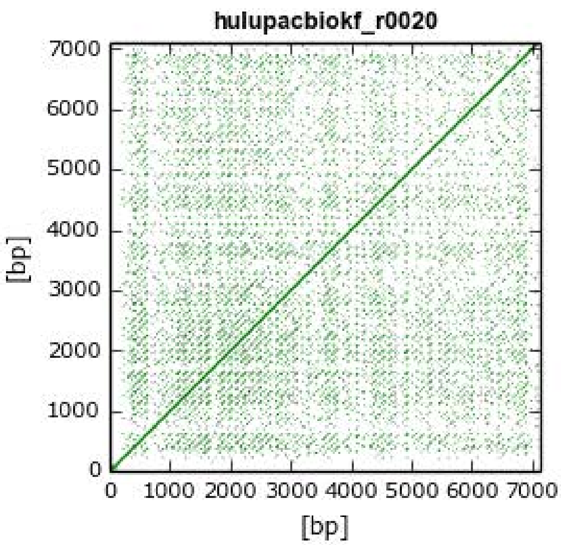
hulupacbiokf_r0020
7000
6000
5000
4000
3000
2000
1000
0
[bp]
0 1000 2000 3000 4000 5000 6000 7000
[bp]

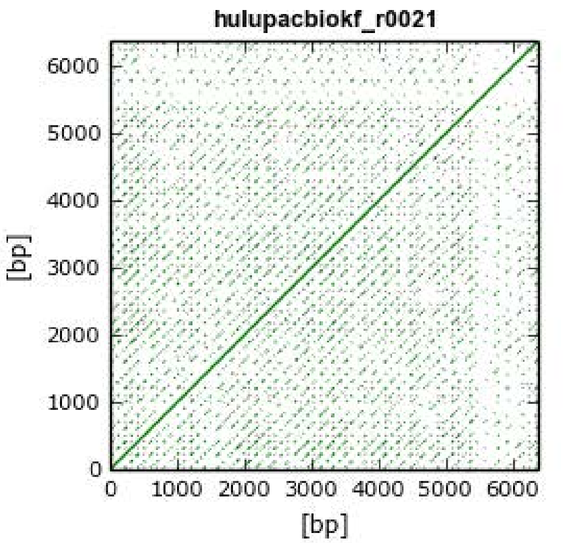
hulupacbiokf_r0021
6000
5000
4000
3000
2000
1000
0
[bp]
0 1000 2000 3000 4000 5000 6000
[bp]

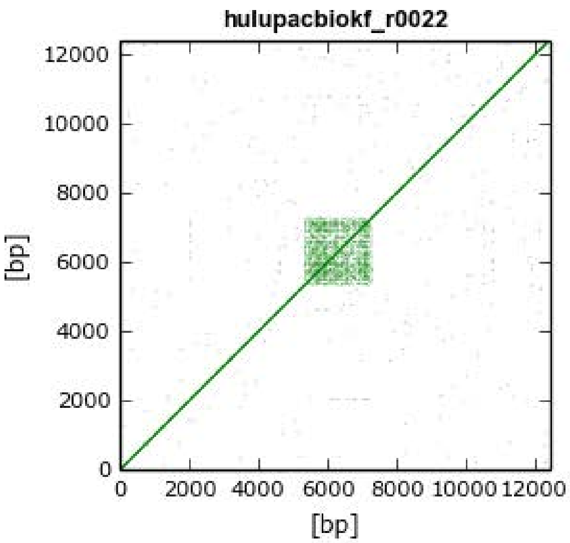
hulupacbiokf_r0022
12000
10000
8000
6000
4000
2000
0
[bp]
0 2000 4000 6000 8000 10000 12000
[bp]

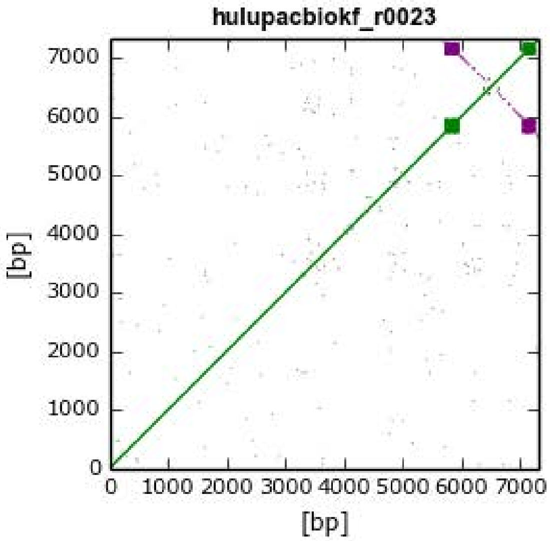
hulupacbiokf_r0023
7000
6000
5000
4000
3000
2000
1000
0
[bp]
0 1000 2000 3000 4000 5000 6000 7000
[bp]

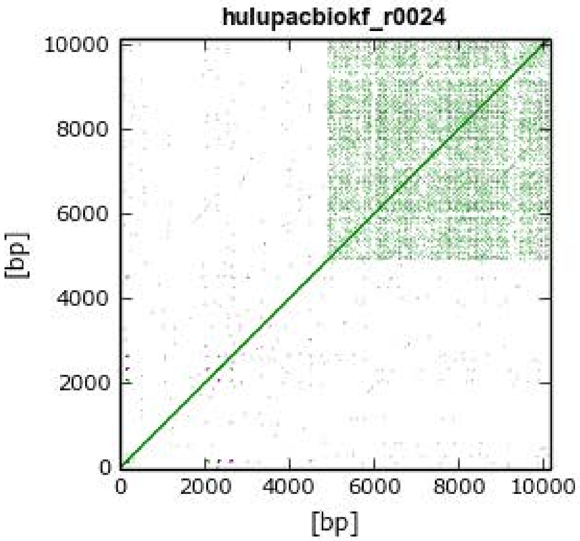
hulupacbiokf_r0024
10000
8000
6000
4000
2000
0
[bp]
0 2000 4000 6000 8000 10000
[bp]

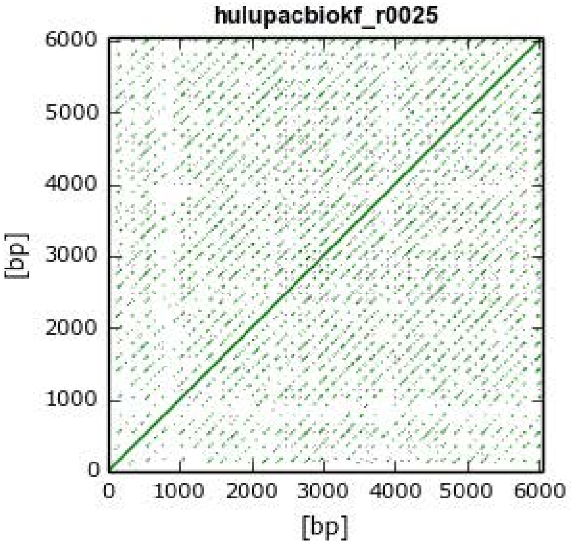
hulupacbiokf_r0025
[bp]
[bp]

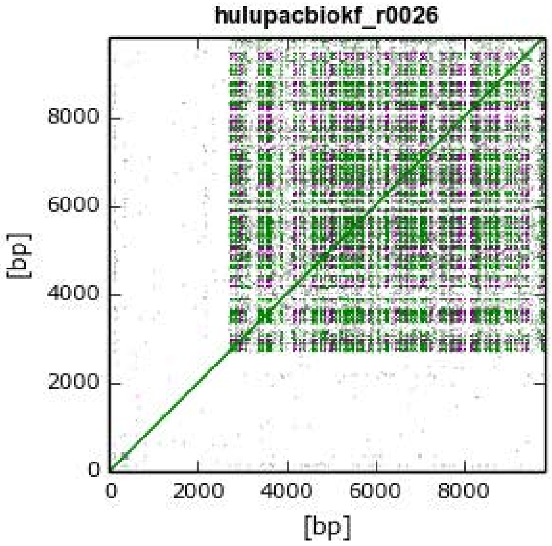
hulupacbiokf_r0026
[bp]
[bp]

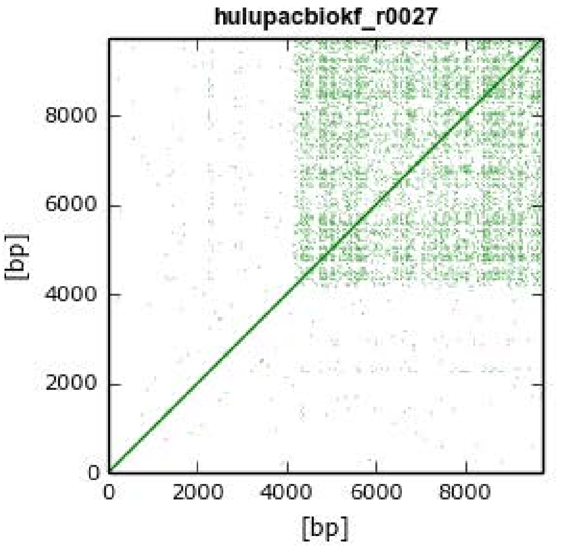
hulupacbiokf_r0027
[bp]
[bp]

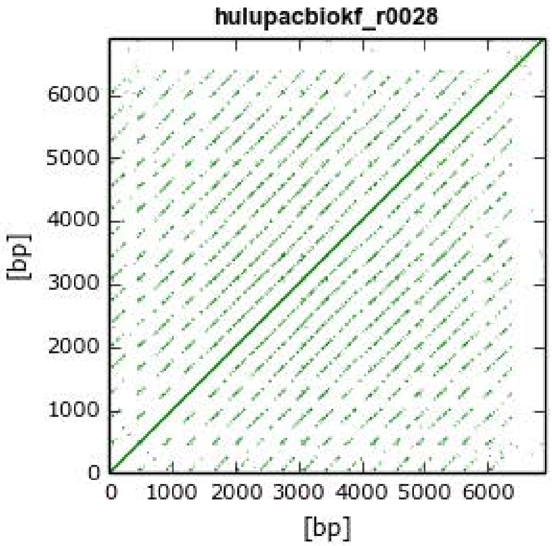
hulupacbiokf_r0028
[bp]
[bp]

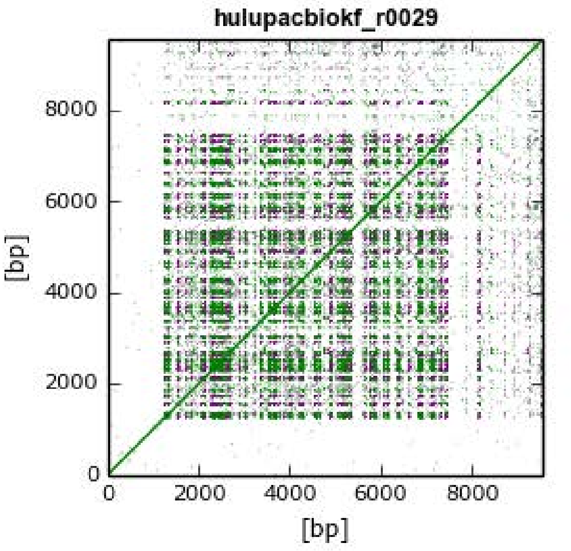
hulupacbiokf_r0029
[bp]
[bp]

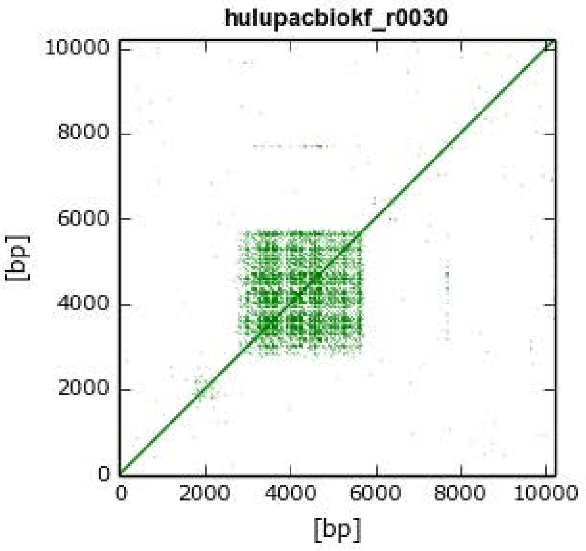
hulupacbiokf_r0030
[bp]
[bp]

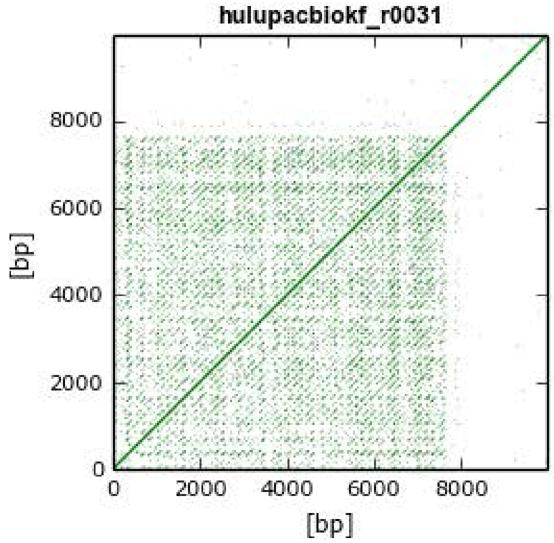

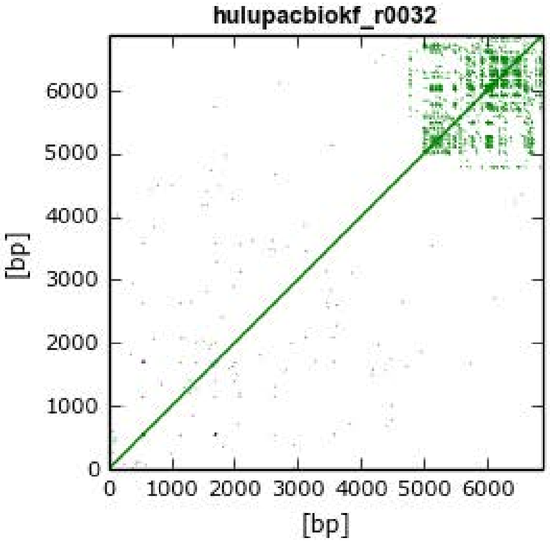

HuluTR050 from read r0033
is in GenBank Acc. MN537565

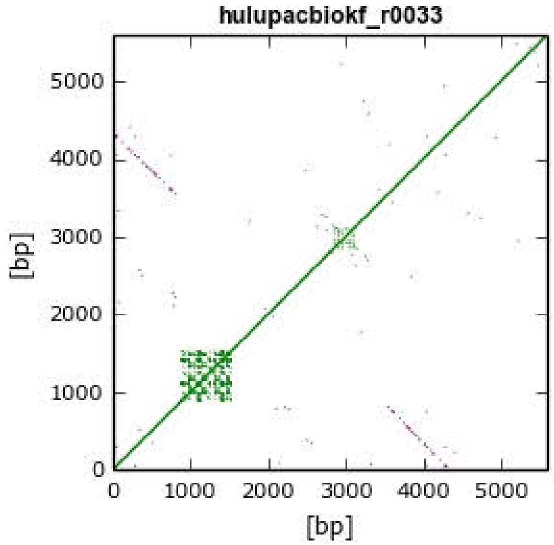

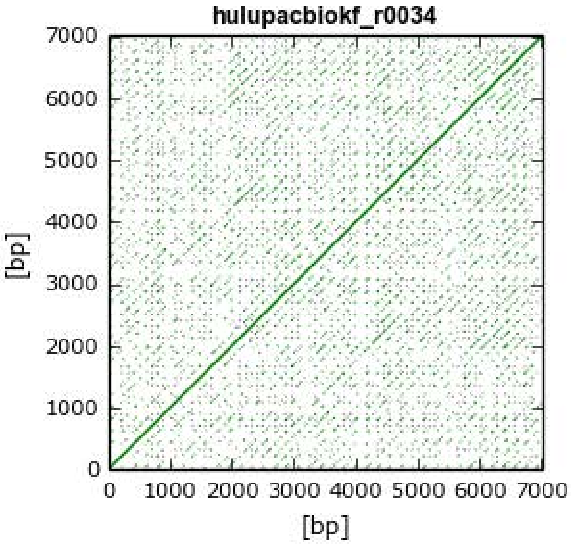

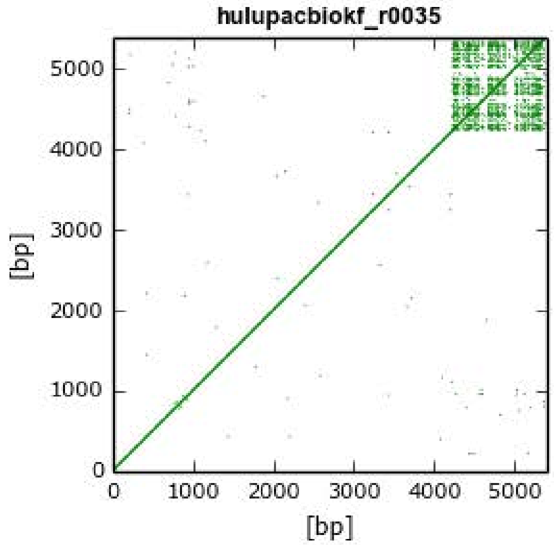

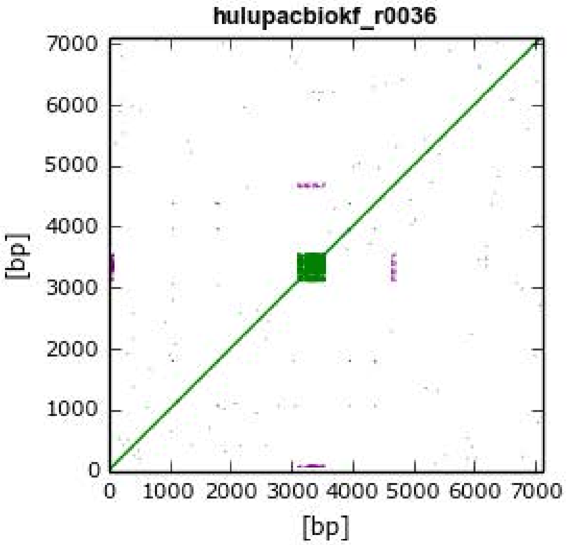

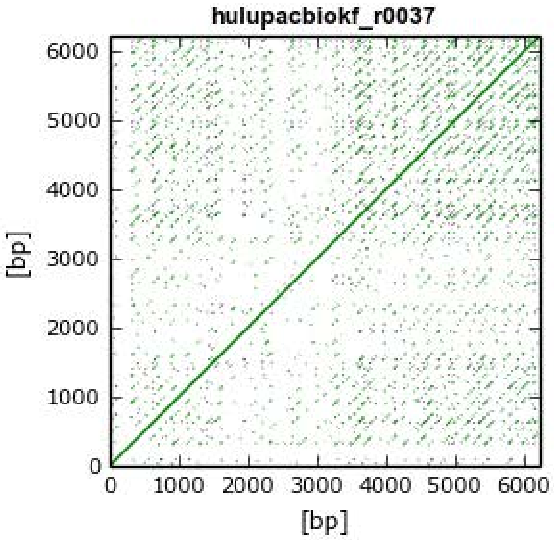
hulupacbiokf_r0037
[bp]
[bp]

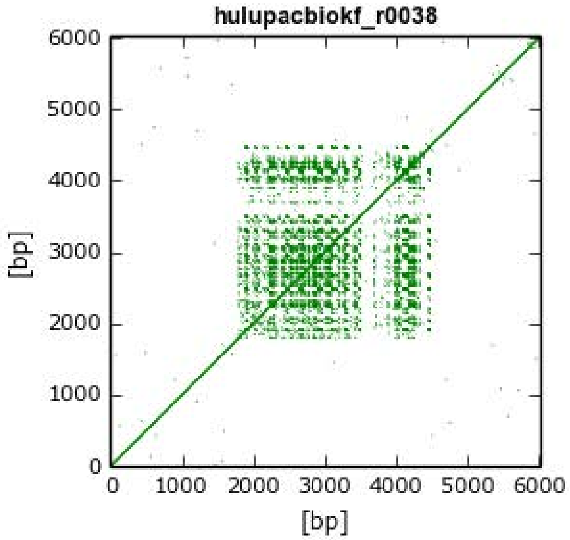
hulupacbiokf_r0038
[bp]
[bp]

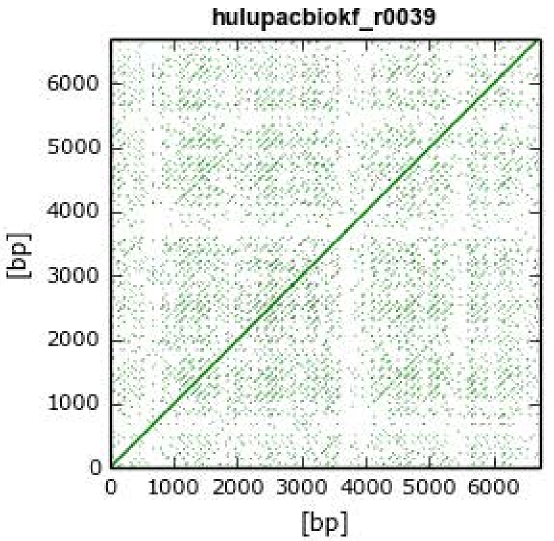
hulupacbiokf_r0039
[bp]
[bp]

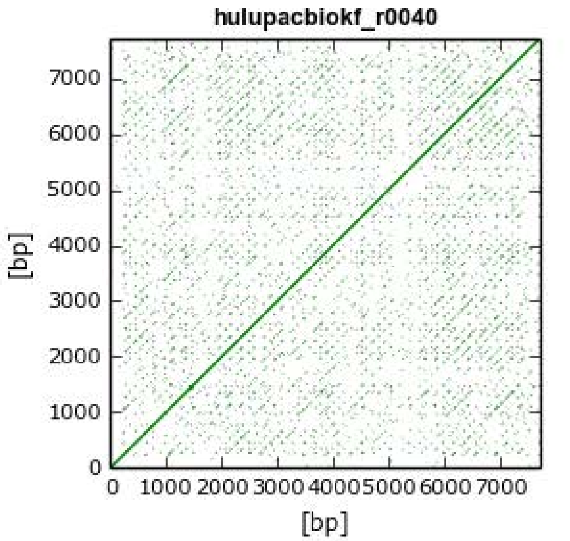
hulupacbiokf_r0040
[bp]
[bp]

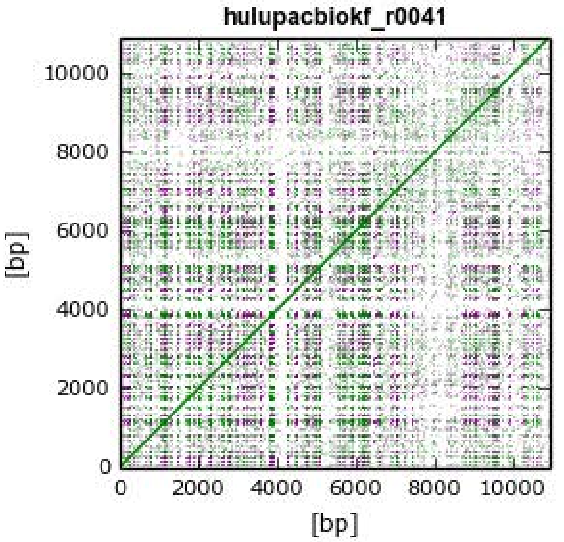
hulupacbiokf_r0041
[bp]
[bp]

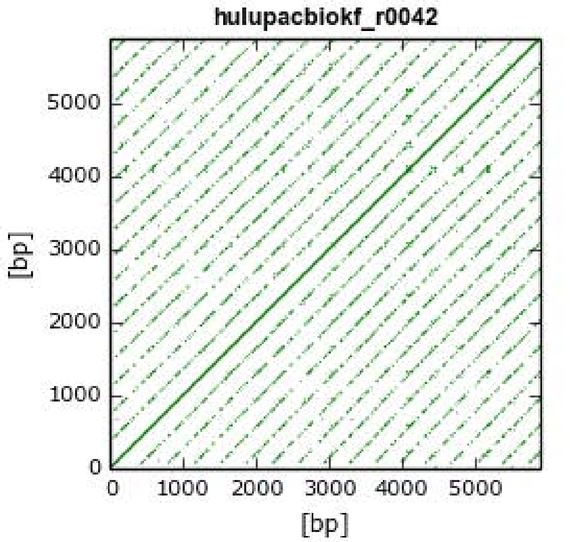
hulupacbiokf_r0042
[bp]
[bp]

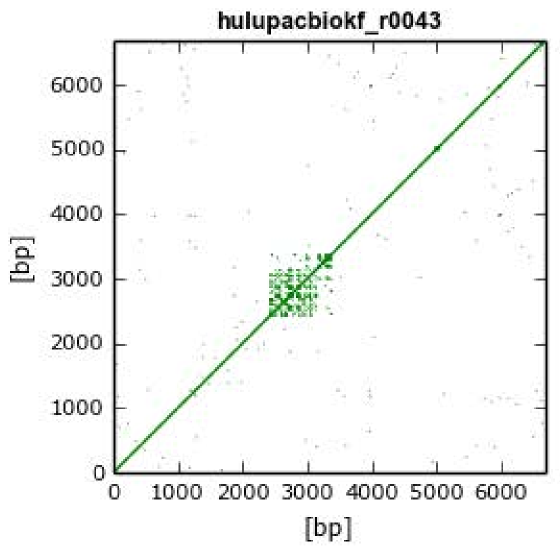
hulupacbiokf_r0043
[bp]
0
1000
2000
3000
4000
5000
6000

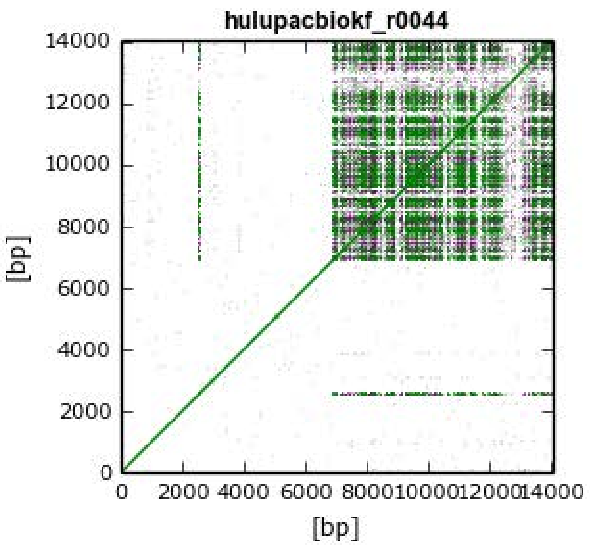
hulupacbiokf_r0044
[bp]
0
2000
4000
6000
8000
10000
12000
14000

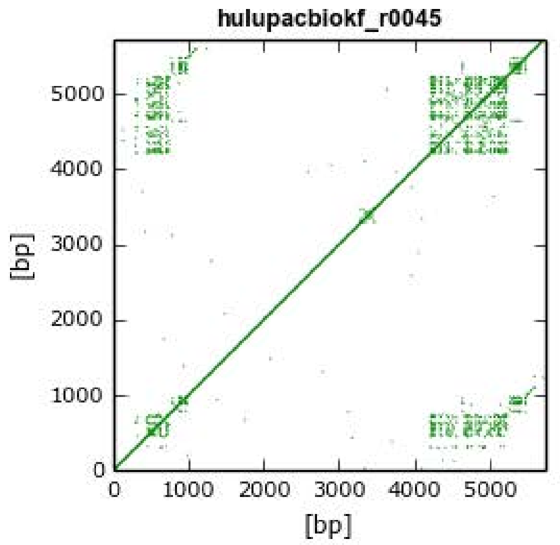
hulupacbiokf_r0045
[bp]
0
1000
2000
3000
4000
5000

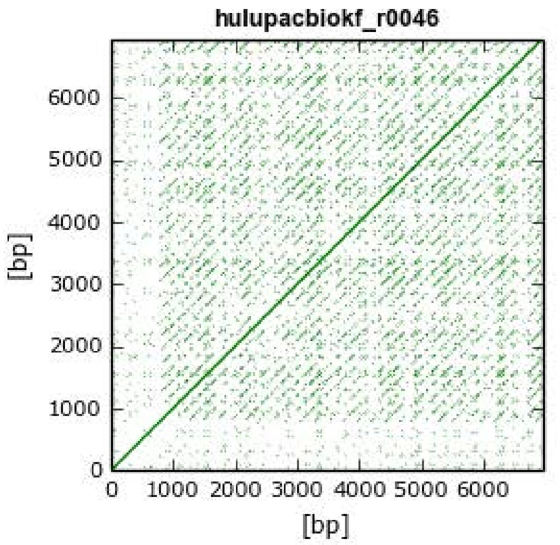
hulupacbiokf_r0046
[bp]
0
1000
2000
3000
4000
5000
6000

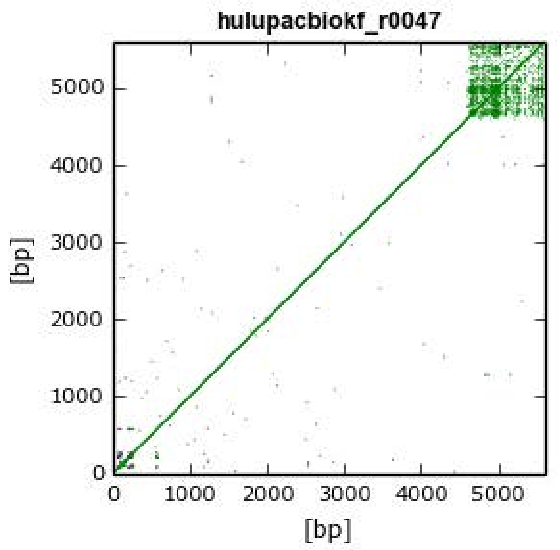
hulupacbiokf_r0047
[bp]
0
1000
2000
3000
4000
5000

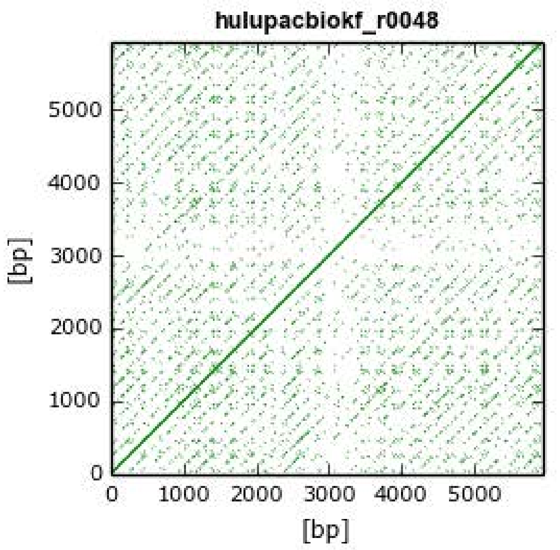
hulupacbiokf_r0048
[bp]
0
1000
2000
3000
4000
5000

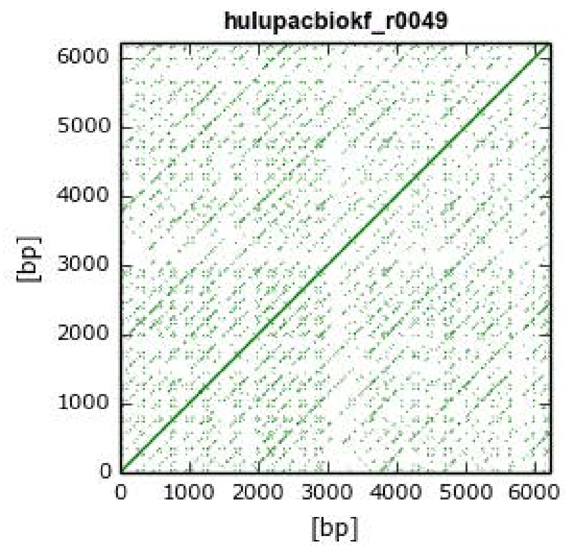
hulupacbiokf_r0049
[bp]
[bp]

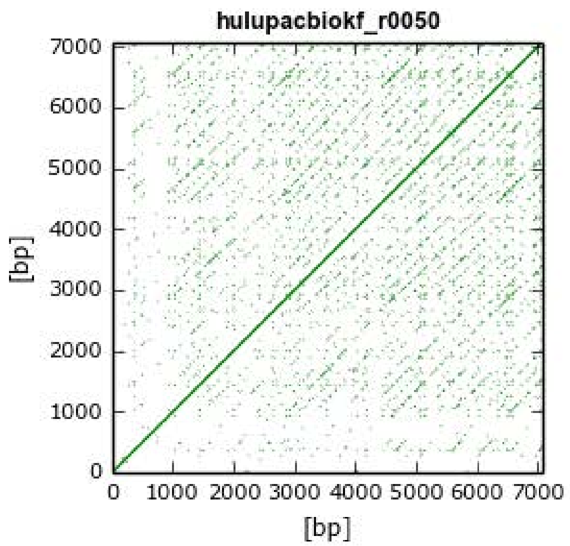
hulupacbiokf_r0050
[bp]
[bp]

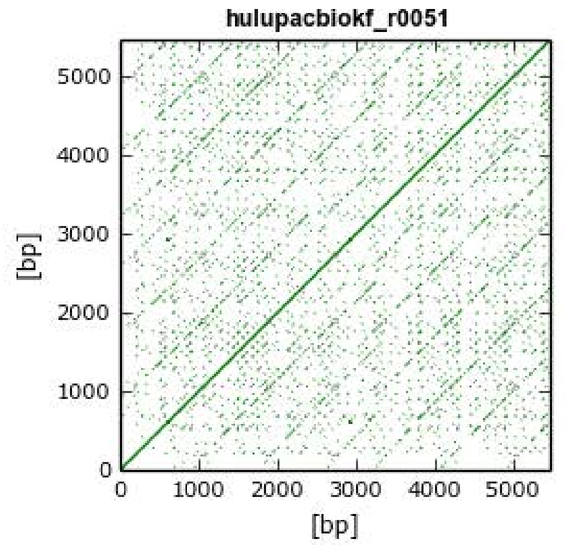
hulupacbiokf_r0051
[bp]
[bp]

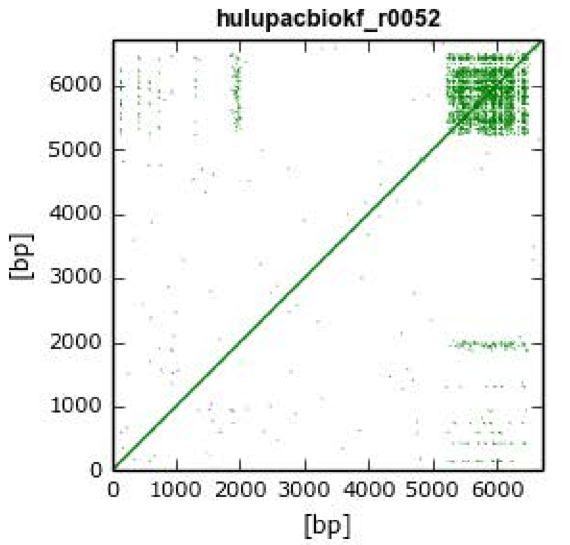
hulupacbiokf_r0052
[bp]
[bp]

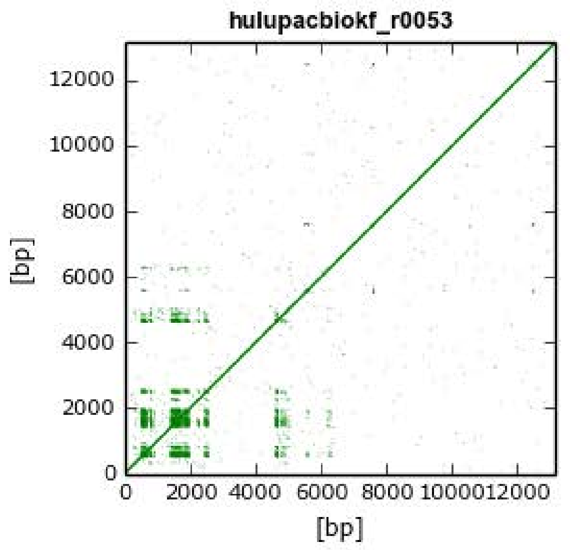
hulupacbiokf_r0053
[bp]
[bp]

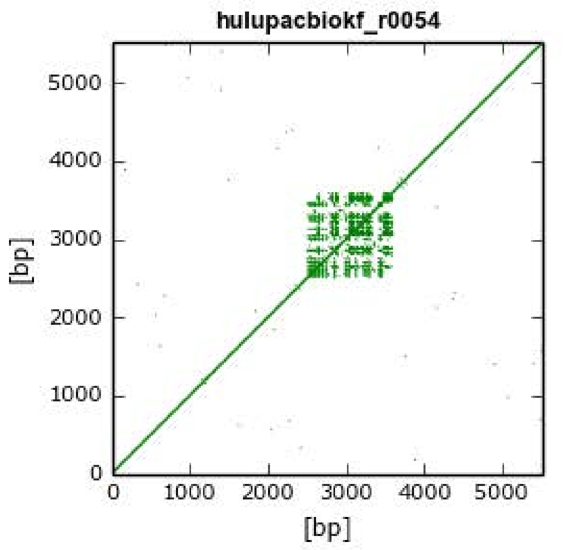
hulupacbiokf_r0054
[bp]
[bp]

HuluTR385 (=HSR1) from r55
matches GenBank Acc. GU831574

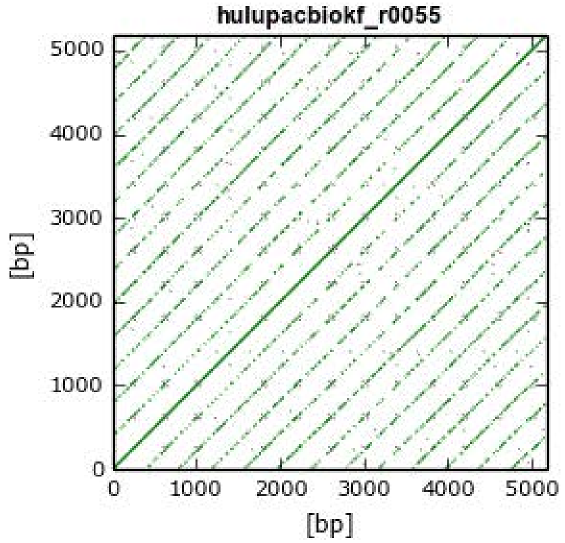

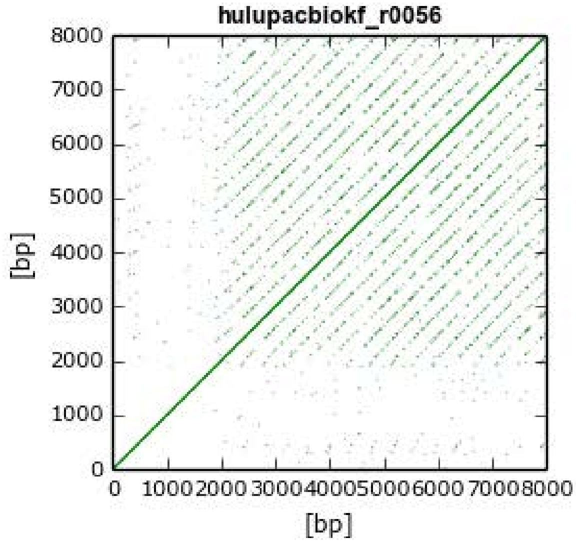

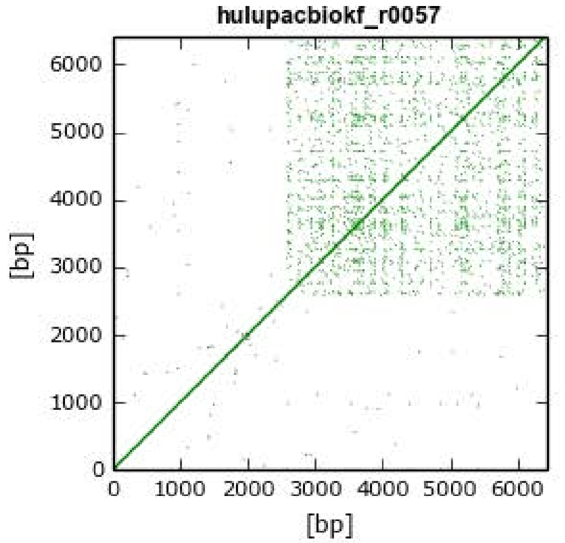

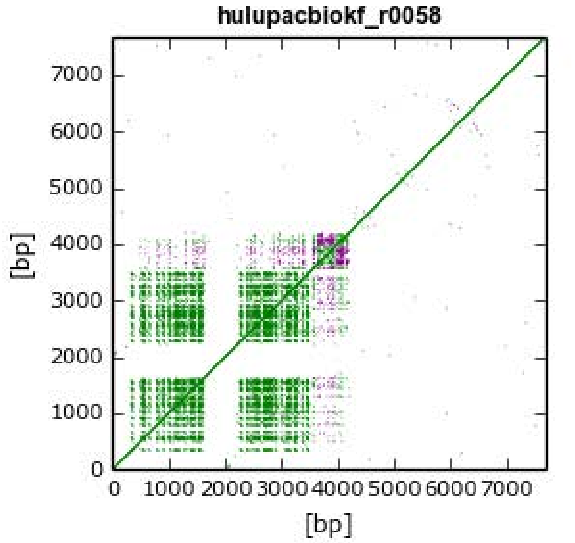

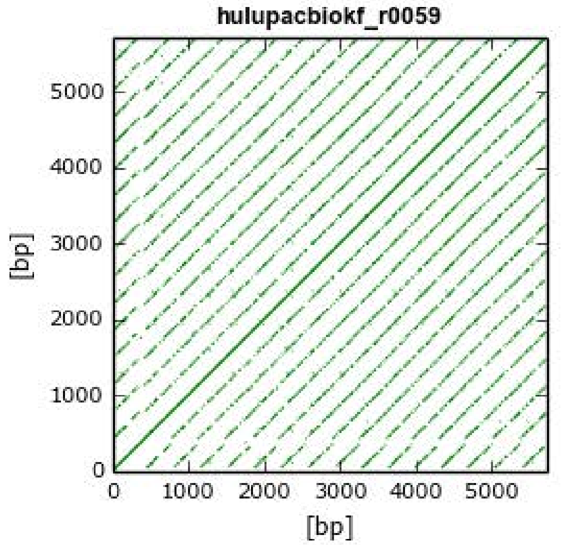

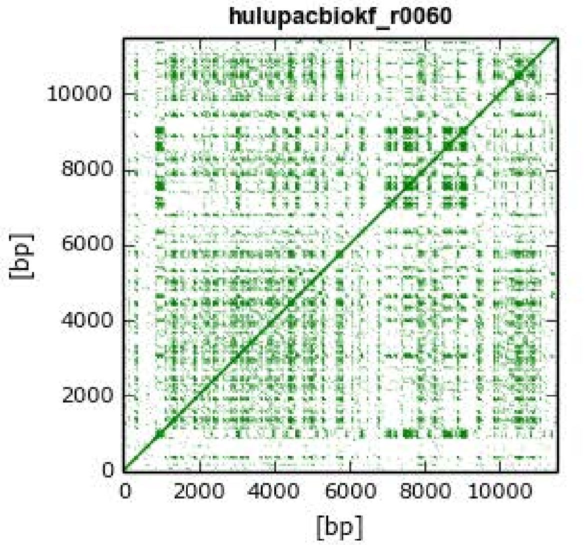

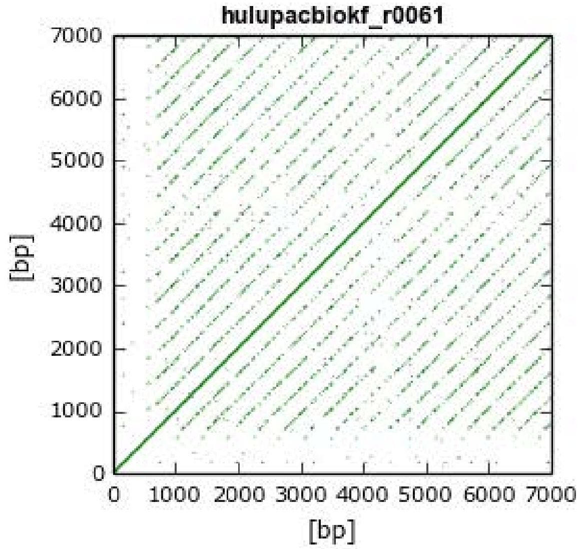
hulupacbiokf_r0061
[bp]
[bp]

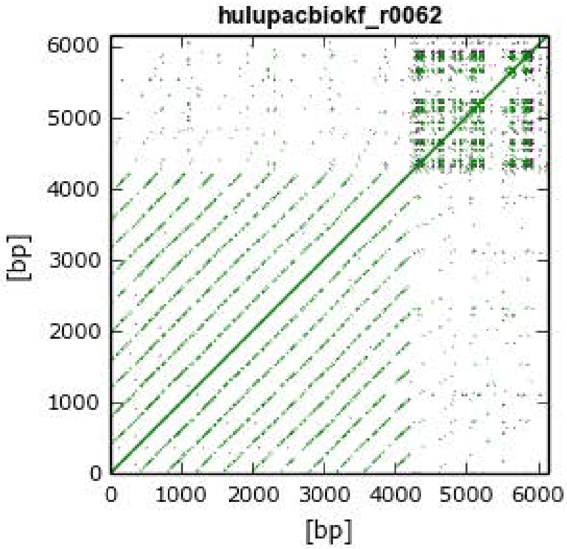
hulupacbiokf_r0062
[bp]
[bp]

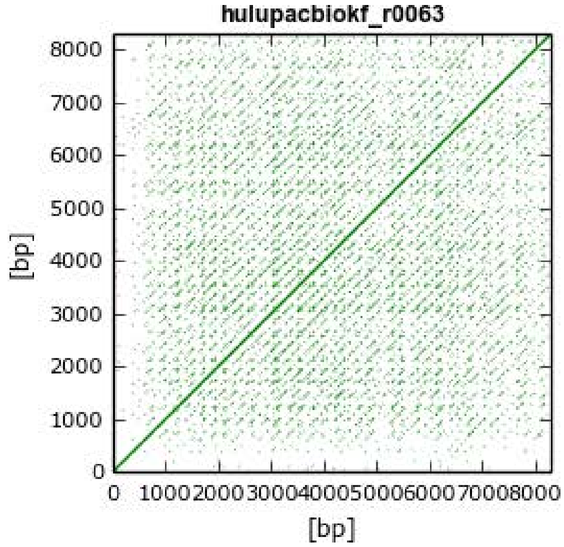
hulupacbiokf_r0063
[bp]
[bp]

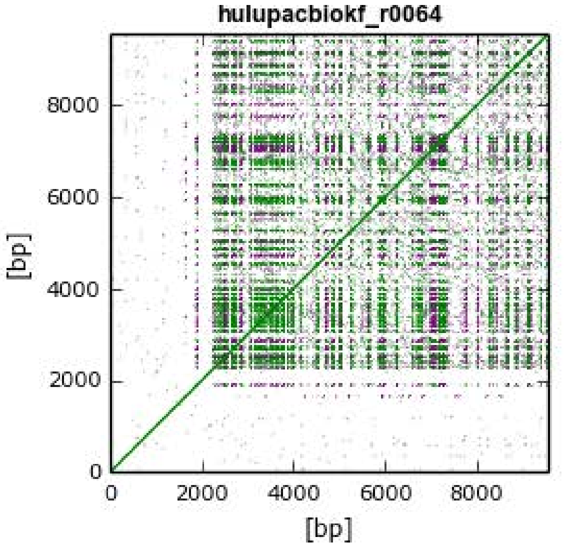
hulupacbiokf_r0064
[bp]
[bp]

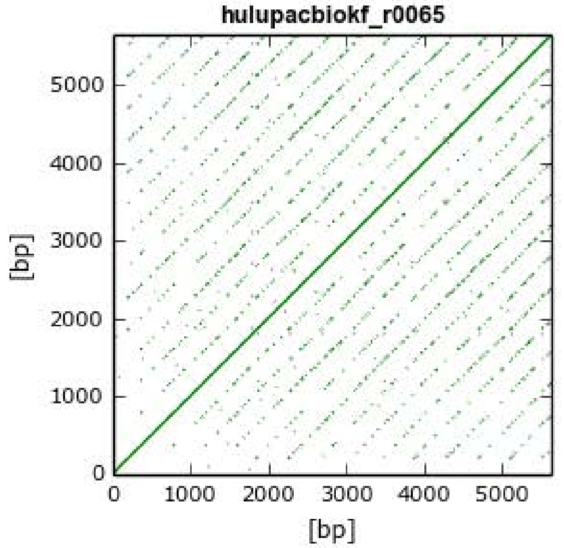
hulupacbiokf_r0065
[bp]
[bp]

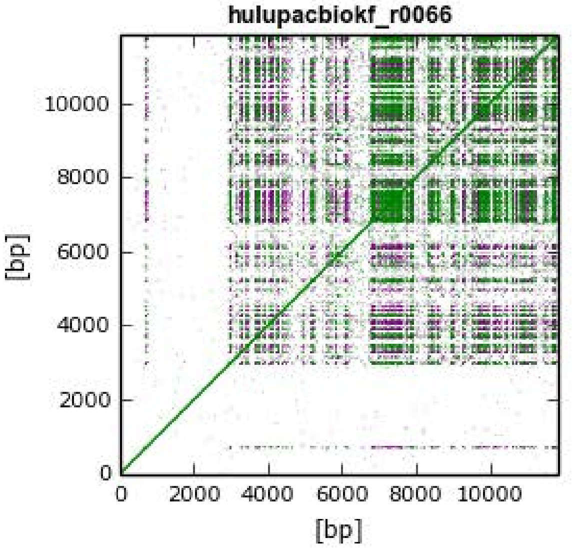
hulupacbiokf_r0066
[bp]
[bp]

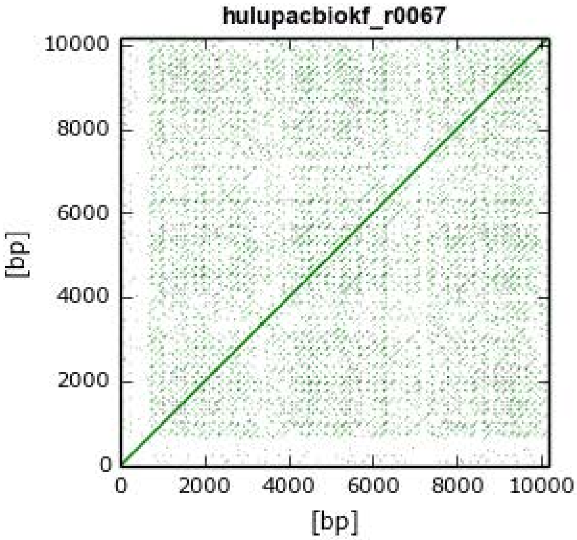
hulupacbiokf_r0067
[bp]
[bp]

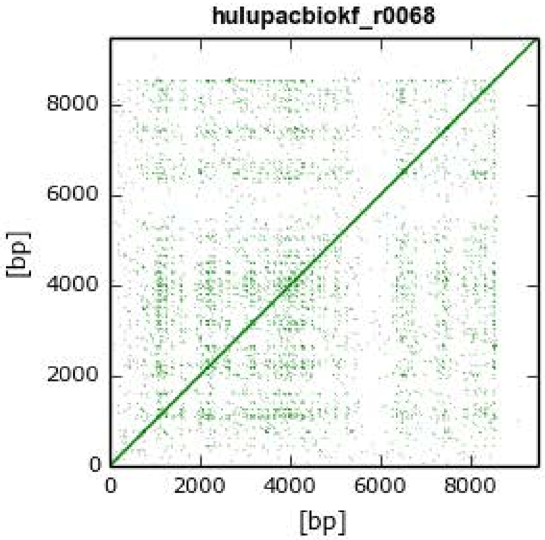
hulupacbiokf_r0068
[bp]
[bp]

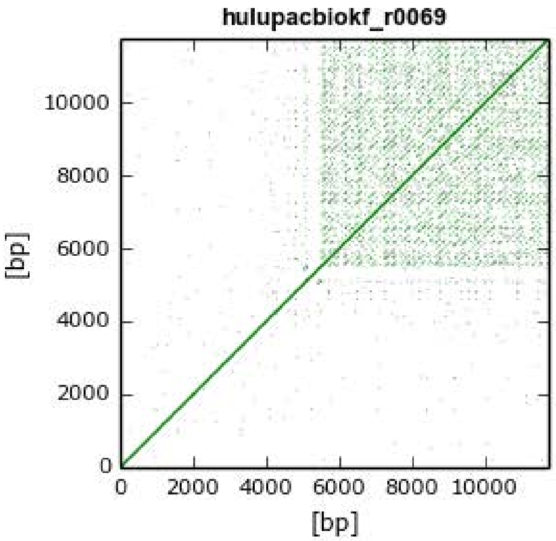
hulupacbiokf_r0069
[bp]
[bp]

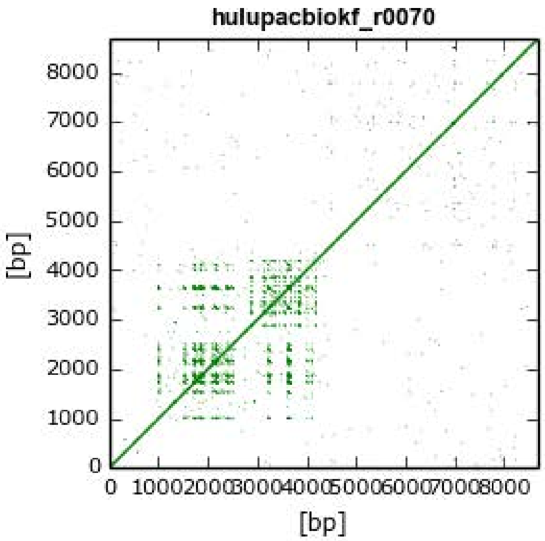
hulupacbiokf_r0070
[bp]
[bp]

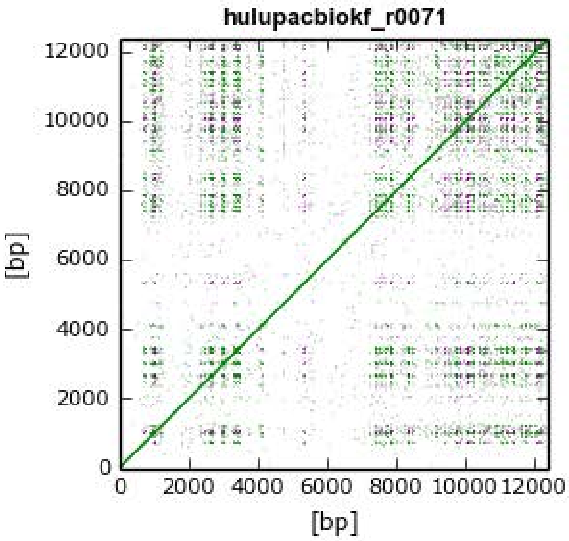
hulupacbiokf_r0071
[bp]
[bp]

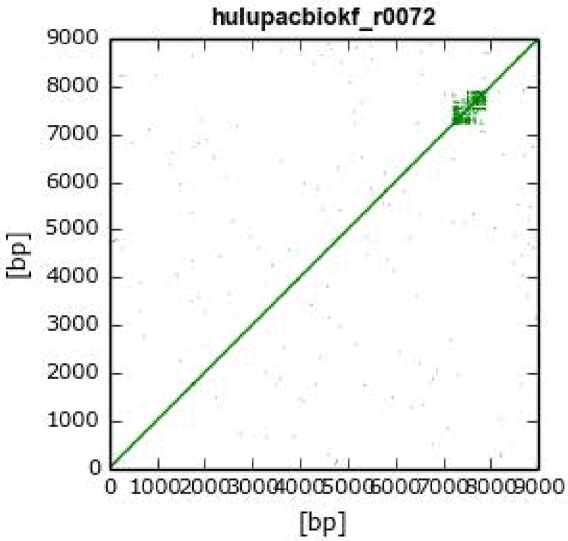
hulupacbiokf_r0072
[bp]
[bp]

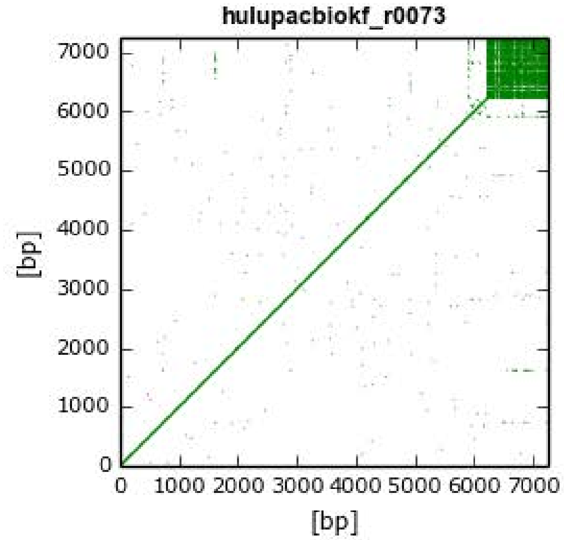
hulupacbiokf_r0073
0
1000
2000
3000
4000
5000
6000
7000
[bp]
[bp]

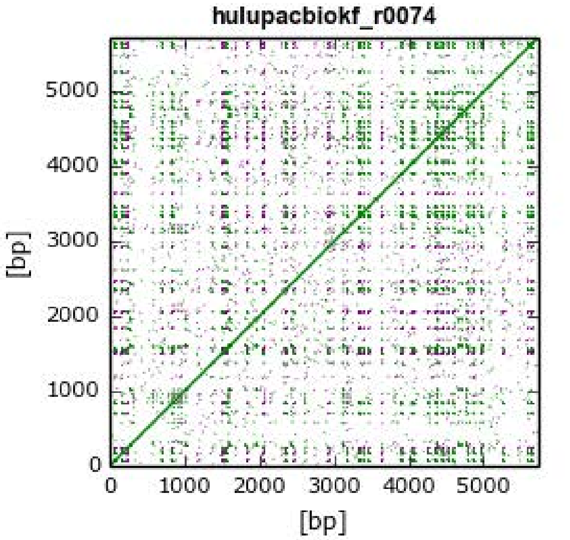
hulupacbiokf_r0074
0
1000
2000
3000
4000
5000
[bp]
[bp]

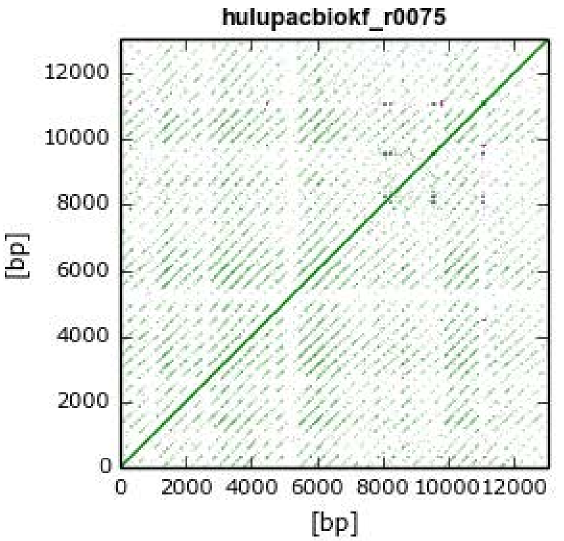
hulupacbiokf_r0075
0
2000
4000
6000
8000
10000
12000
[bp]
[bp]

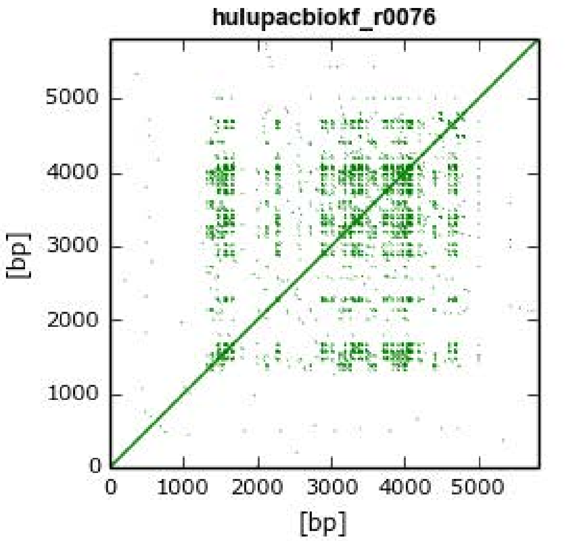
hulupacbiokf_r0076
0
1000
2000
3000
4000
5000
[bp]
[bp]

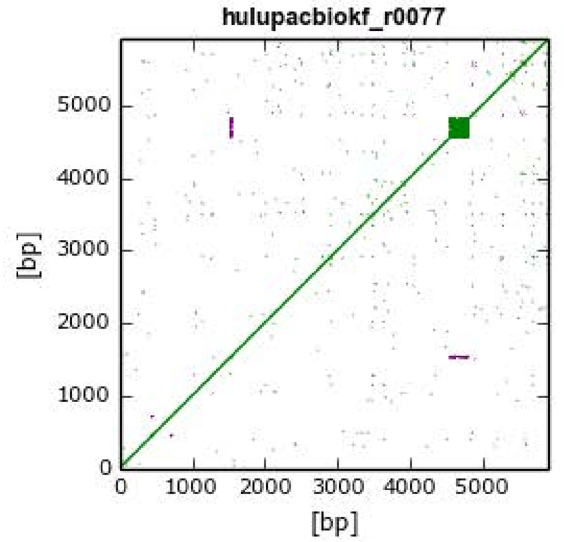
hulupacbiokf_r0077
0
1000
2000
3000
4000
5000
[bp]
[bp]

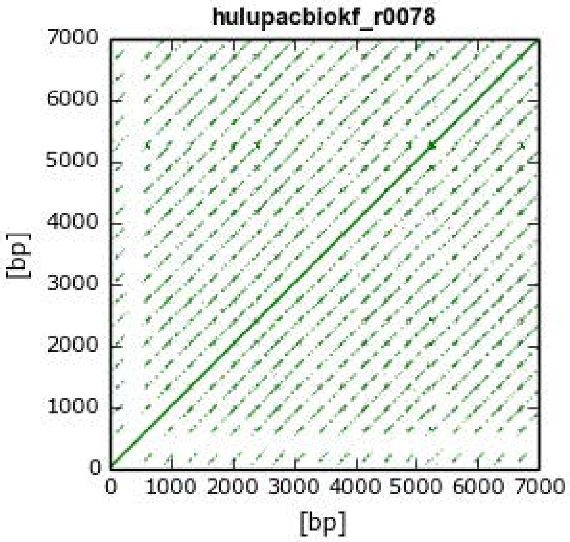
hulupacbiokf_r0078
0
1000
2000
3000
4000
5000
6000
7000
[bp]
[bp]

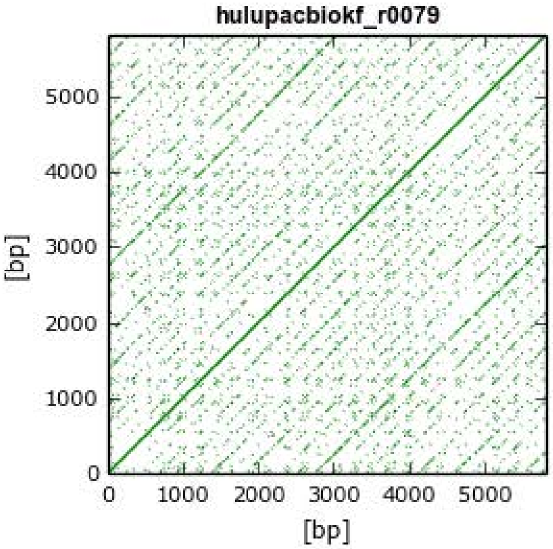
hulupacbiokf_r0079
0
1000
2000
3000
4000
5000
[bp]
[bp]

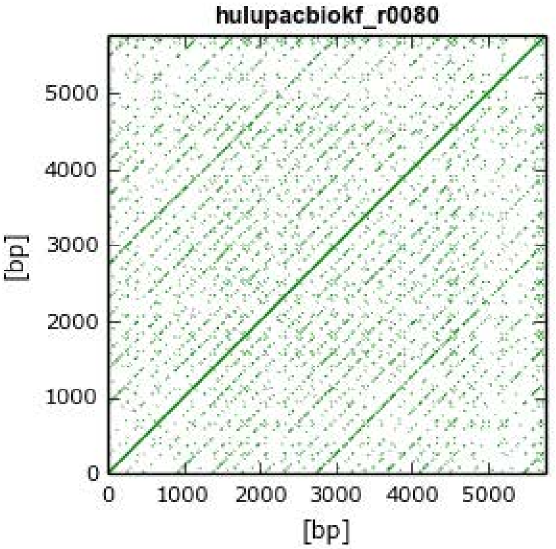
hulupacbiokf_r0080
0
1000
2000
3000
4000
5000
[bp]
[bp]

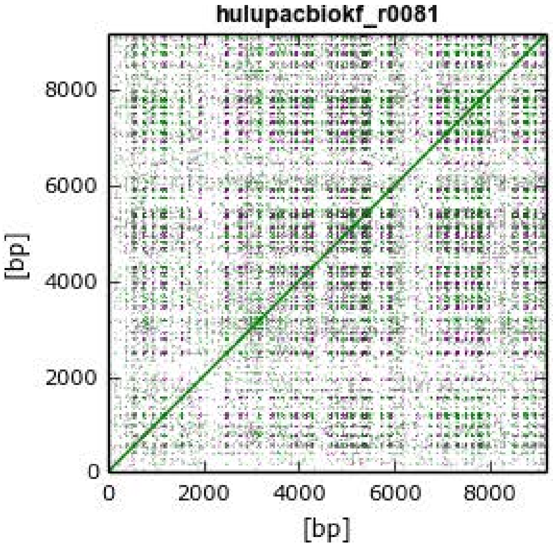
hulupacbiokf_r0081
0
2000
4000
6000
8000
[bp]
[bp]

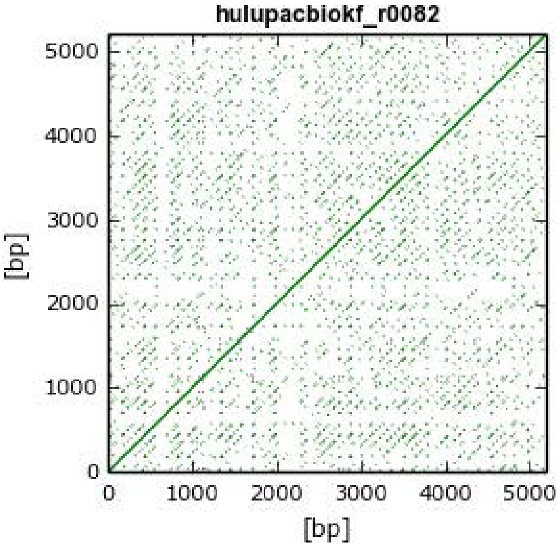
hulupacbiokf_r0082
0
1000
2000
3000
4000
5000
[bp]
[bp]

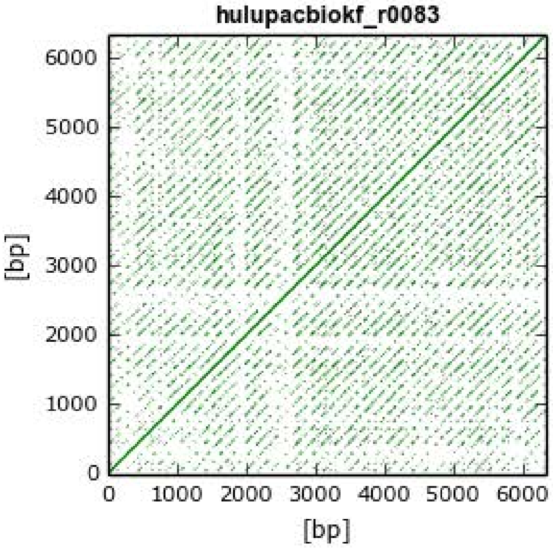
hulupacbiokf_r0083
0
1000
2000
3000
4000
5000
6000
[bp]
[bp]

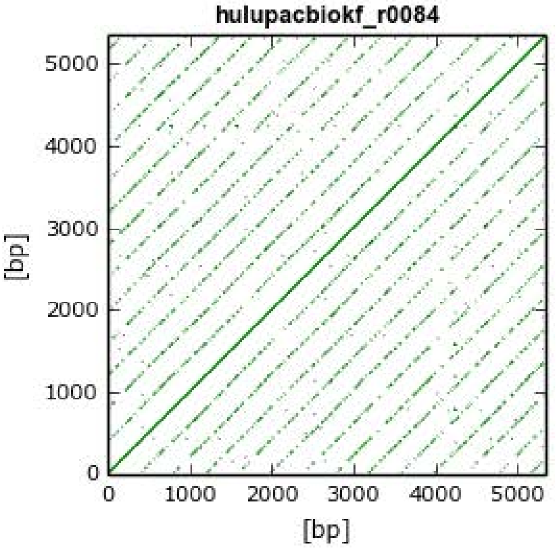
hulupacbiokf_r0084
0
1000
2000
3000
4000
5000
[bp]
[bp]

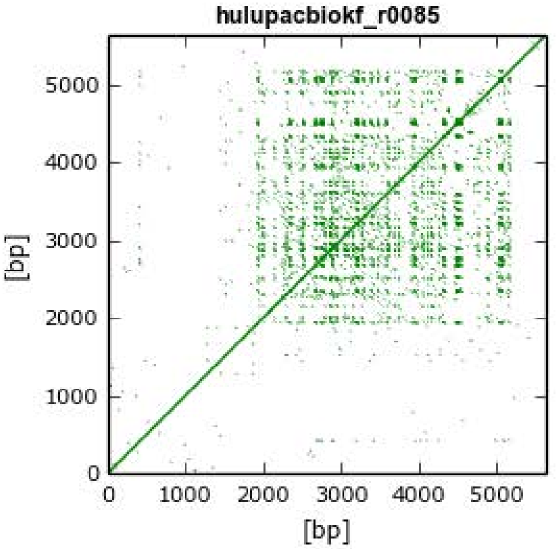
hulupacbiokf_r0085
0
1000
2000
3000
4000
5000
[bp]
[bp]

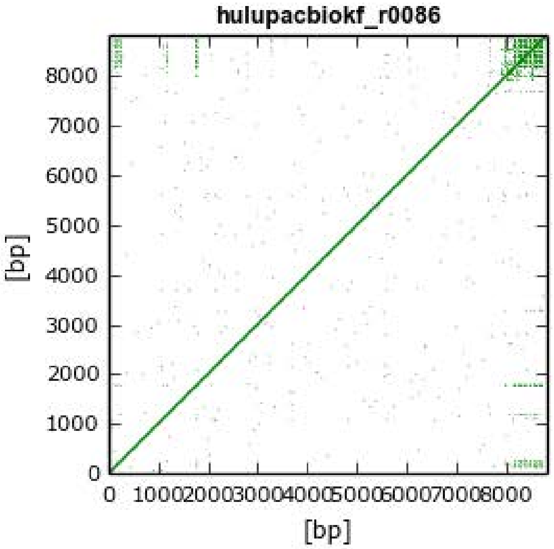
hulupacbiokf_r0086
0
1000
2000
3000
4000
5000
6000
7000
8000
[bp]
[bp]

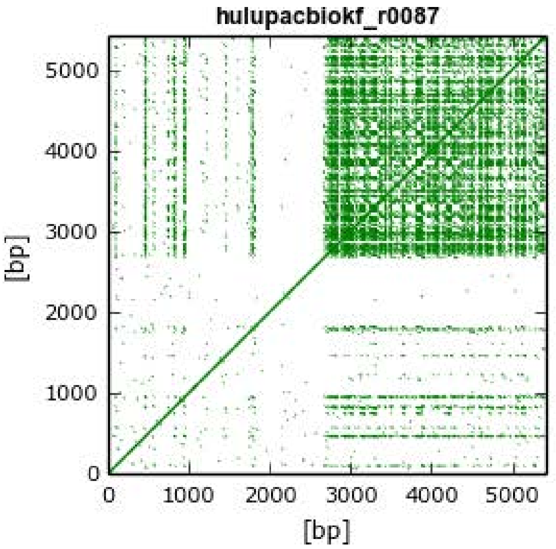
hulupacbiokf_r0087
0
1000
2000
3000
4000
5000
[bp]
[bp]

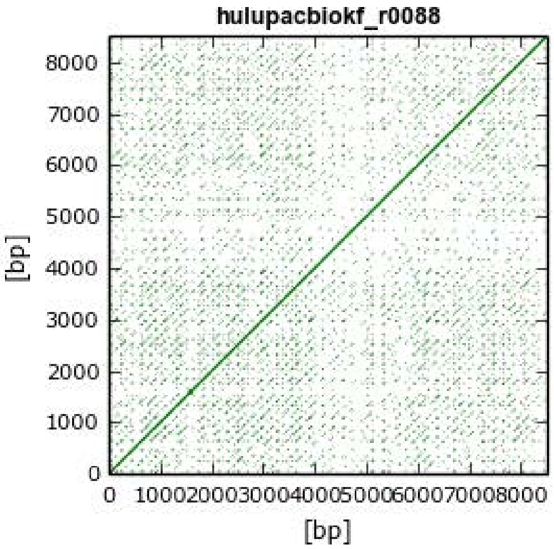
hulupacbiokf_r0088
0
1000
2000
3000
4000
5000
6000
7000
8000
[bp]
[bp]

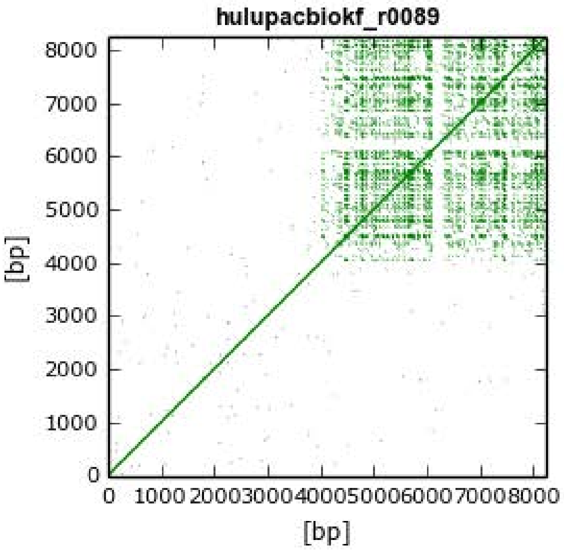
hulupacbiokf_r0089
0
1000
2000
3000
4000
5000
6000
7000
8000
[bp]
[bp]

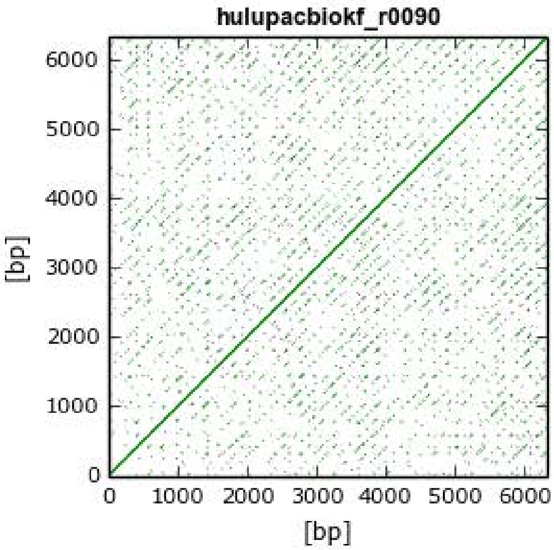
hulupacbiokf_r0090
0
1000
2000
3000
4000
5000
6000
[bp]
[bp]

HuluTR060 from read r0091
is in GenBank Acc. MN537567

hulupacbiokf_r0091

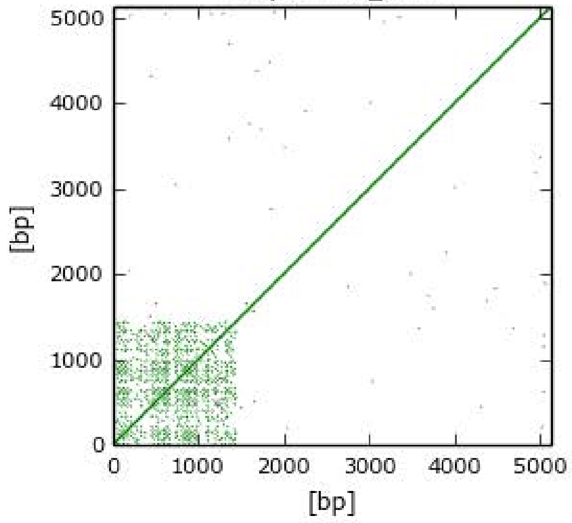

hulupacbiokf_r0092

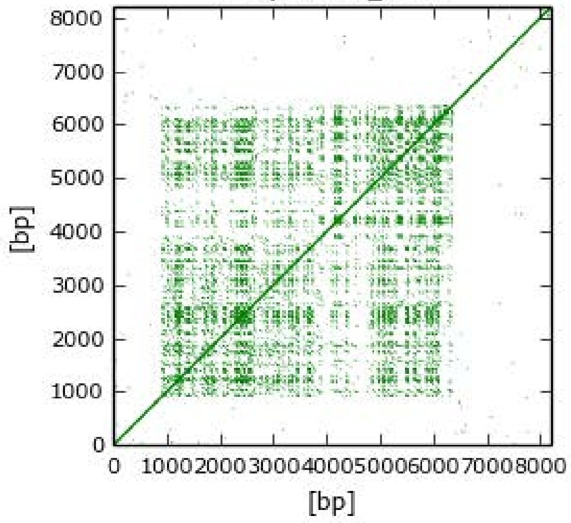

hulupacbiokf_r0093

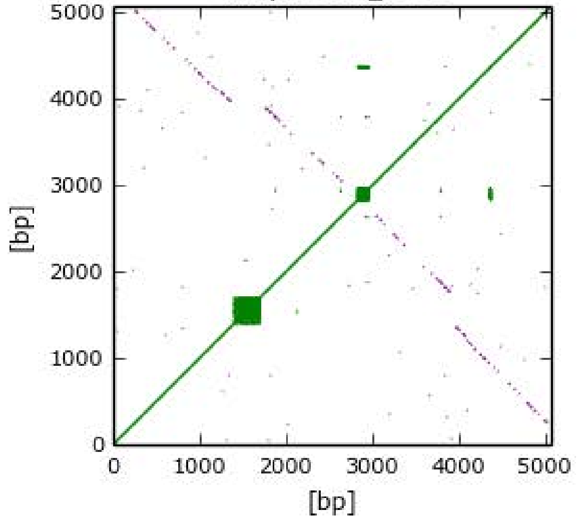

hulupacbiokf_r0094

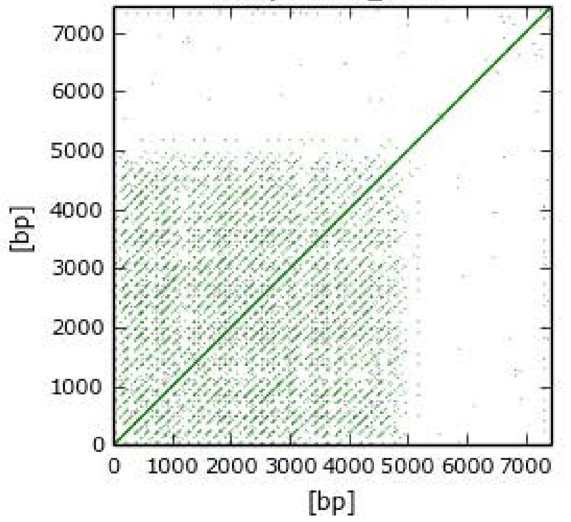

hulupacbiokf_r0095

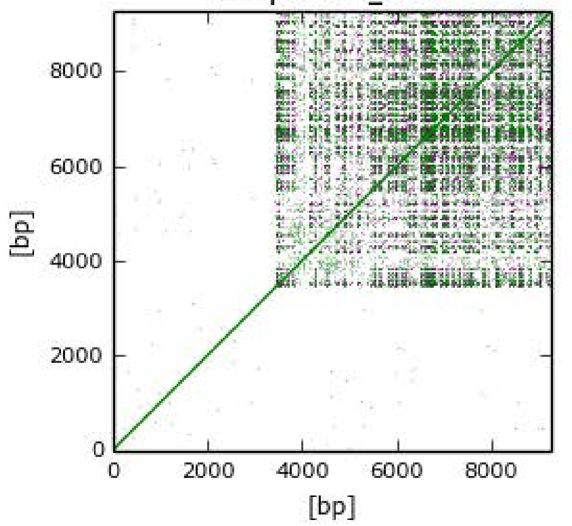

hulupacbiokf_r0096

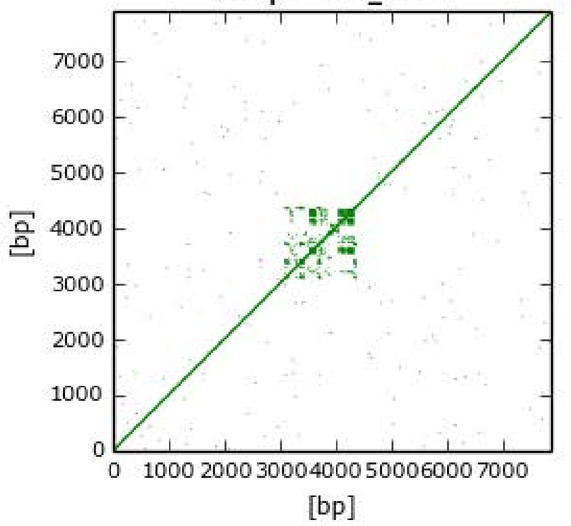

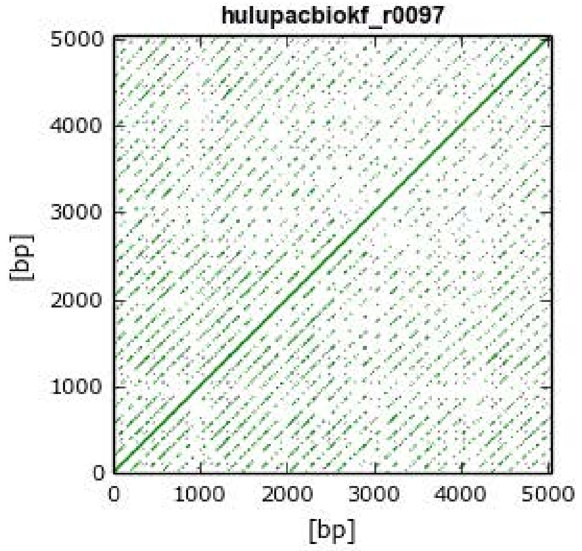
hulupacbiokf_r0097
[bp]
[bp]

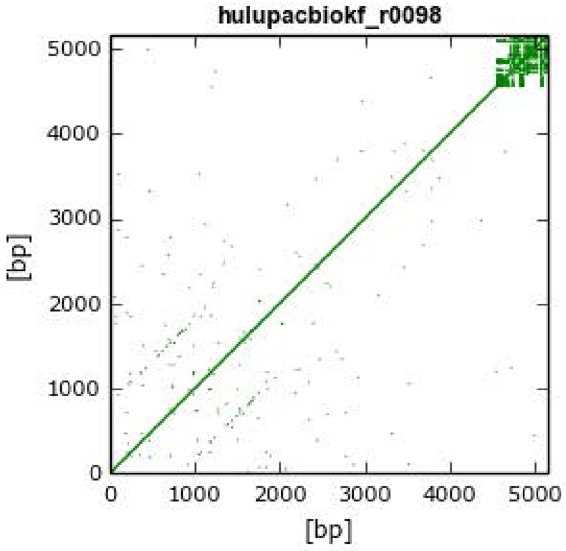
hulupacbiokf_r0098
[bp]
[bp]

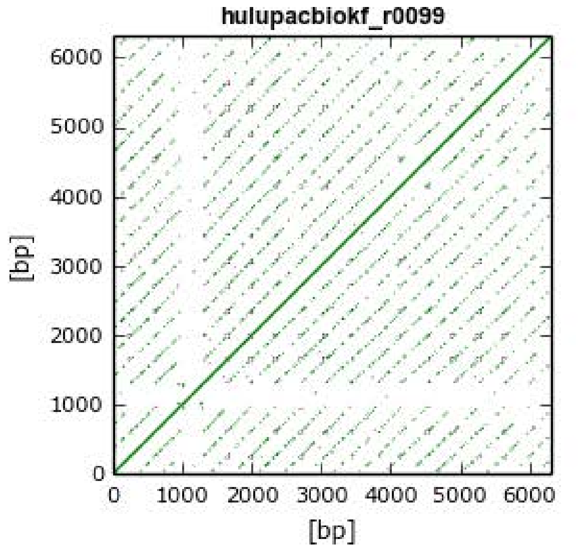
hulupacbiokf_r0099
[bp]
[bp]

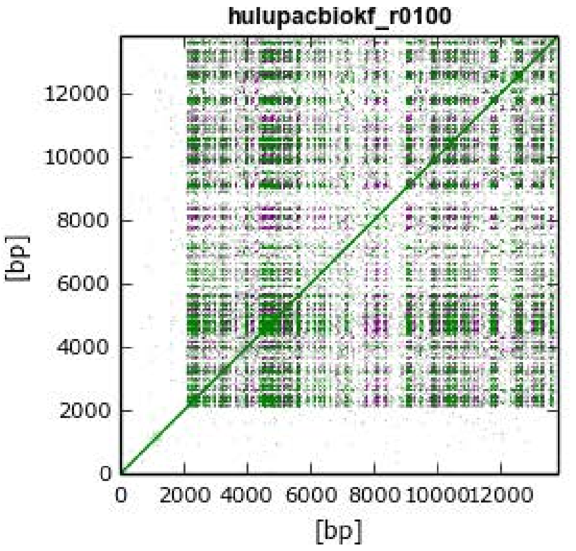
hulupacbiokf_r0100
[bp]
[bp]

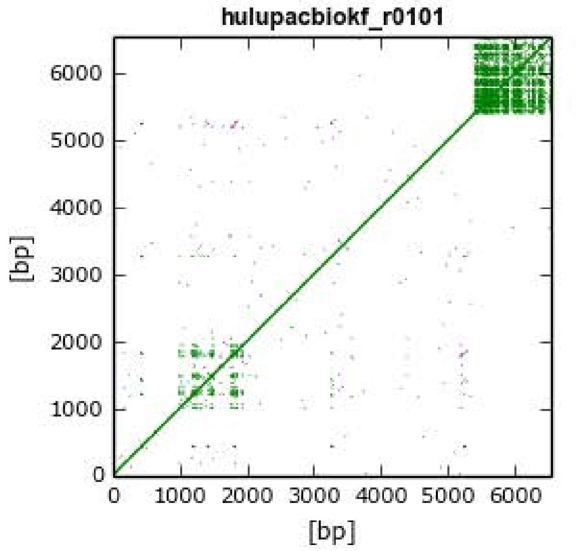
hulupacbiokf_r0101
[bp]
[bp]

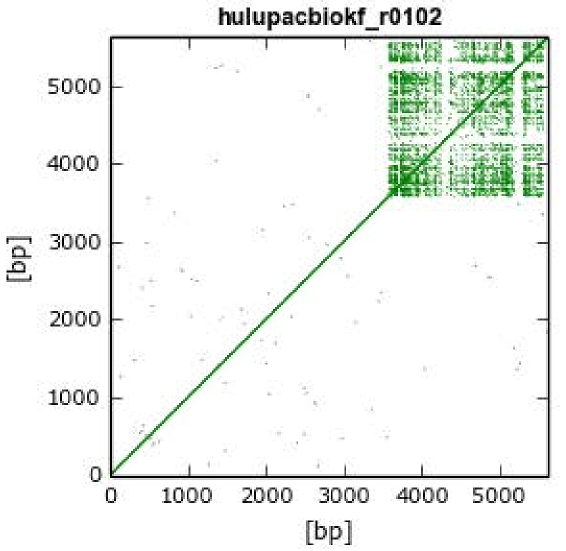
hulupacbiokf_r0102
[bp]
[bp]

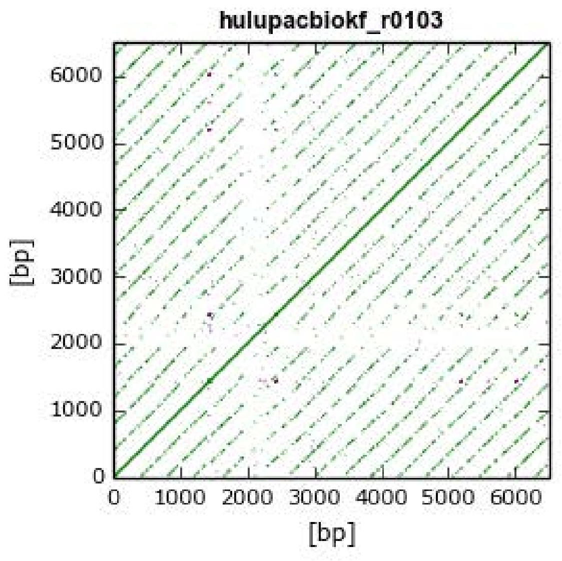
hulupacbiokf_r0103
0
1000
2000
3000
4000
5000
6000
[bp]
[bp]

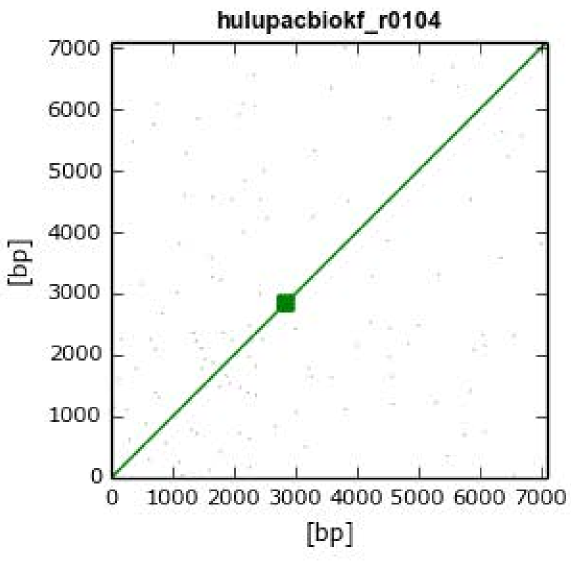
hulupacbiokf_r0104
0
1000
2000
3000
4000
5000
6000
7000
[bp]
[bp]

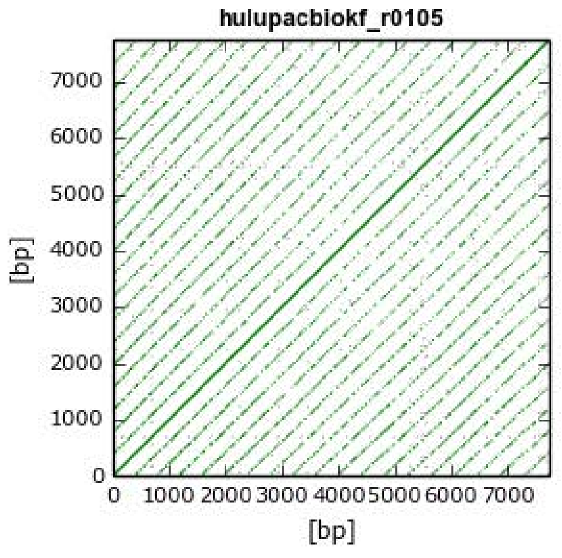
hulupacbiokf_r0105
0
1000
2000
3000
4000
5000
6000
7000
[bp]
[bp]

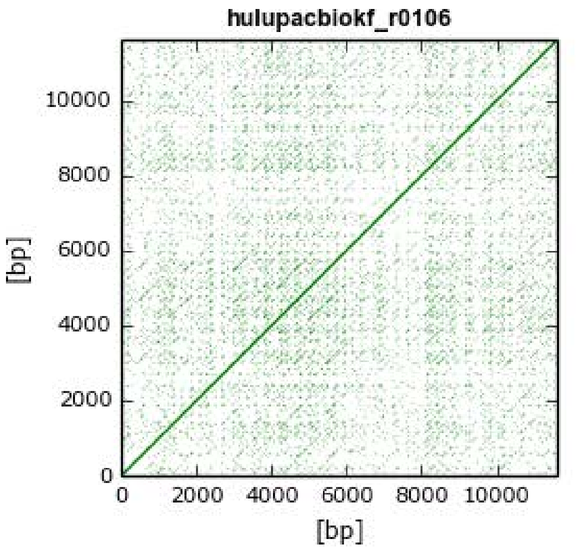
hulupacbiokf_r0106
0
2000
4000
6000
8000
10000
[bp]
[bp]

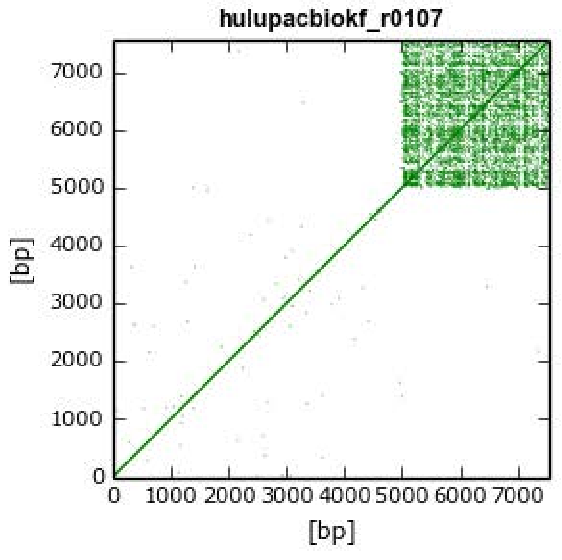
hulupacbiokf_r0107
0
1000
2000
3000
4000
5000
6000
7000
[bp]
[bp]

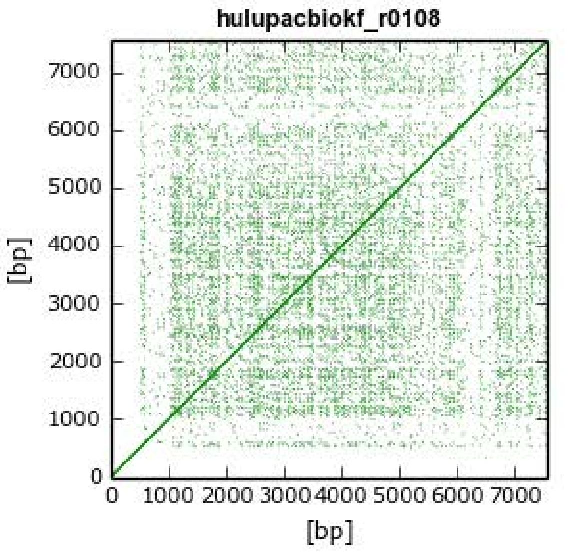
hulupacbiokf_r0108
0
1000
2000
3000
4000
5000
6000
7000
[bp]
[bp]

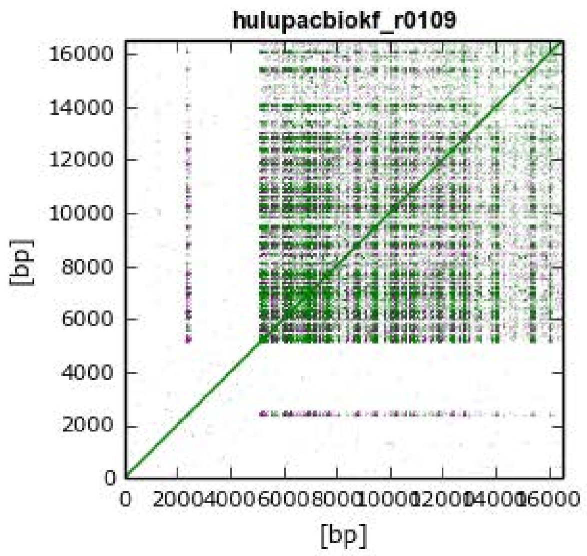
hulupacbiokf_r0109
[bp]
[bp]

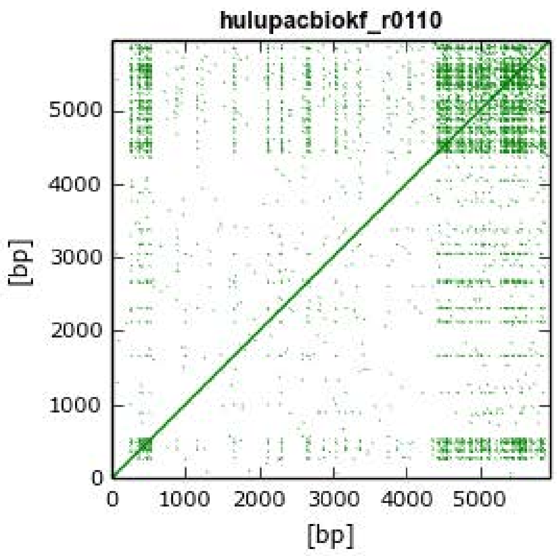
hulupacbiokf_r0110
[bp]
[bp]

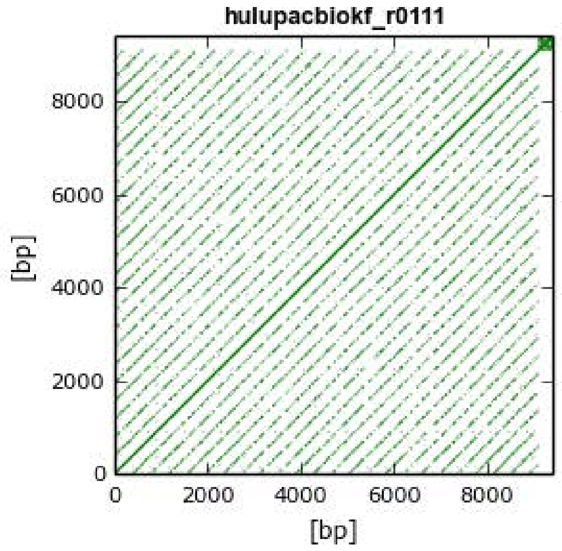
hulupacbiokf_r0111
[bp]
[bp]

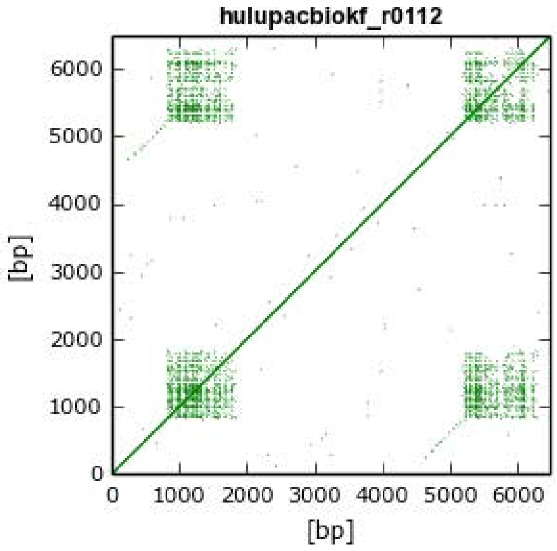
hulupacbiokf_r0112
[bp]
[bp]

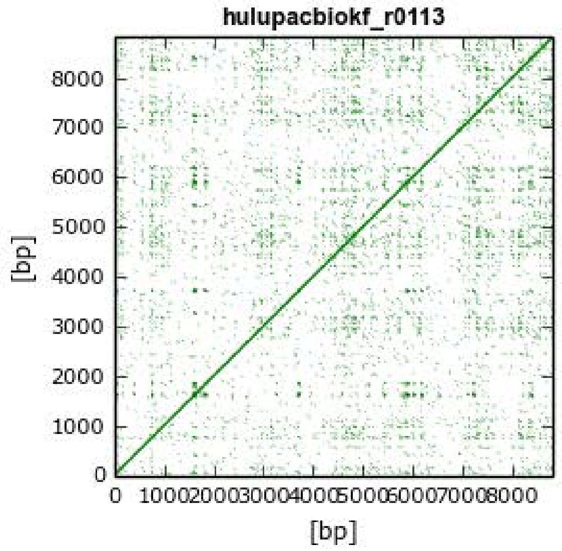
hulupacbiokf_r0113
[bp]
[bp]

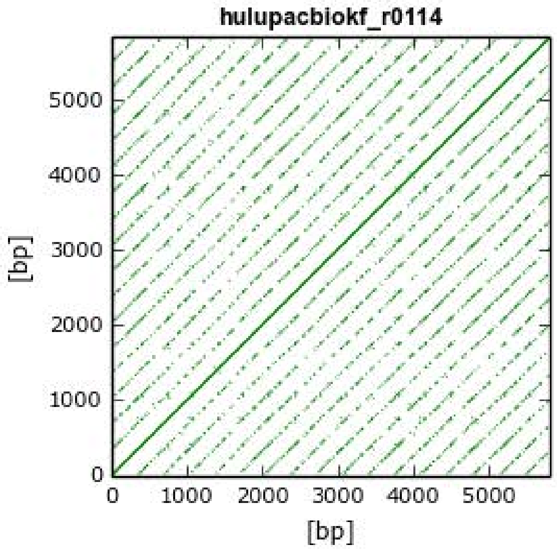
hulupacbiokf_r0114
[bp]
[bp]

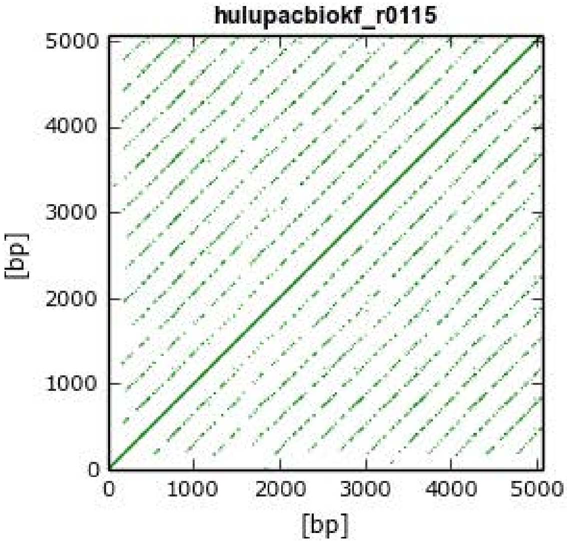

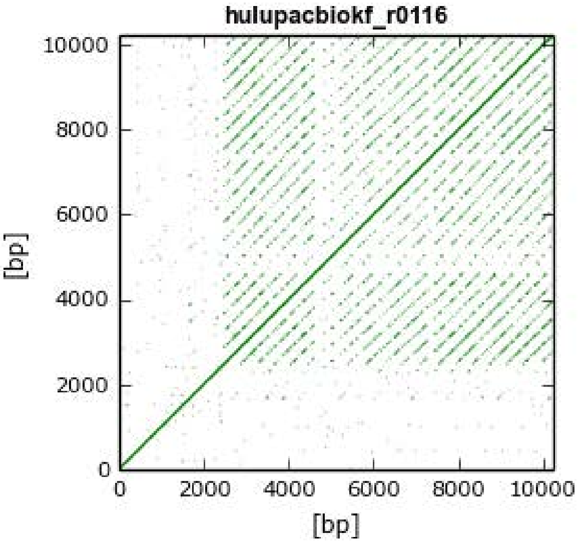

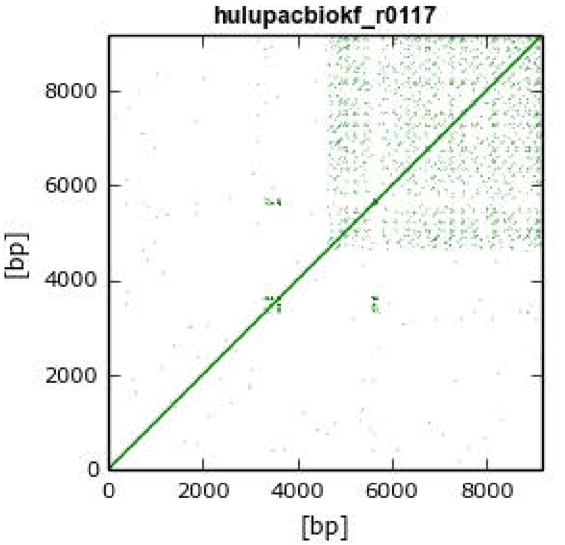

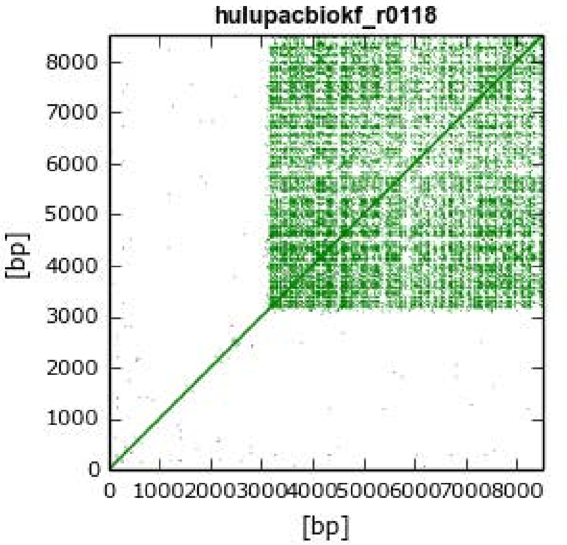

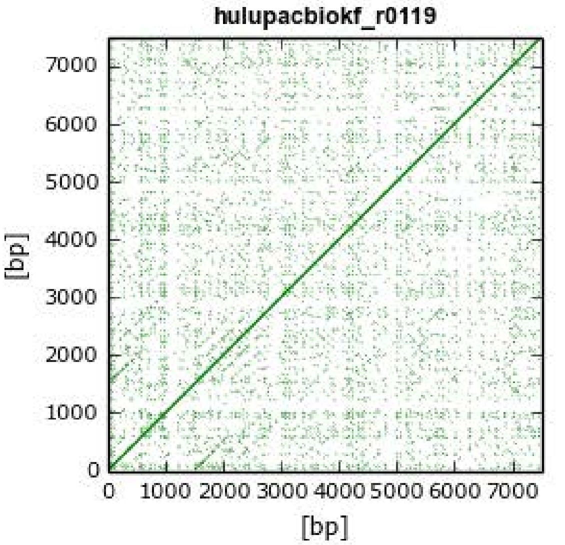

HuluTR180 (=HSR0) from read r0120
is in GenBank Acc. MH188533.1

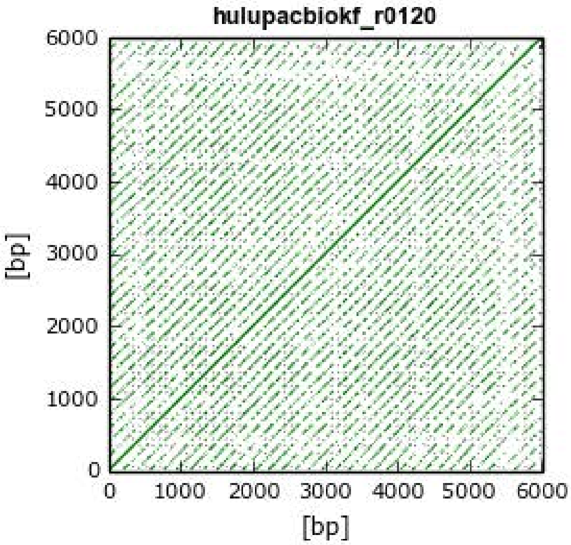

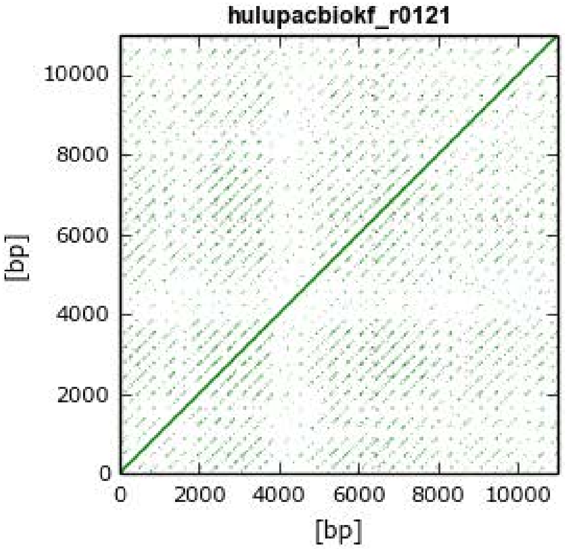
hulupacbiokf_r0121
[bp]
[bp]

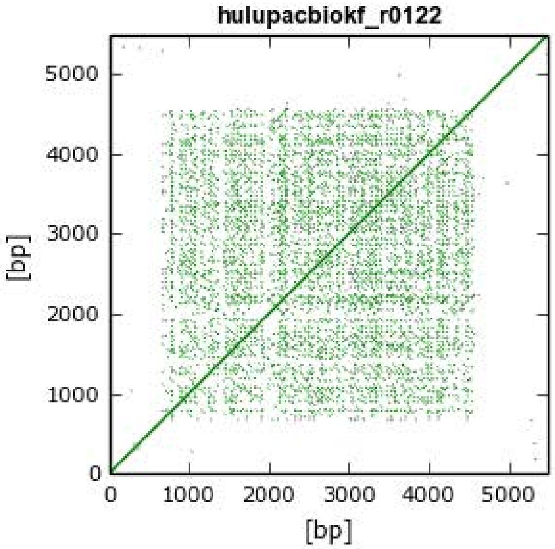
hulupacbiokf_r0122
[bp]
[bp]

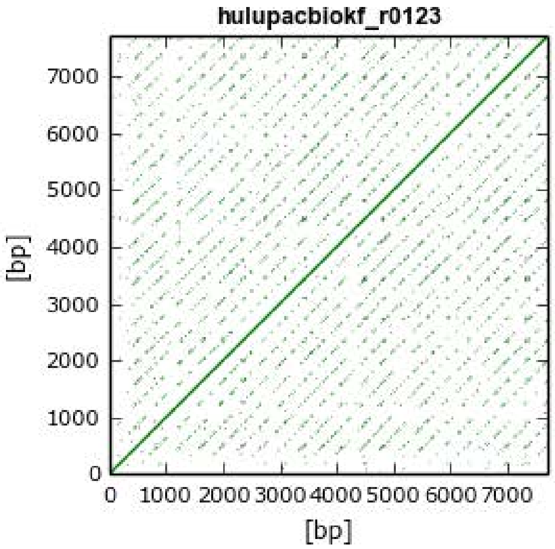
hulupacbiokf_r0123
[bp]
[bp]

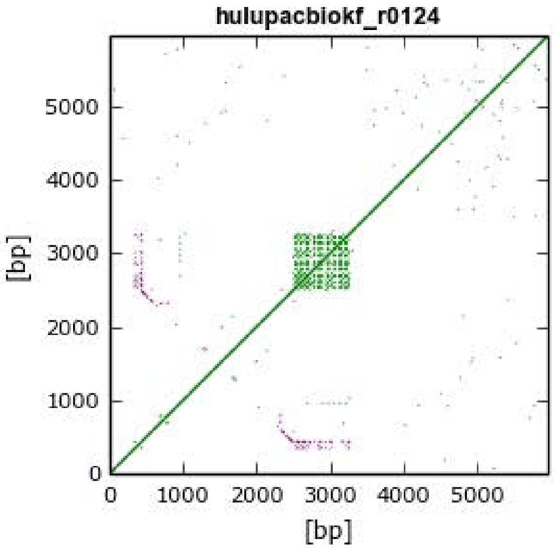
hulupacbiokf_r0124
[bp]
[bp]

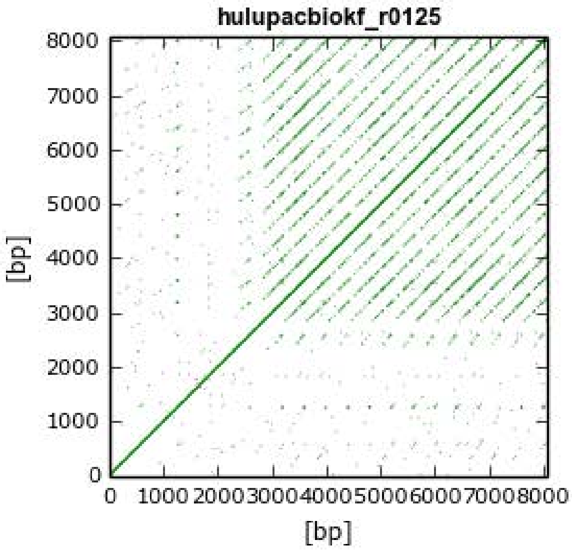
hulupacbiokf_r0125
[bp]
[bp]

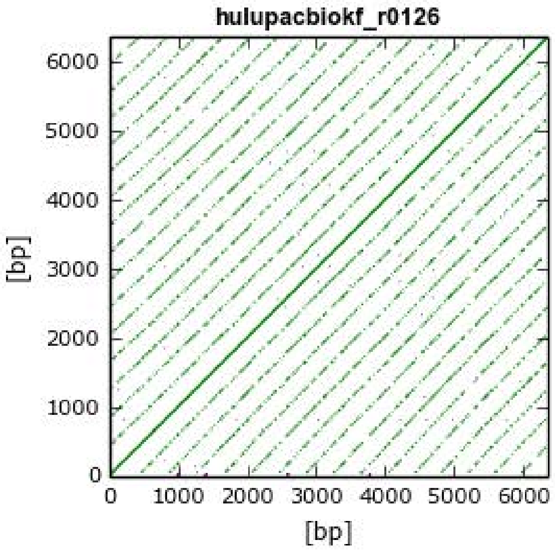
hulupacbiokf_r0126
[bp]
[bp]

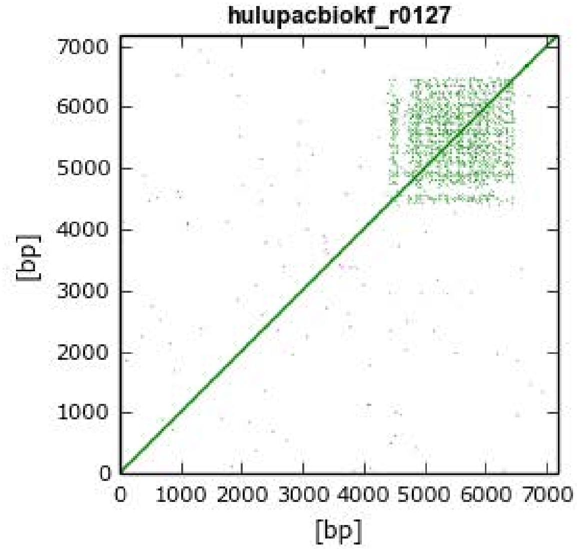
hulupacbiokf_r0127
[bp]
[bp]

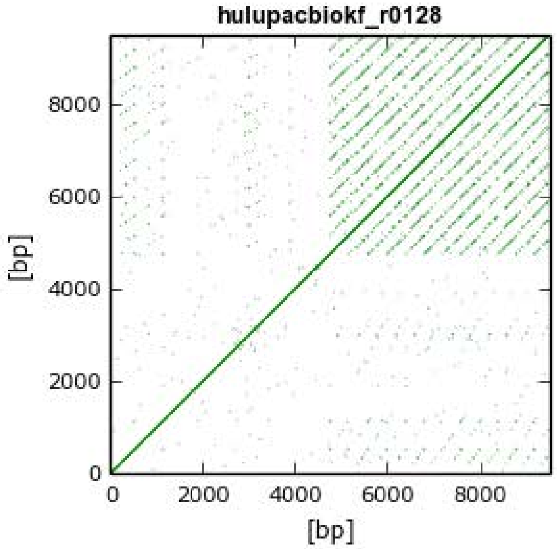
hulupacbiokf_r0128
[bp]
[bp]

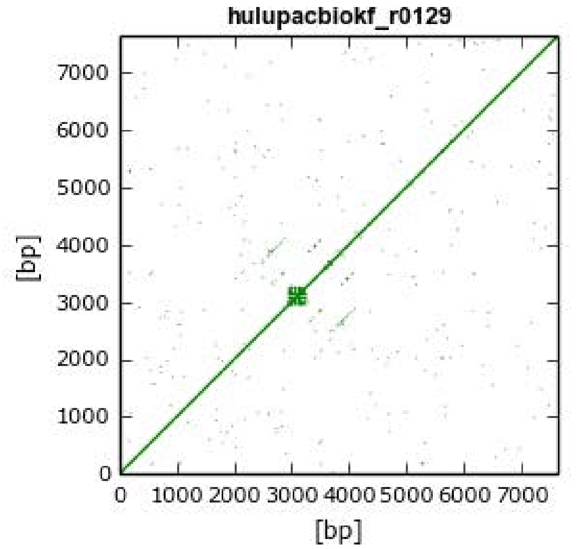
hulupacbiokf_r0129
[bp]
[bp]

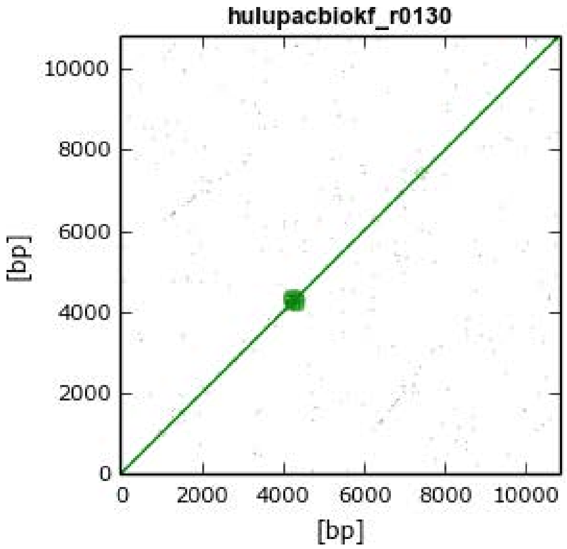
hulupacbiokf_r0130
[bp]
[bp]

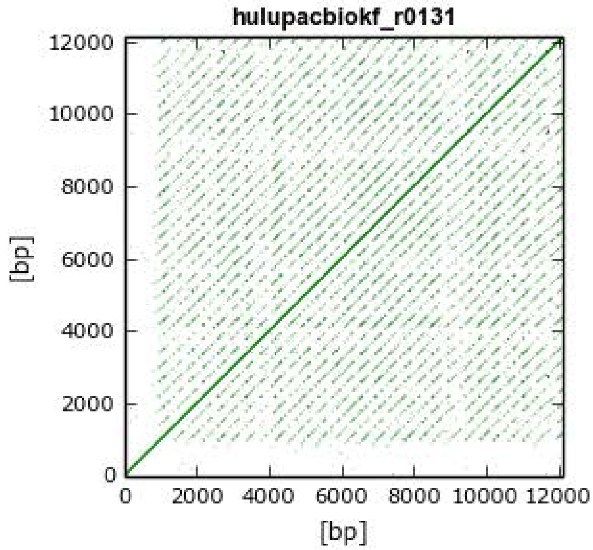
hulupacbiokf_r0131
[bp]
[bp]

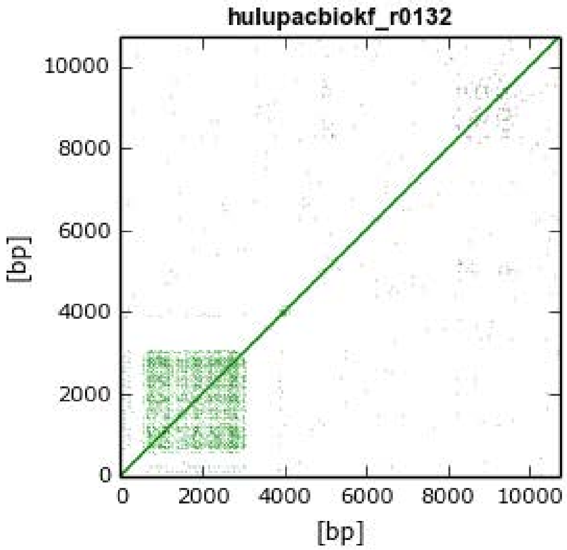
hulupacbiokf_r0132
[bp]
[bp]

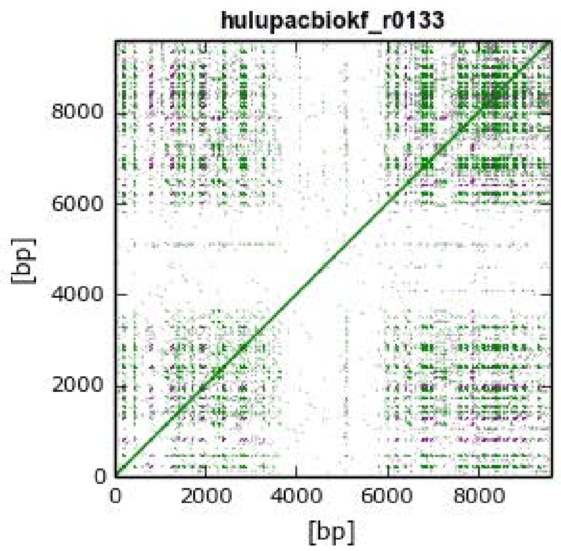
hulupacbiokf_r0133
[bp]
[bp]

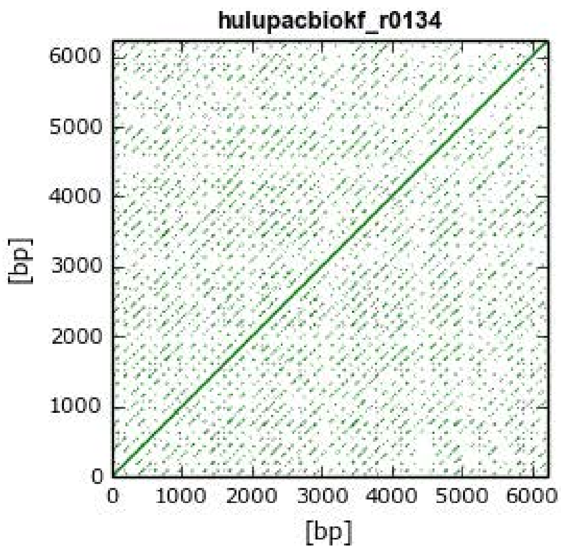
hulupacbiokf_r0134
[bp]
[bp]

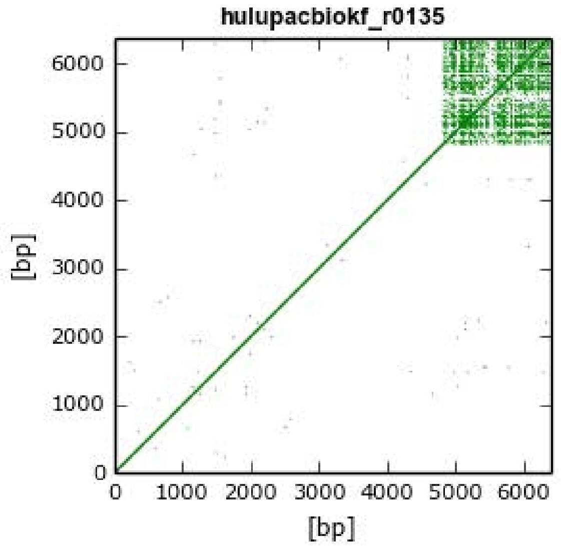
hulupacbiokf_r0135
[bp]
[bp]

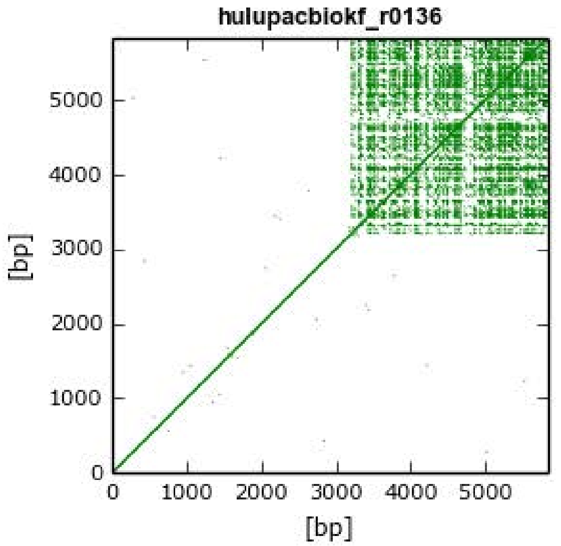
hulupacbiokf_r0136
[bp]
[bp]

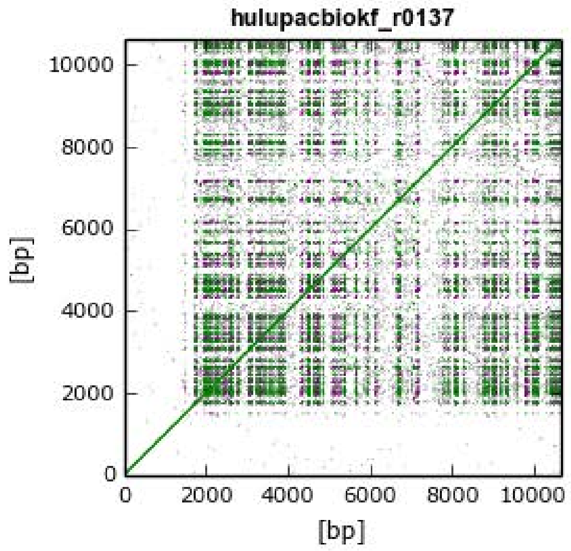
hulupacbiokf_r0137
[bp]
[bp]

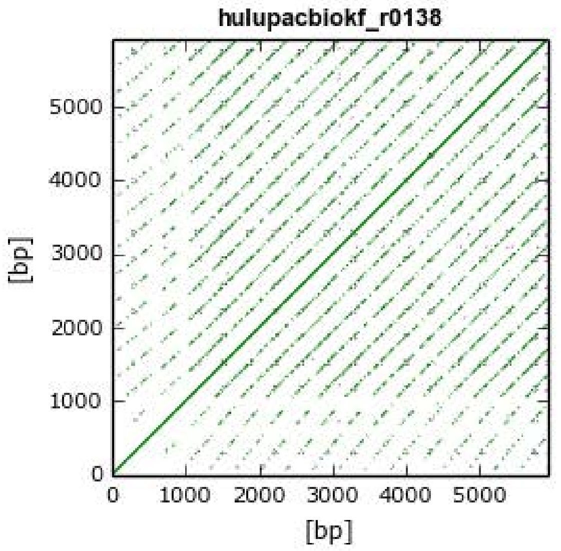
hulupacbiokf_r0138
[bp]
[bp]

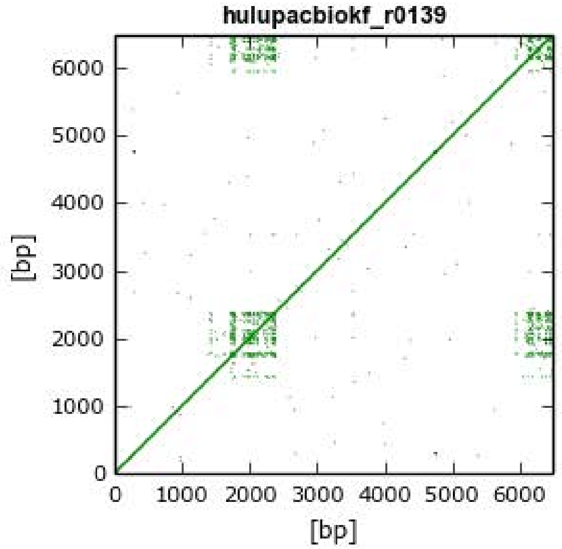
hulupacbiokf_r0139
[bp]
[bp]

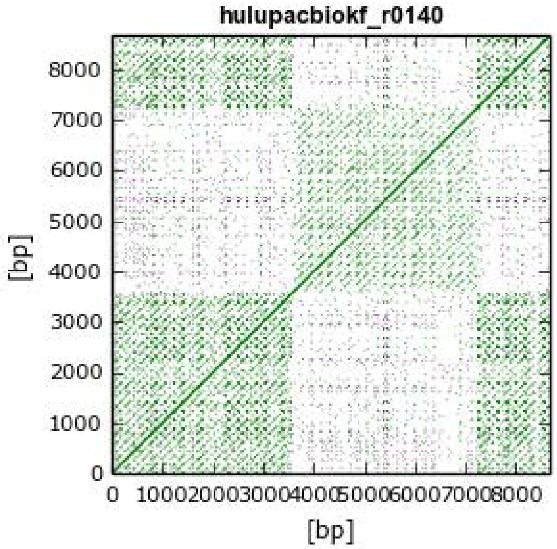
hulupacbiokf_r0140
[bp]
[bp]

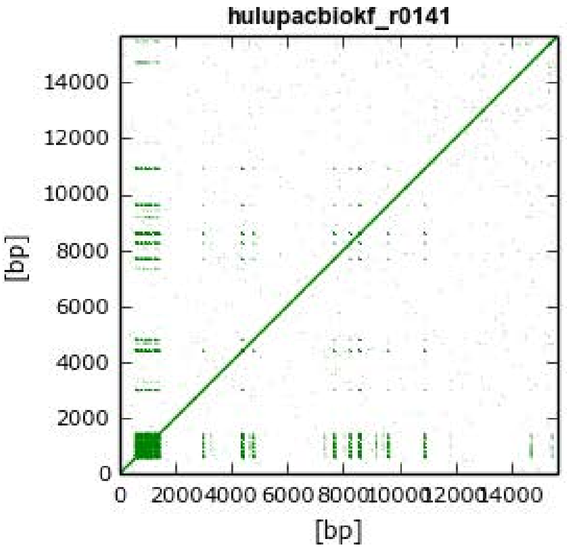
hulupacbiokf_r0141
[bp]
[bp]

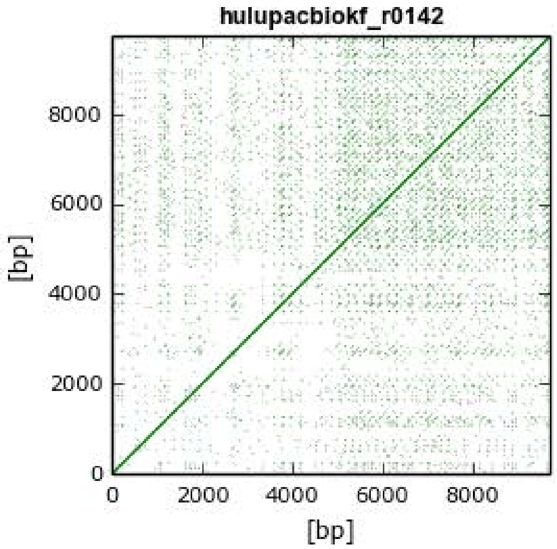
hulupacbiokf_r0142
[bp]
[bp]

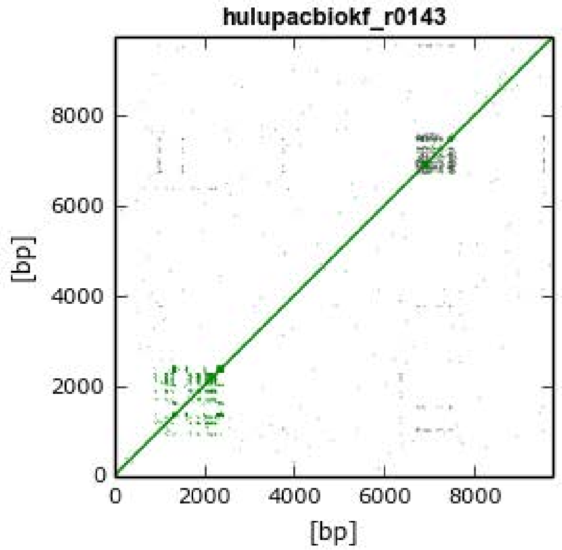
hulupacbiokf_r0143
[bp]
[bp]

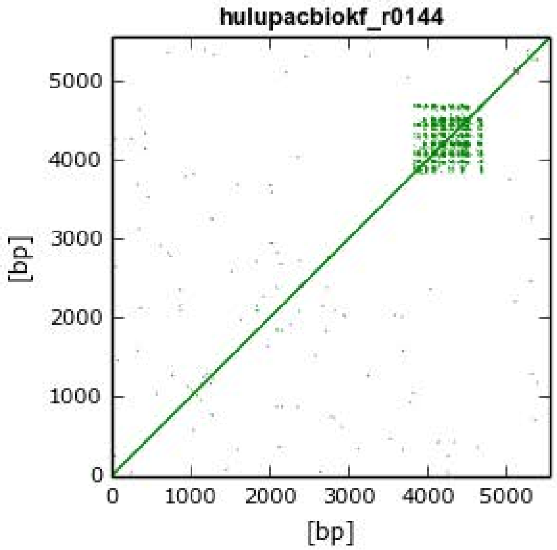
hulupacbiokf_r0144
[bp]
[bp]

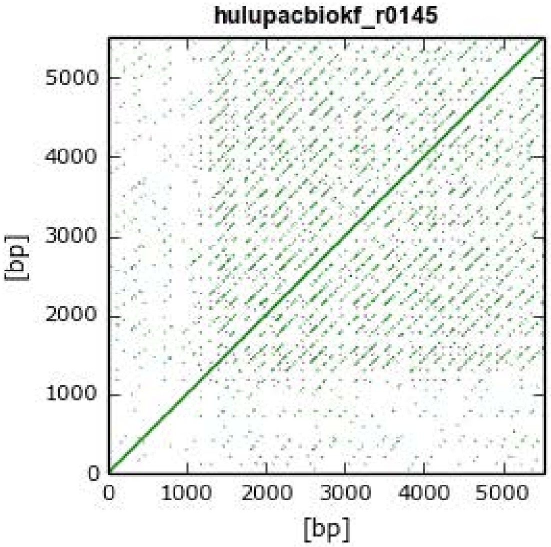
hulupacbiokf_r0145
[bp]
0
1000
2000
3000
4000
5000
[bp]

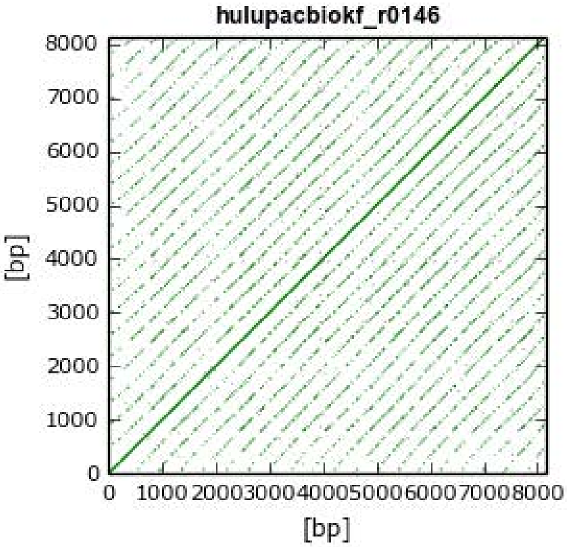
hulupacbiokf_r0146
[bp]
0
1000
2000
3000
4000
5000
6000
7000
8000
[bp]

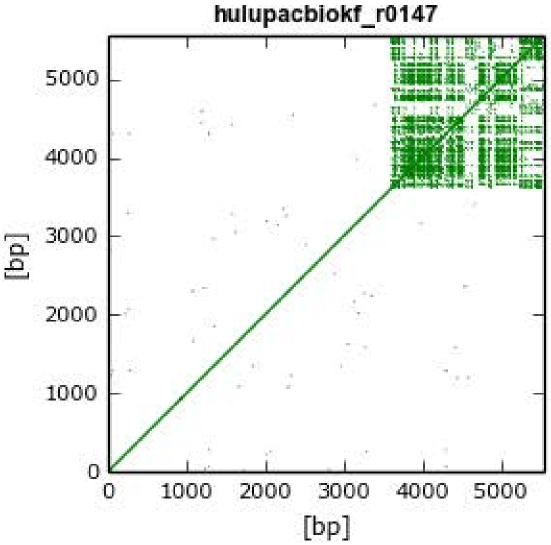
hulupacbiokf_r0147
[bp]
0
1000
2000
3000
4000
5000
[bp]

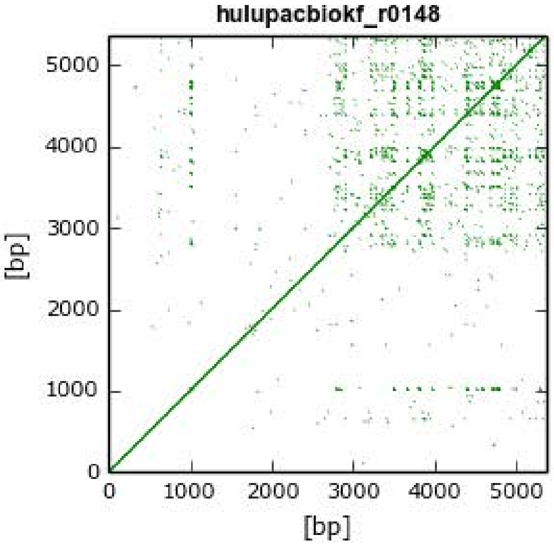
hulupacbiokf_r0148
[bp]
0
1000
2000
3000
4000
5000
[bp]

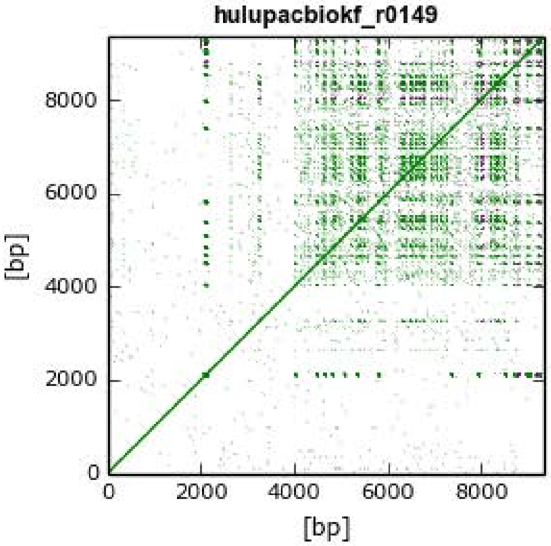
hulupacbiokf_r0149
[bp]
0
2000
4000
6000
8000
[bp]

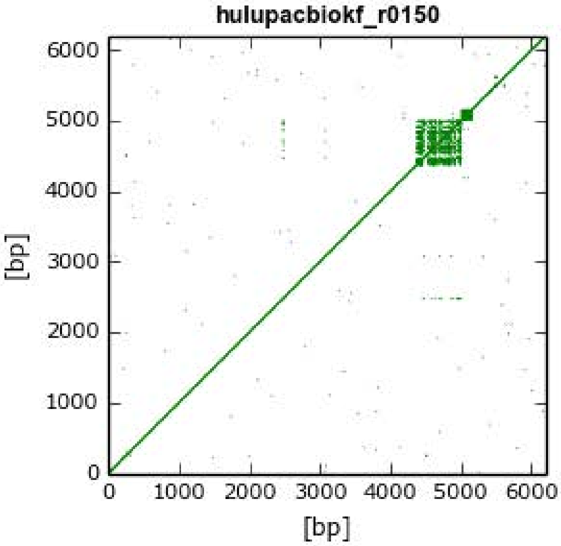
hulupacbiokf_r0150
[bp]
0
1000
2000
3000
4000
5000
6000
[bp]

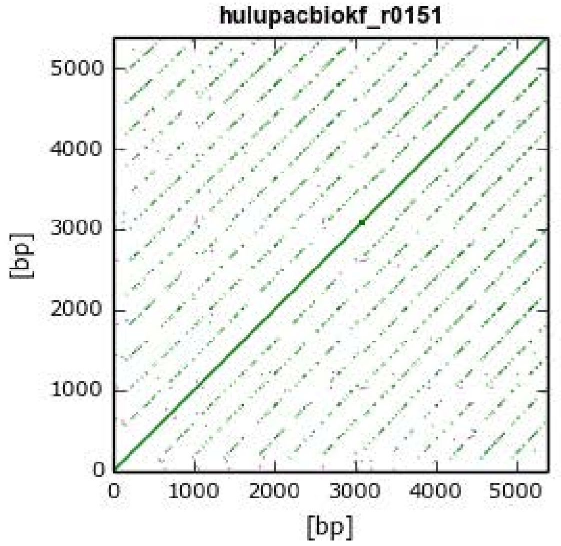
hulupacbiokf_r0151
[bp]
[bp]

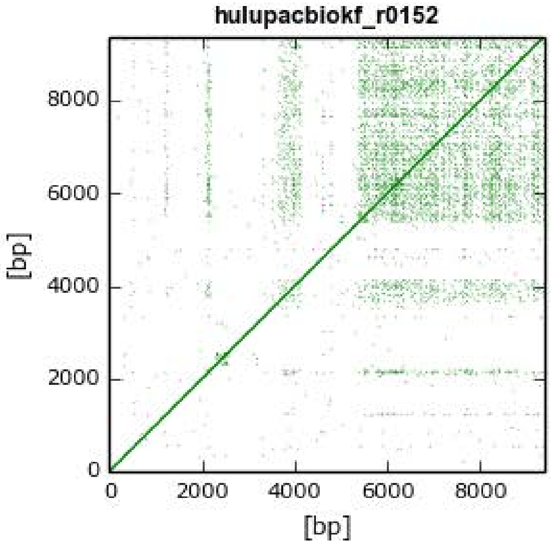
hulupacbiokf_r0152
[bp]
[bp]

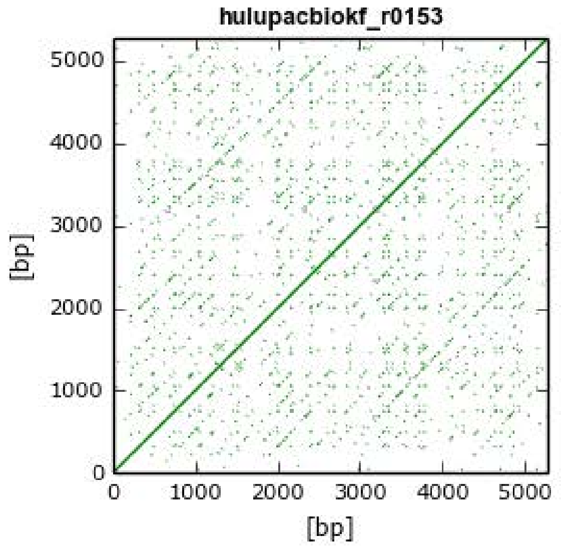
hulupacbiokf_r0153
[bp]
[bp]

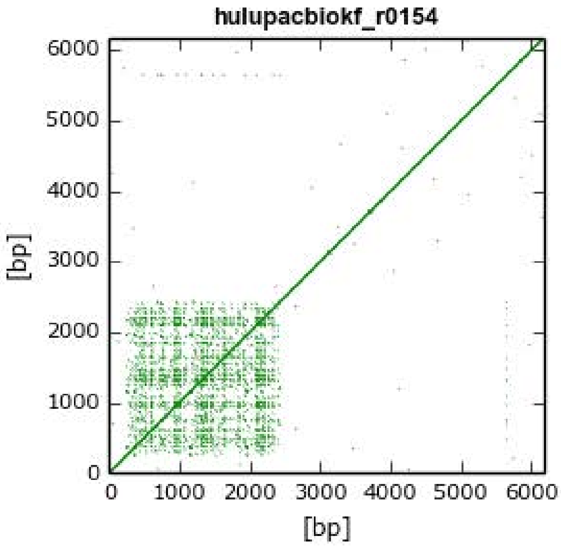
hulupacbiokf_r0154
[bp]
[bp]

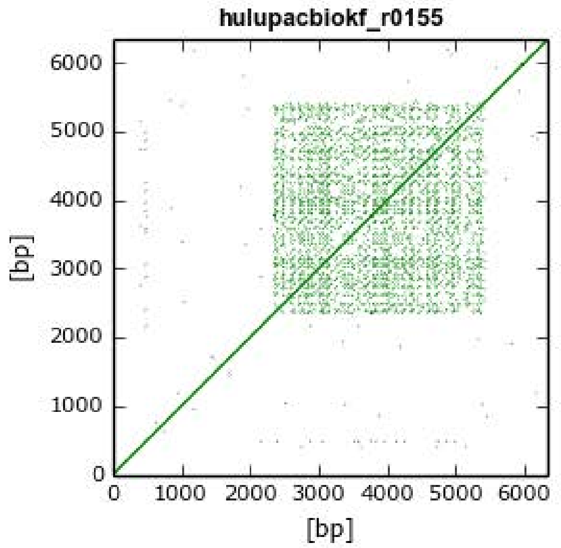
hulupacbiokf_r0155
[bp]
[bp]

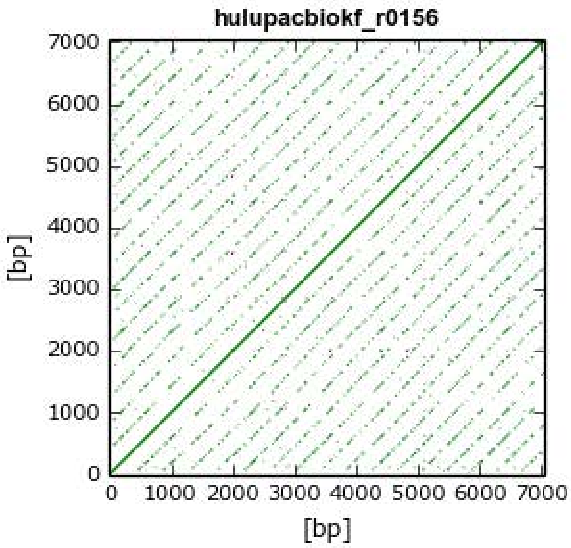
hulupacbiokf_r0156
[bp]
[bp]

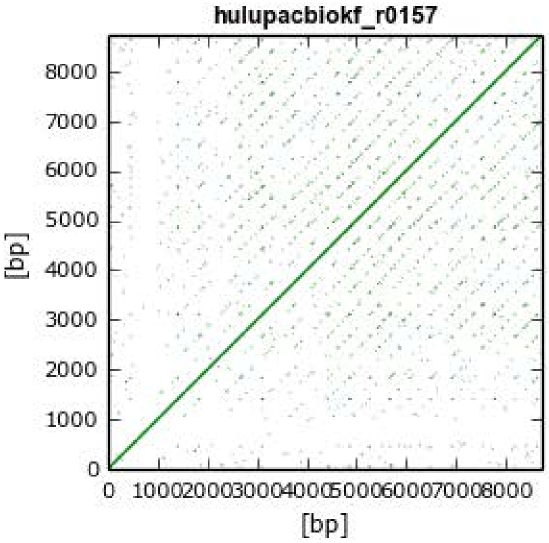
hulupacbiokf_r0157
8000
7000
6000
5000
4000
3000
2000
1000
0
[bp]
0 1000 2000 3000 4000 5000 6000 7000 8000
[bp]

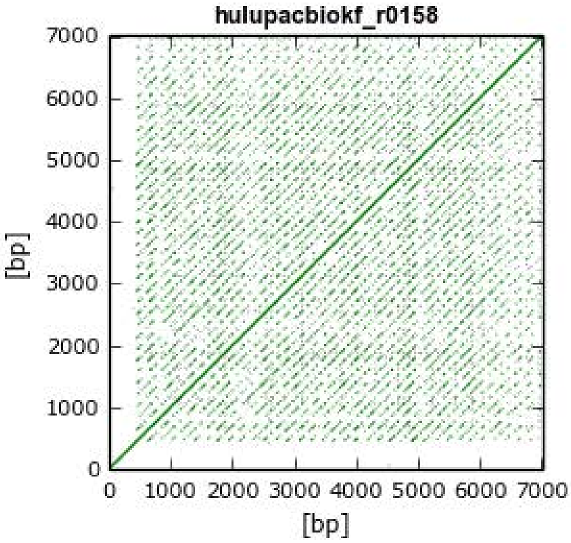
hulupacbiokf_r0158
7000
6000
5000
4000
3000
2000
1000
0
[bp]
0 1000 2000 3000 4000 5000 6000 7000
[bp]

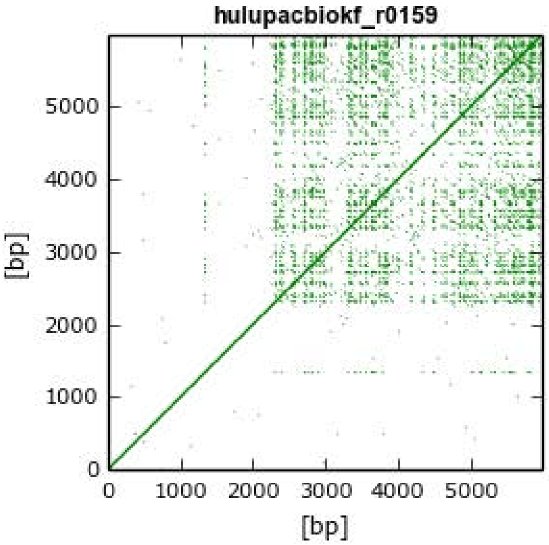
hulupacbiokf_r0159
5000
4000
3000
2000
1000
0
[bp]
0 1000 2000 3000 4000 5000
[bp]

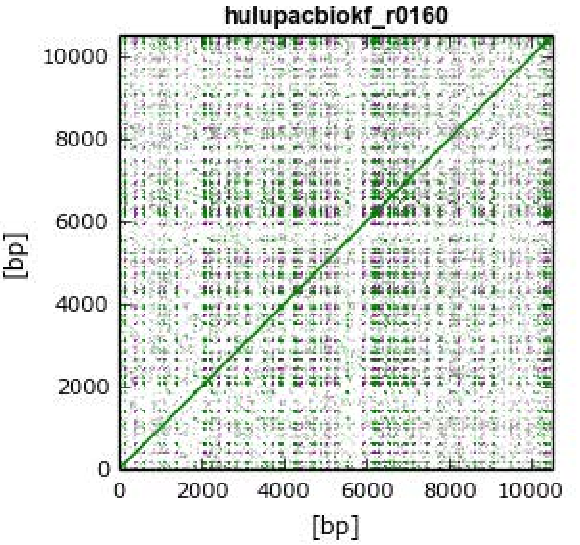
hulupacbiokf_r0160
10000
8000
6000
4000
2000
0
[bp]
0 2000 4000 6000 8000 10000
[bp]

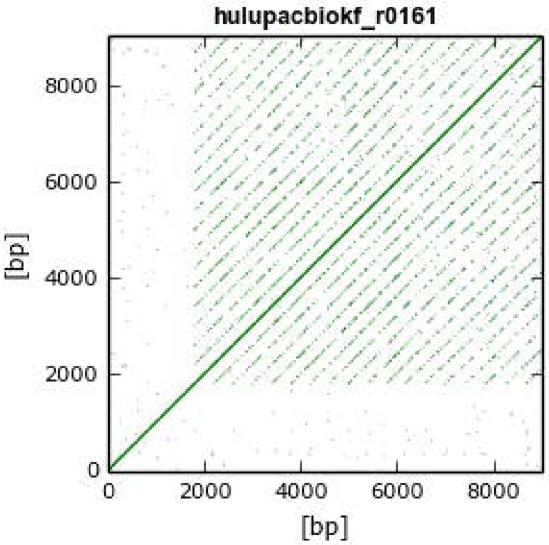
hulupacbiokf_r0161
8000
6000
4000
2000
0
[bp]
0 2000 4000 6000 8000
[bp]

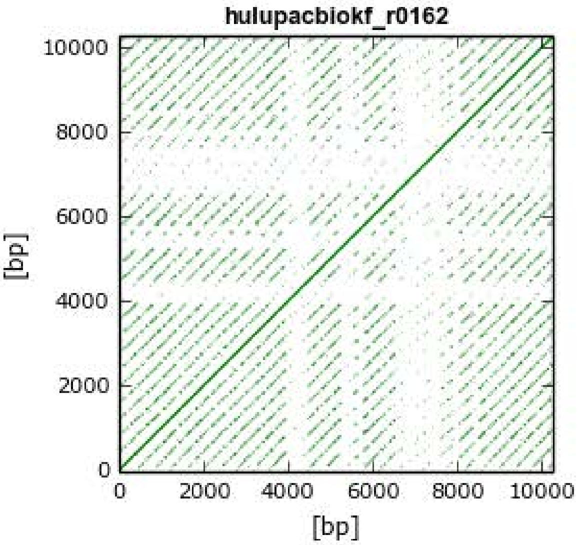
hulupacbiokf_r0162
10000
8000
6000
4000
2000
0
[bp]
0 2000 4000 6000 8000 10000
[bp]

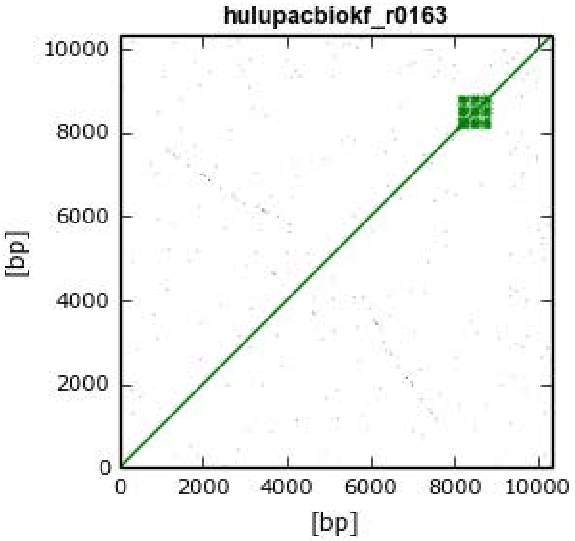
hulupacbiokf_r0163
[bp]
[bp]

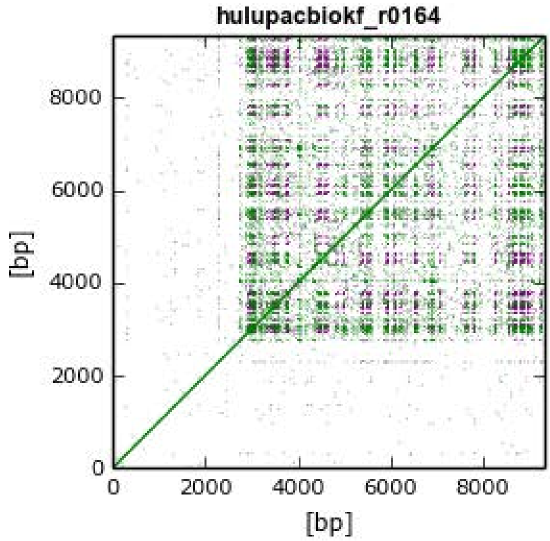
hulupacbiokf_r0164
[bp]
[bp]

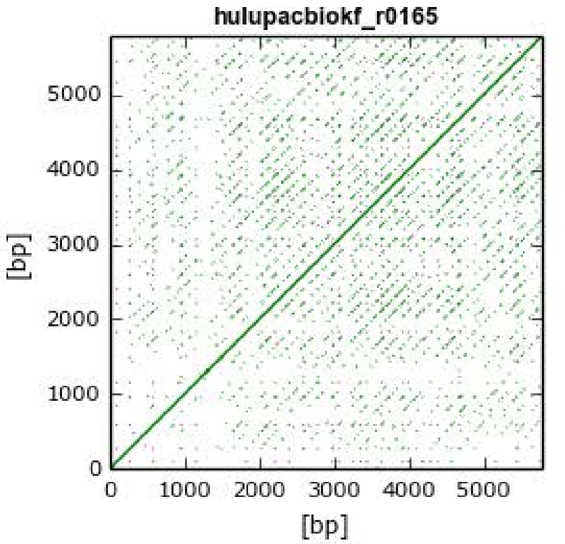
hulupacbiokf_r0165
[bp]
[bp]

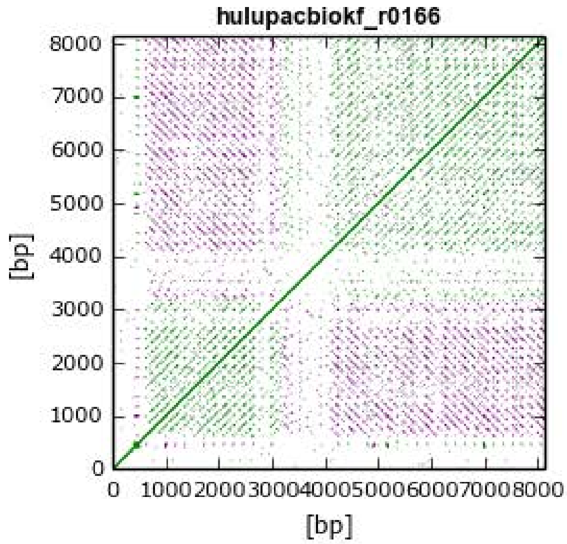
hulupacbiokf_r0166
[bp]
[bp]

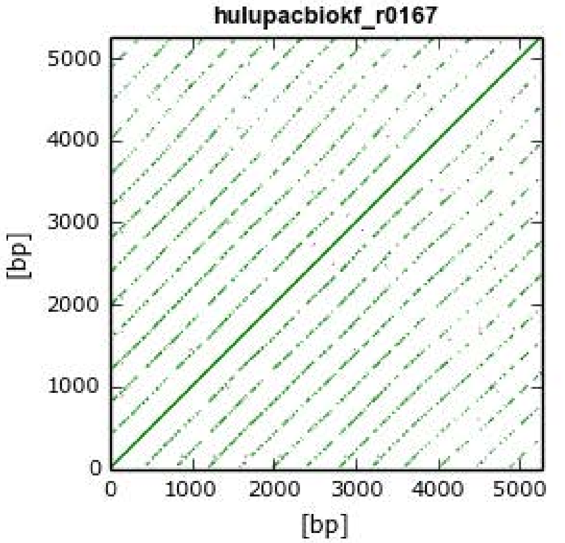
hulupacbiokf_r0167
[bp]
[bp]

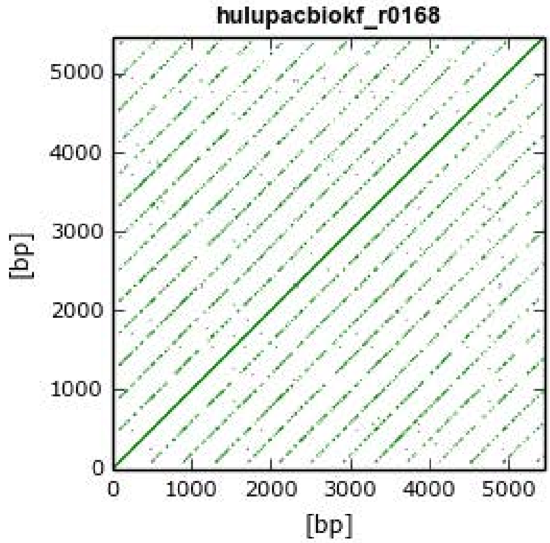
hulupacbiokf_r0168
[bp]
[bp]

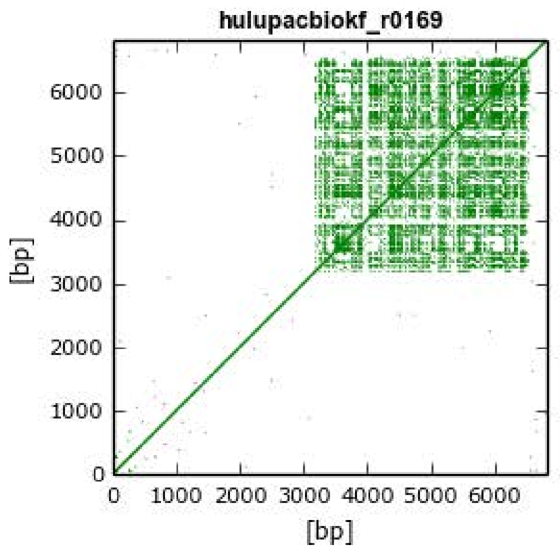
hulupacbiokf_r0169
[bp]
[bp]

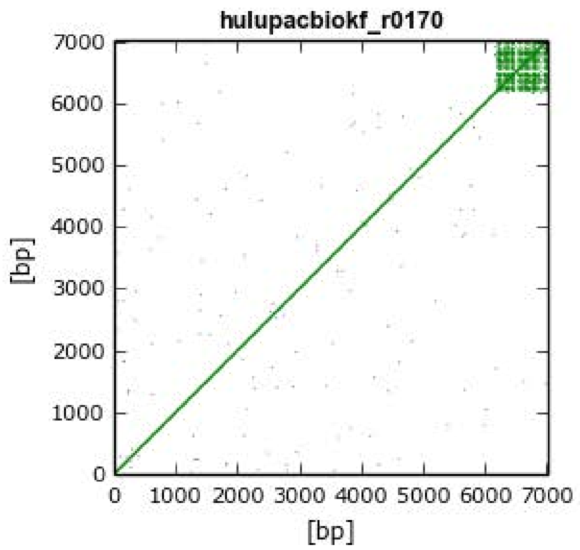
hulupacbiokf_r0170
[bp]
[bp]

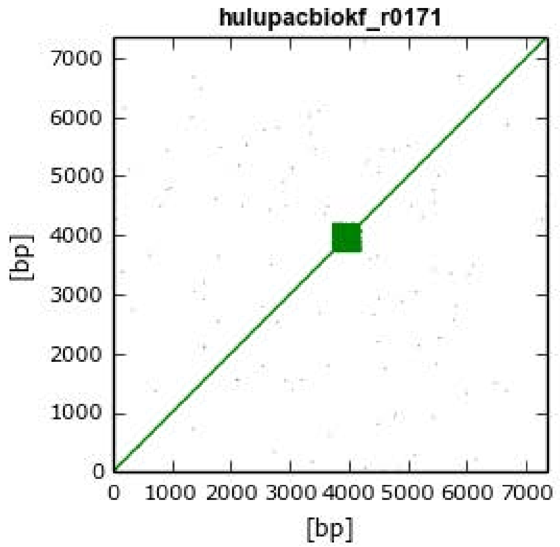
hulupacbiokf_r0171
[bp]
[bp]

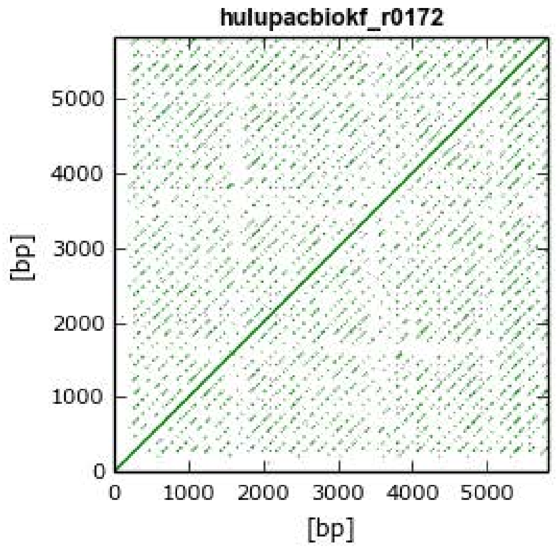
hulupacbiokf_r0172
[bp]
[bp]

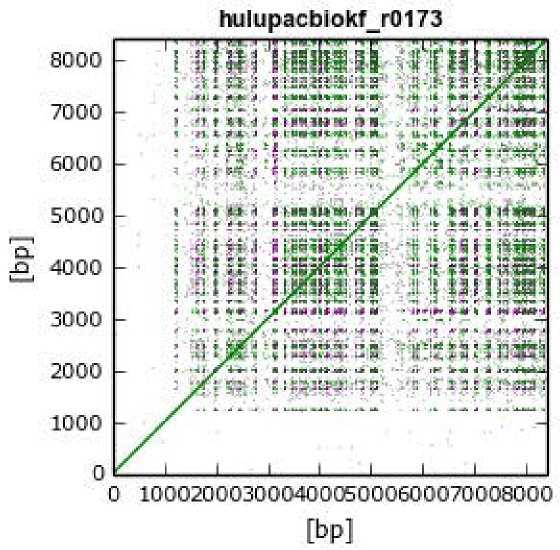
hulupacbiokf_r0173
[bp]
[bp]

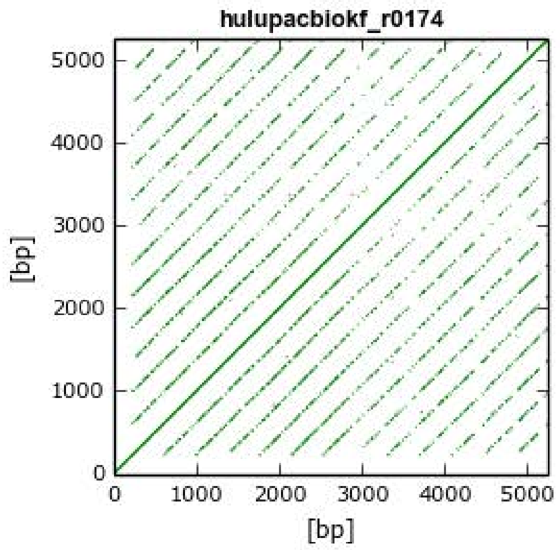
hulupacbiokf_r0174
[bp]
[bp]

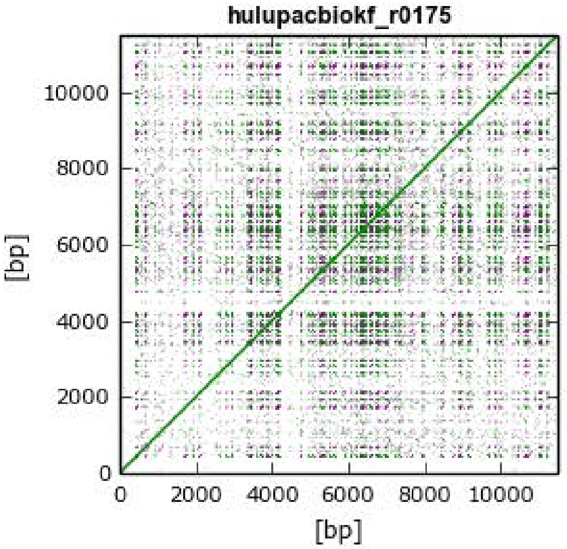
hulupacbiokf_r0175
0
2000
4000
6000
8000
10000
[bp]
[bp]

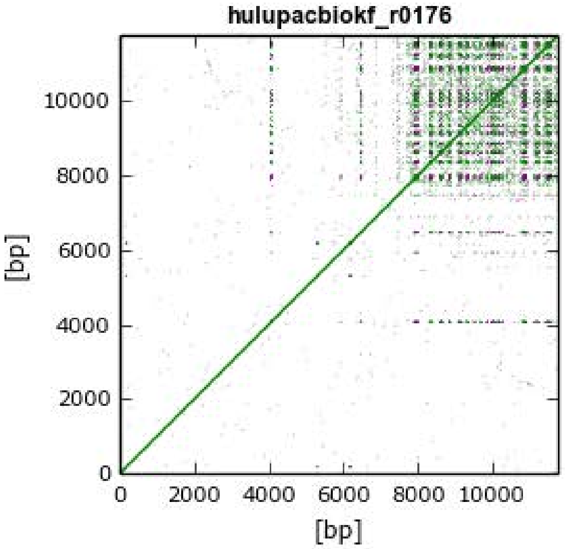
hulupacbiokf_r0176
0
2000
4000
6000
8000
10000
[bp]
[bp]

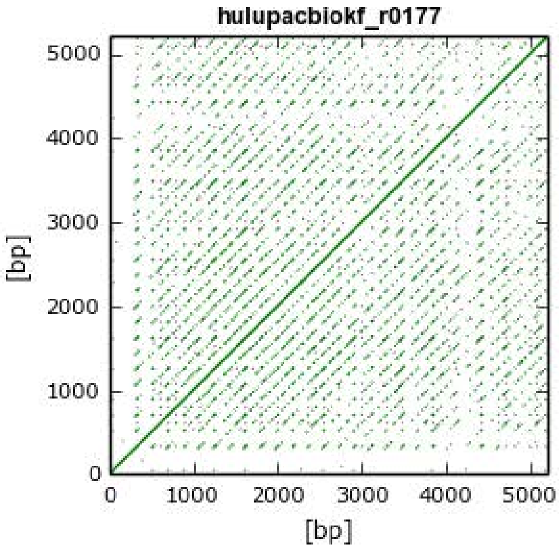
hulupacbiokf_r0177
0
1000
2000
3000
4000
5000
[bp]
[bp]

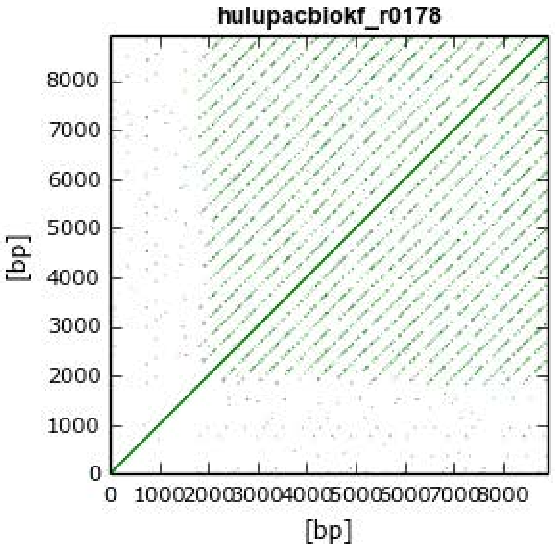
hulupacbiokf_r0178
0
1000
2000
3000
4000
5000
6000
7000
8000
[bp]
[bp]

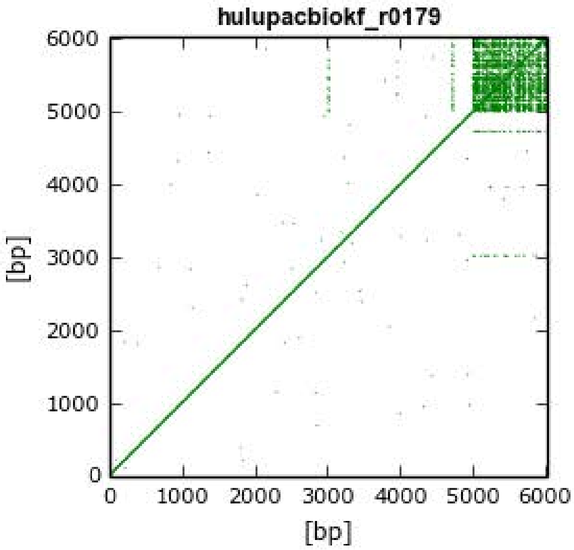
hulupacbiokf_r0179
0
1000
2000
3000
4000
5000
6000
[bp]
[bp]

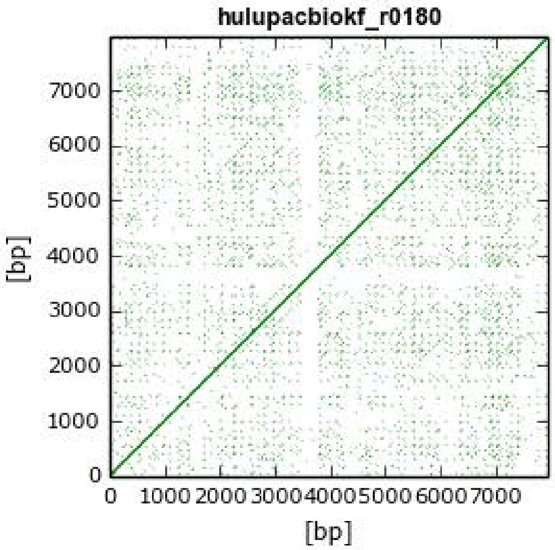
hulupacbiokf_r0180
0
1000
2000
3000
4000
5000
6000
7000
[bp]
[bp]

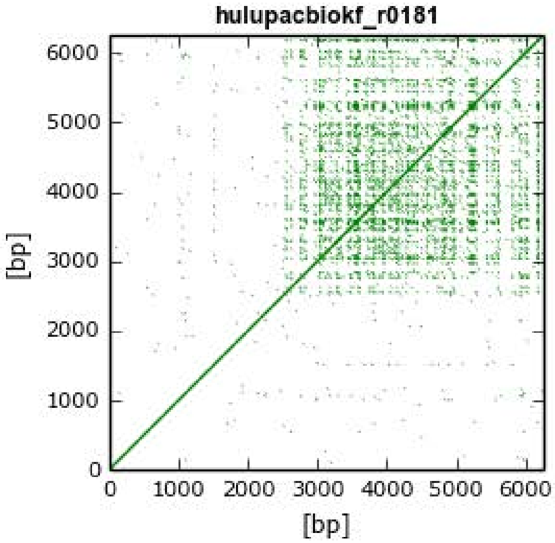
hulupacbiokf_r0181
[bp]
0
1000
2000
3000
4000
5000
6000
[bp]

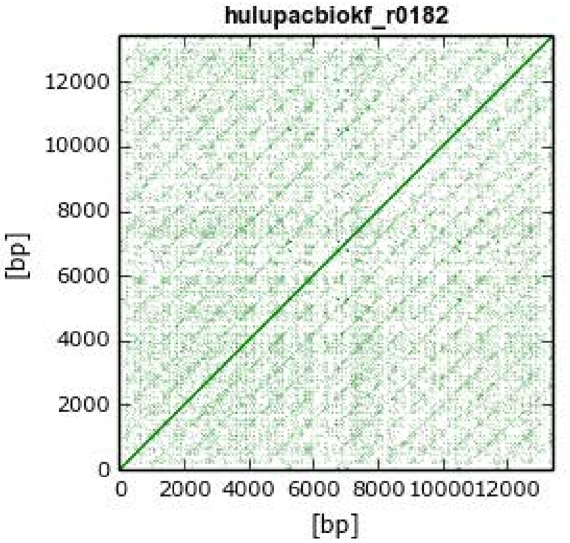
hulupacbiokf_r0182
[bp]
0
2000
4000
6000
8000
10000
12000
[bp]

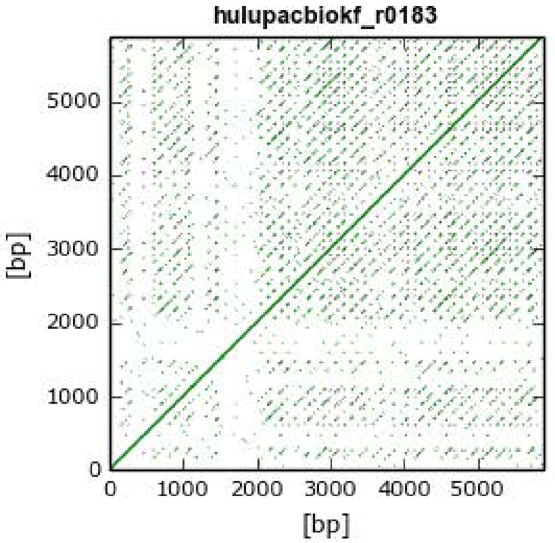
hulupacbiokf_r0183
[bp]
0
1000
2000
3000
4000
5000
[bp]

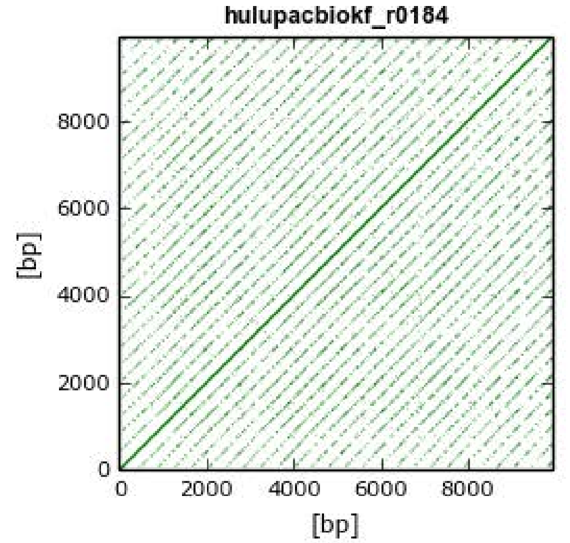
hulupacbiokf_r0184
[bp]
0
2000
4000
6000
8000
[bp]

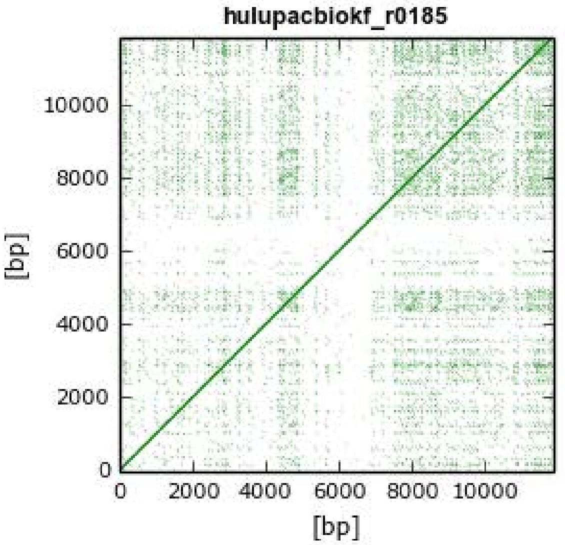
hulupacbiokf_r0185
[bp]
0
2000
4000
6000
8000
10000
[bp]

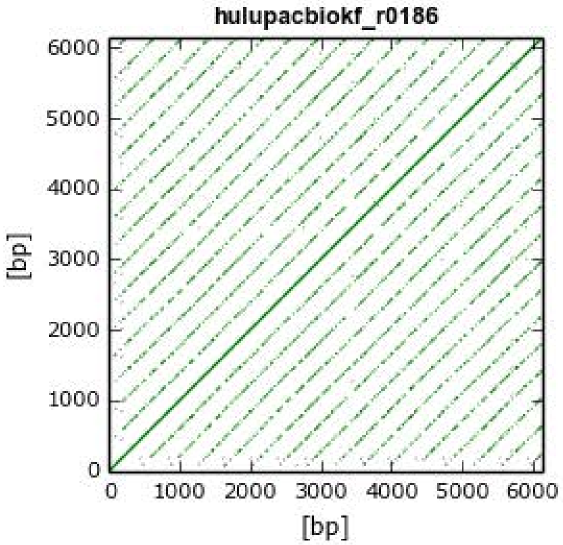
hulupacbiokf_r0186
[bp]
0
1000
2000
3000
4000
5000
6000
[bp]

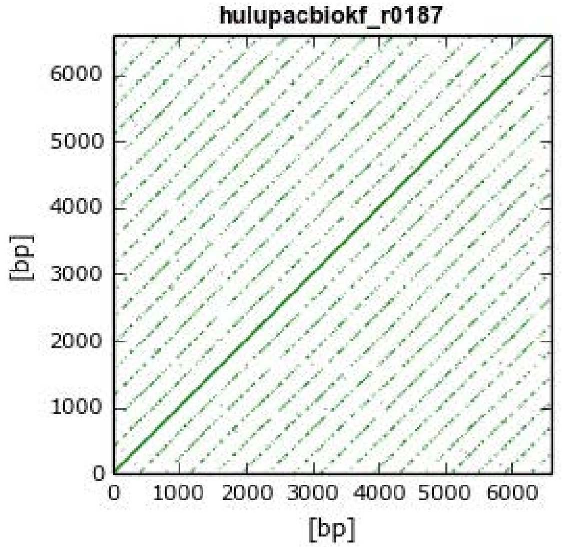
hulupacbiokf_r0187
[bp]
[bp]

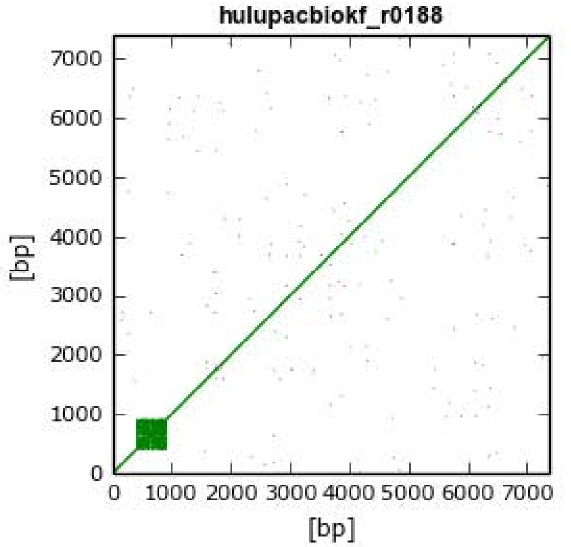
hulupacbiokf_r0188
[bp]
[bp]

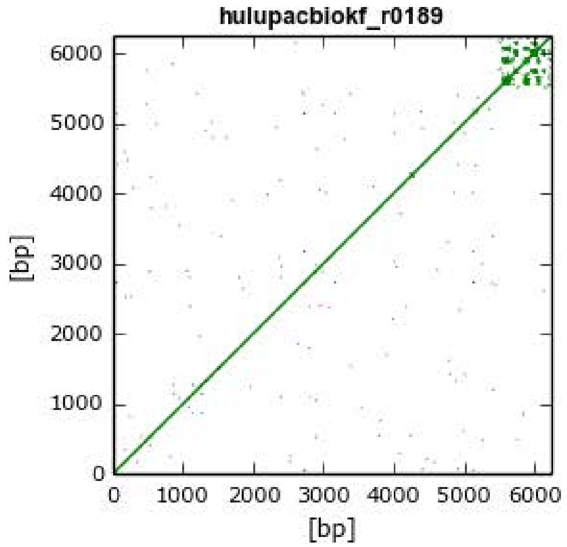
hulupacbiokf_r0189
[bp]
[bp]

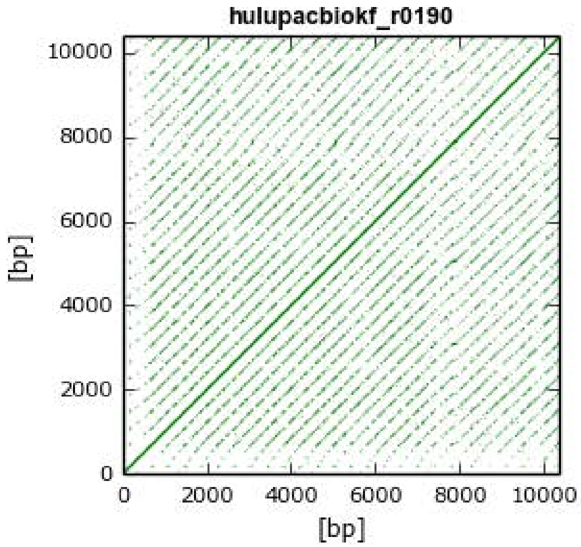
hulupacbiokf_r0190
[bp]
[bp]

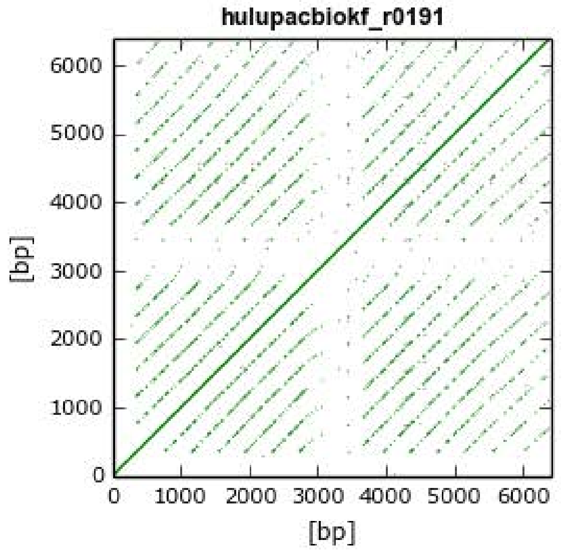
hulupacbiokf_r0191
[bp]
[bp]

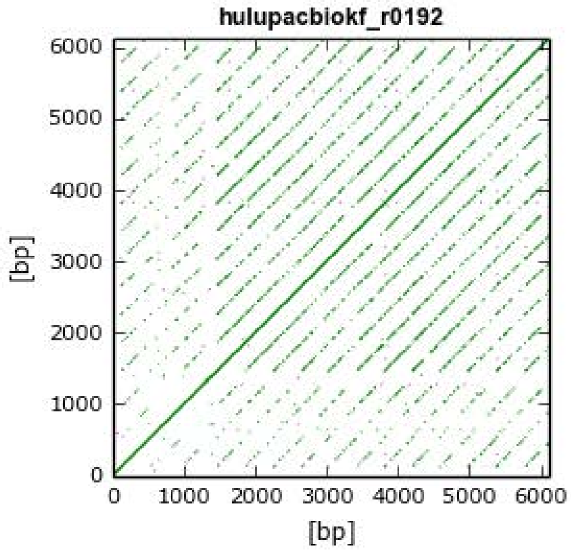
hulupacbiokf_r0192
[bp]
[bp]

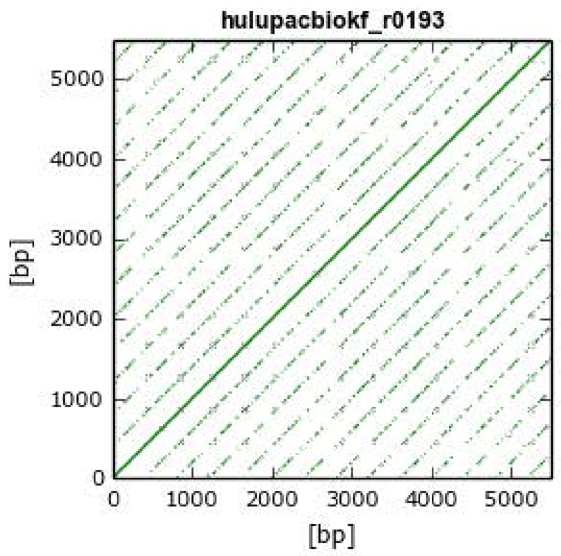
hulupacbiokf_r0193
[bp]
[bp]

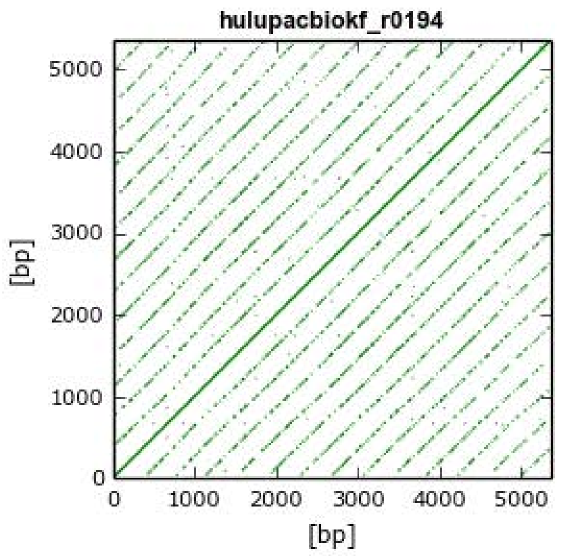
hulupacbiokf_r0194
[bp]
[bp]

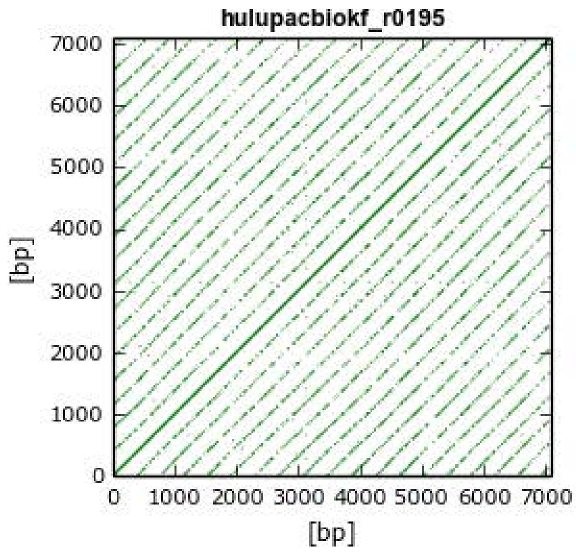
hulupacbiokf_r0195
[bp]
[bp]

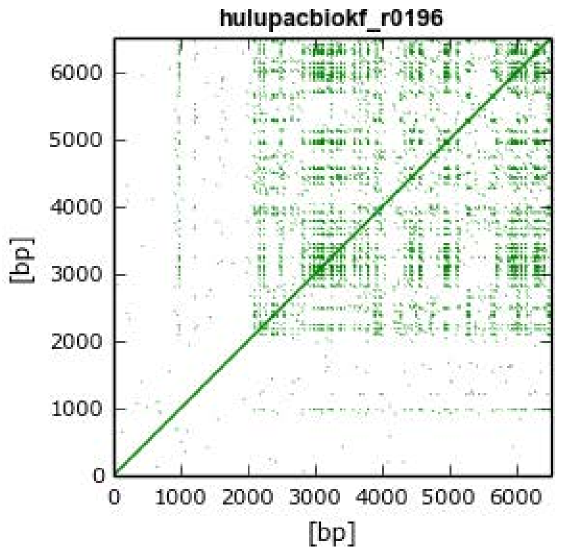
hulupacbiokf_r0196
[bp]
[bp]

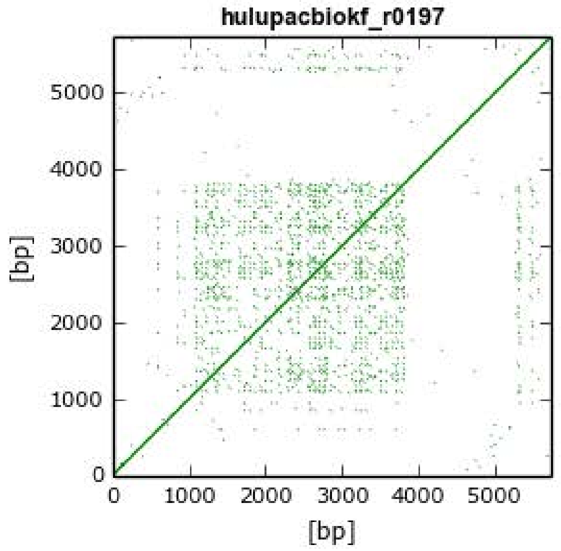
hulupacbiokf_r0197
[bp]
[bp]

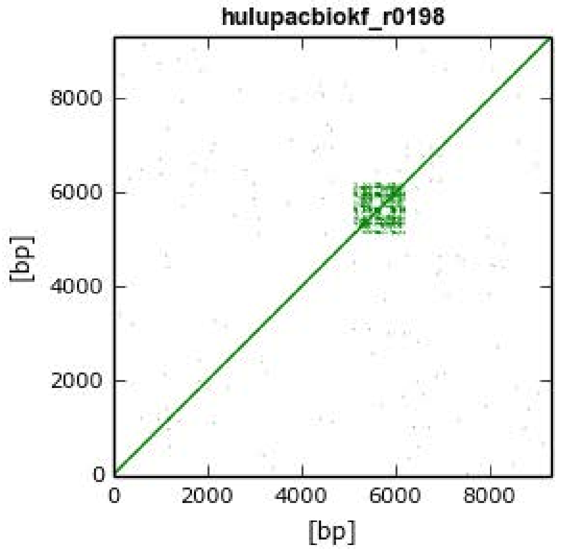
hulupacbiokf_r0198
[bp]
[bp]

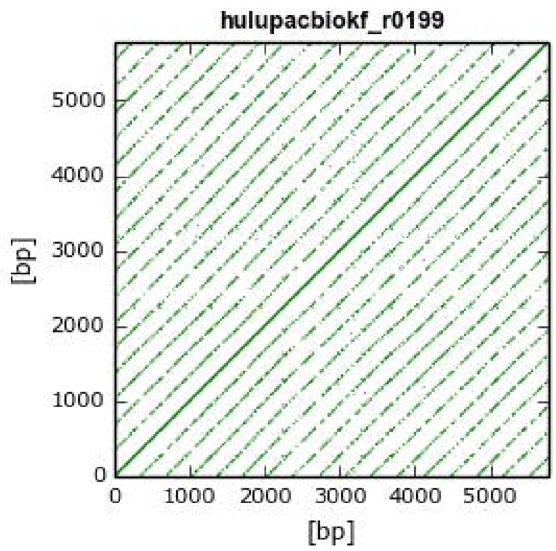
hulupacbiokf_r0199
[bp]
[bp]

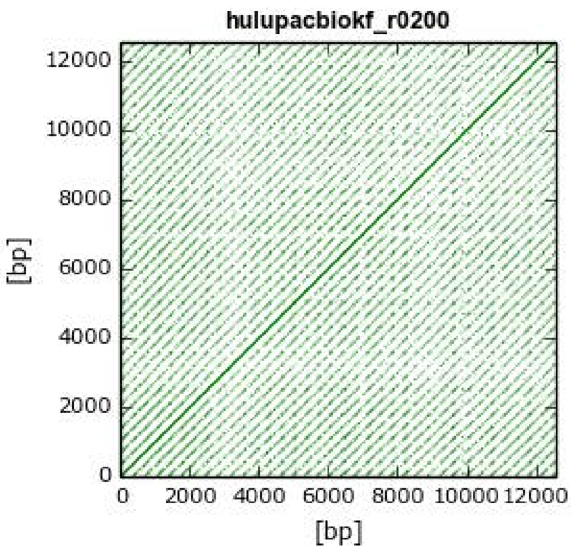
hulupacbiokf_r0200
[bp]
[bp]

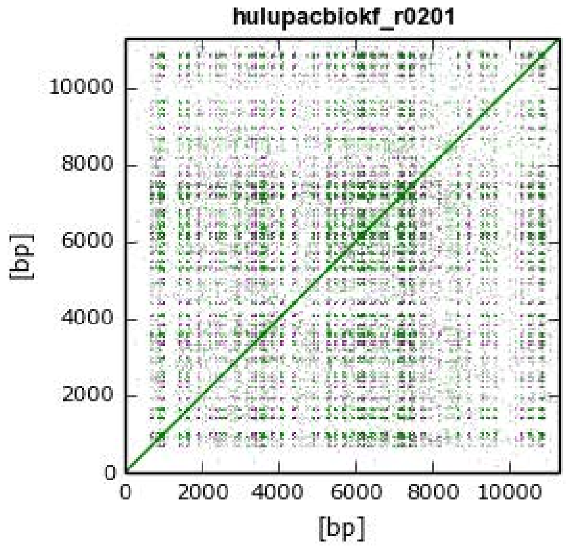
hulupacbiokf_r0201
[bp]
[bp]

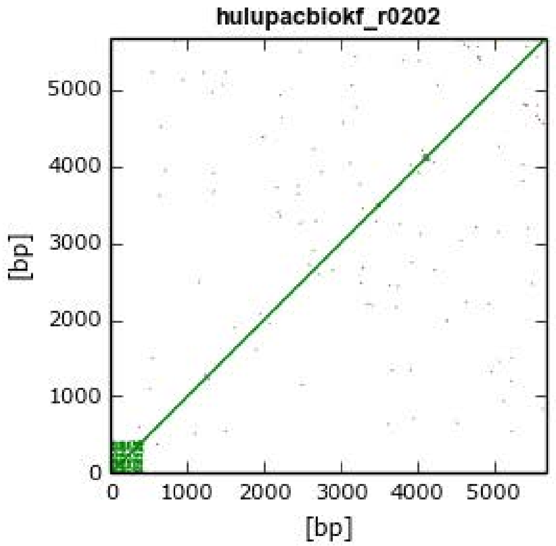
hulupacbiokf_r0202
[bp]
[bp]

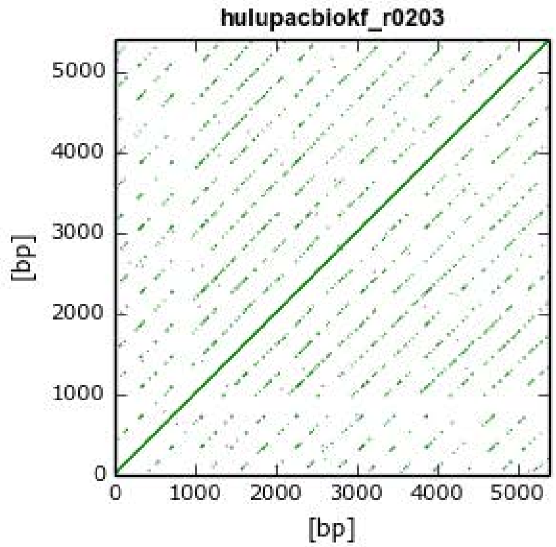
hulupacbiokf_r0203
[bp]
[bp]

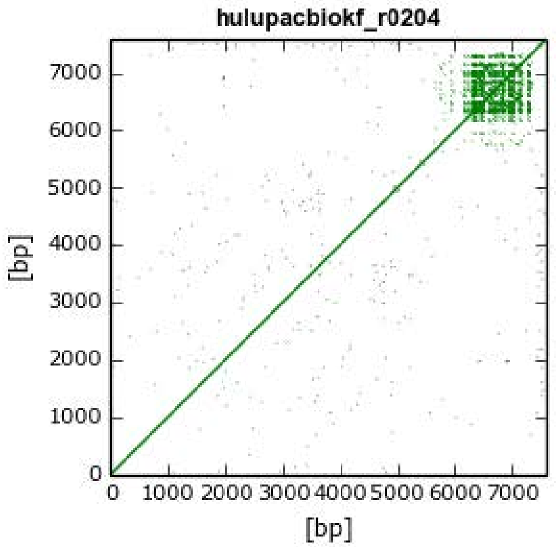
hulupacbiokf_r0204
[bp]
[bp]

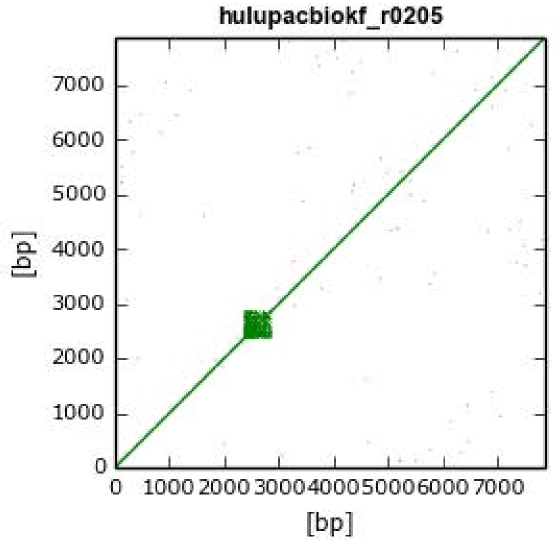
hulupacbiokf_r0205
[bp]
[bp]

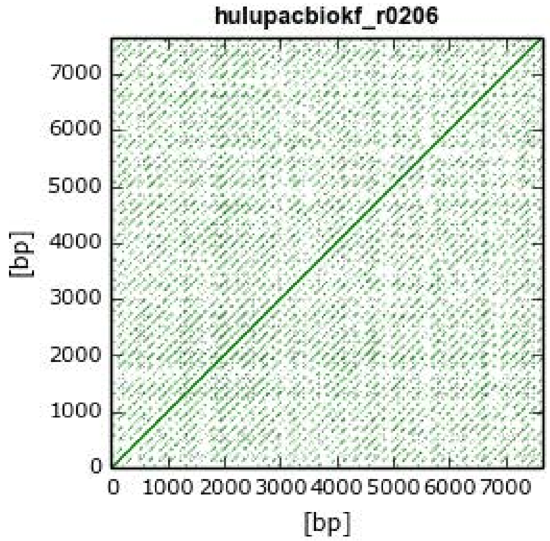
hulupacbiokf_r0206
[bp]
[bp]

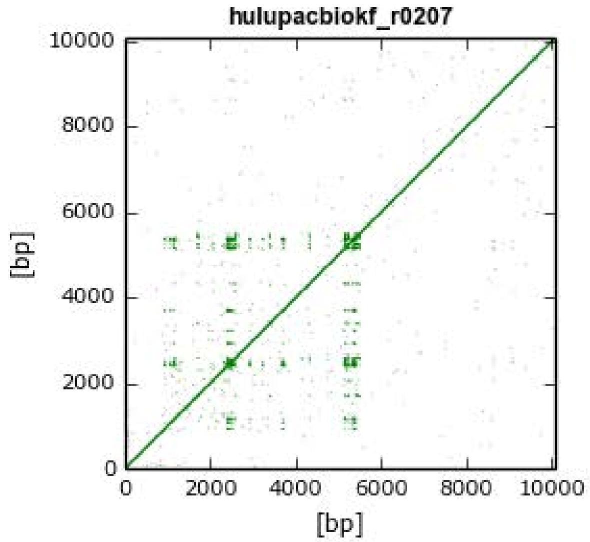
hulupacbiokf_r0207
[bp]
[bp]

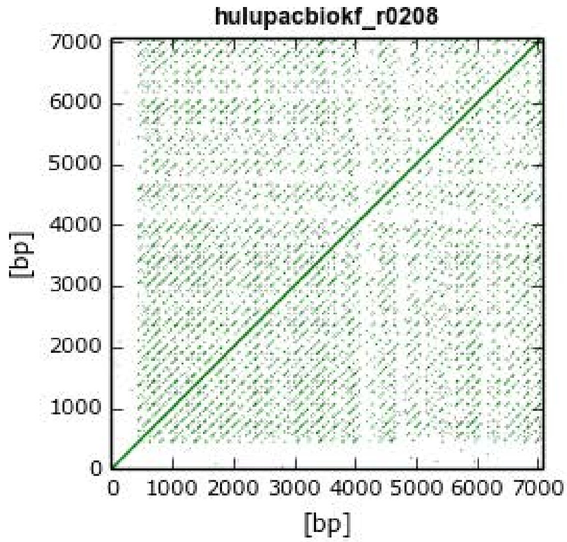
hulupacbiokf_r0208
[bp]
[bp]

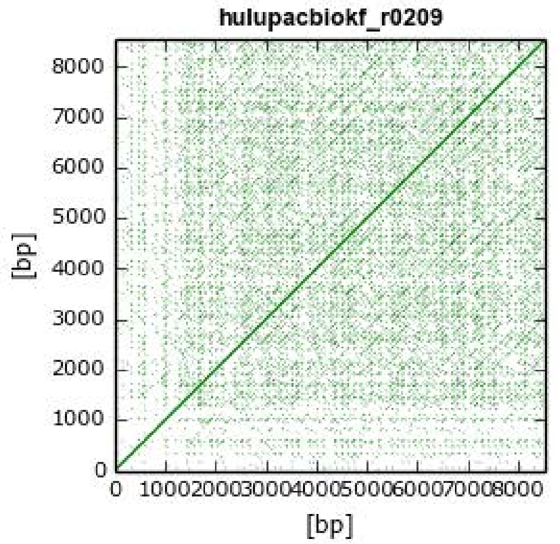
hulupacbiokf_r0209
[bp]
[bp]

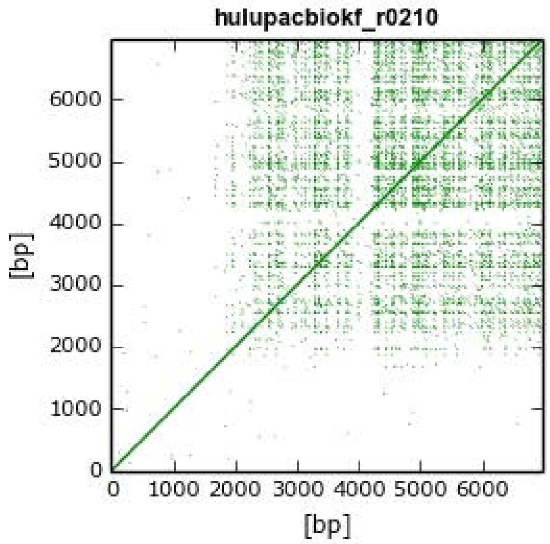
hulupacbiokf_r0210
[bp]
[bp]

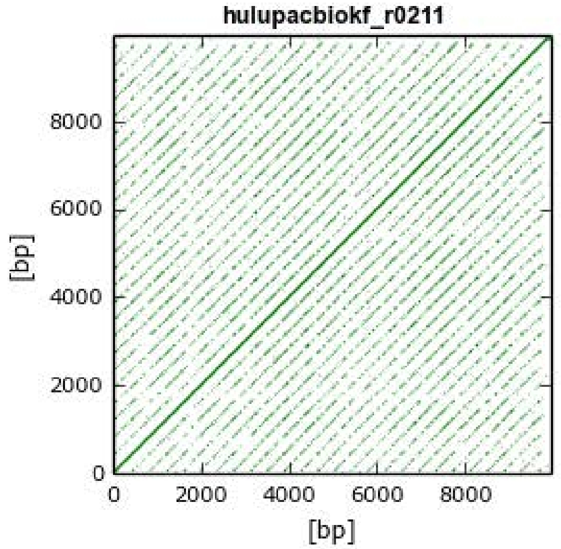
hulupacbiokf_r0211
0
2000
4000
6000
8000
[bp]
[bp]

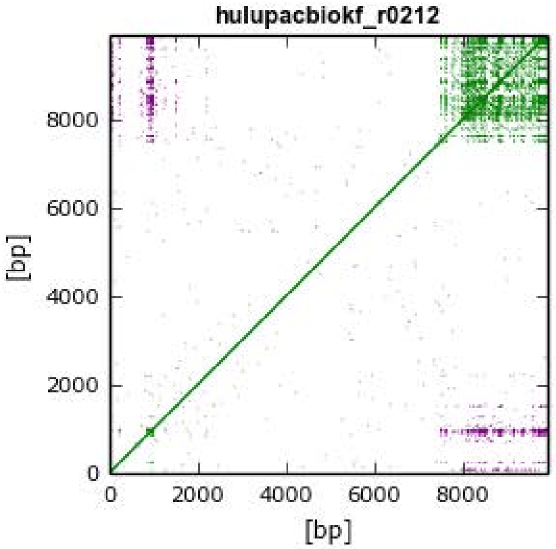
hulupacbiokf_r0212
0
2000
4000
6000
8000
[bp]
[bp]

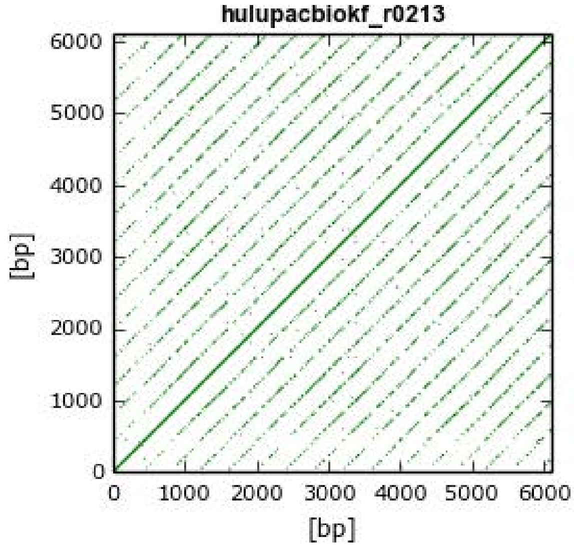
hulupacbiokf_r0213
0
1000
2000
3000
4000
5000
6000
[bp]
[bp]

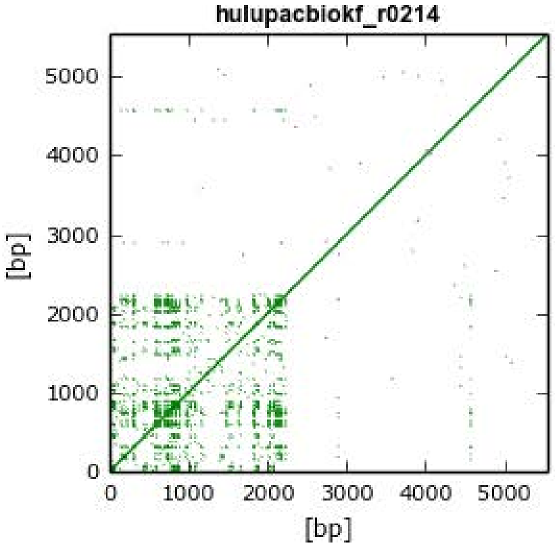
hulupacbiokf_r0214
0
1000
2000
3000
4000
5000
[bp]
[bp]

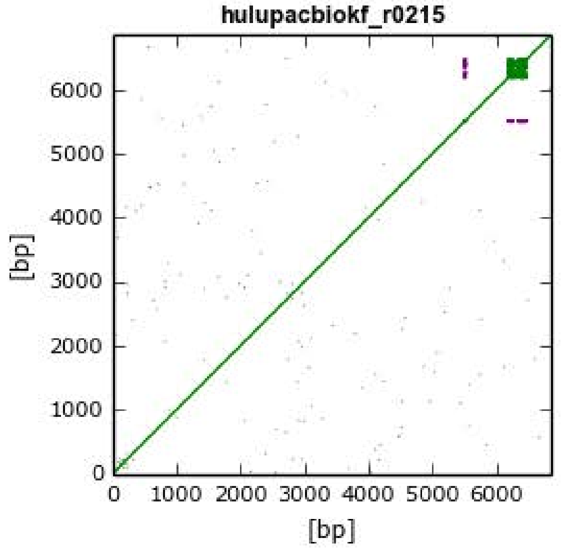
hulupacbiokf_r0215
0
1000
2000
3000
4000
5000
6000
[bp]
[bp]

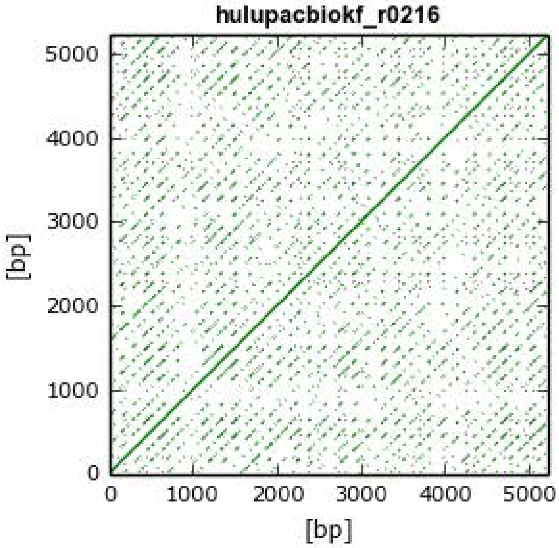
hulupacbiokf_r0216
0
1000
2000
3000
4000
5000
[bp]
[bp]

hulupacbiokf_r0217

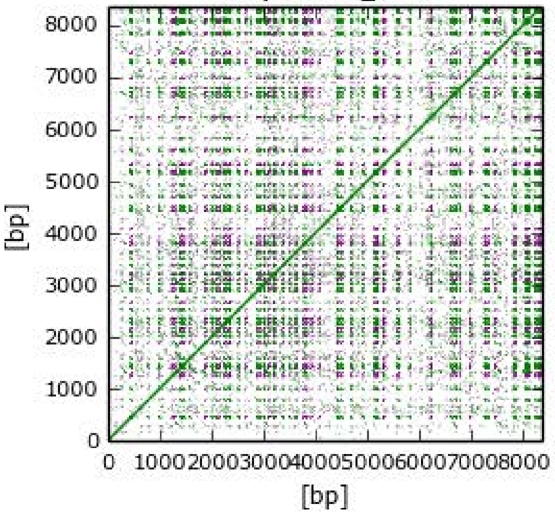

hulupacbiokf_r0218

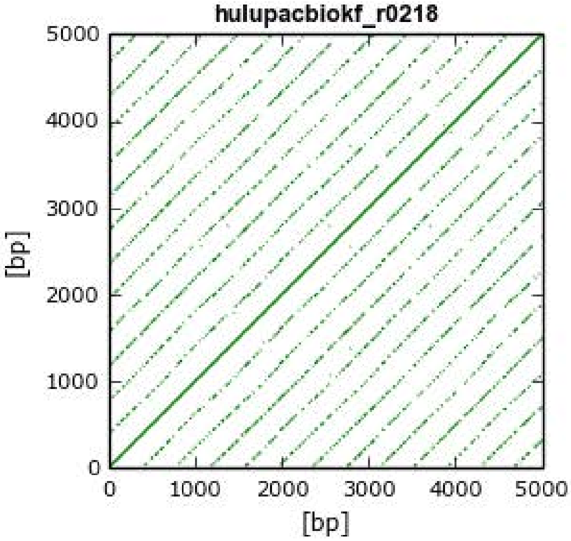

hulupacbiokf_r0219

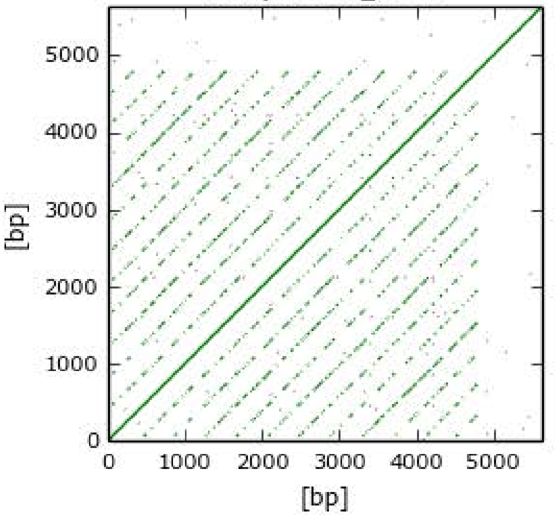

hulupacbiokf_r0220

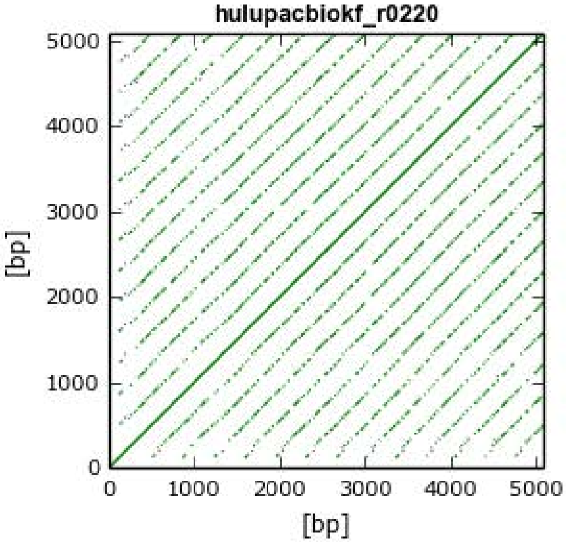

hulupacbiokf_r0221

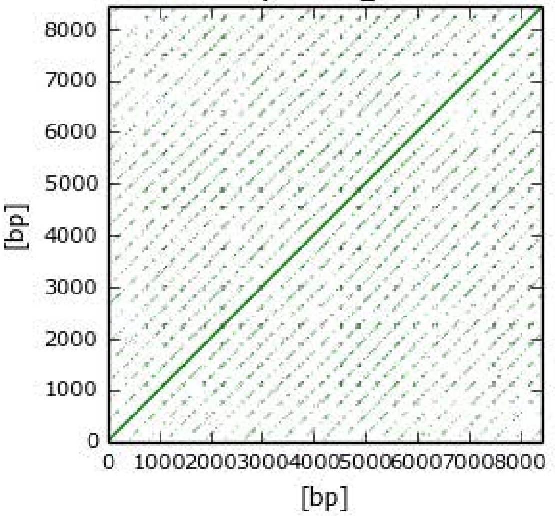

hulupacbiokf_r0222

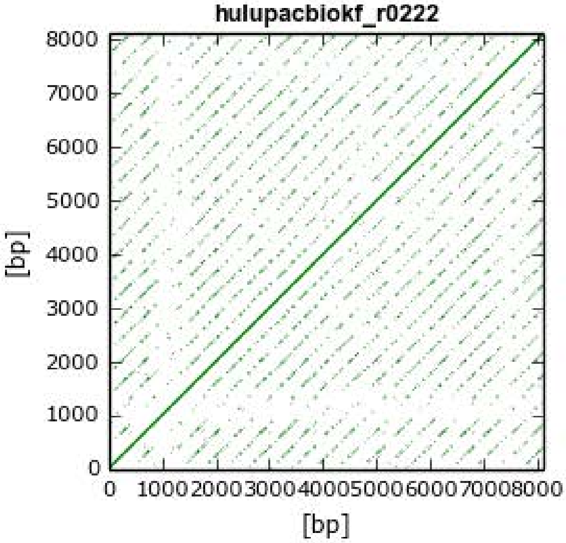

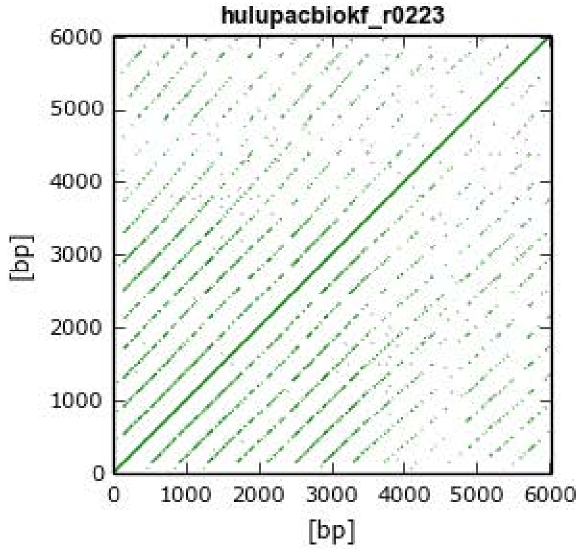
hulupacbiokf_r0223
[bp]
0
1000
2000
3000
4000
5000
6000
[bp]

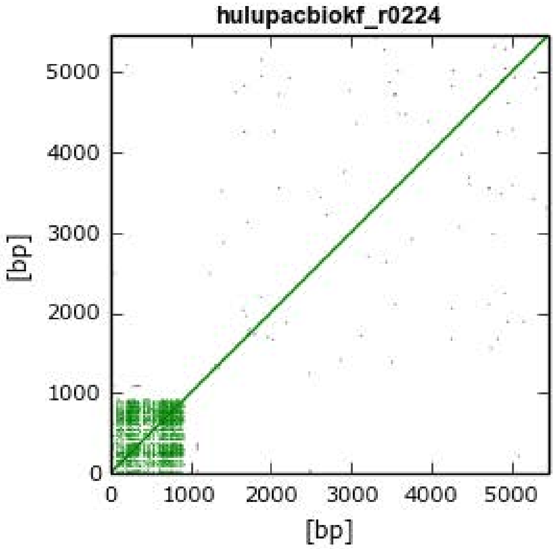
hulupacbiokf_r0224
[bp]
0
1000
2000
3000
4000
5000
[bp]

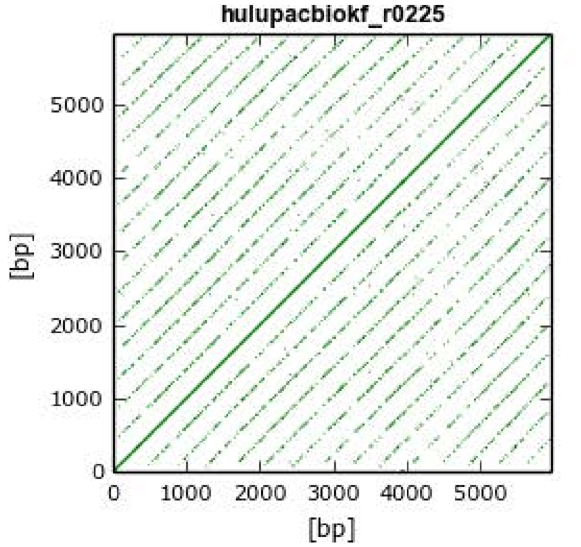
hulupacbiokf_r0225
[bp]
0
1000
2000
3000
4000
5000
[bp]

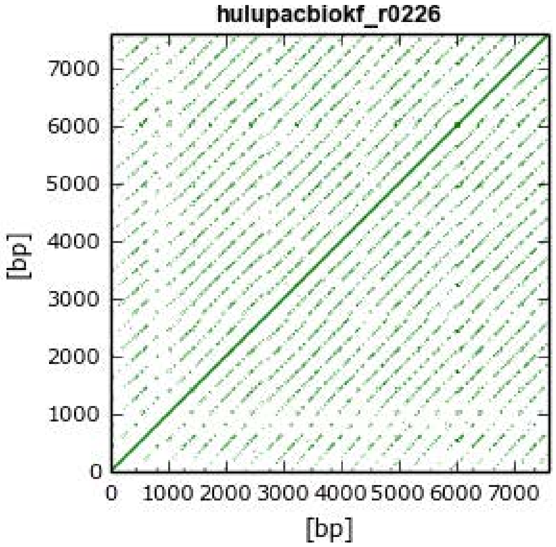
hulupacbiokf_r0226
[bp]
0
1000
2000
3000
4000
5000
6000
7000
[bp]

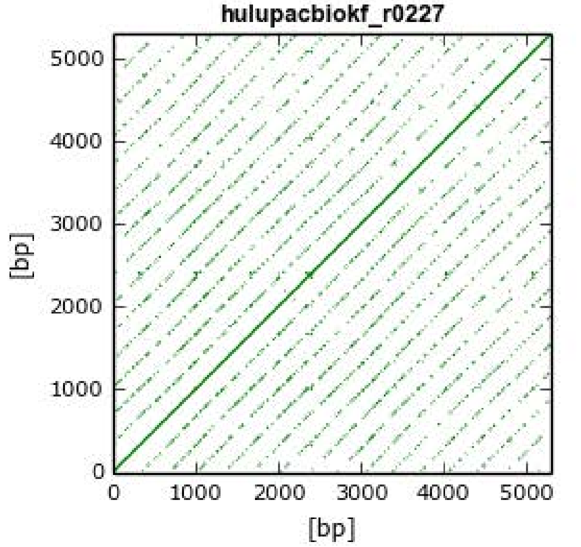
hulupacbiokf_r0227
[bp]
0
1000
2000
3000
4000
5000
[bp]

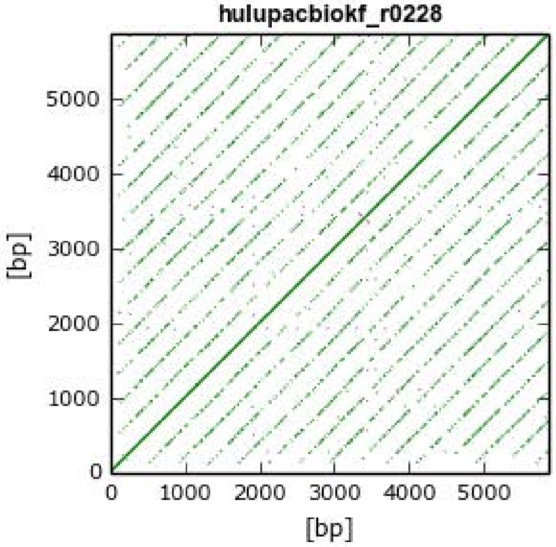
hulupacbiokf_r0228
[bp]
0
1000
2000
3000
4000
5000
[bp]

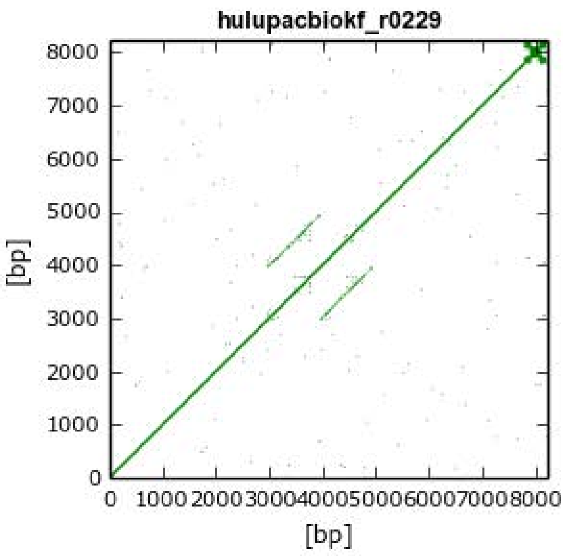
hulupacbiokf_r0229
[bp]
[bp]

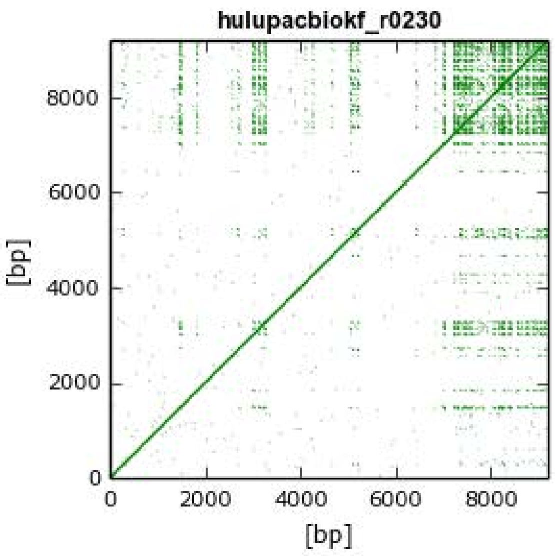
hulupacbiokf_r0230
[bp]
[bp]

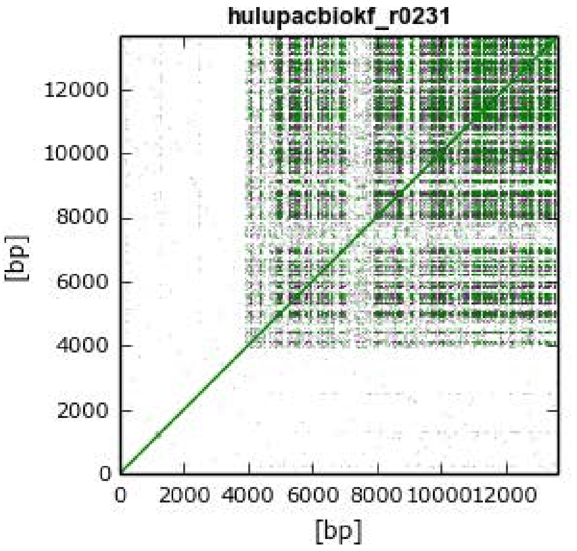
hulupacbiokf_r0231
[bp]
[bp]

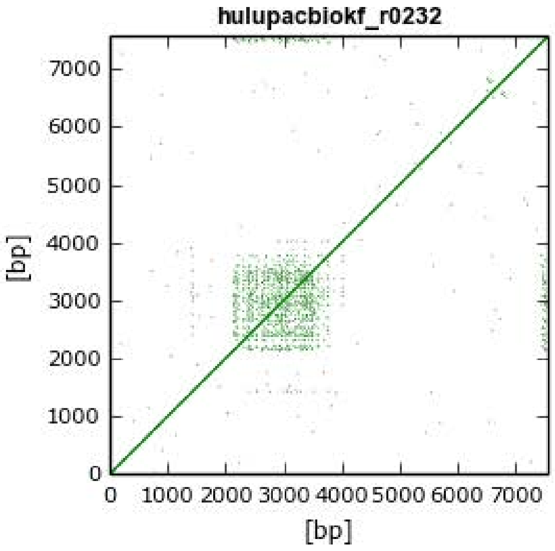
hulupacbiokf_r0232
[bp]
[bp]

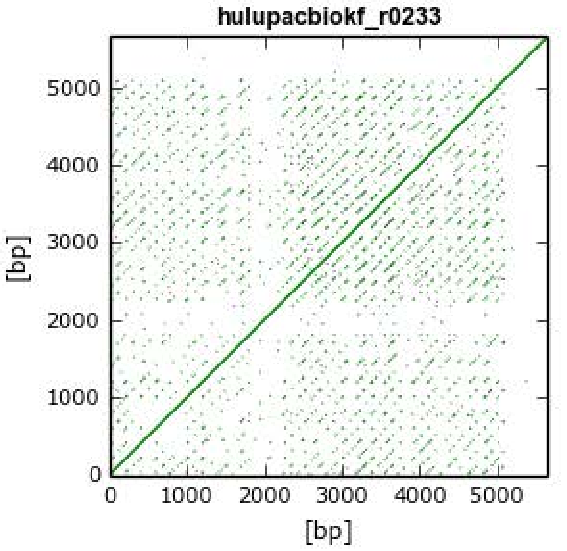
hulupacbiokf_r0233
[bp]
[bp]

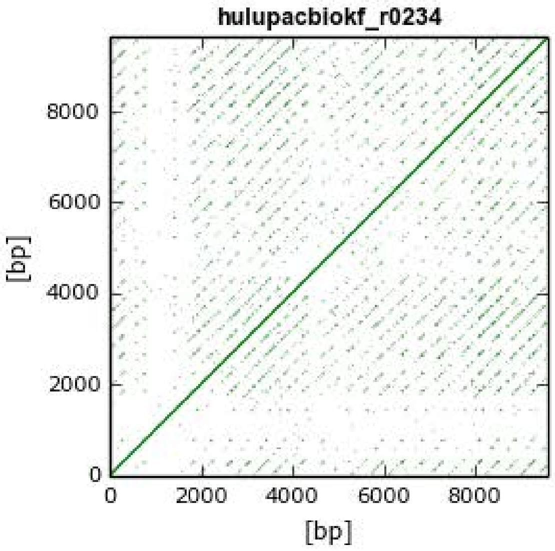
hulupacbiokf_r0234
[bp]
[bp]

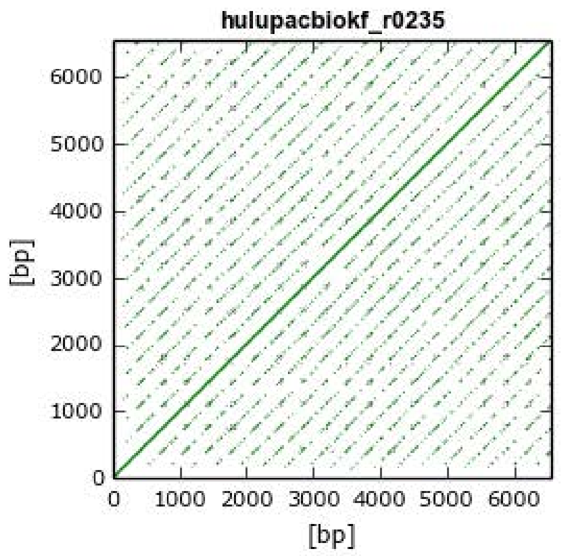
hulupacbiokf_r0235
[bp]
[bp]

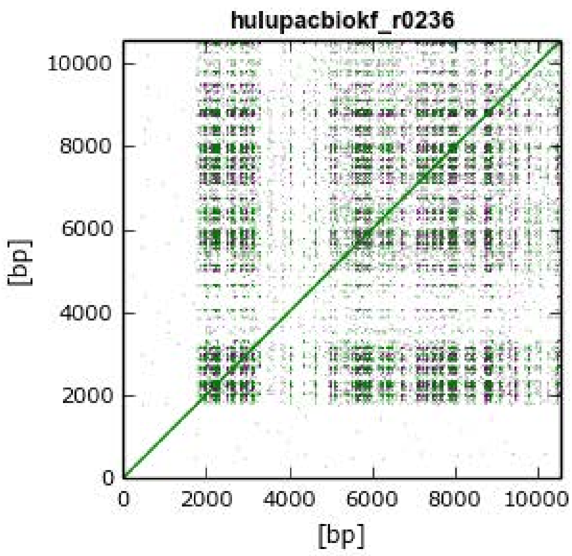
hulupacbiokf_r0236
[bp]
[bp]

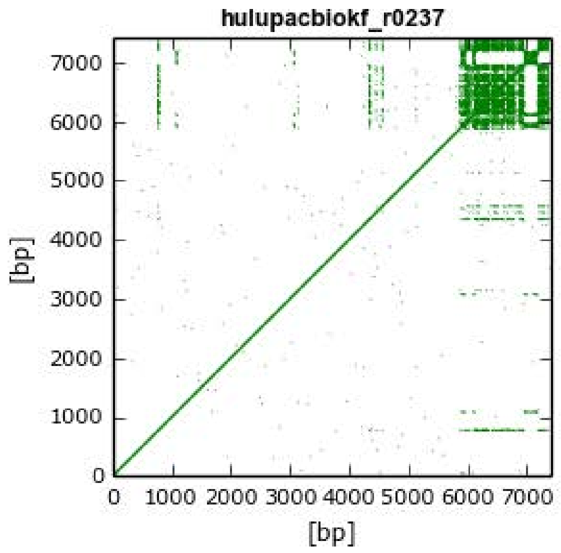
hulupacbiokf_r0237
[bp]
[bp]

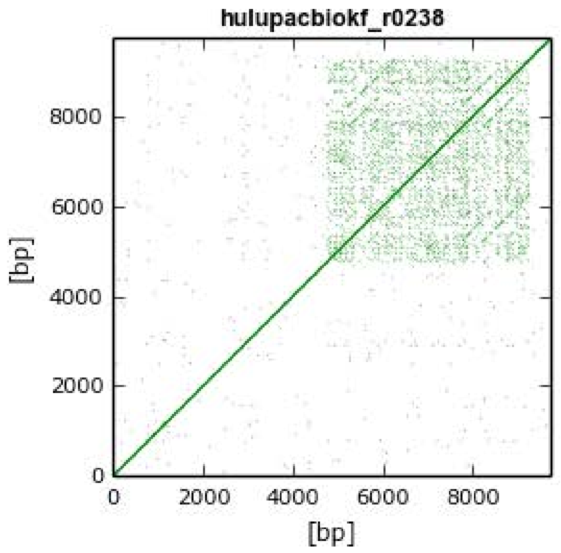
hulupacbiokf_r0238
[bp]
[bp]

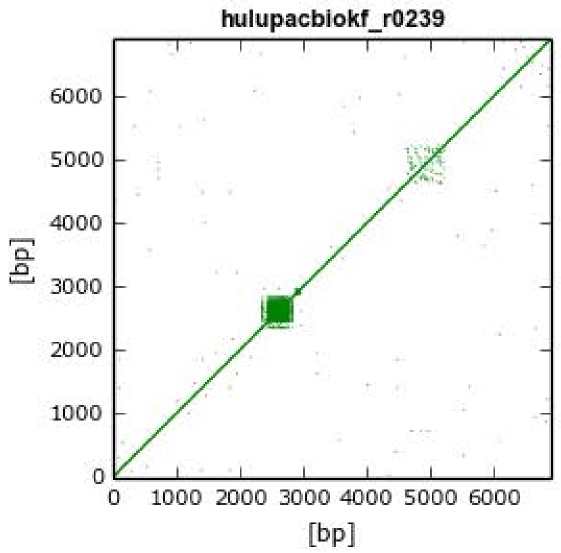
hulupacbiokf_r0239
[bp]
[bp]

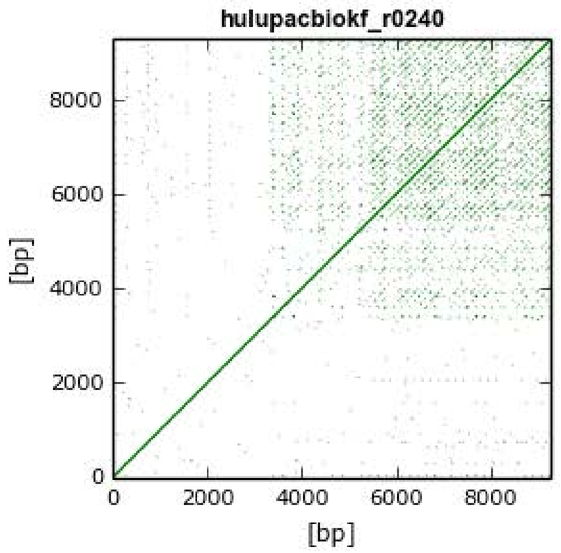
hulupacbiokf_r0240
[bp]
[bp]

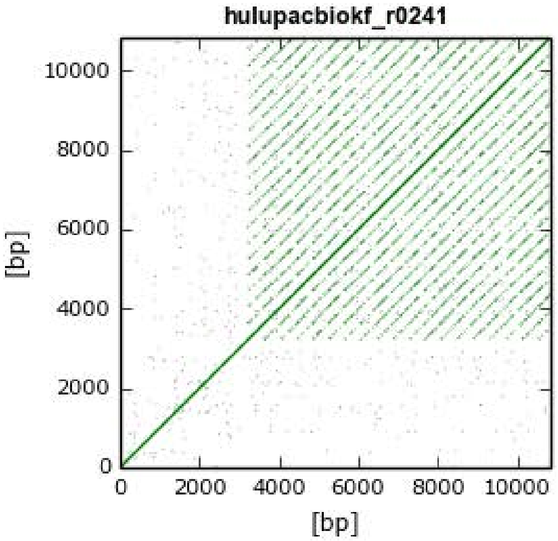

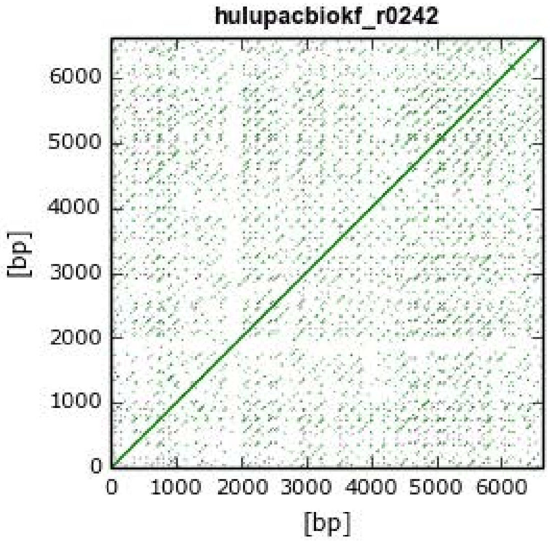

HuluTR335 (=5SrDNA) from read r0243
is in GenBank Acc. MN537579

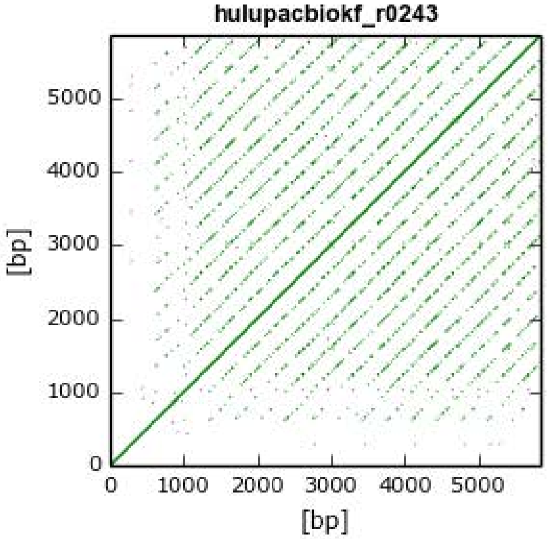

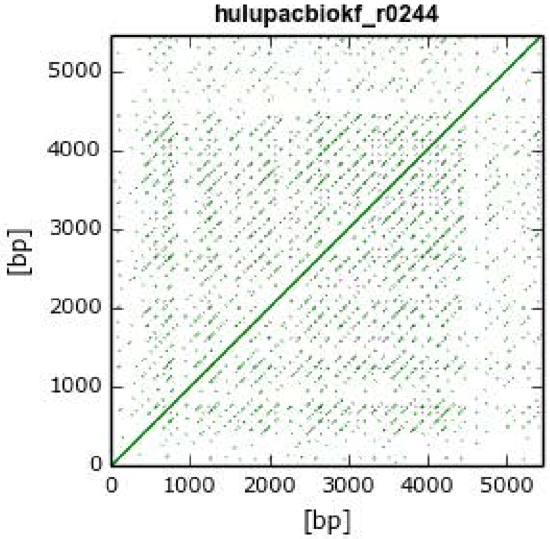

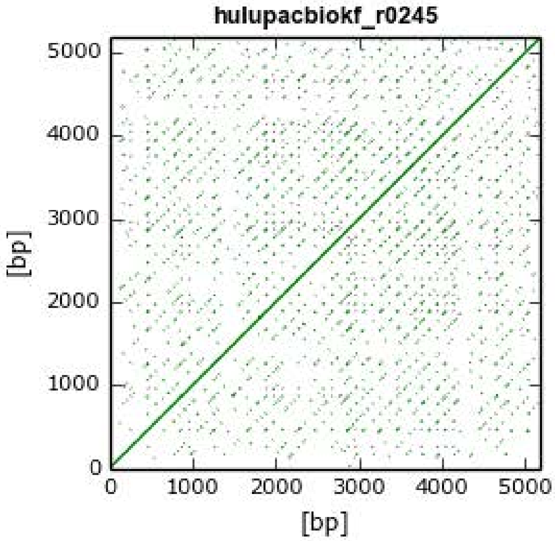

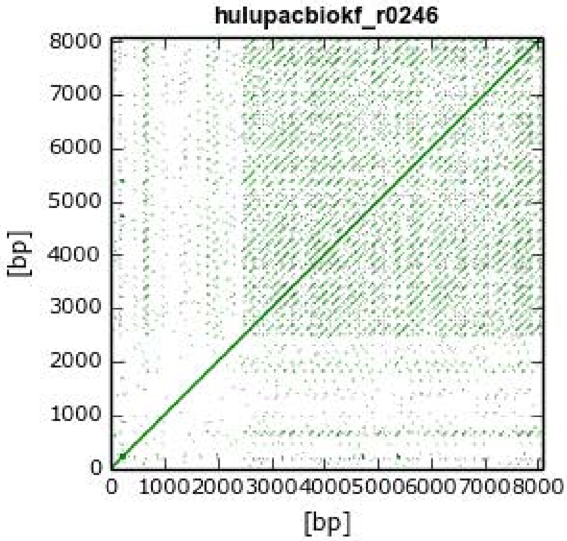

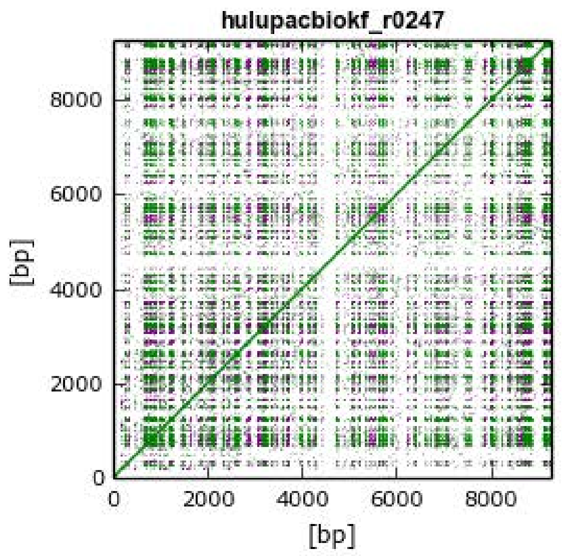
hulupacbiokf_r0247
[bp]
[bp]

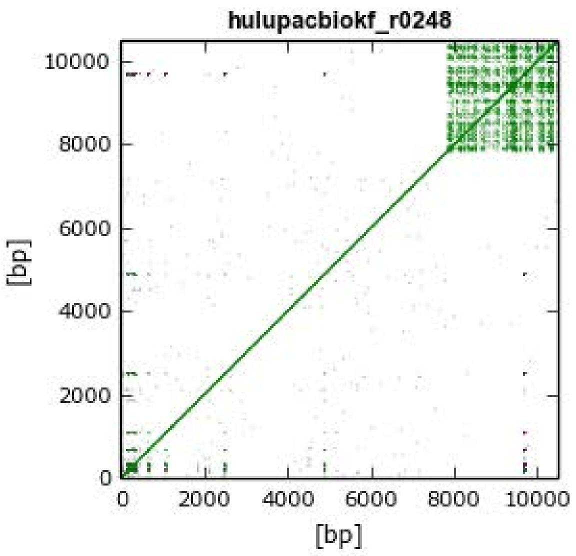
hulupacbiokf_r0248
[bp]
[bp]

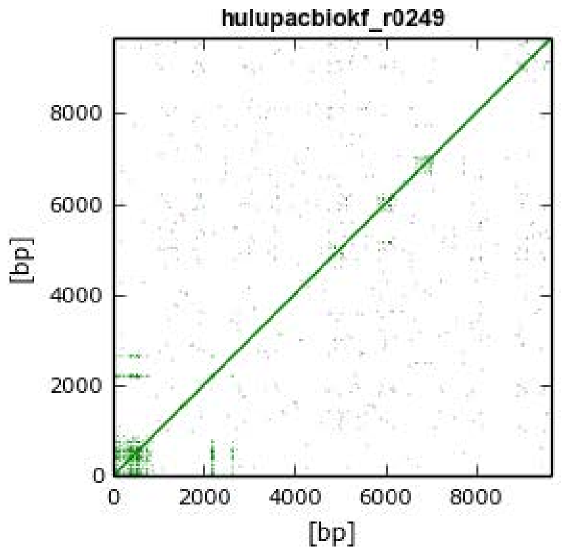
hulupacbiokf_r0249
[bp]
[bp]

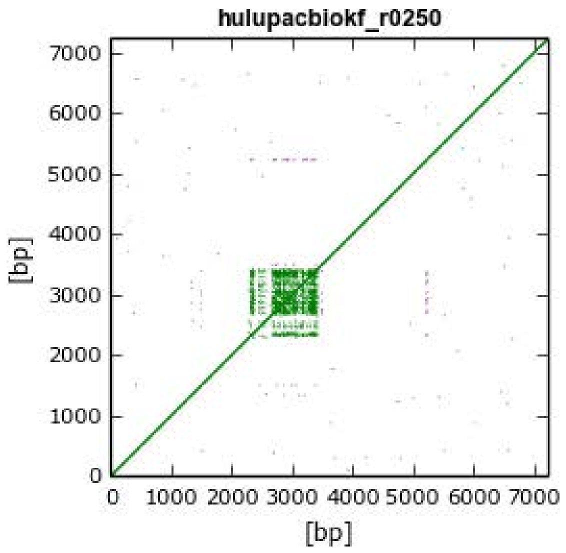
hulupacbiokf_r0250
[bp]
[bp]

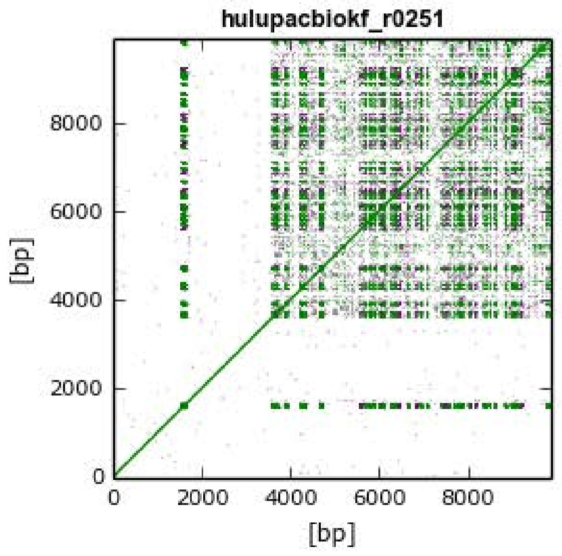
hulupacbiokf_r0251
[bp]
[bp]

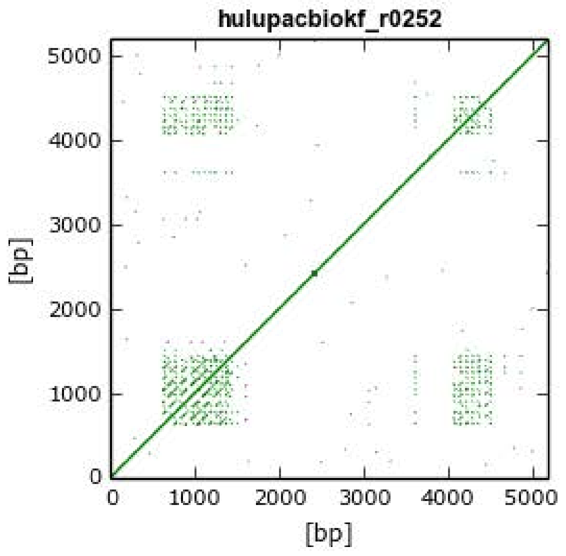
hulupacbiokf_r0252
[bp]
[bp]

HuluTR135 from read r0253
is in GenBank Acc. MN537571

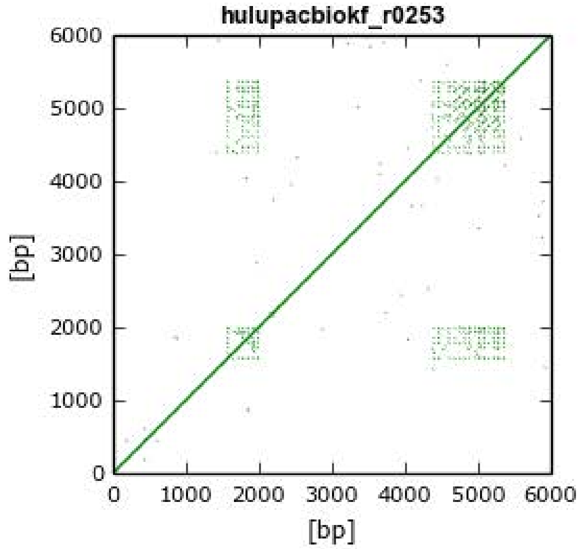

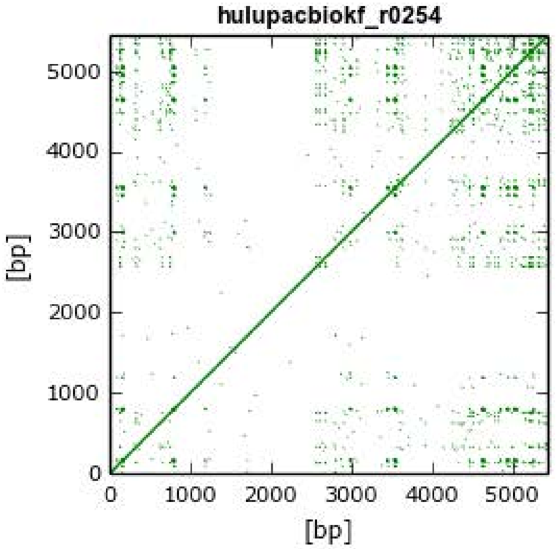

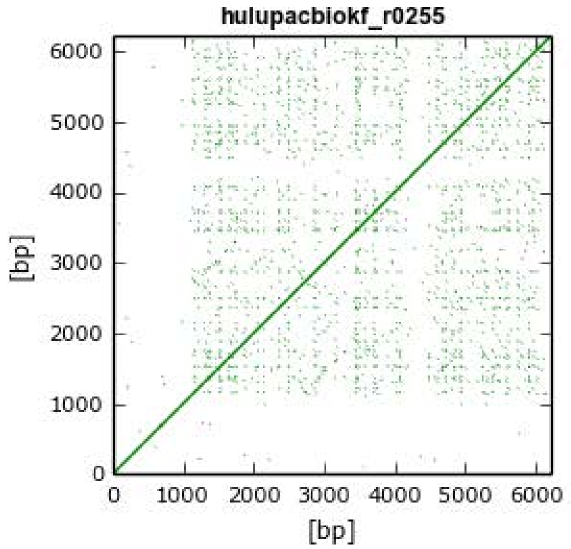

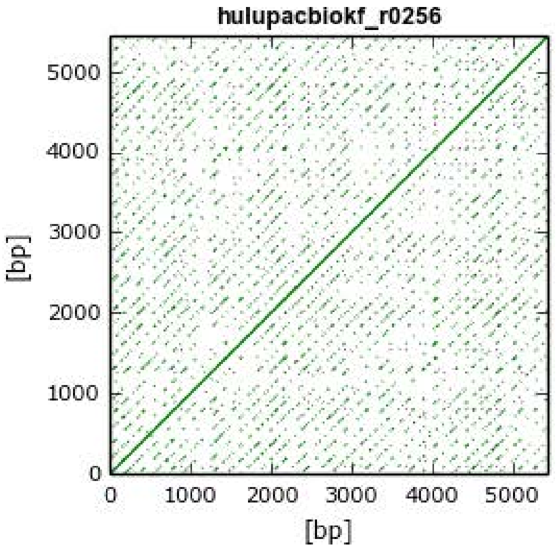

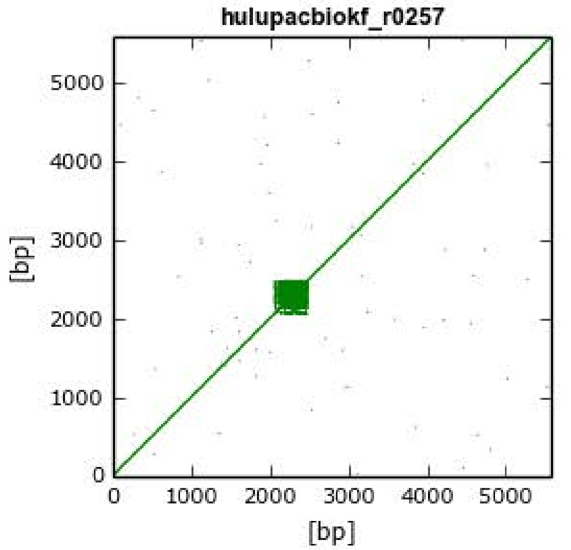

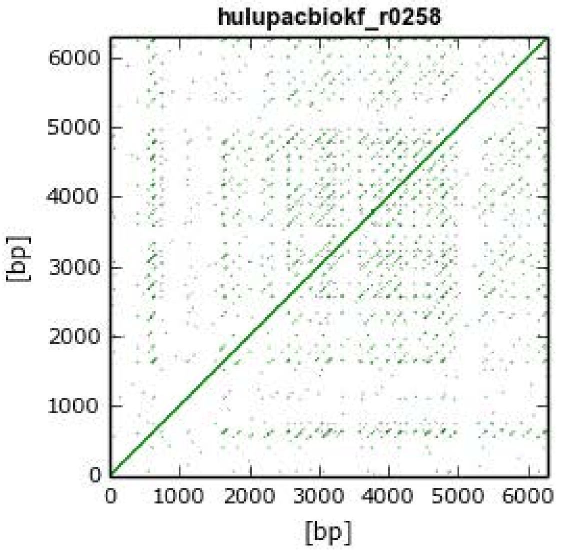

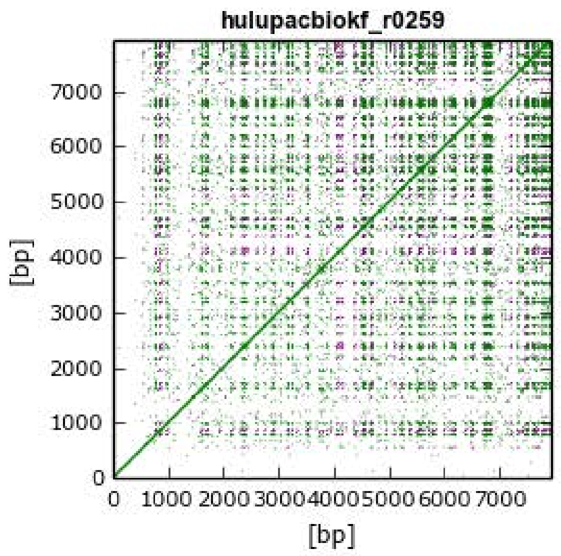
hulupacbiokf_r0259
[bp]
[bp]

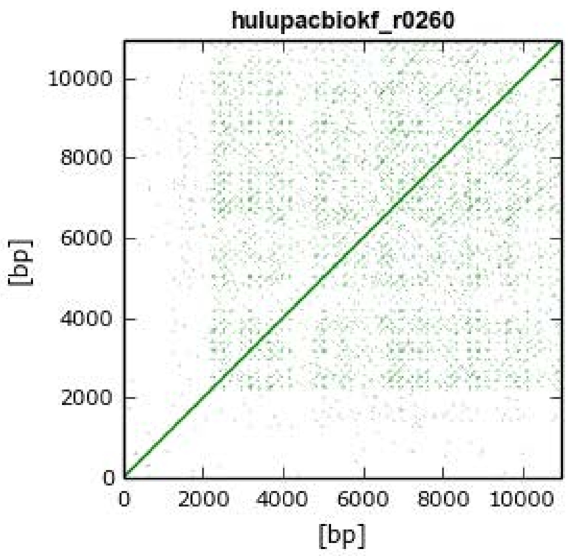
hulupacbiokf_r0260
[bp]
[bp]

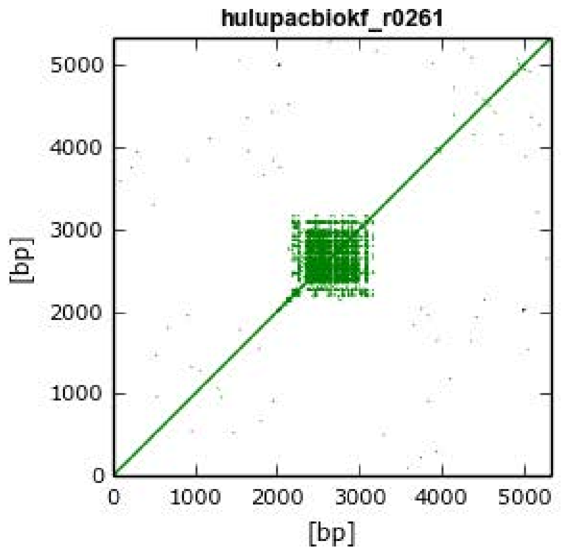
hulupacbiokf_r0261
[bp]
[bp]

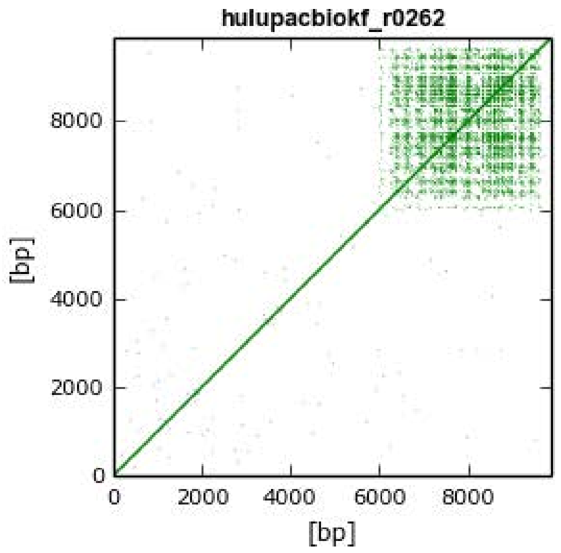
hulupacbiokf_r0262
[bp]
[bp]

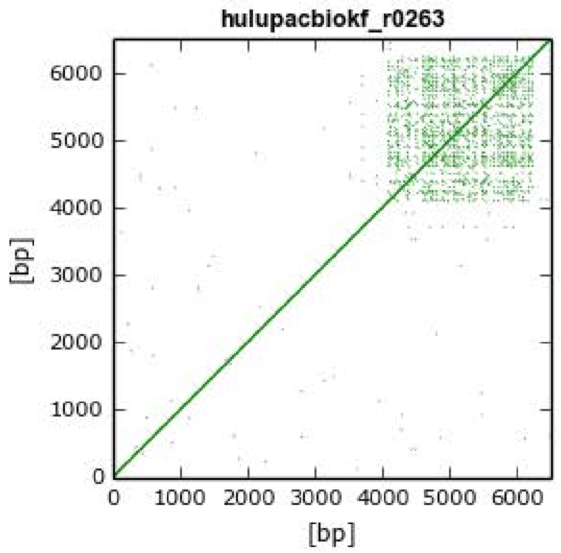
hulupacbiokf_r0263
[bp]
[bp]

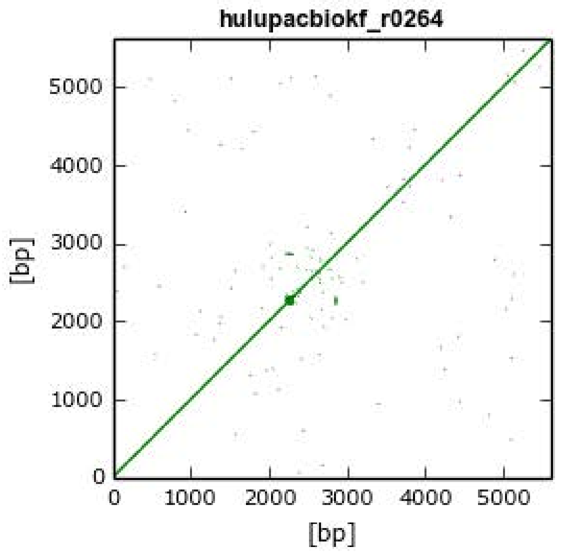
hulupacbiokf_r0264
[bp]
[bp]

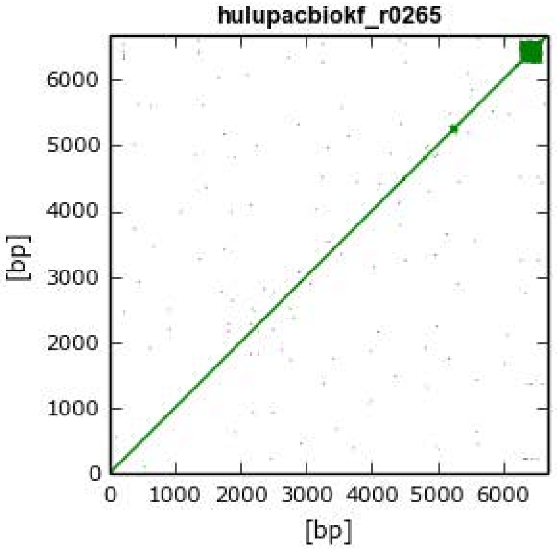
hulupacbiokf_r0265
[bp]
[bp]

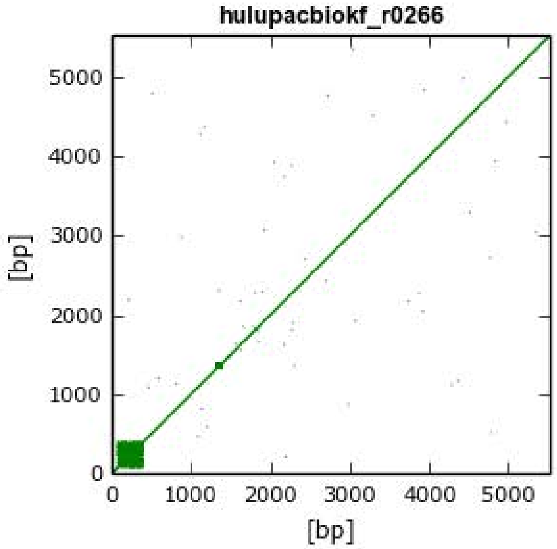
hulupacbiokf_r0266
[bp]
[bp]

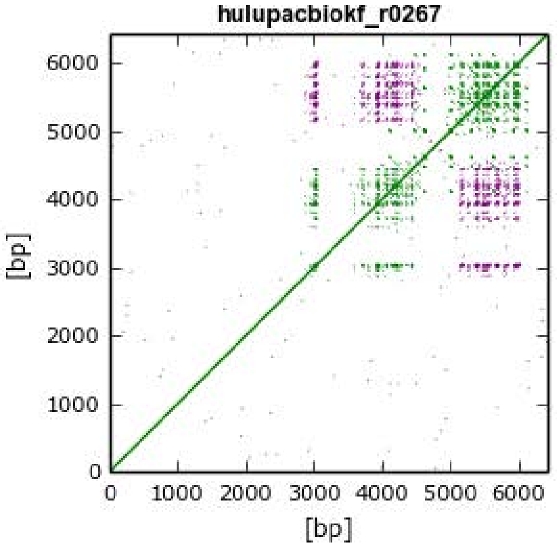
hulupacbiokf_r0267
[bp]
[bp]

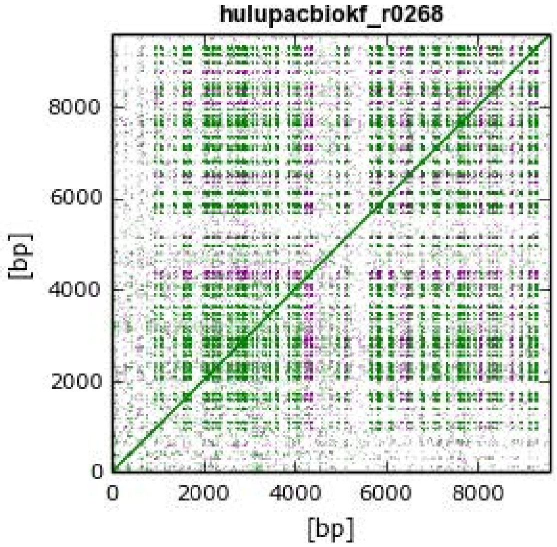
hulupacbiokf_r0268
[bp]
[bp]

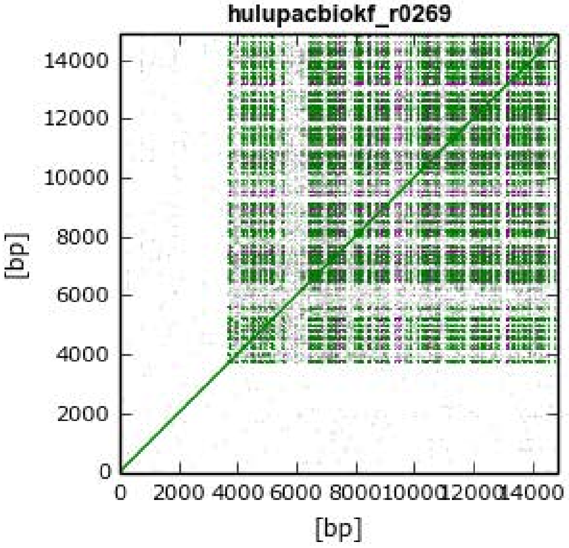
hulupacbiokf_r0269
[bp]
[bp]

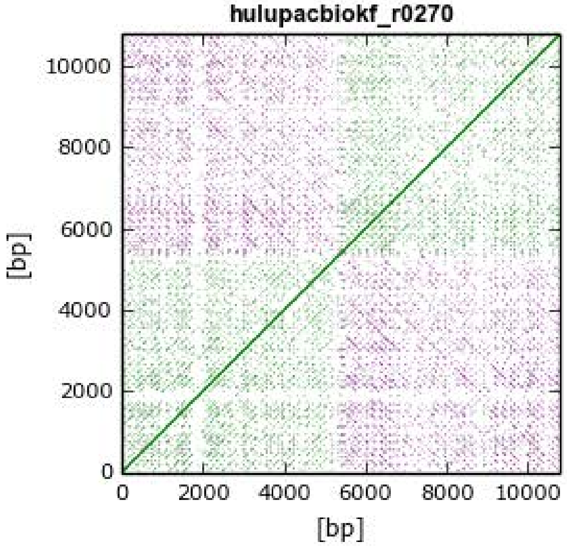
hulupacbiokf_r0270
[bp]
[bp]

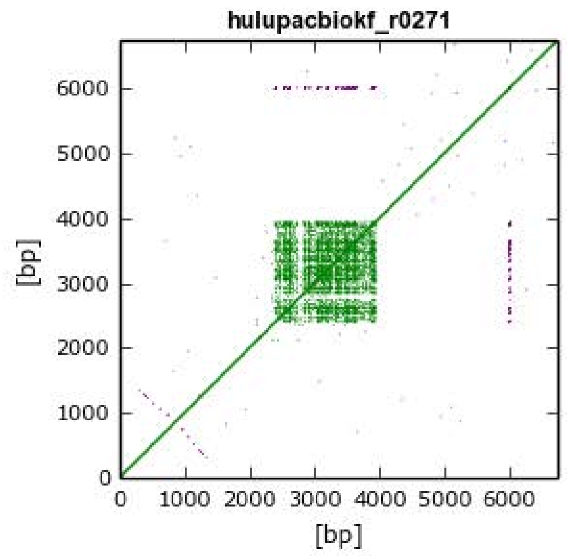
hulupacbiokf_r0271
[bp]
[bp]

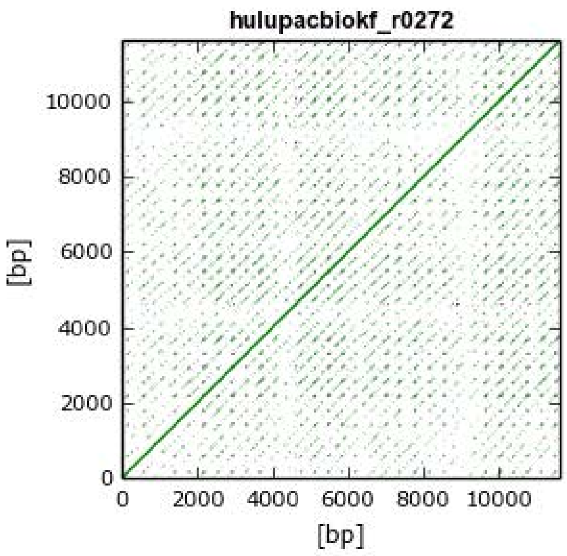
hulupacbiokf_r0272
[bp]
[bp]

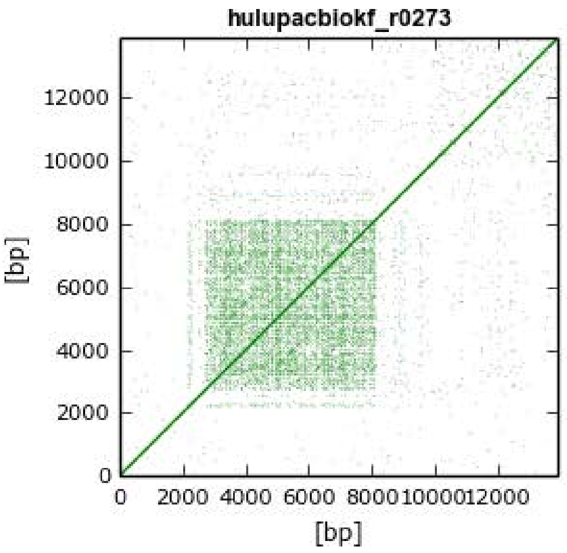
hulupacbiokf_r0273
[bp]
[bp]

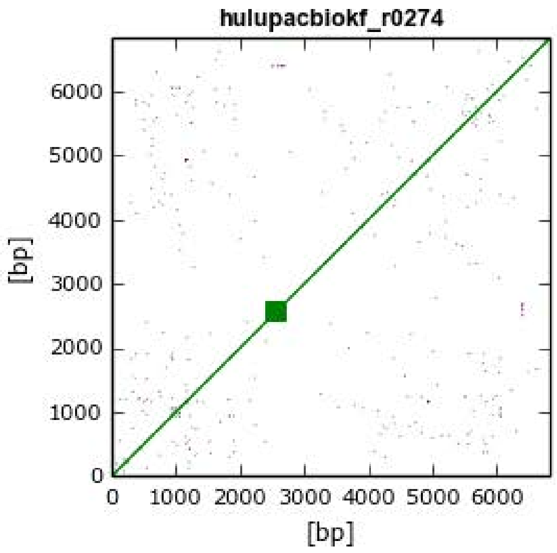
hulupacbiokf_r0274
[bp]
[bp]

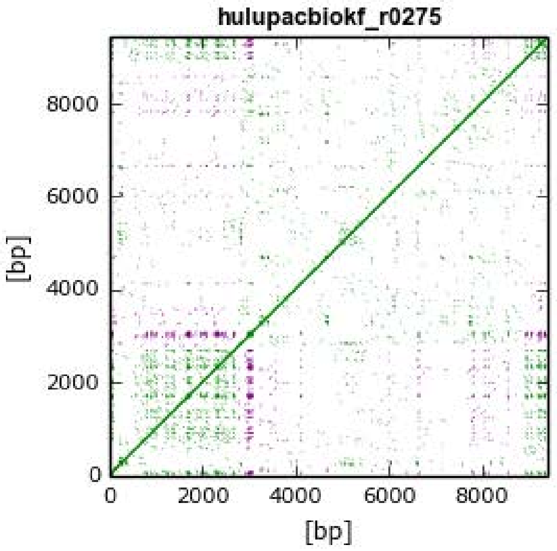
hulupacbiokf_r0275
[bp]
[bp]

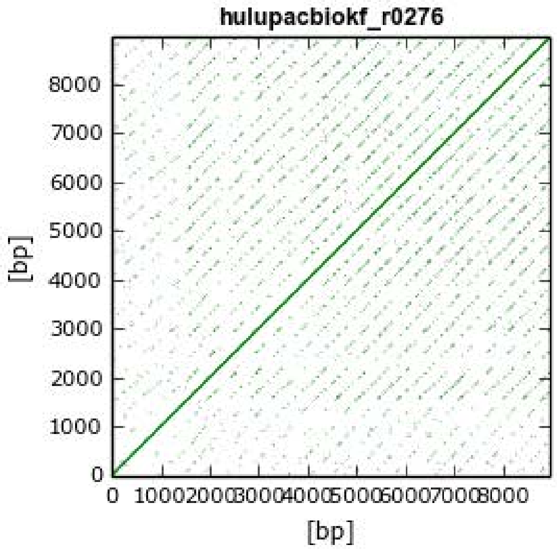
hulupacbiokf_r0276
[bp]
[bp]

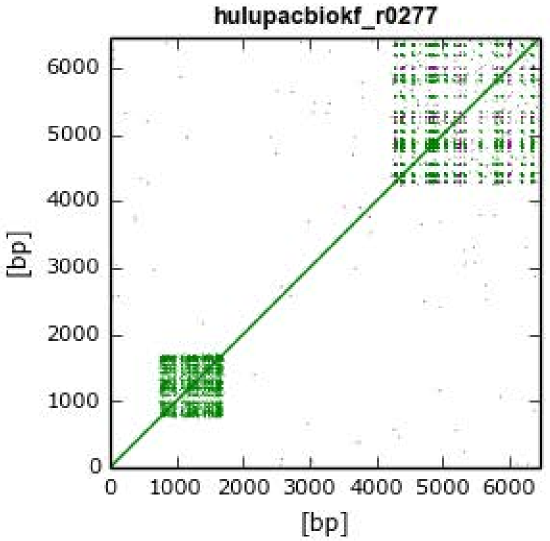
hulupacbiokf_r0277
[bp]
[bp]

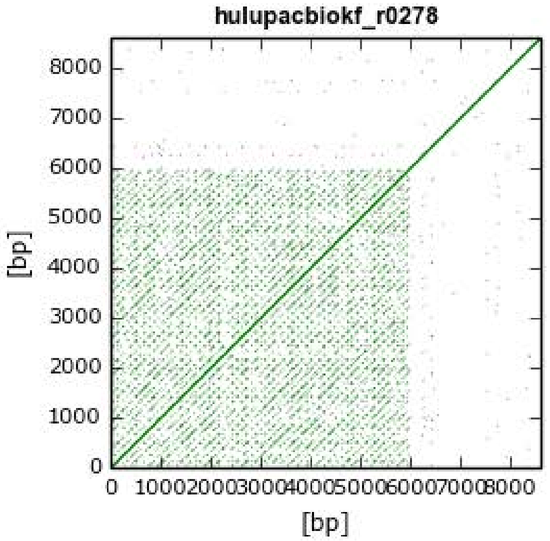
hulupacbiokf_r0278
[bp]
[bp]

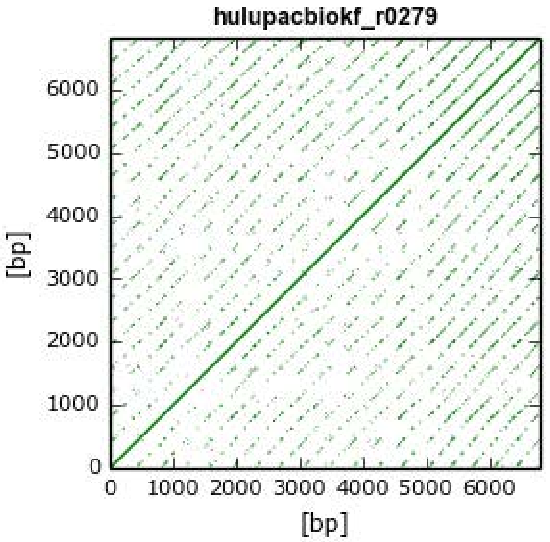
hulupacbiokf_r0279
[bp]
[bp]

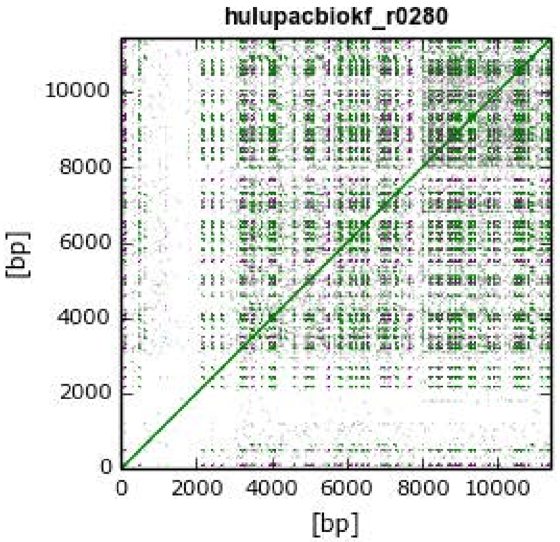
hulupacbiokf_r0280
[bp]
[bp]

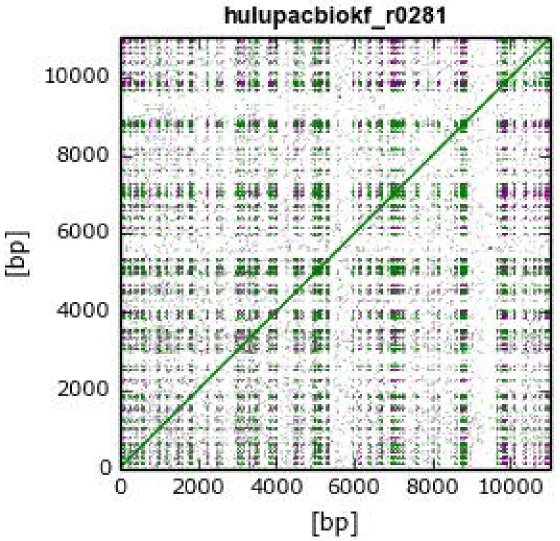
hulupacbiokf_r0281
[bp]
[bp]

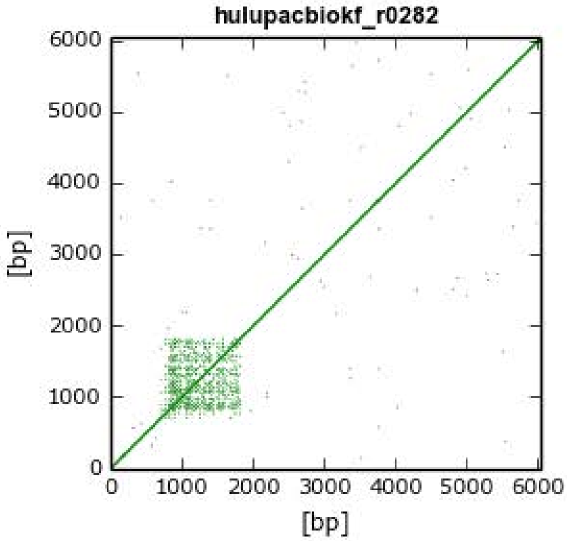
hulupacbiokf_r0282
[bp]
[bp]

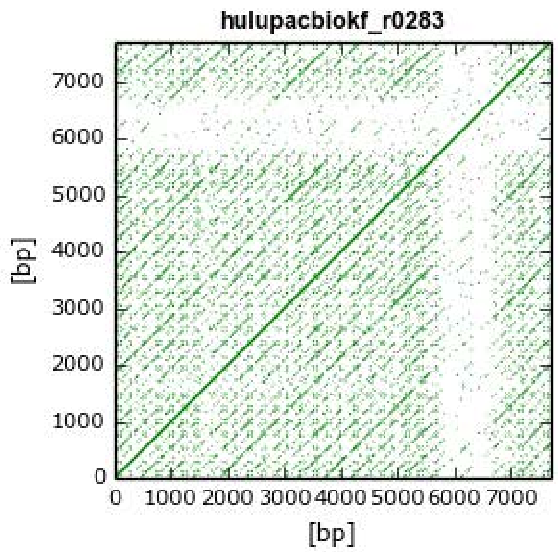
hulupacbiokf_r0283
[bp]
[bp]

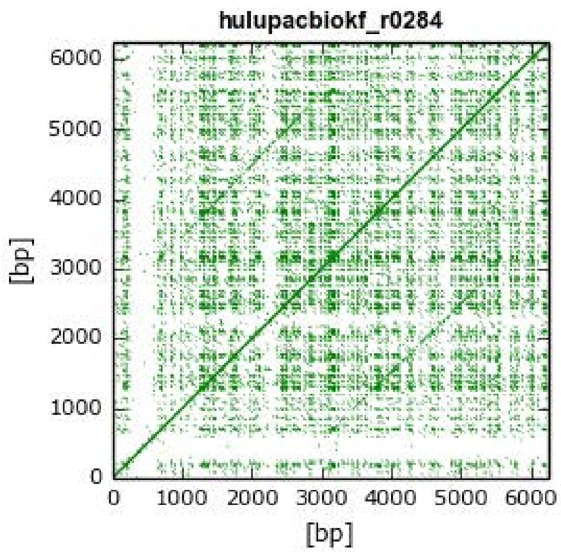
hulupacbiokf_r0284
[bp]
[bp]

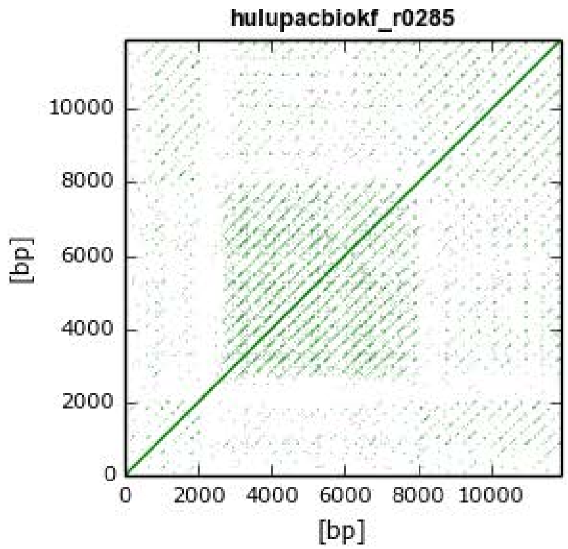
hulupacbiokf_r0285
[bp]
[bp]

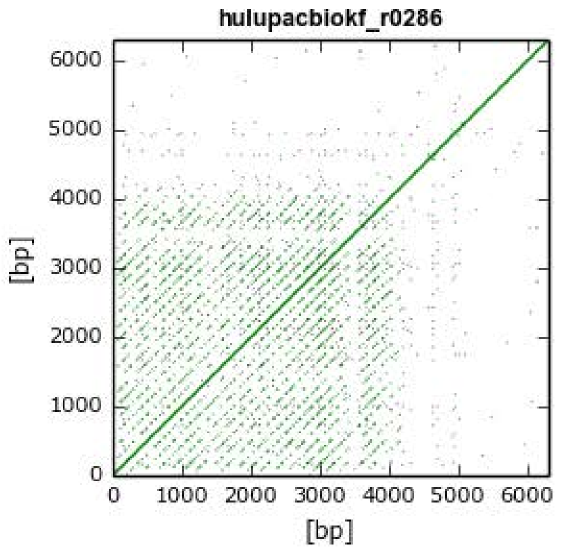
hulupacbiokf_r0286
[bp]
[bp]

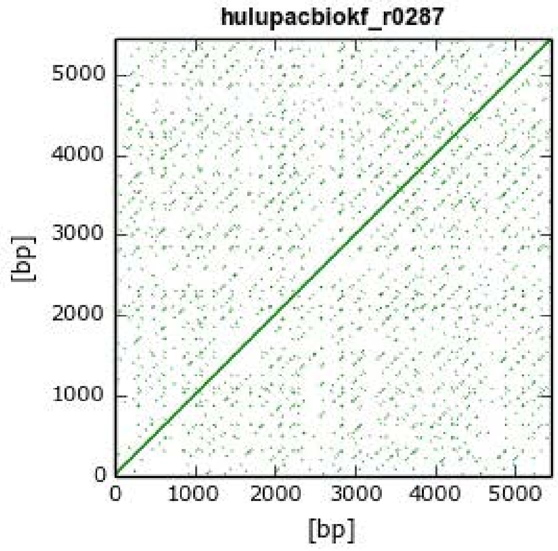
hulupacbiokf_r0287
[bp]
[bp]

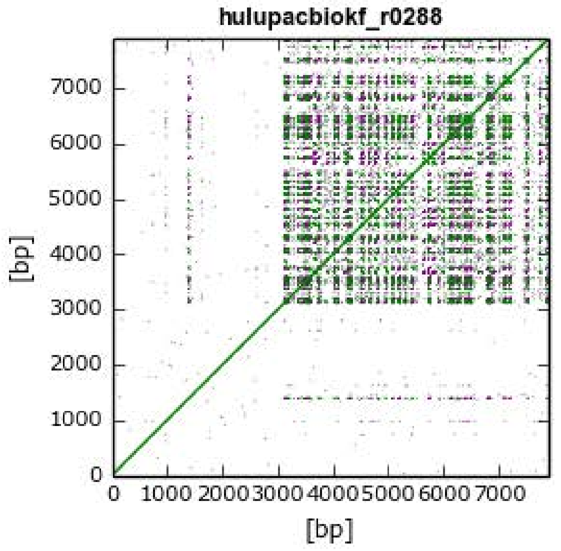
hulupacbiokf_r0288
[bp]
[bp]

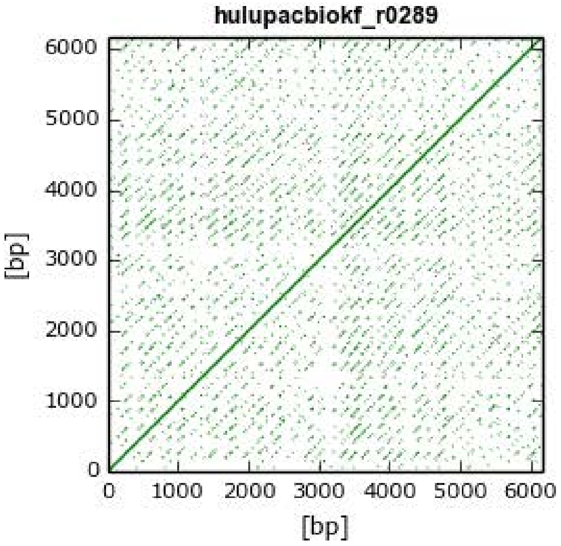

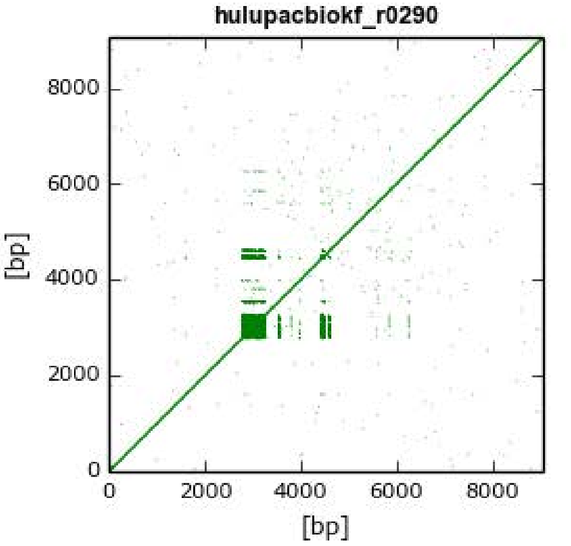

HuluTR055 from read r0292
is in GenBank Acc. MN537566

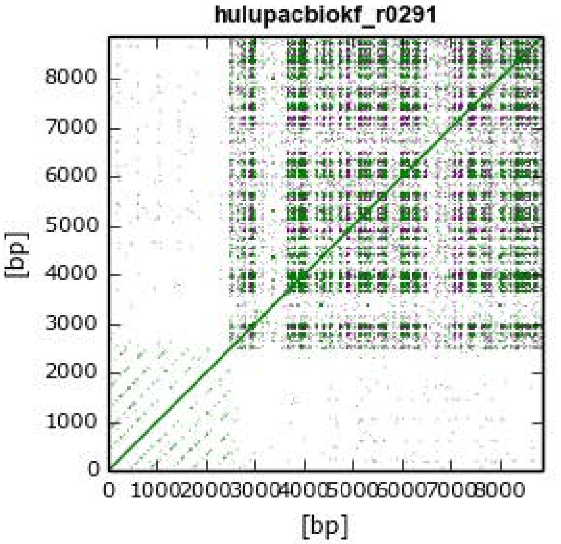

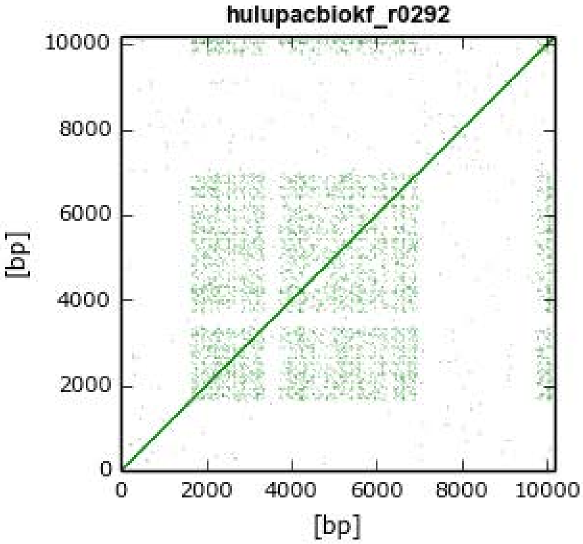

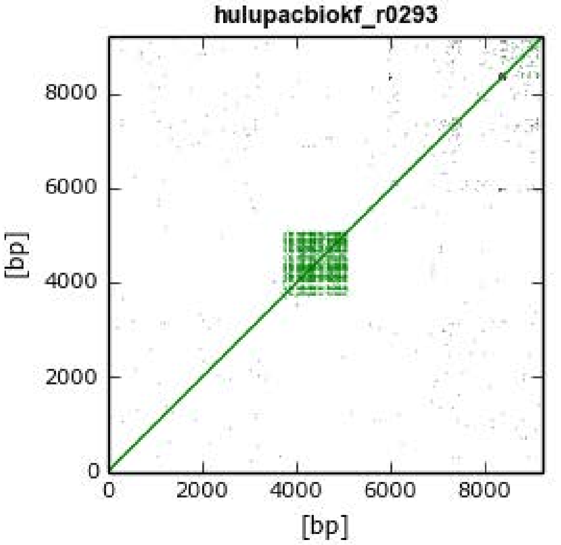

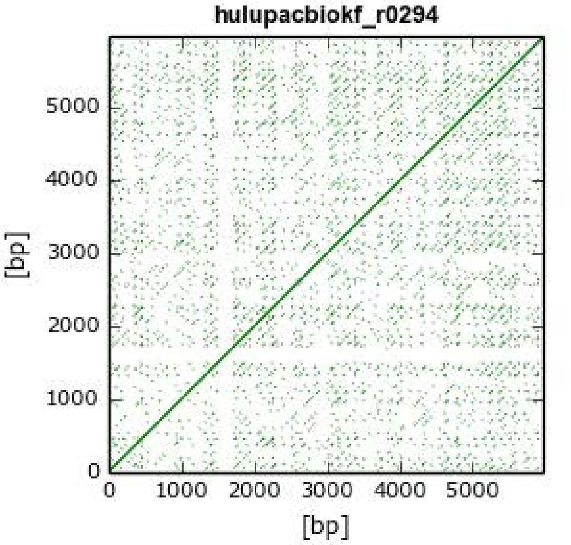

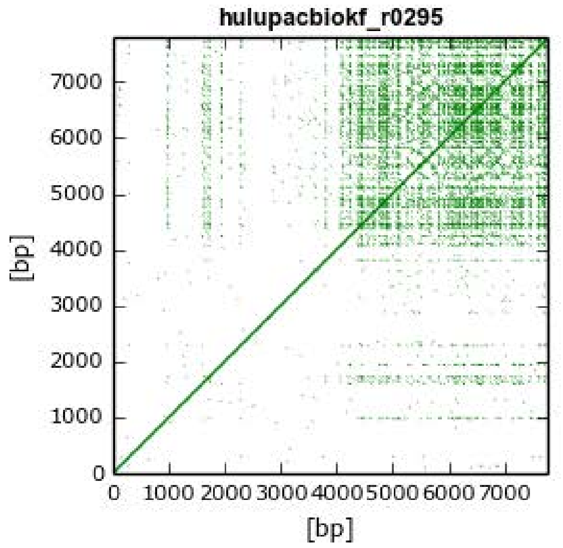
hulupacbiokf_r0295
0
1000
2000
3000
4000
5000
6000
7000
[bp]
0 1000 2000 3000 4000 5000 6000 7000
[bp]

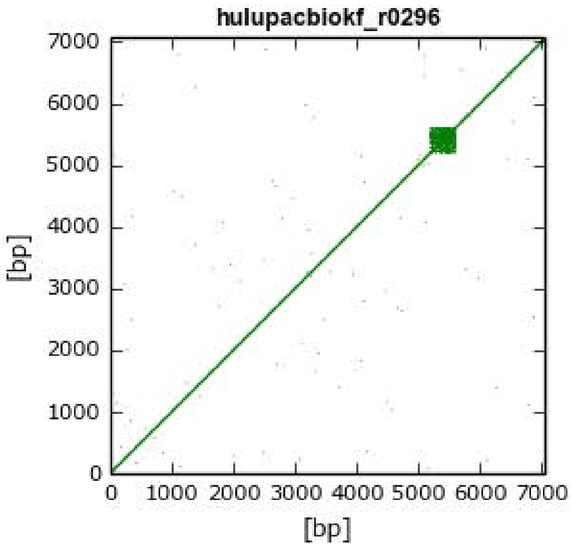
hulupacbiokf_r0296
0
1000
2000
3000
4000
5000
6000
7000
[bp]
0 1000 2000 3000 4000 5000 6000 7000
[bp]

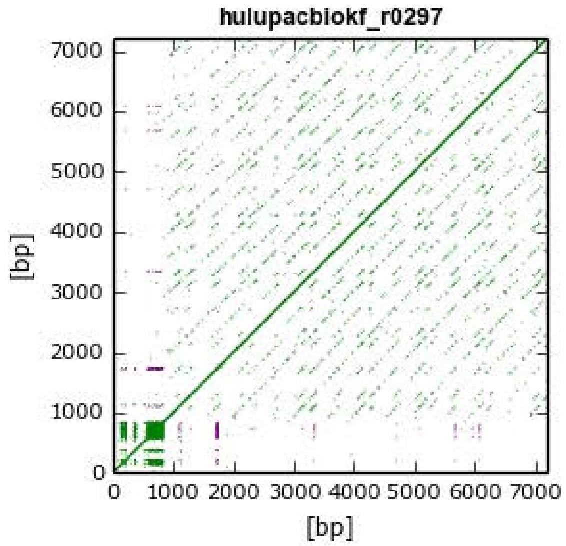
hulupacbiokf_r0297
0
1000
2000
3000
4000
5000
6000
7000
[bp]
0 1000 2000 3000 4000 5000 6000 7000
[bp]

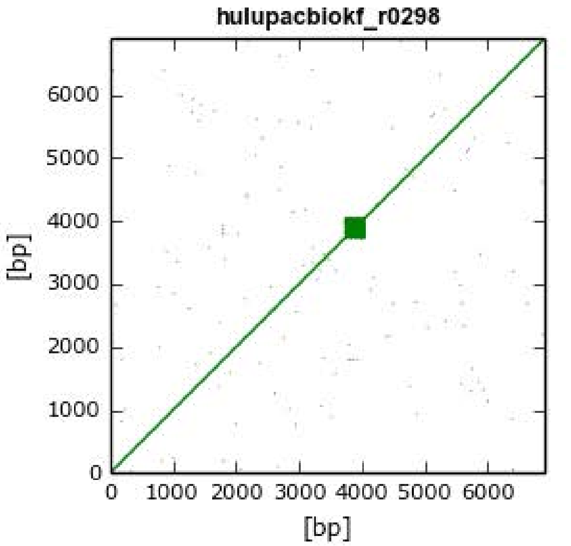
hulupacbiokf_r0298
0
1000
2000
3000
4000
5000
6000
[bp]
0 1000 2000 3000 4000 5000 6000
[bp]

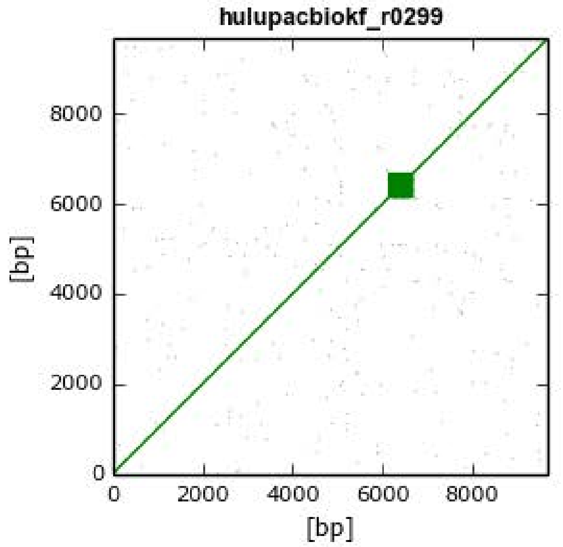
hulupacbiokf_r0299
0
2000
4000
6000
8000
[bp]
0 2000 4000 6000 8000
[bp]

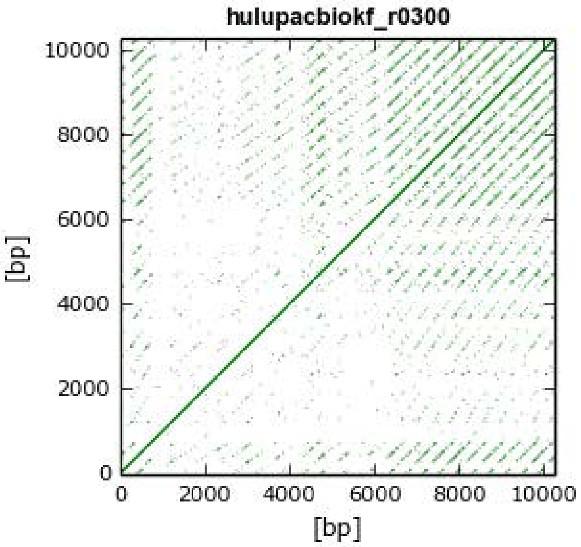
hulupacbiokf_r0300
0
2000
4000
6000
8000
10000
[bp]
0 2000 4000 6000 8000 10000
[bp]

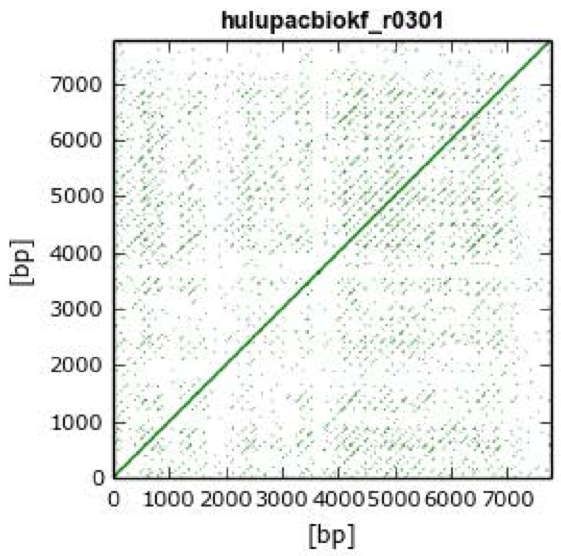
hulupacbiokf_r0301
[bp]
[bp]

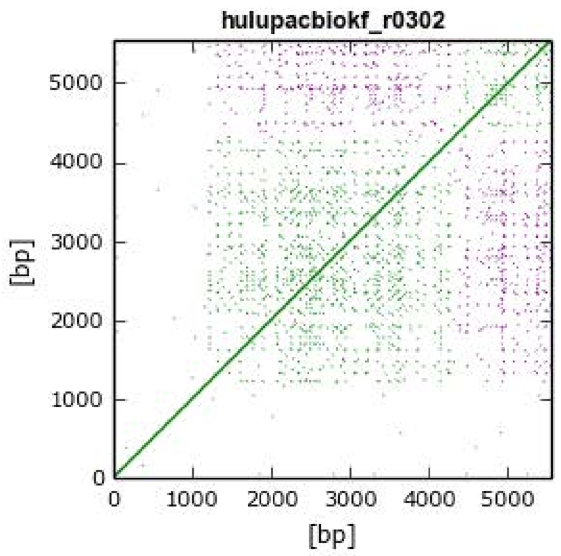
hulupacbiokf_r0302
[bp]
[bp]

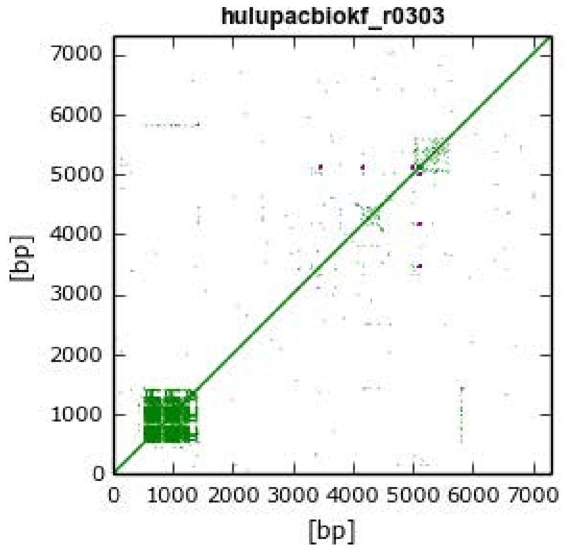
hulupacbiokf_r0303
[bp]
[bp]

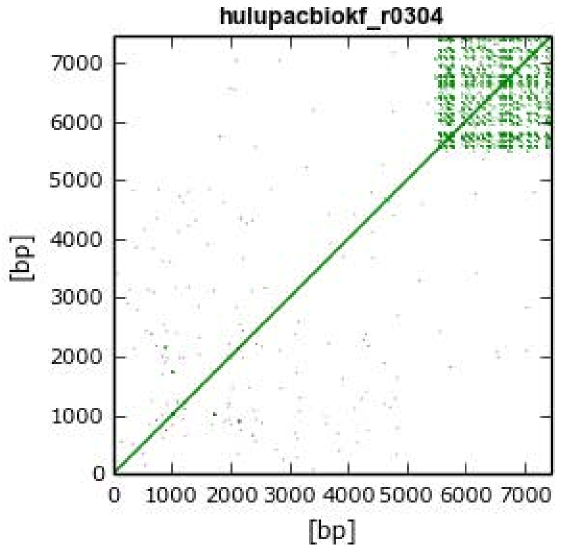
hulupacbiokf_r0304
[bp]
[bp]

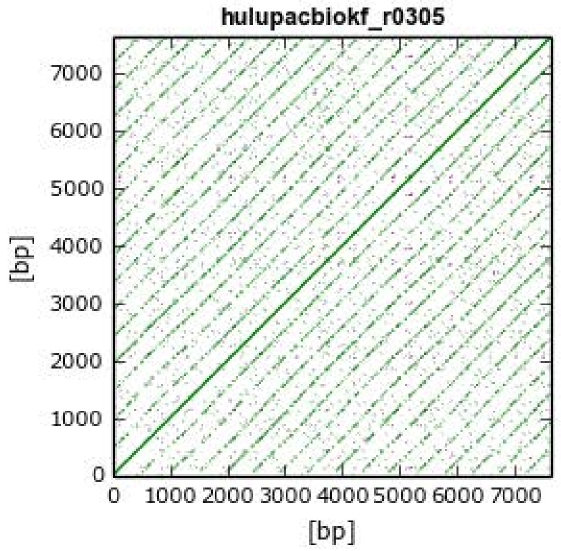
hulupacbiokf_r0305
[bp]
[bp]

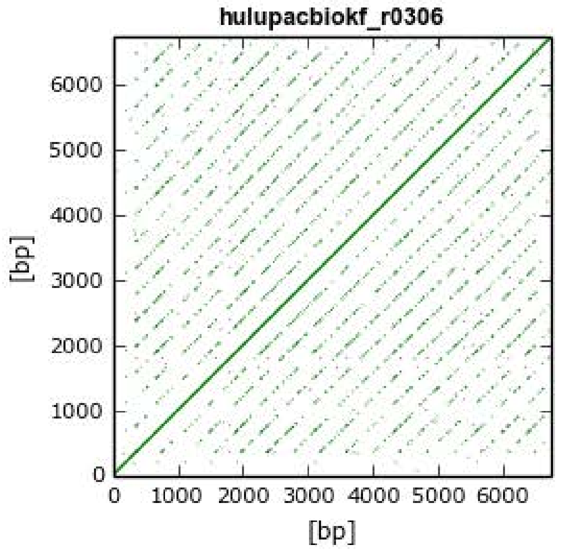
hulupacbiokf_r0306
[bp]
[bp]

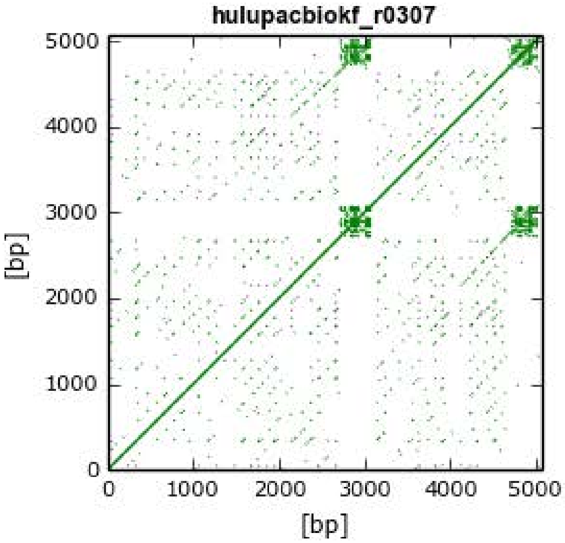
hulupacbiokf_r0307
[bp]
[bp]

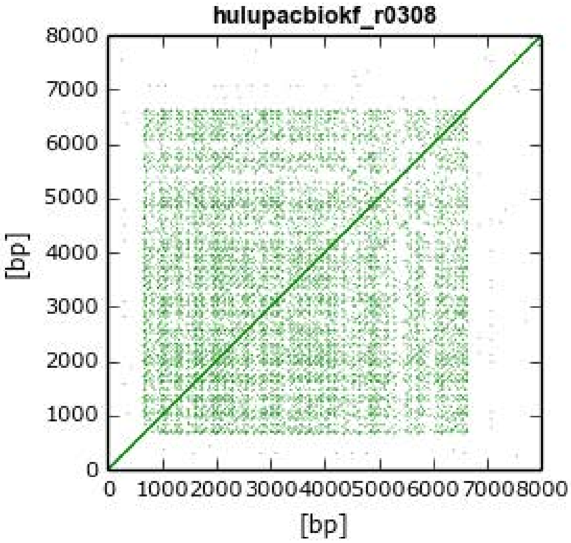
hulupacbiokf_r0308
[bp]
[bp]

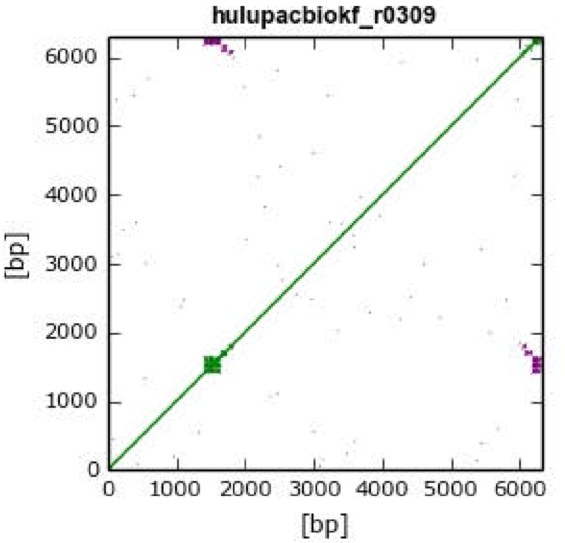
hulupacbiokf_r0309
[bp]
[bp]

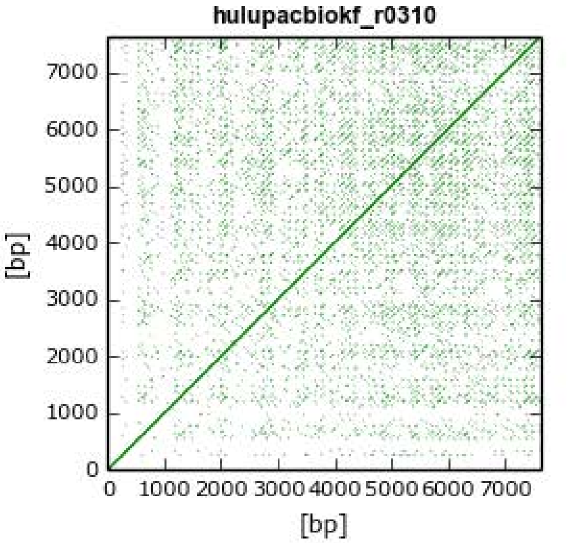
hulupacbiokf_r0310
[bp]
[bp]

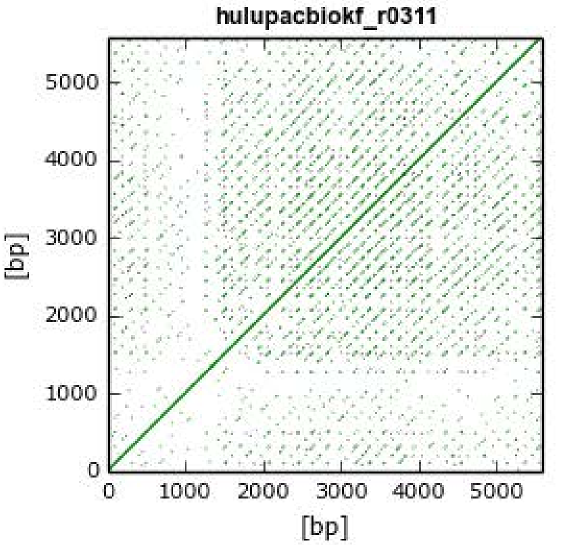
hulupacbiokf_r0311
[bp]
[bp]

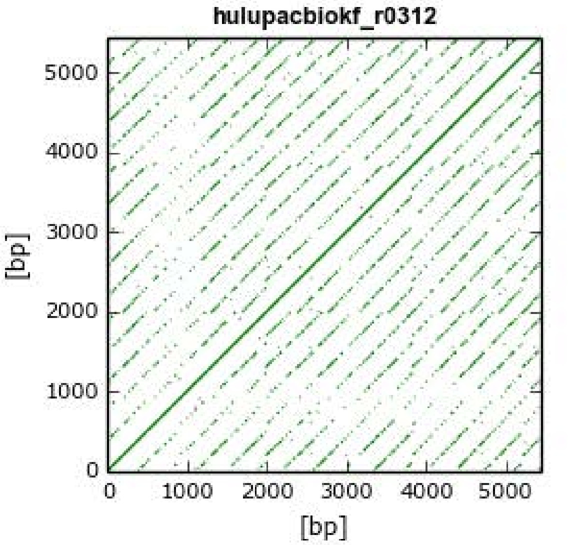
hulupacbiokf_r0312
[bp]
[bp]

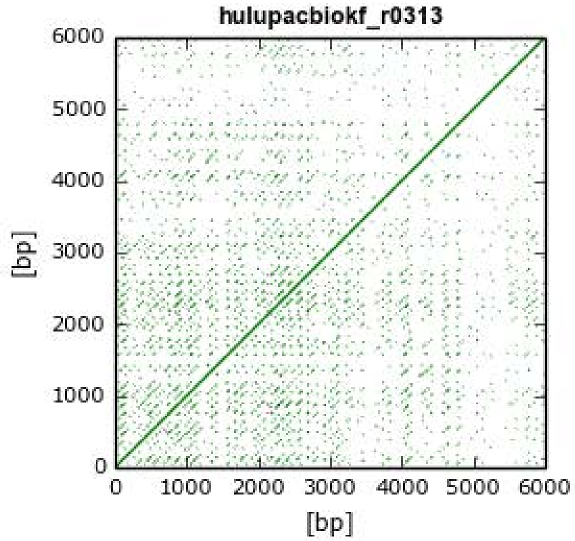
hulupacbiokf_r0313
[bp]
[bp]

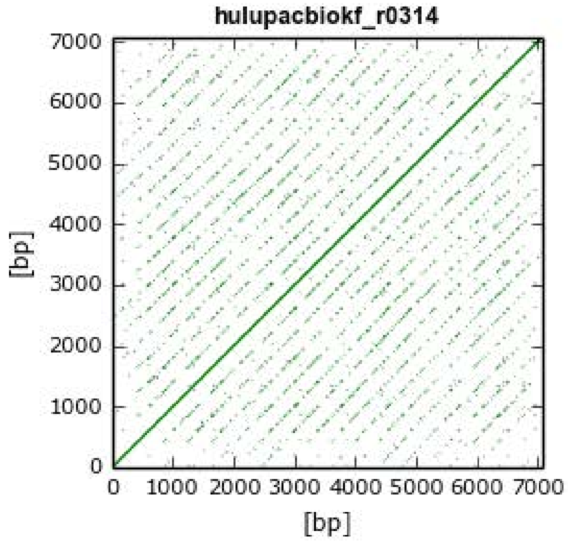
hulupacbiokf_r0314
[bp]
[bp]

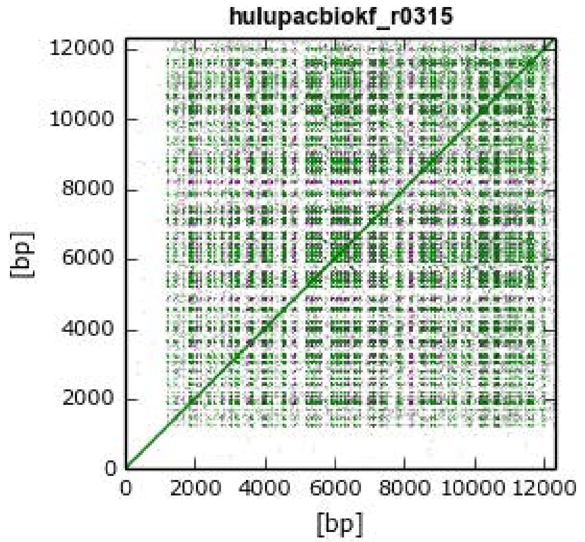
hulupacbiokf_r0315
[bp]
[bp]

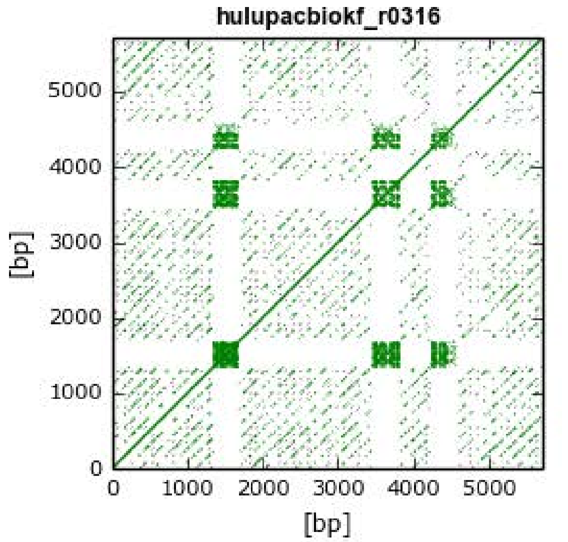
hulupacbiokf_r0316
[bp]
[bp]

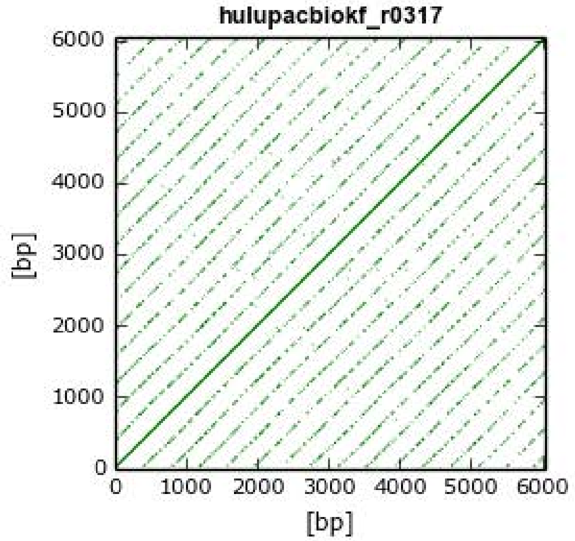
hulupacbiokf_r0317
[bp]
[bp]

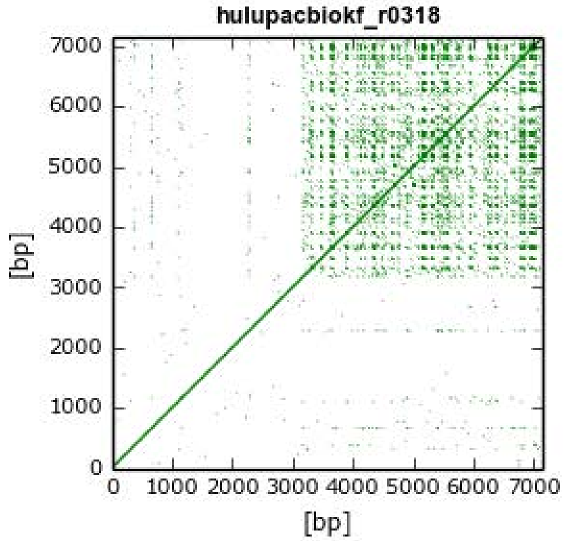
hulupacbiokf_r0318
[bp]
[bp]

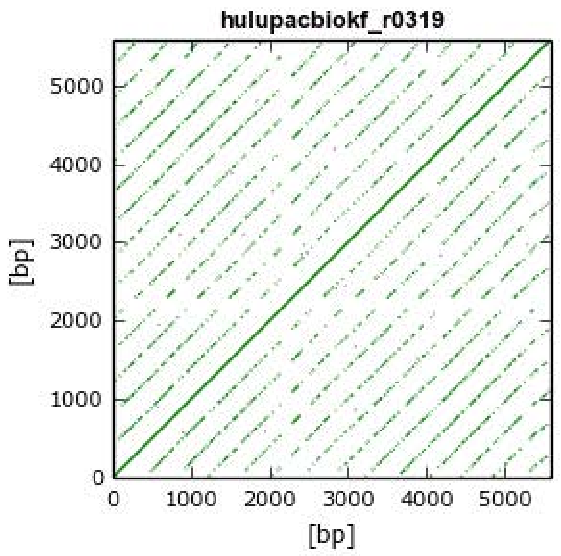
hulupacbiokf_r0319
[bp]
[bp]

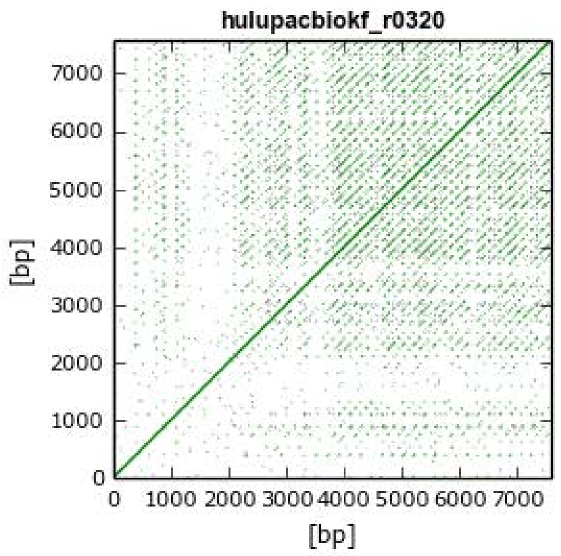
hulupacbiokf_r0320
[bp]
[bp]

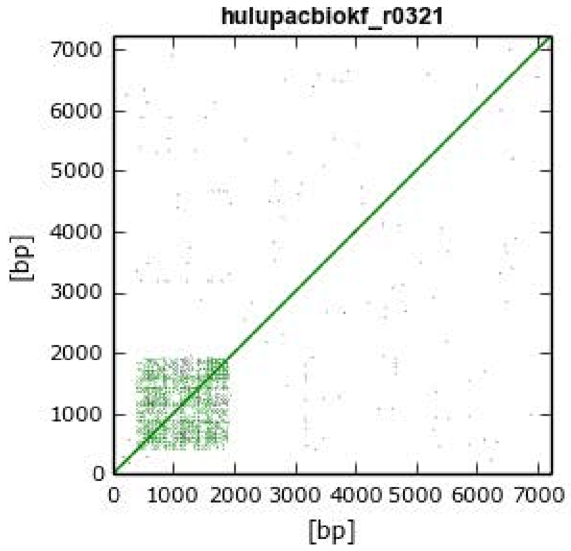
hulupacbiokf_r0321
[bp]
[bp]

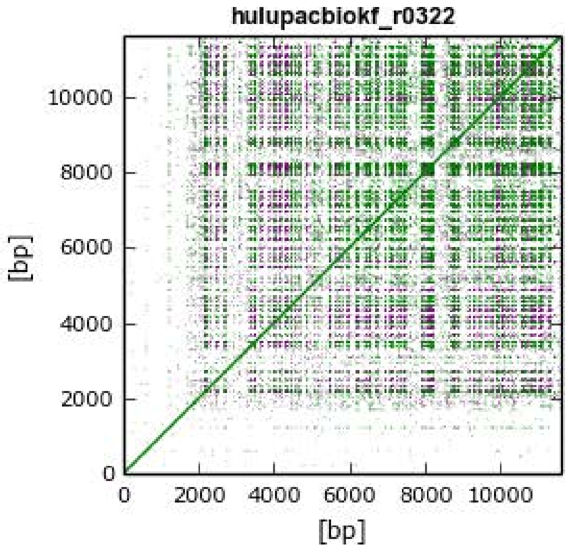
hulupacbiokf_r0322
[bp]
[bp]

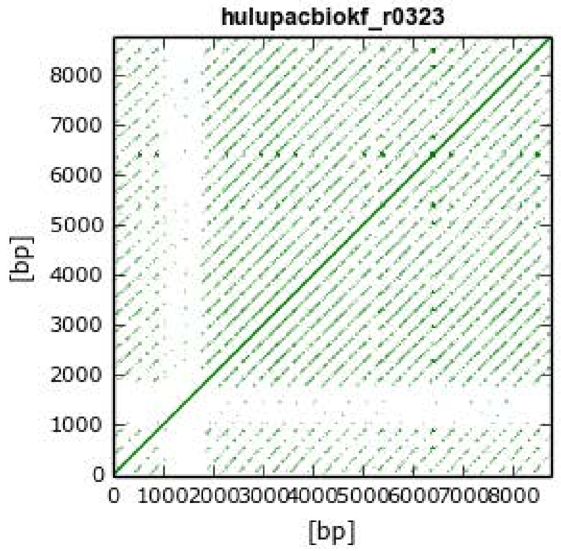
hulupacbiokf_r0323
[bp]
[bp]

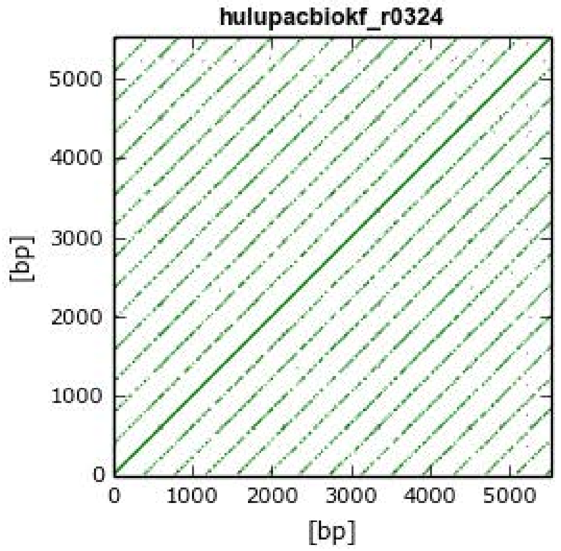
hulupacbiokf_r0324
[bp]
[bp]

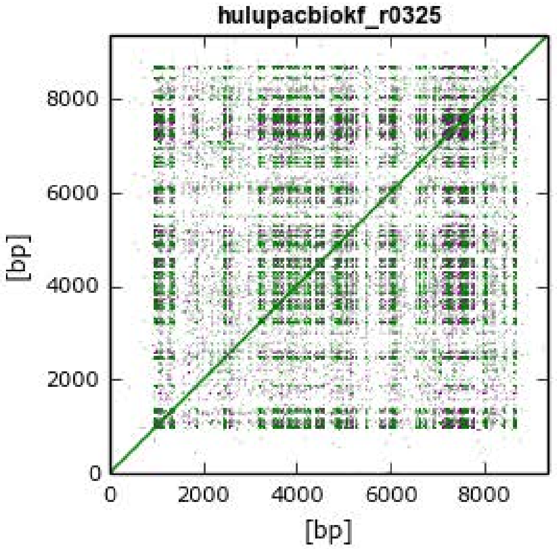
hulupacbiokf_r0325
[bp]
[bp]

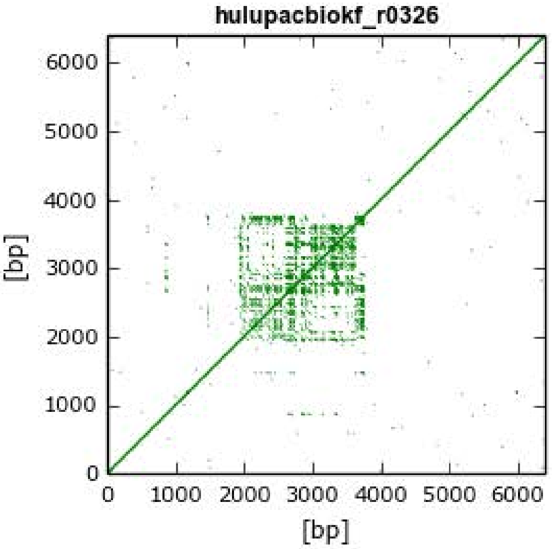
hulupacbiokf_r0326
[bp]
[bp]

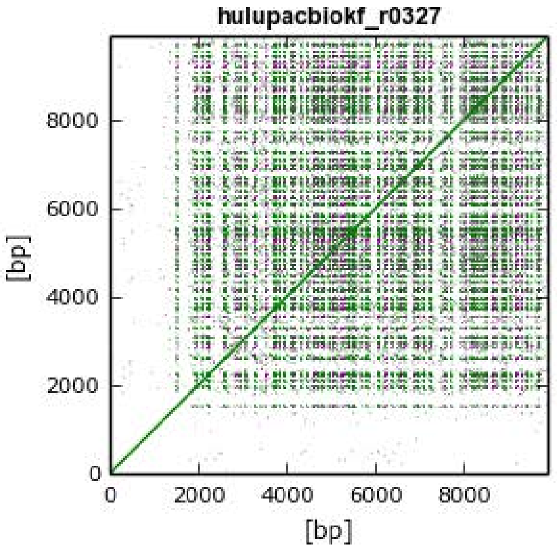
hulupacbiokf_r0327
[bp]
[bp]

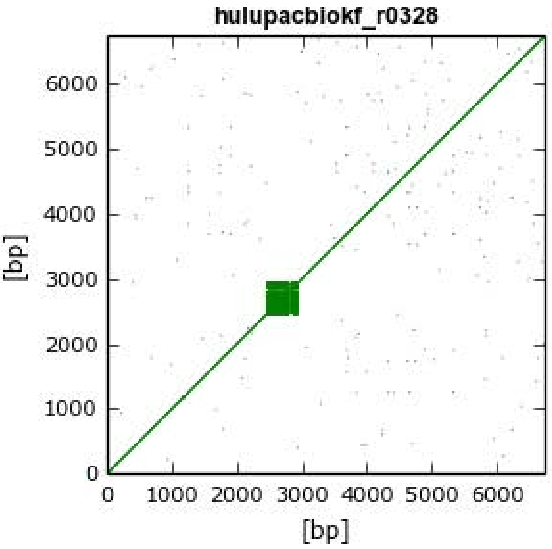
hulupacbiokf_r0328
[bp]
[bp]

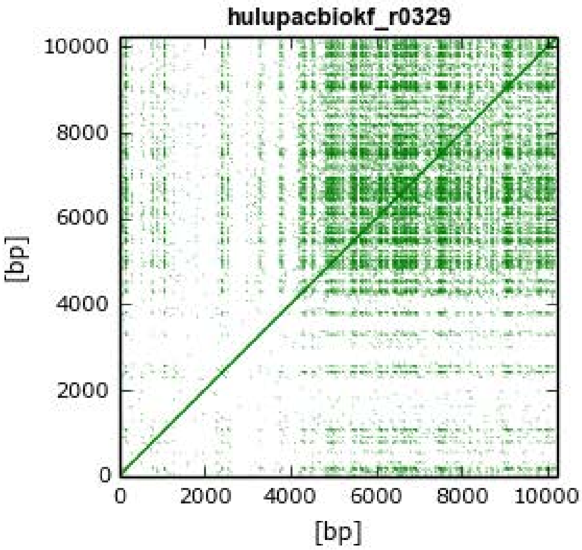
hulupacbiokf_r0329
[bp]
[bp]

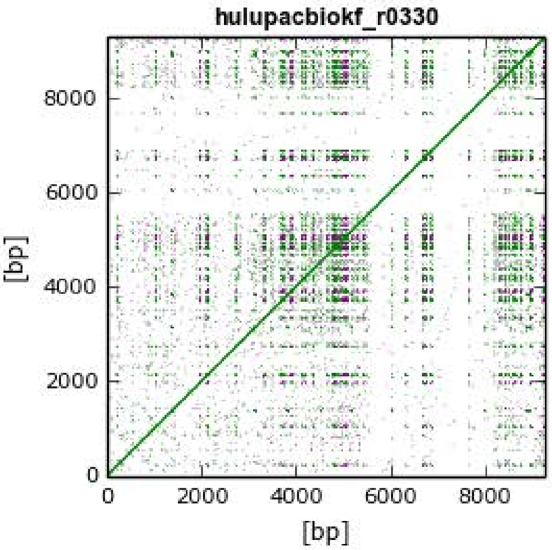
hulupacbiokf_r0330
[bp]
[bp]

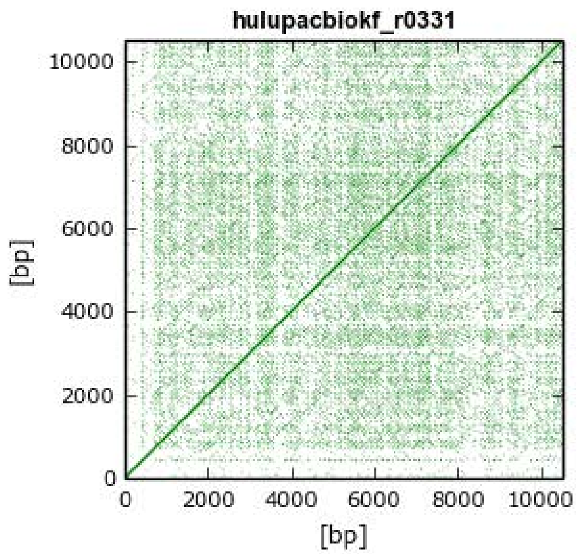
hulupacbiokf_r0331
[bp]
[bp]

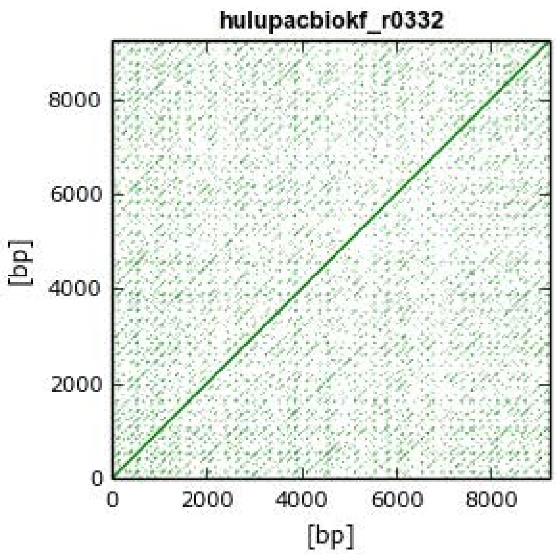
hulupacbiokf_r0332
[bp]
[bp]

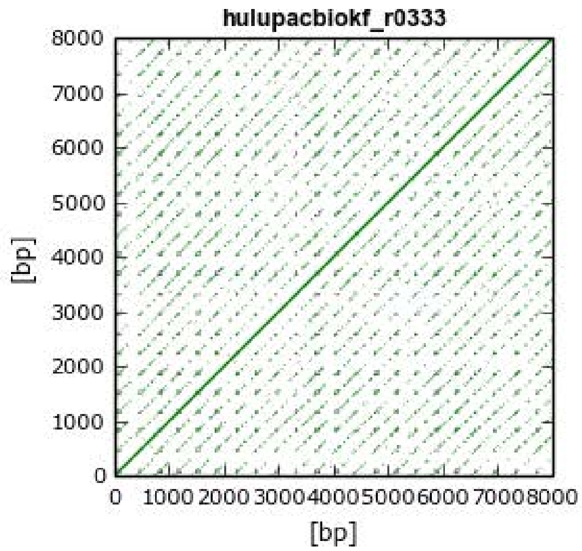
hulupacbiokf_r0333
[bp]
[bp]

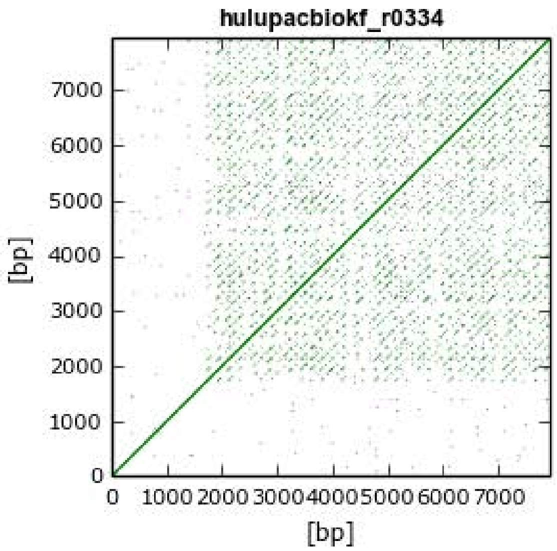
hulupacbiokf_r0334
[bp]
[bp]

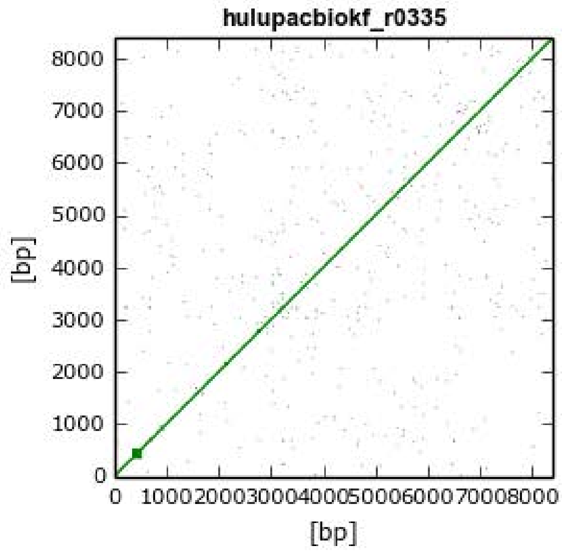
hulupacbiokf_r0335
[bp]
[bp]

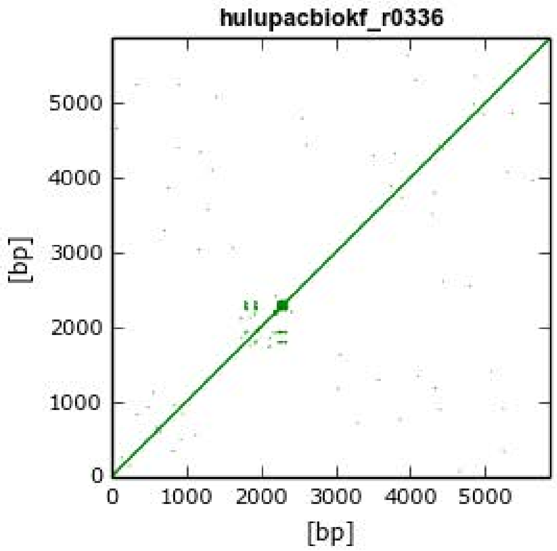
hulupacbiokf_r0336
[bp]
[bp]

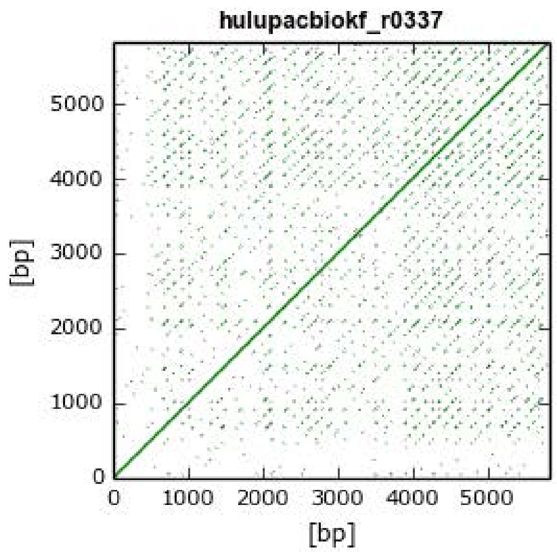
hulupacbiokf_r0337
[bp]
0
1000
2000
3000
4000
5000

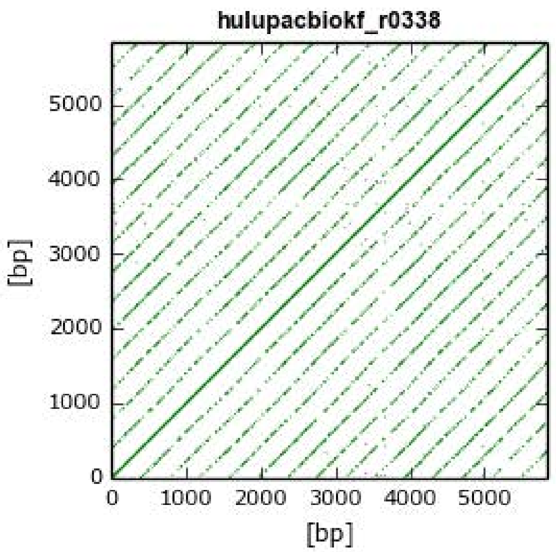
hulupacbiokf_r0338
[bp]
0
1000
2000
3000
4000
5000

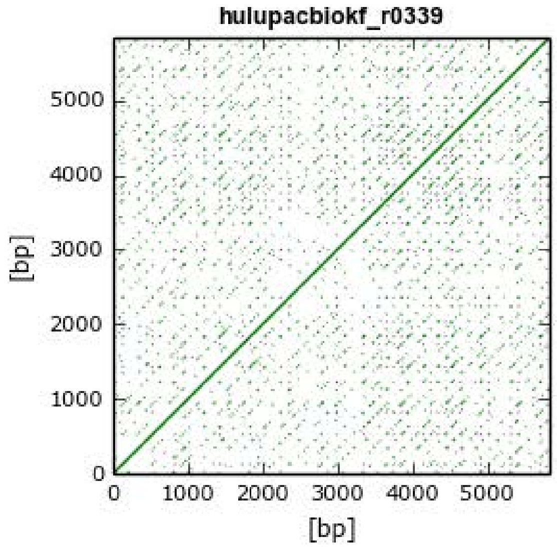
hulupacbiokf_r0339
[bp]
0
1000
2000
3000
4000
5000

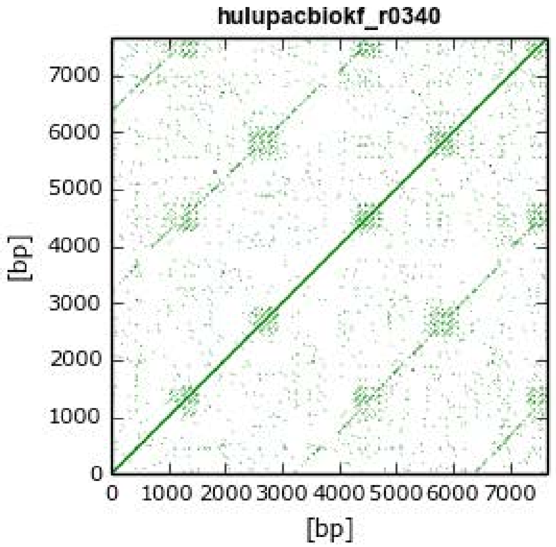
hulupacbiokf_r0340
[bp]
0
1000
2000
3000
4000
5000
6000
7000

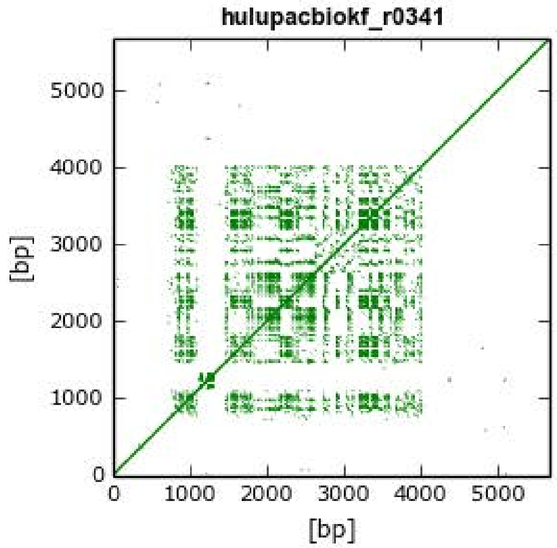
hulupacbiokf_r0341
[bp]
0
1000
2000
3000
4000
5000

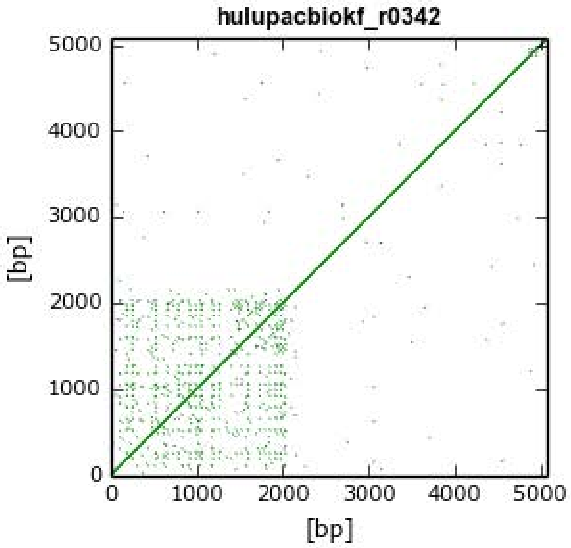
hulupacbiokf_r0342
[bp]
0
1000
2000
3000
4000
5000

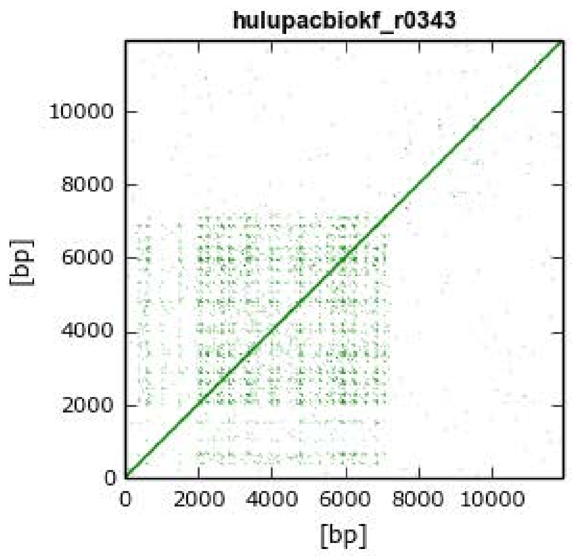
hulupacbiokf_r0343
[bp]
[bp]

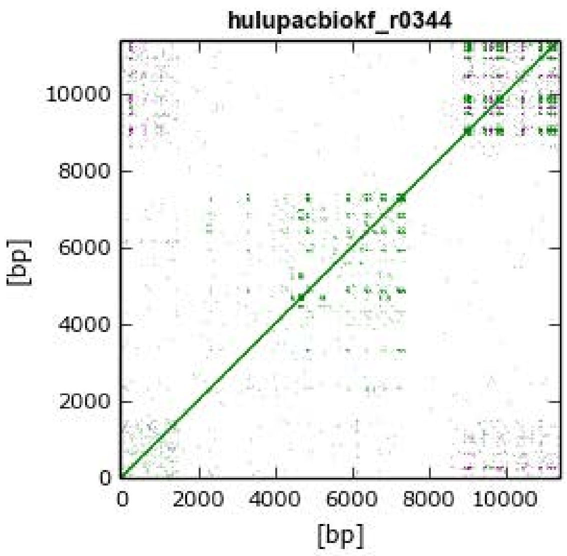
hulupacbiokf_r0344
[bp]
[bp]

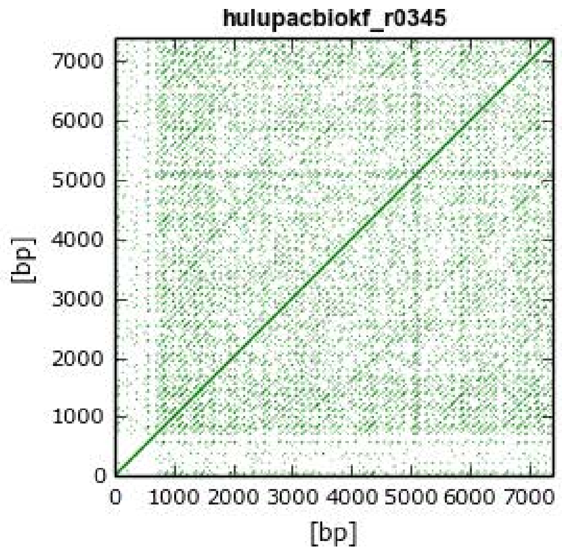
hulupacbiokf_r0345
[bp]
[bp]

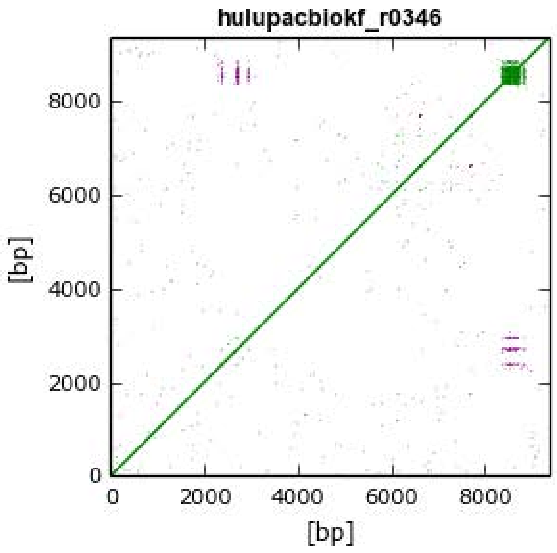
hulupacbiokf_r0346
[bp]
[bp]

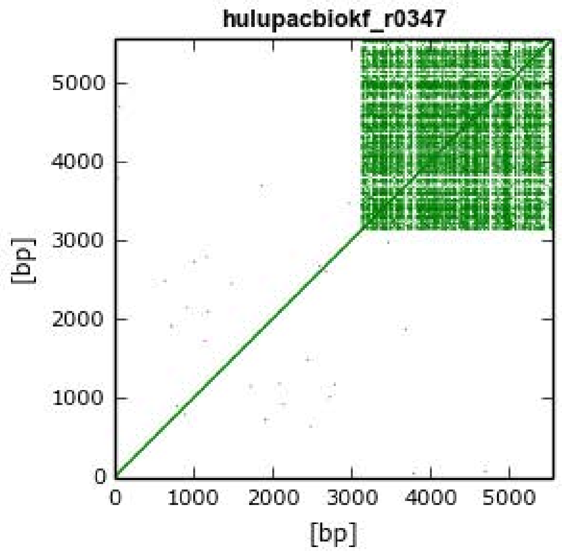
hulupacbiokf_r0347
[bp]
[bp]

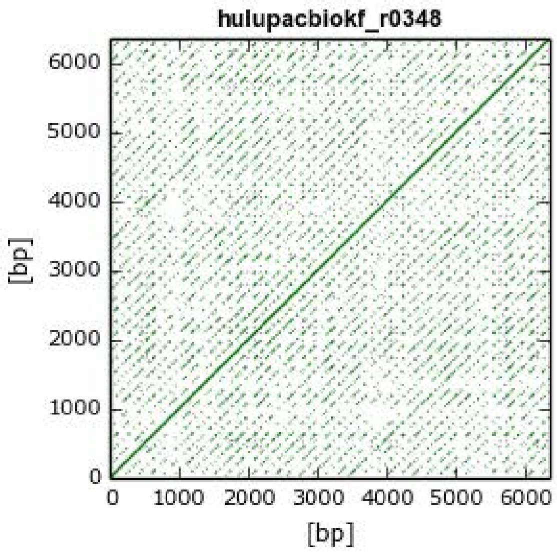
hulupacbiokf_r0348
[bp]
[bp]

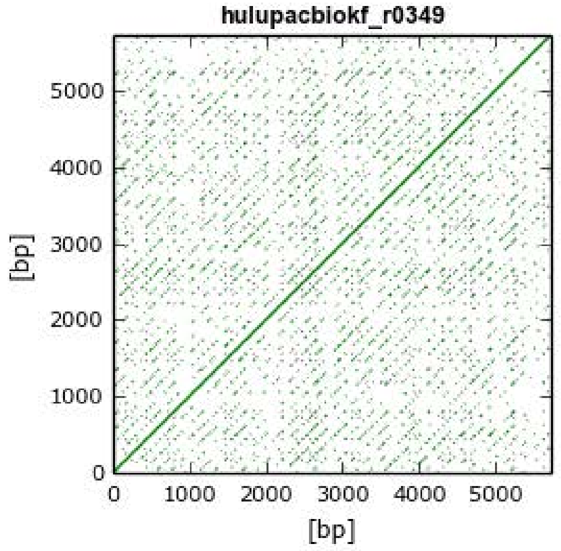
hulupacbiokf_r0349
[bp]
[bp]

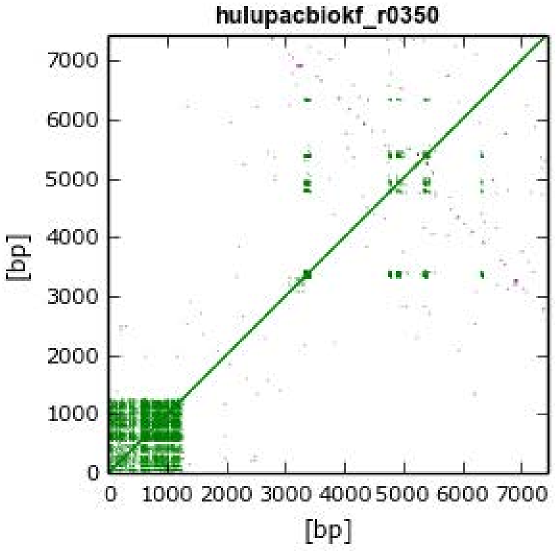
hulupacbiokf_r0350
[bp]
[bp]

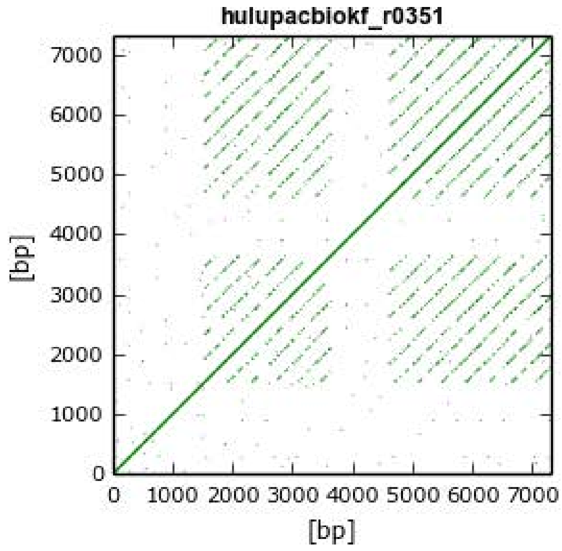
hulupacbiokf_r0351
[bp]
[bp]

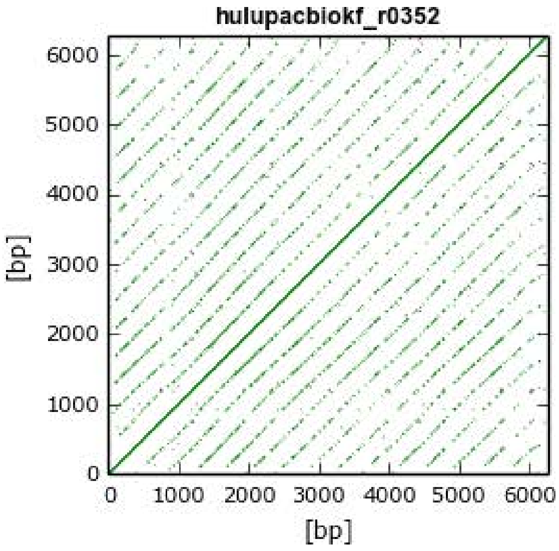
hulupacbiokf_r0352
[bp]
[bp]

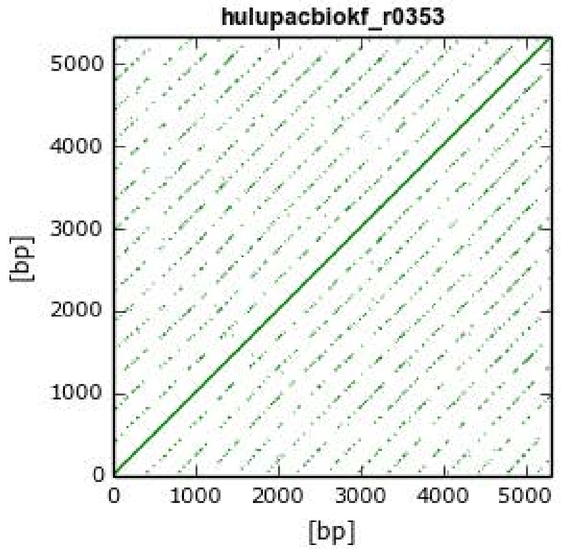
hulupacbiokf_r0353
[bp]
[bp]

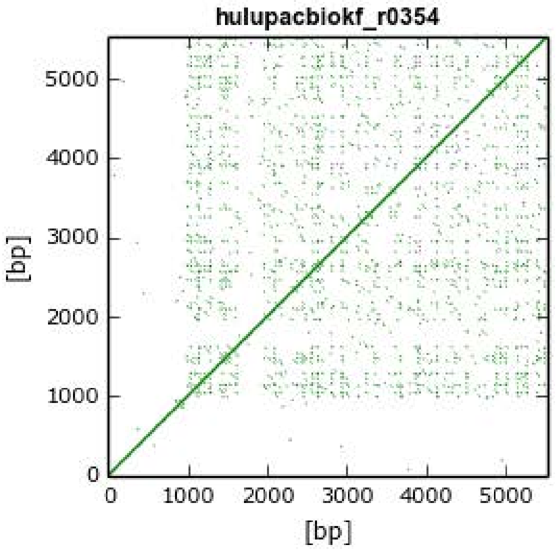
hulupacbiokf_r0354
[bp]
[bp]

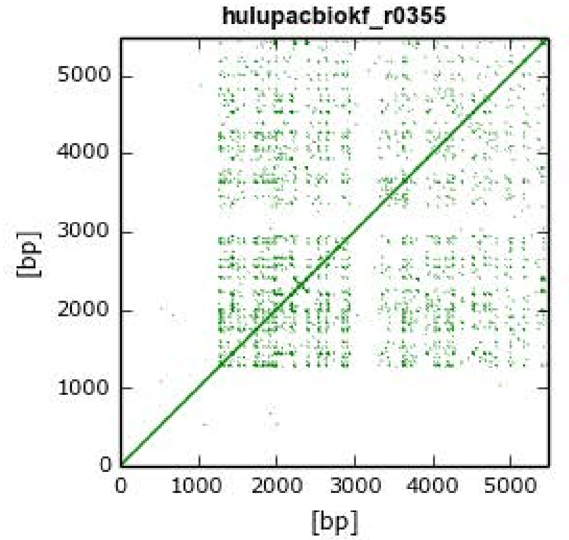
hulupacbiokf_r0355
[bp]
[bp]

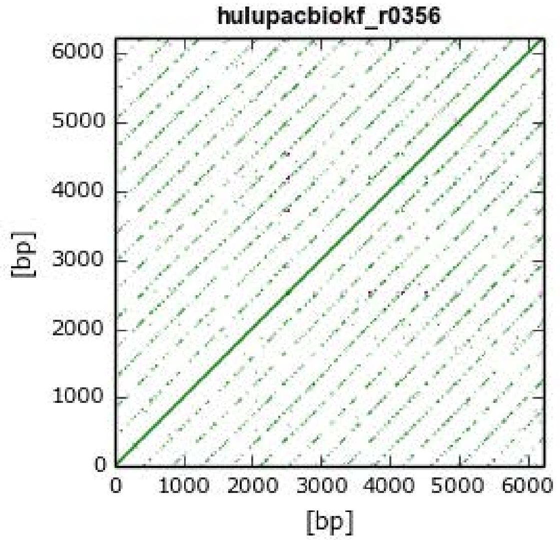
hulupacbiokf_r0356
[bp]
[bp]

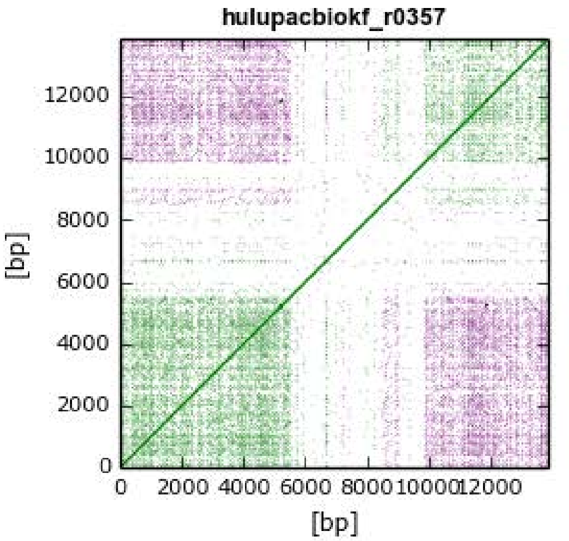
hulupacbiokf_r0357
[bp]
[bp]

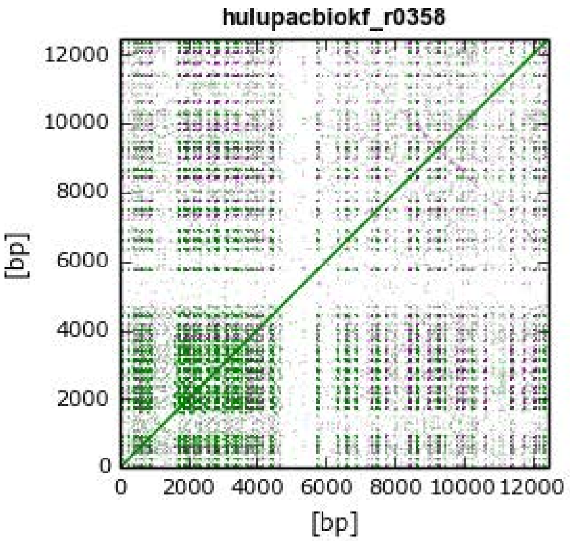
hulupacbiokf_r0358
[bp]
[bp]

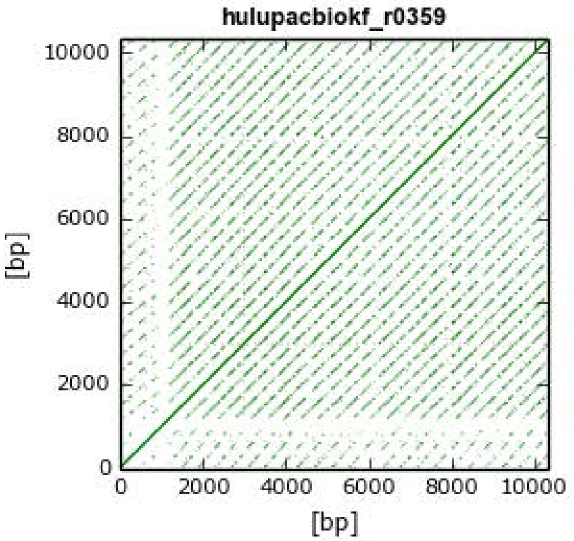
hulupacbiokf_r0359
[bp]
[bp]

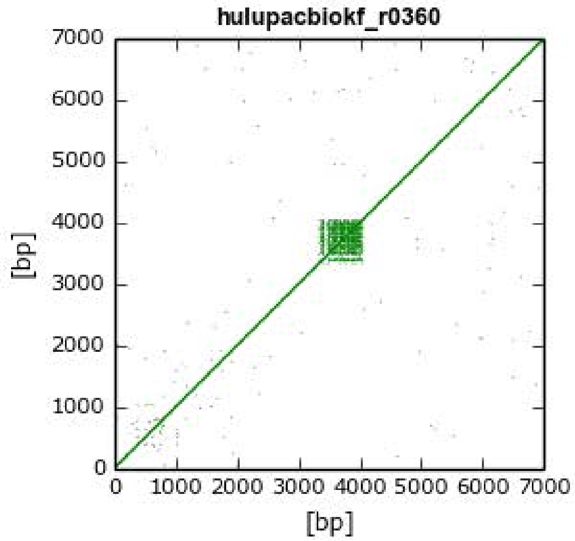
hulupacbiokf_r0360
[bp]
[bp]

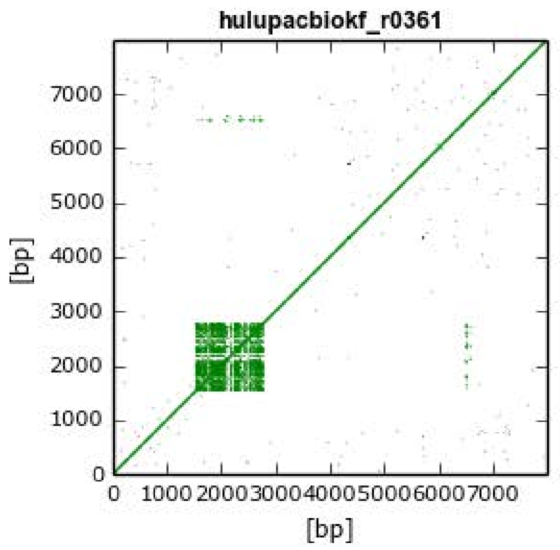
hulupacbiokf_r0361
[bp]
[bp]

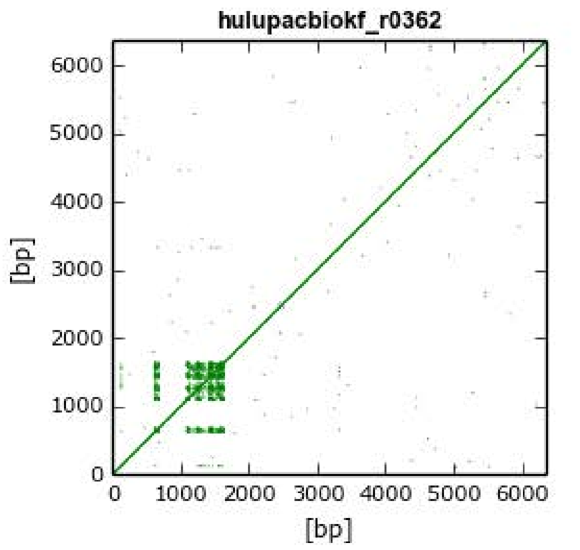
hulupacbiokf_r0362
[bp]
[bp]

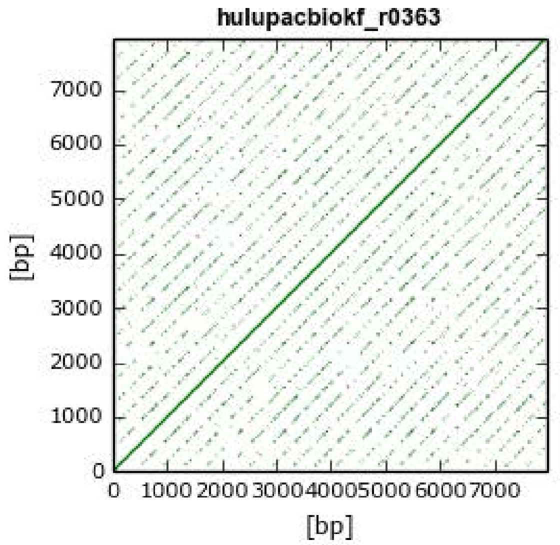
hulupacbiokf_r0363
[bp]
[bp]

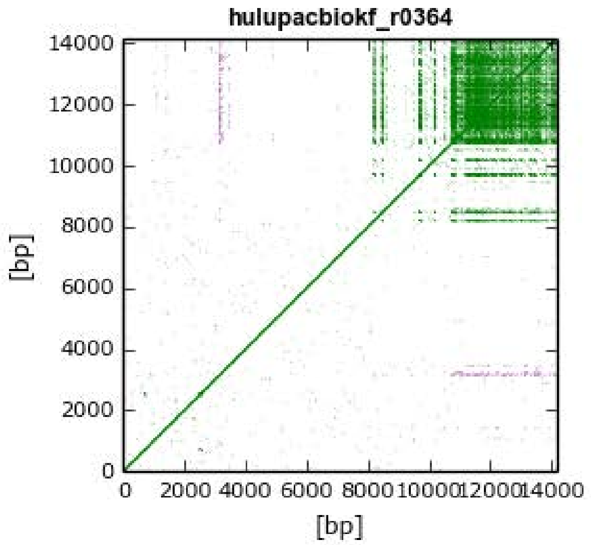
hulupacbiokf_r0364
[bp]
[bp]

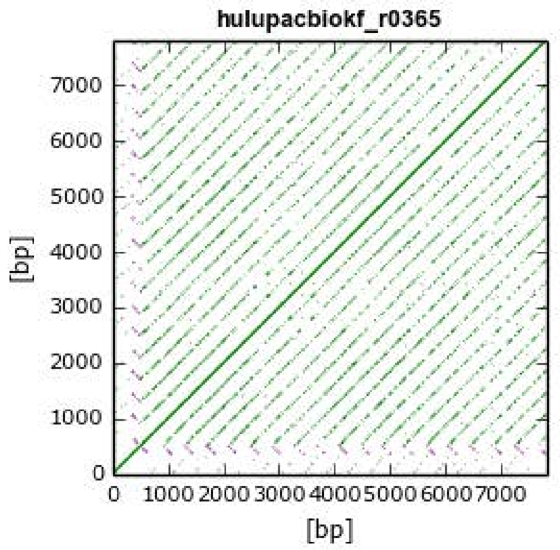
hulupacbiokf_r0365
[bp]
[bp]

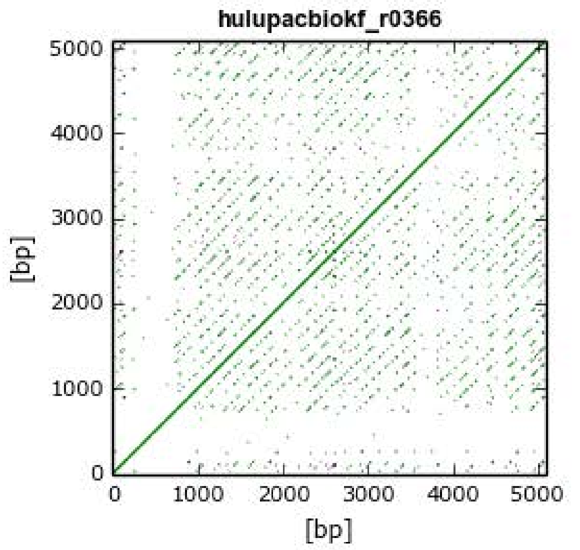
hulupacbiokf_r0366
[bp]
[bp]

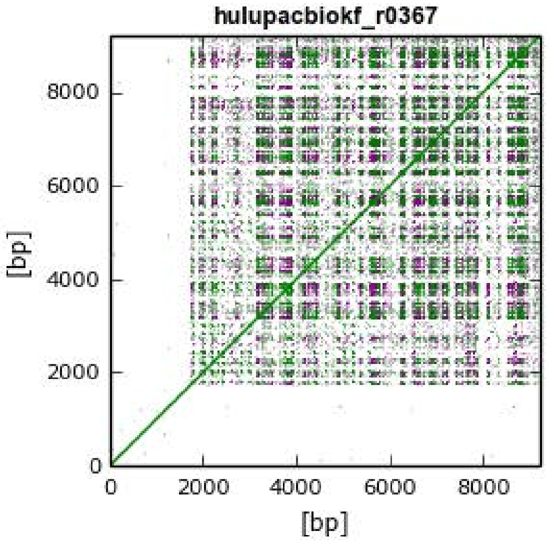
hulupacbiokf_r0367
8000
6000
4000
2000
0
[bp]
0
2000
4000
6000
8000
[bp]

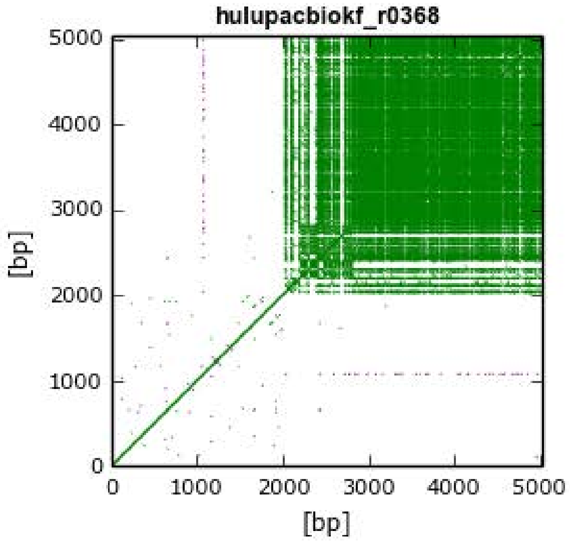
hulupacbiokf_r0368
5000
4000
3000
2000
1000
0
[bp]
0
1000
2000
3000
4000
5000
[bp]

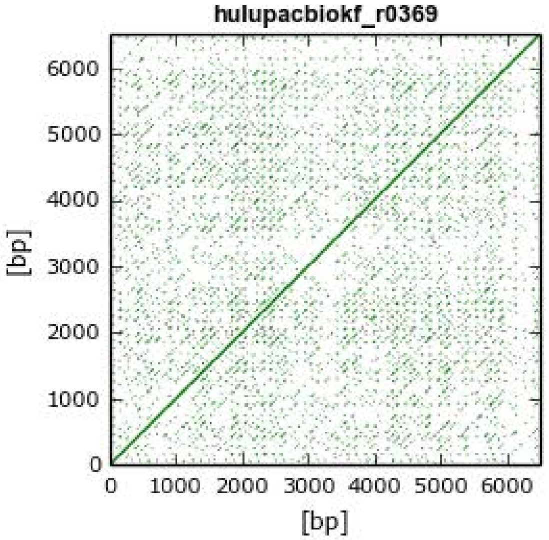
hulupacbiokf_r0369
6000
5000
4000
3000
2000
1000
0
[bp]
0
1000
2000
3000
4000
5000
6000
[bp]

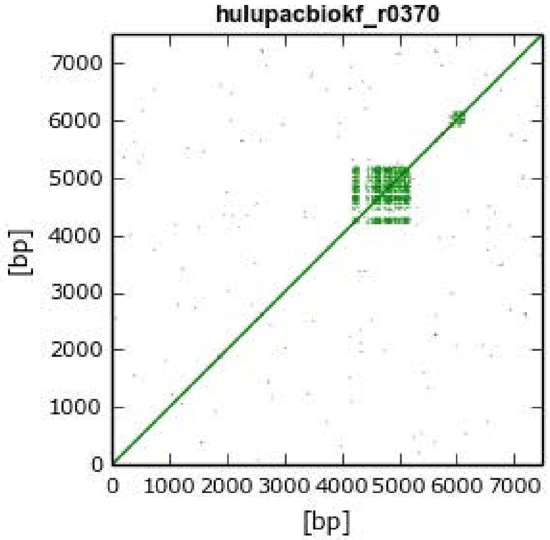
hulupacbiokf_r0370
7000
6000
5000
4000
3000
2000
1000
0
[bp]
0
1000
2000
3000
4000
5000
6000
7000
[bp]

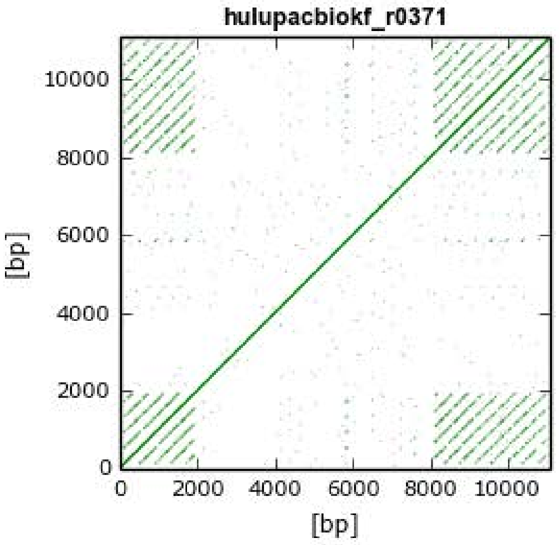
hulupacbiokf_r0371
10000
8000
6000
4000
2000
0
[bp]
0
2000
4000
6000
8000
10000
[bp]

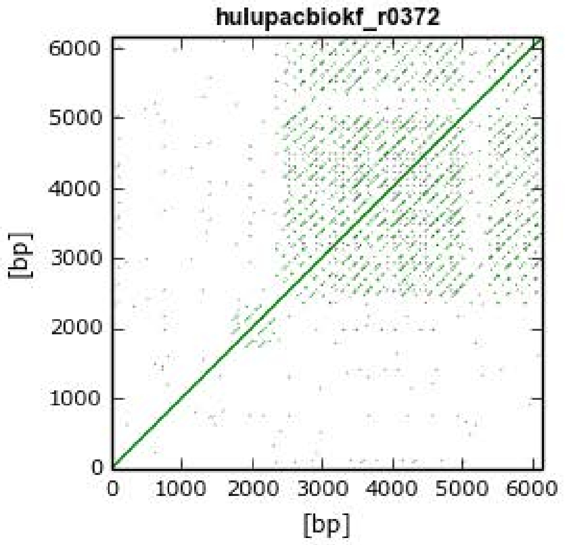
hulupacbiokf_r0372
6000
5000
4000
3000
2000
1000
0
[bp]
0
1000
2000
3000
4000
5000
6000
[bp]

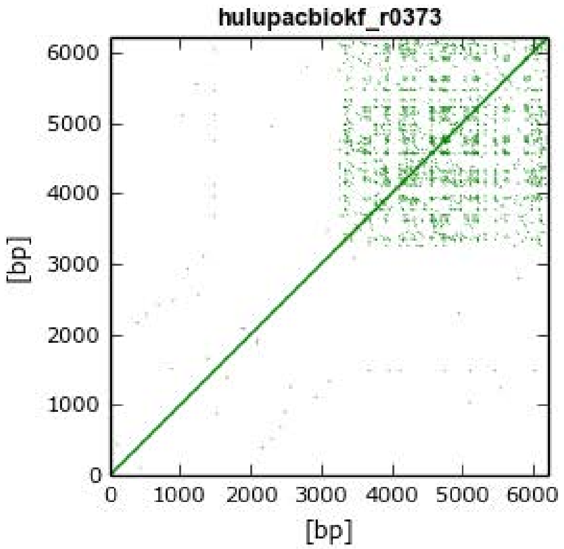
hulupacbiokf_r0373
[bp]
[bp]

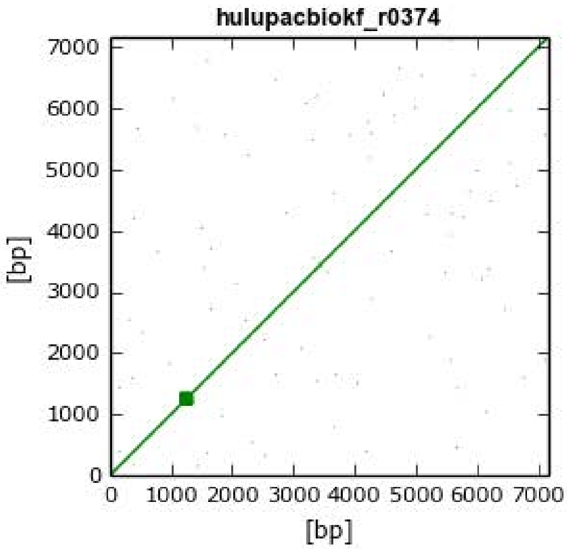
hulupacbiokf_r0374
[bp]
[bp]

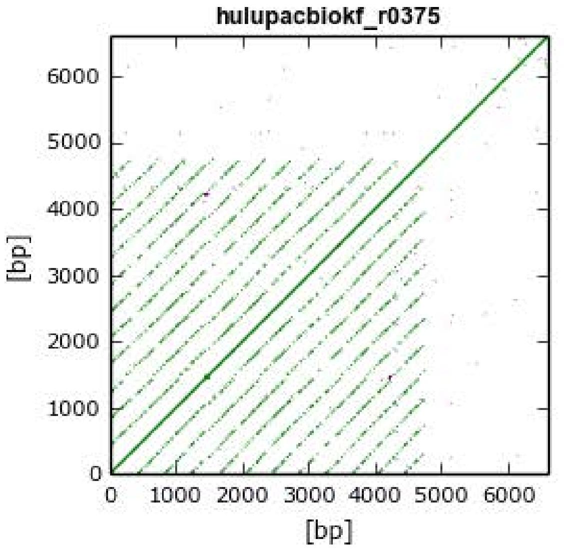
hulupacbiokf_r0375
[bp]
[bp]

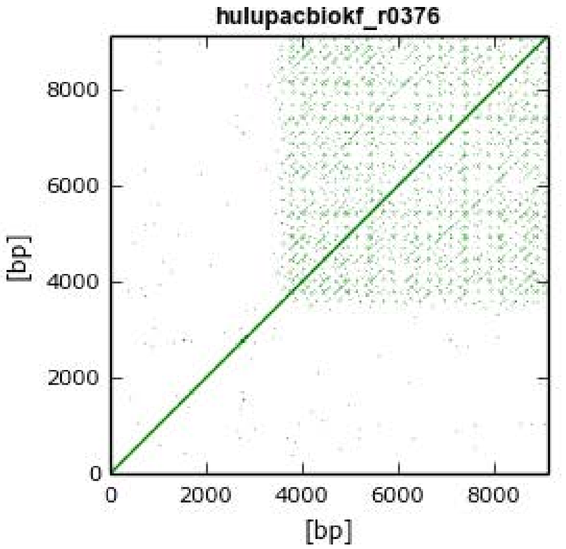
hulupacbiokf_r0376
[bp]
[bp]

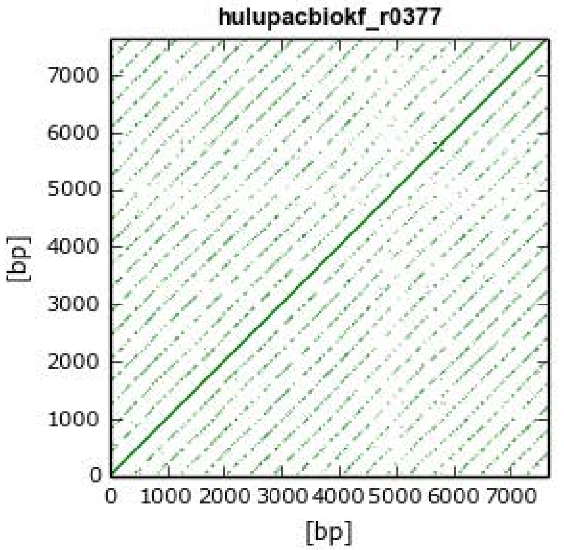
hulupacbiokf_r0377
[bp]
[bp]

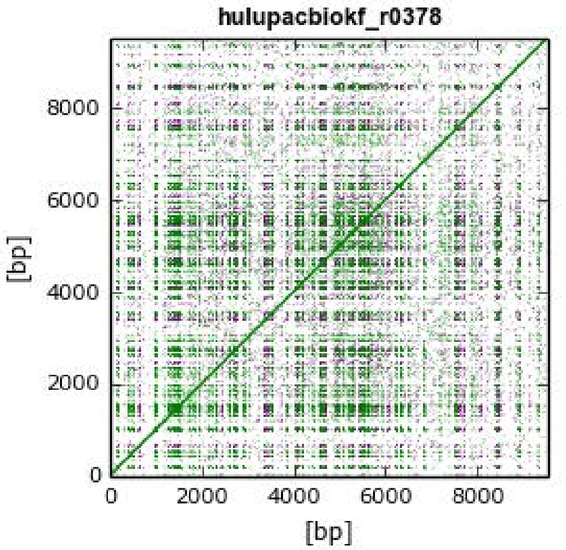
hulupacbiokf_r0378
[bp]
[bp]

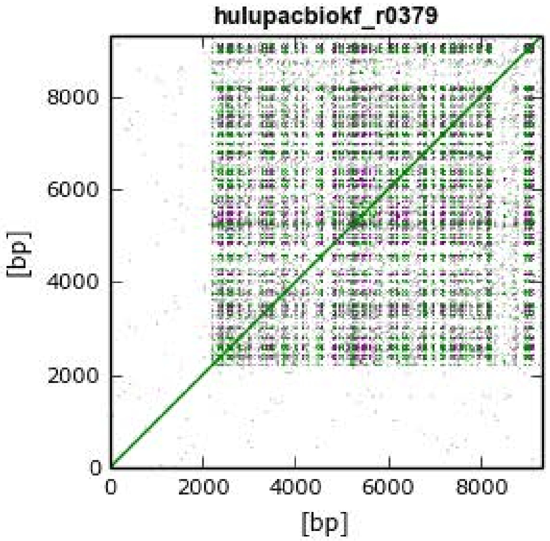
hulupacbiokf_r0379
[bp]
[bp]

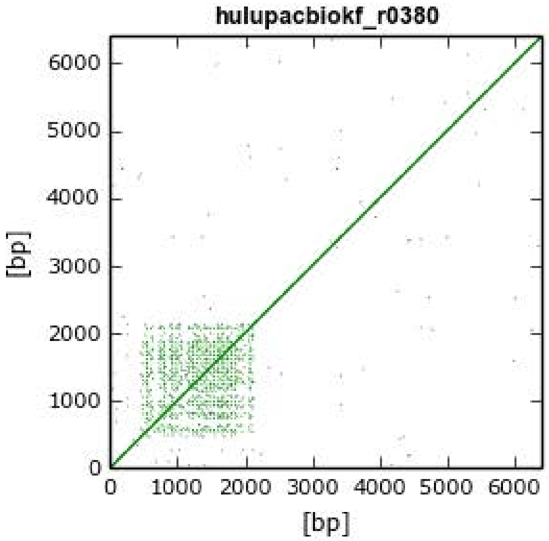
hulupacbiokf_r0380
[bp]
[bp]

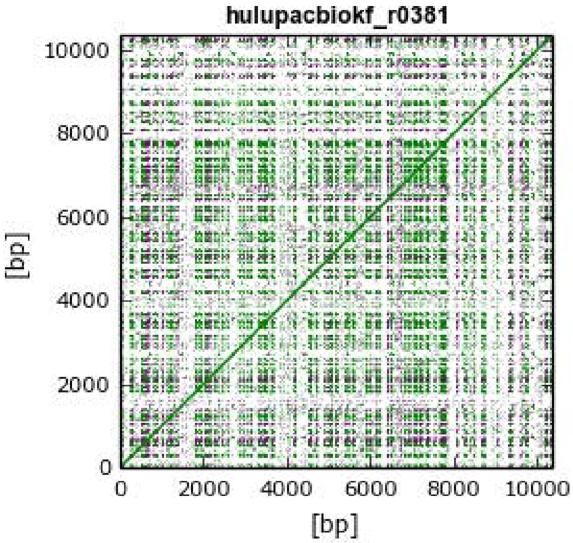
hulupacbiokf_r0381
[bp]
[bp]

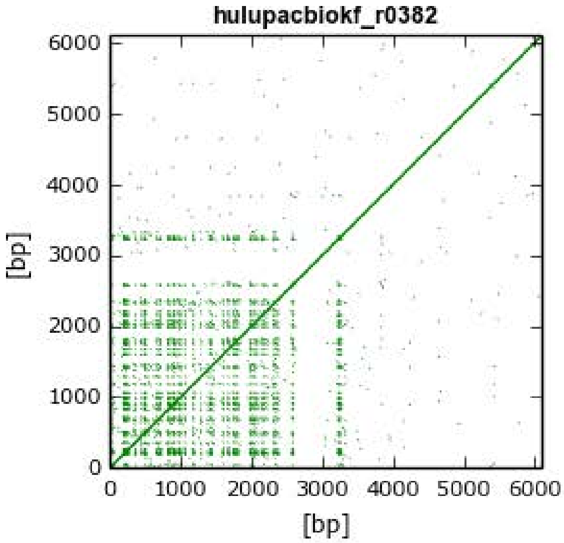
hulupacbiokf_r0382
[bp]
[bp]

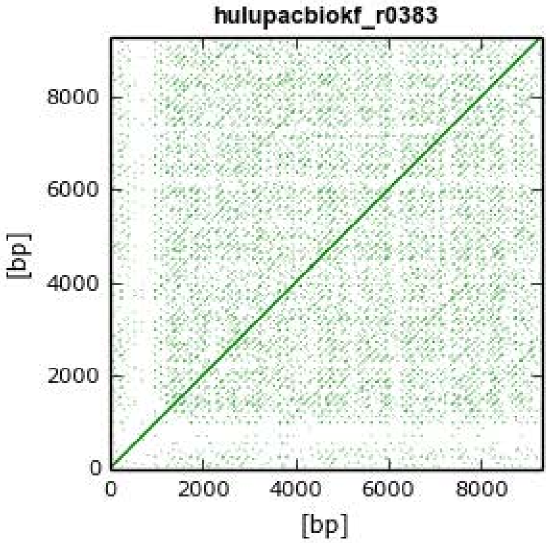
hulupacbiokf_r0383
[bp]
[bp]

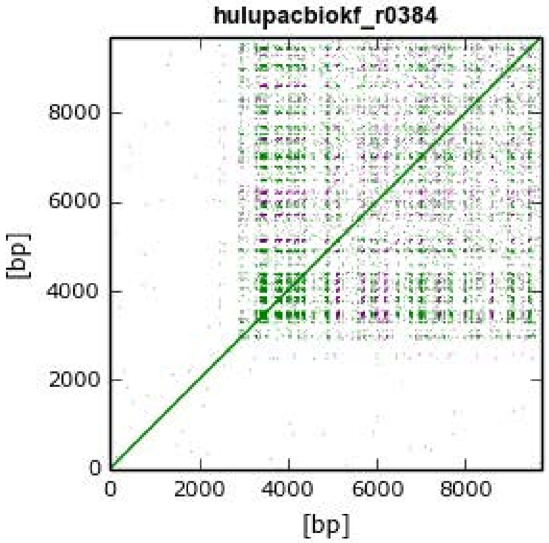
hulupacbiokf_r0384
[bp]
[bp]

hulupacbiokf_r0385

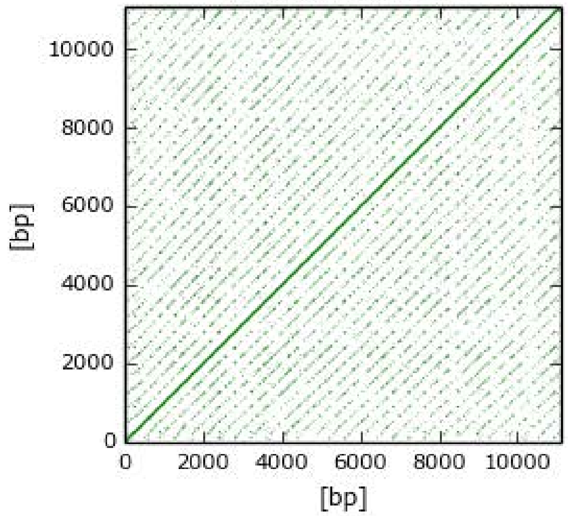

hulupacbiokf_r0386

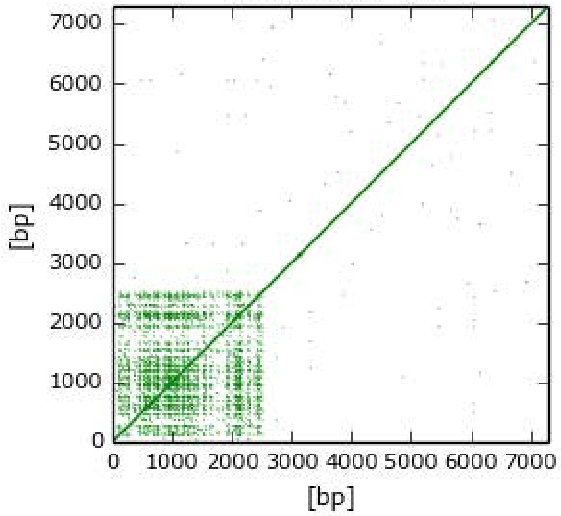

hulupacbiokf_r0387

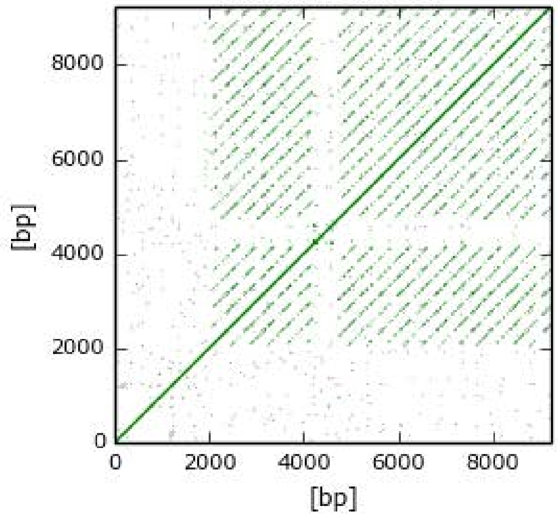

hulupacbiokf_r0388

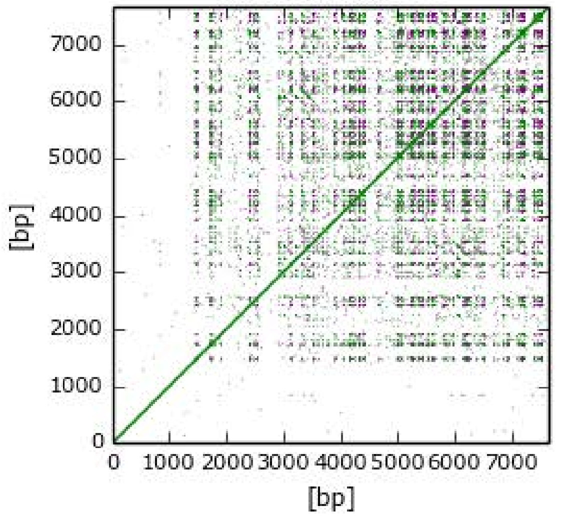

hulupacbiokf_r0389

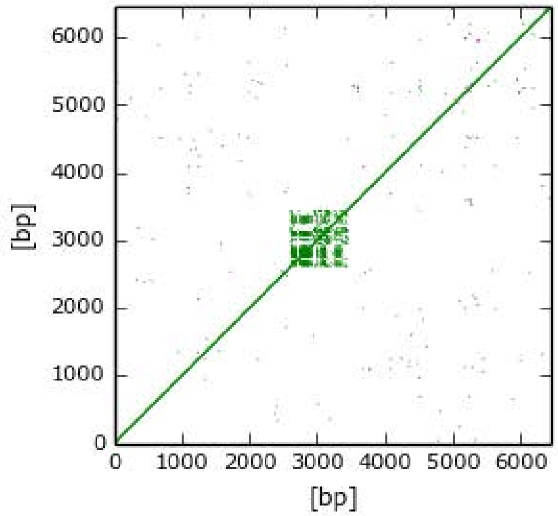

HuluTR150 from read r0390
is in GenBank Acc. MN537572

hulupacbiokf_r0390

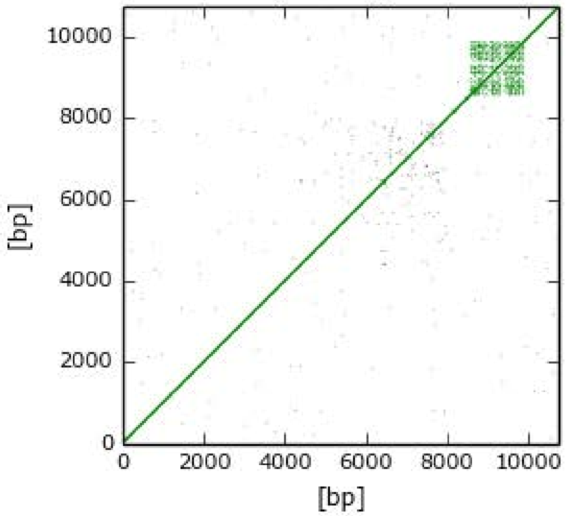

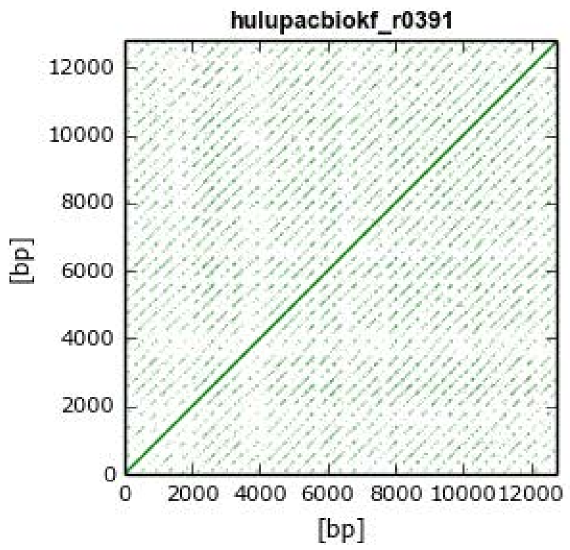
hulupacbiokf_r0391
[bp]
[bp]

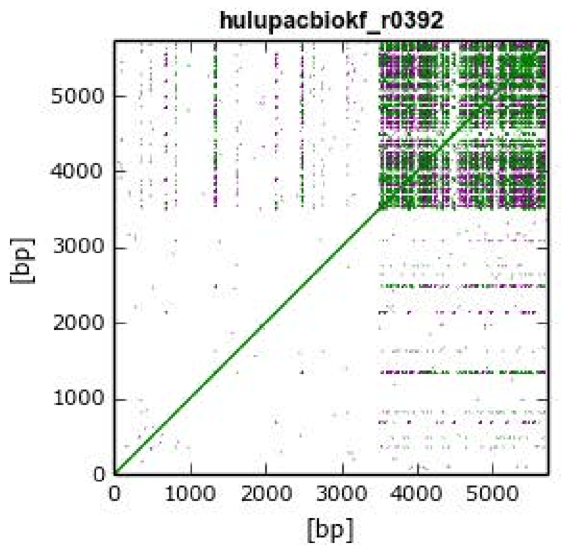
hulupacbiokf_r0392
[bp]
[bp]

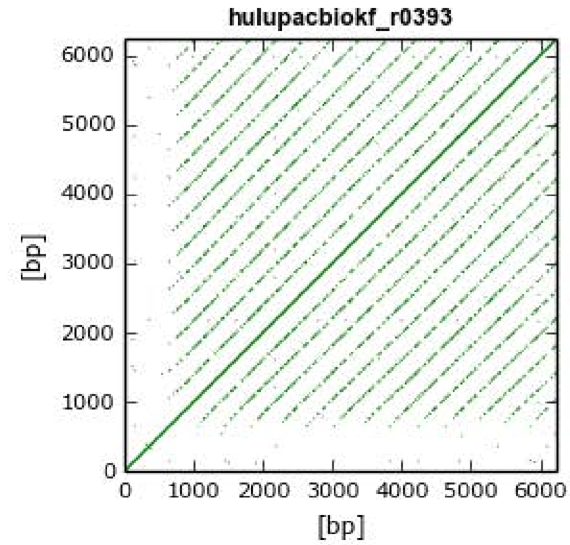
hulupacbiokf_r0393
[bp]
[bp]

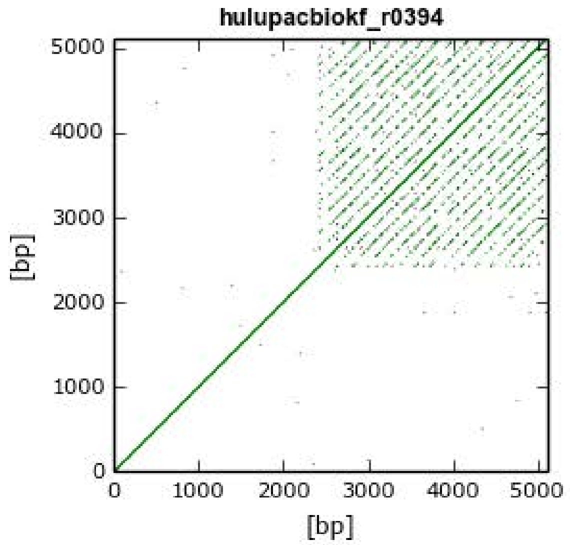
hulupacbiokf_r0394
[bp]
[bp]

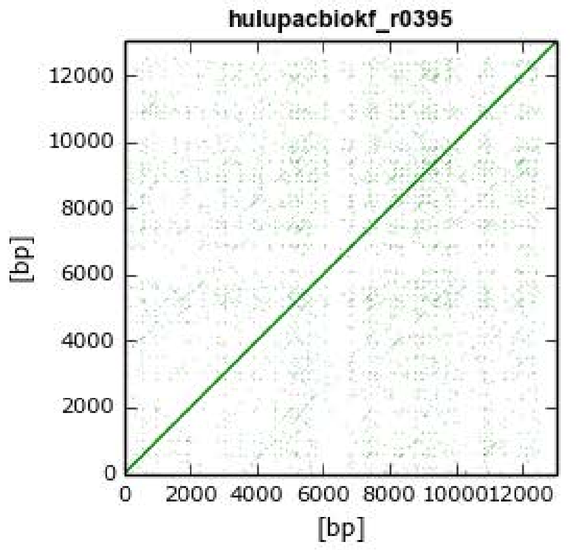
hulupacbiokf_r0395
[bp]
[bp]

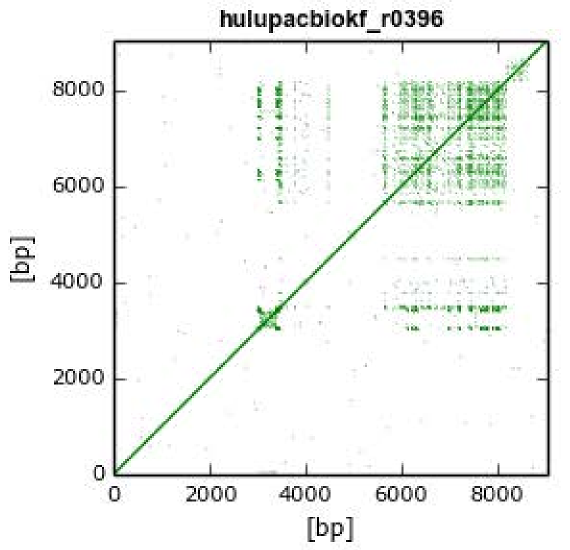
hulupacbiokf_r0396
[bp]
[bp]

HuluTR225 from read r0397 is in GenBank Acc. MN537574

hulupacbiokf_r0397

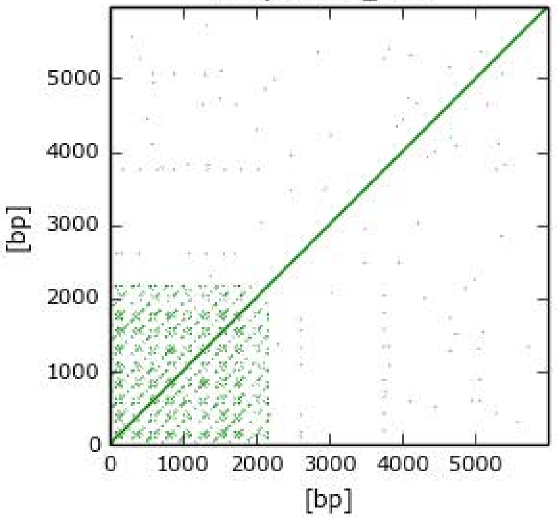

hulupacbiokf_r0398

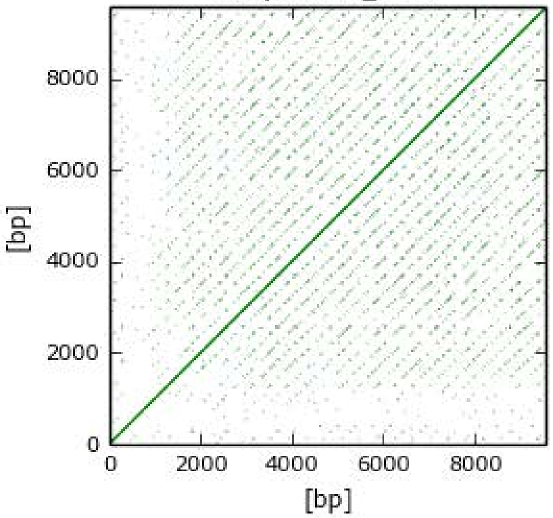

hulupacbiokf_r0399

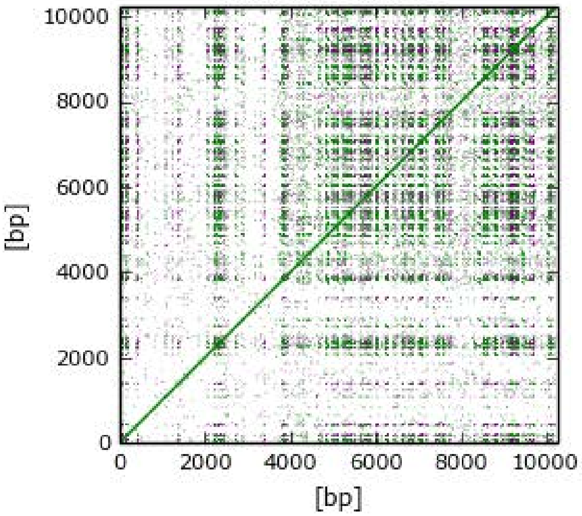

hulupacbiokf_r0400

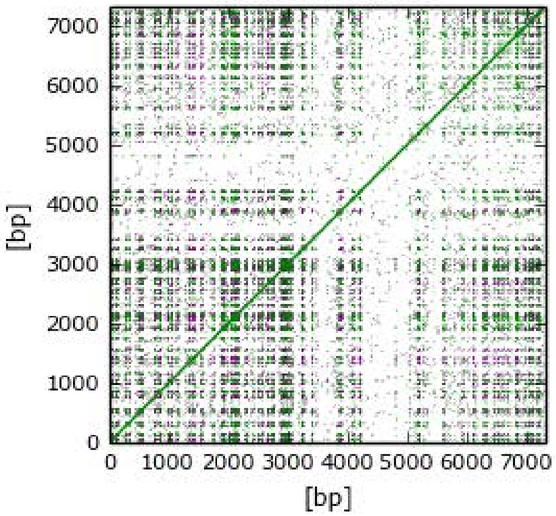

hulupacbiokf_r0401

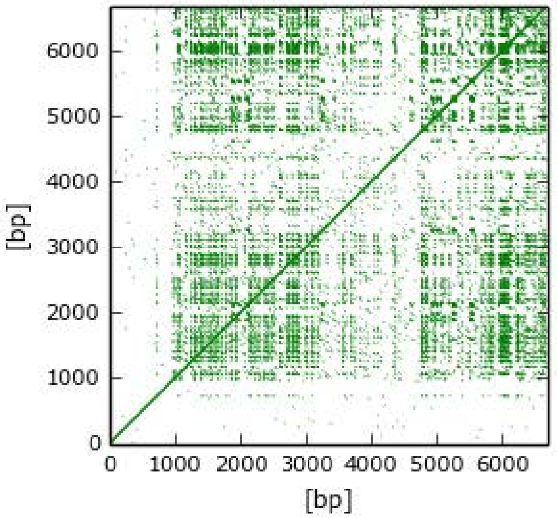

hulupacbiokf_r0402

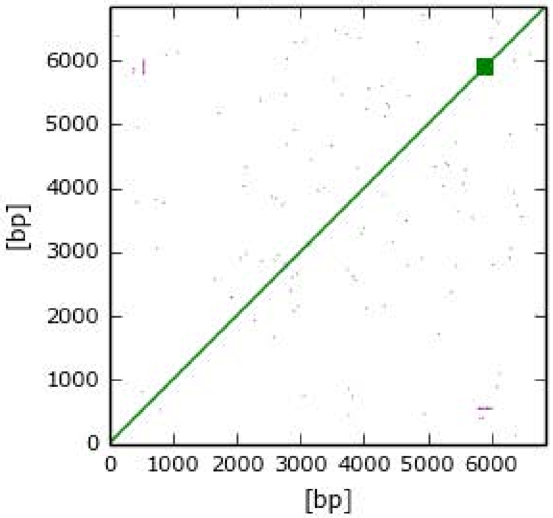

hulupacbiokf_r0403

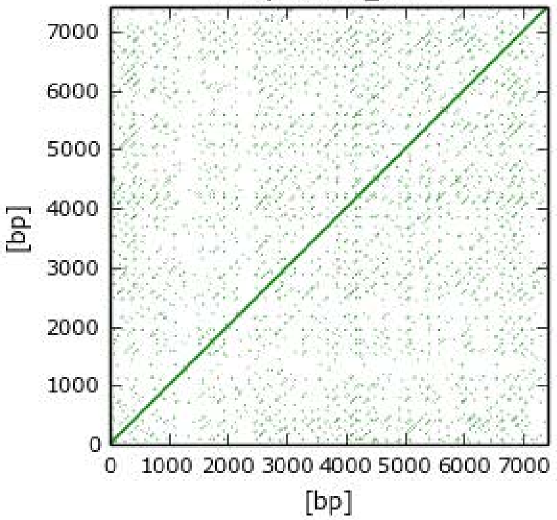

hulupacbiokf_r0404

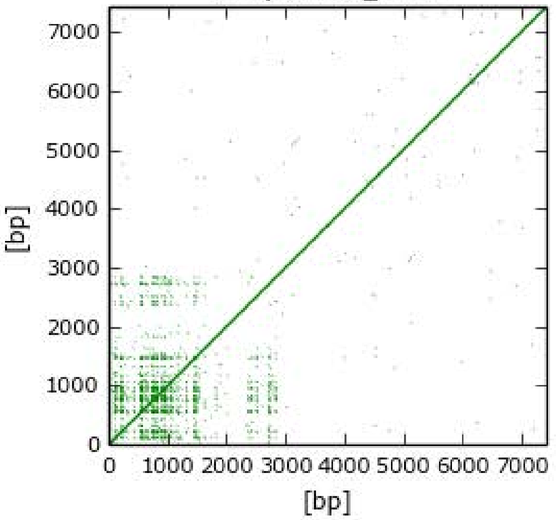

hulupacbiokf_r0405

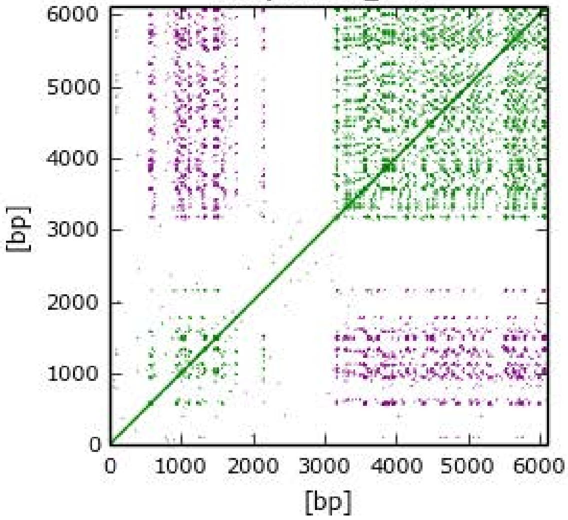

hulupacbiokf_r0406

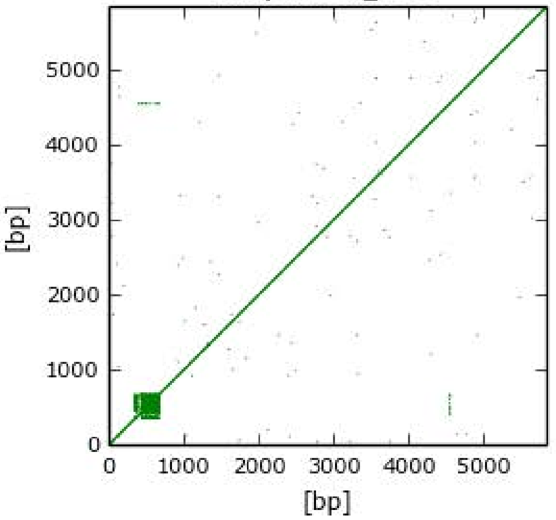

hulupacbiokf_r0407

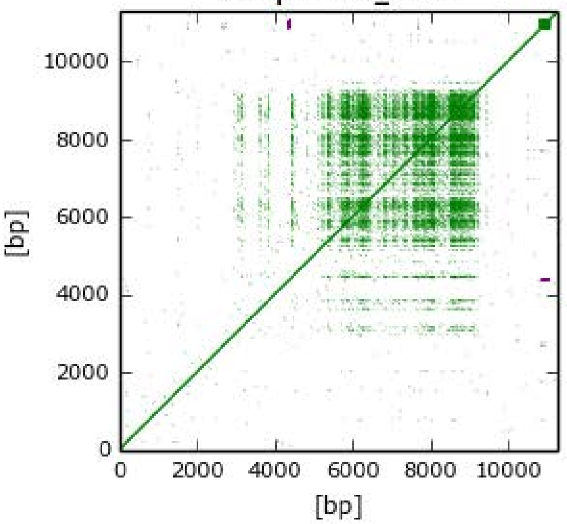

hulupacbiokf_r0408

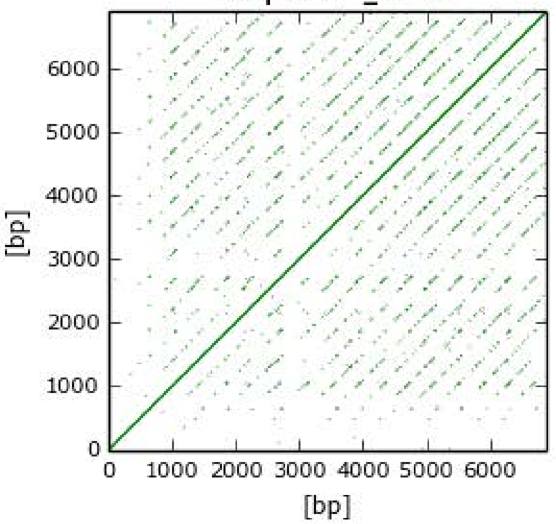

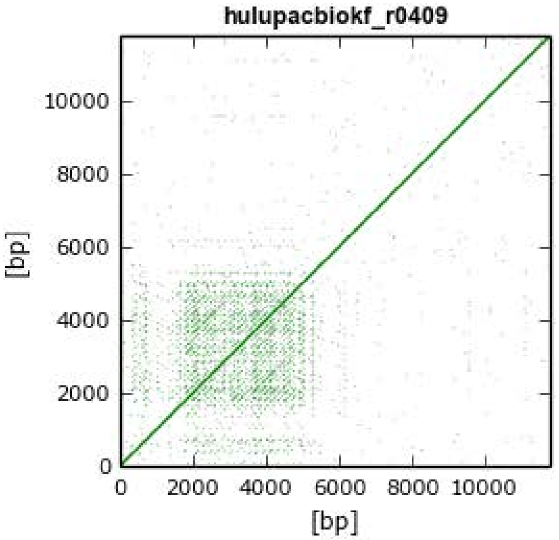
hulupacbiokf_r0409
[bp]
0
2000
4000
6000
8000
10000
[bp]

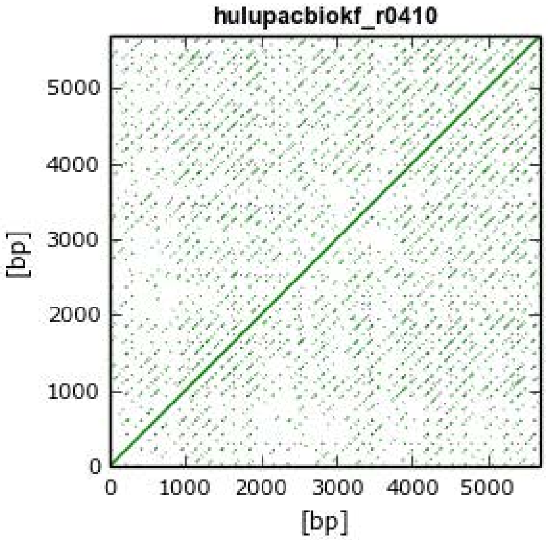
hulupacbiokf_r0410
[bp]
0
1000
2000
3000
4000
5000
[bp]

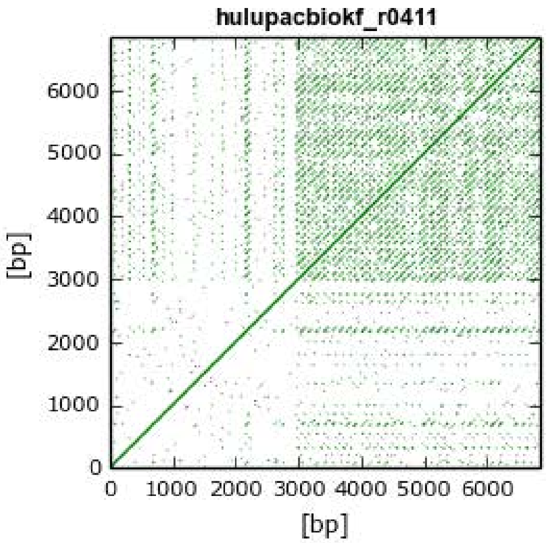
hulupacbiokf_r0411
[bp]
0
1000
2000
3000
4000
5000
6000
[bp]

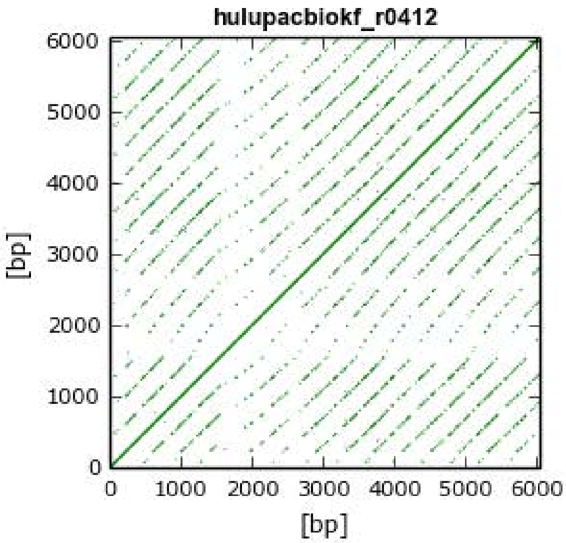
hulupacbiokf_r0412
[bp]
0
1000
2000
3000
4000
5000
6000
[bp]

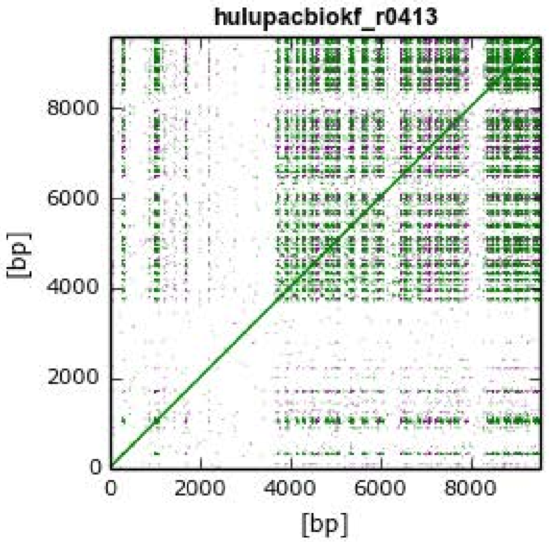
hulupacbiokf_r0413
[bp]
0
2000
4000
6000
8000
[bp]

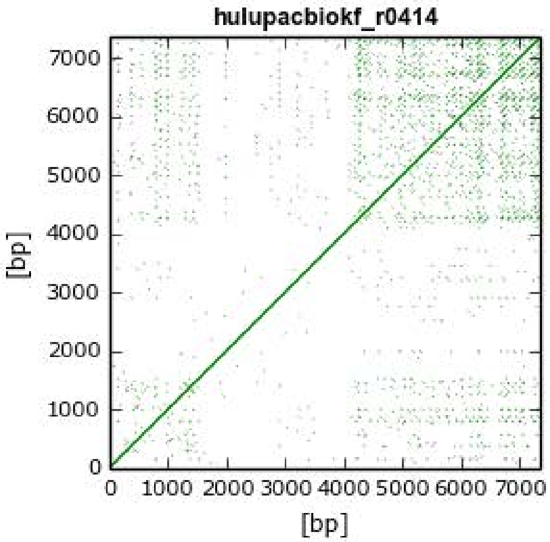
hulupacbiokf_r0414
[bp]
0
1000
2000
3000
4000
5000
6000
7000
[bp]

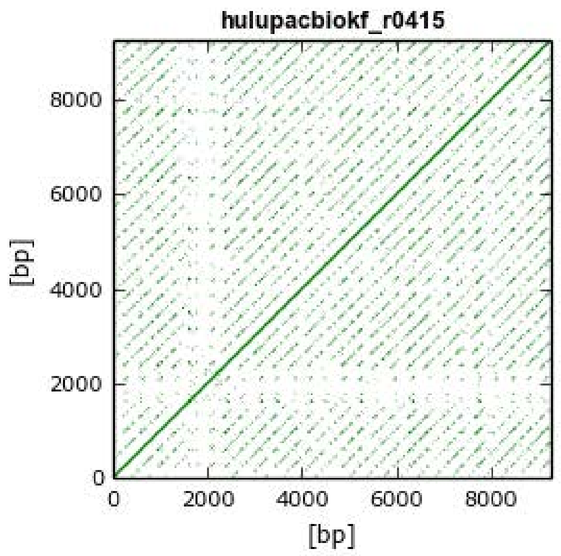
hulupacbiokf_r0415
[bp]
[bp]

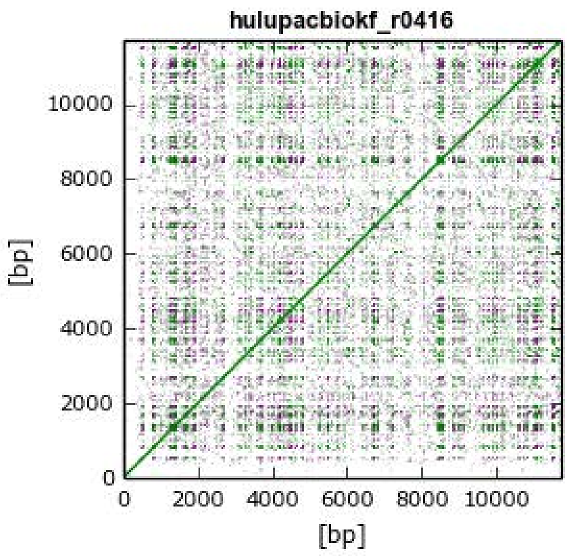
hulupacbiokf_r0416
[bp]
[bp]

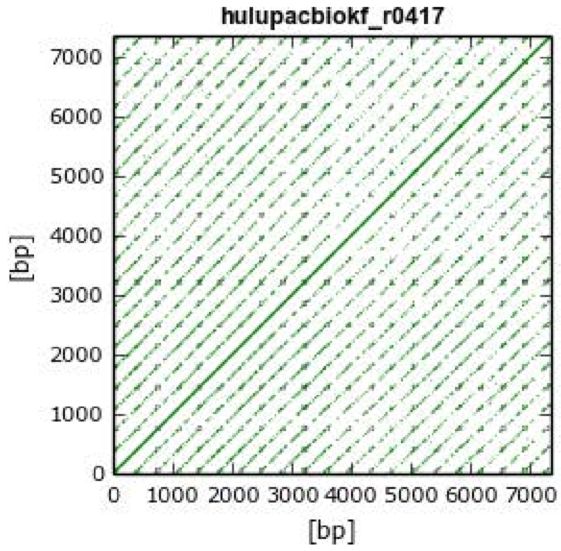
hulupacbiokf_r0417
[bp]
[bp]

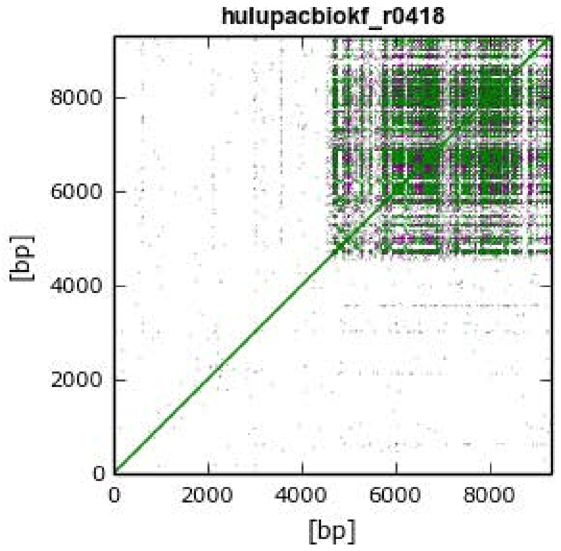
hulupacbiokf_r0418
[bp]
[bp]

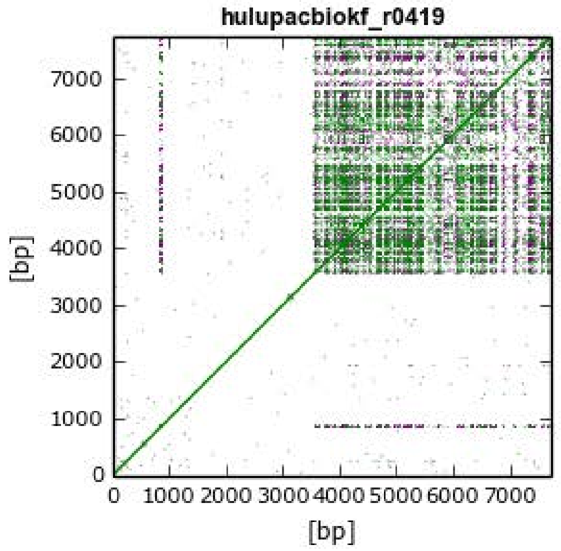
hulupacbiokf_r0419
[bp]
[bp]

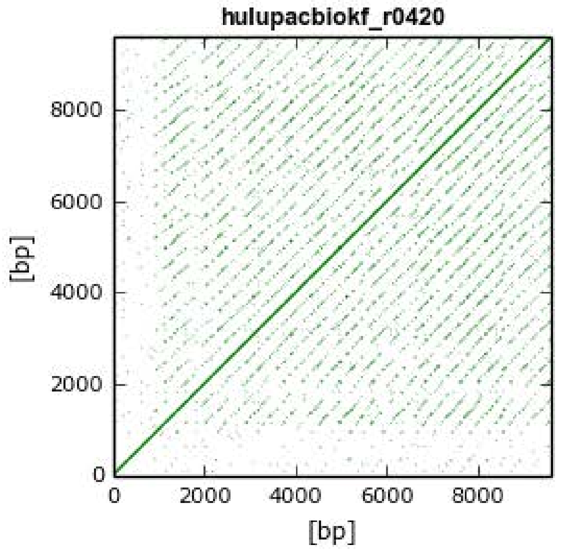
hulupacbiokf_r0420
[bp]
[bp]

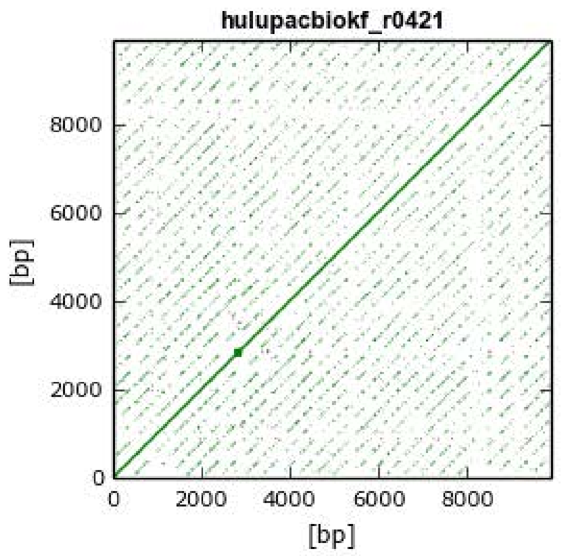

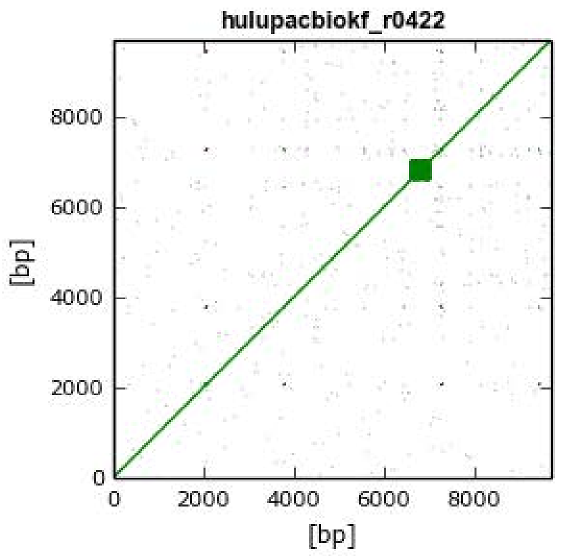

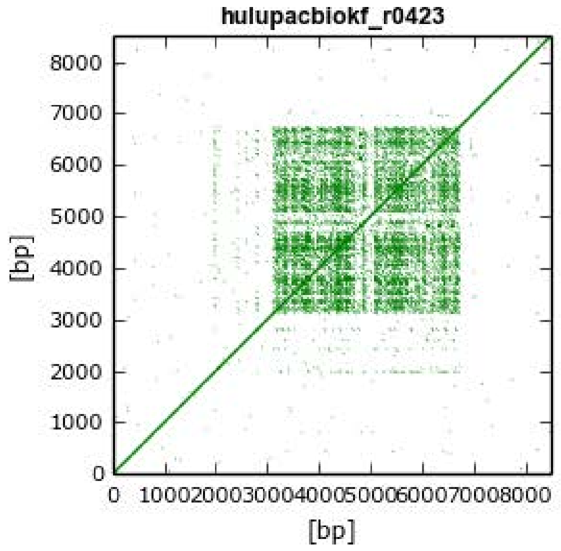

HuluTR185 from read r0424
is in GenBank Acc. MN537573

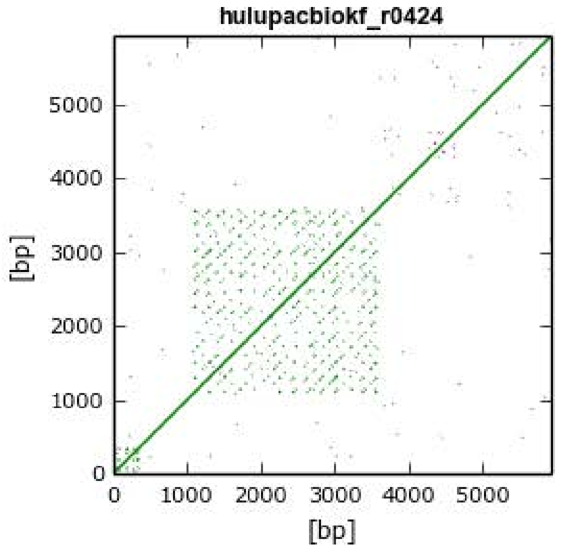

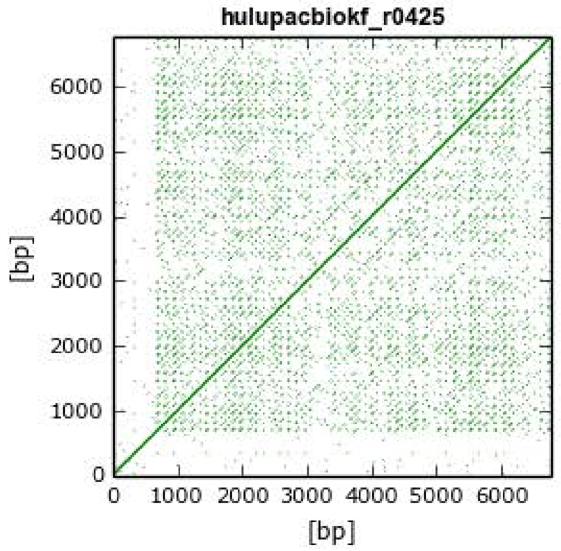

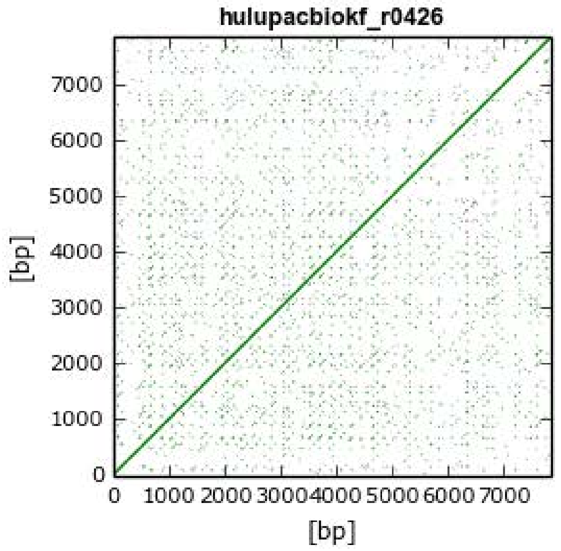

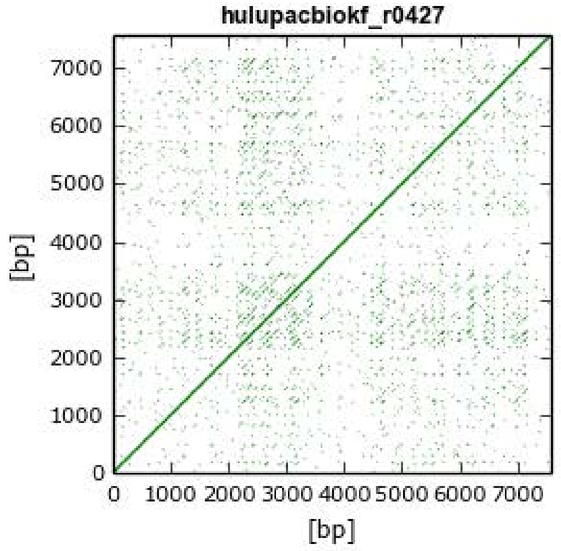
hulupacbiokf_r0427
[bp]
[bp]

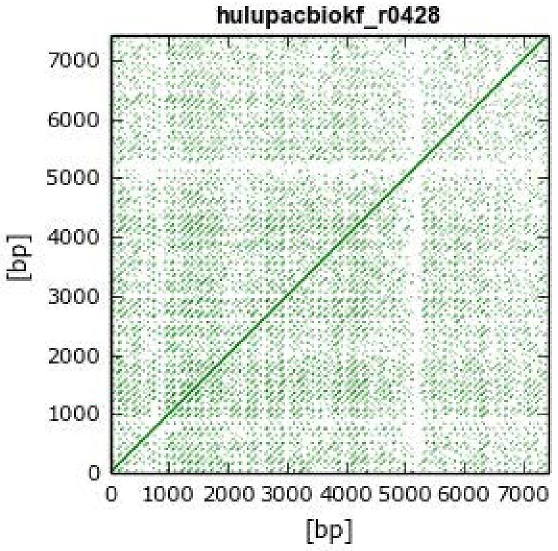
hulupacbiokf_r0428
[bp]
[bp]

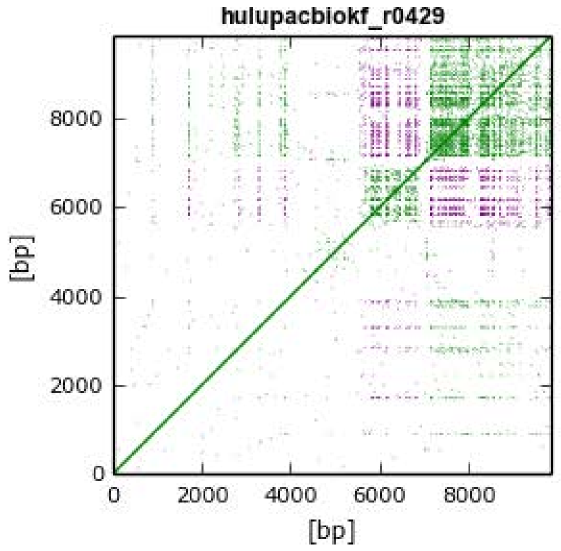
hulupacbiokf_r0429
[bp]
[bp]

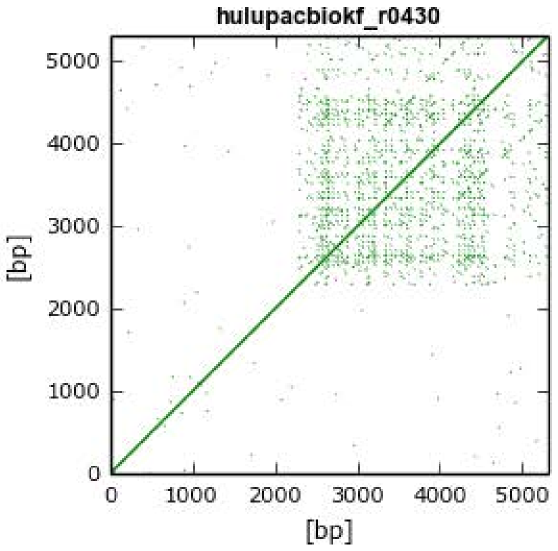
hulupacbiokf_r0430
[bp]
[bp]

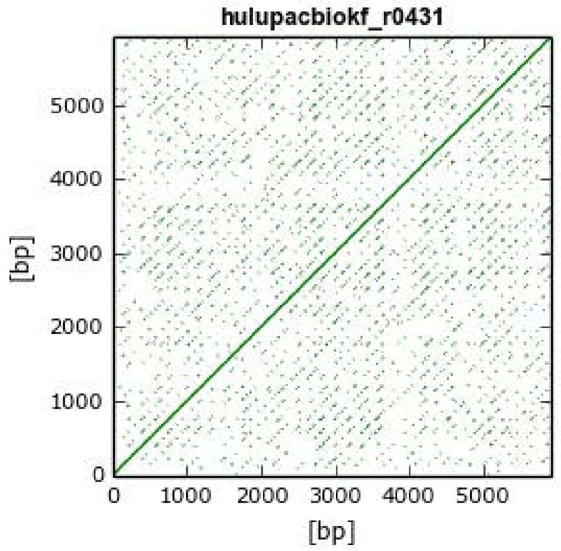
hulupacbiokf_r0431
[bp]
[bp]

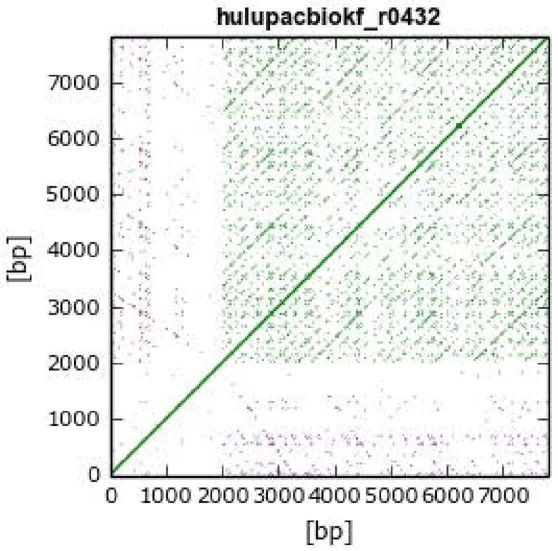
hulupacbiokf_r0432
[bp]
[bp]

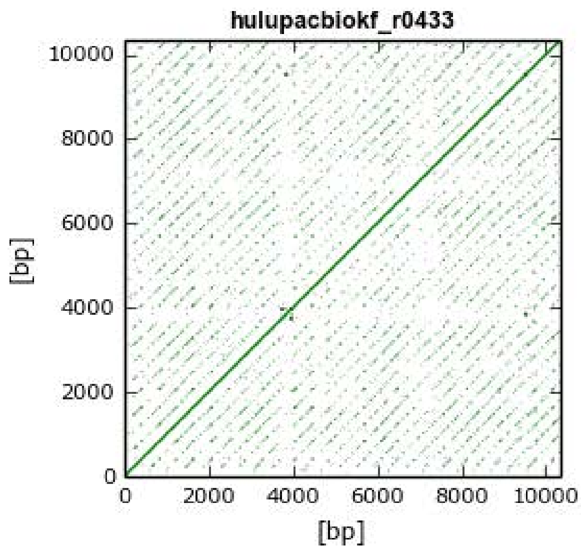
hulupacbiokf_r0433
[bp]
[bp]

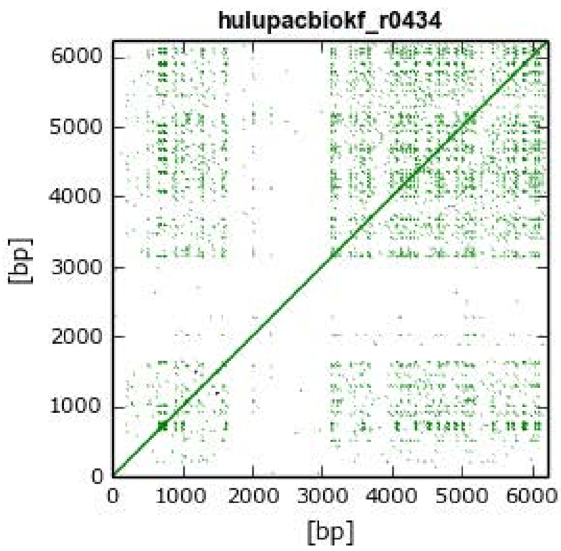
hulupacbiokf_r0434
[bp]
[bp]

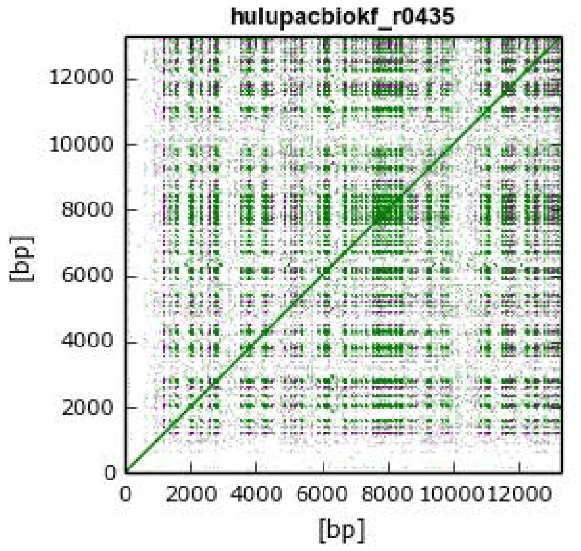
hulupacbiokf_r0435
[bp]
[bp]

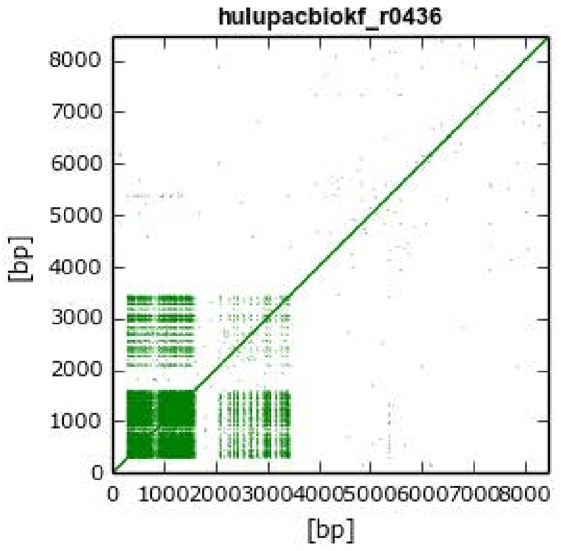
hulupacbiokf_r0436
[bp]
[bp]

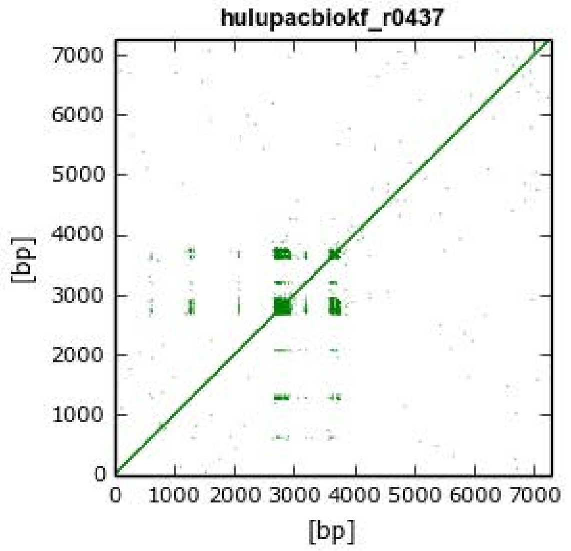
hulupacbiokf_r0437
[bp]
[bp]

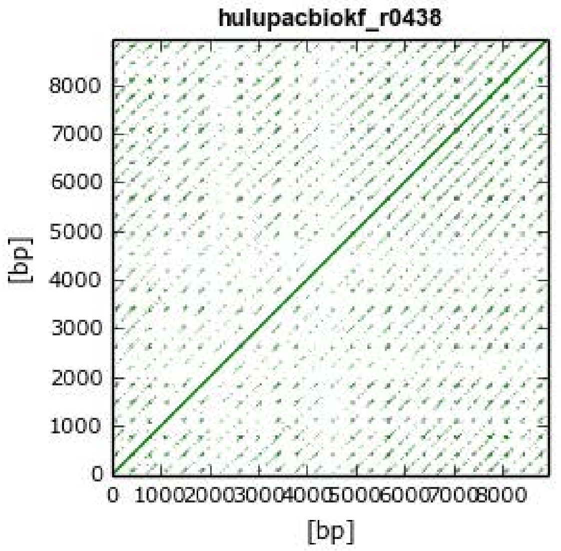
hulupacbiokf_r0438
[bp]
[bp]

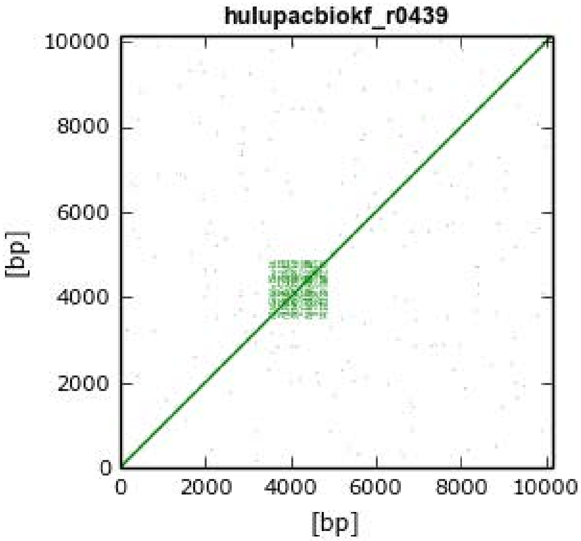
hulupacbiokf_r0439
10000
8000
6000
4000
2000
0
[bp]
0
2000
4000
6000
8000
10000
[bp]

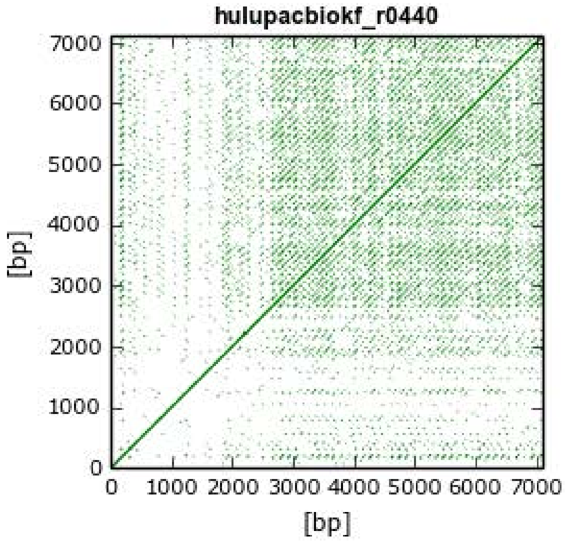
hulupacbiokf_r0440
7000
6000
5000
4000
3000
2000
1000
0
[bp]
0
1000
2000
3000
4000
5000
6000
7000
[bp]

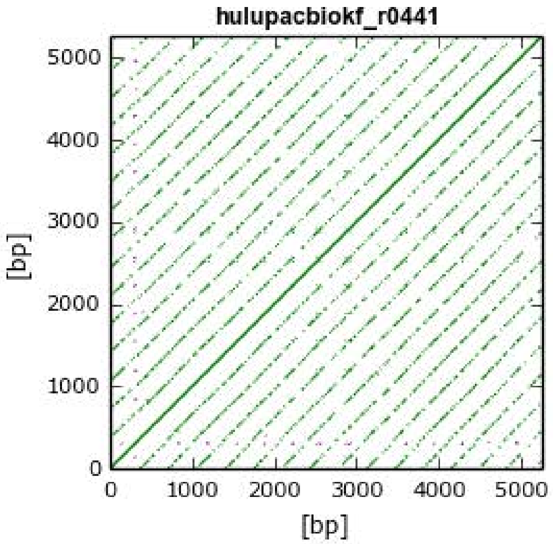
hulupacbiokf_r0441
5000
4000
3000
2000
1000
0
[bp]
0
1000
2000
3000
4000
5000
[bp]

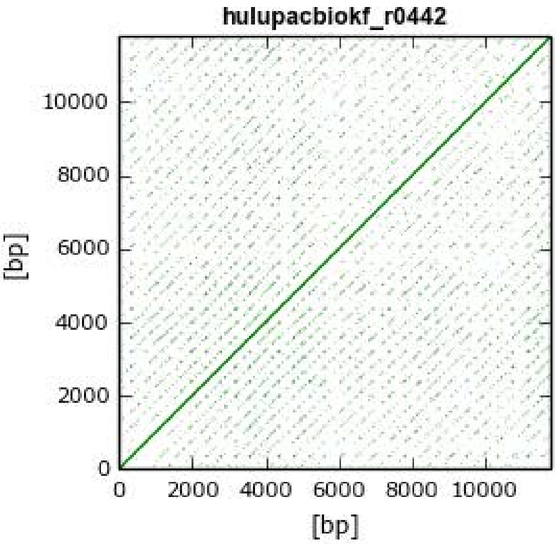
hulupacbiokf_r0442
10000
8000
6000
4000
2000
0
[bp]
0
2000
4000
6000
8000
10000
[bp]

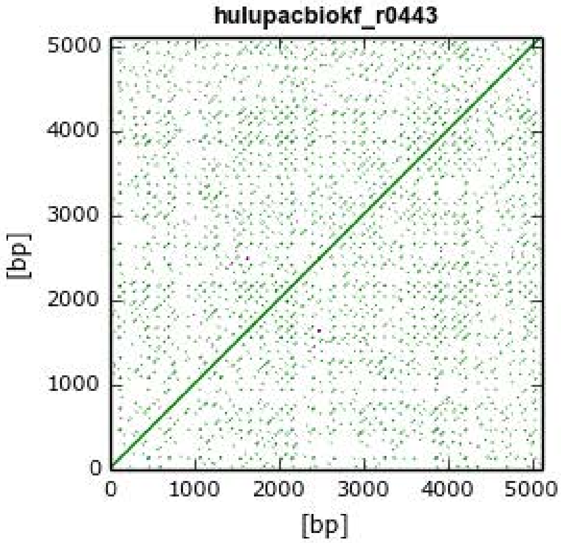
hulupacbiokf_r0443
5000
4000
3000
2000
1000
0
[bp]
0
1000
2000
3000
4000
5000
[bp]

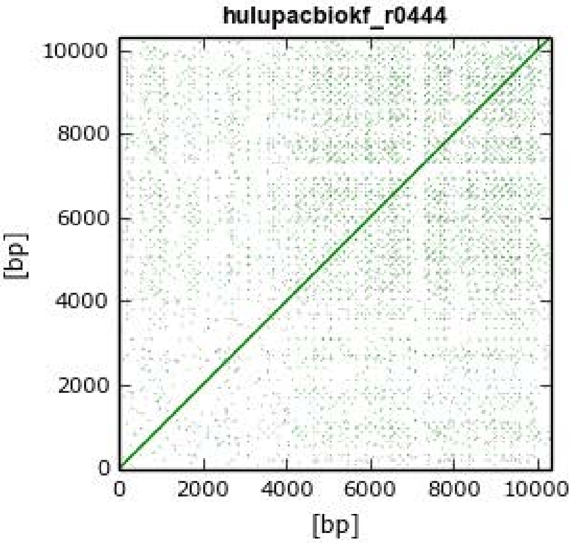
hulupacbiokf_r0444
10000
8000
6000
4000
2000
0
[bp]
0
2000
4000
6000
8000
10000
[bp]

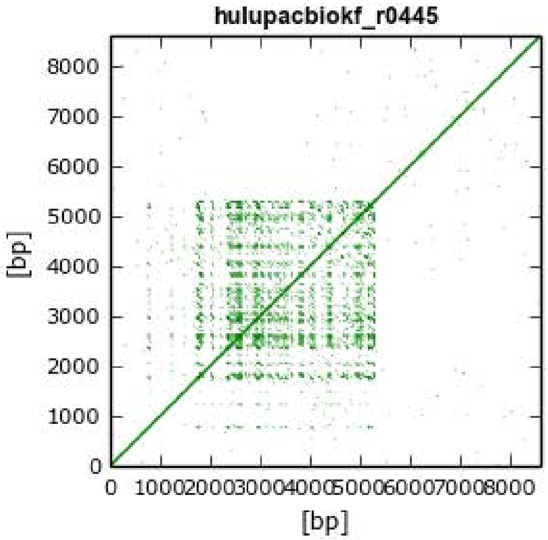
hulupacbiokf_r0445
[bp]
0
1000
2000
3000
4000
5000
6000
7000
8000
[bp]

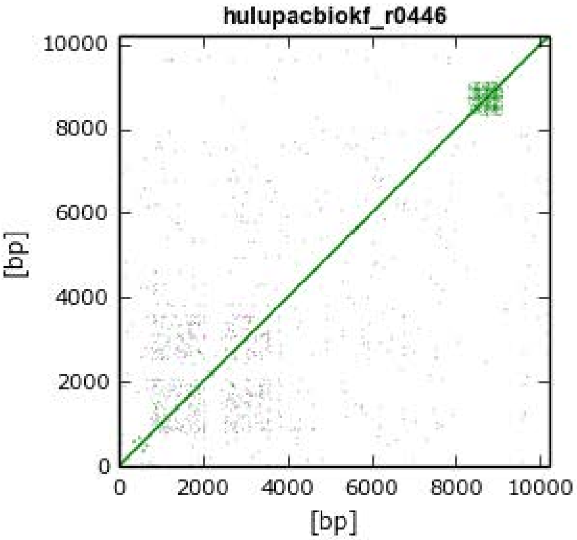
hulupacbiokf_r0446
[bp]
0
2000
4000
6000
8000
10000
[bp]

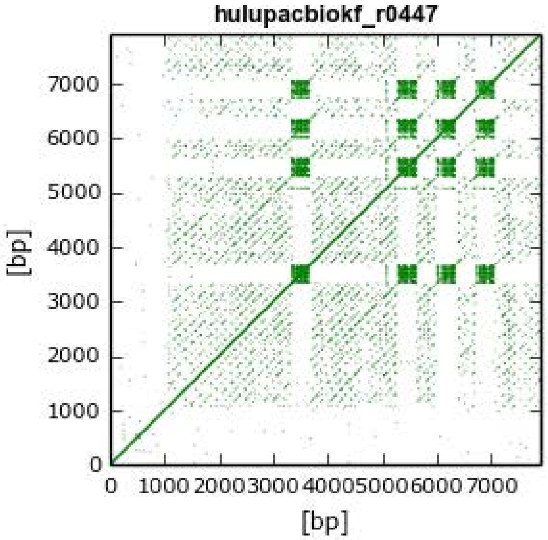
hulupacbiokf_r0447
[bp]
0
1000
2000
3000
4000
5000
6000
7000
[bp]

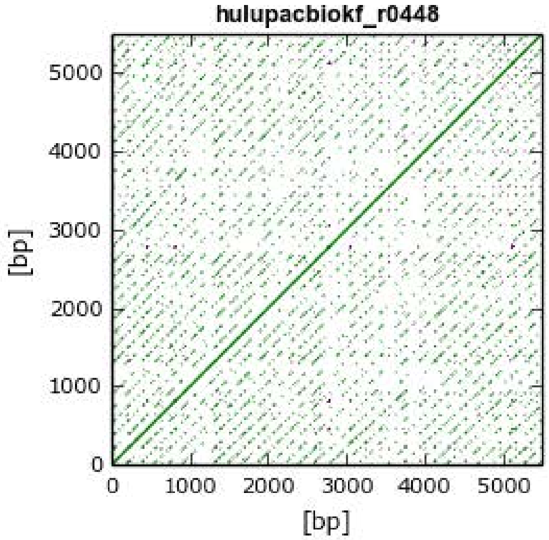
hulupacbiokf_r0448
[bp]
0
1000
2000
3000
4000
5000
[bp]

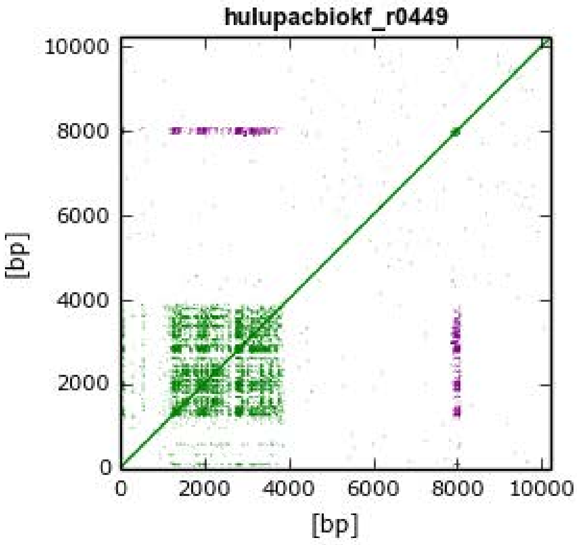
hulupacbiokf_r0449
[bp]
0
2000
4000
6000
8000
10000
[bp]

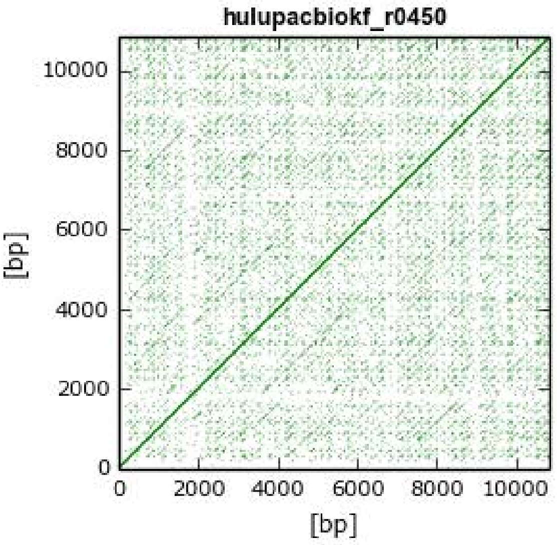
hulupacbiokf_r0450
[bp]
0
2000
4000
6000
8000
10000
[bp]

hulupacbiokf_r0451

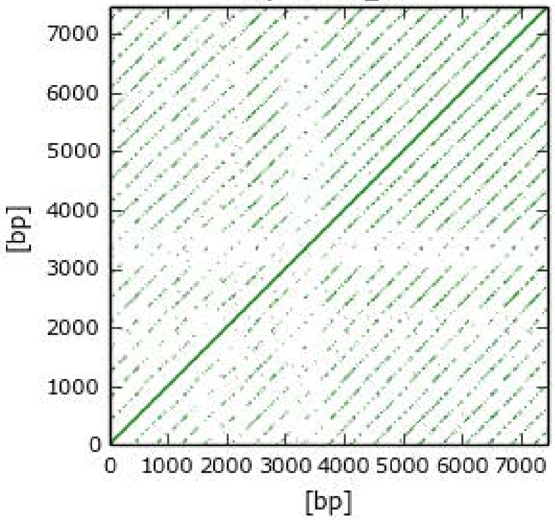

hulupacbiokf_r0452

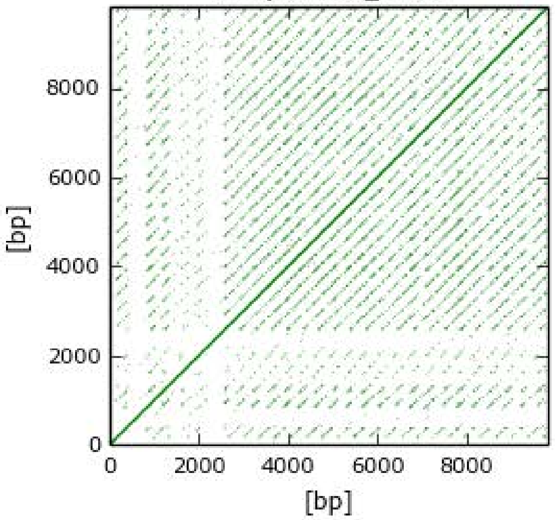

hulupacbiokf_r0453

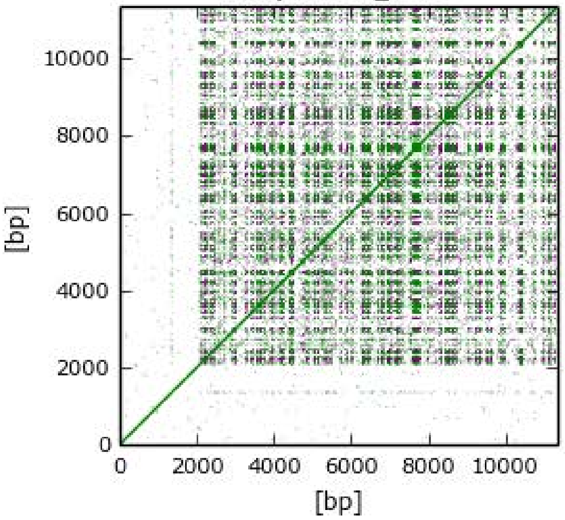

hulupacbiokf_r0454

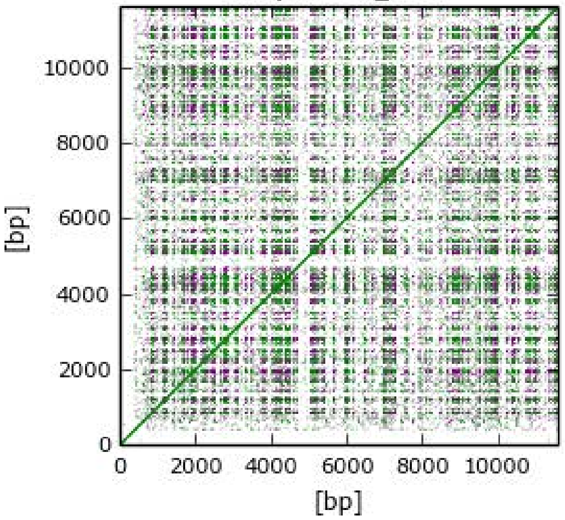

hulupacbiokf_r0455

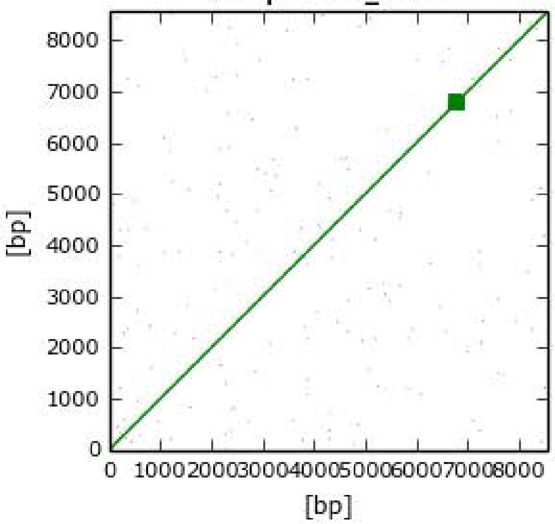

hulupacbiokf_r0456

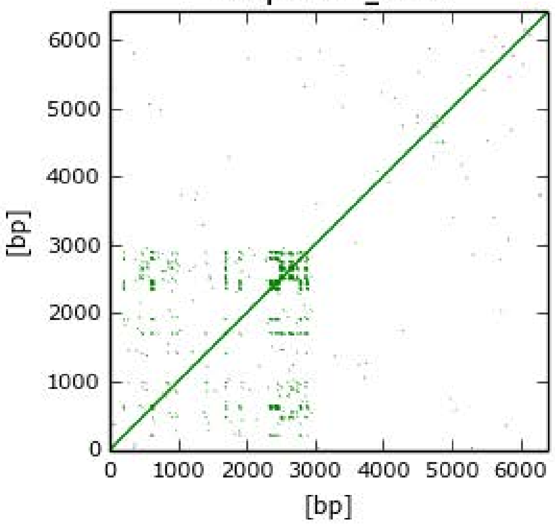

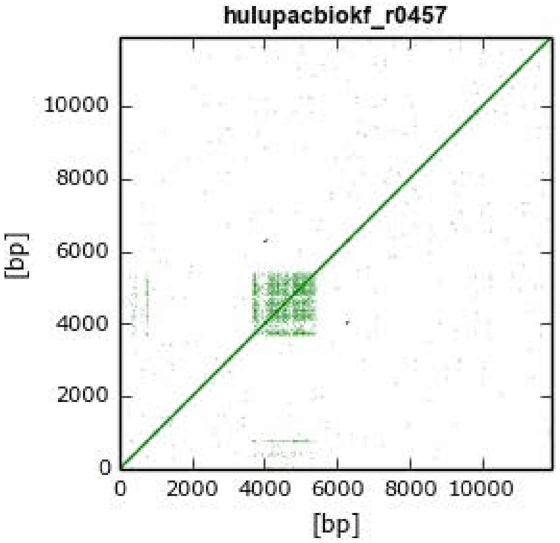
hulupacbiokf_r0457
[bp]
[bp]

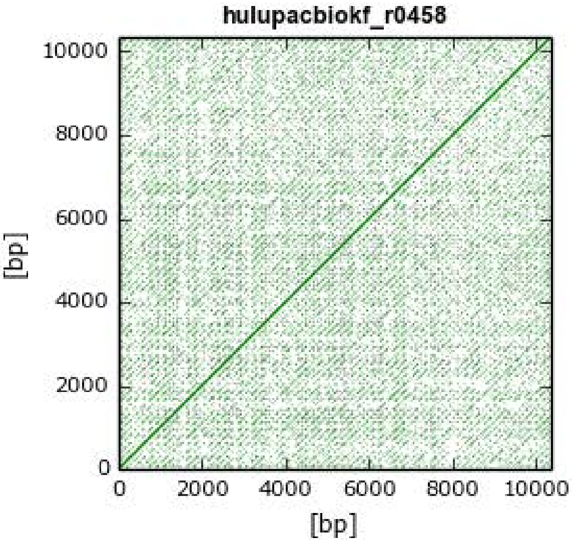
hulupacbiokf_r0458
[bp]
[bp]

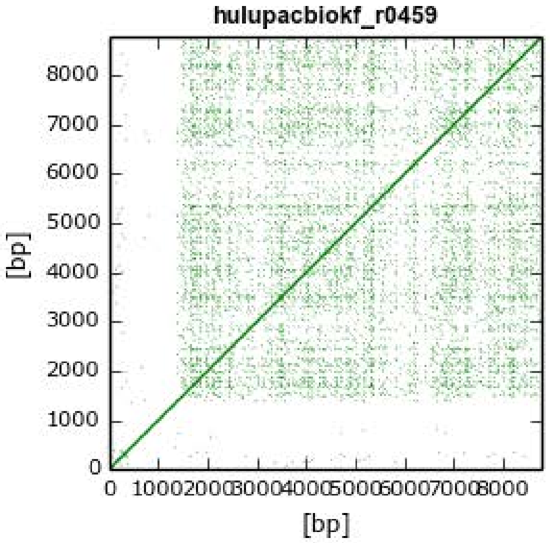
hulupacbiokf_r0459
[bp]
[bp]

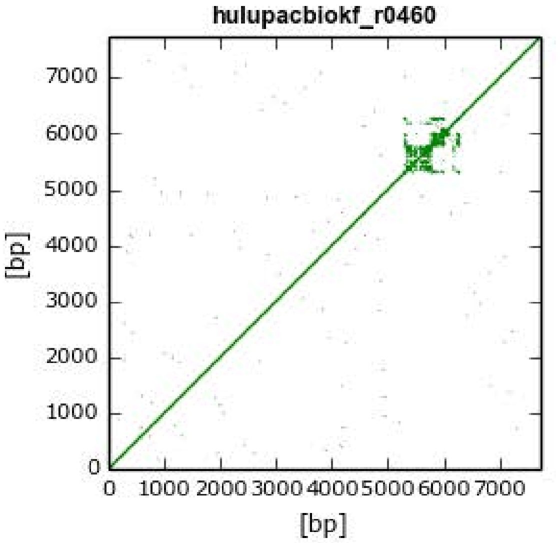
hulupacbiokf_r0460
[bp]
[bp]

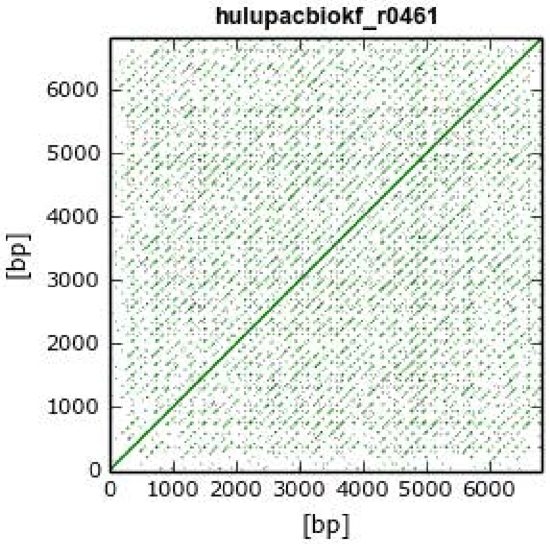
hulupacbiokf_r0461
[bp]
[bp]

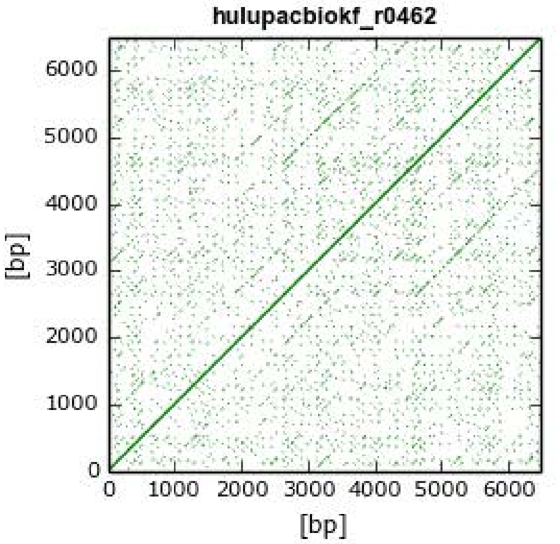
hulupacbiokf_r0462
[bp]
[bp]

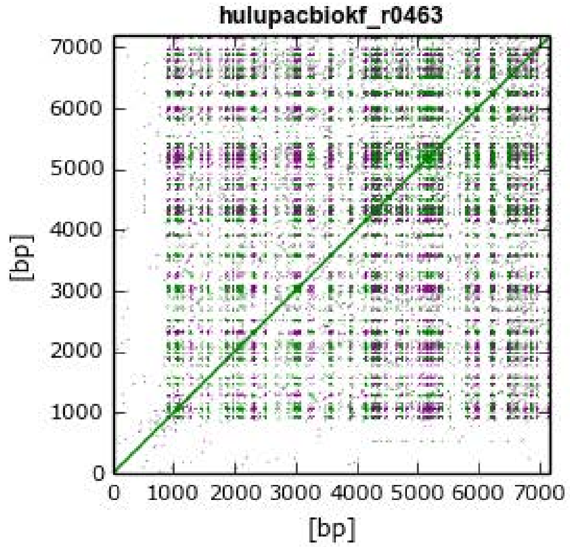
hulupacbiokf_r0463
[bp]
[bp]

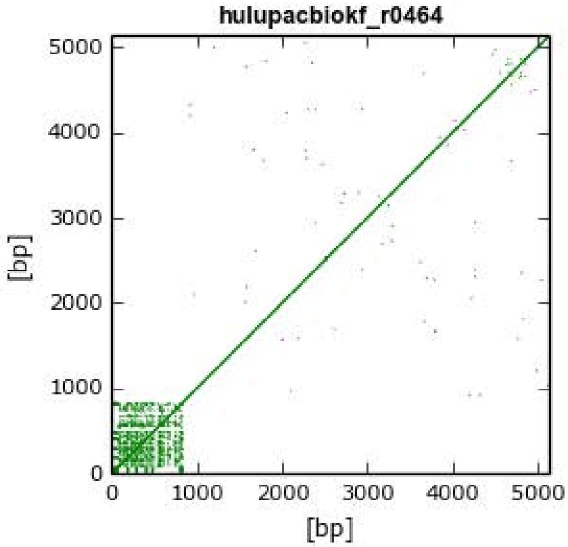
hulupacbiokf_r0464
[bp]
[bp]

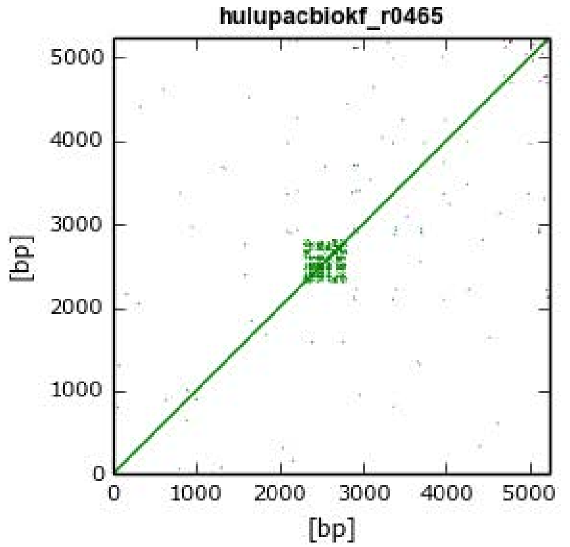
hulupacbiokf_r0465
[bp]
[bp]

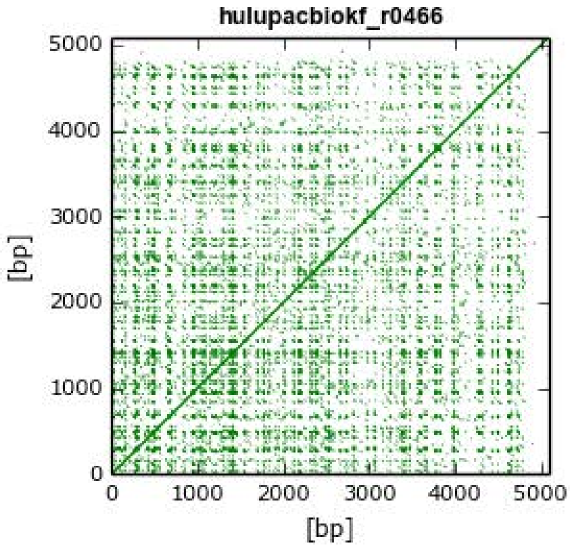
hulupacbiokf_r0466
[bp]
[bp]

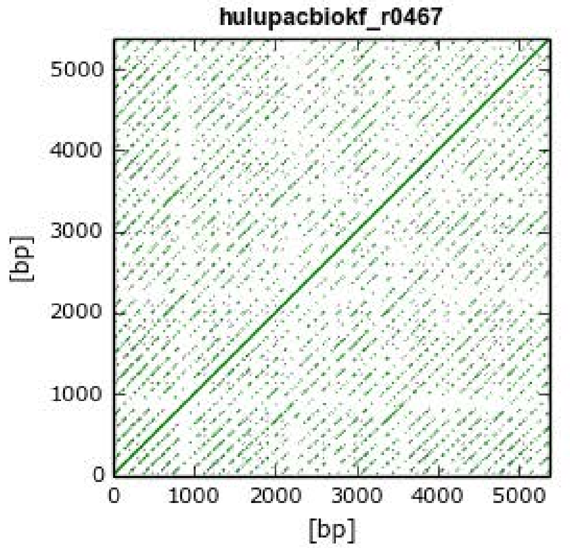
hulupacbiokf_r0467
[bp]
[bp]

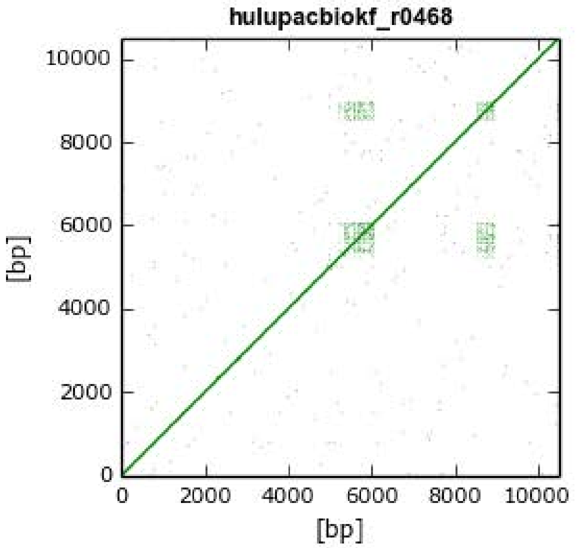
hulupacbiokf_r0468
[bp]
[bp]

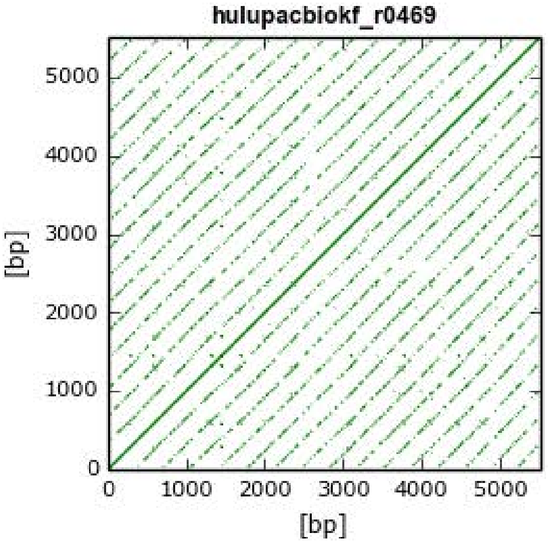
hulupacbiokf_r0469
[bp]
[bp]

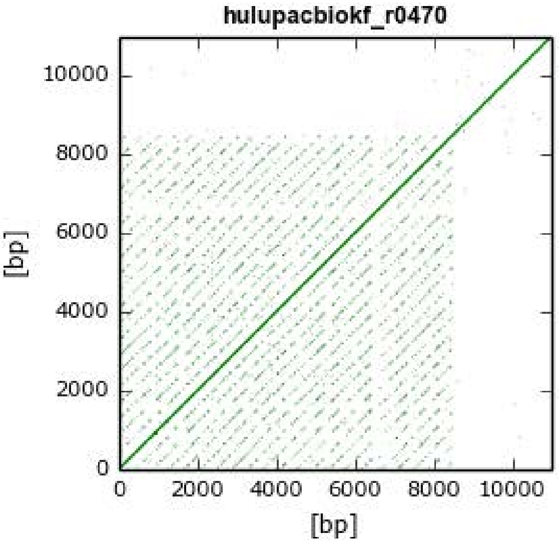
hulupacbiokf_r0470
[bp]
[bp]

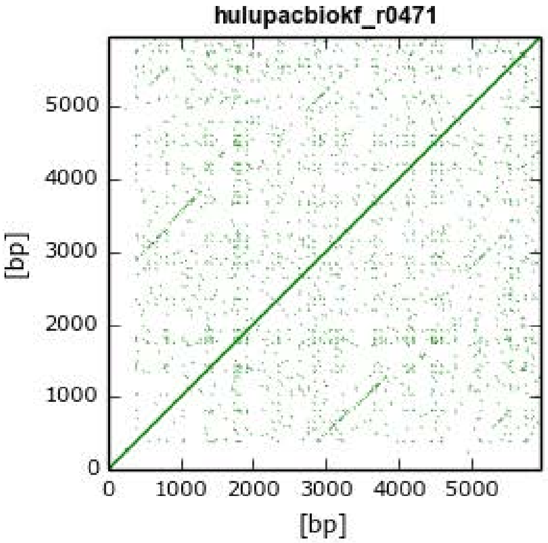
hulupacbiokf_r0471
[bp]
[bp]

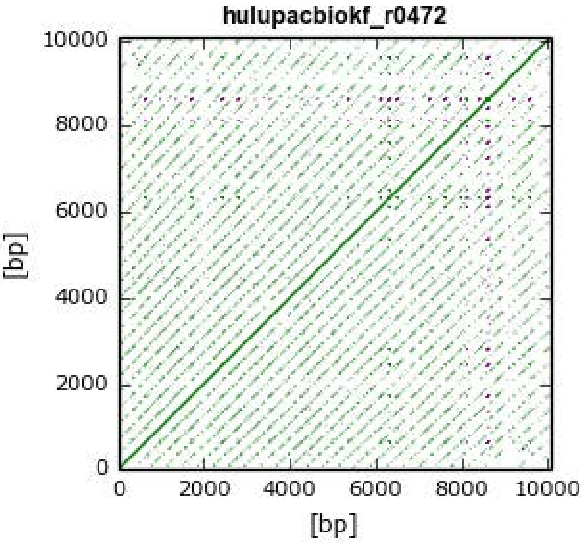
hulupacbiokf_r0472
[bp]
[bp]

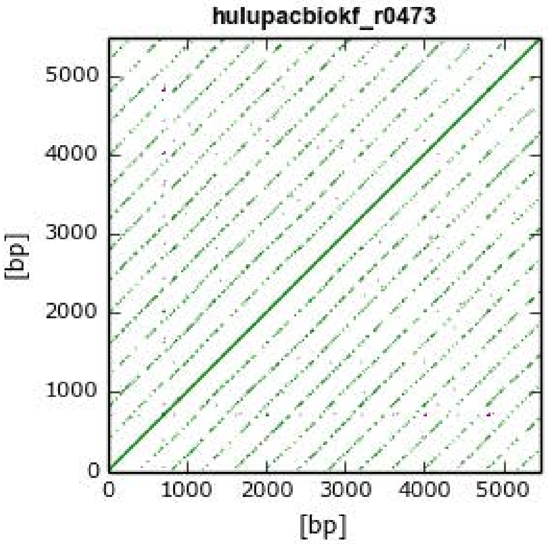
hulupacbiokf_r0473
[bp]
[bp]

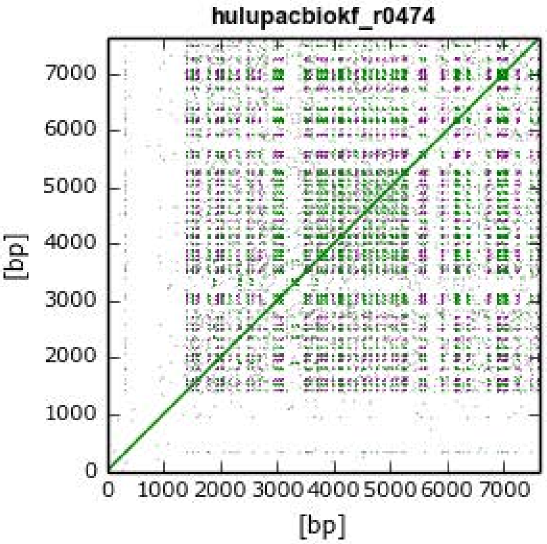
hulupacbiokf_r0474
[bp]
[bp]

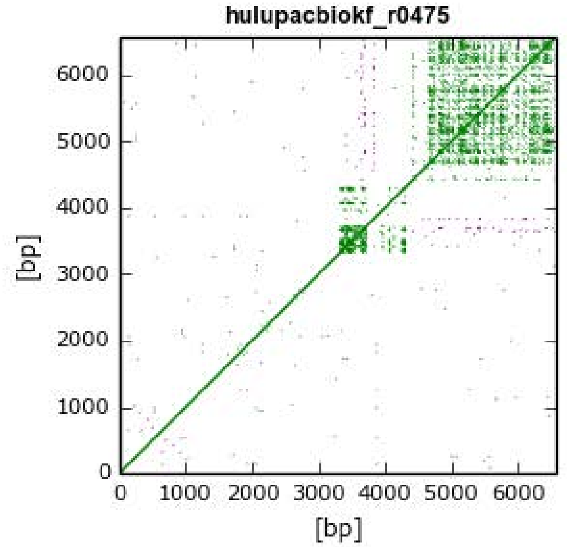
hulupacbiokf_r0475
[bp]
[bp]

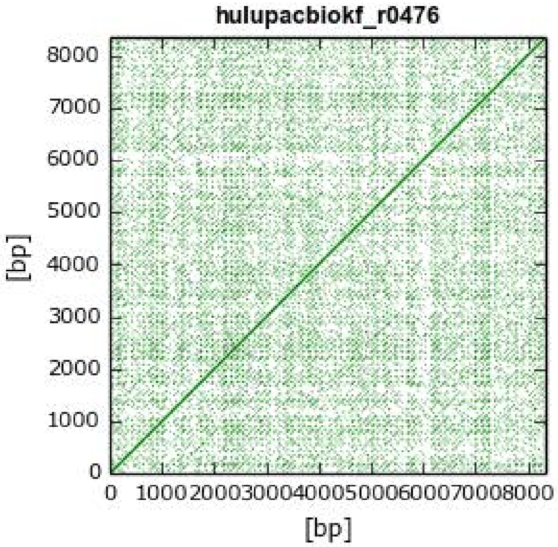
hulupacbiokf_r0476
[bp]
[bp]

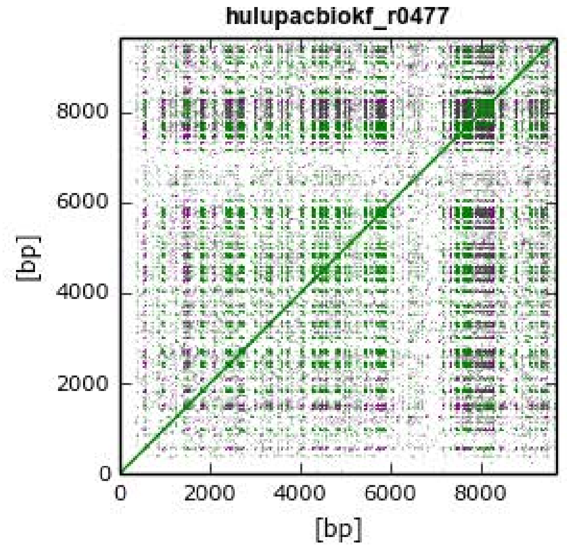
hulupacbiokf_r0477
[bp]
[bp]

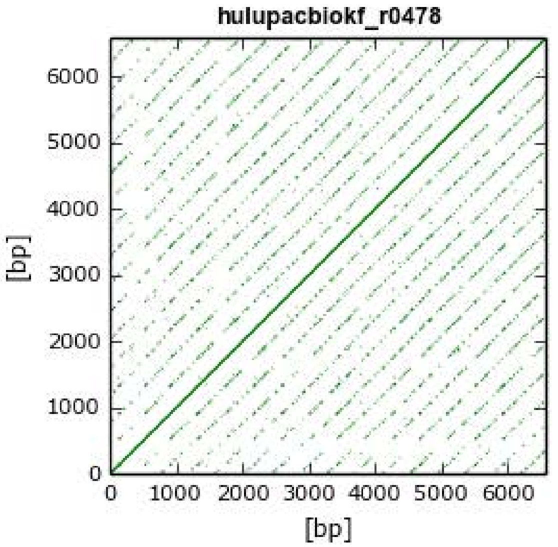
hulupacbiokf_r0478
[bp]
[bp]

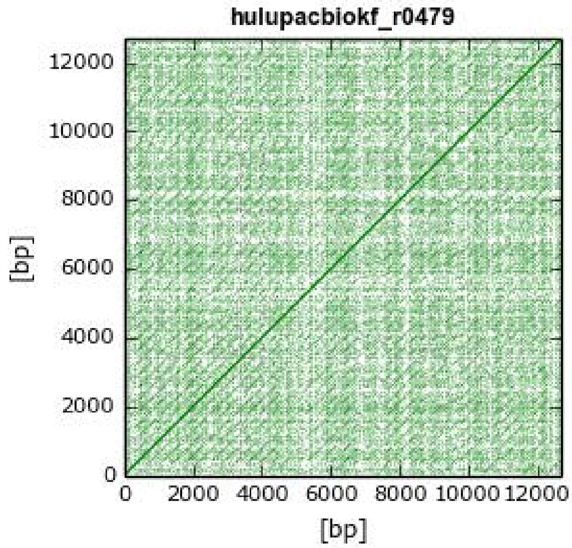
hulupacbiokf_r0479
[bp]
[bp]

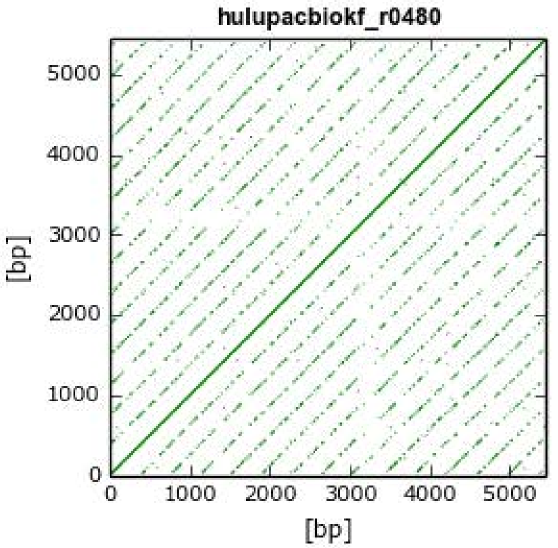
hulupacbiokf_r0480
[bp]
[bp]

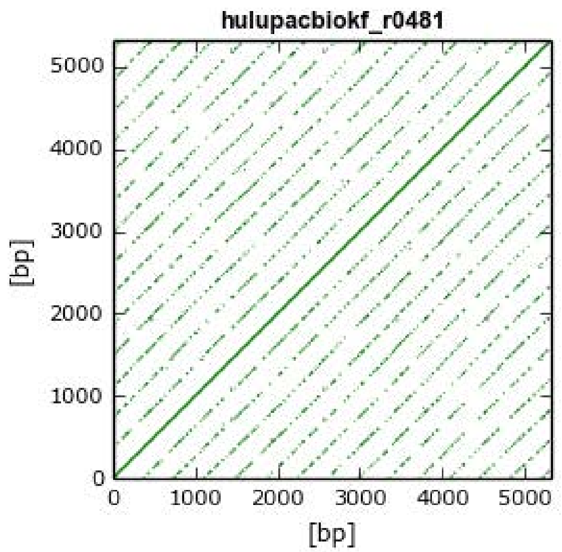
hulupacbiokf_r0481
[bp]
[bp]

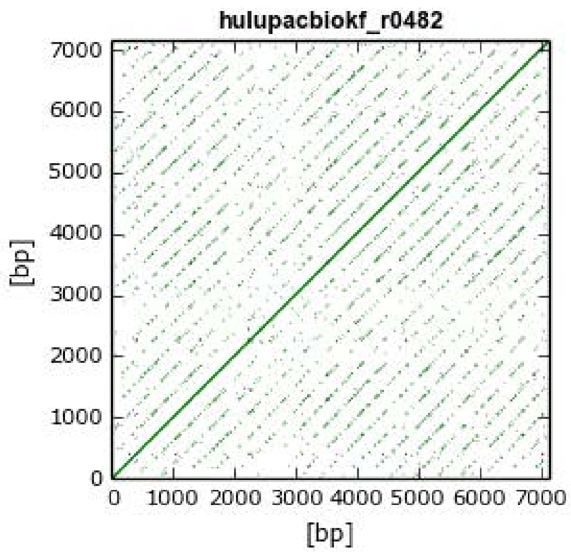
hulupacbiokf_r0482
[bp]
[bp]

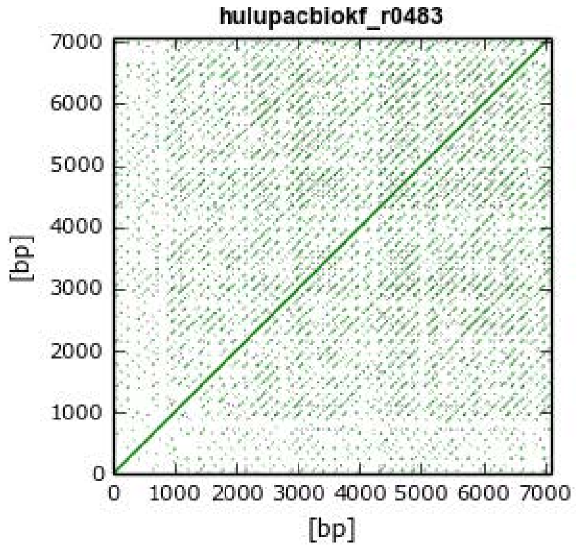
hulupacbiokf_r0483
[bp]
[bp]

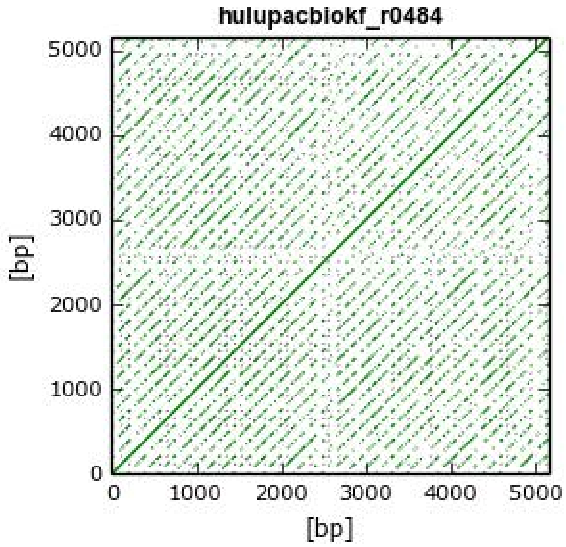
hulupacbiokf_r0484
[bp]
[bp]

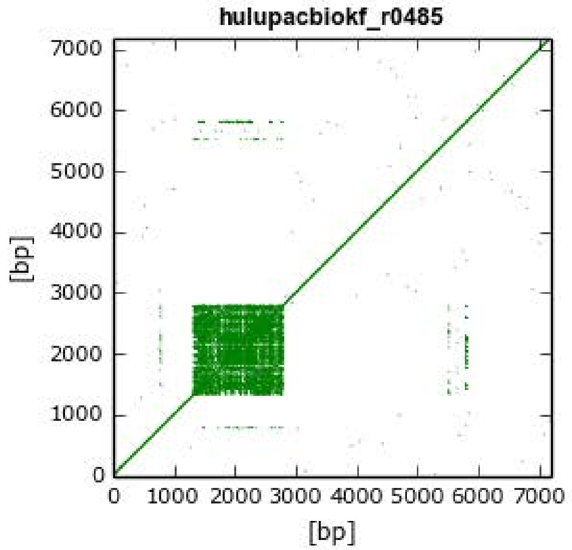
hulupacbiokf_r0485
[bp]
[bp]

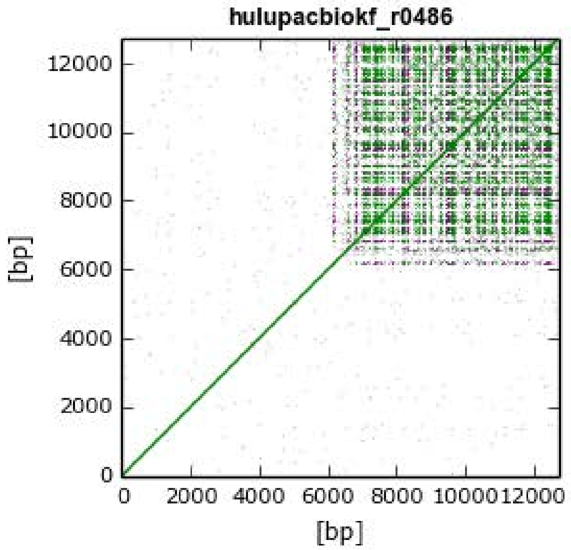
hulupacbiokf_r0486
[bp]
[bp]

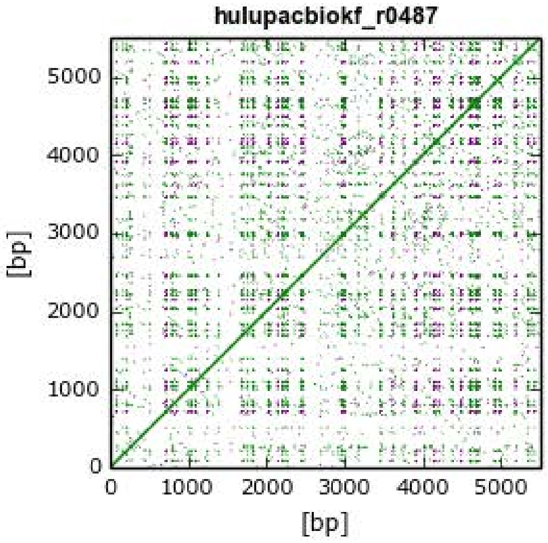
hulupacbiokf_r0487
[bp]
[bp]

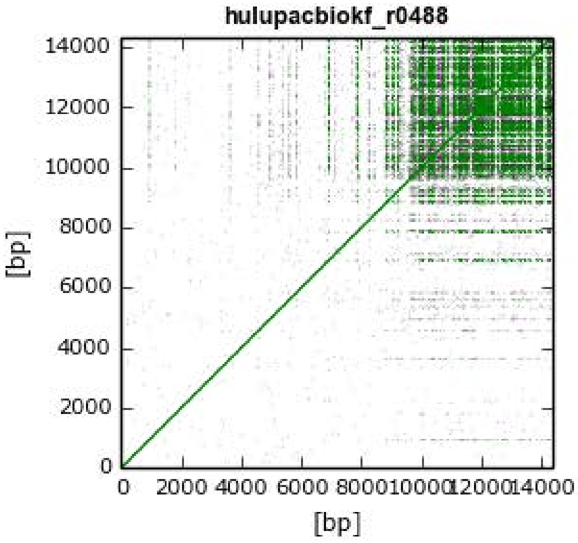
hulupacbiokf_r0488
[bp]
[bp]

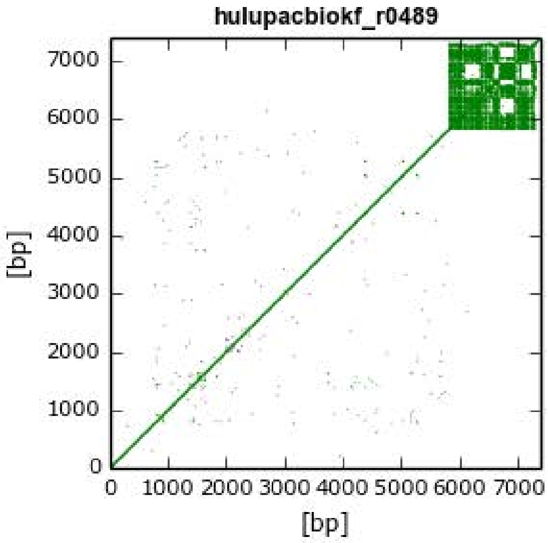
hulupacbiokf_r0489
[bp]
[bp]

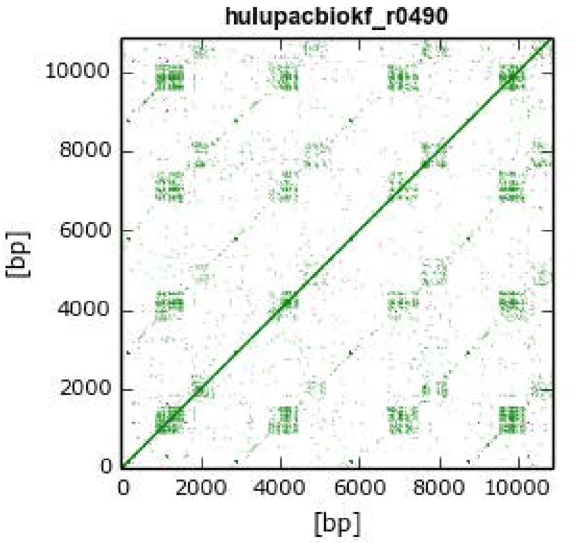
hulupacbiokf_r0490
[bp]
[bp]

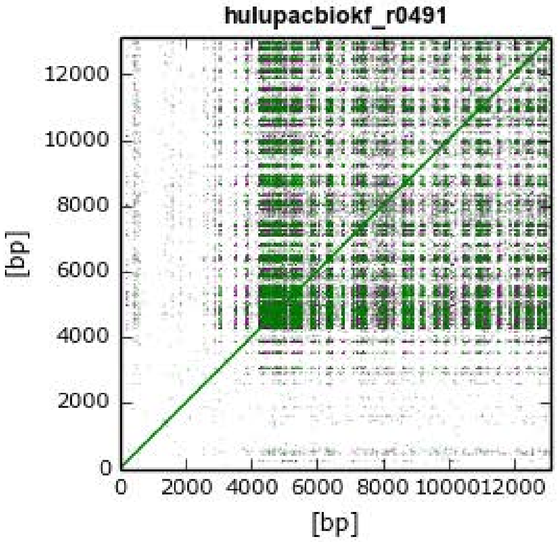
hulupacbiokf_r0491
[bp]
[bp]

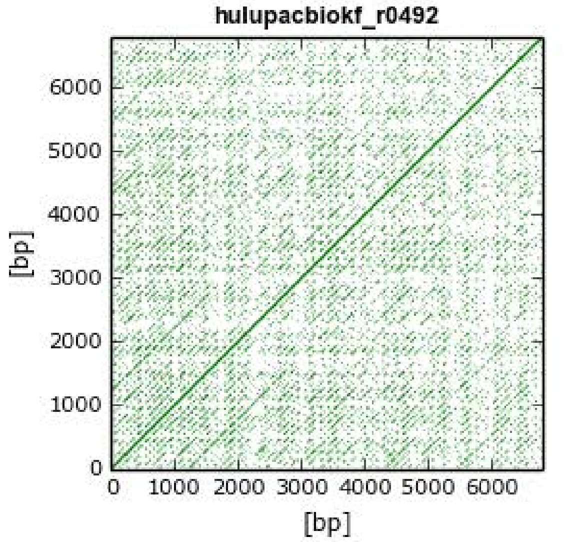
hulupacbiokf_r0492
[bp]
[bp]

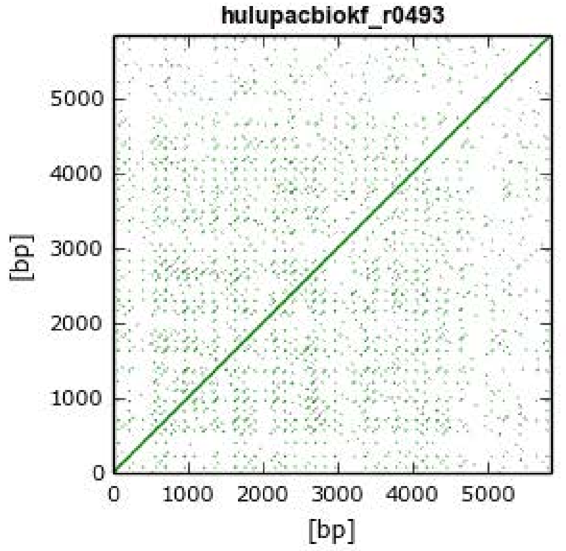
hulupacbiokf_r0493
[bp]
[bp]

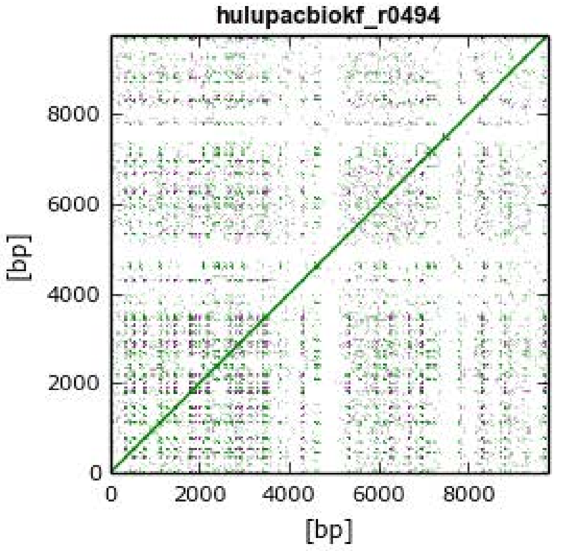
hulupacbiokf_r0494
[bp]
[bp]

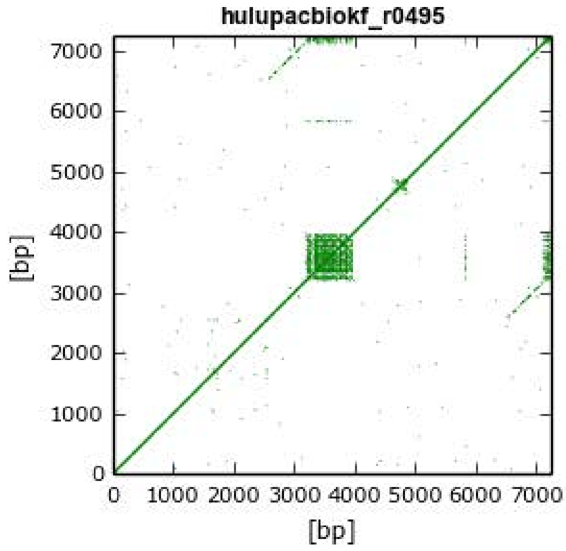
hulupacbiokf_r0495
[bp]
[bp]

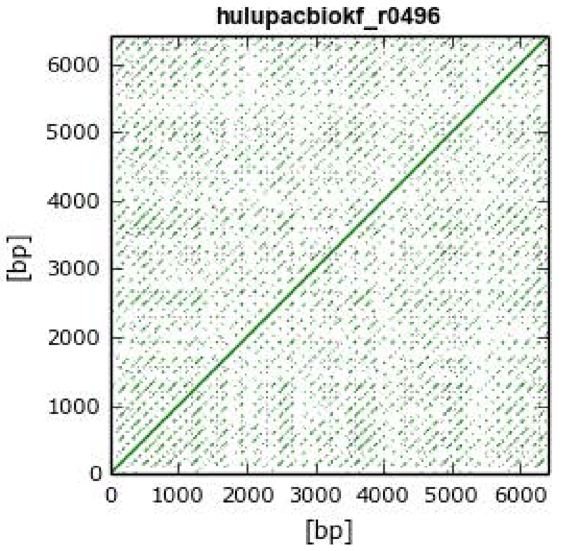
hulupacbiokf_r0496
[bp]
[bp]

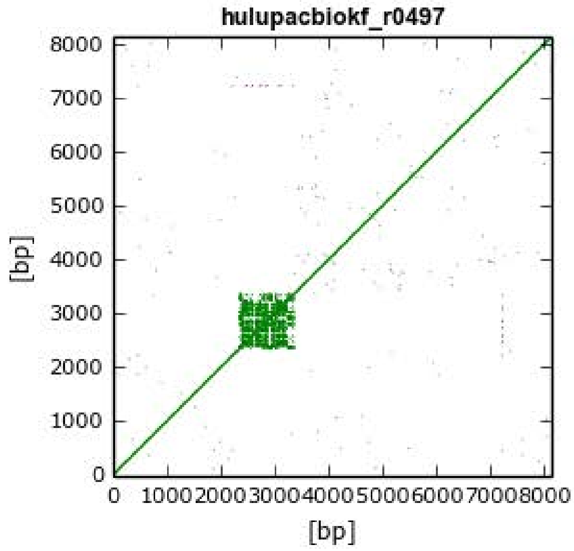
hulupacbiokf_r0497
[bp]
[bp]

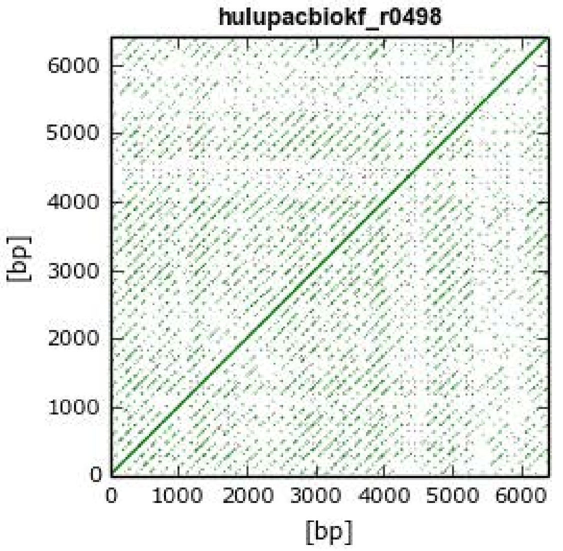
hulupacbiokf_r0498
[bp]
[bp]

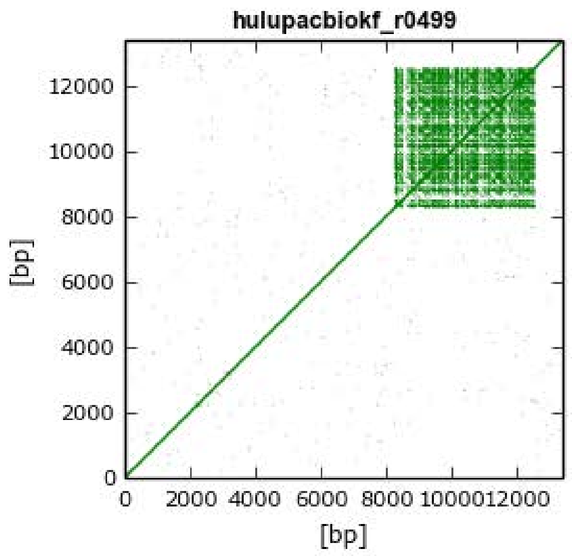
hulupacbiokf_r0499
[bp]
[bp]

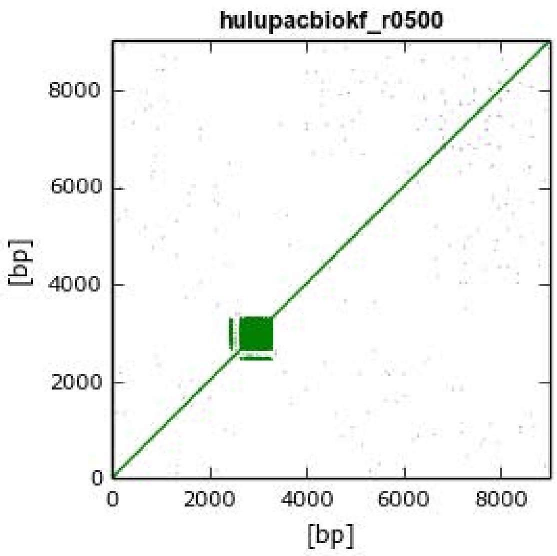
hulupacbiokf_r0500
[bp]
[bp]

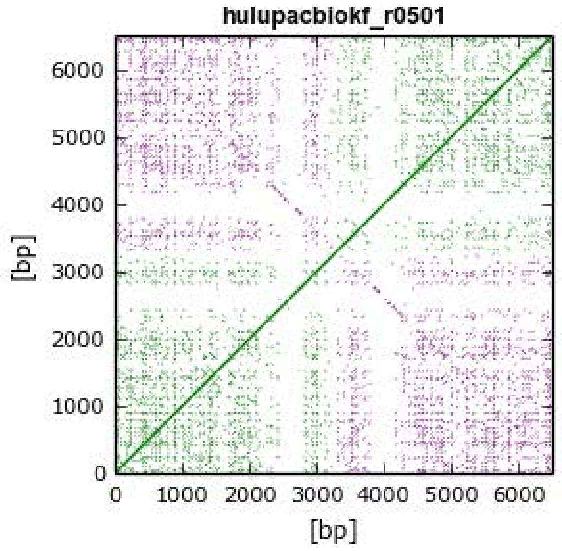
hulupacbiokf_r0501
[bp]
[bp]

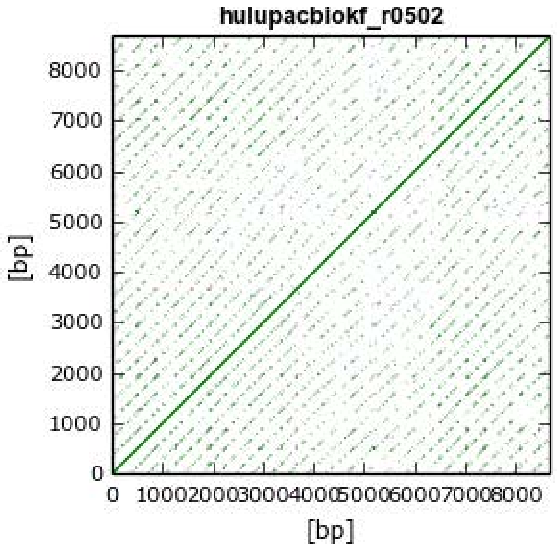
hulupacbiokf_r0502
[bp]
[bp]

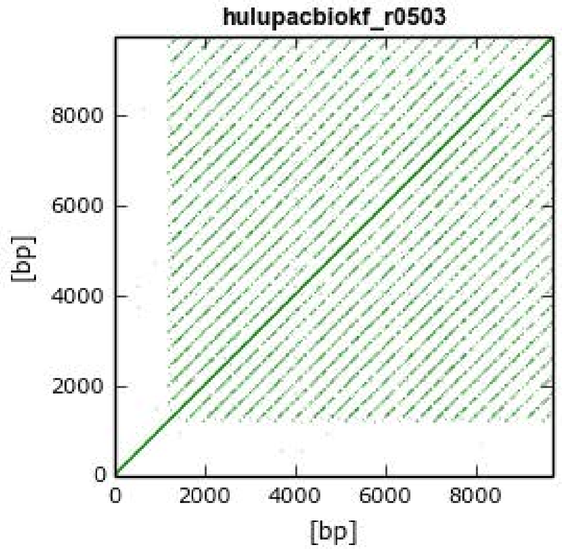
hulupacbiokf_r0503
[bp]
[bp]

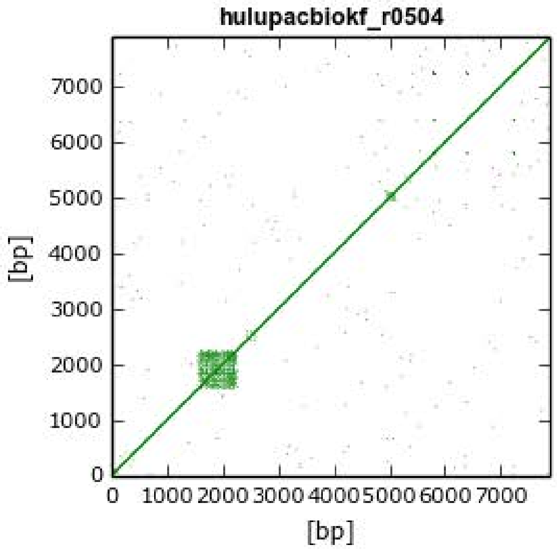
hulupacbiokf_r0504
[bp]
[bp]

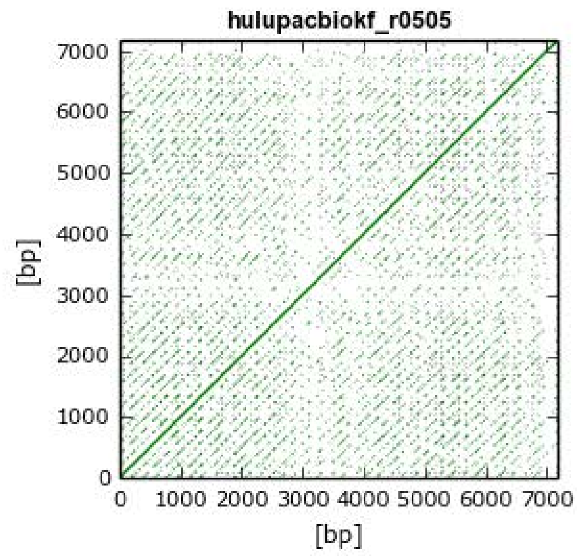
hulupacbiokf_r0505
[bp]
[bp]

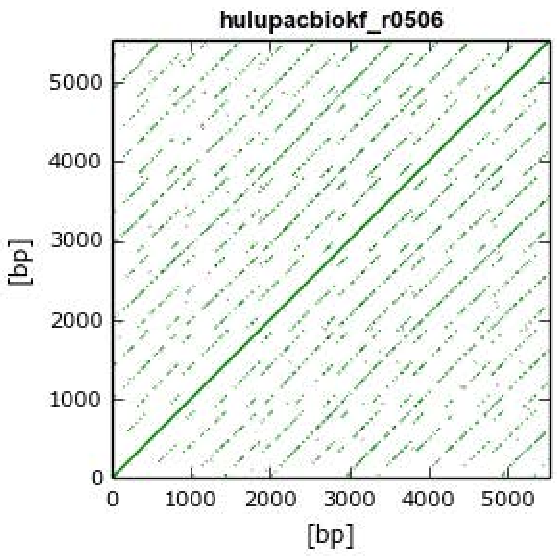
hulupacbiokf_r0506
[bp]
[bp]

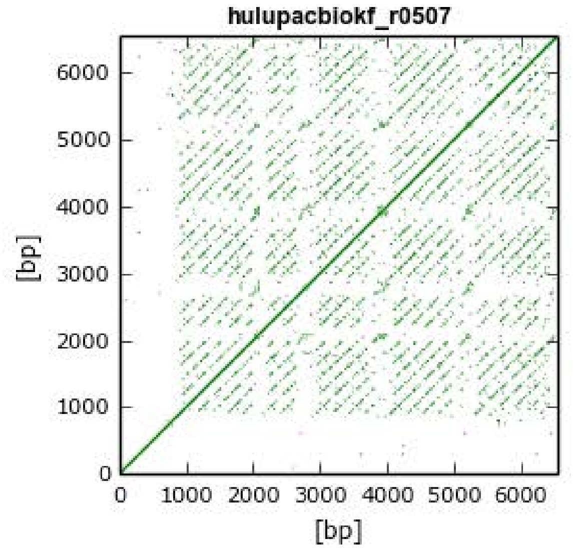
hulupacbiokf_r0507
[bp]
[bp]

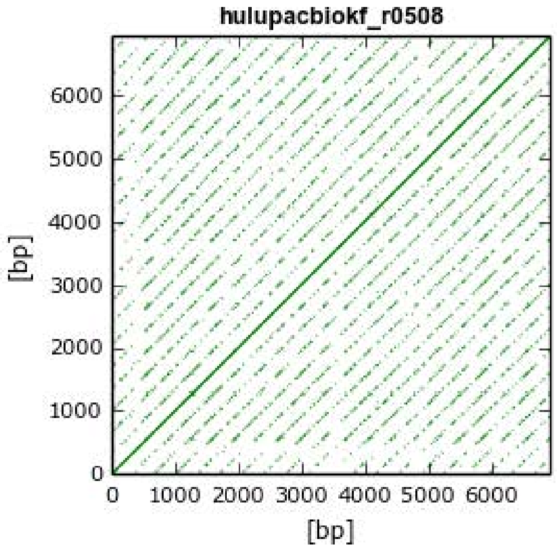
hulupacbiokf_r0508
[bp]
[bp]

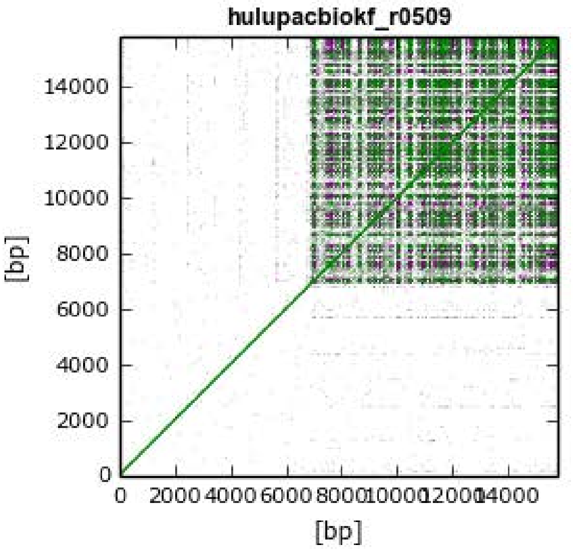
hulupacbiokf_r0509
[bp]
[bp]

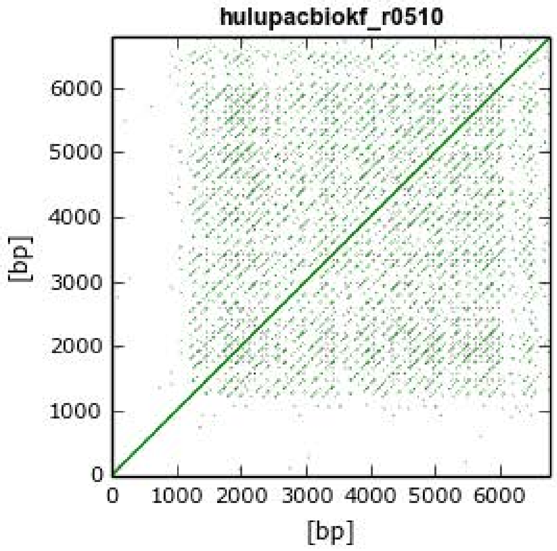
hulupacbiokf_r0510
[bp]
[bp]

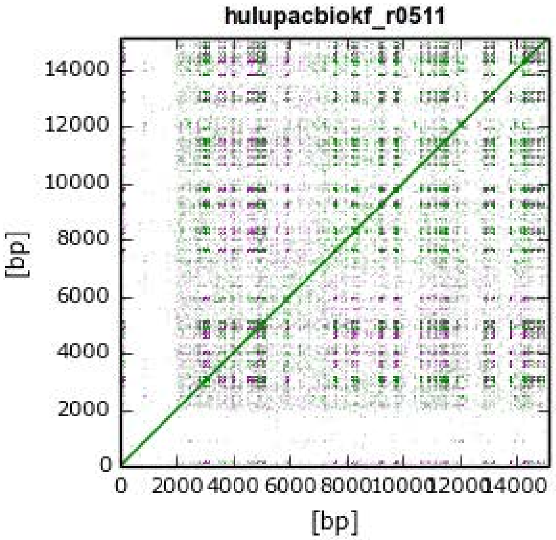
hulupacbiokf_r0511
[bp]
[bp]

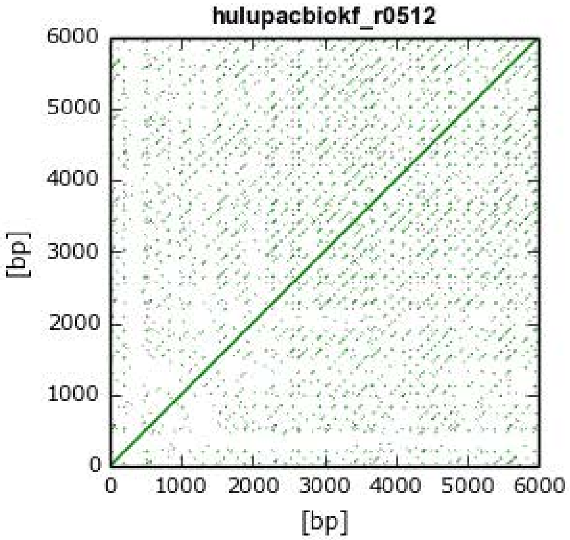
hulupacbiokf_r0512
[bp]
[bp]

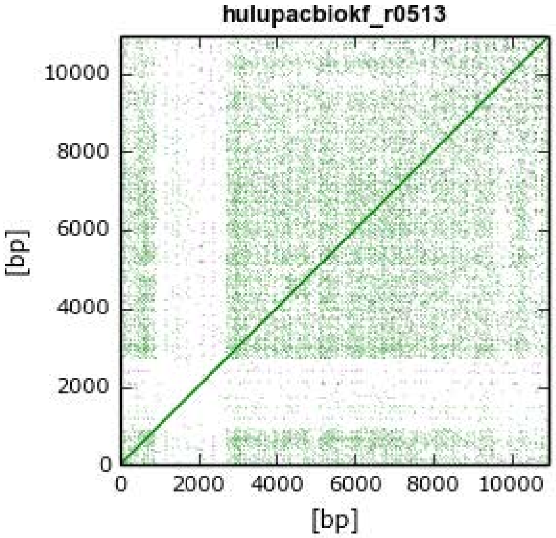
hulupacbiokf_r0513
[bp]
[bp]

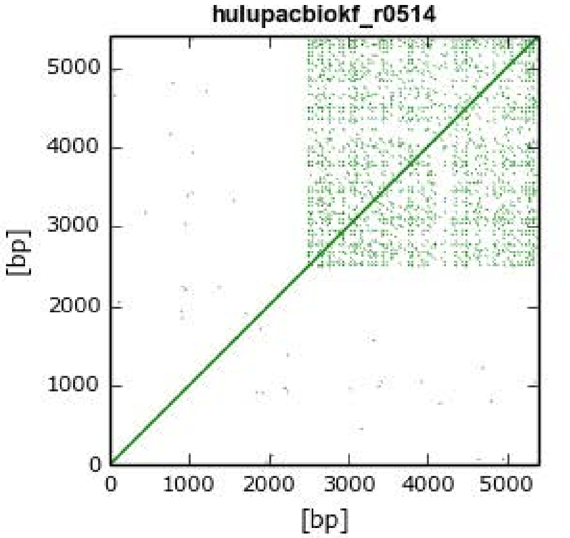
hulupacbiokf_r0514
[bp]
[bp]

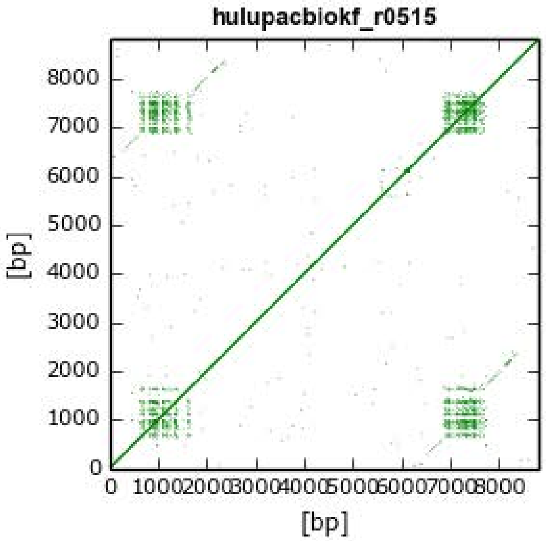
hulupacbiokf_r0515
[bp]
[bp]

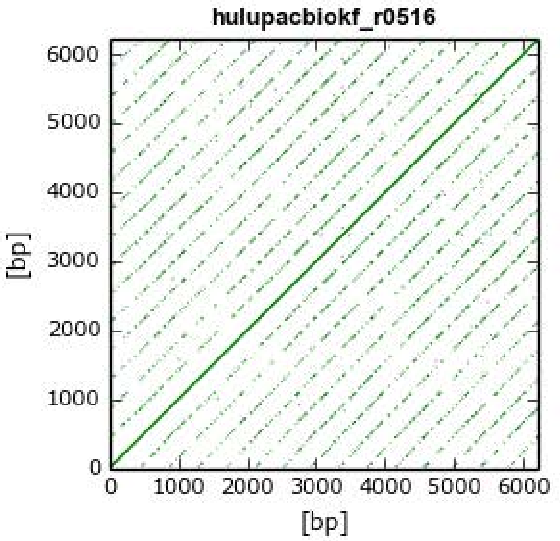
hulupacbiokf_r0516
[bp]
[bp]

hulupacbiokf_r0517

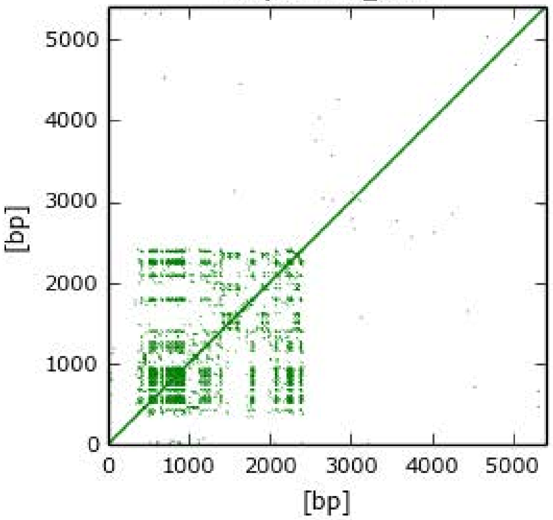

hulupacbiokf_r0518

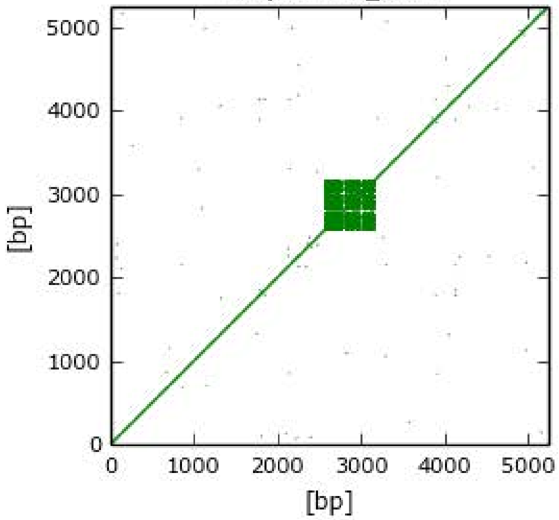

hulupacbiokf_r0519

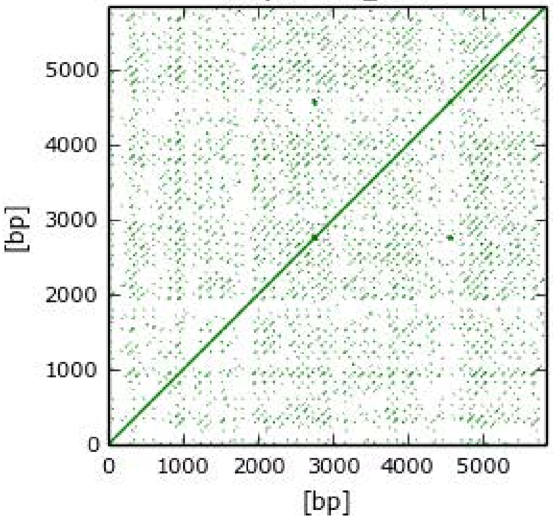

hulupacbiokf_r0520

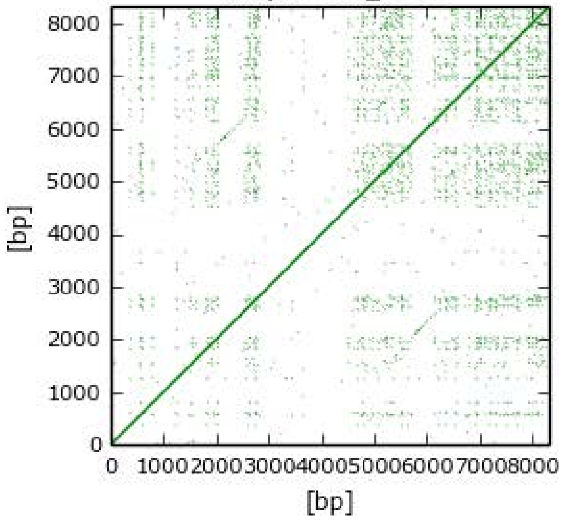

hulupacbiokf_r0521

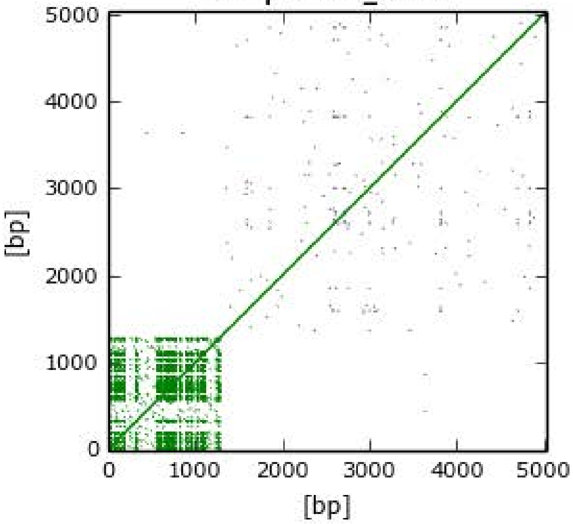

hulupacbiokf_r0522

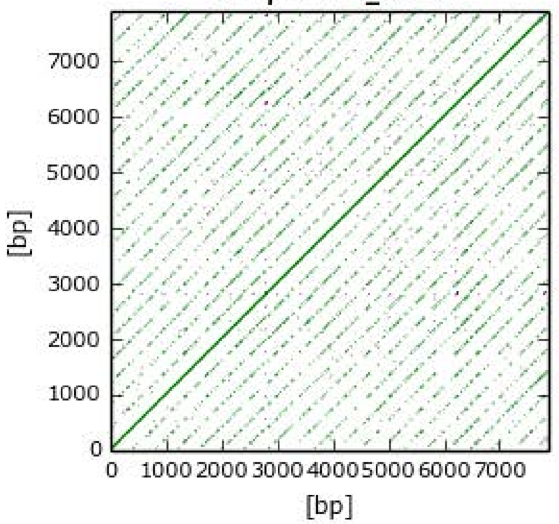

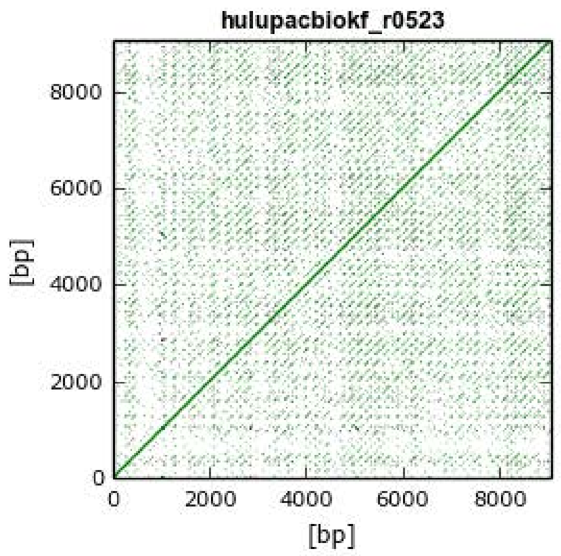
hulupacbiokf_r0523
[bp]
[bp]

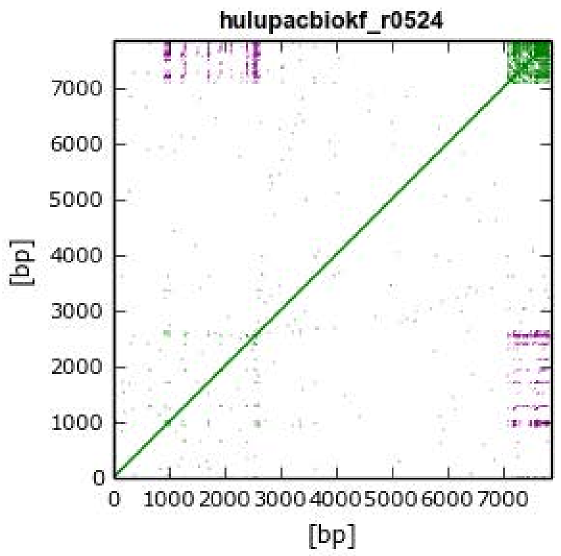
hulupacbiokf_r0524
[bp]
[bp]

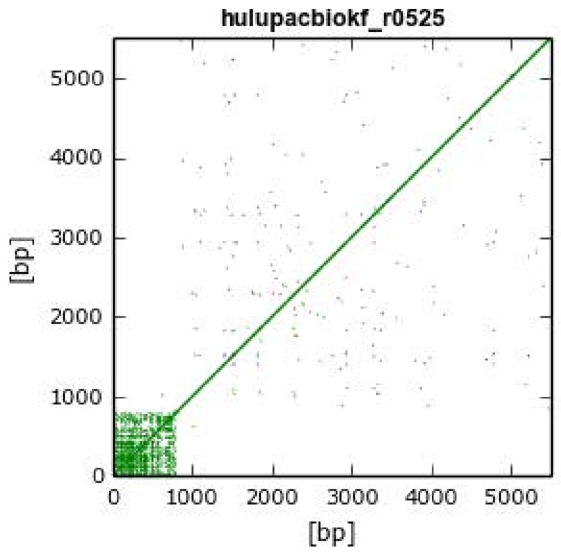
hulupacbiokf_r0525
[bp]
[bp]

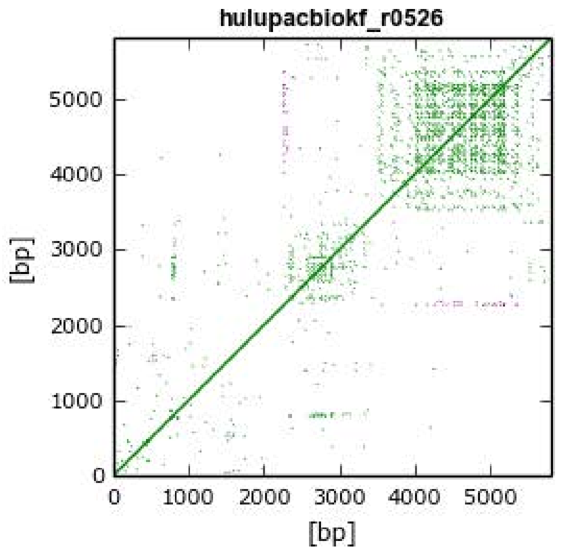
hulupacbiokf_r0526
[bp]
[bp]

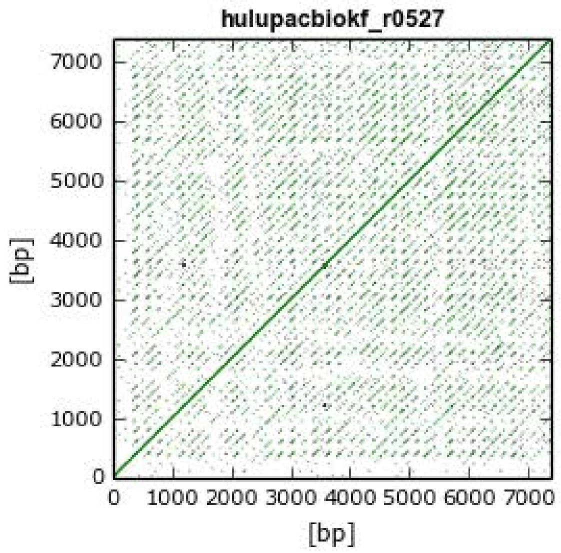
hulupacbiokf_r0527
[bp]
[bp]

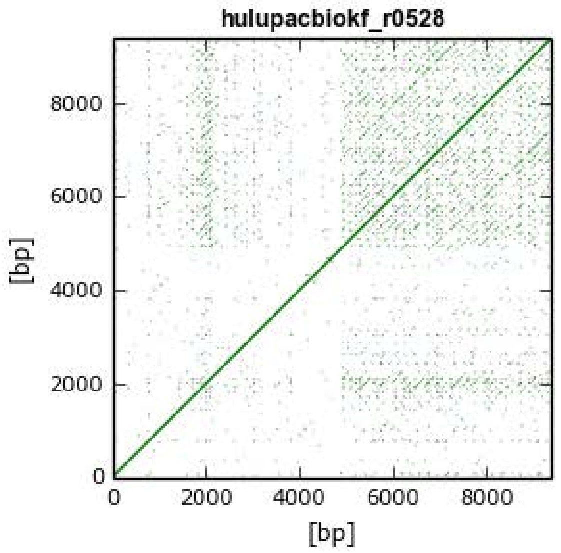
hulupacbiokf_r0528
[bp]
[bp]

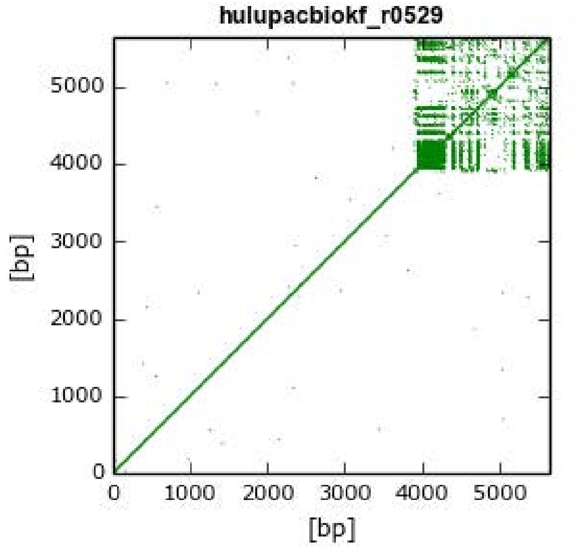
hulupacbiokf_r0529
[bp]
[bp]

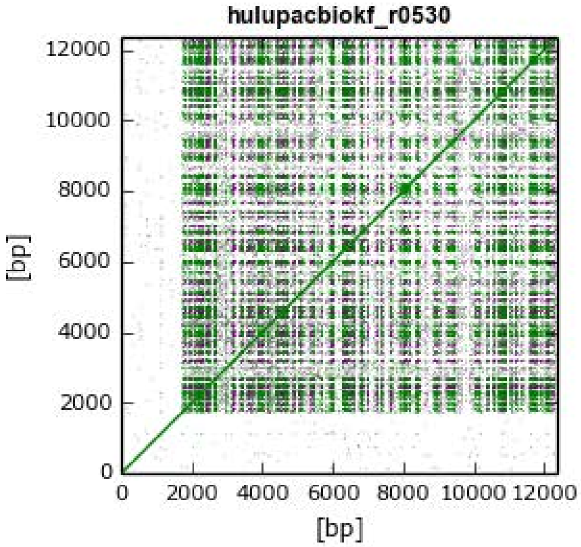
hulupacbiokf_r0530
[bp]
[bp]

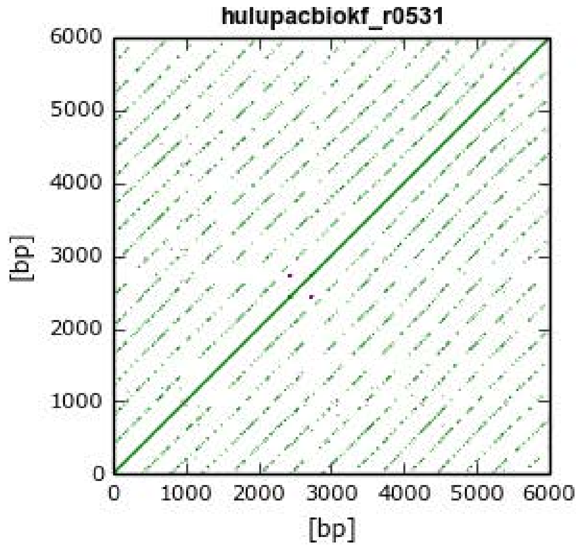
hulupacbiokf_r0531
[bp]
[bp]

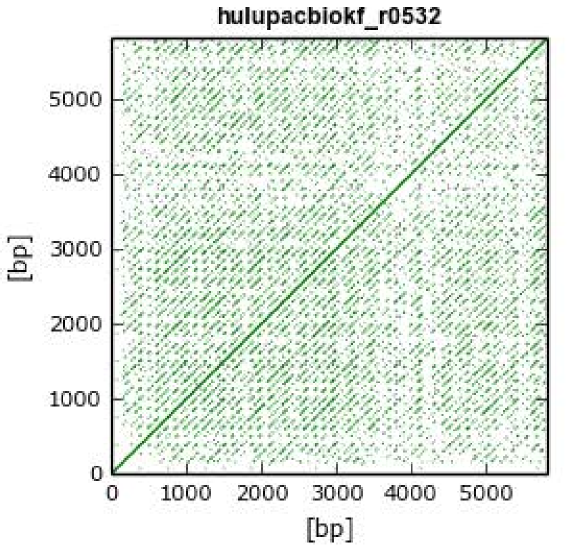
hulupacbiokf_r0532
[bp]
[bp]

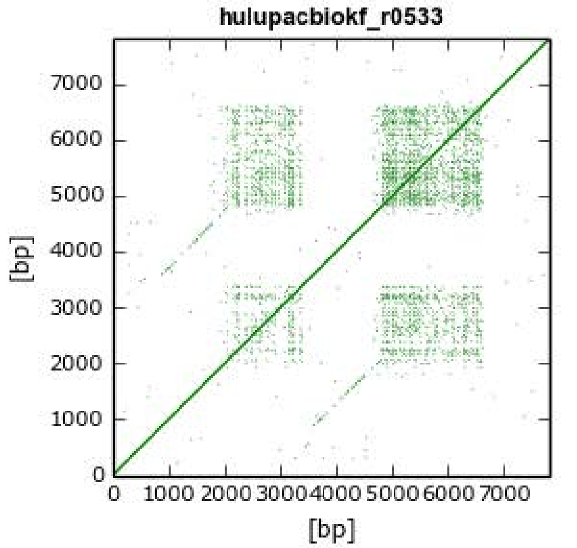
hulupacbiokf_r0533
[bp]
[bp]

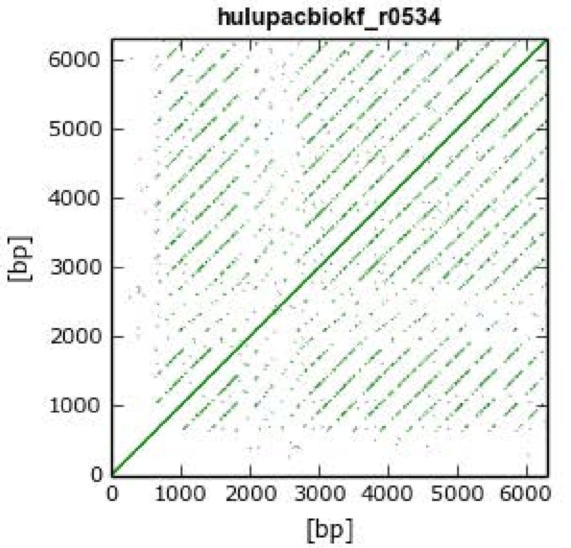
hulupacbiokf_r0534
[bp]
[bp]

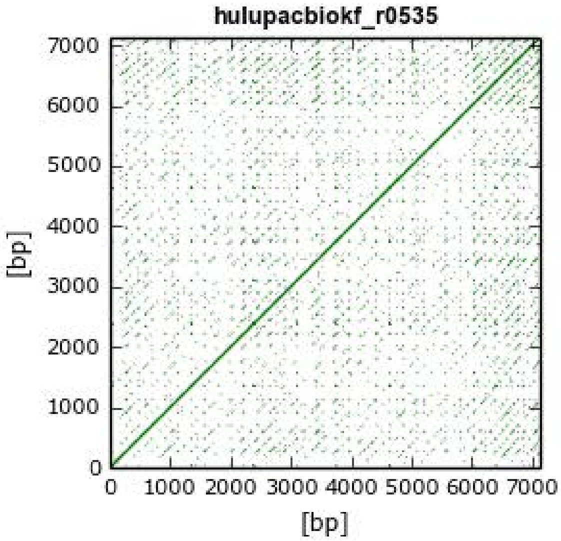
hulupacbiokf_r0535
[bp]
[bp]

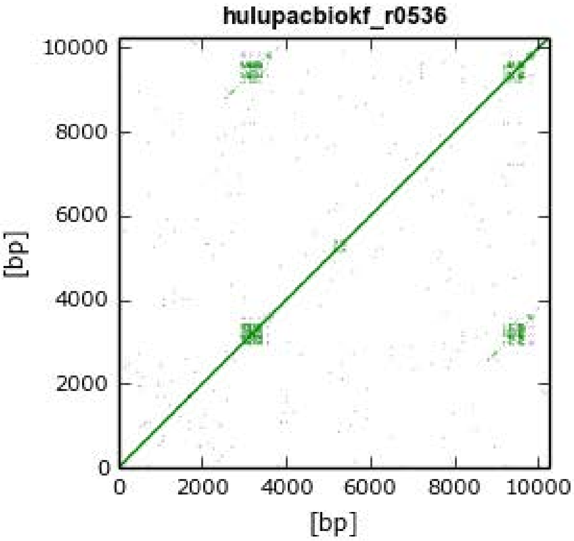
hulupacbiokf_r0536
[bp]
[bp]

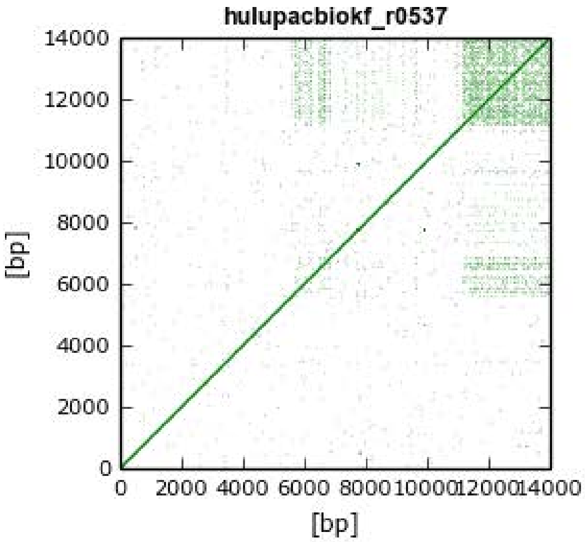
hulupacbiokf_r0537
[bp]
[bp]

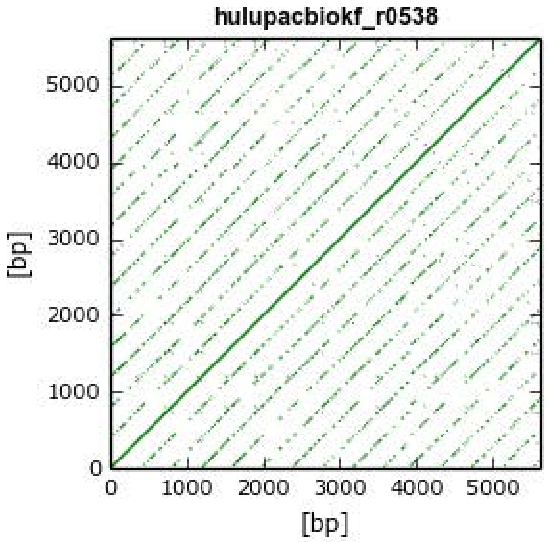
hulupacbiokf_r0538
[bp]
[bp]

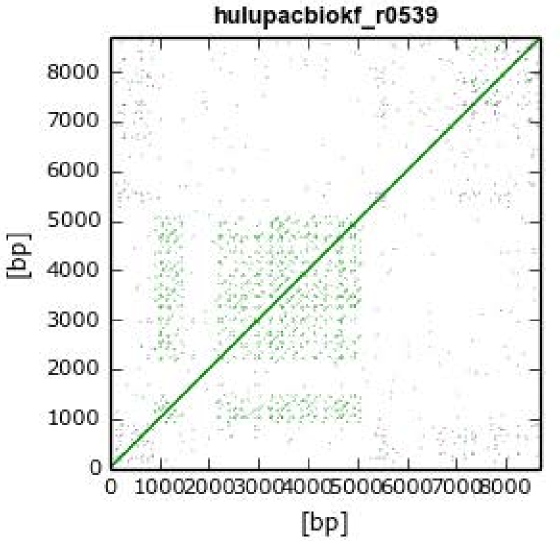
hulupacbiokf_r0539
[bp]
[bp]

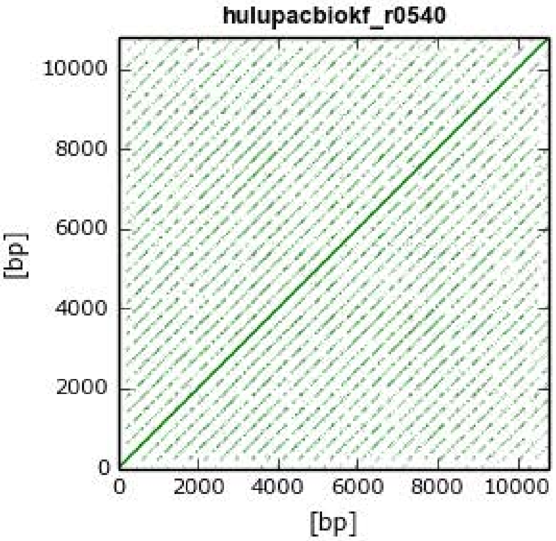
hulupacbiokf_r0540
[bp]
[bp]

HuluTR070 from read r0541
is in GenBank Acc. MN537568

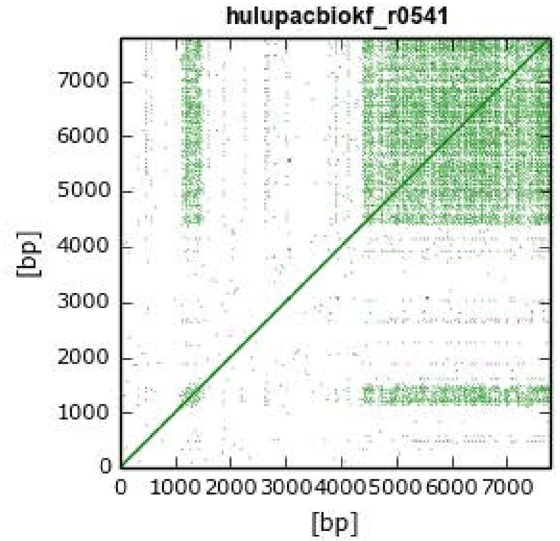

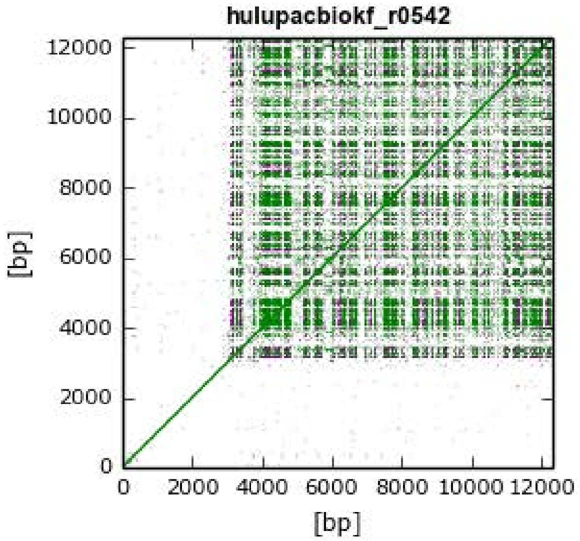

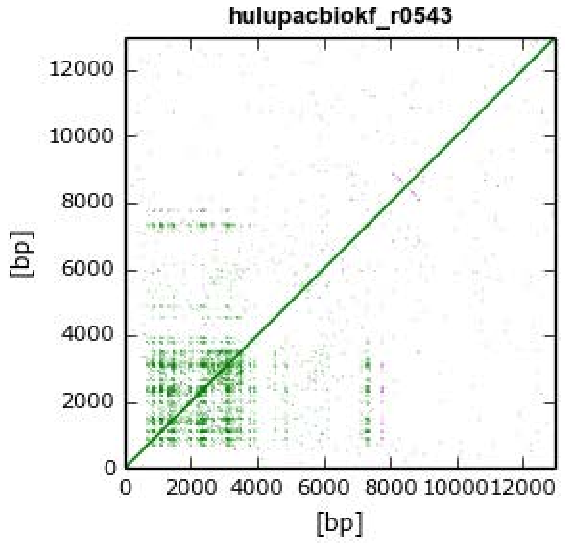

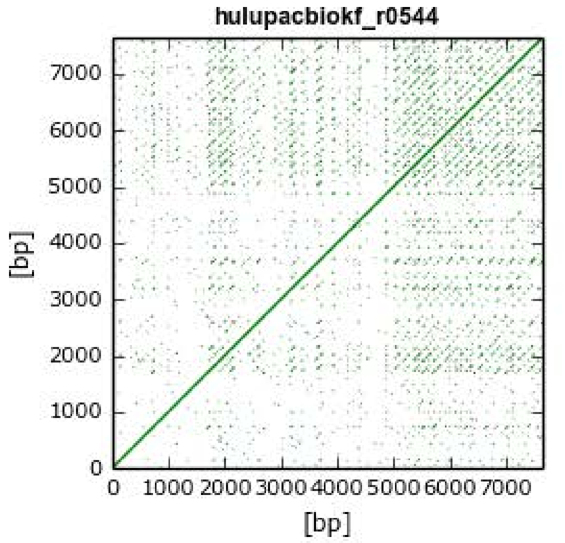

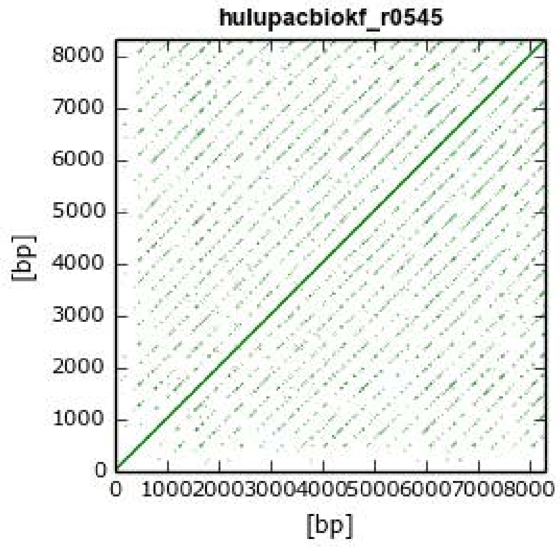

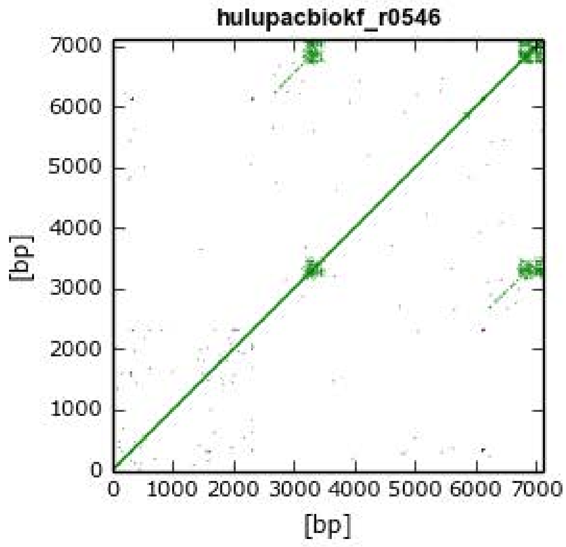

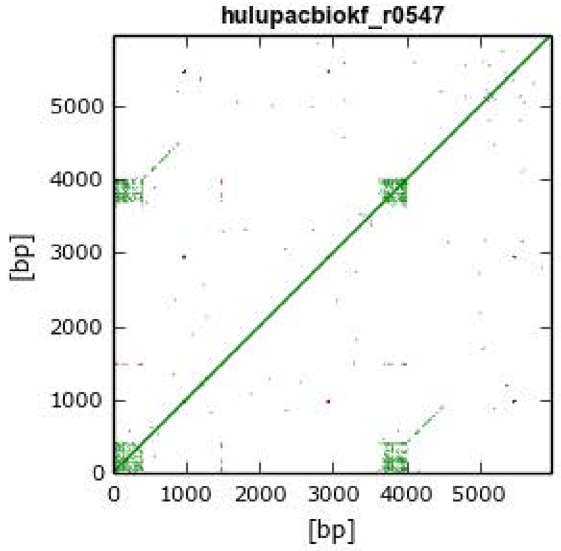
hulupacbiokf_r0547
[bp]
[bp]

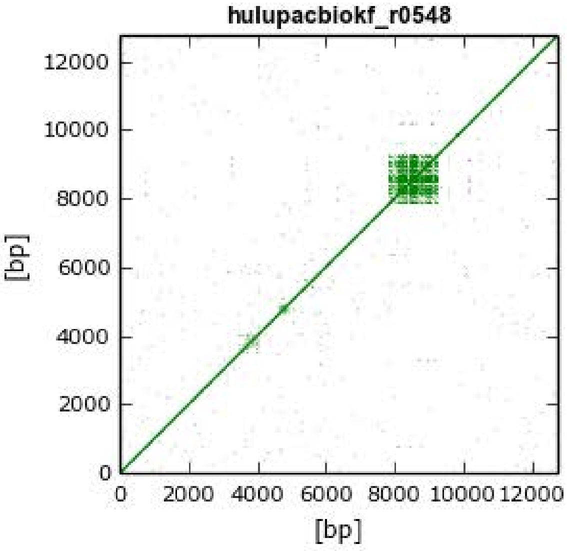
hulupacbiokf_r0548
[bp]
[bp]

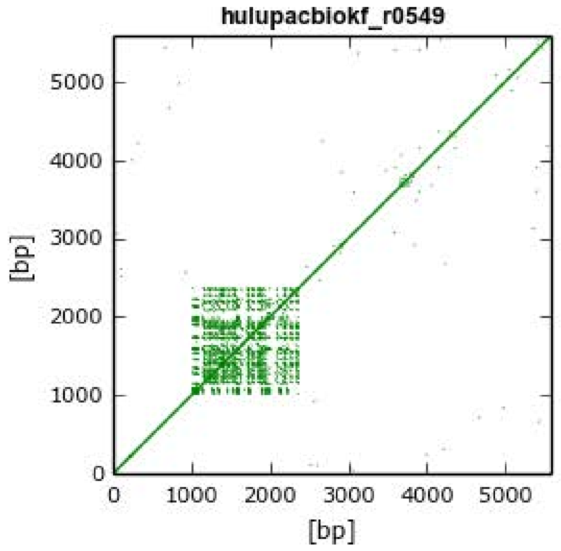
hulupacbiokf_r0549
[bp]
[bp]

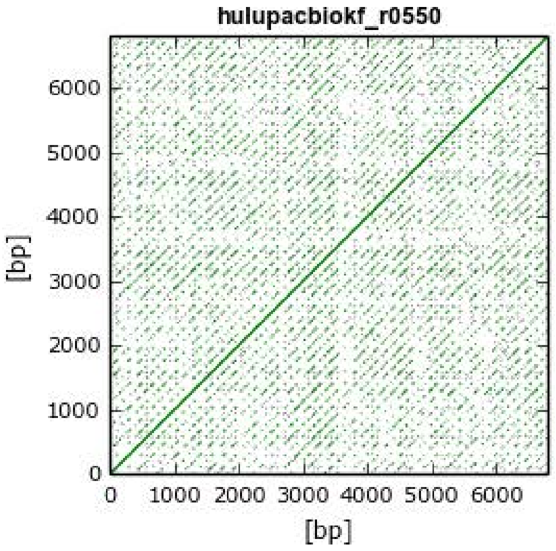
hulupacbiokf_r0550
[bp]
[bp]

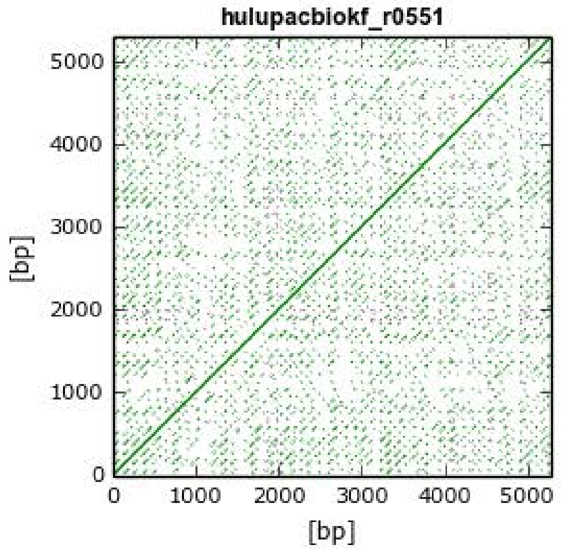
hulupacbiokf_r0551
[bp]
[bp]

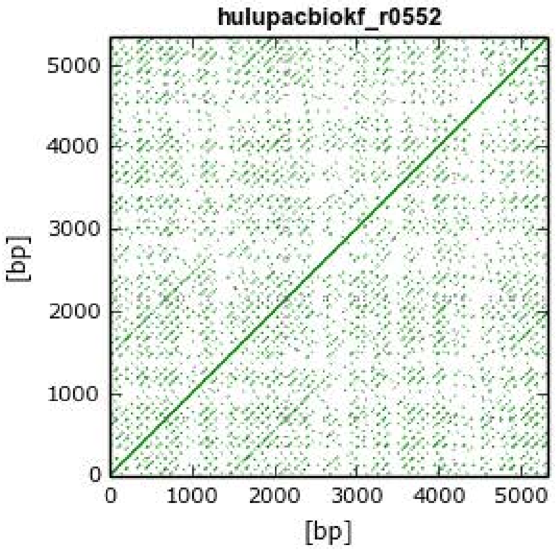
hulupacbiokf_r0552
[bp]
[bp]

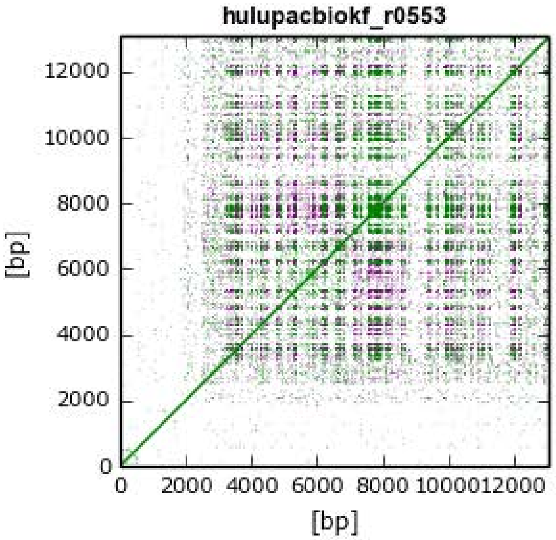
hulupacbiokf_r0553
[bp]
[bp]

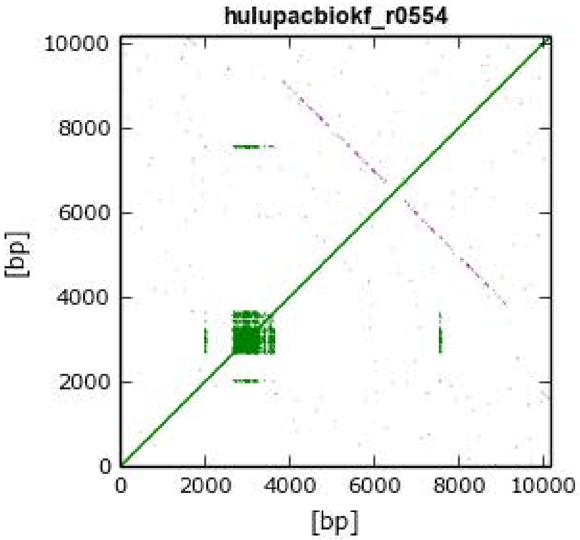
hulupacbiokf_r0554
[bp]
[bp]

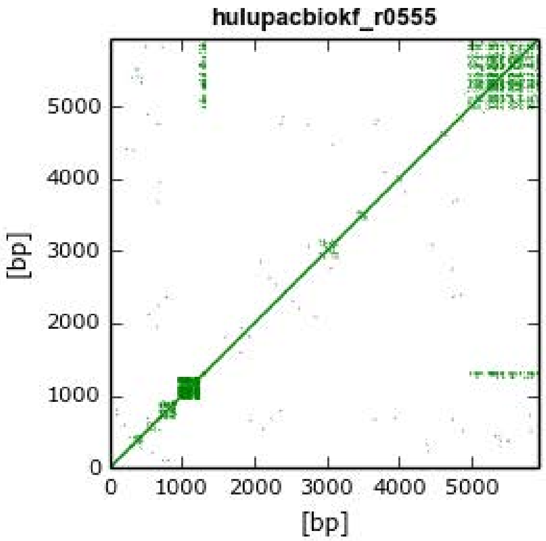
hulupacbiokf_r0555
[bp]
[bp]

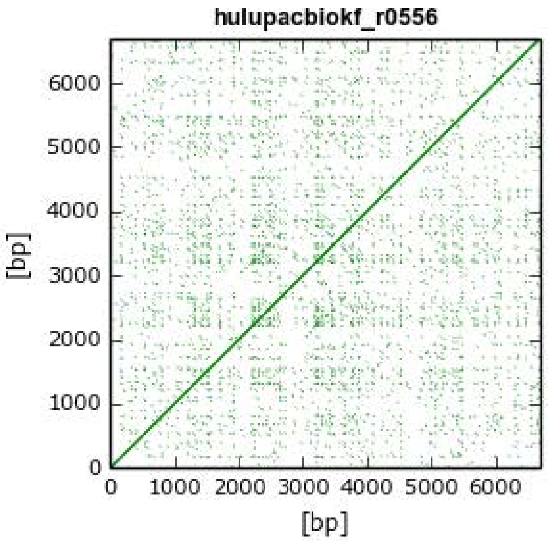
hulupacbiokf_r0556
[bp]
[bp]

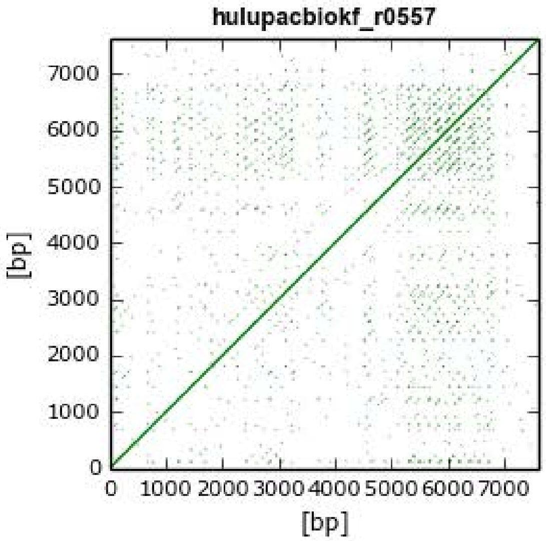
hulupacbiokf_r0557
[bp]
[bp]

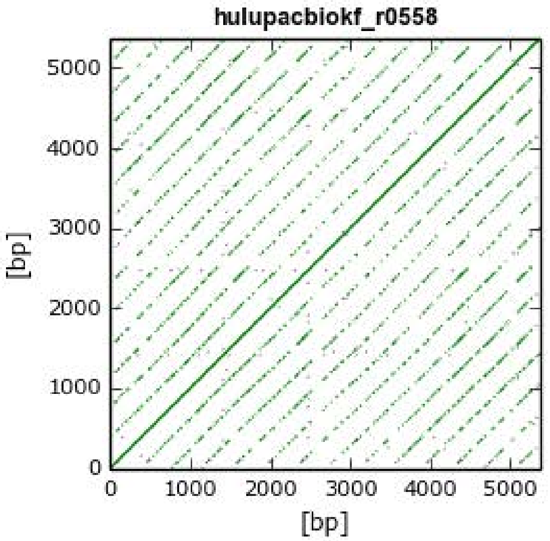
hulupacbiokf_r0558
[bp]
[bp]

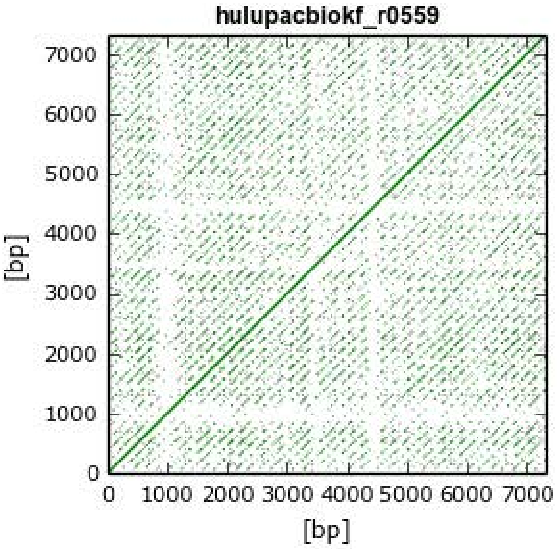
hulupacbiokf_r0559
[bp]
[bp]

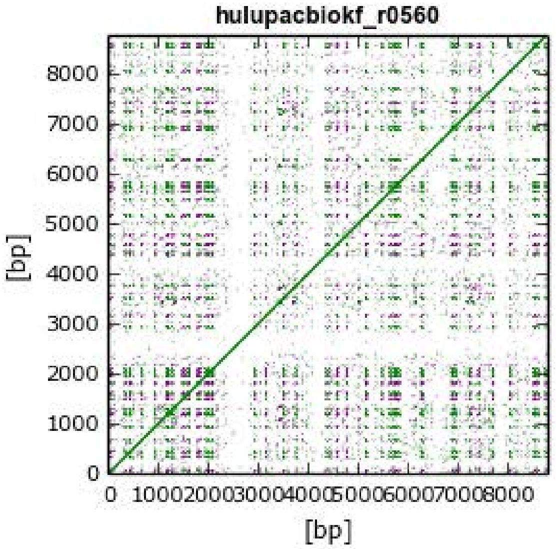
hulupacbiokf_r0560
[bp]
[bp]

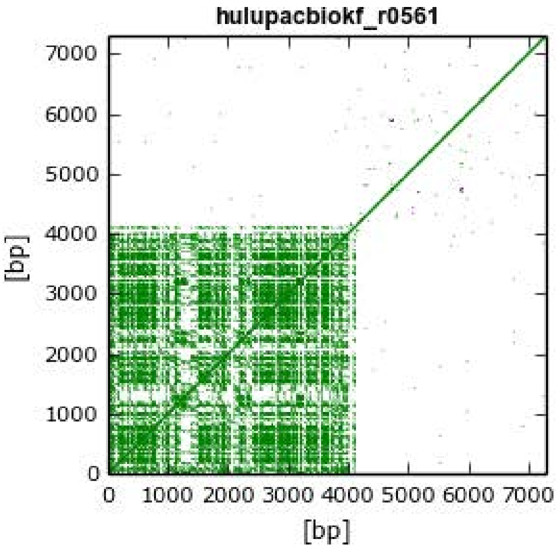
hulupacbiokf_r0561
[bp]
[bp]

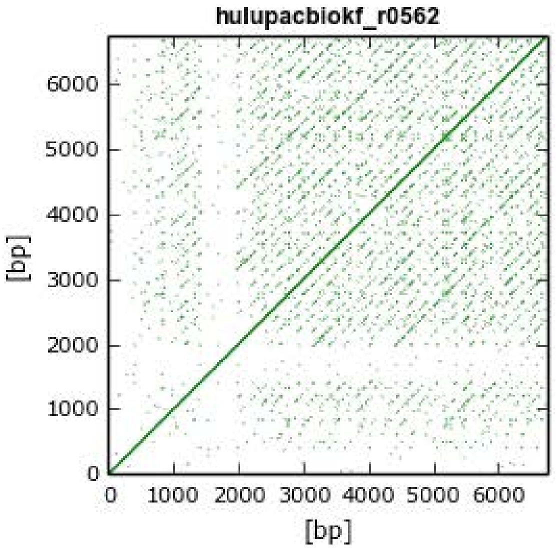
hulupacbiokf_r0562
[bp]
[bp]

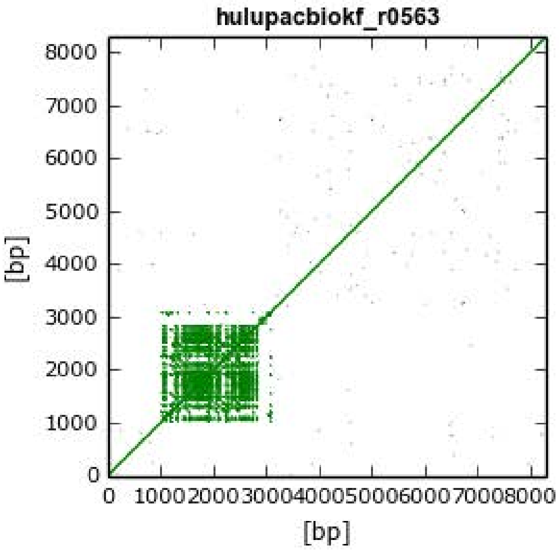
hulupacbiokf_r0563
[bp]
[bp]

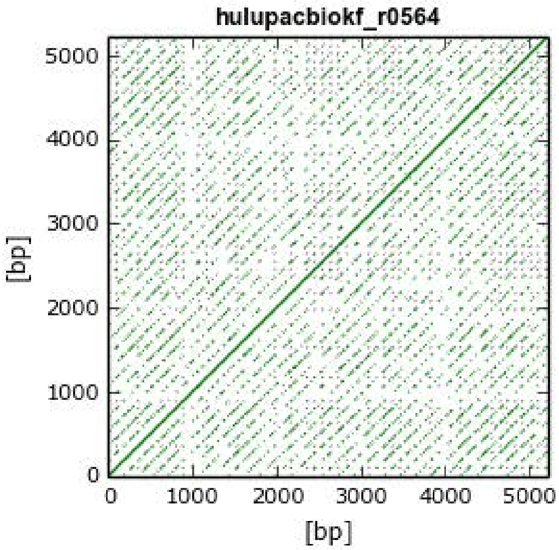
hulupacbiokf_r0564
[bp]
[bp]

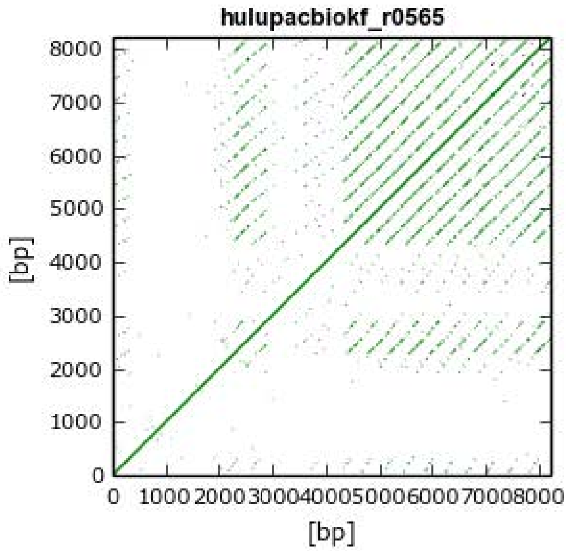
hulupacbiokf_r0565
8000
7000
6000
5000
4000
3000
2000
1000
0
[bp]
0 1000 2000 3000 4000 5000 6000 7000 8000
[bp]

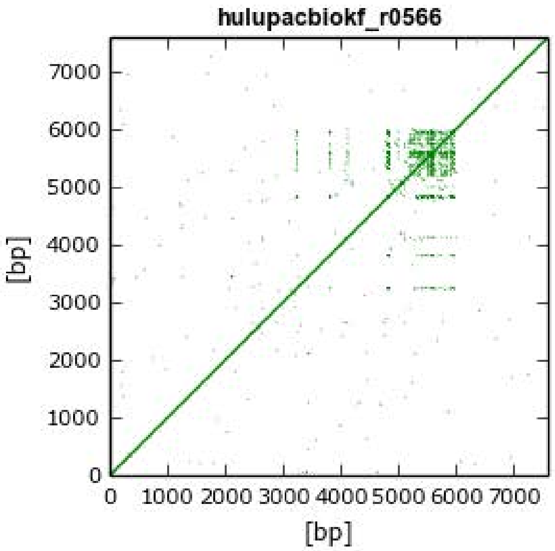
hulupacbiokf_r0566
7000
6000
5000
4000
3000
2000
1000
0
[bp]
0 1000 2000 3000 4000 5000 6000 7000
[bp]

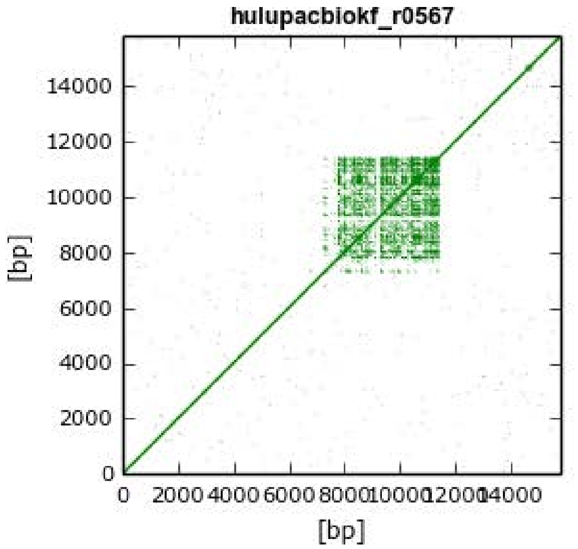
hulupacbiokf_r0567
14000
12000
10000
8000
6000
4000
2000
0
[bp]
0 2000 4000 6000 8000 10000 12000 14000
[bp]

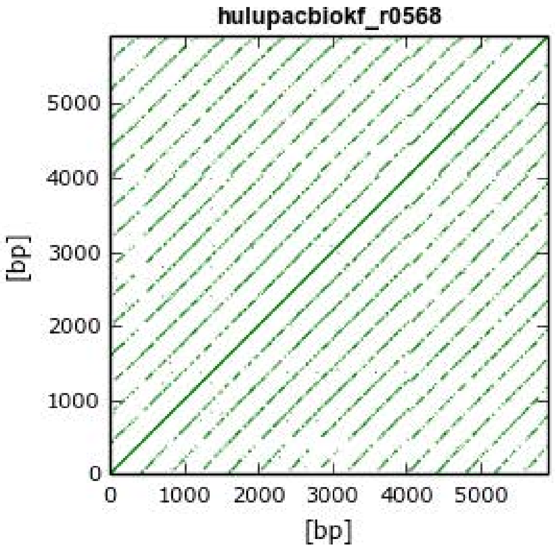
hulupacbiokf_r0568
5000
4000
3000
2000
1000
0
[bp]
0 1000 2000 3000 4000 5000
[bp]

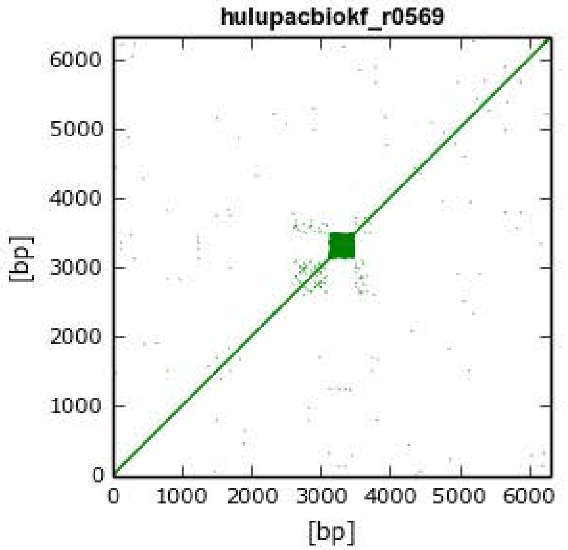
hulupacbiokf_r0569
6000
5000
4000
3000
2000
1000
0
[bp]
0 1000 2000 3000 4000 5000 6000
[bp]

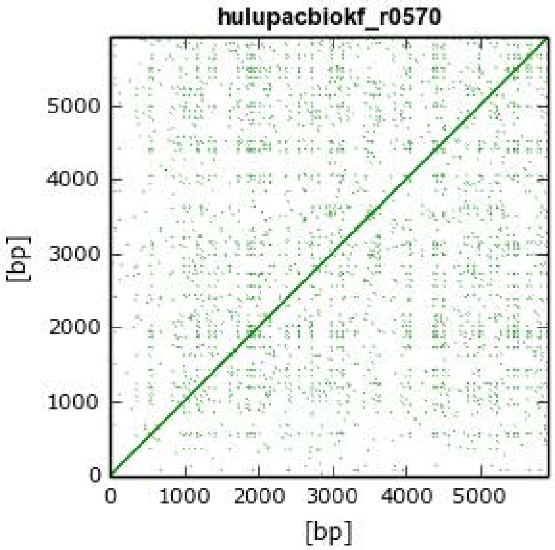
hulupacbiokf_r0570
5000
4000
3000
2000
1000
0
[bp]
0 1000 2000 3000 4000 5000
[bp]

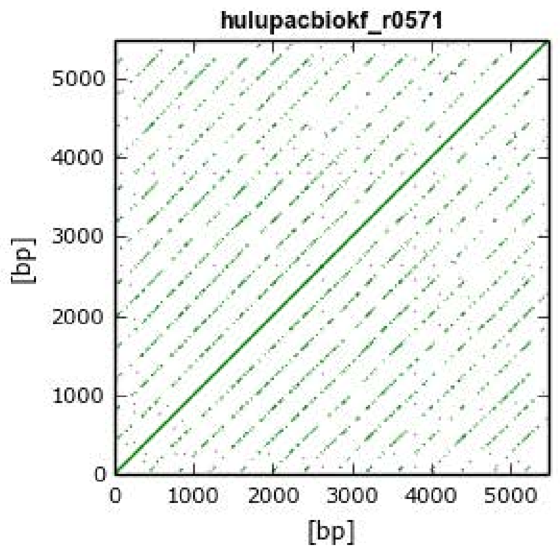
hulupacbiokf_r0571
[bp]
[bp]

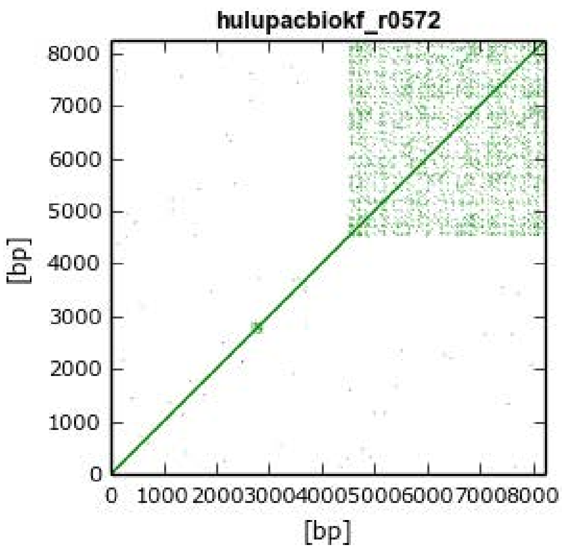
hulupacbiokf_r0572
[bp]
[bp]

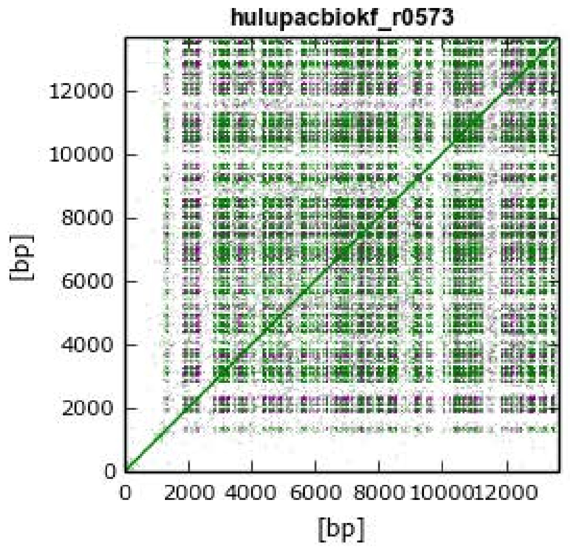
hulupacbiokf_r0573
[bp]
[bp]

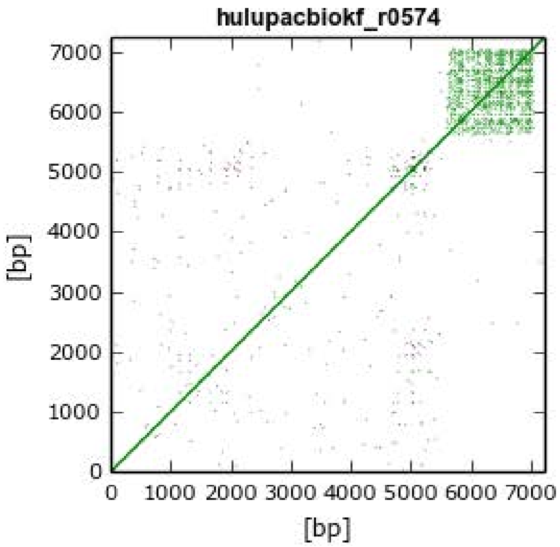
hulupacbiokf_r0574
[bp]
[bp]

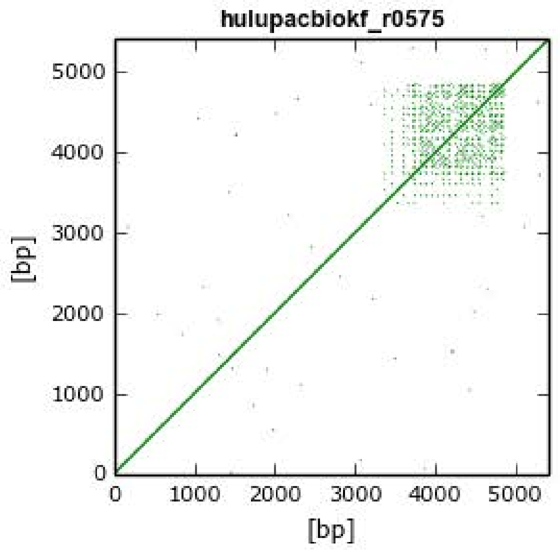
hulupacbiokf_r0575
[bp]
[bp]

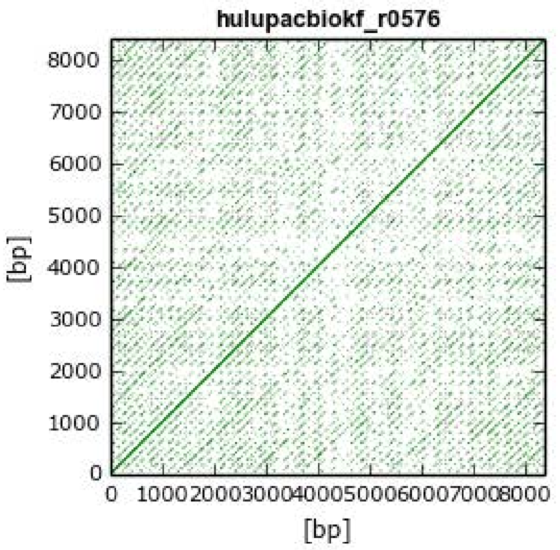
hulupacbiokf_r0576
[bp]
[bp]

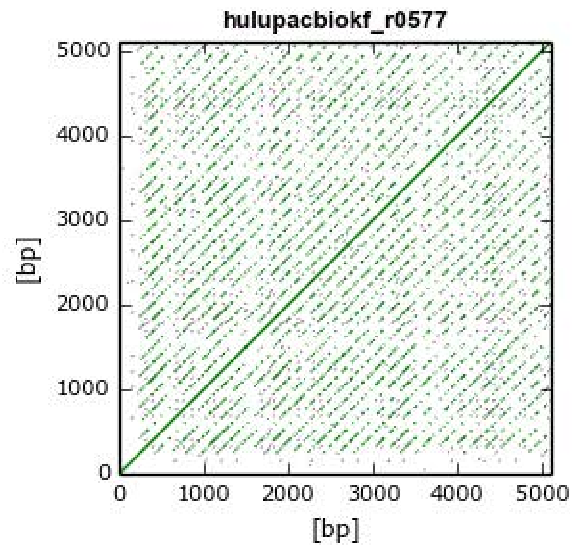
hulupacbiokf_r0577
5000
4000
3000
2000
1000
0
[bp]
0 1000 2000 3000 4000 5000
[bp]

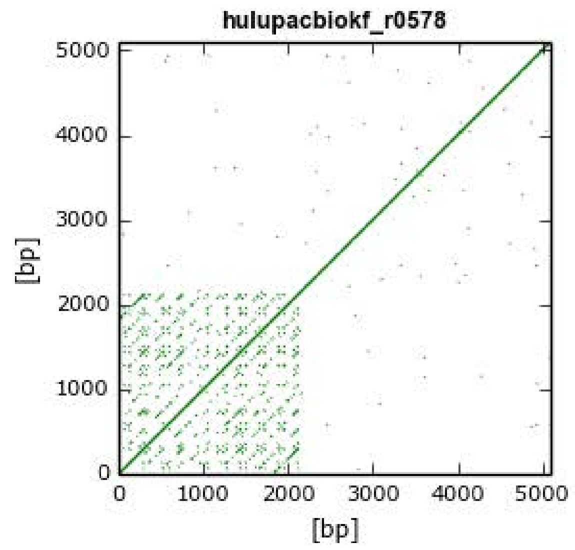
hulupacbiokf_r0578
5000
4000
3000
2000
1000
0
[bp]
0 1000 2000 3000 4000 5000
[bp]

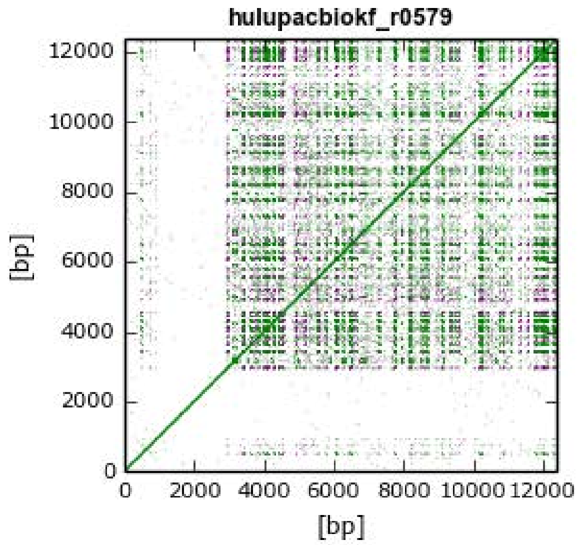
hulupacbiokf_r0579
12000
10000
8000
6000
4000
2000
0
[bp]
0 2000 4000 6000 8000 10000 12000
[bp]

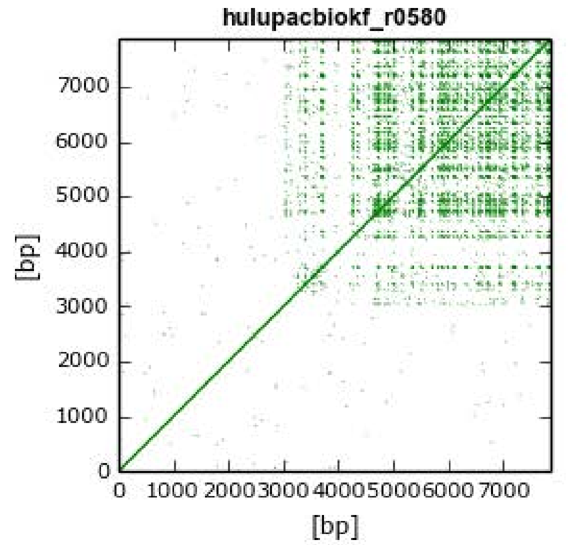
hulupacbiokf_r0580
7000
6000
5000
4000
3000
2000
1000
0
[bp]
0 1000 2000 3000 4000 5000 6000 7000
[bp]

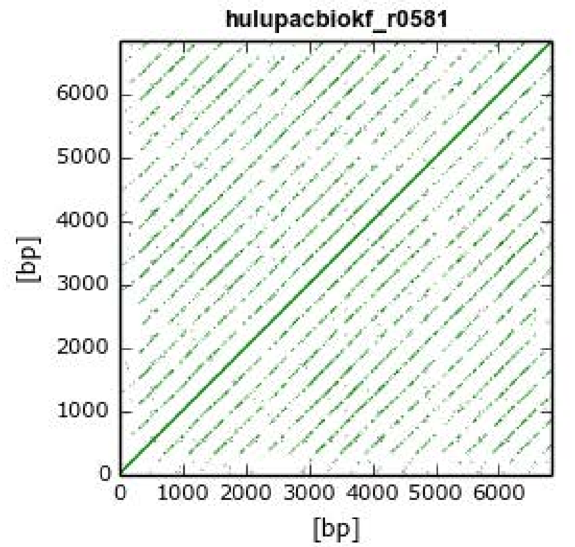
hulupacbiokf_r0581
6000
5000
4000
3000
2000
1000
0
[bp]
0 1000 2000 3000 4000 5000 6000
[bp]

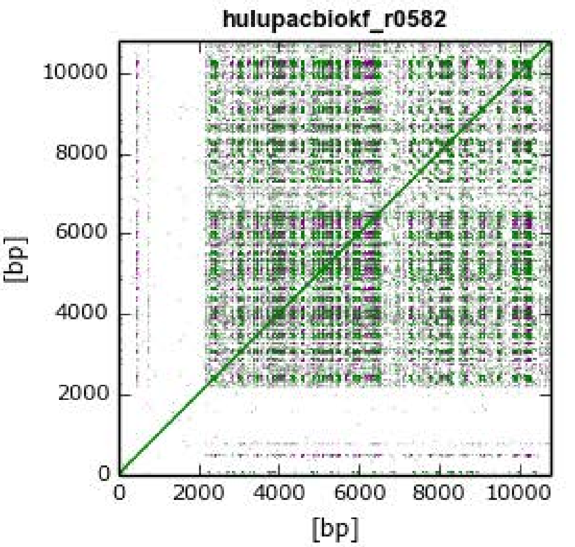
hulupacbiokf_r0582
10000
8000
6000
4000
2000
0
[bp]
0 2000 4000 6000 8000 10000
[bp]

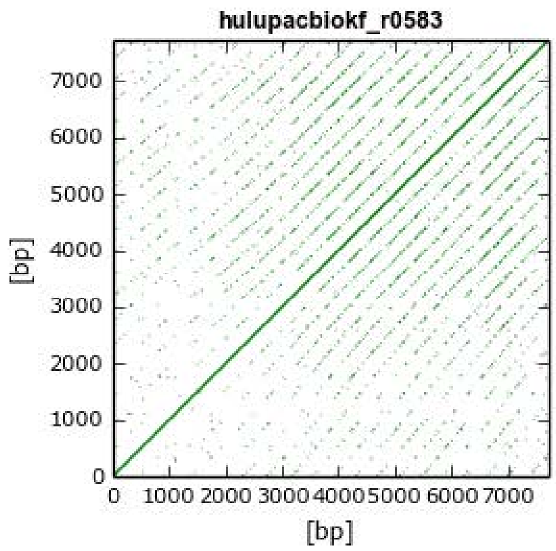
hulupacbiokf_r0583
[bp]
[bp]

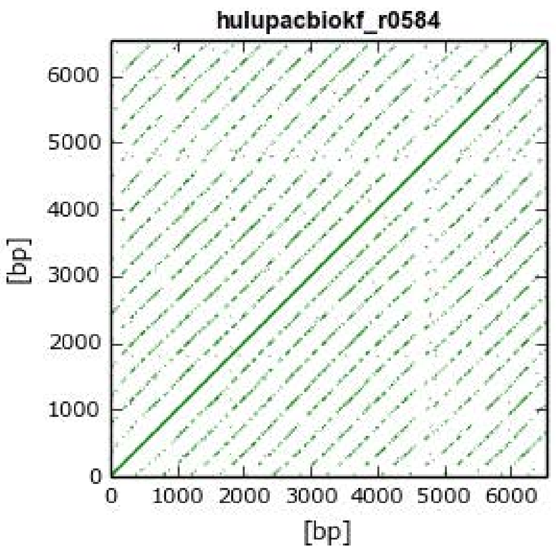
hulupacbiokf_r0584
[bp]
[bp]

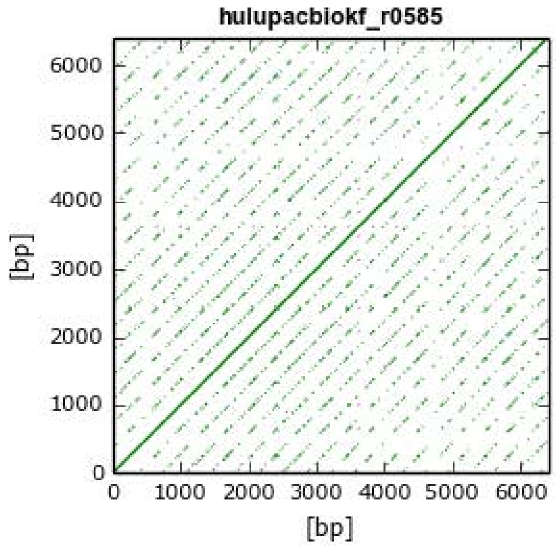
hulupacbiokf_r0585
[bp]
[bp]

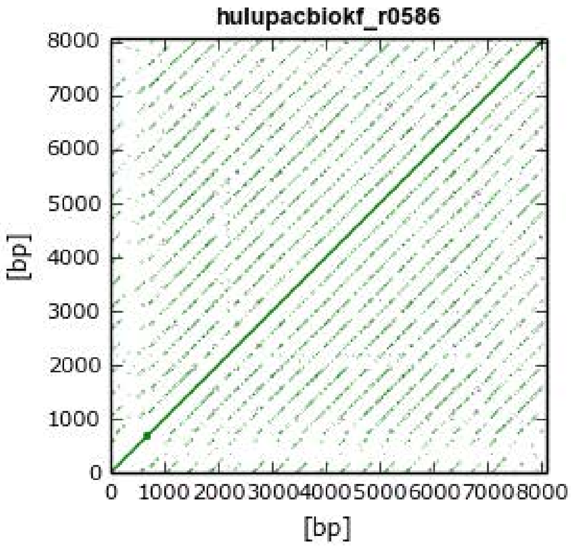
hulupacbiokf_r0586
[bp]
[bp]

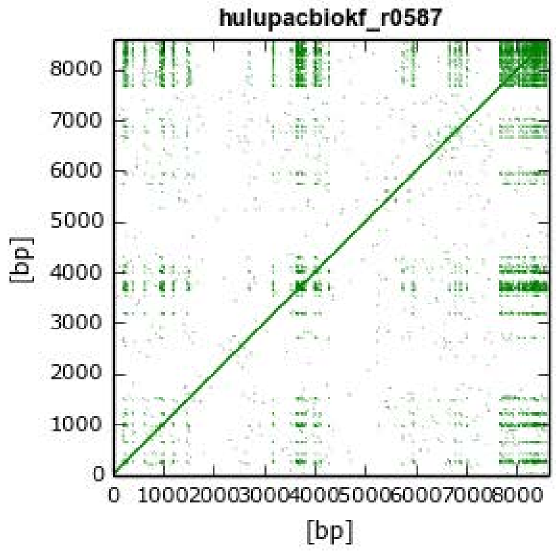
hulupacbiokf_r0587
[bp]
[bp]

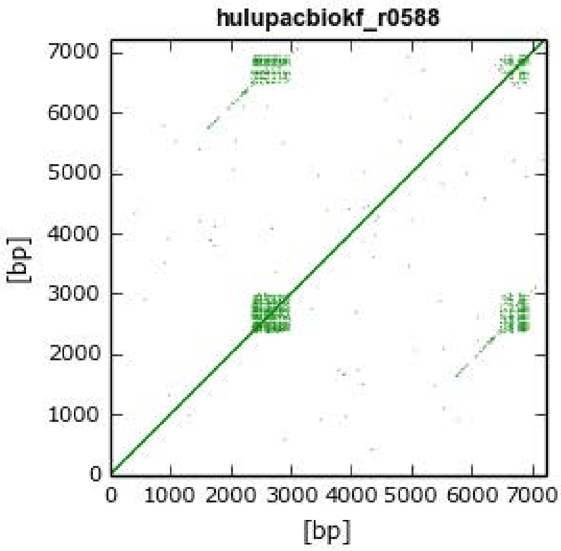
hulupacbiokf_r0588
[bp]
[bp]

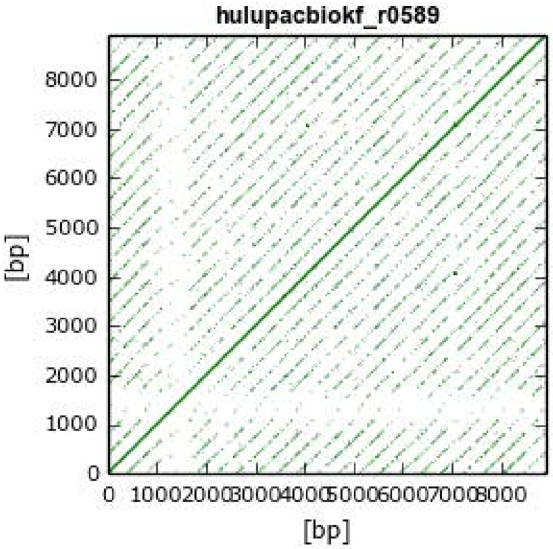
hulupacbiokf_r0589
0
1000
2000
3000
4000
5000
6000
7000
8000
[bp]
0 10002000300040005000600070008000
[bp]

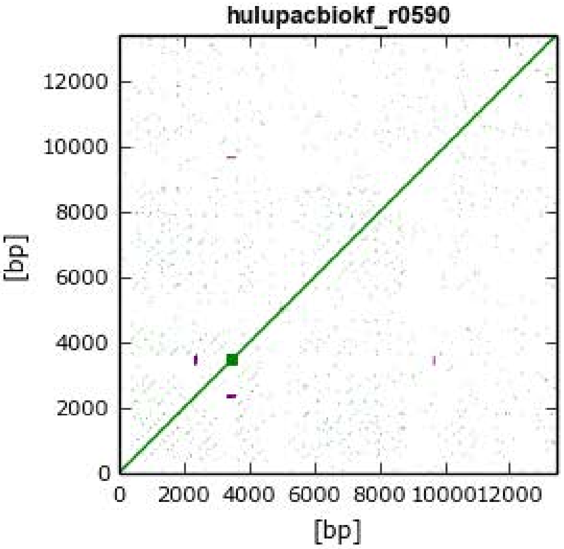
hulupacbiokf_r0590
0
2000
4000
6000
8000
10000
12000
[bp]
0 2000 4000 6000 8000 1000012000
[bp]

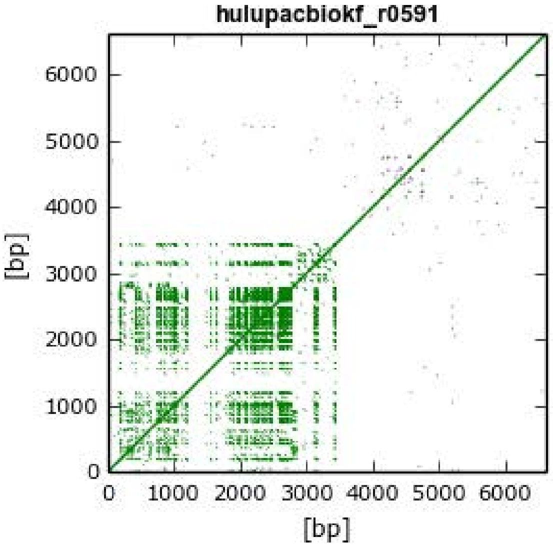
hulupacbiokf_r0591
0
1000
2000
3000
4000
5000
6000
[bp]
0 1000 2000 3000 4000 5000 6000
[bp]

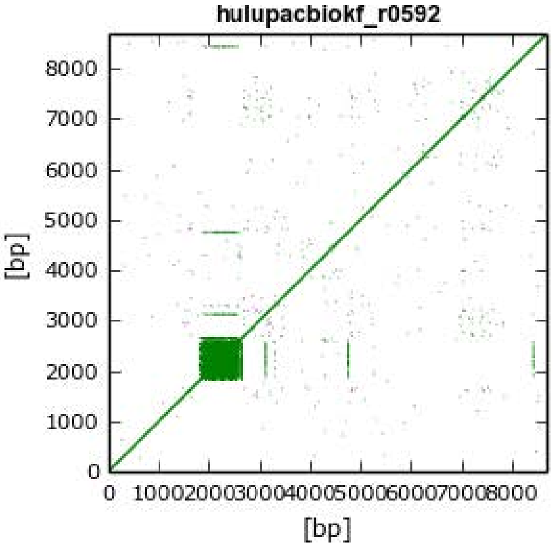
hulupacbiokf_r0592
0
1000
2000
3000
4000
5000
6000
7000
8000
[bp]
0 10002000300040005000600070008000
[bp]

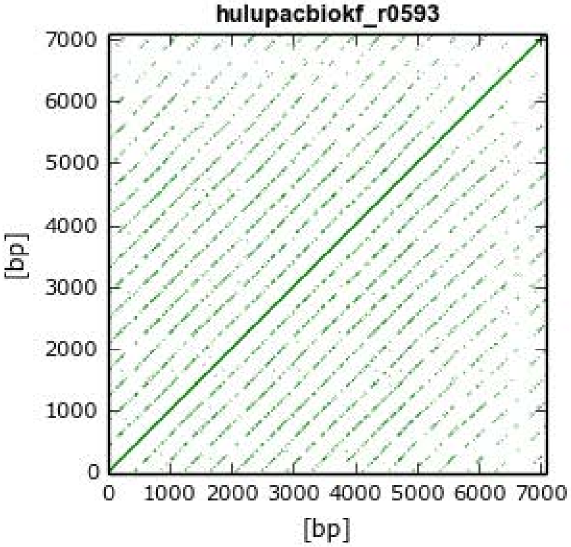
hulupacbiokf_r0593
0
1000
2000
3000
4000
5000
6000
7000
[bp]
0 1000 2000 3000 4000 5000 6000 7000
[bp]

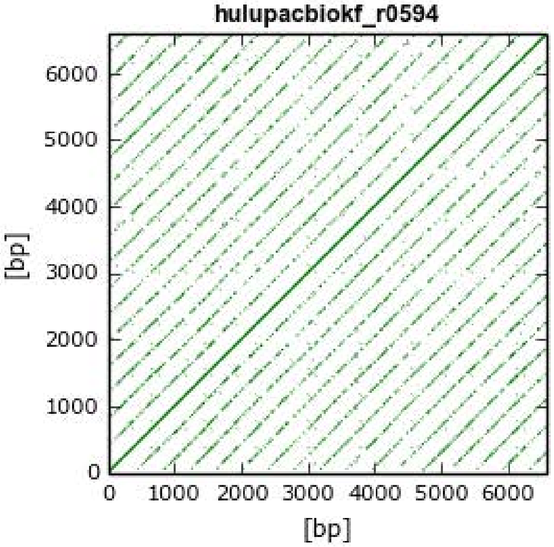
hulupacbiokf_r0594
0
1000
2000
3000
4000
5000
6000
[bp]
0 1000 2000 3000 4000 5000 6000
[bp]

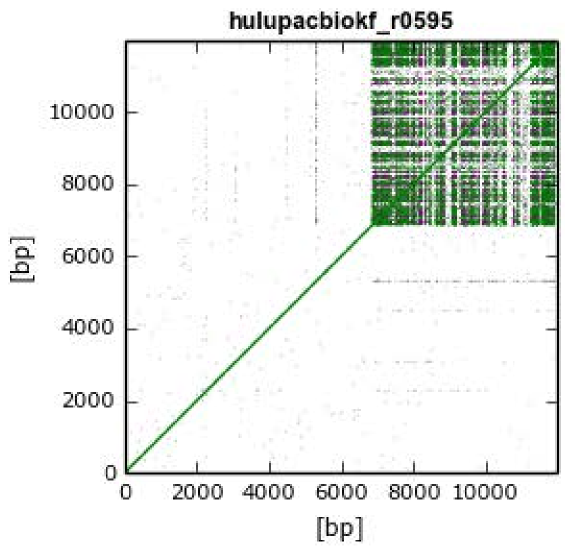
hulupacbiokf_r0595
[bp]
[bp]

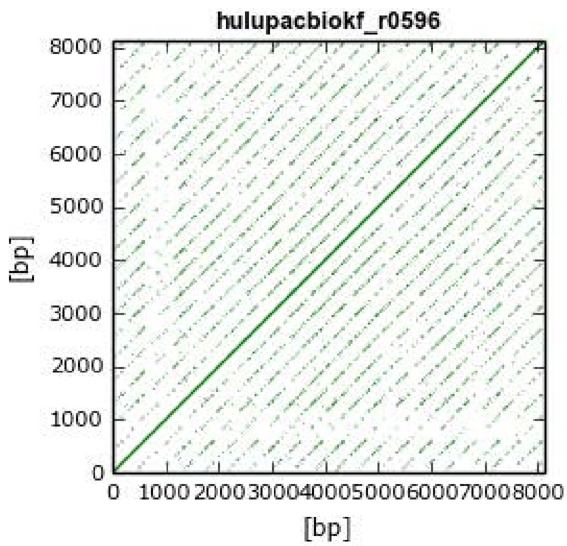
hulupacbiokf_r0596
[bp]
[bp]

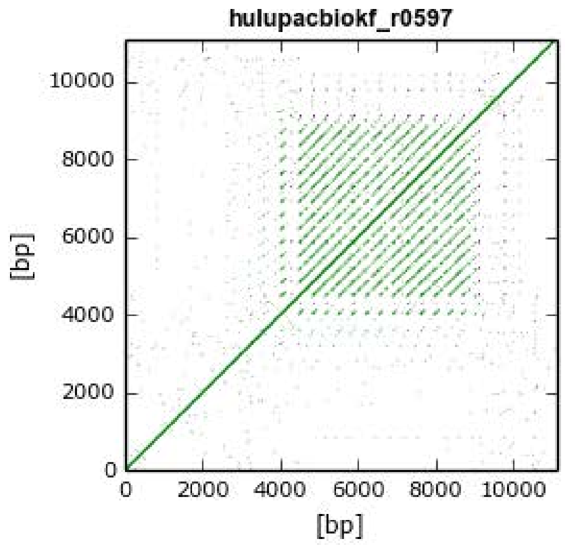
hulupacbiokf_r0597
[bp]
[bp]

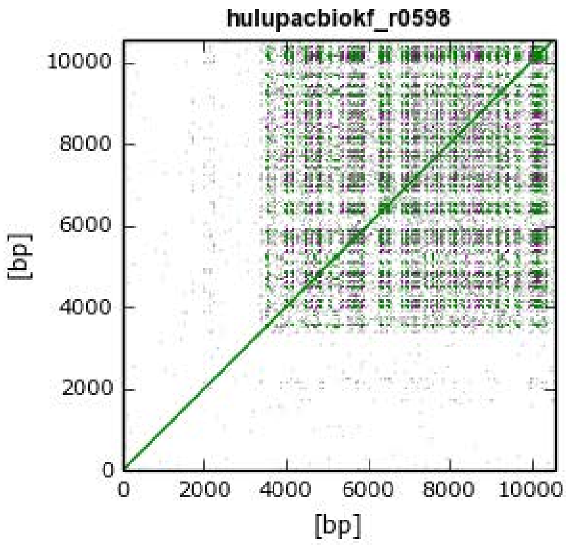
hulupacbiokf_r0598
[bp]
[bp]

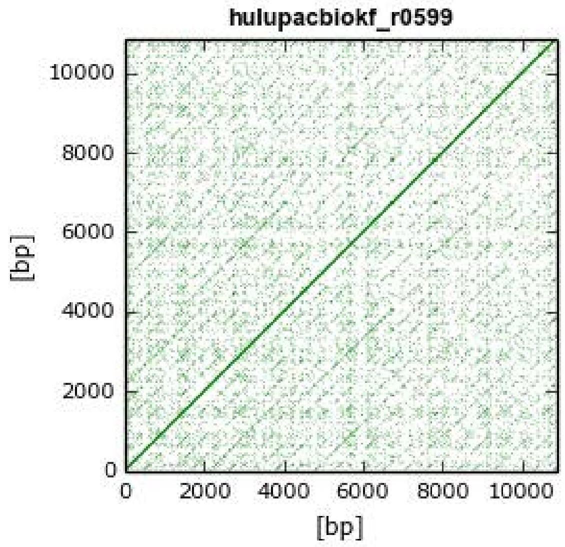
hulupacbiokf_r0599
[bp]
[bp]

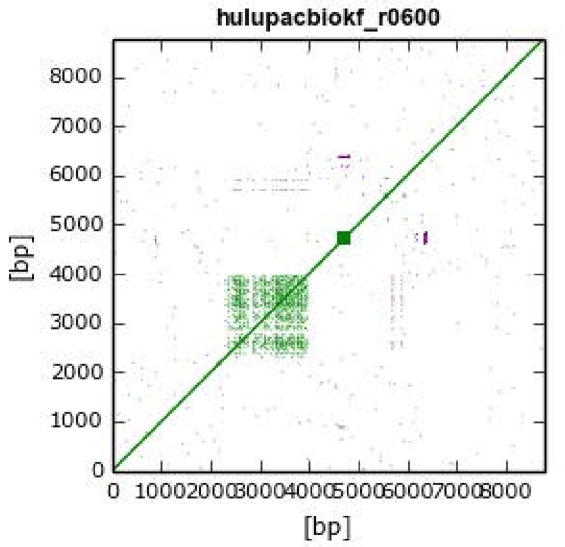
hulupacbiokf_r0600
[bp]
[bp]

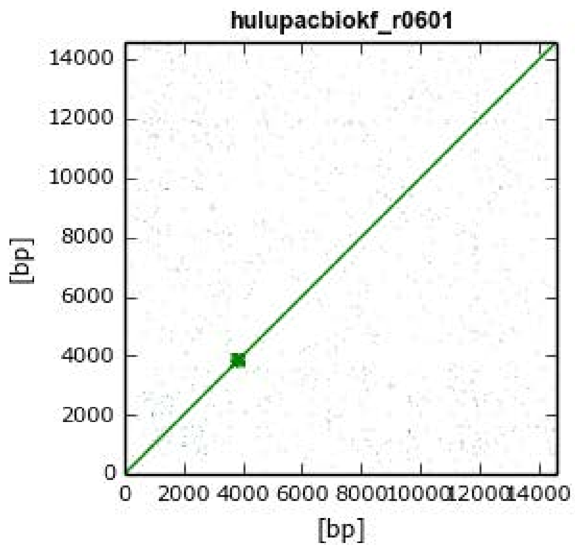
hulupacbiokf_r0601
[bp]
[bp]

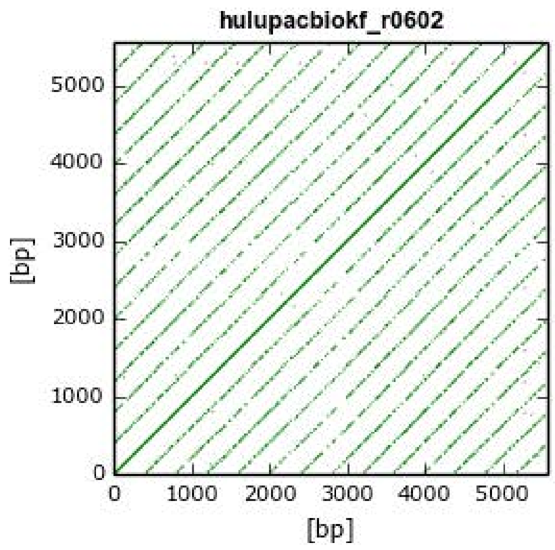
hulupacbiokf_r0602
[bp]
[bp]

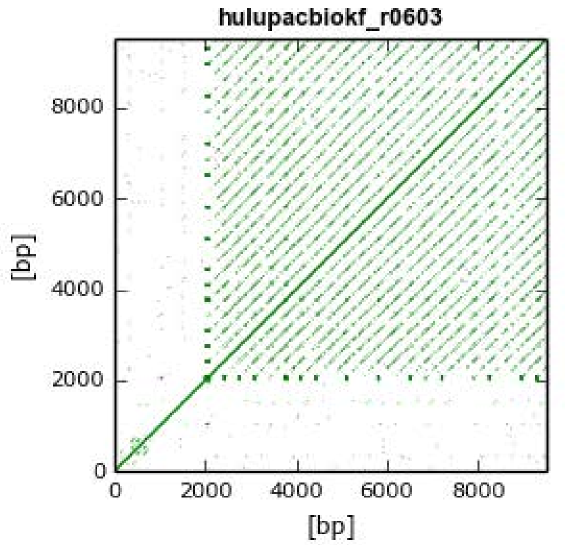
hulupacbiokf_r0603
[bp]
[bp]

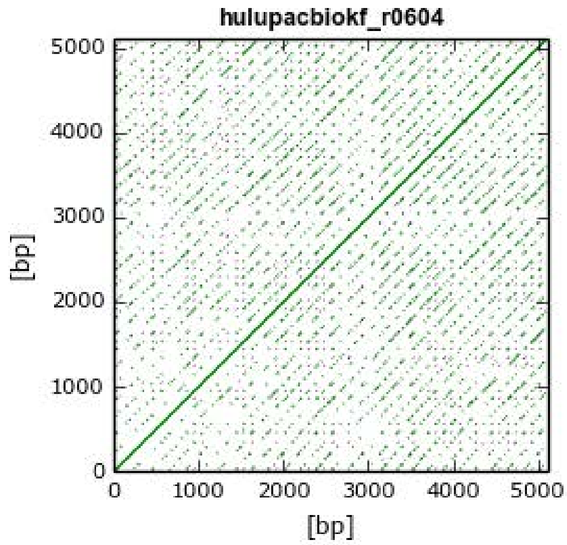
hulupacbiokf_r0604
[bp]
[bp]

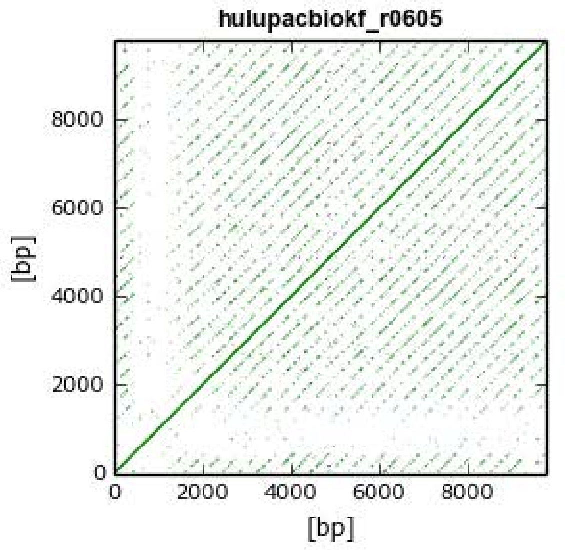
hulupacbiokf_r0605
[bp]
[bp]

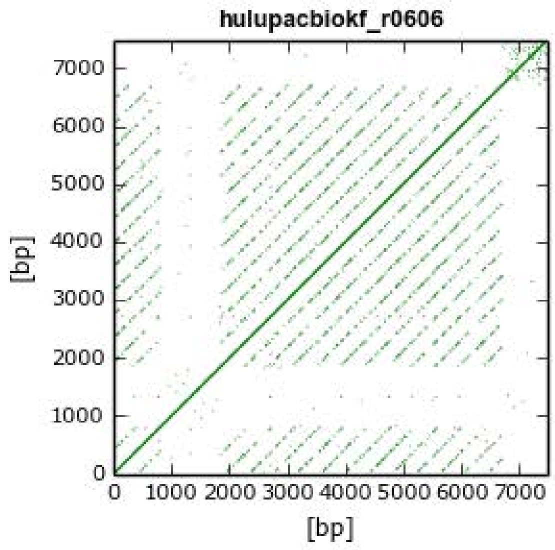
hulupacbiokf_r0606
[bp]
[bp]

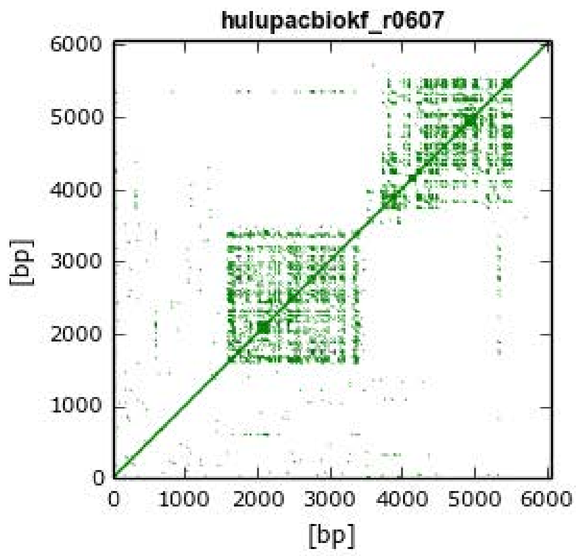
hulupacbiokf_r0607
[bp]
[bp]

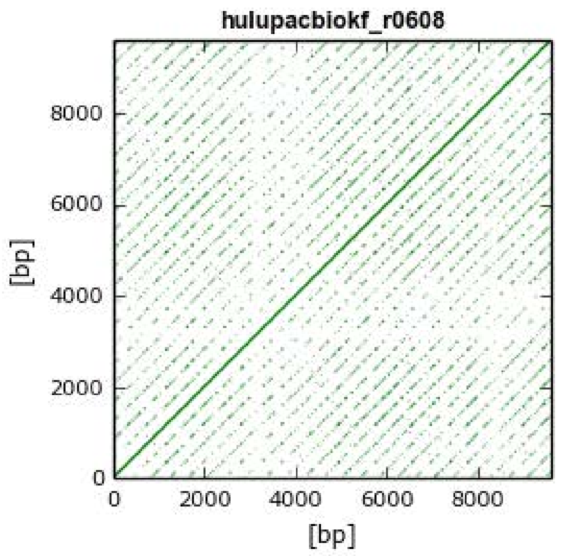
hulupacbiokf_r0608
[bp]
[bp]

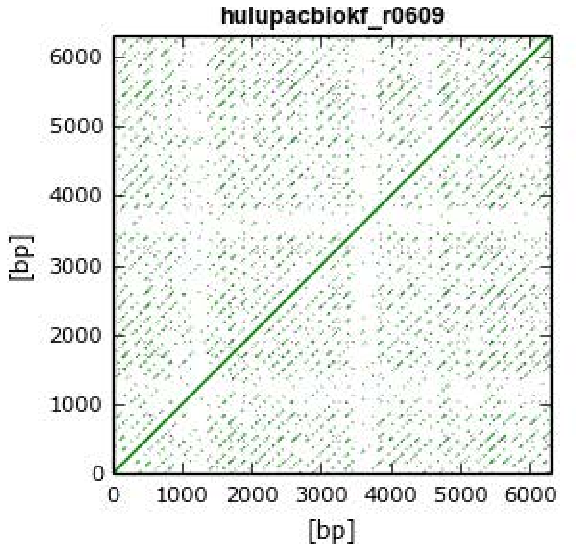
hulupacbiokf_r0609
[bp]
[bp]

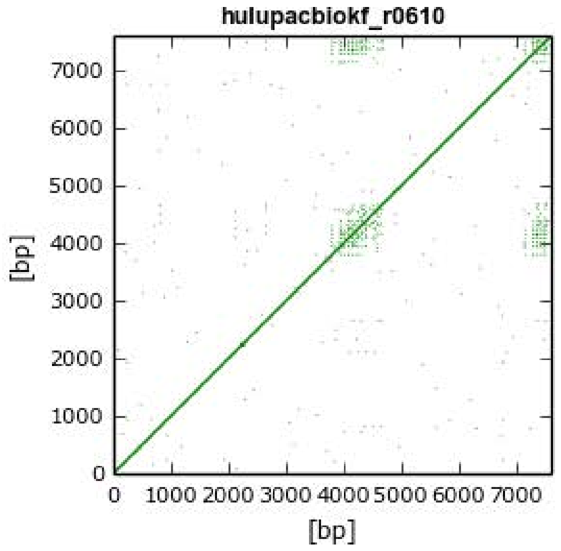
hulupacbiokf_r0610
[bp]
[bp]

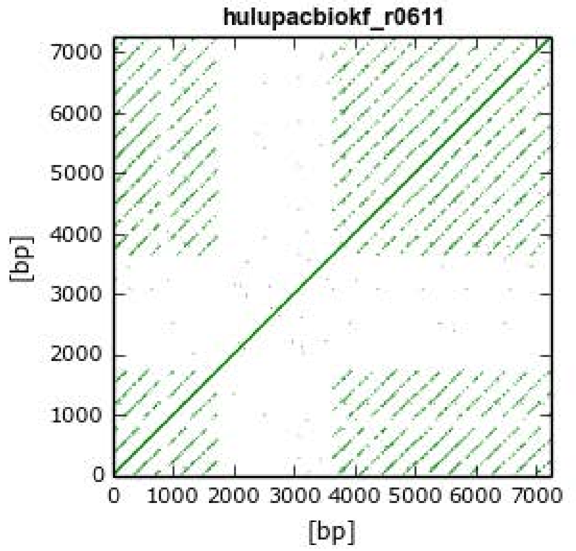
hulupacbiokf_r0611
[bp]
[bp]

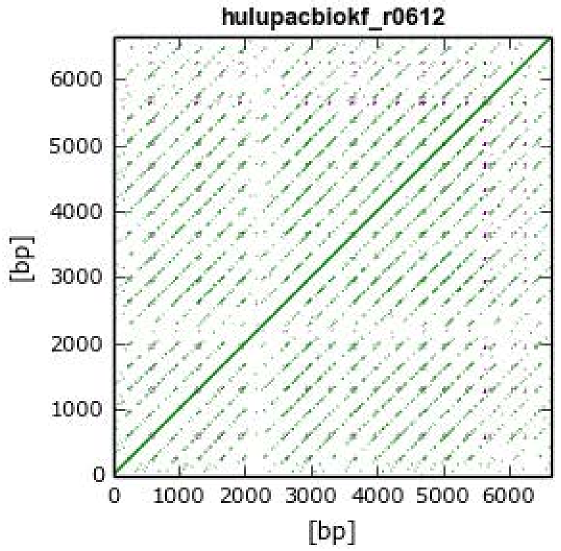
hulupacbiokf_r0612
[bp]
[bp]

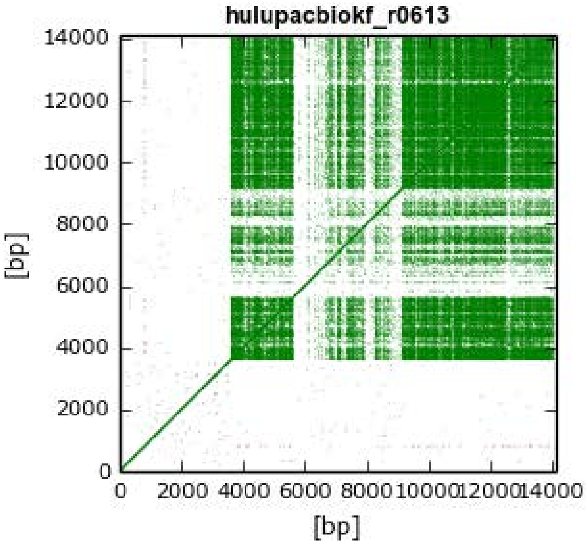
hulupacbiokf_r0613
[bp]
[bp]

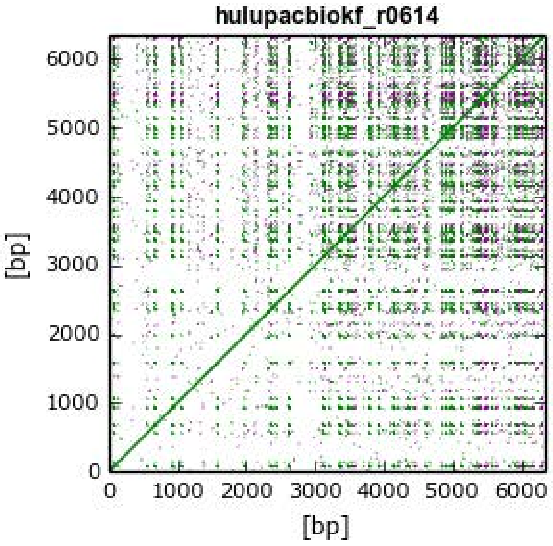
hulupacbiokf_r0614
[bp]
[bp]

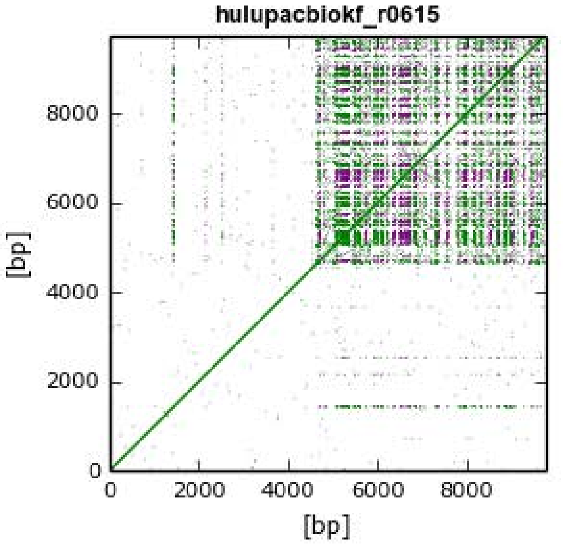
hulupacbiokf_r0615
[bp]
[bp]

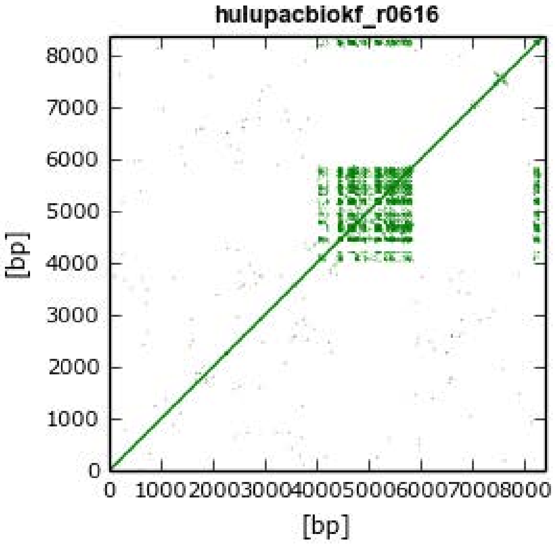
hulupacbiokf_r0616
[bp]
[bp]

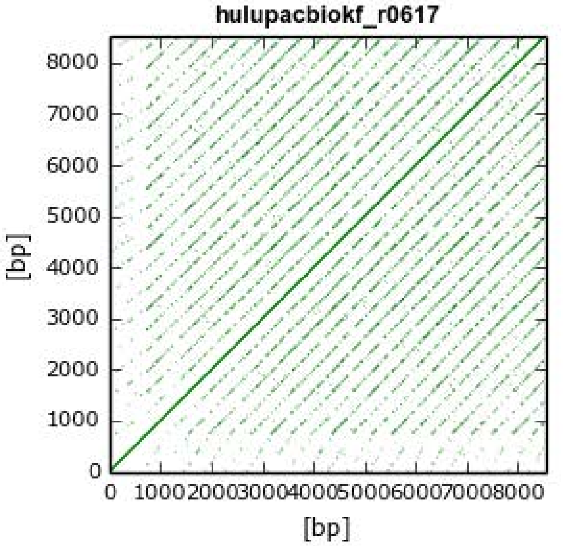
hulupacbiokf_r0617
[bp]
[bp]

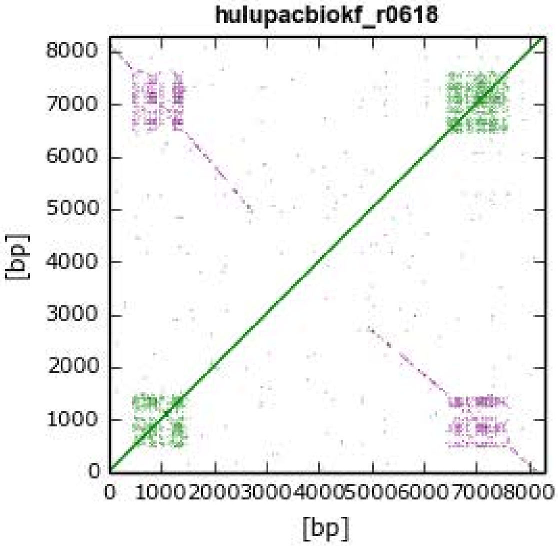
hulupacbiokf_r0618
[bp]
[bp]

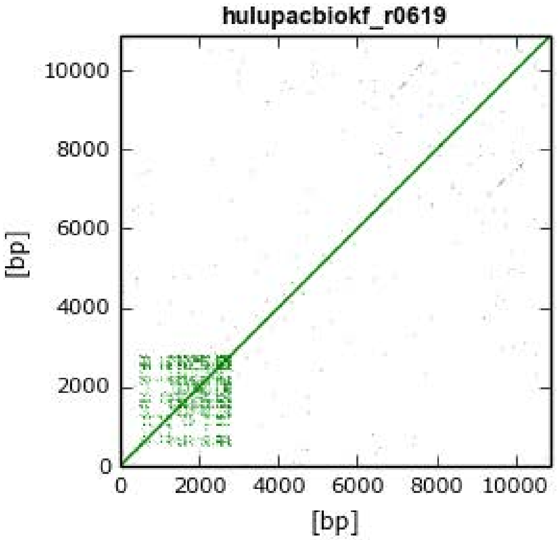
hulupacbiokf_r0619
[bp]
[bp]

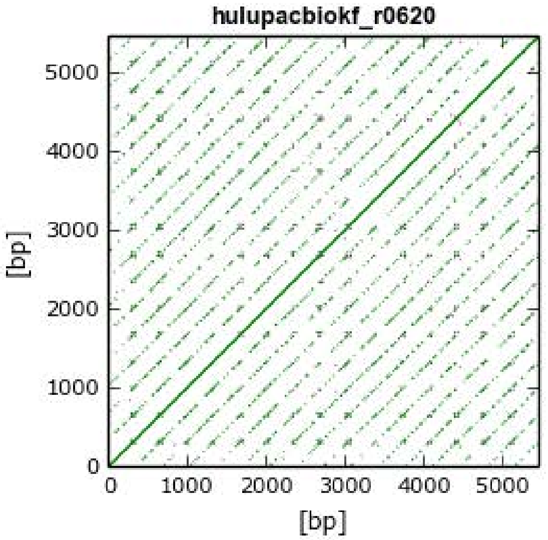
hulupacbiokf_r0620
[bp]
[bp]

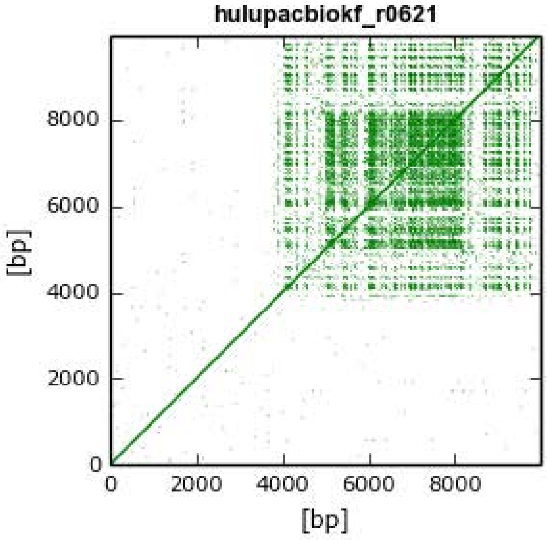
hulupacbiokf_r0621
[bp]
[bp]

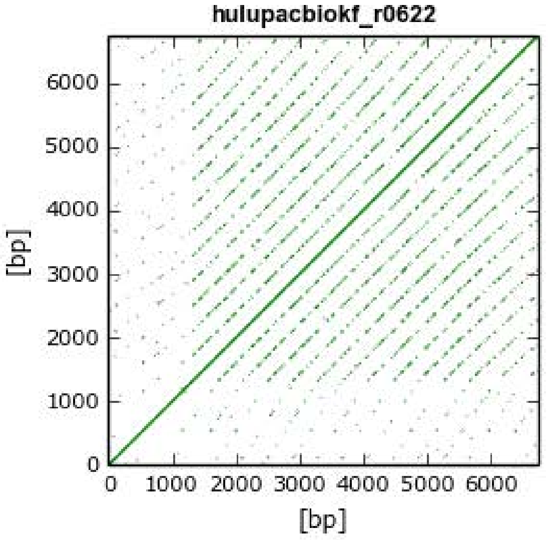
hulupacbiokf_r0622
[bp]
[bp]

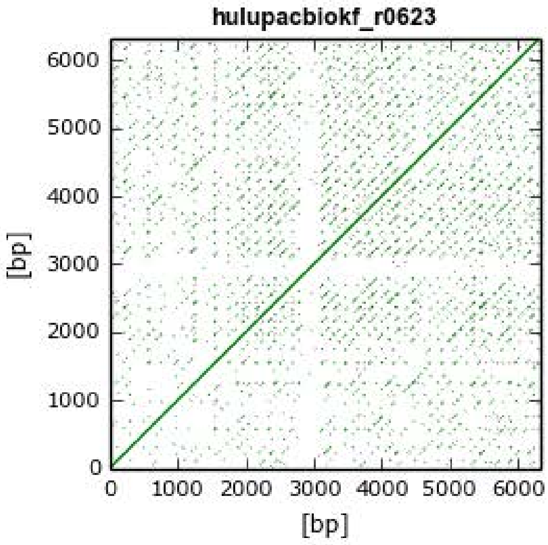
hulupacbiokf_r0623
[bp]
[bp]

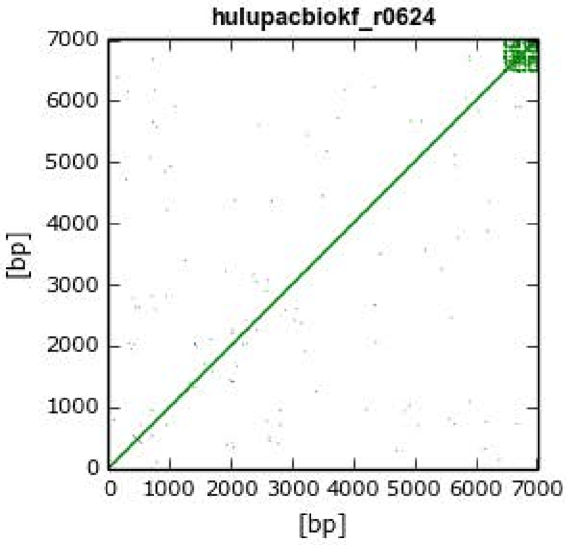
hulupacbiokf_r0624
[bp]
[bp]

HuluTR350 from read r0625
is in GenBank Acc. MN537577

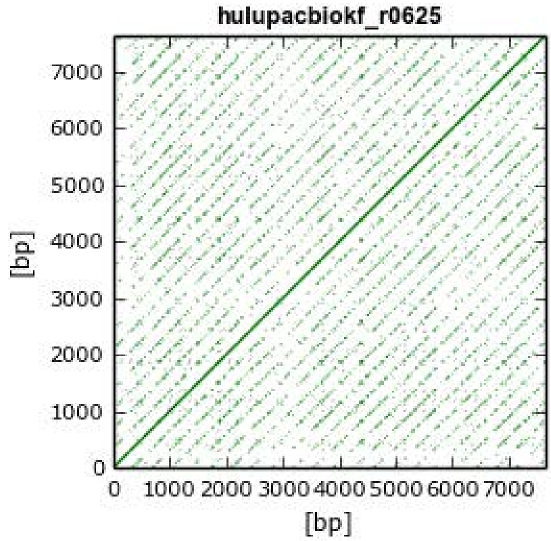

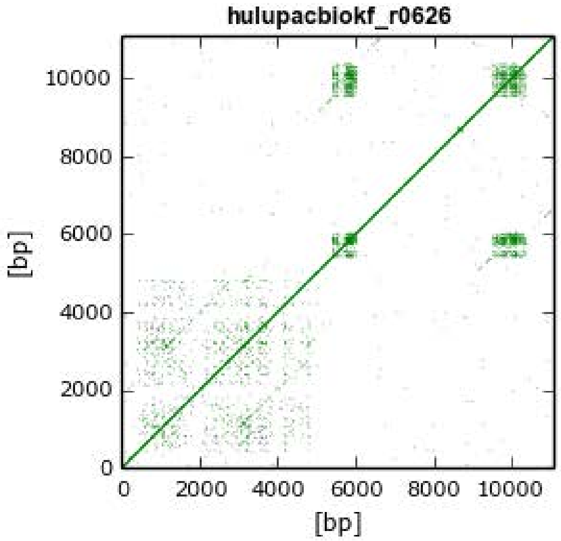

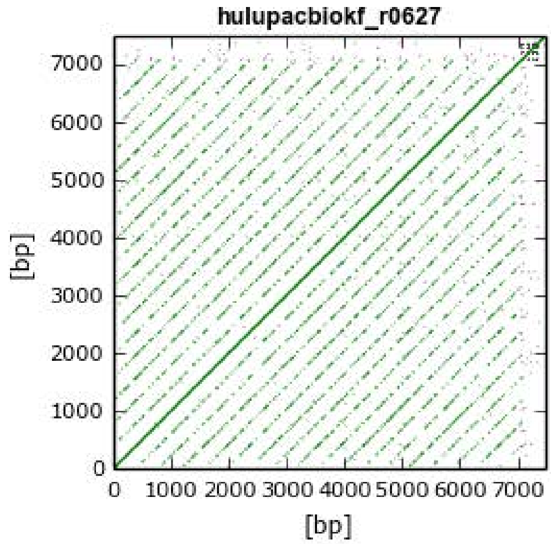

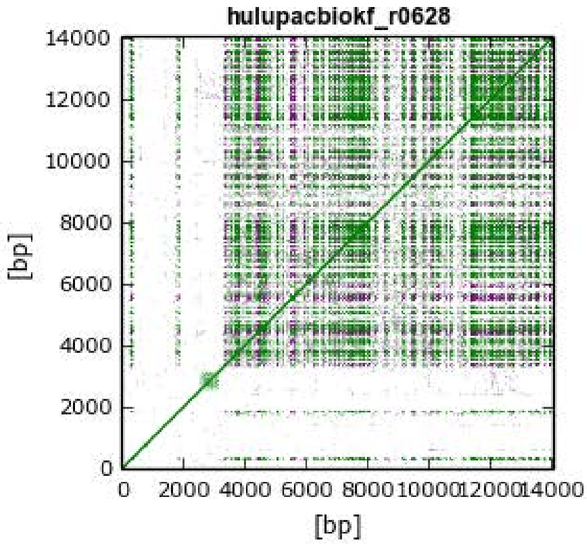

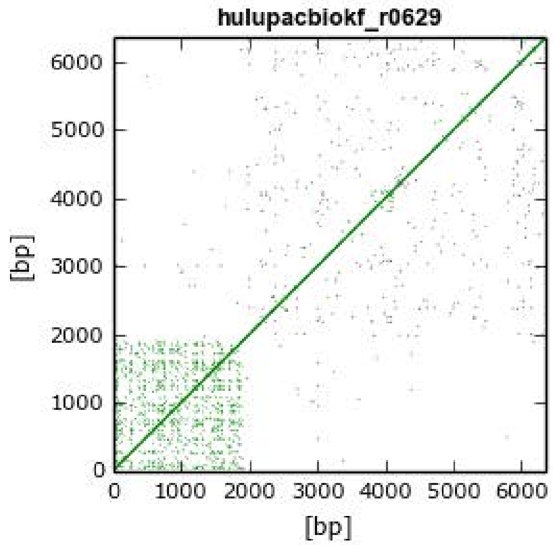

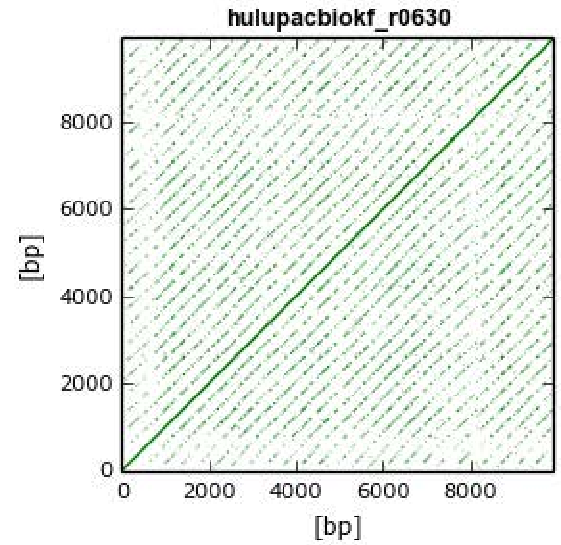

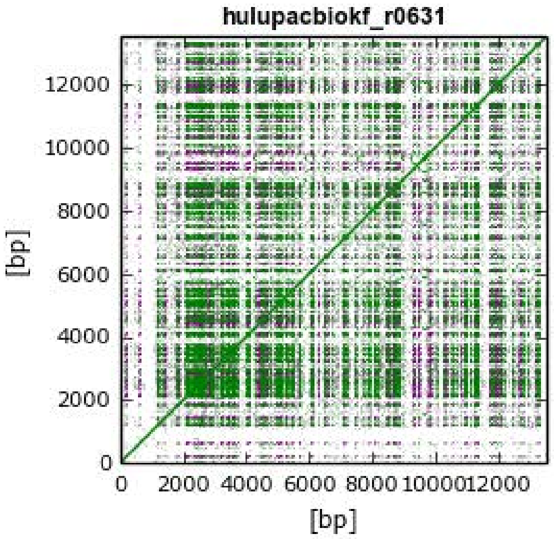
hulupacbiokf_r0631
[bp]
[bp]

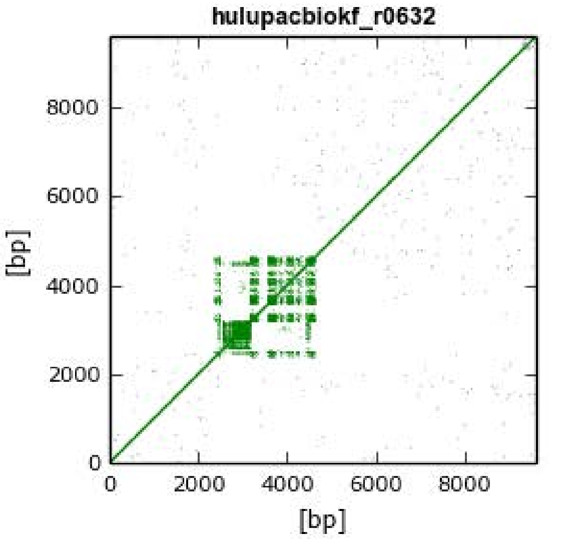
hulupacbiokf_r0632
[bp]
[bp]

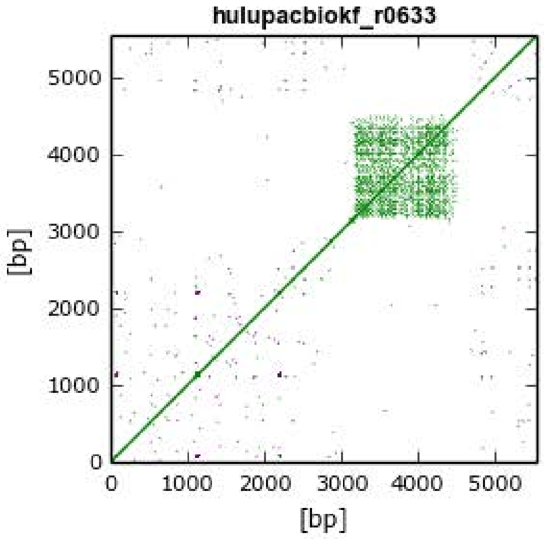
hulupacbiokf_r0633
[bp]
[bp]

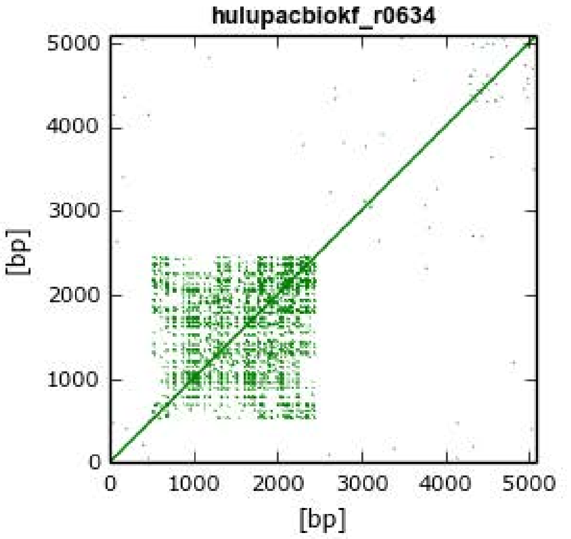
hulupacbiokf_r0634
[bp]
[bp]

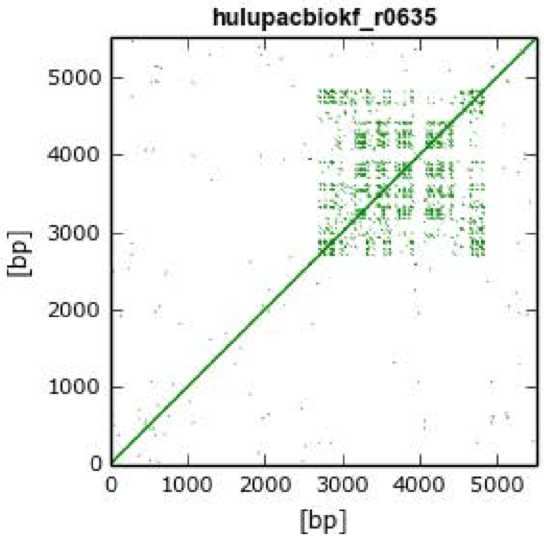
hulupacbiokf_r0635
[bp]
[bp]

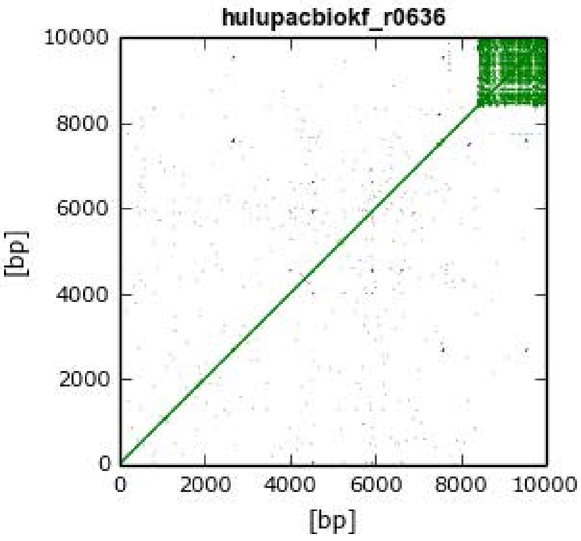
hulupacbiokf_r0636
[bp]
[bp]

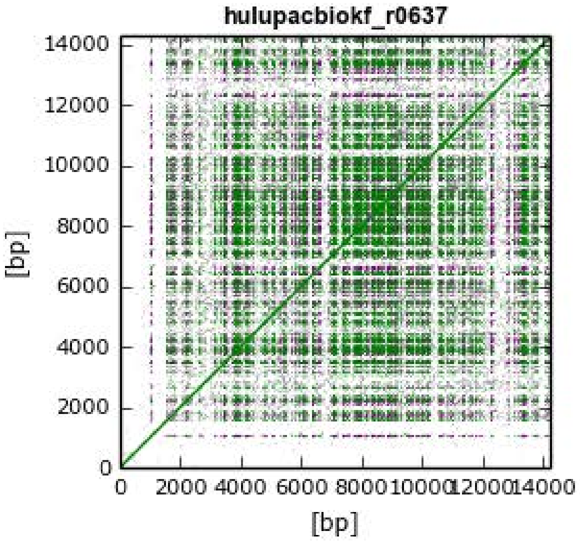

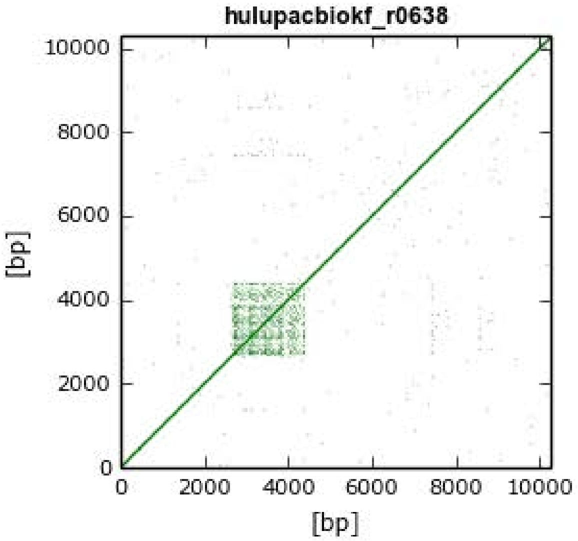

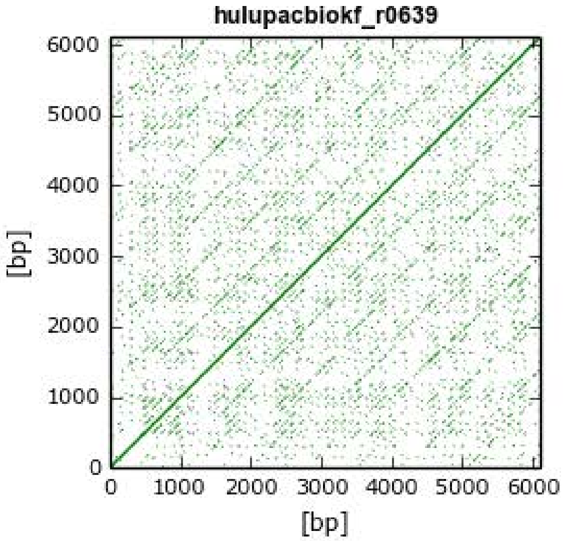

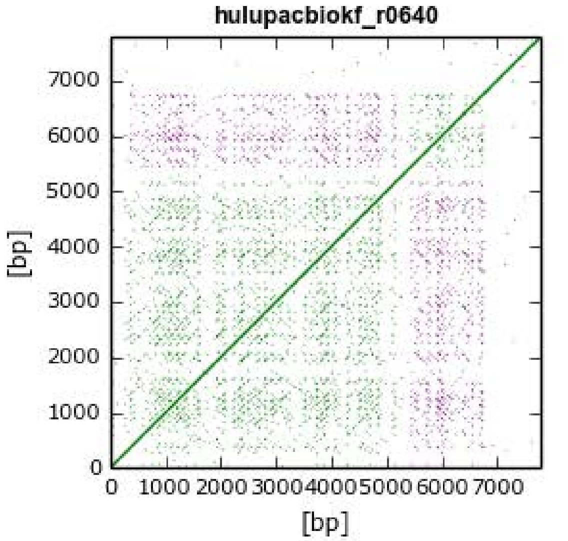

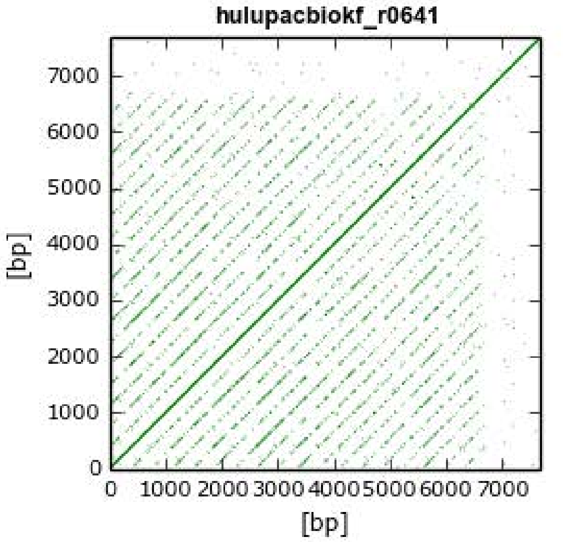

HuluTR360 from read r0642
is in GenBank Acc. MN537578

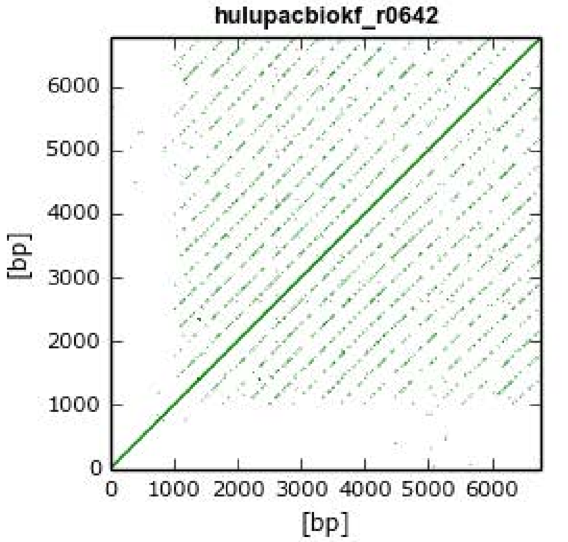

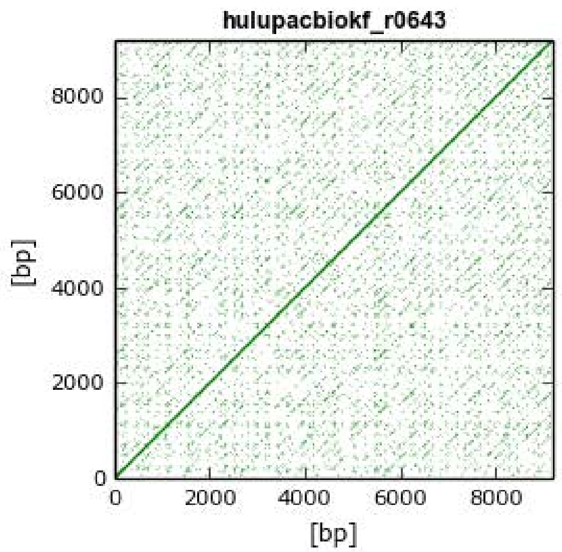
hulupacbiokf_r0643
[bp]
[bp]

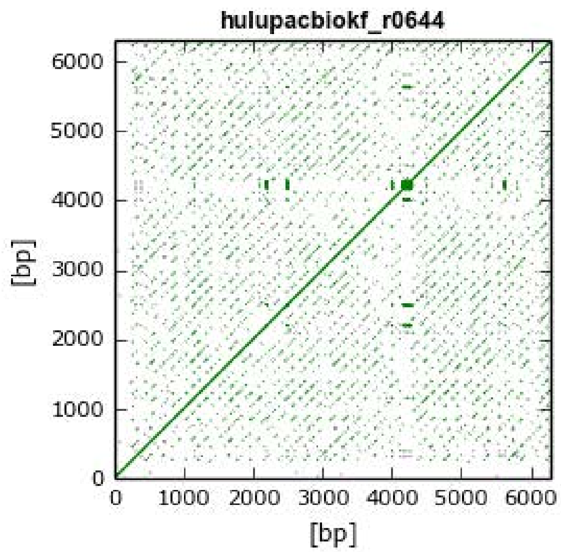
hulupacbiokf_r0644
[bp]
[bp]

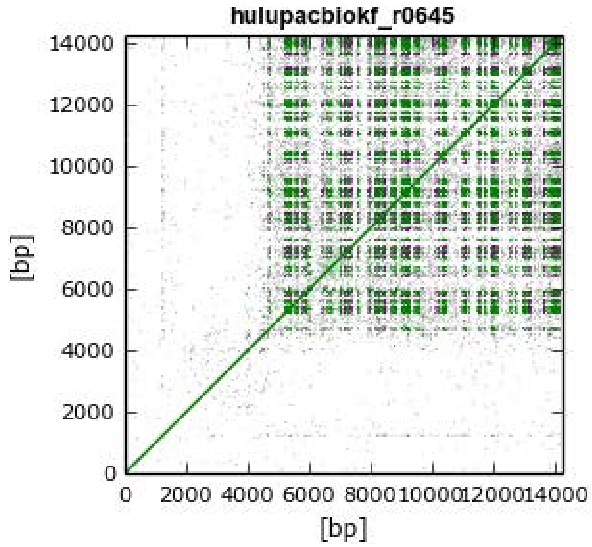
hulupacbiokf_r0645
[bp]
[bp]

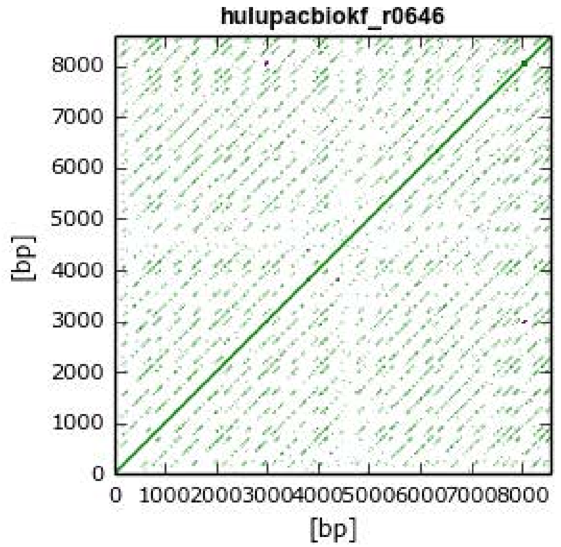
hulupacbiokf_r0646
[bp]
[bp]

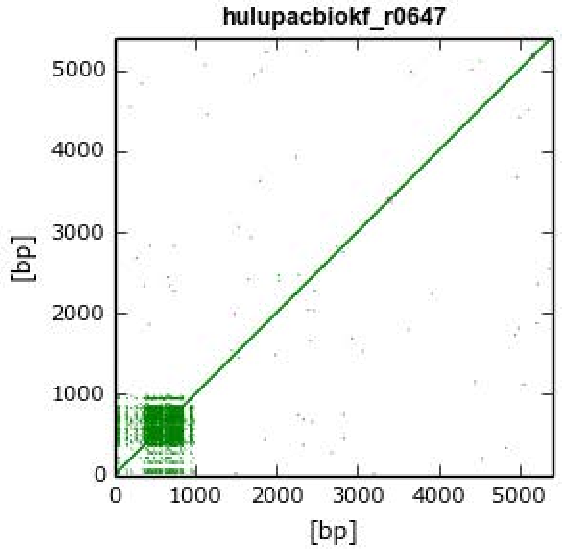
hulupacbiokf_r0647
[bp]
[bp]

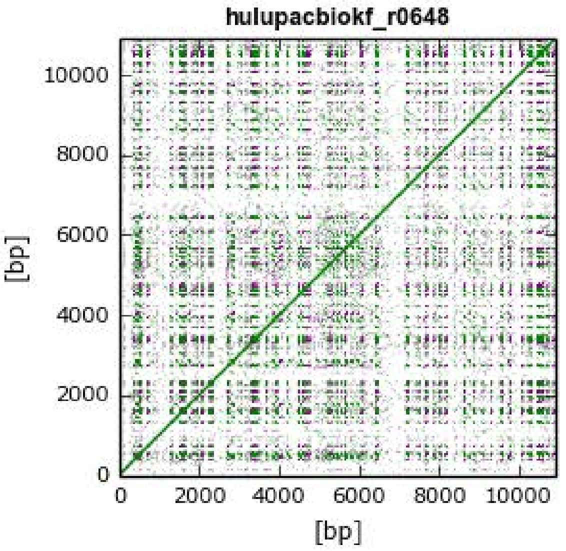
hulupacbiokf_r0648
[bp]
[bp]

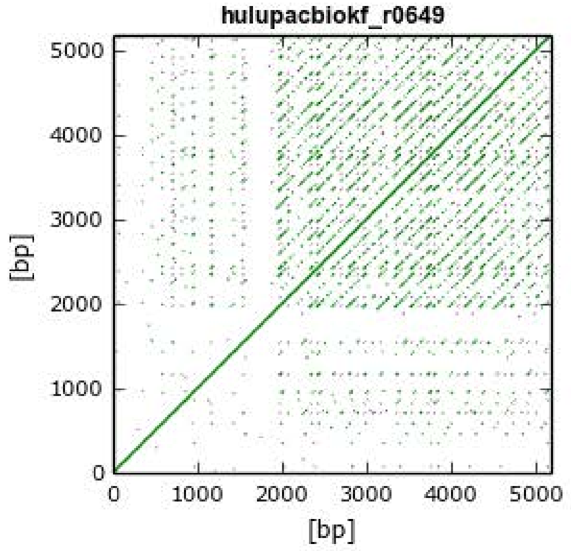
hulupacbiokf_r0649
[bp]
[bp]

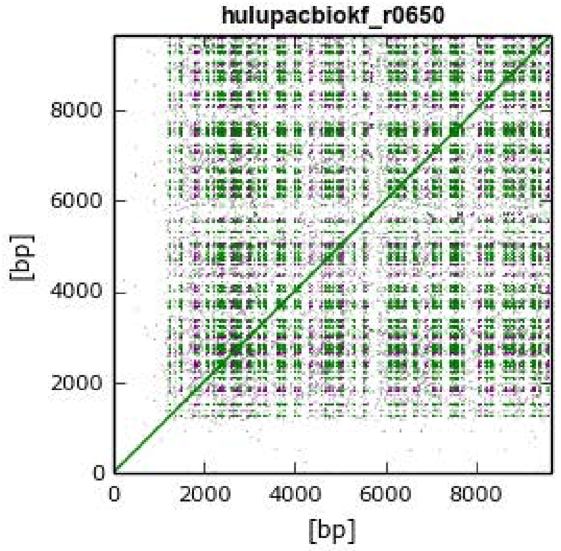
hulupacbiokf_r0650
[bp]
[bp]

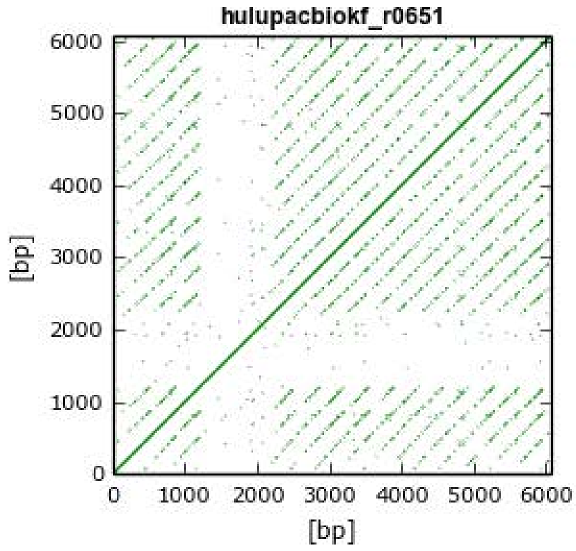
hulupacbiokf_r0651
[bp]
[bp]

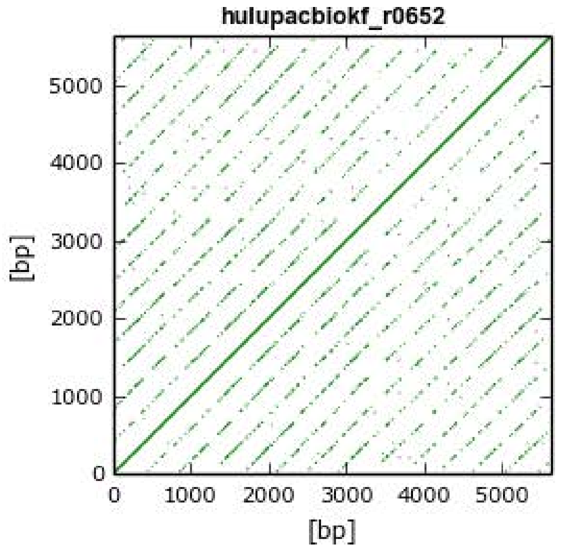
hulupacbiokf_r0652
[bp]
[bp]

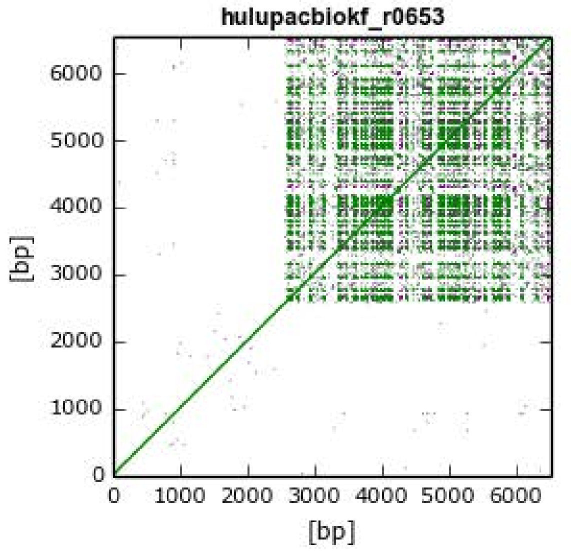
hulupacbiokf_r0653
[bp]
[bp]

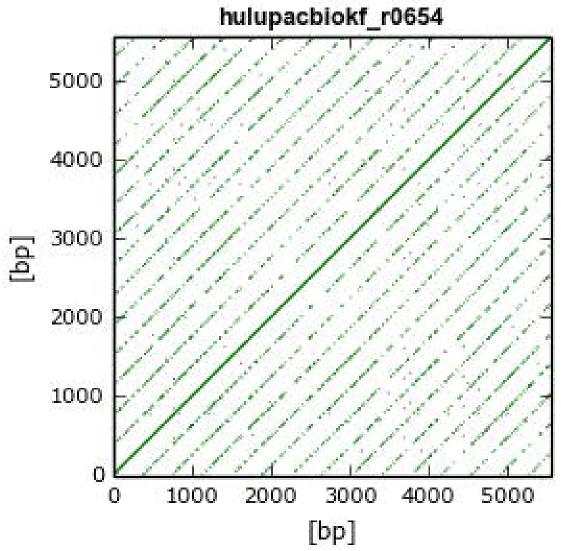
hulupacbiokf_r0654
[bp]
[bp]

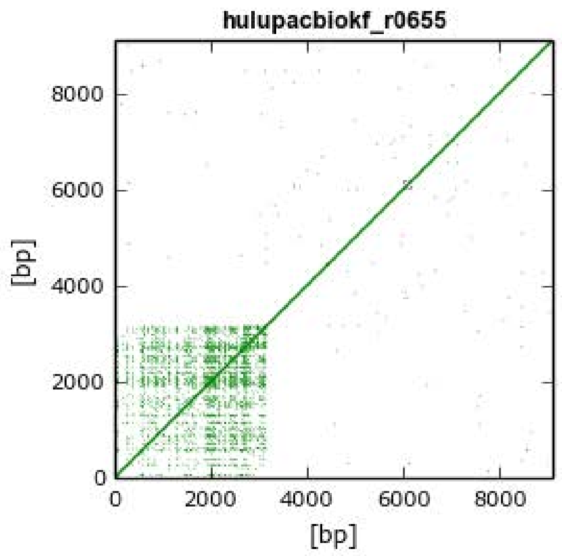
hulupacbiokf_r0655
[bp]
0
2000
4000
6000
8000
[bp]

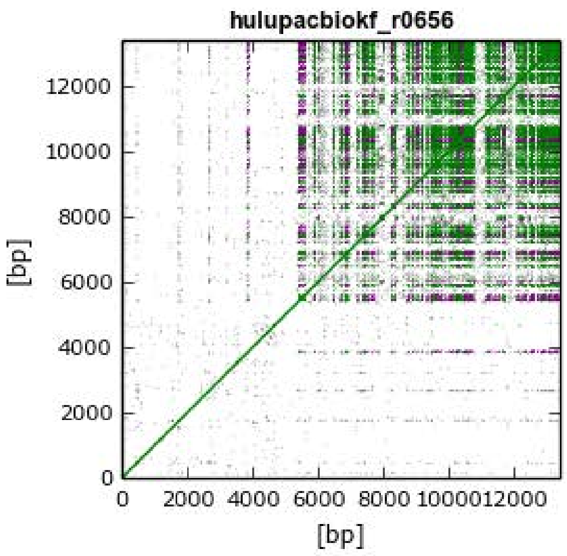
hulupacbiokf_r0656
[bp]
0
2000
4000
6000
8000
10000
12000
[bp]

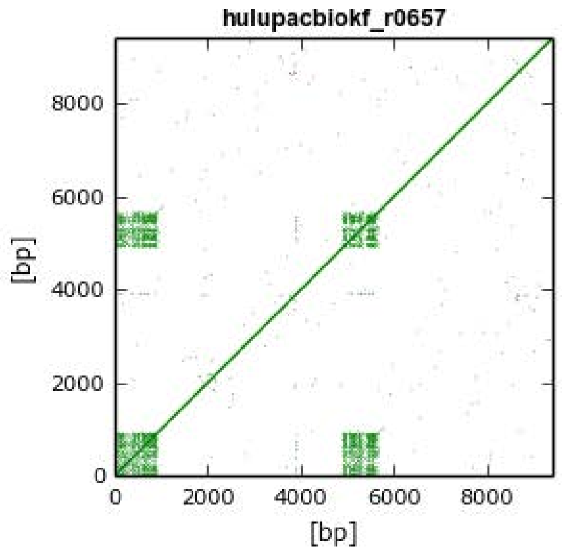
hulupacbiokf_r0657
[bp]
0
2000
4000
6000
8000
[bp]

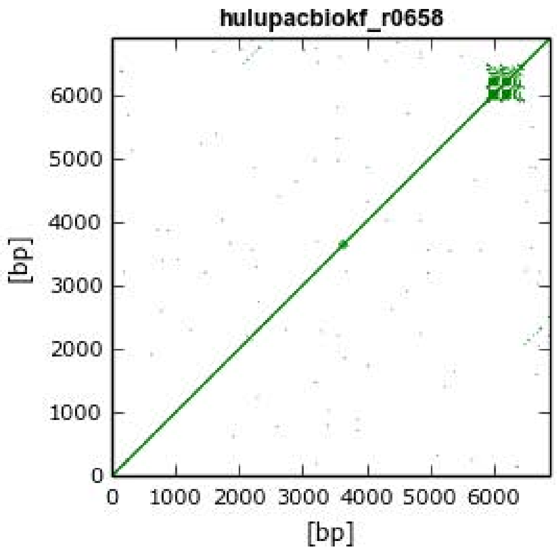
hulupacbiokf_r0658
[bp]
0
1000
2000
3000
4000
5000
6000
[bp]

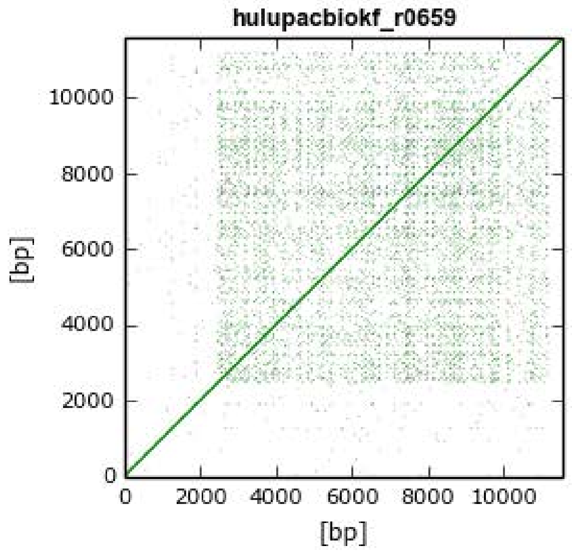
hulupacbiokf_r0659
[bp]
0
2000
4000
6000
8000
10000
[bp]

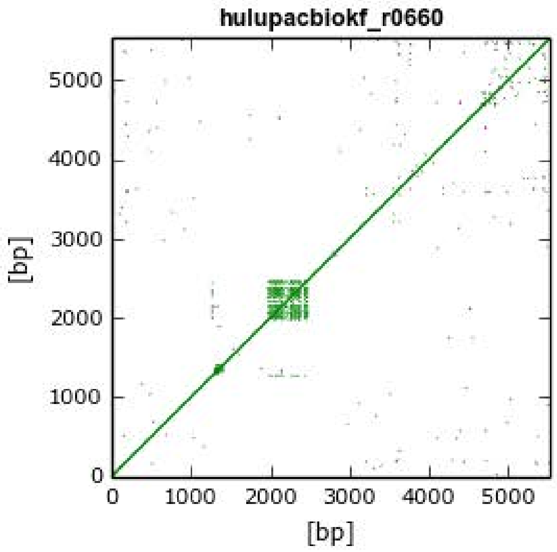
hulupacbiokf_r0660
[bp]
0
1000
2000
3000
4000
5000
[bp]

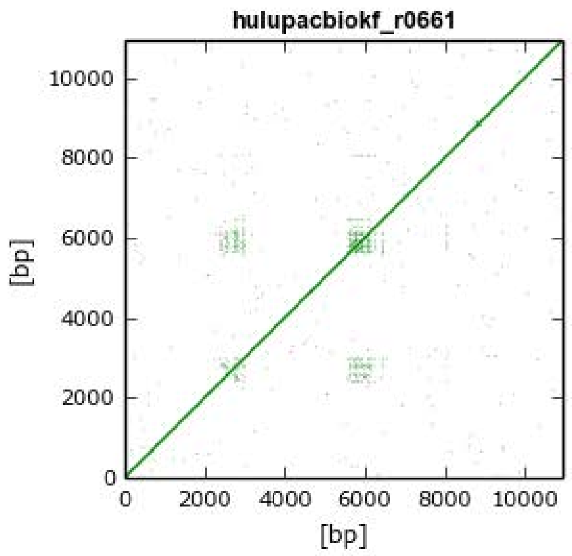
hulupacbiokf_r0661
[bp]
[bp]

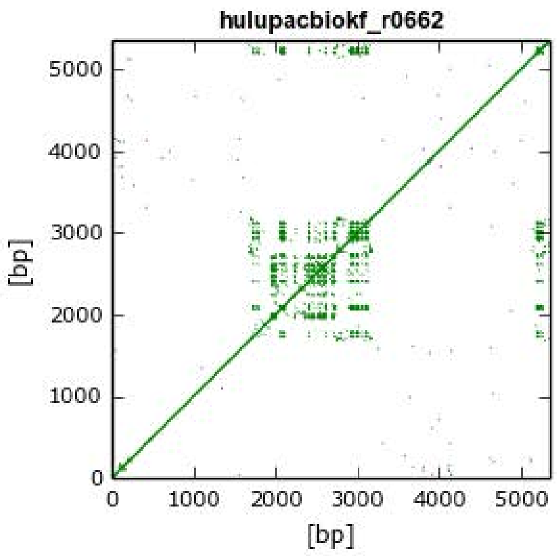
hulupacbiokf_r0662
[bp]
[bp]

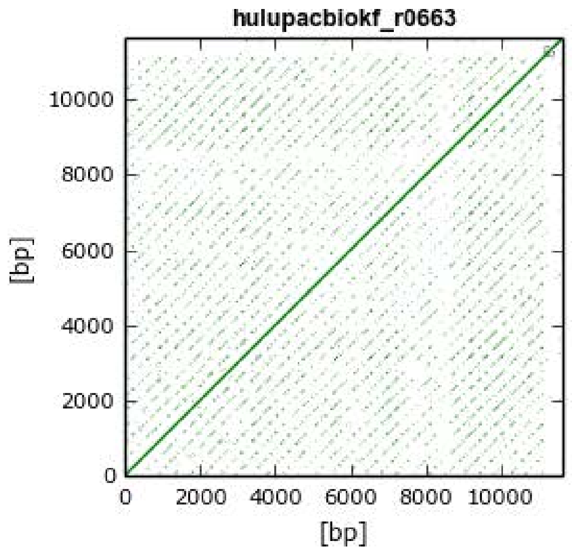
hulupacbiokf_r0663
[bp]
[bp]

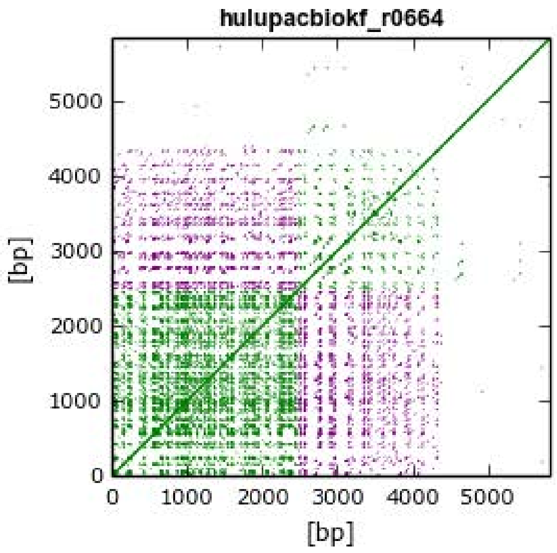
hulupacbiokf_r0664
[bp]
[bp]

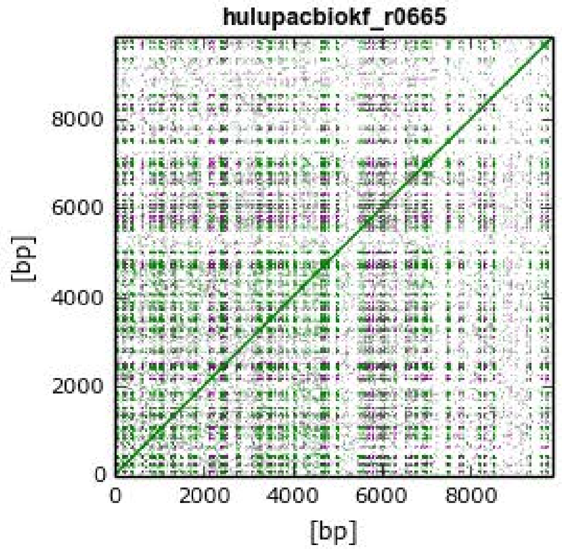
hulupacbiokf_r0665
[bp]
[bp]

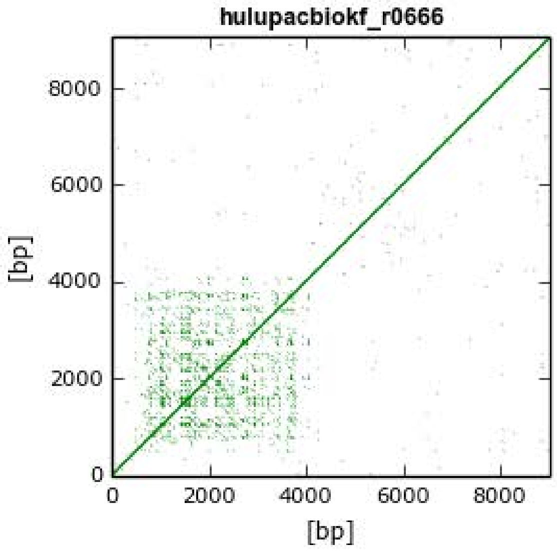
hulupacbiokf_r0666
[bp]
[bp]

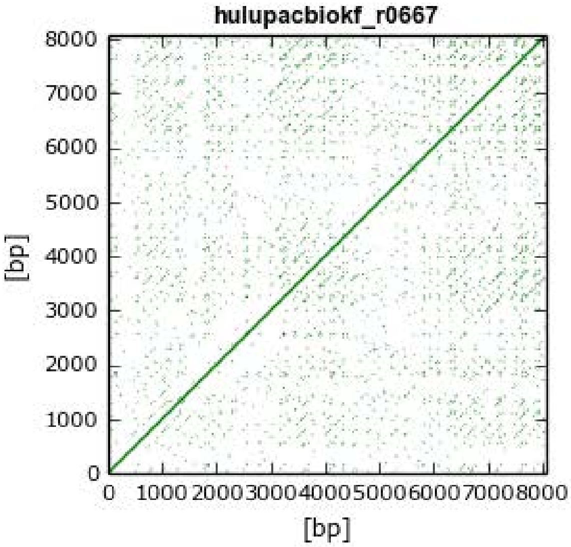
hulupacbiokf_r0667
[bp]
[bp]

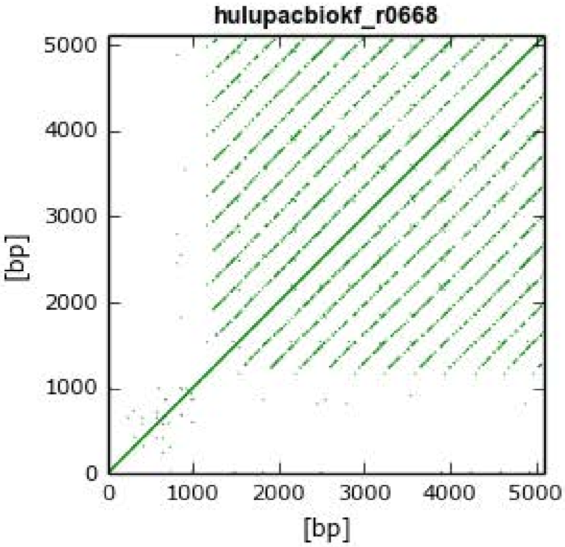
hulupacbiokf_r0668
[bp]
[bp]

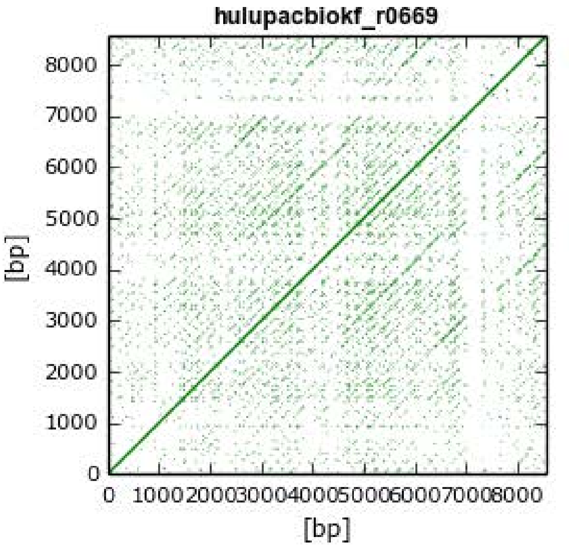
hulupacbiokf_r0669
[bp]
[bp]

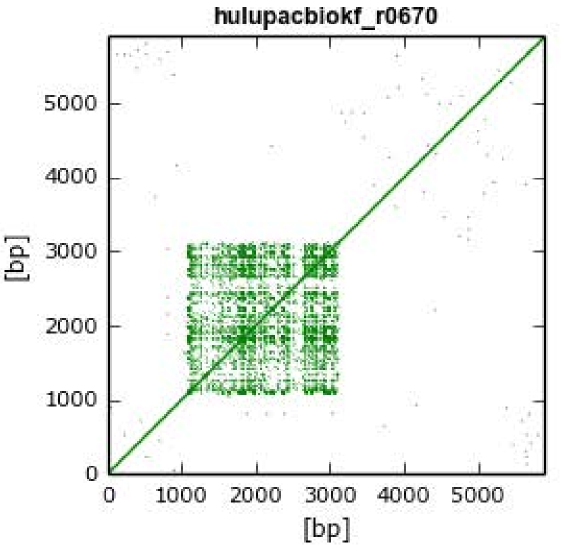
hulupacbiokf_r0670
[bp]
[bp]

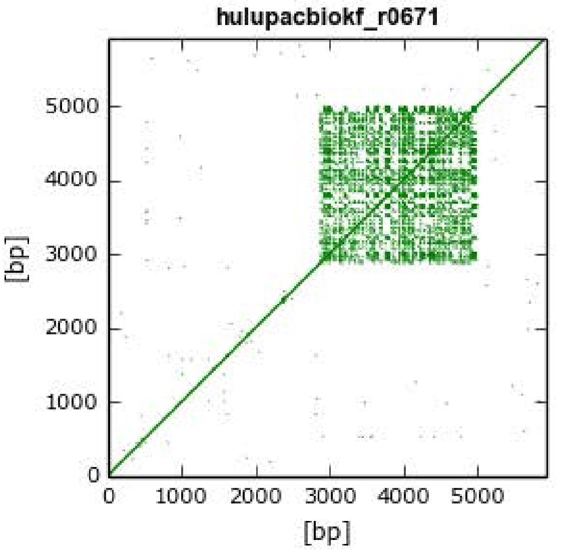
hulupacbiokf_r0671
[bp]
[bp]

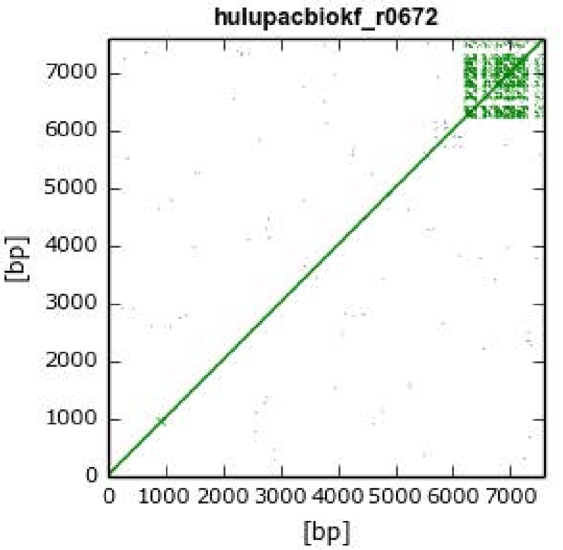
hulupacbiokf_r0672
[bp]
[bp]

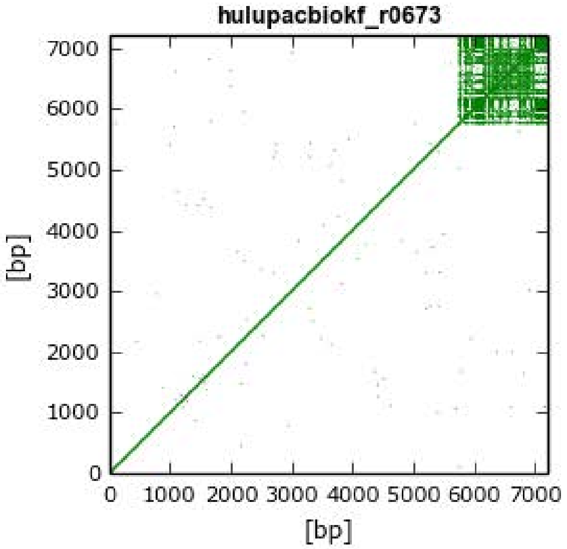
hulupacbiokf_r0673
[bp]
[bp]

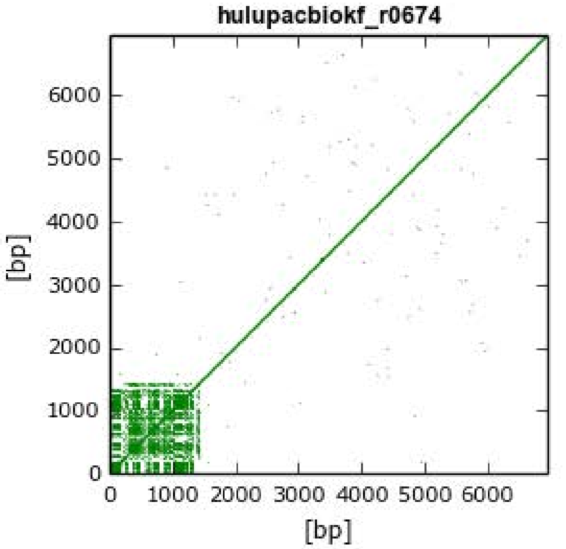
hulupacbiokf_r0674
[bp]
[bp]

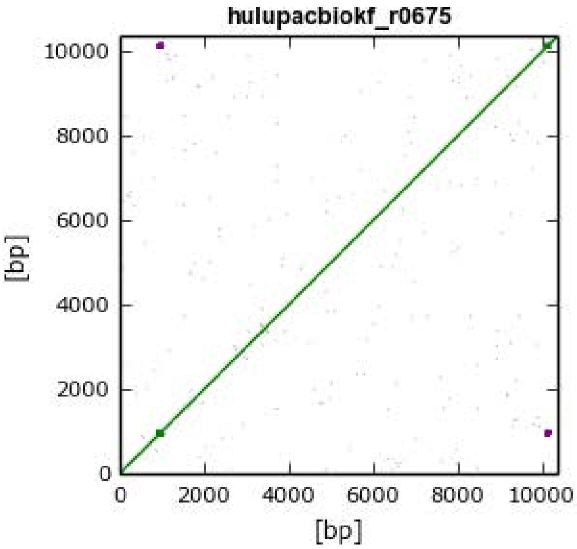
hulupacbiokf_r0675
[bp]
[bp]

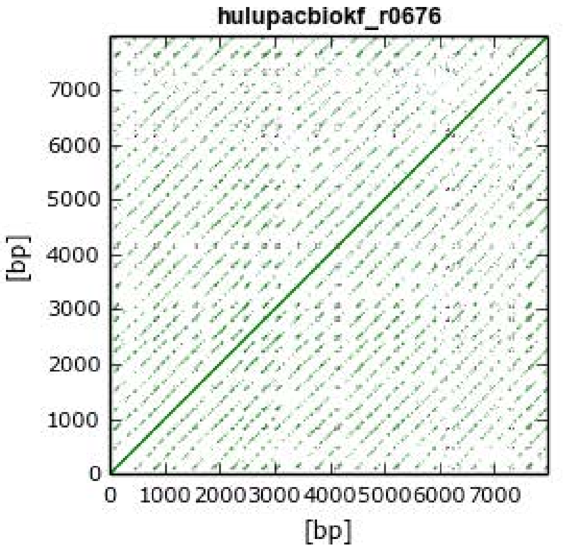
hulupacbiokf_r0676
[bp]
[bp]

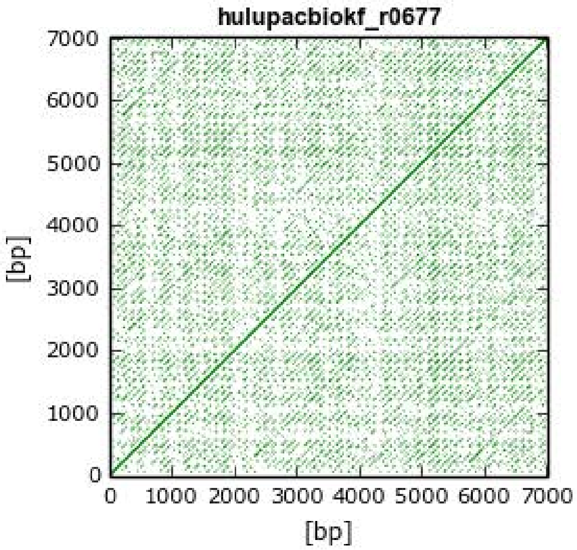
hulupacbiokf_r0677
[bp]
[bp]

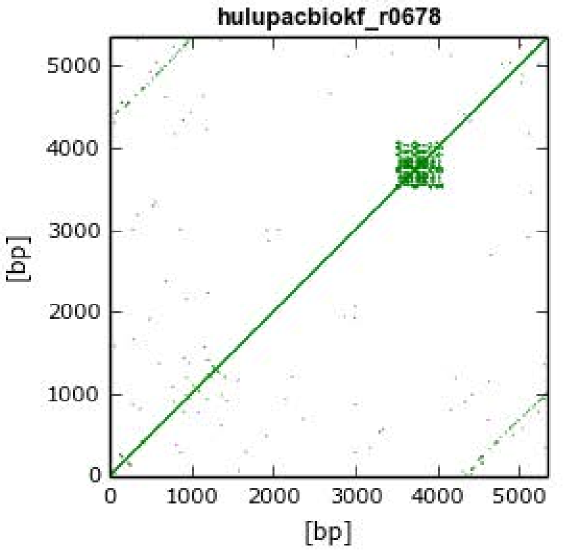
hulupacbiokf_r0678
[bp]
[bp]

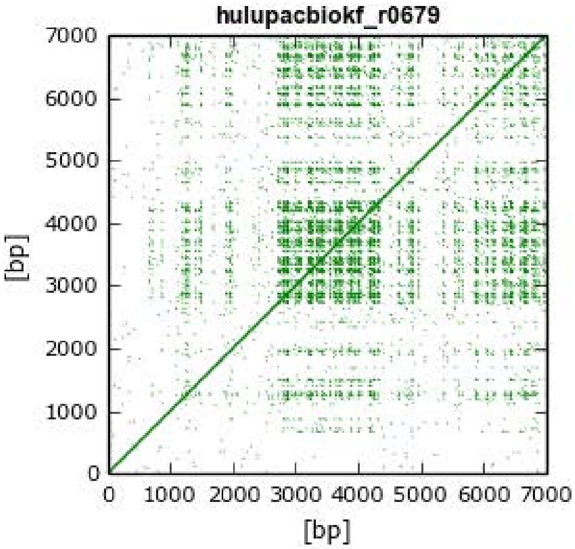
hulupacbiokf_r0679
[bp]
[bp]

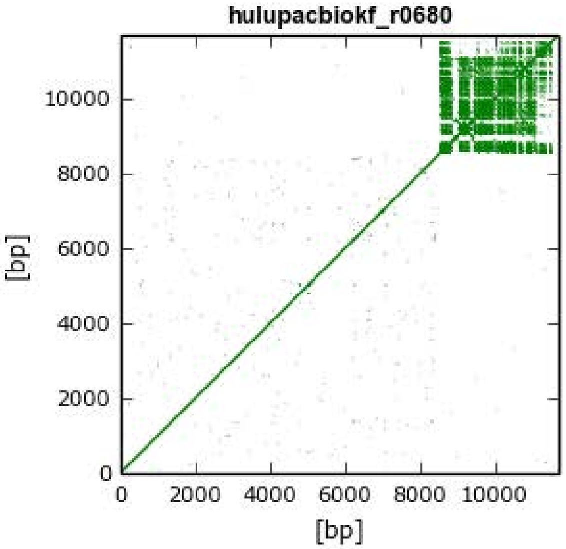
hulupacbiokf_r0680
[bp]
[bp]

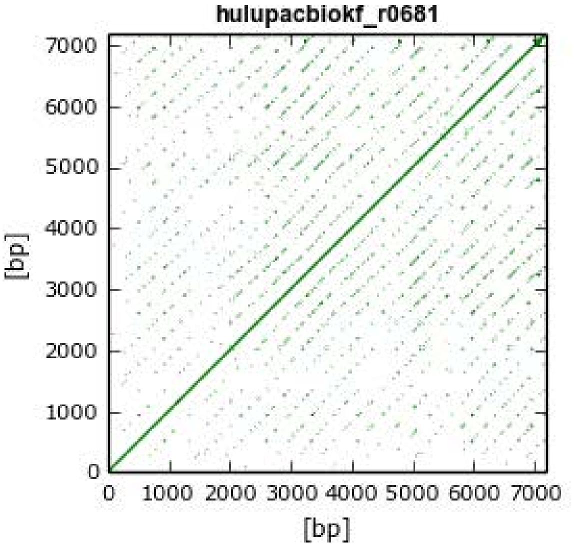
hulupacbiokf_r0681
[bp]
[bp]

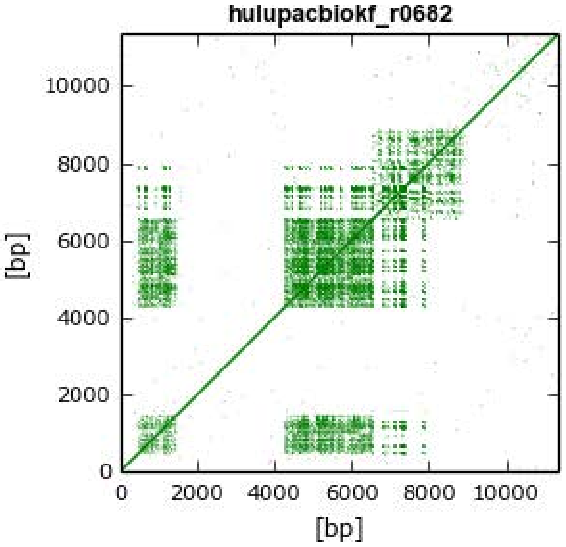
hulupacbiokf_r0682
[bp]
[bp]

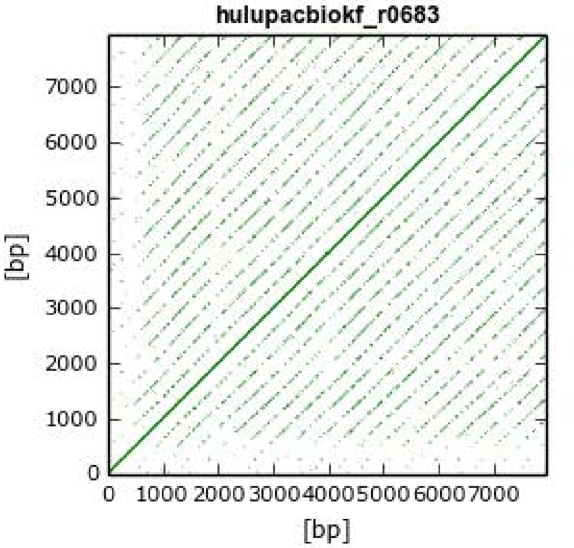
hulupacbiokf_r0683
[bp]
[bp]

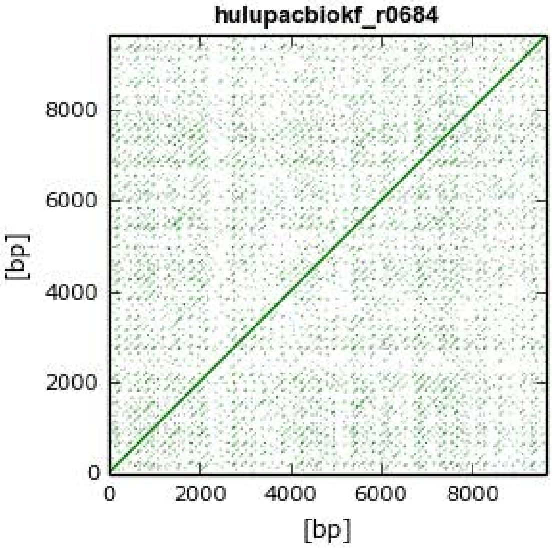
hulupacbiokf_r0684
[bp]
[bp]

hulupacbiokf_r0685

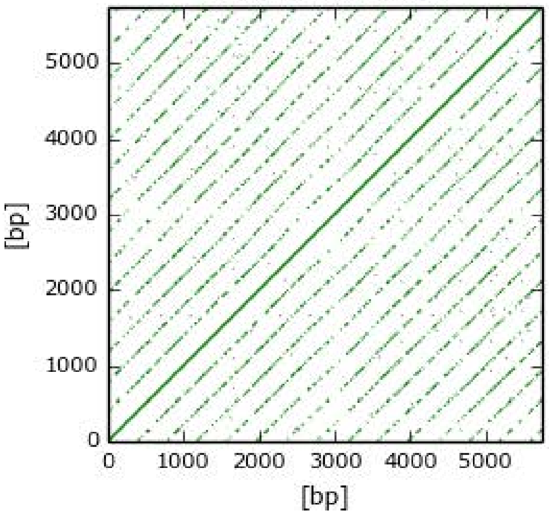

hulupacbiokf_r0686

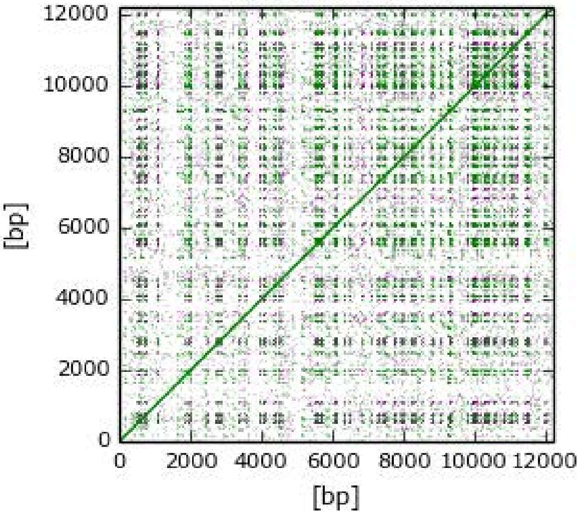

hulupacbiokf_r0687

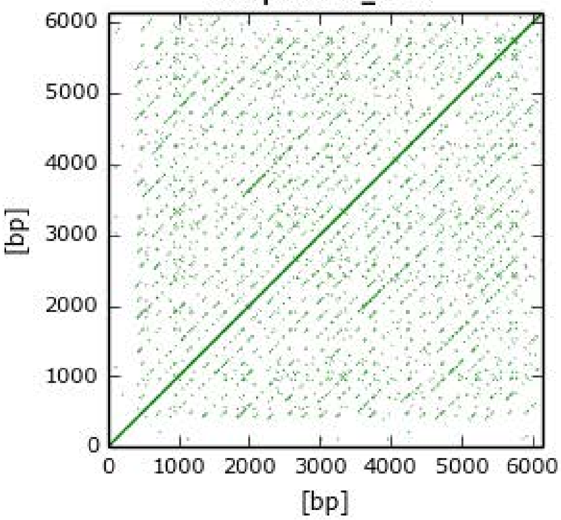

hulupacbiokf_r0688

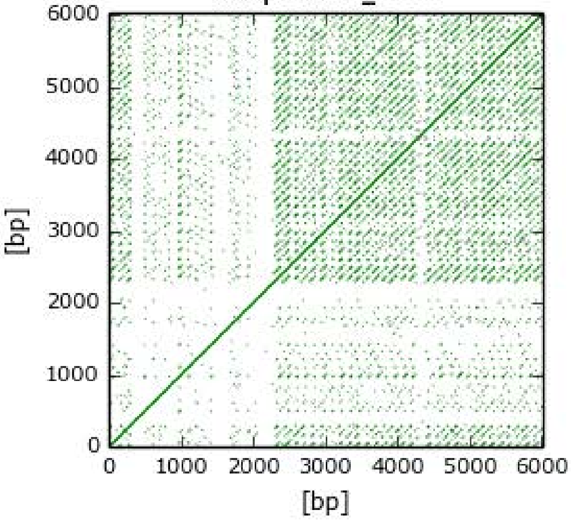

hulupacbiokf_r0689

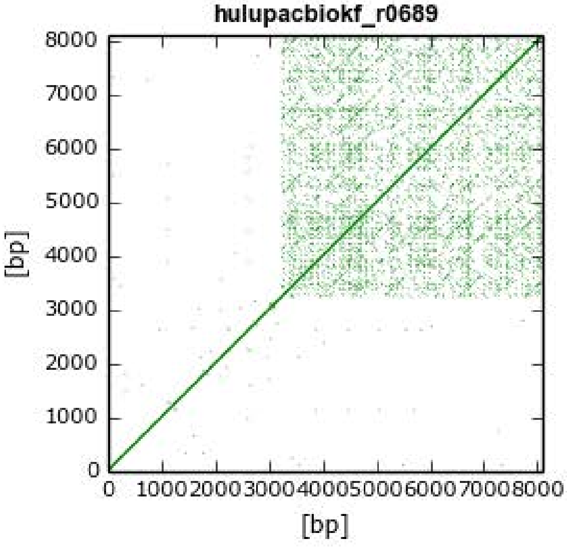

hulupacbiokf_r0690

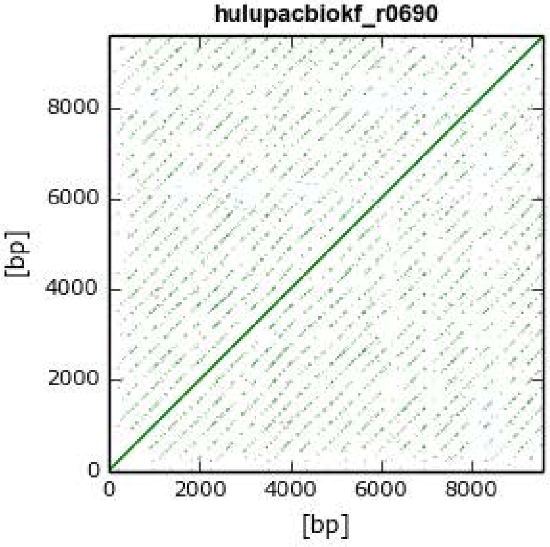

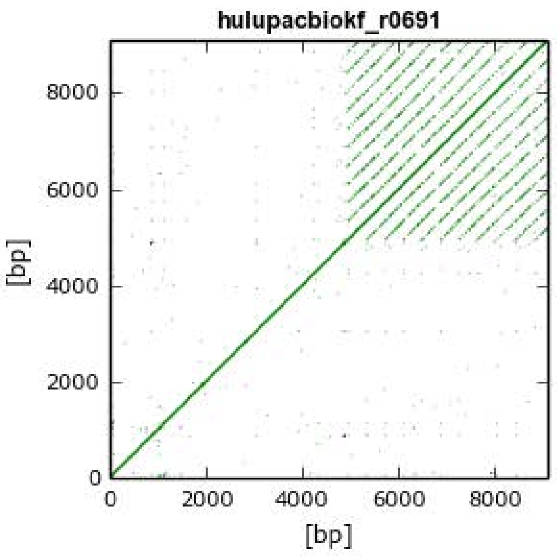
hulupacbiokf_r0691
0
2000
4000
6000
8000
[bp]
0
2000
4000
6000
8000
[bp]

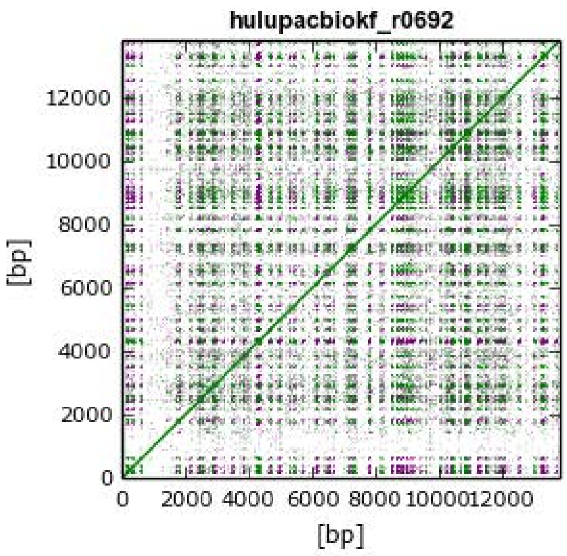
hulupacbiokf_r0692
0
2000
4000
6000
8000
10000
12000
[bp]
0
2000
4000
6000
8000
10000
12000
[bp]

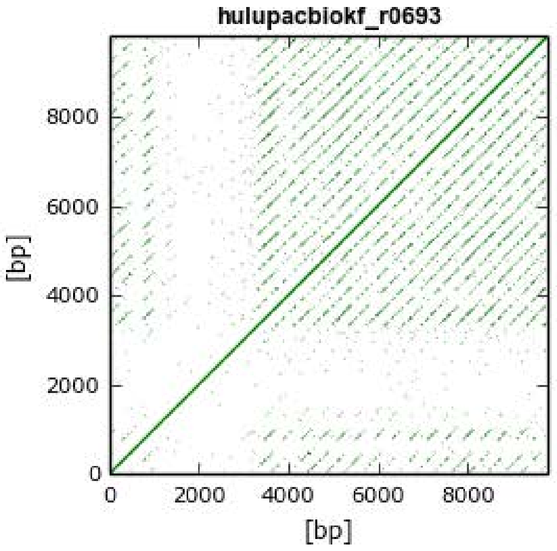
hulupacbiokf_r0693
0
2000
4000
6000
8000
[bp]
0
2000
4000
6000
8000
[bp]

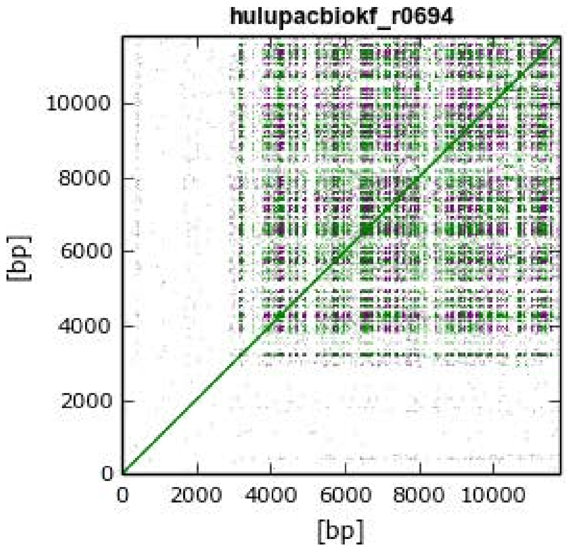
hulupacbiokf_r0694
0
2000
4000
6000
8000
10000
[bp]
0
2000
4000
6000
8000
10000
[bp]

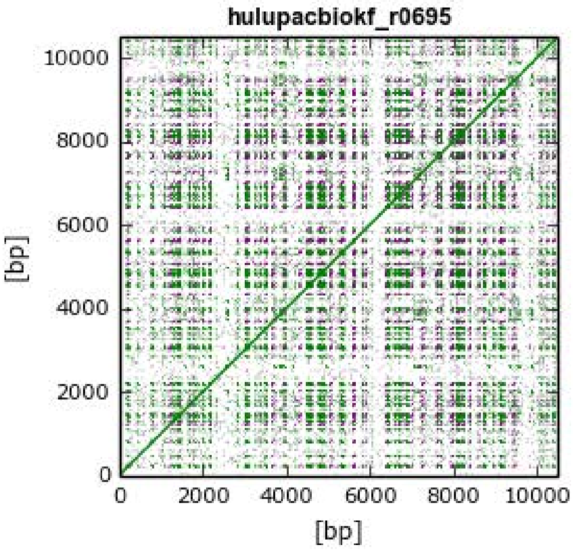
hulupacbiokf_r0695
0
2000
4000
6000
8000
10000
[bp]
0
2000
4000
6000
8000
10000
[bp]

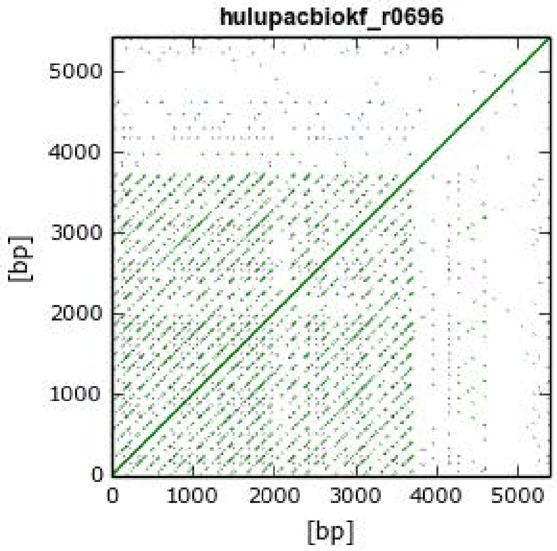
hulupacbiokf_r0696
0
1000
2000
3000
4000
5000
[bp]
0
1000
2000
3000
4000
5000
[bp]

hulupacbiokf_r0697

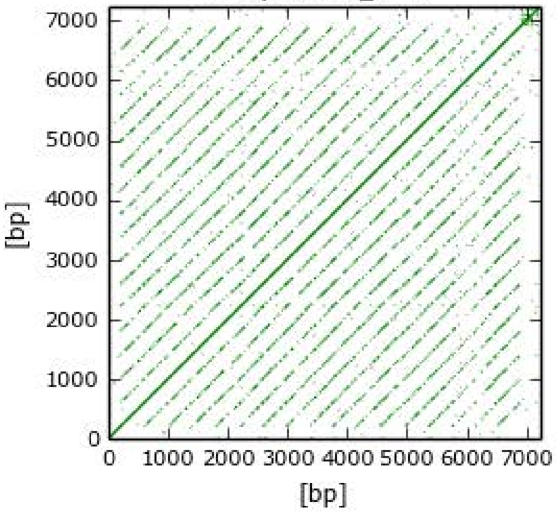

hulupacbiokf_r0698

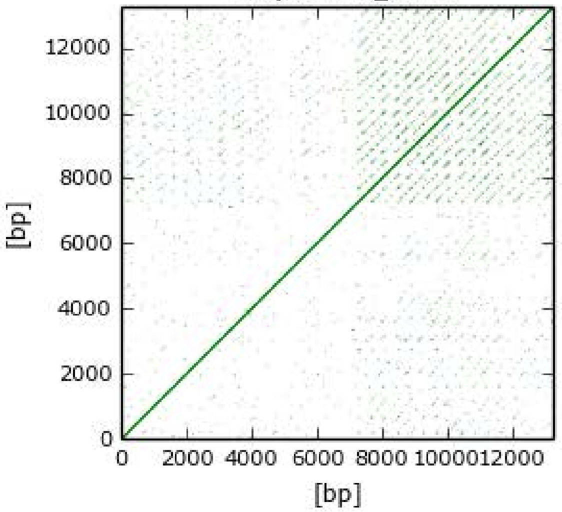

hulupacbiokf_r0699

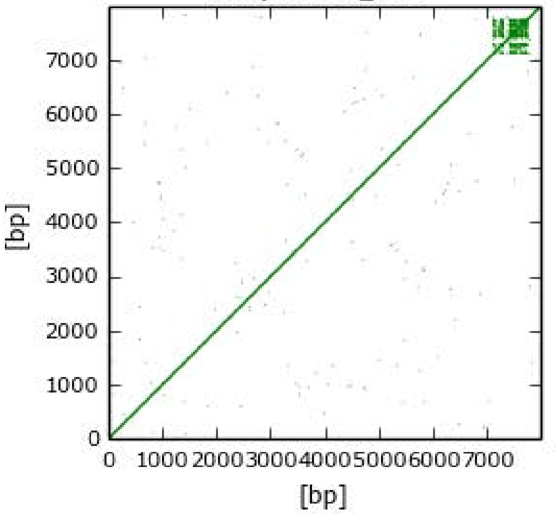

hulupacbiokf_r0700

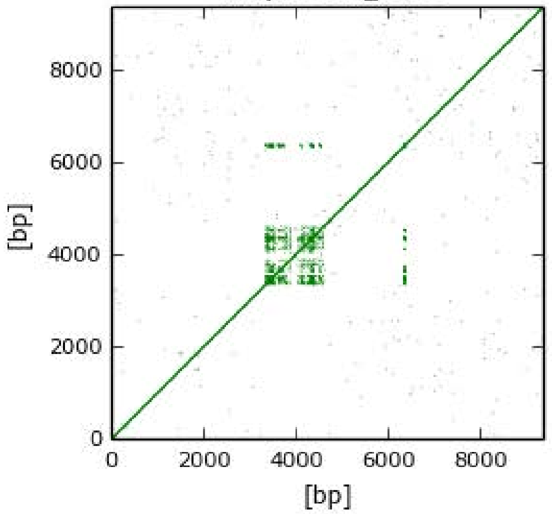

hulupacbiokf_r0701

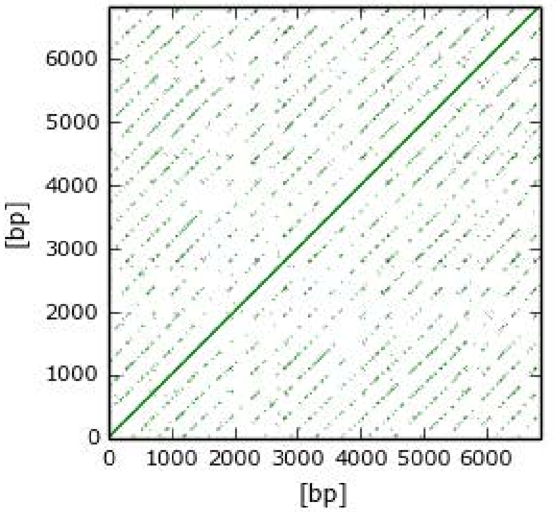

hulupacbiokf_r0702

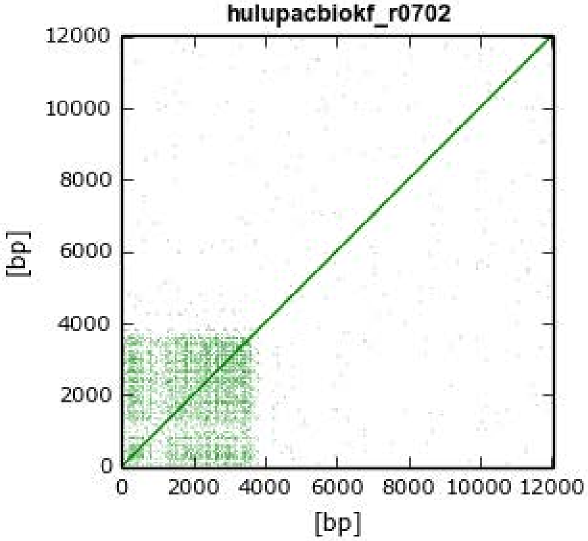

hulupacbiokf_r0703

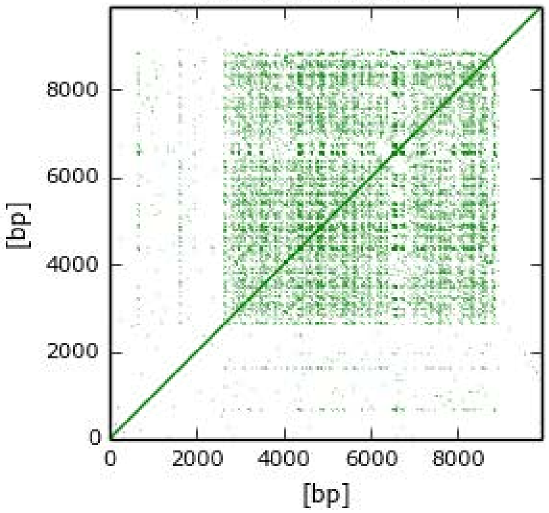

hulupacbiokf_r0704

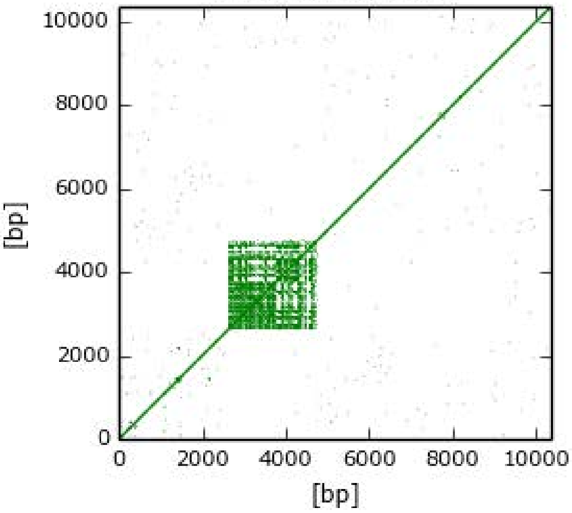

hulupacbiokf_r0705

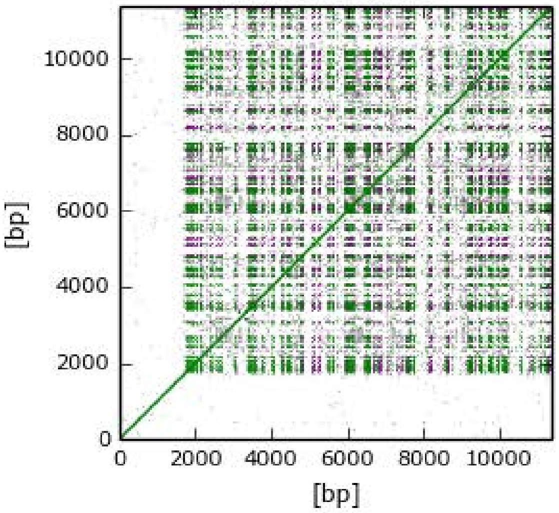

hulupacbiokf_r0706

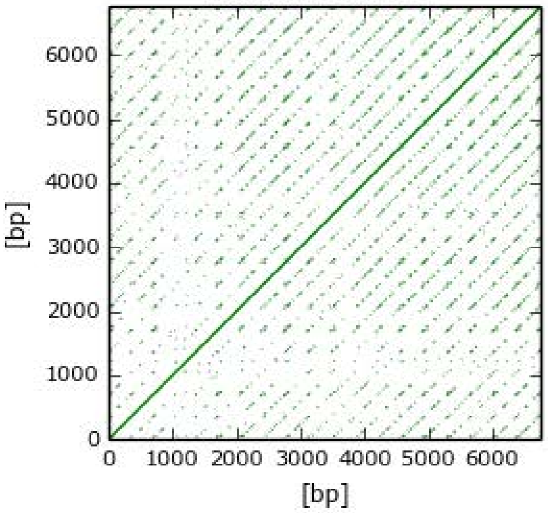

hulupacbiokf_r0707

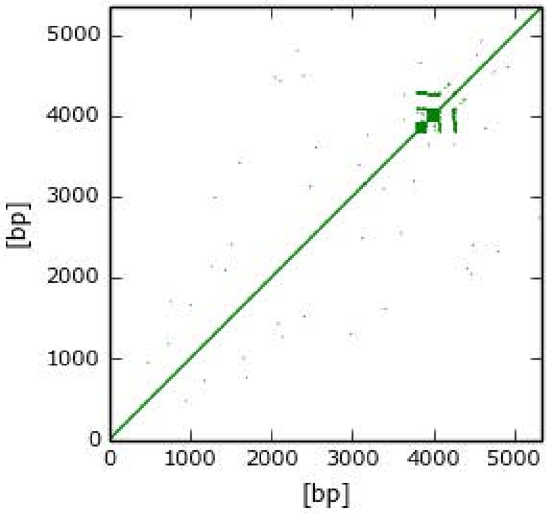

hulupacbiokf_r0708

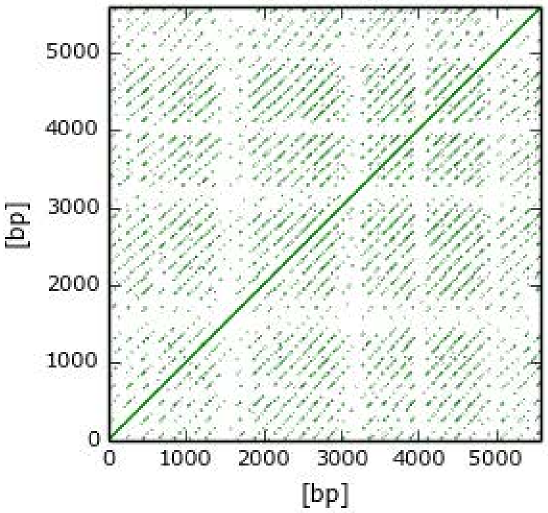

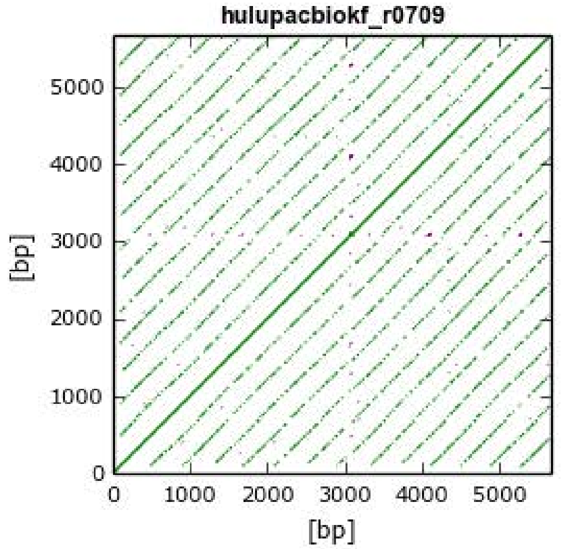
hulupacbiokf_r0709
[bp]
0
1000
2000
3000
4000
5000
[bp]

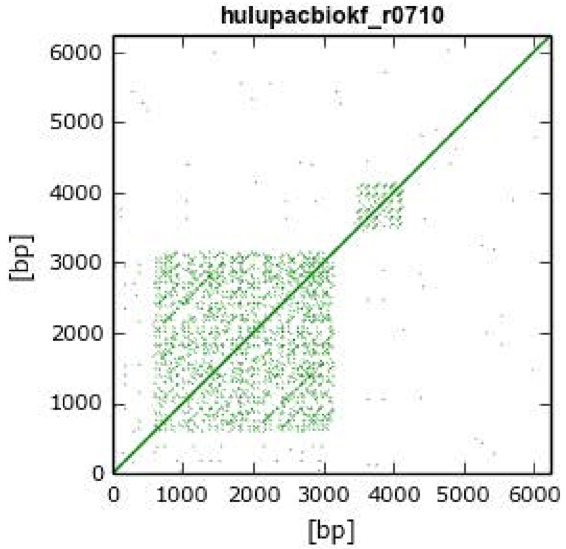
hulupacbiokf_r0710
[bp]
0
1000
2000
3000
4000
5000
6000
[bp]

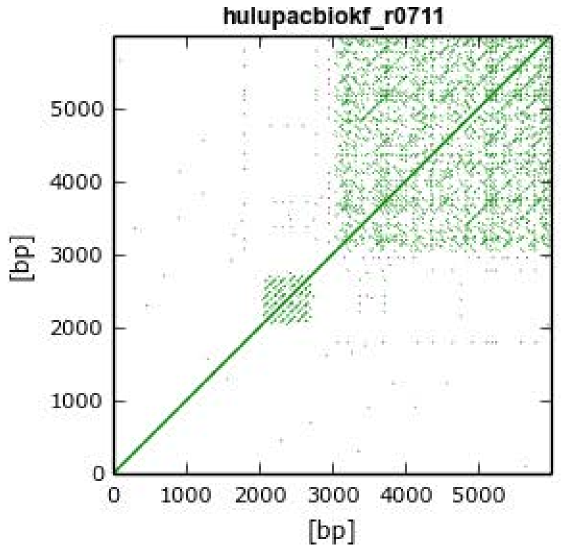
hulupacbiokf_r0711
[bp]
0
1000
2000
3000
4000
5000
[bp]

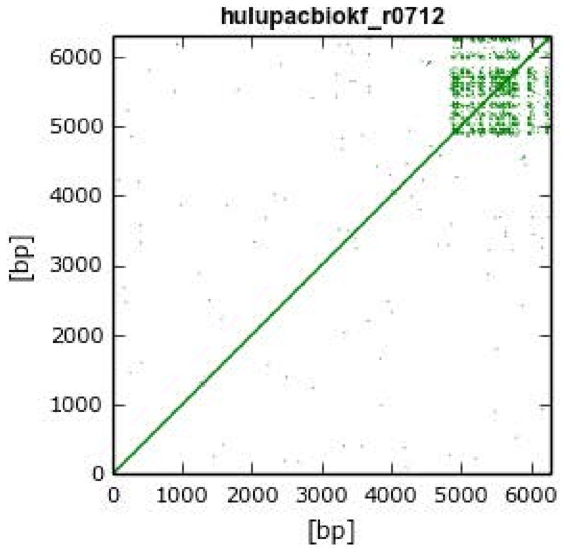
hulupacbiokf_r0712
[bp]
0
1000
2000
3000
4000
5000
6000
[bp]

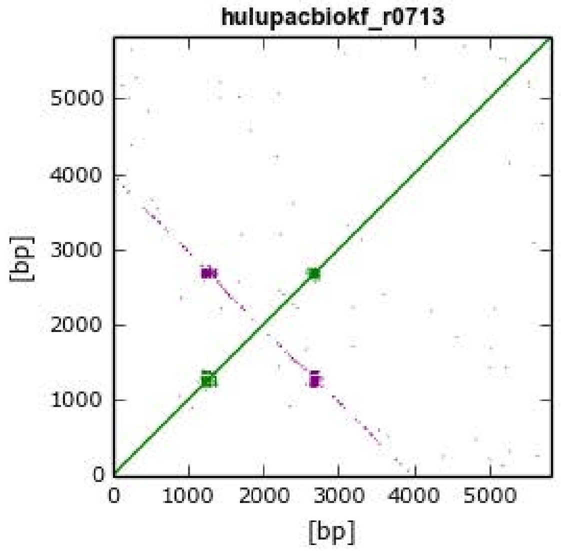
hulupacbiokf_r0713
[bp]
0
1000
2000
3000
4000
5000
[bp]

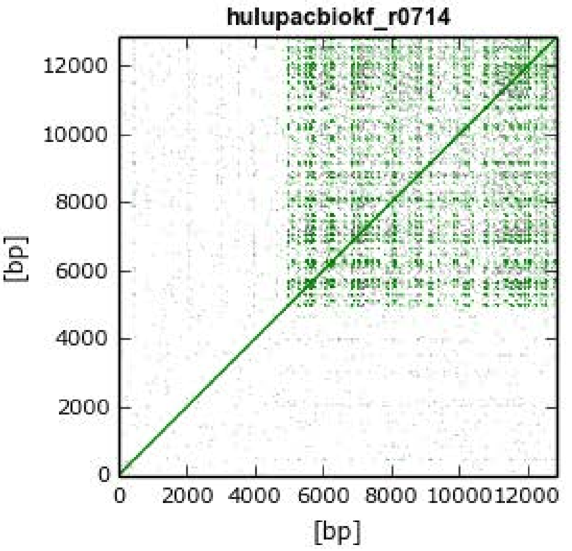
hulupacbiokf_r0714
[bp]
0
2000
4000
6000
8000
10000
12000
[bp]

hulupacbiokf_r0715

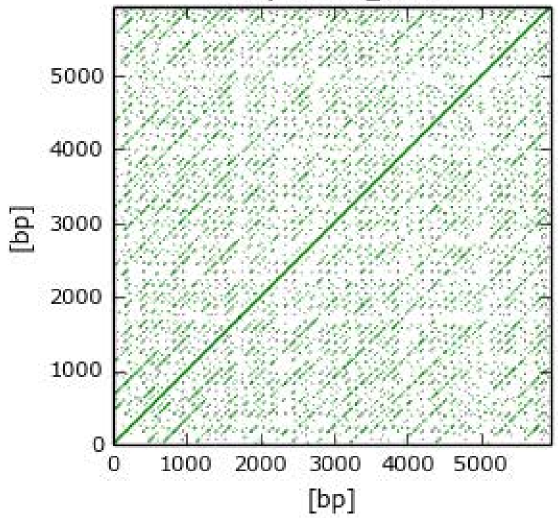

hulupacbiokf_r0716

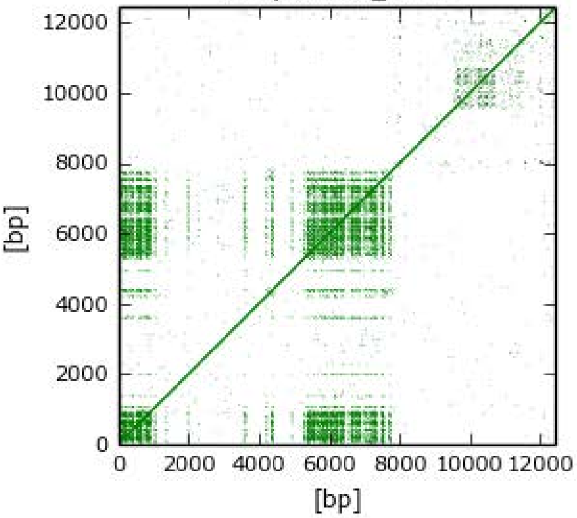

hulupacbiokf_r0717

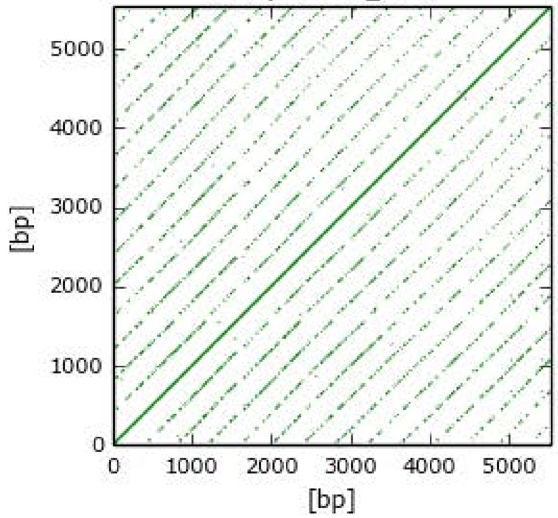

hulupacbiokf_r0718

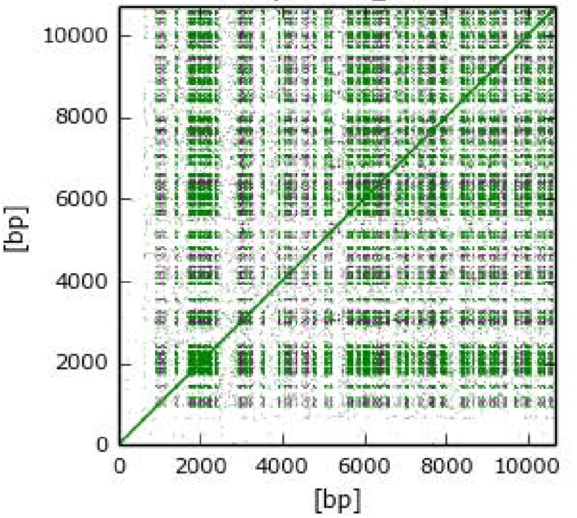

hulupacbiokf_r0719

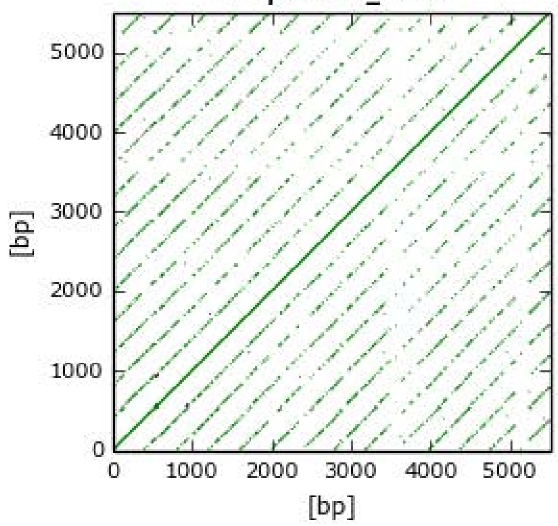

hulupacbiokf_r0720

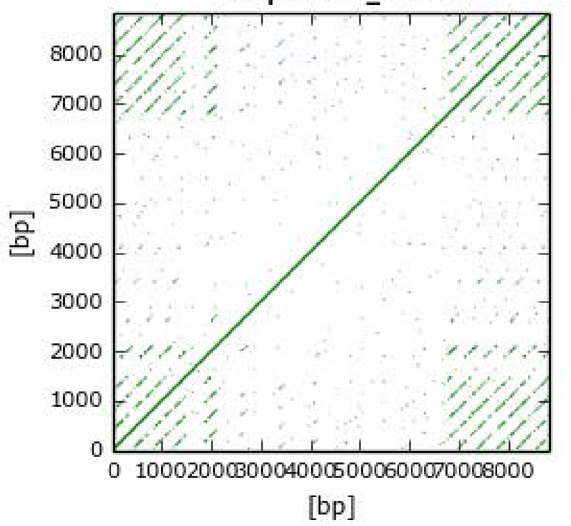

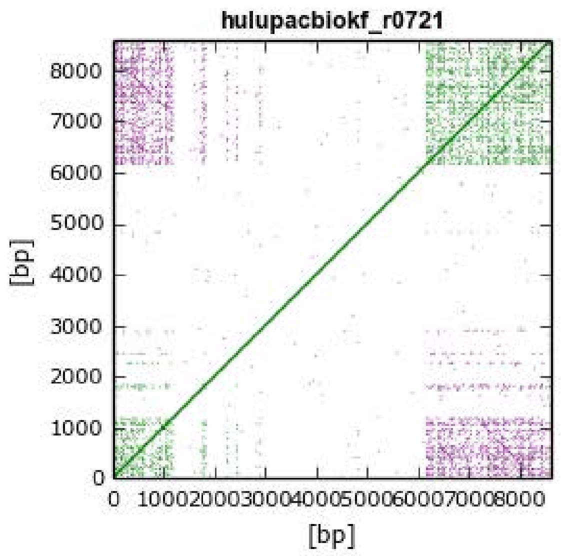
hulupacbiokf_r0721
[bp]
[bp]

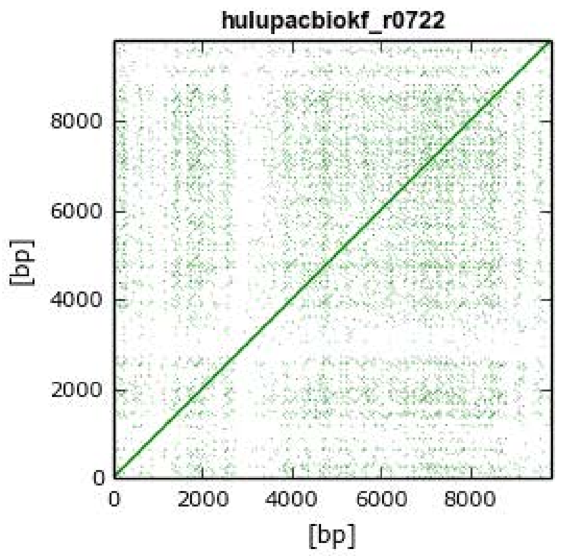
hulupacbiokf_r0722
[bp]
[bp]

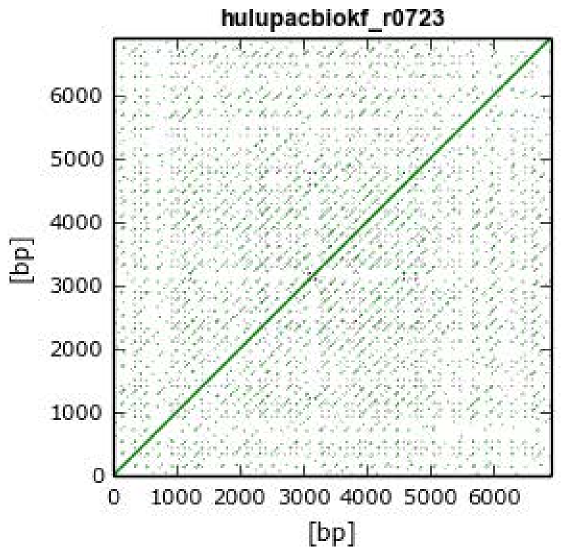
hulupacbiokf_r0723
[bp]
[bp]

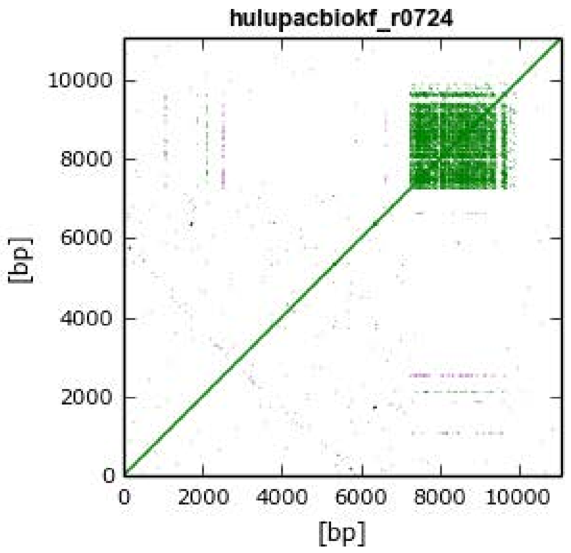
hulupacbiokf_r0724
[bp]
[bp]

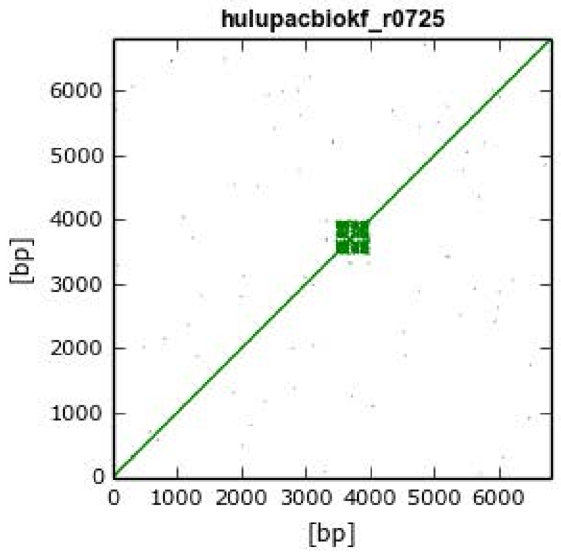
hulupacbiokf_r0725
[bp]
[bp]

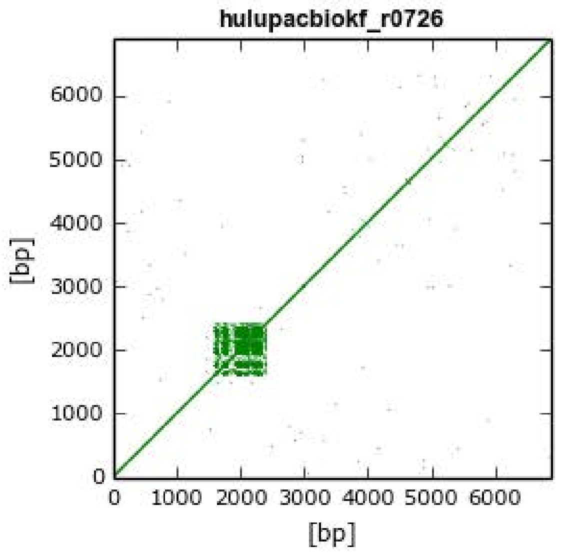
hulupacbiokf_r0726
[bp]
[bp]

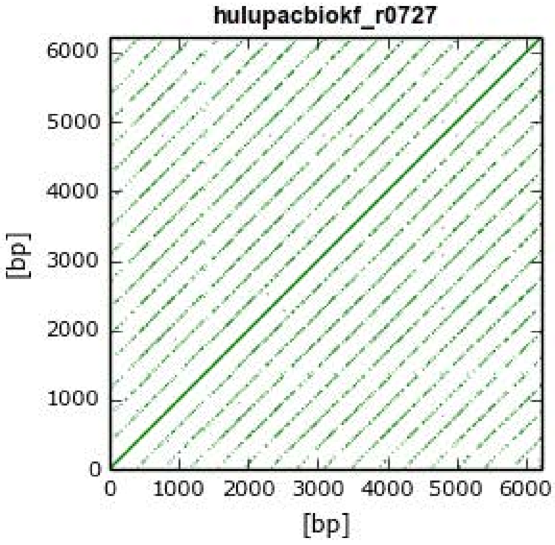
hulupacbiokf_r0727
[bp]
[bp]

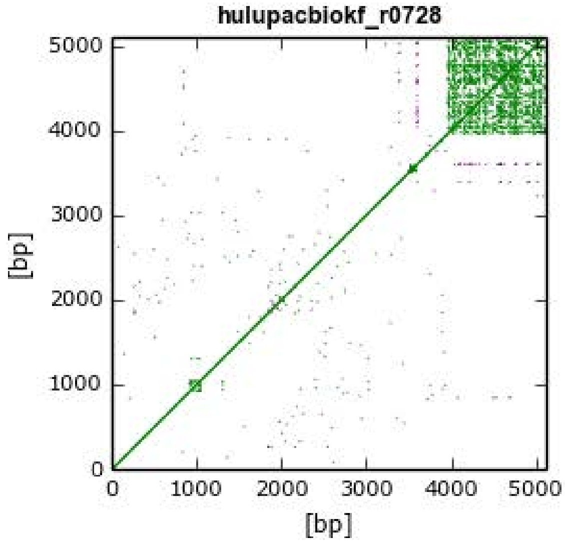
hulupacbiokf_r0728
[bp]
[bp]

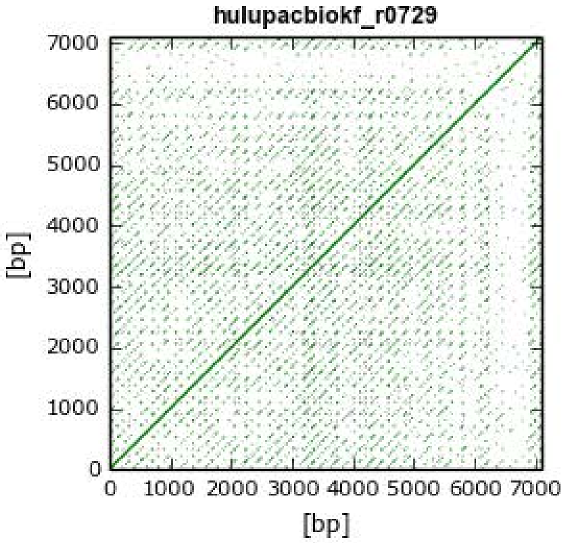
hulupacbiokf_r0729
[bp]
[bp]

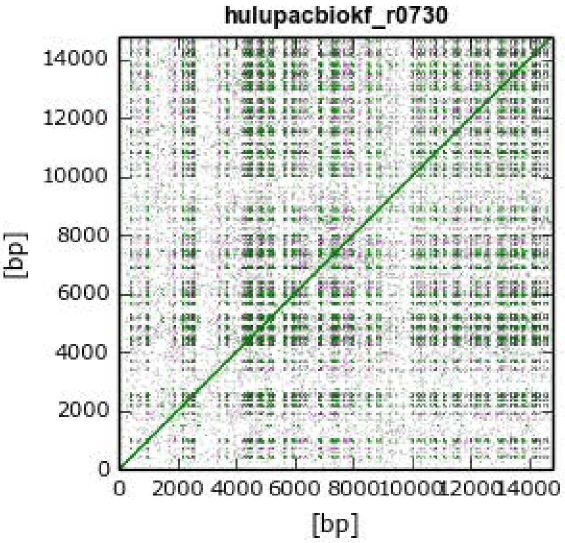
hulupacbiokf_r0730
[bp]
[bp]

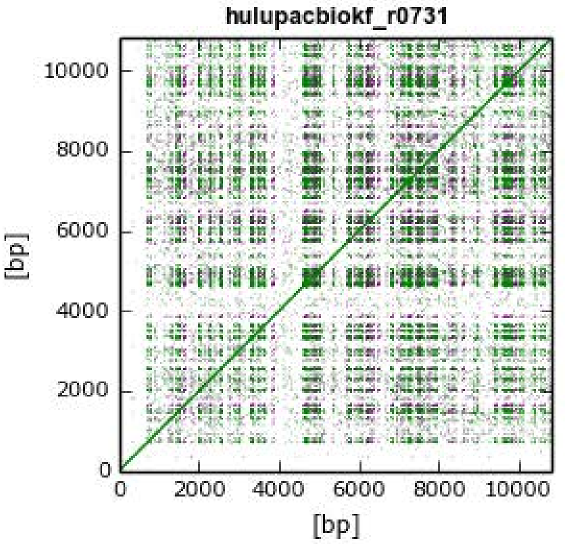
hulupacbiokf_r0731
[bp]
[bp]

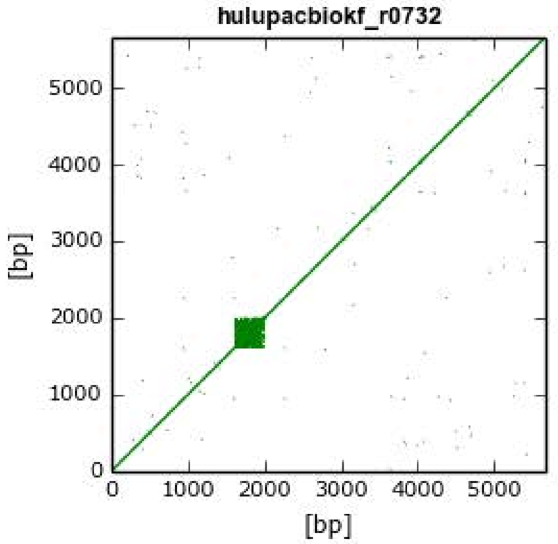
hulupacbiokf_r0732
[bp]
[bp]

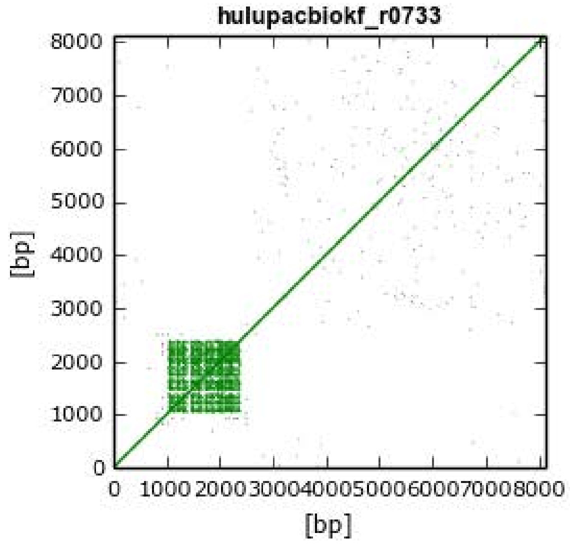
hulupacbiokf_r0733
[bp]
[bp]

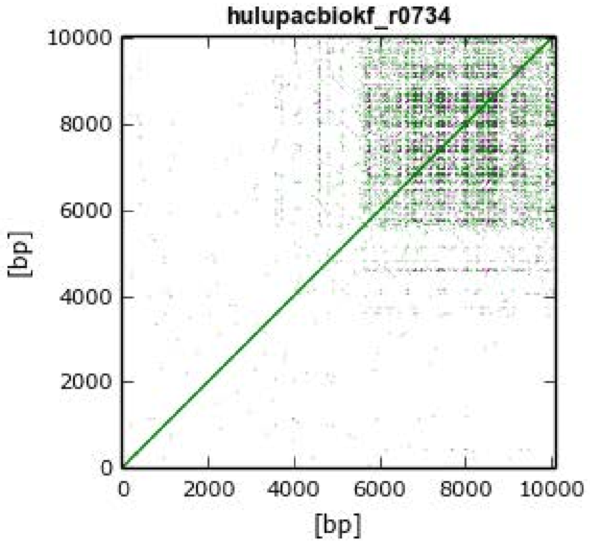
hulupacbiokf_r0734
[bp]
[bp]

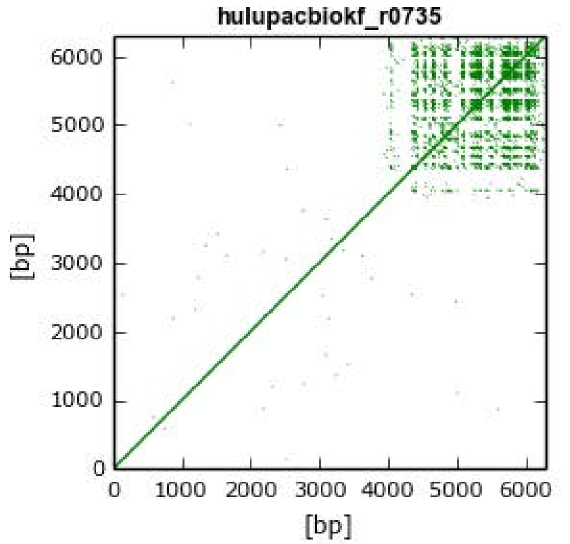
hulupacbiokf_r0735
[bp]
[bp]

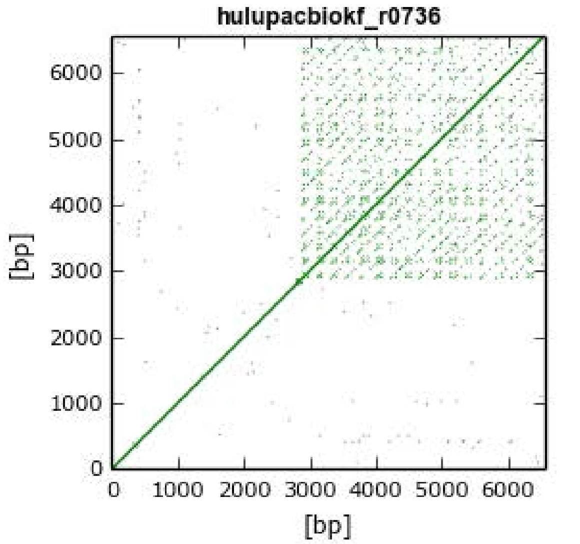
hulupacbiokf_r0736
[bp]
[bp]

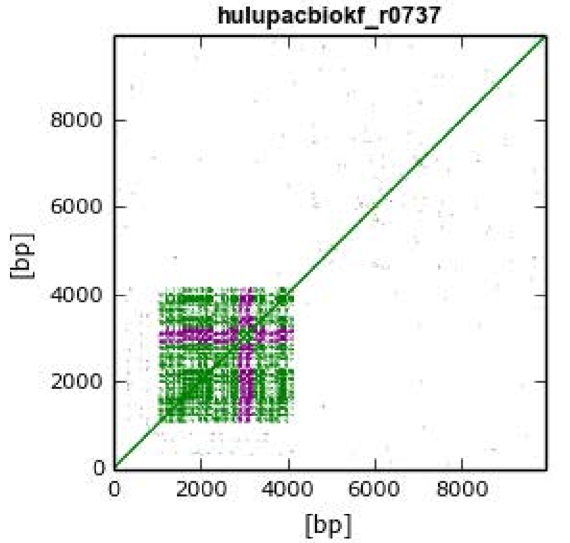
hulupacbiokf_r0737
[bp]
[bp]

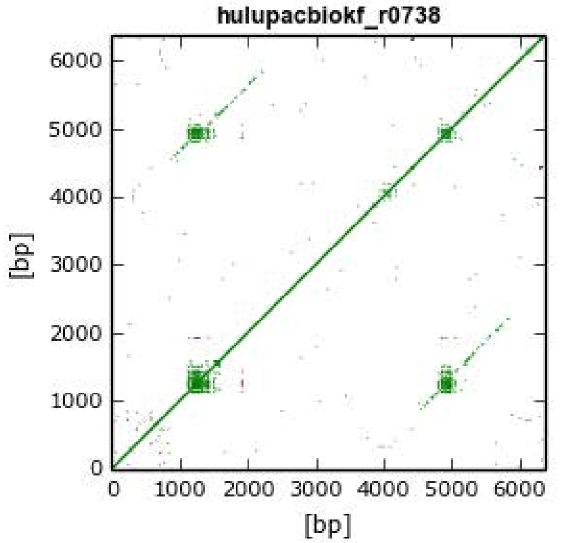
hulupacbiokf_r0738
[bp]
[bp]

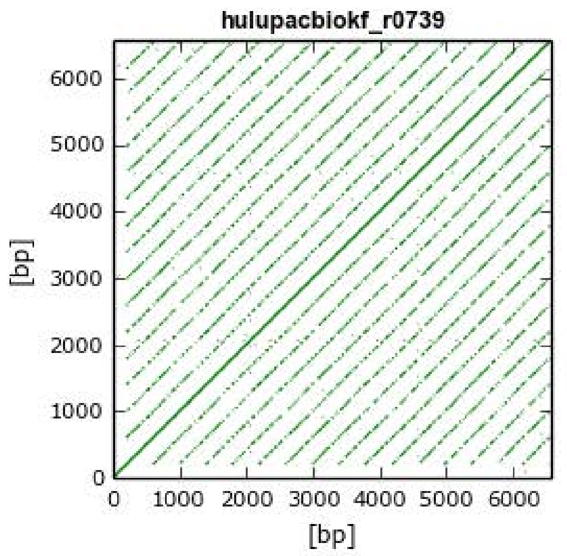
hulupacbiokf_r0739
6000
5000
4000
3000
2000
1000
0
0 1000 2000 3000 4000 5000 6000
[bp]
[bp]

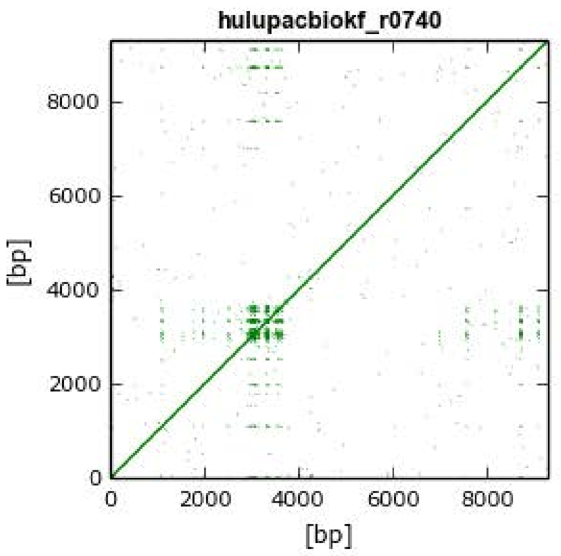
hulupacbiokf_r0740
8000
6000
4000
2000
0
0 2000 4000 6000 8000
[bp]
[bp]

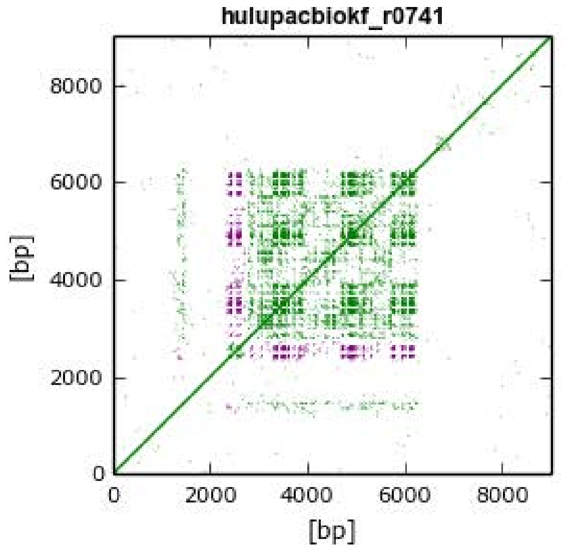
hulupacbiokf_r0741
8000
6000
4000
2000
0
0 2000 4000 6000 8000
[bp]
[bp]

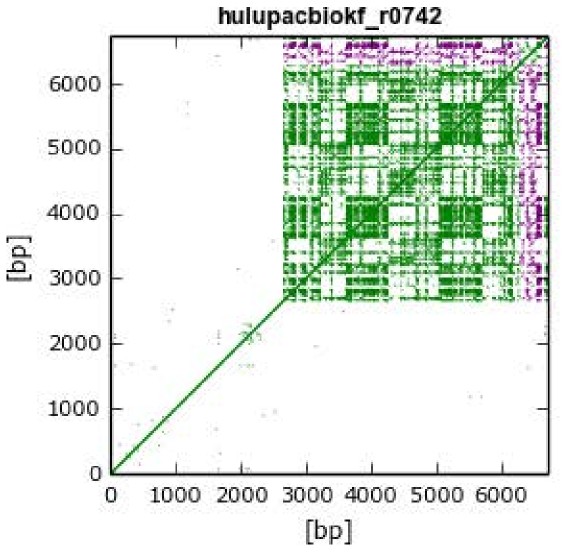
hulupacbiokf_r0742
6000
5000
4000
3000
2000
1000
0
0 1000 2000 3000 4000 5000 6000
[bp]
[bp]

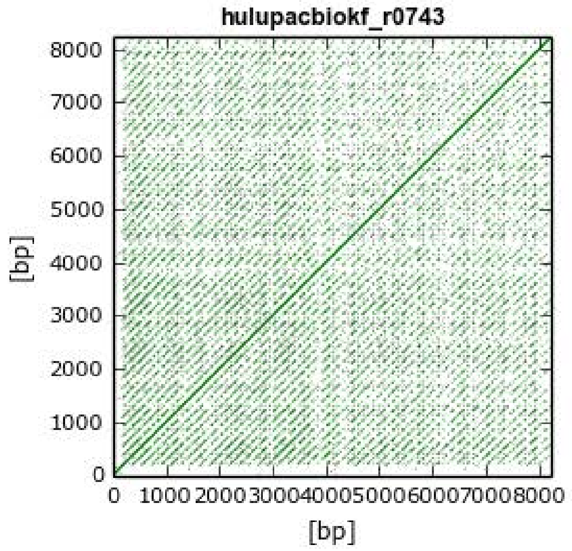
hulupacbiokf_r0743
8000
7000
6000
5000
4000
3000
2000
1000
0
0 1000 2000 3000 4000 5000 6000 7000 8000
[bp]
[bp]

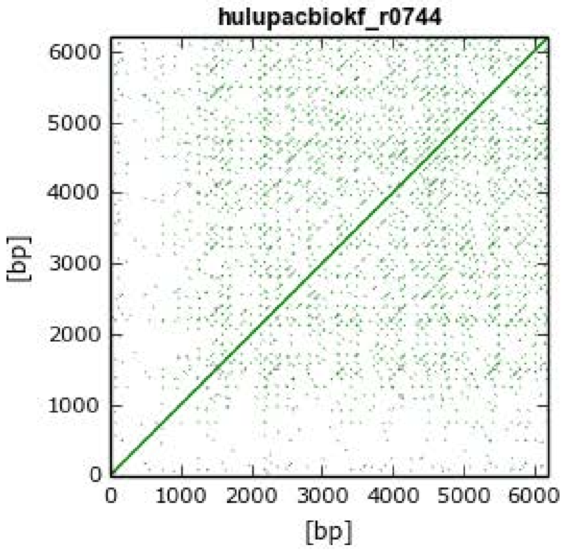
hulupacbiokf_r0744
6000
5000
4000
3000
2000
1000
0
0 1000 2000 3000 4000 5000 6000
[bp]
[bp]

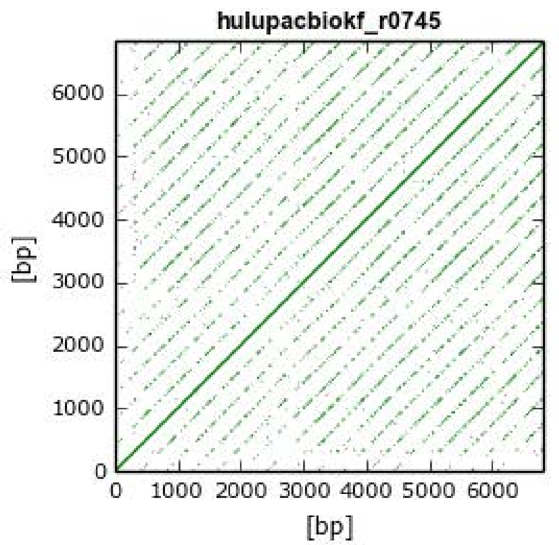
hulupacbiokf_r0745
[bp]
0
1000
2000
3000
4000
5000
6000
[bp]

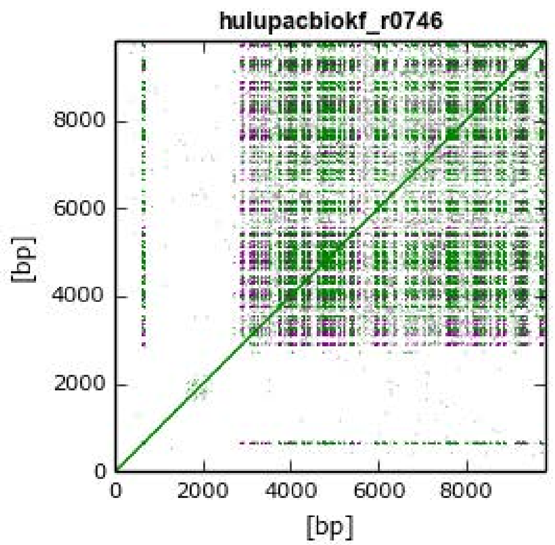
hulupacbiokf_r0746
[bp]
0
2000
4000
6000
8000
[bp]

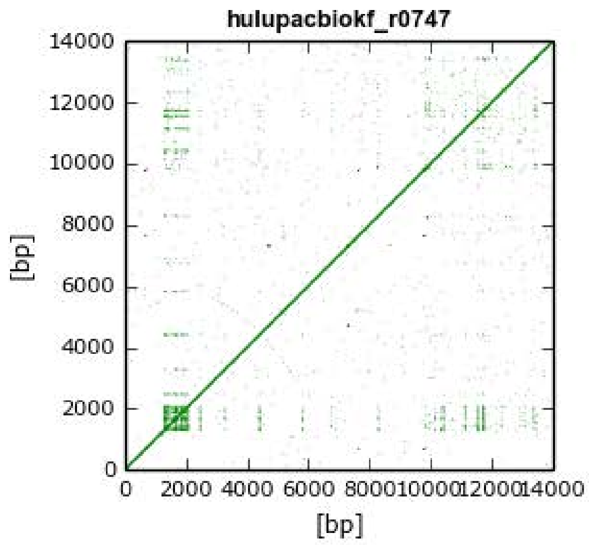
hulupacbiokf_r0747
[bp]
0
2000
4000
6000
8000
10000
12000
14000
[bp]

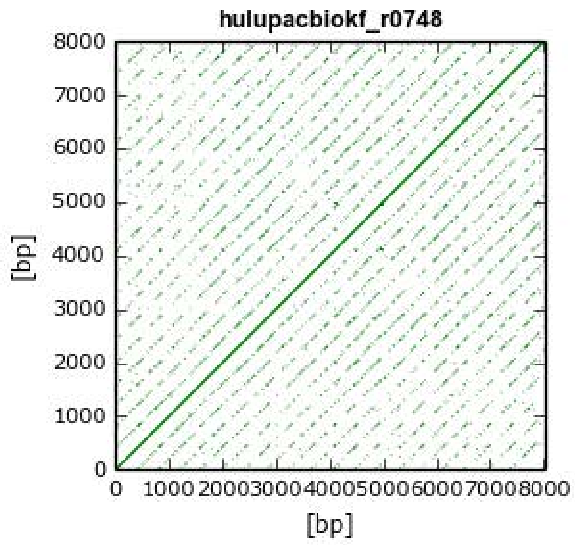
hulupacbiokf_r0748
[bp]
0
1000
2000
3000
4000
5000
6000
7000
8000
[bp]

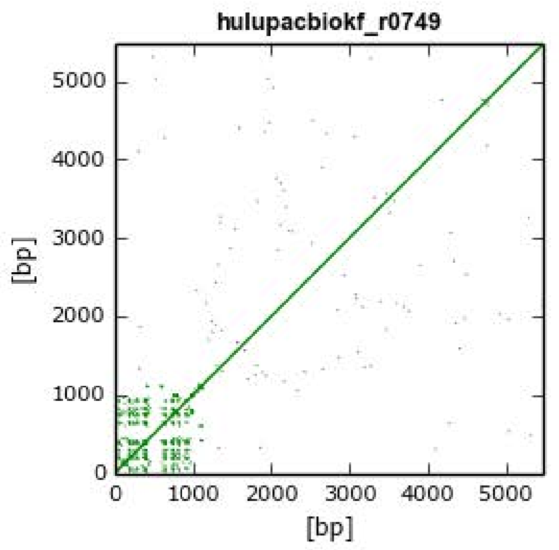
hulupacbiokf_r0749
[bp]
0
1000
2000
3000
4000
5000
[bp]

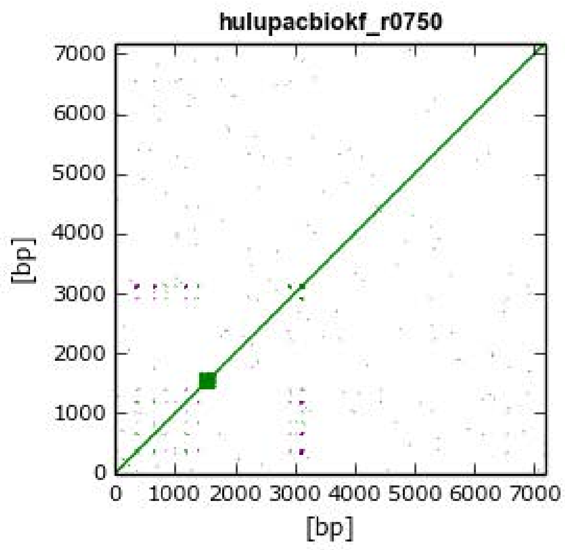
hulupacbiokf_r0750
[bp]
0
1000
2000
3000
4000
5000
6000
7000
[bp]

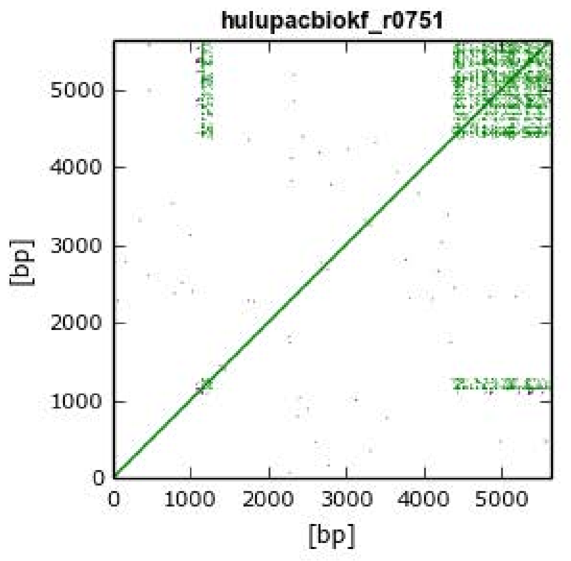
hulupacbiokf_r0751
[bp]
[bp]

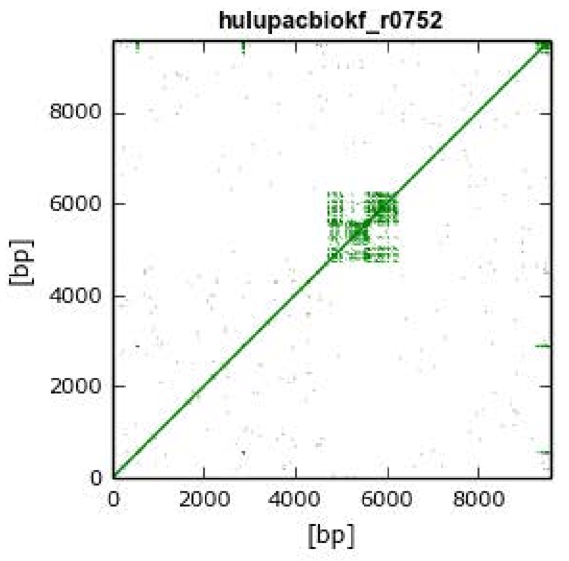
hulupacbiokf_r0752
[bp]
[bp]

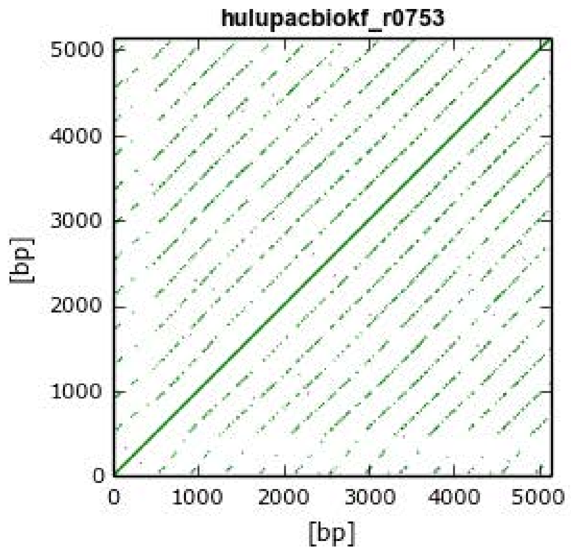
hulupacbiokf_r0753
[bp]
[bp]

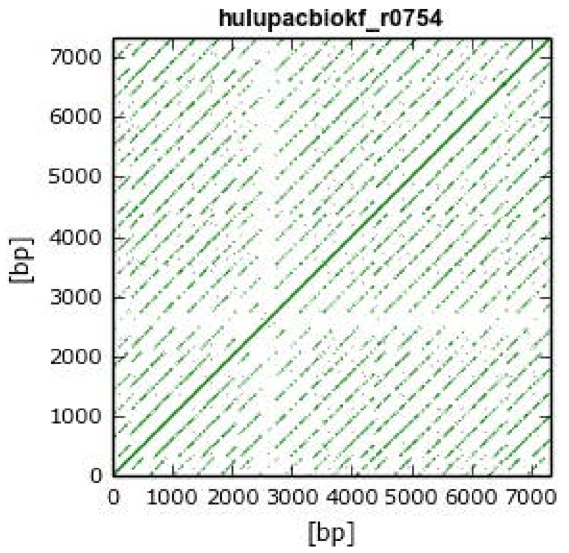
hulupacbiokf_r0754
[bp]
[bp]

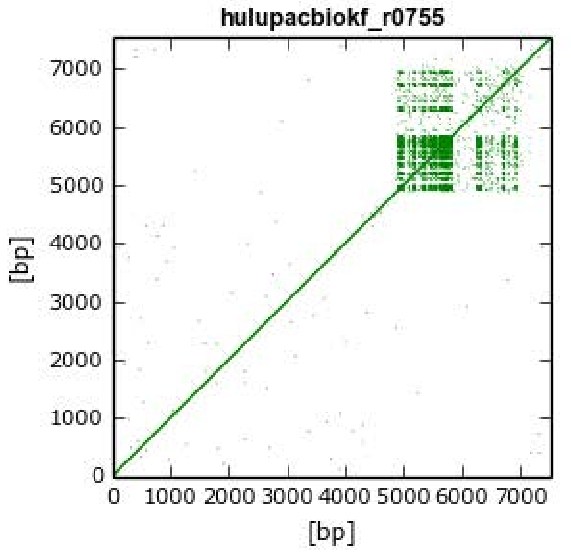
hulupacbiokf_r0755
[bp]
[bp]

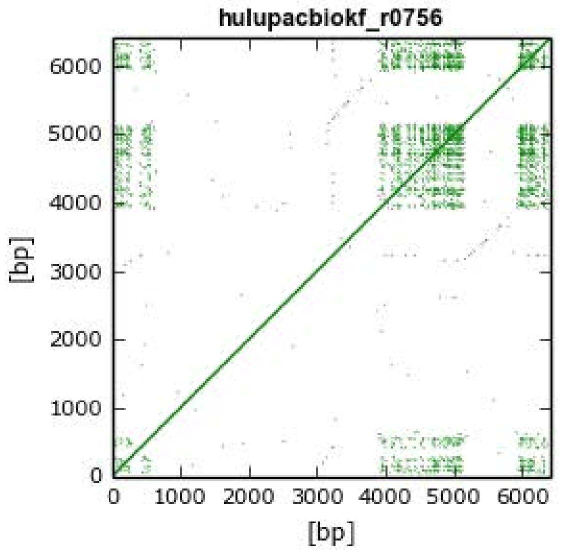
hulupacbiokf_r0756
[bp]
[bp]

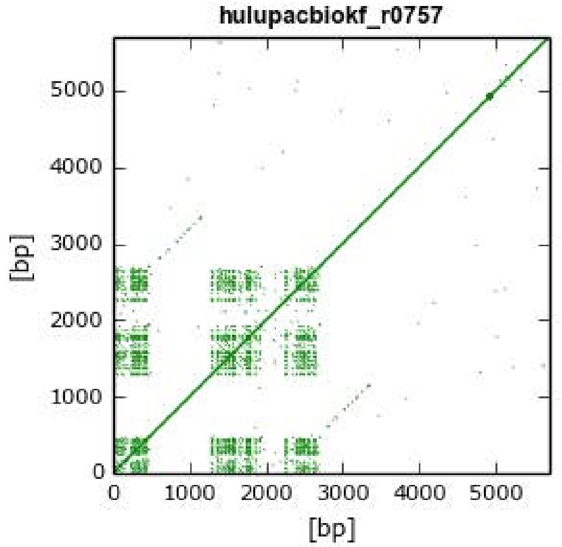
hulupacbiokf_r0757
[bp]
[bp]

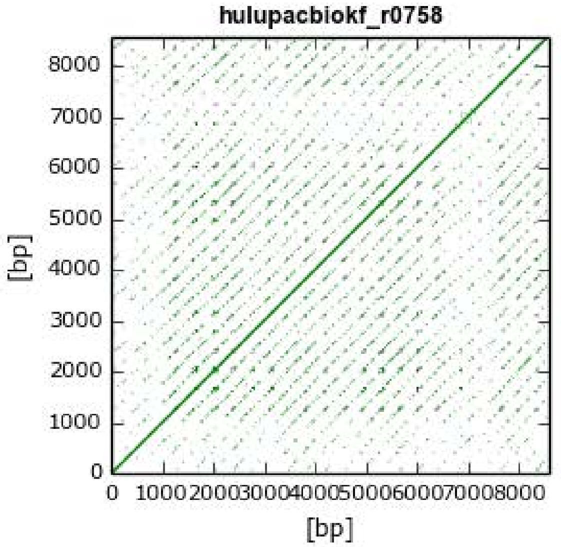
hulupacbiokf_r0758
[bp]
[bp]

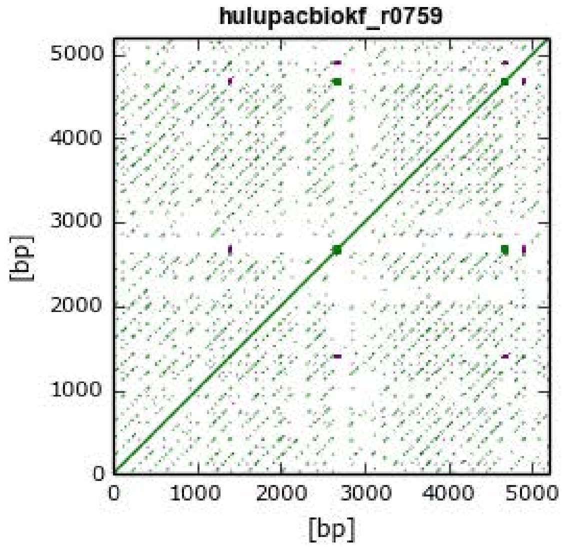
hulupacbiokf_r0759
[bp]
[bp]

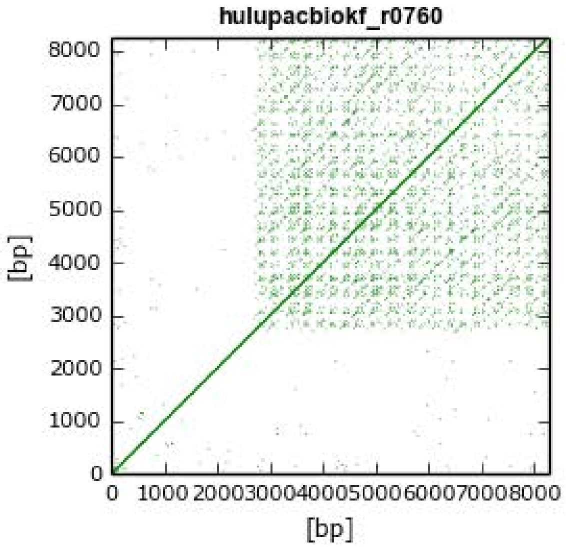
hulupacbiokf_r0760
[bp]
[bp]

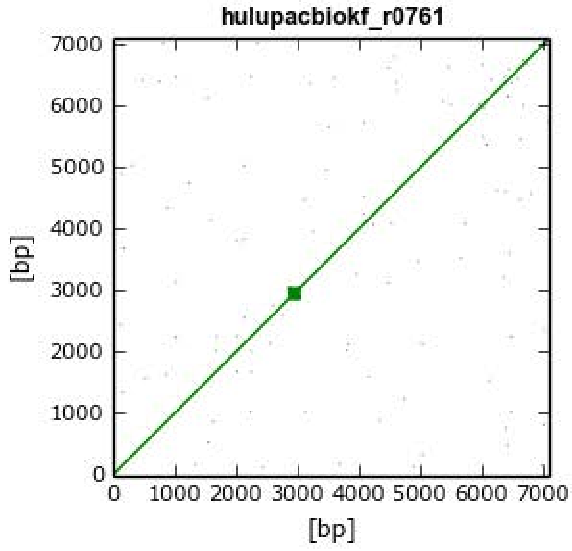
hulupacbiokf_r0761
[bp]
[bp]

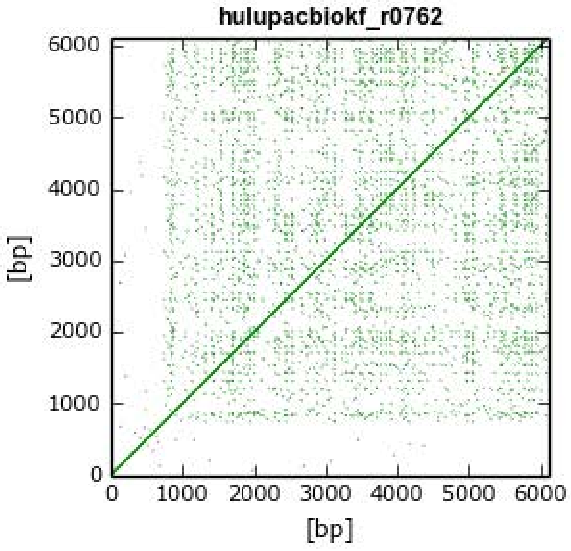
hulupacbiokf_r0762
[bp]
[bp]

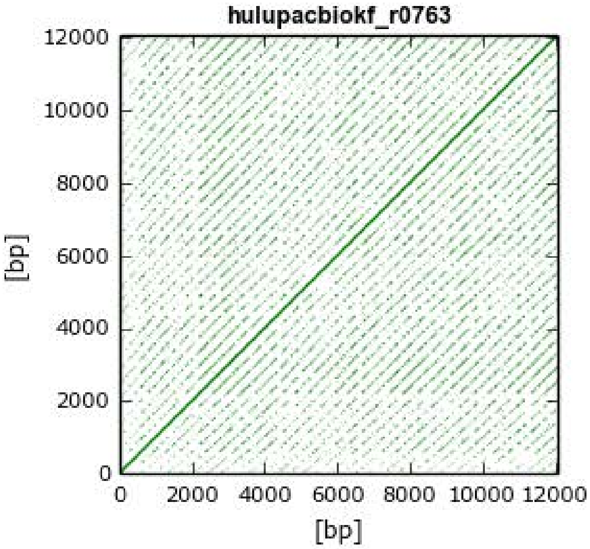
hulupacbiokf_r0763
[bp]
[bp]

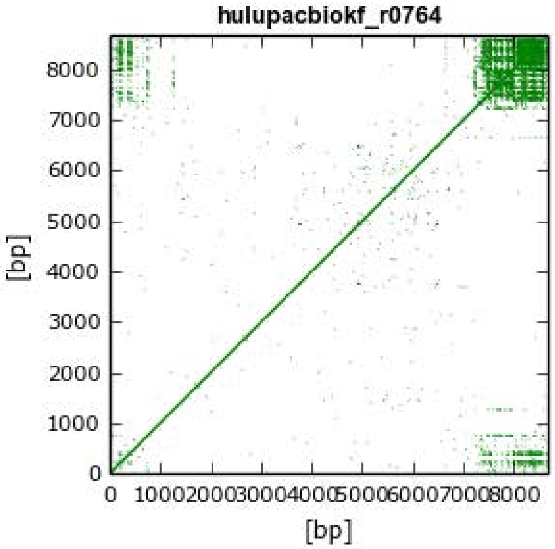
hulupacbiokf_r0764
[bp]
[bp]

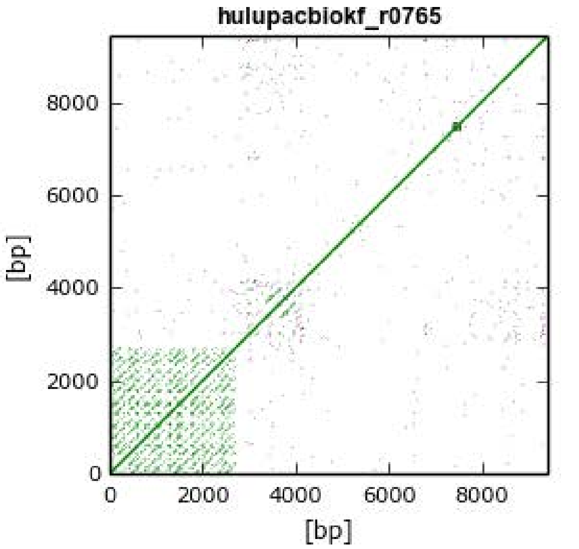
hulupacbiokf_r0765
[bp]
[bp]

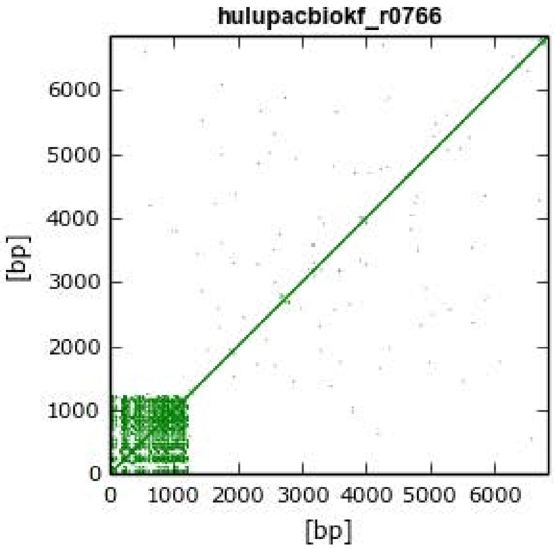
hulupacbiokf_r0766
[bp]
[bp]

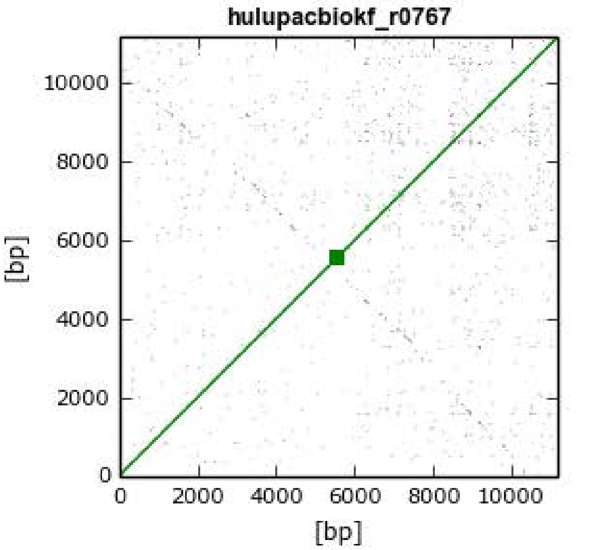
hulupacbiokf_r0767
[bp]
[bp]

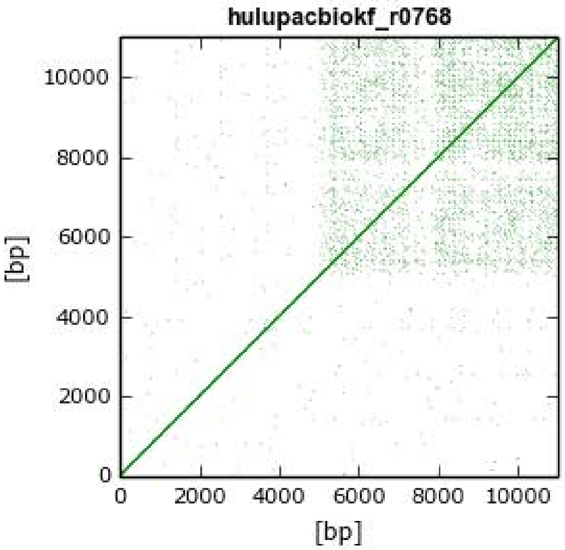
hulupacbiokf_r0768
[bp]
[bp]

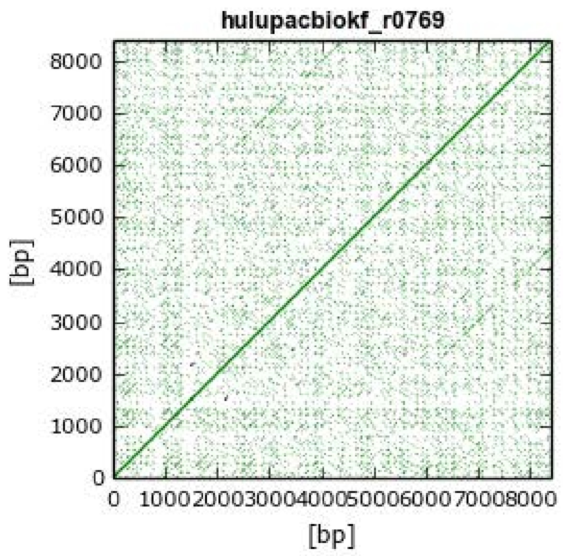
hulupacbiokf_r0769
8000
7000
6000
5000
4000
3000
2000
1000
0
[bp]
0 1000 2000 3000 4000 5000 6000 7000 8000
[bp]

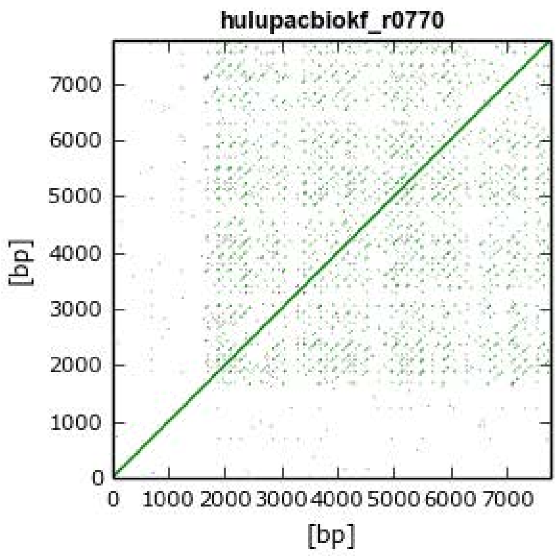
hulupacbiokf_r0770
7000
6000
5000
4000
3000
2000
1000
0
[bp]
0 1000 2000 3000 4000 5000 6000 7000
[bp]

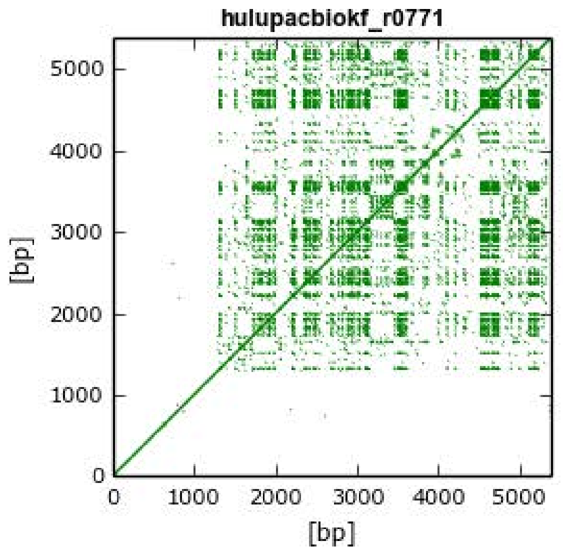
hulupacbiokf_r0771
5000
4000
3000
2000
1000
0
[bp]
0 1000 2000 3000 4000 5000
[bp]

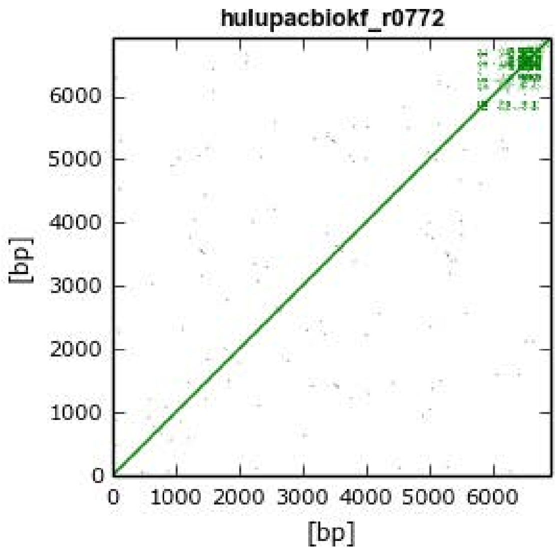
hulupacbiokf_r0772
6000
5000
4000
3000
2000
1000
0
[bp]
0 1000 2000 3000 4000 5000 6000
[bp]

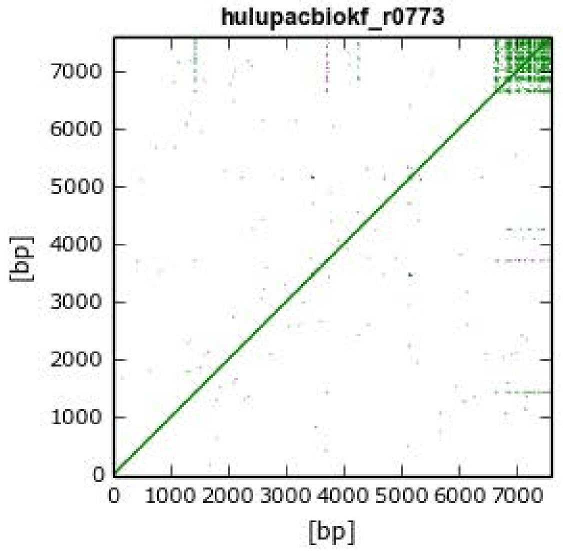
hulupacbiokf_r0773
7000
6000
5000
4000
3000
2000
1000
0
[bp]
0 1000 2000 3000 4000 5000 6000 7000
[bp]

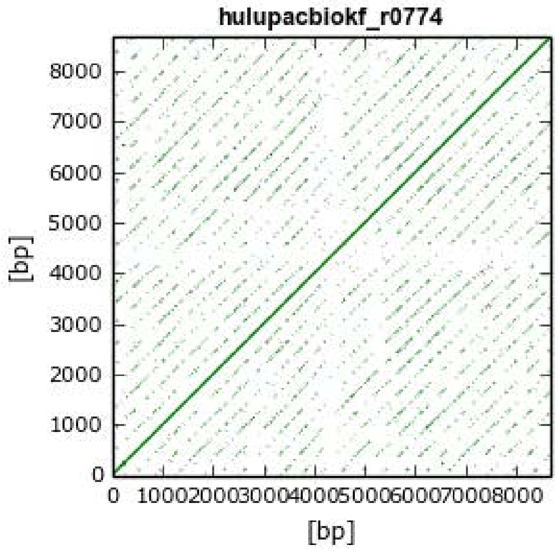
hulupacbiokf_r0774
8000
7000
6000
5000
4000
3000
2000
1000
0
[bp]
0 1000 2000 3000 4000 5000 6000 7000 8000
[bp]

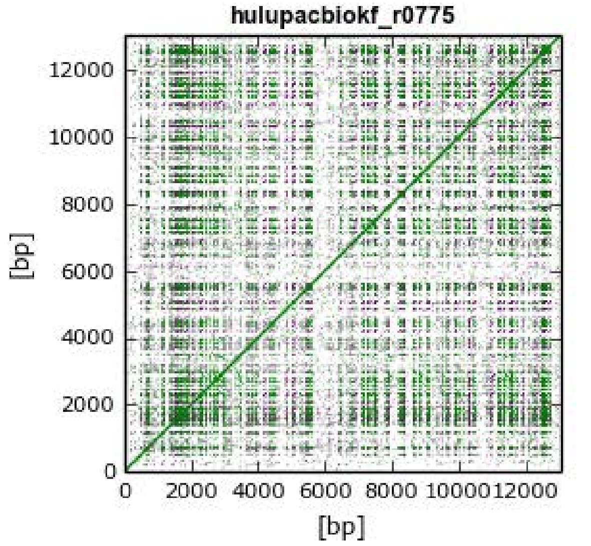
hulupacbiokf_r0775
[bp]
[bp]

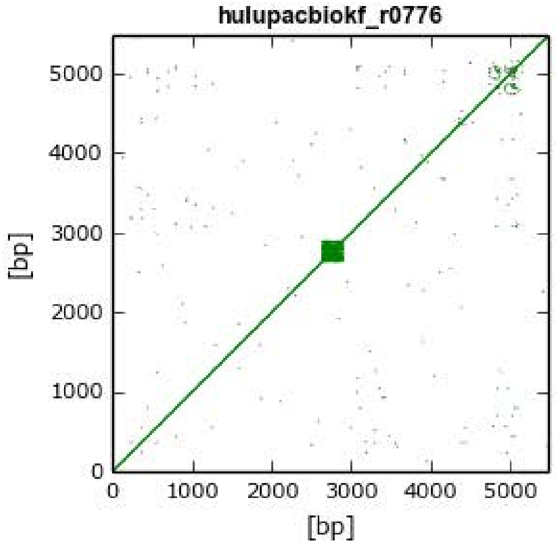
hulupacbiokf_r0776
[bp]
[bp]

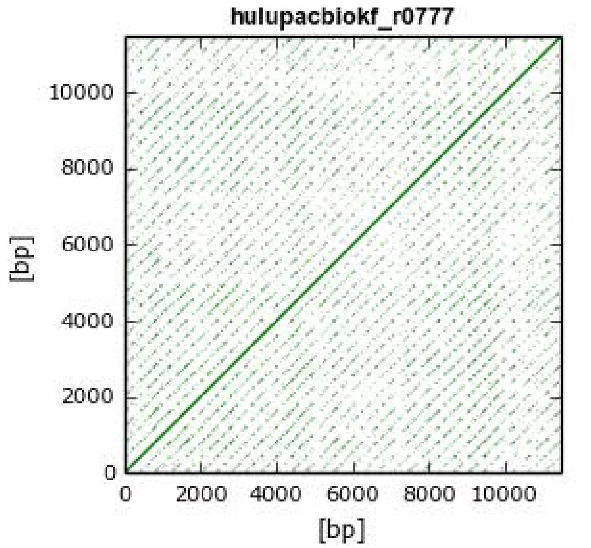
hulupacbiokf_r0777
[bp]
[bp]

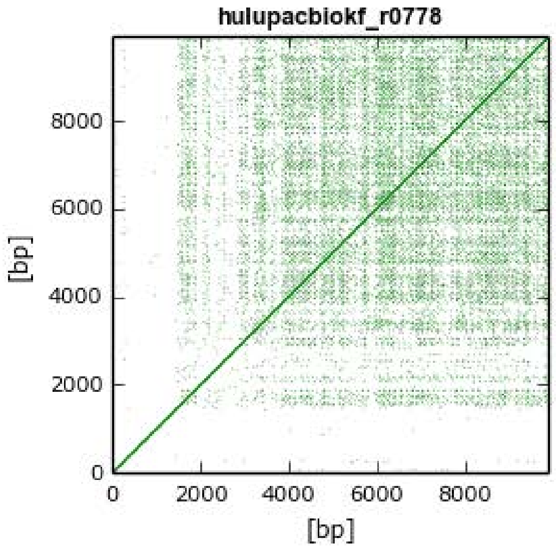
hulupacbiokf_r0778
[bp]
[bp]

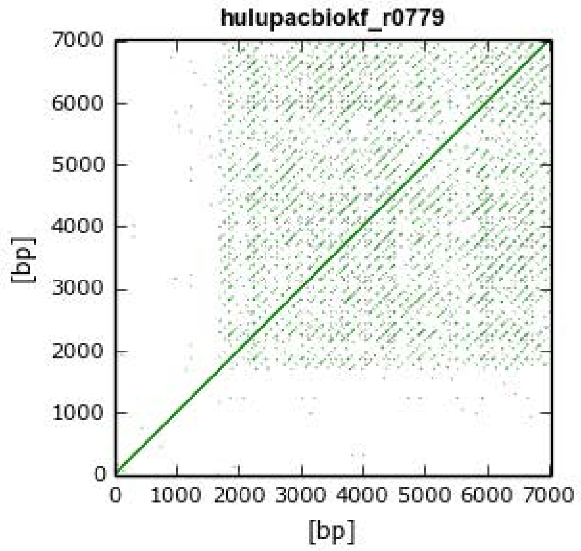
hulupacbiokf_r0779
[bp]
[bp]

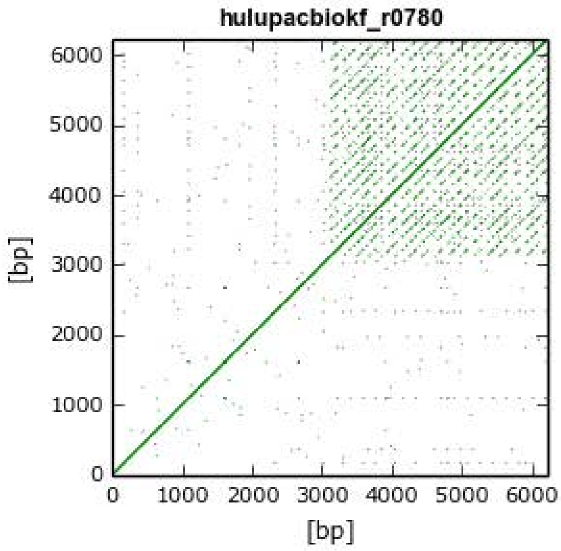
hulupacbiokf_r0780
[bp]
[bp]

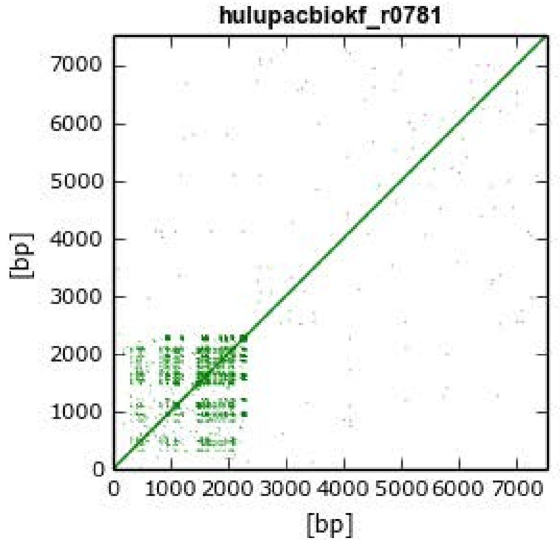

HuluTR120 from read r0782 is in GenBank Acc. MN537570

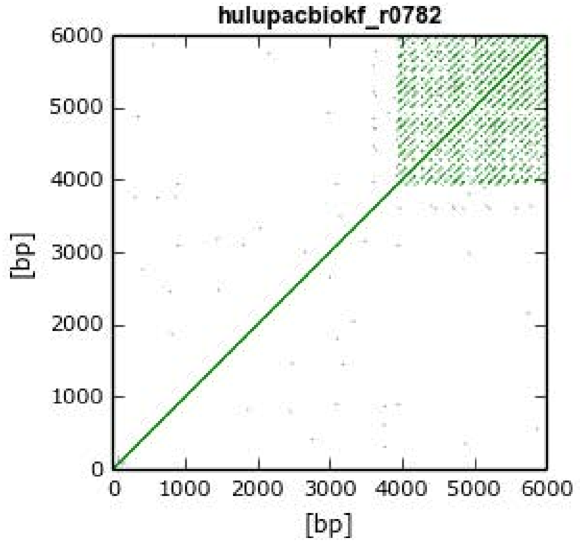

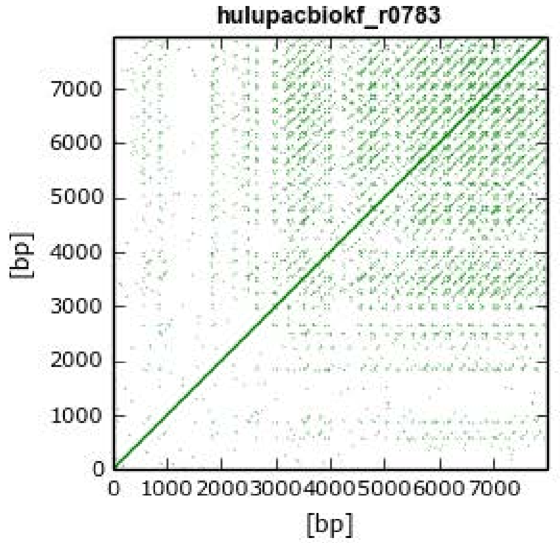

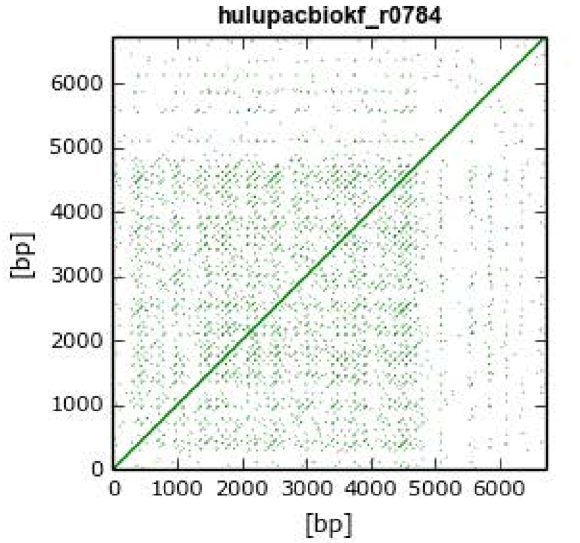

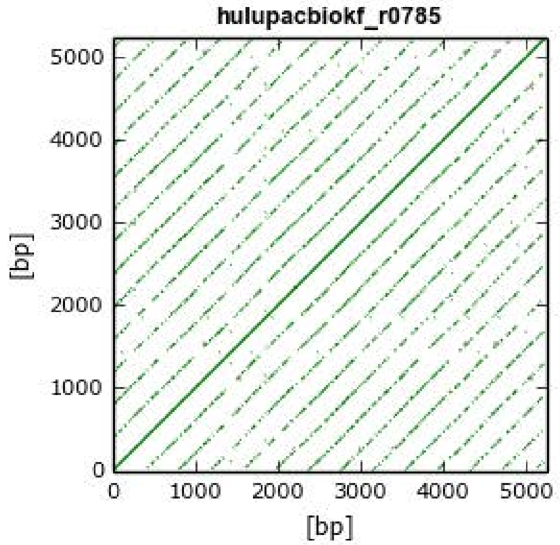

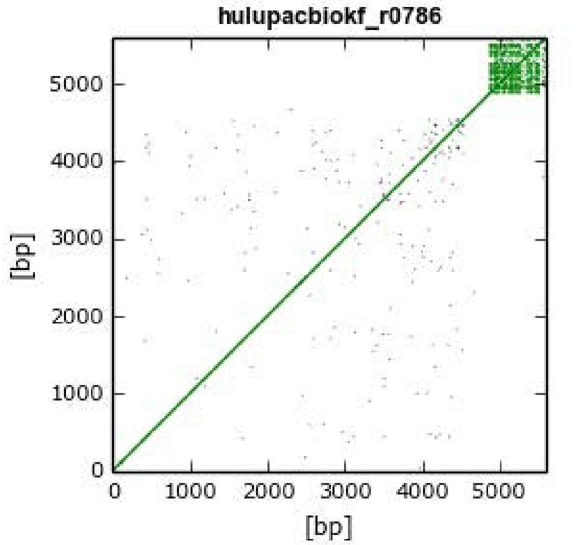

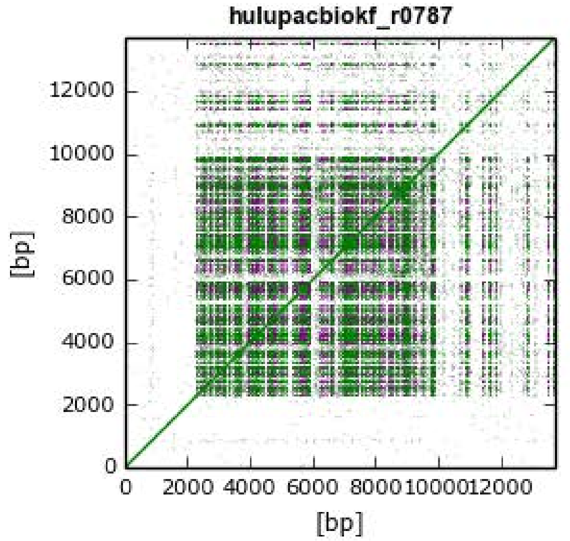
hulupacbiokf_r0787
[bp]
[bp]

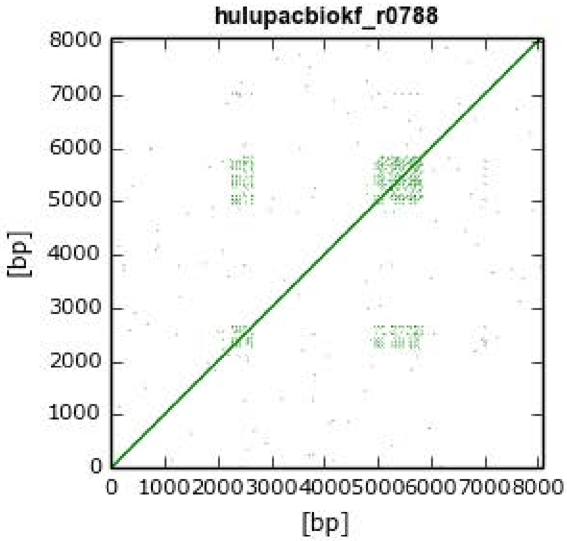
hulupacbiokf_r0788
[bp]
[bp]

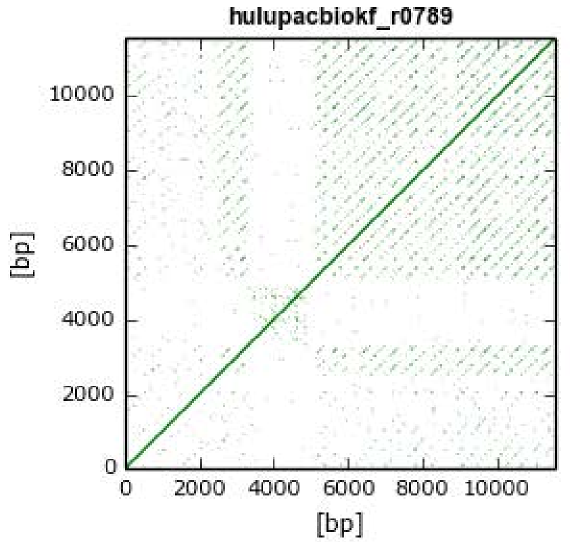
hulupacbiokf_r0789
[bp]
[bp]

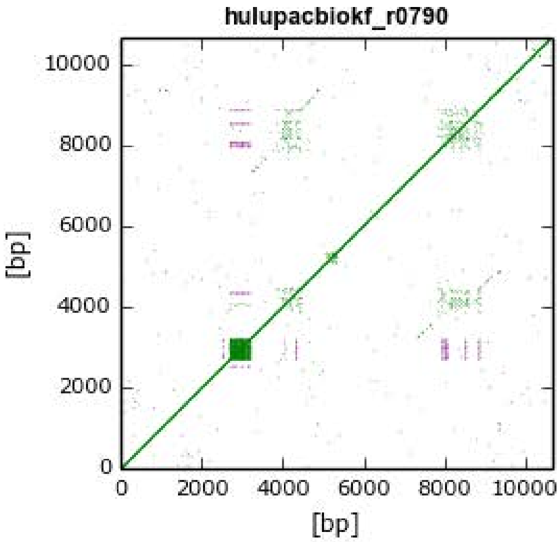
hulupacbiokf_r0790
[bp]
[bp]

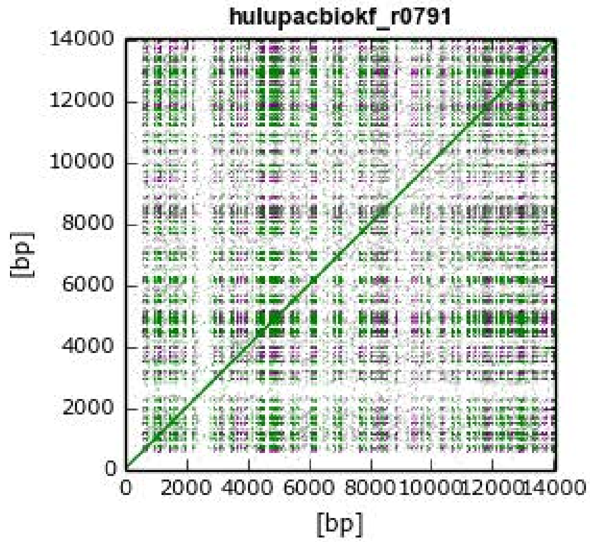
hulupacbiokf_r0791
[bp]
[bp]

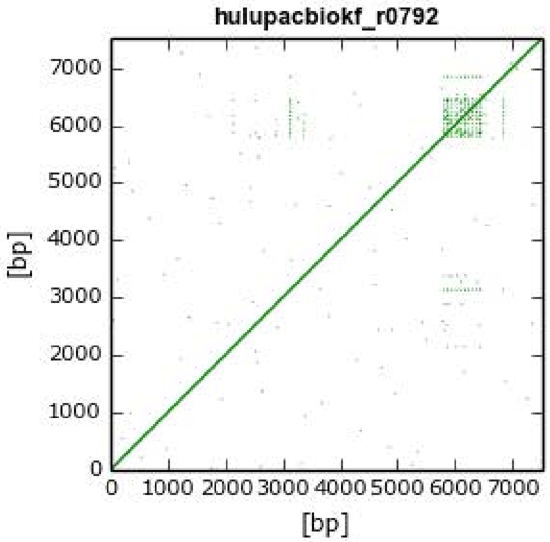
hulupacbiokf_r0792
[bp]
[bp]

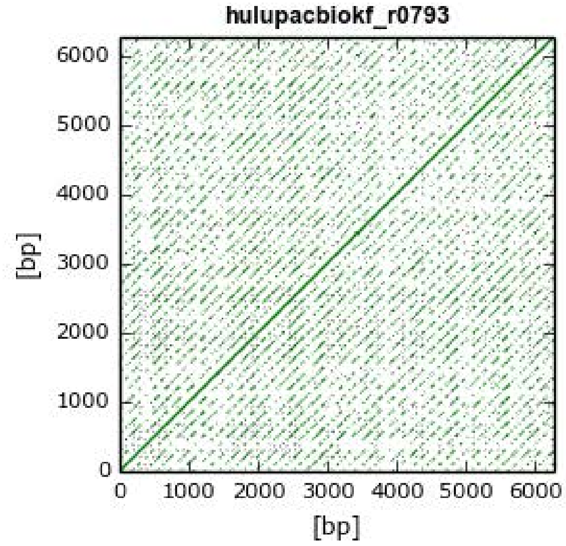
hulupacbiokf_r0793
[bp]
[bp]

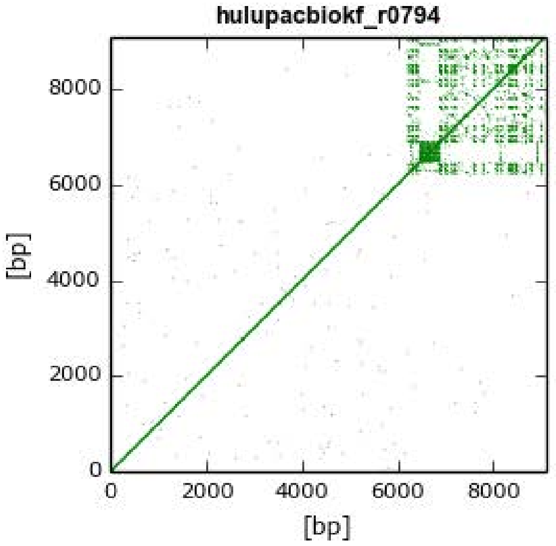
hulupacbiokf_r0794
[bp]
[bp]

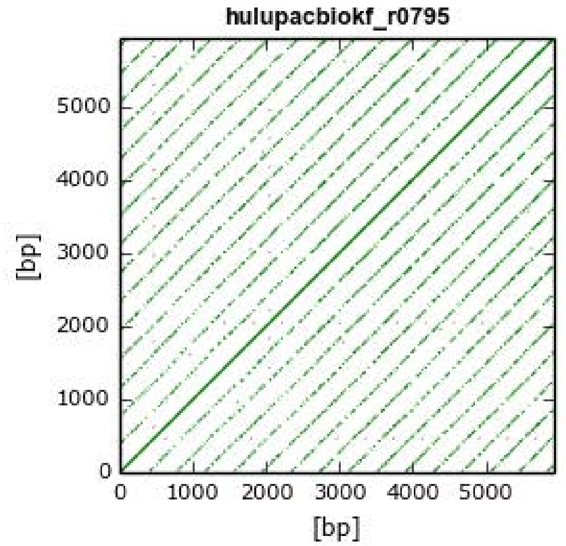
hulupacbiokf_r0795
[bp]
[bp]

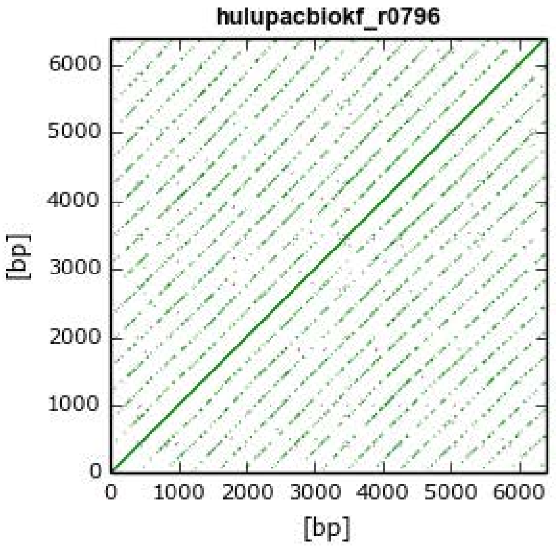
hulupacbiokf_r0796
[bp]
[bp]

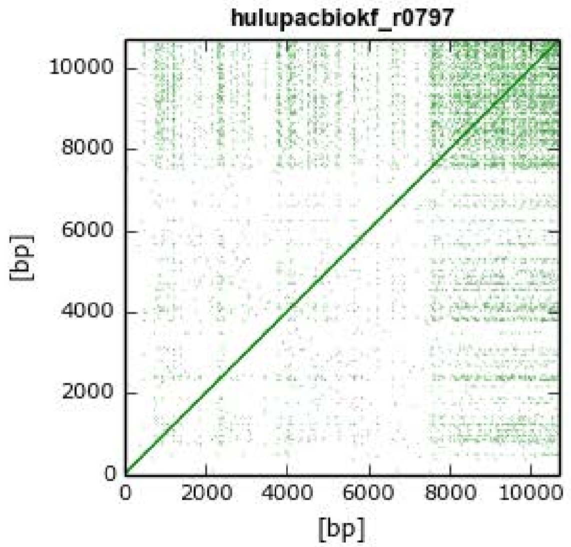
hulupacbiokf_r0797
[bp]
[bp]

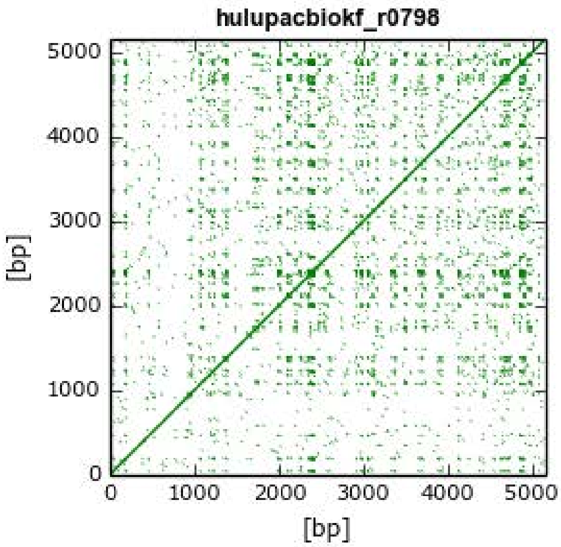
hulupacbiokf_r0798
[bp]
[bp]

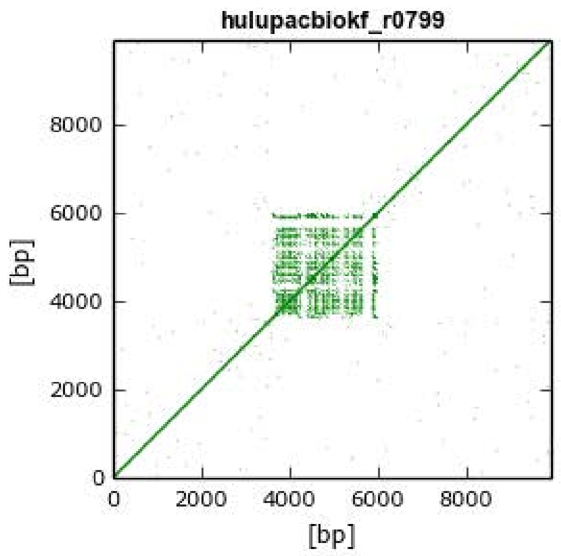
hulupacbiokf_r0799
[bp]
[bp]

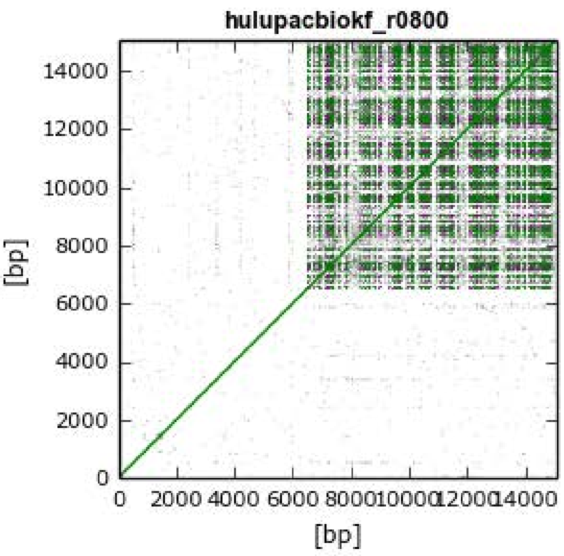
hulupacbiokf_r0800
[bp]
[bp]

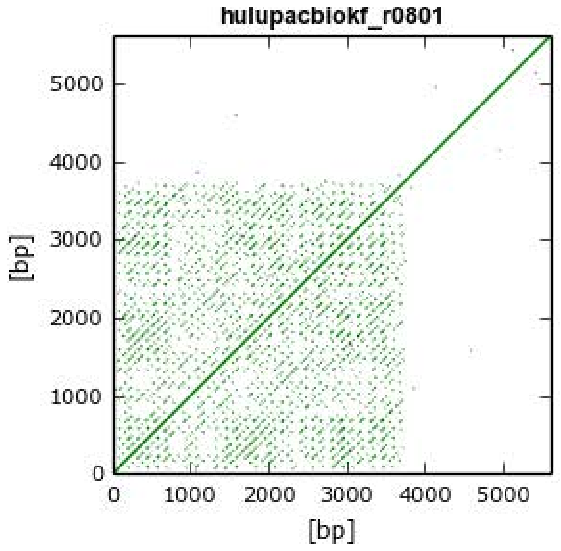
hulupacbiokf_r0801
[bp]
[bp]

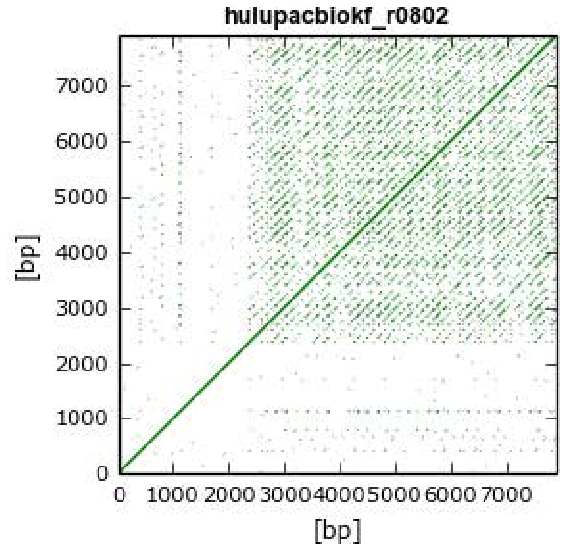
hulupacbiokf_r0802
[bp]
[bp]

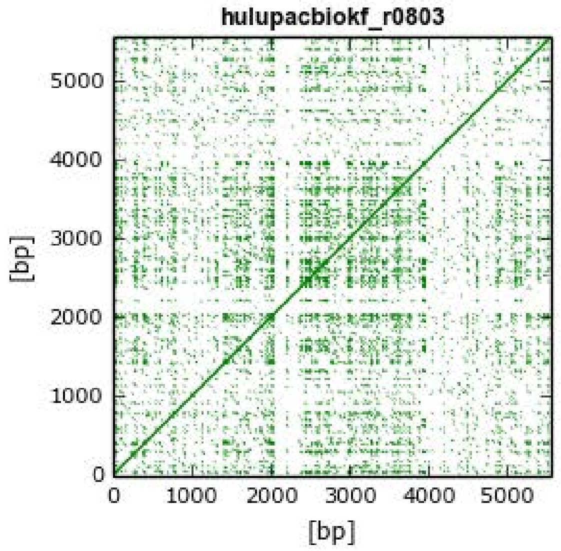
hulupacbiokf_r0803
[bp]
[bp]

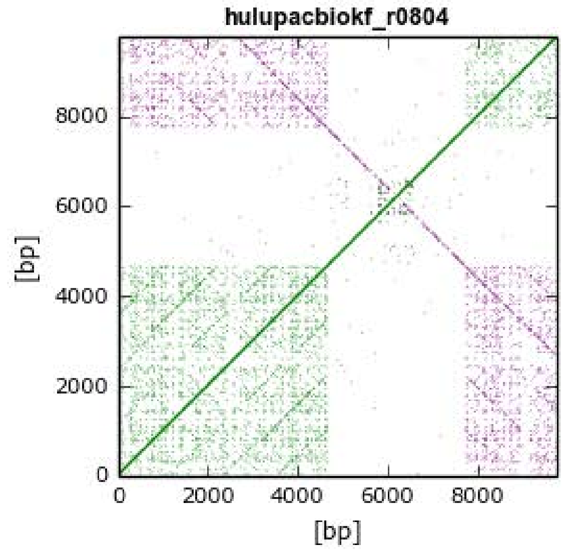
hulupacbiokf_r0804
[bp]
[bp]

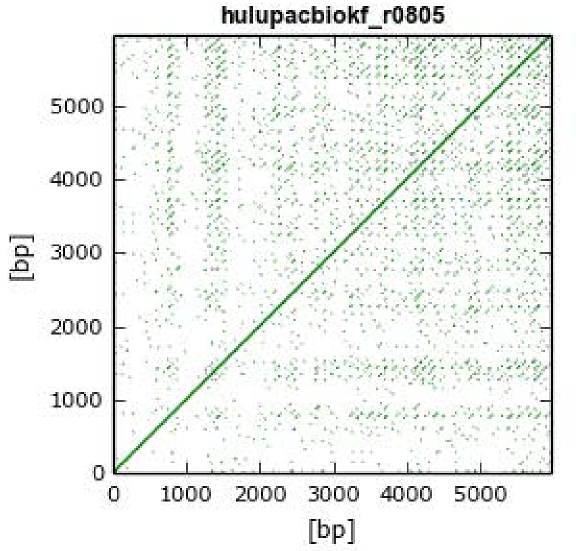
hulupacbiokf_r0805
[bp]
[bp]

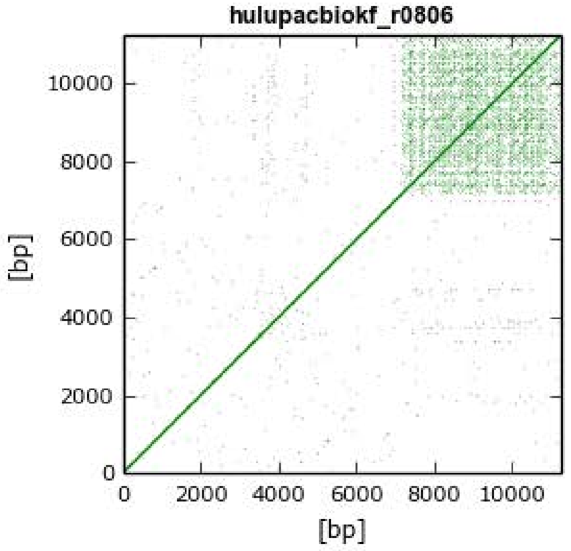
hulupacbiokf_r0806
[bp]
[bp]

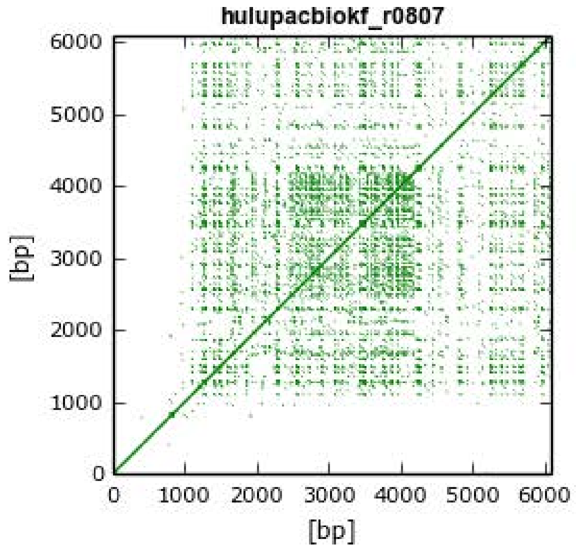
hulupacbiokf_r0807
[bp]
[bp]

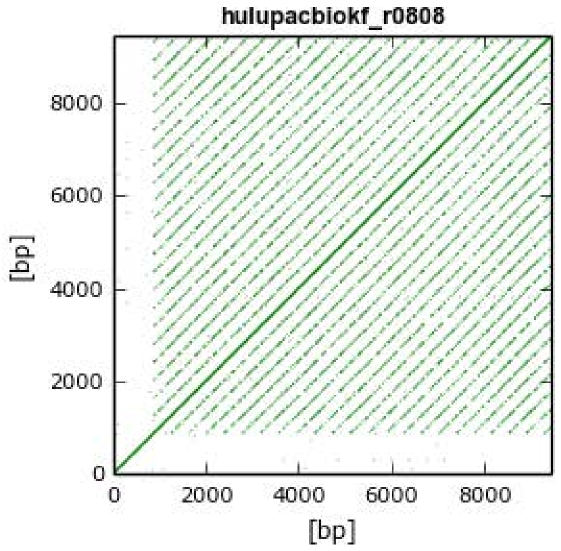
hulupacbiokf_r0808
[bp]
[bp]

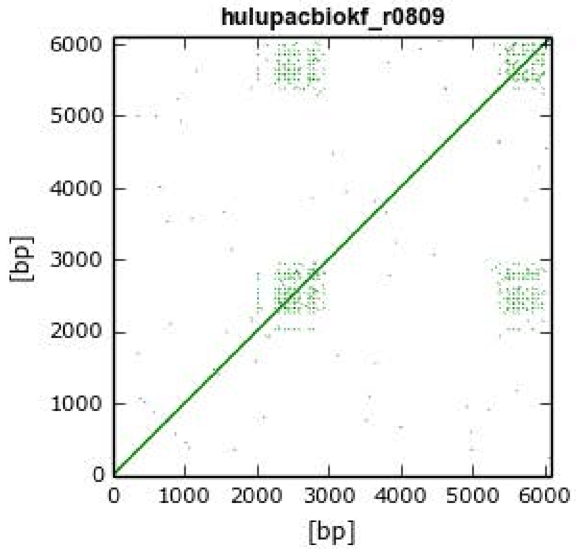
hulupacbiokf_r0809
[bp]
[bp]

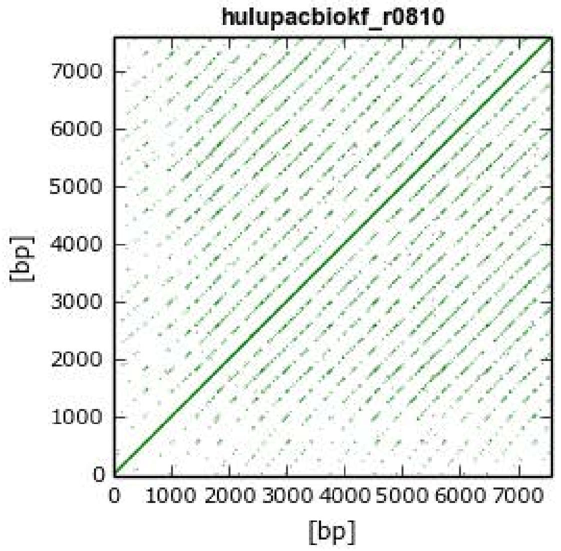
hulupacbiokf_r0810
[bp]
[bp]

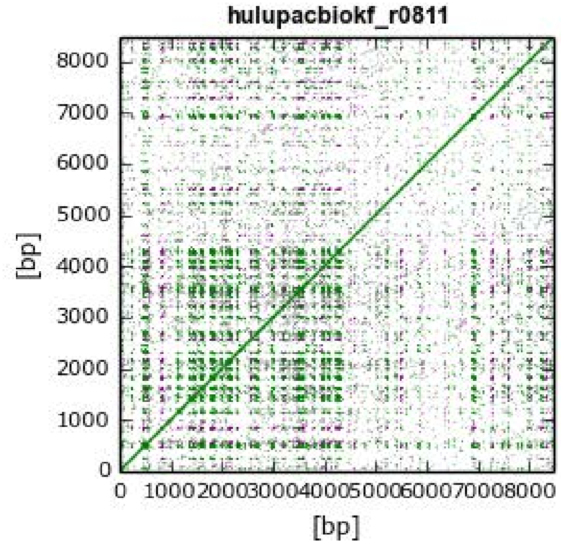
hulupacbiokf_r0811
[bp]
[bp]

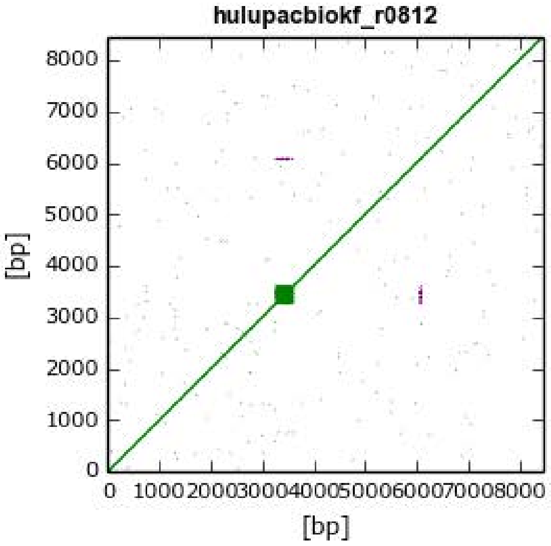
hulupacbiokf_r0812
[bp]
[bp]

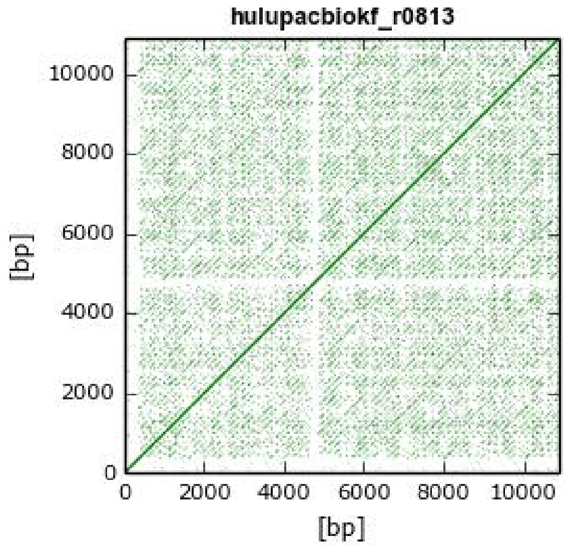
hulupacbiokf_r0813
[bp]
[bp]

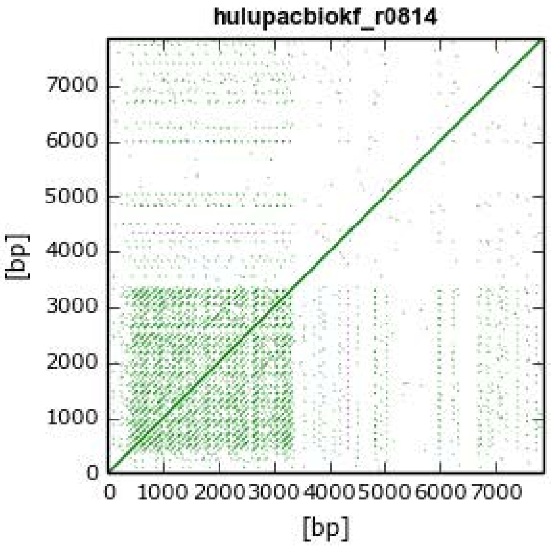
hulupacbiokf_r0814
[bp]
[bp]

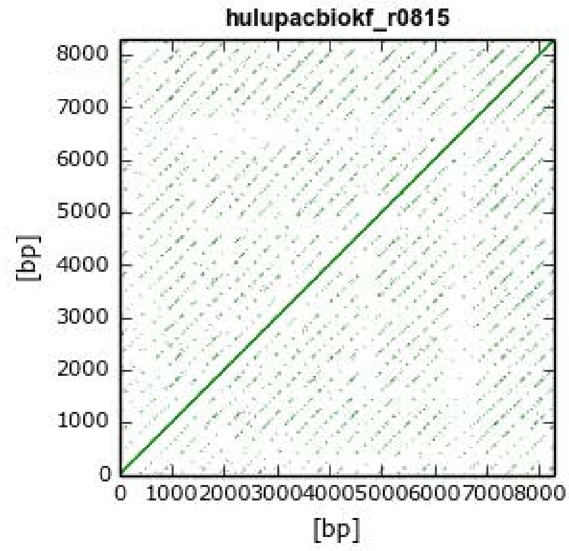
hulupacbiokf_r0815
[bp]
[bp]

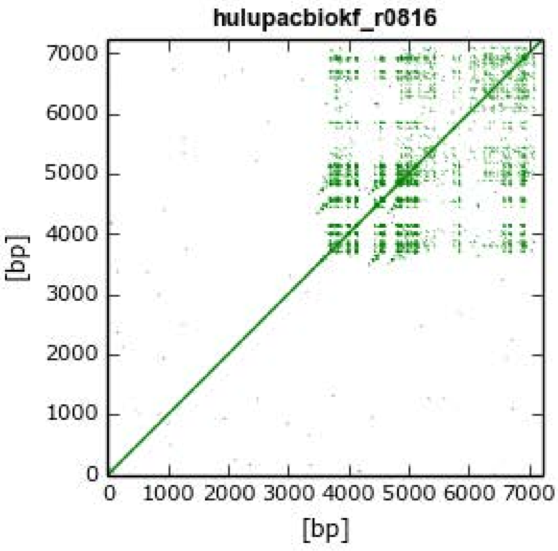
hulupacbiokf_r0816
[bp]
[bp]

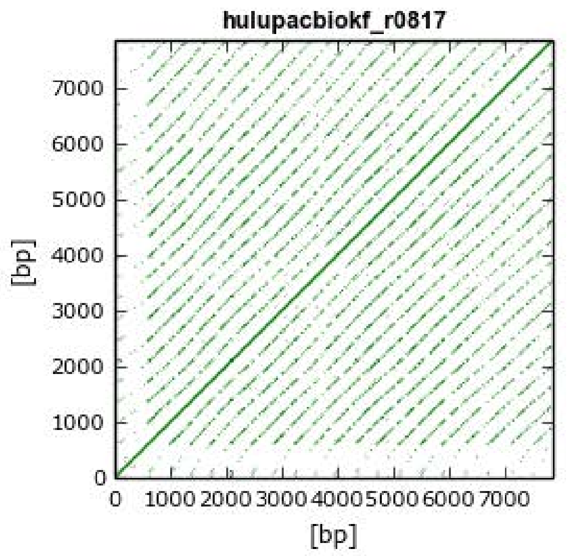
hulupacbiokf_r0817
[bp]
[bp]

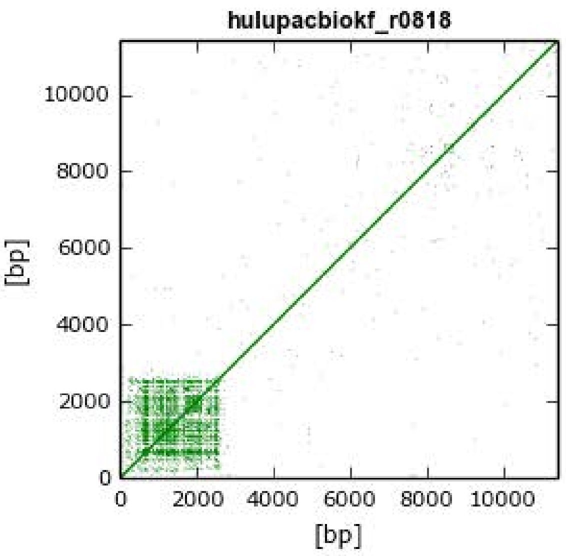
hulupacbiokf_r0818
[bp]
[bp]

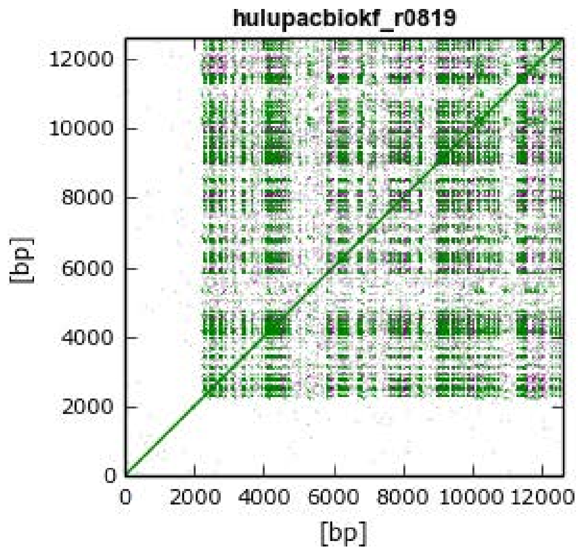
hulupacbiokf_r0819
[bp]
[bp]

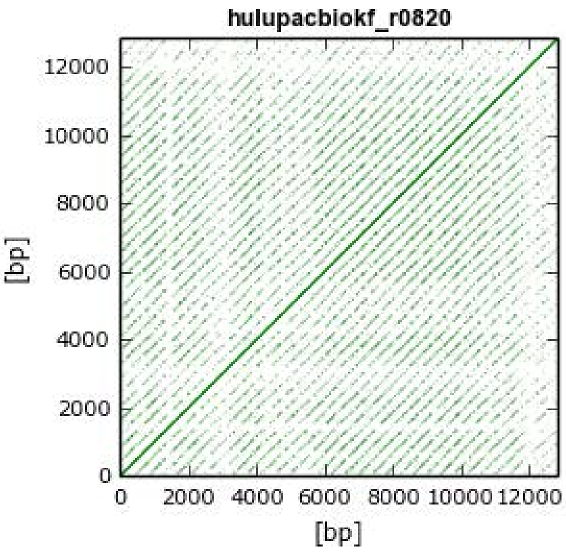
hulupacbiokf_r0820
[bp]
[bp]

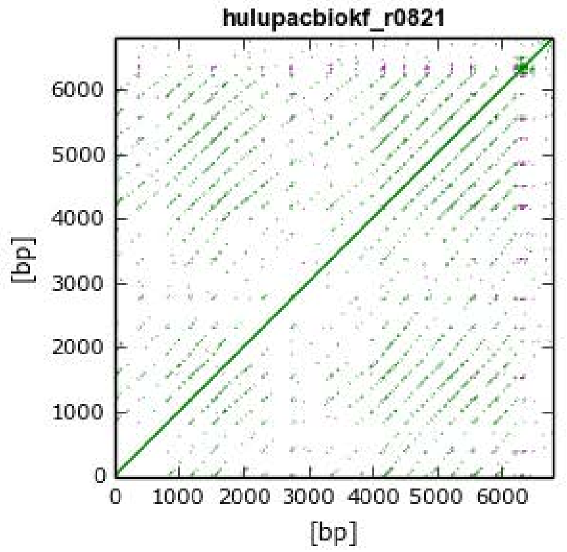
hulupacbiokf_r0821
[bp]
[bp]

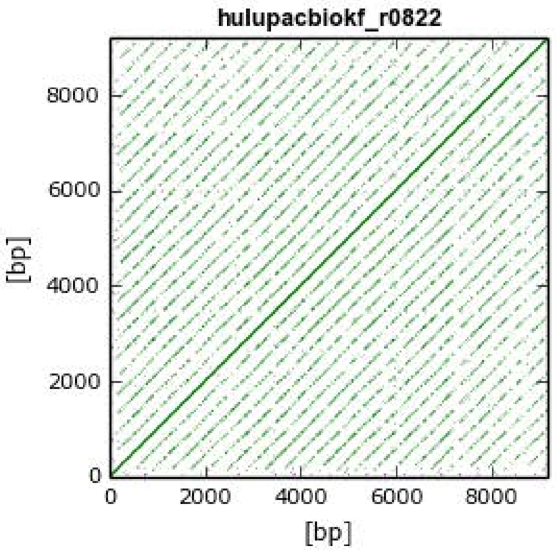
hulupacbiokf_r0822
[bp]
[bp]

HuluTR600 from read r0823
is in GenBank Acc. MN537582

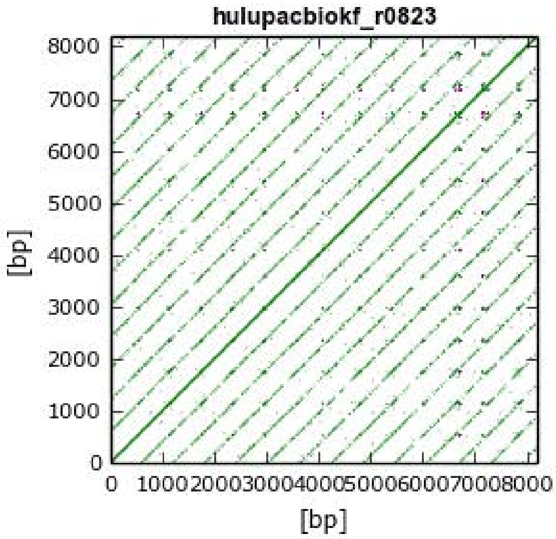

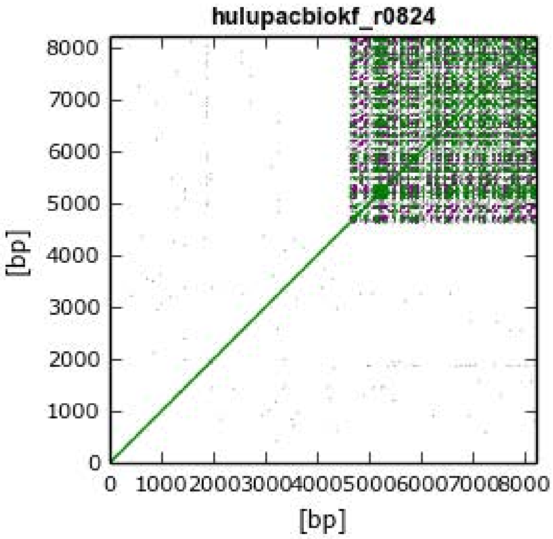

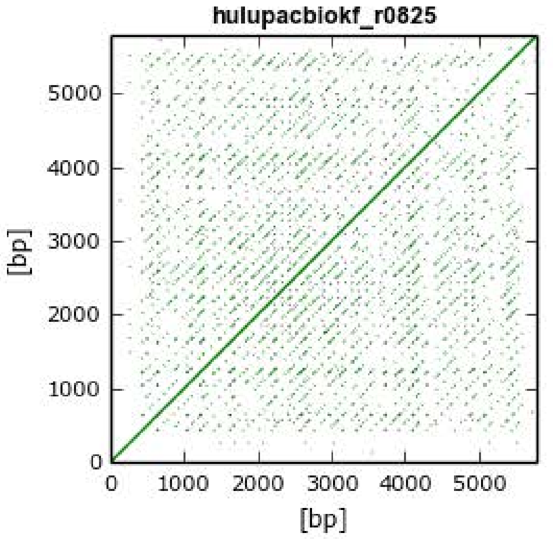

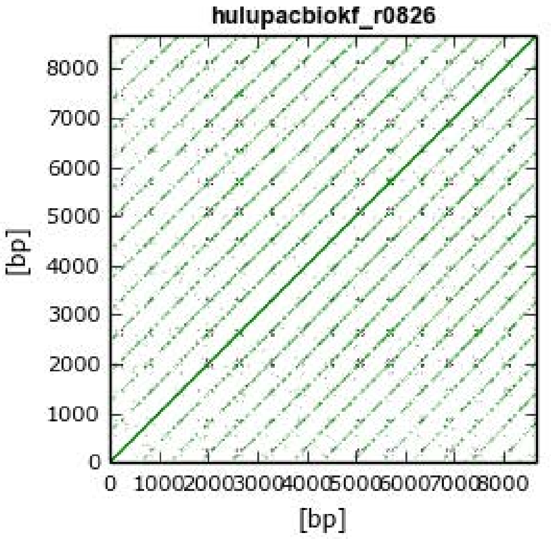

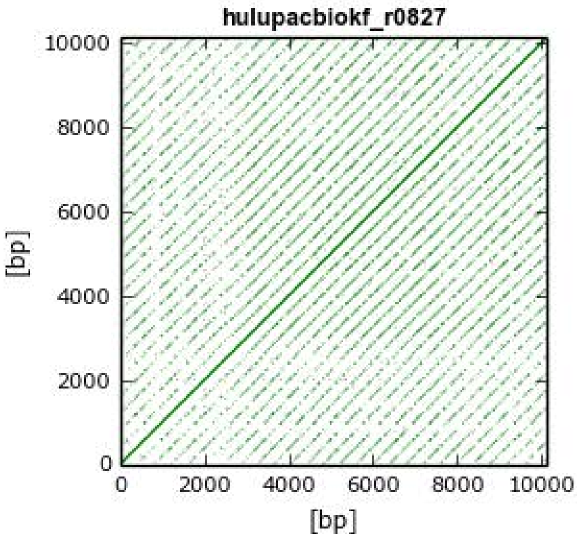

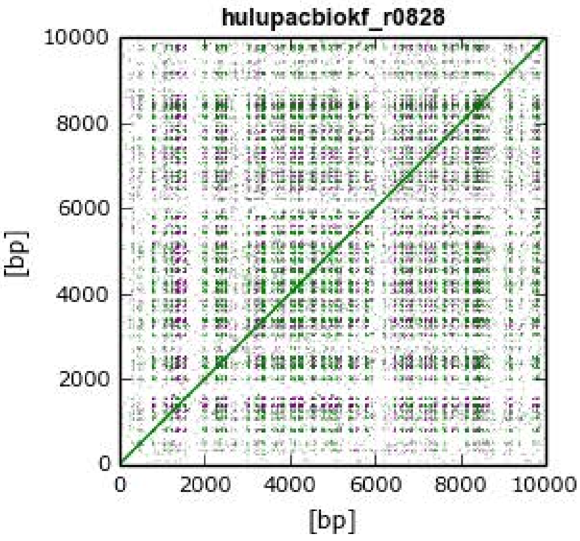

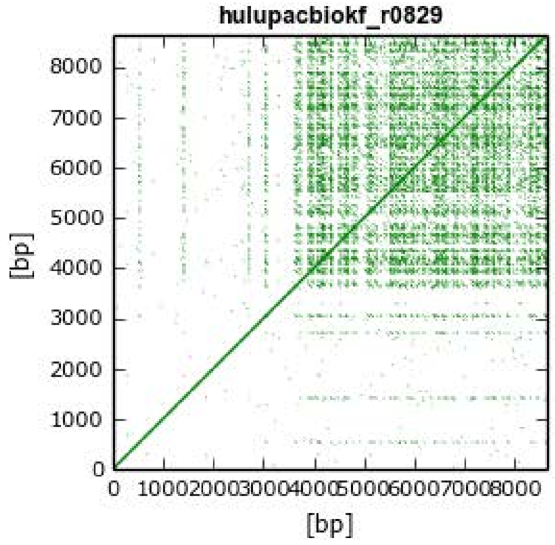
hulupacbiokf_r0829
[bp]
[bp]

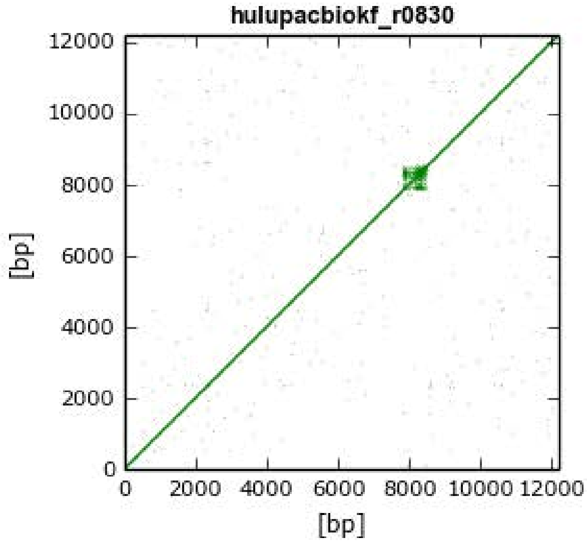
hulupacbiokf_r0830
[bp]
[bp]

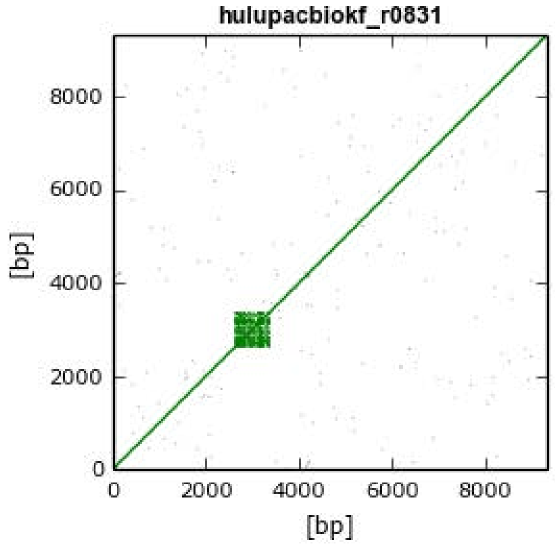
hulupacbiokf_r0831
[bp]
[bp]

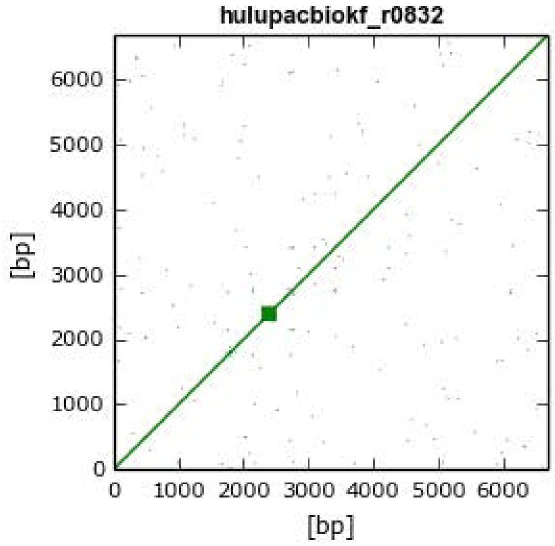
hulupacbiokf_r0832
[bp]
[bp]

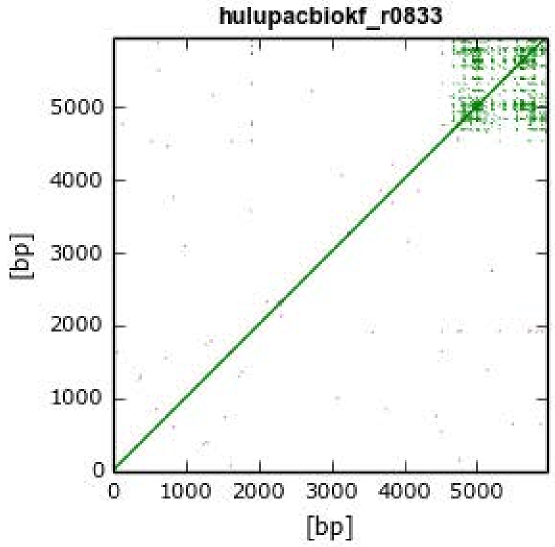
hulupacbiokf_r0833
[bp]
[bp]

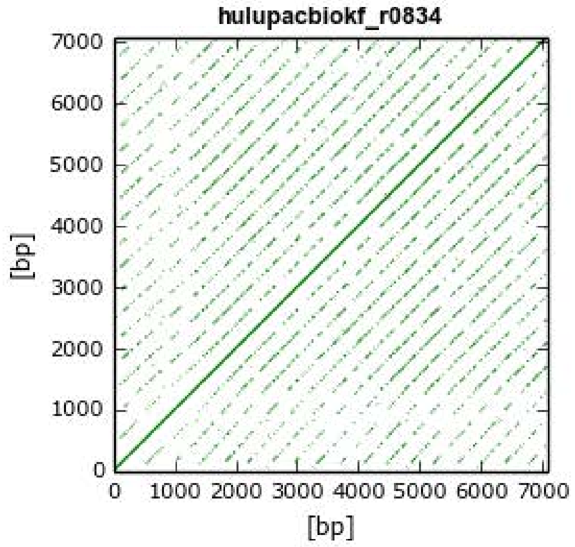
hulupacbiokf_r0834
[bp]
[bp]

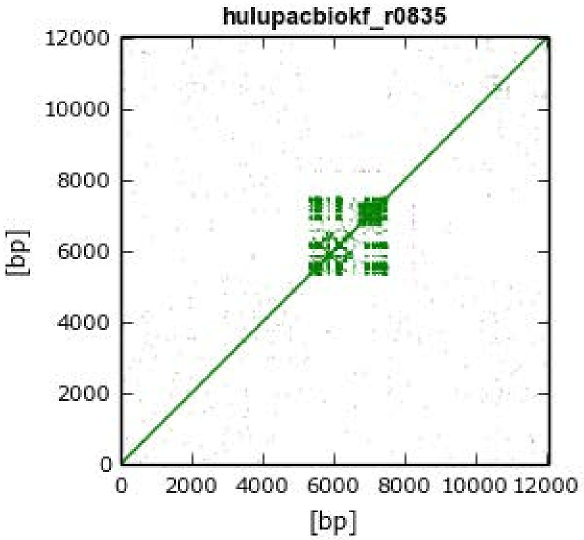
hulupacbiokf_r0835
[bp]
[bp]

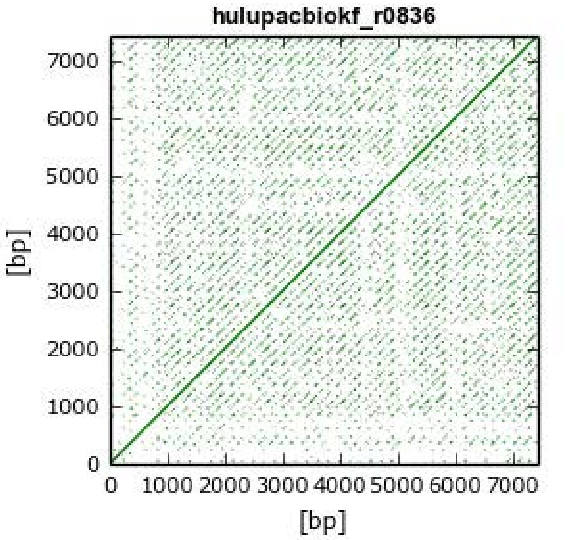
hulupacbiokf_r0836
[bp]
[bp]

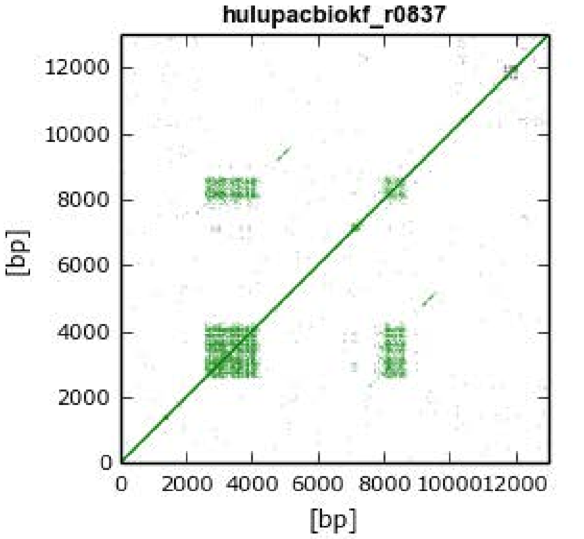
hulupacbiokf_r0837
[bp]
[bp]

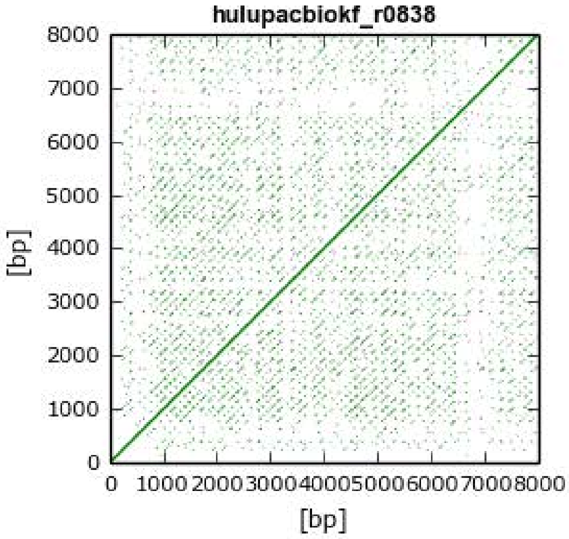
hulupacbiokf_r0838
[bp]
[bp]

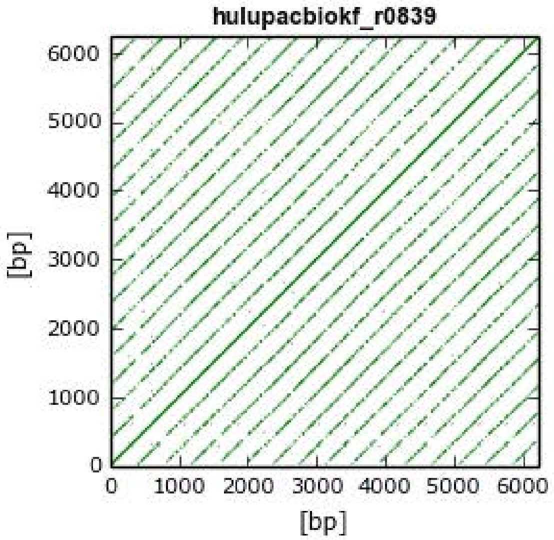
hulupacbiokf_r0839
[bp]
[bp]

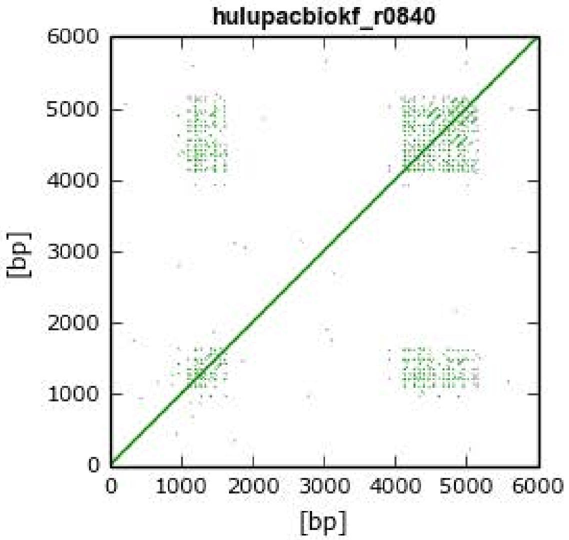
hulupacbiokf_r0840
[bp]
[bp]

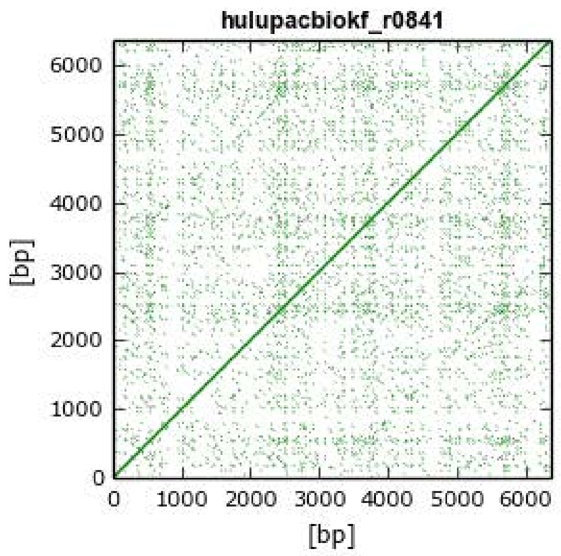
hulupacbiokf_r0841
[bp]
[bp]

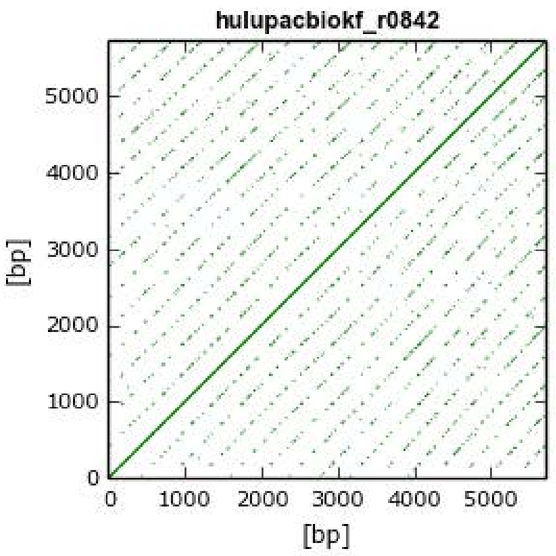
hulupacbiokf_r0842
[bp]
[bp]

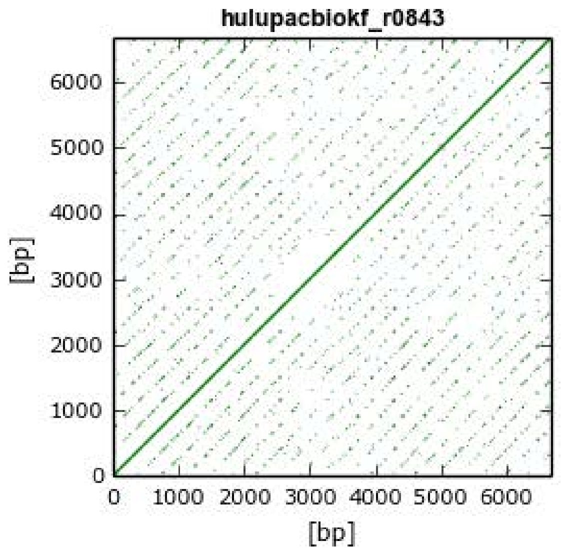
hulupacbiokf_r0843
[bp]
[bp]

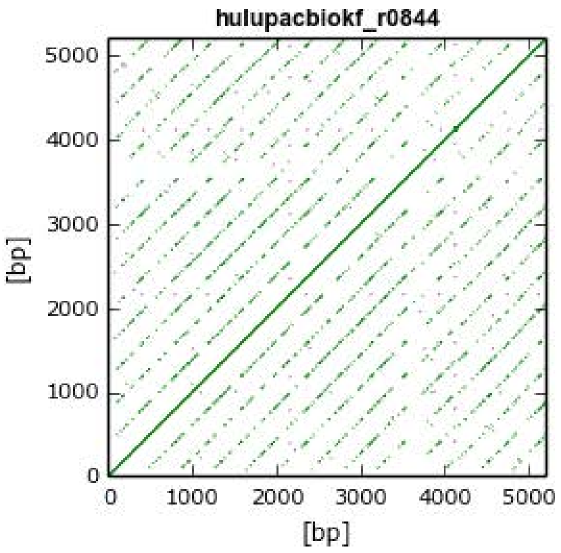
hulupacbiokf_r0844
[bp]
[bp]

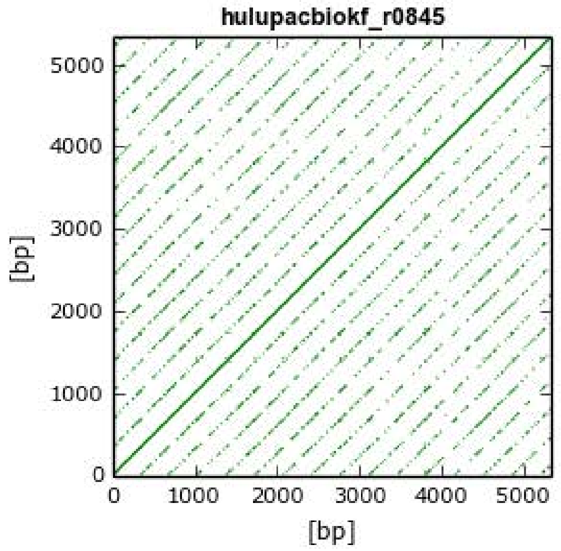
hulupacbiokf_r0845
[bp]
[bp]

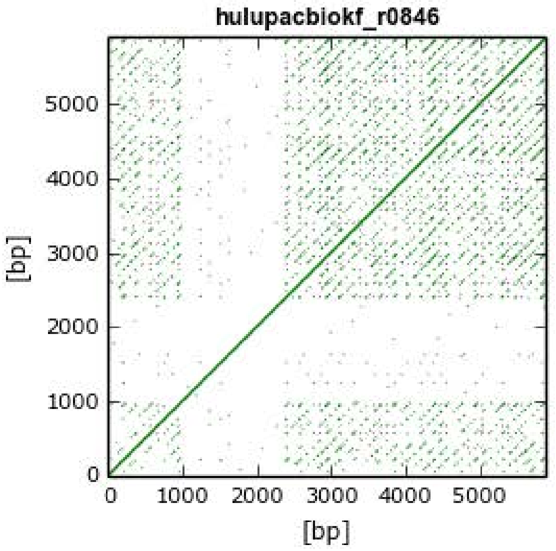
hulupacbiokf_r0846
[bp]
[bp]

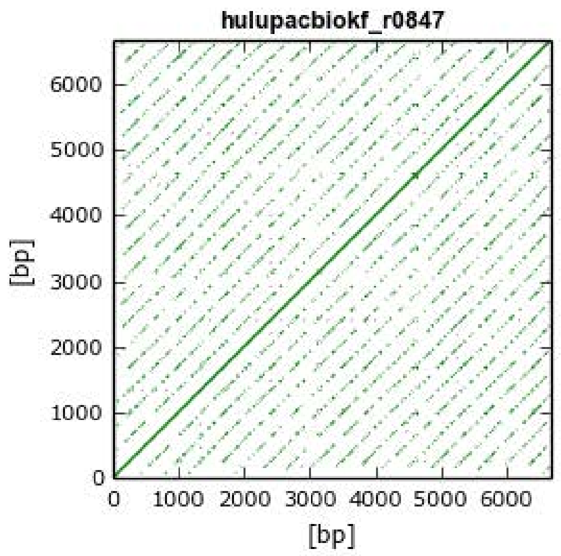
hulupacbiokf_r0847
[bp]
[bp]

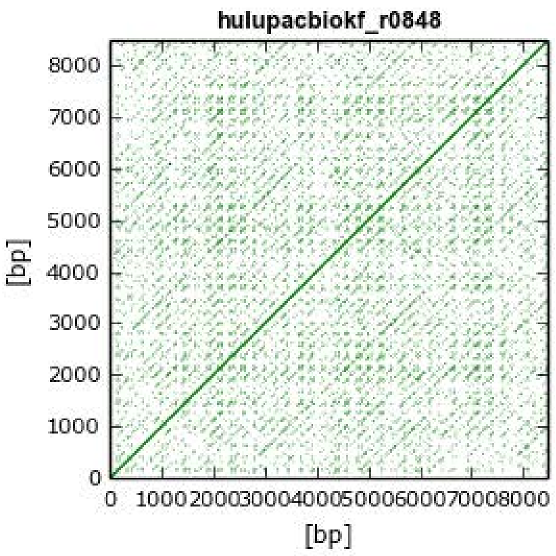
hulupacbiokf_r0848
[bp]
[bp]

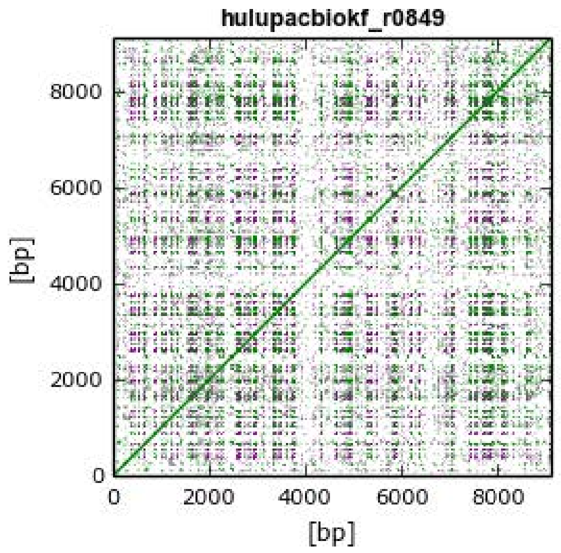
hulupacbiokf_r0849
[bp]
[bp]

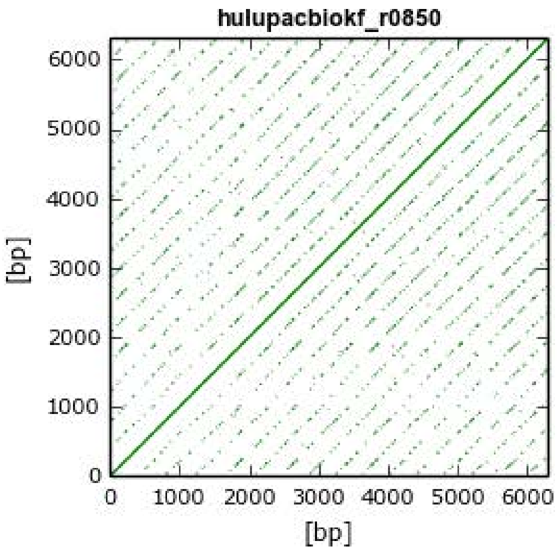
hulupacbiokf_r0850
[bp]
[bp]

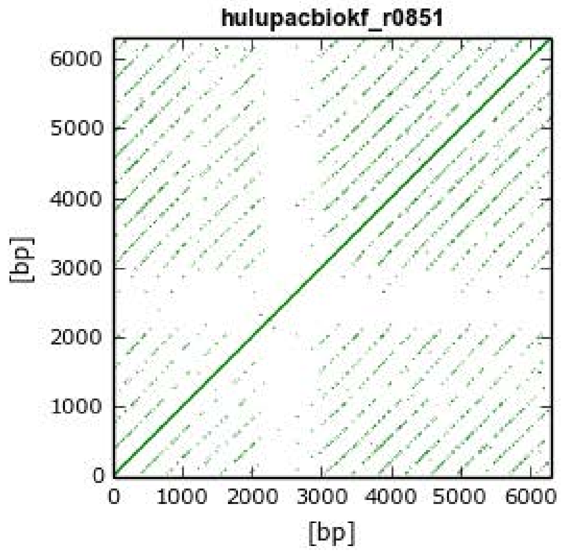
hulupacbiokf_r0851
[bp]
[bp]

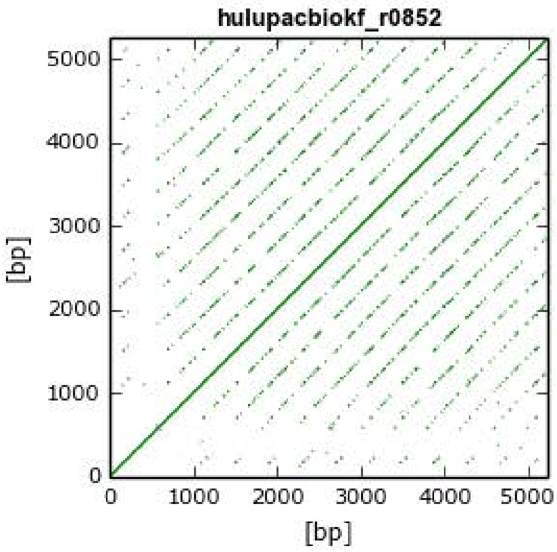
hulupacbiokf_r0852
[bp]
[bp]

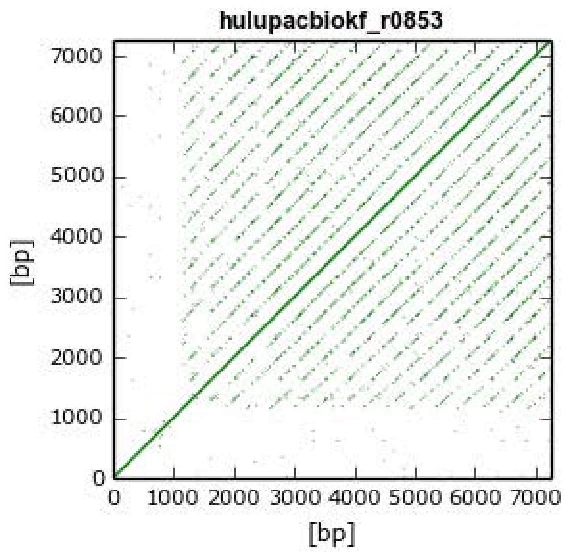
hulupacbiokf_r0853
[bp]
[bp]

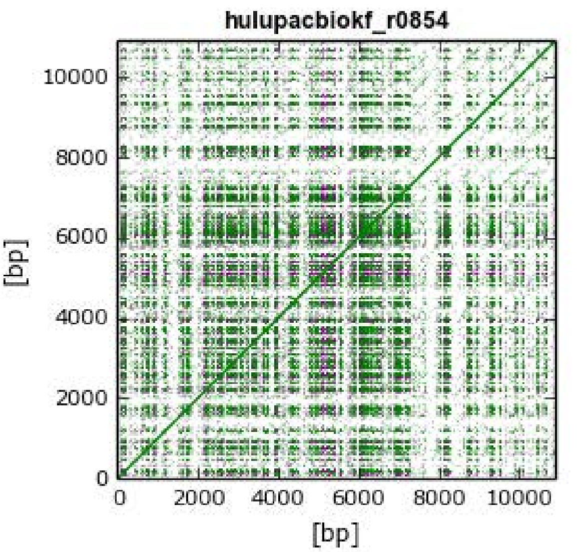
hulupacbiokf_r0854
[bp]
[bp]

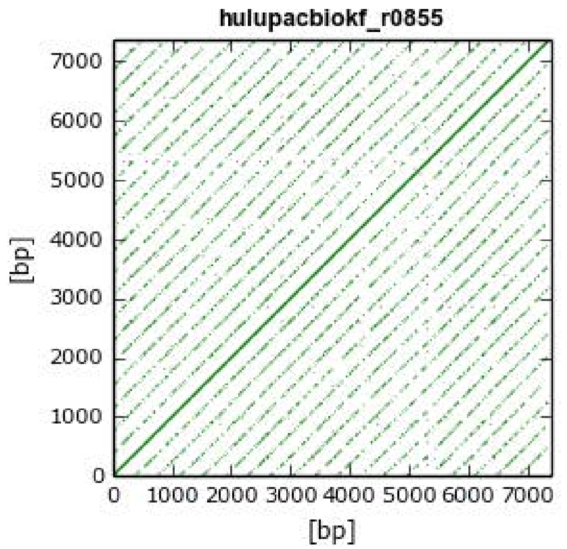
hulupacbiokf_r0855
[bp]
[bp]

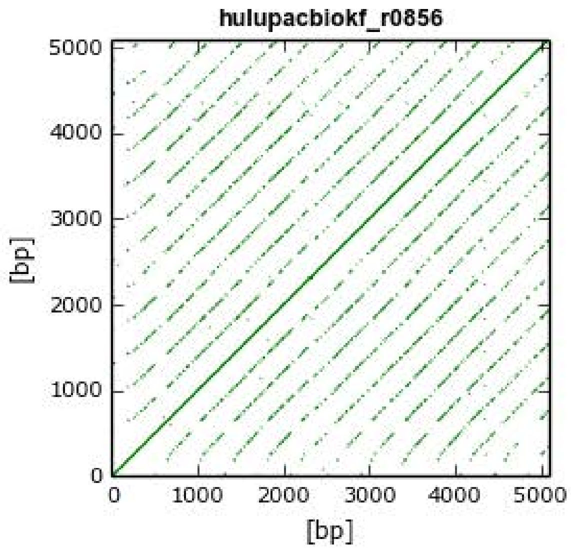
hulupacbiokf_r0856
[bp]
[bp]

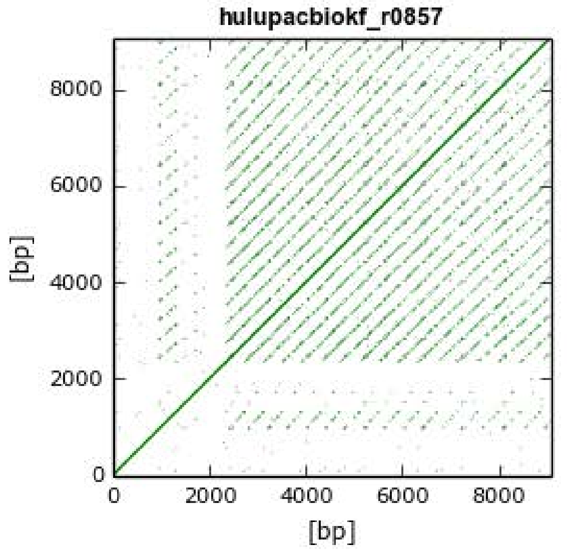
hulupacbiokf_r0857
[bp]
[bp]

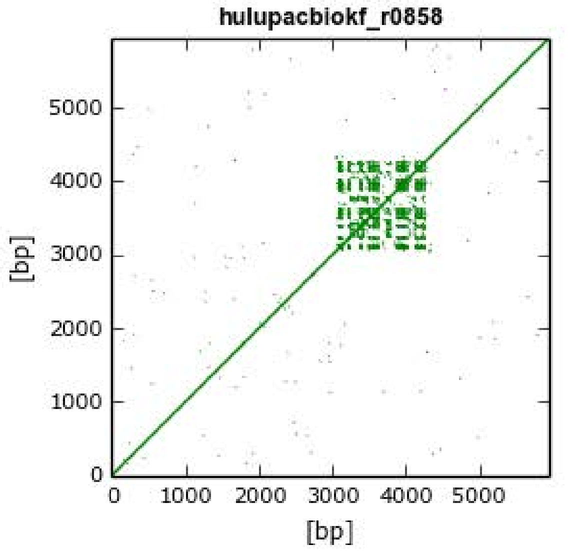
hulupacbiokf_r0858
[bp]
[bp]

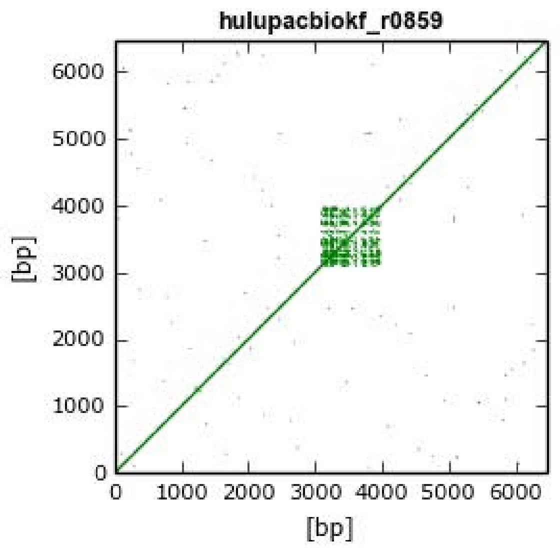
hulupacbiokf_r0859
[bp]
[bp]

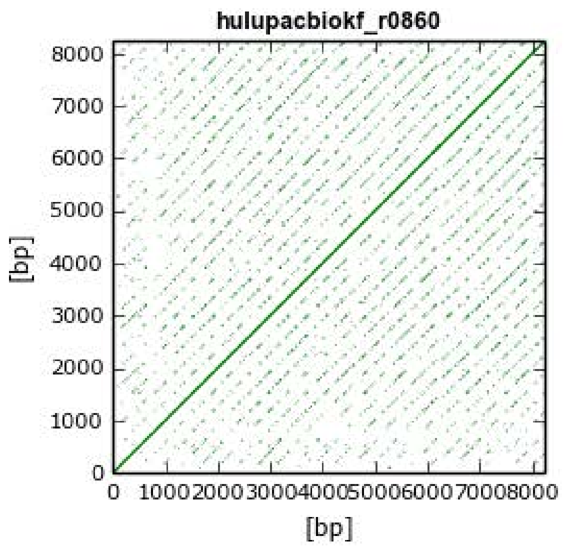
hulupacbiokf_r0860
[bp]
[bp]

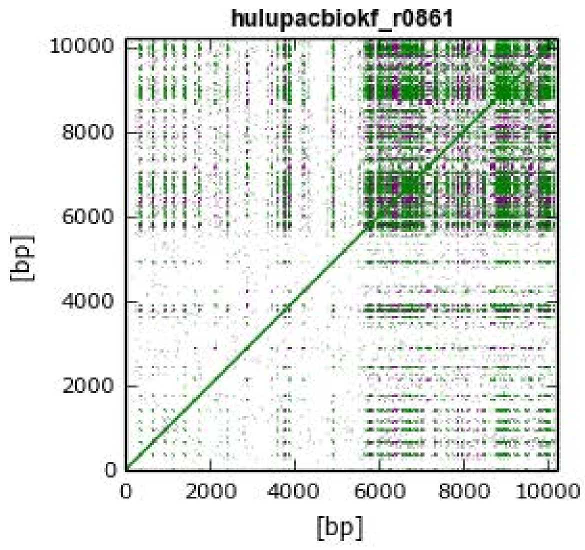
hulupacbiokf_r0861
[bp]
[bp]

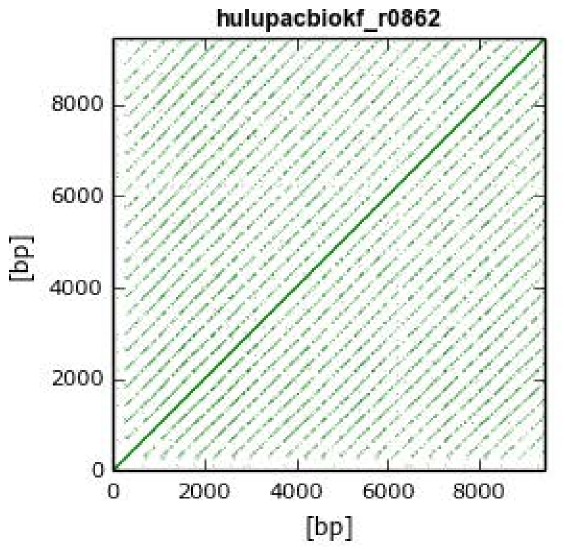
hulupacbiokf_r0862
[bp]
[bp]

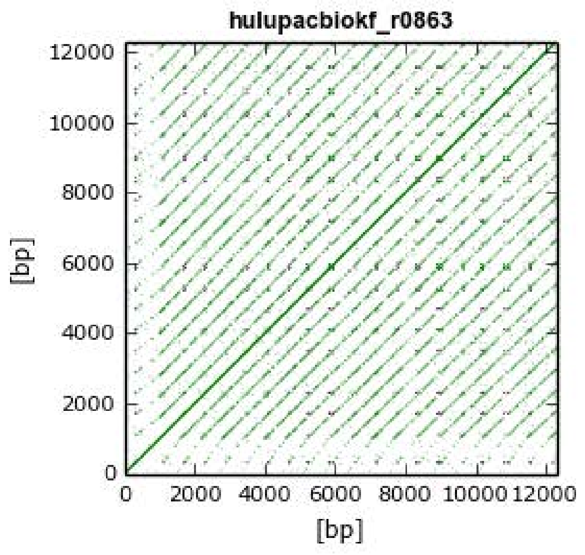
hulupacbiokf_r0863
[bp]
[bp]

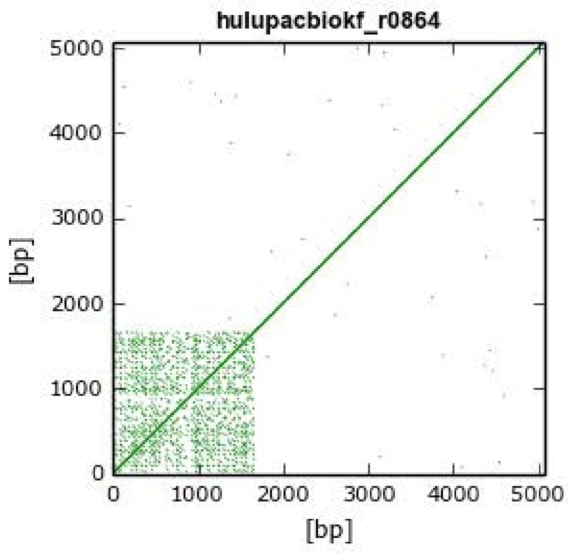
hulupacbiokf_r0864
[bp]
[bp]

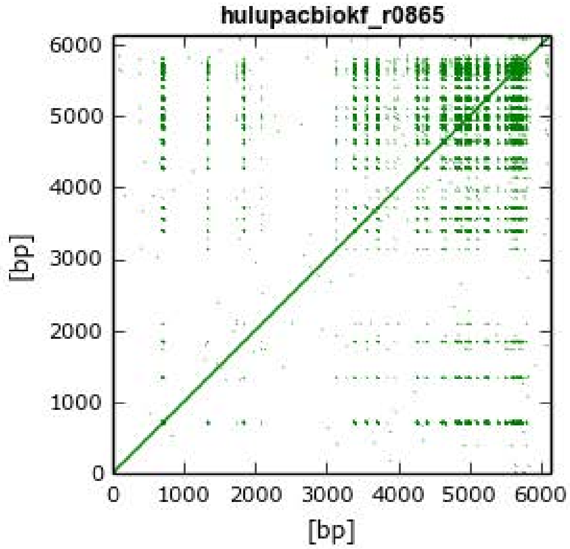
hulupacbiokf_r0865
6000
5000
4000
3000
2000
1000
0
[bp]
0 1000 2000 3000 4000 5000 6000
[bp]

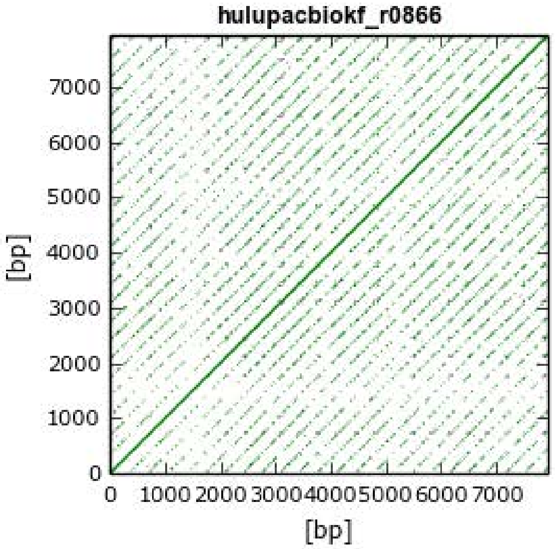
hulupacbiokf_r0866
7000
6000
5000
4000
3000
2000
1000
0
[bp]
0 1000 2000 3000 4000 5000 6000 7000
[bp]

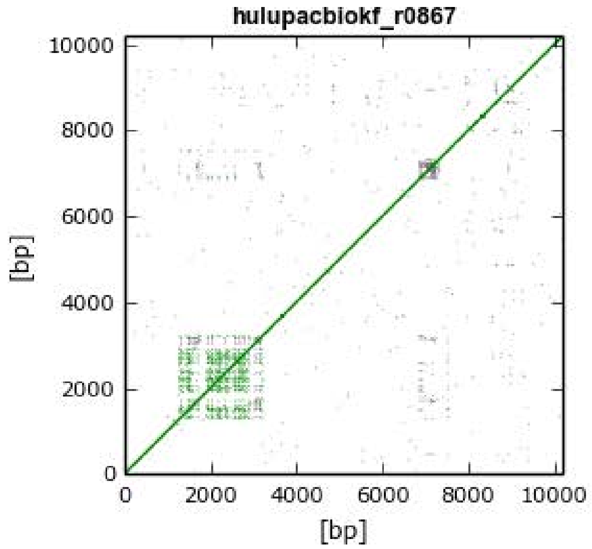
hulupacbiokf_r0867
10000
8000
6000
4000
2000
0
[bp]
0 2000 4000 6000 8000 10000
[bp]

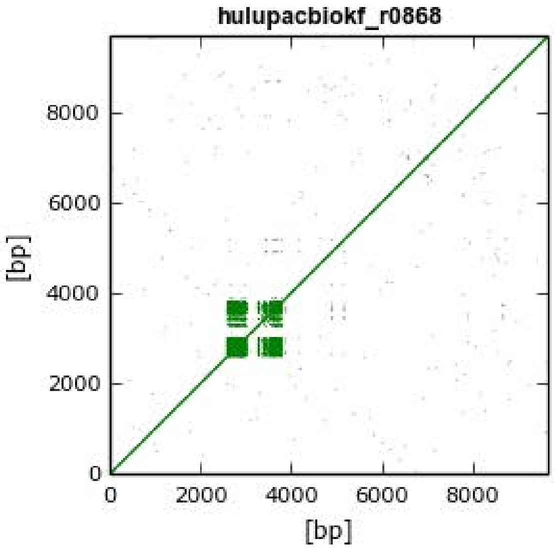
hulupacbiokf_r0868
8000
6000
4000
2000
0
[bp]
0 2000 4000 6000 8000
[bp]

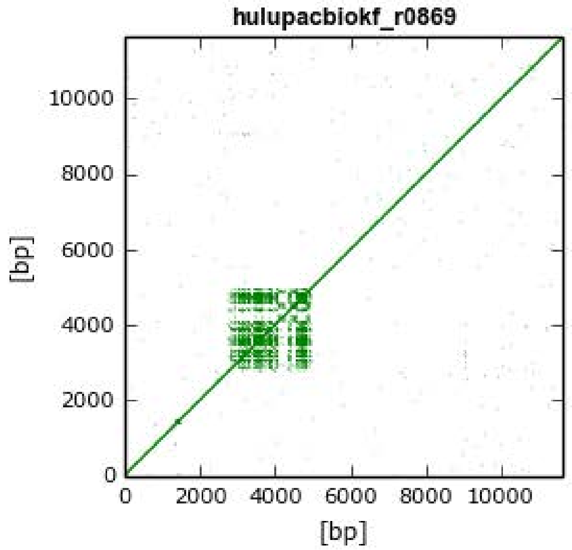
hulupacbiokf_r0869
10000
8000
6000
4000
2000
0
[bp]
0 2000 4000 6000 8000 10000
[bp]

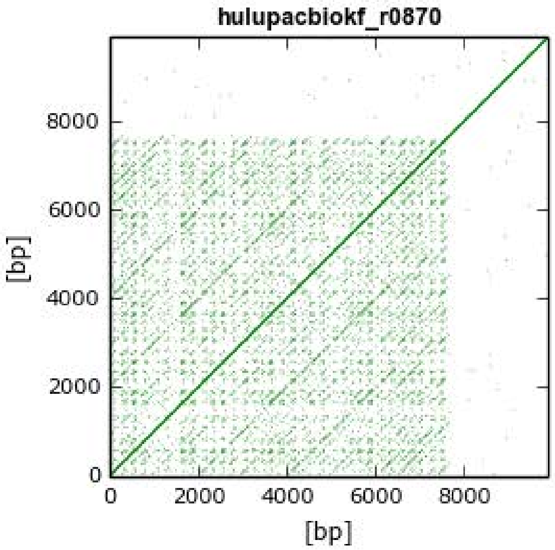
hulupacbiokf_r0870
8000
6000
4000
2000
0
[bp]
0 2000 4000 6000 8000
[bp]

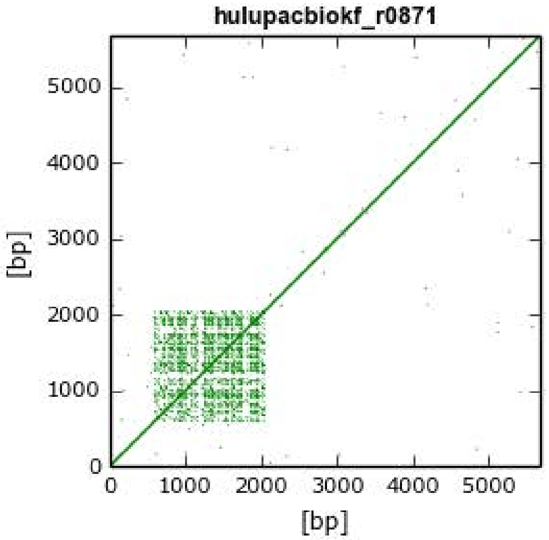

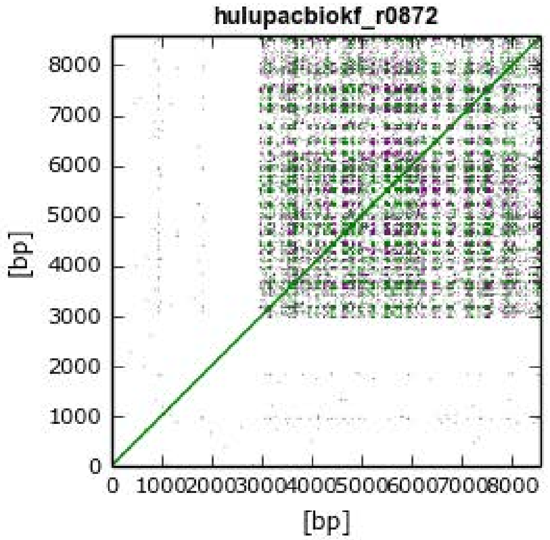

HuluTR450 from read r0873
is in GenBank Acc. MN537581

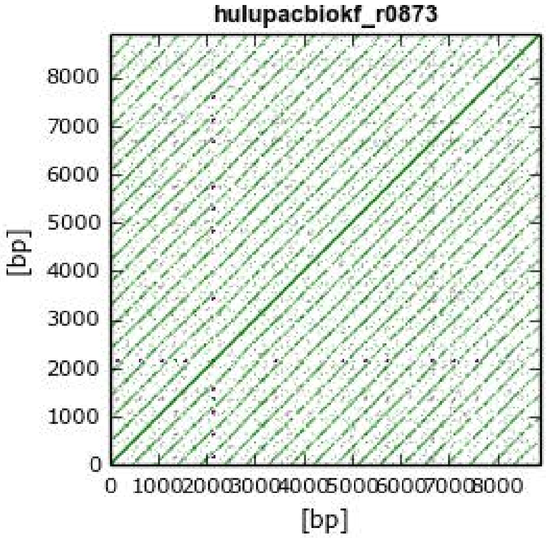

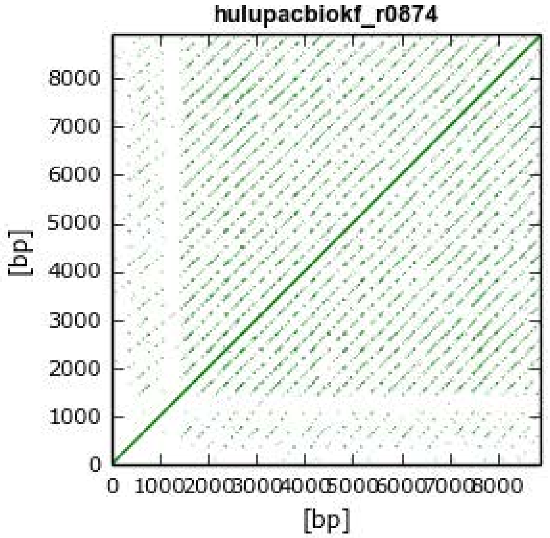

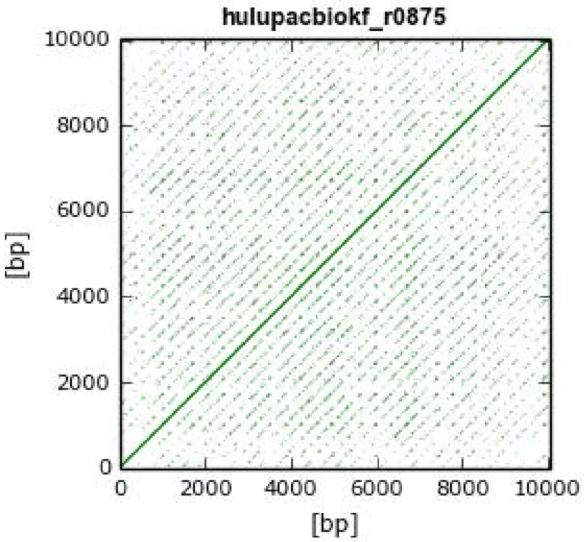

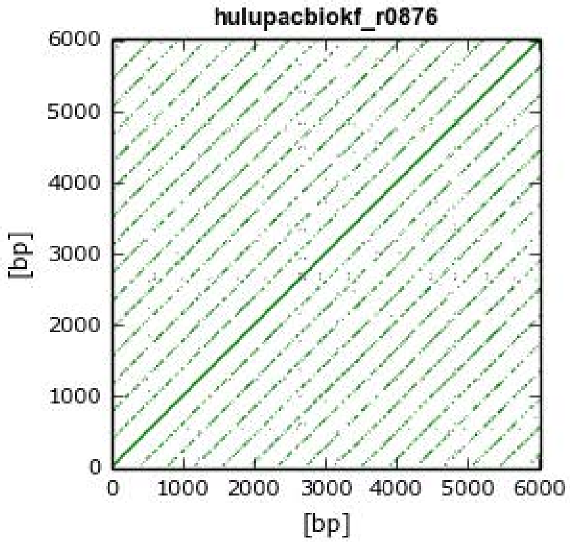

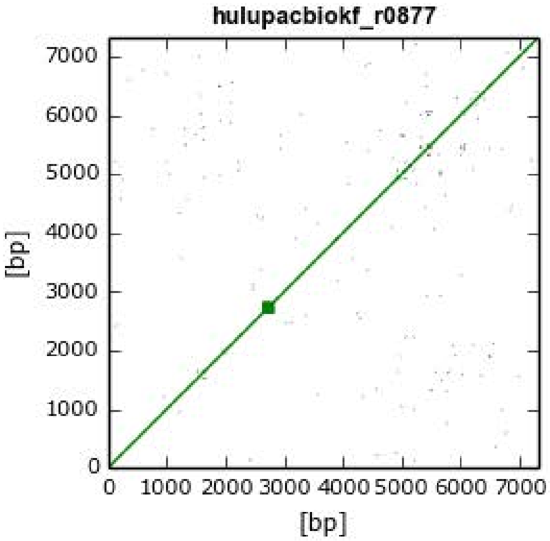
hulupacbiokf_r0877
[bp]
0
1000
2000
3000
4000
5000
6000
7000
[bp]

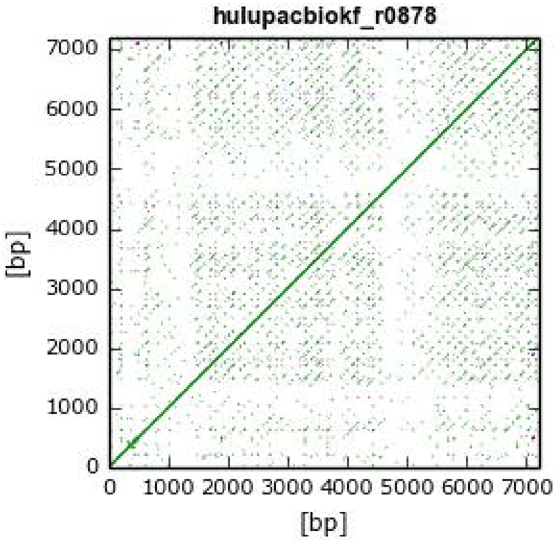
hulupacbiokf_r0878
[bp]
0
1000
2000
3000
4000
5000
6000
7000
[bp]

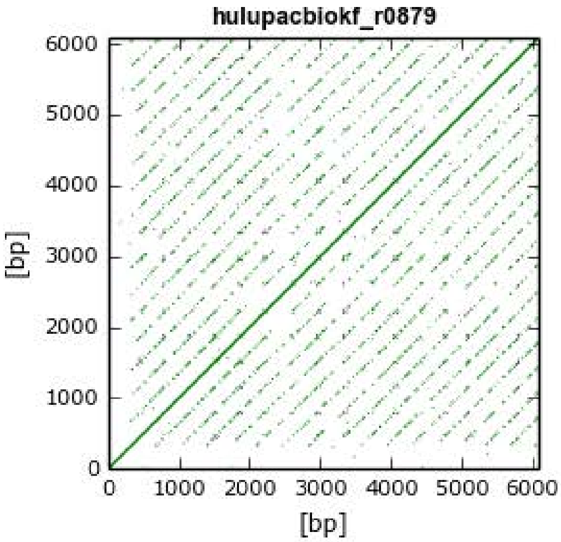
hulupacbiokf_r0879
[bp]
0
1000
2000
3000
4000
5000
6000
[bp]

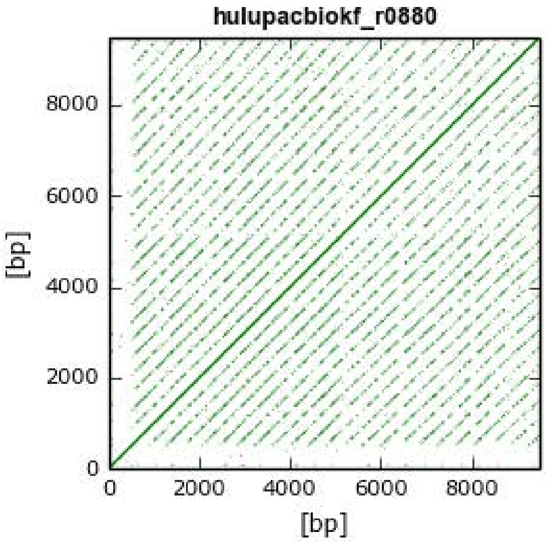
hulupacbiokf_r0880
[bp]
0
2000
4000
6000
8000
[bp]

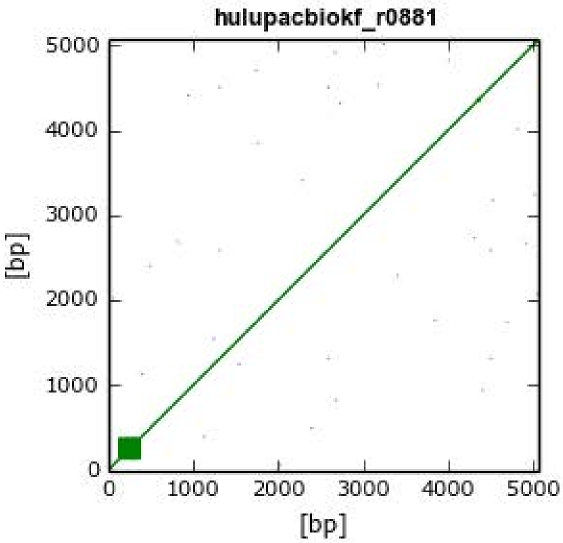
hulupacbiokf_r0881
[bp]
0
1000
2000
3000
4000
5000
[bp]

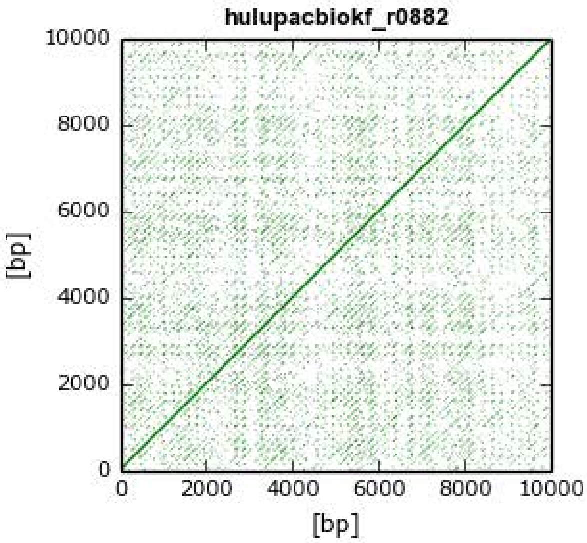
hulupacbiokf_r0882
[bp]
0
2000
4000
6000
8000
10000
[bp]

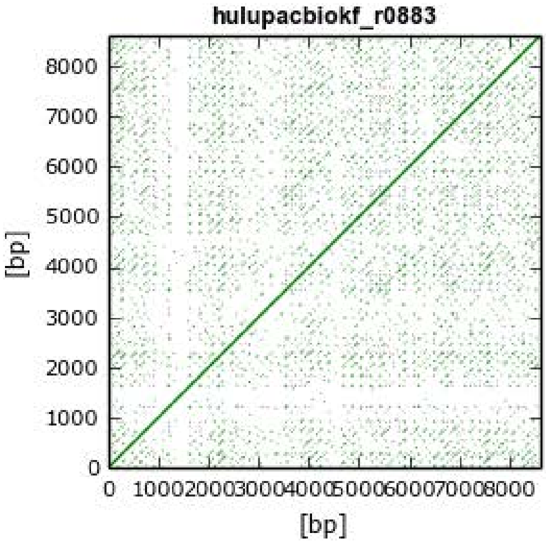
hulupacbiokf_r0883
[bp]
[bp]

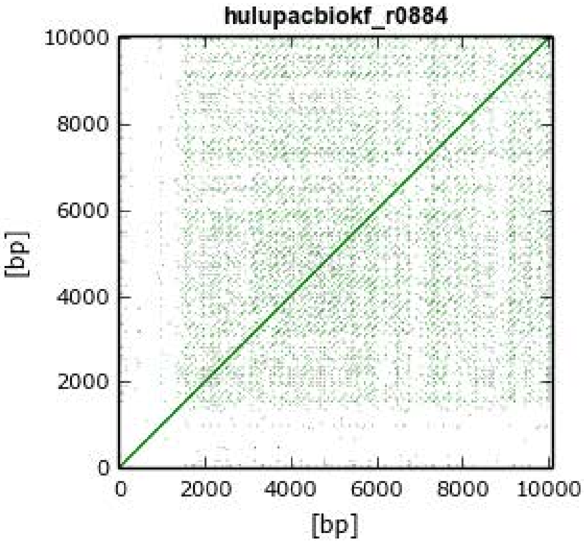
hulupacbiokf_r0884
[bp]
[bp]

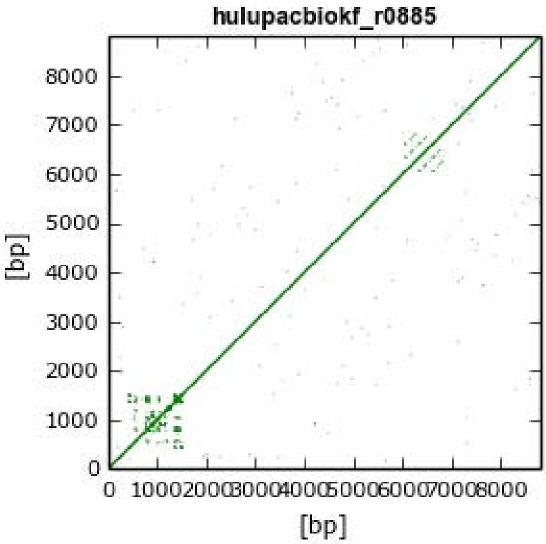
hulupacbiokf_r0885
[bp]
[bp]

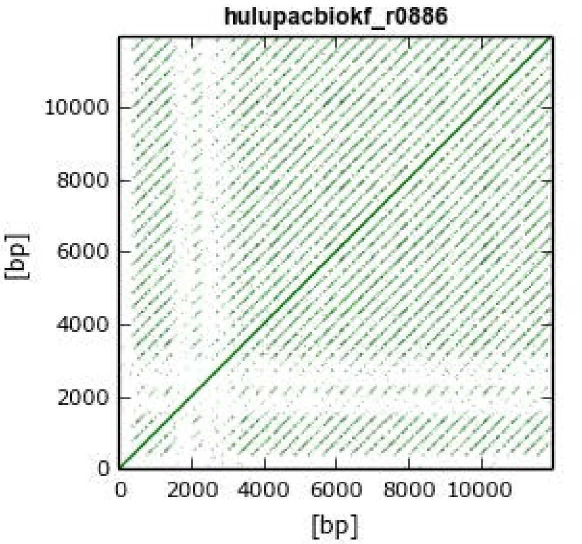
hulupacbiokf_r0886
[bp]
[bp]

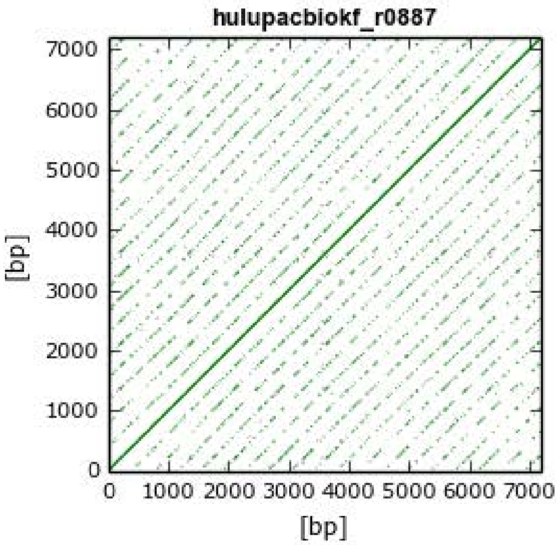
hulupacbiokf_r0887
[bp]
[bp]

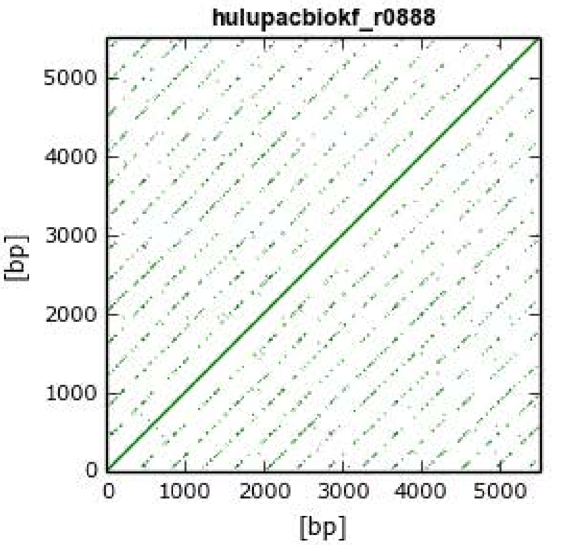
hulupacbiokf_r0888
[bp]
[bp]

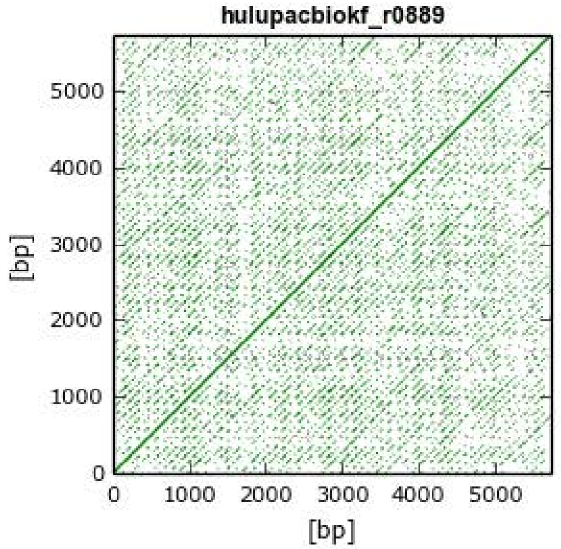
hulupacbiokf_r0889
[bp]
[bp]

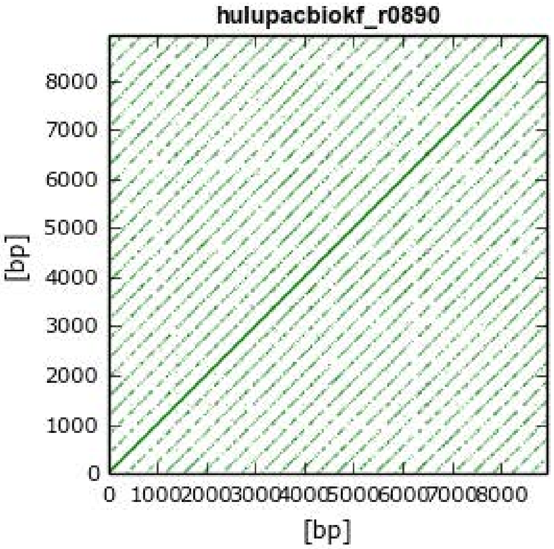
hulupacbiokf_r0890
[bp]
[bp]

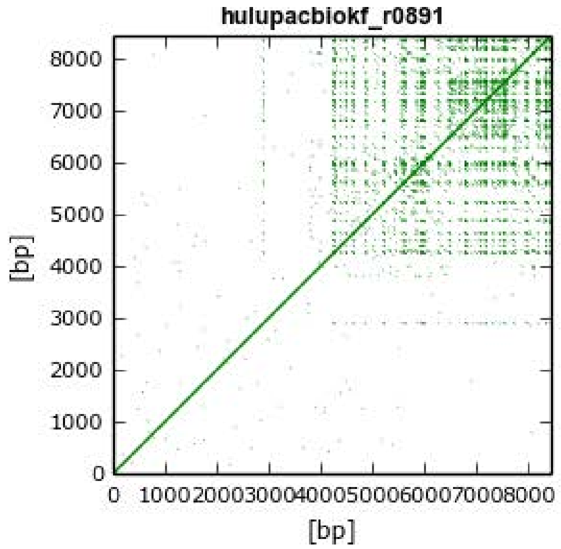
hulupacbiokf_r0891
[bp]
[bp]

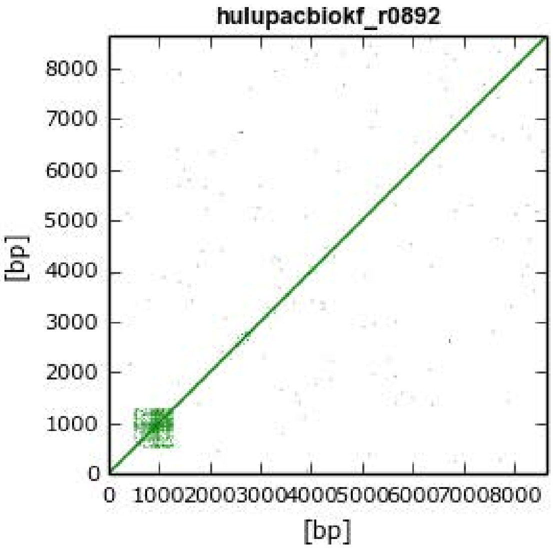
hulupacbiokf_r0892
[bp]
[bp]

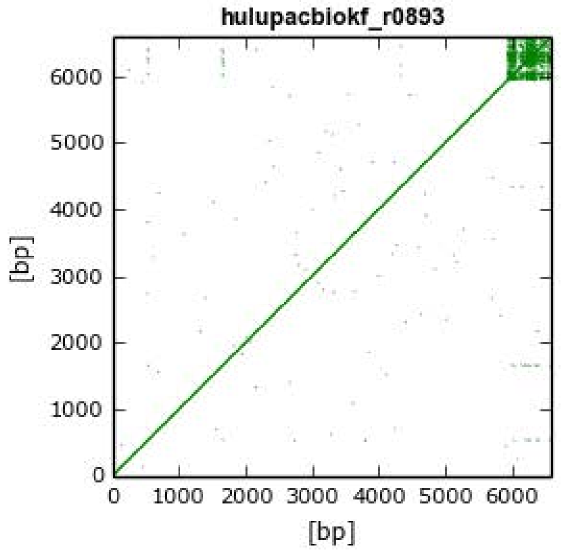
hulupacbiokf_r0893
[bp]
[bp]

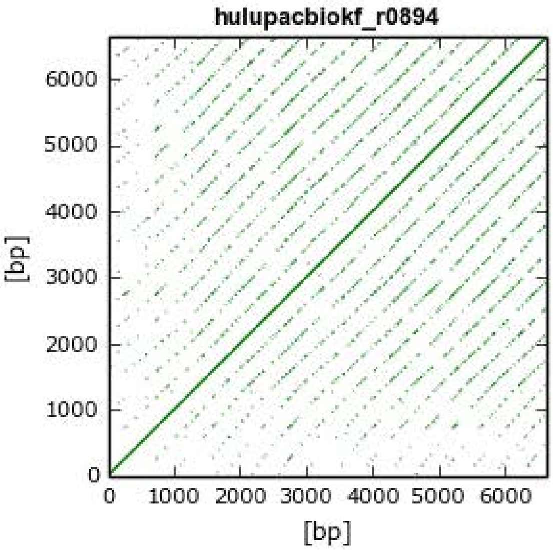
hulupacbiokf_r0894
[bp]
[bp]

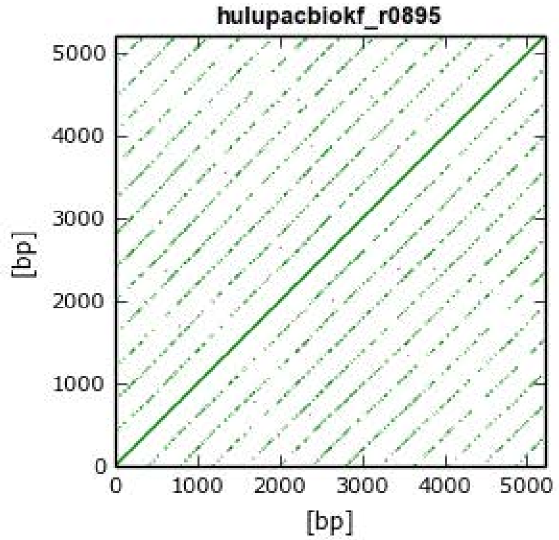
hulupacbiokf_r0895
[bp]
[bp]

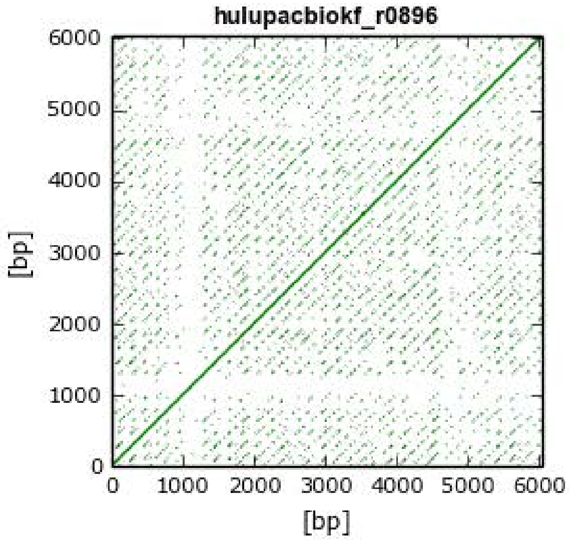
hulupacbiokf_r0896
[bp]
[bp]

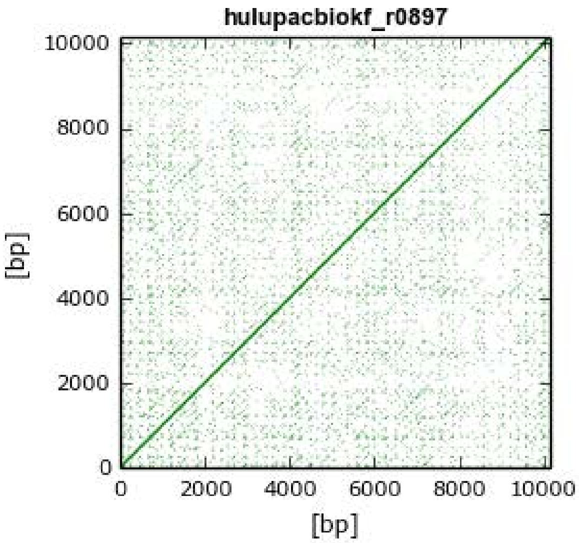
hulupacbiokf_r0897
[bp]
[bp]

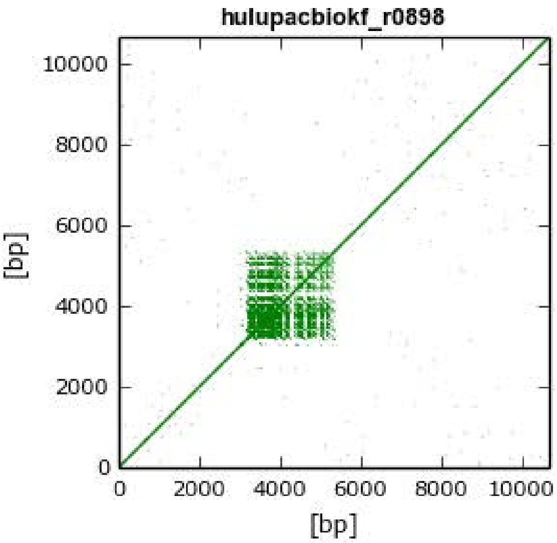
hulupacbiokf_r0898
[bp]
[bp]

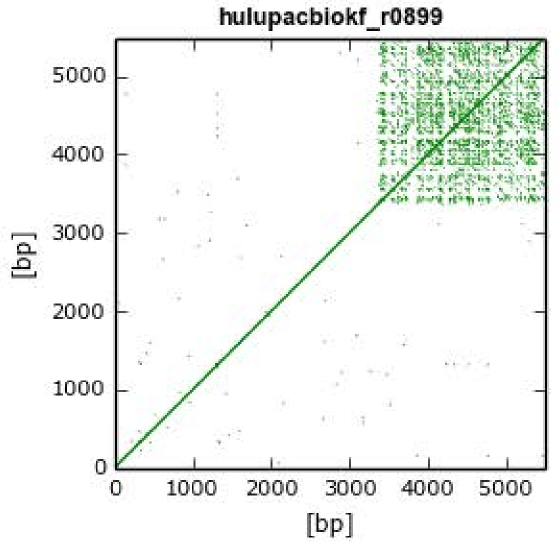
hulupacbiokf_r0899
[bp]
[bp]

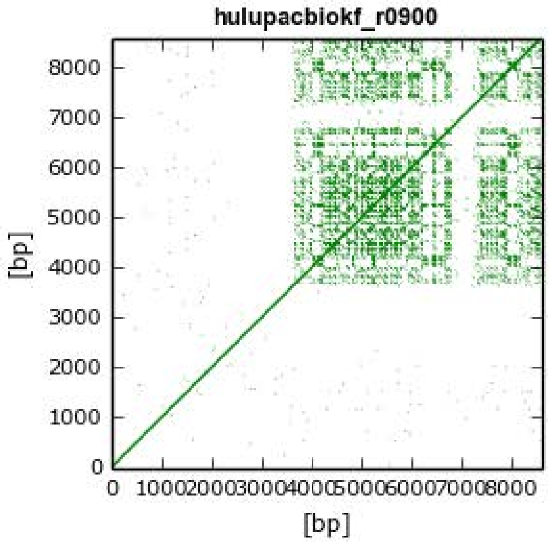
hulupacbiokf_r0900
[bp]
[bp]

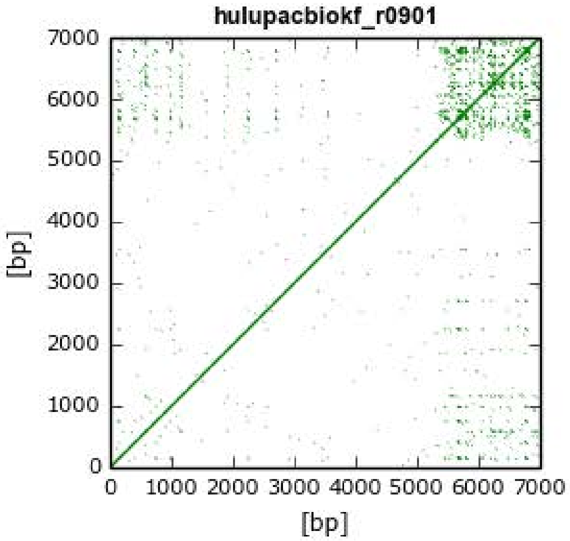
hulupacbiokf_r0901
[bp]
[bp]

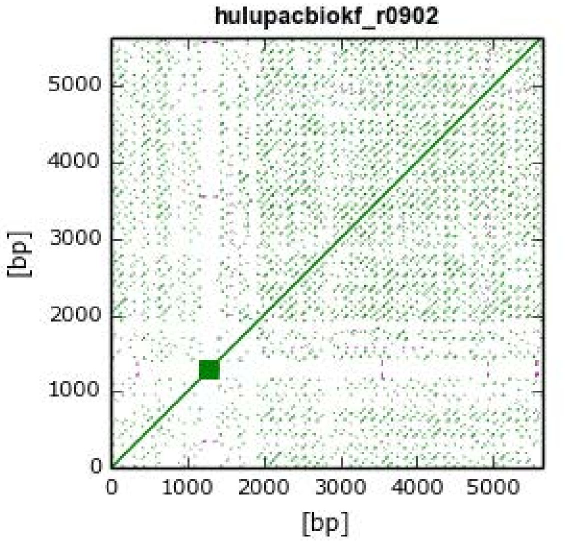
hulupacbiokf_r0902
[bp]
[bp]

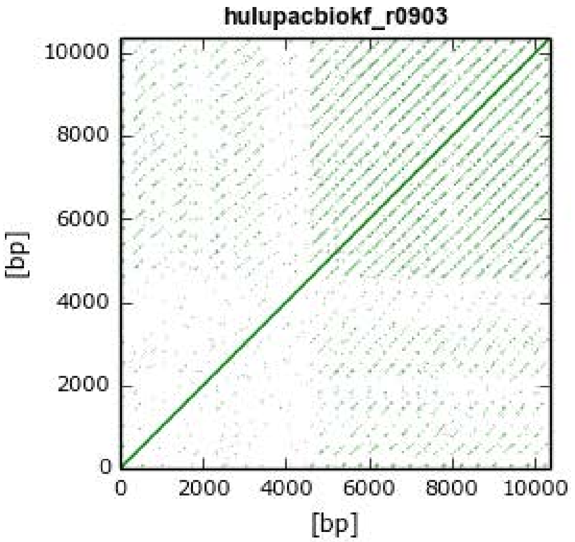
hulupacbiokf_r0903
[bp]
[bp]

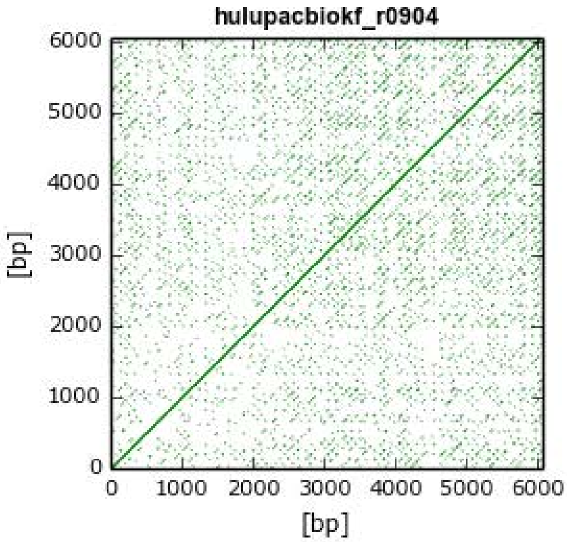
hulupacbiokf_r0904
[bp]
[bp]

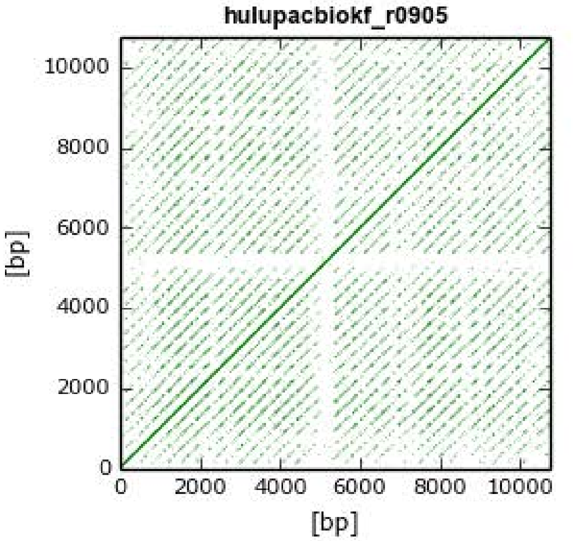
hulupacbiokf_r0905
[bp]
[bp]

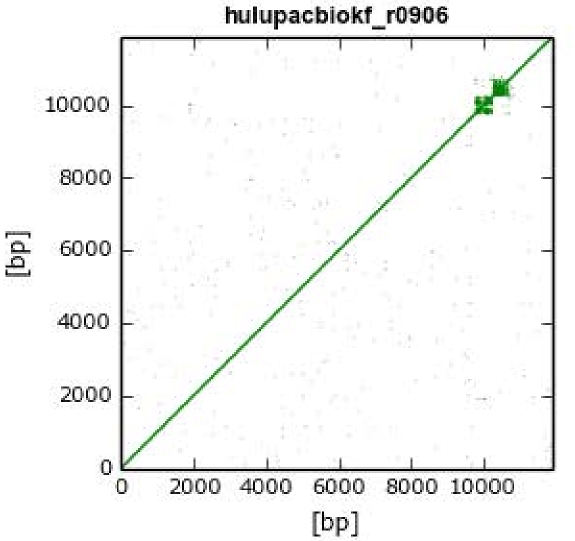
hulupacbiokf_r0906
[bp]
[bp]

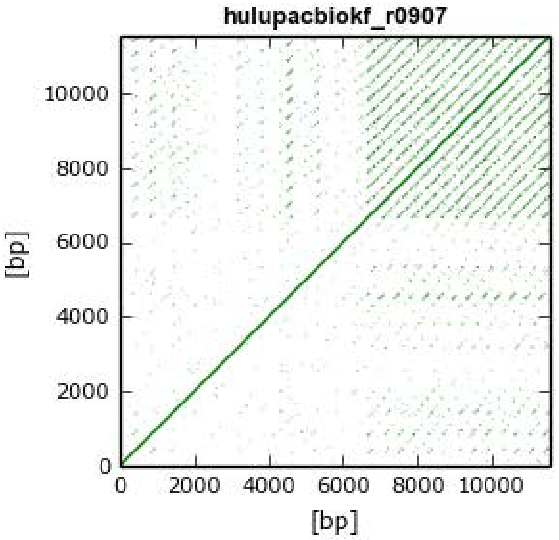
hulupacbiokf_r0907
[bp]
0
2000
4000
6000
8000
10000
[bp]

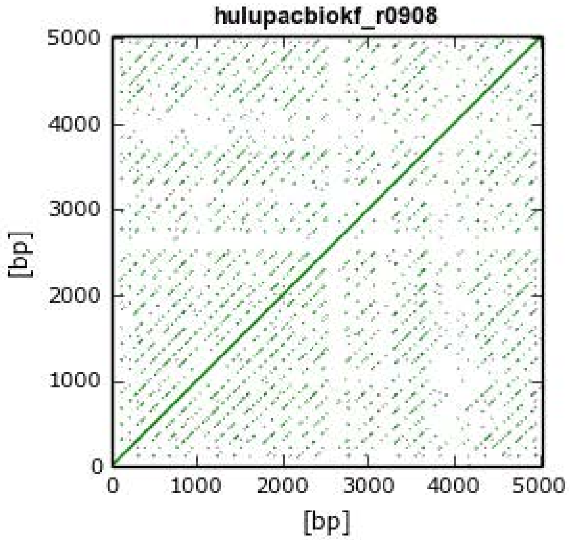
hulupacbiokf_r0908
[bp]
0
1000
2000
3000
4000
5000
[bp]

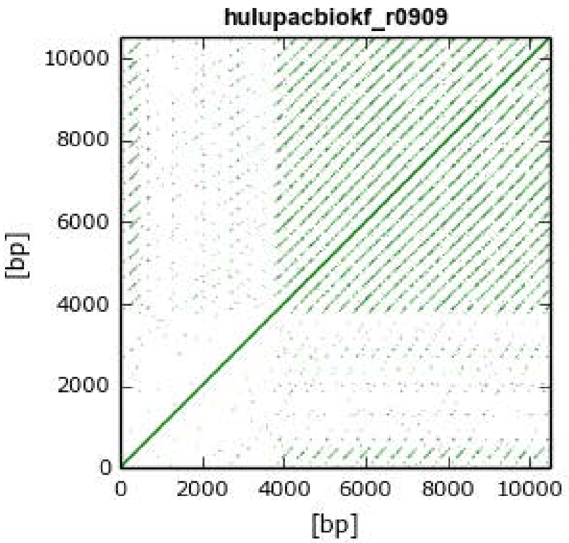
hulupacbiokf_r0909
[bp]
0
2000
4000
6000
8000
10000
[bp]

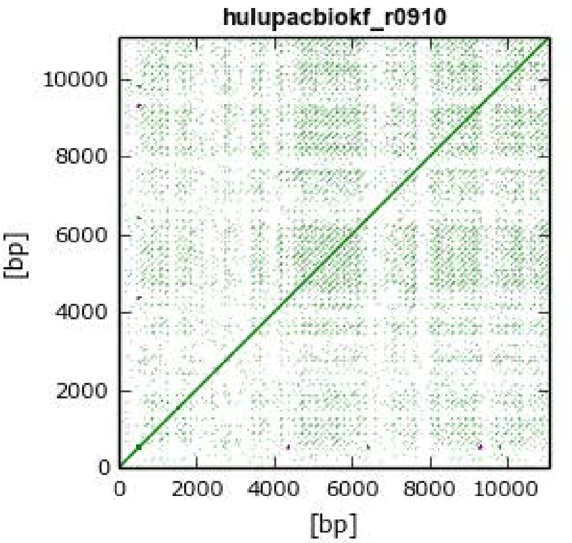
hulupacbiokf_r0910
[bp]
0
2000
4000
6000
8000
10000
[bp]

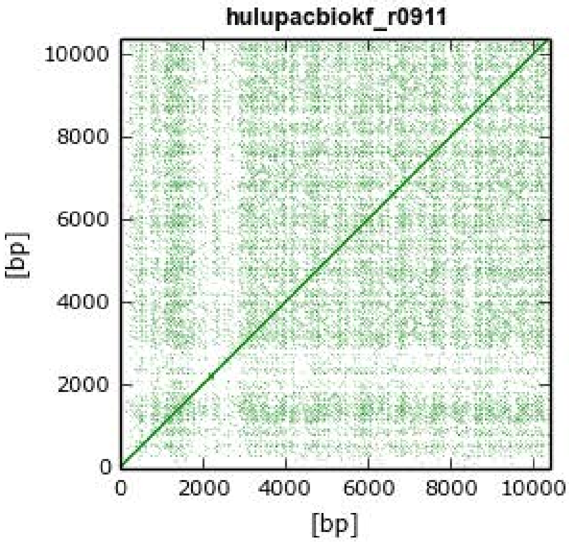
hulupacbiokf_r0911
[bp]
0
2000
4000
6000
8000
10000
[bp]

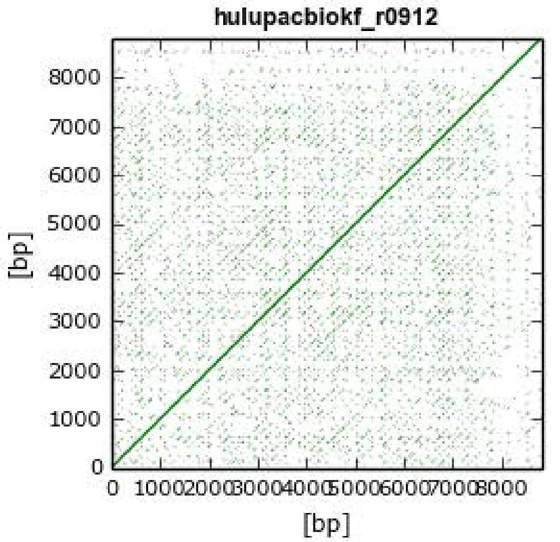
hulupacbiokf_r0912
[bp]
0
1000
2000
3000
4000
5000
6000
7000
8000
[bp]

hulupacbiokf_r0913

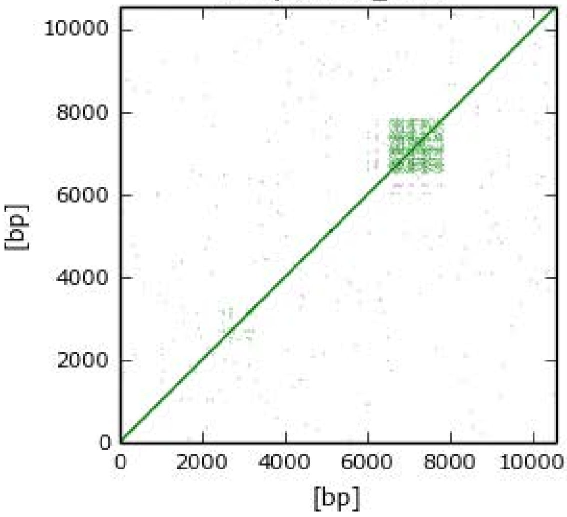

hulupacbiokf_r0914

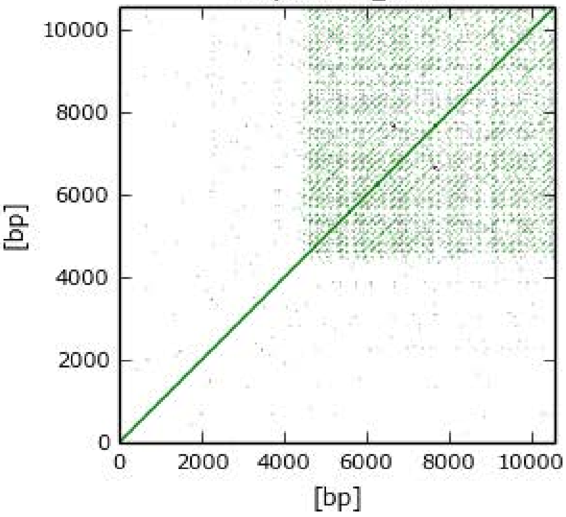

hulupacbiokf_r0915

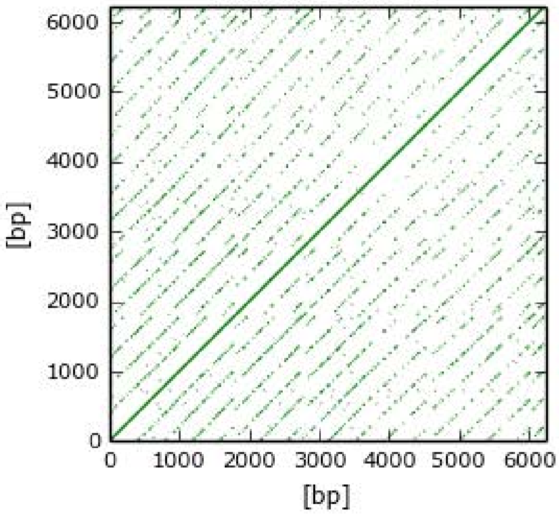

hulupacbiokf_r0916

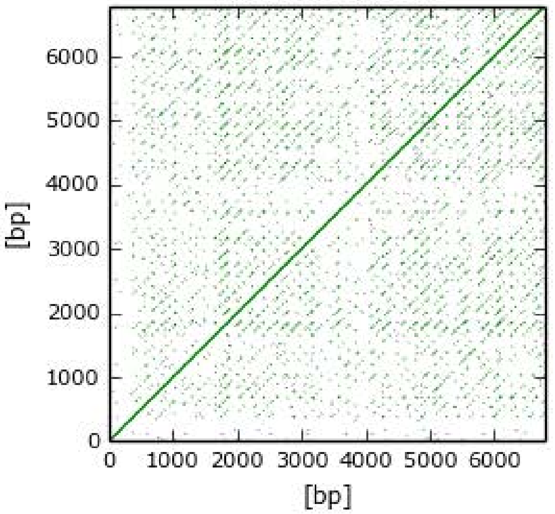

hulupacbiokf_r0917

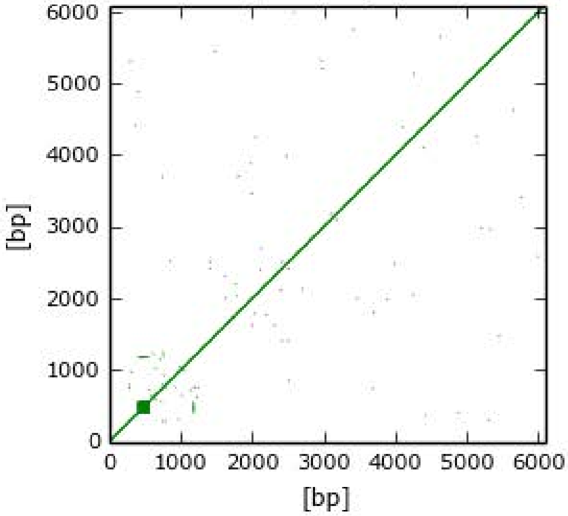

hulupacbiokf_r0918

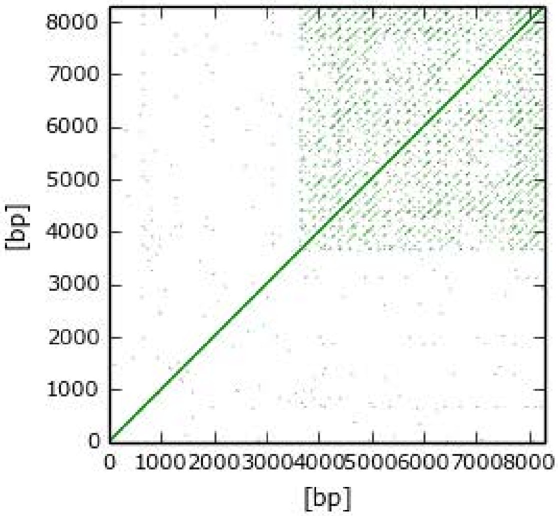

hulupacbiokf_r0919

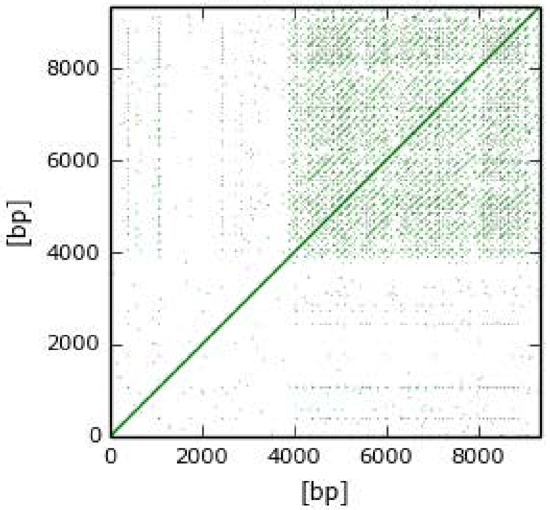

hulupacbiokf_r0920

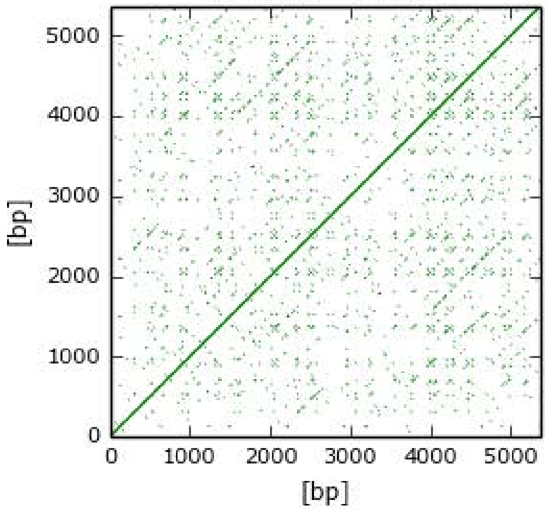

hulupacbiokf_r0921

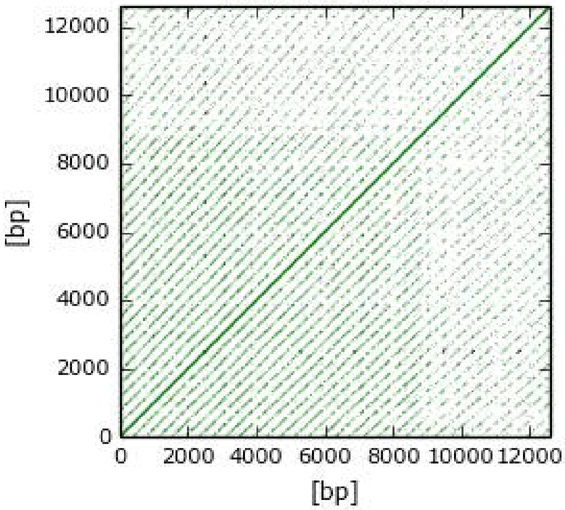

hulupacbiokf_r0922

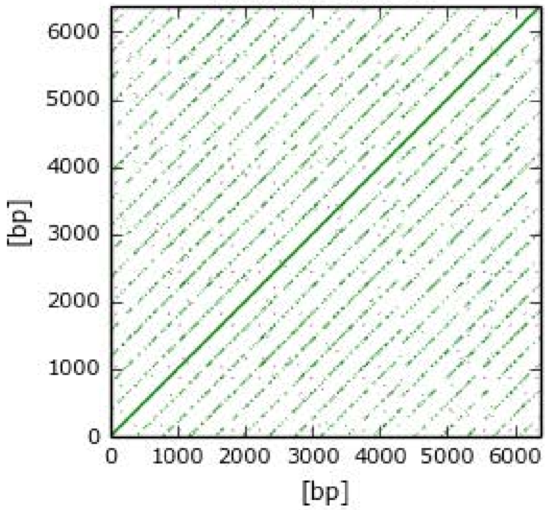

hulupacbiokf_r0923

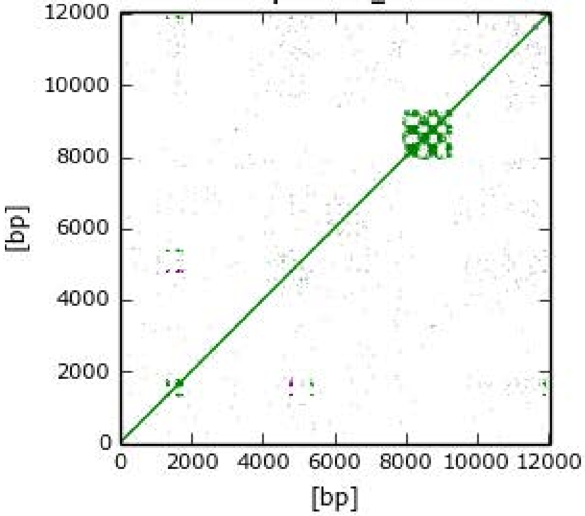

hulupacbiokf_r0924

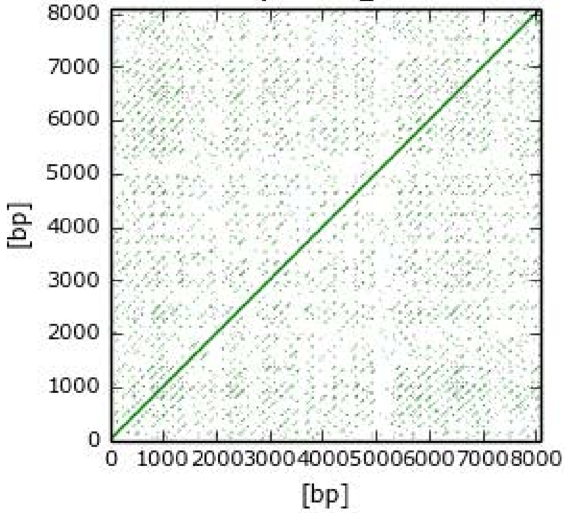

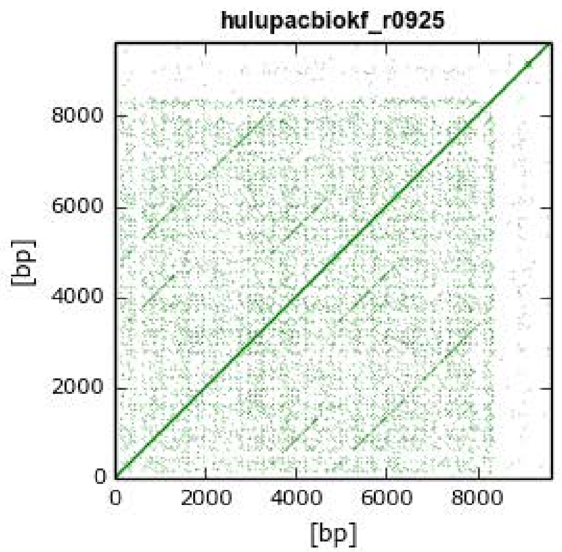
hulupacbiokf_r0925
[bp]
[bp]

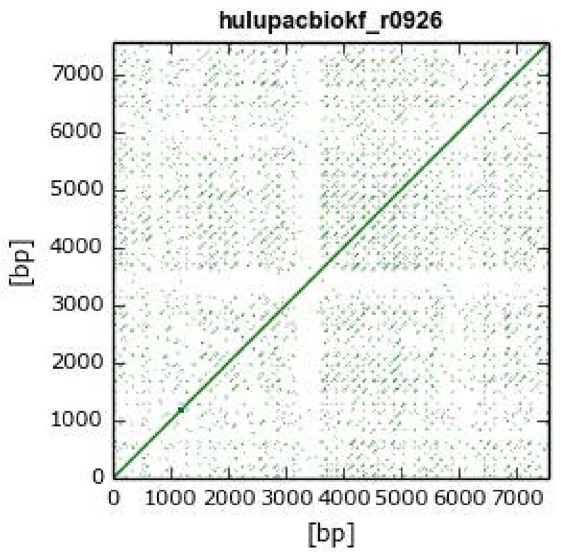
hulupacbiokf_r0926
[bp]
[bp]

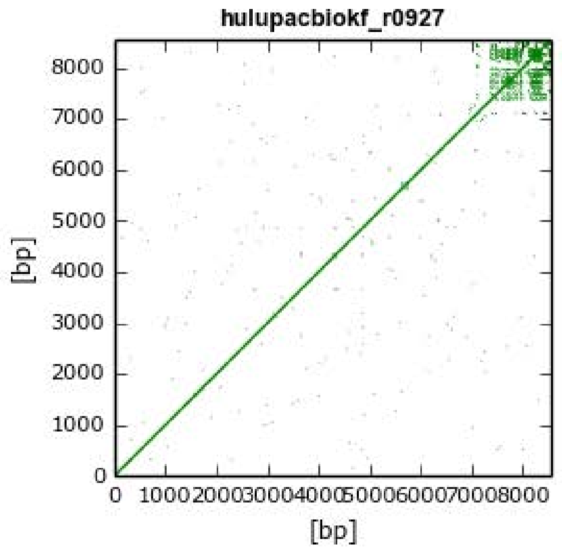
hulupacbiokf_r0927
[bp]
[bp]

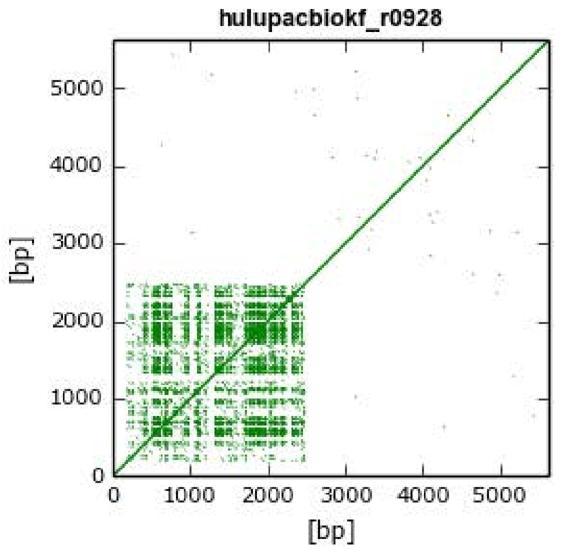
hulupacbiokf_r0928
[bp]
[bp]

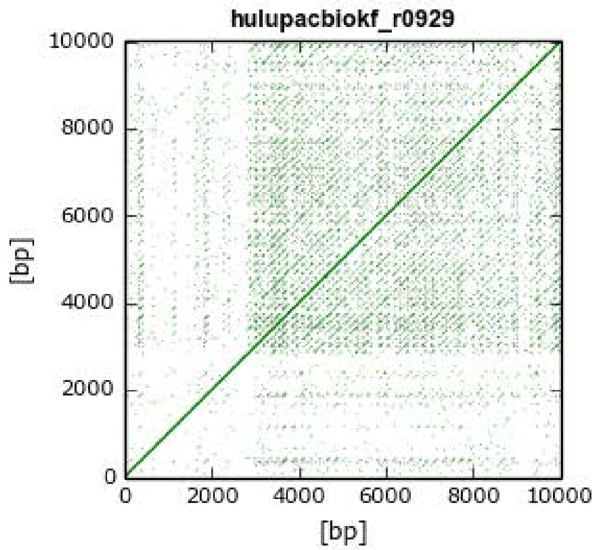
hulupacbiokf_r0929
[bp]
[bp]

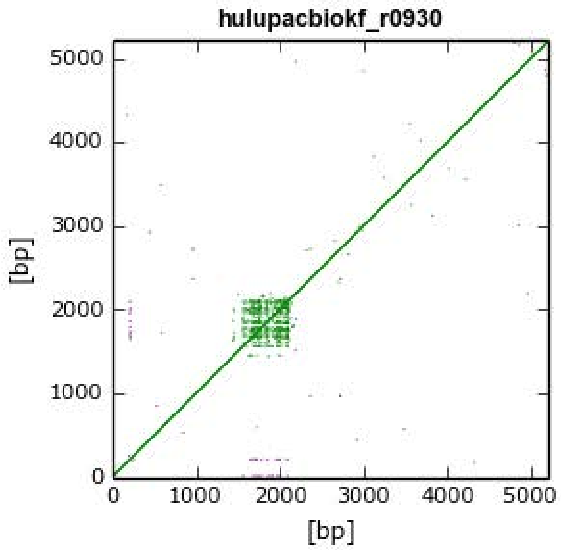
hulupacbiokf_r0930
[bp]
[bp]

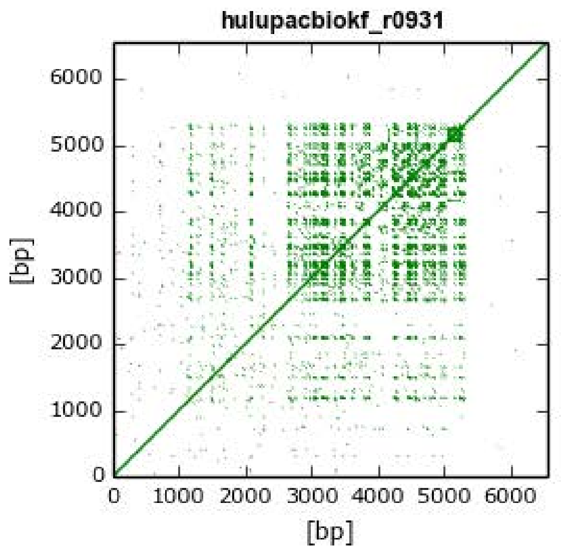

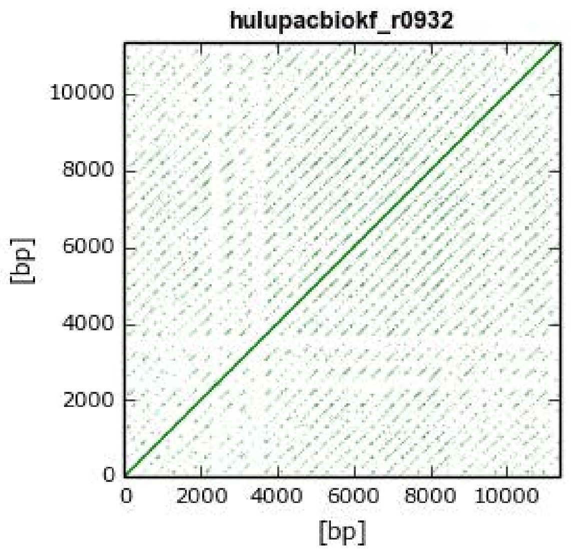

HuluTR280 from read r0934
is in GenBank Acc. MN537576

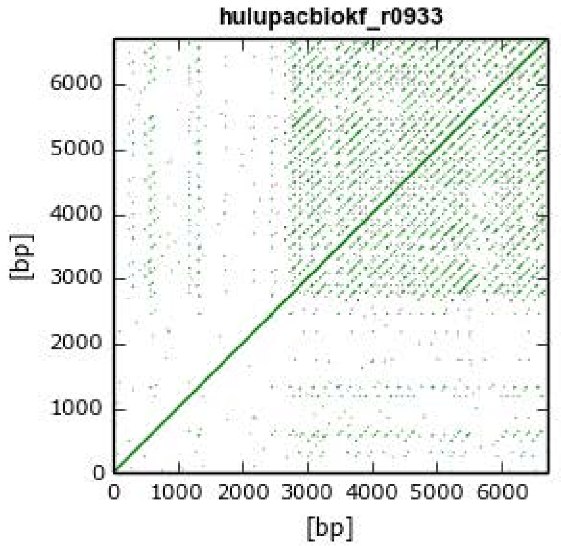

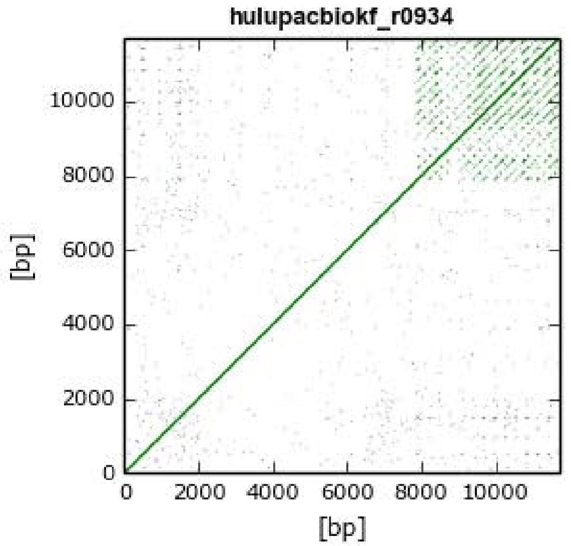

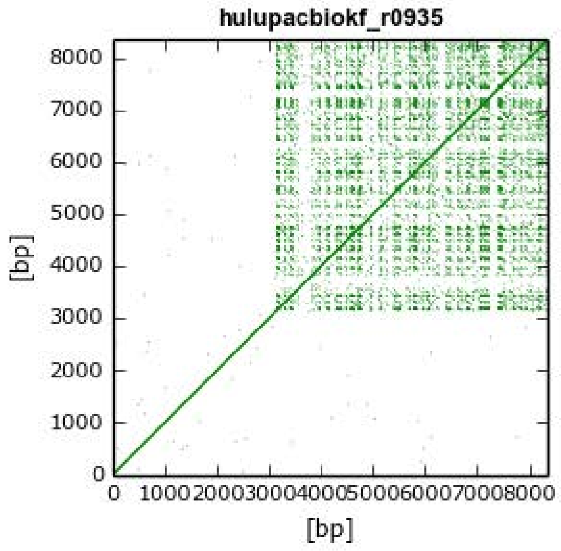

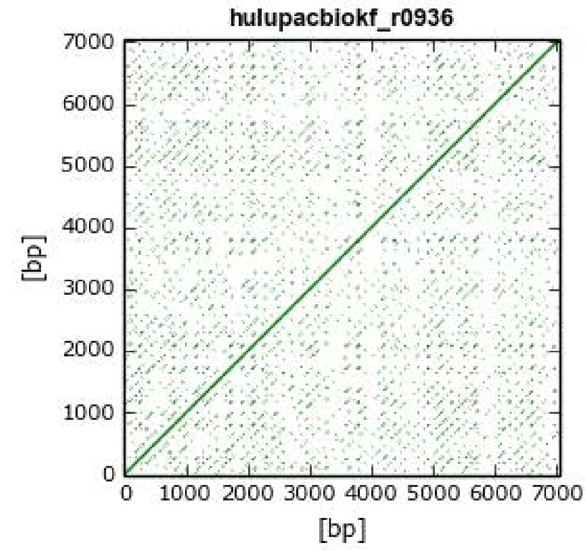

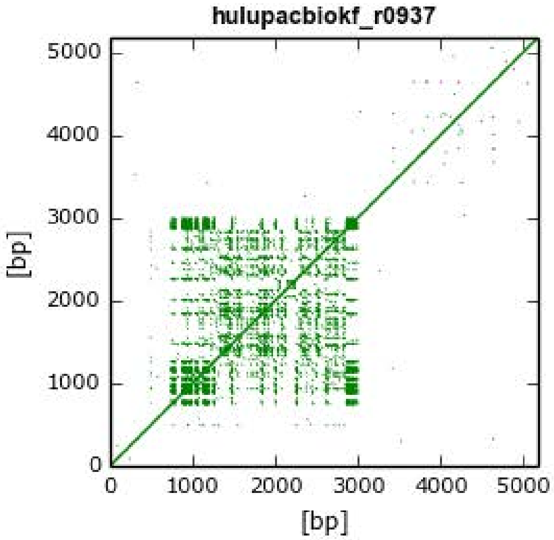
hulupacbiokf_r0937
[bp]
[bp]

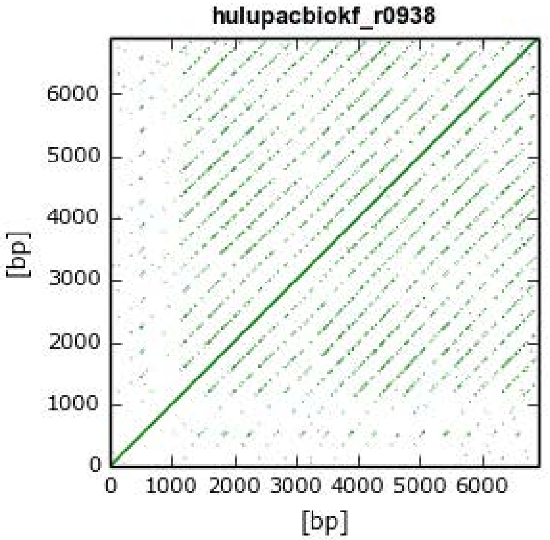
hulupacbiokf_r0938
[bp]
[bp]

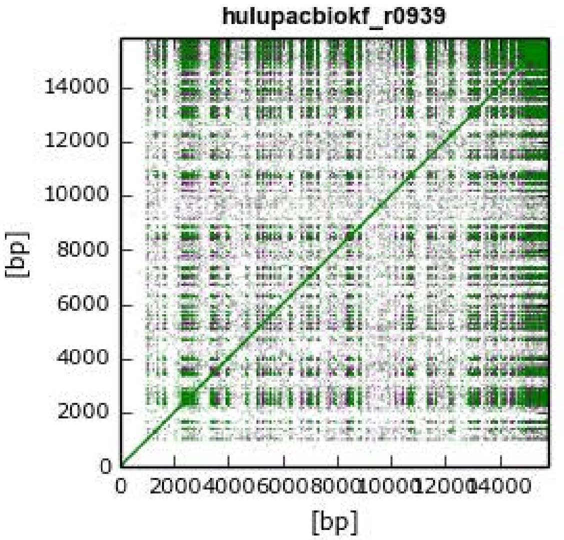
hulupacbiokf_r0939
[bp]
[bp]

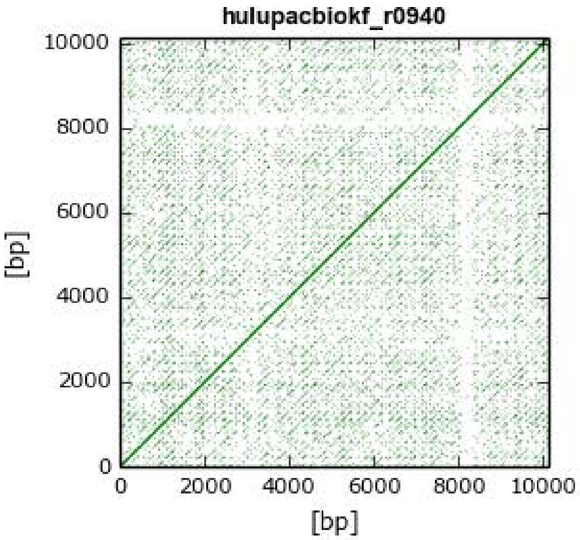
hulupacbiokf_r0940
[bp]
[bp]

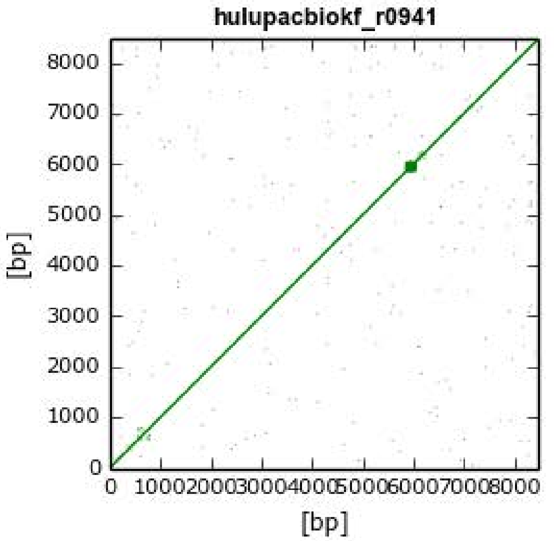
hulupacbiokf_r0941
[bp]
[bp]

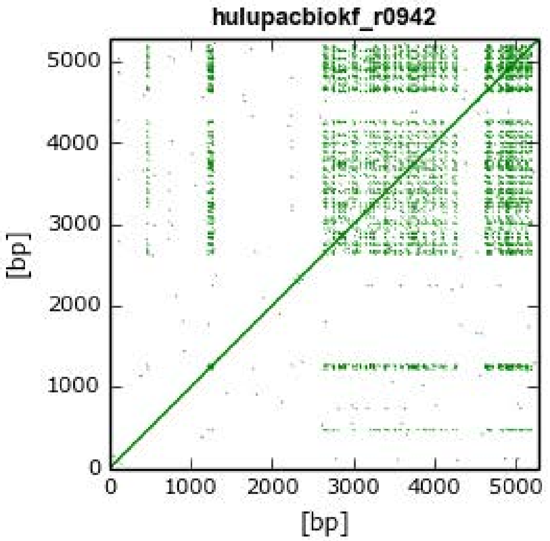
hulupacbiokf_r0942
[bp]
[bp]

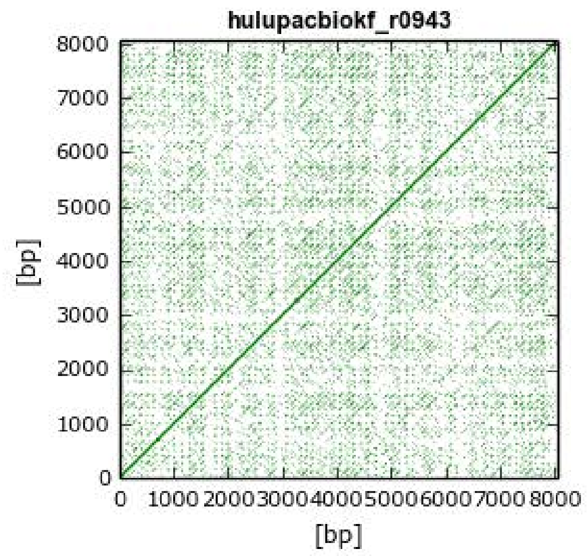
hulupacbiokf_r0943
[bp]
[bp]

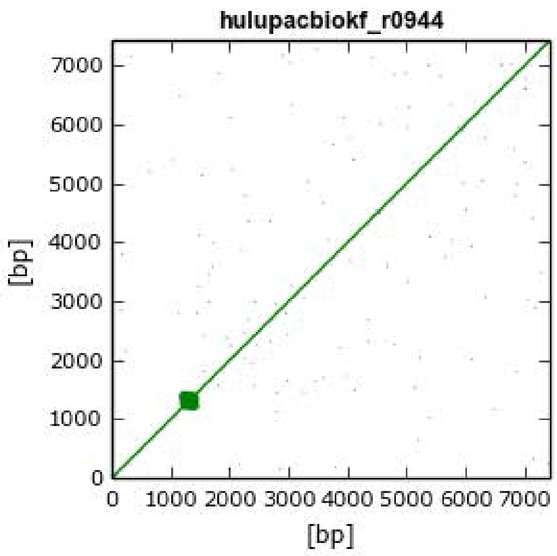
hulupacbiokf_r0944
[bp]
[bp]

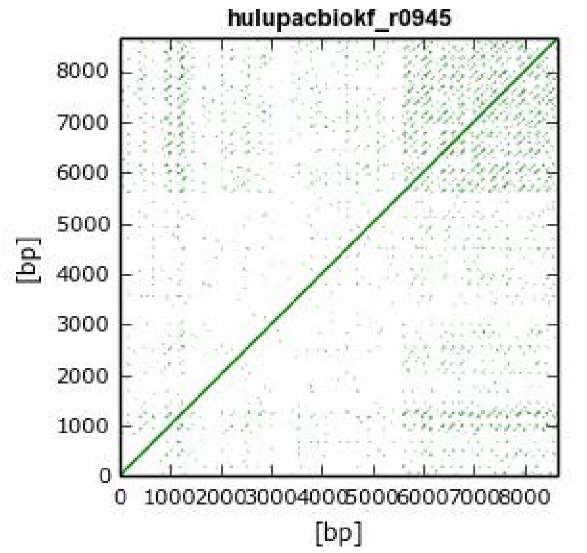
hulupacbiokf_r0945
[bp]
[bp]

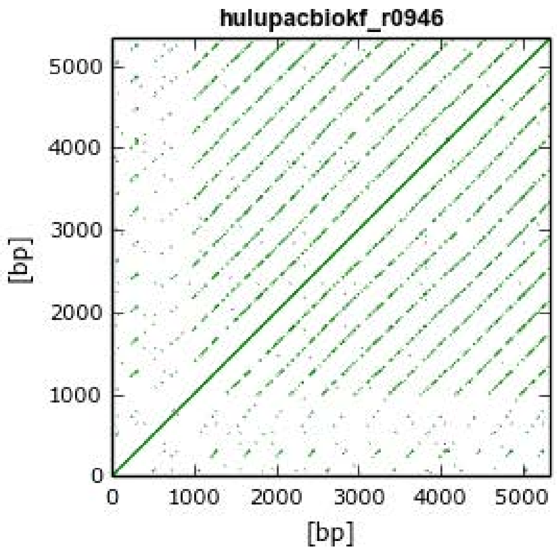
hulupacbiokf_r0946
[bp]
[bp]

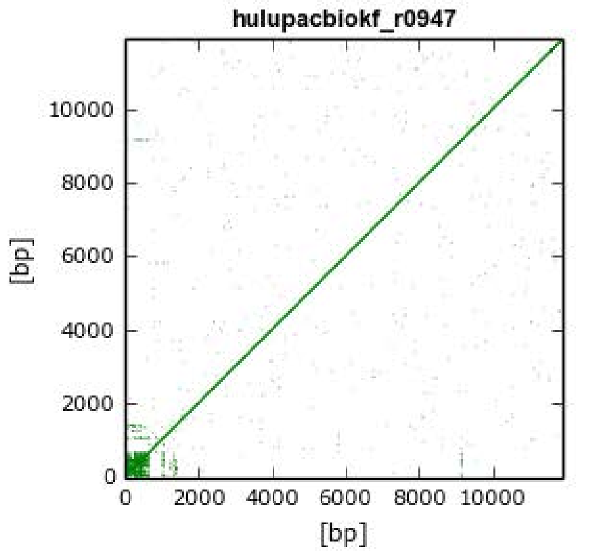
hulupacbiokf_r0947
[bp]
[bp]

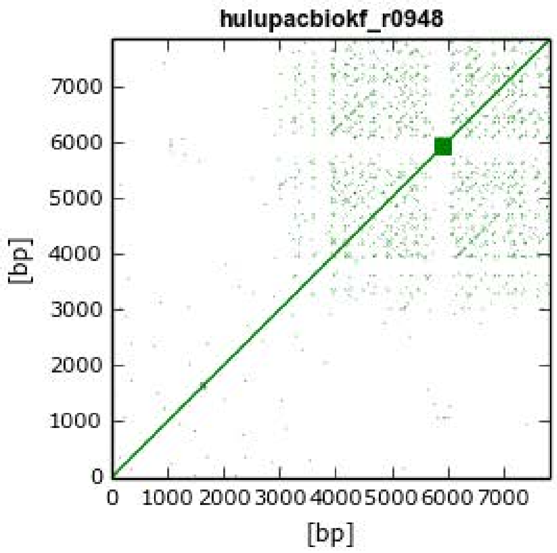
hulupacbiokf_r0948
[bp]
[bp]

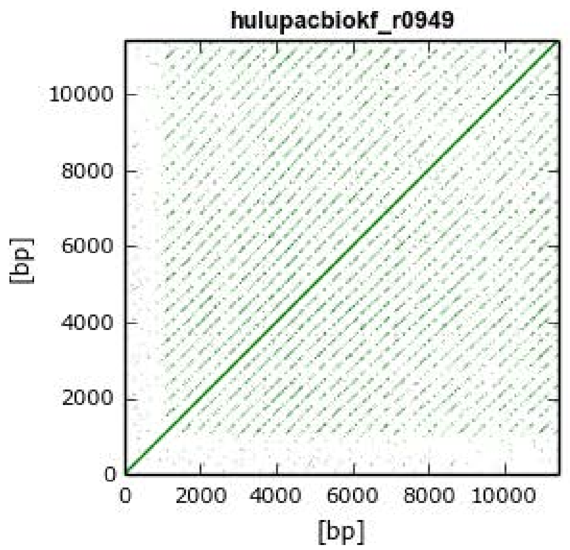
hulupacbiokf_r0949
10000
8000
6000
4000
2000
0
[bp]
0 2000 4000 6000 8000 10000
[bp]

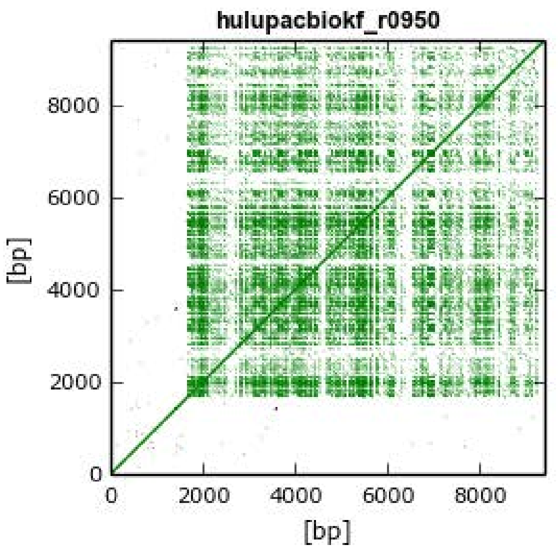
hulupacbiokf_r0950
8000
6000
4000
2000
0
[bp]
0 2000 4000 6000 8000
[bp]

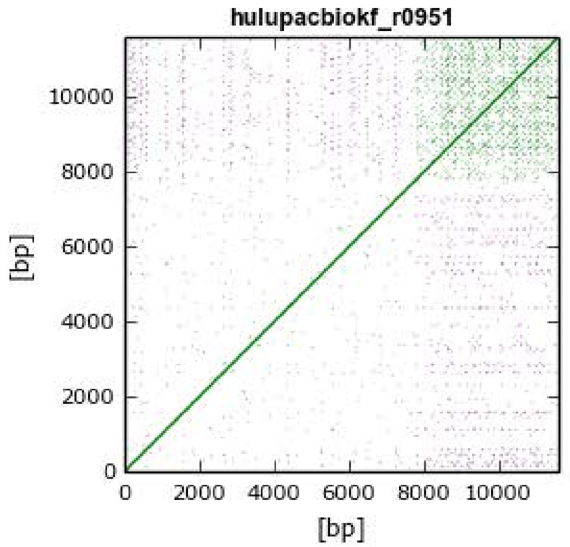
hulupacbiokf_r0951
10000
8000
6000
4000
2000
0
[bp]
0 2000 4000 6000 8000 10000
[bp]

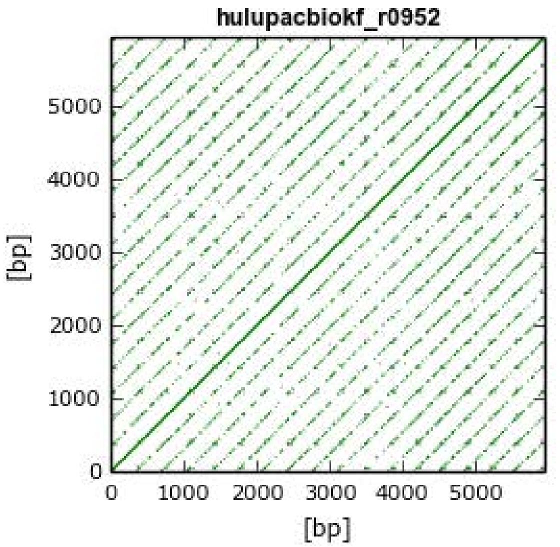
hulupacbiokf_r0952
5000
4000
3000
2000
1000
0
[bp]
0 1000 2000 3000 4000 5000
[bp]

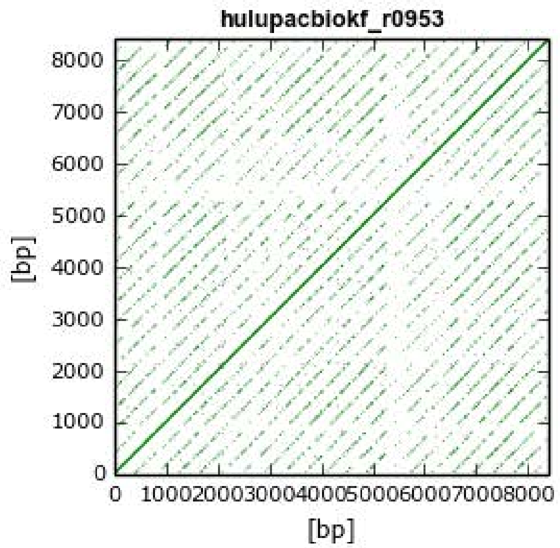
hulupacbiokf_r0953
8000
7000
6000
5000
4000
3000
2000
1000
0
[bp]
0 10002000300040005000600070008000
[bp]

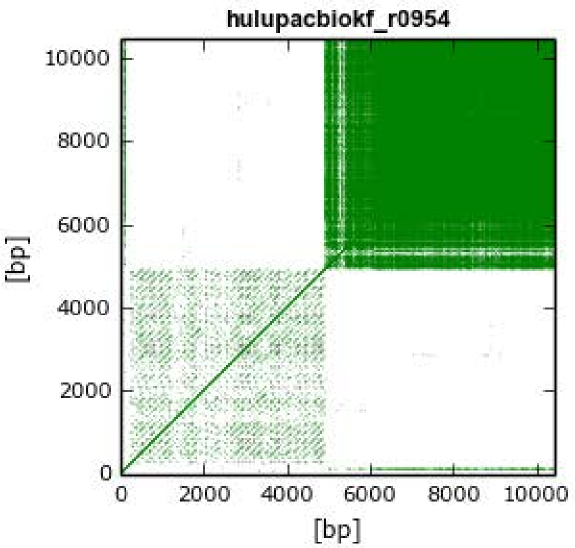
hulupacbiokf_r0954
10000
8000
6000
4000
2000
0
[bp]
0 2000 4000 6000 8000 10000
[bp]

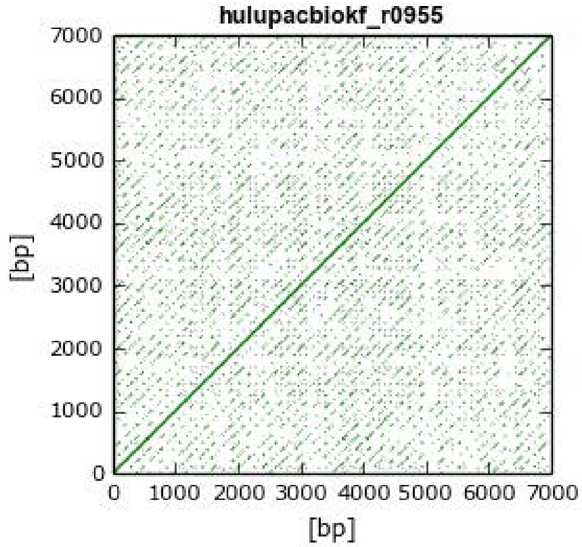
hulupacbiokf_r0955
[bp]
[bp]

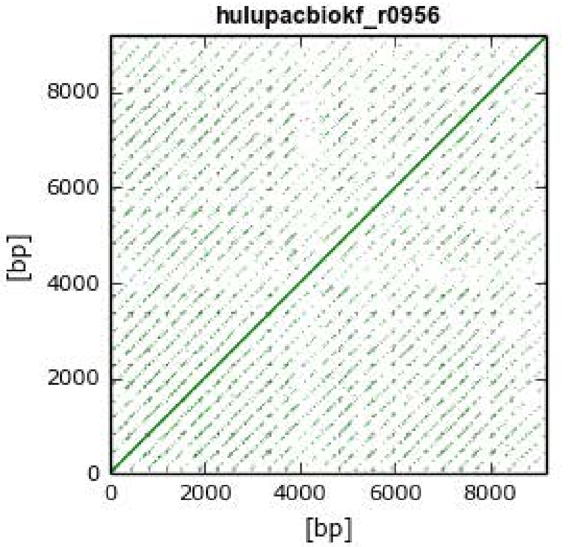
hulupacbiokf_r0956
[bp]
[bp]

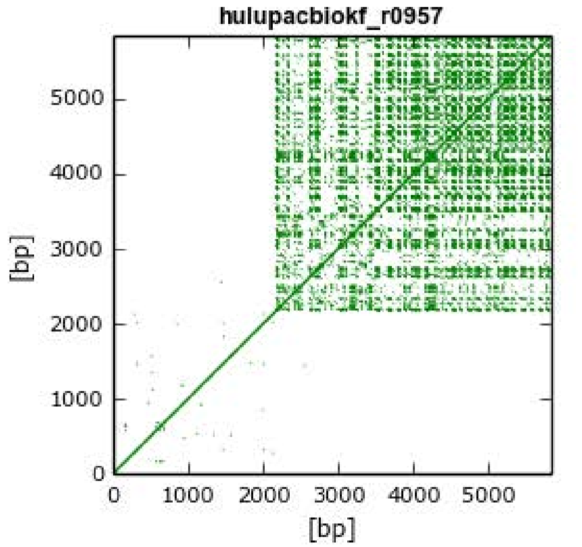
hulupacbiokf_r0957
[bp]
[bp]

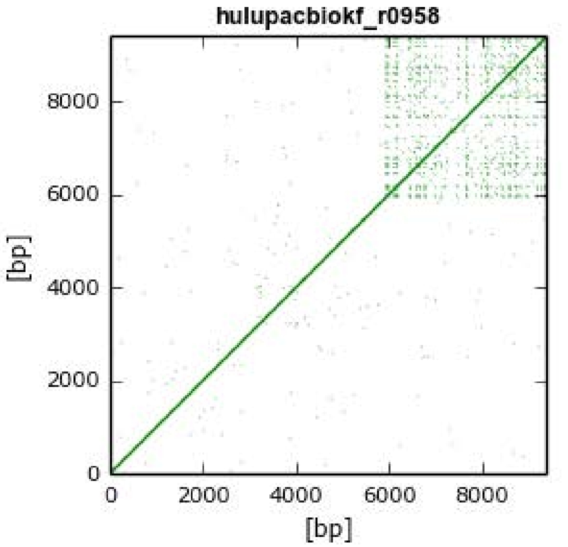
hulupacbiokf_r0958
[bp]
[bp]

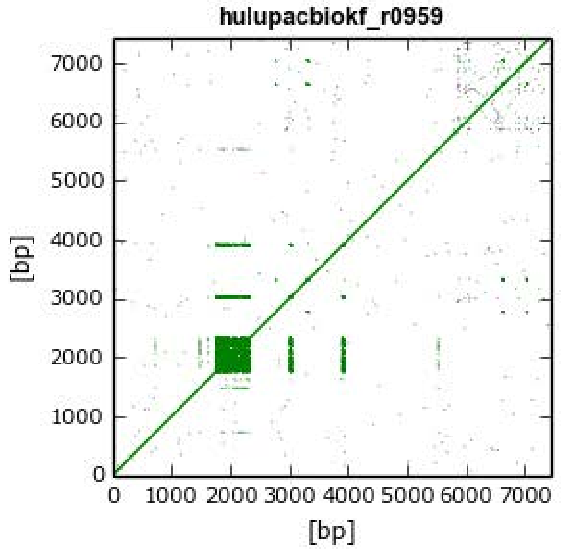
hulupacbiokf_r0959
[bp]
[bp]

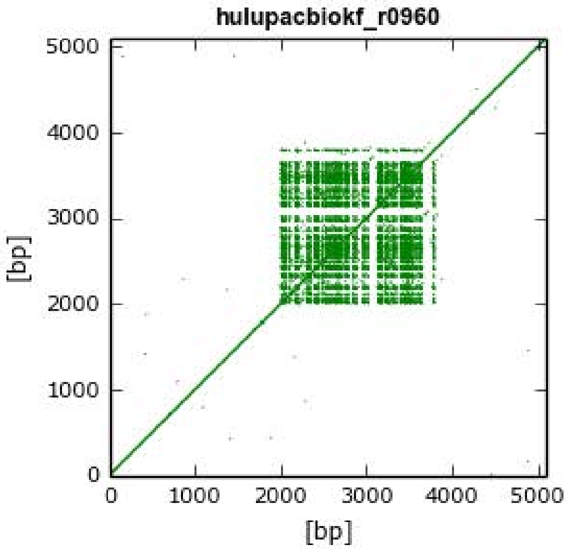
hulupacbiokf_r0960
[bp]
[bp]

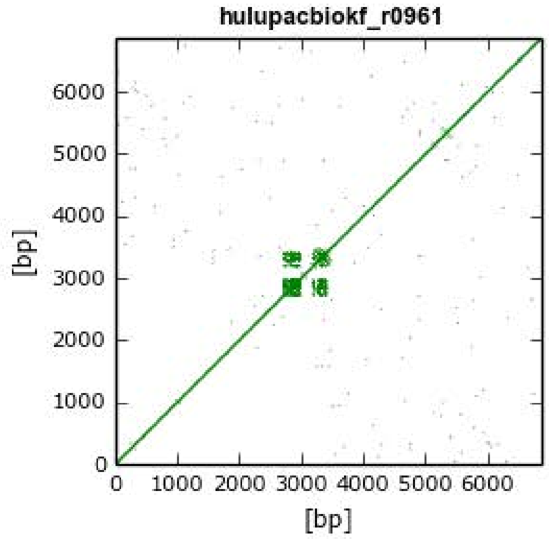
hulupacbiokf_r0961
[bp]
[bp]

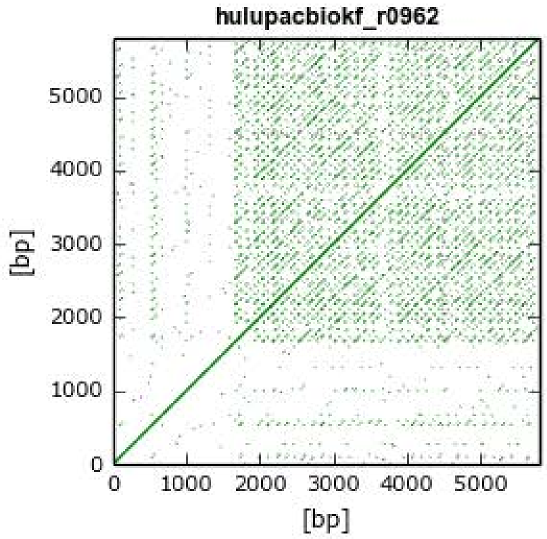
hulupacbiokf_r0962
[bp]
[bp]

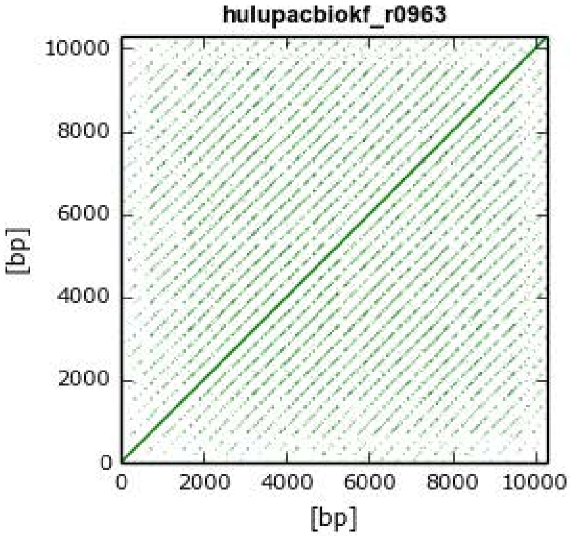
hulupacbiokf_r0963
[bp]
[bp]

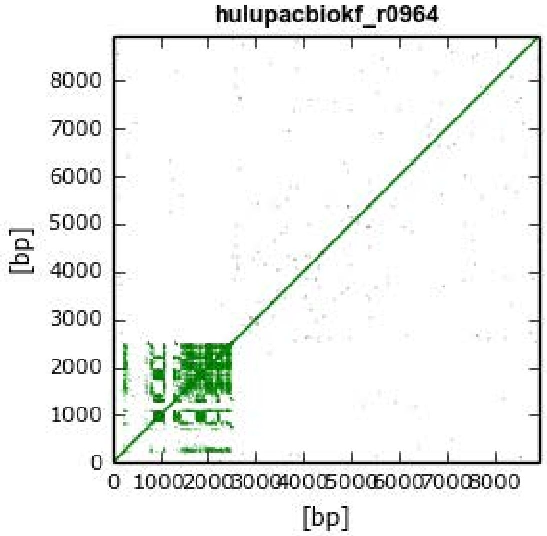
hulupacbiokf_r0964
[bp]
[bp]

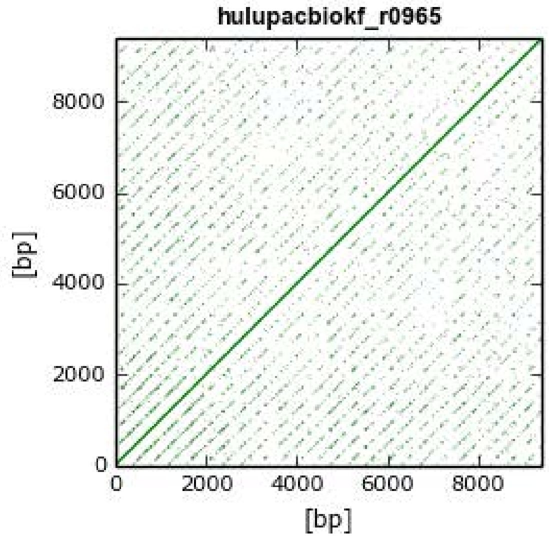
hulupacbiokf_r0965
[bp]
[bp]

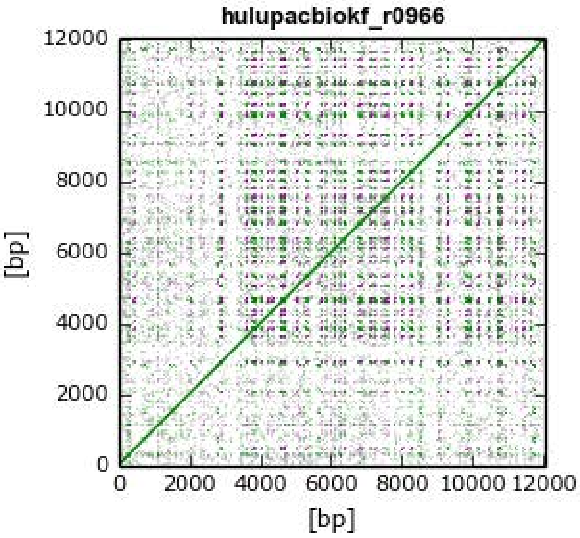
hulupacbiokf_r0966
[bp]
[bp]

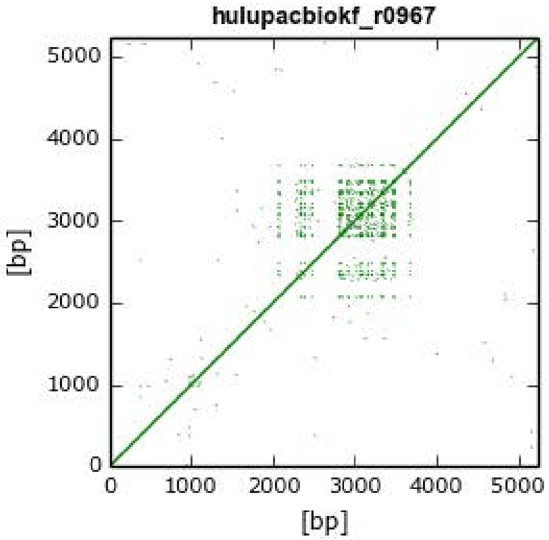
hulupacbiokf_r0967
5000
4000
3000
2000
1000
0
[bp]
0 1000 2000 3000 4000 5000
[bp]

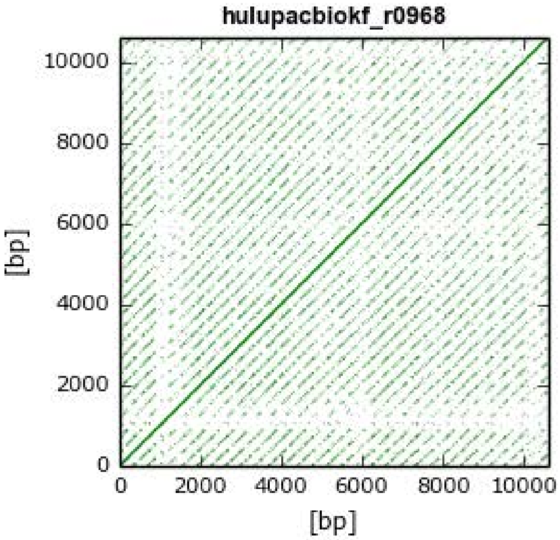
hulupacbiokf_r0968
10000
8000
6000
4000
2000
0
[bp]
0 2000 4000 6000 8000 10000
[bp]

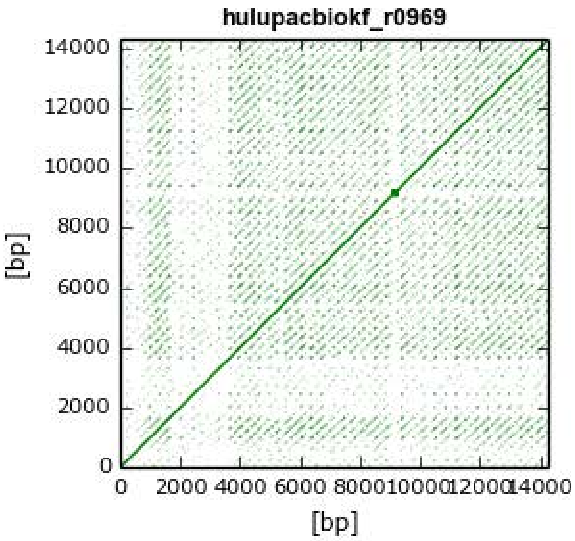
hulupacbiokf_r0969
14000
12000
10000
8000
6000
4000
2000
0
[bp]
0 2000 4000 6000 8000 10000 12000 14000
[bp]

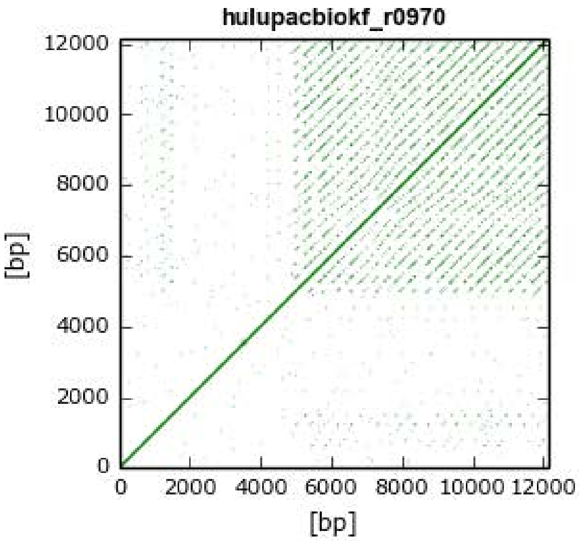
hulupacbiokf_r0970
12000
10000
8000
6000
4000
2000
0
[bp]
0 2000 4000 6000 8000 10000 12000
[bp]

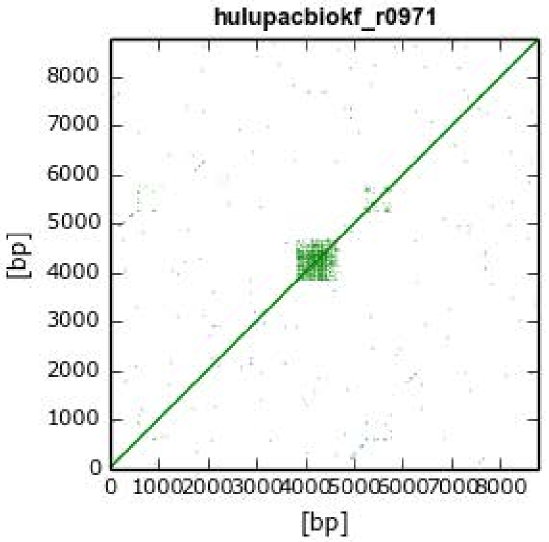
hulupacbiokf_r0971
8000
7000
6000
5000
4000
3000
2000
1000
0
[bp]
0 1000 2000 3000 4000 5000 6000 7000 8000
[bp]

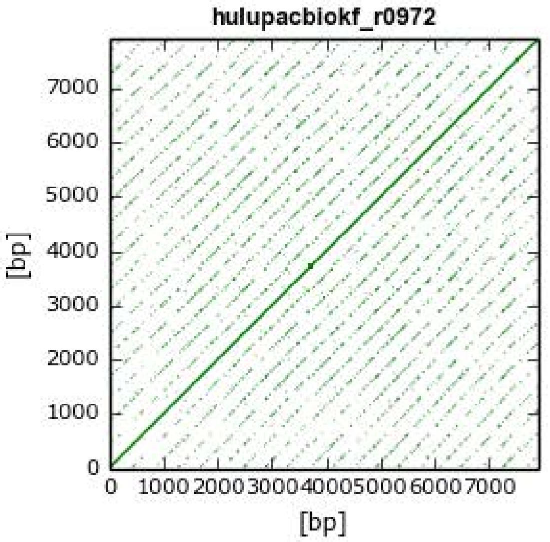
hulupacbiokf_r0972
7000
6000
5000
4000
3000
2000
1000
0
[bp]
0 1000 2000 3000 4000 5000 6000 7000
[bp]

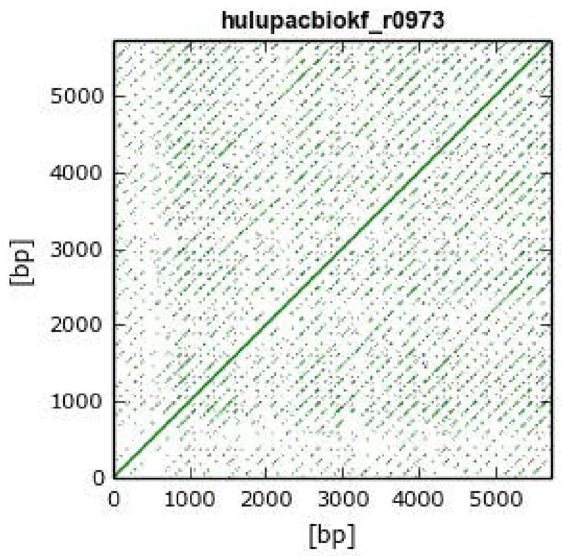
hulupacbiokf_r0973
[bp]
[bp]

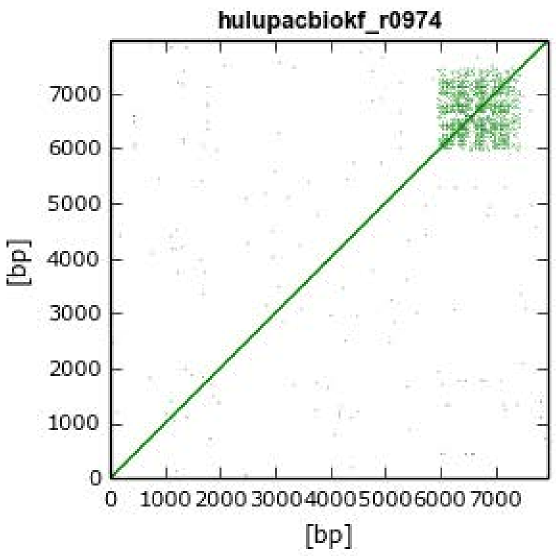
hulupacbiokf_r0974
[bp]
[bp]

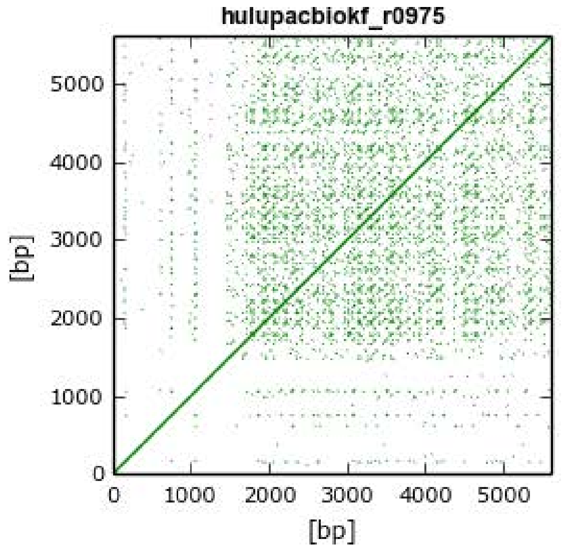
hulupacbiokf_r0975
[bp]
[bp]

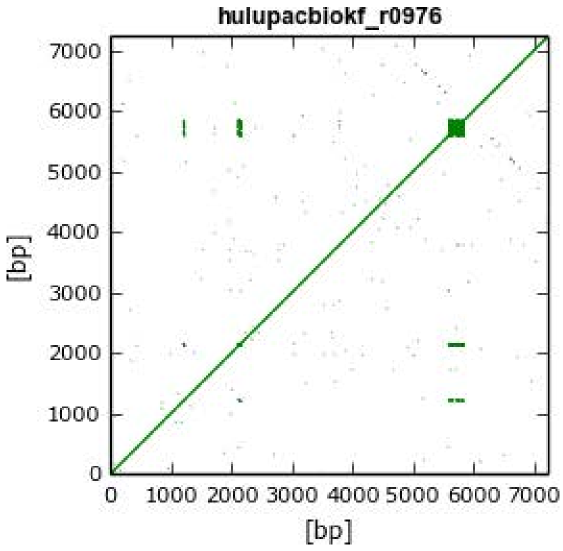
hulupacbiokf_r0976
[bp]
[bp]

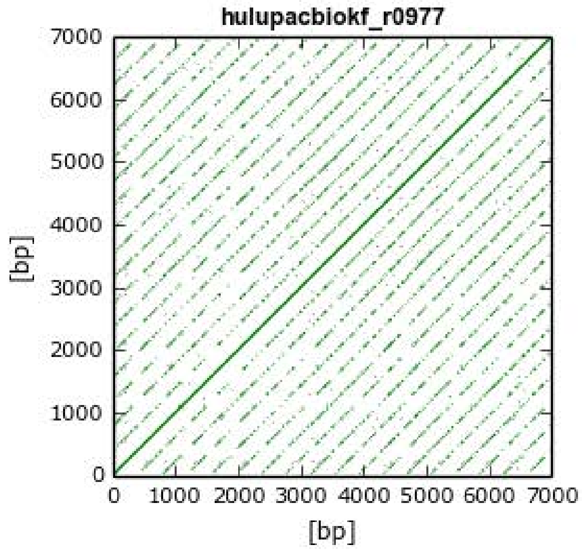
hulupacbiokf_r0977
[bp]
[bp]

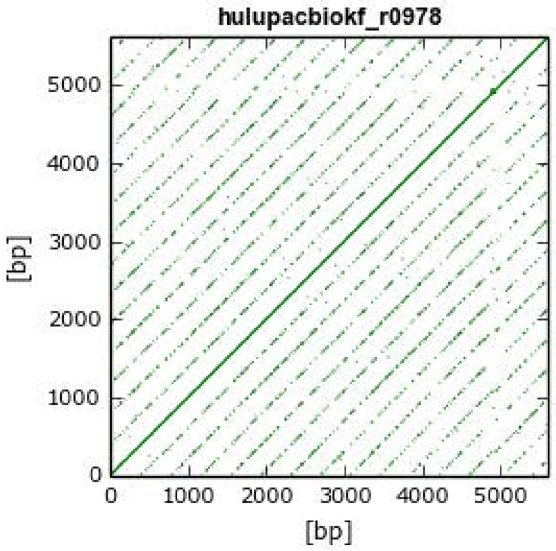
hulupacbiokf_r0978
[bp]
[bp]

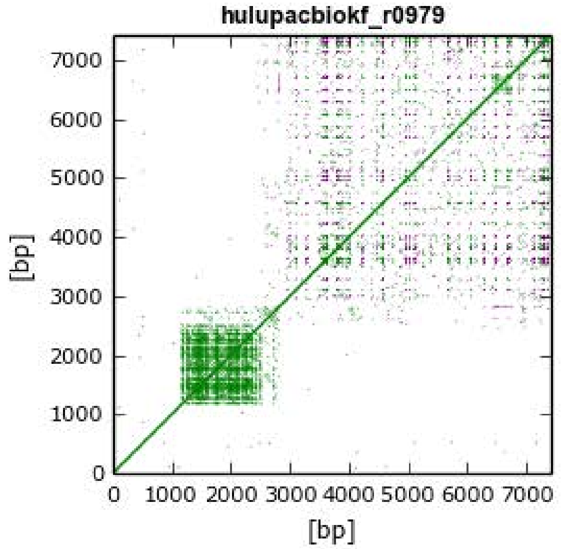

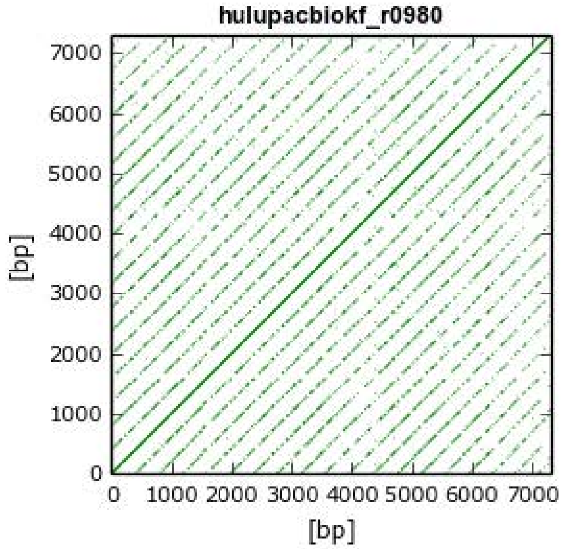

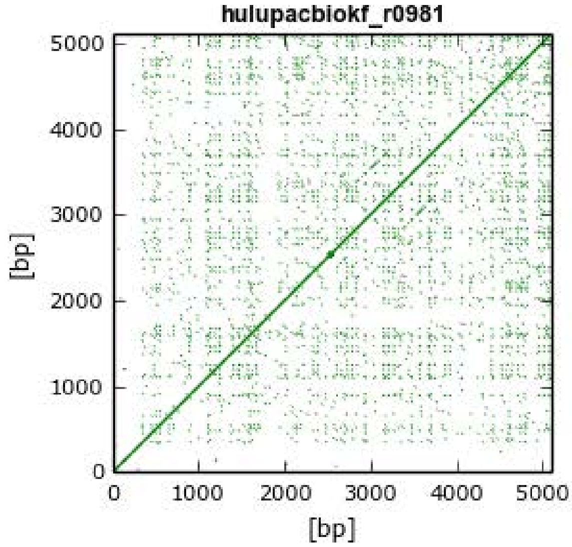

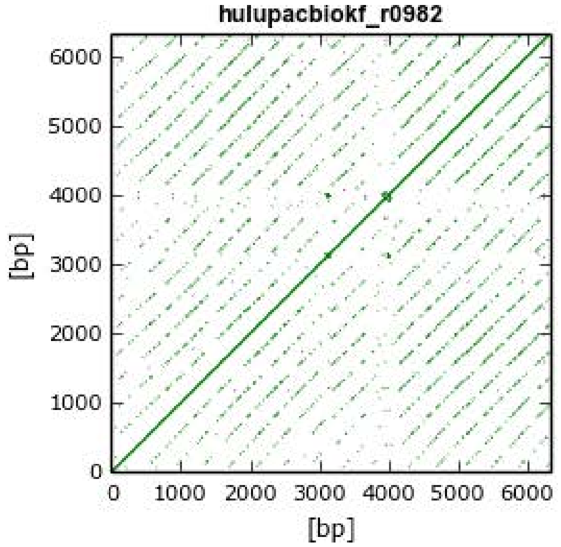

HuluTR100 from read r0983
is in GenBank Acc. MN537569

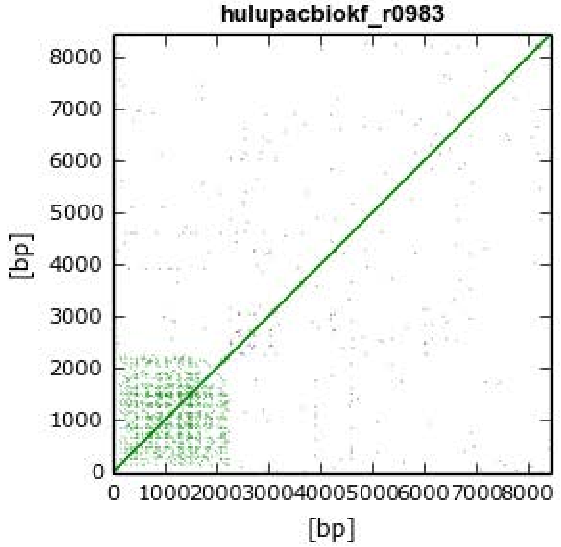

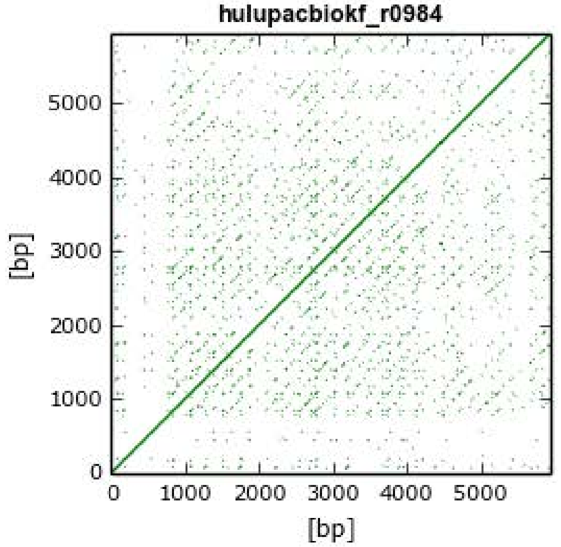

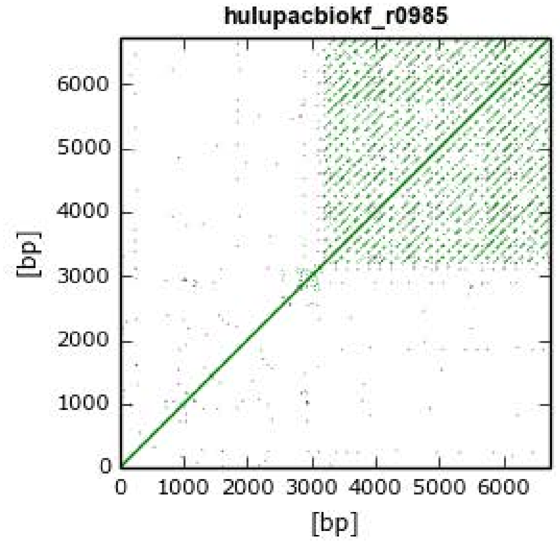
hulupacbiokf_r0985
[bp]
[bp]
0 1000 2000 3000 4000 5000 6000

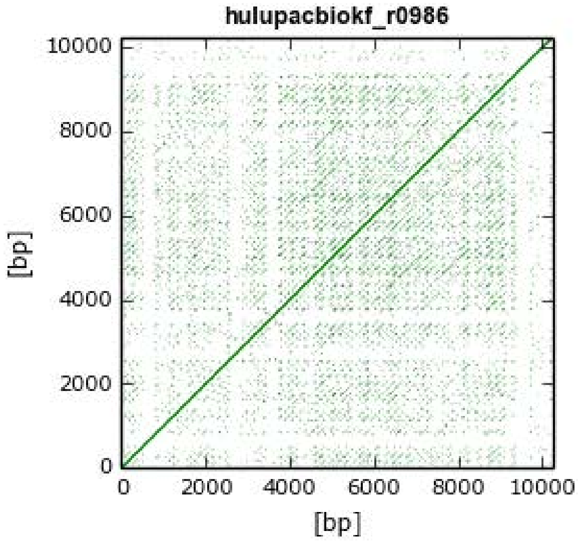
hulupacbiokf_r0986
[bp]
[bp]
0 2000 4000 6000 8000 10000

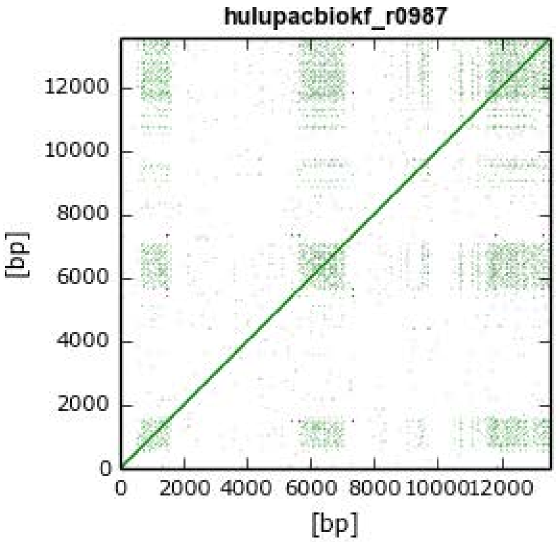
hulupacbiokf_r0987
[bp]
[bp]
0 2000 4000 6000 8000 10000 12000

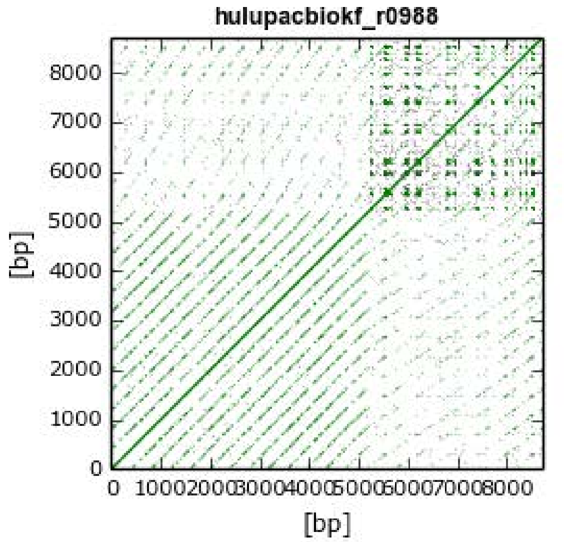
hulupacbiokf_r0988
[bp]
[bp]
0 1000 2000 3000 4000 5000 6000 7000 8000

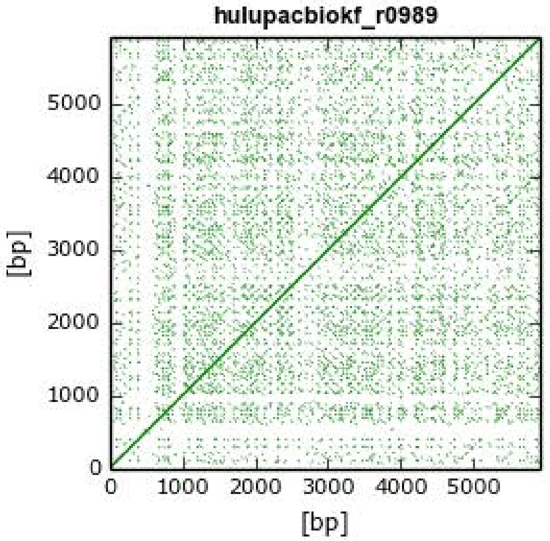
hulupacbiokf_r0989
[bp]
[bp]
0 1000 2000 3000 4000 5000

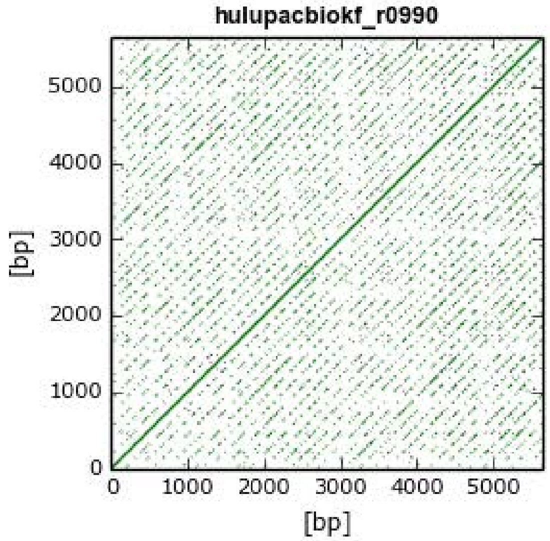
hulupacbiokf_r0990
[bp]
[bp]
0 1000 2000 3000 4000 5000

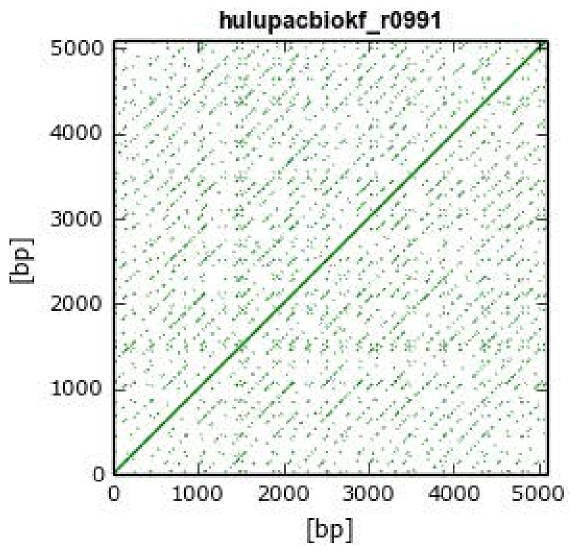
hulupacbiokf_r0991
[bp]
[bp]

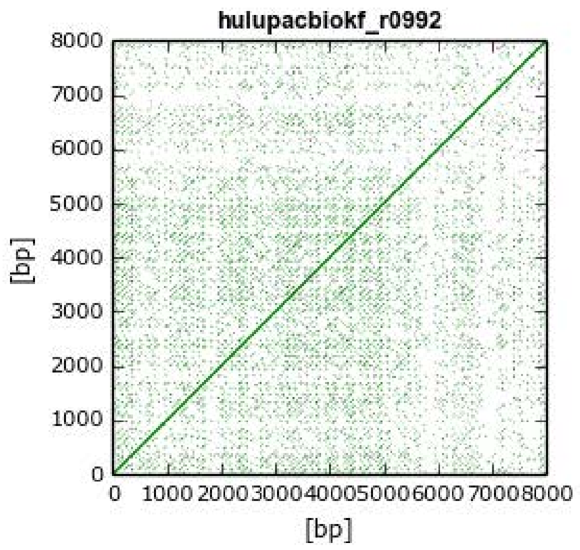
hulupacbiokf_r0992
[bp]
[bp]

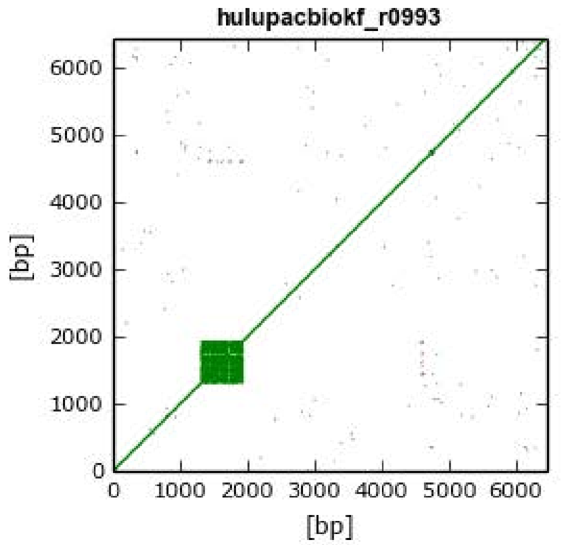
hulupacbiokf_r0993
[bp]
[bp]

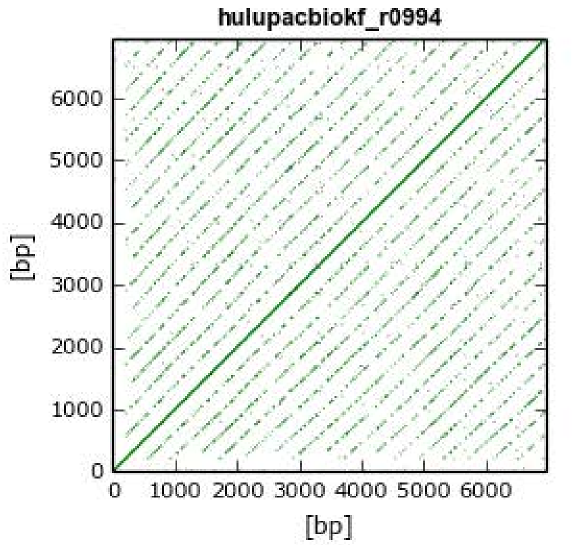
hulupacbiokf_r0994
[bp]
[bp]

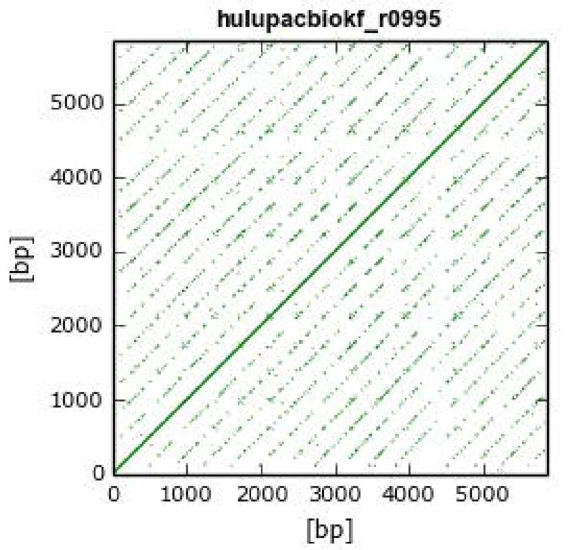
hulupacbiokf_r0995
[bp]
[bp]

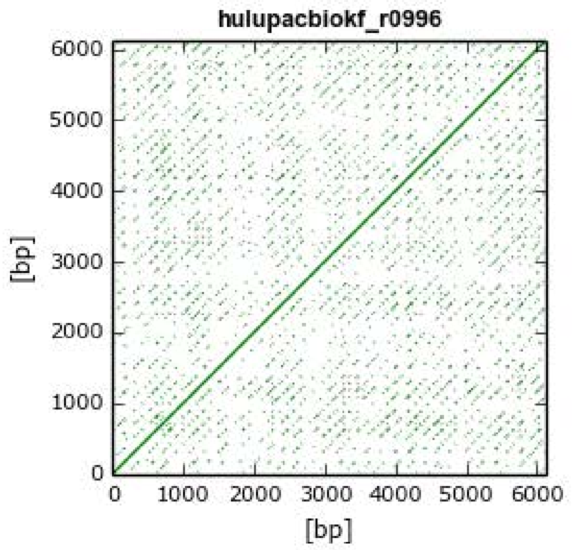
hulupacbiokf_r0996
[bp]
[bp]

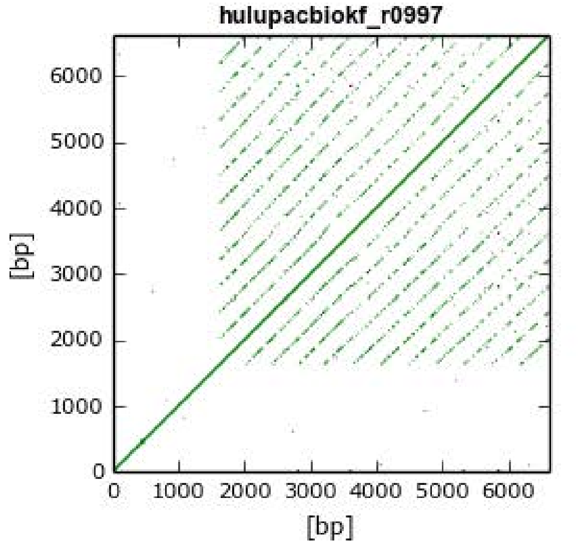

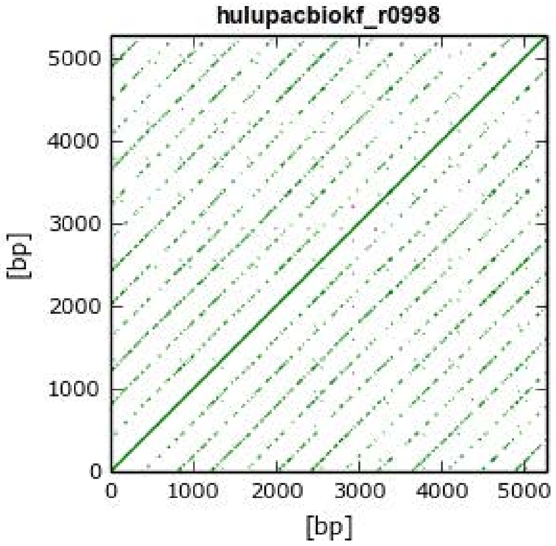

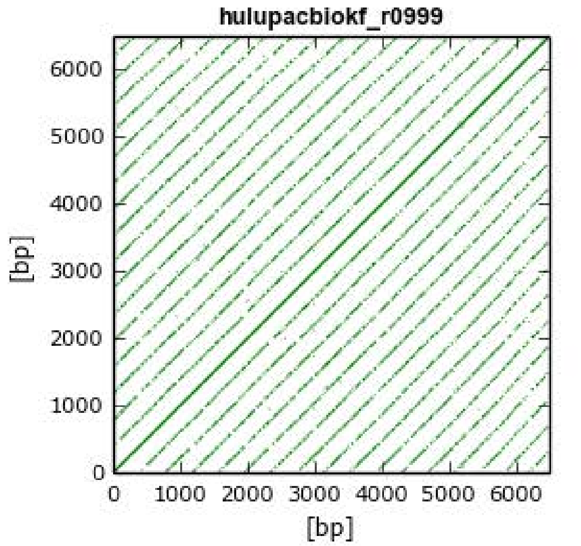

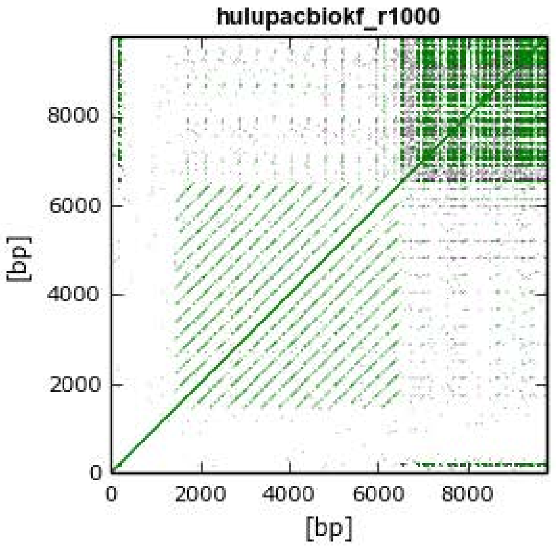

HuluTR240 from read r1001
is in GenBank Acc. MN537575

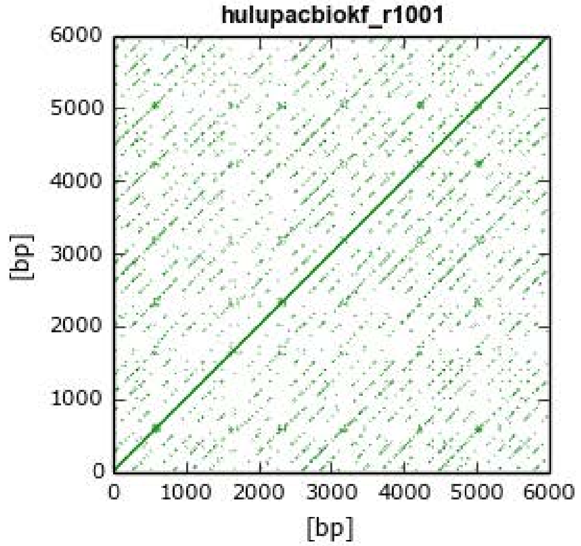

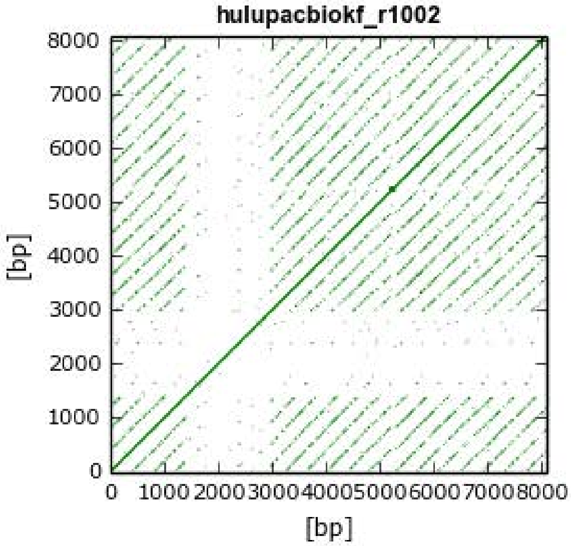

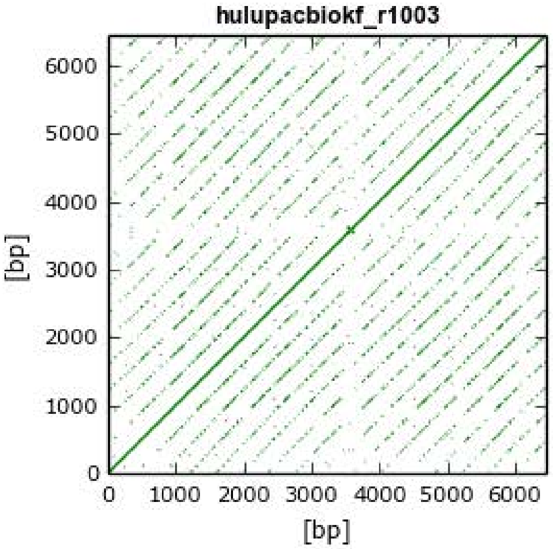
hulupacbiokf_r1003
0 1000 2000 3000 4000 5000 6000
[bp]
[bp]

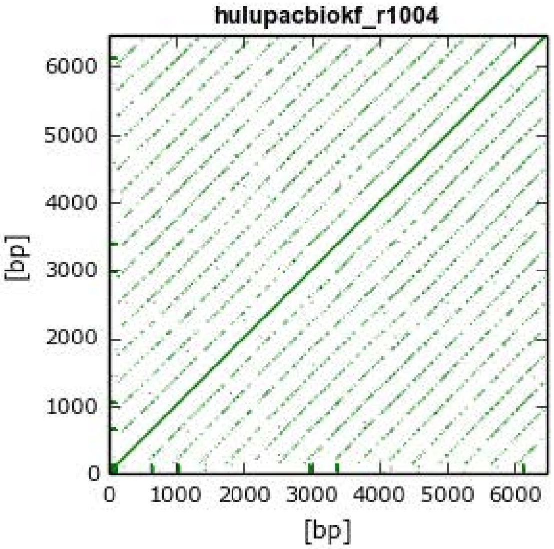
hulupacbiokf_r1004
0 1000 2000 3000 4000 5000 6000
[bp]
[bp]

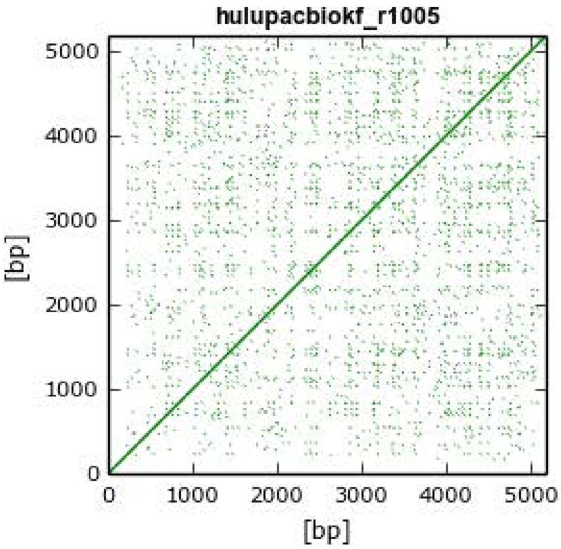
hulupacbiokf_r1005
0 1000 2000 3000 4000 5000
[bp]
[bp]

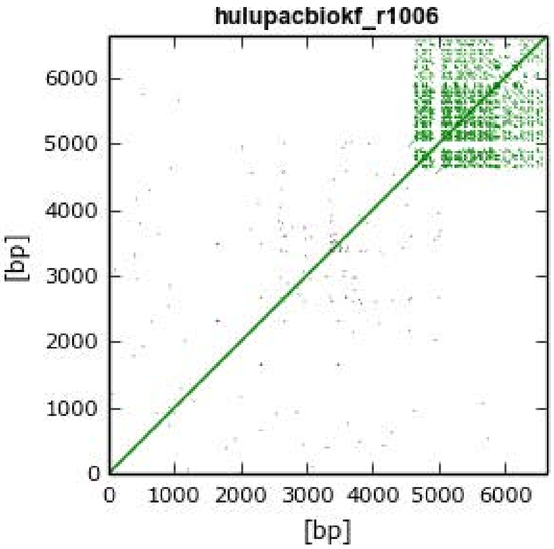
hulupacbiokf_r1006
0 1000 2000 3000 4000 5000 6000
[bp]
[bp]

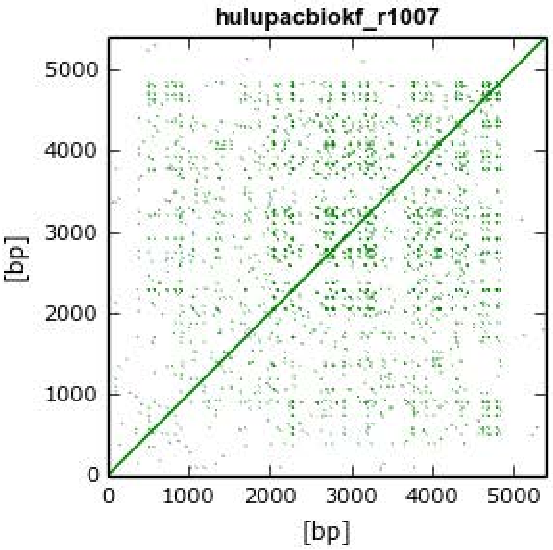
hulupacbiokf_r1007
0 1000 2000 3000 4000 5000
[bp]
[bp]

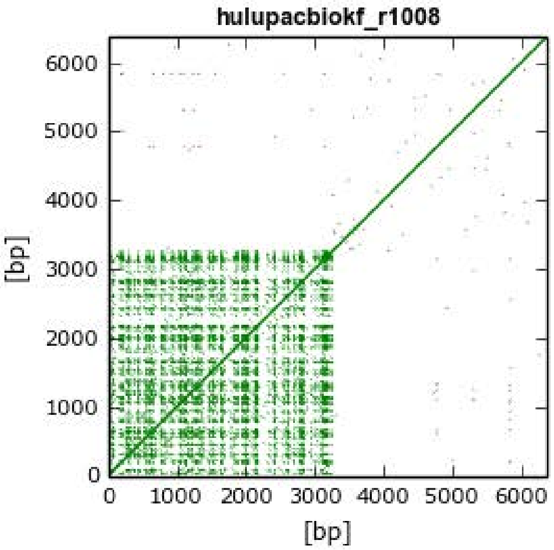
hulupacbiokf_r1008
0 1000 2000 3000 4000 5000 6000
[bp]
[bp]

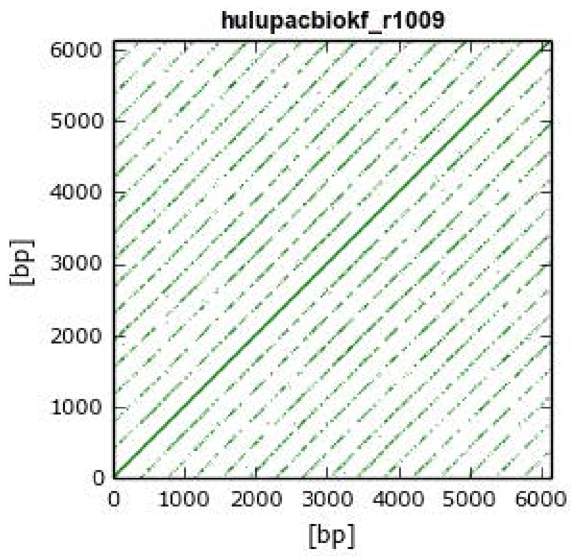
hulupacbiokf_r1009
[bp]
0
1000
2000
3000
4000
5000
6000
[bp]

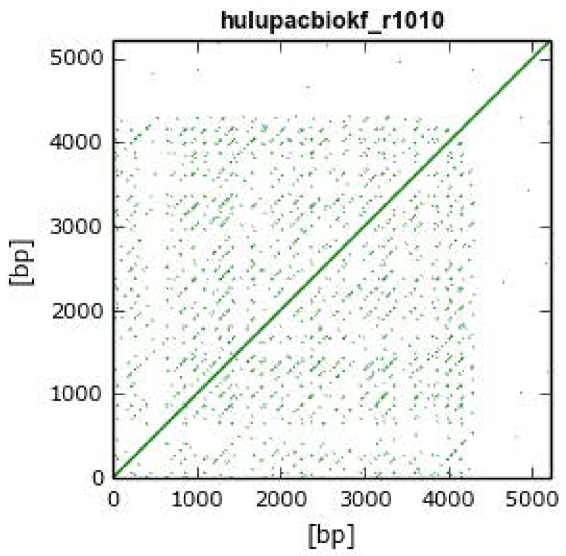
hulupacbiokf_r1010
[bp]
0
1000
2000
3000
4000
5000
[bp]

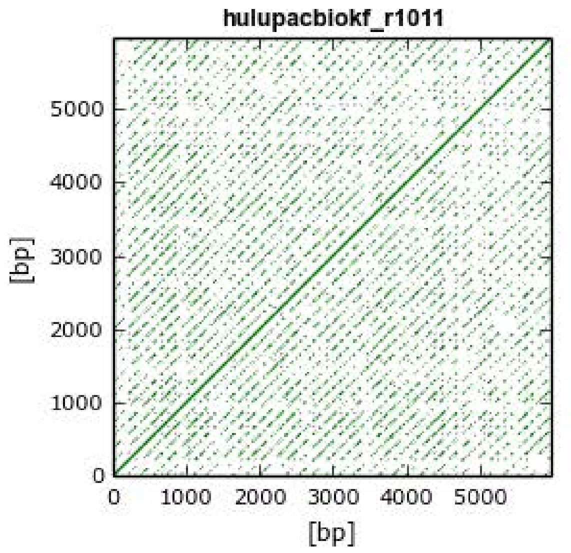
hulupacbiokf_r1011
[bp]
0
1000
2000
3000
4000
5000
[bp]

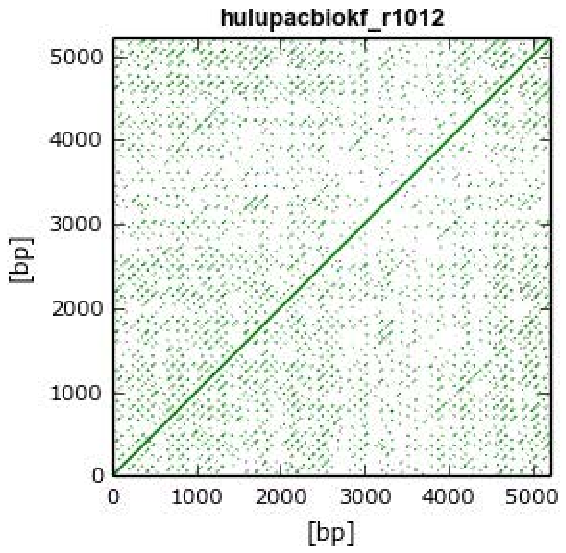
hulupacbiokf_r1012
[bp]
0
1000
2000
3000
4000
5000
[bp]

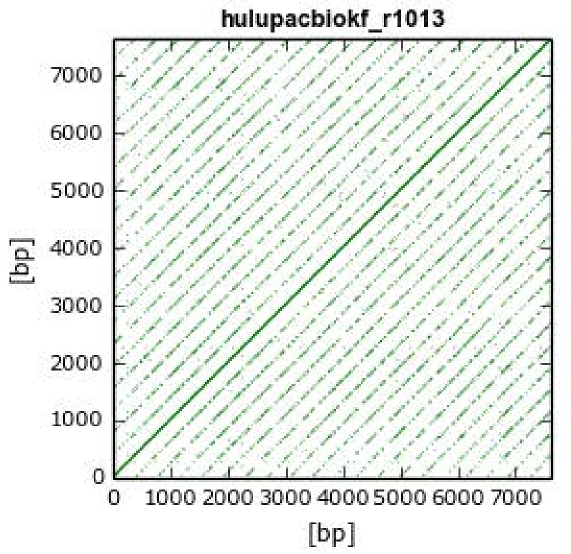
hulupacbiokf_r1013
[bp]
0
1000
2000
3000
4000
5000
6000
7000
[bp]

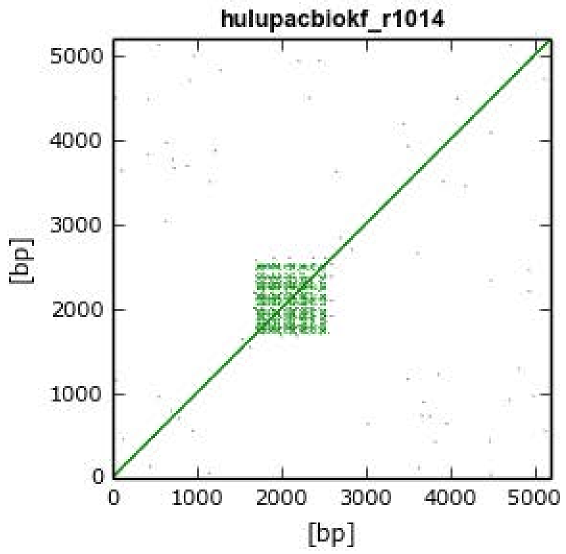
hulupacbiokf_r1014
[bp]
0
1000
2000
3000
4000
5000
[bp]

hulupacbiokf_r1015

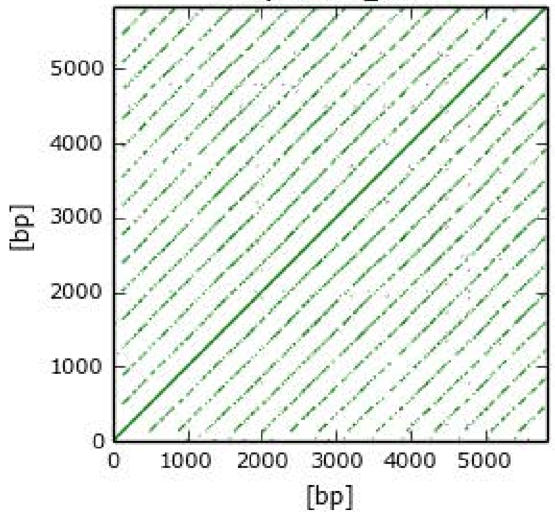

hulupacbiokf_r1016

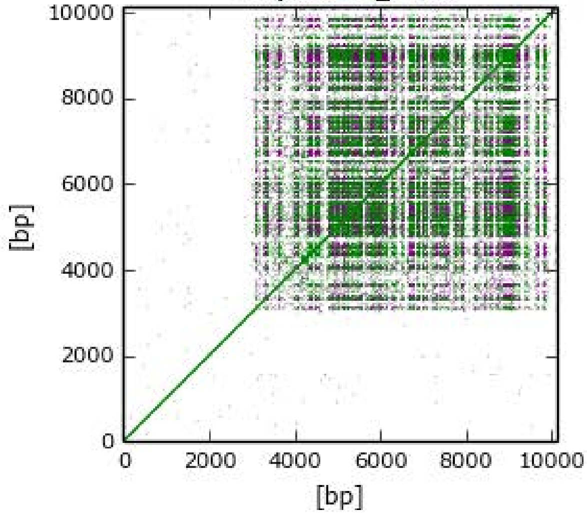

hulupacbiokf_r1017

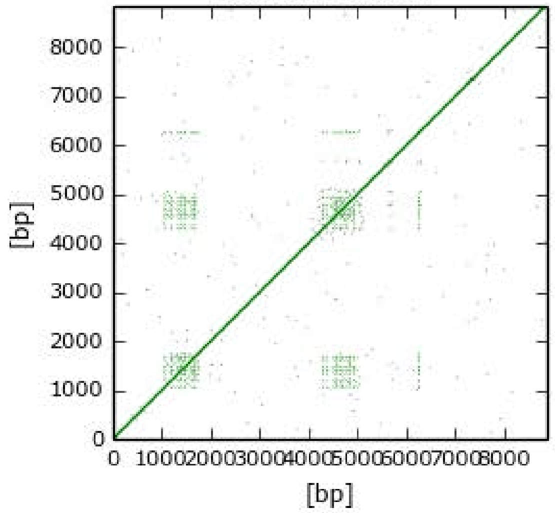

hulupacbiokf_r1018

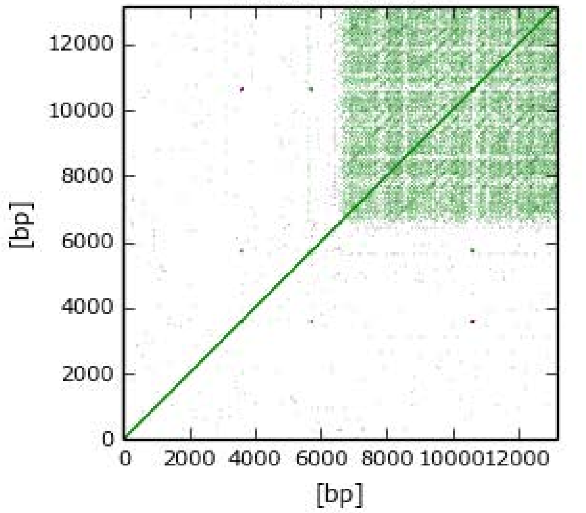

hulupacbiokf_r1019

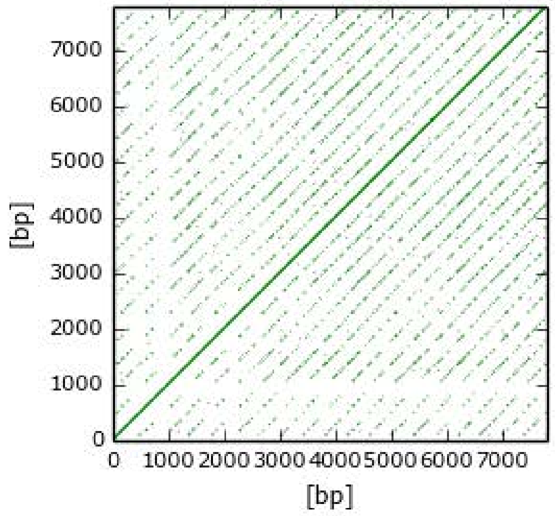

hulupacbiokf_r1020

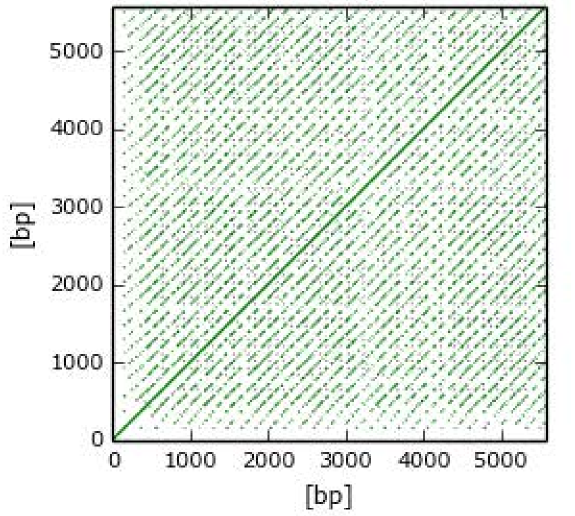

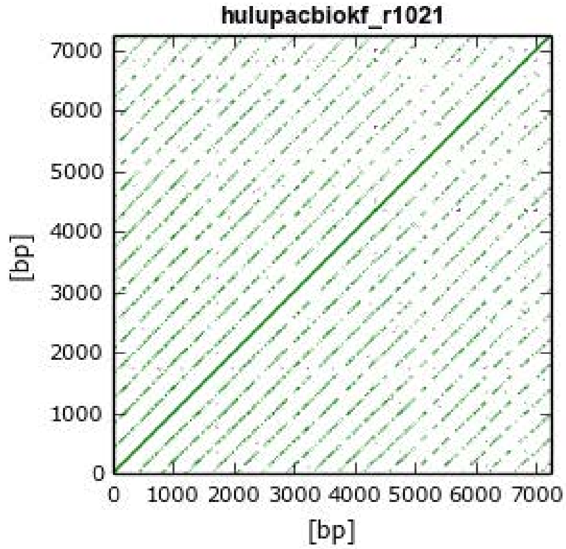
hulupacbiokf_r1021
[bp]
[bp]

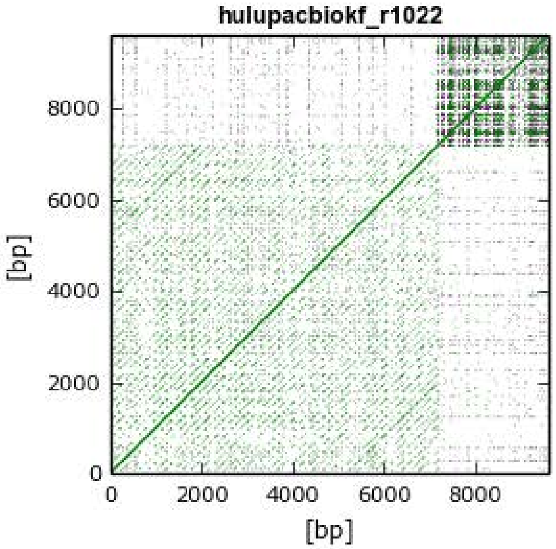
hulupacbiokf_r1022
[bp]
[bp]

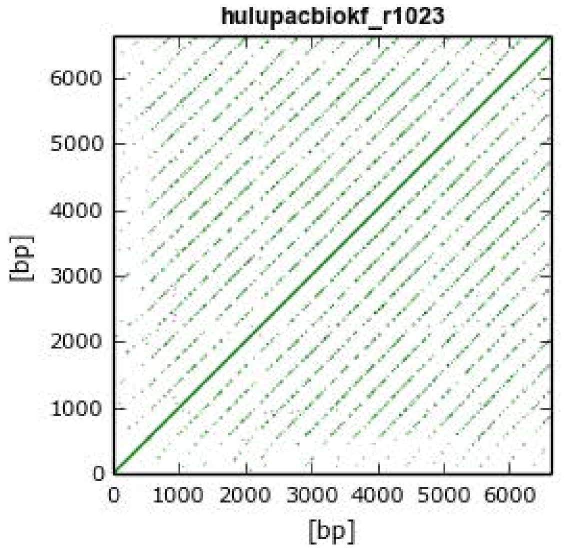
hulupacbiokf_r1023
[bp]
[bp]

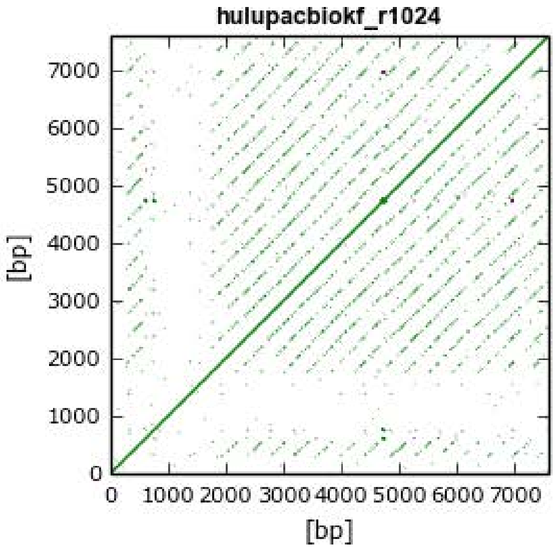
hulupacbiokf_r1024
[bp]
[bp]

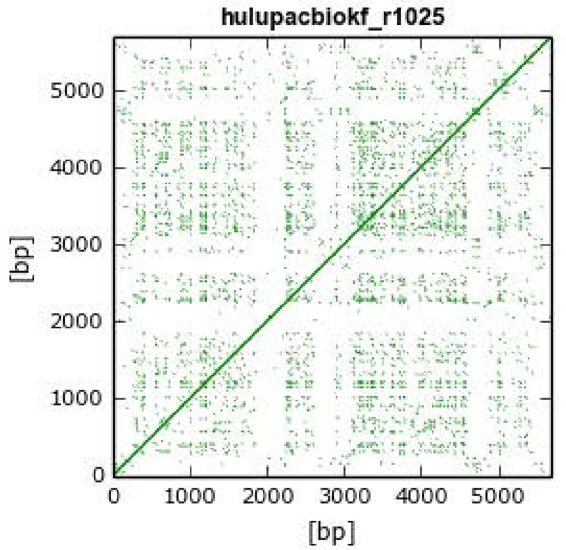
hulupacbiokf_r1025
[bp]
[bp]

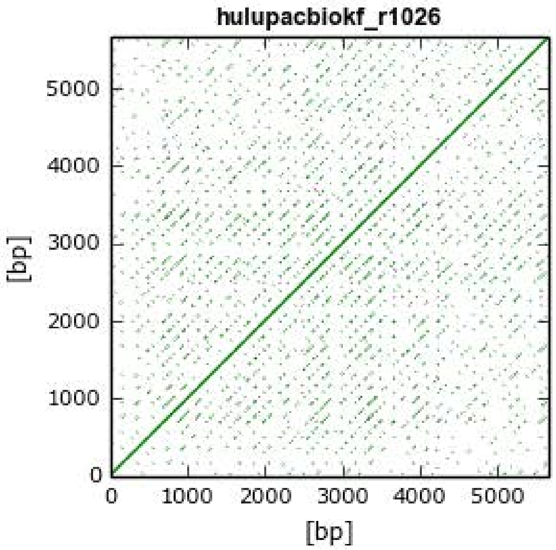
hulupacbiokf_r1026
[bp]
[bp]

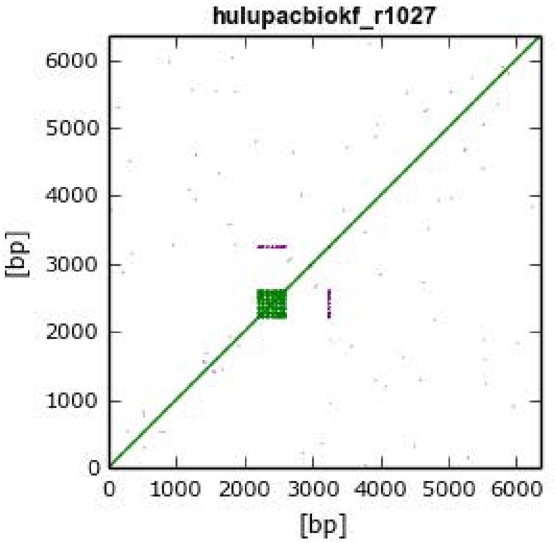
hulupacbiokf_r1027
[bp]
0
1000
2000
3000
4000
5000
6000
0 1000 2000 3000 4000 5000 6000
[bp]

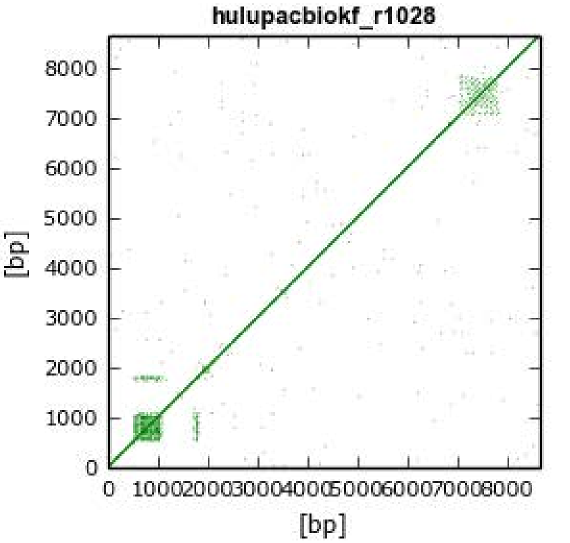
hulupacbiokf_r1028
[bp]
0
1000
2000
3000
4000
5000
6000
7000
8000
0 1000 2000 3000 4000 5000 6000 7000 8000
[bp]

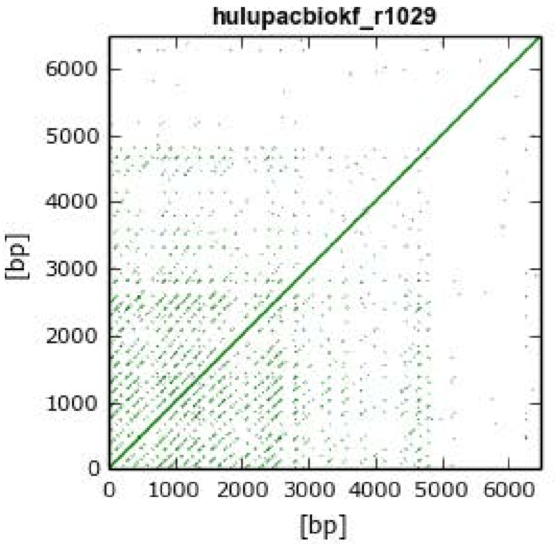
hulupacbiokf_r1029
[bp]
0
1000
2000
3000
4000
5000
6000
0 1000 2000 3000 4000 5000 6000
[bp]

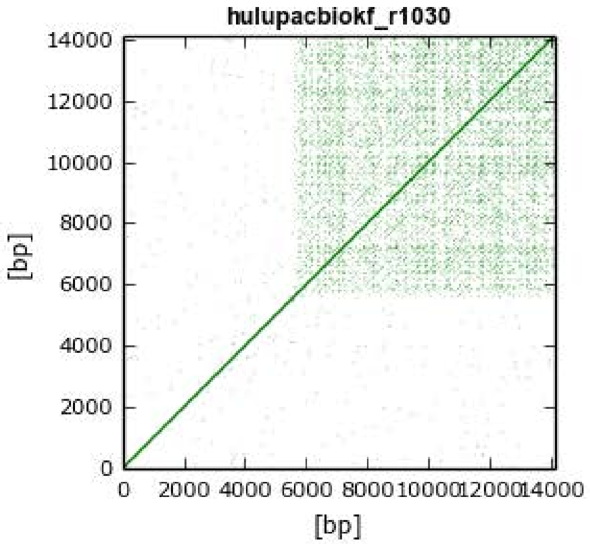
hulupacbiokf_r1030
[bp]
0
2000
4000
6000
8000
10000
12000
14000
0 2000 4000 6000 8000 10000 12000 14000
[bp]

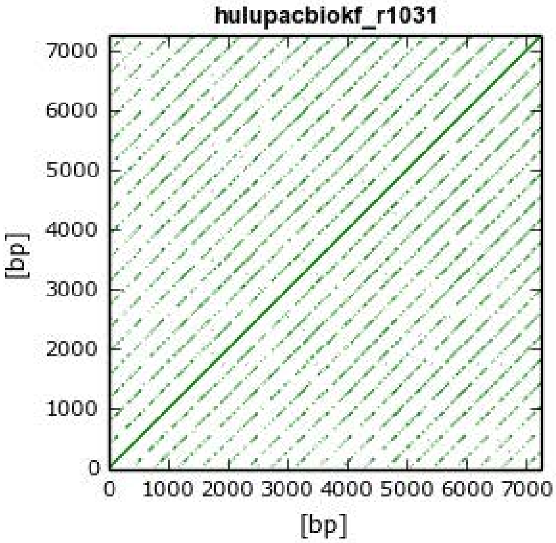
hulupacbiokf_r1031
[bp]
0
1000
2000
3000
4000
5000
6000
7000
0 1000 2000 3000 4000 5000 6000 7000
[bp]

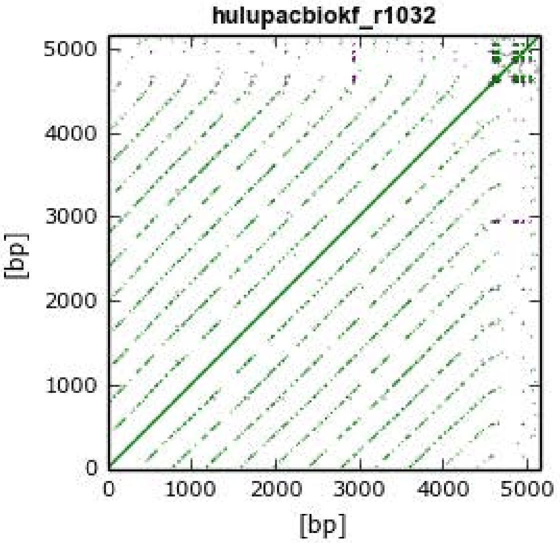
hulupacbiokf_r1032
[bp]
0
1000
2000
3000
4000
5000
0 1000 2000 3000 4000 5000
[bp]

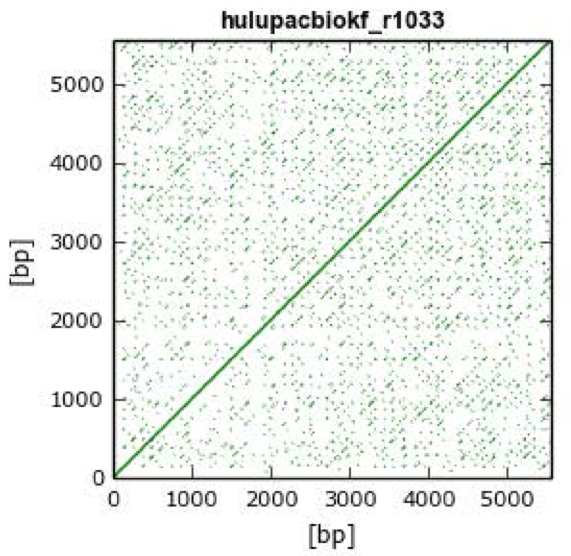
hulupacbiokf_r1033
[bp]
[bp]

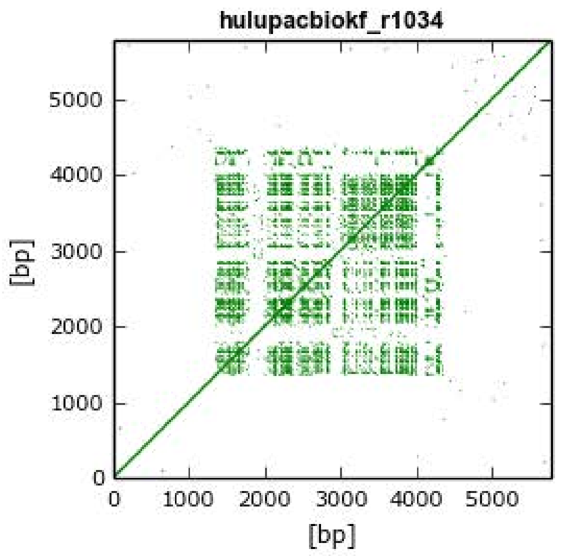
hulupacbiokf_r1034
[bp]
[bp]

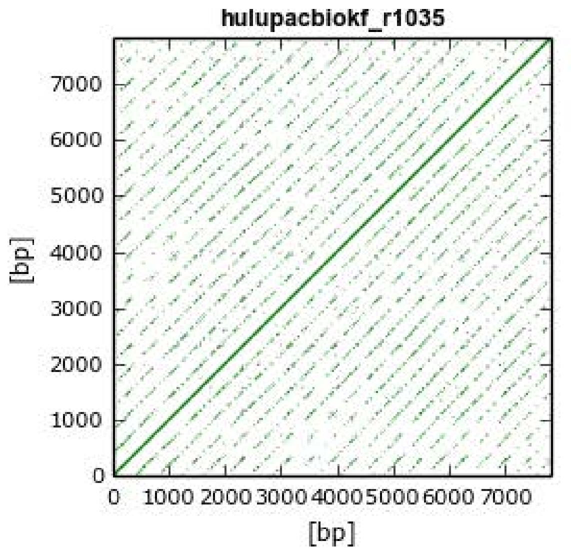
hulupacbiokf_r1035
[bp]
[bp]

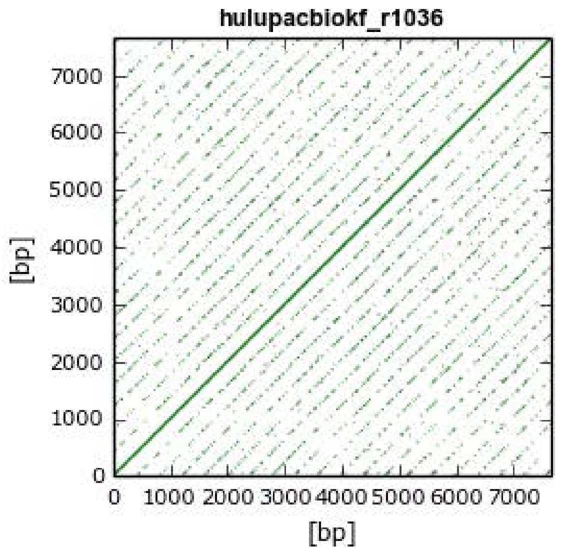
hulupacbiokf_r1036
[bp]
[bp]

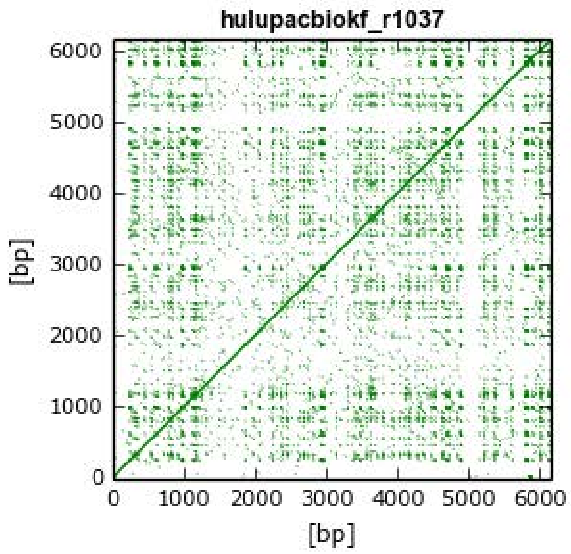
hulupacbiokf_r1037
[bp]
[bp]

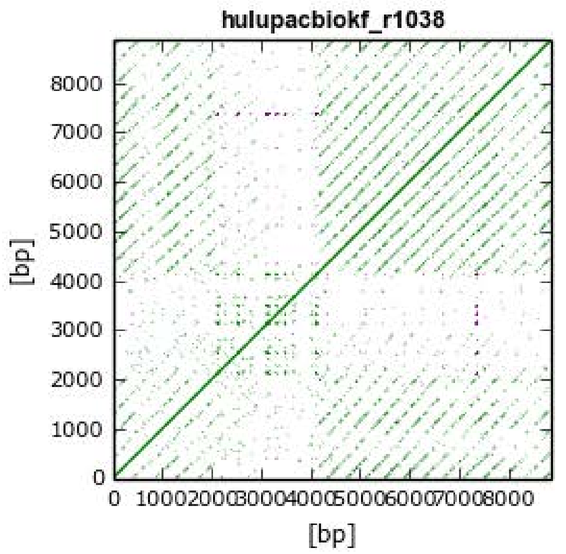
hulupacbiokf_r1038
[bp]
[bp]

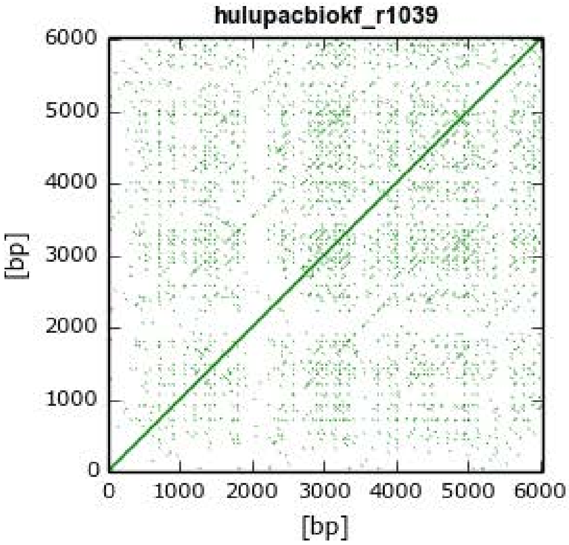
hulupacbiokf_r1039
[bp]
[bp]

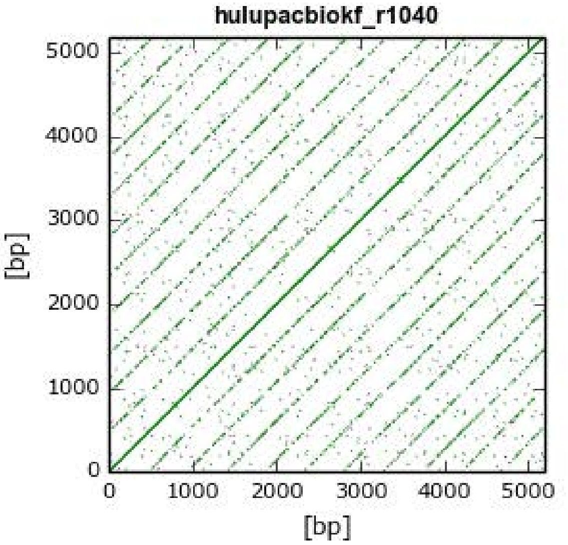
hulupacbiokf_r1040
[bp]
[bp]

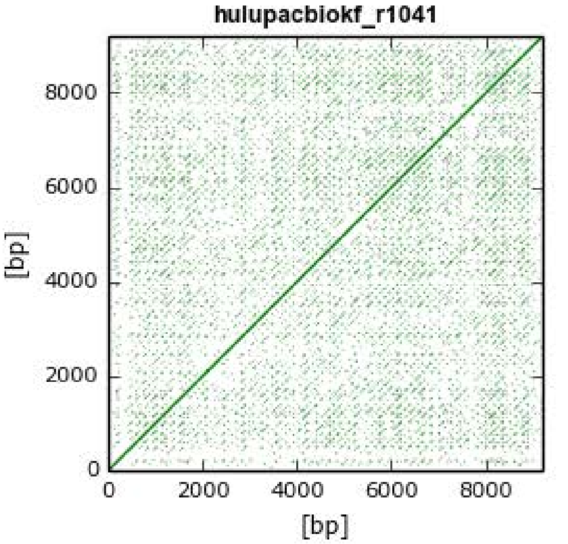
hulupacbiokf_r1041
[bp]
[bp]

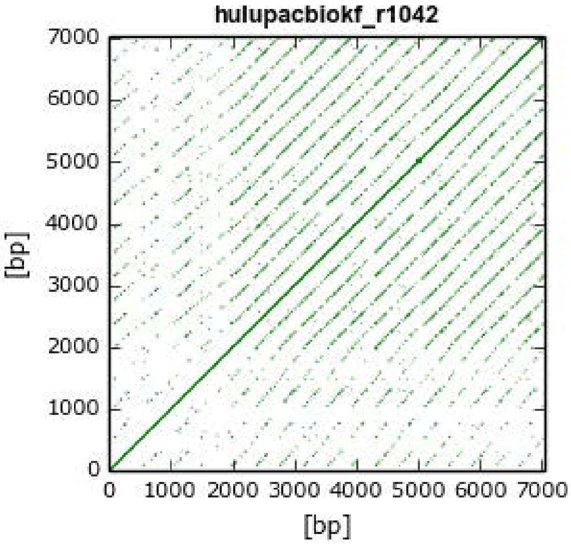
hulupacbiokf_r1042
[bp]
[bp]

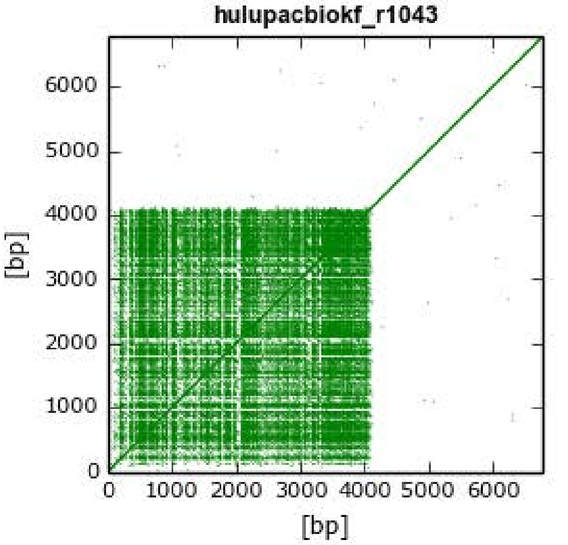
hulupacbiokf_r1043
[bp]
[bp]

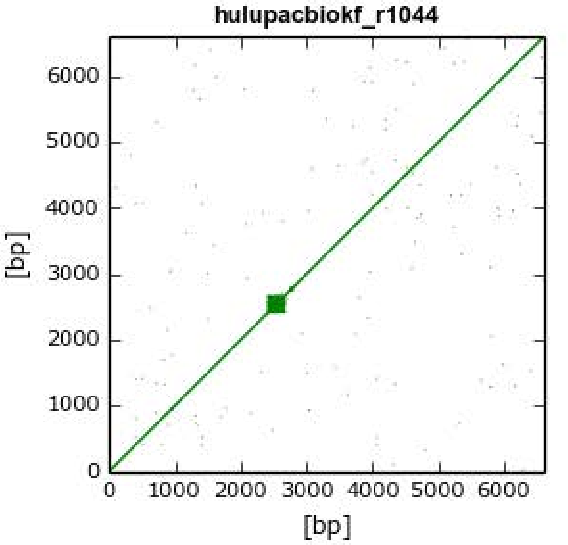
hulupacbiokf_r1044
[bp]
[bp]

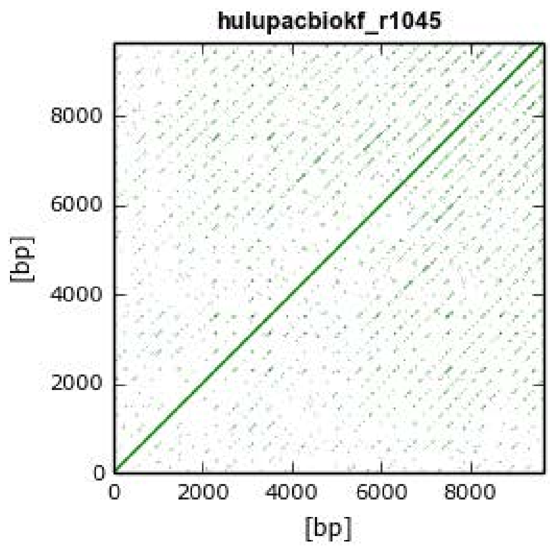
hulupacbiokf_r1045
[bp]
0
2000
4000
6000
8000

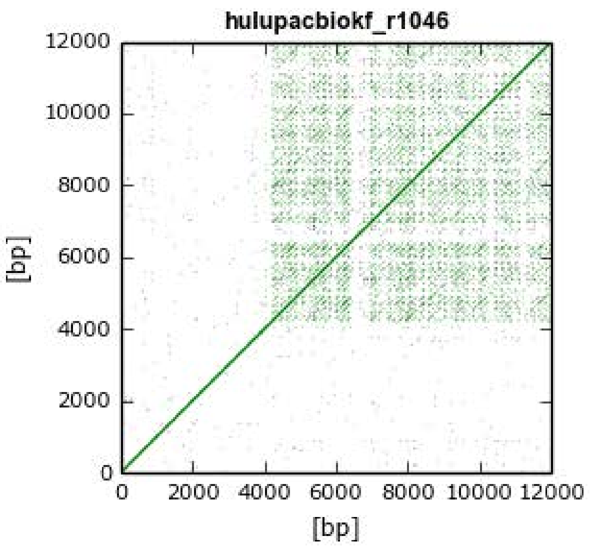
hulupacbiokf_r1046
[bp]
0
2000
4000
6000
8000
10000
12000

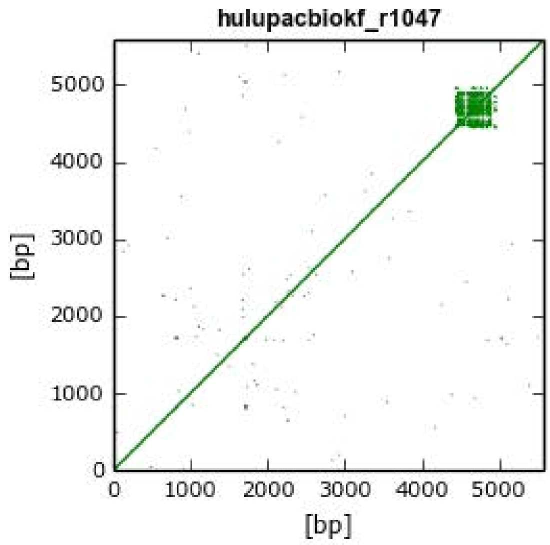
hulupacbiokf_r1047
[bp]
0
1000
2000
3000
4000
5000

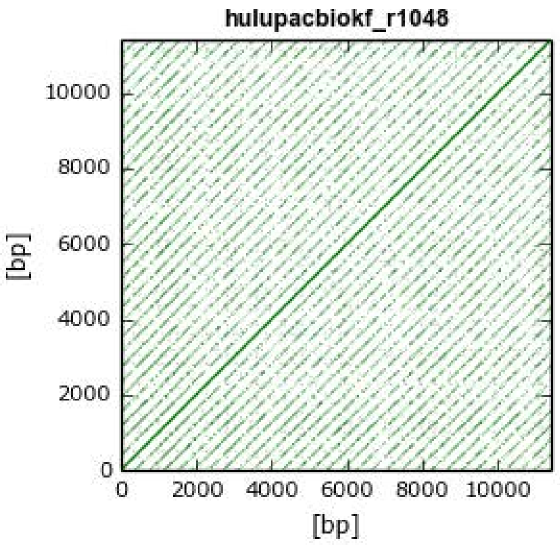
hulupacbiokf_r1048
[bp]
0
2000
4000
6000
8000
10000

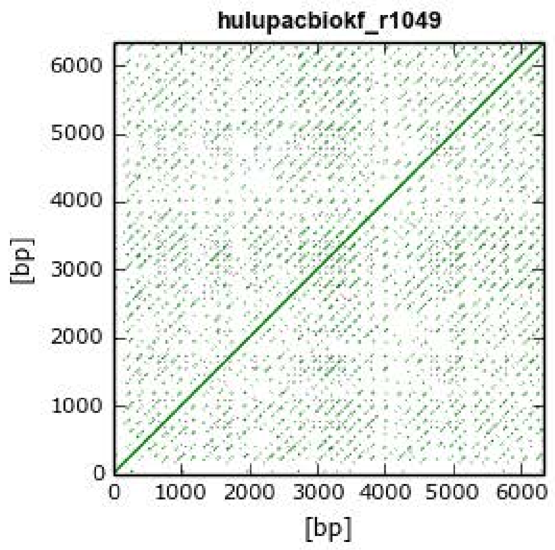
hulupacbiokf_r1049
[bp]
0
1000
2000
3000
4000
5000
6000

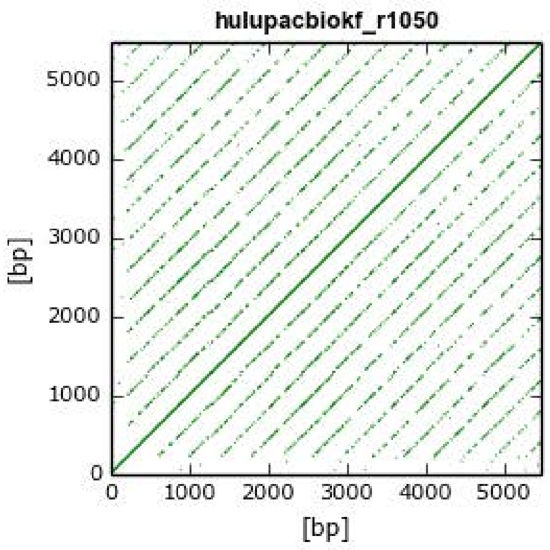
hulupacbiokf_r1050
[bp]
0
1000
2000
3000
4000
5000

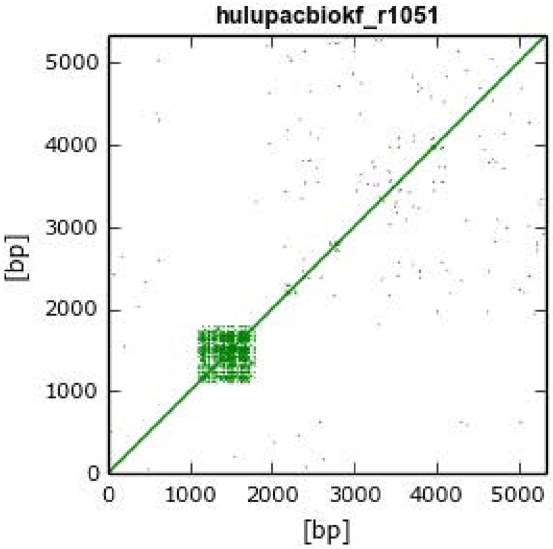
hulupacbiokf_r1051
0
1000
2000
3000
4000
5000
[bp]
0
1000
2000
3000
4000
5000
[bp]

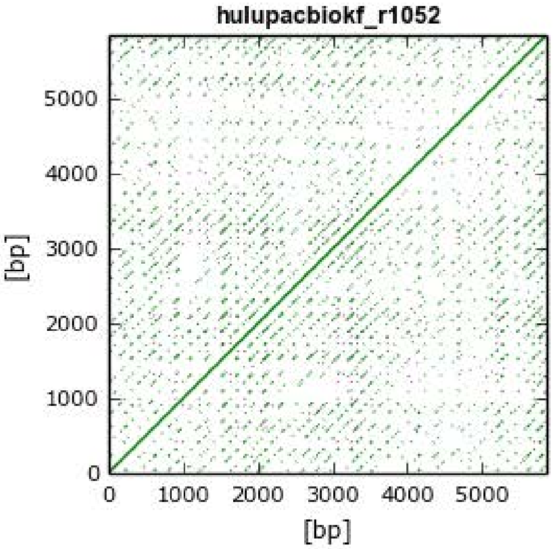
hulupacbiokf_r1052
0
1000
2000
3000
4000
5000
[bp]
0
1000
2000
3000
4000
5000
[bp]

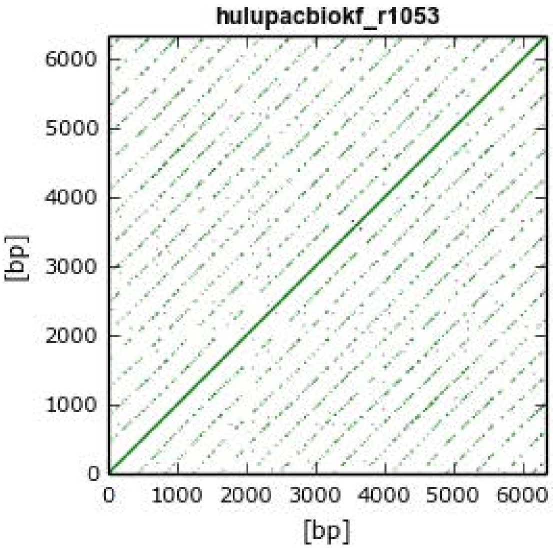
hulupacbiokf_r1053
0
1000
2000
3000
4000
5000
6000
[bp]
0
1000
2000
3000
4000
5000
6000
[bp]

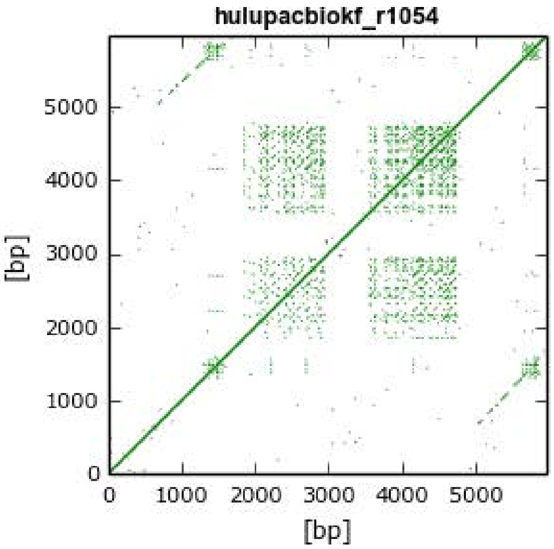
hulupacbiokf_r1054
0
1000
2000
3000
4000
5000
[bp]
0
1000
2000
3000
4000
5000
[bp]

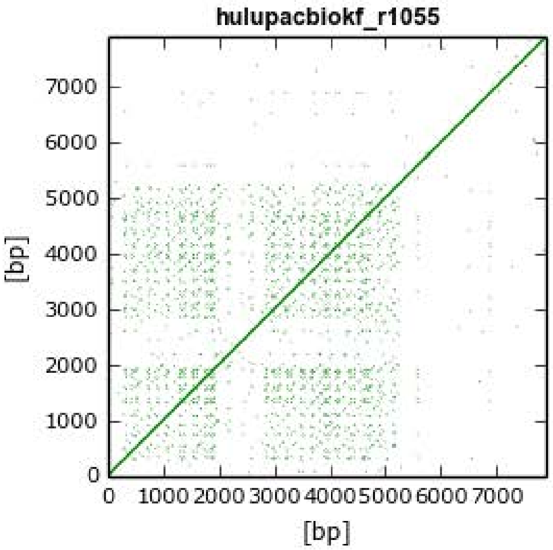
hulupacbiokf_r1055
0
1000
2000
3000
4000
5000
6000
7000
[bp]
0
1000
2000
3000
4000
5000
6000
7000
[bp]

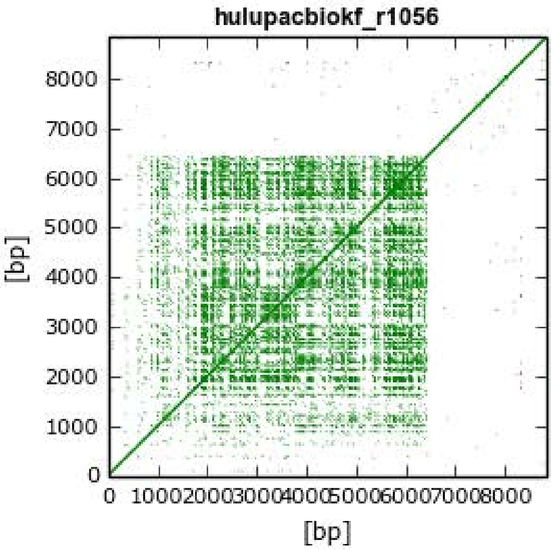
hulupacbiokf_r1056
0
1000
2000
3000
4000
5000
6000
7000
8000
[bp]
0
1000
2000
3000
4000
5000
6000
7000
8000
[bp]

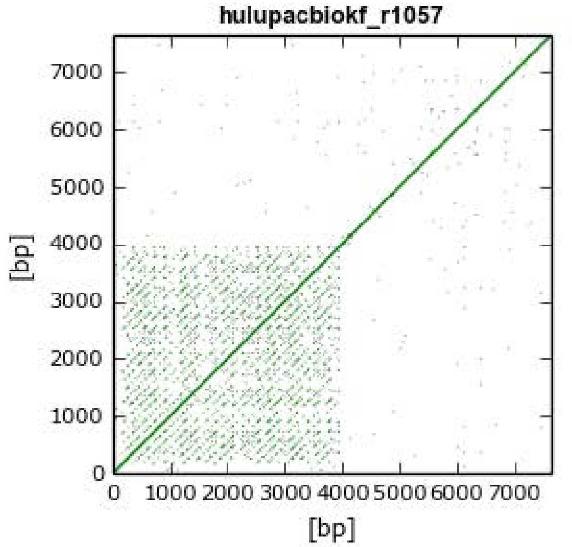
hulupacbiokf_r1057
[bp]
[bp]
0
1000
2000
3000
4000
5000
6000
7000

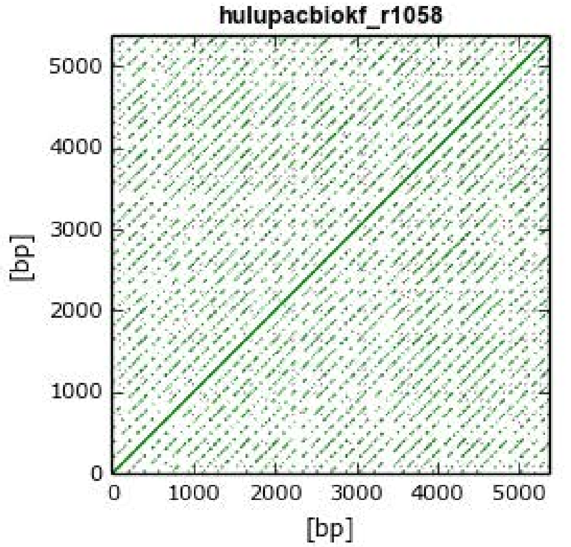
hulupacbiokf_r1058
[bp]
[bp]
0
1000
2000
3000
4000
5000

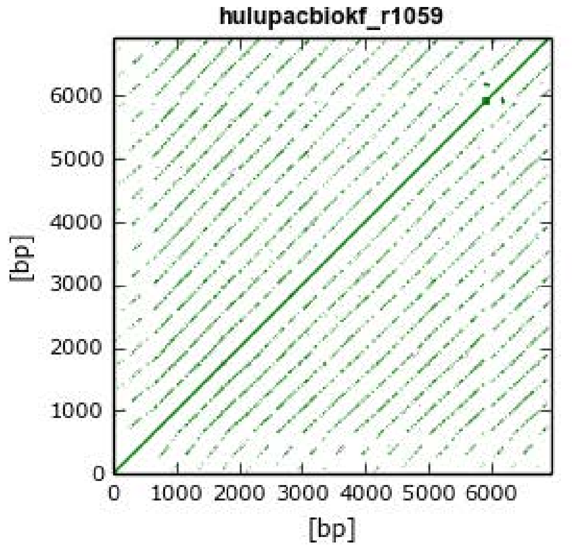
hulupacbiokf_r1059
[bp]
[bp]
0
1000
2000
3000
4000
5000
6000

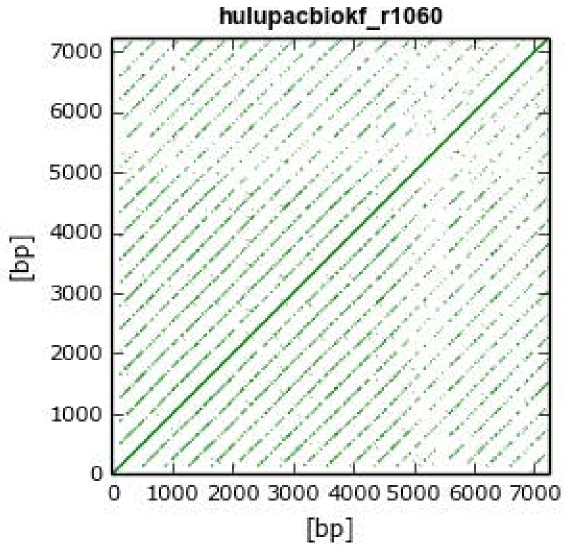
hulupacbiokf_r1060
[bp]
[bp]
0
1000
2000
3000
4000
5000
6000
7000

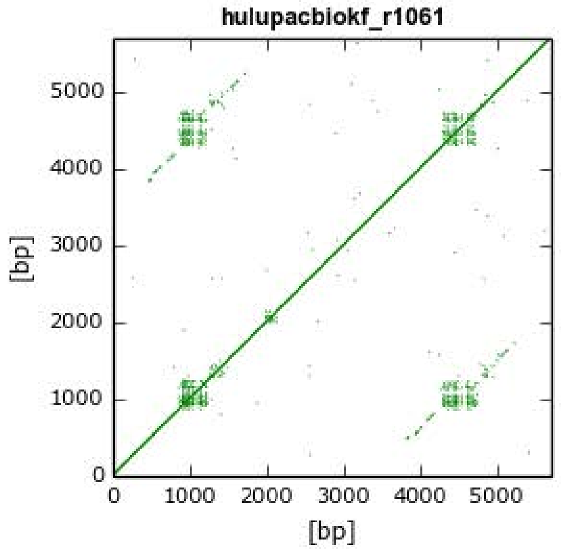
hulupacbiokf_r1061
[bp]
[bp]
0
1000
2000
3000
4000
5000

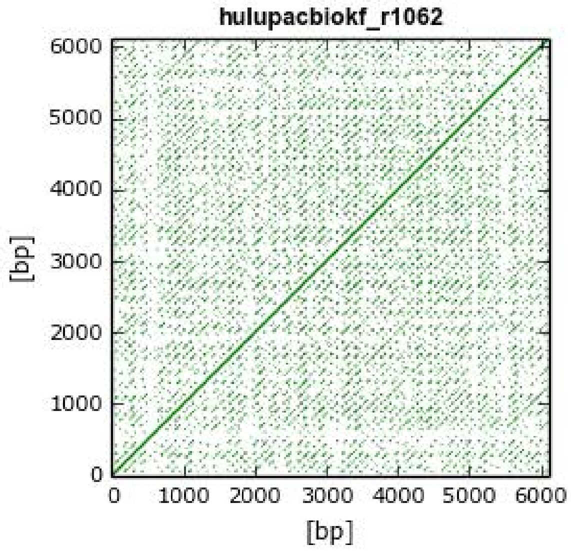
hulupacbiokf_r1062
[bp]
[bp]
0
1000
2000
3000
4000
5000
6000

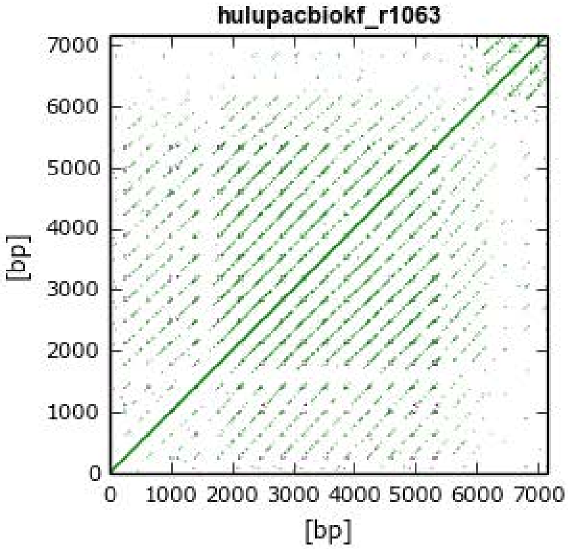
hulupacbiokf_r1063
7000
6000
5000
4000
3000
2000
1000
0
0 1000 2000 3000 4000 5000 6000 7000
[bp]
[bp]

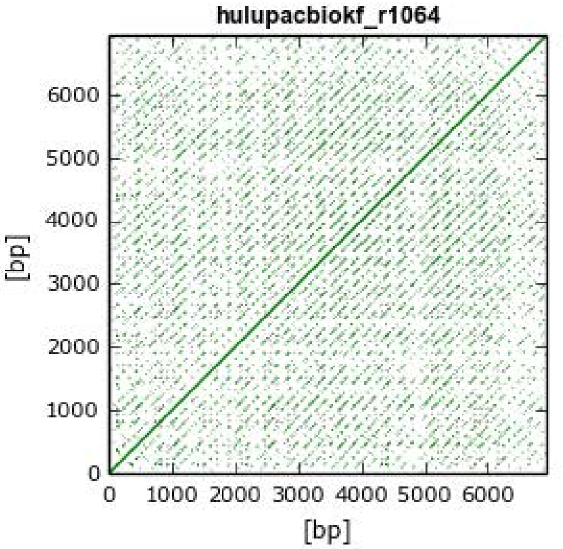
hulupacbiokf_r1064
6000
5000
4000
3000
2000
1000
0
0 1000 2000 3000 4000 5000 6000
[bp]
[bp]

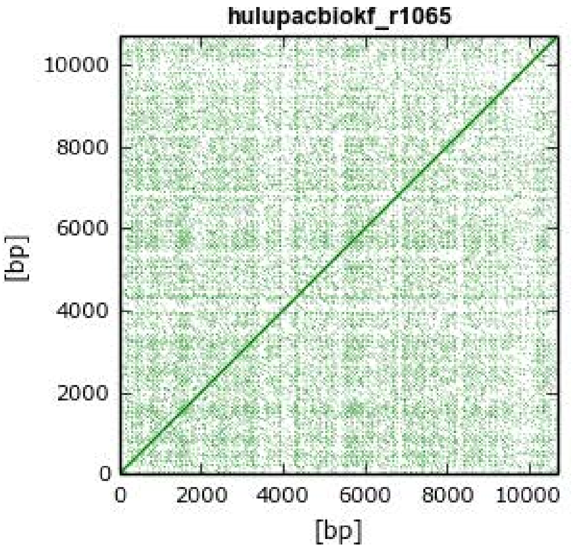
hulupacbiokf_r1065
10000
8000
6000
4000
2000
0
0 2000 4000 6000 8000 10000
[bp]
[bp]

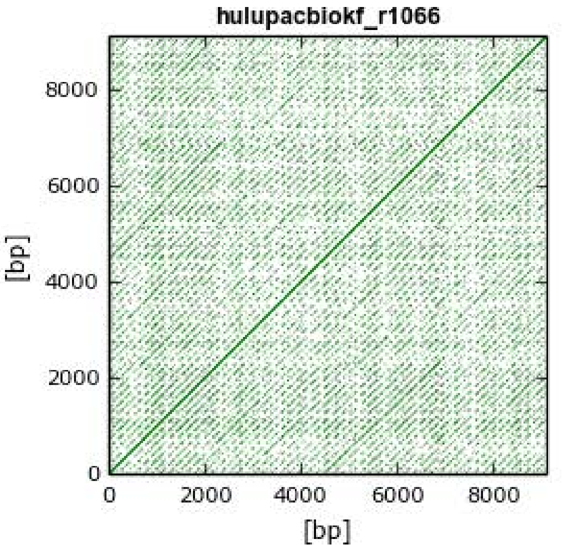
hulupacbiokf_r1066
8000
6000
4000
2000
0
0 2000 4000 6000 8000
[bp]
[bp]

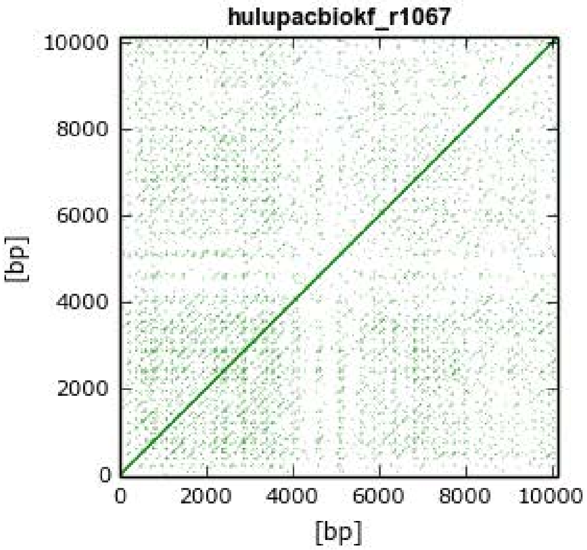
hulupacbiokf_r1067
10000
8000
6000
4000
2000
0
0 2000 4000 6000 8000 10000
[bp]
[bp]

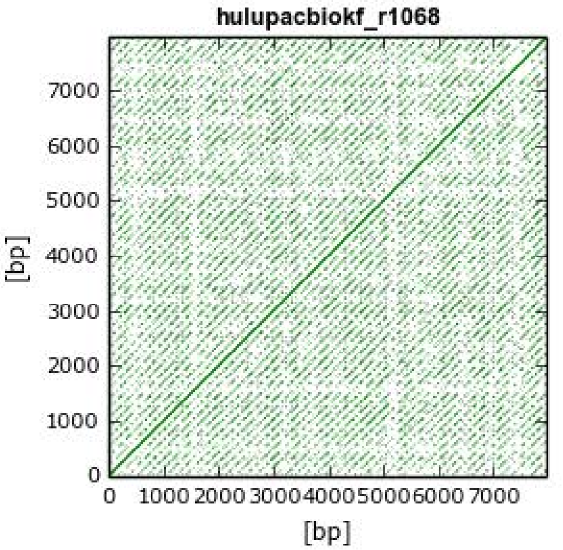
hulupacbiokf_r1068
7000
6000
5000
4000
3000
2000
1000
0
0 1000 2000 3000 4000 5000 6000 7000
[bp]
[bp]

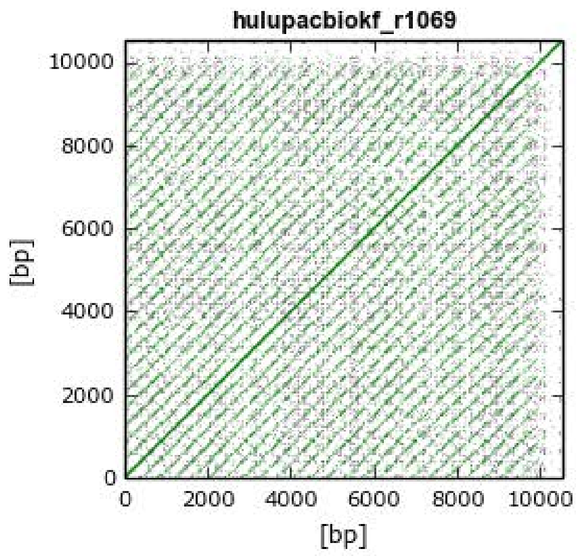
hulupacbiokf_r1069
[bp]
0
2000
4000
6000
8000
10000

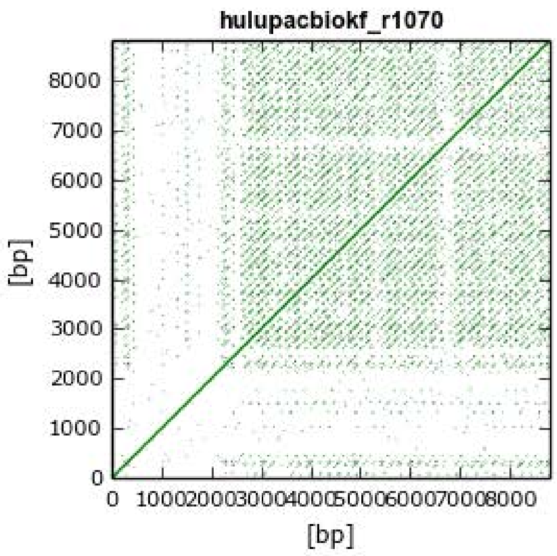
hulupacbiokf_r1070
[bp]
0
1000
2000
3000
4000
5000
6000
7000
8000

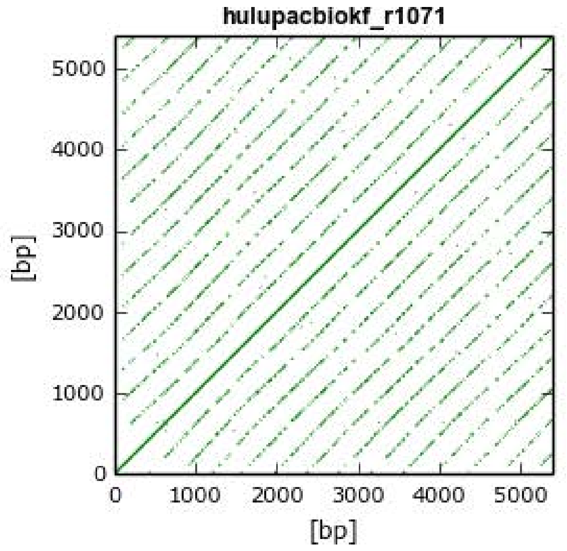
hulupacbiokf_r1071
[bp]
0
1000
2000
3000
4000
5000

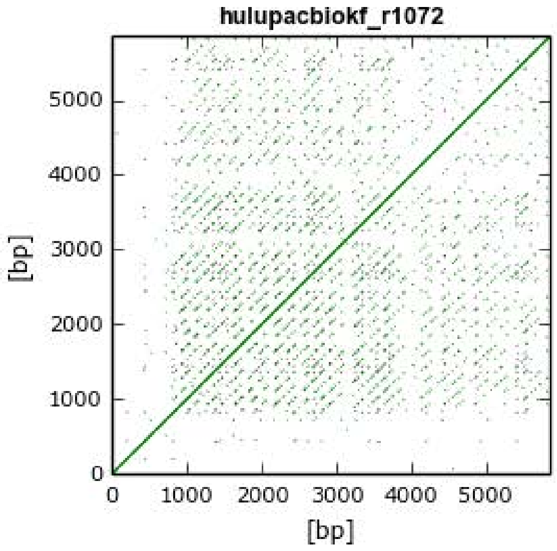
hulupacbiokf_r1072
[bp]
0
1000
2000
3000
4000
5000

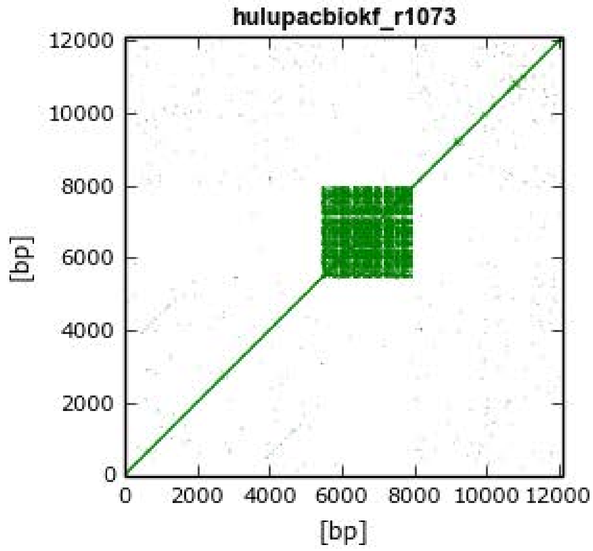
hulupacbiokf_r1073
[bp]
0
2000
4000
6000
8000
10000
12000

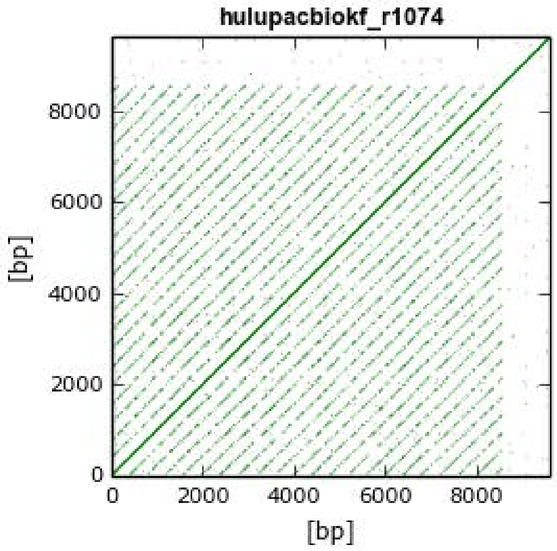
hulupacbiokf_r1074
[bp]
0
2000
4000
6000
8000

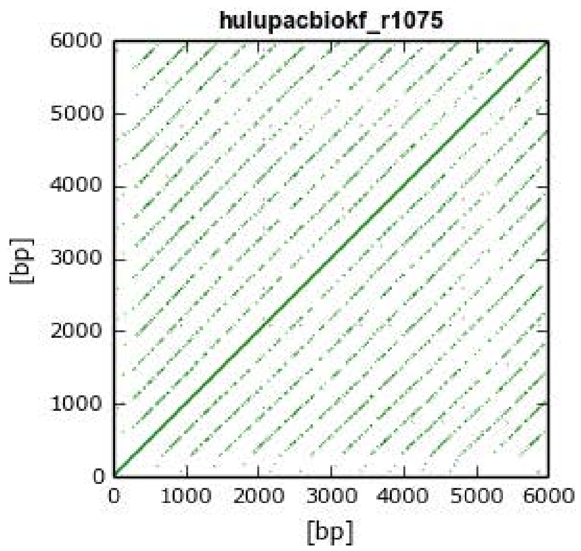
hulupacbiokf_r1075
[bp]
[bp]

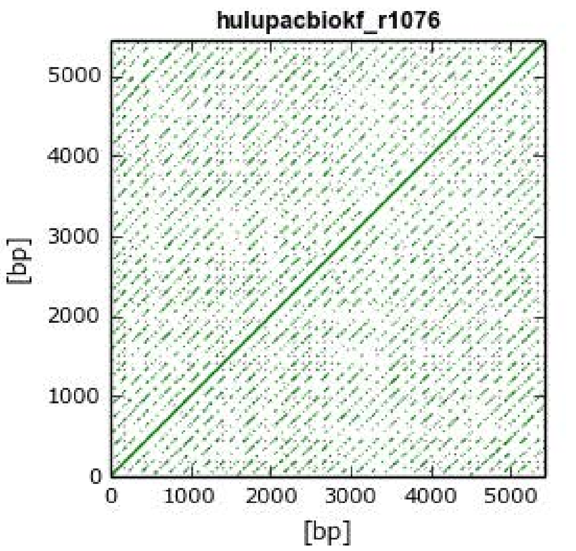
hulupacbiokf_r1076
[bp]
[bp]

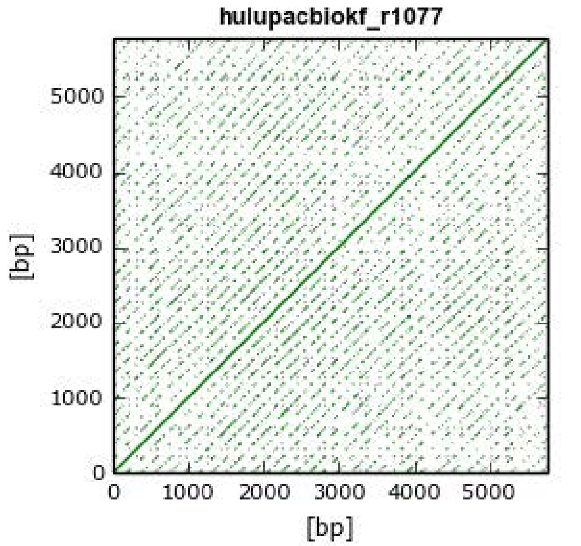
hulupacbiokf_r1077
[bp]
[bp]

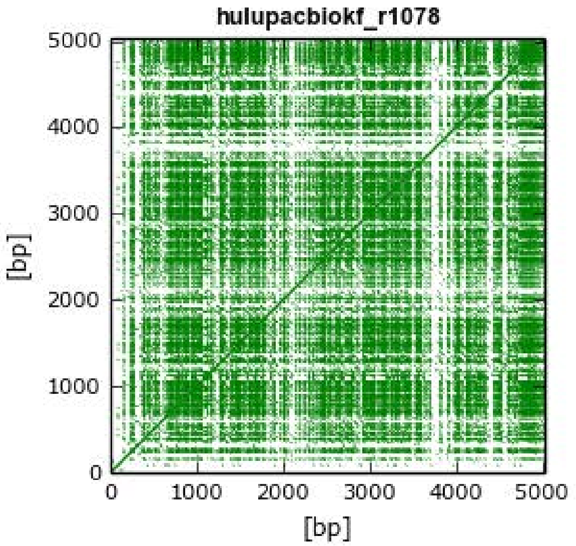
hulupacbiokf_r1078
[bp]
[bp]

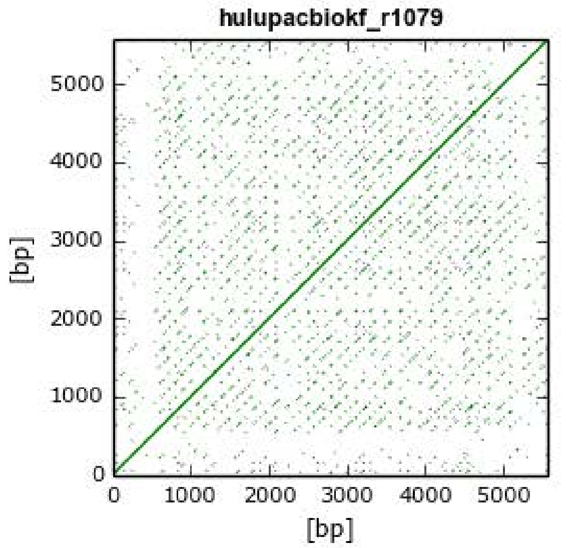
hulupacbiokf_r1079
[bp]
[bp]

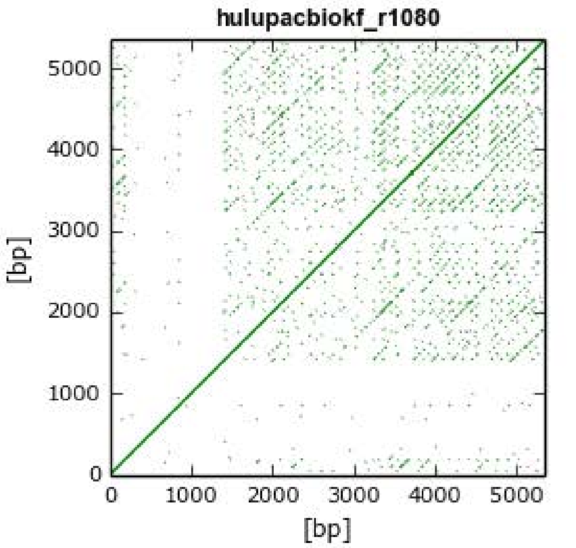
hulupacbiokf_r1080
[bp]
[bp]

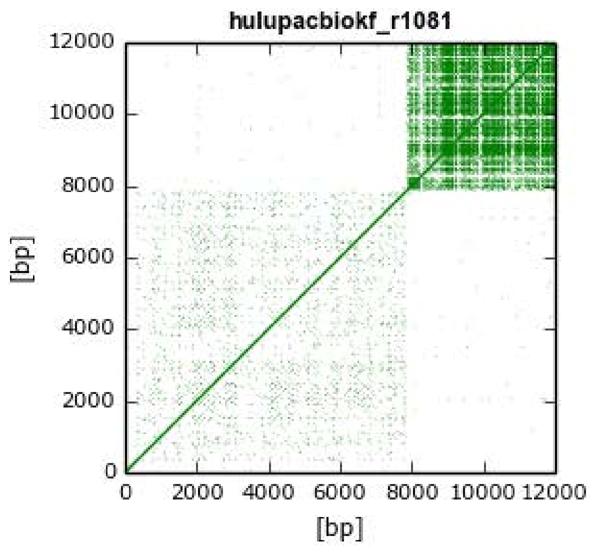
hulupacbiokf_r1081
[bp]
[bp]

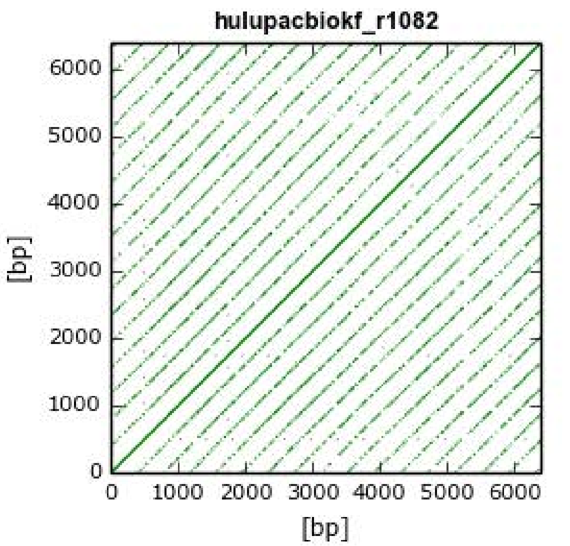
hulupacbiokf_r1082
[bp]
[bp]

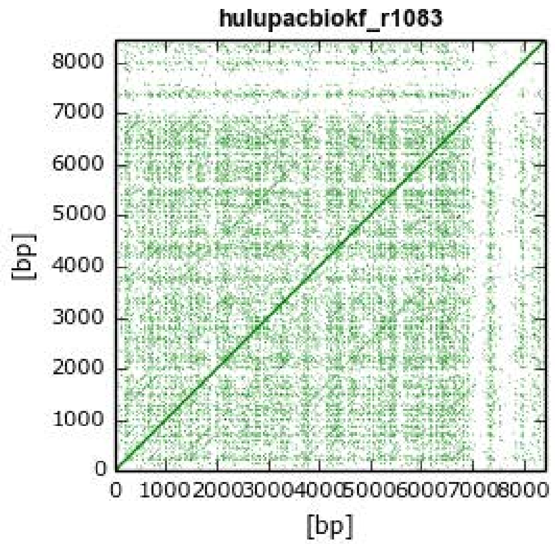
hulupacbiokf_r1083
[bp]
[bp]

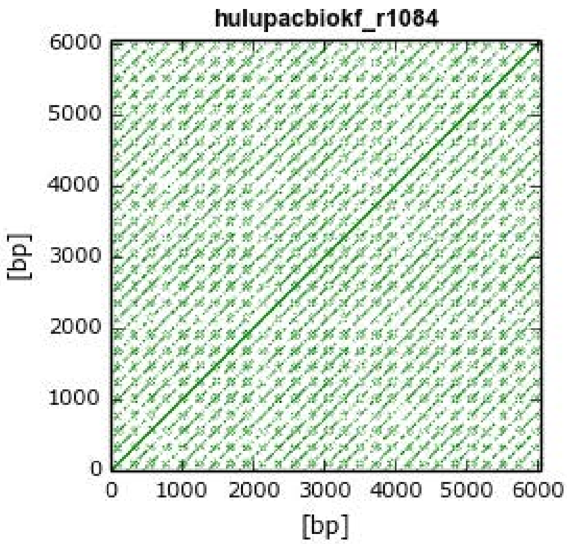
hulupacbiokf_r1084
[bp]
[bp]

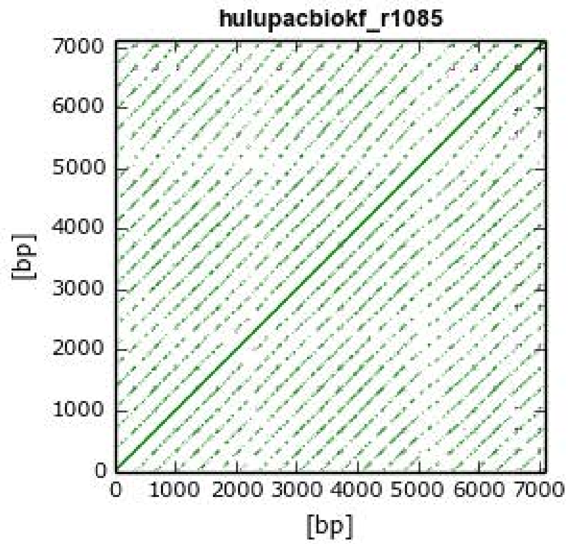
hulupacbiokf_r1085
[bp]
[bp]

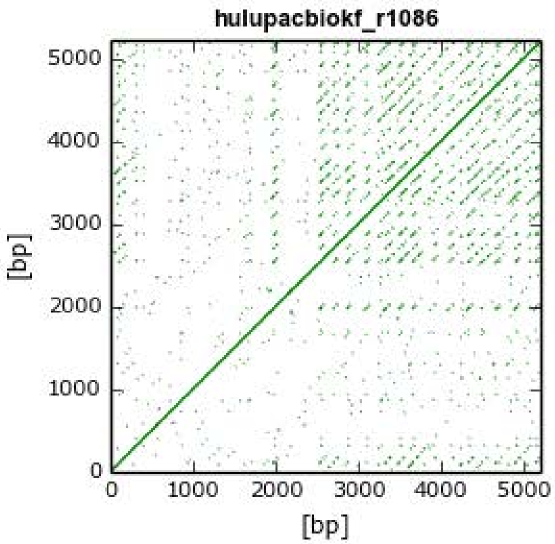
hulupacbiokf_r1086
[bp]
[bp]

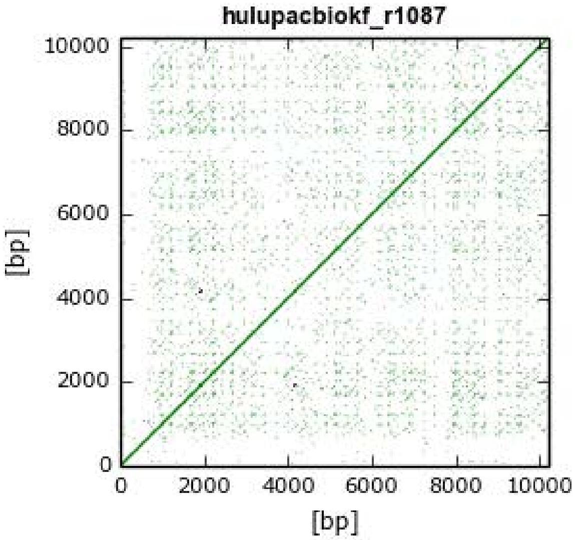
hulupacbiokf_r1087
10000
8000
6000
4000
2000
0
[bp]
0
2000
4000
6000
8000
10000
[bp]

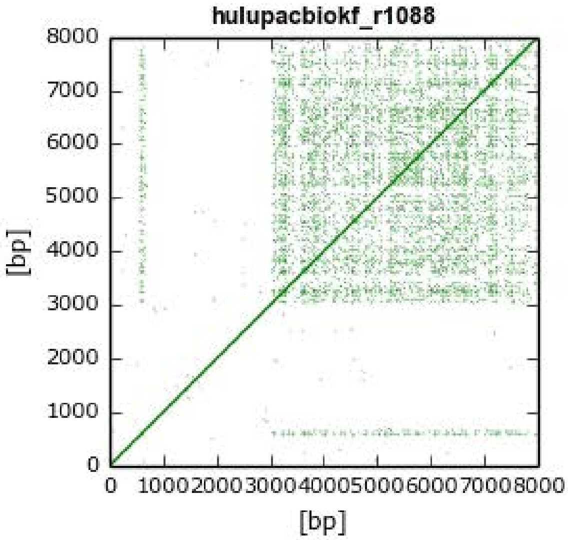
hulupacbiokf_r1088
8000
7000
6000
5000
4000
3000
2000
1000
0
[bp]
0
1000
2000
3000
4000
5000
6000
7000
8000
[bp]

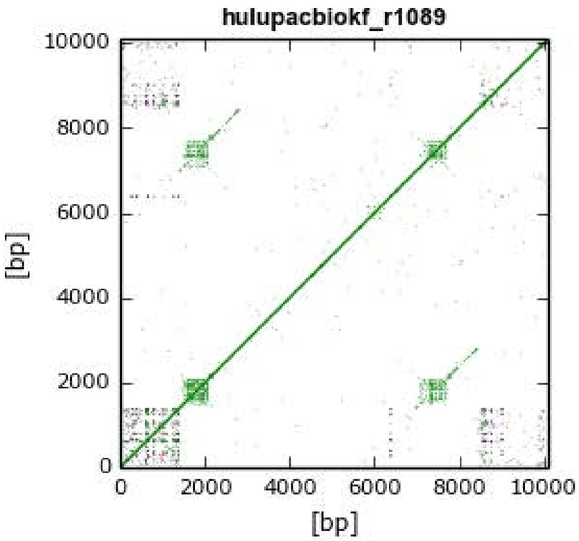
hulupacbiokf_r1089
10000
8000
6000
4000
2000
0
[bp]
0
2000
4000
6000
8000
10000
[bp]

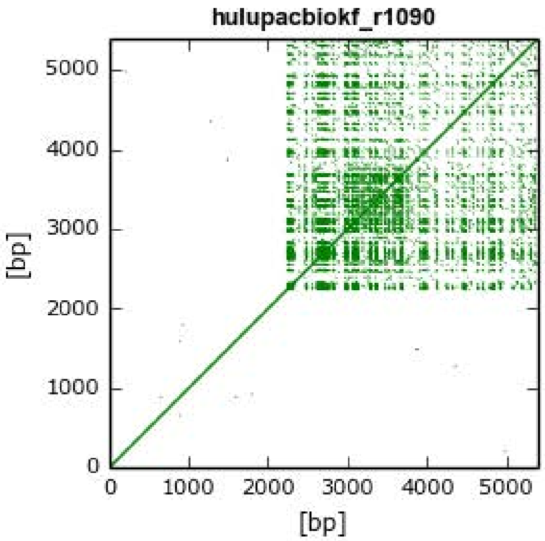
hulupacbiokf_r1090
5000
4000
3000
2000
1000
0
[bp]
0
1000
2000
3000
4000
5000
[bp]

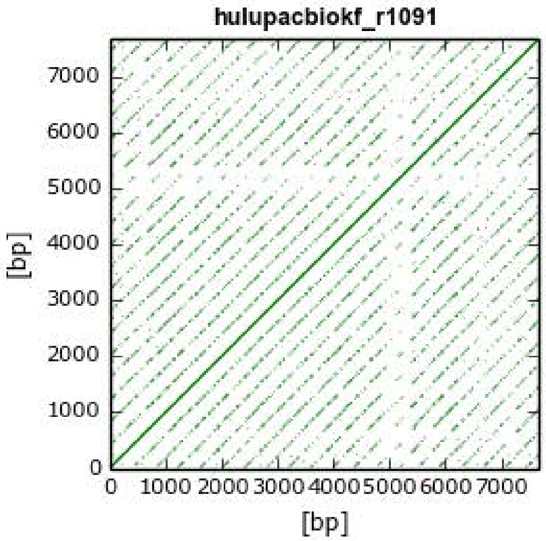
hulupacbiokf_r1091
7000
6000
5000
4000
3000
2000
1000
0
[bp]
0
1000
2000
3000
4000
5000
6000
7000
[bp]

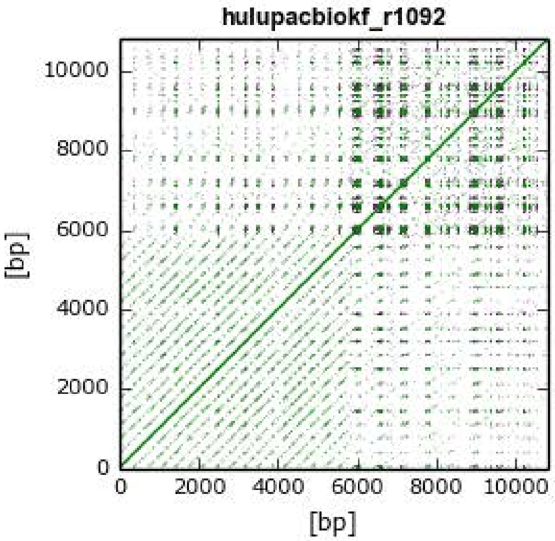
hulupacbiokf_r1092
10000
8000
6000
4000
2000
0
[bp]
0
2000
4000
6000
8000
10000
[bp]

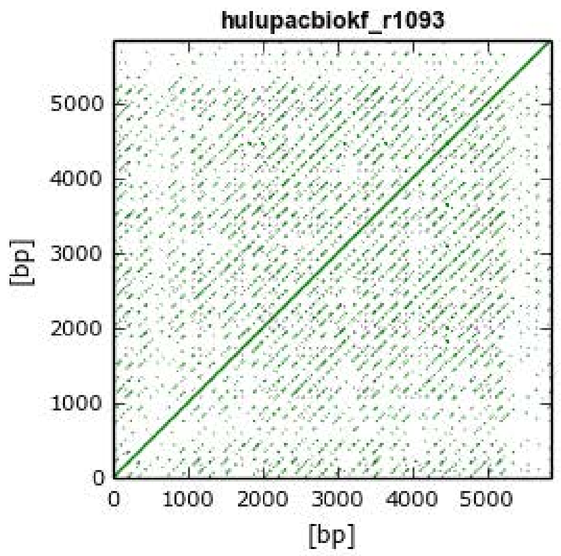
hulupacbiokf_r1093
[bp]
[bp]

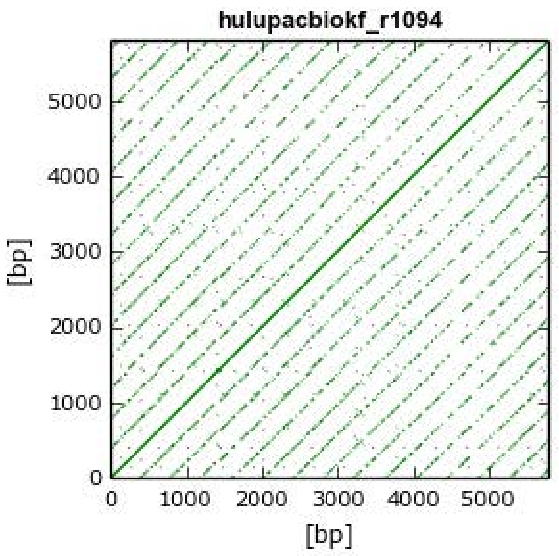
hulupacbiokf_r1094
[bp]
[bp]

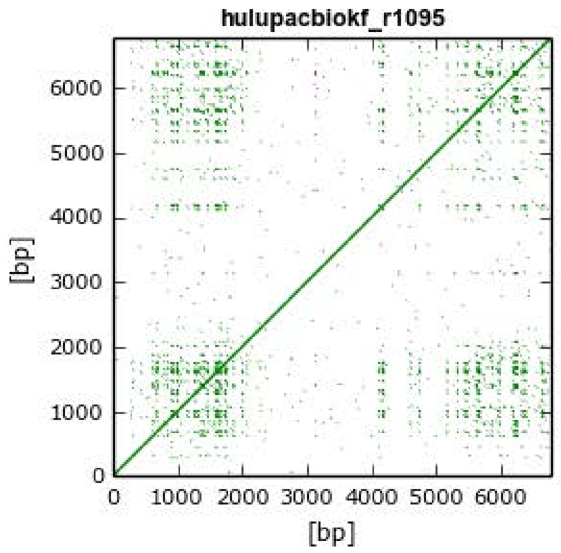
hulupacbiokf_r1095
[bp]
[bp]

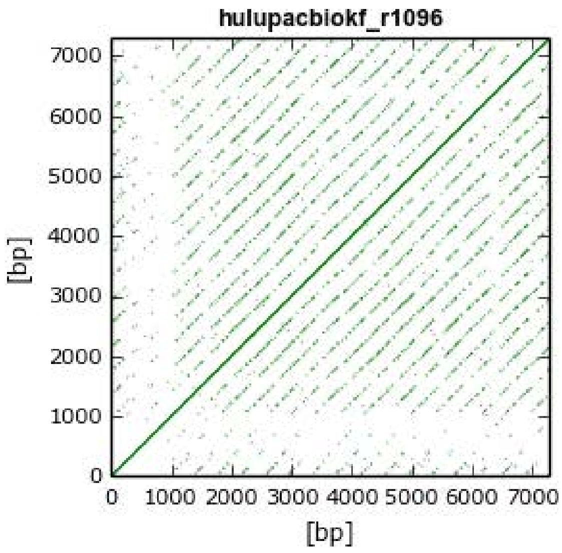
hulupacbiokf_r1096
[bp]
[bp]

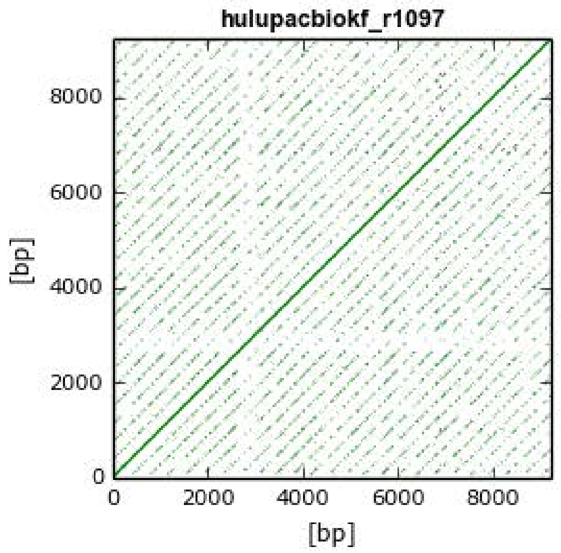
hulupacbiokf_r1097
[bp]
[bp]

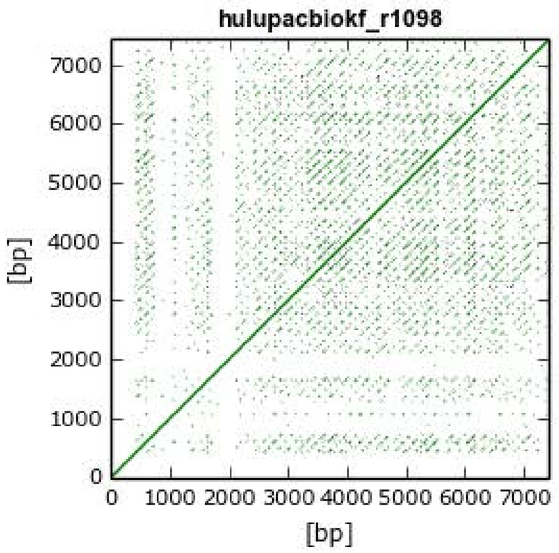
hulupacbiokf_r1098
[bp]
[bp]

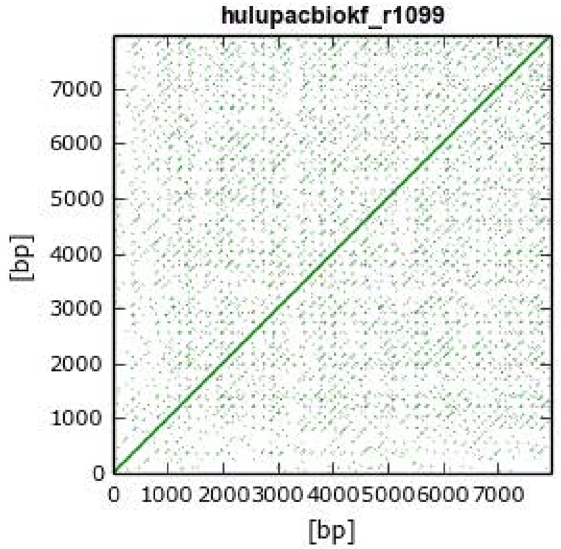
hulupacbiokf_r1099
[bp]
[bp]

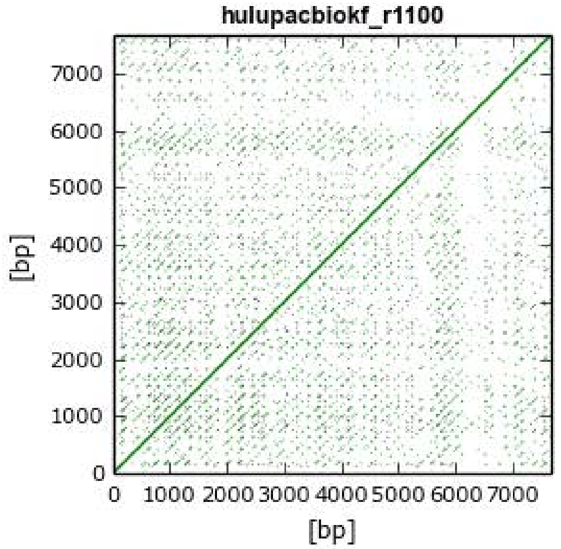
hulupacbiokf_r1100
[bp]
[bp]

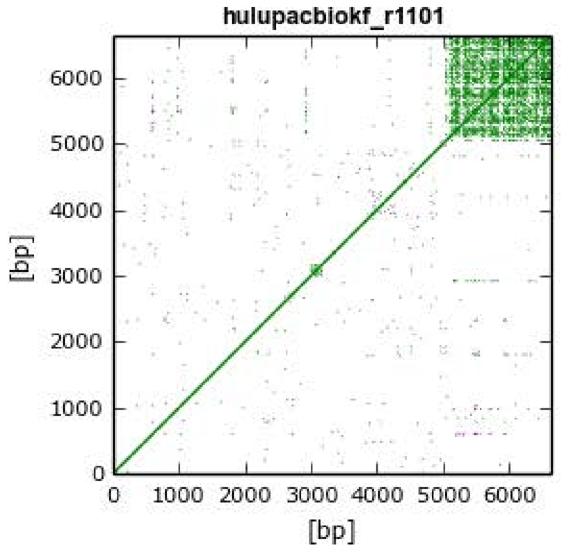
hulupacbiokf_r1101
[bp]
[bp]

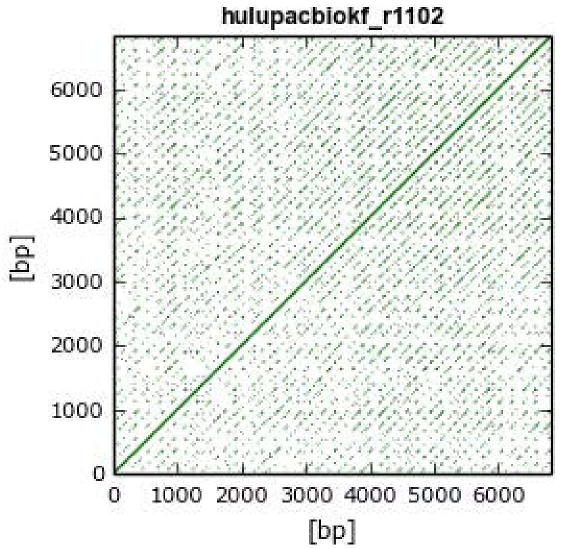
hulupacbiokf_r1102
[bp]
[bp]

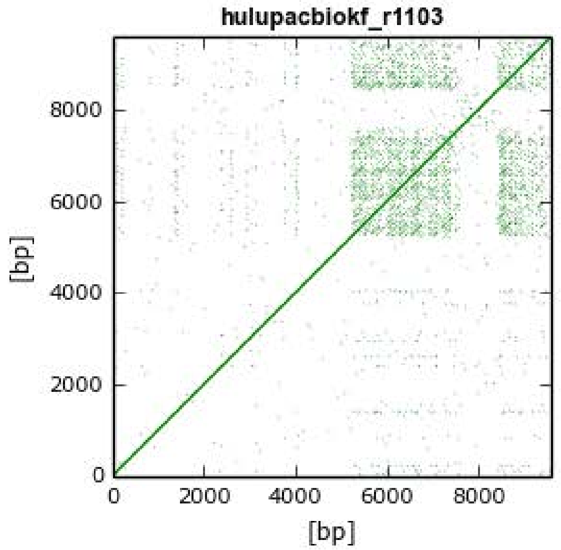
hulupacbiokf_r1103
[bp]
[bp]

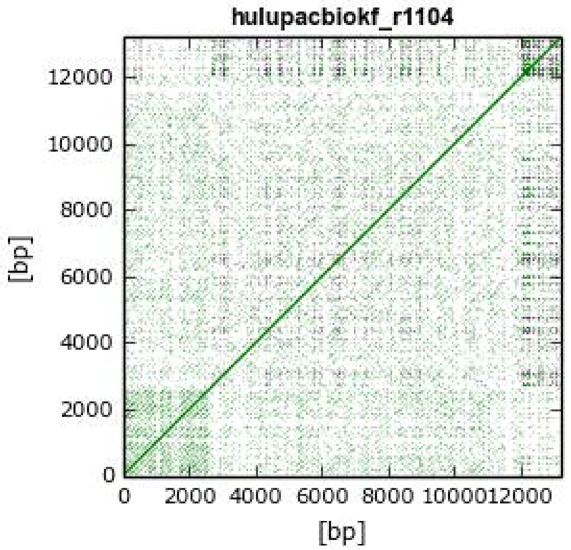
hulupacbiokf_r1104
[bp]
[bp]

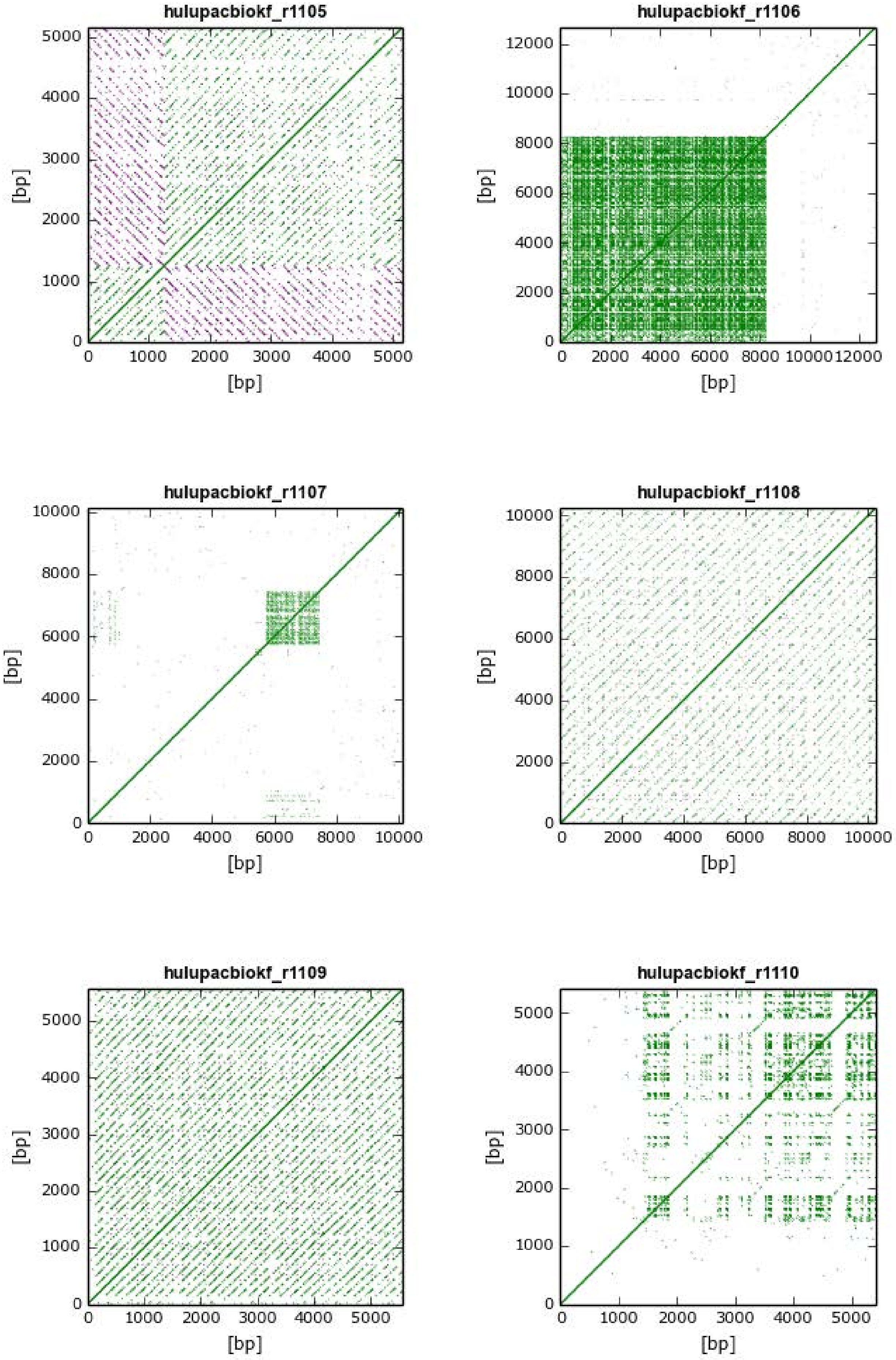
hulupacbiokf_r1105
hulupacbiokf_r1106
hulupacbiokf_r1107
hulupacbiokf_r1108
hulupacbiokf_r1109
hulupacbiokf_r1110
[bp]

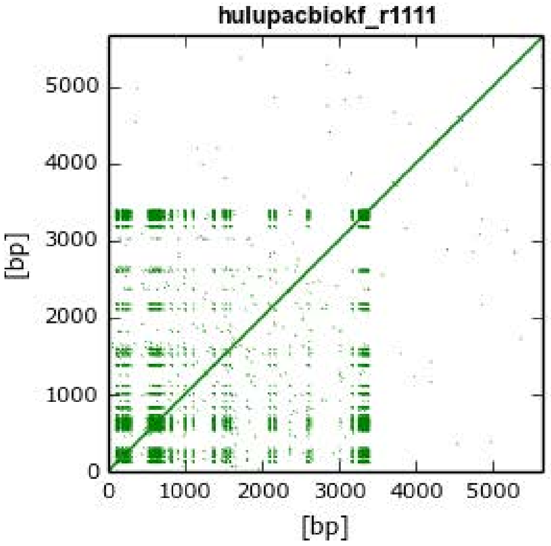
hulupacbiokf_r1111
[bp]
[bp]

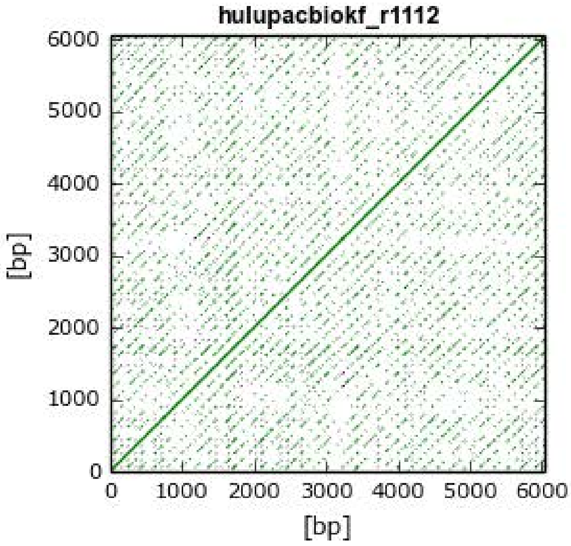
hulupacbiokf_r1112
[bp]
[bp]

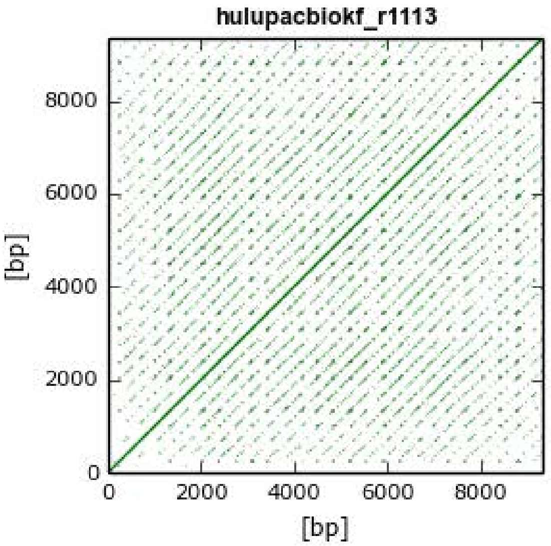
hulupacbiokf_r1113
[bp]
[bp]

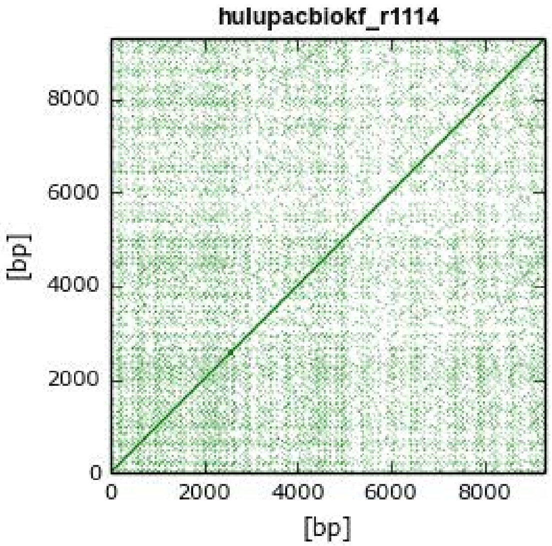
hulupacbiokf_r1114
[bp]
[bp]

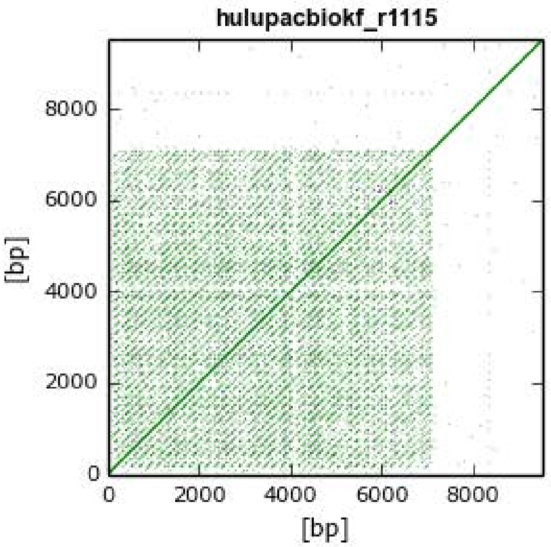
hulupacbiokf_r1115
[bp]
[bp]

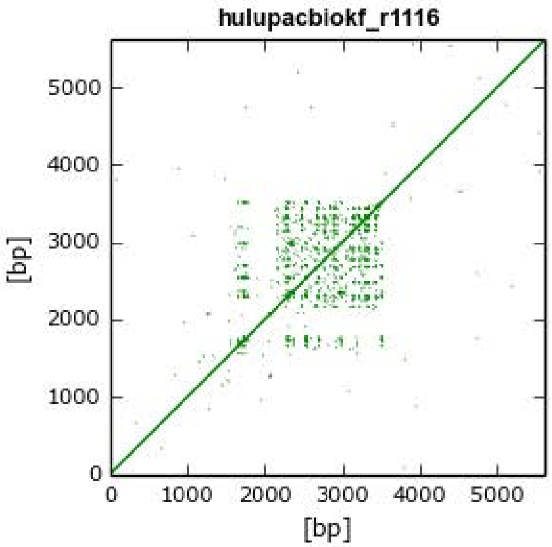
hulupacbiokf_r1116
[bp]
[bp]

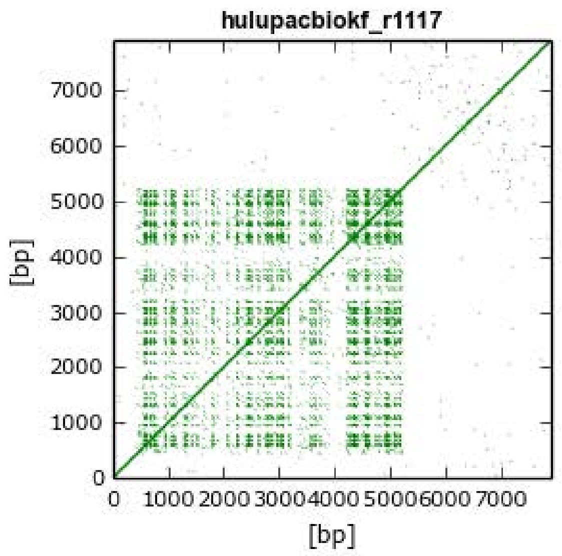
hulupacbiokf_r1117
[bp]
[bp]

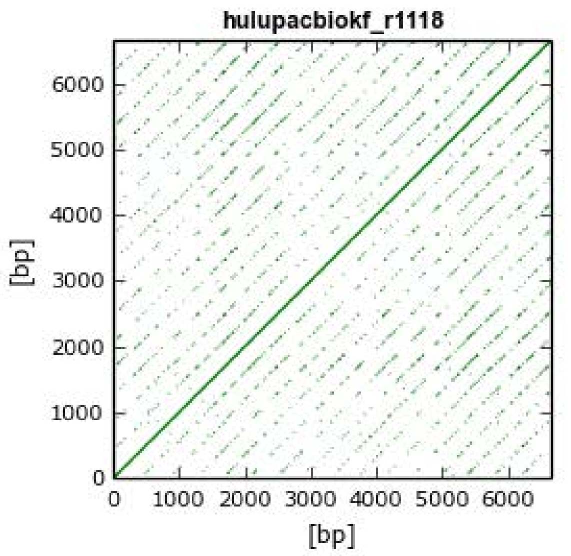
hulupacbiokf_r1118
[bp]
[bp]

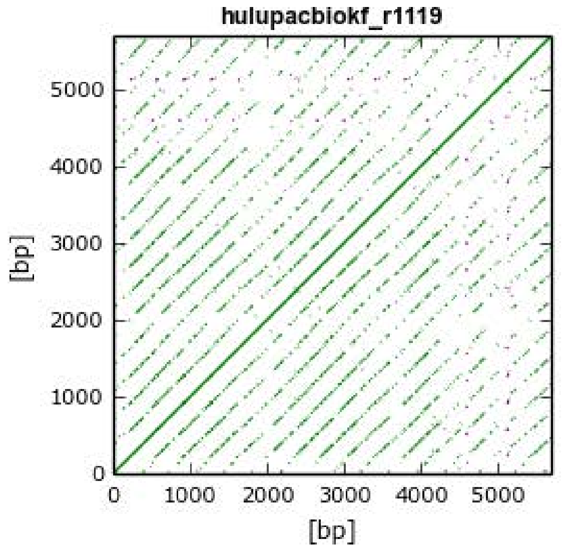
hulupacbiokf_r1119
[bp]
[bp]

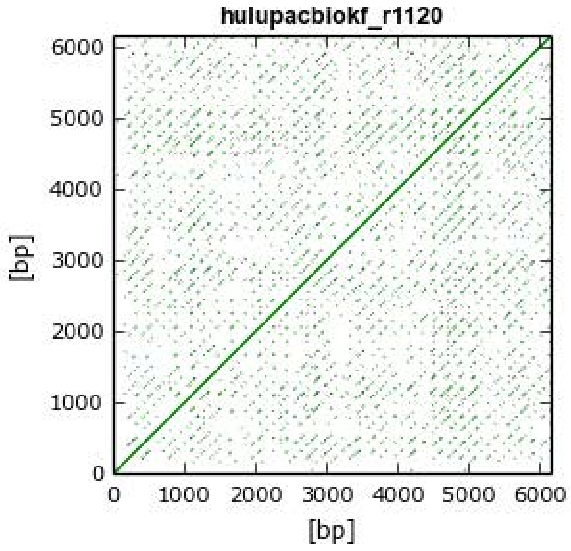
hulupacbiokf_r1120
[bp]
[bp]

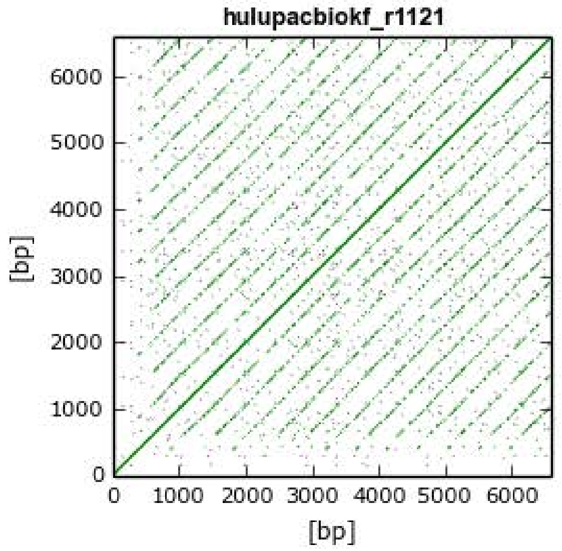
hulupacbiokf_r1121
[bp]
[bp]
