# Supplementary material for: Identification of tandem repeat families from long-read sequences of Humulus lupulus
Source: PLoS One. 2020 Jun 5;15(6):e0233971. doi: 10.1371/journal.pone.0233971 (PMC7274563; doi:10.1371/journal.pone.0233971)

Supplemental Material – Data Sheet 3

- **PolySeq34** for YASS dot plot alignments with hop tandem repeat sequences.
- Table of synonyms for locating HuluTR families at specified 1KB blocks
- PolySeq by consensus sequence dot-plots as drawn by YASS (simple plot)

Easterling, Bass, et al. 2020
Note: embedded are non-sequence notes indicating tke "kb" location (##k) the HuluTR length/ID for 34 sequences (###) and some spacer characters (n). These embedded notes will generate "errors" not DNA sequence reports in the YASS genome server dot-plot output, which can be ignored, but the utility will treat the entire document as a single FASTA file and the resulting dot plot will be generated. Most TRs are in 1 kb blocks, but a few of the larger TRs are in 2 kb blocks. In total the polySeq34 contains 34 blocks of hop TR in 38 kbp of sequence space.

```
>HuluTR_PolySeq34_v7
nn00kTEn
TTTAGGGTTTAGGGTTTAGGGTTTAGGGTTTAGGGTTTAGGGTTTAGGGTTTAGGGTTTAGGGTTTAGGG
TTTAGGGTTTAGGGTTTAGGGTTTAGGGTTTAGGGTTTAGGGTTTAGGGTTTAGGGTTTAGGGTTTAGGG
TTTAGGGTTTAGGGTTTAGGGTTTAGGGTTTAGGGTTTAGGGTTTAGGGTTTAGGGTTTAGGGTTTAGGG
TTTAGGGTTTAGGGTTTAGGGTTTAGGGTTTAGGGTTTAGGGTTTAGGGTTTAGGGTTTAGGGTTTAGGG
TTTAGGGTTTAGGGTTTAGGGTTTAGGGTTTAGGGTTTAGGGTTTAGGGTTTAGGGTTTAGGGTTTAGGG
TTTAGGGTTTAGGGTTTAGGGTTTAGGGTTTAGGGTTTAGGGTTTAGGGTTTAGGGTTTAGGGTTTAGGG
TTTAGGGTTTAGGGTTTAGGGTTTAGGGTTTAGGGTTTAGGGTTTAGGGTTTAGGGTTTAGGGTTTAGGG
TTTAGGGTTTAGGGTTTAGGGTTTAGGGTTTAGGGTTTAGGGTTTAGGGTTTAGGGTTTAGGGTTTAGGG
TTTAGGGTTTAGGGTTTAGGGTTTAGGGTTTAGGGTTTAGGGTTTAGGGTTTAGGGTTTAGGGTTTAGGG
TTTAGGGTTTAGGGTTTAGGGTTTAGGGTTTAGGGTTTAGGGTTTAGGGTTTAGGGTTTAGGGTTTAGGG
TTTAGGGTTTAGGGTTTAGGGTTTAGGGTTTAGGGTTTAGGGTTTAGGGTTTAGGGTTTAGGGTTTAGGG
TTTAGGGTTTAGGGTTTAGGGTTTAGGGTTTAGGGTTTAGGGTTTAGGGTTTAGGGTTTAGGGTTTAGGG
TTTAGGGTTTAGGGTTTAGGGTTTAGGGTTTAGGGTTTAGGGTTTAGGGTT
nn01k038nn
GCGCGGGGGCGACGTAAGGGCGAACGGGCTCCATGCGCCGGCGGCCGAGCGAGGGCGACGGGGCTCCATGGCGGGGG
CTAGCGAGGTGAGCGGGGCTCCATGCGCGCGGGGCCGAGGCGAGGCCATGGGGCCGATGCGCGGGAGCCGAGCGAAA
CGGCATGGGGCCTGCGCGGGGCCAGAGCGGAGGCGACGGGGCTCCATGTGCGGGGGGCCGACAGGTGACGGGGGGCT
CCATGCGCGGGGGCCGAGCGCCGGCGACGGGGTCCATGTGCGGGGCGAGCGAGGTGGCGGGGCTCCATGCGCGGGGG
CCCGAGCGAAGGTCATGGGGCATGCGCGGGCGGCGCGCGGGGGCGACGTAAGGGCGAACGGGCTCCATGCGCCGGCG
GCCGAGCGAGGGCGACGGGGCTCCATGGCGGGGGCTAGCGAGGTGAGCGGGGCTCCATGCGCGCGGGGCCGAGGCGA
GGCCATGGGGCCGATGCGCGGGAGCCGAGCGAAACGGCATGGGGCCTGCGCGGGGCCAGAGCGGAGGCGACGGGGCT
CCATGTGCGGGGGGCCGACAGGTGACGGGGGGCTCCATGCGCGGGGGCCGAGCGCCGGCGACGGGGTCCATGTGCGG
GGCGAGCGAGGTGGCGGGGCTCCATGCGCGGGGGCCCGAGCGAAGGTCATGGGGCATGCGCGGGCGGCGCGCGGGGG
CGACGTAAGGGCGAACGGGCTCCATGCGCGCGCGGGGGCGACGTAAGGGCGAACGGGCTCCATGCGCGCGCGGGGGC
GACGTAAGGGCGAACGGGCTCCATGCGCGCGCGGGGGCGACGTAAGGGCGAACGGGCTCCATGCGCGCGCGGGGGCG
ACGTAAGGGCGAACGGGCTCCATGCGCGCGCGGGGGCGACGTAAGGGCGAACGGGCTCCATGCGCGCGCGGGGGCGA
CGTAAGGGCGAACGGGCTCCATGCGCGCGCGGGGGCGACGTAAGGGCGAACGGGCTCCATGCGCGC
nn02k120nn
TTTCTGGATATTAACCGGTTCATAAAAAAAGAAGTTATTTAAATTGATTATCGTTGTTTTTCTAAAGAGCACAAGAT
ATTGTTAAAATAACGCGTATAAGTTTTCAAAAGTTCCTCGACATAAACCAGGGTCATAAAACTTAGAAGTTAATTTA
AATCAATTGATCCGTTGTTTTCTTAAAGAGCACGAGATATTGATAAAATTACGCAAACTAAGTTTTCGACTAAGTTC
CAGGGACATAACCAGGTCAGTAAATCCTAGAAGTTAATTTAATCATTGATCATTGTTTTTCTTAAAGAGAACGAATA
CCCCTGATAAAAGTTACGCAGAACTTAAAGGTTTTCAAACTAAGTTCCTGGACATAACAGGTCATAAAACTTAGAAG
TTCATTTAAATCATTGAATCGTTTTTTTTTTTCTTAGAGCACGAGATATTGAAAAAATTAACGCGAACTAAGTTTCA
```

AACTAAAGTTCCTGGATATAACCAGATCATAAAACTTAGAAGTTATTTAAATGGATTGATGTTGTTTTTCTTAAAGC
ACGGATAATTGATAAAAATTAACGCGAACTAAGTTTTTAAACTATGTTCTGGACATAACCAGGTCATAAACTTAGAG
TTTAATTTAAATCAATTGGATCGTTGTTTCTTAAAGAGGCATGATAATTATAAAATAATGCGAACAAGTTTTTCAAA
CTAATGTTCCTGGACATAACCAGGTCCTCAAACTTAGAAGTTTTTAAATCAATTGATCGTTGTTTTTCTTAAAGAGC
ATGATATATTGATAAAATAATGCGAACTAAGGTTTTTCAAACTATGTTCCTGGAATACCGGTCATAAAACTTAGAG
TTAATTAAAATCATTCGATCTTGTTTTTTCTTAAAGAAGCCAACGAGACATTGATAAAATTAACGCGAACCTAAGTT
TTCAATTAAGTTCCTGGATA
nn**03k180**nnnnnnnnnnnnnnnnnnnnnnnnnnnnnnnnnnnnnnnnnnnnnnnn
TCTCTCTAAAATGCTCTAATTTTTTTGATCCATTCTCCATCTCAATAATATTGATATAGGCTATTATTGACCATGAA
TTGGGGGTCCTAATATTAACAAACAAAAAAATTGACACTTTAGGCAAATTCCTAGGCAGCTGCTAGGGCTTCCGAAG
CGATTTCGTAATCACATCATATCTTCACCTAAAGCTCTAAGTCTTTTGCACCTTATTTTCCATCTCAAAATATTATA
TAGGGCTATTATTGACGCATGAATTGGGGTCATAATTTAACCAAGAAAAAAAAAATGGCCTTGGTCCGGCCAAATCTC
TCAGGAGCTGCTAGGGCCCTCGCGAGCGATTTCGTAATTCAGCGTCATATTTCTCTCTAACAAGCTGCTAATTTTTT
GTGACCTTTTCCCTCTACAATATATAGGTTATGATAGGGCTGAGATTATATCACCCATGAATTGGGGTCGCTAATTG
CAAGCCAACAAAAAATGTGACTTTCAGGCCAATCTCCTAGGCAGCTGGGCCTAAGGCTCGAGCGATGTTCGGTATTC
ACGAAGGCATATTTCTCTCTAACAAGCTATACTTTTTTTGTAACTTTTCCTTATCCATATATTATATTAGGCTAATT
TATTGACATGAATTGGGGTCTAATTAAGCAAACAAAAAAAAAATTGACCTTTCAGGCCCAAGATCTCCTAGGCAGCTG
CCTAAAGGCACGAAGCGTTTCGTATTCACTCTATTTCTTCTCTACAGCAAGCTTAATTTTTATTGGAACGCTTTTCC
CTCTCAATAATCAATTATACTAGGGCTATTATTGACATGAATTGGGGTCCTACCTTTAACCAACAAAAAAATTGACT
TTCGGCCAAATCTCTCGGCAGGACTGCCTAAGGCACCGACAGCGTTTAGTAATTCACTCATATTTCTCTCTAACAAC
TCTAATTTTTTTGAACTATTCCGCATCGTCAATACATATTATACATAG
nn**04k185**nnnnnnnnnnnnnnnnnnnn
TAATTCGTGCAGGGCGGCATCCATCTGTCTTGATCGGTACACTTGACCGCGAAGCGTGTGTCTCGGTGAACTGGAAA
CGAGTCCAGATCTTGTGTAAATCATCCCCCAACGAGTGGCCTCGTCGCTCGCAATGATTGGATGTAAGTCGGTGCAC
CCGAAAGGGTTTTAATCACACAAATGGTCAGTGCCTCGGTGCATCCGAACGAGATCTAGCAAGATACTCCTAGCCAG
GATCCCGTGTAGTTGGTGAATGAAATGAGTACAAATCTAGTTTAATCACCCTACACCAAGTGCCCGTGACCCGAAAT
GATTCTGCATGGAAGTTCGGCGCACCCGAAGAGCGCCGGAAAGGTGTTAATCACACCTATTGGGGGAGGGGGCCGTG
CCGGTGCATCCGGATTGGTCTATCCGCGGACTTCTGACCTAAACGCGGTGCTCAGTTAATCGAAATGAGCCTCTAGA
TCTTGTGATACCTCTAACGAAGTGCCACAGTTCACCCGGTAAATGATCTGCATGGAAGTCGGTGCACCCCCGAAAGA
CGCGGACGGGTGTATAATCACAGCTAATTACTCGTGCTCGGTGCATTCGCCAAGTCTAGTCGCGGGACAGTCTGACC
CCAGACGCATATCTCAGTTAATCGAAACGAGTCAGATCTGCGTAAGCAACCCTAACCGAGTTCCCTCGTTTAGCCCG
TAAAATCAGATTTCATGGAAGTCGGGCACCGAAAAGAGCGGAAAGGAGTTTAATCACACTAATGCGTTACTCAATGC
TACCGAACGAGTCTAGCTTCGAGTACATCCCTGGCCCAGATGTCGTGTCTTGGTGAGATTGAATGGTACAAAGCATG
GTGTAATCACCCTAACGAGTGCCTCGTCCCCAACCCCCGTAAAAGATTTTCATTGAAGTCCGGTGCACCCGAAGAGC
GCTGAAAGGGGAATCACCACTATTGCGTGACACGGATGCCATC
nn**05k335**nnnnnnnnnnnnnnnnnnnnnnnnn
CTTGGGGCGAGGAGTAAGTACTAGGTGGGTGACCTCACTGGGAAAGTCCTCGGTGTTGCACCCCTGAAAACCATCAT
TTTTTTTTTCCCTTTAACGAAATCCACCCTACGGCTCCAAAATTGTTTTTCTGTTTTAAATCATTATAAAAAATCAT
TTATTTCGTTTCTGTGAGGGGTTAATACCGTGGTCACTAAAACGCAGCGAATAGAGAAGTCATAGTCAAATCCGGGG
GGAAAAGCGGAGGACGACCACGCGCTCGCTATAAATGAATATTGCGAACAAAACCATTCCGGTCGATCATACCAGCA
CTAATGCACCGGATCCCATCAGAACTCCCGCAGTTAAGCGTGCTTGGCGAGAGTAGTACTAGGTGGGTCGACCTCAC
TGGGAAGTCCTCGTGTTGCACCCTGAAAACAATCATTTTTTTTTTTCTTTAAAAATCCAACCTACGGCTCAAAAAGTT
TGTATTATTTGTTTTAAATCTTTAAAAACTCATTTATTTCGTTTTCCTTGAGGGGGTTAAATACGTGGGTCCTGACA
AGGCAGCGAATAAGAGATTCCTAGTCGAATCCGGGGGAGAAAAGGGAGGACACACGCGCTCGCTATAAATGAAGTAA
CTTGCGGACCAAACTTCCGGGTGCGAATCATCACCAGCACTAATGCACCGGATCCCATCAGAACTCCGCAGTTAGAG
CTGCTTGGGCGAGATAGTATAGGATGGTGACCTCCTGGGAGTCCTCGTGTTGCCACCCCGAAAACATCATTTTTTTT
TTCCCTTTAAAAATCCACCTACGGCTCAAAAGTTTGTATTTTTTGTTTTAAATCTTTAAAAAATCATTTATTTTCGT
TTTCCTTGAGGGGTTAATACCGTGGTCACTAACAAGAGCAGCGAATAAGAAGTTCCTAGTCATGGGGAGTAAAGGGA
AGGACAGCCACGCGCTCGTATAAATGATATTTCGCGGAACAAACATTCCGGGTGCGATCATA
nn**06k385**nnnnnn

TAGGGGATACAGTCAAAATTATGAGAGATGTACGAATTATAAGGAAAAAACTTGCGAAAATTGCATCAAAAAGGGGA
ATTTTTTTTGAAAATGTTTGAATAAAGTCCTTGATCGAATCCAACATATTCCATTTACCCATGAGATCTCTTGGTGA
AATTATATCGAACGAGTTGAAACGAGTAATACCATTTCCGAGAAATTATTGAAATCTTCCAATTCACCAAAGGGGTA
CCCCTTTCAGAAATCTTCGATTTCTCCTAATAGGTTCTGGAGAAATGTTTTGATAGTGAATTCTAAAGAATATGCTG
AGGGTCATGTCCAAAAGTTGTAGCCCTATGTCTAAGGGTTCTATGTATAAAAATTTCGTGCAATCCATGGTGGCGTA
ATAGGGTGATAACAGTCAAAATACTGAGACATGTACGAATATAGGAAAAAACTTGCGAAAATTGCCCTCAAAACGGG
GAATTCTTTTTTGAAAATGTTTGGAATTAAGGCTCGTTTCGACTCCAACATATTTATTTACCCATGAGATCTCTTGG
GGGAAAATATCGAAGAGTTTGAACACGAGGGTAATACATTTCCGCGAGAAATTATTGAAAATCTCCAATTCACGAAG
GGTACCCCTTTCAGAAATCTTCGATTTCTTAATATGGATTCGAGCAATGTTTTCAAAATAGTGAATAACTAAAGAAT
ATCGAGGGTCAATTGGGTCACAAAAGTTGTAAGCCTATGTCTAAGGGTTCTATGTATAAAATTTCGTGCAAATCCCG
AGGTGCCGTATAGGGTGAATACAAGCAAAATAGTGAGAGATGTACGAAATATAAGGAAAAAACATGACGACAATATG
CCCTCAAAAAGGGGAATTTTTTTTTGAAATGTTTGAATTAAGGCTCCGTTTCGACTCCAACATATTTATTTACCCAT
GAGATCTCATTGGGGAAAAATATCGGAACCCGAGTTTGAACACGATGGTAATAC

nn**07k450**nnnnnnnnnnnnnn

TGTTTTTTTTTTTGCAAATATTCTTAAATATTGTATTGAGAATTGGGAAATTTAATTCATTGGCAAATAATTAGGGC
CTACCCTAAAGTTAGTTTGAATAATTAAAATATTGAATATACGTTAAATATTAAATGCACCTCTTCATATTCAAAGA
CAATAACCAACTATCTAAACCCTAATTTCAAAAATTATAATTAGAGTGTAAAATTTATTATATACAAACCCTAATTT
CGGCTATGCCAATGTGGTTTACAATTATTATTGAATTTTCTAAGAATTAAAAATACAAAATAATTCACATCTTAAAA
TATGGTATGTAAGAAAGTGGGAATTGTTAATGCACCTCTTCAATTTGAAAGACCTCCAAAGAATAATACTATAGTCC
TAGCATTTGTAAATATAATTATTATATAAAAATATATTTTTTATCATTAATTTCGGCTACAACAATATAGGGTTAAT
TATTGGGTTTTTTTTCAAAGATTATAAATATGGAATTGAGAATTGGGAATTTTTAATTCATTGCATAATTATGGCCT
ACACAATAGTTAGTTTGAATAATTAAATATTGAATATACGTTAAATATAAATGTACTCCTTCATGATTCAAGAATTT
TAACCAACTATCTAAACCCTAATTTCAAAAATTATAATTAGATGTGTAAAATTTATTAATATGAGCAAACCTAATTT
CGGTATGCAATGTGGTTTACAATTATATGTGAAGATTTTTTAGCATTAAAAATACAAAATAAATTGCACATCTTAAA
TATGGTATTGAGAAGTGGGAATTGTTAATGCACCTCTTCAATTTGAAAGACTCAAAGAATATATATATCCTAGCCAT
TTGTAAATAATAATTATTATATAAATATATTTTTTATCATTAATTTTGGCTACCAACATATAGGGTTAATTATTGGG
TTTTTTTTTCAAAGATTATAAAATATGGTATTGAGAATTGGAAATTTAAATTCATTTCATAATTAG

nn**08kJ19**nn

TTGAGAAATAACCTATGTAAATTGACAAAATTATGATGATCACCATTTTTCCCAGTATGTTTTCACAAGAAACTGGA
CTATTACCAGCTTCCAAGAATACGTCGAAAGTAGGTTAGTGGCTCAATTAAGGATCTCGACCCCTTAGTACAACCAT
CGCTGTTGAGAAATAACACTAATCGTGAAACCTAACCCATAGCGTCATCGGCTATACTTGCTAAAGTTTCACATAGA
TCGGCCAATCAGATTCCAATGGTTAGTGAATACCGTGATAAAATGAGTAGGGAAAGCTCAAATTTAGGATCCTCGTT
GCGAGATTCGGATTCAGCCAATAAGTAGTAATTTTCTCCTTTGAAAAATAACACTAATTGGGTGAGACCTGACAATA
TTGTGATGGTATCCTTGGAGACCCCCGTCACAAGTTCGAACAATTCAAACTGTCTGAAATACACGTAAATGGTGAGG
TAGGTCGTTTAGGAATCTCACTAAGAGATCGGATTCAGCTCTAAGTTGTAACCGTCGCTTTGAGAATAACACTTATT
GATGAAACGGACACCAAGTGTGGATCGCCTTTTTCCCATATGTTTTCACAATATCTTTTGACTGTTCTAAGCAGTCC
AGAATACCCCTAAATGAGTAAGGTGGCTAAATTTAGGATCCTAACTGTAAATCGGAGTCGGCCCTAGAATTATTCAC
TTTGAGAAATAACACTAATCGTGAAACCTGATCATAGTGTGAATCGACAATCCTCGTAAACTTTCAAAGATCAGAAC
AATTCCAACGACTAAGTAATAACCTGATAAATTGGTGGACCAGGCTCAATTTAGGTTCTCAGTTGCGAGATCAAATT
TGCCCCTTAGTAGTACTTTCGCTTTGAAAAAATAACACTAA

nn**09kJ26**nnnnnnnnnnnnnnnnnnnnnnnnnnnnnnnnnnnnnnnnnnnnnnnnnnnnnnnnnnnnnnnnnnnnn
nnnnnnnnnnnnnnnnnnnnnnnnnnnnnnnnnnn

AAGGGGAAGAACCAATCTCTTCAAACATACCAATGCAGGCTCAGATAAGCCAAAGGCTACACTCAGAGACCGAAGAG
TATTGATTAATGAATAAAACAATTTTCTTTTGTGGCCATCGATTGAAAATCCATTCCGCACACACATCCTAATCATC
TCCATAGCGCCAAACACACAACCCACACAAACACACACAAAAACACAAGCACAGCACACAAAACACTCAACCCCACA
GAAAAACACCACAACCACACCAAACAAAACCAAACCAACACACAAACACAAACAACACAAATCGGGACACAAACAAC
ACTCAAACCACAACAAATCTAATAACAAAAACTACAAACAAACACACACAAGAACCACAAAGACCAACACACAACAC
ACAACACACCACCAAACACACACCAAAATCAGACCACCAAACATCAACACATCAGAACACAAAACACACACCACACA
AAACACAACACAACAAAACACACCAAACAGCAACCAAAAAAACCACAACCACAAAACCACACACACCACAACACACC
CCAACAAACACCACAAATAACCAAACGAAGAACACACACAAAACAAAAGAAAAACCCACAACAAACACACACAACCA
ACCAAACCCACAACACGAACAACCATACCAAACACTATACTCTCTCAACACAACATACAAACACACACTACACATCA

AAATACCACACAACCACAACAACAACACGCAAACAAACAAAAACACGACAAAACACACCCAACACAACAACAAAACG
ACAACGGACGAAAACAGAGGAAGGAAGAGAGAGATAAAAAAGATCAGTAAGCGATAGGCAGCTATGGAATAGAGGGG
TGGGCGATGATTATCAAAACAACACCACAACACTACAACCACACCACACGAAACACACACCACAAAAAACAGAACAC
CAAAACAAAACATCAACAAACACAAACACAATGGCCACCAAAAAACAACAAAAGAAAATG
nn**10kJ01**nnnnnnnn
AGATAAAGAAGCTGTTAGAGAGAATAAAACAGAATCGGGCCCTAATACATTCTTAAAATCTGGAAATAAGCACATTA
GATTCCAAATAGTGGACACTTTAACATTTCTCTTTTCATATTTGATTAATAAATCTCGAACTAGGGACTTGTGGGTT
AACACTAGTGAAAAGAAATGTATAGGGATAGATGAAAAACCAATTGTCTTGACCCAATACATTAAAAAATGTTCAAA
GATGTTAGTTGCATTCCATCAAGTGGTTATTAACCGATTTCTGCACAAATATTGAATTAATAAGGTCCGAGGTAGTG
AGTAAGAAGTATAACGAGAGACAAGAAAGATATTAGAGAGACATAAAACGAATCGGGACCCGCTACATTCTTATATA
CACGAATAAGCCATTAGAATCAAATAGTGGATATTTTCAACCATTATTCTTTCAATATTGAATTCATTAAATCCGAA
CAAGCCGACTTGTGGTTAGATAAACCAATTGTTGCCTTACAGCCATCCAAAAATGTTCCAAAGAAGTAACTAGCGTT
CCAACTAGGGGATATTTTACAGATTAGTTCGAAAGTTTAGAATTAATAAAGGTCCGAGGATAGTGAGTTGTGAGTAT
AACGAGGAGATAAAAGAAAGCGTTAGAGAGACATAAAACAAATCTAAGGCCCTATACATTCTTAAAATCTGGAAATA
GGCCATTAGAATTCCAAATGGTGGAAAACTTTAACAAGTTCTCTTTATATTTGATTCAATAATCTCGACCCTTGACT
TGTGGTGACAAGTAAGTAAAAAGAAATGTTGTAGGGATATGACAAACCAATTGTTGCCTCATAACATTTAAAAAAT
GTTCAAAGATGTTAGTAAGCCATTCCAGTCAAGTGGTTATTTTAGCCGATTCTGCACCAAATTTTGAATTAAGTAAT
GGTCGAGGTAGTGAAGTGAGAGTTAAGGCGAGAGTAAAGAAAGATATTAGAGAGACATAAAACGAAT
nn**11k225**n
GATACATTGCACTAAGCAGGACCTAACTCTGAATCAATGAATCGTTCCACACGTAAACTAGTAAACATTAATACTAC
CCGCTAGGCCTCAATATTATTTGACGTCTGCTGAGTAAGATAGAGTAGTGAATTAGAAGGCGATCCCGATCCCGGAA
GAAATATTACTAGATATAATTGCAGCTAATAAATTGTAGTTAATTTAGAATAGGCATCCCGCATCCCGAAACAAATA
TGACTAGATAACATTGGCCACTAGCACACACGCAGACCTAATTATGACATAATGAACTCGTTCAATGTGCACTAGTA
AACAATACTAACGTAGGGTTTCCATATAGTGGTCATGCTGAGTAGATAGAAGTAGCTGAATTTAGAAGGGGATCCGA
TCCCGGAAAAAATATTACTAGATAACATTTGCACTAAGATATAGTAGTTAATTAAGAATGGCATCCGATCCCGAAAAA
TTATGGACTAGATAACATTGCACTACGAAAGACCTAATTATGACATAATGAATCGTTCCAAAGTACAGCTAGTAAAC
CCAACTACTACCGCTAGGGTCTCGATAATAGTGGTCATGCTGAGTAGAAAGAGTAAAGTGAATTTAGAAGGGGATCC
CGATCCGGAAAATATTACTAGATAGACACTTGAACAACAATAATAGTAGTTAGATTTAGAATGGCATCCGATCTCGA
AAATTATGACTAGATACACATTGCACTCCAGCAGACCTAAATTATTGACATAATGAATCGTTCCAATGTACATAGTA
AACCAATACTACCGCCTATGGTGCTCATATAGTTGTCAAAGCTGAGTAAAGATAGAGTAGCTGAGTTTAGAAGGGGA
TCCGATCCCGGAAAAAATATTACTAGATAACATTGCACTAAGATATAGTATTATTGAGAATGGCATCCGATCCCCAAA
AATTATGACTCAGATAACATTGACTAAGCAGGACCTTAATTCTGCATAATGAATCGTTCCAACGTAA
nn**12kJ05**n
TTTTGTGATCTCAAGTGAGGTTAGCATGTCCTTTTGGCCTTGCTTTTGTGATCTTTCAAGTGAGGTTATCATGTCCC
TTGTTGGCCTTGCTCTTGTGAACCCTCAAGTGAGGTTAACATGTCCCTTTTGGCCTTGGCTTCTGTGATCTCCAAAG
TGAAGGTAACATGTCCCTTGTGGGCCTTGAAATCTATGTGACCTCAAGCTGAGGTTAAGACGTCCCTTCTAGCCTTG
CTCTTGTTGACCTCAAGTGAGGTTAACACGTCCCCTTTGGCCTTTGCTGTTTTGTGACTCAAGGTGAGGTTAACTAC
GCCCCTTTTGGCAATTCCCCTTTTGTGACCTCAAGTGAGGTTAACACGTCCCACTGGCATTCAATTAGTGATTTCAA
GTGAGGGTTAACCTTTTGTCCGCCTTGTTGGATTGTGGGTAATGTAAAATTTTGTGGTGTGTTTCAAGTGAGTTAAC
ATGTTGTCCCTTTTGGCGTTGTTGGATAGGTGGGCTGGGTCAAGTGTAAAATTTTTTGTGTGCTTGAAGTGAGGTTA
ACATGATCCTTTTGTGATCTCAAGTGAGGTTAGCATGTCCTTTTGGCCTTGCTTTTGTGATCTTTCAAGTGAGGTTA
TCATGTCCCTTGTTGGCCTTGCTCTTGTGAACCCTCAAGTGAGGTTAACATGTCCCTTTTGGCCTTGGCTTCTGTGA
TCTCCAAAGTGAAGGTAACATGTCCCTTGTGGGCCTTGAAATCTATGTGACCTCAAGCTGAGGTTAAGACGTCCCTT
CTAGCCTTGCTCTTGTTGACCTCAAGTGAGGTTAACACGTCCCCTTTGGCCTTTGCTGTTTTGTGACTCAAGGTGAG
GTTAACTACGCCCCTTTTGGCAATTCCCCTTTTGTGACCTCAAGTGAGGTTAACACGTCCCACTGGCATTCAATTAG
TGATTTCAAGTGAGGGTTAACCTTTTGTCCGCCTTGTTGGATTGTGGGTAATGTAAAATTTTGTGGT
nn**13kJ11**n
AGGTGGTGGGTCCGCGGGAACCGTGGCGCCAGCACCTGTGGCGCTGCAAGGTGTGGGTTCAAGCAGGACCTGGTGCC
AGGCGCTGCTTACGCACGACTGAGGTGGTGGGTTGCAGGACCTGGCATGAGCACTGTTGCACCGCGAGGTGGGGTCT
GCGCGGACTTGGCGTGAGGCGTTGTTACACGAACCGACGGTGGATCTGCGCGAACTTGAGCGTGAGGCATTGTTGTG
GATGGCTGGTGGTGTCTGCGAGACCTGGCGCGAGGCGTTGCTGGCGCGGCCGAGGTGGTGGGTTTCGGCGGACCTGG

CGCGAGGCCTGGCTACACGACCGAAAGCTGGTGGGTCGCGGGCACTGGCGCGAGGCGCGCTGCGCCGGTCAGGAGCG
GTCCGCGGGACTGGTGCGAGGCTGATGCGGCCGCAGCCGAGGATGTGTCTAGAGGAACCCACAGGTGCGAGTGCTGC
TACGCGGCAACGGTGTTGGGCTTCACGGGACTTGTGCCTGAGTCGCTGCTGTGTGTACCGAGGTGGTGGGTCTCCGA
GACCGCTGGCACCGAGGCGCTGTTTGCGCGCGCAACCCGGTGGATGGGTCCACGGGACCGCTTAGTGCGAGGCGTTG
CTATGCGGCTGAGTGGTGGTCTTGCGAGACCTGGGCGAGGTGTTGCGGTCCACGGACCCCCACCCCCCTGCGCGTCG
CCACGCGGCACTGCCTAGCGCGACCCCGAGGTTGTGTTGCAGGGACCTTGGCACCGAGATGCCTGCTCGCACGTGGC
CCGCAGTGCCGTGGGCTCTCGCGGACTTCGAGAGACGCTACCTCGCGCGTCAGGCTGGTGGGGTAACACGGACCGTG
GCGCGAGGCGTTTCTGCGGACCCATCGAGGTGGTGGGTCAACGGGACCTGTCGTGAGCGTGCTGCTCGCGCCGAGGT
GGTGGGTTCCCGGGACCATGGCGCTGAGGCGCTAGGCGTTCTCGTGGCACCTGGTGCGAGGGTTCCT

nn**14k060**n

GGTGGGACCGCCTTGGGCTCCGGTCCTCTTTATTTATAACTCCGGACATCGTTTCGTCCGGGTGGGACCAGCCTTGG
GCTTGGTCCTCTTTATTTATACCCCCGGACATCGTTTCGTCTGGGGTGGGACCGGCCTTGGGCGTCGGTGTTCCCTT
TATTTATAACCCCCGGACTTTGTTCGTCCCGGGGTGGGACCGGCCTTGGAACTCGGTCTCTTTATTTATACCGGACA
TCGTTCGTCACGGAGGTGGGACCGGCCTTGGCTTGGTCCTCTTTATTATACCACCTCAGGACATCGTTCGTCGTCGG
GGTGGGACCGGCCTTGGGCCCGGTTCCTTTATTTATAACCATCCCGGACATCGCTTCGTCGGGGTTGTGACCAGCCT
TGGGGCTCGGCCCTCTTTATTTATACCCCCGGACATCGTTCGTCCGGGGTGGGACCGGCCTTTGGGCTCGGTCCTCT
TTATGTTATACTCCGACATGCGTTCGCCGGGTGGGACCGGCTTGGGCTCGGTCCTCTTTATTTATACTCCCGGACAT
CGTTCGTTCGGGTGGGAACCGGCCTTGGGCTCGTTCCCGTTTATTTATACCTCCGGACATCGTTCAGTCCGGGTGGG
ACCGGCCTTAAAGGGCCTCGGTCCTCTTGTATTTATACCCCCGGACATCGTTCAGTCCGAGGTGGGACCGGCTTGGG
CTCGGTCCTGCTTTTATTTATACTACCCGGACATCGTTCGTTCGGGTGGGAACCGGCCTTGGGCTCGTTCCCGTTTA
TTTATACCTCCGGACATCGTTCAGTCCGGGTGGGACCGGCCTTAAAGGGCCTCGGTCCTCTTGTATTTATACCCCCG
GACATCGTTCAGTCCGAGGTGGGACCGGCTTGGGCTCGGTCCTGCTTTTATTTATACTACCCGGACATCGTTGGACA
TCGTTCGTTCGGGTGGGAACCGGCCTTGGGCTCGTTCCCGTTTATTTATACCTCCGGACATCGTTCA

nn**15kJ08**n

CTAAGGATAAGTGGTAGTAGTGGTGGTGTAGTAGATGGTCGGTAGAGTAGGTGGTAGTAGTAGTAGTCAGCTAGTCA
GTGGTAAGCTAAGGATAAGTGGTAGTAGTGGTGGTGTAGTAGATGGTCGGTAGAGTAGGTGGTGGTGTGGGTGGTAG
TAGATGGTGGTAGTAGTAGTGGTGGTACGTAGCCTCGCTGGTGGTGCGTGGTGGTAGTAGTGGTGGTAGTGGTAGTA
GAAGTAAGAAGTAAAGTTAGAAGTGACAGTAGAAGTAGAAGAACTGAAGTAGGAAGCTAGGAAGATAGTAGCTAGAC
TGGATAGTAGTAGTAGTGAGTAAGTAGATAGTGGTTGGAGTAGTCCAGATAGTGGTGGCGTAGTAGTAAGTAGTAGT
AGTCAGTCGGATAGTAGTCAAGTGGTACGTCAGTAGTGGTCAGTAGCCTGGGGTCAGTAAGCTAGTGGACTGGTAGT
AAGTAGCTTGTGGTGGTGGTGGTAGTAGTGGCTGTCAGTACGATGGTAGTAAGTGAACAGAAGTAGAAGCCTAGTAG
TGGTAGTCGGATATAGTCAATAGTAGCTAGTAGTGTAGTCAGAAGCATGAAAAGTAGAAGTAGTGAGTAGAAGGTAG
AAGTAGAGATGTAACCCGTGAGTAGCTAGTAGAAGTGGTAGTAGTAGTGGTGTAGTAGTAGTAAGTAAAGTCGTAGA
TAGTCTGGTGGTAGTAGTAGTAGTAGTAGTAGTAGATGGTAGTAGTACGTGGTAGTAGTAGGCGTGGCTAATAAAAGCTG
GGTGGTAAGTAGATAGTGGTGGTAAGTAGTAGTGGTGGTCGGTGGTGGTAGTAGATGGTGGATAGTAGGTGGTAGTA
GGTGAAGTAGAAGGGTAAGAAGTAGAAGTAGAAGGTAAGAGATAGAAGATAGGAAGTAGGAAGTAGAAAGTAGAAGC
TAAGAAGTAGAAAGTAGAAGCTAGGAGTGGGAAGTGGAAGTGGAAGTGAACACTGGAAGTGGAAGTC

nn**16k135**n

CTTTCGTTTCGCAAGCTCACAGCTCCGGGTCCCGTAAAAGGCGACAAGATGGCACAGATCAGCGACCCCGTGTATAG
CTTTTGCTTCGCAAGCTCAAGCTTCCGGATCTCACAGAGAATATACGTGGGGCAACGACCAAGCGACTCGATGGATG
CCGTTTCTTCCGCAAGCTTCAGCTCCGGGTCGCGTAAGGCAGGTAAATGCATTCGATCCGCAGACCGCTATGTATAA
GCTTTCTGCTTTTCGCAAAGCTTCAAGCCTCCCGGATCCAAACAGAGTACACCATGGCACAAACTCGACCGTCTGGA
TGCTTTTTGCTTCGGCAAGCTCCCCAGCTTCCCCGGAGTCCCGTAGGCAGCGTAATGCATGATCCGCGGACCGCTGT
TAAGCCTTGCTCTCGCAAGCTCAGCTCGGATCTCAACAGAGCAATTCATGGCATATCCGCGACCCTATTAGCTTTTG
CTTCGCAACACTTCAGCTCCGGATCCCACACAGAGTAAACAACCAGGGTGATCCGCGACCACCTGGACTGCCTTTGC
TTCGCAAGCTCCCAGCCTCCGGCGTCCCGTAAGGCGAGGTAATGCATGATCCCCGCGACCGCCTGTTAGCCCTTGCT
TTCGCAGCTTTCAGTTCCAGATCTAAACAGAAGCAAATTCATGGATGAGCCCACCCGACCGCTTTTACCTTTTGCTT
TCGCAAGCTTTCACTCCCAGAGTCCCCAACAGAGTAAACACATGCGTGATCCCGCGACCTCCTTGGATGCACTGGCT
TCGCAAGCTCCCAGCCTCCGGCGTCCCGTAAGGCGAGGTAATGCATGATCCCCGCGACCGCCTGTTAGCCCTTGCTT
TCGCAGCTTTCAGTTCCAGATCTAAACAGAAGCAAATTCATGGATGAGCCCACCCGACCGCTTTTACCTTTTGCTCT
TCGCAAGCTCCCAGCCTCCGGCGTCCCGTAAGGCGAGGTAATGCATGATCCCCGCGACCGCCTGTTA

nn**17kJ14**n
CTCCCAAGCCTCGGCATGGCCTCGCTGGAGTTCCCTAATCGGCGTCCGCGCCCATGGCCTTCGCTTAGAGTGCCCAT
GGCCTTCGCTGGATGCCTCTTCAAGCGTCGGCGCCCGTGGCCCCAAATATGGCCTCGCATGGCCTCGCCCAAGTATA
ATGGCCTCGCCCAAGTTGGCCTCCCATGGCCTTCGCCGAGTATGGCCTCGCATGGCCTCGCCAAAGCATAACGCCAT
CCACGTGGCCCTCGCATTGGCACATCGCCCAGTATGGCCTTGCGTAGCATCAGCAAAAATAATGGCCTCCGCATGGC
CTCGCCCAAGCATAATGGCCCCACATGACCTCGCTGAAGTTGGCCTCCGCATGGAACTTCGTCAAAGCAATACCTCA
CGTGGCCCTCGTATGGCCTCGCGTTAAGCCTCACCCAAACATGGACTCTGCATGGCCTGCGCACACGATCATGGCCA
TCGGCGTGGCGTCAAGCCAAGTGTTGGGCCTCCATGGCCTCGCCCAAAAAAAGCATGCCTTCAACCGTGGCATCCGC
GTTAGCCTCTCAGCCAAATATGGCCCTTCGCTGGCCTCAGCACCAAGTATGGACCCTGGCGTGGCTCAGCCATTGTG
GCCCCAACATGGCCTCGCCCAAGTATGGCCTCCGCATGGTCGCCAAGTTATAAGGCCTCGCGTAGCTCAGCCAAATA
TGGCCTCGCCCAAAAAAAGCATGCCTTCAACCGTGGCATCCGCGTTAGCCTCTCAGCCAAATATGGCCCTTCGCTGG
CCTCAGCACCAAGTATGGACCCTGGCGTGGCTCAGCCATTGTGGCCCCAACATGGCCTCGCCCAAGTATGGCCTCCG
CATGGTCGCCAAGTTATAAGGCCTCGCGTAGCTCAGCCAAATATGGCCTCGCCCAAAAAAAGCATGCCTTCAACCGT
GGCATCCGCGTTAGCCTCTCAGCCAAATATGGCCCTTCGCTGGCCTCAGCACCAAGTATGGACCCTG
nn**18kJ17**n
AGGAACCAAGCAAaTATGATGCTCAAGCTTCCATGGACATCAAGGAAACCAAGAAAGAGGATGCTCAAAAGTTTTGA
AGGAACCTAAGTTCCAACCAAGATGAATGTCTAGGGTTTCCGAGGACACCTCAAGGAAAAAAGCAAGATGAATTTGC
CTCAAAGGAATTCCGAGGAGCTCGAGGAACCAAGCAAAAGGAATCTACAAGGTTCCTAGGACAATCTAGGAACAAAG
CCAAATAAATAATCAGGTTTCGAGGACCTCTAGGAACCAGAAAAAAAGAATGCTTGAGGTTCCTCAGGACCTCAAGA
ACCAAGCAAGTGAATTCTCAAGGTTCTGAGGACACTTCCGAAGAACCAAGCAAAGTTTGAATACTAAAGGTTCCGAG
GAATGCTGAAACCGAGCAAGATGAATTACTGAGGGACCTGTGAGGACCAAAGAACAATGAATGCTCCAAGCCTCATC
GACCATCAAGGAACCCAAGCAAAAGGAATGGCTCAAGGTTCTGAGGACCTCTAGGAAAGAAAGCAAGATGAAAGCTC
CAGGTTCTGAAGGACCATAGATACACAAAGCAAGATGAATGCTCCAAGTTTTTGGGACCTCTAGGAACAAACAAAAT
GAATGCTTCAGGTTCTCCGAGGAACTGAGGAACAGCAAGAGAAATTTCAAGGTTCCTAGAACCTCCTAGGATCAAAG
AGAGATGAGTTCTCAAGGTTCCAAGGACCGTCGAAGGACCAAGCAAAGGAATGCTCAAGATTCTACAGGACCTCTAG
AGAACAAAGAAAAATTGAATGCTCAAGGTTCTGAGCGCCTCTAAGAACCAAGCATGAGGAATACGCTCAAGGTTCTG
AGGACCCTTAGGAACCAAGCAAGAGGATGCTTCAAGGTTCTTACAGGCCTTCTAGGAACCAAGCAATGATGAATGCT
CAAGGGTTCCGAGGACCATTGAGGAACCAAGCAAAAGGAATGCTTAAAGGTTTTGAGGACCTCCTAG
nn**19kJ35**n
TGGTCAAGTTCCTAAACTTACCCAAAAGCCTGTATGAGGCCCCCATCCCGATCTATATCGATGCCTATCGAGGTATT
CCCTAAACTACCCCAACAAGTCCTGTGCCATCGAGTGCACAACATCTATATCCAATGCCTAATCGAGGGGCGCATTC
CCTAAACTACCCATAAAGACGGACCATCGAGGCCCCAATCGATTCTTCTATCGATGCCTAAGCGAGGTATCCCTAAA
TAACCCCAAAAGCCTGTGCCACGAGGCCCCAATCGATCTATACCGATGCACAATCGAGGATTTCCCTAAAACTACCC
AAAAAATACAGACCATTCGAGGCCTCCAATCGATCTCTATGATGCCTACAGAGGTATTTCCCTAAACTACCCAAAAG
CCCTGTCCATCGAGGCCCCATTCGATCCTATCGATCCTATCGAGTATTTCCCAAGCTTACCCAAAAGACGACCCAGC
GAGCCTATCGATCTCTATAGATGGCTATCGACGGTATTCCTATAACCTTACCCAAAAAGCCTGGCCCATCGAAGGCC
CCATCGATCTATATCGAGCATCAATACTTCCCTAGTAACTACCCAAAAAATCCTGTACACATAAAAGAGGCACCAGC
ATCCCGATCTCCATACCGATGCCGATCGAGGTAATTCCCAAACTACACAAAAAGCCGTACATCGAGGGCCCATCGAT
CTCTATCGATGCCTATGCGAGTAAATTTCCTTAAGCTACCCAAAACCTGTGCATCGAGGCCCCATCACGGATCTATA
TCGAGTGCCTATCGAGTACTCCCTAAACTACCCAAAACCCCGTACCATCAAAAAGGCACCATCGATCTCATCGATGC
CCTATCGAGAGATTCCCTAAACGACCCAAAAAGCCCATACCATCGAGGCACCCATCGATTATCTATCGATGCCCCTA
TCCCGAGGGTATGCTATAAAATGTTCCCCAAAGTCTGGATCATCGAGTCAACCCATGCGATCTCTAT
nn**20k150**n
TGGGTTATCAATAGGGTTTCGATAGGTAGGTGGTTTGGGTTCATGGGTTCAATGGGGTTCGATAGGGGTTTCGAGTA
GTGGTTCGATGGGGGTTCGATAGGCAGATGGGTTTGGGTTCCGATGGGCGTTCGATAGGGATTCAATAGTGGTTCGA
ATAGGGGGTTTCAATAGTGTTCGATCGAGGTTTATAGGGGGTTCGATAGGGGTTCAAGTAGTGTTCGATAGGGGTTT
CGTATAGTGGTTCAACTGGGGTTCGATAGGCAGATGGTTTGGGTTCGAATTGGGGGTTTGATGGGGTTCGATAGGGA
TTCGATAGTGGGTCGATTAGGGGTTTCCAATAGTTGCTTCGATCGGGGTTTATAGGAGGGTTCGATAGGCAGATGTT
TGGGTTTCATGGGGTTCGAATATGGTTCGATGAGGTTCGATAGGCAGGTGTTTAGGTTCATGGGTTCGATTGGGGGT
TCGATGGGGGTTCATGGGGTTCGATAGTGGTTCAATGGGGGTTTTATAGGGTTTTCGATAGGCTGATTTGGGTTATC
AATAGGGTTTCGATAGGTAGGTGGTTTGGGTTCATGGGTTCAATGGGGTTCGATAGGGGTTTCGAGTAGTGGTTCGA

TGGGGGTTCGATAGGCAGATGGGTTTGGGTTCCGATGGGCGTTCGATAGGGATTCAATAGTGGTTCGAATAGGGGGT
TTCAATAGTGTTCGATCGAGGTTTATAGGGGGTTCGATAGGGGTTCAAGTAGTGTTCGATAGGGGTTTCGTATAGTG
GTTCAACTGGGGTTCGATAGGCAGATGGTTTGGGTTCGAATTGGGGGTTTGATGGGGTTCGATAGGGATTCGATAGT
GGGTCGATTAGGGGTTTCCAATAGTTGCTTCGATCGGGGTTTATAGGAGGGTTCGATAGGCAGATGTTTGGGTTTCA
TGGGGTTCGAATATGGTTCGATGAGGTTCGATAGGCAGGTGTTTAGGTTCATGGGTTCGATTGGGGG

nn**21kJ44**n

GTGGCTGGACATCTTGCGAGGCCCACTTCGGGCCGGTGGTGCCTAAGTTCCGCAGAGGCCCACTTCGGGGAGGCATG
GCCTAGGGCCCCCAACTCGCGAGGCCACTTTGGGAGGTGGTGGCTTAGGCGTCTCGCGAAGGCCCTTGAGAGGTTGG
GTGGCCTAGGCAACTCGCGAAGGCAACTTGGTGGCATGGCCTAACCATCTCGCGAGGCCACTTGGGTGGTGTGCTCT
TAGGCGTCTCGCGAGGCTACTTGAGAGGTGTGGCCTAGGCACTCGCGAGGCCACTGGGTGGCATGGCCTAAGCAATC
TCGCGAGGCCACTTGGGTGGCATGGCCTAGCATCTCCGAGGGCCACTTAGGGAGGCATGGCCTGAGGCAACTCGCGA
GGTCCCACTTGGGTAGGCGCATGGCCTAAAGGCATCTCGCGGCCACTTTTTGAGGTGTGGCCTAAGAACAACTCGCA
AGAGCCACTTGGGTGACATGGCCAAGCATCGCCCCCCCCCCTCGCGAGGTCACTTTTGGGAGGCATGGCCTAGGCAT
TCGTGGAGGCCACTTAGGAGGCATGCCTAGAATATCACGAGCCACTTTGGGAGGTGTGGCTTGCATCTCGCGGAGGA
CCAATTGGGAGTGTGGCCTACGAAAAAACTCCGAGGCCCACTTGGGTGGCCCGTGGTCTAGGGCATCTCGCGAGGCC
ACTTTGGGGAGGCGTGGCCTAGGCGTCTCACCGAGGCCACTTAAAGAGGTGTGGCCTAGACACTCGCCGAGGCCACT
TTGGGGAGGCGTGGCCTAGGCGTCTCACCGAGGCCACTTAAAGAGGTGTGGCCTAGACACTCGCCGAGGCCACTTTG
GGGAGGCGTGGCCTAGGCGTCTCACCGAGGCCACTTAAAGAGGTGTGGCCTAGACACTCGCCGAGGCCACTTTGGGA
AGGCGATGGCCTAGGCGCTCCGCGAGAGCAGTGGGGCCGAGTTGCATTGAGCGGATGGGTCTCGC

nn**22k390**nnn

TTTATTTTAAATTATCACTATTATAAATTAAAATAATTTCTTGTTTATAAAAATGAAATAAGTTTATTTAATTAAAA
AATAATTTATTAATTTATTCATAATTTCAAAAATTATAATATATGTAGTATTAAAATTAATATATATATTATACTTT
ATTTCTACCCAAAAAAACTTTTATATATGTATTGTTTAATAAATTAATATATTTTAAATTTTCAATAATATAAACAA
ATTAGAATATATTTAATAATGGTATTCTTTATAATGACAATTGGTAGGTCGGTTCAACTCAAACGAGACCTACCATG
GTCACATTGAGAAGTCATCTTCCACAAAACCGACCCTACATGTCCTCTAAAAAATTATTAAAGAGATACACTAAATT
ATATTATAAATTTTTTTTTATTTTTAAATTATCACTATTAATAAATATTAAAATATTTTCTTTTTTTATAAAATGAA
ATAAATTTATTTAATTATAAATAATTTATTAATTTTTCATATTTAAATTATAATATTATGTAGTATTAATAATTATA
TATTTTTAACTTATTTCCATACCCAAATAAATTTTATATAAGTTTGTTTAAATAAATAAATATATTTTAATTTTCAA
TAATATAACATATTAGAGTATATTTAATAATGTATTTTTATAATAATGGGTAGGCTCGGTTCTACACAACAGTCCTA
ACCATGTCCATGAGAAGTCATTTTCCACACCAACCGACCTACAATGTCCTCTAAAAAATTATTATAGAATAGACAAA
TTATGTTATAATTTTTTTATTTAAATTATCACAATTATTAAATTAAAAATTTTCTTGTTATAAAAATGAAATAAATT
CATTTAATTATTAAAAATAATTTTAATTCATTTATTCATTTTTCAAAAATTATAATATATGTGTATTAATAATTAAT
ATATTTTTATACTTATTTCATACCCAAATAAATTTTATATAGTATTGTTTAAATAAATTATATATTTTAAATTTTTC
AATAATATAAACAAAGTAGAAATTTATTTAATAATGTATTTTTCTAATGACATGGGTAGGTTCGTTCAACACAACCG
ACCTAACCATGTCACATGTGAAGTCCTTTCACACTCACCGCCTAACCATCGTCTTCTAAAAAATATTAAAGAGATTA
CACTAAATTATGTTATAAATTTTTTTATTTTAAATTCATCATATTATTAAATTAAAATATTTTCTTGTTATAAATGA
ATAAATTCATTTAATTATAAATAATTTTTTTTTTTTTTTAAAAATTTATTCATATTTCAAAAATTATAATATATGT
AGTATTATAATTATTATTTTTTATACTTATTTCATACCAAATAAATTTTATATATATATTGTTTAAATAATTAATAT
ATTTTAAATTTTCAATAATATAAACAAATTTGAGTATATTTAAATAATTGTAATTTTTATGATGACATGGTAGGTCG
GTTCAACACAACCGACCTACCATGTCACATGTGATAGTCCTTTCCCACACAACCGACCTACATGCTCTAAAAATTAT
TAAAGAAGATACACTAATATTATGTTATAAATTTTTTTATTTAAATTATCTCAATTATTAAATTAAAATATTTTCTT
GTTTATAAAAATGAAATAAATCATTTCATTATAAATAATTTATTCATTATATCATTTTTCAAAAATTATAATATATG
TTAGTATTAATAATTATATATTTTTTATACTTTATTTCATACCCCAAATAAATTTTATTATAGTTTGTTTAAATAAT
TAATAATTTTATATTATCAATATATAAACAAATTAGAATATATTTAAGTAATGTATTTTTATAATGACATGCGTAGG
TCGTTCAACACATACCGGACCTACCATGTCAACATGTGAAGGTCCTTTCCCACAAACCGACCTACCATGTCTTCCTA
AAAAAATTTTAAAGAGATACACTAATTATGTTAAAAATTTTTTTTTTTAAATTATCAACTATTT

nn**24k600**nnn

TACTCAACTAAACAACTAGAGCCAACAAACCTCGATCAAGCATAATTCATTAAACTTAAGCACCACCAGCAAGATAG
ACCGTACTTTCAAAGCATCAAAAAATTATTTTCTCAAATTGACTTAAATTGAAATGCGCCACAGCAGAAGTATTTTT
GAATTGAATACCAACACAATTCCCATGAGAGACATGTTAGGTGTAGACATTAGAAAATATCTCTTTATATATGCAAA
GAATGTGTTTAATCTCAACATAGTCAAATTGCTTTATCAAATTACTTTTCCATTAACTTCAGCTTTTCAAAGAATAT

ATAAATATAAGGAATAGAAATTGAAGGGGGTTTTACTCCTTTTTTTTTGAAAAATAAAAAAAAACAAGAAGAAAAGA
AGGAAATTTGTCTTTCATACAGTGCATCTTCACAAATATGAAAATAAAATGGGAGTTAATTCTCCGGTAAATAGTAA
AAAAAAACAAAAAAAAAAGCAAAGTATTGCCCATAAATTATTCAGTTGTAACCATAACAAAAAATTAGAGTGATGGA
AGGGTATATAGAAAATAAATAAGCTAGTTGCCAAGAGATCTATTTATTCTGCAACATACGGGCAACAAAGCCAATGG
TTTCATCAAGCTAACTTCCTTTTATAGCAATACTCCAACTAACAACTAGCAAGCAACAAACATAGCTCAAGCATAAT
TCATTAAACTTAAGCACCACCAGCAAAATTAGACCTACTTTCAAATAATCAAAAATTATTTTCTTCAATGATTAAAT
AAATGCCACAGCAGAAGATTGAAAATACCACACAATTCCCAATGAGAGTACATGGTTAGGTGTAGACTAGAAAATAC
ATCATTTAGTAACAAGAGAATGTGTTTATCTCCAATAGTCAAGTTTGCTTATCAAATAAACTTTTCCATAAGCTTAG
CTTTCAAAGAATATATAAAAATAAGGAGAAGAATAAAGGGGGGTTTTTCCTCTTTTTTTTTTTAAATTAAAAGAAAA
CAAGAAGAAAGAAGAAAATTTGTCCTTCATAAGTCATCGTCACAAATATGAAAAAGAAAATGTAAATTCTCATTAAA
TAGAAAAAAAAAAAAAAGCAAATAGTGTGCCATAATTTTCAGTTGTAACAATTAACGAAAAATTAAGATATTGGAAGG
GTATTATAGAAAATAAATAAGCTAGTTTGCAAGAATCTAATTTATTCTGCATACATCCGGGCAACAAAAGCCAATGG
TTCAATCAAGCCTACTTCCTTTTATAGCAGATACTCAACTATACAACTAGCAAAGCAACAAACACTCGATCAAGCAT
AATTTGCATTAAACCTTAAGCACCACCAGGCAAATTTAGACCTACTTTTCAAAGCATCAAAAATTATTTTCTTCAAT
TGATTAAACTTAAATGCCACCCCAGCAGAAAGATTTGAATGAAATACCACACCAATTCCCAATGGAGGACATGTTAG
GTGTAGACTAGAAAAATACAATCATTTATTAAGCAAAGAATGTGTTTTAAATCTCAATAGTGCAATTGGCTATCAAA
ATTACTTTTCCATAAGCTTACTTTCAAAGAATAATAGAAATAAGGAATGAAATGAAGGAGGGGTTTTTTACTCTTTT
TTTTGAAAATAAAAAAAAAACAAAGAAGAAAGAAGAAATTTGTTTCATAAGTCATCTTCACAAATATGAAAAATAAA
ATGGAGTAATTCTCGTAAATTAGAAAAAAAAACAAAAAAAAAAGCAAGTAGTGTGGCCATAAGATTTTCAGGTTGTA
ACCAATAACAAAATTAGATTATGGAAGGTATATTTAAGAAAATAAATAAGCTAGTTGCAAGAATCCTATTTTATTCT
GCAACATCCCGGCAACAAAAGCCAATGTTCAATCAGCCTACTTCCTTTTAATAGCATACATTCAACTAACAACCTAG
CAAGCAACAAAACTACTAAGCTCAAGCATAATTCCAATTAAACTTAAGCACACTGAATACTATAGC

nn**26k050**n

TATGTCGCCTTATAGTCCTTAGGCATATGACTTGATTTAACAACAAGCCCAAGAAATATGTTGTTCAGTCACTTGGG
CATATGACTTGCTGTAGATAAGCAAGCCCTGAGAATTTATGATTGGGCATATGACTTGTTAACCGATAGCAAGCCGC
CATAATTTATGAAGTGGGCGTATGACTTGCTTAGATAAGGAGCCAAGAAGTTATATTGGGGGCATATGACTTGGCTG
TAGATATGCCAAGCCCCACAAATTTTATGGGCCATATGACTTGCTTAGGCAACAAGCCCAAGAATCTATGATGGGCA
TTATTATTATAGTCCTAATATGGATATATGTTTTTATATAGTCAACGTTTTTATAGTACATGATTATGATCAATAGT
CCTTTATGCCTTATGAAGATAGTGATGTTGTTGTTAGGTAGATTTTCCTTACTGGGCAGAGCTCACTCCTTTATTTT
TAGAGTCTGATGCAGAAATGAAATTATGGAAGGCCAGAAGGATTCTTTGGTAAGCTTGGTTGGTGTTTGAAGAATGA
ATGGATTCAATGGACTGGCGTGTCGAATCGACGGAACGACGTTATTATGTCGCCTTATAGTCCTTAGGCATATGACT
TGATTTAACAACAAGCCCAAGAAATATGTTGTTCAGTCACTTGGGCATATGACTTGCTGTAGATAAGCAAGCCCTGA
GAATTTATGATTGGGCATATGACTTGTTAACCGATAGCAAGCCGCCATAATTTATGAAGTGGGCGTATGACTTGCTT
AGATAAGGAGCCAAGAAGTTATATTGGGGGCATATGACTTGGCTGTAGATATGCCAAGCCCCACAAATTTTATGGGC
CATATGACTTGCTTAGGCAACAAGCCCAAGAATCTATGATGGGCATTATTATTATAGTCCTAATATGGATATATGTT
TTTATATAGTCAACGTTTTTATAGTACATGATTATGATCAATAGTCCTTTATGCCTTATGAAGATAG

nn**27kJ82**n

ATAACCCTTCCCCCATGCGCCTGCCAAGGCCACGCAGGGTCCTCATTAAGCGAGGAACCTTCCCCAGCATGCATGGC
TACCATTTCCCCCGCATGCGCCTGCCAAGGCCACGAGGGTCGACTATGCGAGGGGTACCCTTGCCCCCGCATGTGAG
GTTACCCTCCCCCCGCATGCGCTCCCAACCCACGAAGGGTCTCATTACTGCGAGGTGCCCTTTCCCCCACATGCGCC
TGCCAAGGCCACGAGGGTCTCACGTATGAGTACCTTCTTCCGCATCGCGAGGCTACCTTCCCCGCTGCGCACTCGCG
AAGGCGACGAGGCGTCTCACCTATGCAGGGTACCCTTCCCCGCATGCCGAGCTACCGCTCTTCACCGCATGCGCCTG
CCAAGGCACAAGGGTCTCCACTATGCGAAGGGGTACCCCATCCCGCATGCGTGGCTACCTTCCCCGCATGCGCTGCA
AGGCACGAGGTCCTCACTAATGCGAGGTACCTTCCCCCGCATGGGAGGACTACCCTTCCCCCGCACGCGCCTGCAAA
AGGCCACGAGGGTTCTCATTATGCGAGGGTCCCTTCCCCGCATGCTATGGCTACCTTCCCGCATGCGCTGCCACGGC
ACGGGGTCTCACTATGCGAGGGTACCCTTCCTCGCATGTGTTTACCTTCCCCGCATGCGCCTGCCAAAAGCCACCGA
GGGTCTCATTATGTCGAGGGTATCTTCCCCCCGCATGCGTGGCTACCCTTCCCCGCATAGGCCTAACCAAGGCCACG
AGGGTCTCACTATGCGAGGGTACCCTTCCCAGCATGTGAGGCTACCCTCCCCCGCATGCCCTGCAAGGCCACCAGGG
GTCTCACTTACTGCGAGGGTAACCCTTCCCCCTGCATGGTGGCTACGCTTCCTCCGCATGCGCCTGCCAAGCCACGA
GGGTCTCACTATGTTAGGTACCCTTTCCCGAATGTGAGGTACCTTCCCCCGAATGCGCCTGCCAGGG

nn**28k055**n

TATTCTGAAATGACCAAGTAACTTTTTATTGTGTGTTTAGTATAATCATTACTCATTGCTGTGTCACTAGGTCACTT
GTAAGTGTGGTGTTTAAGTAGAATCATTACTCGTACTATGTCACTAATGATCAACTTCTTATAGTGAGATTAGAAGA
TCATTACTAGTTTAGAGTCACTGAGGTTGCTAGTTTGTGTGAGAATAGTACATCATTTGTACTCATTCCAATCTATA
TGTCGCTTGTTTGTGTACATTAGTAAGATCATTACTCTTTTGAGTAAGTAGGTGACGTGTTAGTGTGTGTTTAGTAA
TCATTACTCGTATCAAGTCCTAGGTCAATTAATAAAATACGAGATTAGCATAATCTTACCTAGTTTTTGGTGTCACT
TGGGTTTCTAGTTGGTGGTGGAGATTAGTACATCATTACTCATTTCCGACAACACTAGGTCACTTGTTAGTGTTGAA
ATTAGTGGATATTACTAGTTATGAGTCACCAGGTGACCTATTTGTGTCCATAGTAGATTATTACTCCGTTCAGAAGA
TGATAGGCGACTTGTTAGTGTGACATTAGTAATTATAACTCGTTACAATGCACTAAGGTCACTTGTTTGTGTGAGAT
TAGTAGATTCTACCATTTTTAGTCATTAGGTGATTTGTTAGTGTGTGTTTAACTAGATCATTACACGTTCTGCTGAA
GCACTAGGTAACTAGATCACCTAGTTAAGTGCTGATTAGTACATCATTTCCTCTTCAGAGTCACTCAGTCAATTGGT
AGTGTGTTAGATTAGTAAACATCTTACTTGTTCAGAGTCACTAGGTGAATTTTTTATTGTGGTGTTTAGCATATCAT
TTCTCATTCCGATCACTAGGTGATTTGCTAATGTGTGTTTAGTAGAAAATTACTCAGTACTGATTCTCTAGGTCACT
TATTAGTGCGAGATTAGCAGATTATTACTTGTTAGAGTCACTAGGATGCTAGTTAGTGGTGAGATTA

nn**29k240**n

AAGGATATGAATTAAGATCCAATTCTACATGAGTAAAGTGTTGTCTCTAATACTATAAACACATCATCATAATAGTA
CCTTCAAAGCTATCTACCCTCAAGATCATTGATCATCATAGTAAGTTTGATTGAAATATTACCACTCTAAGTCTGTA
ATCATCATAAATATCATAAAGATAAGTTTTTGGTTTAACTTTATAAATATGTTAGACAAGTGTCGAAATCTGCTAGA
TTGTTCAAAAATGTATAATAGAGTTAAGATAATTATTCGACATGCAAATGTGTGTCTAATACTATAAACCAACAACC
ATCATAATAGTACCTCAATATATTATACCACCAAAATCCTTGATCATCATAGTAATTTGATTGAAATATGTACACCA
ATCGTAATCATCATAAGCTCATAAAGATTAAGTTATGTTTAACTTTATTAAAATGTGAGACAGTTGTCCGAAATTCA
TGGCTAATTGATCAAAAAGTAATAATGATTAAGATTCCAATTCGAATGATAAAGTGTTTTCTAATACTATTAAACAC
AATCAATCATAATAAGTACCTCAATAATTATCTACCACTAAACCTTGATCATCATATGATTTGATTGAAATATGTAC
ACTCAAATCATGTAATCATATAATCTCATAAGATTAATTTATATTAACTTATAAAATGTTAGACAAGTGTCGAAATT
CATGGTCTAATTGTCAAAAATGTATAATGGAATTAGATCCAATTCGACATGATAAAAGTGTTGTTCTATAAATAACA
CAGTCATCATAATAGTACCTCAATAAGATTATCTTACACTAATCATTGATCATCAATAGTCGTTGATTGAAATATGT
ACCACTCAAATCTGTAATCATCATAAATCTCATAAAGATTAAGTTTGTTTAACTTTAAATTTTAGACAAGTTGTCAA
AATTCATGCAAAATGTCAATATGTATAATGAATTAAGATCCAAATTCGACATGAAAAAGGTTAATTC

nn**30k070**n

TTCTATAATAATATTTAATGAAAAACCATCCACTCCATAGAATATTATAACCAATTGTTCTCCACTAATTCTCTTCT
ATAATAATATTTTAATGAAAAACATCACTTTCCAATAGATAGTTACCAATTGTTTCCTCCACTAATTCTTTCTATAA
TATATTTTTAATGAAAACATCCACTCCATAGATATTACCAATTGTTCTCCACTAATTCTCTTCTATAATATATTTTT
AATGAAACATTCCACTCATAGATATTAACCAATTGTTCTTCCACTAATTTCTTCTATAATAATATTTTATGAAAAAC
ATACAAGTCCAATAGACTATTAACCAATTGTTCTCCACTAATTCCTCTTCTATAATAATATTTTAAATGAAAAAACA
TCCCACCCTTCCATAGATATTAAACCAATTCGTTCTCCACTATTCTCTTCTATAATAATATTTTAATGAAAAACATC
ACTCCATAGATATTAACCAATGTGTTCTCCATAATTCTCTTCTATAATAATATTTTAAATGAAAAACATCCACTACC
GATAGATTTAACCAATTGGTTCTCCACGTAATTCCTGCTTCTATAAGTAATATTTTATGAAACAAACATCCACTCCA
TAAGATATTAAACCAATTGTTCTCGCTAATTCTCTTCTATAATAATATTTTATGAAAACATCCACTCATAGTATTAA
CCCAGTTTGTTCTCCACTAATTTCTTCTATATATAATATTTTAATGAAAAAATCACTCCATAGATATTGAAACCCAA
TGGGTTCTCCACTATTCTTTCTAATAATGAATTTTTAATGAAAAACATCCACTCCATAGATTATTAACCAATTGTTC
TACCACTAATTCTCTTCTATAATAATATTTTATGAAAAAGCTCCACTCCCATAGATATTAACCAATTGTTATCCGAT
CTAATTCATCTTCTATAATAATATTTTAGATGAAAAACATCTCAATAGATATTACCAATTGTTTCCC

nn**31k350**n

TAGTGAATAAAAACACCAAGTTAAACGCAAACATTCATGTGAATACCGCCAGGGTTAAAGCAAACATTCGTGTGAAA
TAACATTAAGCTCCAAAATTTAAGCAATAAGAGTGTGAGTTTAGTATAATTAGTAGGACTTATGAAATATATAGAAT
TTTTAAGAGTTAGAACACTAAGTACTCAAGCGAAAATATAAGGCATATATTAGAGTGACTTTACTCATTCAGCAAAG
ACTTATAATGTTTTAAGTCTAGAAAGCTTAGAATTTTCAGTTACGCAAAGAAGAGTAAAGTCTAATTGGCCAACAGC
TCGATGTACGAGCAAATATAATAAACTCTGCATAGTGAAATAAAATCCACGCAAGTTAAAAGCAAAATTCATGTGAA
ATCACCAGGTTAAAGCAAACATTCGTGGTGAAATAAAACATTAAGCTCAGAAAATTTTAGAGCAATAAGCGTGTGAG
TGAGTATAATTAGTTAGGAACTTATGAAATATCTAGAATTTTCTAAGAGTTAGTGAACACAAGTACTCAGCGACCAA
TATAGGATATATCAAGAGTGACTTTACTATTCACAAAAGACTTTAATGTTTTAAGTTCTAGAGATTAGAATTTTCAA
GTTATGCAAGAAAGTAAGTCTTAATGCACAGCAACGACTGTAGAGCACATATAATTTTTTAAACCTTTGGTTAACCT

GCATAGTGAAATAAATACGACCAAGTTAAAAGCAACATTCTGTGAATACCCGCCAGGCTTAAAAGCAACAATTCGTG
TGATAGAAAAATTAGCTCCAAATTTTAGAGCAATAAGAGTGTGGAGTTTAGTATAATTAGTACGCGAACTTATGAAA
TATCTAGAATTATTCTAAGAGTTAGGAACACTAAGTCCCTCAGGAAAATATAAGGCATATATAAGAGTGCTATTACT
ATACAGCAAAAGAATATAATGTTTGTCAGTCTTACGAAAGACTATAGAATTTTCAAGTTAGCAAAGAAAAGTAAAGT
CTAAATGCAAGCACGATGTCGAGCAAAAAATATACATAACTAATGCATATGGTGAAATAAAATACCACAGTTAAAAG
CAACATTGTTTGTGAATACCGCCAGGTTAAAAGCAAACATTCGTGTGAATAAAACATTAAGCTCCCAAAATTTTTGA
GAGCATAAGGGTGTGAGTTACGTATAATTAGTAGGACTTATGAAATAGTCTAGAATTTCTAAGAGTTAGAACCACTA
GATACCTCAAGCGAAAATGATAAGGCAATATATCGAGTGACTTTACTATTCACAAAGACTTATAATGTTTTAAGTCT
AGAAGATTGAATTTTCAAGGTTAGCAAAGAAAGTAAAGTCTAAAATCCAACGCTCGCTCGCTAGCAAAACGAGCAAA
TATAATAACTATGCATAGTGAAATAAACACACCAAGTTAAAAGCAAACATTCATGTGAATACCACCAGGGTGAAAAG
CAACATTGTGGTGGAAATAACATTAAGCTCAAAATTTTAGAGCATAAAGAGTGTGAGTTAGTATATTAGTAGAATTG
ATGAAATATCCTAGGATTCTAAGATTACGAACCTAAGTAGCTTCCAAGGCGACAAATATAAGGGCATATAAATCAGC
AGTGAGCTTTATATTCCACAAGACTATATAATGTTTTAAGTCTAGAAGAGCTCTAGAATTTTCAAGTTAGCAAAAGA
AAGTAAAGTCTGAAATTGACCAACAGCACGACTGCTACGGCAAAATATAATAACATATGCATAGTGAAATAAAATAC
CGACCAAAGTTAAGAAGCAACATTTCATGTGAAATACGGGCCAGGTTAAAAGCAAACATTCGTAGTAGAATAAAAAT
TAAAAGACCACAGCTCACAAGAATTTTAGAGGCAATAAGAGTGTGAGTTAGTACCATTAGTAGGAACTTTATGAATC
ATCTAGAATTTCTAAGAGTTGAGAACACTAGAGGTAGCTCAAGCGAAAATAAGGCATATATCAAGA

nn**33k360**n

ACACAAAGGATAGCTCGATCAAGGAAGGTACGTGAAGAATGGCACAACGCGTTAAGGATTTGATGAGGACTTACCAT
ACCGTCCACGTCACTTTTGTTAACATTAGAATTTTAACTGAGATATATCTTAAAATCTTTACTTAGACTTTCTTGAA
GATATACAAACTTTTGTTTTCTAAATATATTACAATGTCGATTCTATGTTATGAGGATATGAAAGACTTTCGTGGCA
AATGAGTTTTCCAGATGCGATAGCTAACTAGTAACTTTAGTGAAAATATTCAATTATTAAAGTTCTAGATTTACCTT
AAAATTAAGTATATTAATTCAACTCATGCAATGATTTTAATTTTAGGTGTGGCGGAAAAGAACACTTACAACAAAGG
ATGTGAATCAATGATGTACGTGAGAGAAGTGGCACAATGCGTTAAGGATTTATGAGTAATACGTAGGTCCTGCTAAC
TTTTGTTAACATACAACTTATAACGAGATATACTTACAATGTTAACTTTAGATTTTCTTGAAGATATACACACTTTT
GATTTCTAACTATAGTATAGAGTGGATTCTATGTATATGGATGATGACAGACTTGTGACAAATGAGTTTTCAATGCG
ATAGCTAACTATTAATGTATGTGAAAATATTCATTATTAATGTTCTAGGTTTAACCTTCCAGATTATTATTTTATTC
AAACTCATGCAATGAATTTTGTGTTAGTATGCCGGGAAGAATCTTATATAAGATGAGATCATGAAGTACGTGAAGAA
TGGGCACAATGCGTCAAGGACTCTGTGCATGAGTACCACATACAACCCACTCACTTTTGCTGTAATTAGACTTCTTA
CGAGATATACTTACAATCTTAACTTAGACTTTCTTGAAGATATACACAACTTTTTTTCTGTTTTCTAAATATAGTAC
AAATGTAGATTCTATGTTACTGAGGATGATGGAAAGACTTCGTGGCAAATGATTTCATACGATAGCTAACTAGTAAC
GTAAGTGAAAATATTCATTATTAAAGTTCTAGCTTACCTAAAAAATTAAGTATATTAATTCAAGTCAATGCAATGAC
TCTTATTTAGTGTGGCGGGAAGAACACTTACACCAAAGGATGGTGATCAATGAAAGTACGTGAAGAATGGGCCACAA
TGCGTTATGGATTTGATGAGTACTTACATAGGTCCGCTCACTTTGTTAGACATACACTTAGTAACGAGATATACTCT
CCATGTTAACTTAGACTTTCTTGAAAGATATAAACATTTTGATTTCTAACTATAGTATAGAGTGGATTCTATGTTAT
GAGGATGATGACAGACTTCGTGACAAATGAGTTTTTTTCAATGCGATGGCTAACTAGTAAACGTATGTGAAAATATT
CATTATTAAAGTTCCTAGGTTTACCTTAAGATTAAATTATATTATTCAACTCATGCAATGAATTTTGGTTTAGTATG
CCGGGAAGAACCTTATACAAAAGGATGAGATCAAAGAAGTACGTGAAAAAATGGAGCAACGCGTTAAGGATTTGGATG
AGACTACATACGTCCTCACTGGGCTTTGTTACTTAGAACTTTTAACCGAGATAATACTTACAATCTTTACTTAGAGC
TTTCTTGAAGATATAAAACTTTTGTTTTCTAAATATATTACAATGTAATTCTATGTTAATGAGGATATGAAAGACTT
CGTGGCAAAATGAGTCTTTCAAAACGATAGCTAACAAGTAACGTAAGTGAAAATATTATTATTAAAGTTCCTAGCTC
TTACCATTAAAATTAAGTATATTAATTCAAGTCATGCAATGACTTCTTATTTTAGTGTGGCGGGAAGAACACTTAAC
ACAAAGGGAATGTGATCATGAGTACGTGAAGAATGGGGCCACAATGCCGTTATGGATTTGATGAGTACTACATAGGT
CCGCTCCACTTTTGTTTAACAATAACACTTATAACGAGATATACTATCCAATGTTAACTTAGACTT

nn**35k100**n

ACTCGATTATTCCTCATTCCAAGTCCACCAAGTGCTATGTTAAGTCGTAAAATAGTGCGATTATTCCTAATTCCGAG
TGACCAAGTATCAAGTTTGATCTGAAACAACTCAATTATTCCTCATTCTGAGCCCCAACAAGTAAACTAAGTTTAGT
GTTAAATAAATGTGATTAATCCTAATTCCGAGTAATCCAAGTACTATGTAGTGTGAAACAACTCGATTATTCCCATT
CCAAGTCACCAAGTGAATGTTACTTGTGAAATAATGCAATTAATTCCTCATAACGAGTCCACCAAGTACAAATTAAG
TGTGGAAACAACTCAAGACCTATTCCTCATTCCAAGTCCACCAAAGTACCAATGTTAGTATGAAACAACTCGATTAT
TGCCAAATTTTGGAGTGACCAAGTTGGCAAAGTTAAGTGTGAAACAACTCGACTATTCCTCAATTTTAGTCACCAAG

TACTATGTTAGTGTGAAACGAACTCCGATTATTCTTCATTCCGAGTCACCAAGTTCTATGTTAGTGTGAAATAATGG
CTTATTATTCCTAATTCCAATGTGACCAAGTACCAAGTTGGCGTAAACAAGTCGATAATTCCATTATTCTGAGTTAA
AAGTACTAAATGTTTAGTGTGAAACAACTCGATTATTCCTCCATGTCCGAGTTCCAAGTACCAAGTTAGGGTGAAAC
TAACTCAATTATTCTAATTCCCTAATGAACAAGTACAAAGTAAGTGTGAAACAATCGATTATCCTCATTCCTGAGTC
ACCAAGTACTATGCTCAGTTGTGAAAGCAACTTGATTATTCCTCATTCTGAGTACCAAGTGCTATGTTAAGTGGTGA
AAATTAGTGGTGAAACAACTCCCGATCATTCATCATTATGAGTTCAACCAAGTACTATATTAGTGTGAAACAACTCG
ATTATTCCTAAATTTTGAGTGAACCAGGTATAAATTTTTGTGTGAAATAGTGTGAATTAATCCTCAT
nn**36k280**n
CAACATCATAATTCTTTTAAACTTCATTTAATTTCCCTTTTCTTTTCTCCATATCATCTTCTTTTCTTCTCTTTCTC
TTCTCTCTCGTCATCATCATCATCAATCATGTCGGGCCTGAAAGTGATGATAAAAGTAATATTATTGTATTATTATT
ACAATATTTTTCAAAAAATAATCTCACTCCATGAACTTGTAATTACACATCACTCTATTTCACATTAAATTTATGAA
TCATCTCCACATTTAACGAACTAGAGCACACCTATAAAAATAACGAGATTATCTGATCAACATCATAATTCTTTTAA
ACTTCATTTACTTTCCCTTTTCTTTCTCCCATATCTTCTTTTCCTTTTTCTTTCTCTTTCTCTTTCTCCTCTCGATC
ATCAATCATCATCATCATGCGCCATGAAAGTTGATGATCAAAGGTAAATTAGTGTTTATTATTATAATAATTTATTT
TTTCAAATAATAATCTCACTCATGAACTCTGTATTACACAAGTCACTTCCTGTTTCACAATGTAAAGTTATGATCAT
CACTCACATTTACGAACTACTAGCATCACTGTAAAATAATAACCAGATAACTGTCAAGATCATATTCTTTAAACCTT
CCATTTATTCCTTTCTAACTCCCATATCATCTTTCTCTTTCTCTCGATCATGCTCATCATCATCGATGTCCGGCCAT
GAAAGTTGATTGATCACTGGTAATATATTGTTATTATTATAATAATATTTTTCAAAATAAATAATCTCACTCATGAA
CTCTGTTTAACACAAGTCAACCTGTTTCATAATTATAATTAATGATATCACTCACCATTTTAACGACTATAGCCATC
ACACTGTAAAAAAACGCATAACTGATCCAAGATCATTAATTCTTTTAACTTCATTTACTTTCCTTTTCTAACTCCAT
CTCATCTTTCTTTTTCTTTCTATTTCTCTTTCTCTCTCGATCATCGTCTCAATCATCATGTCGGCCC
nn**37J94**nn
CCTCAAAAATTTCAAAAAGAGTCTCAAAAGTGAGACGCTTGCAAAATGGTCTCAAAAGTTAACGCTTTTACAAAAGT
GTTCCAAAAGTAGACACTTCAAAAAGGGGTCTCAAAAGTATACGCTGCAAAAAGCGGTCTCAAAAGTAGATGCCTAT
GAAAATGGTCTCCAAAATCAGTGCTCTTGCAAAAGGGTCTCAAAATGTAGGCTCTTGCAAATAAAGGTCTCAAAAGT
AGATGCCTGCAAAAAGGGTCTCAAGTAGACTCCTGCAAAAATGGTCTCAAAAGTAACGCTTACGAAAAGGGGTCTCA
AAAGTAGACGCTTGCAAAAAGGGTTCAAGTAGACCGCTGCAAAAAGGGTCTCAAAAGTAGACGCTTGAAAAAGGGTC
TCAAAGTAGTACGTCTACAAAAAGGGTTCAAAAAGTAGACGCTTGCAAAAAGGGTCTCAAAAGTAGATCGCCTGCAA
AATGGTCTCAAAAGTATACCTTACAAAAGGGGTCTCAAAAGTAGACGCTTGCAAAAGGGTCTCAAAAGTAGACGCCT
GCAAAAAGGGTCGCAAAAAAGTAGGATGCTTGCAAAAGAGTCTCAAAAGTAGACGCCTGCAAAAAAGGTCTCAAAAG
TAGACGCTTGCAAAAATAGTCTCATAGTACGACGCTTGCAAAAAGAGTCTCAAAATACACGCTGCCTCAAAAATTTC
AAAAAGAGTCTCAAAAGTGAGACGCTTGCAAAATGGTCTCAAAAGTTAACGCTTTTACAAAAGTGTTCCAAAAGTAG
ACACTTCAAAAAGGGGTCTCAAAAGTATACGCTGCAAAAAGCGGTCTCAAAAGTAGATGCCTATGAAAATGGTCTCC
AAAATCAGTGCTCTTGCAAAAGGGTCTCAAAATGTAGGCTCTTGCAAATAAAGGTCTCAAAAGTAGATGCCTGCAAA
AAGGGTCTCAAGTAGACTCCTGCAAAAATGGTCTCAAAAGTAACGCTTACGAAAAGGGGTCTCAAAA
nnnnnnnnn

Table of Synonyms and TR locations

HuluTR (Table 1 name)	Location in PolySeq34 (in Kbp)	Name embedded in PolySeq34
**HuluTR385 (HSR1)**	6-7	06k385
**HuluTR180 (HSR0)**	3-4	03k180
**HuluTR120**	2-3	02k120
**HuluTR335 (5SrDNA)**	5-6	05k335
**HuluTR225**	11-12	11k225
**HuluTR060**	14-15	14k060
**HuluTR450**	7-8	07k450
**HuluTR135**	16-17	16k135
**HuluTR600**	24-26	24k600
**HuluTR390**	22-24	22k390
**HuluTR360**	33-35	33k360
**HuluTR240**	29-30	29k240
**HuluTR185**	4-5	04k185
**HuluTR100**	35-36	35k100
**HuluTR350**	31-33	31k350
**HuluTR280**	36-37	36k280
**HuluTR150**	20-21	20k150
**HuluTR070**	30-31	30k070
**HuluTR055**	28-29	28k055
**HuluTR050**	26-27	26k050

YASS dot-plots of polySeq34 vs. consensus sequences (pasted 1-3X depending on repeat size) from Representative HuluTRs (Table 1). TR family sequence blocks are spaced in 1kb or 2kb groups, starting with 00kb = 0-1,000bp, 01kb = 1,001-2000 bp, and so on.

>HuluTR385-r55_HSR1_TRFconsensus

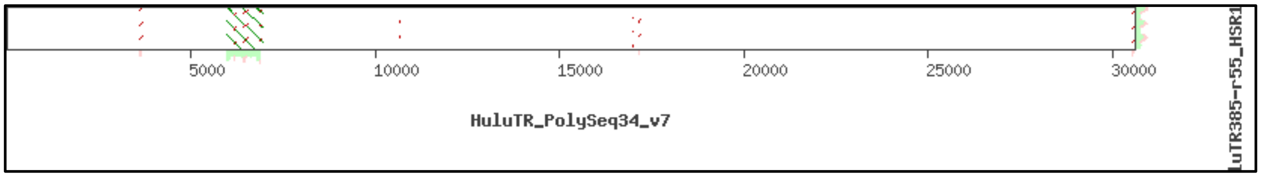

>HuluTR180-r120_HSR0_TRFconsensus

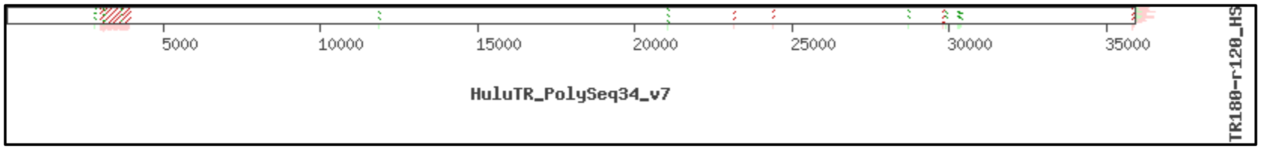

>HuluTR120-r782_TRFconsensus

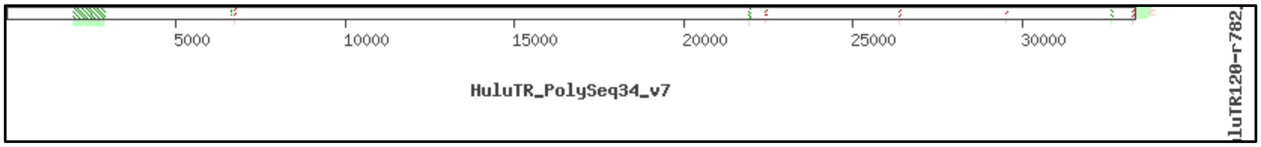

>HuluTR335-r243_5SrDNA_TRFconsensus

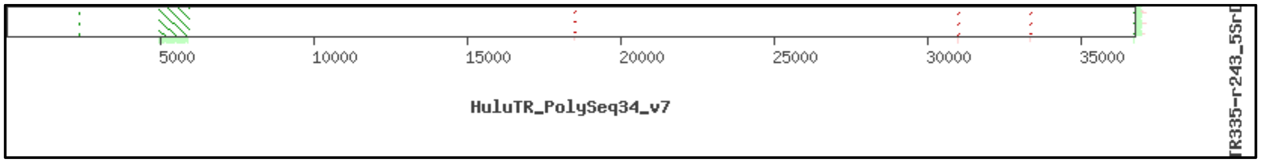

>HuluTR225-r397_TRFconsensus

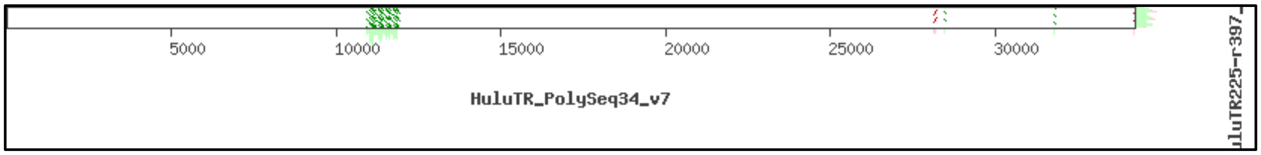

>HuluTR060-r91_TRFconsensus

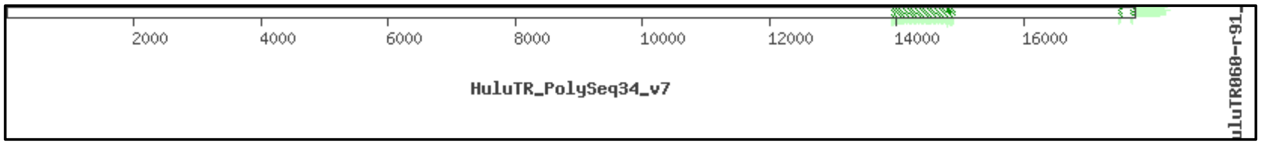

>HuluTR450-r873_TRFconsensus

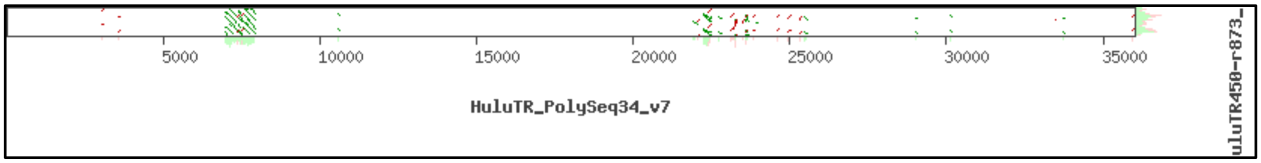

>HuluTR135-r253_TRFconsensus

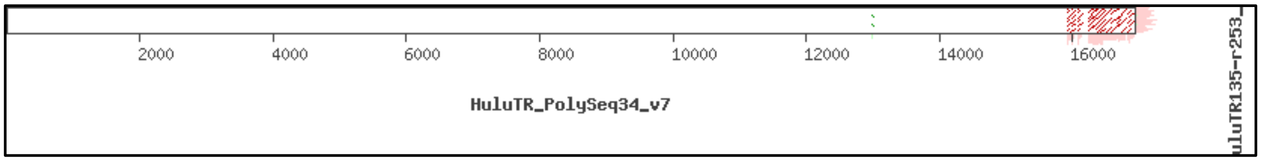

>HuluTR600-r823_TRFconsensus

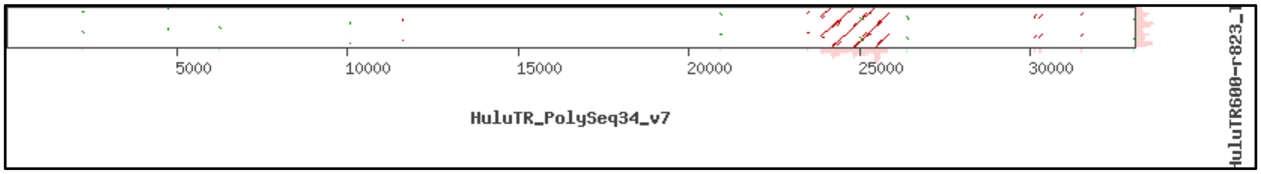

>**HuluTR390-r15**_TRFconsensus

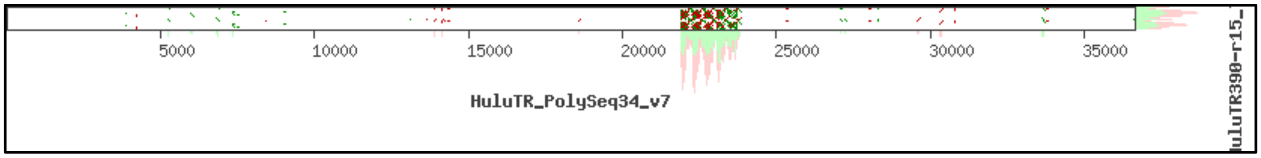

>HuluTR360-r642_TRFconsensus

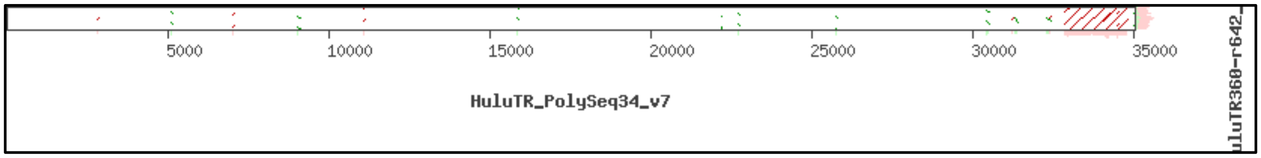

>HuluTR240-r1001_TRFconsensus

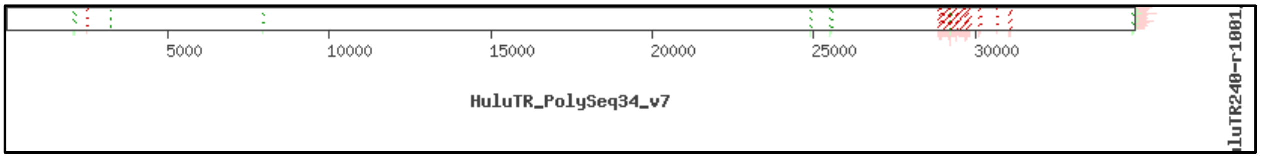

>HuluTR185-r424_TRFconsensus

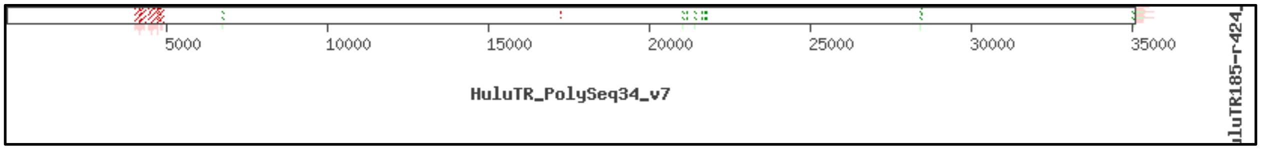

>HuluTR100-r983_TRFconsensus

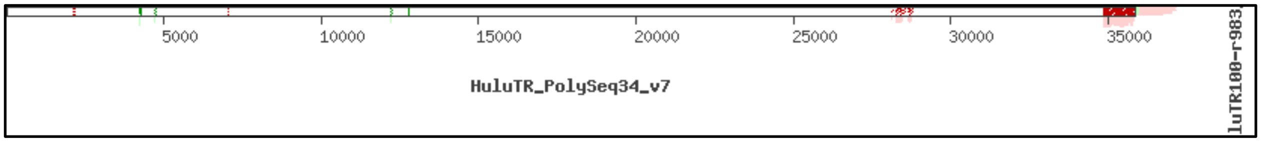

>HuluTR350-r625_TRFconsensus

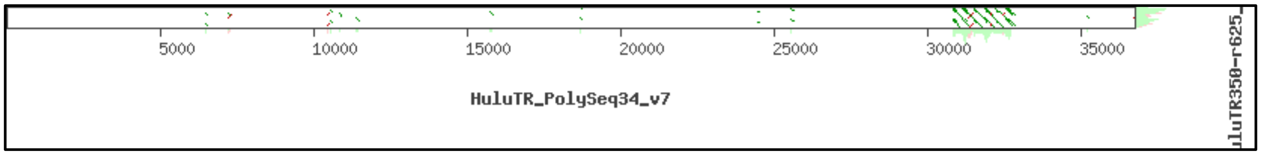

>HuluTR280-r934_TRFconsensus

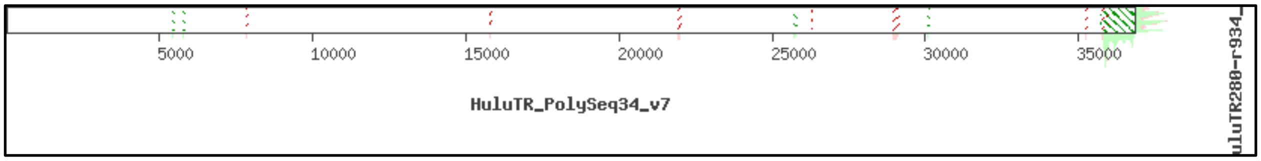

>HuluTR150-r390_TRFconsensus

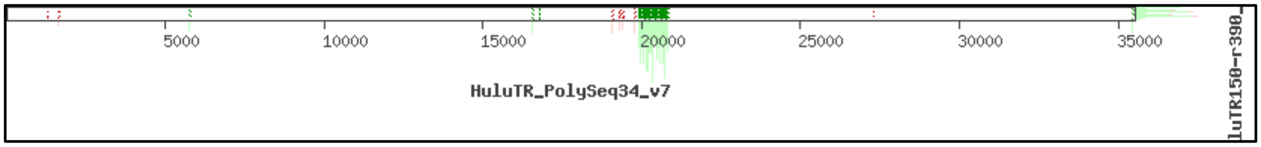

>HuluTR070-r541_TRFconsensus

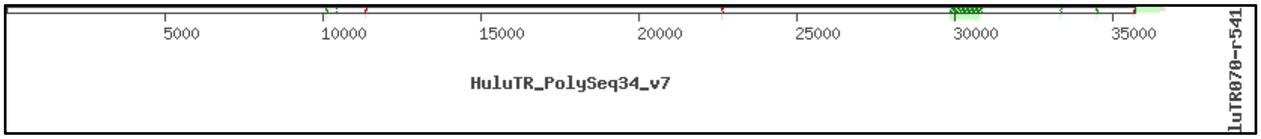

>HuluTR055-r292_TRFconsensus

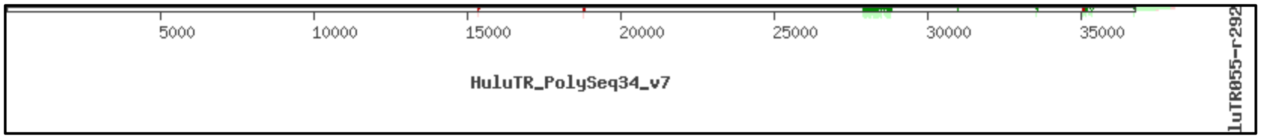

>HuluTR050-r33_TRFconsensus

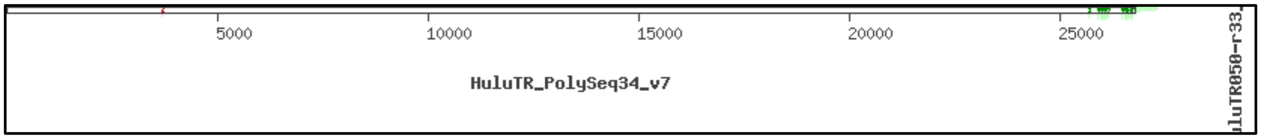

Supplement: S2 Fig — (PDF) [file pone.0233971.s005.pdf]
