# Supplementary material for: Identification of tandem repeat families from long-read sequences of Humulus lupulus
Source: PLoS One. 2020 Jun 5;15(6):e0233971. doi: 10.1371/journal.pone.0233971 (PMC7274563; doi:10.1371/journal.pone.0233971)
Supplement: S3 Fig — (PDF) [file pone.0233971.s006.pdf]

Supplemental Material - Data Sheet 4.

List of consensus sequences[1] for hop tandem repeat families. Easterling KA, et al. assembled 2019, updated 2020.

>HuluTR385-r55_HSR1_TRFconsensus
AAATACTGAGAGATGTACGAATATAAGGCAAAAACTTGTGAAAATTTCCAACAAAAAGGGTAATTTTTTT
TCGAATGTAGAATTAAGGCTCCTTTTCAACTCCAATAGATGCATTTCCCATGATATCTCCTGGGGAAGAA
ATTTTGGAACGAGTTGTACACCAGTGTAATACATTTCGGAGAAATTAGTGAAATCTCCAATTCACCAAAA
GGGGTACCCCTTTCAGAAATATTCGATTTCTCTAGTATGAATTGGAGCAATGTTTTGAAAGTGAATACTA
AGGAAATCCGAGGGTCATGGGCACAAAAGTTGTAGCCCTATATCTTAGGCTTCTATGTATAAAATATCGT
GCATATCCGAGGTGTTTAGAGGGTGATACGGTCA

>HuluTR180-r120_HSR0_TRFconsensus
ATATAATATTATTGAAATGGGAAAAGGTTCAAAAAAATTAGAGCTTGTTAGAGAGAAATATGAGTGAATT
ACGAGATCGCTTCGGAGCCCTAGGCAGCTGCCTAGGAGATTTGGCAGGAAAGGCCAATTTTTTTGTTTGG
TTGAATTAGCATCCCAATTCATGGTCAATAGGAGCCCT

>HuluTR120-r782_TRFconsensus
TTAACACGAACTAAGTTTTCAAAATAAGTTCCTGGTTTTAACCAGGTCATAAAACTTAGAAGTTAACTTA
AATTTATTGATCGTAGTTCTTCCTTTAAGAGCTCGGGATATGGATAAAAG

>HuluTR335-r243_5SrDNA_TRFconsensus
GGGGAGAAAAGGAGGACACACGCGCTCGCTATAAATGAATATTGCGGACCAAACATTCCGGGTGCGATCA
TACCAGCACTAATGCACCGGATCCCATCAGAACTCCGCAGTTAAGCGTGCTTGGGCGAGAGTAGTACTAG
GATGGGTGACCTCCTGGGAAGTCCTCGTGTTGCACCCTGAAGACATCATTTTTTTTTTCCCTTTAAAAAT
CCACCTACGGCTCAAAAGTTTGTATTTTTTTGTTTTAAATCTTAAAAACTCATTTATTTCGTTTTCCTTGA
GGGGTTAATACCGTGGTCACTAACAAGCAGCGAATAAGAAGTTCCTAGTCAATCC

>HuluTR225-r397_TRFconsensus
CGTACCCTAGTAAACCAATACTACAGCTAGAGTCTCAAATAGTTTTCATGCTGAGTAAGATAGAGTAGTG
AATTTAGAAGGGGATCCGATCCCGAAAAATATTACTAGATAACATTGCACTAAGATATAGTAGTGAATTT
AGAAGGGGATCCGATCCCGAAAAATTATTACTAGATAAACATTGCACTAAGCAGACCTAATTATGACATA
ATGAACCGTTCCA

>HuluTR060-r91_TRFconsensus
CCCCGGACGTCGTTCGTCCGGGGTGGACCGGCCTGGGCTCGGTCCTCTTTGTTATAA

[1] For each newly discovered HuluTR family, a single consensus sequence was obtained from the representative clone (Table 1) using the Tandem Repeats Finder server on Jan. 18, 2020 at https://tandem.bu.edu/trf/trf.html (Benson, G. (1999). *Nucleic Acids Res*. 27, 573–580.)

>HuluTR450-r873_TRFconsensus
AAATGCACCTCTTCATATTCAAAGACAATAACCAACTATCTAAACCCTAATTTCAAAATTATAATTAGAT
GTGTAAAATTTATTAATATTAAAACCCTAATTTCGGCTATGCCAATTGAGGTTTACAATTAATATTGAAA
TTTTTTAGCATTAAAATACAAAATAATTGCACATCTTAAATATGGTATTGAGAAGTGGGAATTGTTAATG
CACCTCTTCAAATTGAAAGACTCAAAGACTATATATATTCCTAGCATTTGGCAAATAATTAATTATTATA
TAAAATATATTTTTTATCATTAATTTCGGCTACAAAAATATAGGGTTAATTATTGTGTTTTTTTTTTCAA
TATTATAAATATGGTATTGAGAATTGGGAAATTTAATTCATTTGCATAATTATGGCCTACACTAATAGTT
AGTTTGAATAATTAAATATTGAATATACGTTAAATATT

>HuluTR135-r253_TRFconsensus
CCCGGAGCTGGAGCTTGCGAAGCAAAGCTCCAGCAGTCGCAGGTCATGCATTATTTTGGGAGATCCGGAG
CTGAAGCTTGCGAAGCAAAGCTAGCAGCGGTCGCGGATCTCGCCATCAGCTCGTCATGCAGGA

>HuluTR600-r823_TRFconsensus
TGAAAGCTAAGCTTATGGAAAAGTAATTTGATAGCCAATTGACTATTGAGATTAAACACACTCTTTGCTT
ACTAAATGATGTATTTTCTAGTCTACACCTAACATGTCTCTCATTGGGAATTGTCTGGTATTCATTCAAA
TCTTCTGCTGTGGCATTTAATTTAATCATTGAGAAAATAATTTTTGATGCTTTGAAAGTAGATCTATTTG
CTGGTGGTGCTTAAGTTTAATGAATTATGCTTGAGCTAGTGTTTGTTGCTTGCTGGTTGTTAGTTGAGTA
TTGCTATAAAAGGAAGTAGGCTTGATTGAACCATTGGCTTTTGTTGCCCGGATGTTGCAGAATAAATAGA
TTCTTGCAACTAGCTTATTTATTTTCTATATACCCTTCCATATCTAATTTTTGTTATTGTTACAACTGAA
AATTATGGCAACTACTCTGCTTTTCTTTTCCTATTTATGAGAATTACTCCATTTTCCTTTTCATATTTGT
GAAGATGACTTATGAAGACAAATTTCTTCTTTCTTCTTTTTTTTTTTATTAAAAAAAAAAAGAGGAAAAC
CCCCTTCAATTTCTTTTCCTTATTTTATATATTCTT

>**HuluTR390-r15**_TRFconsensus
AATGACATGGTAGGTCGGTTCAACACAAACCGACCTACCATGTCACATGAGAAGTCATTTCCCACACAAC
CGACCTACCATGTCCTCTAAAAAATTATTAAAGAGATACACTAAATTATGTTATAAATTTTTTTATTTTA
AATTATCACTATTATTAAATTAAAATATTTTCTTTTTTATAAAAATGAAATAAATTTATTTAATTATAAA
TAATTTATTAATTTATTCATATTTAAAATTATAATATATGTAGTATTAATAATTATATATTTTTTATACT
TATTTCATACCCAAATAAATTTTATATATGTATTGTTTAAATAAATTAATATATTTTAAATTTTCAATAA
TATAAACAAATTAGAATATATTTAATAATGTATTTTTAT

>HuluTR360-r642_TRFconsensus
GTTAGAAAACAAAAGTTTGTATATCTTCAAGAAAGTCTAAGTTAACATTGTAAGTATATCTCGTAATAAG
TCTAAGTAAACAAAAGTGAGTGACCTATGTAGTACTCATCAAATCCTTAACGCATTGTGCCCATTCTTCA
CGTACTTCATTGATCTCATCTTTTTGTAAGAGTTCTTCCCGCCCACACTAAAATAAAAATTCATTGCATG
AGTTGAATTAATATAATTAATCTTAAGGTAAACCTAGAACTTTAATAATGAATATTTTCACTTACGTTAC
TAGTTAGCTATCGCATTGAAAACTCATTTGTCACGAAGTCTTCATCATCCTCATAACATAGAATCAACAT
TGTACTAATA

>HuluTR240-r1001_TRFconsensus
AACATATTTCAATCAACTACTATGATGATCAAGGATTGAGTGGTAAATAATCTATTGAGGTACTATTATG
ATGATTGTGTTATAGTATTGAAAAAAACAACTTTTATCCATGTACGAATTGGAATCTTAAGTCATCATGA
CATTTTTGACAATTAGCCATGAATTTCGACAACTTGTCTAACATTTATAAGTTAACAAAACTTAATCTTT
ATGAGATTATGATGATTACAGTTAAGTGGT

>HuluTR185-r424_TRFconsensus
GGCACTCGTTAGGGTGATTACACAAGATTGGACTCGTTTCGATTCACCGAGACACGGTTCGGTATCAGGA
TTGCCAGCTGGAACTCGTGCTGGATTCACCGAGTCATCGCCAATAGTGTGATTAACACCATCTCGATGCT
CTCTTCGGTGGCACCGACTTCCATGAAATCATTATGGGTGGCCGA

>HuluTR100-r983_TRFconsensus
TAATCGAGTTGTTTCACACTAACTTGGTACTTGGTGACTCGGAATGAGGAATAATCAGTTGTTTCAAACT
AACTTGGTACTTGGTGACTCAGAATGAGGAA

>HuluTR350-r625_TRFconsensus
TTCAAGTTAGCAAAGAAAAGTAAAGTCTAAATGCAACAGCACGACTGTACGAGCAAAATATAATAACTAC
TGCATAGTGAAATAAAATACACCAAGTTAAAAGCAAACATTCATGTGAAATACCGCCAGGTTAAAAGCAA
ACATTCGTGTGAAATAAAACATTAAGCTCACAAAATTTTAGAGCAATAAGAGTGTGAGTTAGTATAATTA
GTAGGAACTTATGAAATATCTAGAATTTCTAAGAGTTAGAACACTAAGTACTCAAGCGAAAATATAAGGC
ATATATCAGAGTGACTTTACTATTCACAAAAGACTTATAATGTTTTAAGTCTAGAAAGACTTAGAATT

>HuluTR280-r934_TRFconsensus
GGCCATGAAAGTTGATGATCAAGGTAATATTATTGTTATTATTATTATATATTTTCAAATAATAATCTCA
CTCATGAACTCTGTATTACACAAGTCACTCTGTTTCACAATTAAATTTATGATATCACTCACATTTAACG
AACTAGAGCATCACTATAATAATAACCAGATTACTGTCAAGATCATAATTCTTTTAAACTTCATTTACTT
TCCCTTTTCTTTCTCCATATCATCTTTCTCTTTCTCTTTCTCTCTCGATCATCATCATCATCATCATGTC

>HuluTR150-r390_TRFconsensus
TTCGATGCATGCTCGATGGGGTTCGATAGGGTAGGCATTAGGGGTTCGATGGGGTAGGCCCATTATGGGT
TTCCAGGTTTAAAACTAAGAAATTAGATGCATGCTCGATGGGGGAGTTCGATGTACTGCTCGATGGTTGG
TCTTTGTTGGG

>HuluTR070-r541_TRFconsensus
GATATTAAACCAATTGTTCTCCACTAATTCTCTTTCTATAATAATATTTTAATGAAAACATCCACTCCAT
A

>HuluTR055-r292_TRFconsensus
GTCACTAGGTGACTTGTGAGTGTGTGTTCAGTAGATCATTACTTCGTTCCAA

>HuluTR050-r33_TRFconsensus
TTGGGCATATGACTTGCTGTAGATAAGCAAGCCGCCAGAATTTATGAA
