# Supplementary material for: Identification of tandem repeat families from long-read sequences of Humulus lupulus
Source: PLoS One. 2020 Jun 5;15(6):e0233971. doi: 10.1371/journal.pone.0233971 (PMC7274563; doi:10.1371/journal.pone.0233971)
Supplement: S4 Fig — (PDF) [file pone.0233971.s007.pdf]

Supplemental Material - Data Sheet 5.

Pairwise sequence alignment (PacBio read vs. Perfect Repeat)
Using tools from https://www.ebi.ac.uk/Tools/psa/

Repeat	PacBio read	Comparison	% Identity (Global Alignment)	% Identity (Local Alignment)
TELOMERE	>MH188536.1 Humulus lupulus cultivar Apollo clone HuluTR180TEL-954 satellite HSR0 and telomere repeat sequence. **Terminal 4kb region of original read.**	Synthetic (gold standard) of **4kb of perfect TTTAGGG** repeats	**90.2 %**<br><br>EMBOSS - NEEDLE<br><br>DNA, Default parameters	**90.4 %**<br><br>EMBOSS - WATER<br><br>DNA, Default parameters

PacBio (error-prone single molecule technology read)
>MH188536.1 Humulus lupulus cultivar Apollo clone HuluTR180TEL-954 satellite HSR0 and telomere repeat sequence

```
>MH188536_HuluTR180TEL_954_terminalTeloRepeatRegion_4kbp
GGTTTAGGGTTTAGGCGTTCTGGGTTTAGGGTTTAGGTTTAGGGTTTGAGGGTTTAGGTTAGGGTTTAGGGTTTAGGTTTAGGGTT
AGGGTTTAGGGTTTAGGGTTTAGGTTTAGGGTTAGGGTTTAGGGTTTAAGGGTTTTAGGGTCCTTAGAGGGTTACGGGTTTAGGGT
TTAGGGTTTAGGGTTTAGGTTTAGGGTTTAGGTTTAGGGTTTAGGGTTTAGGGTTTAGGGTTTAGGGGTTTAGGTTTAGGGTTTAG
GTTTAGGGTTTAGGGTTTAGGGTTTAGGGTTTAGGGTTGTAGGGTTAGGGTTTAGGTTTAGGGTTTAGGTTTAGGGTTTAAGGGTT
TAGGGTTAGGGTTTAGGGTTTAAGGGTTTAGGGTTAGGGTTTAGGTTTAGGGTTTAGGGTTTAGGGTTTAGGGTTTAGGTTTAGGG
TTGTTTAGGGTTAGGGTTAGGTTTAGGGTTTAGGGTTTAGGGTTTTTAGGGTTTAGGGTTTAGGGTTTAGGGTTAGGGTTAGGGTT
TAGGGTTTCAGGGTTTAGGGTTTTAGGGTTTAGGTTCAAGGTTTAGGGTGTTAGGGTTTAGGGTTTAGGGTTTAGGGTTTAGGGTT
TAGGGTTTAGGTTTTAGGGTTTTAGGGTTTAGGGTTTAGGGTTTAGGGTTTAGGGTTTCACGGGTTAGGTTAGGGCTTTCAGGGTT
TAGGGTTTAGGGTTTAAGGTTTAGGGTTTAGTTTAGGGTTTAGGGTTTAGGGTTTAGGGTTAGGTTTAGGGTTTGGGTTTAGGGTT
GGGTTTAGGGTTTAGGGTTTACGCGGTTTAGGGTTTTTAGGGTTTAGGGTTTAGGGTTTAGGTTTTAGGGTTTAGGGTTTAGGGTT
TCAGGGTTTGGGTTTGGGTTTAGGGTTTCAGGGTTTAGGGTTTACGGGTTTAGCGGTTTAGGGTTTAGGGTTTAAGGTTAGGGTTT
AGGTTTTAAGGGTTTAGGGTTTAAGGGTTTAGGGTTAGTTTAGGGTTTAGGGTTTAGGTTCAGGGTTAGGGGTTTCAGGGTTTAGG
GTTTGGGGTTTAGGGTTTAGGTTAGGTTTTATGGGTTTAGGGTTCAGGGTTTAGGGTTTAGGGTTTAAGGGTTTAGGGTTTTAGGG
TTTAGGTTTAGGGTTTAGGTTTAGGGTTTAGGGTTTGAGGGTTTAGGGTTTGGGTTTAGGGTTTAGGGTTAGGGTTGGTTTTAGGG
TTTAGGTTTAGGGTTTTAGGGTTTAGGGTTCAGGGTTTTAGGGTTTAGGGTTTAGGGTTTAGGGTTTAGGGTTTAGGGTTTAGGGT
TTAGGGTTTACGGTTTAGTTTAGGGTTTTAGGGTTTAGGGTTTAGGTTTAGGTTAGGTTTGGGTTTCAGGTTTCTAGGGTTTAGGG
TTTTAGGGTTAGGGTTTAGGGTTTAGGGTCTGGTCTAGGGTTTAGGGTTTAGGGTTTAGGGTTTAGGGTTTAGGGTTTTAGGGTTT
AGGGTTTAGGGTTTTAGGGTTTTAGGTTTAGGGTTTAGGGTCTTAGGGTTTAGGGTTTTAGGGTTTAGGGTTTAAGGGTTGGTTTA
GGTTTCAGGGTTTAGGGTTTAAGGGTTTAGGGTTTAGGGTTTAGGGTTTTAGGTTTAGGTTTAGGGTTTAGGTTTAAGGGTTTAGG
GTTTGGGTTTAGGGTTTACCGGGTTTGAGGGTTTTAGGGTTTAGGGTTTAGGGTTTAGGGTTTAGGGGTTTTAGGGTTTTAGGGTT
TAGGGGTTTAGGGTTTAGGGTTTAGGGTTTAGGTTTAGGTTTAGGTTTAGGTTTAGGGTTTAAGGGTTAGGGTTAGGTTTAGGGTT
TAGGGTTTACGGGTGAGGGTTTACGGGTTTAGGGTTAAGGGTTTAGGGTTTAGGGTTTAGGGTTTAGGGTTTAAGGTTTAGGGTTT
AGGGTTTAGGGTTTAGGGTTTAGGGTTTAGGGTTTAGGTAGGGTTTAGGGTTAGGGTTTAGGGTTTTTACGGGTTTAGGTTTAGGG
```

TTAGGGTTTAGGGCTTTAGGGTTTAGGGTTTCAGGGTTATAGGGTTAGGGTTTTAGGGTTTGGGTTAGGGTTTGGGGTTTAAGGGT
TTAGGGTTTAGGTTTAGGGTTTAGGGTTTTAGGGTTTGGGTTTTAGGGTTAGGTTTAGGGTTTAGGGTTTGGGTTTAGGGTTTCGG
TTTAGGGTTTAGGGTTTAGGTTTTGAGGTTTAGGTTCAGGGTTTAGGTTTTGAGGTTTACGGGCGTTTAGGGTTTGAGGTTAGGGT
TTCAGGGTTTAGGGTTTAAGGGTTTAGGTTTCAGGGTTTAGGGTTTAGGGCTTTAGGGTTTCAGGGTTAGGGTTTGGGTTTAGGTT
TAGGTTTTAGGGTTTTAGGGTTTAGGTTTAGGGTTTAGGGTTTAGGGTTTTAGGTTTTAGGGTTTAGGGTTTAGGGTTGTATACGG
TTTAGGGTTAGGGTTTAGGGTTTACGGGTTTCAGGGTTTAGGGTTTACGGGTTTAGAGGTTTAGGTTTAGCGTTTTAGGGTTTAGG
GTTTAGGGTTTAGGGTTTAGGGTTTAGCGGTTTAGGGTGTTACGGGTTTAAGGTTTAGGGTTTAGGGTTTAGGGGTTTTAAGGTTA
GGGTTTTAGGGTTTGAGGGTTTAGGGGTTTAGCGGTTTAGGGTTTAGGAGTTAGGGTTTAGGTCTTAGGTTTAGGGTTTAGGGTTT
CAGGTTTAGGTTTTAGGGTTTAGGGTTTTAGGGTTAGGGTTTGGGTTTAGGGTTTTAGGGTTTAGGGTTTAGGGTTTAGGTTAGGG
TTTAGGGTTTAGGGTTAGGTTTGGGTTTAGGGTTTAGGTTTAGGGTTAGGTTAGGGTTAGGGTTTAGGGTTTAGGGTTTAGGGTTA
AGGGTTTGGGTTTAGGGTTTATGGGTTTAGGGTTTAGGGTTAGGTTTAGGGTTTAGGGTTTAGTTTAGGGTTTGGGTTGGGTTTAG
GGTTAGGGTTTTTGGGTTTAGGGTTTAGGGTTTAGGGTTTAGGGTTTAGGGTTTAGGGTTTAGGTTTAGGGTTTAGGGTTAGGGTT
TAGGGTTTAGGGTTTAGGGTTTAGTTGTGTTTAGGGTTTAGGTTTAGGGTTTACGGGTTTAGGGTTTAGGTTTACGGGTTTAGGGT
TTTAGGGTTTAGGGTTTAGGGTTAGGGTTTGGGTTGAGGGGTTTAGGGTTTGGGTTTAGGGTTTAGGTTTAGCGGTTTAGGTTTAG
GGTTTTGGTTTGGGTTTAGGGTTTAGGTTTAGGGTTTAGGGTTTAGGGTTTAGGGTTTAGGGTTTAGGGTTTAGGGTTTAGGGTTT
AGGTTTAGGGTTTTAGGGTTTAGGGTTTAGGGTTTAACAGGTTTAGGGTTAGGGTTTAGGGTTTAGGGTTTAGGGTTTAGGGTTTA
GGGTTTAGGTTTAGGGTTTAGGGTTTAGGTTTAGGGTTTAGGGGGGTTTTAGGTAGGGTTTAGGGTTTAGGGTTCTAGGGTTAGG
GTTCTAGGGTTTAGGGTTTAGGGTTTAGGGTTTTAAGGGTTTTAGGGTTTAGGGTTTAGGGTTTAGGGTTTAGGGTTAGGTTTAGG
GTTTAGGGTTAGGGTTTTAGGGTTTAGGGTTTAGGGTTTTAGGGTTAGGGTTTAGGGTTTAGGGTTTAGGGTTTCAGGGTTTAGGG
TTTGGGTTTAGGGTTTAGGGTTTTAGGGTTTAGGGTTTAGGTTTAGGGTTTTTAGGGTTTAGGGTTTAGGGTTTAGGGTTTAAGGG
TTTTAGGGTTTAGGGTTTAGGGTTTTGGGTTTAGGGTTTAGGGTTTAAGGTTAGGGTTTTAGGGTTAGGTTTAGGGTTTAGGGTTT
AGCGGTTTCAGGGTTTAAGGGTTTAGGGTTTCCAGGGTTTCAGGGTTTAGGGATTTCAGGGTTTTCAGGGTTTAGGGTTTAGGGTT
TAGGTTTAGGGTTTAGGGTTTCAGGGTTTTTTAGGGGTATTTAGGGTTTAGGGTTTAGGGTTTAGGGTTTTAGGCGTTTAGGTTT
AGGGTTTTAGGGTTTTAGGGTTTAGGGTTTTAGGGTTTAGGTTT

>SyntheticTTTAGGG_TelomereRepeat_4kbp
TTTAGGGTTTAGGGTTTAGGGTTTAGGGTTTAGGGTTTAGGGTTTAGGGTTTAGGGTTTAGGGTTTAGGGTTTAGGGTTTAGGGTT
TAGGGTTTAGGGTTTAGGGTTTAGGGTTTAGGGTTTAGGGTTTAGGGTTTAGGGTTTAGGGTTTAGGGTTTAGGGTTTAGGGTTTA
GGGTTTAGGGTTTAGGGTTTAGGGTTTAGGGTTTAGGGTTTAGGGTTTAGGGTTTAGGGTTTAGGGTTTAGGGTTTAGGGTTTAGG
GTTTAGGGTTTAGGGTTTAGGGTTTAGGGTTTAGGGTTTAGGGTTTAGGGTTTAGGGTTTAGGGTTTAGGGTTTAGGGTTTAGGGT
TTAGGGTTTAGGGTTTAGGGTTTAGGGTTTAGGGTTTAGGGTTTAGGGTTTAGGGTTTAGGGTTTAGGGTTTAGGGTTTAGGGTTT
AGGGTTTAGGGTTTAGGGTTTAGGGTTTAGGGTTTAGGGTTTAGGGTTTAGGGTTTAGGGTTTAGGGTTTAGGGTTTAGGGTTTAG
GGTTTAGGGTTTAGGGTTTAGGGTTTAGGGTTTAGGGTTTAGGGTTTAGGGTTTAGGGTTTAGGGTTTAGGGTTTAGGGTTTAGGG
TTTAGGGTTTAGGGTTTAGGGTTTAGGGTTTAGGGTTTAGGGTTTAGGGTTTAGGGTTTAGGGTTTAGGGTTTAGGGTTTAGGGTT
TAGGGTTTAGGGTTTAGGGTTTAGGGTTTAGGGTTTAGGGTTTAGGGTTTAGGGTTTAGGGTTTAGGGTTTAGGGTTTAGGGTTTA
GGGTTTAGGGTTTAGGGTTTAGGGTTTAGGGTTTAGGGTTTAGGGTTTAGGGTTTAGGGTTTAGGGTTTAGGGTTTAGGGTTTAGG
GTTTAGGGTTTAGGGTTTAGGGTTTAGGGTTTAGGGTTTAGGGTTTAGGGTTTAGGGTTTAGGGTTTAGGGTTTAGGGTTTAGGGT
TTAGGGTTTAGGGTTTAGGGTTTAGGGTTTAGGGTTTAGGGTTTAGGGTTTAGGGTTTAGGGTTTAGGGTTTAGGGTTTAGGGTTT
AGGGTTTAGGGTTTAGGGTTTAGGGTTTAGGGTTTAGGGTTTAGGGTTTAGGGTTTAGGGTTTAGGGTTTAGGGTTTAGGGTTTAG
GGTTTAGGGTTTAGGGTTTAGGGTTTAGGGTTTAGGGTTTAGGGTTTAGGGTTTAGGGTTTAGGGTTTAGGGTTTAGGGTTTAGGG
TTTAGGGTTTAGGGTTTAGGGTTTAGGGTTTAGGGTTTAGGGTTTAGGGTTTAGGGTTTAGGGTTTAGGGTTTAGGGTTTAGGGTT
TAGGGTTTAGGGTTTAGGGTTTAGGGTTTAGGGTTTAGGGTTTAGGGTTTAGGGTTTAGGGTTTAGGGTTTAGGGTTTAGGGTTTA
GGGTTTAGGGTTTAGGGTTTAGGGTTTAGGGTTTAGGGTTTAGGGTTTAGGGTTTAGGGTTTAGGGTTTAGGGTTTAGGGTTTAGG
GTTTAGGGTTTAGGGTTTAGGGTTTAGGGTTTAGGGTTTAGGGTTTAGGGTTTAGGGTTTAGGGTTTAGGGTTTAGGGTTTAGGGT
TTAGGGTTTAGGGTTTAGGGTTTAGGGTTTAGGGTTTAGGGTTTAGGGTTTAGGGTTTAGGGTTTAGGGTTTAGGGTTTAGGGTTT
AGGGTTTAGGGTTTAGGGTTTAGGGTTTAGGGTTTAGGGTTTAGGGTTTAGGGTTTAGGGTTTAGGGTTTAGGGTTTAGGGTTTAG
GGTTTAGGGTTTAGGGTTTAGGGTTTAGGGTTTAGGGTTTAGGGTTTAGGGTTTAGGGTTTAGGGTTTAGGGTTTAGGGTTTAGGG
TTTAGGGTTTAGGGTTTAGGGTTTAGGGTTTAGGGTTTAGGGTTTAGGGTTTAGGGTTTAGGGTTTAGGGTTTAGGGTTTAGGGTT
TAGGGTTTAGGGTTTAGGGTTTAGGGTTTAGGGTTTAGGGTTTAGGGTTTAGGGTTTAGGGTTTAGGGTTTAGGGTTTAGGGTTTA
GGGTTTAGGGTTTAGGGTTTAGGGTTTAGGGTTTAGGGTTTAGGGTTTAGGGTTTAGGGTTTAGGGTTTAGGGTTTAGGGTTTAGG
GTTTAGGGTTTAGGGTTTAGGGTTTAGGGTTTAGGGTTTAGGGTTTAGGGTTTAGGGTTTAGGGTTTAGGGTTTAGGGTTTAGGGT
TTAGGGTTTAGGGTTTAGGGTTTAGGGTTTAGGGTTTAGGGTTTAGGGTTTAGGGTTTAGGGTTTAGGGTTTAGGGTTTAGGGTTT
AGGGTTTAGGGTTTAGGGTTTAGGGTTTAGGGTTTAGGGTTTAGGGTTTAGGGTTTAGGGTTTAGGGTTTAGGGTTTAGGGTTTAG
GGTTTAGGGTTTAGGGTTTAGGGTTTAGGGTTTAGGGTTTAGGGTTTAGGGTTTAGGGTTTAGGGTTTAGGGTTTAGGGTTTAGGG
TTTAGGGTTTAGGGTTTAGGGTTTAGGGTTTAGGGTTTAGGGTTTAGGGTTTAGGGTTTAGGGTTTAGGGTTTAGGGTTTAGGGTT
TAGGGTTTAGGGTTTAGGGTTTAGGGTTTAGGGTTTAGGGTTTAGGGTTTAGGGTTTAGGGTTTAGGGTTTAGGGTTTAGGGTTTA

GGGTTTAGGGTTTAGGGTTTAGGGTTTAGGGTTTAGGGTTTAGGGTTTAGGGTTTAGGGTTTAGGGTTTAGGGTTTAGGGTTTAGGGTTTAGG
GTTTAGGGTTTAGGGTTTAGGGTTTAGGGTTTAGGGTTTAGGGTTTAGGGTTTAGGGTTTAGGGTTTAGGGTTTAGGGTTTAGGGTTTAGGGT
TTAGGGTTTAGGGTTTAGGGTTTAGGGTTTAGGGTTTAGGGTTTAGGGTTTAGGGTTTAGGGTTTAGGGTTTAGGGTTTAGGGTTTAGGGTTT
AGGGTTTAGGGTTTAGGGTTTAGGGTTTAGGGTTTAGGGTTTAGGGTTTAGGGTTTAGGGTTTAGGGTTTAGGGTTTAGGGTTTAGGGTTTAG
GGTTTAGGGTTTAGGGTTTAGGGTTTAGGGTTTAGGGTTTAGGGTTTAGGGTTTAGGGTTTAGGGTTTAGGGTTTAGGGTTTAGGGTTTAGGG
TTTAGGGTTTAGGGTTTAGGGTTTAGGGTTTAGGGTTTAGGGTTTAGGGTTTAGGGTTTAGGGTTTAGGGTTTAGGGTTTAGGGTTTAGGGTT
TAGGGTTTAGGGTTTAGGGTTTAGGGTTTAGGGTTTAGGGTTTAGGGTTTAGGGTTTAGGGTTTAGGGTTTAGGGTTTAGGGTTTAGGGTTTA
GGGTTTAGGGTTTAGGGTTTAGGGTTTAGGGTTTAGGGTTTAGGGTTTAGGGTTTAGGGTTTAGGGTTTAGGGTTTAGGGTTTAGGGTTTAGG
GTTTAGGGTTTAGGGTTTAGGGTTTAGGGTTTAGGGTTTAGGGTTTAGGGTTTAGGGTTTAGGGTTTAGGGTTTAGGGTTTAGGGTTTAGGGT
TTAGGGTTTAGGGTTTAGGGTTTAGGGTTTAGGGTTTAGGGTTTAGGGTTTAGGGTTTAGGGTTTAGGGTTTAGGGTTTAGGGTTTAGGGTTT
AGGGTTTAGGGTTTAGGGTTTAGGGTTTAGGGTTTAGGGTTTAGGGTTTAGGGTTTAGGGTTTAGGGTTTAGGGTTTAGGGTTTAGGGTTTAG
GGTTTAGGGTTTAGGGTTTAGGGTTTAGGGTTTAGGGTTTAGGGTTTAGGGTTTAGGGTTTAGGGTTTAGGGTTTAGGGTTTAGGGTTTAGGG
TTTAGGGTTTAGGGTTTAGGGTTTAGGGTTTAGGGTTTAGGGTTTAGGGTTTAGGGTTTAGGGTTTAGGGTTTAGGGTTTAGGGTTTAGGGTT
TAGGGTTTAGGGTTTAGGGTTTAGGGTTTAGGGTTTAGGGTTTAGGGTTTAGGGTTTAGGGTTTAGGGTTTAGGGTTTAGGGTTTAGGGTTTA
GGGTTTAGGGTTTAGGGTTTAGGGTTTAGGGTTTAGGGTTTAGGGTTTAGGGTTTAGGGTTTAGGGTTTAGGGTTTAGGGTTTAGGGTTTAGG
GTTTAGGGTTTAGGGTTTAGGGTTTAGGGTTTAGGGTTTAGGGTTTAGGGTTTAGGGTTTAGGGTTTAGGGTTTAGGGTTTAGGGTTTAGGGT
TTAGGGTTTAGGGTTTAGGGTTTAGGGTTTAGGTTTAGGTTTAG

```
########################################
# Program: needle
# Rundate: Fri 31 Jan 2020 20:16:33
# Commandline: needle
#    -auto
#    -stdout
#    -asequence emboss_needle-I20200131-201631-0624-13360819-p1m.asequence
#    -bsequence emboss_needle-I20200131-201631-0624-13360819-p1m.bsequence
#    -datafile EDNAFULL
#    -gapopen 10.0
#    -gapextend 0.5
#    -endopen 10.0
#    -endextend 0.5
#    -aformat3 pair
#    -snucleotide1
#    -snucleotide2
# Align_format: pair
# Report_file: stdout
########################################

#=======================================
#
# Aligned_sequences: 2
# 1: MH188536_HuluTR180TEL_954_terminalTeloRepeatRegion_4kbp
# 2: SyntheticTTTAGGG_TelomereRepeat_4kbp
# Matrix: EDNAFULL
# Gap_penalty: 10.0
# Extend_penalty: 0.5
#
# Length: 4156
# Identity:     3749/4156 (90.2%)
# Similarity:   3749/4156 (90.2%)
# Gaps:          312/4156 ( 7.5%)
# Score: 16048.0
#
#
#=======================================

MH188536_Hulu      1 GGTTTAGGGTTTAGGCGTTCTGGGTTTAGGGTTTA-GGTTTAGGGTTTGA     49
                       ||||||||||||| |||..|||||||||||||| |||||||||||| |
SyntheticTTTA      1 --TTTAGGGTTTAGG-GTTTAGGGTTTAGGGTTTAGGGTTTAGGGTTT-A     46

MH188536_Hulu     50 GGGTTTAGG--TTAGGGTTTAGGGTTTA-GGTTTAGGG-TTAGGGTTTAG     95
                     |||||||||  ||||||||||||||||| ||||||||| |||||||||||
SyntheticTTTA     47 GGGTTTAGGGTTTAGGGTTTAGGGTTTAGGGTTTAGGGTTTAGGGTTTAG     96

MH188536_Hulu     96 GGTTTAGGGTTTA-GGTTTAGGG-TTAGGGTTTAGGGTTTAAGGGTTTTA    143
                     ||||||||||||| ||||||||| |||||||||||||||||  ||.||||
SyntheticTTTA     97 GGTTTAGGGTTTAGGGTTTAGGGTTTAGGGTTTAGGGTTTA--GGGTTTA    144

MH188536_Hulu    144 GGGTCCTTAGAGGGTTACGGGTTTAGGGTTTAGGGTTTAGGGTTTA-GGT    192
                     ||||  ||  ||||||..|||||||||||||||||||||||||||| |||
SyntheticTTTA    145 GGGT--TT--AGGGTTTAGGGTTTAGGGTTTAGGGTTTAGGGTTTAGGGT    190

MH188536_Hulu    193 TTAGGGTTTA-GGTTTAGGGTTTAGGGTTTAGGGTTTAGGGTTTAGGGGT    241
                     |||||||||| |||||||||||||||||||||||||||||||||| ||||
```

```
SyntheticTTTA    191 TTAGGGTTTAGGGTTTAGGGTTTAGGGTTTAGGGTTTAGGGTTTA-GGGT    239

MH188536_Hulu    242 TTA-GGTTTAGGGTTTA-GGTTTAGGGTTTAGGGTTTAGGGTTTAGGGTT    289
                     ||| ||||||||||||| ||||||||||||||||||||||||||||||||
SyntheticTTTA    240 TTAGGGTTTAGGGTTTAGGGTTTAGGGTTTAGGGTTTAGGGTTTAGGGTT    289

MH188536_Hulu    290 TAGGGTTGTAGGG-TTAGGGTTTA-GGTTTAGGGTTTA-GGTTTAGGGTT    336
                     ||||||| ||||| |||||||||| ||||||||||||| |||||||||||
SyntheticTTTA    290 TAGGGTT-TAGGGTTTAGGGTTTAGGGTTTAGGGTTTAGGGTTTAGGGTT    338

MH188536_Hulu    337 TAAGGGTTTAGGG-TTAGGGTTTAGGGTTTAAGGGTTTAGGG-TTAGGGT    384
                     | ||||||||||| ||||||||||||||| ||||||||||| |||||||
SyntheticTTTA    339 T-AGGGTTTAGGGTTTAGGGTTTAGGGTTT-AGGGTTTAGGGTTTAGGGT    386

MH188536_Hulu    385 TTA-GGTTTAGGGTTTAGGGTTTAGGGTTTAGGGTTTA-GGTTTAGGGTT    432
                     ||| |||||||||||||||||||||||||||||||||| |||||||||||
SyntheticTTTA    387 TTAGGGTTTAGGGTTTAGGGTTTAGGGTTTAGGGTTTAGGGTTTAGGGTT    436

MH188536_Hulu    433 ----GTTTAGGG-TTAGGGTT--AGGTTTAGGGTTTAGGGTTTAGGGTTT    475
                         |||||||| |||||||  .||||||||||||||||||||||  |
SyntheticTTTA    437 TAGGGTTTAGGGTTTAGGGTTTAGGGTTTAGGGTTTAGGGTTTAGGG--T    484

MH188536_Hulu    476 TTAGGGTTTAGGGTTTAGGGTTTAGGG-TTAGGG-TTAGGGTTTAGGGTT    523
                     ||||||||||||||||||||||||||| |||||| ||||||||||||||
SyntheticTTTA    485 TTAGGGTTTAGGGTTTAGGGTTTAGGGTTTAGGGTTTAGGGTTTAGGGTT    534

MH188536_Hulu    524 TCAGGGTTTAGGGTTTTAGGGTTTA-GGTTCAAGGTTTAGGGTGTTAGGG    572
                     | ||||||||||| ||||||||||| ||||.|.|||||||||| ||||||
SyntheticTTTA    535 T-AGGGTTTAGGG-TTTAGGGTTTAGGGTTTAGGGTTTAGGGT-TTAGGG    581

MH188536_Hulu    573 TTTAGGGTTTAGGGTTTAGGGTTTAGGGTTTAGGGTTTAGGTTTTAGGGT    622
                     |||||||||||||||||||||||||||||||||||||||||.|||||||
SyntheticTTTA    582 TTTAGGGTTTAGGGTTTAGGGTTTAGGGTTTAGGGTTTAGGGTTTAGGG-    630

MH188536_Hulu    623 TTTAGGGTTTAGGGTTTAGGGTTTAGGGTTTAGGGTTTCACGGGTTAGG-    671
                     ||||||||||||||||||||||||||||||||||||| |.||.|||||
SyntheticTTTA    631 TTTAGGGTTTAGGGTTTAGGGTTTAGGGTTTAGGGTTT-AGGGTTTAGGG    679

MH188536_Hulu    672 -TTAGGGCTTTCAGGGTTTAGGGTTTAGGGTTTAAGGTTTAGGGTTTA--    718
                      ||||||  ||.||||||||||||||||||||.|||||||||||||
SyntheticTTTA    680 TTTAGGG--TTTAGGGTTTAGGGTTTAGGGTTTAGGGTTTAGGGTTTAGG    727

MH188536_Hulu    719 GTTTAGGGTTTAGGGTTTAGGGTTTAGGGTT--AGGTTTAGGGTTT-GGG    765
                     |||||||||||||||||||||||||||||||  .|||||||||||| |||
SyntheticTTTA    728 GTTTAGGGTTTAGGGTTTAGGGTTTAGGGTTTAGGGTTTAGGGTTTAGGG    777

MH188536_Hulu    766 TTTAGGGTT--GGGTTTAGGGTTTAGGGTTTACGCGGTTTAGGGTTTTTA    813
                     |||||||||  ||||||||||||||||||||  .|||||||||  ||||
SyntheticTTTA    778 TTTAGGGTTTAGGGTTTAGGGTTTAGGGTTTA--GGGTTTAGGG--TTTA    823

MH188536_Hulu    814 GGGTTTAGGGTTTAGGGTTTAGGTTTTAGGGTTTAGGGTTTAGGGTTTCA    863
                     ||||||||||||||||||||||.|||||||||||||||||||||||| |
SyntheticTTTA    824 GGGTTTAGGGTTTAGGGTTTAGGGTTTAGGGTTTAGGGTTTAGGGTTT-A    872

MH188536_Hulu    864 GGGTTT-GGGTTT-GGGTTTAGGGTTTCAGGGTTTAGGGTTTACGGGTTT    911
                     |||||| |||||| ||||||||||||| ||||||||||||||| ||||||
```

```
SyntheticTTTA    873 GGGTTTAGGGTTTAGGGTTTAGGGTTT-AGGGTTTAGGGTTTA-GGGTTT    920

MH188536_Hulu    912 AGCGGTTTAGGGTTTAGGGTTTAAGG-TTAGGGTTTAGGTTTTAAGGGTT    960
                     || ||||||||||||||||||||.|| ||||||||||||.||| ||||||
SyntheticTTTA    921 AG-GGTTTAGGGTTTAGGGTTTAGGGTTTAGGGTTTAGGGTTT-AGGGTT    968

MH188536_Hulu    961 TAGGGTTTAAGGGTTTAGGGTT---AGTTTAGGGTTTAGGGTTTA-GGTT   1006
                     |||||||| |||||||||||||   .||||||||||||||||||| ||||
SyntheticTTTA    969 TAGGGTTT-AGGGTTTAGGGTTTAGGGTTTAGGGTTTAGGGTTTAGGGTT   1017

MH188536_Hulu   1007 CAGGGTTAGGGGTTTCAGGGTTTAGGGTTTGGGGTTTAGGGTTTAGG--T   1054
                     .||||||..|||||| ||||||||||||||.||||||||||||||||  |
SyntheticTTTA   1018 TAGGGTTTAGGGTTT-AGGGTTTAGGGTTTAGGGTTTAGGGTTTAGGGTT   1066

MH188536_Hulu   1055 TAGGTTTTATGGGTTTAGGGTTCAGGGTTTAGGGTTTAGGGTTTAAGGGT   1104
                     ||||.|||| ||||||||||||.||||||||||||||||||||| |||||
SyntheticTTTA   1067 TAGGGTTTA-GGGTTTAGGGTTTAGGGTTTAGGGTTTAGGGTTT-AGGGT   1114

MH188536_Hulu   1105 TTAGGGTTTTAGGGTTTA-GGTTTAGGGTTTA-GGTTTAGGGTTTAGGGT   1152
                     |||||| ||||||||||| ||||||||||||| |||||||||||||||||
SyntheticTTTA   1115 TTAGGG-TTTAGGGTTTAGGGTTTAGGGTTTAGGGTTTAGGGTTTAGGGT   1163

MH188536_Hulu   1153 TTGAGGGTTTAGGGTTT-GGGTTTAGGGTTTAGGG-TTAGGGTT--GGTT   1198
                     || |||||||||||||| ||||||||||||||||| ||||||||  ||.|
SyntheticTTTA   1164 TT-AGGGTTTAGGGTTTAGGGTTTAGGGTTTAGGGTTTAGGGTTTAGGGT   1212

MH188536_Hulu   1199 TTAGGGTTTA-GGTTTAGGGTTTTAGGGTTTAGGGTTCAGGGTTTTAGGG   1247
                     |||||||||| ||||||||| ||||||||||||||||.|||| |||||||
SyntheticTTTA   1213 TTAGGGTTTAGGGTTTAGGG-TTTAGGGTTTAGGGTTTAGGG-TTTAGGG   1260

MH188536_Hulu   1248 TTTAGGGTTTAGGGTTTAGGGTTTAGGGTTTAGGGTTTAGGGTTTAGGGT   1297
                     ||||||||||||||||||||||||||||||||||||||||||||||||||
SyntheticTTTA   1261 TTTAGGGTTTAGGGTTTAGGGTTTAGGGTTTAGGGTTTAGGGTTTAGGGT   1310

MH188536_Hulu   1298 TTACGGTTTA--GTTTAGGGTTTTAGGGTTTAGGGTTTA-GGTTTAGG--   1342
                     |||.||||||  |||||||| |||||||||||||||||| ||||||||
SyntheticTTTA   1311 TTAGGGTTTAGGGTTTAGGG-TTTAGGGTTTAGGGTTTAGGGTTTAGGGT   1359

MH188536_Hulu   1343 TTAGG--TTTGGGTTTCAGGTTTCTAGGGTTTAGGGTTTTAGGG-TTAGG   1389
                     |||||  ||.||||||..||||  |||||||||||| ||||||| |||||
SyntheticTTTA   1360 TTAGGGTTTAGGGTTTAGGGTT--TAGGGTTTAGGG-TTTAGGGTTTAGG   1406

MH188536_Hulu   1390 GTTTAGGGTTTAGGGTCT--GGTCTAGGGTTTAGGGTTTAGGGTTTAGGG   1437
                     ||||||||||||||||.|  |||.||||||||||||||||||||||||||
SyntheticTTTA   1407 GTTTAGGGTTTAGGGTTTAGGGTTTAGGGTTTAGGGTTTAGGGTTTAGGG   1456

MH188536_Hulu   1438 TTTAGGGTTTAGGGTTTTAGGGTTTAGGGTTTAGGGTTTTAGGGTTTTAG   1487
                     |||||||||||||| ||||||||||||||||||||| ||||||||||..|
SyntheticTTTA   1457 TTTAGGGTTTAGGG-TTTAGGGTTTAGGGTTTAGGG-TTTAGGGTTTAGG   1504

MH188536_Hulu   1488 GTTTAGGGTTTAGGGTCTTAGGGTTTAGGGTTTTAGGGTTTAGGGTTTAA   1537
                     |||||||||||||||| ||||||||||||| ||||||||||||||||| |
SyntheticTTTA   1505 GTTTAGGGTTTAGGGT-TTAGGGTTTAGGG-TTTAGGGTTTAGGGTTT-A   1551

MH188536_Hulu   1538 GGGTT---GGTTTAGGTTTCAGGGTTTAGGGTTTAAGGGTTTAGGGTTTA   1584
                     |||||   ||||||||.||.|||||||||||||| |||||||||||||||
```

```
SyntheticTTTA   1552 GGGTTTAGGGTTTAGGGTTTAGGGTTTAGGGTTT-AGGGTTTAGGGTTTA   1600

MH188536_Hulu   1585 GGGTTTAGGGTTTTAGGTTTA-GGTTTAGGGTTTAGGTTTAAGGGTTTAG   1633
                     |||||||||||||..|||||| |||||||||||||||.||.|||||||||
SyntheticTTTA   1601 GGGTTTAGGGTTTAGGGTTTAGGGTTTAGGGTTTAGGGTTTAGGGTTTAG   1650

MH188536_Hulu   1634 GGTTT-GGGTTTAGGGTTTACCGGGTTTGAGGGTTTTAGGGTTTAGGGTT   1682
                     ||||| ||||||||||||||  |||||| |||| ||||||||||||||||
SyntheticTTTA   1651 GGTTTAGGGTTTAGGGTTTA--GGGTTT-AGGG-TTTAGGGTTTAGGGTT   1696

MH188536_Hulu   1683 TAGGGTTTAGGGTTTAGGGGTTTTAGGGTTTTAGGGTTTAGGGGTTTAGG   1732
                     |||||||||||||||||||  ||||||| ||||||||||| |||||||||
SyntheticTTTA   1697 TAGGGTTTAGGGTTTAGGG--TTTAGGG-TTTAGGGTTTA-GGGTTTAGG   1742

MH188536_Hulu   1733 GTTTAGGGTTTAGGGTTTA-GGTTTA-GGTTTA-GGTTTA-GGTTTAGGG   1778
                     ||||||||||||||||||| |||||| |||||| |||||| |||||||||
SyntheticTTTA   1743 GTTTAGGGTTTAGGGTTTAGGGTTTAGGGTTTAGGGTTTAGGGTTTAGGG   1792

MH188536_Hulu   1779 TTTAAGGGTTAGGGTT--AGGTTTAGGGTTTAGGGTTTACGGGTGAGGGT   1826
                     ||||.||.||||||||  .||||||||||||||||||||.||.|.|||||
SyntheticTTTA   1793 TTTAGGGTTTAGGGTTTAGGGTTTAGGGTTTAGGGTTTAGGGTTTAGGGT   1842

MH188536_Hulu   1827 TTACGGGTTTAGGGTTAAGGGTTTAGGGTTTAGGGTTTAGGGTTTAGGGT   1876
                     ||| ||||||||||||.|||||||||||||||||||||||||||||||||
SyntheticTTTA   1843 TTA-GGGTTTAGGGTTTAGGGTTTAGGGTTTAGGGTTTAGGGTTTAGGGT   1891

MH188536_Hulu   1877 TTAAGGTTTAGGGTTTAGGGTTTAGGGTTTAGGGTTTAGGGTTTAGGGTT   1926
                     |||.||||||||||||||||||||||||||||||||||||||||||||||
SyntheticTTTA   1892 TTAGGGTTTAGGGTTTAGGGTTTAGGGTTTAGGGTTTAGGGTTTAGGGTT   1941

MH188536_Hulu   1927 TAGG---TAGGGTTTAGGG-TTAGGGTTTAGGGTTTTTACGGGTTTA-GG   1971
                     ||||   |||||||||||| ||||||||||||||||   .||||||| ||
SyntheticTTTA   1942 TAGGGTTTAGGGTTTAGGGTTTAGGGTTTAGGGTTT---AGGGTTTAGGG   1988

MH188536_Hulu   1972 TTTAGGG-TTAGGGTTTAGGGCTTTAGGGTTTAGGGTTTCAGGGTTATAG   2020
                     ||||||| ||||||||||||| ||||||||||||||||| |||||| |||
SyntheticTTTA   1989 TTTAGGGTTTAGGGTTTAGGG-TTTAGGGTTTAGGGTTT-AGGGTT-TAG   2035

MH188536_Hulu   2021 GG-TTAGGGTTTTAGGGTTT--GGGTTAGGGTTTGGGGTTTAAGGGTTTA   2067
                     || |||||| ||||||||||  ||.|||||||||.|||||| ||||||||
SyntheticTTTA   2036 GGTTTAGGG-TTTAGGGTTTAGGGTTTAGGGTTTAGGGTTT-AGGGTTTA   2083

MH188536_Hulu   2068 GGGTTTA-GGTTTAGGGTTTAGGGTTTTAGGGTTTGGGTTTTAGGGTT--   2114
                     ||||||| |||||||||||||||| ||||||||||.||.|||||||||
SyntheticTTTA   2084 GGGTTTAGGGTTTAGGGTTTAGGG-TTTAGGGTTTAGGGTTTAGGGTTTA   2132

MH188536_Hulu   2115 AGGTTTAGGGTTTAGGGTTT-GGGTTTAGGGTTT-CGGTTTAGGGTTTAG   2162
                     .||||||||||||||||||| ||||||||||||| .||||||||||||||
SyntheticTTTA   2133 GGGTTTAGGGTTTAGGGTTTAGGGTTTAGGGTTTAGGGTTTAGGGTTTAG   2182

MH188536_Hulu   2163 GGTTTAGGTTTTGAGGTTTA-GGTTCAGGGTTTAGGTTTTGAGGTTTACG   2211
                     ||||||||.|||..|||||| ||||.||||||||||.|||..||||||
SyntheticTTTA   2183 GGTTTAGGGTTTAGGGTTTAGGGTTTAGGGTTTAGGGTTTAGGGTTTA--   2230

MH188536_Hulu   2212 GGCGTTTAGGGTTTGAGG--TTAGGGTTTCAGGGTTTAGGGTTTAAGGGT   2259
                      |.||||||||||| |||  ||||||||| |||||||||||||| |||||
```

```
SyntheticTTTA   2231 -GGGTTTAGGGTTT-AGGGTTTAGGGTTT-AGGGTTTAGGGTTT-AGGGT   2276

MH188536_Hulu   2260 TTAGGTTTCAGGGTTTAGGGTTTAGGGCTTTAGGGTTTCAGGG-TTAGGG   2308
                     |||||.||.|||||||||||||||||| |||||||||| |||| ||||||
SyntheticTTTA   2277 TTAGGGTTTAGGGTTTAGGGTTTAGGG-TTTAGGGTTT-AGGGTTTAGGG   2324

MH188536_Hulu   2309 TTT-GGGTTTA-GGTTTAGGTTTTAGGGTTTTAGGGTTTA-GGTTTAGGG   2355
                     ||| ||||||| ||||||||.||||||| ||||||||||| |||||||||
SyntheticTTTA   2325 TTTAGGGTTTAGGGTTTAGGGTTTAGGG-TTTAGGGTTTAGGGTTTAGGG   2373

MH188536_Hulu   2356 TTTAGGGTTTAGGGTTTTAGGTTTTAGGGTTTAGGGTTTAGGGTTGTATA   2405
                     |||||||||||||| ||||||.|||||||||||||||||||||||   ||
SyntheticTTTA   2374 TTTAGGGTTTAGGG-TTTAGGGTTTAGGGTTTAGGGTTTAGGGTT---TA   2419

MH188536_Hulu   2406 CGGTTTAGGG-TTAGGGTTTAGGGTTTACGGGTTTCAGGGTTTAGGGTTT   2454
                     .||||||||| ||||||||||||||||| |||||| ||||||||||||||
SyntheticTTTA   2420 GGGTTTAGGGTTTAGGGTTTAGGGTTTA-GGGTTT-AGGGTTTAGGGTTT   2467

MH188536_Hulu   2455 ACGGGTTTAGAGGTTTA-GGTTTAGCGTTTTAGGGTTTAGGGTTTAGGGT   2503
                     | |||||||| |||||| ||||||| |.||||||||||||||||||||||
SyntheticTTTA   2468 A-GGGTTTAG-GGTTTAGGGTTTAG-GGTTTAGGGTTTAGGGTTTAGGGT   2514

MH188536_Hulu   2504 TTAGGGTTTAGGGTTTAGCGGTTTAGGGTGTTACGGGTTTAAGGTTTAGG   2553
                     |||||||||||||||||| |||||||||| ||| |||||||.||||||||
SyntheticTTTA   2515 TTAGGGTTTAGGGTTTAG-GGTTTAGGGT-TTA-GGGTTTAGGGTTTAGG   2561

MH188536_Hulu   2554 GTTTAGGGTTTAGGGGTTTTAAGG-TTAGGGTTTTAGGGTTTGAGGGTTT   2602
                     |||||||||||||||  ||||.|| |||||| |||||||||| |||||||
SyntheticTTTA   2562 GTTTAGGGTTTAGGG--TTTAGGGTTTAGGG-TTTAGGGTTT-AGGGTTT   2607

MH188536_Hulu   2603 AGGGGTTTAGCGGTTTAGGGTTTAGGAGTTAGGGTTTAGGTCTTA-GGTT   2651
                     | |||||||| |||||||||||||||..||||||||||||..||| ||||
SyntheticTTTA   2608 A-GGGTTTAG-GGTTTAGGGTTTAGGGTTTAGGGTTTAGGGTTTAGGGTT   2655

MH188536_Hulu   2652 TAGGGTTTAGGGTTTCAGGTTTAGGTTTTAGGGTTTAGGGTTTTAGGG-T   2700
                     |||||||||||||||..||||||||.|||||||||||||| ||||||| |
SyntheticTTTA   2656 TAGGGTTTAGGGTTTAGGGTTTAGGGTTTAGGGTTTAGGG-TTTAGGGTT   2704

MH188536_Hulu   2701 TAGGGTTT-GGGTTTAGGGTTTTAGGGTTTAGGGTTTAGGGTTTAGG--T   2747
                     |||||||| |||||||||| |||||||||||||||||||||||||||  |
SyntheticTTTA   2705 TAGGGTTTAGGGTTTAGGG-TTTAGGGTTTAGGGTTTAGGGTTTAGGGTT   2753

MH188536_Hulu   2748 TAGGGTTTAGGGTTTAGGGTTAGGTTTGGGTTTAGGGTTTA-GGTTTAGG   2796
                     ||||||||||||||||||||    ||.|||||||||||||| ||||||||
SyntheticTTTA   2754 TAGGGTTTAGGGTTTAGGGT----TTAGGGTTTAGGGTTTAGGGTTTAGG   2799

MH188536_Hulu   2797 GTTAGGTTAGGG-TTAGGGTTTAGGGTTTAGGGTTTAGGGTTAAGGGTTT   2845
                     ||    |||||| |||||||||||||||||||||||||||||.|||||||
SyntheticTTTA   2800 GT----TTAGGGTTTAGGGTTTAGGGTTTAGGGTTTAGGGTTTAGGGTTT   2845

MH188536_Hulu   2846 -GGGTTTAGGGTTTATGGGTTTAGGGTTTAGGGTT--AGGTTTAGGGTTT   2892
                      |||||||||||||| |||||||||||||||||||  .||||||||||||
SyntheticTTTA   2846 AGGGTTTAGGGTTTA-GGGTTTAGGGTTTAGGGTTTAGGGTTTAGGGTTT   2894

MH188536_Hulu   2893 AGGGTTTA--GTTTAGGGTTT-GGGTT--GGGTTTAGGG-TTAGGGTTTT   2936
                     ||||||||  ||||||||||| |||||  |||||||||| |||||| |||
```

```
SyntheticTTTA   2895 AGGGTTTAGGGTTTAGGGTTTAGGGTTTAGGGTTTAGGGTTTAGGG-TTT   2943

MH188536_Hulu   2937 TGGGTTTAGGGTTTAGGGTTTAGGGTTTAGGGTTTAGGGTTTAGGGTTTA   2986
                     .|||||||||||||||||||||||||||||||||||||||||||||||||
SyntheticTTTA   2944 AGGGTTTAGGGTTTAGGGTTTAGGGTTTAGGGTTTAGGGTTTAGGGTTTA   2993

MH188536_Hulu   2987 -GGTTTAGGGTTTAGGG-TTAGGGTTTAGGGTTTAGGGTTTAGGGTTTAG   3034
                      |||||||||||||||| ||||||||||||||||||||||||||||||| 
SyntheticTTTA   2994 GGGTTTAGGGTTTAGGGTTTAGGGTTTAGGGTTTAGGGTTTAGGGTTTA-   3042

MH188536_Hulu   3035 TTGTGTTTAGGGTTTA-GGTTTAGGGTTTACGGGTTTAGGGTTTA-GGTT   3082
                       |.|||||||||||| ||||||||||||| |||||||||||||| ||||
SyntheticTTTA   3043 --GGGTTTAGGGTTTAGGGTTTAGGGTTTA-GGGTTTAGGGTTTAGGGTT   3089

MH188536_Hulu   3083 TACGGGTTTAGGGTTTTAGGGTTTAGGGTTTAGGG-TTAGGGTTT-GGGT   3130
                     || |||||||||| ||||||||||||||||||||| ||||||||| ||||
SyntheticTTTA   3090 TA-GGGTTTAGGG-TTTAGGGTTTAGGGTTTAGGGTTTAGGGTTTAGGGT   3137

MH188536_Hulu   3131 TGAGGGGTTTAGGGTTT-GGGTTTAGGGTTTA-GGTTTAGCGGTTTA-GG   3177
                     |.| ||||||||||||| |||||||||||||| ||||||| |||||| ||
SyntheticTTTA   3138 TTA-GGGTTTAGGGTTTAGGGTTTAGGGTTTAGGGTTTAG-GGTTTAGGG   3185

MH188536_Hulu   3178 TTTAGGGTTTTGG--TTTGGGTTTAGGGTTTA-GGTTTAGGGTTTAGGGT   3224
                     ||||||||||.||  ||.|||||||||||||| |||||||||||||||||
SyntheticTTTA   3186 TTTAGGGTTTAGGGTTTAGGGTTTAGGGTTTAGGGTTTAGGGTTTAGGGT   3235

MH188536_Hulu   3225 TTAGGGTTTAGGGTTTAGGGTTTAGGGTTTAGGGTTTAGGGTTTA-GGTT   3273
                     ||||||||||||||||||||||||||||||||||||||||||||| ||||
SyntheticTTTA   3236 TTAGGGTTTAGGGTTTAGGGTTTAGGGTTTAGGGTTTAGGGTTTAGGGTT   3285

MH188536_Hulu   3274 TAGGGTTTTAGGGTTTAGGGTTTAGGGTTTAACAGGTTTAGGG-TTAGGG   3322
                     ||||| |||||||||||||||||||||||||  .||||||||| ||||||
SyntheticTTTA   3286 TAGGG-TTTAGGGTTTAGGGTTTAGGGTTTA--GGGTTTAGGGTTTAGGG   3332

MH188536_Hulu   3323 TTTAGGGTTTAGGGTTTAGGGTTTAGGGTTTAGGGTTTA-GGTTTAGGGT   3371
                     ||||||||||||||||||||||||||||||||||||||| ||||||||||
SyntheticTTTA   3333 TTTAGGGTTTAGGGTTTAGGGTTTAGGGTTTAGGGTTTAGGGTTTAGGGT   3382

MH188536_Hulu   3372 TTAGGGTTTA-GGTTTAGGGTTTAGGGGGGTTTTTAGG---TAGGGTTTA   3417
                     |||||||||| ||||||||||||||||     ||||||   |||||||||
SyntheticTTTA   3383 TTAGGGTTTAGGGTTTAGGGTTTAGGG-----TTTAGGGTTTAGGGTTTA   3427

MH188536_Hulu   3418 GGGTTTAGGGTTCTAGGG-TTAGGGTTCTAGGGTTTAGGGTTTAGGGTTT   3466
                     |||||||||||| ||||| |||||||| ||||||||||||||||||||||
SyntheticTTTA   3428 GGGTTTAGGGTT-TAGGGTTTAGGGTT-TAGGGTTTAGGGTTTAGGGTTT   3475

MH188536_Hulu   3467 AGGGTTTTAAGGGTTTTAGGGTTTAGGGTTTAGGGTTTAGGGTTTAGGGT   3516
                     |||||||  |||| ||||||||||||||||||||||||||||||||||||
SyntheticTTTA   3476 AGGGTTT--AGGG-TTTAGGGTTTAGGGTTTAGGGTTTAGGGTTTAGGGT   3522

MH188536_Hulu   3517 T--AGGTTTAGGGTTTAGGG-TTAGGGTTTTAGGGTTTAGGGTTTAGGGT   3563
                     |  .|||||||||||||||| |||||| ||||||||||||||||||||| 
SyntheticTTTA   3523 TTAGGGTTTAGGGTTTAGGGTTTAGGG-TTTAGGGTTTAGGGTTTAGGG-   3570

MH188536_Hulu   3564 TTTAGGG-TTAGGGTTTAGGGTTTAGGGTTTAGGGTTTCAGGGTTTAGGG   3612
                     ||||||| |||||||||||||||||||||||||||||| |||||||||||
```

```
SyntheticTTTA   3571 TTTAGGGTTTAGGGTTTAGGGTTTAGGGTTTAGGGTTT-AGGGTTTAGGG   3619

MH188536_Hulu   3613 TTT-GGGTTTAGGGTTTAGGGTTTTAGGGTTTAGGGTTTA-GGTTTAGGG   3660
                     ||| ||||||||||||||||| |||||||||||||||||| |||||||||
SyntheticTTTA   3620 TTTAGGGTTTAGGGTTTAGGG-TTTAGGGTTTAGGGTTTAGGGTTTAGGG   3668

MH188536_Hulu   3661 TTTTTAGGGTTTAGGGTTTAGGGTTTAGGGTTTAAGGGTTTTAGGGTTTA   3710
                       ||||||||||||||||||||||||||||||||  ||.|||||||||||
SyntheticTTTA   3669 --TTTAGGGTTTAGGGTTTAGGGTTTAGGGTTTA--GGGTTTAGGGTTTA   3714

MH188536_Hulu   3711 GGGTTTAGGGTTTTGGGTTTAGGGTTTAGGGTTTAAGG-TTAGGGTTTTA   3759
                     |||||||||||||.|||||||||||||||||||||.|| |||||| ||||
SyntheticTTTA   3715 GGGTTTAGGGTTTAGGGTTTAGGGTTTAGGGTTTAGGGTTTAGGG-TTTA   3763

MH188536_Hulu   3760 GGGTT--AGGTTTAGGGTTTAGGGTTTAGCGGTTTCAGGGTTTAAGGGTT   3807
                     |||||  .||||||||||||||||||||| ||||| ||||||| ||||||
SyntheticTTTA   3764 GGGTTTAGGGTTTAGGGTTTAGGGTTTAG-GGTTT-AGGGTTT-AGGGTT   3810

MH188536_Hulu   3808 TAGGGTTTCCAGGGTTTCAGGGTTTAGGGATTTCAGGGTTTTCAGGGTTT   3857
                     ||||||||  ||||||| |||||||||||  ||.|||||||  |||||||
SyntheticTTTA   3811 TAGGGTTT--AGGGTTT-AGGGTTTAGGG--TTTAGGGTTT--AGGGTTT   3853

MH188536_Hulu   3858 AGGGTTTAGGGTTTA-GGTTTAGGGTTTAGGGTTTCAGGGTTT---TTTT   3903
                     ||||||||||||||| ||||||||||||||||||| |||||||   .|||
SyntheticTTTA   3854 AGGGTTTAGGGTTTAGGGTTTAGGGTTTAGGGTTT-AGGGTTTAGGGTTT   3902

MH188536_Hulu   3904 AGGGGTATTTAGGGTTTAGGGTTTAGGGTTTAGGGTTTTAGGCGTTTA-G   3952
                     ||||   |||||||||||||||||||||||||||| |||||| ||||| |
SyntheticTTTA   3903 AGGG---TTTAGGGTTTAGGGTTTAGGGTTTAGGG-TTTAGG-GTTTAGG   3947

MH188536_Hulu   3953 GTTTAGGGTTTTAGGGTTTTAGGGTTTAGGGTTTTAGGGTTTAGGTTT--   4000
                     |||||||| ||||||| |||||||||||||| ||||||||||||||||
SyntheticTTTA   3948 GTTTAGGG-TTTAGGG-TTTAGGGTTTAGGG-TTTAGGGTTTAGGTTTAG   3994

MH188536_Hulu   4001 ------   4000

SyntheticTTTA   3995 GTTTAG   4000

#---------------------------------------
#---------------------------------------
```

```
########################################
# Program: water
# Rundate: Fri 31 Jan 2020 20:21:49
# Commandline: water
#    -auto
#    -stdout
#    -asequence emboss_water-I20200131-202146-0108-43146441-p1m.asequence
#    -bsequence emboss_water-I20200131-202146-0108-43146441-p1m.bsequence
#    -datafile EDNAFULL
#    -gapopen 10.0
#    -gapextend 0.5
#    -aformat3 pair
#    -snucleotide1
#    -snucleotide2
# Align_format: pair
# Report_file: stdout
########################################

#=======================================
#
# Aligned_sequences: 2
# 1: MH188536_HuluTR180TEL_954_terminalTeloRepeatRegion_4kbp
# 2: SyntheticTTTAGGG_TelomereRepeat_4kbp
# Matrix: EDNAFULL
# Gap_penalty: 10.0
# Extend_penalty: 0.5
#
# Length: 4146
# Identity:    3749/4146 (90.4%)
# Similarity:  3749/4146 (90.4%)
# Gaps:         302/4146 ( 7.3%)
# Score: 16048.0
#
#
#=======================================

MH188536_Hulu      3 TTTAGGGTTTAGGCGTTCTGGGTTTAGGGTTTA-GGTTTAGGGTTTGAGG     51
                     ||||||||||||| |||..|||||||||||||| |||||||||||| |||
SyntheticTTTA      1 TTTAGGGTTTAGG-GTTTAGGGTTTAGGGTTTAGGGTTTAGGGTTT-AGG     48

MH188536_Hulu     52 GTTTAGG--TTAGGGTTTAGGGTTTA-GGTTTAGGG-TTAGGGTTTAGGG     97
                     |||||||  ||||||||||||||||| ||||||||| |||||||||||||
SyntheticTTTA     49 GTTTAGGGTTTAGGGTTTAGGGTTTAGGGTTTAGGGTTTAGGGTTTAGGG     98

MH188536_Hulu     98 TTTAGGGTTTA-GGTTTAGGG-TTAGGGTTTAGGGTTTAAGGGTTTTAGG    145
                     ||||||||||| ||||||||| |||||||||||||||||  ||.||||||
SyntheticTTTA     99 TTTAGGGTTTAGGGTTTAGGGTTTAGGGTTTAGGGTTTA--GGGTTTAGG    146

MH188536_Hulu    146 GTCCTTAGAGGGTTACGGGTTTAGGGTTTAGGGTTTAGGGTTTA-GGTTT    194
                     ||  ||  ||||||..|||||||||||||||||||||||||||| |||||
SyntheticTTTA    147 GT--TT--AGGGTTTAGGGTTTAGGGTTTAGGGTTTAGGGTTTAGGGTTT    192

MH188536_Hulu    195 AGGGTTTA-GGTTTAGGGTTTAGGGTTTAGGGTTTAGGGTTTAGGGGTTT    243
                     |||||||| |||||||||||||||||||||||||||||||||| ||||||
```

```
SyntheticTTTA     193 AGGGTTTAGGGTTTAGGGTTTAGGGTTTAGGGTTTAGGGTTTA-GGGTTT    241

MH188536_Hulu     244 A-GGTTTAGGGTTTA-GGTTTAGGGTTTAGGGTTTAGGGTTTAGGGTTTA    291
                      | ||||||||||||| ||||||||||||||||||||||||||||||||||
SyntheticTTTA     242 AGGGTTTAGGGTTTAGGGTTTAGGGTTTAGGGTTTAGGGTTTAGGGTTTA    291

MH188536_Hulu     292 GGGTTGTAGGG-TTAGGGTTTA-GGTTTAGGGTTTA-GGTTTAGGGTTTA    338
                      ||||| ||||| |||||||||| ||||||||||||| ||||||||||||
SyntheticTTTA     292 GGGTT-TAGGGTTTAGGGTTTAGGGTTTAGGGTTTAGGGTTTAGGGTTT-    339

MH188536_Hulu     339 AGGGTTTAGGG-TTAGGGTTTAGGGTTTAAGGGTTTAGGG-TTAGGGTTT    386
                      ||||||||||| |||||||||||||||| ||||||||||| |||||||||
SyntheticTTTA     340 AGGGTTTAGGGTTTAGGGTTTAGGGTTT-AGGGTTTAGGGTTTAGGGTTT    388

MH188536_Hulu     387 A-GGTTTAGGGTTTAGGGTTTAGGGTTTAGGGTTTA-GGTTTAGGGTT--    432
                      | |||||||||||||||||||||||||||||||||| |||||||||||
SyntheticTTTA     389 AGGGTTTAGGGTTTAGGGTTTAGGGTTTAGGGTTTAGGGTTTAGGGTTTA    438

MH188536_Hulu     433 --GTTTAGGG-TTAGGGTT--AGGTTTAGGGTTTAGGGTTTAGGGTTTTT    477
                        |||||||| ||||||||  .|||||||||||||||||||||||  |||
SyntheticTTTA     439 GGGTTTAGGGTTTAGGGTTTAGGGTTTAGGGTTTAGGGTTTAGGG--TTT    486

MH188536_Hulu     478 AGGGTTTAGGGTTTAGGGTTTAGGG-TTAGGG-TTAGGGTTTAGGGTTTC    525
                      ||||||||||||||||||||||||| |||||| ||||||||||||||||
SyntheticTTTA     487 AGGGTTTAGGGTTTAGGGTTTAGGGTTTAGGGTTTAGGGTTTAGGGTTT-    535

MH188536_Hulu     526 AGGGTTTAGGGTTTTAGGGTTTA-GGTTCAAGGTTTAGGGTGTTAGGGTT    574
                      ||||||||||| ||||||||||| ||||.|.|||||||||| ||||||||
SyntheticTTTA     536 AGGGTTTAGGG-TTTAGGGTTTAGGGTTTAGGGTTTAGGGT-TTAGGGTT    583

MH188536_Hulu     575 TAGGGTTTAGGGTTTAGGGTTTAGGGTTTAGGGTTTAGGTTTTAGGGTTT    624
                      |||||||||||||||||||||||||||||||||||||||.||||||| ||
SyntheticTTTA     584 TAGGGTTTAGGGTTTAGGGTTTAGGGTTTAGGGTTTAGGGTTTAGGG-TT    632

MH188536_Hulu     625 TAGGGTTTAGGGTTTAGGGTTTAGGGTTTAGGGTTTCACGGGTTAGG--T    672
                      |||||||||||||||||||||||||||||||||||| |.||.|||||  |
SyntheticTTTA     633 TAGGGTTTAGGGTTTAGGGTTTAGGGTTTAGGGTTT-AGGGTTTAGGGTT    681

MH188536_Hulu     673 TAGGGCTTTCAGGGTTTAGGGTTTAGGGTTTAAGGTTTAGGGTTTA--GT    720
                      |||||  ||.||||||||||||||||||||||.|||||||||||||  ||
SyntheticTTTA     682 TAGGG--TTTAGGGTTTAGGGTTTAGGGTTTAGGGTTTAGGGTTTAGGGT    729

MH188536_Hulu     721 TTAGGGTTTAGGGTTTAGGGTTTAGGGTT--AGGTTTAGGGTTT-GGGTT    767
                      |||||||||||||||||||||||||||||  .|||||||||||| |||||
SyntheticTTTA     730 TTAGGGTTTAGGGTTTAGGGTTTAGGGTTTAGGGTTTAGGGTTTAGGGTT    779

MH188536_Hulu     768 TAGGGTT--GGGTTTAGGGTTTAGGGTTTACGCGGTTTAGGGTTTTTAGG    815
                      |||||||  |||||||||||||||||||||  .|||||||||  ||||||
SyntheticTTTA     780 TAGGGTTTAGGGTTTAGGGTTTAGGGTTTA--GGGTTTAGGG--TTTAGG    825

MH188536_Hulu     816 GTTTAGGGTTTAGGGTTTAGGTTTTAGGGTTTAGGGTTTAGGGTTTCAGG    865
                      |||||||||||||||||||||.|||||||||||||||||||||||| |||
SyntheticTTTA     826 GTTTAGGGTTTAGGGTTTAGGGTTTAGGGTTTAGGGTTTAGGGTTT-AGG    874

MH188536_Hulu     866 GTTT-GGGTTT-GGGTTTAGGGTTTCAGGGTTTAGGGTTTACGGGTTTAG    913
                      |||| |||||| ||||||||||||| ||||||||||||||| ||||||||
```

```
SyntheticTTTA    875 GTTTAGGGTTTAGGGTTTAGGGTTT-AGGGTTTAGGGTTTA-GGGTTTAG    922

MH188536_Hulu    914 CGGTTTAGGGTTTAGGGTTTAAGG-TTAGGGTTTAGGTTTTAAGGGTTTA    962
                      ||||||||||||||||||||.|| ||||||||||||.||| ||||||||
SyntheticTTTA    923 -GGTTTAGGGTTTAGGGTTTAGGGTTTAGGGTTTAGGGTTT-AGGGTTTA    970

MH188536_Hulu    963 GGGTTTAAGGGTTTAGGGTT---AGTTTAGGGTTTAGGGTTTA-GGTTCA   1008
                     |||||| |||||||||||||   .||||||||||||||||||| ||||.|
SyntheticTTTA    971 GGGTTT-AGGGTTTAGGGTTTAGGGTTTAGGGTTTAGGGTTTAGGGTTTA   1019

MH188536_Hulu   1009 GGGTTAGGGGTTTCAGGGTTTAGGGTTTGGGGTTTAGGGTTTAGG--TTA   1056
                     |||||..|||||| ||||||||||||||.||||||||||||||||  |||
SyntheticTTTA   1020 GGGTTTAGGGTTT-AGGGTTTAGGGTTTAGGGTTTAGGGTTTAGGGTTTA   1068

MH188536_Hulu   1057 GGTTTTATGGGTTTAGGGTTCAGGGTTTAGGGTTTAGGGTTTAAGGGTTT   1106
                     ||.|||| ||||||||||||.||||||||||||||||||||| |||||||
SyntheticTTTA   1069 GGGTTTA-GGGTTTAGGGTTTAGGGTTTAGGGTTTAGGGTTT-AGGGTTT   1116

MH188536_Hulu   1107 AGGGTTTTAGGGTTTA-GGTTTAGGGTTTA-GGTTTAGGGTTTAGGGTTT   1154
                     |||| ||||||||||| ||||||||||||| |||||||||||||||||||
SyntheticTTTA   1117 AGGG-TTTAGGGTTTAGGGTTTAGGGTTTAGGGTTTAGGGTTTAGGGTTT   1165

MH188536_Hulu   1155 GAGGGTTTAGGGTTT-GGGTTTAGGGTTTAGGG-TTAGGGTT--GGTTTT   1200
                      |||||||||||||| ||||||||||||||||| ||||||||  ||.|||
SyntheticTTTA   1166 -AGGGTTTAGGGTTTAGGGTTTAGGGTTTAGGGTTTAGGGTTTAGGGTTT   1214

MH188536_Hulu   1201 AGGGTTTA-GGTTTAGGGTTTTAGGGTTTAGGGTTCAGGGTTTTAGGGTT   1249
                     |||||||| ||||||||| ||||||||||||||||.|||| |||||||||
SyntheticTTTA   1215 AGGGTTTAGGGTTTAGGG-TTTAGGGTTTAGGGTTTAGGG-TTTAGGGTT   1262

MH188536_Hulu   1250 TAGGGTTTAGGGTTTAGGGTTTAGGGTTTAGGGTTTAGGGTTTAGGGTTT   1299
                     ||||||||||||||||||||||||||||||||||||||||||||||||||
SyntheticTTTA   1263 TAGGGTTTAGGGTTTAGGGTTTAGGGTTTAGGGTTTAGGGTTTAGGGTTT   1312

MH188536_Hulu   1300 ACGGTTTA--GTTTAGGGTTTTAGGGTTTAGGGTTTA-GGTTTAGG--TT   1344
                     |.||||||  |||||||| |||||||||||||||||| ||||||||  ||
SyntheticTTTA   1313 AGGGTTTAGGGTTTAGGG-TTTAGGGTTTAGGGTTTAGGGTTTAGGGTTT   1361

MH188536_Hulu   1345 AGG--TTTGGGTTTCAGGTTTCTAGGGTTTAGGGTTTTAGGG-TTAGGGT   1391
                     |||  ||.||||||..||||  |||||||||||| ||||||| |||||||
SyntheticTTTA   1362 AGGGTTTAGGGTTTAGGGTT--TAGGGTTTAGGG-TTTAGGGTTTAGGGT   1408

MH188536_Hulu   1392 TTAGGGTTTAGGGTCT--GGTCTAGGGTTTAGGGTTTAGGGTTTAGGGTT   1439
                     ||||||||||||||.|  |||.||||||||||||||||||||||||||||
SyntheticTTTA   1409 TTAGGGTTTAGGGTTTAGGGTTTAGGGTTTAGGGTTTAGGGTTTAGGGTT   1458

MH188536_Hulu   1440 TAGGGTTTAGGGTTTTAGGGTTTAGGGTTTAGGGTTTTAGGGTTTTAGGT   1489
                     |||||||||||| ||||||||||||||||||||| ||||||||||..|||
SyntheticTTTA   1459 TAGGGTTTAGGG-TTTAGGGTTTAGGGTTTAGGG-TTTAGGGTTTAGGGT   1506

MH188536_Hulu   1490 TTAGGGTTTAGGGTCTTAGGGTTTAGGGTTTTAGGGTTTAGGGTTTAAGG   1539
                     |||||||||||||| ||||||||||||| ||||||||||||||||| |||
SyntheticTTTA   1507 TTAGGGTTTAGGGT-TTAGGGTTTAGGG-TTTAGGGTTTAGGGTTT-AGG   1553

MH188536_Hulu   1540 GTT---GGTTTAGGTTTCAGGGTTTAGGGTTTAAGGGTTTAGGGTTTAGG   1586
                     |||   ||||||||.||.|||||||||||||| |||||||||||||||||
```

```
SyntheticTTTA   1554 GTTTAGGGTTTAGGGTTTAGGGTTTAGGGTTT-AGGGTTTAGGGTTTAGG   1602

MH188536_Hulu   1587 GTTTAGGGTTTTAGGTTTA-GGTTTAGGGTTTAGGTTTAAGGGTTTAGGG   1635
                     |||||||||||..|||||| |||||||||||||||.||.|||||||||||
SyntheticTTTA   1603 GTTTAGGGTTTAGGGTTTAGGGTTTAGGGTTTAGGGTTTAGGGTTTAGGG   1652

MH188536_Hulu   1636 TTT-GGGTTTAGGGTTTACCGGGTTTGAGGGTTTTAGGGTTTAGGGTTTA   1684
                     ||| ||||||||||||||  |||||| |||| ||||||||||||||||||
SyntheticTTTA   1653 TTTAGGGTTTAGGGTTTA--GGGTTT-AGGG-TTTAGGGTTTAGGGTTTA   1698

MH188536_Hulu   1685 GGGTTTAGGGTTTAGGGGTTTTAGGGTTTTAGGGTTTAGGGGTTTAGGGT   1734
                     |||||||||||||||||  ||||||| ||||||||||| |||||||||||
SyntheticTTTA   1699 GGGTTTAGGGTTTAGGG--TTTAGGG-TTTAGGGTTTA-GGGTTTAGGGT   1744

MH188536_Hulu   1735 TTAGGGTTTAGGGTTTA-GGTTTA-GGTTTA-GGTTTA-GGTTTAGGGTT   1780
                     ||||||||||||||||| |||||| |||||| |||||| |||||||||||
SyntheticTTTA   1745 TTAGGGTTTAGGGTTTAGGGTTTAGGGTTTAGGGTTTAGGGTTTAGGGTT   1794

MH188536_Hulu   1781 TAAGGGTTAGGGTT--AGGTTTAGGGTTTAGGGTTTACGGGTGAGGGTTT   1828
                     ||.||.||||||||  .||||||||||||||||||||.||.|.|||||||
SyntheticTTTA   1795 TAGGGTTTAGGGTTTAGGGTTTAGGGTTTAGGGTTTAGGGTTTAGGGTTT   1844

MH188536_Hulu   1829 ACGGGTTTAGGGTTAAGGGTTTAGGGTTTAGGGTTTAGGGTTTAGGGTTT   1878
                     | ||||||||||||.|||||||||||||||||||||||||||||||||||
SyntheticTTTA   1845 A-GGGTTTAGGGTTTAGGGTTTAGGGTTTAGGGTTTAGGGTTTAGGGTTT   1893

MH188536_Hulu   1879 AAGGTTTAGGGTTTAGGGTTTAGGGTTTAGGGTTTAGGGTTTAGGGTTTA   1928
                     |.||||||||||||||||||||||||||||||||||||||||||||||||
SyntheticTTTA   1894 AGGGTTTAGGGTTTAGGGTTTAGGGTTTAGGGTTTAGGGTTTAGGGTTTA   1943

MH188536_Hulu   1929 GG---TAGGGTTTAGGG-TTAGGGTTTAGGGTTTTACGGGTTTA-GGTT   1973
                     ||   |||||||||||| ||||||||||||||||   .||||||| ||||
SyntheticTTTA   1944 GGGTTTAGGGTTTAGGGTTTAGGGTTTAGGGTTT---AGGGTTTAGGGTT   1990

MH188536_Hulu   1974 TAGGG-TTAGGGTTTAGGGCTTTAGGGTTTAGGGTTTCAGGGTTATAGGG   2022
                     ||||| ||||||||||||| ||||||||||||||||| |||||| |||||
SyntheticTTTA   1991 TAGGGTTTAGGGTTTAGGG-TTTAGGGTTTAGGGTTT-AGGGTT-TAGGG   2037

MH188536_Hulu   2023 -TTAGGGTTTTAGGGTTT--GGGTTAGGGTTTGGGGTTTAAGGGTTTAGG   2069
                      |||||| ||||||||||  ||.|||||||||.|||||| ||||||||||
SyntheticTTTA   2038 TTTAGGG-TTTAGGGTTTAGGGTTTAGGGTTTAGGGTTT-AGGGTTTAGG   2085

MH188536_Hulu   2070 GTTTA-GGTTTAGGGTTTAGGGTTTTAGGGTTTGGGTTTTAGGGTT--AG   2116
                     ||||| |||||||||||||||| ||||||||||.||.|||||||||  .|
SyntheticTTTA   2086 GTTTAGGGTTTAGGGTTTAGGG-TTTAGGGTTTAGGGTTTAGGGTTTAGG   2134

MH188536_Hulu   2117 GTTTAGGGTTTAGGGTTT-GGGTTTAGGGTTT-CGGTTTAGGGTTTAGGG   2164
                     |||||||||||||||||| ||||||||||||| .||||||||||||||||
SyntheticTTTA   2135 GTTTAGGGTTTAGGGTTTAGGGTTTAGGGTTTAGGGTTTAGGGTTTAGGG   2184

MH188536_Hulu   2165 TTTAGGTTTTGAGGTTTA-GGTTCAGGGTTTAGGTTTTGAGGTTTACGGG   2213
                     ||||||.|||..|||||| ||||.||||||||||.|||..||||||   |
SyntheticTTTA   2185 TTTAGGGTTTAGGGTTTAGGGTTTAGGGTTTAGGGTTTAGGGTTTA---G   2231

MH188536_Hulu   2214 CGTTTAGGGTTTGAGG--TTAGGGTTTCAGGGTTTAGGGTTTAAGGGTTT   2261
                     .||||||||||| |||  ||||||||| |||||||||||||| |||||||
```

```
SyntheticTTTA   2232 GGTTTAGGGTTT-AGGGTTTAGGGTTT-AGGGTTTAGGGTTT-AGGGTTT   2278

MH188536_Hulu   2262 AGGTTTCAGGGTTTAGGGTTTAGGGCTTTAGGGTTTCAGGG-TTAGGGTT   2310
                     |||.||.|||||||||||||||||| |||||||||| |||| ||||||||
SyntheticTTTA   2279 AGGGTTTAGGGTTTAGGGTTTAGGG-TTTAGGGTTT-AGGGTTTAGGGTT   2326

MH188536_Hulu   2311 T-GGGTTTA-GGTTTAGGTTTTAGGGTTTTAGGGTTTA-GGTTTAGGGTT   2357
                     | ||||||| ||||||||.||||||| ||||||||||| |||||||||||
SyntheticTTTA   2327 TAGGGTTTAGGGTTTAGGGTTTAGGG-TTTAGGGTTTAGGGTTTAGGGTT   2375

MH188536_Hulu   2358 TAGGGTTTAGGGTTTTAGGTTTTAGGGTTTAGGGTTTAGGGTTGTATACG   2407
                     |||||||||||| ||||||.|||||||||||||||||||||||   ||.|
SyntheticTTTA   2376 TAGGGTTTAGGG-TTTAGGGTTTAGGGTTTAGGGTTTAGGGTT---TAGG   2421

MH188536_Hulu   2408 GTTTAGGG-TTAGGGTTTAGGGTTTACGGGTTTCAGGGTTTAGGGTTTAC   2456
                     |||||||| ||||||||||||||||| |||||| ||||||||||||||| 
SyntheticTTTA   2422 GTTTAGGGTTTAGGGTTTAGGGTTTA-GGGTTT-AGGGTTTAGGGTTTA-   2468

MH188536_Hulu   2457 GGGTTTAGAGGTTTA-GGTTTAGCGTTTTAGGGTTTAGGGTTTAGGGTTT   2505
                     |||||||| |||||| ||||||| |.||||||||||||||||||||||||
SyntheticTTTA   2469 GGGTTTAG-GGTTTAGGGTTTAG-GGTTTAGGGTTTAGGGTTTAGGGTTT   2516

MH188536_Hulu   2506 AGGGTTTAGGGTTTAGCGGTTTAGGGTGTTACGGGTTTAAGGTTTAGGGT   2555
                     |||||||||||||||| |||||||||| ||| |||||||.||||||||||
SyntheticTTTA   2517 AGGGTTTAGGGTTTAG-GGTTTAGGGT-TTA-GGGTTTAGGGTTTAGGGT   2563

MH188536_Hulu   2556 TTAGGGTTTAGGGGTTTTAAGG-TTAGGGTTTTAGGGTTTGAGGGTTTAG   2604
                     |||||||||||||  ||||.|| |||||| |||||||||| |||||||| 
SyntheticTTTA   2564 TTAGGGTTTAGGG--TTTAGGGTTTAGGG-TTTAGGGTTT-AGGGTTTA-   2608

MH188536_Hulu   2605 GGGTTTAGCGGTTTAGGGTTTAGGAGTTAGGGTTTAGGTCTTA-GGTTTA   2653
                     |||||||| |||||||||||||||..||||||||||||..||| ||||||
SyntheticTTTA   2609 GGGTTTAG-GGTTTAGGGTTTAGGGTTTAGGGTTTAGGGTTTAGGGTTTA   2657

MH188536_Hulu   2654 GGGTTTAGGGTTTCAGGTTTAGGTTTTAGGGTTTAGGGTTTTAGGG-TTA   2702
                     |||||||||||||..||||||||.|||||||||||||| ||||||| |||
SyntheticTTTA   2658 GGGTTTAGGGTTTAGGGTTTAGGGTTTAGGGTTTAGGG-TTTAGGGTTTA   2706

MH188536_Hulu   2703 GGGTTT-GGGTTTAGGGTTTTAGGGTTTAGGGTTTAGGGTTTAGG--TTA   2749
                     |||||| |||||||||| |||||||||||||||||||||||||||  |||
SyntheticTTTA   2707 GGGTTTAGGGTTTAGGG-TTTAGGGTTTAGGGTTTAGGGTTTAGGGTTTA   2755

MH188536_Hulu   2750 GGGTTTAGGGTTTAGGGTTAGGTTTGGGTTTAGGGTTTA-GGTTTAGGGT   2798
                     ||||||||||||||||||    ||.|||||||||||||| ||||||||||
SyntheticTTTA   2756 GGGTTTAGGGTTTAGGGT----TTAGGGTTTAGGGTTTAGGGTTTAGGGT   2801

MH188536_Hulu   2799 TAGGTTAGGG-TTAGGGTTTAGGGTTTAGGGTTTAGGGTTAAGGGTTT-G   2846
                         |||||| |||||||||||||||||||||||||||||.||||||| |
SyntheticTTTA   2802 ----TTAGGGTTTAGGGTTTAGGGTTTAGGGTTTAGGGTTTAGGGTTTAG   2847

MH188536_Hulu   2847 GGTTTAGGGTTTATGGGTTTAGGGTTTAGGGTT--AGGTTTAGGGTTTAG   2894
                     ||||||||||||| |||||||||||||||||||  .||||||||||||||
SyntheticTTTA   2848 GGTTTAGGGTTTA-GGGTTTAGGGTTTAGGGTTTAGGGTTTAGGGTTTAG   2896

MH188536_Hulu   2895 GGTTTA--GTTTAGGGTTT-GGGTT--GGGTTTAGGG-TTAGGGTTTTTG   2938
                     ||||||  ||||||||||| |||||  |||||||||| |||||| |||.|
```

```
SyntheticTTTA   2897 GGTTTAGGGTTTAGGGTTTAGGGTTTAGGGTTTAGGGTTTAGGG-TTTAG   2945

MH188536_Hulu   2939 GGTTTAGGGTTTAGGGTTTAGGGTTTAGGGTTTAGGGTTTAGGGTTTA-G   2987
                     |||||||||||||||||||||||||||||||||||||||||||||||| |
SyntheticTTTA   2946 GGTTTAGGGTTTAGGGTTTAGGGTTTAGGGTTTAGGGTTTAGGGTTTAGG   2995

MH188536_Hulu   2988 GTTTAGGGTTTAGGG-TTAGGGTTTAGGGTTTAGGGTTTAGGGTTTAGTT   3036
                     ||||||||||||||| |||||||||||||||||||||||||||||||
SyntheticTTTA   2996 GTTTAGGGTTTAGGGTTTAGGGTTTAGGGTTTAGGGTTTAGGGTTTA---   3042

MH188536_Hulu   3037 GTGTTTAGGGTTTA-GGTTTAGGGTTTACGGGTTTAGGGTTTA-GGTTTA   3084
                     |.|||||||||||| ||||||||||||| |||||||||||||| ||||||
SyntheticTTTA   3043 GGGTTTAGGGTTTAGGGTTTAGGGTTTA-GGGTTTAGGGTTTAGGGTTTA   3091

MH188536_Hulu   3085 CGGGTTTAGGGTTTTAGGGTTTAGGGTTTAGGG-TTAGGGTTT-GGGTTG   3132
                      |||||||||| ||||||||||||||||||||| ||||||||| |||||.
SyntheticTTTA   3092 -GGGTTTAGGG-TTTAGGGTTTAGGGTTTAGGGTTTAGGGTTTAGGGTTT   3139

MH188536_Hulu   3133 AGGGGTTTAGGGTTT-GGGTTTAGGGTTTA-GGTTTAGCGGTTTA-GGTT   3179
                     | ||||||||||||| |||||||||||||| ||||||| |||||| ||||
SyntheticTTTA   3140 A-GGGTTTAGGGTTTAGGGTTTAGGGTTTAGGGTTTAG-GGTTTAGGGTT   3187

MH188536_Hulu   3180 TAGGGTTTTGG--TTTGGGTTTAGGGTTTA-GGTTTAGGGTTTAGGGTTT   3226
                     ||||||||.||  ||.|||||||||||||| |||||||||||||||||||
SyntheticTTTA   3188 TAGGGTTTAGGGTTTAGGGTTTAGGGTTTAGGGTTTAGGGTTTAGGGTTT   3237

MH188536_Hulu   3227 AGGGTTTAGGGTTTAGGGTTTAGGGTTTAGGGTTTAGGGTTTA-GGTTTA   3275
                     ||||||||||||||||||||||||||||||||||||||||||| ||||||
SyntheticTTTA   3238 AGGGTTTAGGGTTTAGGGTTTAGGGTTTAGGGTTTAGGGTTTAGGGTTTA   3287

MH188536_Hulu   3276 GGGTTTTAGGGTTTAGGGTTTAGGGTTTAACAGGTTTAGGG-TTAGGGTT   3324
                     ||| |||||||||||||||||||||||||  .||||||||| ||||||||
SyntheticTTTA   3288 GGG-TTTAGGGTTTAGGGTTTAGGGTTTA--GGGTTTAGGGTTTAGGGTT   3334

MH188536_Hulu   3325 TAGGGTTTAGGGTTTAGGGTTTAGGGTTTAGGGTTTA-GGTTTAGGGTTT   3373
                     ||||||||||||||||||||||||||||||||||||| ||||||||||||
SyntheticTTTA   3335 TAGGGTTTAGGGTTTAGGGTTTAGGGTTTAGGGTTTAGGGTTTAGGGTTT   3384

MH188536_Hulu   3374 AGGGTTTA-GGTTTAGGGTTTAGGGGGGTTTTTAGG---TAGGGTTTAGG   3419
                     |||||||| ||||||||||||||||     ||||||   |||||||||||
SyntheticTTTA   3385 AGGGTTTAGGGTTTAGGGTTTAGGG-----TTTAGGGTTTAGGGTTTAGG   3429

MH188536_Hulu   3420 GTTTAGGGTTCTAGGG-TTAGGGTTCTAGGGTTTAGGGTTTAGGGTTTAG   3468
                     |||||||||| ||||| |||||||| ||||||||||||||||||||||||
SyntheticTTTA   3430 GTTTAGGGTT-TAGGGTTTAGGGTT-TAGGGTTTAGGGTTTAGGGTTTAG   3477

MH188536_Hulu   3469 GGTTTTAAGGGTTTTAGGGTTTAGGGTTTAGGGTTTAGGGTTTAGGGTT-   3517
                     |||||  |||| |||||||||||||||||||||||||||||||||||||
SyntheticTTTA   3478 GGTTT--AGGG-TTTAGGGTTTAGGGTTTAGGGTTTAGGGTTTAGGGTTT   3524

MH188536_Hulu   3518 -AGGTTTAGGGTTTAGGG-TTAGGGTTTTAGGGTTTAGGGTTTAGGGTTT   3565
                      .|||||||||||||||| |||||| ||||||||||||||||||||| ||
SyntheticTTTA   3525 AGGGTTTAGGGTTTAGGGTTTAGGG-TTTAGGGTTTAGGGTTTAGGG-TT   3572

MH188536_Hulu   3566 TAGGG-TTAGGGTTTAGGGTTTAGGGTTTAGGGTTTCAGGGTTTAGGGTT   3614
                     ||||| |||||||||||||||||||||||||||||| |||||||||||||
```

```
SyntheticTTTA   3573 TAGGGTTTAGGGTTTAGGGTTTAGGGTTTAGGGTTT-AGGGTTTAGGGTT   3621

MH188536_Hulu   3615 T-GGGTTTAGGGTTTAGGGTTTTAGGGTTTAGGGTTTA-GGTTTAGGGTT   3662
                     | ||||||||||||||||| |||||||||||||||||| |||||||||  
SyntheticTTTA   3622 TAGGGTTTAGGGTTTAGGG-TTTAGGGTTTAGGGTTTAGGGTTTAGGG--   3668

MH188536_Hulu   3663 TTTAGGGTTTAGGGTTTAGGGTTTAGGGTTTAAGGGTTTTAGGGTTTAGG   3712
                     ||||||||||||||||||||||||||||||||  ||.|||||||||||||
SyntheticTTTA   3669 TTTAGGGTTTAGGGTTTAGGGTTTAGGGTTTA--GGGTTTAGGGTTTAGG   3716

MH188536_Hulu   3713 GTTTAGGGTTTTGGGTTTAGGGTTTAGGGTTTAAGG-TTAGGGTTTTAGG   3761
                     |||||||||||.|||||||||||||||||||||.|| |||||| ||||||
SyntheticTTTA   3717 GTTTAGGGTTTAGGGTTTAGGGTTTAGGGTTTAGGGTTTAGGG-TTTAGG   3765

MH188536_Hulu   3762 GTT--AGGTTTAGGGTTTAGGGTTTAGCGGTTTCAGGGTTTAAGGGTTTA   3809
                     |||  .||||||||||||||||||||| ||||| ||||||| ||||||||
SyntheticTTTA   3766 GTTTAGGGTTTAGGGTTTAGGGTTTAG-GGTTT-AGGGTTT-AGGGTTTA   3812

MH188536_Hulu   3810 GGGTTTCCAGGGTTTCAGGGTTTAGGGATTTCAGGGTTTTCAGGGTTTAG   3859
                     ||||||  ||||||| |||||||||||  ||.|||||||  |||||||||
SyntheticTTTA   3813 GGGTTT--AGGGTTT-AGGGTTTAGGG--TTTAGGGTTT--AGGGTTTAG   3855

MH188536_Hulu   3860 GGTTTAGGGTTTA-GGTTTAGGGTTTAGGGTTTCAGGGTTT---TTTTAG   3905
                     ||||||||||||| ||||||||||||||||||| |||||||   .|||||
SyntheticTTTA   3856 GGTTTAGGGTTTAGGGTTTAGGGTTTAGGGTTT-AGGGTTTAGGGTTTAG   3904

MH188536_Hulu   3906 GGGTATTTAGGGTTTAGGGTTTAGGGTTTAGGGTTTTAGGCGTTTA-GGT   3954
                     ||   |||||||||||||||||||||||||||| |||||| ||||| |||
SyntheticTTTA   3905 GG---TTTAGGGTTTAGGGTTTAGGGTTTAGGG-TTTAGG-GTTTAGGGT   3949

MH188536_Hulu   3955 TTAGGGTTTTAGGGTTTTAGGGTTTAGGGTTTTAGGGTTTAGGTTT   4000
                     |||||| ||||||| |||||||||||||| ||||||||||||||||
SyntheticTTTA   3950 TTAGGG-TTTAGGG-TTTAGGGTTTAGGG-TTTAGGGTTTAGGTTT   3992


#---------------------------------------
#---------------------------------------
```
