# Supplementary material for: Identification of tandem repeat families from long-read sequences of Humulus lupulus
Source: PLoS One. 2020 Jun 5;15(6):e0233971. doi: 10.1371/journal.pone.0233971 (PMC7274563; doi:10.1371/journal.pone.0233971)

1,121 hits for repetitive sequences
passing 12mer abundance criterion

(Read-Number)
PAGE 1

For Tandem Repeat families of ### bp:
PacBio Read named "HuluTR###-r1"

>m141120_105518_42134_c100732402550000001823141405141504_s1_p0/101868/161_14634

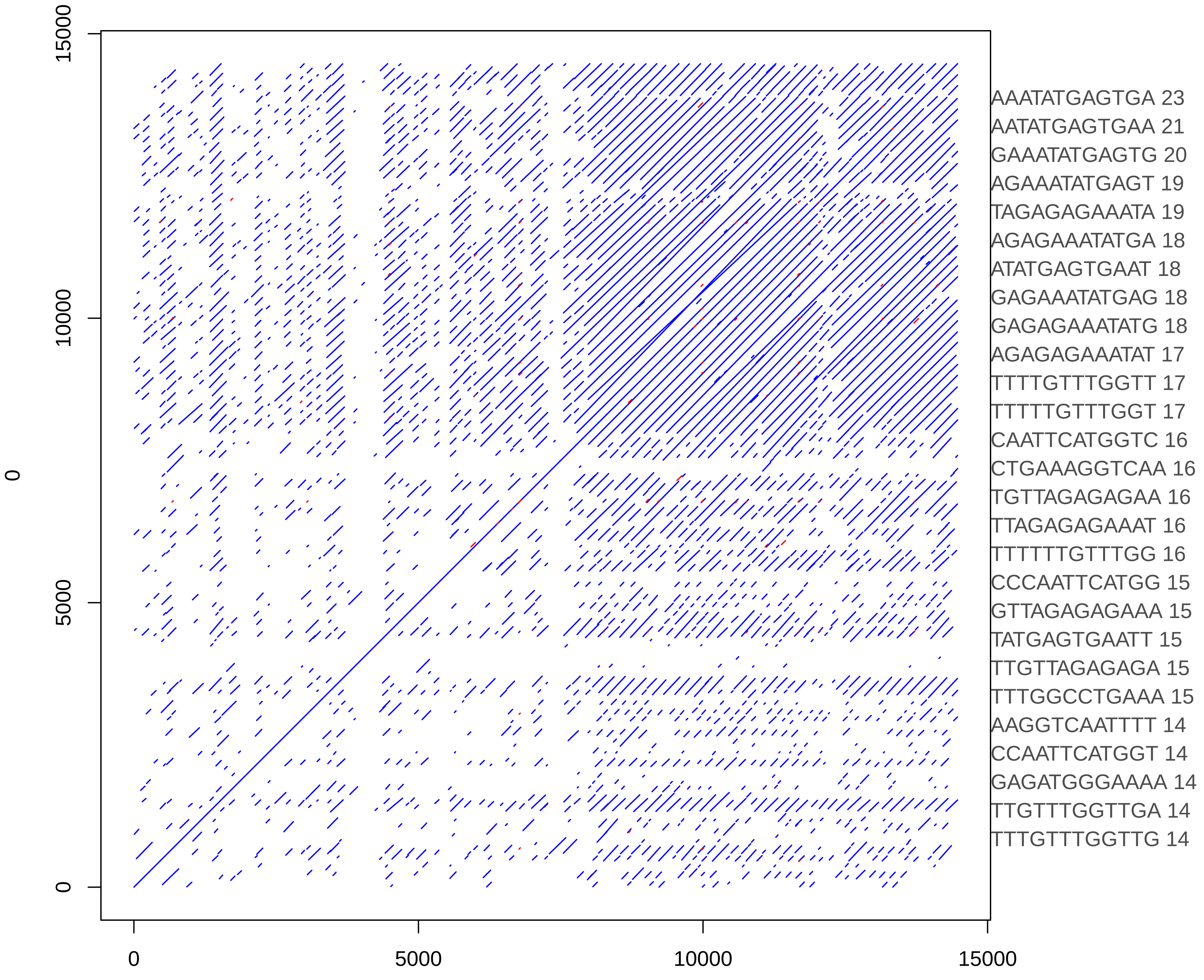

(HW Bass, DL Vera, FSU '17
Hopsteiner PacBio Apollo reads input)

0

Supplemental Material
k-mer screen & YASS dot-plots

KA Easterling et al. 2020

>m141120_105518_42134_c100732402550000001823141405141504_s1_p0/102314/927_10458

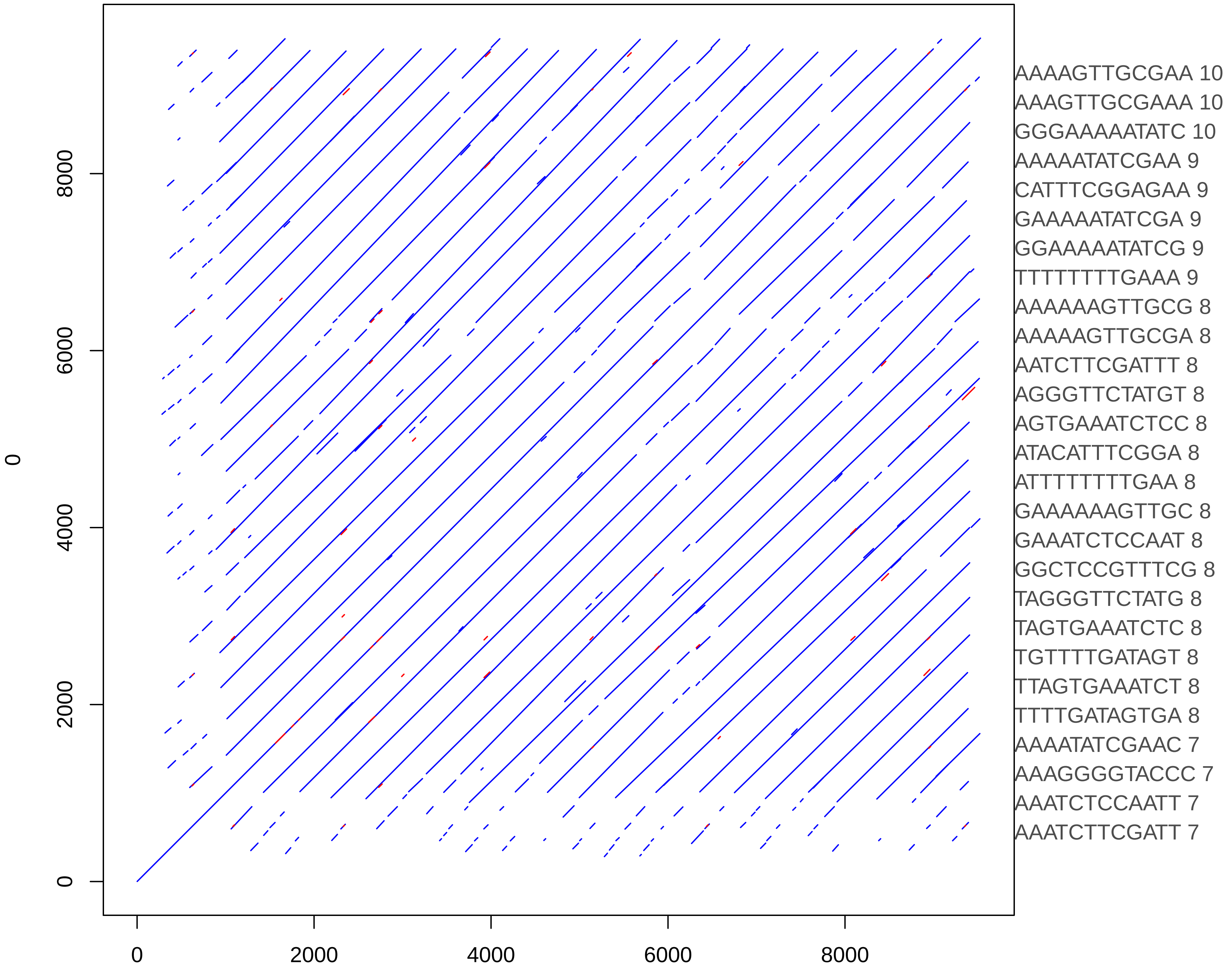

>m141120_105518_42134_c100732402550000001823141405141504_s1_p0/102894/6164_11701

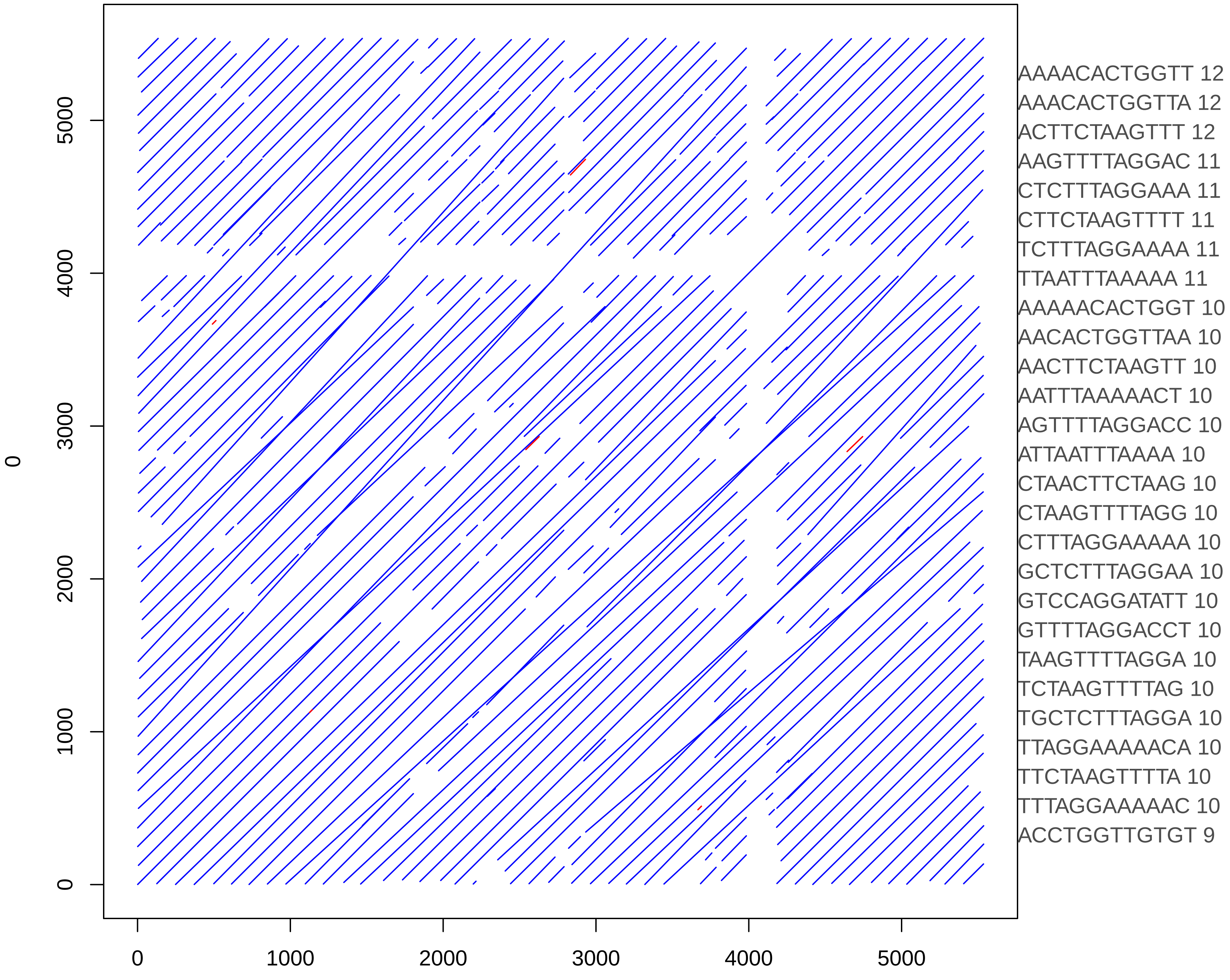

>m141120_105518_42134_c100732402550000001823141405141504_s1_p0/108707/11384_17561

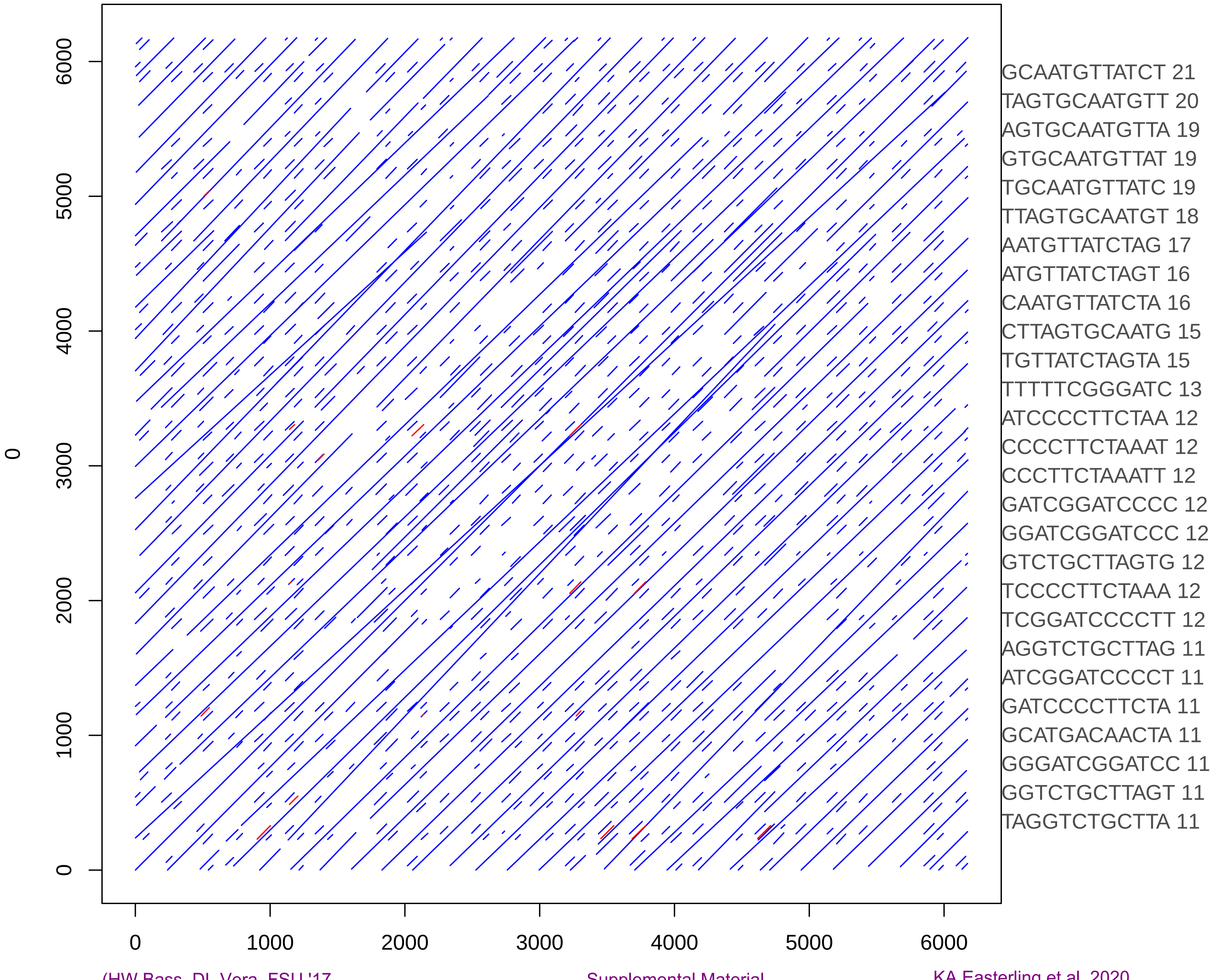

>m141120_105518_42134_c100732402550000001823141405141504_s1_p0/108707/5257_11338

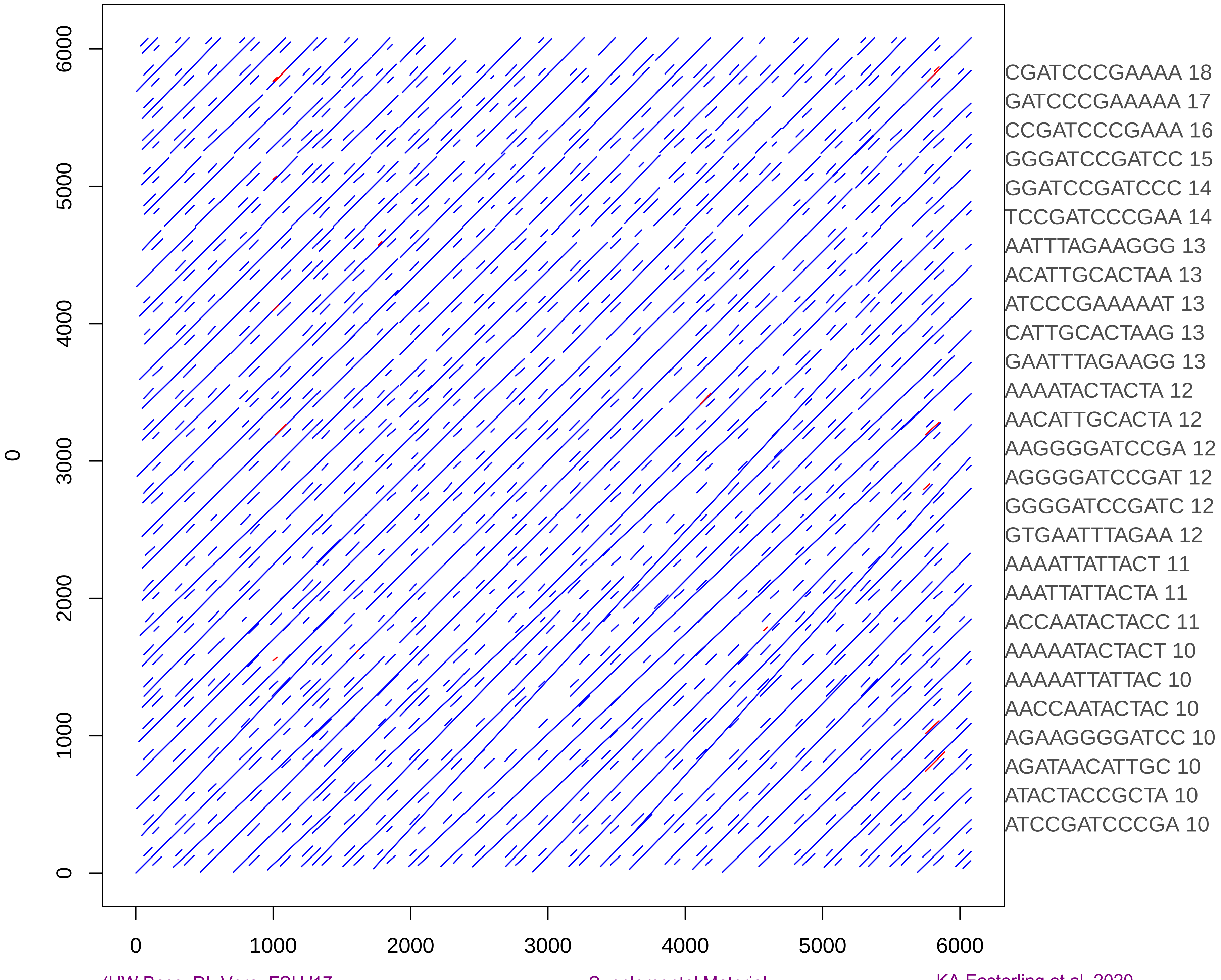

>m141120_105518_42134_c100732402550000001823141405141504_s1_p0/116729/698_7195

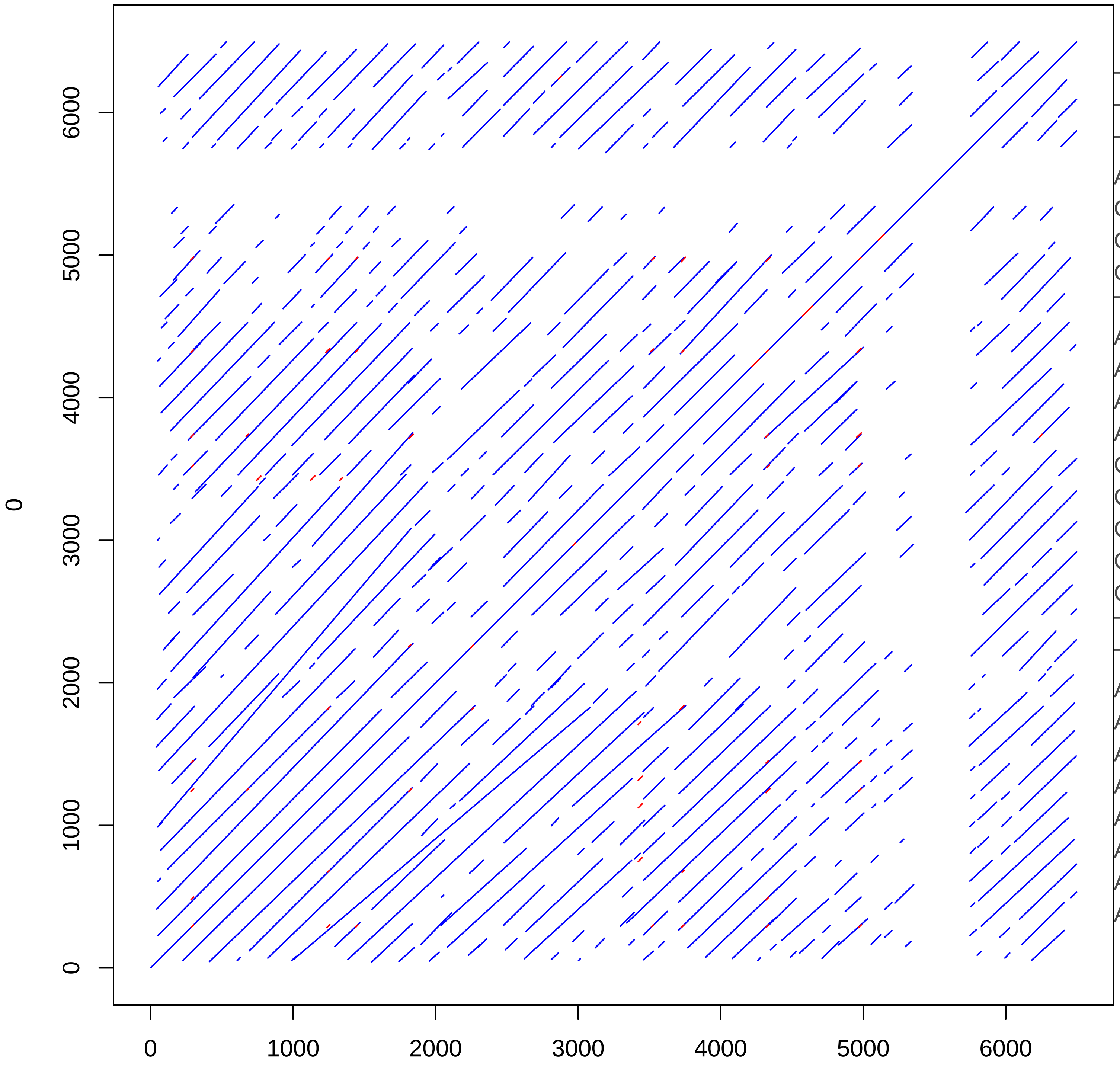

TTGGGGTCCTAA 11
TCTAATTTTTTT 10
TGGGGTCCTAAT 10
ATTGGGGTCCTA 9
CTCTAATTTTTT 9
GAATTGGGGTCC 9
GGGGTCCTAATT 9
TAATTTTTTTGA 9
AATTGGGGTCCT 8
AATTTTTTTGAA 8
ATGAATTGGGGT 8
ATTTCGTAATTC 8
CAAACAAAAAAA 8
CAACCAAACAAA 8
GGGTCCTAATTC 8
GGTCCTAATTCA 8
GTCCTAATTCAA 8
TGAATTGGGGTC 8
TTCAACCAAACA 8
AAAAAAATTGAC 7
AAAAAATTGACC 7
AAATCTCCTAGG 7
AACCAAACAAAA 7
AATCTCCTAGGC 7
AATTCAACCAAA 7
ACCAAACAAAAA 7
ATATTTCTCTCT 7

>m141120_105518_42134_c100732402550000001823141405141504_s1_p0/11980/2707_9505

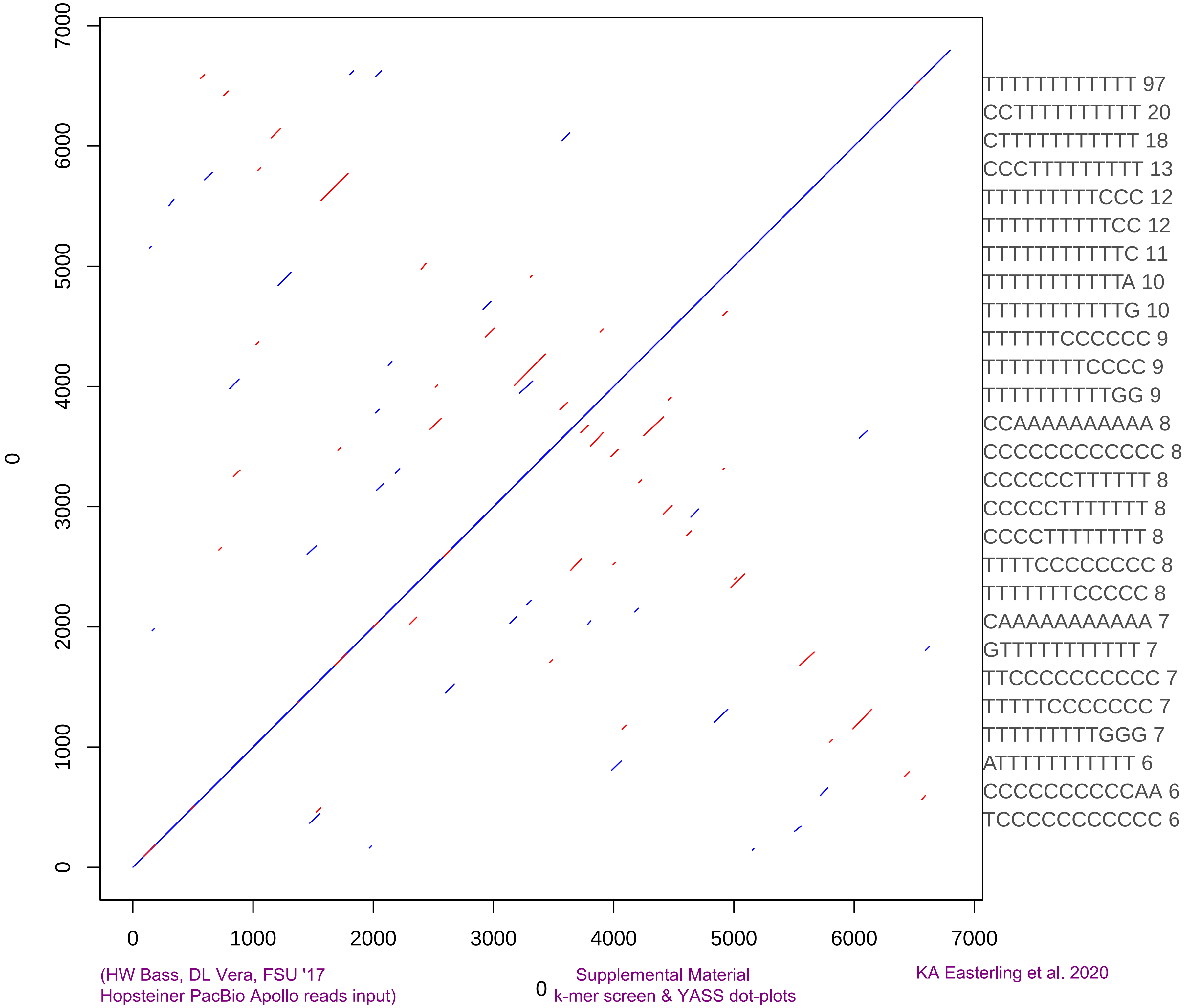

>m141120_105518_42134_c100732402550000001823141405141504_s1_p0/120405/7766_13109

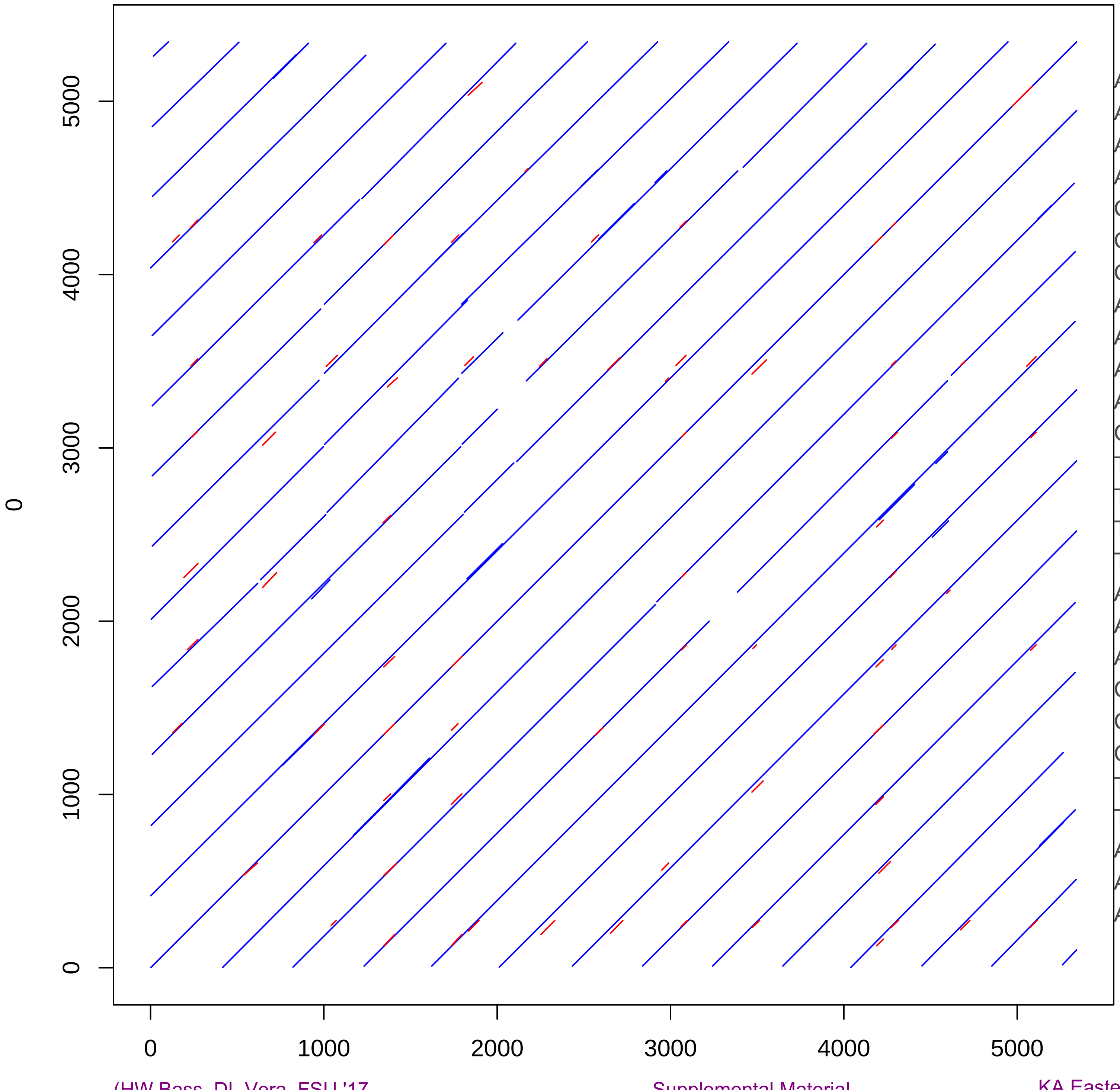

AAAAAAAATTCC 10
AGAGATCTCATG 10
AGATCTCATGGG 10
ATCTCATGGGTA 10
GACTGTATCACC 10
GAGATCTCATGG 10
GATCTCATGGGT 10
AAAAAAAAATTC 9
AAGAGATCTCAT 9
ATATTAGAGAAA 9
ATTTAAAAAAAA 9
CAAGAGATCTCA 9
TCTCATGGGTAA 9
TGACTGTATCAC 9
TTTAAAAAAAAA 9
TTTTGACTGTAT 9
AAAAAAATTCCC 8
ACATTTAAAAAA 8
ACTGTATCACCC 8
CATATTAGAGAA 8
CATTTAAAAAAA 8
CTGTATCACCCT 8
TTGACTGTATCA 8
TTTGACTGTATC 8
AACTCGTTCGAT 7
ATTTCACTAATT 7
ATTTTGACTGTA 7

>m141120_105518_42134_c100732402550000001823141405141504_s1_p0/122511/12020_17126

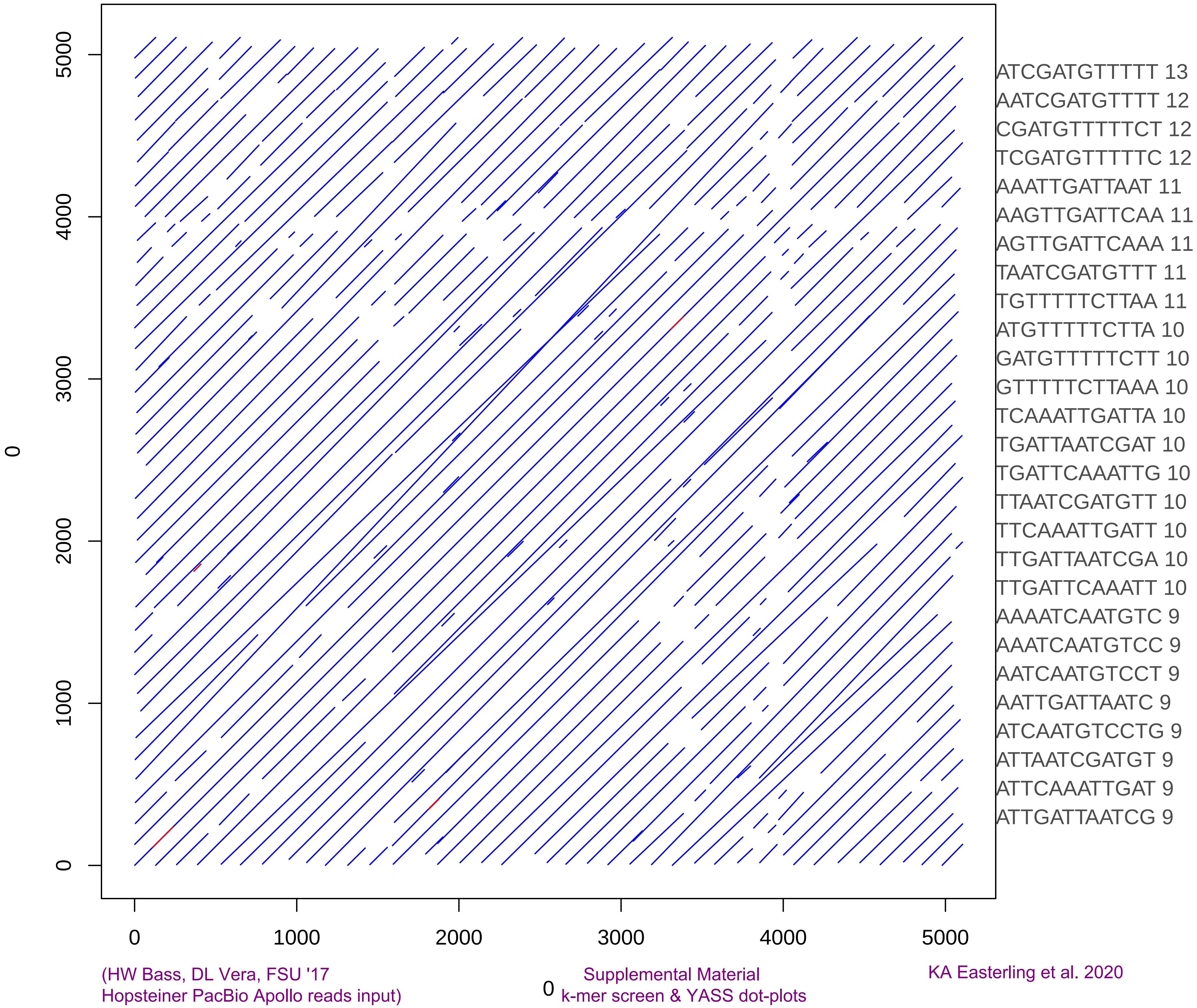

>m141120_105518_42134_c100732402550000001823141405141504_s1_p0/122511/6279_11971

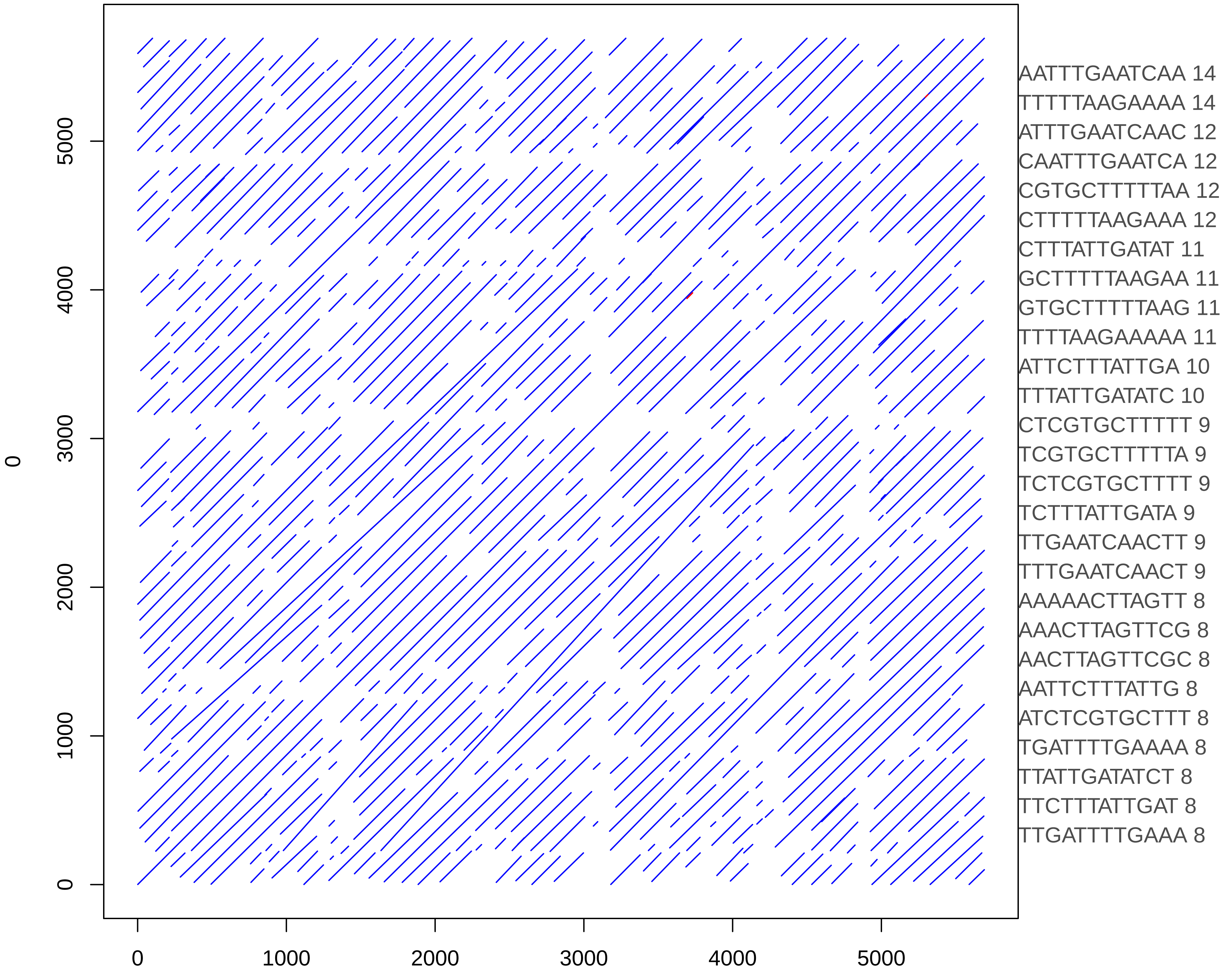

>m141120_105518_42134_c100732402550000001823141405141504_s1_p0/124279/4079_14068

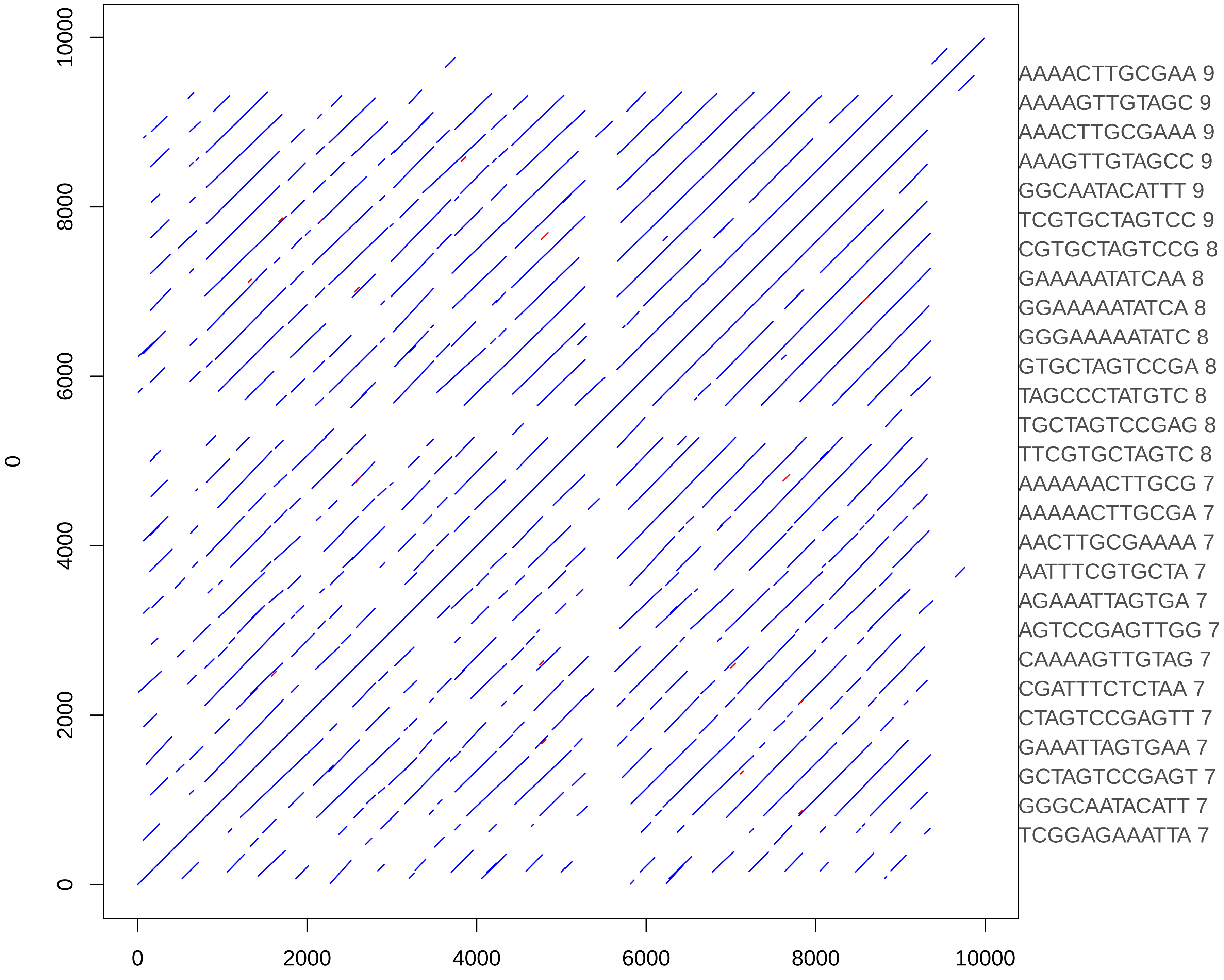

0

>m141120_105518_42134_c100732402550000001823141405141504_s1_p0/126400/13190_20067

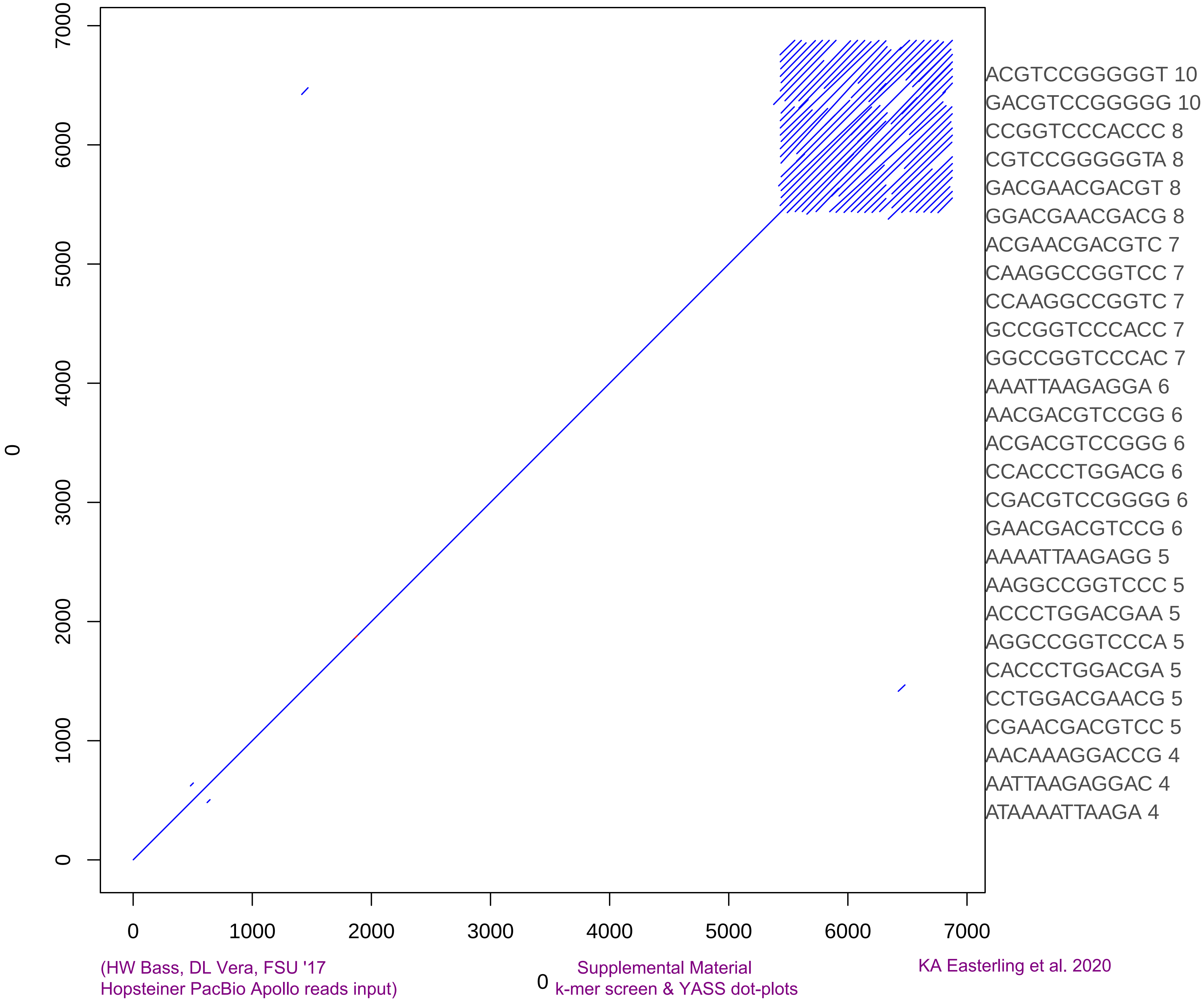

>m141120_105518_42134_c100732402550000001823141405141504_s1_p0/127222/1681_12839

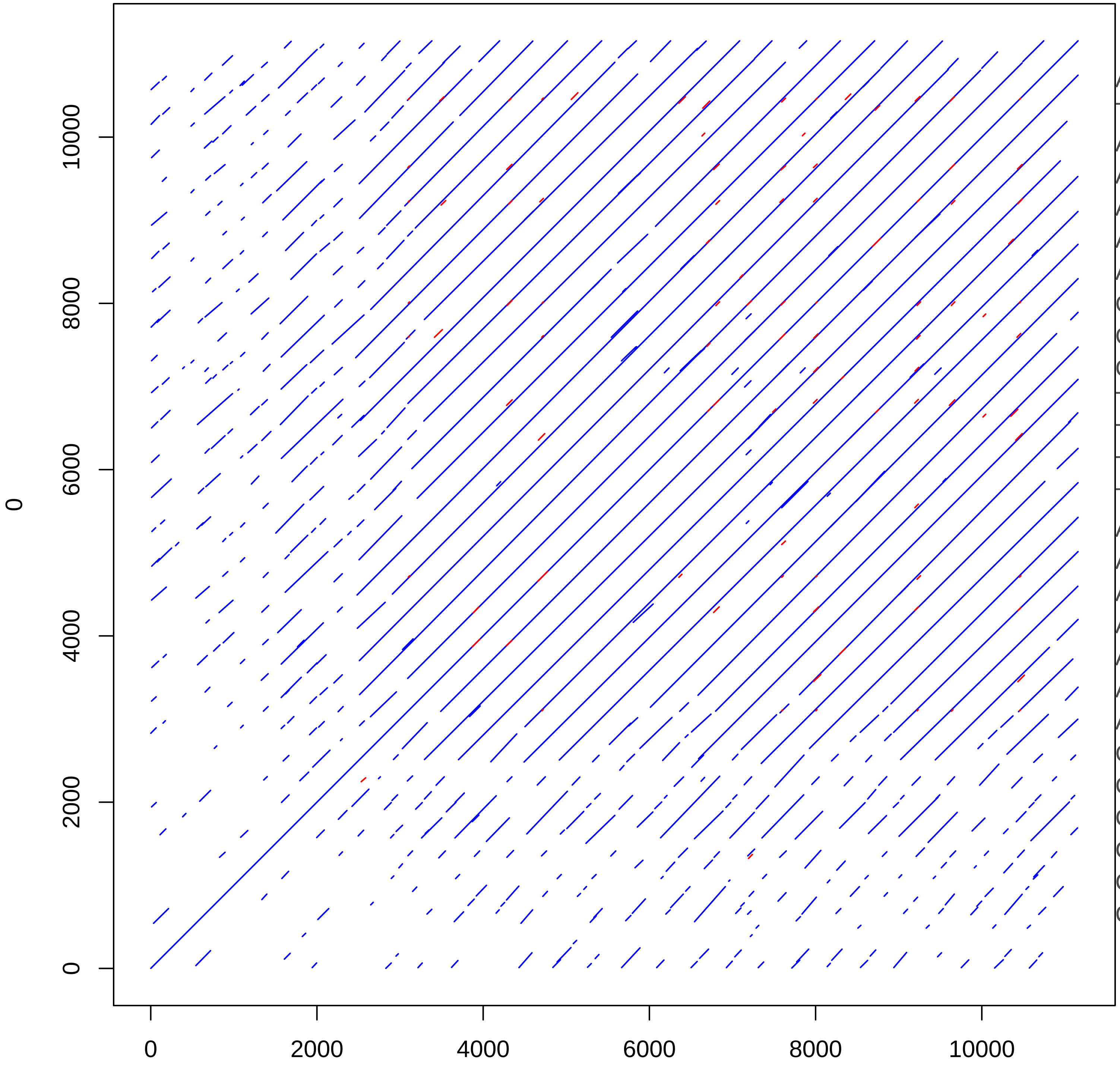

AAATCTTCGATT 11
TTTCAGAAATCT 11
AAAACTTGCGAA 10
AAACTTGCGAAA 10
AAAGGGGTACCC 10
AAGGGGTACCCC 10
AATCTTCGATTT 10
CTCCAATATATT 10
GGTCCGTTTCCA 10
GTCCGTTTCCAC 10
TCAGAAATCTTC 10
TCCGTTTCCACT 10
TCGATTCGAGCA 10
TTCAGAAATCTT 10
AAAAACTTGCGA 9
AAAATTTCGTGC 9
AATTCACCAAAG 9
ACCAAAGGGGTA 9
ACTCCAATATAT 9
AGGGTCCGTTTC 9
ATTCACCAAAGG 9
CAAAGGGGTACC 9
CAATTCACCAAA 9
CACCAAAGGGGT 9
CCGTTTCCACTC 9
CGATTCGAGCAA 9
CGTTTCCACTCC 9

>m141120_105518_42134_c100732402550000001823141405141504_s1_p0/128356/1510_11380

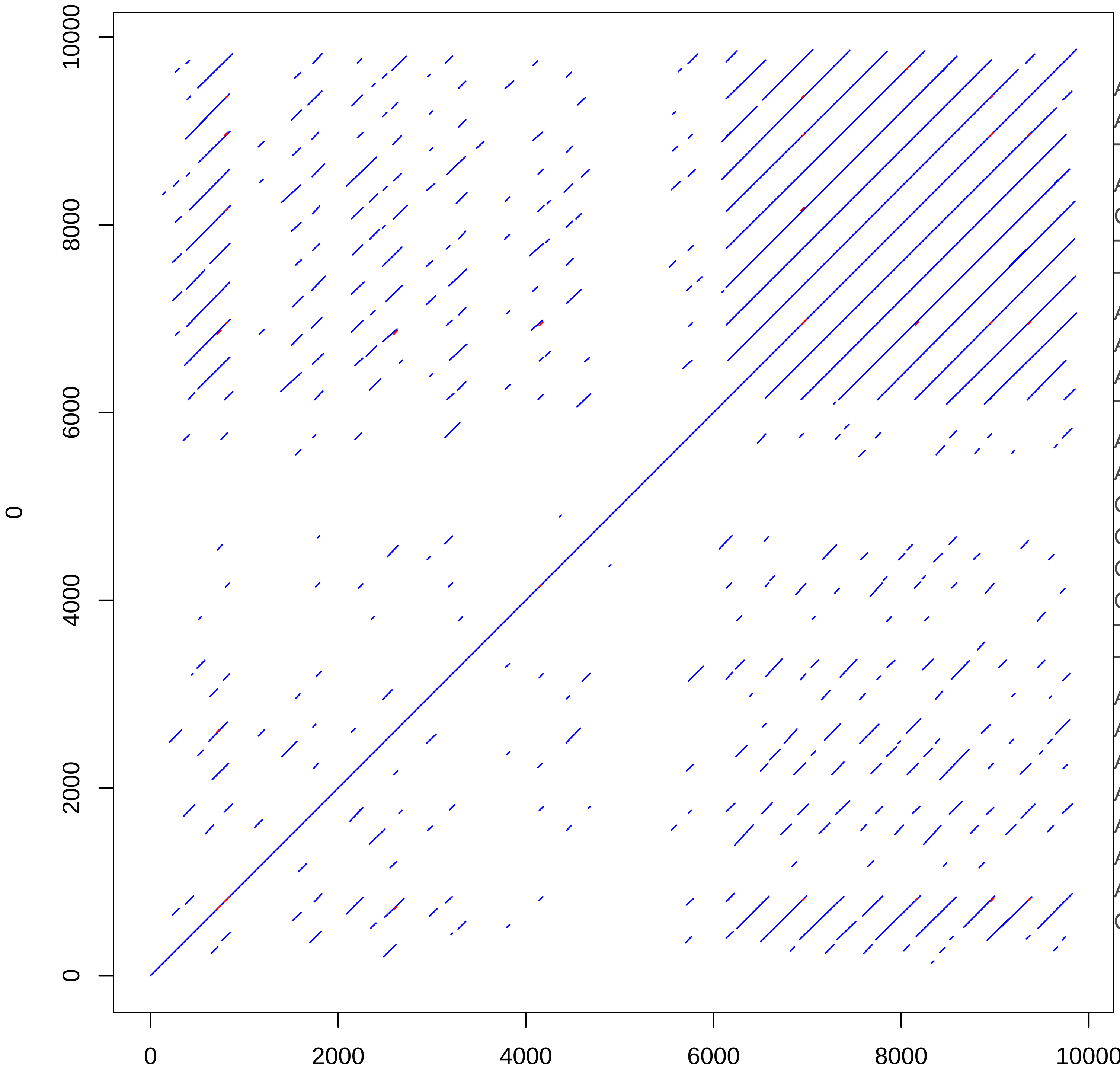

AATCTCCAATTC 10
ATCTCCAATTCA 10
TCTCCAATTCAC 10
AAATCTCCAATT 8
GAAATCTCCAAT 8
TGAAATCTCCAA 8
TTTCAGAAATCT 8
AAACTTGTGAAA 7
AATTGGTGAAAT 7
ATTGGTGAAATC 7
TTCAGAAATCTT 7
AAAAAGGGGATT 6
AAAACTTGTGAA 6
CCTTTCAGAAAT 6
CTCCAATTCACC 6
CTTTCAGAAATC 6
GTGAAATCTCCA 6
TCAGAAATCTTC 6
TCCAATTCACCA 6
AAAAGGGGATTT 5
AAAGGGGATTTT 5
AAATTGGTGAAA 5
AACTTGTGAAAA 5
ACCCATGAGATC 5
ACTCCAATAGAT 5
ACTTGTGAAAAT 5
CAAAAAGGGGAT 5

>m141120_105518_42134_c100732402550000001823141405141504_s1_p0/1316/0_7108

Representative Read (Table 1): HuluTR390-r15, 2 ppm, 79% A+T,
GenBank Acc. MN537580

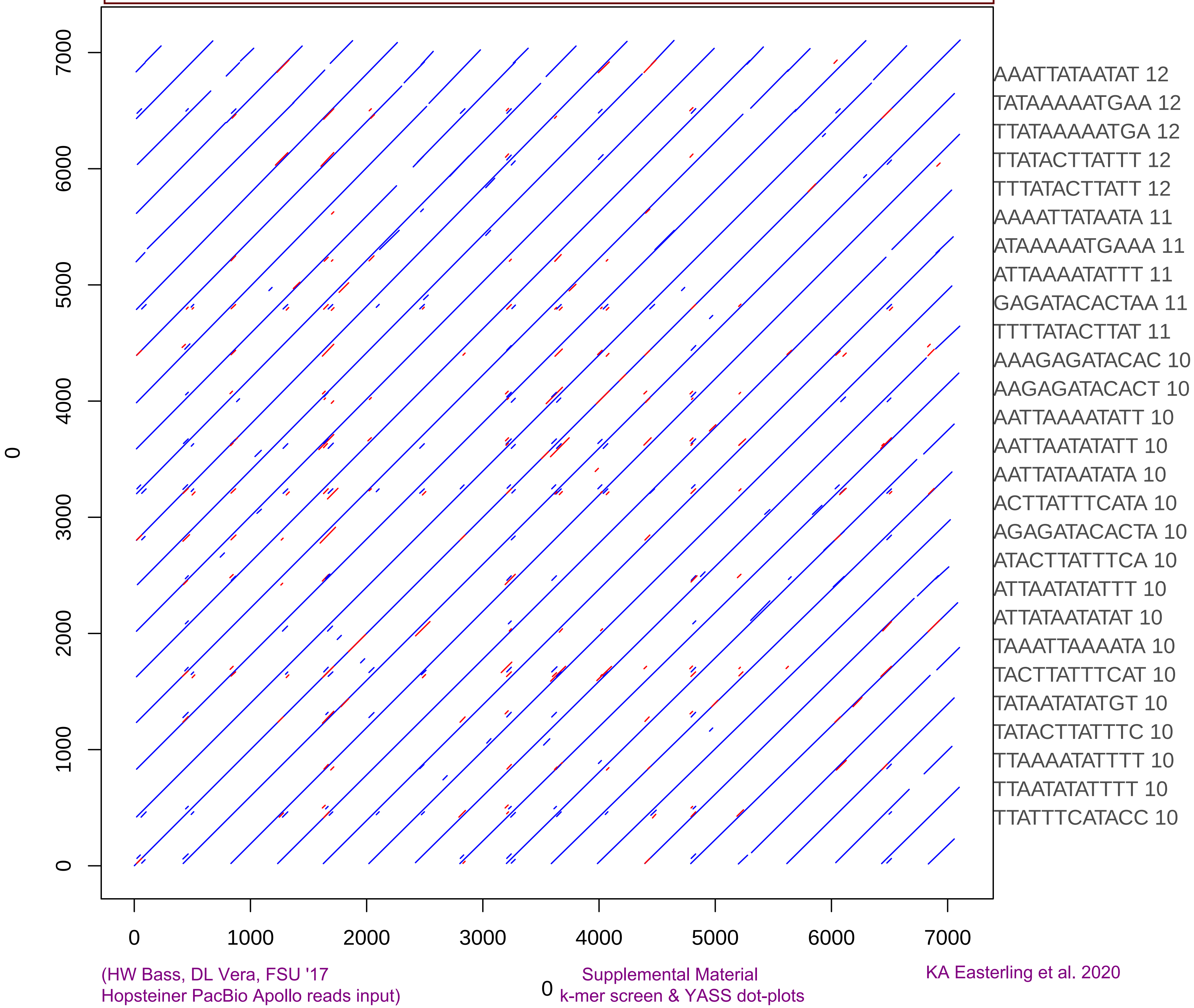

>m141120_105518_42134_c100732402550000001823141405141504_s1_p0/134/0_8933

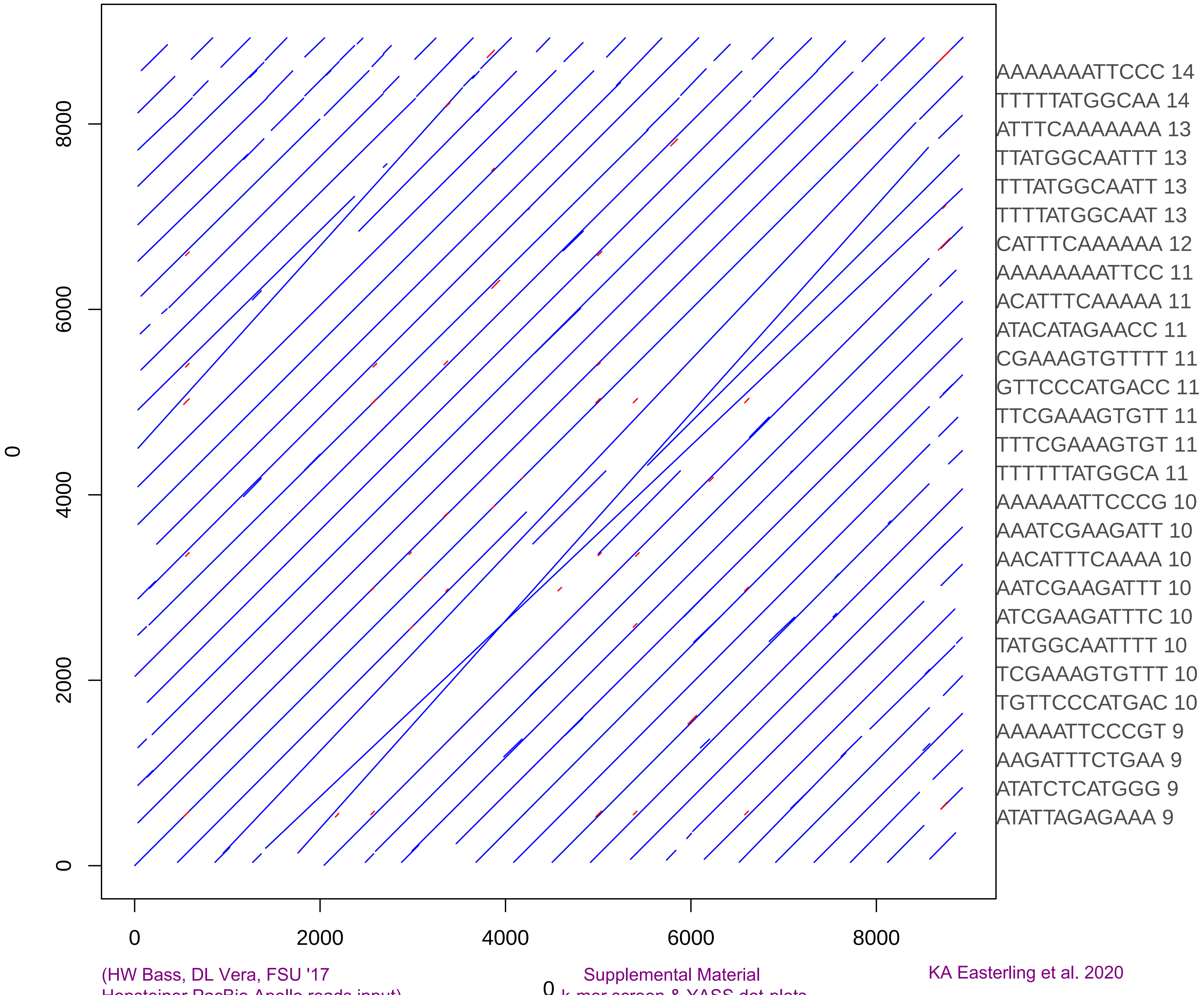

>m141120_105518_42134_c100732402550000001823141405141504_s1_p0/135525/0_11533

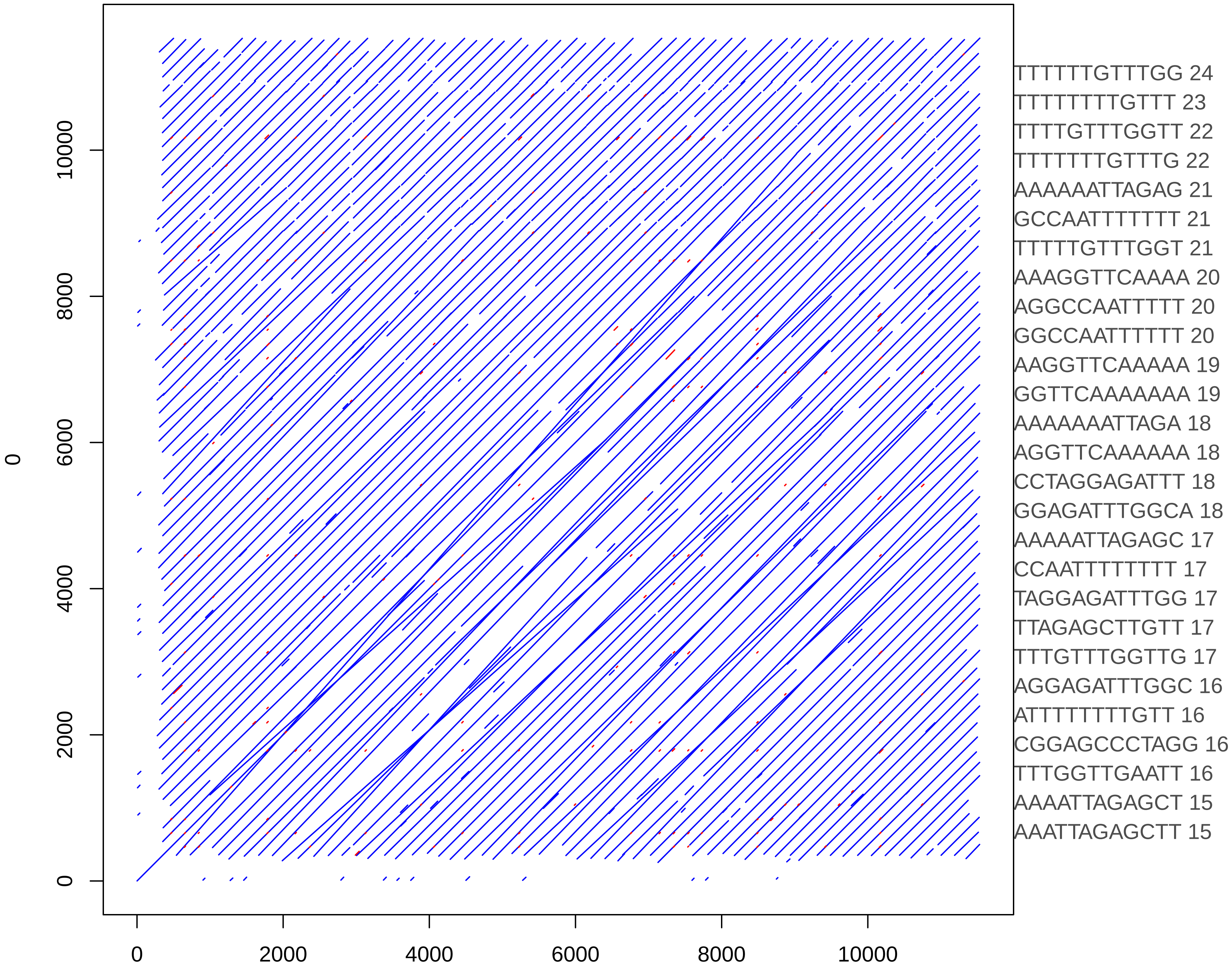

>m141120_105518_42134_c100732402550000001823141405141504_s1_p0/142397/8758_20595

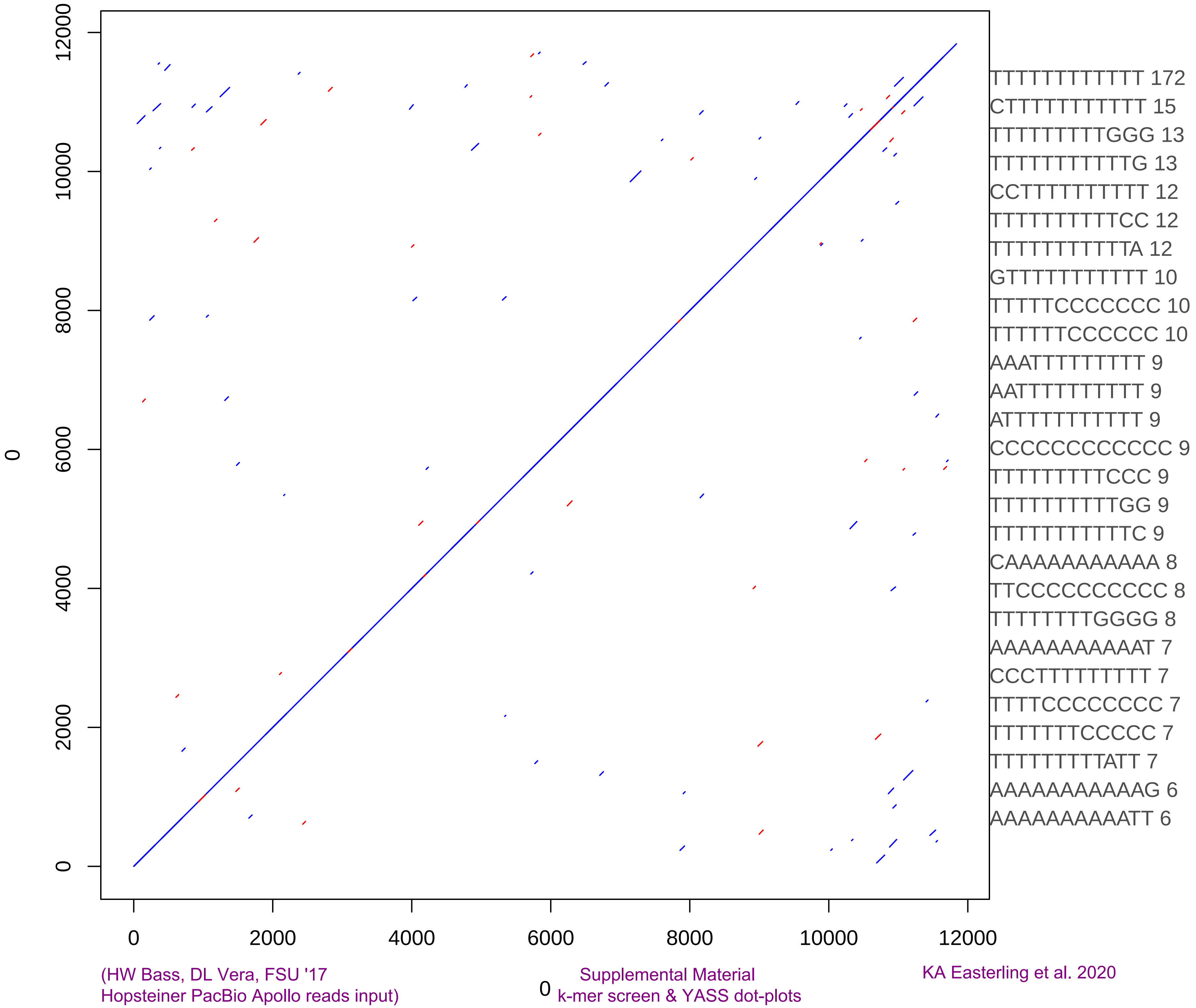

>m141120_105518_42134_c100732402550000001823141405141504_s1_p0/145404/0_10211

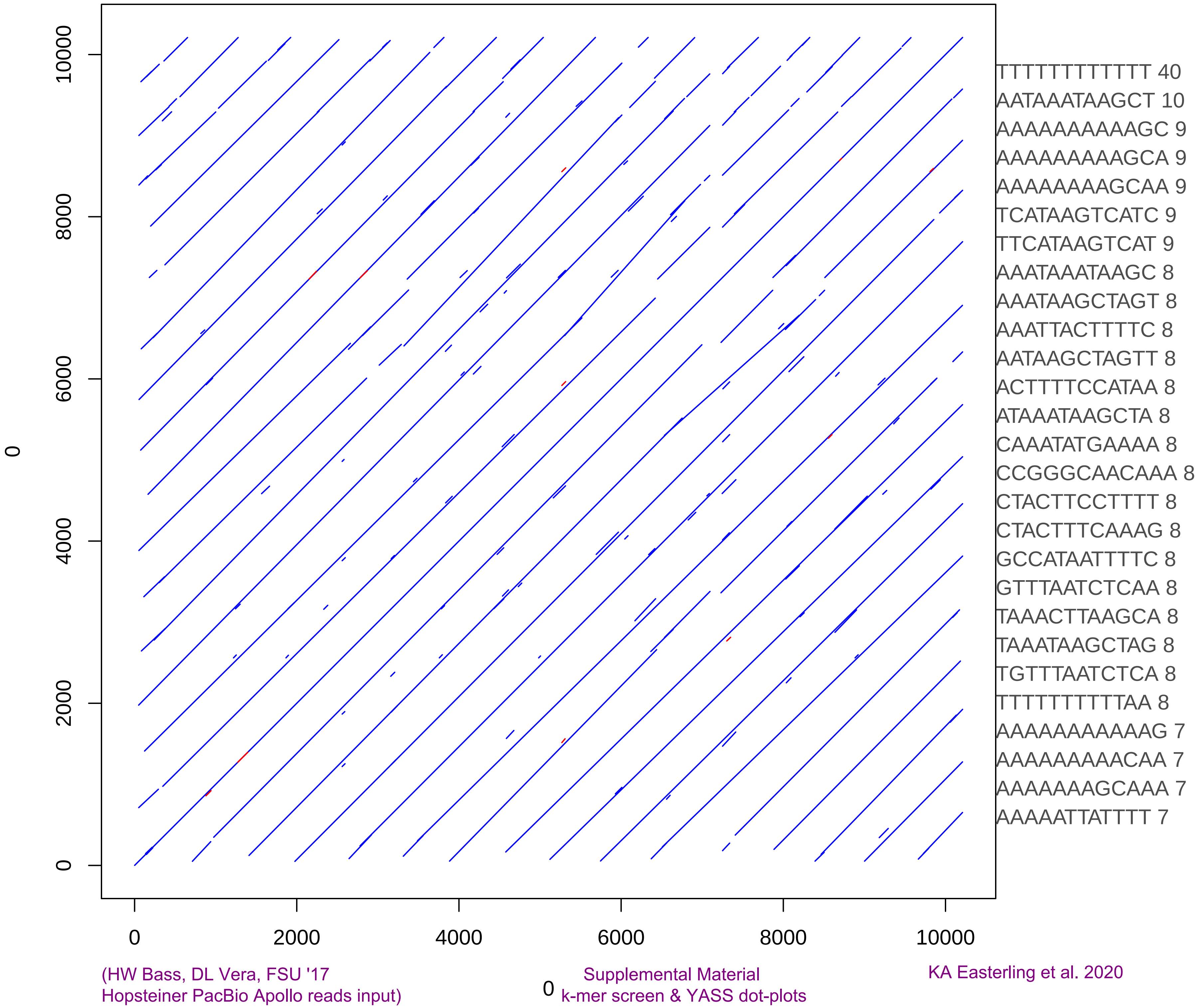

>m141120_105518_42134_c100732402550000001823141405141504_s1_p0/150771/0_7118

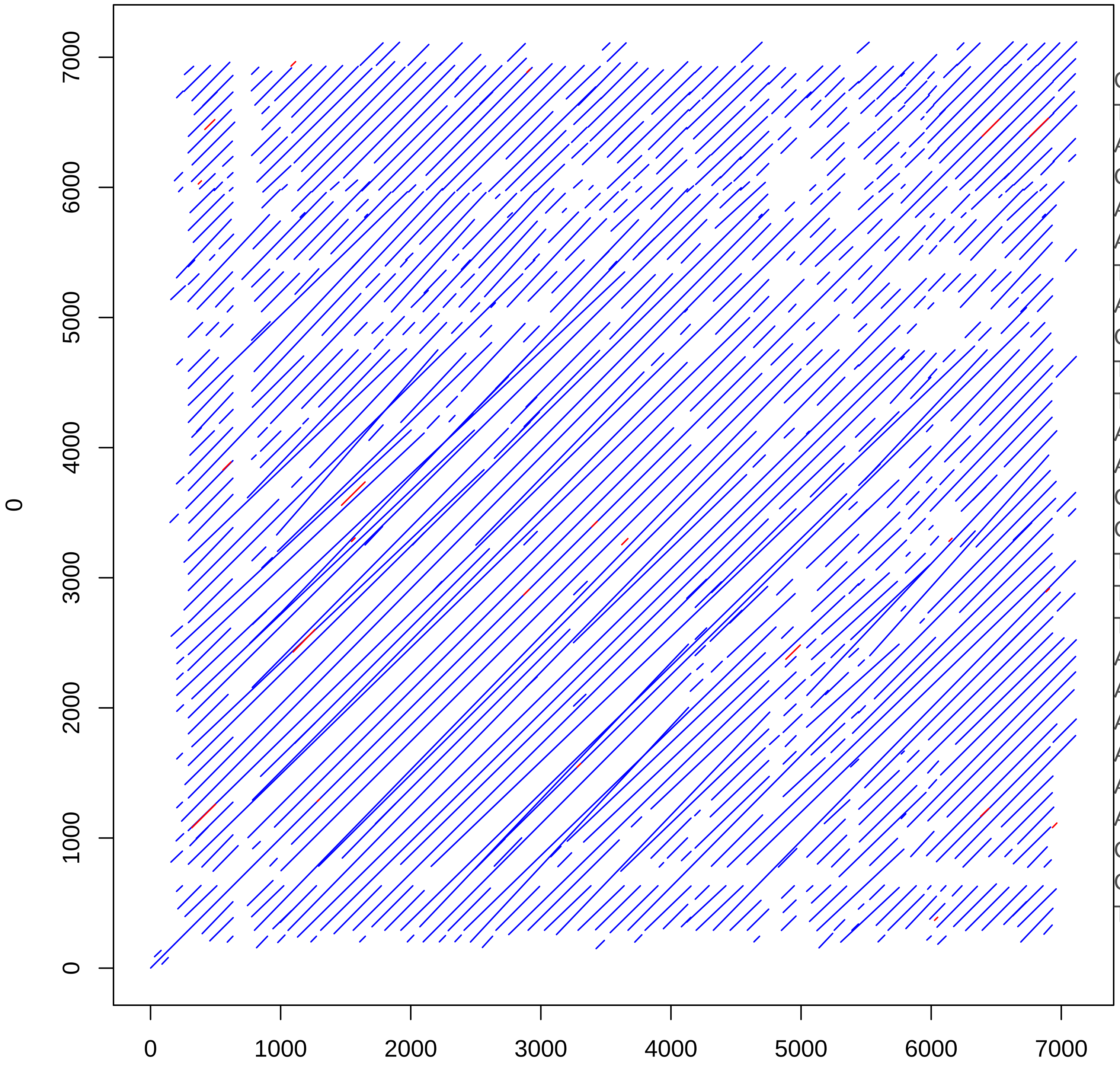

CTTTAAGAAAAA 17
TCTTTAAGAAAA 17
AAACTTAGTTCG 16
CTCTTTAAGAAA 16
AAAACAACGATC 15
AAGTTTTATGAC 15
TTTGAAAACTTA 15
AAAACTTAGTTC 14
GAAAACTTAGTT 14
TTGAAAACTTAG 14
TTTTATCAATAT 14
AAACAACGATCA 13
AGTTTTATGACC 13
CTTCTAAGTTTT 13
GCTCTTTAAGAA 13
TGAAAACTTAGT 13
TTAAGAAAAACA 13
TTAATTTTTATC 13
AACAACGATCAA 12
AACTTAGTTCGC 12
AACTTCTAAGTT 12
ACTTCTAAGTTT 12
ATCAATTGATTT 12
ATTTAAATTAAC 12
CCTGGTTATGTC 12
GATCAATTGATT 12
TAAATTAACTTC 12

>m141120_105518_42134_c100732402550000001823141405141504_s1_p0/152466/6069_12455

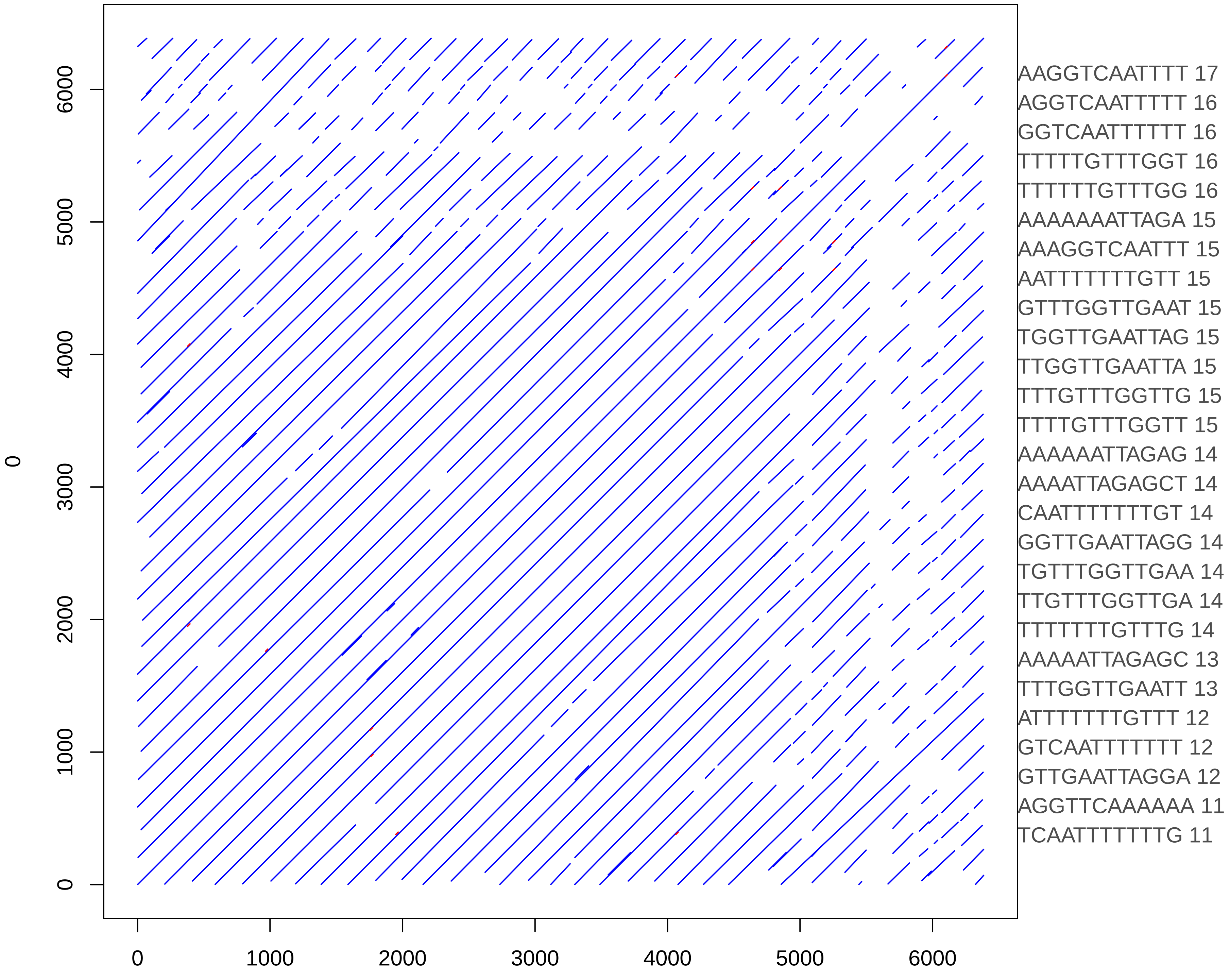

>m141120_105518_42134_c100732402550000001823141405141504_s1_p0/15326/2038_14450

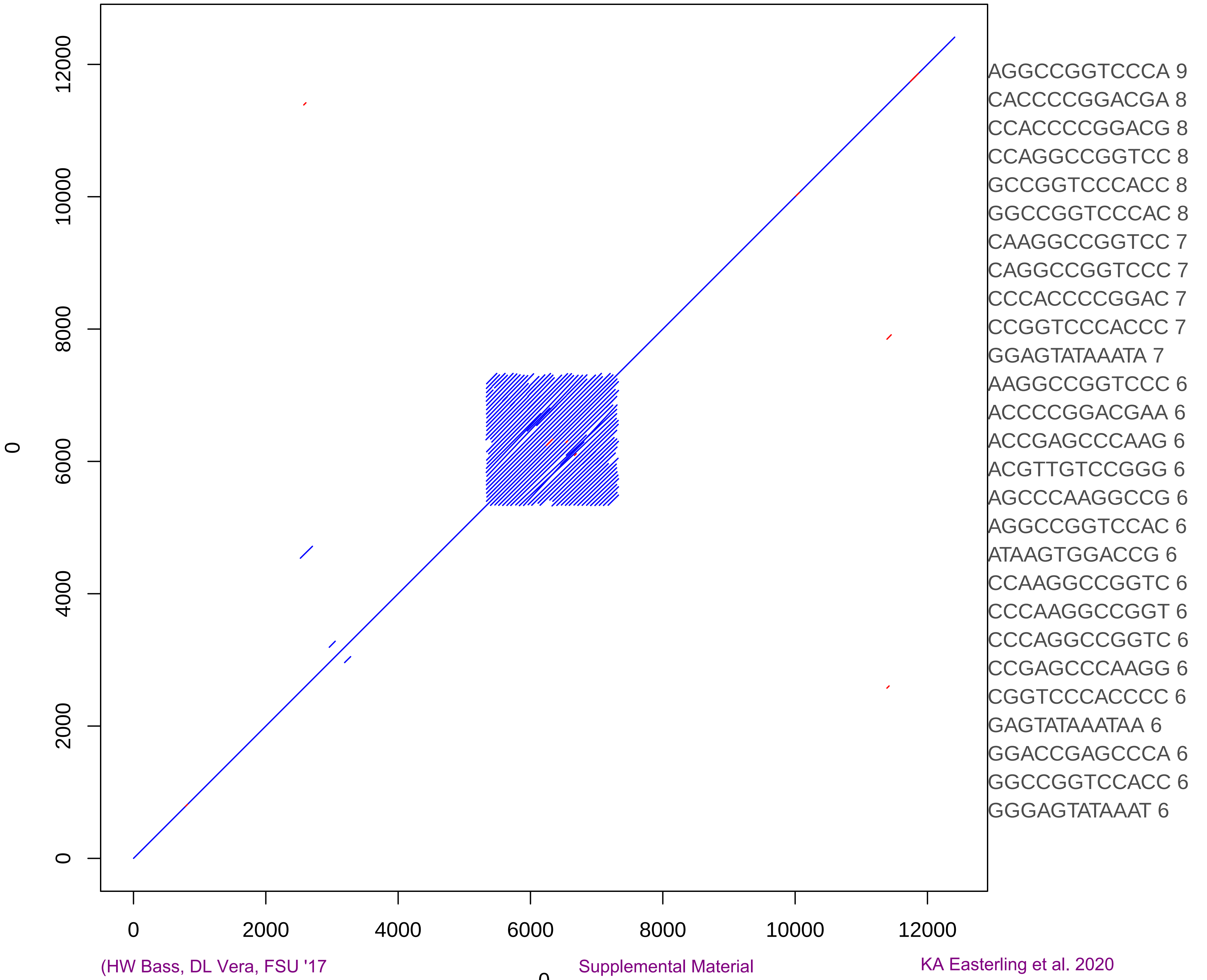

0

>m141120_105518_42134_c100732402550000001823141405141504_s1_p0/153673/6347_13678

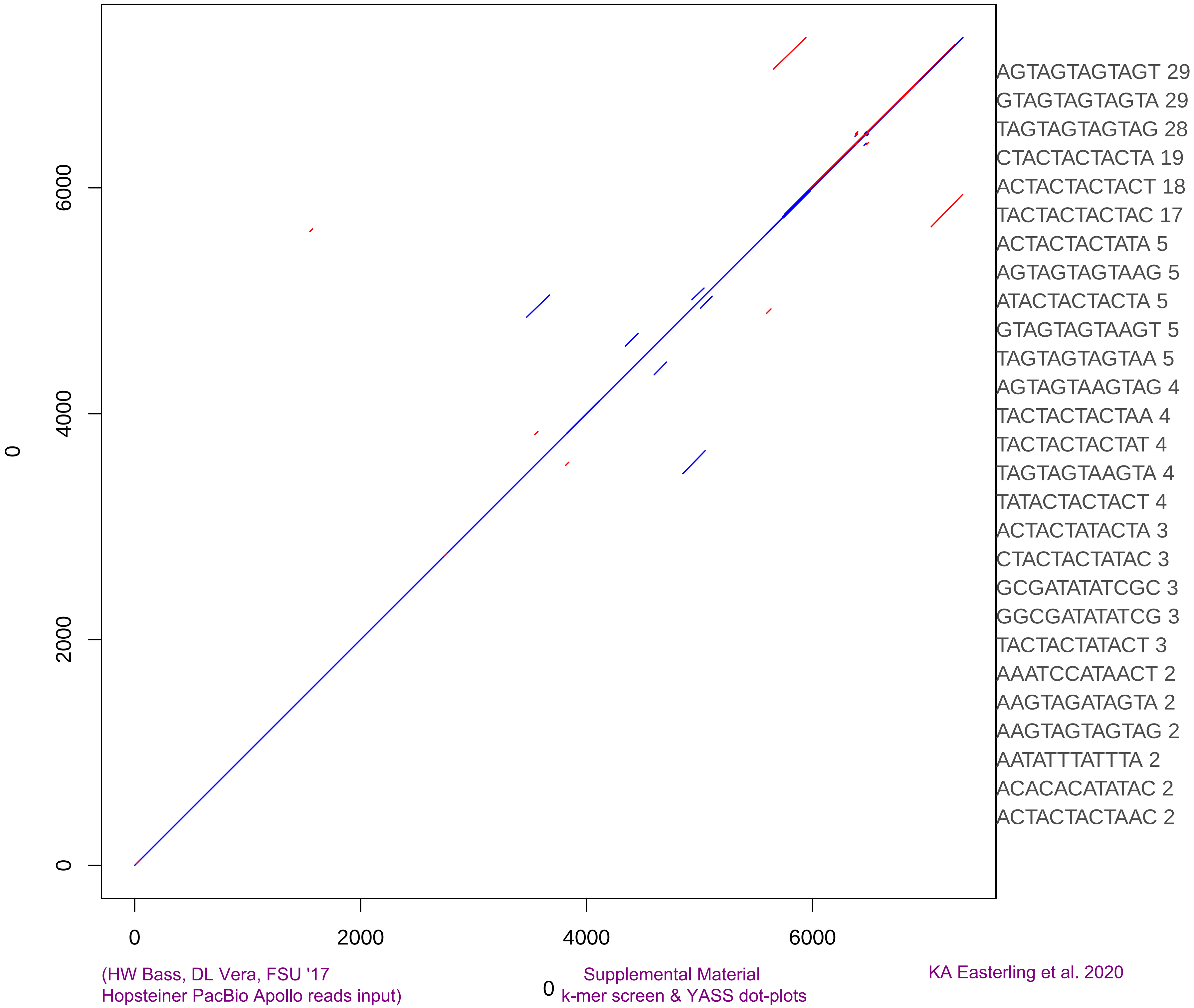

>m141120_105518_42134_c100732402550000001823141405141504_s1_p0/154800/5076_15221

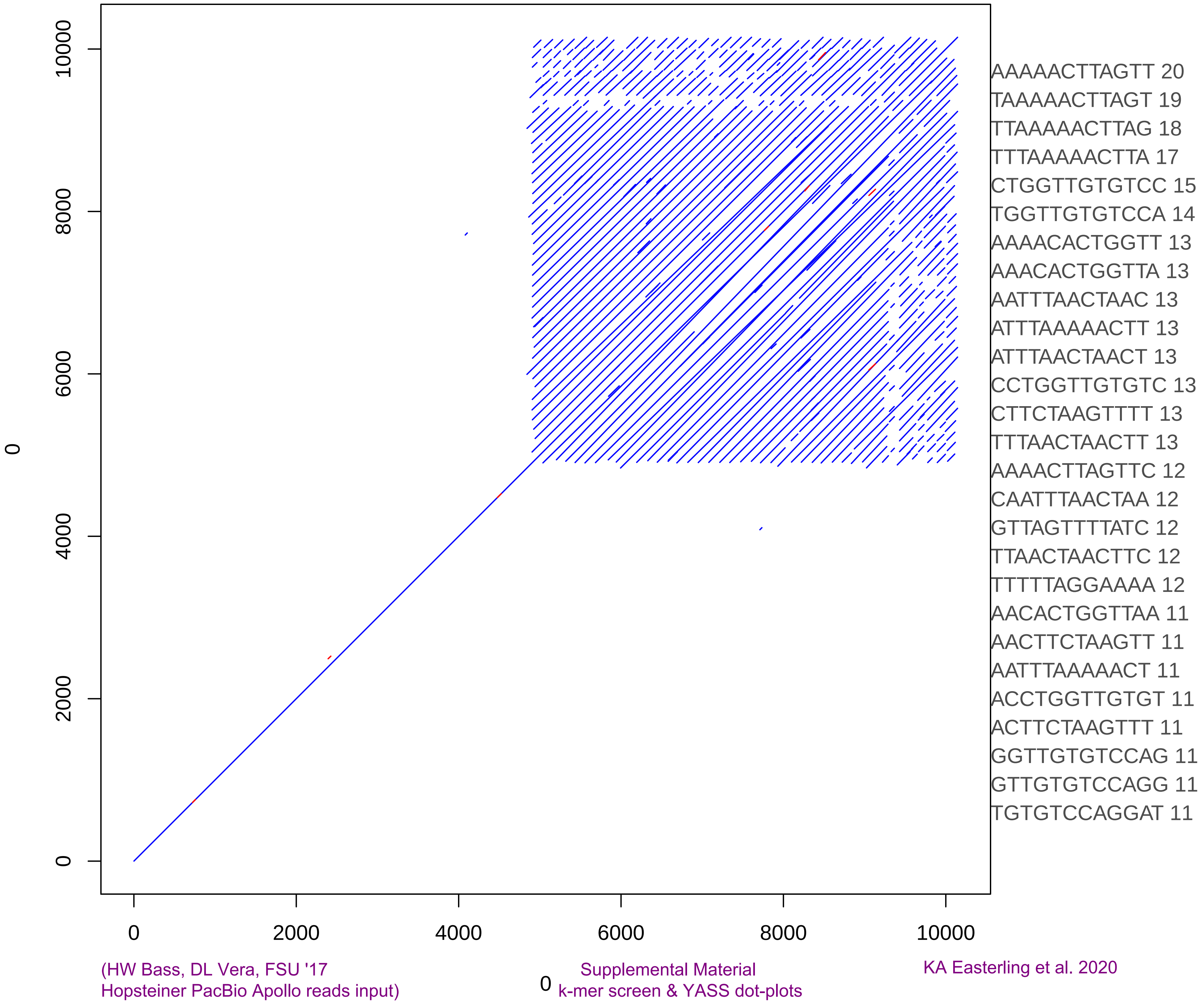

>m141120_105518_42134_c100732402550000001823141405141504_s1_p0/161652/0_6058

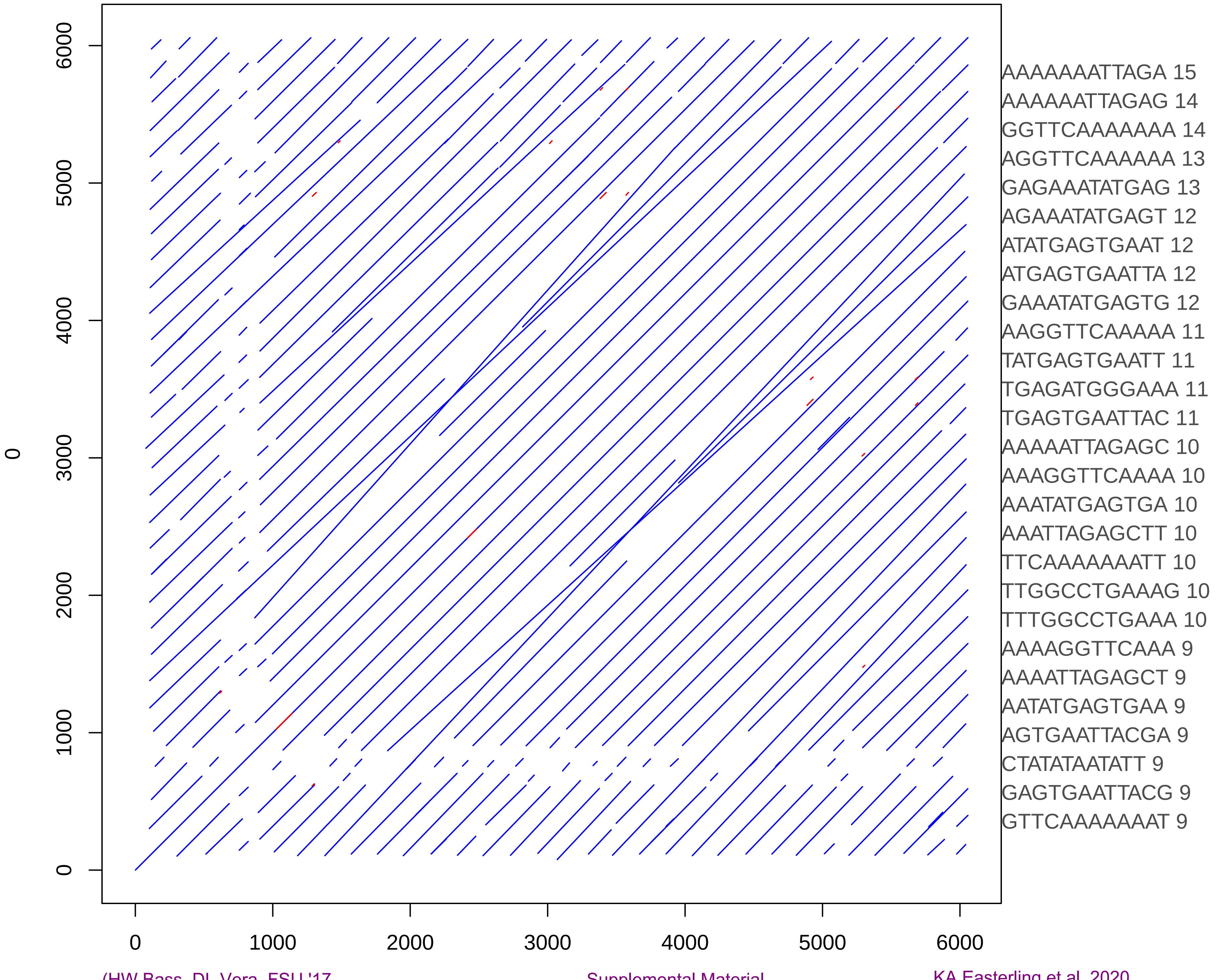

>m141120_105518_42134_c100732402550000001823141405141504_s1_p0/17236/3783_13605

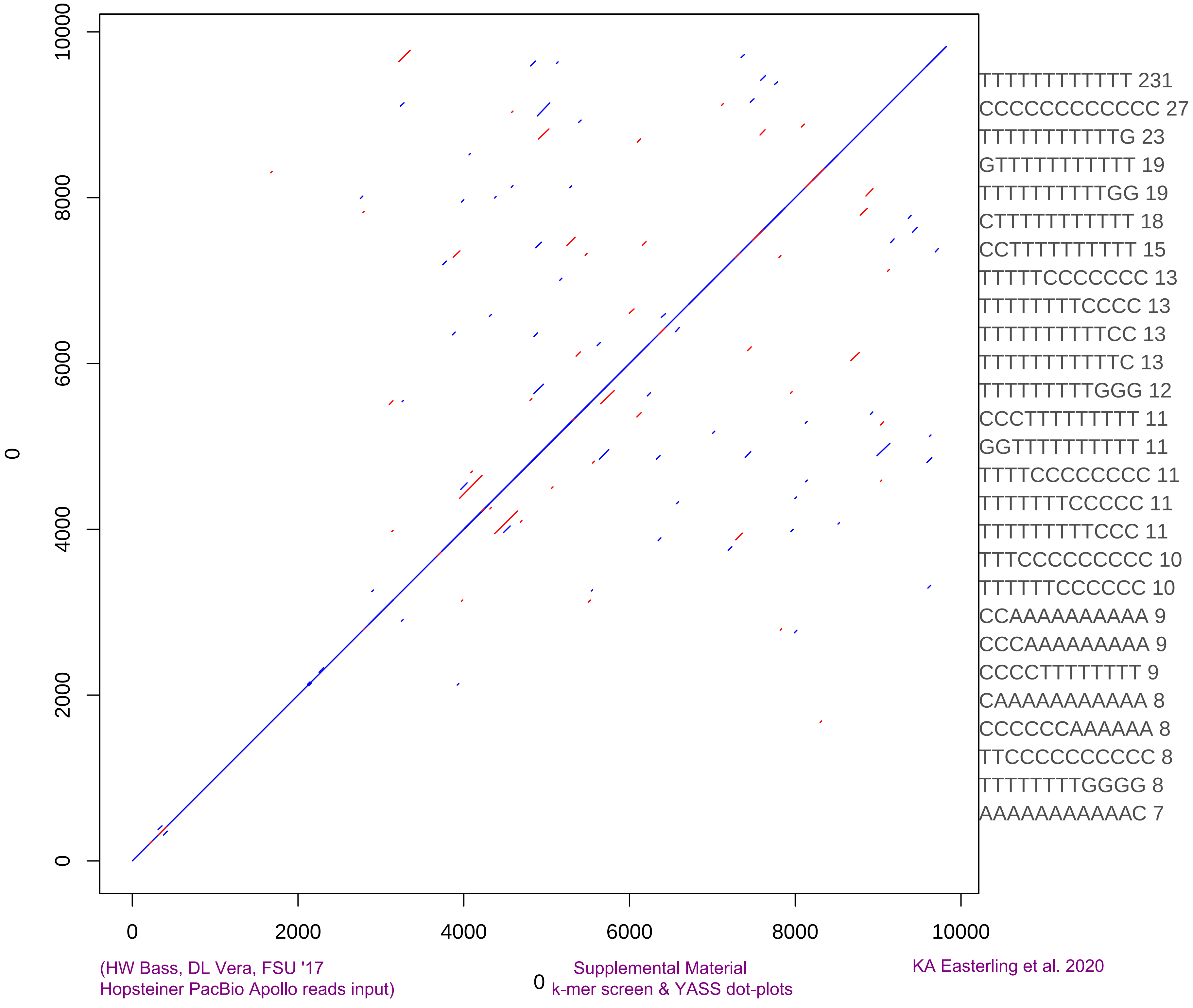

>m141120_105518_42134_c100732402550000001823141405141504_s1_p0/18582/353_10095

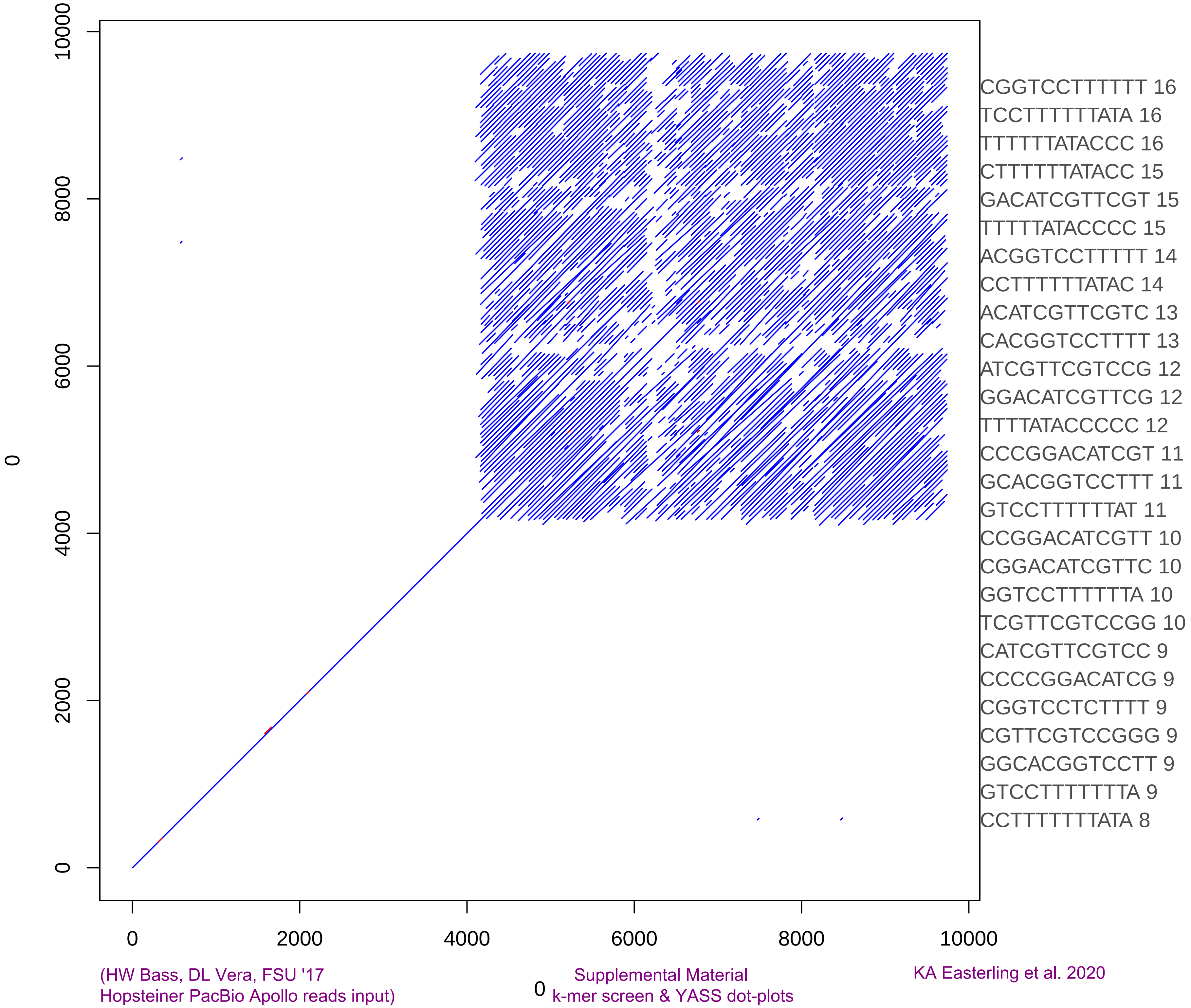

>m141120_105518_42134_c100732402550000001823141405141504_s1_p0/19522/2523_9426

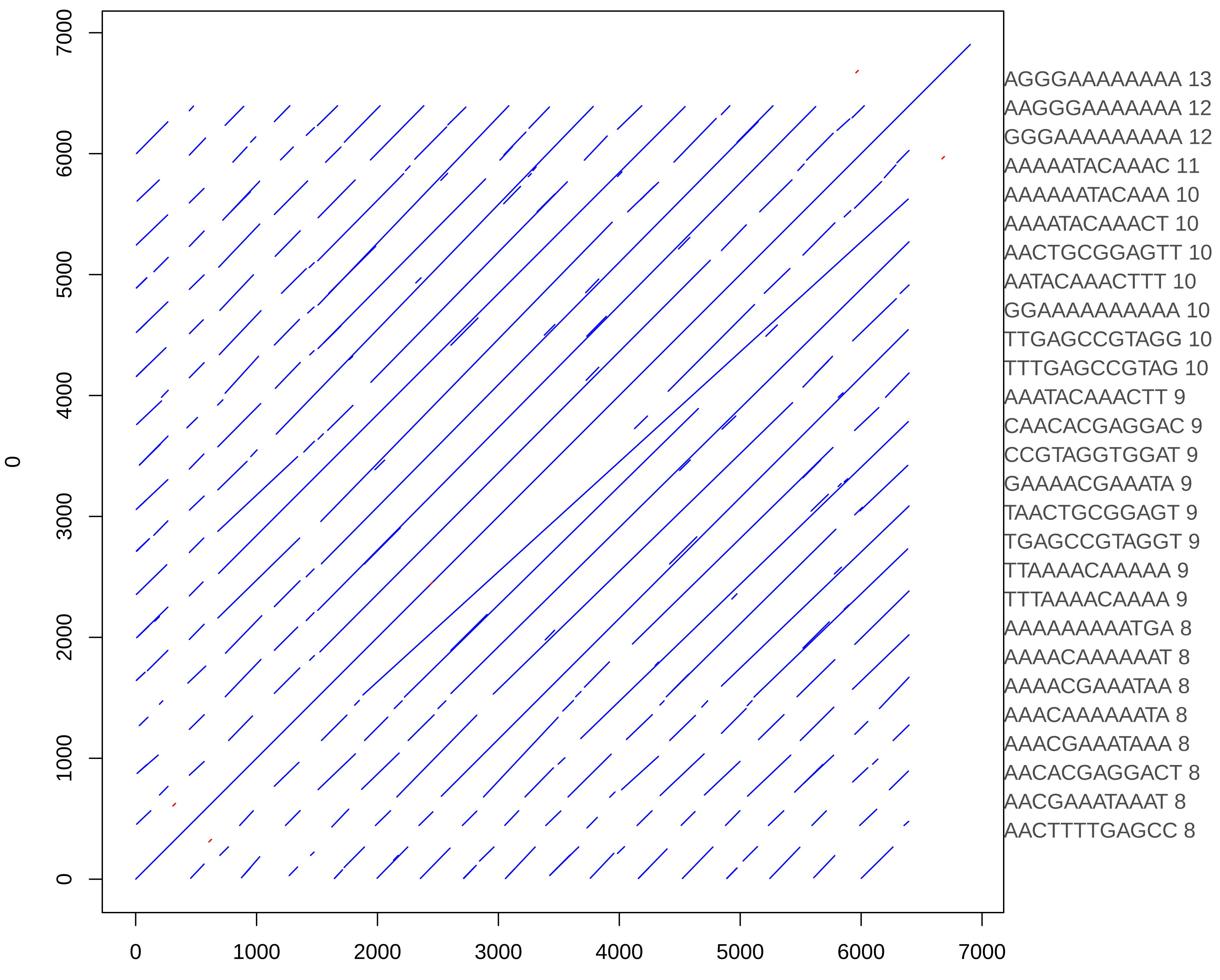

>m141120_105518_42134_c100732402550000001823141405141504_s1_p0/2045/3797_13360

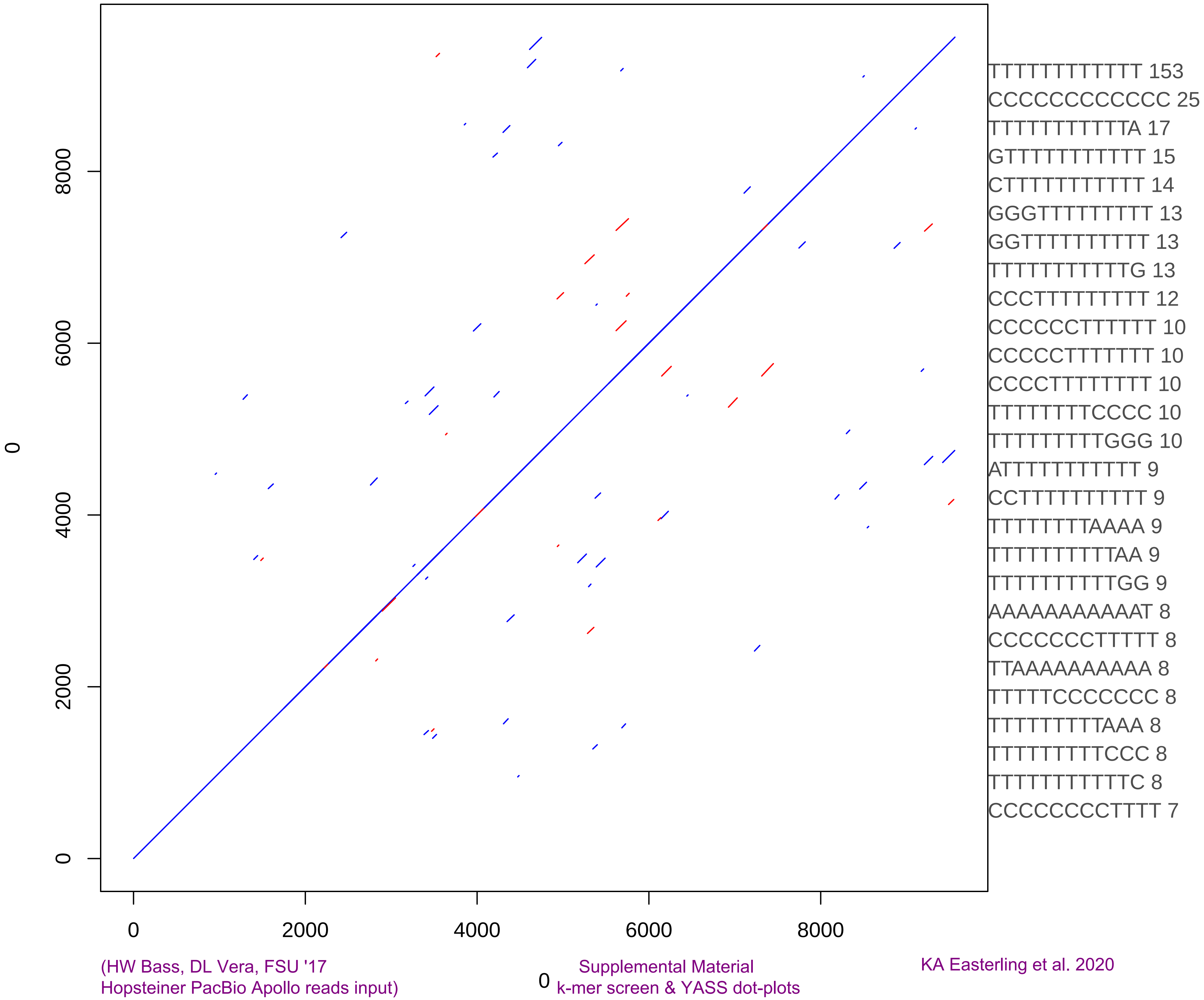

>m141120_105518_42134_c100732402550000001823141405141504_s1_p0/20675/169_10368

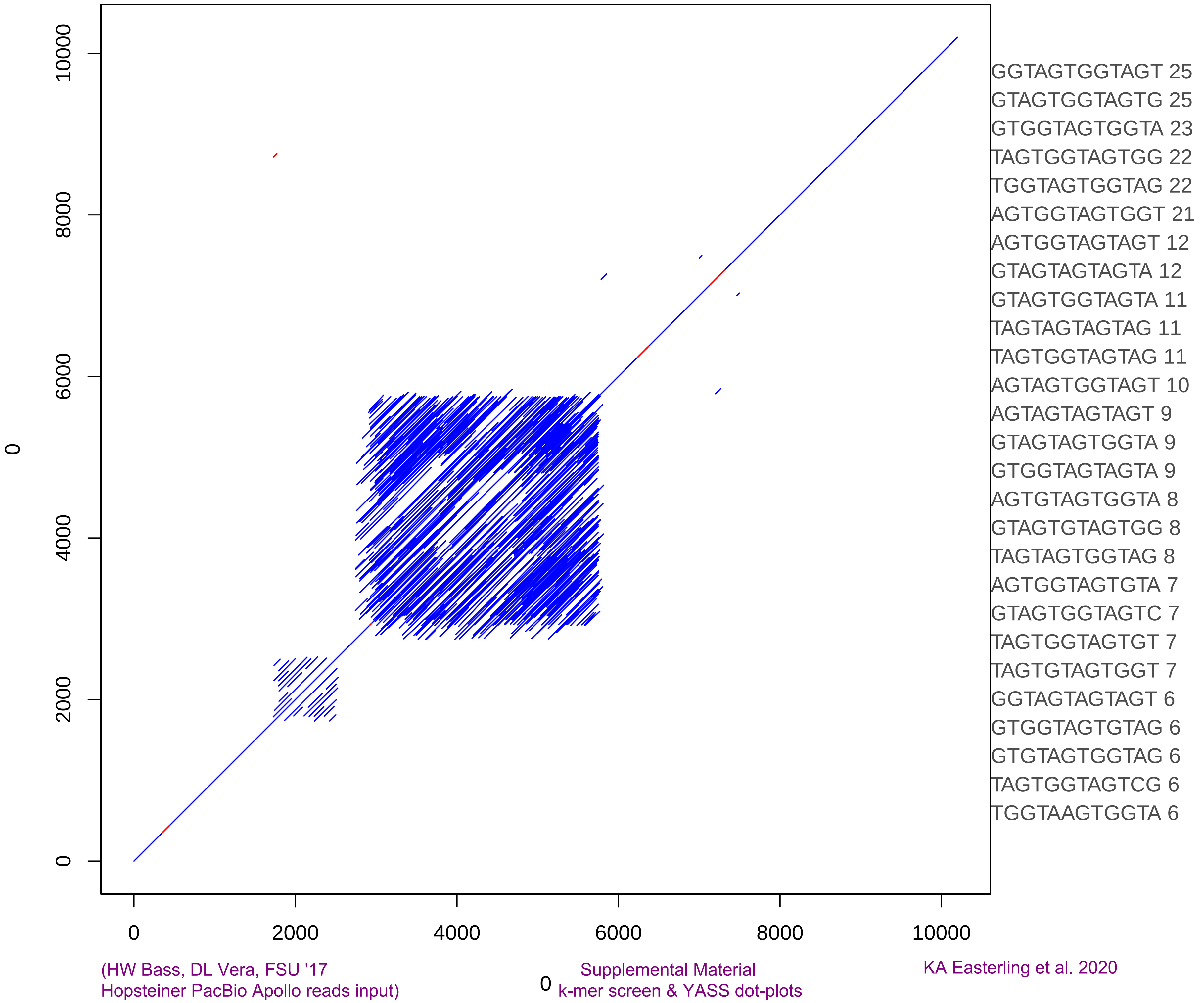

>m141120_105518_42134_c100732402550000001823141405141504_s1_p0/21150/3347_13310

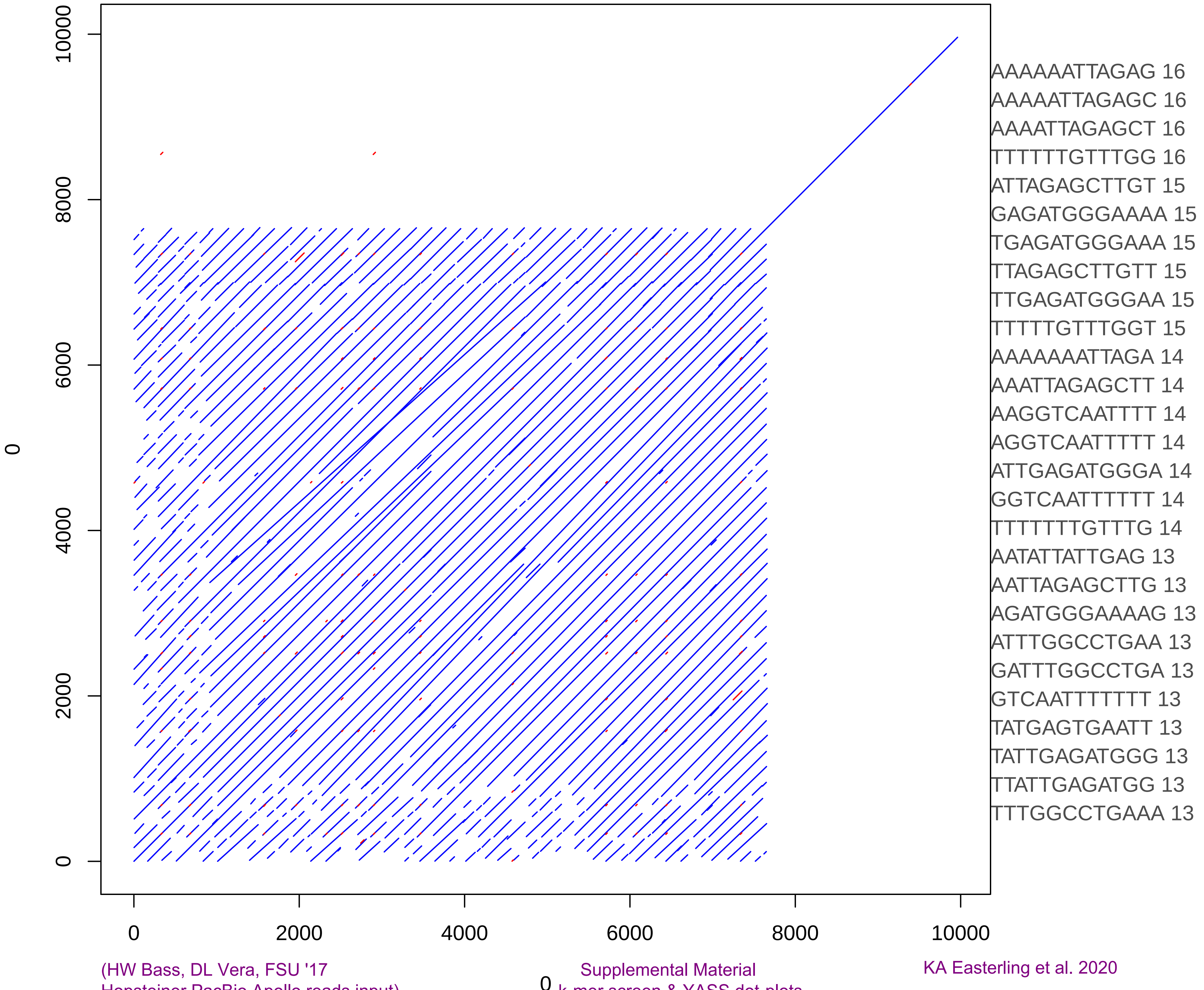

0

>m141120_105518_42134_c100732402550000001823141405141504_s1_p0/32707/14230_21100

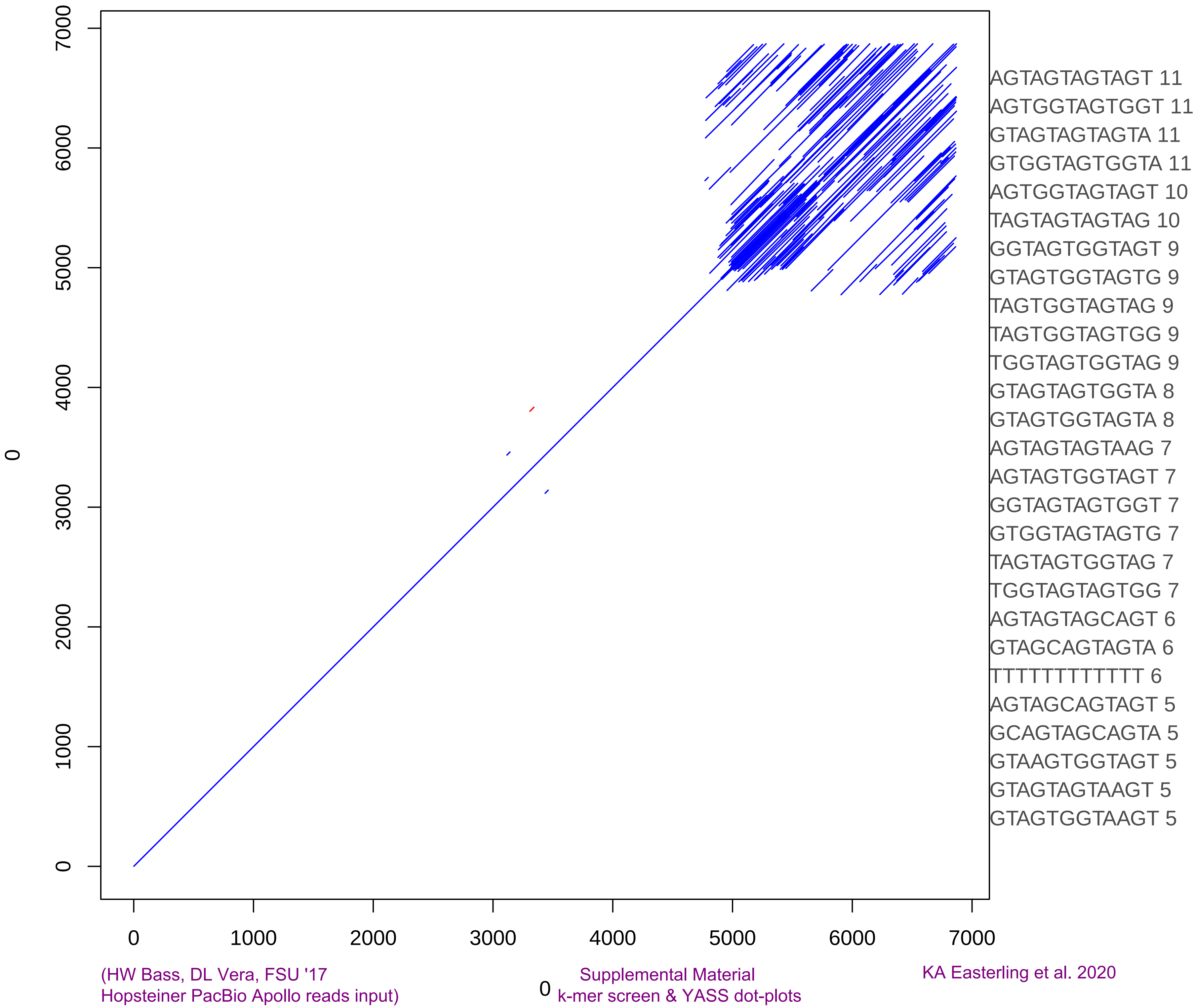

>m141120_105518_42134_c100732402550000001823141405141504_s1_p0/33129/2743_8330

Representative (only one) Read (Table 1): HuluTR050-r33, 1 ppm, 57% A+T, GenBank Acc. MN537565

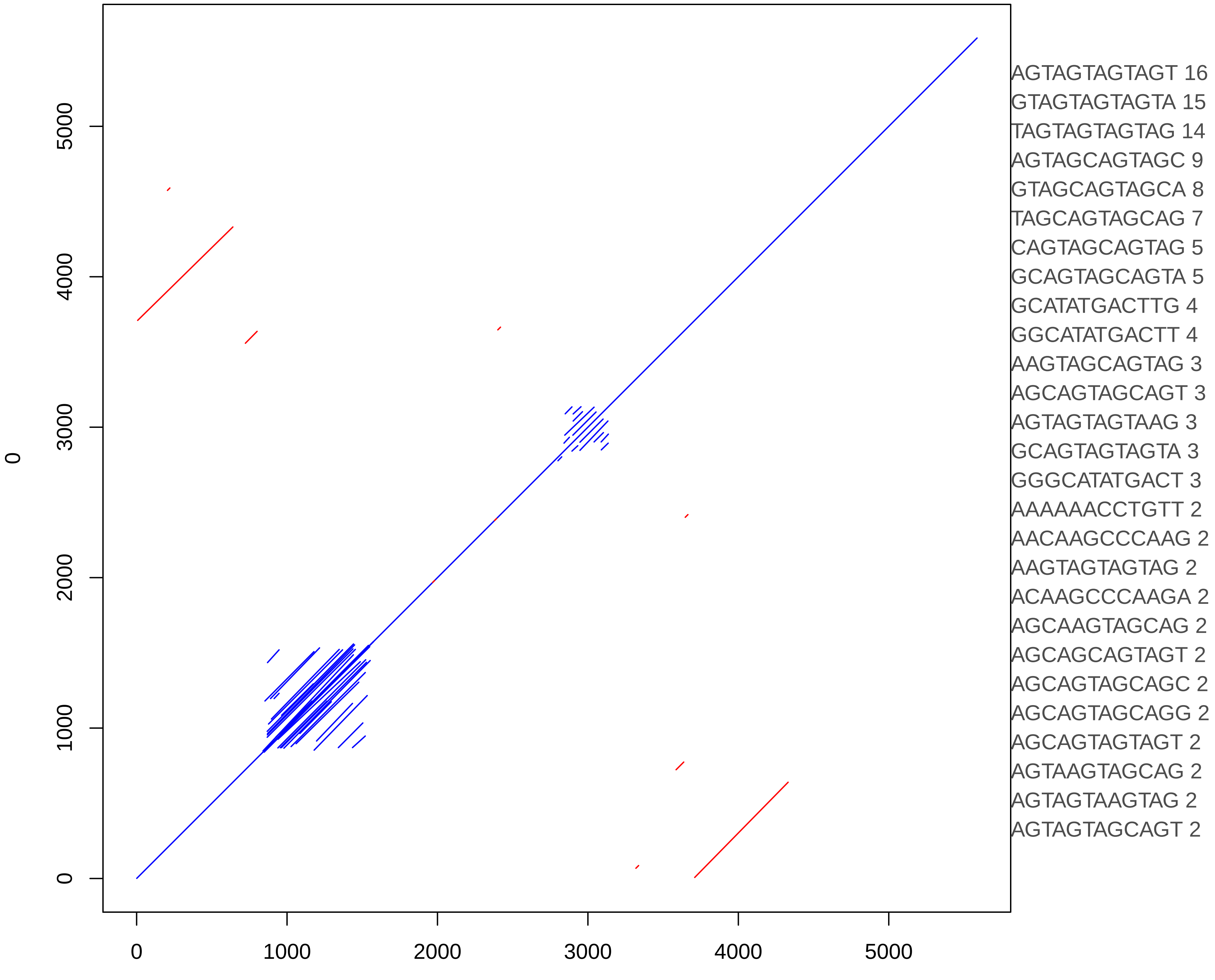

>m141120_105518_42134_c100732402550000001823141405141504_s1_p0/38850/0_7007

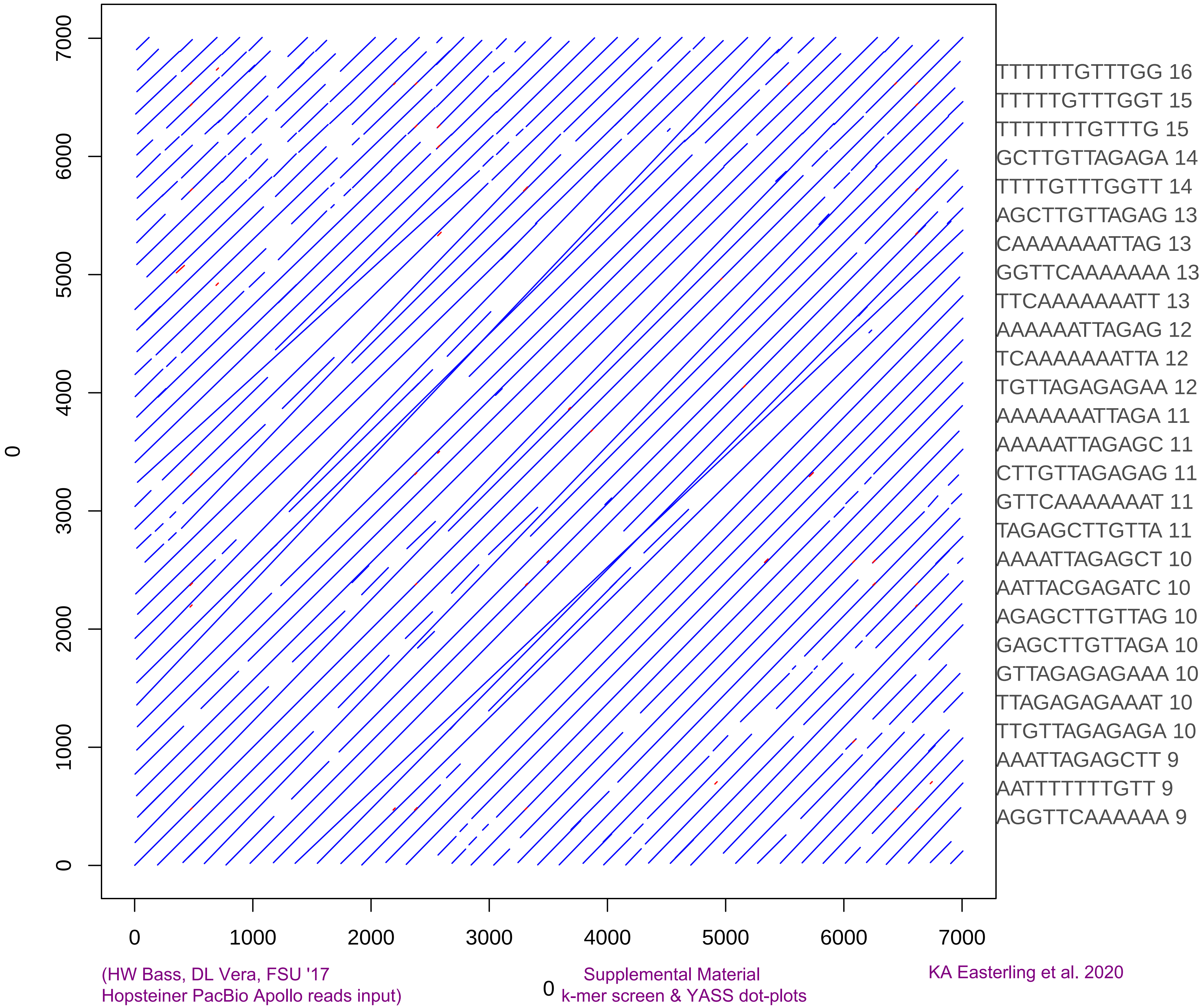

>m141120_105518_42134_c100732402550000001823141405141504_s1_p0/44568/12016_17411

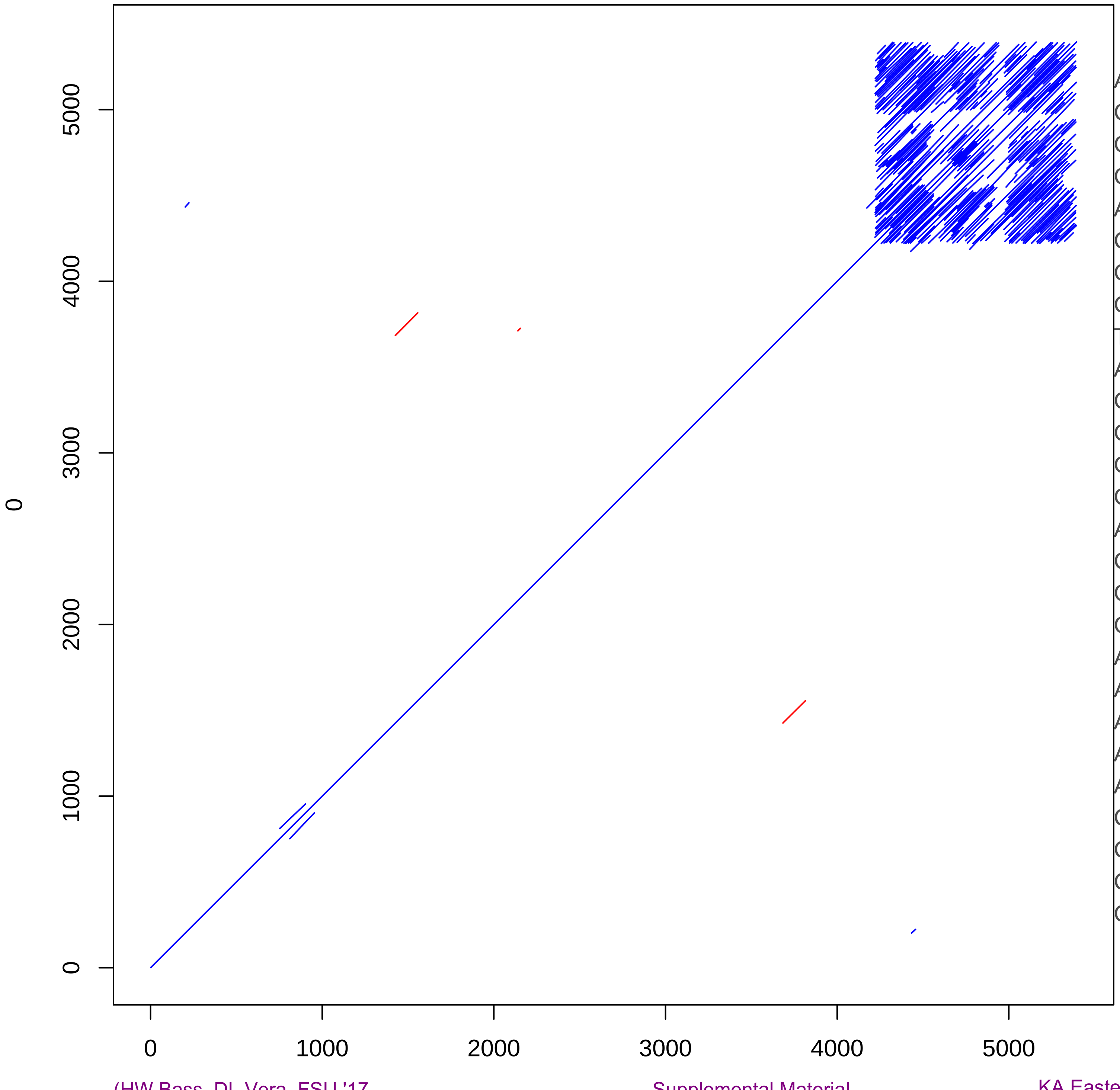

AGGAGGCCATGG 13
CATGGAGGAGGC 13
GAGGAGGCCATG 13
GGAGGAGGCCAT 13
ATGGAGGAGGCC 12
CCATGGAGGAGG 12
GCCATGGAGGAG 12
GGCCATGGAGGA 11
TGGAGGAGGCCA 11
AGGCCATGGAGG 8
GGAGGCCATGGG 8
GAGGCCATGGAG 6
GAGGCCATGGGG 6
GGCCATGGCGAG 5
AGGCCATGGCGA 4
CGAGGCCCATGG 4
GAGGCCATGGCG 4
GGAGGCCATGGC 4
AGGCAGGCCATG 3
AGGCCATGGGCG 3
AGGCCATGGGGG 3
ATGGAGGCAGGC 3
ATGGCAGGAGGC 3
CATGGAGGCAGG 3
CATGGCAGGAGG 3
CCATGGAGGCAG 3
GAGGCAGGCCAT 3

>m141120_105518_42134_c100732402550000001823141405141504_s1_p0/477/355_7463

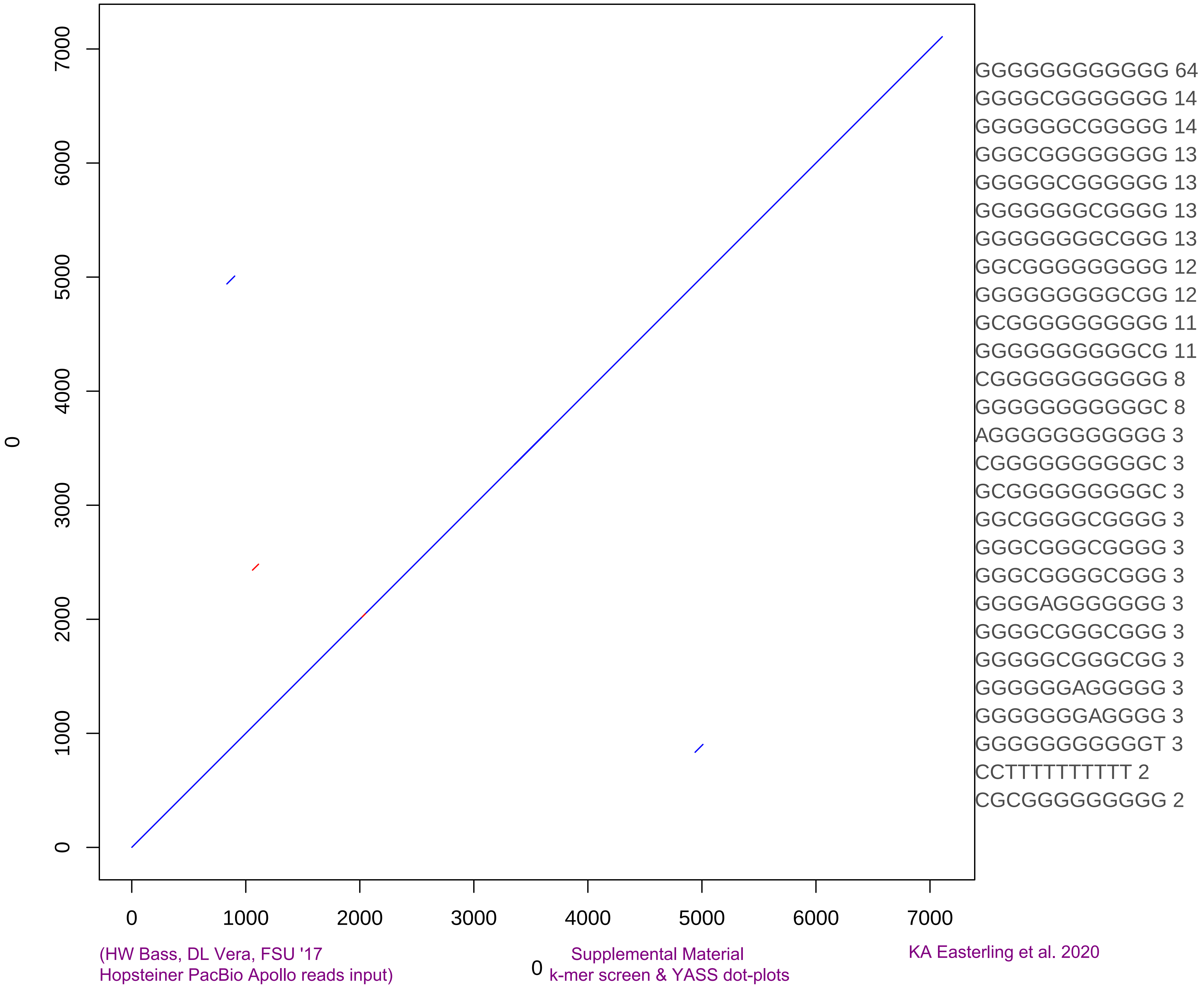

>m141120_105518_42134_c100732402550000001823141405141504_s1_p0/49708/10418_16638

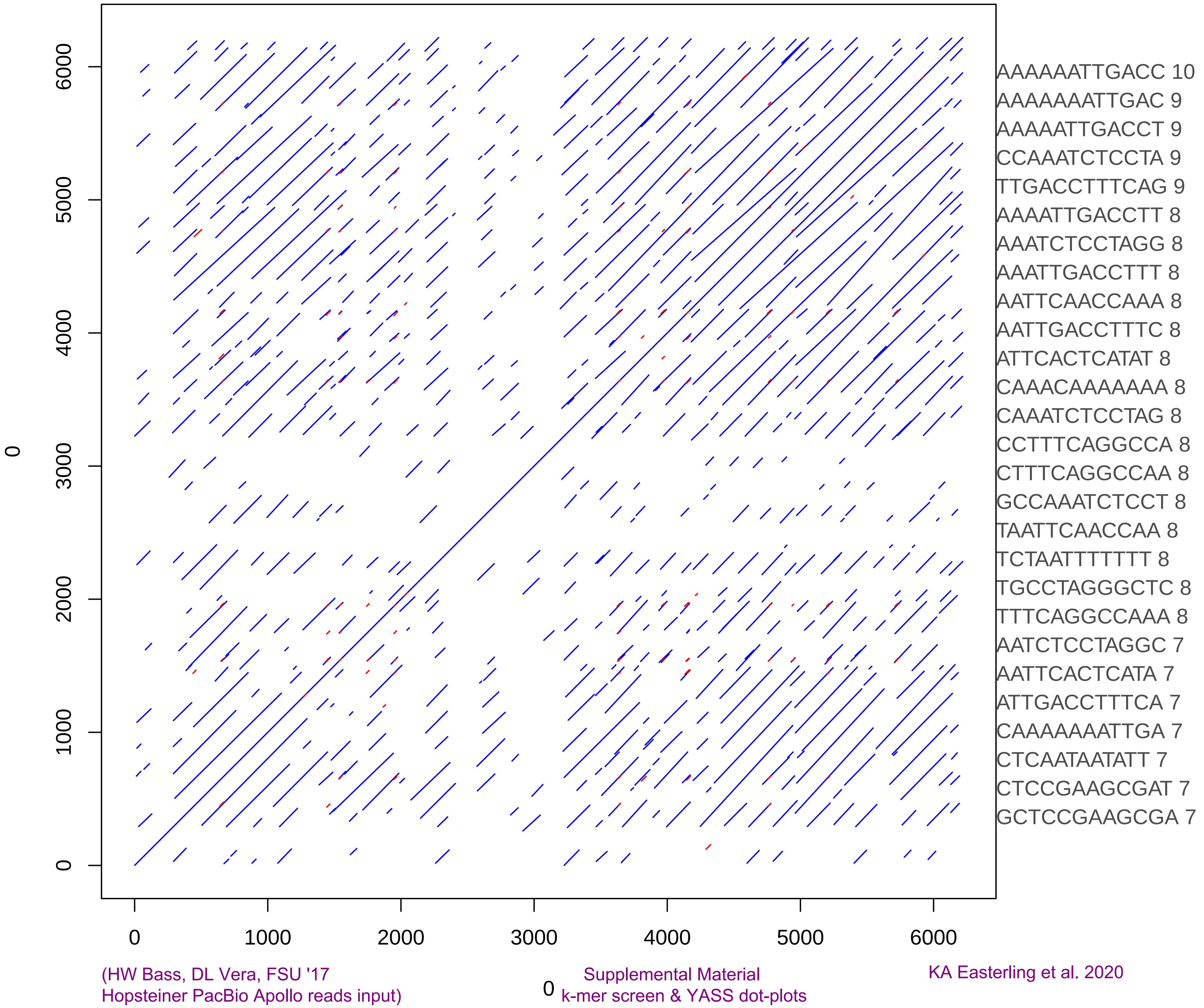

>m141120_105518_42134_c100732402550000001823141405141504_s1_p0/65907/13313_19331

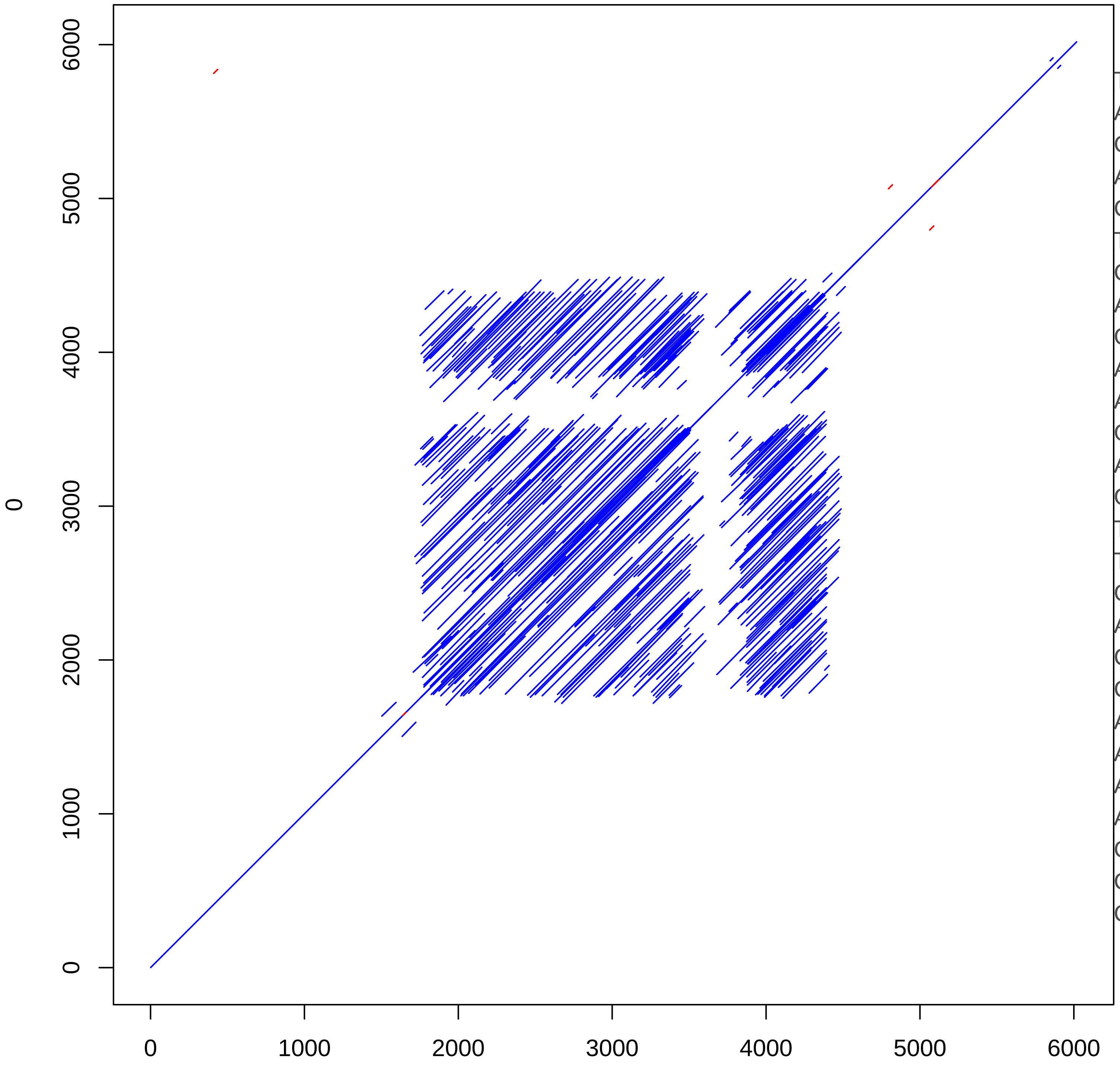

TAGTAGTAGTAG 48
AGTAGTAGTAGT 46
GTAGTAGTAGTA 46
AGTAGCAGTAGC 28
GTAGCAGTAGCA 27
TAGCAGTAGCAG 27
CAGTAGCAGTAG 24
AGCAGTAGCAGT 21
GCAGTAGCAGTA 19
AGTAGTAGCAGT 11
AGCAGTAGCAGC 10
GTAGTAGCAGTA 10
AGTAGCAGTAGT 9
GCAGTAGCAGCA 9
TAGTAGCAGTAG 9
TAGCAGTAGTAG 8
GTAGCAGTAGTA 7
AGTAGCAGCAGT 6
CAGCAGTAGCAG 6
CAGTAGCAGCAG 6
AAGTAGCAGTAG 5
AGCAGTAGTAGC 5
AGTAGTAGTAAG 5
AGTAGTAGTAGC 5
GCAGCAGTAGCA 5
GTAGCAGCAGTA 5
GTAGTAGTAGCA 5

>m141120_105518_42134_c100732402550000001823141405141504_s1_p0/67652/4167_10877

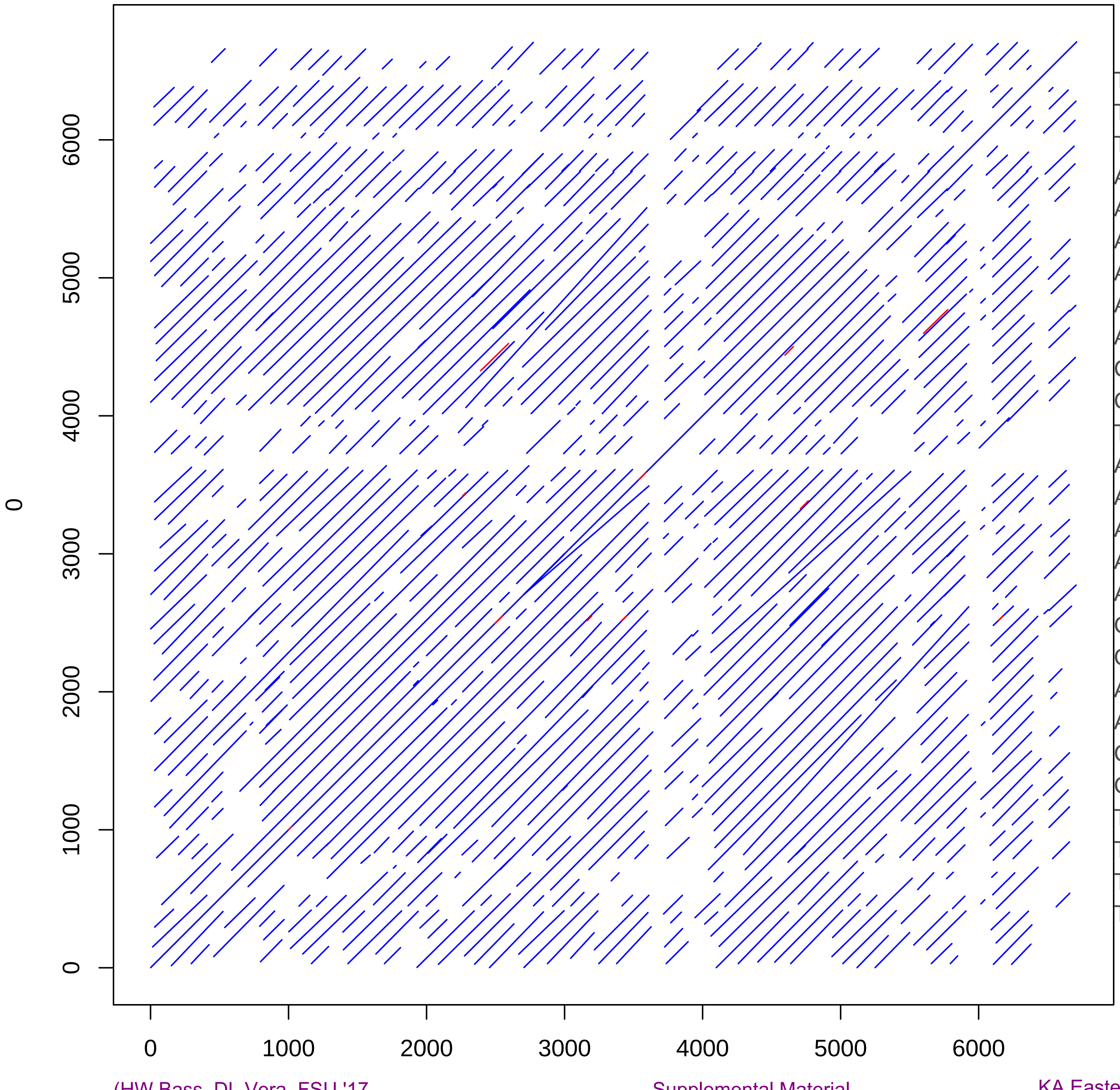

TAAGAAAAACAA 14
TTAAGAAAAACA 14
TTTAAGAAAAAC 13
AAAACTTAGTTC 12
AAAAACAACGAT 11
AAACAACGATCA 11
AAACTTAGTTCG 11
AAGTTTTATGAC 11
AGAAAAACAACG 11
CTCTTTAAGAAA 11
GAAAAACAACGA 11
TCTTTAAGAAAA 11
AAAACAACGATC 10
AACAACGATCAA 10
ACTTCTAAGTTT 10
ATTAACTTCTAA 10
ATTTAAATTAAC 10
CTTTAAGAAAAA 10
GATTTAAATTAA 10
AACTTAGTTCGC 9
AAGAAAAACAAC 9
CTTCTAAGTTTT 9
GCTCTTTAAGAA 9
TTAACTTCTAAG 9
TTGATTTAAATT 9
TTTAAATTAACT 9
TTTTATCAATAT 9

>m141120_105518_42134_c100732402550000001823141405141504_s1_p0/68203/7759_15486

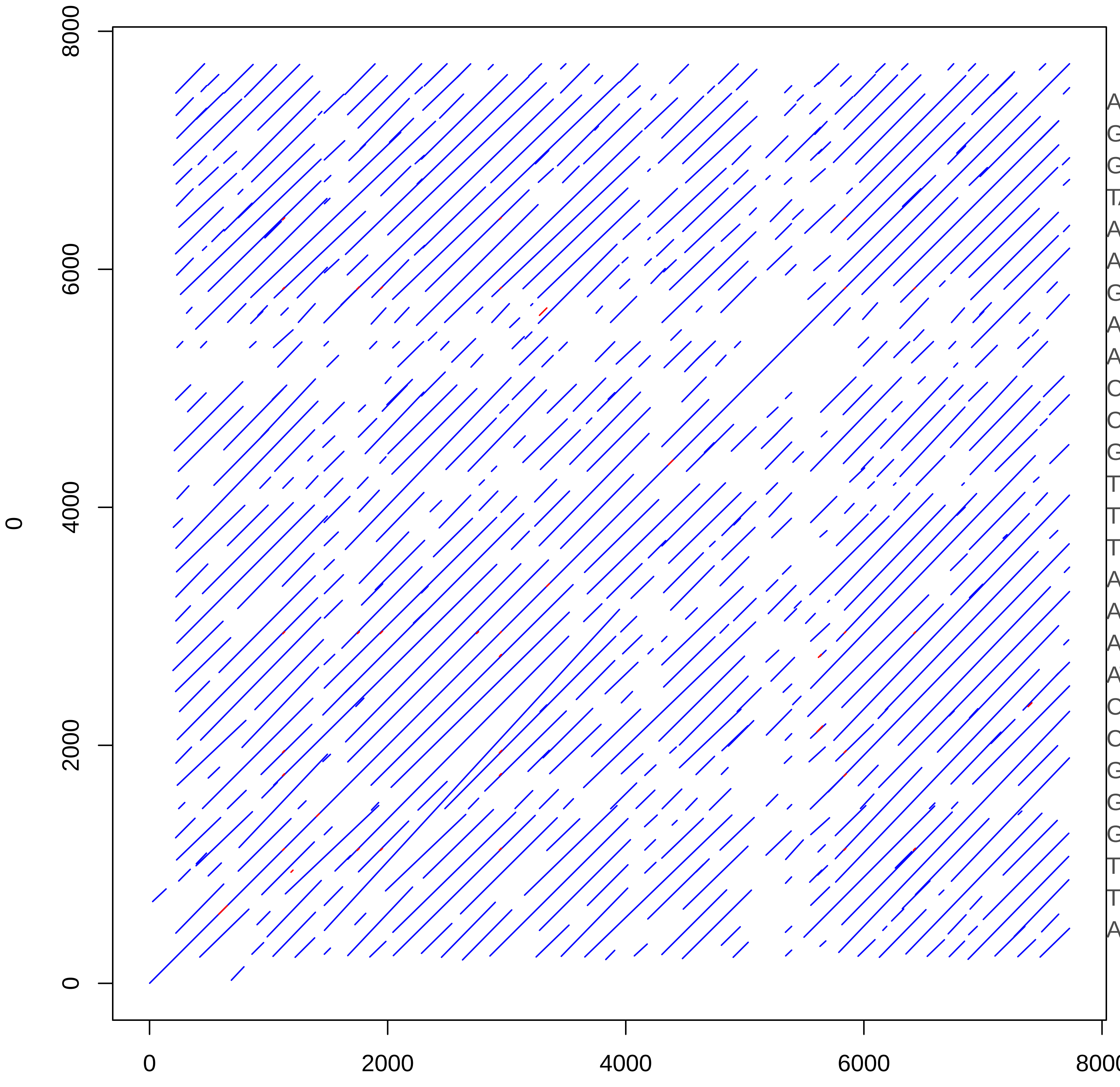

>m141120_105518_42134_c100732402550000001823141405141504_s1_p0/69383/4241_15149

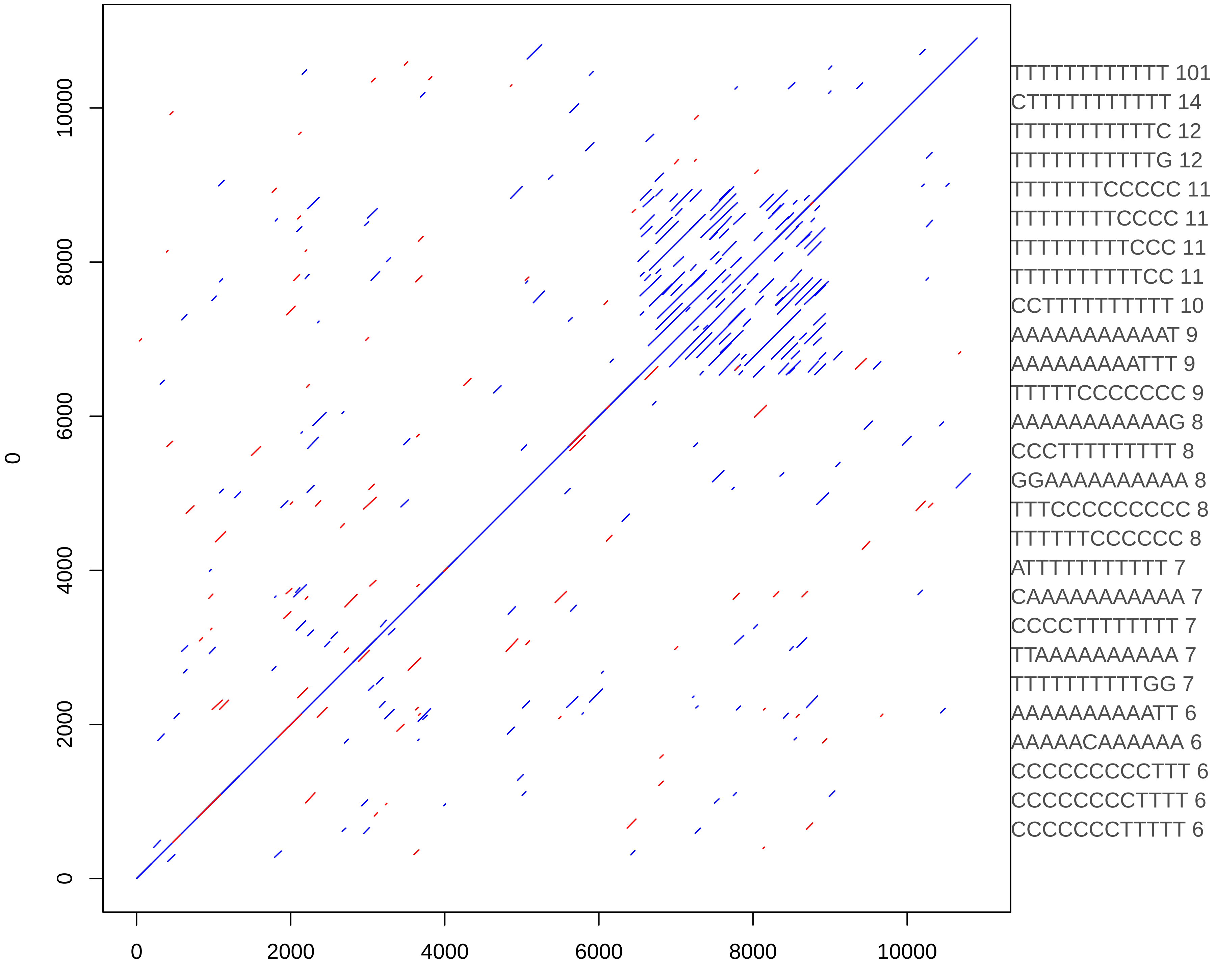

0

>m141120_105518_42134_c100732402550000001823141405141504_s1_p0/71108/0_5898

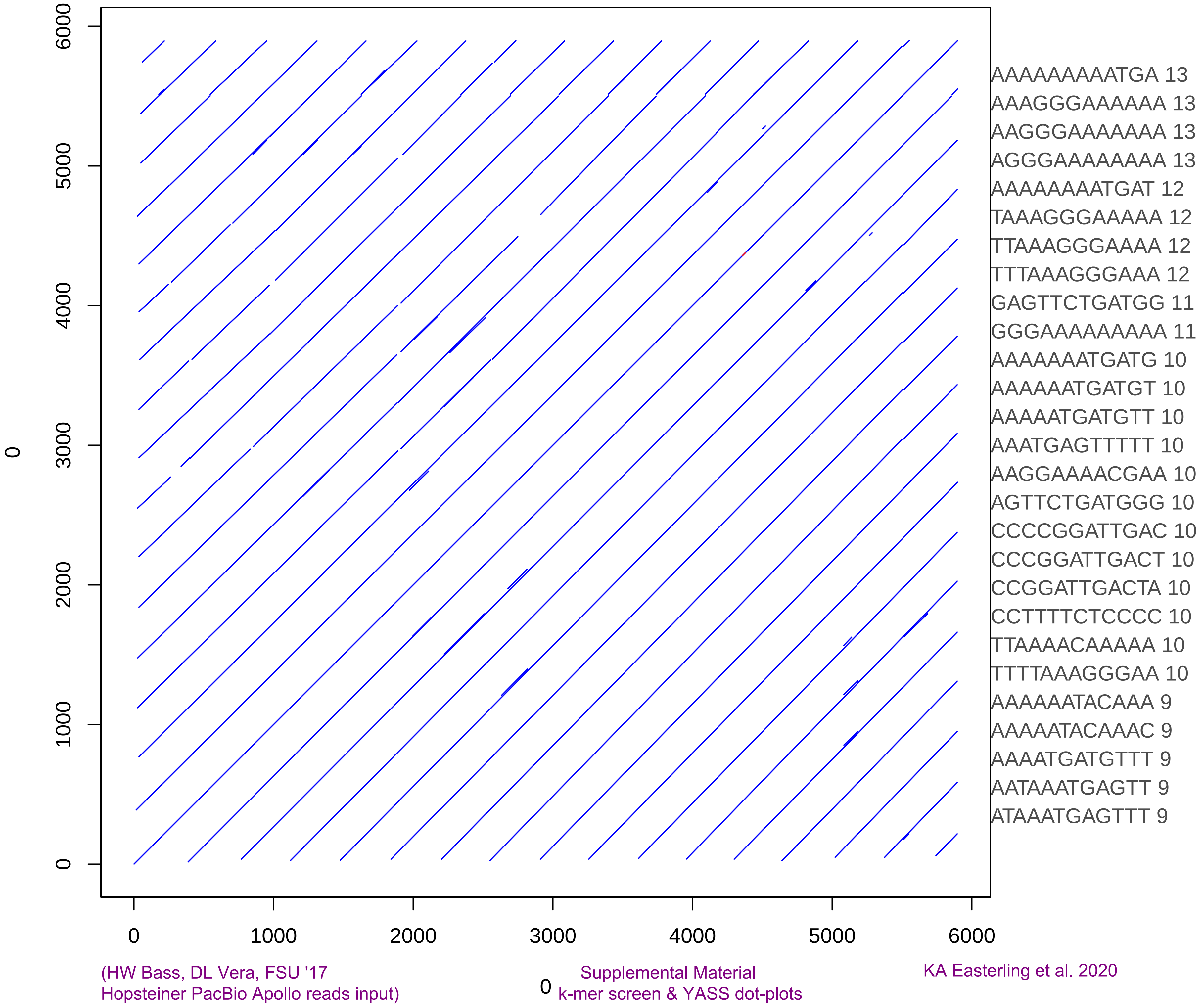

>m141120_105518_42134_c100732402550000001823141405141504_s1_p0/72674/8494_15164

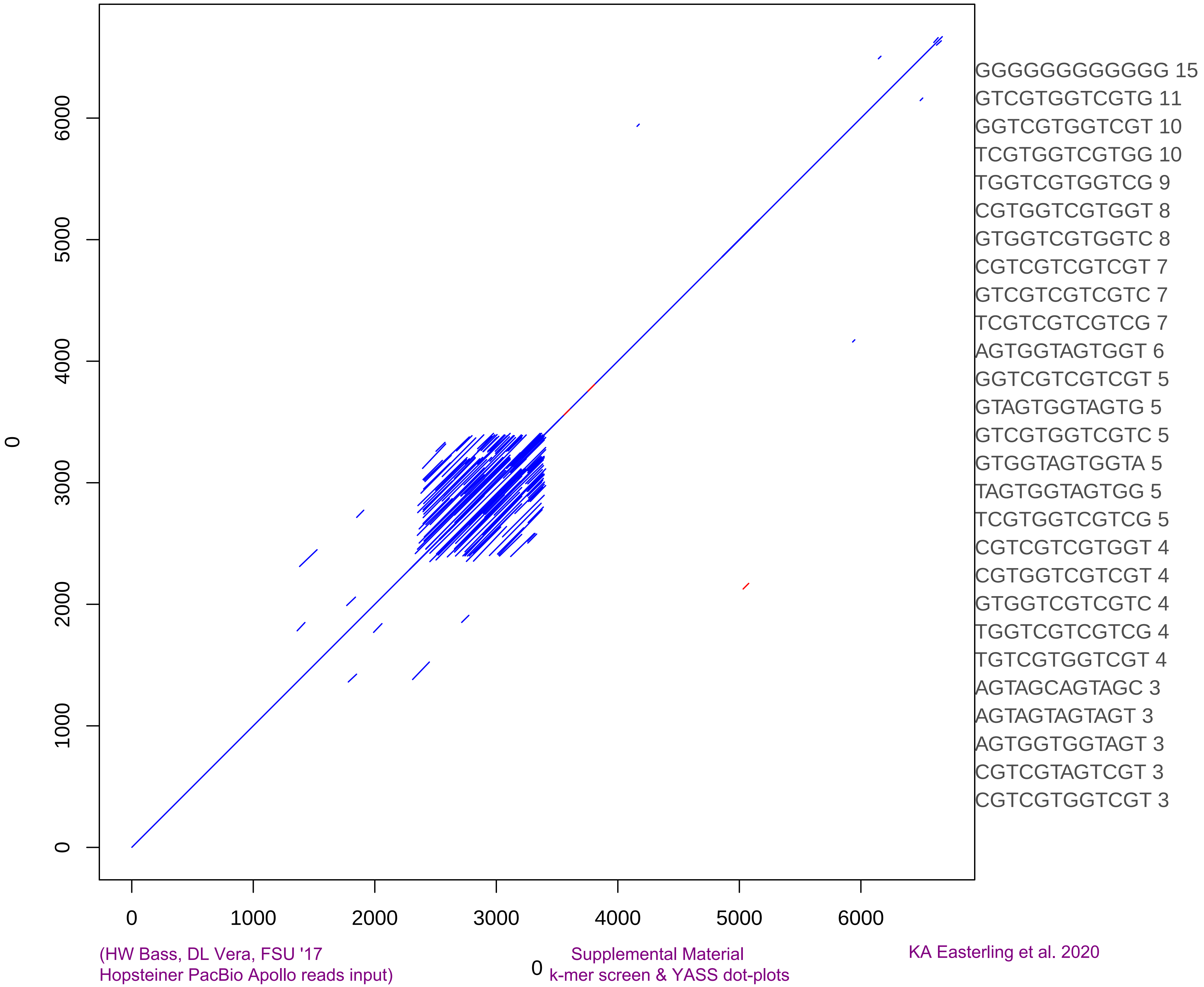

>m141120_105518_42134_c100732402550000001823141405141504_s1_p0/75980/409_14437

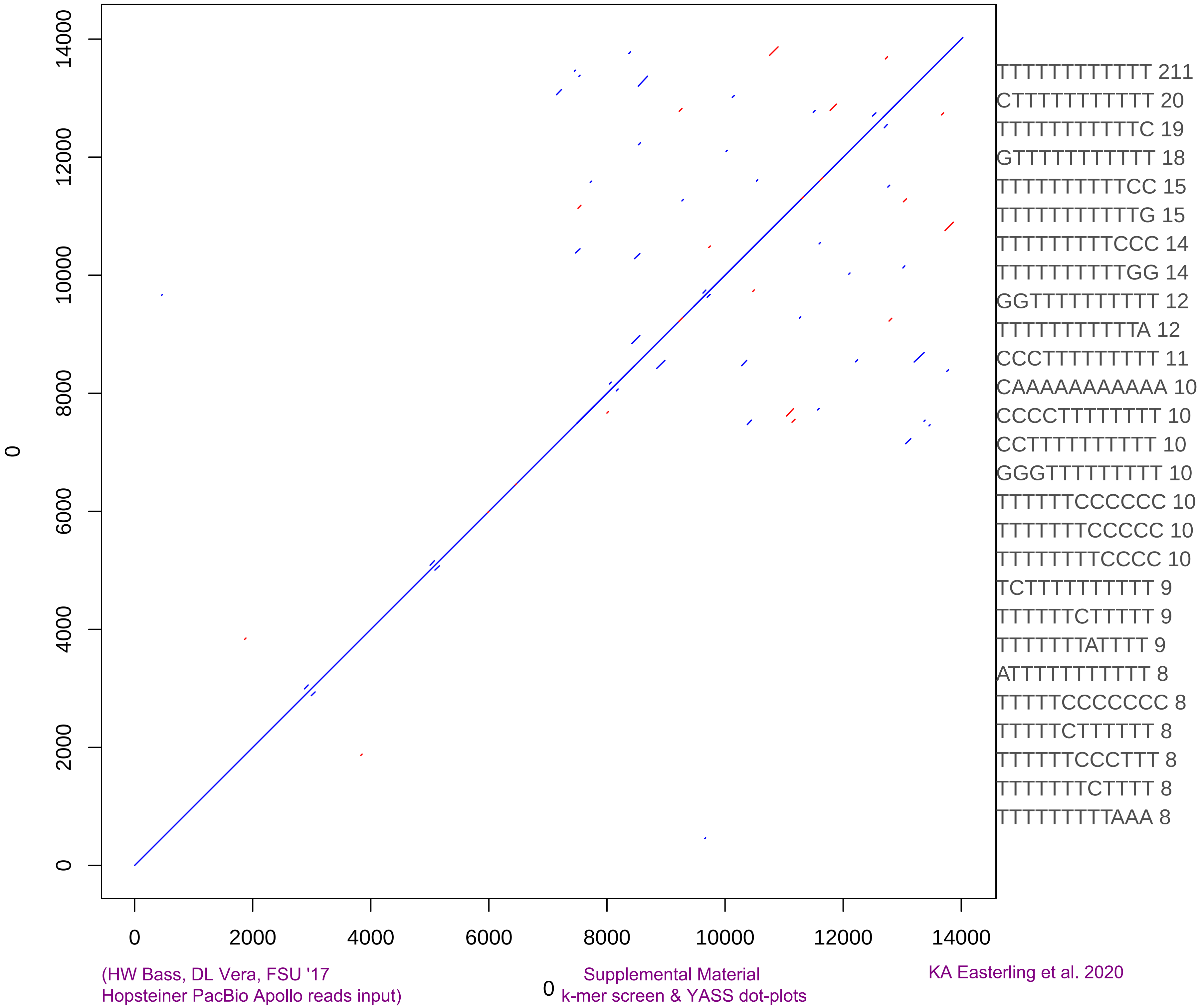

>m141120_105518_42134_c100732402550000001823141405141504_s1_p0/76513/1992_7721

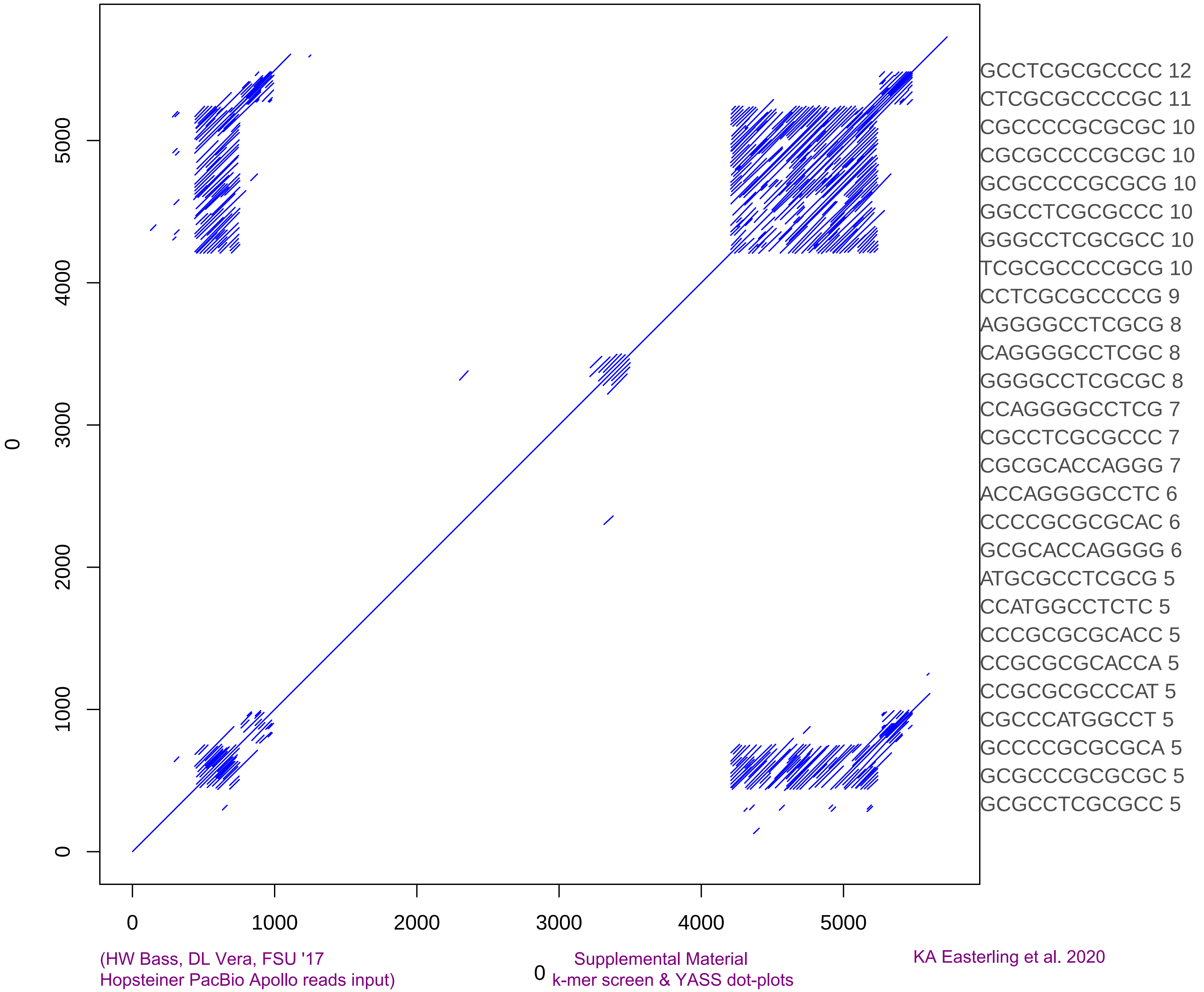

>m141120_105518_42134_c100732402550000001823141405141504_s1_p0/78451/2754_9707

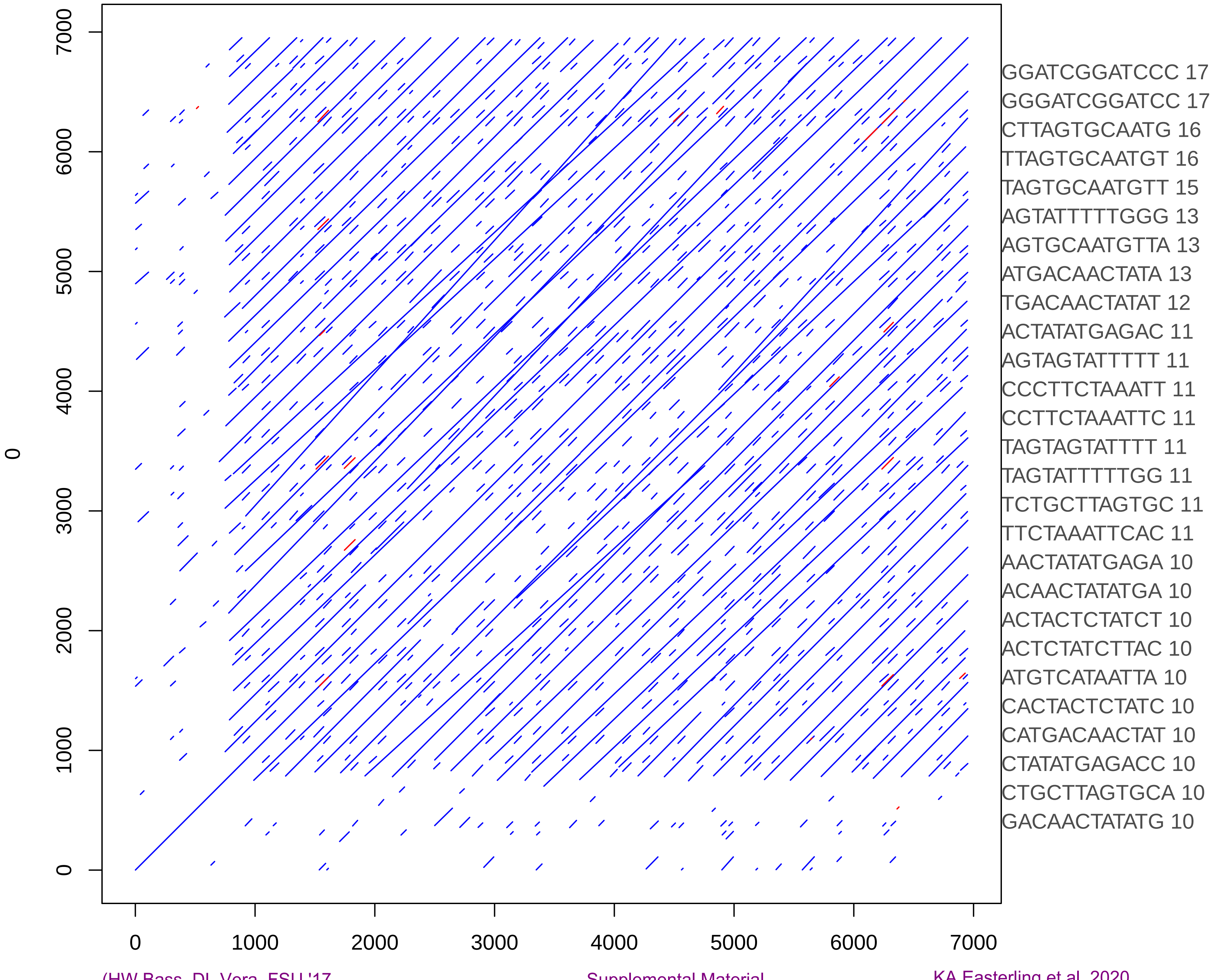

>m141120_105518_42134_c100732402550000001823141405141504_s1_p0/78610/0_5598

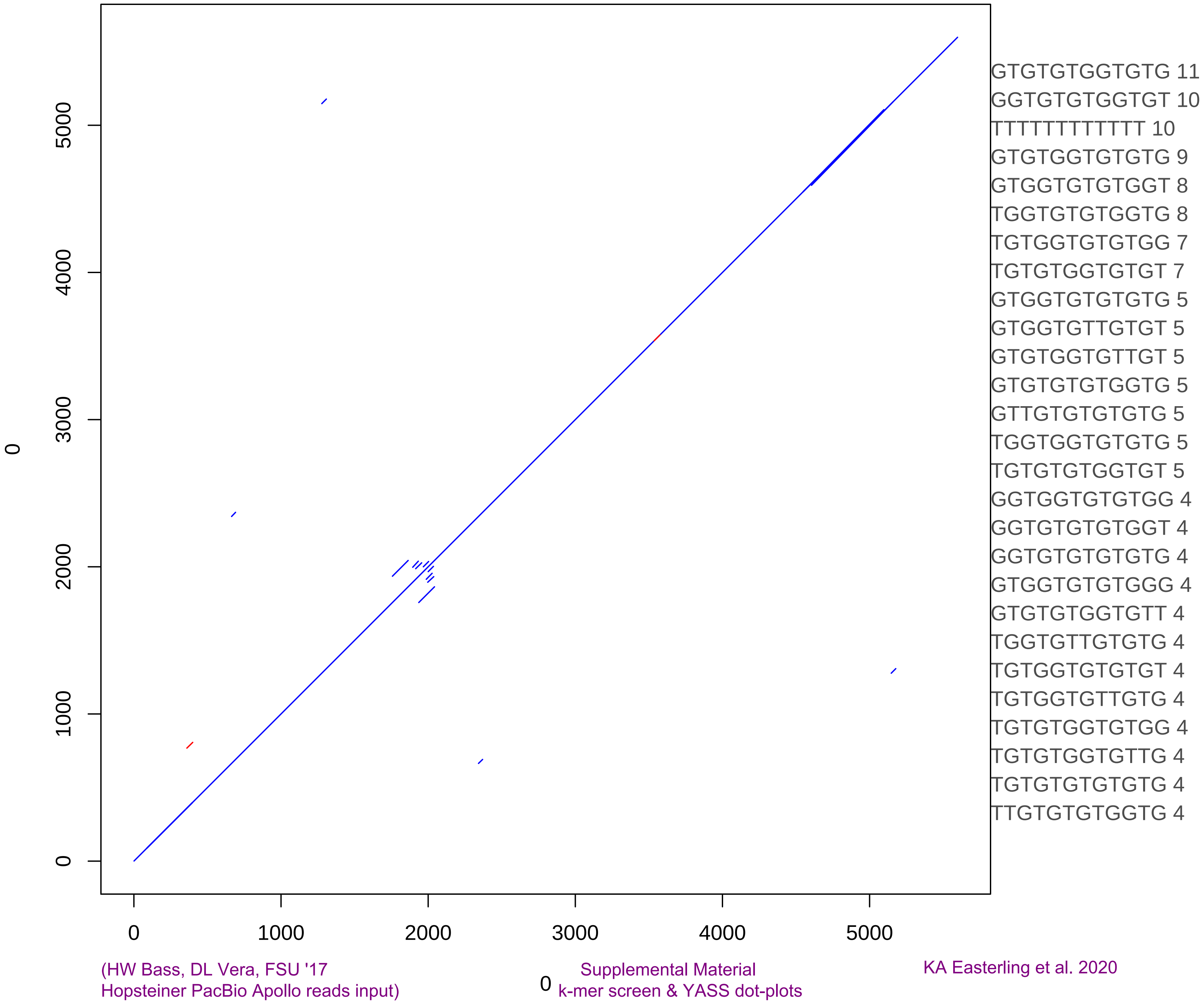

>m141120_105518_42134_c100732402550000001823141405141504_s1_p0/87211/875_6819

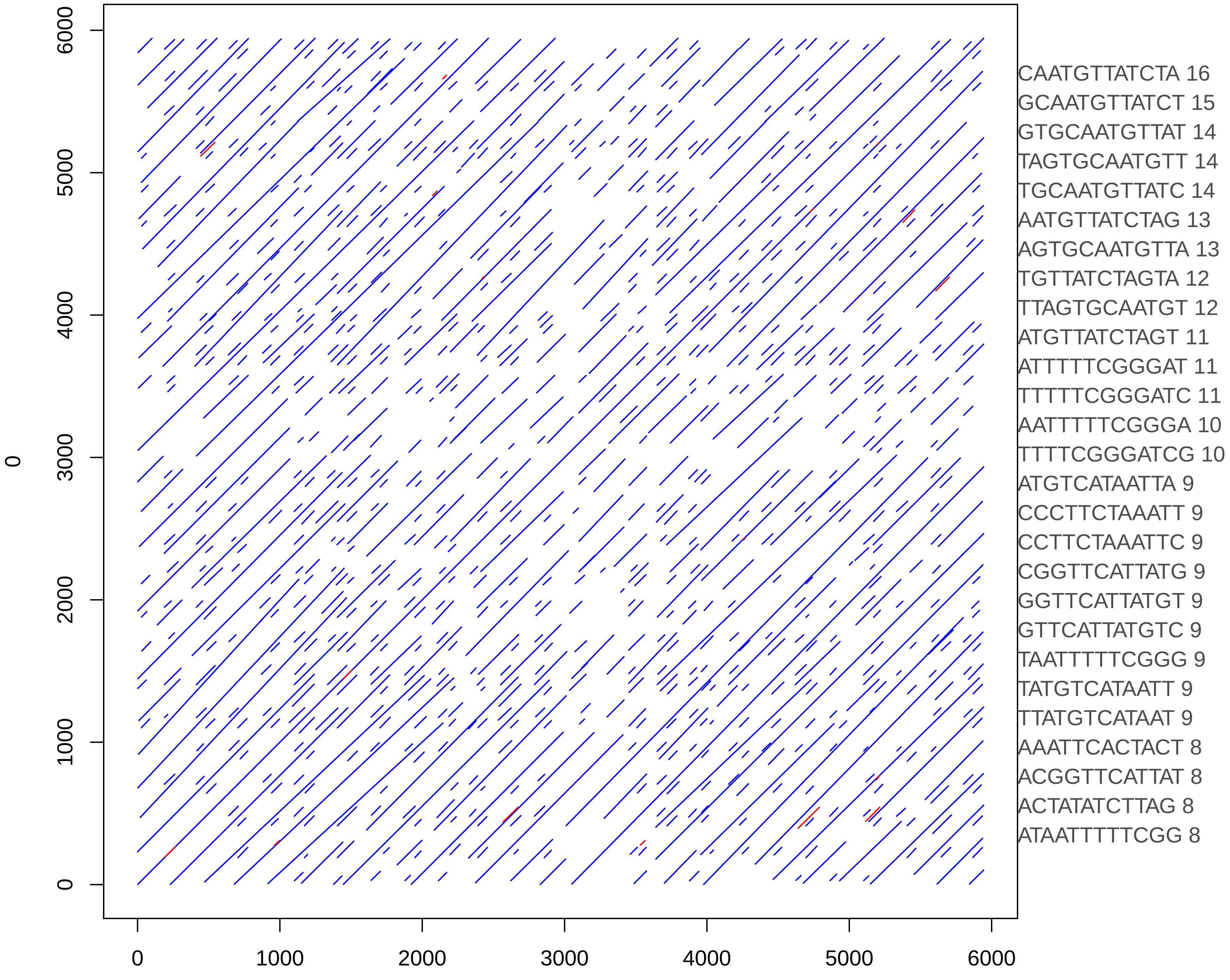

>m141120_105518_42134_c100732402550000001823141405141504_s1_p0/99387/14485_20717

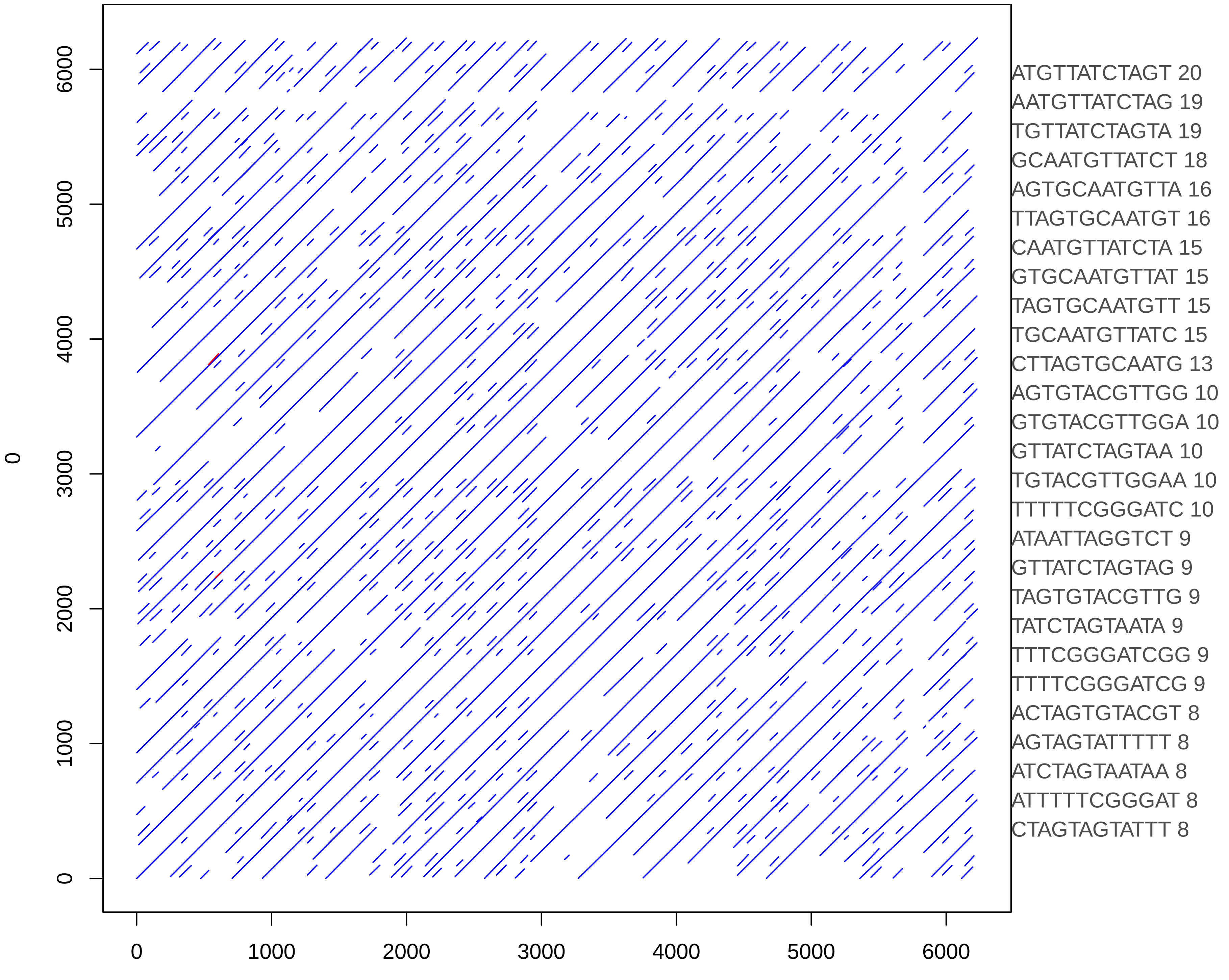

>m141120_105518_42134_c100732402550000001823141405141504_s1_p0/99387/7352_14436

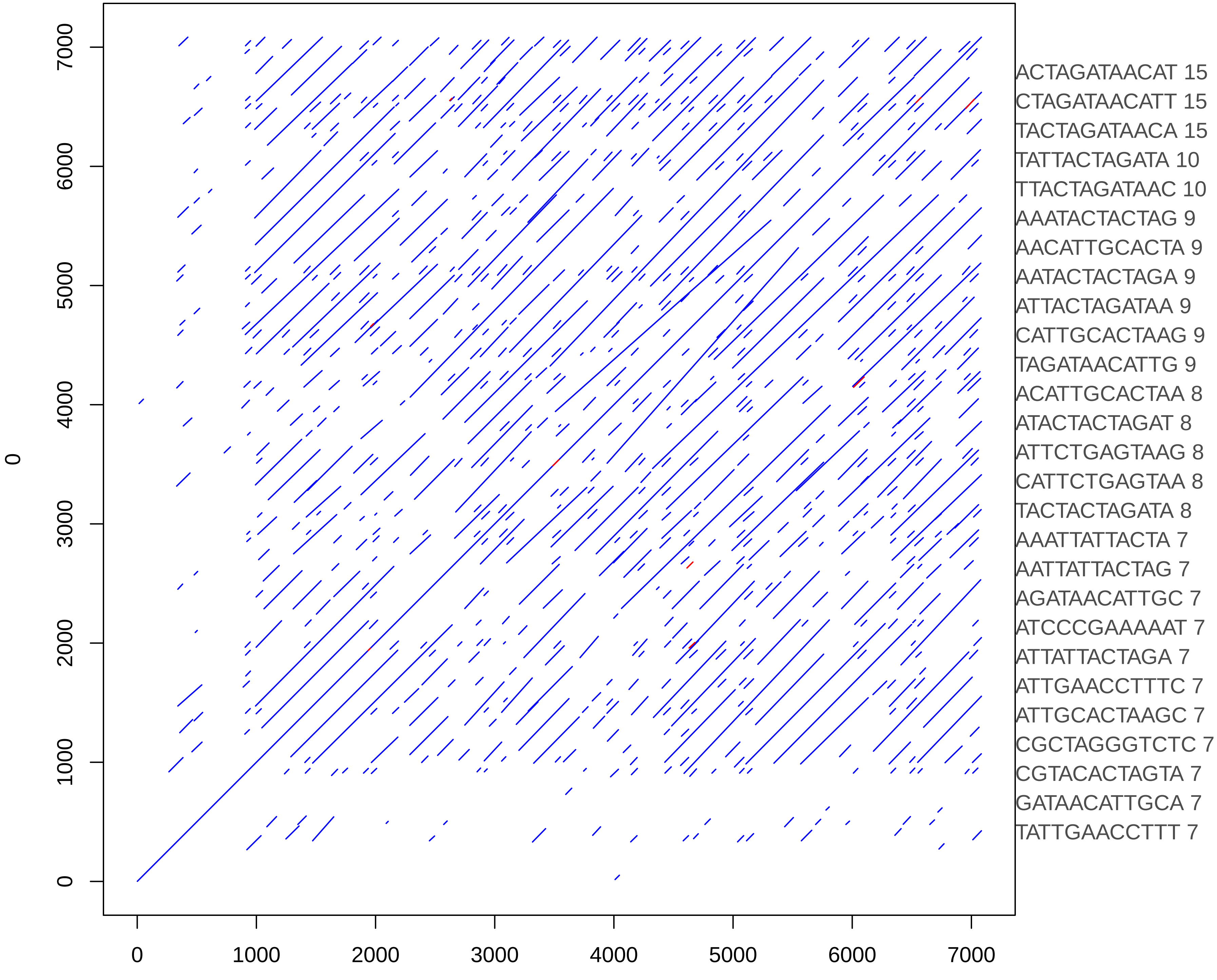

>m141120_131642_42134_c100732402550000001823141405141505_s1_p0/100112/0_5478

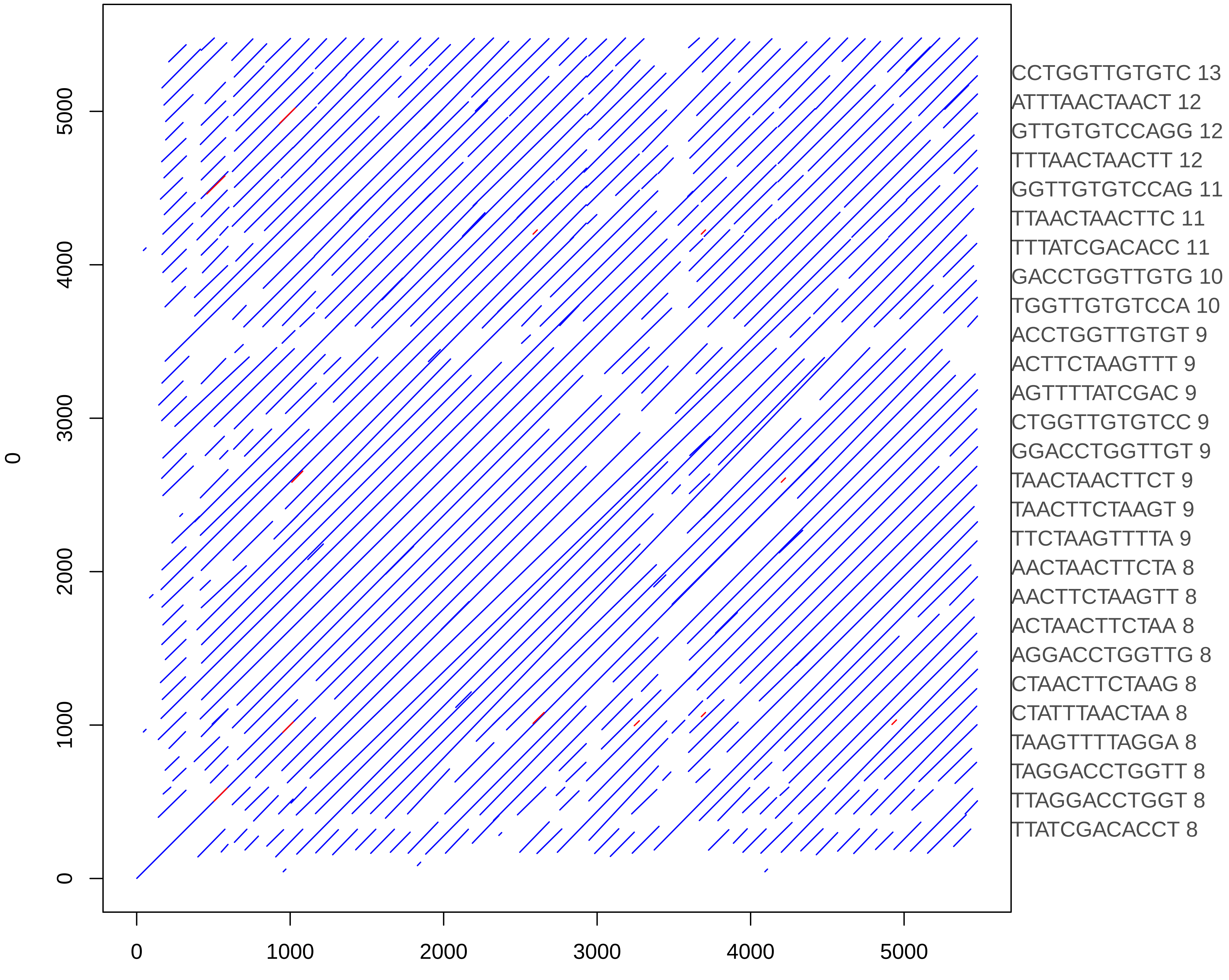

>m141120_131642_42134_c100732402550000001823141405141505_s1_p0/104321/0_6721

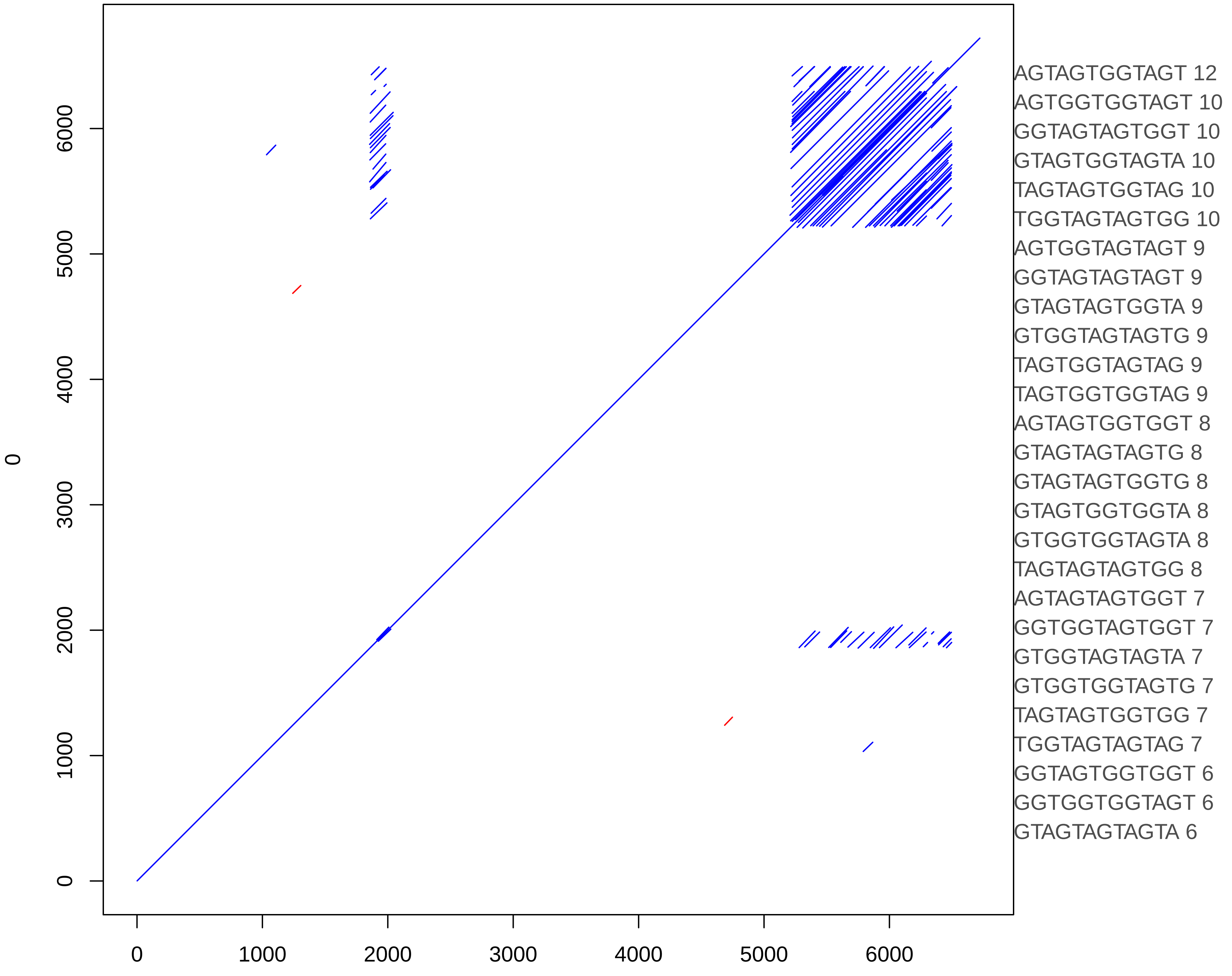

>m141120_131642_42134_c100732402550000001823141405141505_s1_p0/10856/297_13471

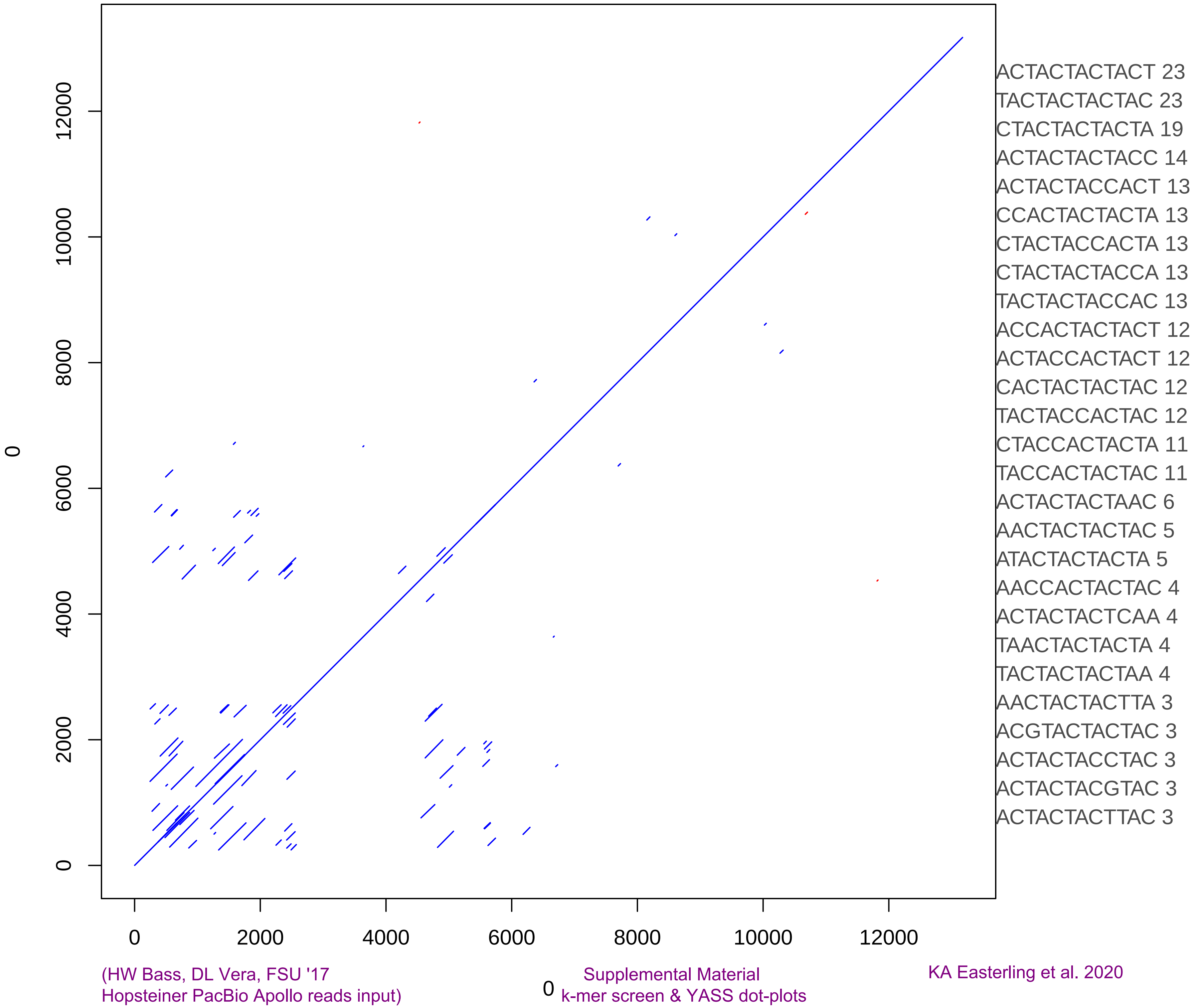

>m141120_131642_42134_c100732402550000001823141405141505_s1_p0/109827/0_5525

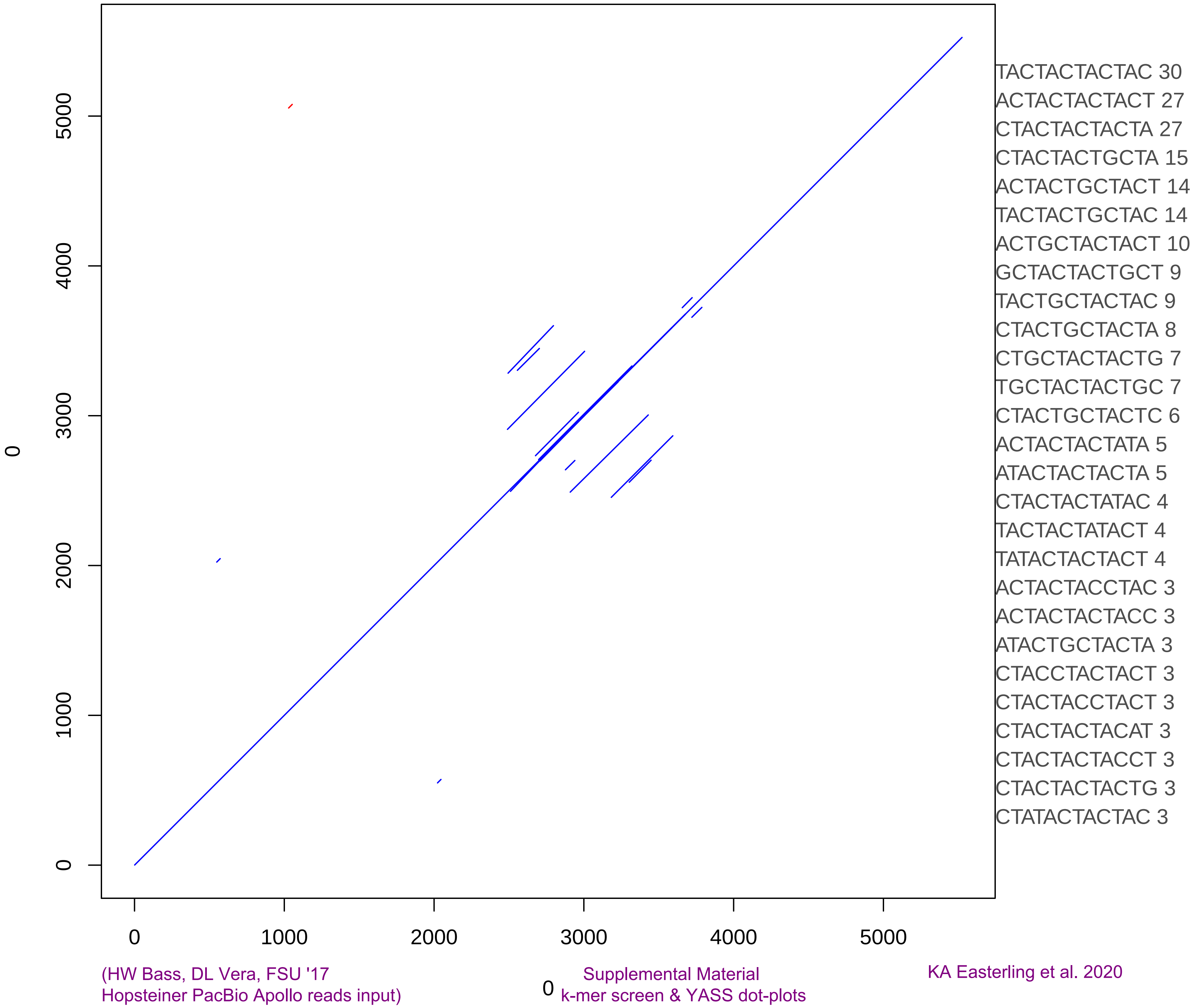

>m141120_131642_42134_c100732402550000001823141405141505_s1_p0/110134/0_5172

Representative Read (Table 1): (HSR1) HuluTR385-r55, 232 ppm, 61% A+T, GenBank Acc. GU831574

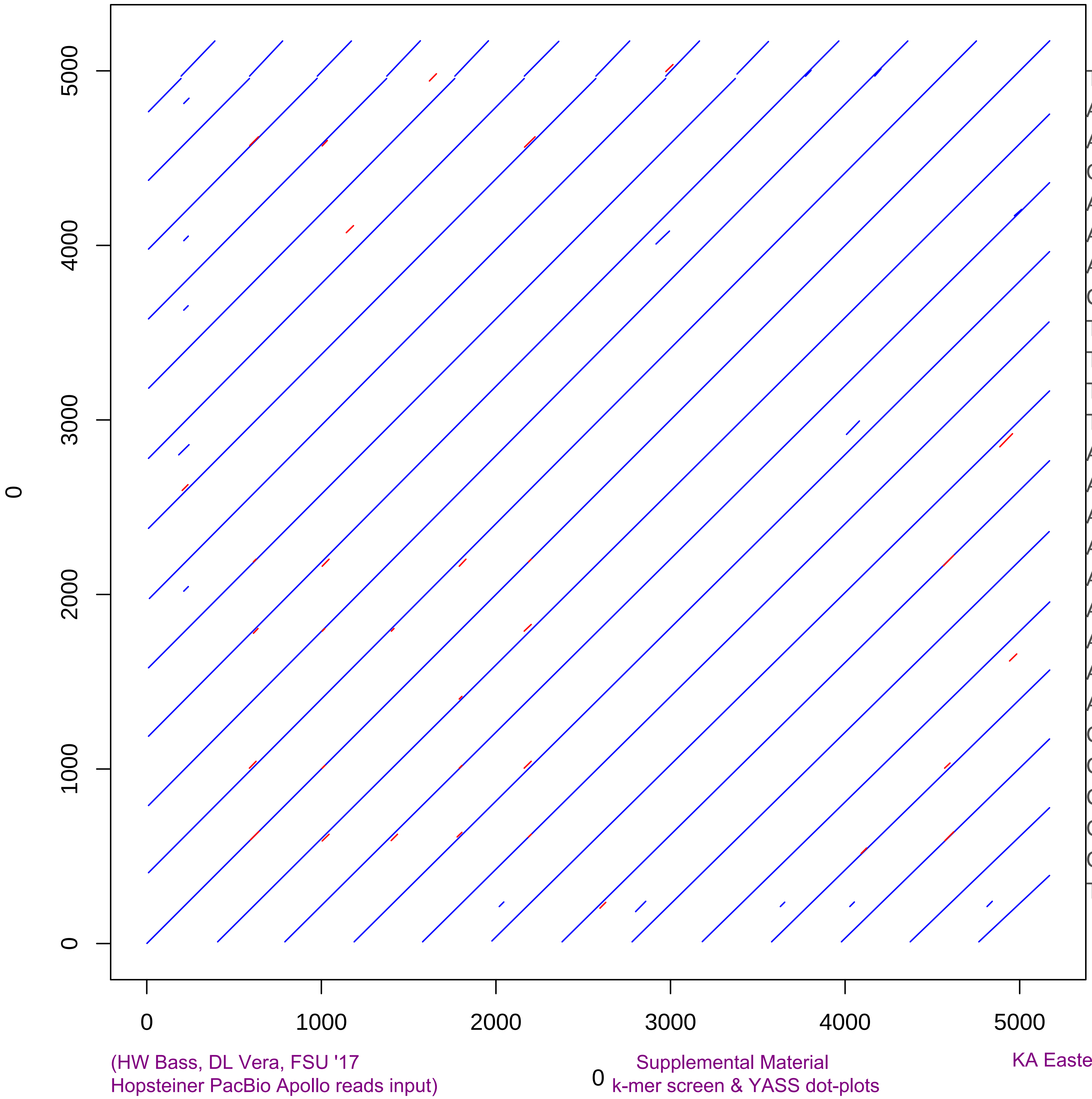

TATGAATTGGAG 11
AATACTGAGAGA 10
ATGAATTGGAGC 10
GTATGAATTGGA 10
AAATACTGAGAG 9
AGTATGAATTGG 9
ATTTCGGAGAAA 9
CAAAATACTGAG 9
TCAAAATACTGA 9
TTTCAGAAATAT 9
TTTCGGAGAAAT 9
TTTTGAAAGTGA 9
AAAATACTGAGA 8
AACTTGTGAAAA 8
AATAGATGCATT 8
AATGTTTTGAAA 8
AATTGGAGCAAT 8
ATACTGAGAGAT 8
ATAGATGCATTT 8
ATGGGCACAAAA 8
ATGTTTTGAAAG 8
CAATAGATGCAT 8
CCAATAGATGCA 8
CCTTTCAGAAAT 8
GAATTGGAGCAA 8
GTTTTGAAAGTG 8
TAGTATGAATTG 8

>m141120_131642_42134_c100732402550000001823141405141505_s1_p0/112540/4430_12421

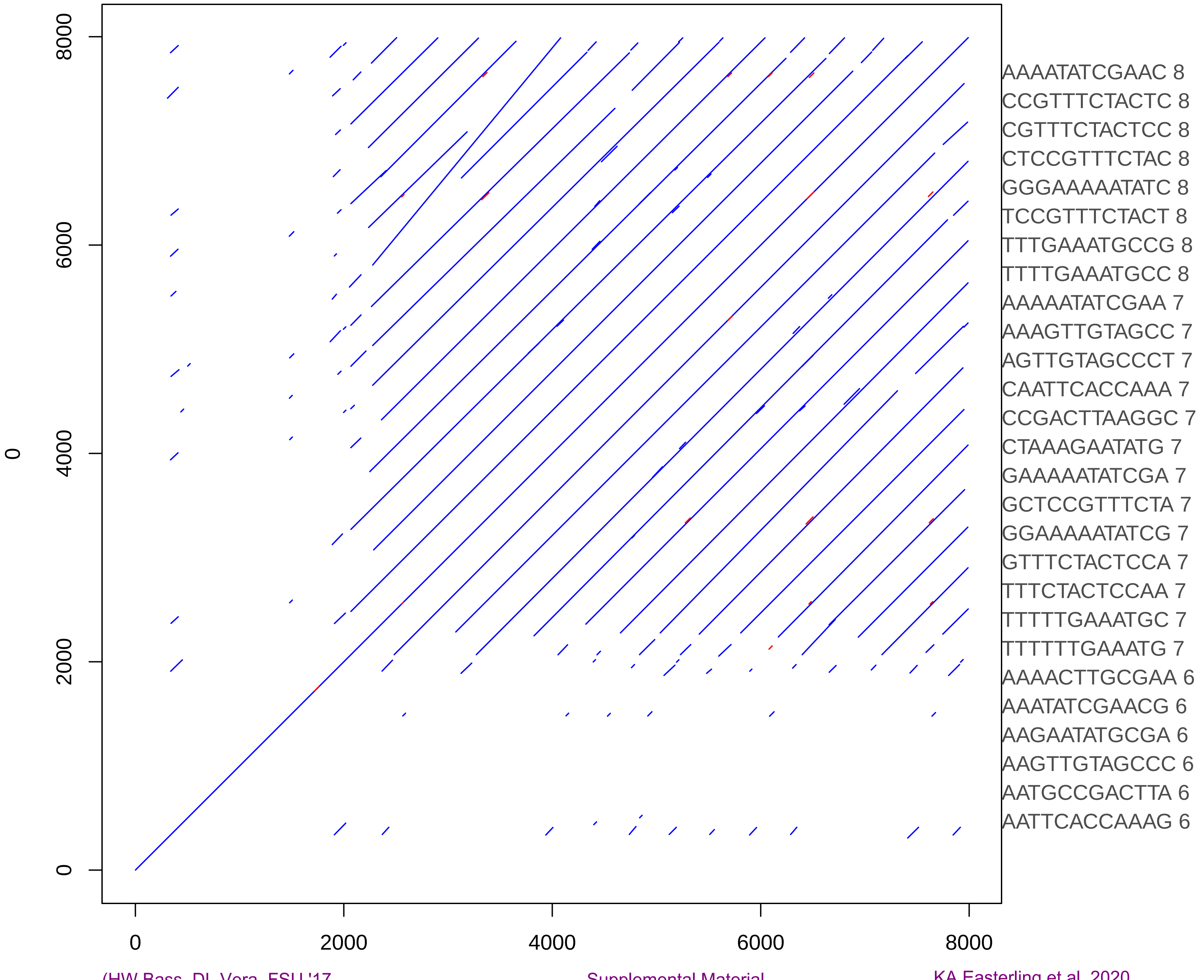

>m141120_131642_42134_c100732402550000001823141405141505_s1_p0/113900/4053_10472

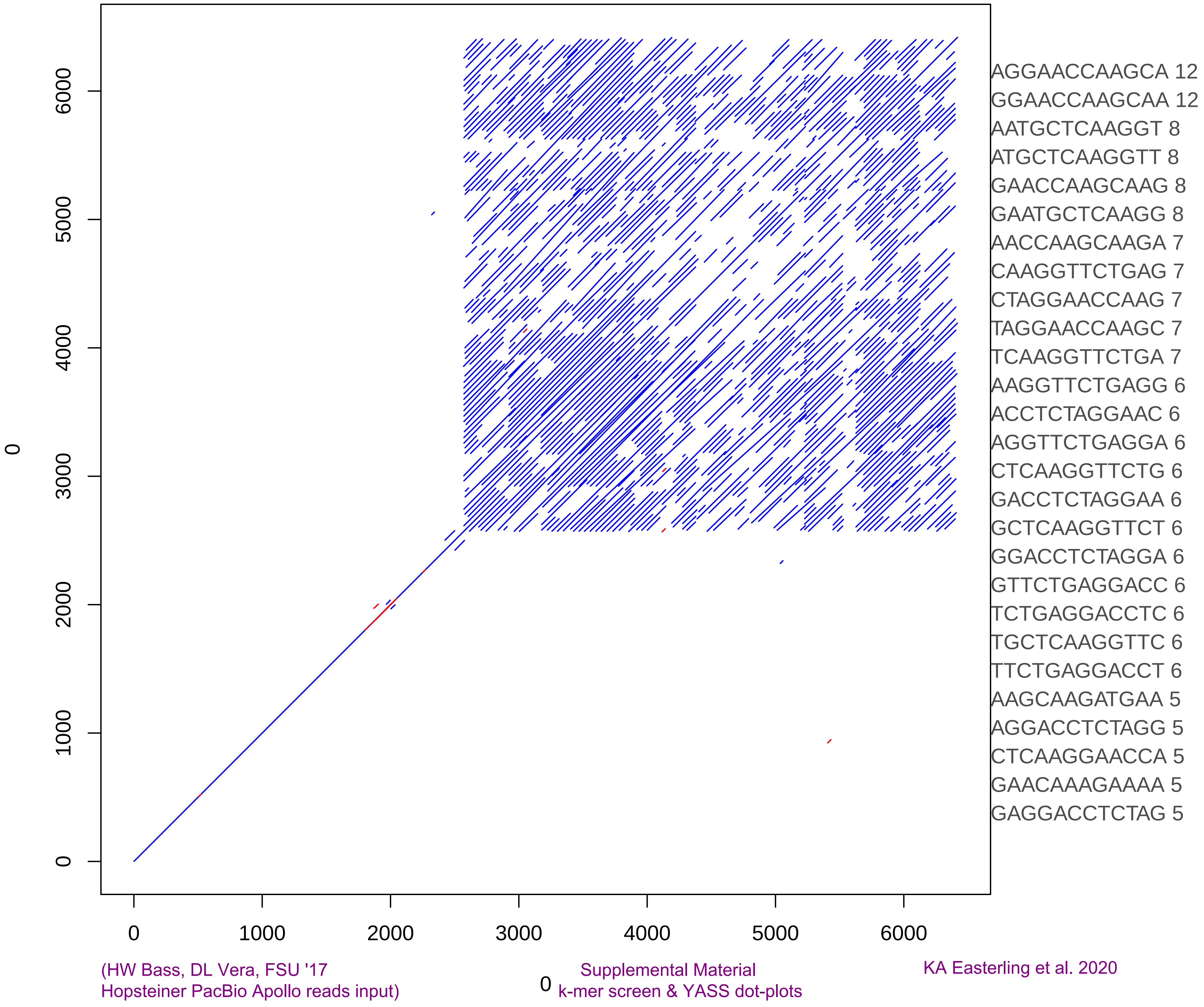

>m141120_131642_42134_c100732402550000001823141405141505_s1_p0/114857/6622_14313

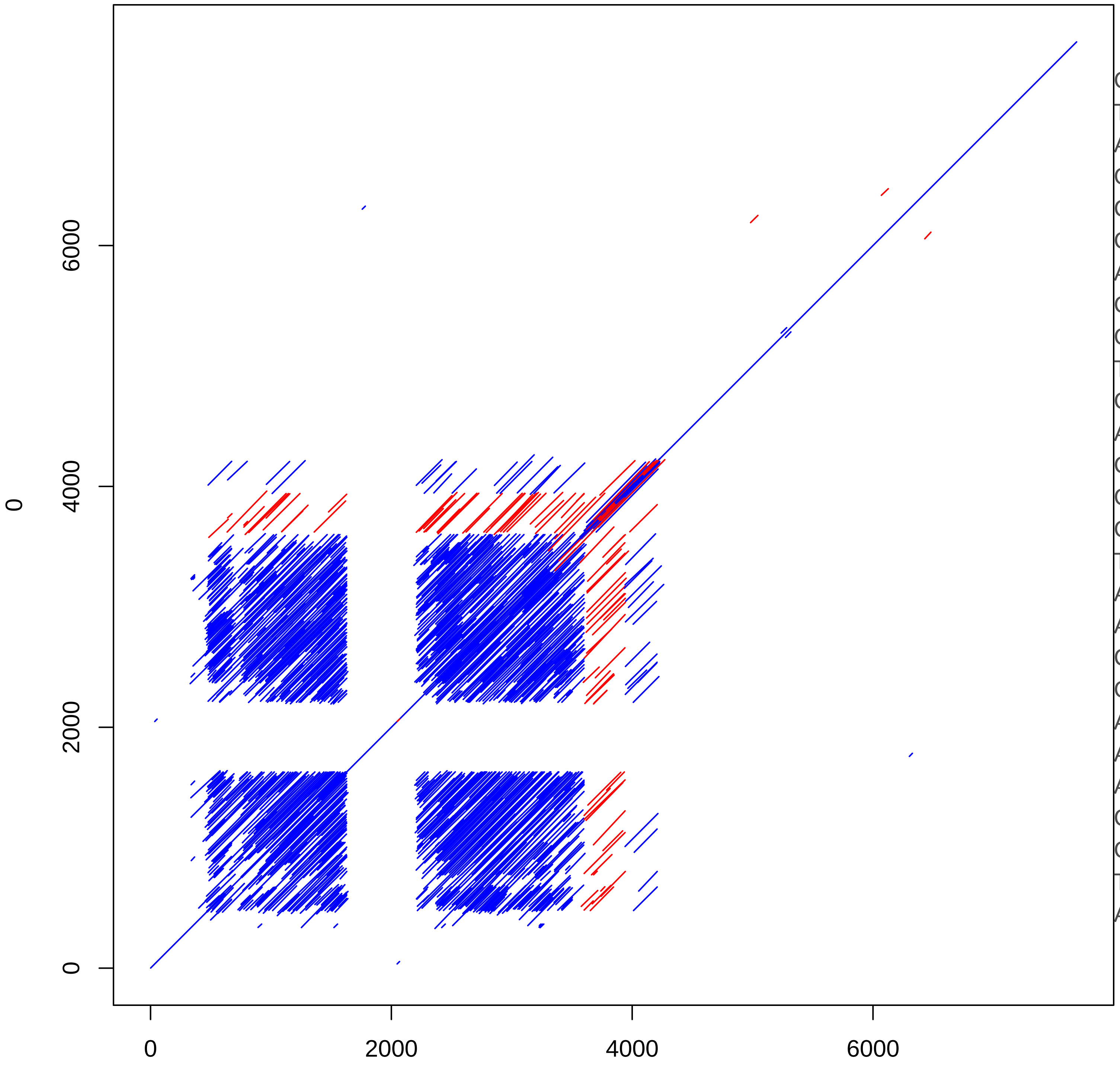

GCAGTGGCAGTG 44
TGGCAGTGGCAG 41
AGTGGCAGTGGC 40
GGCAGTGGCAGT 40
CAGTGGCAGTGG 39
GTGGCAGTGGCA 39
AGCAGTGGCAGT 17
CAGTGGCAGTAG 16
GTAGCAGTGGCA 15
TAGCAGTGGCAG 15
GCAGTGGCAGTA 14
AGTAGCAGTGGC 13
CAGTAGCAGTGG 13
GCAGTAGCAGTG 12
GGCAGTAGCAGT 12
TGGCAGTAGCAG 9
AGTGGCAGTAGC 8
AGTGGCAGTGCA 8
CAGTGGCAGTGC 8
GTGGCAGTAGCA 8
AAGTGGCAGTGG 7
ACTACTACTACT 7
AGTGCAGTGGCA 7
GCAGTGCAGTAG 7
GTGGCAGTGCAG 7
TGGCAAGTGGCA 7
AGTGGCAAGTGG 6

>m141120_131642_42134_c100732402550000001823141405141505_s1_p0/118197/0_5717

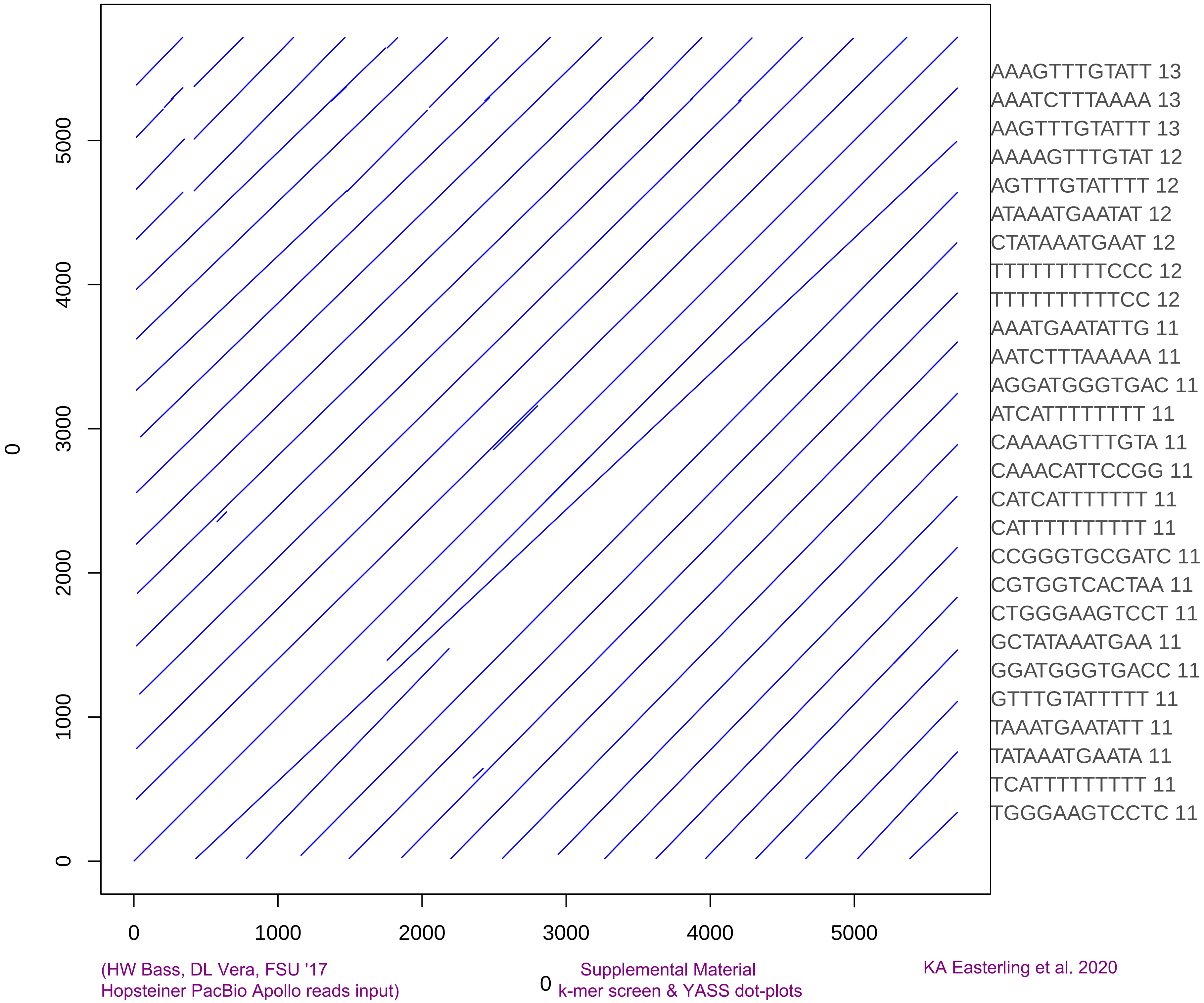

>m141120_131642_42134_c100732402550000001823141405141505_s1_p0/119985/539_12048

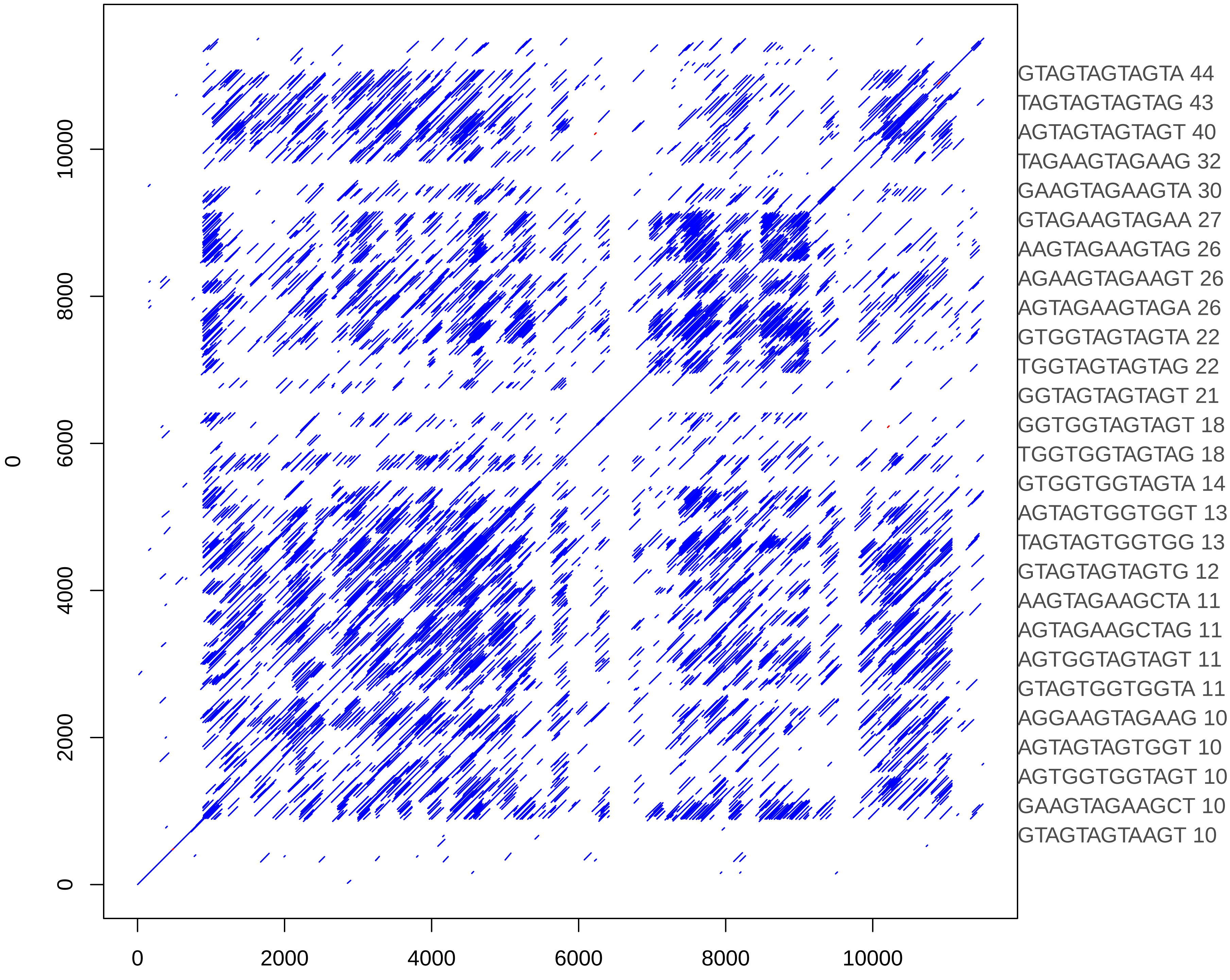

>m141120_131642_42134_c100732402550000001823141405141505_s1_p0/120371/10737_17740

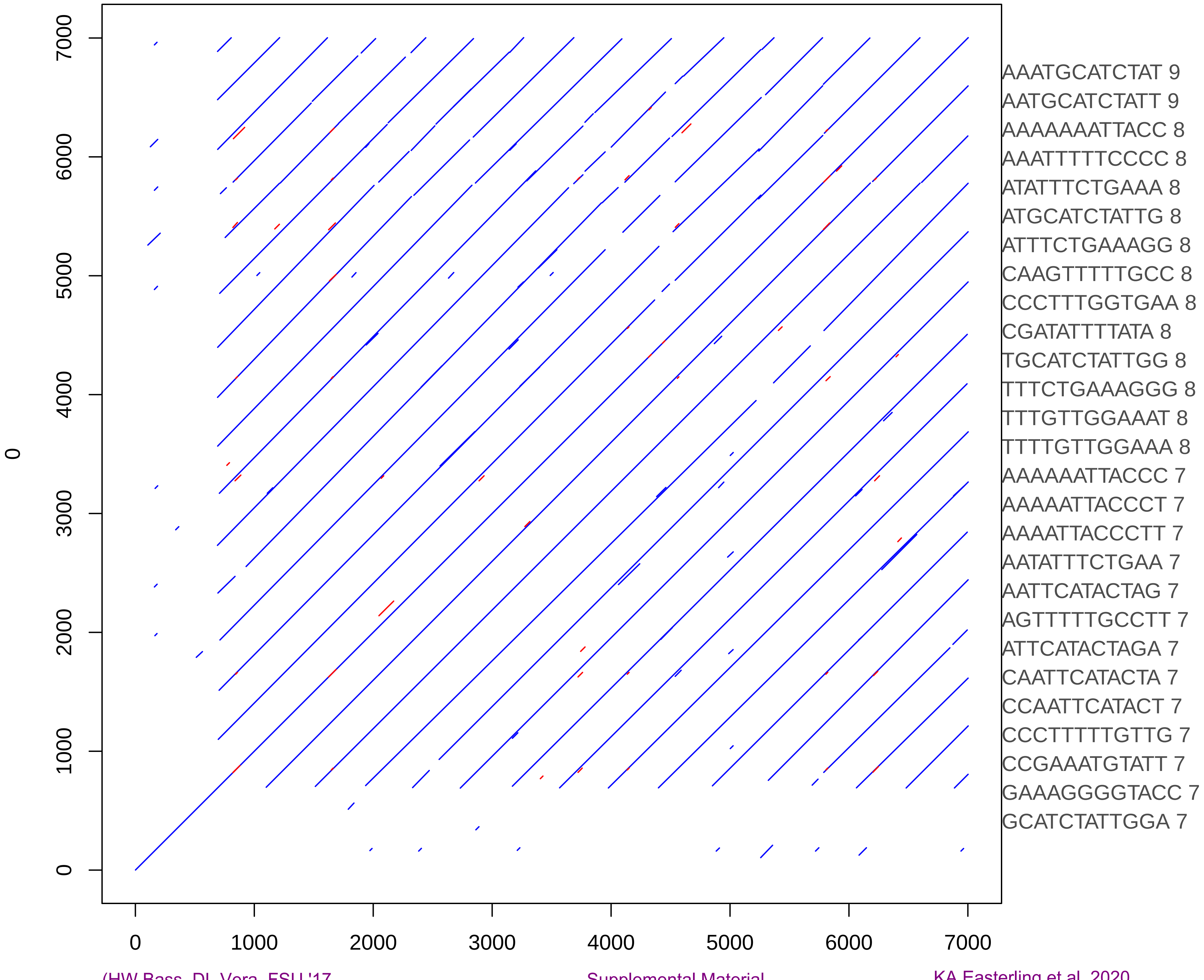

>m141120_131642_42134_c100732402550000001823141405141505_s1_p0/12374/0_6147

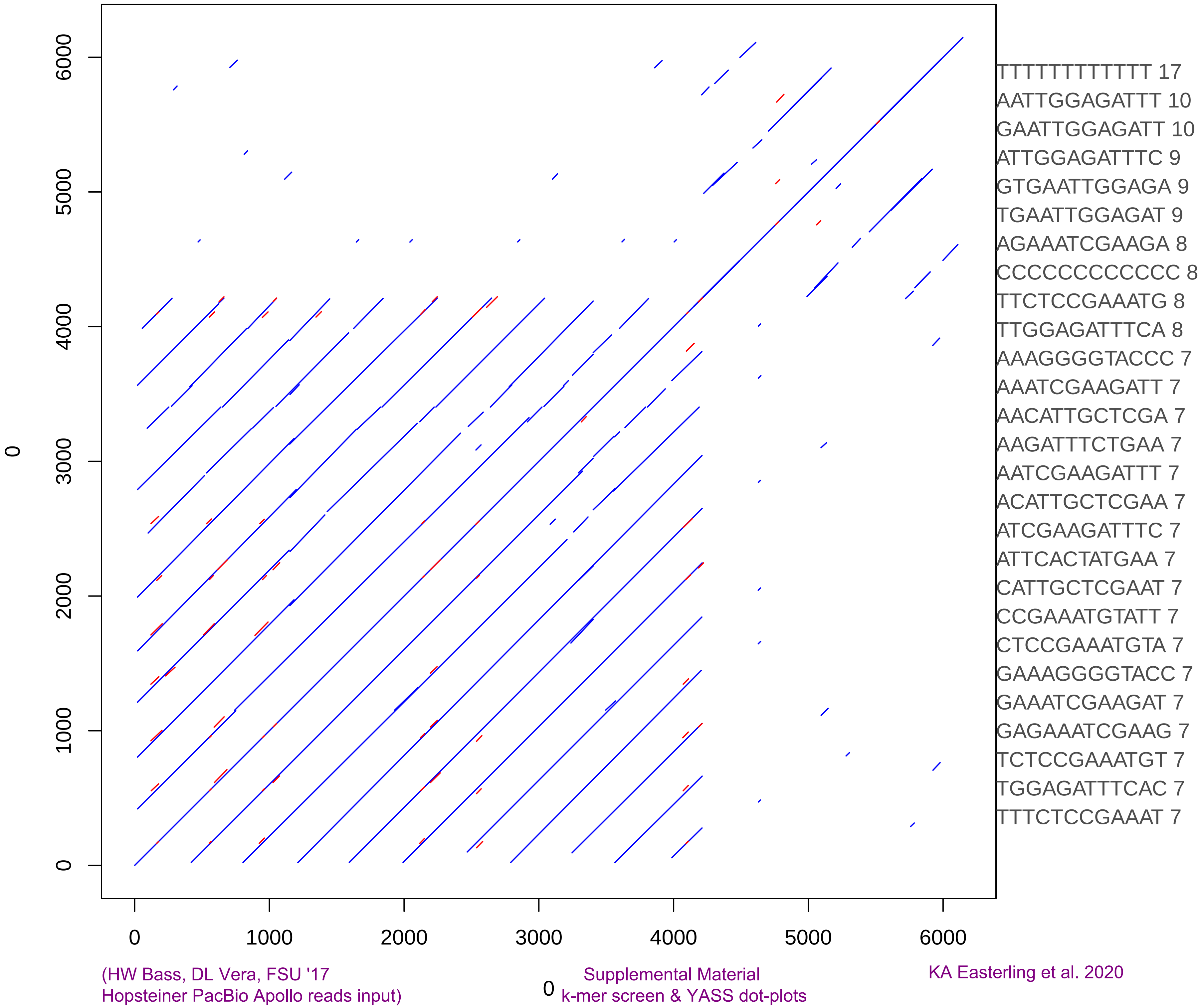

>m141120_131642_42134_c100732402550000001823141405141505_s1_p0/124699/0_8295

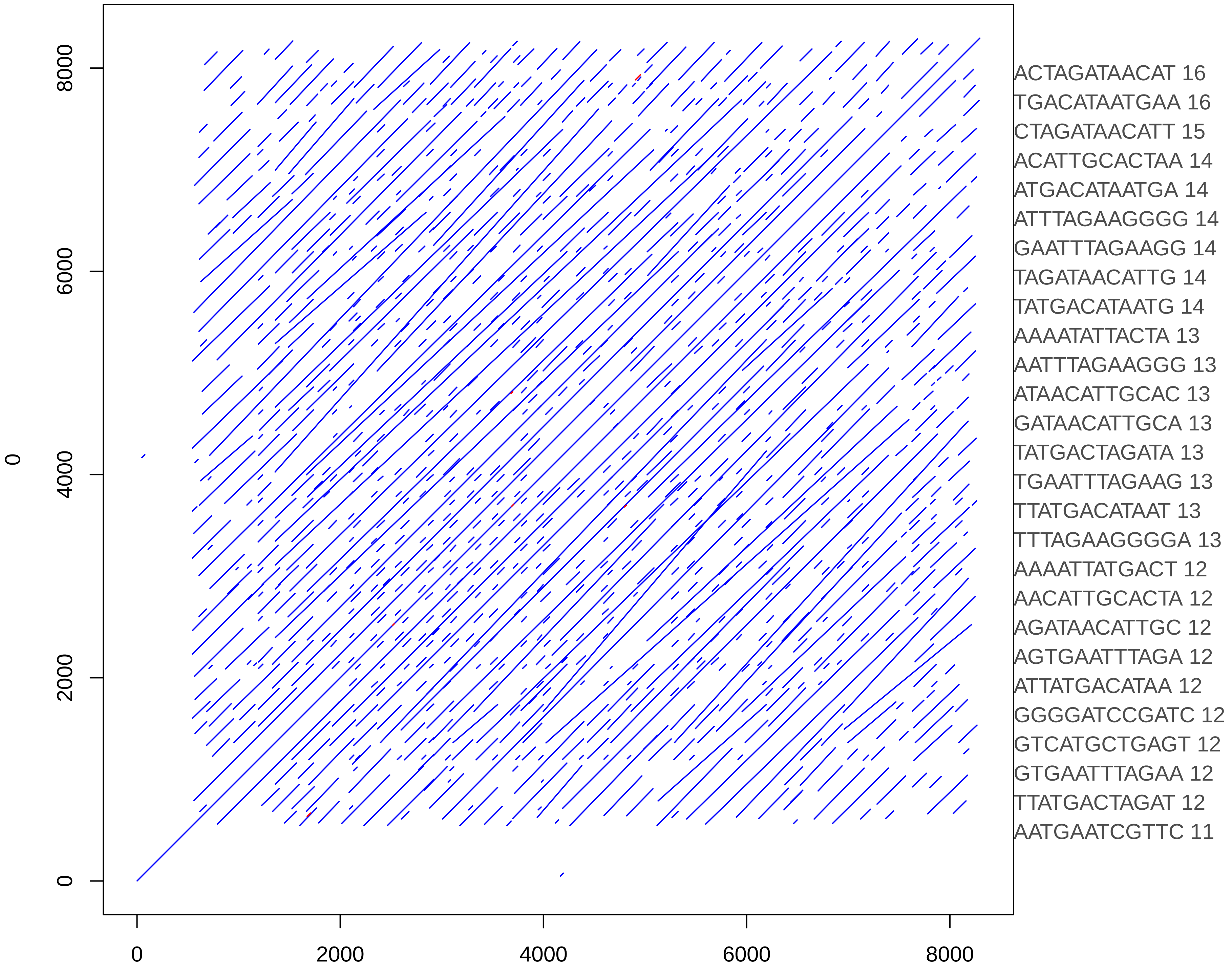

>m141120_131642_42134_c100732402550000001823141405141505_s1_p0/127544/0_9550

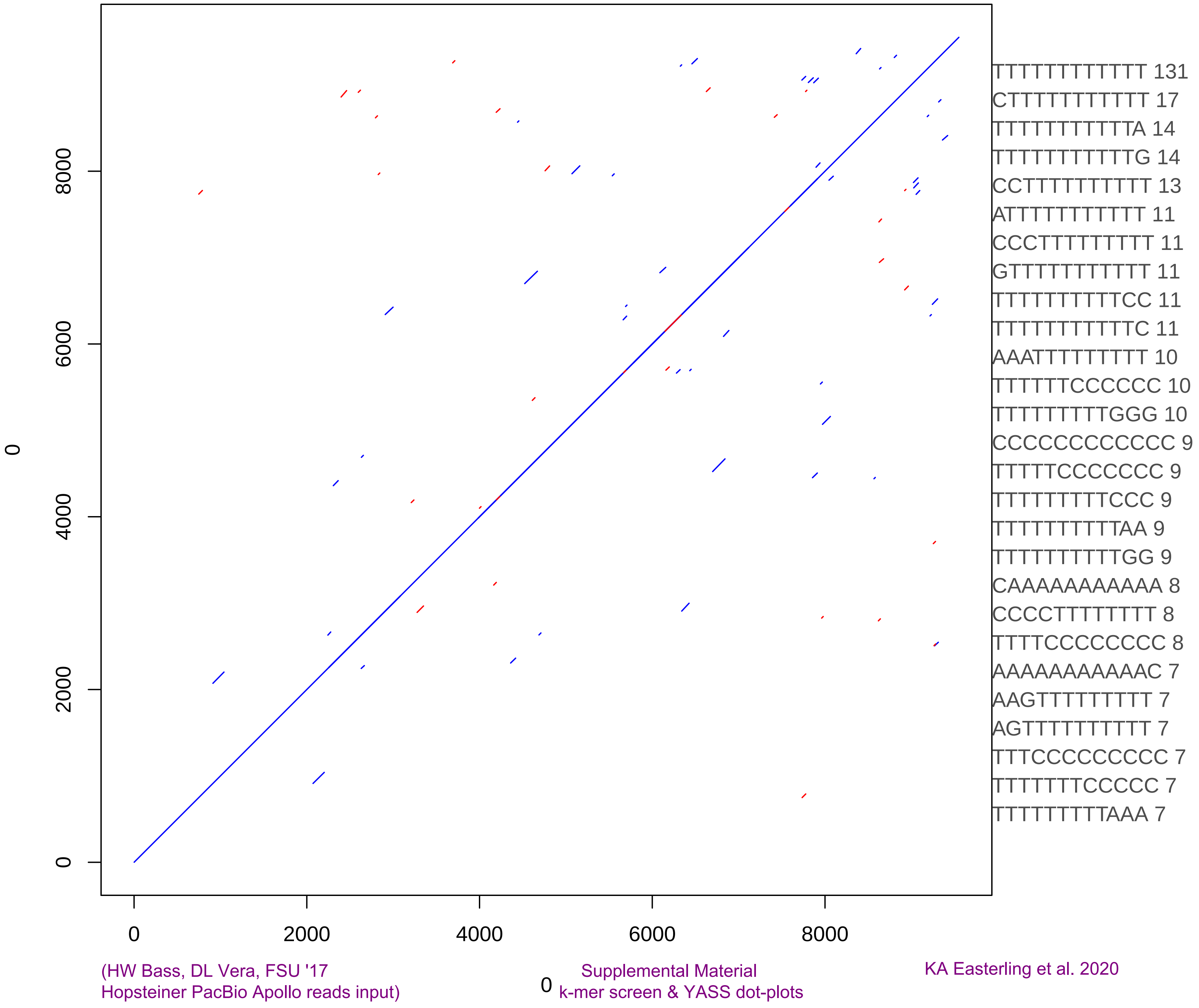

>m141120_131642_42134_c100732402550000001823141405141505_s1_p0/132348/10701_16342

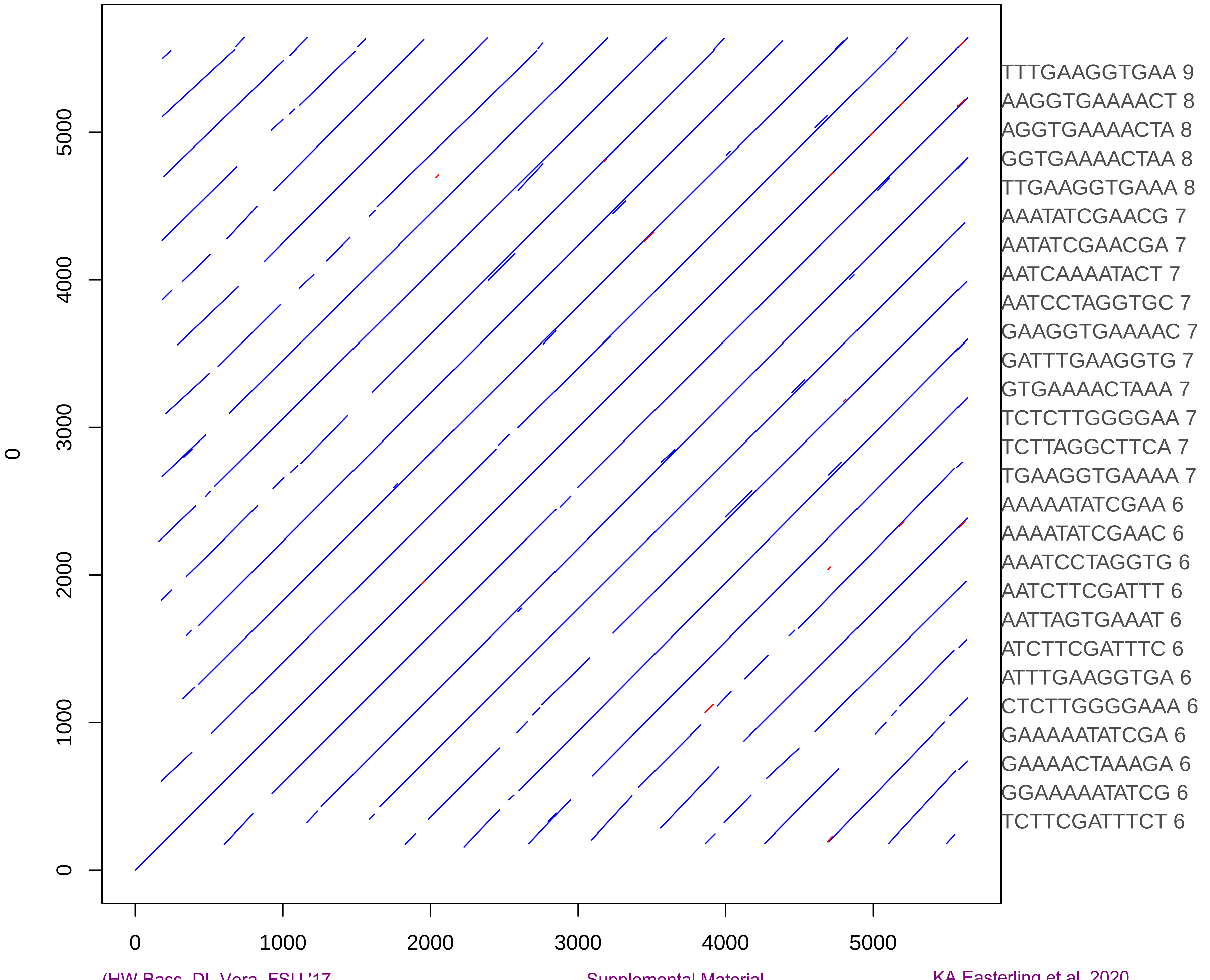

>m141120_131642_42134_c100732402550000001823141405141505_s1_p0/13292/3562_15406

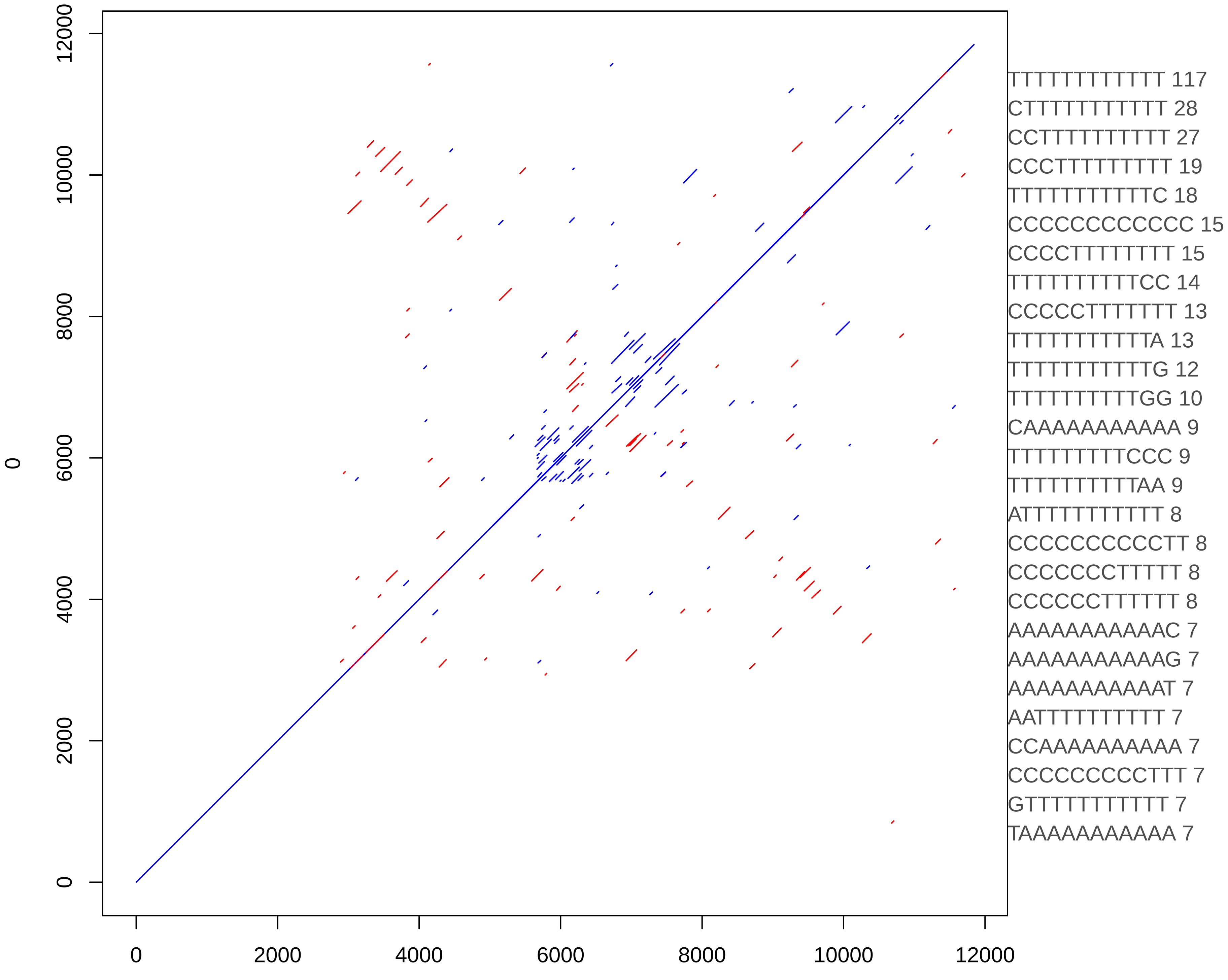

>m141120_131642_42134_c100732402550000001823141405141505_s1_p0/133909/9237_19411

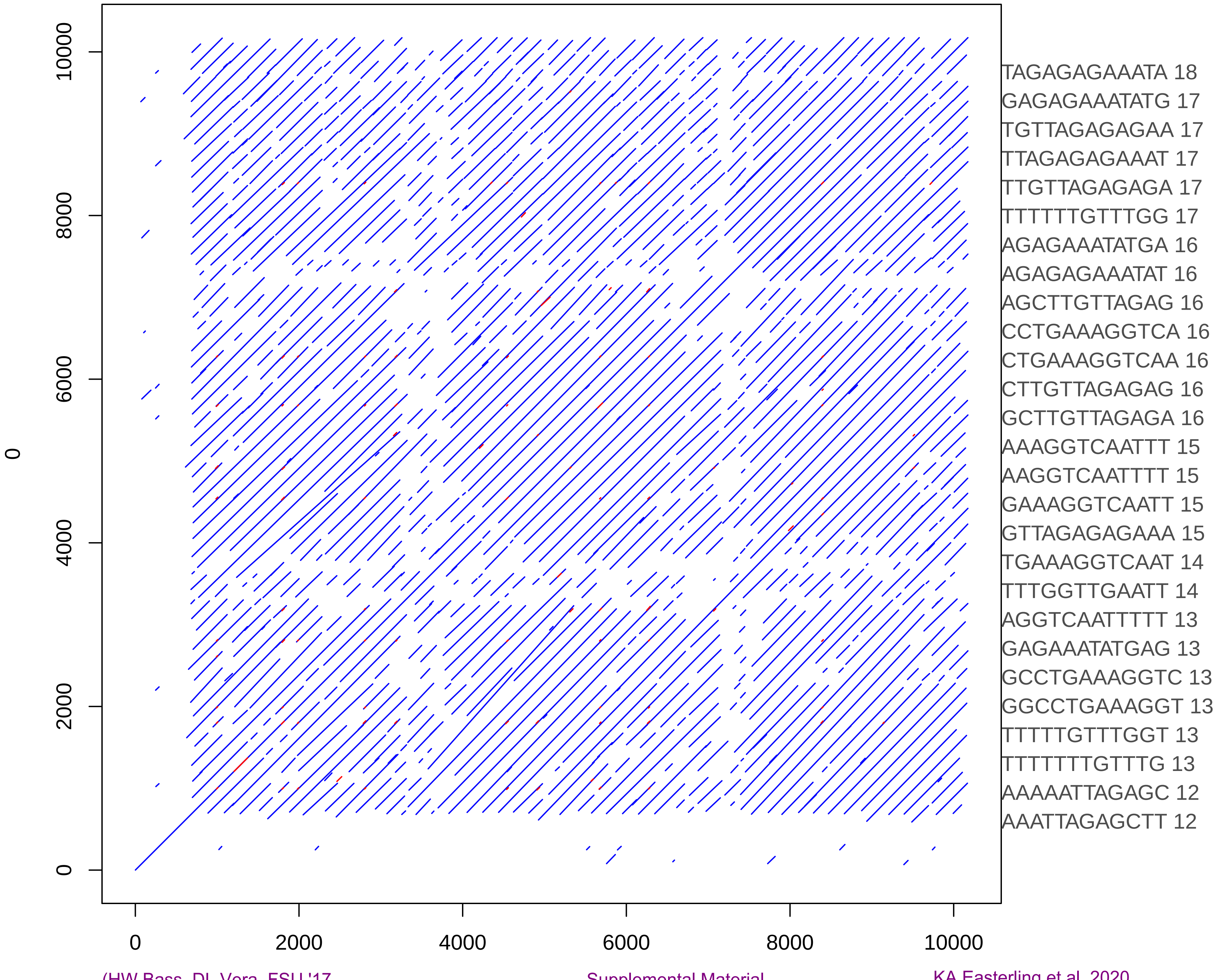

0

>m141120_131642_42134_c100732402550000001823141405141505_s1_p0/135451/306_9817

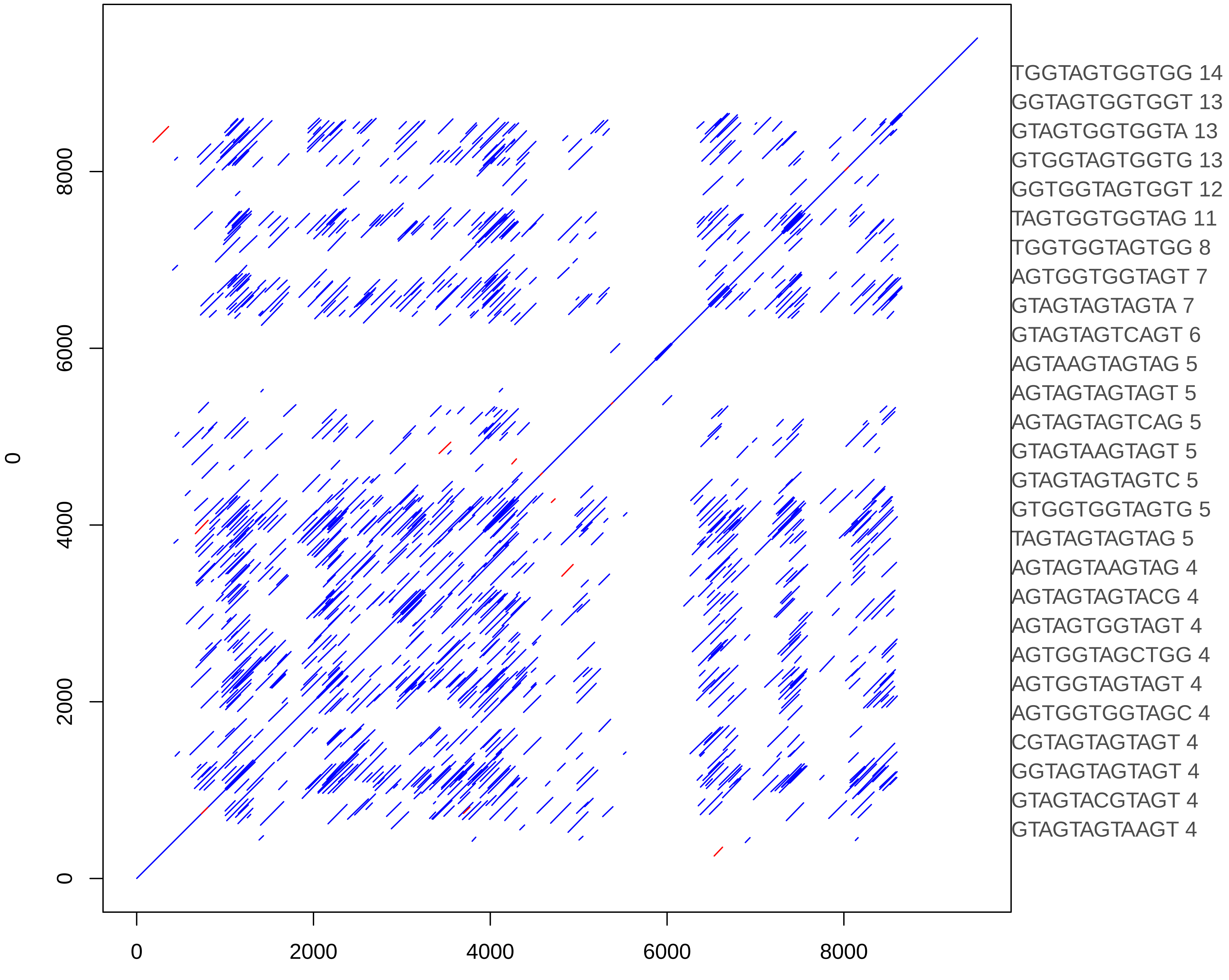

>m141120_131642_42134_c100732402550000001823141405141505_s1_p0/138402/1971_13732

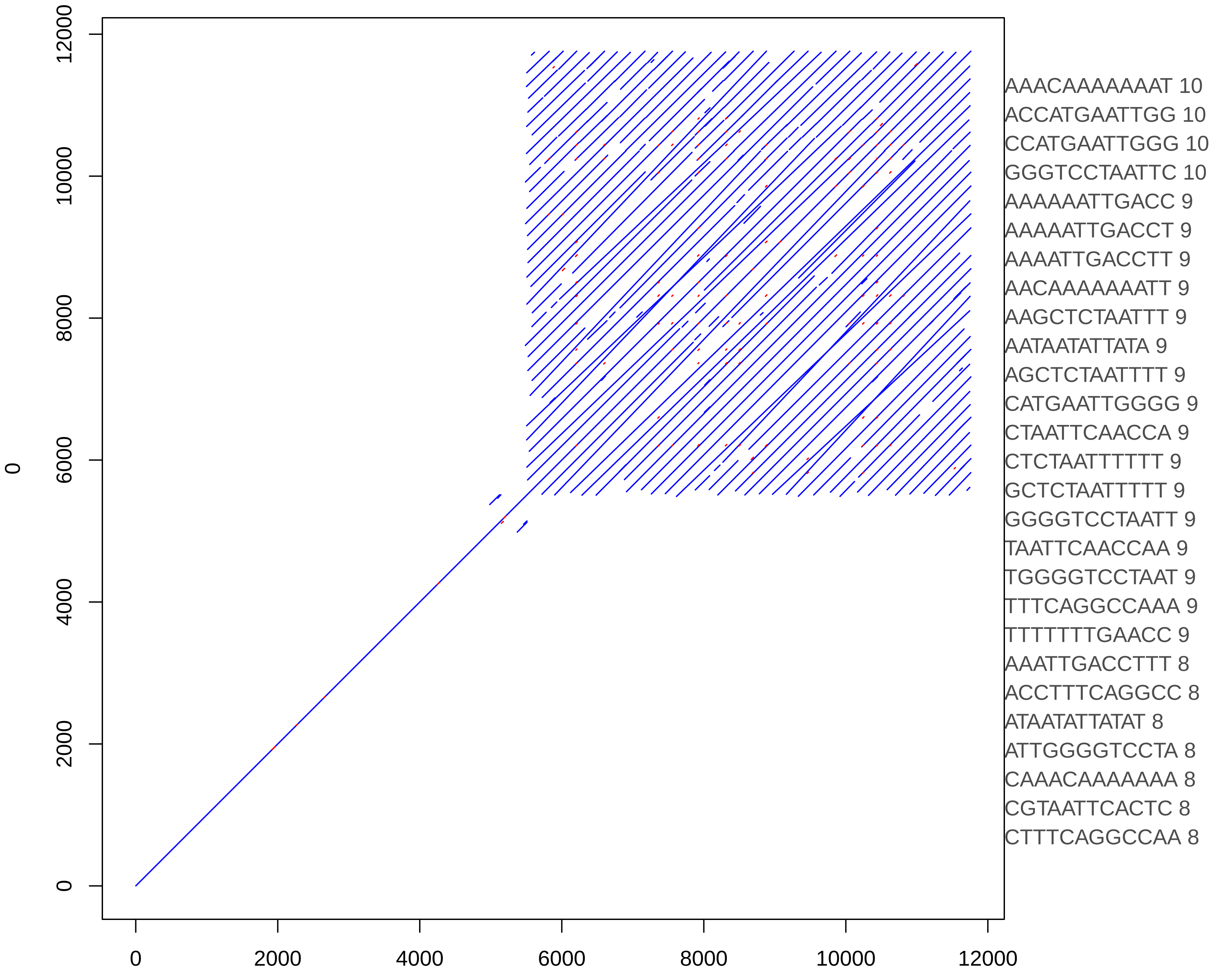

>m141120_131642_42134_c100732402550000001823141405141505_s1_p0/139632/777_9460

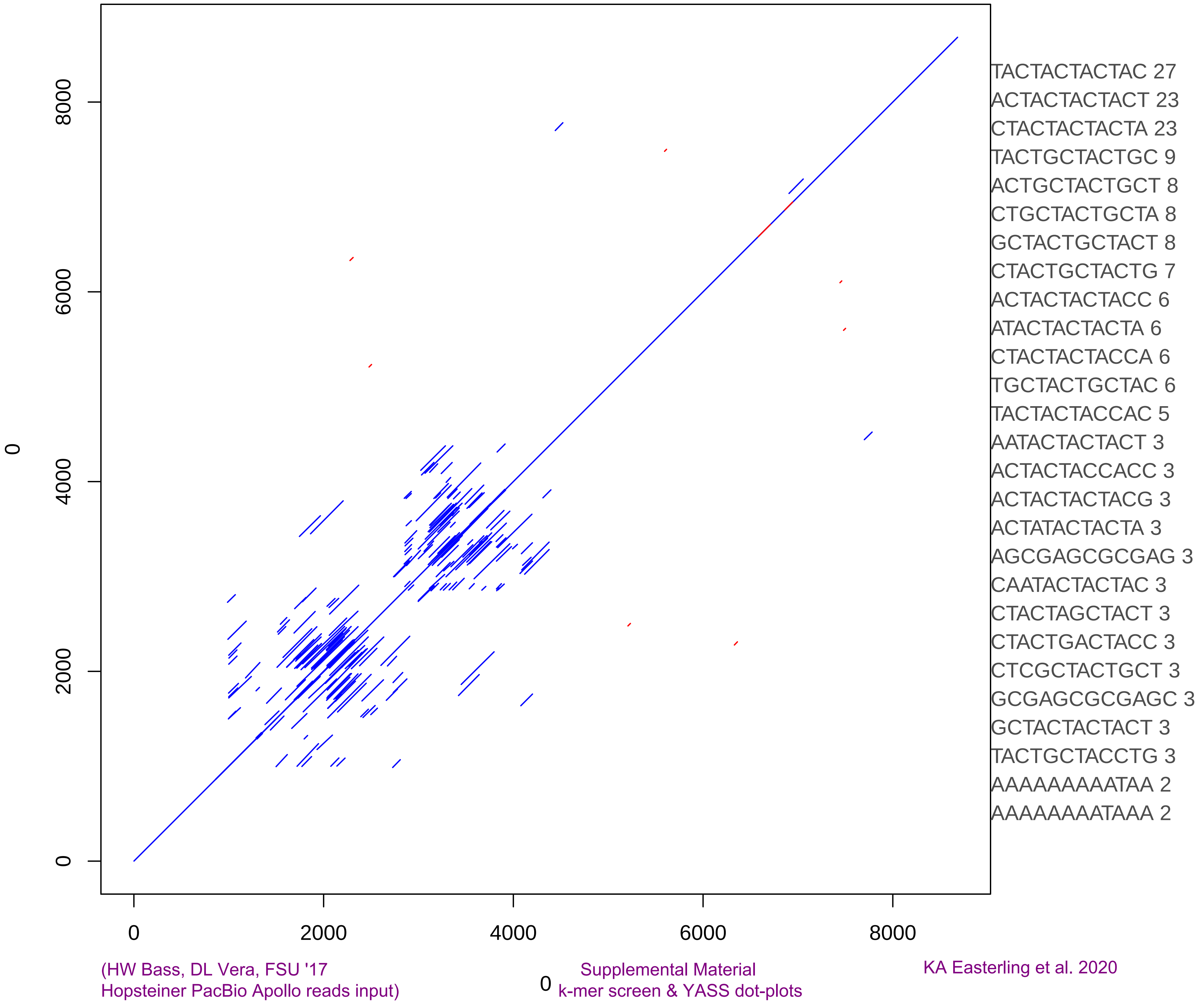

>m141120_131642_42134_c100732402550000001823141405141505_s1_p0/14528/7102_19491

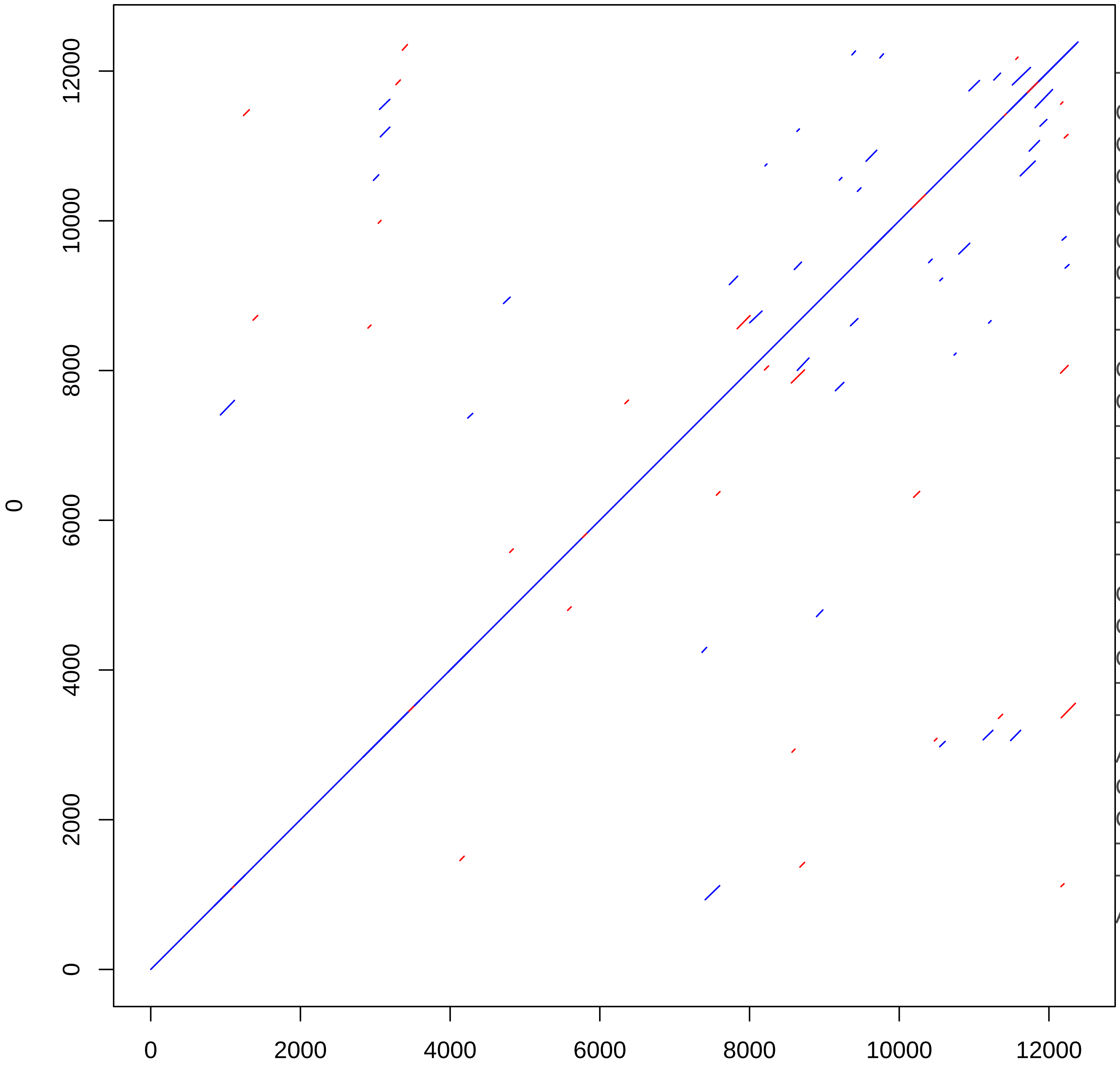

TTTTTTTTTTTT 38
CCCCCCCCCCCC 18
CCCTTTTTTTTT 9
CCCCCCCCCCCG 8
CCTTTTTTTTTT 8
CTTTTTTTTTTT 8
GGGCCCCCCCCC 8
TCCCCCCCCCCC 8
TTTTTTTCCCCC 8
CCCCTTTTTTTT 7
GTTTTTTTTTTT 7
TTTTTTTTCCCC 7
TTTTTTTTTCCC 7
TTTTTTTTTTCC 7
TTTTTTTTTTGG 7
TTTTTTTTTTTC 7
GCCCCCCCCCCC 6
GGCCCCCCCCCC 6
GGTTTTTTTTTT 6
TTTTTTTTTGGG 6
TTTTTTTTTTTG 6
AAGGCCCCCCCC 5
CCCCCCCCCCGG 5
CCCCCTTTTTTT 5
TTTTTTCCCCCC 5
TTTTTTTTTTAT 5
AAAAAAAAAAAG 4

>m141120_131642_42134_c100732402550000001823141405141505_s1_p0/151080/6925_15916

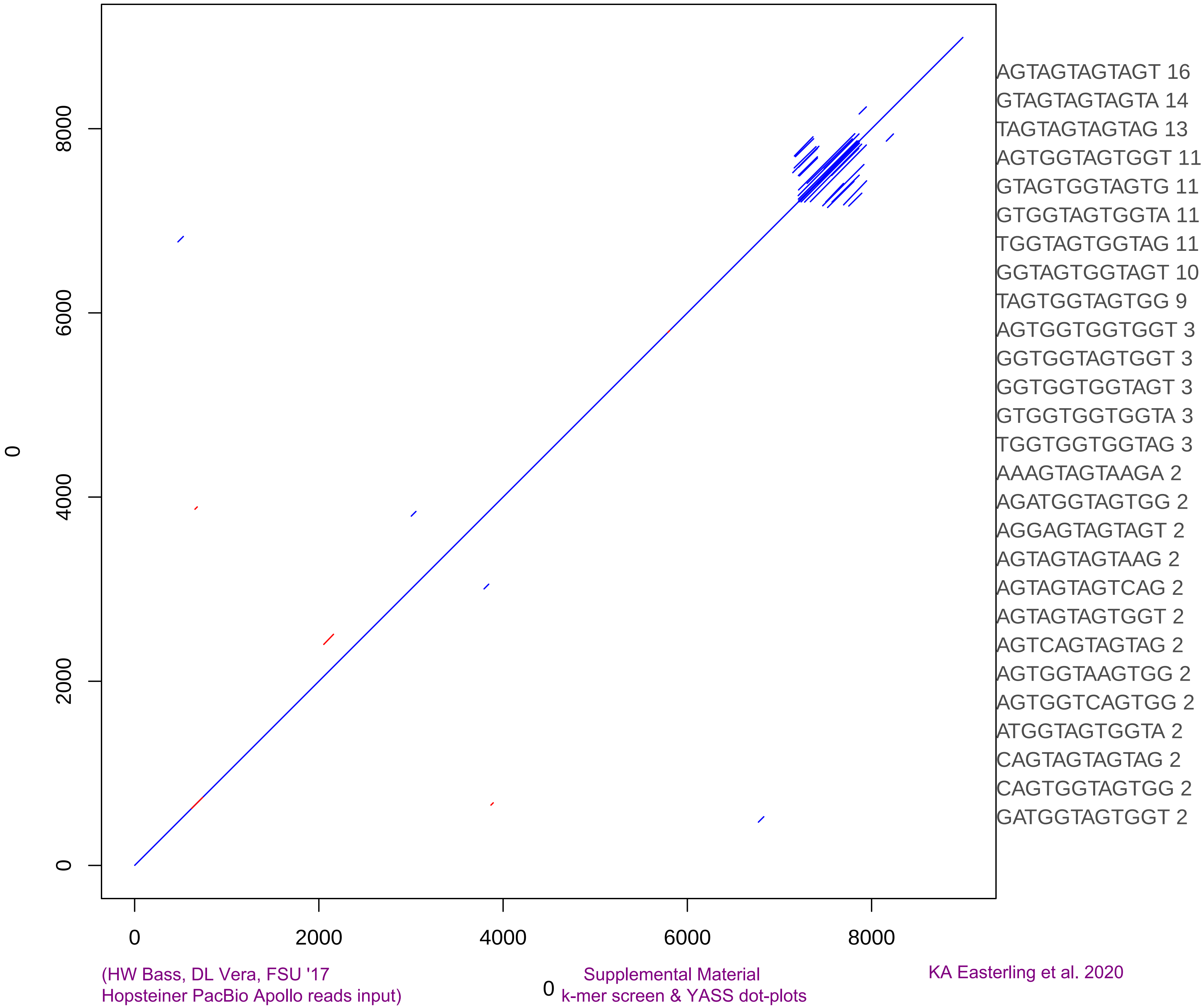

>m141120_131642_42134_c100732402550000001823141405141505_s1_p0/152951/0_7254

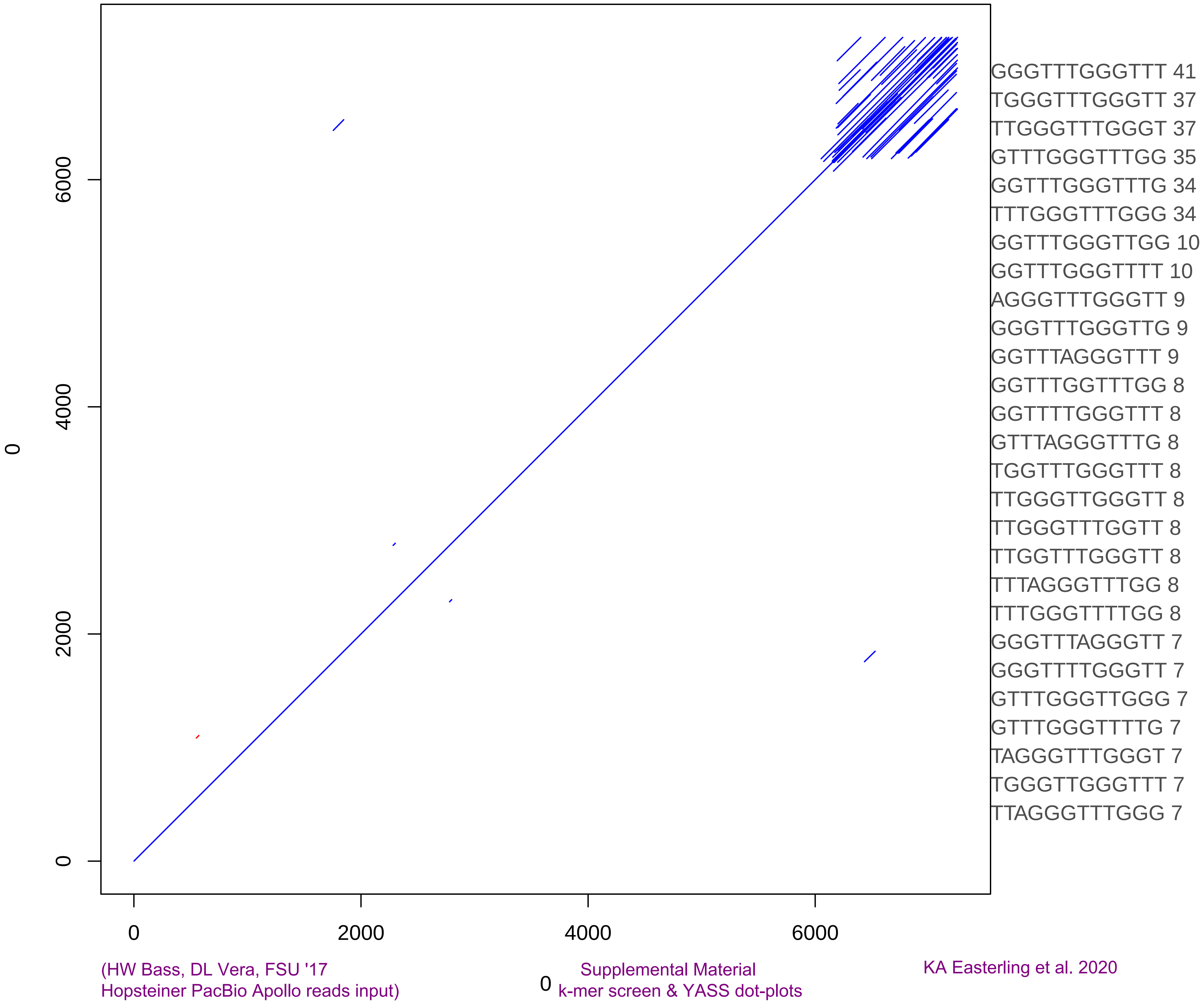

>m141120_131642_42134_c100732402550000001823141405141505_s1_p0/153489/5342_11074

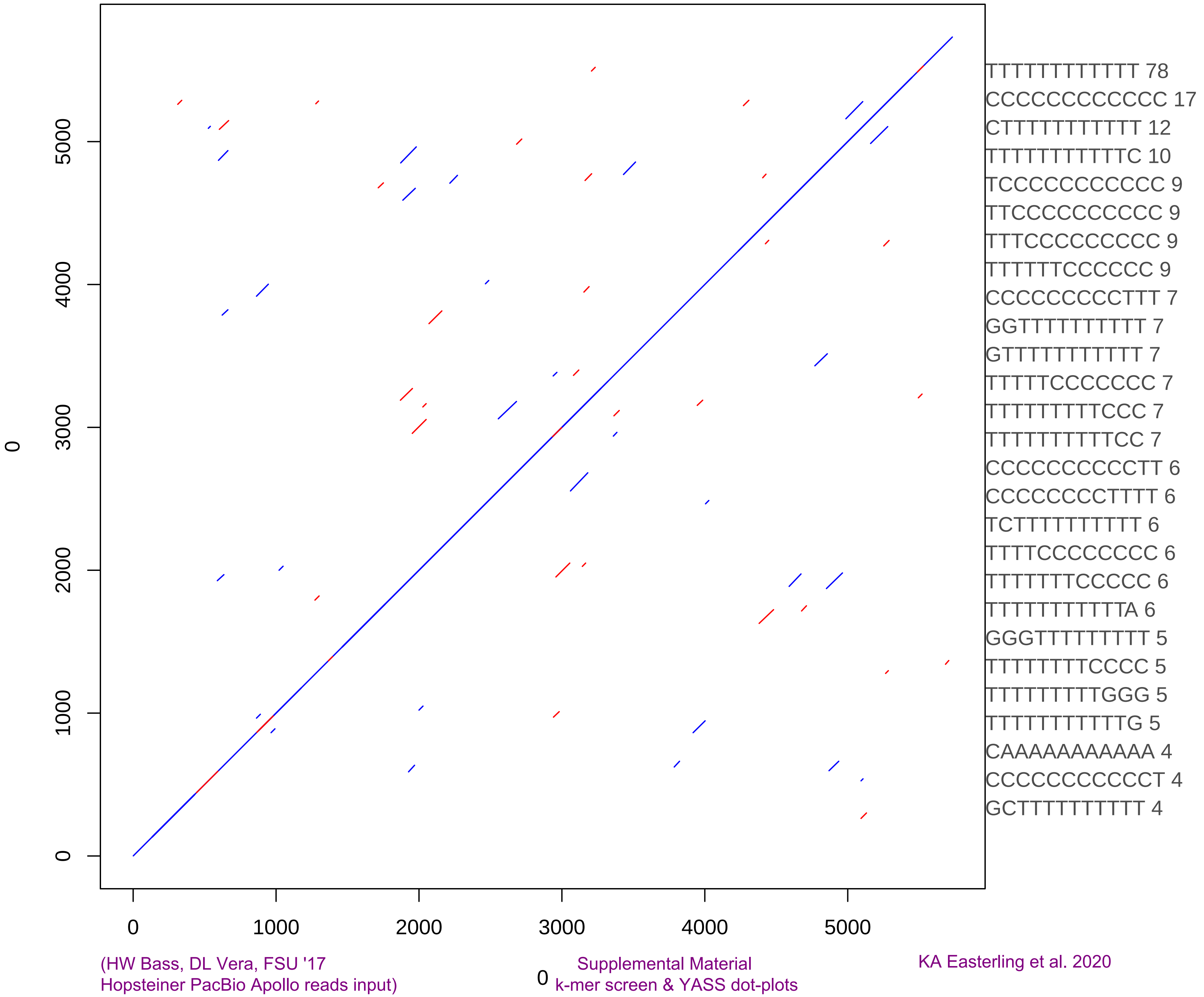

>m141120_131642_42134_c100732402550000001823141405141505_s1_p0/155432/0_13040

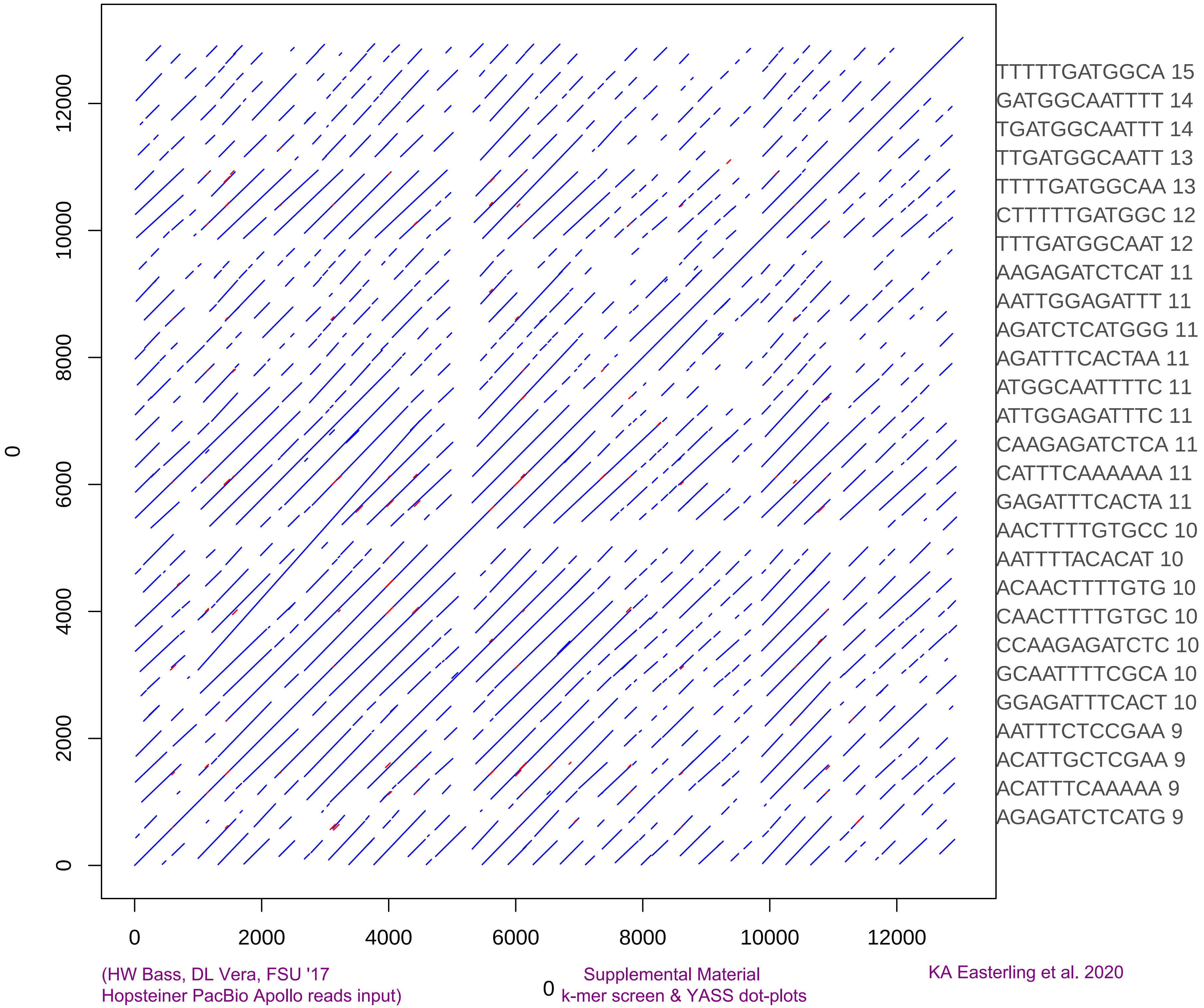

>m141120_131642_42134_c100732402550000001823141405141505_s1_p0/157927/7338_13150

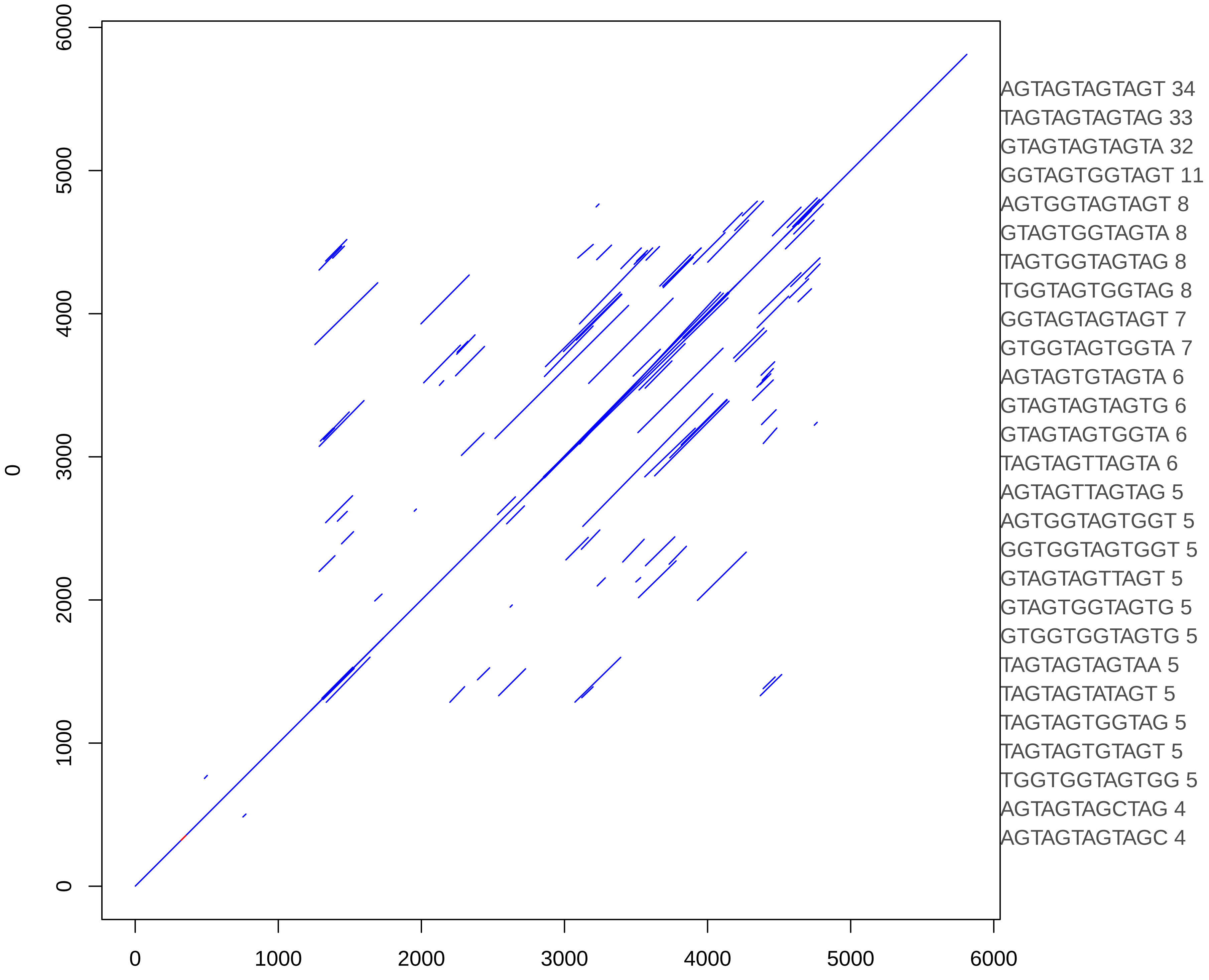

>m141120_131642_42134_c100732402550000001823141405141505_s1_p0/158805/3154_9069

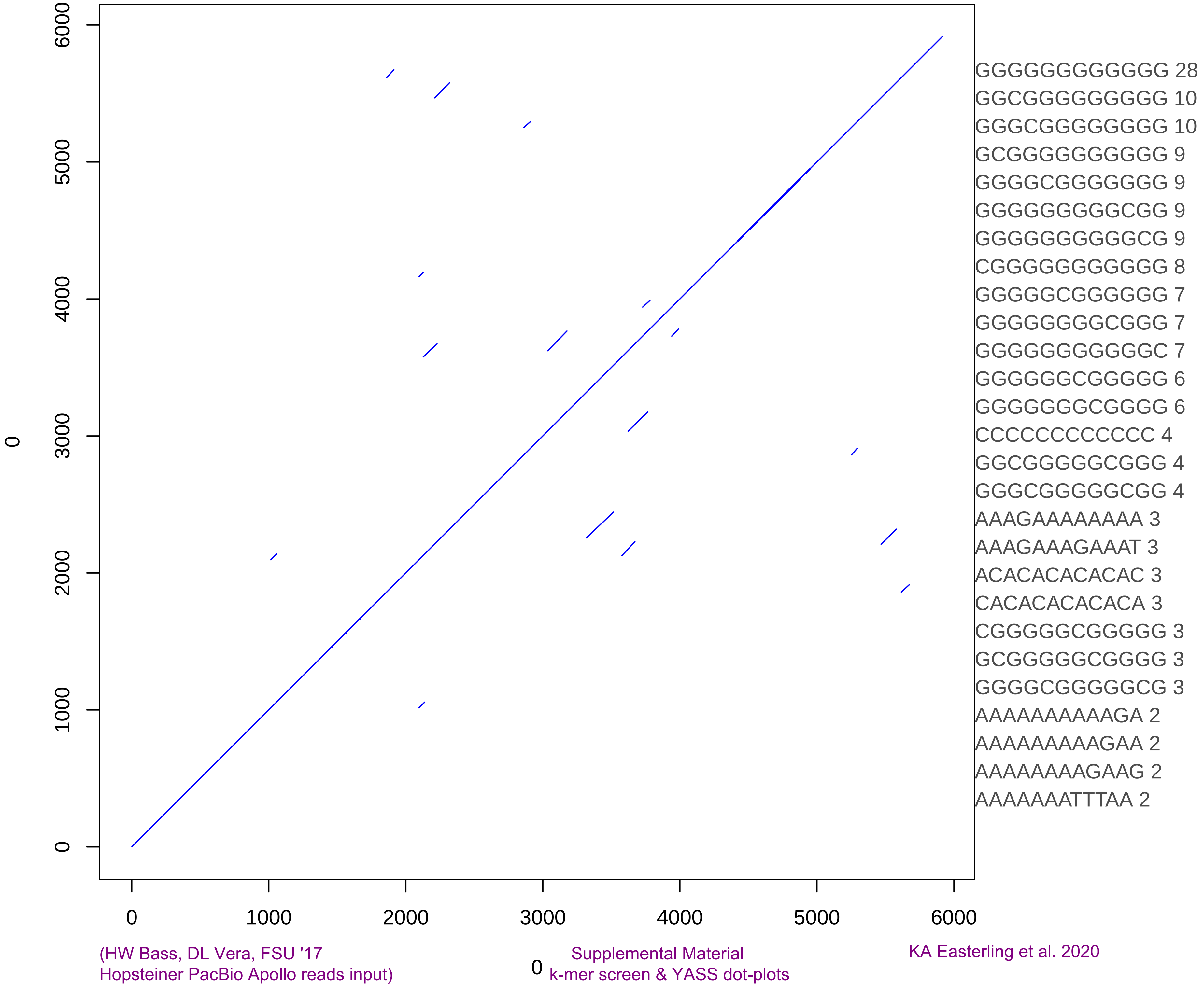

>m141120_131642_42134_c100732402550000001823141405141505_s1_p0/17418/0_6990

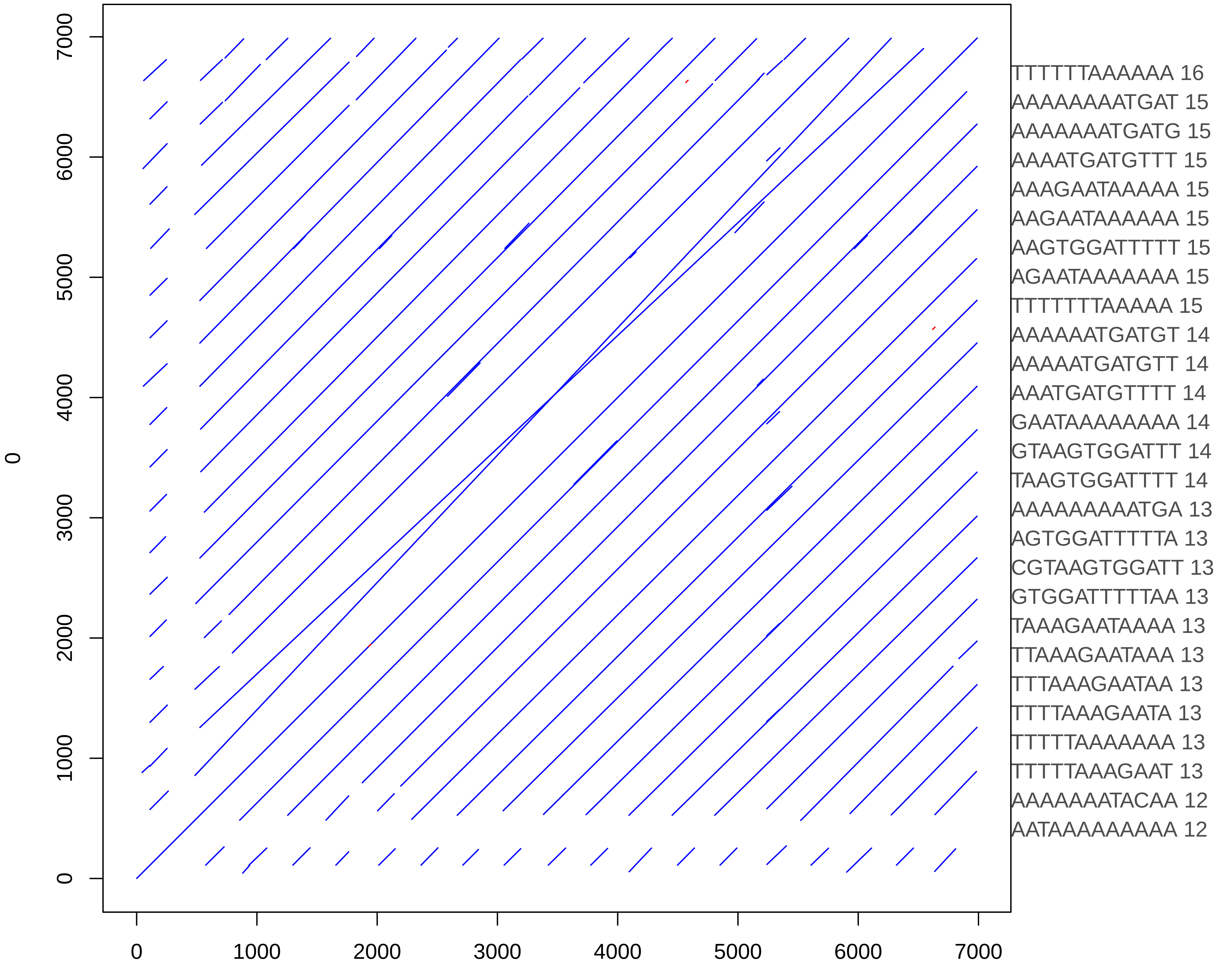

>m141120_131642_42134_c100732402550000001823141405141505_s1_p0/20897/0_5815

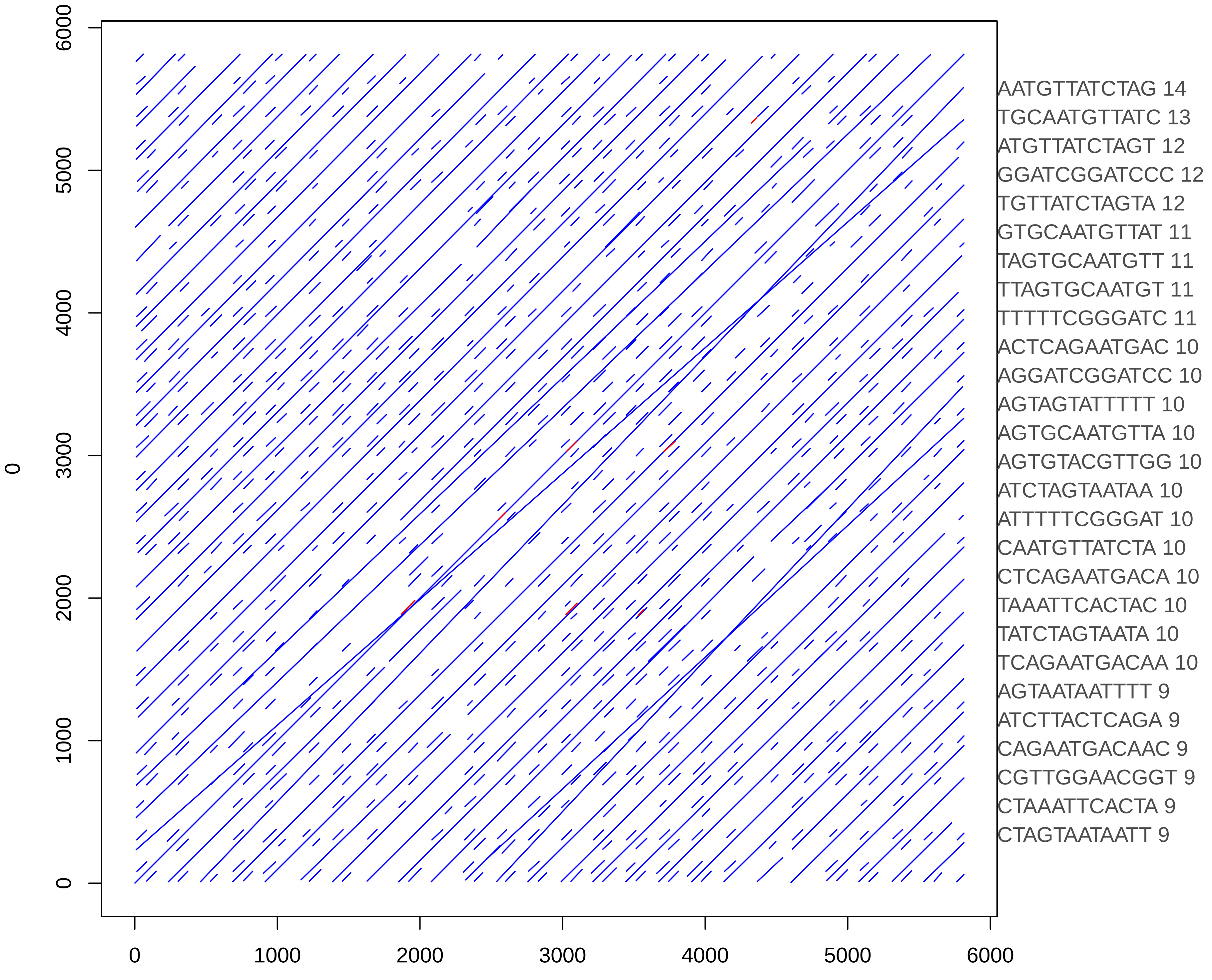

>m141120_131642_42134_c100732402550000001823141405141505_s1_p0/20897/5862_11624

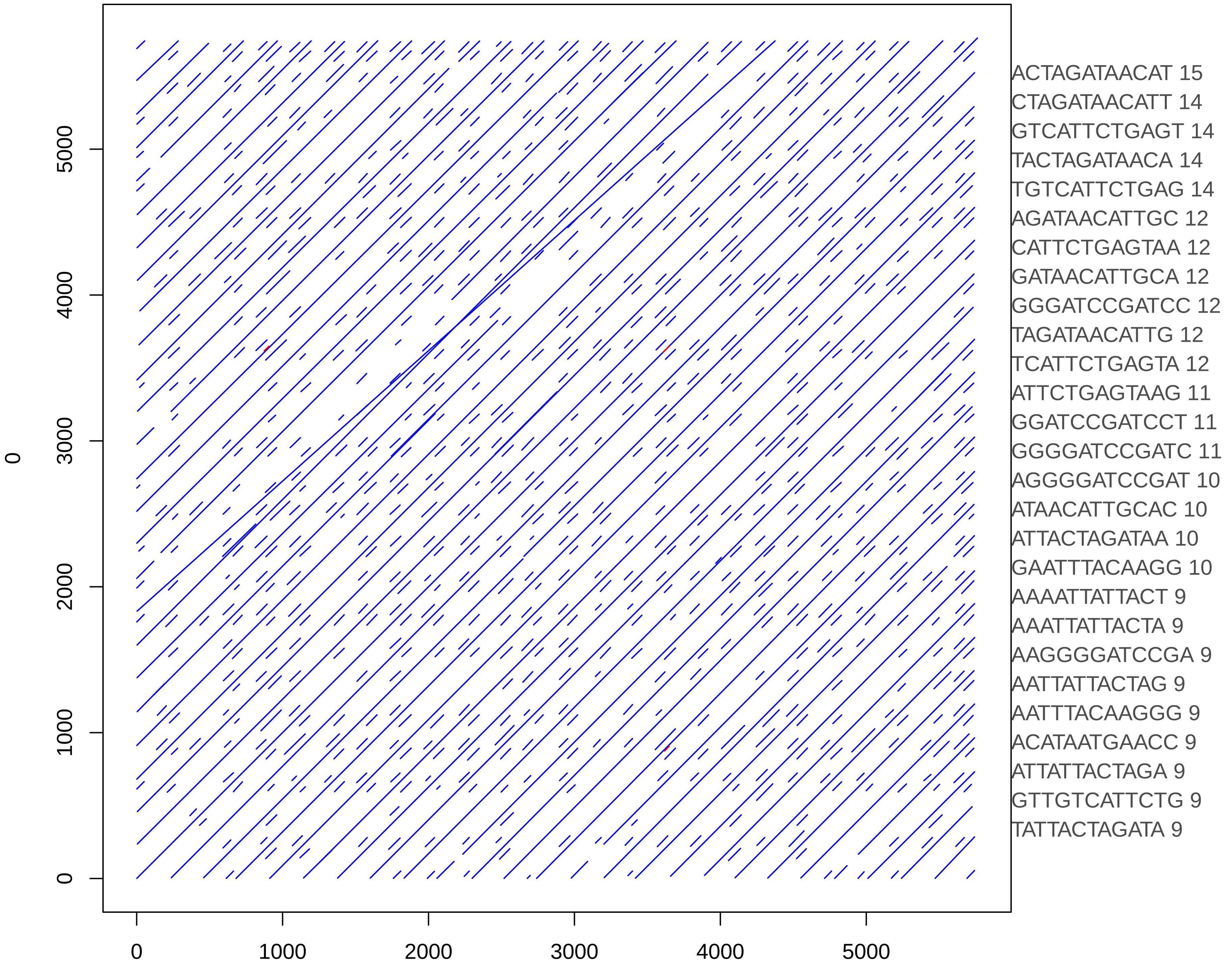

>m141120_131642_42134_c100732402550000001823141405141505_s1_p0/23009/7040_16209

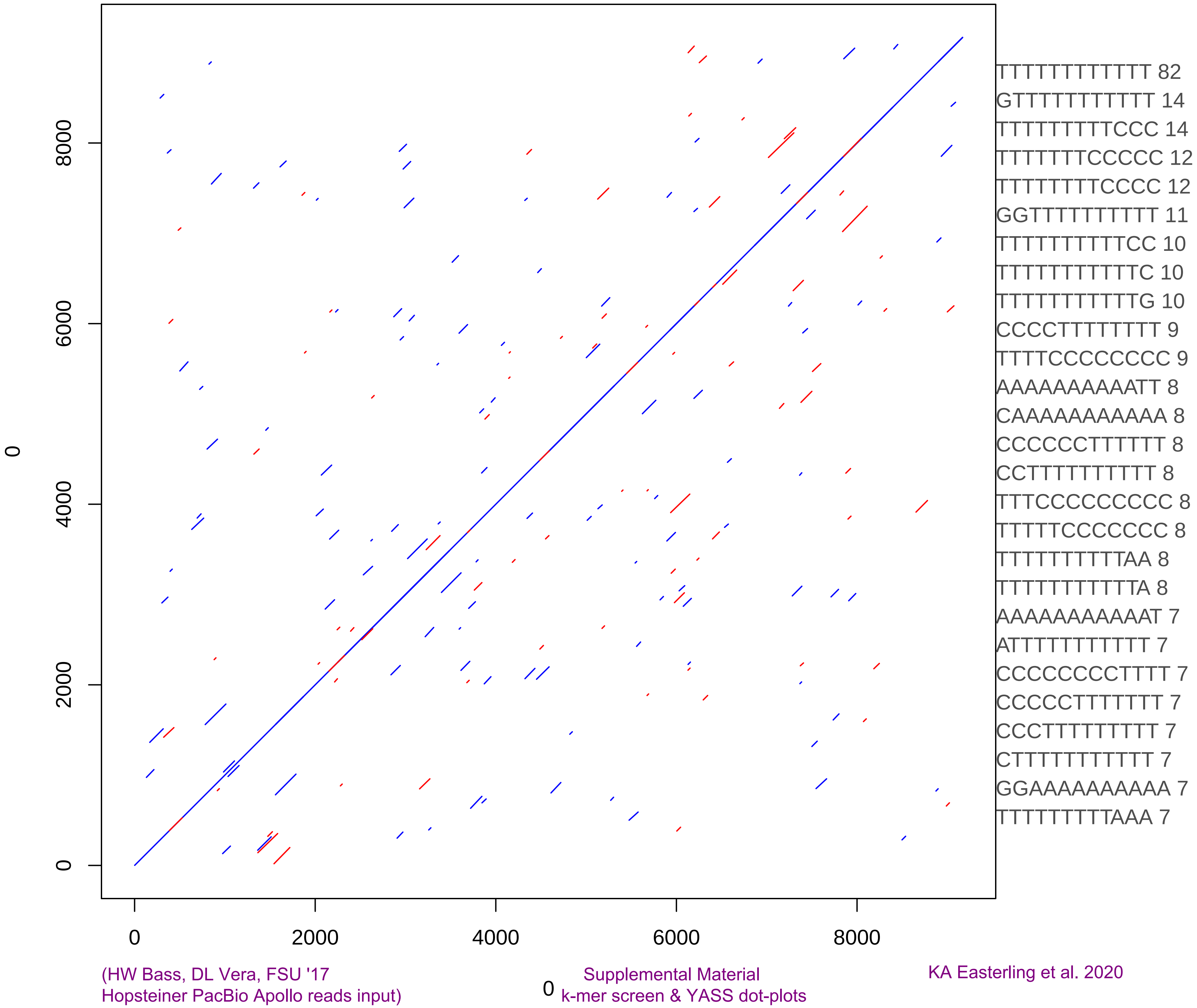

>m141120_131642_42134_c100732402550000001823141405141505_s1_p0/23378/11272_16469

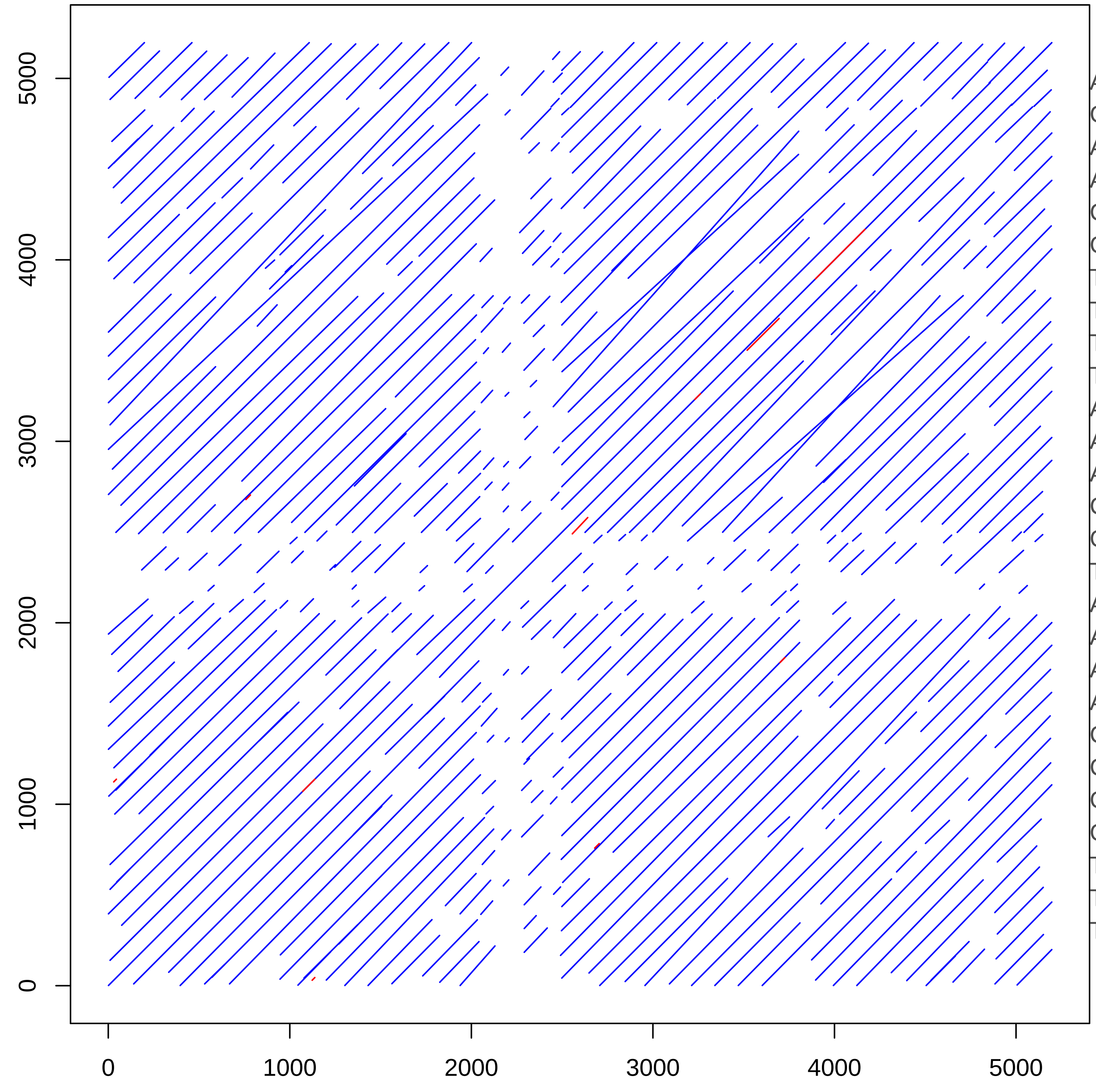

AAGTTAATTTAA 11
GAACTAAGTTTT 11
AGAAGTTAATTT 10
ATTGATCGTTGT 10
CGAACTAAGTTT 10
GAAGTTAATTTA 10
TAGAAGTTAATT 10
TGATCGTTGTTT 10
TTGATCGTTGTT 10
TTTCTTAAAGAG 10
AACTAAGTTTTC 9
ACTAAGTTTTCA 9
AGTTAATTTAAA 9
CAGGTCATAAAA 9
GATCGTTGTTTT 9
TTCTTAAAGAGC 9
AAAACTTAGAAG 8
AAATCAATTGAT 8
AATCAATTGATC 8
AATTGATCGTTG 8
CCAGGTCATAAA 8
CTAAGTTTTCAA 8
GTTAATTTAAAT 8
GTTCCTGGACAT 8
TTCCTGGACATA 8
TTTTCTTAAAGA 8
TTTTTCTTAAAG 8

1,121 hits for repetitive sequences
passing 12mer abundance criterion

(Read-Number)
PAGE 83

For Tandem Repeat families of ### bp:
PacBio Read named "HuluTR###-r83"

>m141120_131642_42134_c100732402550000001823141405141505_s1_p0/2369/0_6334

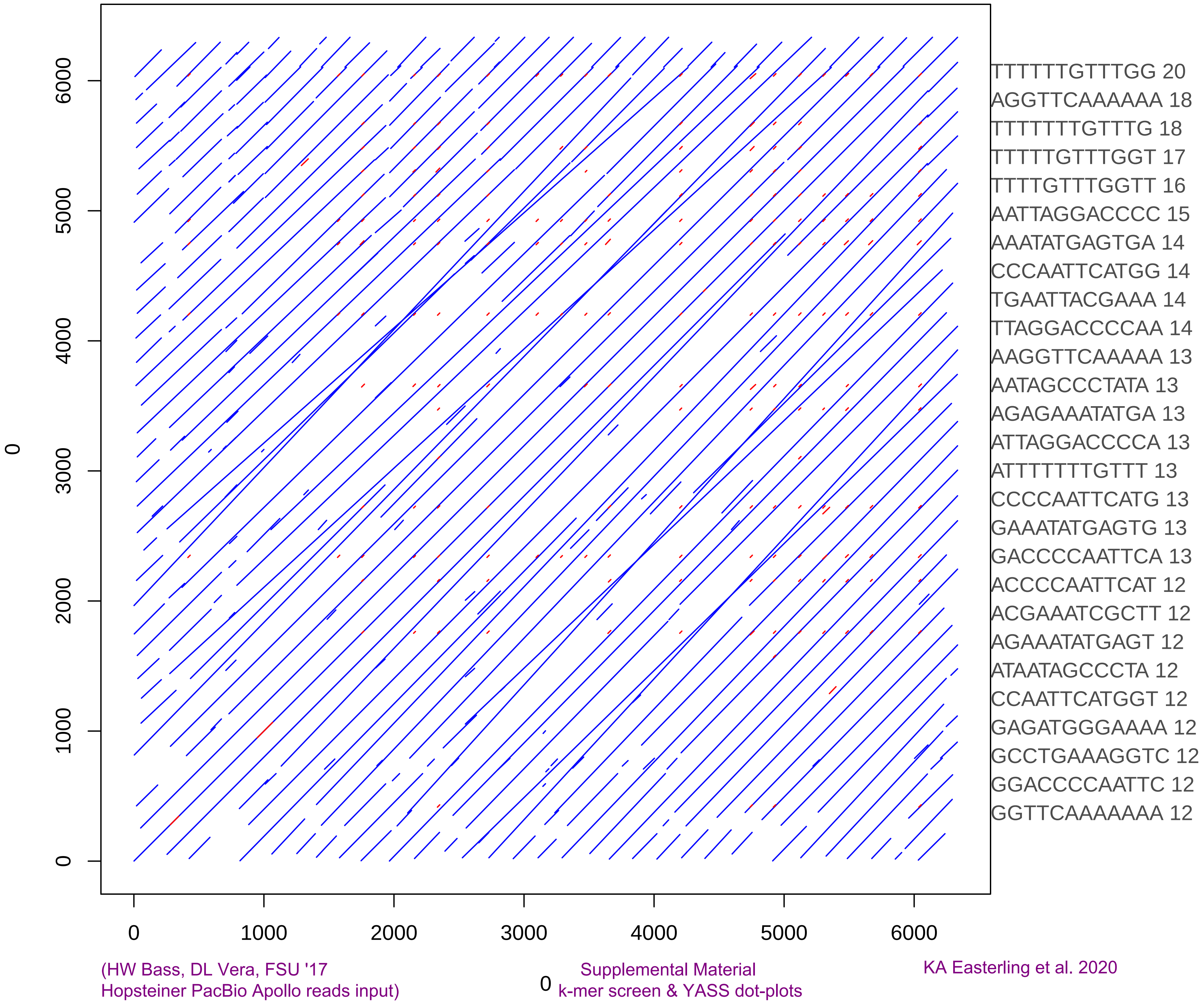

(HW Bass, DL Vera, FSU '17
Hopsteiner PacBio Apollo reads input)

Supplemental Material
k-mer screen & YASS dot-plots

KA Easterling et al. 2020

>m141120_131642_42134_c100732402550000001823141405141505_s1_p0/33587/9272_14622

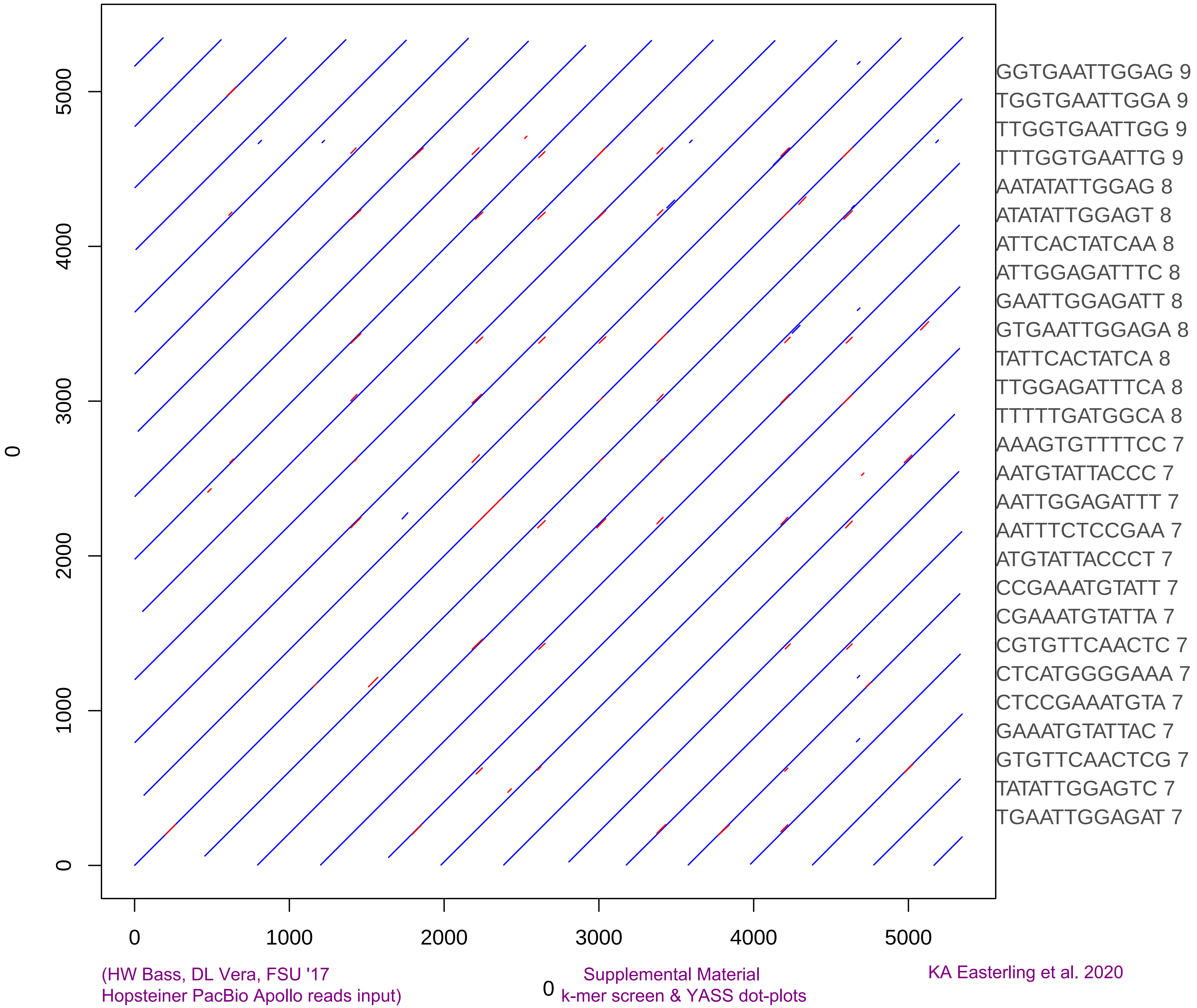

>m141120_131642_42134_c100732402550000001823141405141505_s1_p0/365/0_5634

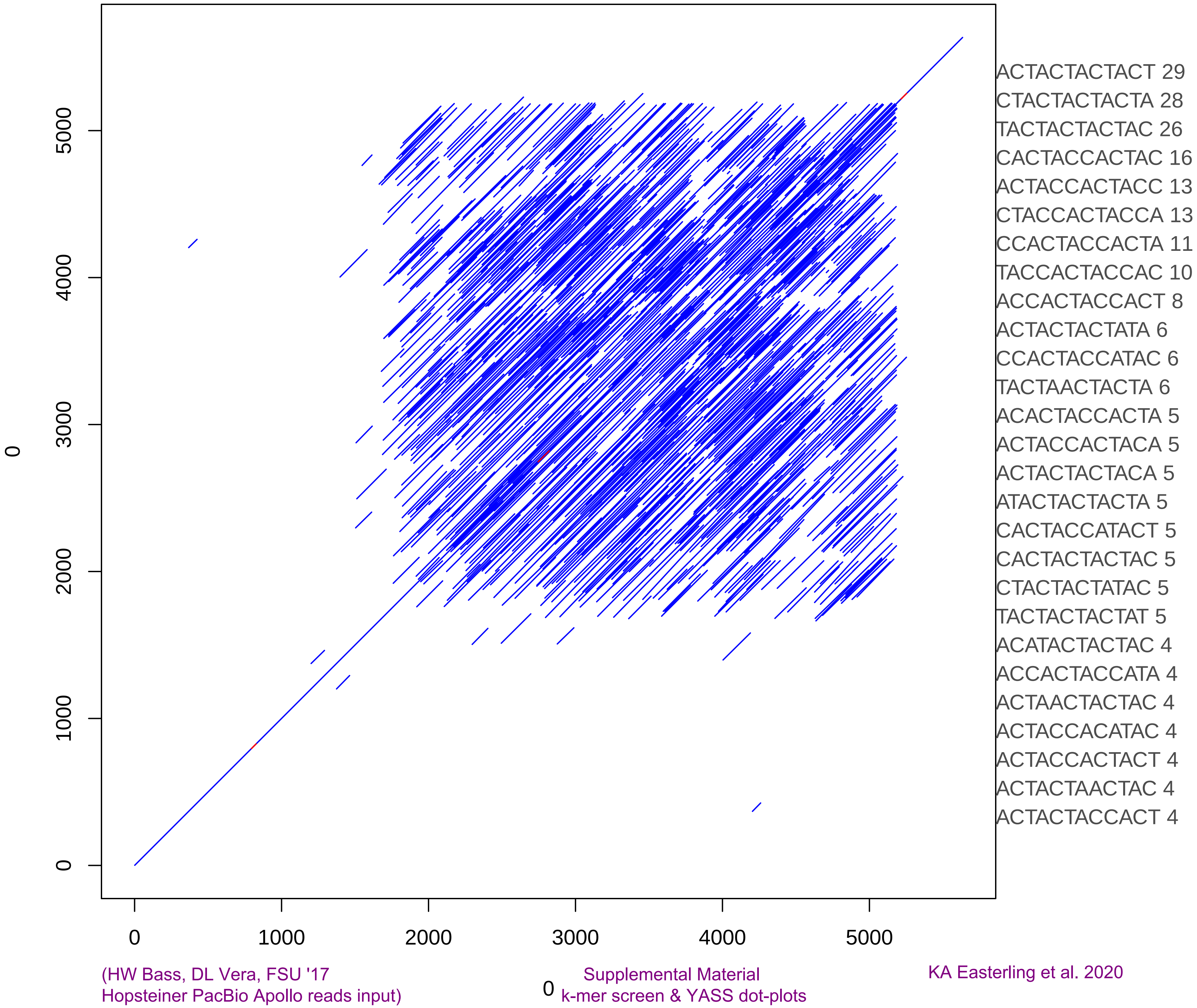

>m141120_131642_42134_c100732402550000001823141405141505_s1_p0/37906/11361_20192

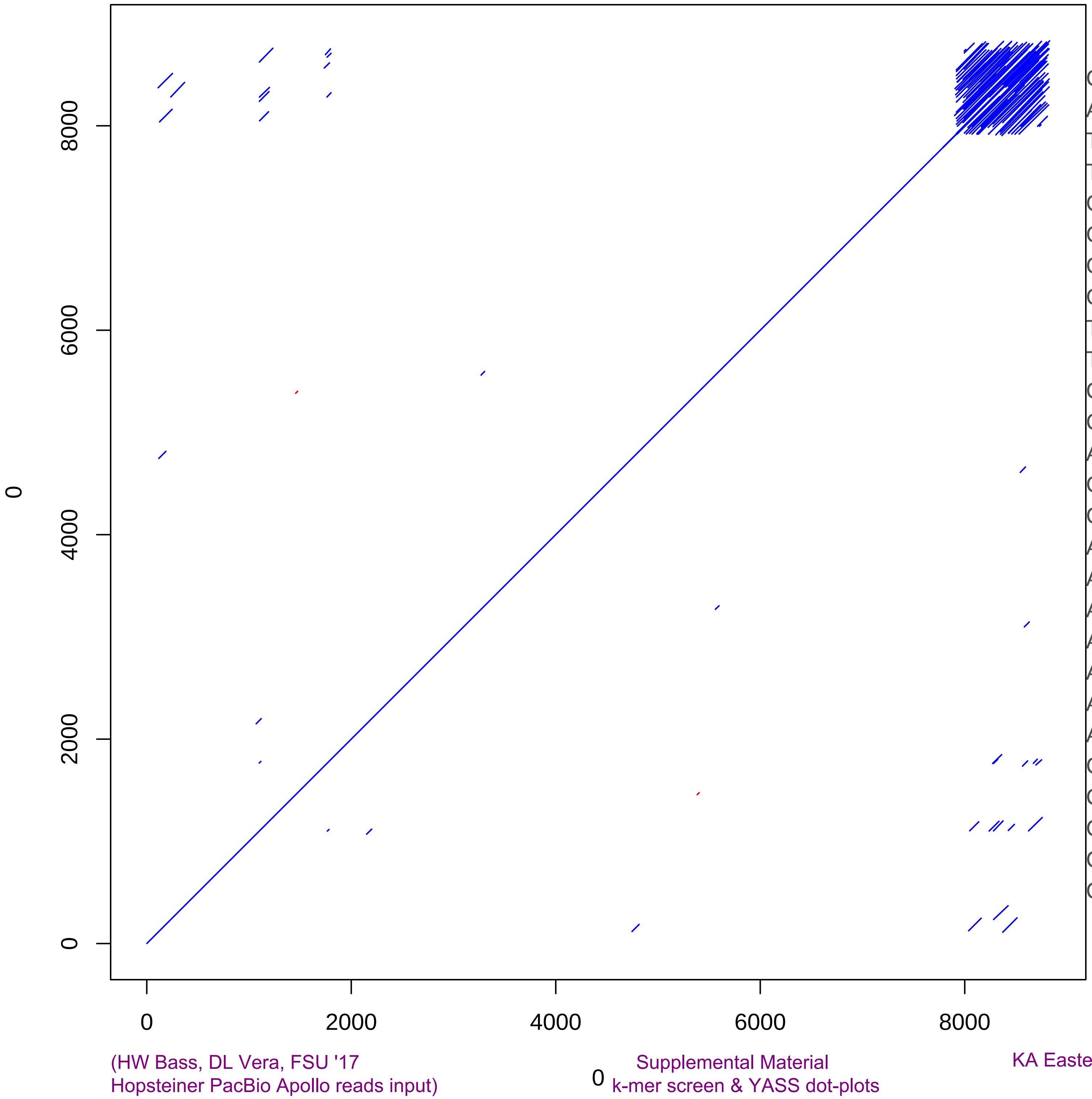

CTGCTACTGCTA 18
ACTGCTACTGCT 15
TACTGCTACTGC 13
TGCTACTGCTAC 12
GCTACTGCTACT 9
CTACTGCTACTG 8
GCTGCTGCTGCT 7
GCTACTGCTATT 6
TGCTACTGCTAT 6
TGCTGCTGCTGC 6
CTGCTGCTGCTA 4
CTGCTGCTGCTG 4
ATACTGCTACTG 3
CTACTGCTACAT 3
CTACTGCTATTG 3
AATAGCTTACTA 2
ACCTGCTACTGC 2
ACTAGAGACTAA 2
ACTGCTGCTGCT 2
ACTTCTACTGCT 2
ATAGCTTACTAG 2
ATGCTGCTGCTG 2
CAATAGCTTACT 2
CATTAGCTGATG 2
CCTGCTACTGCT 2
CTACTACTGCTA 2
CTACTAGAGACT 2

>m141120_131642_42134_c100732402550000001823141405141505_s1_p0/3912/4954_10366

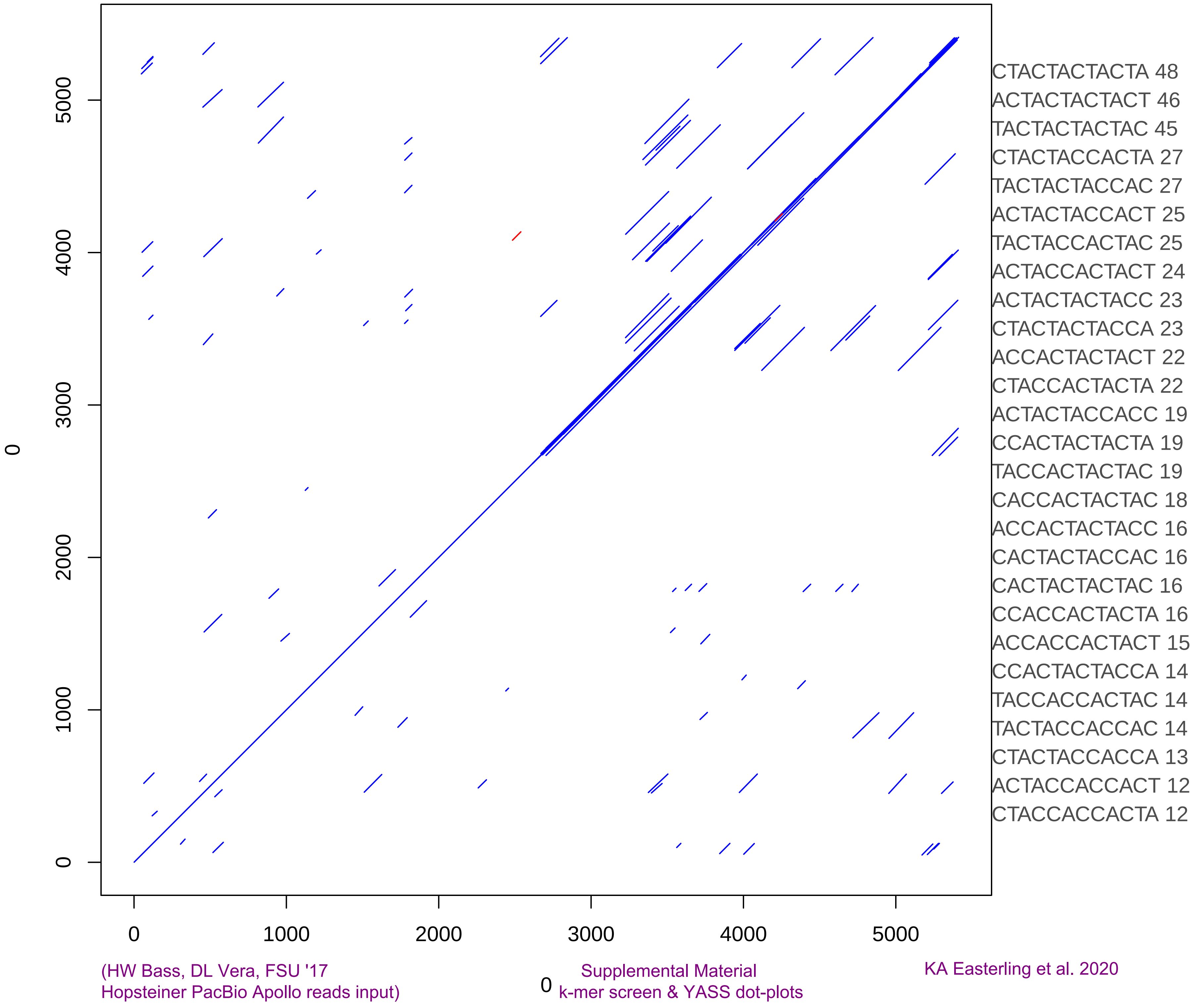

>m141120_131642_42134_c100732402550000001823141405141505_s1_p0/39532/2804_11309

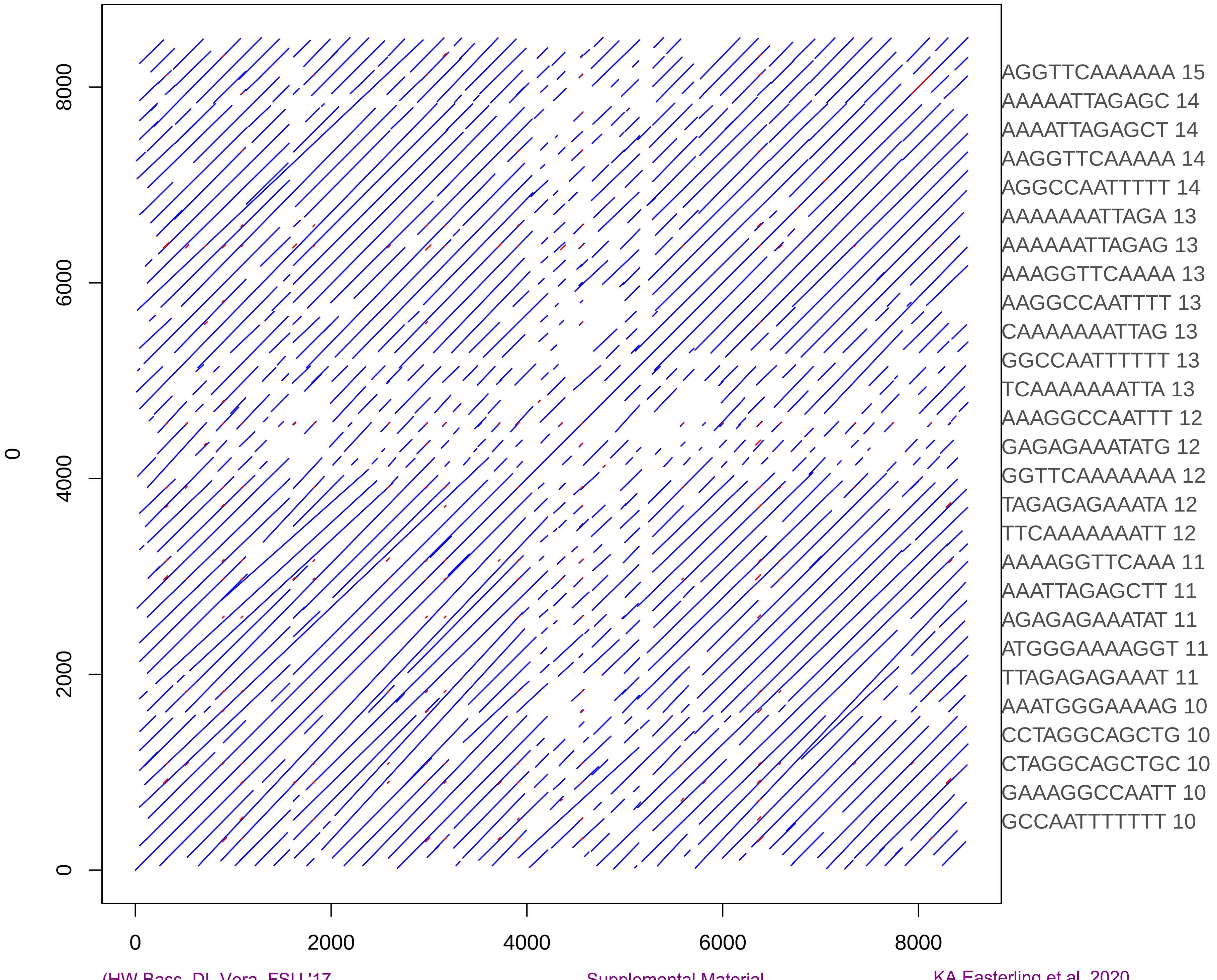

>m141120_131642_42134_c100732402550000001823141405141505_s1_p0/39665/6194_14430

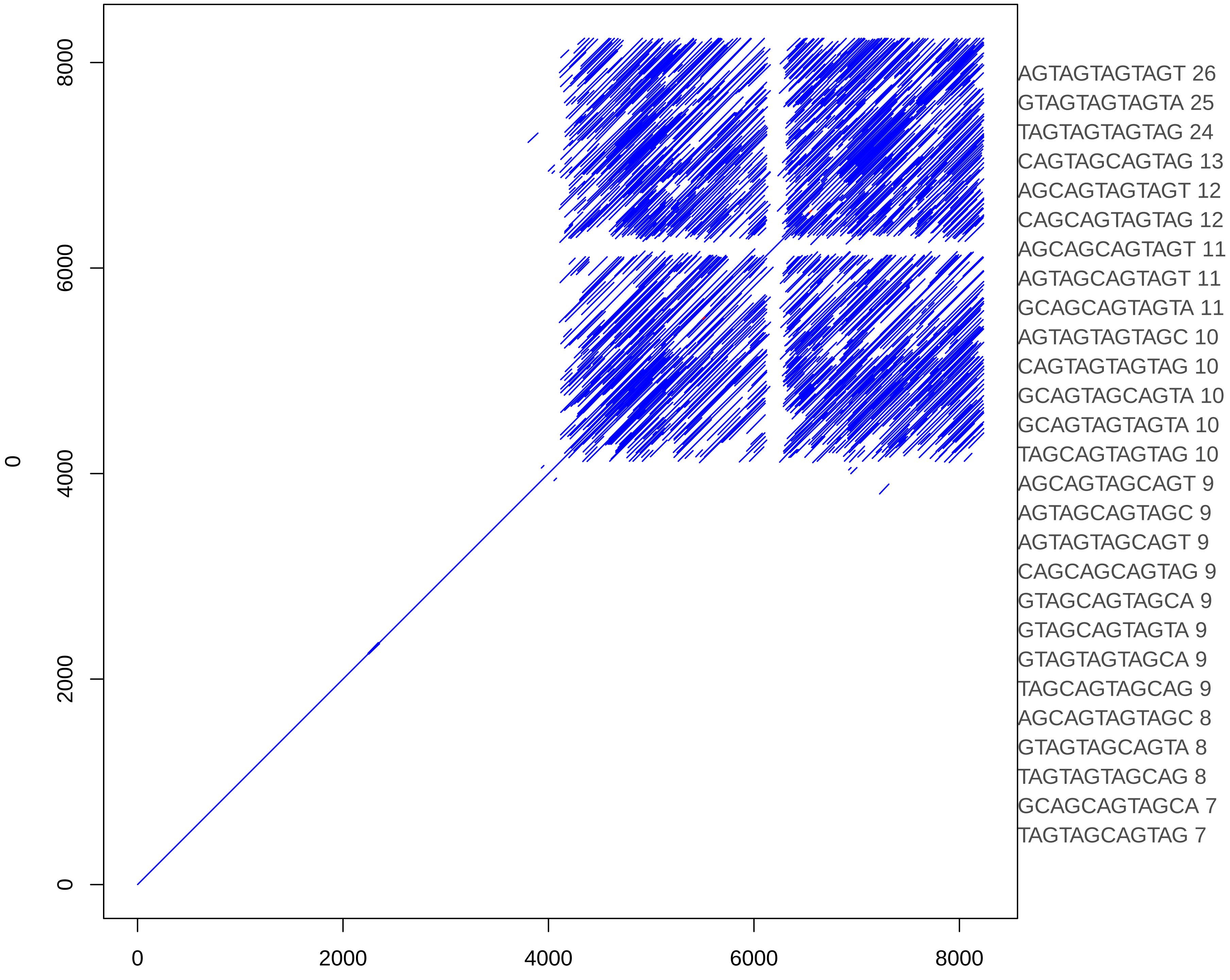

>m141120_131642_42134_c100732402550000001823141405141505_s1_p0/44730/5824_12153

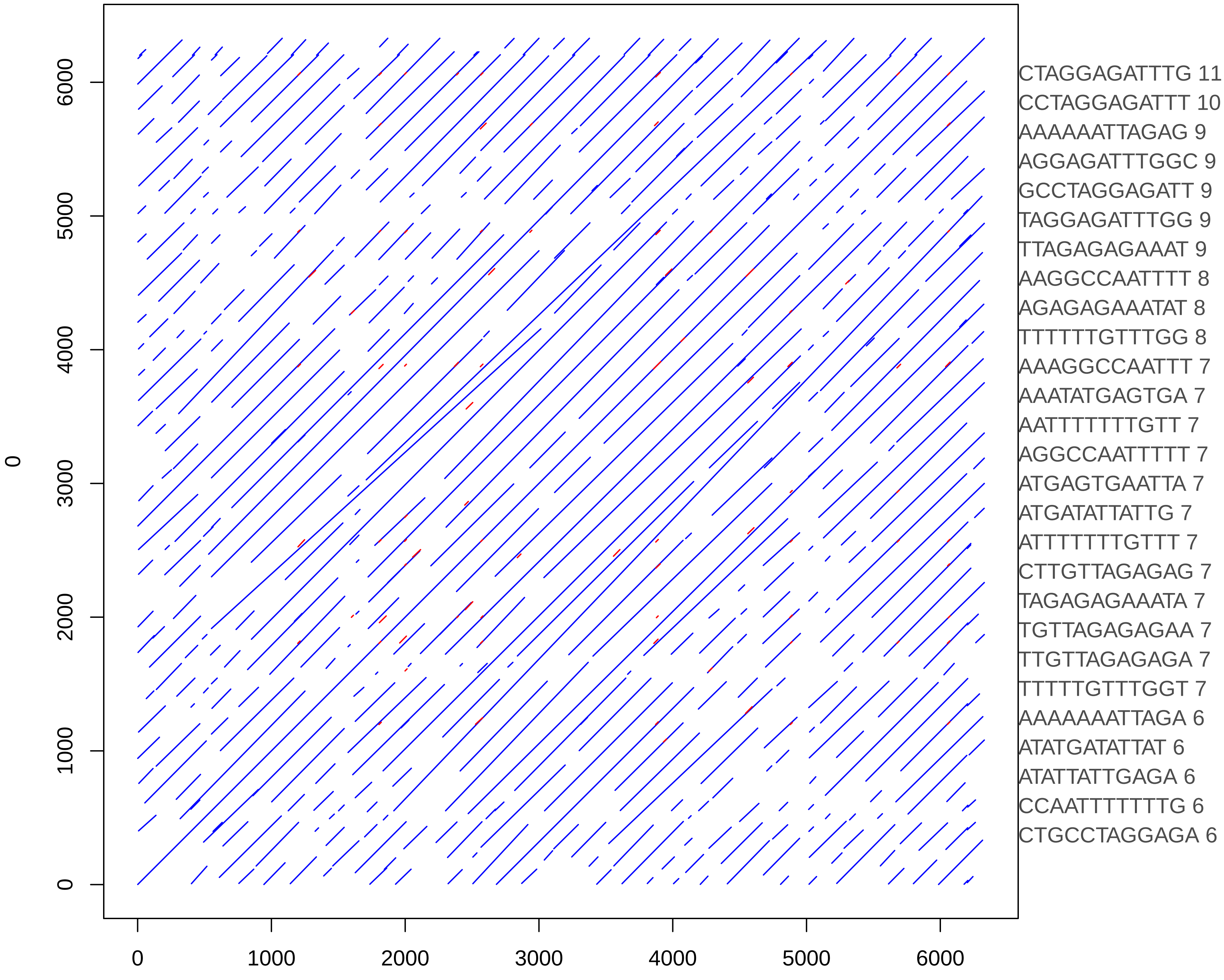

>m141120_131642_42134_c100732402550000001823141405141505_s1_p0/50916/197_5330

Representative Read (Table 1): HuluTR060-r91, 34 ppm, 59% A+T,
GenBank Acc. MN537567

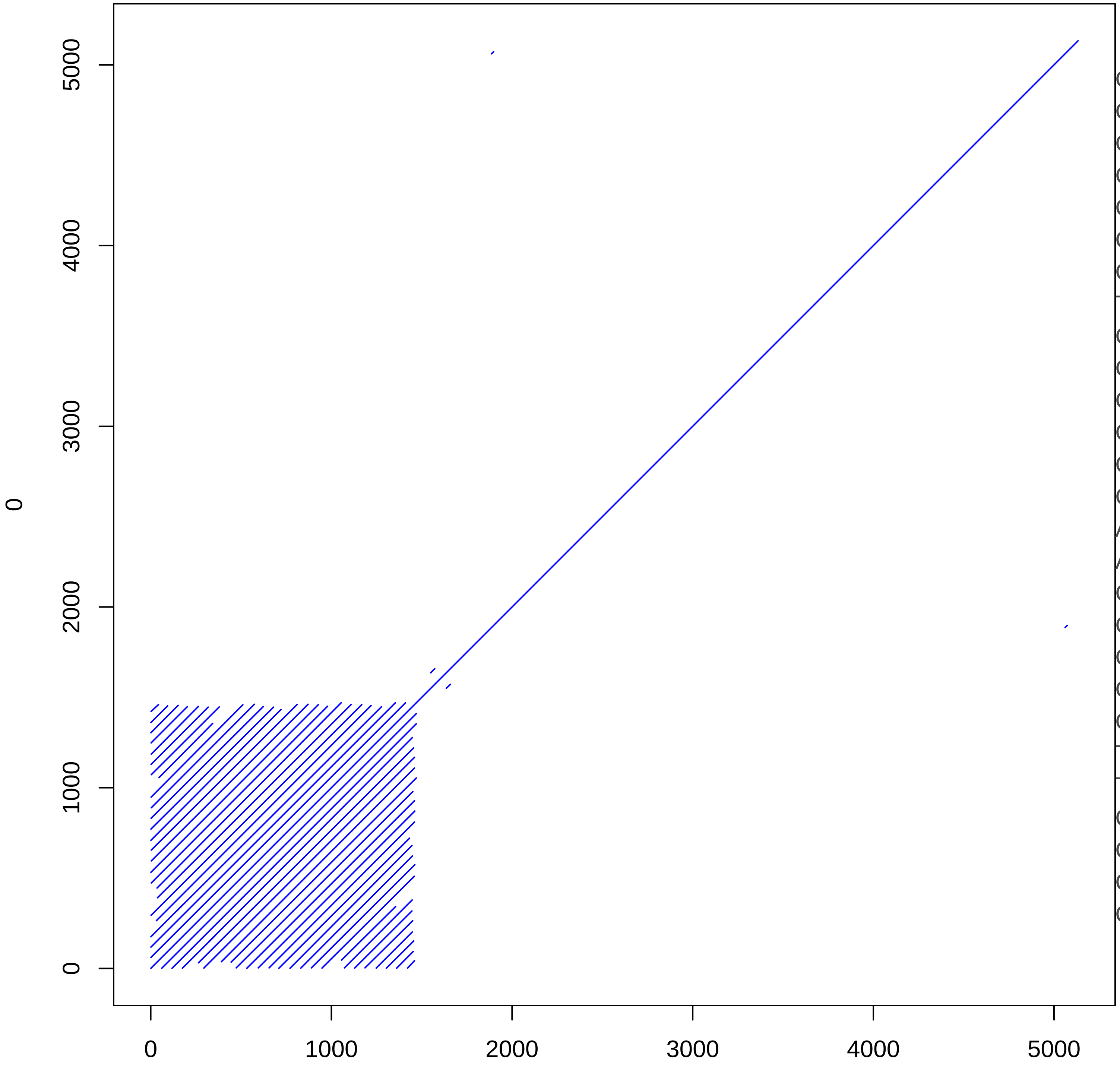

GGGCTCGGTCCT 10
GCTCGGTCCTCT 9
GGCTCGGTCCTC 9
CCCCGGACGTCG 8
CCCGGACGTCGT 8
CCGGACGTCGTT 8
GGACGTCGTTCG 8
TGGGCTCGGTCC 8
CGGACGTCGTTC 7
CTCGGTCCTCTT 7
GACGTCGTTCGT 7
GGGTGGGACCGG 7
GGTGGGACCGGC 7
GTGGGACCGGCC 7
ACGTCGTTCGTC 6
ACCCCGGACGTC 5
CCCCCGGACGTC 5
CGGTCCTCTTAA 5
CGTTCGTCCGGG 5
GGGGTGGGACCG 5
GGTCCTCTTAAT 5
TCGTTCGTCCGG 5
TGGGACCGGCCT 5
CCGGGGTGGGAC 4
CCTTGGGCTCGG 4
CGGGGTGGGACC 4
CGTCGTTCGTCC 4

>m141120_131642_42134_c100732402550000001823141405141505_s1_p0/52292/8871_17084

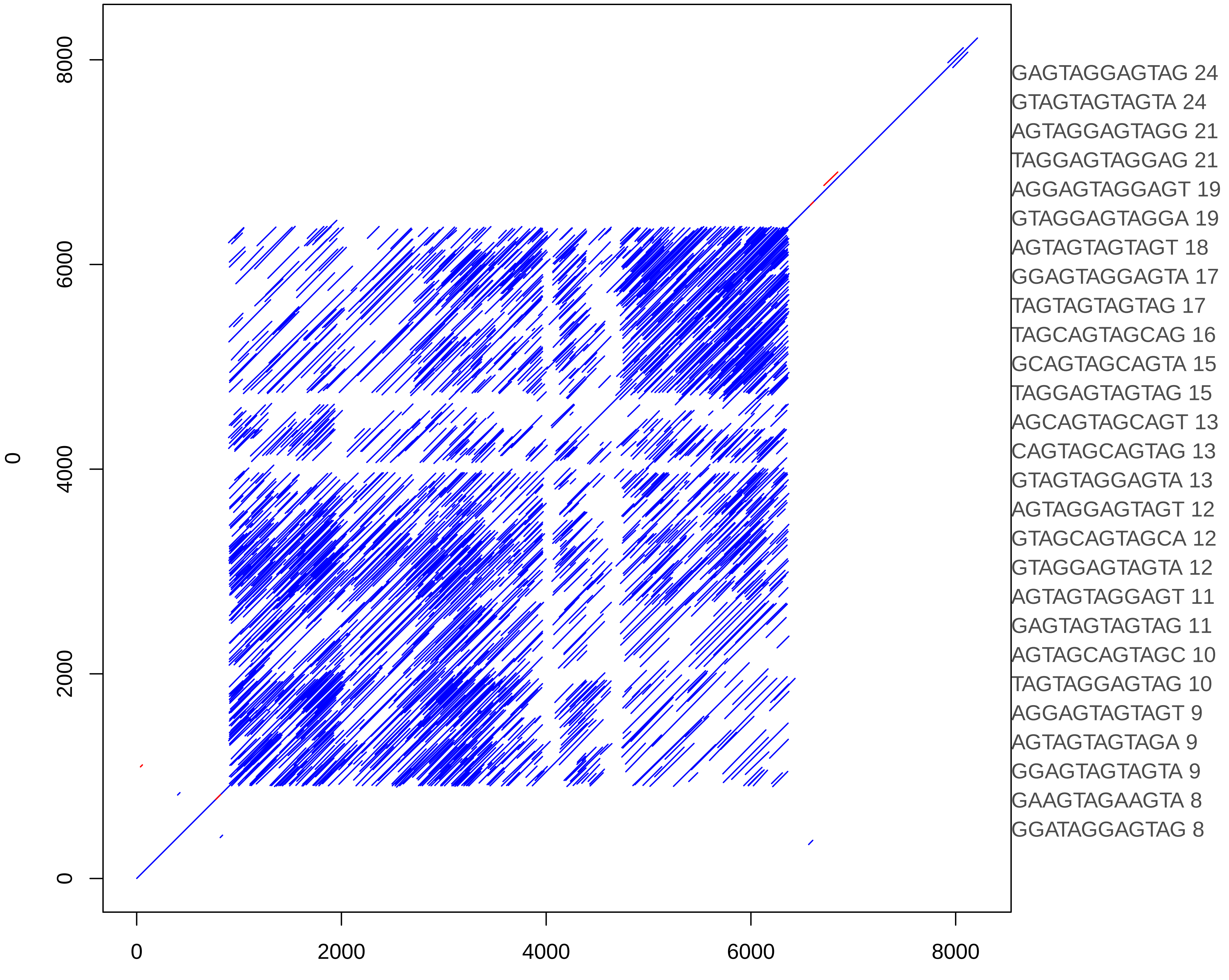

>m141120_131642_42134_c100732402550000001823141405141505_s1_p0/53455/9507_14579

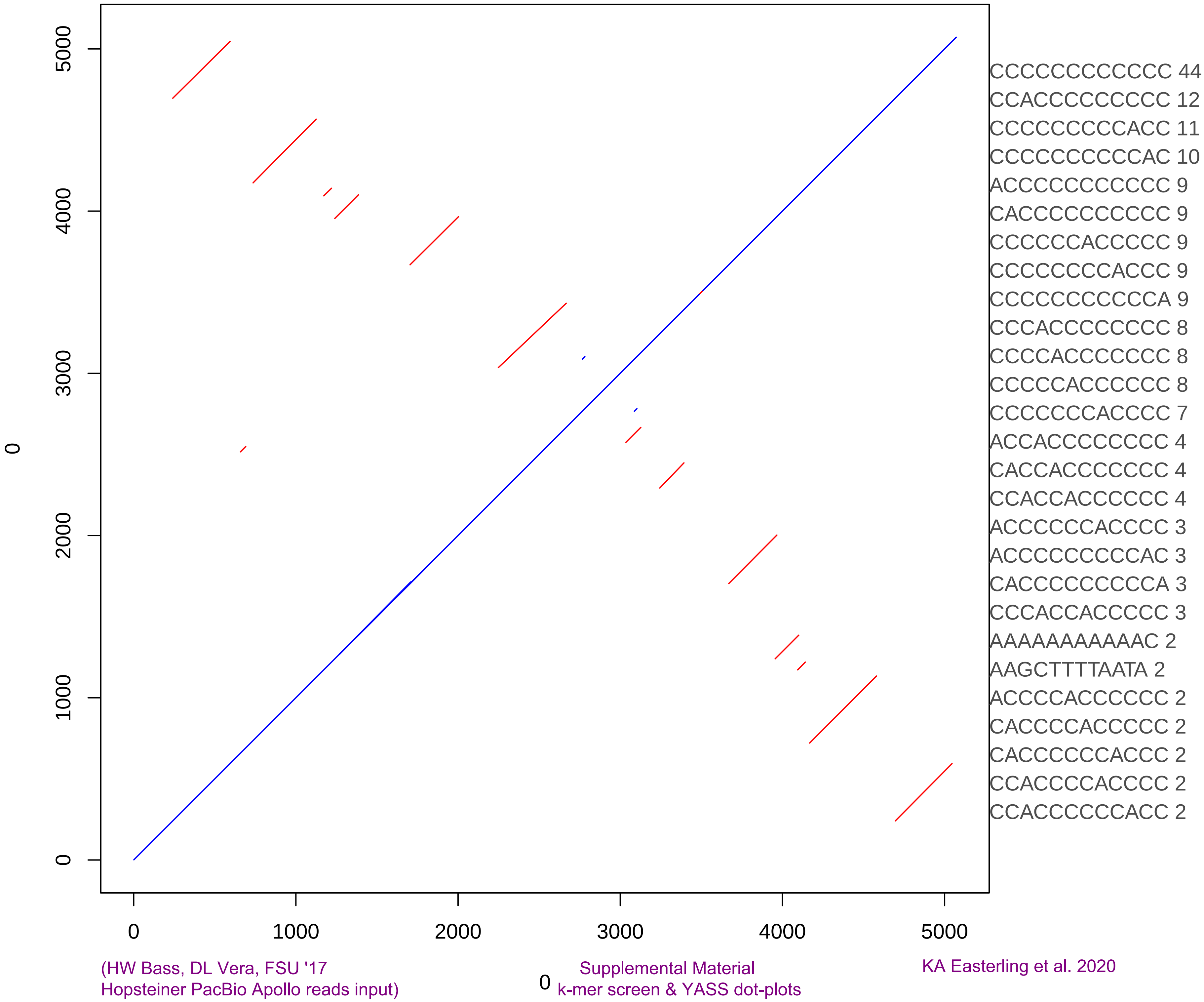

>m141120_131642_42134_c100732402550000001823141405141505_s1_p0/5408/1631_9071

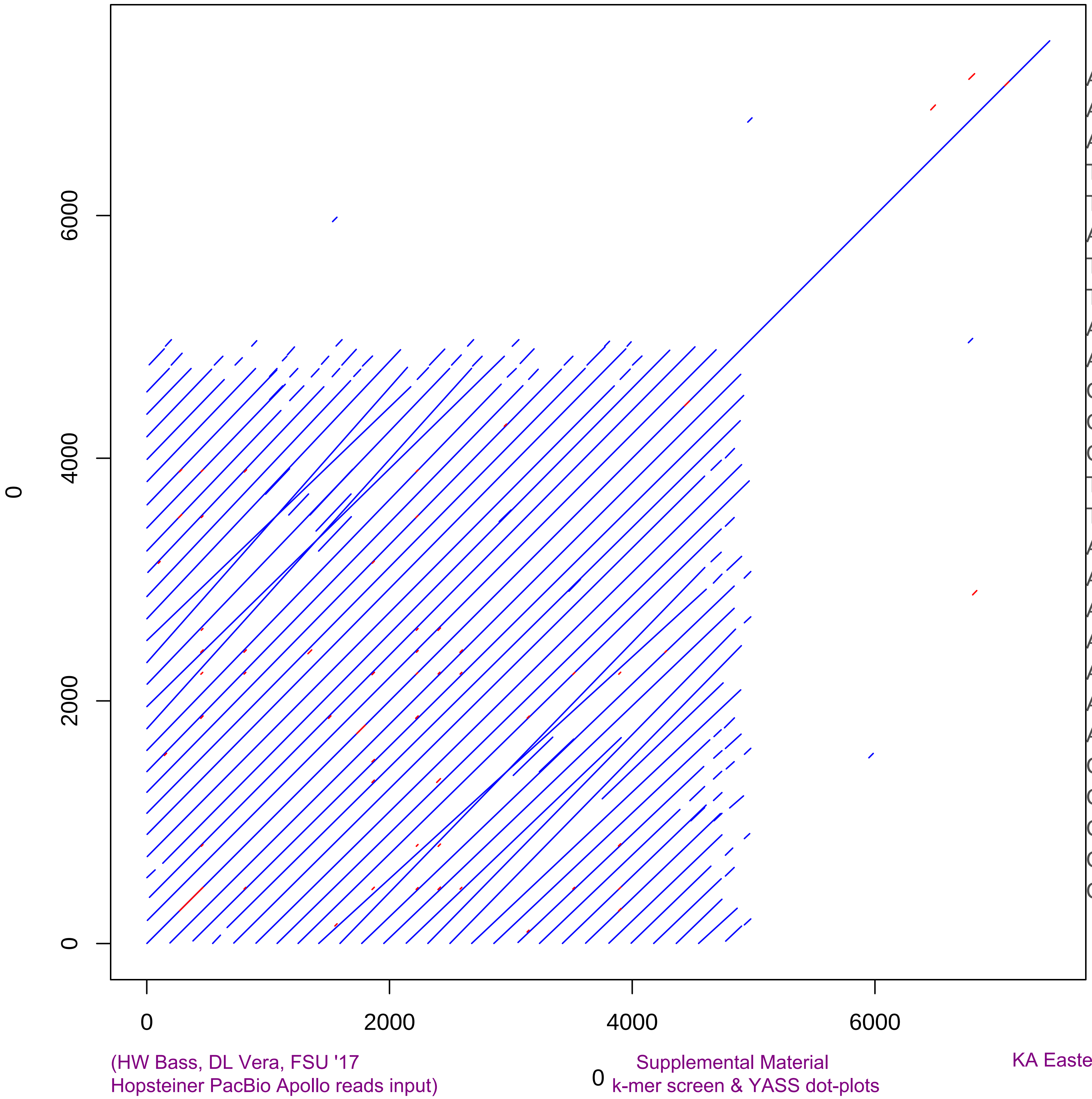

AGGTTCAAAAAA 17
AAAAAATTAGAG 15
AAGGTTCAAAAA 14
TTTTTTGTTTGG 14
TTTTTTTGTTTG 14
ATTTTTTTGTTT 13
TTATTGAGATGG 13
TTTTTGTTTGGT 13
AAAGGTTCAAAA 12
AATTTTTTTGTT 12
GATGGGAAAAGG 12
GGTCAATTTTTT 12
GGTTCAAAAAAA 12
TAGAGAGAAATA 12
TTGAGATGGGAA 12
AAAAATTAGAGC 11
AAGGTCAATTTT 11
AGATGGGAAAAG 11
AGGTCAATTTTT 11
ATGGGAAAAGGT 11
ATTATTGAGATG 11
ATTGAGATGGGA 11
CCTGAAAGGTCA 11
GAAAAGGTTCAA 11
GGAAAAGGTTCA 11
GGGAAAAGGTTC 11
GTCAATTTTTTT 11

>m141120_131642_42134_c100732402550000001823141405141505_s1_p0/60538/6179_15432

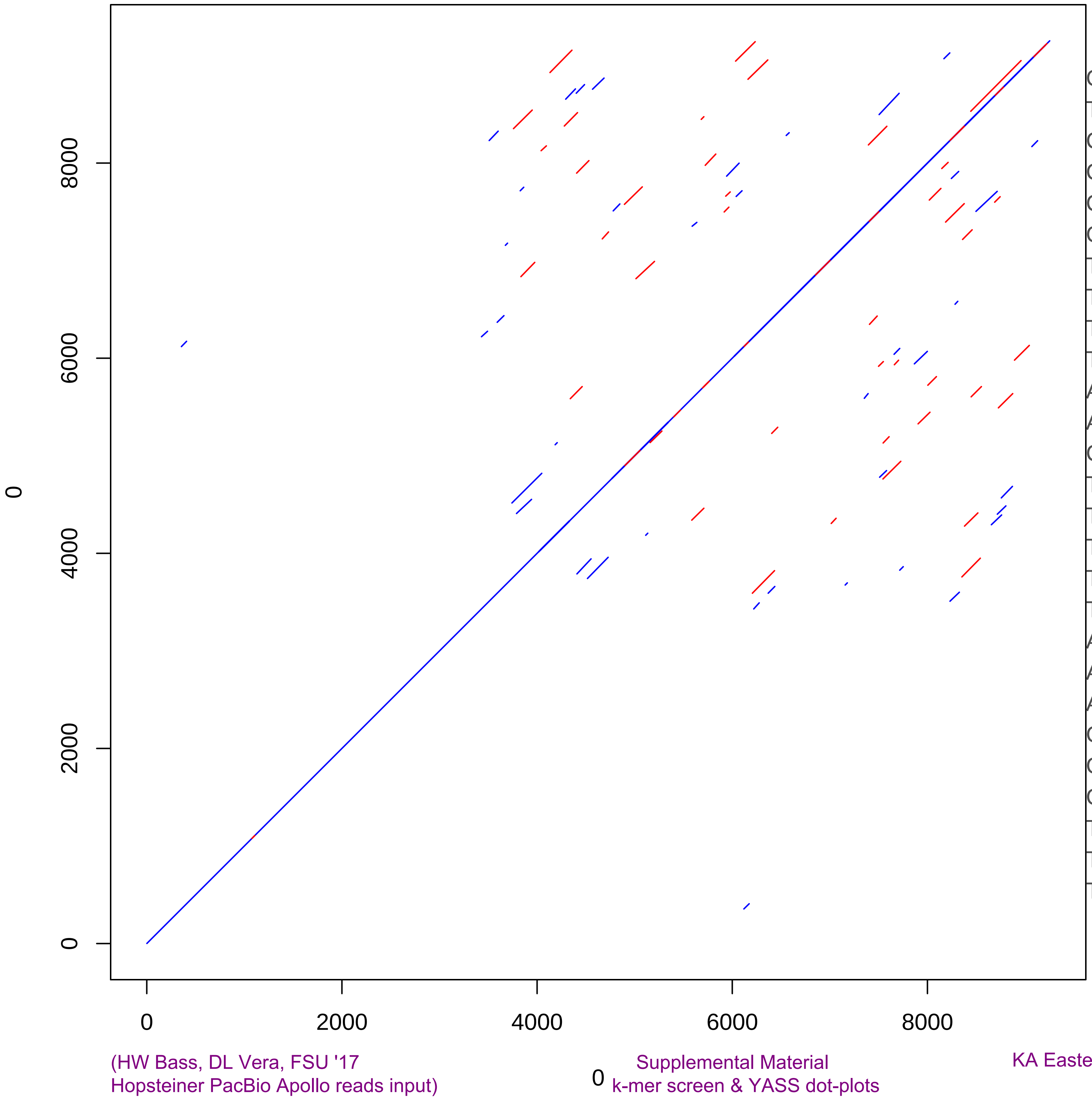

GGGGGGGGGGGG 61
TTTTTTTTTTTT 61
CCCCCCCCCCCC 19
CAAAAAAAAAAA 11
CTTTTTTTTTTT 10
GGGGGGGGGGTT 10
TTCTTTTTTTTT 10
TTTCTTTTTTTT 10
TTTGGGGGGGGG 10
TTTTTTTTTTGG 10
AAAAAAAAAAAC 9
AAAGGGGGGGGG 9
CCAAAAAAAAAA 9
TTTTCTTTTTTT 9
TTTTTTTTTTTG 9
TCTTTTTTTTTT 8
TTTTTGGGGGGG 8
TTTTTTTTTGGG 8
AAAAAGGGGGGG 7
AAAAGGGGGGGG 7
AAGGGGGGGGGG 7
GGGGGGGCCCCC 7
GGGGGGGGGGGC 7
GGGGGGGGGGGT 7
TGGGGGGGGGGG 7
TTTTGGGGGGGG 7
TTTTTCTTTTTT 7

>m141120_131642_42134_c100732402550000001823141405141505_s1_p0/61815/7020_14899

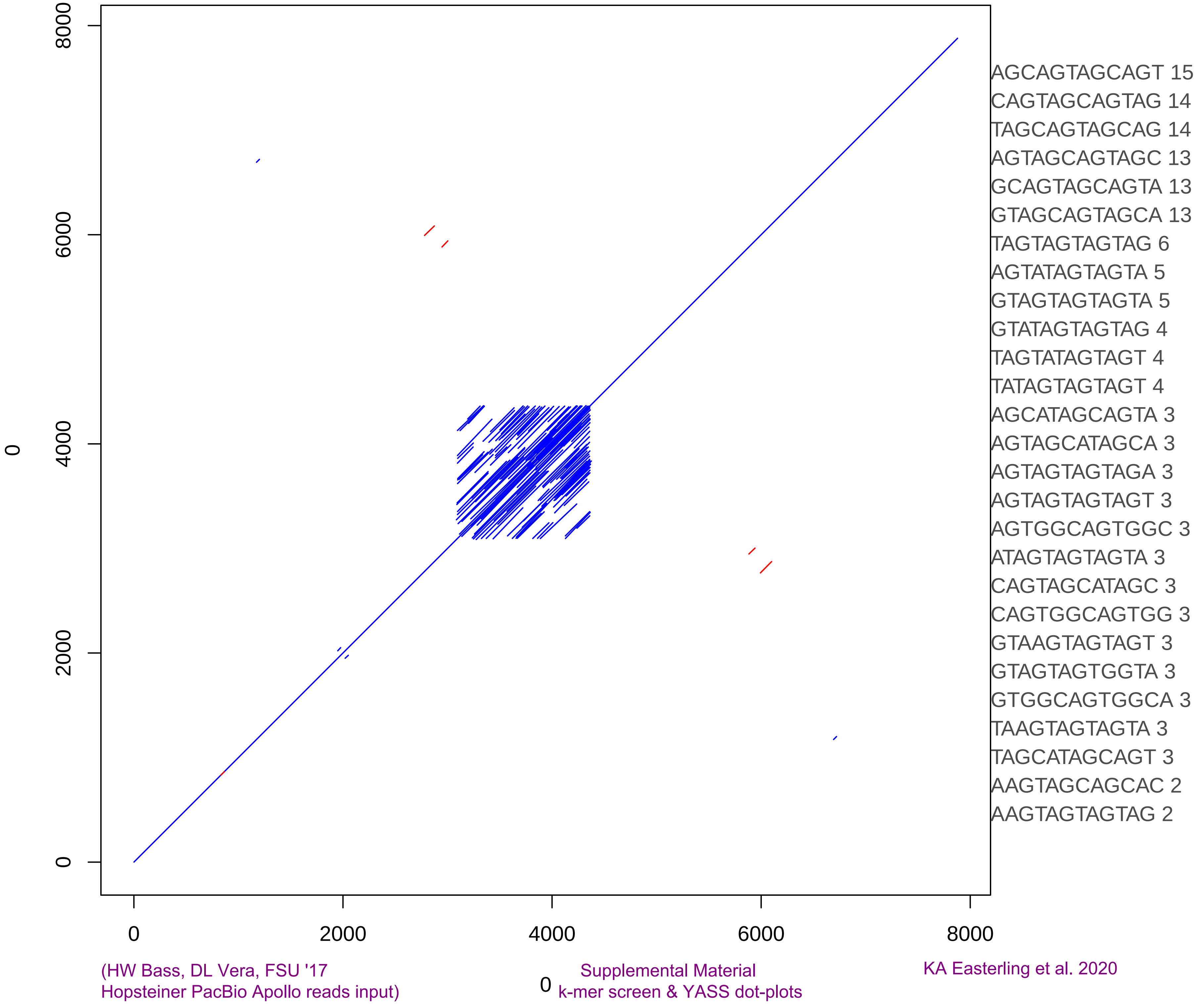

>m141120_131642_42134_c100732402550000001823141405141505_s1_p0/65362/0_5039

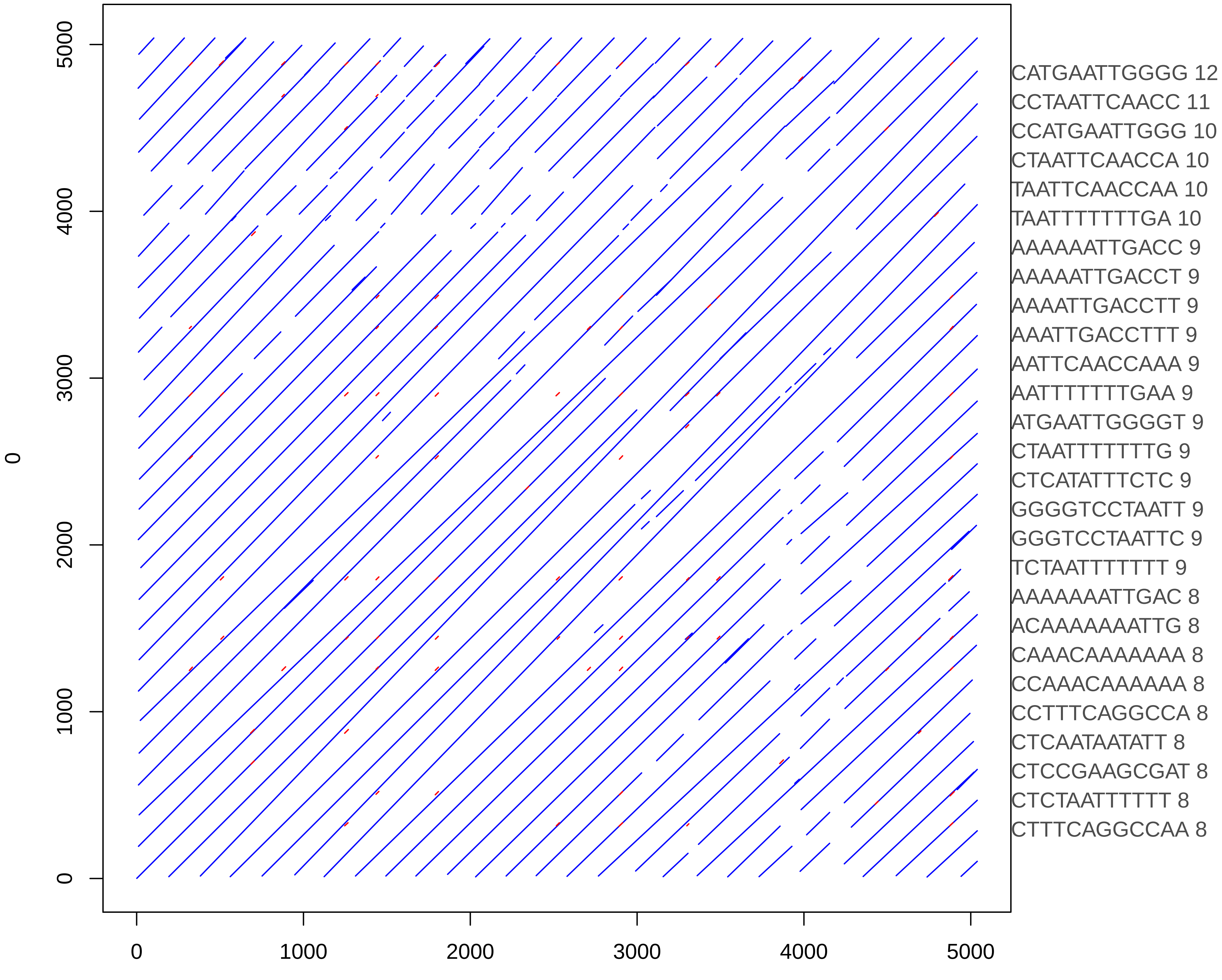

>m141120_131642_42134_c100732402550000001823141405141505_s1_p0/67518/7327_12487

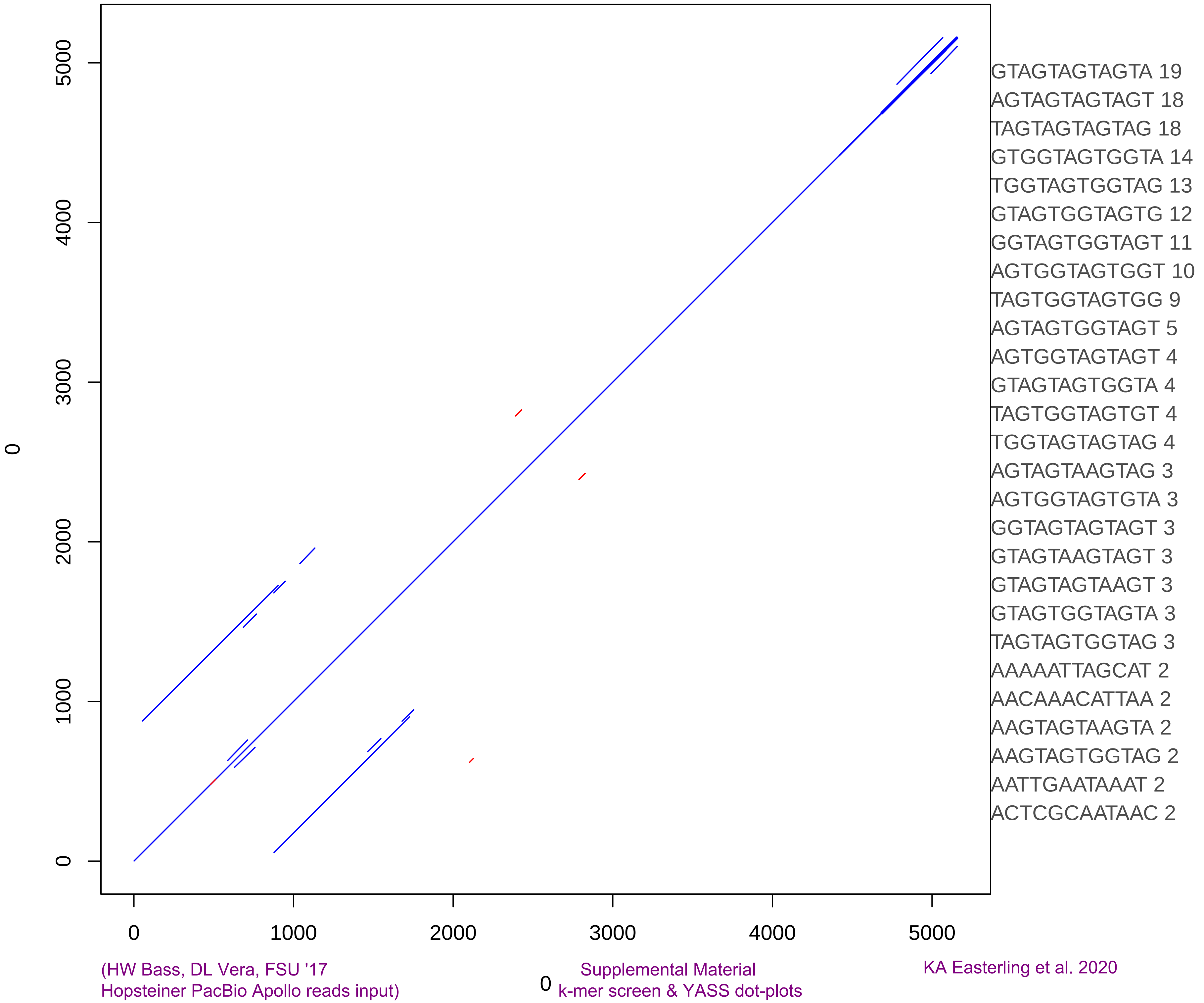

>m141120_131642_42134_c100732402550000001823141405141505_s1_p0/69009/3065_9373

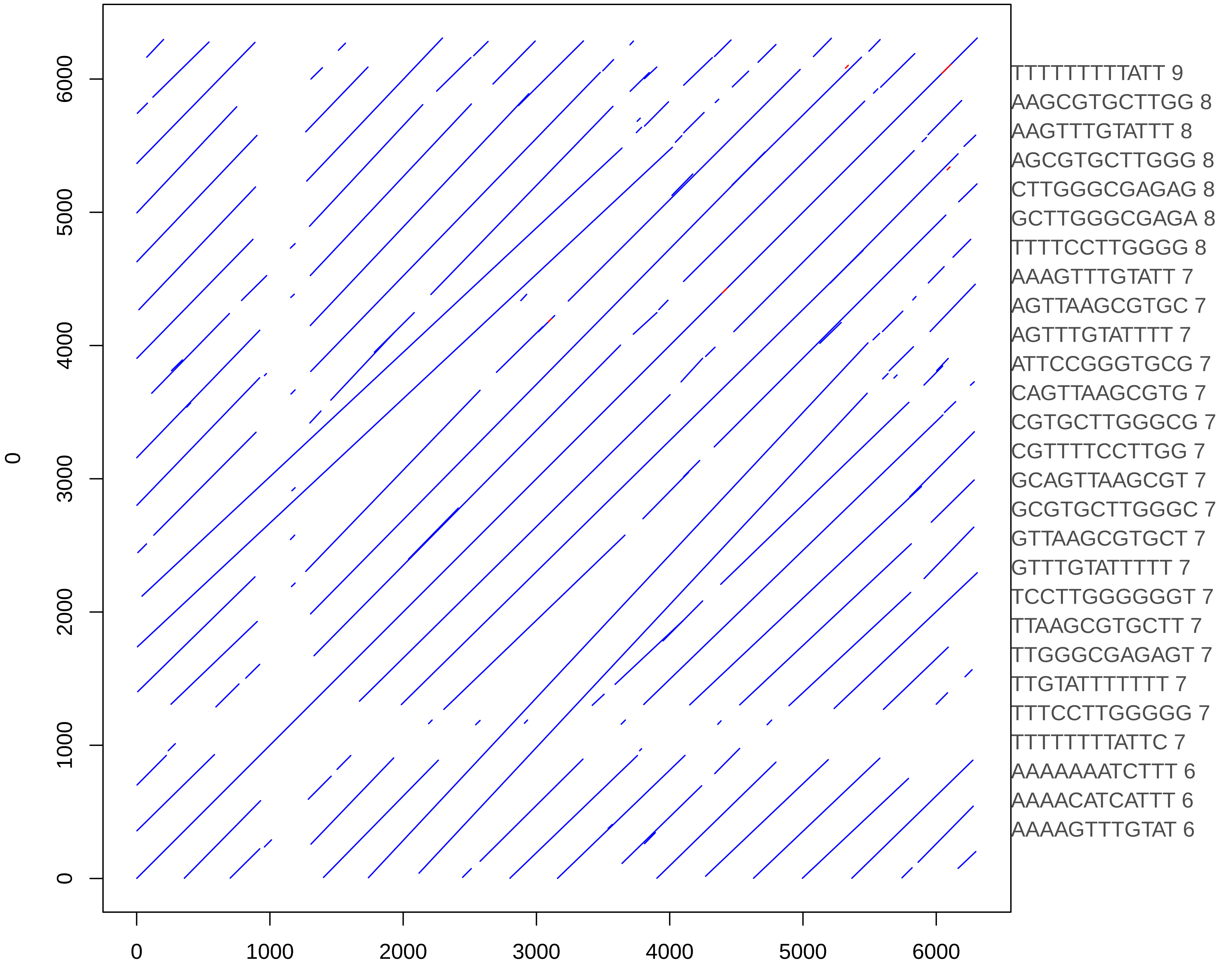

>m141120_131642_42134_c100732402550000001823141405141505_s1_p0/72747/207_14016

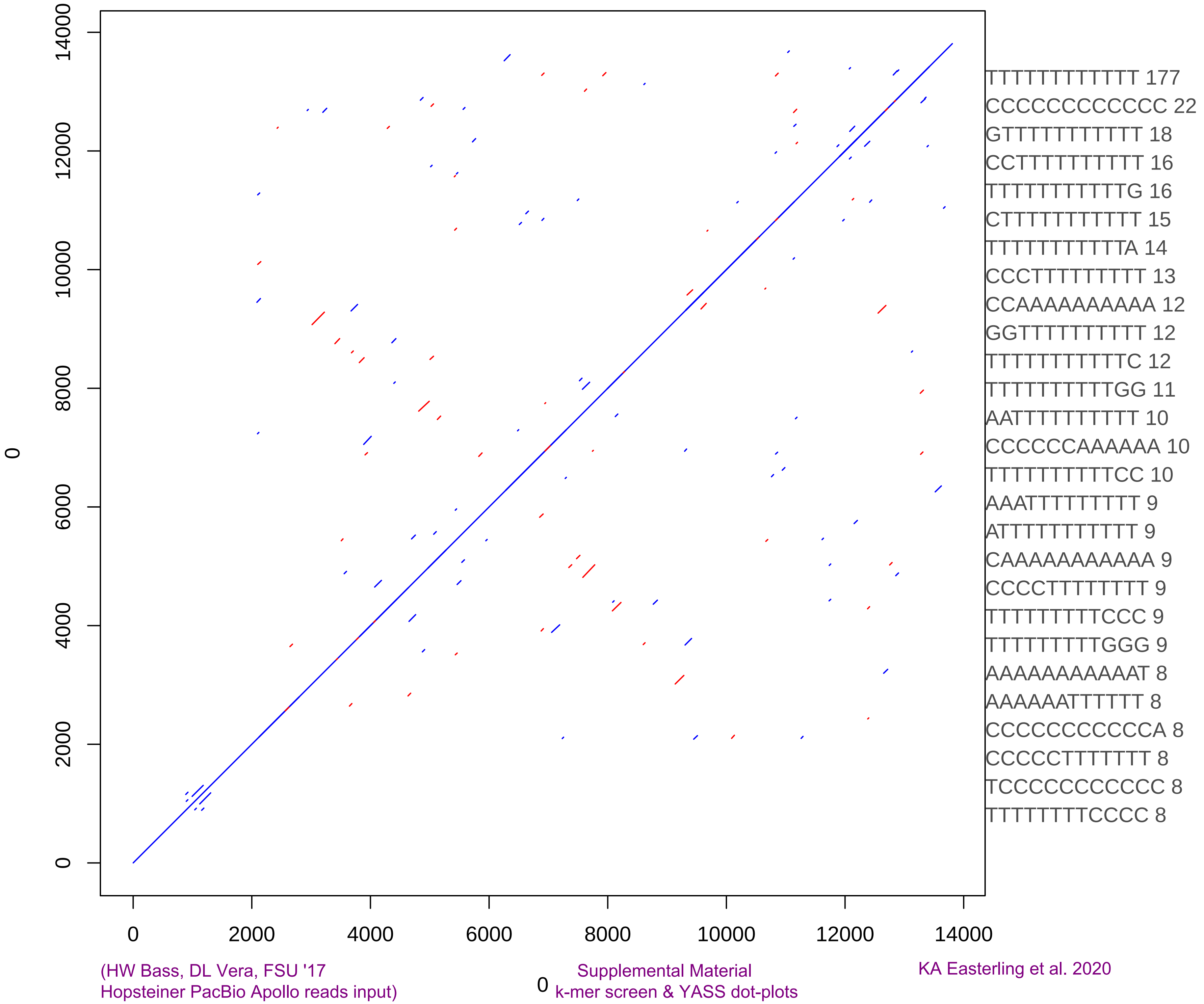

>m141120_131642_42134_c100732402550000001823141405141505_s1_p0/74417/5912_12449

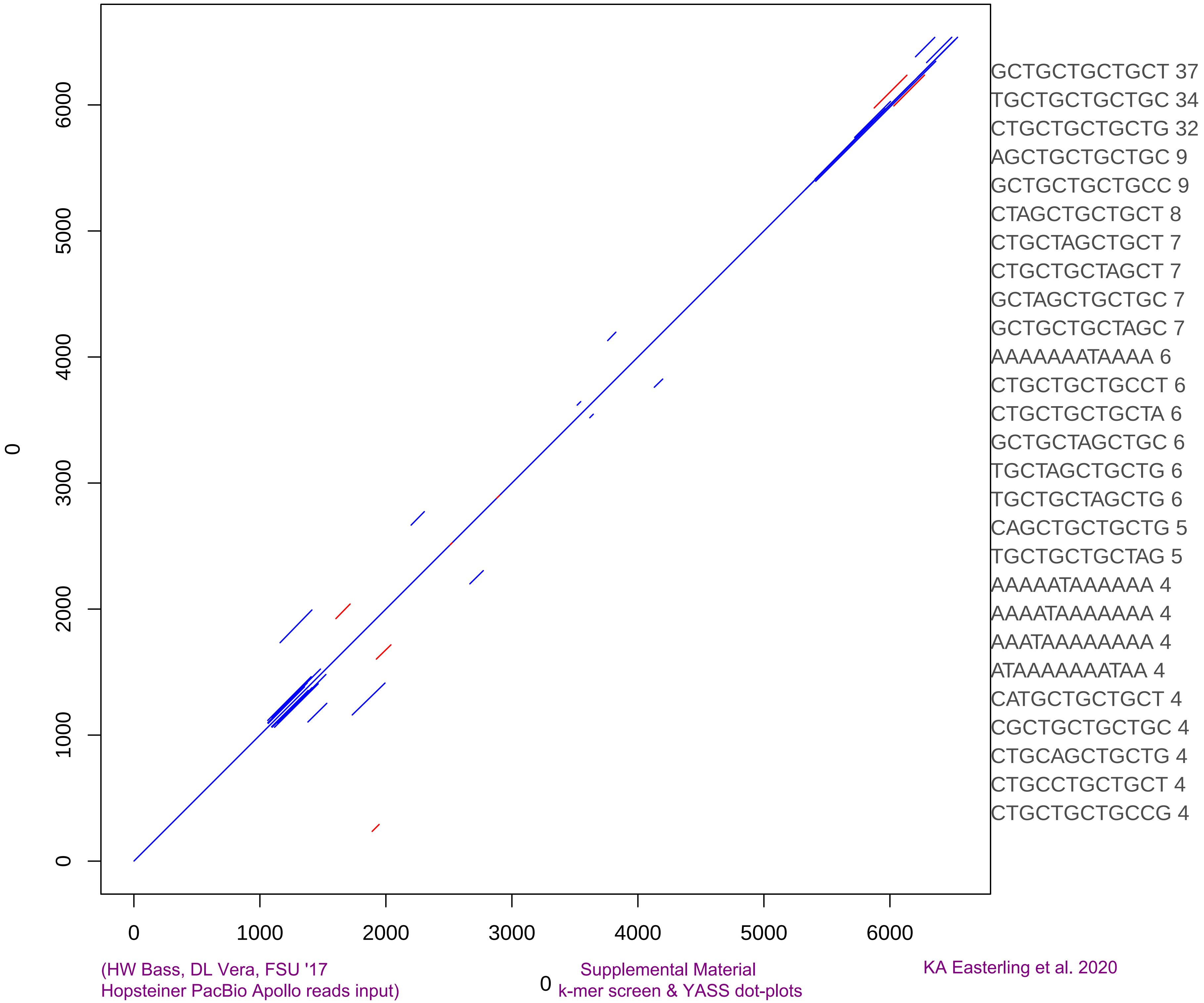

>m141120_131642_42134_c100732402550000001823141405141505_s1_p0/76835/9987_15611

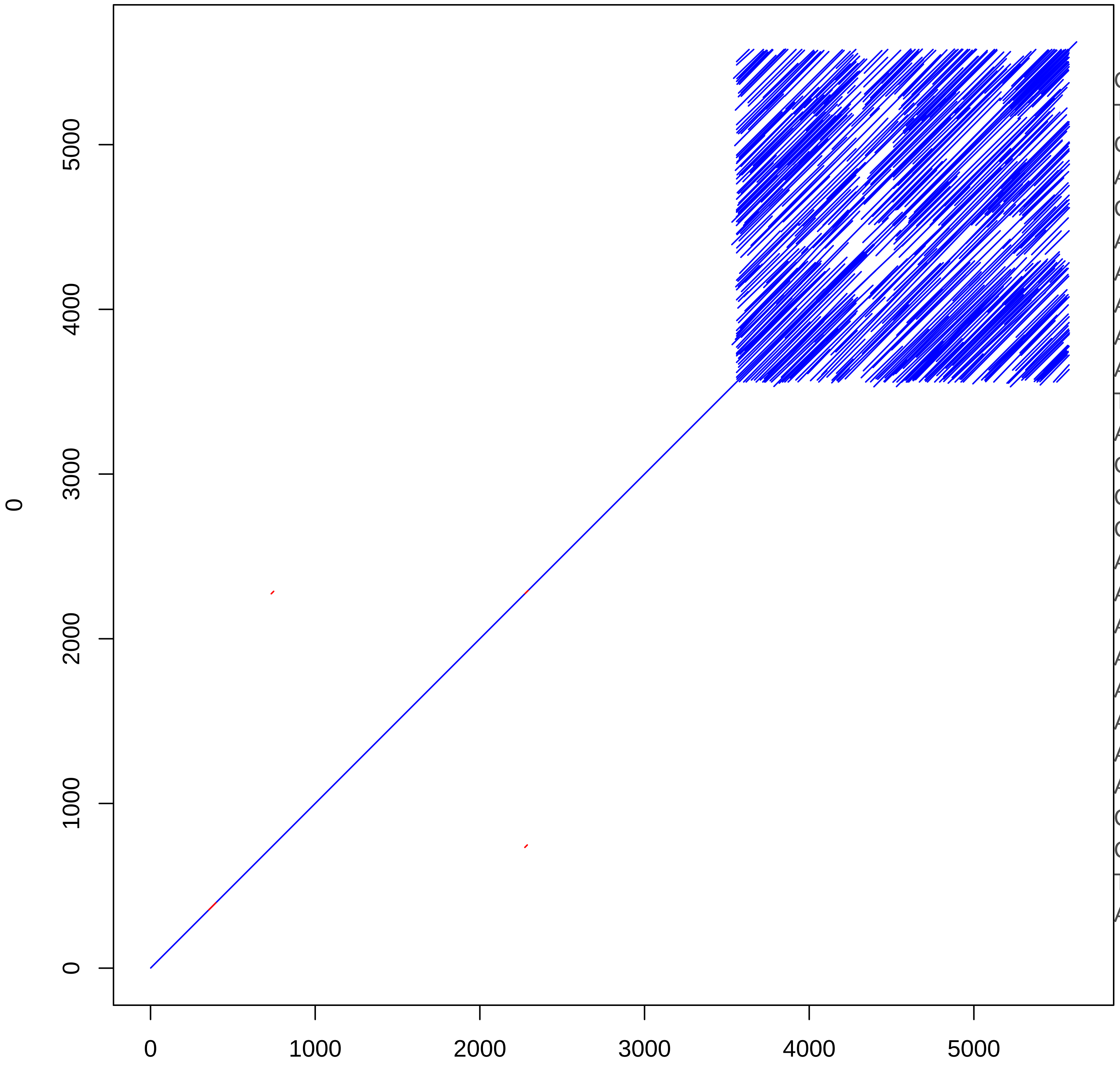

CTAAACCCTAAA 17
TAAACCCTAAAC 14
CCTAAACCCTAA 13
AAACCCTAAACC 12
CTAAACCTAAAC 12
AACCTAAACTAA 10
ACTAAACCCTAA 10
AACCTAAACCCT 9
ACCTAAACCCTA 9
ACCTAAACTAAA 9
TAAACCTAAACC 9
AACTAAACCCTA 8
CCCTAAACCTAA 8
CCTAAACCTAAA 8
CTAAACTAAACC 8
AAACCTAAACCC 7
AAACCTAAACTA 7
AACCCTAAACCC 7
AACCCTAAACCT 7
AACTAAACCTAA 7
ACCCTAAACCTA 7
ACTAAACCTAAA 7
ACTAAACTAAAC 7
CCTAAACTAAAC 7
CTAAACCCTAAC 7
TAAACCCTAACC 7
AAACTAAACCCT 6

>m141120_131642_42134_c100732402550000001823141405141505_s1_p0/77552/0_6510

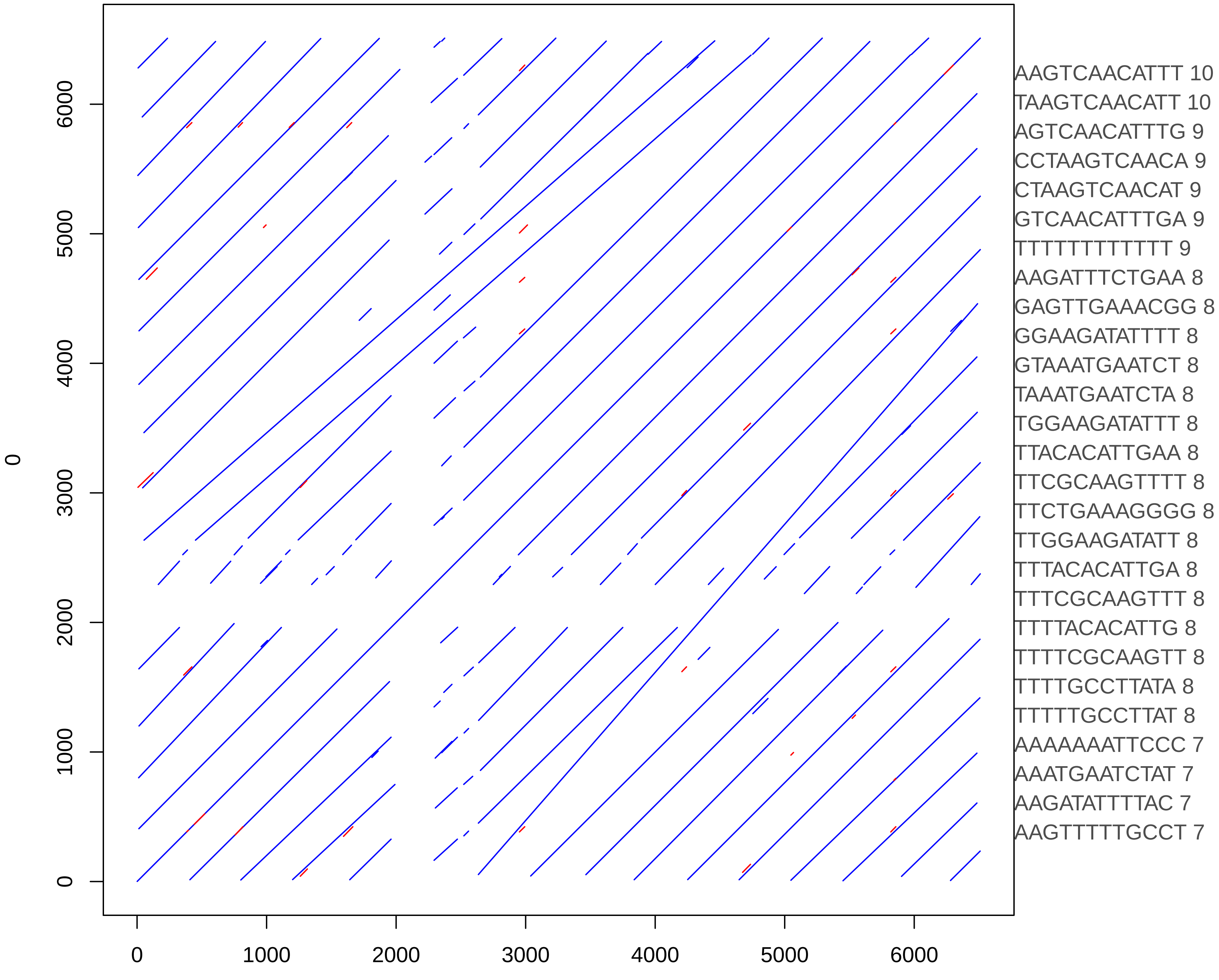

0

>m141120_131642_42134_c100732402550000001823141405141505_s1_p0/80751/11643_18759

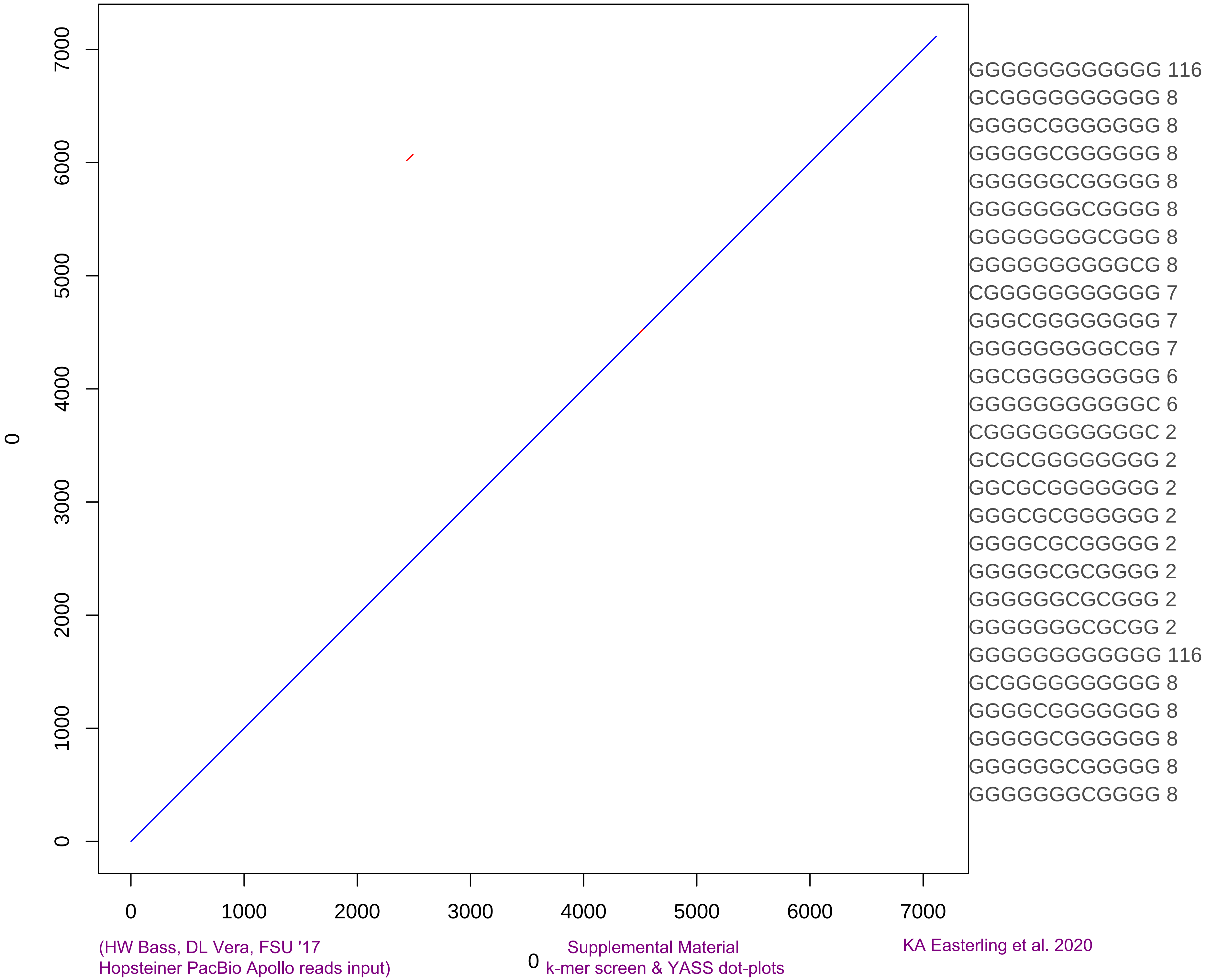

>m141120_131642_42134_c100732402550000001823141405141505_s1_p0/87246/0_7748

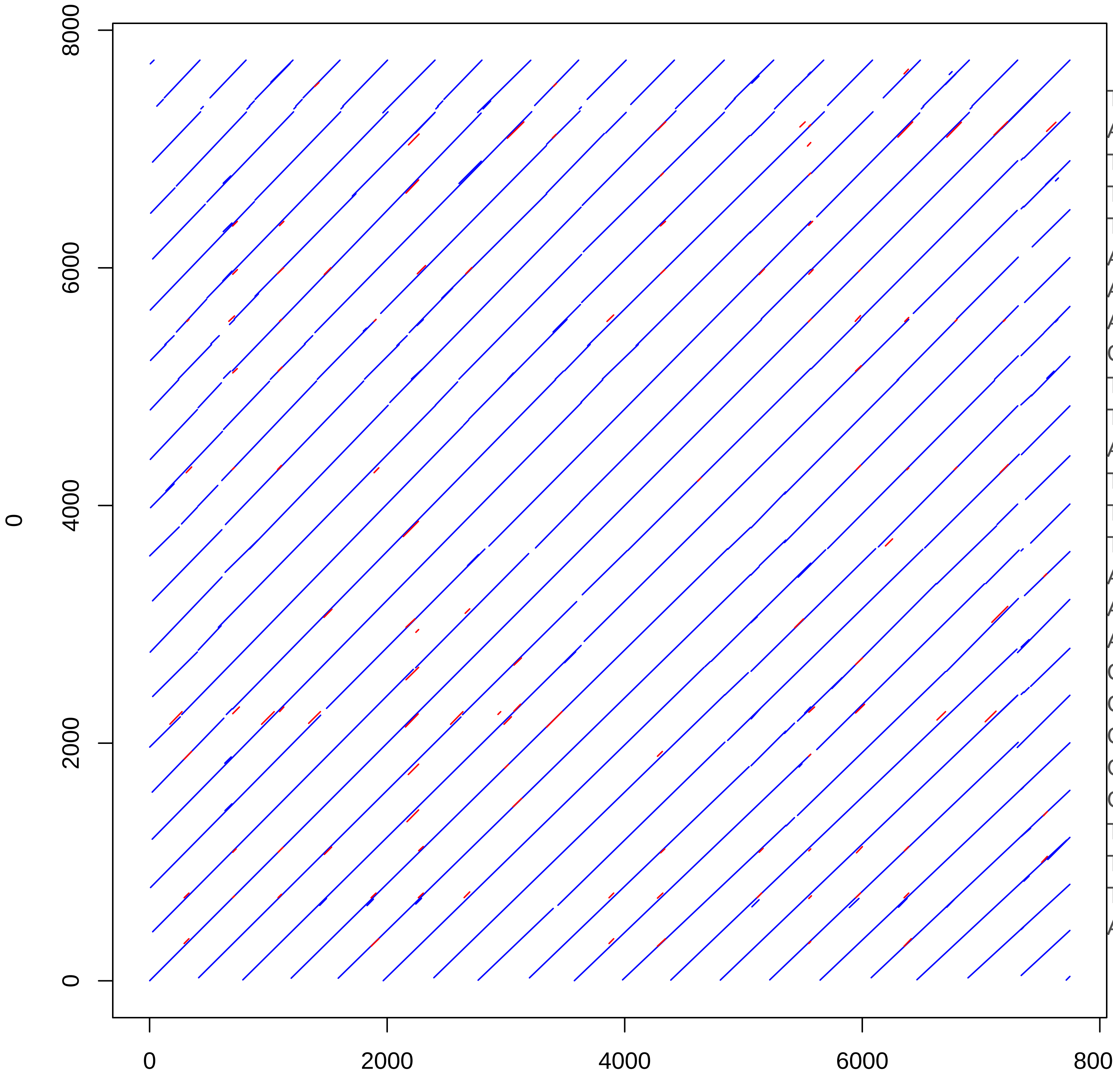

>m141120_131642_42134_c100732402550000001823141405141505_s1_p0/87892/0_11618

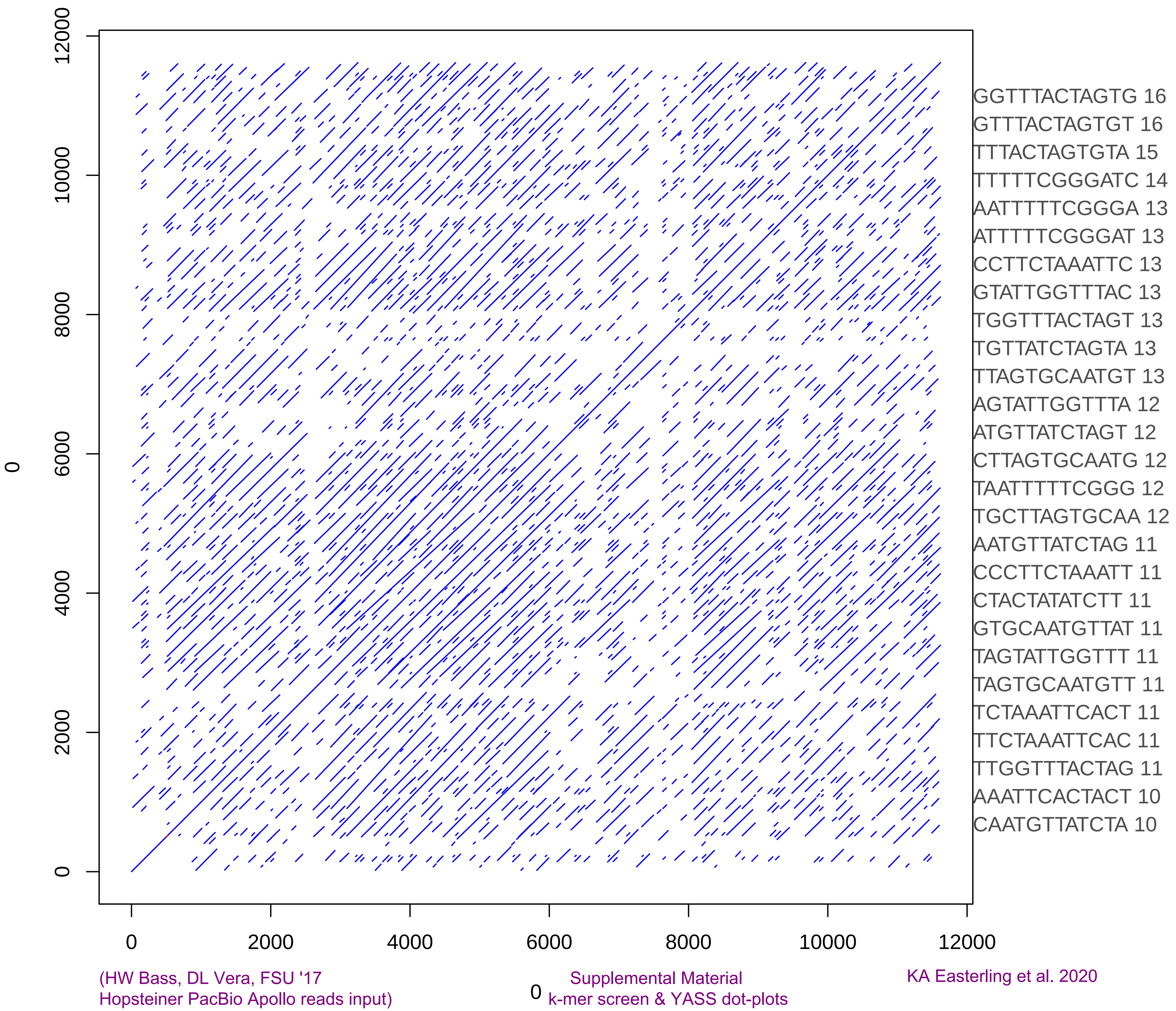

>m141120_131642_42134_c100732402550000001823141405141505_s1_p0/88600/0_7548

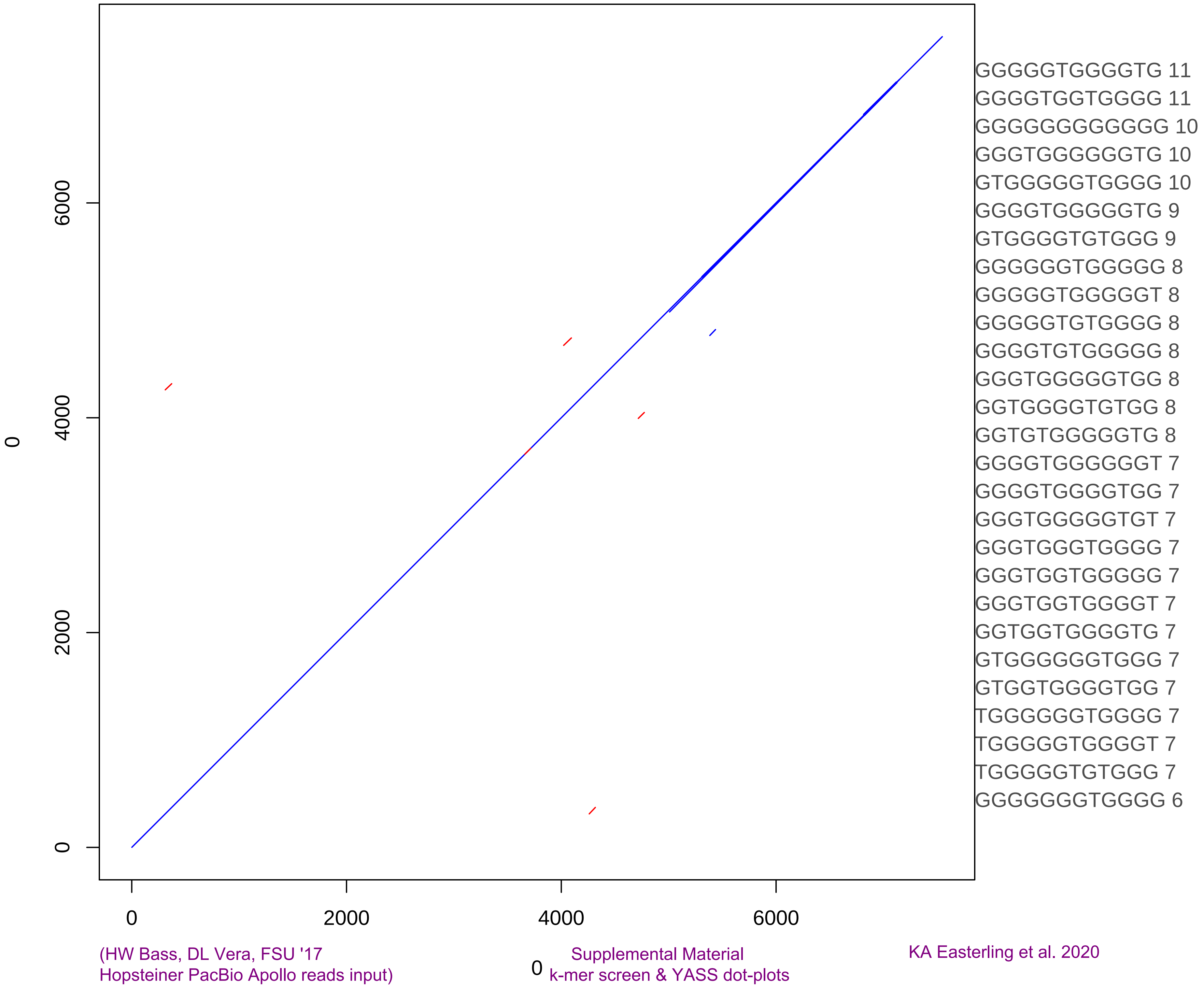

>m141120_131642_42134_c100732402550000001823141405141505_s1_p0/88681/1611_9175

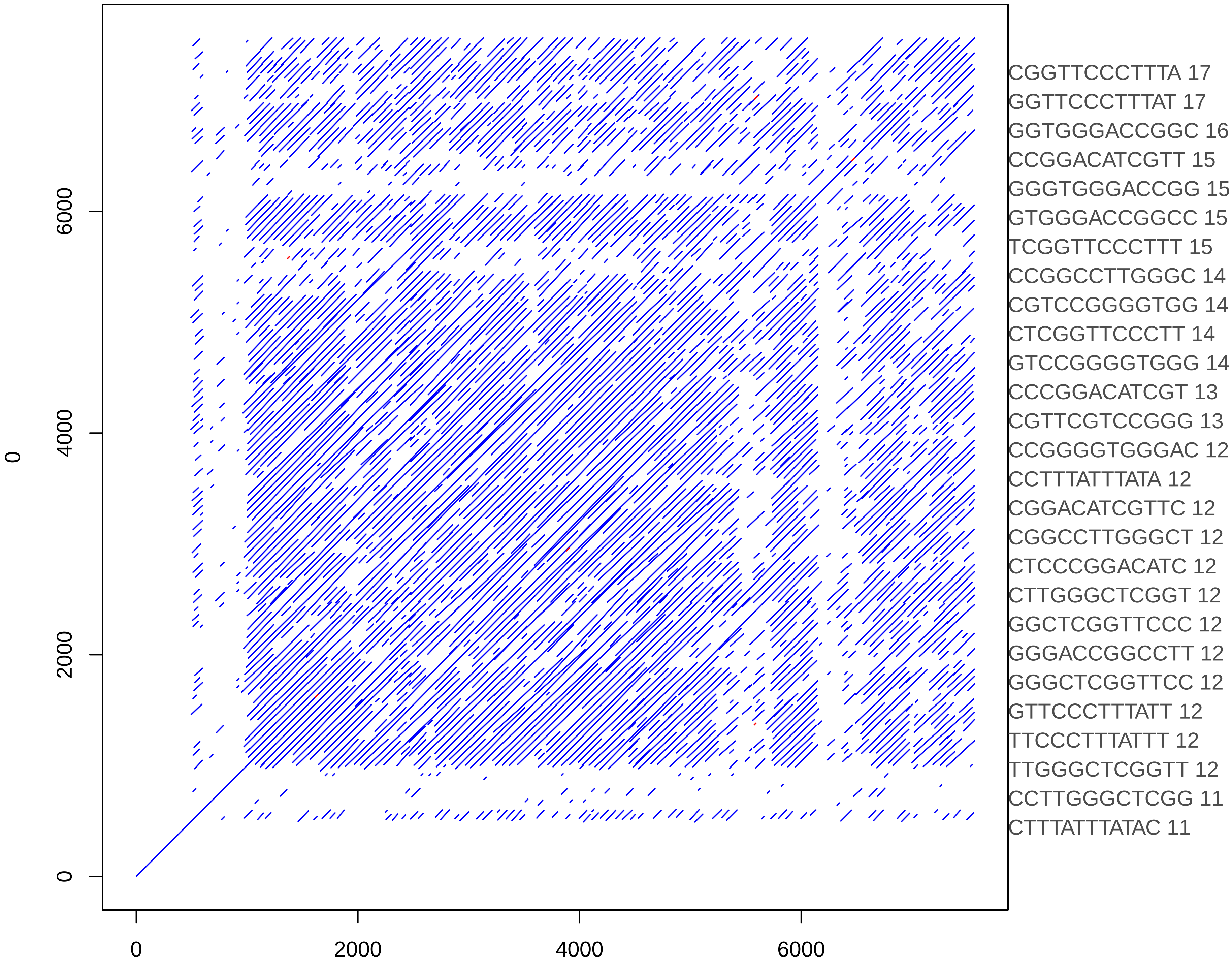

>m141120_131642_42134_c100732402550000001823141405141505_s1_p0/97888/0_16525

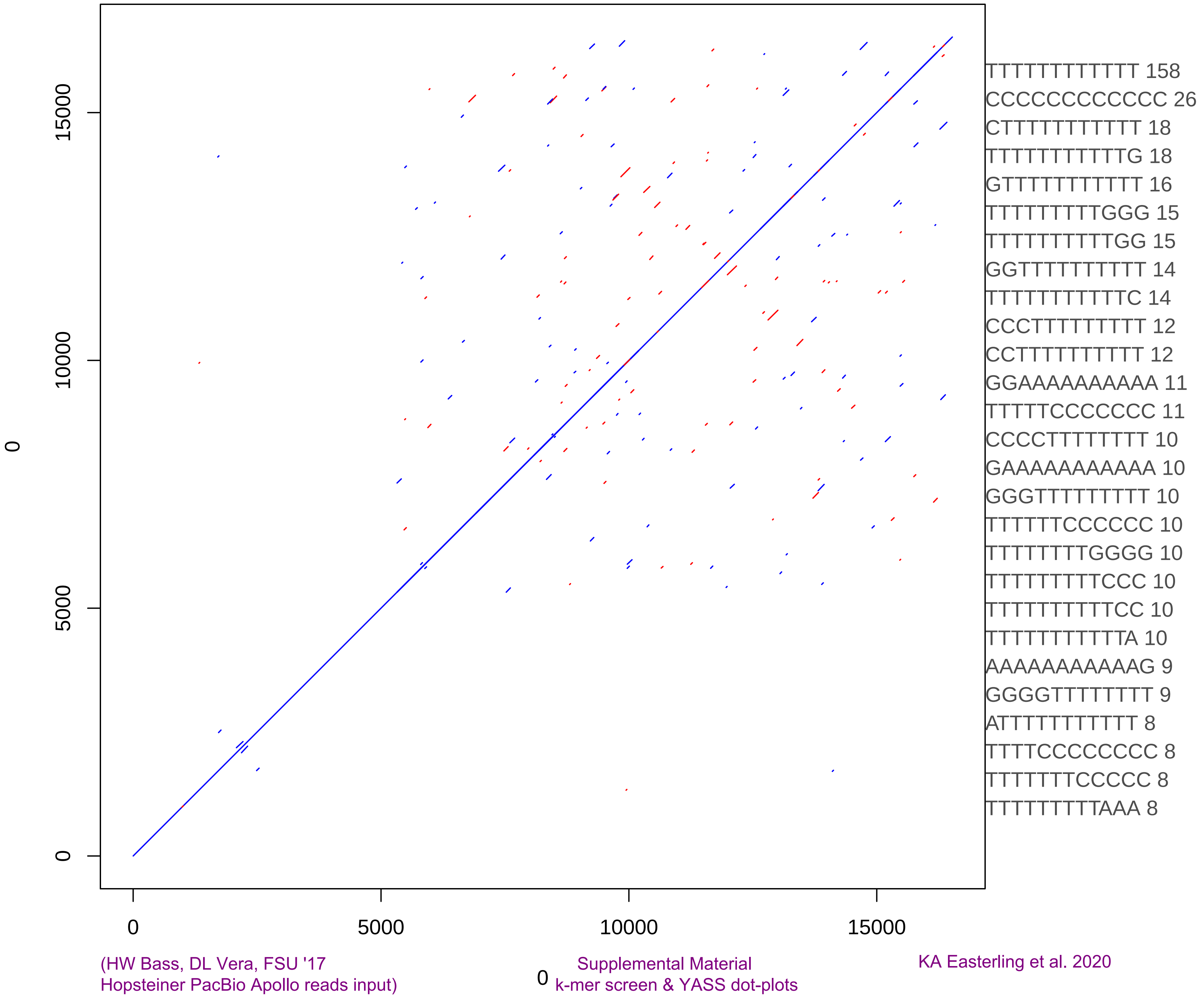

>m141121_210323_42134_c100732402550000001823141405141506_s1_p0/124662/5442_11394

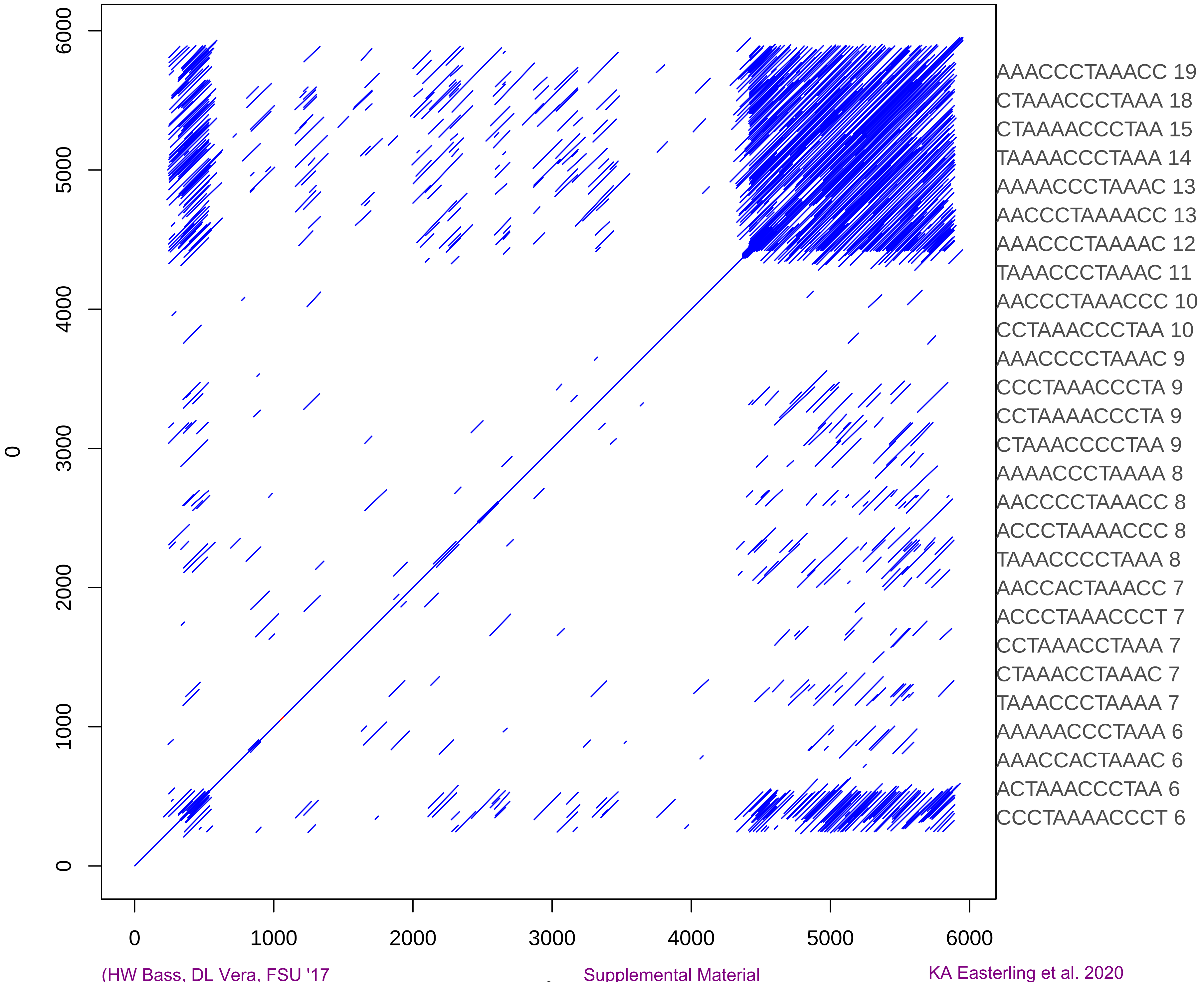

>m141121_210323_42134_c100732402550000001823141405141506_s1_p0/128664/0_9402

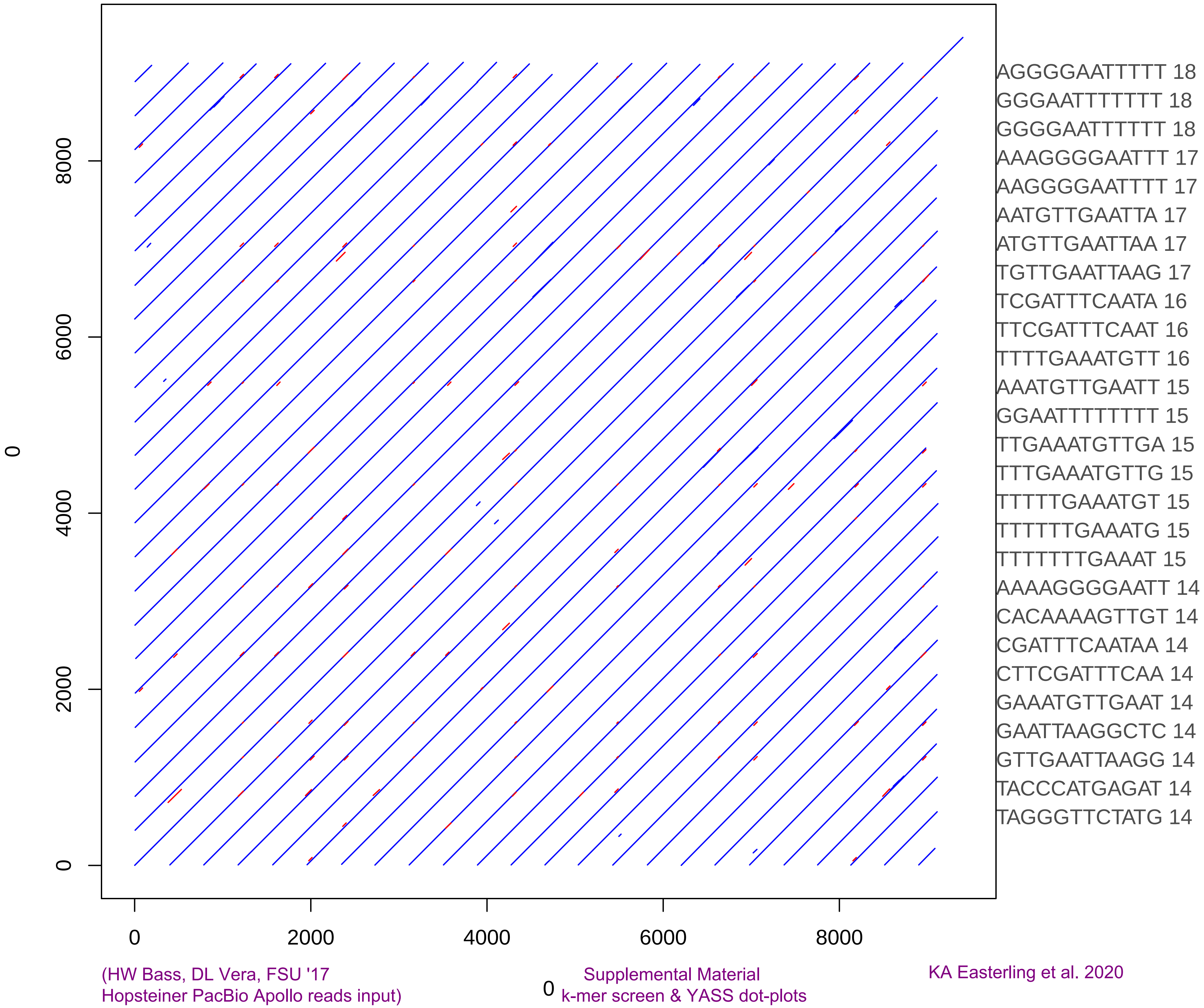

>m141121_210323_42134_c100732402550000001823141405141506_s1_p0/129545/388_6868

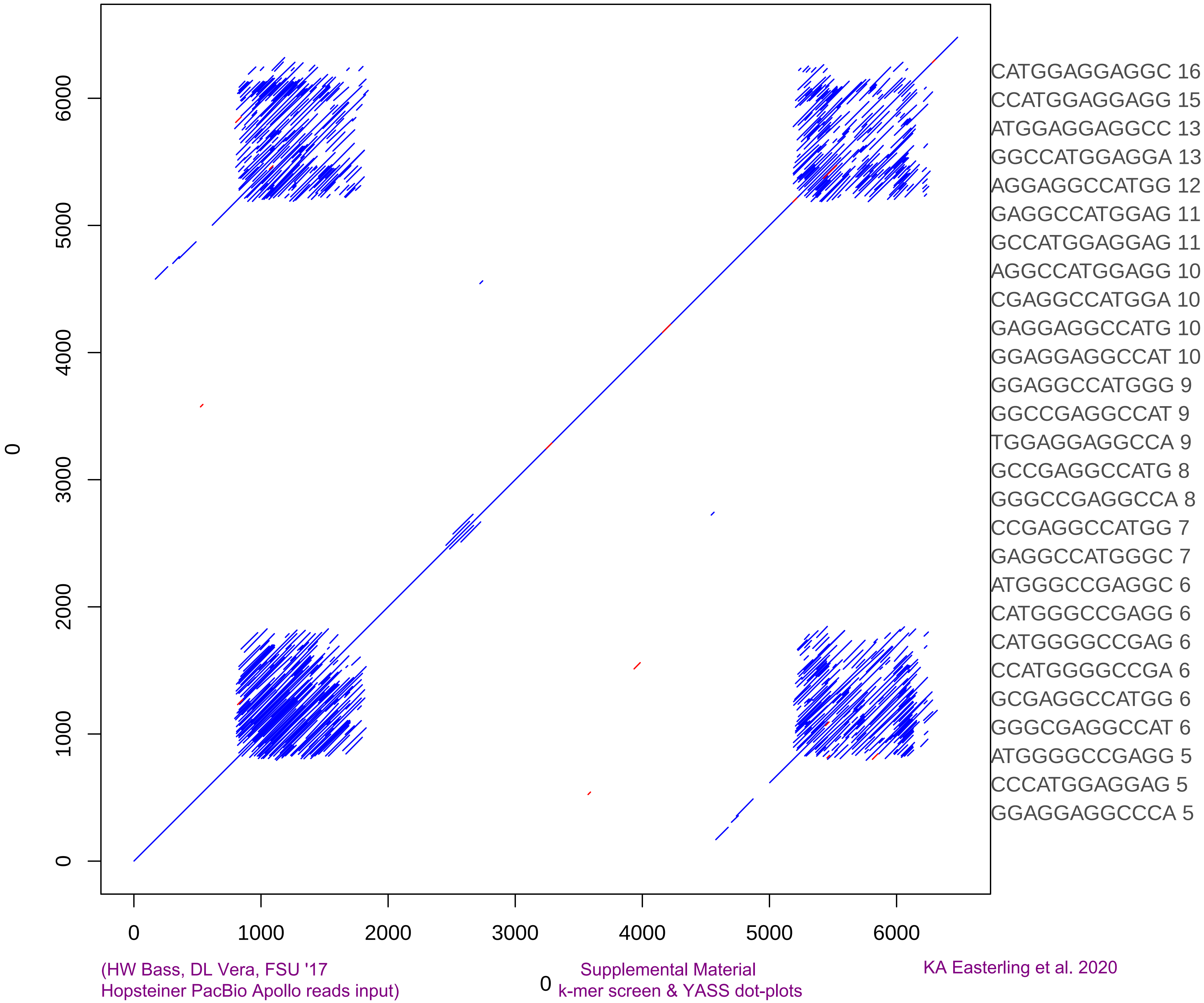

>m141121_210323_42134_c100732402550000001823141405141506_s1_p0/130969/4281_13102

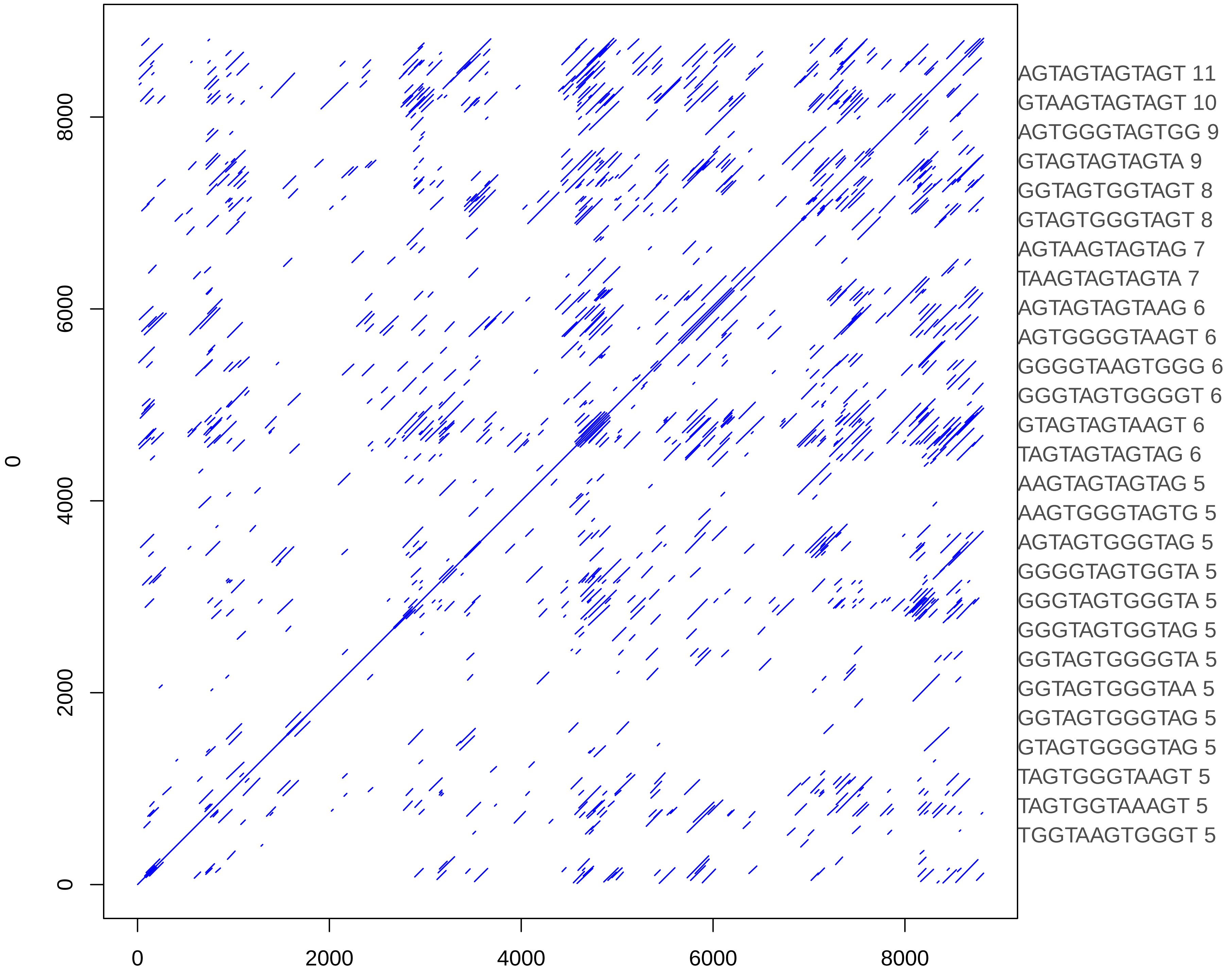

>m141121_210323_42134_c100732402550000001823141405141506_s1_p0/134133/11124_16946

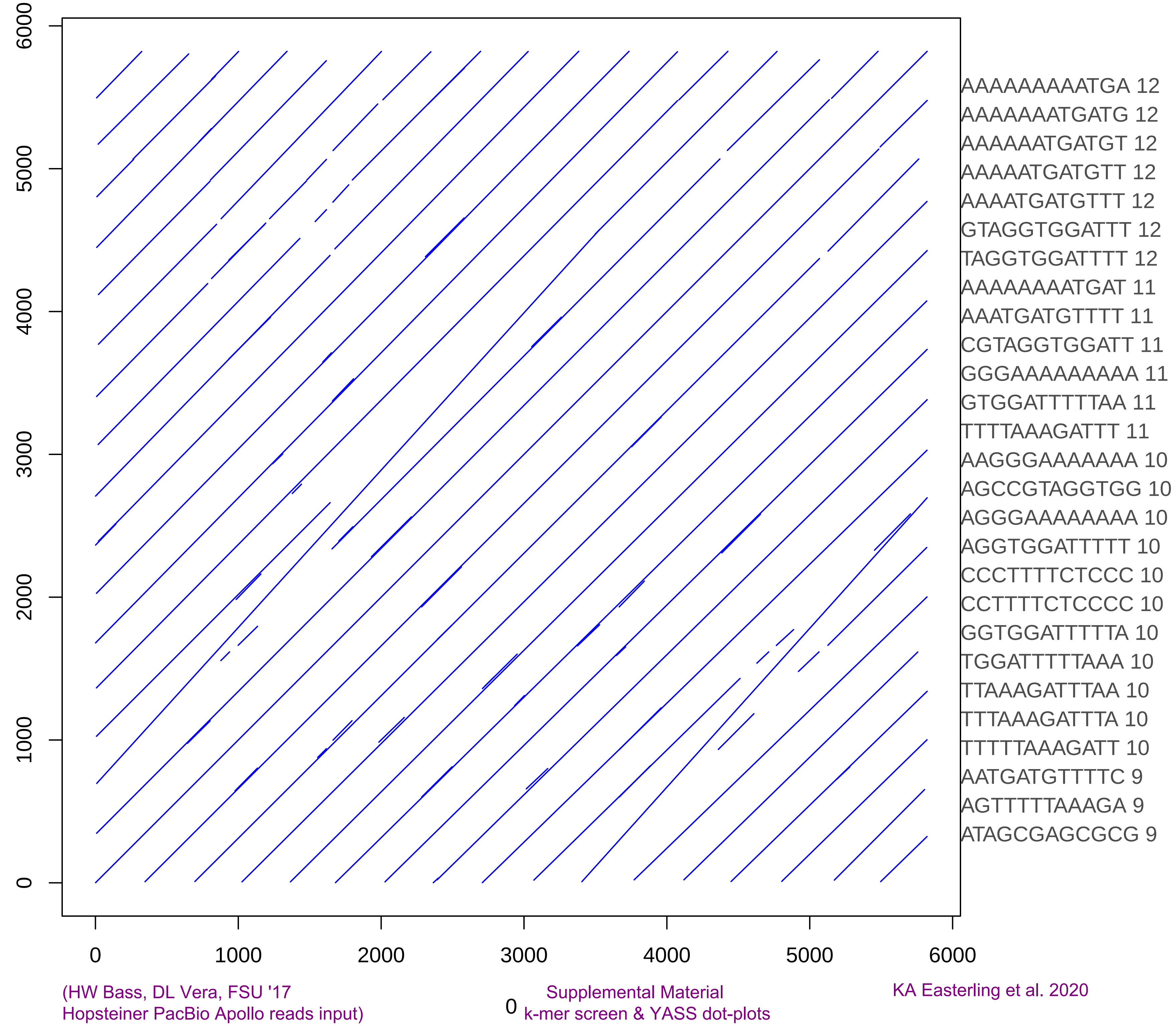

>m141121_210323_42134_c100732402550000001823141405141506_s1_p0/134133/6018_11078

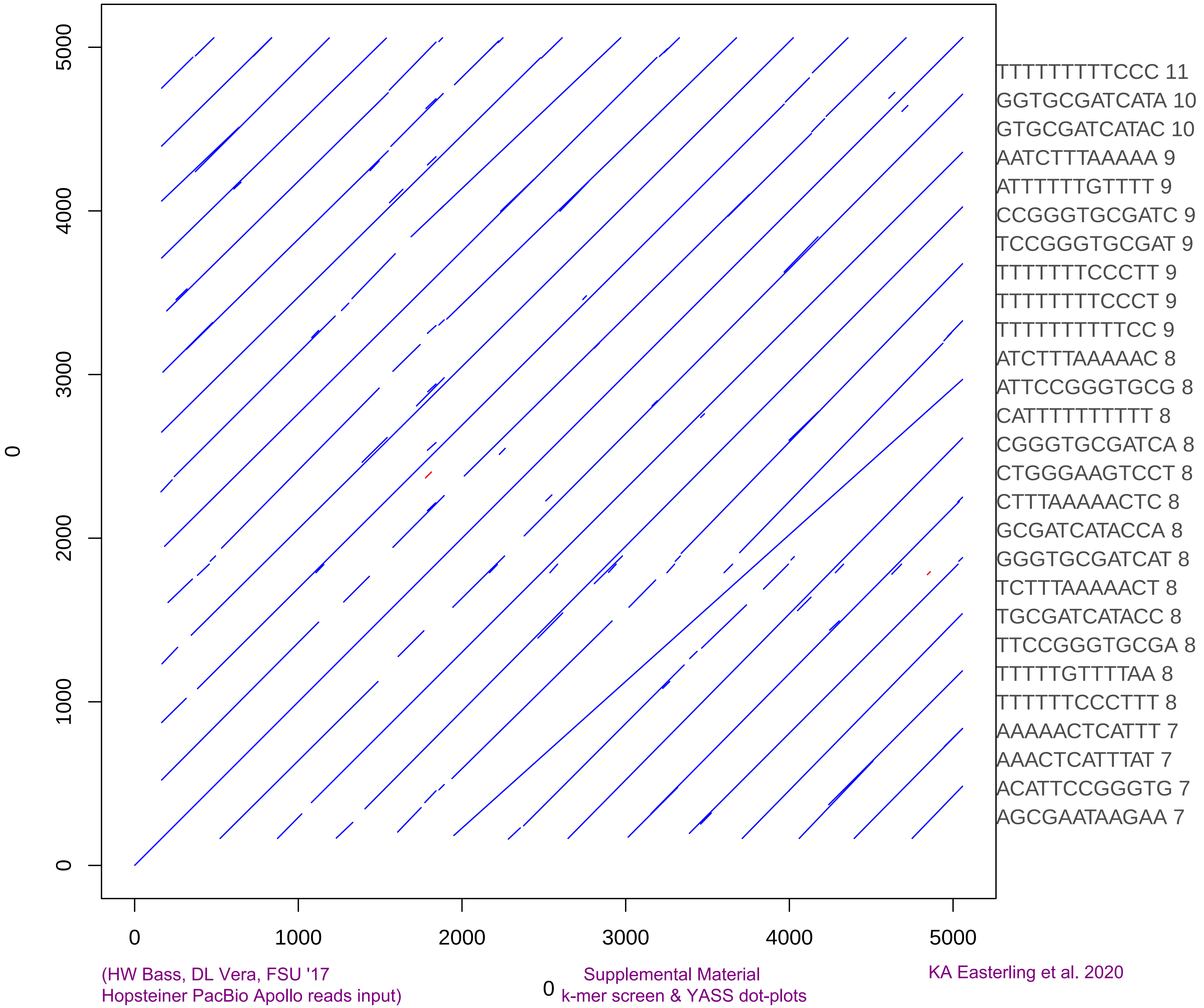

>m141121_210323_42134_c100732402550000001823141405141506_s1_p0/134577/3762_13975

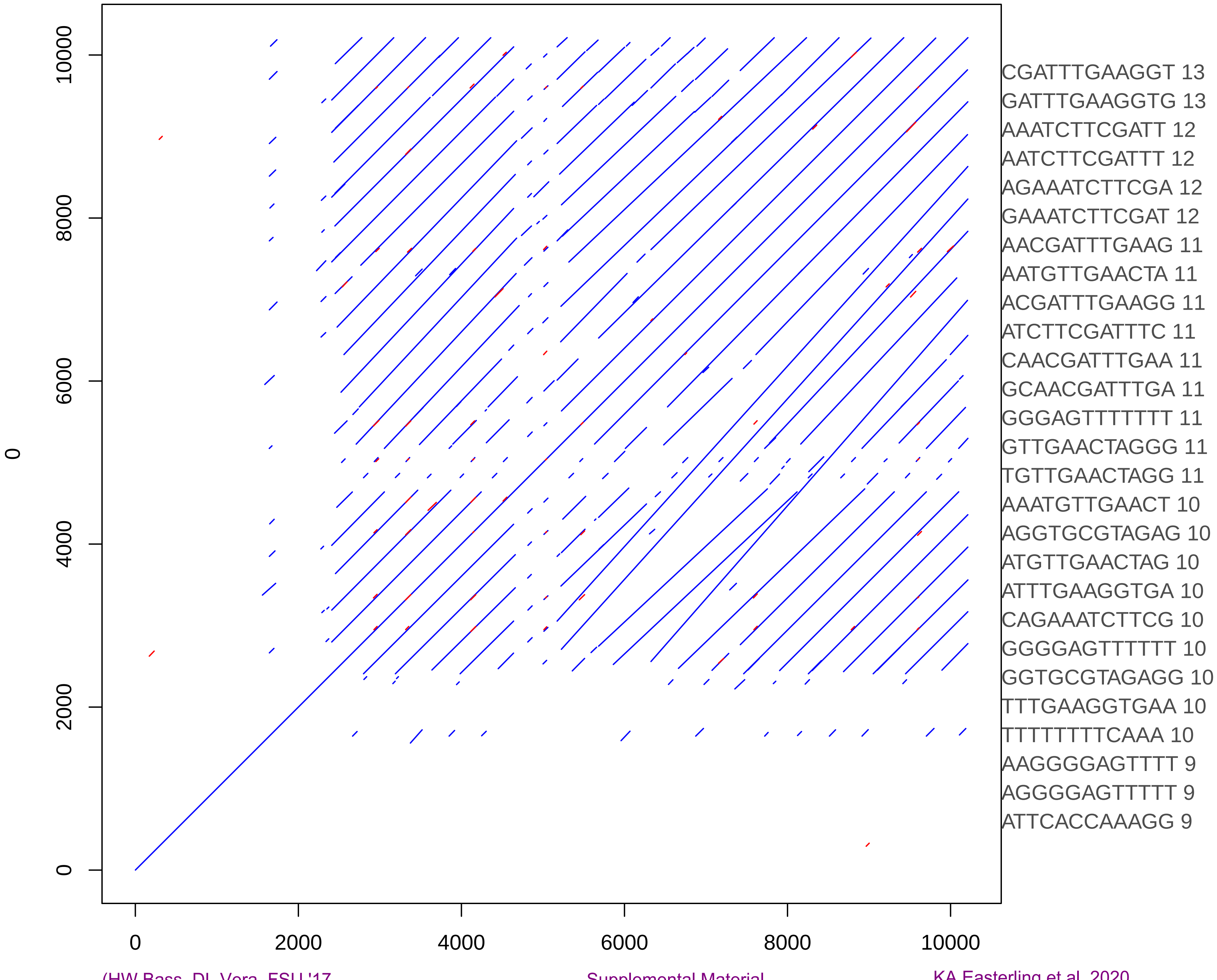

0

>m141121_210323_42134_c100732402550000001823141405141506_s1_p0/135239/9371_18524

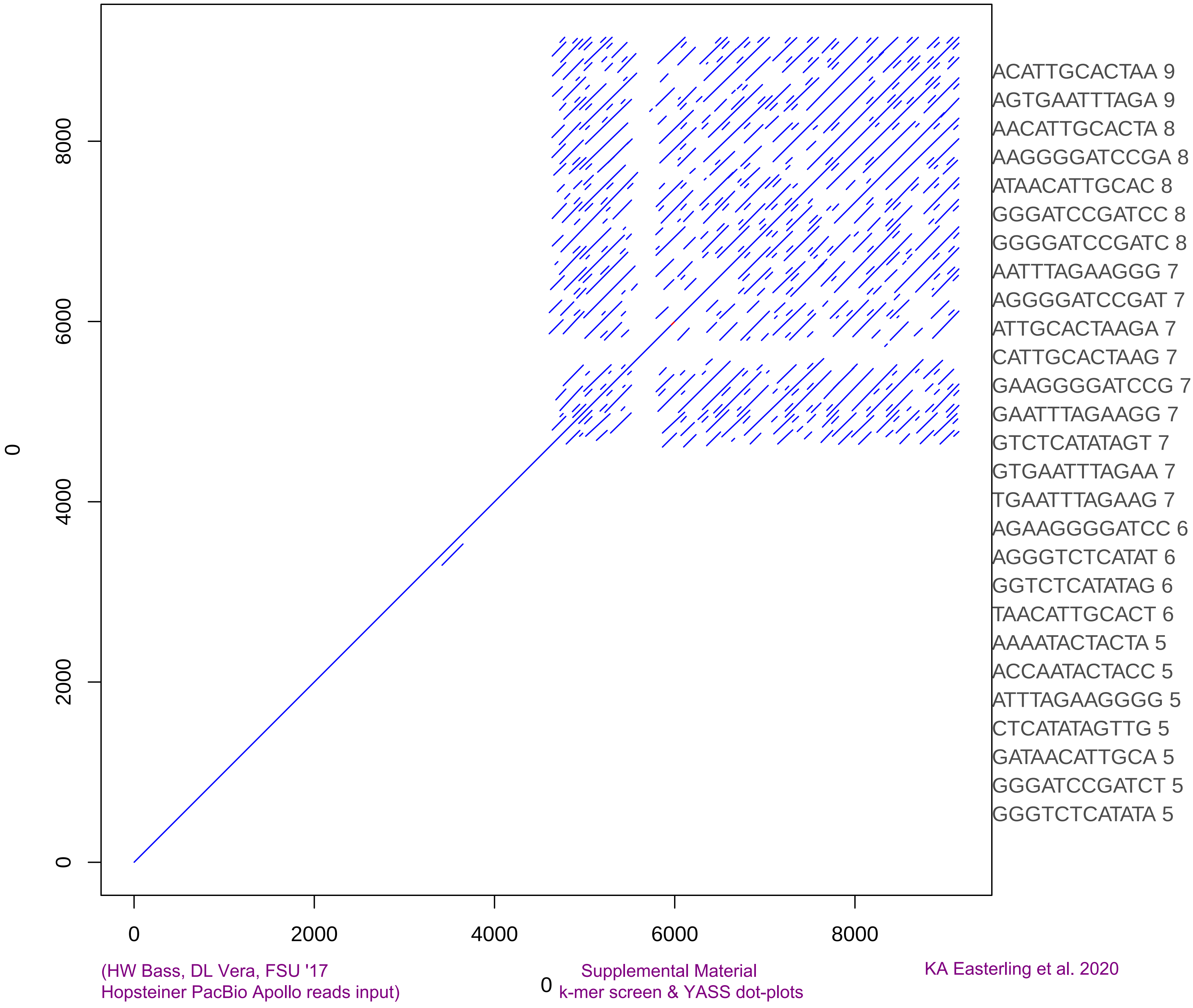

>m141121_210323_42134_c100732402550000001823141405141506_s1_p0/143869/2630_11133

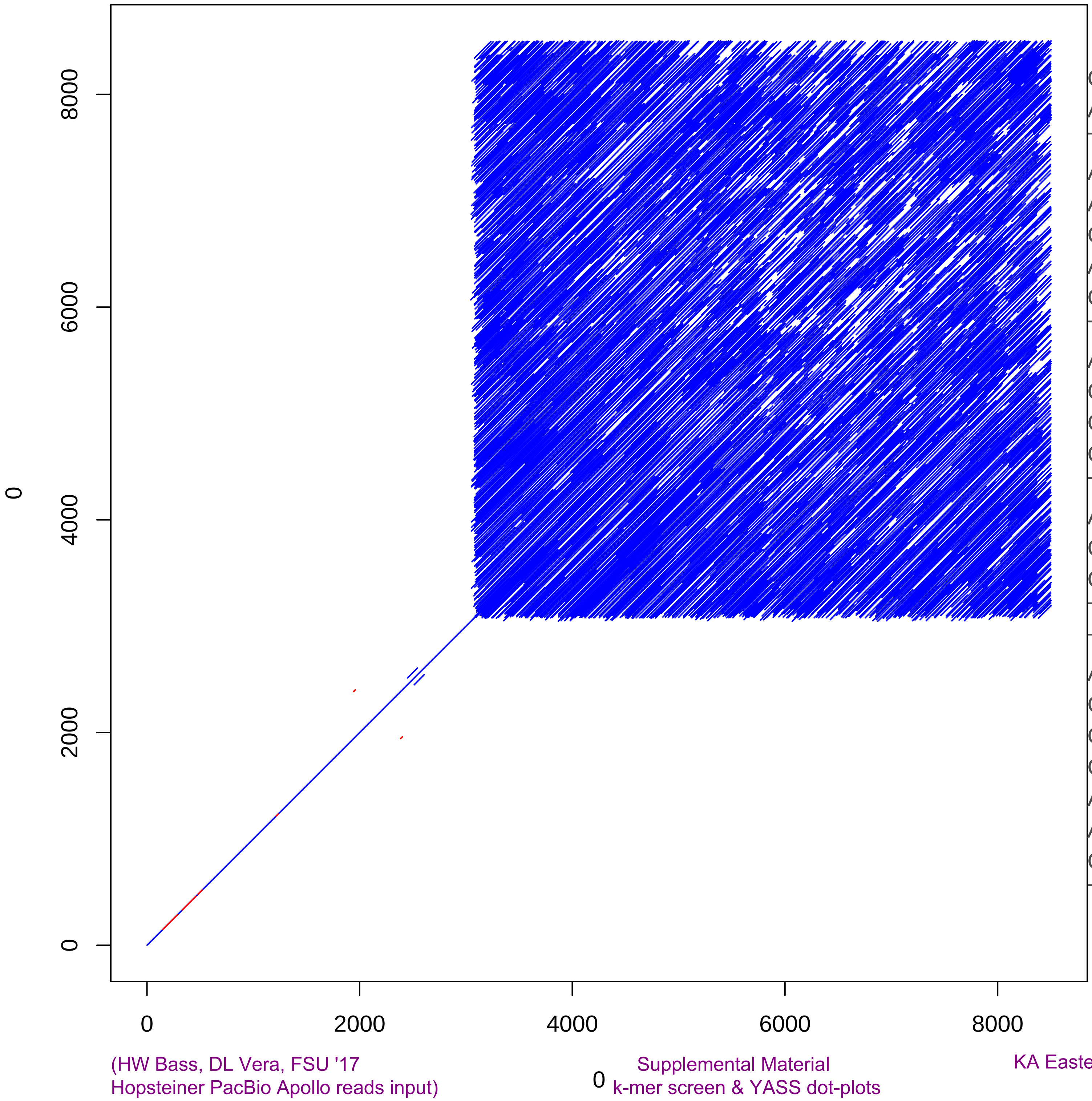

GTAGTAGTAGTA 34
AGTAGTAGTAGT 32
TAGTAGTAGTAG 31
AGTAGCAGTAGT 27
AGTAGCAGTAGC 26
CAGTAGCAGTAG 25
AGTAGTAGCAGT 24
GTAGTAGCAGTA 24
TAGTAGTAGCAG 23
AGCAGTAGCAGT 22
CAGGAGCAGTAG 22
GTAGCAGTAGCA 22
GTAGCAGTAGTA 22
TAGCAGTAGCAG 22
AGTAGTAGTAGC 21
CAGTAGTAGTAG 21
GCAGTAGCAGTA 21
TAGCAGTAGTAG 21
TAGTAGCAGTAG 21
AGCAGGAGCAGT 20
GCAGGAGCAGTA 20
GCAGTAGTAGTA 20
GTAGTAGTAGCA 20
AGCAGTAGTAGT 19
AGGAGCAGTAGC 18
GGAGCAGTAGCA 18
TAGCAGGAGCAG 17

>m141121_210323_42134_c100732402550000001823141405141506_s1_p0/146024/0_7503

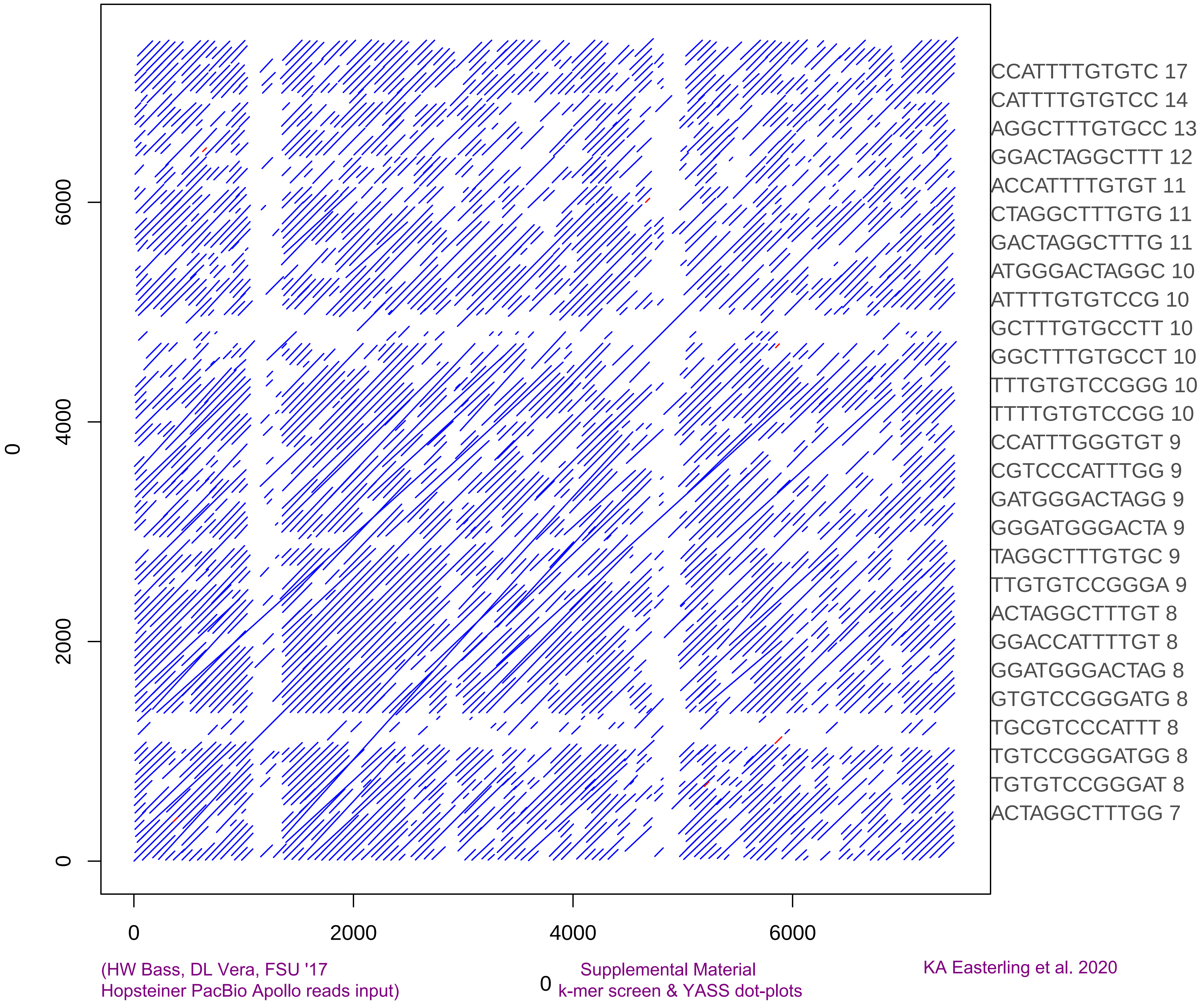

>m141121_210323_42134_c100732402550000001823141405141506_s1_p0/14838/0_6005

Representative Read (Table 1): (HSR0) HuluTR180-r120, 163 ppm,
62% A+T, GenBank Acc. MH188533.1

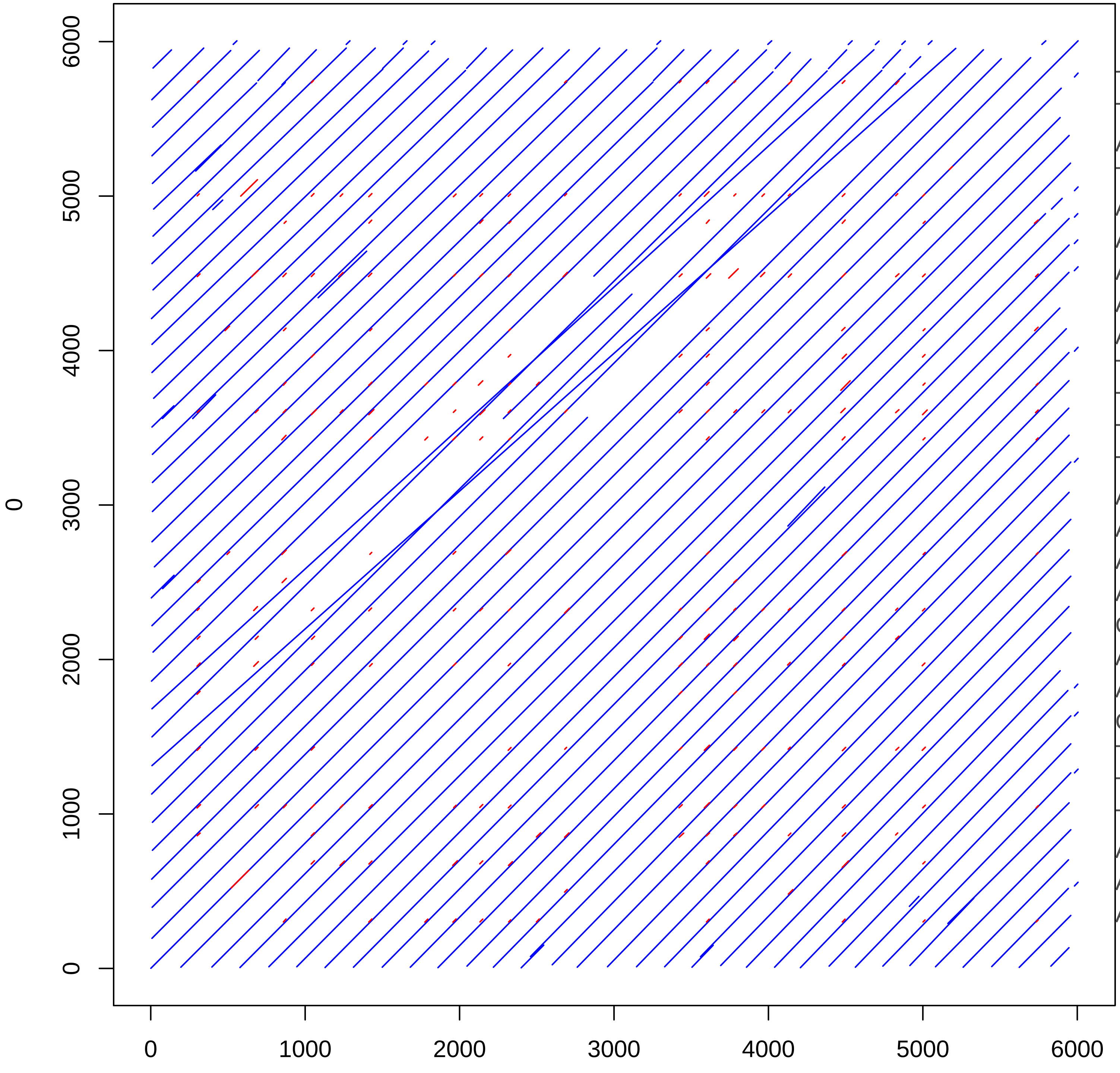

TTTTTTTGTTTG 21
TTTTTTGTTTGG 20
AAAAAATTAGAG 19
TTTTGTTTGGTT 19
AAAAATTAGAGC 18
AAAATTAGAGCT 18
AAGGTTCAAAAA 18
AGGTTCAAAAAA 18
ATTTTTTTGTTT 18
TGTTTGGTTGAA 18
TTGGTTGAATTA 18
TTTGGTTGAATT 18
TTTTTGTTTGGT 18
AAAGGTTCAAAA 17
AAATTAGAGCTT 17
ATATTATTGAAA 17
ATTATTGAAATG 17
GTTTGGTTGAAT 17
AAGGCCAATTTT 16
AATTTTTTTGTT 16
GTGAATTACGAG 16
TATTATTGAAAT 16
TTGTTTGGTTGA 16
TTTGTTTGGTTG 16
AGAGCTTGTTAG 15
AGGCCAATTTTT 15
AGTGAATTACGA 15

>m141121_210323_42134_c100732402550000001823141405141506_s1_p0/153579/772_11761

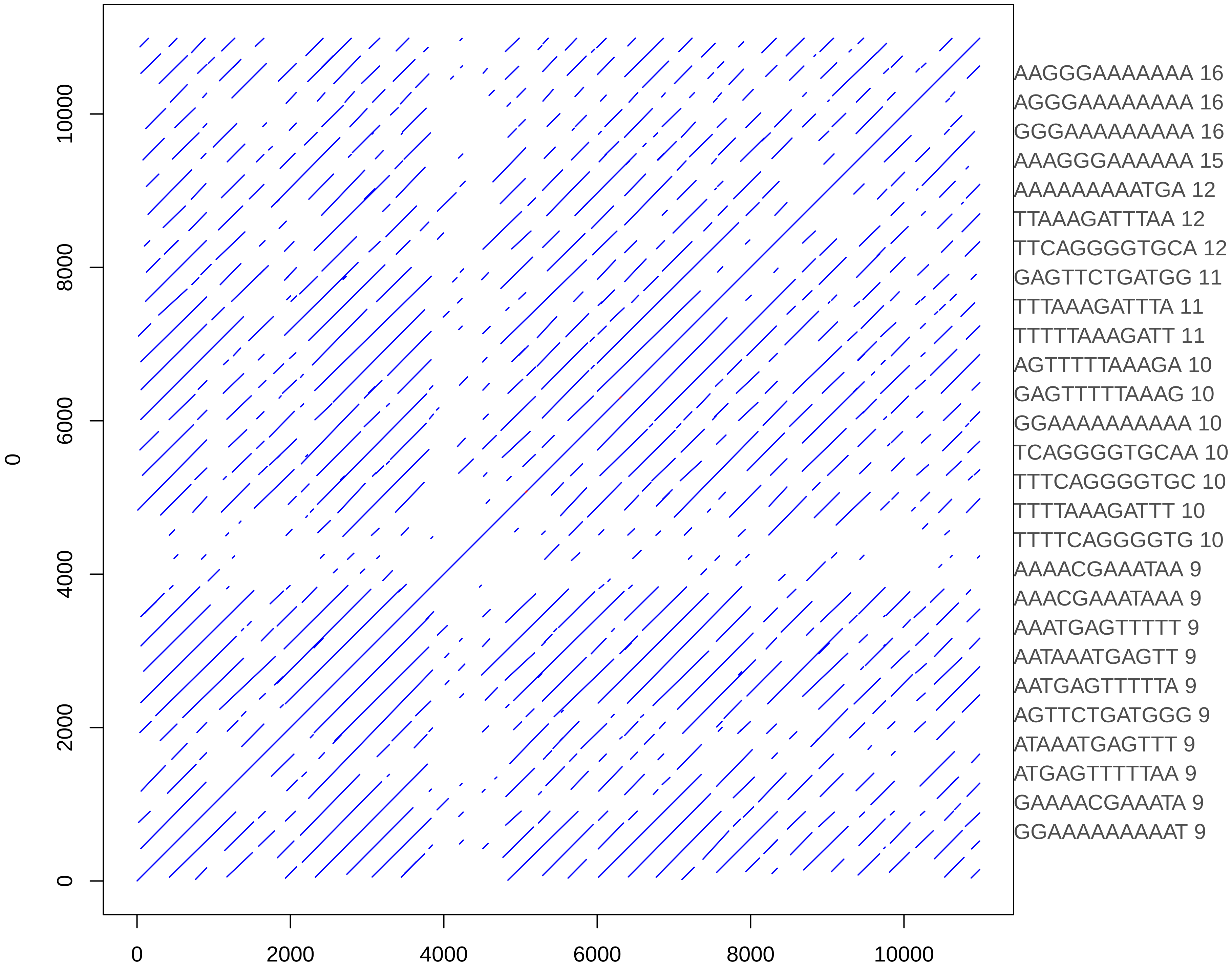

>m141121_210323_42134_c100732402550000001823141405141506_s1_p0/31095/4755_10244

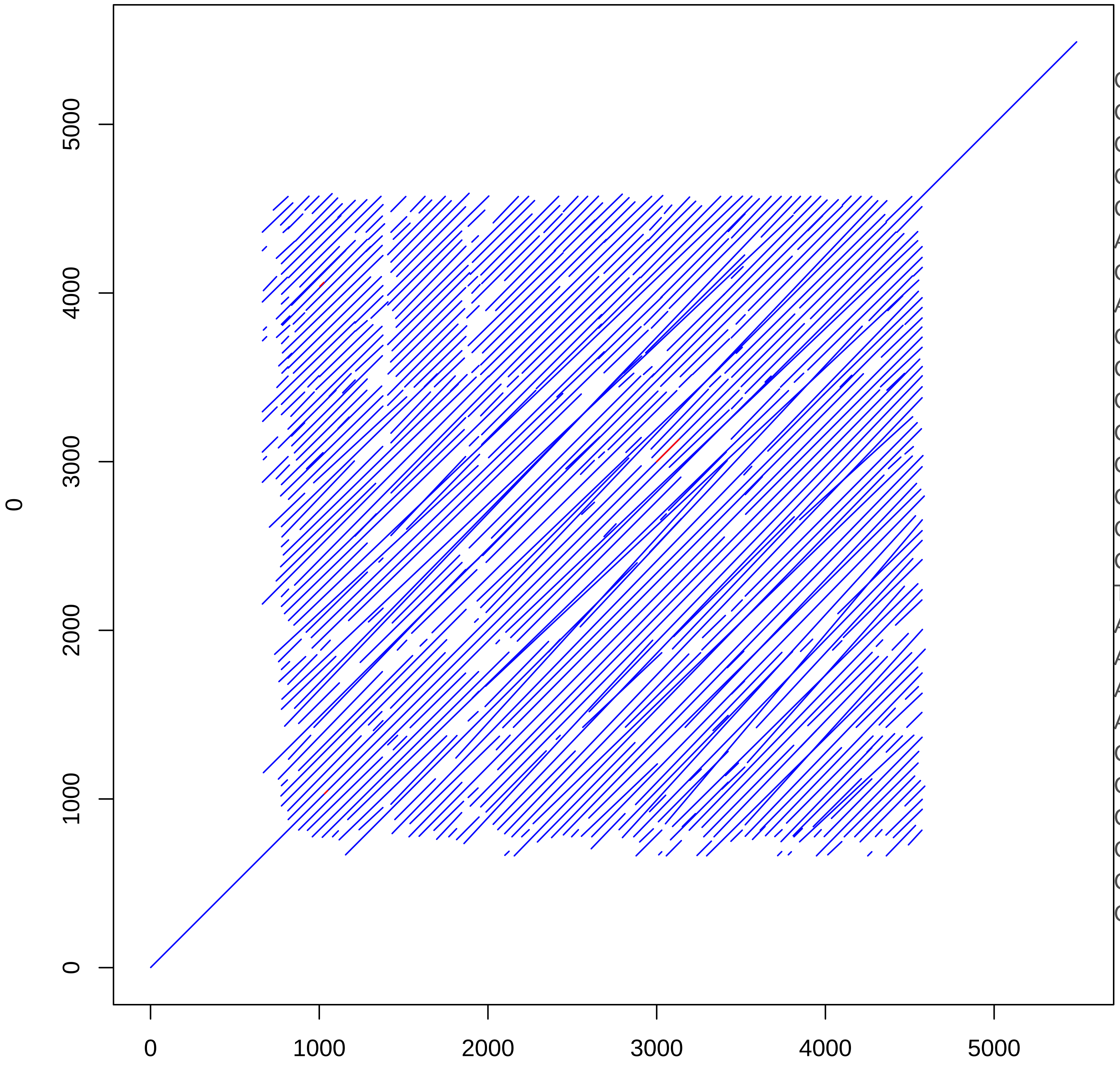

CCAAGGCCGGTC 19
CAAGGCCGGTCC 17
CCCAAGGCCGGT 17
CCCGGACGAACG 15
CCGGACGAACGA 15
AGCCCAAGGCCG 14
GCCCAAGGCCGG 14
AAGGCCGGTCCC 13
CCGGTCCCACCC 13
CGAGCCCAAGGC 13
GAGCCCAAGGCC 13
CACCCCGGACGA 12
CCACCCCGGACG 12
CCCACCCCGGAC 12
GCCGGTCCCACC 12
CCCCGGACGAAC 11
TCCCACCCCGGA 11
AAGGGAACCGAG 10
ACCGAGCCCAAG 10
ACGATGTCCGGG 10
AGGGAACCGAGC 10
CCGGGAGTATAA 10
CGGACGAACGAT 10
CGGTCCCACCCC 10
GGCCGGTCCCAC 10
GGGAACCGAGCC 10
GTCCCACCCCGG 10

>m141121_210323_42134_c100732402550000001823141405141506_s1_p0/32238/3999_11710

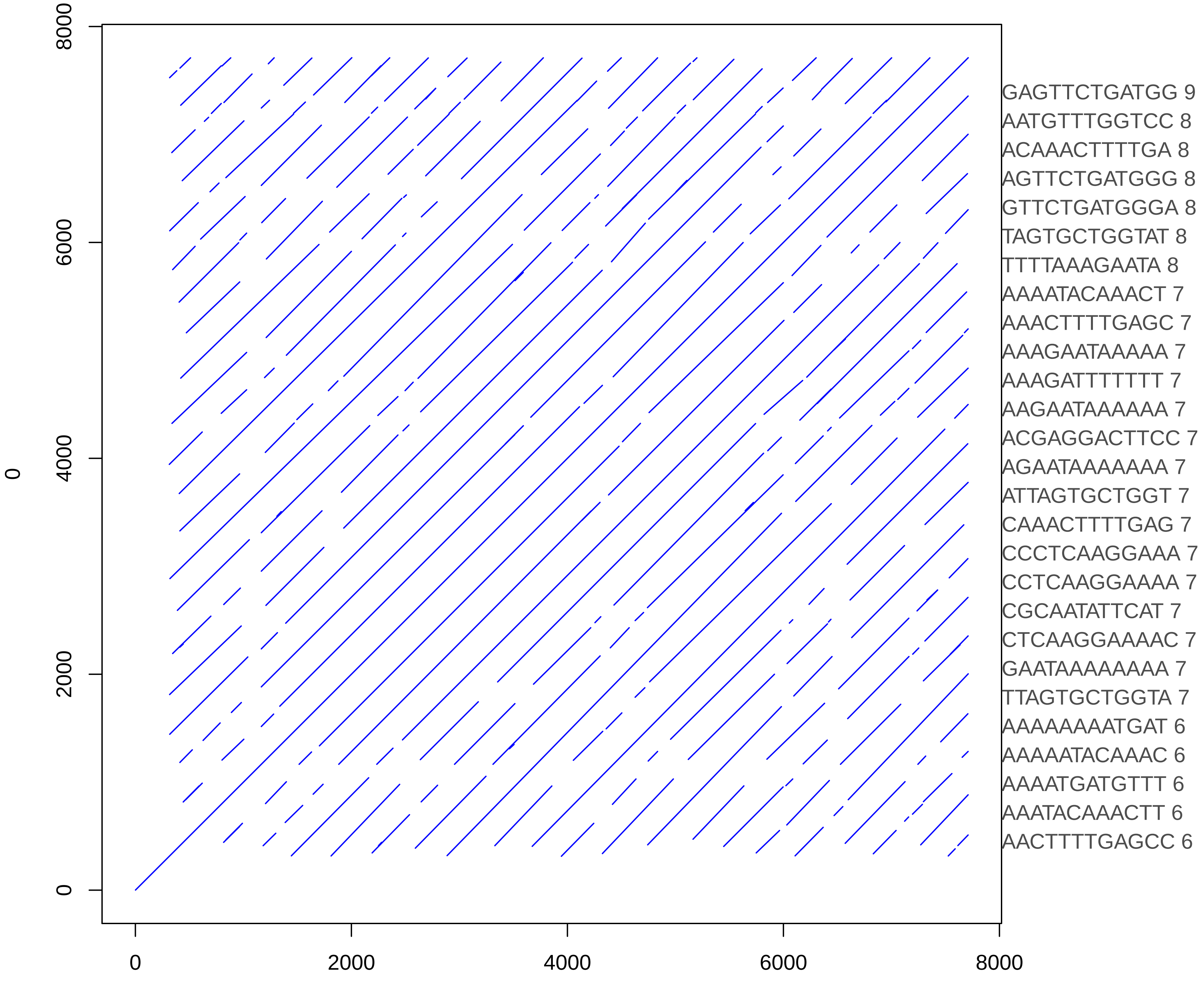

>m141121_210323_42134_c100732402550000001823141405141506_s1_p0/33917/6925_12881

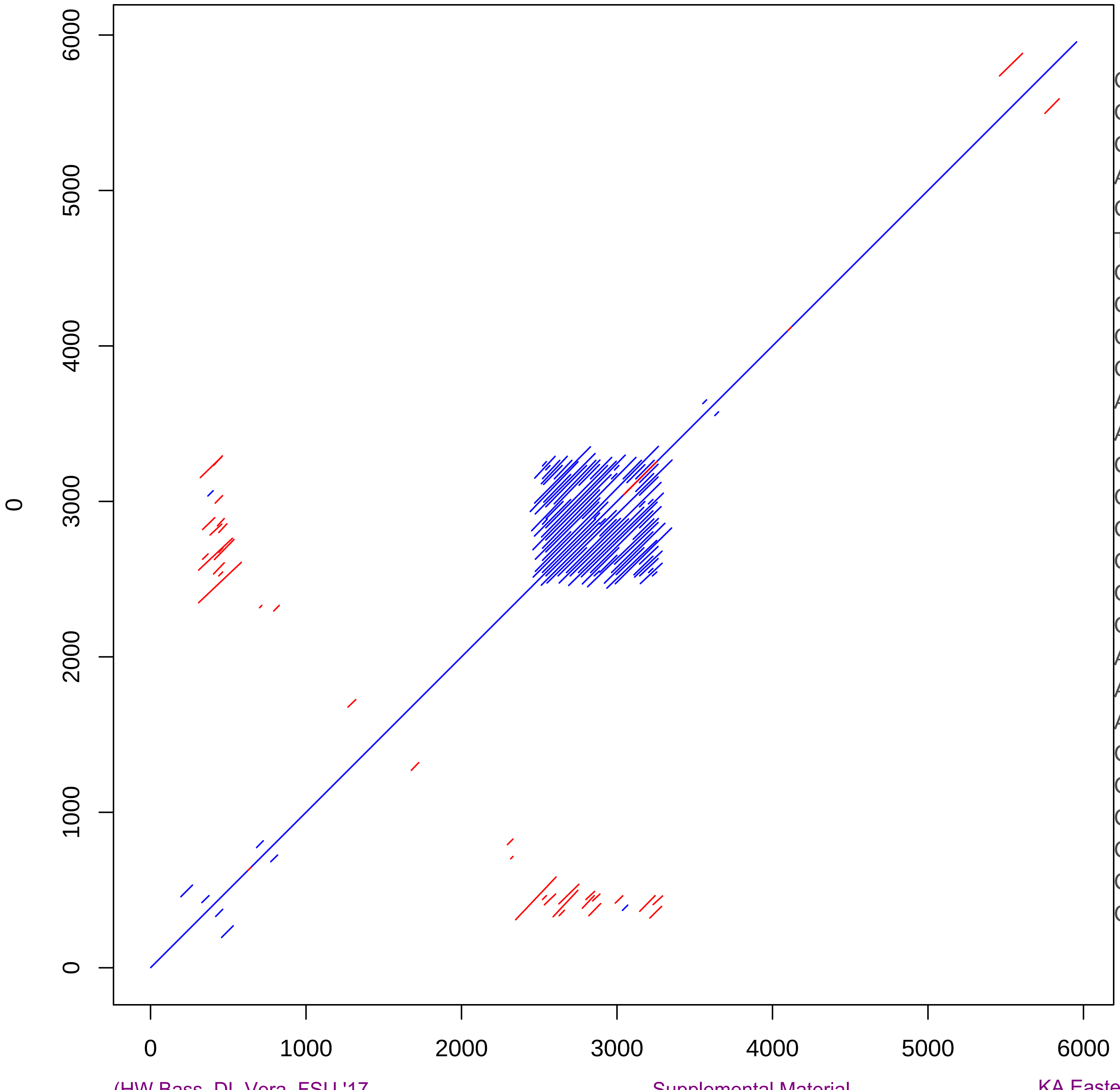

CGCATGCGGGGG 12
GCATGCGGGGGA 12
CATGCGGGGGAA 10
ATGCGGGGGAAG 9
GCGGGGGAAGGG 9
TGCGGGGGAAGG 9
GCGCATGCGGGG 8
GGCGCATGCGGG 6
GGGAAGGGTAGC 6
GGGGAAGGGTAG 6
AAGGGCAGCCTC 5
AGGCGCATGCGG 5
CGGGGGAAGGGT 5
GAGACCCTTGTG 5
GCAGCCTCGCAT 5
GGAAGGGTAGCC 5
GGCAGCCTCGCA 5
GGGGGAAGGGTA 5
AGACCCTTGTGG 4
AGCCTCGCATAG 4
AGCCTCGCATGC 4
CAGCCTCGCATA 4
CAGGCGCATGCG 4
CCTCGCATAGTG 4
CCTCGCATGCGG 4
CTCGCATGCGGG 4
GAAGGGCAGCCT 4

>m141121_210323_42134_c100732402550000001823141405141506_s1_p0/34176/320_8391

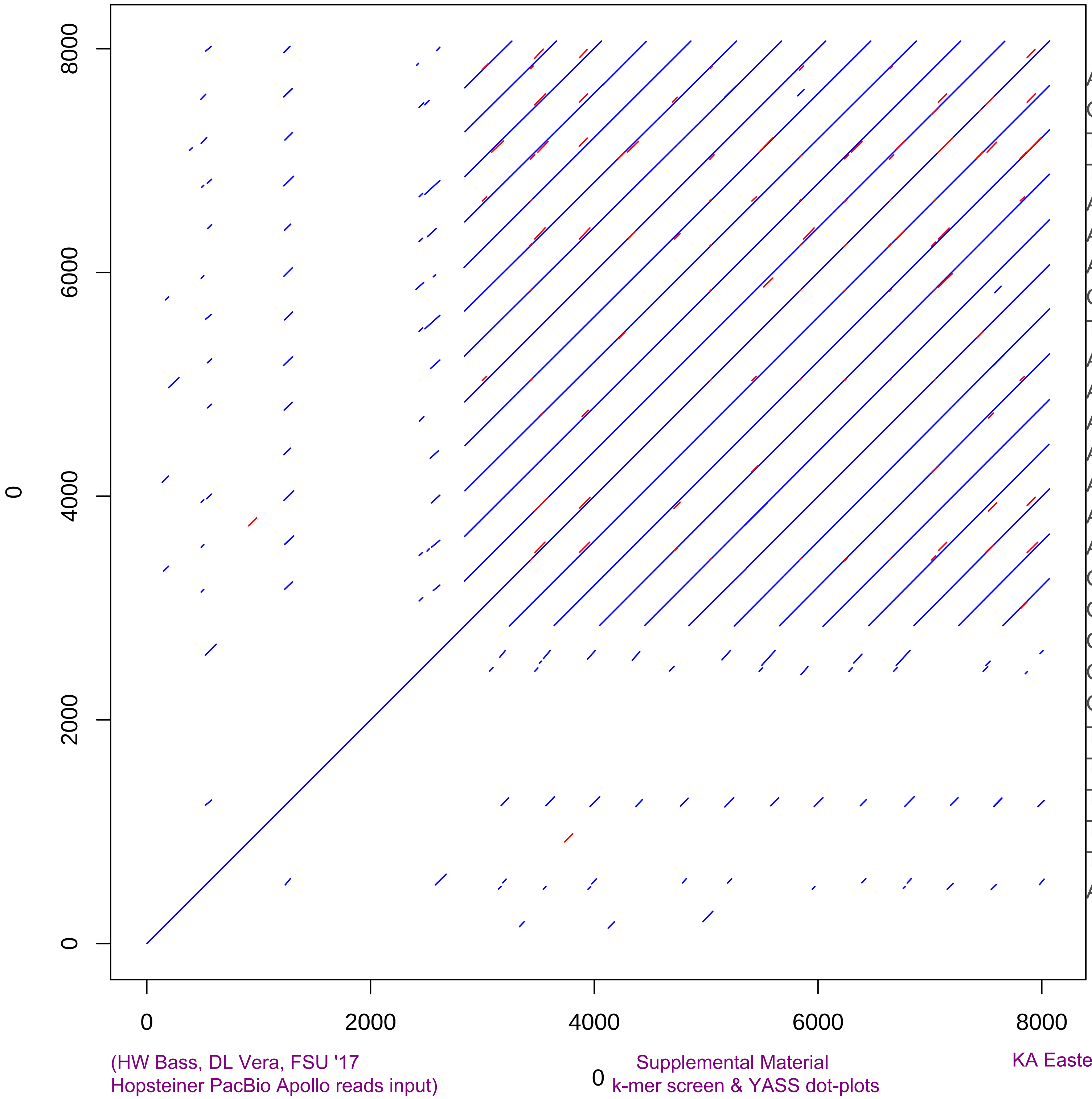

ATTTCAAAAAAA 11
CATTTCAAAAAA 10
TTCAAAAAAAAA 10
TTTCAAAAAAAA 10
AAAAAAAATTCC 9
AAAAAAATTCCC 9
ATATATTGGAGT 9
CCCCTTTGGTGA 9
TTTTTCCTTATA 9
AAAAAATTCCCC 8
AATATATTGGAG 8
AATGTATTACCC 8
ACATTTCAAAAA 8
ACCCCTTTGGTG 8
ATGGCAATTTTC 8
ATTTTGACTGTA 8
CCAAGAGATCTC 8
CCCAAGAGATCT 8
CCCCAAGAGATC 8
CCCTTTGGTGAA 8
GGGTACCCCTTT 8
TATATTGGAGTC 8
TATTTTGACTGT 8
TCAAAAAAAAAT 8
TGGCAATTTTCG 8
TTTTCCTTATAT 8
AAAAAAAAATTC 7

>m141121_210323_42134_c100732402550000001823141405141506_s1_p0/4896/0_6351

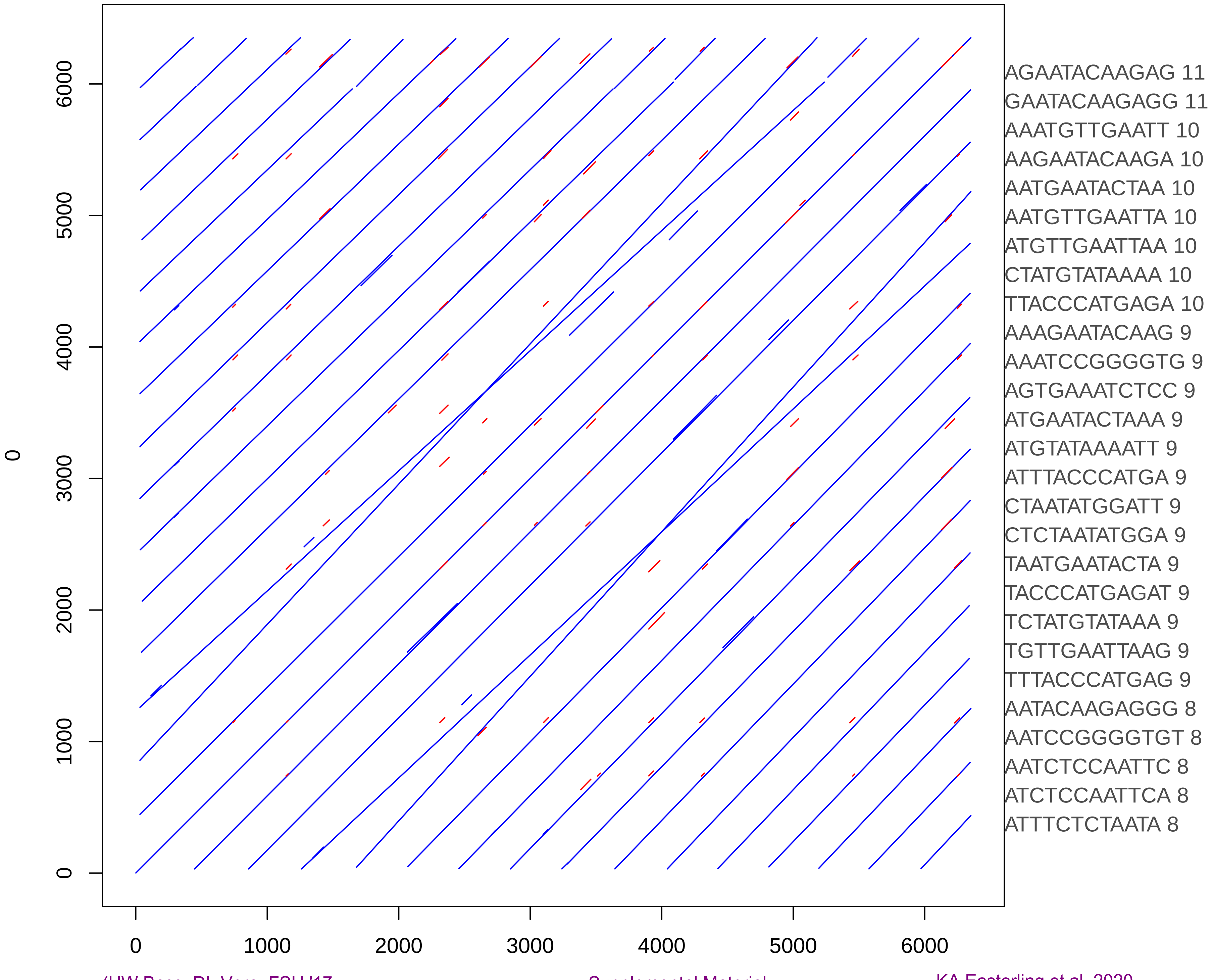

>m141121_210323_42134_c100732402550000001823141405141506_s1_p0/60415/12404_19582

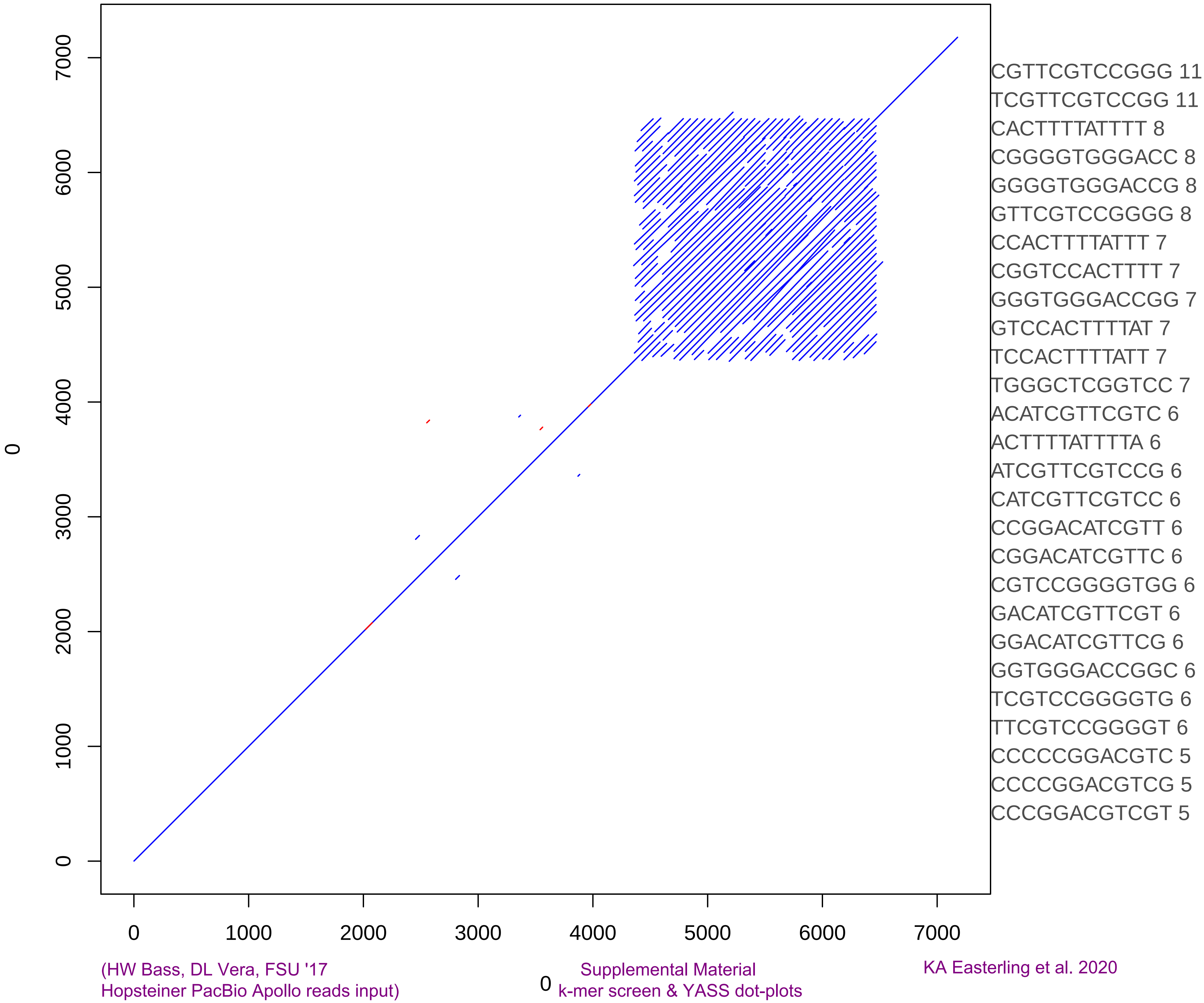

>m141121_210323_42134_c100732402550000001823141405141506_s1_p0/64635/7028_16525

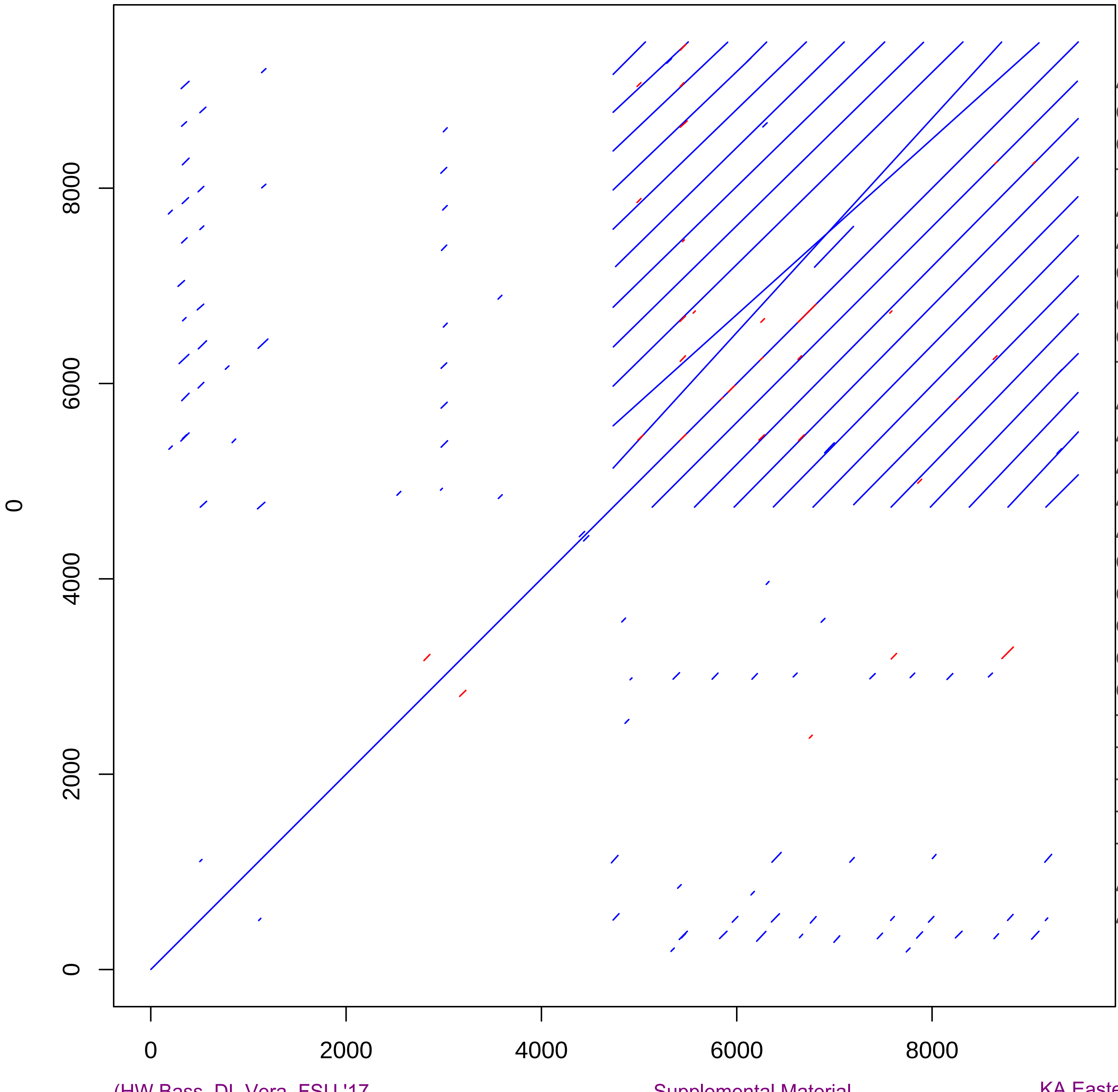

>m141121_210323_42134_c100732402550000001823141405141506_s1_p0/67577/0_7633

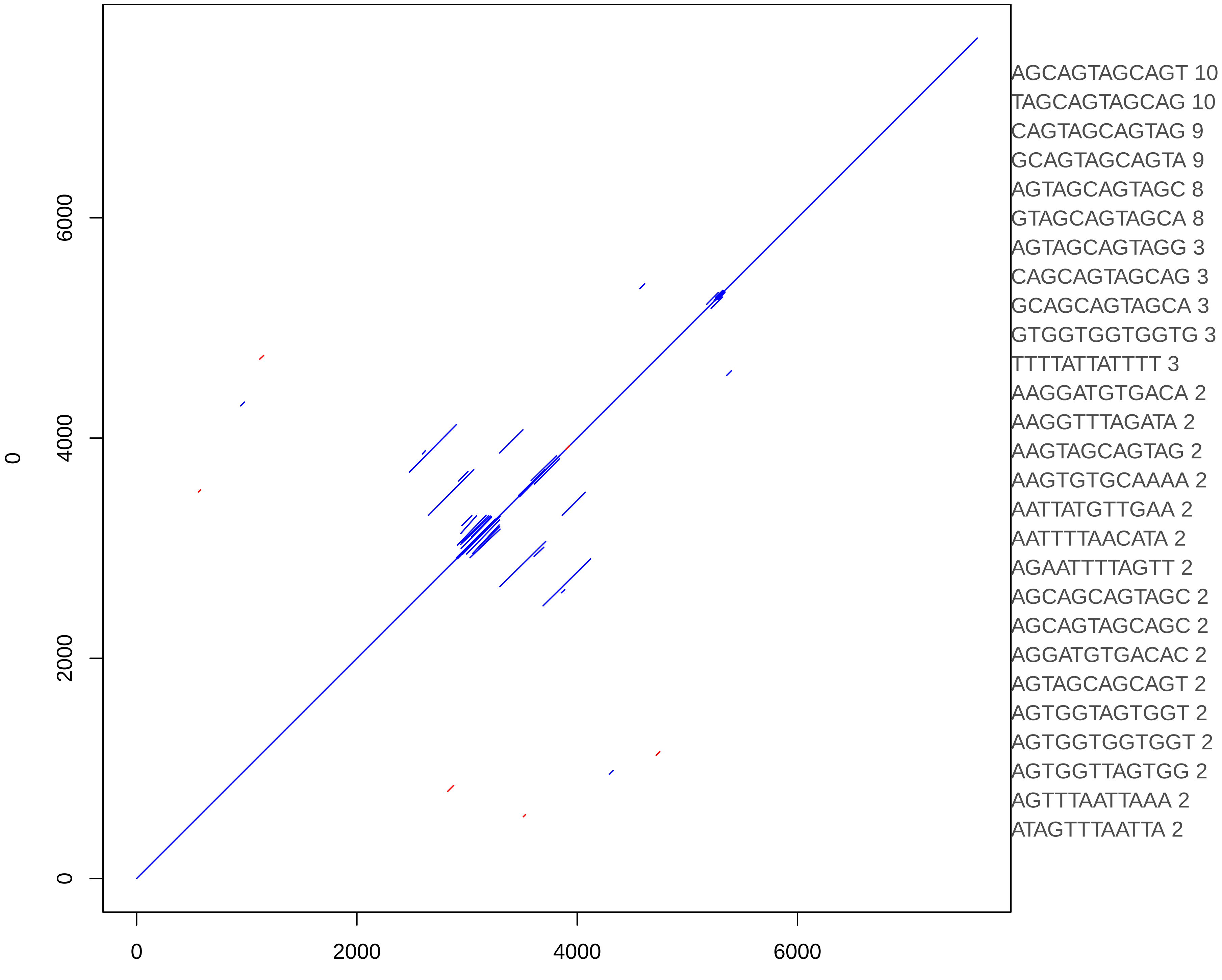

>m141121_210323_42134_c100732402550000001823141405141506_s1_p0/71008/2180_12987

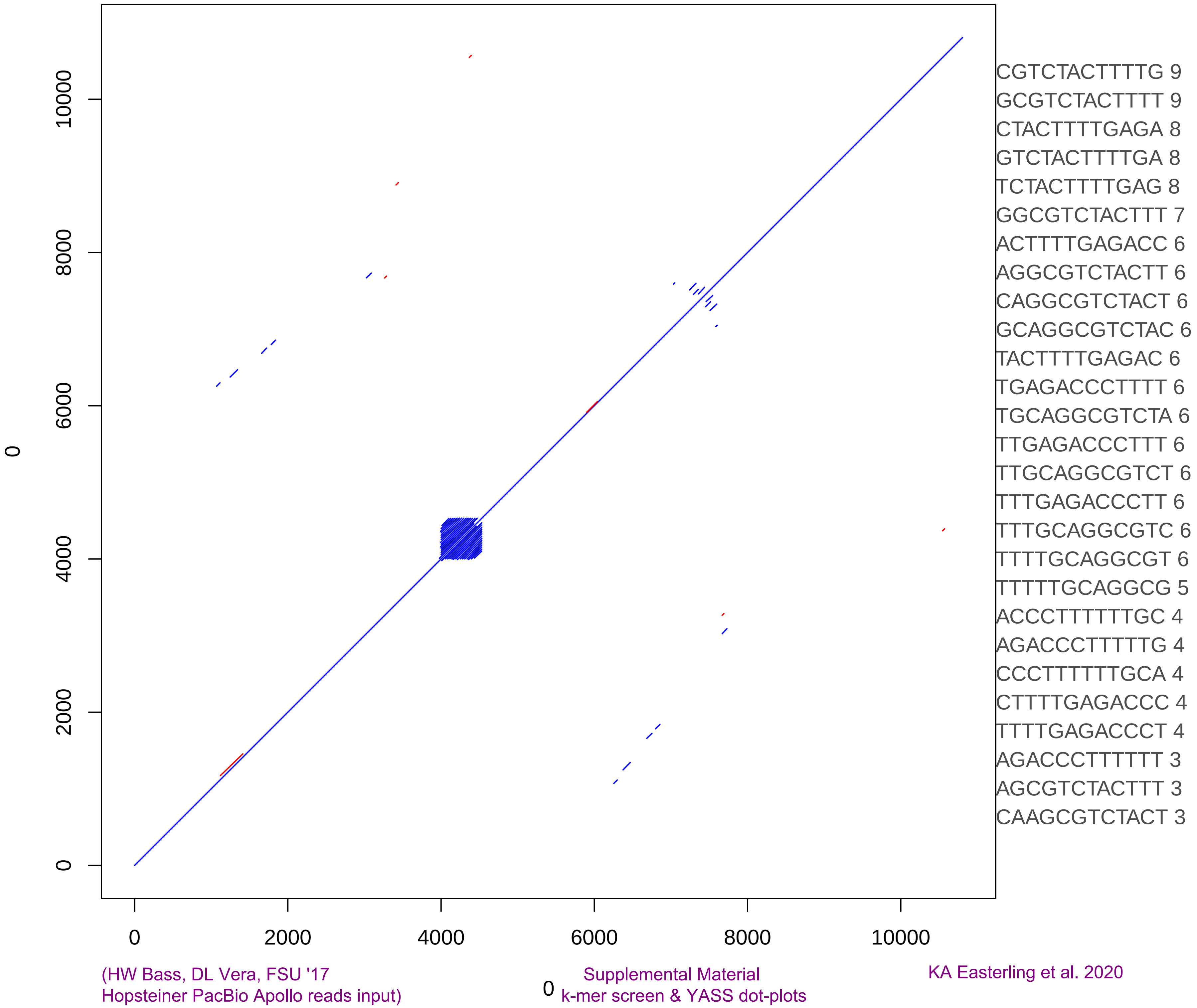

>m141121_210323_42134_c100732402550000001823141405141506_s1_p0/75170/631_12752

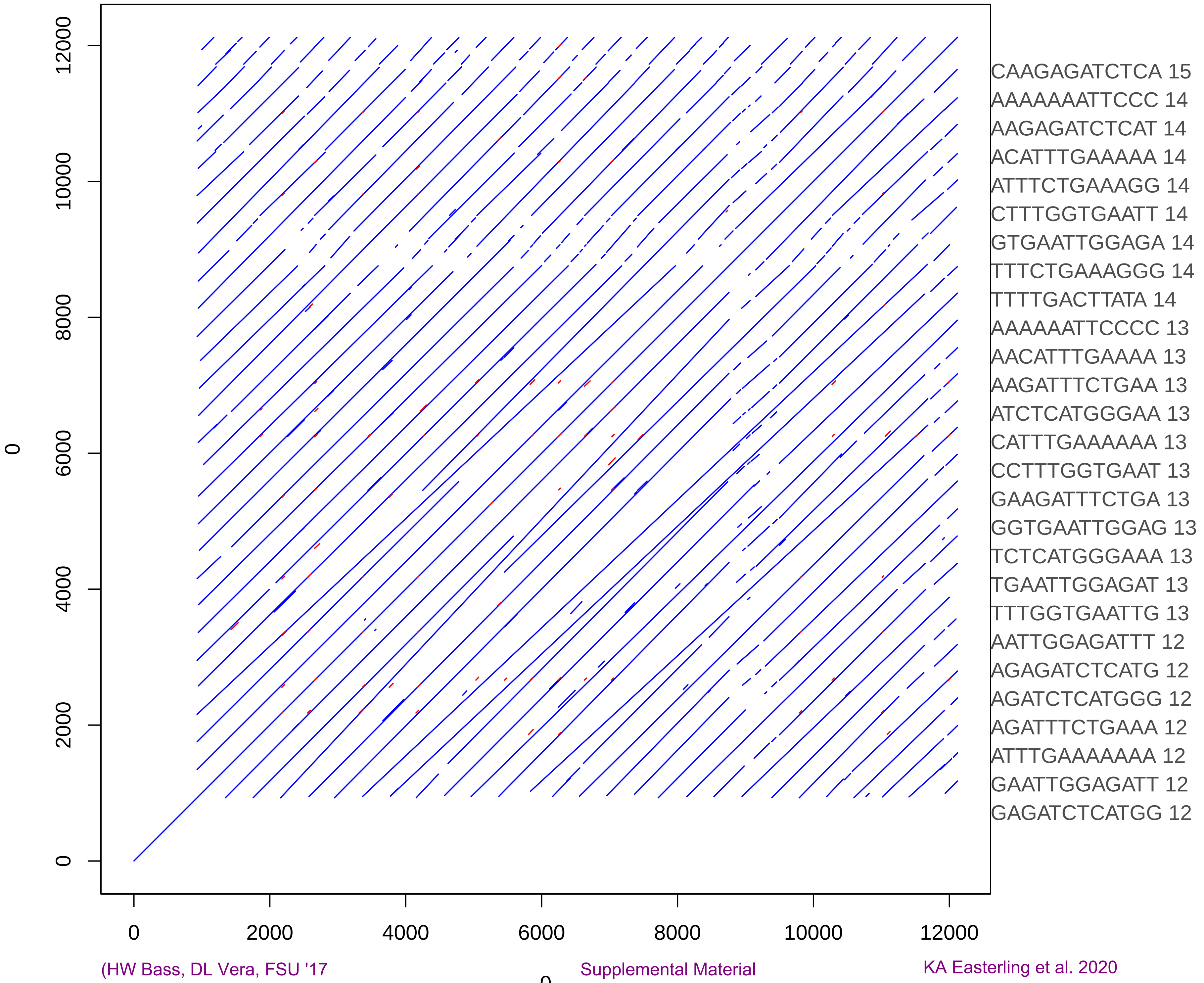

>m141121_210323_42134_c100732402550000001823141405141506_s1_p0/7734/0_10711

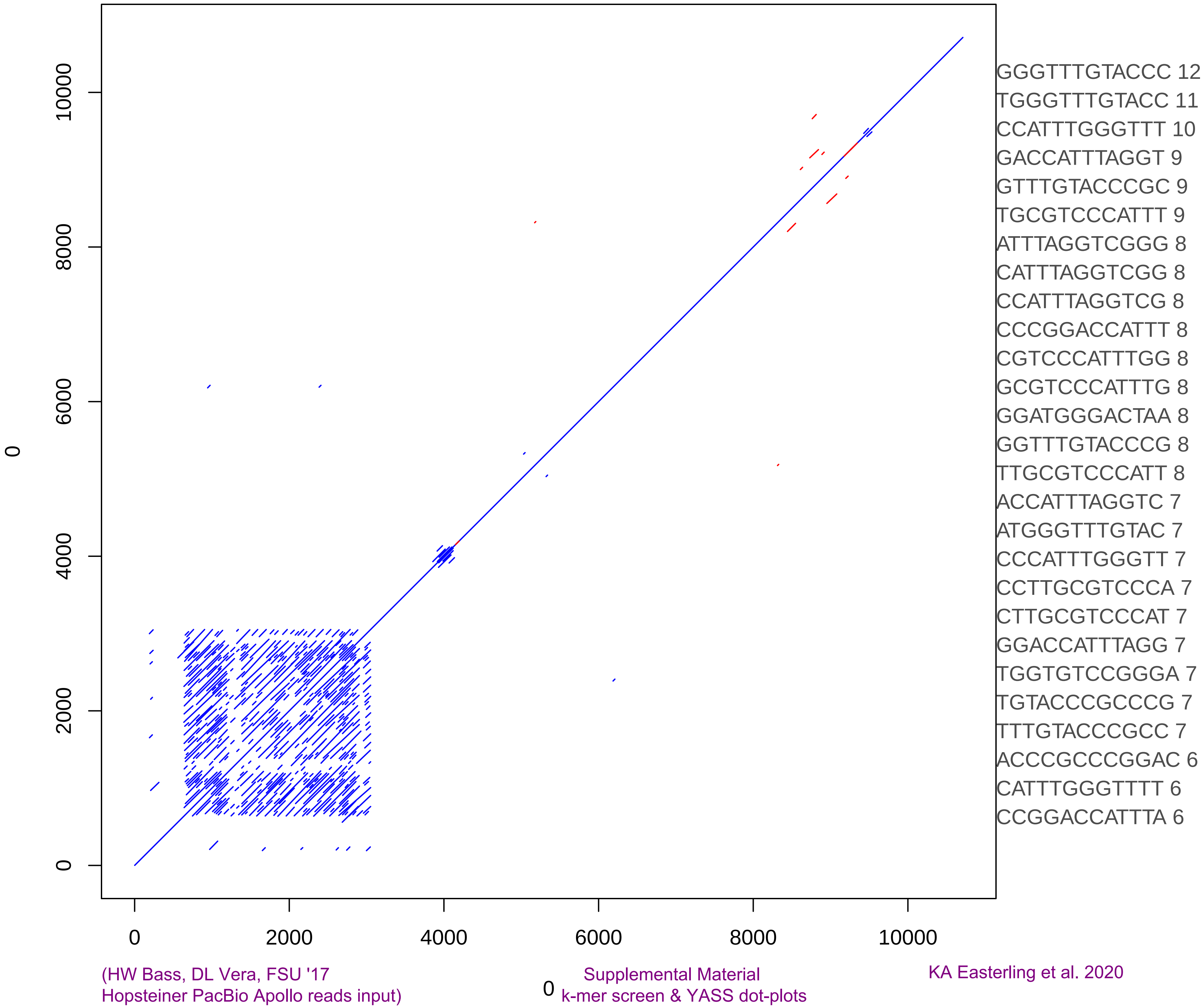

>m141121_210323_42134_c100732402550000001823141405141506_s1_p0/83158/7187_16769

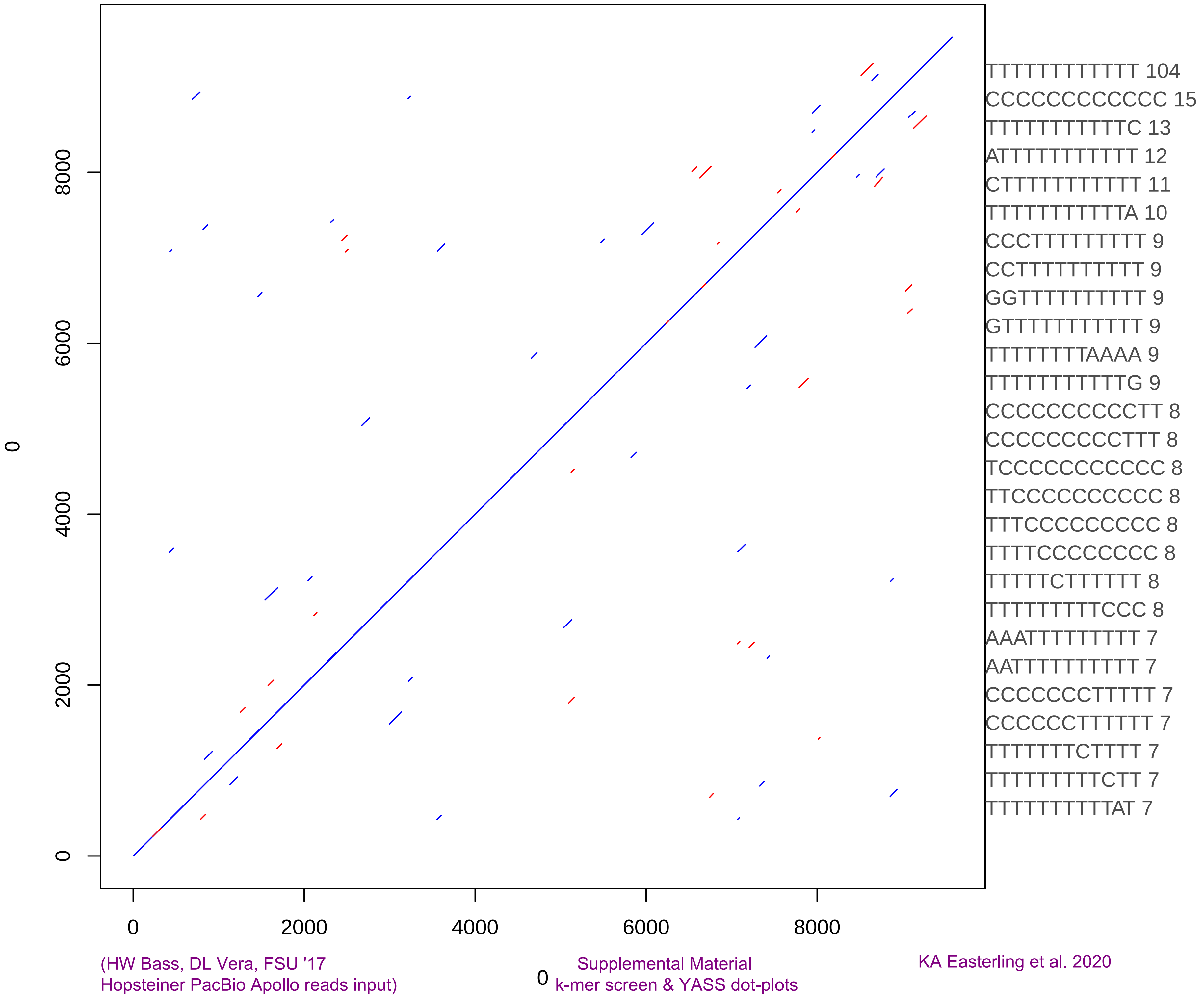

>m141121_210323_42134_c100732402550000001823141405141506_s1_p0/85317/0_6224

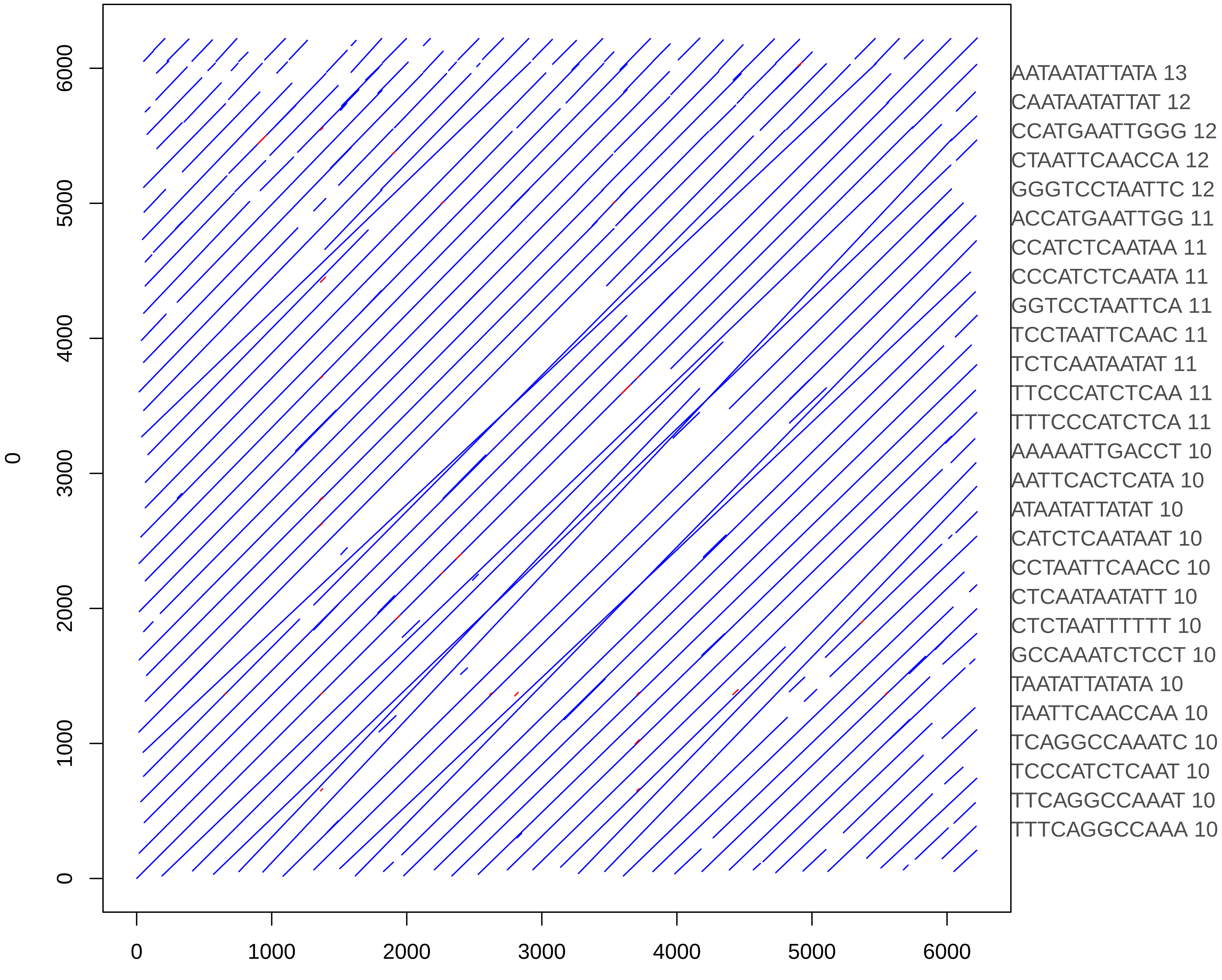

0

>m141121_210323_42134_c100732402550000001823141405141506_s1_p0/89525/4040_10426

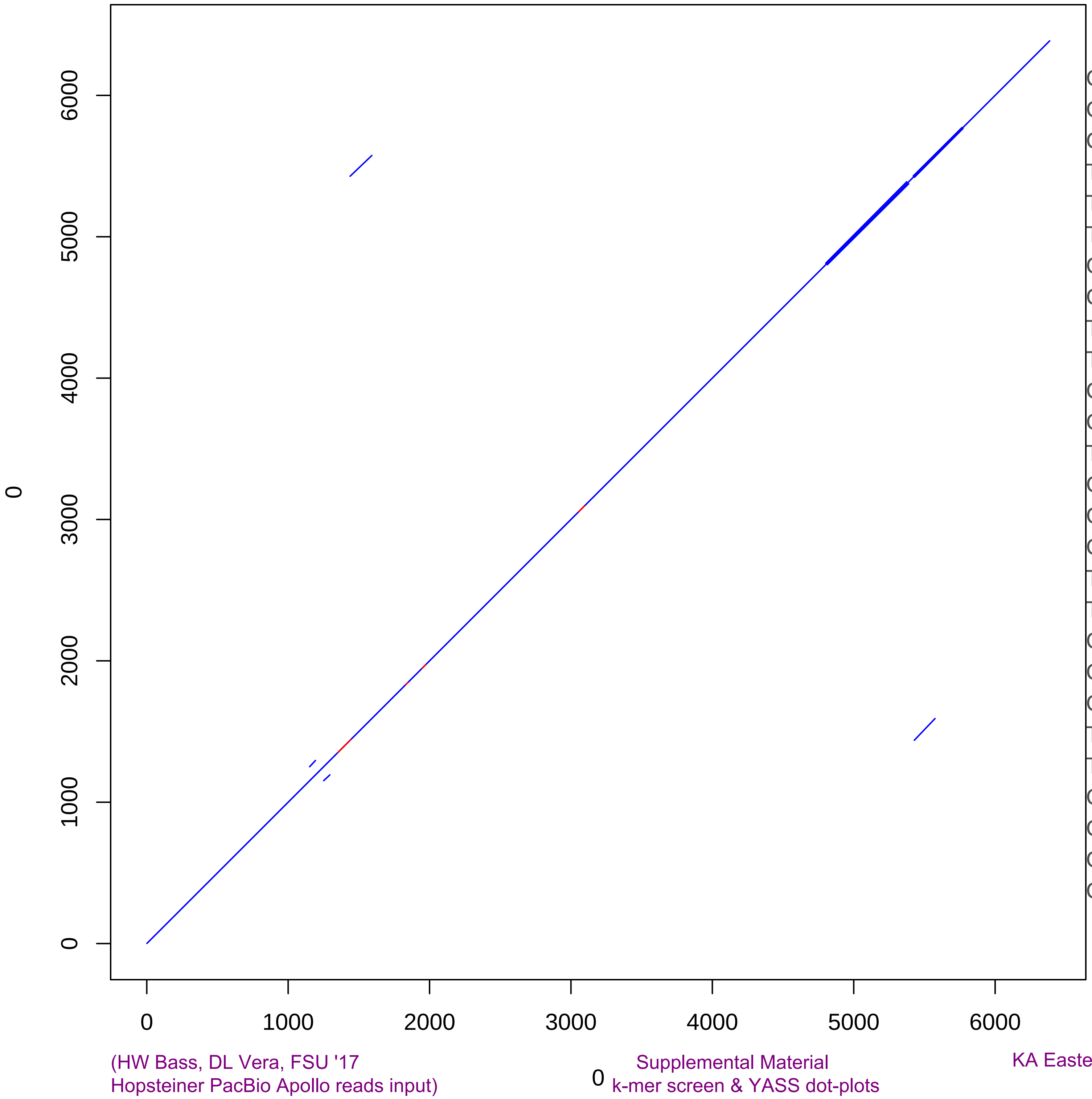

GTGTGTGGTGTG 17
GTGTGGTGTGTG 16
GTGTGTGTGGTG 15
TGTGTGTGGTGT 14
TGTGTGGTGTGT 13
TGTGTGTGTGTG 13
GTGGTGTGTGTG 11
GTGTGTGTGTGG 11
TGTGGTGTGTGT 11
TGTGTGTGTGGT 11
GTGTGTGTGTGT 10
GGTGTGTGTGTG 9
TGTGTGGTGTGG 7
GTGTGTTGTGTG 6
GTGTTGTGTGTG 6
GTTGTGTGTGTG 6
TGGTGTGTGTGG 6
TGGTGTGTGTGT 6
GGTGTGTGTGGT 5
GTGGTGGTGTGT 5
GTGTGGTGGTGT 5
TGTGGGTGTGGT 5
TGTGGTGGTGTG 5
GGTGTGTGGTGT 4
GTGGGTGTGGTG 4
GTGGTGTGGGTG 4
GTGGTGTGGTGG 4

>m141121_210323_42134_c100732402550000001823141405141506_s1_p0/94264/5634_11470

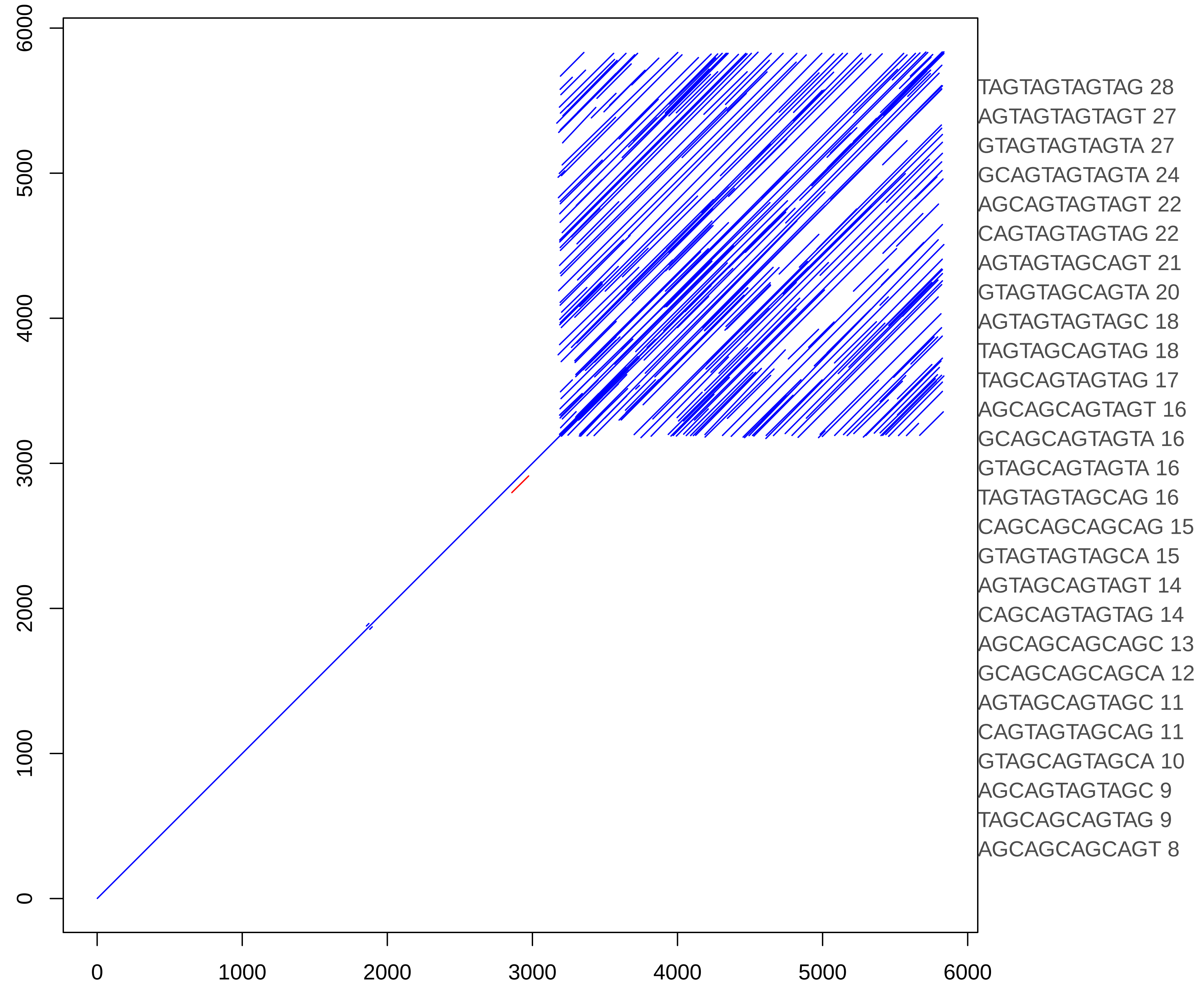

>m141121_210323_42134_c100732402550000001823141405141506_s1_p0/94332/1729_12373

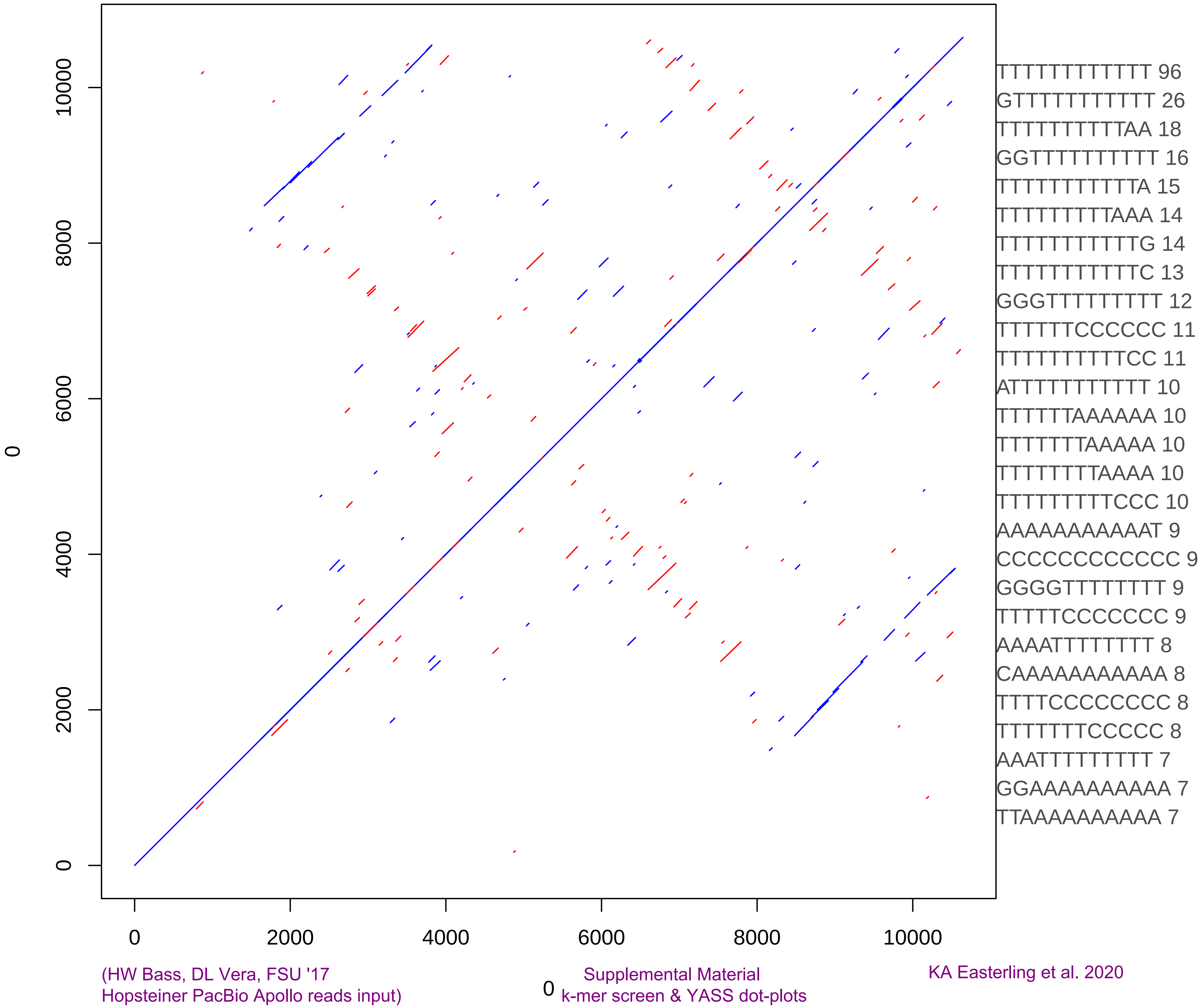

>m141121_232159_42134_c100732402550000001823141405141507_s1_p0/10893/3398_9324

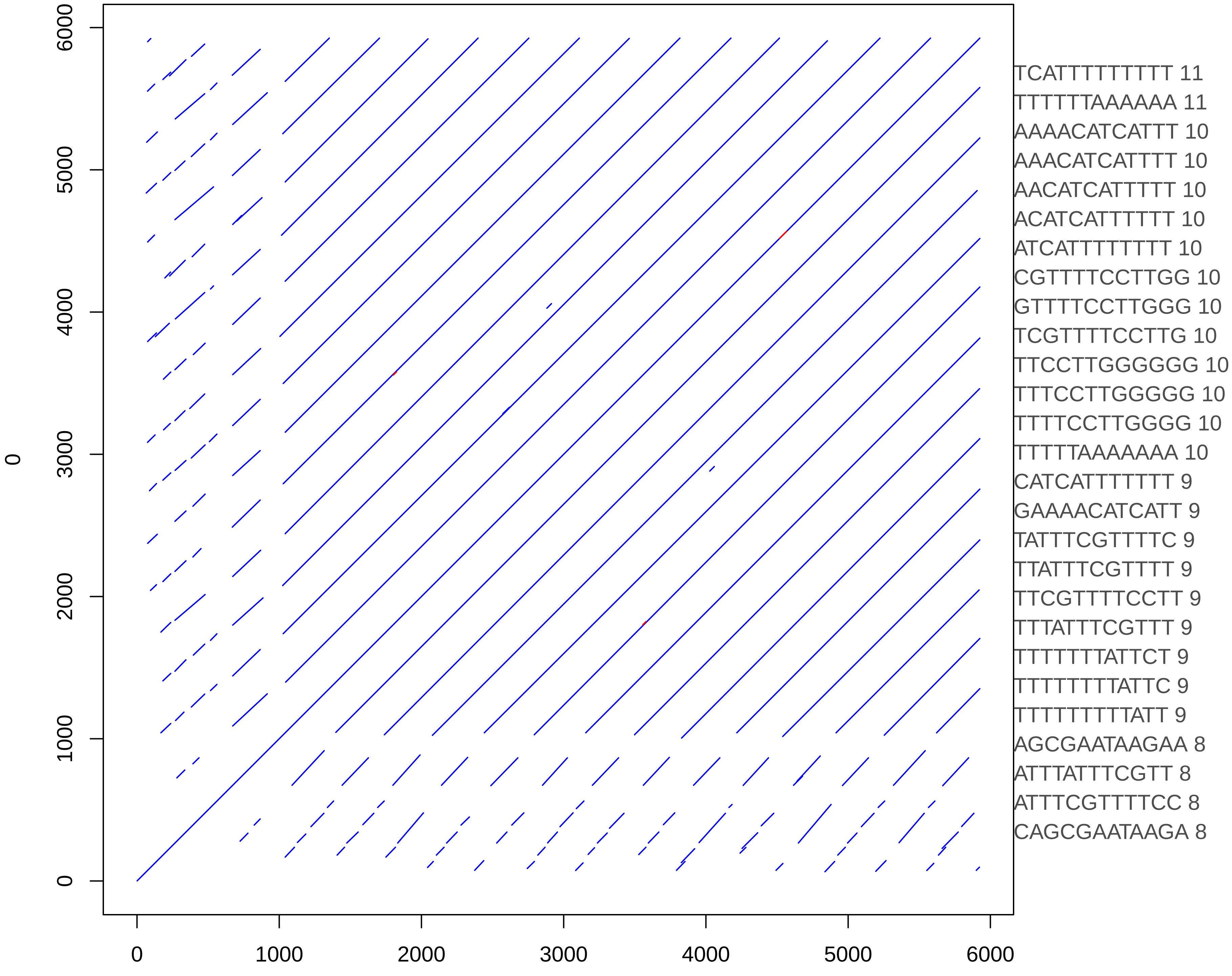

1,121 hits for repetitive sequences
passing 12mer abundance criterion

(Read-Number)
PAGE 139

For Tandem Repeat families of ### bp:
PacBio Read named "HuluTR###-r139"

>m141121_232159_42134_c100732402550000001823141405141507_s1_p0/122340/5808_12292

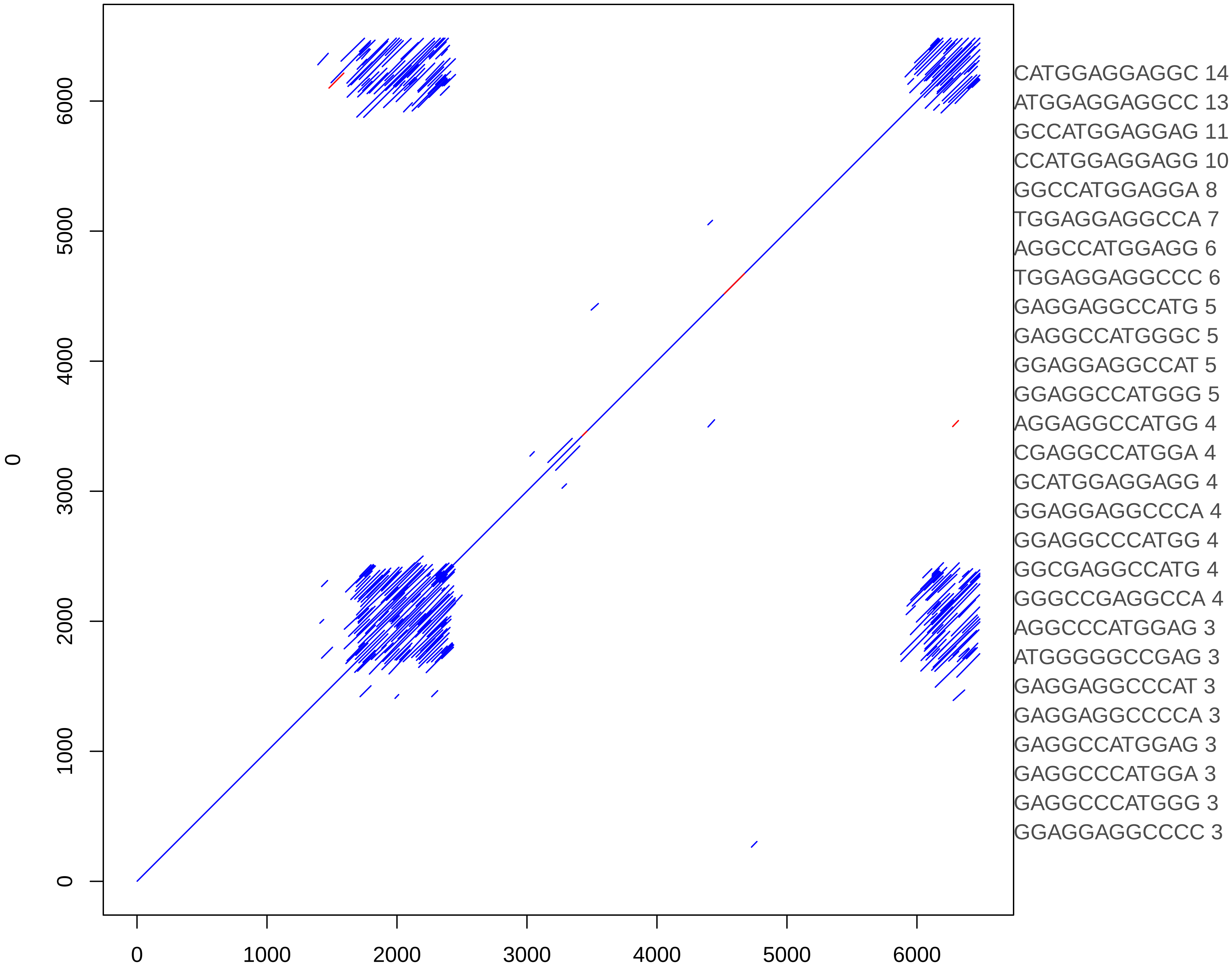

(HW Bass, DL Vera, FSU '17
Hopsteiner PacBio Apollo reads input)

Supplemental Material
k-mer screen & YASS dot-plots

KA Easterling et al. 2020

>m141121_232159_42134_c100732402550000001823141405141507_s1_p0/123465/4790_13466

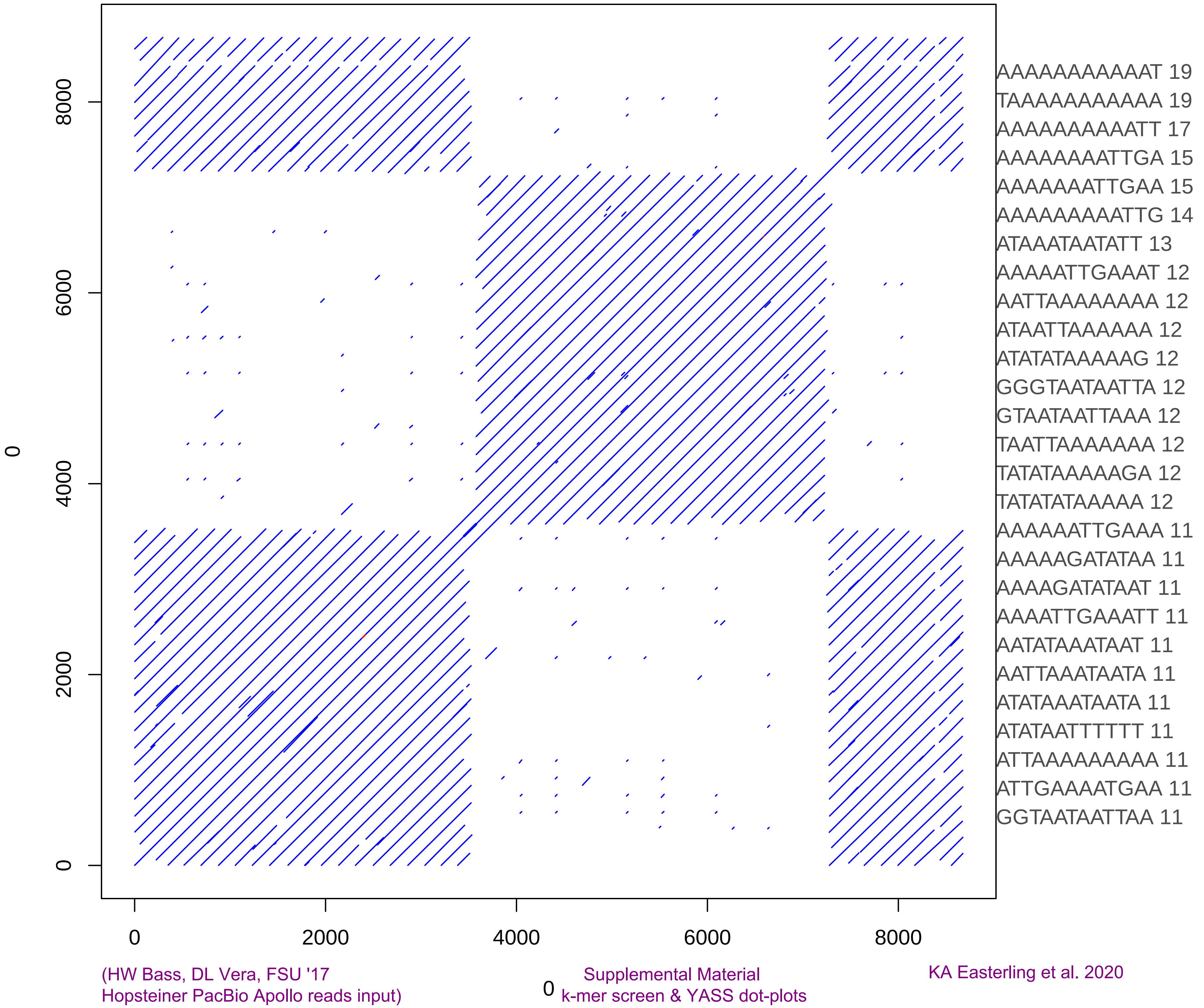

>m141121_232159_42134_c100732402550000001823141405141507_s1_p0/124964/1474_17123

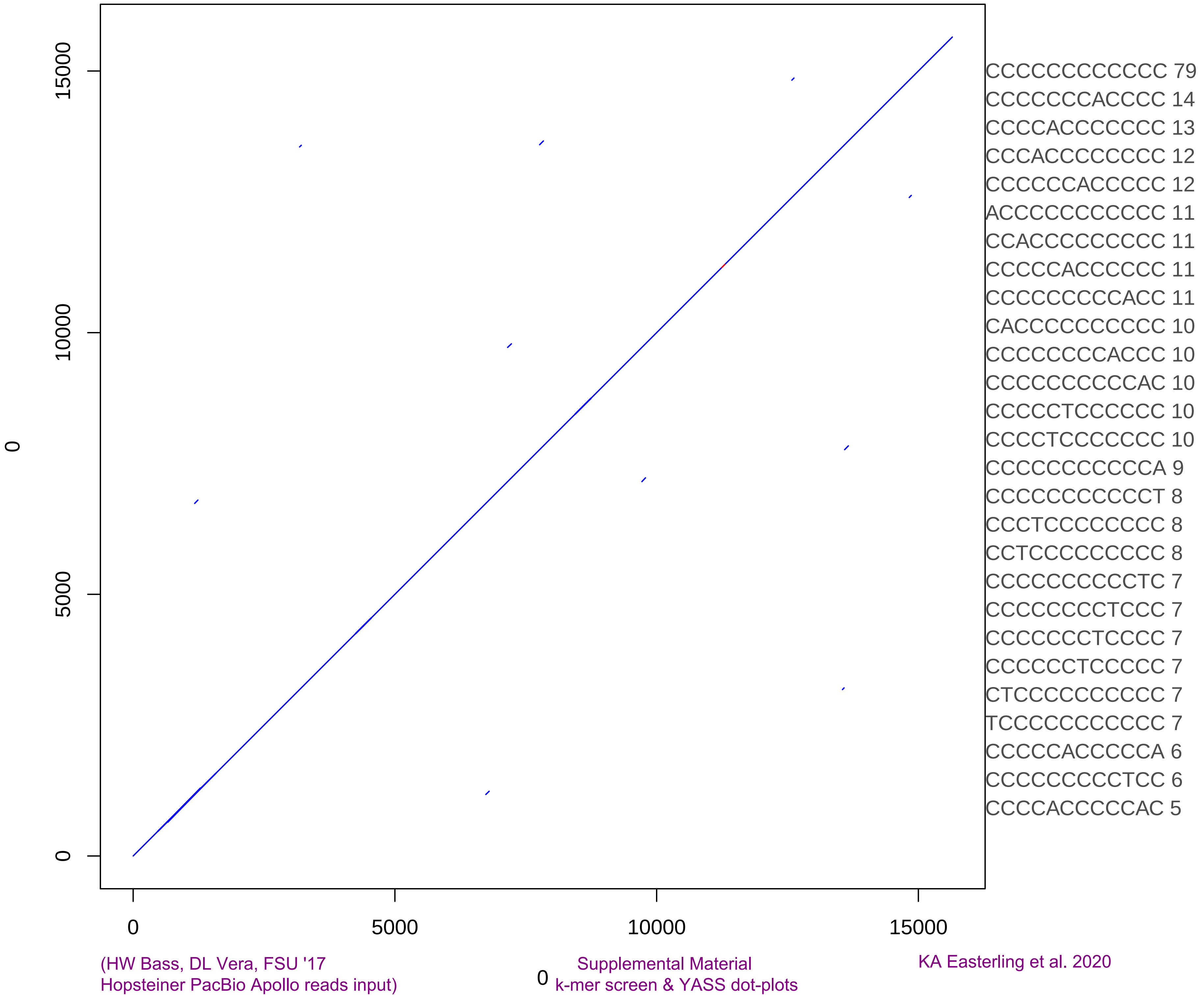

>m141121_232159_42134_c100732402550000001823141405141507_s1_p0/127083/268_9991

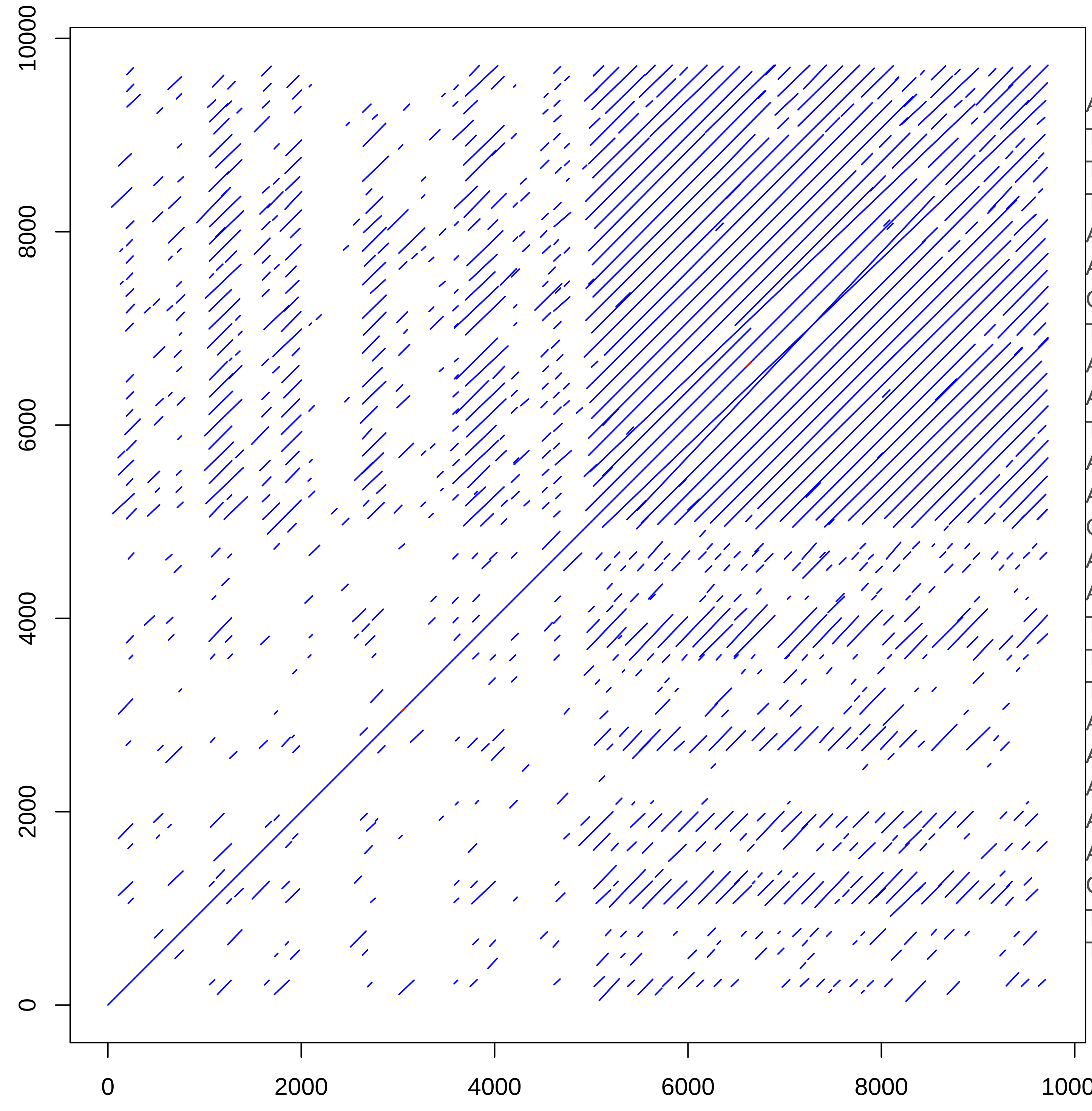

AAATATGAGTGA 15
TTTTTTTGTTTG 15
TTTTTGTTTGGT 14
TTTTTTGTTTGG 14
AATATGAGTGAA 13
ATATGAGTGAAT 13
GAAATATGAGTG 13
TTTTGTTTGGTT 13
AAAAAAATTAGA 12
AAAAAATTAGAG 12
TTAGAGAGAAAT 12
AATTTTTTTGTT 11
AGAAATATGAGT 11
GAGAAATATGAG 11
ATGAGTGAATTA 10
ATTTTTTTGTTT 10
TAGAGAGAAATA 10
TATGAGTGAATT 10
TGTTTGGTTGAA 10
AAAAAAAATTAG 9
AAGGTTAAAAAA 9
AGAGAGAAATAT 9
ATATTATTGAGA 9
ATTATTGAGATG 9
GTTAGAGAGAAA 9
TAGGAGATTTGG 9
TATTATTGAGAT 9

>m141121_232159_42134_c100732402550000001823141405141507_s1_p0/135006/4727_14468

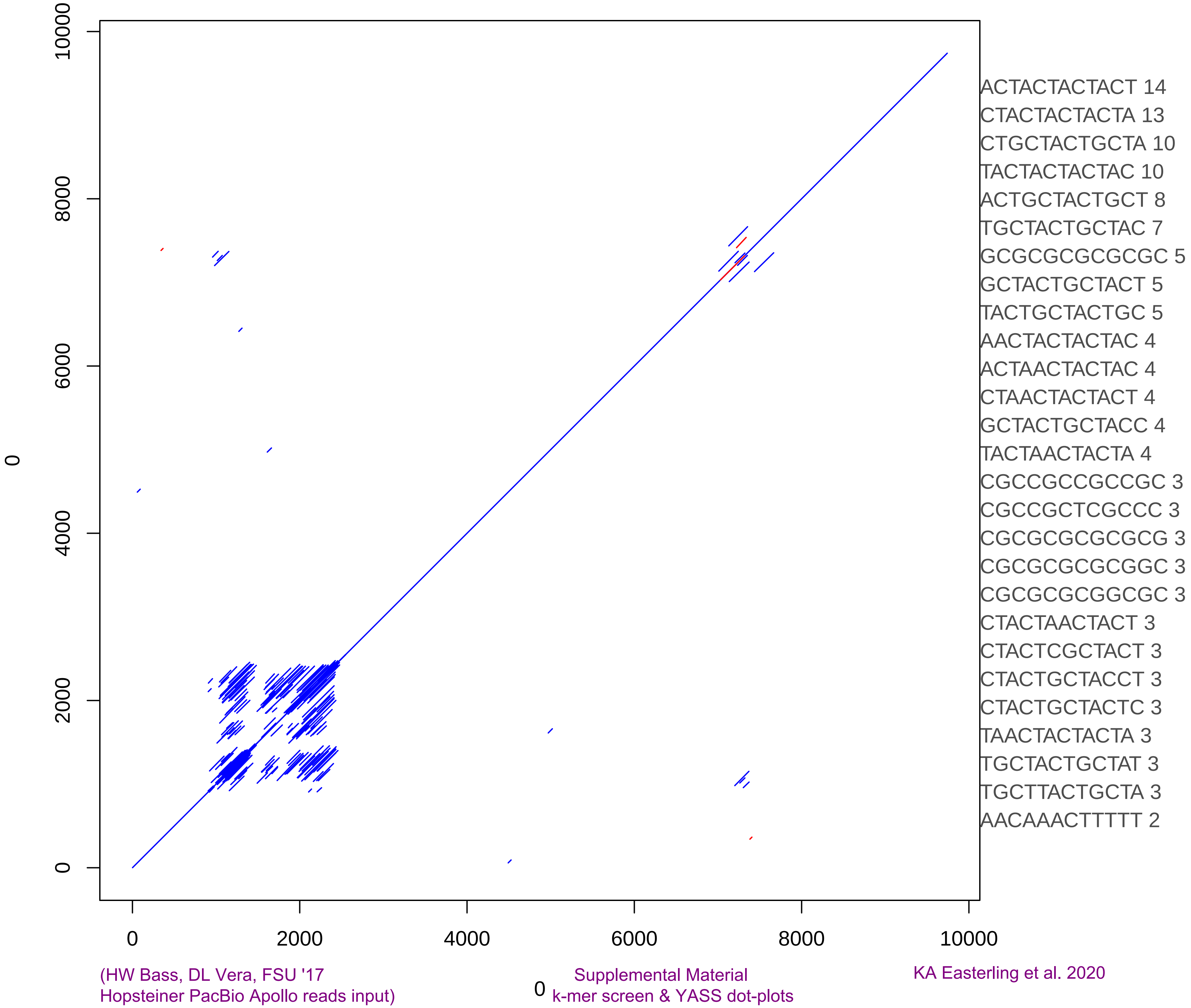

>m141121_232159_42134_c100732402550000001823141405141507_s1_p0/142171/1569_7124

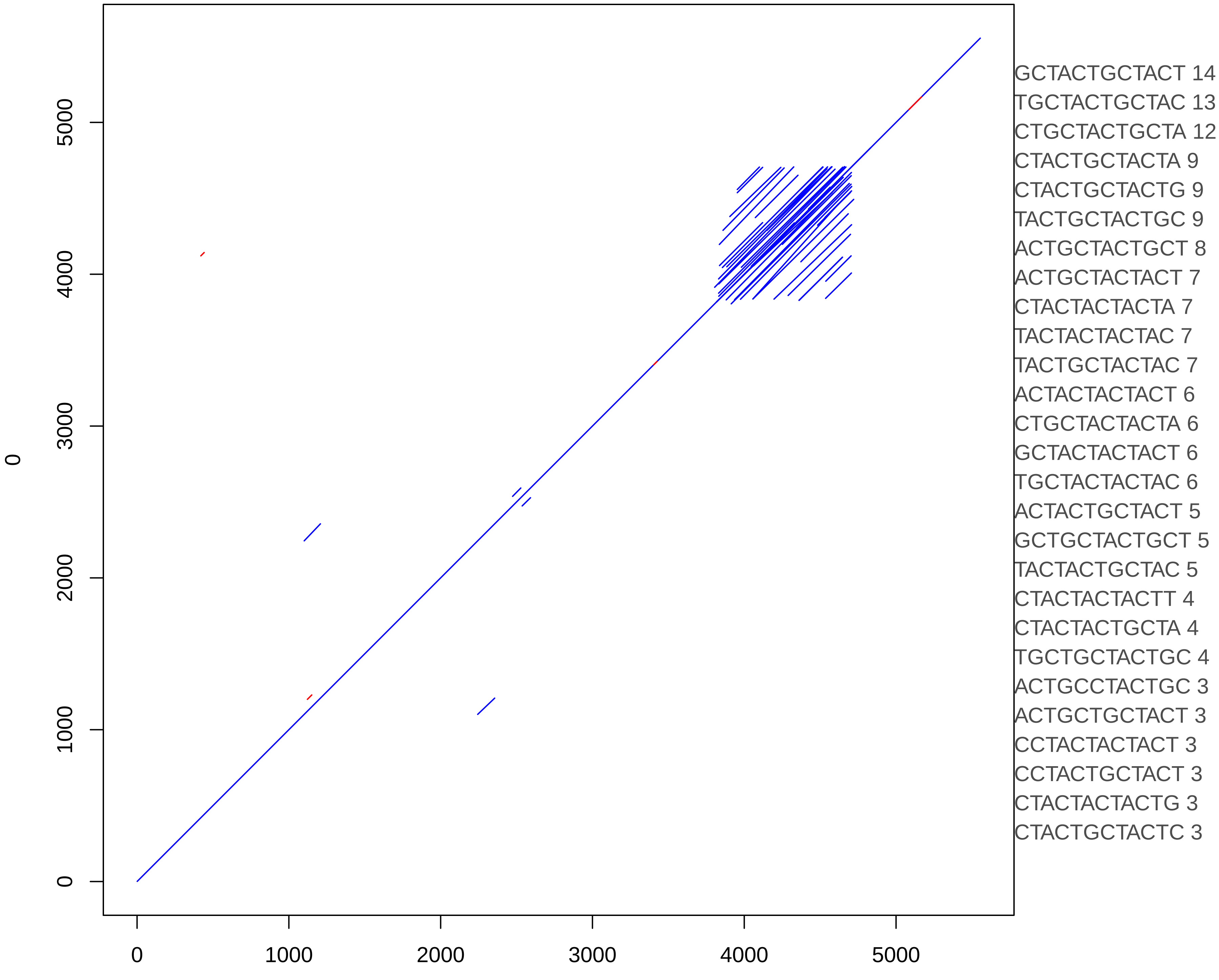

0

>m141121_232159_42134_c100732402550000001823141405141507_s1_p0/14387/6265_11792

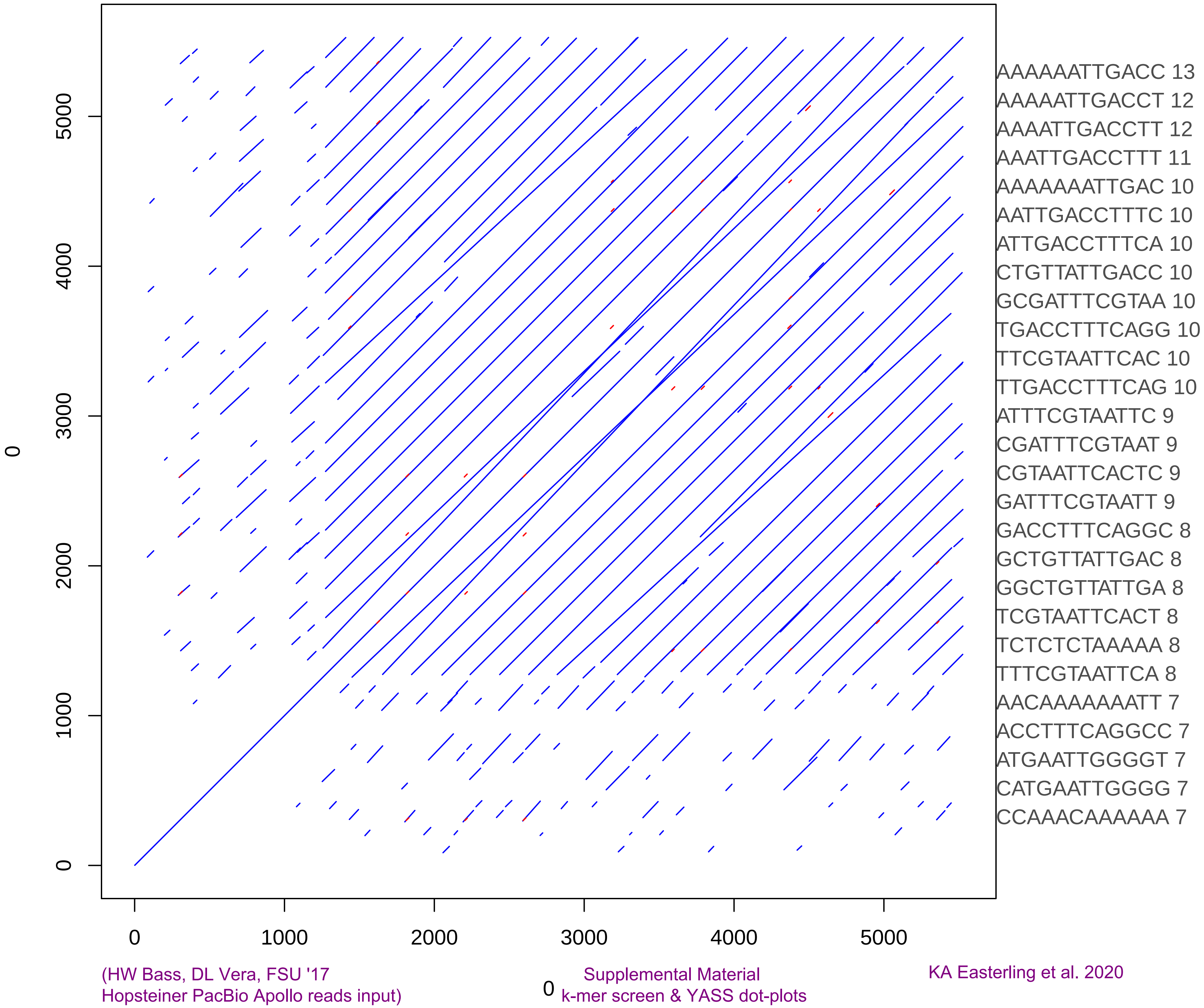

>m141121_232159_42134_c100732402550000001823141405141507_s1_p0/153246/928_9080

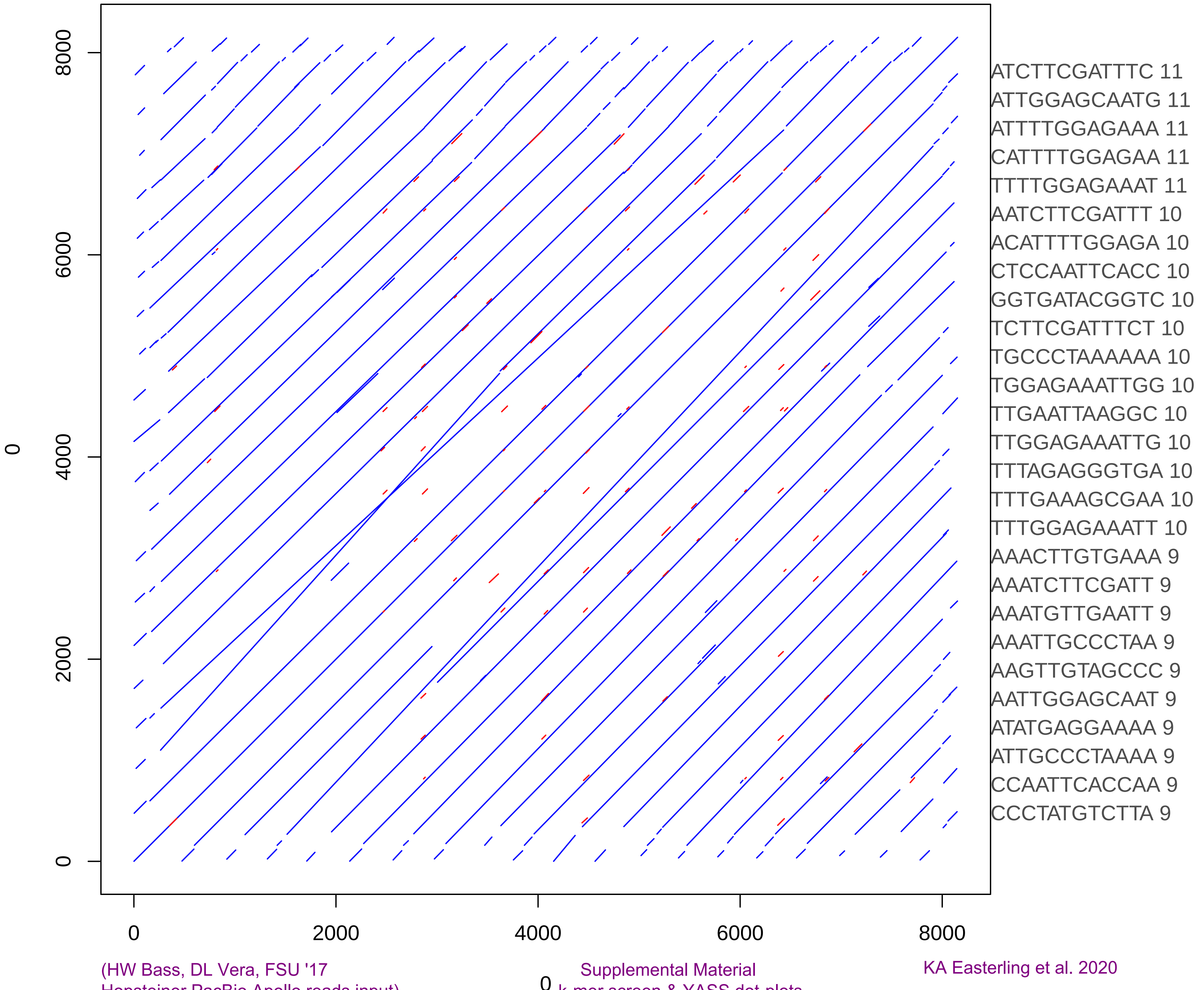

>m141121_232159_42134_c100732402550000001823141405141507_s1_p0/159115/5586_11142

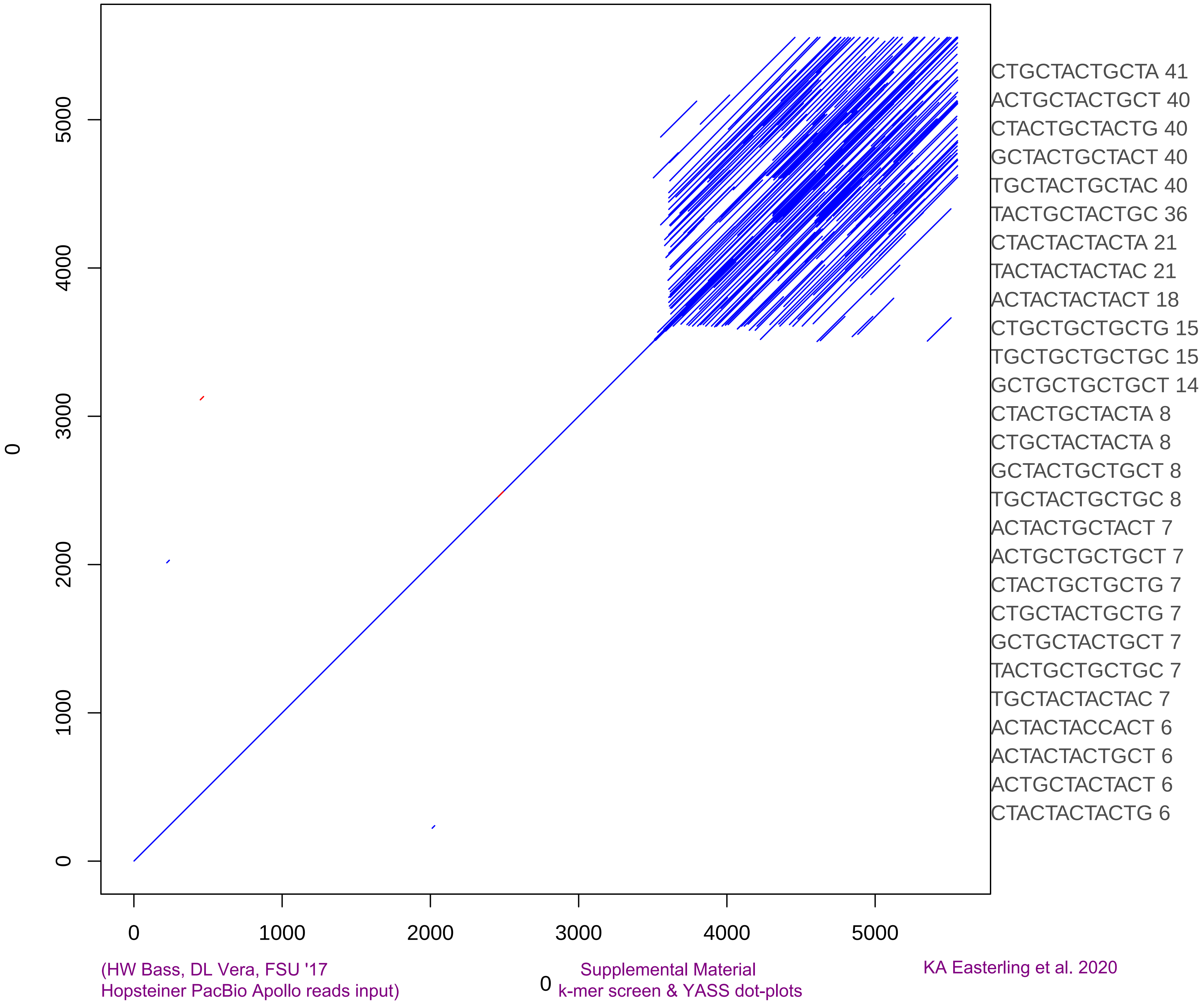

>m141121_232159_42134_c100732402550000001823141405141507_s1_p0/16464/7530_12898

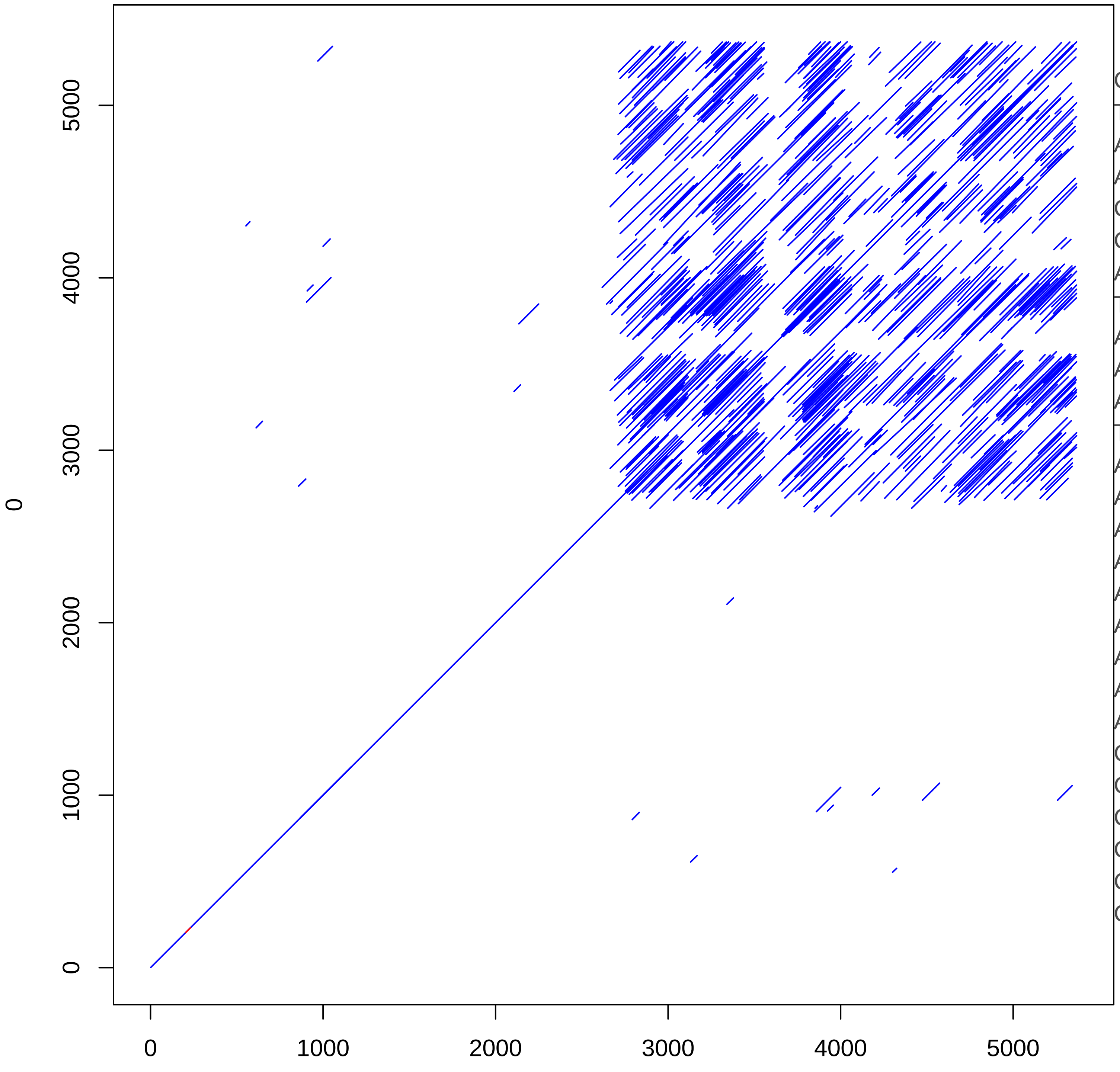

CTACTACTACTA 13
TACTACTACTAC 13
ACTACTACTACT 11
ACCACTACCACT 8
CACTACCACTAC 8
CCACTACCACTA 8
ACTACCACTACC 7
TACCACTACCAC 5
ACCACTACTACT 4
ACTACTACTACA 4
ATACTACTACTA 4
TACCACTACTAC 4
AACCACTACCAC 3
AATACTACTACT 3
ACATACTACATA 3
ACCACCCACTAC 3
ACCACTACTACC 3
ACTAATACTACT 3
ACTACCACCACT 3
ACTACTACCACC 3
ACTACTACTATA 3
CACCACTACCAC 3
CACTACTACCAC 3
CACTACTACTAC 3
CCACTACTACTA 3
CTAATACTACTA 3
CTACCACCACTA 3

>m141121_232159_42134_c100732402550000001823141405141507_s1_p0/16933/1001_10367

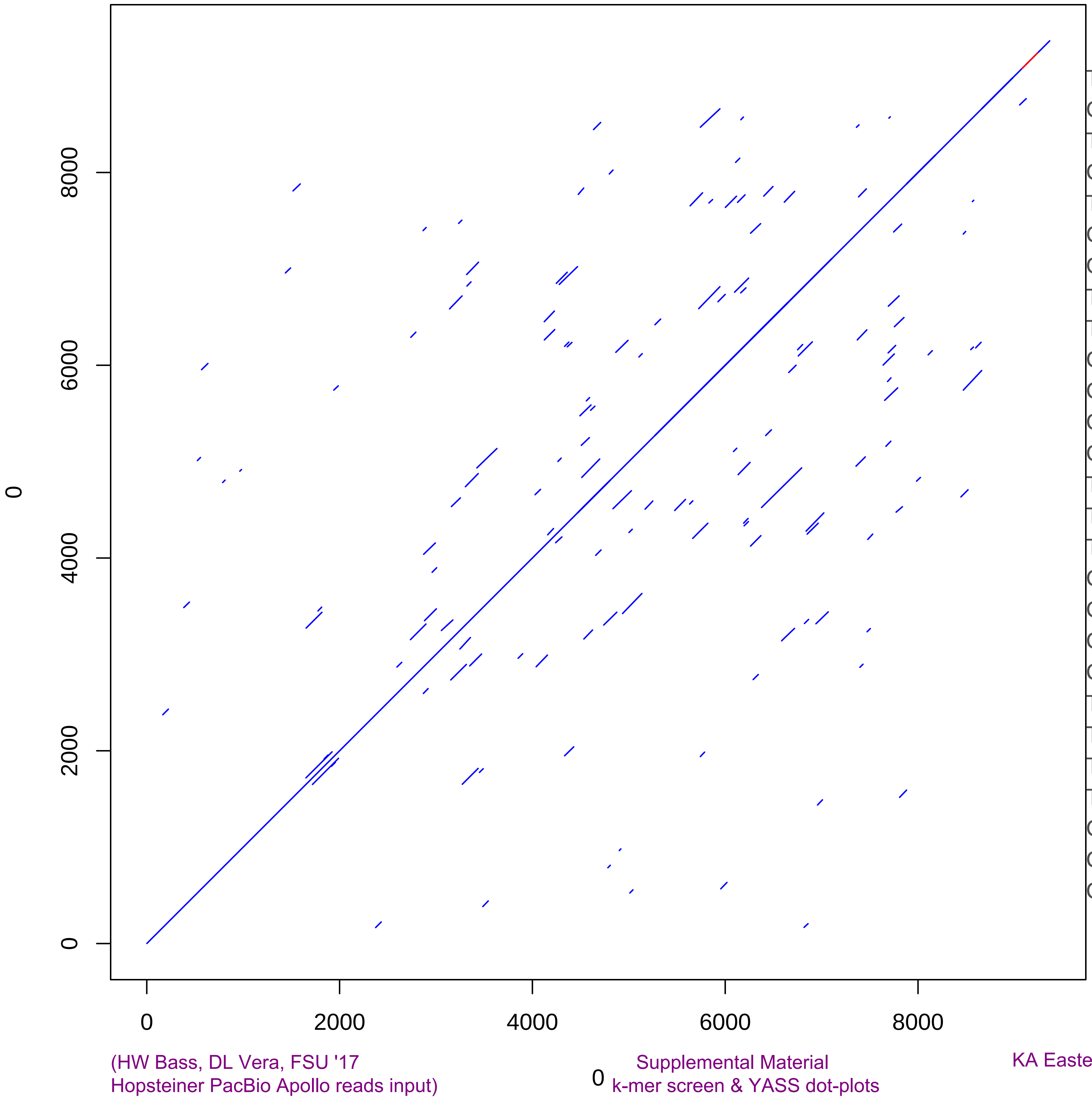

TTTTTTTTTTTT 91
CCCCCCCCCCCC 48
TCCCCCCCCCCC 12
CCCCCCCCCCCA 9
TTCCCCCCCCCC 9
CCCCCCCCTTTT 8
GGGGGGGGGGGG 8
TTTTTCCCCCCC 8
TTTTTTTTTTCC 8
CCCCCCCCCCCG 7
CCCTTTTTTTTT 7
GCCCCCCCCCCC 7
GTTTTTTTTTTT 7
TTTTTTTTCCCC 7
TTTTTTTTTCCC 7
TTTTTTTTTTTC 7
CCCCCCCCCCCT 6
CCCCCCCCCCTT 6
CCCCCCCTTTTT 6
CCCCTTTTTTTT 6
TTTTCCCCCCCC 6
TTTTTTCCCCCC 6
TTTTTTTCCCCC 6
TTTTTTTTTTTG 6
CCCCCCCCCACC 5
CCCCCCCCCCAC 5
CCCCCCCCCTTT 5

>m141121_232159_42134_c100732402550000001823141405141507_s1_p0/17363/7240_13436

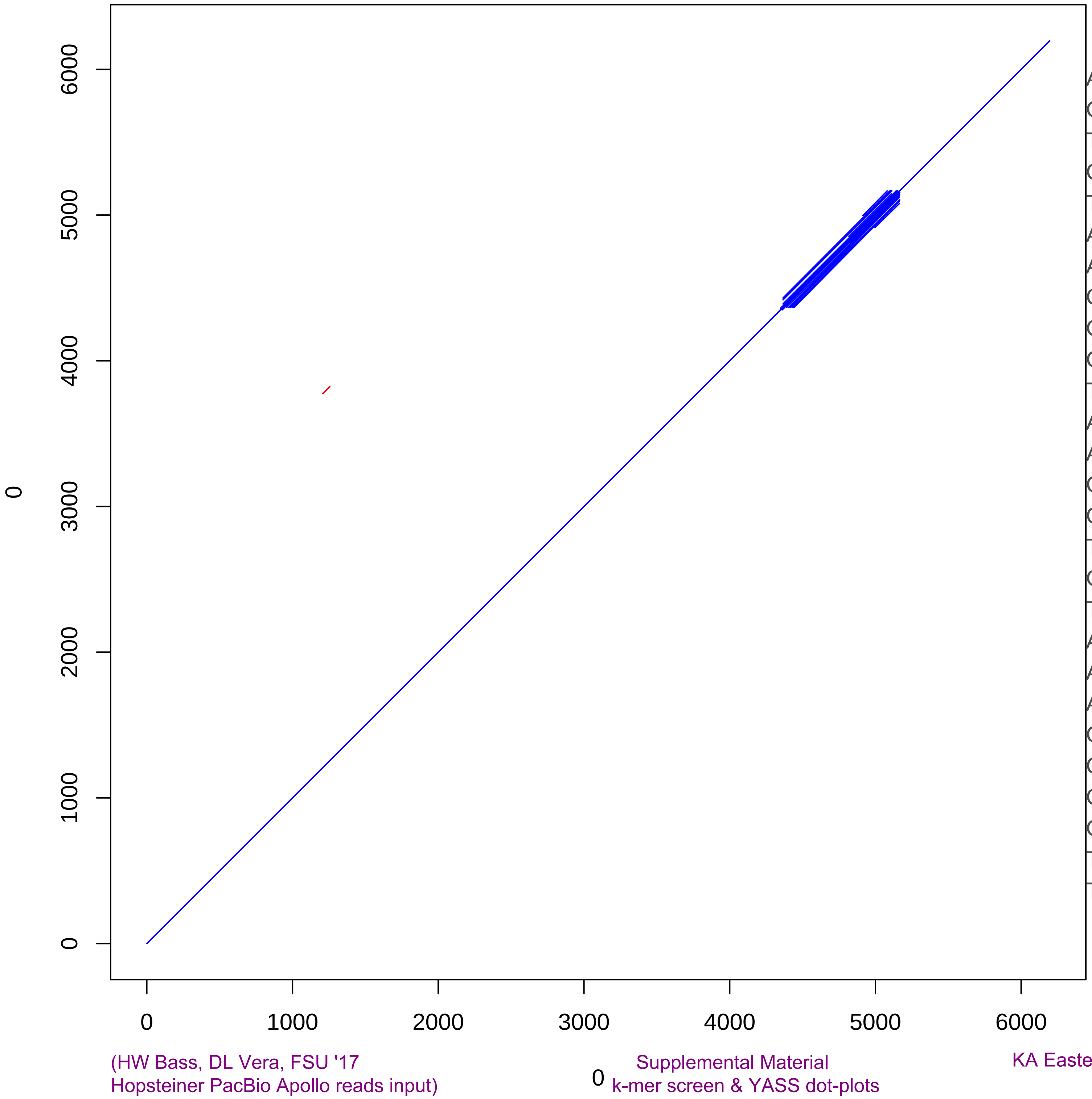

AGTAGTAGTAGT 25
GTAGTAGTAGTA 24
TAGTAGTAGTAG 20
CAGTAGCAGTAG 11
TAGCAGTAGCAG 11
AGCAGTAGCAGT 10
AGTAGCAGTAGC 10
GCAGTAGCAGTA 10
GTAGCAGTAGCA 10
GTAGTGGTAGTG 7
TAGTAAGTAGTA 7
AAGTAGTAGTAG 6
AGTAAGTAGTAG 6
GTAAGTAGTAGT 6
GTAGTAGTAGTG 6
TAAGTAGTAGTA 6
GGTAGTGGTAGT 5
TGGTAGTGGTAG 5
AGTAGTAGTATA 4
AGTAGTGGTAGT 4
AGTGGTAGTGGT 4
GTAGTAGTGGTA 4
GTAGTGGTAGTA 4
GTGGTAGTGGTA 4
GTGTAGTAGTAG 4
TAGTAGTAGTAT 4
TAGTAGTAGTGG 4

>m141121_232159_42134_c100732402550000001823141405141507_s1_p0/25564/8532_13927

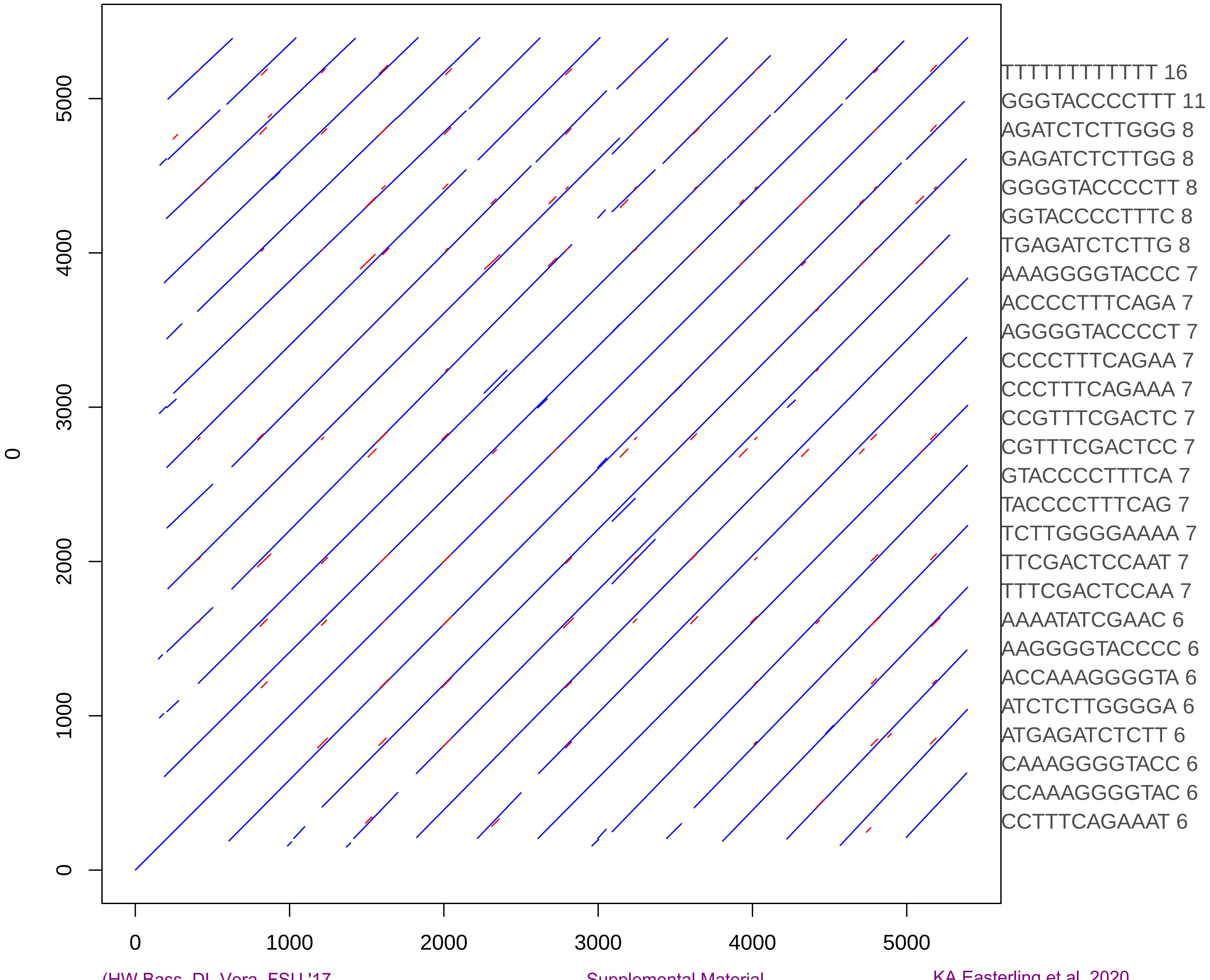

0

>m141121_232159_42134_c100732402550000001823141405141507_s1_p0/28297/6130_15512

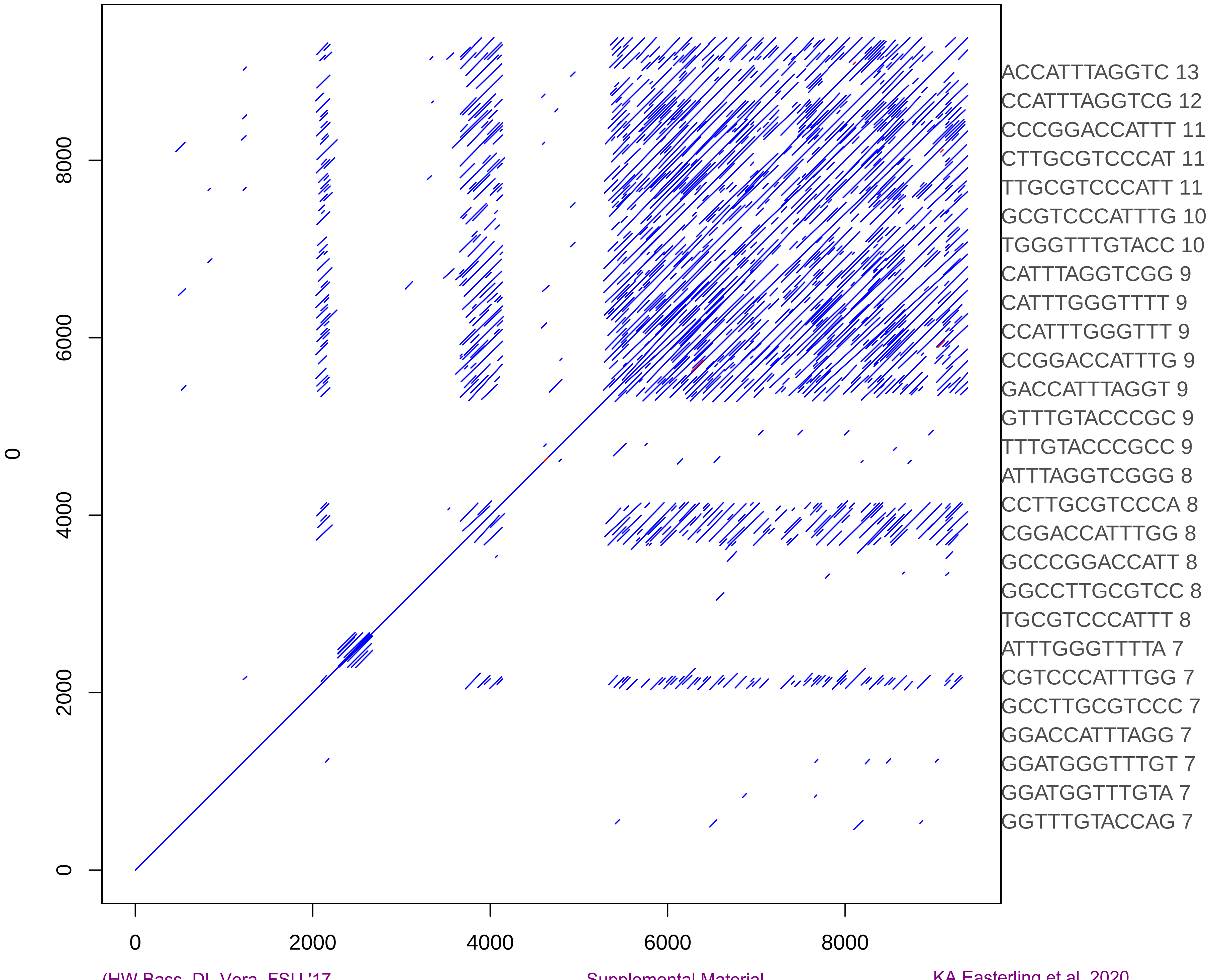

>m141121_232159_42134_c100732402550000001823141405141507_s1_p0/34471/7814_13094

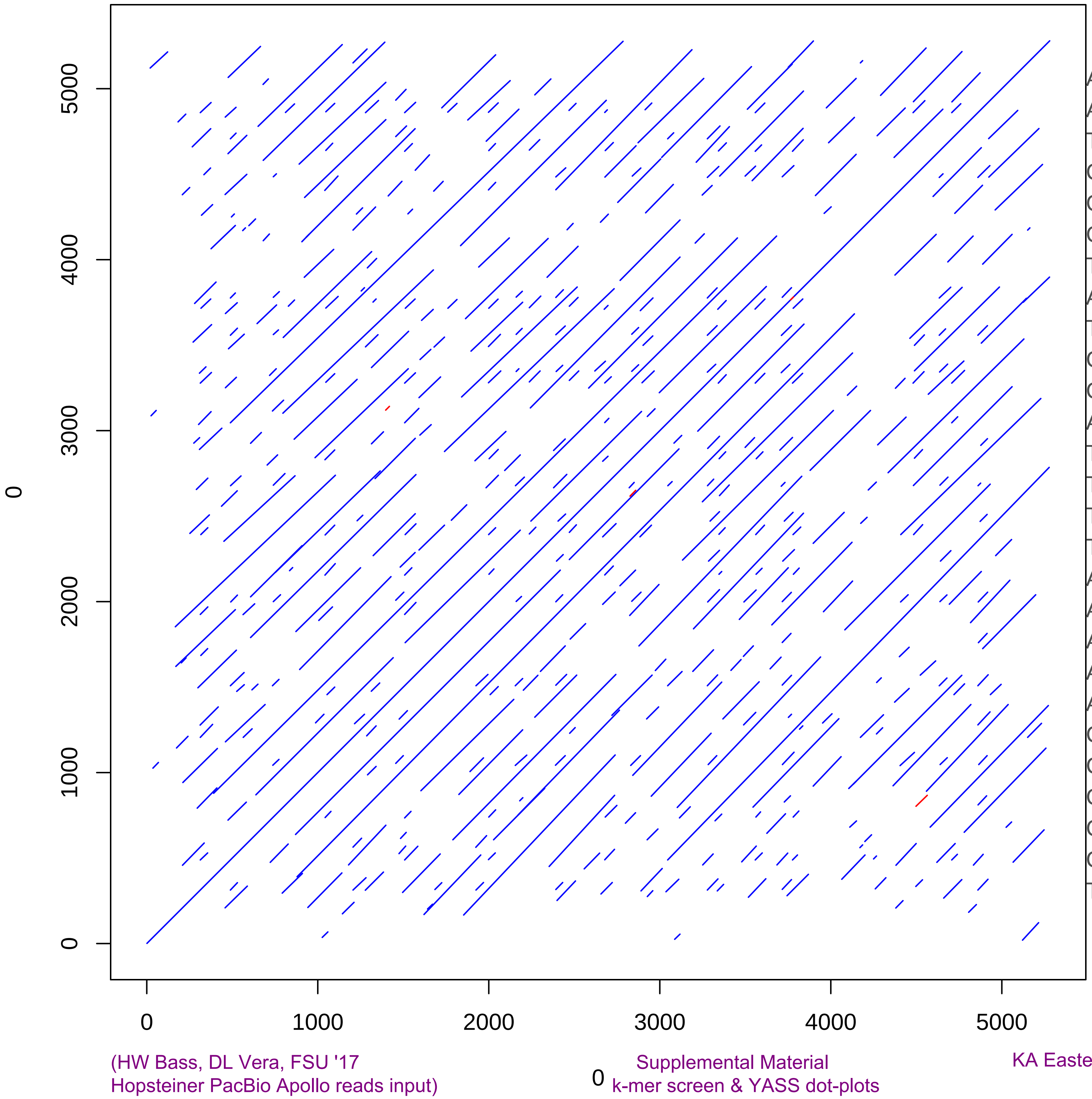

AATGTTATCTAG 13
ATGTTATCTAGT 13
TGCAATGTTATC 13
CAATGTTATCTA 12
GCAATGTTATCT 12
GTGCAATGTTAT 12
TGTTATCTAGTA 12
AGTGCAATGTTA 10
TAGTGCAATGTT 10
GGTTTACTAGTG 8
GTTATCTAGTAA 8
ATGTCATAATTA 6
TATGTCATAATT 6
TGGTTTACTAGT 6
TTAGTGCAATGT 6
TTATCTAGTAAT 6
AAGGTTCAATAT 5
ACGTTGGAAAGG 5
ACTACTATATCT 5
AGGTTCAATATG 5
ATTGGTTTACTA 5
CTAGTAATAATT 5
CTTAGTGCAATG 5
GAAAGGTTCAAT 5
GGAAAGGTTCAA 5
GTTTACTAGTGT 5
TGGAAAGGTTCA 5

>m141121_232159_42134_c100732402550000001823141405141507_s1_p0/36512/3233_9404

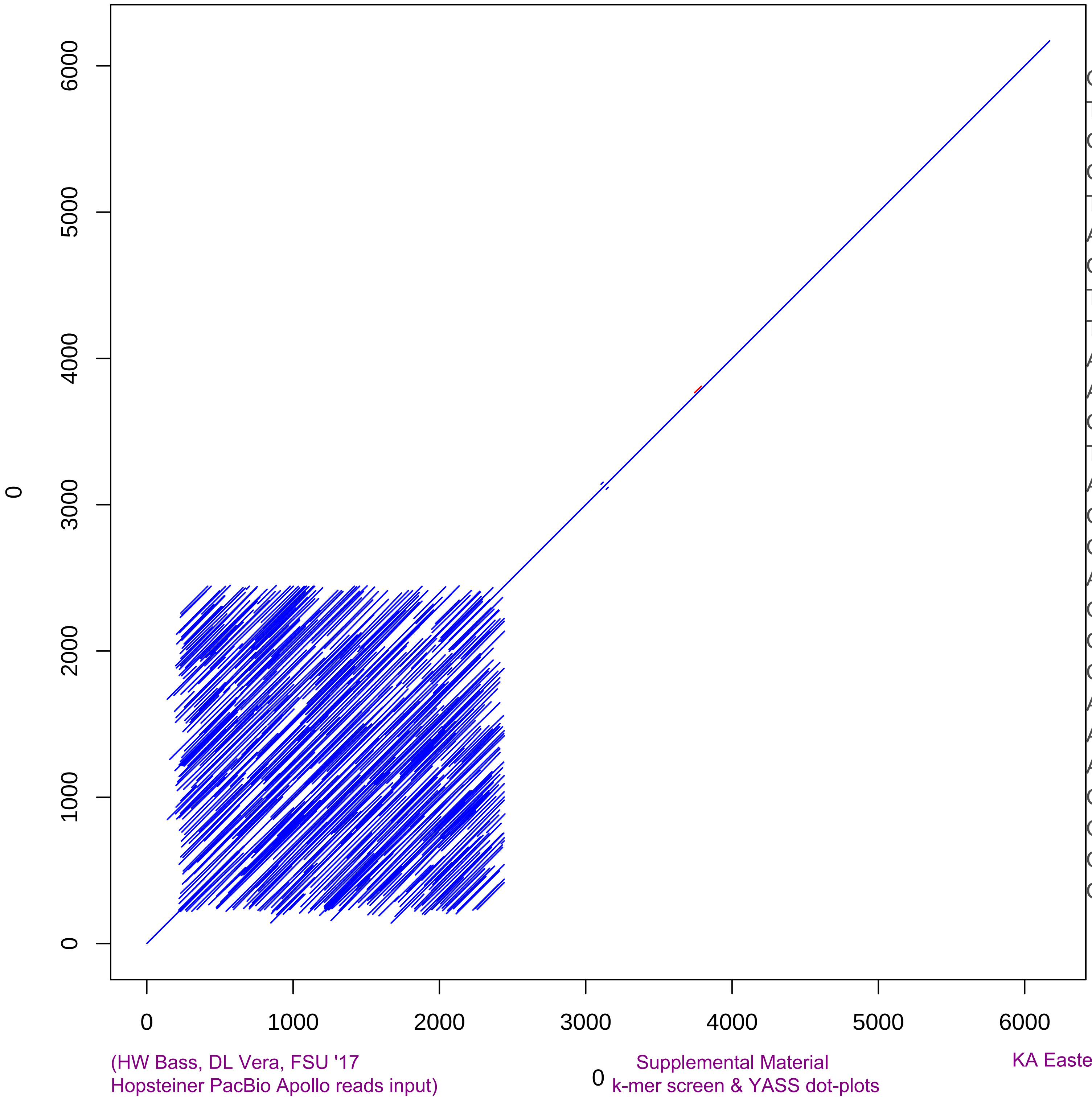

GTAGTAGTGGTA 12
TAGTGGTAGTAG 12
GTAGTGGTAGTA 10
GGTAGTAGTGGT 9
TGGTAGTGGTAG 9
AGTAGTGGTAGT 8
GTGGTAGTGGTA 8
TAGTAGTGGTAG 8
TAGTGGTAGTGG 8
AGTAGTAGTAGT 7
AGTGGTAGTAGT 7
GTGGTAGTAGTG 7
TGGTAGTAGTGG 7
AGTGGTAGTGGT 6
GGTAGTGGTAGT 6
GTAGTGGTAGTG 6
AAGTAGTAGTAG 5
GGTGGTGGTGGT 5
GTAGTAGTAGTG 5
GTGGTGGTGGTG 5
AGTAGTAGTGGT 4
AGTAGTGTAGTA 4
AGTGTAGTAGTA 4
GGTGGTAGTGGT 4
GTAGTAGTAGTA 4
GTGGTGGTAGTG 4
GTGTAGTAGTAG 4

>m141121_232159_42134_c100732402550000001823141405141507_s1_p0/425/0_6342

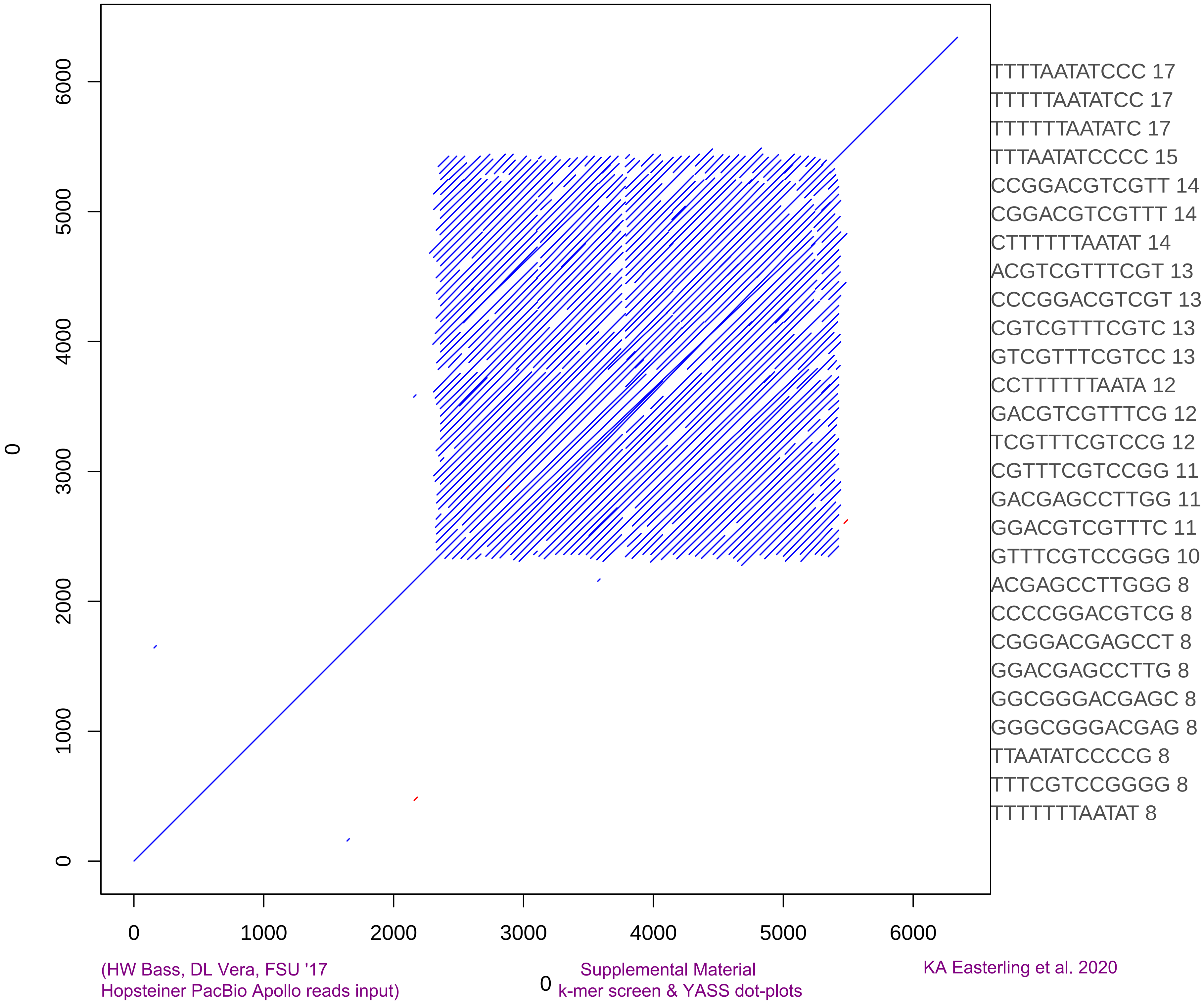

>m141121_232159_42134_c100732402550000001823141405141507_s1_p0/4972/5876_12915

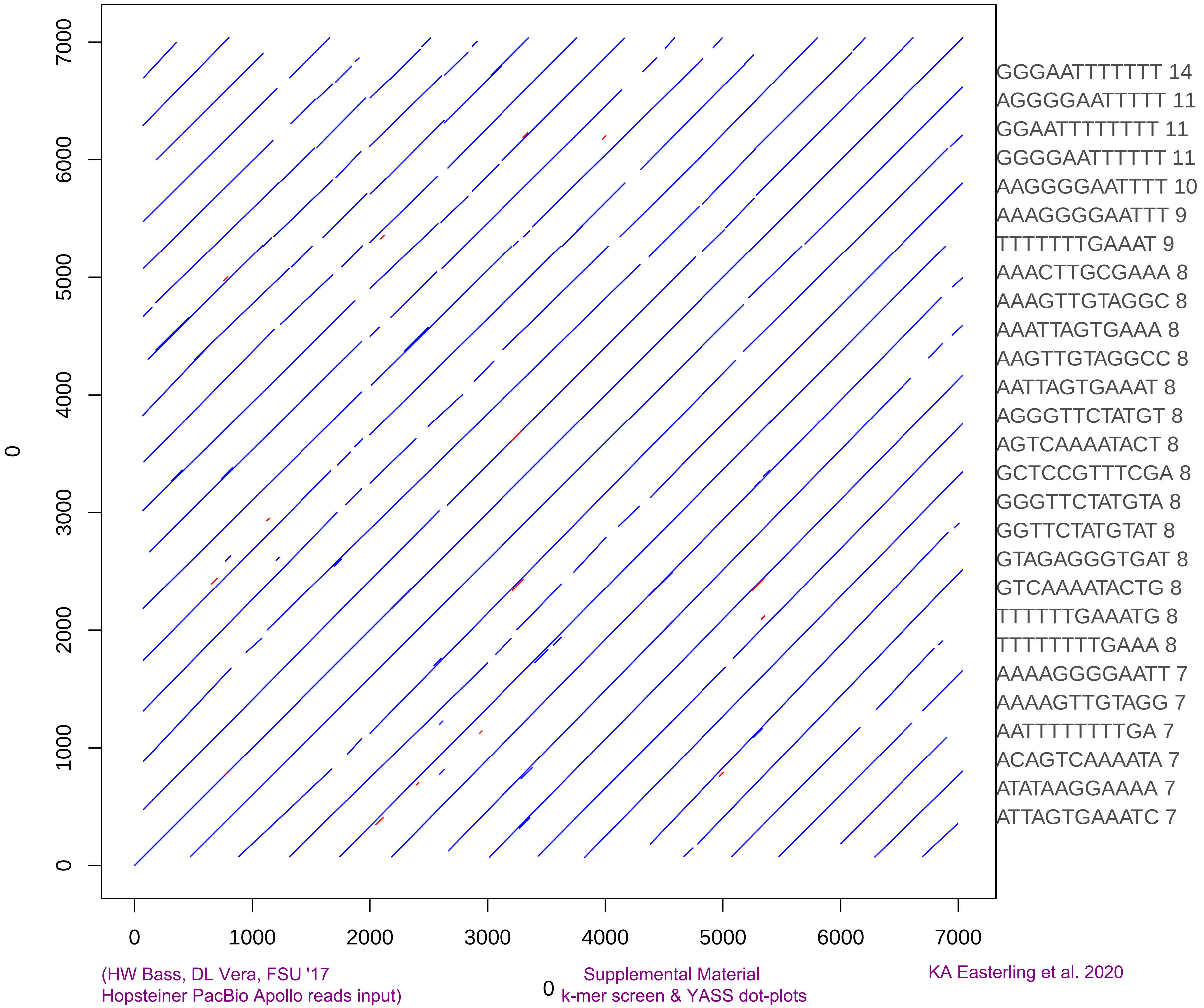

>m141121_232159_42134_c100732402550000001823141405141507_s1_p0/53991/182_8901

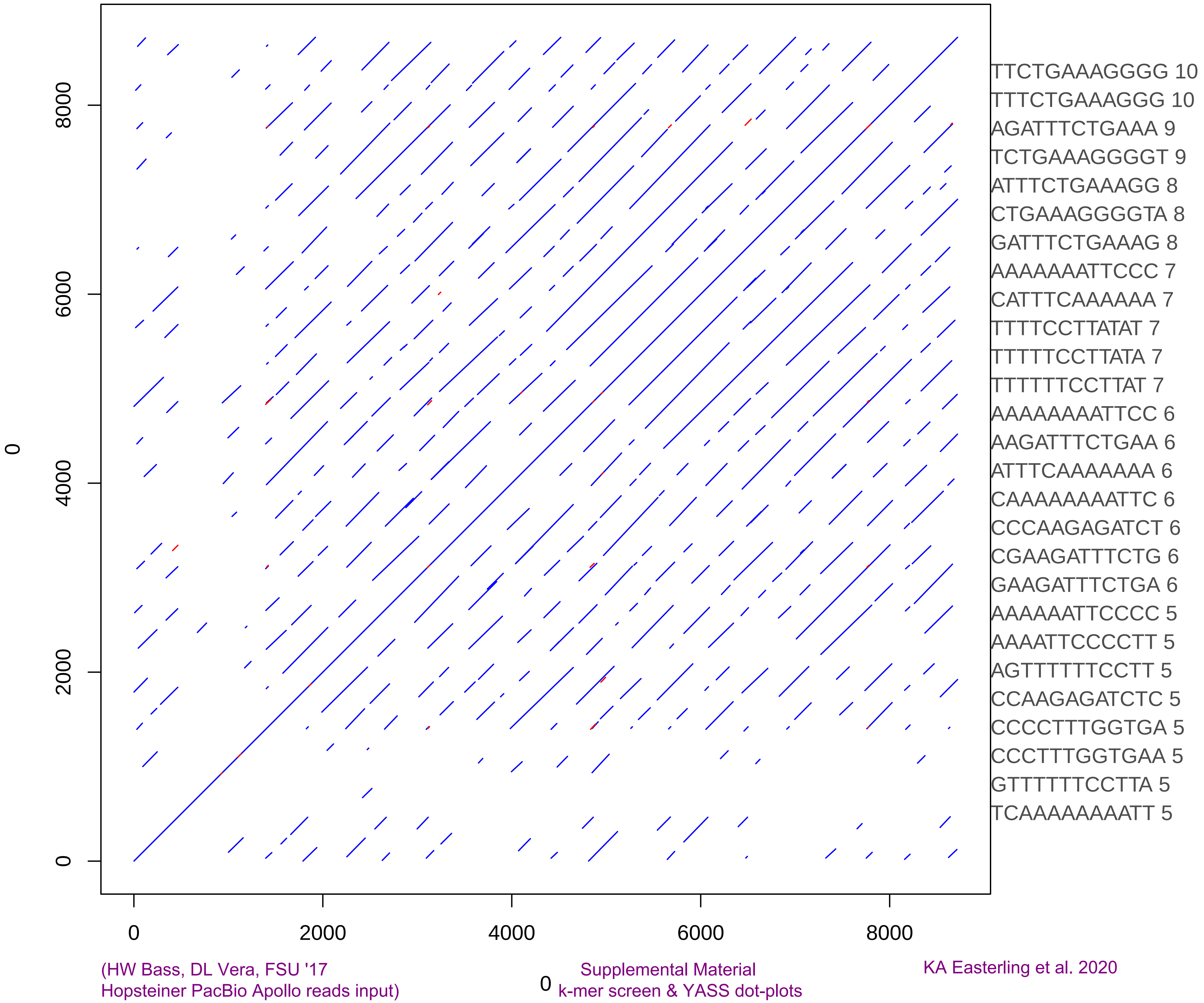

>m141121_232159_42134_c100732402550000001823141405141507_s1_p0/54455/2802_9817

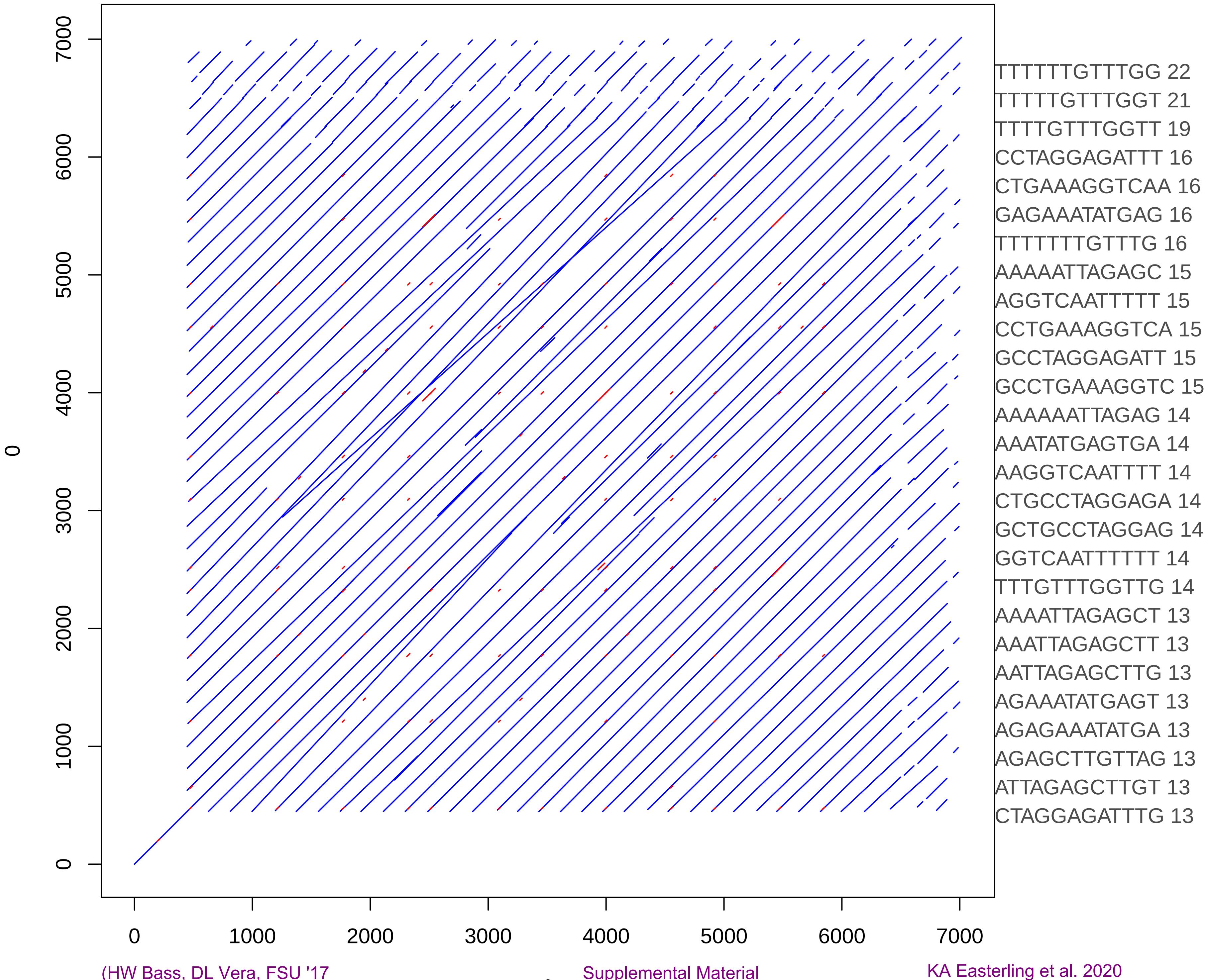

>m141121_232159_42134_c100732402550000001823141405141507_s1_p0/5531/4265_10239

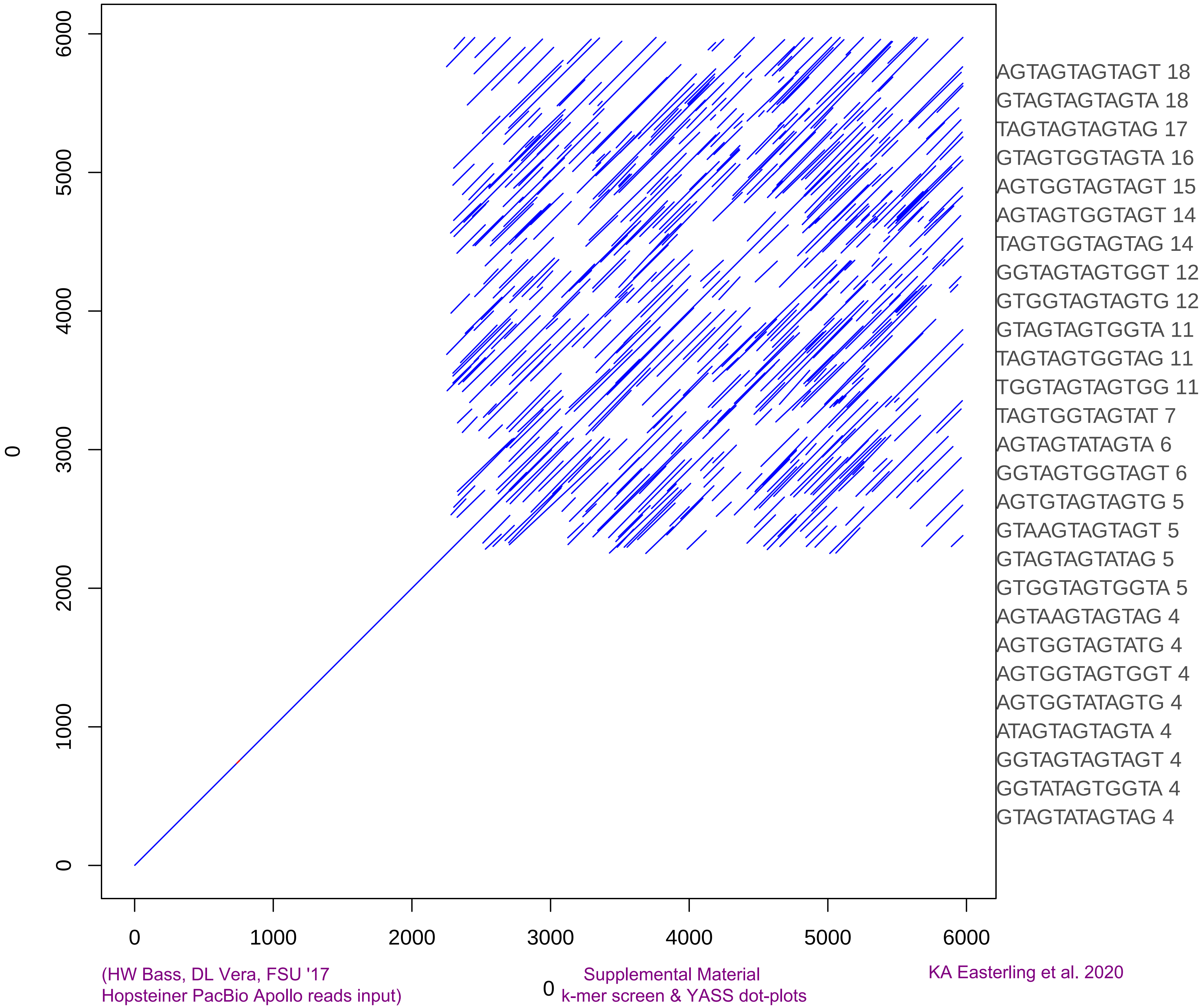

>m141121_232159_42134_c100732402550000001823141405141507_s1_p0/56268/7078_17579

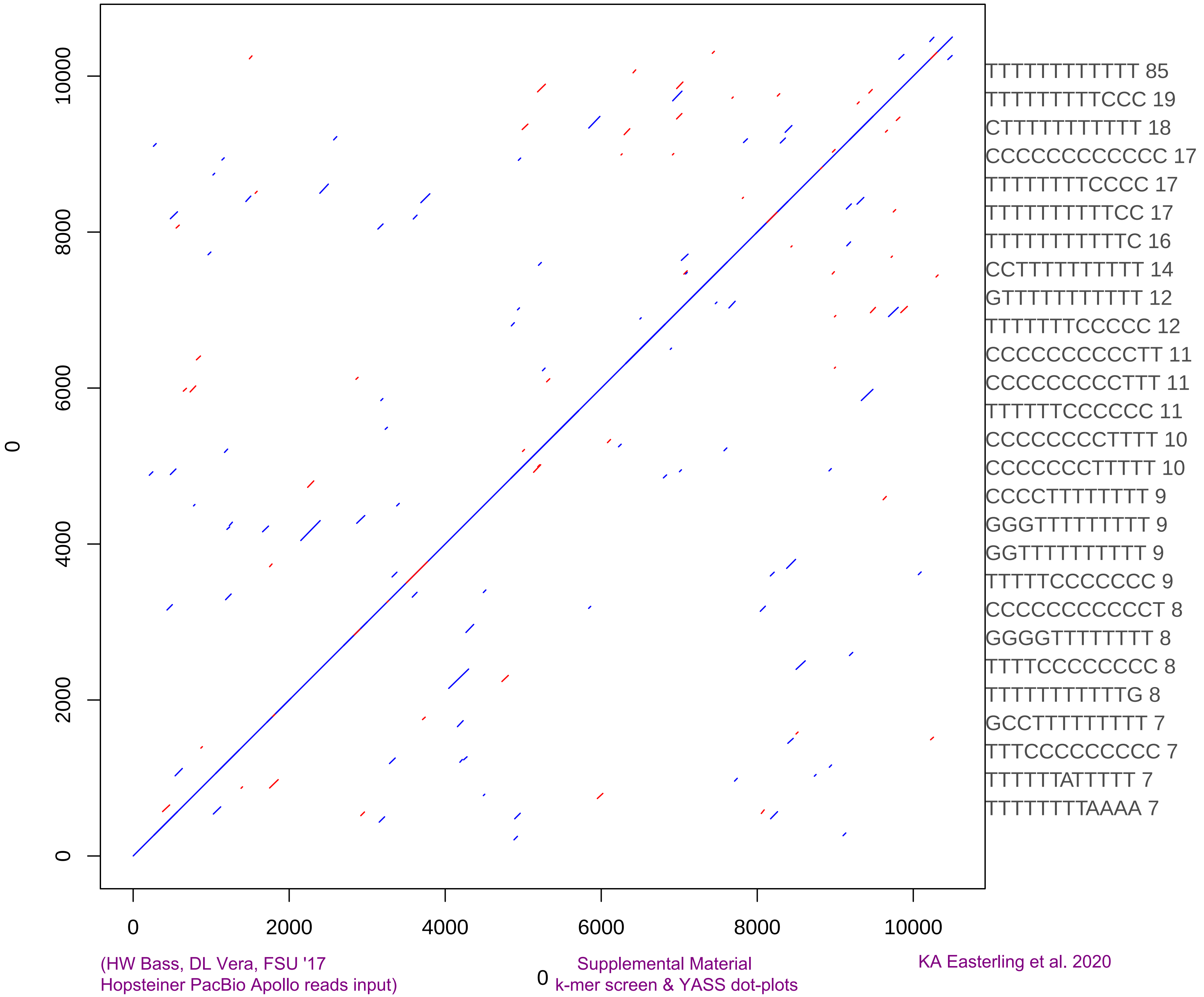

>m141121_232159_42134_c100732402550000001823141405141507_s1_p0/57017/11747_20757

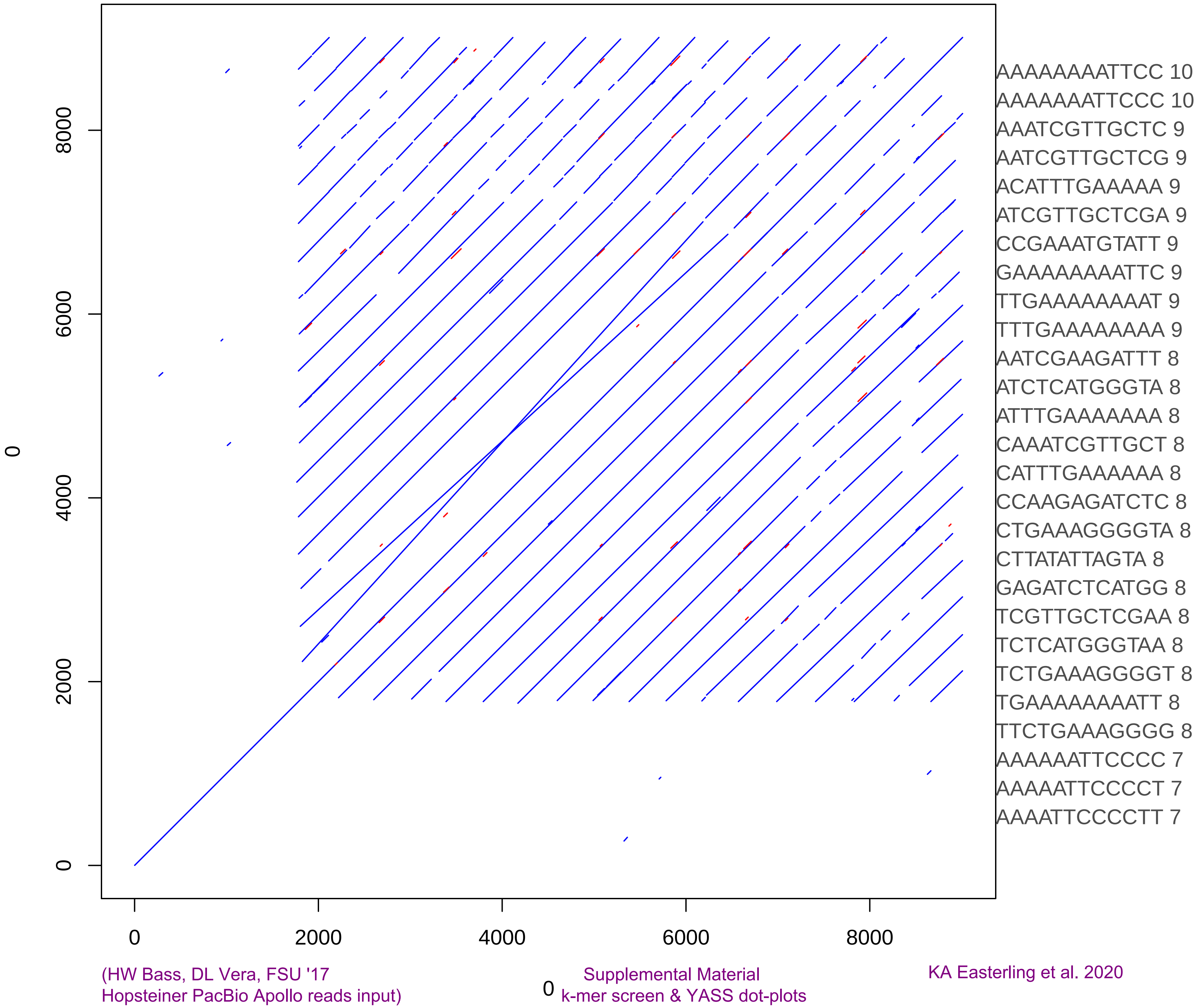

>m141121_232159_42134_c100732402550000001823141405141507_s1_p0/5811/3836_14090

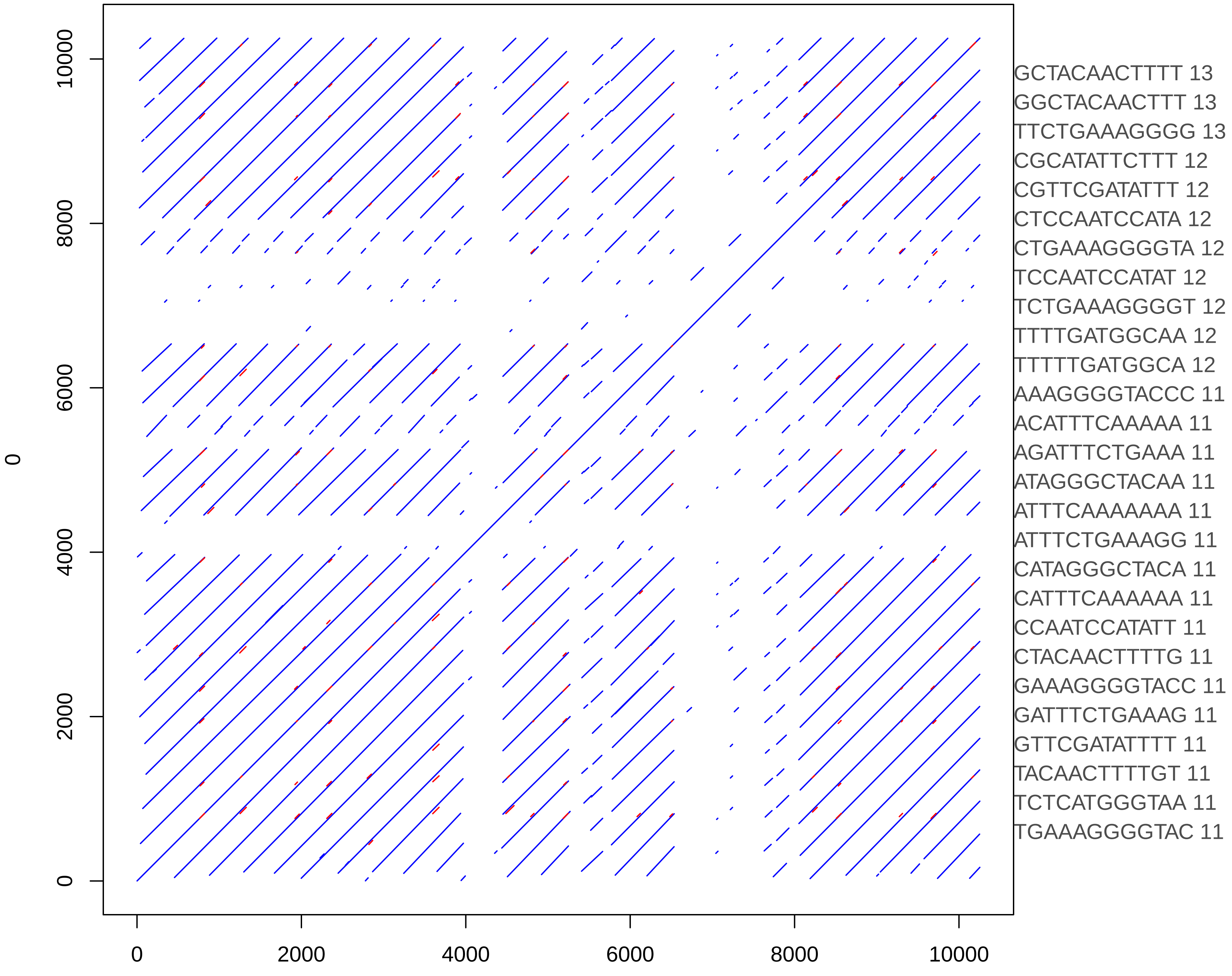

>m141121_232159_42134_c100732402550000001823141405141507_s1_p0/59454/4370_14686

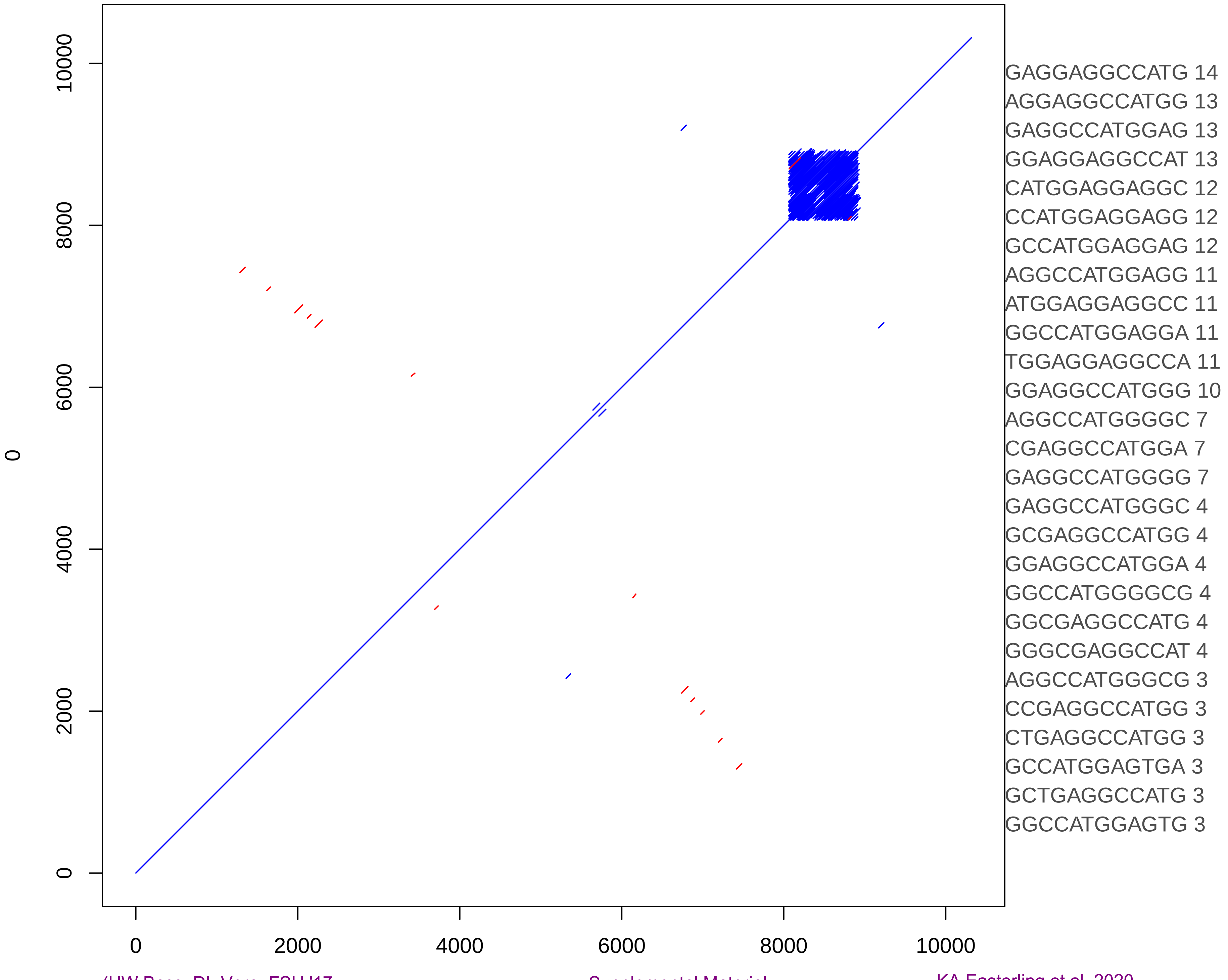

0

>m141121_232159_42134_c100732402550000001823141405141507_s1_p0/62806/5747_15077

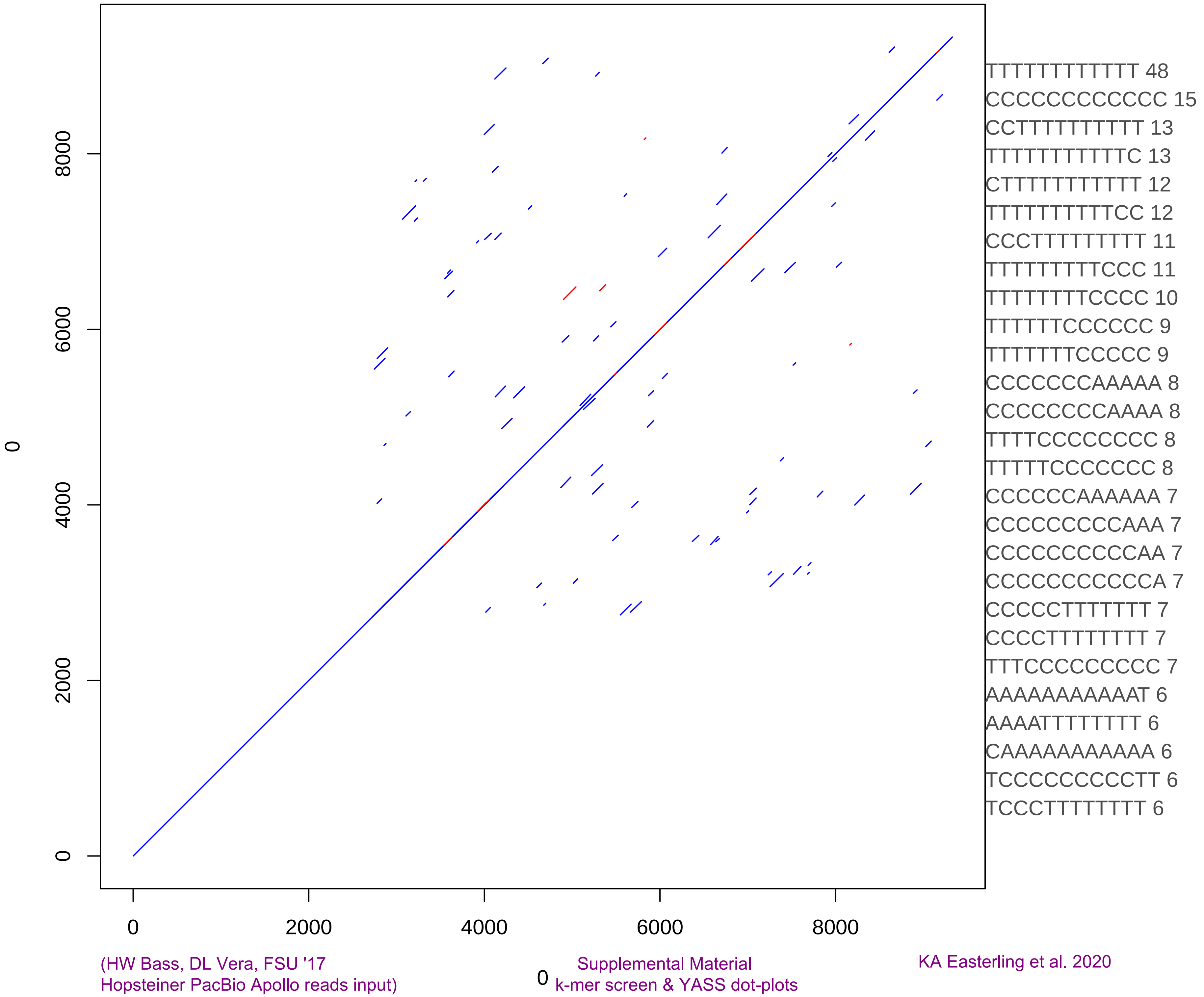

>m141121_232159_42134_c100732402550000001823141405141507_s1_p0/64601/5493_11268

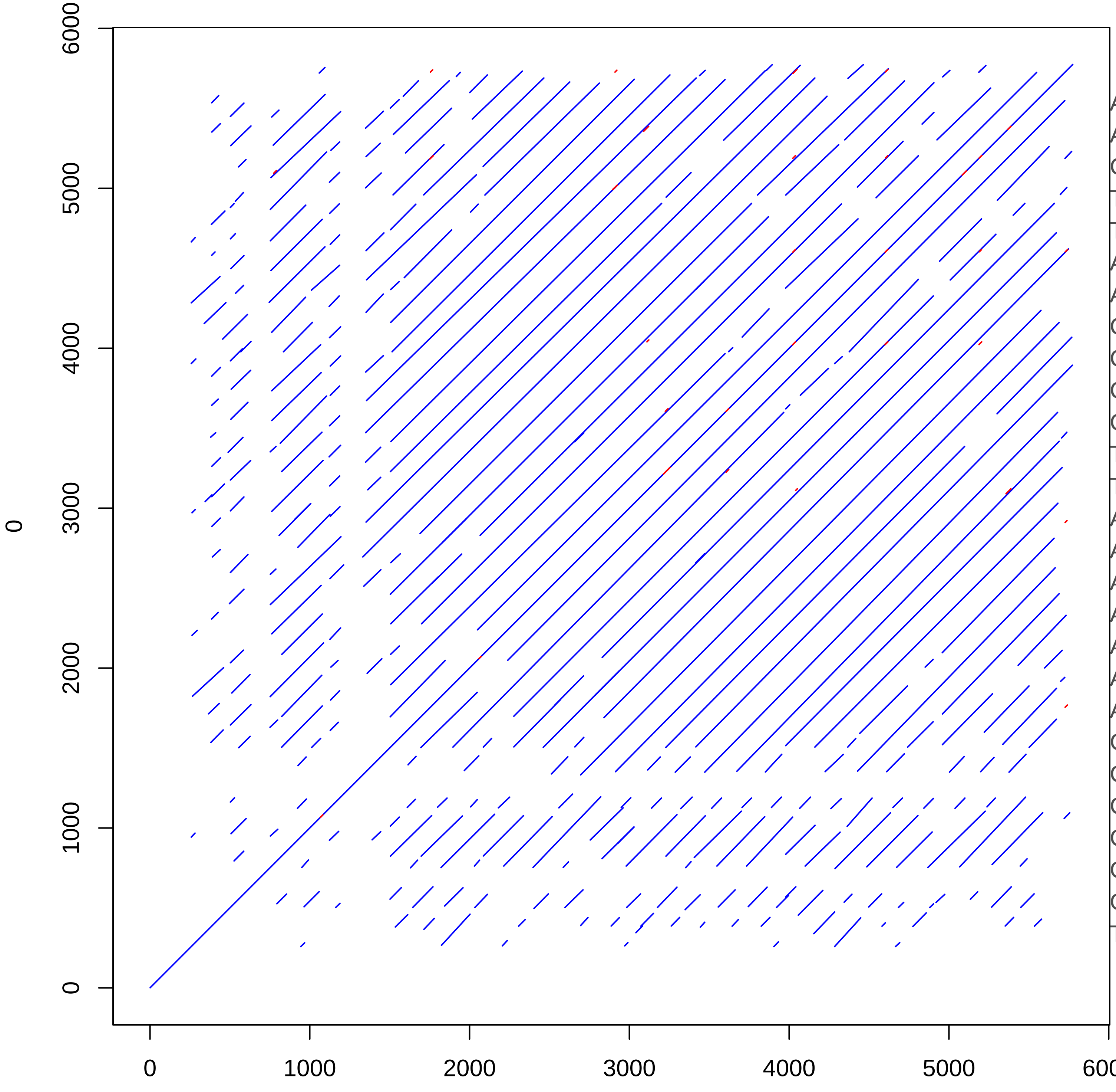

AAAAATTGGCCT 9
AAAATTGGCCTT 9
CTTTCCTGCCAA 9
TCTAATTTTTTT 9
TTTCCTGCCAAA 9
AAAAAATTGGCC 8
AAATTGGCCTTT 8
CATATTTCTCTC 8
CCCATCTCAATA 8
CCTTTCCTGCCA 8
CTCTAATTTTTT 8
TTCCTGCCAAAT 8
TTTCCCATCTCA 8
AAAAAAATTGGC 7
AACAAGCTCTAA 7
AATTGGCCTTTC 7
AATTTTTTTGAA 7
ACAAGCTCTAAT 7
ATTGGCCTTTCC 7
ATTTTTTTGAAC 7
CAAGCTCTAATT 7
CCAAATCTCCTA 7
CCTTTTCCCATC 7
CTTTTCCCATCT 7
GAACCTTTTCCC 7
GCCAAATCTCCT 7
TAATTTTTTTGA 7

>m141121_232159_42134_c100732402550000001823141405141507_s1_p0/65248/11611_19742

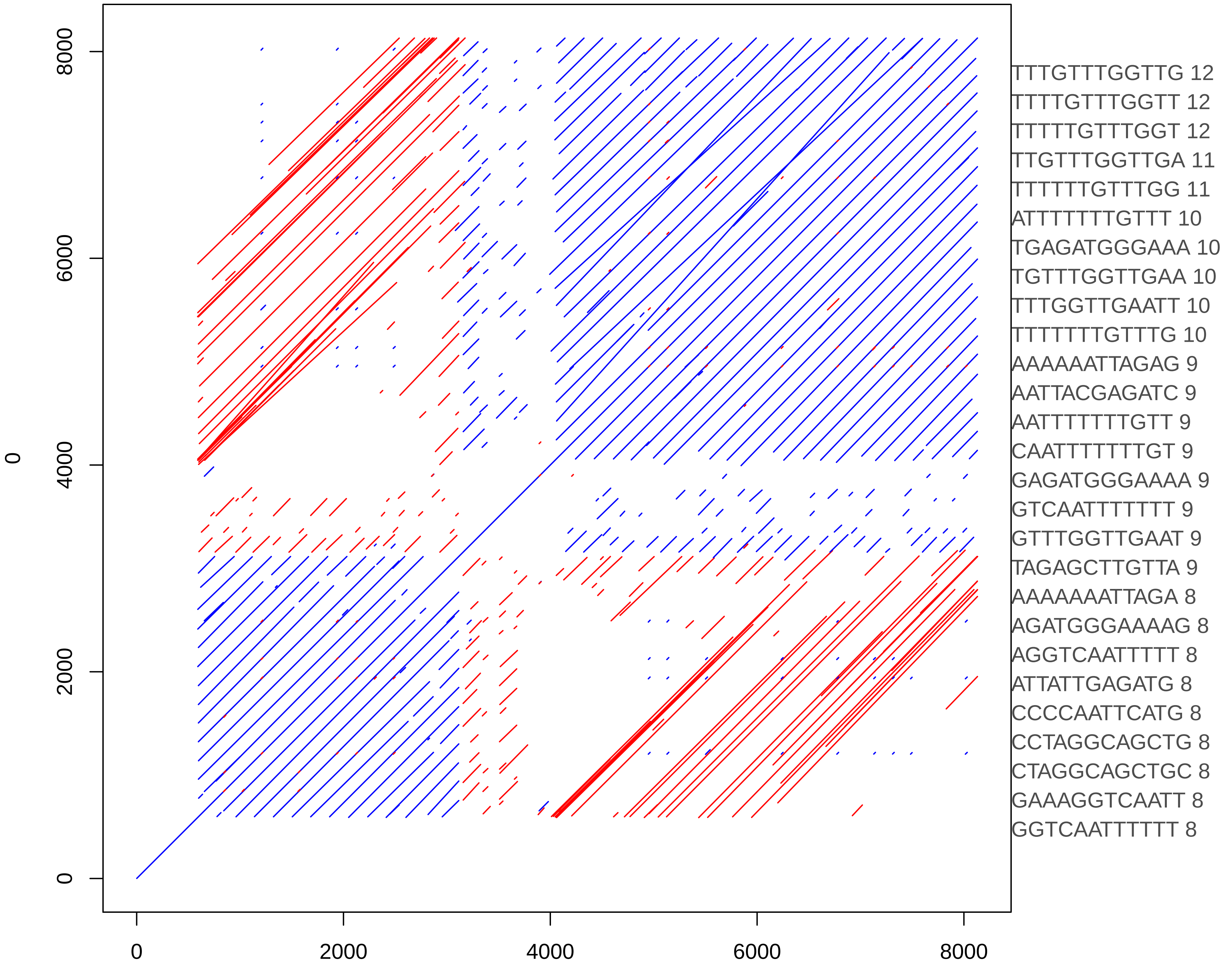

>m141121_232159_42134_c100732402550000001823141405141507_s1_p0/68845/1513_6776

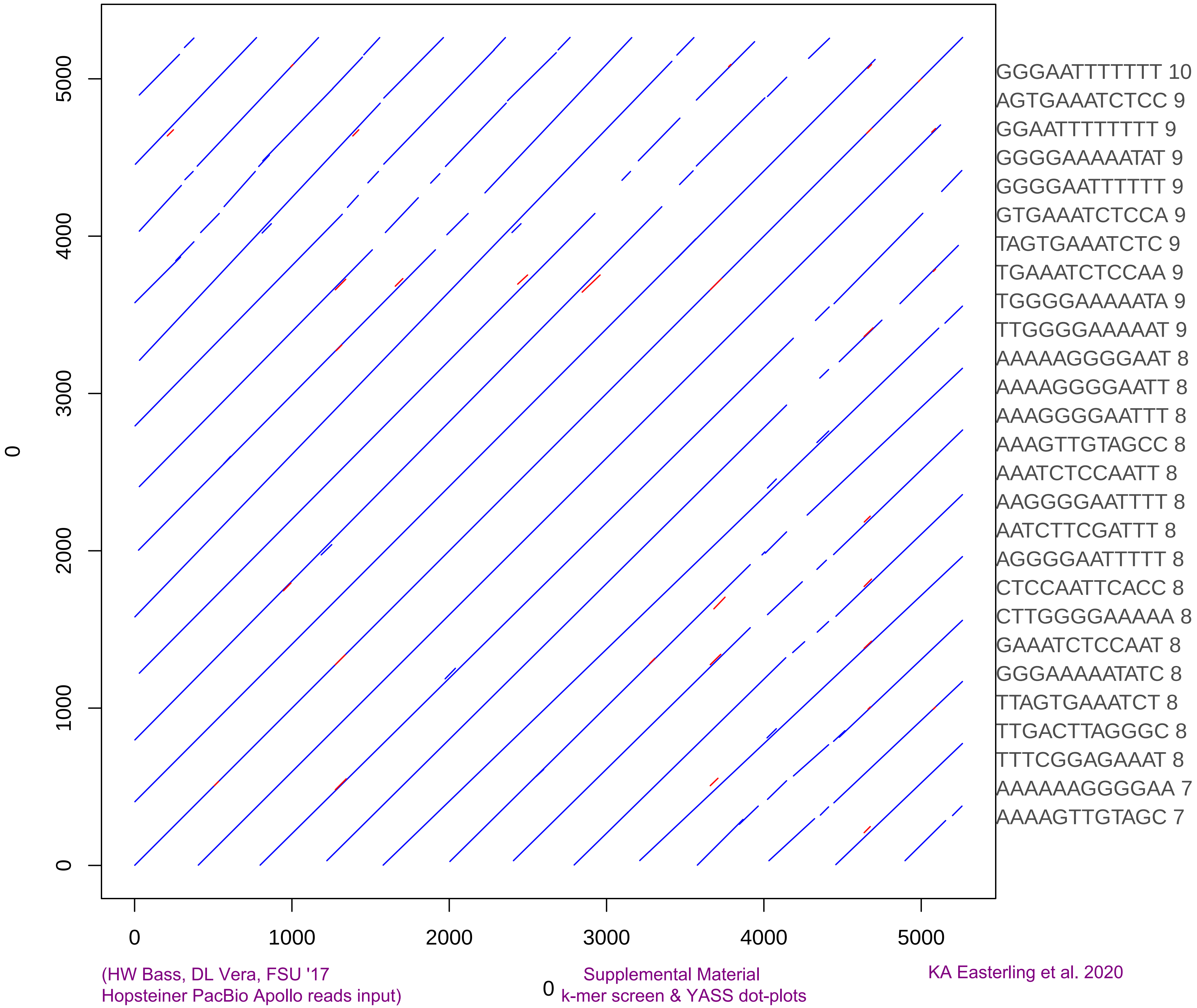

>m141121_232159_42134_c100732402550000001823141405141507_s1_p0/68937/3910_9373

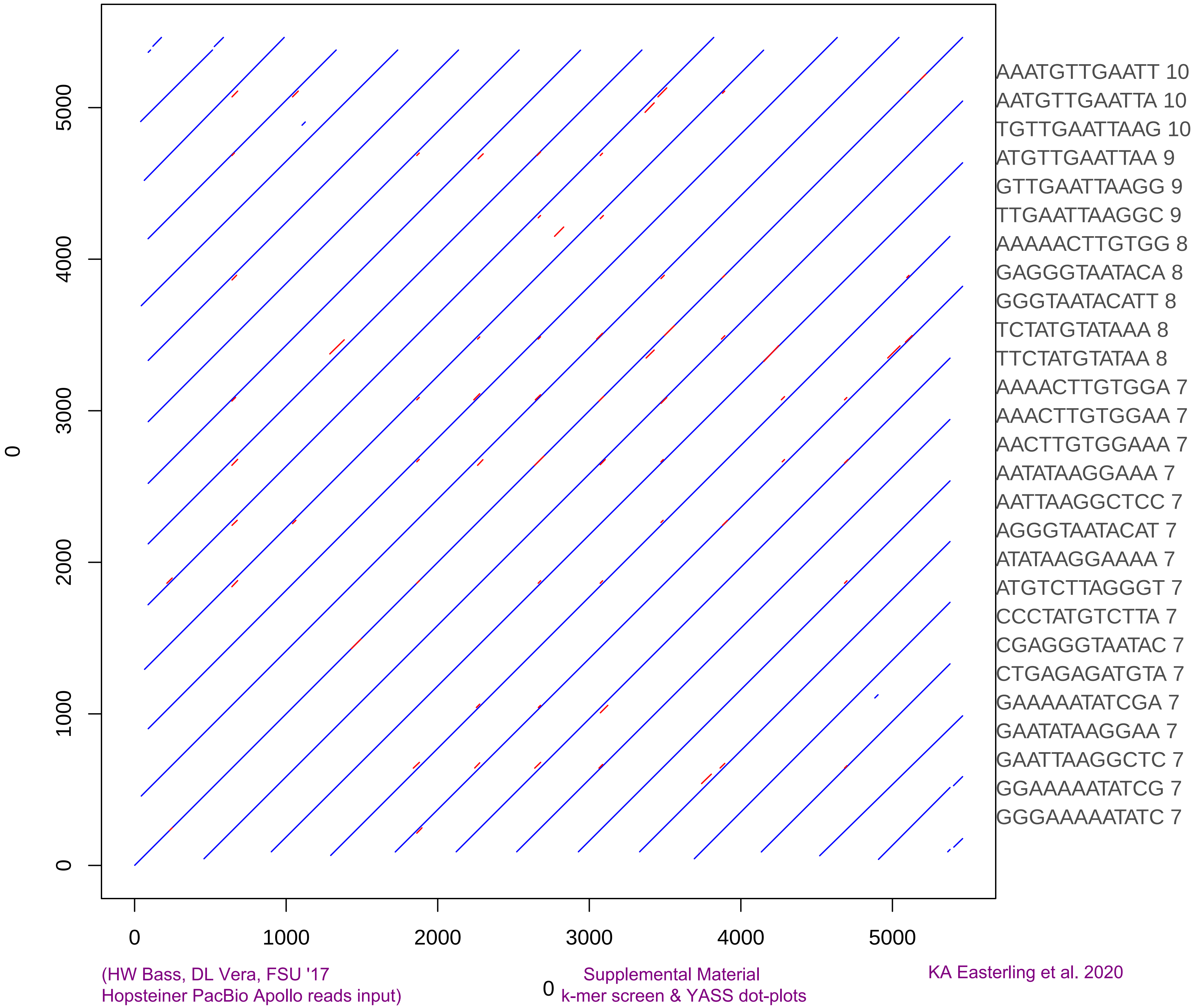

>m141121_232159_42134_c100732402550000001823141405141507_s1_p0/70785/0_6819

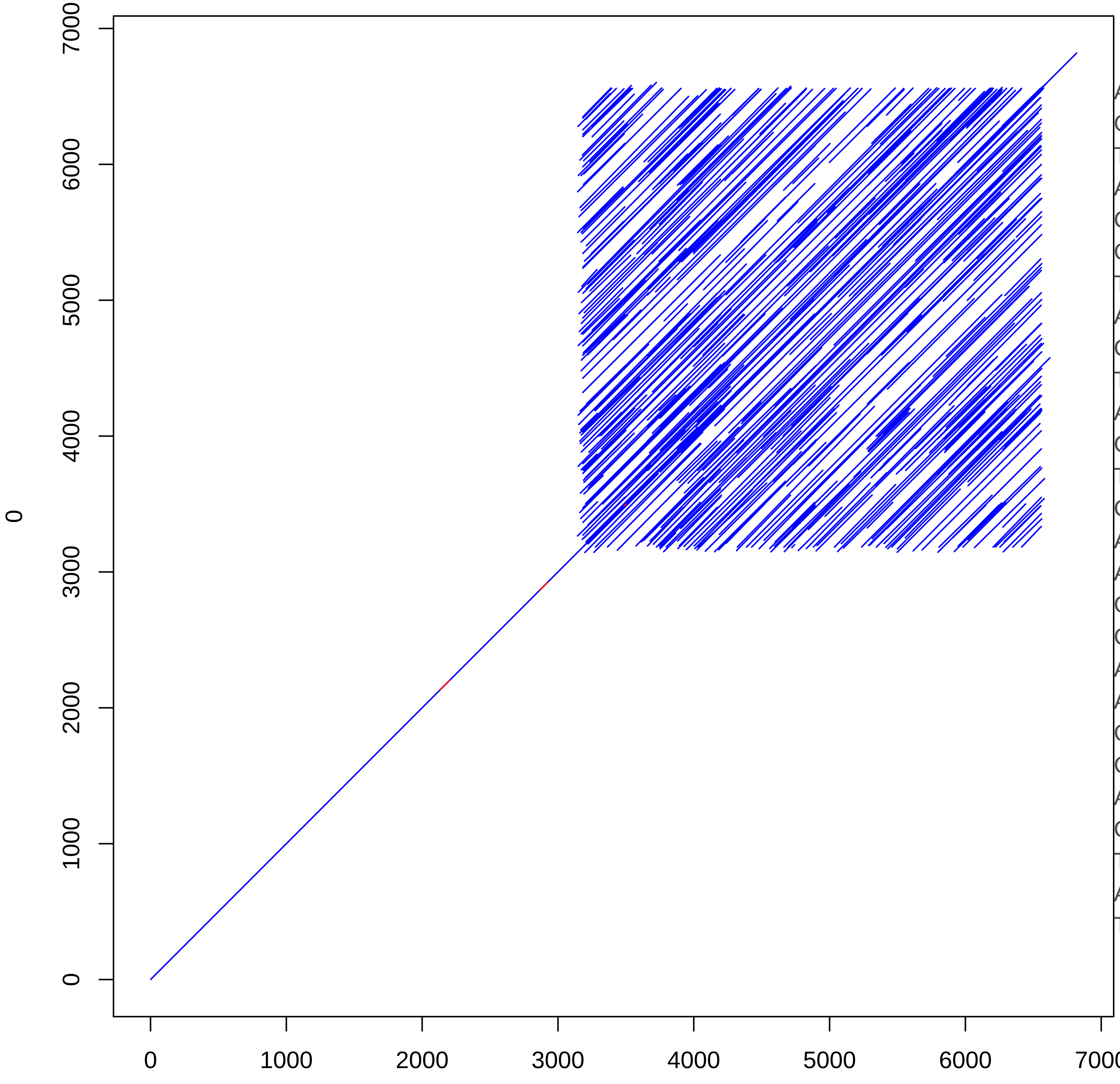

AGCAGTAGCAGT 29
GCAGTAGCAGTA 29
TAGCAGTAGCAG 29
AGTAGCAGTAGC 27
CAGTAGCAGTAG 27
GTAGCAGTAGCA 27
TAGCAGTAGTAG 27
AGTAGCAGTAGT 25
GTAGCAGTAGTA 25
TAGTAGTAGTAG 25
AGTAGTAGTAGT 24
GTAGTAGTAGTA 23
TAGTAGCAGTAG 21
GTAGTAGCAGTA 20
AGTAGTAGCAGT 18
AGCAGTAGTAGT 17
GCAGTAGTAGTA 17
CAGTAGTAGTAG 15
AGCAGTAGTAGC 13
AGTAGTAGTAGA 13
GCAGTAGTAGCA 13
CAGTAGTAGCAG 11
AGTAGTAGATAG 10
GTAGTAGATAGT 10
TAGTAGTAGCAG 10
AGCAGCAGCAGC 9
TAGCAGCAGTAG 9

>m141121_232159_42134_c100732402550000001823141405141507_s1_p0/80462/8042_15061

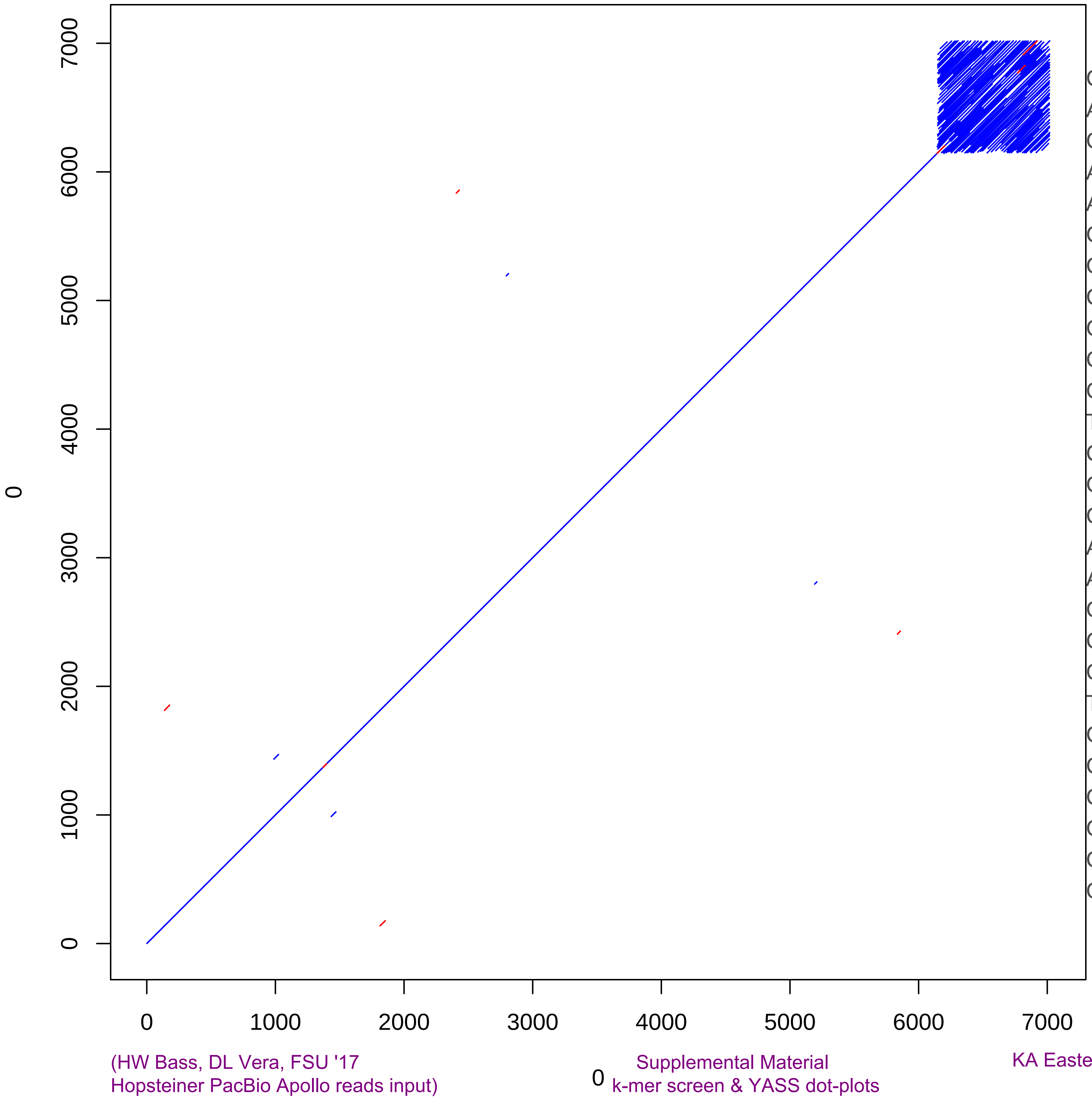

CATGGAGGAGGC 14
AGGCCATGGAGG 11
GAGGCCATGGAG 11
AGGAGGCCATGG 10
ATGGAGGAGGCC 10
CGAGGCCATGGA 10
GGCCATGGAGGA 10
CCATGGAGGAGG 9
GAGGAGGCCATG 9
GCCATGGAGGAG 9
GGAGGCCATGGG 9
TGGAGGAGGCCA 9
CCGAGGCCATGG 8
GAGGCCATGGGG 7
GGGCCGAGGCCA 7
AGGCCATGGGGC 6
ATGGGGCCGAGG 6
GCCGAGGCCATG 6
GGCCATGGGGCC 6
GGCCGAGGCCAT 6
TGGGGCCGAGGC 6
CATGGGGCCGAG 5
CCATGGGGCCGA 5
GCATGGAGGAGG 5
GGCGAGGCCATG 5
GGGGCCGAGGCC 5
GCCATGGGGCCG 4

>m141121_232159_42134_c100732402550000001823141405141507_s1_p0/80860/8021_15368

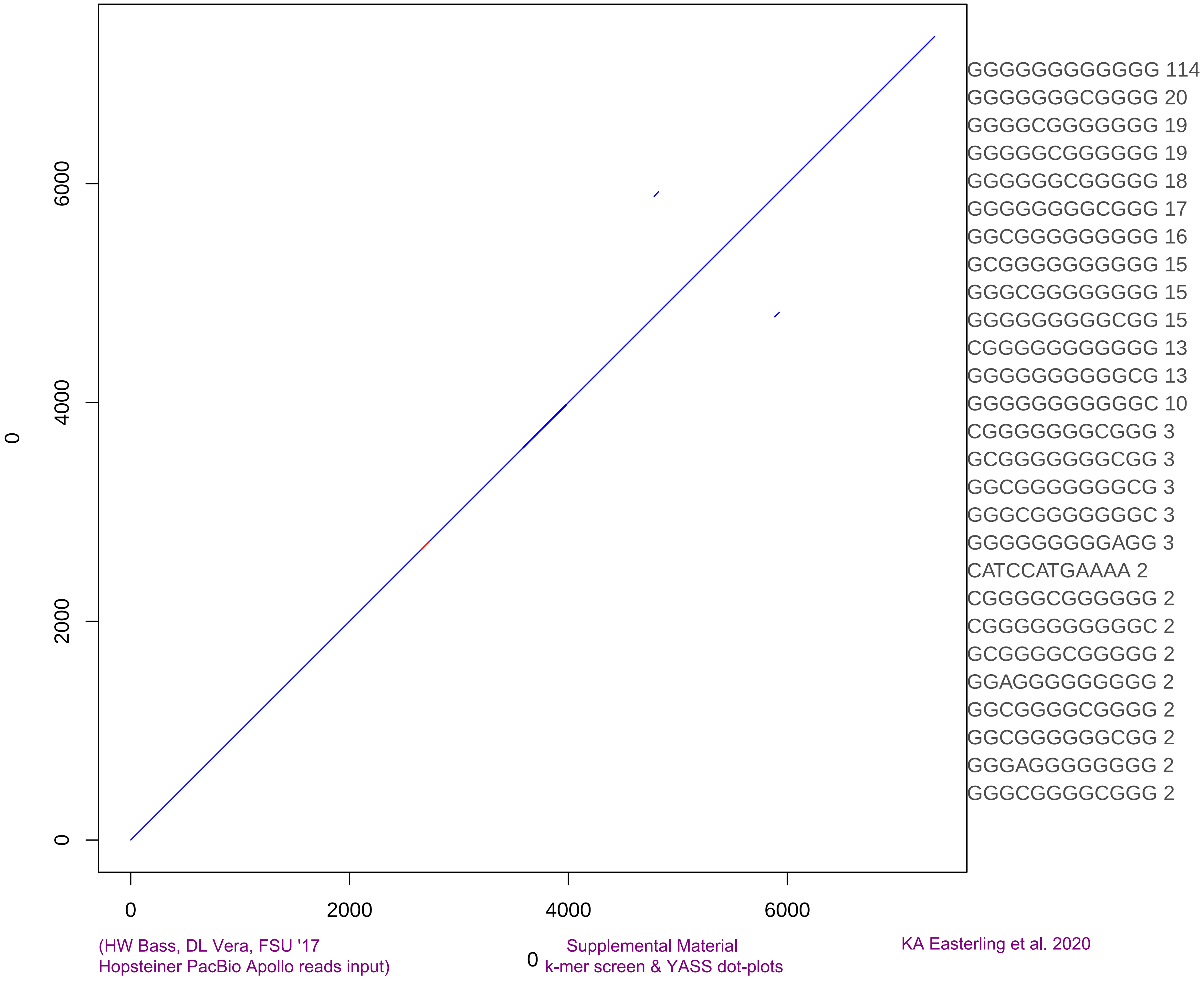

>m141121_232159_42134_c100732402550000001823141405141507_s1_p0/8659/1581_7400

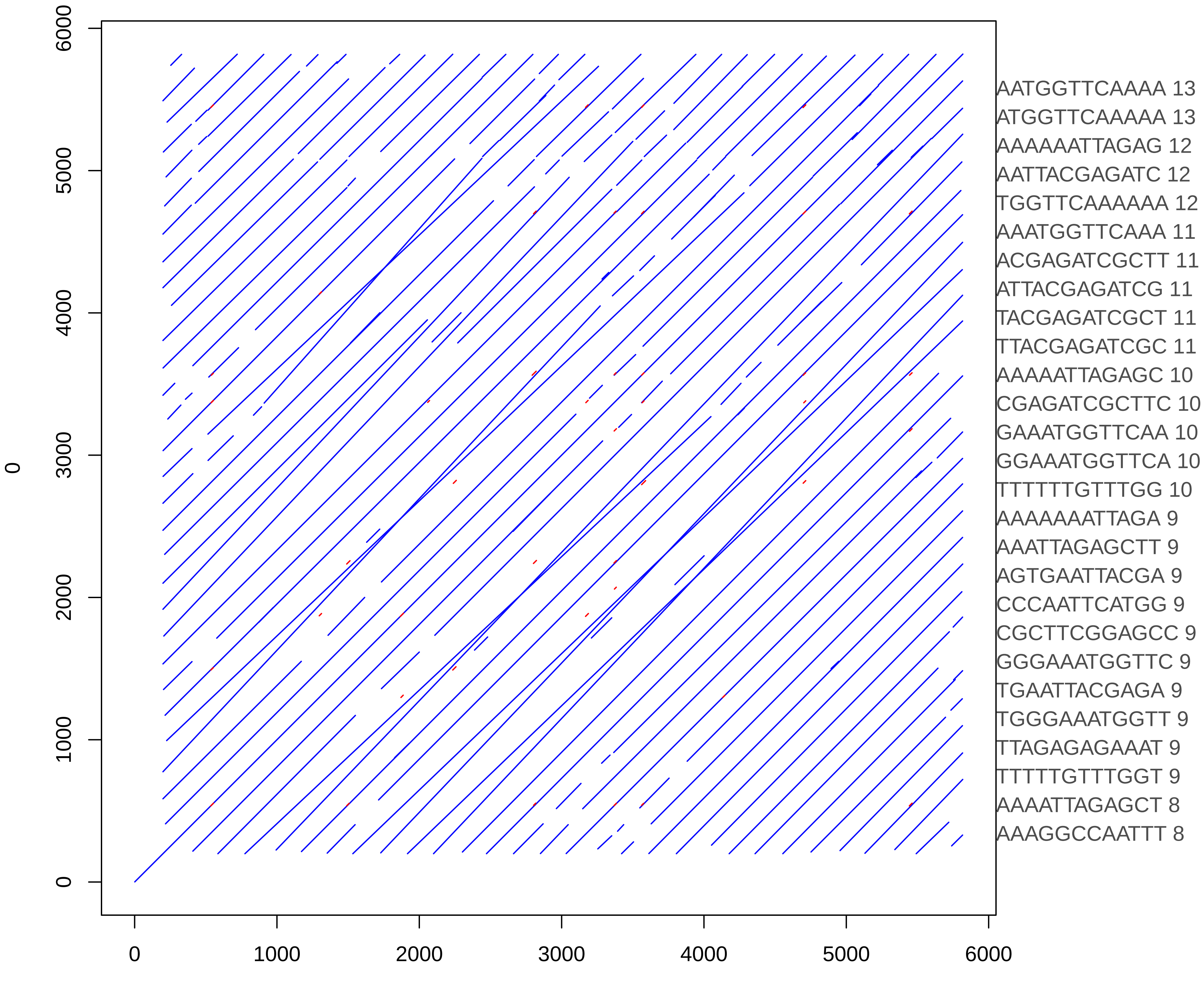

>m141121_232159_42134_c100732402550000001823141405141507_s1_p0/90203/2412_10814

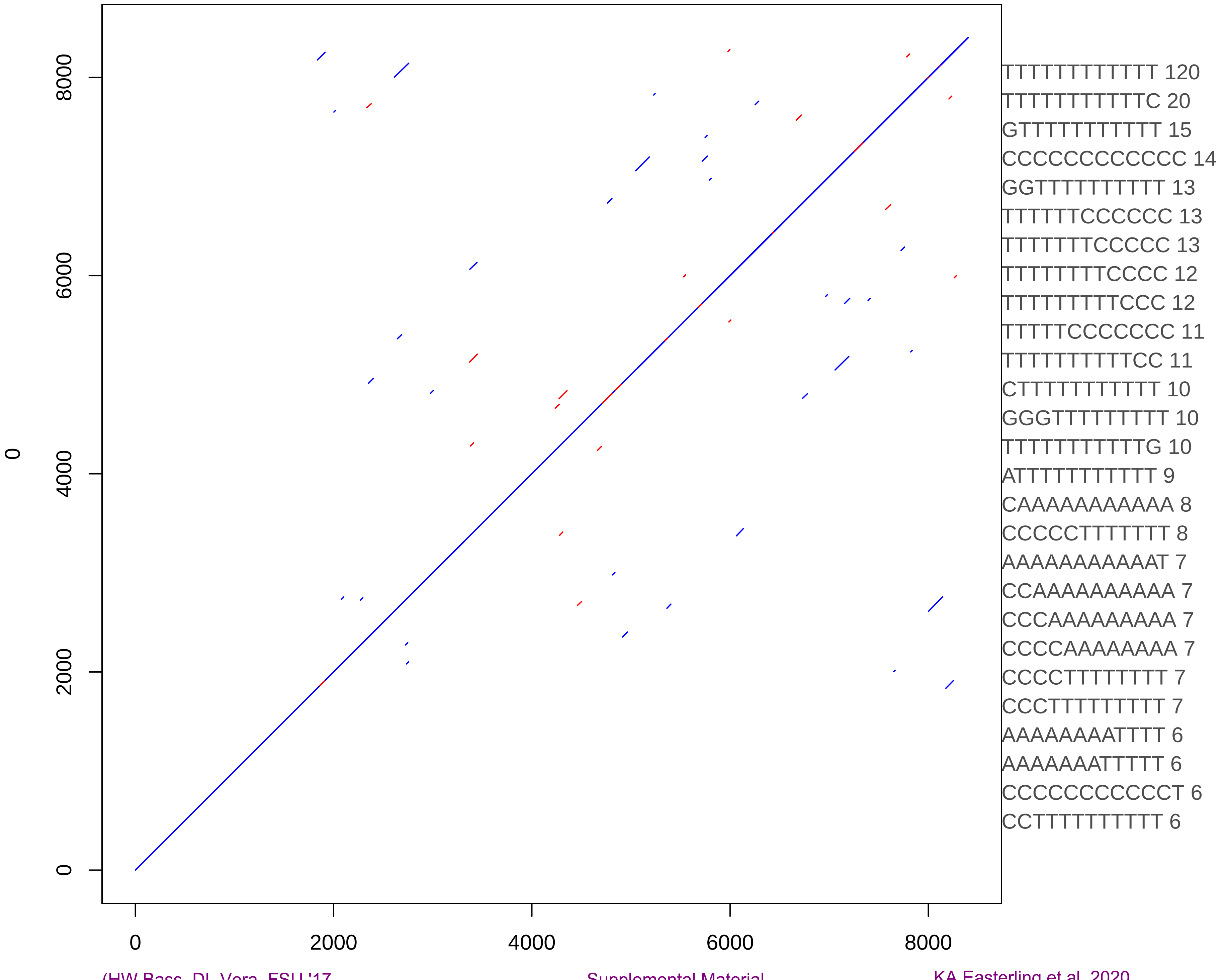

>m141121_232159_42134_c100732402550000001823141405141507_s1_p0/92762/0_5247

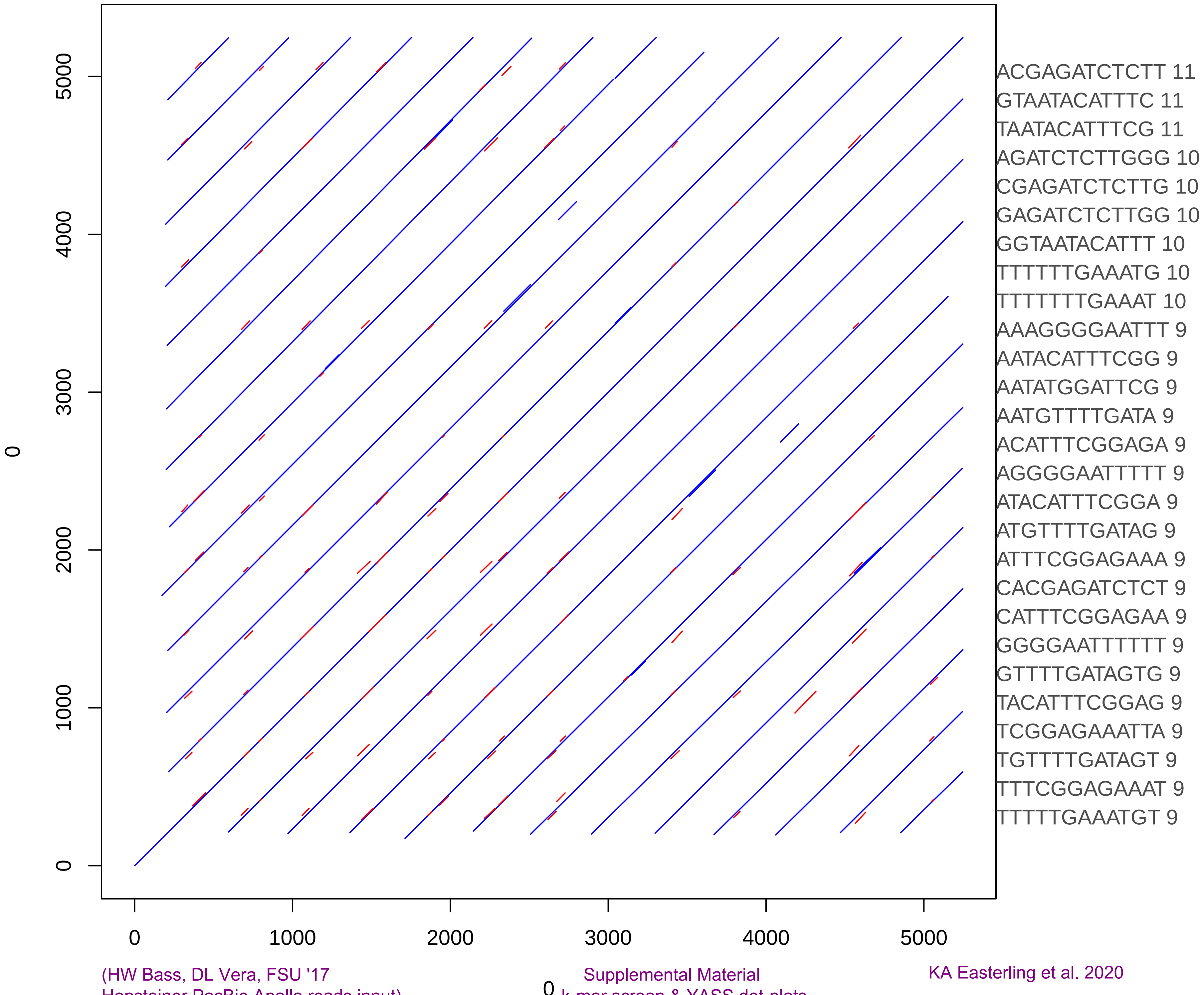

>m141121_232159_42134_c100732402550000001823141405141507_s1_p0/93462/3284_14773

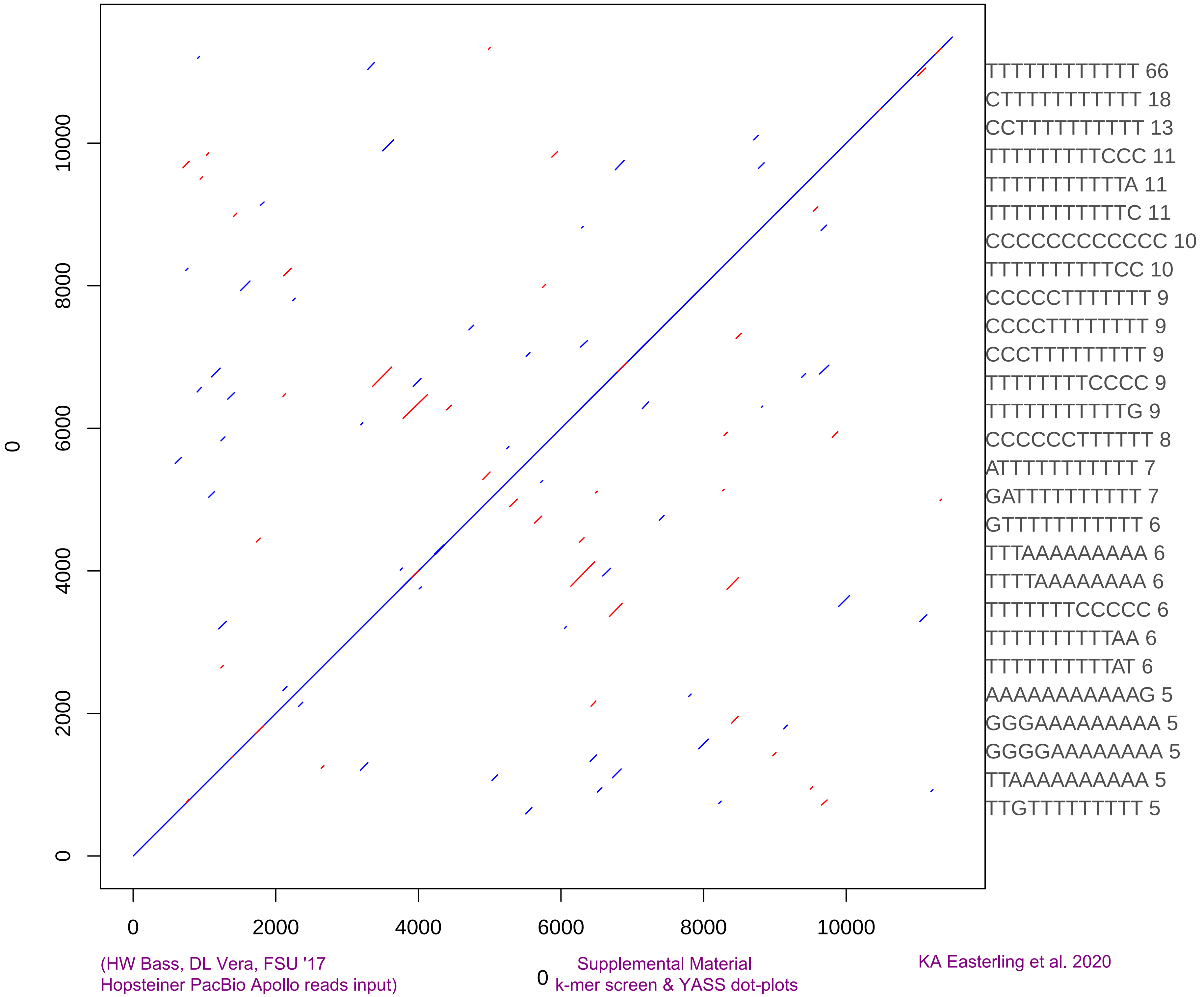

>m141121_232159_42134_c100732402550000001823141405141507_s1_p0/95562/10119_21892

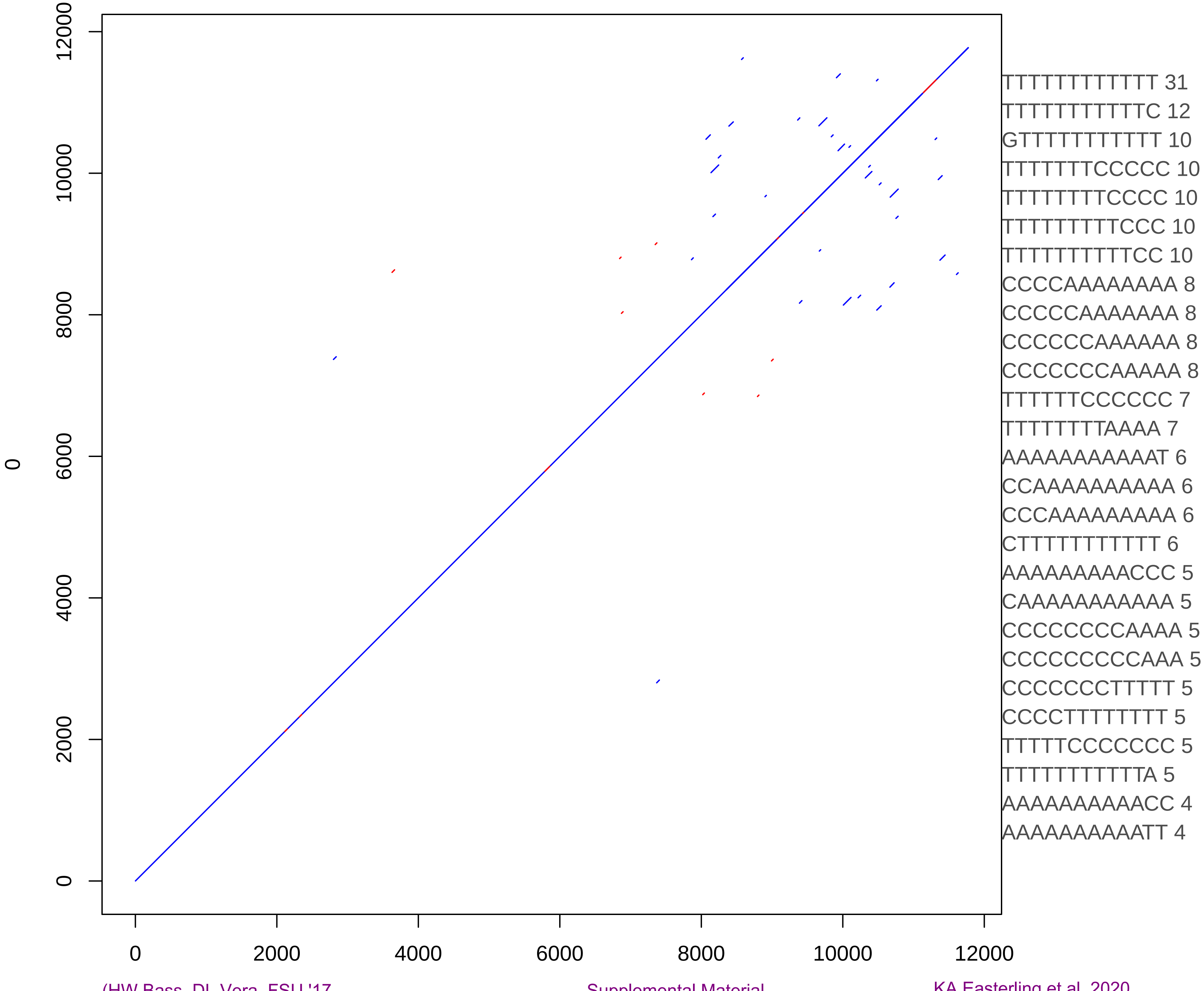

0

>m141121_232159_42134_c100732402550000001823141405141507_s1_p0/95679/6247_11460

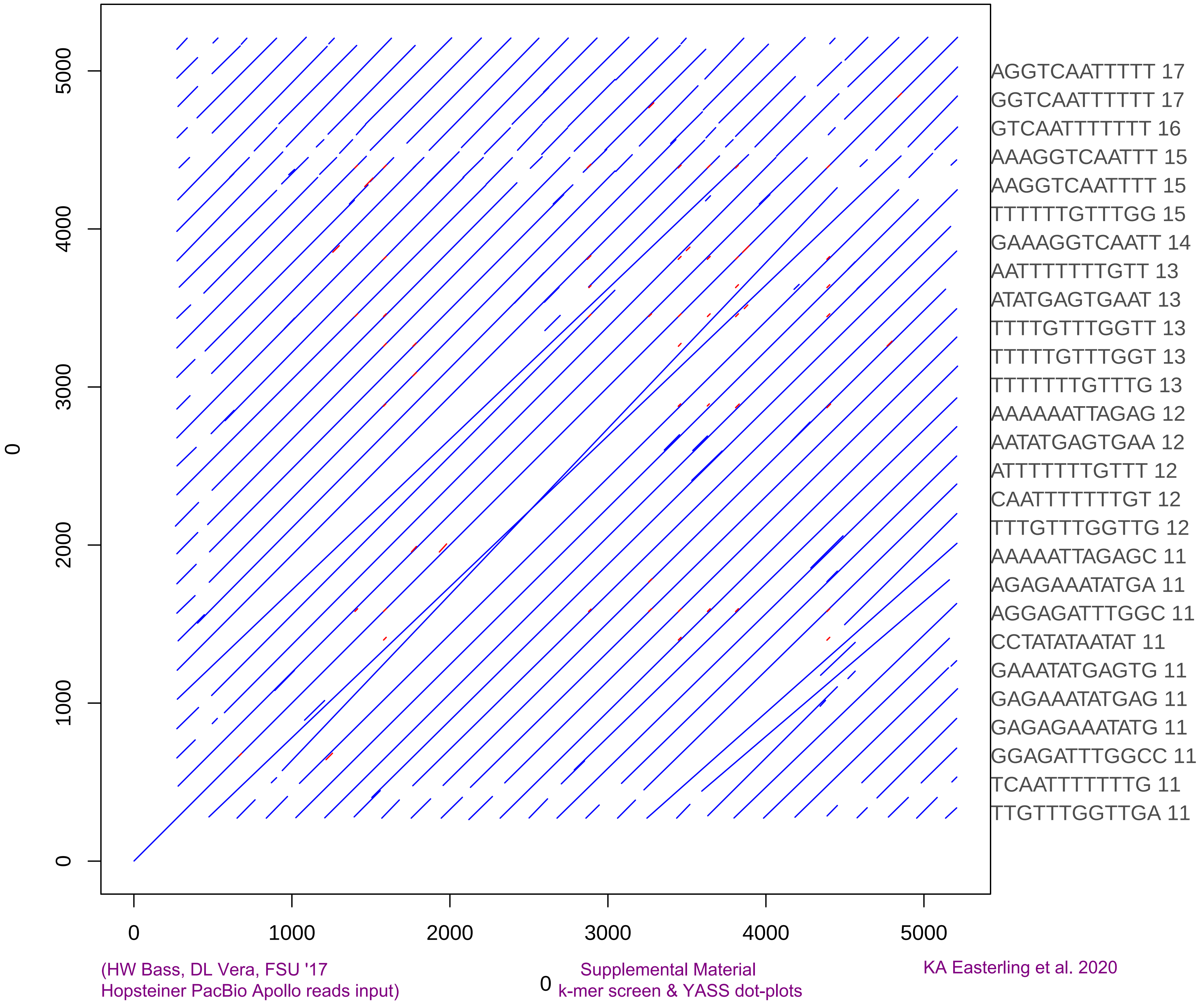

>m141122_014521_42134_c100732202550000001823141405141560_s1_p0/101479/8146_17057

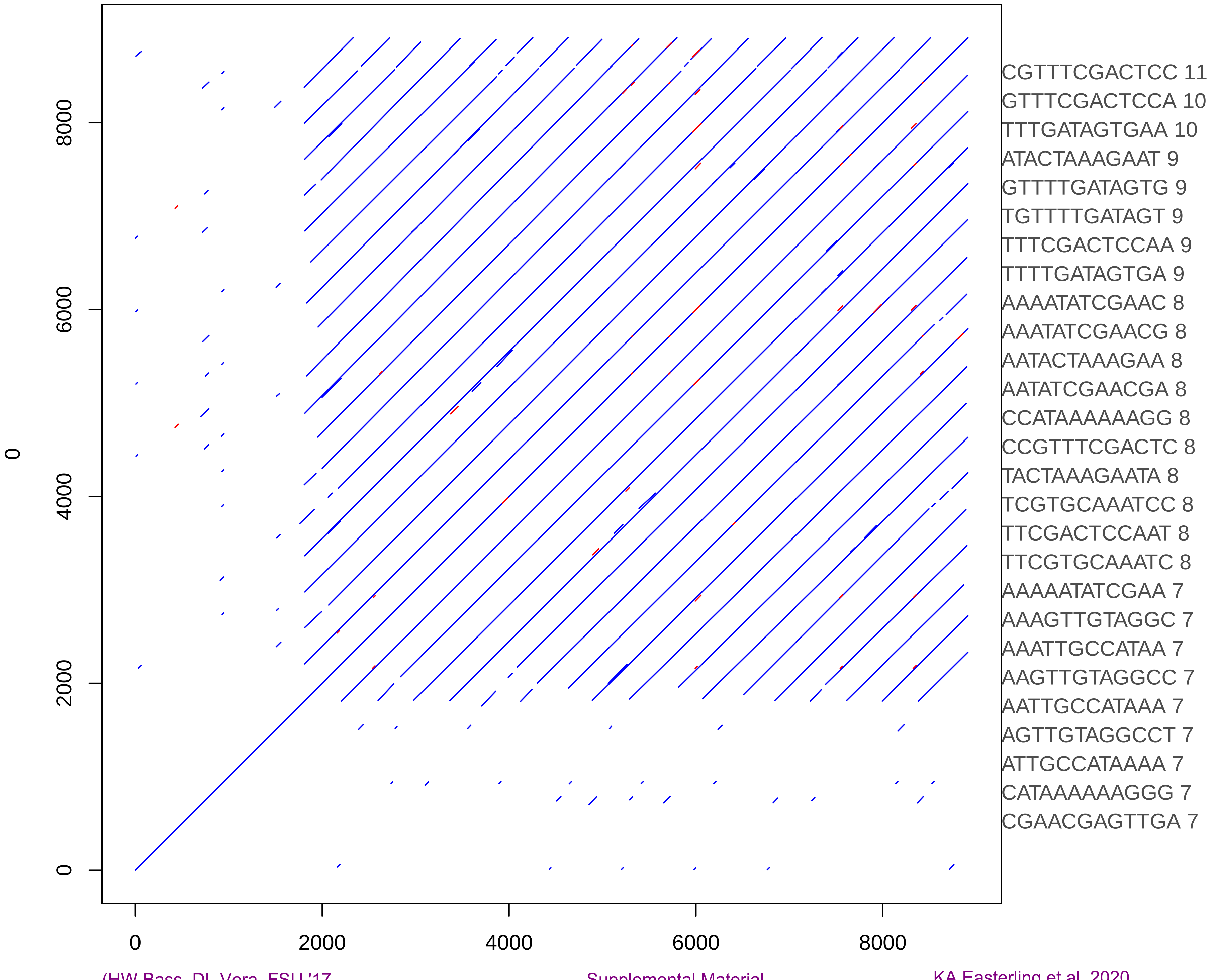

>m141122_014521_42134_c100732202550000001823141405141560_s1_p0/105534/0_6028

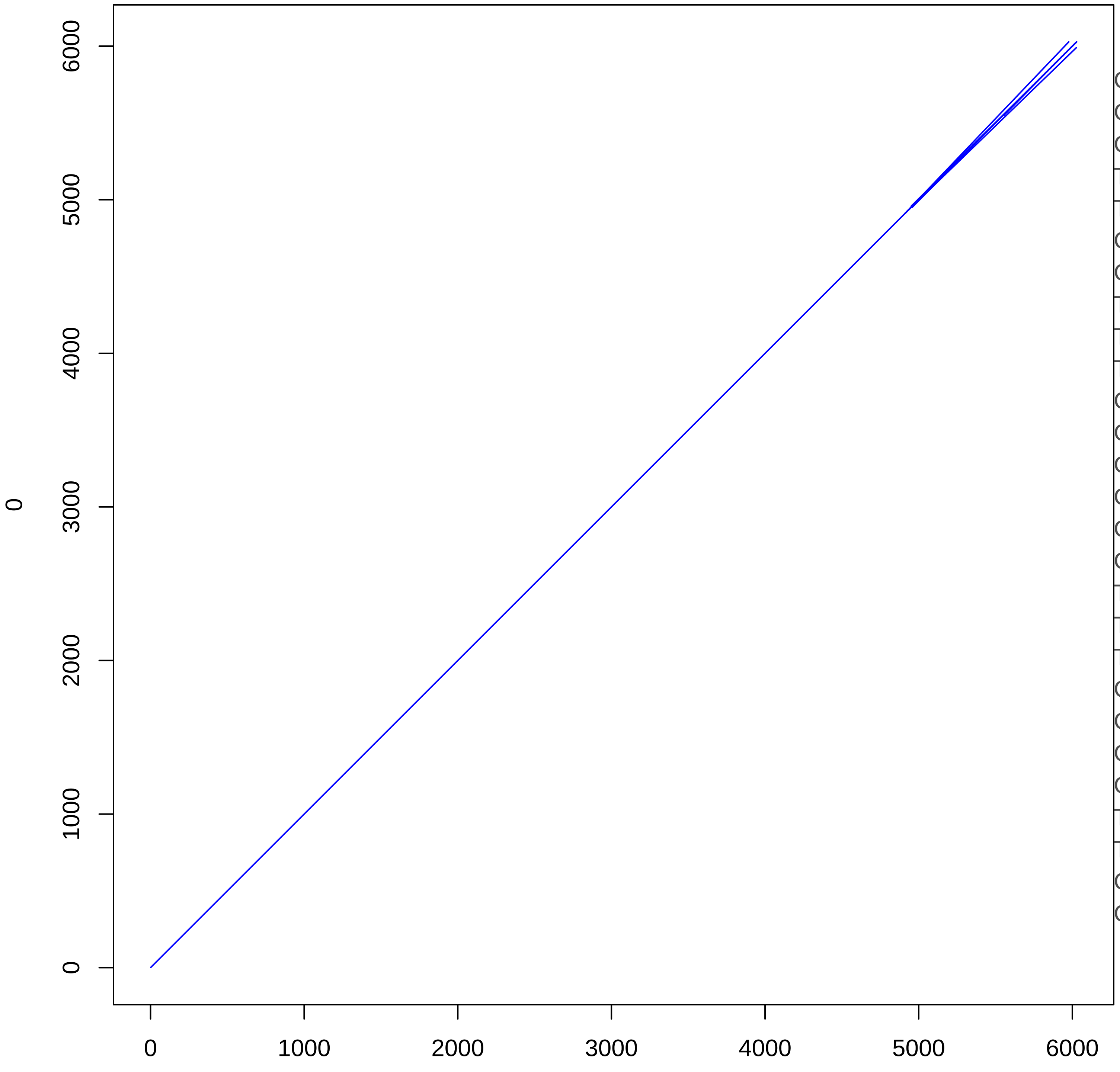

GTGGTGTGTGTG 18
GTGTGTGTGTGT 16
GGTGTGTGTGTG 15
TGGTGTGTGTGT 15
TGTGTGTGTGTG 14
GTGTGTGTGGTG 12
GGTGGTGTGTGT 10
TGTGGTGTGTGT 10
TGGTGGTGTGTG 9
TGTGTGTGGTGT 9
GTGTGTGTGTGG 8
GTGGTGGTGTGT 7
GTGTGGTGGTGT 7
GTGTGGTGTGTG 7
GTGTGTGGTGTG 7
GTGTGTGTGTTG 7
TGTGGTGGTGTG 7
TGTGTGTGTGGT 7
TGTGTGTGTTGT 7
GGTGTGGTGGTG 6
GTGGTGTGGTGG 6
GTGTGGTGTGGT 6
GTGTGTGTTGTG 6
TGGTGTGGTGGT 6
TGTGGTGTGGTG 6
GGTGTGTGTGGT 5
GTGGGTGGTGTG 5

>m141122_014521_42134_c100732202550000001823141405141560_s1_p0/106662/2616_10574

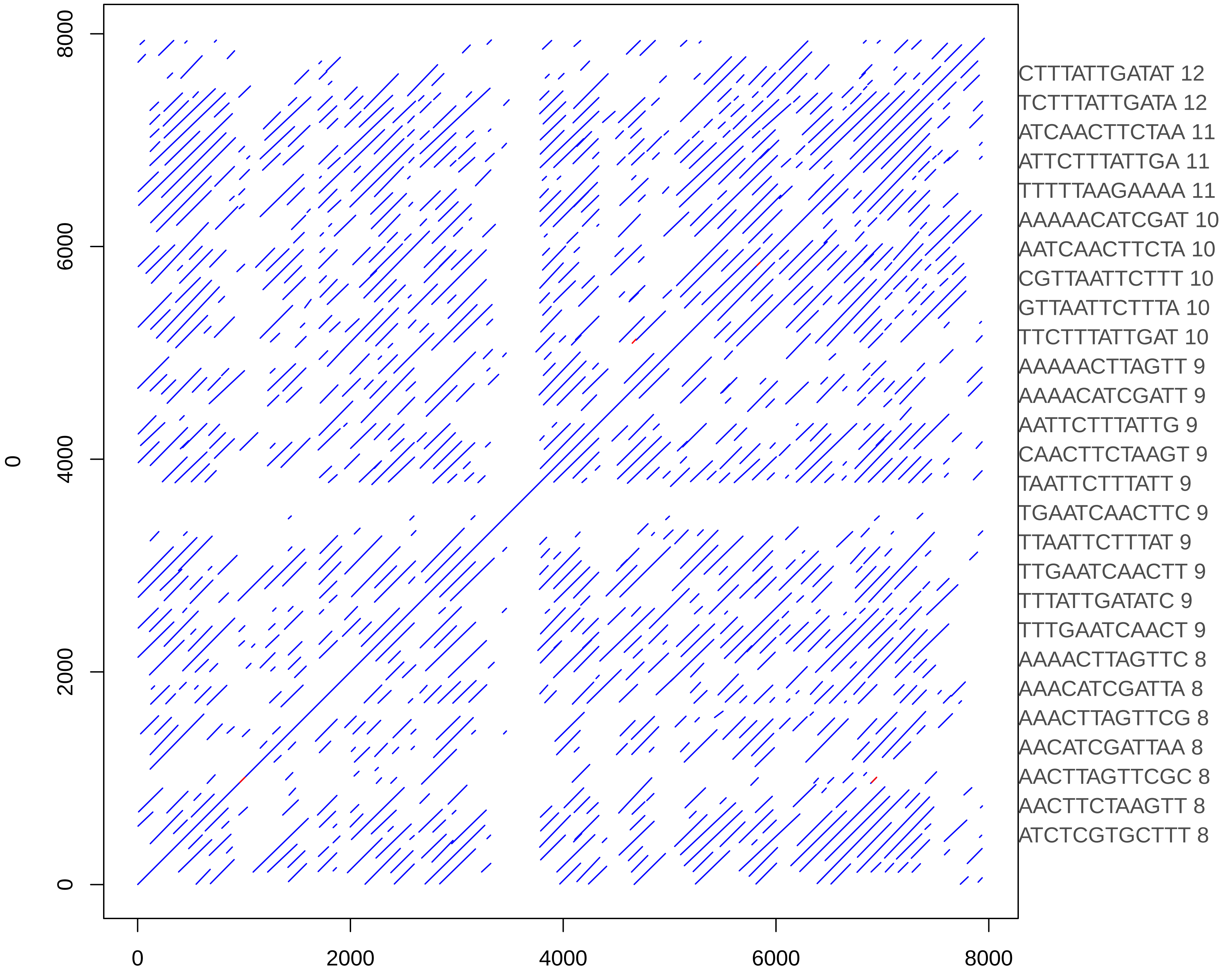

>m141122_014521_42134_c100732202550000001823141405141560_s1_p0/109291/1969_8215

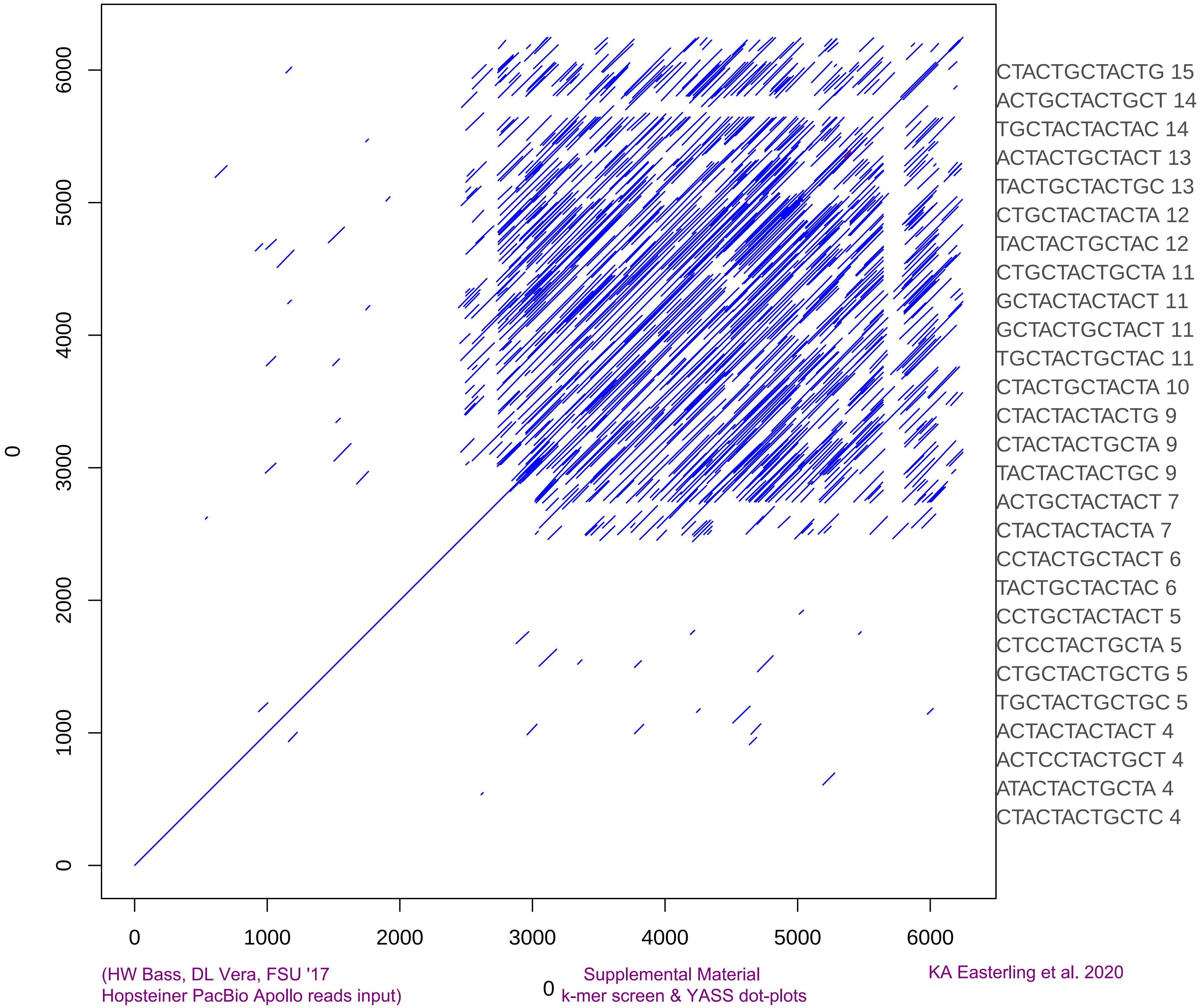

>m141122_014521_42134_c100732202550000001823141405141560_s1_p0/111276/2544_15960

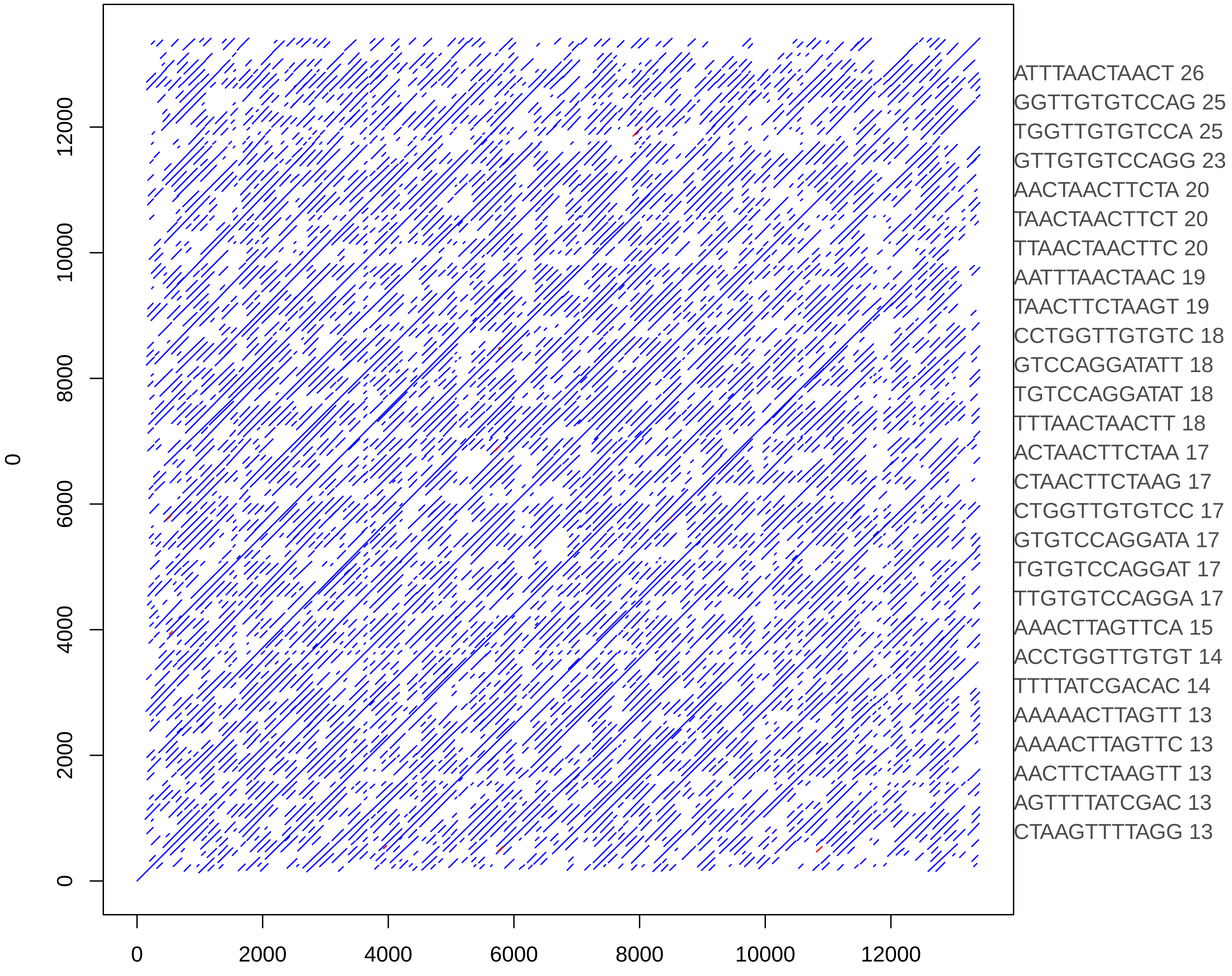

>m141122_014521_42134_c100732202550000001823141405141560_s1_p0/118648/0_5885

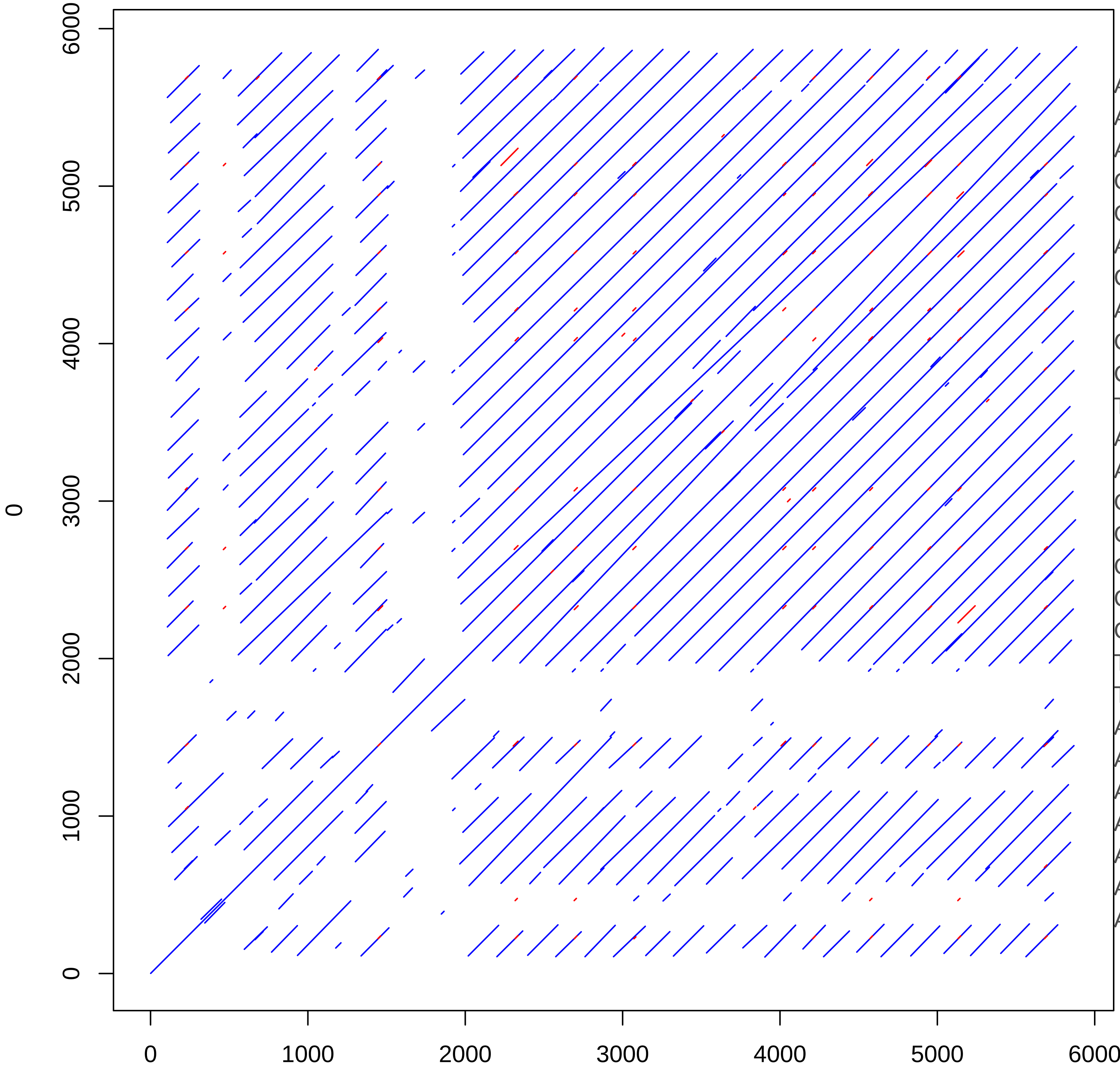

AAAGGTCAATTT 15
AAGGTCAATTTT 14
AGGTCAATTTTT 14
GAAAGGTCAATT 14
GGTCAATTTTTT 14
AATTTTTTTGTT 13
CTGAAAGGTCAA 13
ATTTTTTTGTTT 12
CAATTTTTTTGT 12
CCTGAAAGGTCA 12
TGAAAGGTCAAT 12
AAAAGGTTCAAA 10
AAAGGTTCAAAA 10
GAAAAGGTTCAA 10
GATGGGAAAAGG 10
GGAAAAGGTTCA 10
GGTTCAAAAAAA 10
GTCAATTTTTTT 10
TCAATTTTTTTG 10
TTTTTTGTTTGG 10
AAAAAAATTAGA 9
AAAAAATTAGAG 9
AAGGTTCAAAAA 9
AGGCAGCTGCCT 9
AGGTTCAAAAAA 9
ATGGGAAAAGGT 9
ATTATTGAGATG 9

>m141122_014521_42134_c100732202550000001823141405141560_s1_p0/1189/0_9956

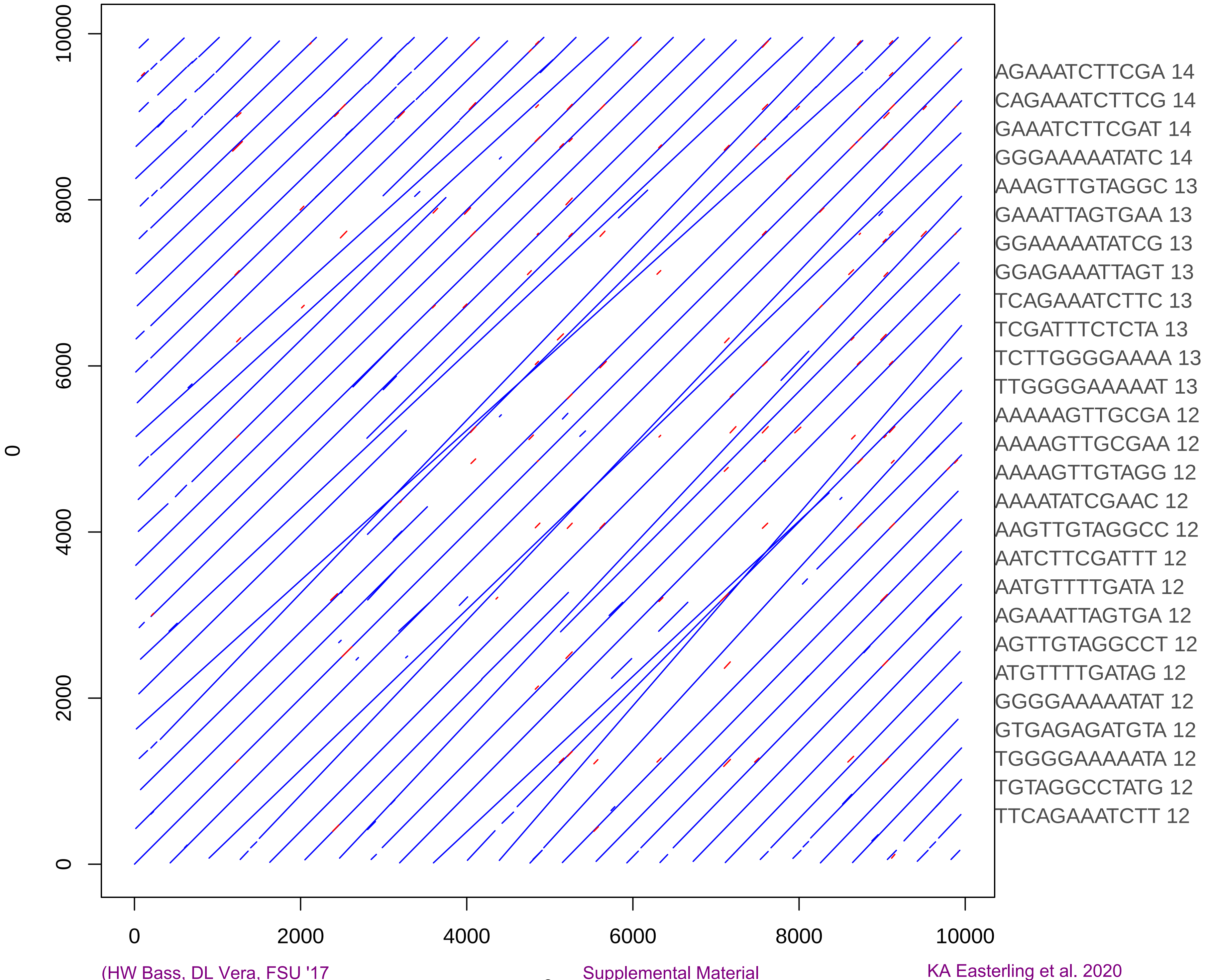

>m141122_014521_42134_c100732202550000001823141405141560_s1_p0/120181/0_11846

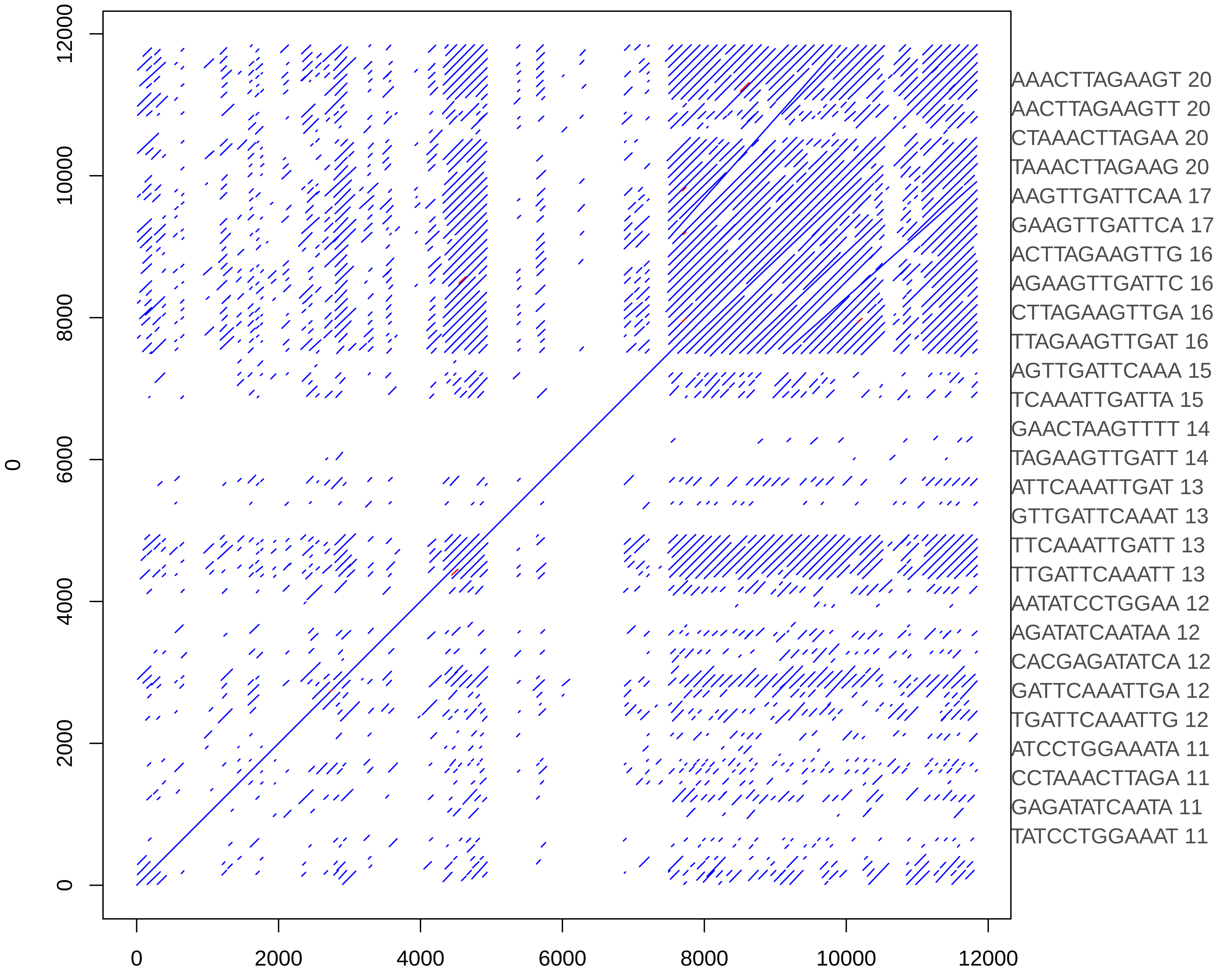

>m141122_014521_42134_c100732202550000001823141405141560_s1_p0/12096/0_6154

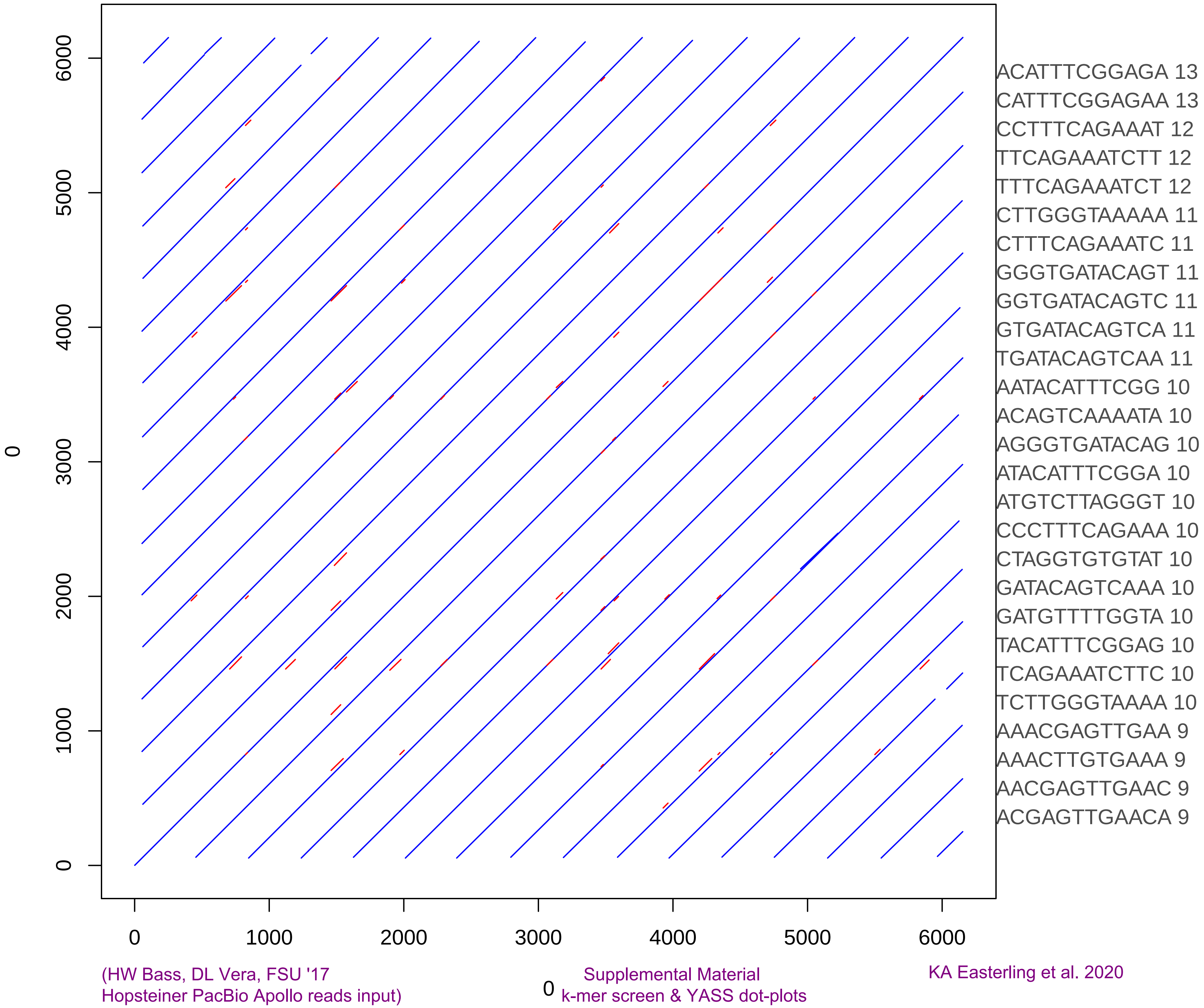

>m141122_014521_42134_c100732202550000001823141405141560_s1_p0/121672/9680_16266

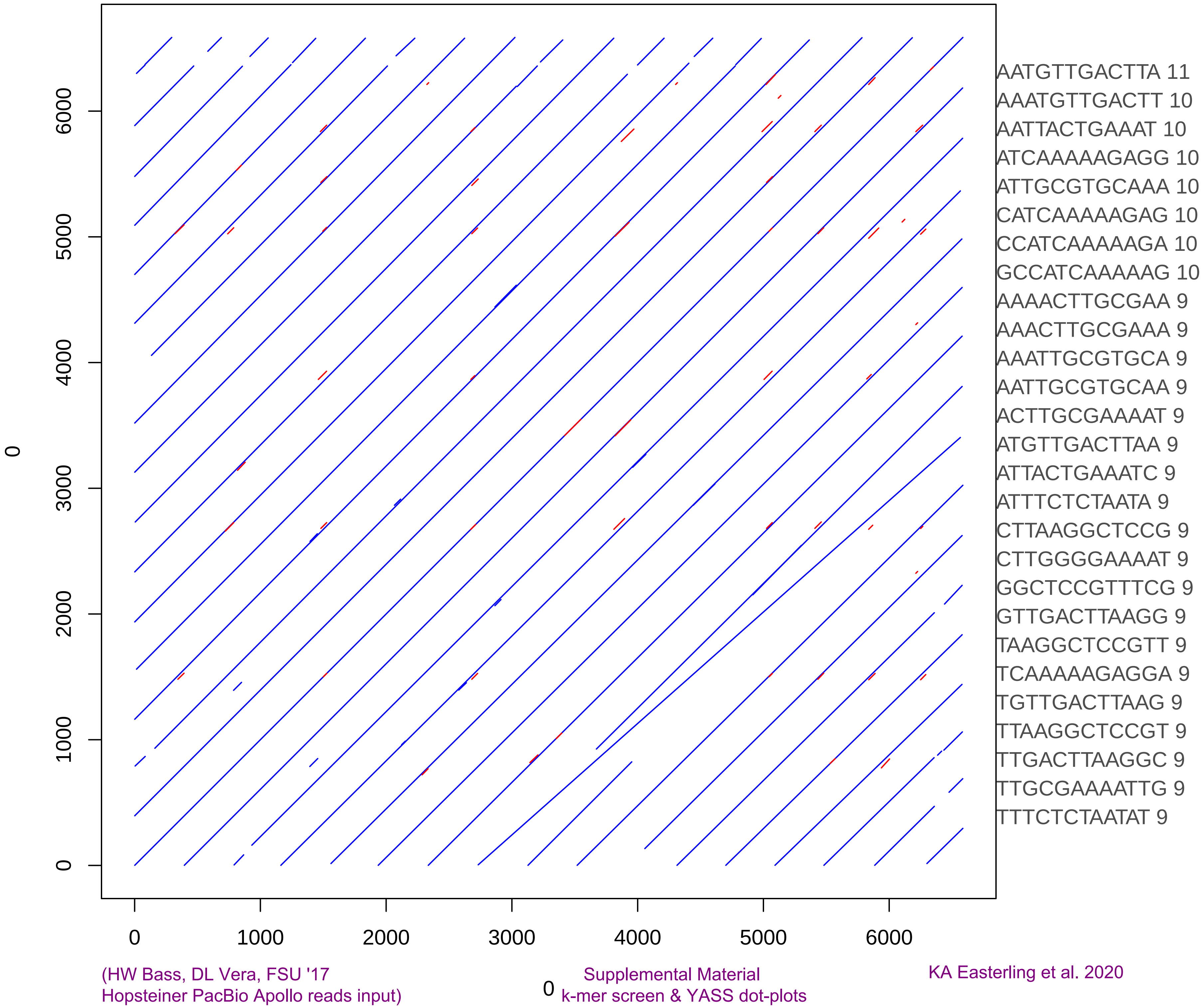

>m141122_014521_42134_c100732202550000001823141405141560_s1_p0/122288/4893_12286

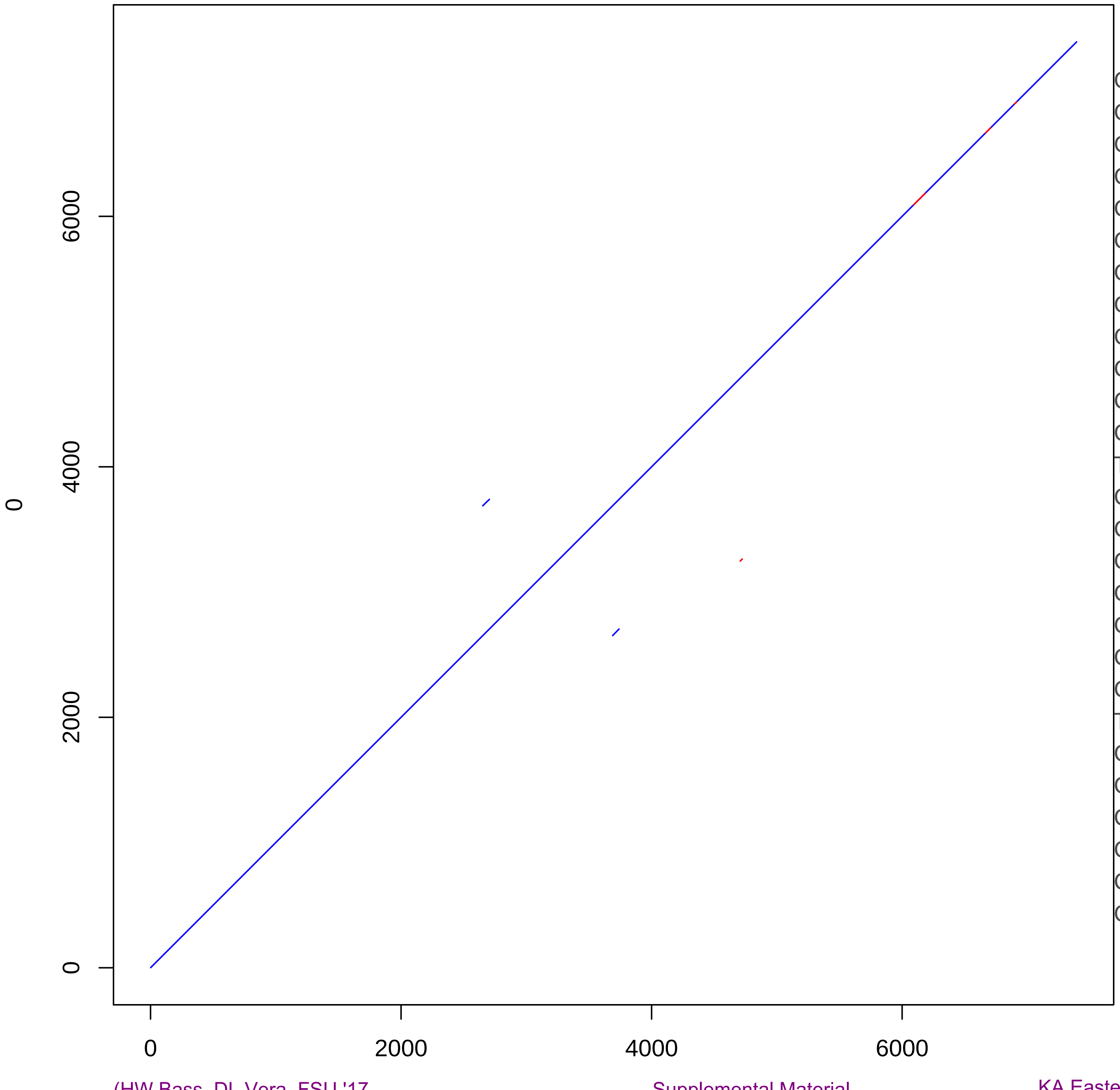

GGGGGGGGGGGG 40
GGGGGGGGTGGG 12
GGGGGGGTGGGG 12
GGGGGGTGGGGG 11
GGGGGTGGGGGG 11
GGGGTGGGGGGG 11
GGGTGGGGGGGG 11
GGTGGGGGGGGG 11
GGGGGGGGGTGG 10
GGGGGGGGGGTG 8
GTGGGGGGGGGG 7
GGGGGGGGGGGT 6
TGGGGGGGGGGG 6
GGGGGGTGGGGT 5
GGGGGTGGGGTG 5
GGGGTGGGGTGG 5
GGGTGGGGTGGG 5
GGTGGGGTGGGG 5
GTGGGGGGTGGG 5
GTGGGGTGGGGG 5
TGGGGTGGGGGG 5
GGGGGTGGTGG 4
GGGGTGGGGGGT 4
GGGTGGGGGGTG 4
GGTGGGGGGGGT 4
GGTGGGGGGTGG 4
GTGGGGGGGGGT 4

>m141122_014521_42134_c100732202550000001823141405141560_s1_p0/123417/14610_20842

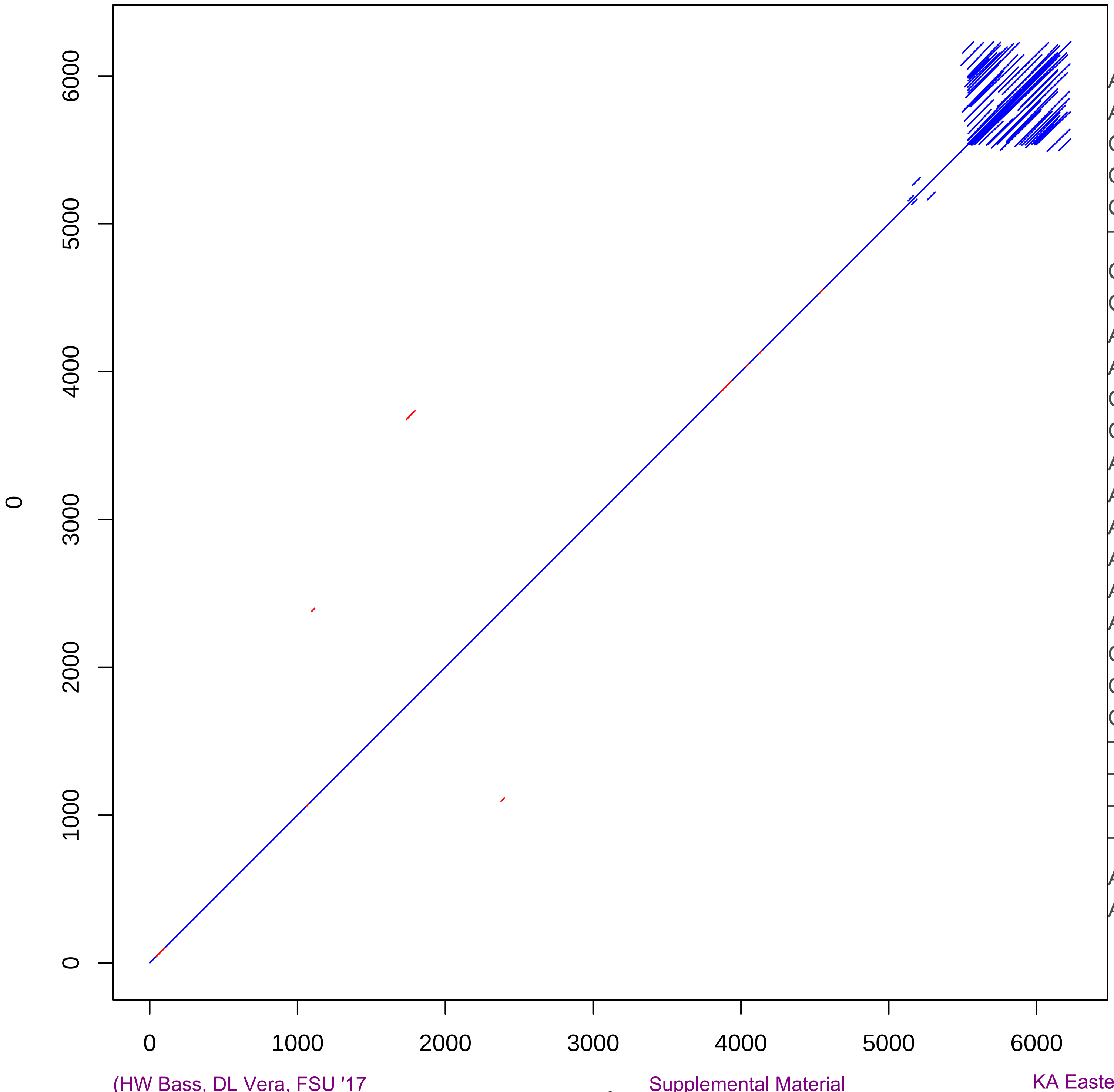

ACTACTACTACT 15
ACCACCACCACC 14
CACCACCACCAC 14
CTACTACTACTA 14
CCACCACCACCA 13
TACTACTACTAC 12
CACTACCACCAC 5
CCACCACCACTA 5
ACCACCACCACT 4
ACTACCACCACC 4
CACCACCACTAC 4
CCACTACCACCA 4
AATTATTTAATT 3
ACCACTACCACC 3
ACTAACTACTAC 3
ACTACTAACTAC 3
ATTATTTAATTA 3
ATTGCTAGGAAT 3
CACCACTACCAC 3
CTAACTACTACT 3
CTACTAACTACT 3
TAACTACTACTA 3
TACCACCACCAC 3
TACTAACTACTA 3
TTGCTAGGAATT 3
AAATCACAAATA 2
AACTACTACTAC 2

>m141122_014521_42134_c100732202550000001823141405141560_s1_p0/124459/0_10373

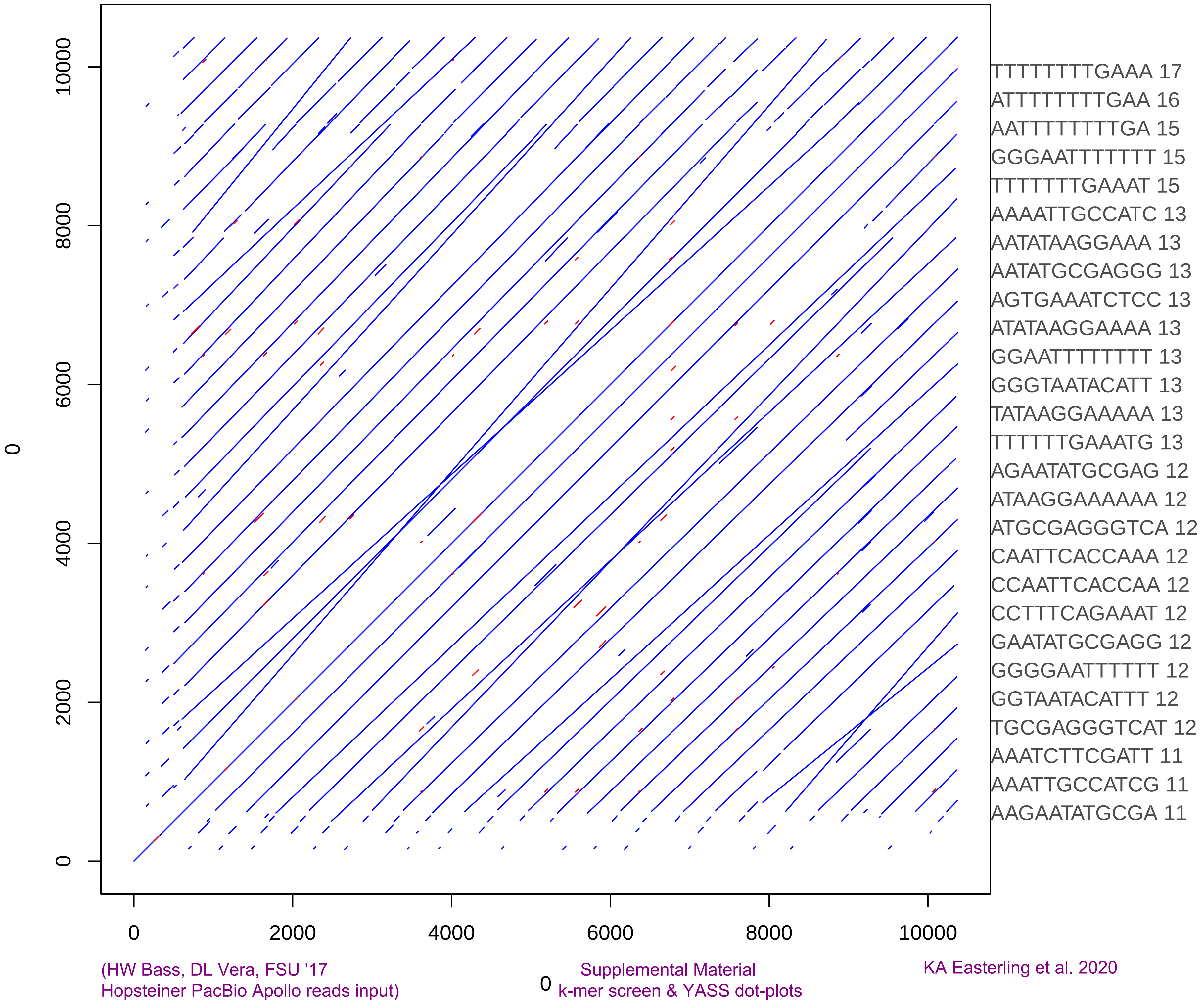

>m141122_014521_42134_c100732202550000001823141405141560_s1_p0/126506/1802_8211

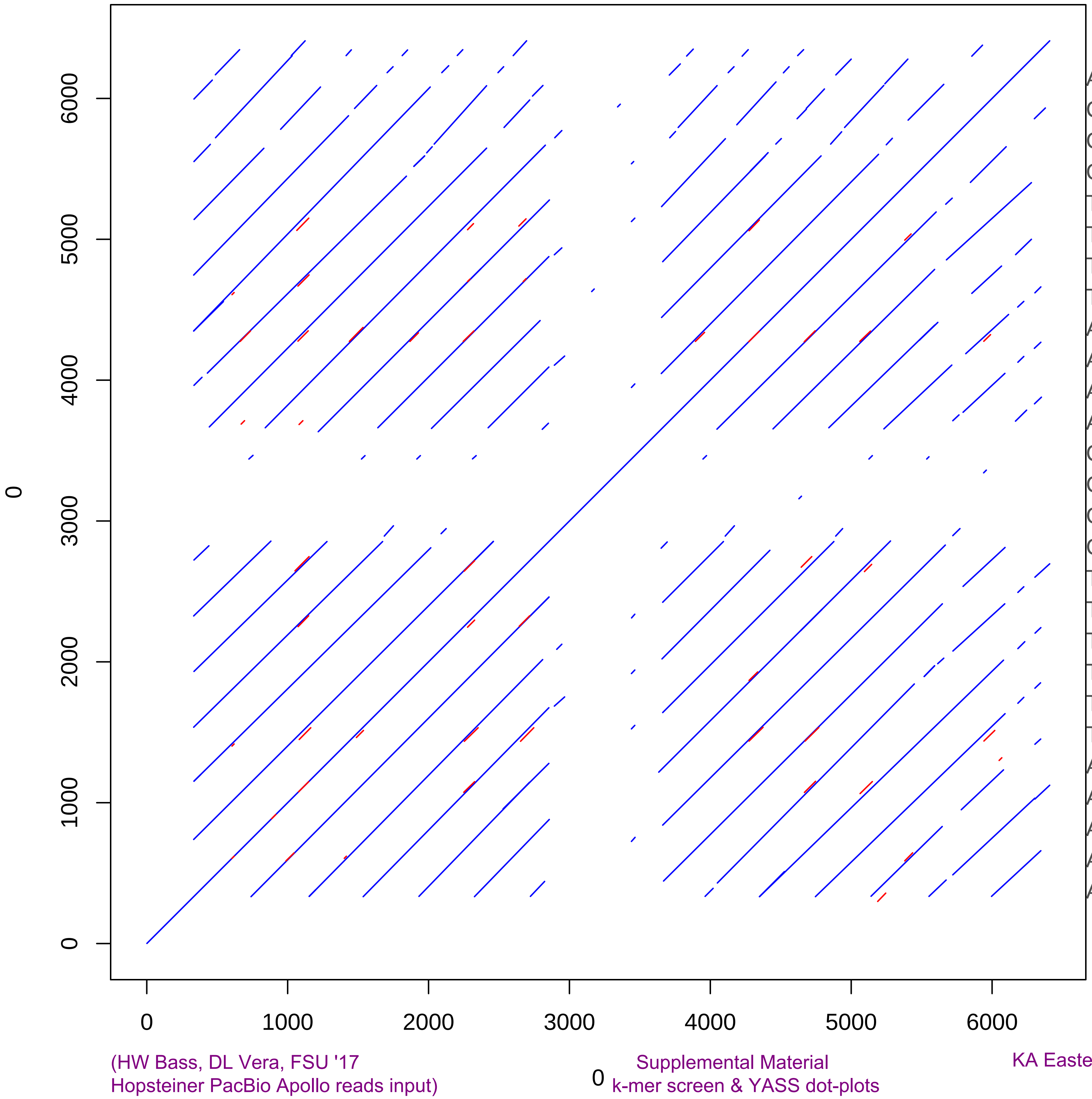

AATCGAAGATTT 9
CATTTGAAAAAA 9
GAGTTGAAACGG 9
GGTGAATTGGAG 9
TGGTGAATTGGA 9
TTGGAGTTGAAA 9
TTGGTGAATTGG 9
TTTGGTGAATTG 9
AAATCGAAGATT 8
ATATTCTTTAGT 8
ATTGGAGTTGAA 8
ATTTGAAAAAAA 8
CATATTCTTTAG 8
CCCTTTTTGATG 8
GATGGCAATTTT 8
GGAGTTGAAACG 8
TATTGGAGTTGA 8
TGATGGCAATTT 8
TGGAGTTGAAAC 8
TTGATGGCAATT 8
TTTGATGGCAAT 8
TTTGGAAGATAT 8
AAAAAAATTCCC 7
AACATTTGAAAA 7
AAGAGATCTCAT 7
AATCTATTGGAG 7
ACATTTGAAAAA 7

>m141122_014521_42134_c100732202550000001823141405141560_s1_p0/131333/0_6132

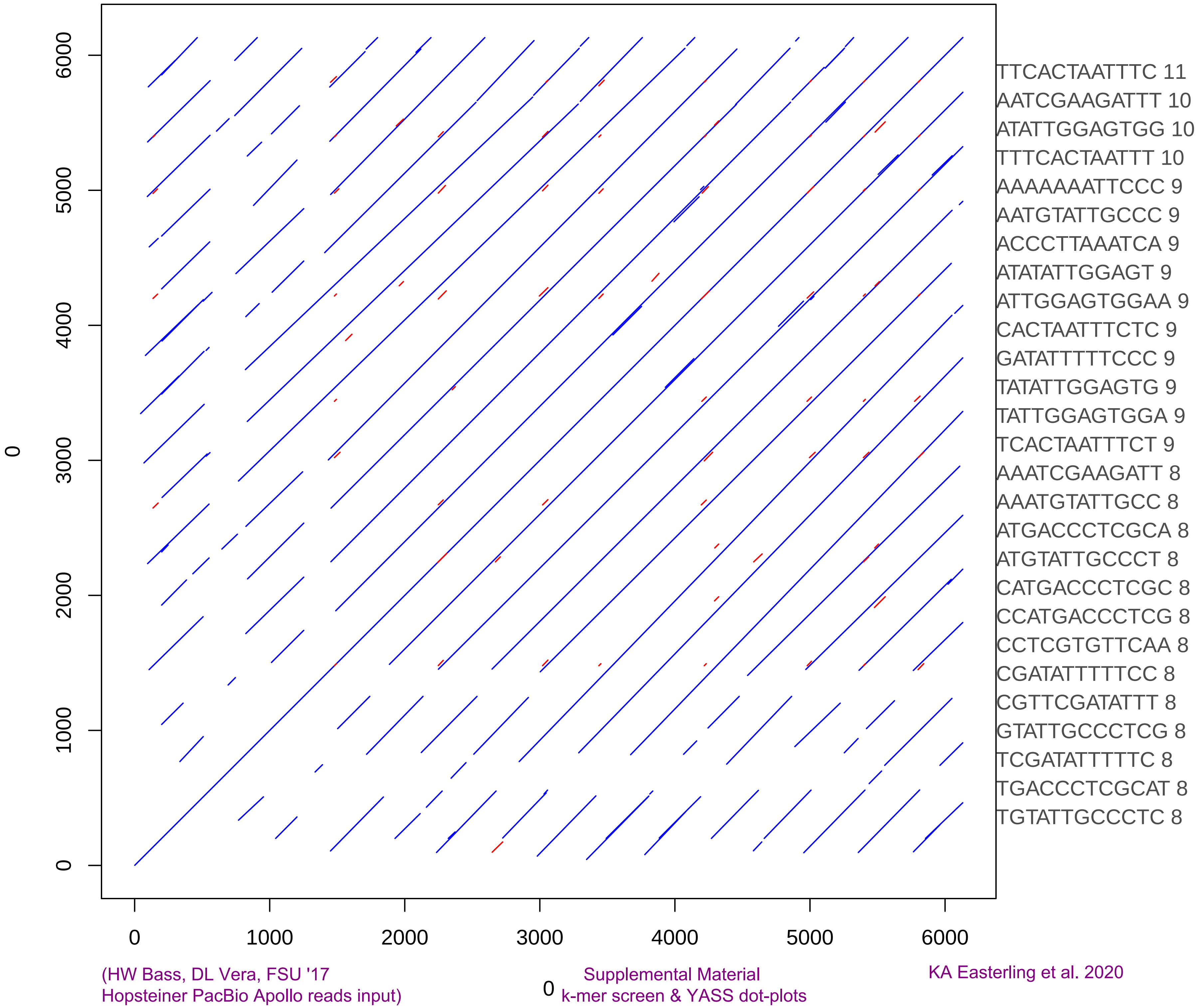

>m141122_014521_42134_c100732202550000001823141405141560_s1_p0/136676/13135_18627

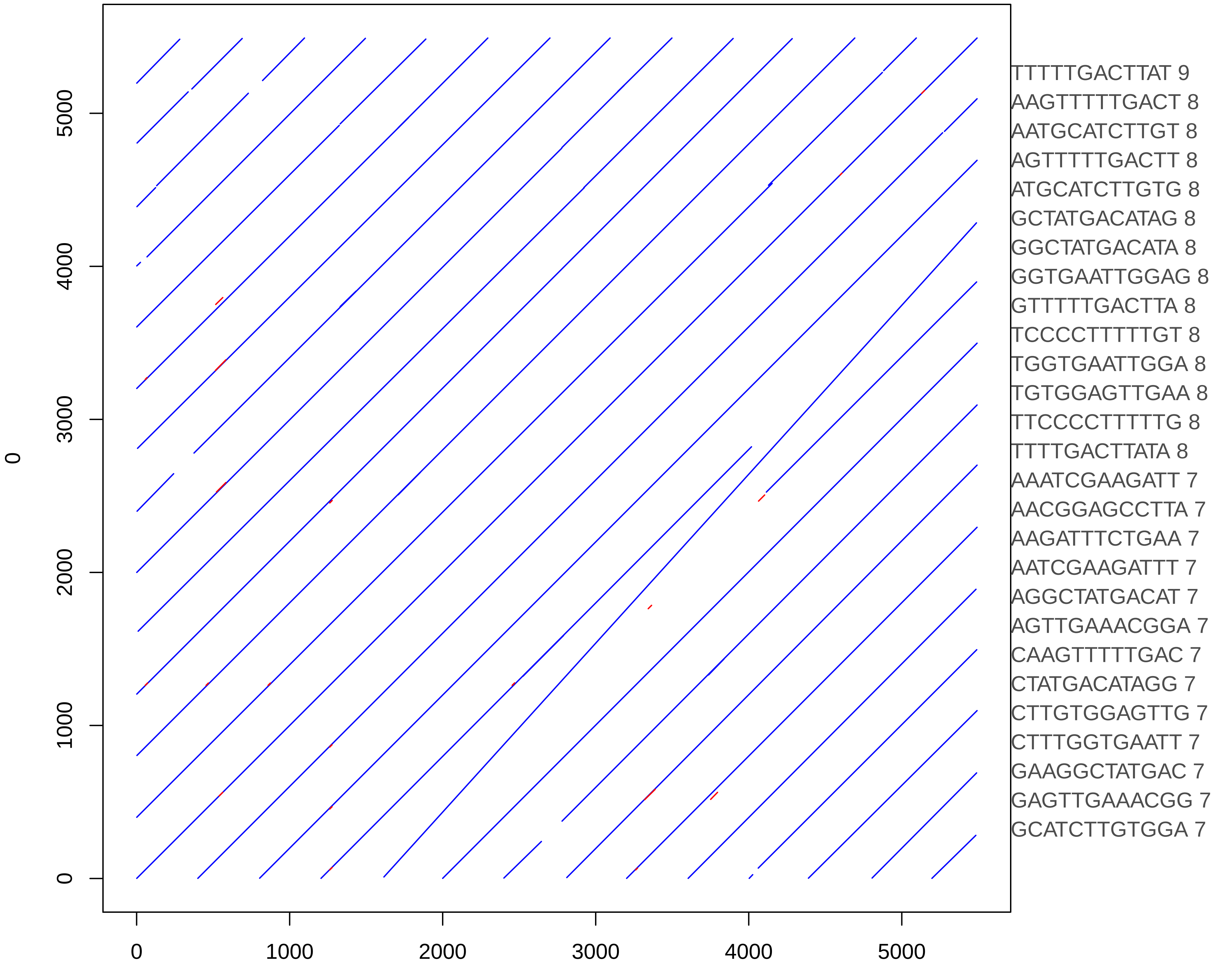

>m141122_014521_42134_c100732202550000001823141405141560_s1_p0/137302/0_5363

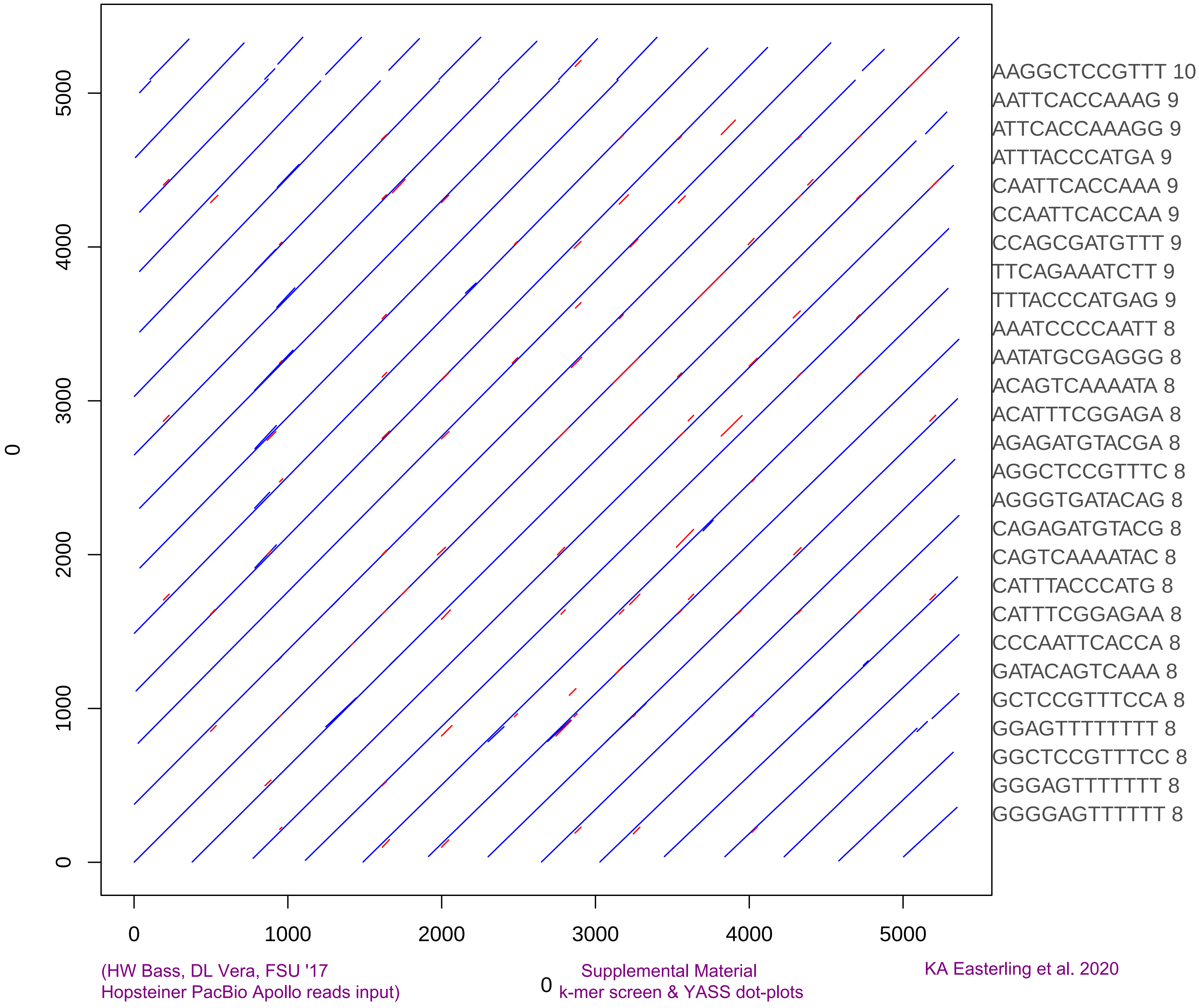

>m141122_014521_42134_c100732202550000001823141405141560_s1_p0/140079/14866_21949

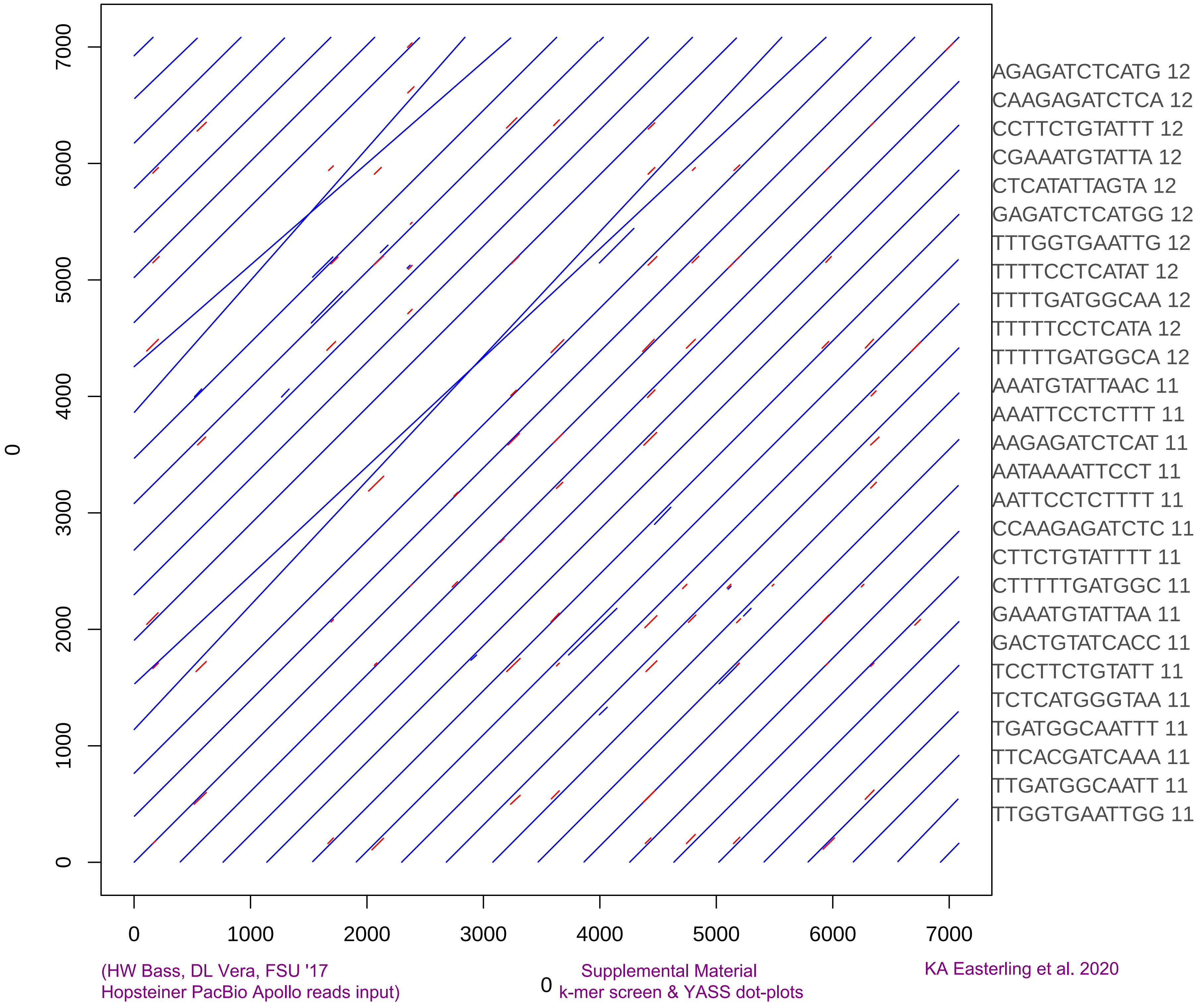

>m141122_014521_42134_c100732202550000001823141405141560_s1_p0/143273/1561_8082

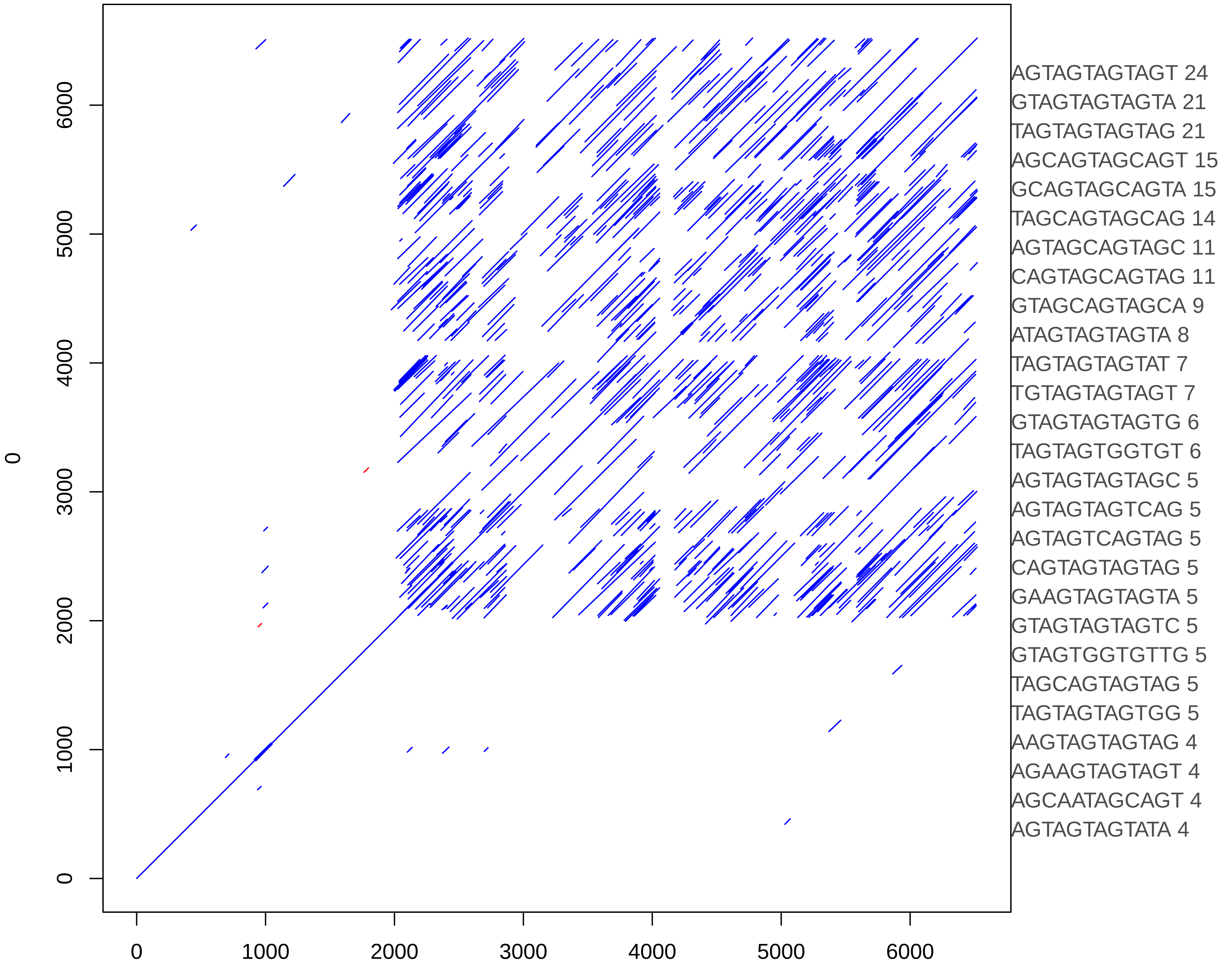

>m141122_014521_42134_c100732202550000001823141405141560_s1_p0/14837/12540_18269

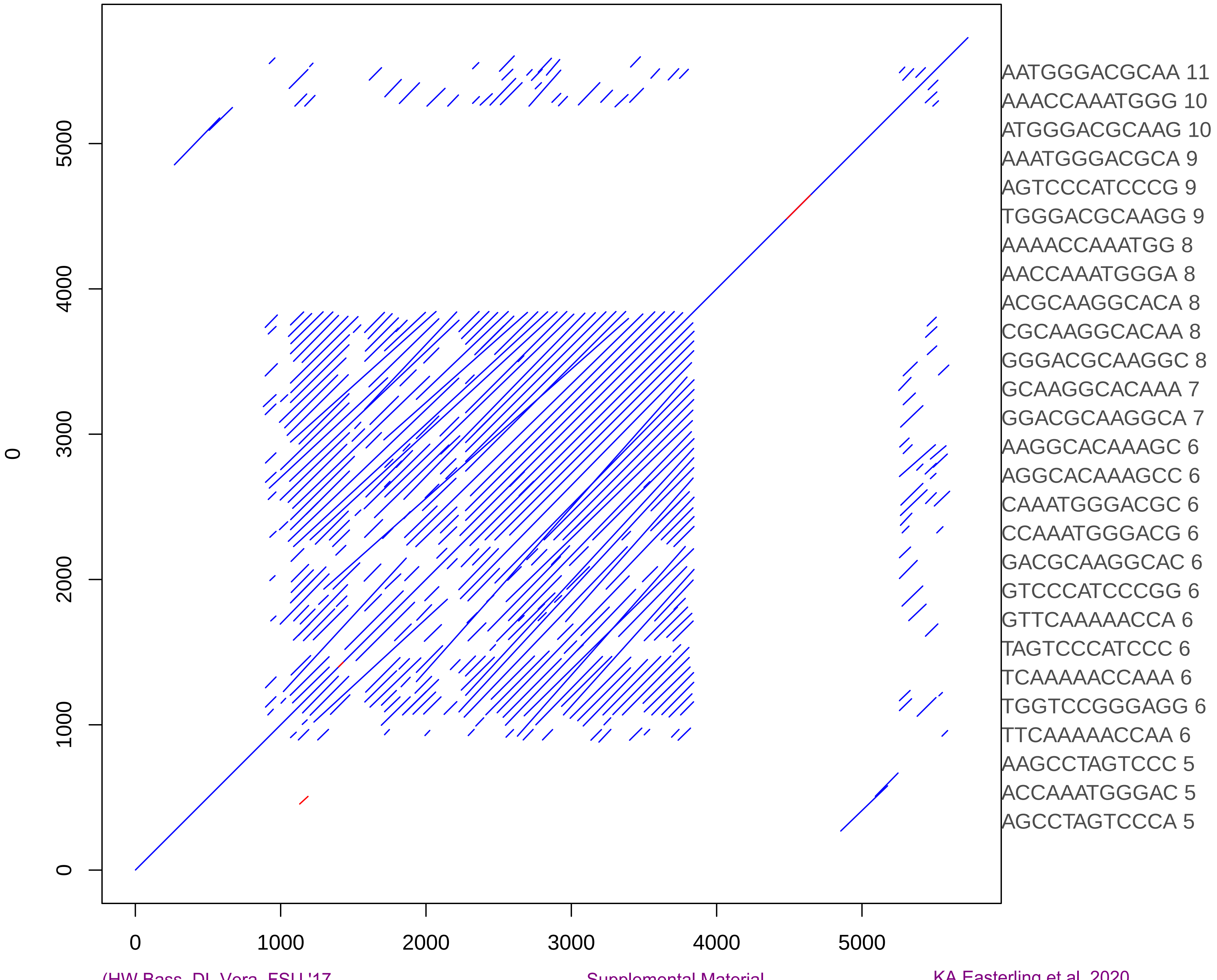

1,121 hits for repetitive sequences
passing 12mer abundance criterion

(Read-Number)
PAGE 198

For Tandem Repeat families of ### bp:
PacBio Read named "HuluTR###-r198"

>m141122_014521_42134_c100732202550000001823141405141560_s1_p0/148605/256_9558

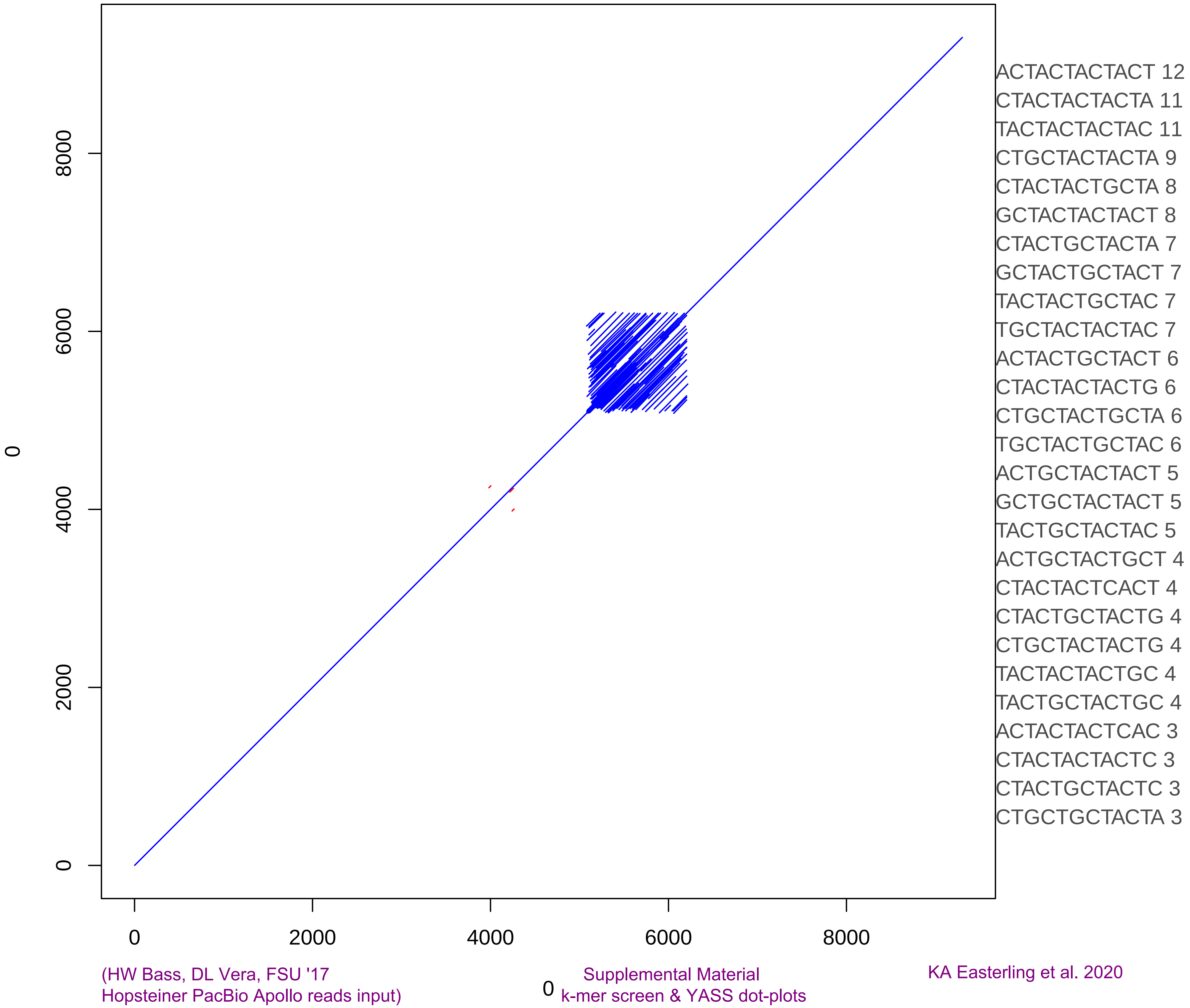

(HW Bass, DL Vera, FSU '17
Hopsteiner PacBio Apollo reads input)

Supplemental Material
k-mer screen & YASS dot-plots

KA Easterling et al. 2020

>m141122_014521_42134_c100732202550000001823141405141560_s1_p0/149280/292_6074

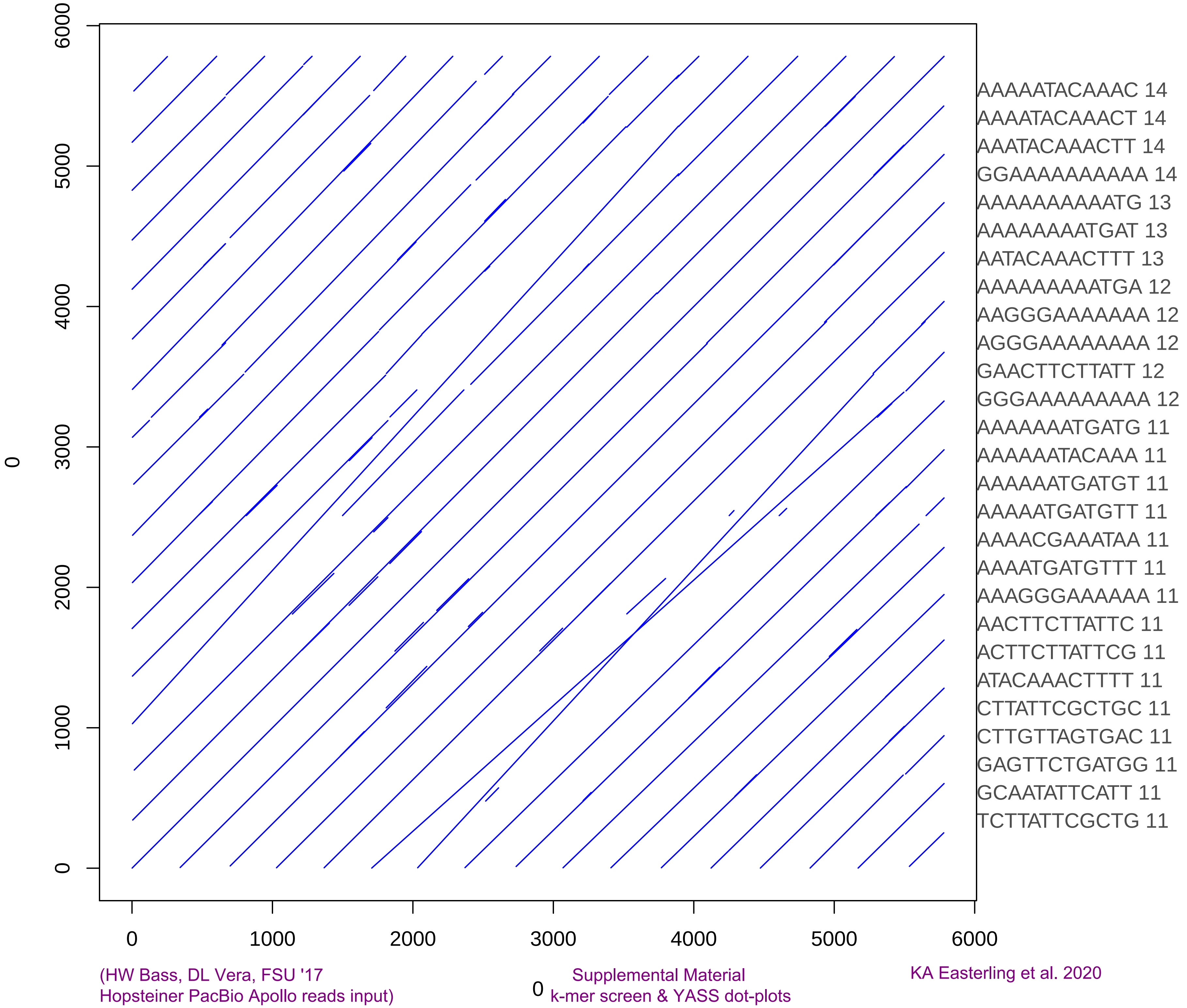

>m141122_014521_42134_c100732202550000001823141405141560_s1_p0/150912/0_12573

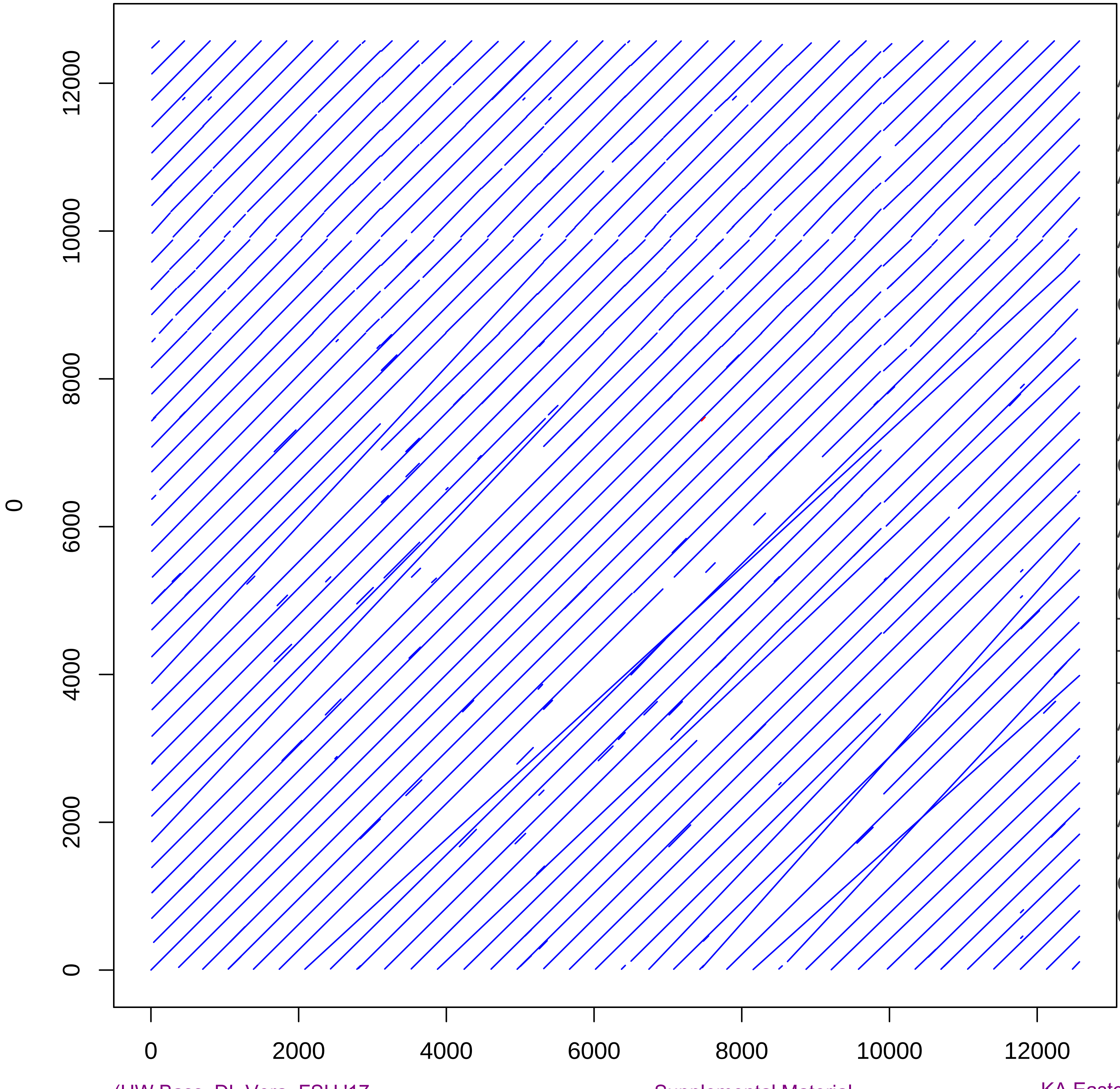

AAAAAAAATGAT 26
AAAAAAATGATG 25
AAAAAATGATGT 25
AAGGGAAAAAAA 25
AGGGAAAAAAAA 25
AAAAAAAAATGA 24
GGAAAAAAAAAA 23
GGGAAAAAAAAA 23
AAAGGGAAAAAA 22
AAAAAAAAAATG 20
AAAGATTTAAAA 20
AGGAGGTCACCC 20
CAGGAGGTCACC 20
AAAAATGATGTT 19
AAAATGATGTTT 19
AGGAAAACGAAA 19
CCAGGAGGTCAC 19
TAAAGGGAAAAA 19
TTAAAGGGAAAA 19
TTTAAAGGGAAA 19
AAAAATACAAAC 18
AAATACAAACTT 18
AAGATTTAAAAC 18
AAGGAAAACGAA 18
AATACAAACTTT 18
CGTAGGTGGATT 18
GAGTTCTGATGG 18

>m141122_014521_42134_c100732202550000001823141405141560_s1_p0/152325/1221_12516

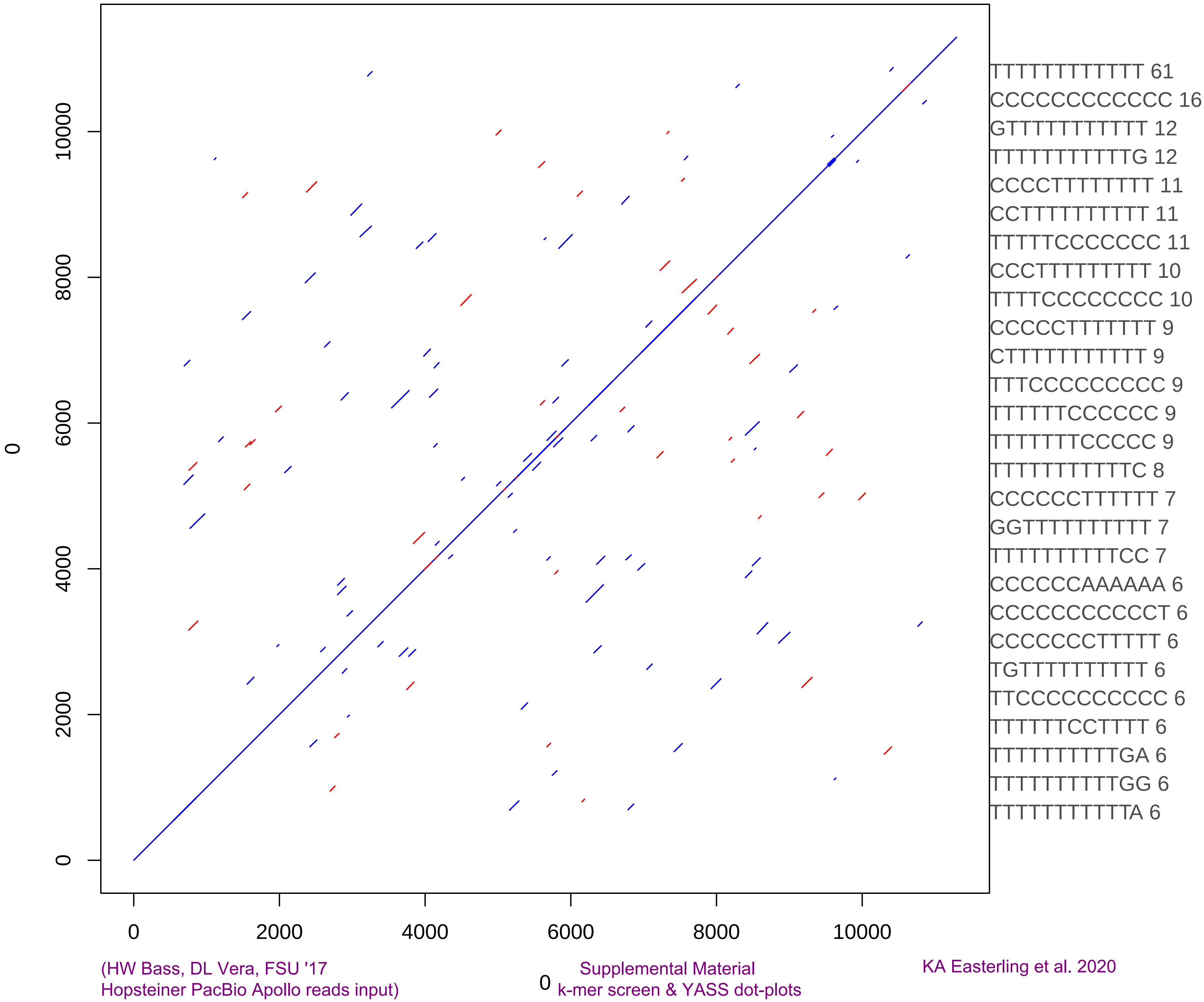

>m141122_014521_42134_c100732202550000001823141405141560_s1_p0/155405/0_5677

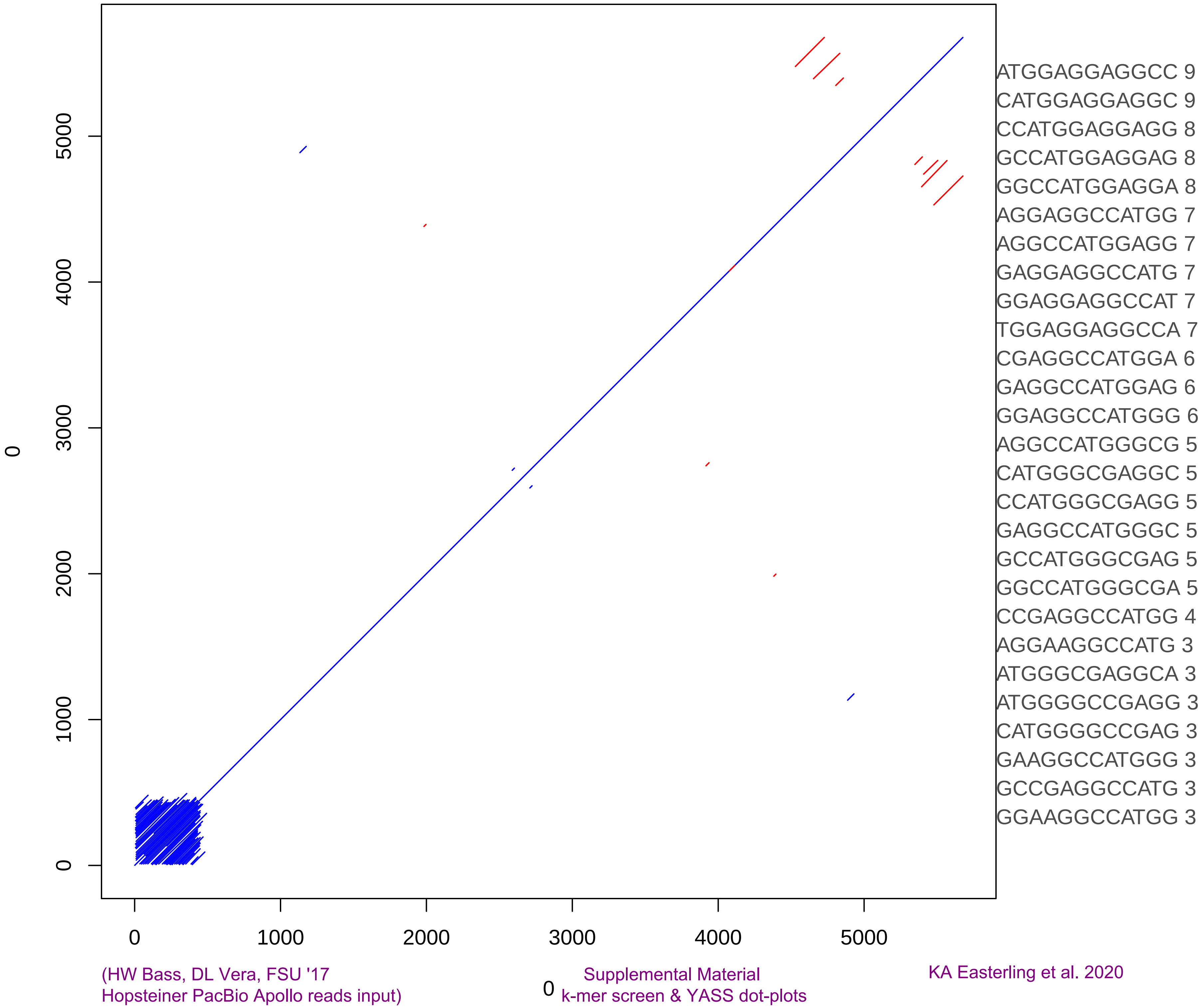

>m141122_014521_42134_c100732202550000001823141405141560_s1_p0/155425/0_5398

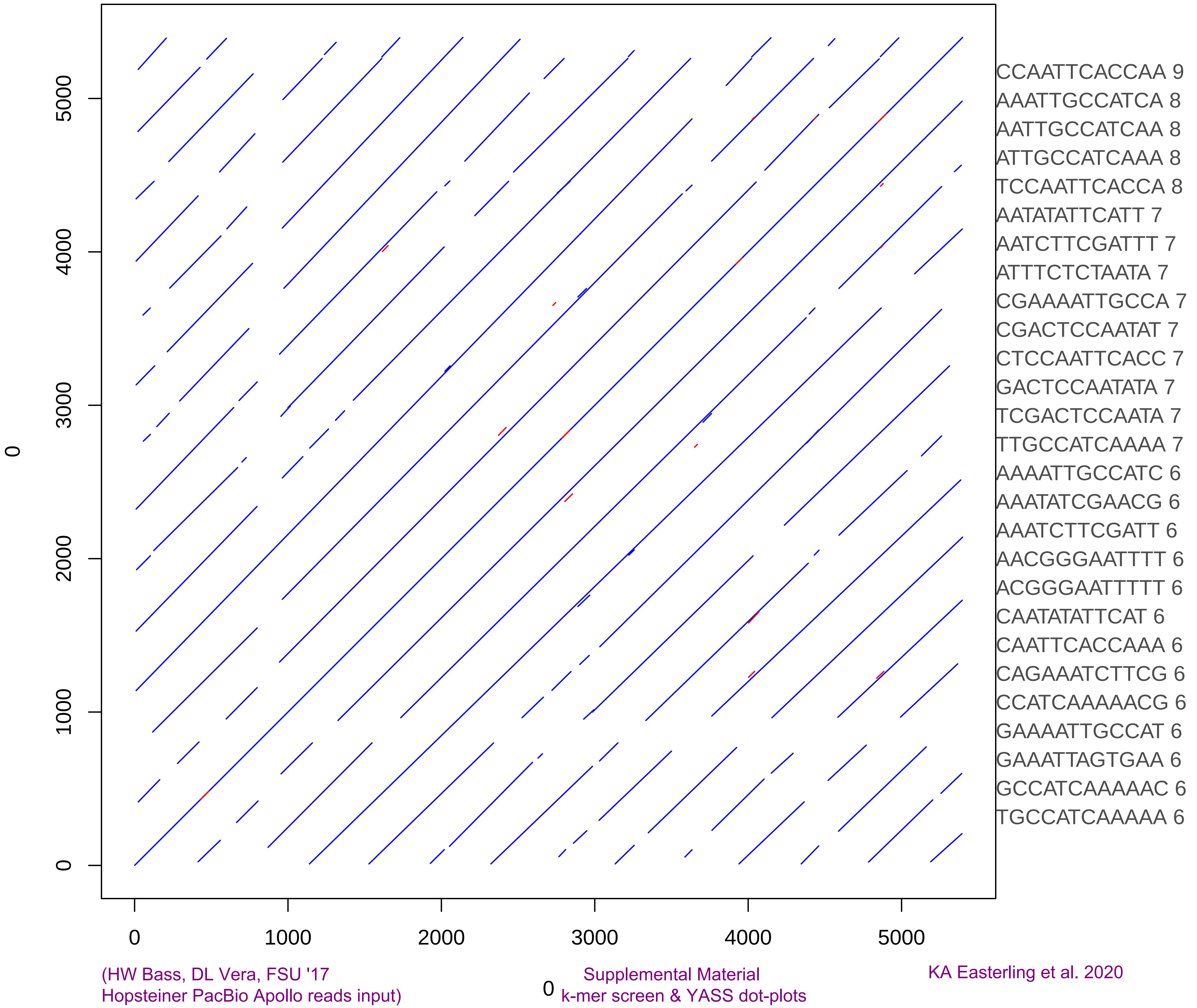

>m141122_014521_42134_c100732202550000001823141405141560_s1_p0/157696/8154_15740

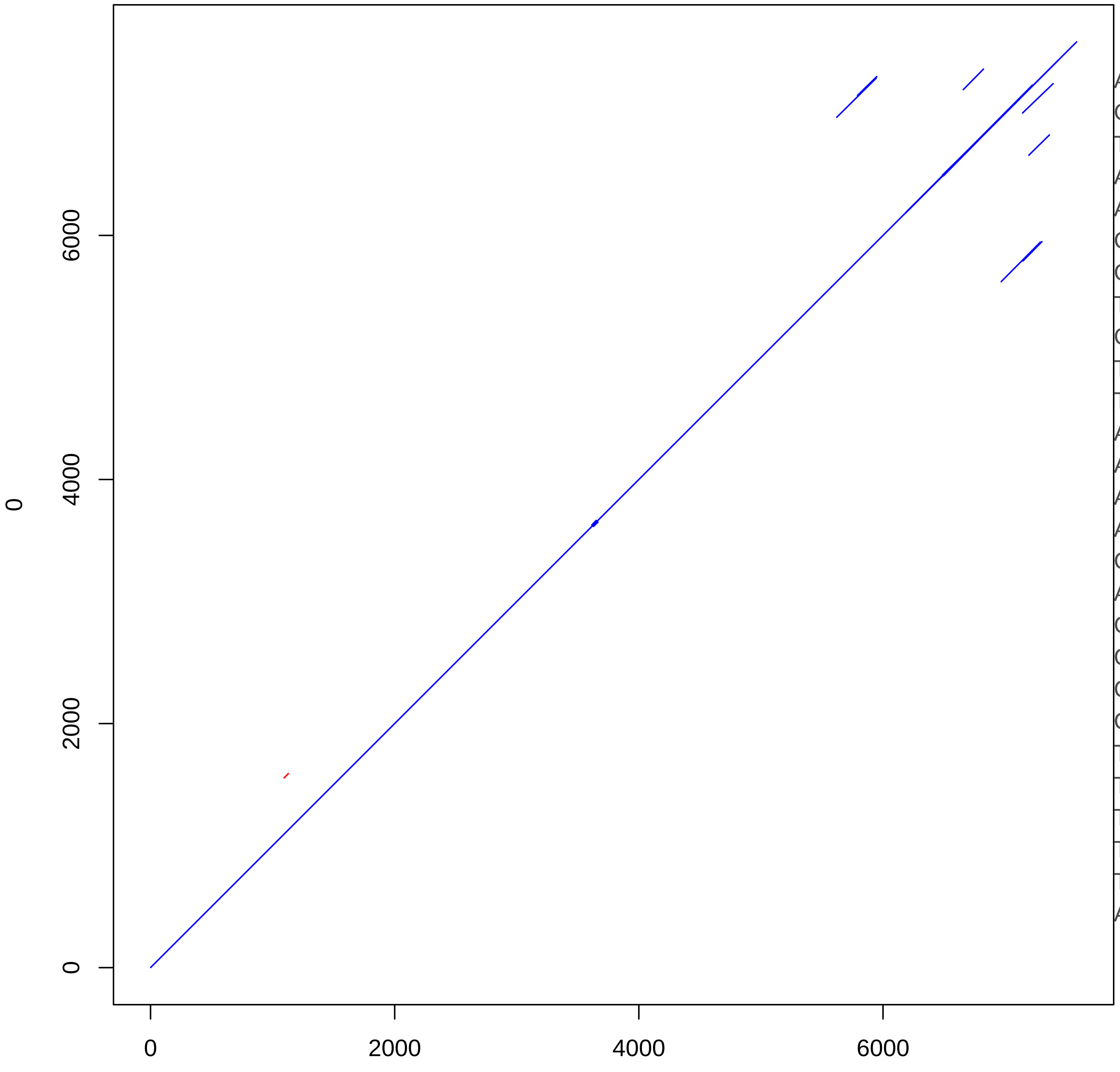

AGTAGTAGTAGT 23
GTAGTAGTAGTA 23
TAGTAGTAGTAG 20
AGTAGTAGTAAG 8
AGTGGTAGTAGT 8
GTAGTAGTAAGT 8
GTAGTAGTGGTA 8
TAGTAGTAAGTA 8
GTGGTAGTAGTG 7
TAGTAGTAGTAA 7
TAGTAGTGGTAG 7
AAGTAGTAGTAG 6
AGTAAGTAGTAG 6
AGTAGTAAGTAG 6
ATAGTAGTAGTA 6
GTAAGTAGTAGT 6
AGTAGTGGTAGT 5
GGTAGTAGTGGT 5
GTAGTAAGTAGT 5
GTAGTGGTAGTA 5
GTATAGTAGTAG 5
TAAGTAGTAGTA 5
TAGTAAGTAGTA 5
TAGTAGTAGTAT 5
TATAGTAGTAGT 5
TGGTAGTAGTGG 5
AGTAGTAGTGTA 4

>m141122_014521_42134_c100732202550000001823141405141560_s1_p0/159514/2078_9962

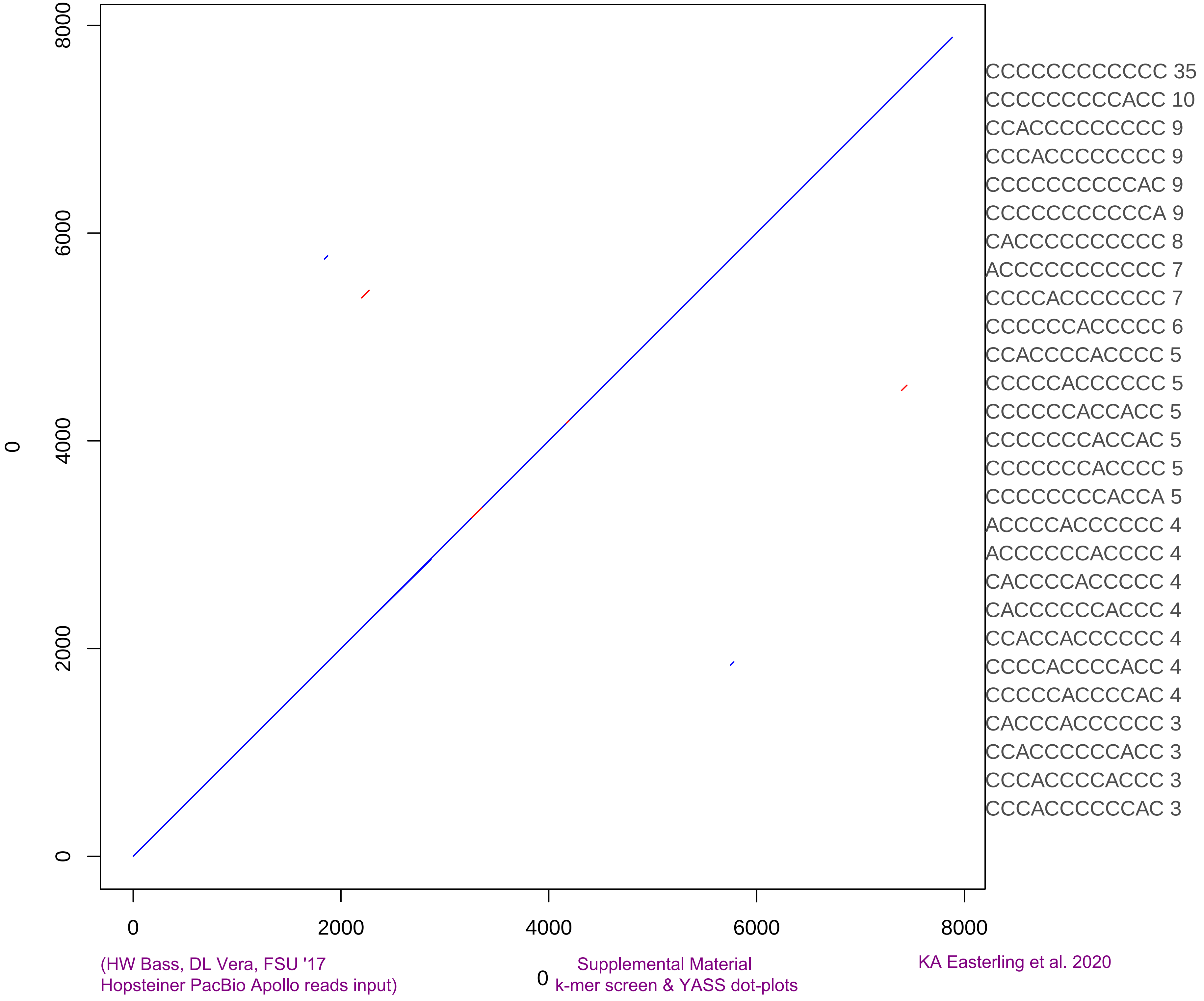

>m141122_014521_42134_c100732202550000001823141405141560_s1_p0/160154/0_7654

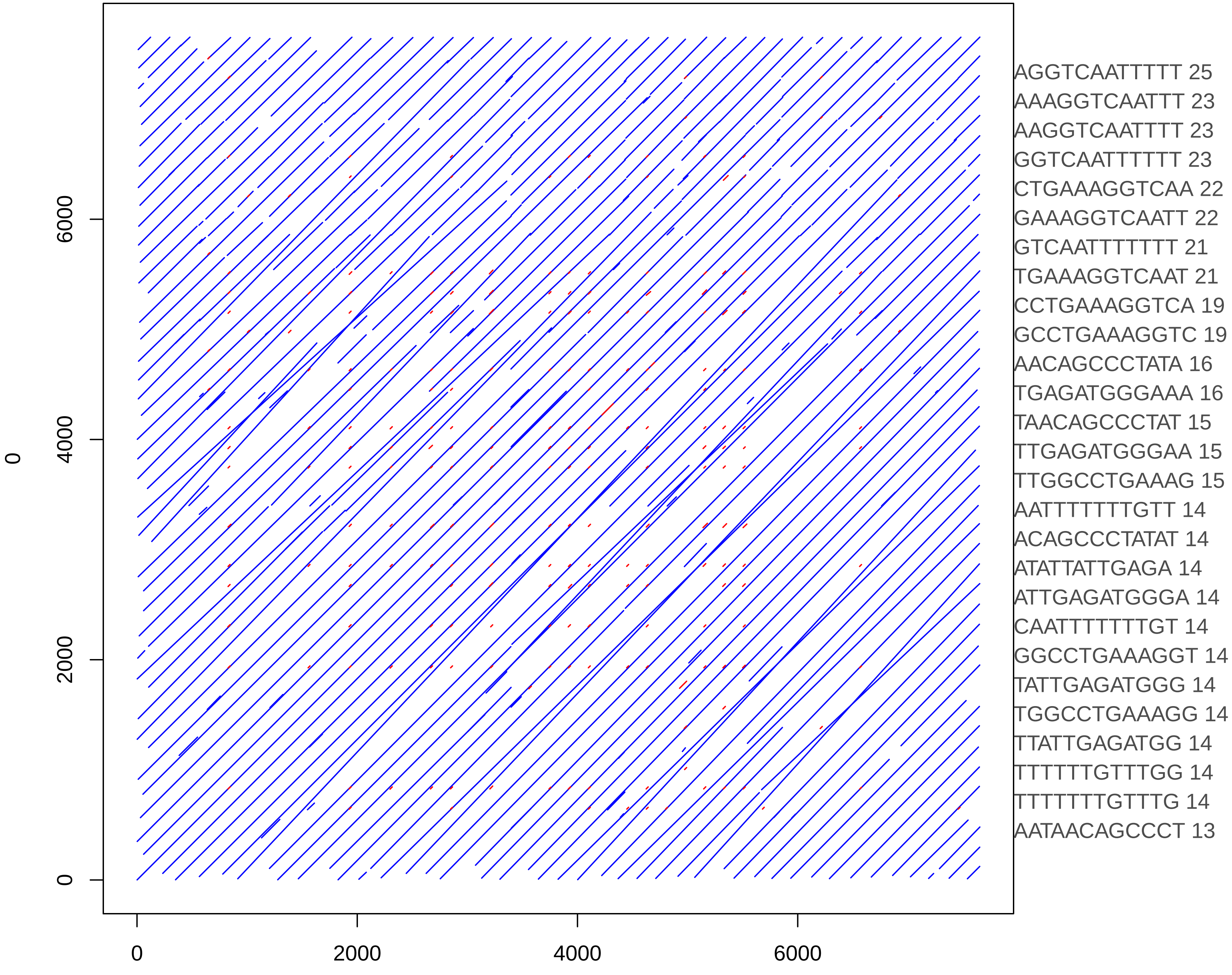

>m141122_014521_42134_c100732202550000001823141405141560_s1_p0/160975/2695_12773

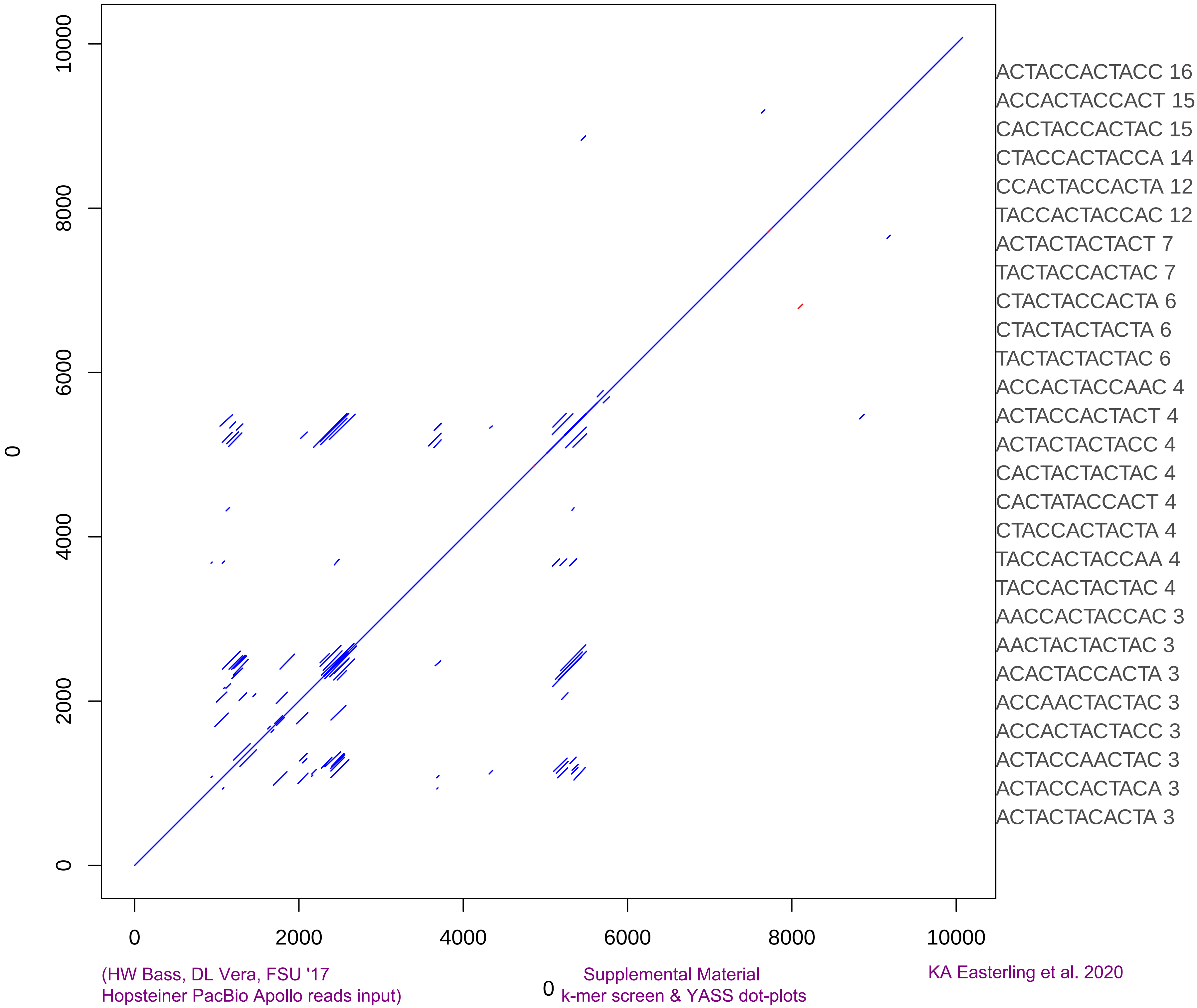

>m141122_014521_42134_c100732202550000001823141405141560_s1_p0/161024/0_7076

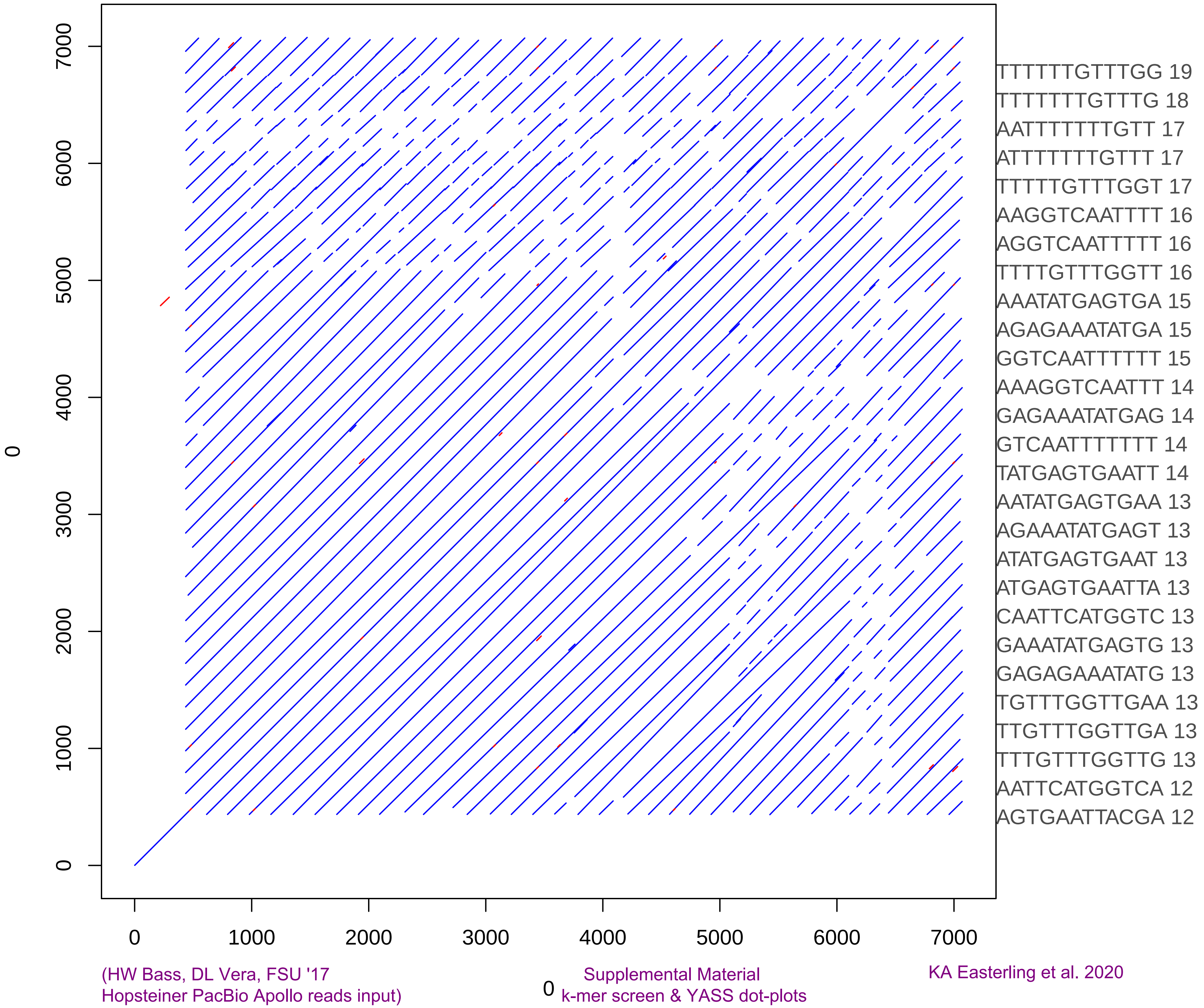

>m141122_014521_42134_c100732202550000001823141405141560_s1_p0/161832/0_8540

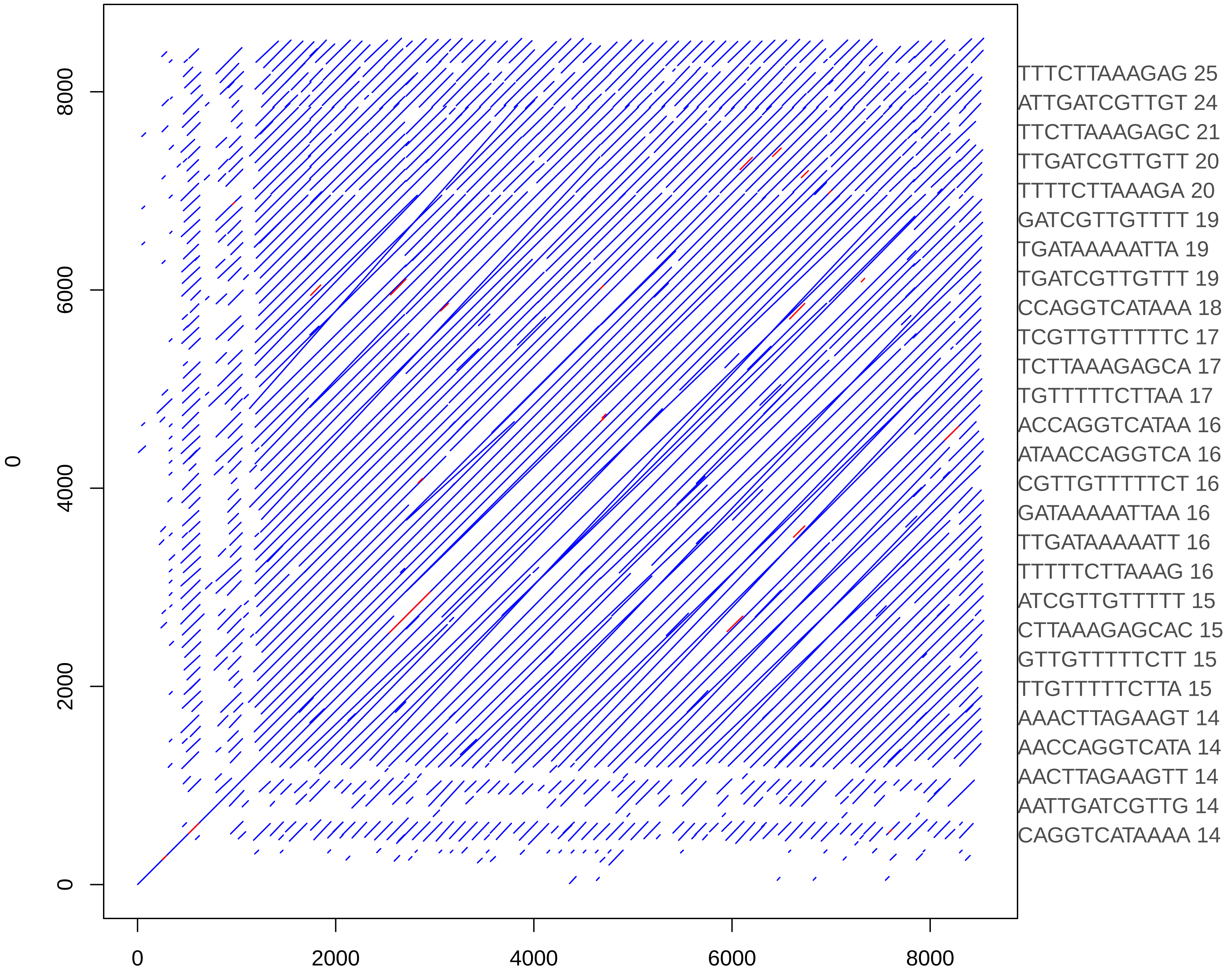

>m141122_014521_42134_c100732202550000001823141405141560_s1_p0/17374/8516_15487

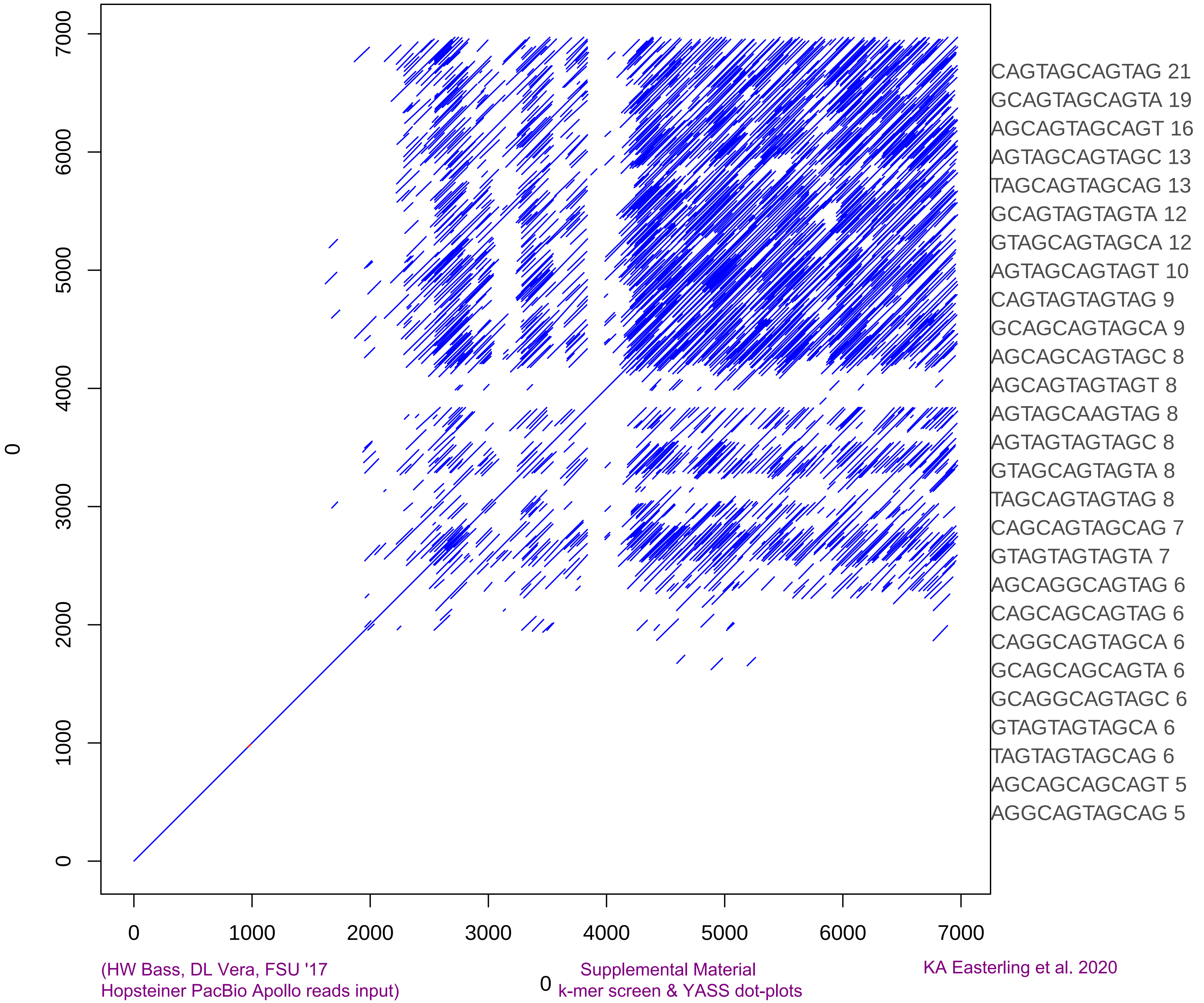

>m141122_014521_42134_c100732202550000001823141405141560_s1_p0/2097/439_10387

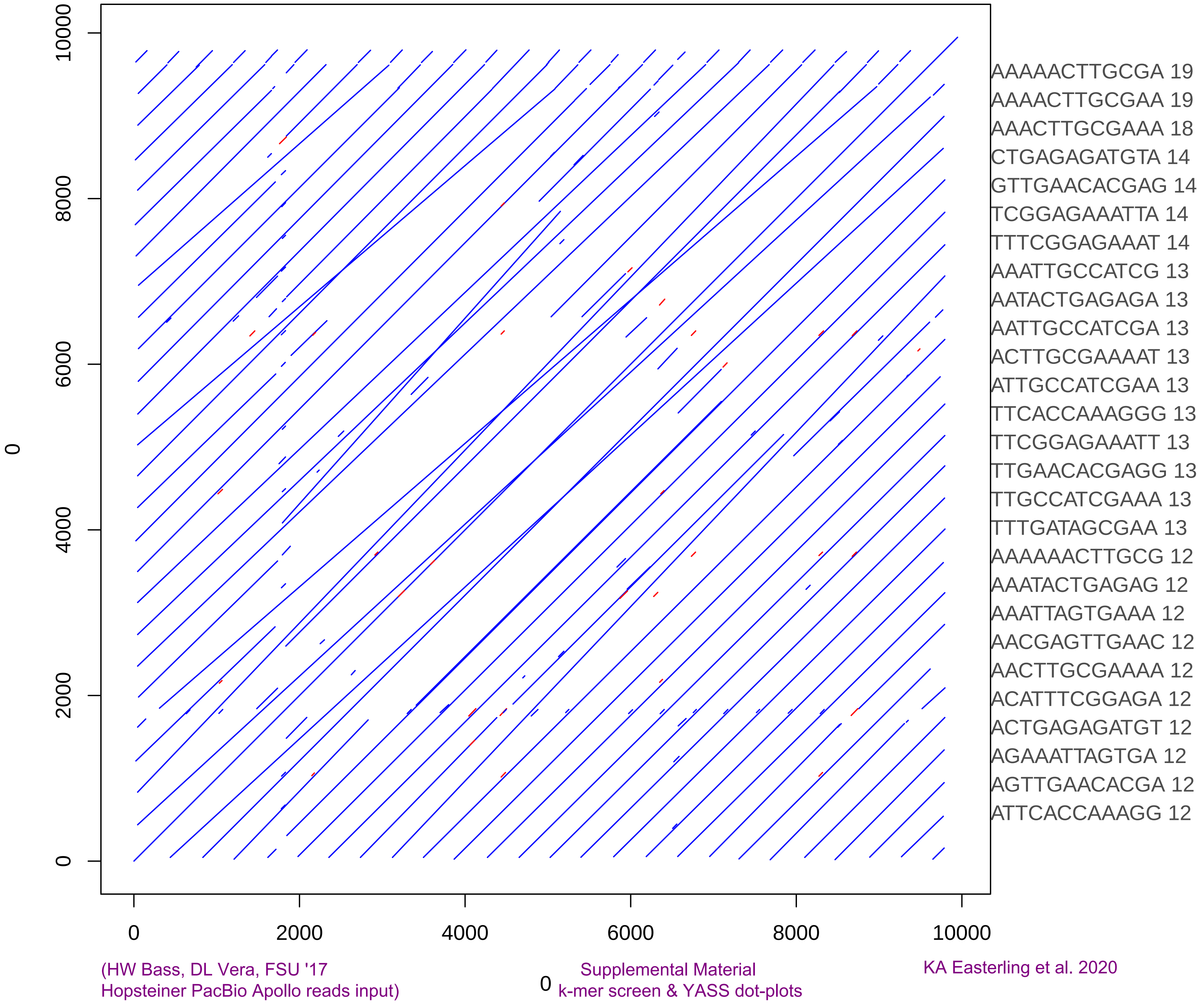

>m141122_014521_42134_c100732202550000001823141405141560_s1_p0/22204/1505_11409

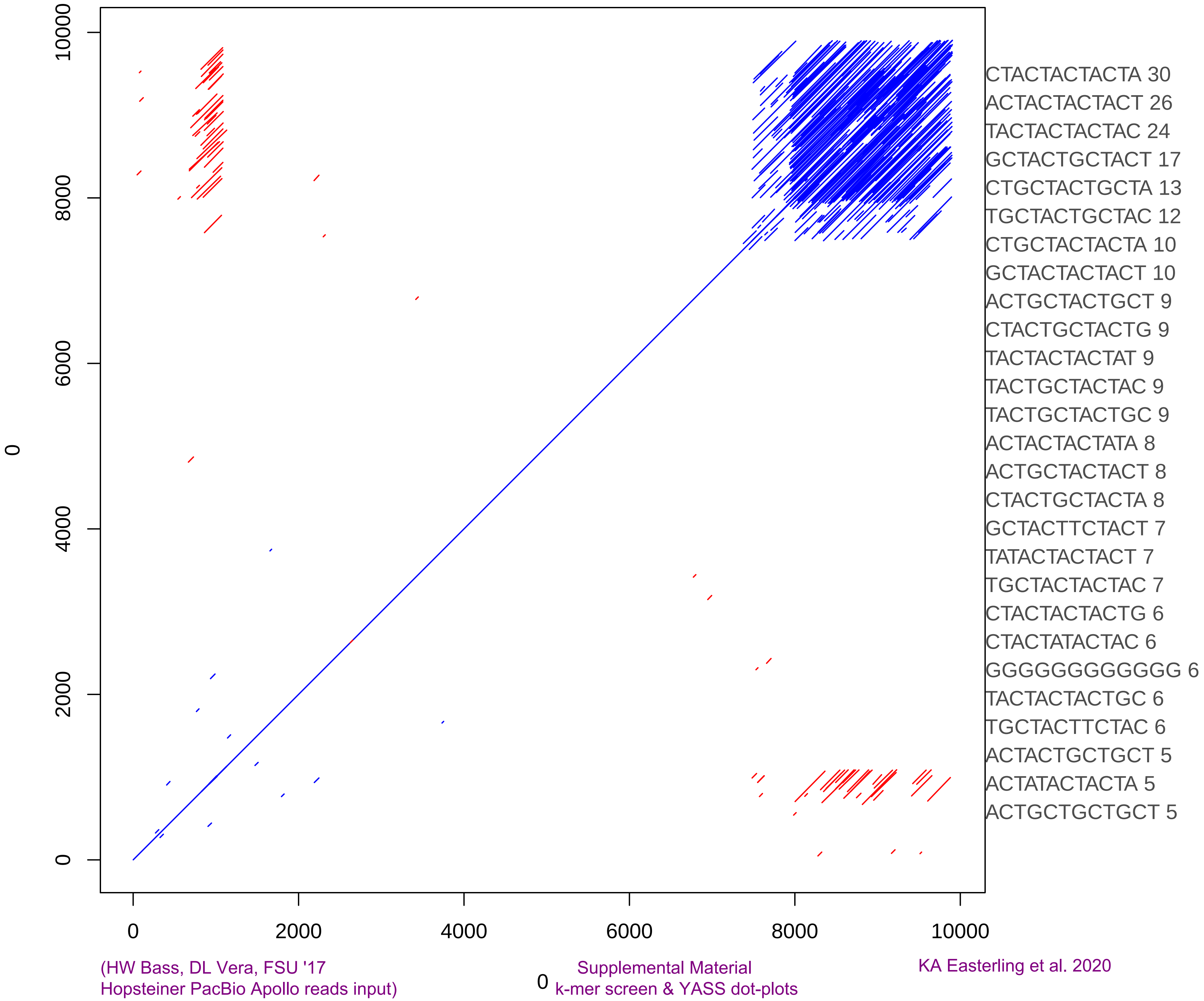

>m141122_014521_42134_c100732202550000001823141405141560_s1_p0/25879/3598_9700

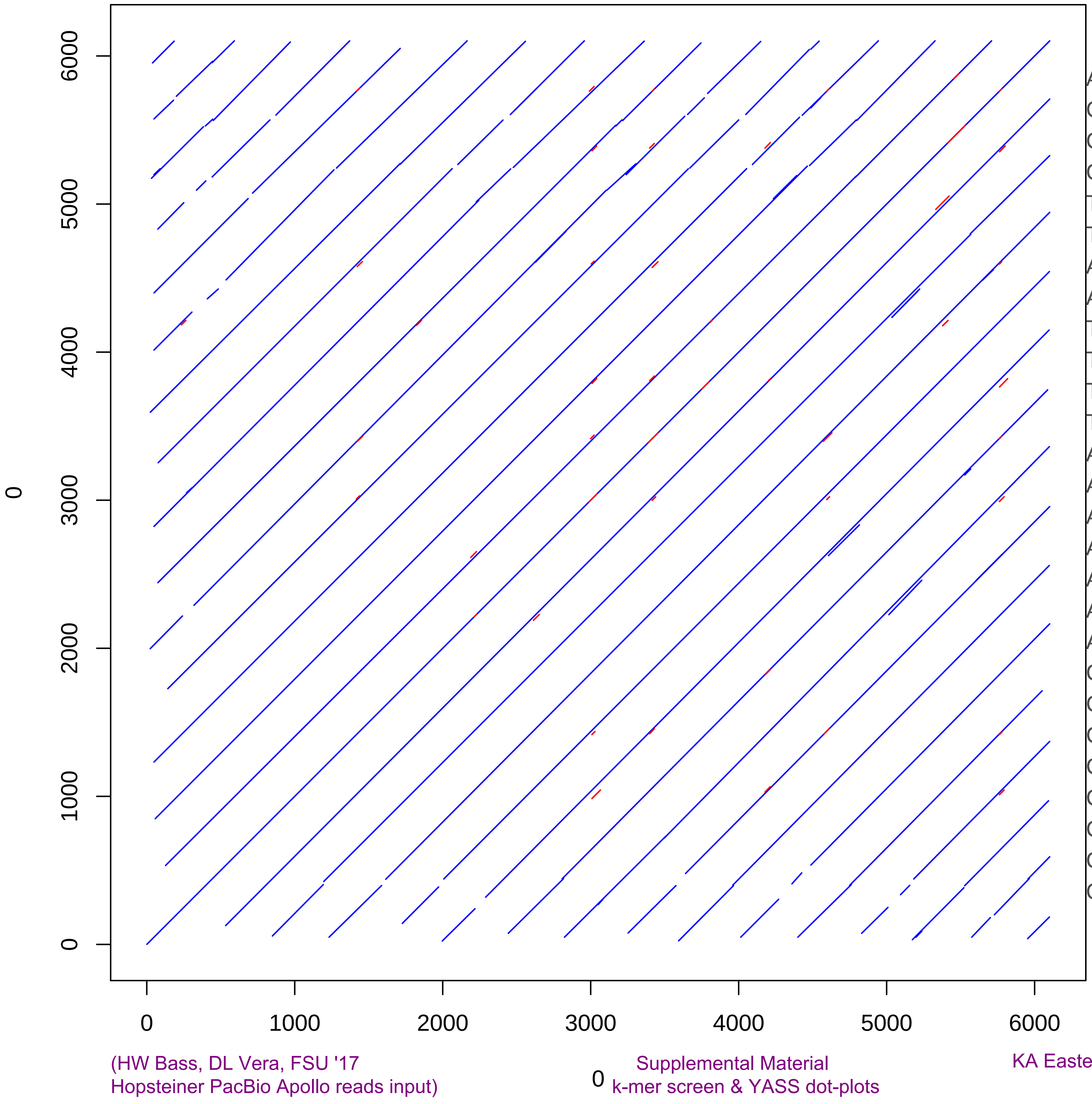

>m141122_014521_42134_c100732202550000001823141405141560_s1_p0/26079/5138_10673

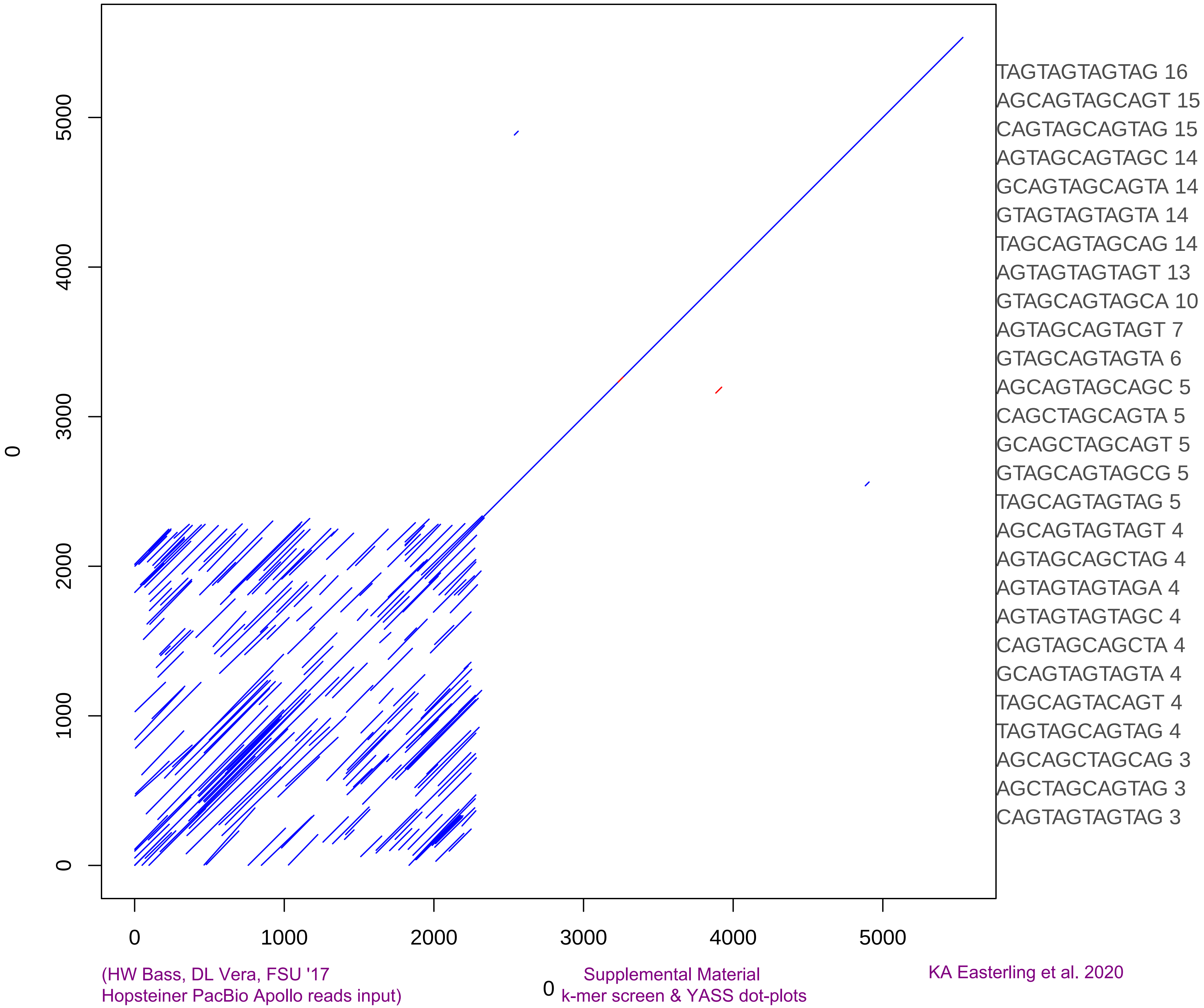

>m141122_014521_42134_c100732202550000001823141405141560_s1_p0/27829/5704_12550

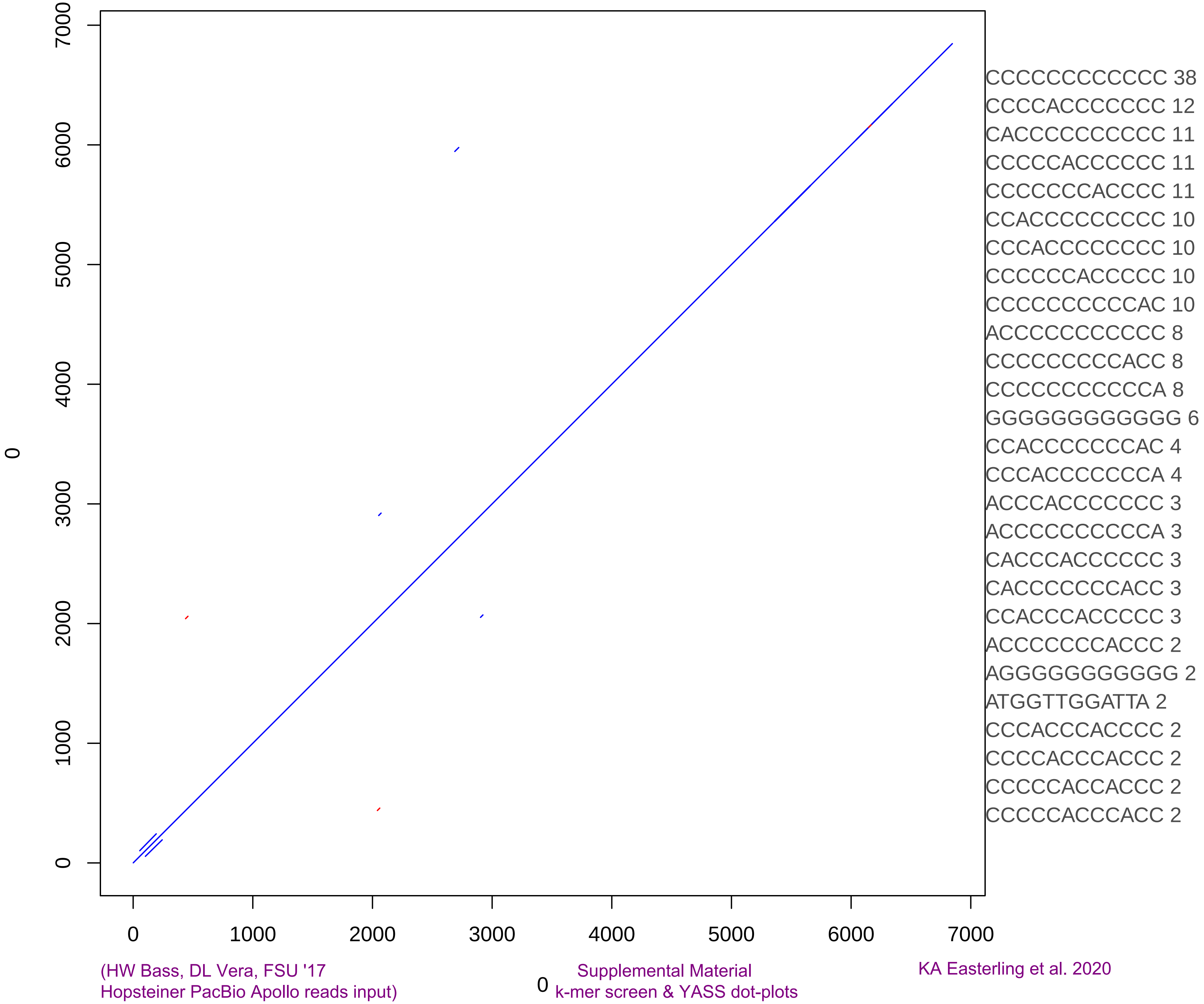

>m141122_014521_42134_c100732202550000001823141405141560_s1_p0/30423/9903_15122

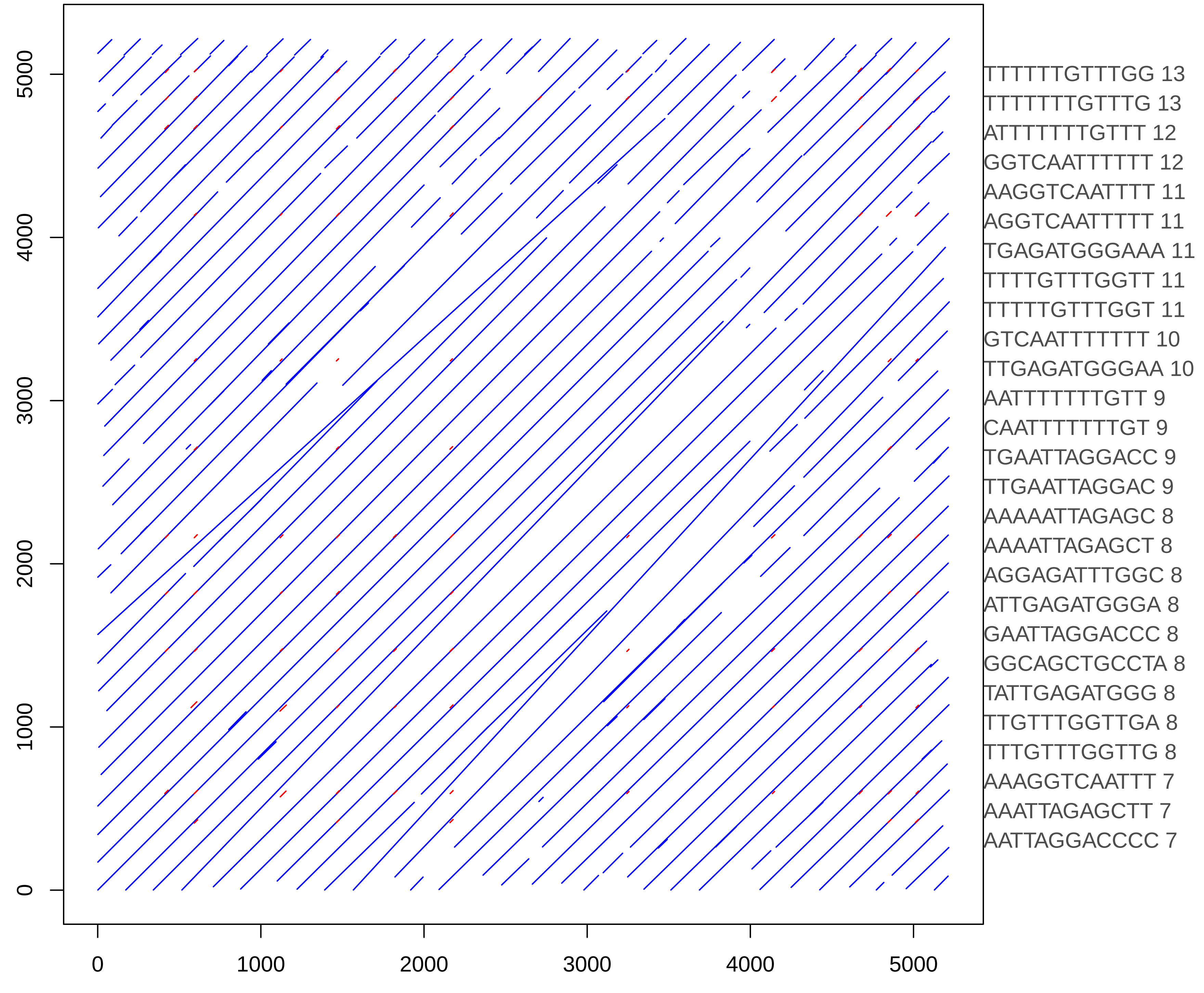

>m141122_014521_42134_c100732202550000001823141405141560_s1_p0/31381/6166_14514

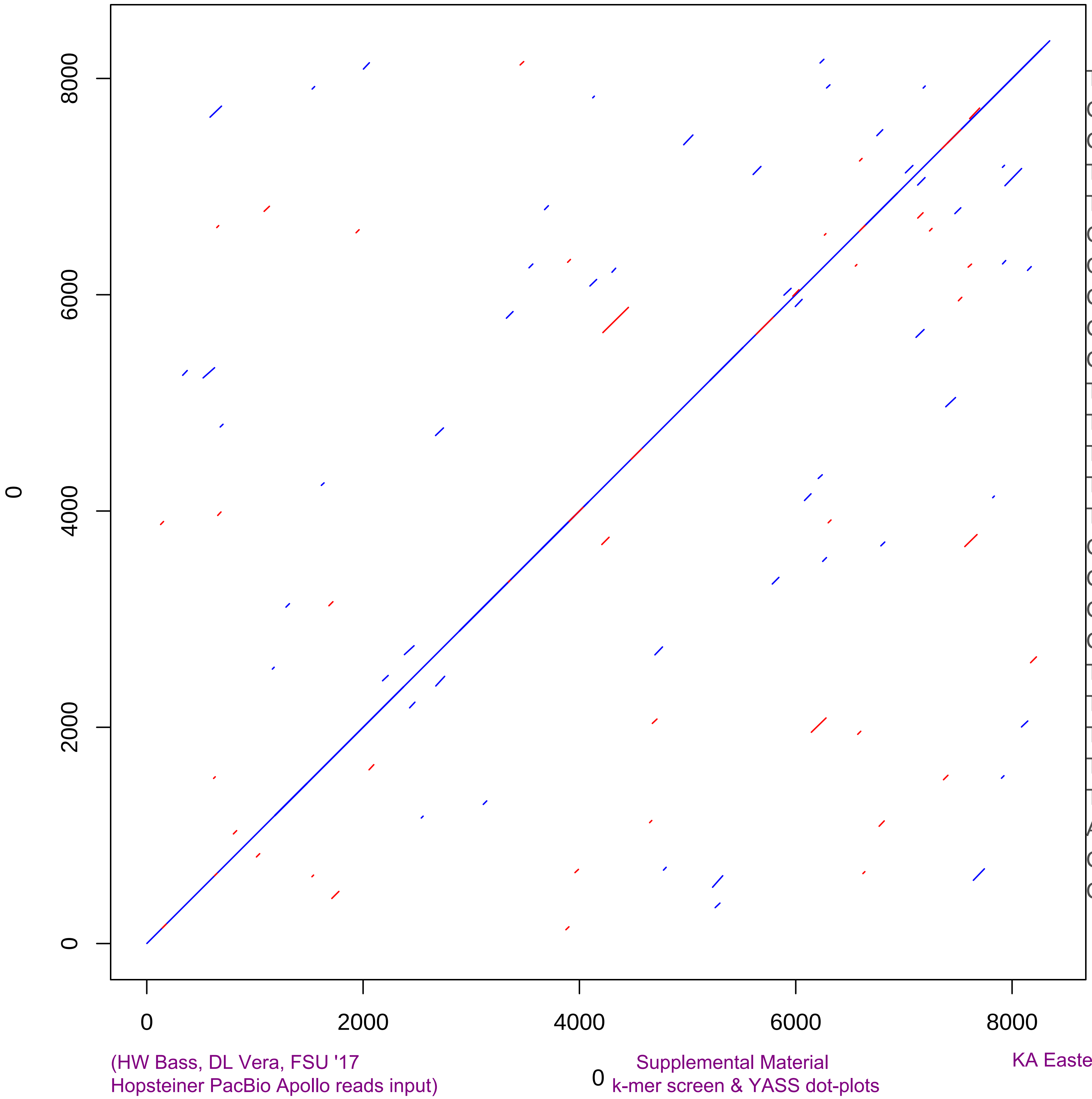

>m141122_014521_42134_c100732202550000001823141405141560_s1_p0/32571/0_5007

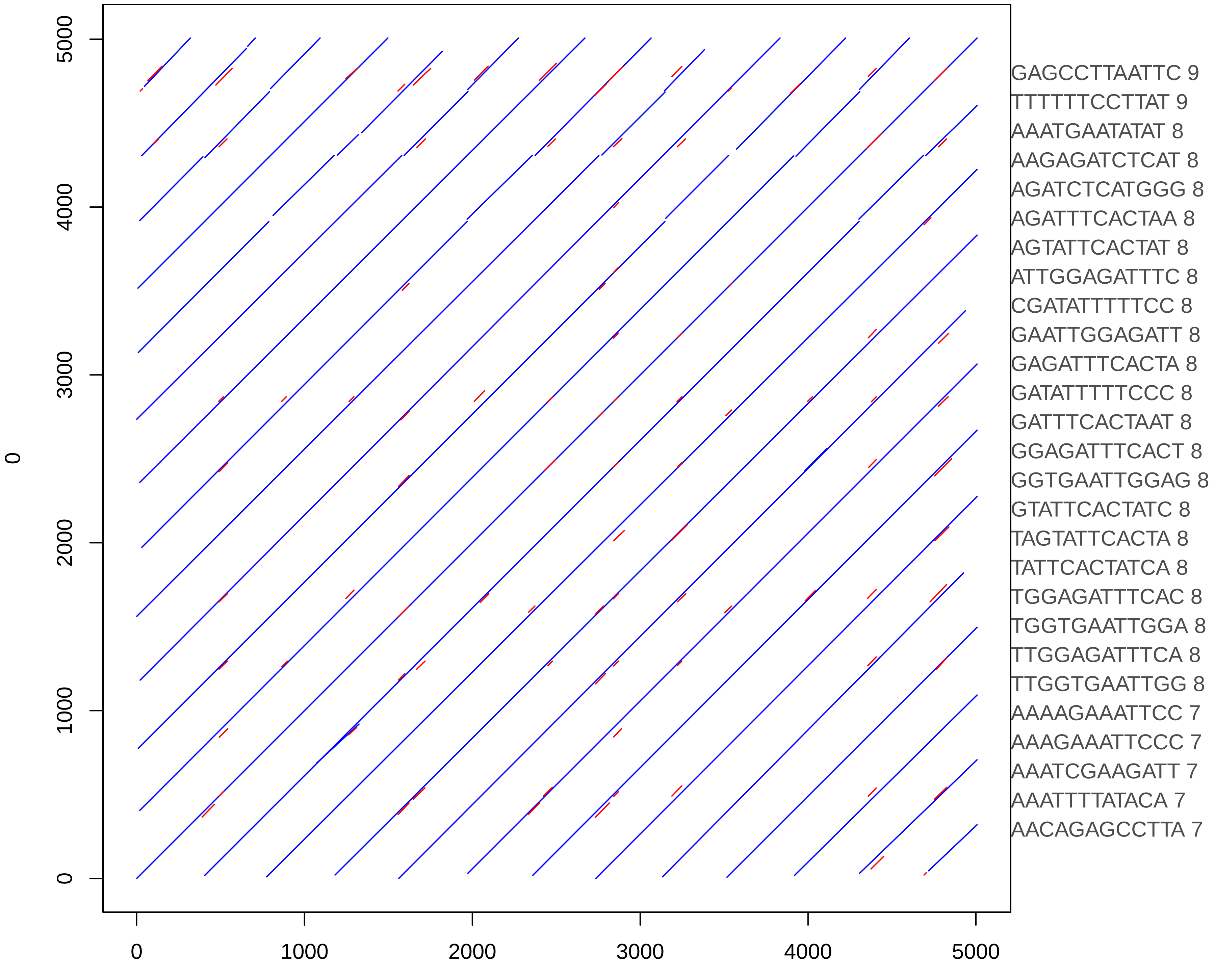

>m141122_014521_42134_c100732202550000001823141405141560_s1_p0/36733/10088_15712

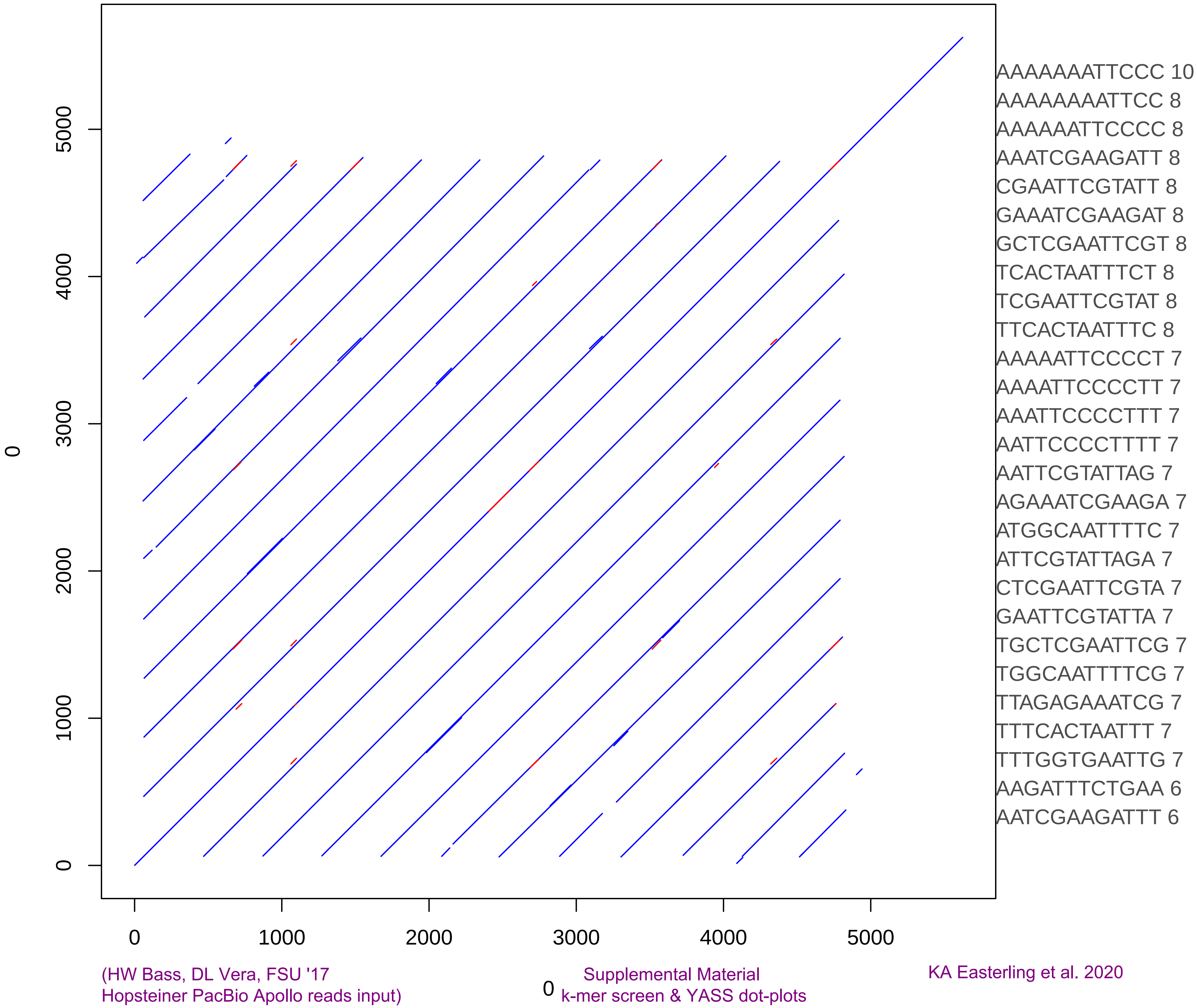

>m141122_014521_42134_c100732202550000001823141405141560_s1_p0/37699/962_6042

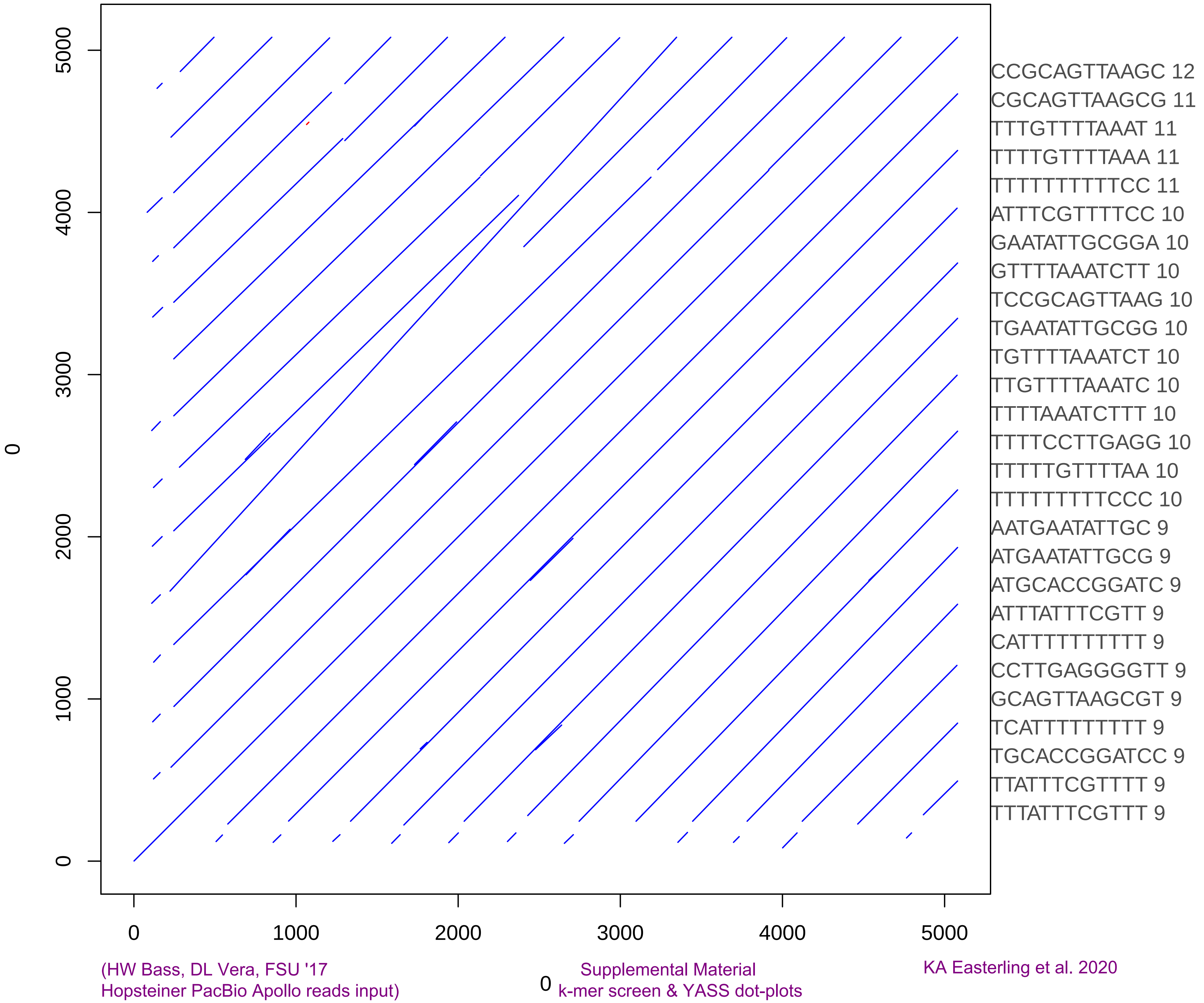

>m141122_014521_42134_c100732202550000001823141405141560_s1_p0/38653/8152_16568

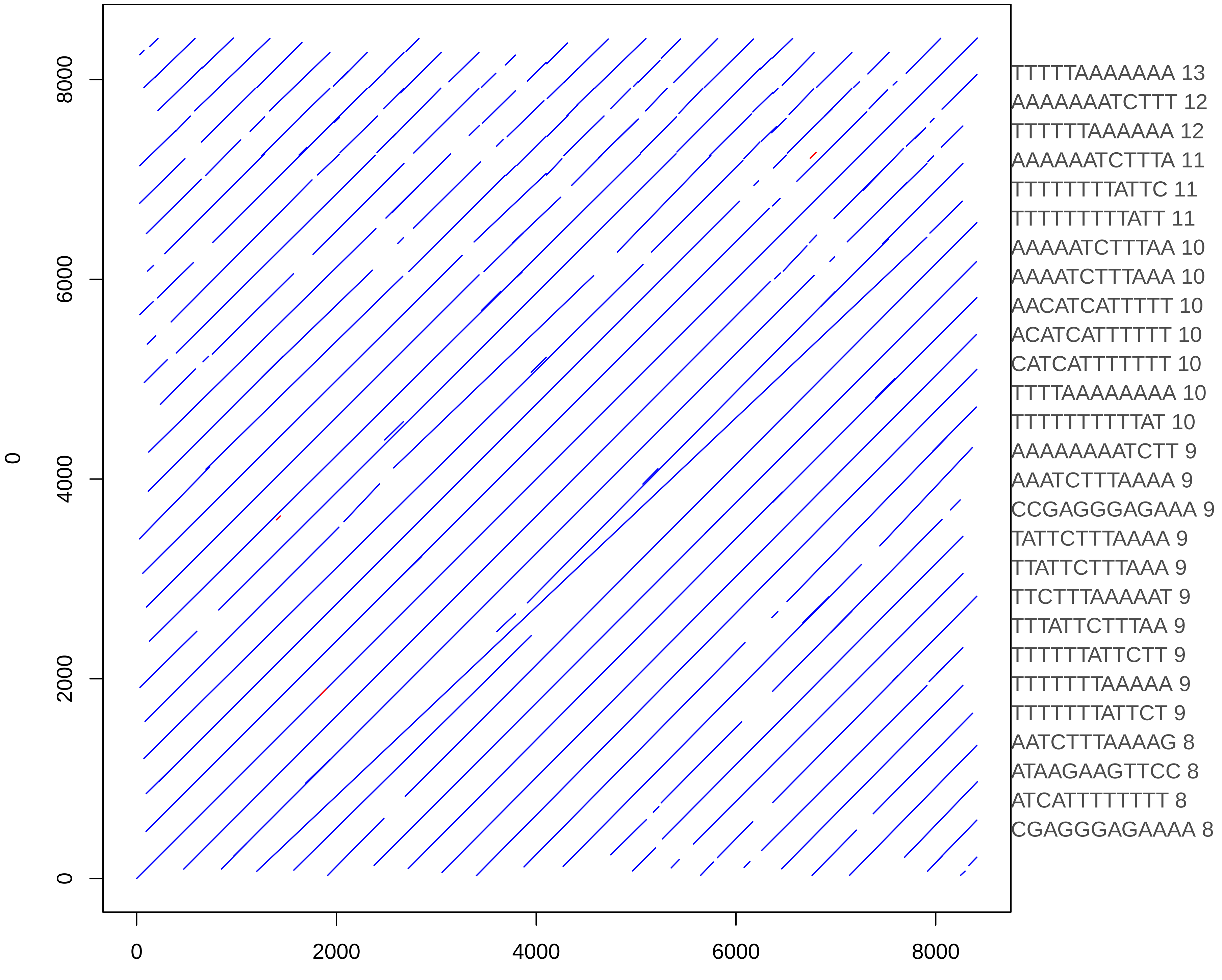

>m141122_014521_42134_c100732202550000001823141405141560_s1_p0/40005/314_8421

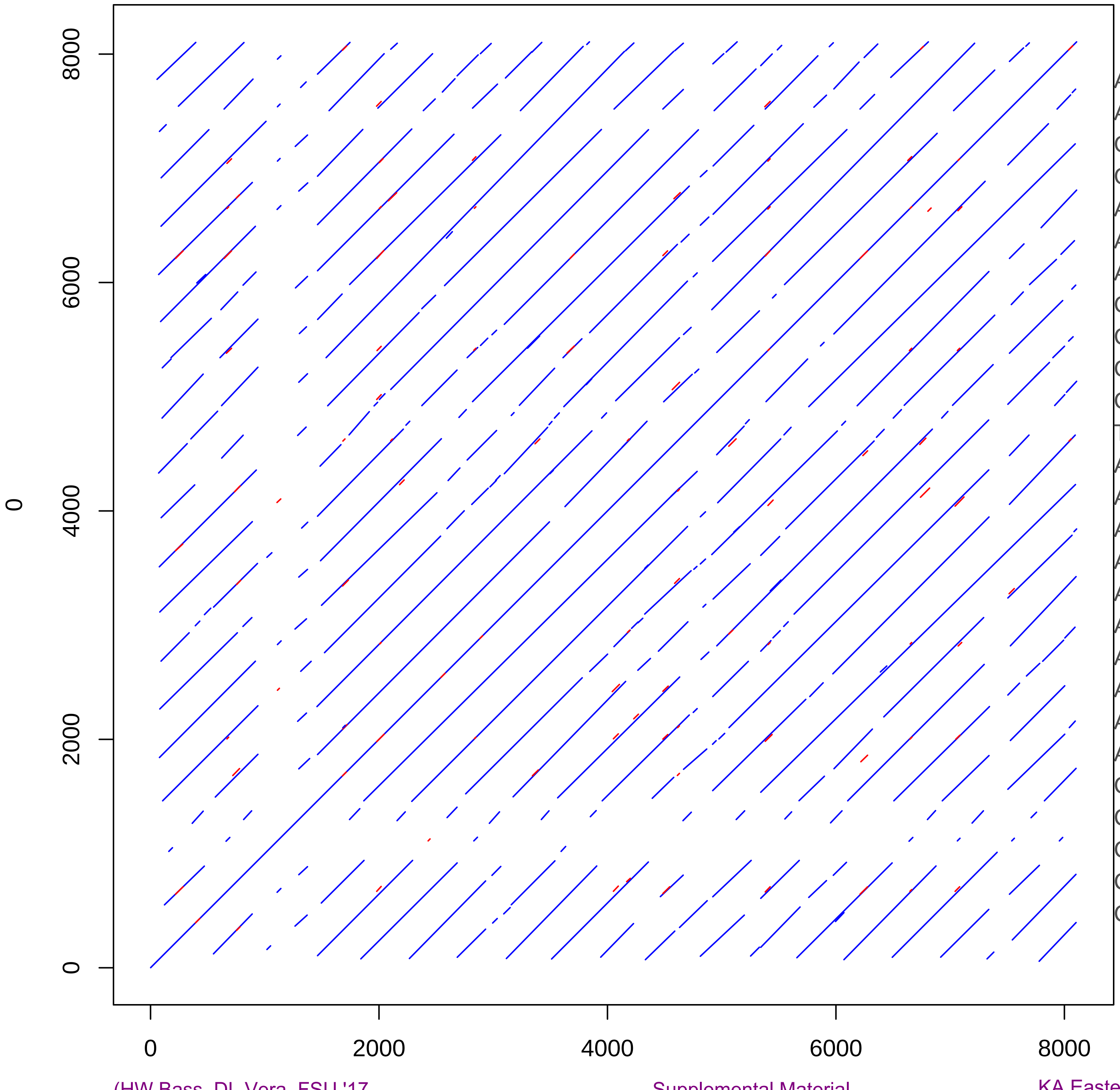

AAAGGGGTACCC 11
AACATTGCTCGA 10
GAAAGGGGTACC 9
GAAATGCATTAC 9
AAAAATTCCACT 8
AAAACATTGCTC 8
AAATGCATTACC 8
CAATTTTCGCAA 8
CGAAATGCATTA 8
GATTTCACTAAT 8
GCAATTTTCGCA 8
TGAAAGGGGTAC 8
AAAAAATTCCAC 7
AAAATTCCACTT 7
AAACATTGCTCG 7
AAATCGAAGATT 7
AATTTTCGCAAG 7
ACATTGCTCGAA 7
AGATTTCACTAA 7
ATATTCTTTAGT 7
ATTTCACTAATT 7
ATTTTCGCAAGT 7
CAAAACATTGCT 7
CCCAAGAGATCT 7
CCCTAAGACACA 7
CCCTCGTGTTCA 7
CCTAAGACACAG 7

>m141122_014521_42134_c100732202550000001823141405141560_s1_p0/43043/8634_14649

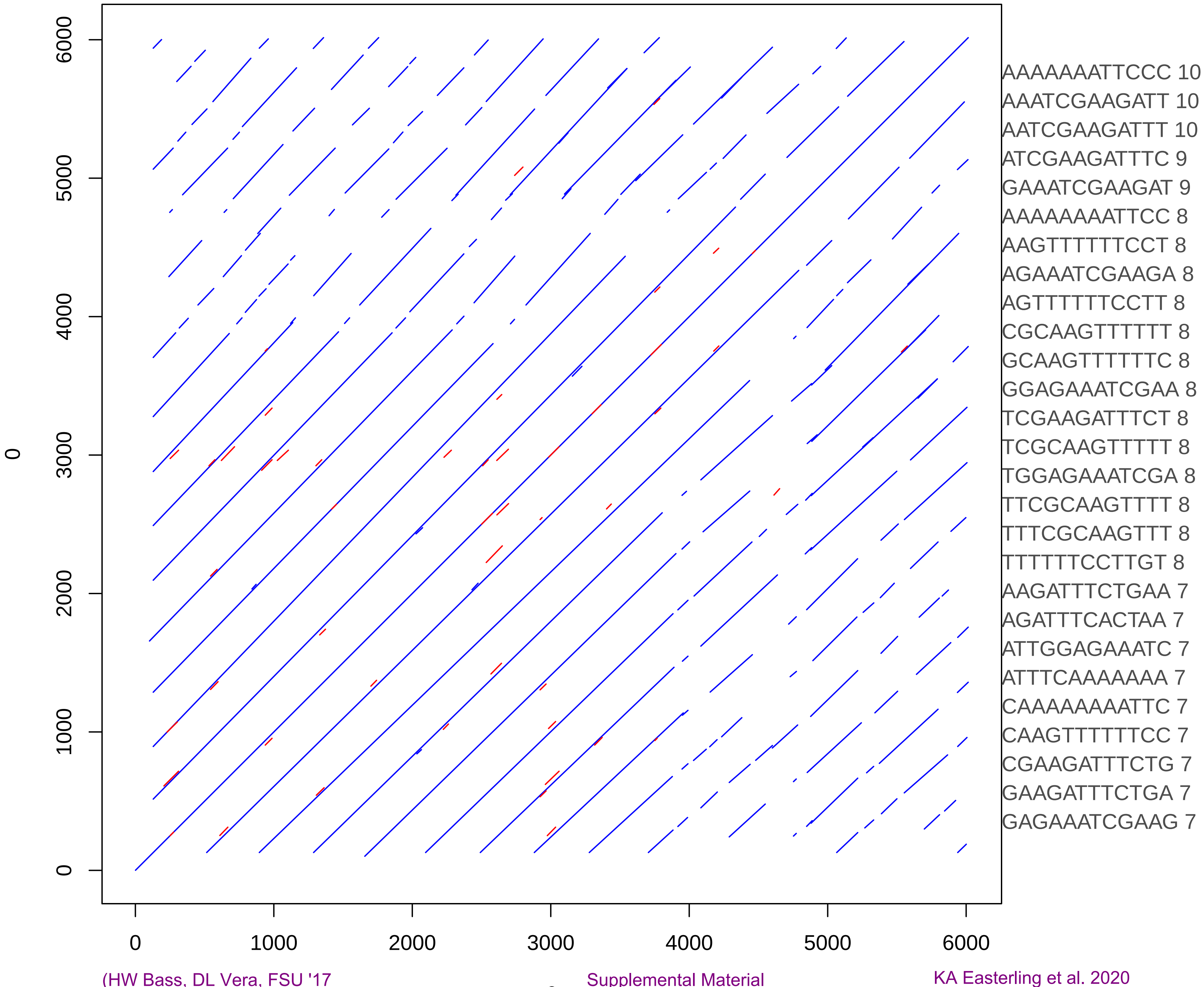

>m141122_014521_42134_c100732202550000001823141405141560_s1_p0/45837/6221_11679

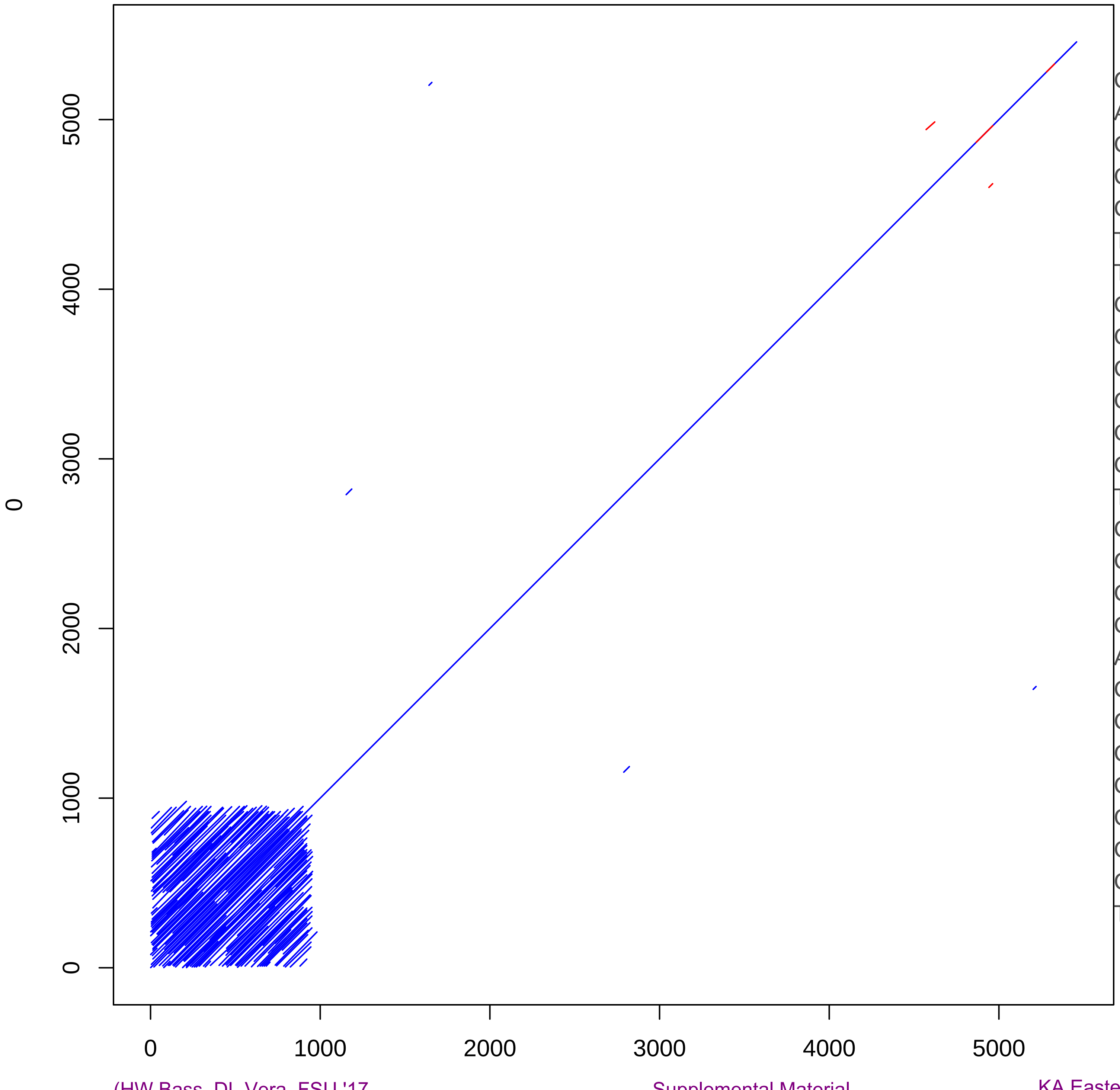

CTCCATGGCCTC 14
ATGGCCTCCTCC 13
CATGGCCTCCTC 13
CCATGGCCTCCT 12
CTCCTCCATGGC 10
TGGCCTCCTCCA 10
TCCTCCATGGCC 9
CCCATGGCCTCC 8
CCTCCATGGCCT 8
CCTCCTCCATGG 8
GCCTCCTCCATG 8
GGCCTCCTCCAT 8
CCATGGCCTCGC 6
TCCATGGCCTCC 6
CATGGCCTCGCC 5
CCCCATGGCCTC 5
CCTCCGTGGCCT 5
CGCCCATGGCCT 5
ATGGCCTCGCCC 4
CCTCCTCCGTGG 4
CTCCGTGGCCTC 4
CTCCTCCGTGGC 4
CTCGCCCATGGC 4
GCCCATGGCCTC 4
GCCTCCTCCGTG 4
GGCCTCCTCCGT 4
TCCTCCGTGGCC 4

>m141122_014521_42134_c100732202550000001823141405141560_s1_p0/45994/0_5950

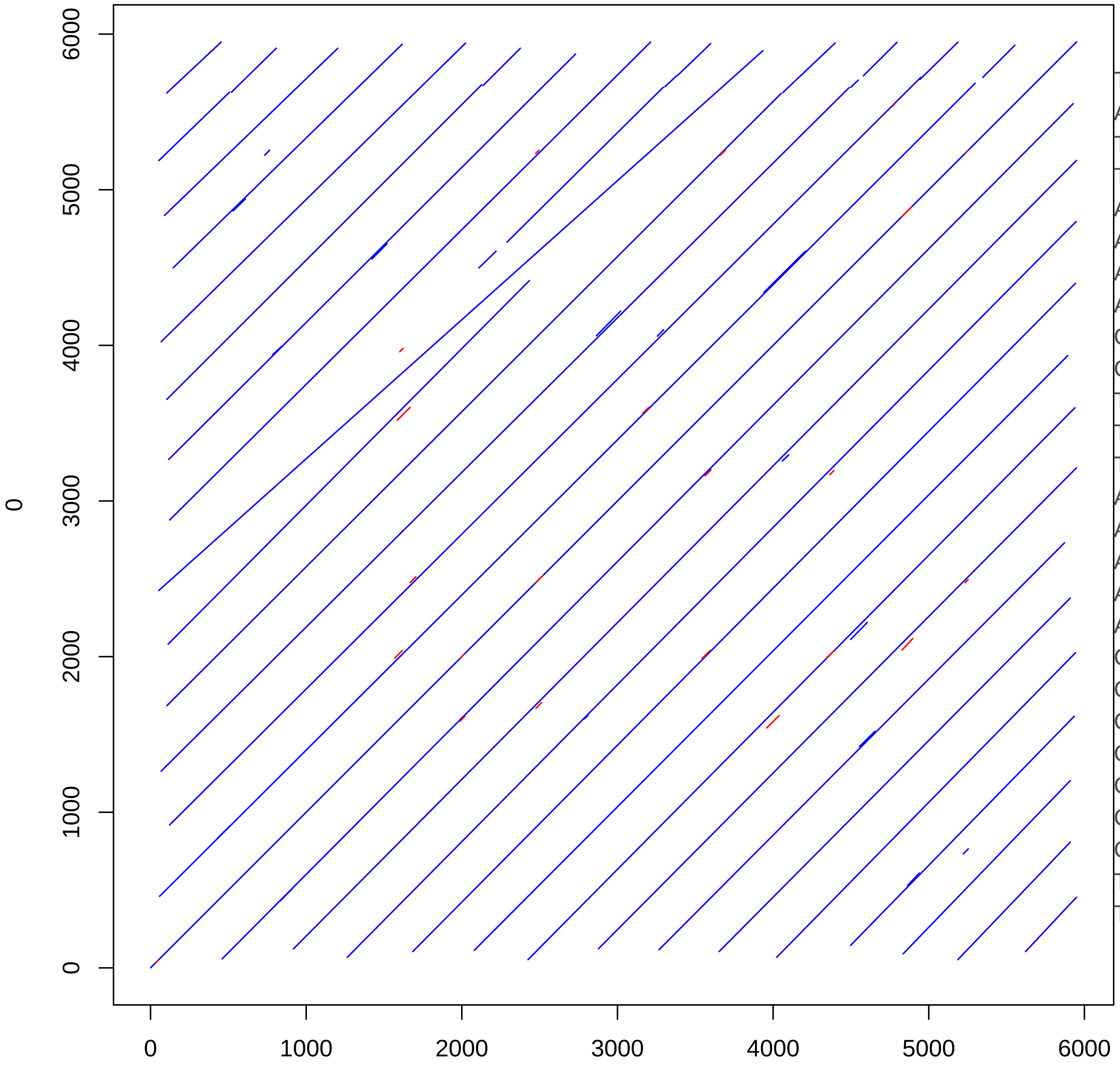

TGCGAAAATTGC 11
AGTGAATACTAA 10
TTCTATGTATAA 10
TTGCGAAAATTG 10
AAAAACTTGCGA 9
AAAACTTGCGAA 9
AAACTTGCGAAA 9
AACTTGCGAAAA 9
GCGAAAATTGCC 9
GTGAATACTAAA 9
TAGTGAATACTA 9
TCTATGTATAAA 9
TGATAGTGAATA 9
ACTTGCGAAAAT 8
ATAGTGAATACT 8
ATCGAAAAGGGG 8
ATGCGAGGGTCA 8
ATGTATAAAATT 8
CAAAATACTGAG 8
CTATGTATAAAA 8
CTTGCGAAAATT 8
GAACGAGTTGAA 8
GATAGTGAATAC 8
GCAAAATACTGA 8
GGTTCTATGTAT 8
TATGCGAGGGTC 8
TATGTATAAAAT 8

>m141122_014521_42134_c100732202550000001823141405141560_s1_p0/50118/7907_15513

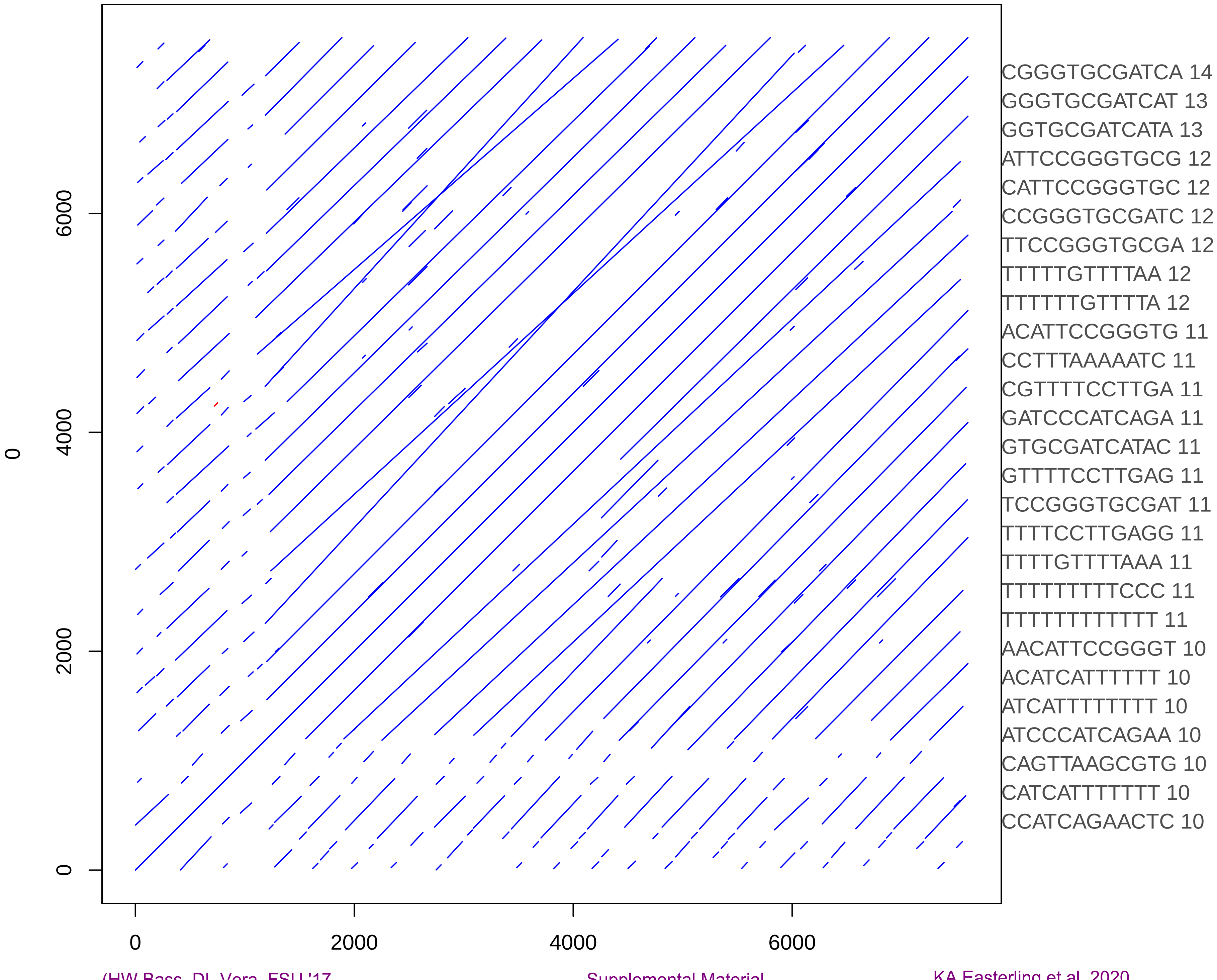

0

>m141122_014521_42134_c100732202550000001823141405141560_s1_p0/53125/12667_17964

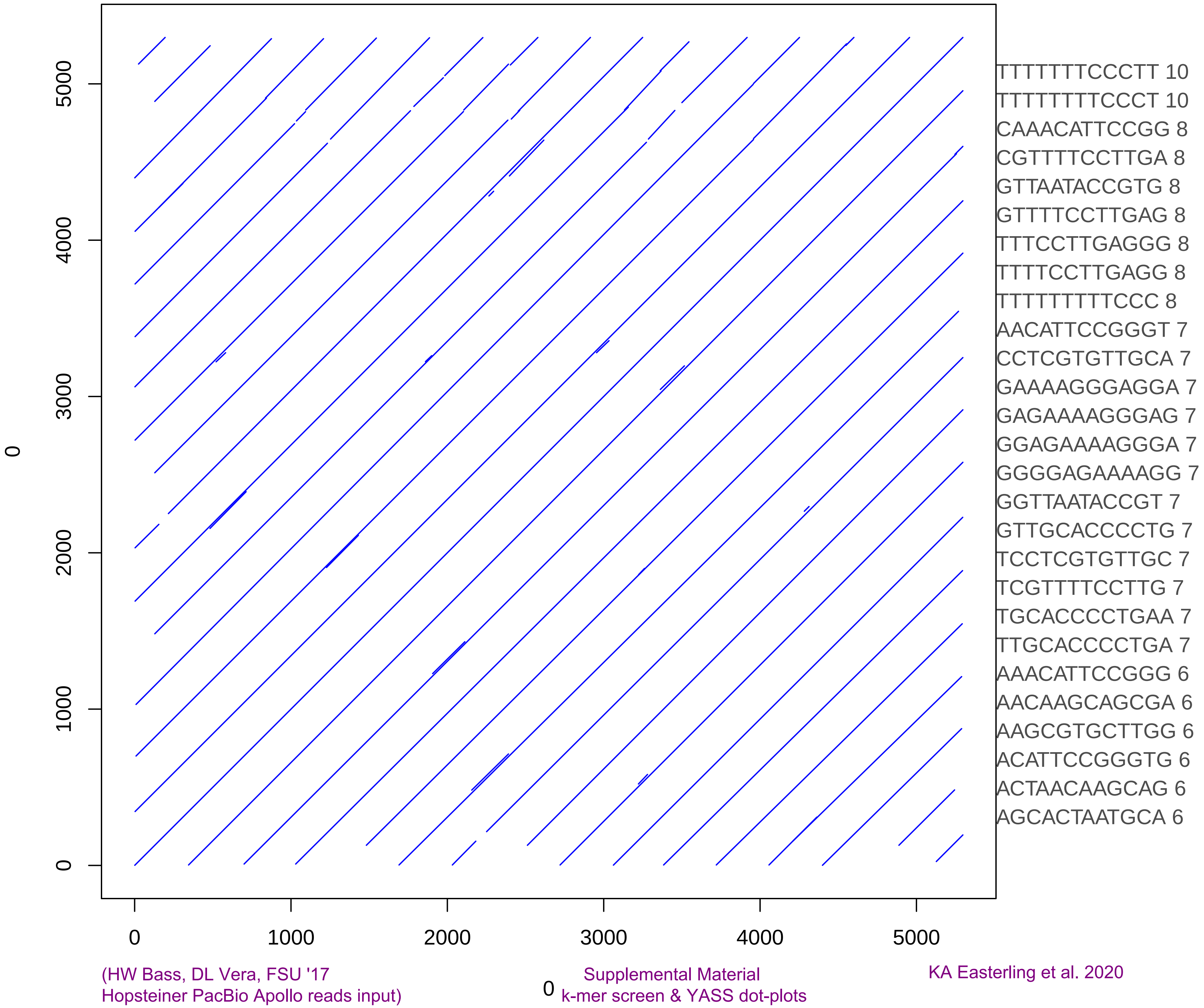

>m141122_014521_42134_c100732202550000001823141405141560_s1_p0/57038/5979_11842

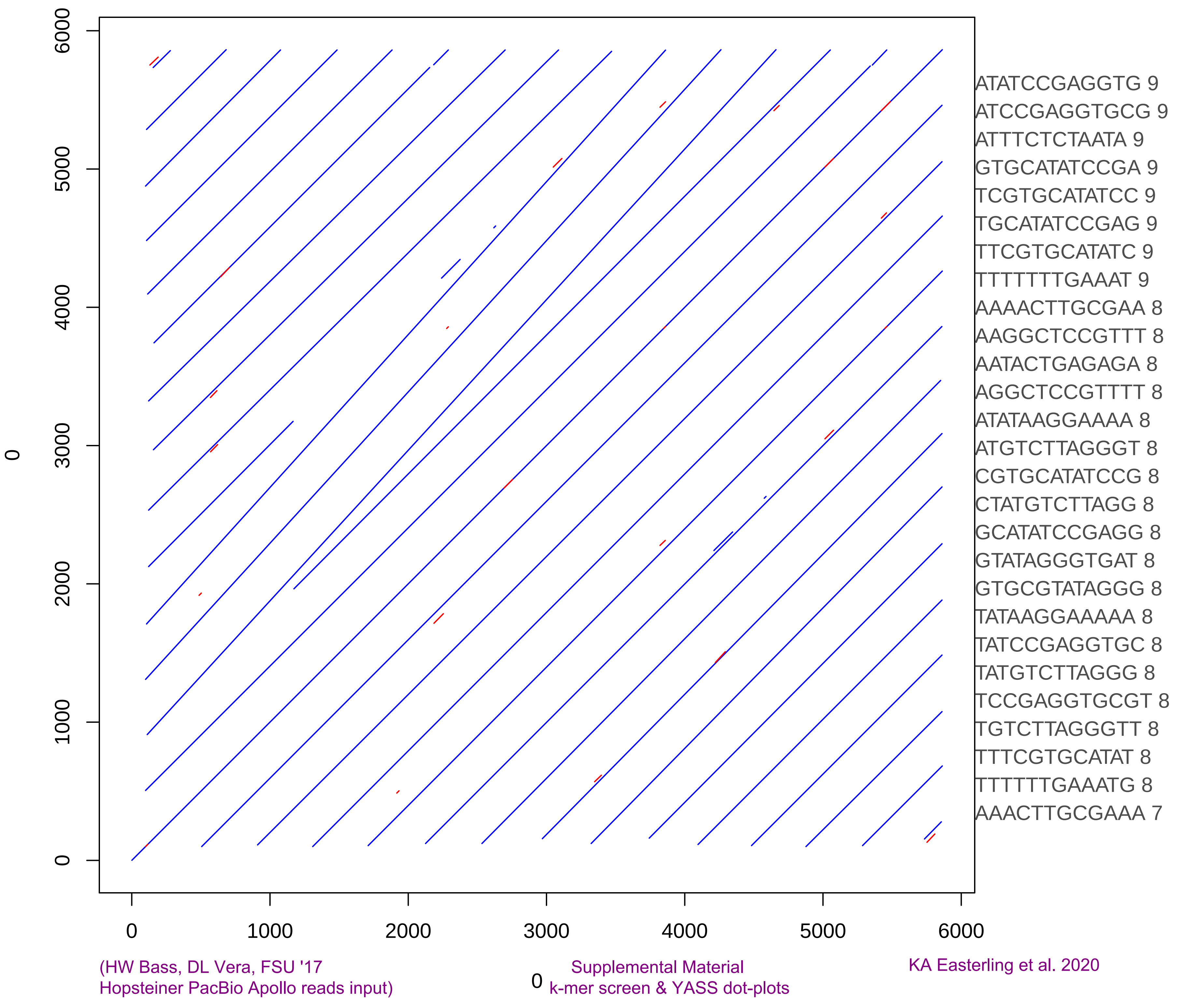

>m141122_014521_42134_c100732202550000001823141405141560_s1_p0/57114/0_8228

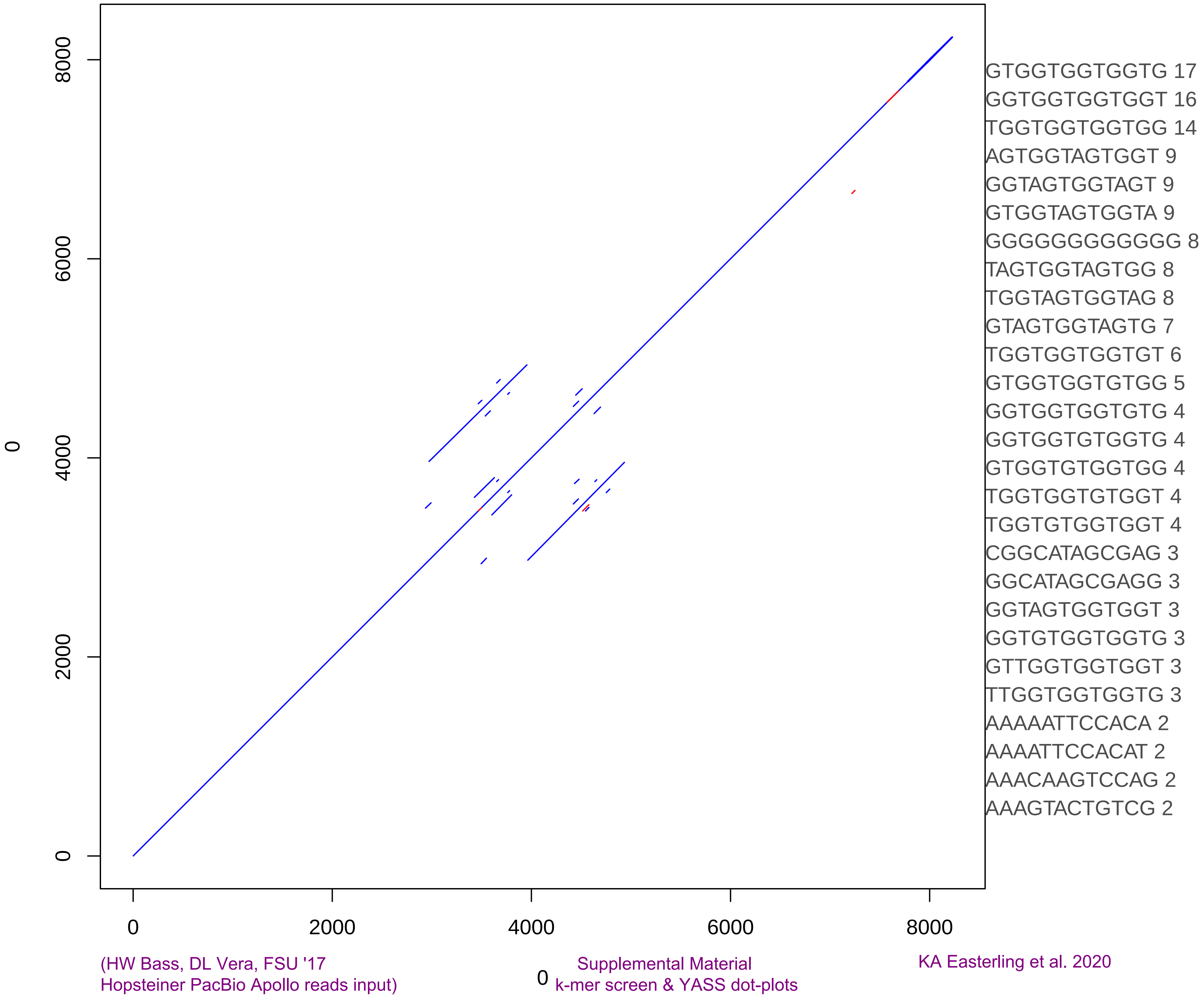

>m141122_014521_42134_c100732202550000001823141405141560_s1_p0/64/1631_10830

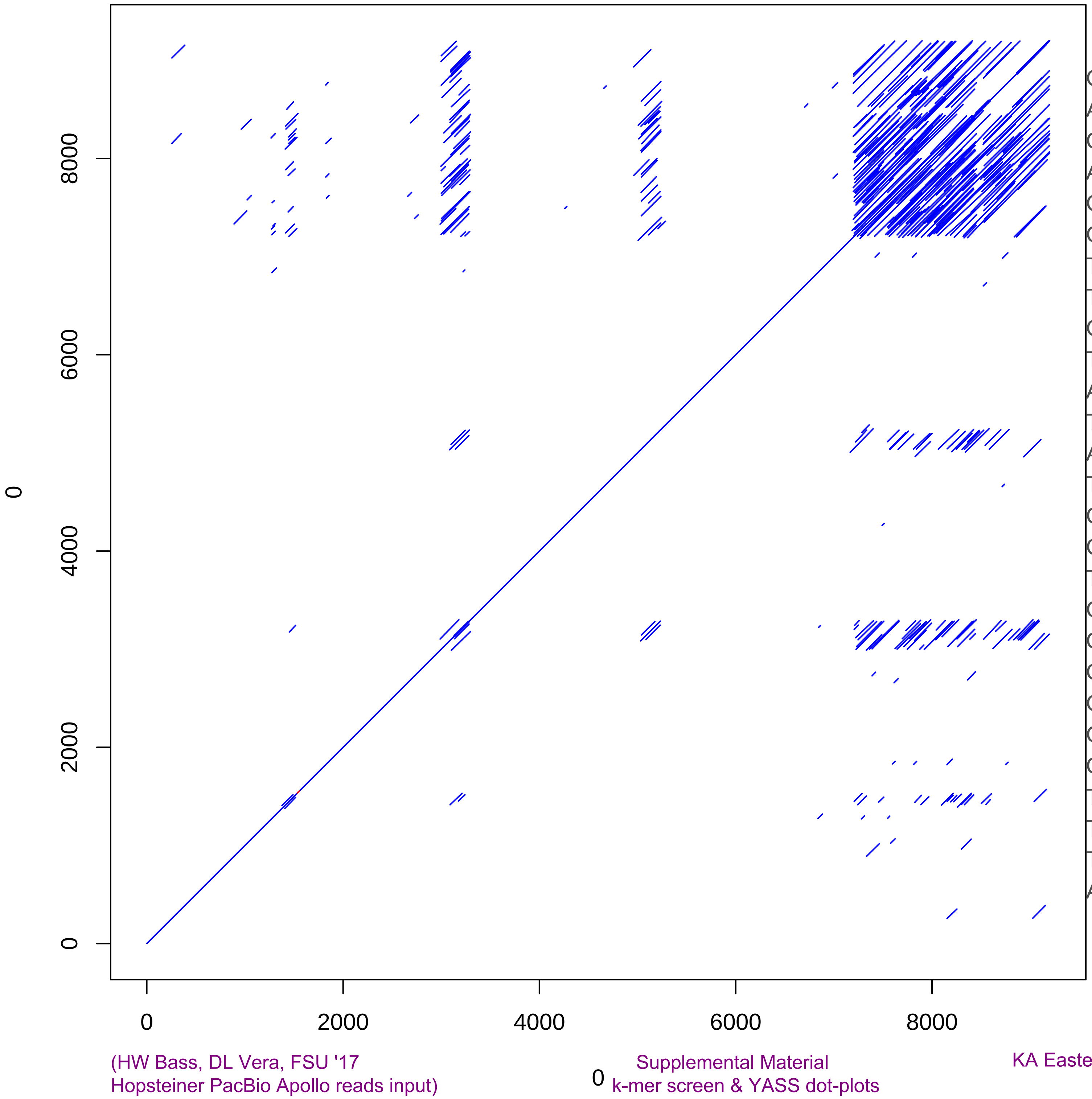

GTAGTGGTAGTG 16
AGTAGTGGTAGT 15
GTAGTGGTAGTA 14
AGTGGTAGTGGT 13
GTGGTAGTGGTA 13
GTAGTAGTGGTA 12
TAGTAGTGGTAG 12
TAGTGGTAGTAG 12
GGTAGTGGTAGT 11
TAGTGGTAGTGG 11
AGTAGTAGTAGT 10
TGGTAGTGGTAG 10
AGTGGTAGTAGT 9
TAGTAGTAGTAG 9
GTAGTAGTAGTA 8
GTGGTAGTAGTG 7
TGTAGTGGTAGT 7
GTAGTGTAGTAG 6
GTGGTAGTAGTA 6
GGTAGTAGTGGT 5
GTAGTAGTAGTG 5
GTAGTAGTATGG 5
GTGTAGTGGTAG 5
TAGTAGTAGTGG 5
TGGTAGTAGTGT 5
TGGTAGTATAGT 5
AGTAGTAGTGGT 4

>m141122_014521_42134_c100732202550000001823141405141560_s1_p0/64706/316_13955

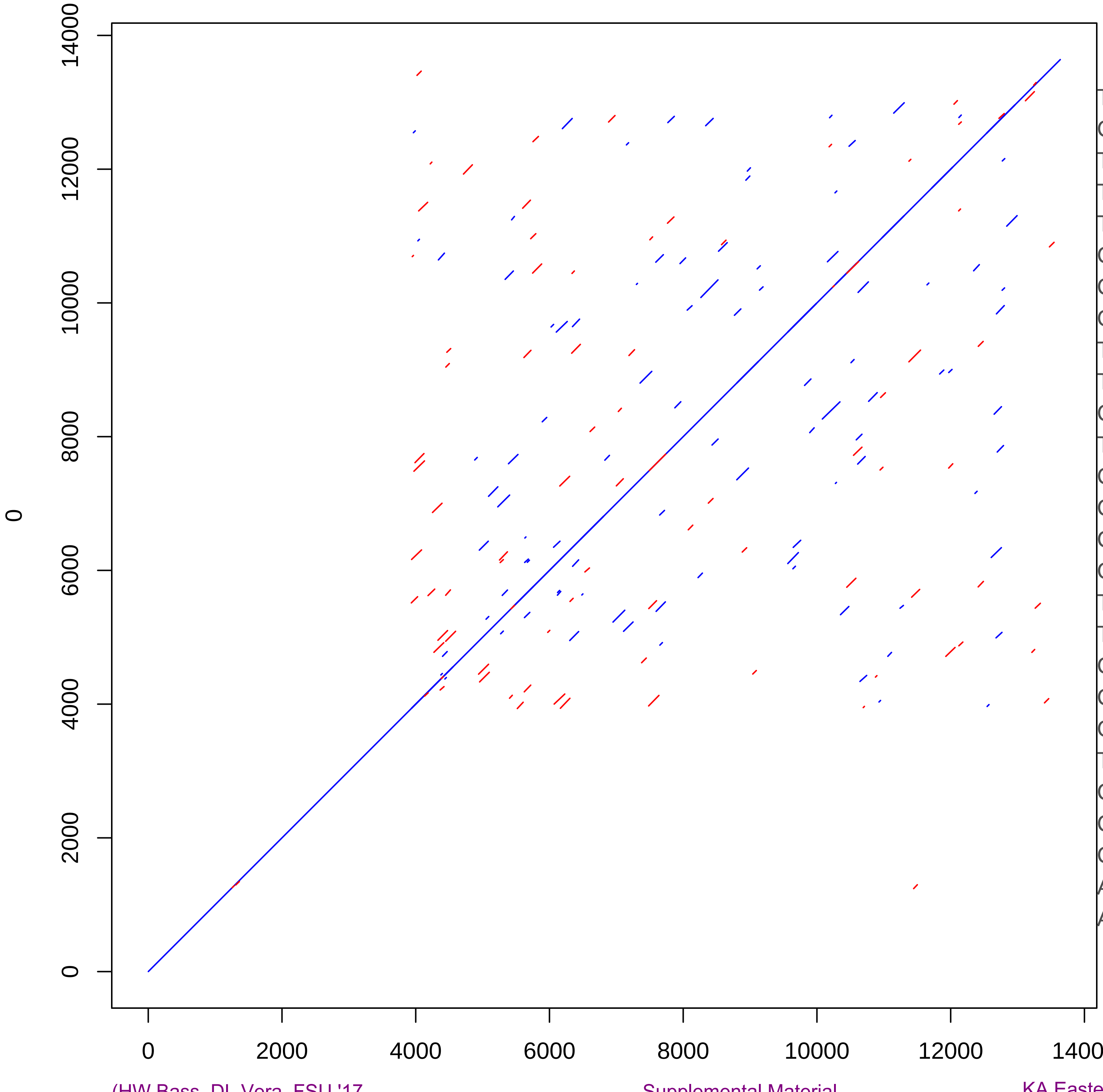

TTTTTTTTTTTT 155
CTTTTTTTTTTT 30
TTTTTTTTTTCC 25
TTTTTTTTTTTC 25
TTTTTTTTTCCC 18
CAAAAAAAAAAA 15
CCTTTTTTTTTT 15
GTTTTTTTTTTT 15
TTTTTTTTTTTA 14
TTTTTTTTTTTG 14
CCCCCCCCCTTT 13
TTTTTTTTTTAA 13
CCAAAAAAAAAA 12
CCCCCCCCTTTT 11
CCCCCTTTTTTT 11
GCTTTTTTTTTT 11
TTTTTTTTTAAA 11
TTTTTTTTTTGG 11
CCCCTTTTTTTT 10
CCCTTTTTTTTT 10
GGTTTTTTTTTT 10
TTTTTTTTCCCC 10
CCCAAAAAAAAA 9
CCCCAAAAAAAA 9
CCCCCAAAAAAA 9
AAAAAAAAAAAG 8
AAAAAAAAAAAT 8

1,121 hits for repetitive sequences
passing 12mer abundance criterion

(Read-Number)
PAGE 232

For Tandem Repeat families of ### bp:
PacBio Read named "HuluTR###-r232"

>m141122_014521_42134_c100732202550000001823141405141560_s1_p0/64948/10593_18158

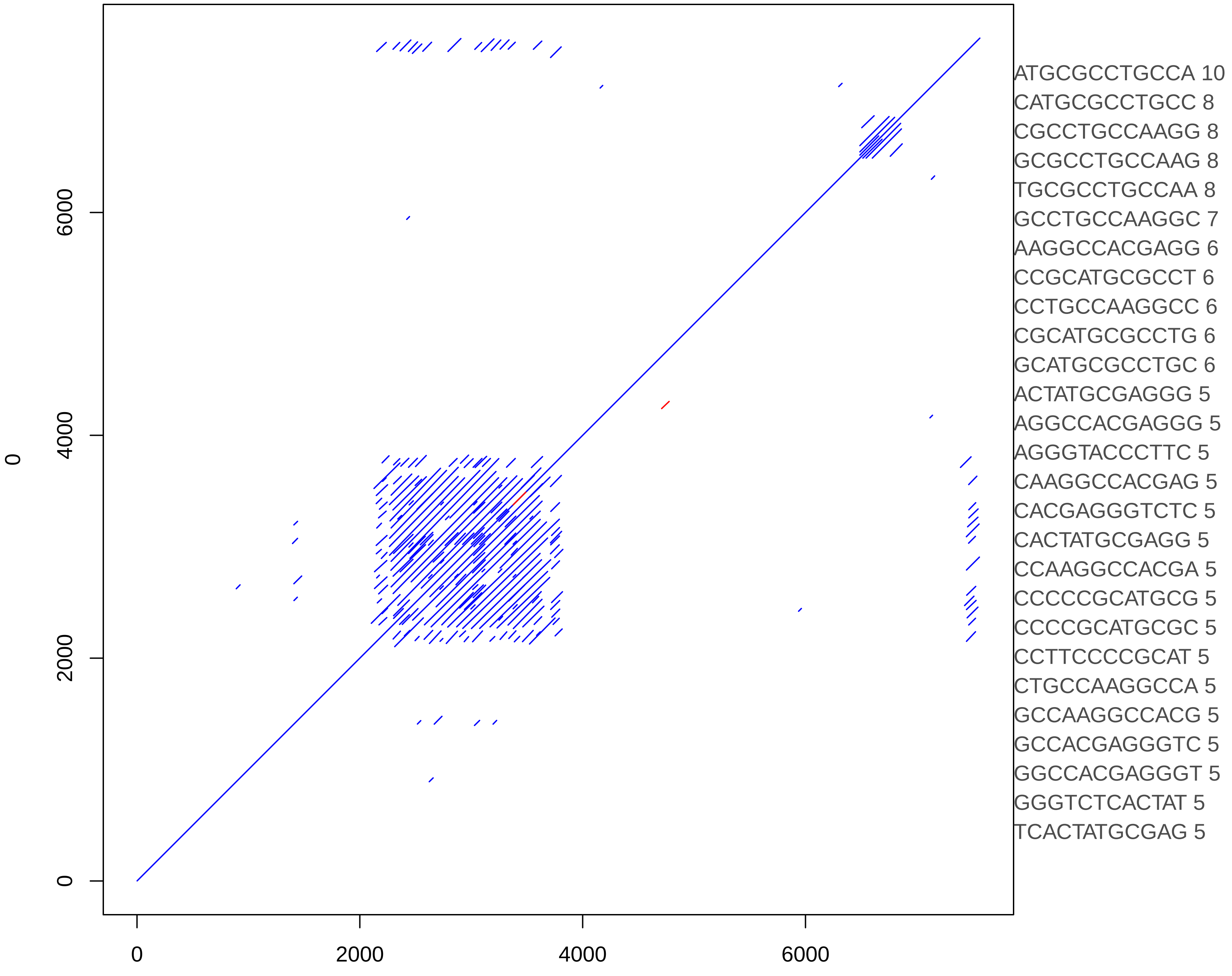

(HW Bass, DL Vera, FSU '17
Hopsteiner PacBio Apollo reads input)

0

Supplemental Material
k-mer screen & YASS dot-plots

KA Easterling et al. 2020

>m141122_014521_42134_c100732202550000001823141405141560_s1_p0/66819/9172_14823

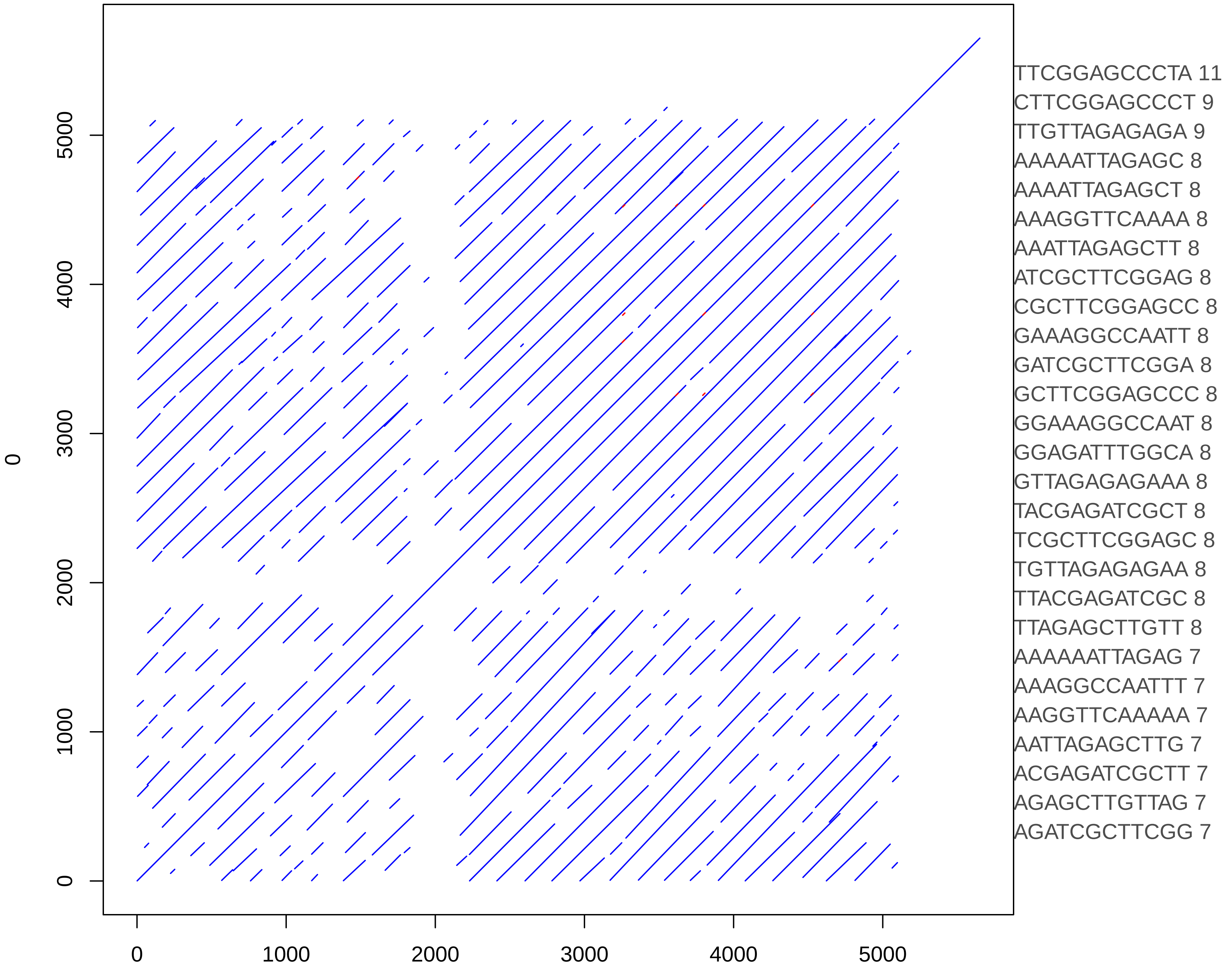

>m141122_014521_42134_c100732202550000001823141405141560_s1_p0/6726/496_10105

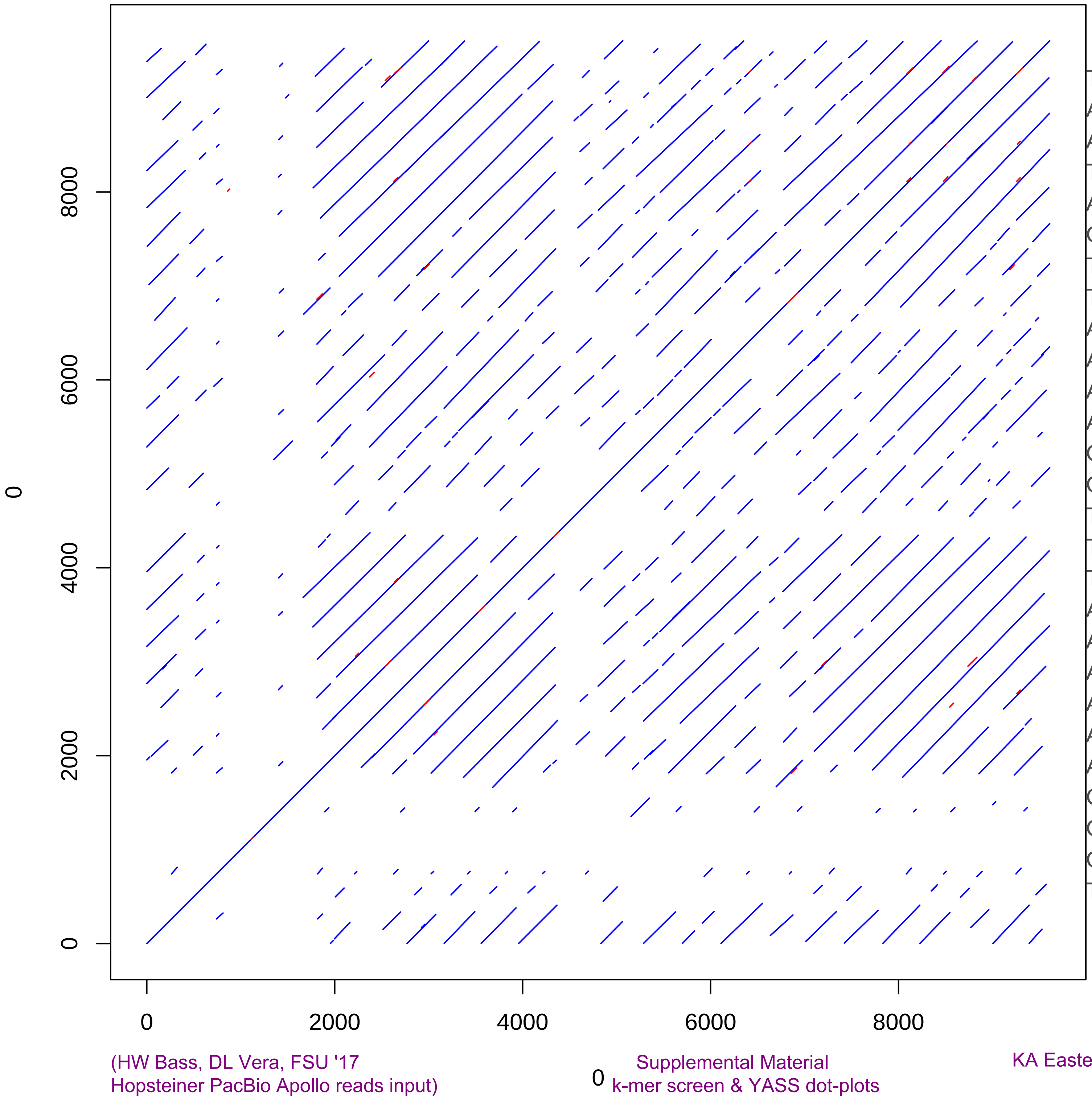

TTTCATAGTGAA 13
AAATCTCCAATT 10
AATCTCCAATTC 10
TGAAATCTCCAA 10
AGTGAAATCTCC 9
GAAATCTCCAAT 9
TTCATAGTGAAT 9
TTTTCATAGTGA 9
AAAATATCGAAC 8
AAATTAGTGAAA 8
ATAGTGAATACT 8
ATCTCCAATTCA 8
GTGAAATCTCCA 8
GTTTTCATAGTG 8
TAGTGAAATCTC 8
TCATAGTGAATA 8
TTTTTTTTTAAA 8
AATTAGTGAAAT 7
AGAAATTAGTGA 7
AGTGAATACTAA 7
ATGAGATCTCTT 7
ATTCACCAAAGG 7
ATTTTTTTTTAA 7
CATAGTGAATAC 7
GAAATTAGTGAA 7
GAGAAATTAGTG 7
TAGTGAATACTA 7

>m141122_014521_42134_c100732202550000001823141405141560_s1_p0/71429/0_6553

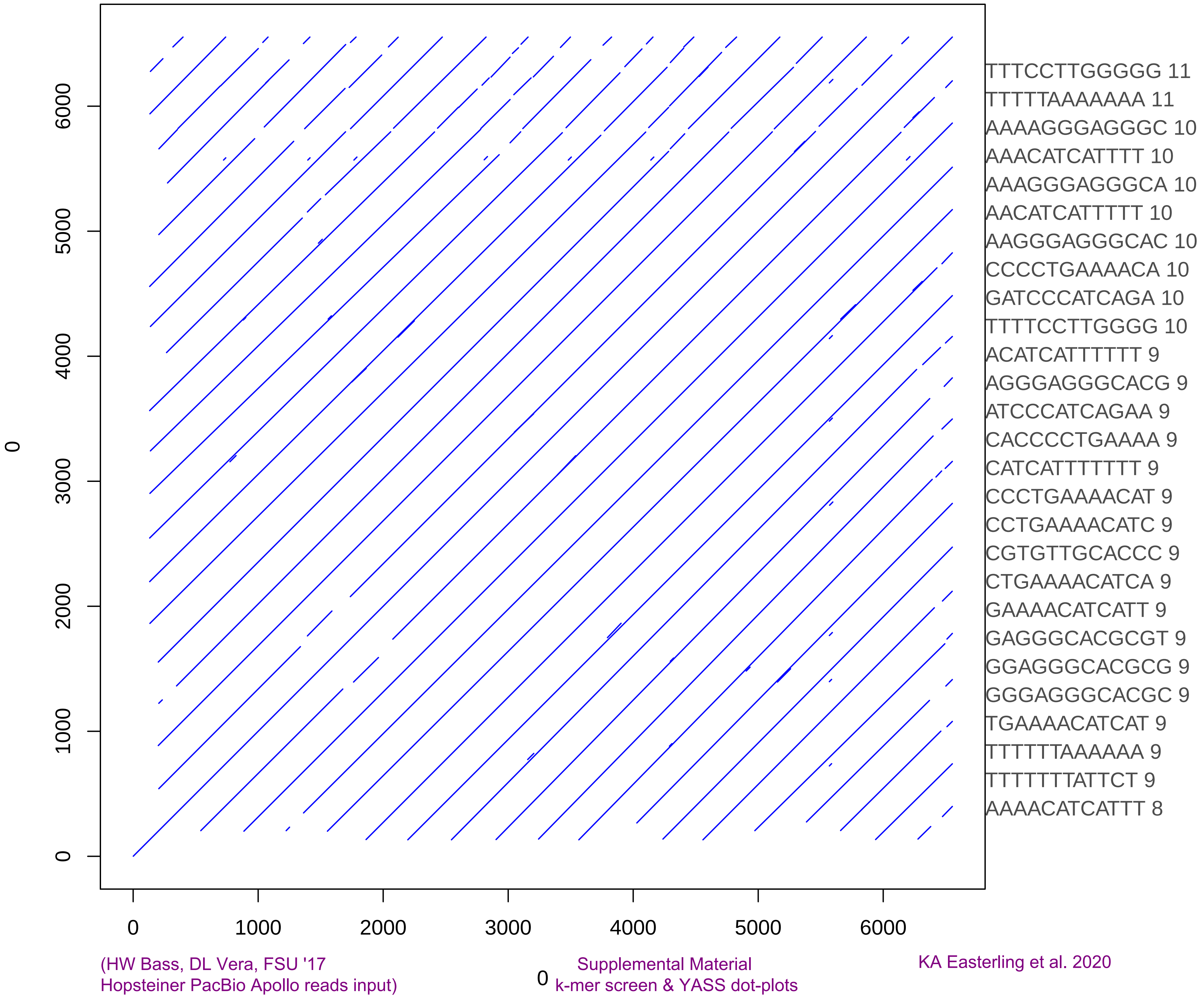

>m141122_014521_42134_c100732202550000001823141405141560_s1_p0/71689/7020_17581

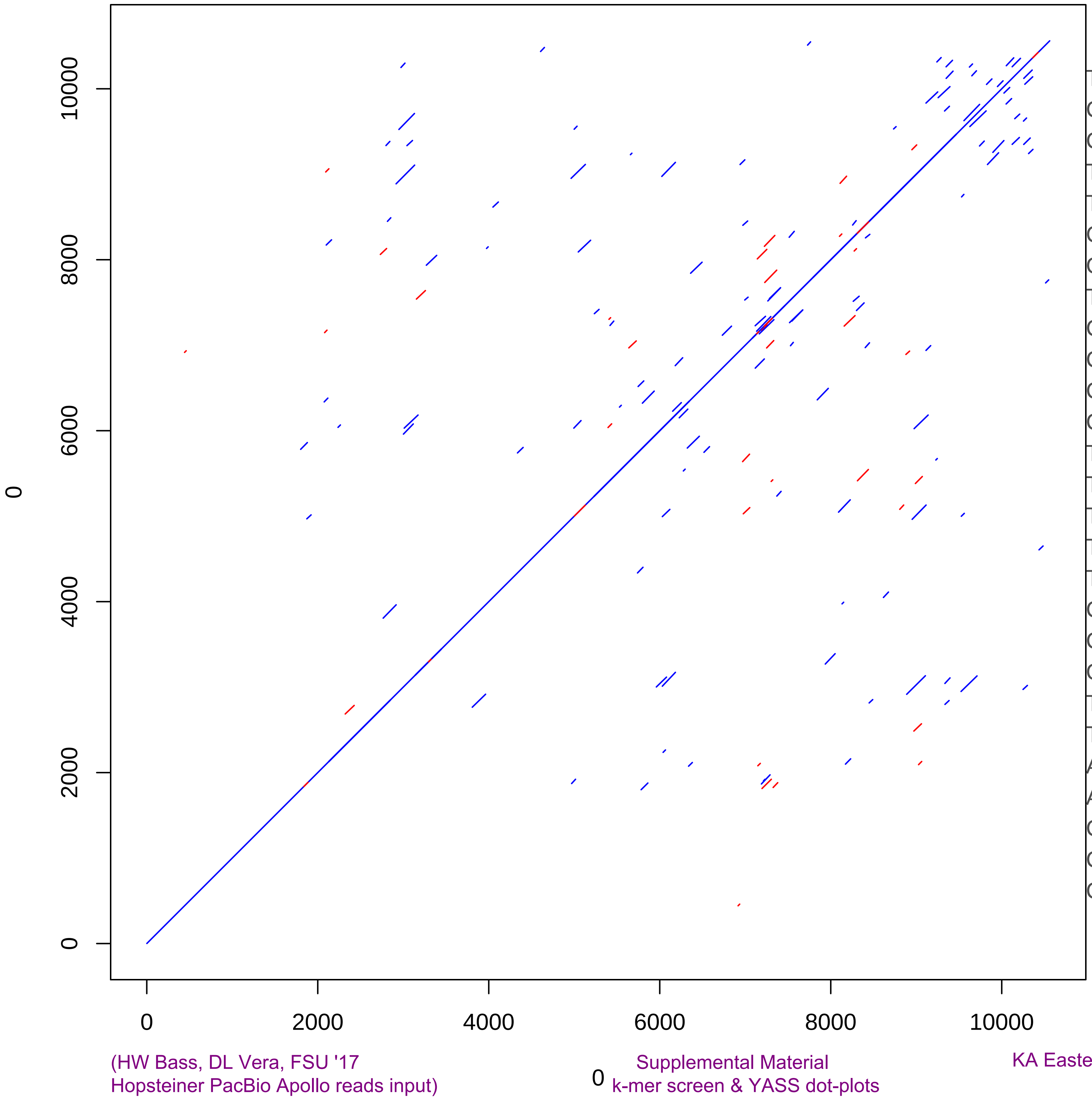

TTTTTTTTTTTT 89
CTTTTTTTTTTT 14
CCTTTTTTTTTT 13
TTTTTTTTTTCC 13
TTTTTTTTTTTC 13
CCCTTTTTTTTT 11
GTTTTTTTTTTT 11
TTTTTTTTTTTG 11
CAAAAAAAAAAA 10
CCCCCCTTTTTT 10
CCCCTTTTTTTT 10
CCCCCCCCCCCC 9
TTCCCCCCCCCC 9
TTTTCCCCCCCC 9
TTTTTTTTCCCC 9
TTTTTTTTTCCC 9
TTTTTTTTTGGG 9
CCAAAAAAAAAA 8
CCCCCCCTTTTT 8
GGTTTTTTTTTT 8
TTTCCCCCCCCC 8
TTTTTTTTTTGG 8
AAAAAAAAAAAC 7
AAAAAAAAAATT 7
CCCAAAAAAAAA 7
CCCCCCCCTTTT 7
CCCCCTTTTTTT 7

>m141122_014521_42134_c100732202550000001823141405141560_s1_p0/72645/0_7409

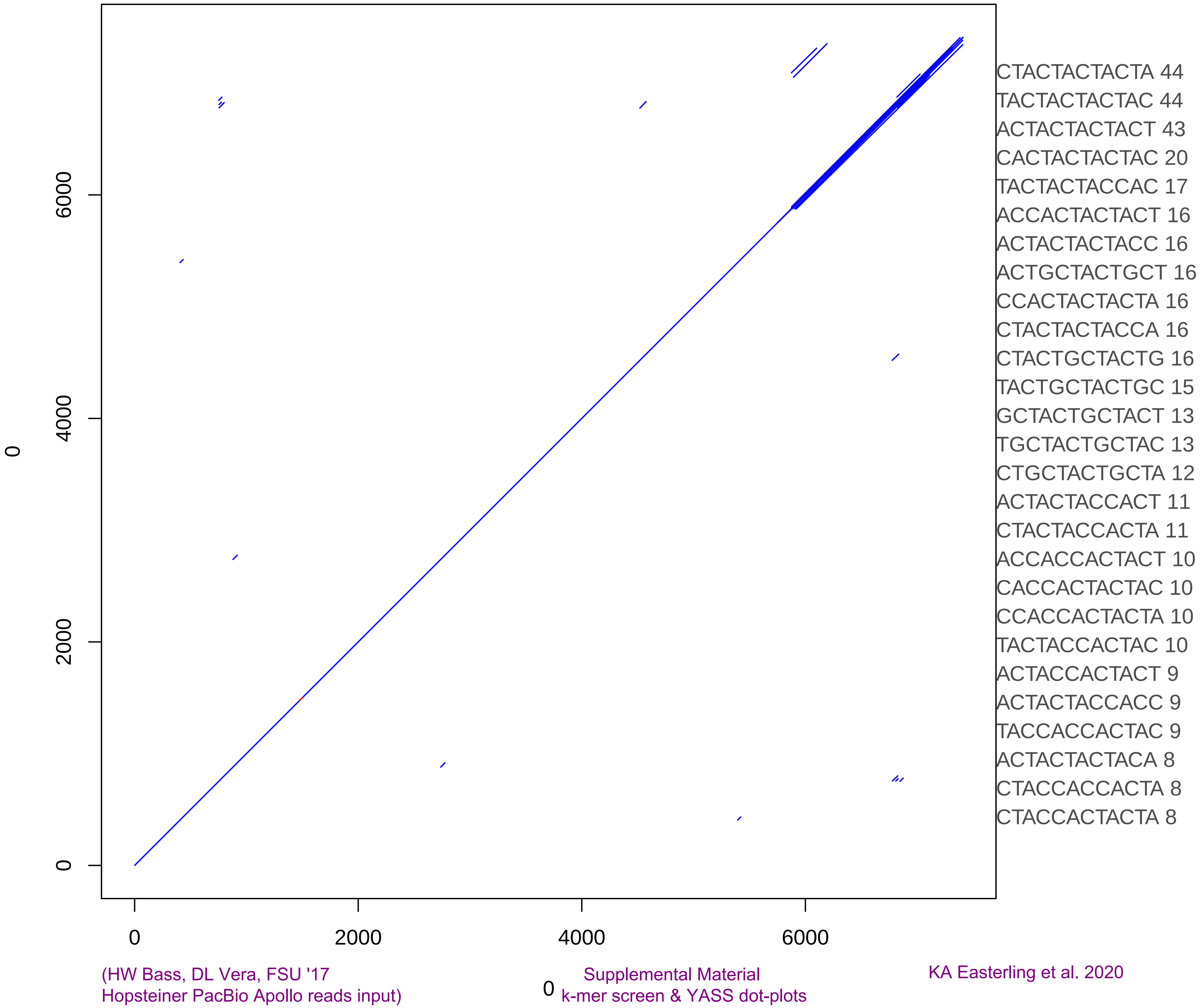

>m141122_014521_42134_c100732202550000001823141405141560_s1_p0/74776/3223_12964

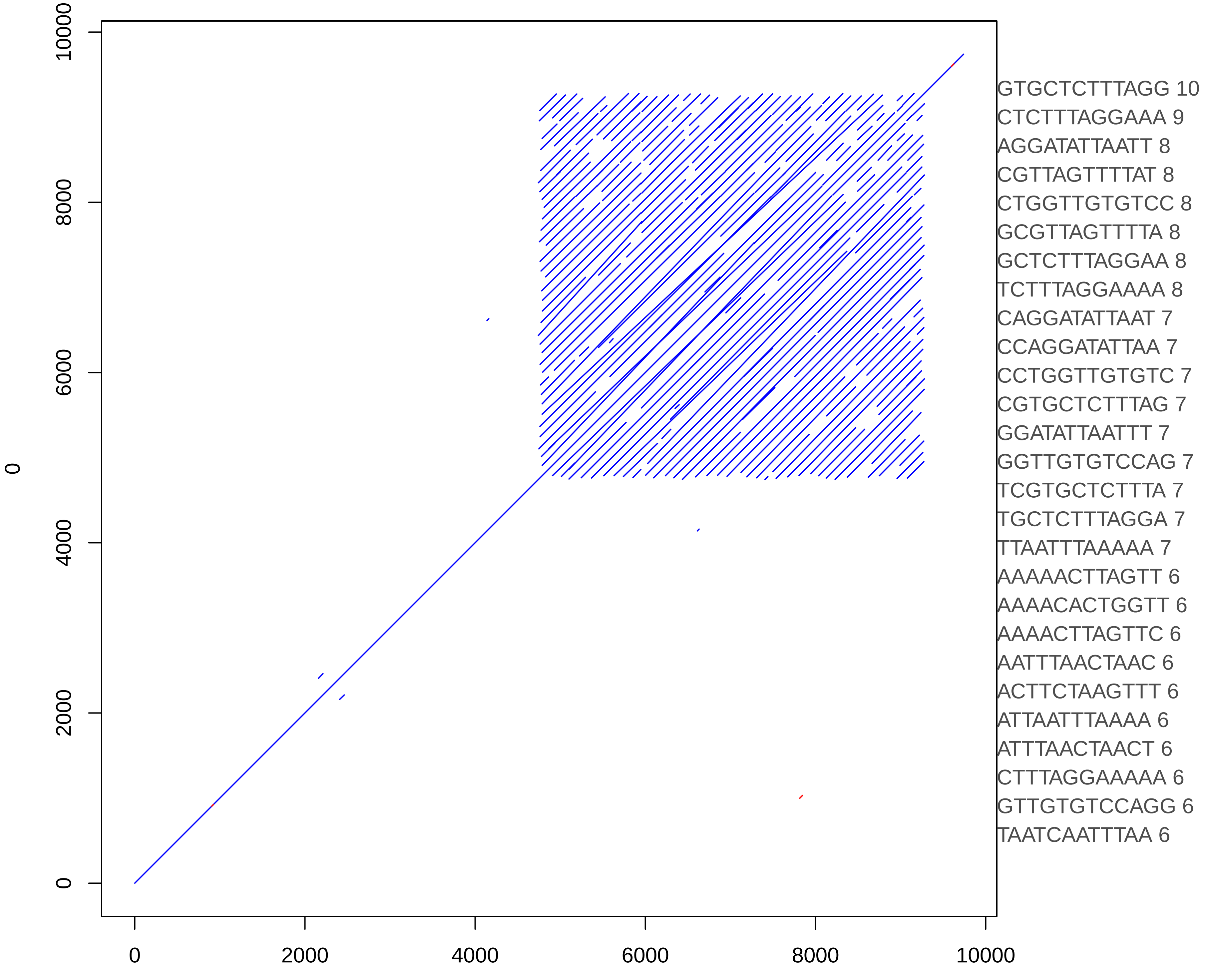

>m141122_014521_42134_c100732202550000001823141405141560_s1_p0/75081/7588_14482

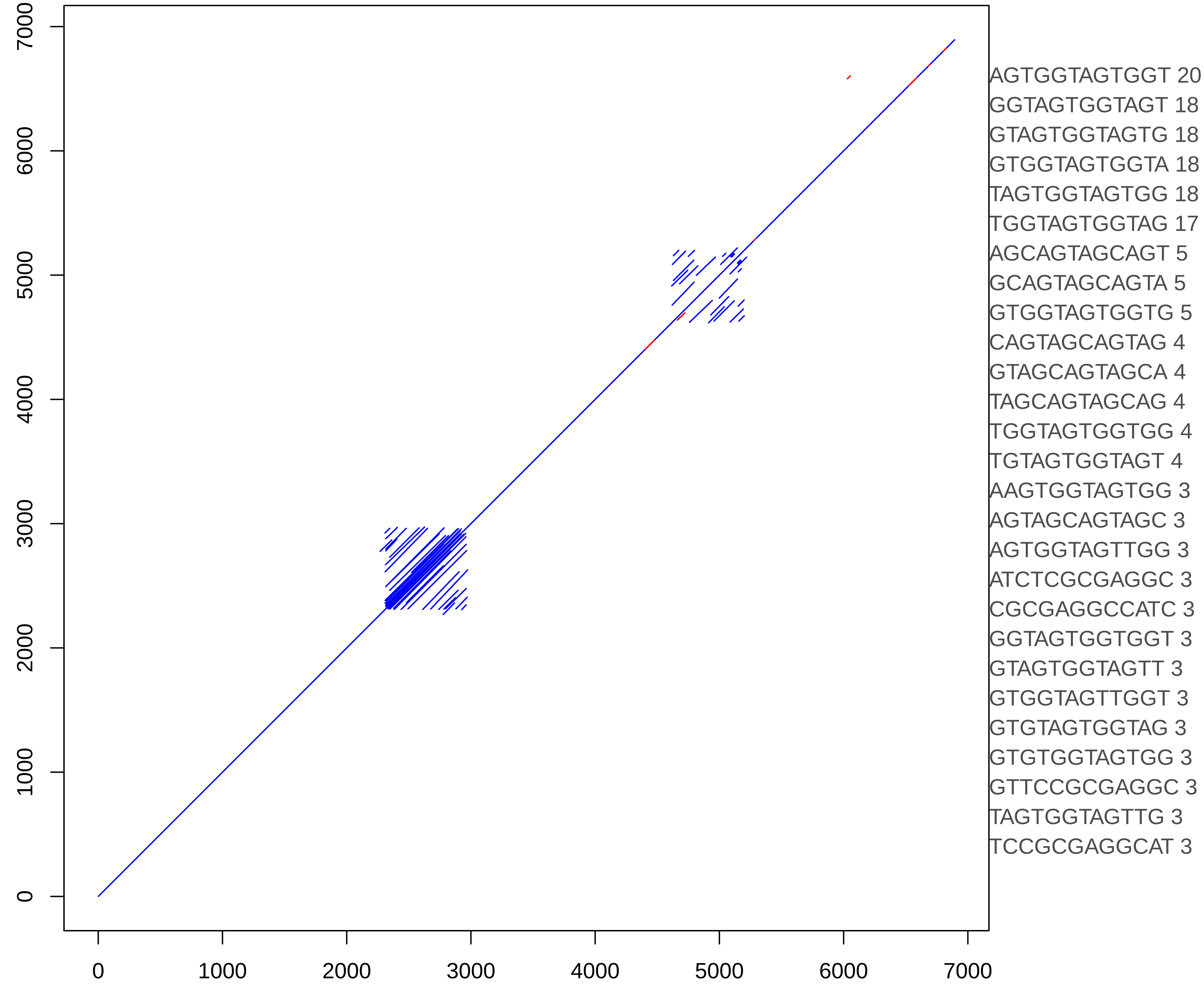

>m141122_014521_42134_c100732202550000001823141405141560_s1_p0/75709/5853_15140

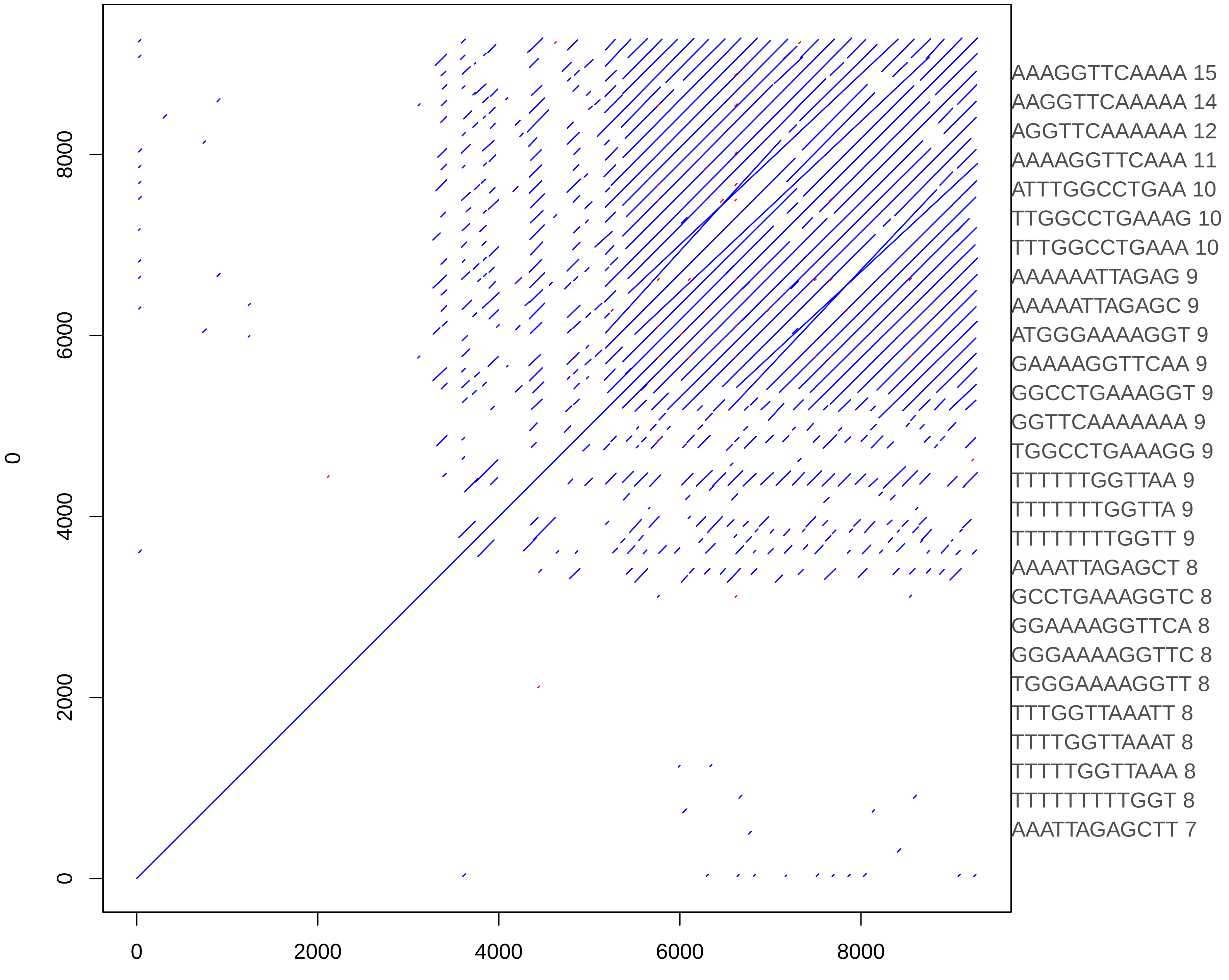

>m141122_014521_42134_c100732202550000001823141405141560_s1_p0/76256/0_10852

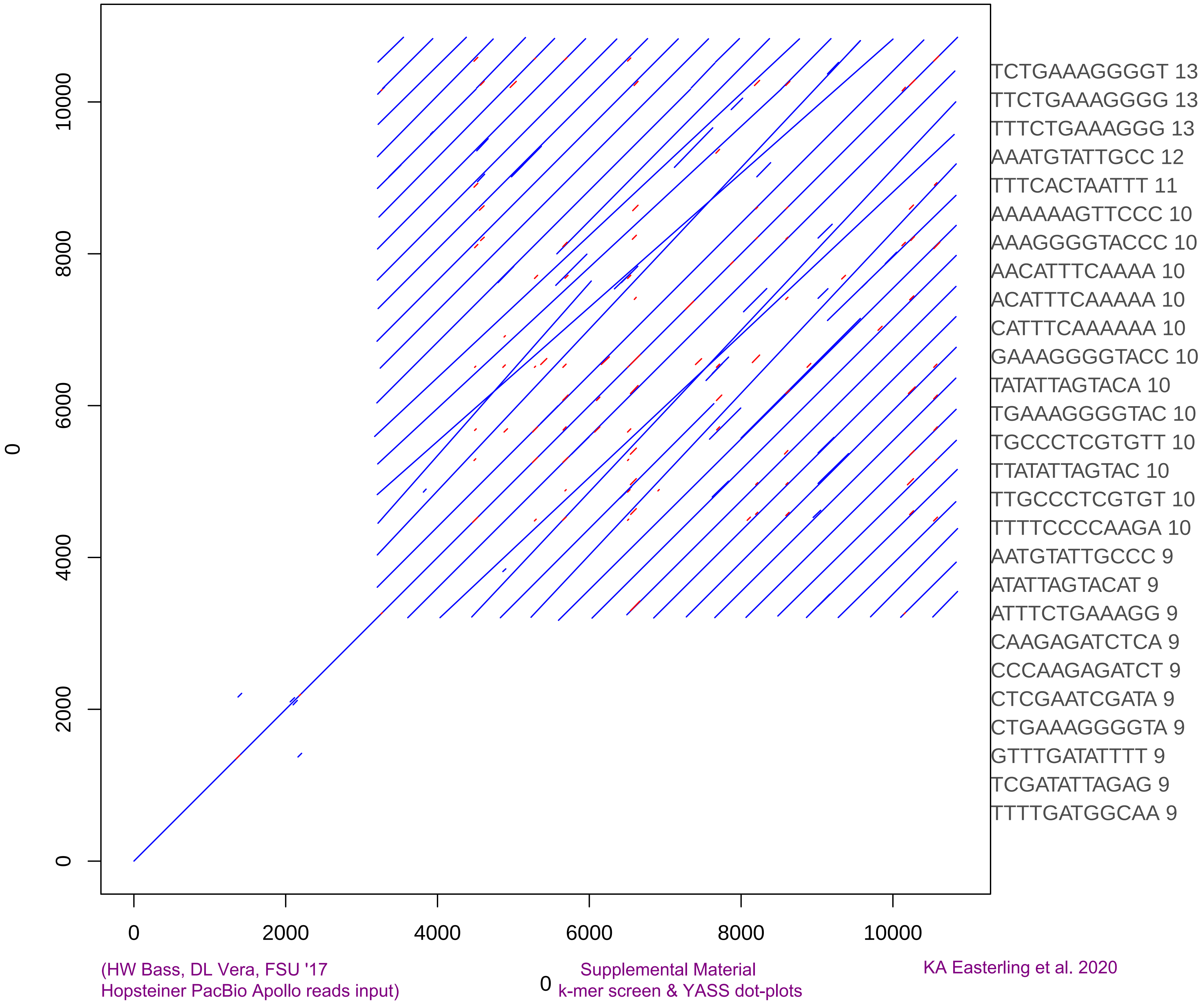

>m141122_014521_42134_c100732202550000001823141405141560_s1_p0/76666/0_6625

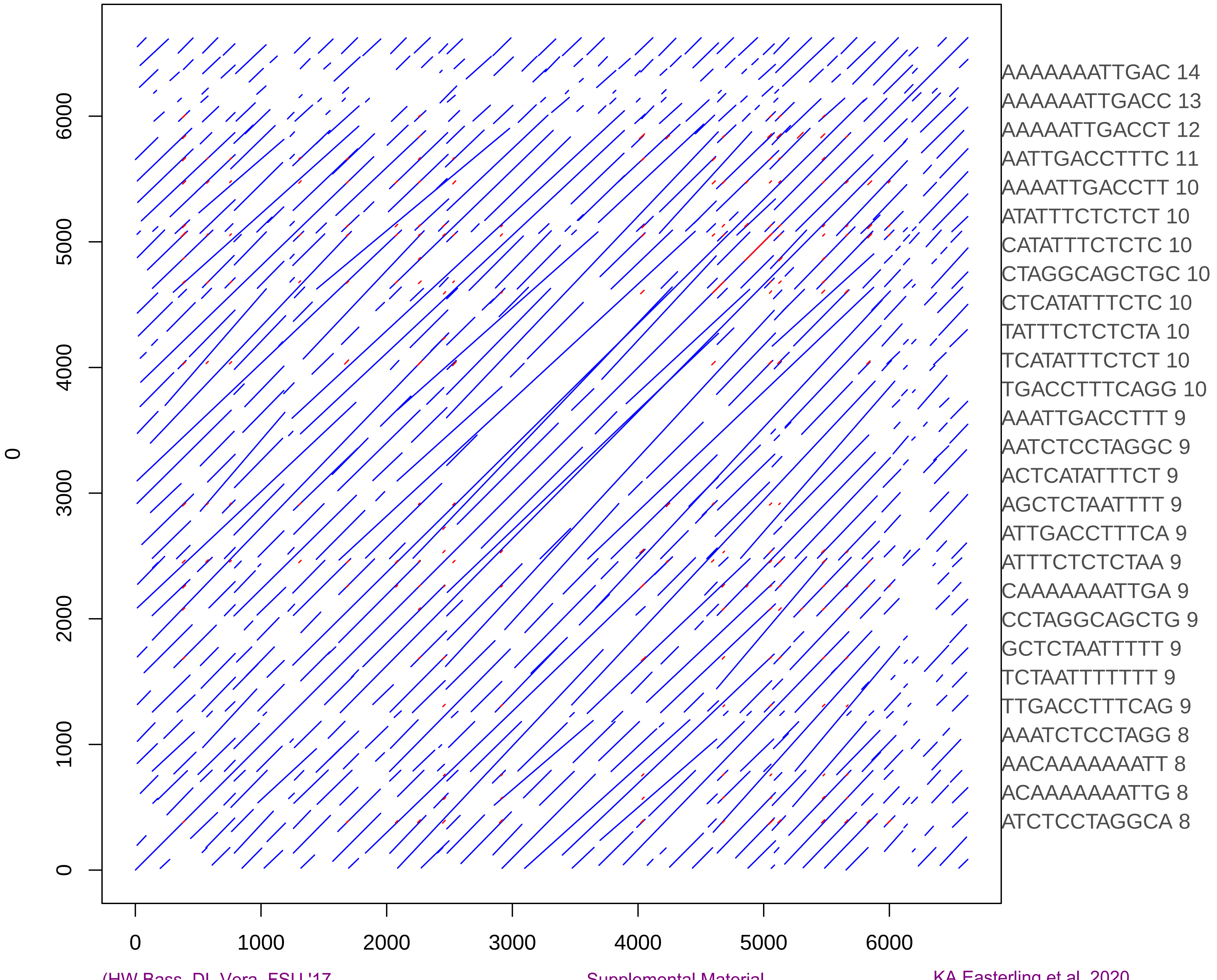

>m141122_014521_42134_c100732202550000001823141405141560_s1_p0/79931/3239_9088

Representative Read (Table 1): (5S rDNA) HuluTR335-r243, 63 ppm, 55% A+T, GenBank Acc. MN537579

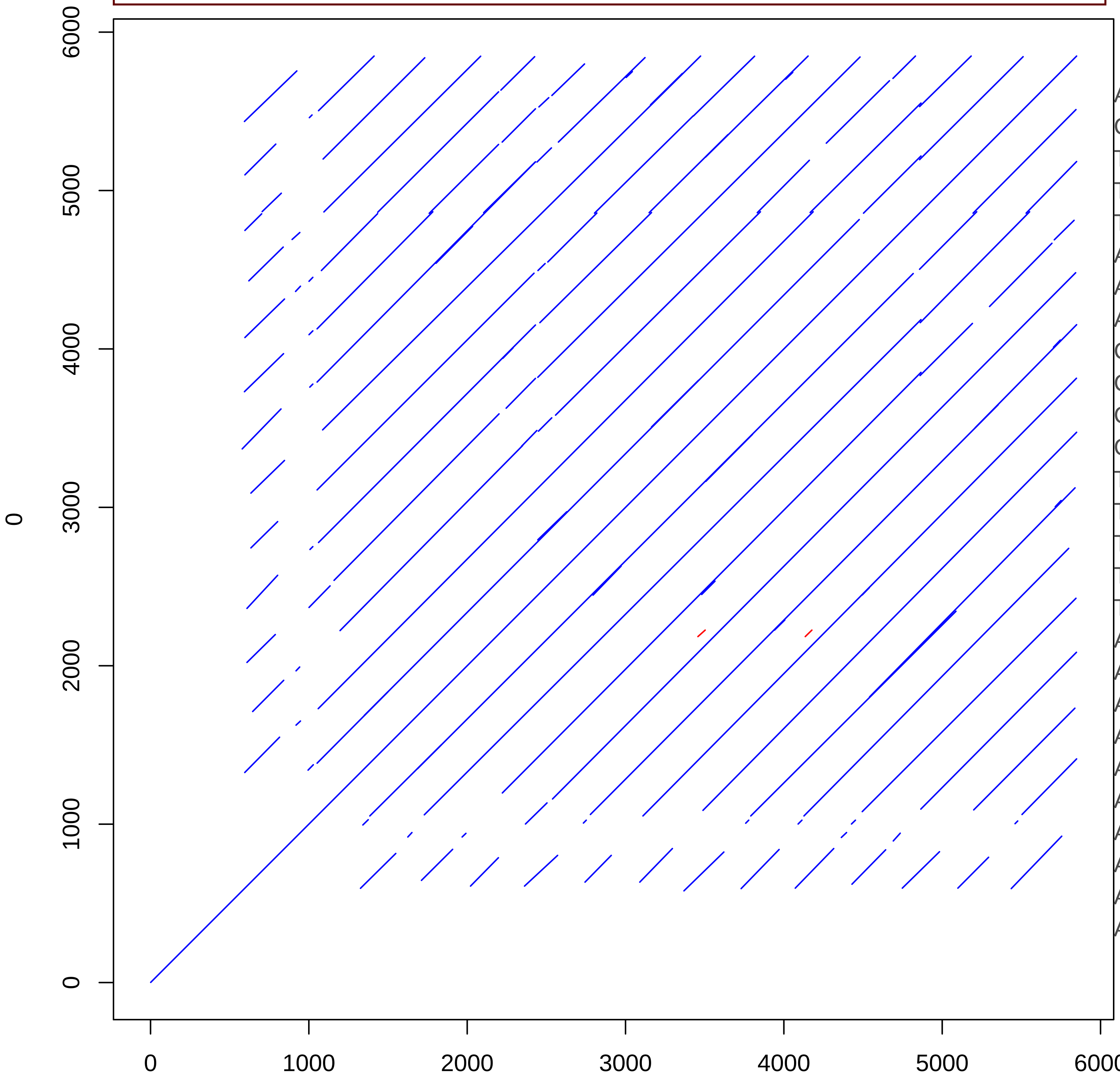

ATCATTTTTTTT 10
CATCATTTTTTT 9
TCATTTTTTTTT 9
TTCGTTTTCCTT 9
TTTTTTTCCCTT 9
AAGCAGCGAATA 8
ACATCATTTTTT 8
AGCAGCGAATAA 8
CGAATAAGAAGT 8
CGCAGTTAAGCG 8
CTAGGATGGGTG 8
GTCAATCCGGGG 8
TAGGATGGGTGA 8
TCGTTTTCCTTG 8
TTAAAAATCCAC 8
TTTCCTTGAGGG 8
TTTTTTTTCCCT 8
AAAAGTTTGTAT 7
AAAATCCACCTA 7
AAAGTTTGTATT 7
AACAAGCAGCGA 7
AAGTTTGTATTT 7
AATCCACCTACG 7
ACAAGCAGCGAA 7
AGCGAATAAGAA 7
AGGATGGGTGAC 7
AGTCAATCCGGG 7

>m141122_014521_42134_c100732202550000001823141405141560_s1_p0/85098/460_5914

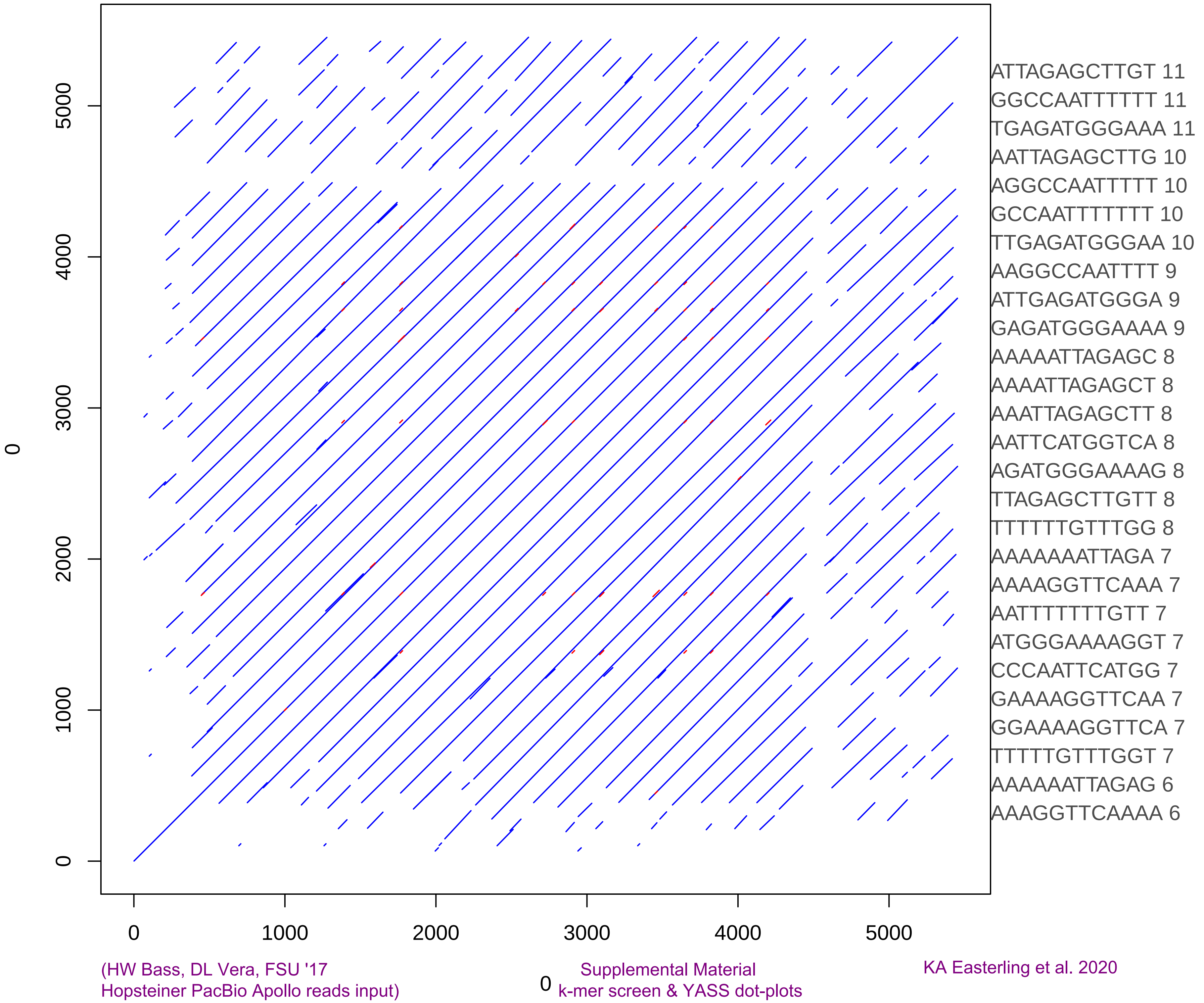

>m141122_014521_42134_c100732202550000001823141405141560_s1_p0/87595/3355_8536

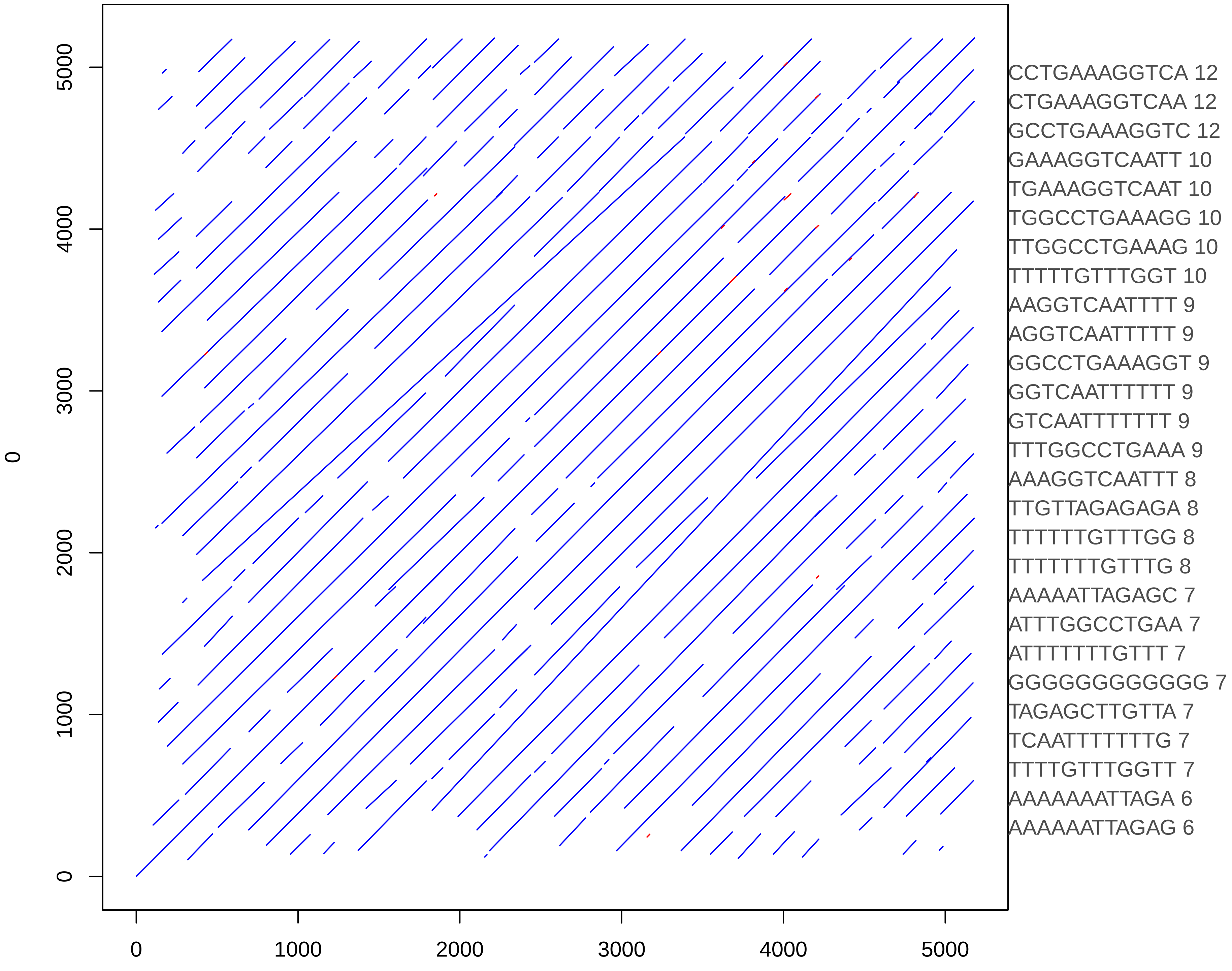

>m141122_014521_42134_c100732202550000001823141405141560_s1_p0/98769/6368_14448

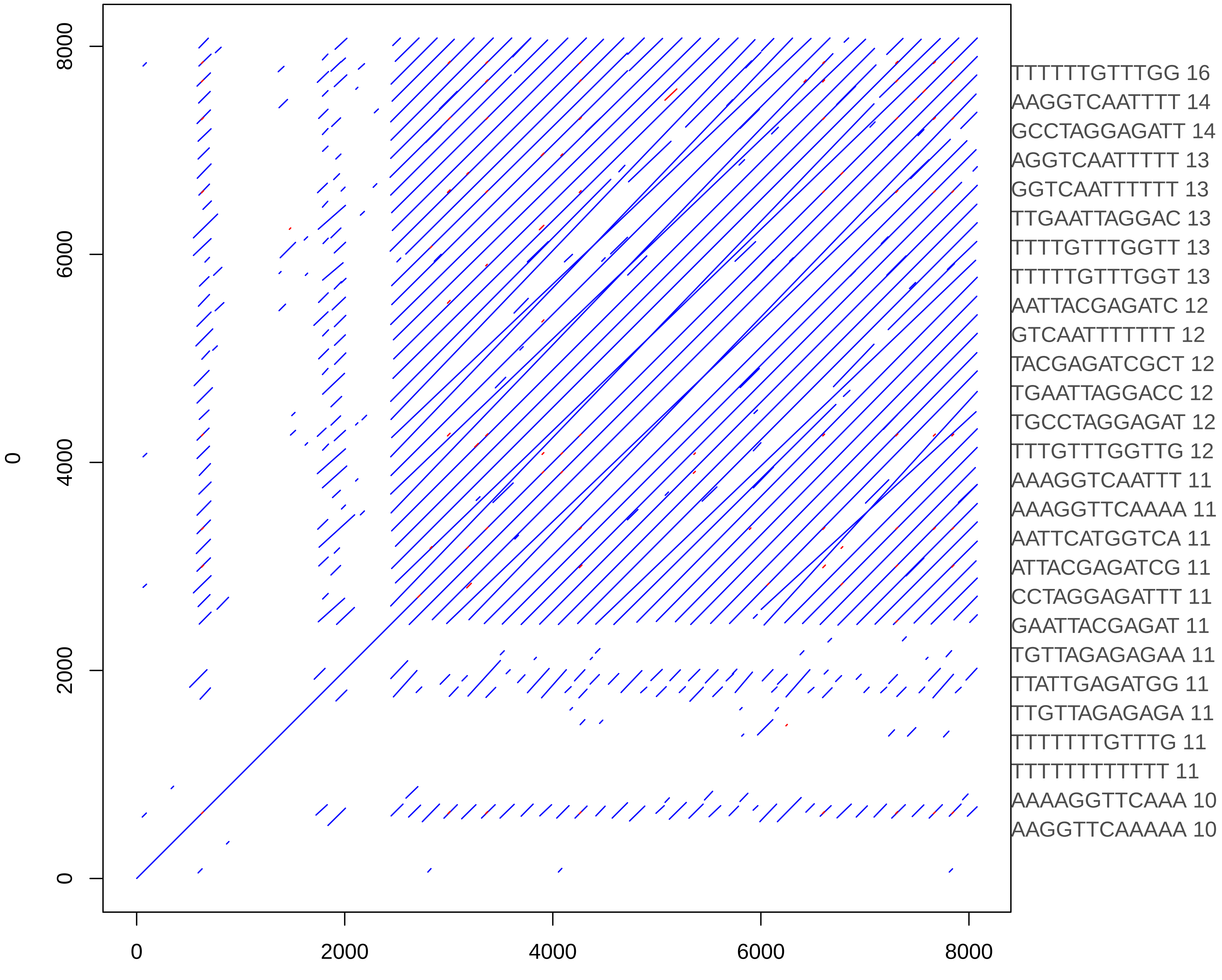

>m141122_040740_42134_c100732202550000001823141405141561_s1_p0/101800/5965_15235

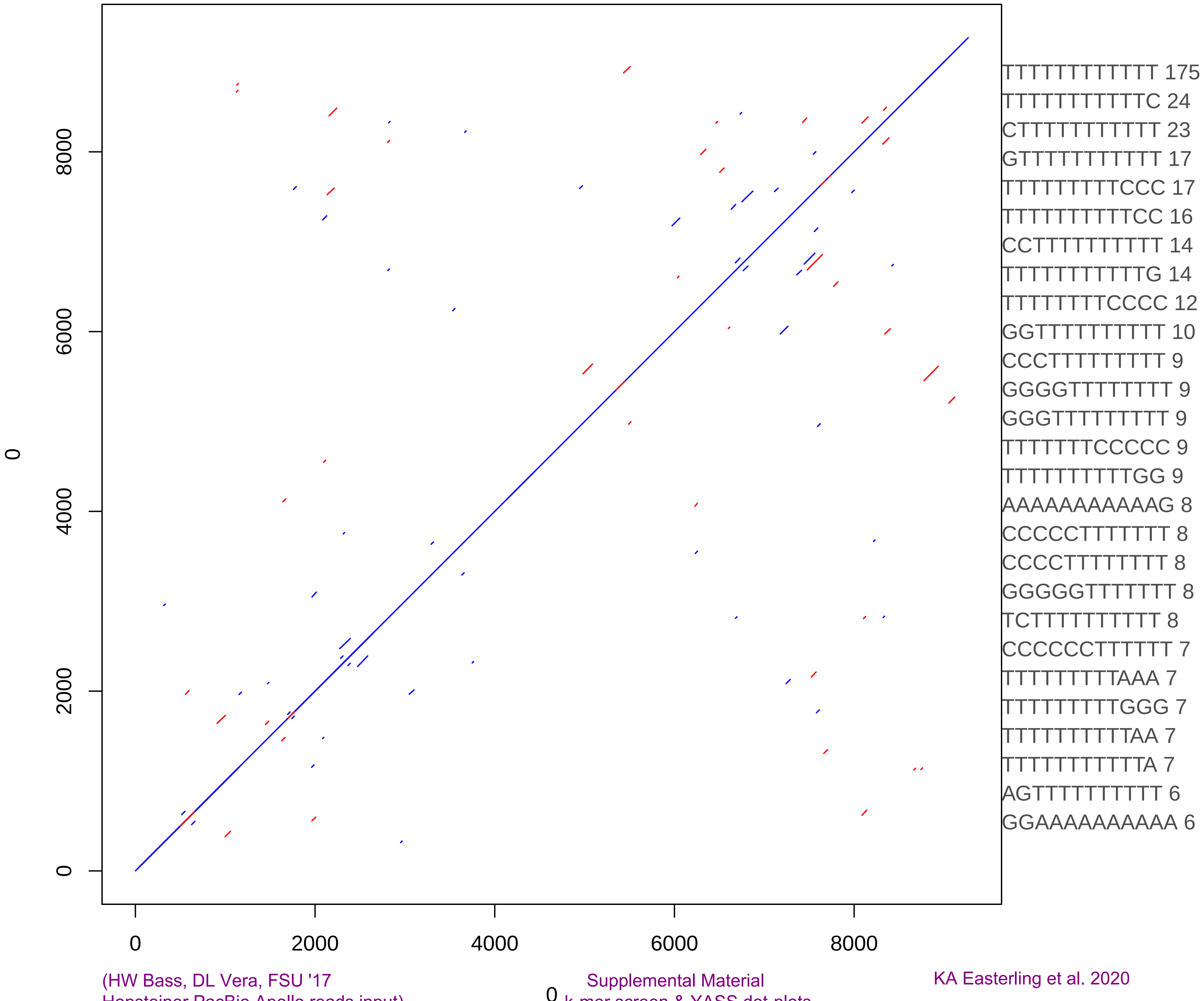

>m141122_040740_42134_c100732202550000001823141405141561_s1_p0/103079/7060_17567

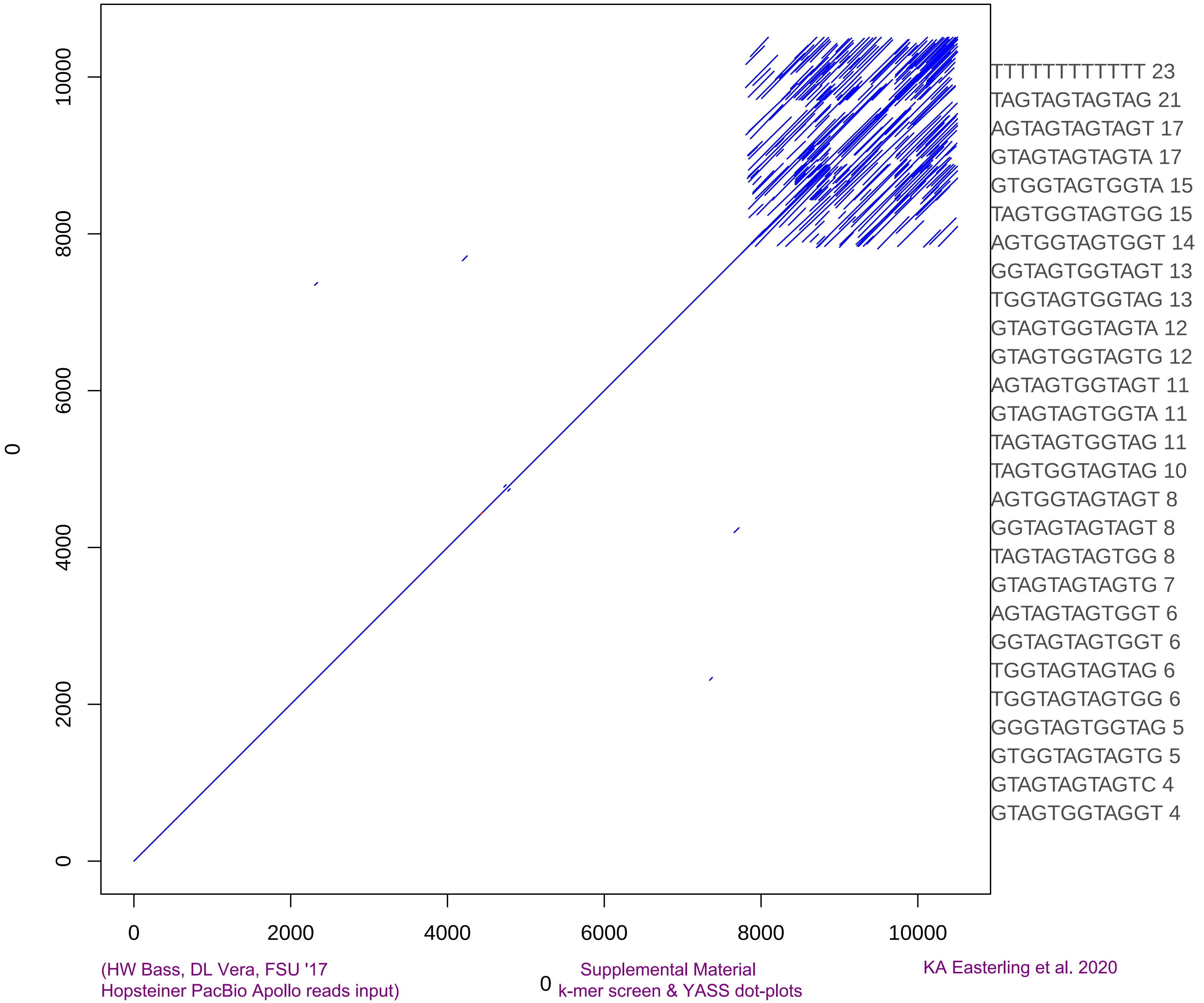

>m141122_040740_42134_c100732202550000001823141405141561_s1_p0/10448/0_9656

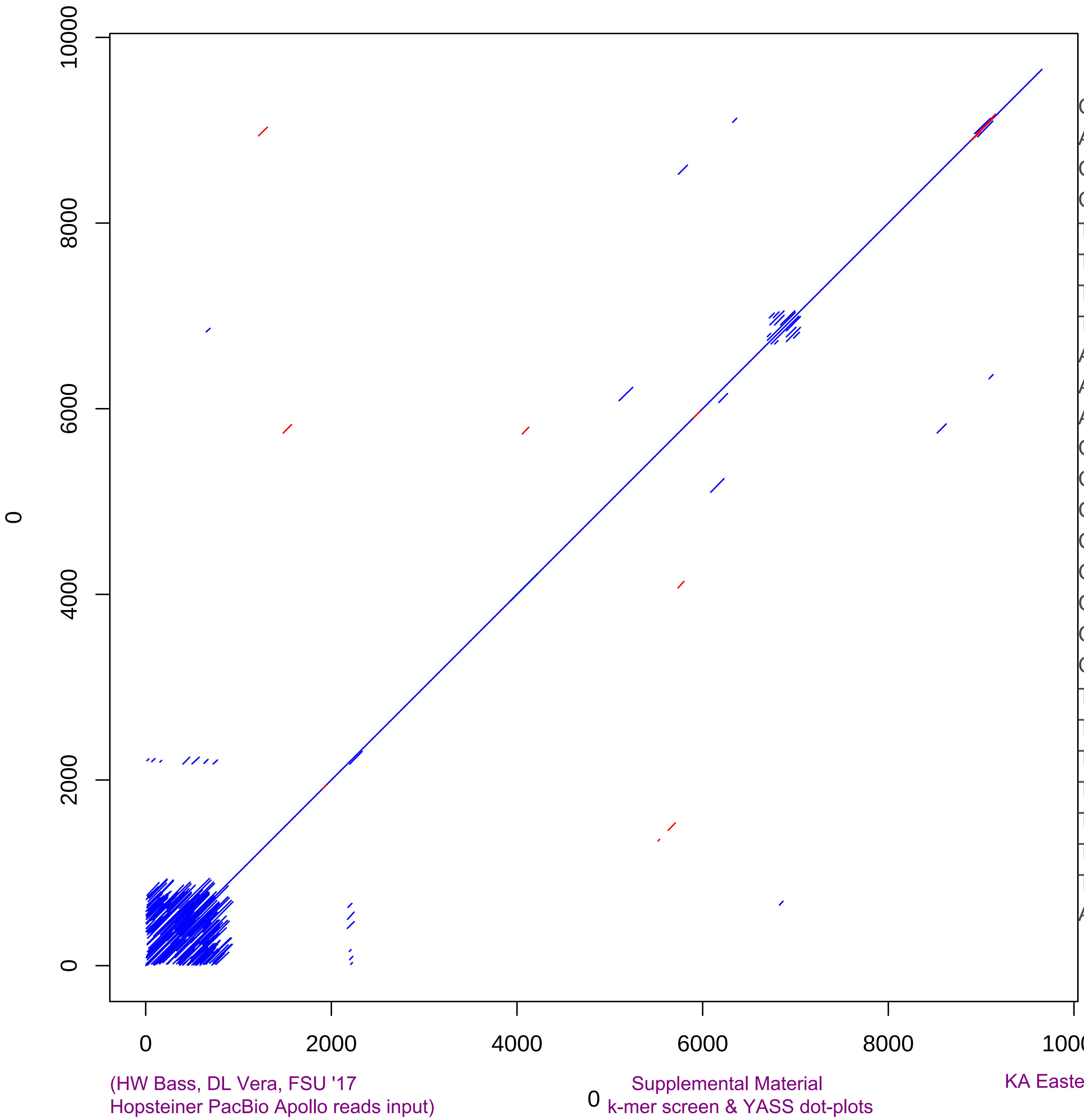

GGTTTAGGGTTT 14
AGGGTTTAGGGT 8
GGGTTTAGGGTT 8
GTTTAGGGTTTA 8
TTAGGGTTTAGG 8
TTTAGGGTTTAG 8
TAGGGTTTAGGG 7
TTTTTTTTTTTT 4
AGGGTTTCAGGG 3
AGGTTTAGGGTT 3
ATTAGGGTTTAG 3
GGGTTTCAGGGT 3
GGGTTTTAGGGT 3
GGTTTCAGGGTT 3
GGTTTTAGGGTT 3
GTGGTTTAGGGT 3
GTTTAGGGTTTC 3
GTTTAGGGTTTT 3
GTTTCAGGGTTT 3
TAGGTTTAGGGT 3
TATTAGGGTTTA 3
TGGTTTAGGGTT 3
TTAGGTTTAGGG 3
TTTAGGGTTTCT 3
TTTAGGGTTTTC 3
TTTAGGTTTAGG 3
AAGTAGGTTCTG 2

>m141122_040740_42134_c100732202550000001823141405141561_s1_p0/105101/9179_16425

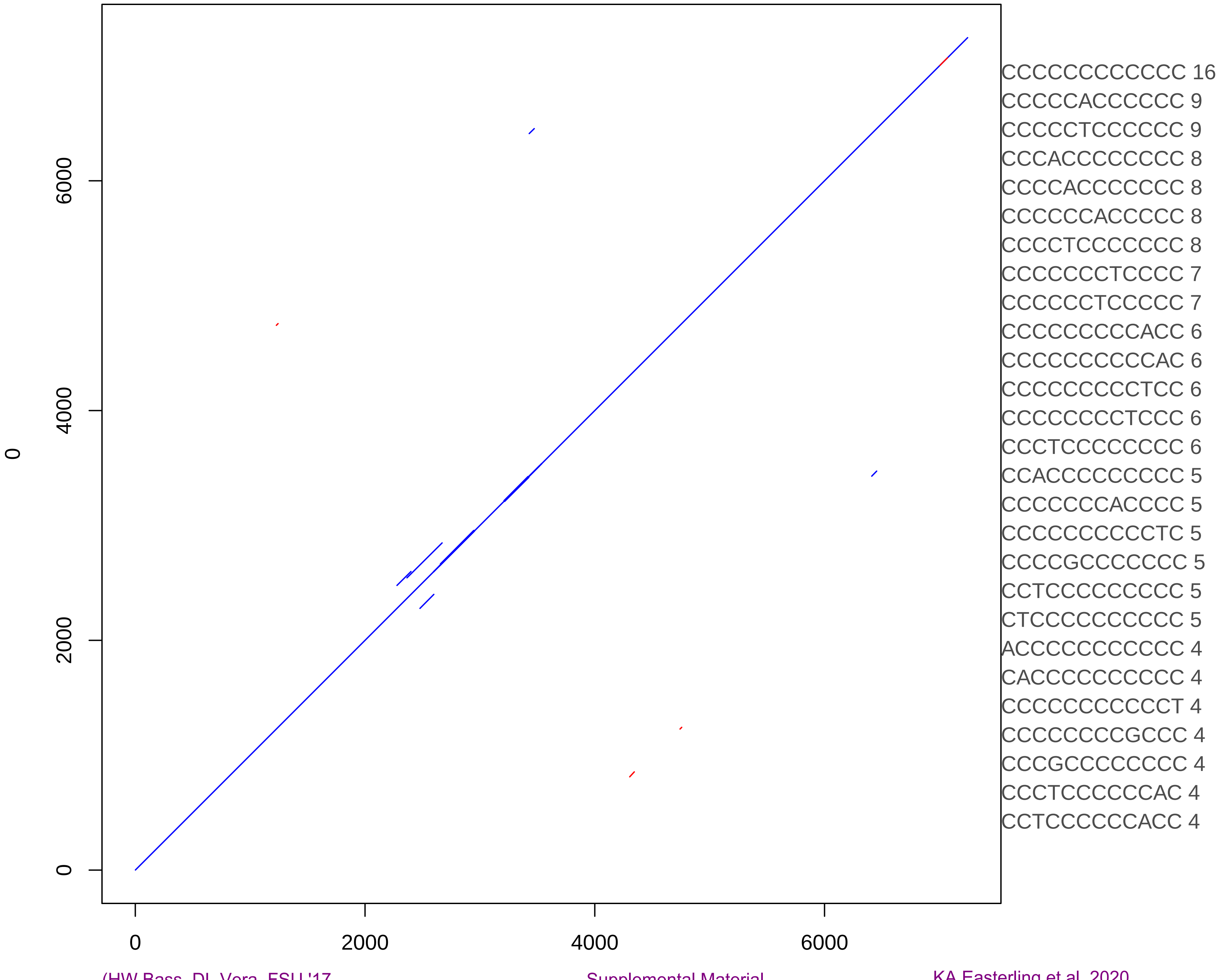

>m141122_040740_42134_c100732202550000001823141405141561_s1_p0/107971/251_10129

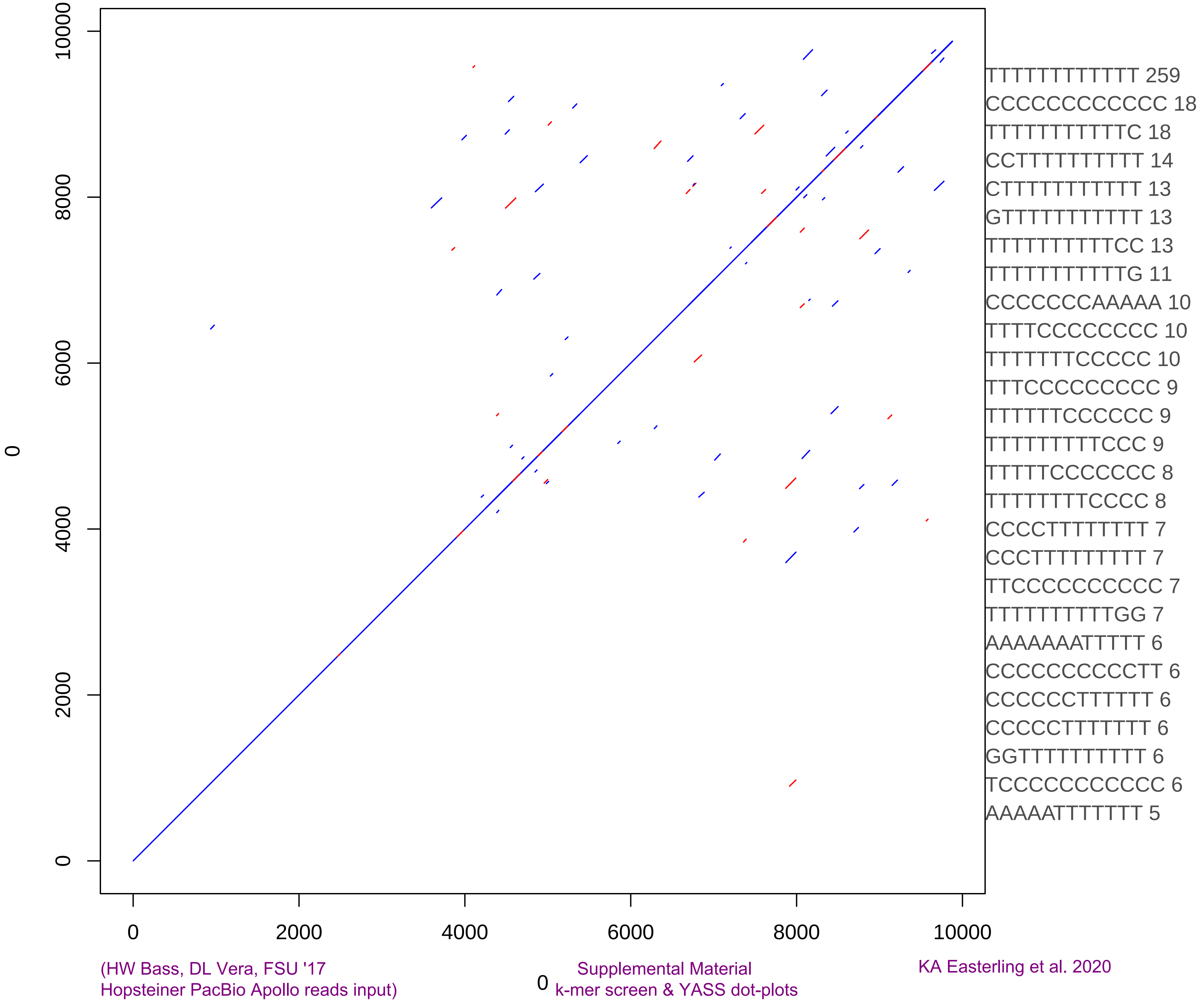

>m141122_040740_42134_c100732202550000001823141405141561_s1_p0/121058/12804_18002

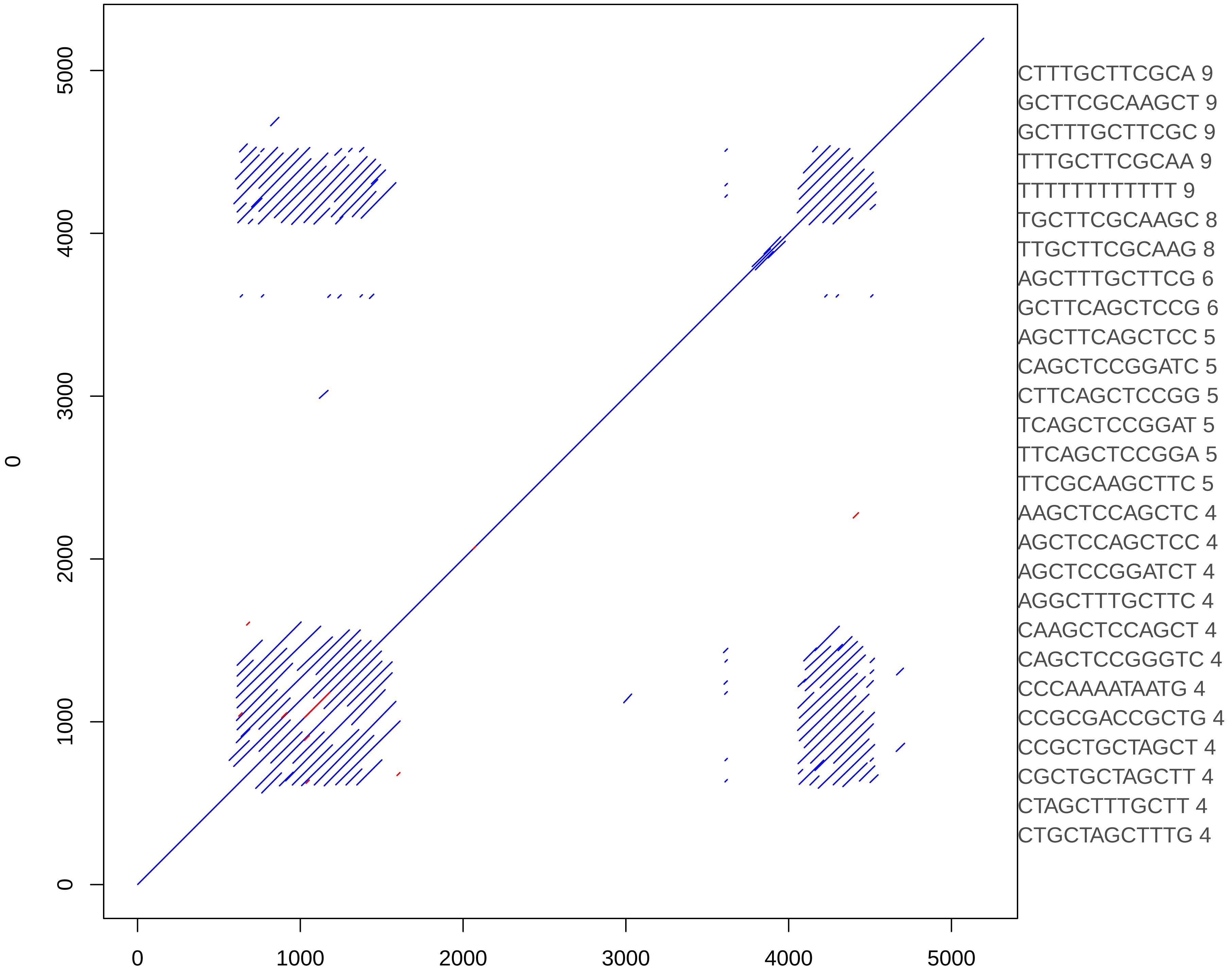

>m141122_040740_42134_c100732202550000001823141405141561_s1_p0/121058/6761_12760

Representative Read (Table 1): HuluTR135-r253, 5 ppm, 42% A+T,
GenBank Acc. MN537571

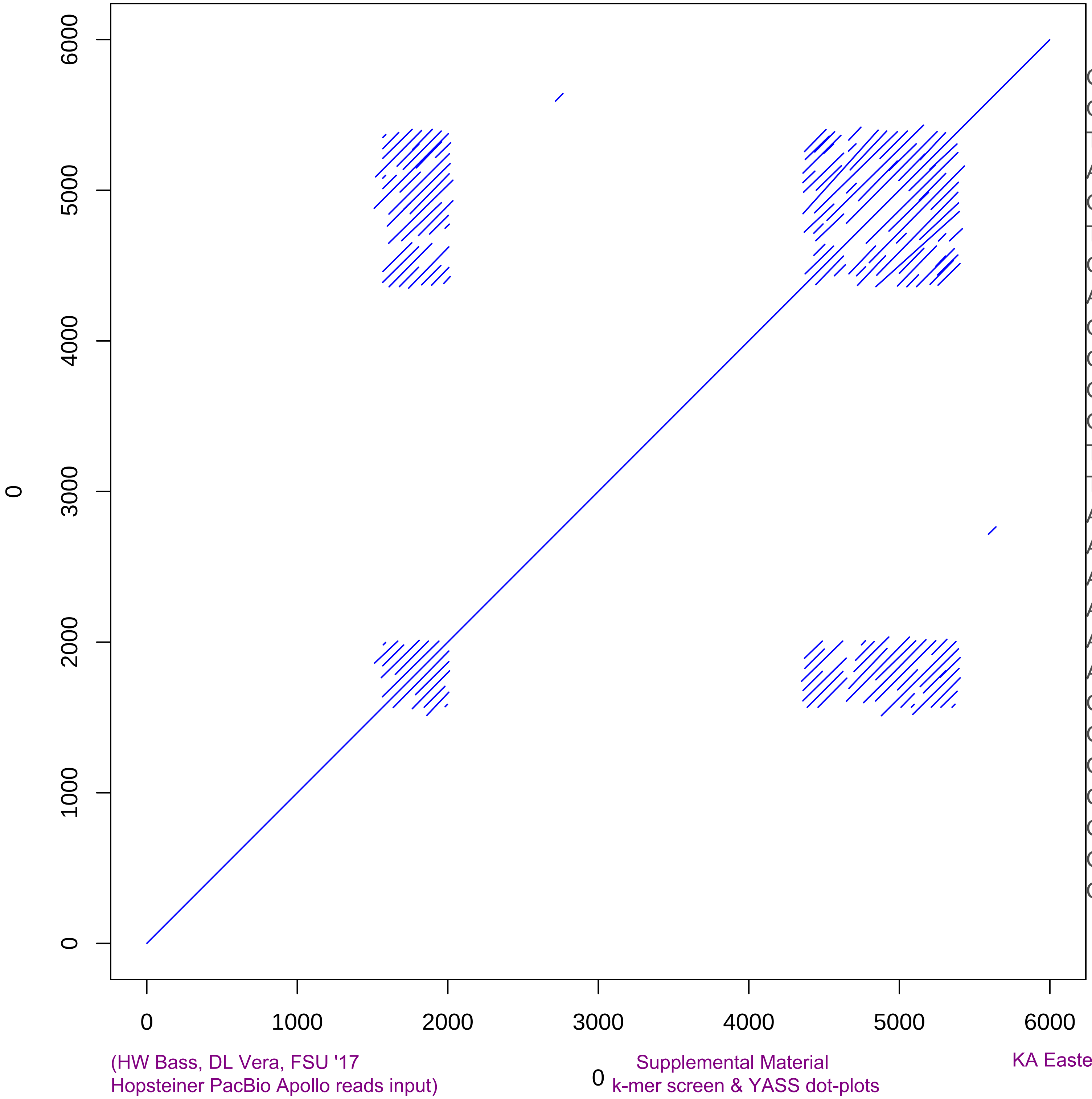

CTTGCGAAGCAA 12
GCTTGCGAAGCA 11
TTGCGAAGCAAA 10
AGCTTGCGAAGC 9
GCGAAGCAAAGC 8
TGCGAAGCAAAG 8
CGAAGCAAAGCT 7
AAGCTTGCGAAG 5
GAAGCTTGCGAA 5
CGCAGGTCATGC 4
GAGCTTGCGAAG 4
GGAGCTTGCGAA 4
TGAAGCTTGCGA 4
TGGAGCTTGCGA 4
AAGCAAAGCTCC 3
AAGCTAGCAGCG 3
AGCAAAGCTAGC 3
AGCAAAGCTCCA 3
AGCGGTCGCGGA 3
AGGTCATGCATT 3
CAAAGCTAGCAG 3
CAAAGCTCCAGC 3
CAGCGGTCGCGG 3
CAGGTCATGCAT 3
CCGGAGCTGAAG 3
CGGAGCTGAAGC 3
CGTCATGCAGGA 3

>m141122_040740_42134_c100732202550000001823141405141561_s1_p0/124123/19302_24745

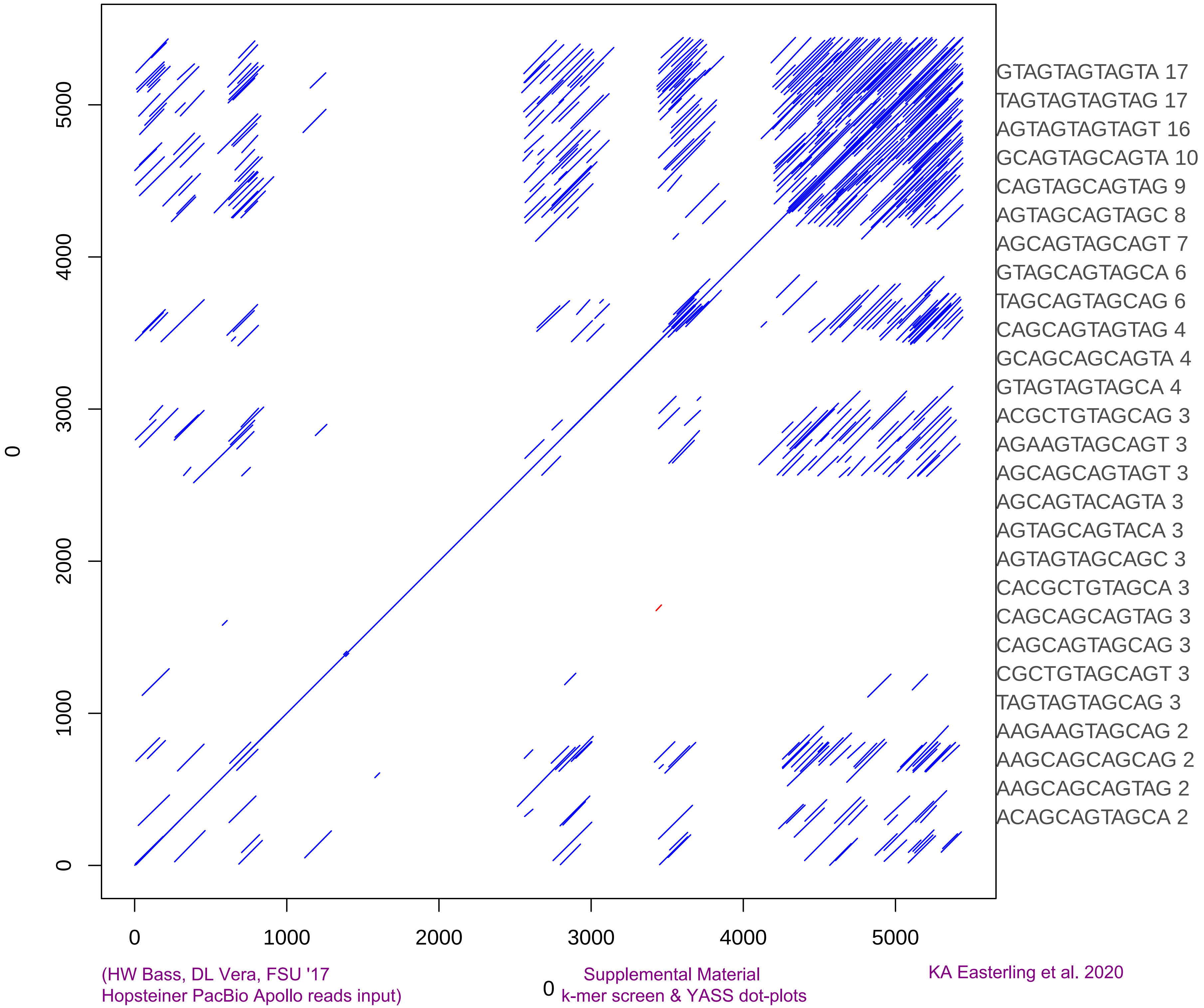

>m141122_040740_42134_c100732202550000001823141405141561_s1_p0/135602/5719_11934

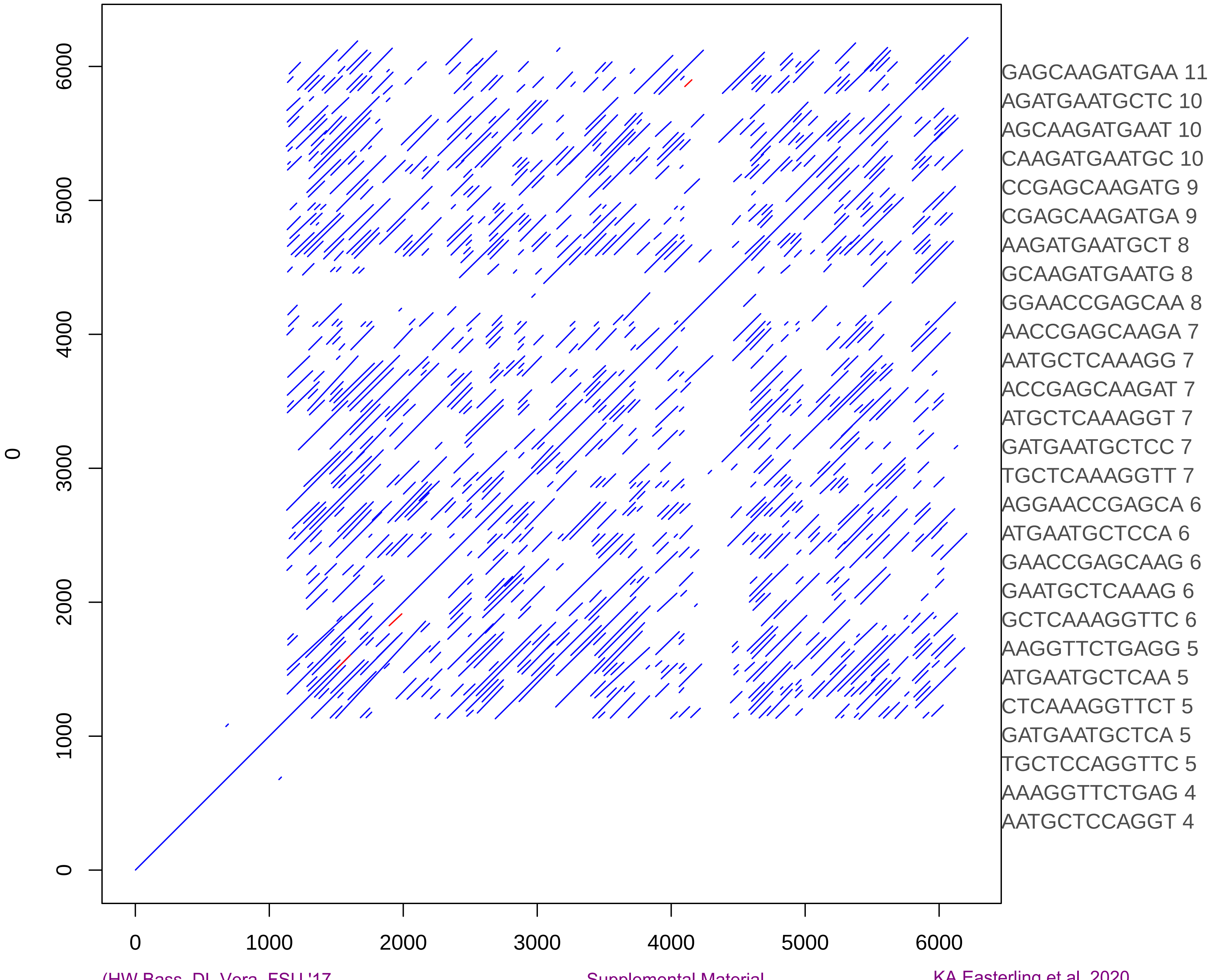

1,121 hits for repetitive sequences
passing 12mer abundance criterion

For Tandem Repeat families of ### bp:
PacBio Read named "HuluTR###-r256"

>m141122_040740_42134_c100732202550000001823141405141561_s1_p0/139816/8213_13650

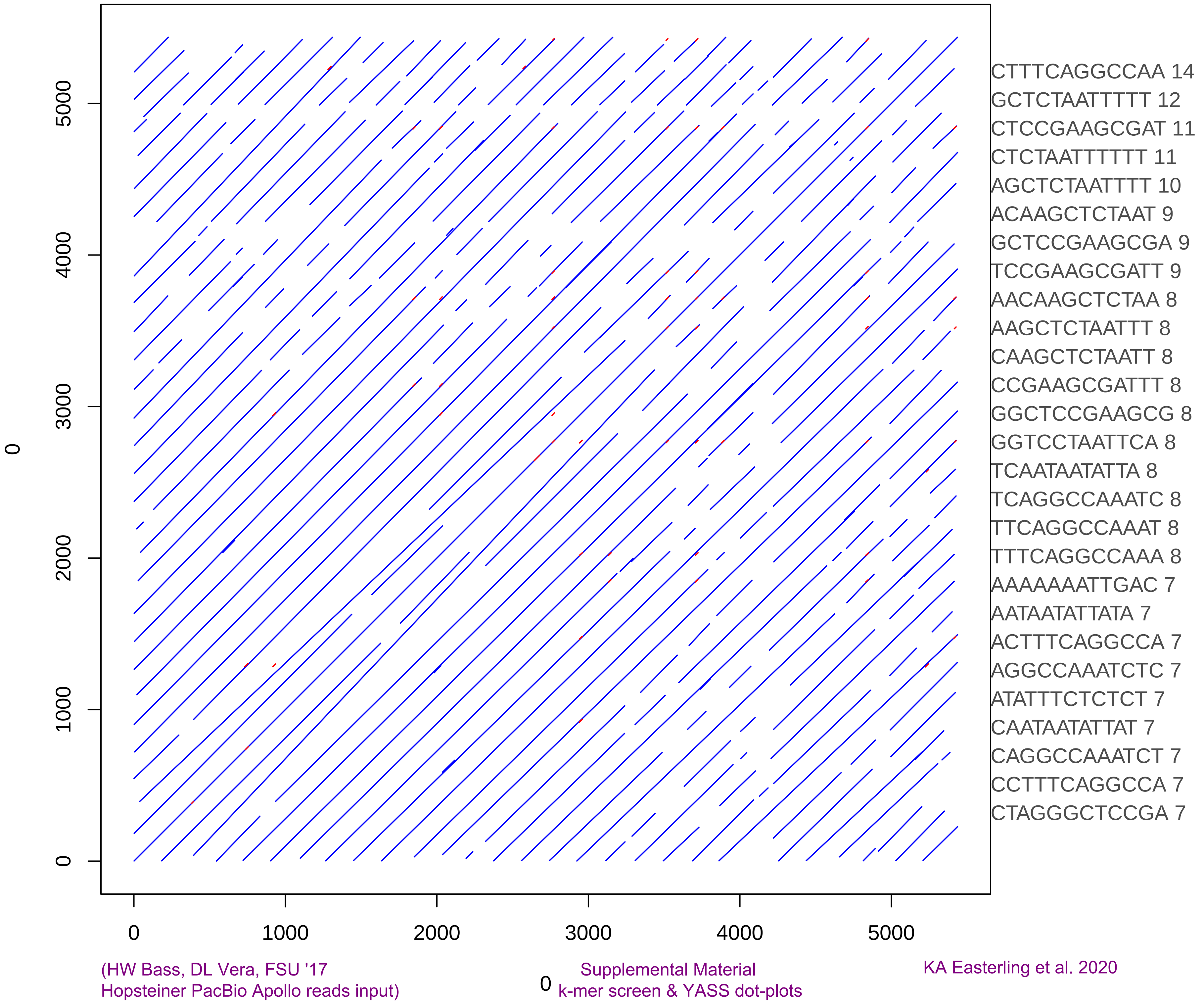

(HW Bass, DL Vera, FSU '17
Hopsteiner PacBio Apollo reads input)

Supplemental Material
k-mer screen & YASS dot-plots

KA Easterling et al. 2020

>m141122_040740_42134_c100732202550000001823141405141561_s1_p0/140135/4549_10122

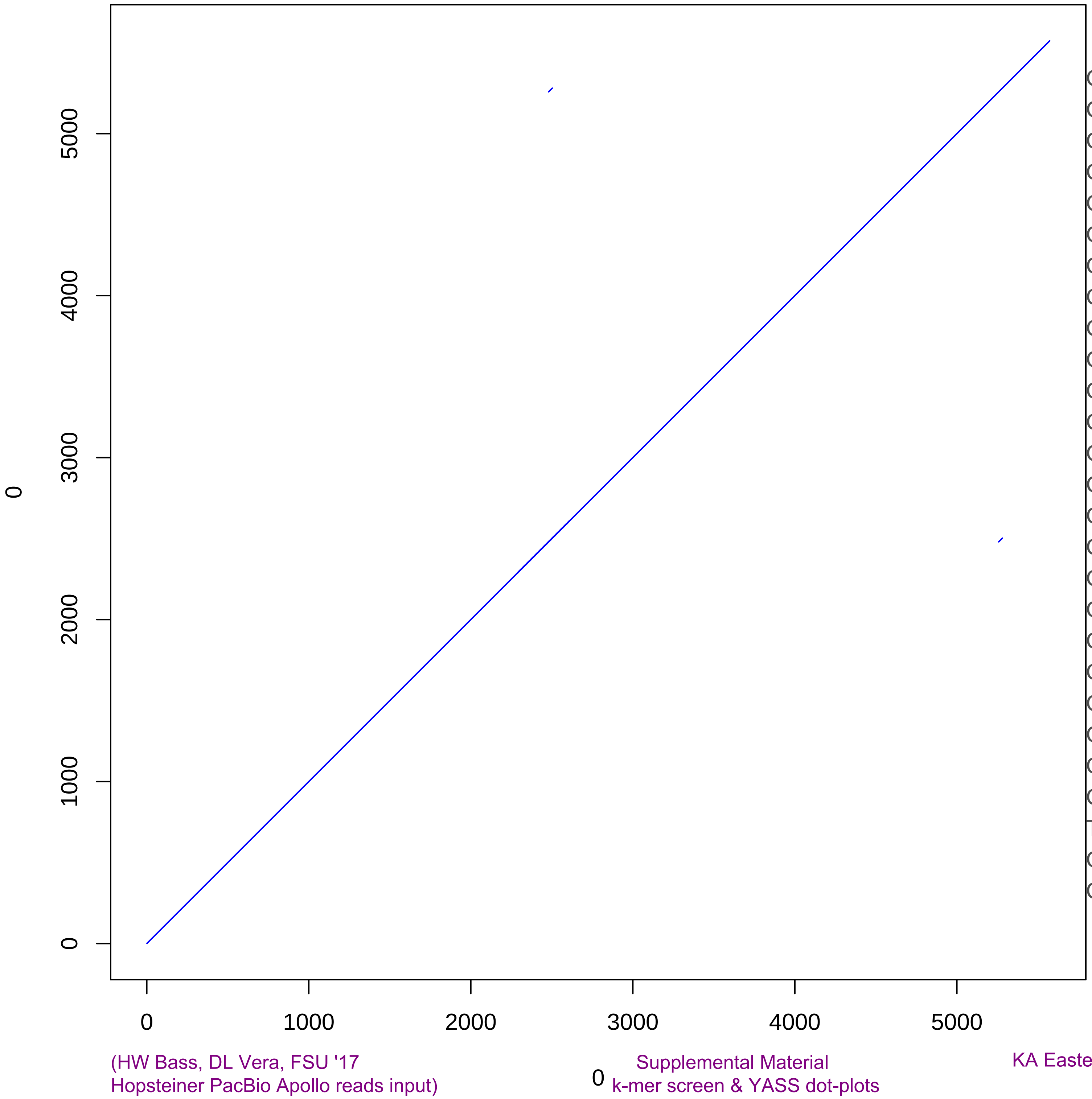

GGGGGGGGGGGG 156
CGGGGGGGGGGG 9
GGGGGGGGGGCG 9
GGGGGGGGGGGC 9
GGGGCGGGGGGG 8
GGGGGCGGGGGG 8
GCGGGGGGGGGG 7
GGGGGGCGGGGG 7
GGGGGGGCGGGG 7
GGGGGGGGGCGG 7
GGCGGGGGGGGG 6
GGGCGGGGGGGG 6
GGGGGGGGCGGG 6
CCGGGGGGGGGG 2
GCCGGGGGGGGG 2
GGCCGGGGGGGG 2
GGGCCGGGGGGG 2
GGGGCCGGGGGG 2
GGGGGCCGGGGG 2
GGGGGCGGGGAG 2
GGGGGGCCGGGG 2
GGGGGGGCCGGG 2
GGGGGGGGCCGG 2
GGGGGGGGGCGA 2
TTCCGTGACAAA 2
GGGGGGGGGGGG 156
CGGGGGGGGGGG 9

>m141122_040740_42134_c100732202550000001823141405141561_s1_p0/142963/579_6869

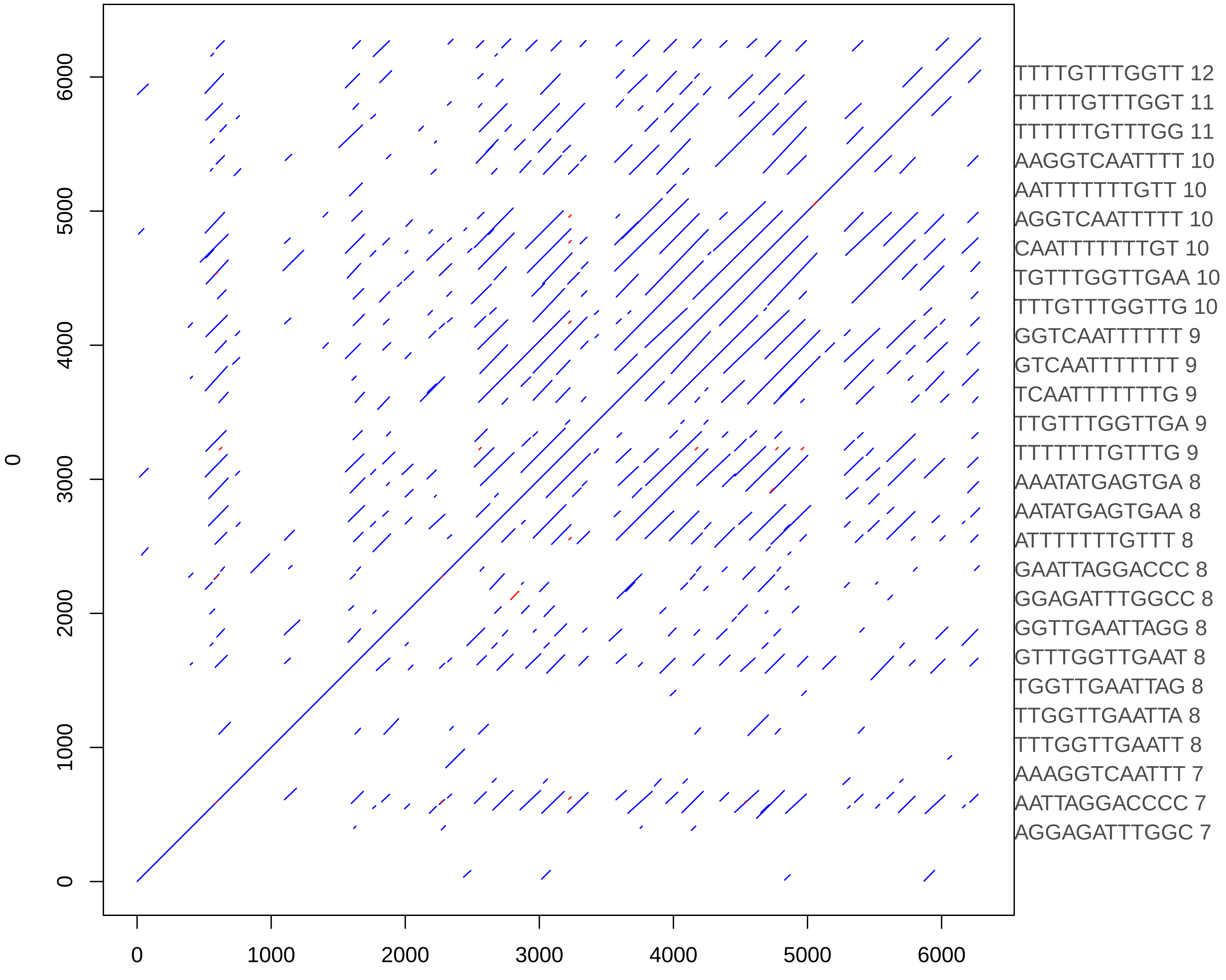

>m141122_040740_42134_c100732202550000001823141405141561_s1_p0/148827/11486_19412

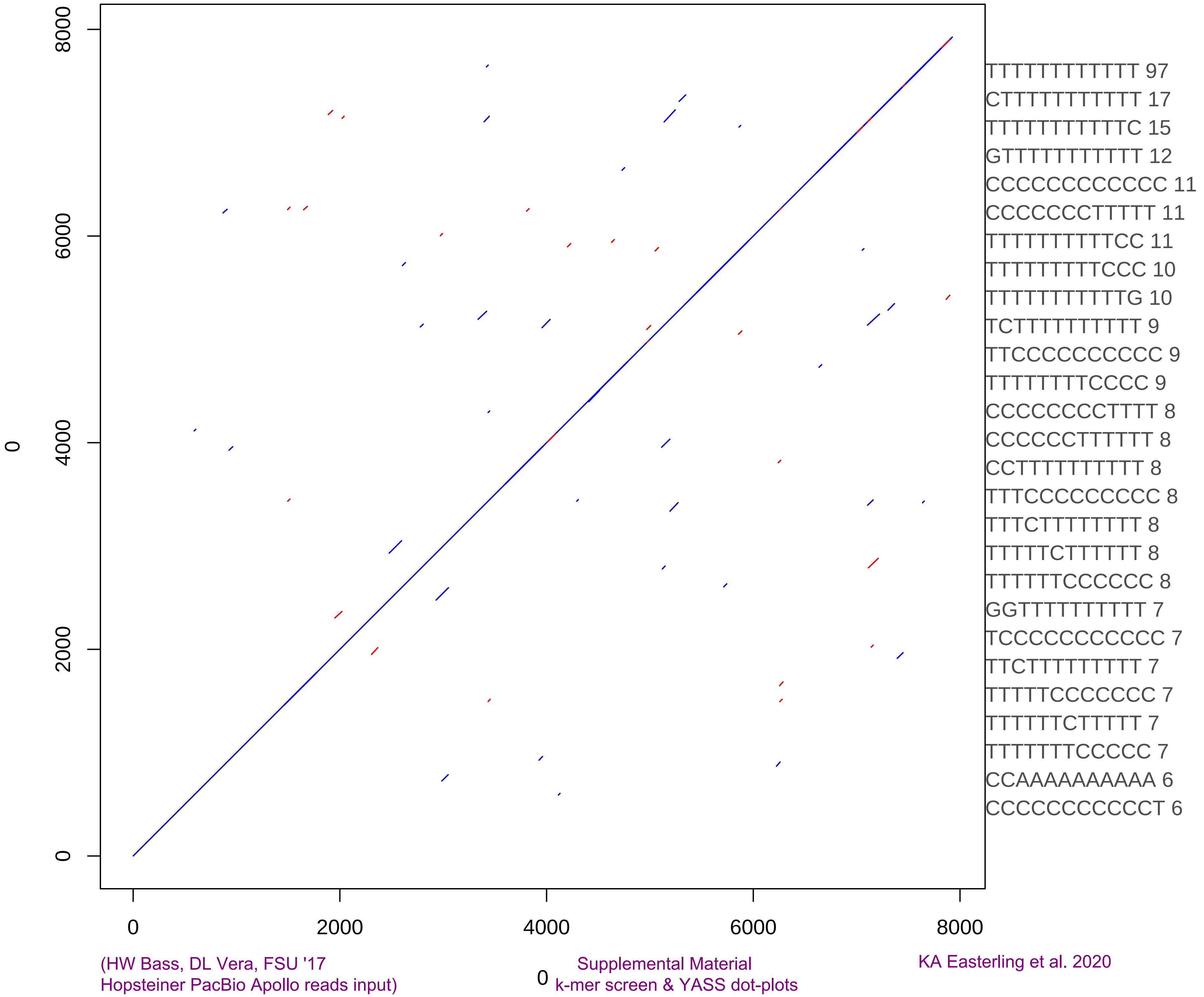

>m141122_040740_42134_c100732202550000001823141405141561_s1_p0/149699/4250_15193

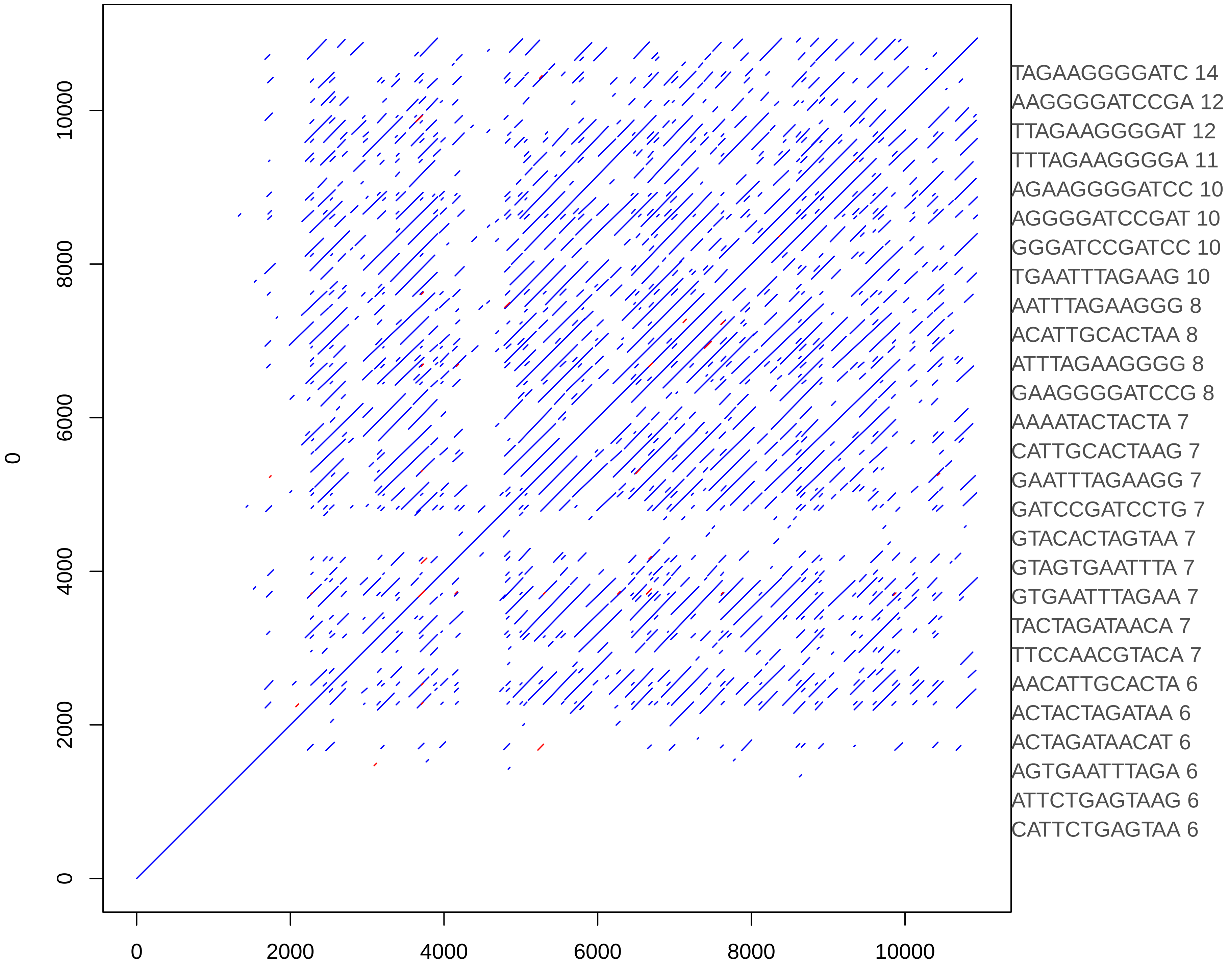

>m141122_040740_42134_c100732202550000001823141405141561_s1_p0/160921/7999_13326

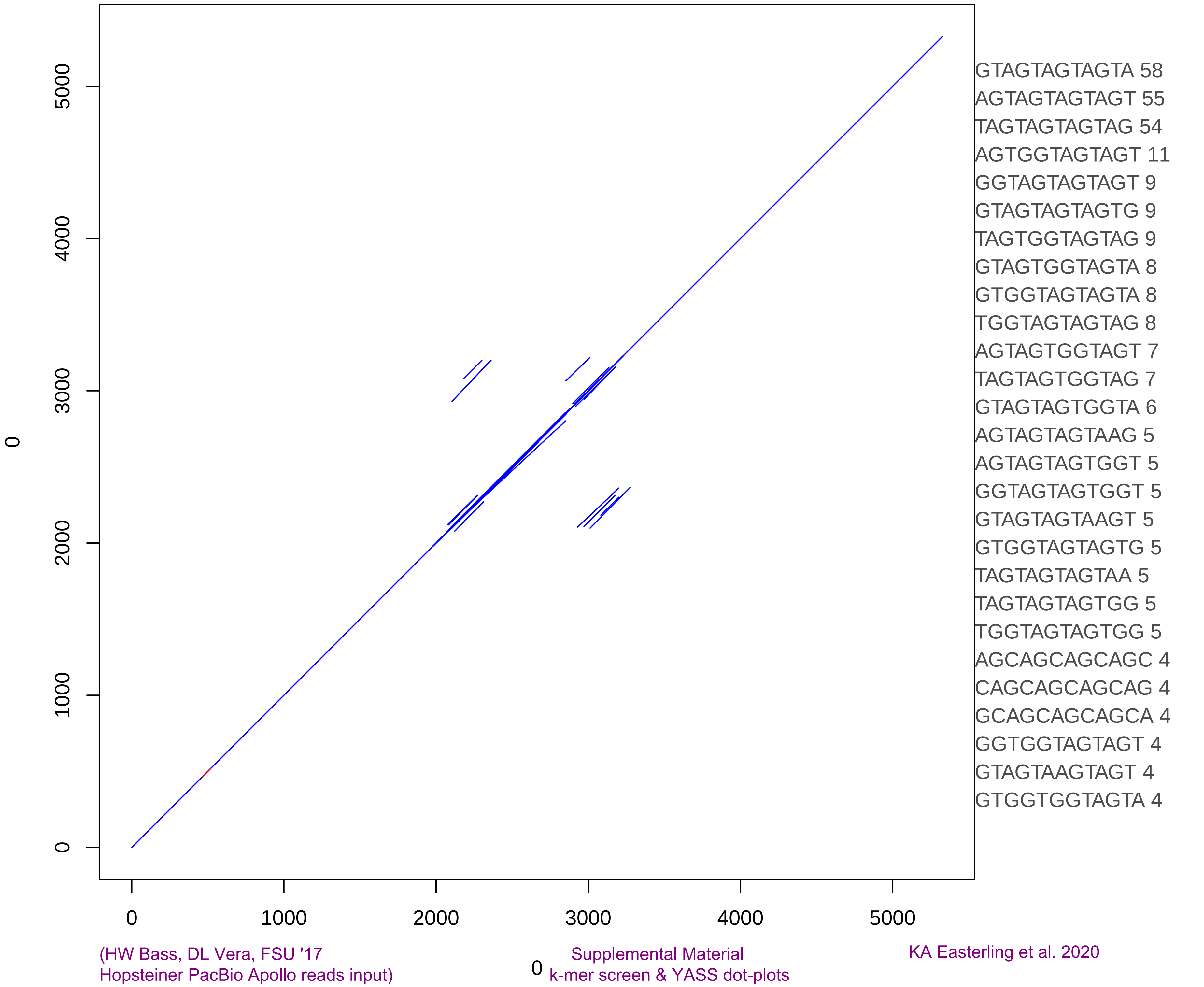

>m141122_040740_42134_c100732202550000001823141405141561_s1_p0/1651/6426_16298

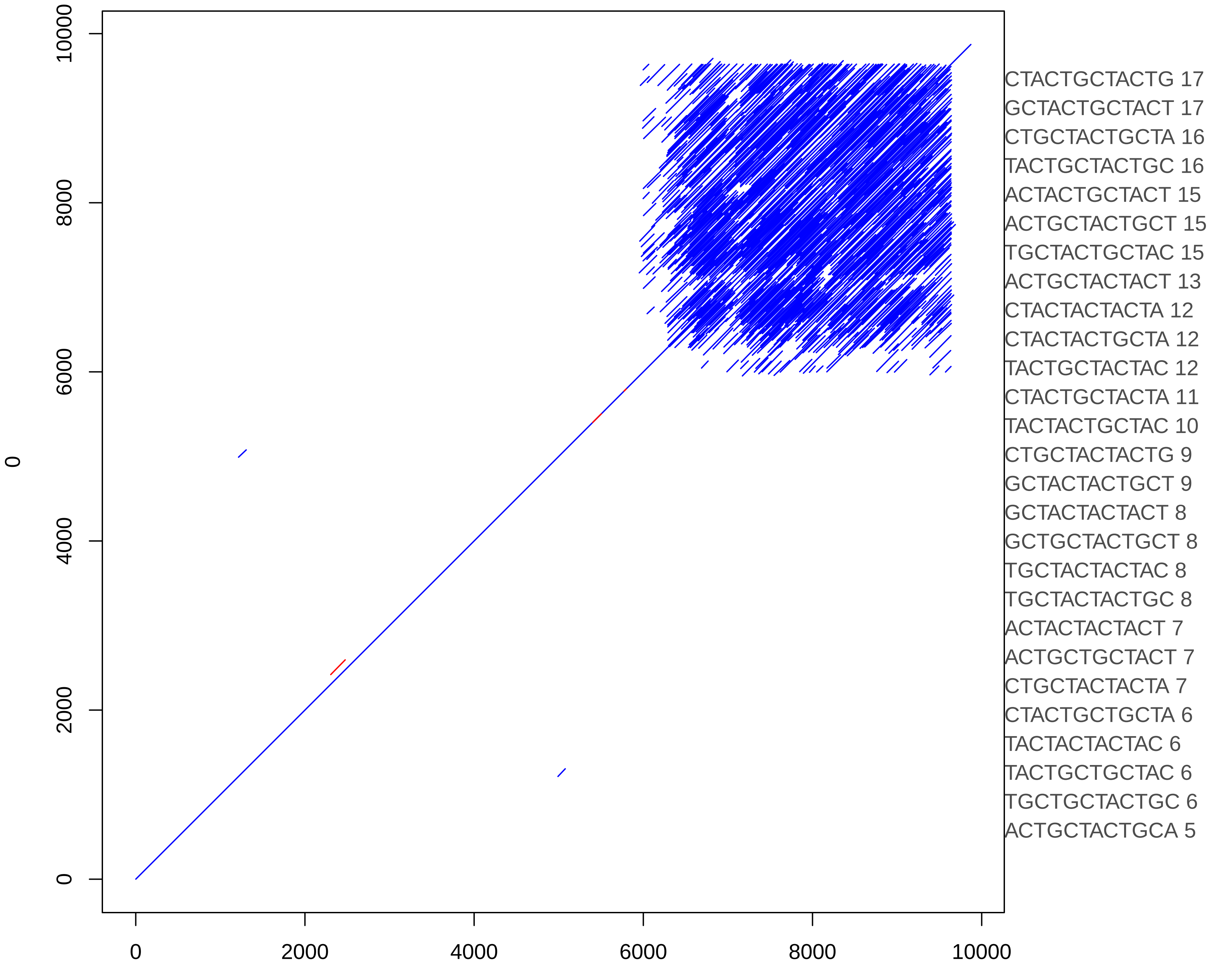

>m141122_040740_42134_c100732202550000001823141405141561_s1_p0/19151/14389_20885

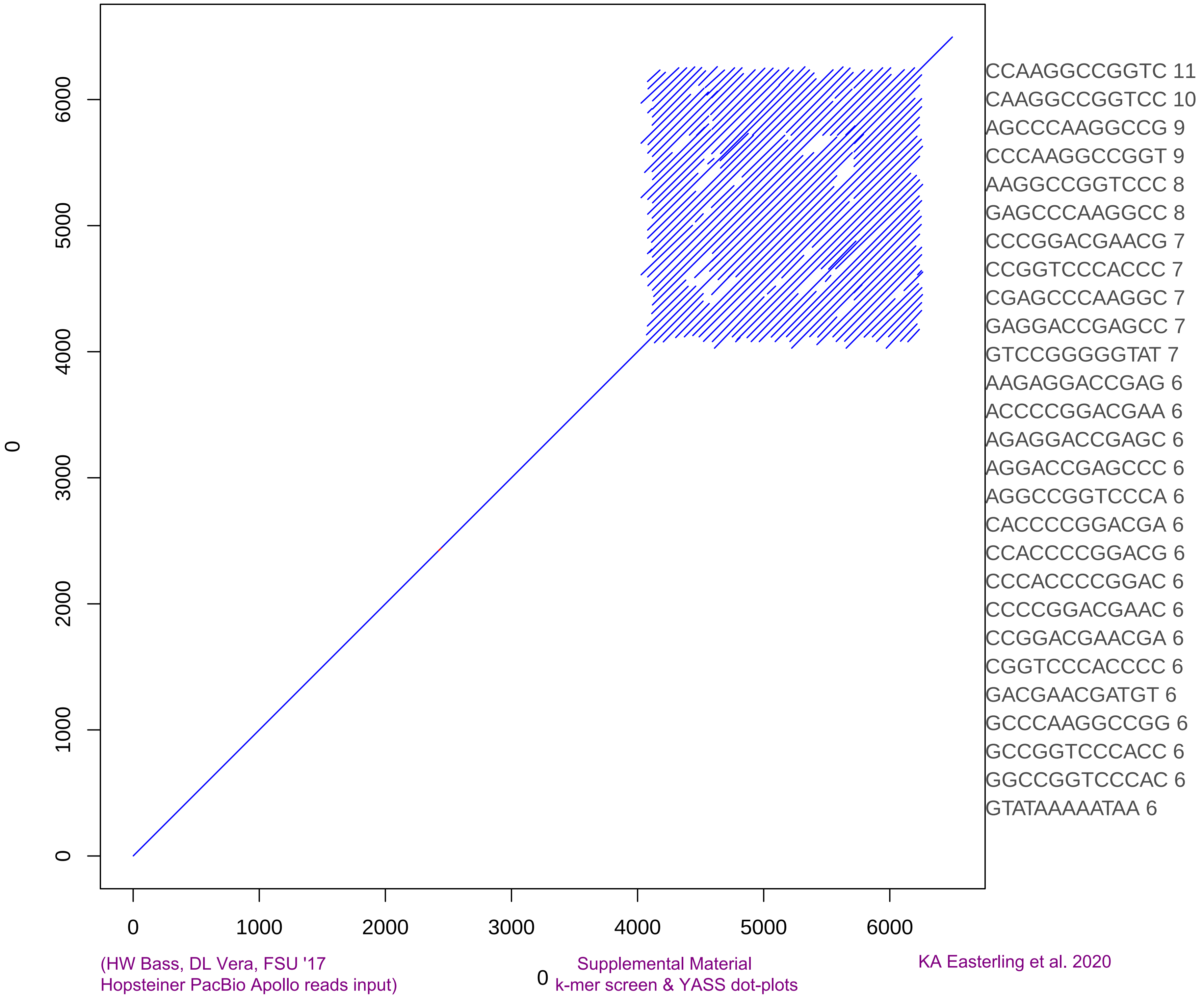

>m141122_040740_42134_c100732202550000001823141405141561_s1_p0/1974/11132_16743

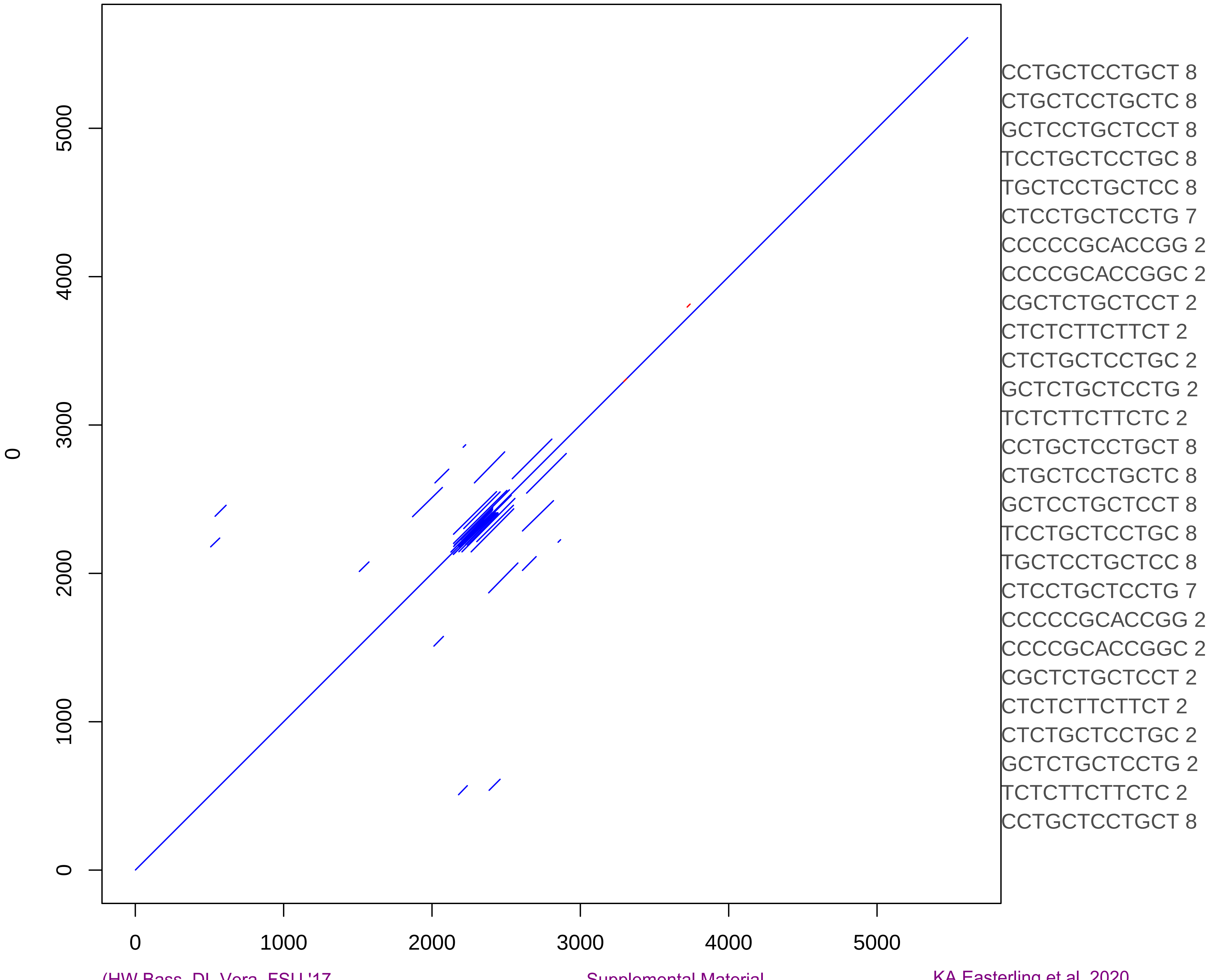

>m141122_040740_42134_c100732202550000001823141405141561_s1_p0/2483/0_6680

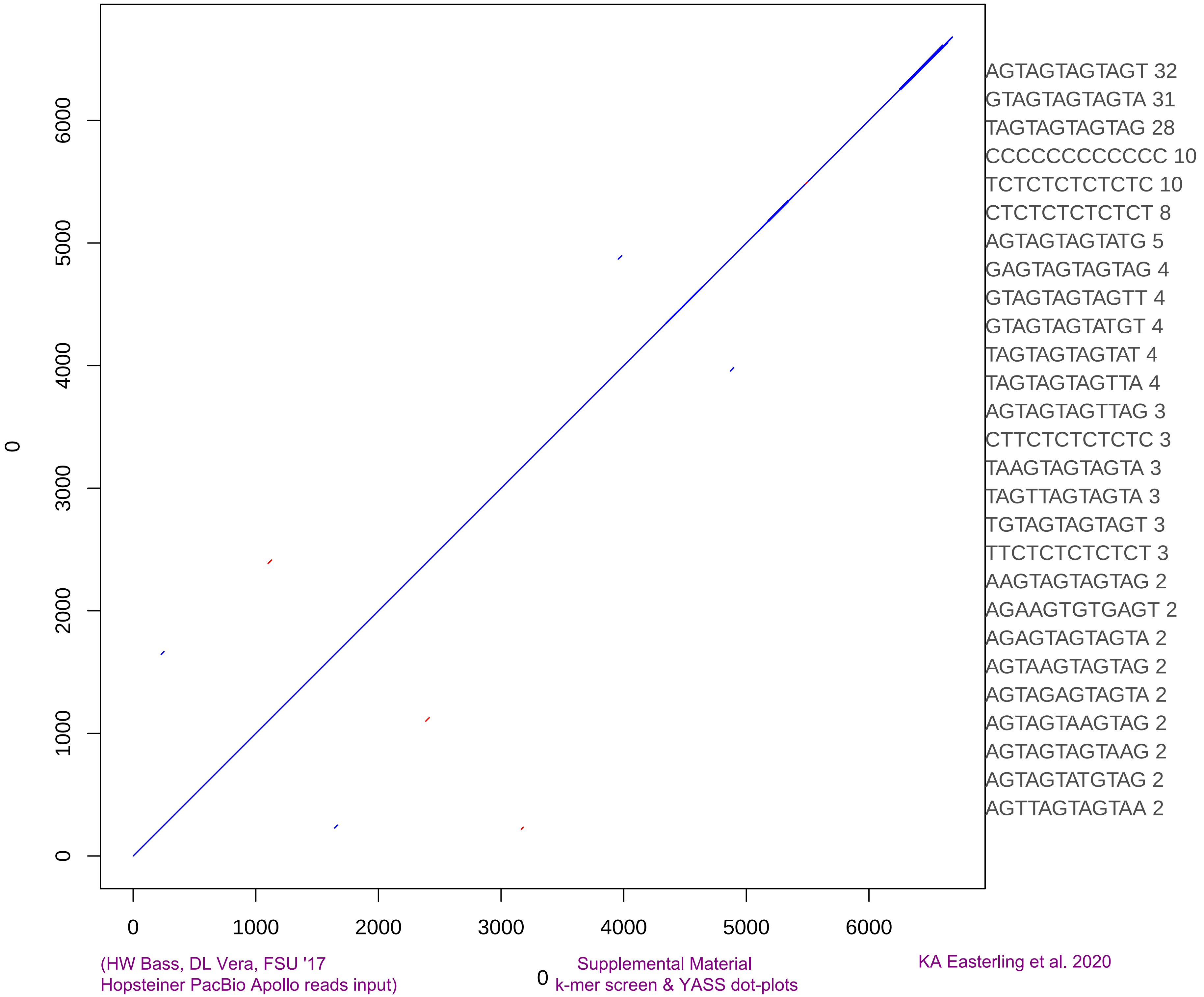

>m141122_040740_42134_c100732202550000001823141405141561_s1_p0/2483/6719_12245

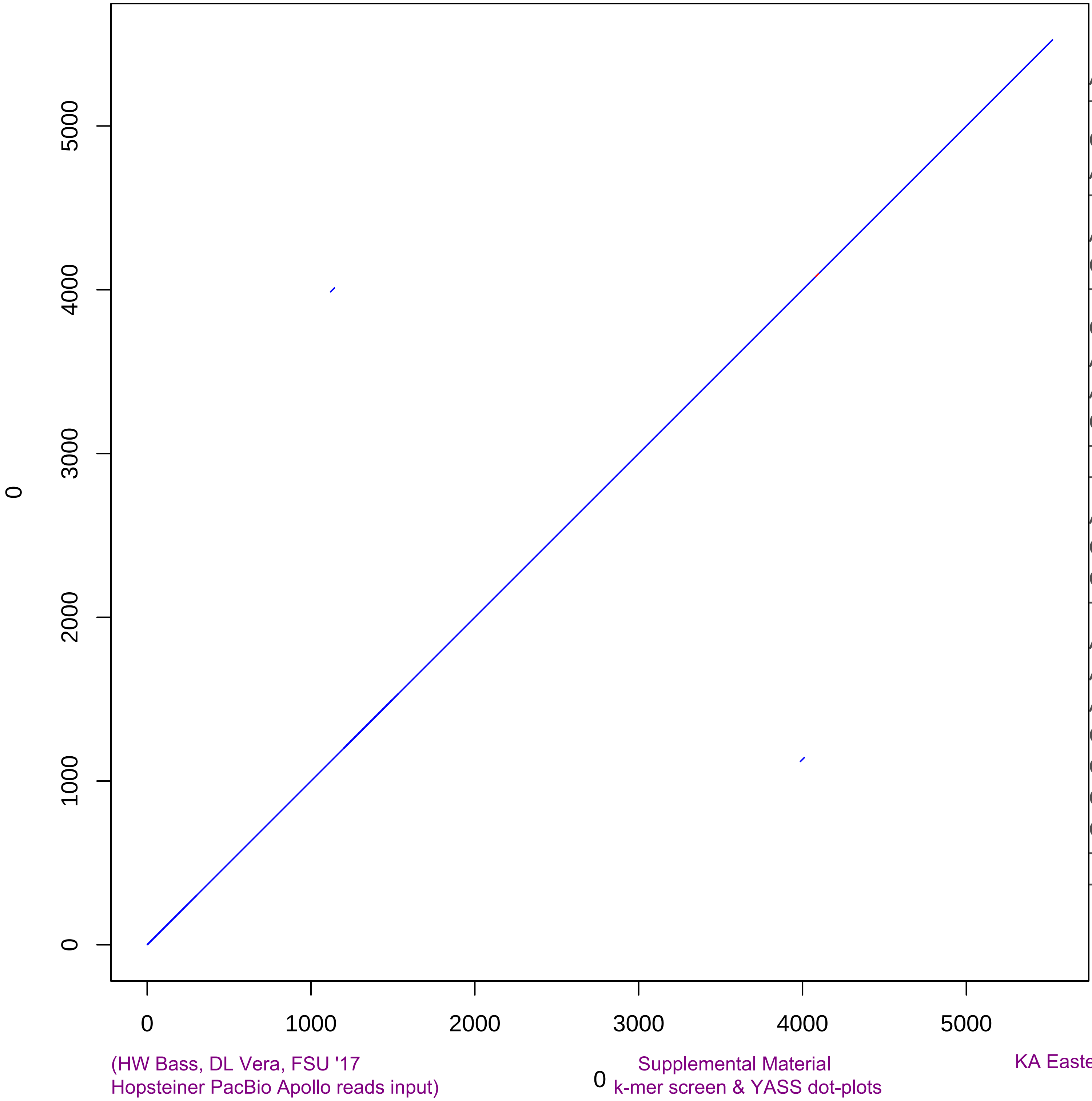

ACTACTACTACT 24
TACTACTACTAC 24
CTACTACTACTA 19
ATACTACTACTA 11
TATACTACTACT 11
ACTATACTACTA 10
CTATACTACTAC 10
TACTATACTACT 10
GAGAGAGAGAGA 9
ACTACTATACTA 8
AGAGAGAGAGAG 8
CTACTATACTAC 8
TACTACTATACT 7
TACTACTACTAT 6
ACTACTACTATA 5
CTACTACTATAC 5
CTACTACTACTT 4
TACTACTTACTA 4
ACTACTACTCTA 3
ACTACTACTTAC 3
ACTACTTACTAC 3
CTACTACTACTC 3
CTACTACTTACT 3
CTACTTACTACT 3
CTTACTACTACT 3
TACTACTACTCT 3
TACTACTACTTA 3

>m141122_040740_42134_c100732202550000001823141405141561_s1_p0/27294/11981_18410

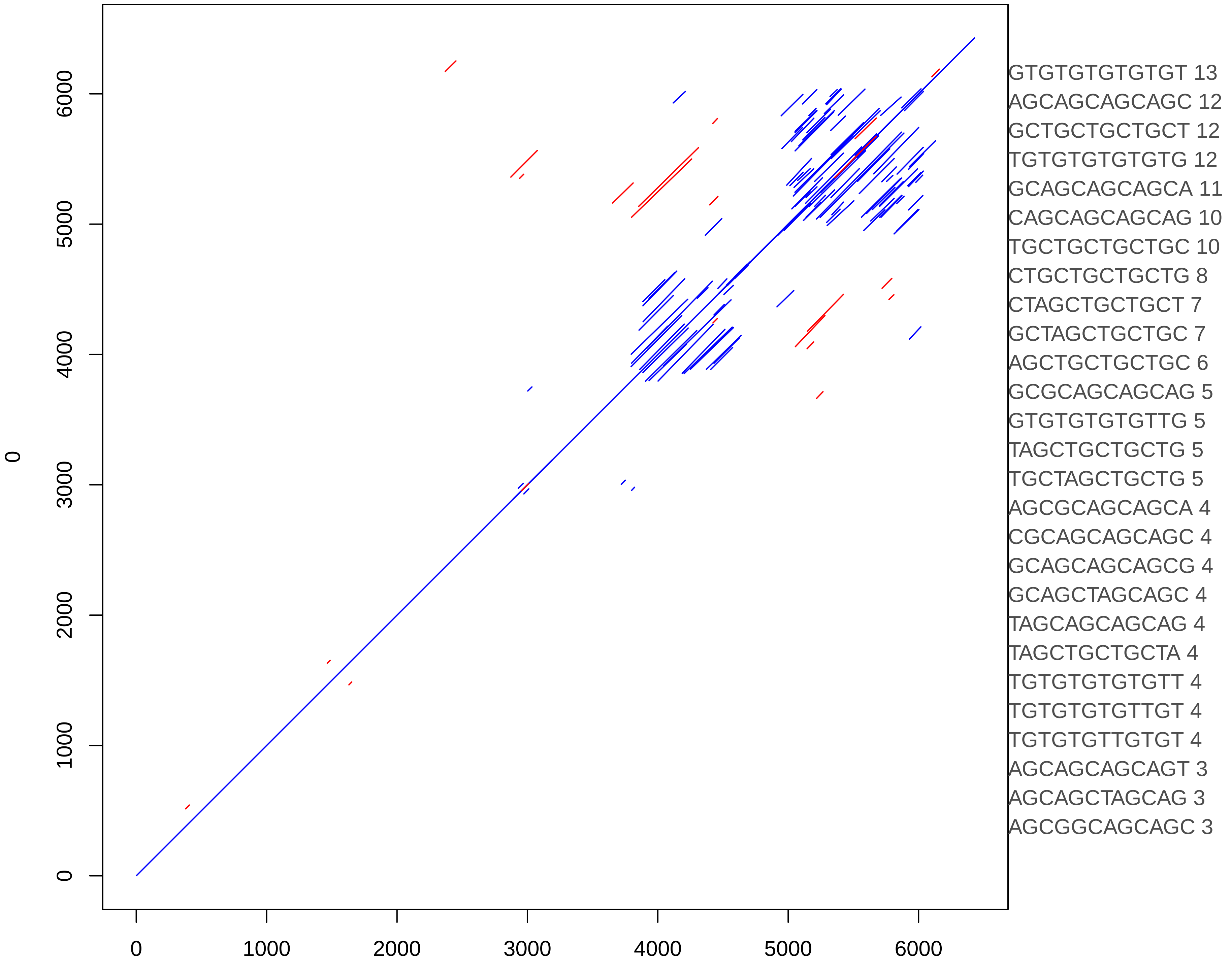

>m141122_040740_42134_c100732202550000001823141405141561_s1_p0/27504/10520_20105

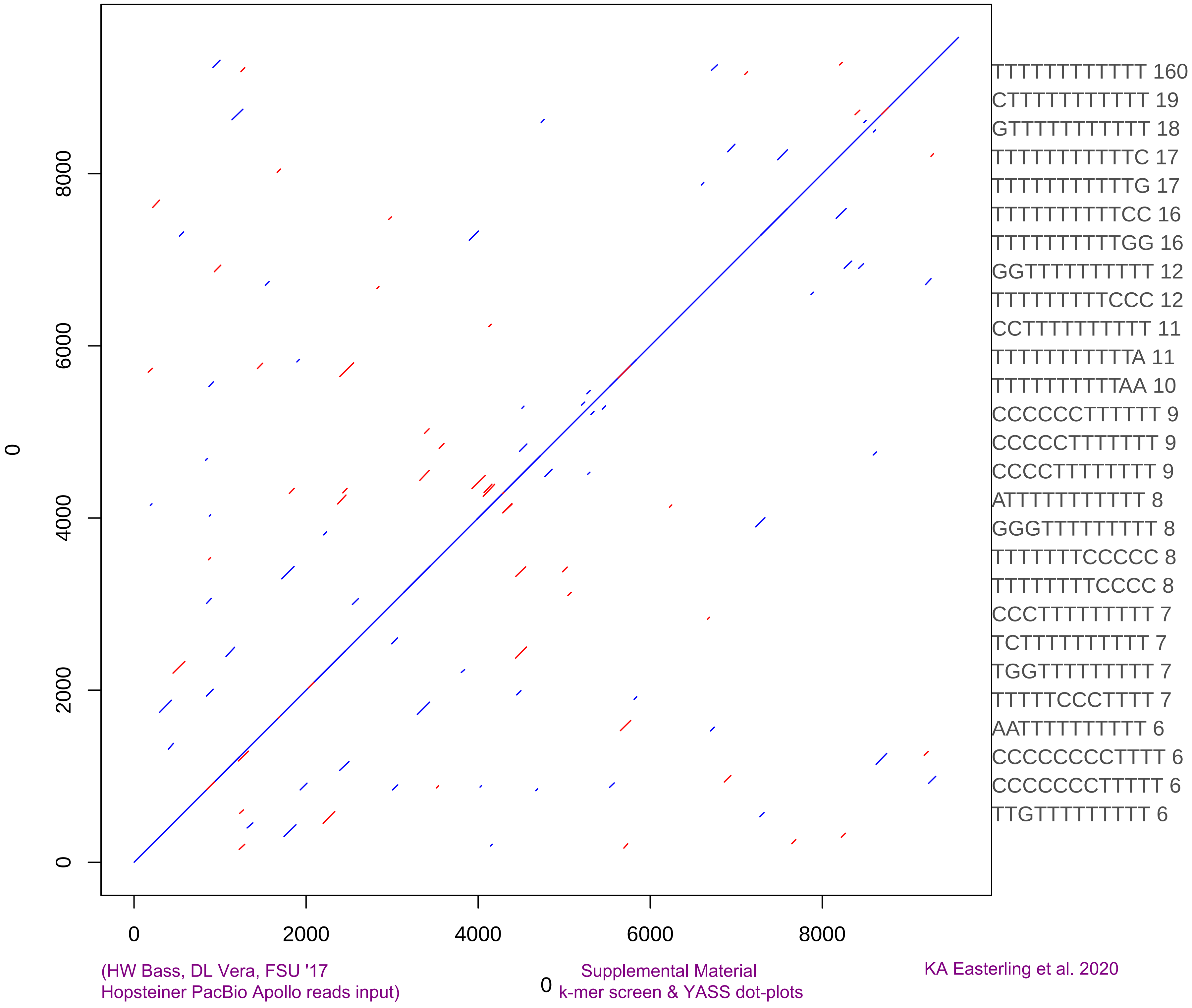

>m141122_040740_42134_c100732202550000001823141405141561_s1_p0/278/0_14870

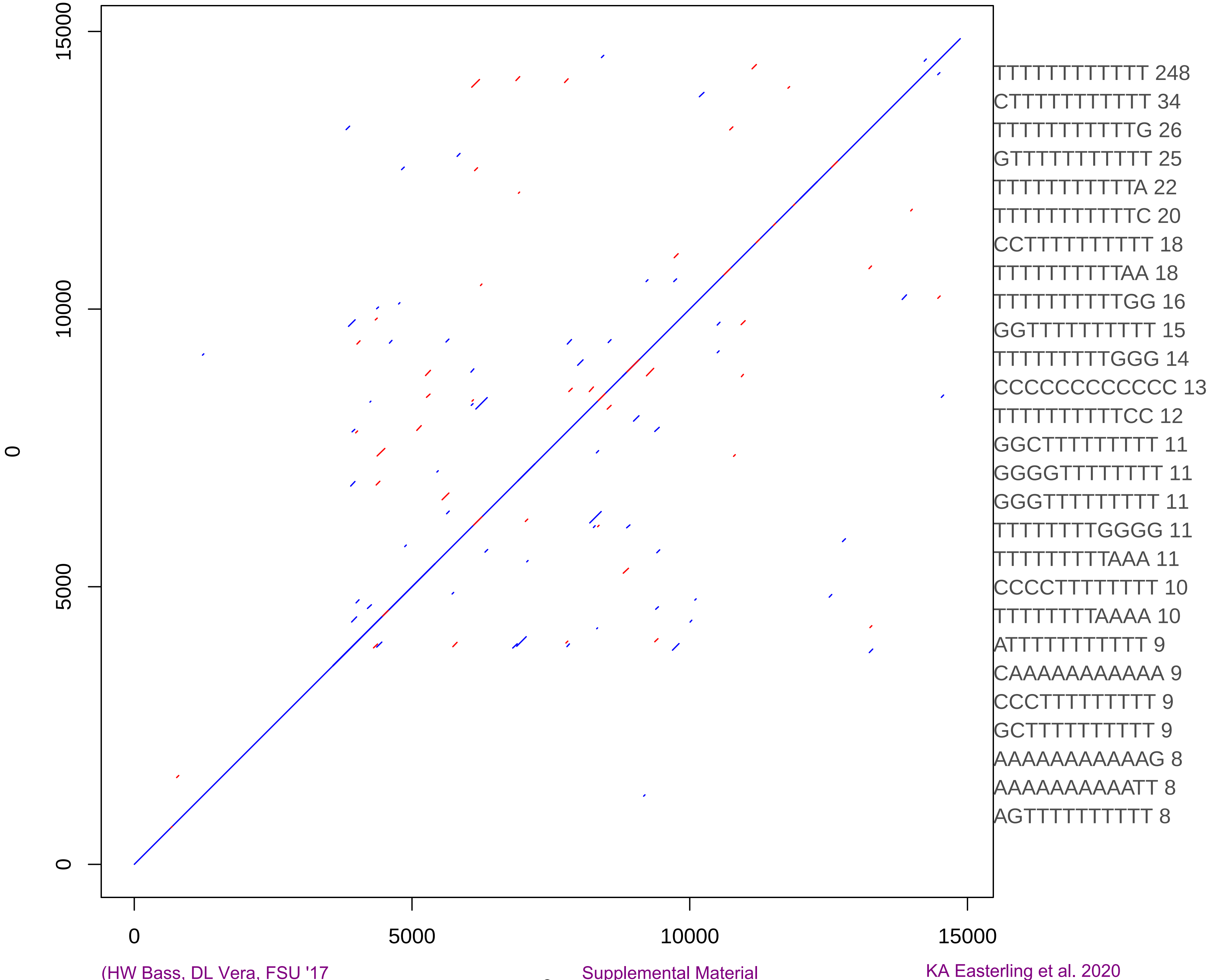

>m141122_040740_42134_c100732202550000001823141405141561_s1_p0/29712/3793_14587

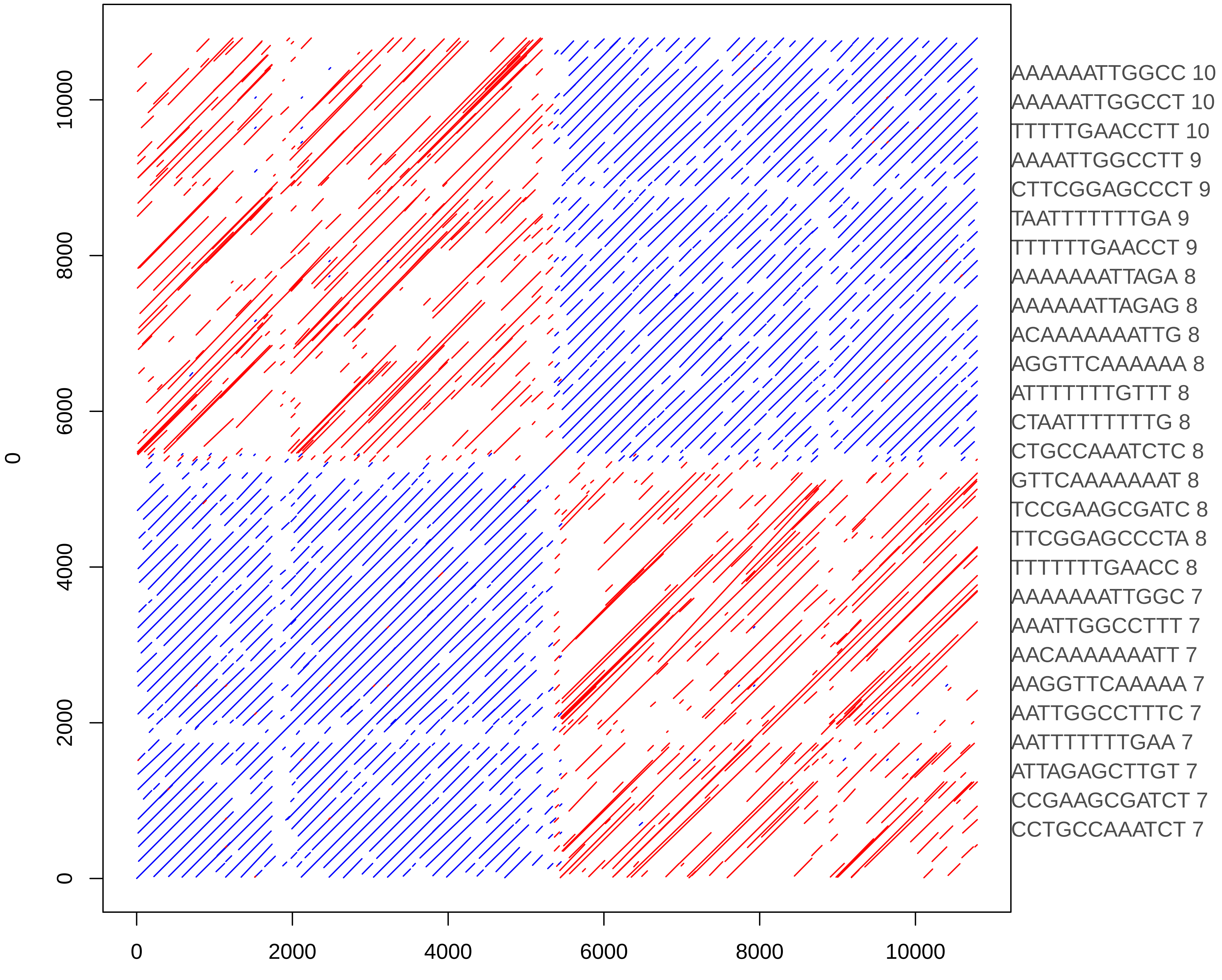

>m141122_040740_42134_c100732202550000001823141405141561_s1_p0/3501/7450_14187

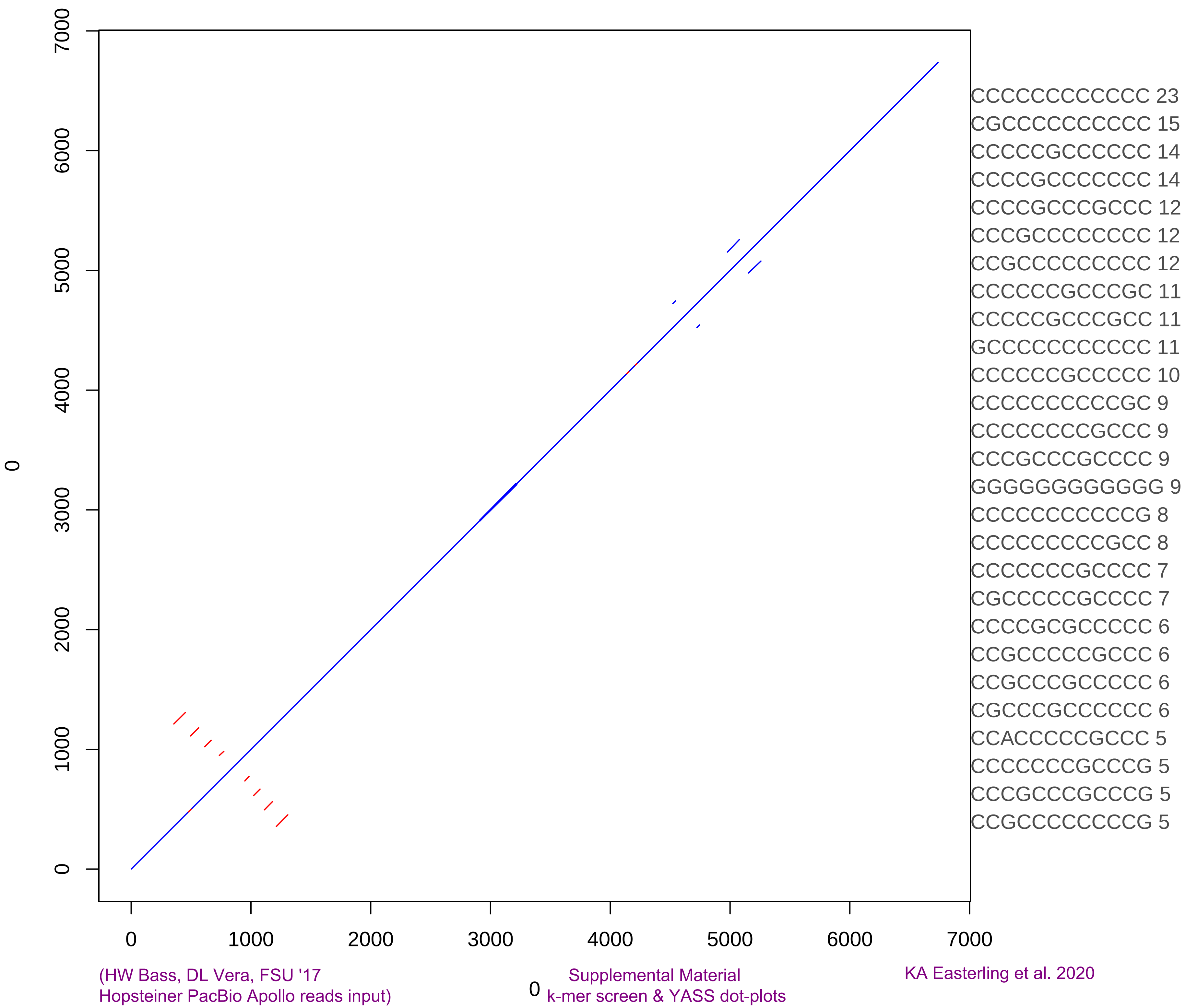

>m141122_040740_42134_c100732202550000001823141405141561_s1_p0/36738/1980_13611

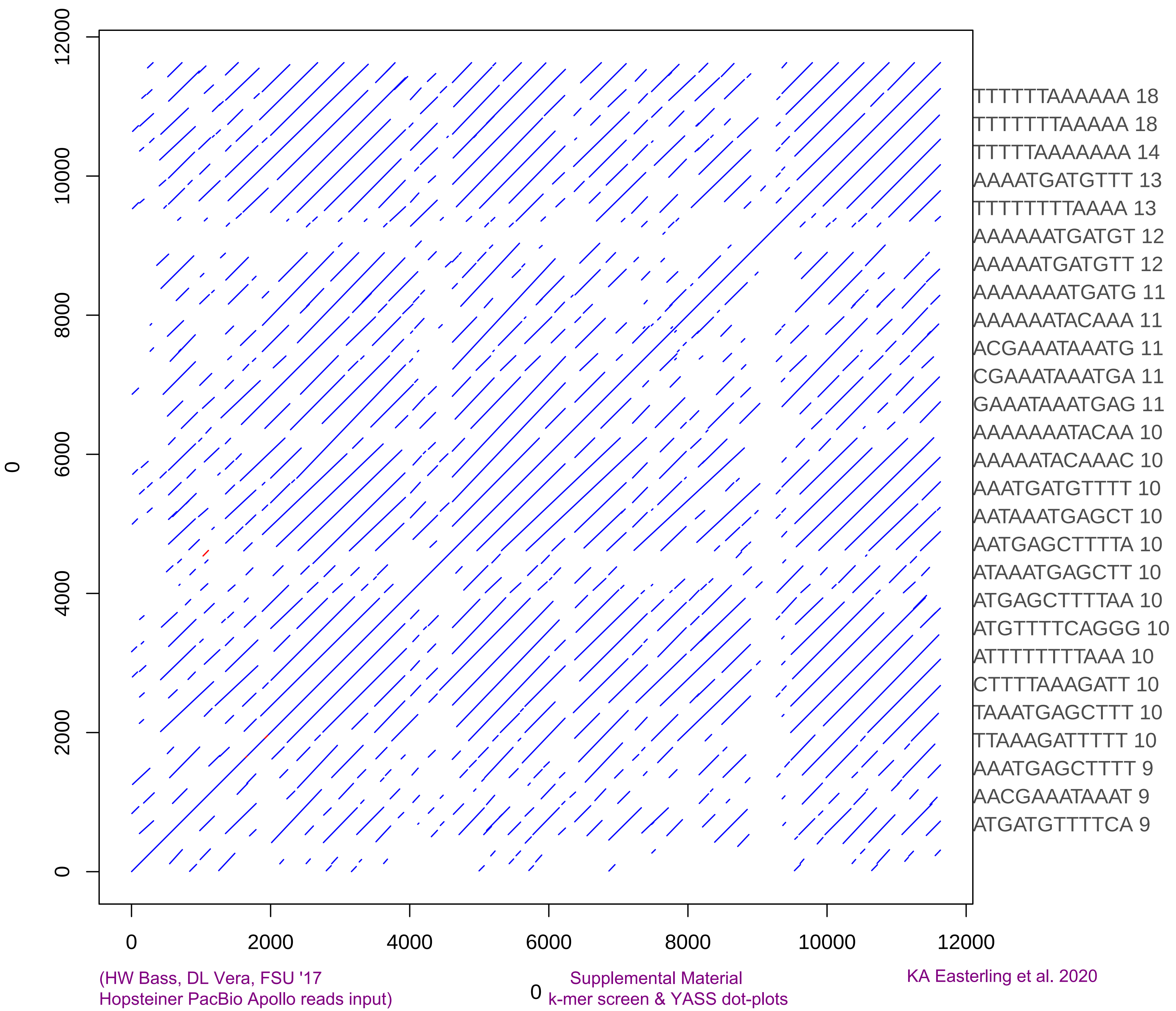

>m141122_040740_42134_c100732202550000001823141405141561_s1_p0/38587/0_13884

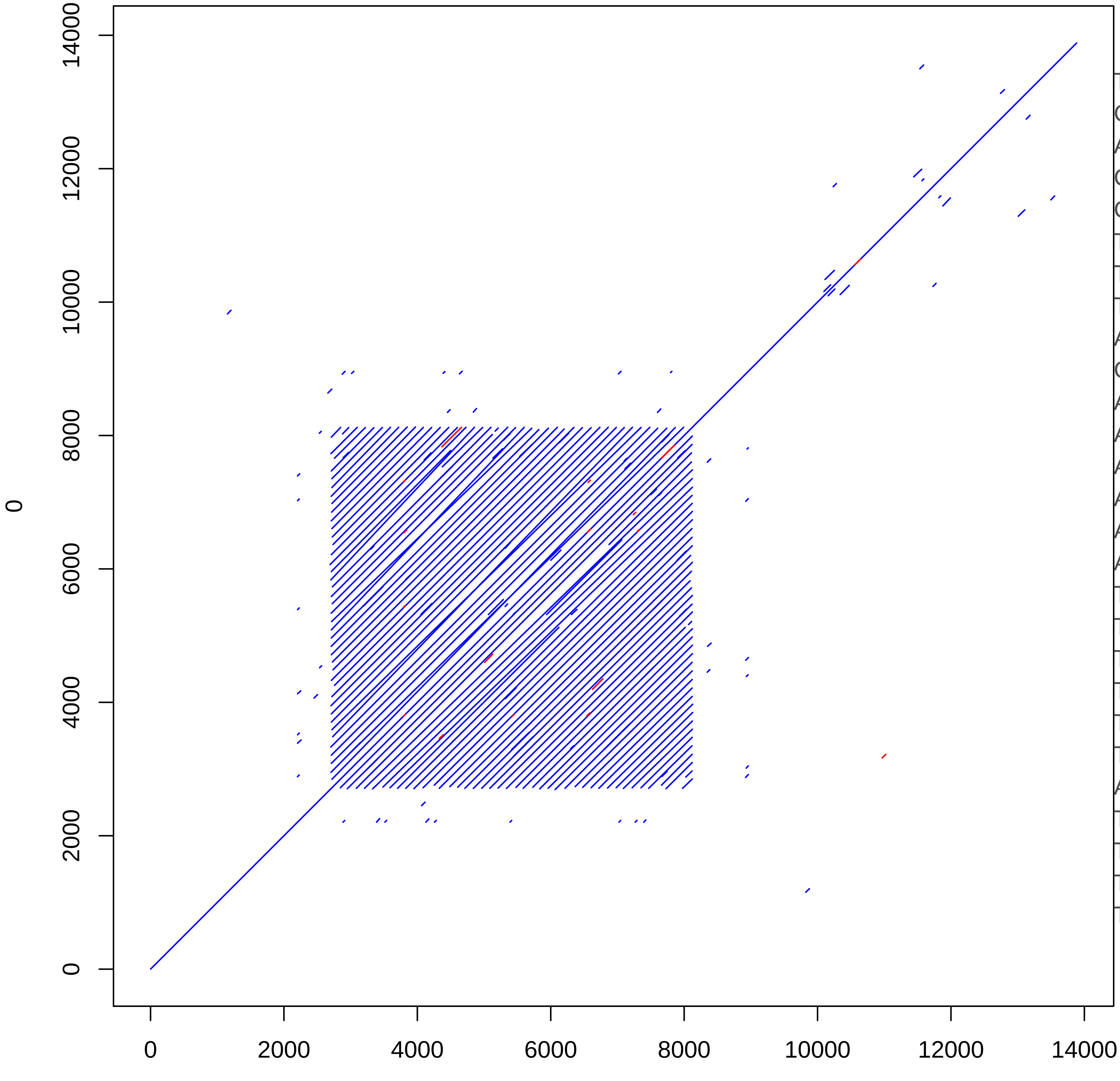

>m141122_040740_42134_c100732202550000001823141405141561_s1_p0/3894/7109_13939

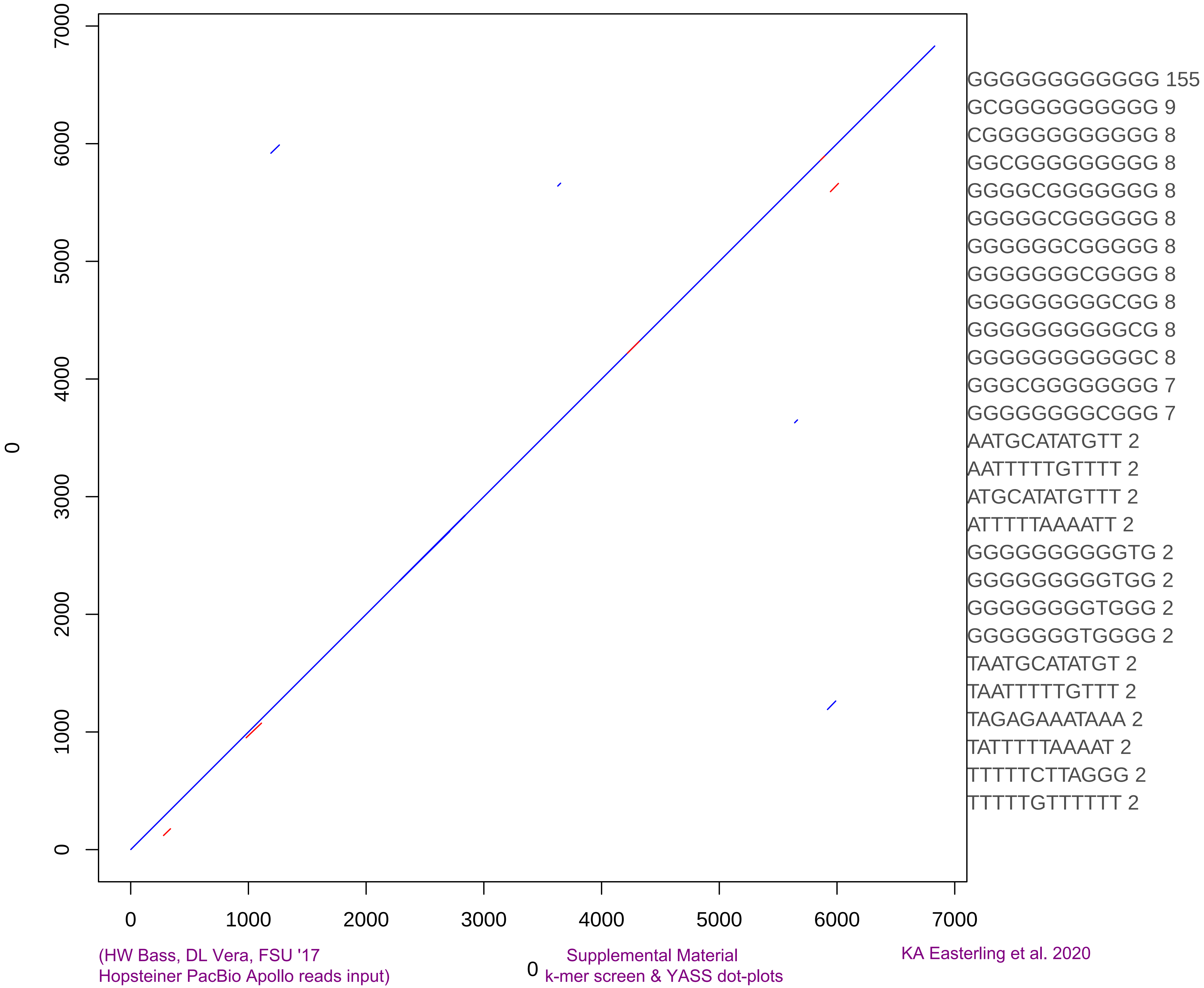

>m141122_040740_42134_c100732202550000001823141405141561_s1_p0/42794/10420_19829

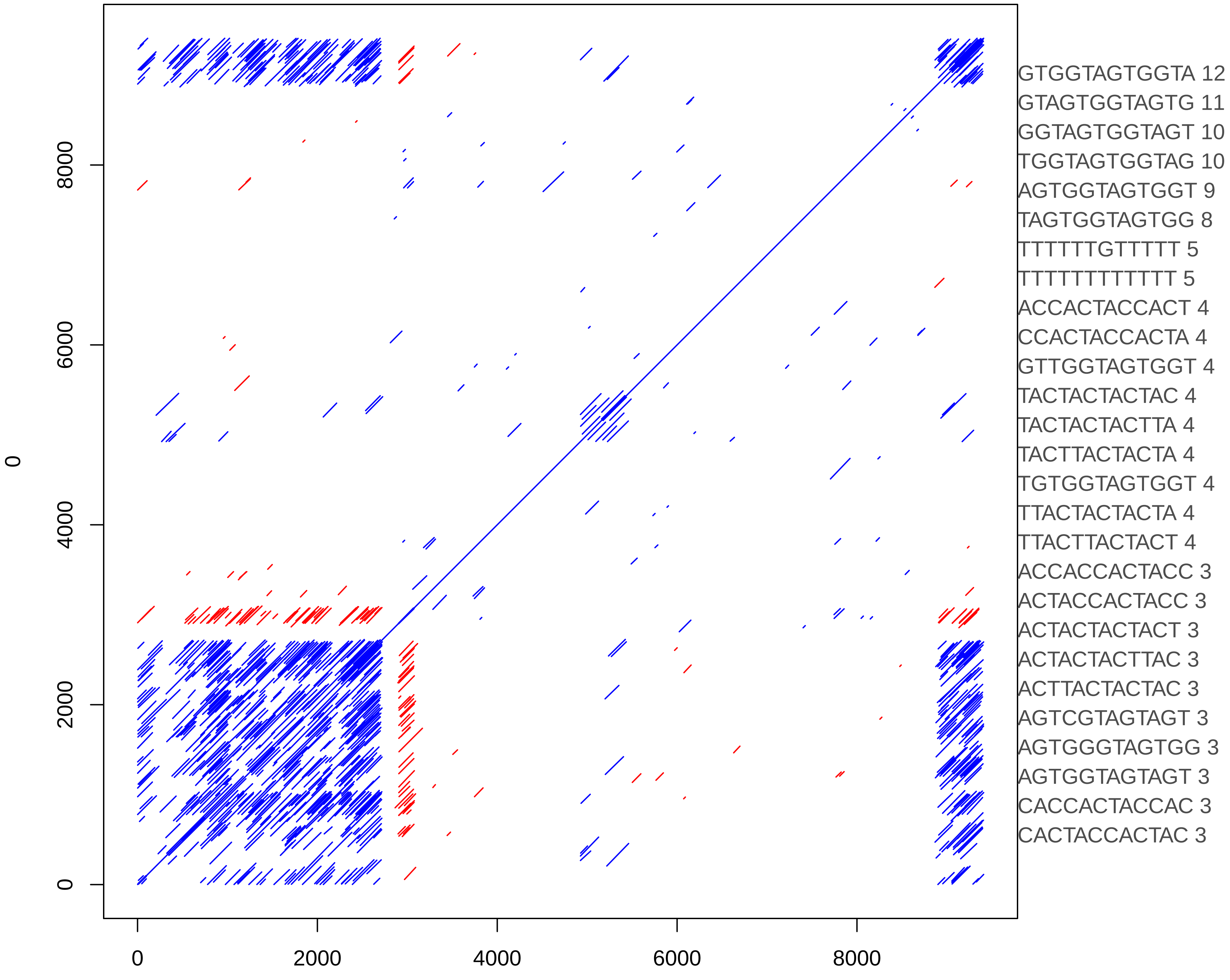

>m141122_040740_42134_c100732202550000001823141405141561_s1_p0/43017/7738_16703

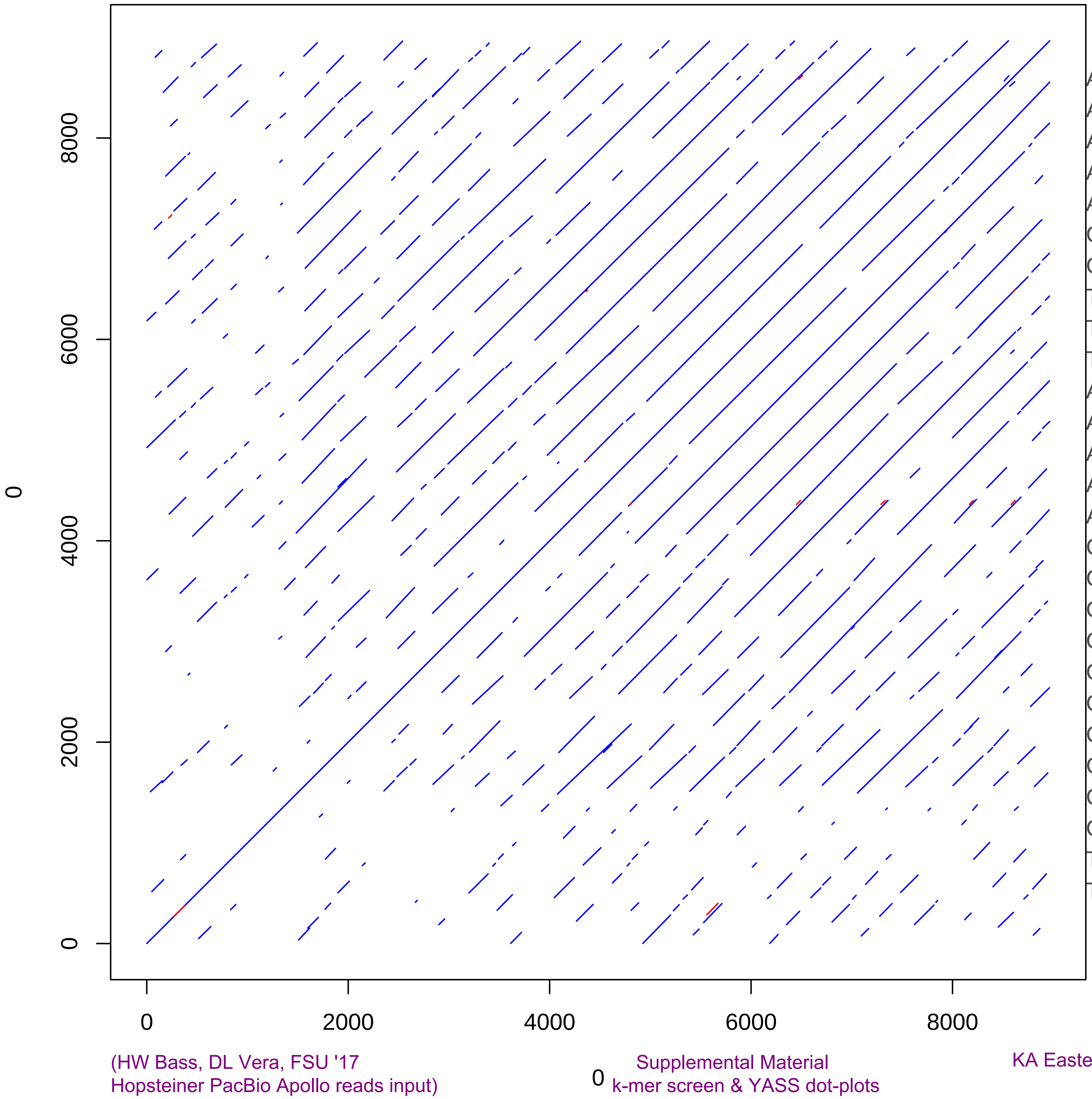

AAACTTGCGAAA 9
AAAAACTTGCGA 8
AAAATATCGAAC 8
AACTTGCGAAAA 8
ACTTGCGAAAAT 8
CCTTTCAGAAAT 8
CTTTCAGAAATC 8
TCTTCGATTTCT 8
TTTTGAAATGTT 8
TTTTTGAAATGT 8
AAAACTTGCGAA 7
AATCCGAGGTGT 7
AGAAATTAGTGA 7
AGGTGTGTAGAG 7
ATCCGAGGTGTG 7
CCGAGGTGTGTA 7
CGAGGTGTGTAG 7
CGGAGAAATTAG 7
GAAAAATATCGA 7
GAAATTAGTGAA 7
GAGGTGTGTAGA 7
GGAAAAATATCG 7
GGGAAAAATATC 7
GGTGTGTAGAGG 7
GTGTGTAGAGGG 7
TCAGAAATCTTC 7
TCCGAGGTGTGT 7

>m141122_040740_42134_c100732202550000001823141405141561_s1_p0/48977/5414_11878

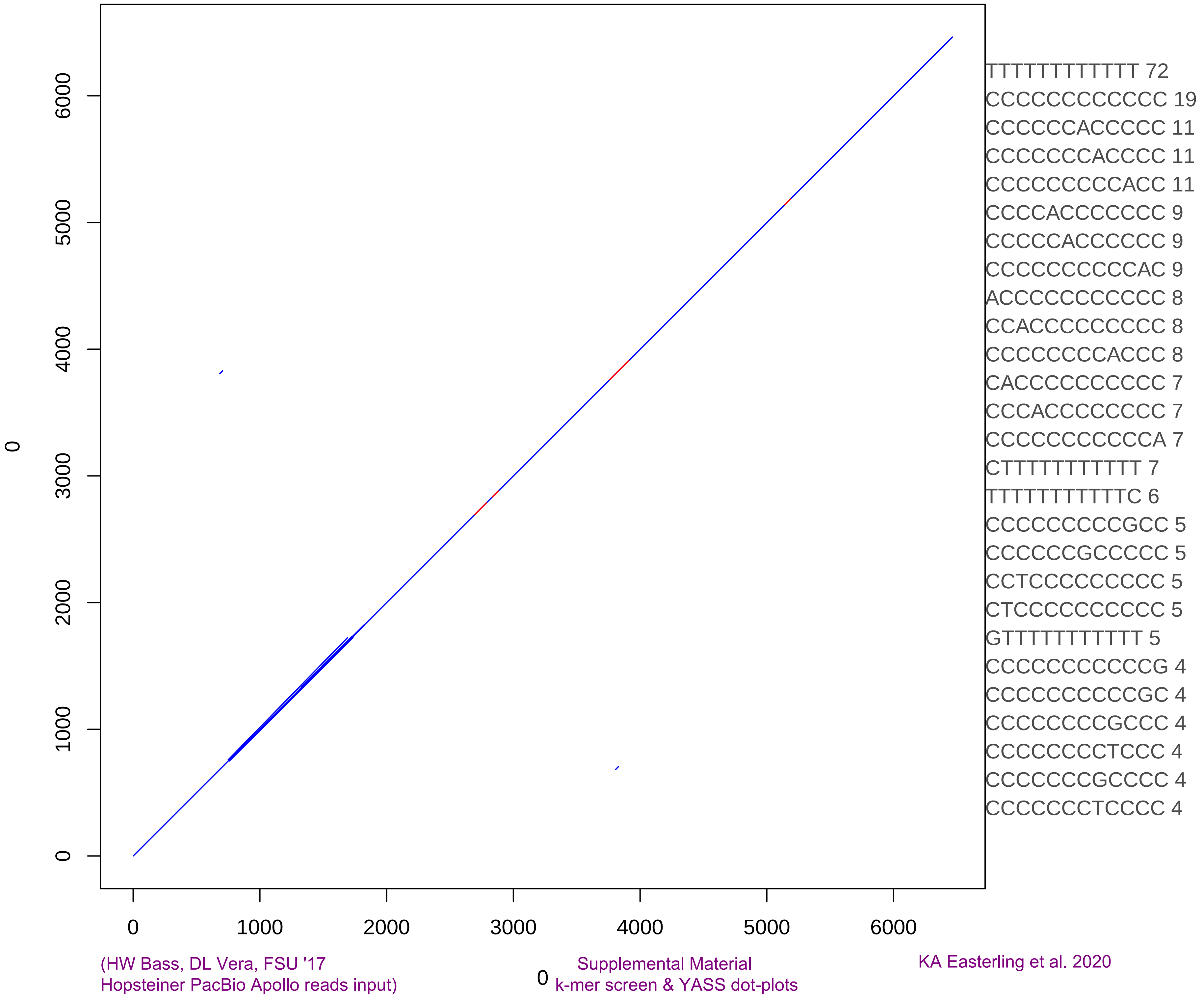

>m141122_040740_42134_c100732202550000001823141405141561_s1_p0/50138/1600_10232

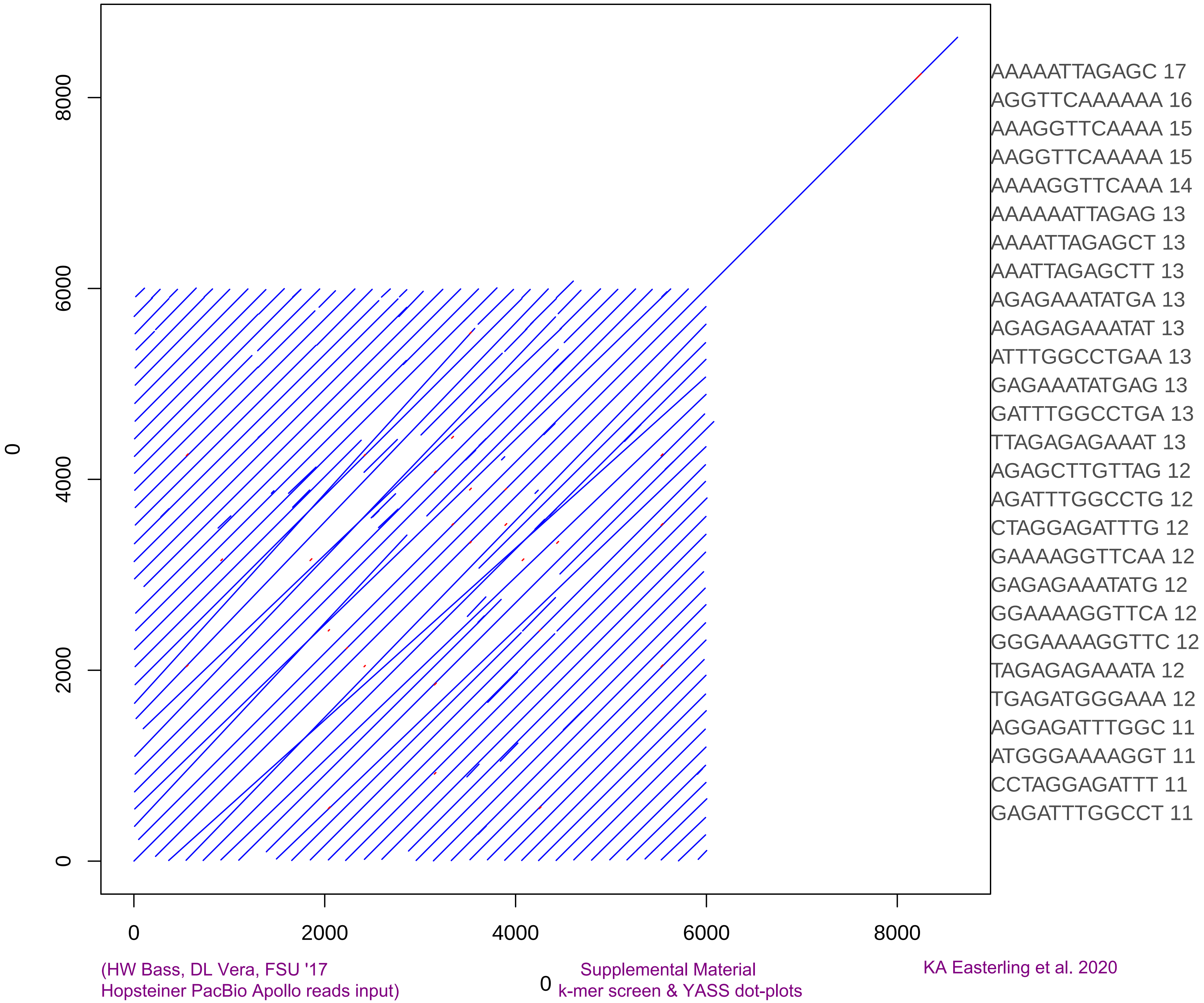

>m141122_040740_42134_c100732202550000001823141405141561_s1_p0/54137/2590_9395

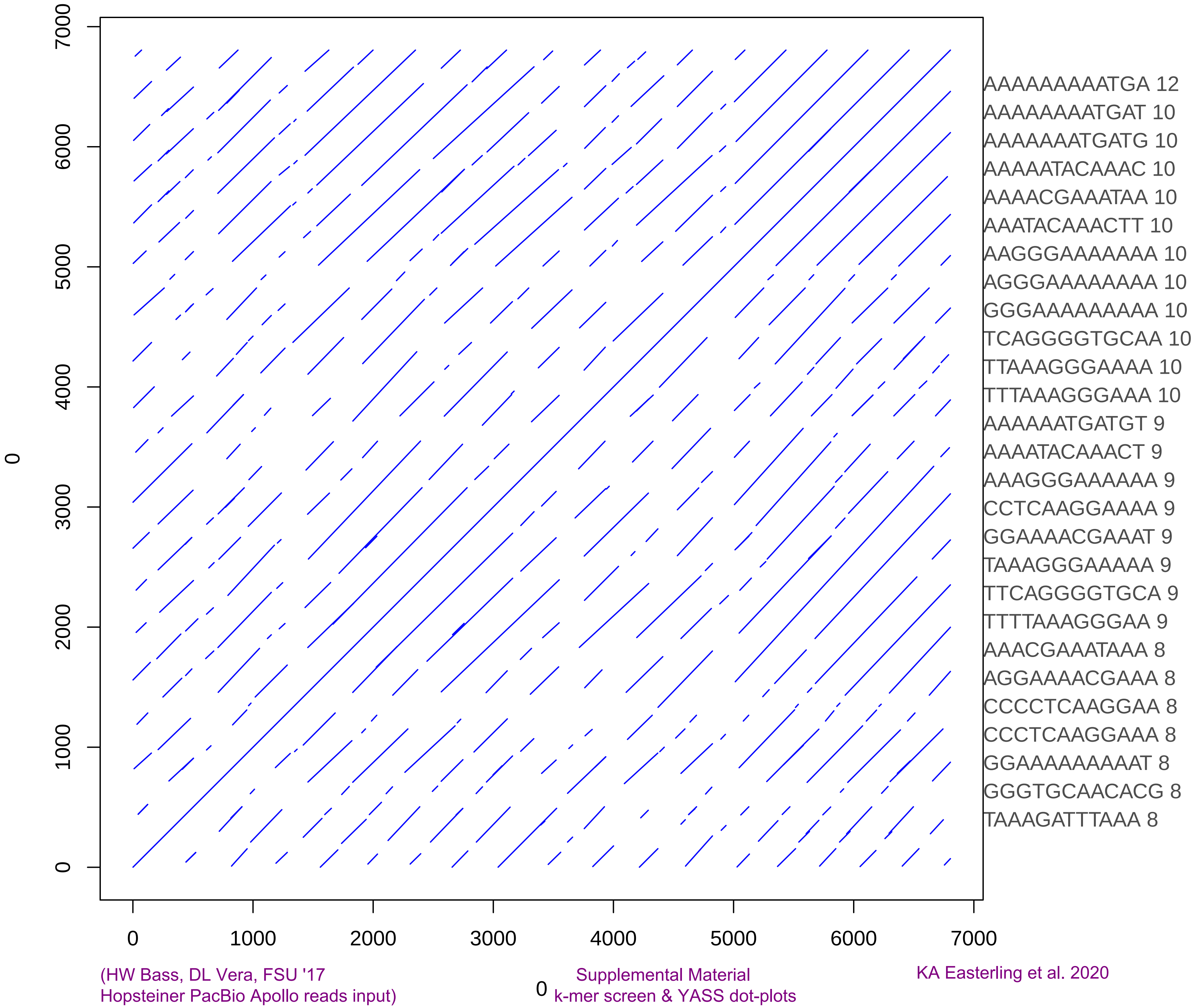

>m141122_040740_42134_c100732202550000001823141405141561_s1_p0/54277/5022_16458

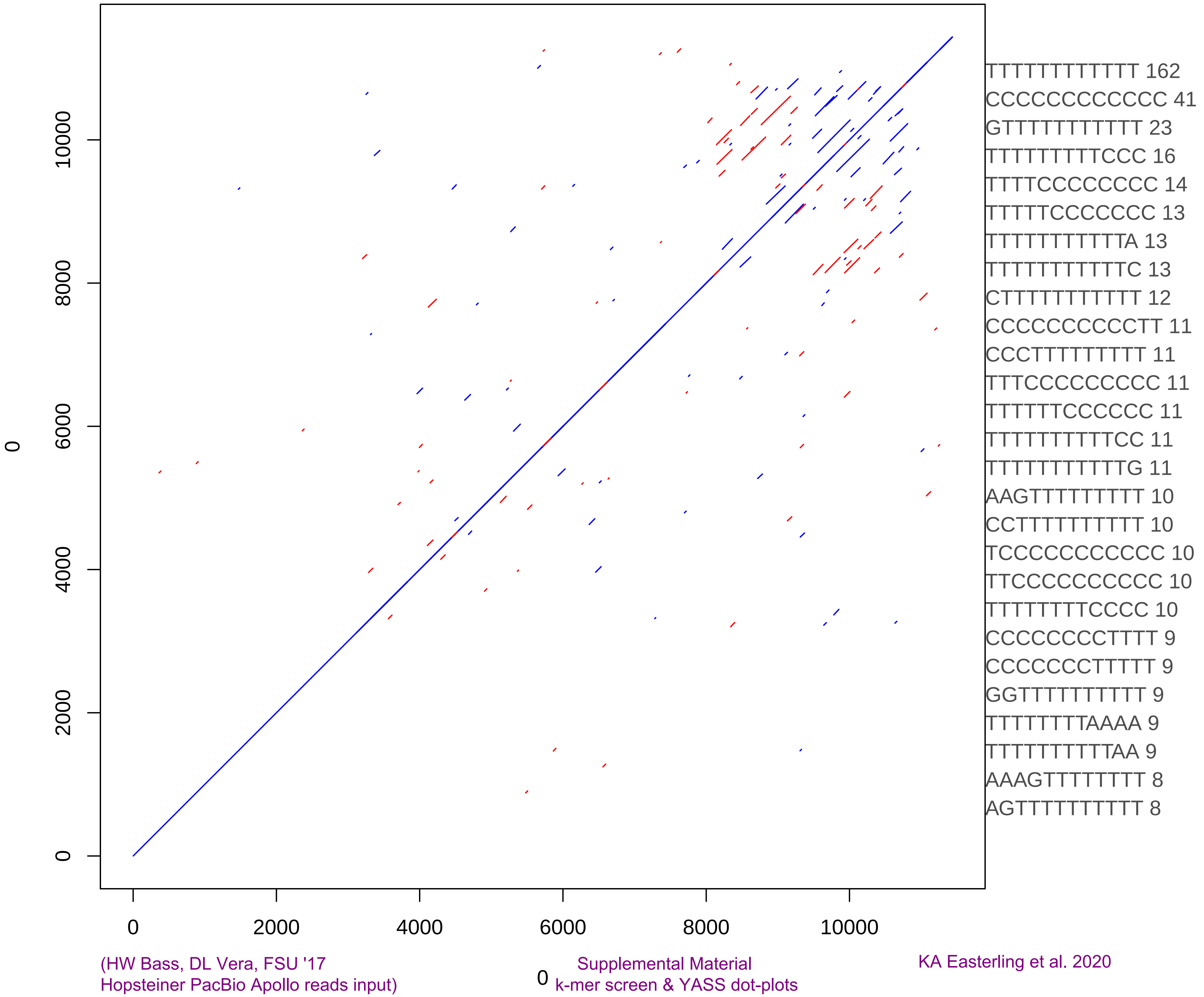

1,121 hits for repetitive sequences
passing 12mer abundance criterion

For Tandem Repeat families of ### bp:
PacBio Read named "HuluTR###-r281"

>m141122_040740_42134_c100732202550000001823141405141561_s1_p0/54856/12527_23542

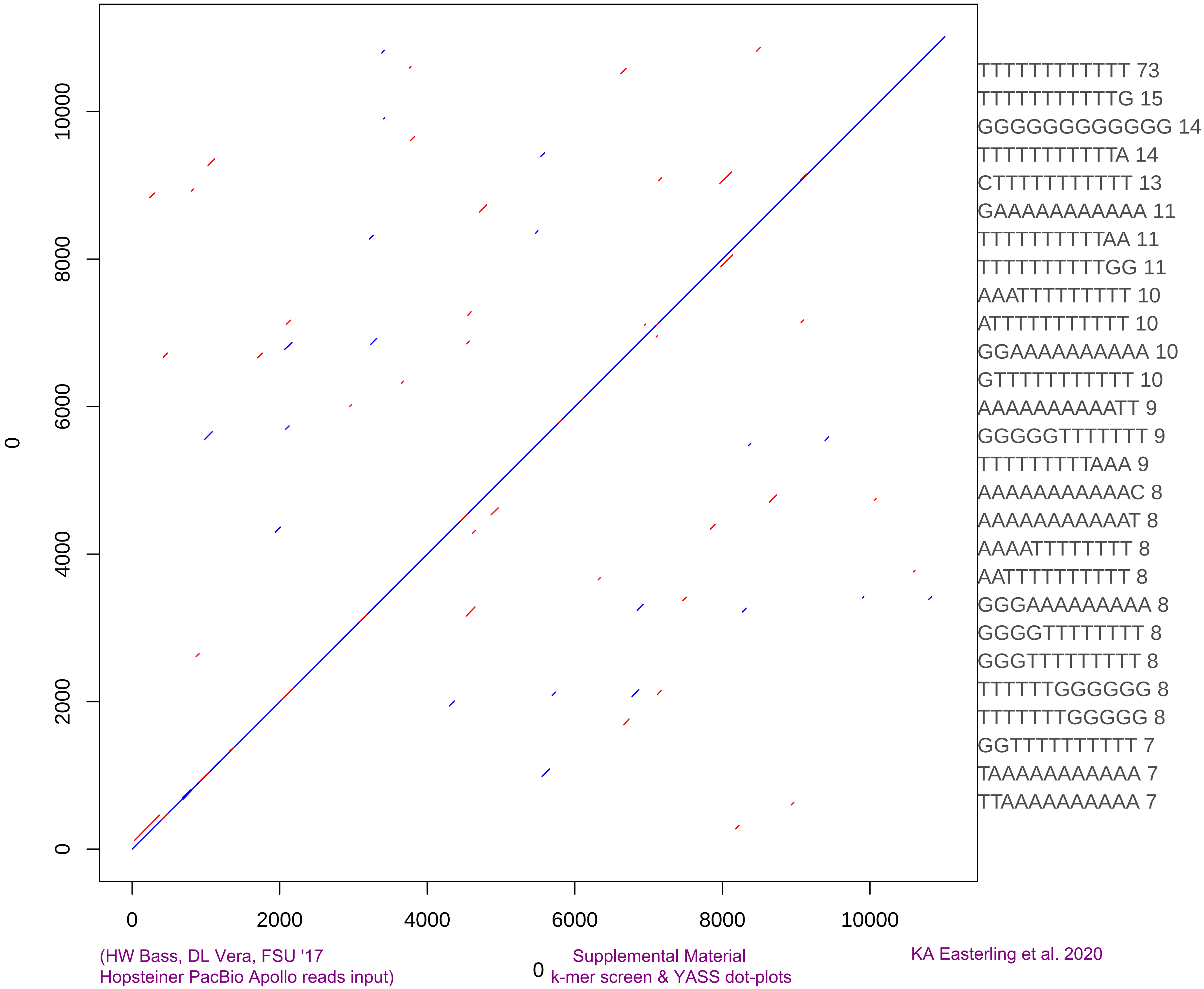

(HW Bass, DL Vera, FSU '17
Hopsteiner PacBio Apollo reads input)

Supplemental Material
k-mer screen & YASS dot-plots

KA Easterling et al. 2020

>m141122_040740_42134_c100732202550000001823141405141561_s1_p0/54925/0_6055

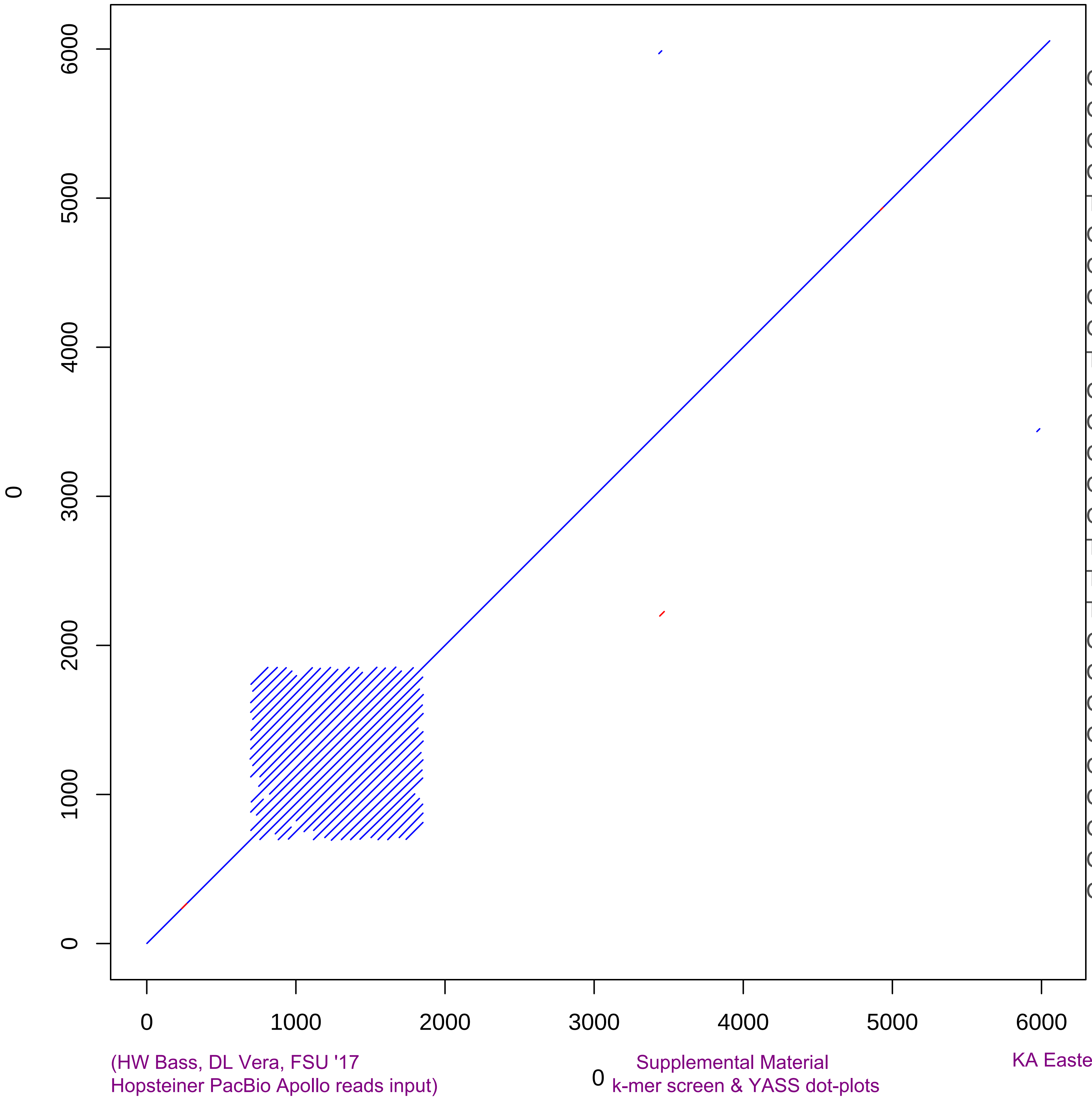

CGTCCGGGGTGG 9
CGTTCGTCCGGG 9
GTCCGGGGTGGG 9
CCGGGGTGGGAC 8
TCGTTCGTCCGG 8
CGGGGTGGGACC 7
GGCTCGGTCCTC 7
GGGGTGGGACCG 7
GGGTGGGACCGG 7
TCCGGGGTGGGA 7
CGGTCCTCTTAT 6
CTCGGTCCTCTT 6
GCTCGGTCCTCT 6
GGTCCTCTTATT 6
GTTCGTCCGGGG 6
TCGGTCCTCTTA 6
TCGTCCGGGGTG 6
TTCGTCCGGGGT 6
CGTCGTTCGTCC 5
CTCTTATTTTTA 5
CTTATTTTTACT 5
GGGACCGGCCTT 5
GGTGGGACCGGC 5
GTCGTTCGTCCG 5
CCCGGACGTCGT 4
CCGGACGTCGTT 4
CCTCTTATTTTT 4

>m141122_040740_42134_c100732202550000001823141405141561_s1_p0/67264/0_7713

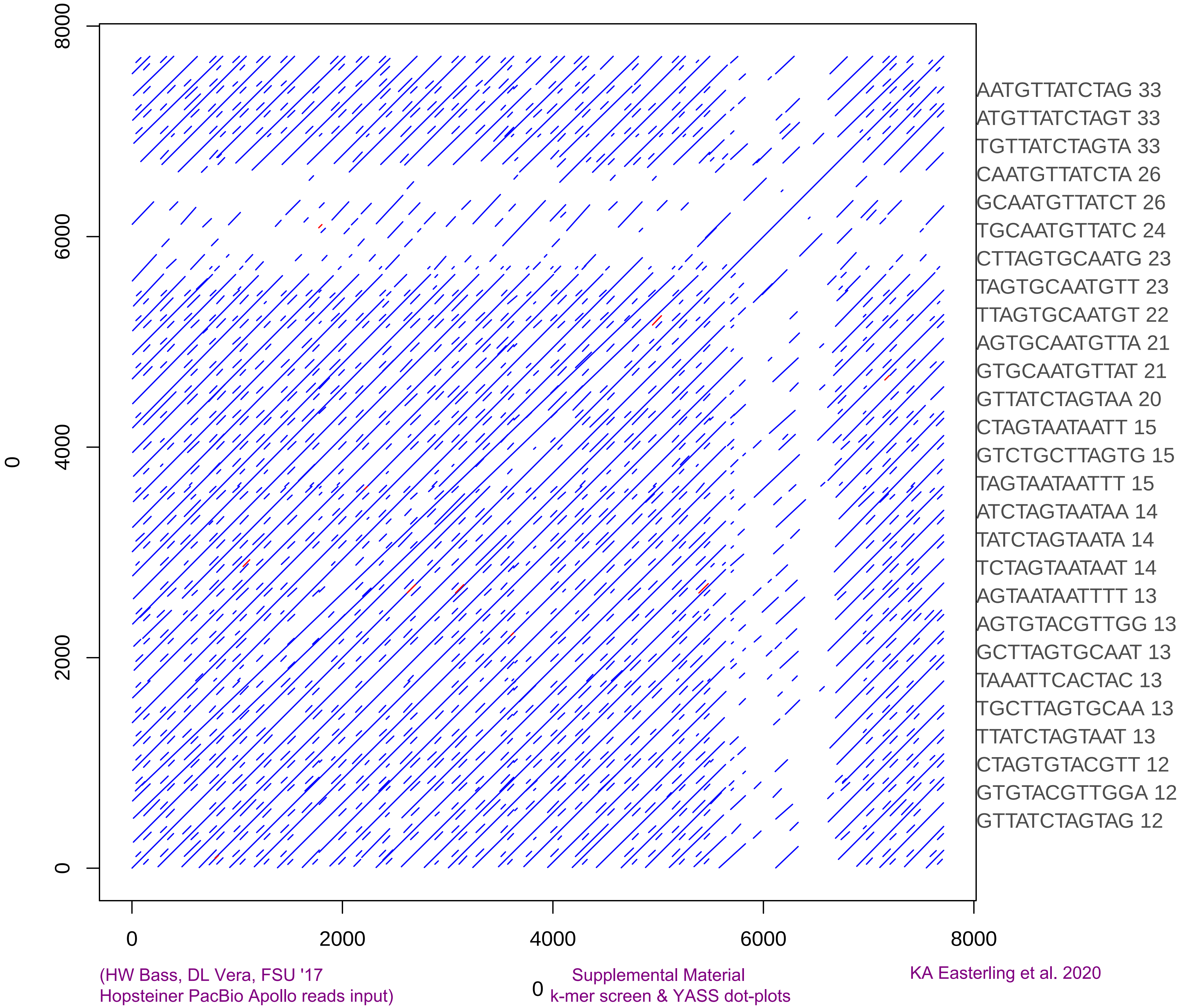

>m141122_040740_42134_c100732202550000001823141405141561_s1_p0/72716/0_6253

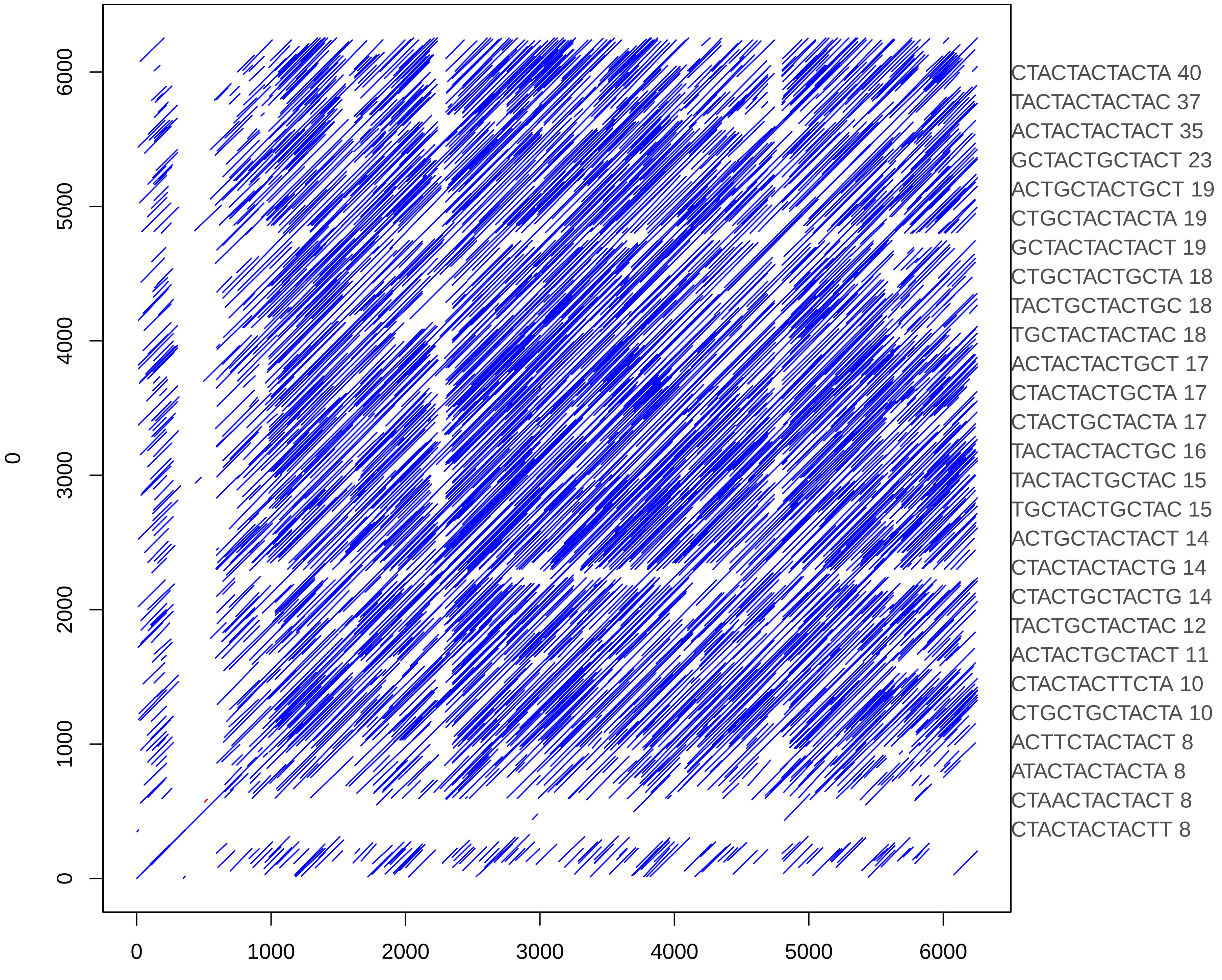

>m141122_040740_42134_c100732202550000001823141405141561_s1_p0/75306/0_11890

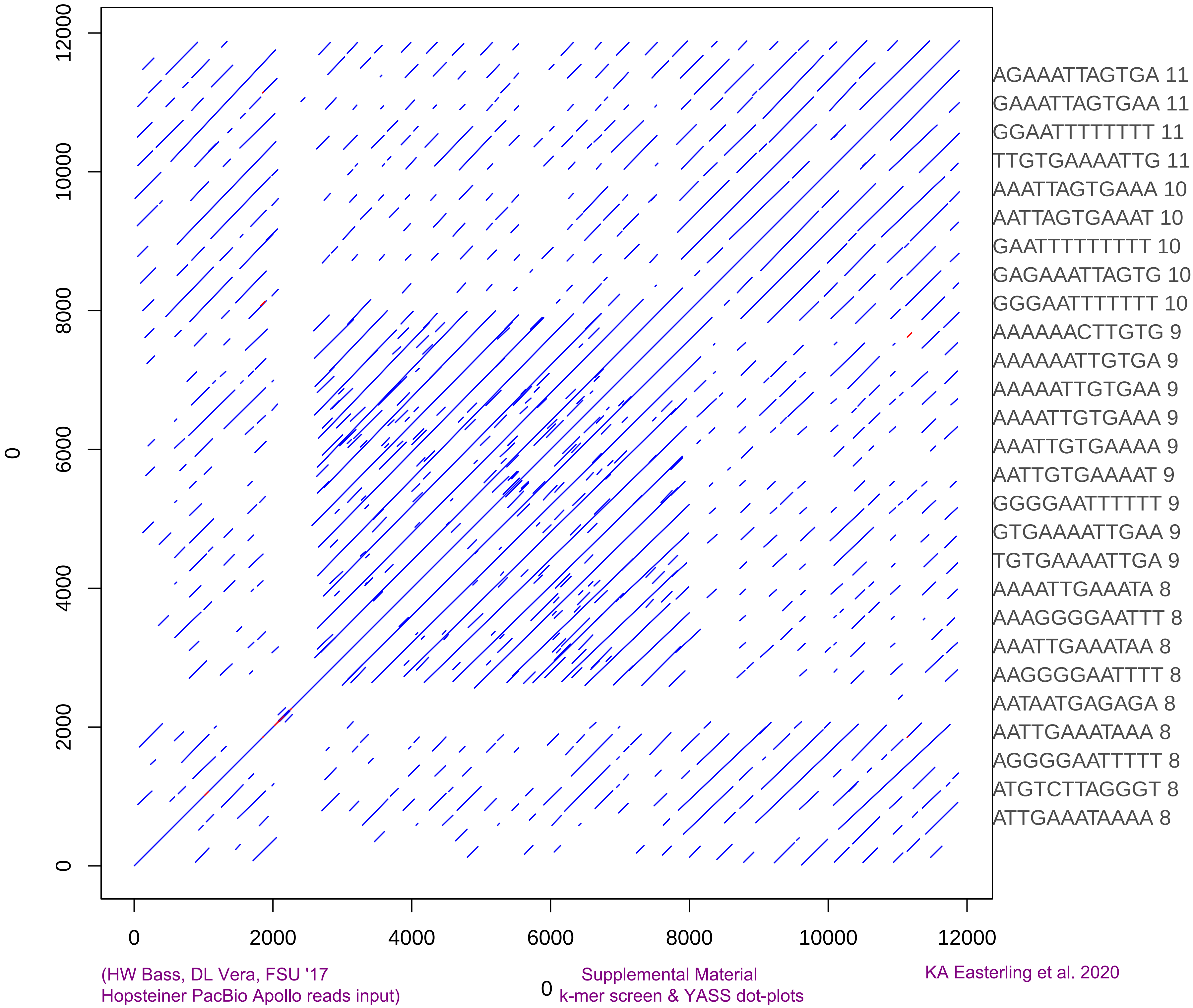

>m141122_040740_42134_c100732202550000001823141405141561_s1_p0/7964/4132_10435

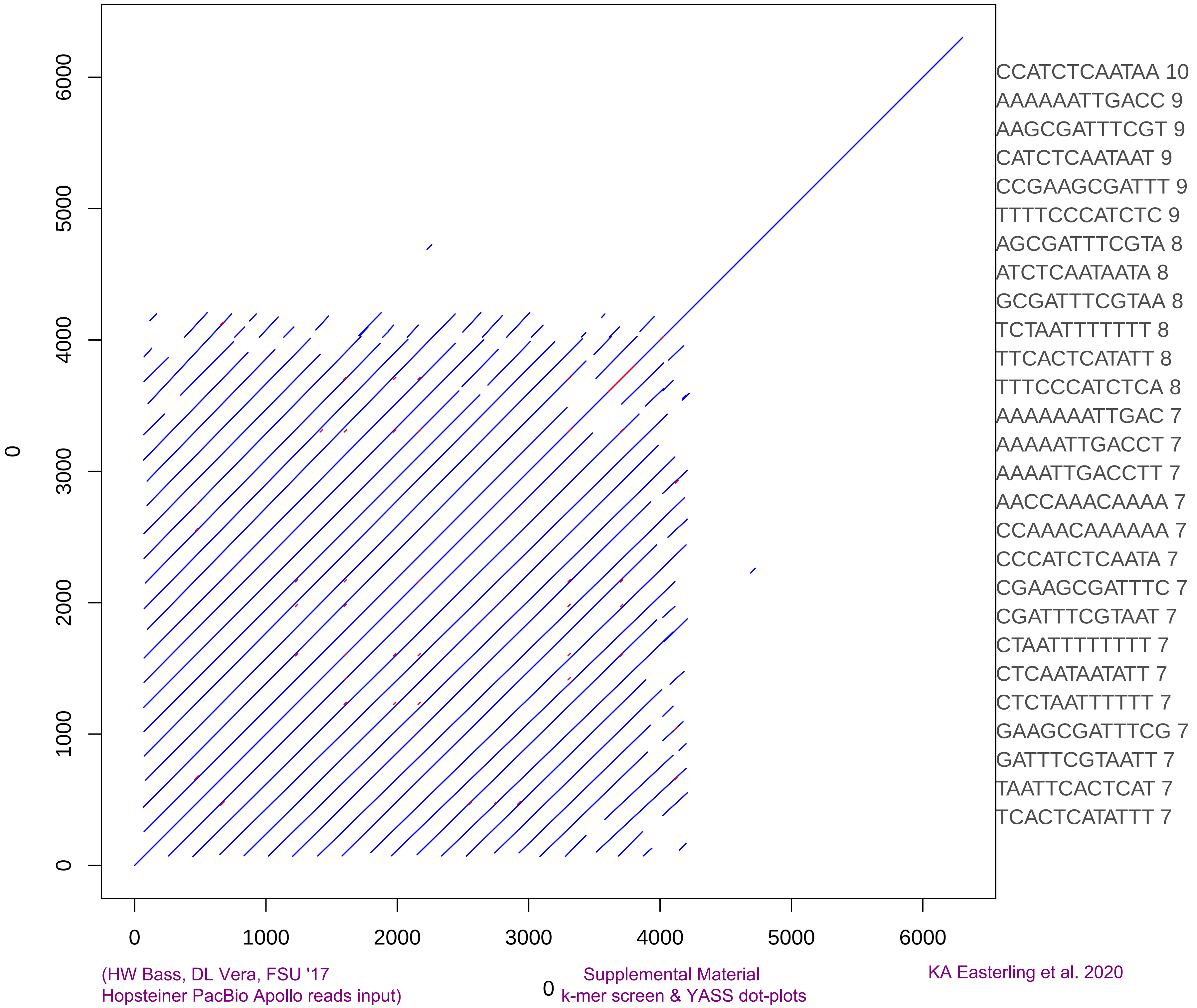

>m141122_040740_42134_c100732202550000001823141405141561_s1_p0/82939/8045_13490

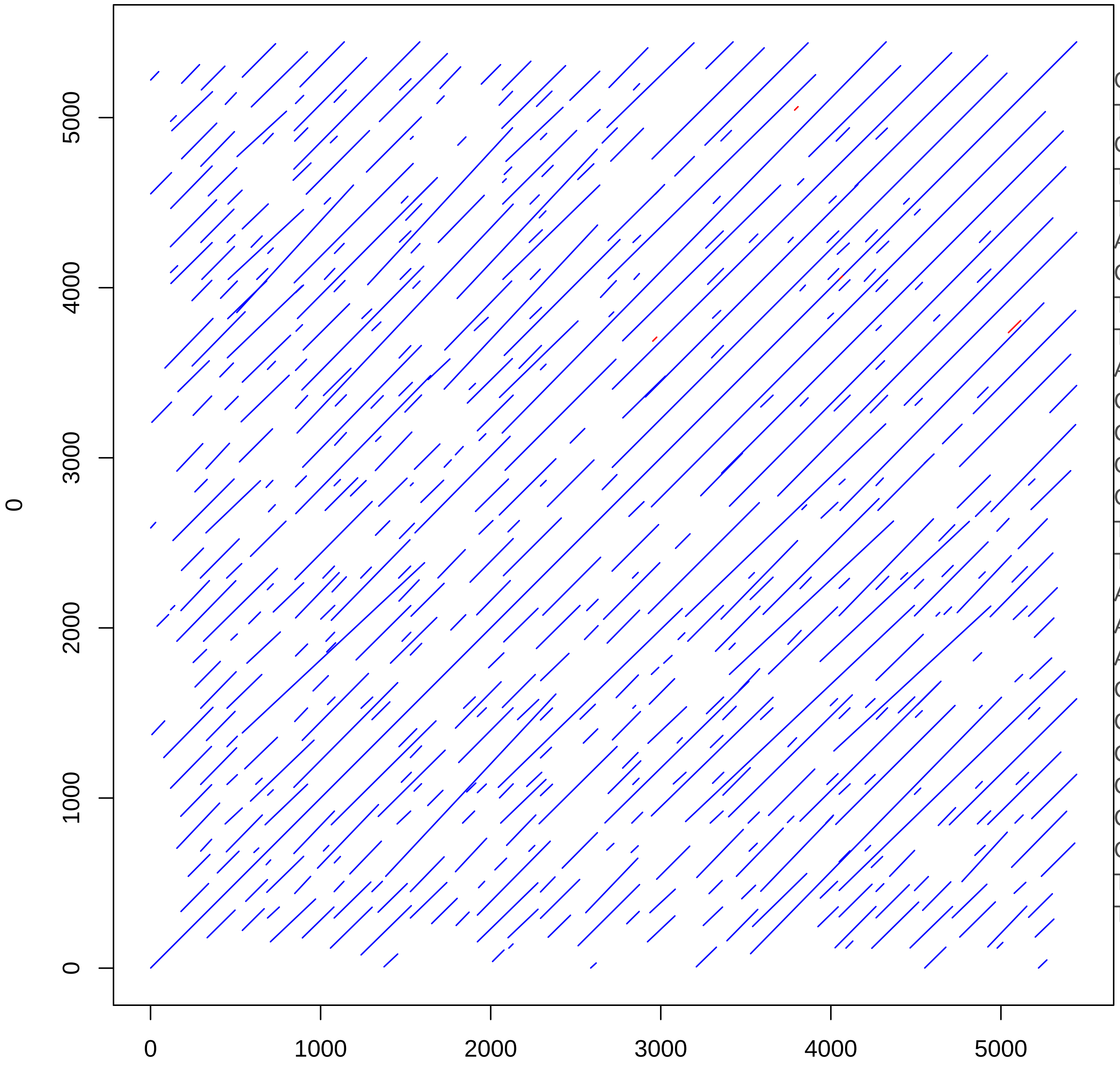

>m141122_040740_42134_c100732202550000001823141405141561_s1_p0/85685/8400_16301

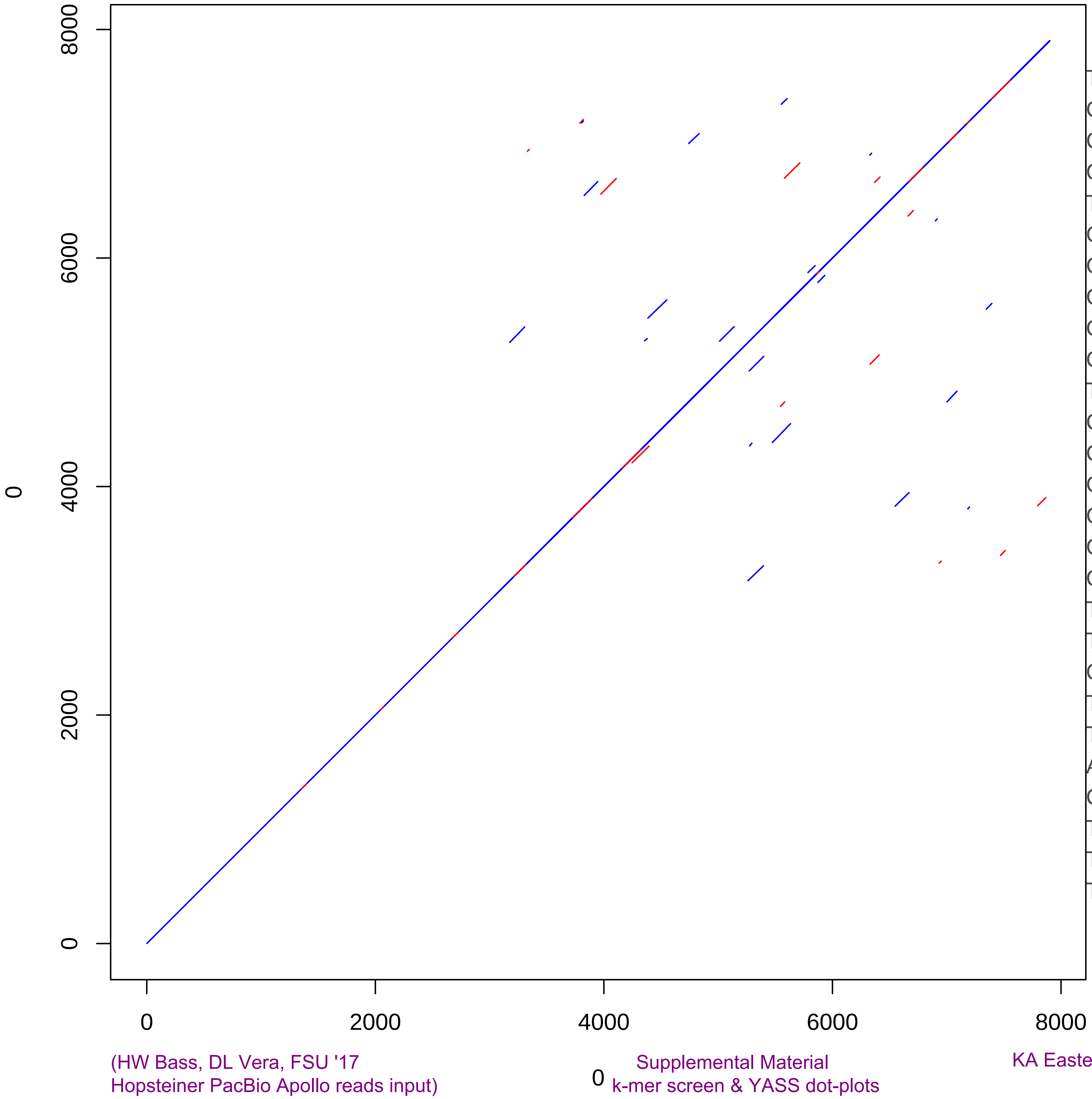

TTTTTTTTTTTT 91
GGGGGGGGGGGG 37
CCCCCCCCCCCC 12
GTTTTTTTTTTT 12
TTTTTTTTTTTA 12
GGTTTTTTTTTT 11
GAAAAAAAAAAA 10
GGGGGGGTTTTT 10
GGGGGTTTTTTT 10
GGGTTTTTTTTT 10
TTTTTTTTTTAA 10
CTTTTTTTTTTT 9
GGGGGGGGGGTT 9
GGGGGGGGTTTT 9
GGGGGGTTTTTT 9
GGGGGGGGGTTT 8
GGGGTTTTTTTT 8
TTTTTTTTTAAA 8
TTTTTTTTTTTC 8
GGGGGGGGGGGT 7
TGGGGGGGGGGG 7
TTTTTTTTTCCC 7
AAAAAAAAAAAC 6
CCTTTTTTTTTT 6
TTTTCTTTTTTT 6
TTTTTTTTTTCC 6
TTTTTTTTTTTG 6

>m141122_040740_42134_c100732202550000001823141405141561_s1_p0/9189/13042_19218

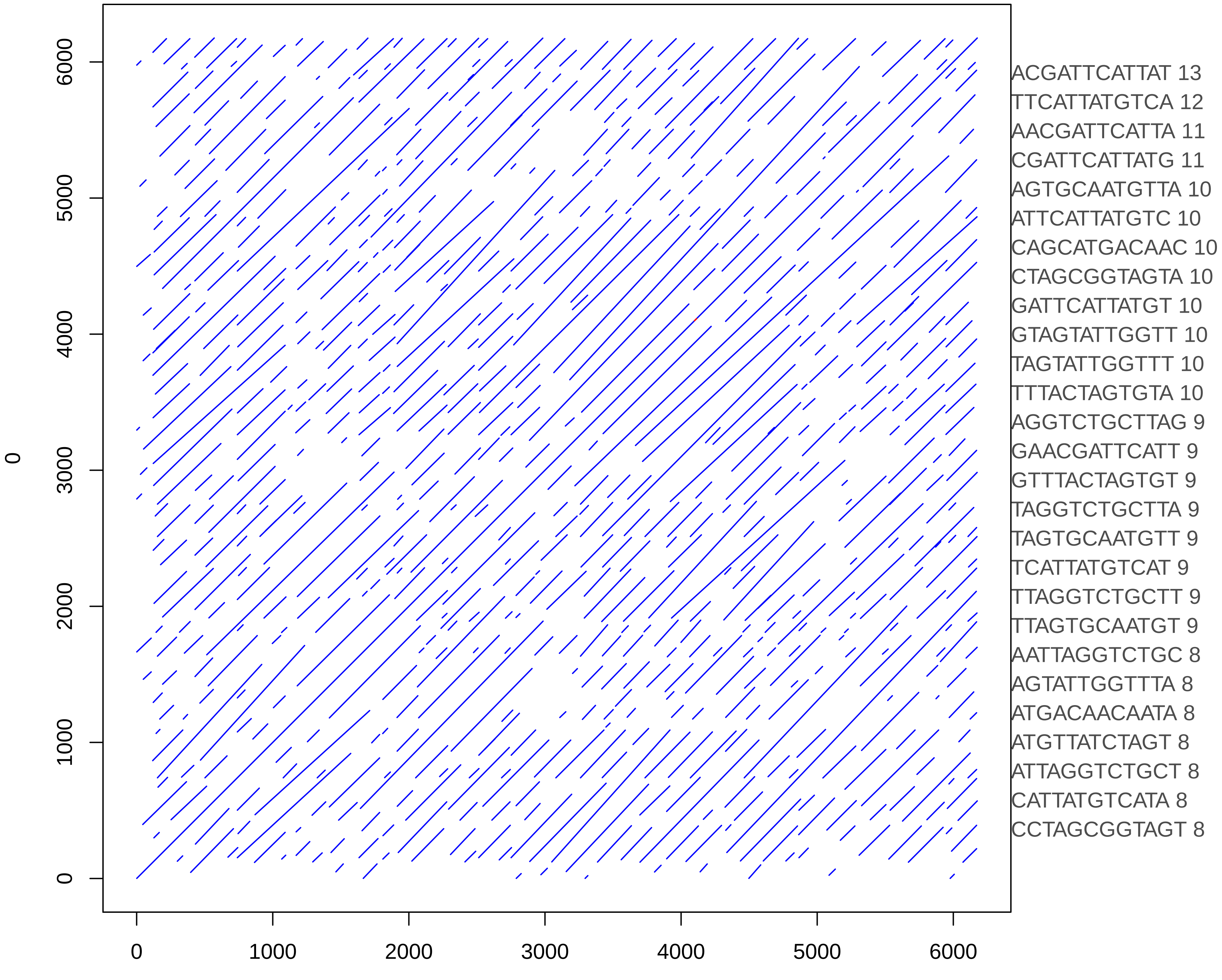

>m141122_040740_42134_c100732202550000001823141405141561_s1_p0/97529/0_9071

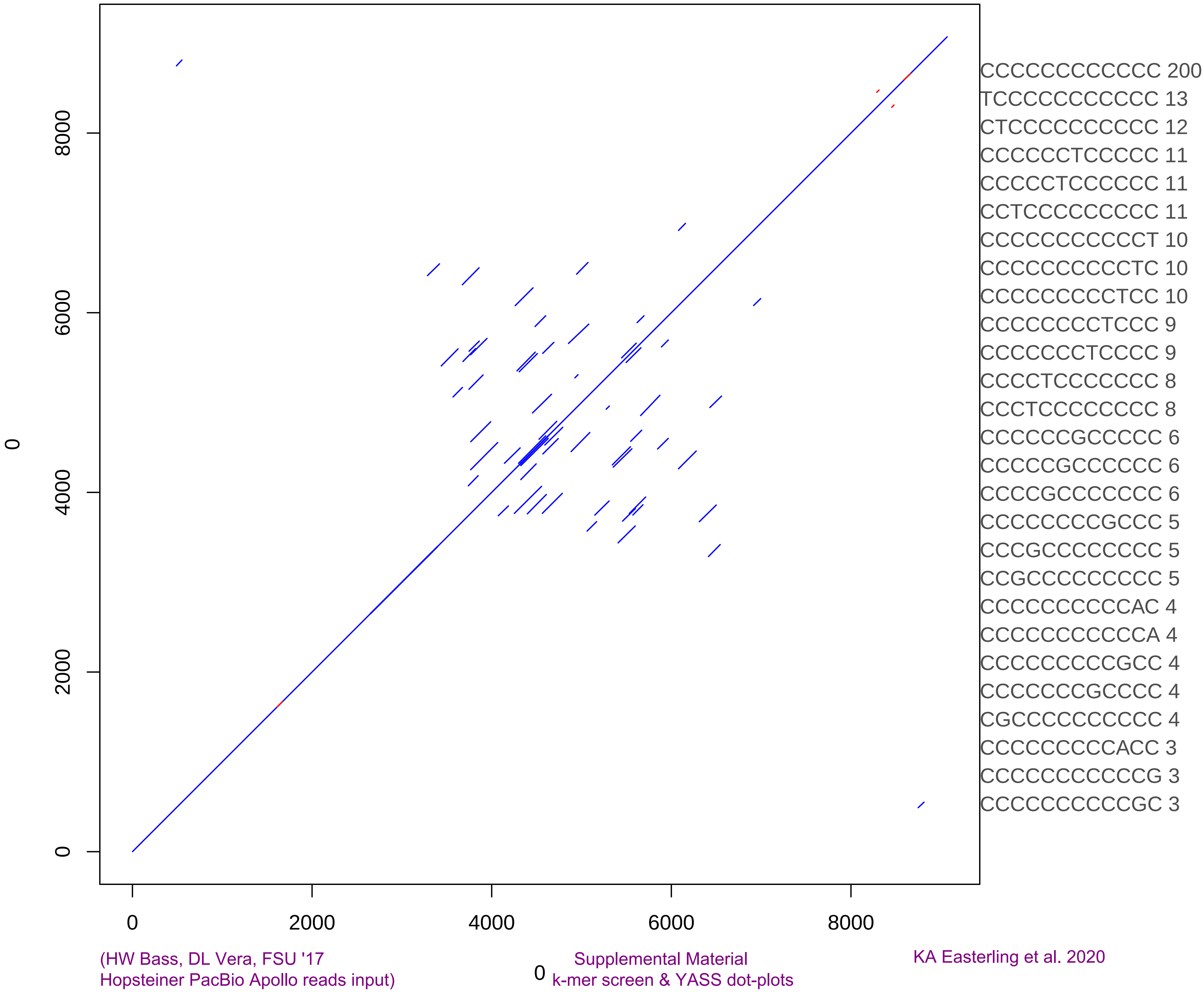

>m141122_040740_42134_c100732202550000001823141405141561_s1_p0/99726/4250_13118

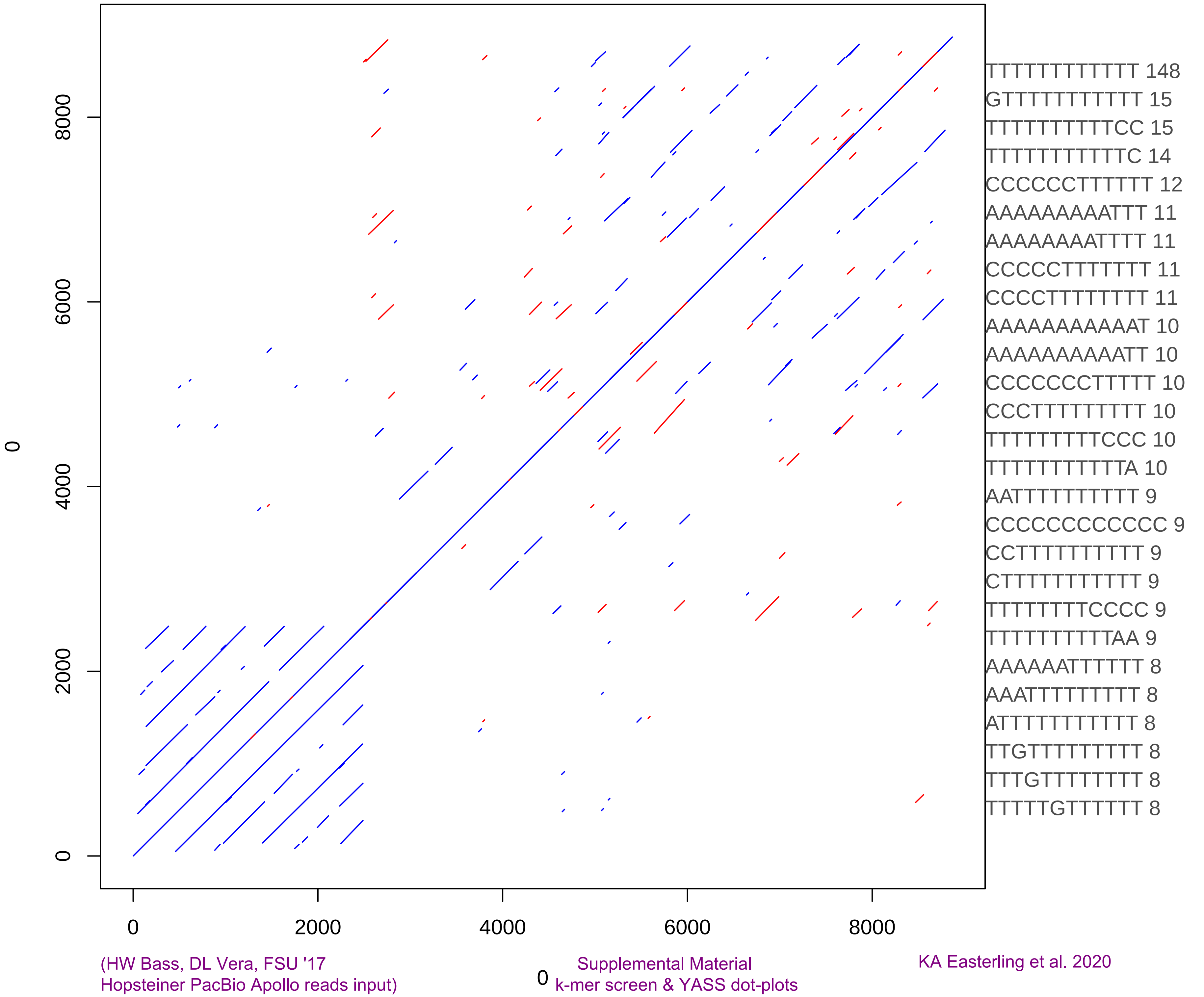

>m141122_062923_42134_c100732202550000001823141405141562_s1_p0/102924/0_10184

Representative (only one) Read (Table 1): HuluTR055-r292, 1 ppm, 64% A+T, GenBank Acc. MN537566

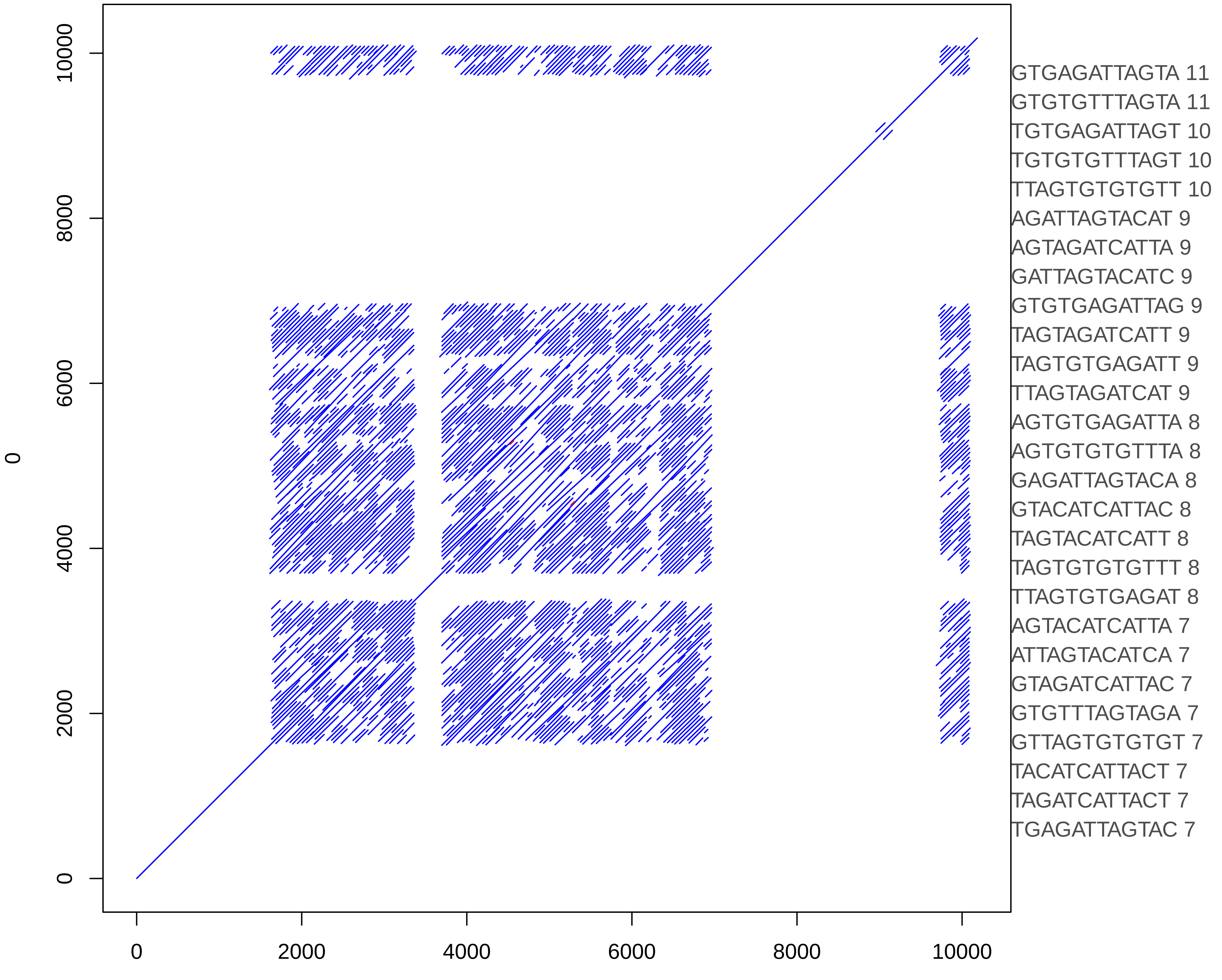

>m141122_062923_42134_c100732202550000001823141405141562_s1_p0/105460/3717_12942

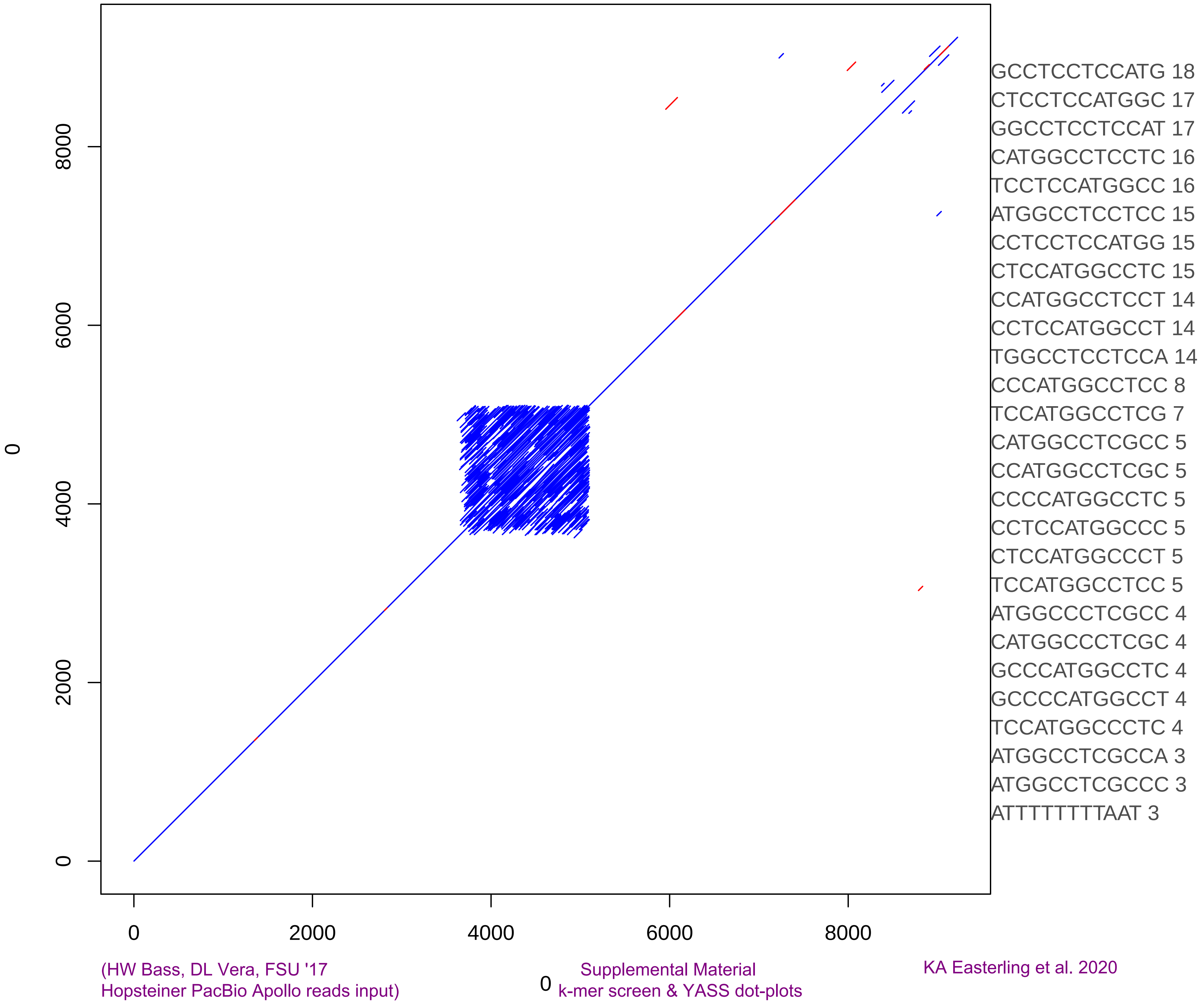

>m141122_062923_42134_c100732202550000001823141405141562_s1_p0/106632/12891_18868

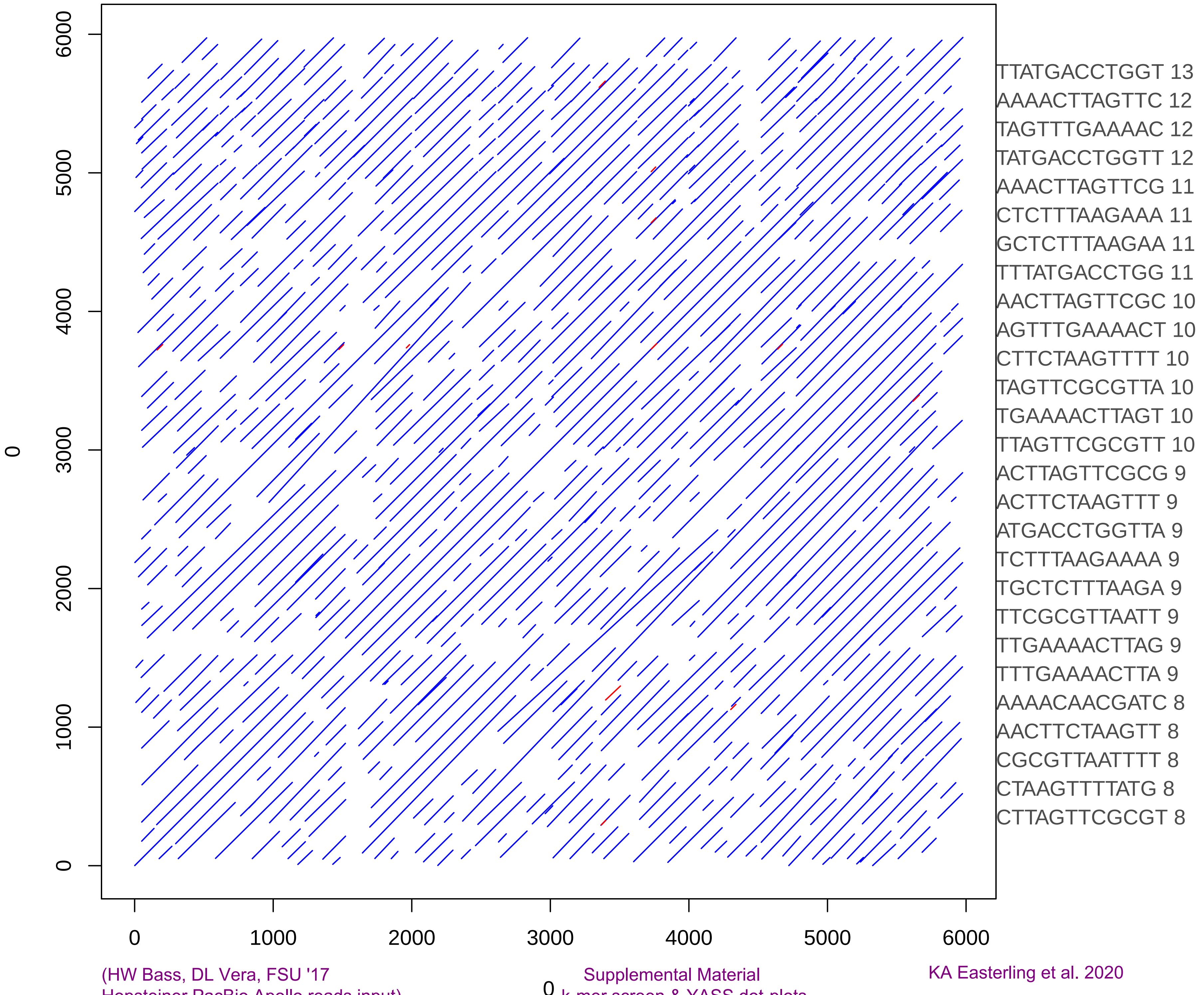

>m141122_062923_42134_c100732202550000001823141405141562_s1_p0/107661/195_7978

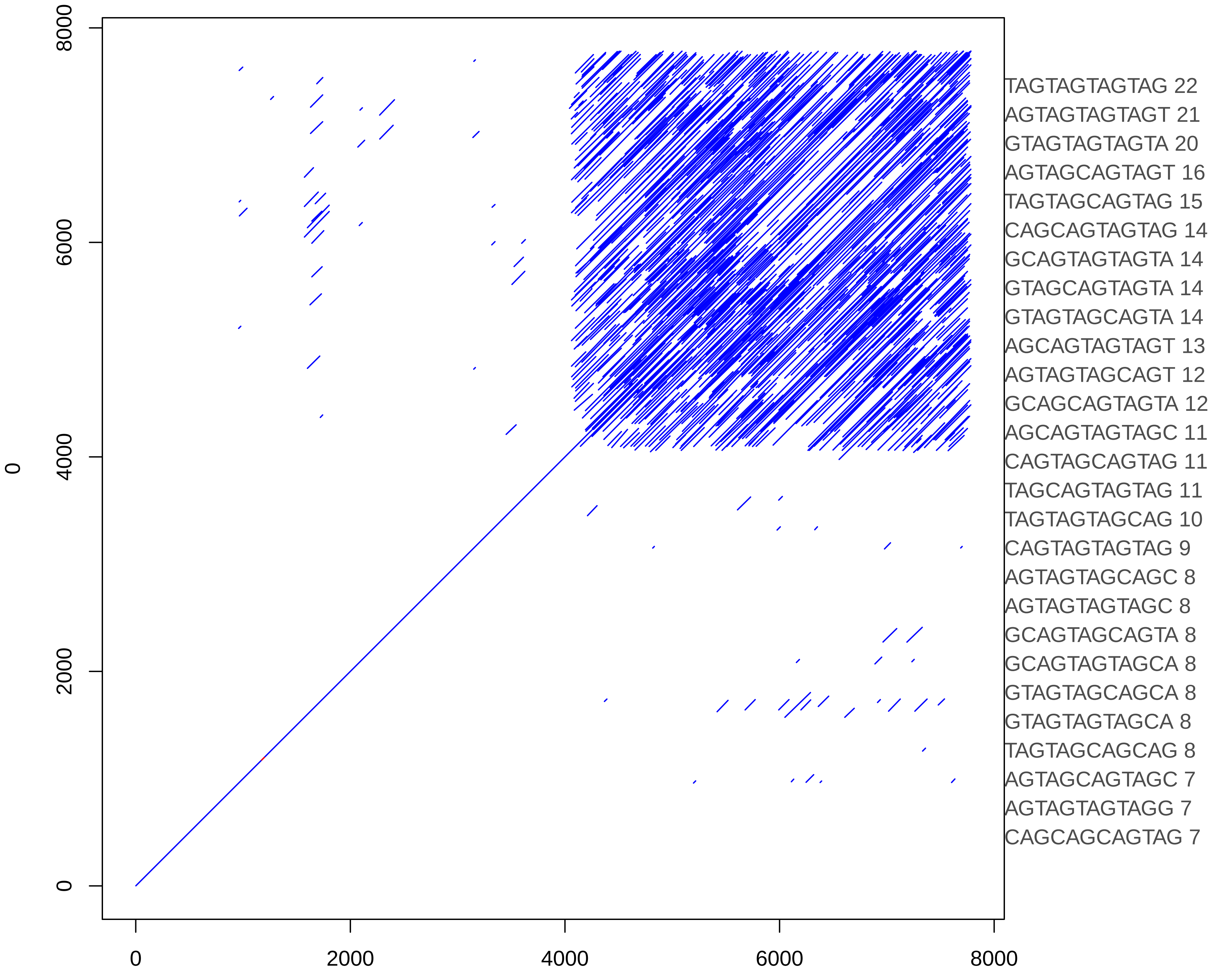

>m141122_062923_42134_c100732202550000001823141405141562_s1_p0/110710/11518_18579

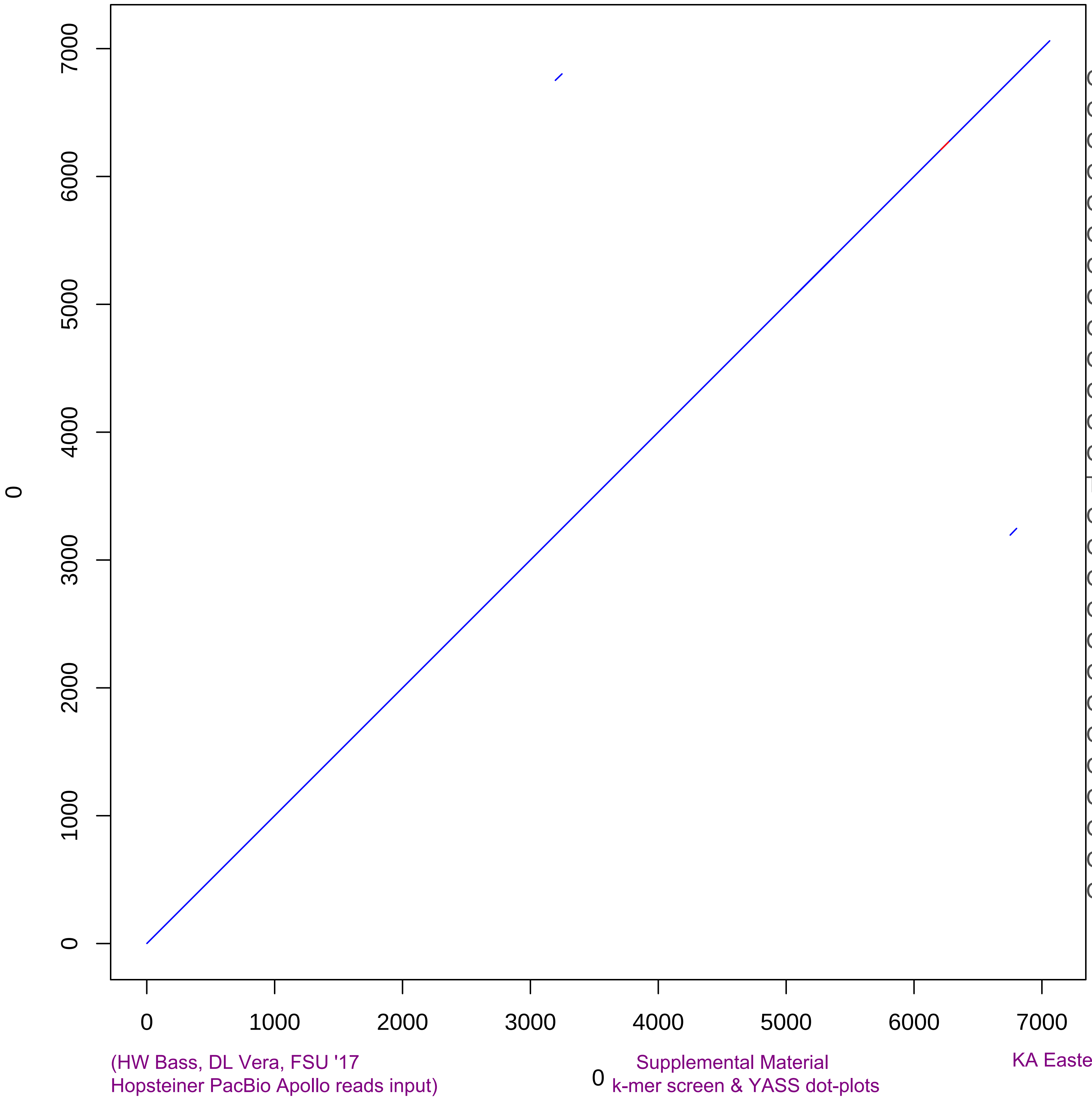

GGGGGGGGGGGG 49
GGGGGGCGGGGG 9
GGGGGGGCGGGG 9
GGGGGGGGCGGG 8
GGGGGGGGGGGC 8
GGGGGCGGGGGG 7
GGGGGGGGGCGG 7
CGGGGGGGGGGG 6
GGCGGGGGGGGG 6
GGGCGGGGGGGG 6
GGGGCGGGGGGG 6
GGGGGGGGGGCG 6
GCGGGGGGGGGG 5
TGGGGGGGGGGG 4
GCGGGGGGGCGG 3
GGGCGGGGGGGC 3
GGGGGGGGTGGG 3
GTGGGGGGGGGG 3
CGGGGGGGCGGG 2
GCGCGGGGGGGG 2
GGCGCGGGGGGG 2
GGGCGCGGGGGG 2
GGGCGGGGGCGG 2
GGGGCGGGGGCG 2
GGGGGCGGGGGC 2
GGGGGGGGGTGG 2
GGGGGGGTGGGG 2

>m141122_062923_42134_c100732202550000001823141405141562_s1_p0/112866/6932_14153

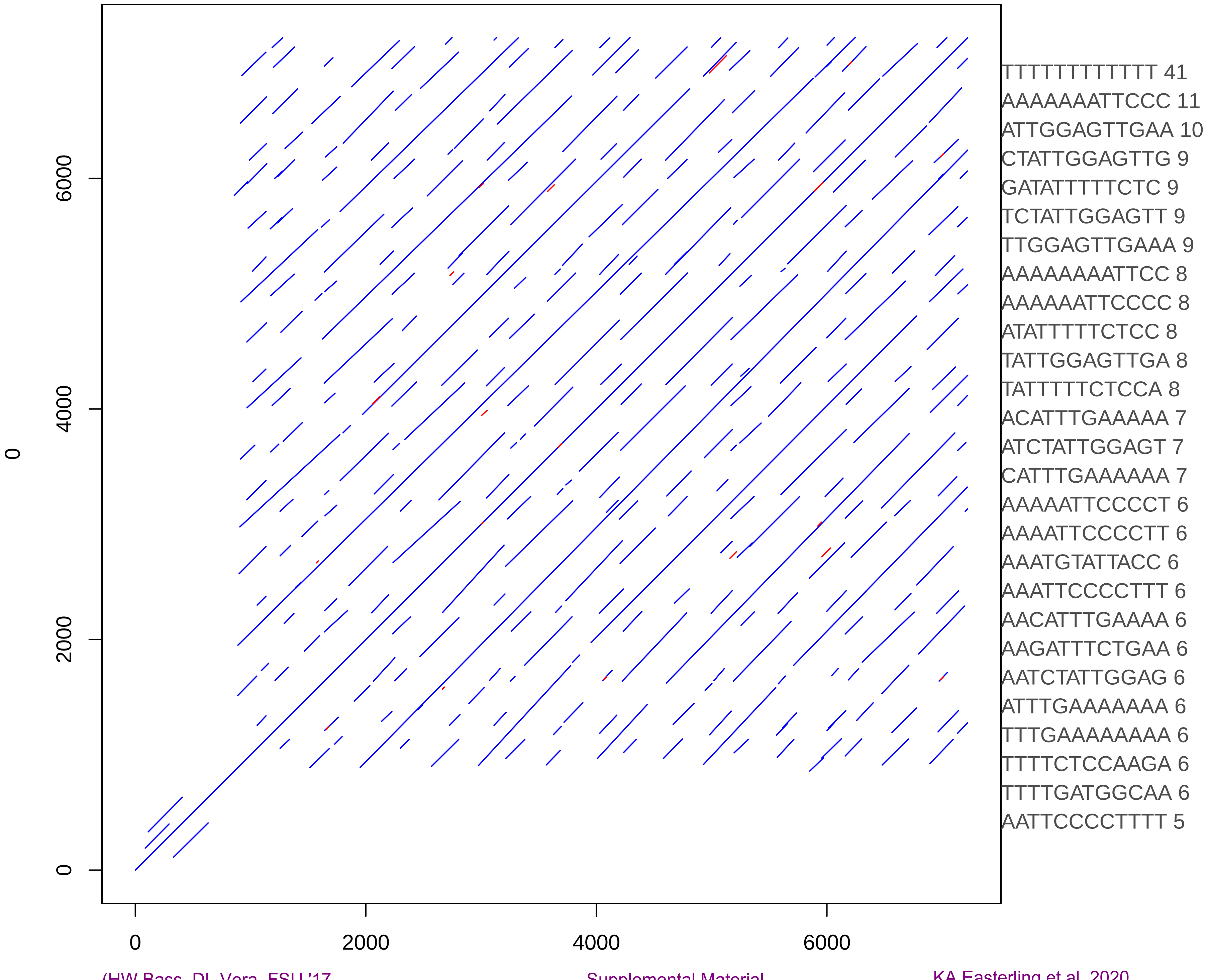

>m141122_062923_42134_c100732202550000001823141405141562_s1_p0/114436/9915_16829

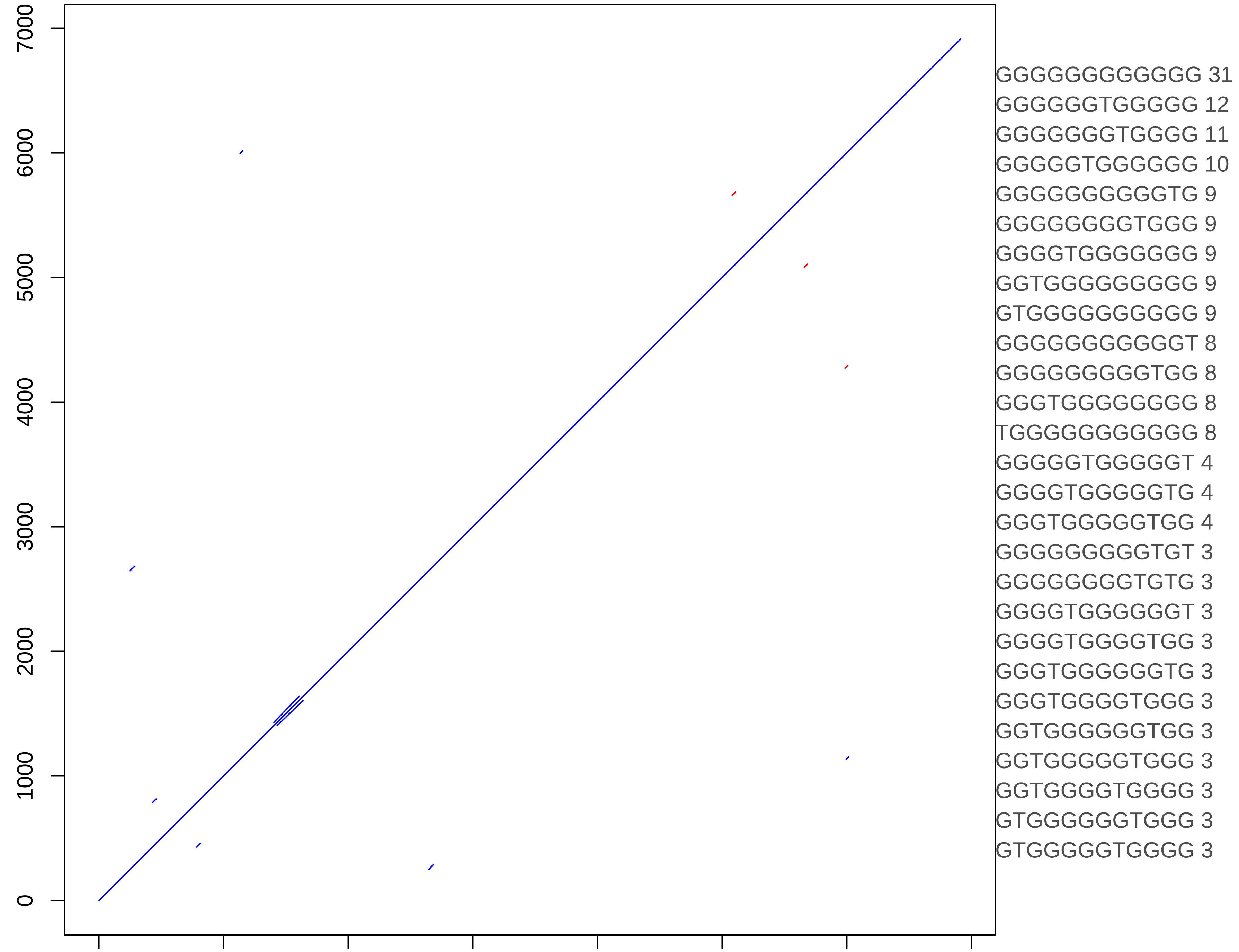

>m141122_062923_42134_c100732202550000001823141405141562_s1_p0/126666/0_9694

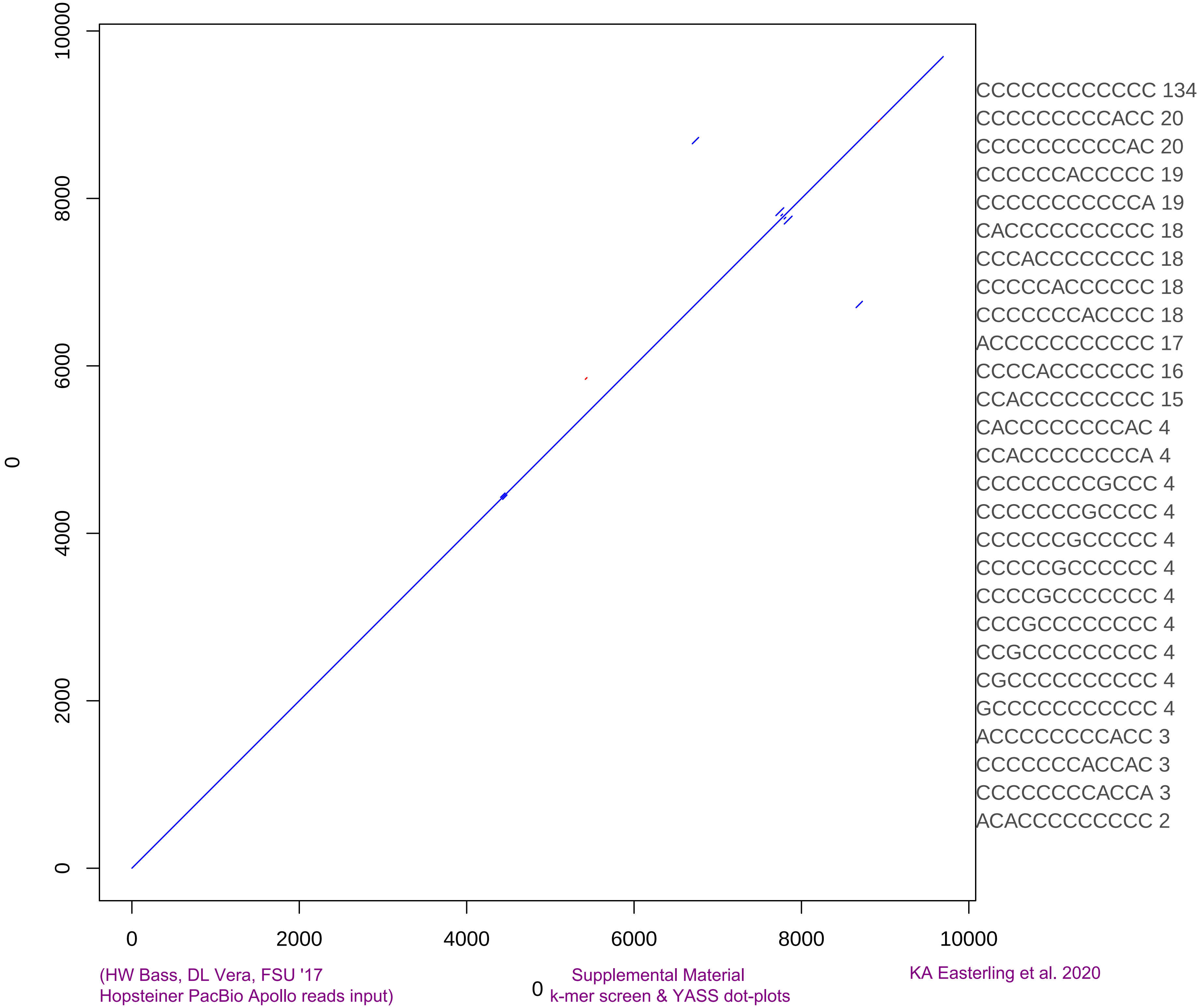

>m141122_062923_42134_c100732202550000001823141405141562_s1_p0/129318/4906_15189

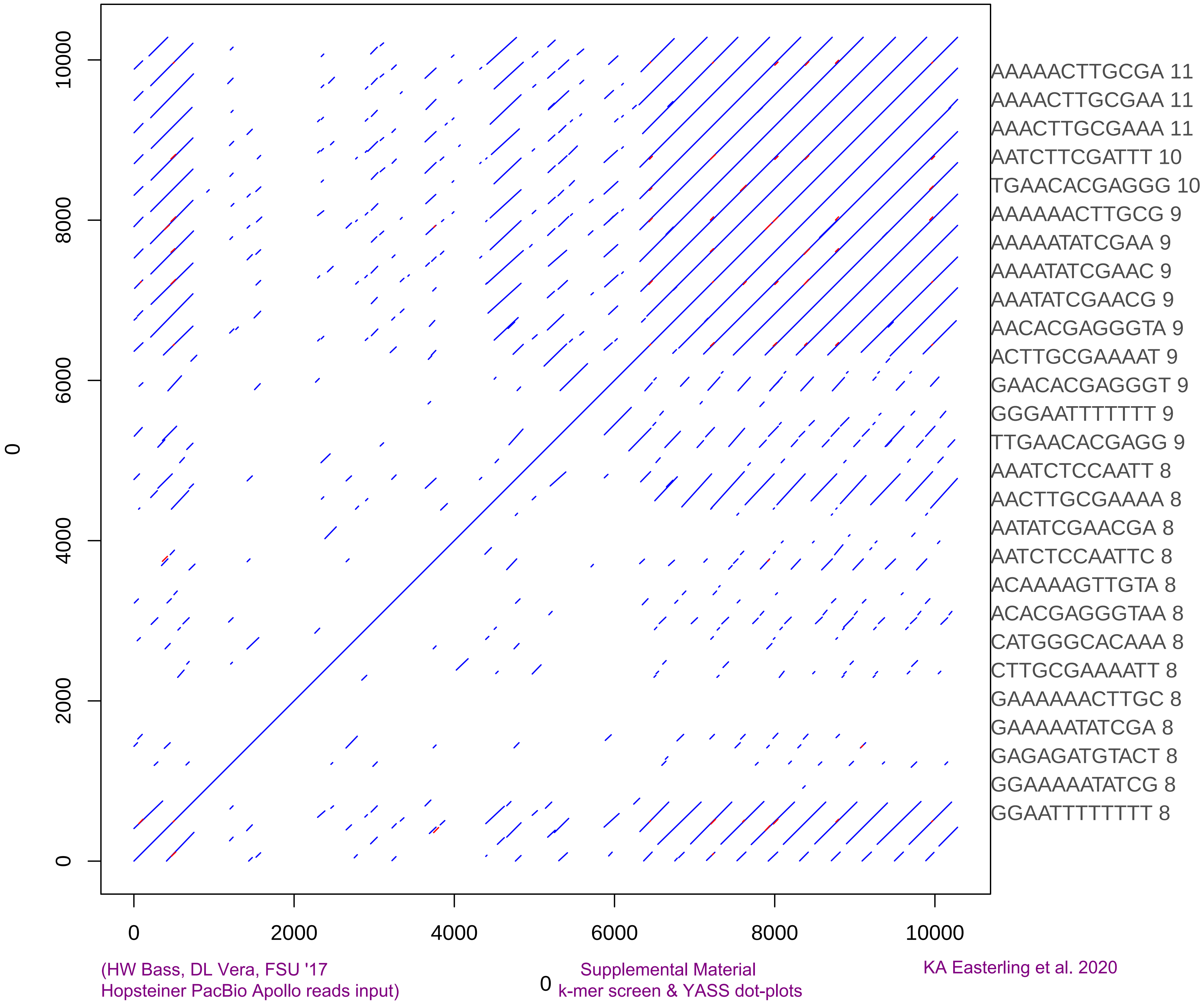

>m141122_062923_42134_c100732202550000001823141405141562_s1_p0/131226/0_7768

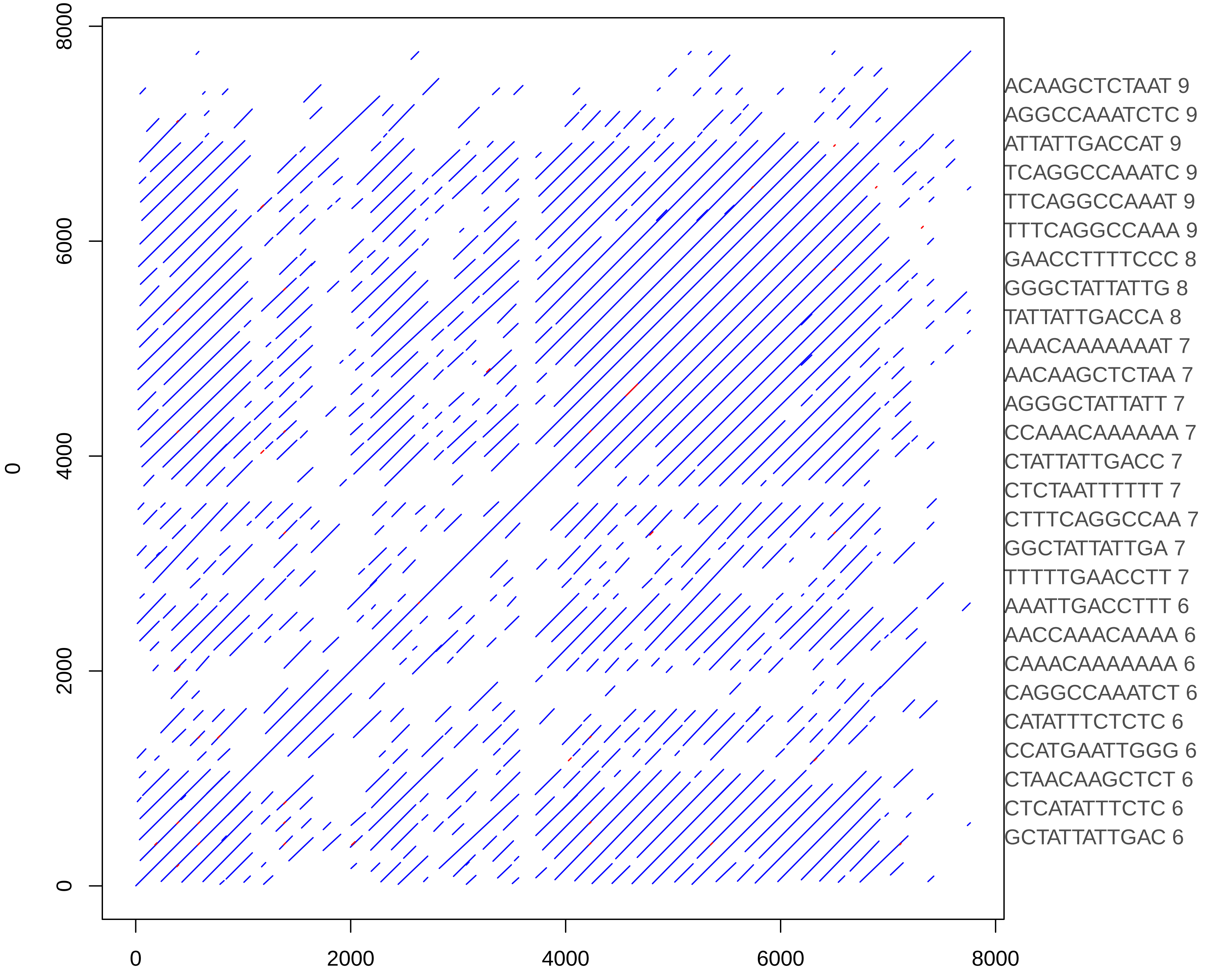

>m141122_062923_42134_c100732202550000001823141405141562_s1_p0/133330/13120_18663

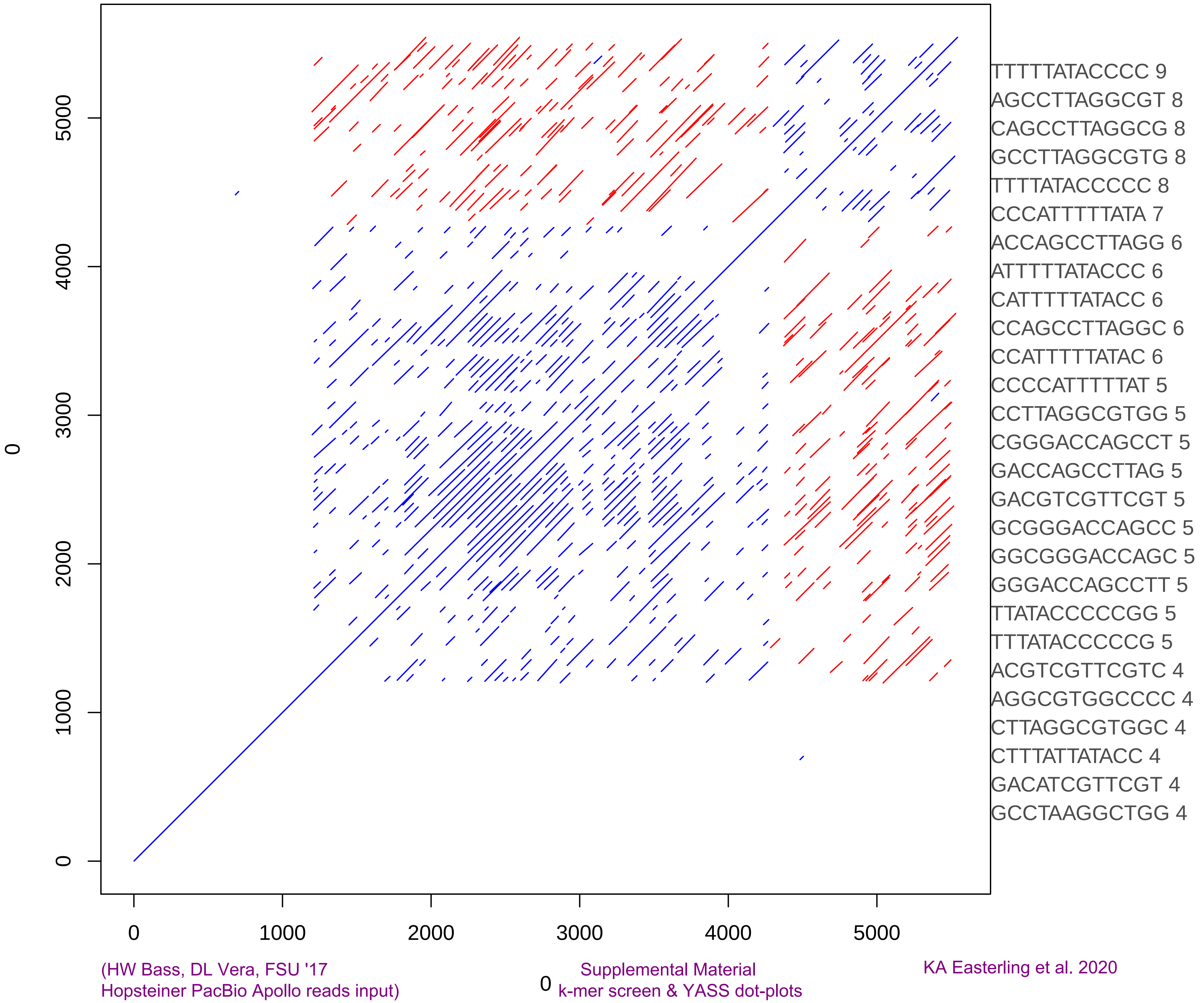

>m141122_062923_42134_c100732202550000001823141405141562_s1_p0/134367/14983_22280

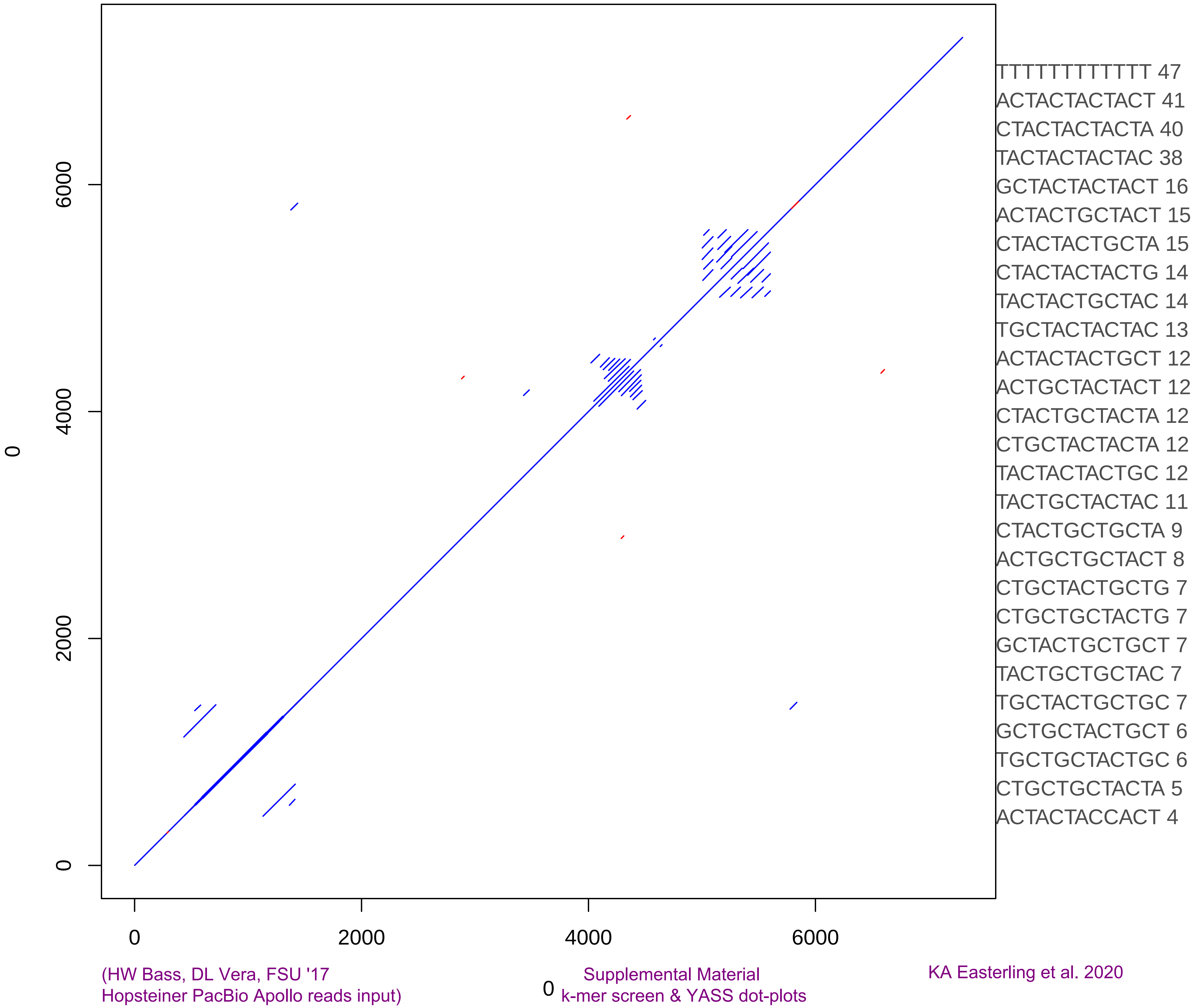

>m141122_062923_42134_c100732202550000001823141405141562_s1_p0/134912/17578_25028

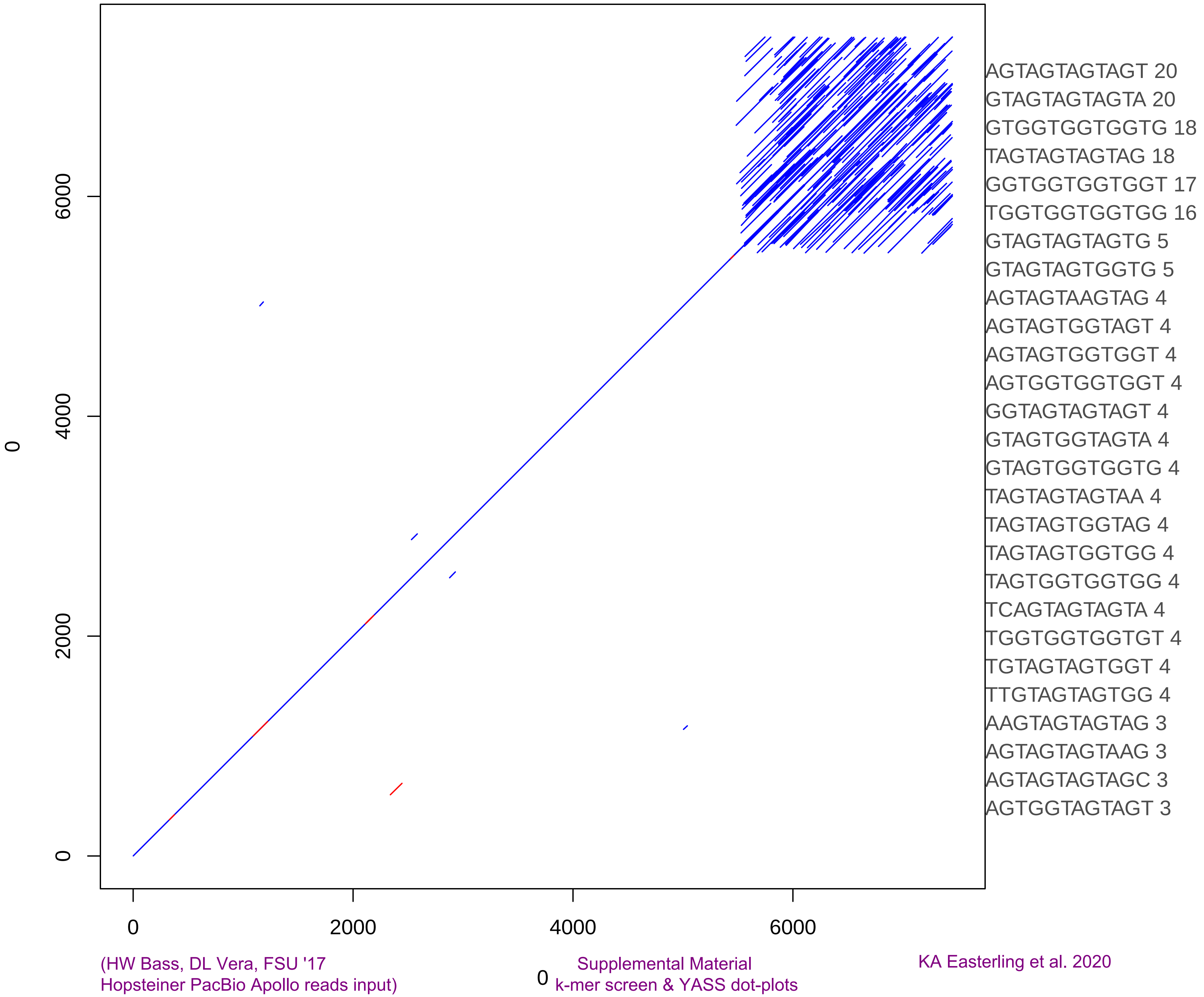

>m141122_062923_42134_c100732202550000001823141405141562_s1_p0/135186/261_7892

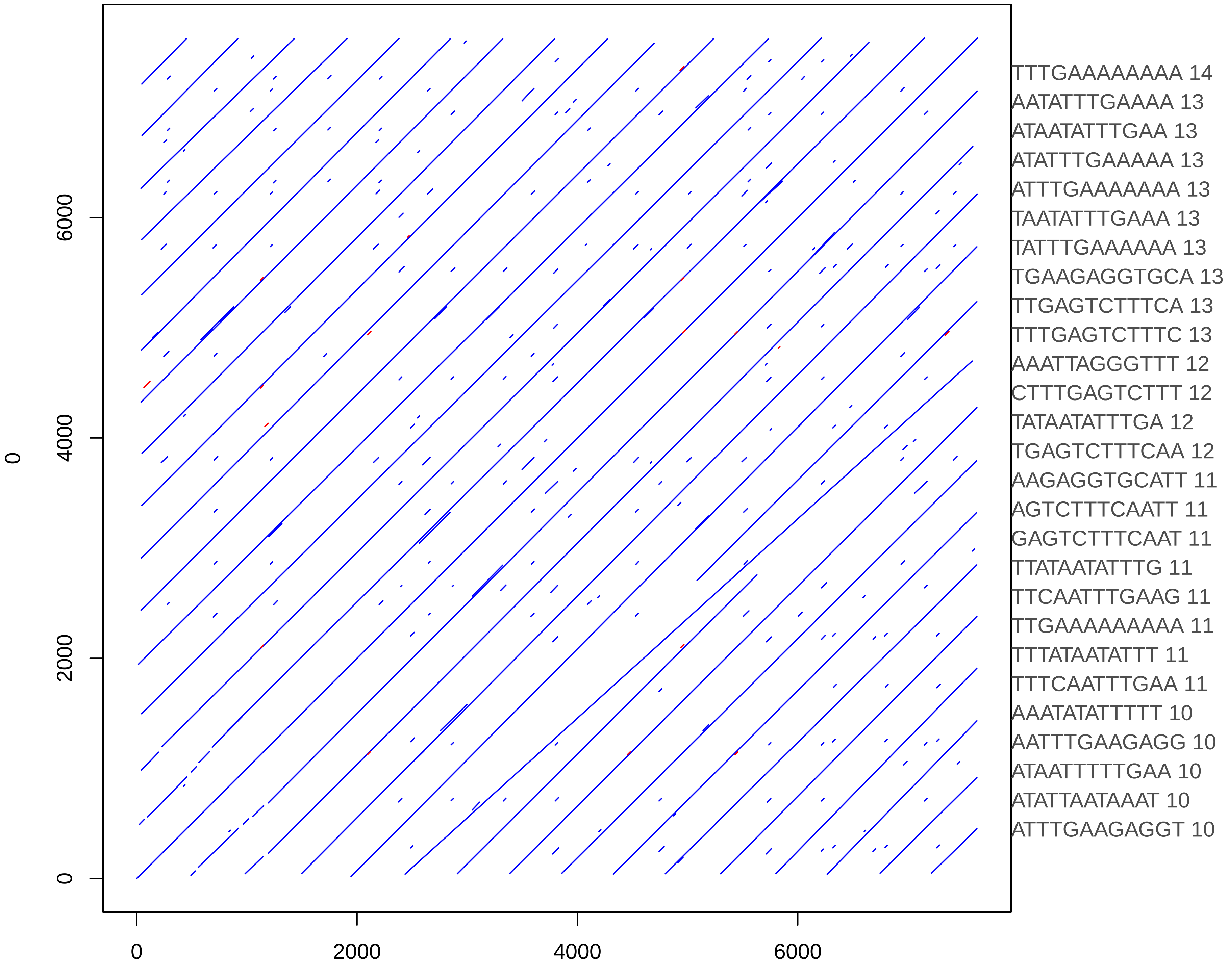

>m141122_062923_42134_c100732202550000001823141405141562_s1_p0/135514/4559_11293

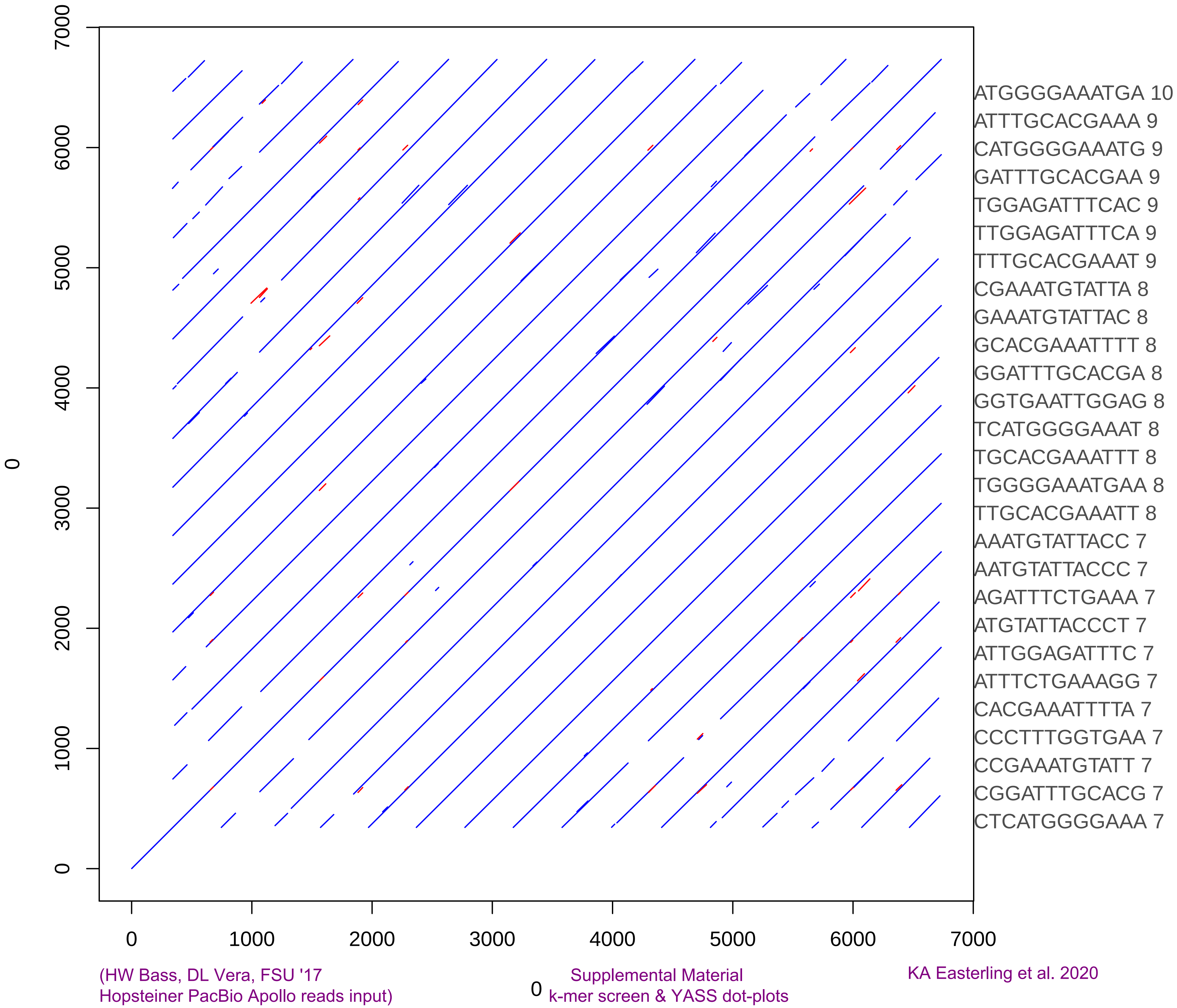

>m141122_062923_42134_c100732202550000001823141405141562_s1_p0/140960/10145_15204

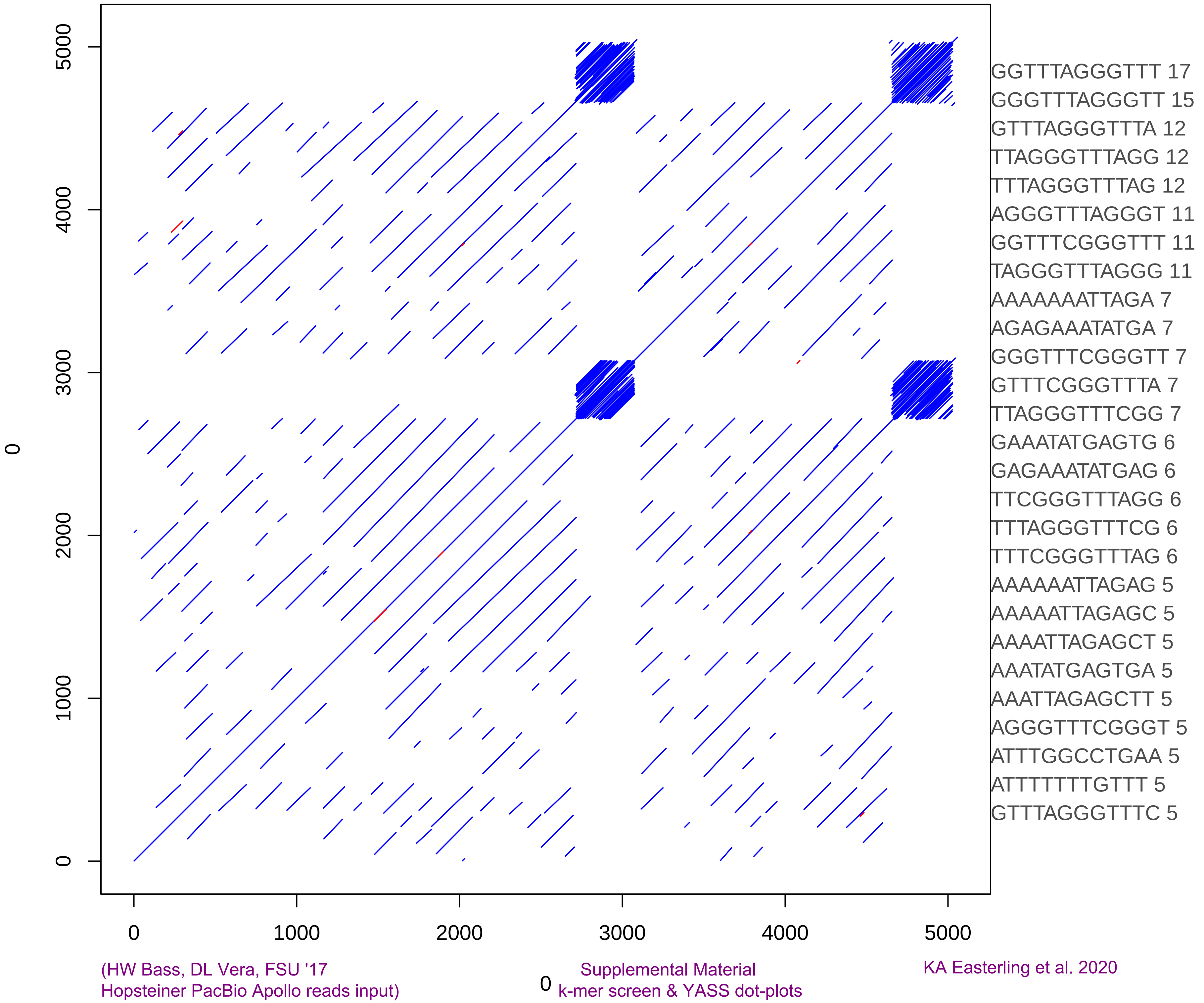

>m141122_062923_42134_c100732202550000001823141405141562_s1_p0/142455/1335_9327

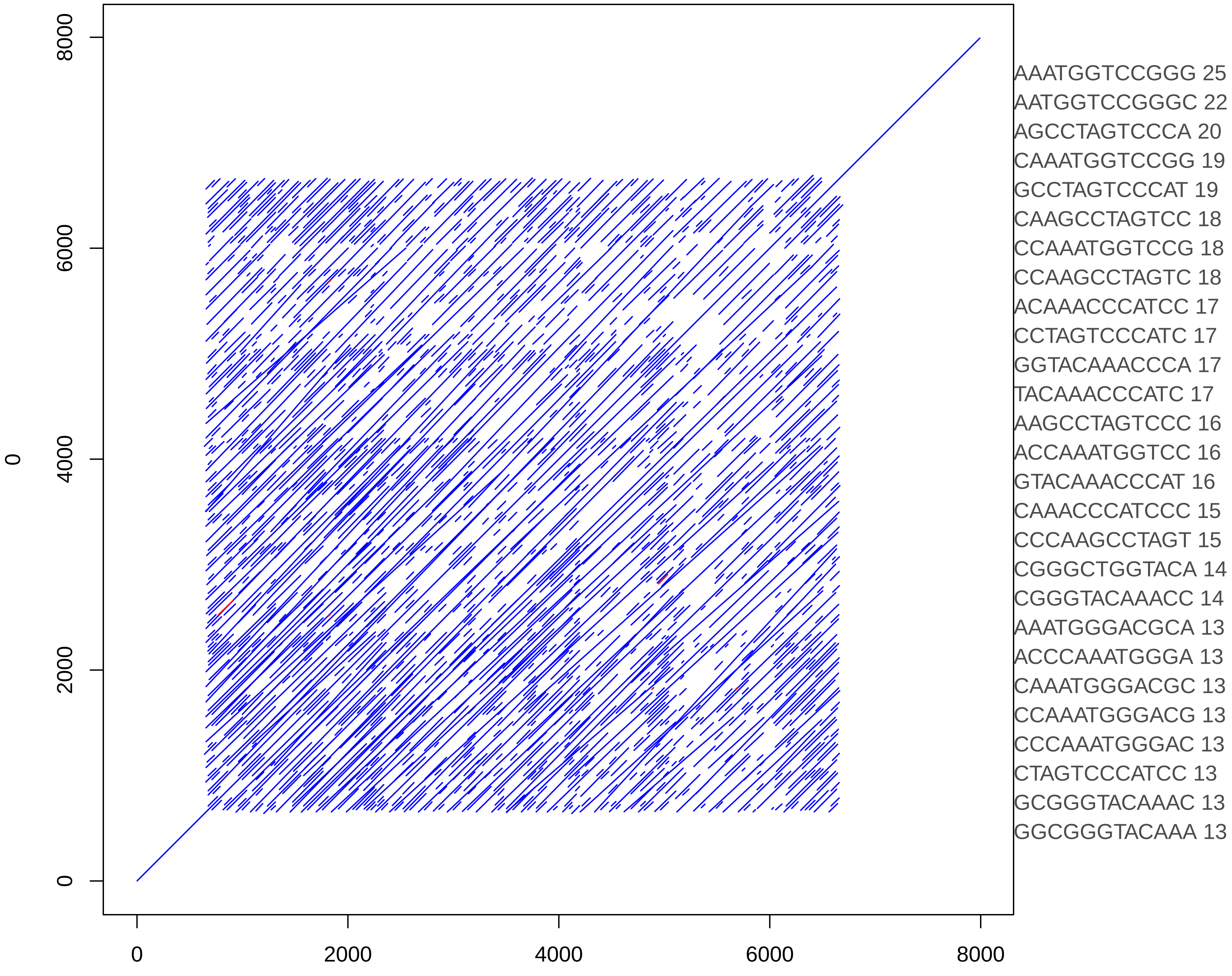

>m141122_062923_42134_c100732202550000001823141405141562_s1_p0/144329/16218_22528

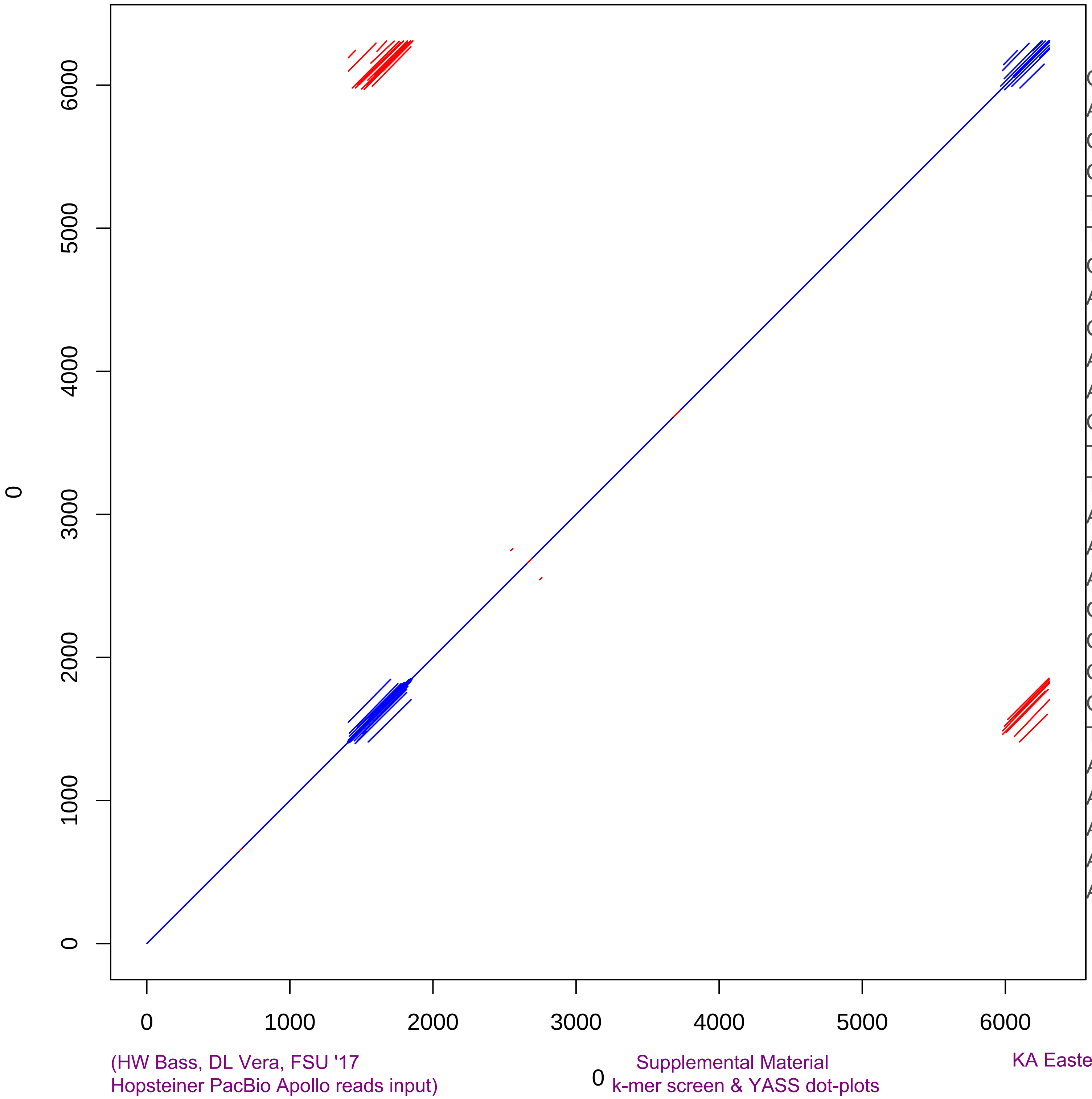

GCTACTGCTACT 11
ACTGCTACTGCT 9
CTACTGCTACTG 9
CTGCTACTGCTA 9
TGCTACTGCTAC 8
TACTGCTACTGC 7
GTAGCAGTAGCA 6
AGTAGCAGTAGC 5
CACTACCACTAC 5
ACCACTACCACT 4
AGCAGTAGCAGT 4
CCACTACCACTA 4
TACCACTACCAC 4
TAGCAGTAGCAG 4
ACTACCACTACC 3
AGTAGCAAGTAG 3
AGTAGTAGTAGT 3
CAGTAGCAGTAG 3
CTACCACTACCA 3
GCAGTAGCAGTA 3
GTAGCAAGTAGC 3
TAGCAAGTAGCA 3
AAGTAGCAGTAG 2
ACCACTACTACC 2
ACTACCACTACT 2
ACTACTACATAC 2
ACTACTACTACA 2

>m141122_062923_42134_c100732202550000001823141405141562_s1_p0/151719/16140_23782

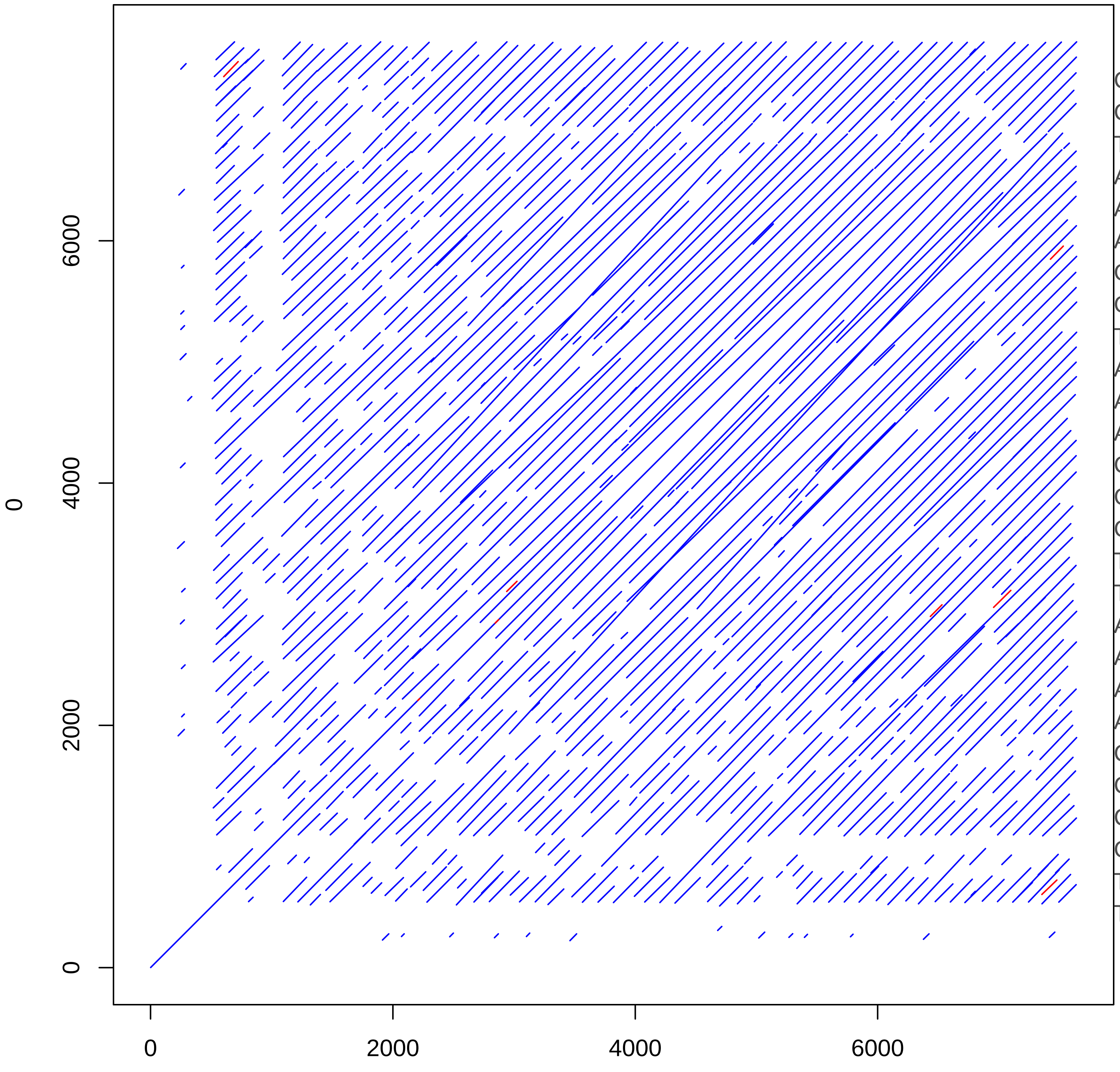

CGAACTAAGTTT 12
CTATGTTCCTGG 12
TGATCGTTGTTT 12
AAAACTTAGAAG 11
AAACTTAGAAGT 11
AACTAAGTTTTC 11
GATCGTTGTTTT 11
GCGAACTAAGTT 11
TATGTTCCTGGA 11
AACTTAGAAGTT 10
ACATAACCAGGT 10
ATTGATCGTTGT 10
CATAACCAGGTC 10
GATAAAAATTAA 10
GGACATAACCAG 10
TGTTTTTCTTAA 10
TTGATCGTTGTT 10
ACTAAGTTTTCA 9
ACTTAGAAGTTA 9
ATCGTTGTTTTT 9
ATGTTCCTGGAC 9
CCTGGACATAAC 9
CTAAGTTTTCAA 9
CTGGACATAACC 9
GTTGTTTTTCTT 9
TAAGTTTTCAAA 9
TAGAAGTTAATT 9

>m141122_062923_42134_c100732202550000001823141405141562_s1_p0/152576/5626_11199

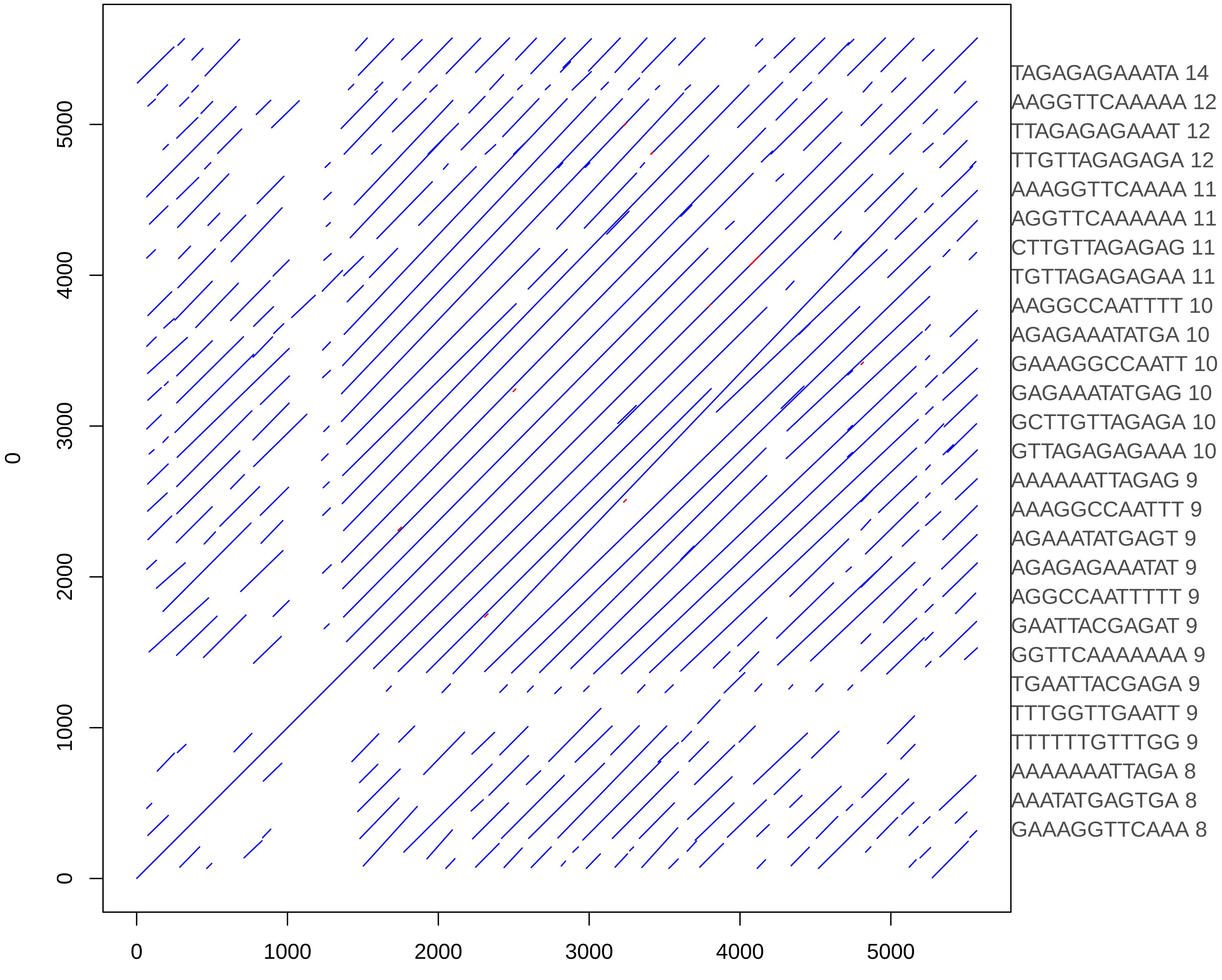

>m141122_062923_42134_c100732202550000001823141405141562_s1_p0/152845/8567_14011

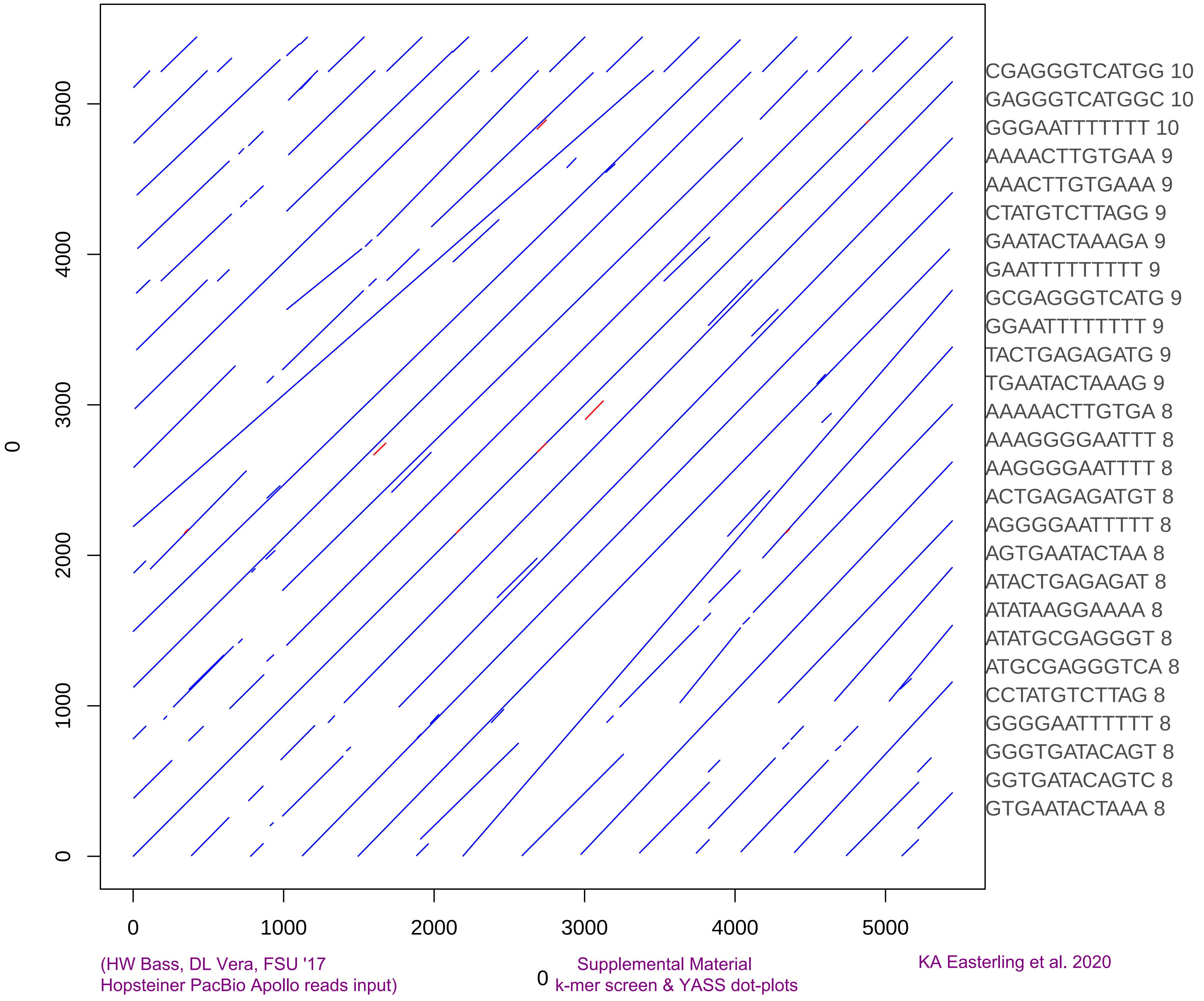

>m141122_062923_42134_c100732202550000001823141405141562_s1_p0/157113/0_6009

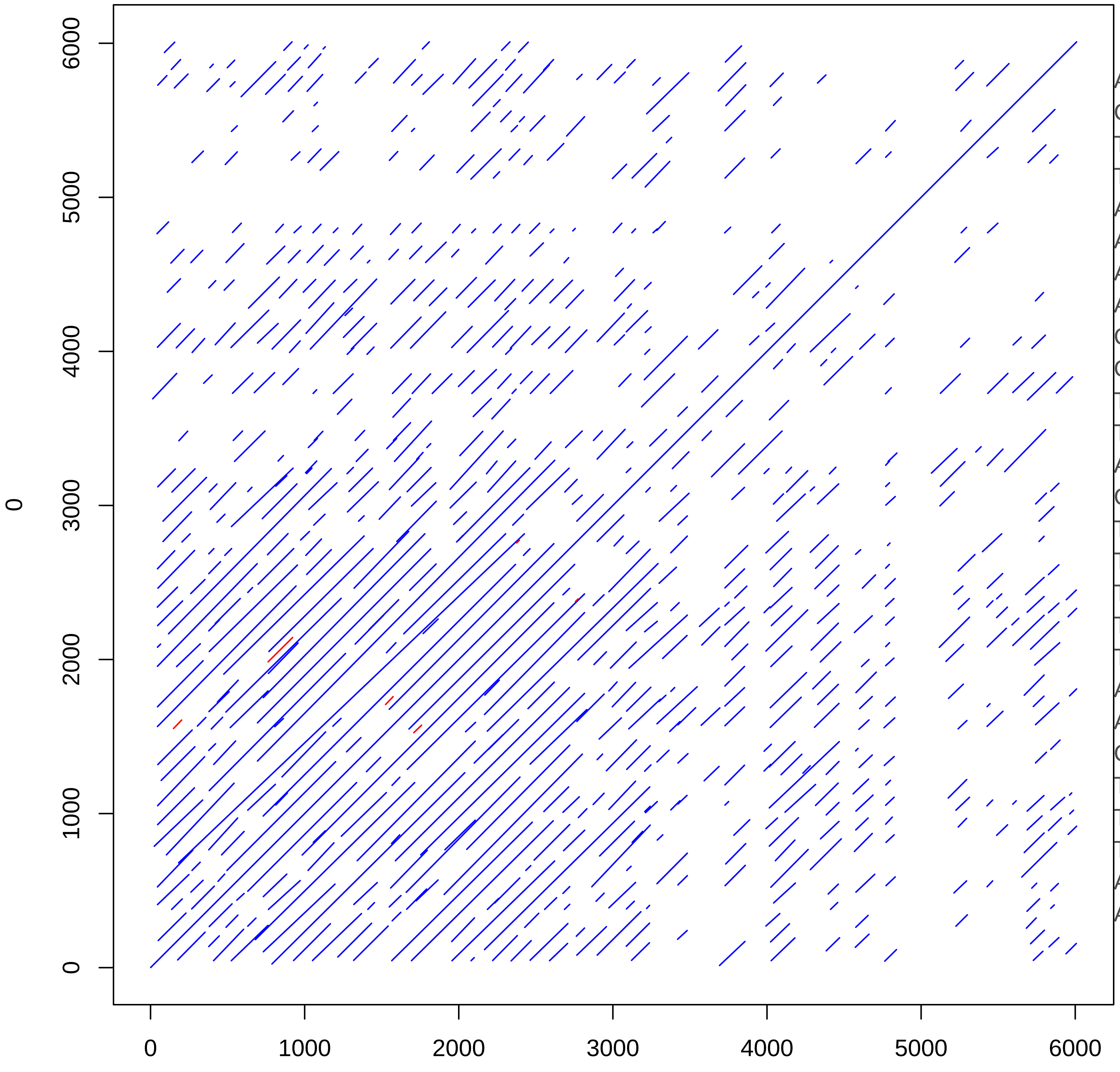

>m141122_062923_42134_c100732202550000001823141405141562_s1_p0/159402/0_7081

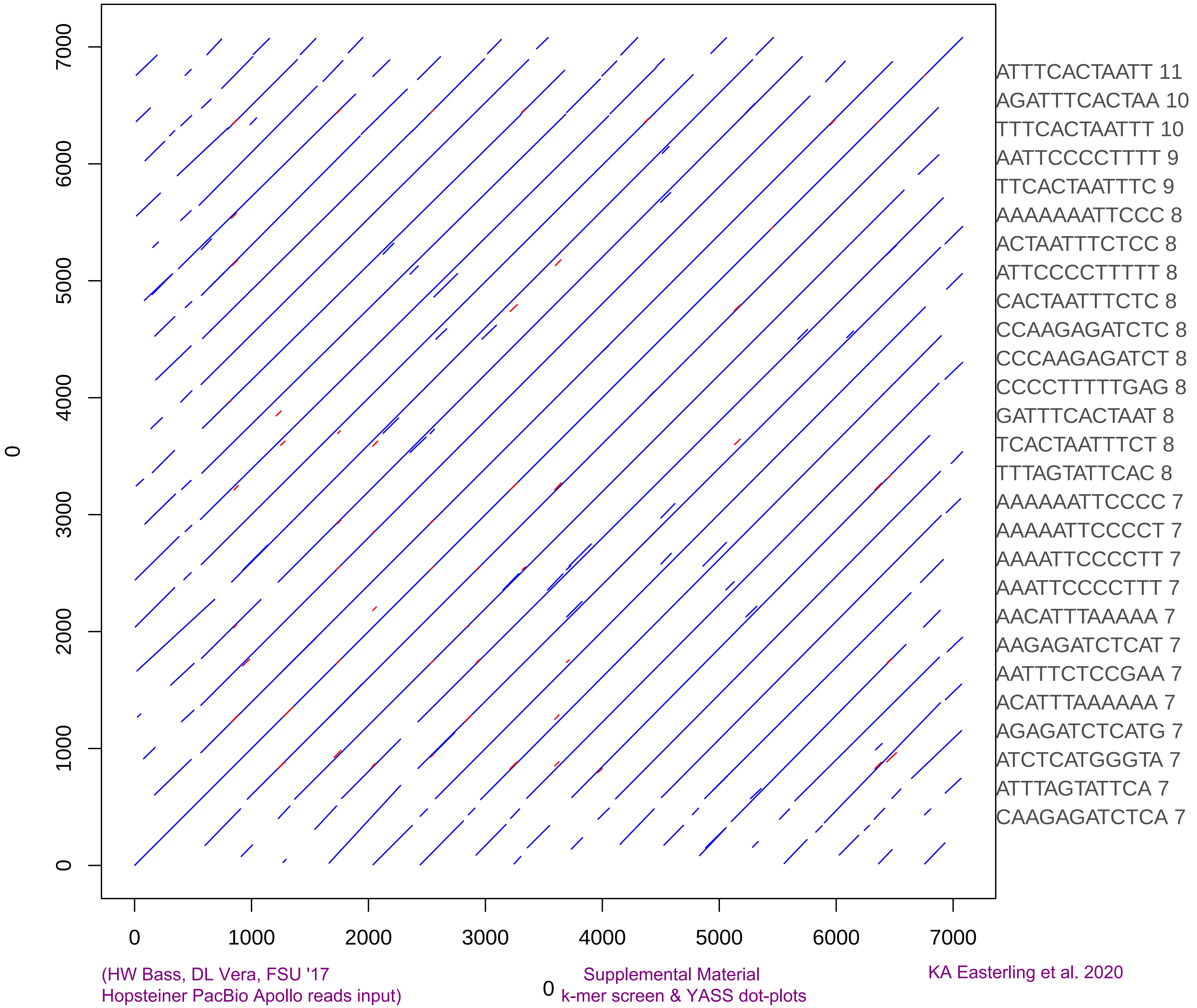

>m141122_062923_42134_c100732202550000001823141405141562_s1_p0/160155/0_12335

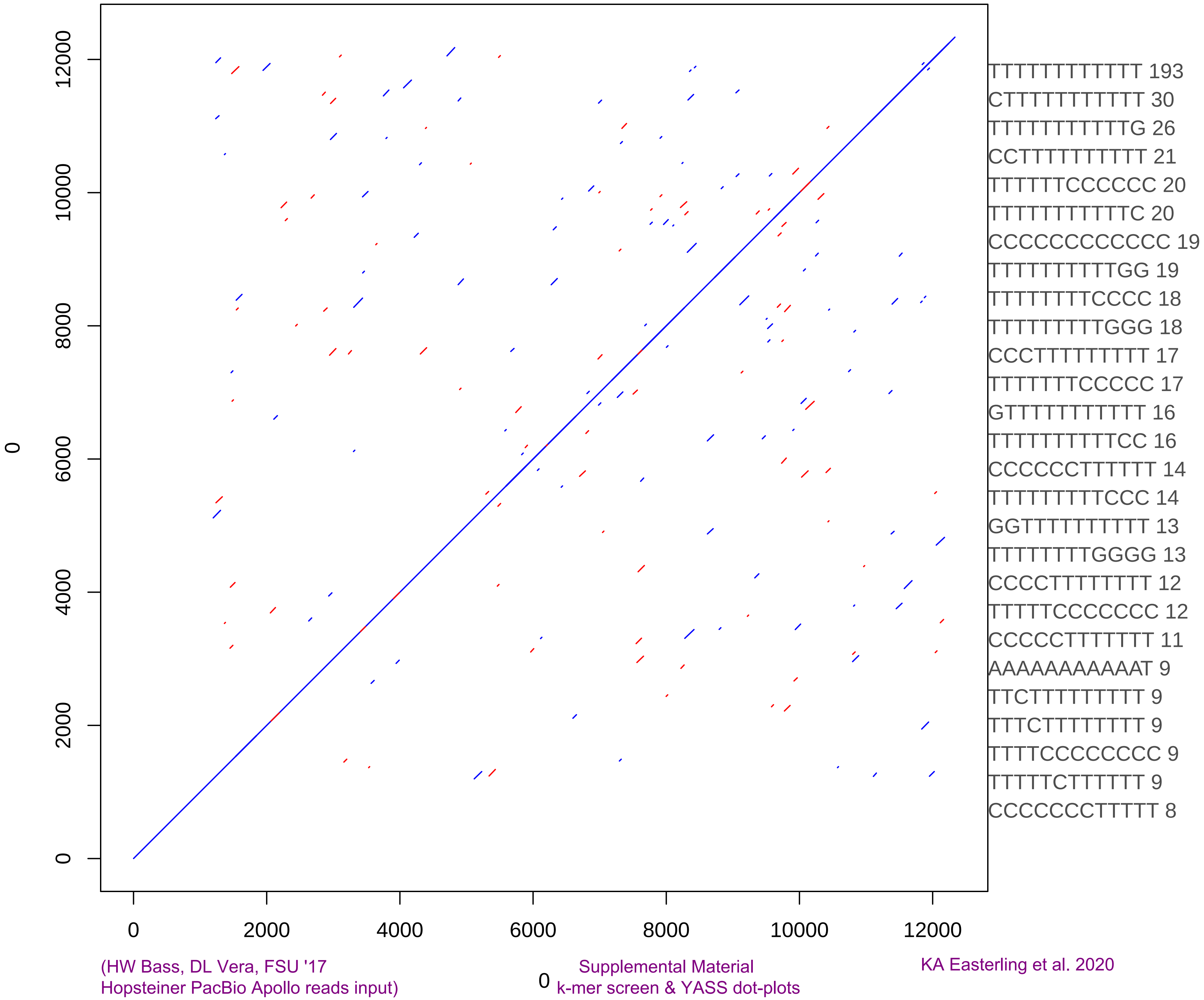

>m141122_062923_42134_c100732202550000001823141405141562_s1_p0/162003/0_5717

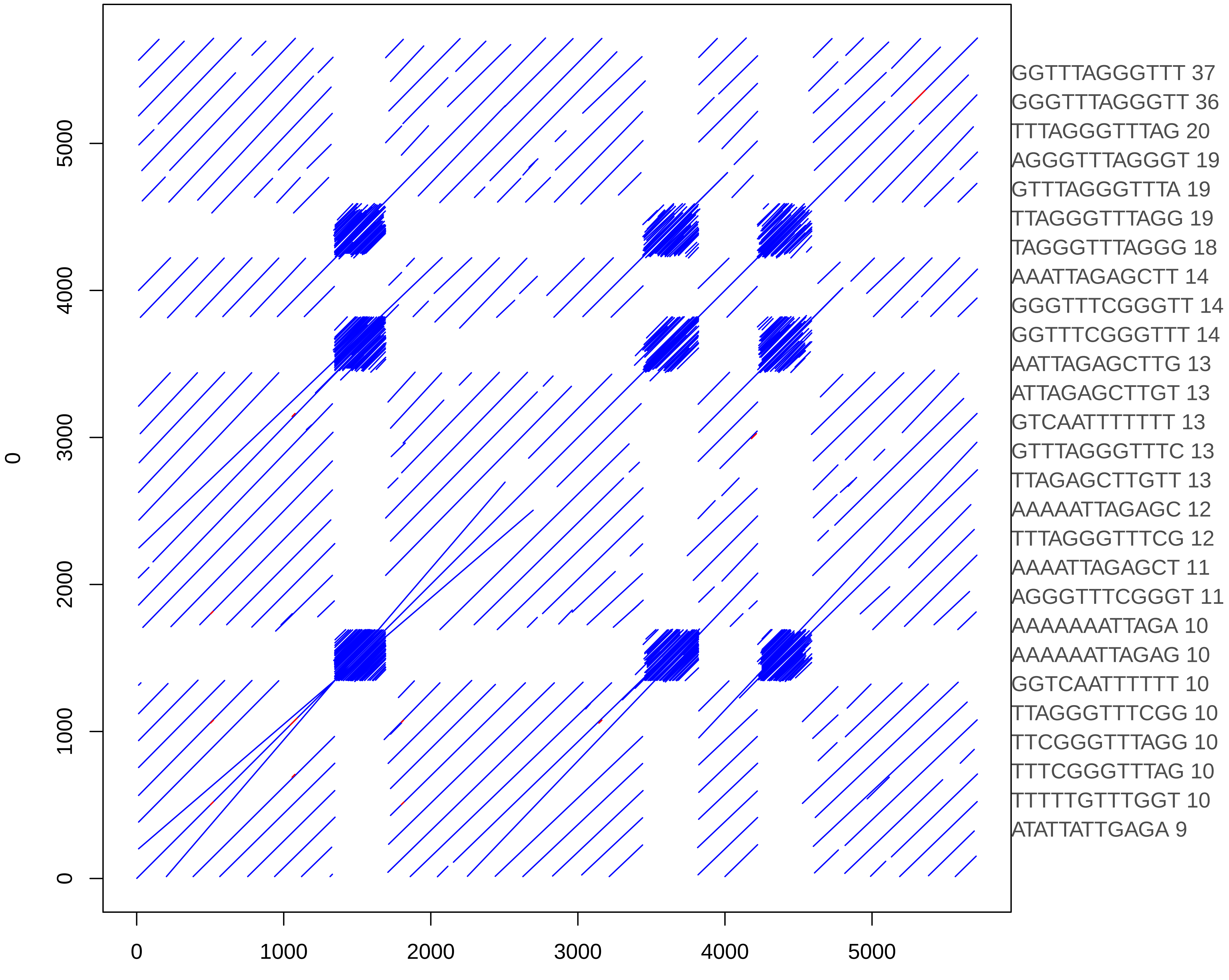

>m141122_062923_42134_c100732202550000001823141405141562_s1_p0/162632/0_6048

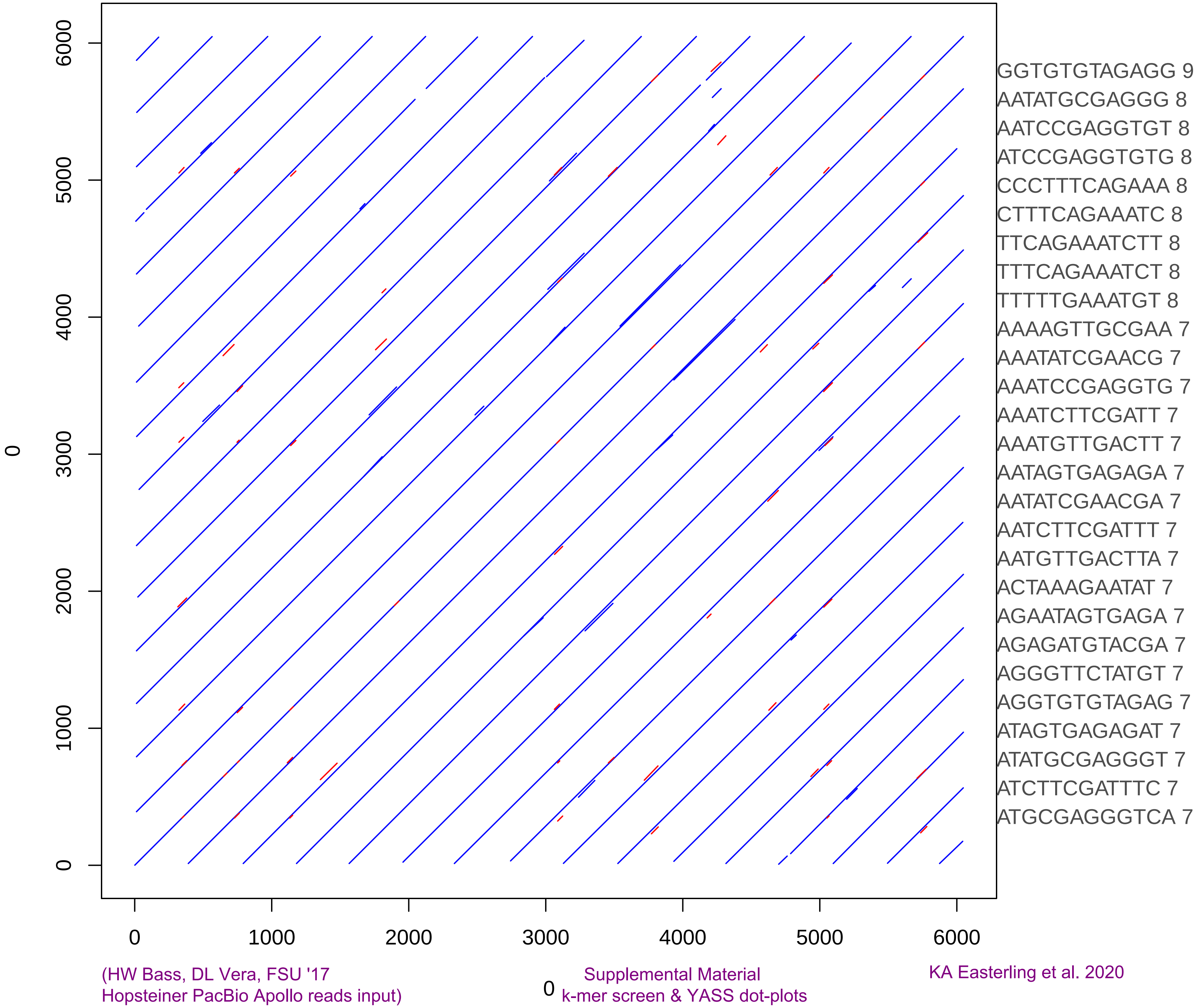

>m141122_062923_42134_c100732202550000001823141405141562_s1_p0/163036/1279_8429

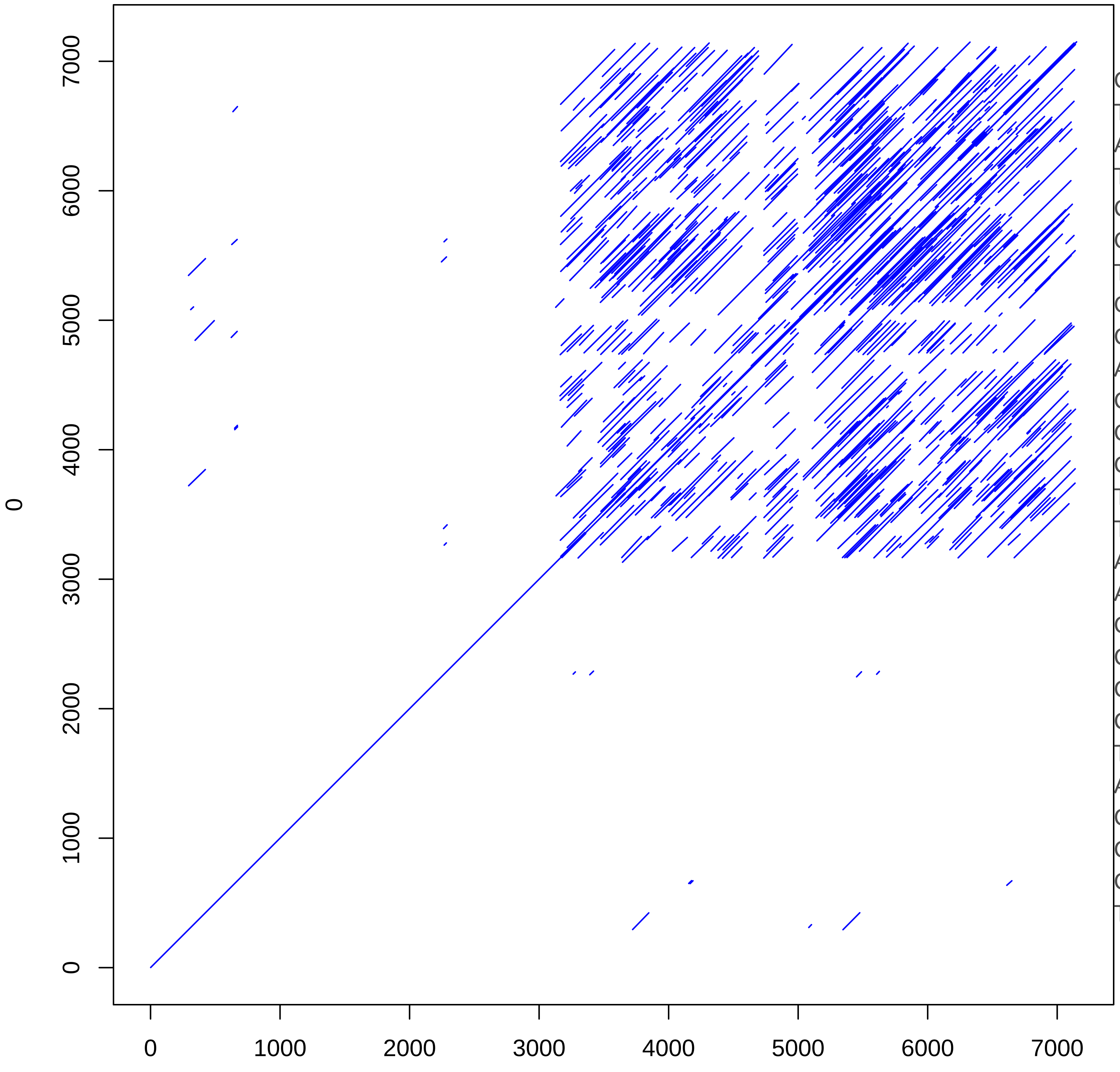

CTACTACTACTA 39
TACTACTACTAC 35
ACTACTACTACT 33
TGCTACTGCTAC 14
CTGCTACTGCTA 12
GCTACTACTACT 12
TGCTACTACTAC 11
GCTACTGCTACT 10
CTGCTACTACTA 9
ACTGCTACTACT 7
CTACTCCTACTA 7
CTACTGCTACTG 7
CTACTGGCTACT 7
TACTACTACTAA 7
TACTGCTACTGC 7
ACTACTGCTACT 6
ACTGCTACTGCT 6
CCTACTACTACT 6
CTACTACTAACT 6
CTACTACTACTG 6
CTACTGCTACTA 6
TACTGCTACTAC 6
ACTACTACTACA 5
CTACTACTACTC 5
CTACTTCCTACT 5
GCTACTGCTACA 5
TACTACTAACTA 5

>m141122_062923_42134_c100732202550000001823141405141562_s1_p0/163375/195_5775

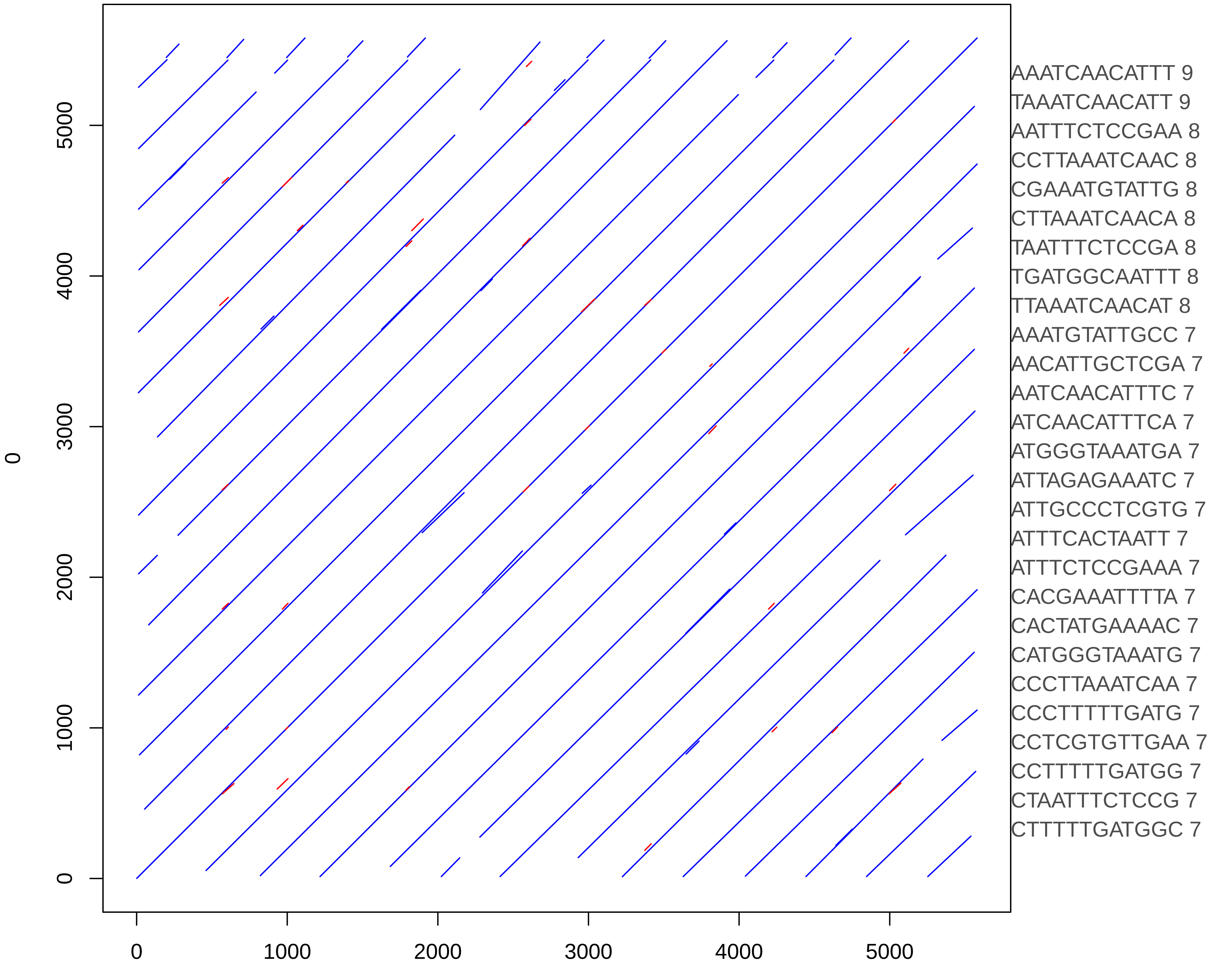

>m141122_062923_42134_c100732202550000001823141405141562_s1_p0/1896/11425_19003

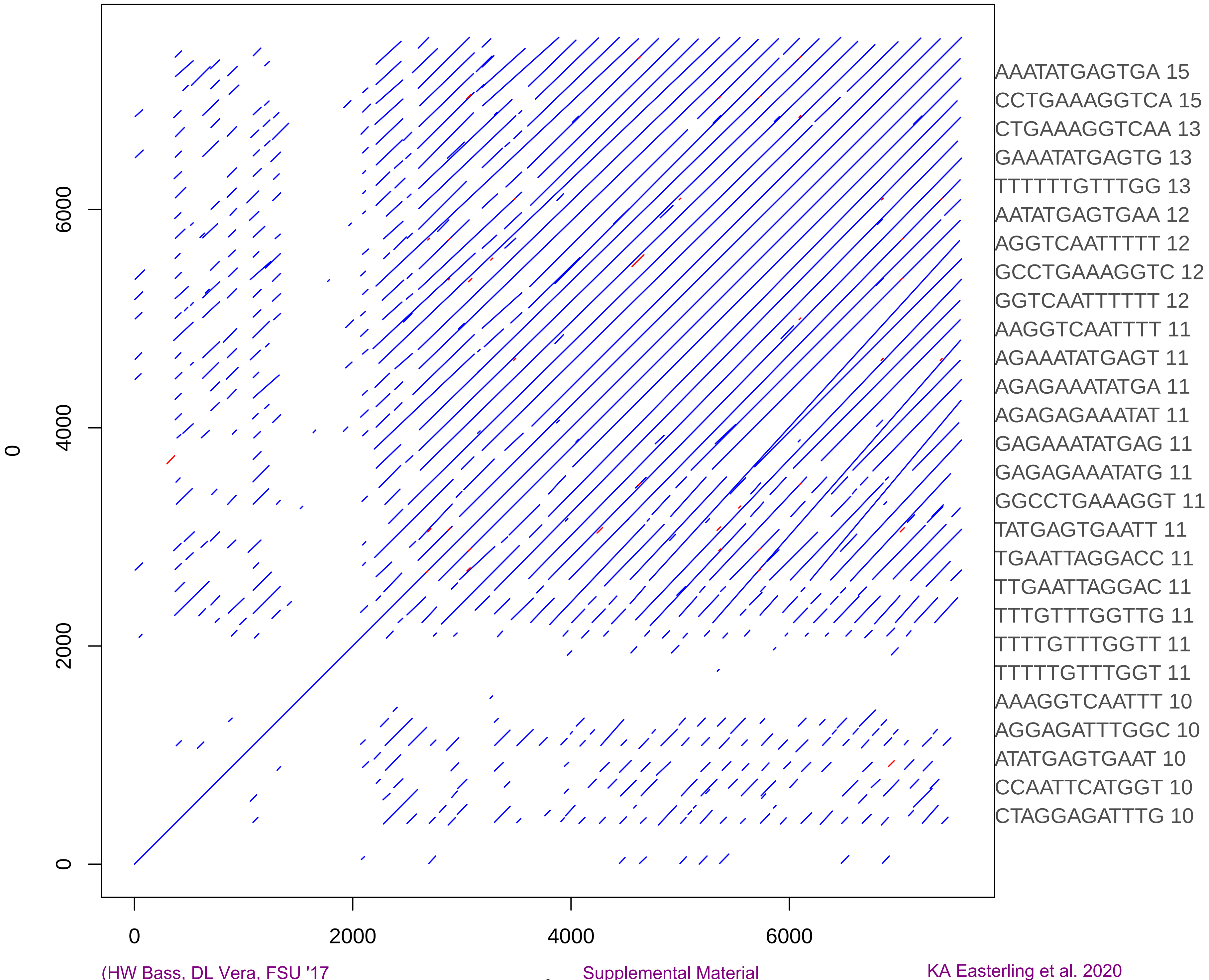

>m141122_062923_42134_c100732202550000001823141405141562_s1_p0/195/0_7220

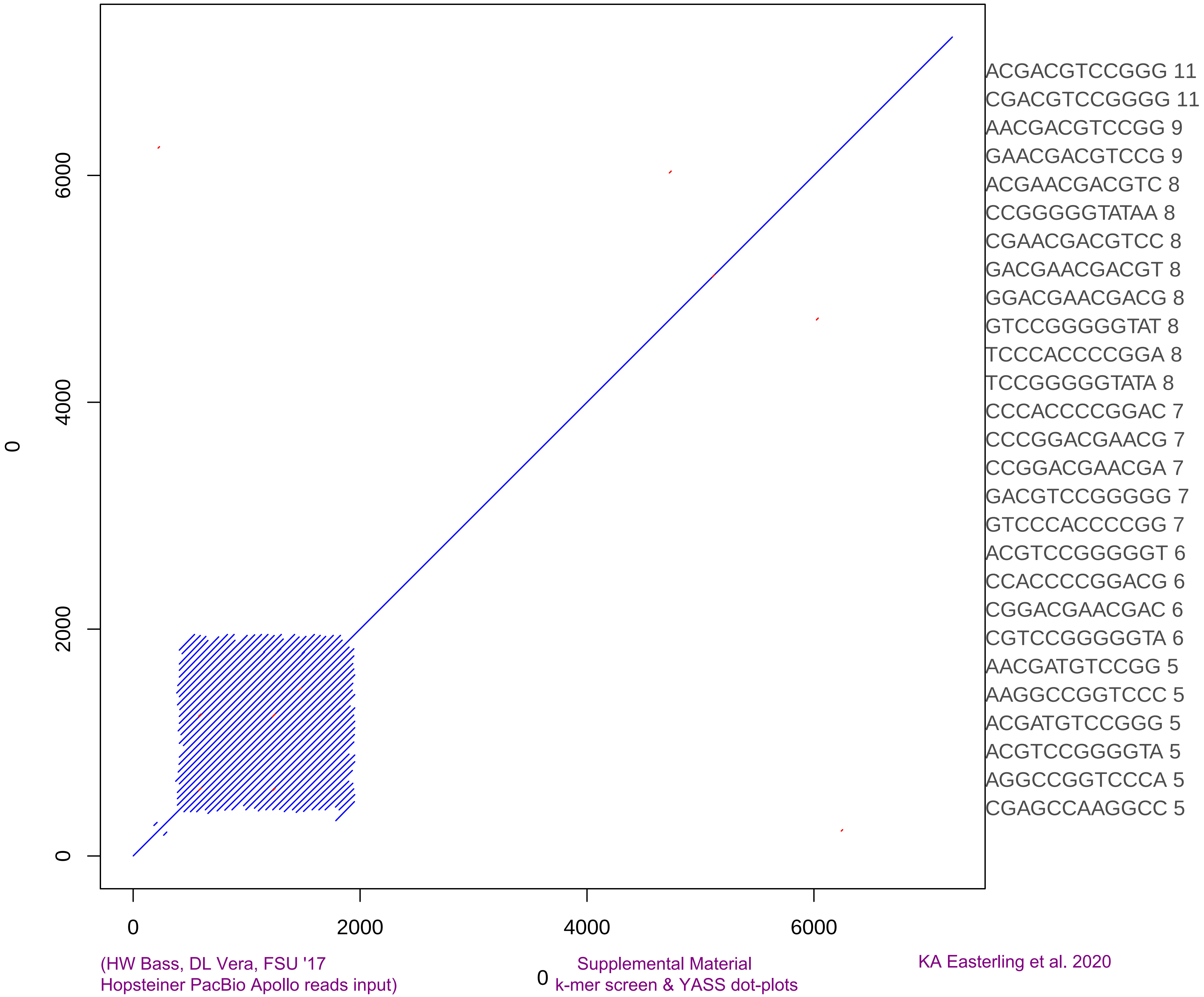

>m141122_062923_42134_c100732202550000001823141405141562_s1_p0/20307/4230_15826

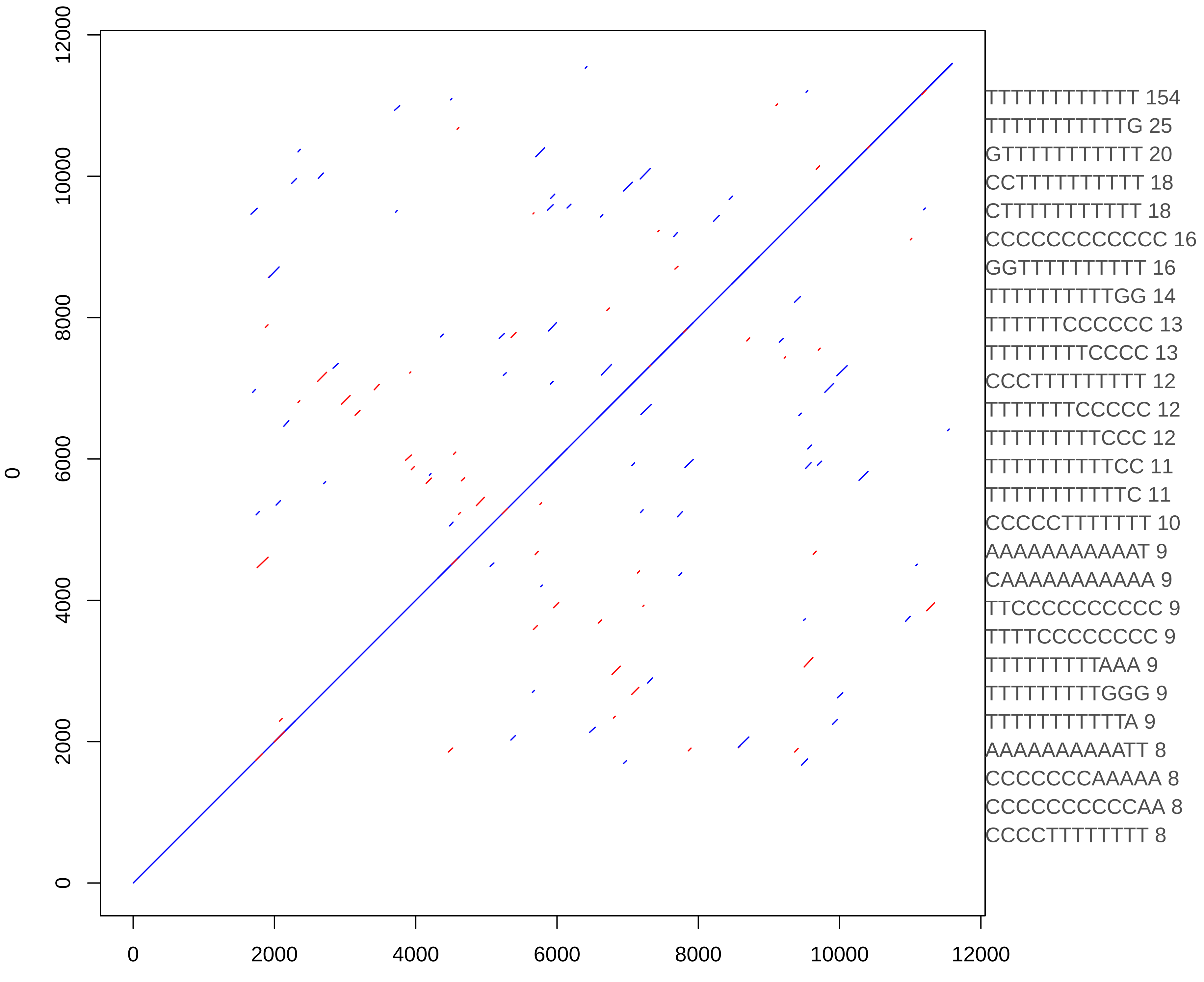

>m141122_062923_42134_c100732202550000001823141405141562_s1_p0/22007/10972_19726

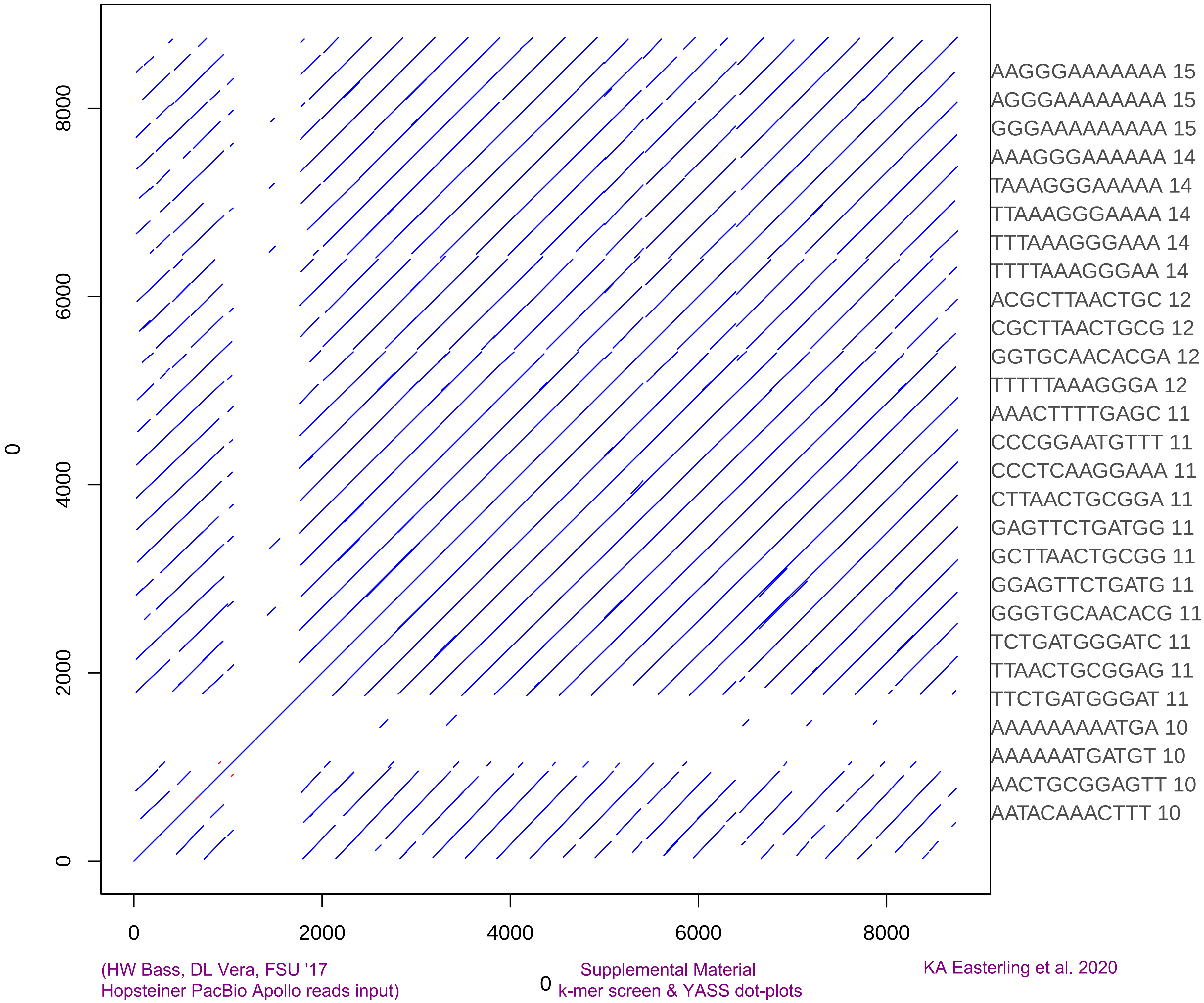

>m141122_062923_42134_c100732202550000001823141405141562_s1_p0/23836/351_5876

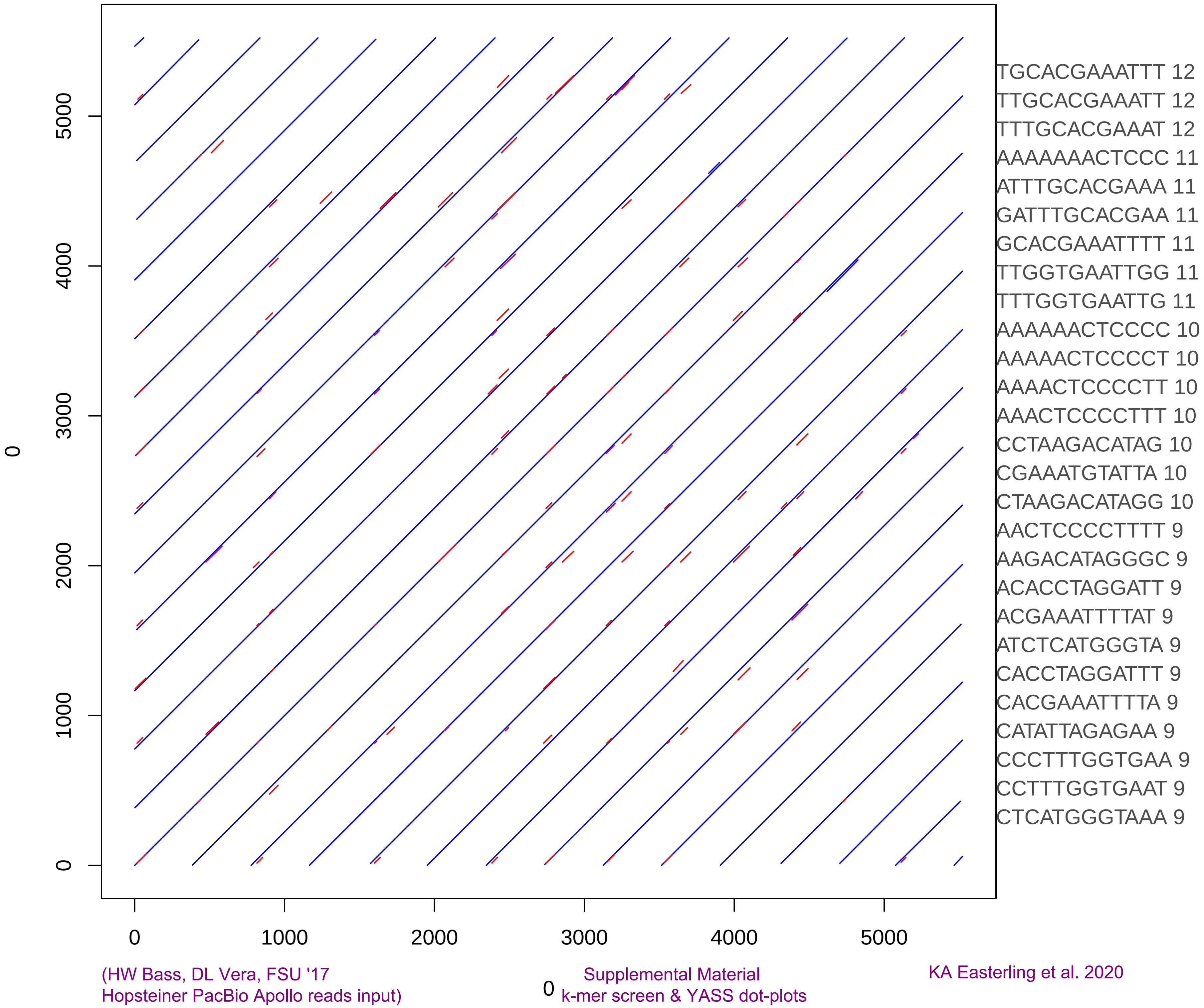

>m141122_062923_42134_c100732202550000001823141405141562_s1_p0/28541/849_10200

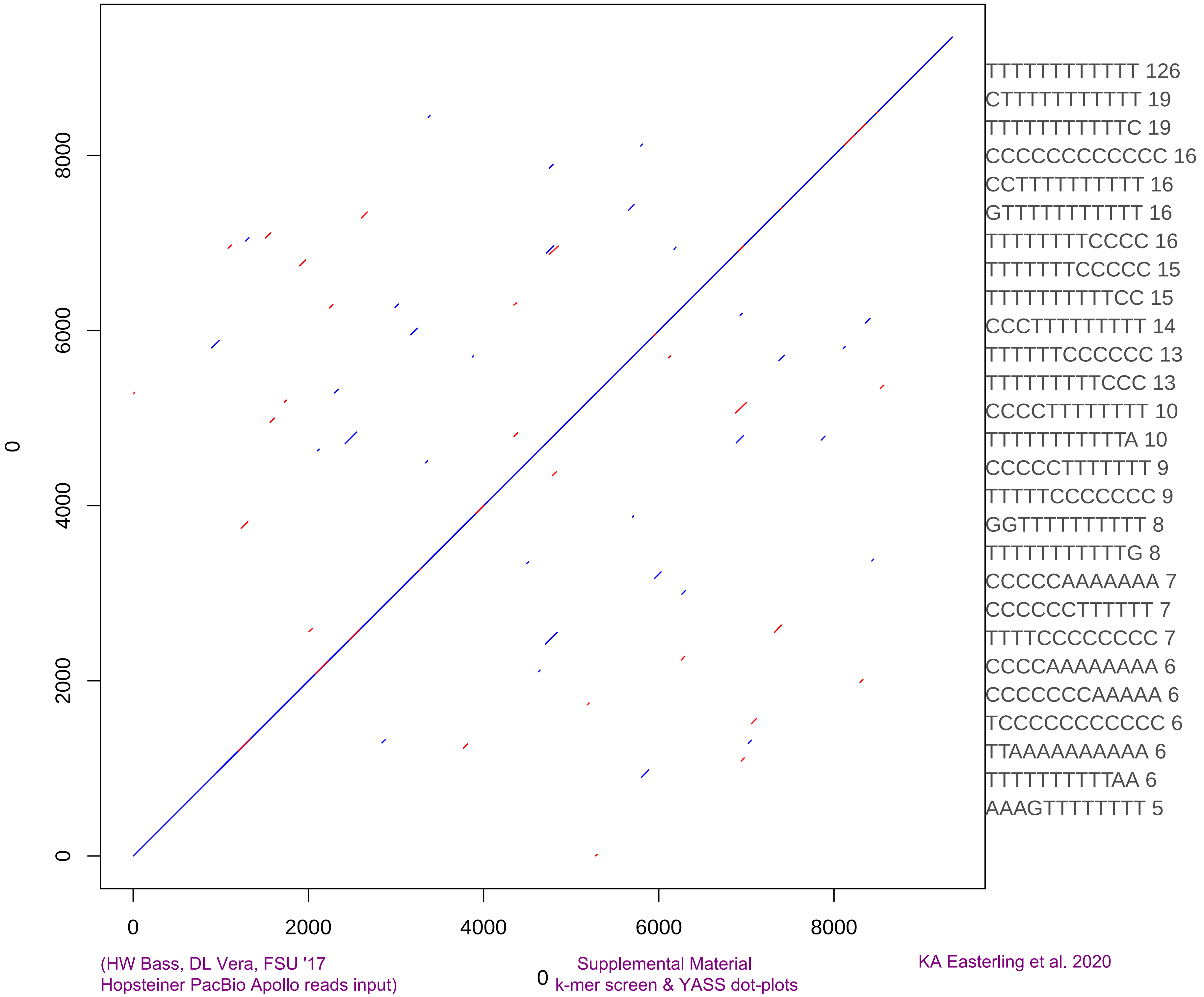

>m141122_062923_42134_c100732202550000001823141405141562_s1_p0/40372/0_6406

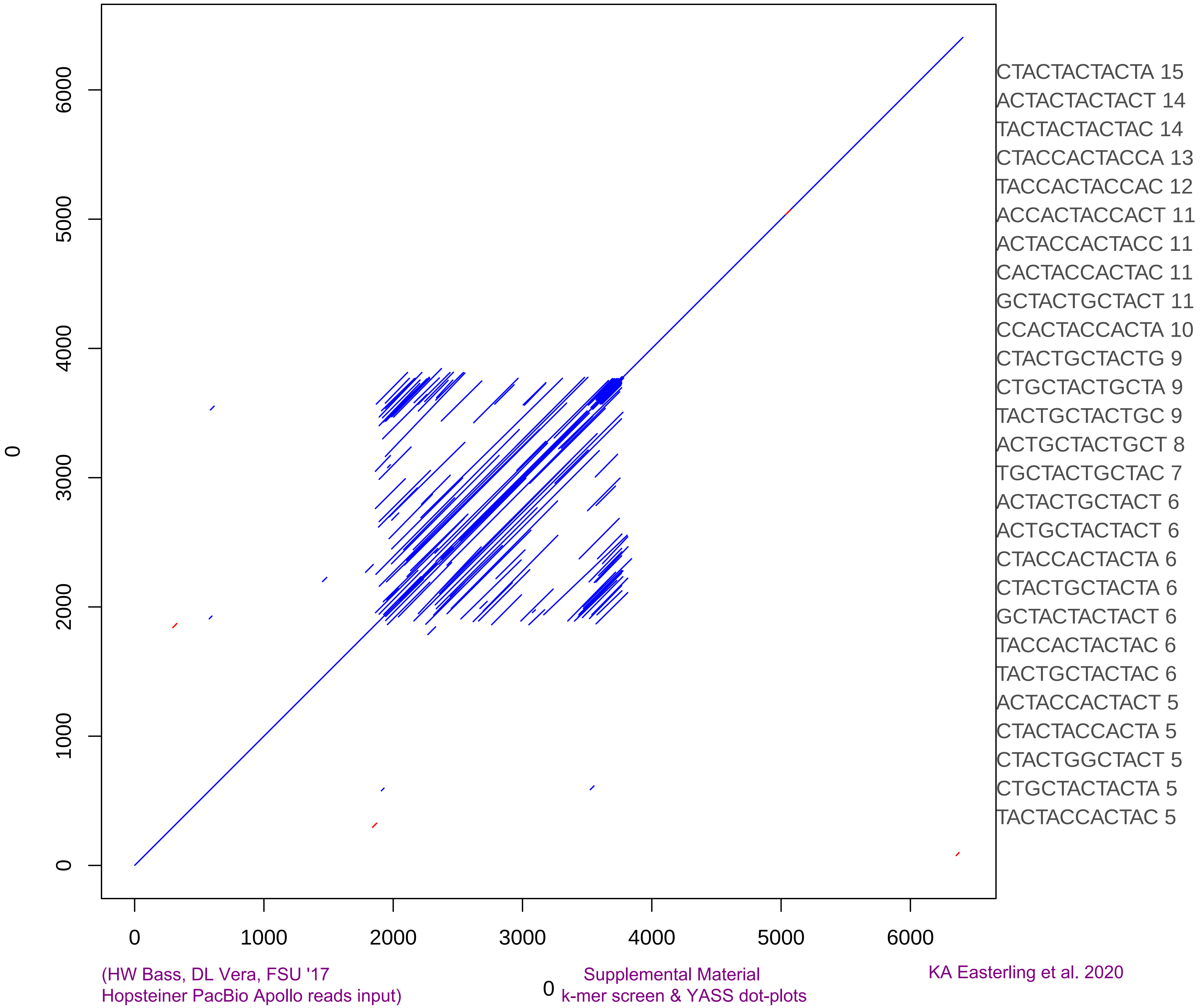

>m141122_062923_42134_c100732202550000001823141405141562_s1_p0/41414/5545_15427

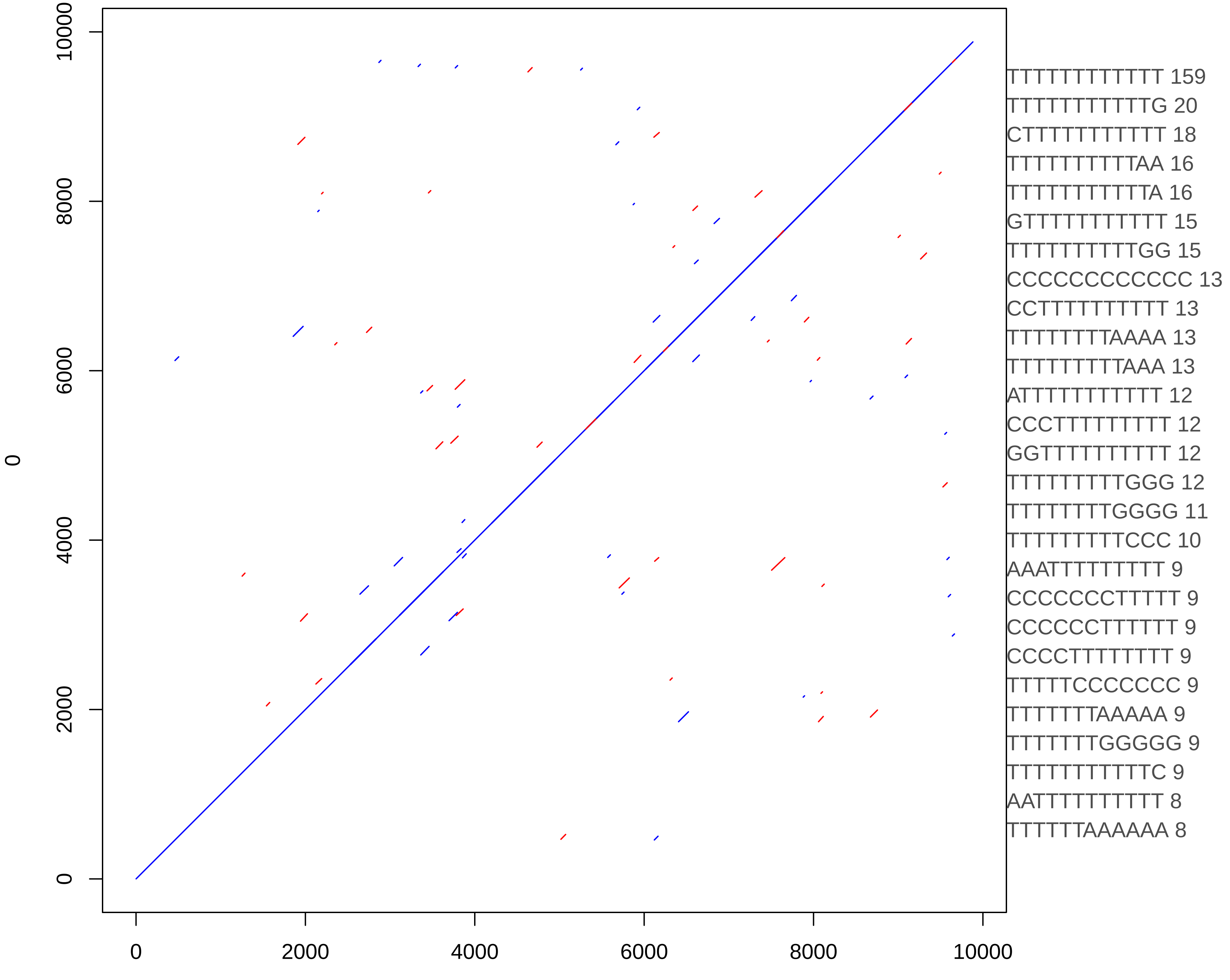

>m141122_062923_42134_c100732202550000001823141405141562_s1_p0/453/0_6741

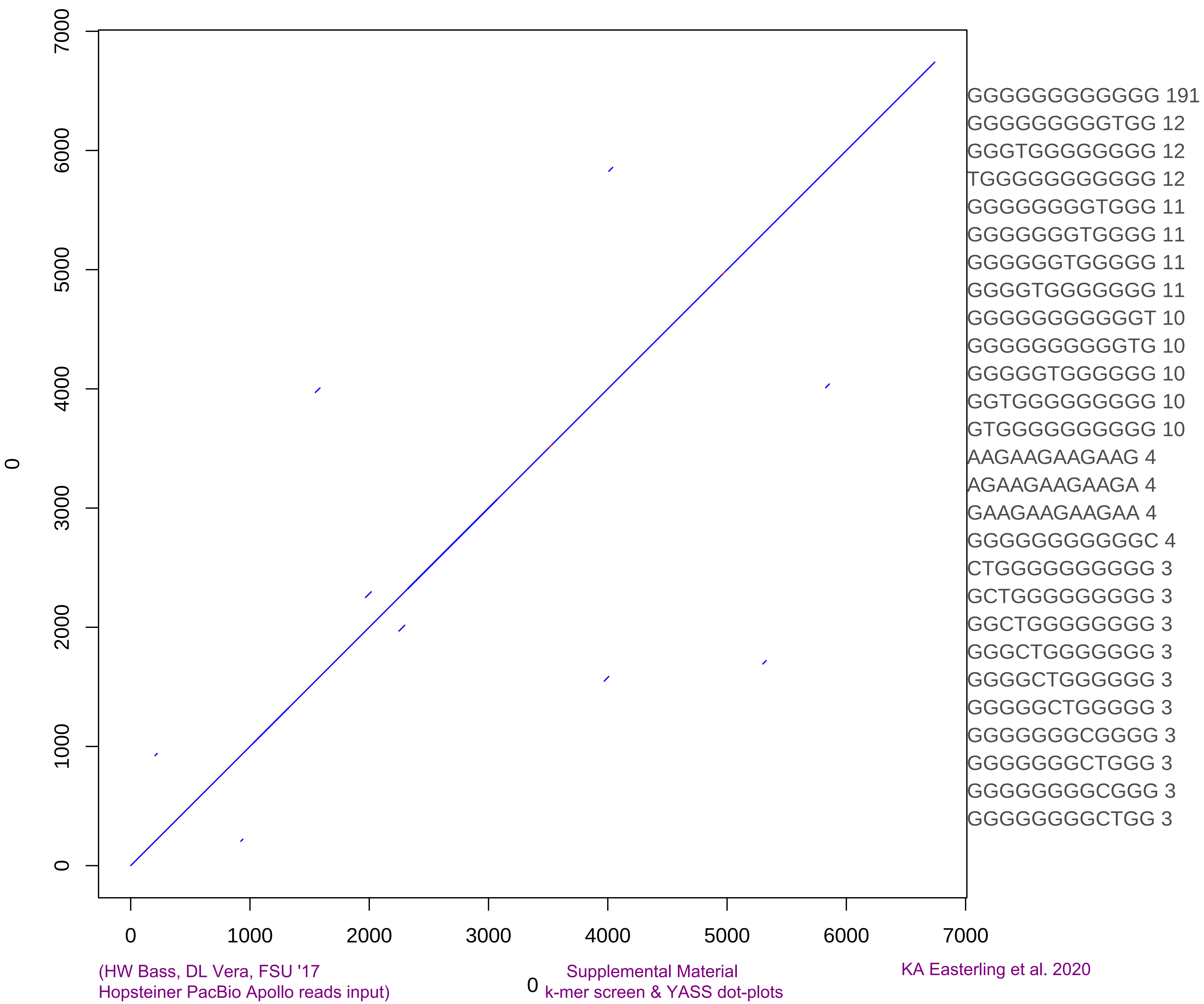

>m141122_062923_42134_c100732202550000001823141405141562_s1_p0/46109/906_11122

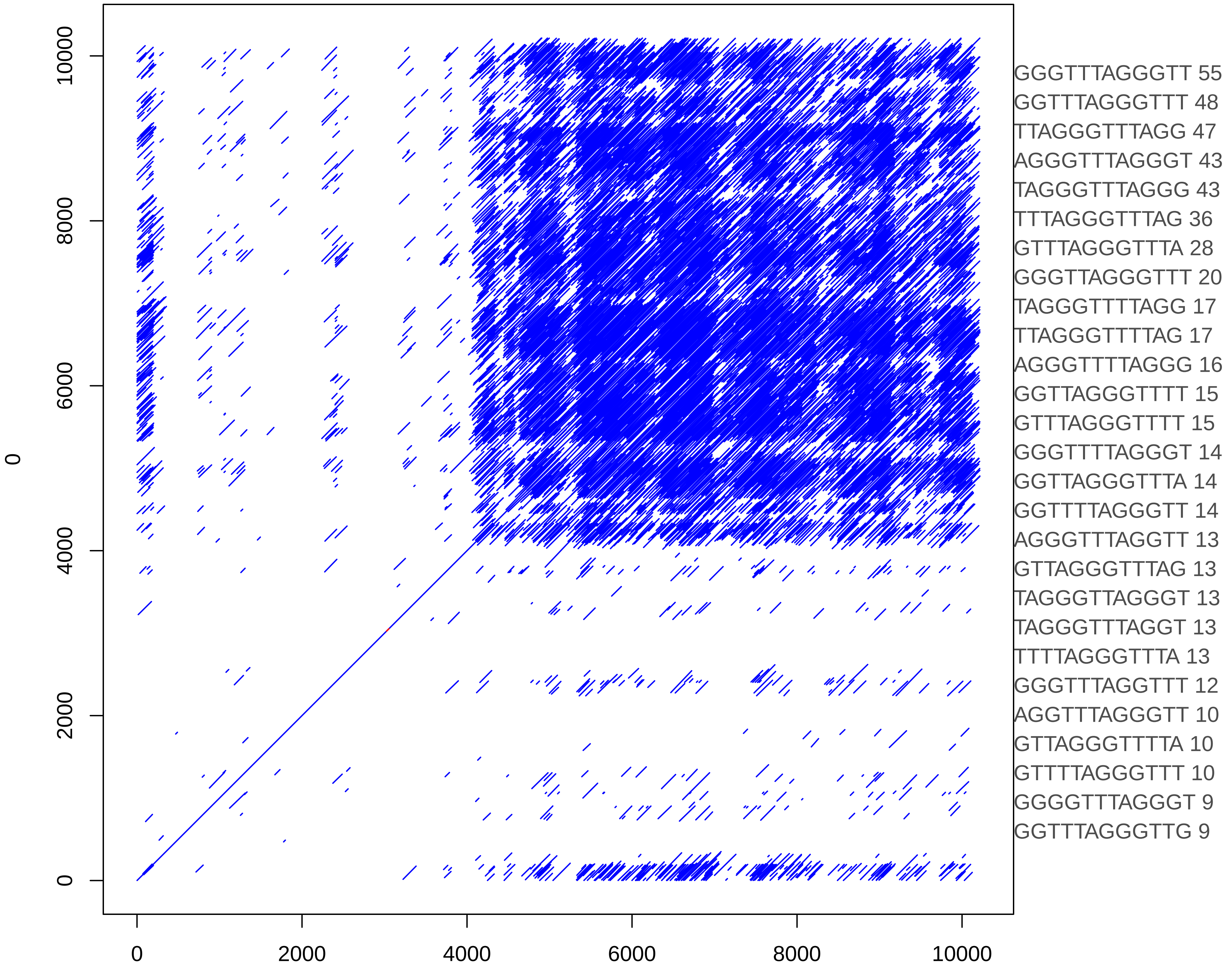

1,121 hits for repetitive sequences passing 12mer abundance criterion

(Read-Number)
PAGE 330

For Tandem Repeat families of ### bp:
PacBio Read named "HuluTR###-r330"

>m141122_062923_42134_c100732202550000001823141405141562_s1_p0/59611/7440_16712

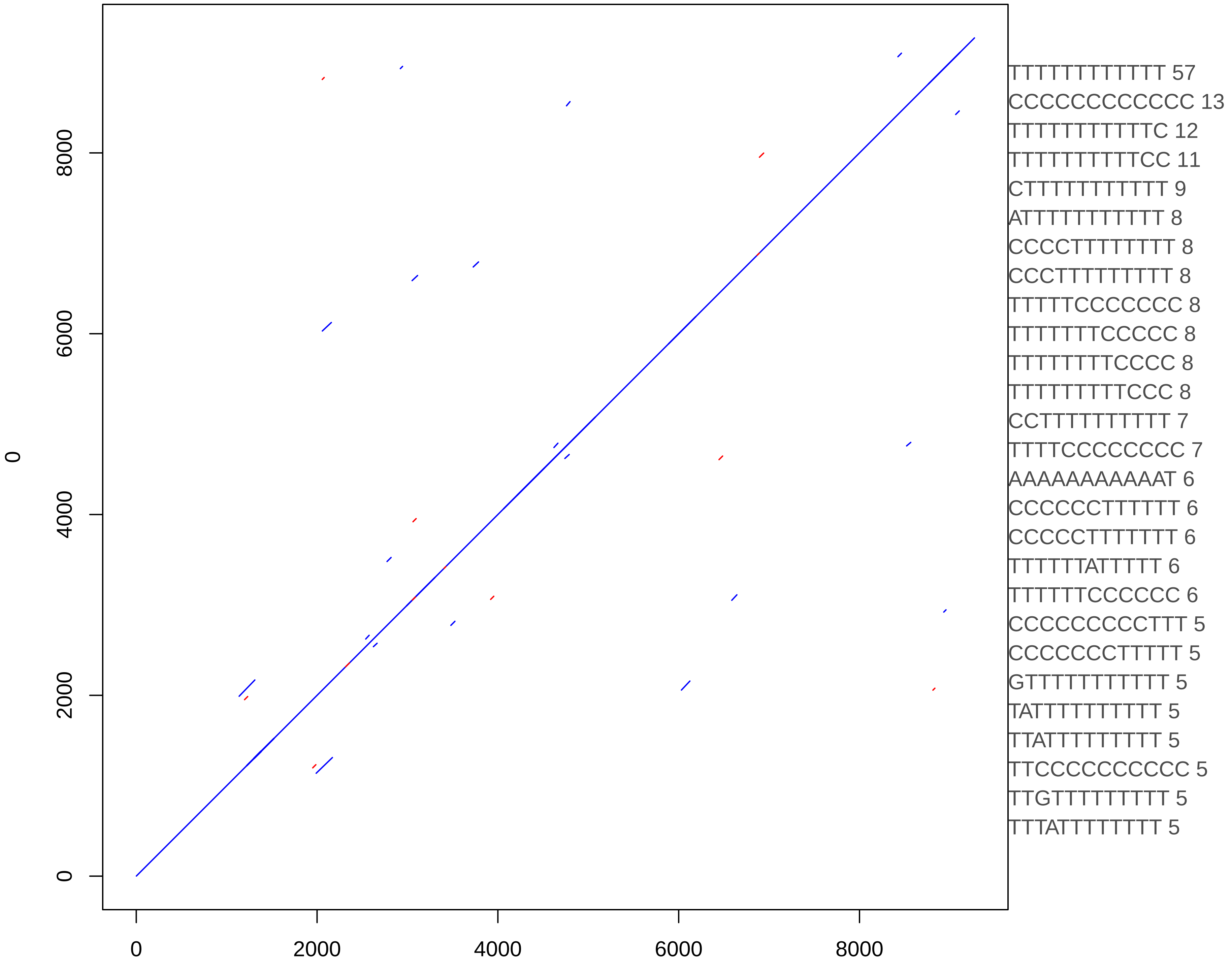

(HW Bass, DL Vera, FSU '17
Hopsteiner PacBio Apollo reads input)

0

Supplemental Material
k-mer screen & YASS dot-plots

KA Easterling et al. 2020

>m141122_062923_42134_c100732202550000001823141405141562_s1_p0/60471/1454_11969

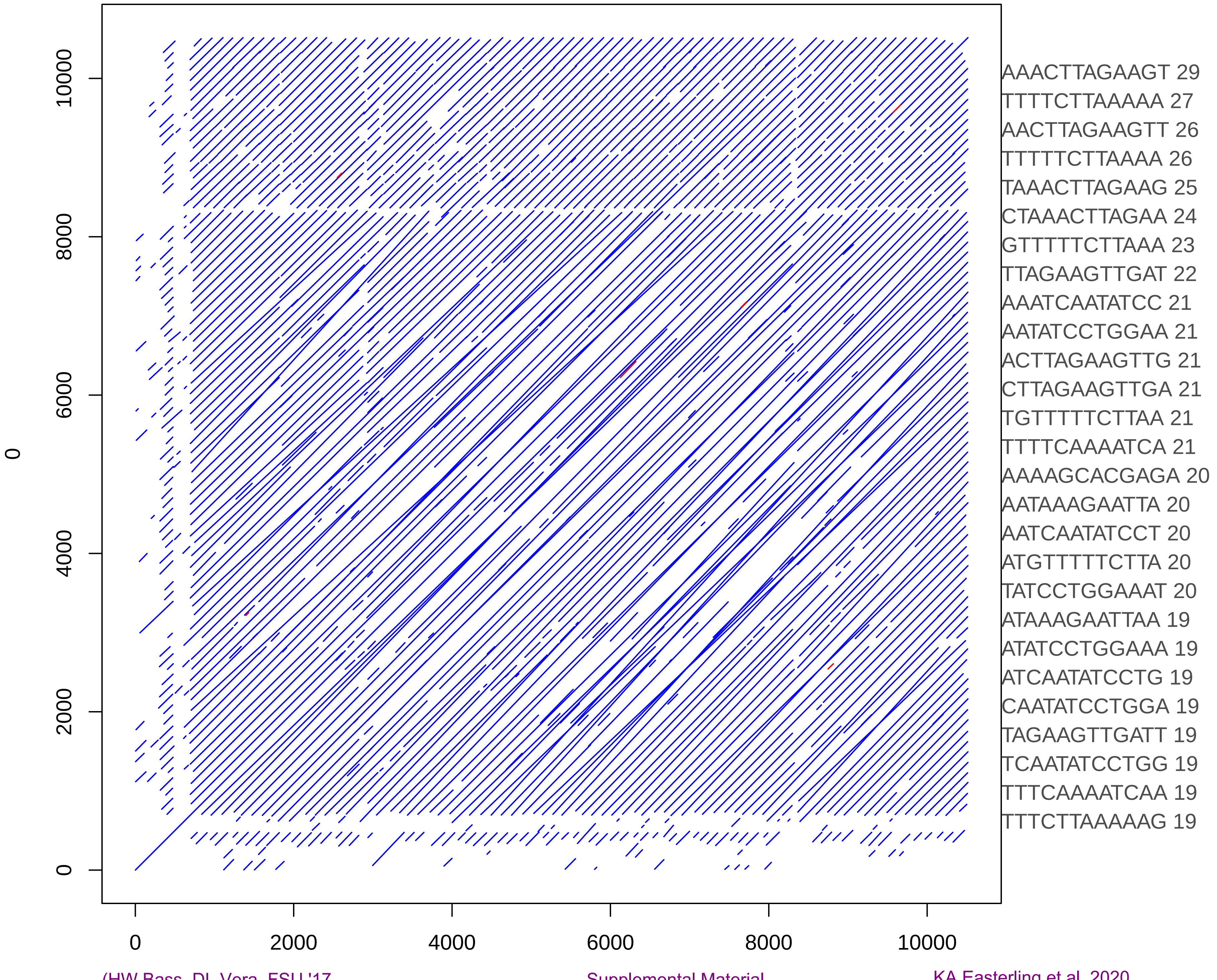

>m141122_062923_42134_c100732202550000001823141405141562_s1_p0/62161/0_9246

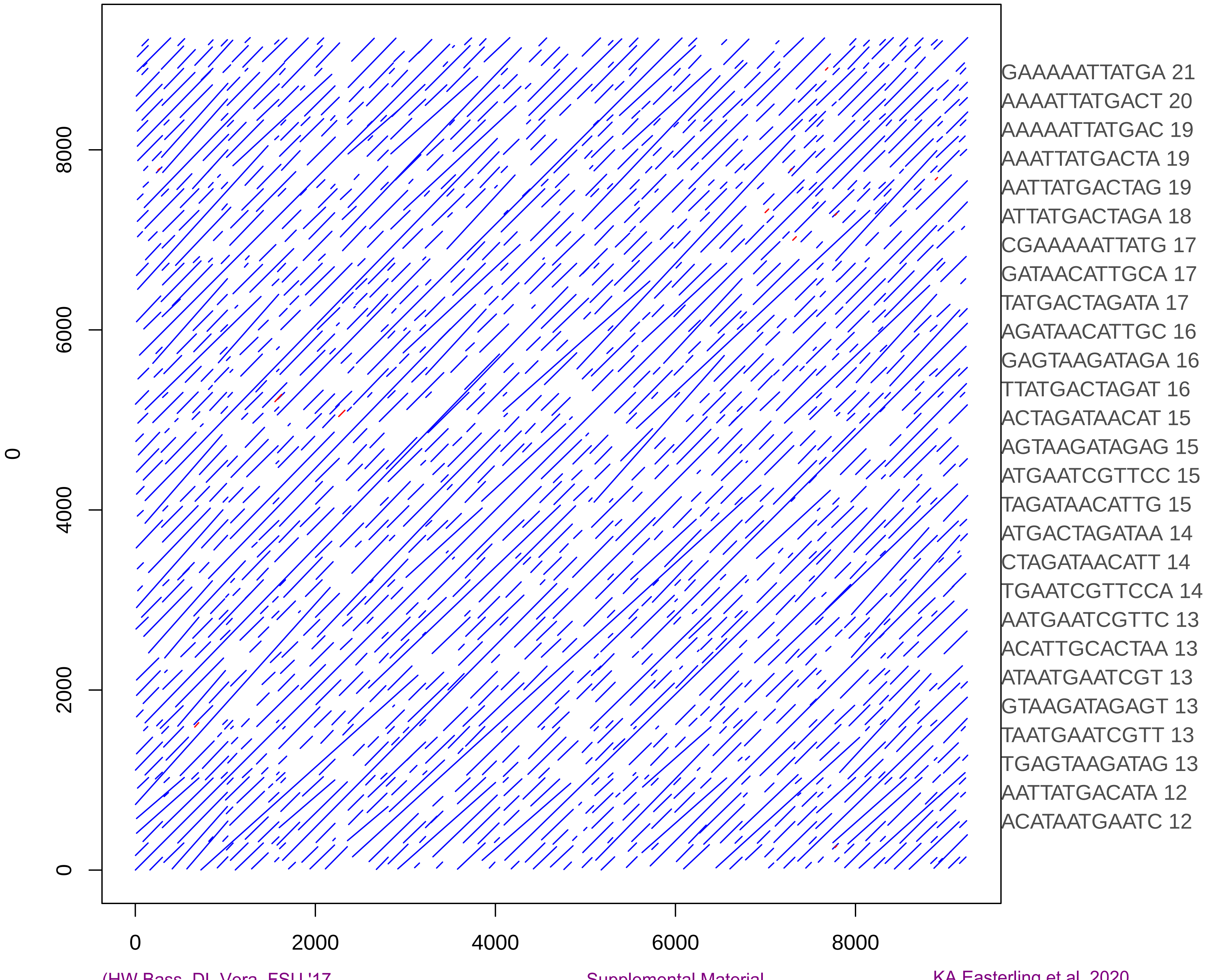

>m141122_062923_42134_c100732202550000001823141405141562_s1_p0/64552/1788_9793

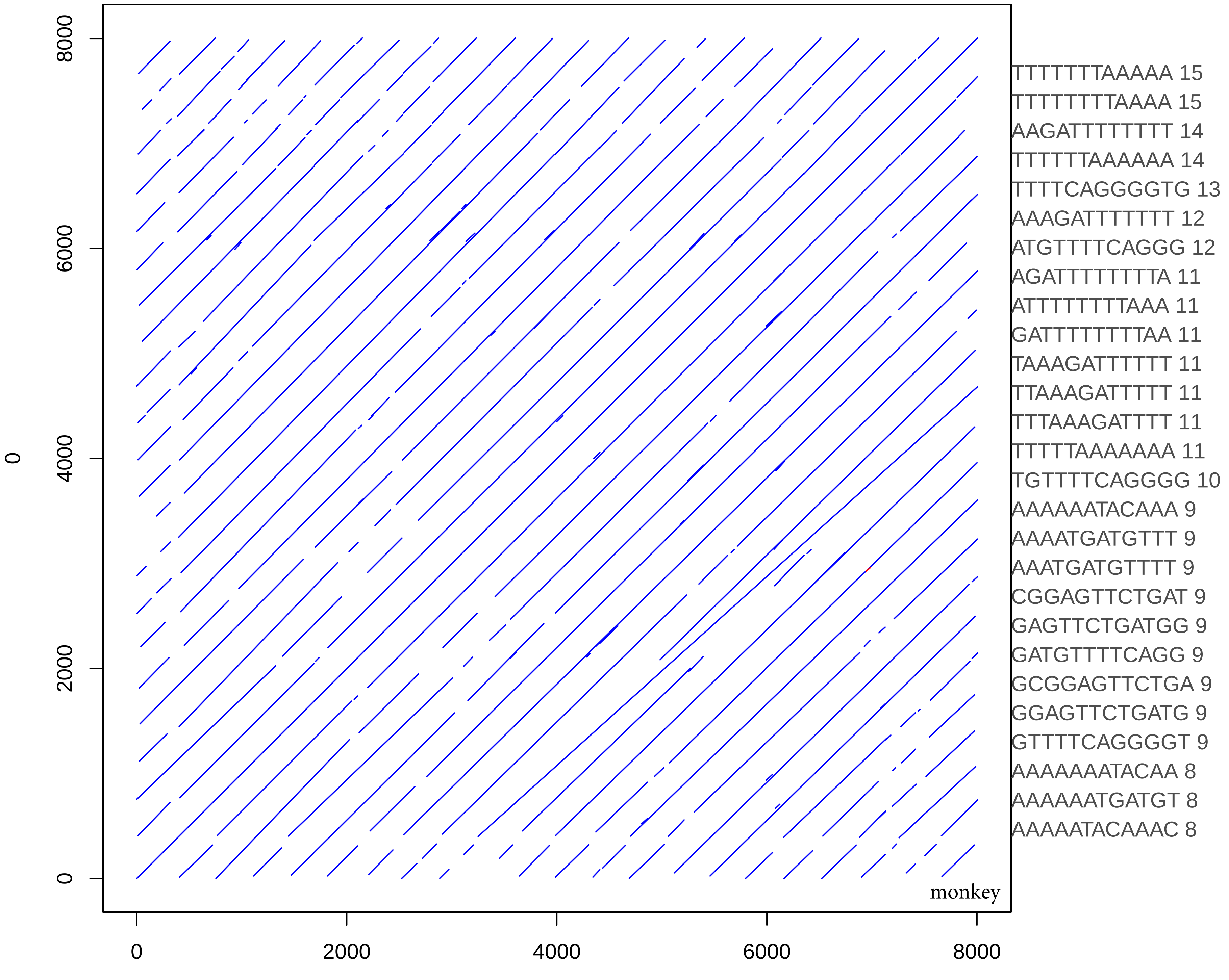

>m141122_062923_42134_c100732202550000001823141405141562_s1_p0/68207/6915_14863

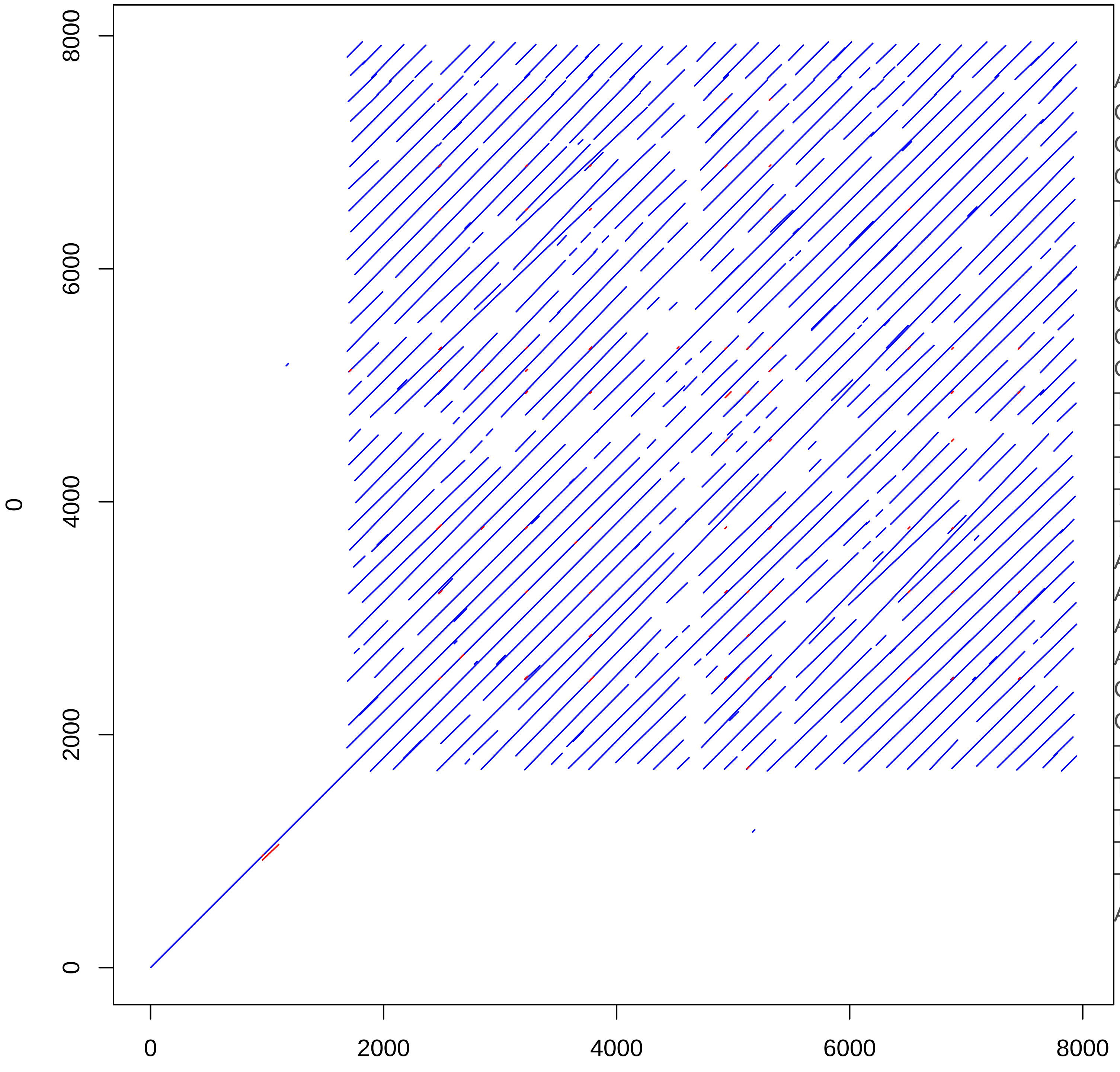

AGAGAAATATGA 11
GAGAAATATGAG 11
GAGAGAAATATG 10
GTCAATTTTTTT 10
TGTTAGAGAGAA 10
AAGGTCAATTTT 9
AGAAATATGAGT 9
GAAAGGTCAATT 9
GGCCTGAAAGGT 9
GGTCAATTTTTT 9
TCAATTTTTTTT 9
TGAAAGGTCAAT 9
TGGCCTGAAAGG 9
TTGTTAGAGAGA 9
TTTGGCCTGAAA 9
AAAAAAATTAGA 8
AAAGGTCAATTT 8
AGAGAGAAATAT 8
AGGTCAATTTTT 8
CCCAATTCATGG 8
GCCTGAAAGGTC 8
TAGAGAGAAATA 8
TCAAAAAAATTA 8
TTCAAAAAAATT 8
TTGGCCTGAAAG 8
TTTTTTTTGTTT 8
AAATATGAGTGA 7

>m141122_062923_42134_c100732202550000001823141405141562_s1_p0/71787/2121_10509

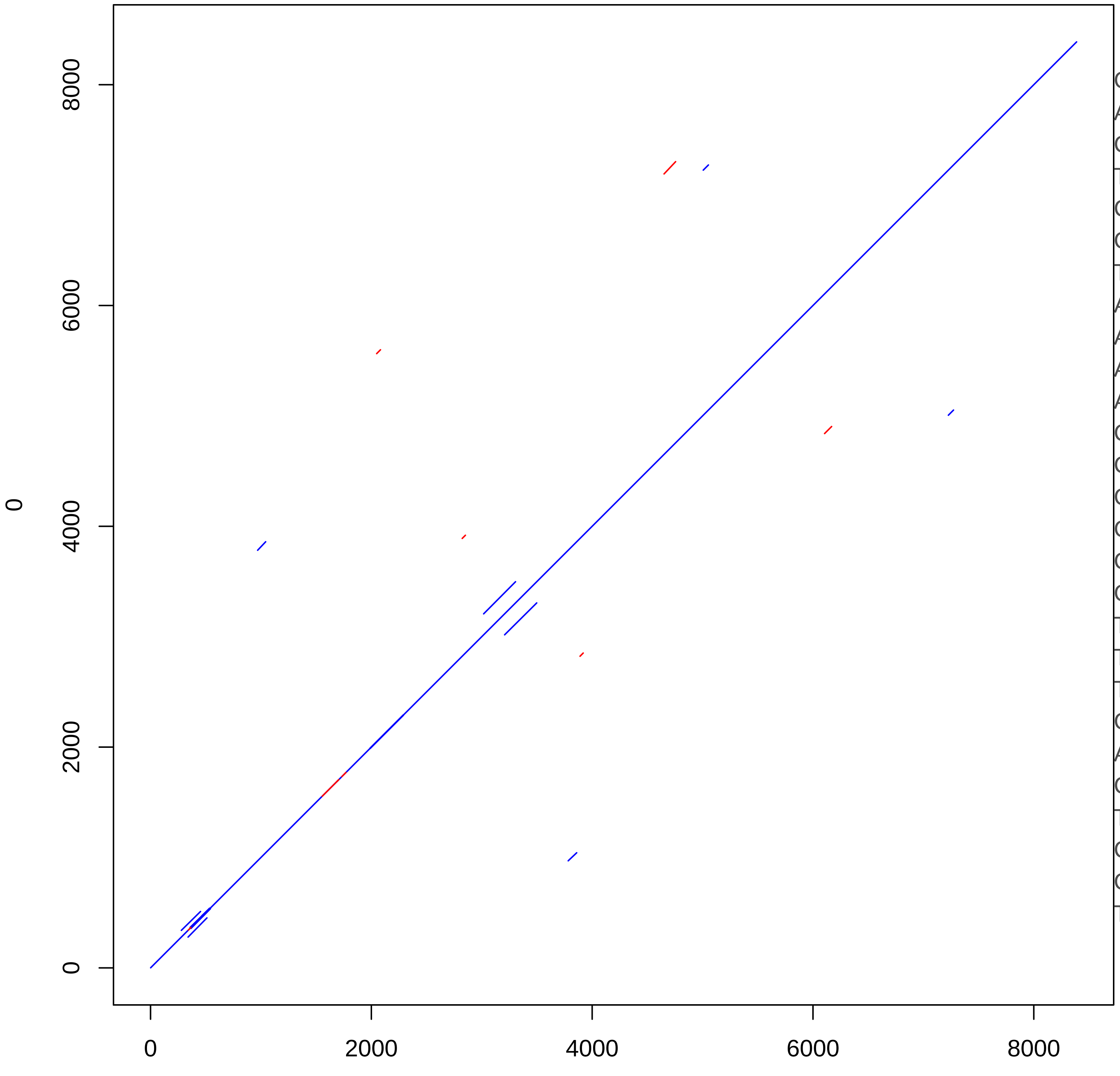

GCAGTAGCAGTA 13
AGCAGTAGCAGT 12
CAGTAGCAGTAG 12
TAGCAGTAGCAG 12
CCCCCCCCCCCC 11
GTAGCAGTAGCA 11
TTTTTTTTTTTT 3
AAATATTTTTTT 2
AATATTTTAATG 2
AATCATATACTT 2
ACAAATTTTCAT 2
CCAACAAAGTTT 2
CCATGAAATTTT 2
GAAACTTTGAAA 2
GCAGATTTTAAG 2
GGAAACTTTGAA 2
GTAGCAGTAGCG 2
TAATATTTTAAT 2
TATTTTAAATTT 2
TTTCATAATTTT 2
GCAGTAGCAGTA 13
AGCAGTAGCAGT 12
CAGTAGCAGTAG 12
TAGCAGTAGCAG 12
CCCCCCCCCCCC 11
GTAGCAGTAGCA 11
TTTTTTTTTTTT 3

0

>m141122_062923_42134_c100732202550000001823141405141562_s1_p0/72283/734_6606

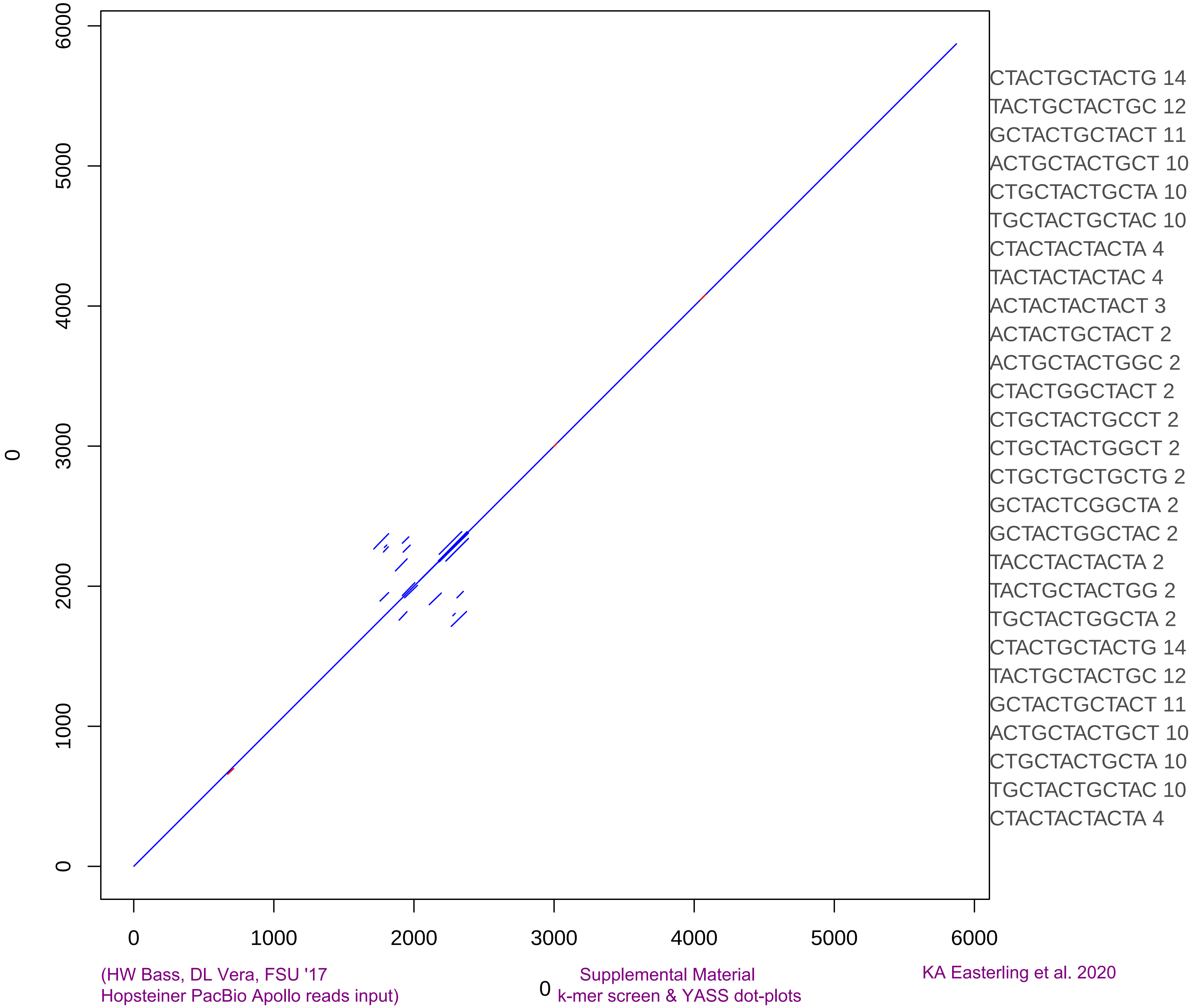

>m141122_062923_42134_c100732202550000001823141405141562_s1_p0/76312/335_6172

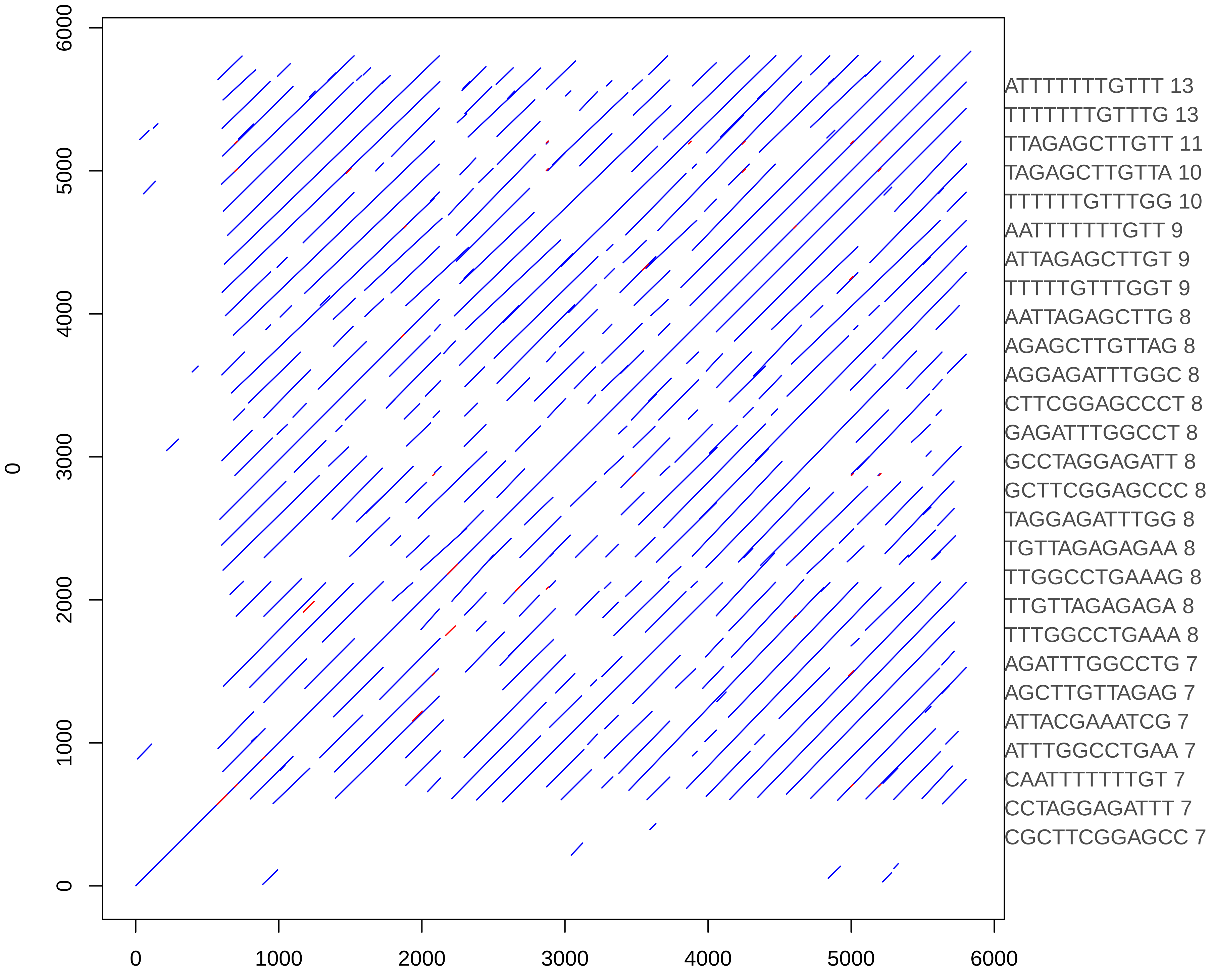

>m141122_062923_42134_c100732202550000001823141405141562_s1_p0/85611/12276_18118

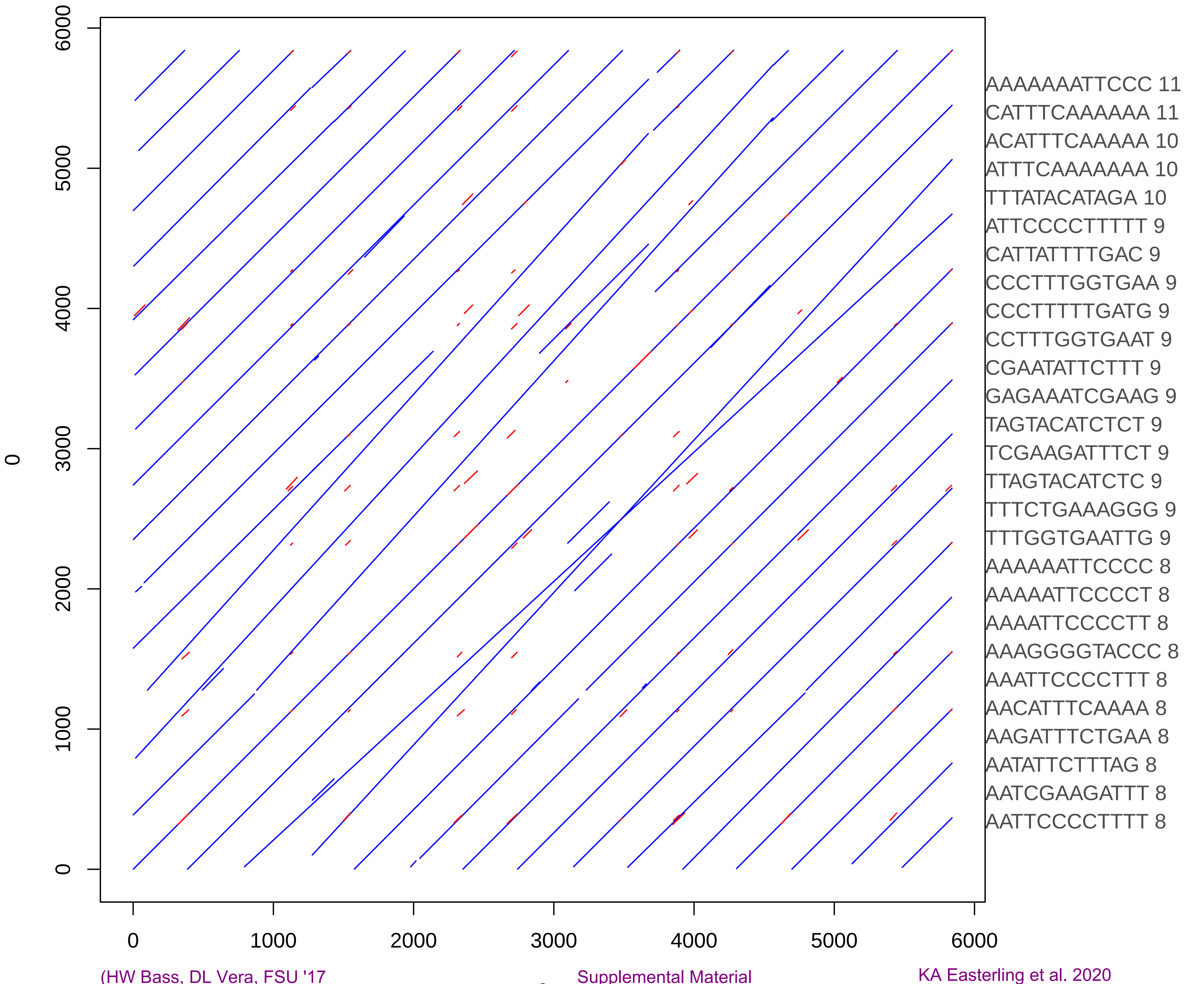

>m141122_062923_42134_c100732202550000001823141405141562_s1_p0/8682/2754_8592

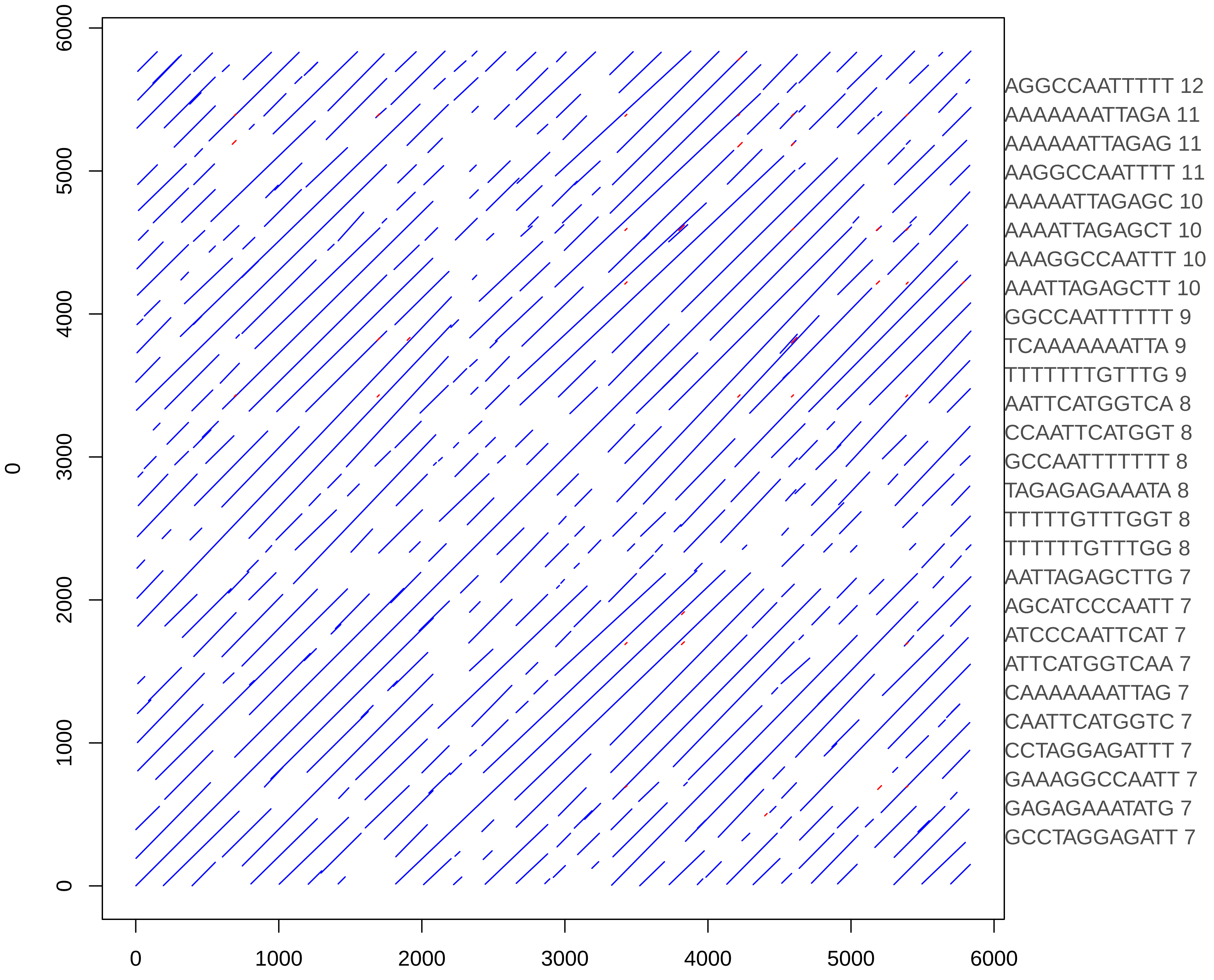

>m141122_062923_42134_c100732202550000001823141405141562_s1_p0/88775/12330_19993

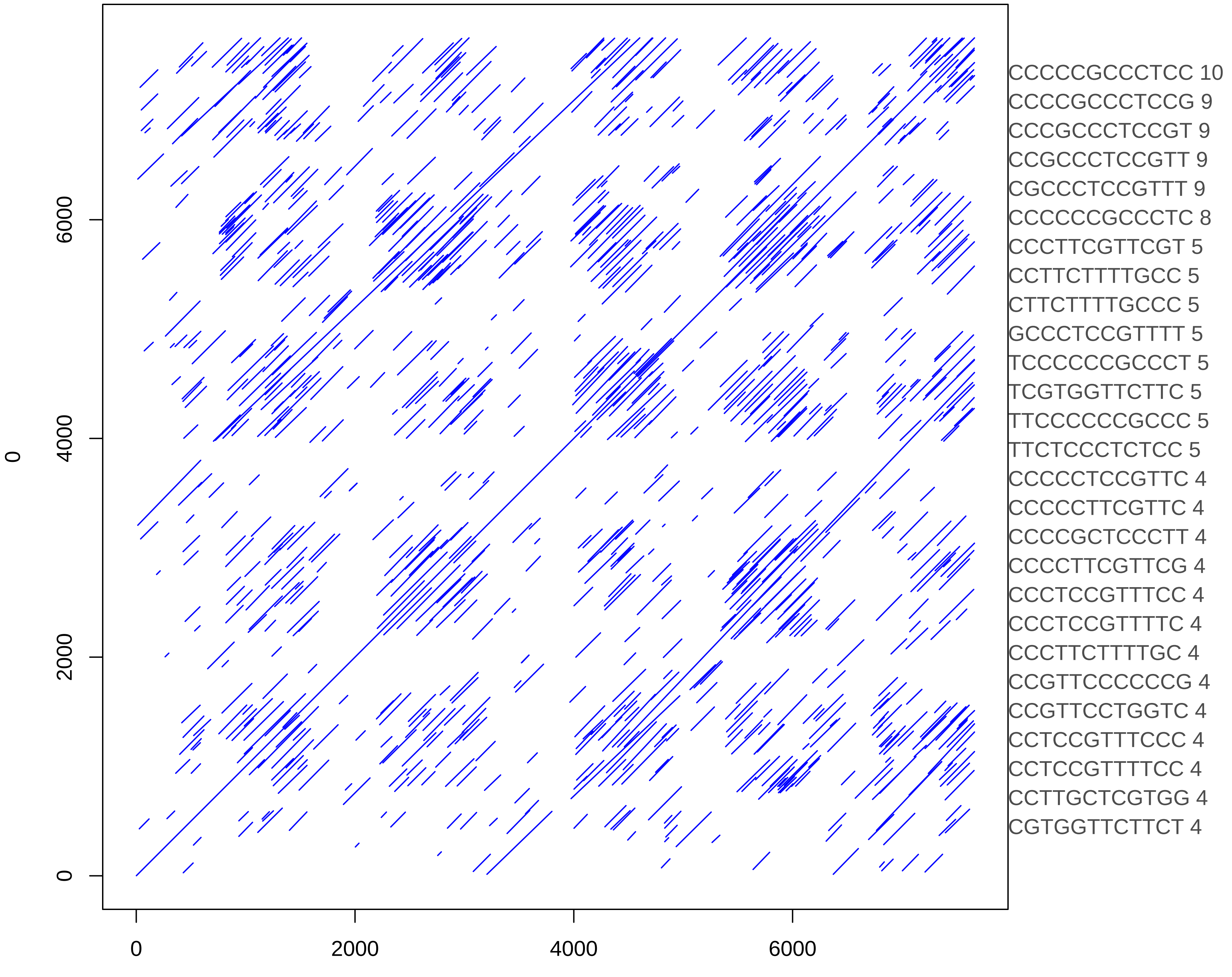

>m141122_062923_42134_c100732202550000001823141405141562_s1_p0/94342/8414_14094

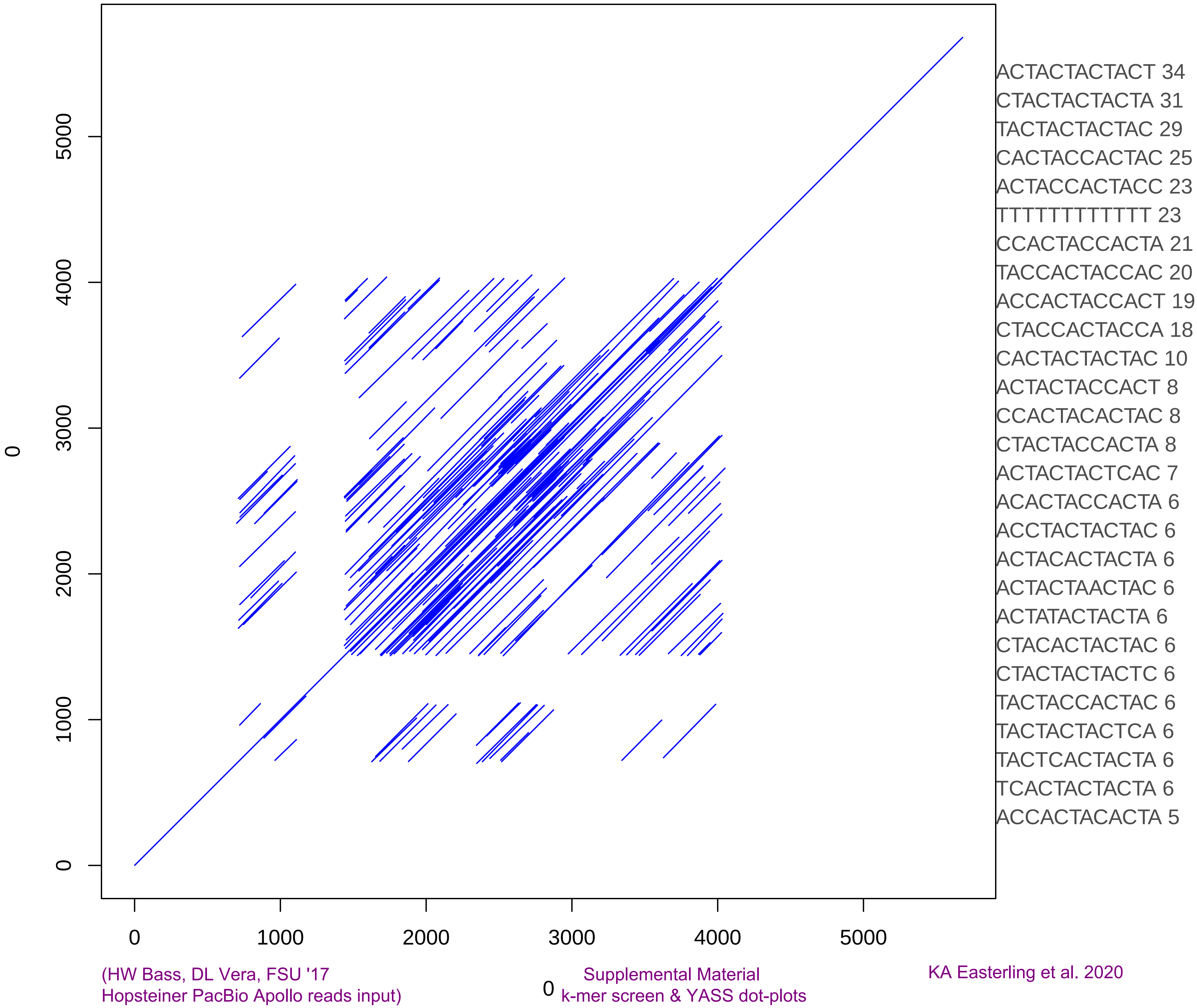

>m141122_062923_42134_c100732202550000001823141405141562_s1_p0/94834/5262_10342

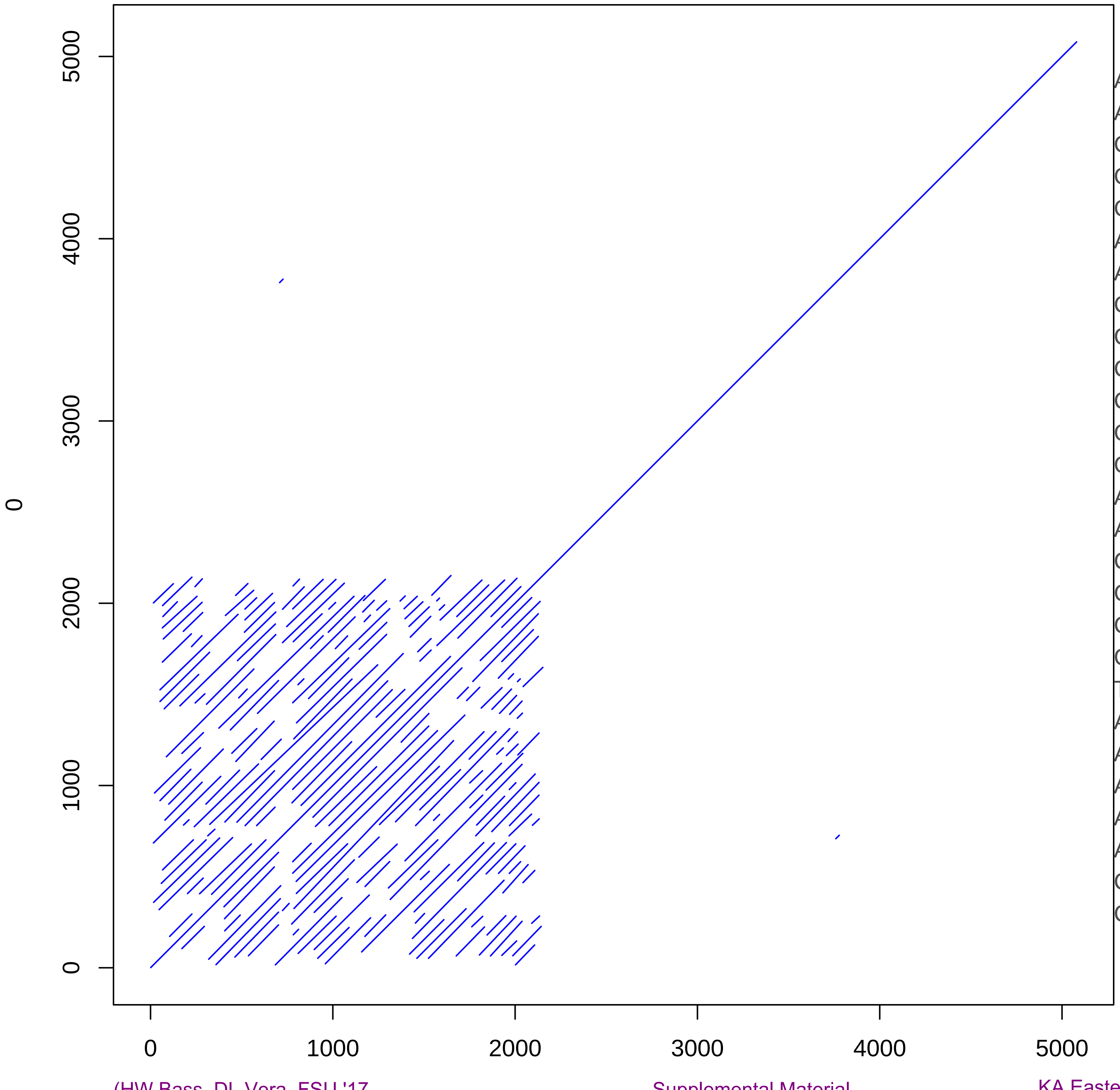

ACGAACGATGTC 11
AACGATGTCCGG 9
CGAACGATGTCC 9
GAACGATGTCCG 9
GACGAACGATGT 9
ACGATGTCCGGG 6
AGGCCGGTCCCA 5
CCCGGACGAACG 5
CCGGACGAACGA 5
CCGGTCCCACCC 5
CGGTCCCACCCC 5
GCCGGTCCCACC 5
GGCCGGTCCCAC 5
AAATAAAAGAGG 4
AATAAAAGAGGA 4
CGAACGATGTCG 4
CGATGTCCGGGA 4
CGGACGAACGAT 4
GGACGAACGATG 4
TAAATAAAAGAG 4
AAATAAAAGTGG 3
AATAAAAGTGGA 3
ACCGAGCCAAGG 3
ATAAAAGAGGAC 3
ATGTCCGGGAGT 3
CAGGCCGGTCCC 3
CCAGGCCGGTCC 3

>m141122_062923_42134_c100732202550000001823141405141562_s1_p0/99682/9607_21521

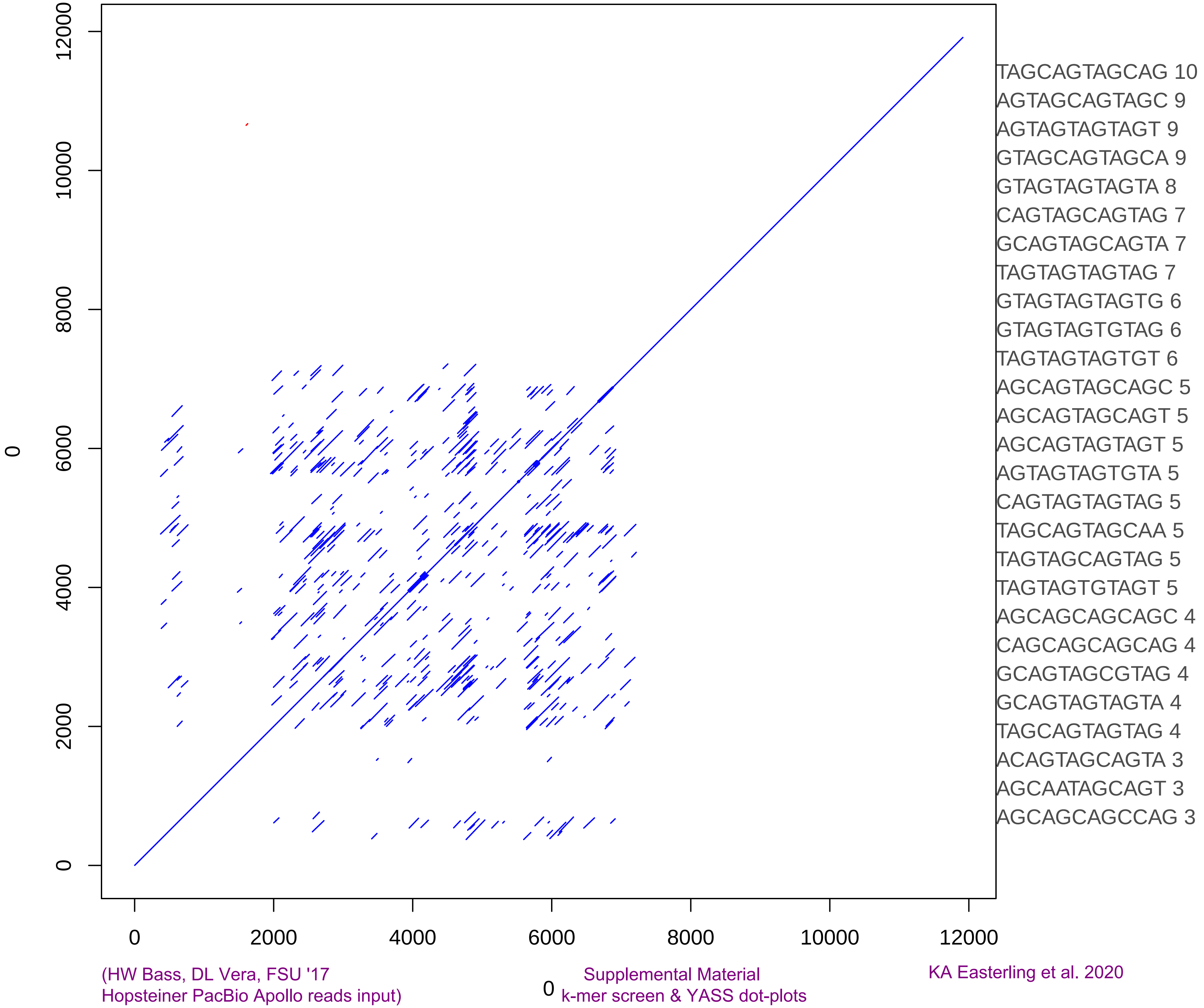

>m141122_085117_42134_c100732202550000001823141405141563_s1_p0/10516/6141_17516

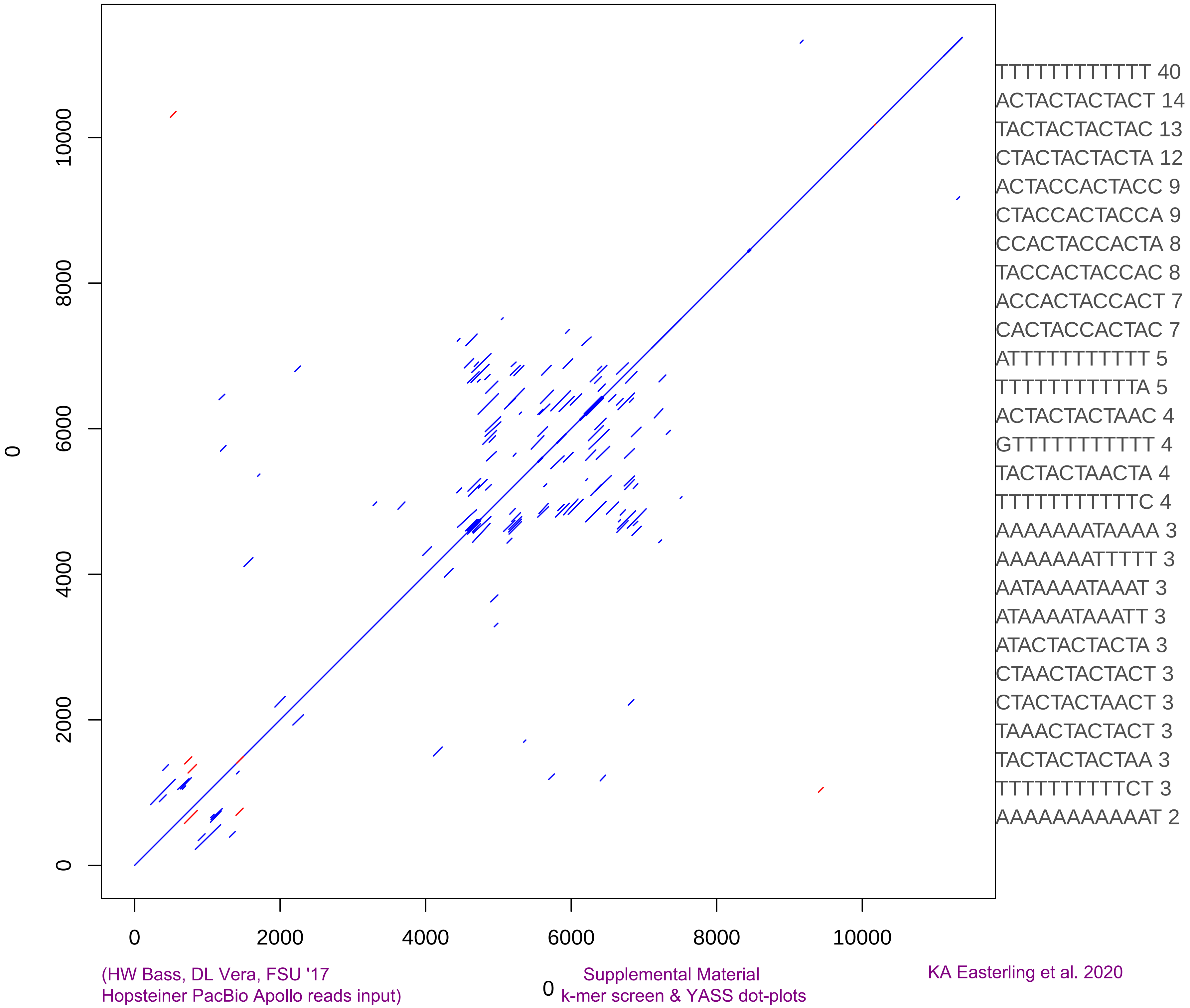

>m141122_085117_42134_c100732202550000001823141405141563_s1_p0/107421/10531_17923

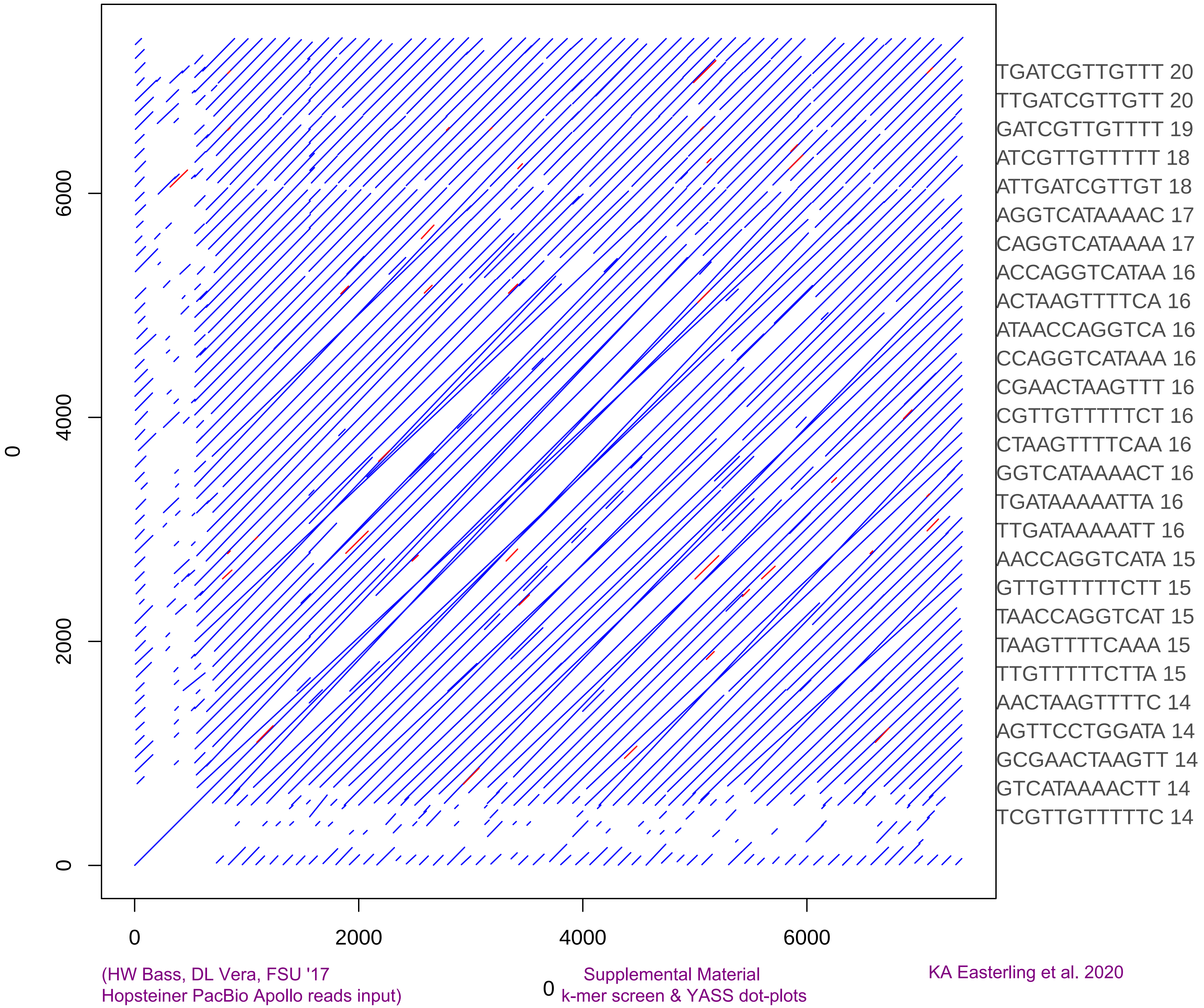

>m141122_085117_42134_c100732202550000001823141405141563_s1_p0/110275/15084_24448

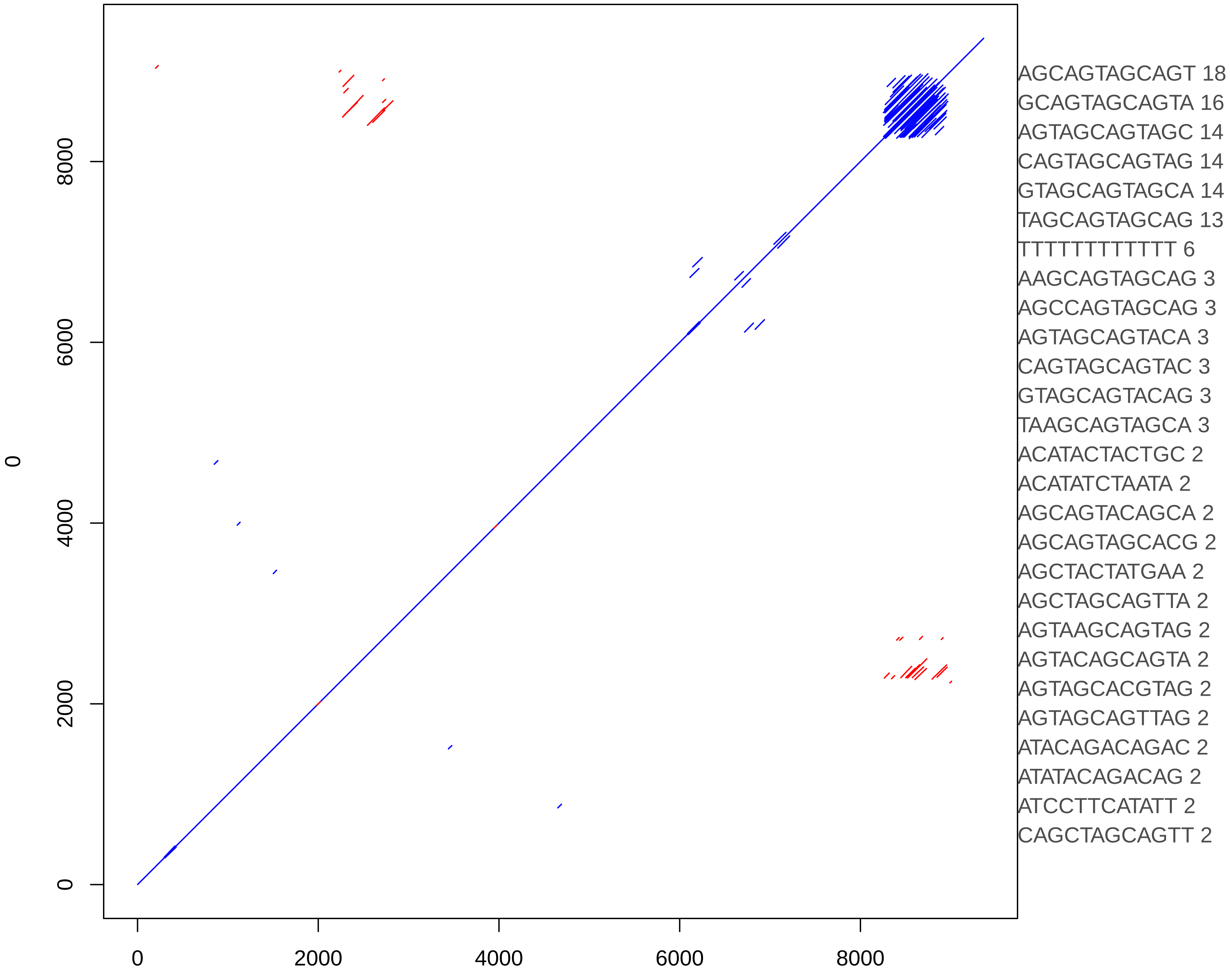

>m141122_085117_42134_c100732202550000001823141405141563_s1_p0/119917/6046_11597

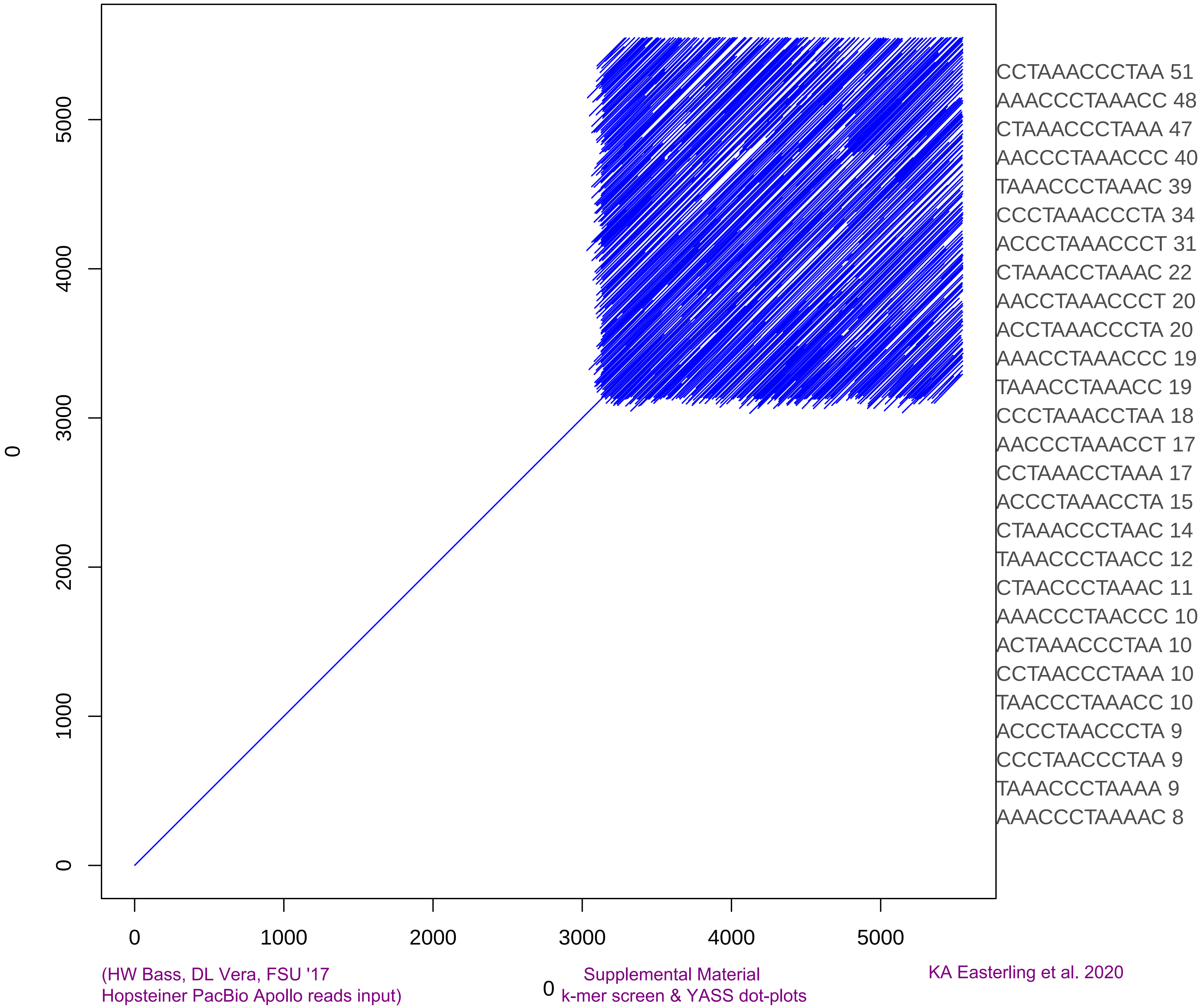

>m141122_085117_42134_c100732202550000001823141405141563_s1_p0/1266/0_6337

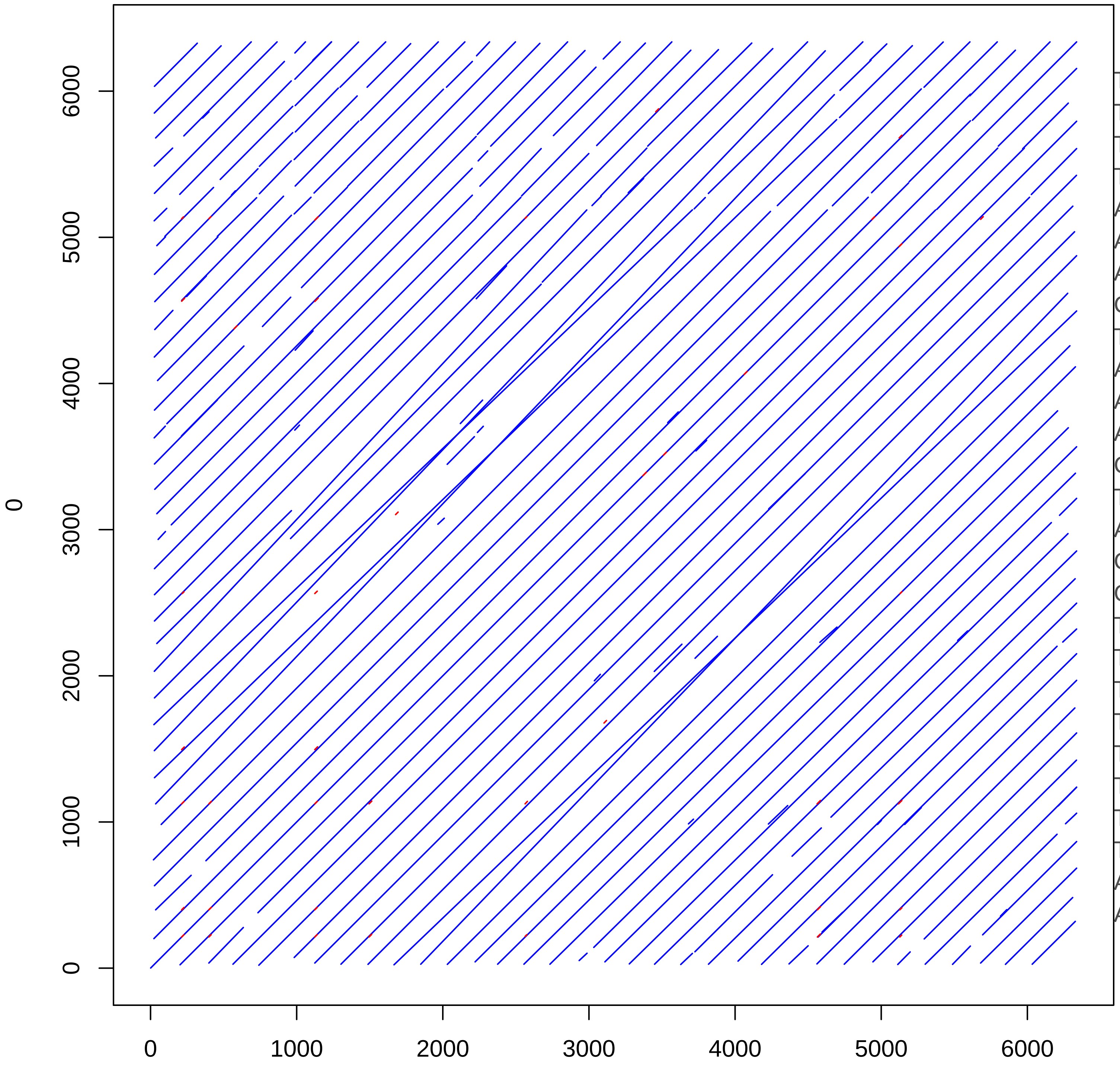

>m141122_085117_42134_c100732202550000001823141405141563_s1_p0/1266/6387_12111

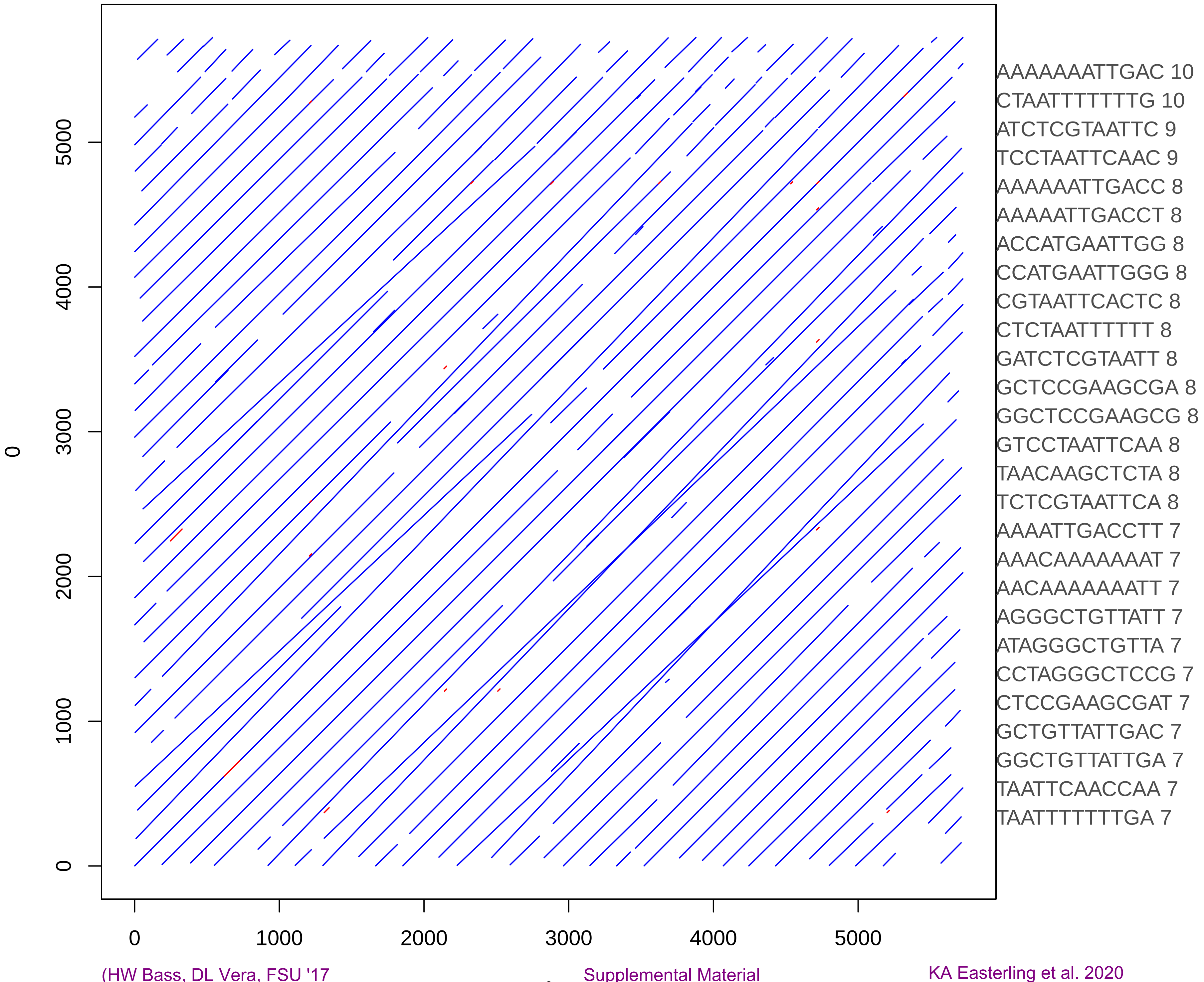

>m141122_085117_42134_c100732202550000001823141405141563_s1_p0/130265/8255_15686

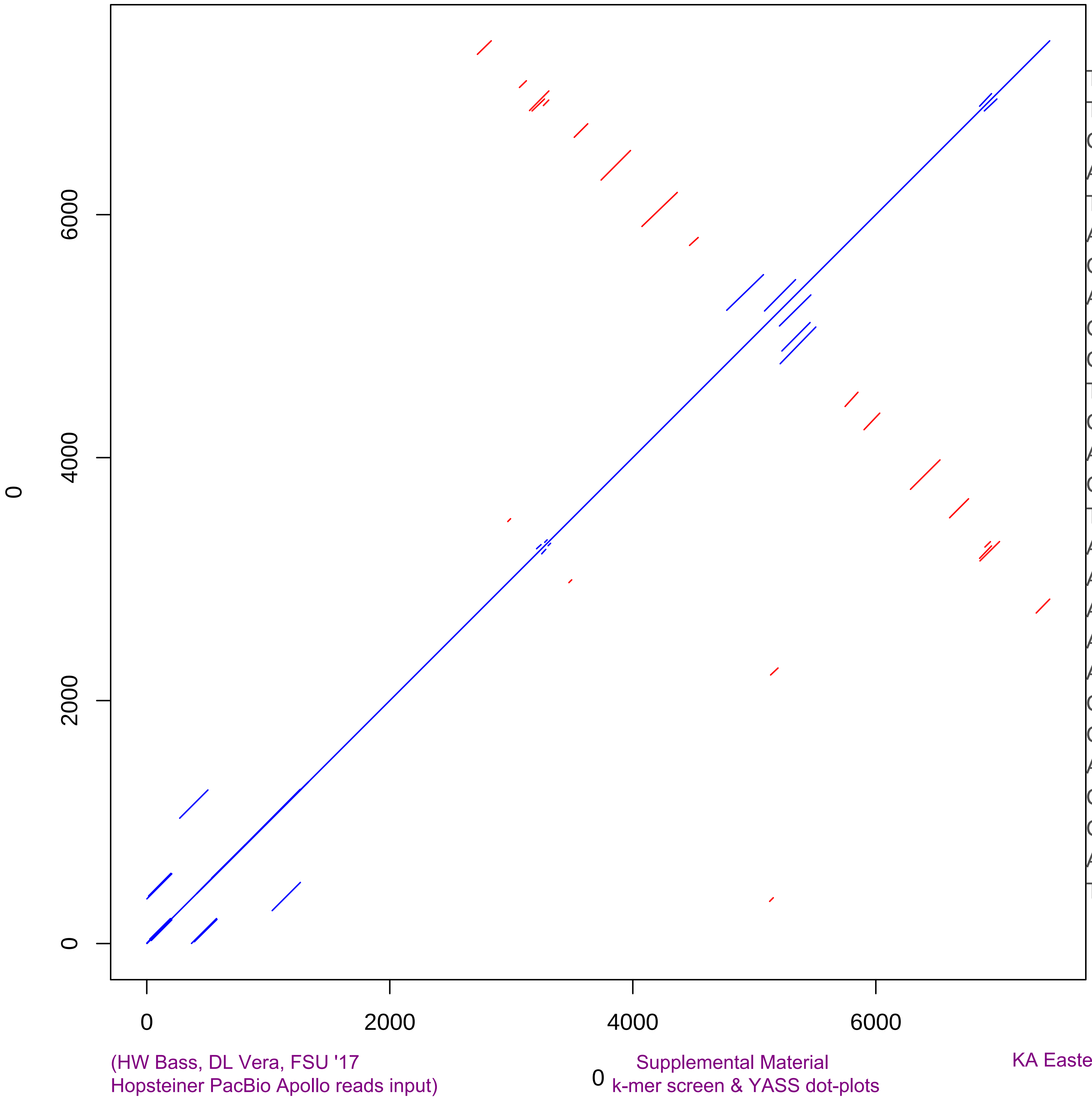

TTTTTTTTTTTT 62
TACTACTACTAC 33
CTACTACTACTA 32
ACTACTACTACT 30
TACCACTACTAC 16
ACTACTACTACC 15
CACTACTACTAC 14
ACTACTACCACT 13
CTACCACTACTA 13
CTACTACCACTA 13
TACTACCACTAC 13
CTACTACTACCA 12
ACTACCACTACT 11
CCACTACTACTA 10
TACTACTACCAC 10
ACCACTACTACT 9
ACTACACTACTA 8
ACCACTACTACC 7
ACACTACTACTA 6
ACTACTACACTA 6
CCACTACTACCA 6
CTACACTACTAC 6
ACTACTACTACA 5
CACTACTACCAC 5
CTACTACACTAC 5
ATTTTTTTTTTT 4
TACACTACTACT 4

>m141122_085117_42134_c100732202550000001823141405141563_s1_p0/131396/15718_23022

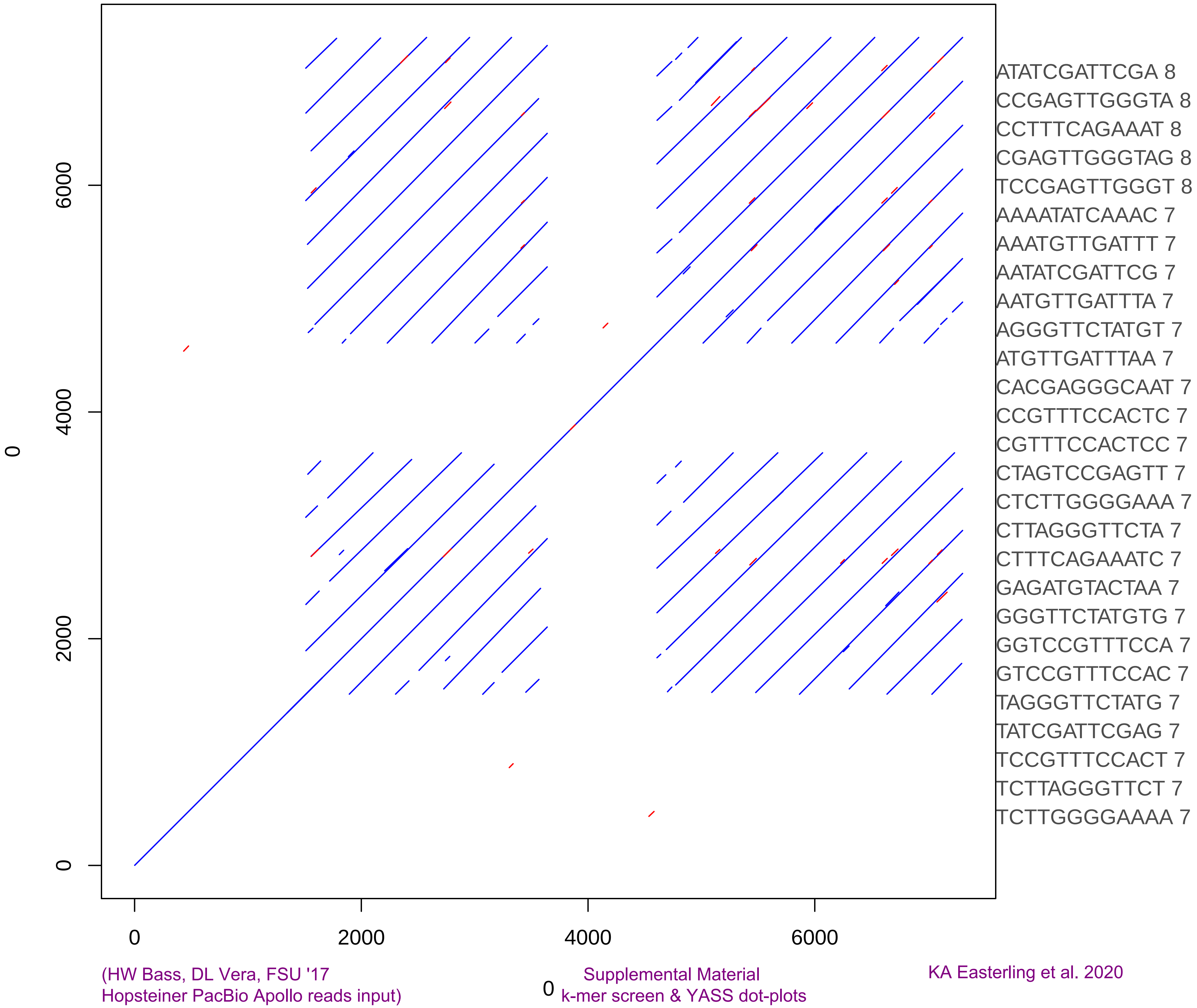

>m141122_085117_42134_c100732202550000001823141405141563_s1_p0/143485/4625_10883

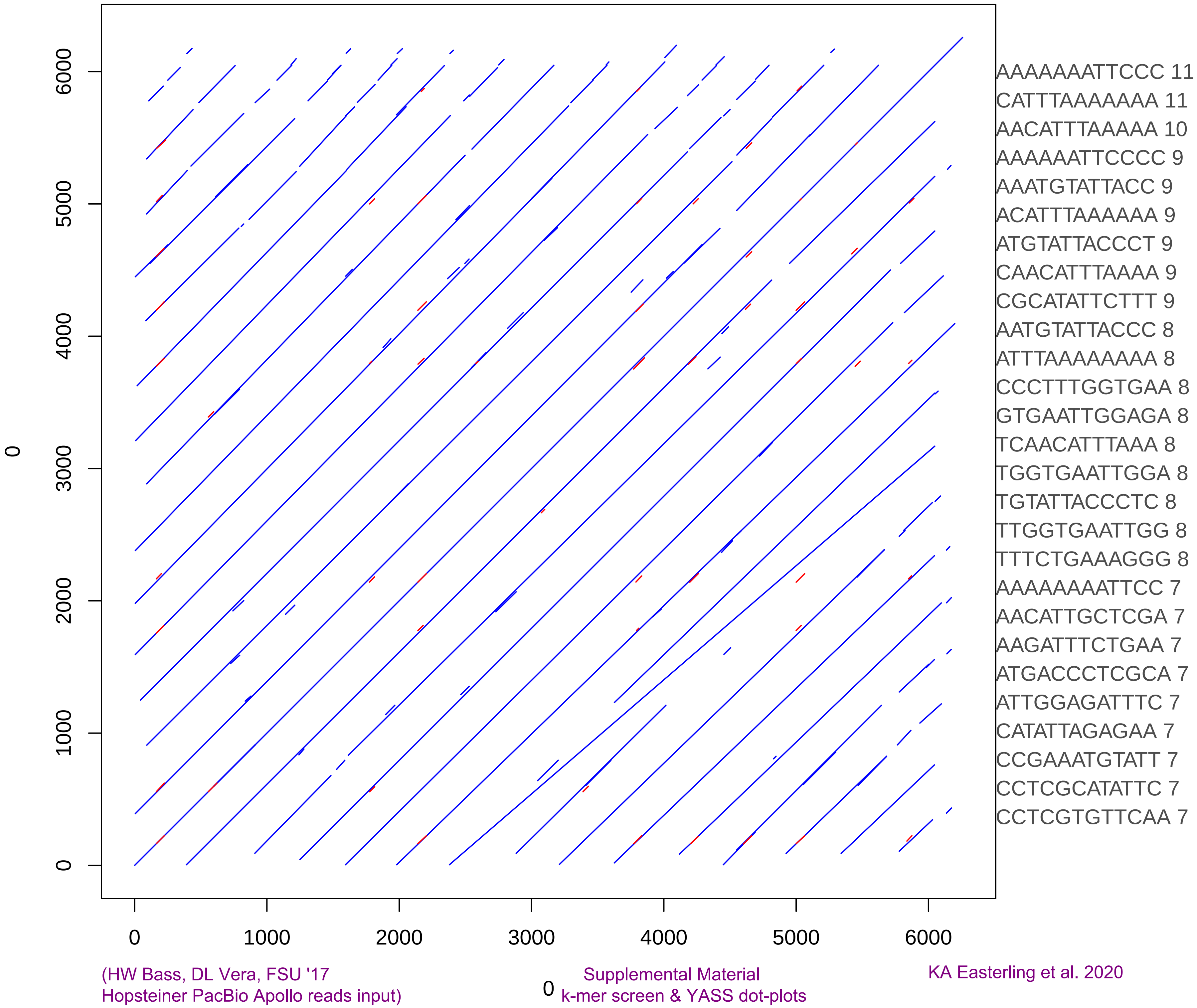

>m141122_085117_42134_c100732202550000001823141405141563_s1_p0/151569/8371_13678

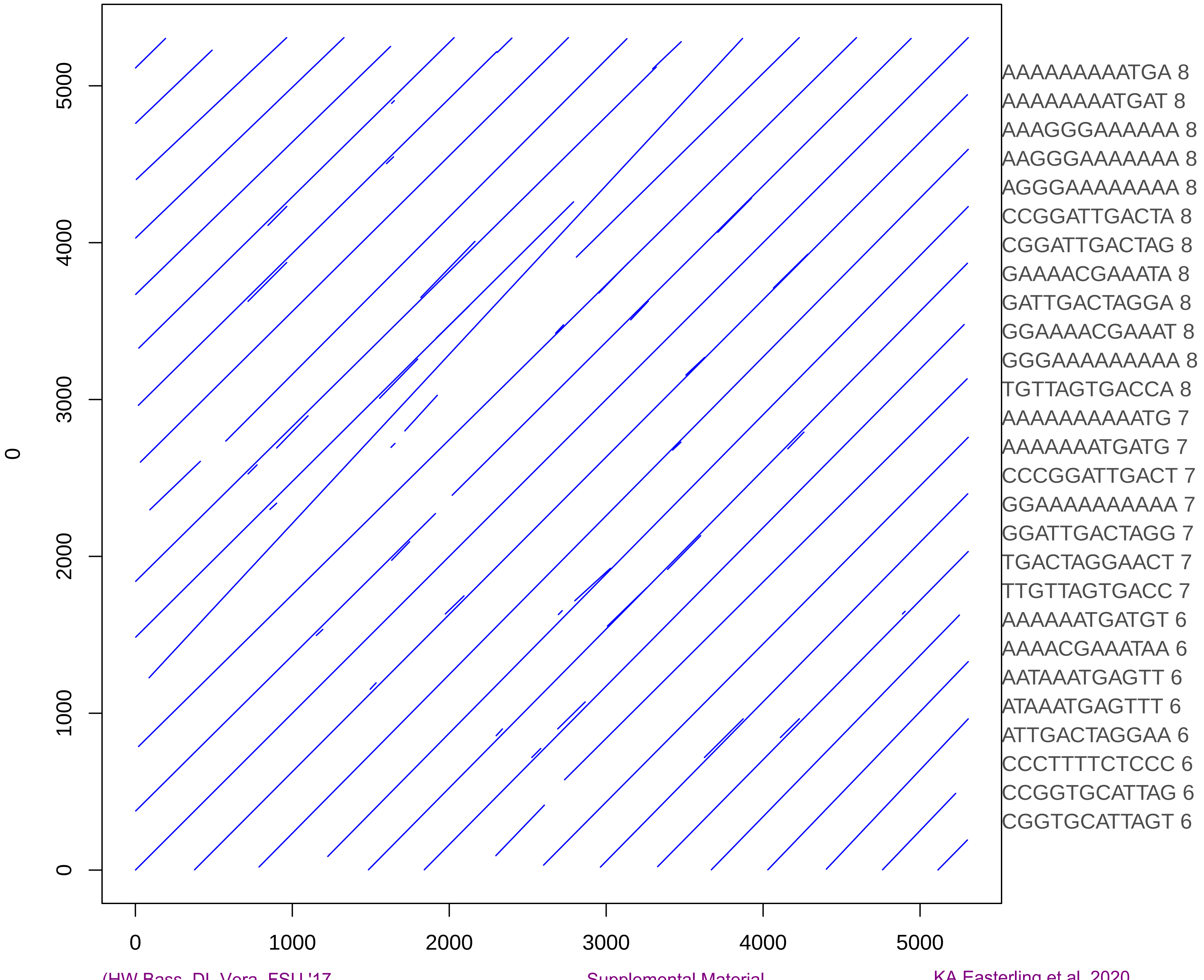

>m141122_085117_42134_c100732202550000001823141405141563_s1_p0/152677/7520_13040

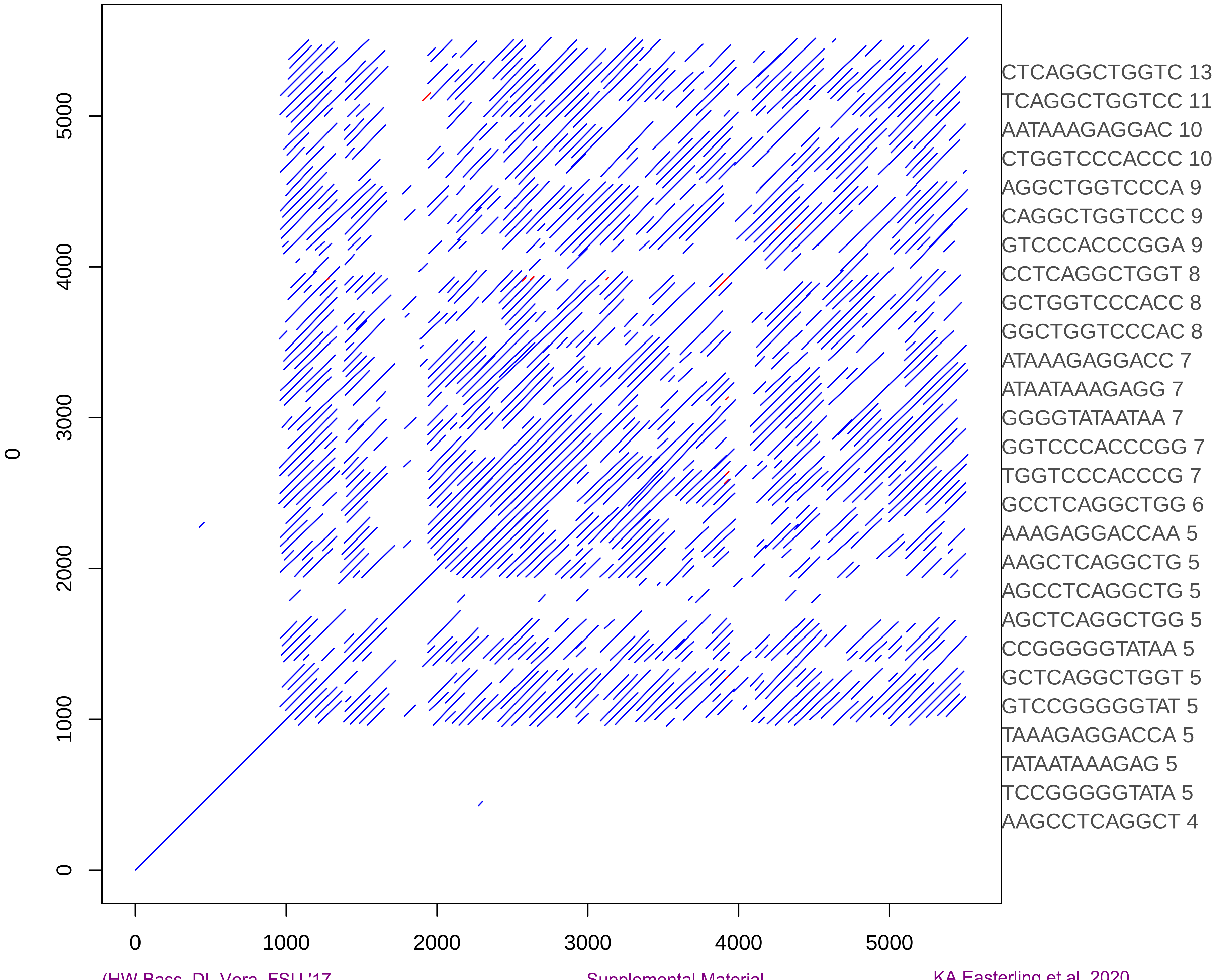

>m141122_085117_42134_c100732202550000001823141405141563_s1_p0/155193/12703_18186

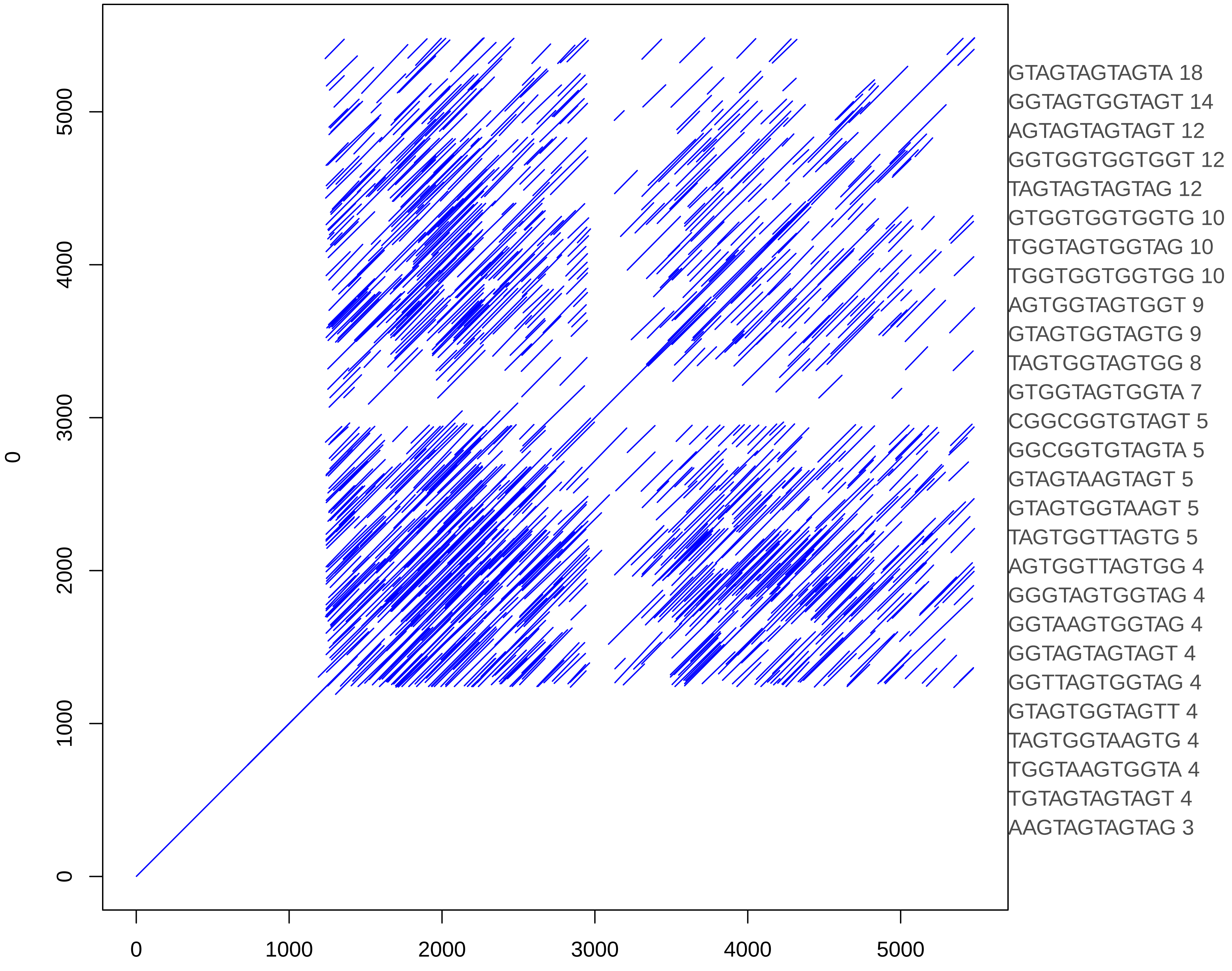

>m141122_085117_42134_c100732202550000001823141405141563_s1_p0/1576/3628_9864

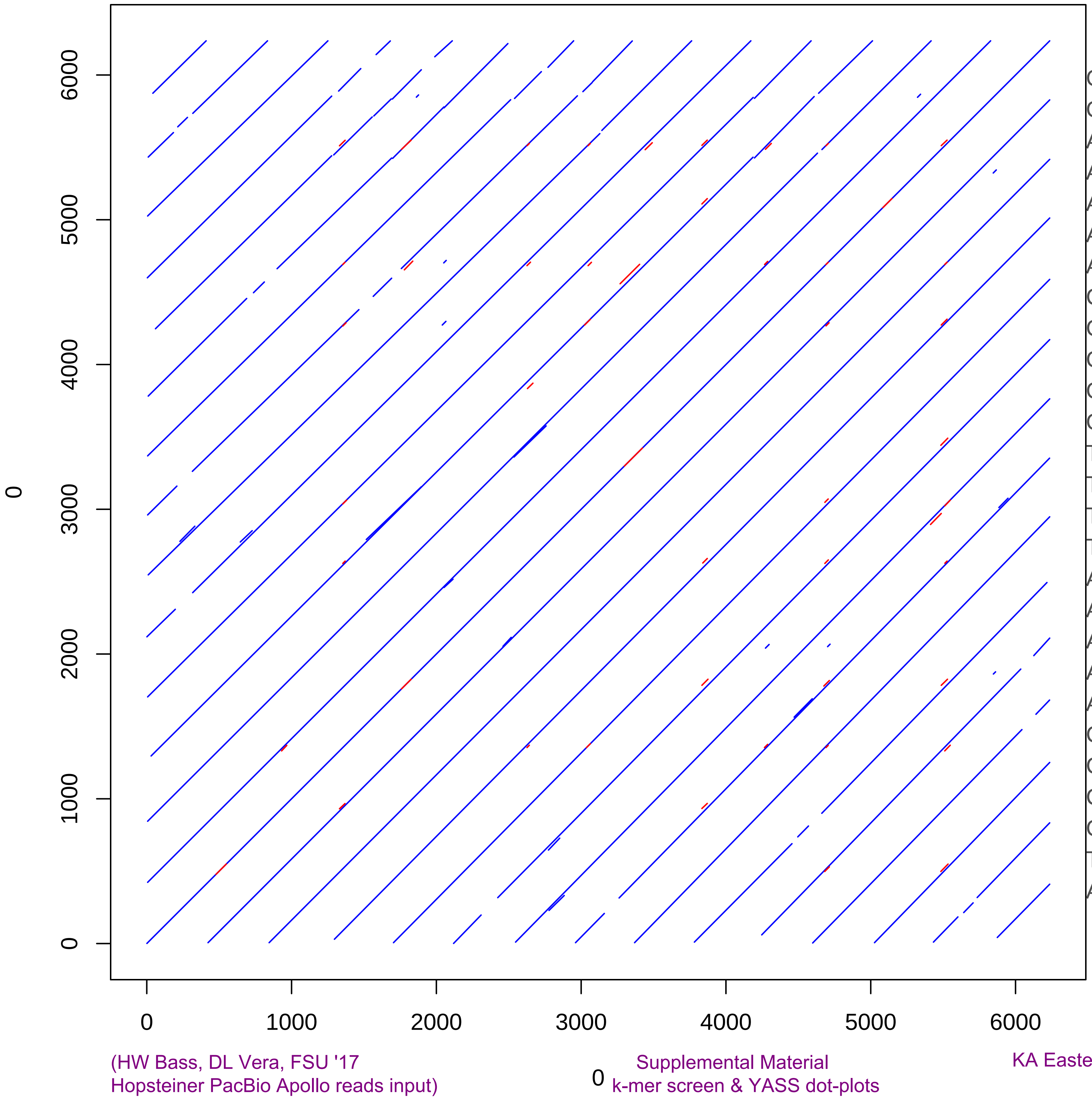

GGAGTTTTTTTT 10
GGGAGTTTTTTT 10
AATAGATTCATT 8
AATGTGTAAAAT 8
AGGGCTCCGTTT 8
ATAGATTCATTT 8
ATGTGTAAAATT 8
CTAGGGCTCCGT 8
GCTCCGTTTCAA 8
GGCTCCGTTTCA 8
GGGCTCCGTTTC 8
GGGGAGTTTTTT 8
TAGATTCATTTA 8
TAGGGCTCCGTT 8
TGTGTAAAATTT 8
TTTTTTTTCAAA 8
AAATGTTGAACT 7
AACGATTTGAAG 7
AGATTCATTTAA 7
AGGGGAGTTTTT 7
ATTTCTCTAATA 7
CAACGATTTGAA 7
CATTTAACCATG 7
CGATTTCTCTAA 7
GATTTCTCTAAT 7
TTTCTCTAATAT 7
AAAACTAAAGAA 6

>m141122_085117_42134_c100732202550000001823141405141563_s1_p0/160103/4796_18661

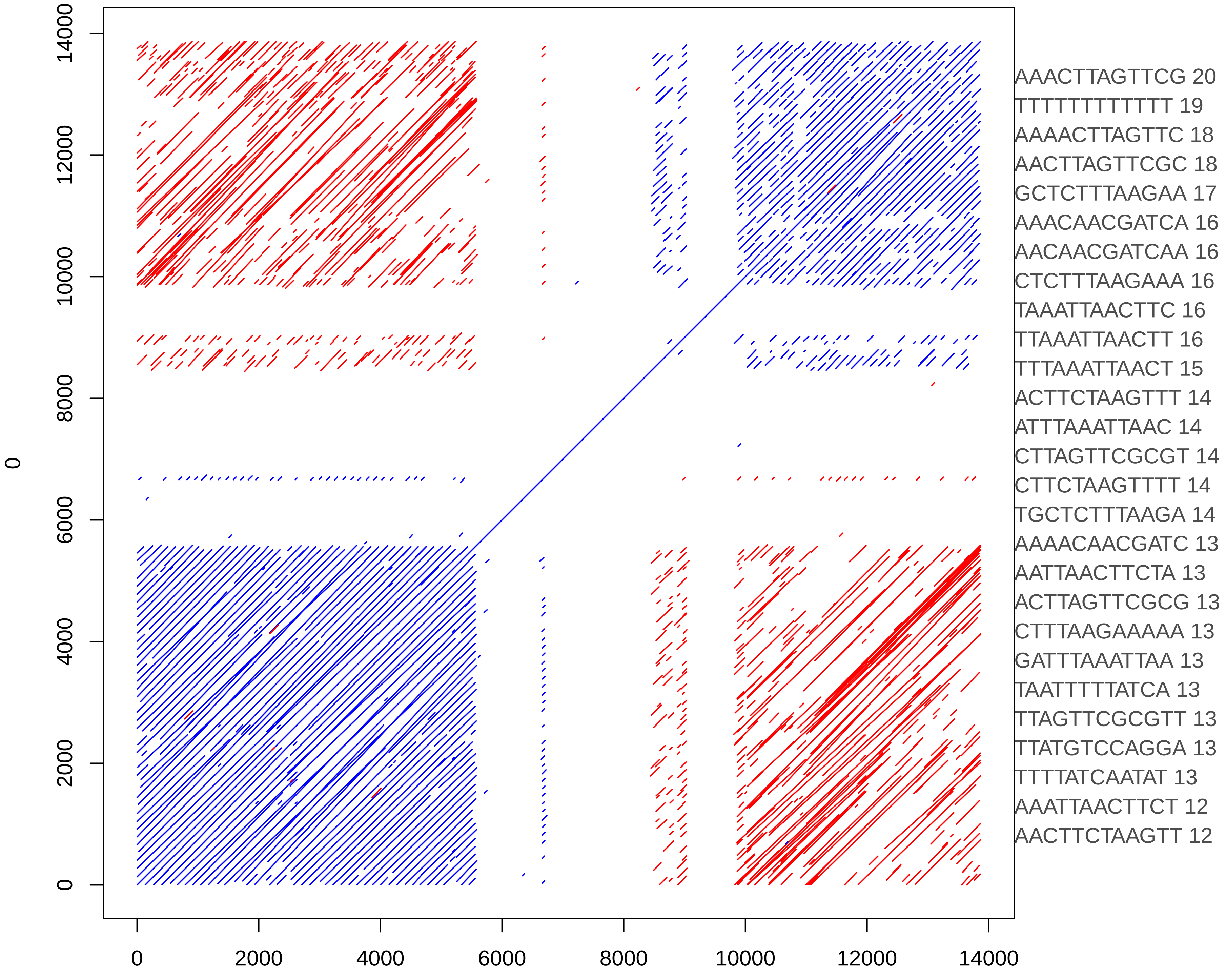

>m141122_085117_42134_c100732202550000001823141405141563_s1_p0/16919/1513_13983

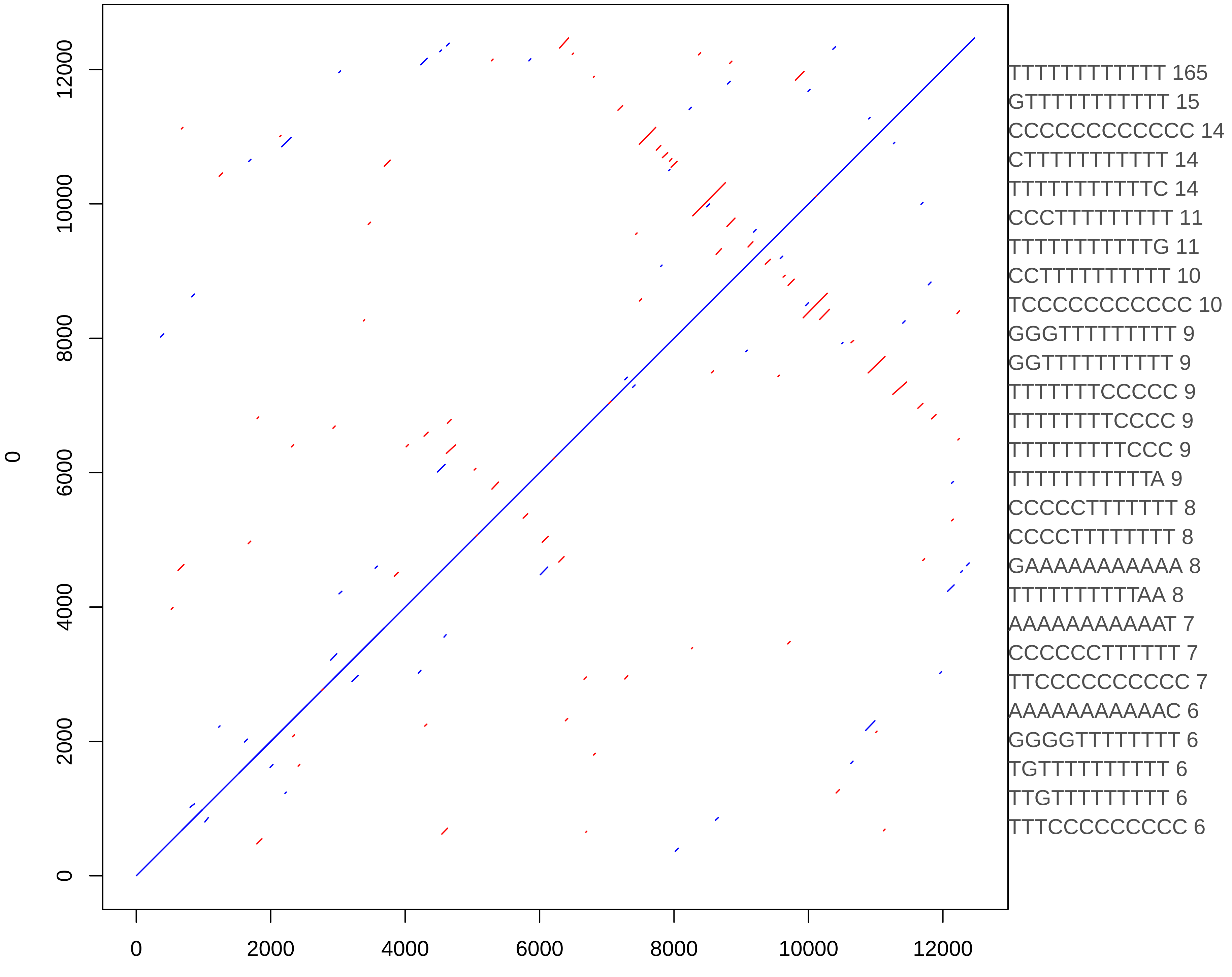

>m141122_085117_42134_c100732202550000001823141405141563_s1_p0/2015/4582_14914

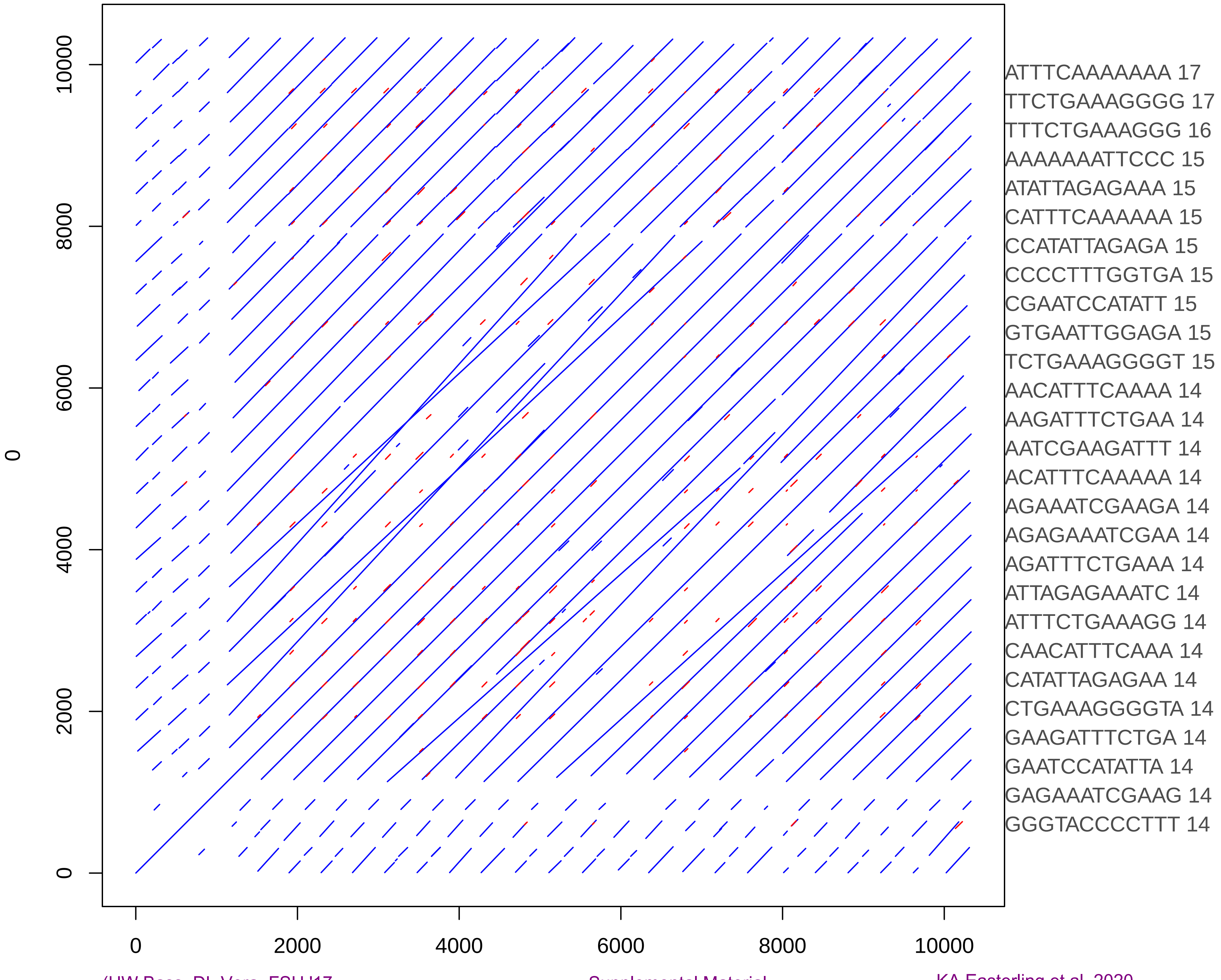

0

>m141122_085117_42134_c100732202550000001823141405141563_s1_p0/25839/8078_15071

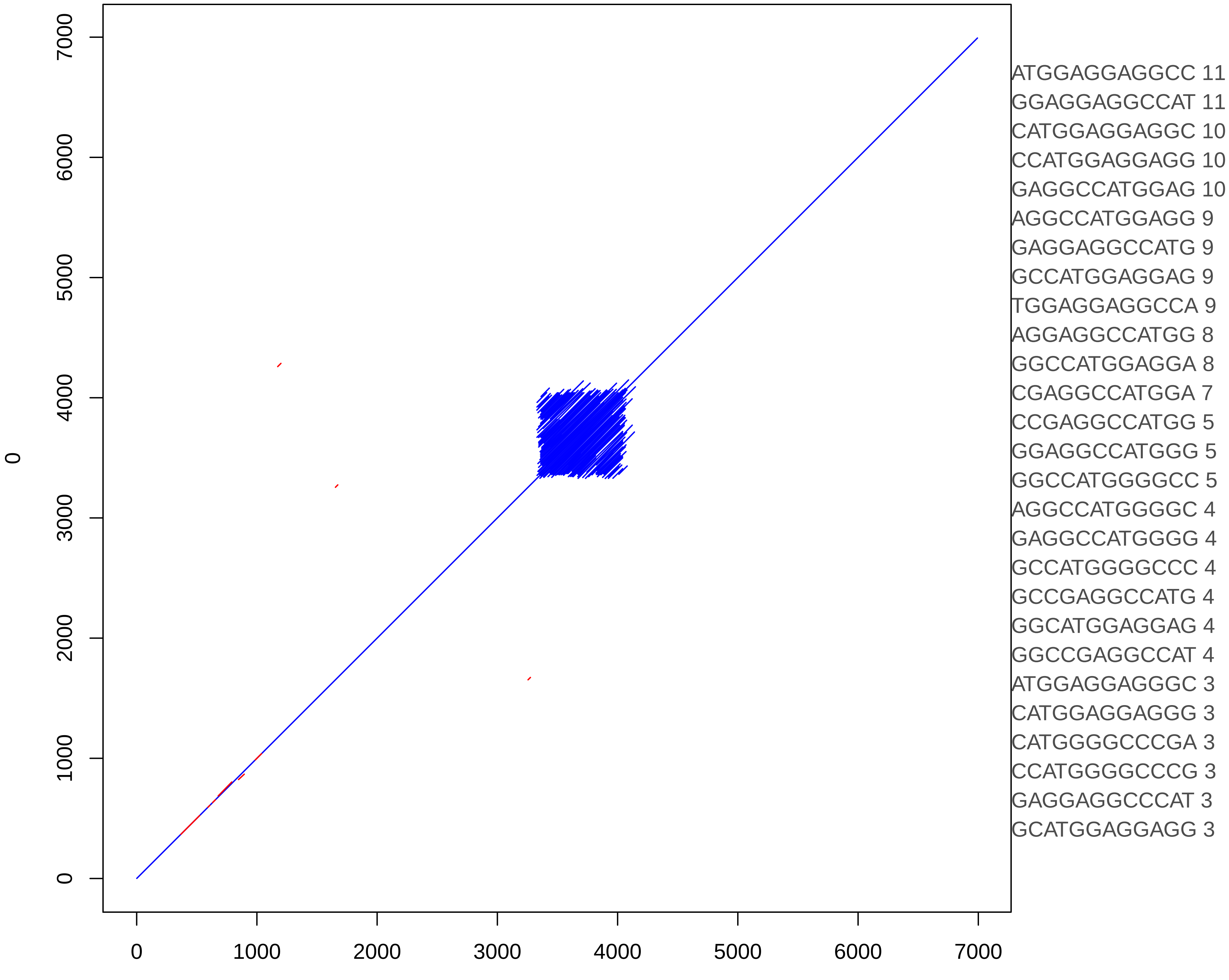

>m141122_085117_42134_c100732202550000001823141405141563_s1_p0/26822/5613_13588

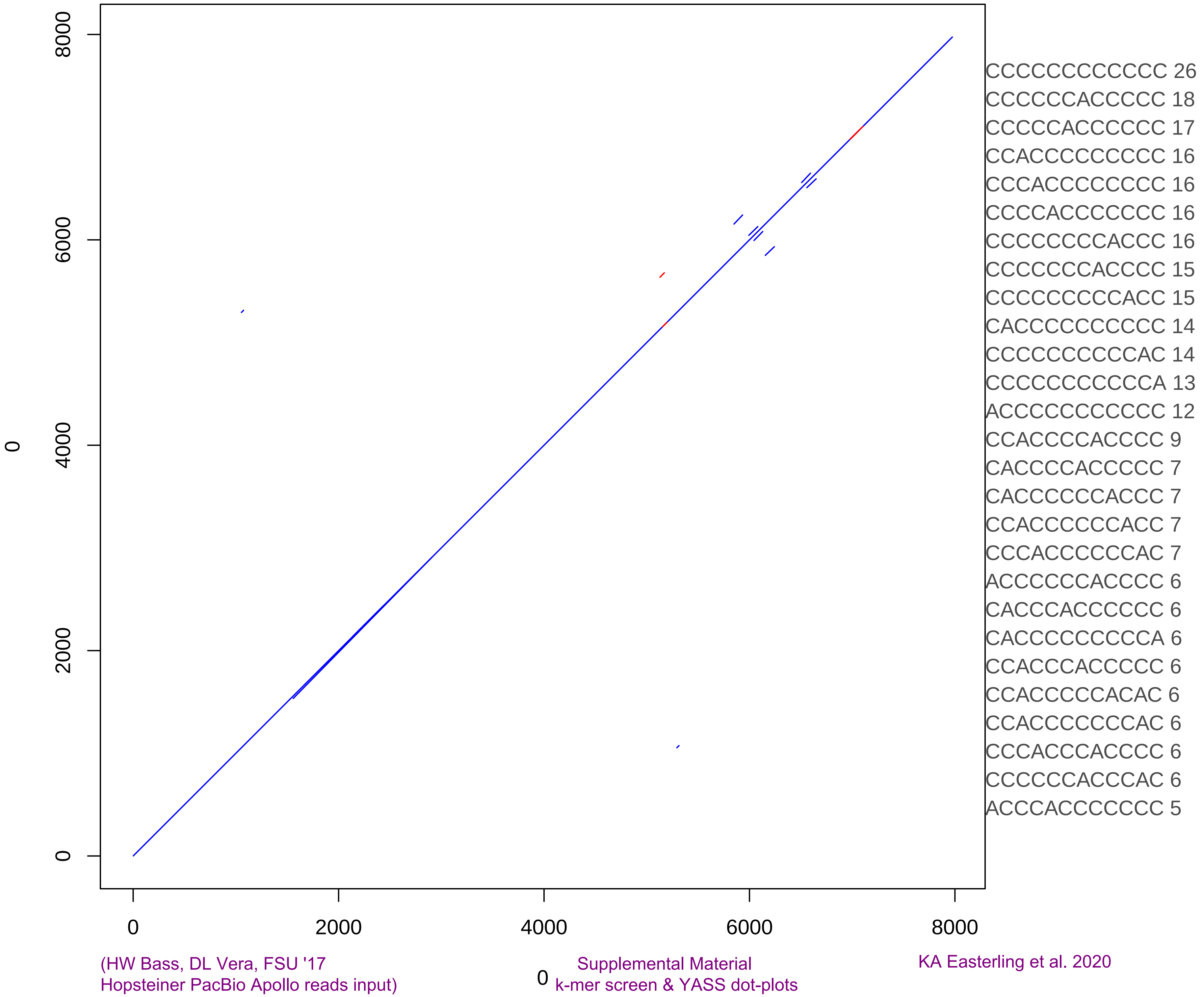

>m141122_085117_42134_c100732202550000001823141405141563_s1_p0/27668/1372_7726

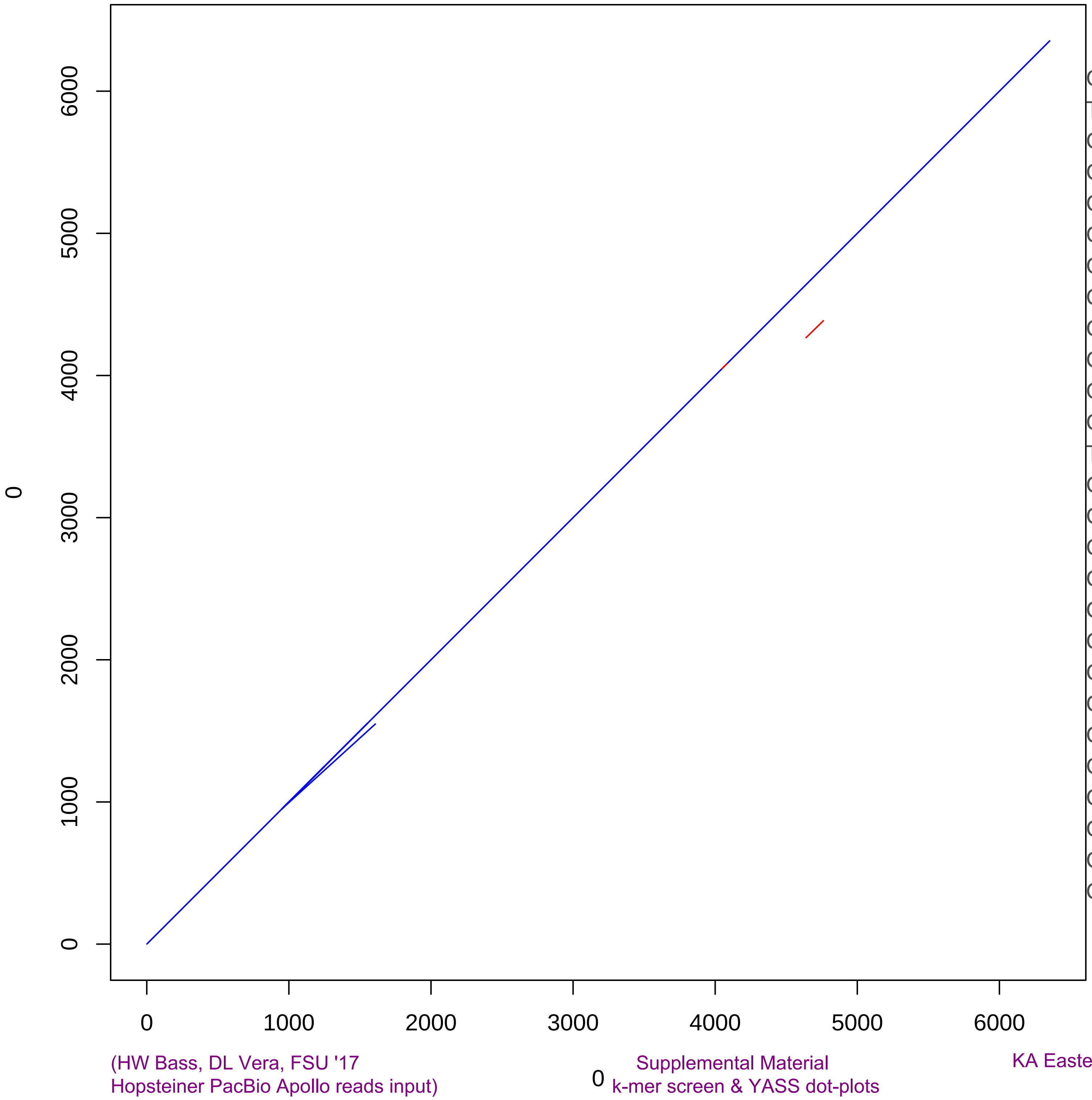

GGGGGGGGGGGG 21
TGGGGGGGGGGG 10
GTGGGGGGGGGG 9
GGGGGGGGGGGT 8
GGTGGGGGGGGG 8
GGGTGGGGGGGG 7
GGGGGGGGGGTG 5
GGGGGGGTGGGG 5
GGGGGGTGGGGG 5
GGGGGTGGGGGG 5
GGGGTGGGGGGG 5
GGGGGGGGTGGG 4
TGGGTGGGGGGG 4
GAGGGGTGGGGG 3
GGGGGGGGGAGG 3
GGGGGGGGGTGG 3
GGGGGTGGGTGG 3
GGGGTGAGGGGT 3
GGGGTGGGTGGG 3
GGGTGAGGGGTG 3
GGGTGGGTGGGG 3
GGTGAGGGGTGG 3
GGTGGGTGGGGG 3
GGTGGTGTGGGG 3
GTGAGGGGTGGG 3
GTGGGTGGGGGG 3
GTGGTGTGGGGG 3

>m141122_085117_42134_c100732202550000001823141405141563_s1_p0/27861/2114_10062

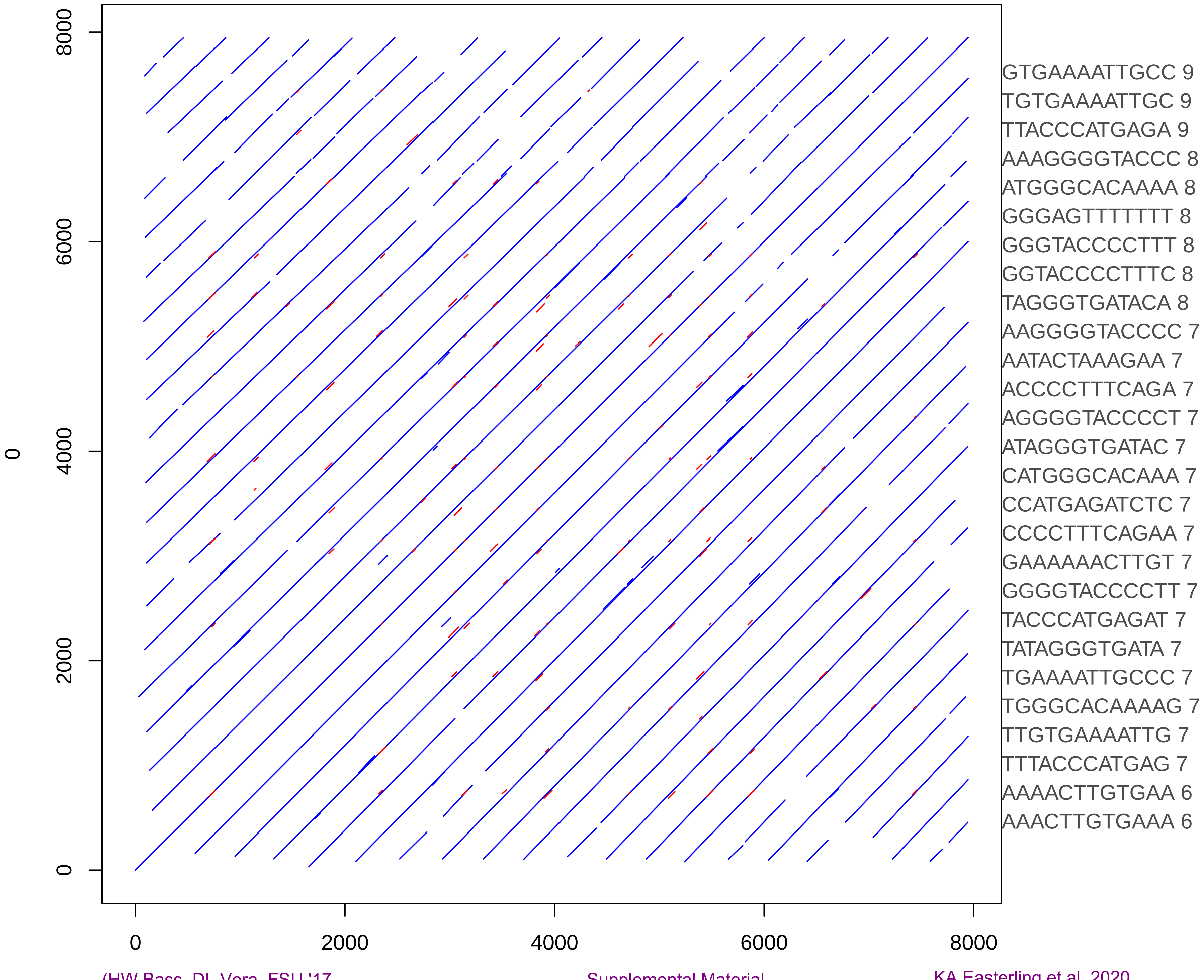

>m141122_085117_42134_c100732202550000001823141405141563_s1_p0/31139/5466_19642

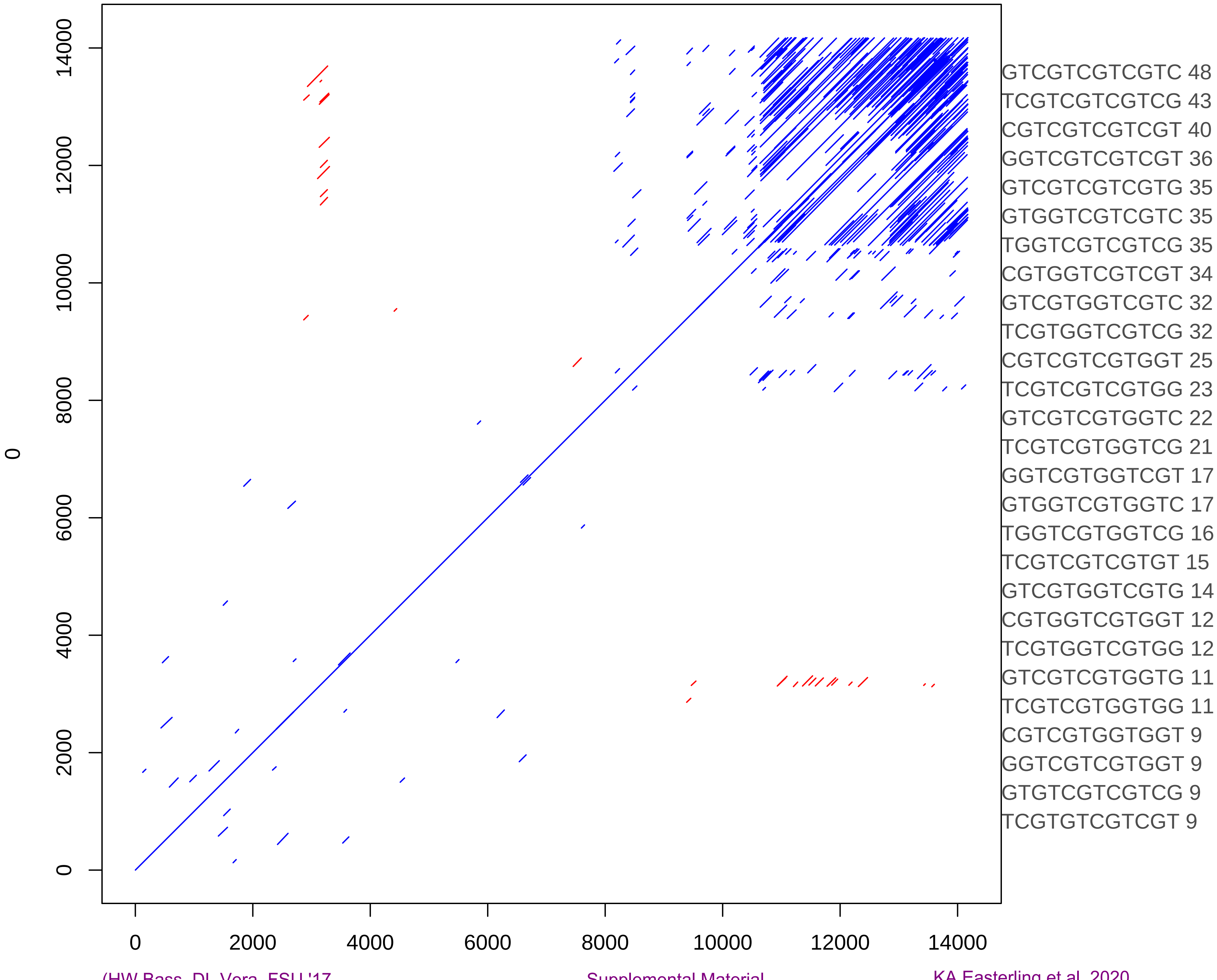

>m141122_085117_42134_c100732202550000001823141405141563_s1_p0/3259/818_8635

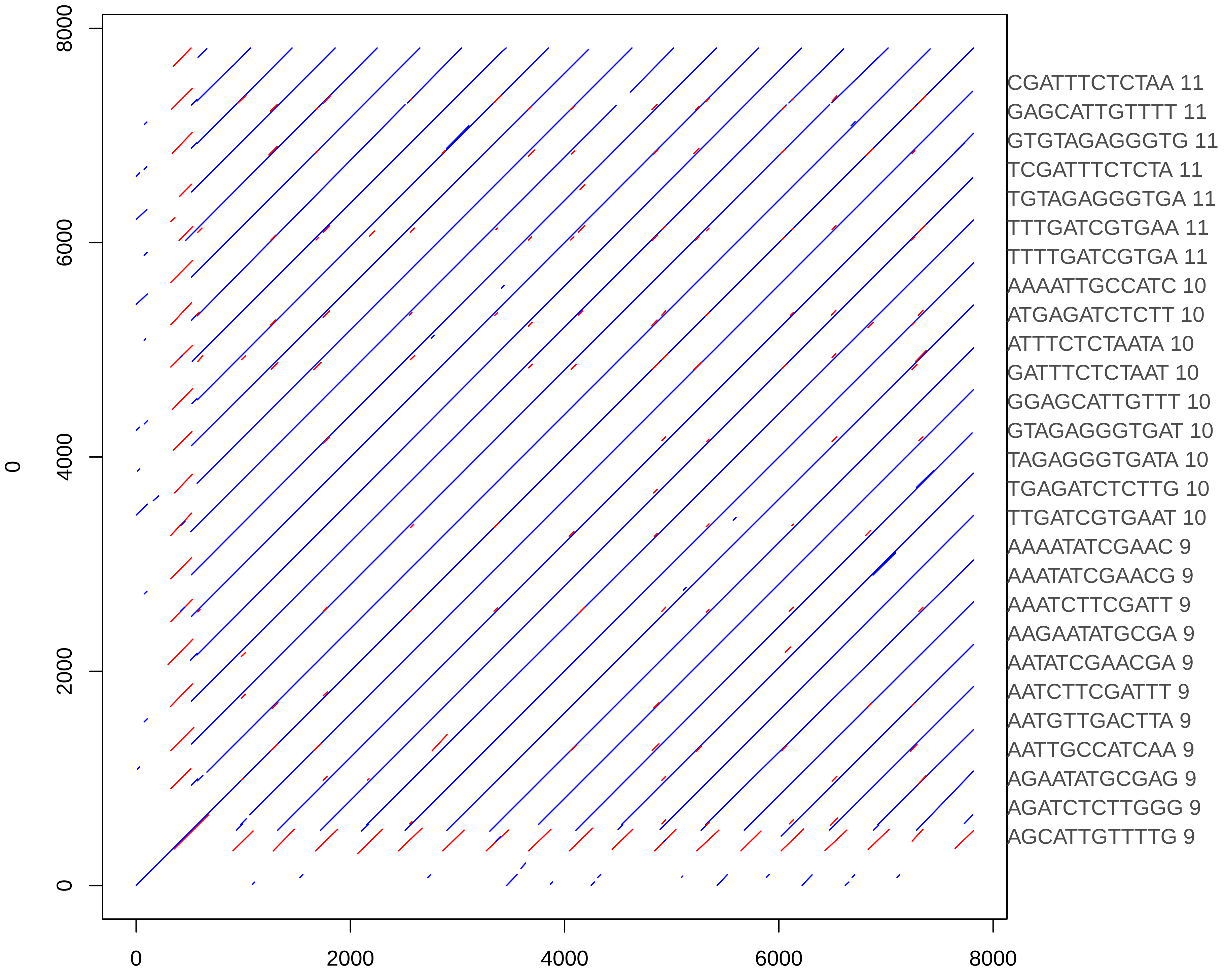

>m141122_085117_42134_c100732202550000001823141405141563_s1_p0/3732/13618_18714

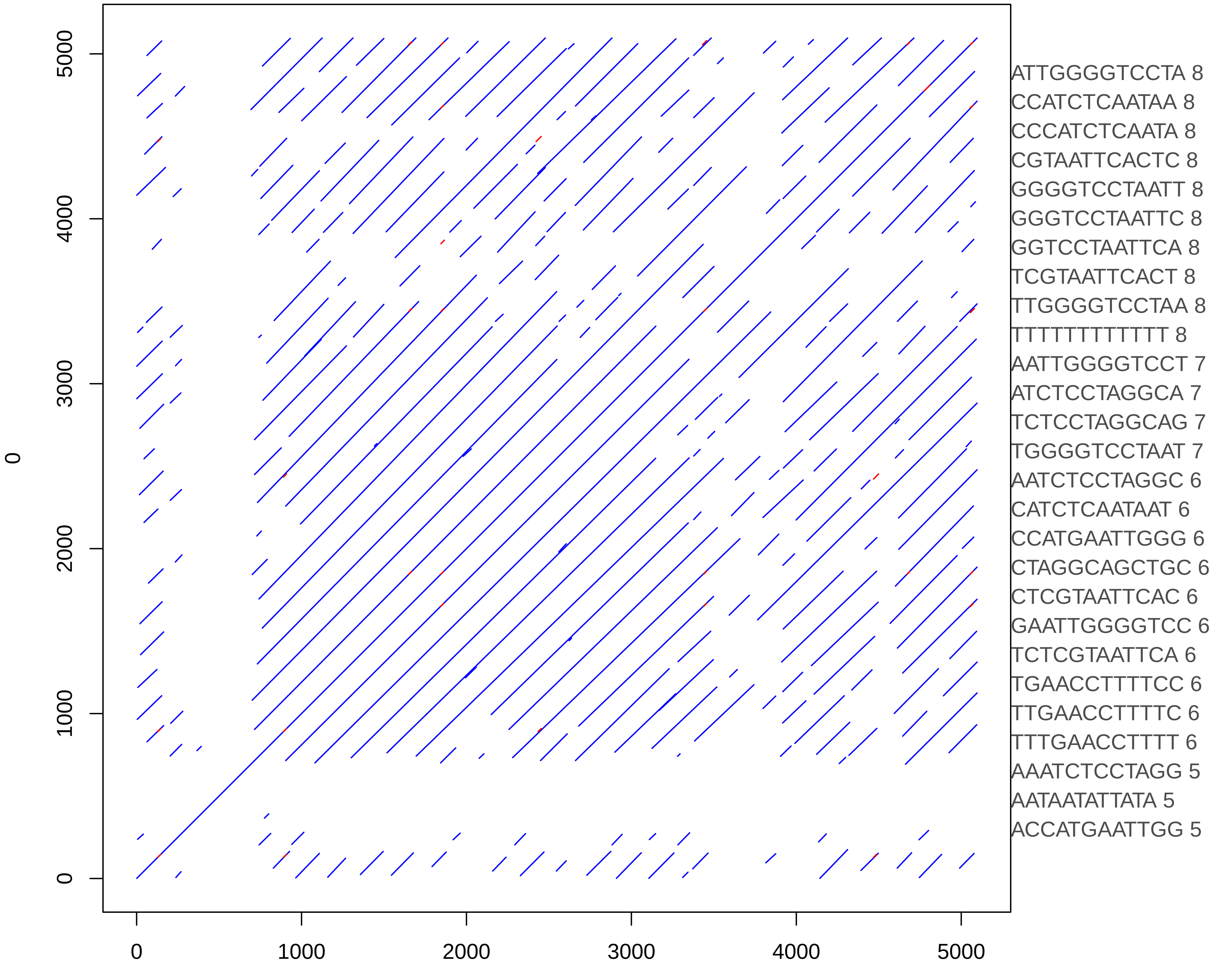

>m141122_085117_42134_c100732202550000001823141405141563_s1_p0/40591/9064_18278

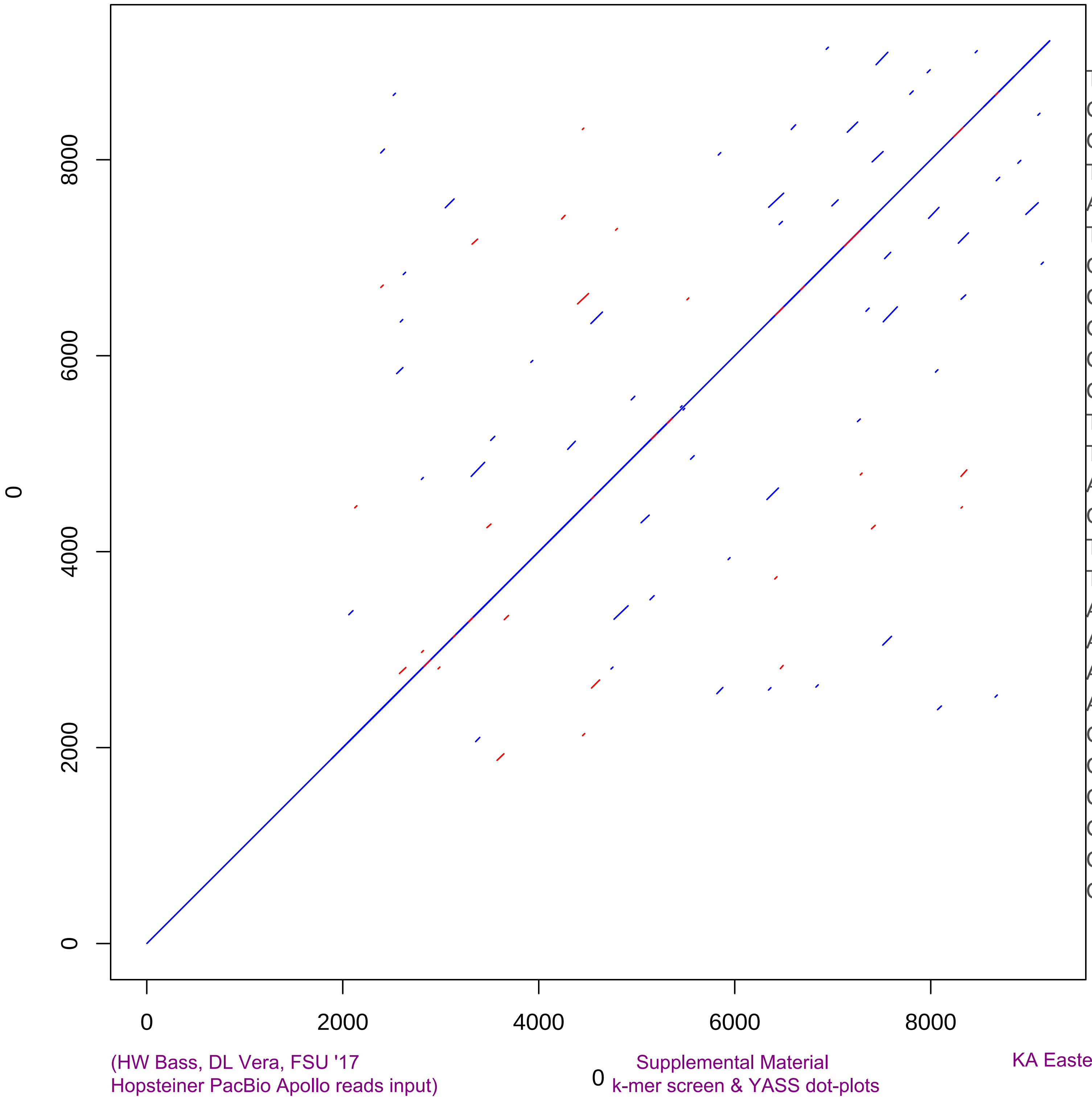

TTTTTTTTTTTT 97
CCCCCCCCCCCC 23
GTTTTTTTTTTT 15
TTTTTTTTTTTG 15
AAAAAAAAAAAG 12
TTTTTTTTTTGG 12
GGTTTTTTTTTT 11
CAAAAAAAAAAA 10
CCAAAAAAAAAA 10
CCCAAAAAAAAA 10
CTTTTTTTTTTT 10
TTTTTTTGGGGG 10
TTTTTTTTGGGG 10
AAAAAAATTTTT 9
GAAAAAAAAAAA 9
TTTTTTGGGGGG 9
TTTTTTTTTTTC 9
AAAAAAAAAAAT 8
AAAAAAAAATTT 8
AAAAAAAATTTT 8
AAAAAATTTTTT 8
CCCCAAAAAAAA 8
CCCCCCAAAAAA 8
CCCCCCCCCCCT 8
CCCTTTTTTTTT 8
CCTTTTTTTTTT 8
GGGTTTTTTTTT 8

>m141122_085117_42134_c100732202550000001823141405141563_s1_p0/46354/660_5693

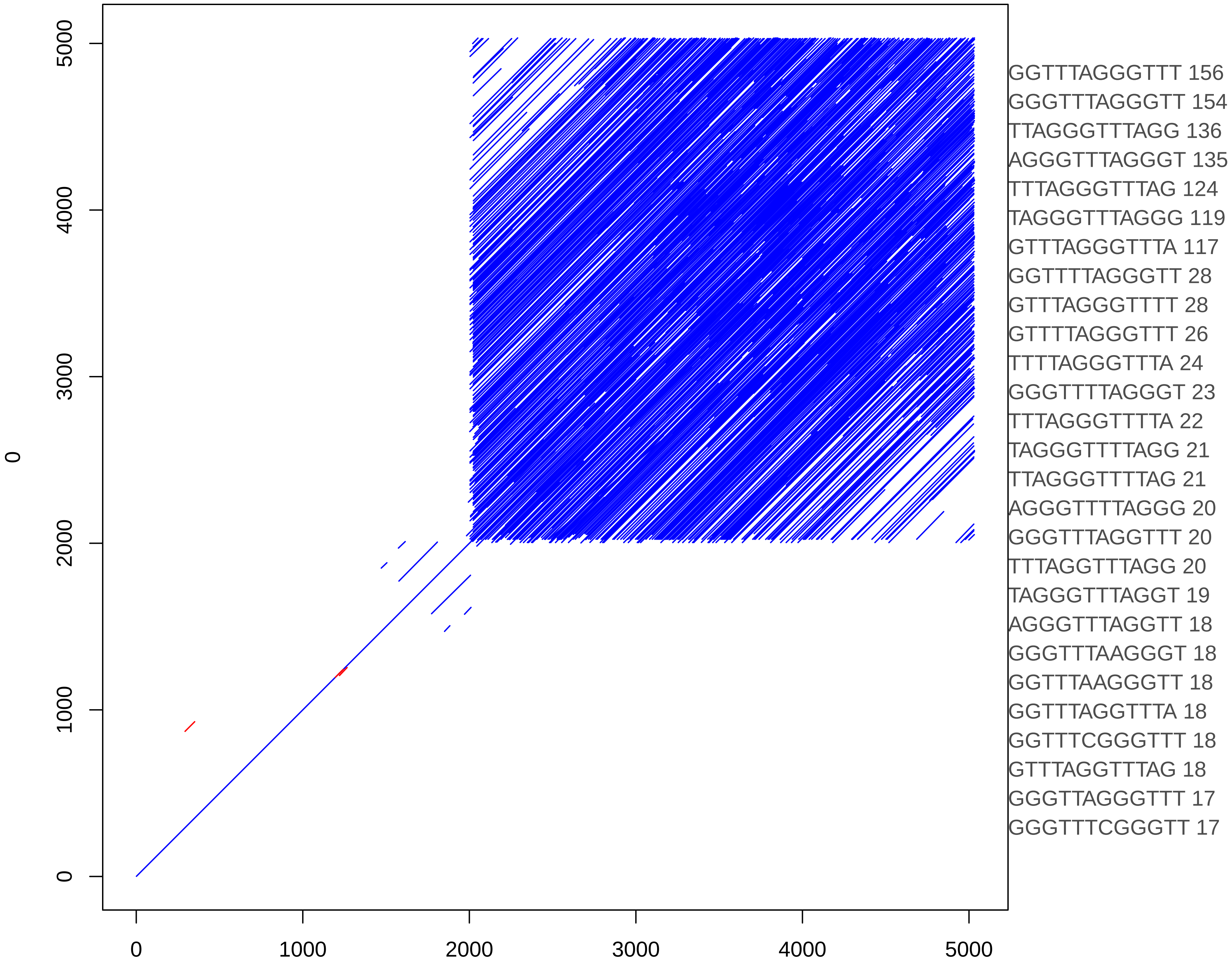

0

>m141122_085117_42134_c100732202550000001823141405141563_s1_p0/5223/6925_13429

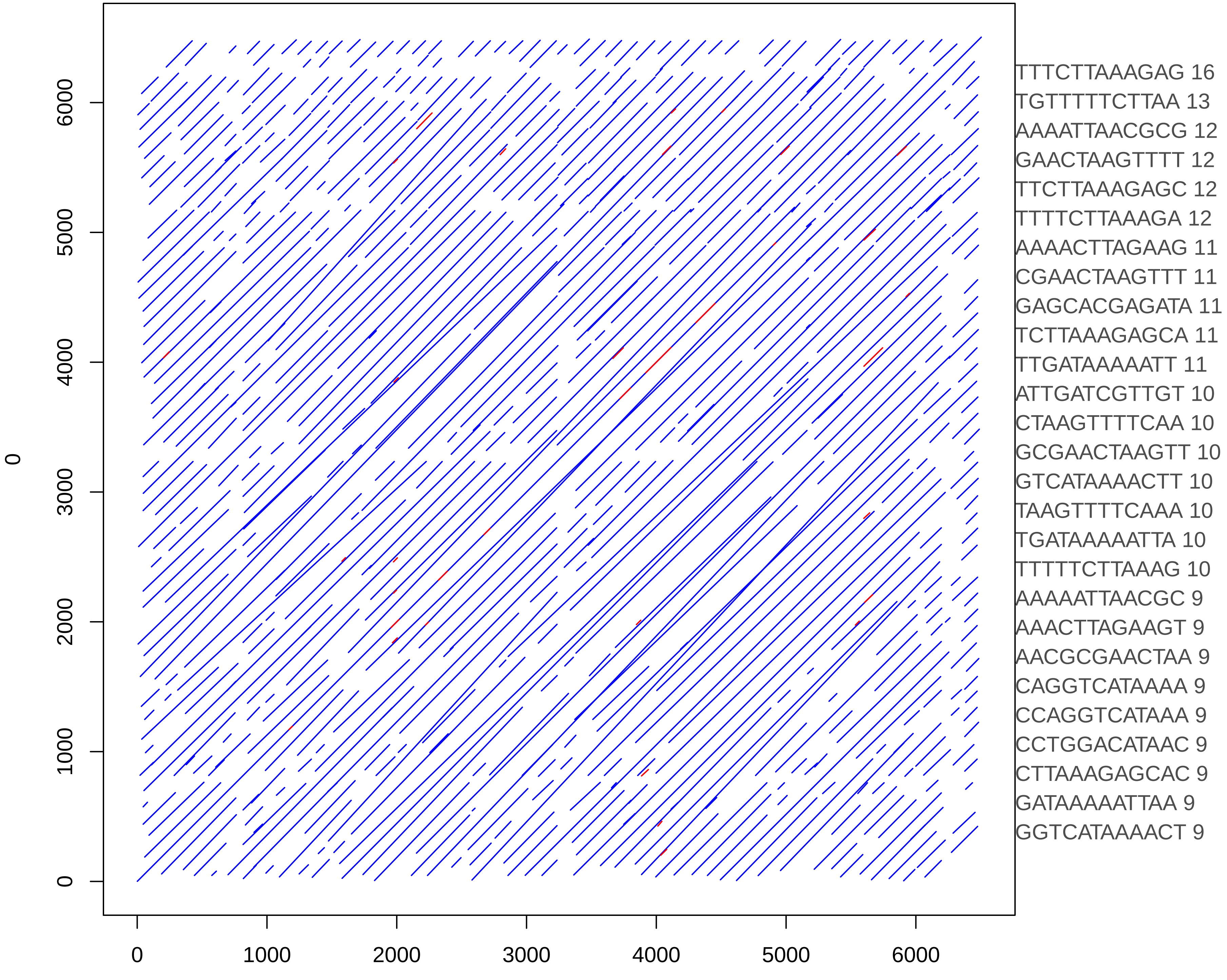

>m141122_085117_42134_c100732202550000001823141405141563_s1_p0/5496/6777_14266

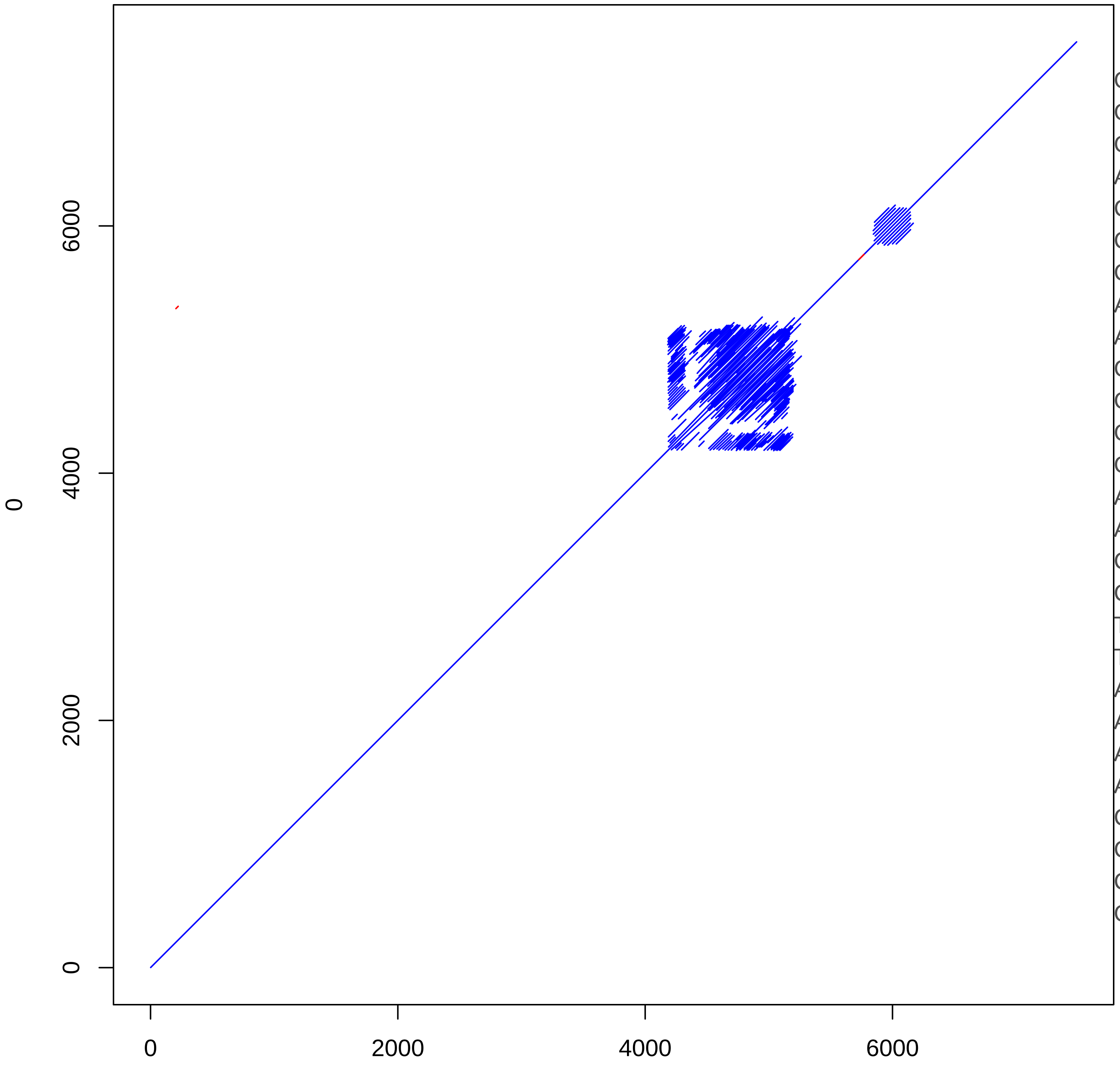

GCCATGGAGGAG 12
GAGGCCATGGAG 11
GGCCATGGAGGA 11
AGGCCATGGAGG 10
CATGGAGGAGGC 10
CCATGGAGGAGG 10
CGAGGCCATGGA 10
AGGAGGCCATGG 7
ATGGAGGAGGCC 6
GAGGAGGCCATG 6
GAGGCCATGGGC 6
GCGAGGCCATGG 6
GGAGGCCATGGG 6
ACTTTGGAGGCC 5
ATGGAGGAGGCA 5
CCGAGGCCATGG 5
CTACTTTGGAGG 5
TACTTTGGAGGC 5
TGGAGGAGGCCA 5
AGGCCATGGGCG 4
AGGCCATGGGGC 4
AGTGAGGCCATG 4
ATGGAGGAGCCA 4
CATGGAGGAGCC 4
CATGGGCGAGGC 4
CCATGGAGGAGC 4
GAGGCCATGGGG 4

>m141122_085117_42134_c100732202550000001823141405141563_s1_p0/58355/0_11085

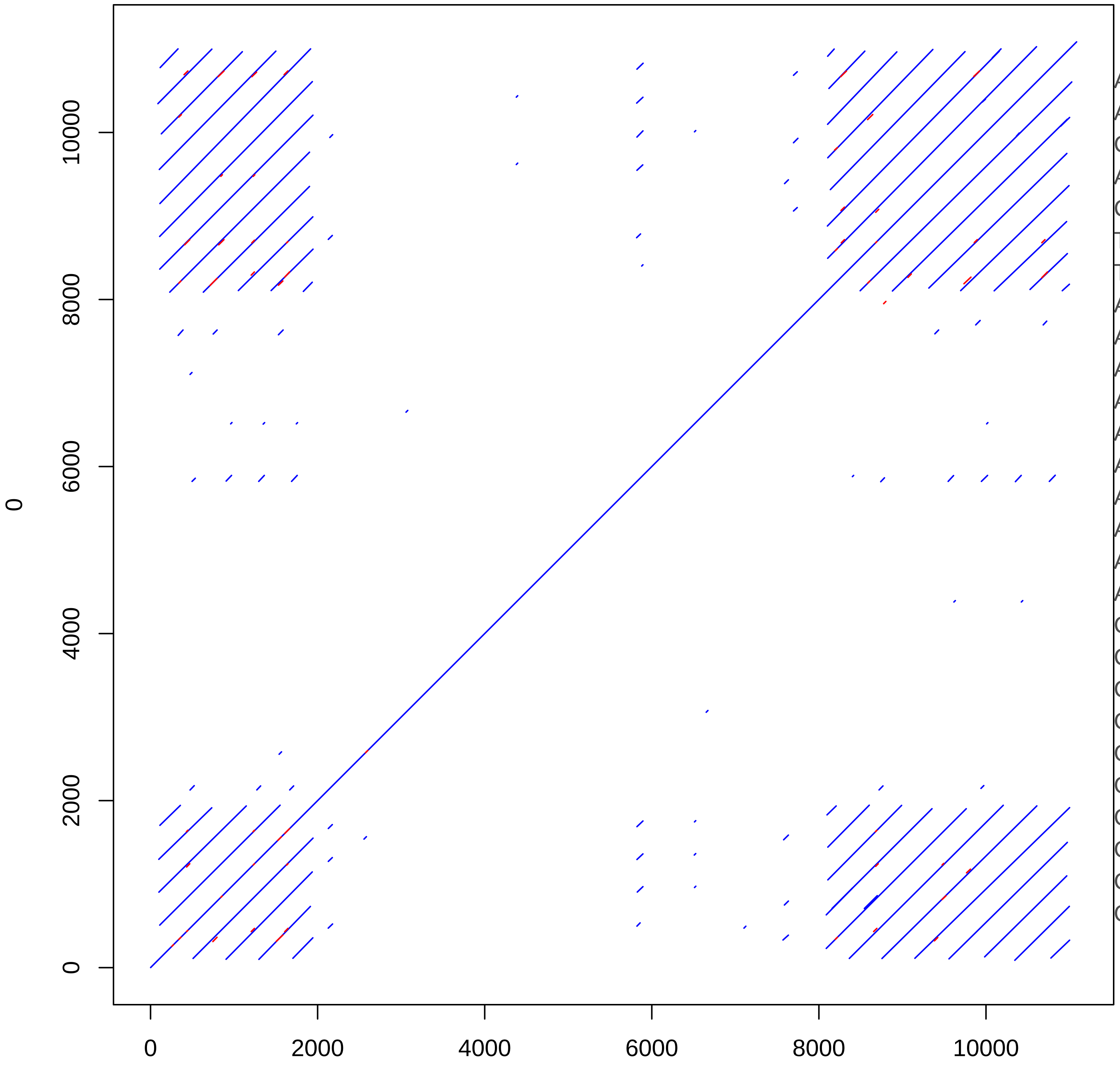

AAATACTCAGAG 10
AATACTCAGAGA 10
CTTAGGGTTCTA 10
ATACATTTCGGA 9
CATTTCGGAGAA 9
TGAATTAAGGCT 9
TTGAATTAAGGC 9
AAAATACTCAGA 8
AAATTAGTGAAA 8
AATACATTTCGG 8
AATATAAGGAAA 8
ACATTTCGGAGA 8
AGAAATTAGTGA 8
AGGGTAATACAT 8
AGTCAAAATACT 8
ATACTCAGAGAT 8
ATTTCGGAGAAA 8
CAAAAGTTGTAG 8
CAAAATACTCAG 8
CTCTTGGGTAAA 8
GAAATTAGTGAA 8
GAATTAAGGCTC 8
GAGAAATTAGTG 8
GGAGAAATTAGT 8
GGGAGTTTTTTT 8
GGGGAGTTTTTT 8
GGGTAATACATT 8

>m141122_085117_42134_c100732202550000001823141405141563_s1_p0/6039/4794_10937

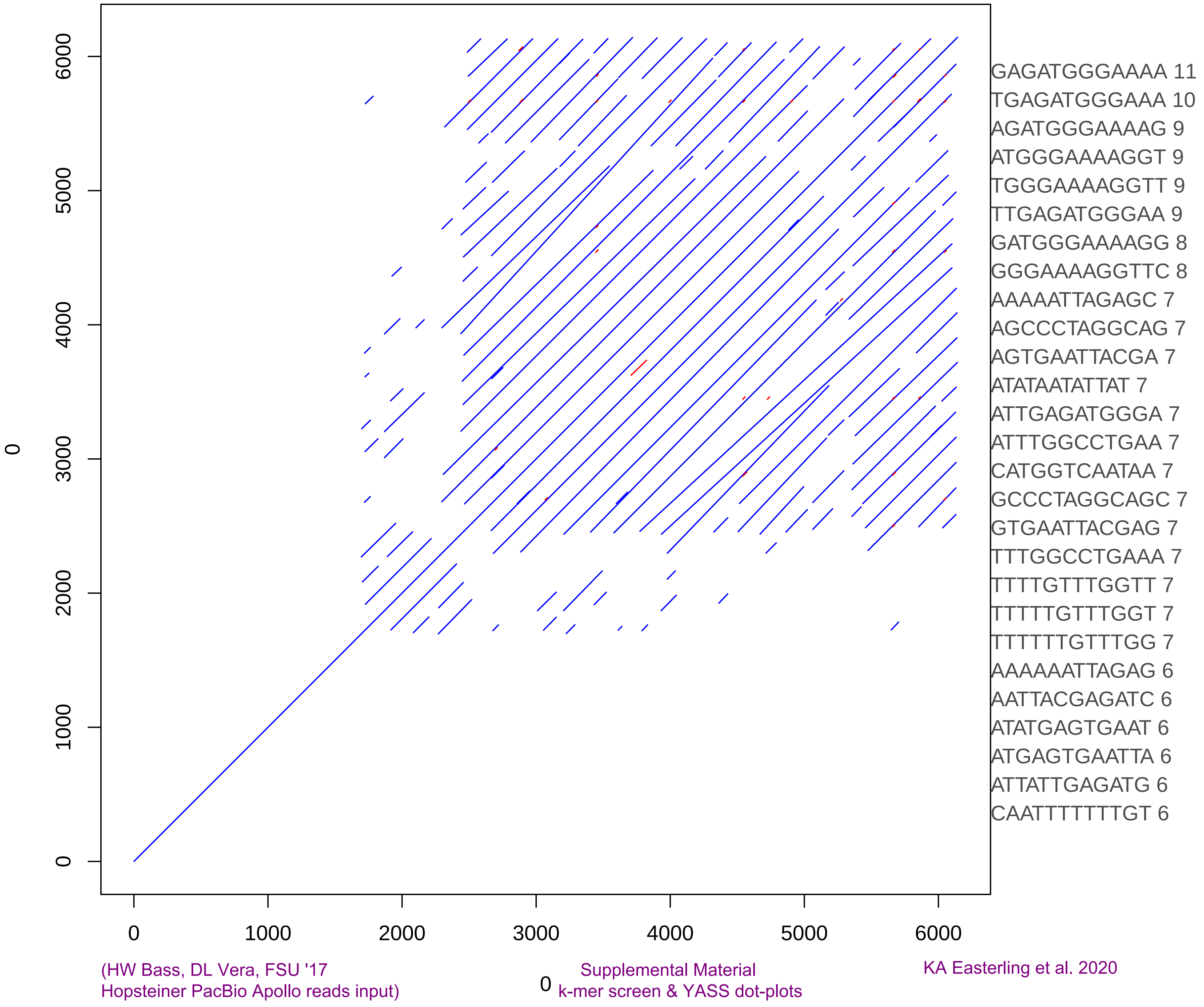

>m141122_085117_42134_c100732202550000001823141405141563_s1_p0/69146/6090_12305

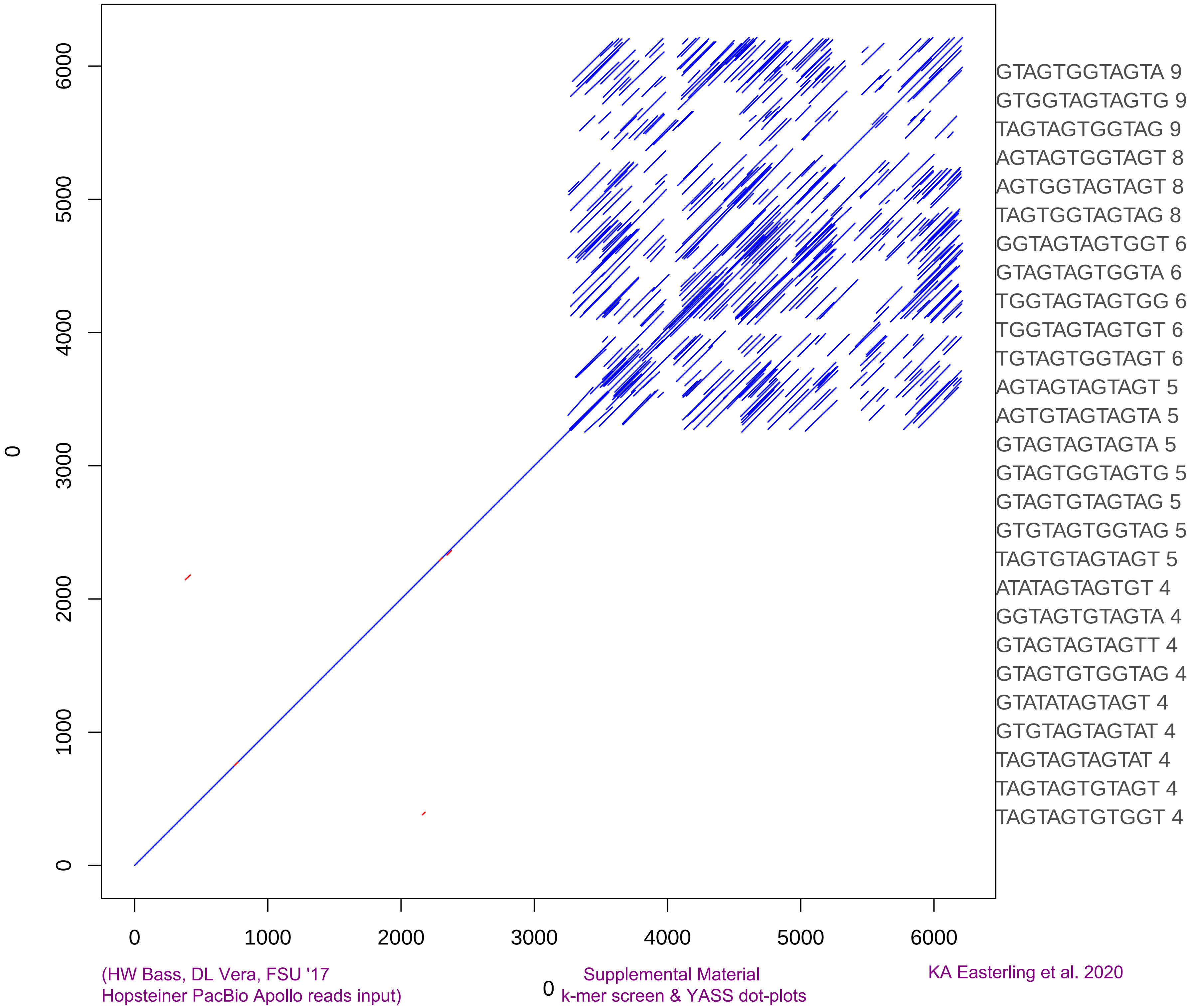

>m141122_085117_42134_c100732202550000001823141405141563_s1_p0/69649/1815_8978

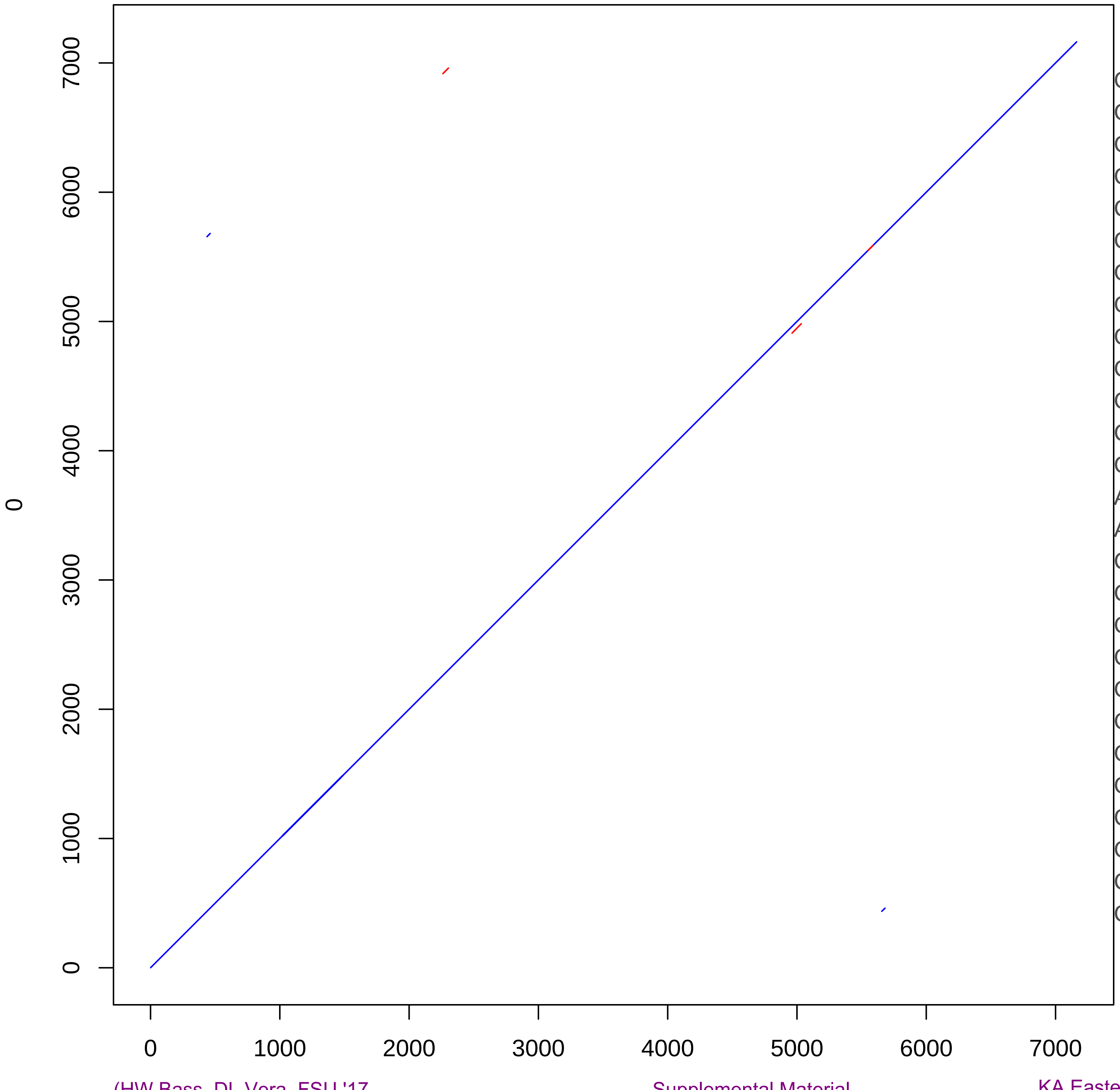

GGGGGGGGGGGG 49
GGGGCGGGGGGG 9
GGGGGCGGGGGG 9
GGCGGGGGGGGG 8
GGGCGGGGGGGG 8
GGGGGGCGGGGG 8
GGGGGGGCGGGG 8
GGGGGGGGGCGG 8
GCGGGGGGGGGG 7
GGGGGGGGCGGG 7
GGGGGGGGGGCG 7
CGGGGGGGGGGG 6
GGGGGGGGGGGC 6
AACCACGCCCAC 2
ACCACGCCCACG 2
CCACGCCCACGA 2
CCGAGCTCCGGG 2
GCGGGGGCGGGG 2
GGCGGGGGCGGG 2
GGGCGGGGGCGG 2
GGGGCGGGGGCG 2
GGGGGCGGGGGC 2
GGGGGGGGGGGG 49
GGGGCGGGGGGG 9
GGGGGCGGGGGG 9
GGCGGGGGGGGG 8
GGGCGGGGGGGG 8

>m141122_085117_42134_c100732202550000001823141405141563_s1_p0/7754/676_7277

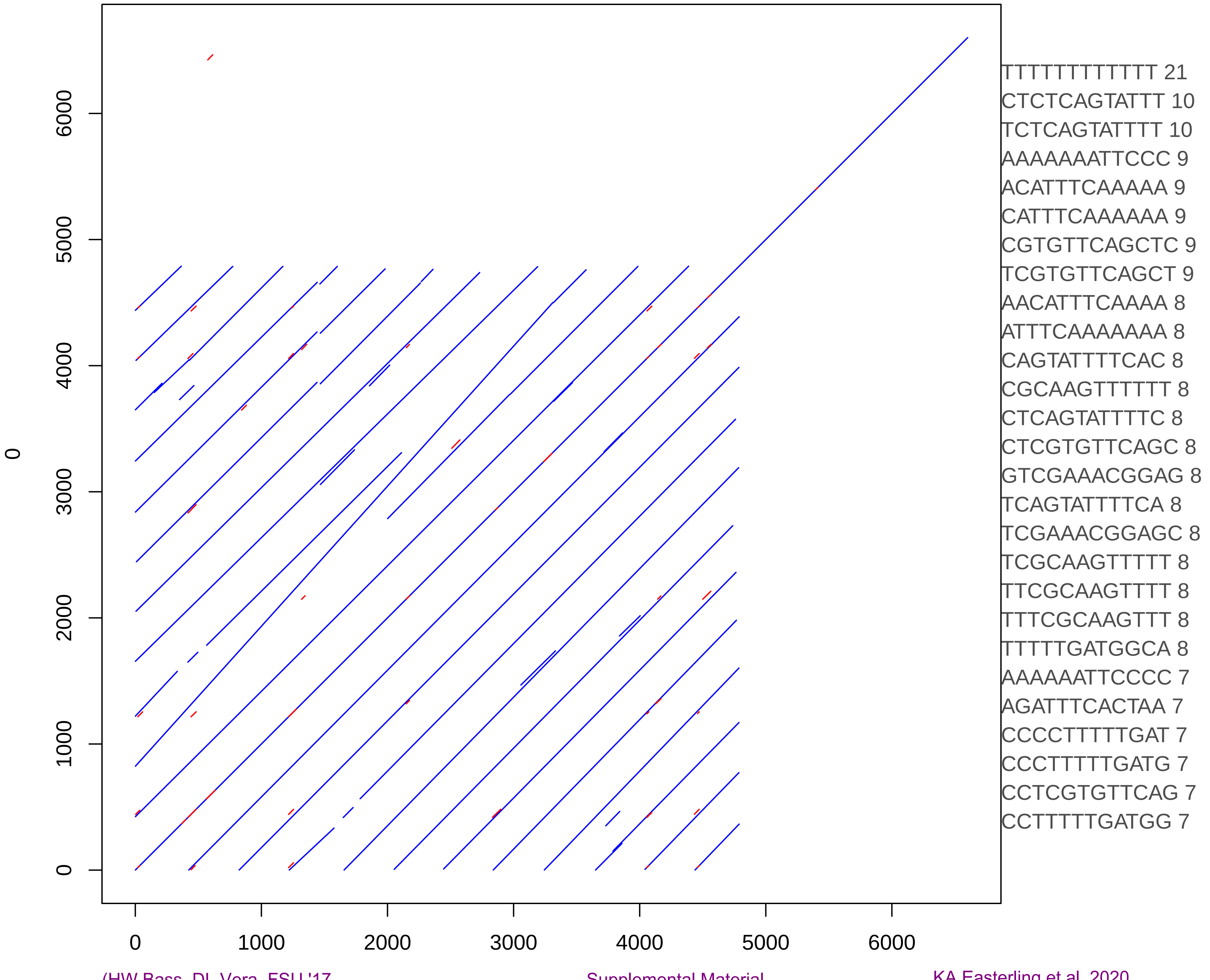

0

>m141122_085117_42134_c100732202550000001823141405141563_s1_p0/86232/4518_13635

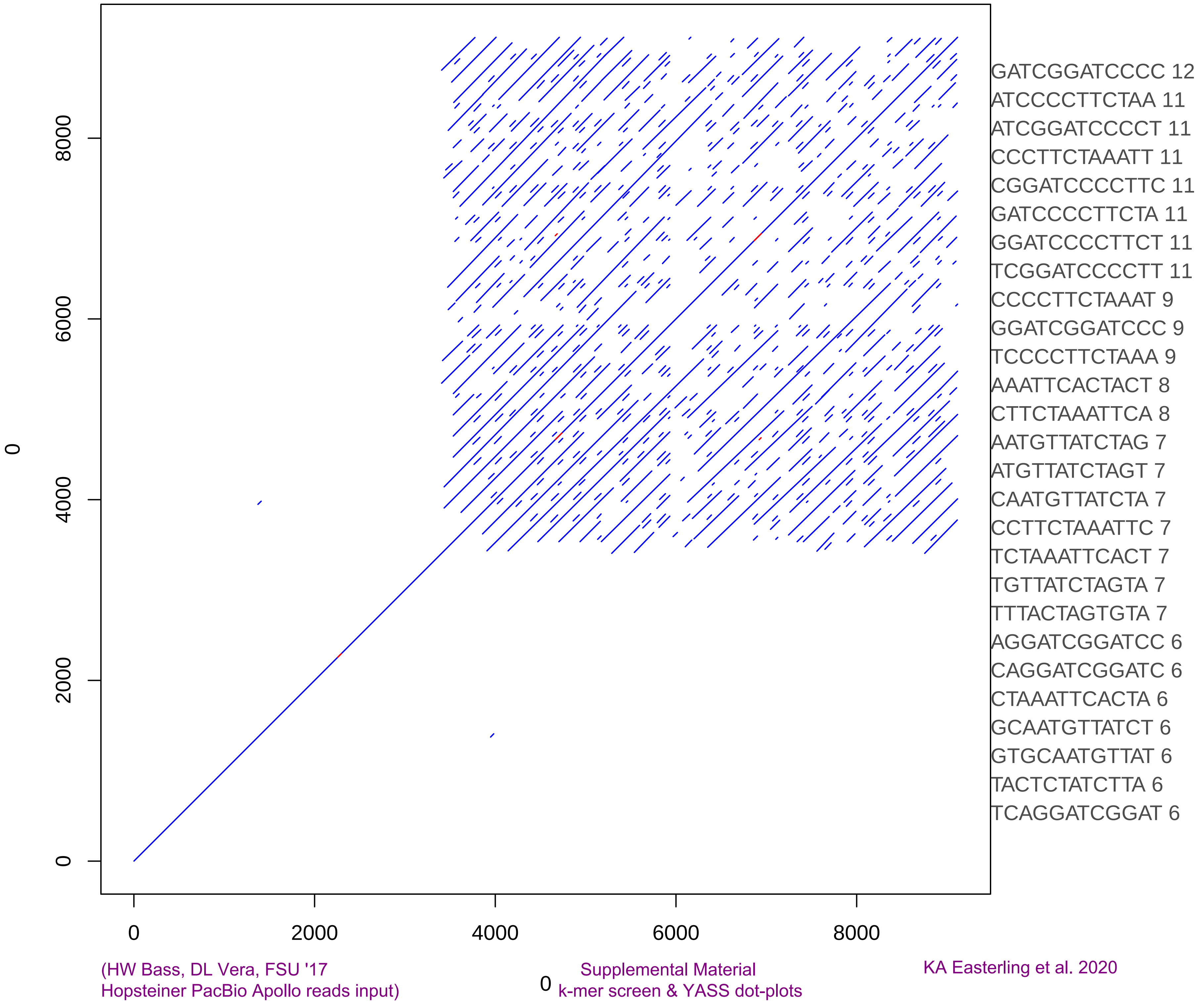

>m141122_085117_42134_c100732202550000001823141405141563_s1_p0/91897/3568_11222

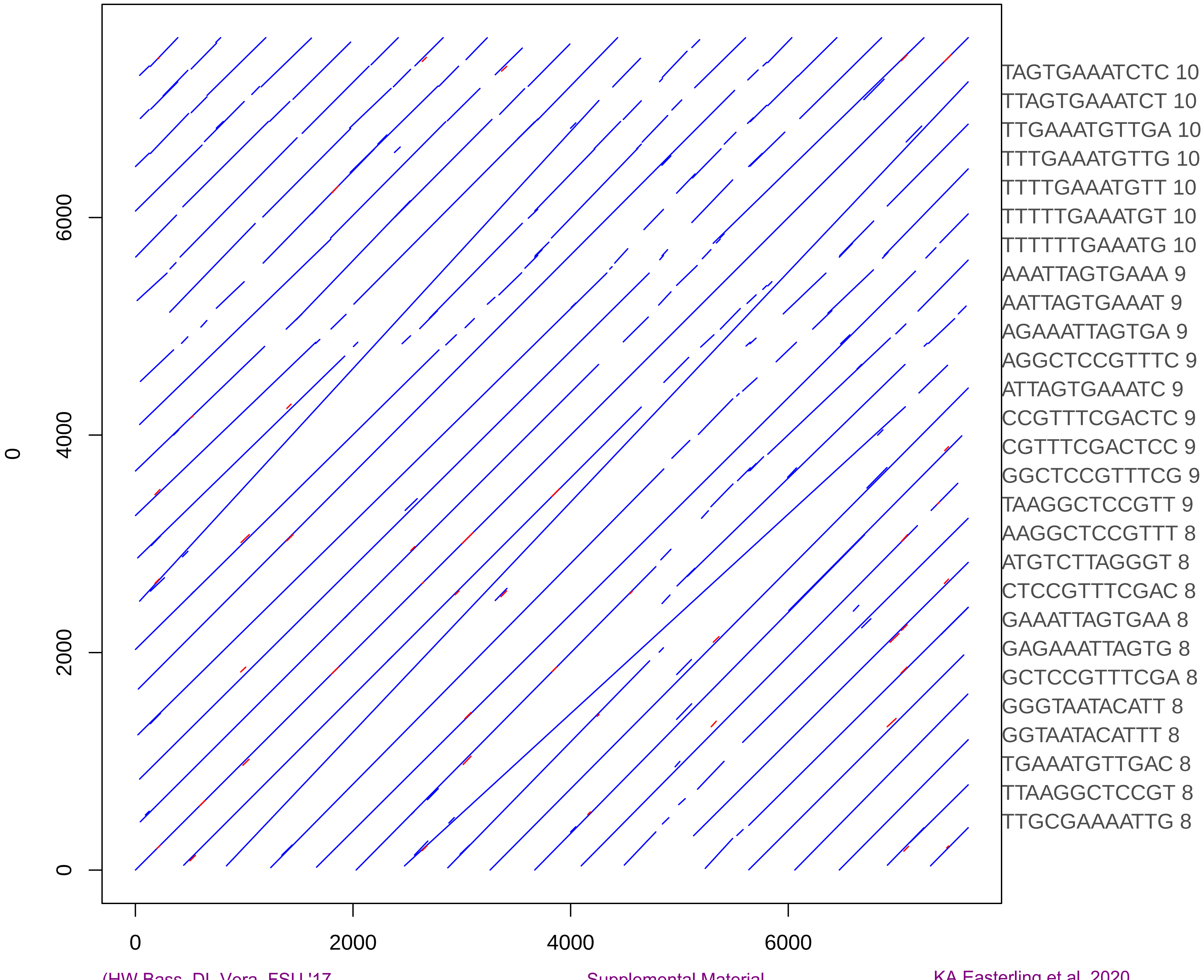

>m141122_085117_42134_c100732202550000001823141405141563_s1_p0/91977/10515_20034

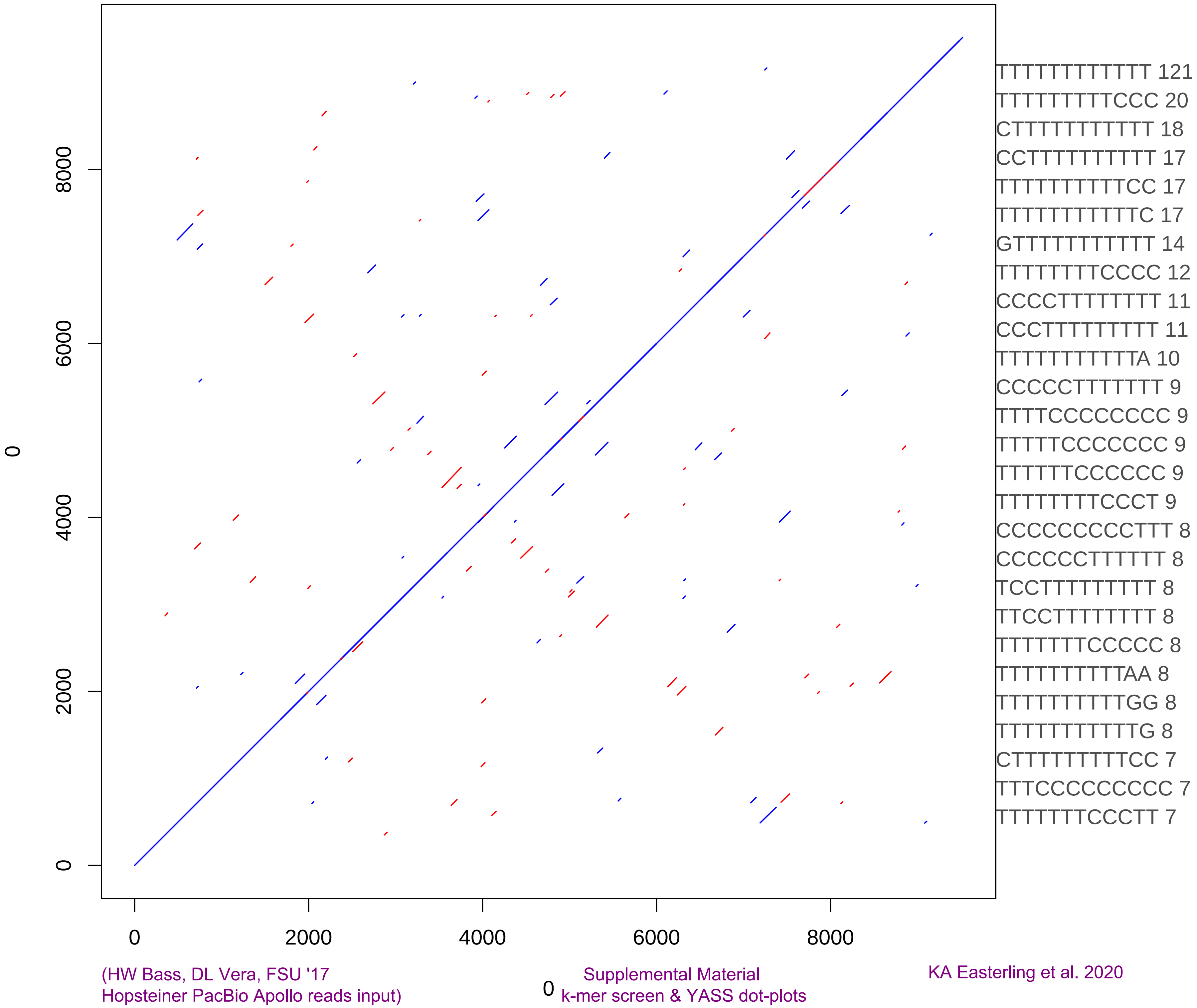

>m141122_085117_42134_c100732202550000001823141405141563_s1_p0/92285/12556_21863

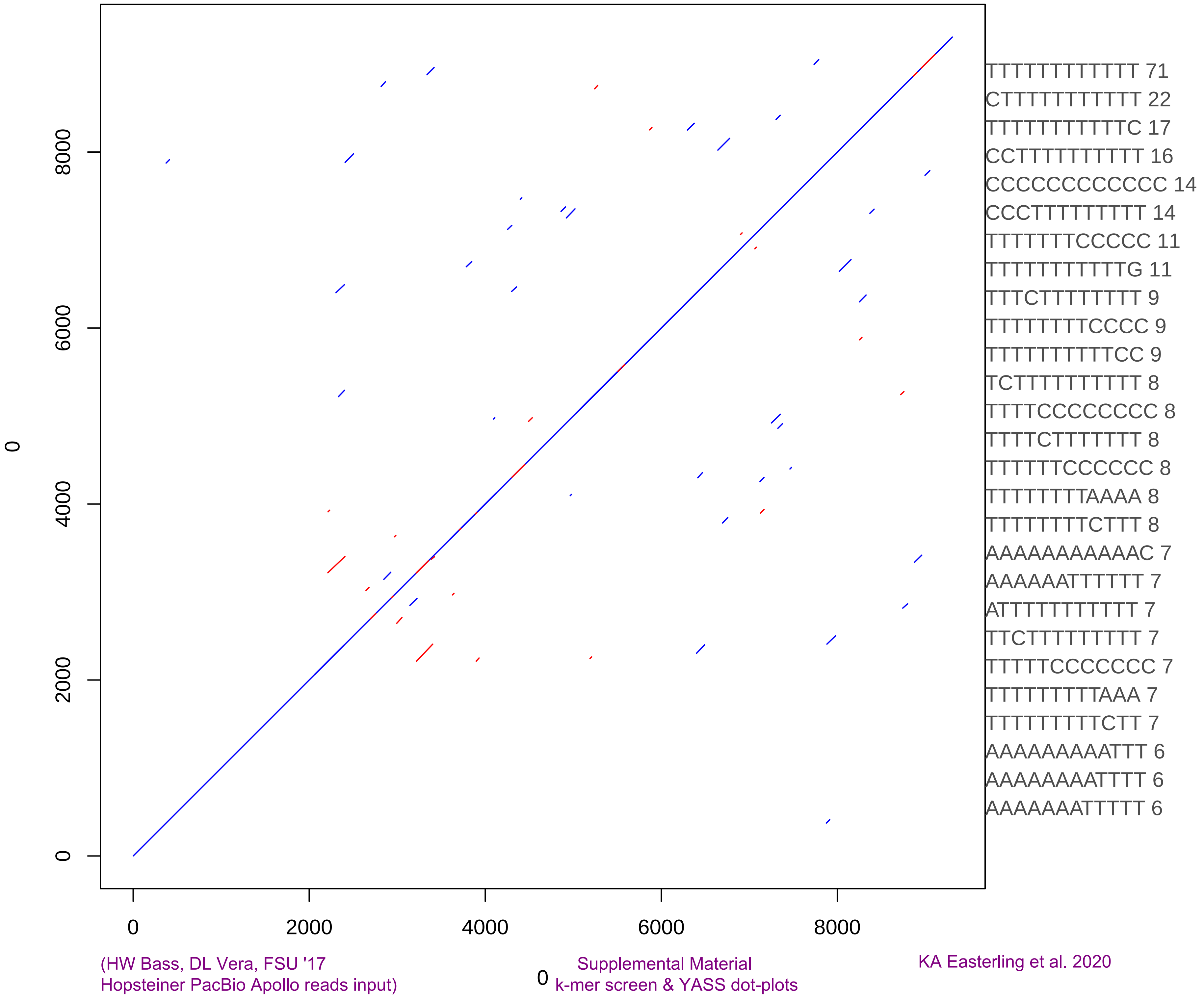

1,121 hits for repetitive sequences
passing 12mer abundance criterion

(Read-Number)
PAGE 380

For Tandem Repeat families of ### bp:
PacBio Read named "HuluTR###-r380"

>m141122_111549_42134_c100732202550000001823141405141564_s1_p0/106380/0_6404

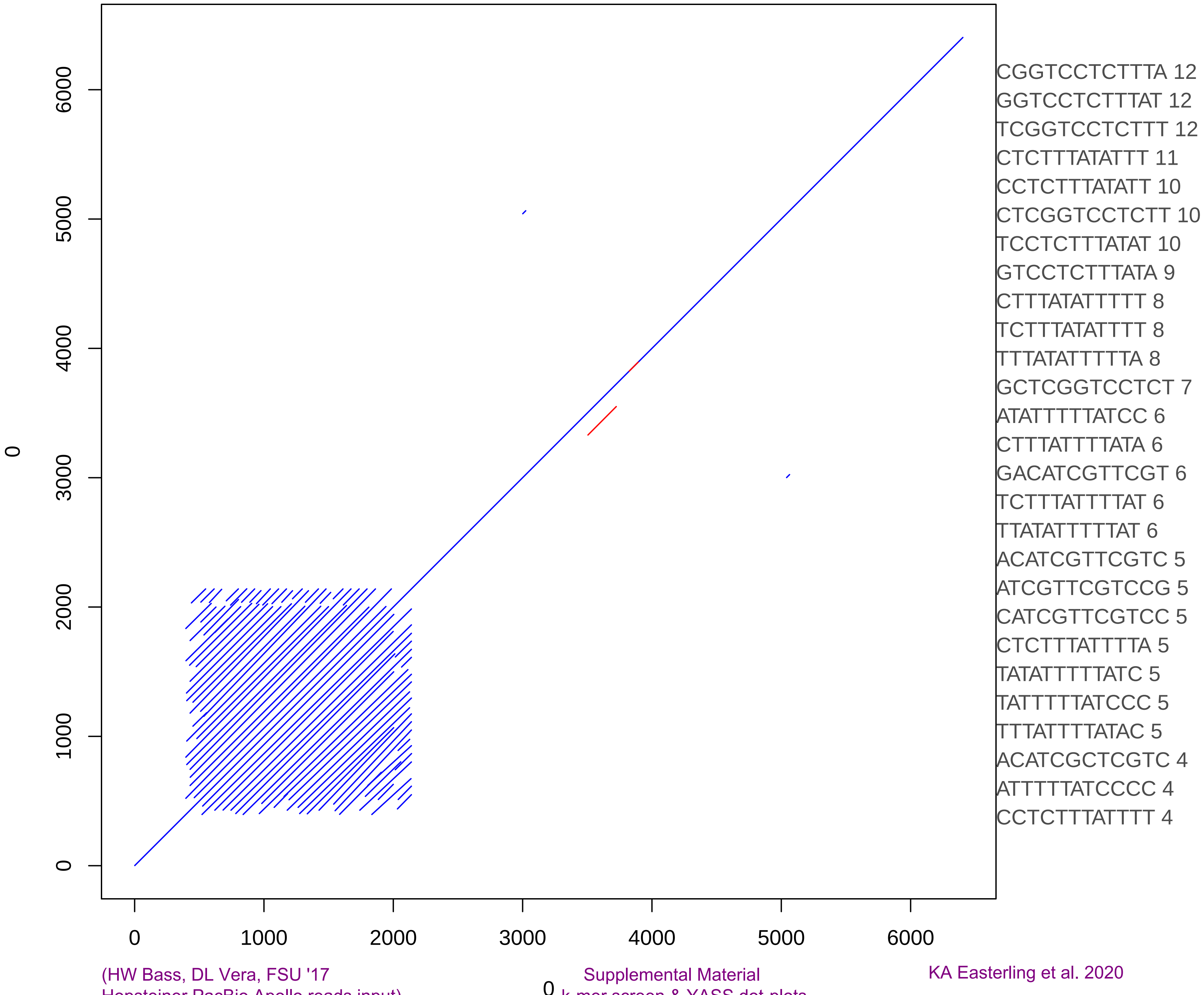

(HW Bass, DL Vera, FSU '17
Hopsteiner PacBio Apollo reads input)

Supplemental Material
k-mer screen & YASS dot-plots

KA Easterling et al. 2020

>m141122_111549_42134_c100732202550000001823141405141564_s1_p0/111016/7743_18085

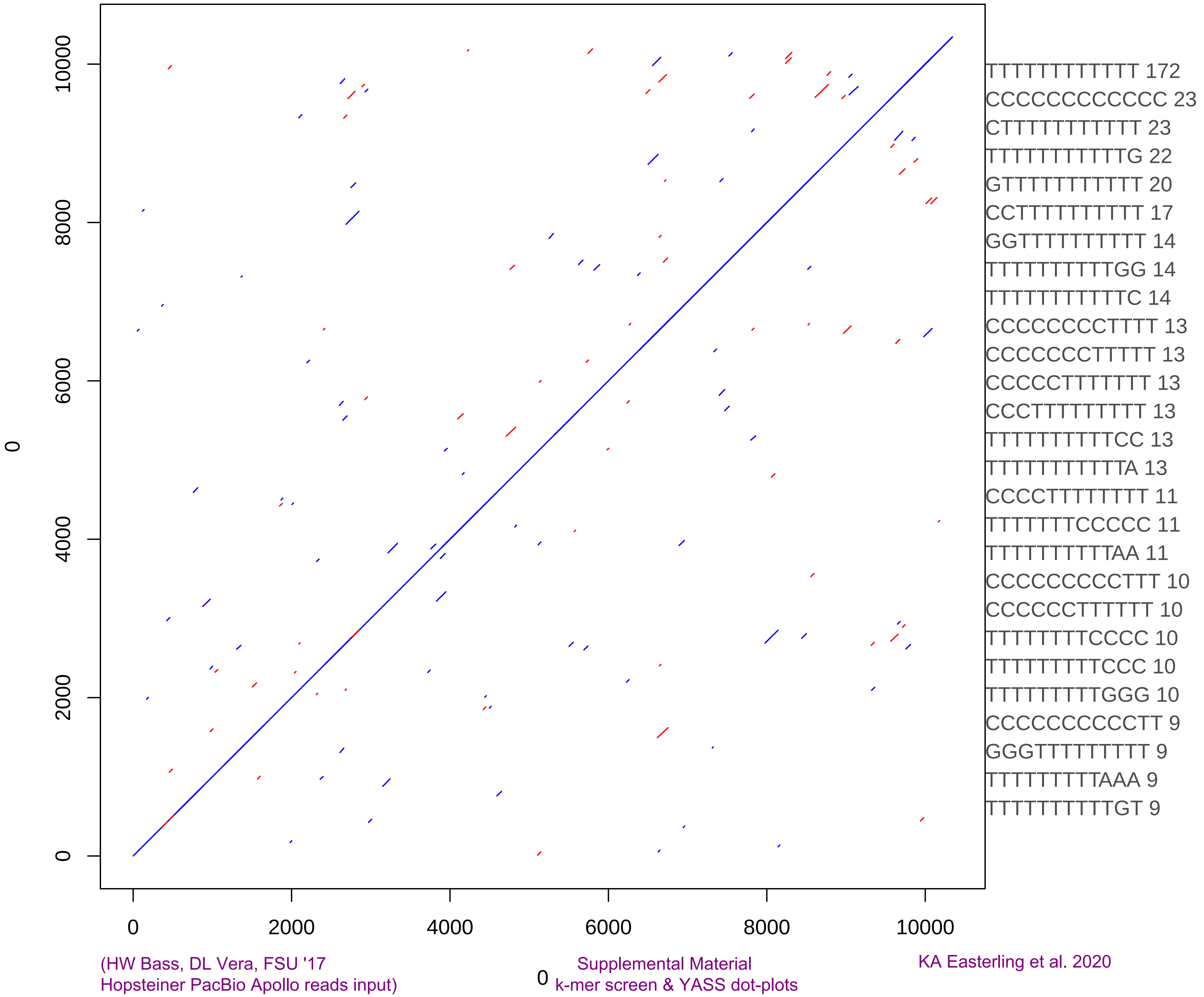

>m141122_111549_42134_c100732202550000001823141405141564_s1_p0/120860/14368_20468

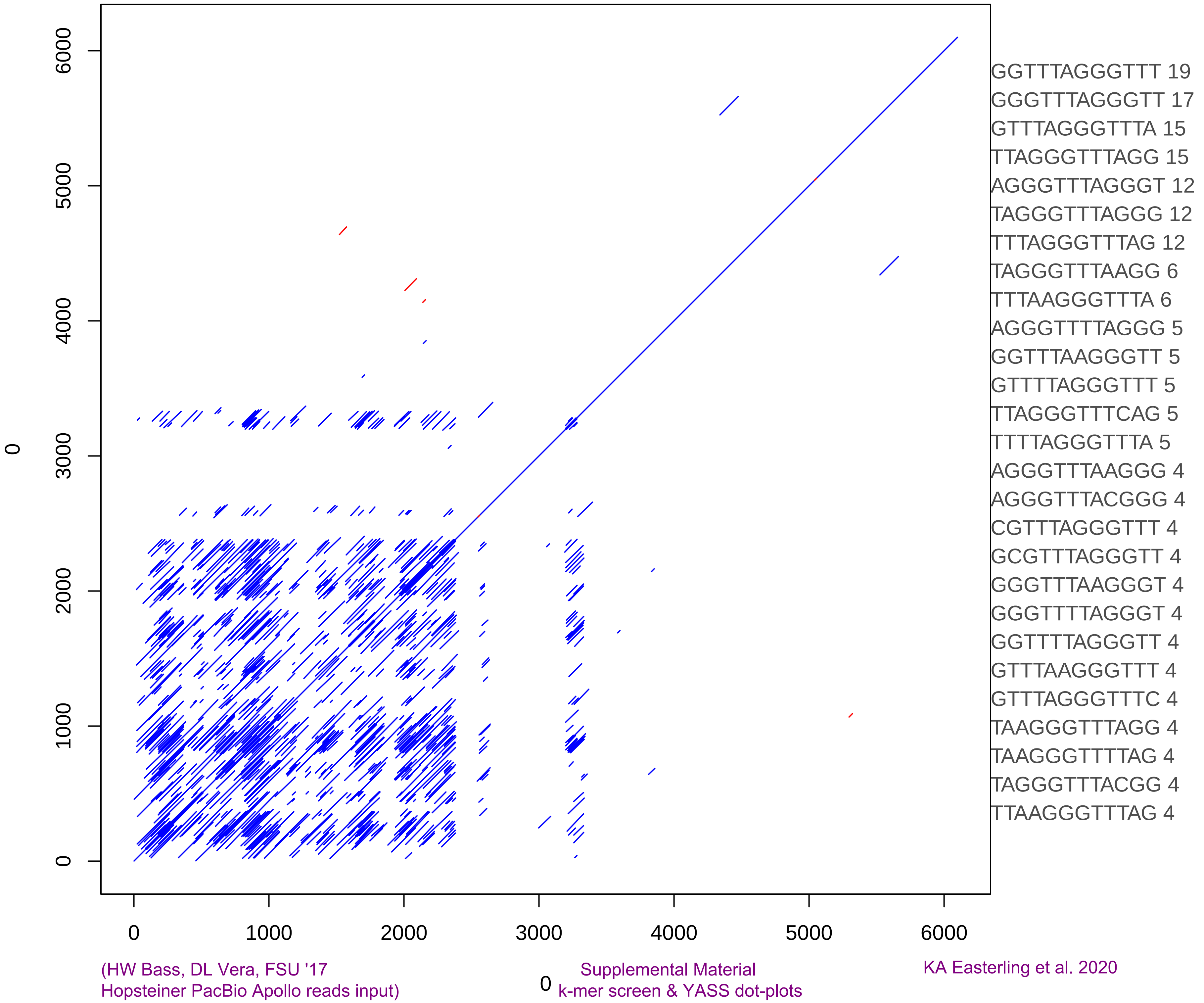

>m141122_111549_42134_c100732202550000001823141405141564_s1_p0/12155/0_9305

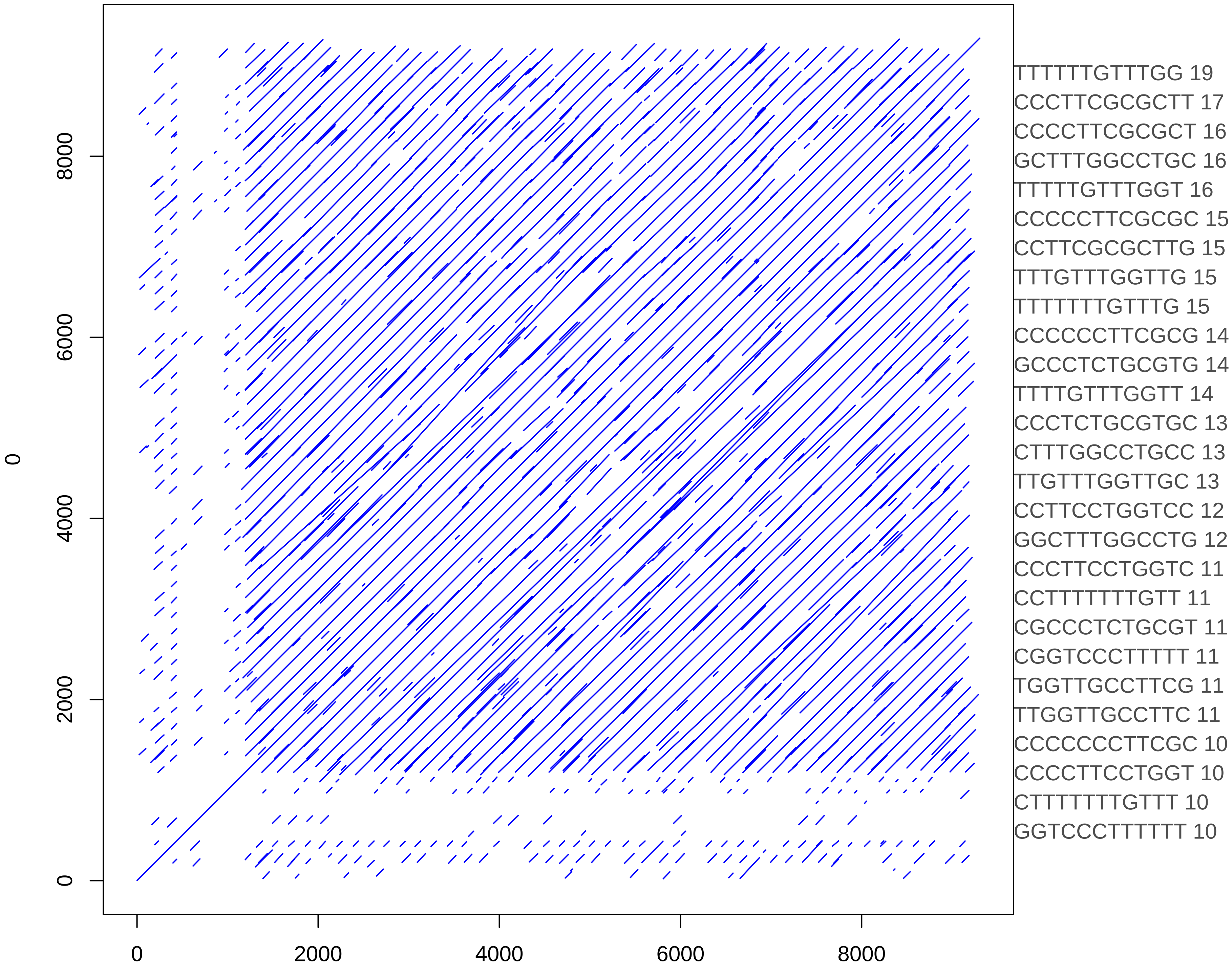

>m141122_111549_42134_c100732202550000001823141405141564_s1_p0/121633/12129_21849

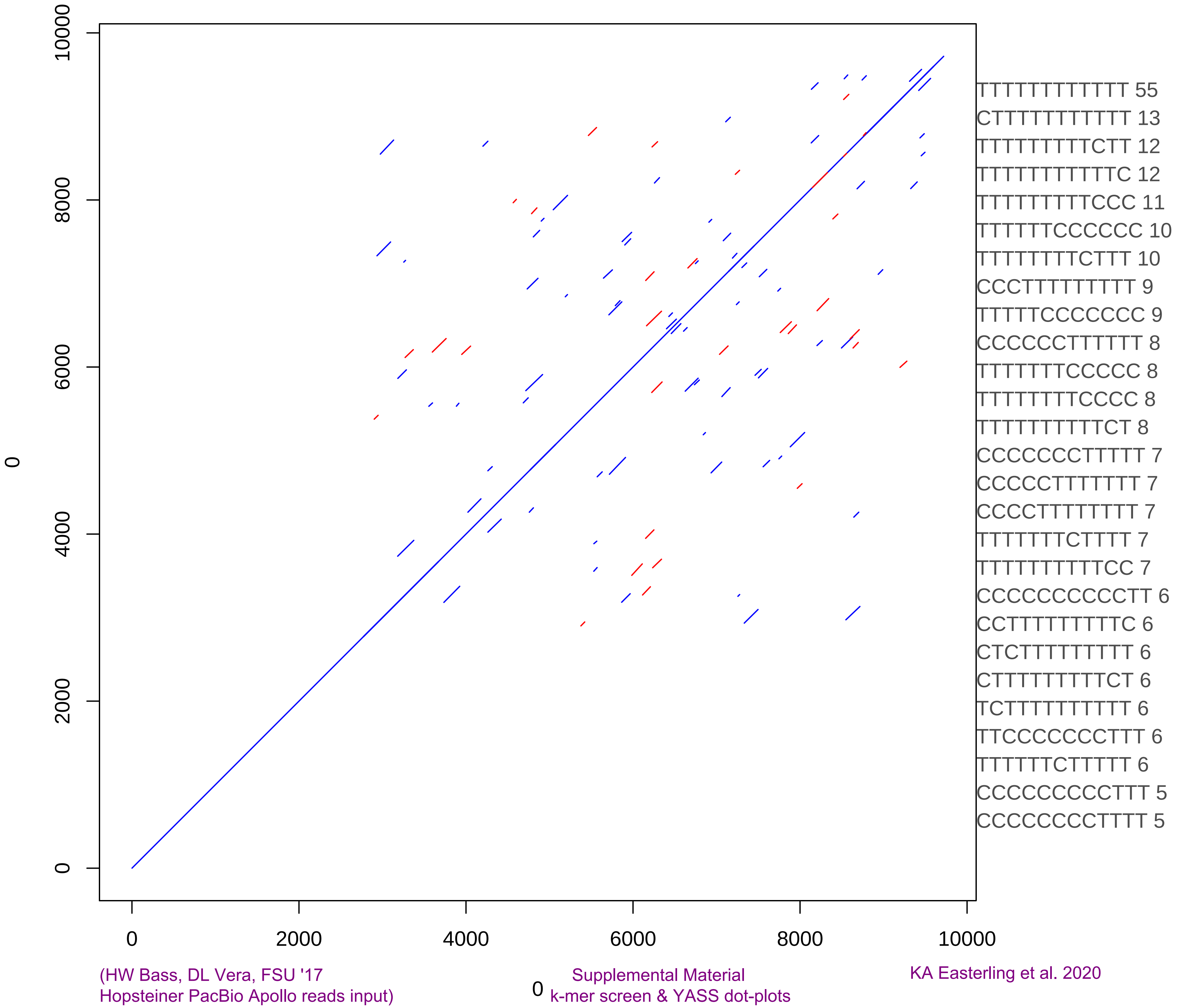

>m141122_111549_42134_c100732202550000001823141405141564_s1_p0/125366/5827_16934

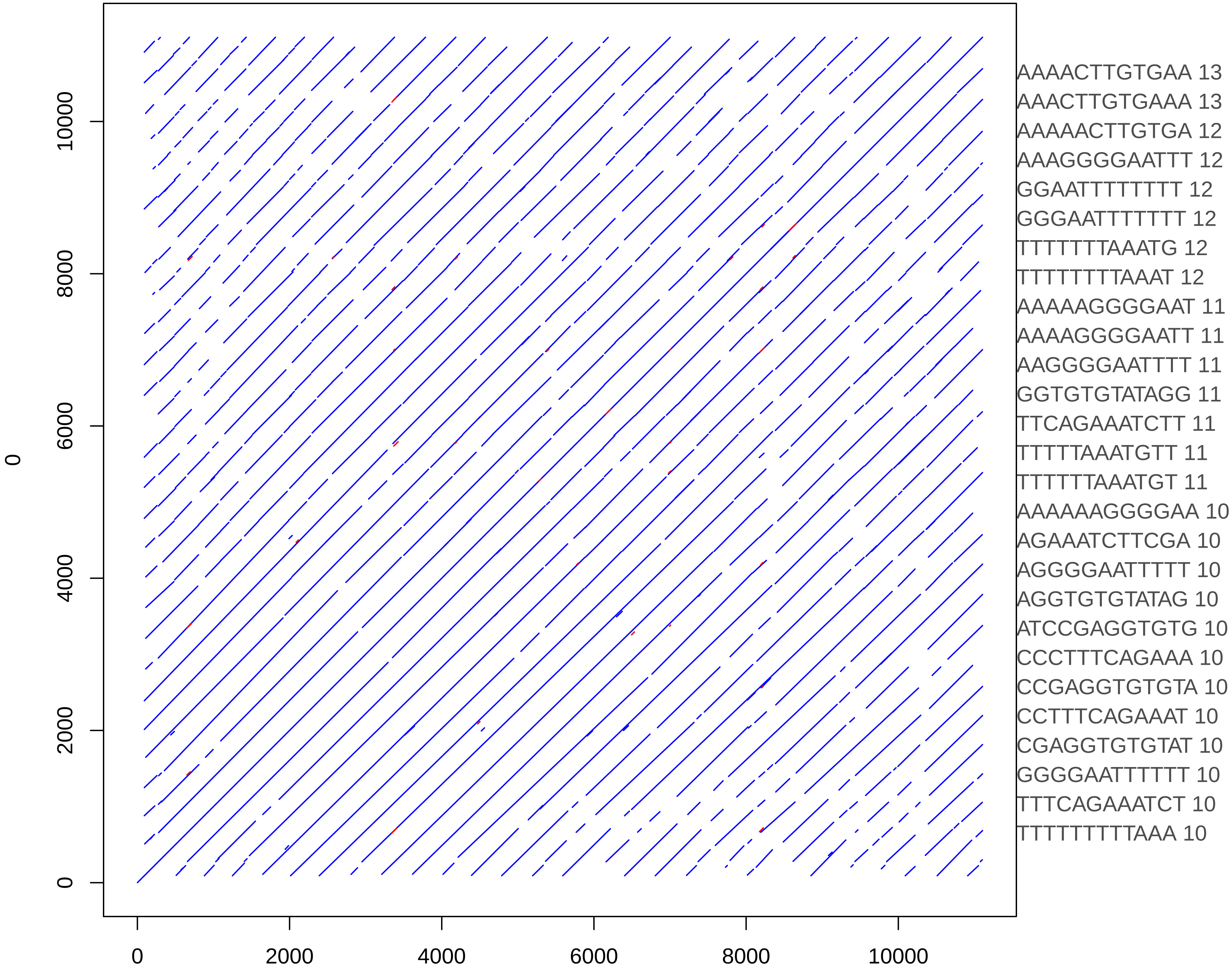

>m141122_111549_42134_c100732202550000001823141405141564_s1_p0/129548/12640_19943

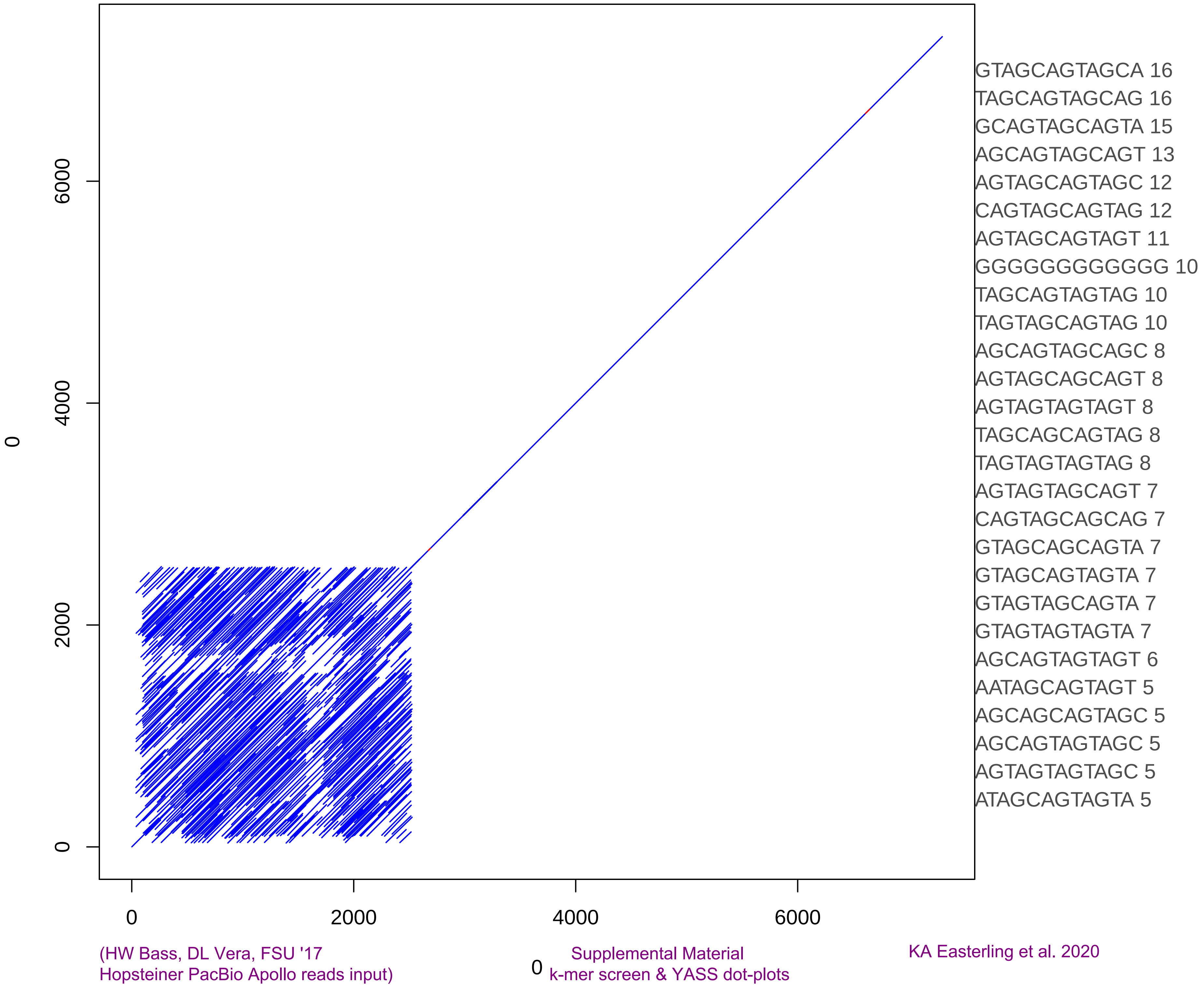

>m141122_111549_42134_c100732202550000001823141405141564_s1_p0/130089/580_9812

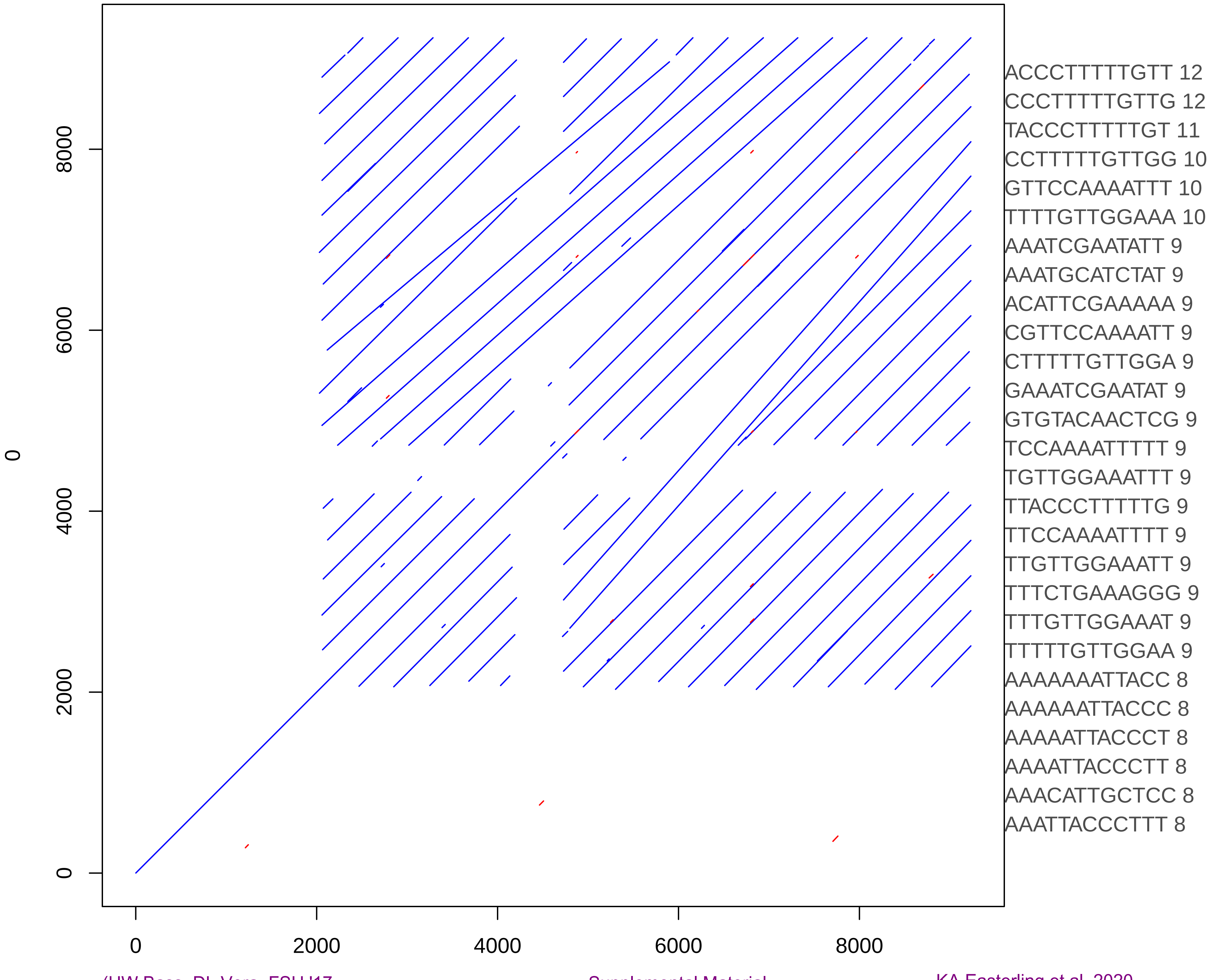

>m141122_111549_42134_c100732202550000001823141405141564_s1_p0/143004/8152_15809

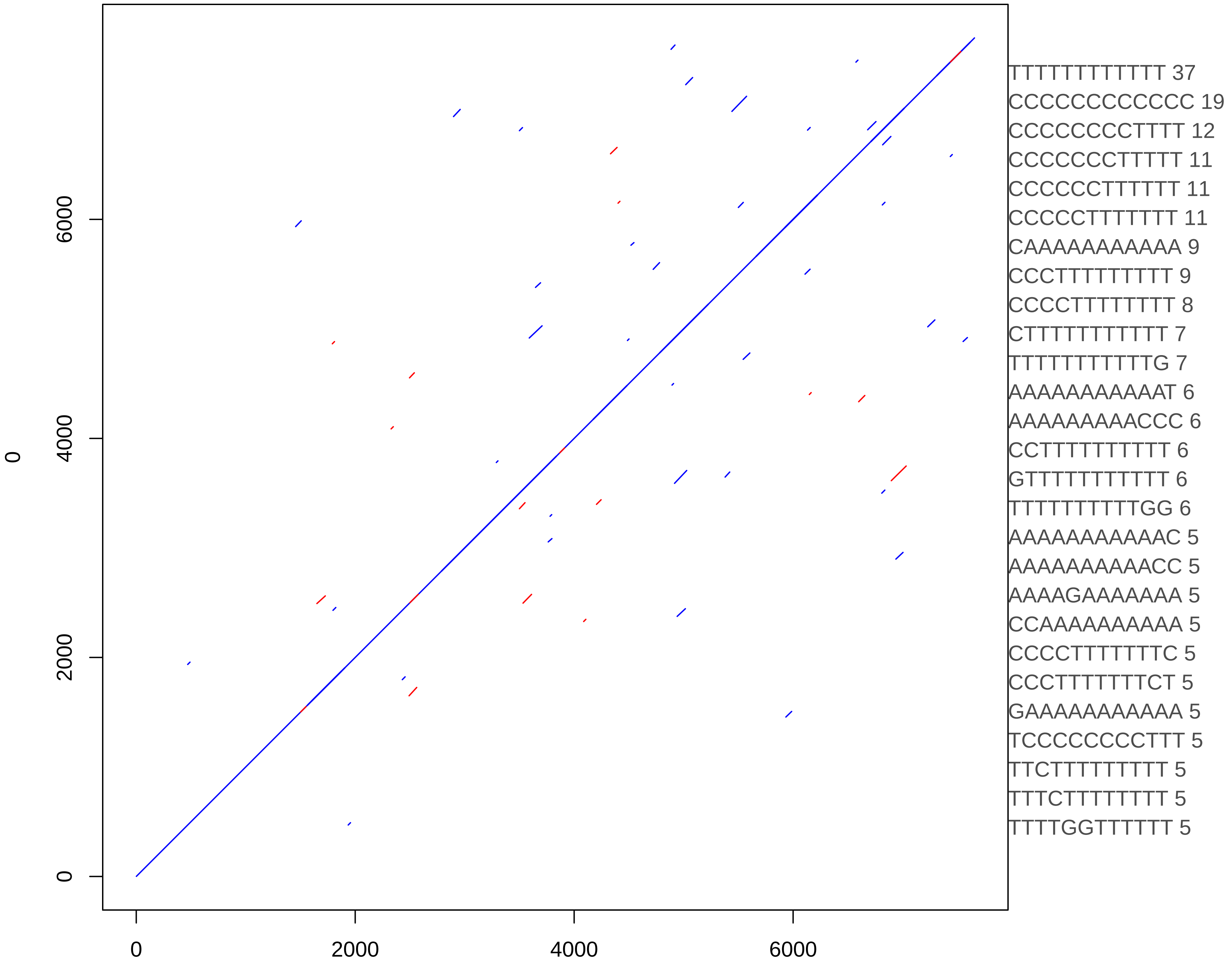

0

>m141122_111549_42134_c100732202550000001823141405141564_s1_p0/147751/3459_9914

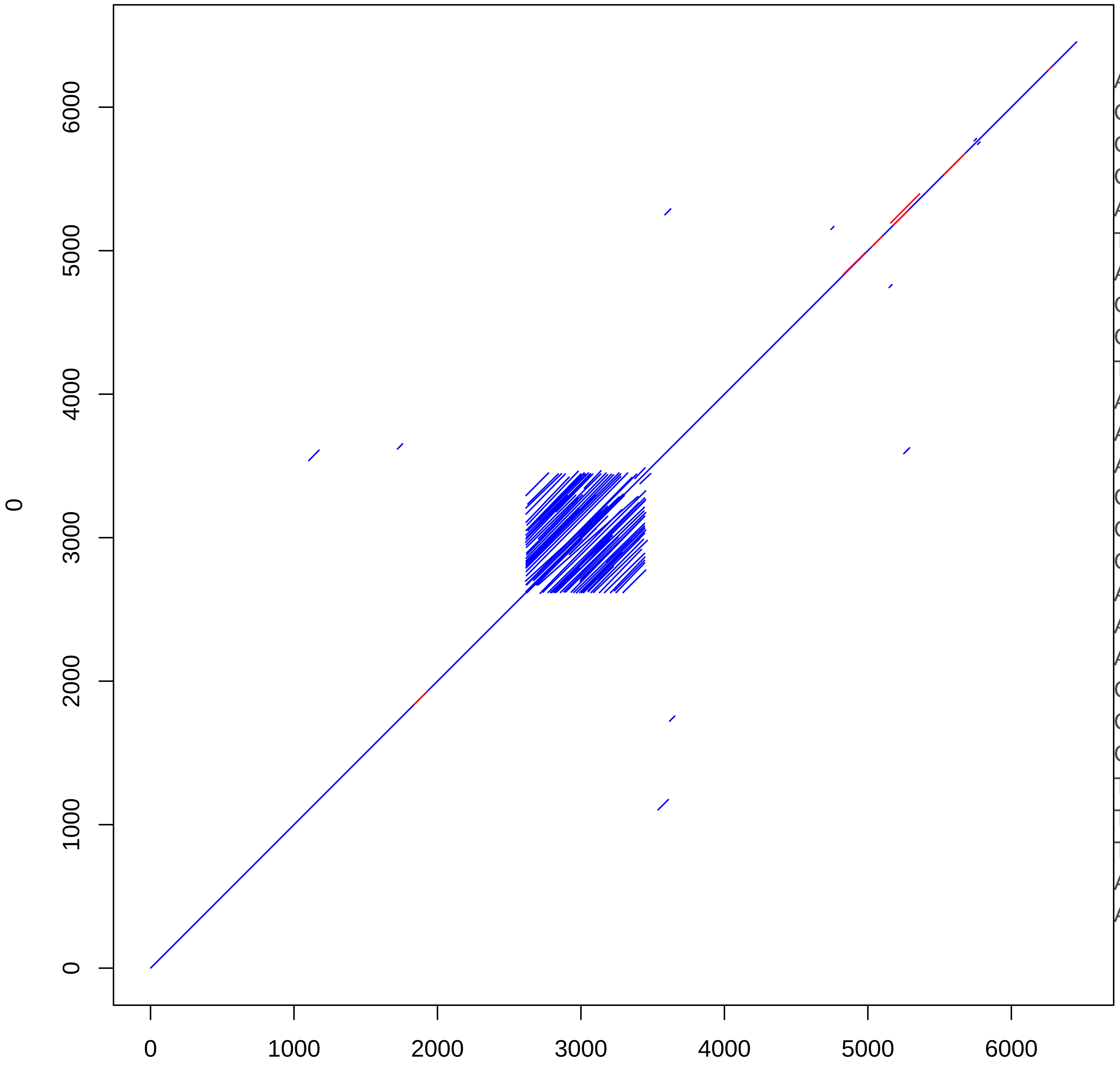

AGTAGCAGTAGC 17
CAGTAGCAGTAG 16
GCAGTAGCAGTA 15
GTAGCAGTAGCA 15
AGCAGTAGCAGT 14
TAGCAGTAGCAG 14
AGCAGCAGCAGC 8
GCAGCAGCAGCA 8
CAGCAGCAGCAG 6
TAGTAGTAGTAG 6
AGTAGTAGCAGC 5
AGTAGCAGCAGC 4
AGTAGCAGTAAG 4
GTAGCAGCAGCA 4
GTAGTAGCAGCA 4
GTAGTAGTAGTA 4
ACAGTAGCAGTA 3
AGTAGTAGTAGC 3
AGTAGTAGTAGT 3
GATAGCAGTAGC 3
GTAGCAGTAAGT 3
GTAGTAGTAGCA 3
TAGCAGCAGCAG 3
TAGTAGCAGCAG 3
TAGTAGTAGCAG 3
AACAACAGATAG 2
AAGTAAGTAGTA 2

>m141122_111549_42134_c100732202550000001823141405141564_s1_p0/14995/6551_17296

Representative (only one) Read (Table 1): HuluTR150-r390, 1 ppm, 60% A+T, GenBank Acc. MN537572

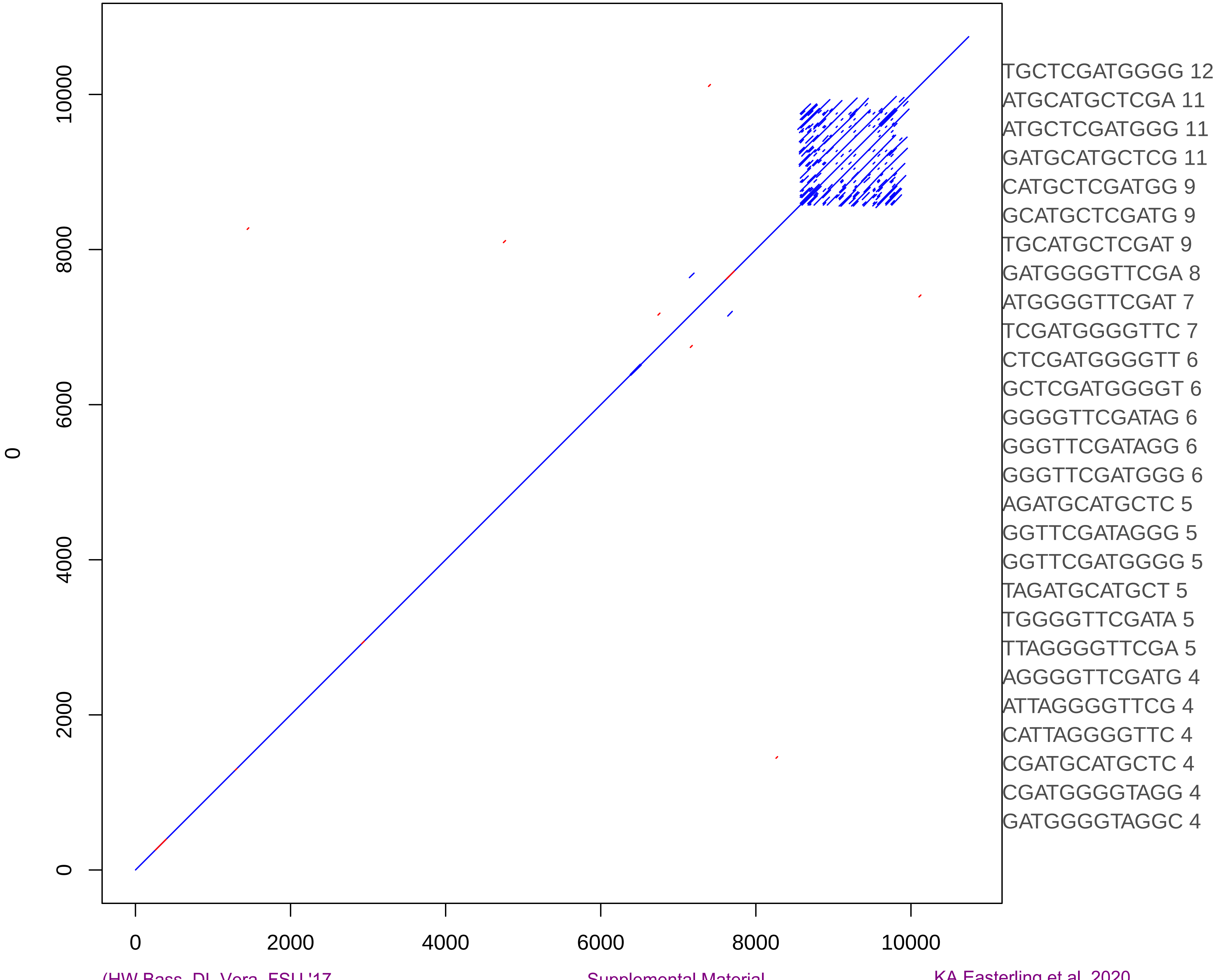

0

>m141122_111549_42134_c100732202550000001823141405141564_s1_p0/162110/1020_13796

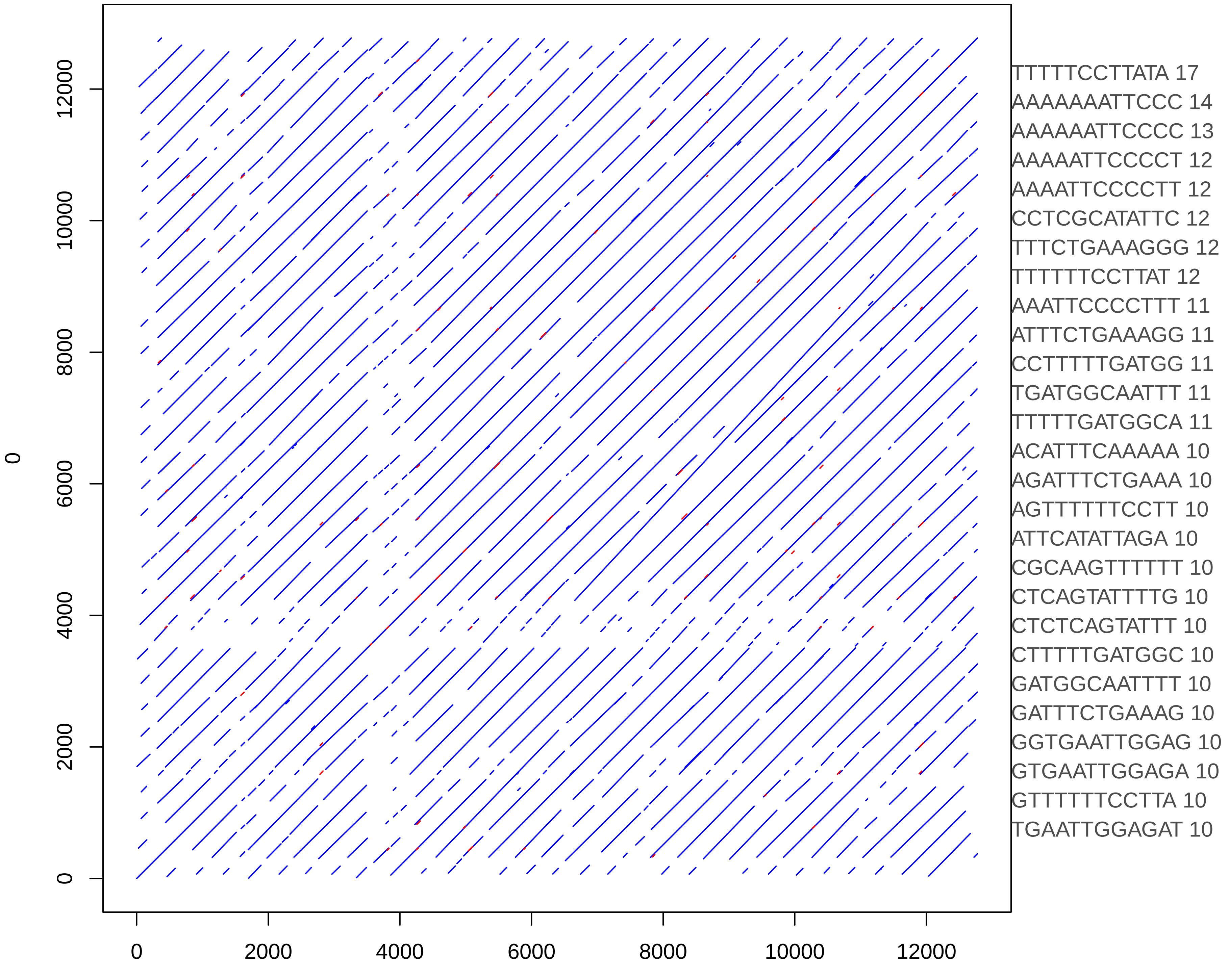

>m141122_111549_42134_c100732202550000001823141405141564_s1_p0/163344/4563_10287

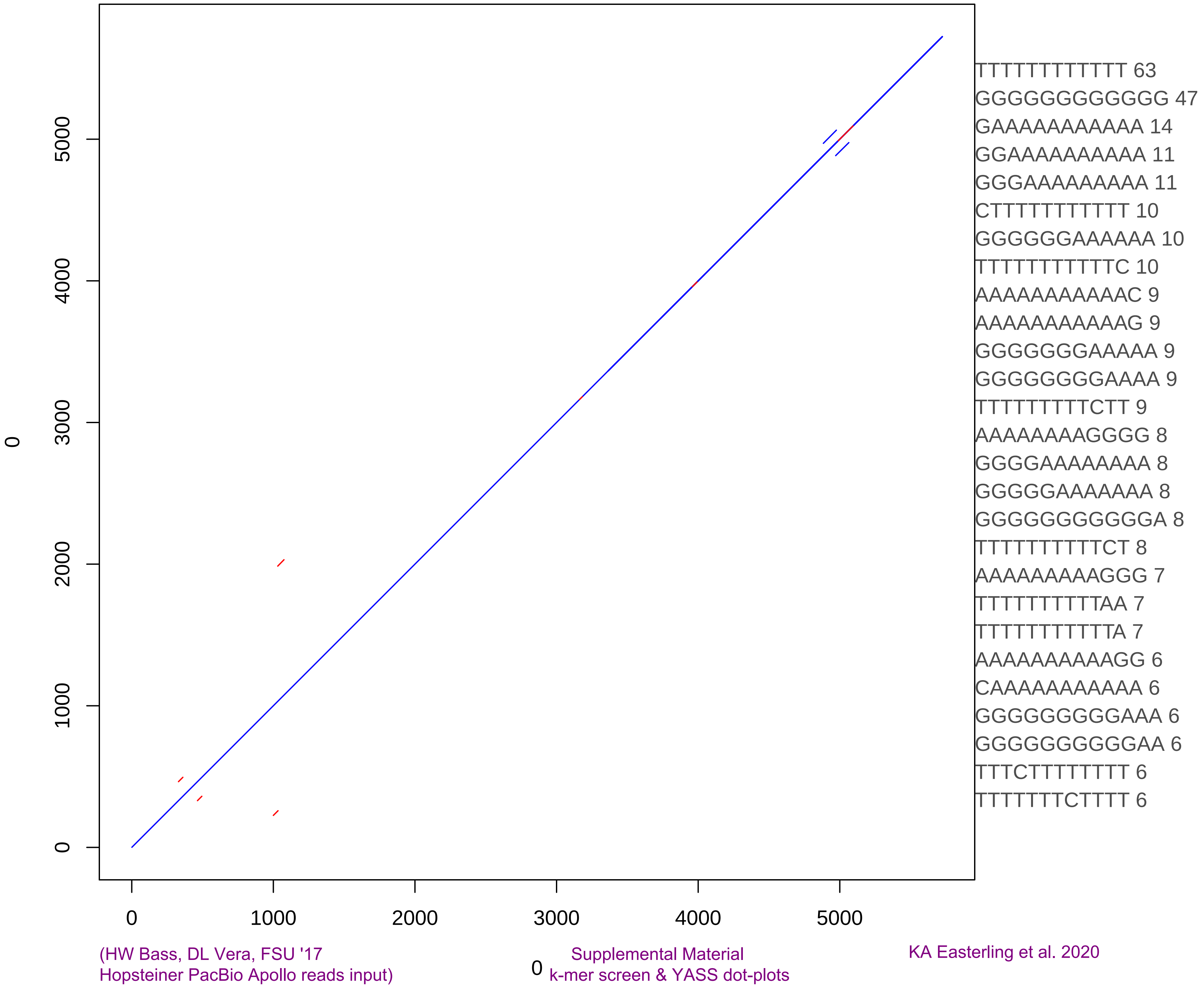

>m141122_111549_42134_c100732202550000001823141405141564_s1_p0/18281/2903_9150

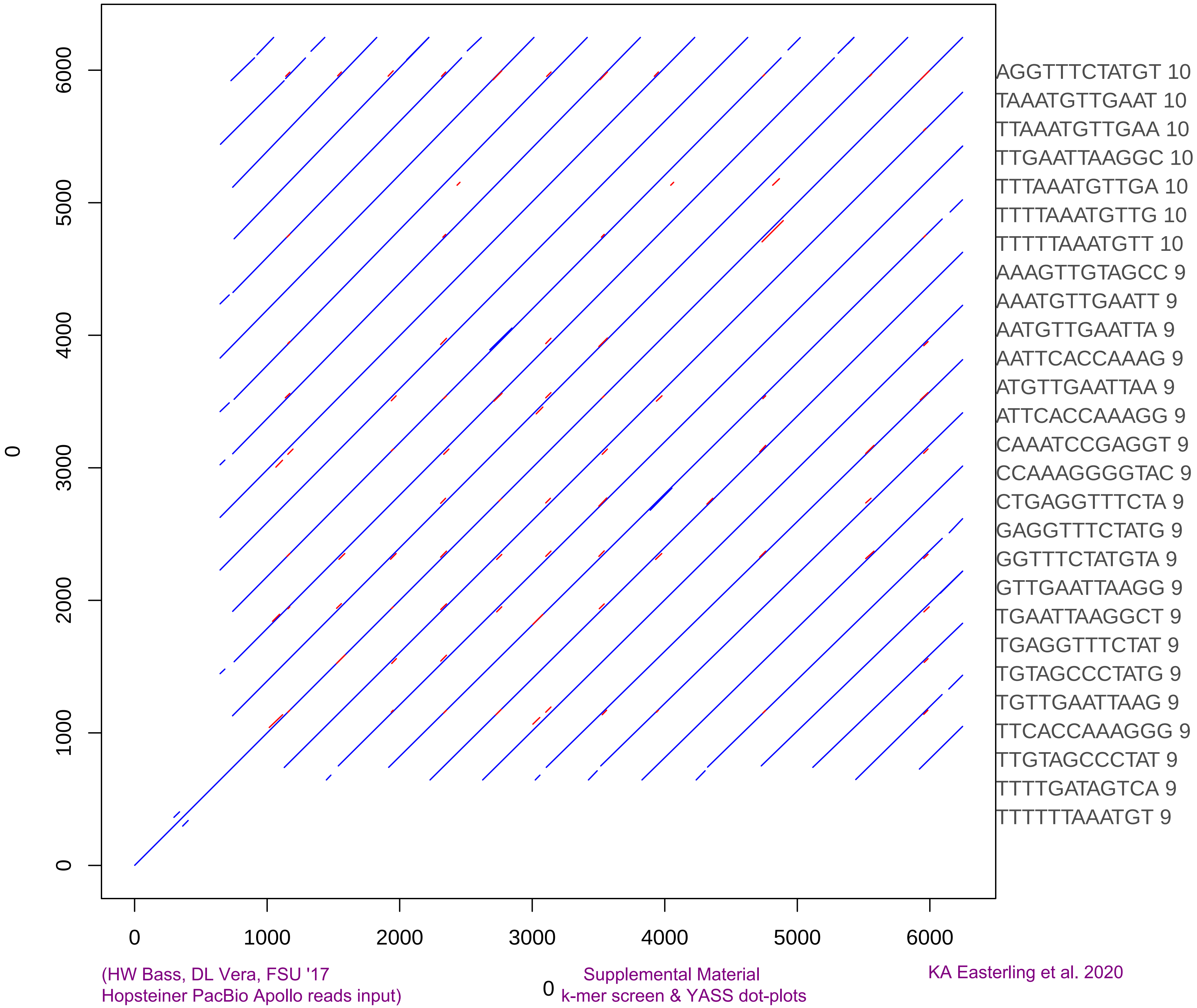

>m141122_111549_42134_c100732202550000001823141405141564_s1_p0/29792/5242_10361

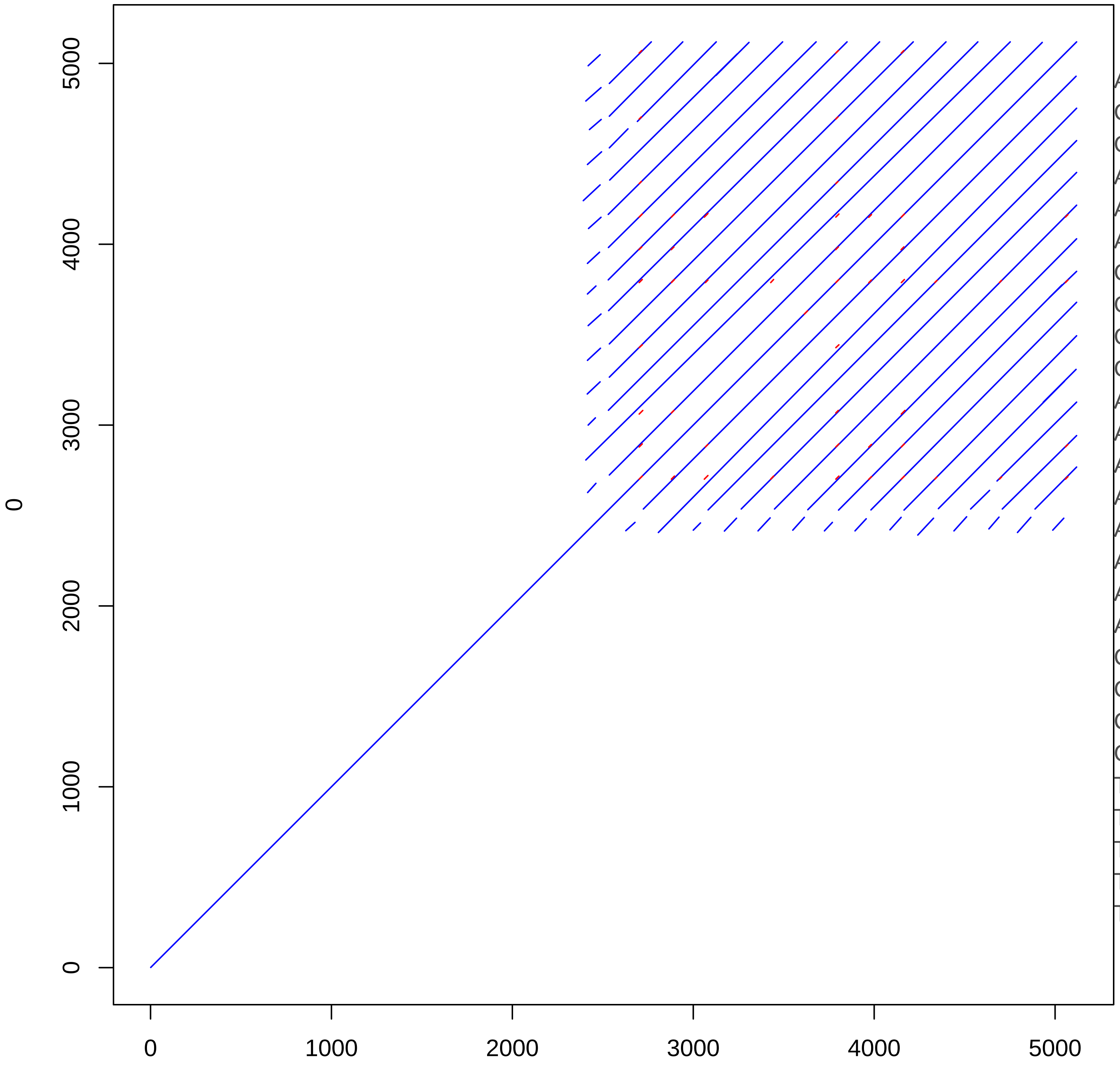

AGCTGCCTAGGA 9
CAGCTGCCTAGG 9
GCAGCTGCCTAG 9
AATATTATTGAG 8
AATTACGAAATC 8
ATATTATTGAGA 8
CGAAATCGCTTC 8
GAAATCGCTTCG 8
GCCCTATATAAT 8
GCTGCCTAGGAG 8
AAATCGCTTCGG 7
ACGAAATCGCTT 7
AGAGAAATATGA 7
AGCCCTATATAA 7
ATAATATTATTG 7
ATATAATATTAT 7
ATTACGAAATCG 7
ATTGAGATGGGA 7
CCCTATATAATA 7
CCTATATAATAT 7
CTATATAATATT 7
GAATTACGAAAT 7
TAATATTATTGA 7
TATAATATTATT 7
TATATAATATTA 7
TTACGAAATCGC 7
TTGAGATGGGAA 7

>m141122_111549_42134_c100732202550000001823141405141564_s1_p0/3993/5869_18932

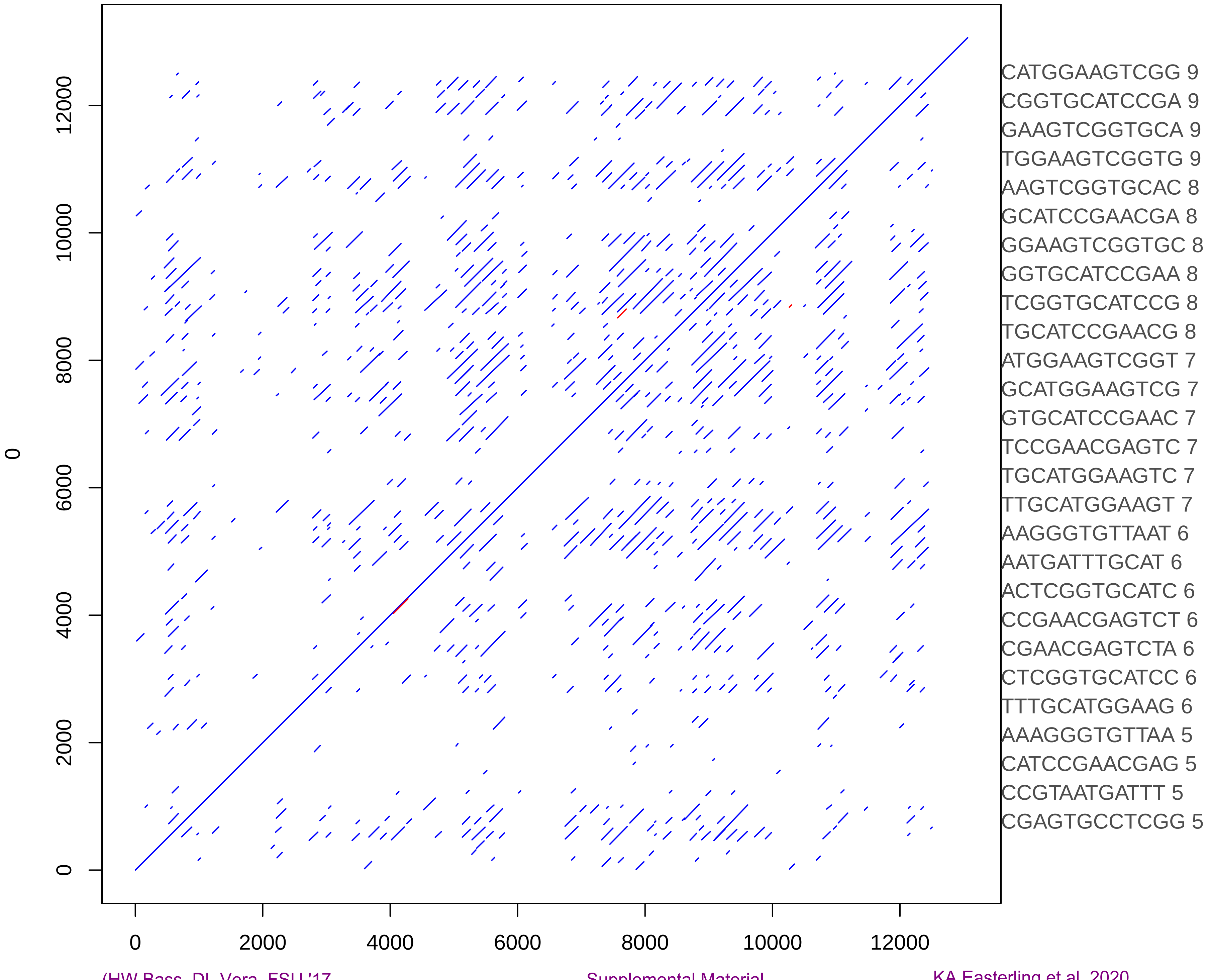

>m141122_111549_42134_c100732202550000001823141405141564_s1_p0/40370/5314_14360

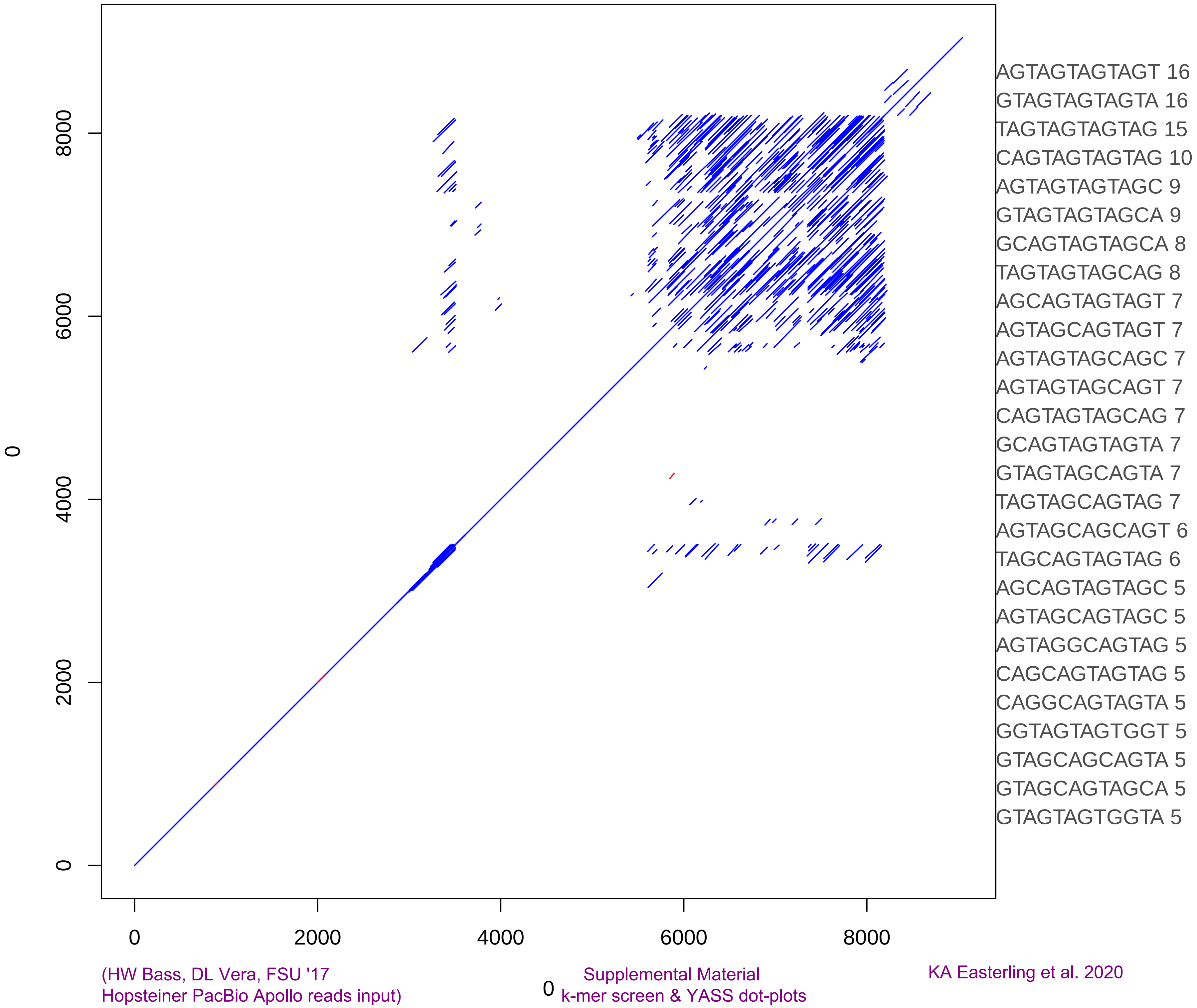

>m141122_111549_42134_c100732202550000001823141405141564_s1_p0/4423/4132_10105

Representative Read (Table 1): HuluTR225-r397, 46 ppm, 64% A+T, GenBank Acc. MN537574

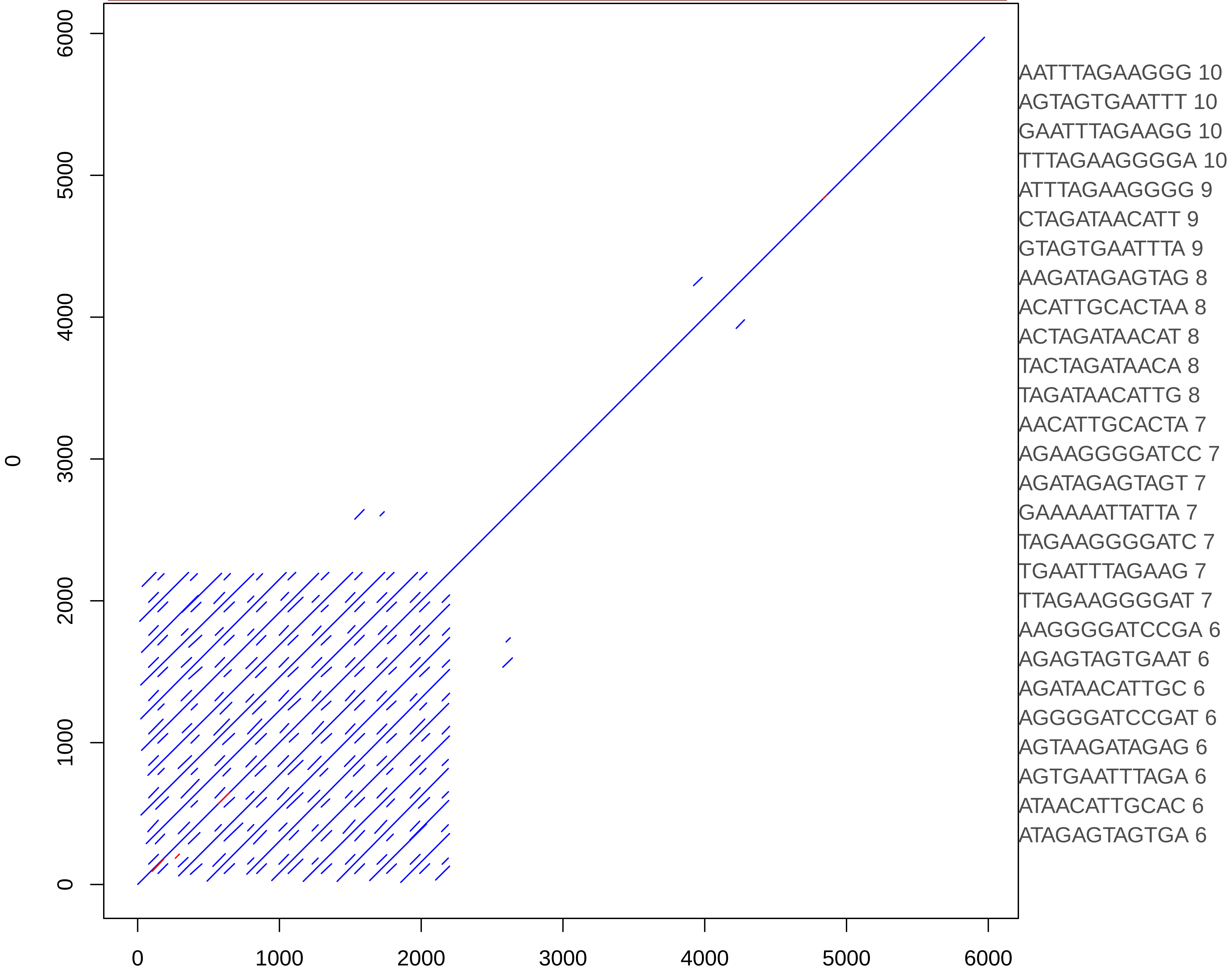

>m141122_111549_42134_c100732202550000001823141405141564_s1_p0/66993/8354_17918

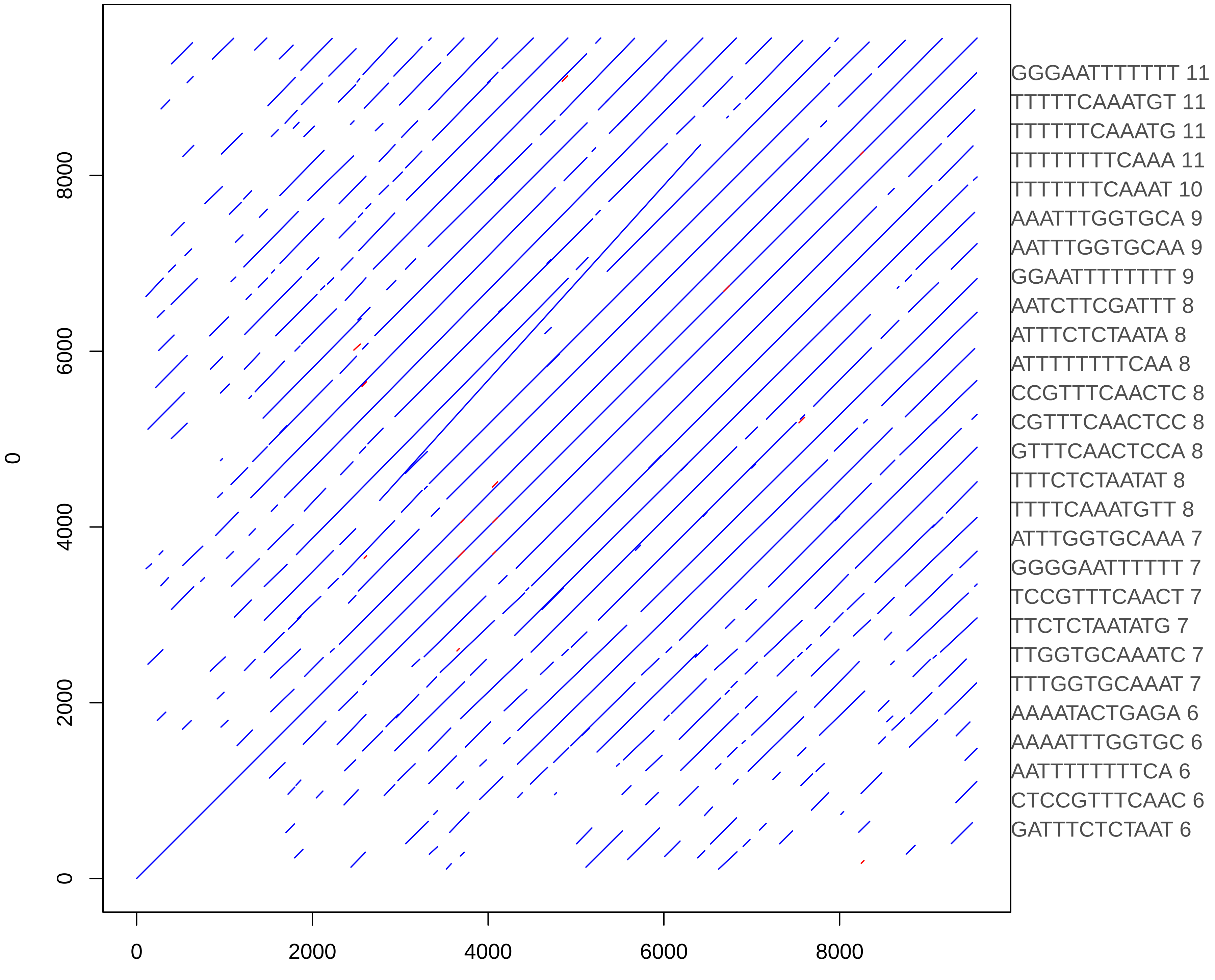

>m141122_111549_42134_c100732202550000001823141405141564_s1_p0/6948/9270_19525

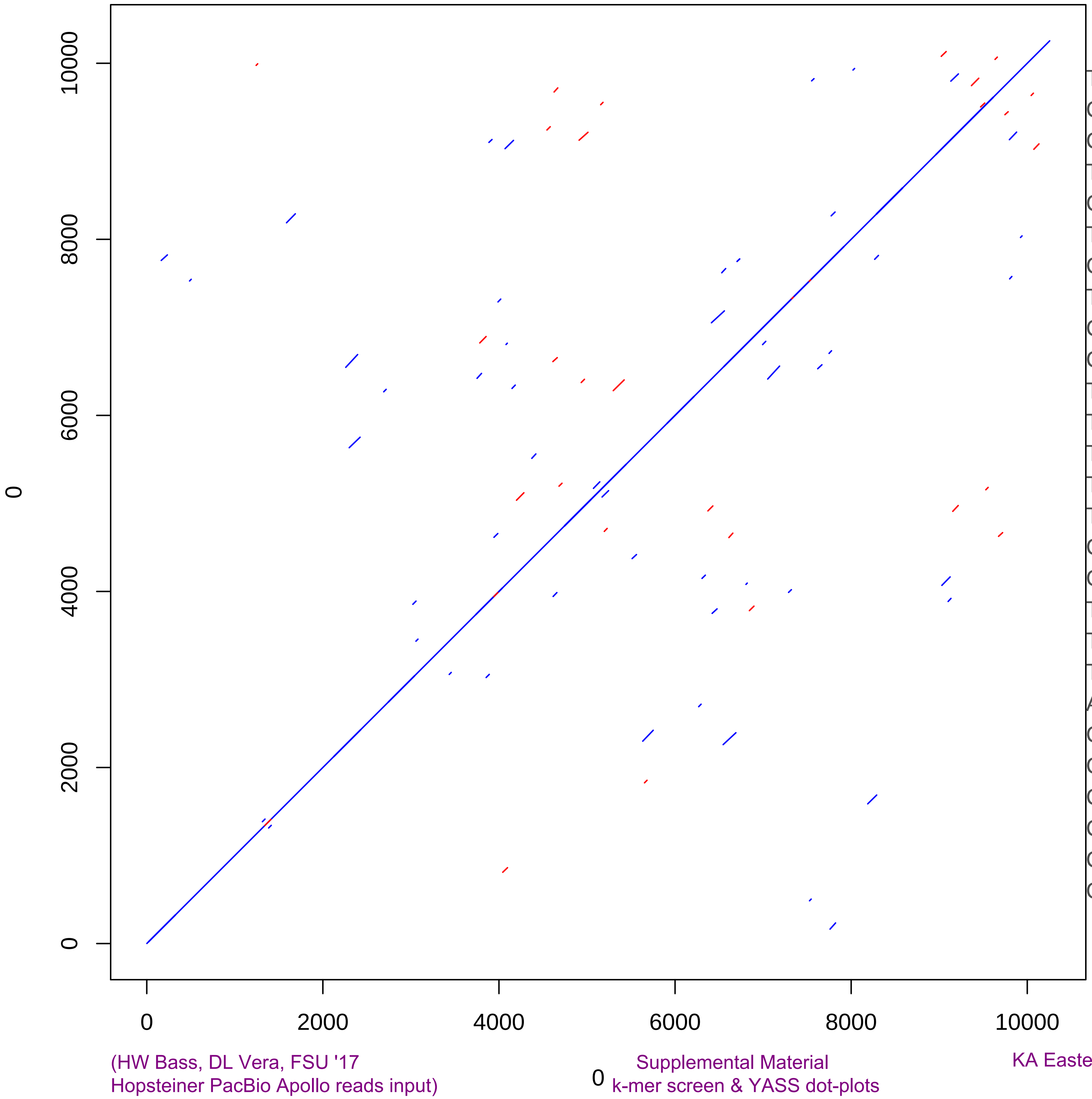

TTTTTTTTTTTT 95
CCCCCCCCCCCC 24
CTTTTTTTTTTT 15
TTTTTTTTTTGG 15
CCCCCCAAAAAA 11
TTTTTTTTTGGG 11
CCTTTTTTTTTT 10
TTTTTTTTTTTG 10
CCCTTTTTTTTT 9
CCCCCCCAAAAA 8
TTTTTCCCCCCC 8
TTTTTTCCCCCC 8
TTTTTTTTCCCC 8
TTTTTTTTTTAA 8
TTTTTTTTTTTA 8
CCCCTTTTTTTT 7
GGTTTTTTTTTT 7
TTTTTTTCCCCC 7
TTTTTTTTTCCC 7
TTTTTTTTTTTC 7
ACTTTTTTTTTT 6
CAAAAAAAAAAA 6
CCCCCAAAAAAA 6
CCCCCCCCAAAA 6
CCCCCCCCCCTT 6
CCCCCTTTTTTT 6
GTTTTTTTTTTT 6

>m141122_111549_42134_c100732202550000001823141405141564_s1_p0/98525/6603_13946

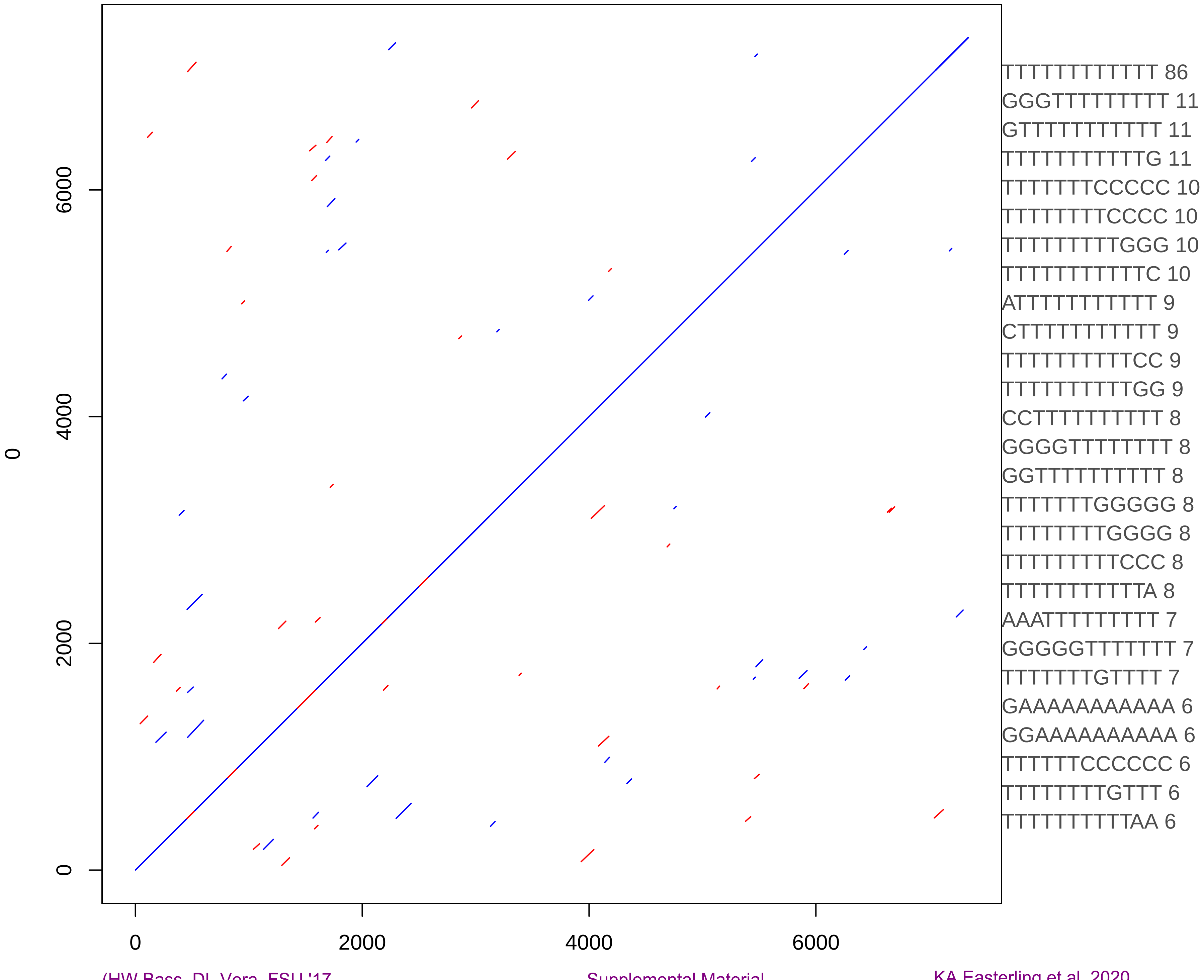

>m141122_133804_42134_c100732202550000001823141405141565_s1_p0/105818/6709_13395

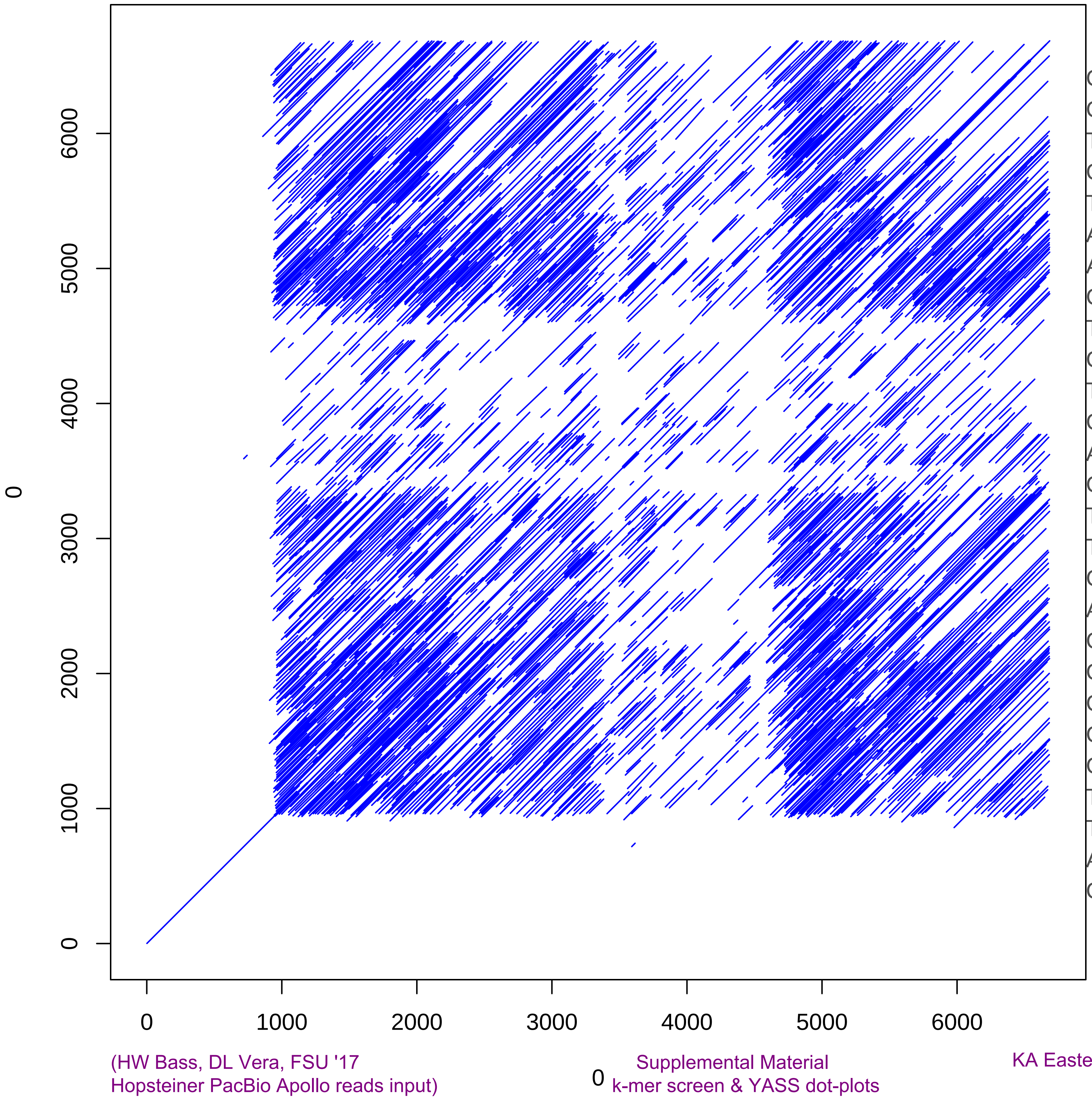

GTAGTGGTAGTG 38
GTGGTAGTGGTA 38
TGGTAGTGGTAG 38
GGTAGTGGTAGT 35
TAGTGGTAGTGG 35
AGTGGTAGTGGT 34
AGTAGTAGTAGT 32
GTAGTAGTAGTA 30
TAGTAGTAGTAG 29
GTGGTGGTGGTG 27
TGGTGGTGGTGG 25
GGTGGTGGTGGT 21
AGTGGTAGTAGT 19
GTAGTGGTAGTA 19
TAGTGGTAGTAG 17
TAGTAGTGGTAG 16
GTAGTAGTGGTA 15
AGTAGTGGTAGT 14
GTGGTAGTAGTA 11
GGTAGTAGTAGT 10
GTAGTAGTAGTG 10
GTAGTGTAGTGG 10
GTGGTAGTAGTG 10
TGGTAGTAGTAG 10
TGGTAGTGTAGT 10
AGTGGTAGTGTA 9
GGTAGTGGTATG 9

>m141122_133804_42134_c100732202550000001823141405141565_s1_p0/118685/7717_14574

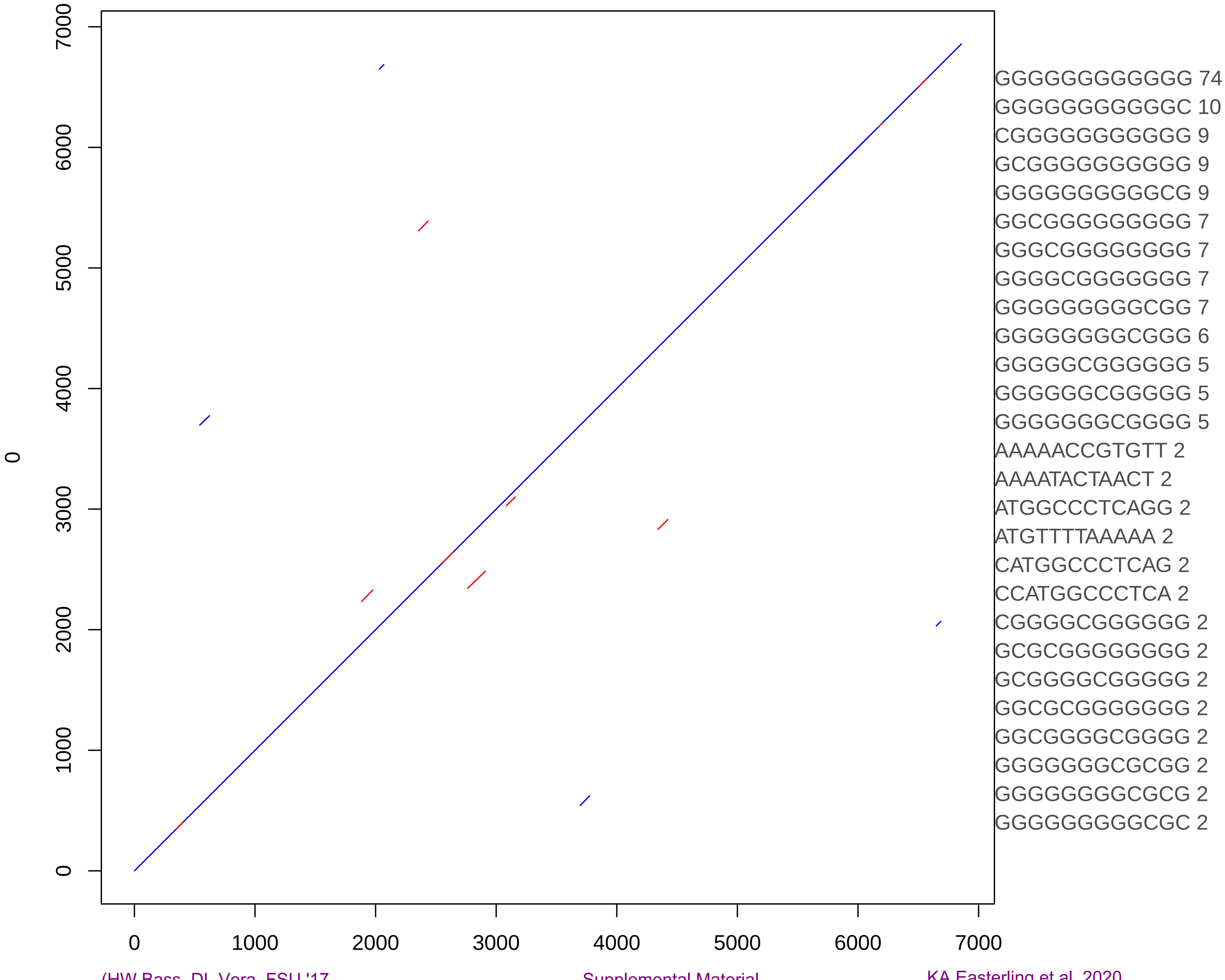

>m141122_133804_42134_c100732202550000001823141405141565_s1_p0/121022/6904_14319

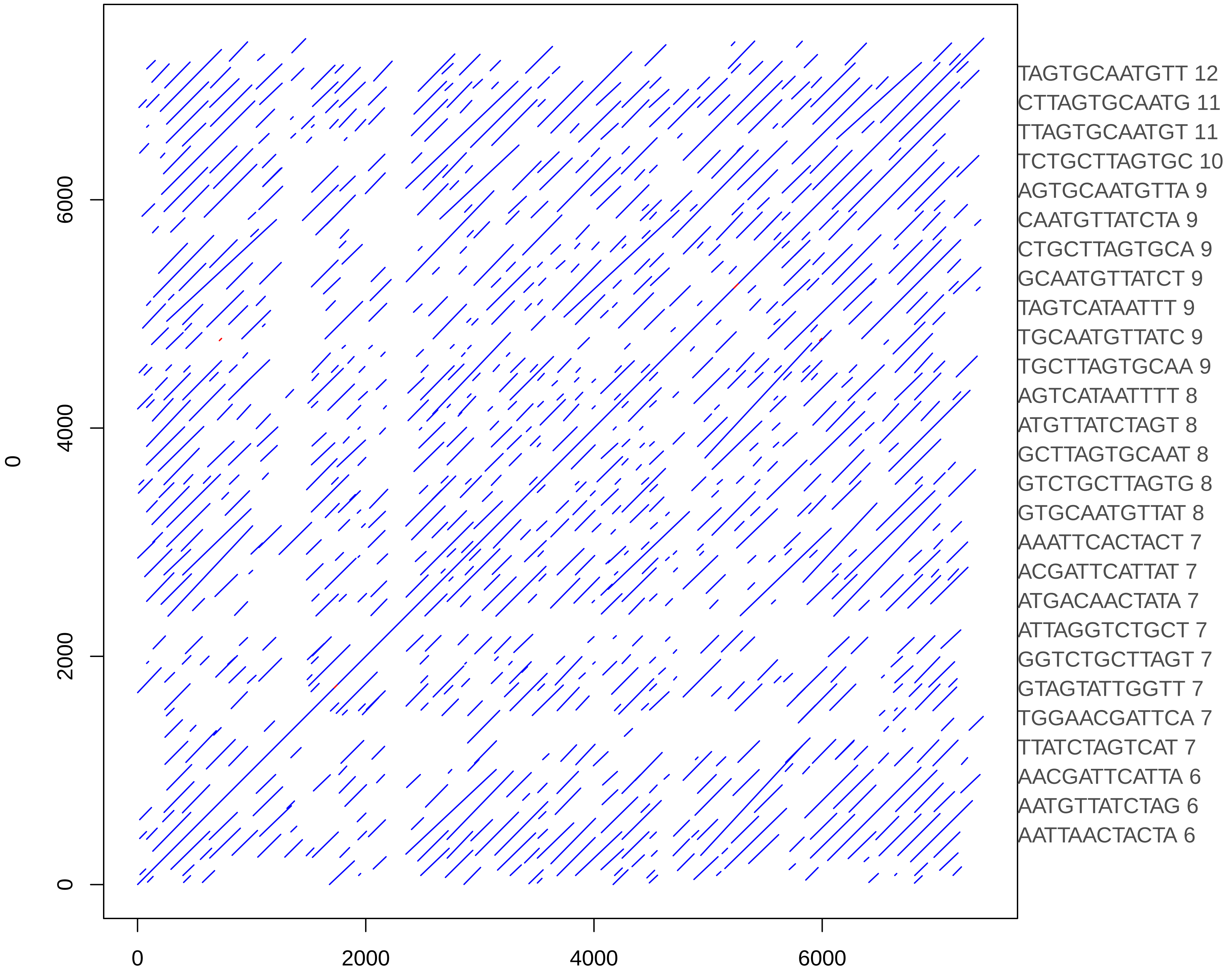

>m141122_133804_42134_c100732202550000001823141405141565_s1_p0/121253/3138_10566

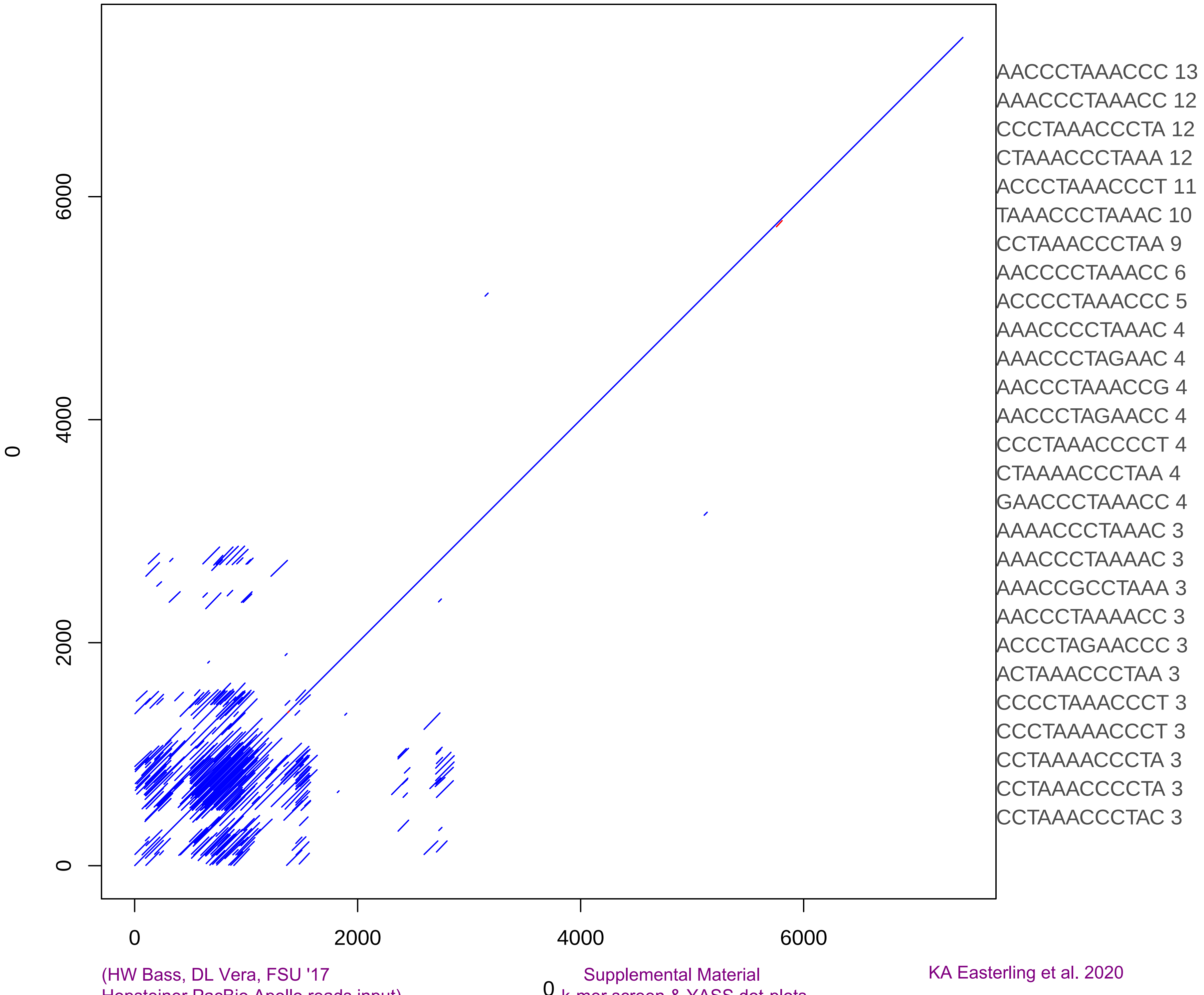

>m141122_133804_42134_c100732202550000001823141405141565_s1_p0/125694/3561_9670

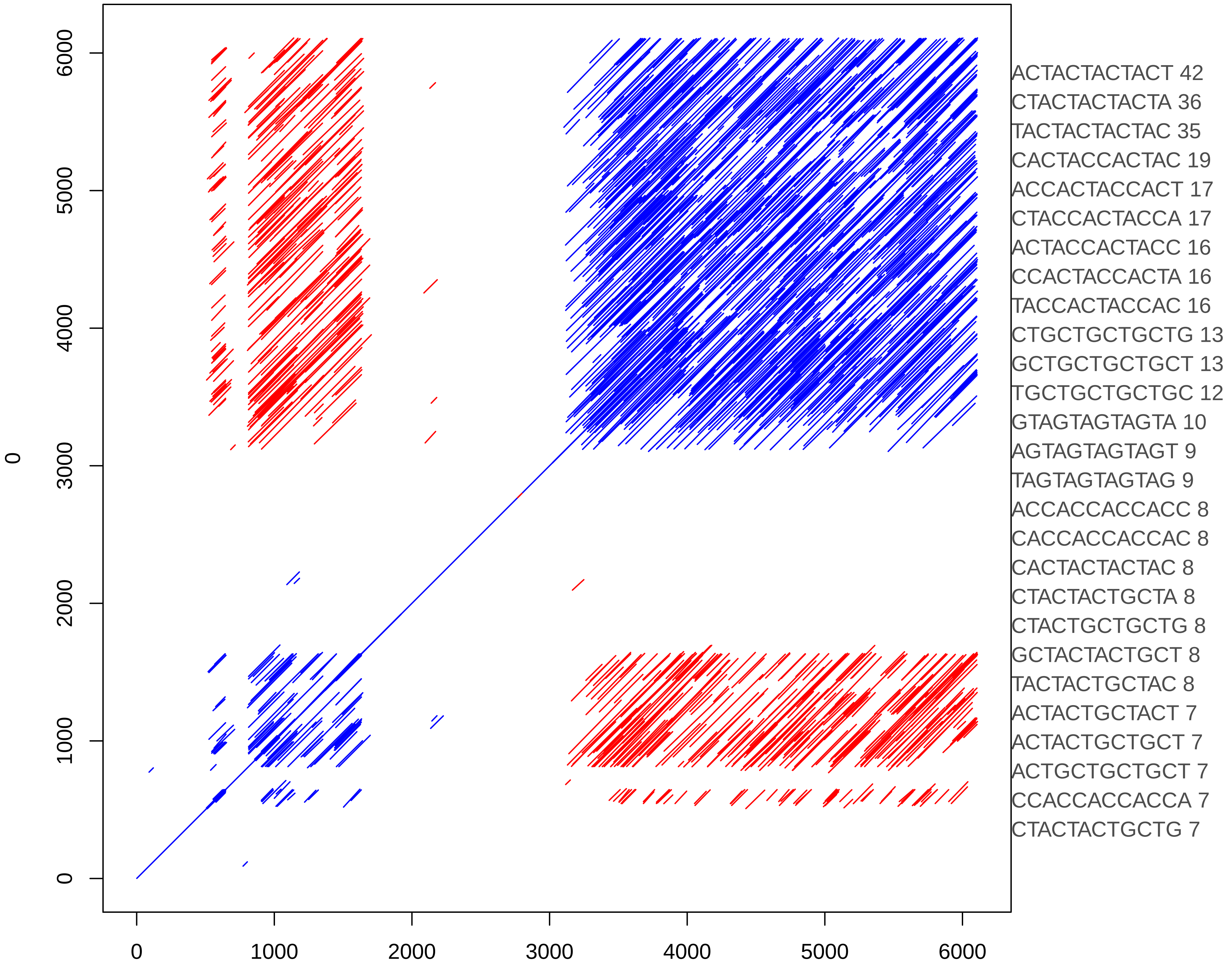

>m141122_133804_42134_c100732202550000001823141405141565_s1_p0/127505/13012_18850

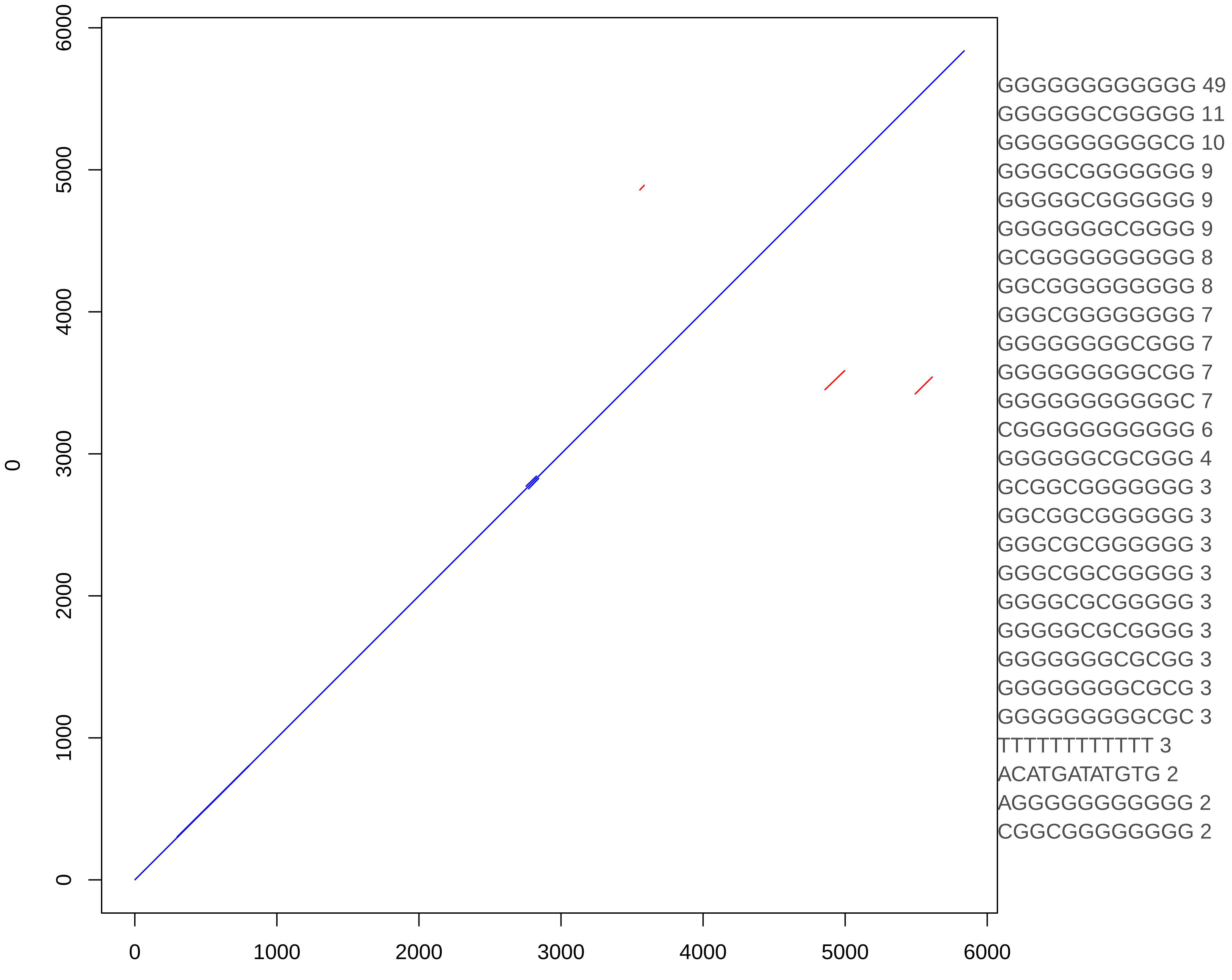

>m141122_133804_42134_c100732202550000001823141405141565_s1_p0/128023/7325_18594

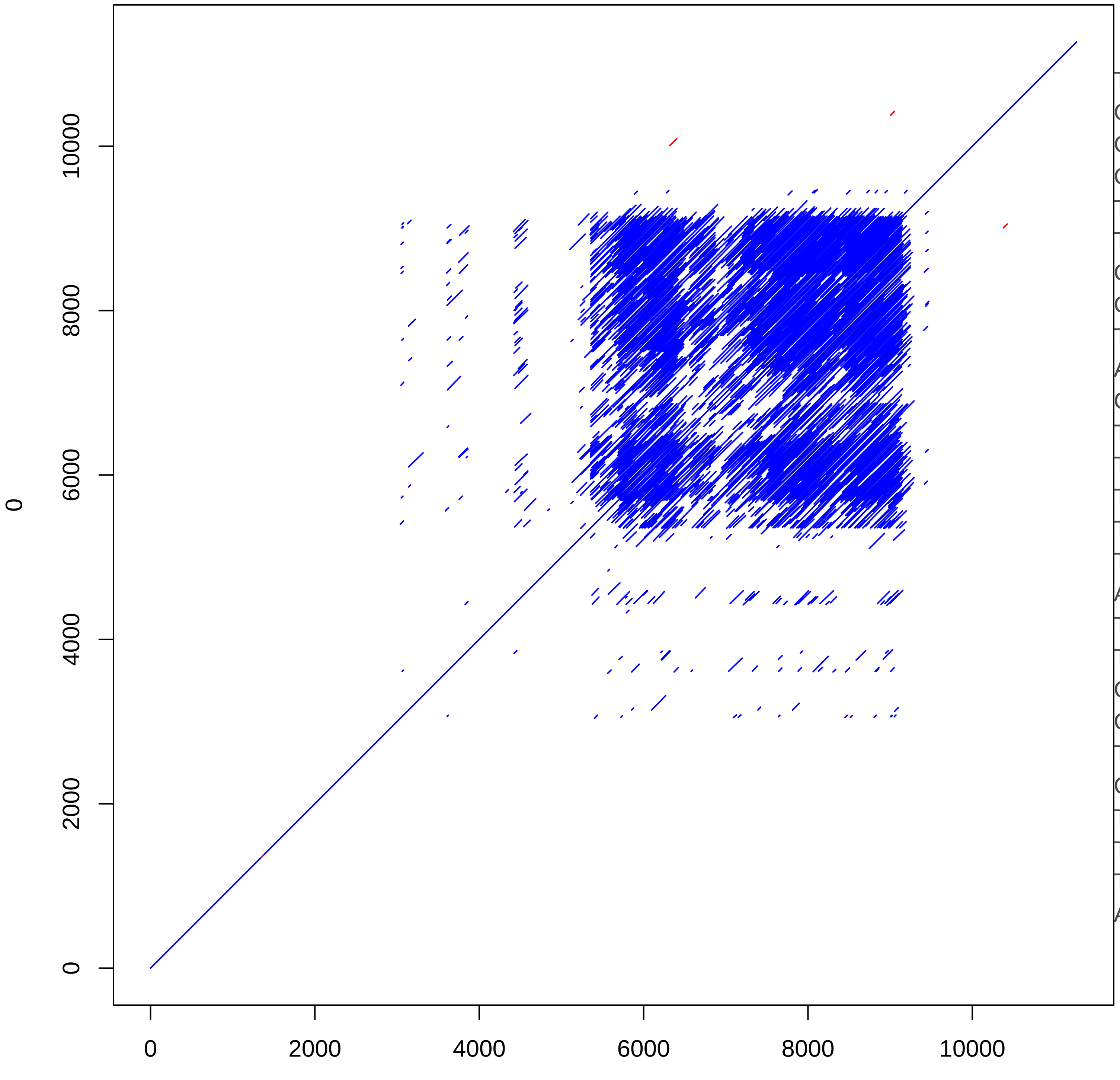

TTTTTTTTTTTT 152
GGTTTAGGGTTT 27
GGTTTTAGGGTT 24
GGGTTTAGGGTT 23
TTTTAGGGTTTT 21
TTTAGGGTTTTA 20
GTTTAGGGTTTT 19
GTTTTAGGGTTT 19
TTTAGGGTTTTT 19
AGGGTTTAGGGT 18
GGGTTTTAGGGT 18
TAGGGTTTAGGG 18
TAGGGTTTTAGG 18
TTTTTAGGGTTT 18
TTAGGGTTTAGG 17
TTTTAGGGTTTA 17
AGGGTTTTAGGG 16
TTAGGGTTTTAG 16
TTTAGGGTTTAG 16
GGTTTTTAGGGT 14
GTTTTTAGGGTT 14
TTAGGGTTTTTA 13
GGGTTTTTAGGG 11
TTAGGGTTTTTT 11
TAGGGTTTTTAG 10
TAGGGTTTTTTT 10
AGGGTTTTTAGG 9

>m141122_133804_42134_c100732202550000001823141405141565_s1_p0/128581/2118_9008

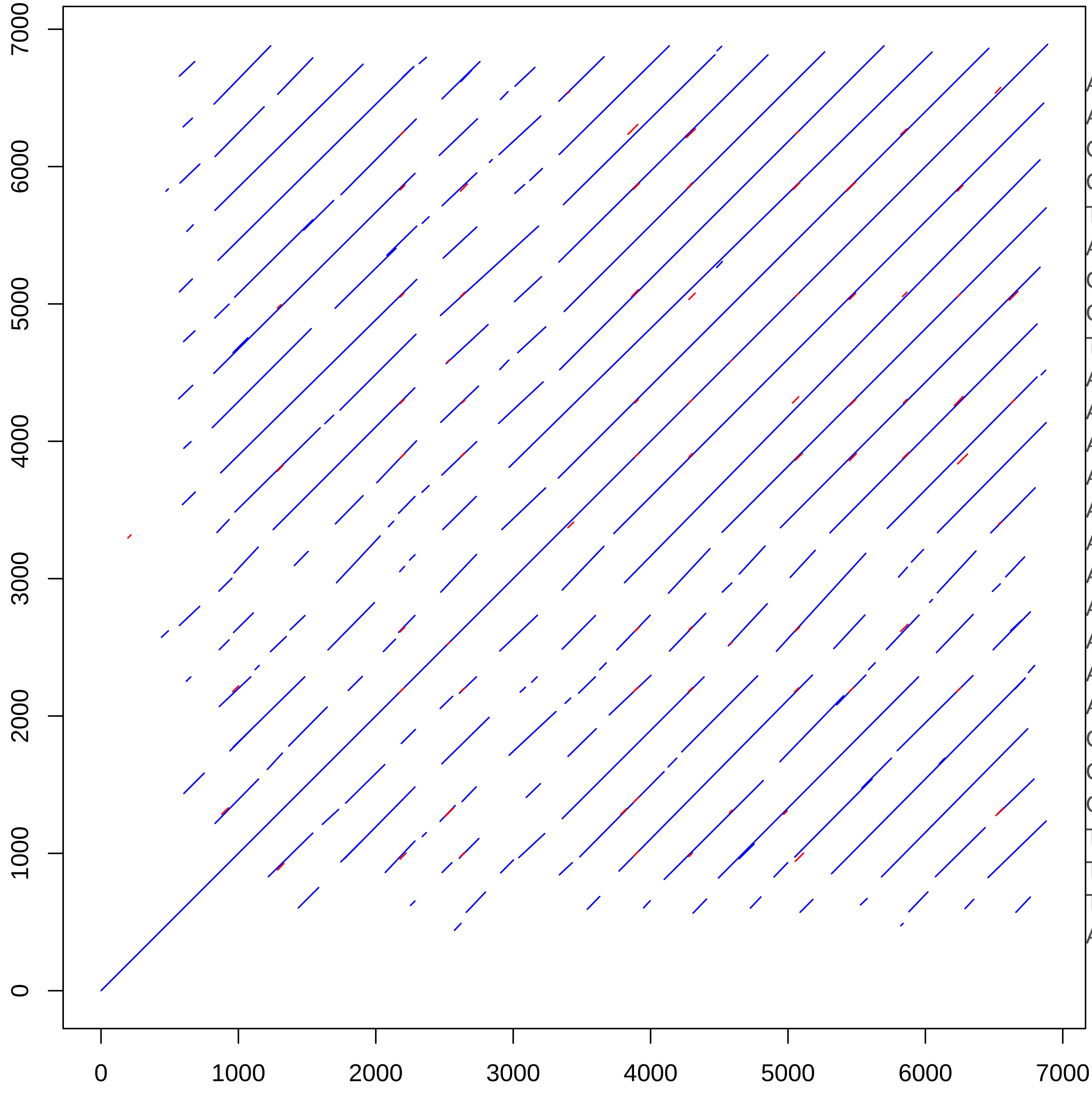

>m141122_133804_42134_c100732202550000001823141405141565_s1_p0/133100/3828_15600

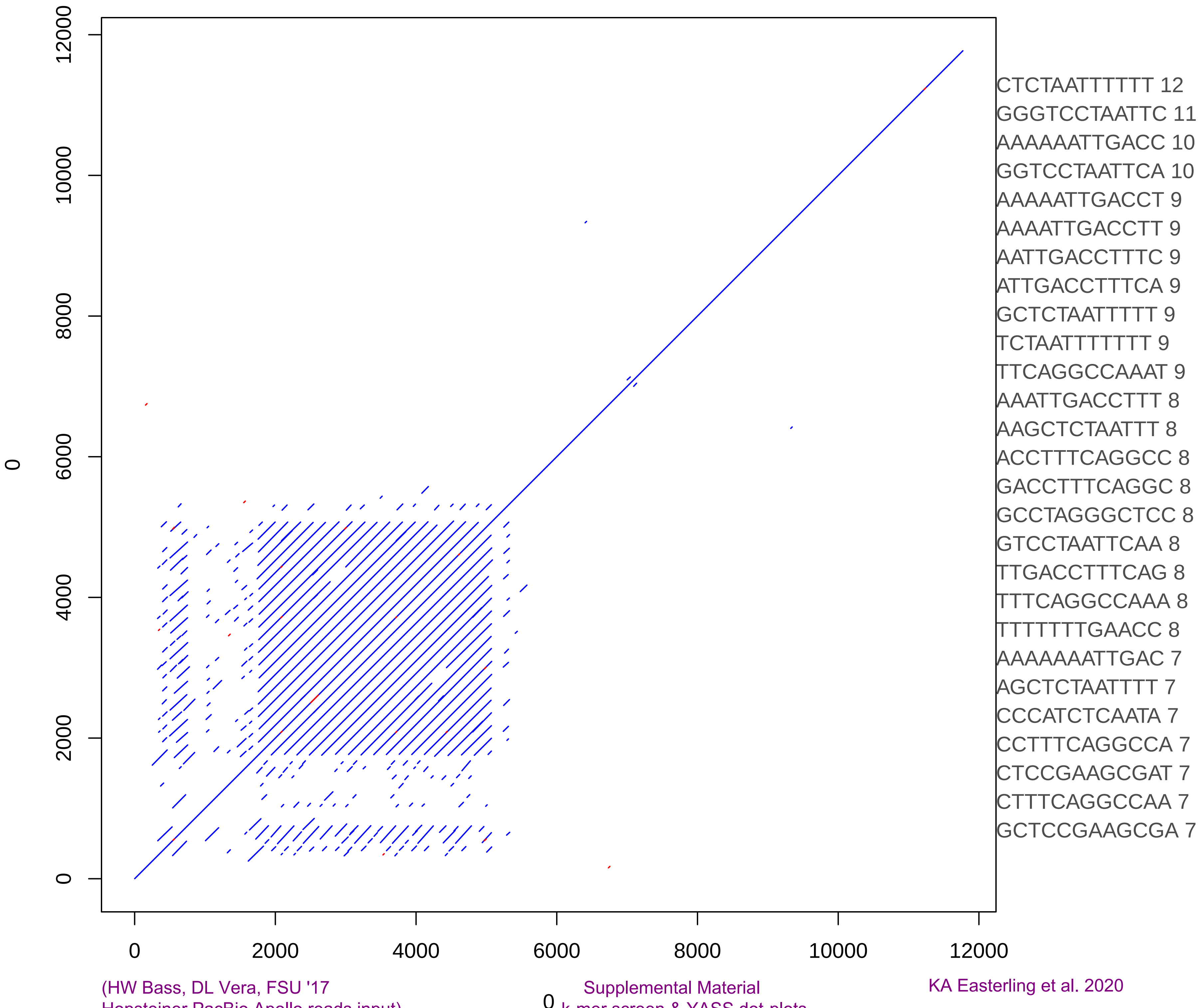

>m141122_133804_42134_c100732202550000001823141405141565_s1_p0/145778/12789_18480

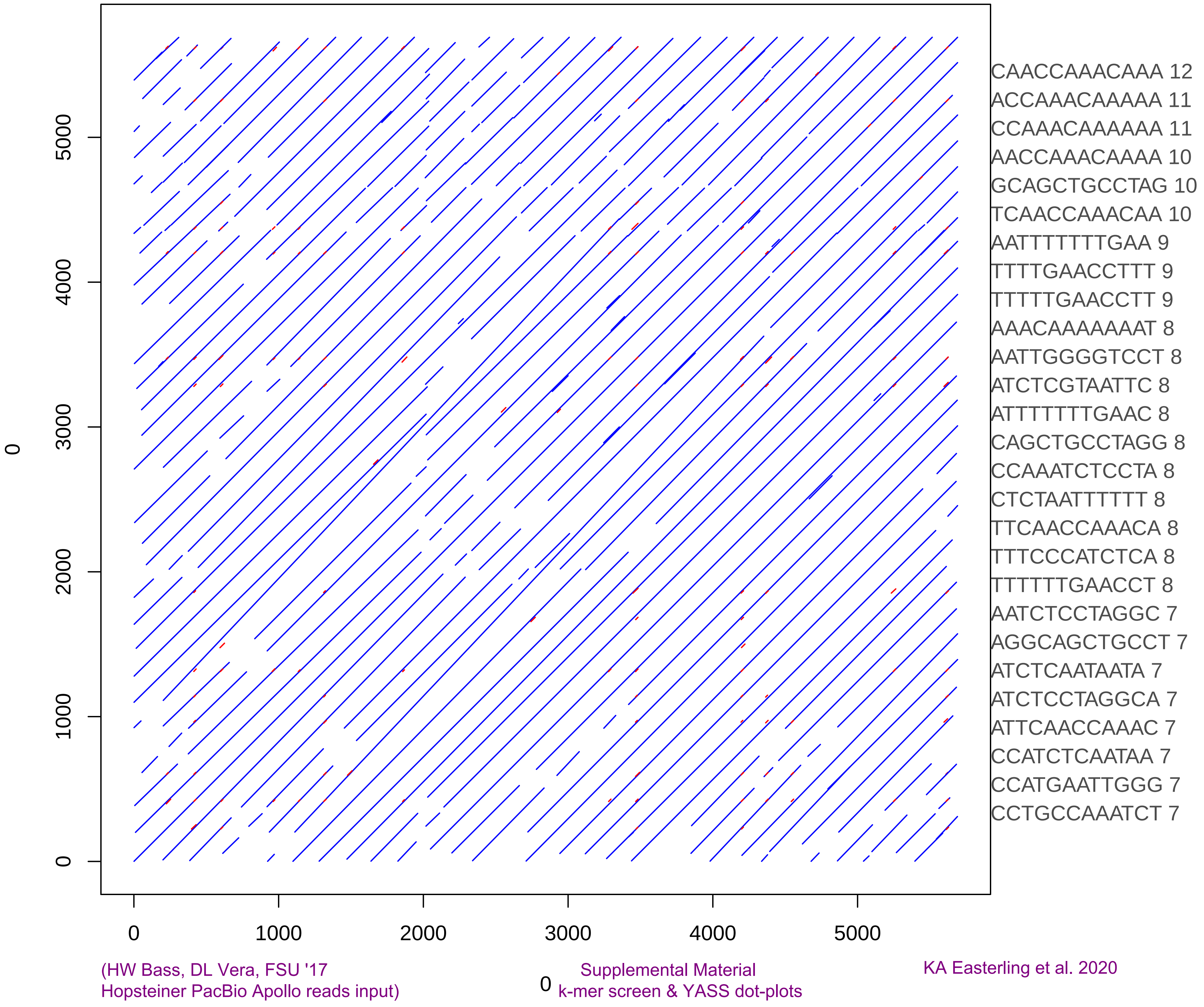

>m141122_133804_42134_c100732202550000001823141405141565_s1_p0/146582/600_7438

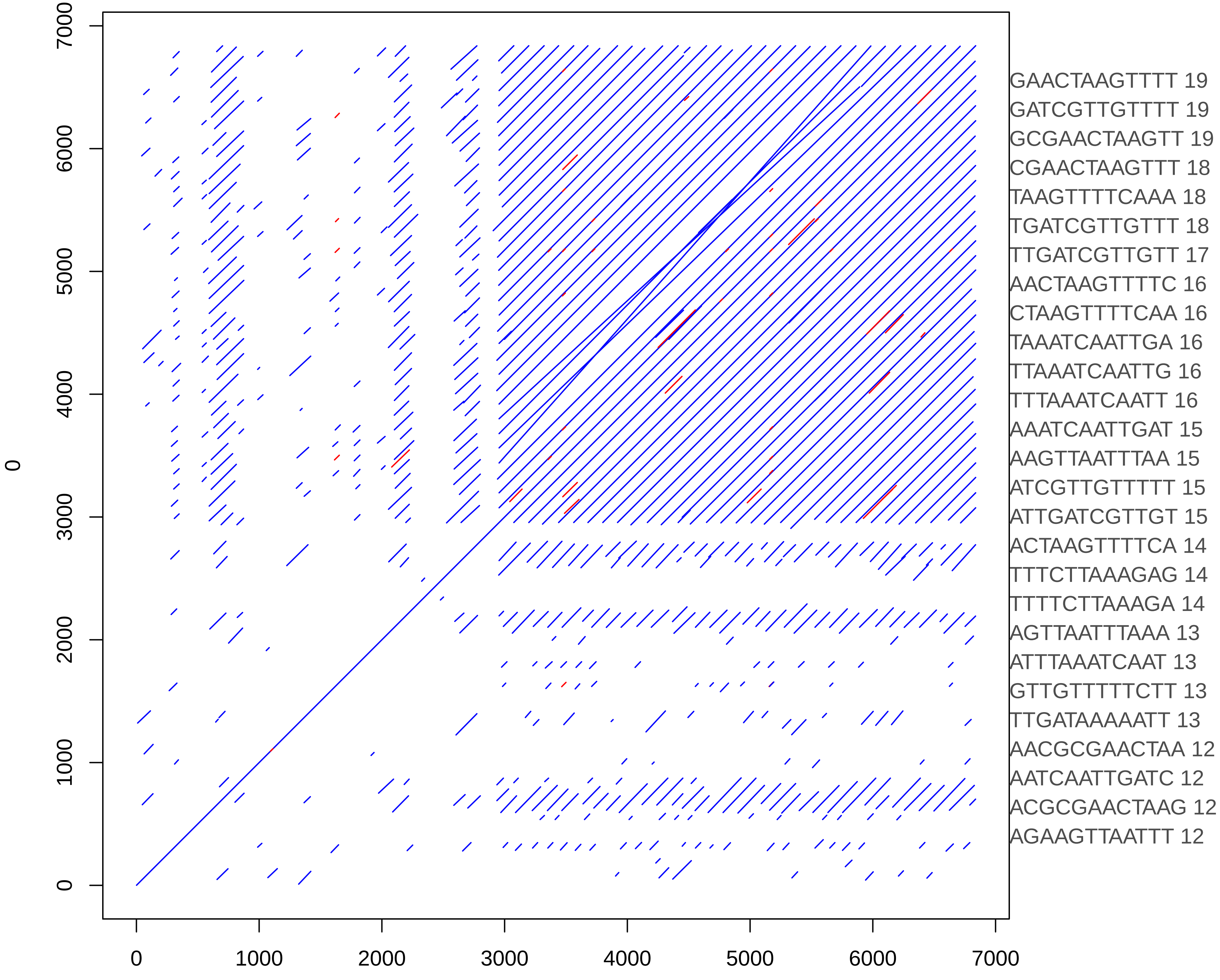

>m141122_133804_42134_c100732202550000001823141405141565_s1_p0/146821/3543_9584

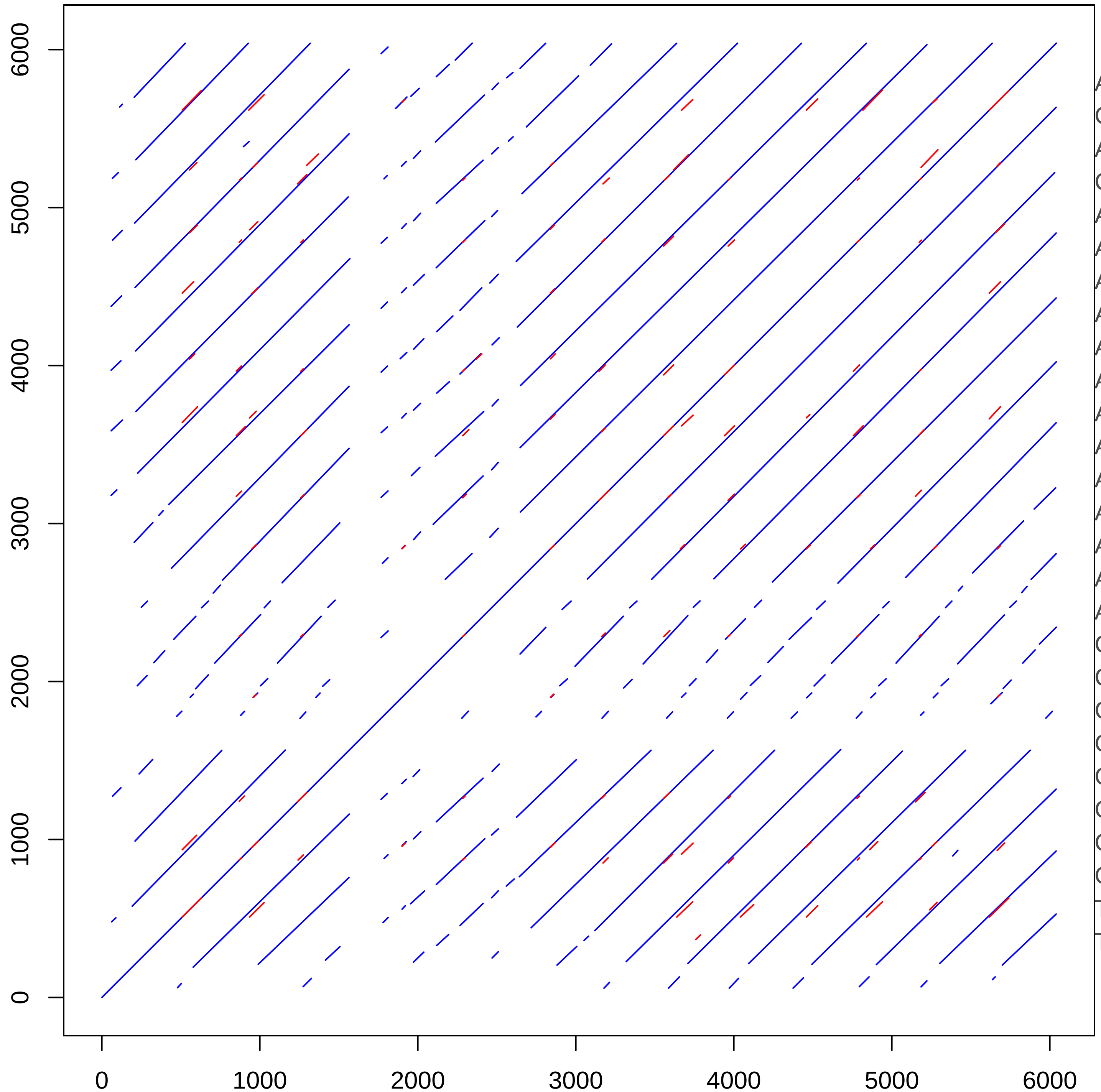

>m141122_133804_42134_c100732202550000001823141405141565_s1_p0/147679/7820_17366

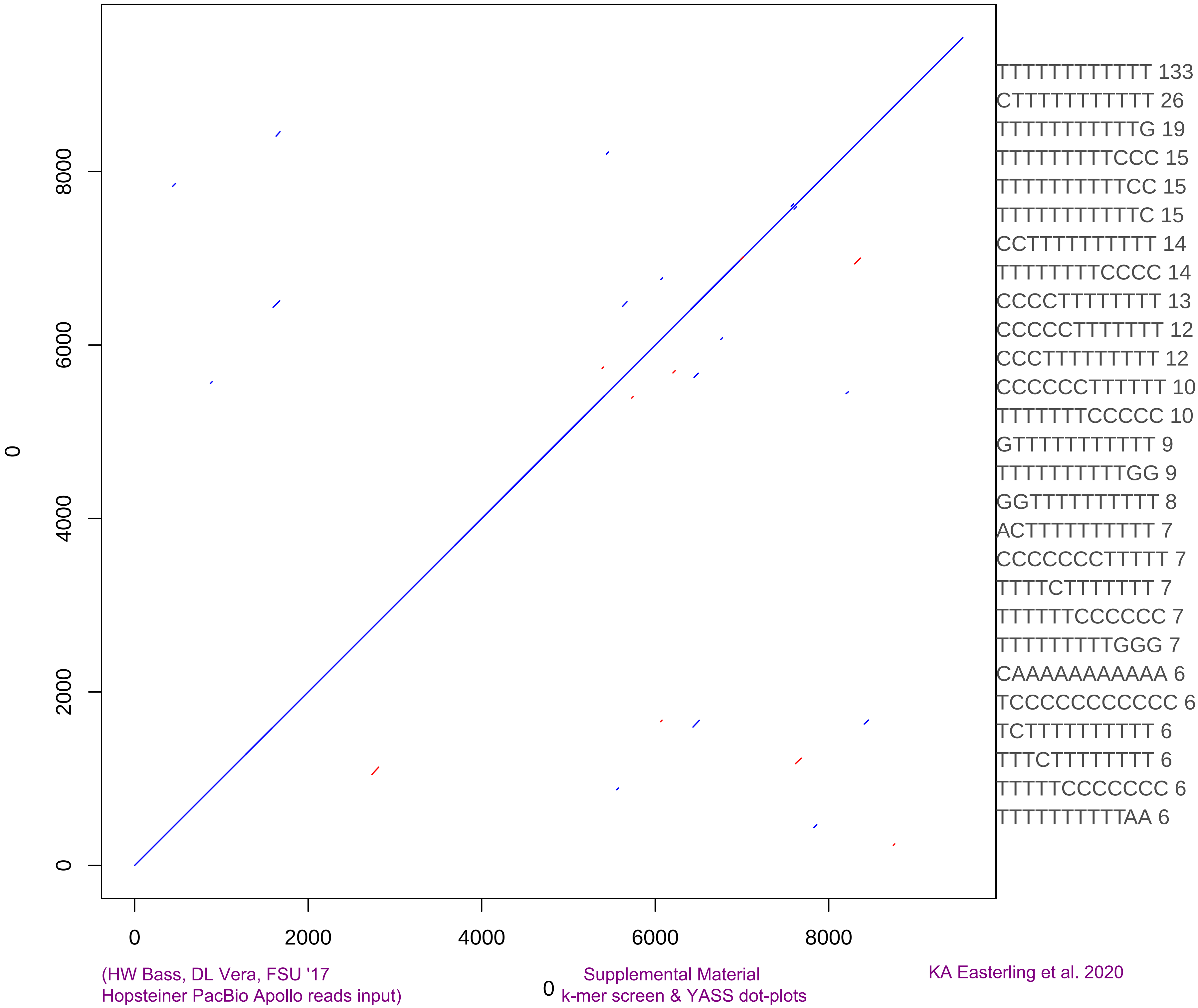

>m141122_133804_42134_c100732202550000001823141405141565_s1_p0/151308/14982_22327

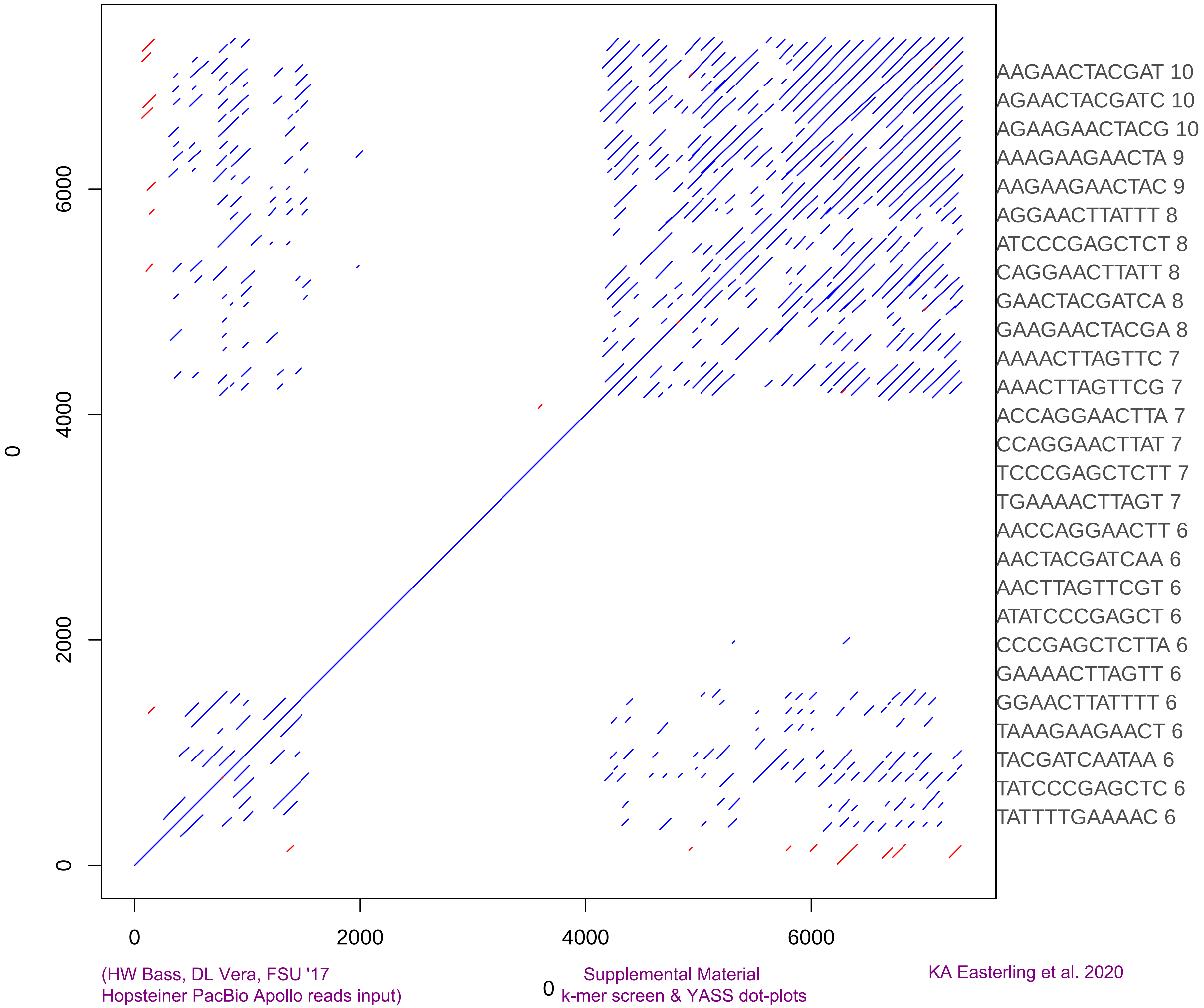

>m141122_133804_42134_c100732202550000001823141405141565_s1_p0/151945/8768_18025

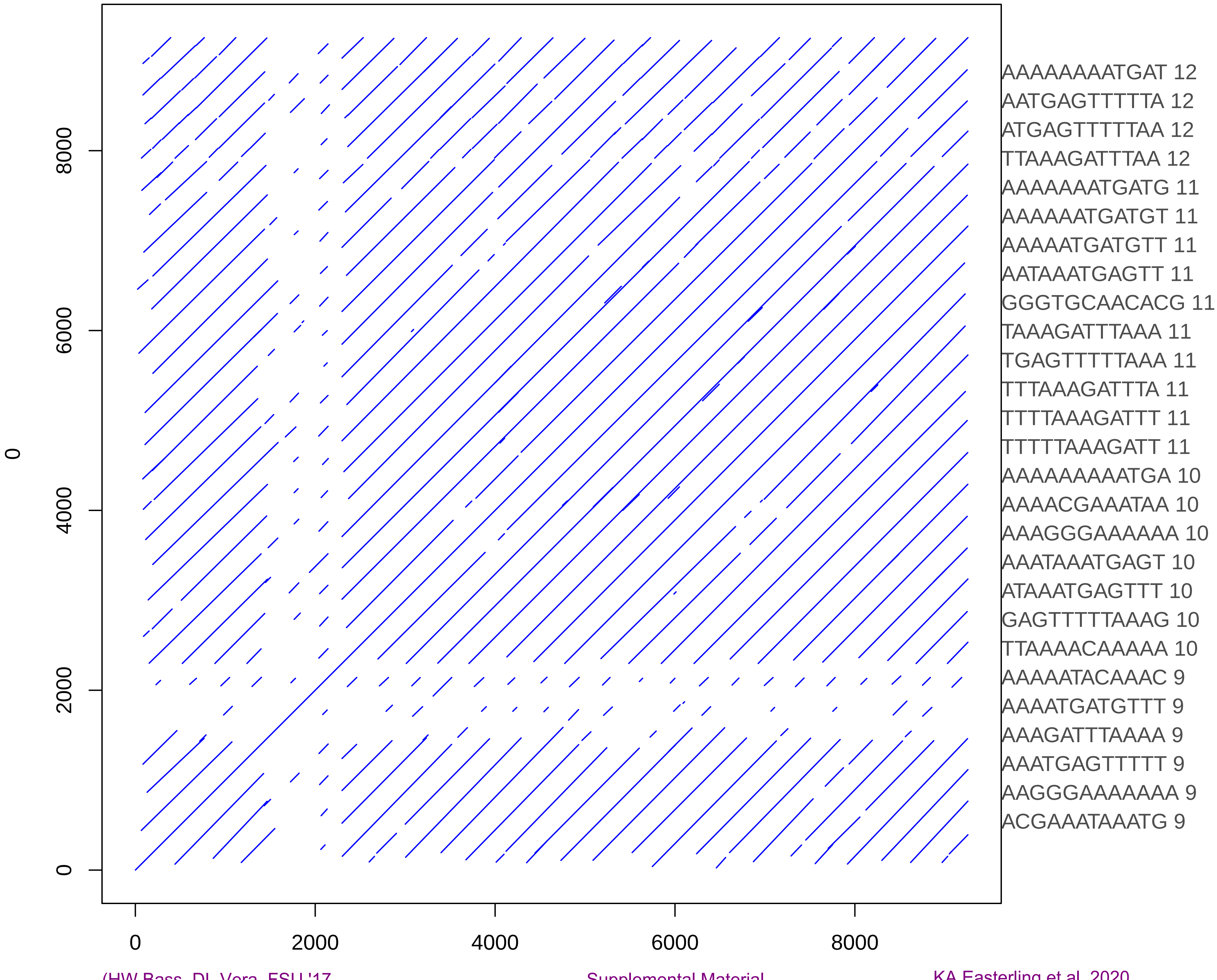

>m141122_133804_42134_c100732202550000001823141405141565_s1_p0/157022/5061_16782

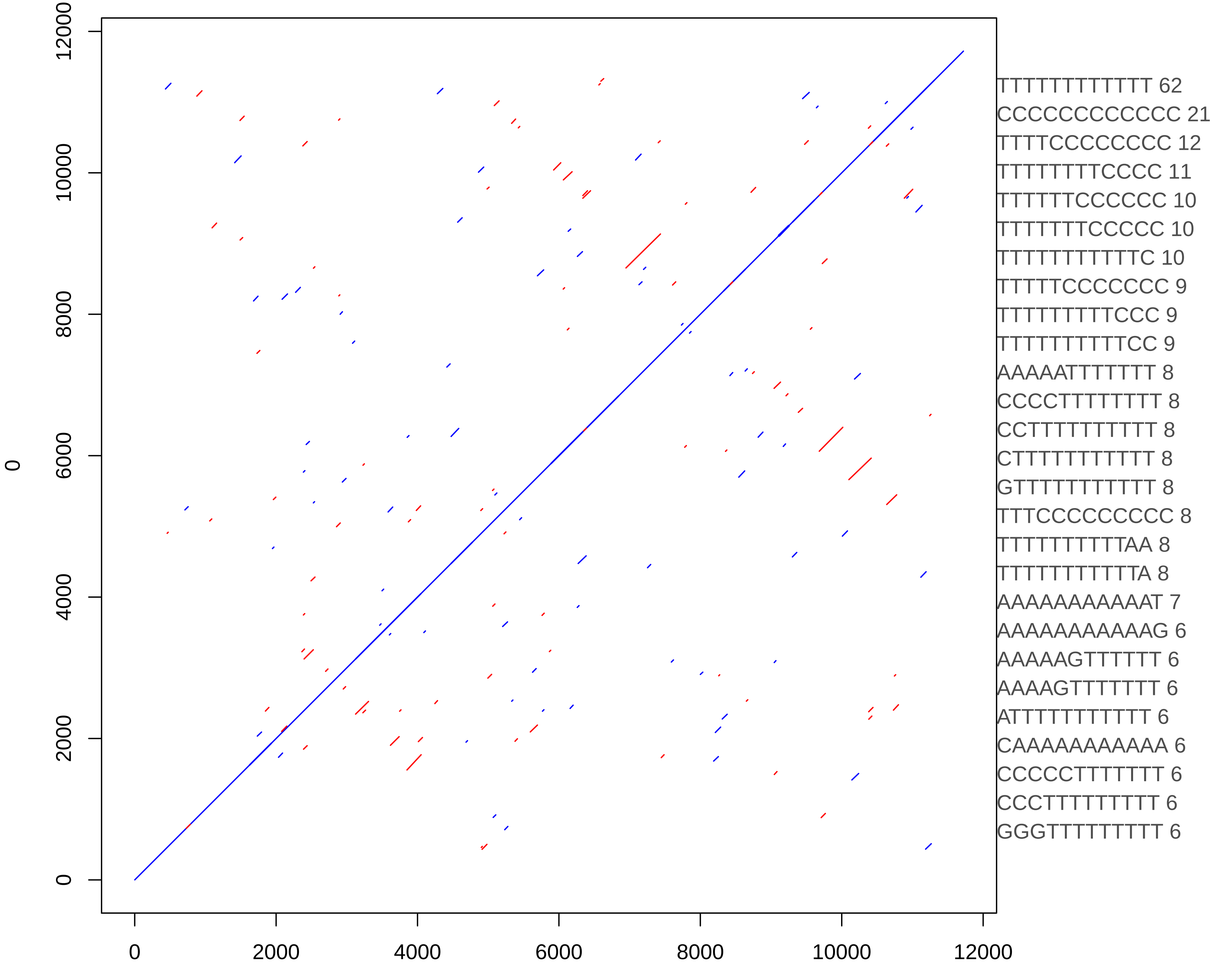

>m141122_133804_42134_c100732202550000001823141405141565_s1_p0/159117/0_7343

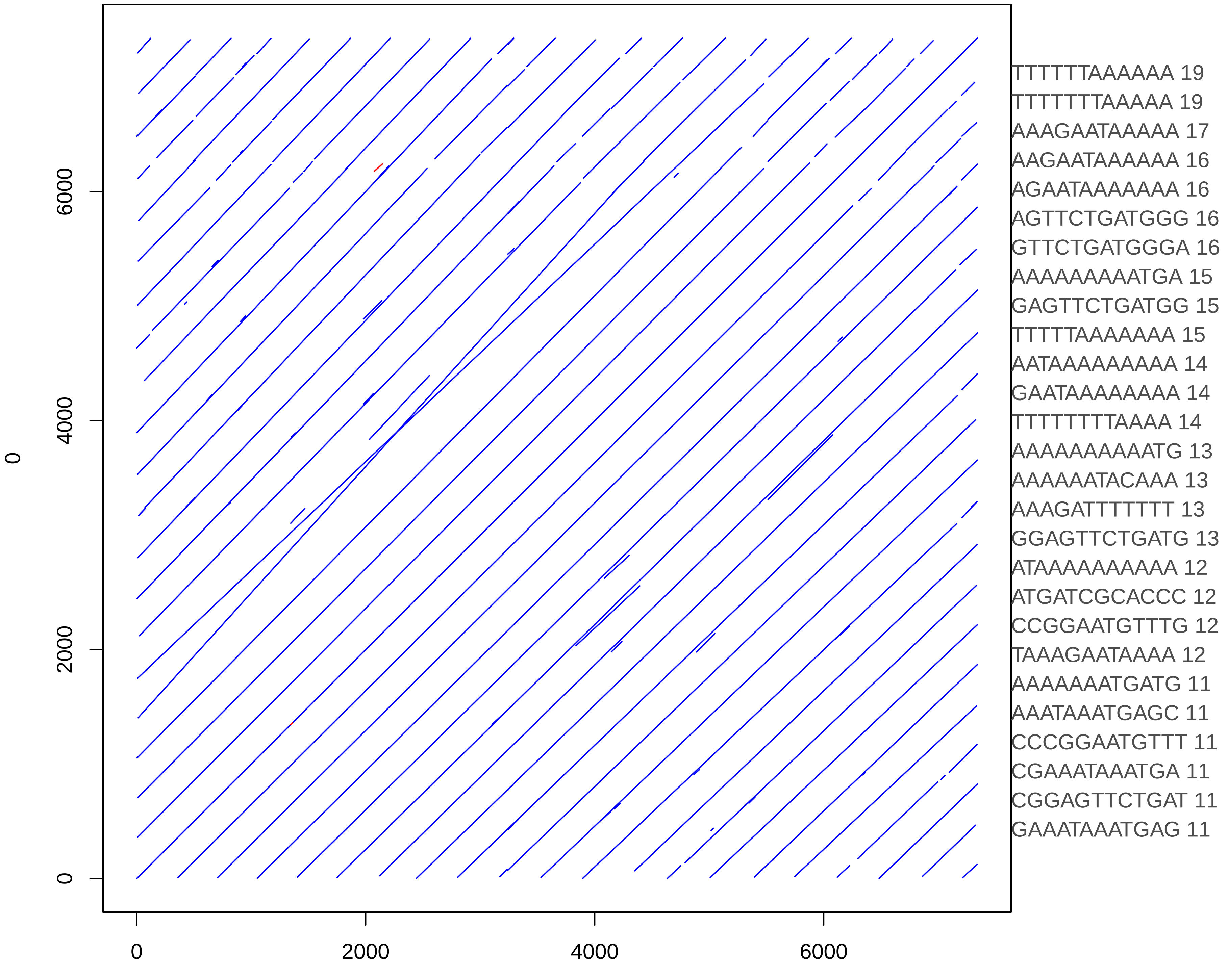

>m141122_133804_42134_c100732202550000001823141405141565_s1_p0/162292/3611_12929

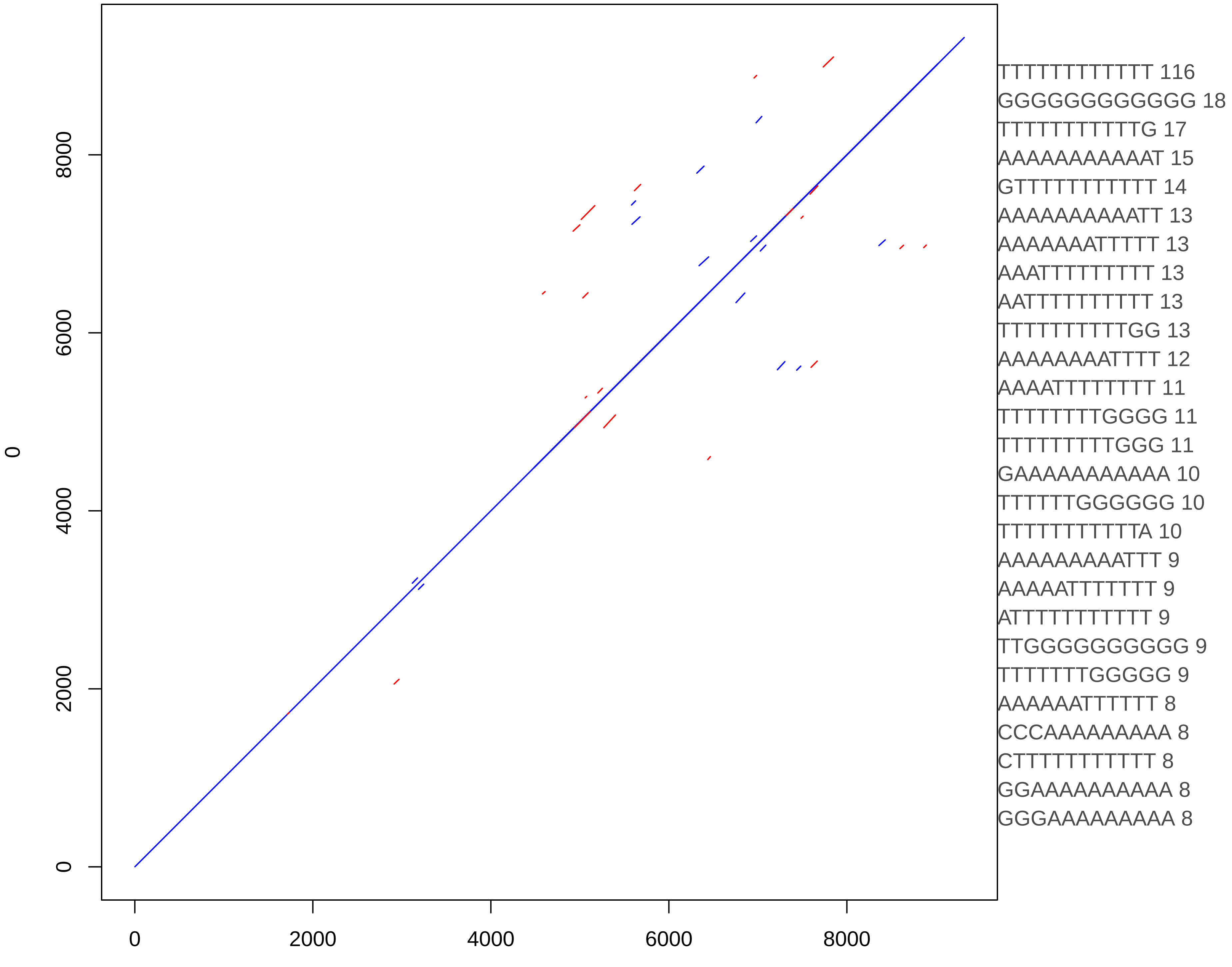

1,121 hits for repetitive sequences
passing 12mer abundance criterion

(Read-Number)
PAGE 419

For Tandem Repeat families of ### bp:
PacBio Read named "HuluTR###-r419"

>m141122_133804_42134_c100732202550000001823141405141565_s1_p0/17170/0_7724

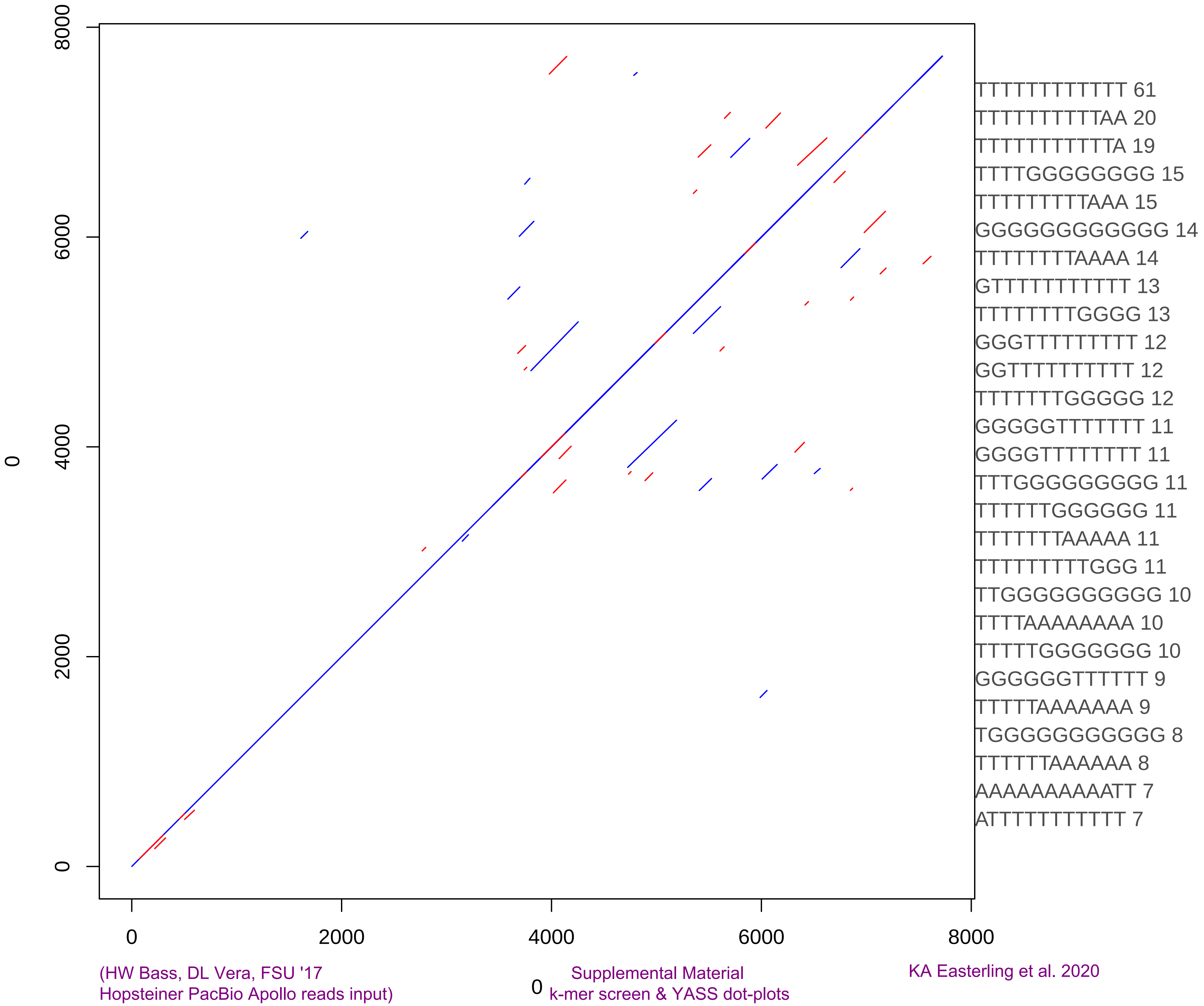

(HW Bass, DL Vera, FSU '17
Hopsteiner PacBio Apollo reads input)

Supplemental Material
k-mer screen & YASS dot-plots

KA Easterling et al. 2020

>m141122_133804_42134_c100732202550000001823141405141565_s1_p0/19471/8936_18531

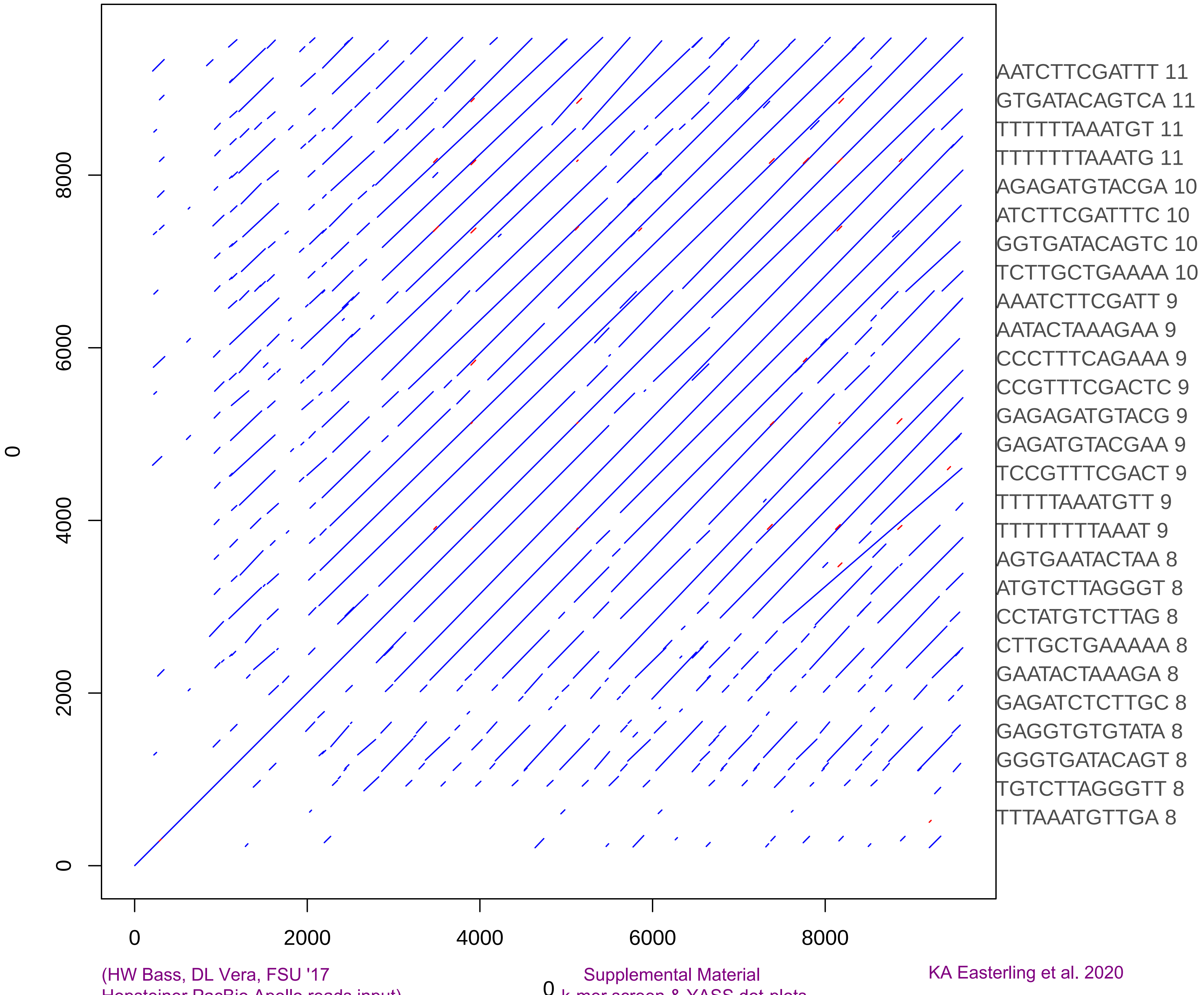

>m141122_133804_42134_c100732202550000001823141405141565_s1_p0/22688/4145_14043

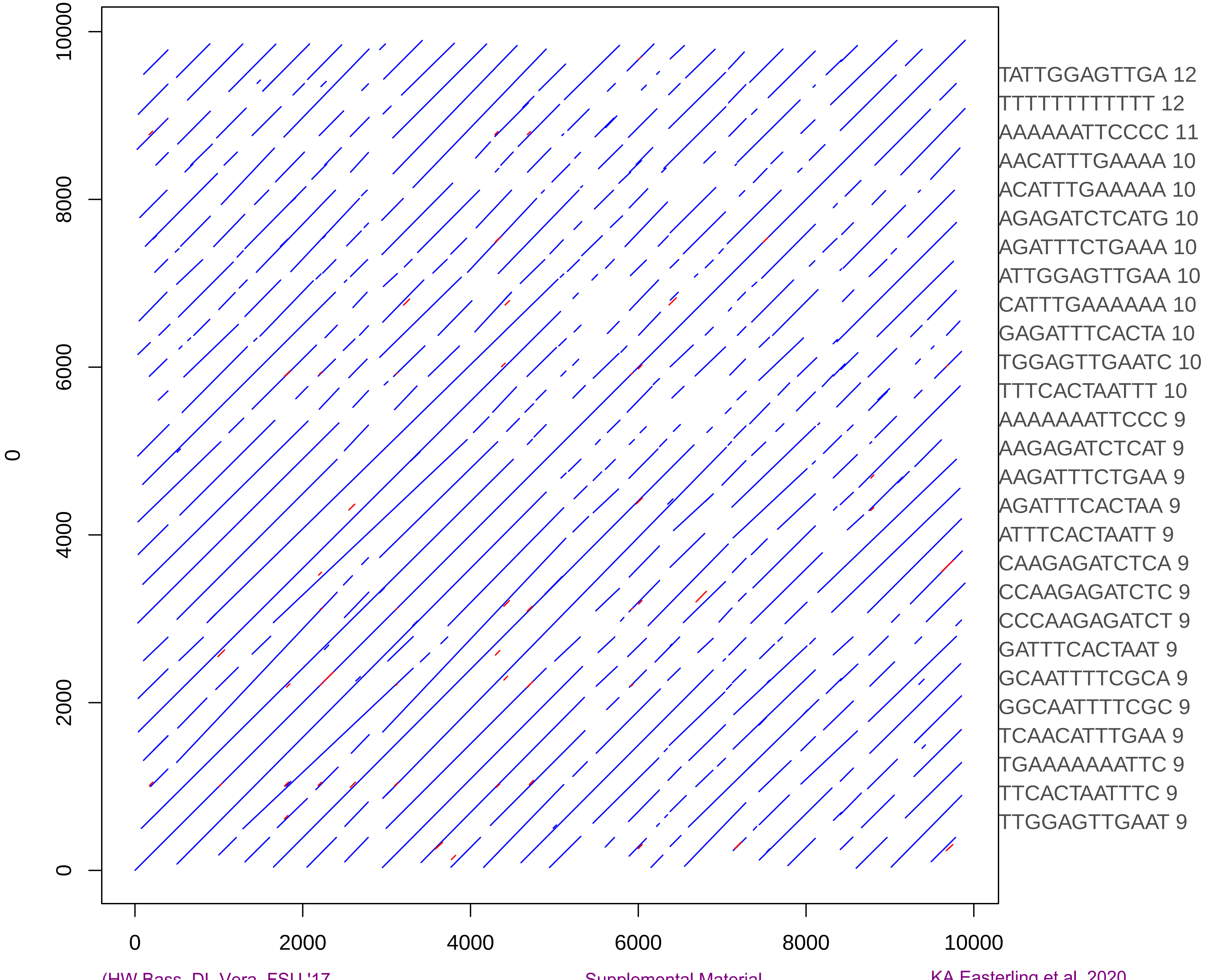

>m141122_133804_42134_c100732202550000001823141405141565_s1_p0/27/709_10418

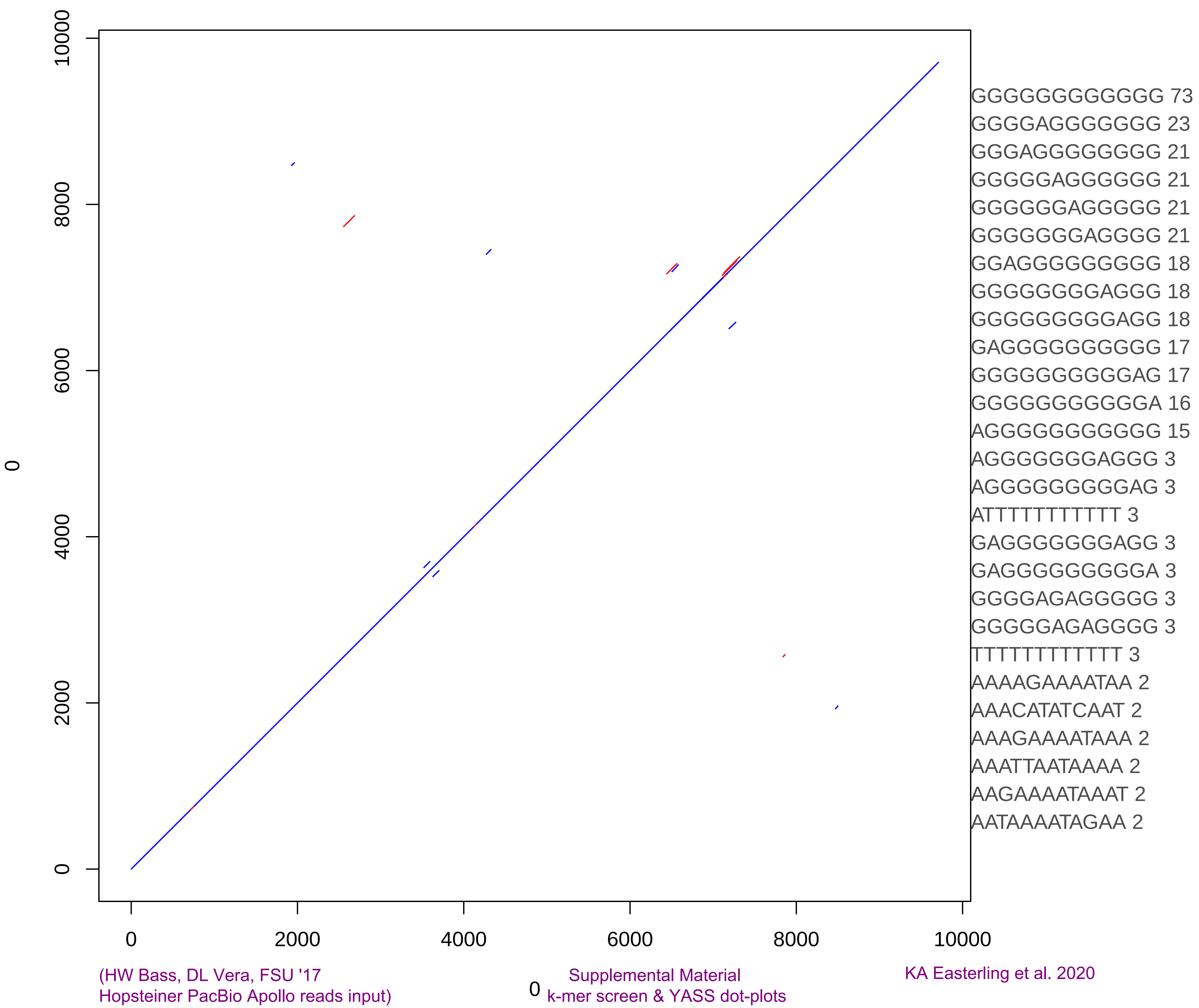

>m141122_133804_42134_c100732202550000001823141405141565_s1_p0/33739/9287_17786

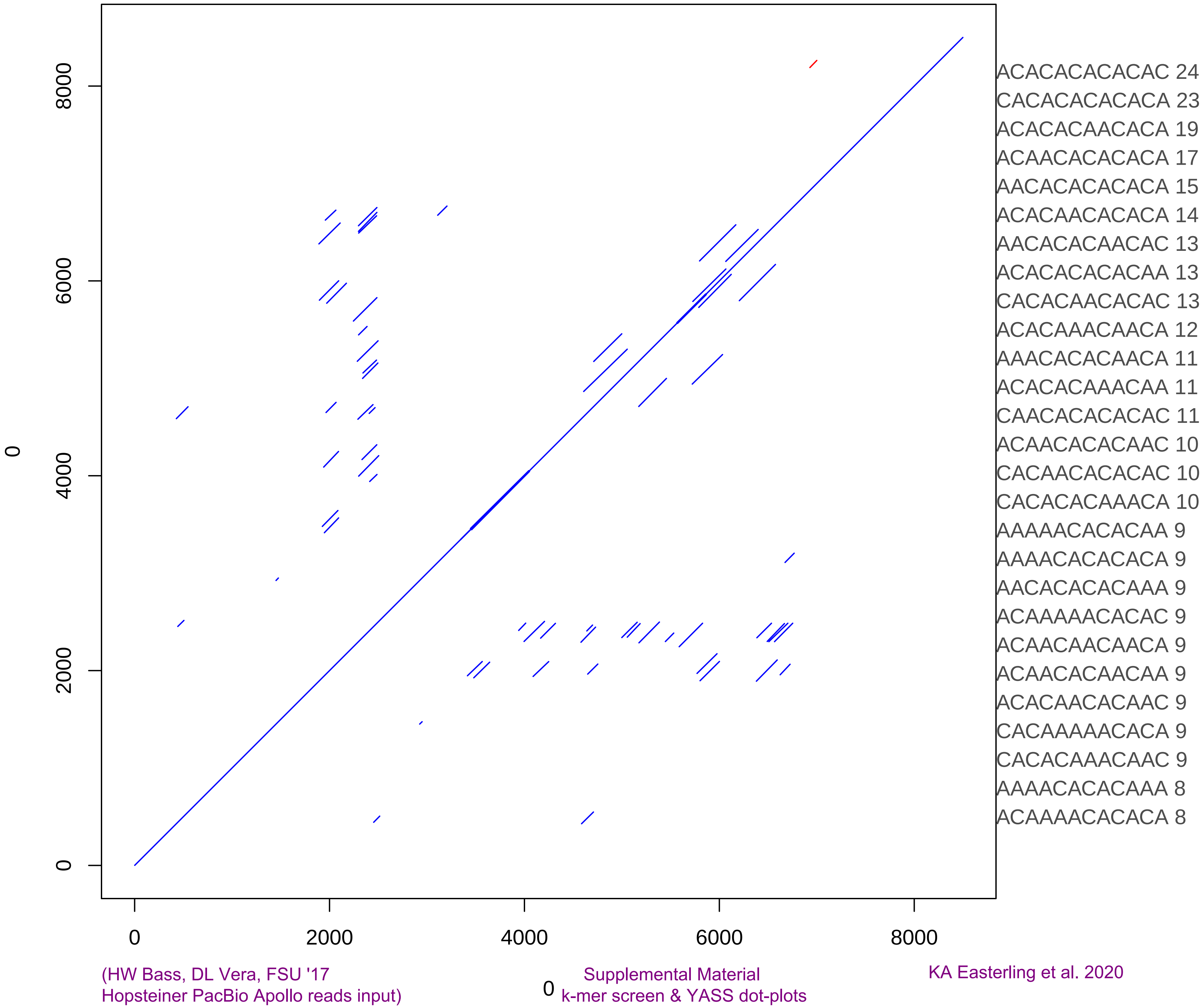

>m141122_133804_42134_c100732202550000001823141405141565_s1_p0/43351/7597_13525

Representative Read (Table 1): HuluTR185-r424, 2 ppm, 49% A+T,
GenBank Acc. MN537573

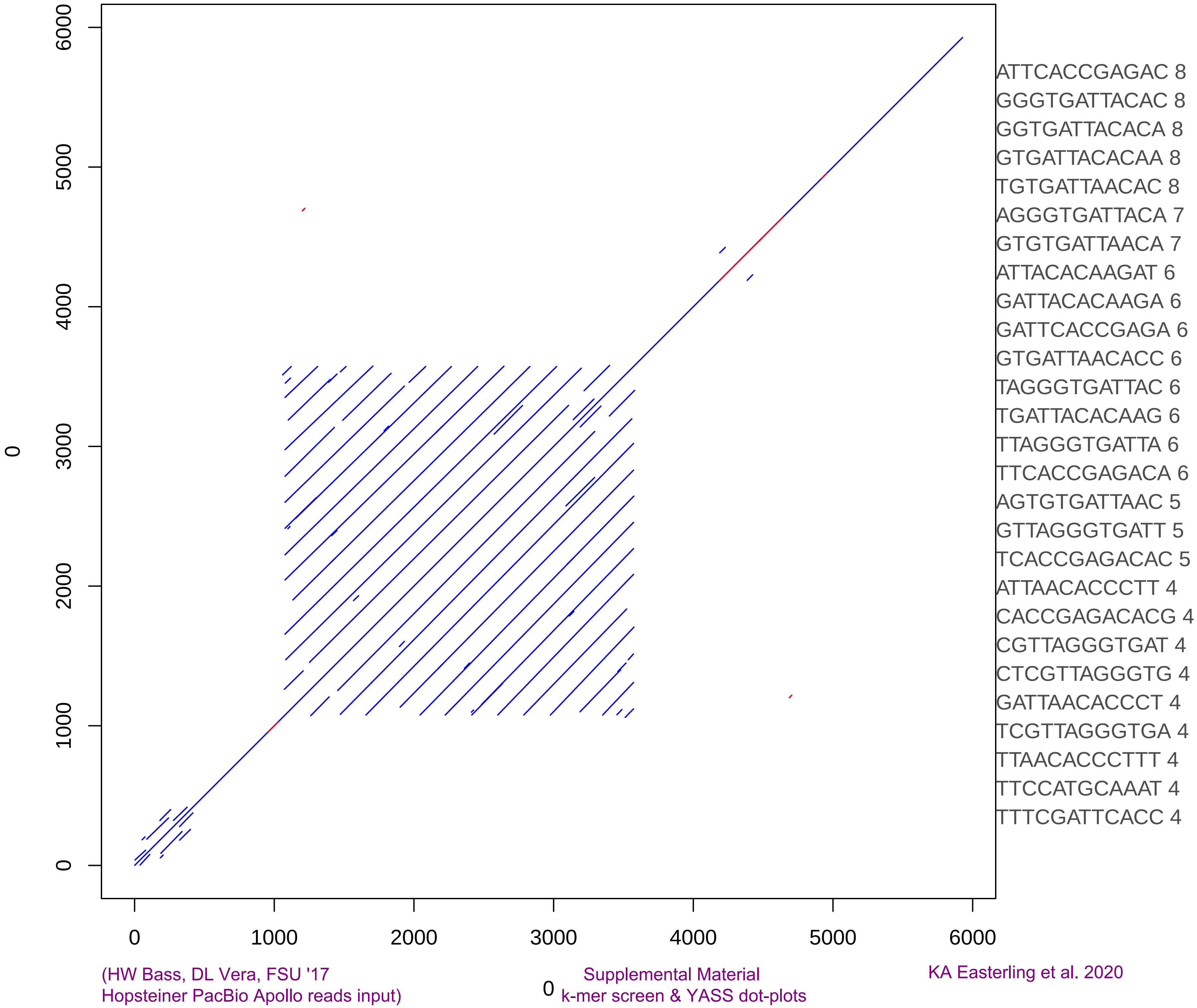

1,121 hits for repetitive sequences passing 12mer abundance criterion

(Read-Number) PAGE 425

For Tandem Repeat families of ### bp: PacBio Read named "HuluTR###-r425"

>m141122_133804_42134_c100732202550000001823141405141565_s1_p0/43937/15511_22279

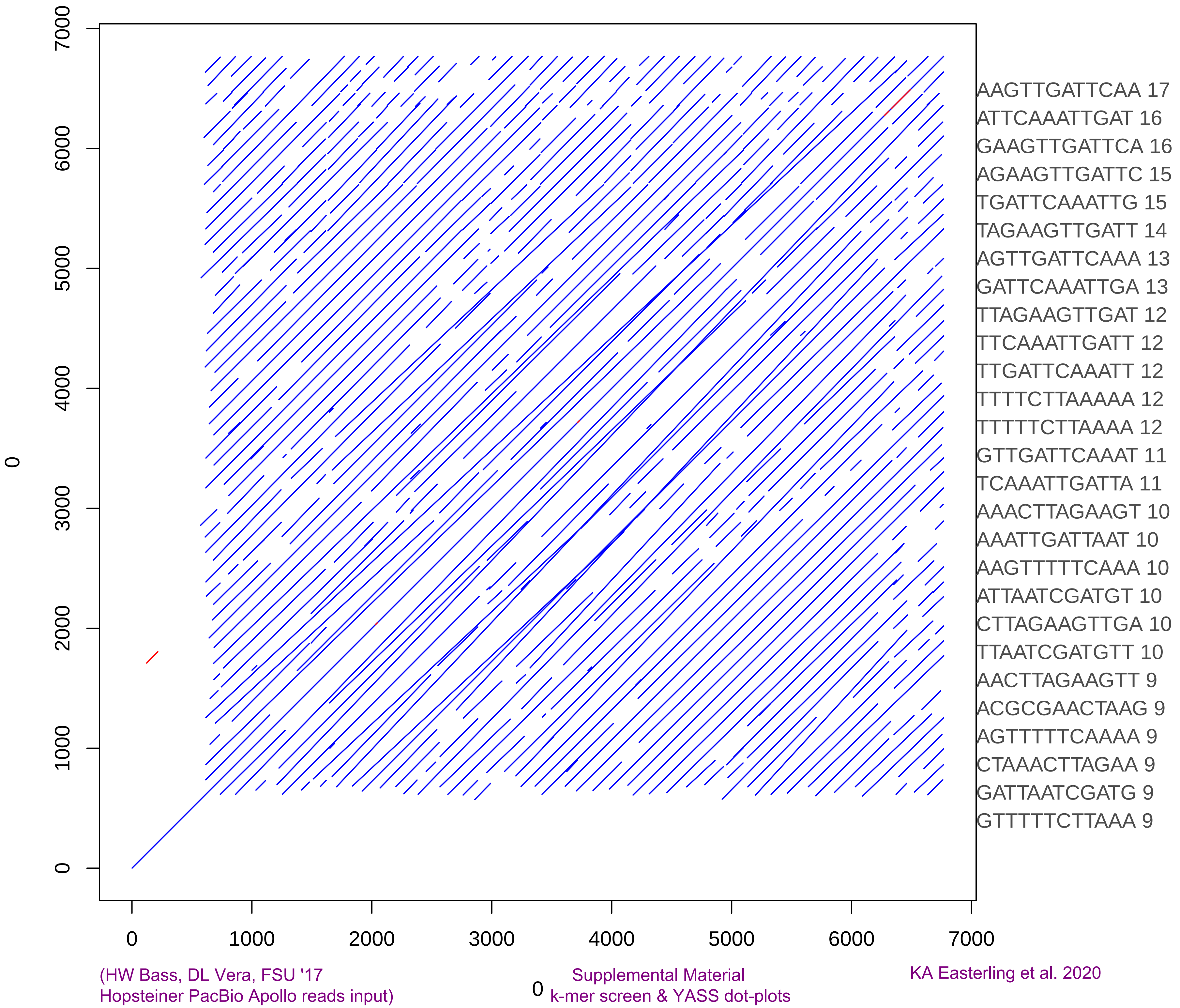

(HW Bass, DL Vera, FSU '17 Hopsteiner PacBio Apollo reads input)

0

Supplemental Material k-mer screen & YASS dot-plots

KA Easterling et al. 2020

>m141122_133804_42134_c100732202550000001823141405141565_s1_p0/47213/3116_10955

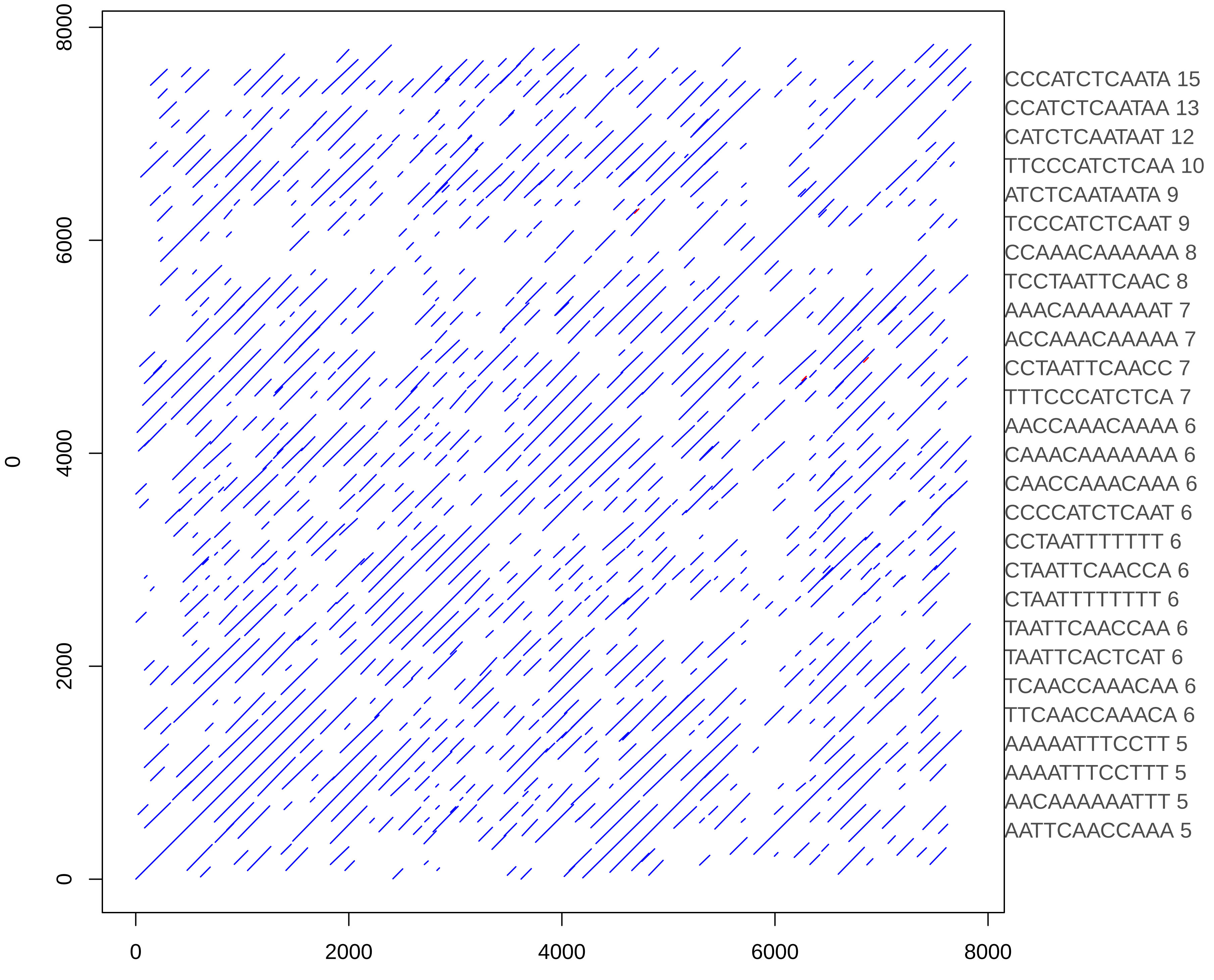

>m141122_133804_42134_c100732202550000001823141405141565_s1_p0/50758/9383_16953

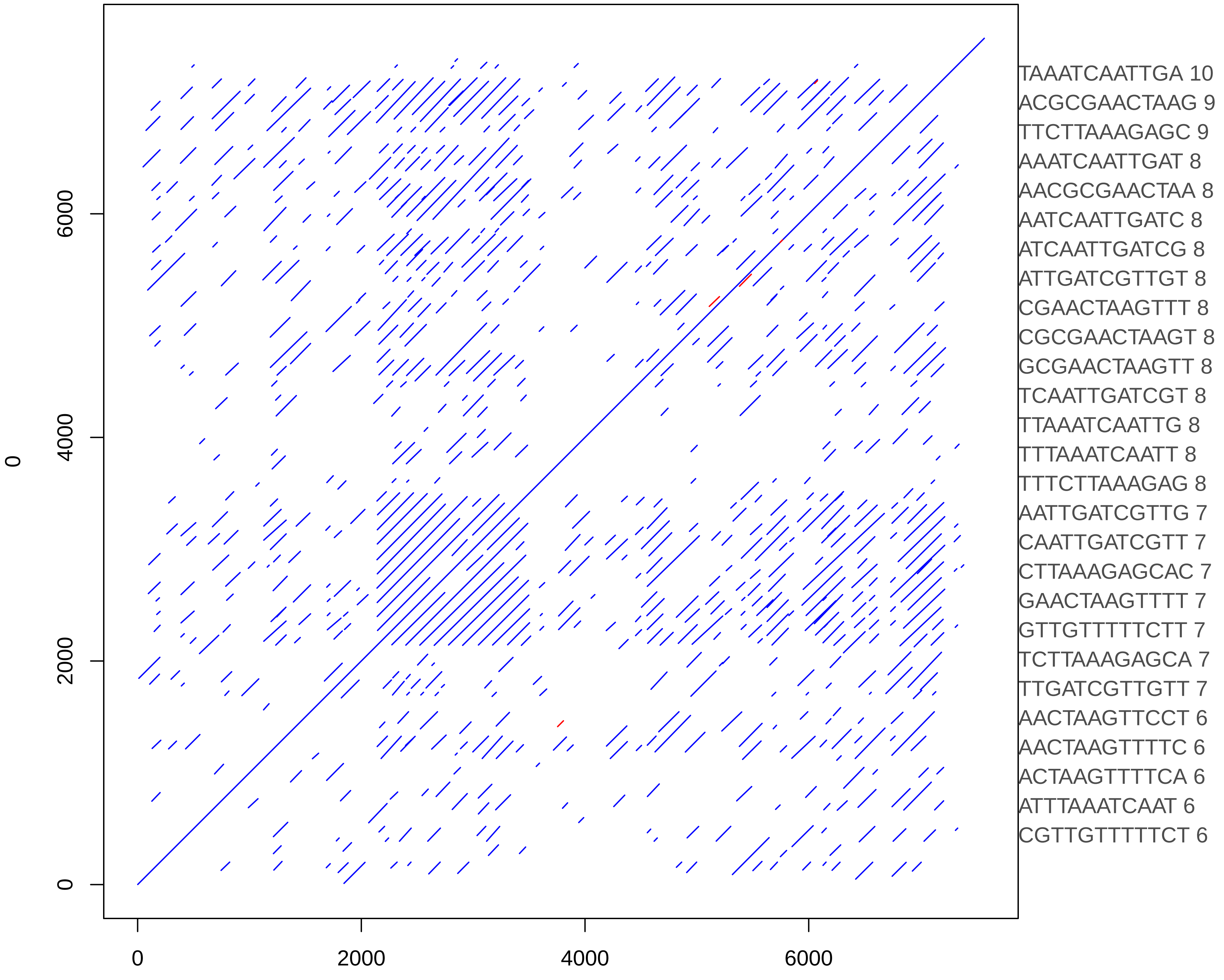

>m141122_133804_42134_c100732202550000001823141405141565_s1_p0/51827/236_7657

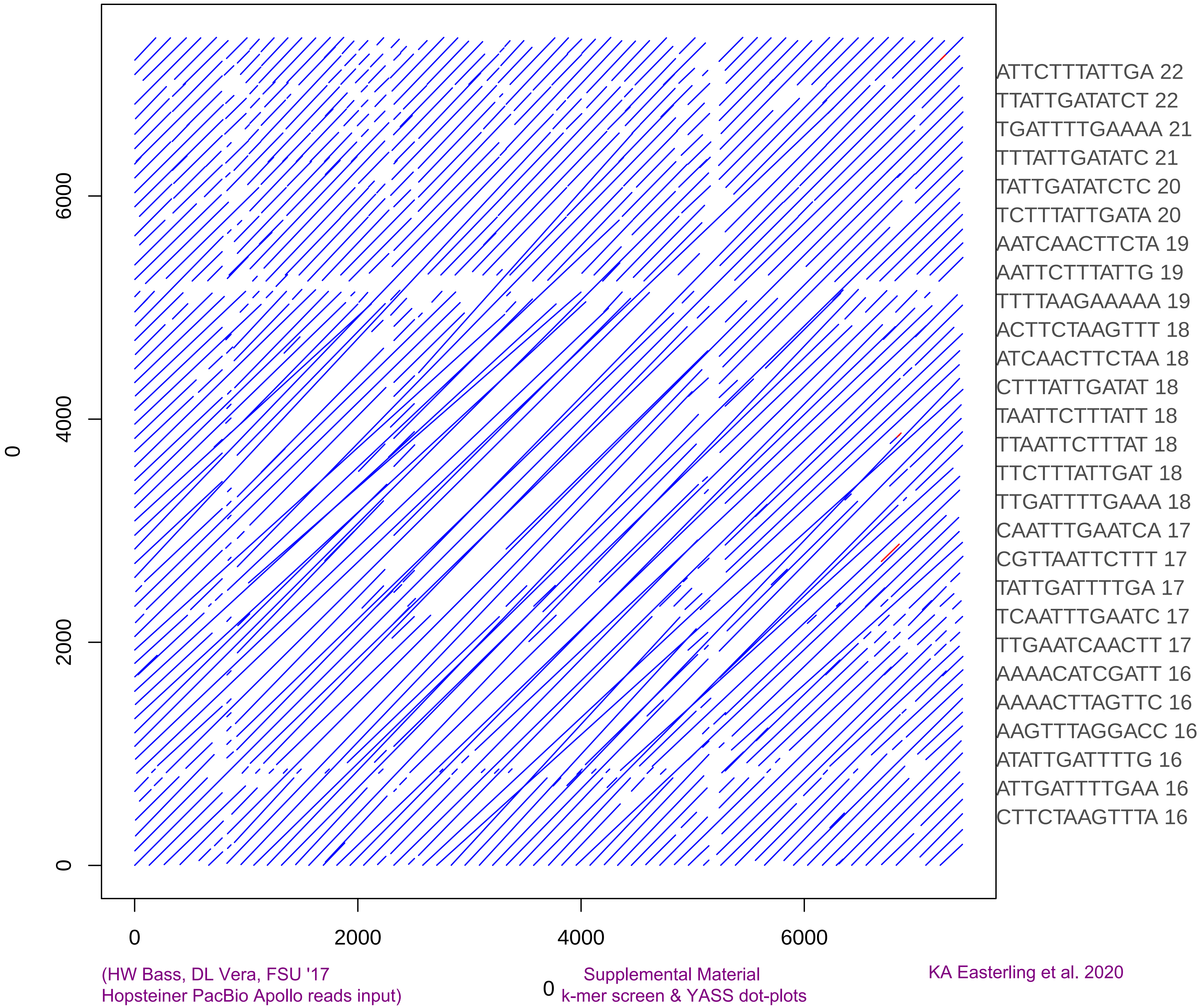

>m141122_133804_42134_c100732202550000001823141405141565_s1_p0/52522/10352_20200

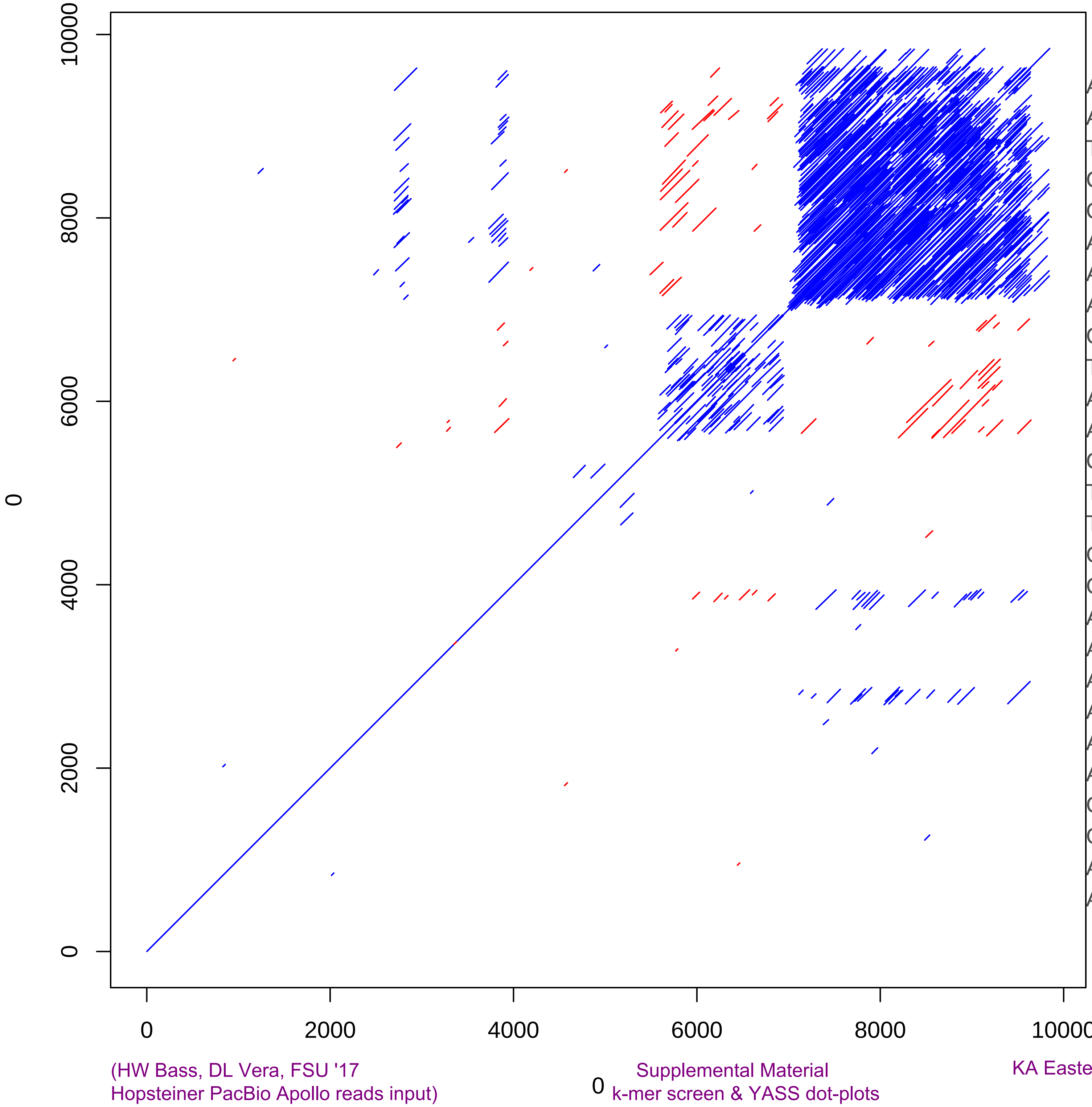

AAGTAGTAGTAG 11
AGAAGTAGTAGT 11
TAGAAGTAGTAG 11
CTACTACTACTA 10
GTAGAAGTAGTA 10
ACTACTACTACT 9
AGTAGAAGTAGT 9
AGTAGTAGTAGT 9
GTAGTAGTAGTA 9
TACTACTACTAC 9
AGAAGAAGTAGT 8
AGTAGTAGAAGT 8
GTAGTAGAAGTA 8
TAGTAGAAGTAG 8
TAGTAGTAGTAG 8
GAAGAAGTAGTA 7
GAAGTAGTAGTA 7
AAGAAGAAGTAG 6
AAGTAGAAGTAG 6
AAGTAGTAGAAG 6
AGAAGAAGAAGT 6
AGAAGTAGAAGT 6
AGTAGTAGTAGA 6
GAAGAAGAAGTA 6
GAAGTAGAAGTA 6
AAGAAGTAGTAG 5
AGAAGTAGTAGA 5

>m141122_133804_42134_c100732202550000001823141405141565_s1_p0/53365/0_5307

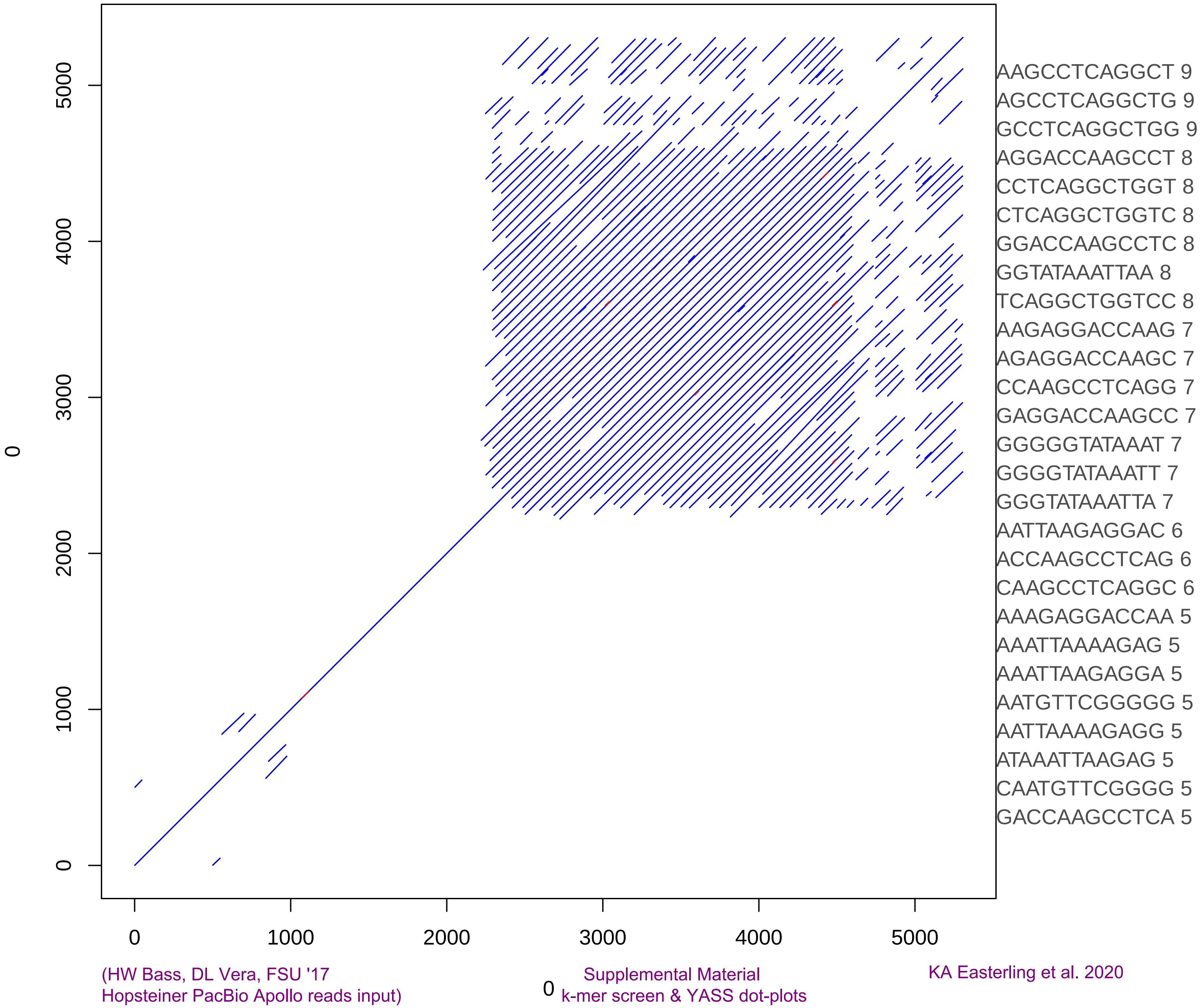

>m141122_133804_42134_c100732202550000001823141405141565_s1_p0/62510/9336_15248

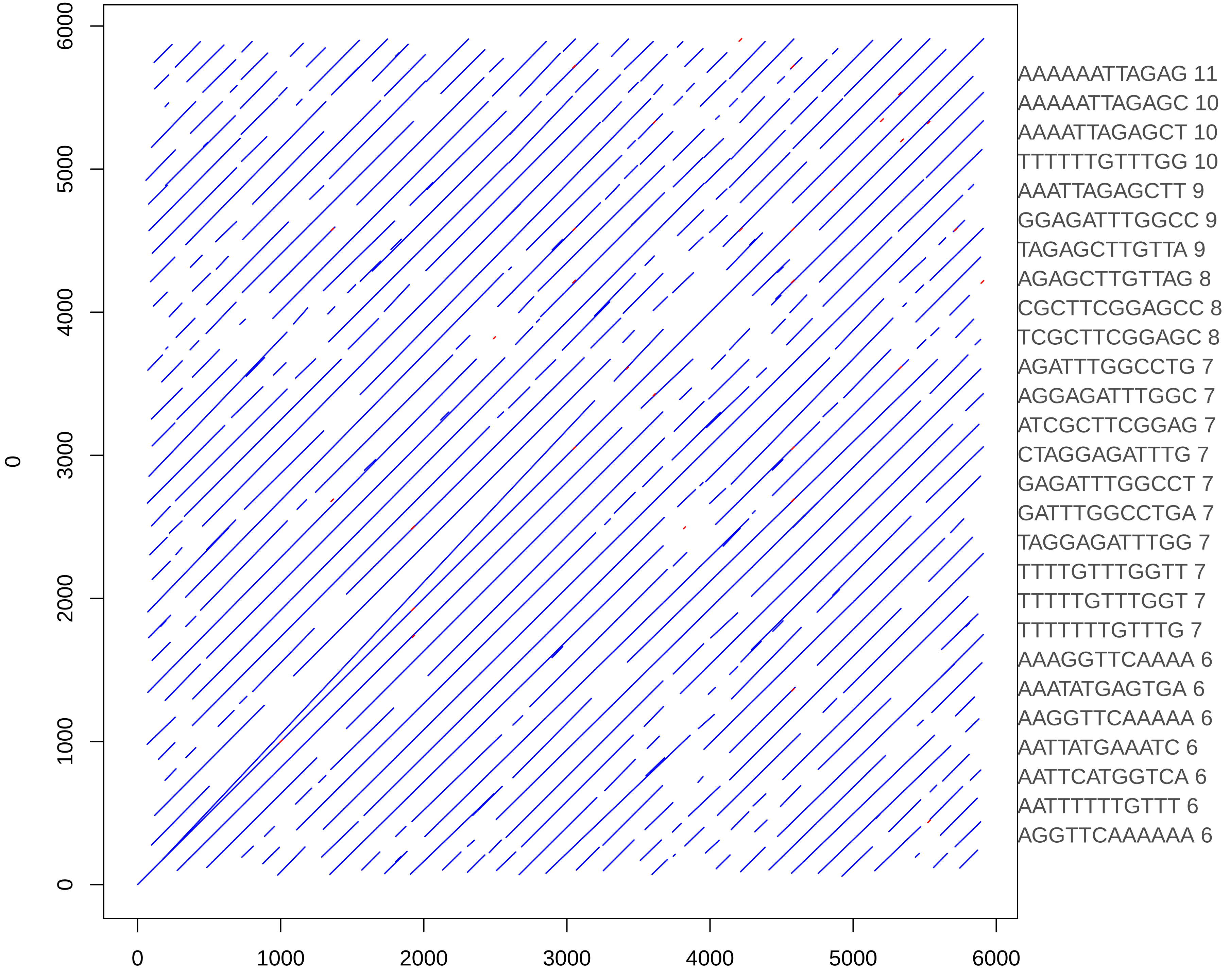

>m141122_133804_42134_c100732202550000001823141405141565_s1_p0/73109/16553_24347

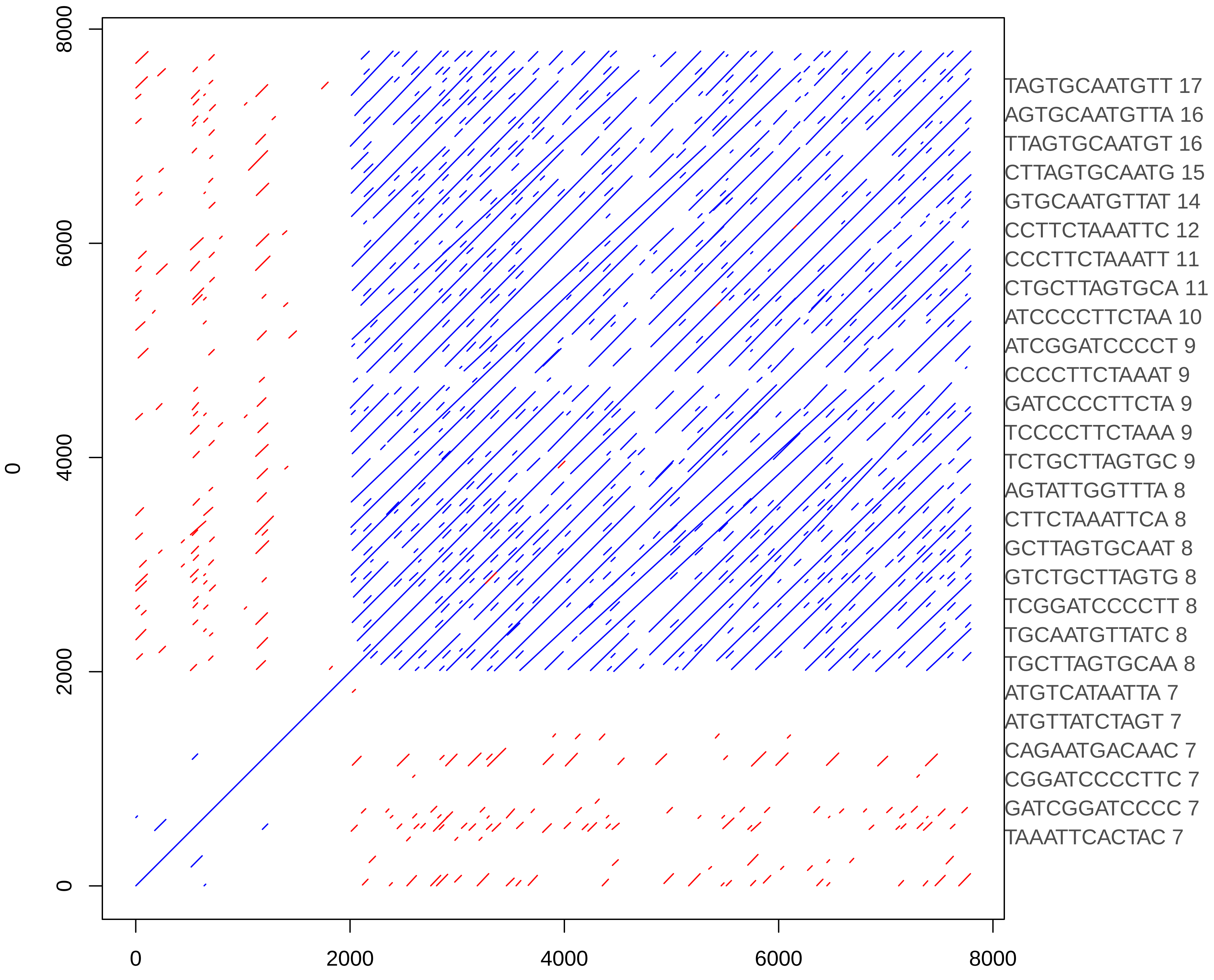

0

1,121 hits for repetitive sequences passing 12mer abundance criterion

For Tandem Repeat families of ### bp: PacBio Read named "HuluTR###-r433"

>m141122_133804_42134_c100732202550000001823141405141565_s1_p0/83834/7703_18033

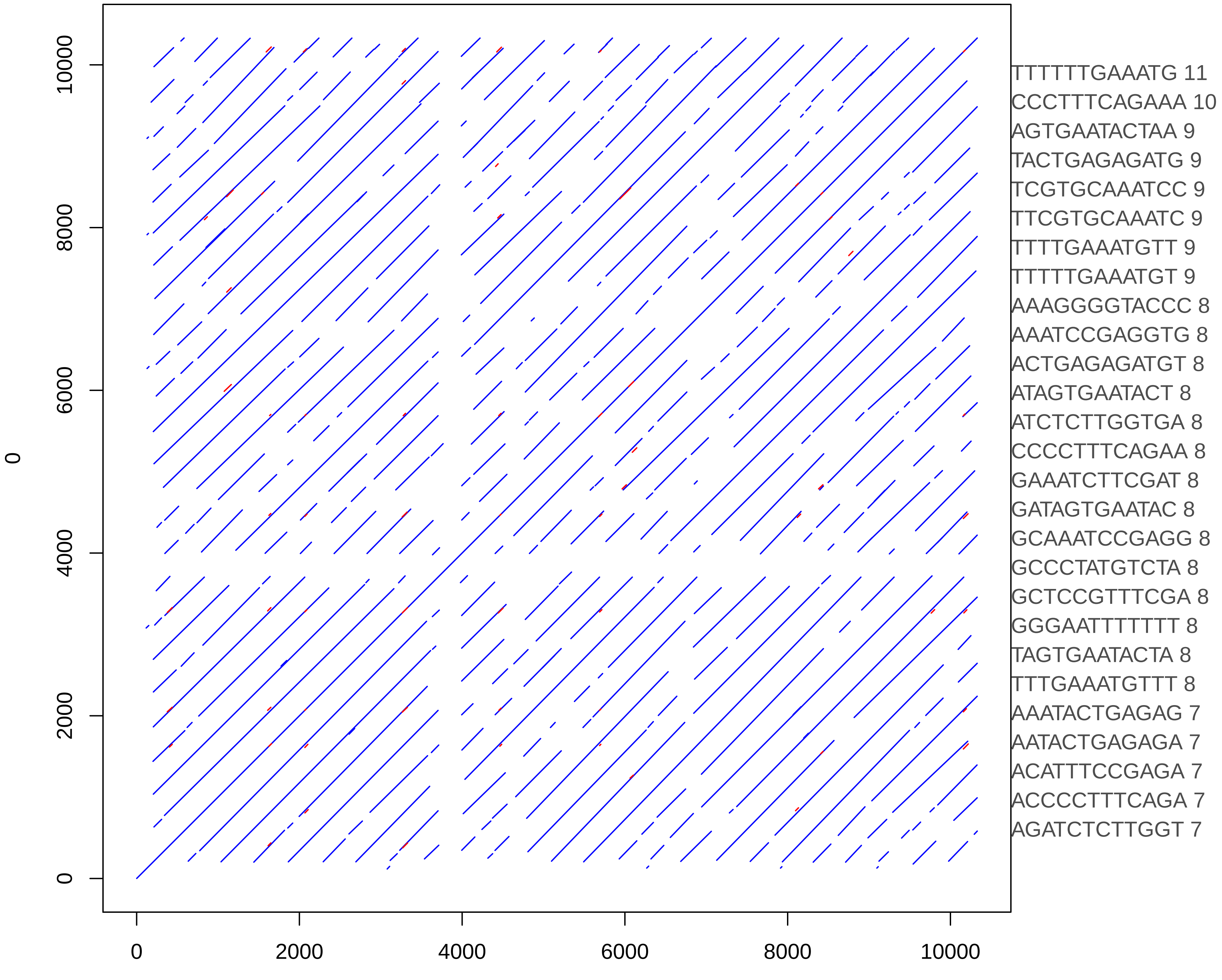

(HW Bass, DL Vera, FSU '17
Hopsteiner PacBio Apollo reads input)

0

Supplemental Material
k-mer screen & YASS dot-plots

KA Easterling et al. 2020

>m141122_133804_42134_c100732202550000001823141405141565_s1_p0/87106/3340_9570

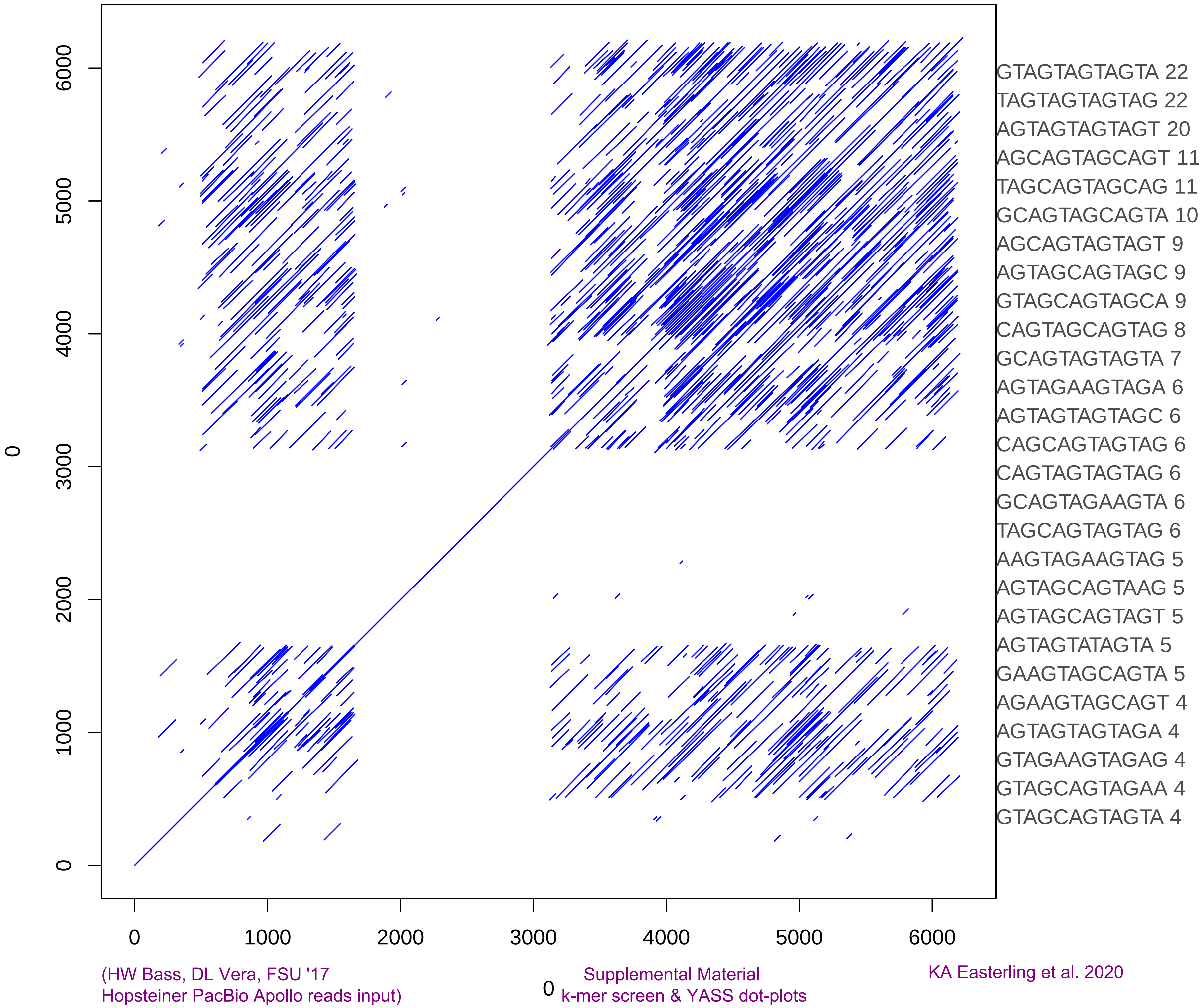

>m141122_133804_42134_c100732202550000001823141405141565_s1_p0/88068/0_13242

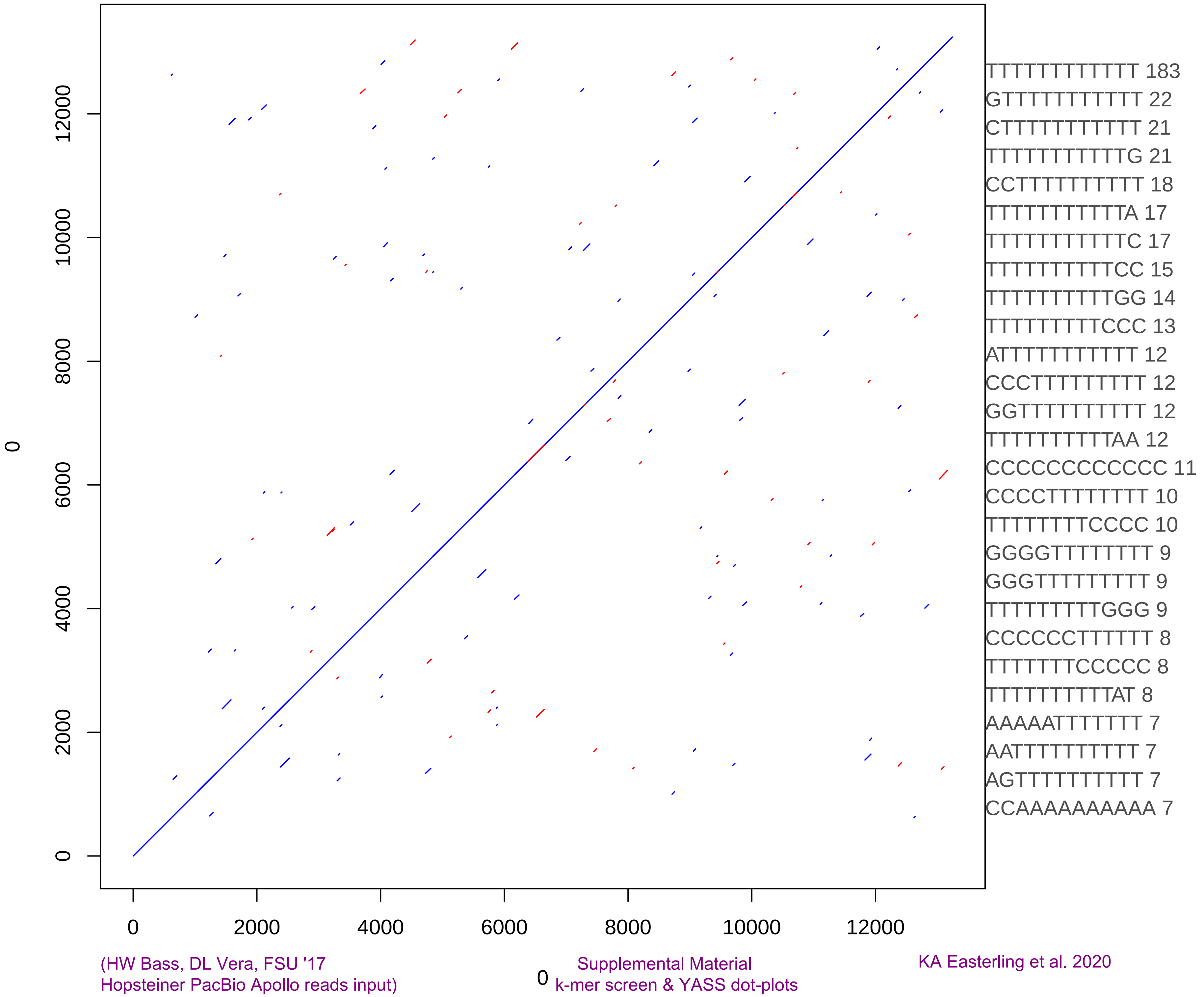

>m141122_133804_42134_c100732202550000001823141405141565_s1_p0/88171/4789_13239

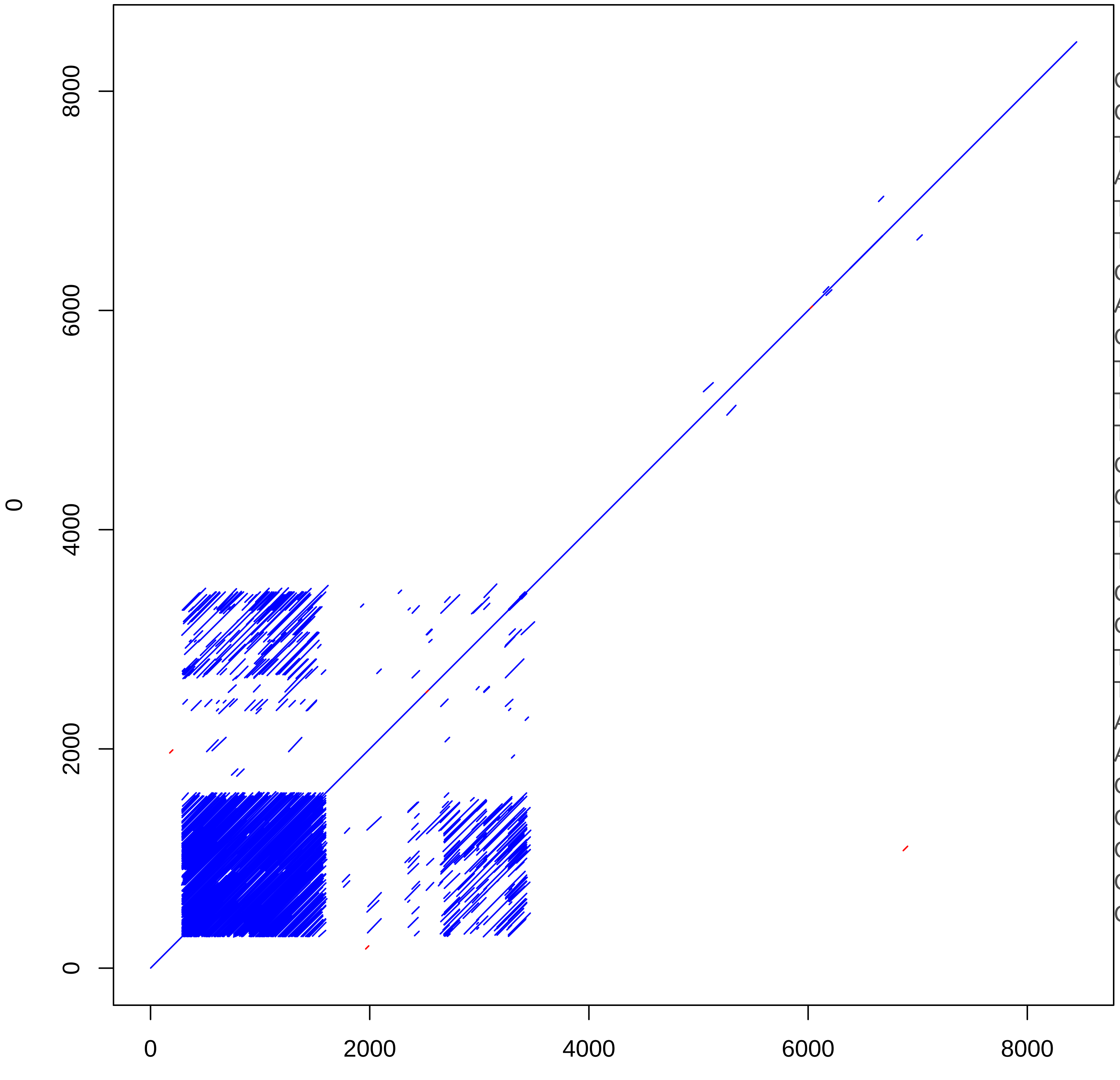

GGGTTTAGGGTT 60
GGTTTAGGGTTT 60
TTAGGGTTTAGG 58
AGGGTTTAGGGT 56
TAGGGTTTAGGG 52
TTTAGGGTTTAG 52
GTTTAGGGTTTA 50
AGGGTTTAGGTT 12
GGTTTTAGGGTT 12
TAGGGTTAGGGT 12
TAGGGTTTAGGT 12
TTAGGGTTTTAG 12
GGGTTTAGGTTT 11
GGGTTTTAGGGT 11
TAGGGTTTTAGG 11
TTAGGGTTAGGG 11
GGGTTAGGGTTT 10
GTTTTAGGGTTT 10
TTTAGGGTTAGG 10
TTTAGGGTTTTA 10
AGGGTTTTAGGG 9
AGGTTTAGGGTT 9
GGTTTAGGGTTA 9
GTTTAGGGTTAG 9
GGTTAGGGTTTA 8
GTTAGGGTTTAG 8
GTTTAGGGTTTT 8

>m141126_012232_42134_c100732202550000001823141405141566_s1_p0/101943/5865_13133

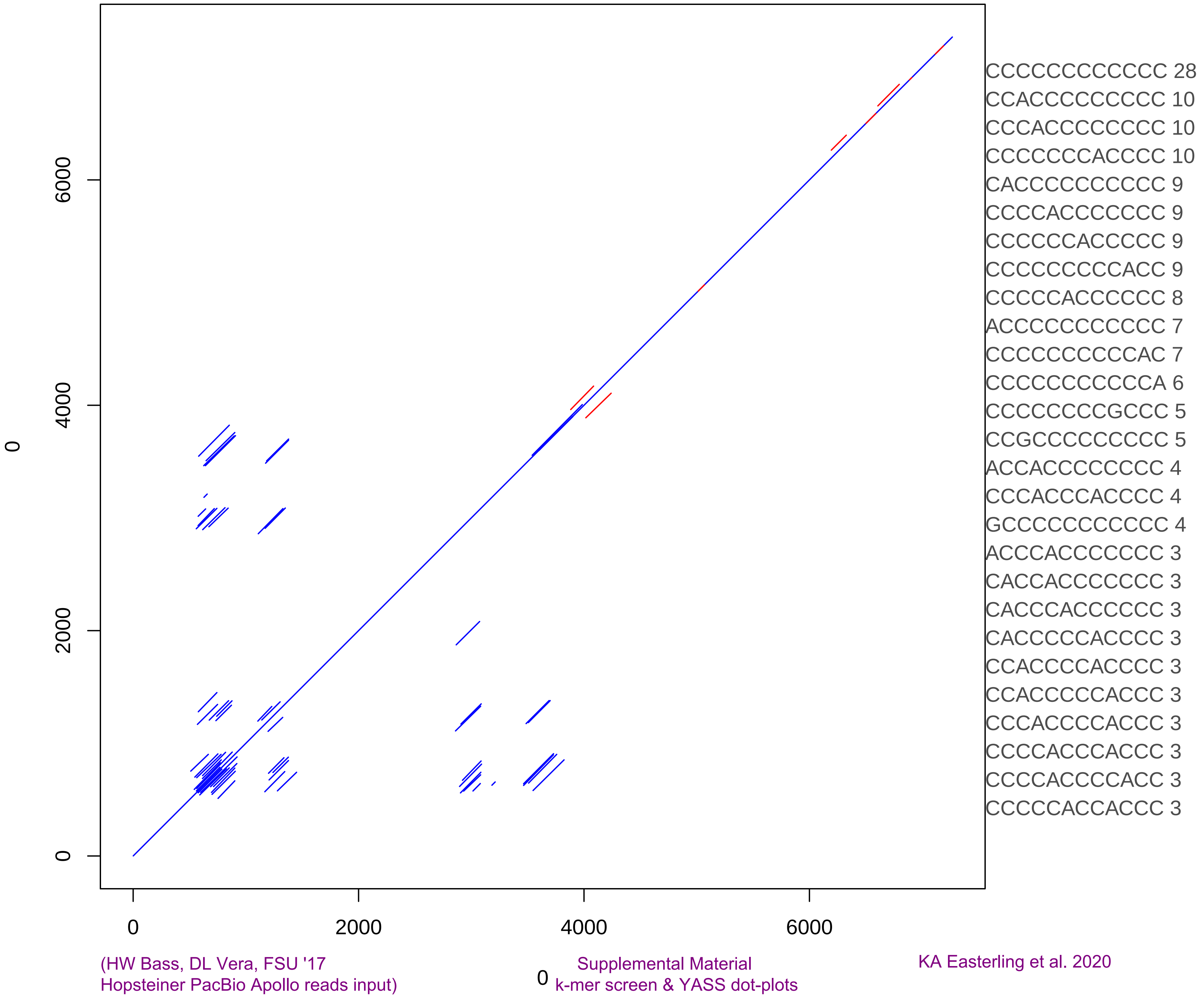

>m141126_012232_42134_c100732202550000001823141405141566_s1_p0/103483/5135_14089

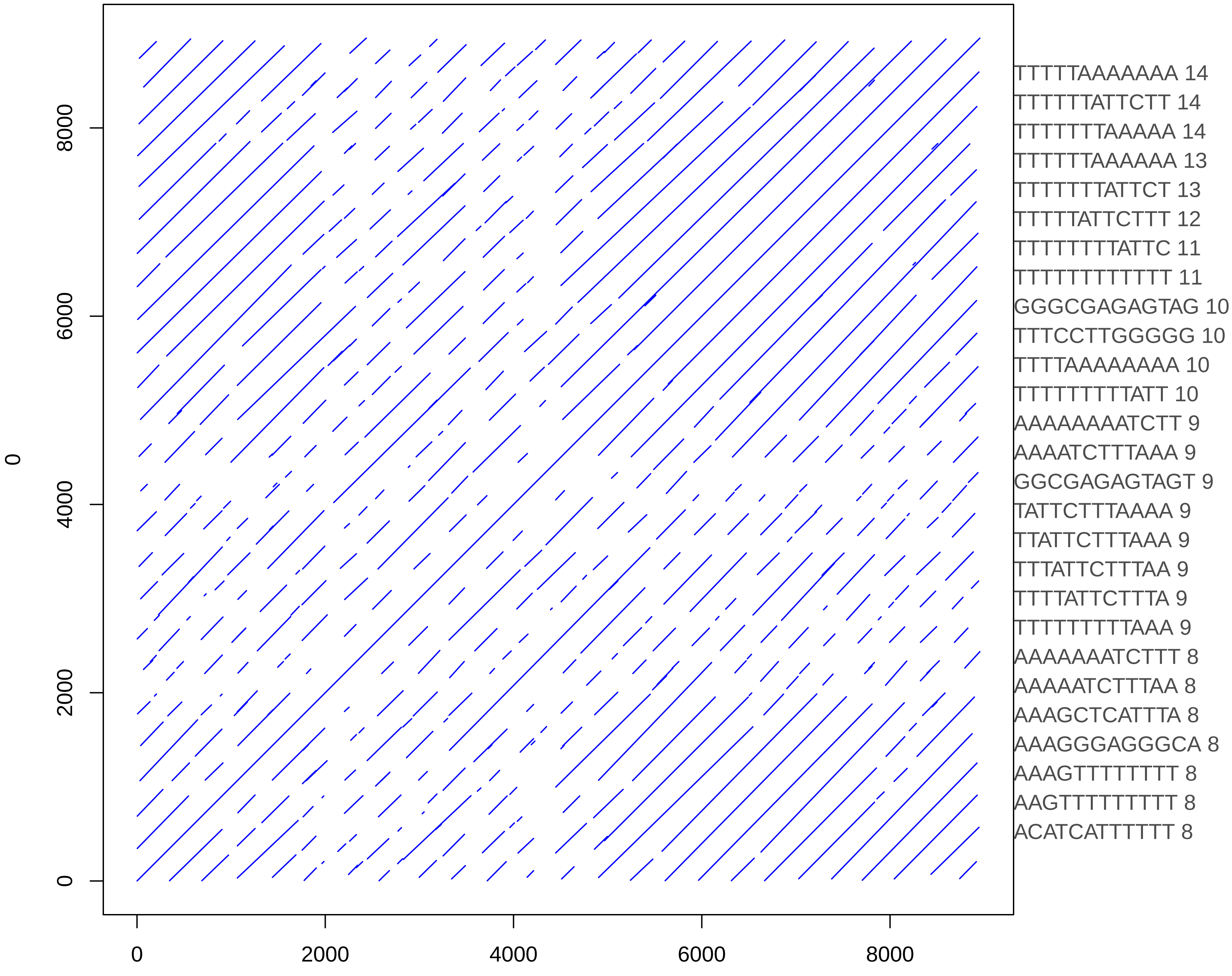

>m141126_012232_42134_c100732202550000001823141405141566_s1_p0/109973/0_10143

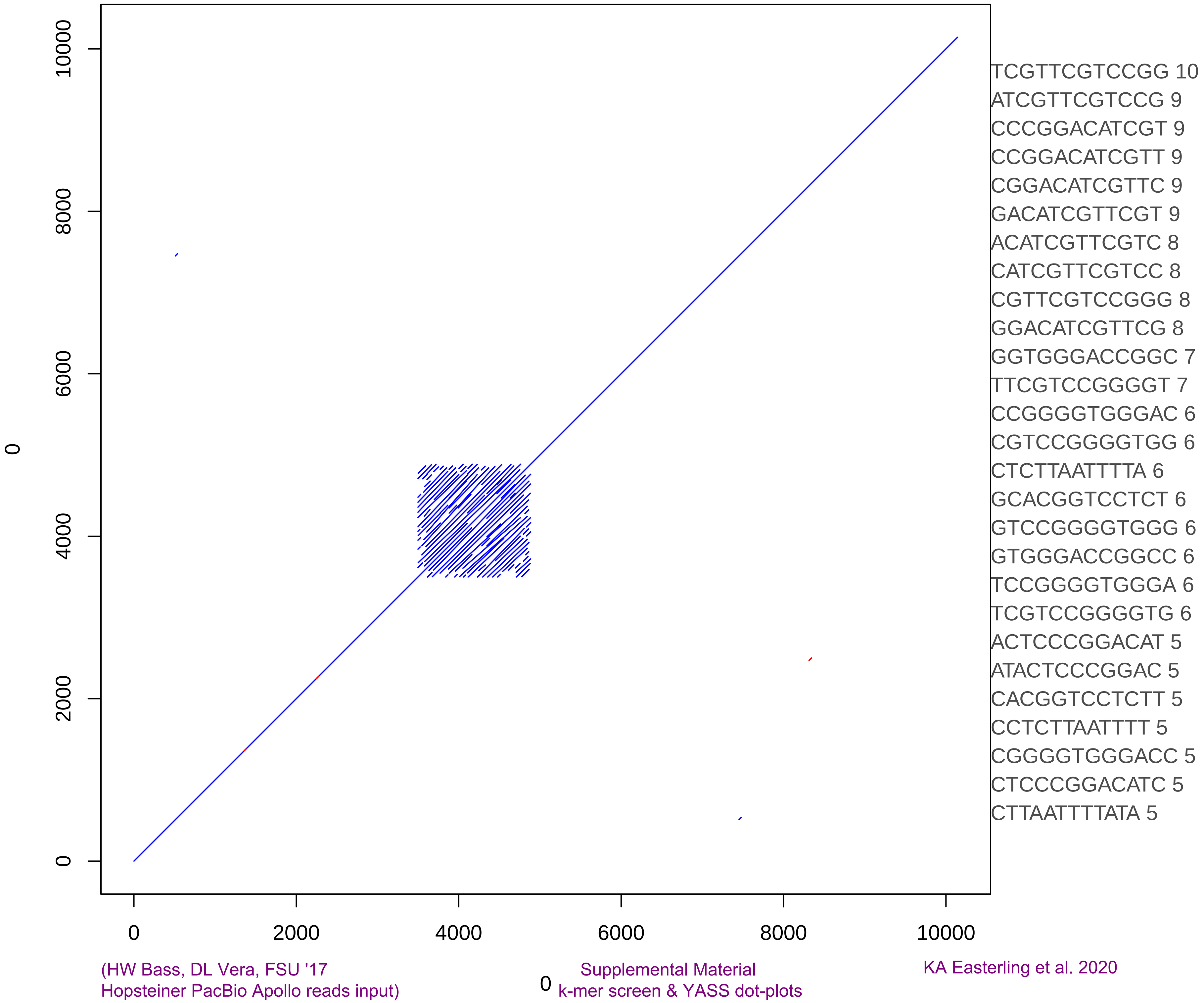

>m141126_012232_42134_c100732202550000001823141405141566_s1_p0/111583/0_7113

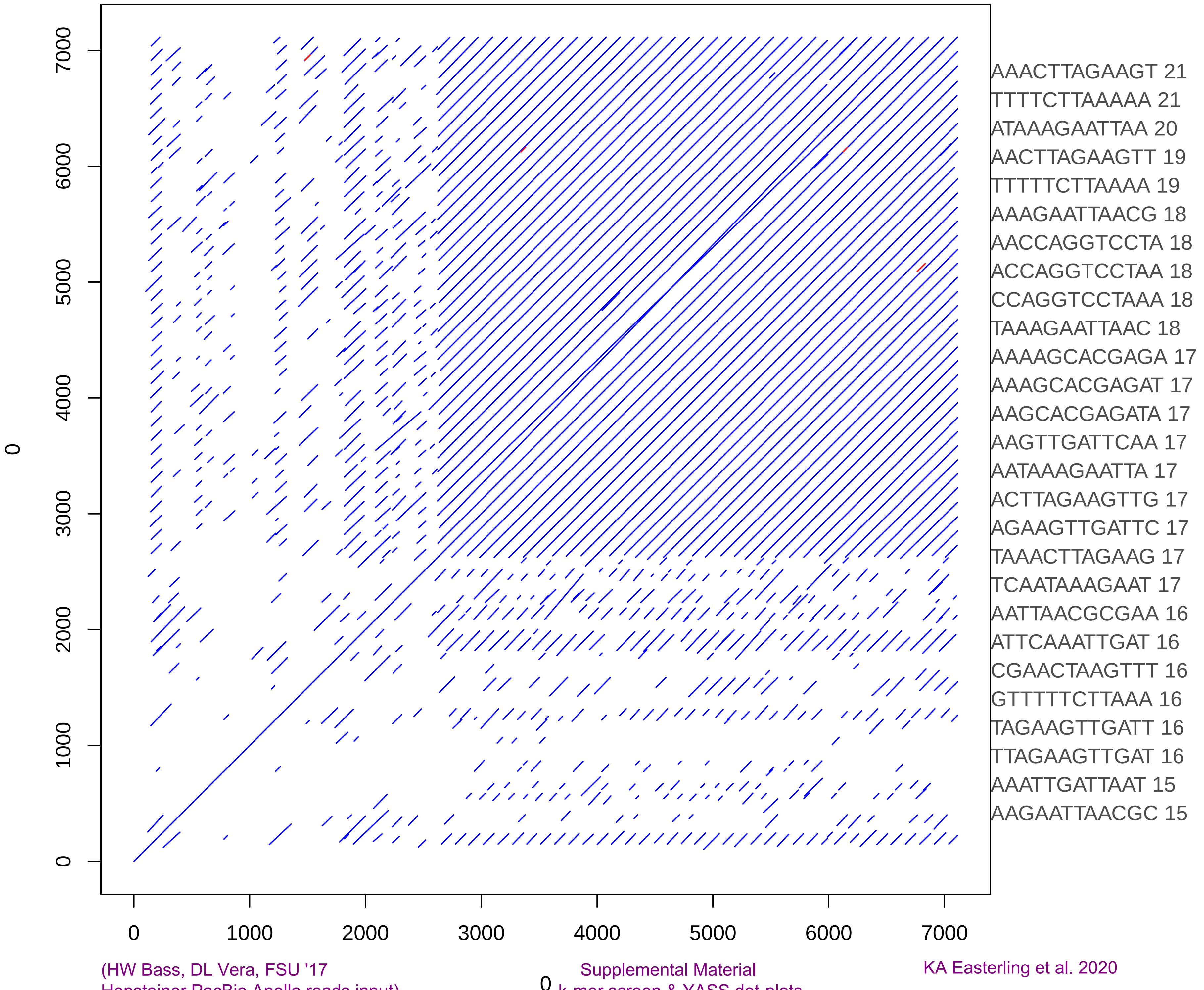

>m141126_012232_42134_c100732202550000001823141405141566_s1_p0/112754/8963_14209

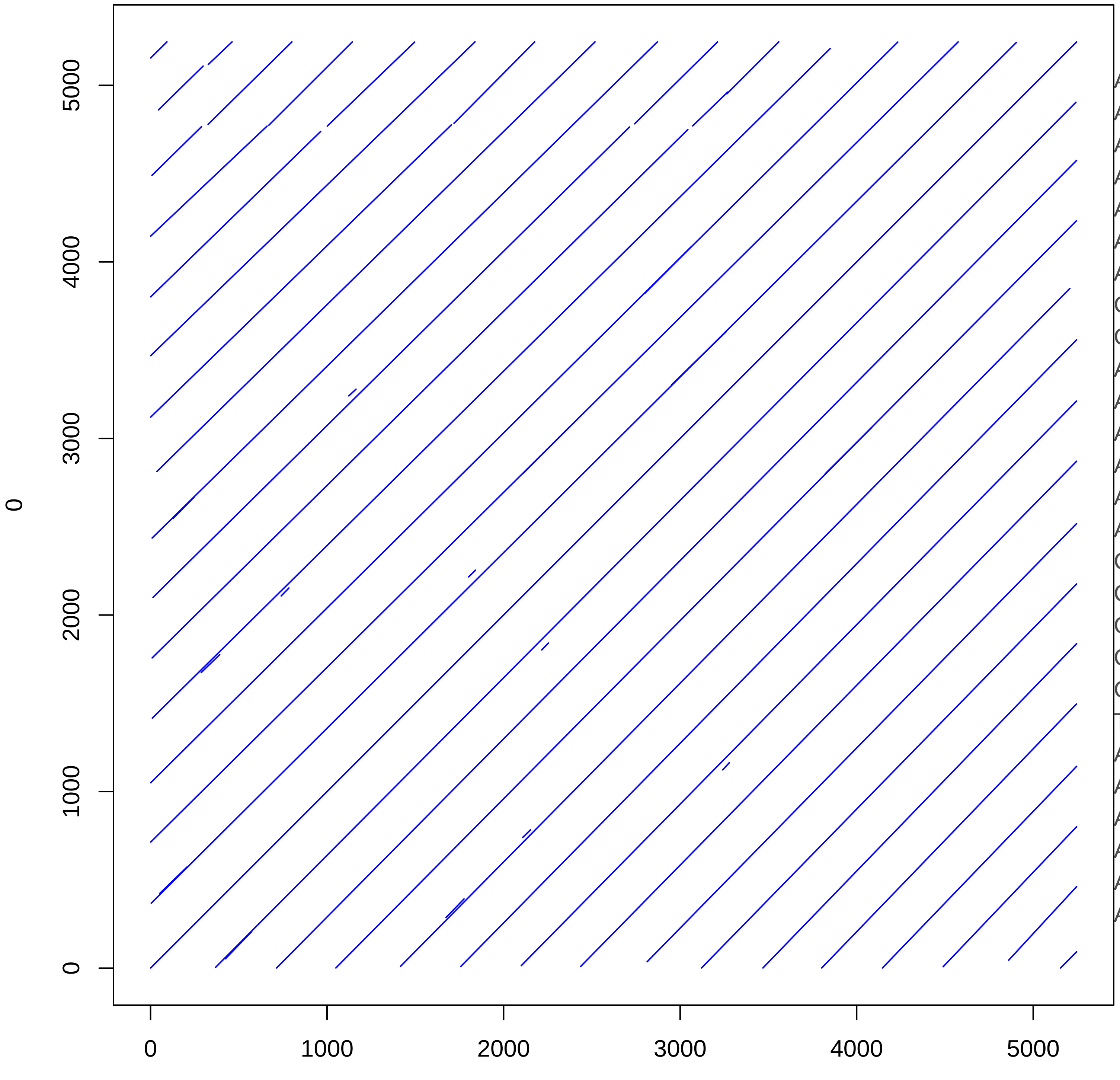

AAACGAAATAAA 12
AACGAAATAAAT 11
AAAATACAAACT 10
AAATACAAACTT 10
AATAAATGAGTT 10
AATACAAACTTT 10
ACGAAATAAATG 10
CGAAATAAATGA 10
GAAATAAATGAG 10
AAAAAAAAATGA 9
AAAACGAAATAA 9
AACACGAGGACT 9
ACGGTATTAACC 9
AGCGAGCGCGTG 9
ATAAATGAGTTT 9
CAACACGAGGAC 9
CACGGTATTAAC 9
GAAAACGAAATA 9
GGAAAAAAAAAA 9
GGAAAACGAAAT 9
TAAATGAGTTTT 9
AAAAAAAAAATG 8
AAAAAATACAAA 8
AAAAATACAAAC 8
AAAGATTTAAAA 8
AAGGAAAACGAA 8
AATATTCATTTA 8

0

>m141126_012232_42134_c100732202550000001823141405141566_s1_p0/113304/5331_17109

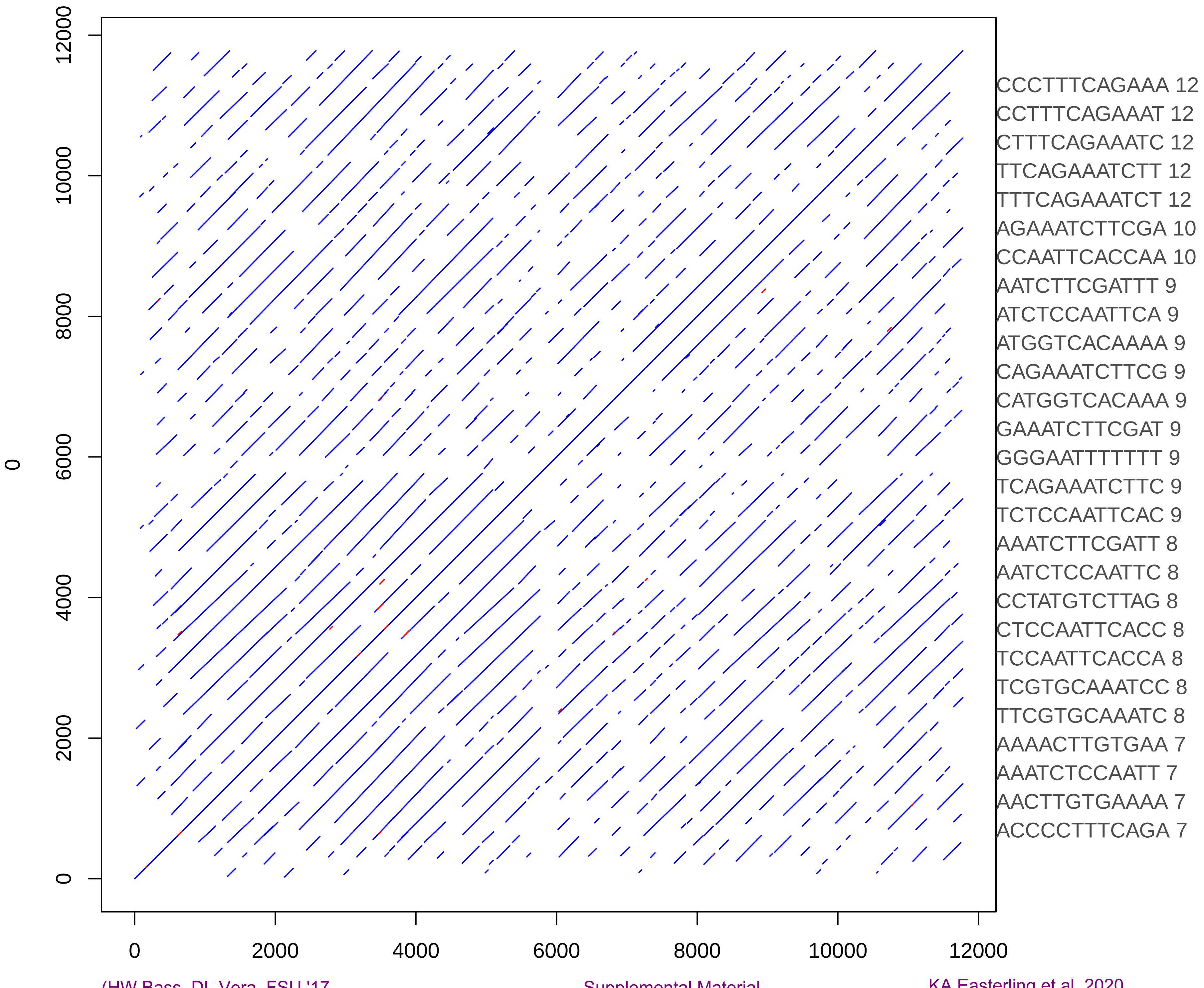

>m141126_012232_42134_c100732202550000001823141405141566_s1_p0/113690/5729_10844

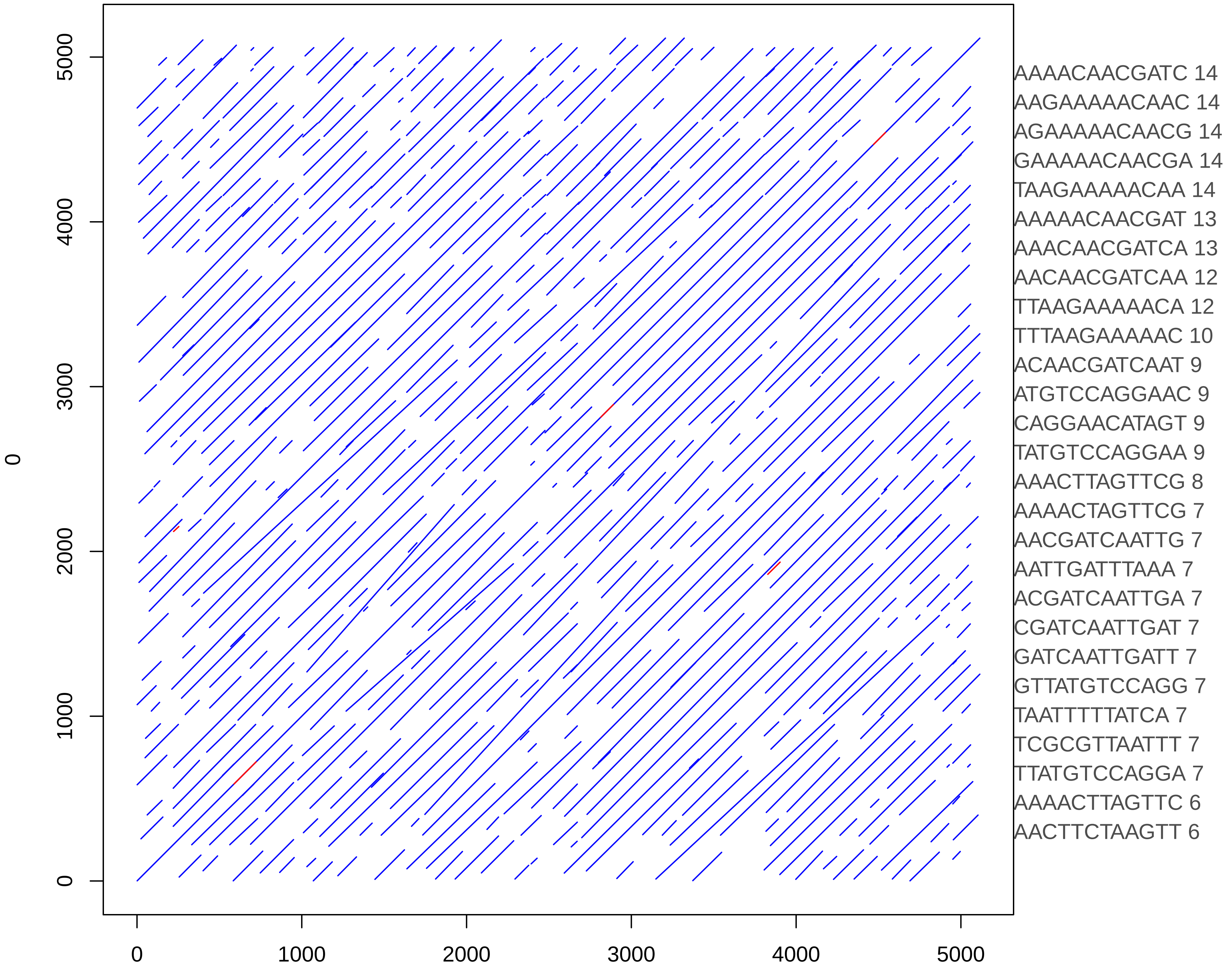

>m141126_012232_42134_c100732202550000001823141405141566_s1_p0/114767/3995_14324

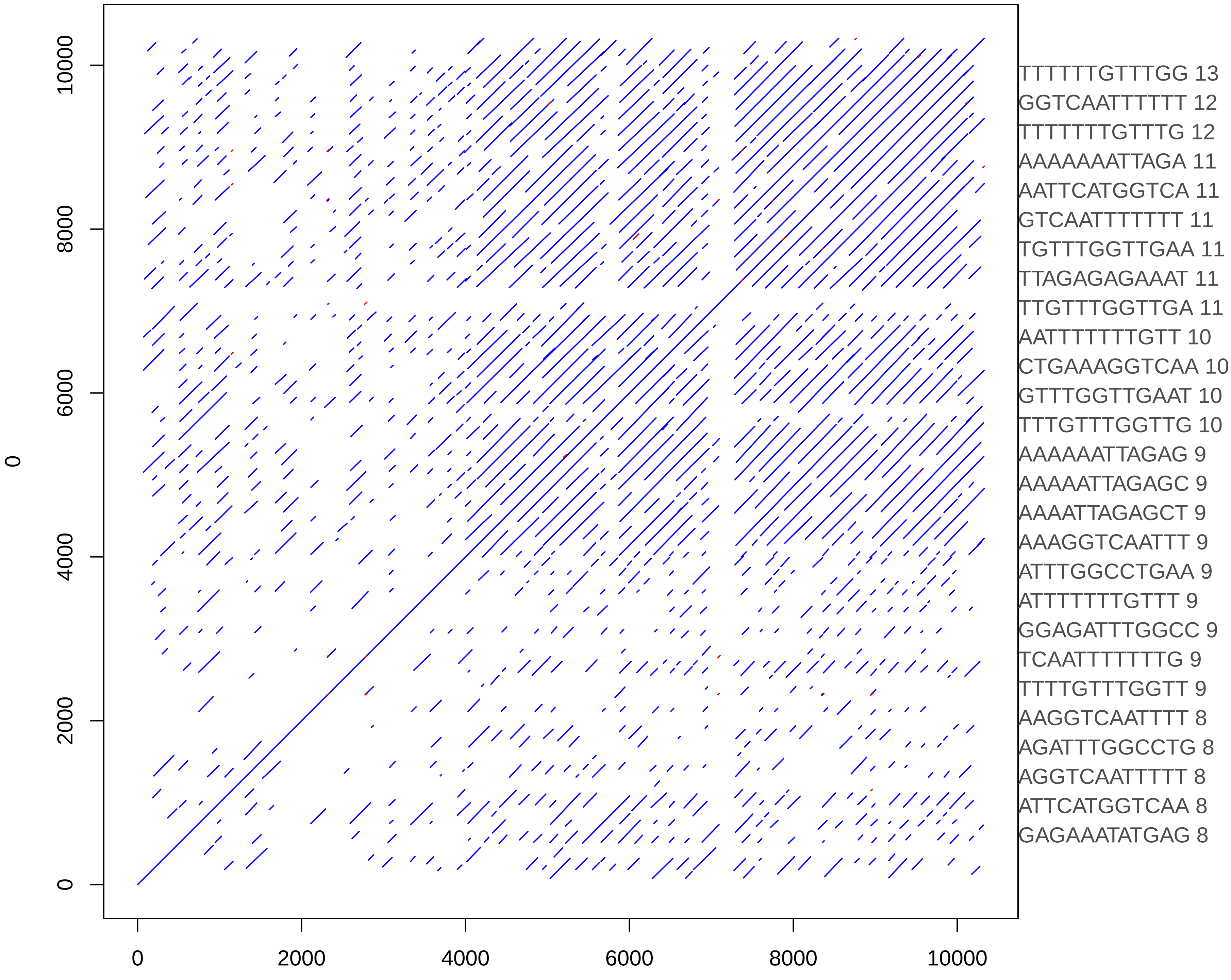

>m141126_012232_42134_c100732202550000001823141405141566_s1_p0/118763/3603_12216

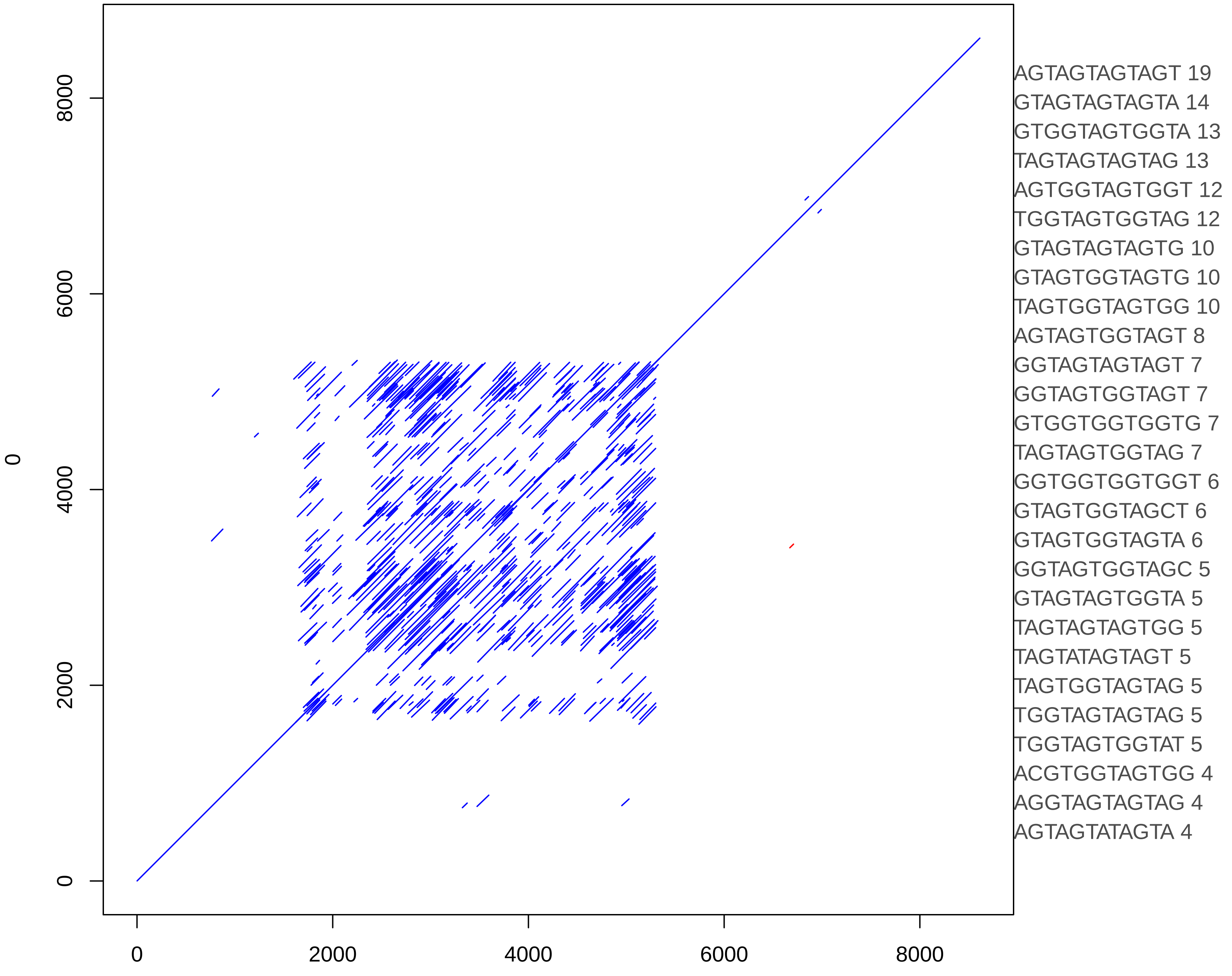

>m141126_012232_42134_c100732202550000001823141405141566_s1_p0/12783/0_10206

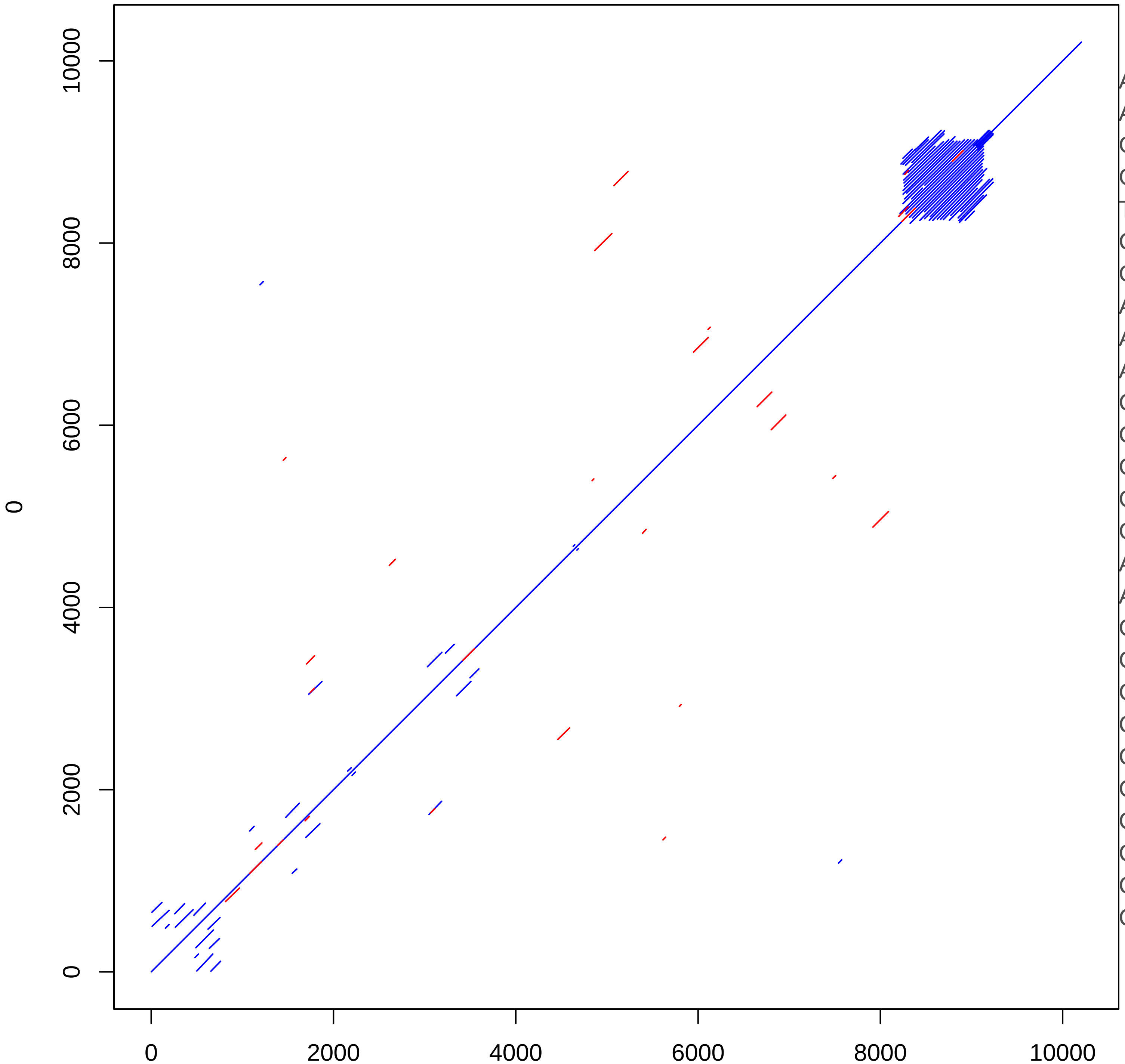

AGATGCCTAGGC 9
ATGCCTAGGCCA 9
GATGCCTAGGCC 9
GAGATGCCTAGG 8
TGCCTAGGCCAC 8
CGAGATGCCTAG 6
GCGAGATGCCTA 6
ACACCTCCCAAG 5
ACCTCCCAAGTG 5
AGGCCACACCTC 5
CACCCAAAGGGC 5
CACCTCCCAAGT 5
CCTAGGCCACAC 5
GCCTAGGCCACA 5
GGCCACACCTCC 5
AAAGGGCCTCGC 4
ACCCAAAGGGCC 4
CACACCTCCCAA 4
CCAAGTGGCCTC 4
CCACACCTCCCA 4
CCCAAAGGGCCT 4
CCTCCCAAGTGG 4
CCTCGCAAGATG 4
CCTCGCGAGATG 4
CGCGAGATGCCT 4
CTAGGCCACACC 4
CTCGCGAGATGC 4

1,121 hits for repetitive sequences passing 12mer abundance criterion

(Read-Number)
PAGE 447

For Tandem Repeat families of ### bp:
PacBio Read named "HuluTR###-r447"

>m141126_012232_42134_c100732202550000001823141405141566_s1_p0/132994/5050_12955

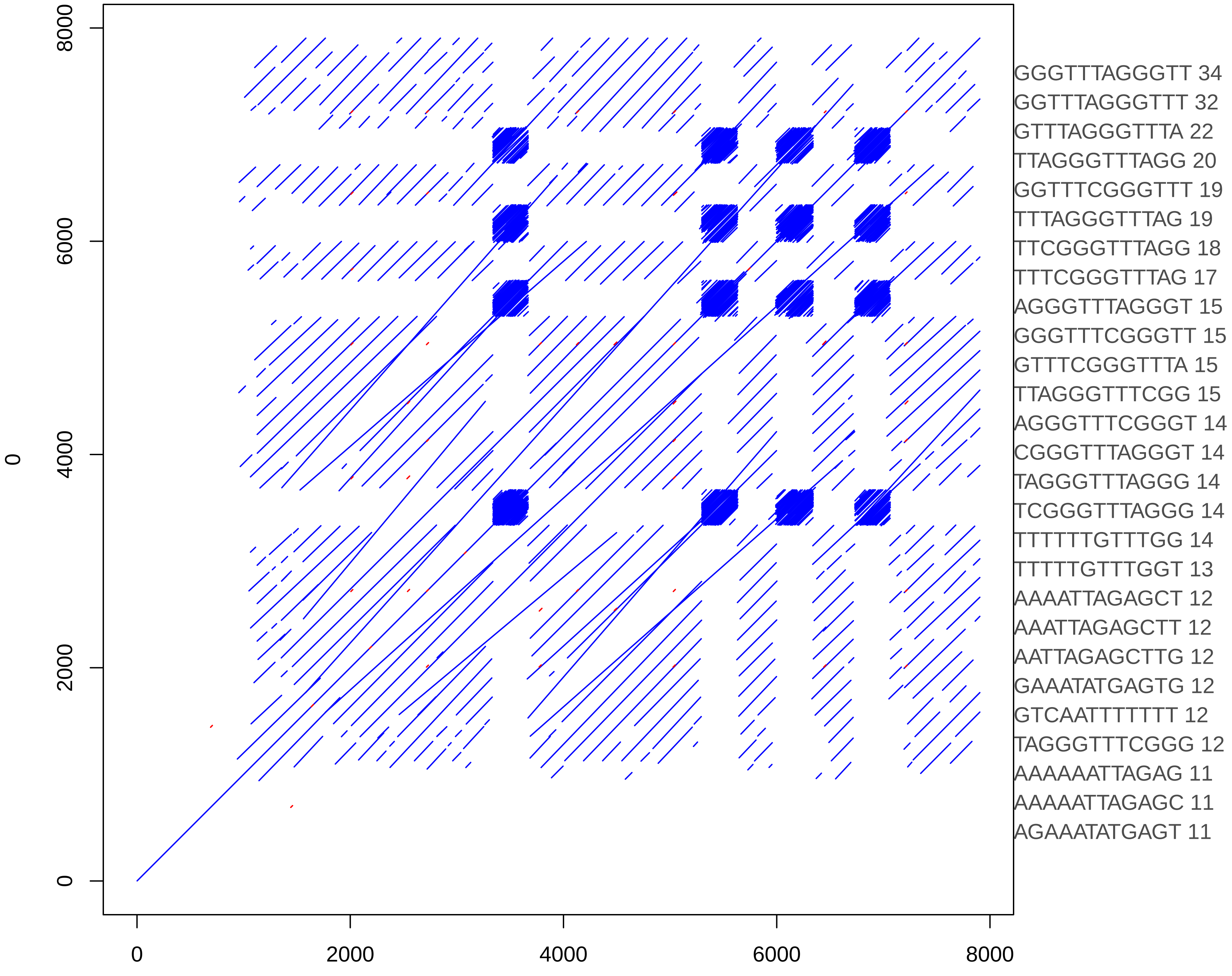

(HW Bass, DL Vera, FSU '17
Hopsteiner PacBio Apollo reads input)

0

Supplemental Material
k-mer screen & YASS dot-plots

KA Easterling et al. 2020

>m141126_012232_42134_c100732202550000001823141405141566_s1_p0/14378/246_5734

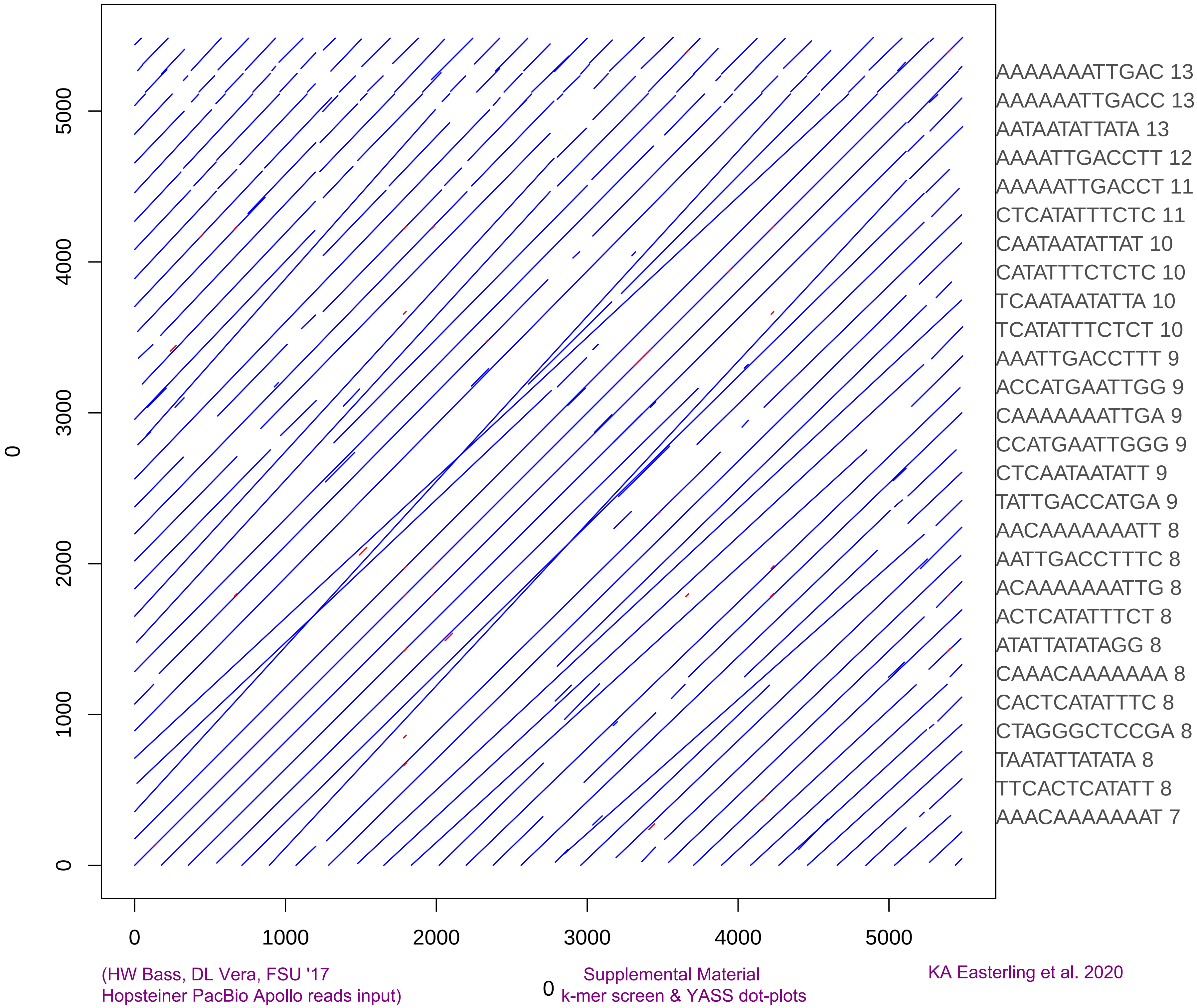

>m141126_012232_42134_c100732202550000001823141405141566_s1_p0/144343/5378_15580

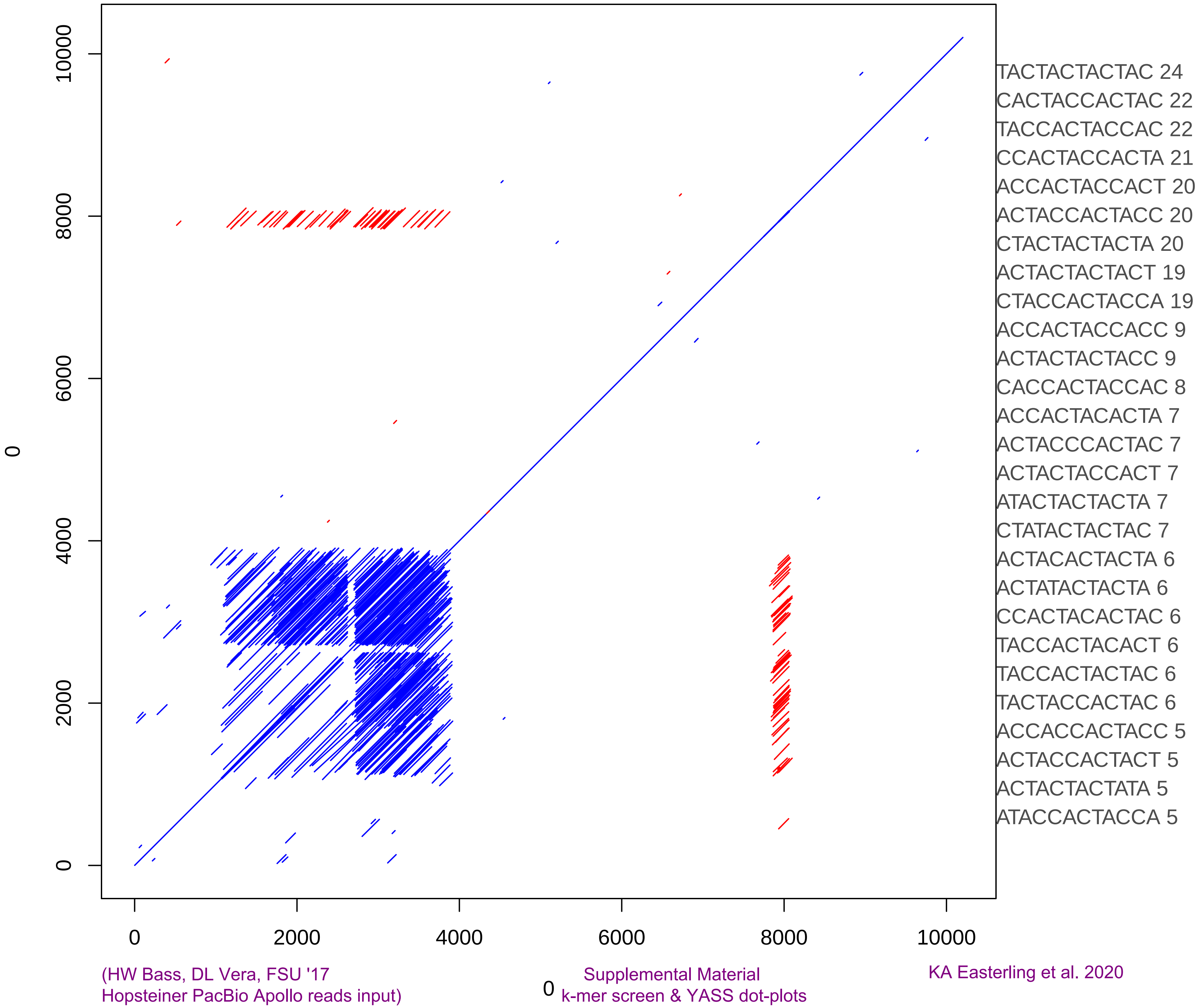

>m141126_012232_42134_c100732202550000001823141405141566_s1_p0/145468/5296_16121

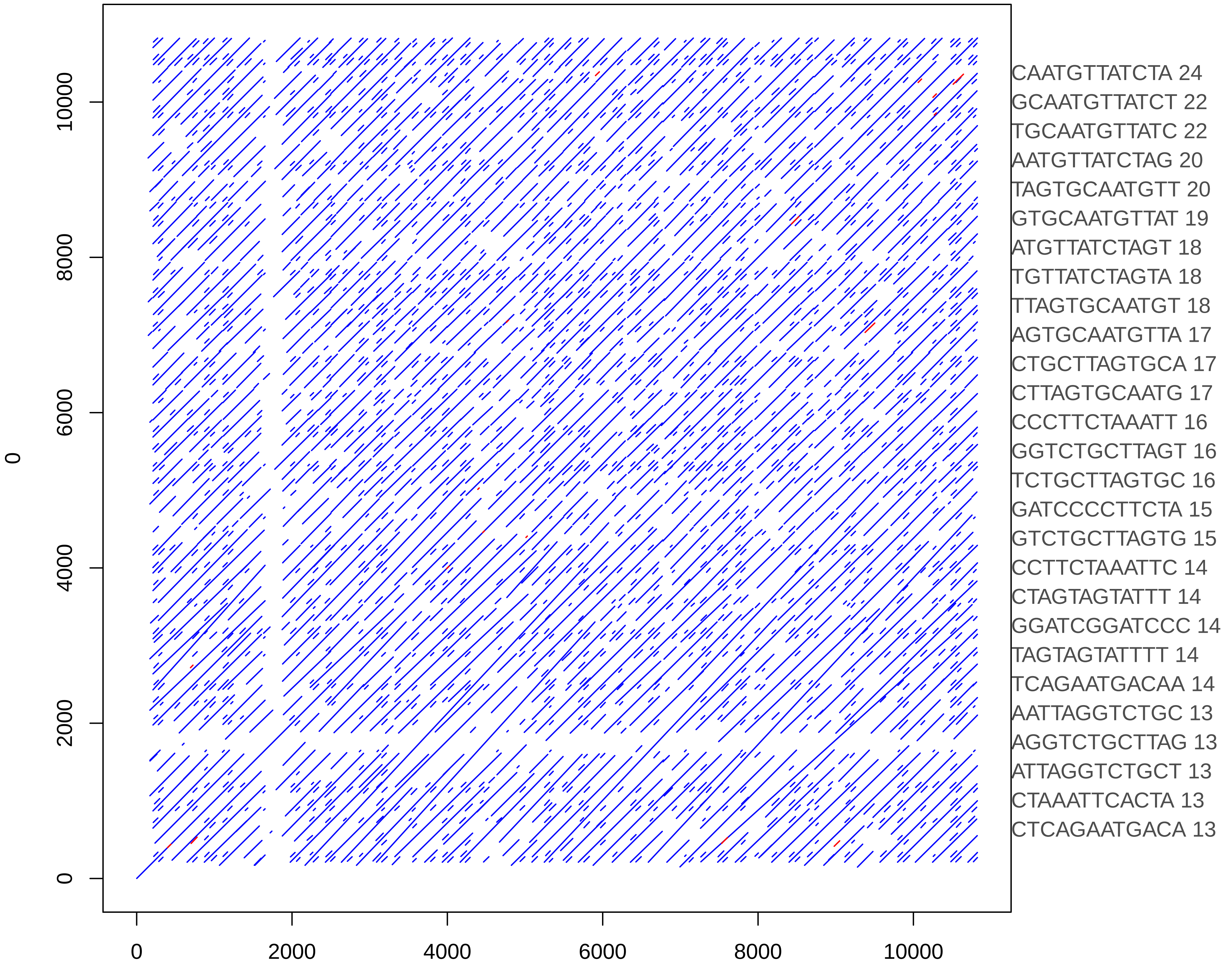

>m141126_012232_42134_c100732202550000001823141405141566_s1_p0/150639/295_7765

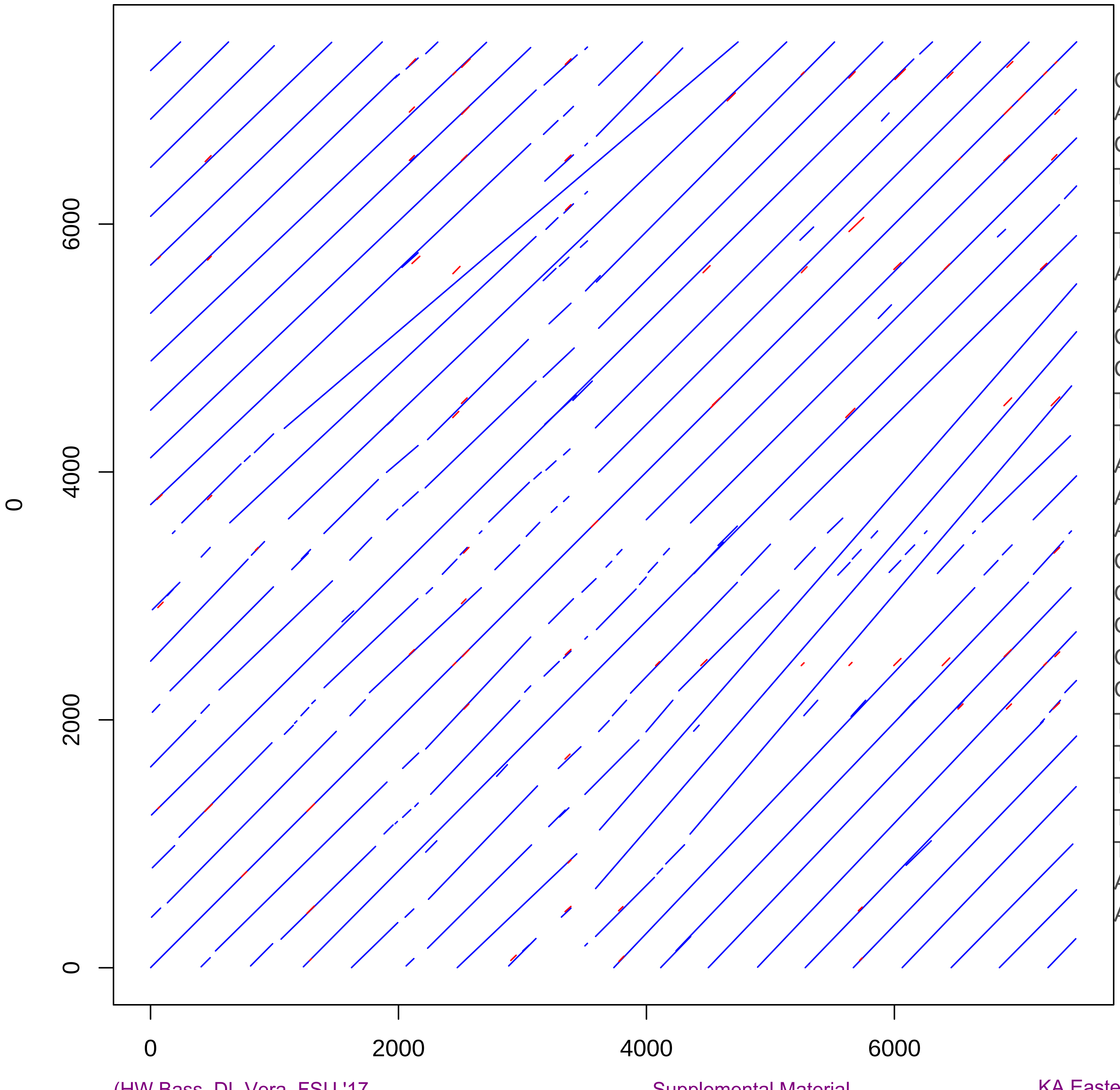

CCTTTCAGAAAT 12
ATTCACCAAAGG 11
CCCTTTCAGAAA 11
TTCACCAAAGGG 11
TTCAGAAATCTT 11
TTTCAGAAATCT 11
AGATCTCTTGGG 10
ATATCCAACGAG 10
CCGTTTCGACTC 10
CTTTCAGAAATC 10
TATCCAACGAGT 10
TCCAACGAGTAG 10
AATATCCAACGA 9
AGTGAAATCTCC 9
ATCCAACGAGTA 9
CGTTTCGACTCC 9
GAGATCTCTTGG 9
GGAATTTTTTTT 9
GGGAAAAATATC 9
GGTTCTATGTAT 9
TCAGAAATCTTC 9
TCCGTTTCGACT 9
TGAGATCTCTTG 9
TTTGATAGTGAA 9
TTTTTTTAAATG 9
AAAATATCCAAC 8
AAACTTGTGAAA 8

>m141126_012232_42134_c100732202550000001823141405141566_s1_p0/151837/239_10072

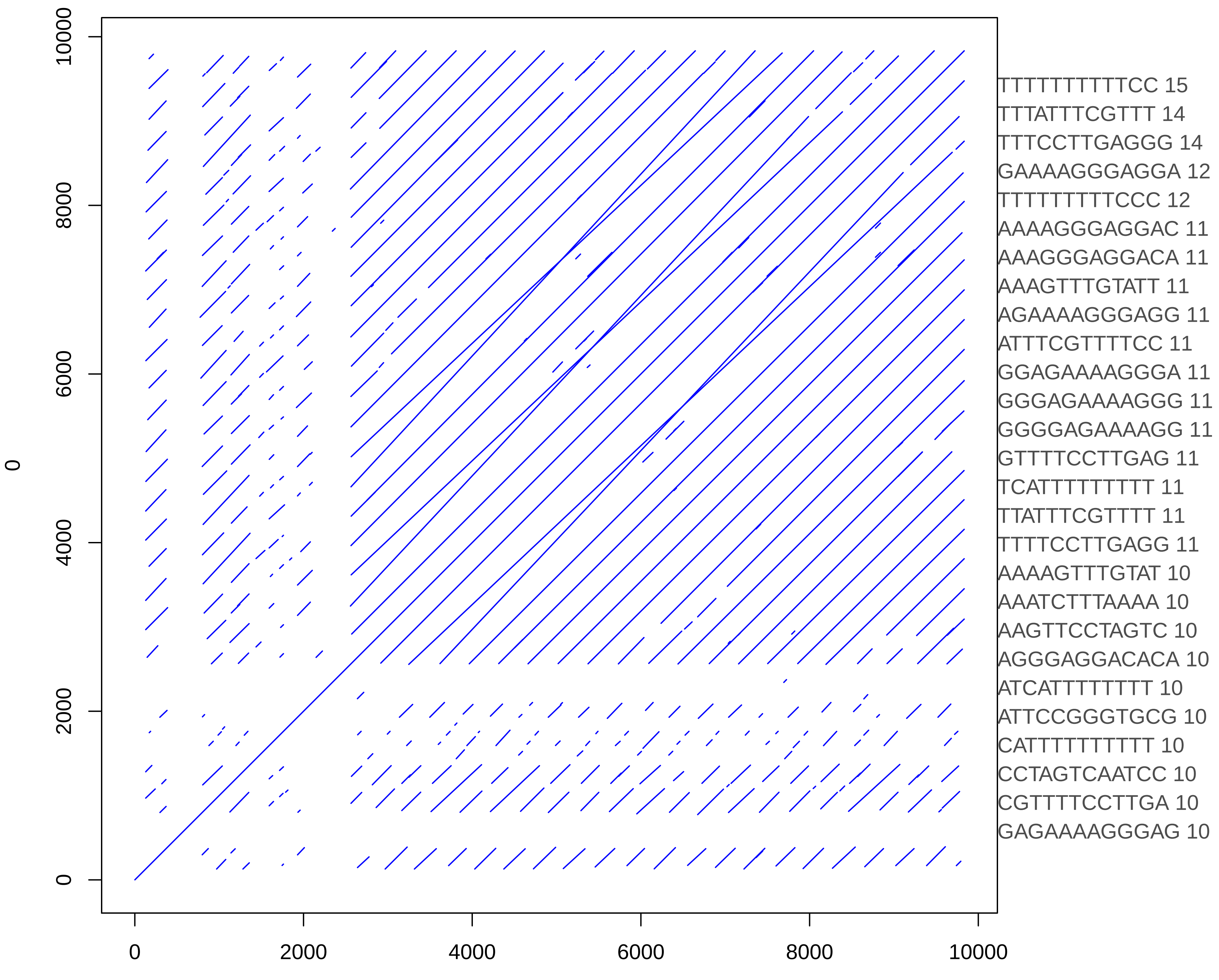

>m141126_012232_42134_c100732202550000001823141405141566_s1_p0/153439/5435_16780

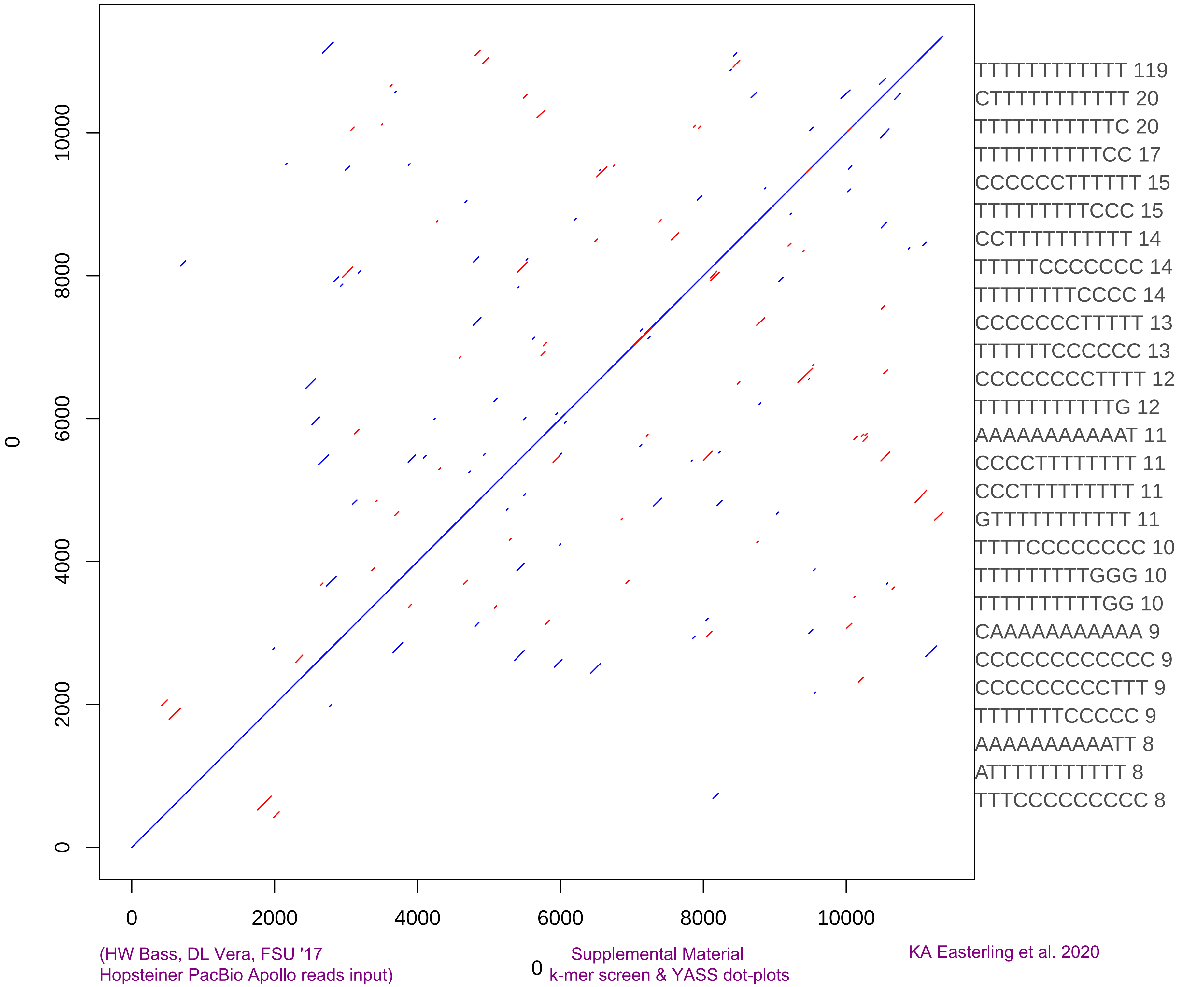

>m141126_012232_42134_c100732202550000001823141405141566_s1_p0/153890/1681_13277

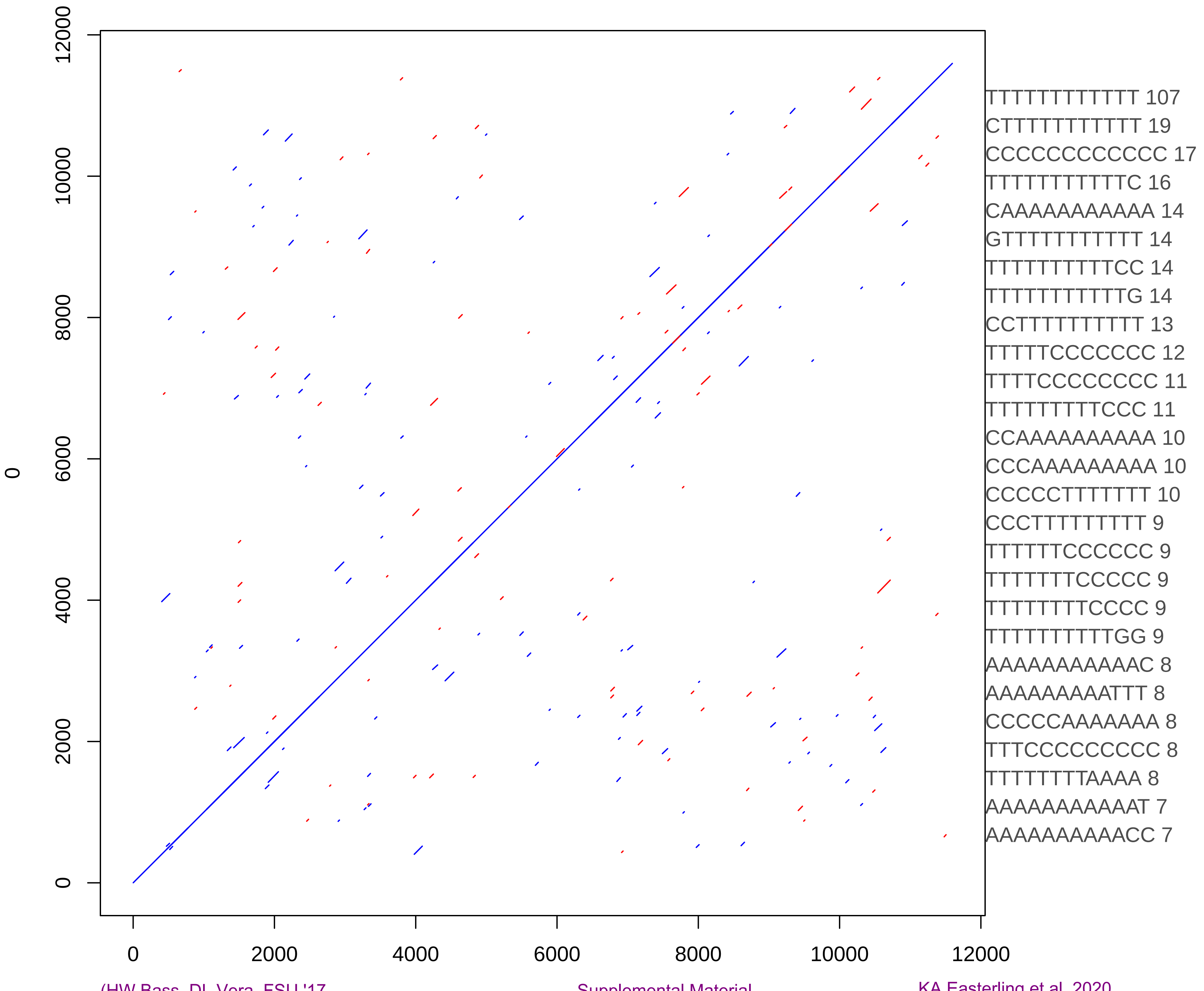

>m141126_012232_42134_c100732202550000001823141405141566_s1_p0/160121/9121_17685

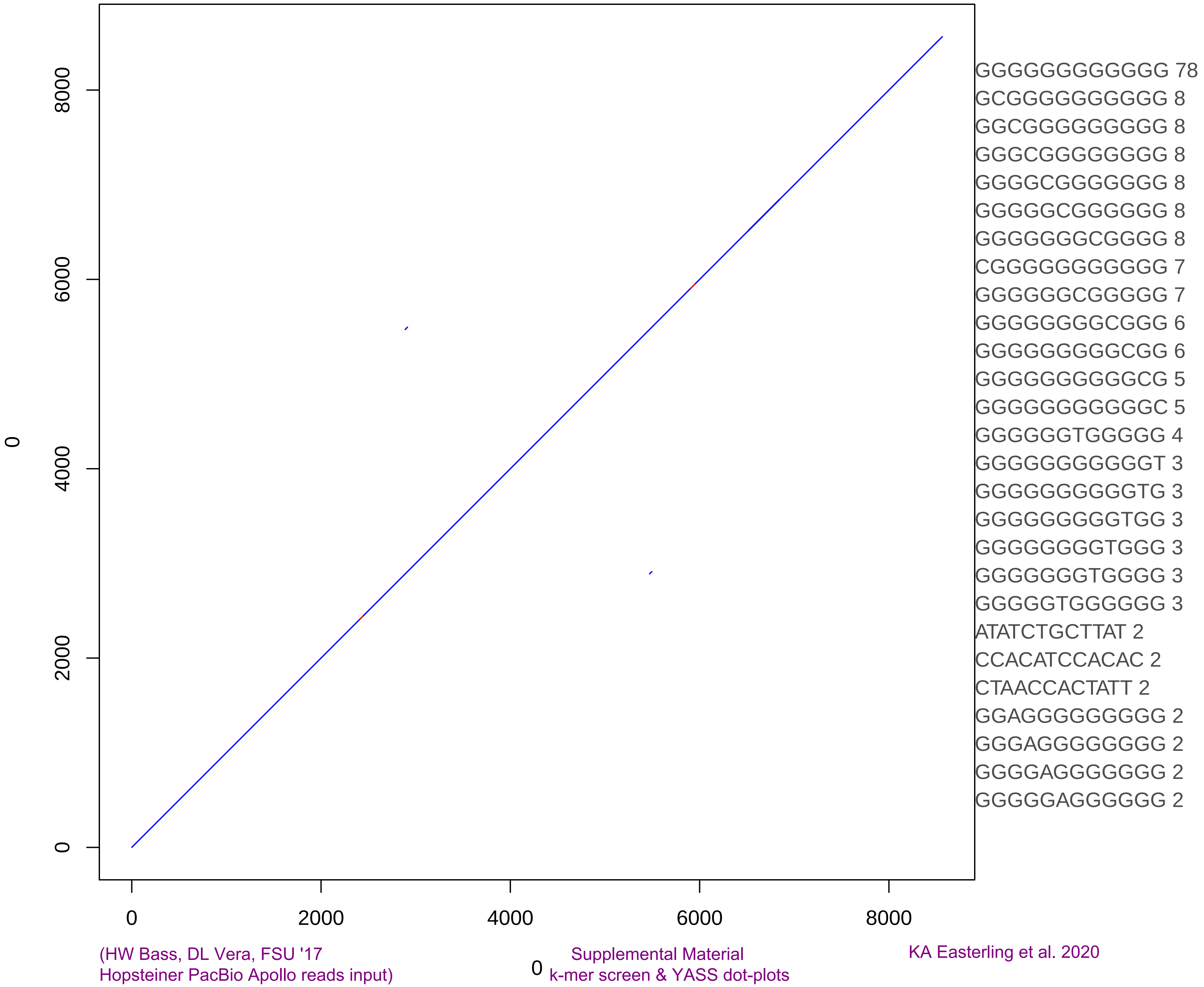

>m141126_012232_42134_c100732202550000001823141405141566_s1_p0/161362/3889_10300

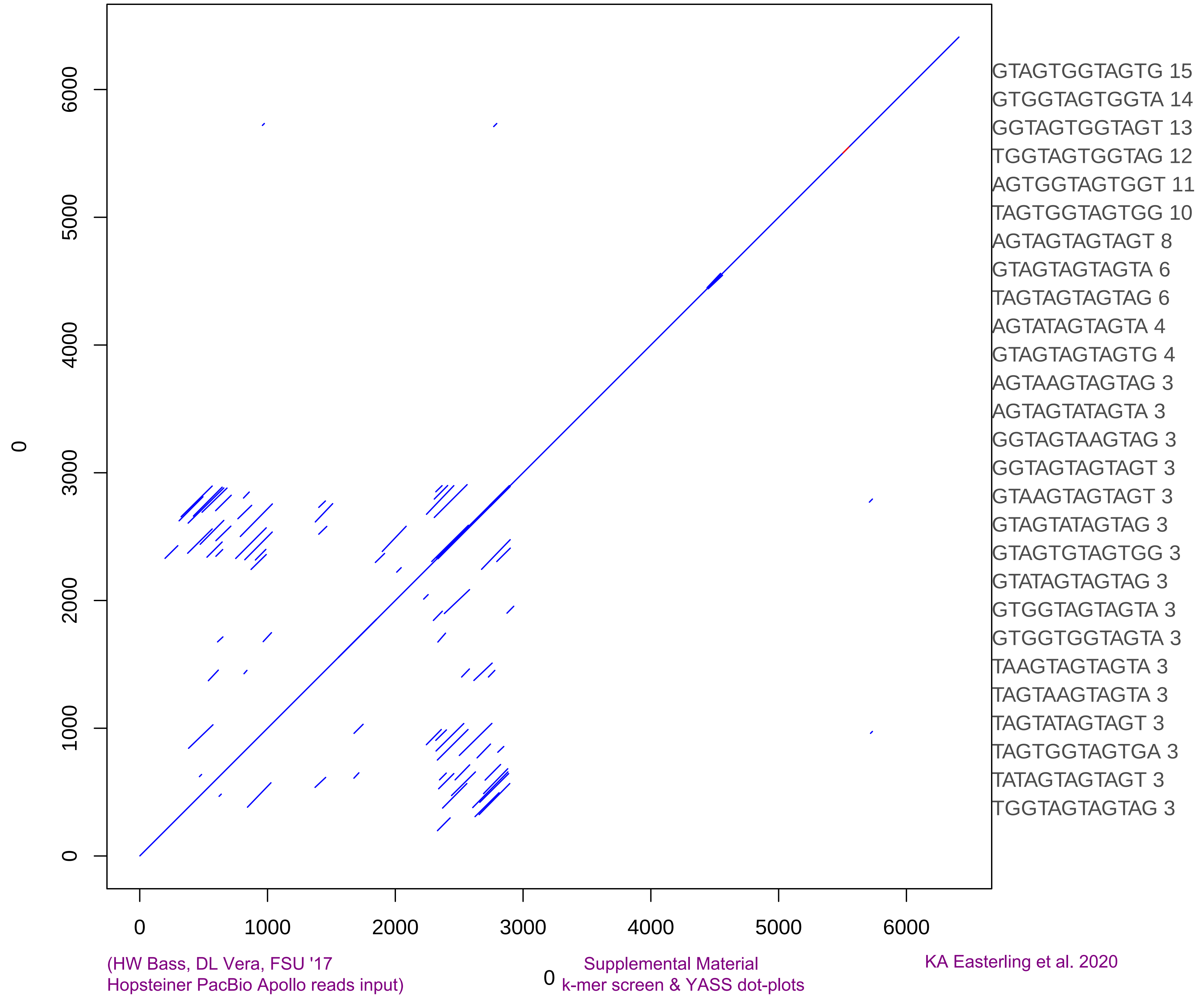

>m141126_012232_42134_c100732202550000001823141405141566_s1_p0/162666/1146_13057

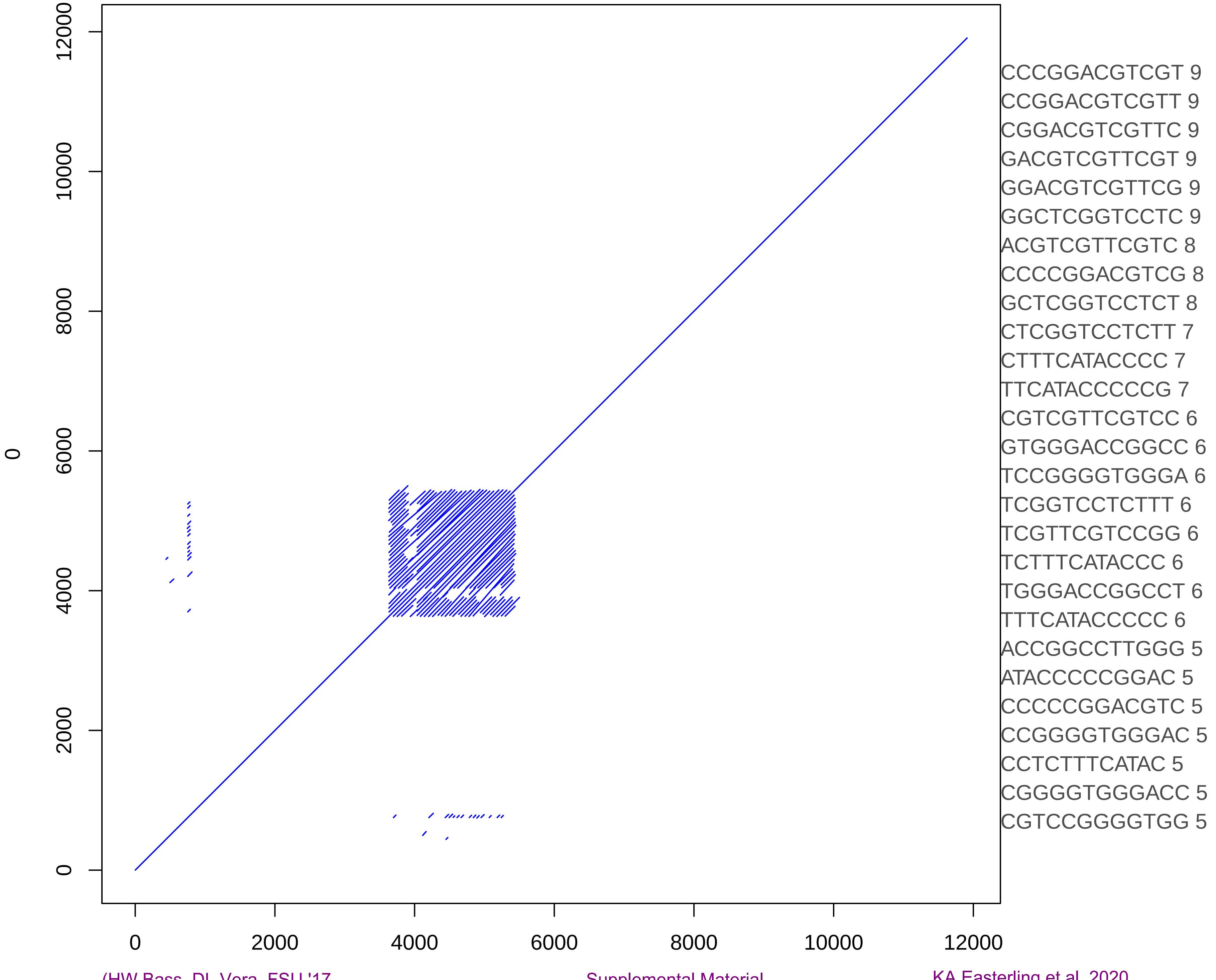

>m141126_012232_42134_c100732202550000001823141405141566_s1_p0/163132/0_10347

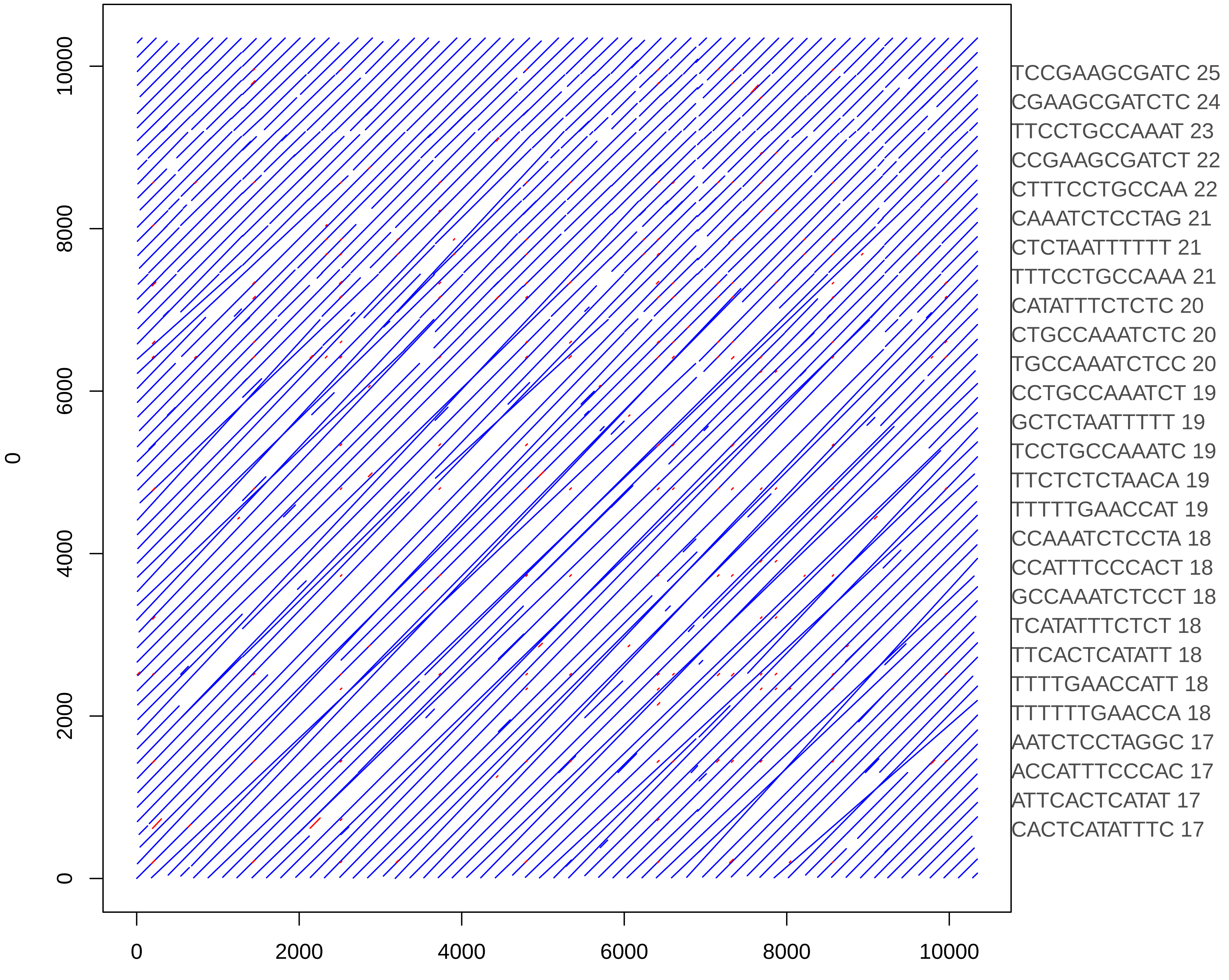

0

>m141126_012232_42134_c100732202550000001823141405141566_s1_p0/17206/0_8781

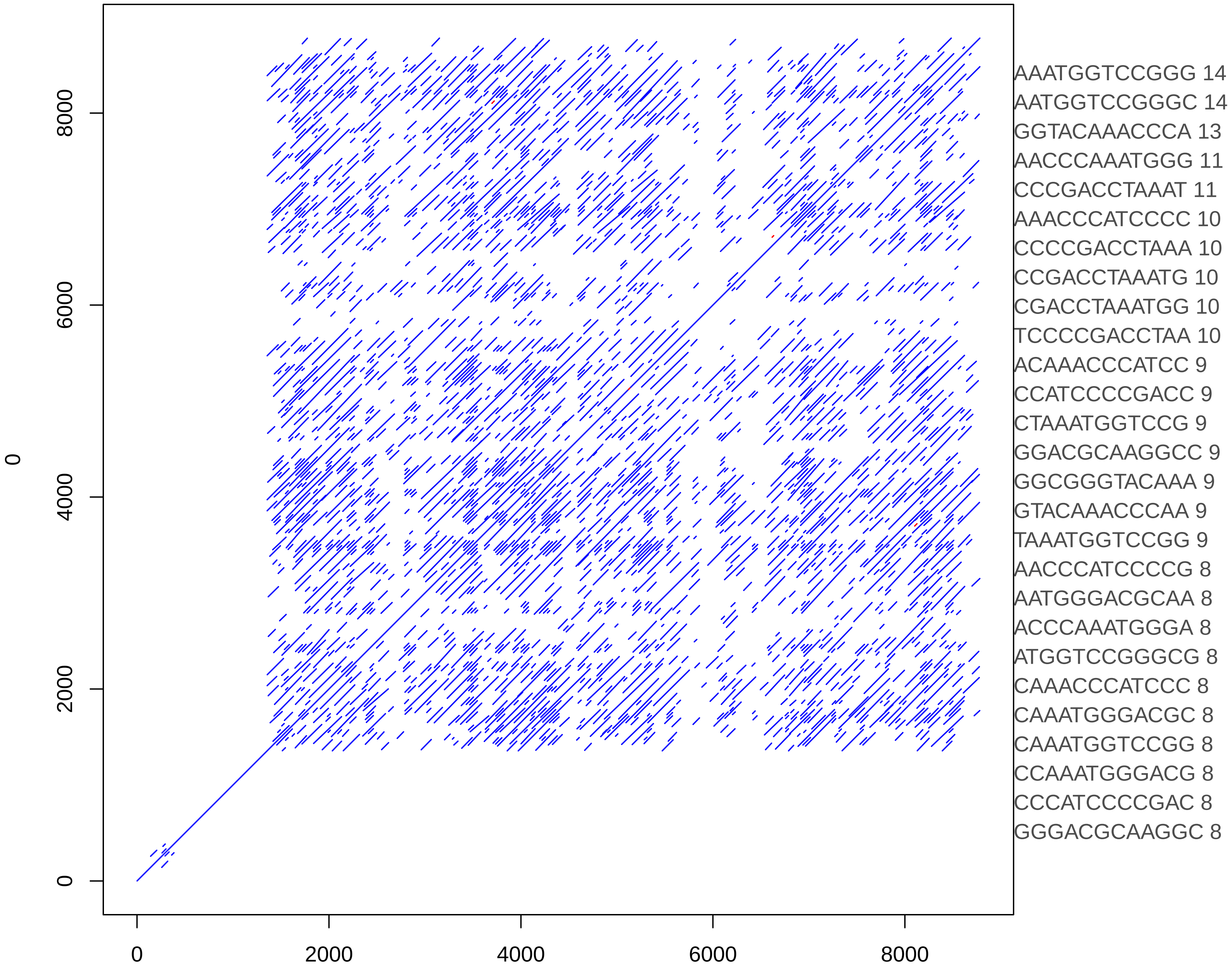

>m141126_012232_42134_c100732202550000001823141405141566_s1_p0/1782/1524_9252

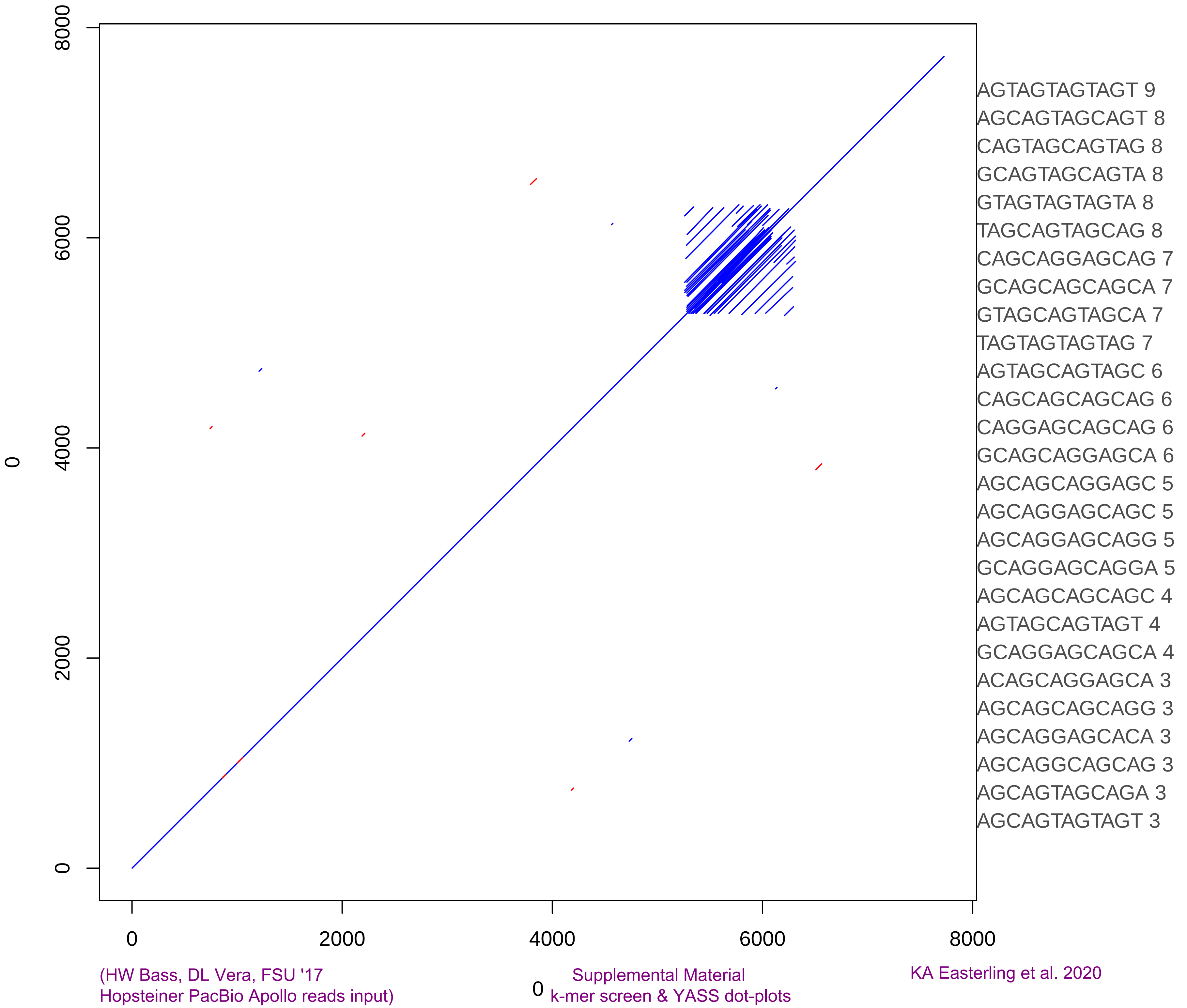

>m141126_012232_42134_c100732202550000001823141405141566_s1_p0/2214/0_6824

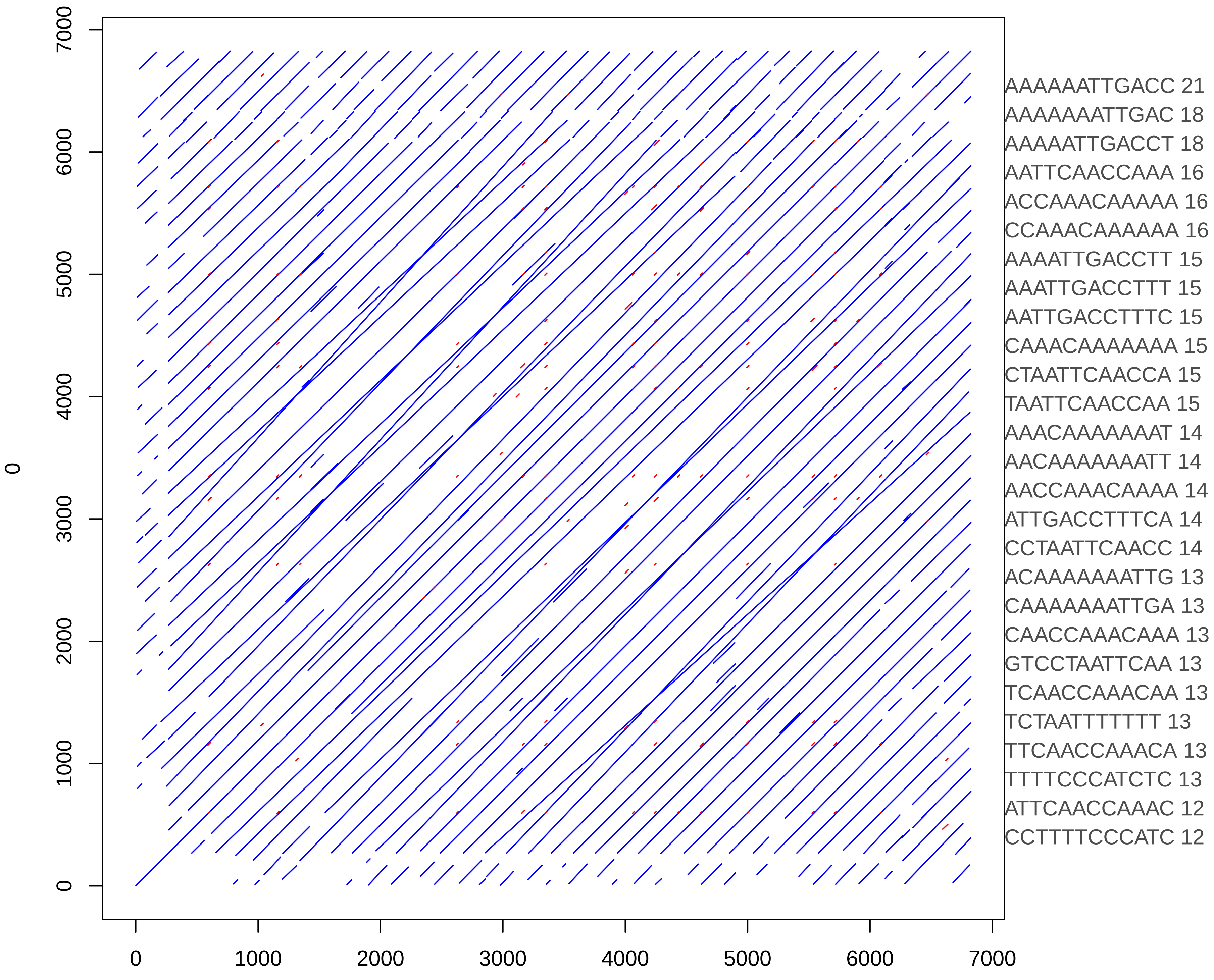

>m141126_012232_42134_c100732202550000001823141405141566_s1_p0/22774/3988_10471

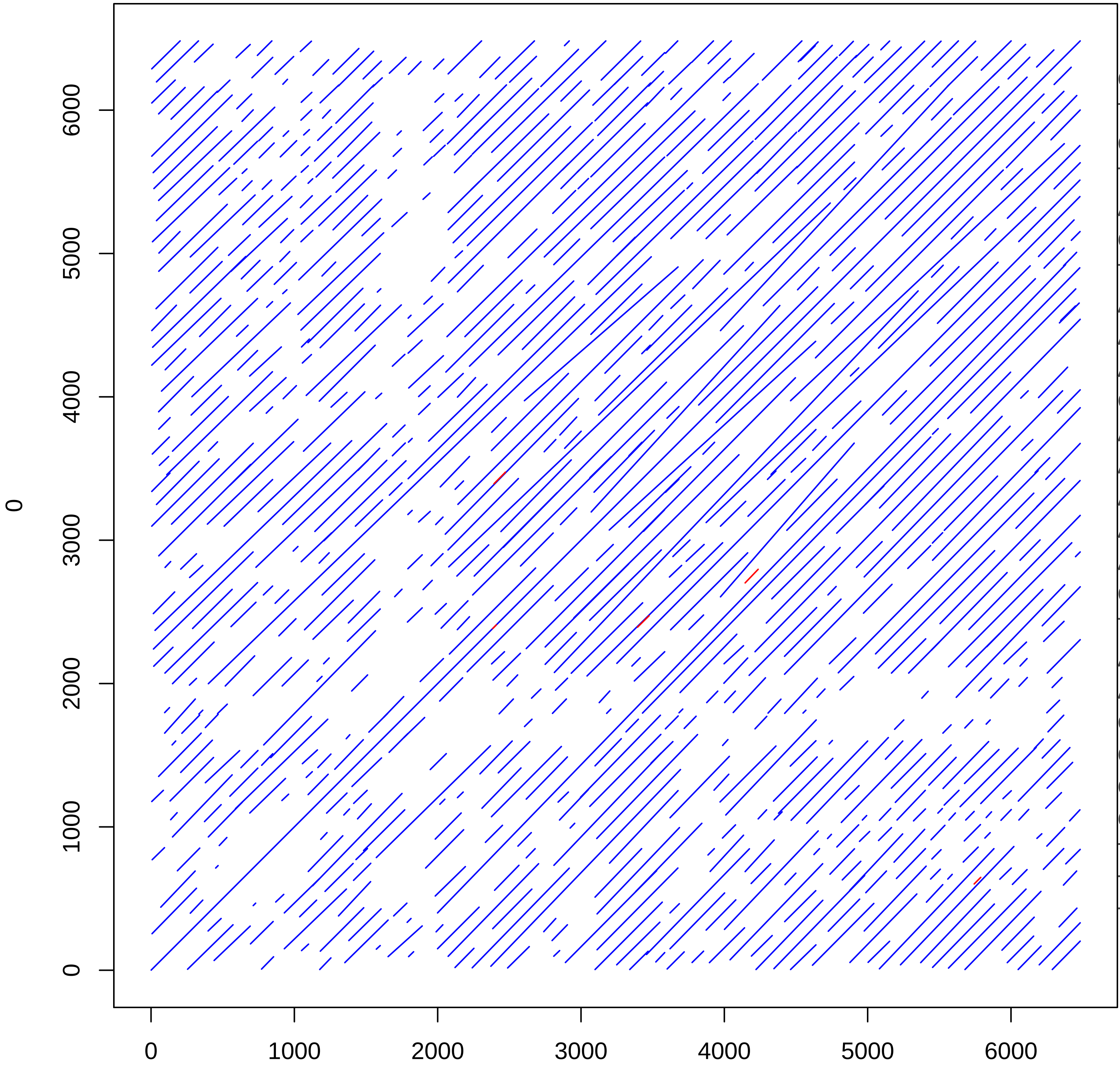

>m141126_012232_42134_c100732202550000001823141405141566_s1_p0/25855/7324_14509

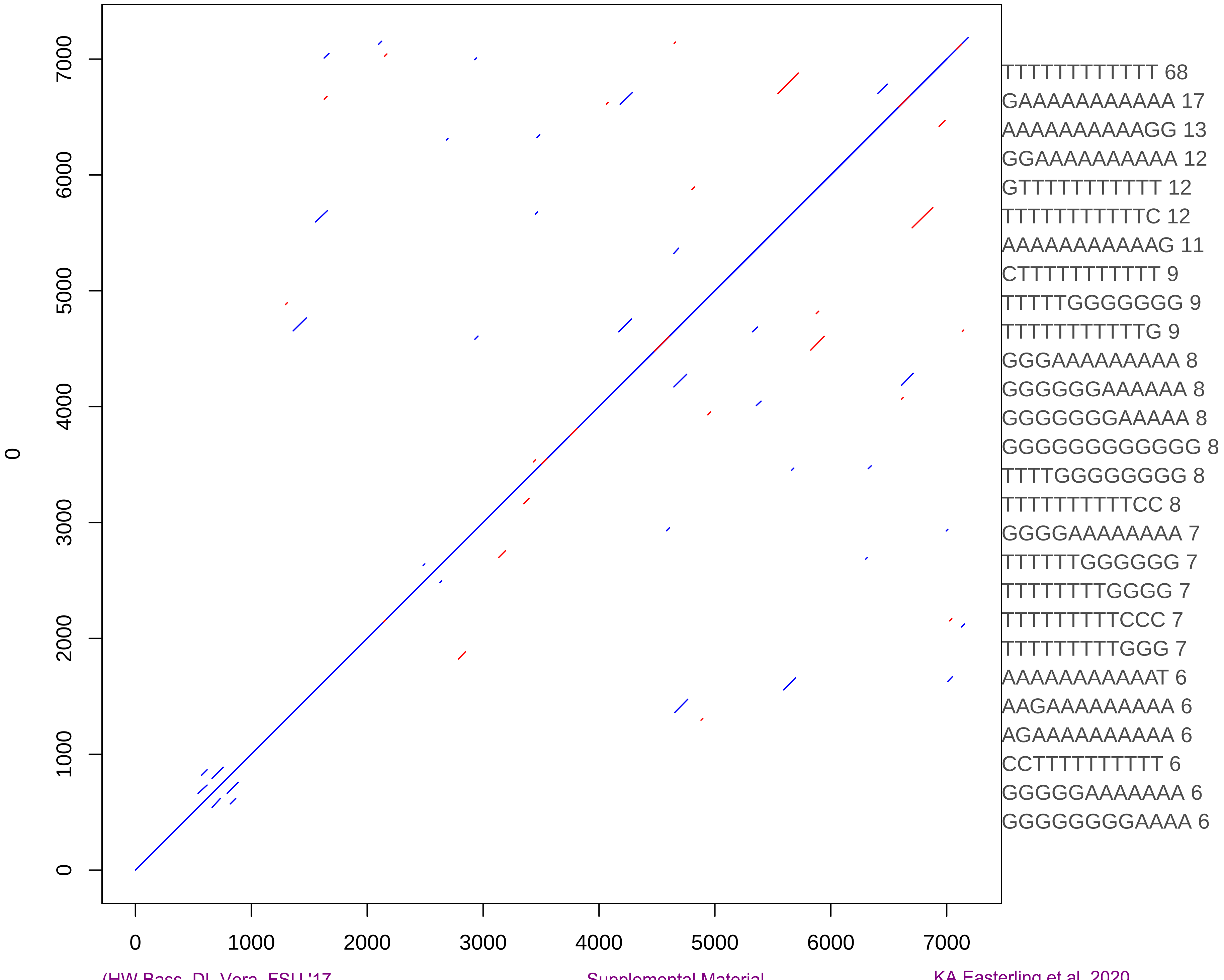

0

>m141126_012232_42134_c100732202550000001823141405141566_s1_p0/26669/9799_14934

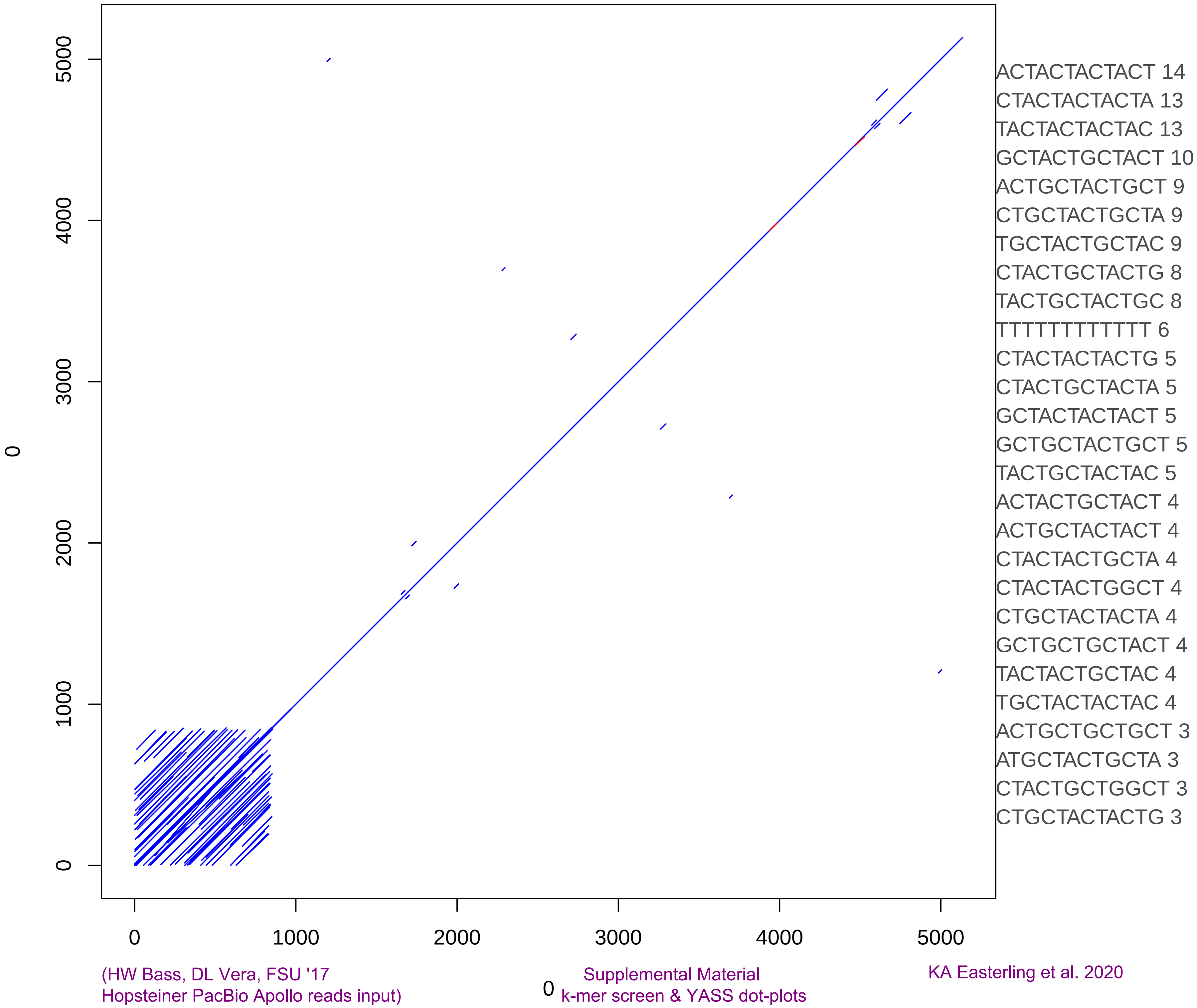

>m141126_012232_42134_c100732202550000001823141405141566_s1_p0/27606/0_5247

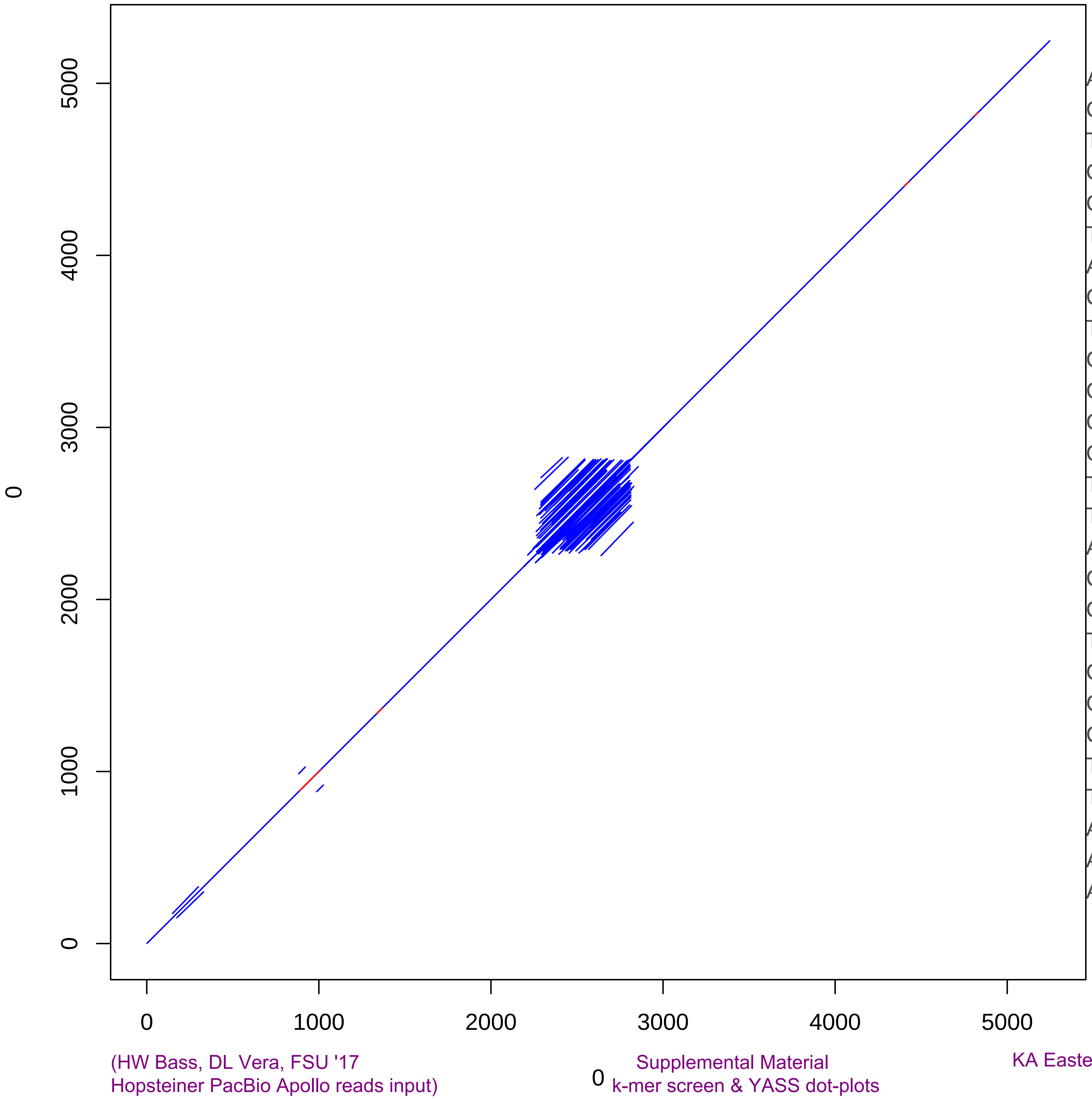

ACTGCTACTGCT 11
CTACTGCTACTG 11
TACTGCTACTGC 11
GCTACTGCTACT 10
CTGCTACTGCTA 9
TGCTACTGCTAC 9
ACTACTACTACT 7
CTACTACTACTA 6
TACTACTACTAC 6
CTGCTACTGCTC 5
CTGCTCCTGCTA 5
GCTACTGCTCCT 5
GCTCCTGCTACT 5
TGCTACTGCTCC 5
TGCTCCTGCTAC 5
ACTGCTCCTGCT 4
CCTGCTACTGCT 4
CTCCTGCTACTG 4
TCCTGCTACTGC 4
CTAACTACTACT 3
CTACTGCTCCTG 3
CTGCTCCTGCTC 3
TAACTACTACTA 3
TACTGCTCCTGC 3
AACTACTACTAC 2
AATAAAAACTAT 2
ACTACTACTAAC 2

>m141126_012232_42134_c100732202550000001823141405141566_s1_p0/29616/0_5095

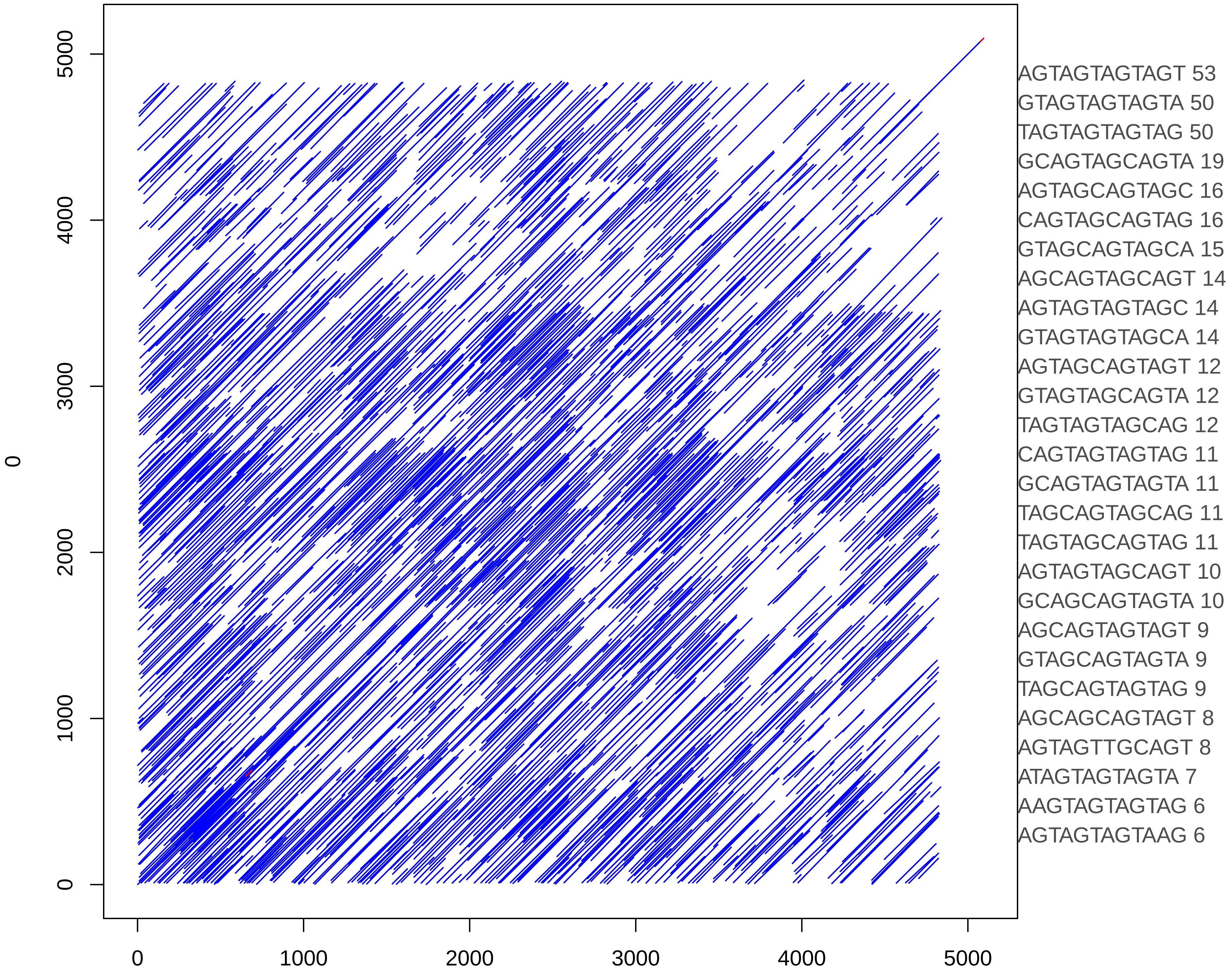

0

>m141126_012232_42134_c100732202550000001823141405141566_s1_p0/35449/8020_13405

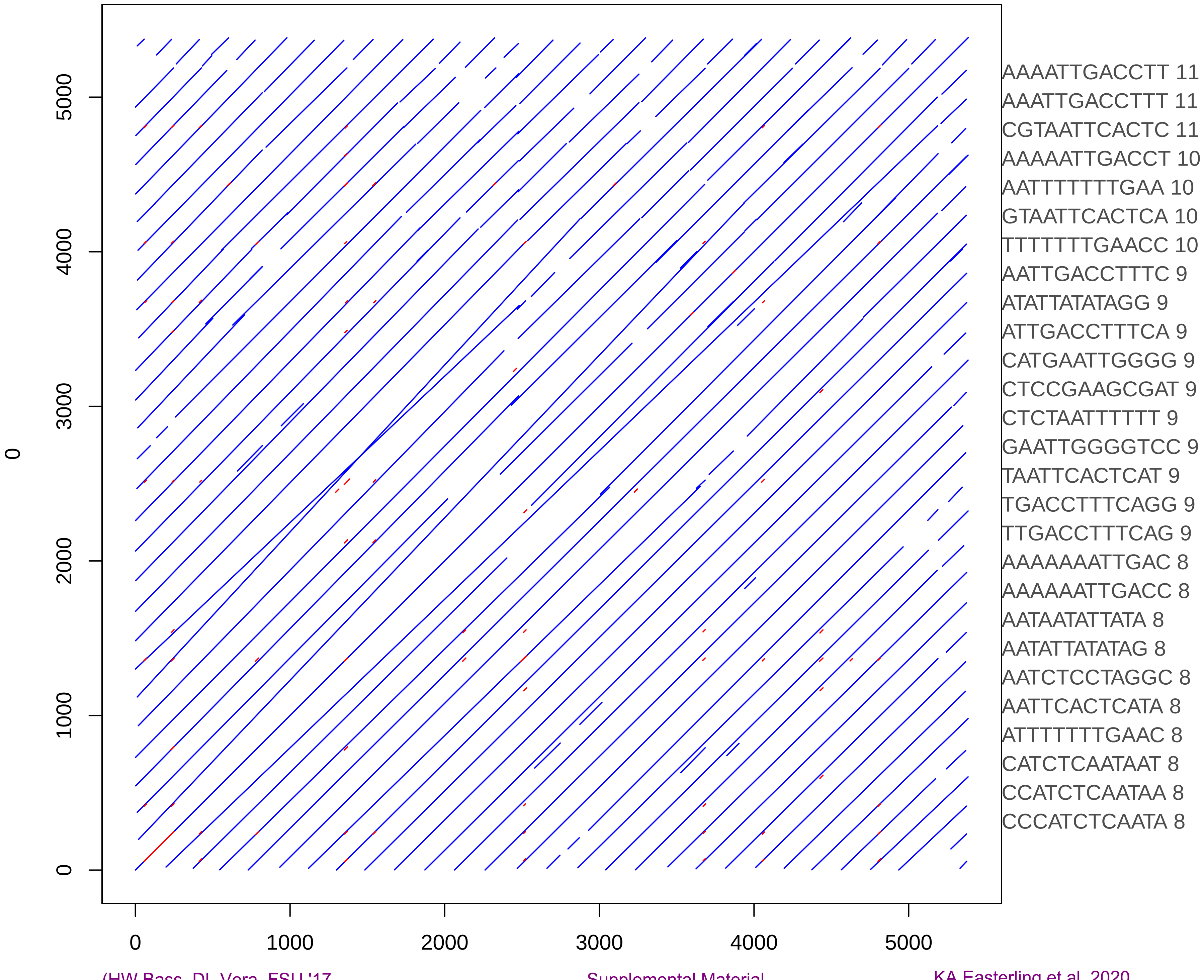

>m141126_012232_42134_c100732202550000001823141405141566_s1_p0/3578/8435_18941

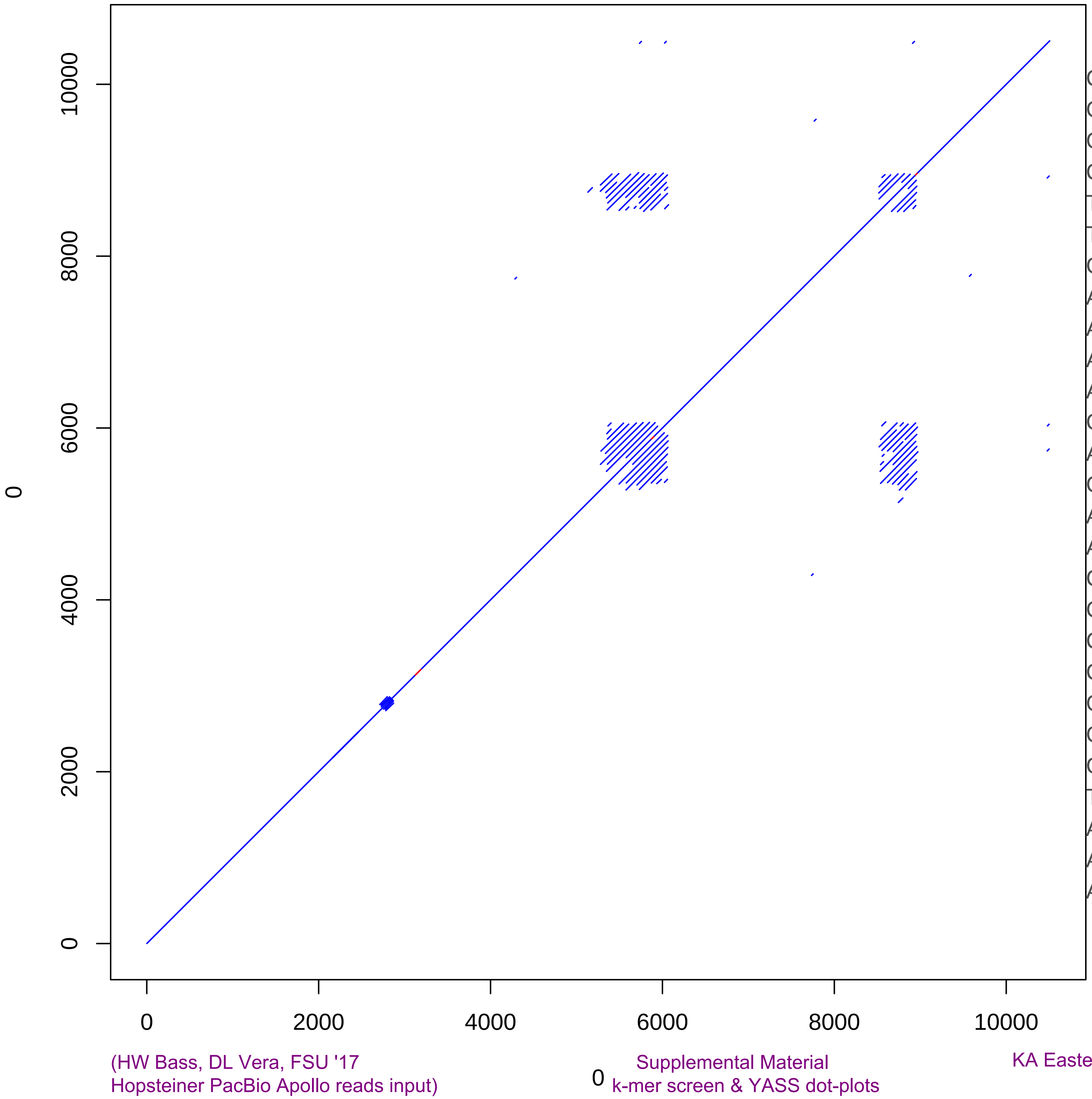

CGAAGCAAAGCT 10
GCGAAGCAAAGC 10
GAAGCAAAGCTT 9
CTTGCGAAGCAA 8
TGCGAAGCAAAG 8
TTGCGAAGCAAA 8
GCTTGCGAAGCA 7
AAGCAAAGCTTC 5
AAGCAAAGCTTT 5
AGCAAAGCTTCC 5
AGCTTGCGAAGC 5
GGGGGGGGGGGG 5
AGCAAAGCTTTA 4
GCAAAGCTTCCA 4
ACAGCGGGTCGC 3
AGCGGGTCGCGG 3
CAAAGCTTCCAG 3
CAAAGCTTTACA 3
CAGCGGGTCGCG 3
CATGCCATGCAC 3
GAGCTTGCGAAG 3
GCAAAGCTTTAC 3
GGAGCTTGCGAA 3
TCATGCCATGCA 3
AAAGCTTCCAGC 2
AAAGCTTTACAG 2
AAAGCTTTGACA 2

>m141126_012232_42134_c100732202550000001823141405141566_s1_p0/36645/0_5516

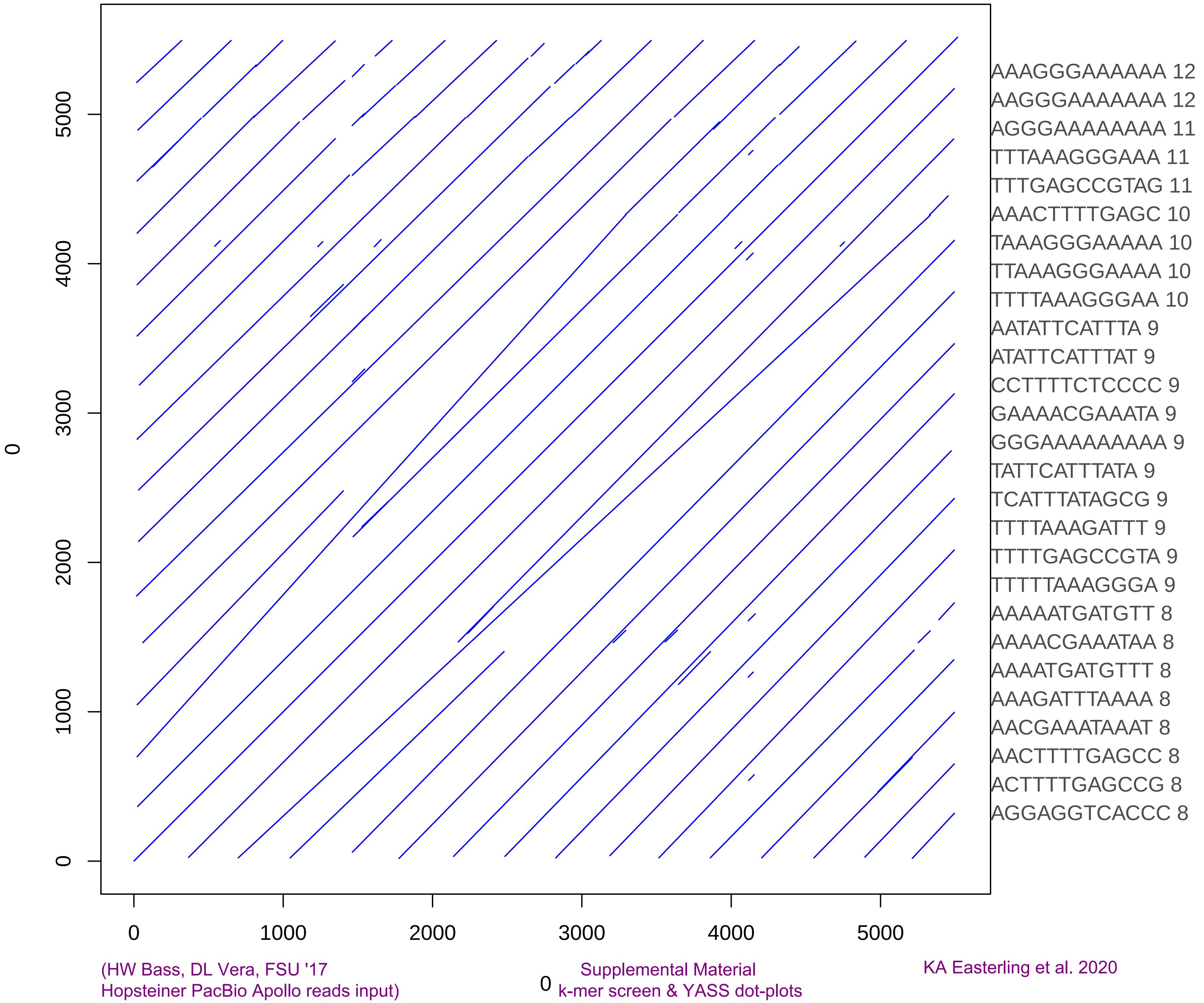

>m141126_012232_42134_c100732202550000001823141405141566_s1_p0/39773/213_11174

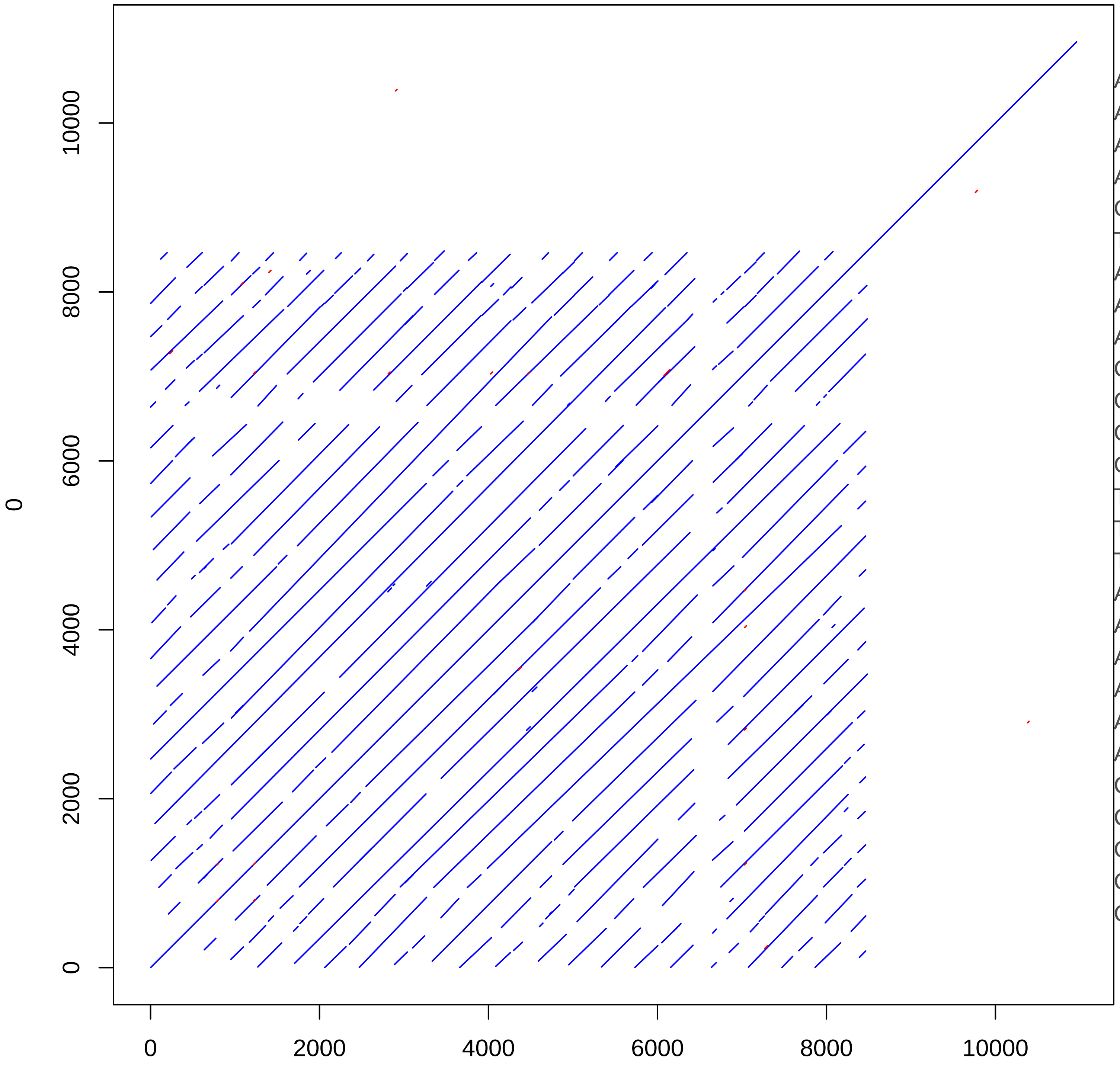

AATCTTCGATTT 10
AGGAAAAAACTT 9
ATACTGAGAGAT 9
ATCTTCGATTTC 9
GTTGAGGGTGAA 9
TGACTTAAGTCT 9
AAATCTTCGATT 8
AAGTTTCGTACG 8
AATACTGAGAGA 8
CGTACGAATCCG 8
GAAATCTTCGAT 8
GACTTAAGTCTC 8
GTACGAATCCGA 8
TGTTGAGGGTGA 8
TTGAGGGTGAAA 8
TTTCGTACGAAT 8
AATCCGAGGTGT 7
ACTGAGAGATGT 7
AGAAATCTTCGA 7
AGTTTCGTACGA 7
ATCTCCAATTCA 7
ATGTATAAAGTT 7
CAGAAATCTTCG 7
CTGAGAGATGTA 7
GTGAATACTAAA 7
GTGTGTTGAGGG 7
GTGTTGAGGGTG 7

>m141126_012232_42134_c100732202550000001823141405141566_s1_p0/40040/0_5950

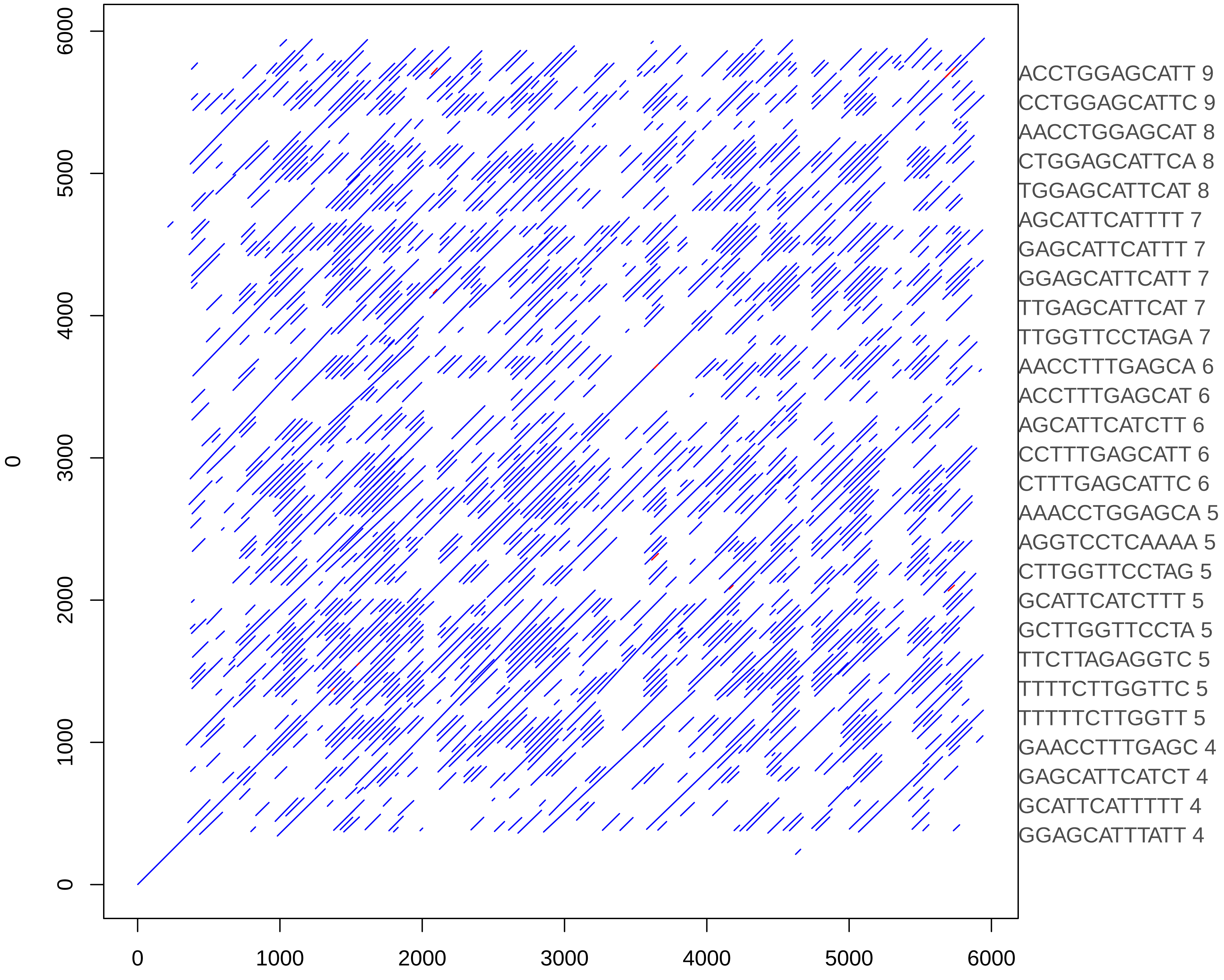

>m141126_012232_42134_c100732202550000001823141405141566_s1_p0/40075/213_10282

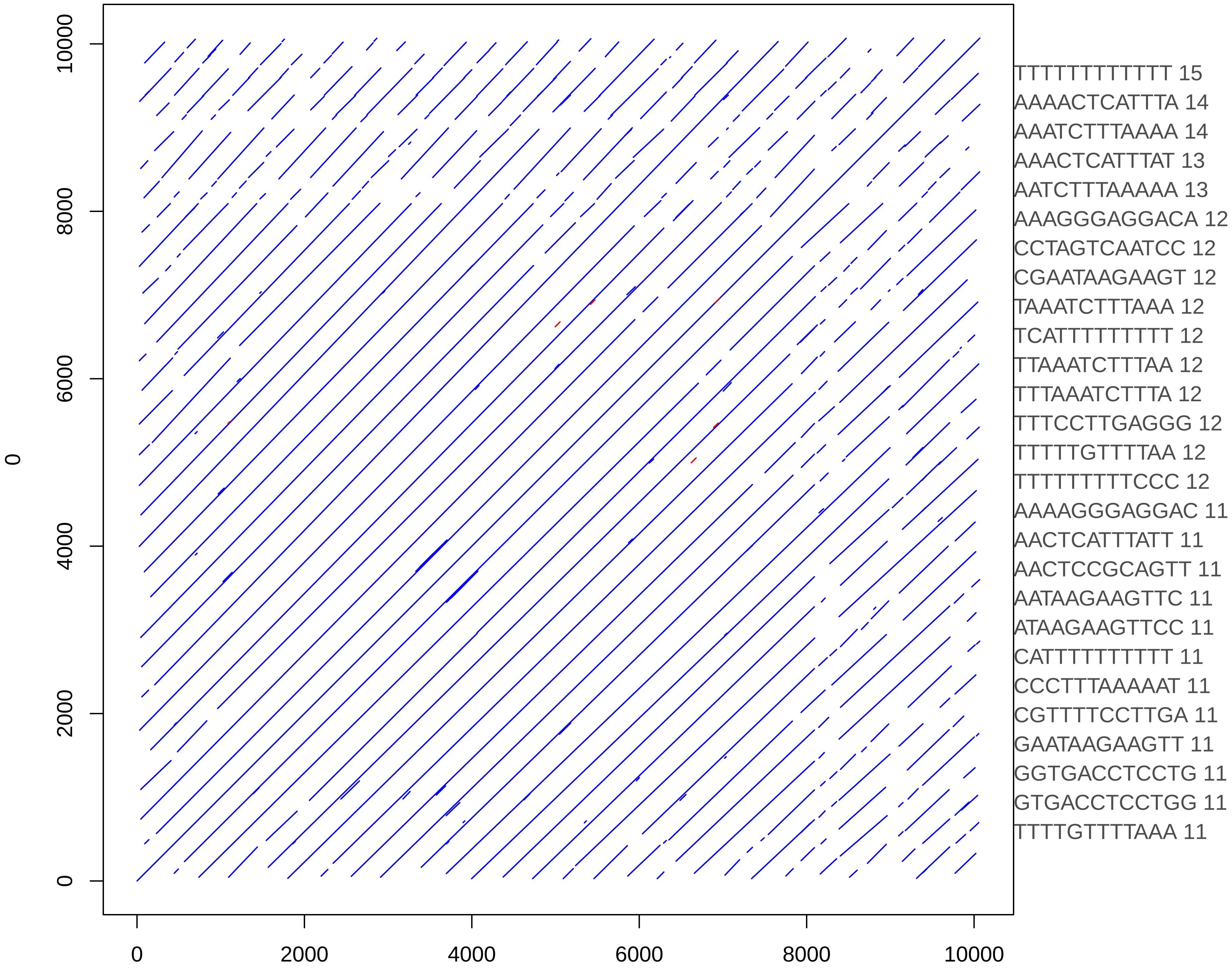

>m141126_012232_42134_c100732202550000001823141405141566_s1_p0/42709/0_5473

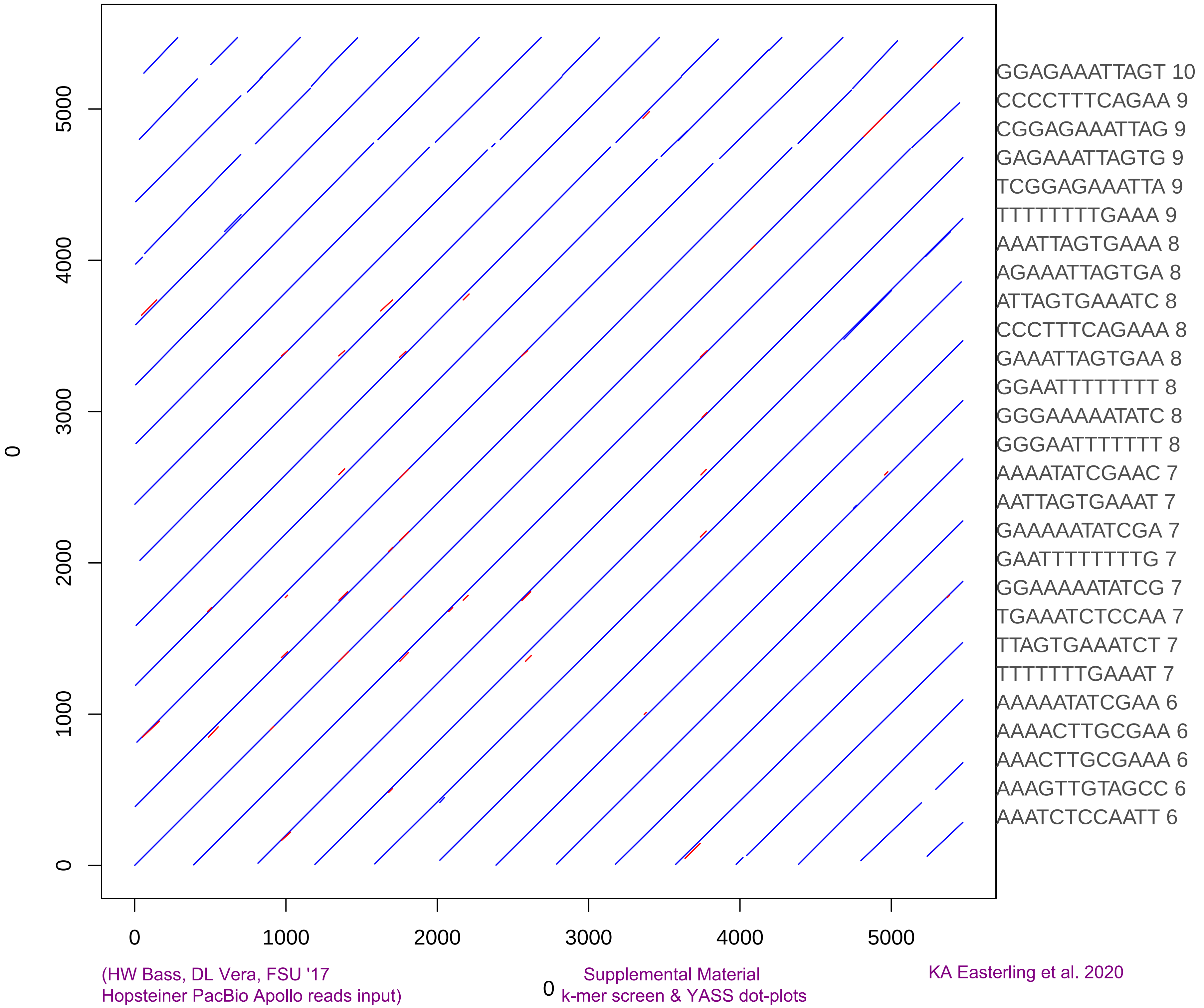

>m141126_012232_42134_c100732202550000001823141405141566_s1_p0/45733/2256_9876

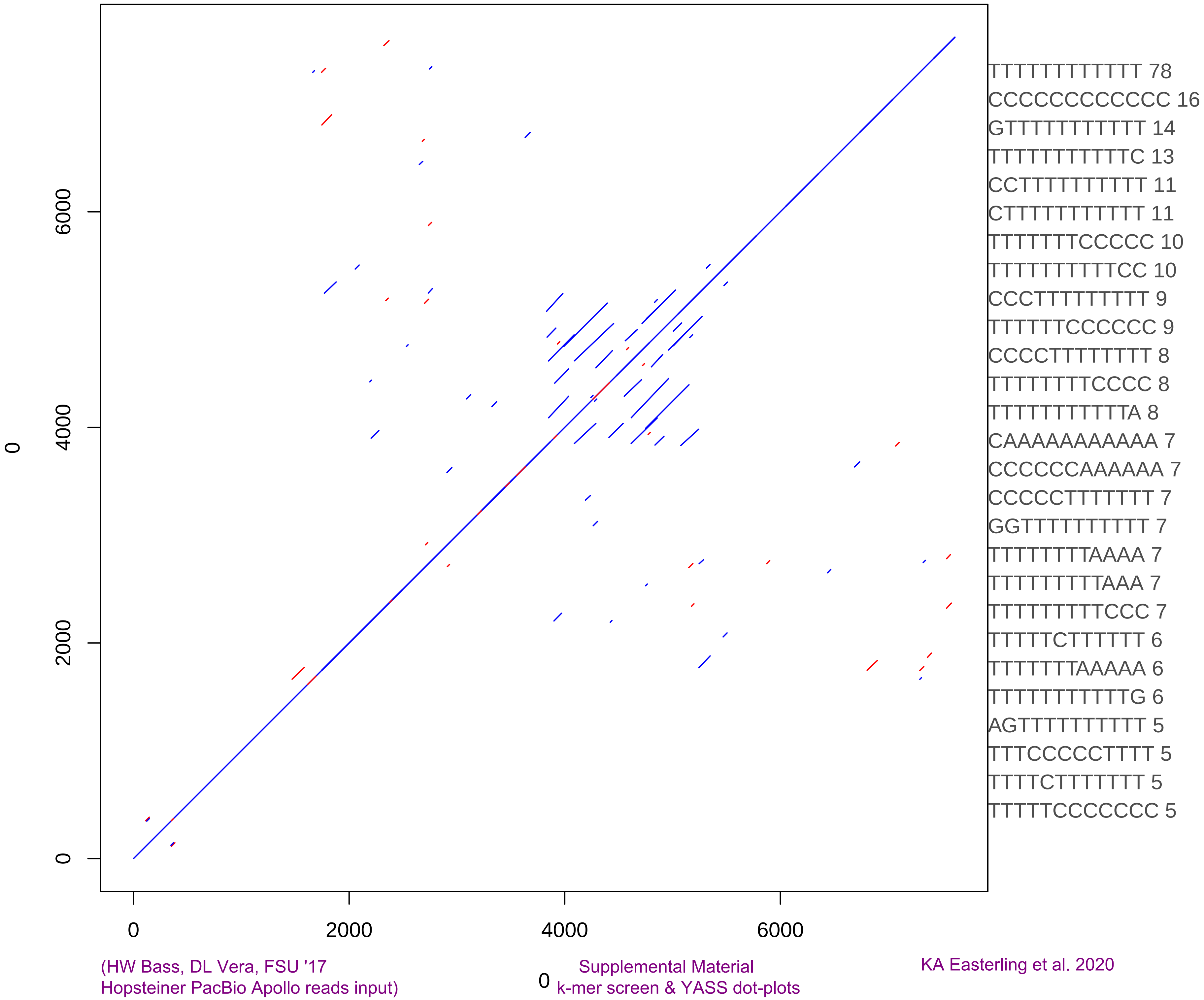

>m141126_012232_42134_c100732202550000001823141405141566_s1_p0/48349/6624_13203

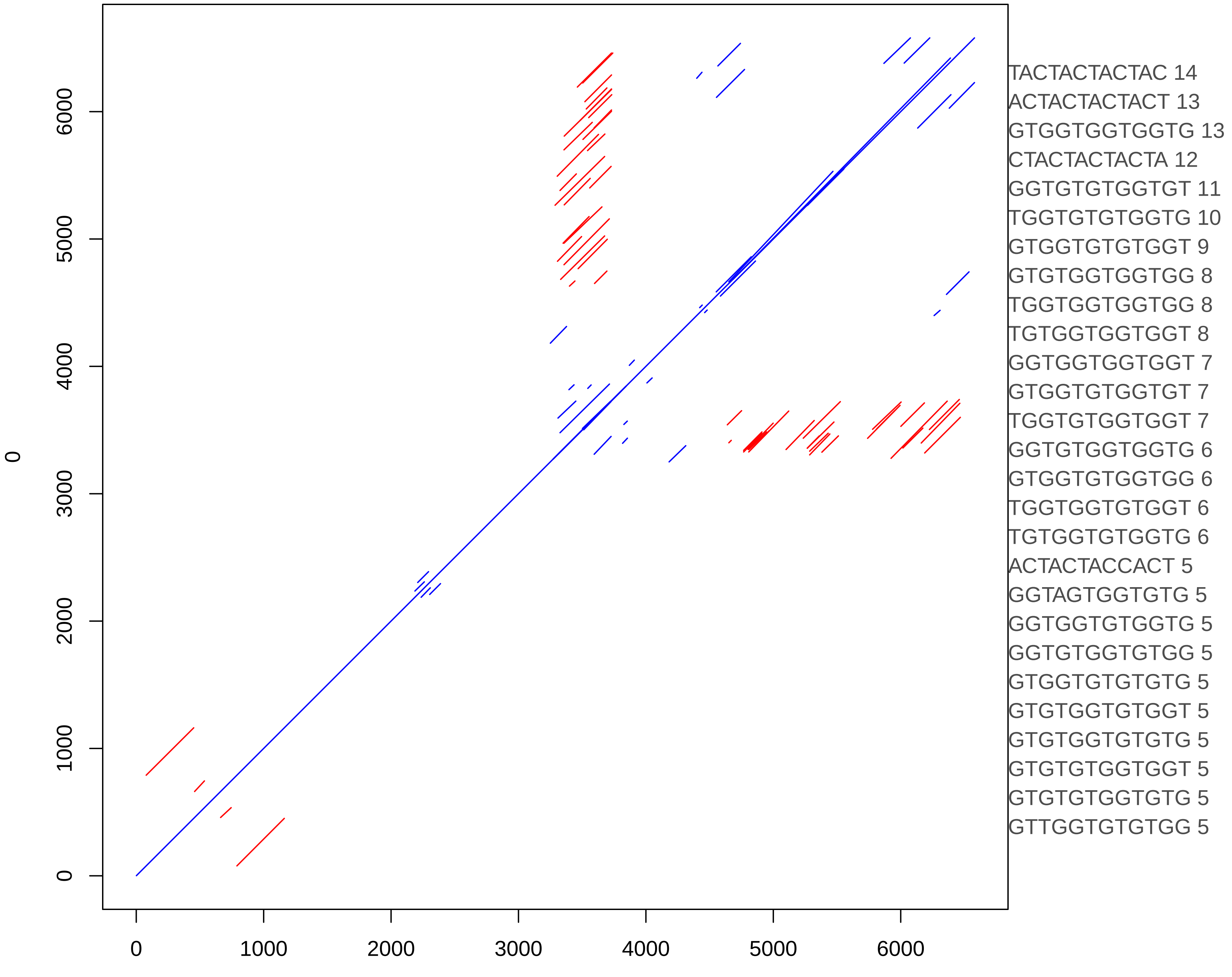

>m141126_012232_42134_c100732202550000001823141405141566_s1_p0/5026/0_8364

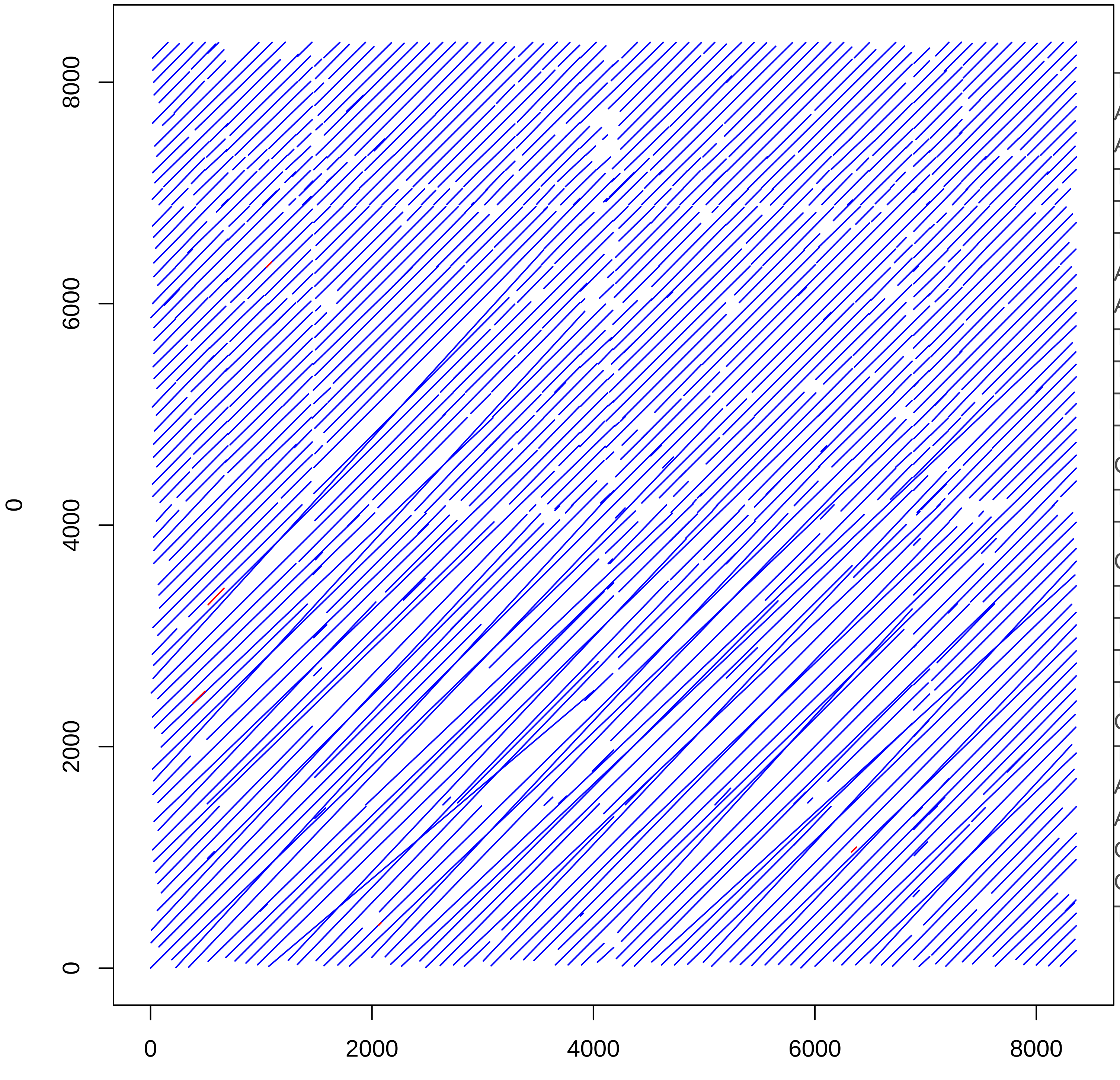

TTTATTGATCGT 35
AATTTATTGATC 33
ATTTATTGATCG 33
TAAATTTATTGA 28
TTATTGATCGTA 28
TATTGATCGTAG 27
AAATTTATTGAT 26
ATTGATCGTAGT 26
TTGATCGTAGTT 26
TTAAATTTATTG 25
TTAAGAGCTCGG 25
TTTAAGAGCTCG 25
CTTTAAGAGCTC 23
TCTTTAAGAGCT 22
TTCTTTAAGAGC 22
CTTCTTTAAGAG 21
TAAGAGCTCGGG 21
TGATCGTAGTTC 21
TCTTCTTTAAGA 20
TTCTTCTTTAAG 20
GATCGTAGTTCT 19
TAACTTAAATTT 19
AGAGCTCGGGAT 18
AGTTCTTCTTTA 18
GTAGTTCTTCTT 18
GTTCTTCTTTAA 18
TTAACTTAAATT 18

>m141126_012232_42134_c100732202550000001823141405141566_s1_p0/5485/5482_15149

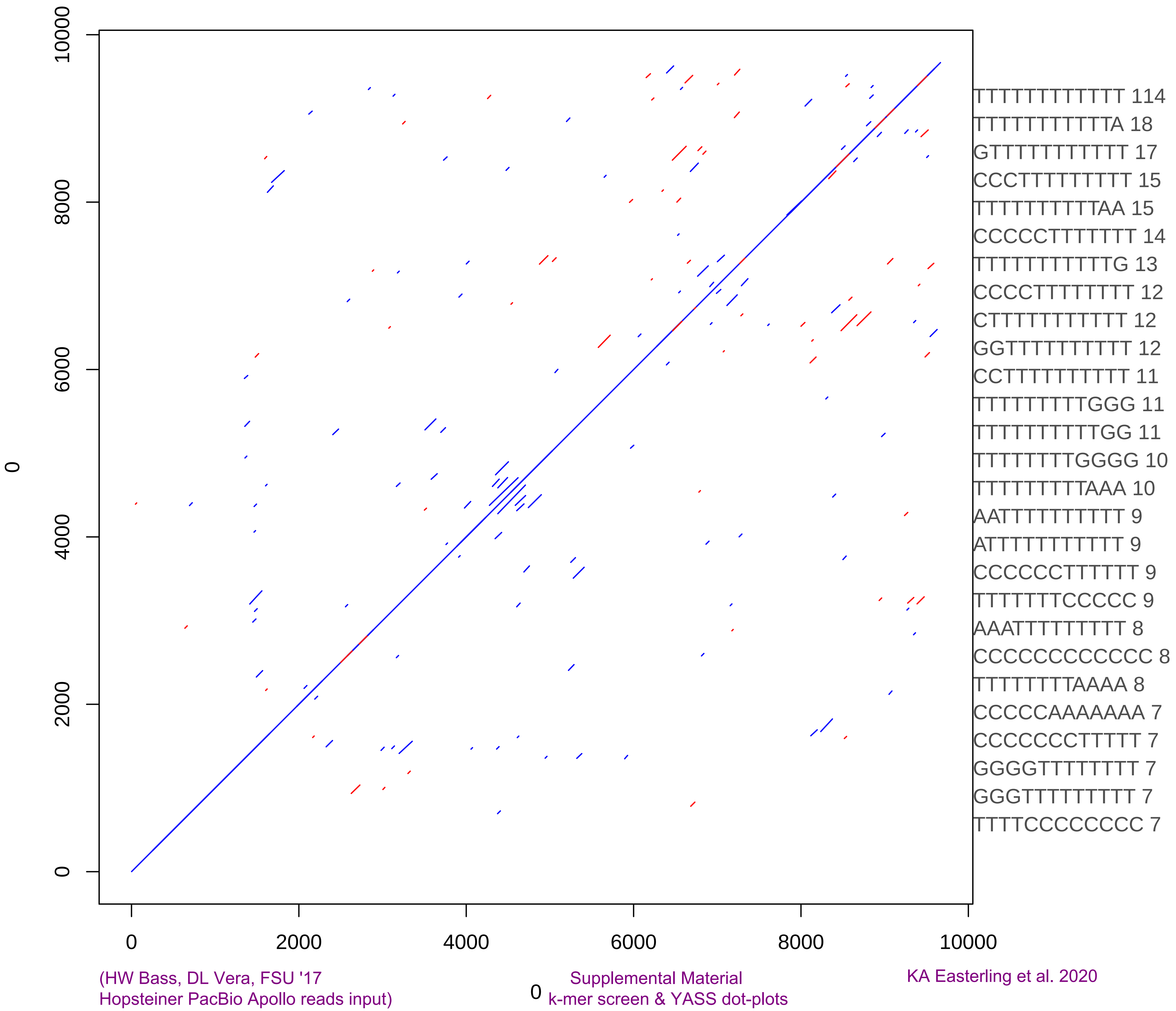

>m141126_012232_42134_c100732202550000001823141405141566_s1_p0/57791/11132_17723

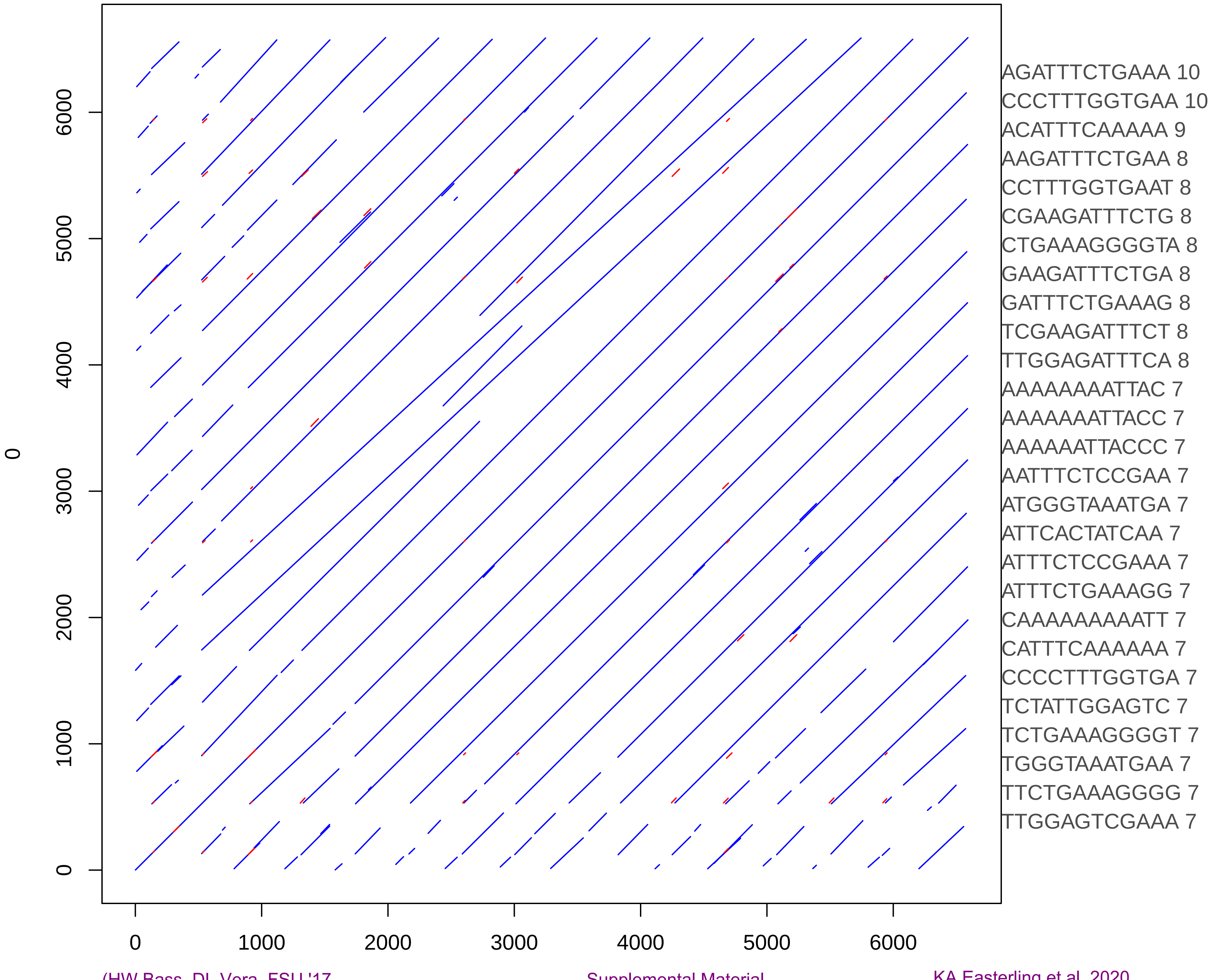

0

>m141126_012232_42134_c100732202550000001823141405141566_s1_p0/60404/0_12697

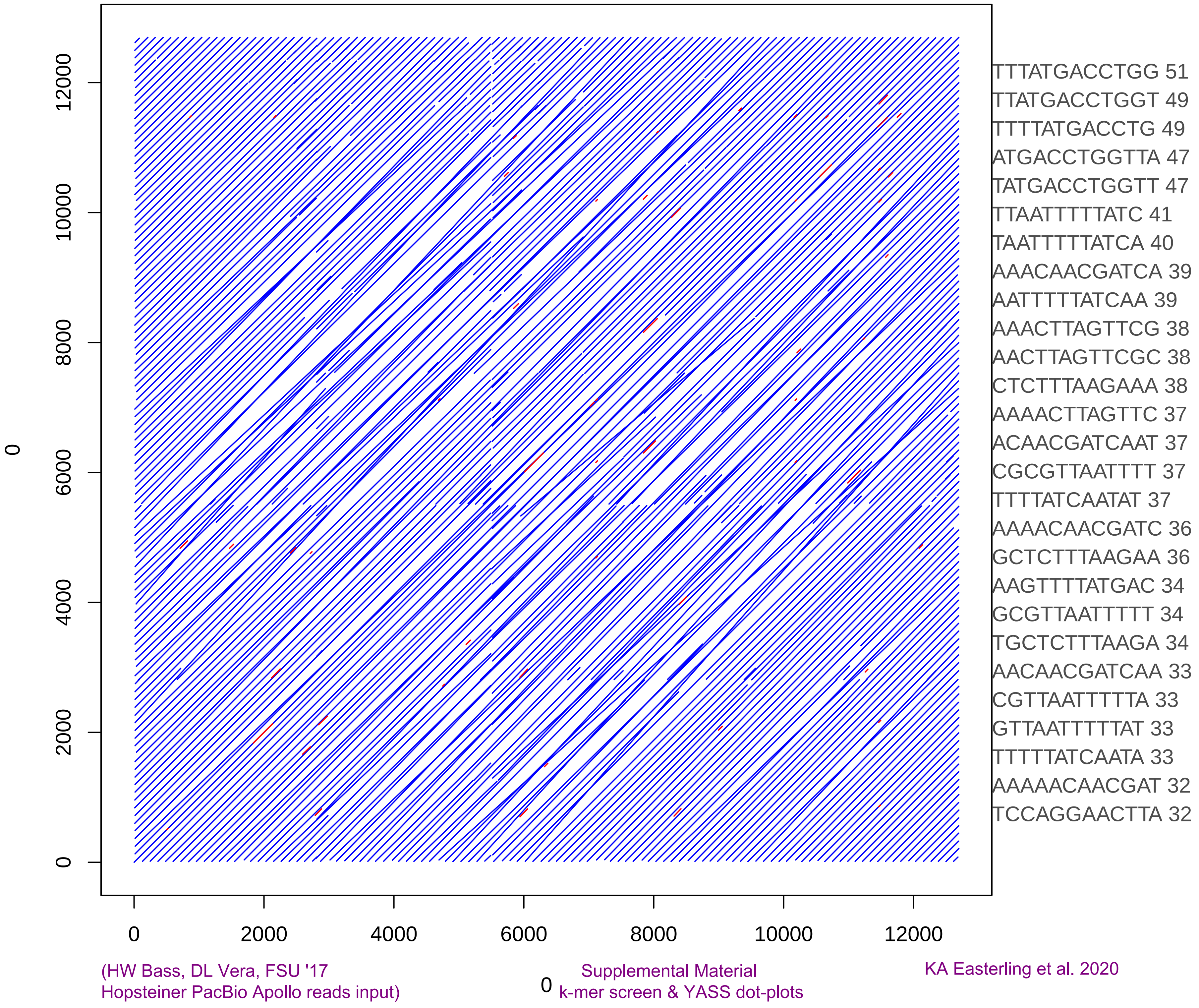

>m141126_012232_42134_c100732202550000001823141405141566_s1_p0/6082/0_5455

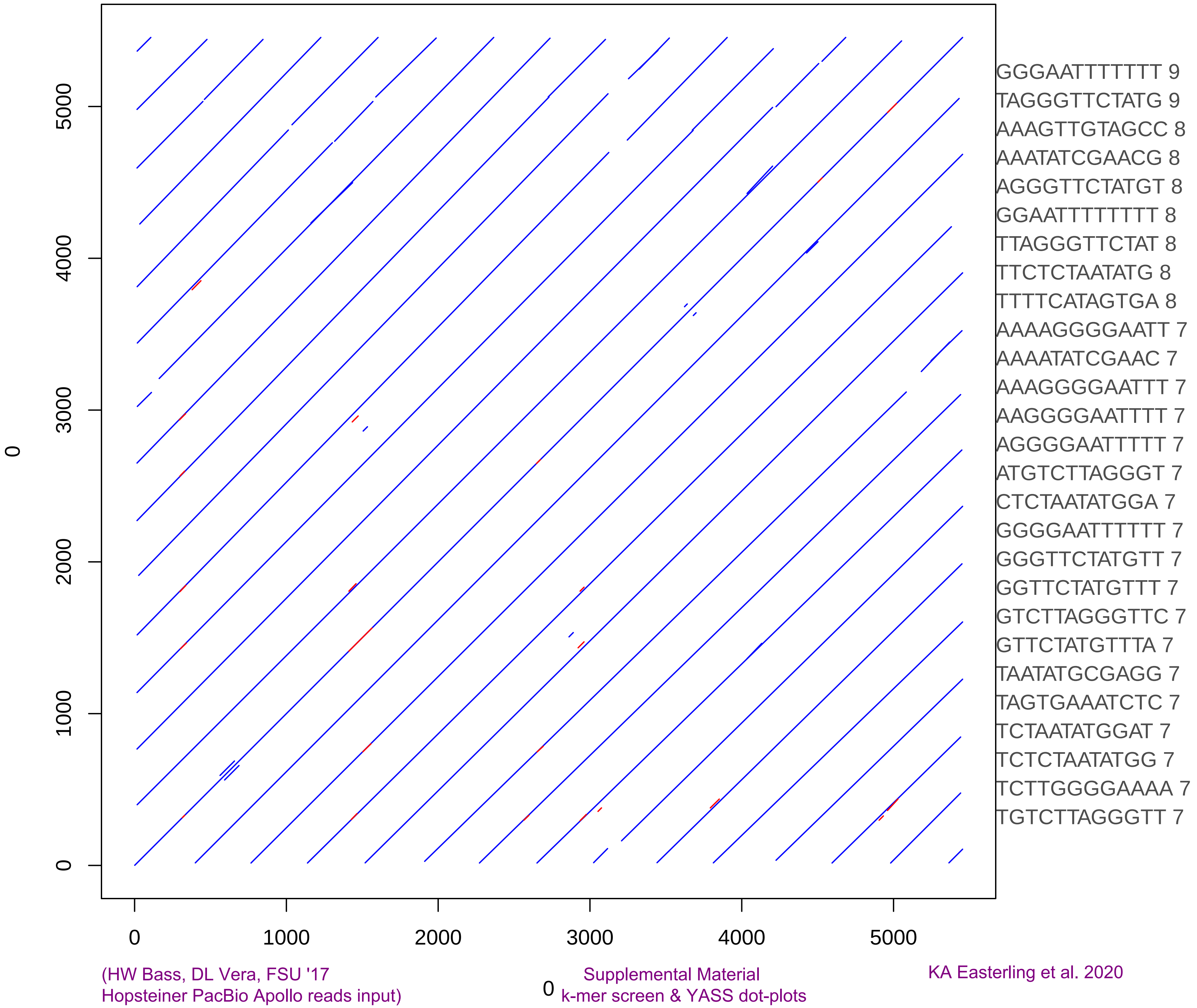

>m141126_012232_42134_c100732202550000001823141405141566_s1_p0/6082/5498_10809

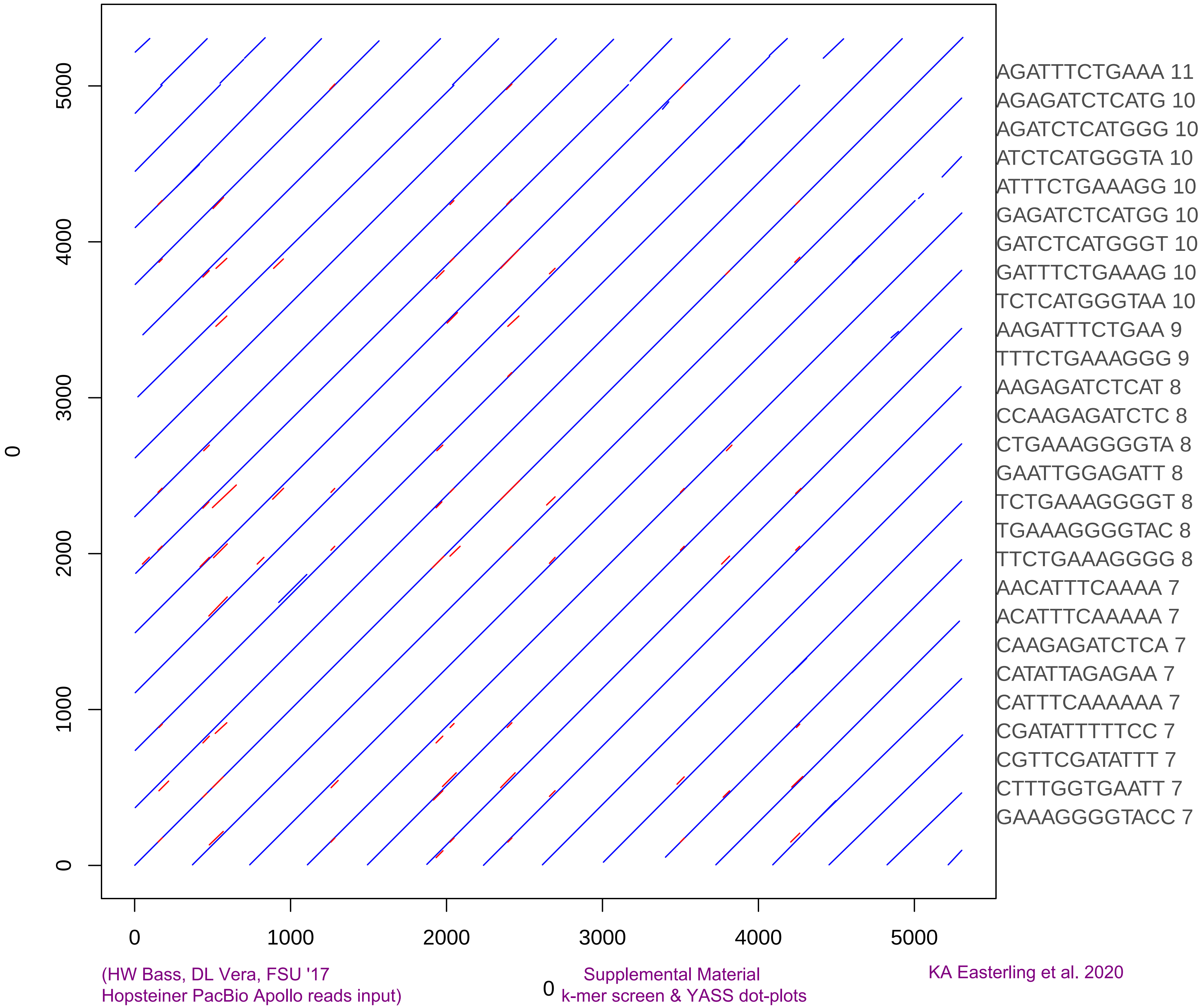

>m141126_012232_42134_c100732202550000001823141405141566_s1_p0/6319/7575_14719

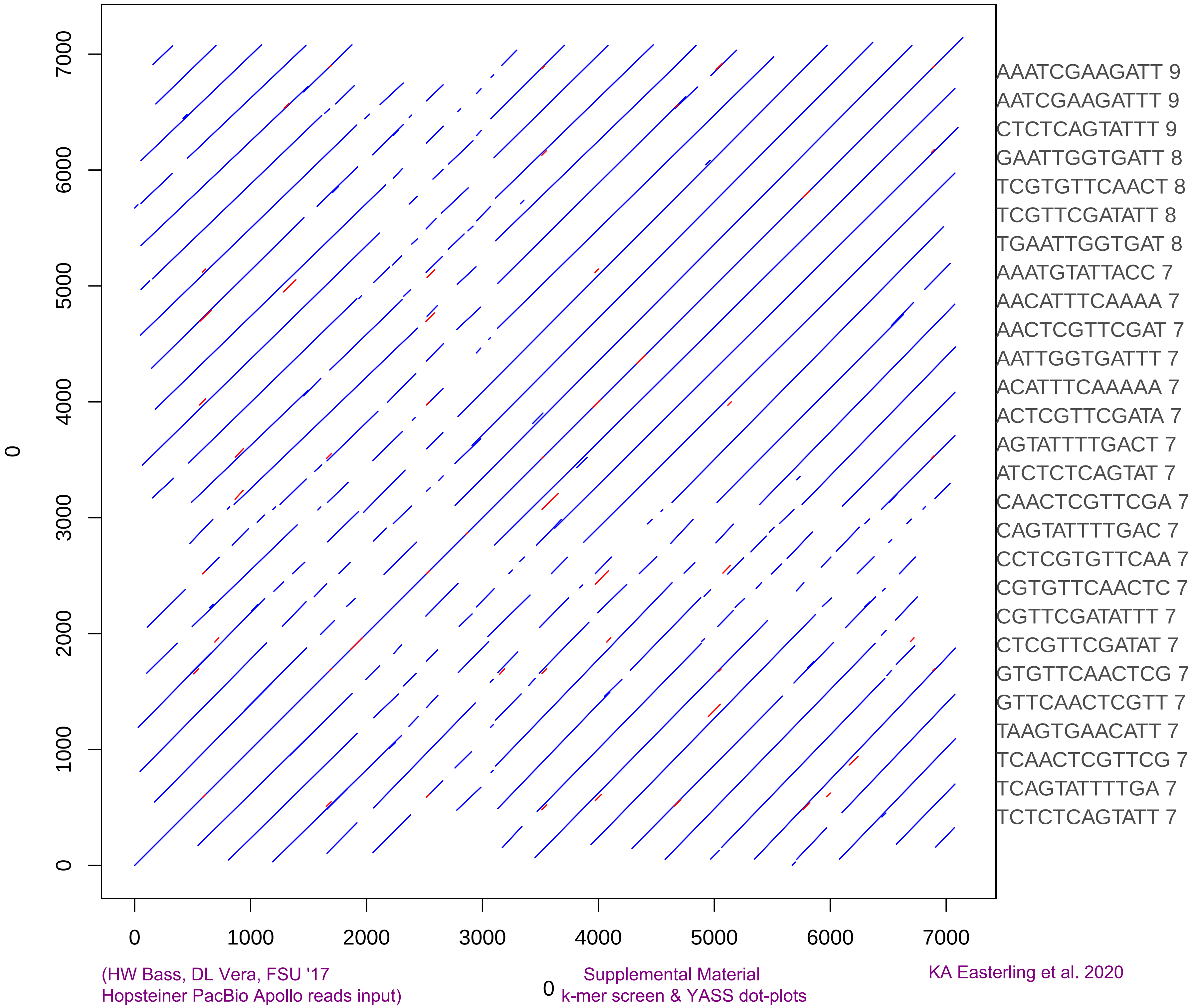

>m141126_012232_42134_c100732202550000001823141405141566_s1_p0/64051/0_7086

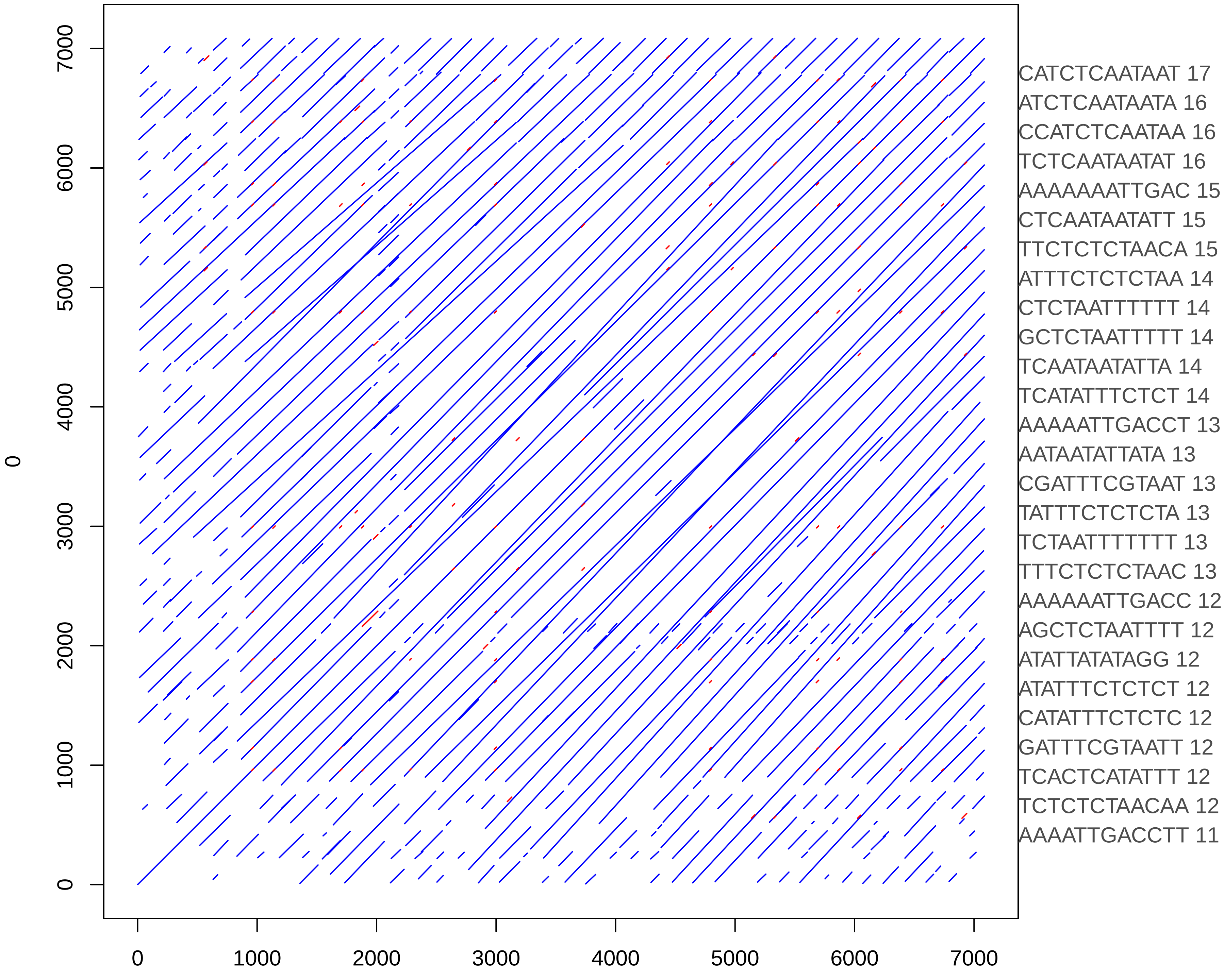

>m141126_012232_42134_c100732202550000001823141405141566_s1_p0/64861/3055_8215

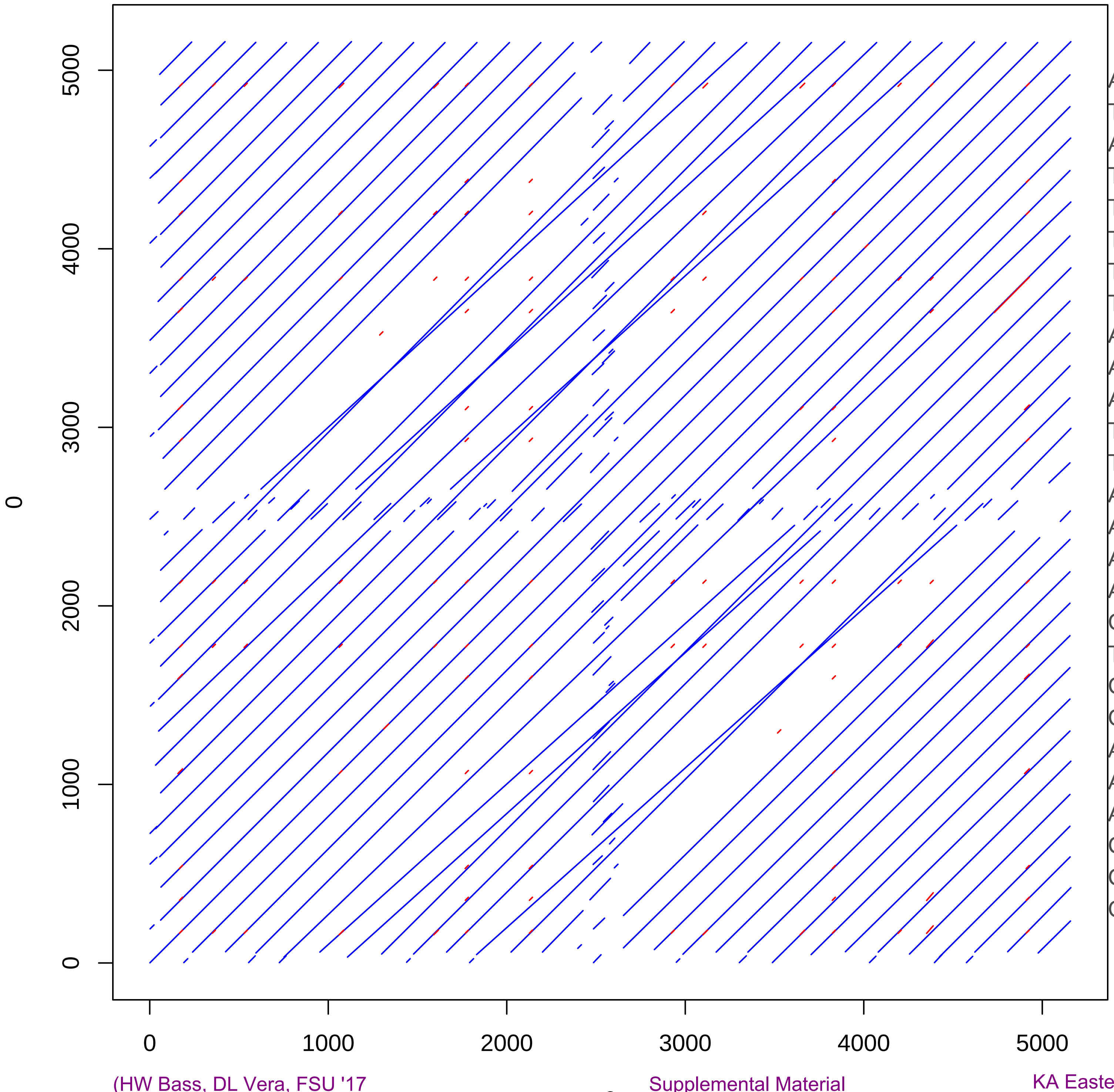

>m141126_012232_42134_c100732202550000001823141405141566_s1_p0/65964/5952_13137

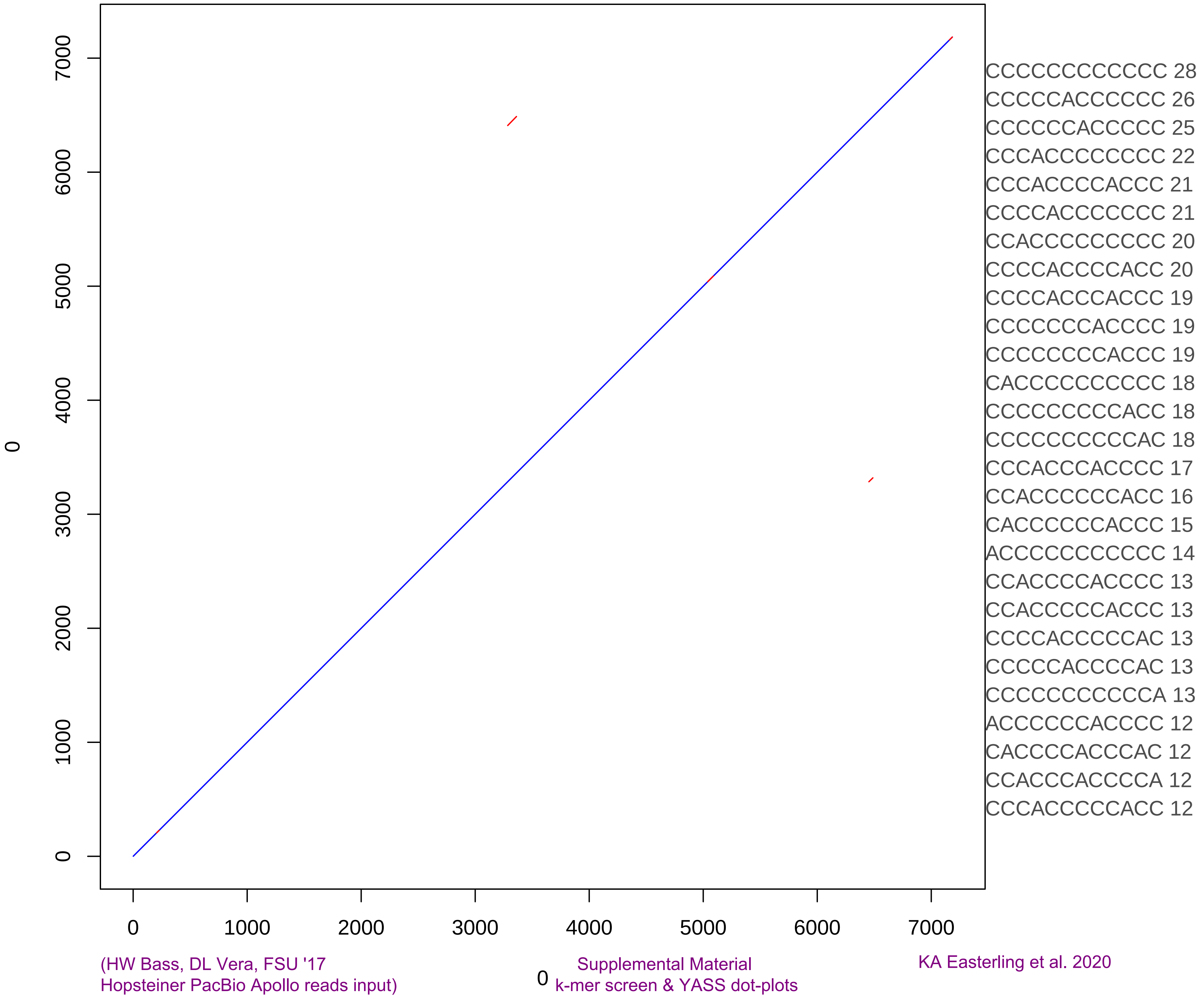

>m141126_012232_42134_c100732202550000001823141405141566_s1_p0/67884/4578_17315

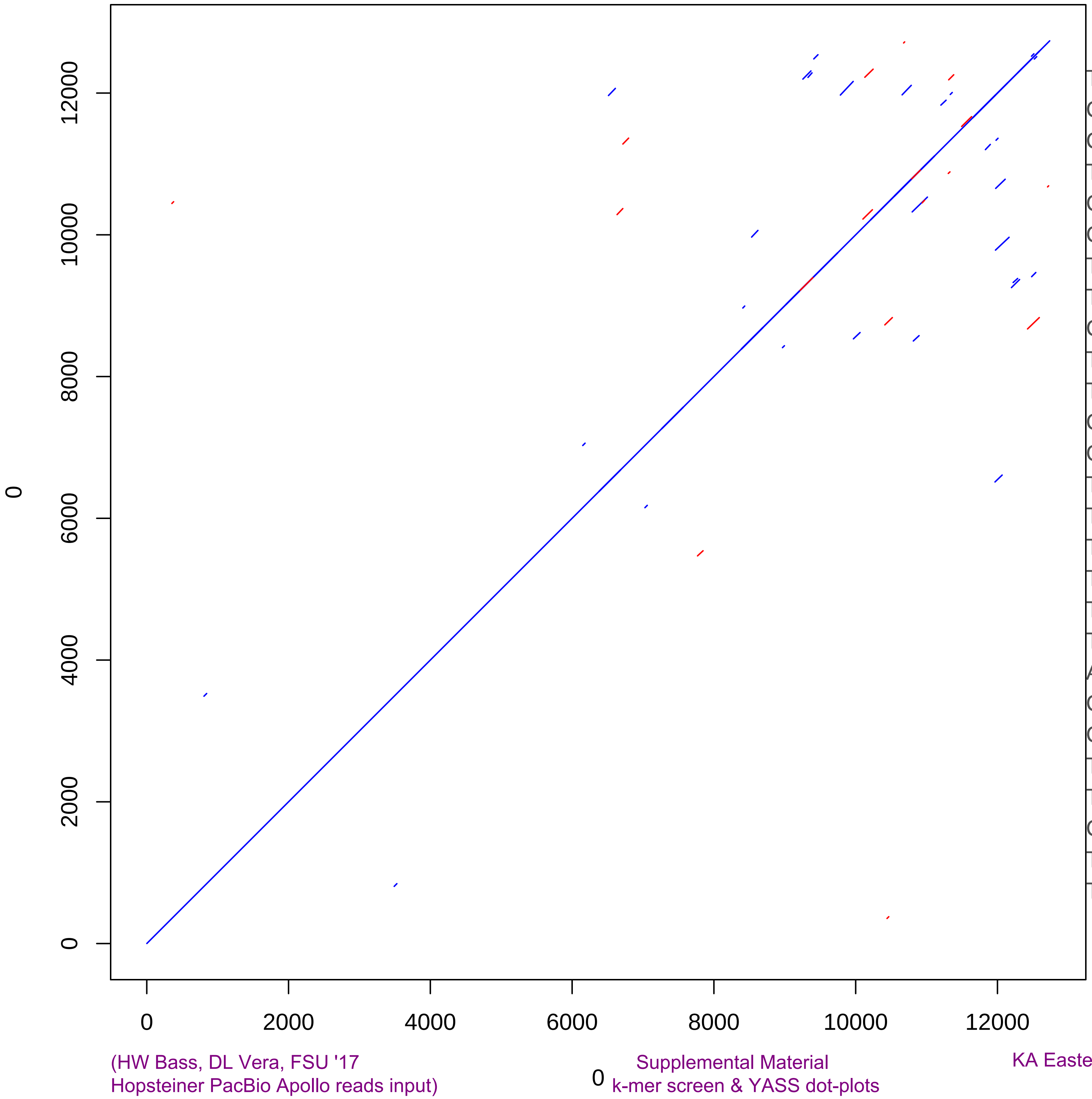

TTTTTTTTTTTT 91
CTTTTTTTTTTT 15
CCCCCCCCCCCC 14
TTTTTTTTTTTC 14
CCCCCCTTTTTT 13
CCTTTTTTTTTT 12
TTTTTTTTCCCC 12
TTTTTTTTTTCC 12
CCCCCTTTTTTT 11
TTTGTTTTTTTT 11
TTTTTTTCCCCC 11
CCCCTTTTTTTT 10
GTTTTTTTTTTT 10
TTTTGTTTTTTT 10
TTTTTTCCCCCC 10
TTTTTTTTTCCC 10
TTTTTTTTTTGT 10
TTTTTTTTTTTG 10
TTTTTTTTTGTT 9
AAAAAAAAAAAC 8
CCCCAAAAAAAA 8
CCCTTTTTTTTT 8
TGTTTTTTTTTT 8
TTTTTGTTTTTT 8
CCCCCCCTTTTT 7
TCCCCCCCCCCC 7
TTGTTTTTTTTT 7

>m141126_012232_42134_c100732202550000001823141405141566_s1_p0/73377/0_5514

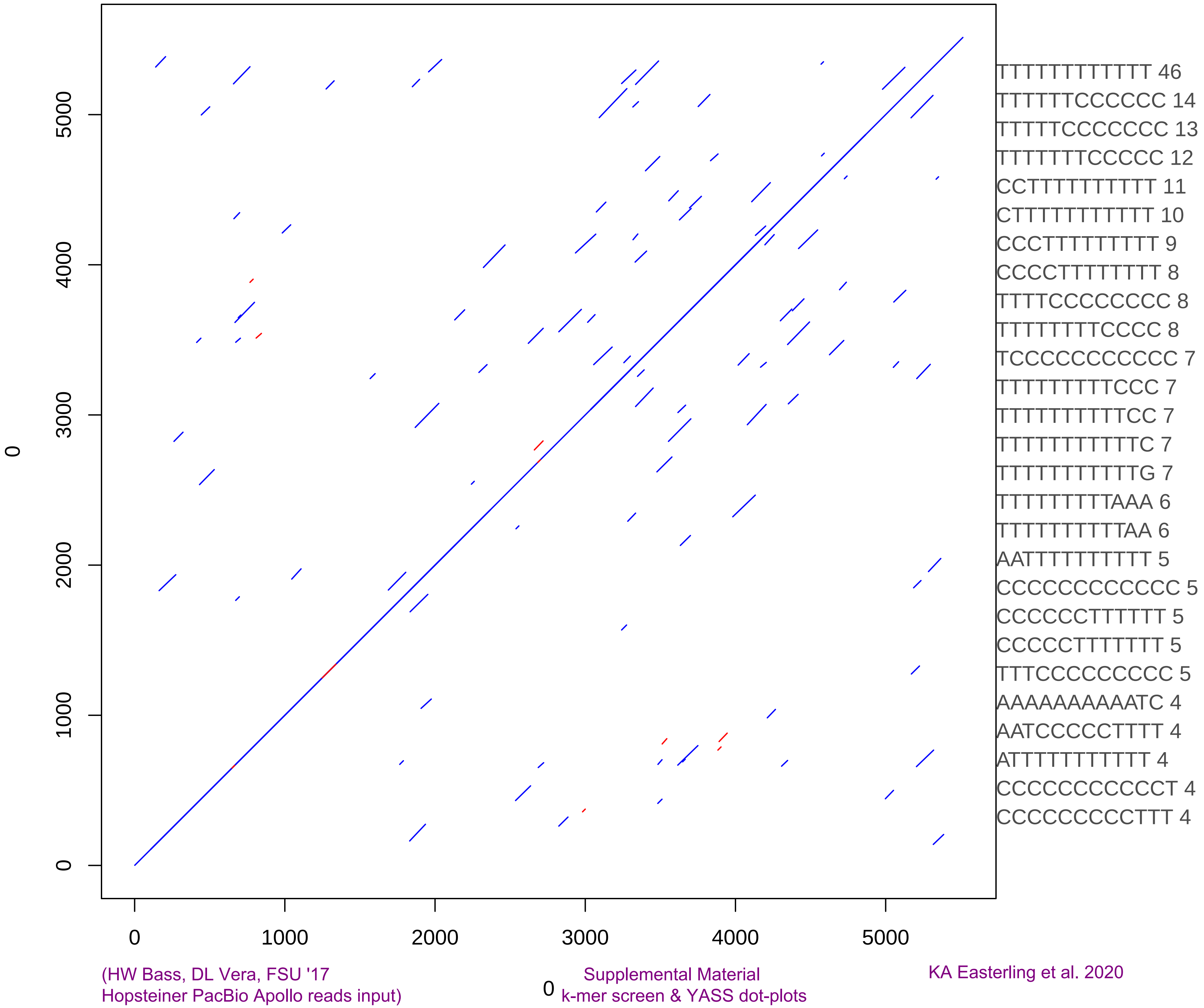

>m141126_012232_42134_c100732202550000001823141405141566_s1_p0/75741/0_14336

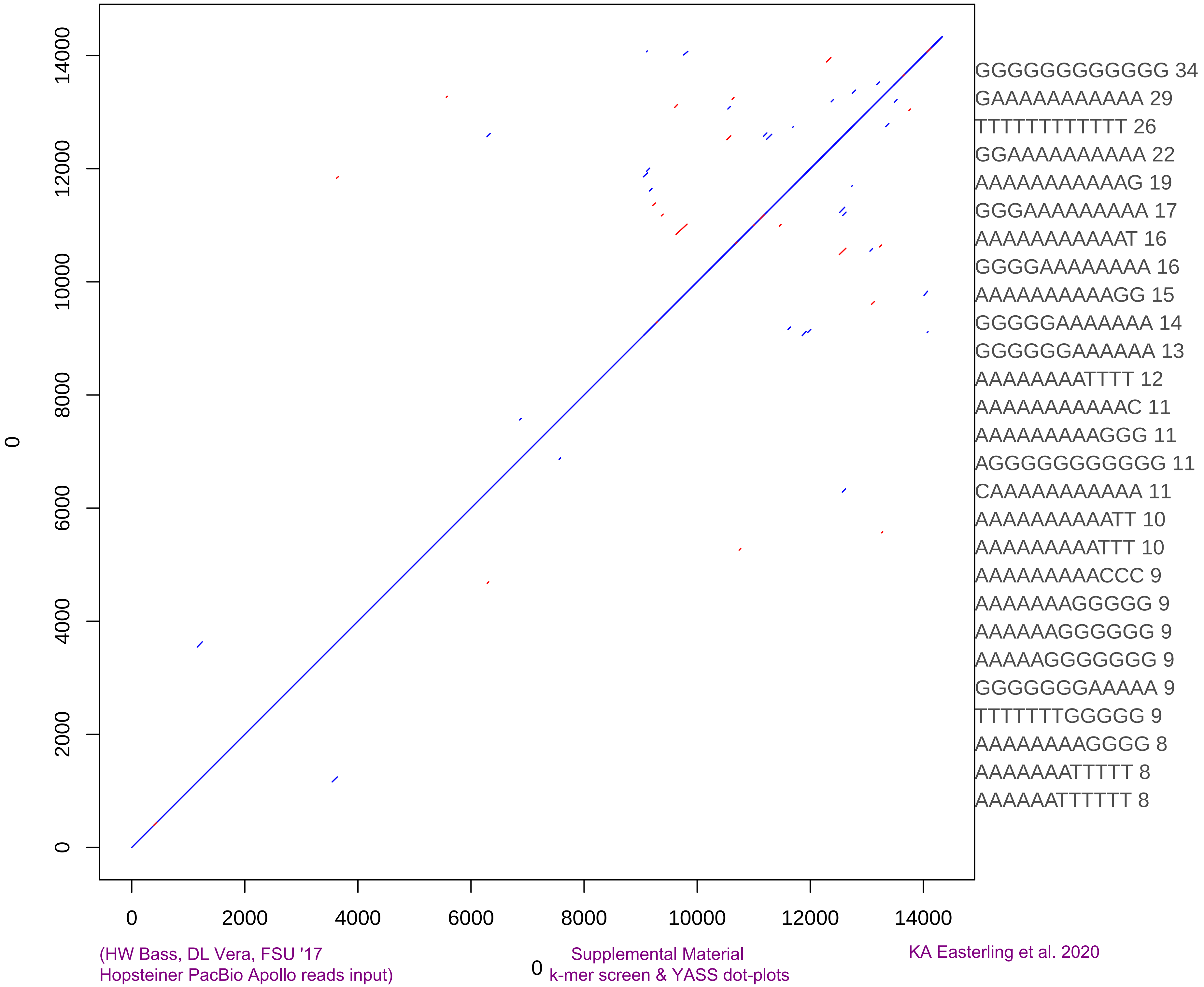

>m141126_012232_42134_c100732202550000001823141405141566_s1_p0/78825/4986_12382

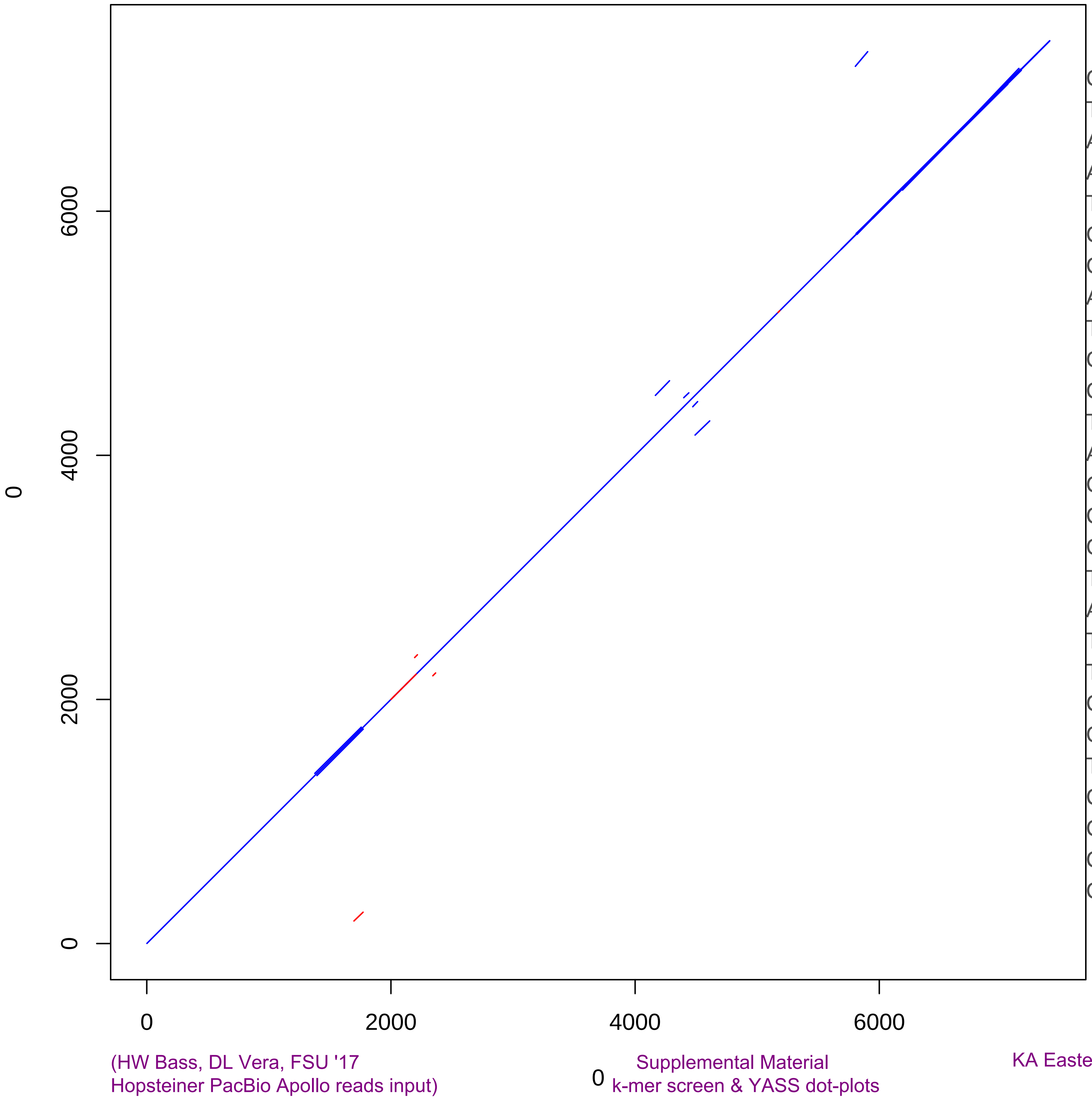

CTACTACTACTA 46
TACTACTACTAC 44
ACTACTACTACT 42
ACTACTGCTACT 32
TACTACTGCTAC 32
CTACTGCTACTA 31
CTACTACTGCTA 30
ACTGCTACTACT 29
TACTGCTACTAC 29
GCTACTACTGCT 21
CTGCTACTACTG 20
TGCTACTACTGC 20
ACTACTACTGCT 12
CTACTGCTACTG 12
CTGCTACTGCTA 12
GCTACTGCTACT 12
TACTGCTACTGC 12
ACTGCTACTGCT 11
TACTACTACTGC 11
TGCTACTGCTAC 11
CTGCTACTACTA 10
GCTACTACTACT 10
TGCTACTACTAC 10
CTACTACTACTG 9
CTAACTACTACT 6
CTACTAACTACT 6
CTACTACTACCT 6

>m141126_012232_42134_c100732202550000001823141405141566_s1_p0/82410/5796_16653

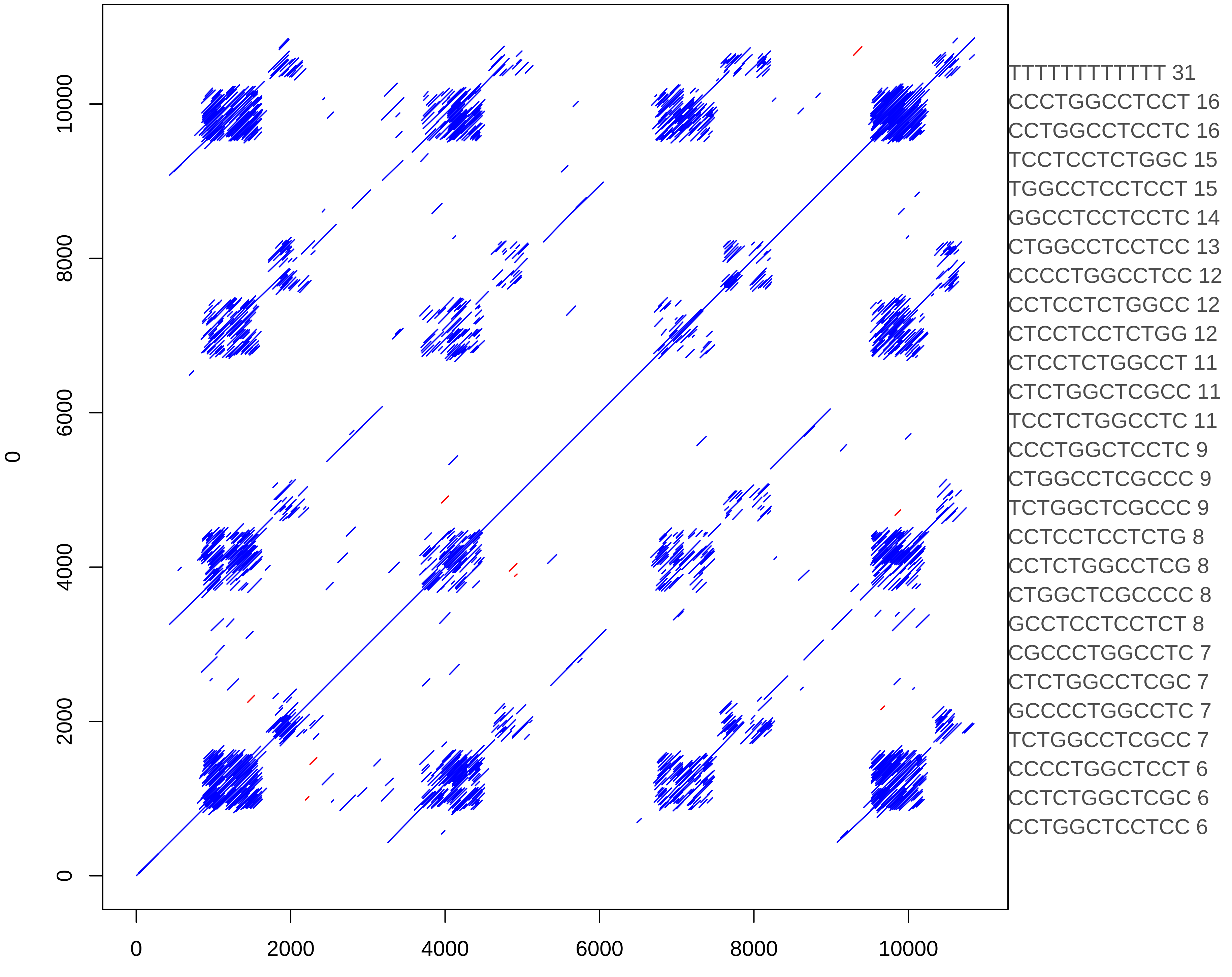

>m141126_012232_42134_c100732202550000001823141405141566_s1_p0/82412/480_13603

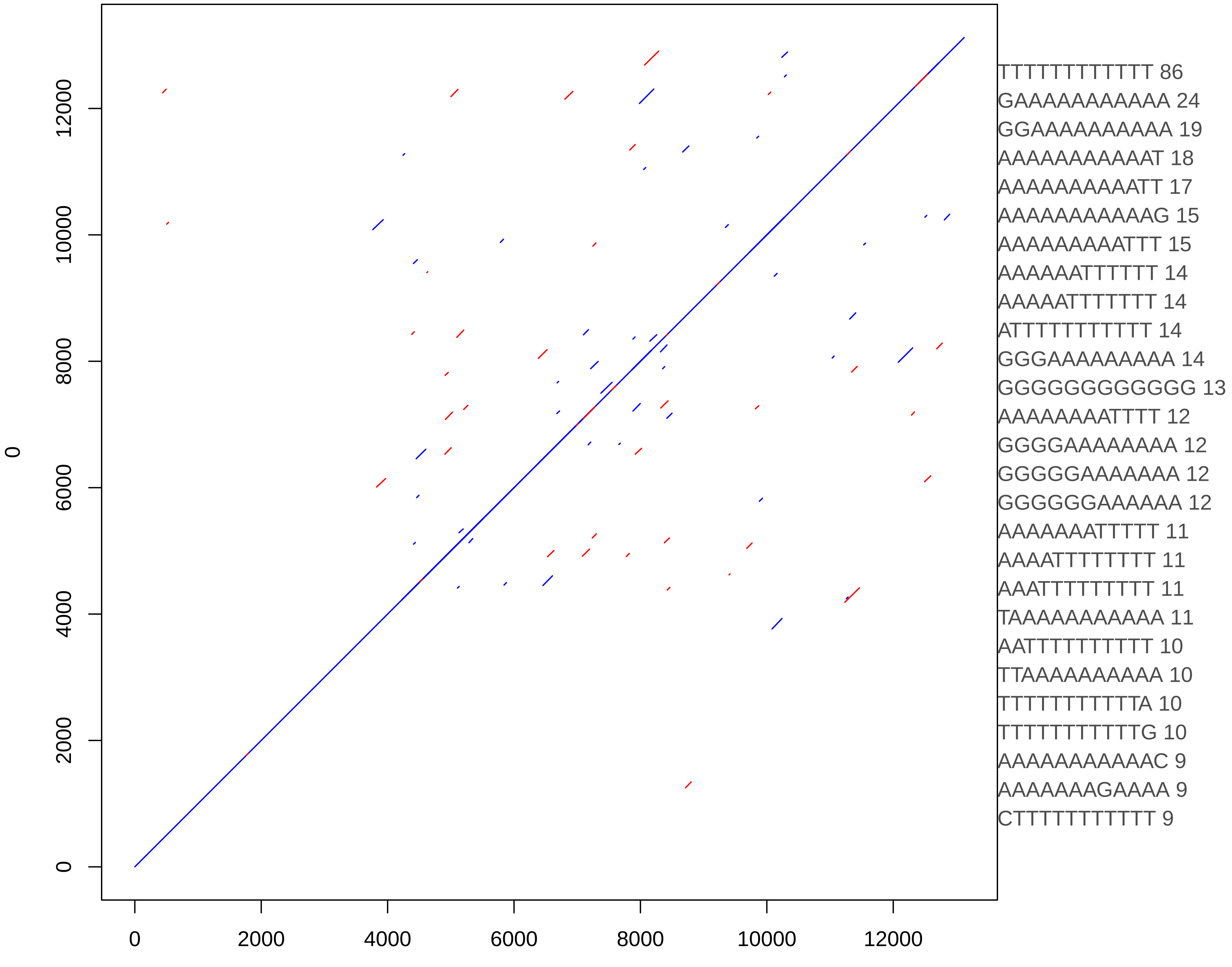

>m141126_012232_42134_c100732202550000001823141405141566_s1_p0/8436/896_7688

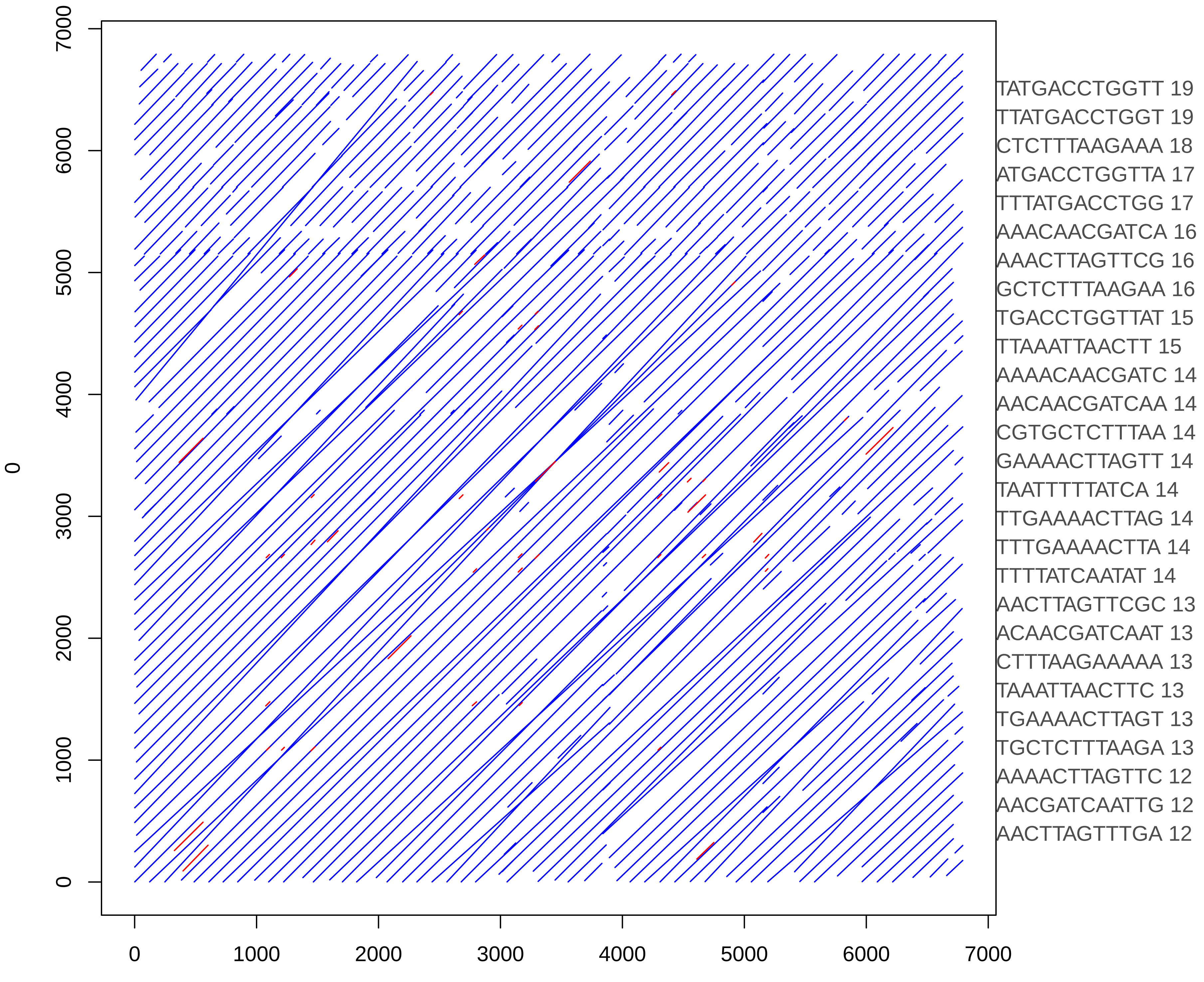

>m141126_012232_42134_c100732202550000001823141405141566_s1_p0/8613/9958_15804

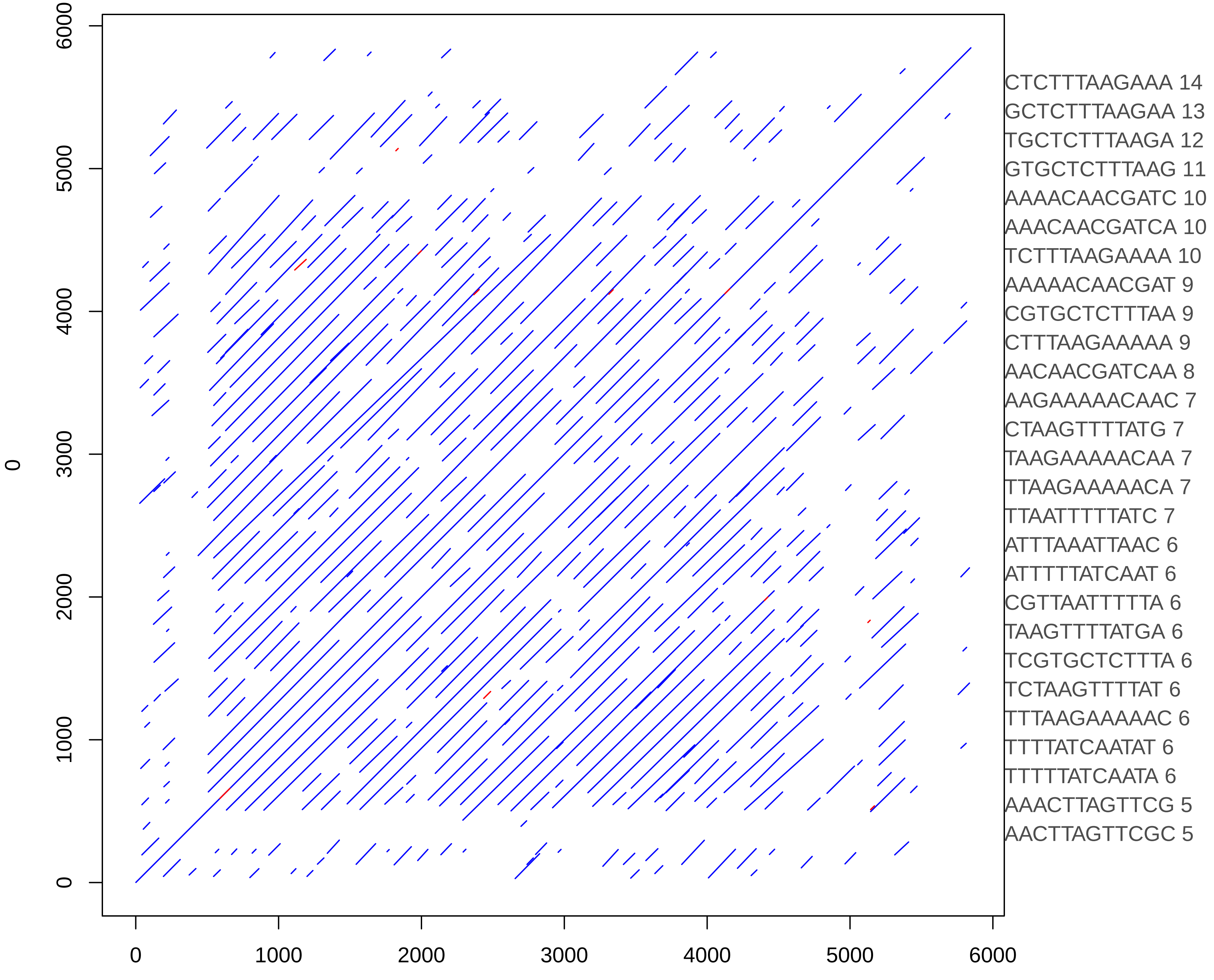

>m141126_012232_42134_c100732202550000001823141405141566_s1_p0/87132/6759_16534

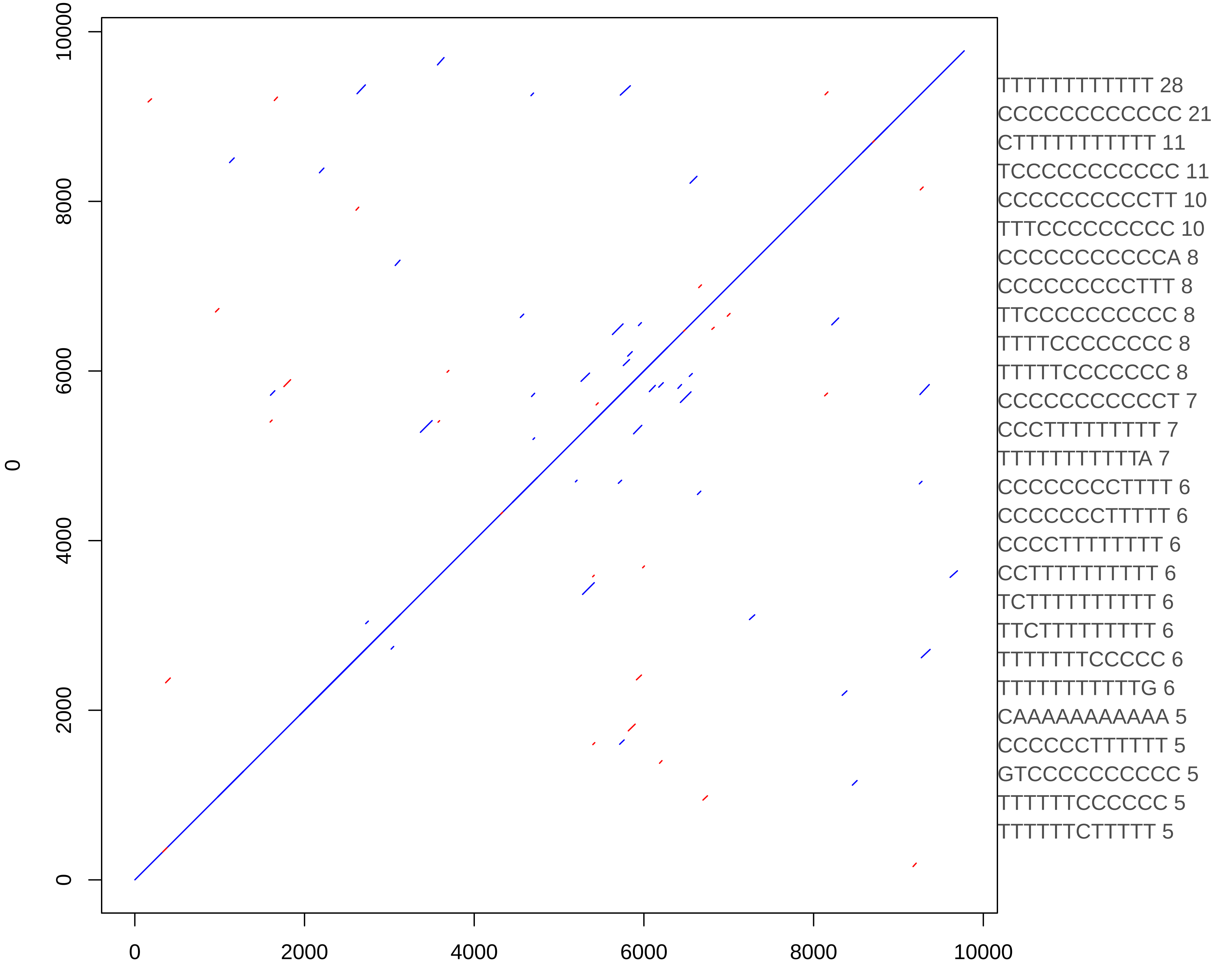

>m141126_012232_42134_c100732202550000001823141405141566_s1_p0/91064/2488_9727

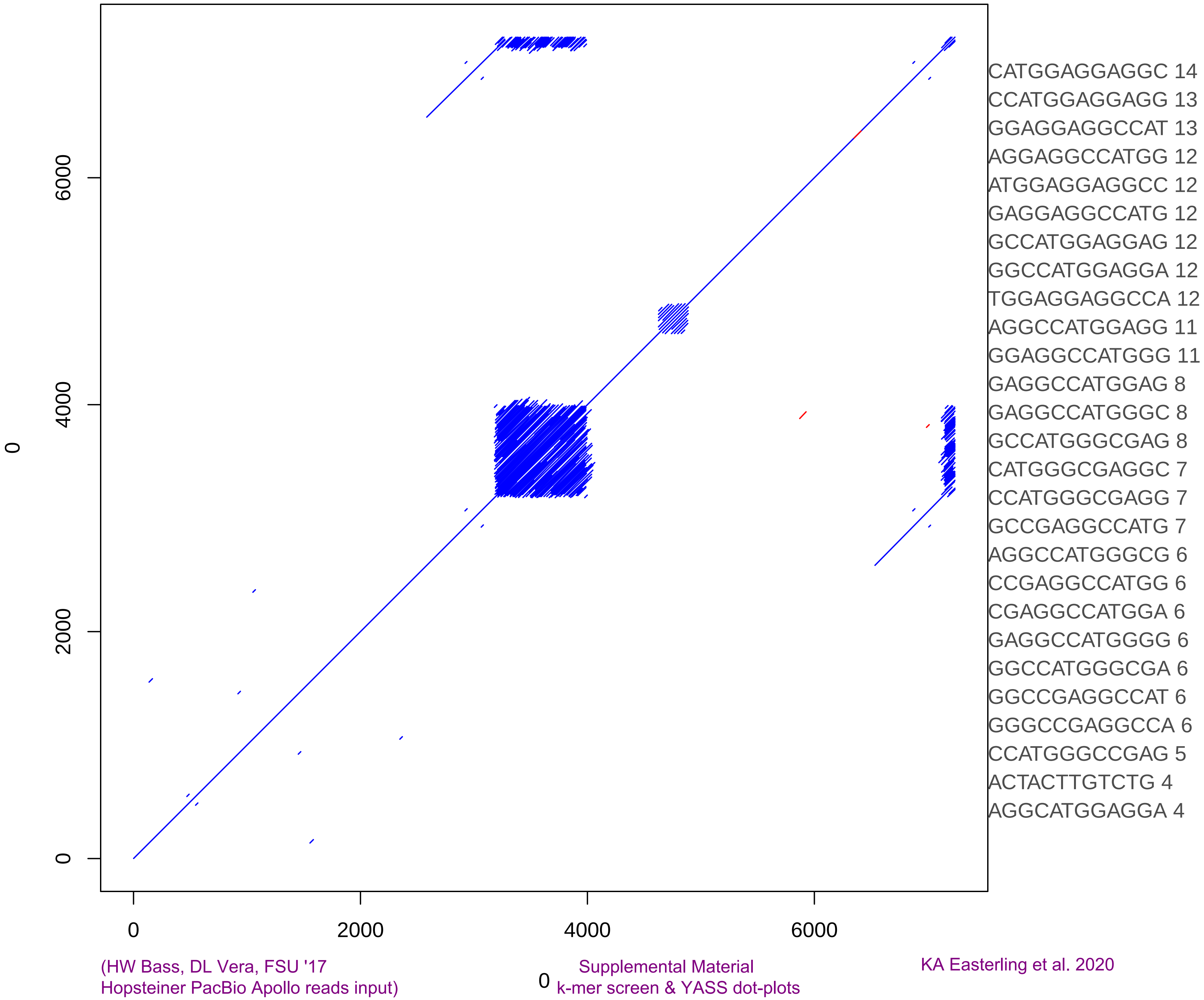

1,121 hits for repetitive sequences
passing 12mer abundance criterion

(Read-Number)
PAGE 496

For Tandem Repeat families of ### bp:
PacBio Read named "HuluTR###-r496"

>m141126_012232_42134_c100732202550000001823141405141566_s1_p0/949/0_6406

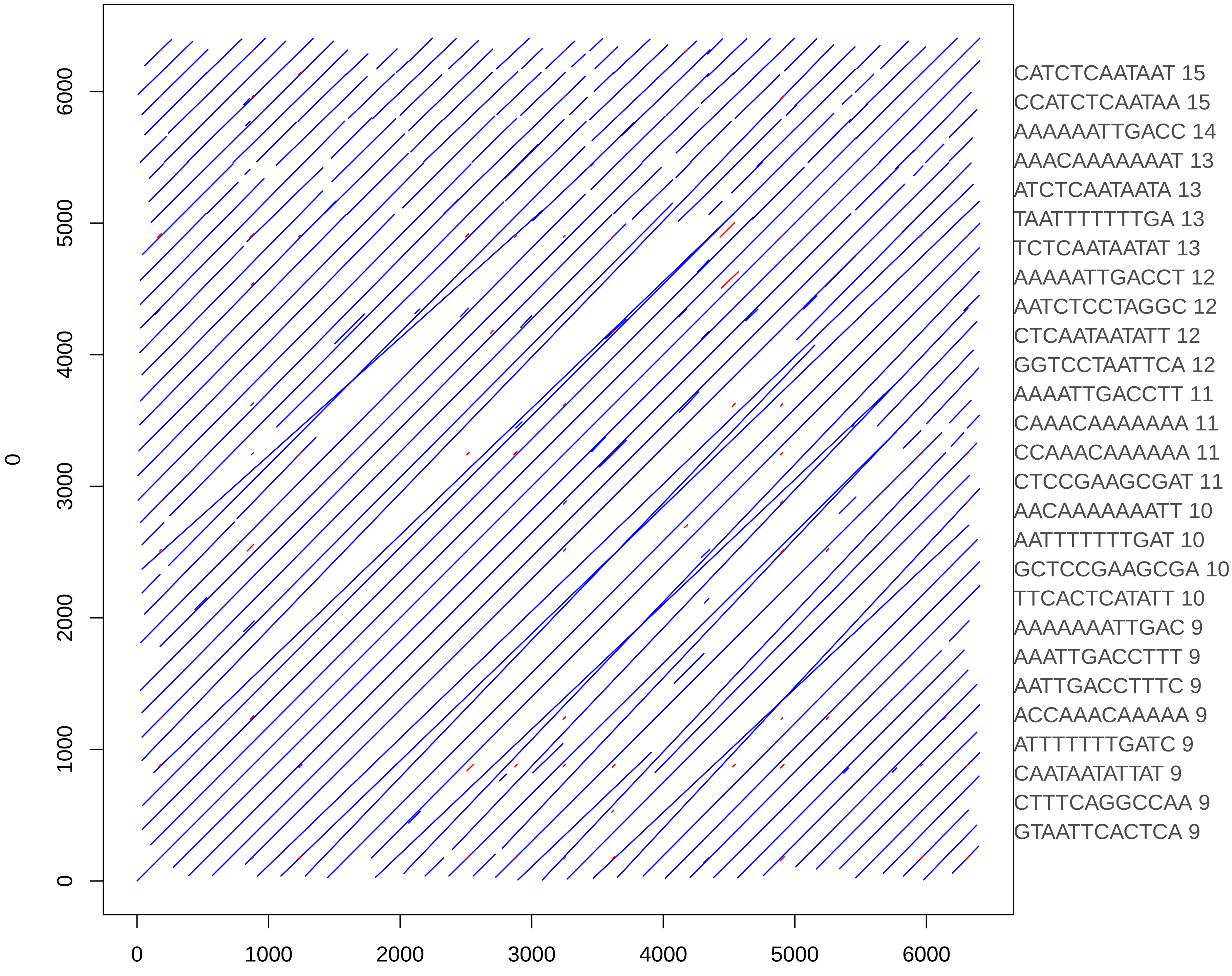

(HW Bass, DL Vera, FSU '17
Hopsteiner PacBio Apollo reads input)

0

Supplemental Material
k-mer screen & YASS dot-plots

KA Easterling et al. 2020

>m141126_012232_42134_c100732202550000001823141405141566_s1_p0/96874/0_8135

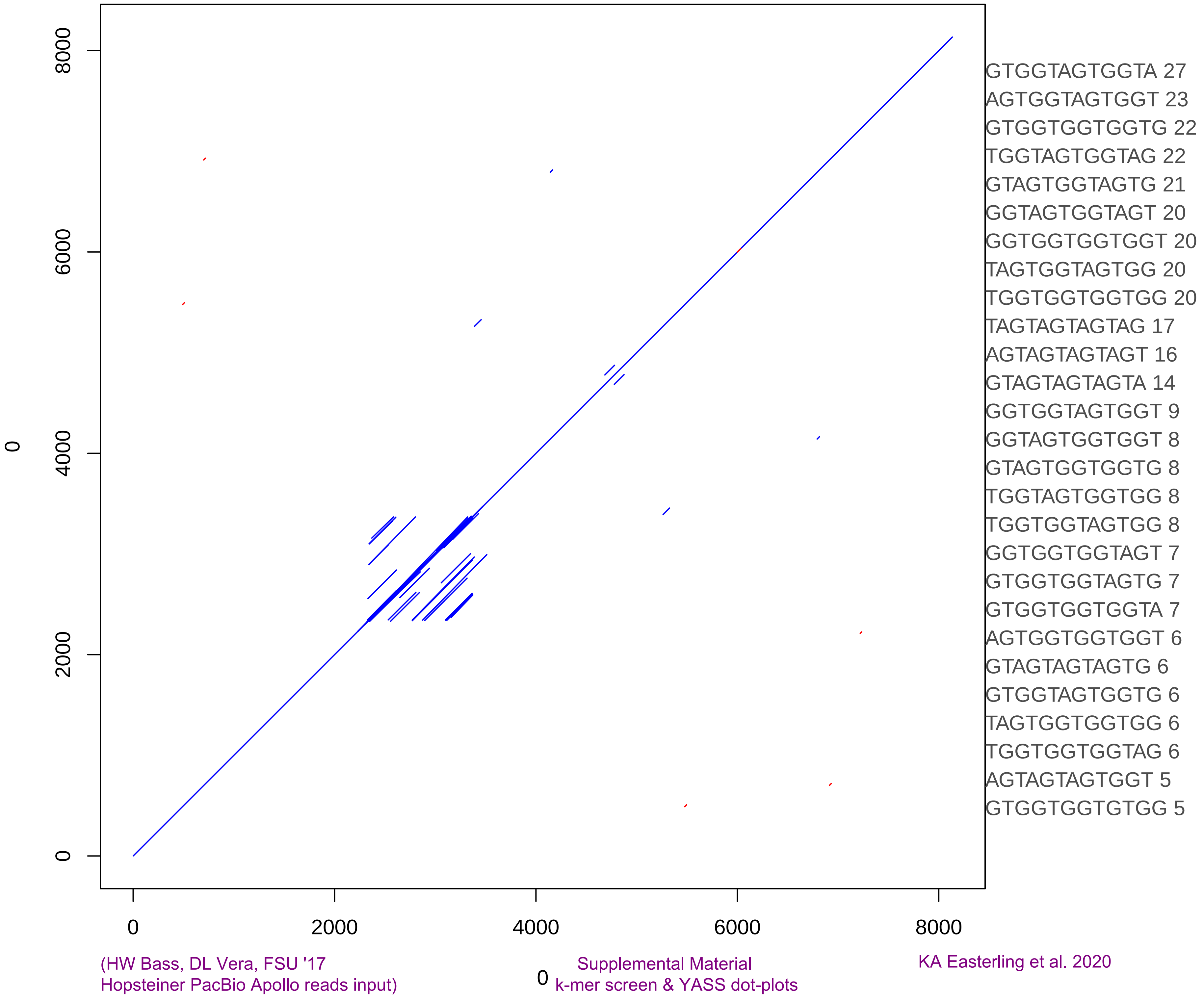

>m141126_034130_42134_c100732202550000001823141405141567_s1_p0/102292/0_6401

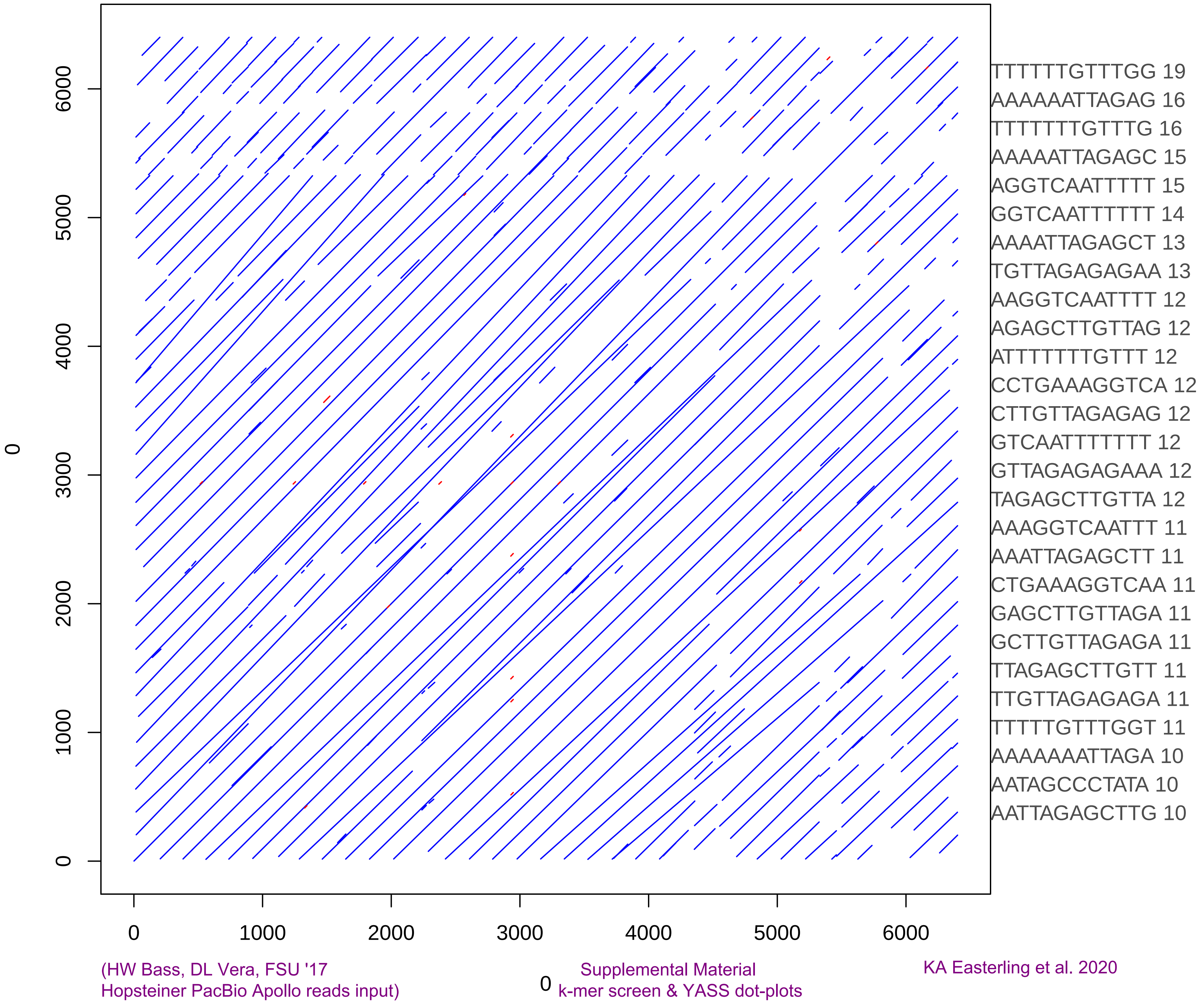

>m141126_034130_42134_c100732202550000001823141405141567_s1_p0/102395/0_13383

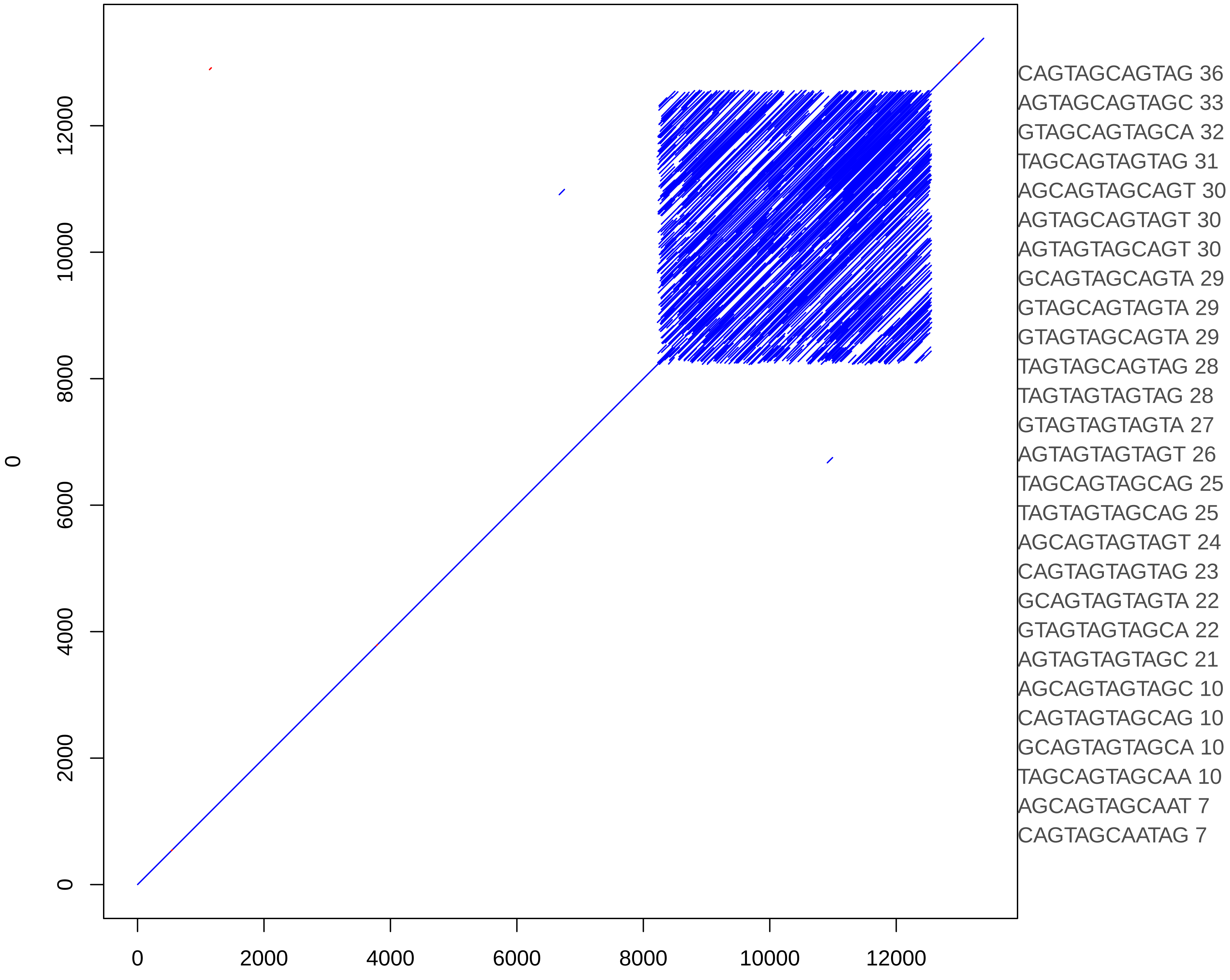

>m141126_034130_42134_c100732202550000001823141405141567_s1_p0/107084/3310_12337

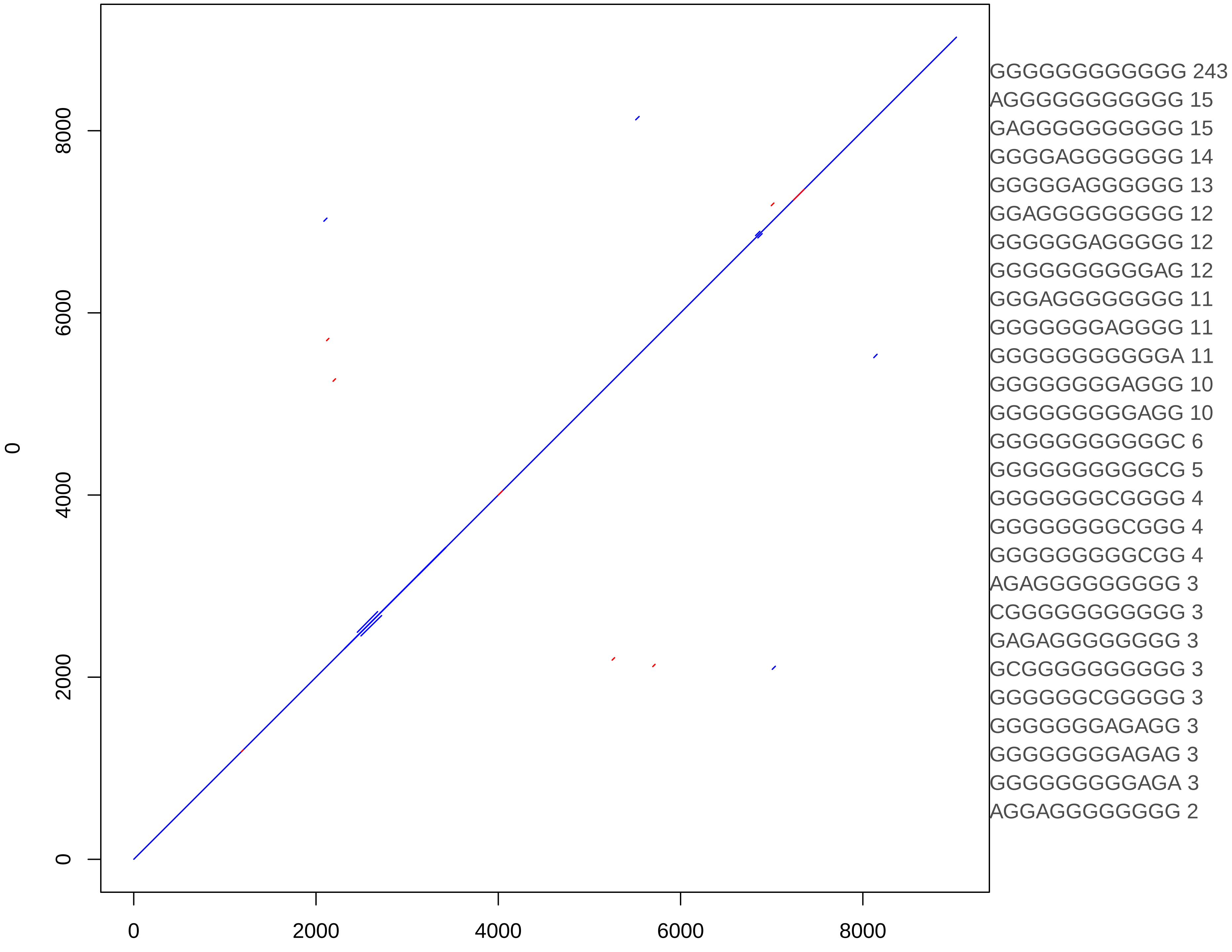

>m141126_034130_42134_c100732202550000001823141405141567_s1_p0/108924/7036_13540

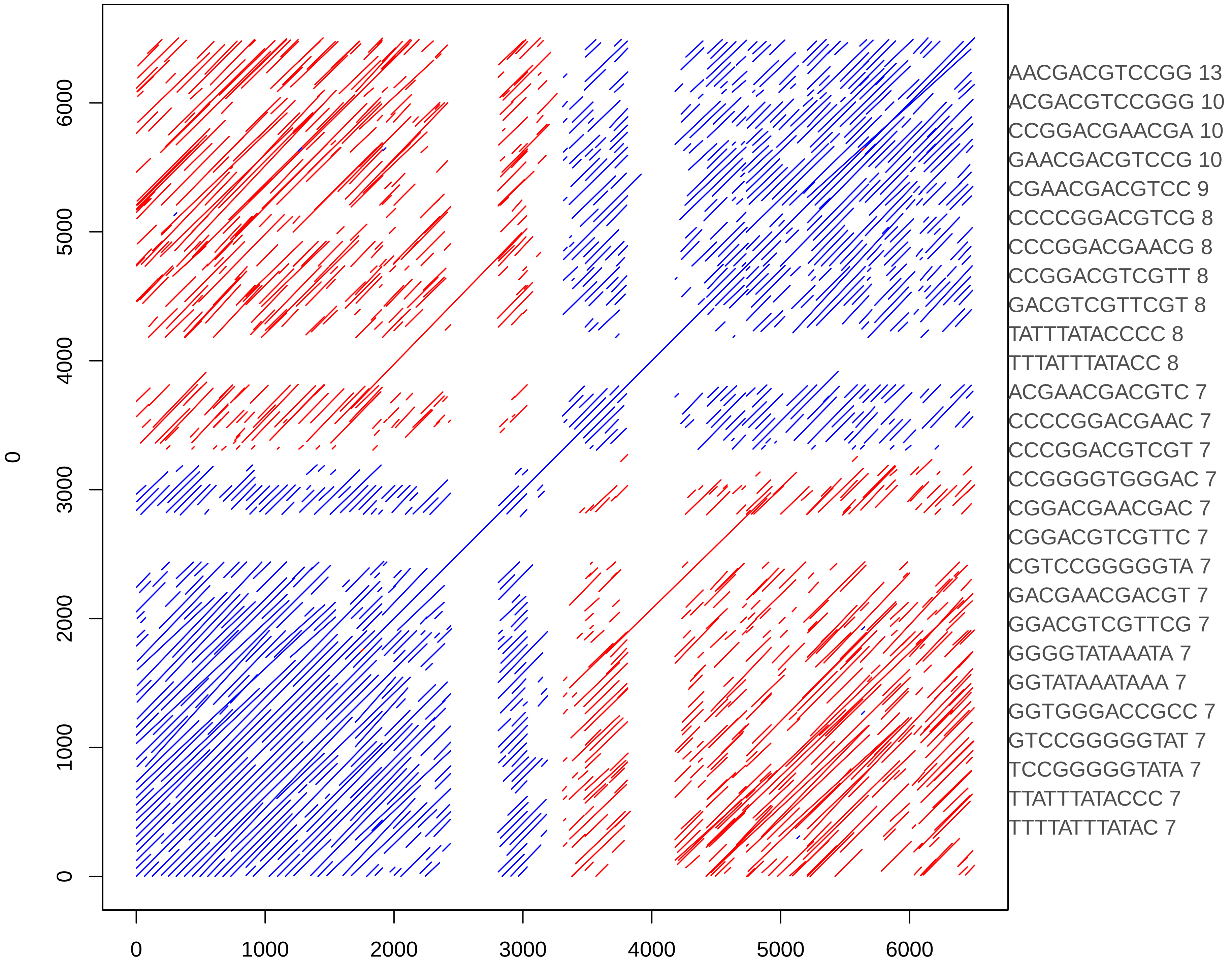

>m141126_034130_42134_c100732202550000001823141405141567_s1_p0/111270/7286_15968

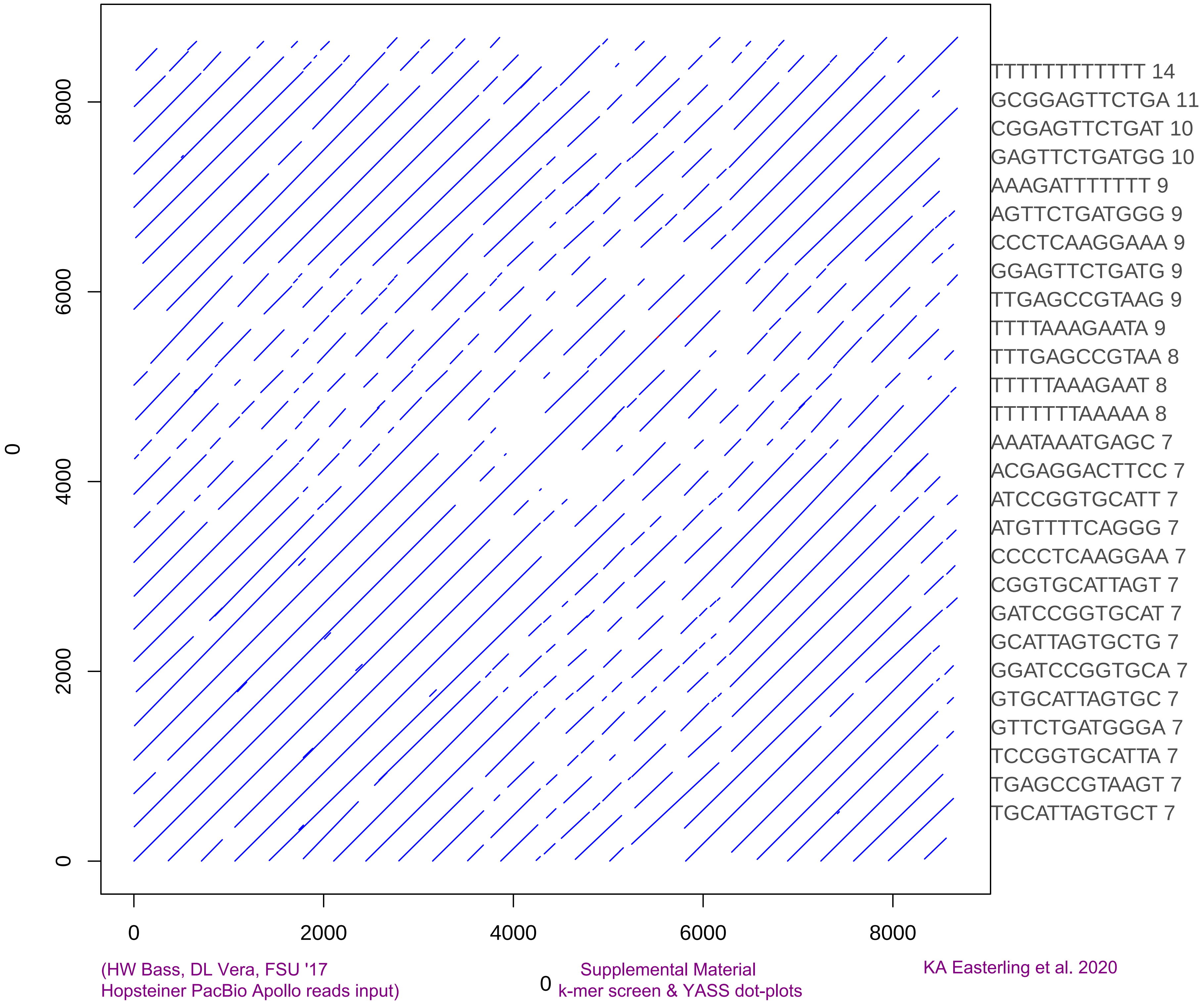

>m141126_034130_42134_c100732202550000001823141405141567_s1_p0/113794/5284_15004

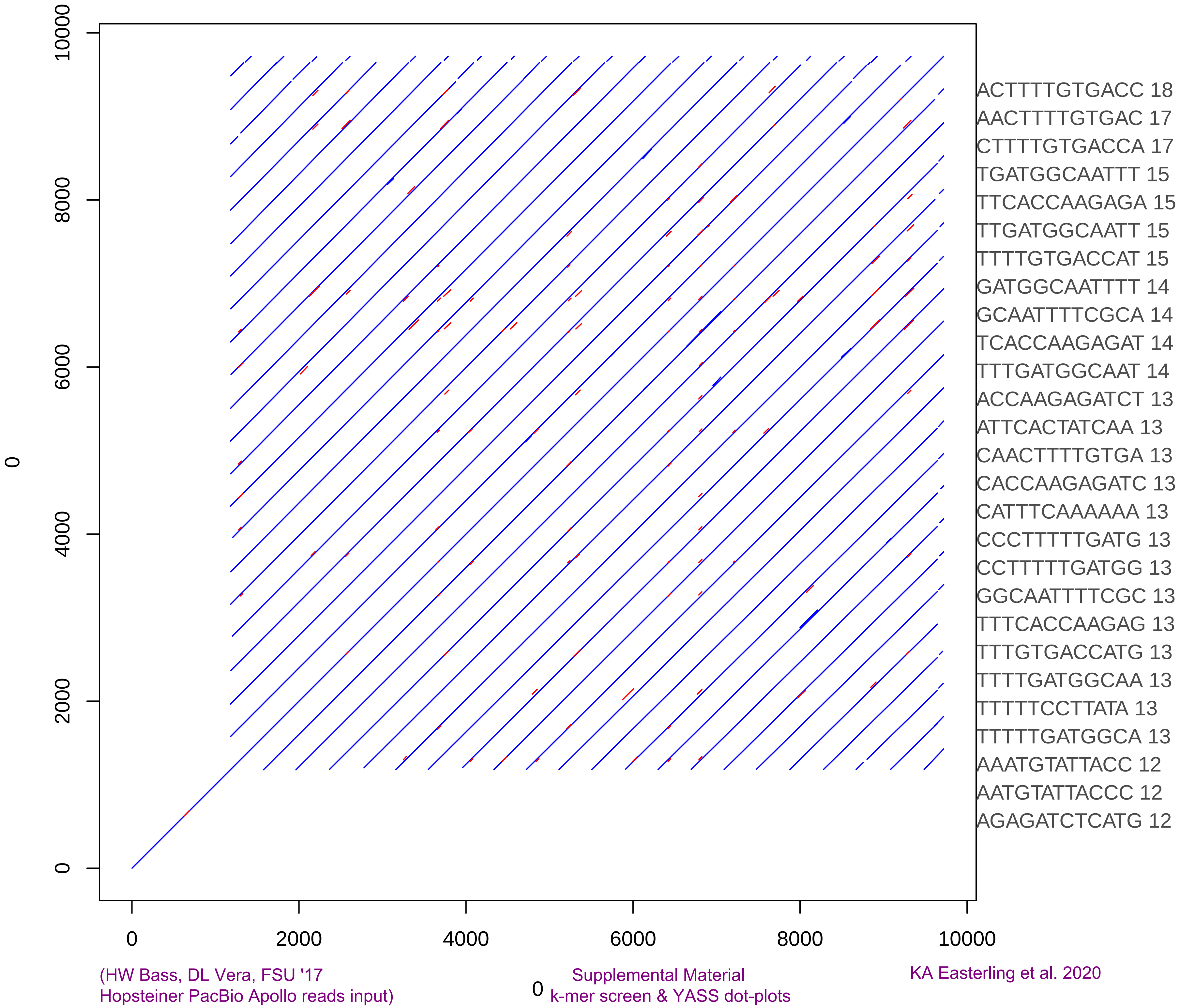

>m141126_034130_42134_c100732202550000001823141405141567_s1_p0/113873/1200_9093

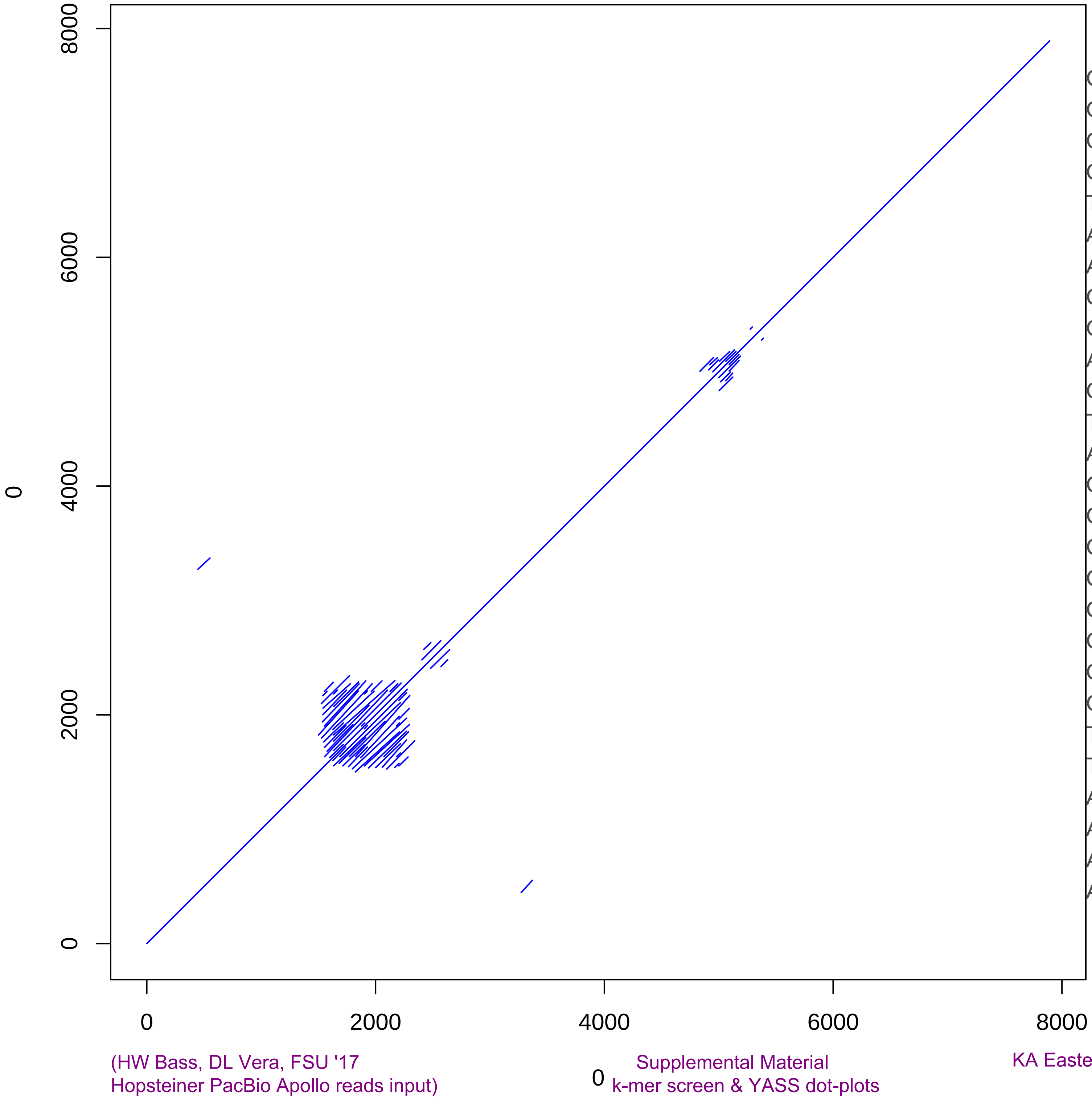

CGAGGCTACCCT 8
GAGGCTACCCTT 8
GCTACCCTTCCC 8
GGCTACCCTTCC 8
TGCGAGGCTACC 8
AGGCTACCCTTC 7
ATGCGAGGCTAC 7
CTACCCTTCCCC 7
GCGAGGCTACCC 7
ACTATGCGAGGC 6
CTATGCGAGGCT 6
TCACTATGCGAG 6
ATGCGCCTGCCA 5
CACTATGCGAGG 5
CATGCGCCTGCC 5
CCCCGCATGCGC 5
CCGCATGCGCCT 5
CCTGCCAAGGCC 5
CGCATGCGCCTG 5
GCATGCGCCTGC 5
GCCTGCCAAGGC 5
TATGCGAGGCTA 5
TGCGCCTGCCAA 5
AAGGCCACAAGG 4
AAGGGTCTCACT 4
ACCCTTCCCCCG 4
AGGCCACAAGGG 4

>m141126_034130_42134_c100732202550000001823141405141567_s1_p0/122795/1180_8356

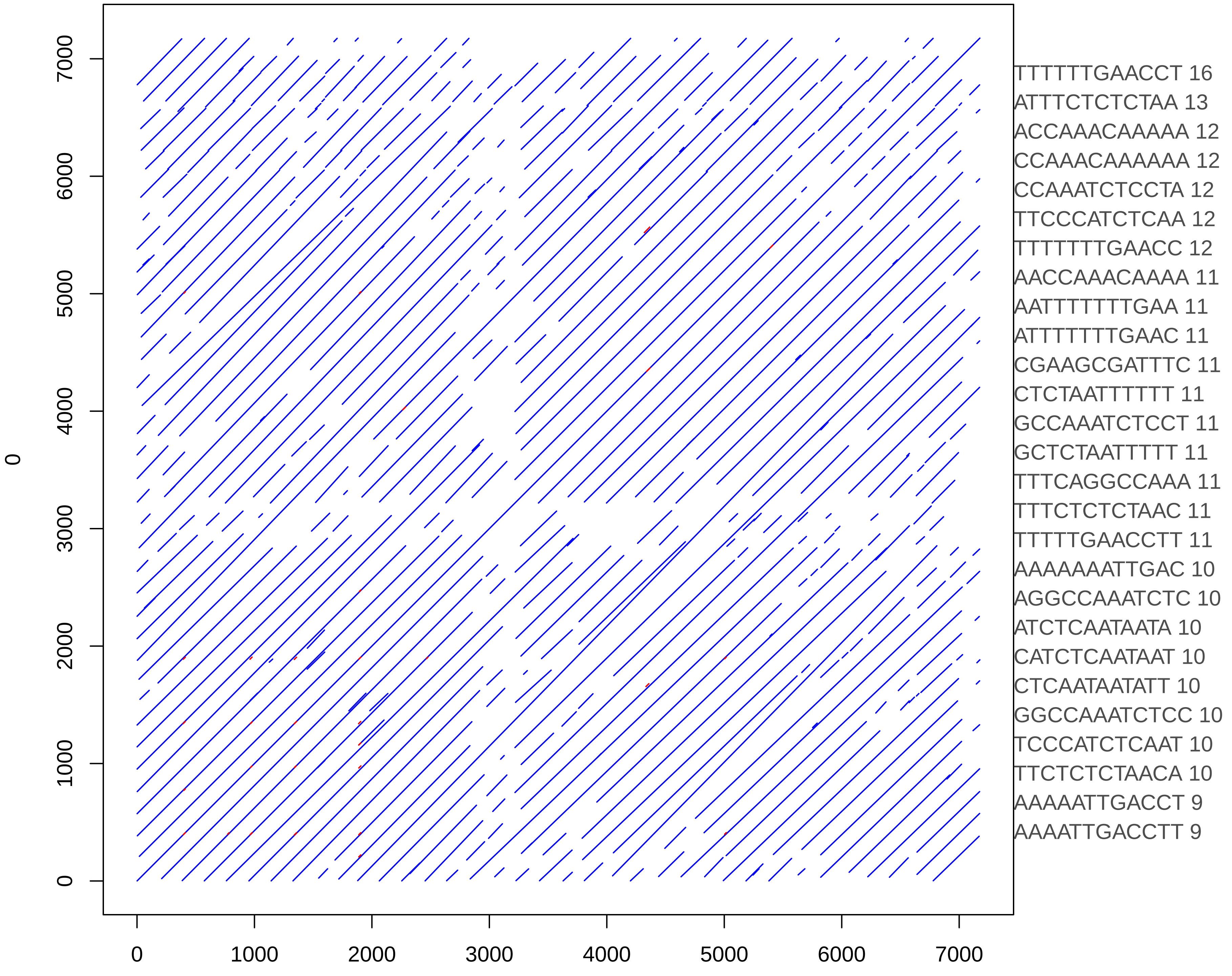

>m141126_034130_42134_c100732202550000001823141405141567_s1_p0/1272/0_5536

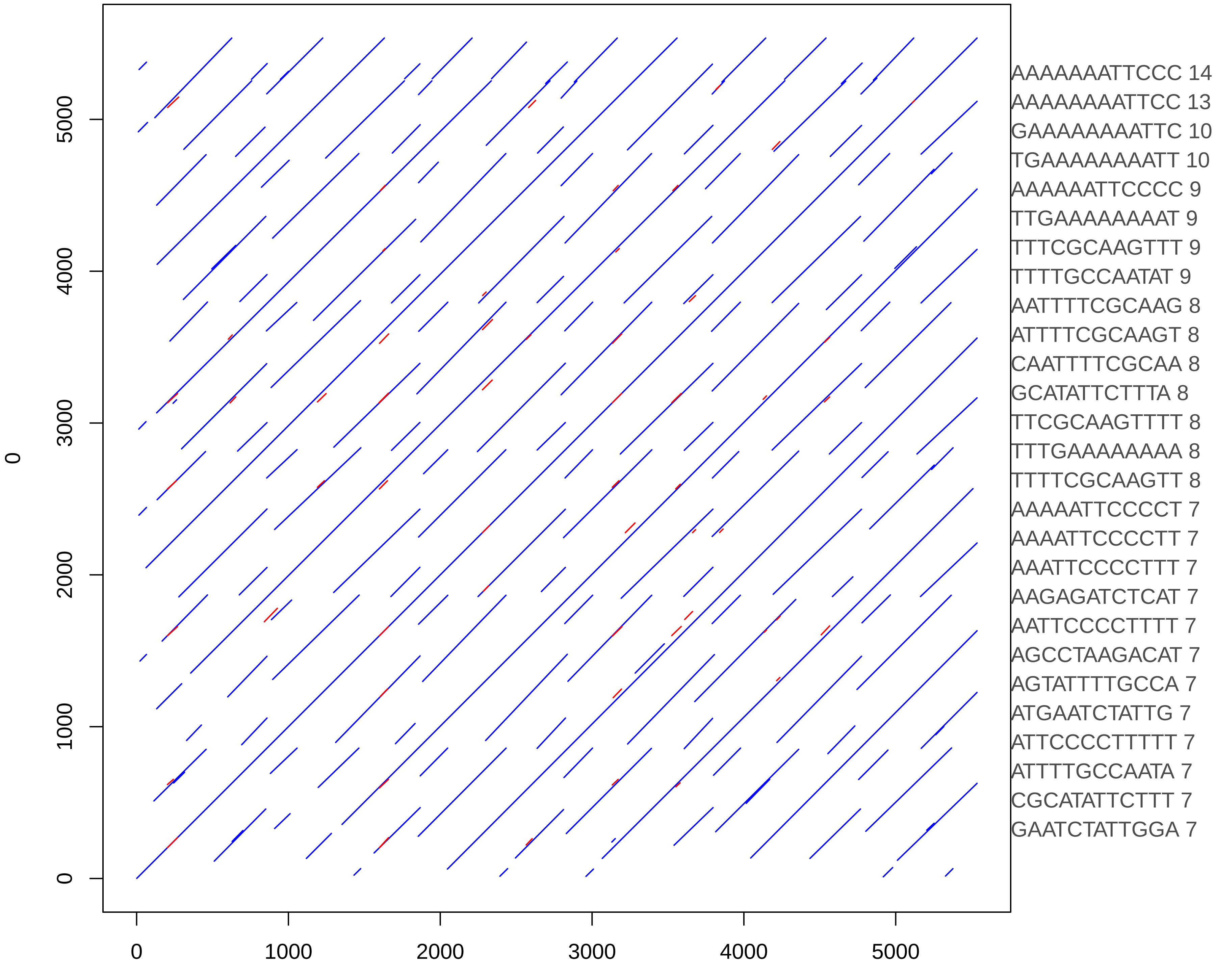

>m141126_034130_42134_c100732202550000001823141405141567_s1_p0/12994/6197_12745

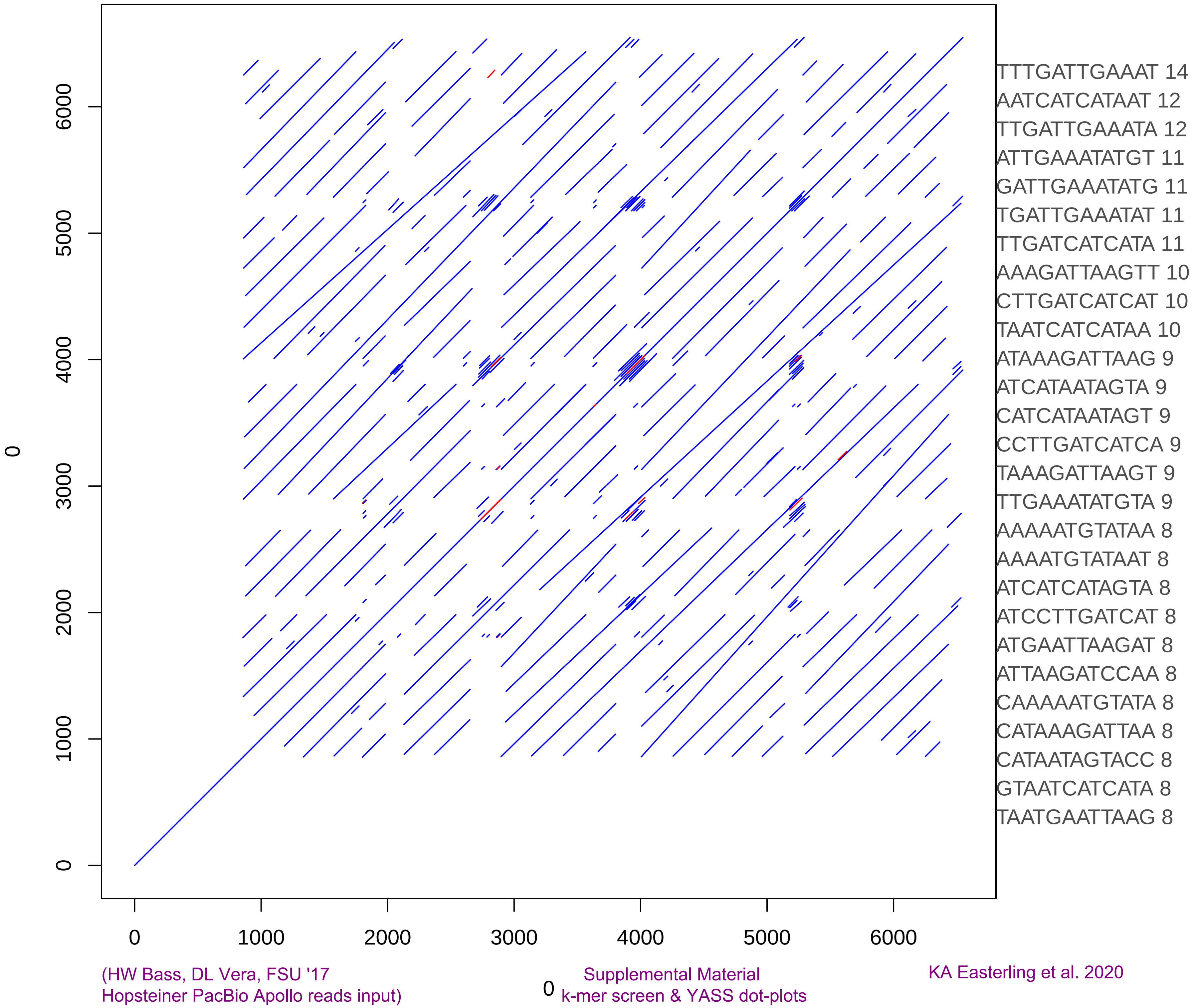

>m141126_034130_42134_c100732202550000001823141405141567_s1_p0/132055/0_6932

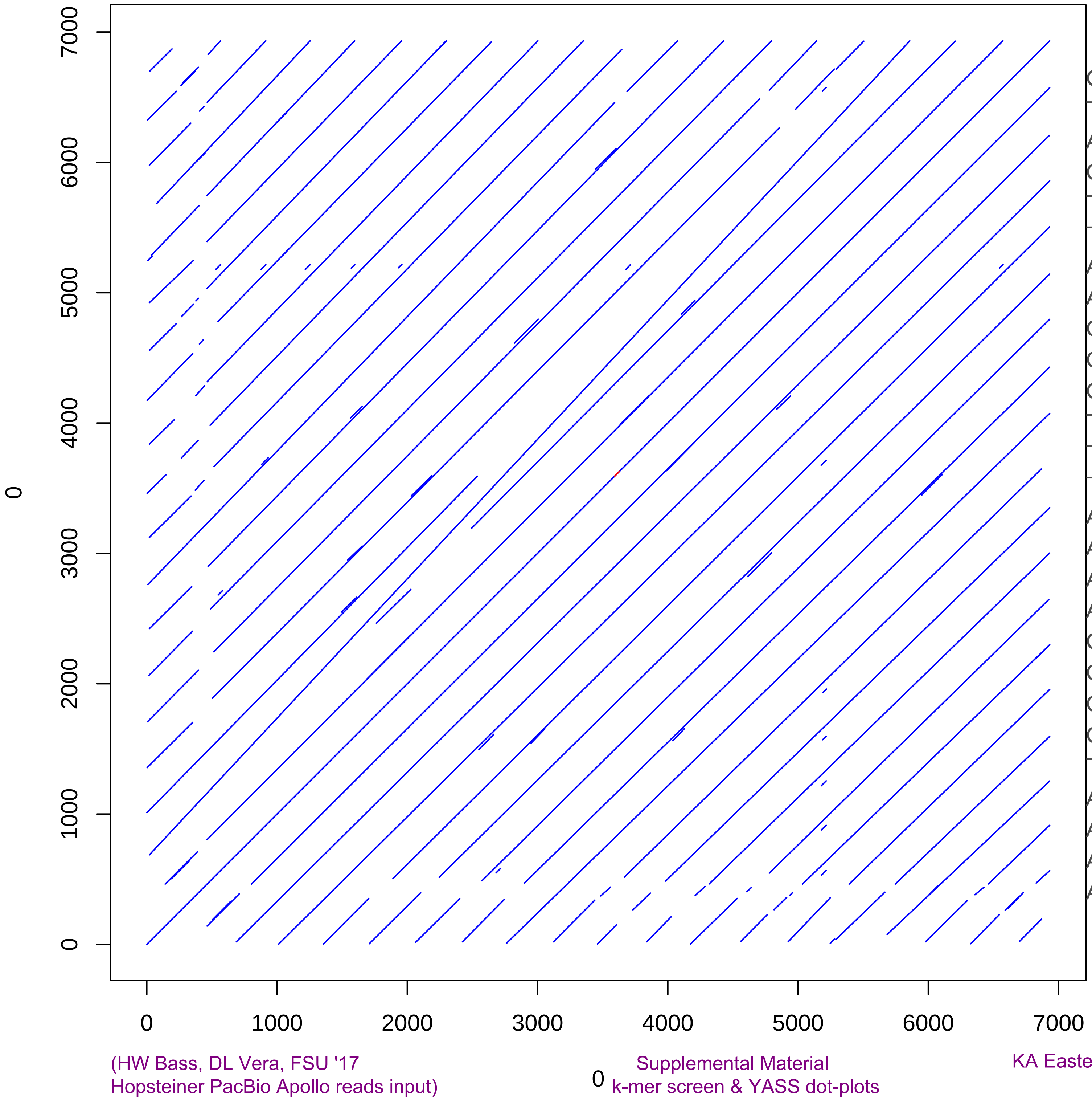

CAGCACTAATGC 13
TTTTTTTCCCTT 13
ATCATTTTTTTT 12
CATCATTTTTTT 12
TTTTTTTTCCCT 12
TTTTTTTTTCCC 12
ACATCATTTTTT 11
AGCACTAATGCA 11
CAGTTAAGCGTG 11
CCAGCACTAATG 11
GCAGTTAAGCGT 11
TCATTTTTTTTT 11
TTTATTTCGTTT 11
TTTTTTTTTTCC 11
AAATCTTTAAAA 10
ACTCCGCAGTTA 10
AGTTAAGCGTGC 10
ATTTATTTCGTT 10
CATTTATTTCGT 10
CATTTTTTTTTT 10
CGCAGTTAAGCG 10
CTCCGCAGTTAA 10
TAGTCAATCCGG 10
AAACATCATTTT 9
AACATCATTTTT 9
AACTCCGCAGTT 9
ACCAGCACTAAT 9

>m141126_034130_42134_c100732202550000001823141405141567_s1_p0/134379/0_15789

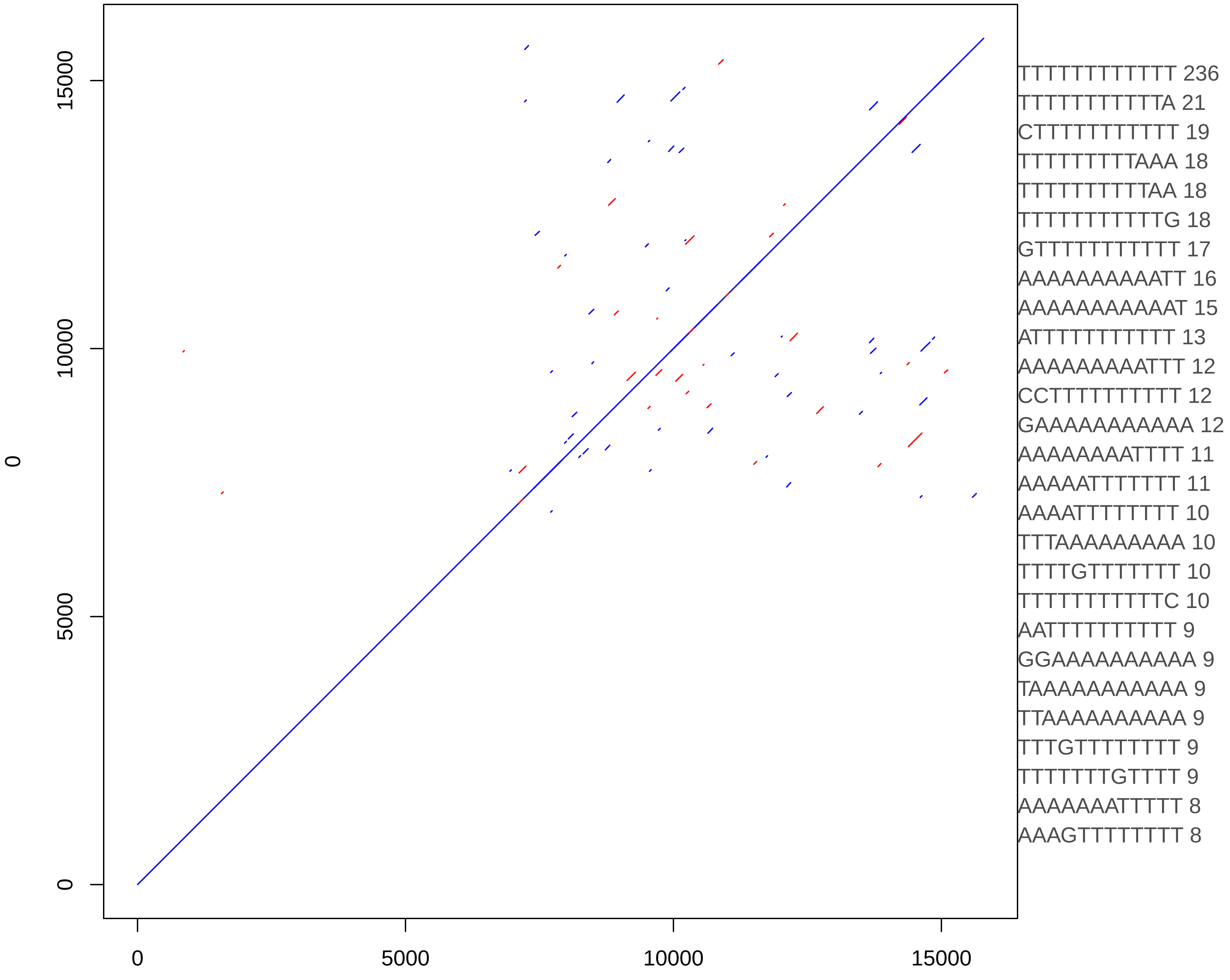

>m141126_034130_42134_c100732202550000001823141405141567_s1_p0/134708/2226_9007

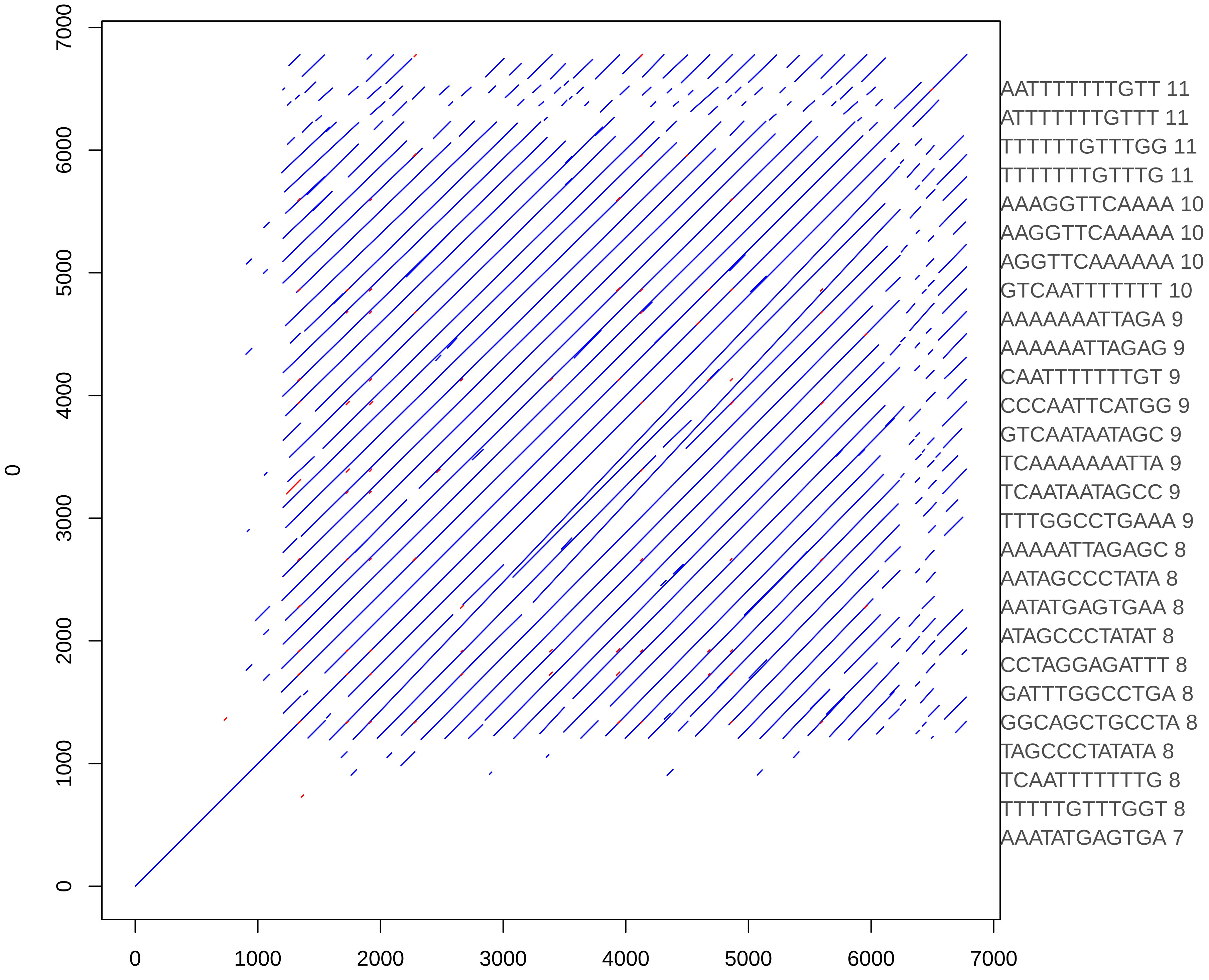

>m141126_034130_42134_c100732202550000001823141405141567_s1_p0/137742/0_15157

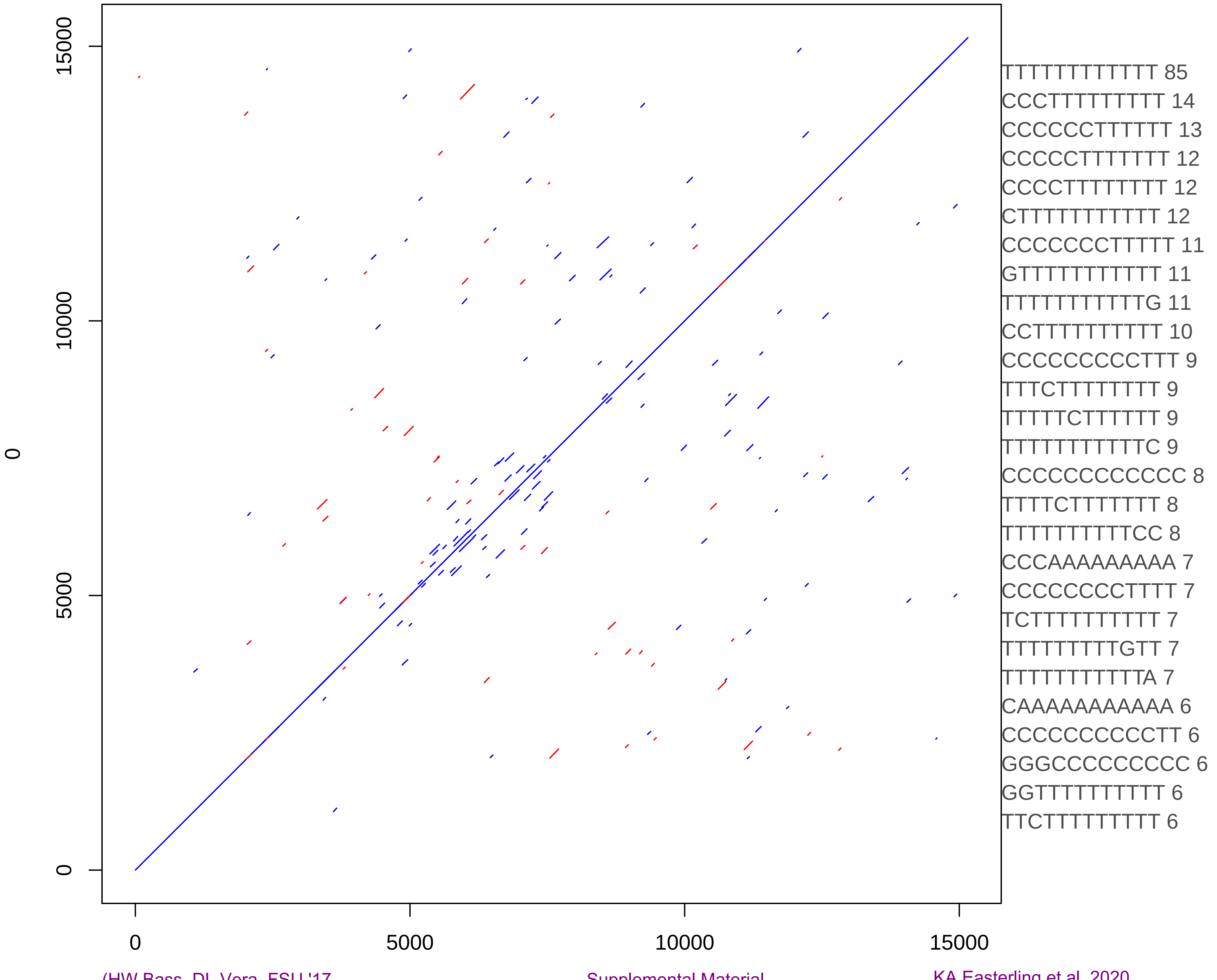

>m141126_034130_42134_c100732202550000001823141405141567_s1_p0/137975/1309_7306

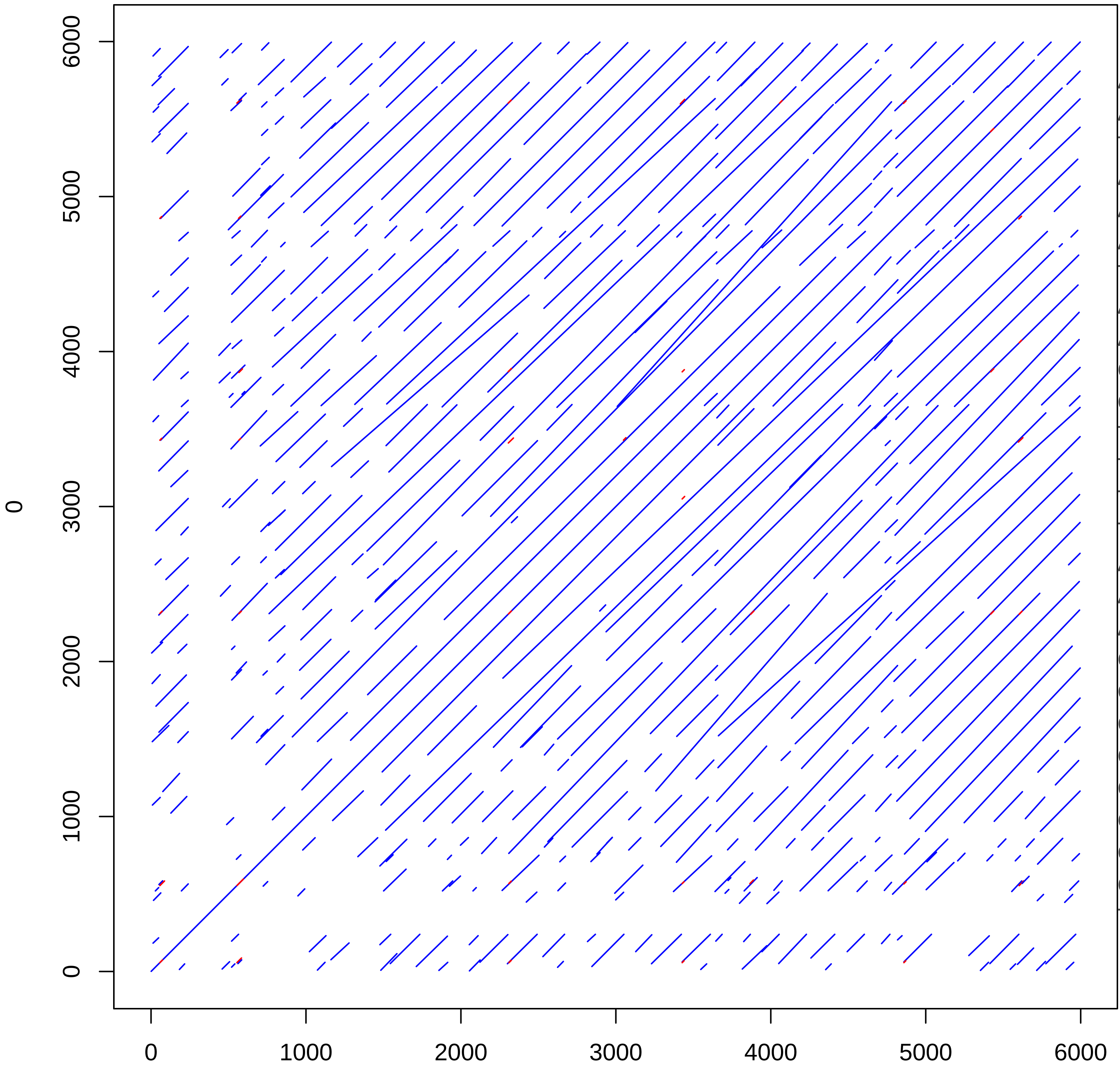

1,121 hits for repetitive sequences
passing 12mer abundance criterion

(Read-Number)
PAGE 513

For Tandem Repeat families of ### bp:
PacBio Read named "HuluTR###-r513"

>m141126_034130_42134_c100732202550000001823141405141567_s1_p0/140501/1443_12409

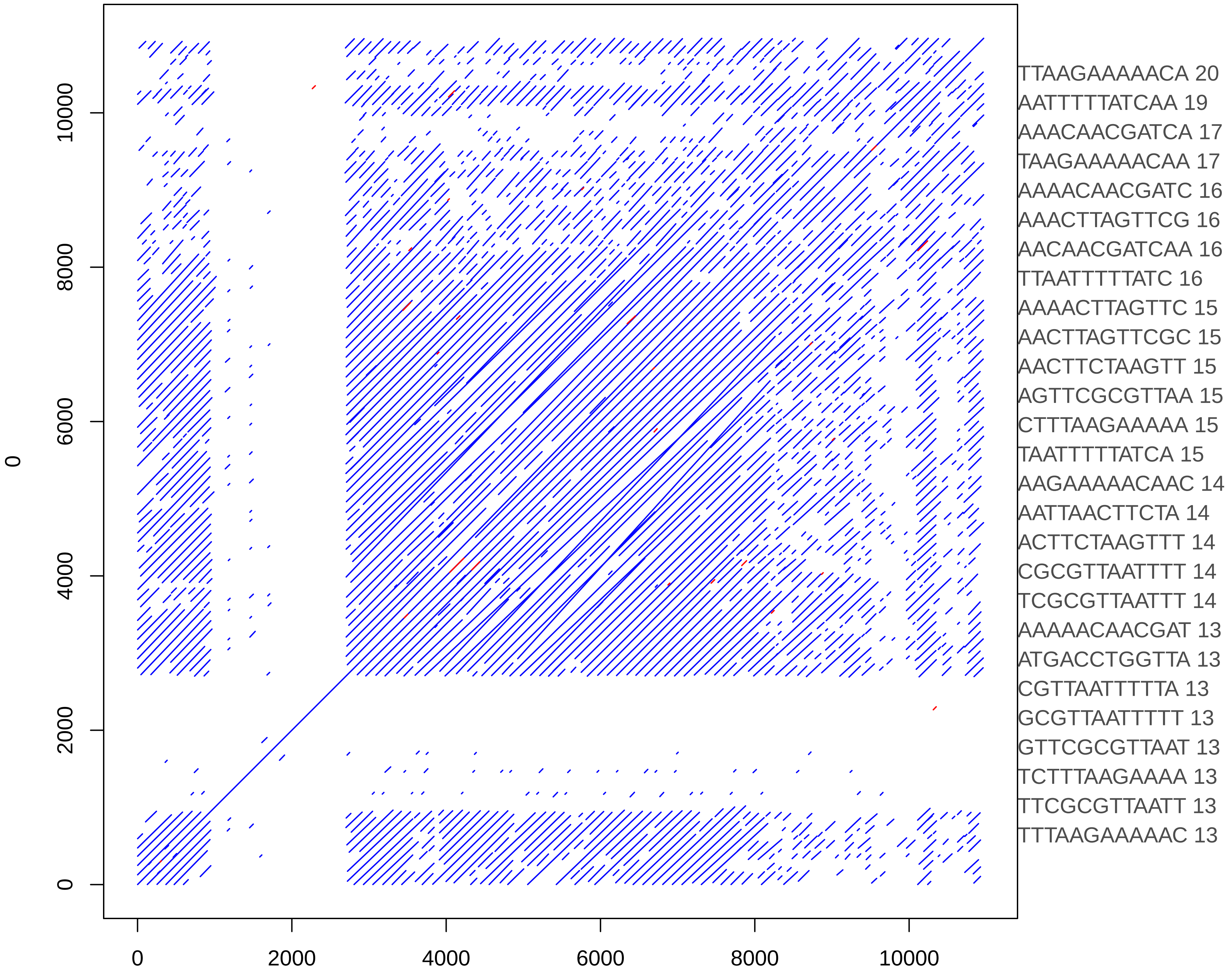

(HW Bass, DL Vera, FSU '17
Hopsteiner PacBio Apollo reads input)

0
Supplemental Material
k-mer screen & YASS dot-plots

KA Easterling et al. 2020

>m141126_034130_42134_c100732202550000001823141405141567_s1_p0/141938/6599_11999

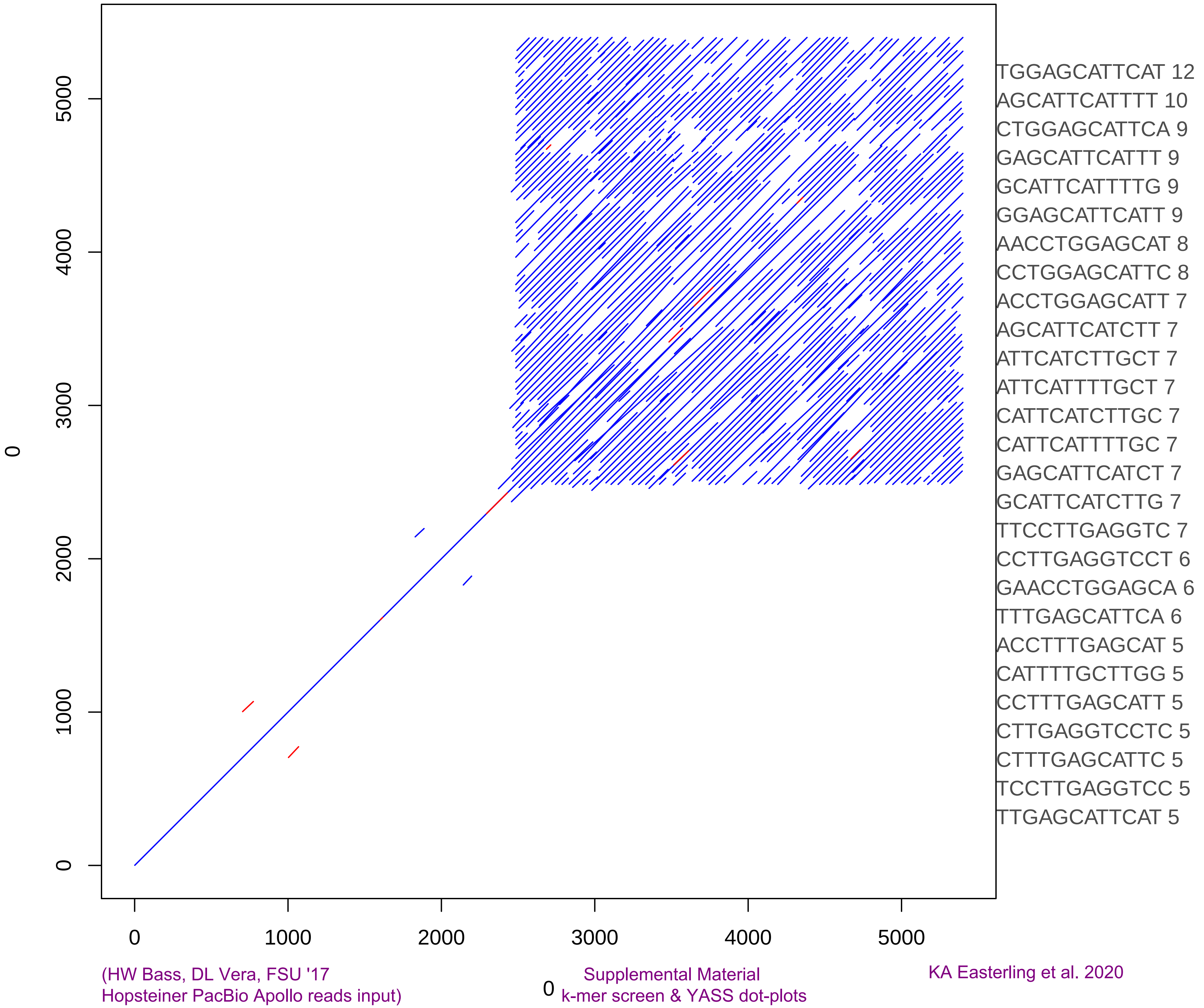

1,121 hits for repetitive sequences
passing 12mer abundance criterion

(Read-Number)
PAGE 515

For Tandem Repeat families of ### bp:
PacBio Read named "HuluTR###-r515"

>m141126_034130_42134_c100732202550000001823141405141567_s1_p0/146845/186_9005

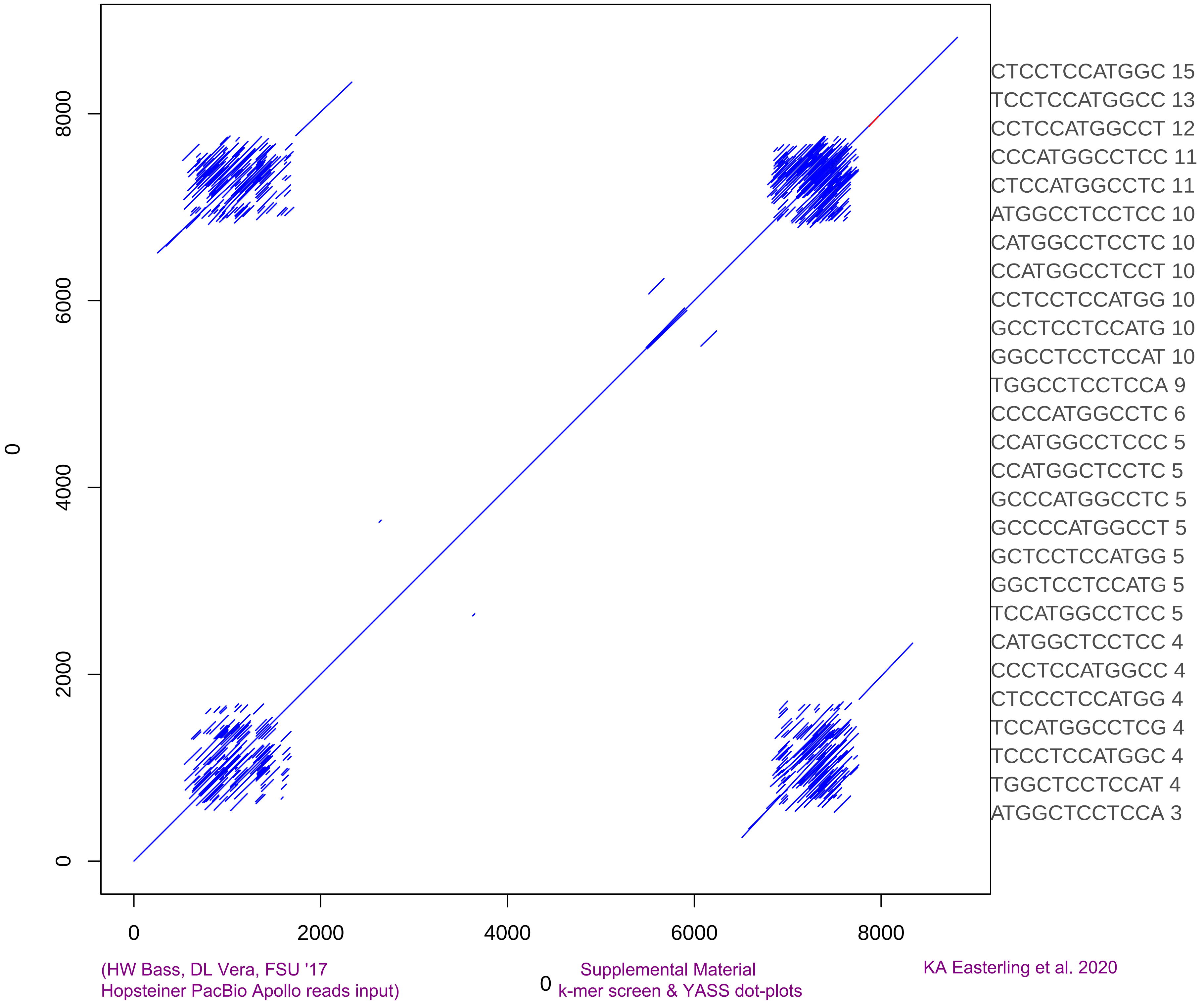

(HW Bass, DL Vera, FSU '17
Hopsteiner PacBio Apollo reads input)

Supplemental Material
k-mer screen & YASS dot-plots

KA Easterling et al. 2020

>m141126_034130_42134_c100732202550000001823141405141567_s1_p0/149811/4312_10540

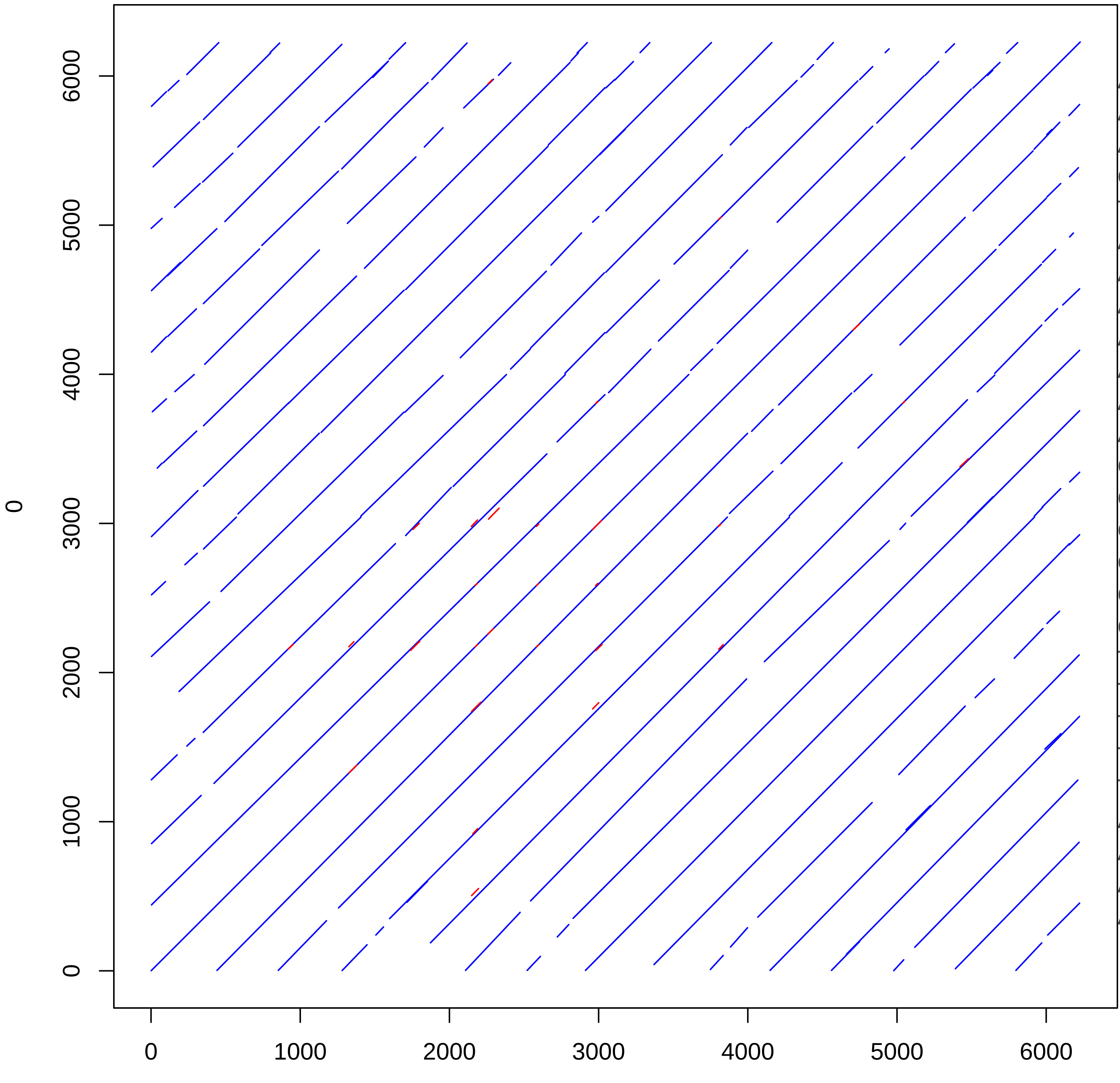

>m141126_034130_42134_c100732202550000001823141405141567_s1_p0/152130/7228_12632

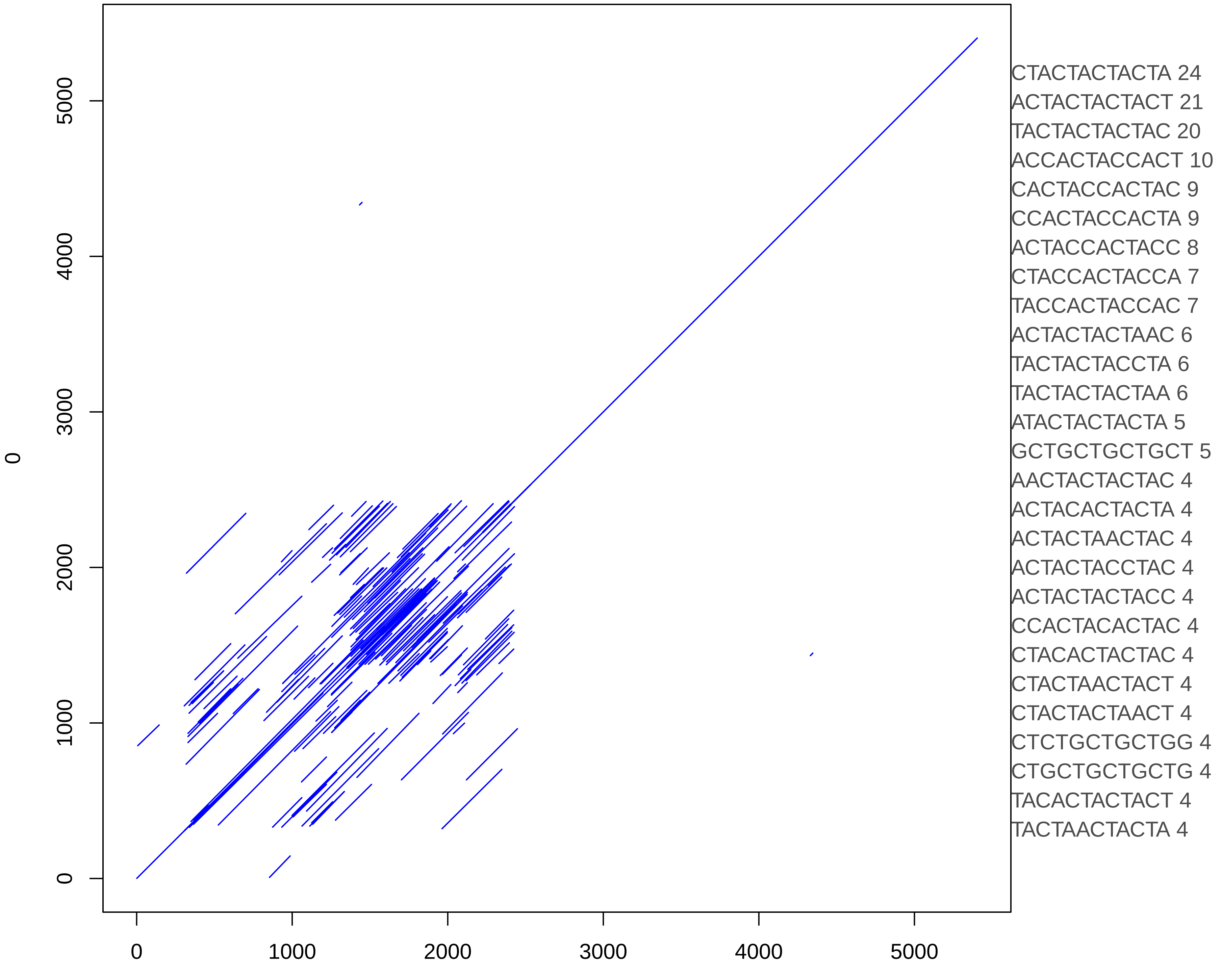

>m141126_034130_42134_c100732202550000001823141405141567_s1_p0/152147/349_5596

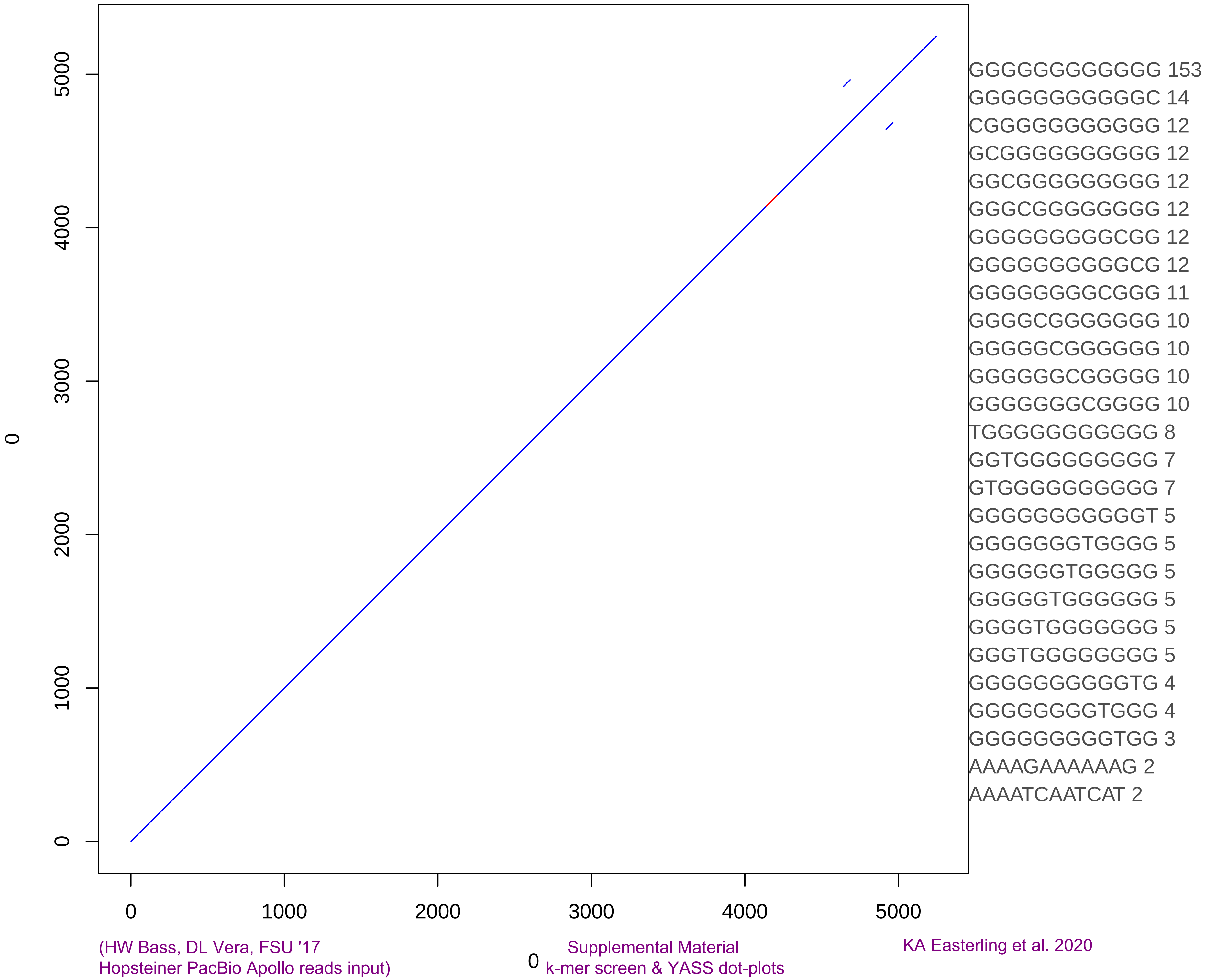

>m141126_034130_42134_c100732202550000001823141405141567_s1_p0/155093/506_6346

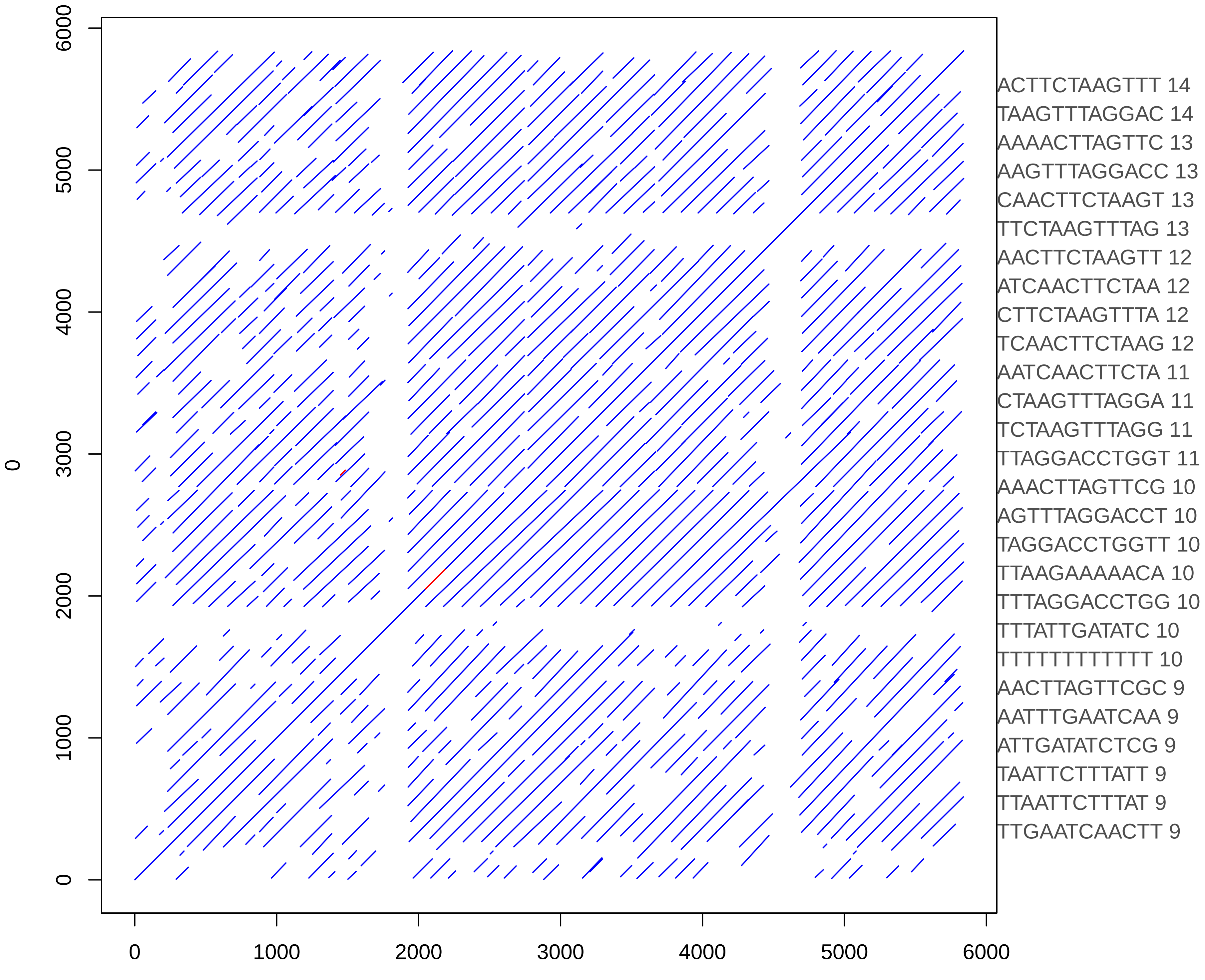

>m141126_034130_42134_c100732202550000001823141405141567_s1_p0/160384/8223_16532

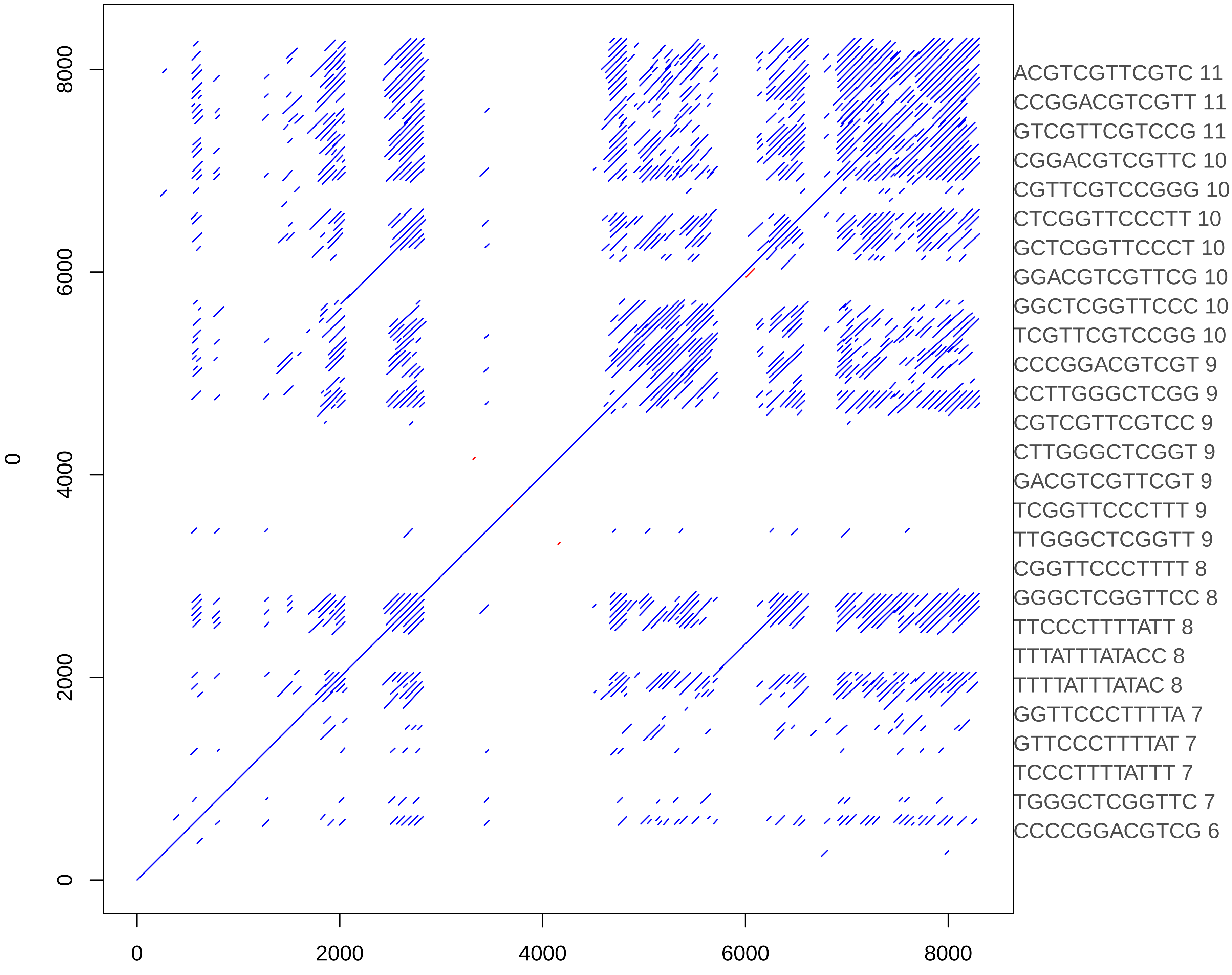

0

>m141126_034130_42134_c100732202550000001823141405141567_s1_p0/161585/10497_15527

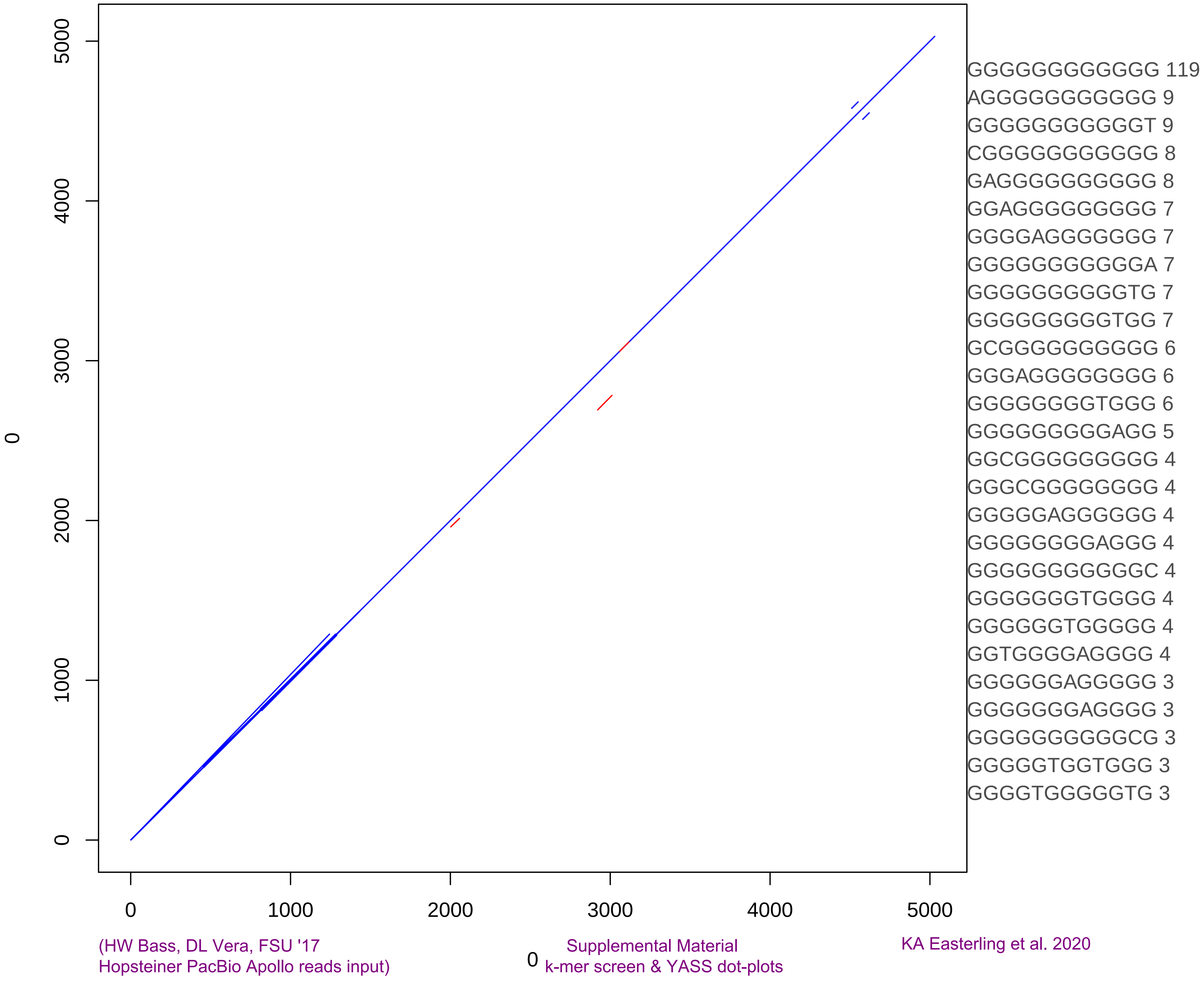

>m141126_034130_42134_c100732202550000001823141405141567_s1_p0/1660/0_7876

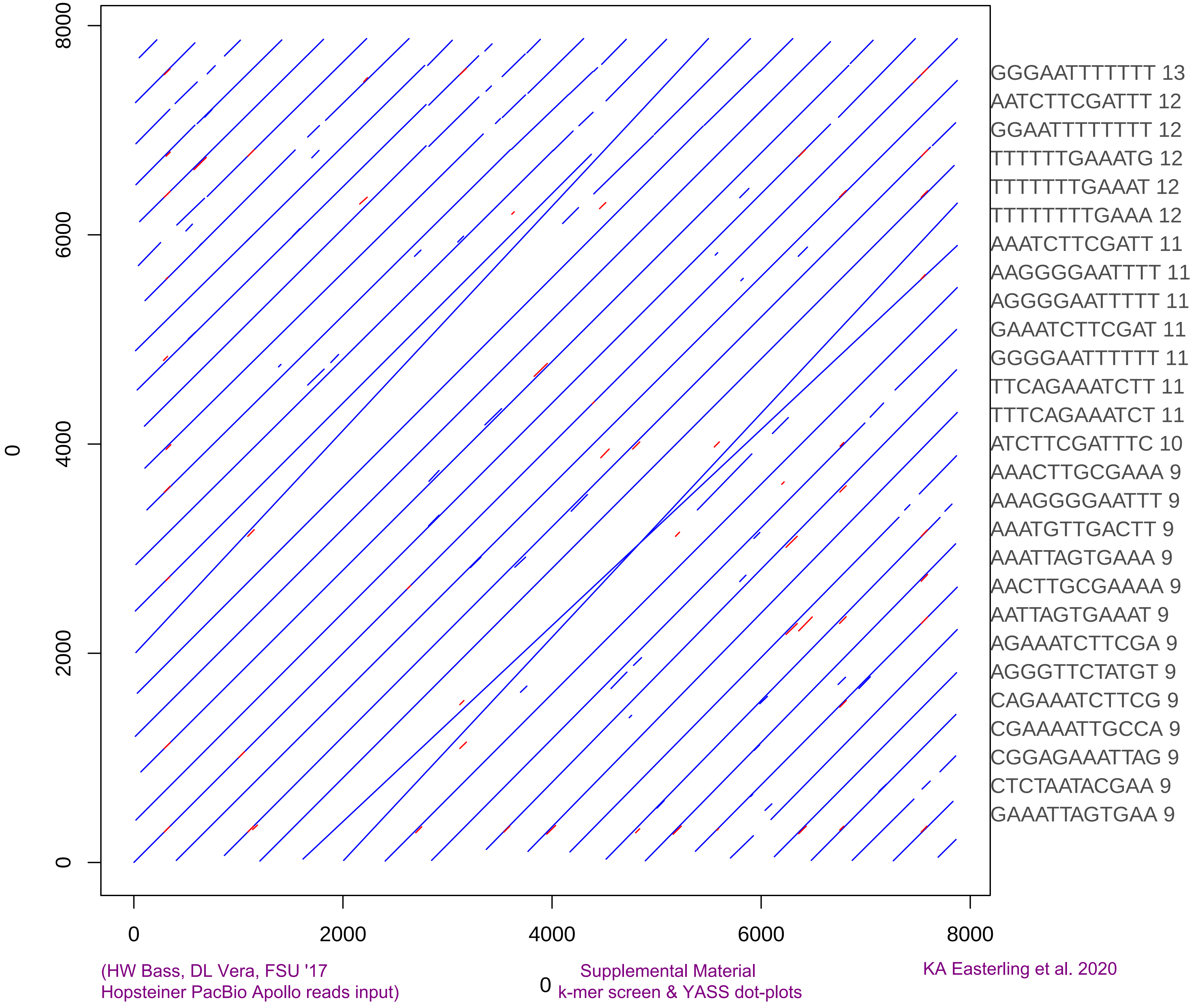

>m141126_034130_42134_c100732202550000001823141405141567_s1_p0/212/0_9063

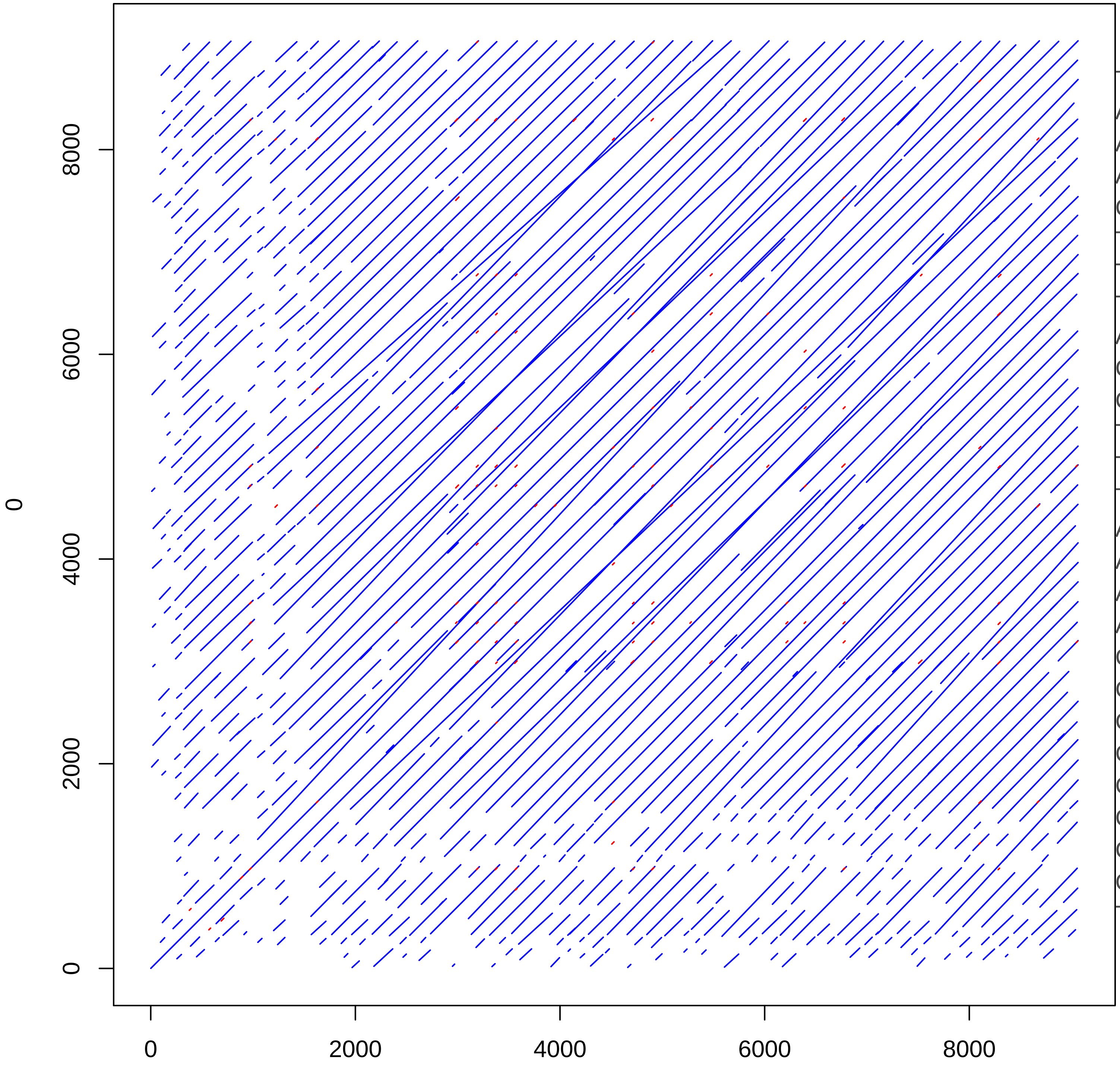

TTTCTCTCTAAC 16
AAAAAAATTGGC 15
ATTCACTCATAT 15
ATTTCTCTCTAA 15
CTCTAACAAGCT 15
TATTTCTCTCTA 15
TCTCTAACAAGC 15
TTCTCTCTAACA 15
ATATTTCTCTCT 14
CATATTTCTCTC 14
CTAACAAGCTCT 14
TCTCCTAGGCAG 14
TCTCTCTAACAA 14
TTCACTCATATT 14
AAATCTCCTAGG 13
AATCTCCTAGGC 13
AATTCACTCATA 13
ATCTCCTAGGCA 13
CAAACAAAAAAA 13
CAAATCTCCTAG 13
CCAAATCTCCTA 13
CCATCTCAATAA 13
CCCATCTCAATA 13
CTCCTAGGCAGC 13
CTCTCTAACAAG 13
GCCAAATCTCCT 13
TAATTCACTCAT 13

>m141126_034130_42134_c100732202550000001823141405141567_s1_p0/21818/10167_18037

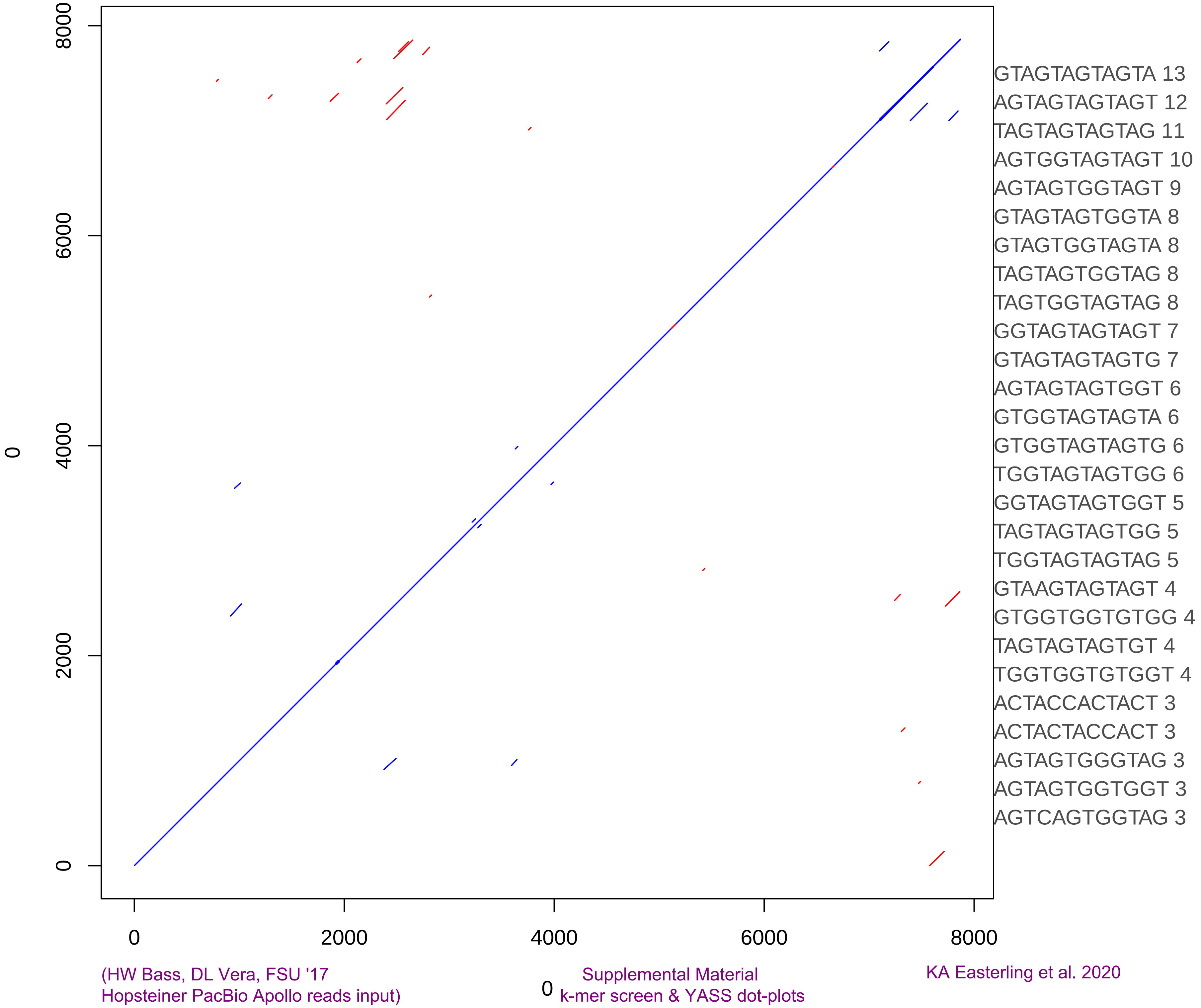

>m141126_034130_42134_c100732202550000001823141405141567_s1_p0/22520/7632_13136

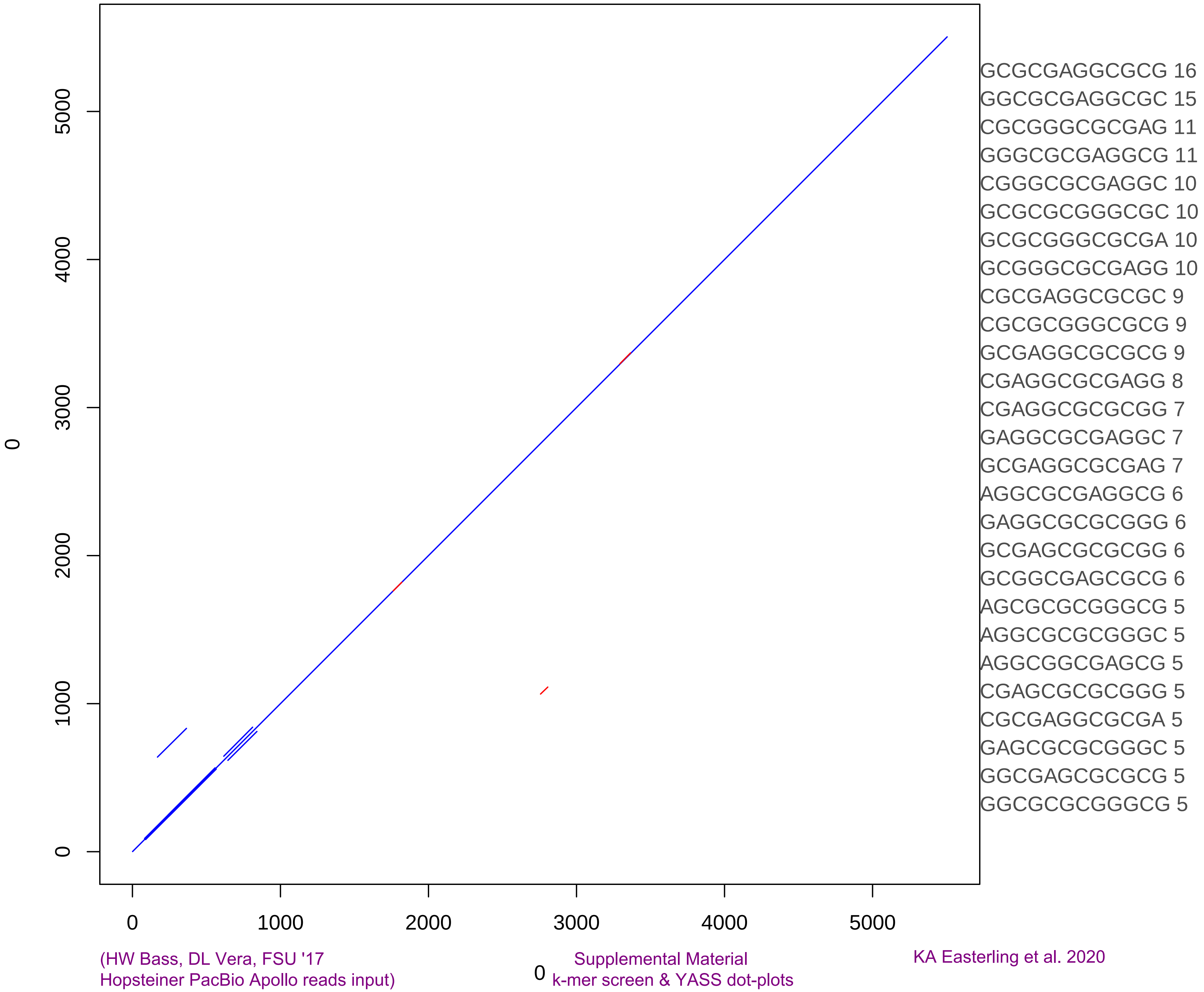

>m141126_034130_42134_c100732202550000001823141405141567_s1_p0/24741/3139_8943

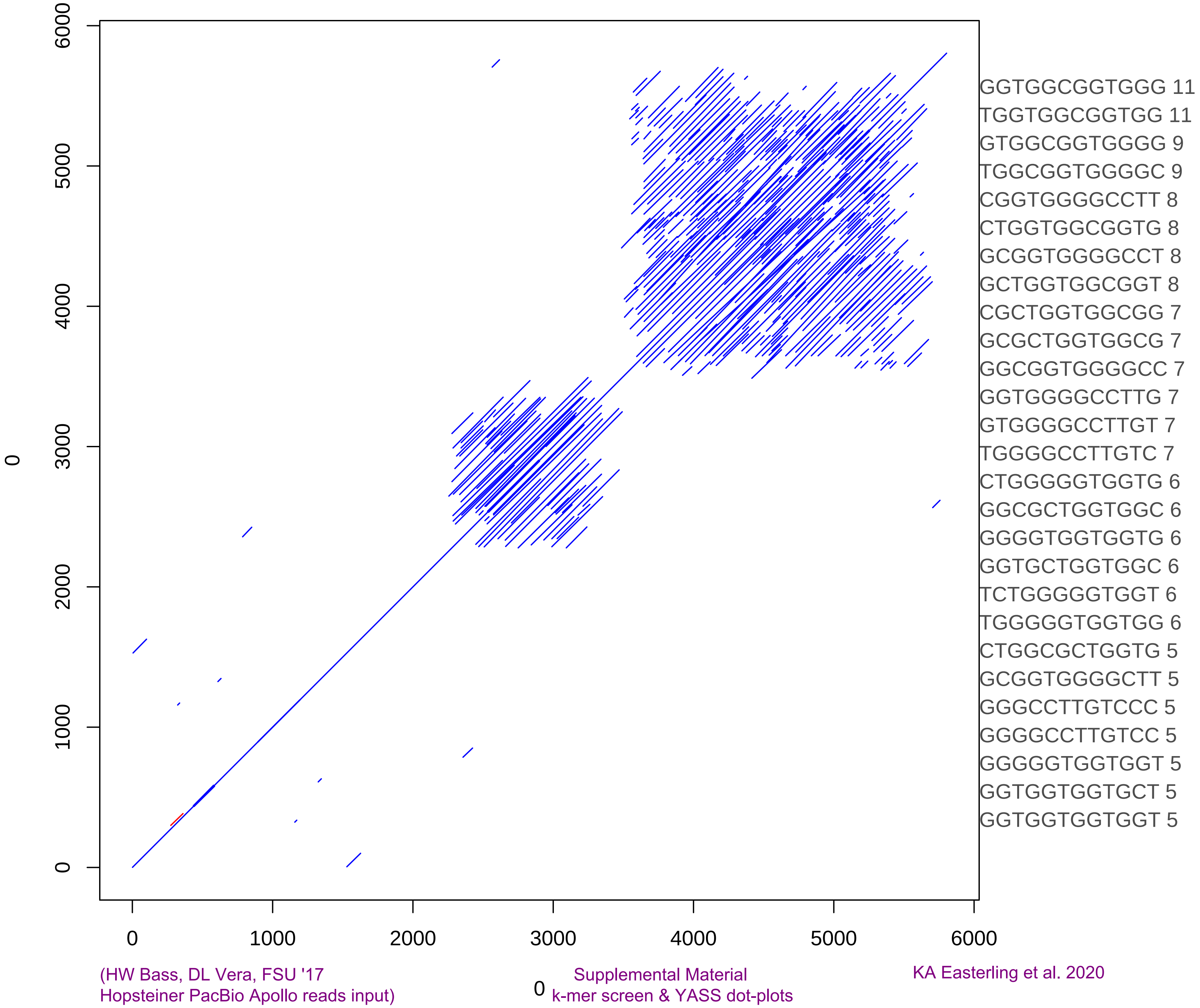

>m141126_034130_42134_c100732202550000001823141405141567_s1_p0/27788/892_8274

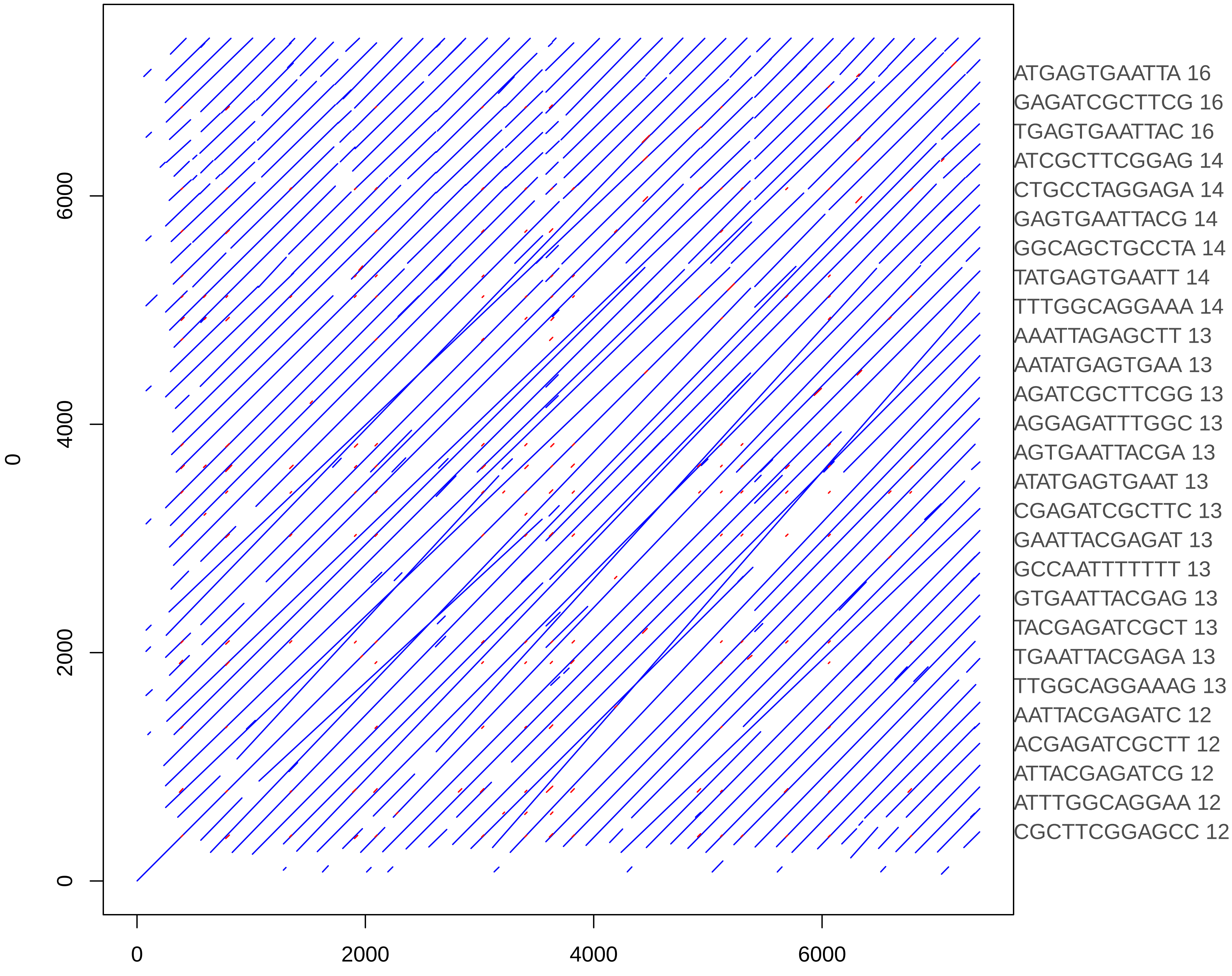

>m141126_034130_42134_c100732202550000001823141405141567_s1_p0/2791/9222_18593

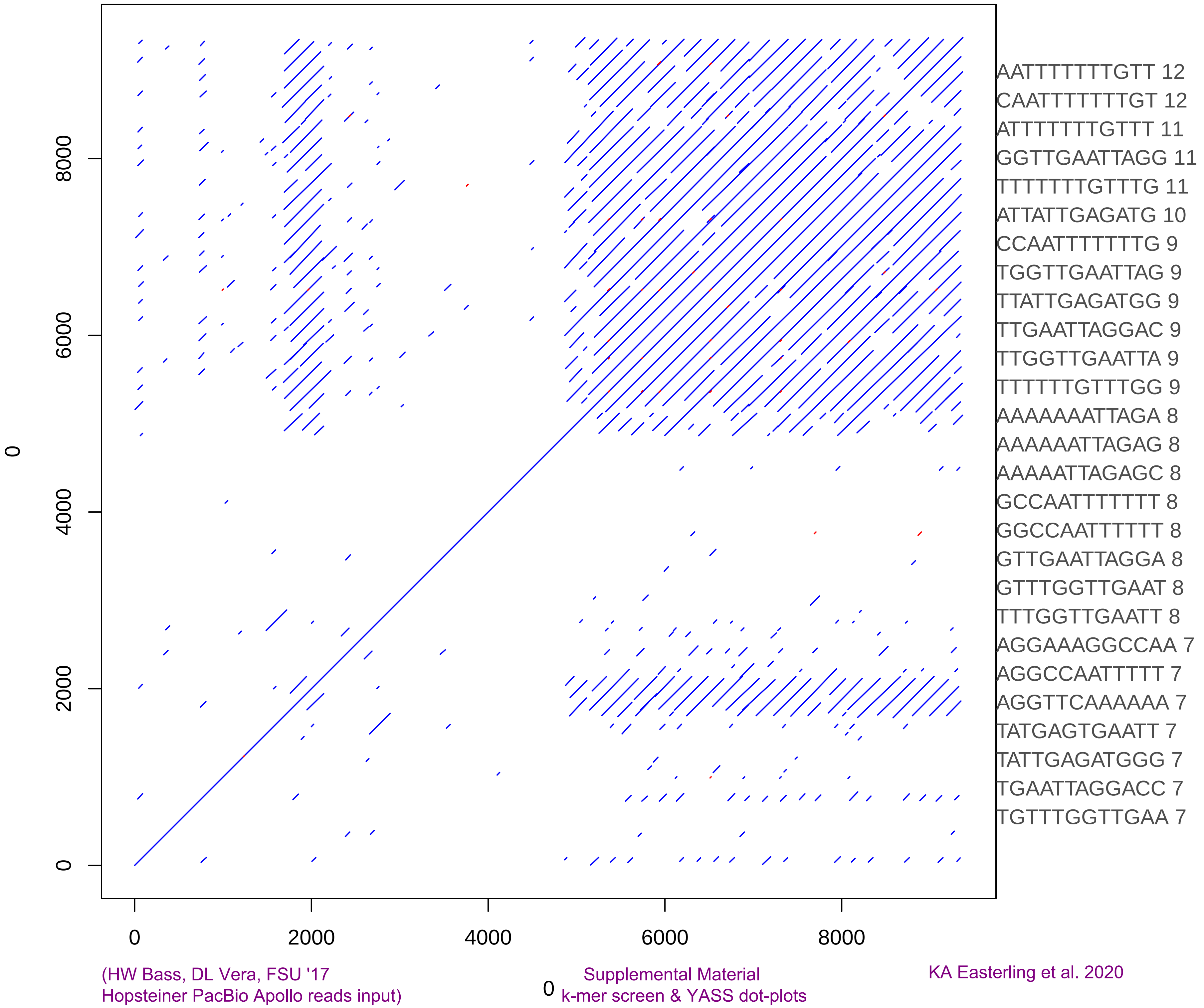

>m141126_034130_42134_c100732202550000001823141405141567_s1_p0/28311/12205_17858

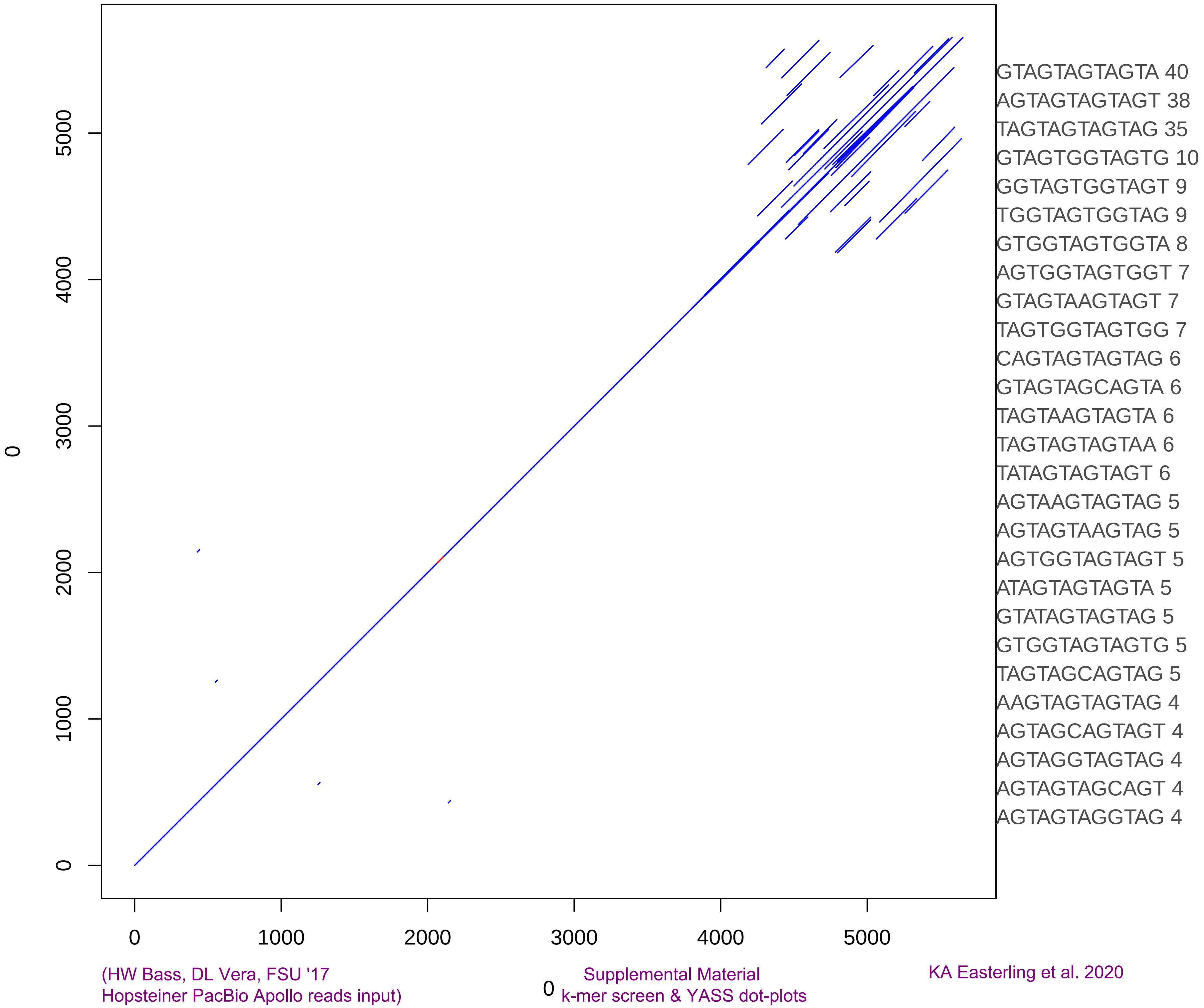

>m141126_034130_42134_c100732202550000001823141405141567_s1_p0/30705/5518_17895

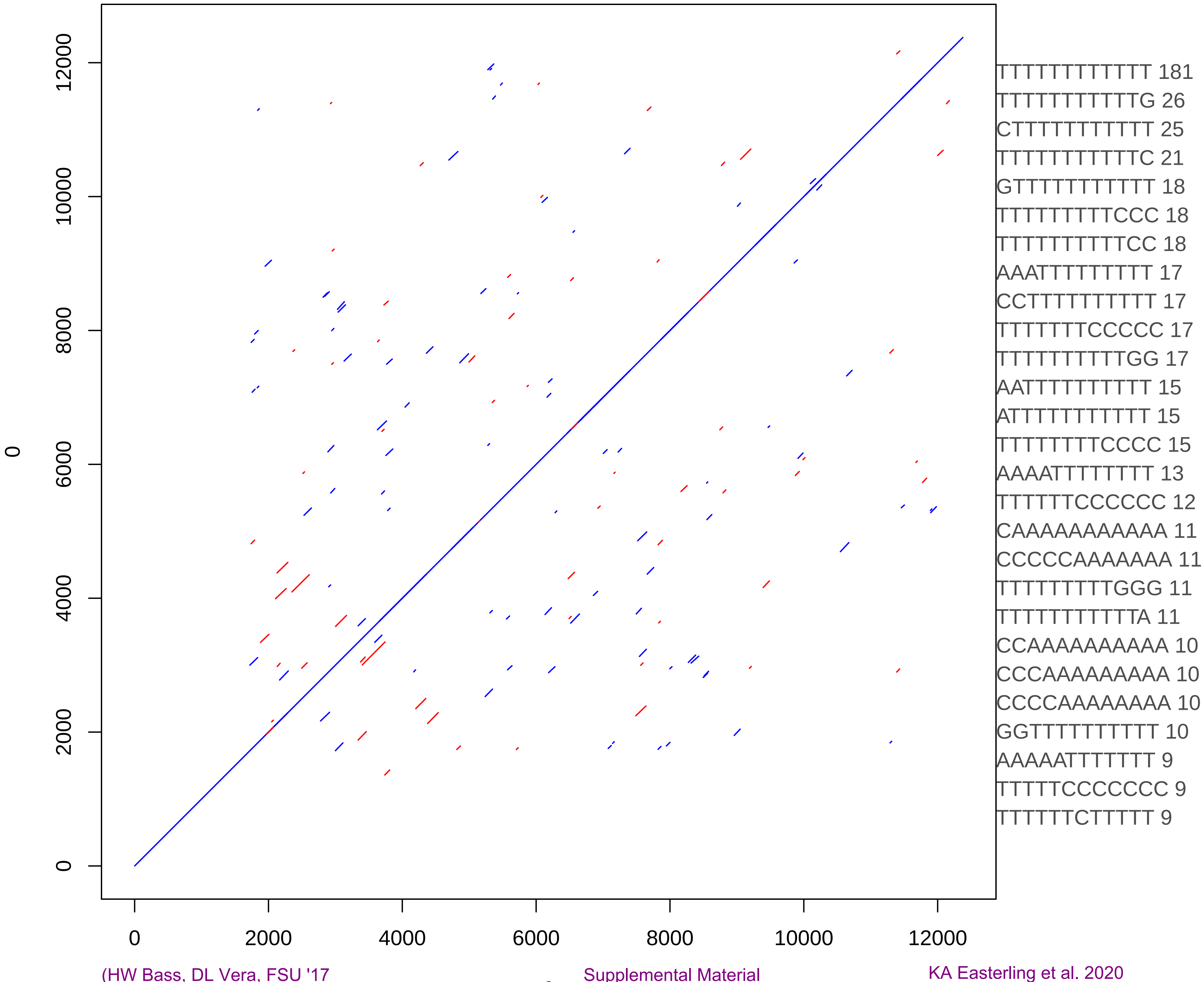

>m141126_034130_42134_c100732202550000001823141405141567_s1_p0/31658/7425_13432

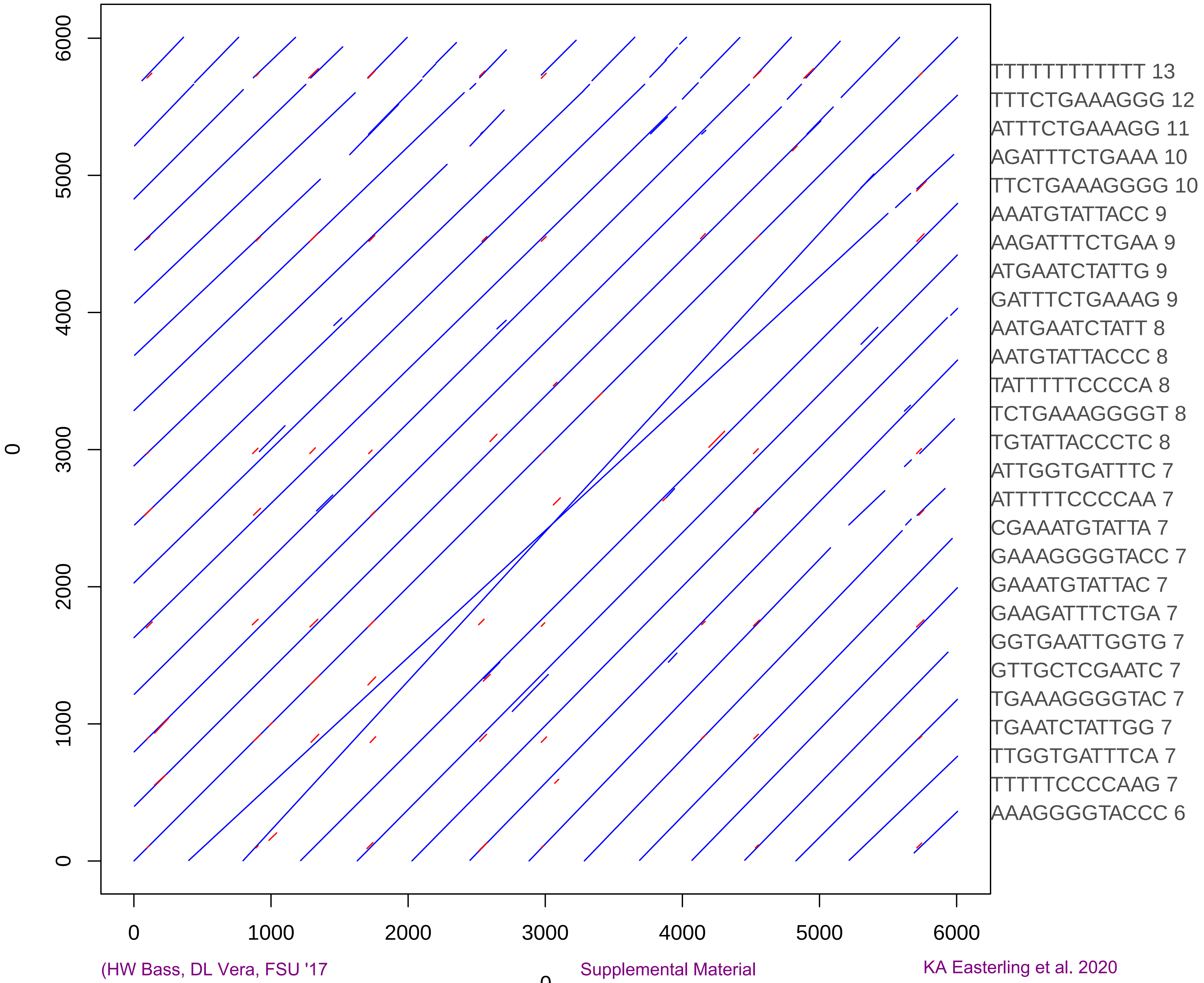

>m141126_034130_42134_c100732202550000001823141405141567_s1_p0/39485/5734_11564

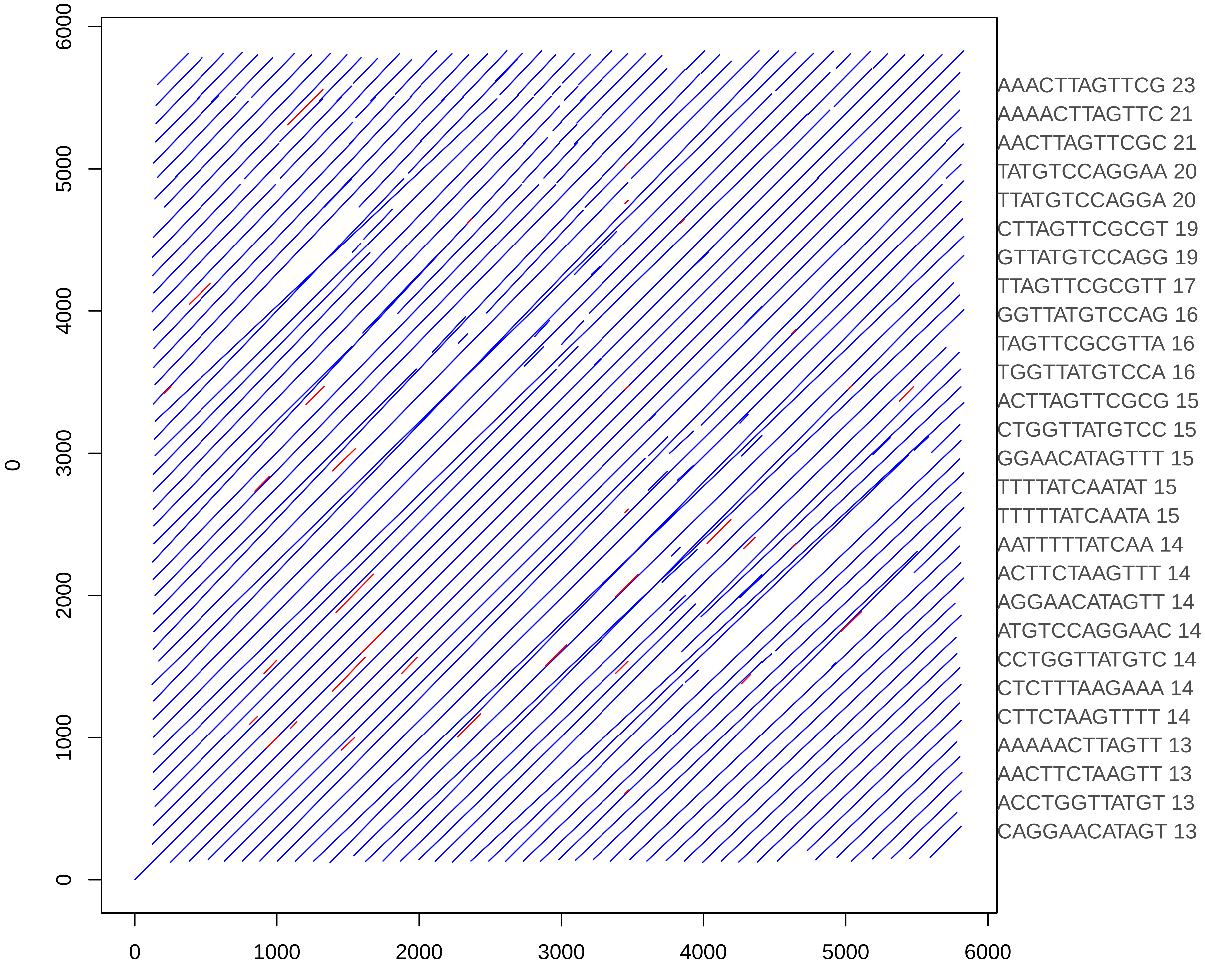

>m141126_034130_42134_c100732202550000001823141405141567_s1_p0/39849/448_8272

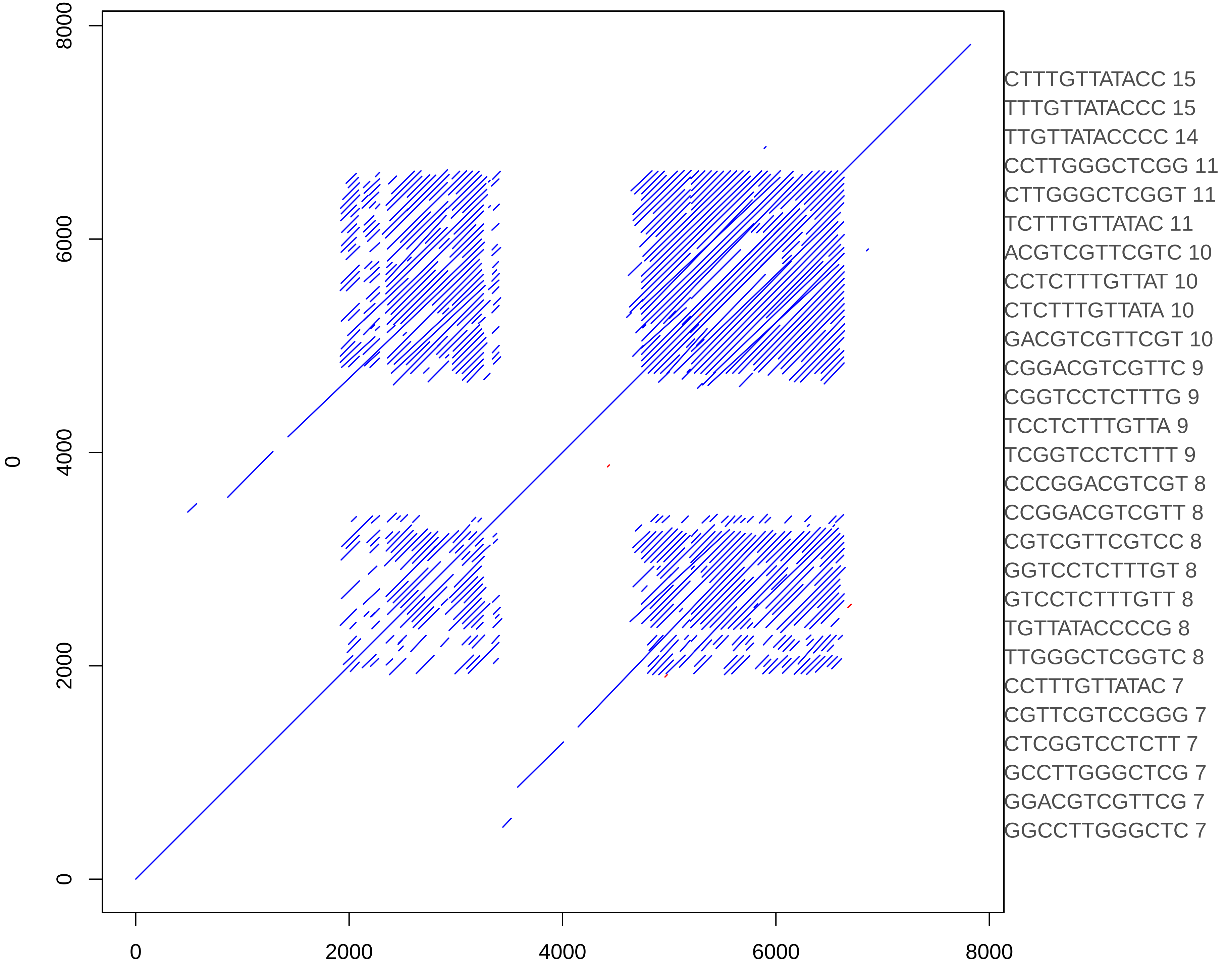

>m141126_034130_42134_c100732202550000001823141405141567_s1_p0/43086/6176_12492

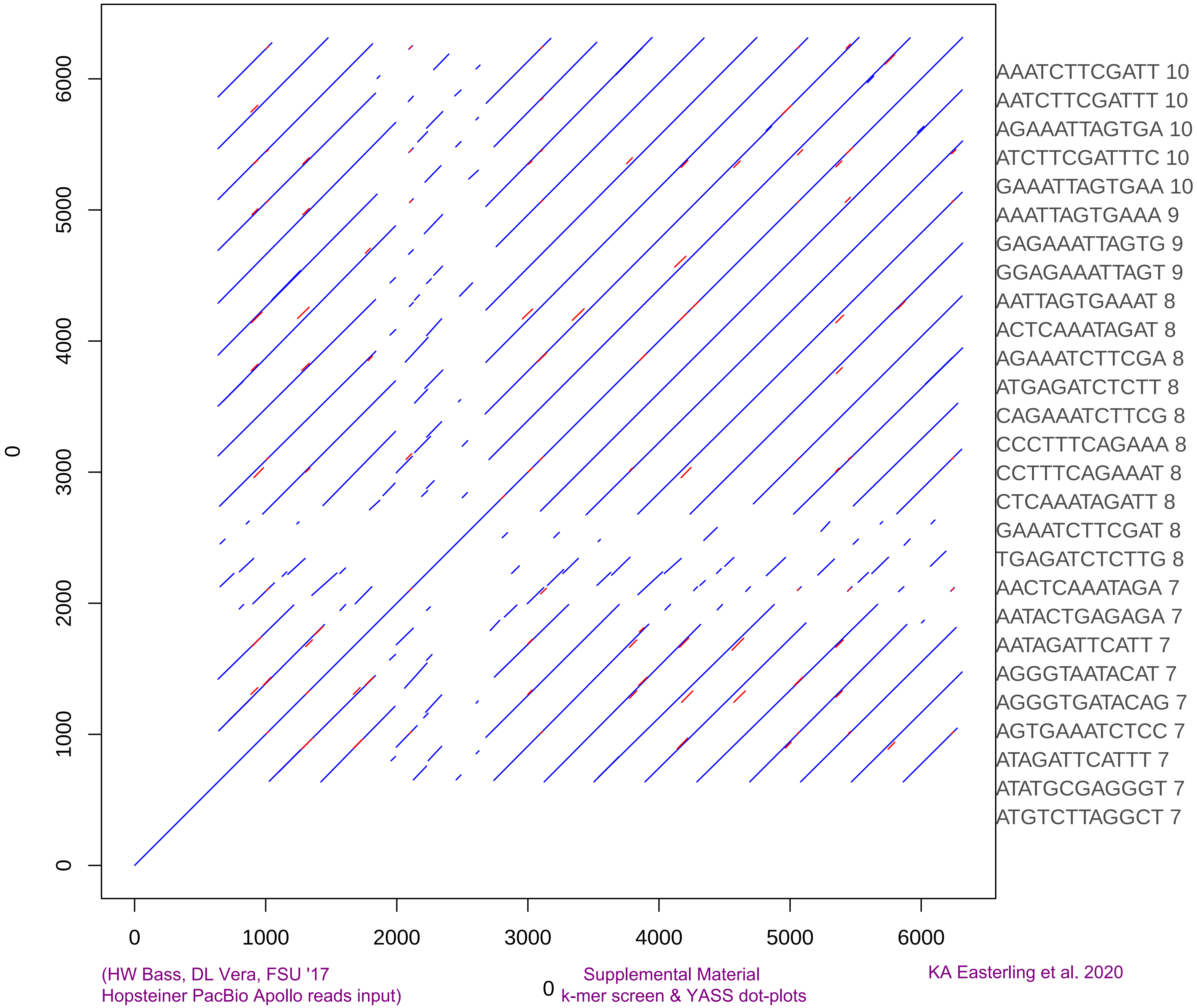

>m141126_034130_42134_c100732202550000001823141405141567_s1_p0/5022/0_7146

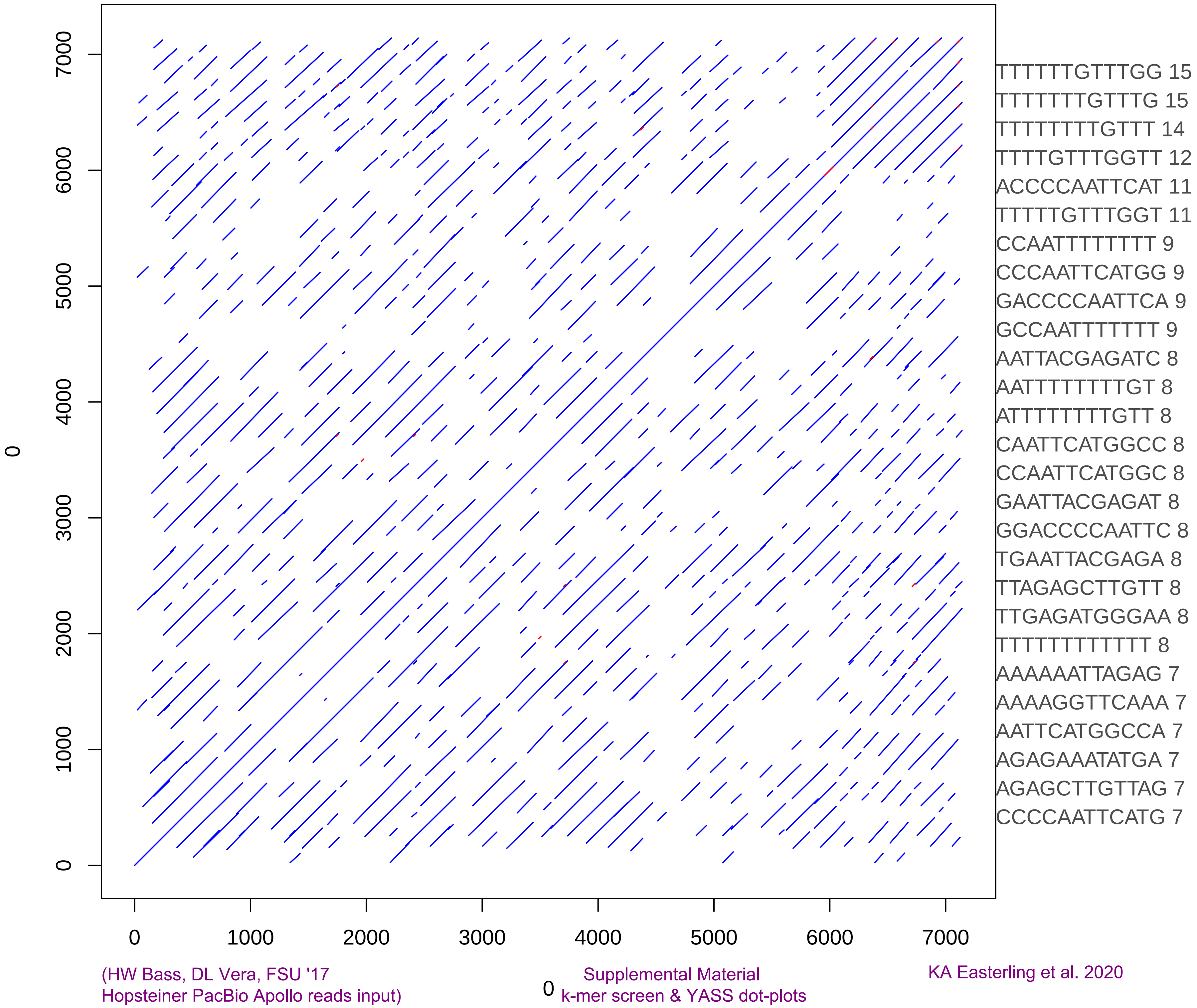

>m141126_034130_42134_c100732202550000001823141405141567_s1_p0/5716/0_10271

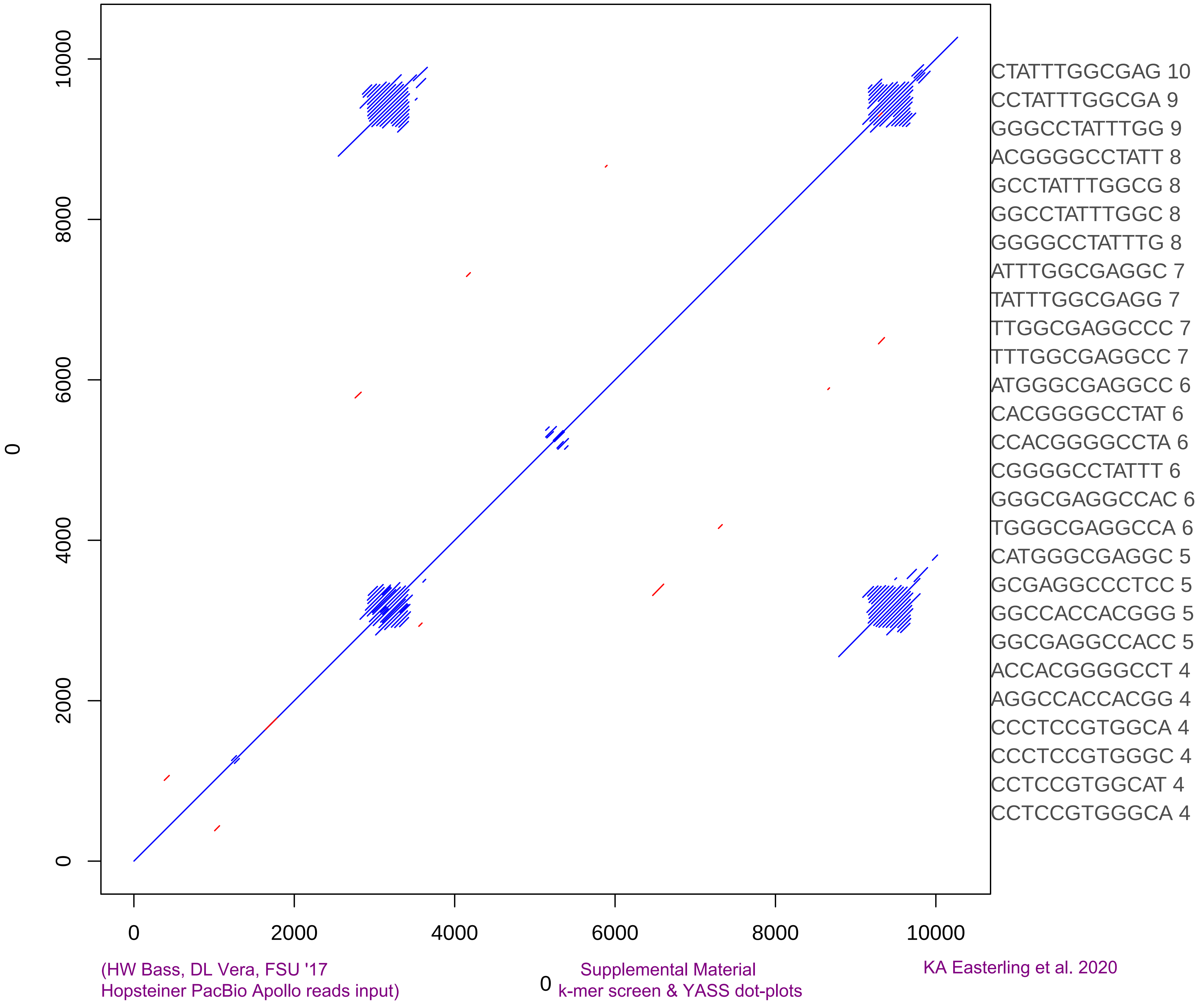

>m141126_034130_42134_c100732202550000001823141405141567_s1_p0/57173/4320_18364

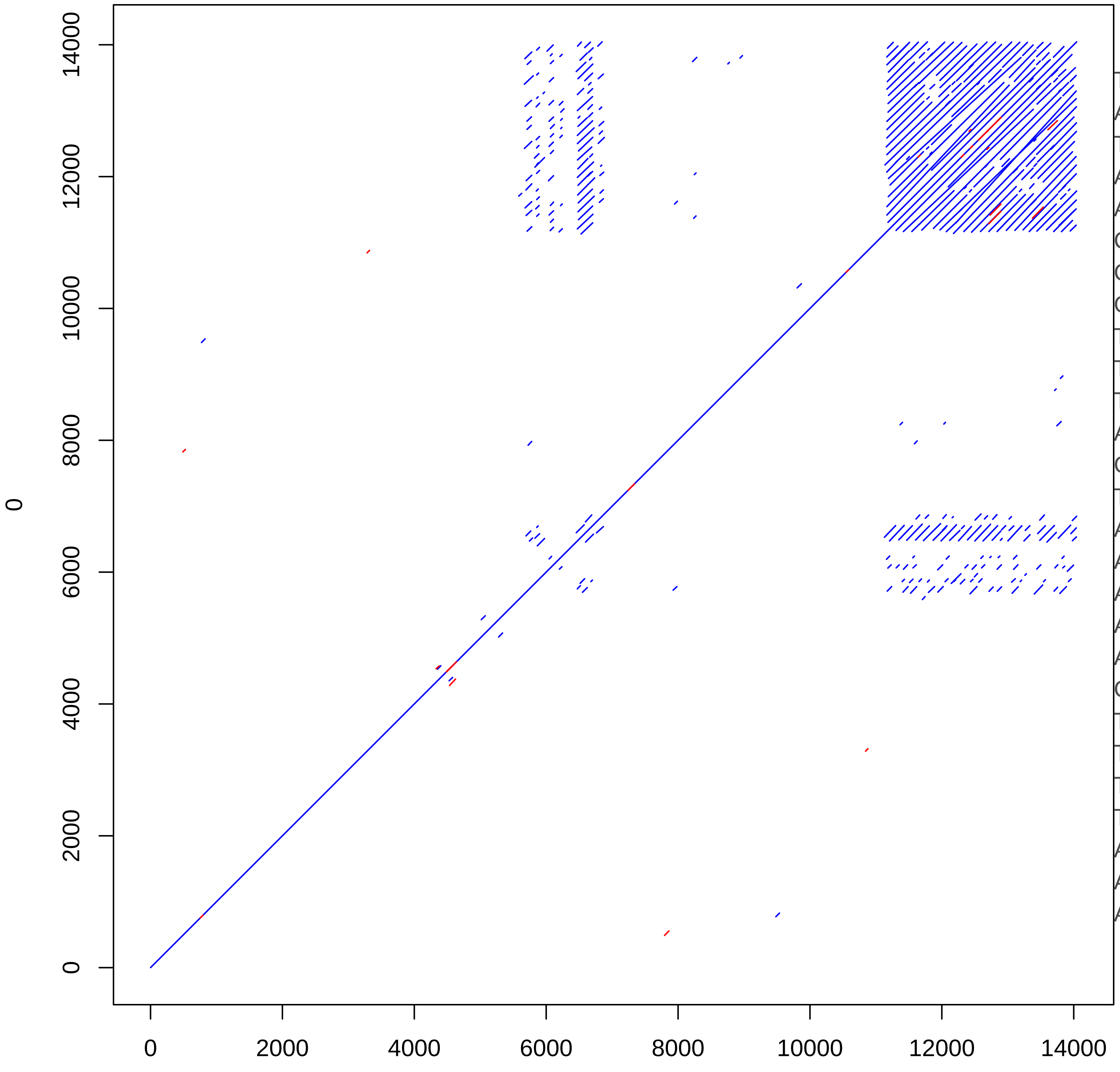

>m141126_034130_42134_c100732202550000001823141405141567_s1_p0/62478/0_5626

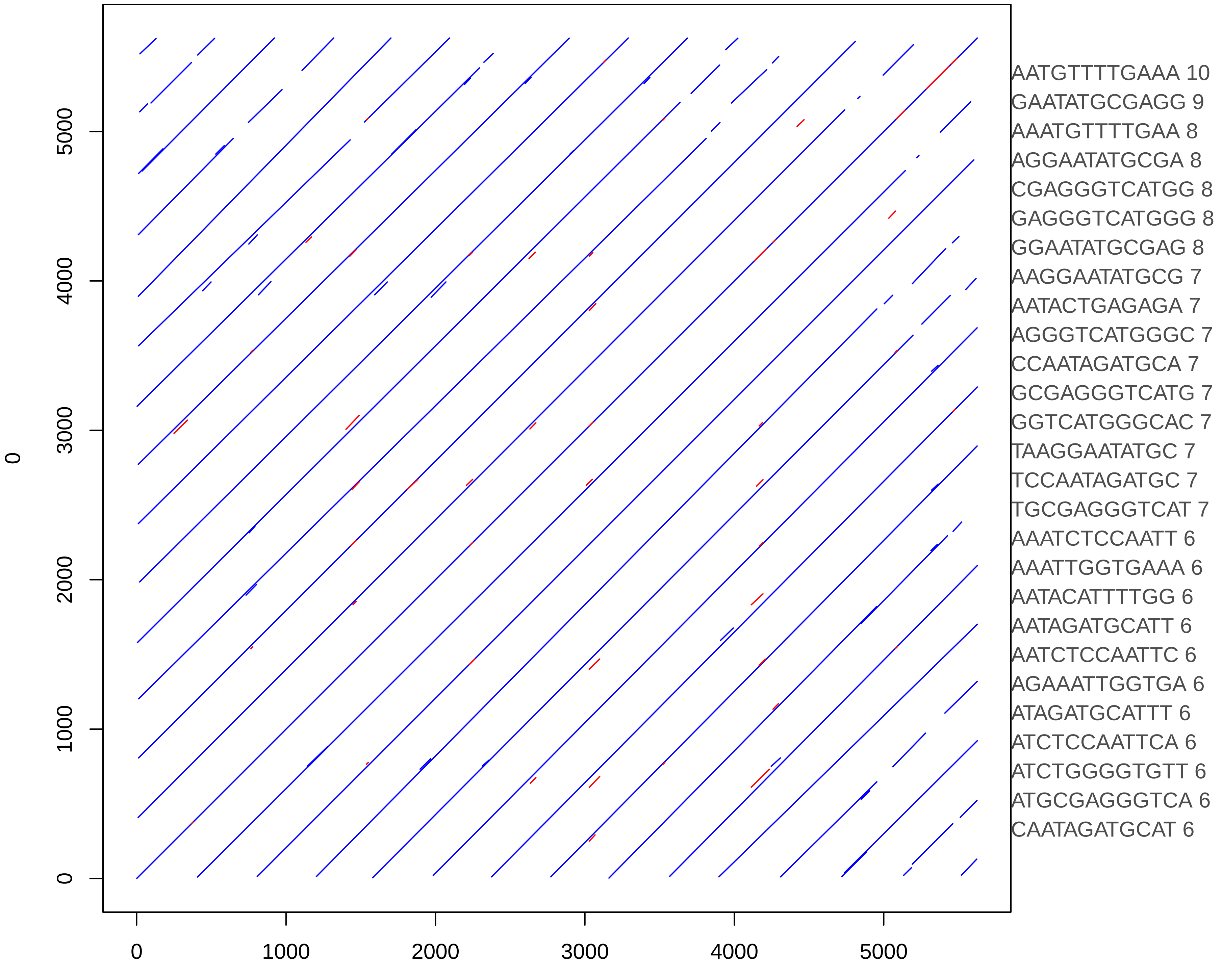

>m141126_034130_42134_c100732202550000001823141405141567_s1_p0/64354/5864_14576

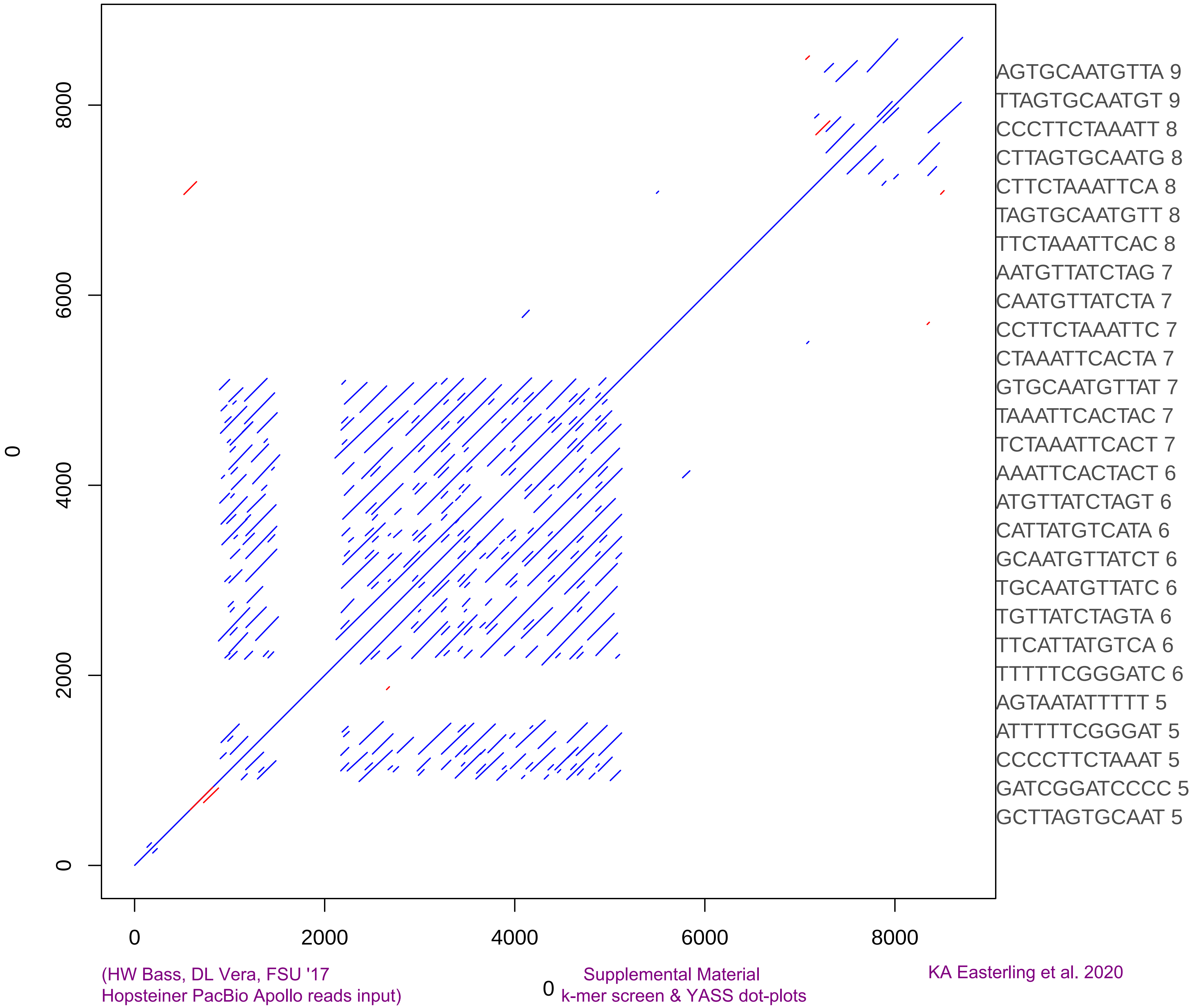

1,121 hits for repetitive sequences
passing 12mer abundance criterion

(Read-Number)
PAGE 540

For Tandem Repeat families of ### bp:
PacBio Read named "HuluTR###-r540"

>m141126_034130_42134_c100732202550000001823141405141567_s1_p0/65957/771_11581

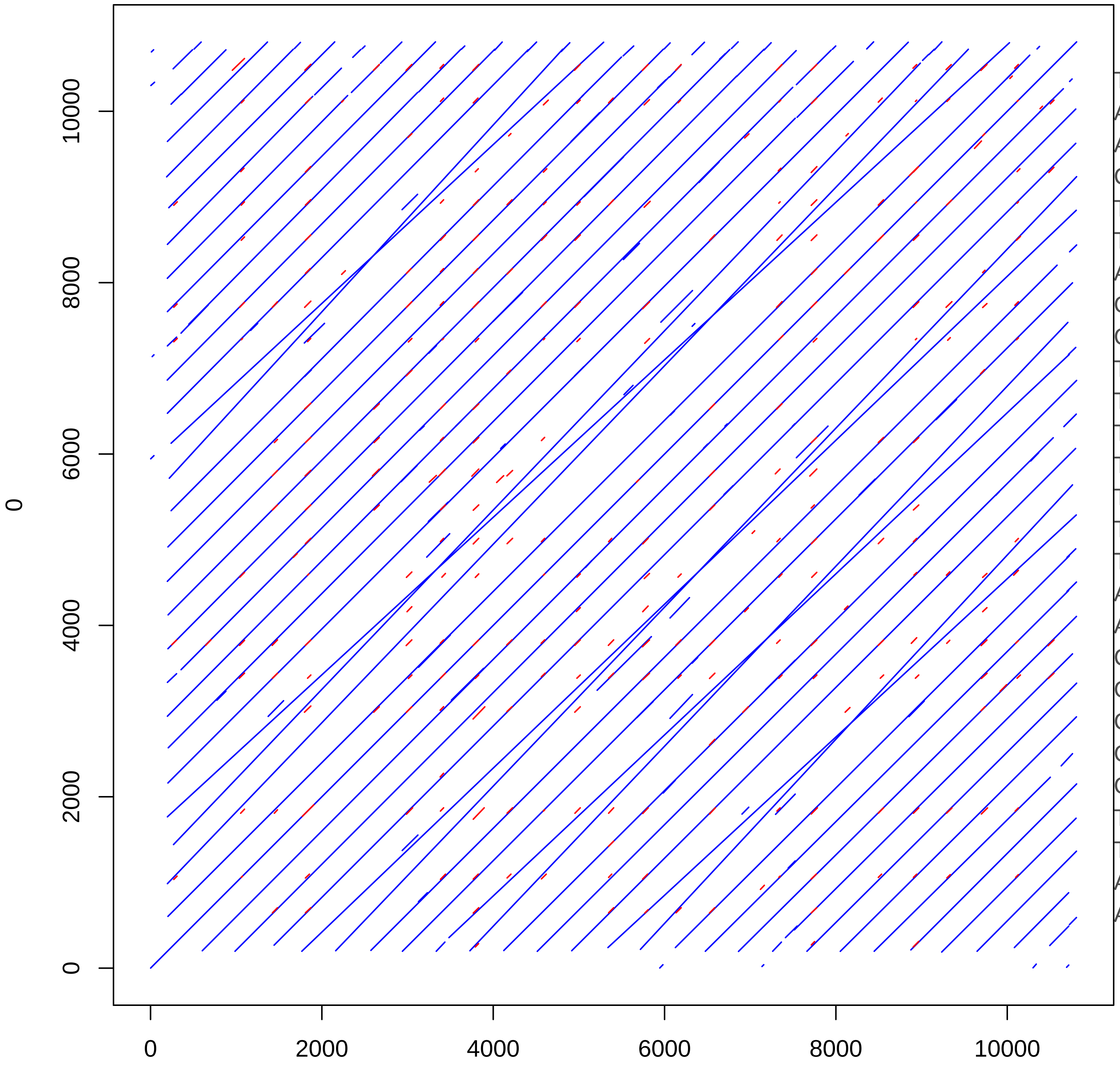

(HW Bass, DL Vera, FSU '17
Hopsteiner PacBio Apollo reads input)

Supplemental Material
k-mer screen & YASS dot-plots

KA Easterling et al. 2020

>m141126_034130_42134_c100732202550000001823141405141567_s1_p0/6856/6361_14156

Representative (only one) Read (Table 1): HuluTR070-r541, 1 ppm,
73% A+T, GenBank Acc. MN537568

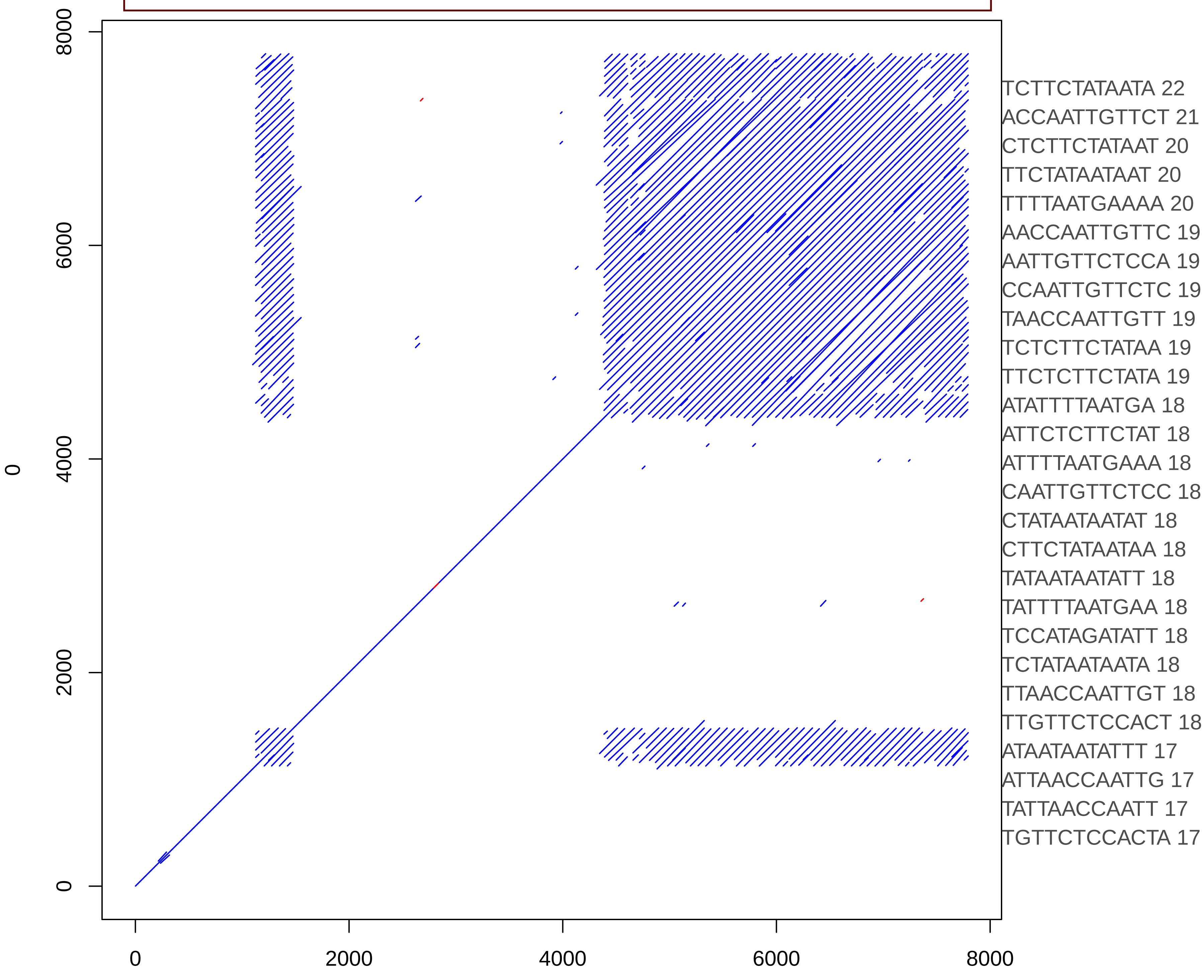

>m141126_034130_42134_c100732202550000001823141405141567_s1_p0/70162/0_12312

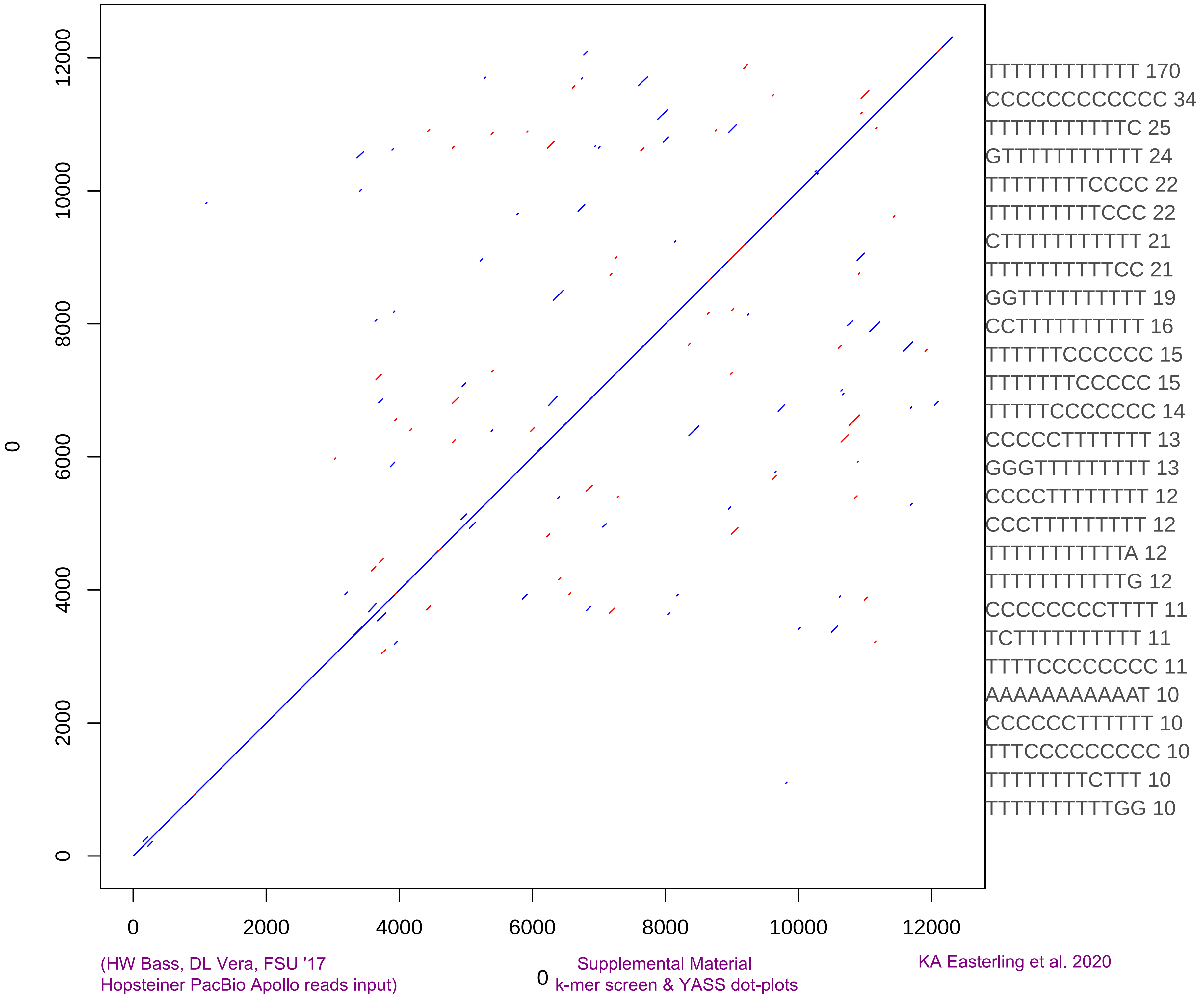

>m141126_034130_42134_c100732202550000001823141405141567_s1_p0/769/1739_14718

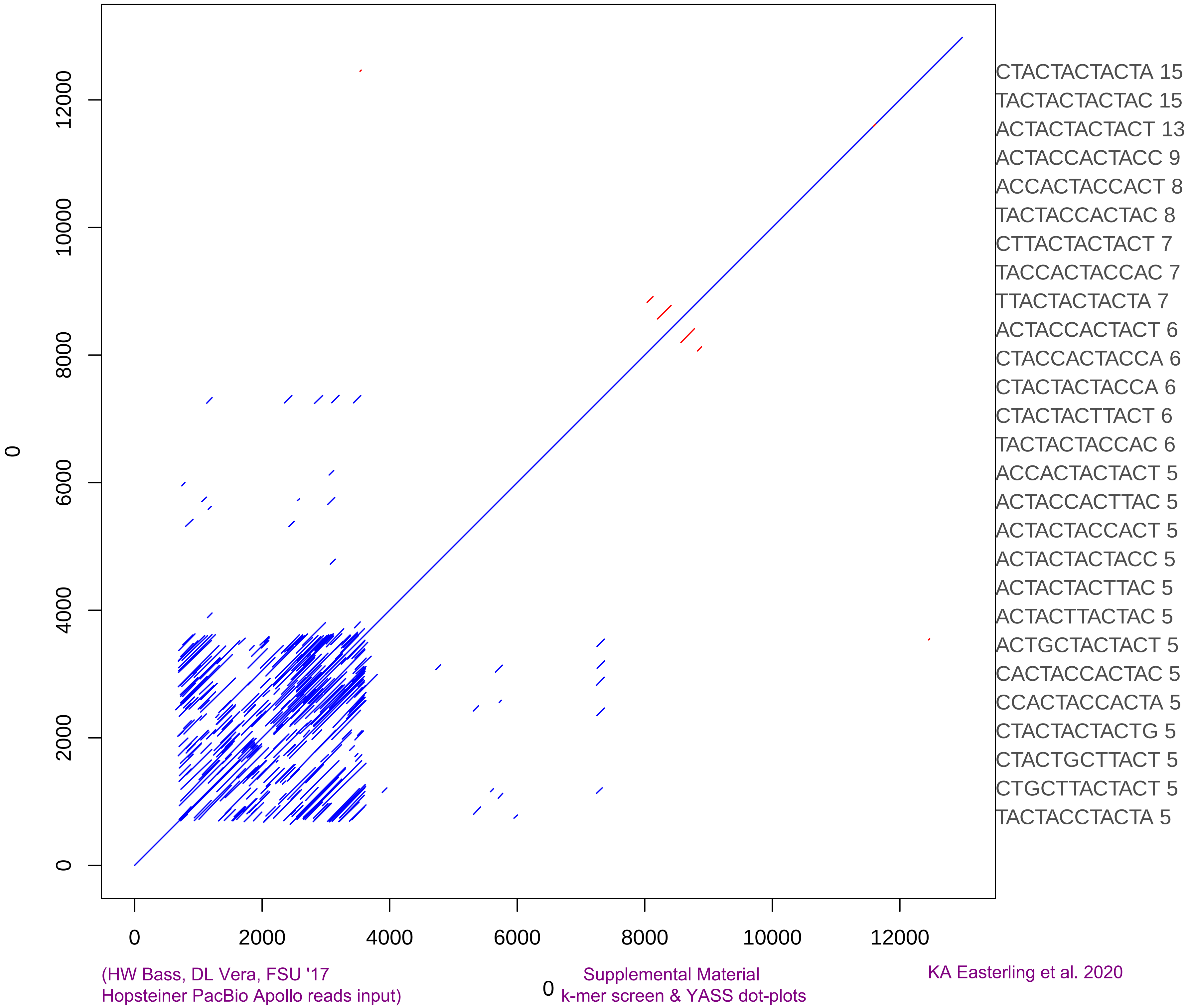

>m141126_034130_42134_c100732202550000001823141405141567_s1_p0/77317/4861_12516

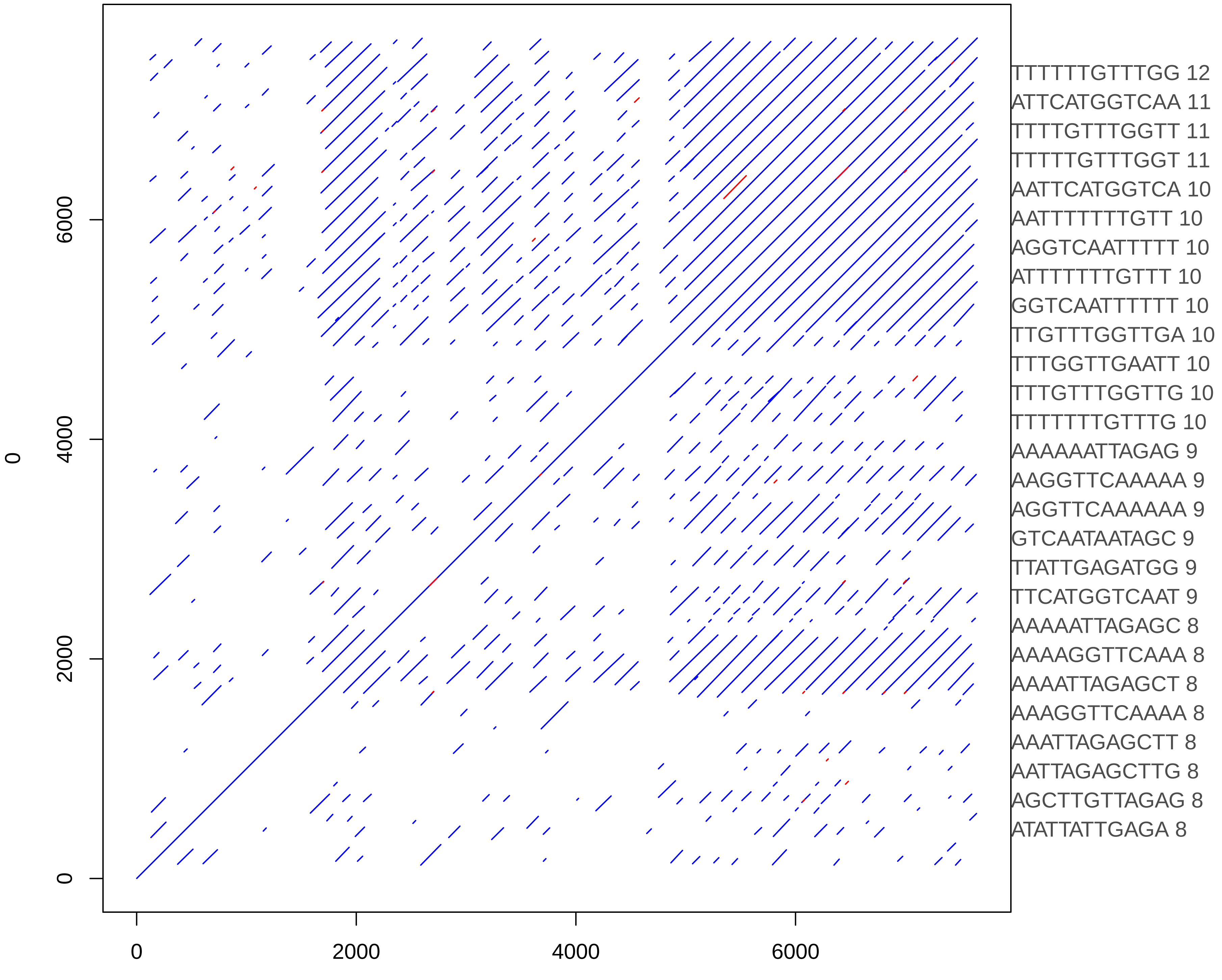

>m141126_034130_42134_c100732202550000001823141405141567_s1_p0/83456/872_9196

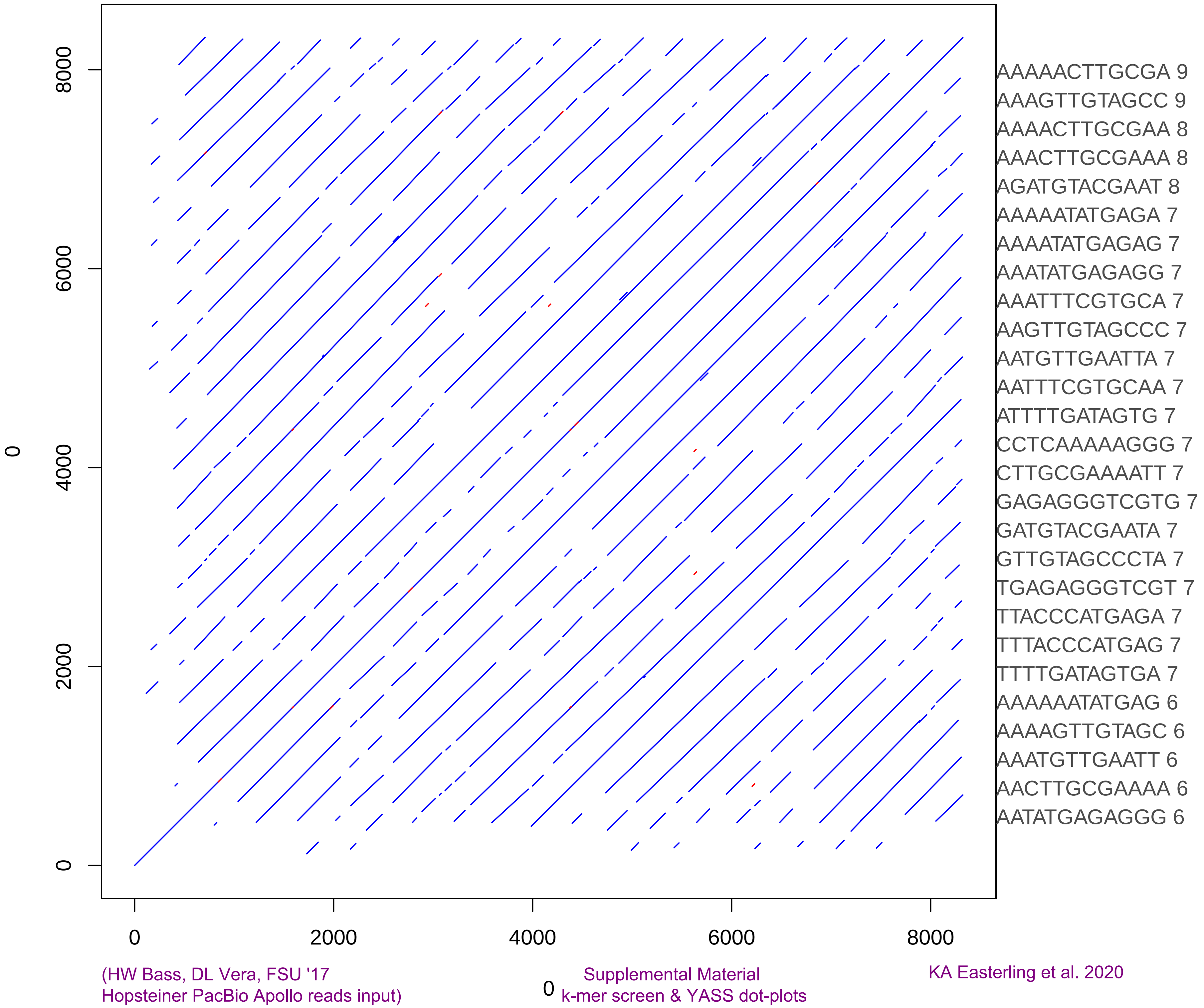

>m141126_034130_42134_c100732202550000001823141405141567_s1_p0/83594/0_7116

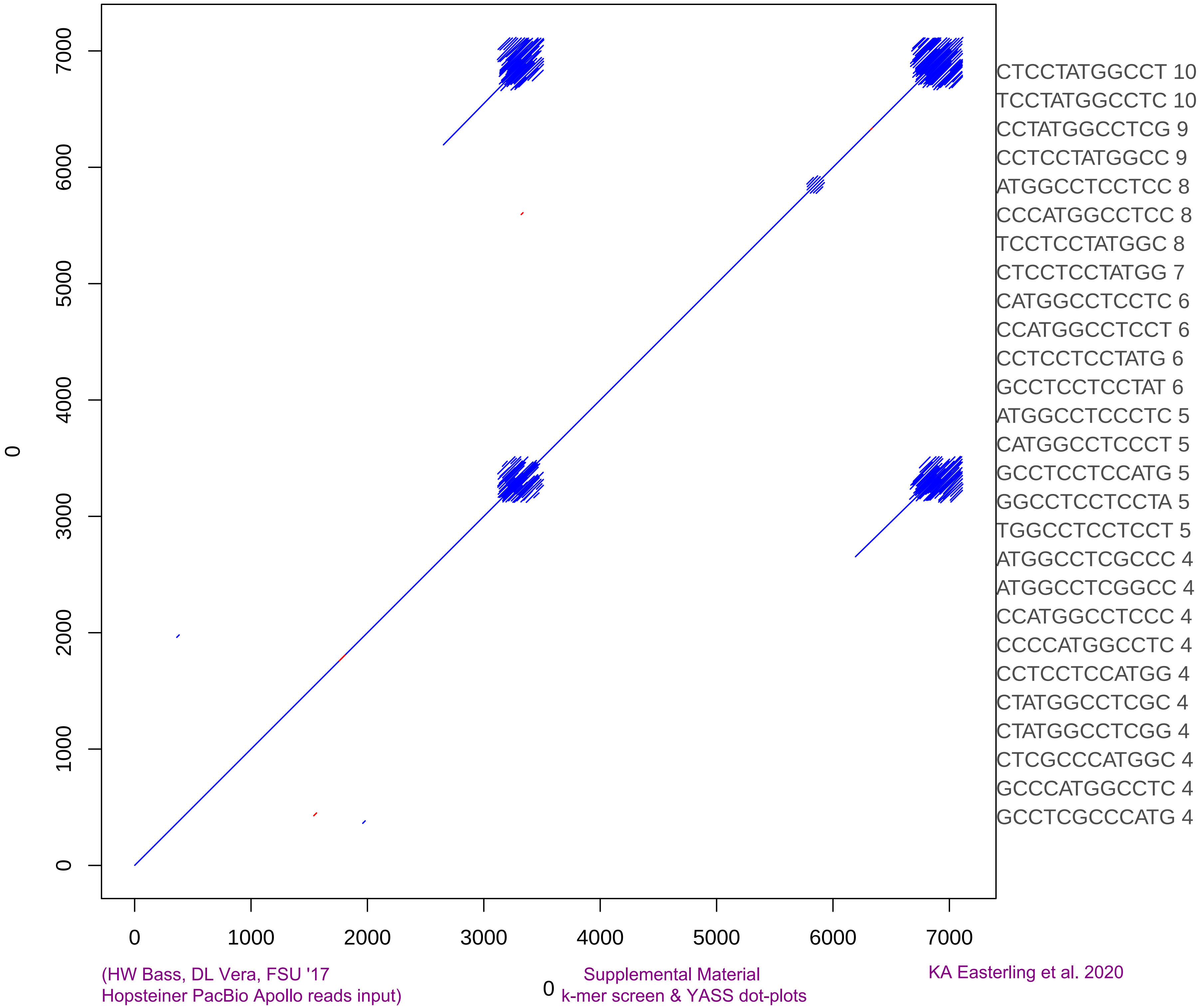

>m141126_034130_42134_c100732202550000001823141405141567_s1_p0/83594/7161_13130

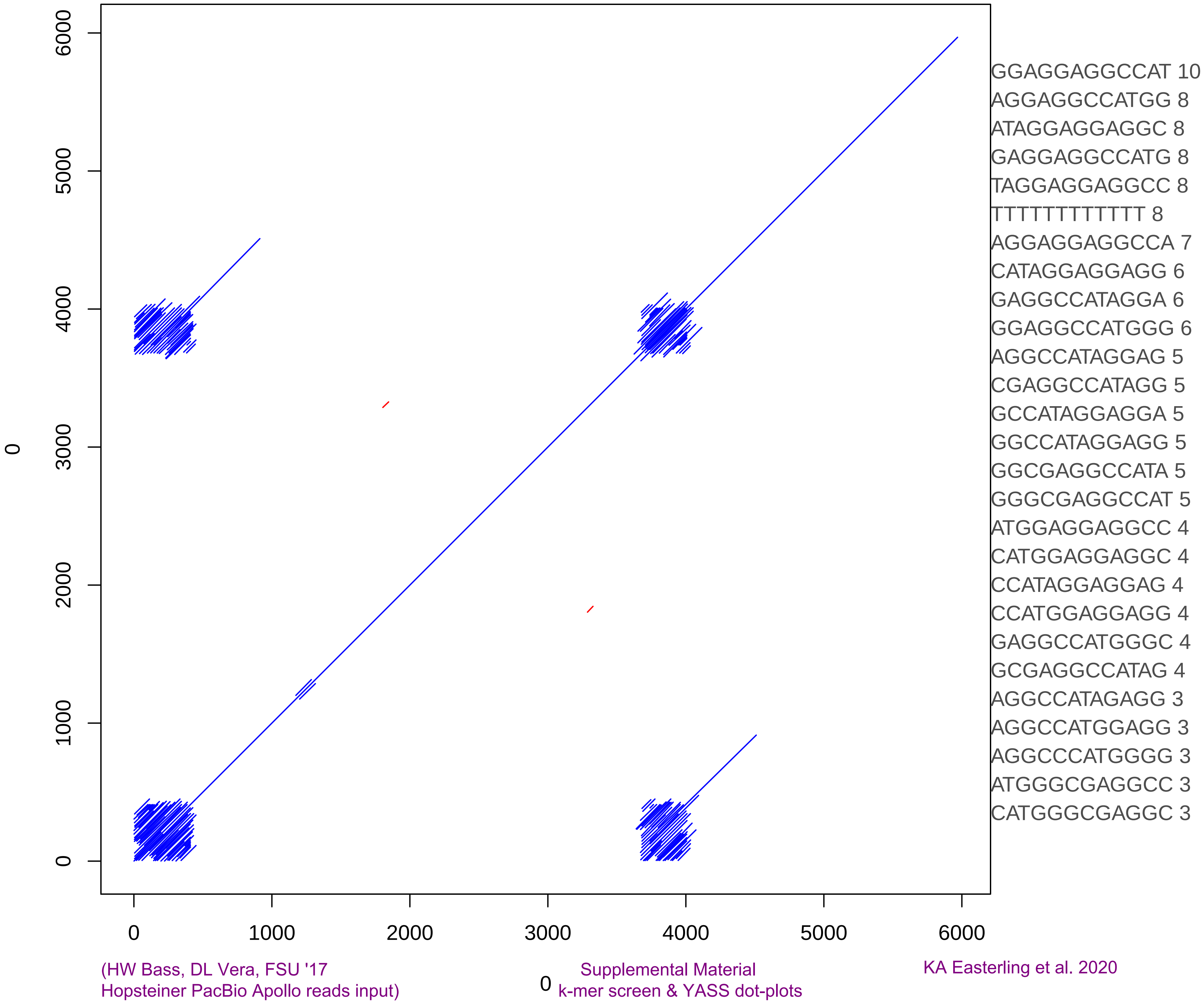

1,121 hits for repetitive sequences
passing 12mer abundance criterion

(Read-Number)
PAGE 548

For Tandem Repeat families of ### bp:
PacBio Read named "HuluTR###-r548"

>m141126_034130_42134_c100732202550000001823141405141567_s1_p0/85115/2809_15577

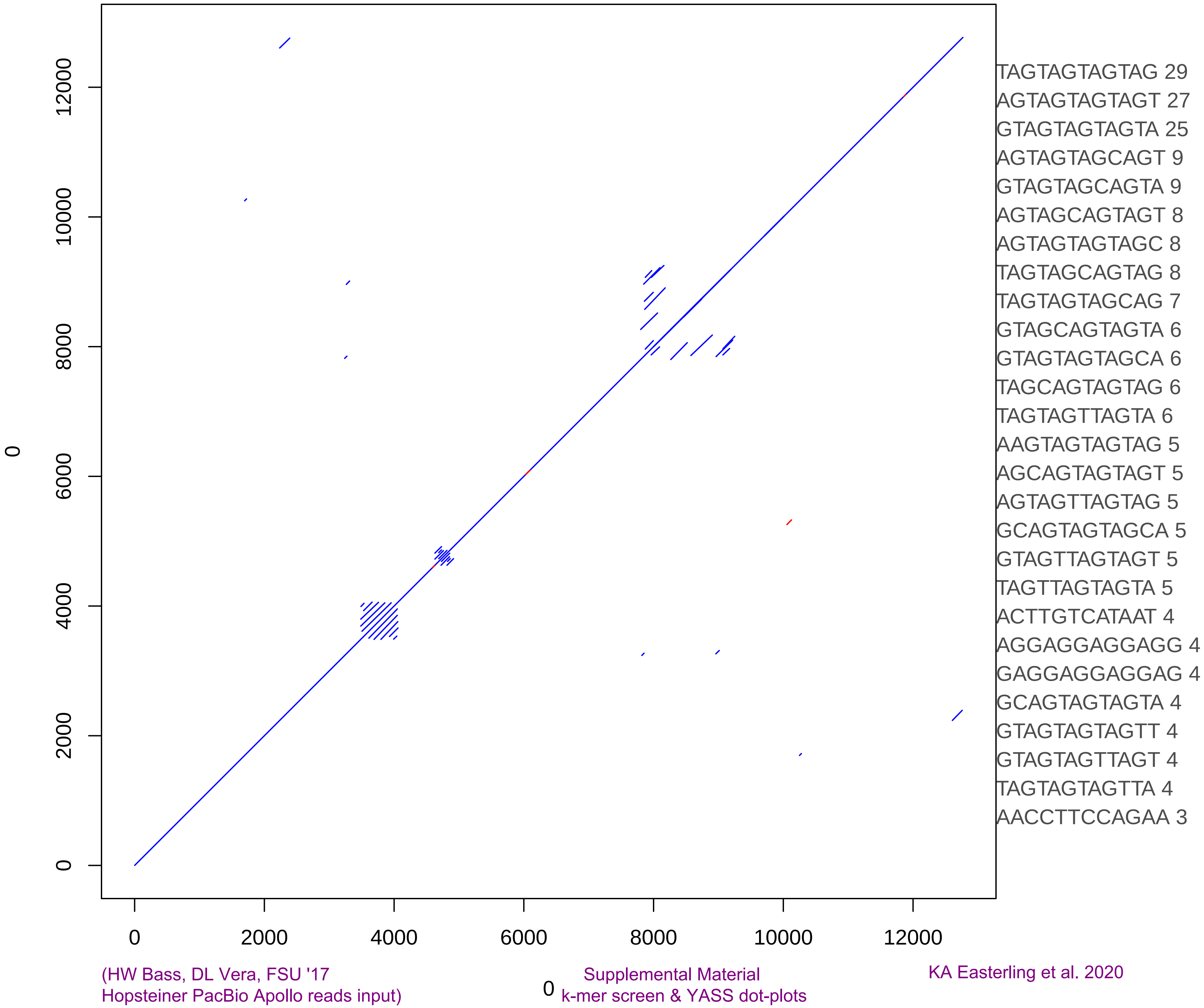

(HW Bass, DL Vera, FSU '17
Hopsteiner PacBio Apollo reads input)

Supplemental Material
k-mer screen & YASS dot-plots

KA Easterling et al. 2020

>m141126_034130_42134_c100732202550000001823141405141567_s1_p0/86923/0_5574

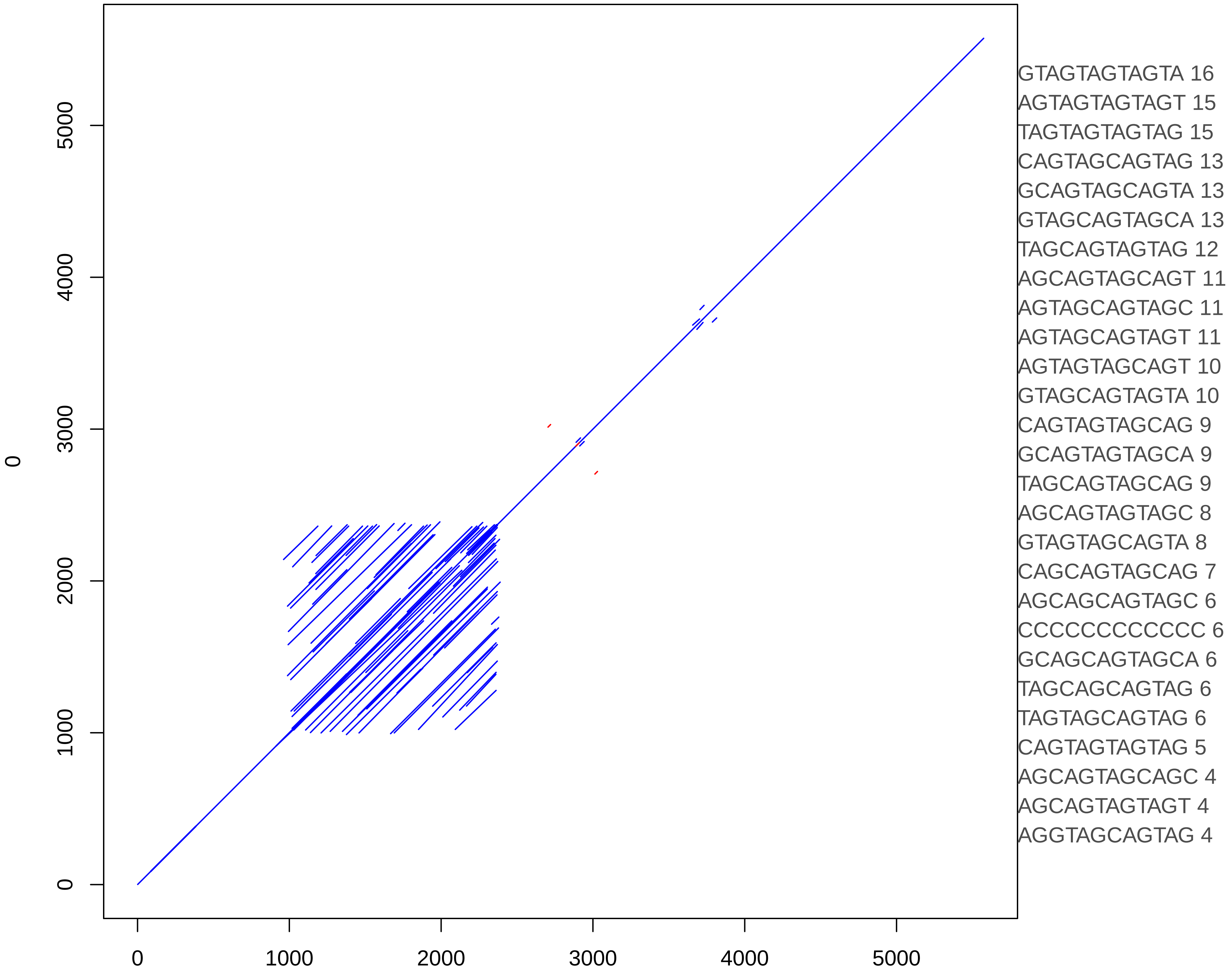

>m141126_034130_42134_c100732202550000001823141405141567_s1_p0/86967/0_6811

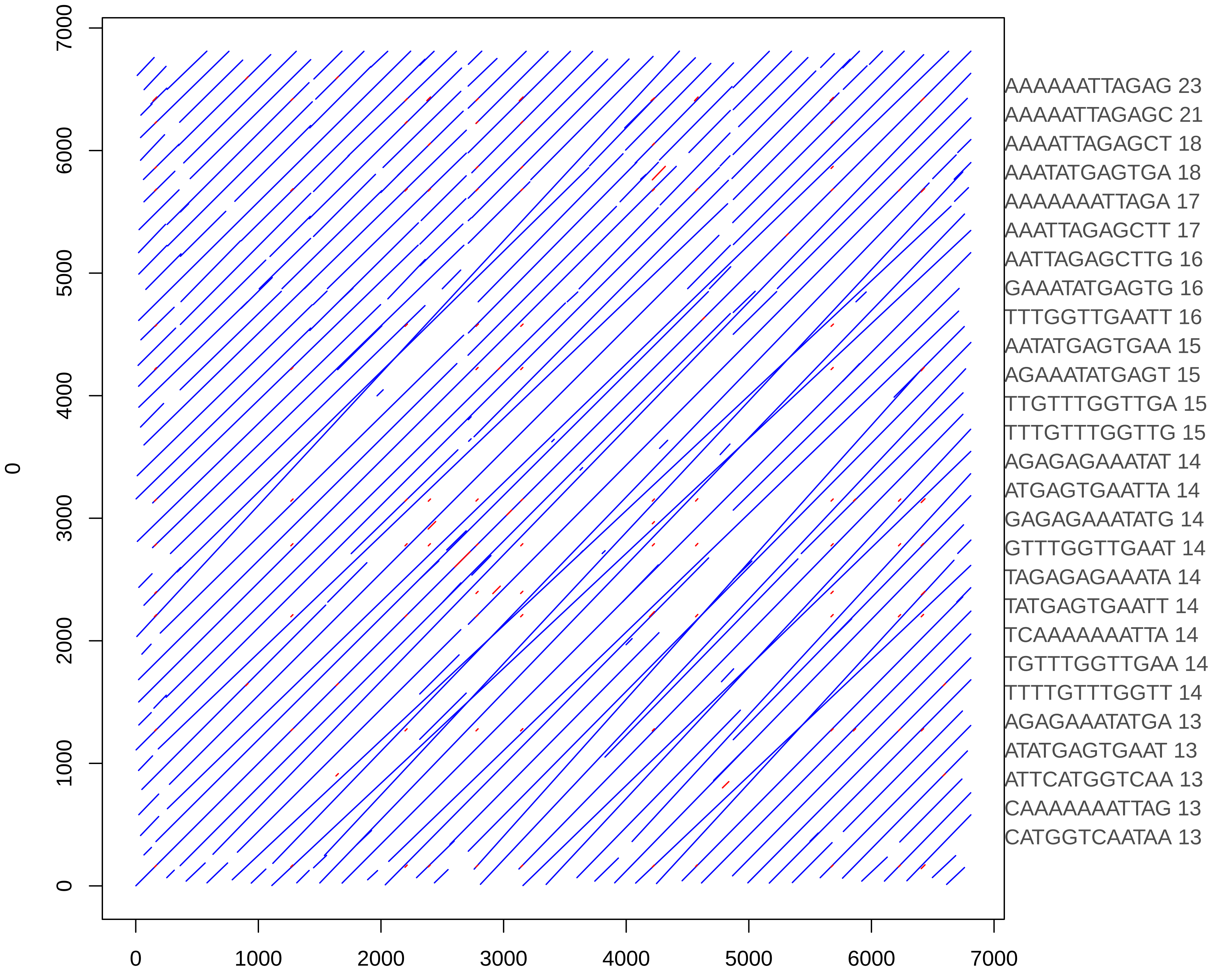

>m141126_034130_42134_c100732202550000001823141405141567_s1_p0/89674/13194_18477

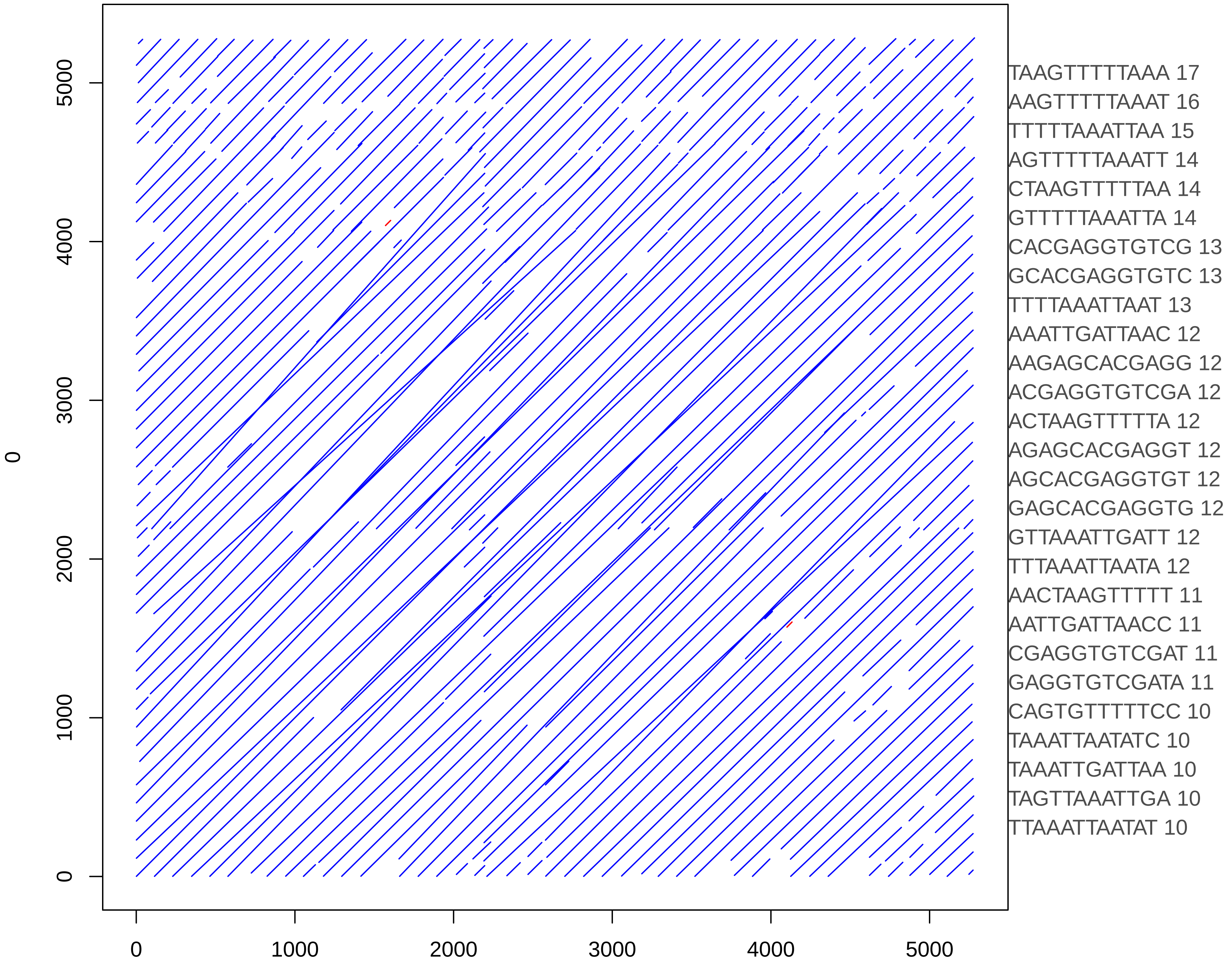

>m141126_034130_42134_c100732202550000001823141405141567_s1_p0/89674/7815_13149

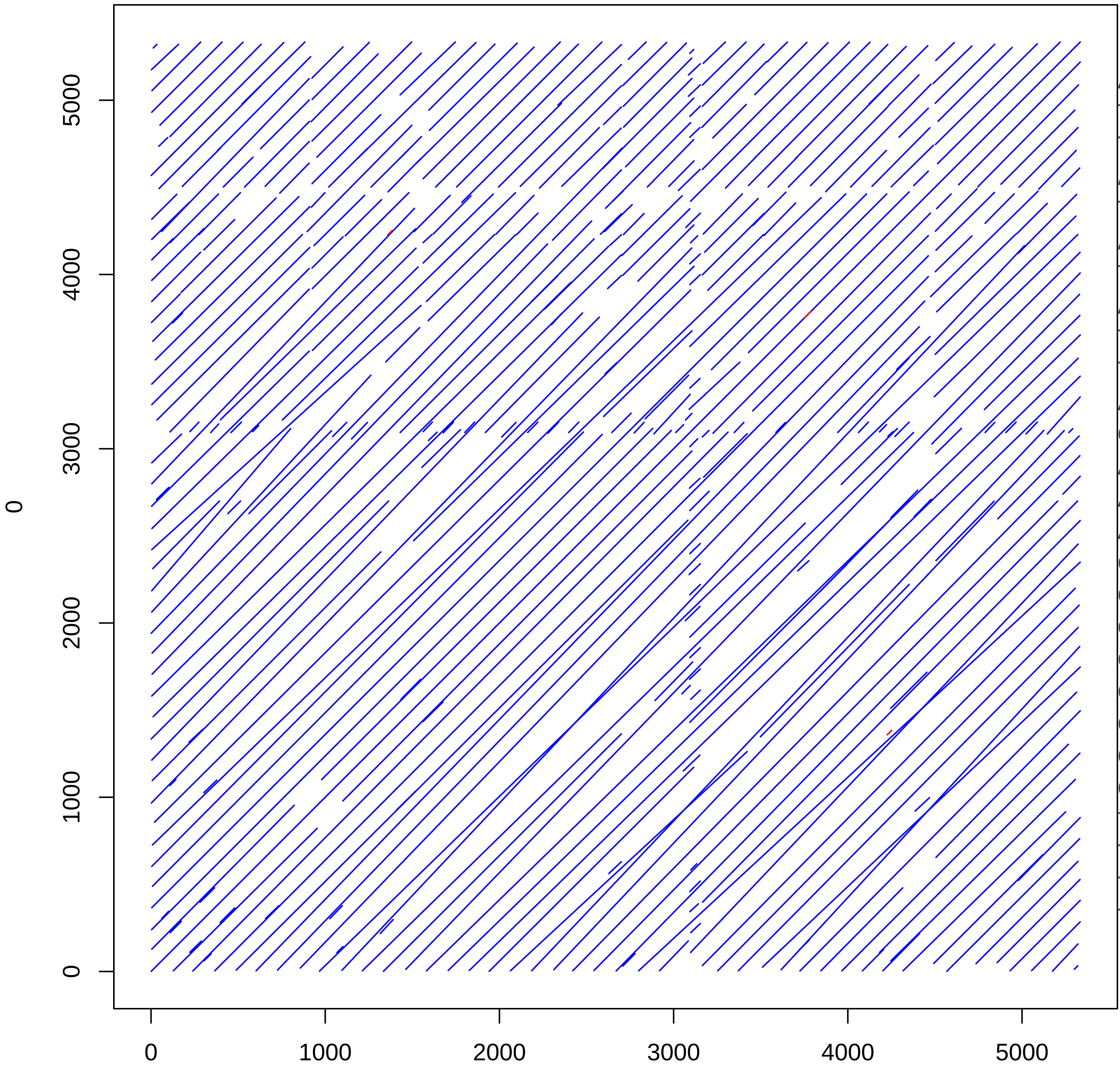

>m141126_034130_42134_c100732202550000001823141405141567_s1_p0/91646/5816_18891

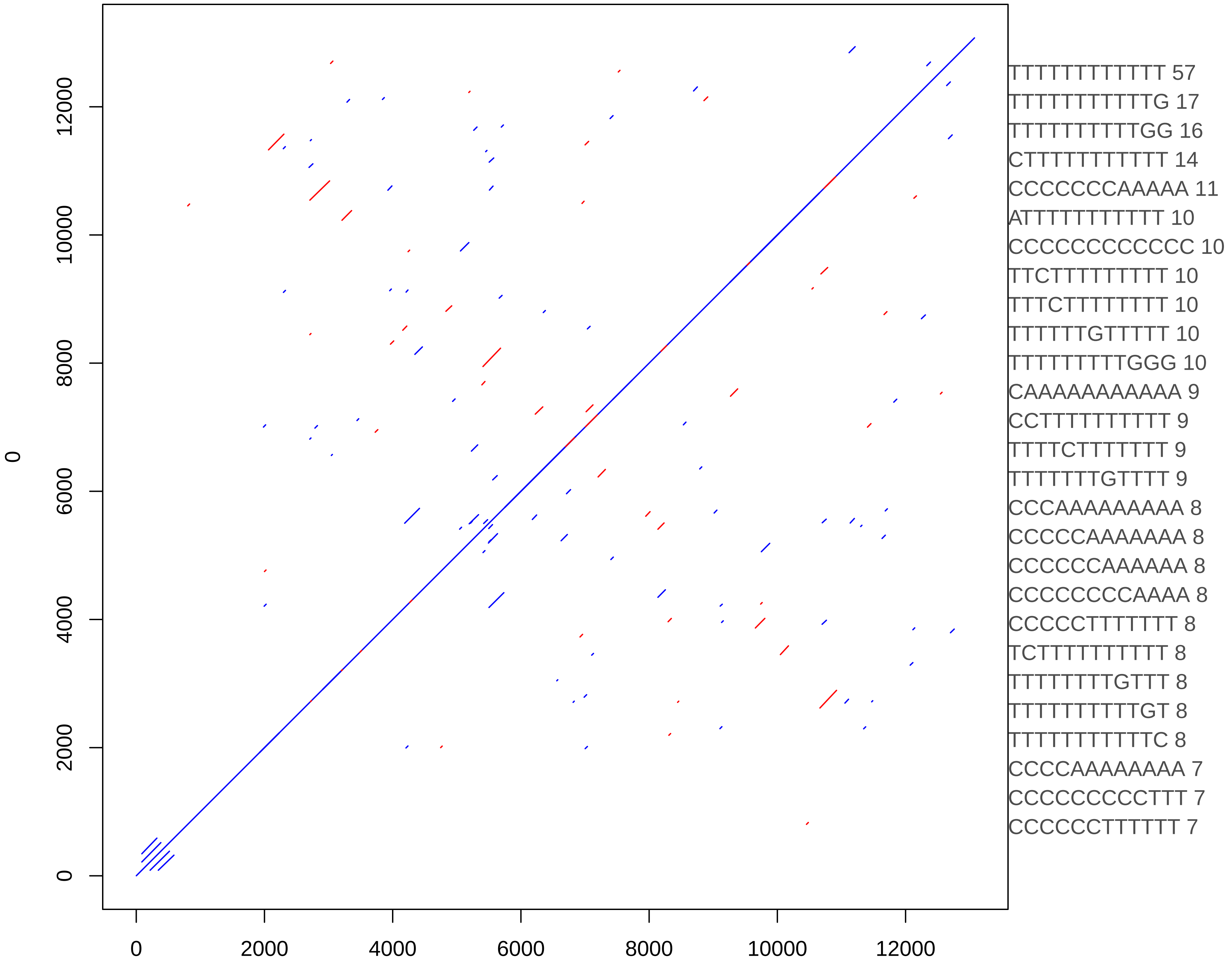

0

>m141126_034130_42134_c100732202550000001823141405141567_s1_p0/92126/0_10165

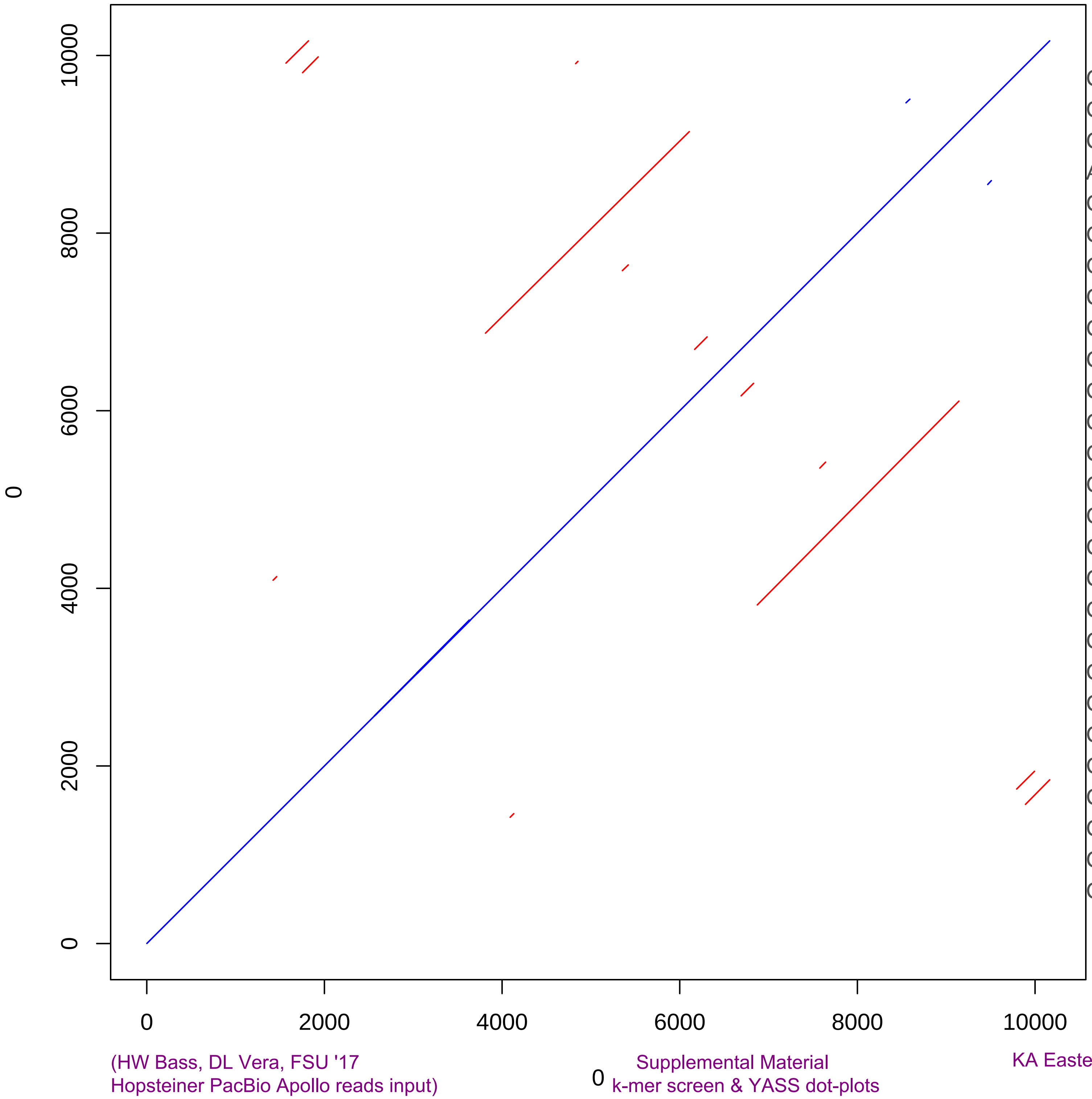

CCCCCCCCCCCC 64
CACCCCCCCCCC 16
CCCCCCCACCCC 15
ACCCCCCCCCCC 14
CCCACCCCCCCC 14
CCCCCCCCCCAC 14
CCCCACCCCCCC 13
CCCCCCCCCACC 13
CCACCCCCCCCC 12
CCCCCCACCCCC 12
CCCCCACCCCCC 11
CCCCCCCCCCCA 11
CCCTCCCCCCCC 6
CGCCCCCCCCCC 6
CCCCCCCCCCCG 5
CCCCCCCCCCCT 5
CCCCCCCCCCTC 5
CCCCCCCCCGCC 5
CCCCCCCCGCCC 5
CCCCCCCCTCCC 5
CCCCCCCTCCCC 5
CCCCCCTCCCCC 5
CCCCCTCCCCCC 5
CCGCCCCCCCCC 5
CCTCCCCCCCCC 5
CCCCCCCCCCGC 4
CCCCCCCGCCCC 4

>m141126_034130_42134_c100732202550000001823141405141567_s1_p0/949/4656_10596

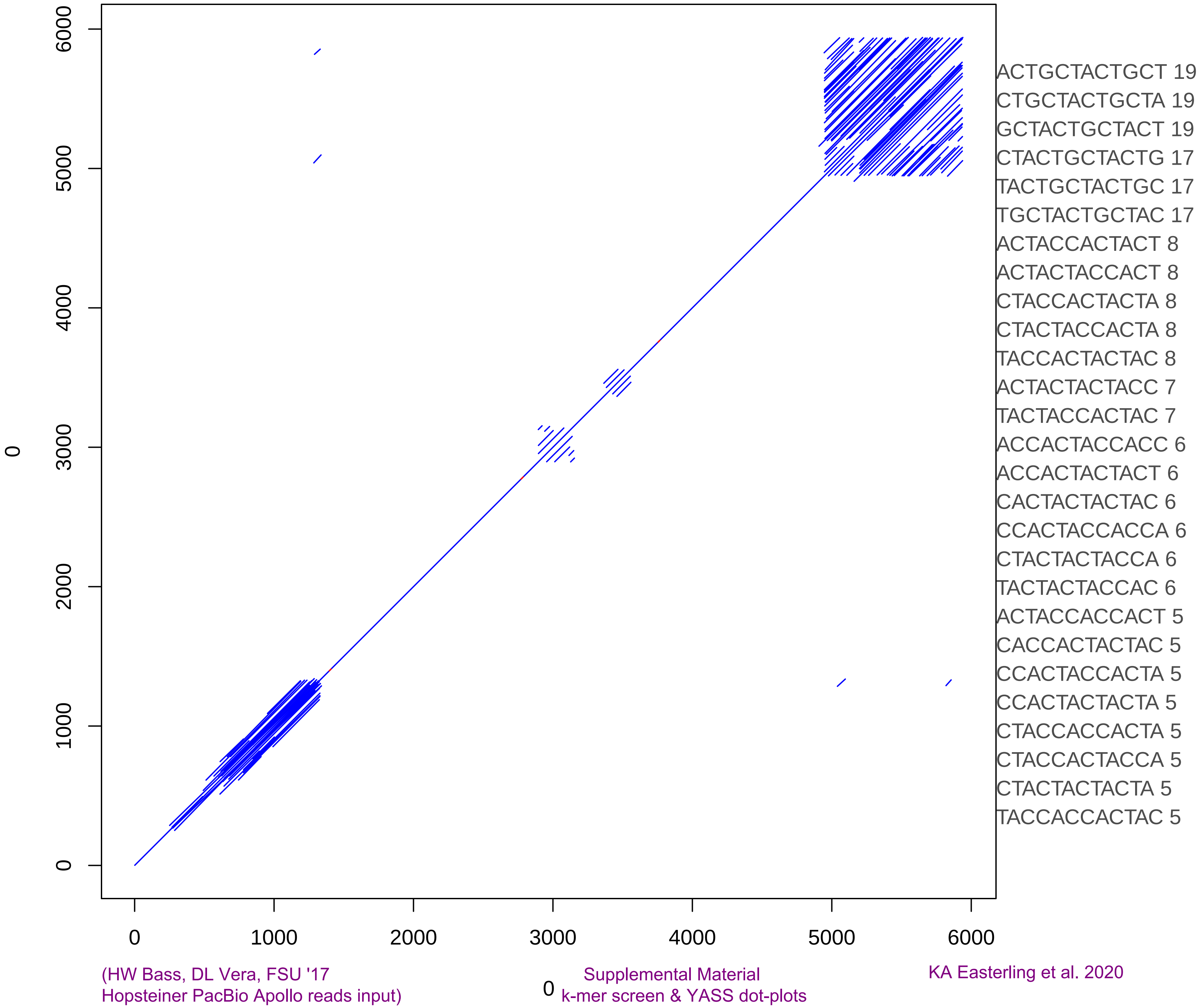

>m141126_034130_42134_c100732202550000001823141405141567_s1_p0/95174/0_6702

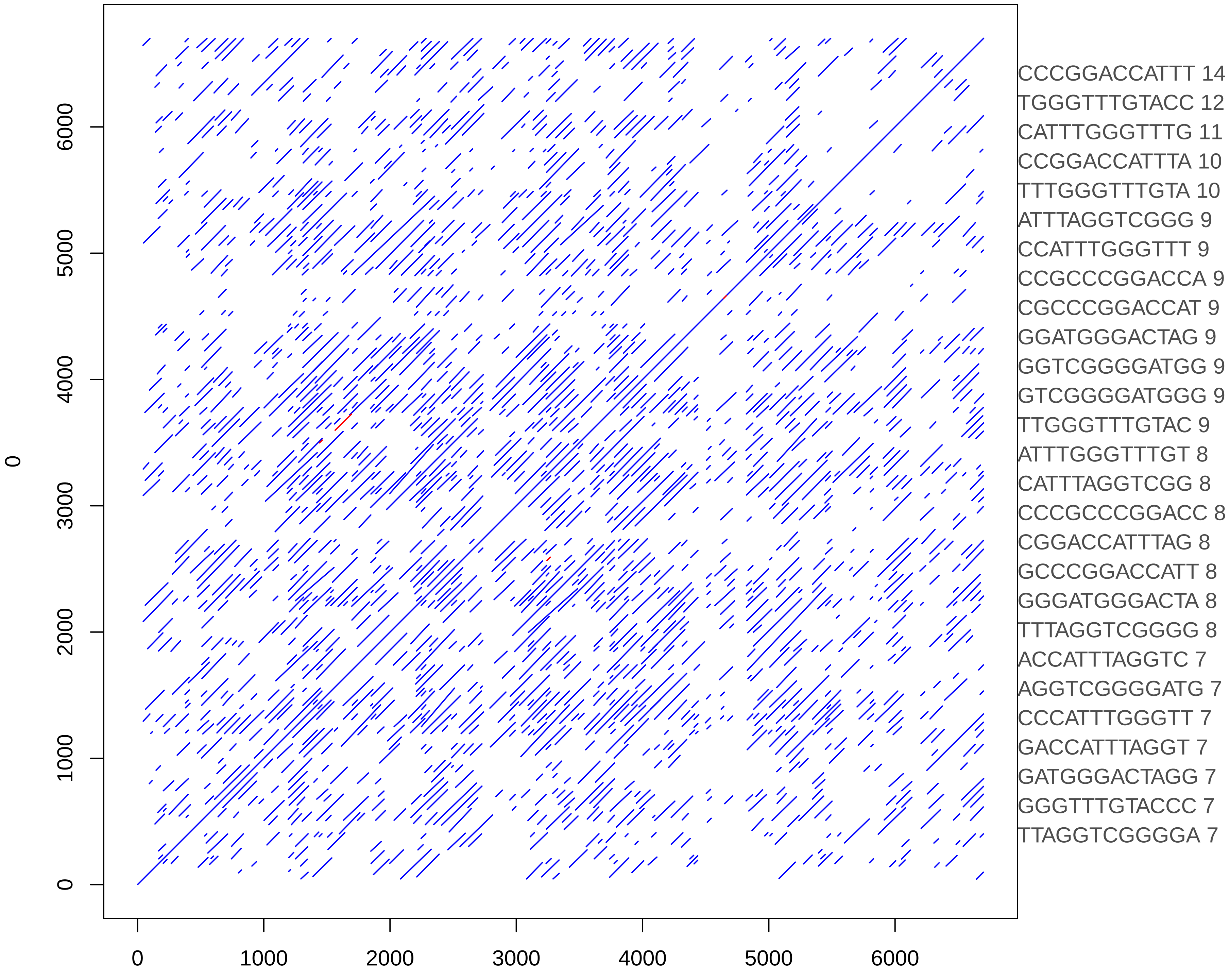

>m141126_034130_42134_c100732202550000001823141405141567_s1_p0/97941/0_7636

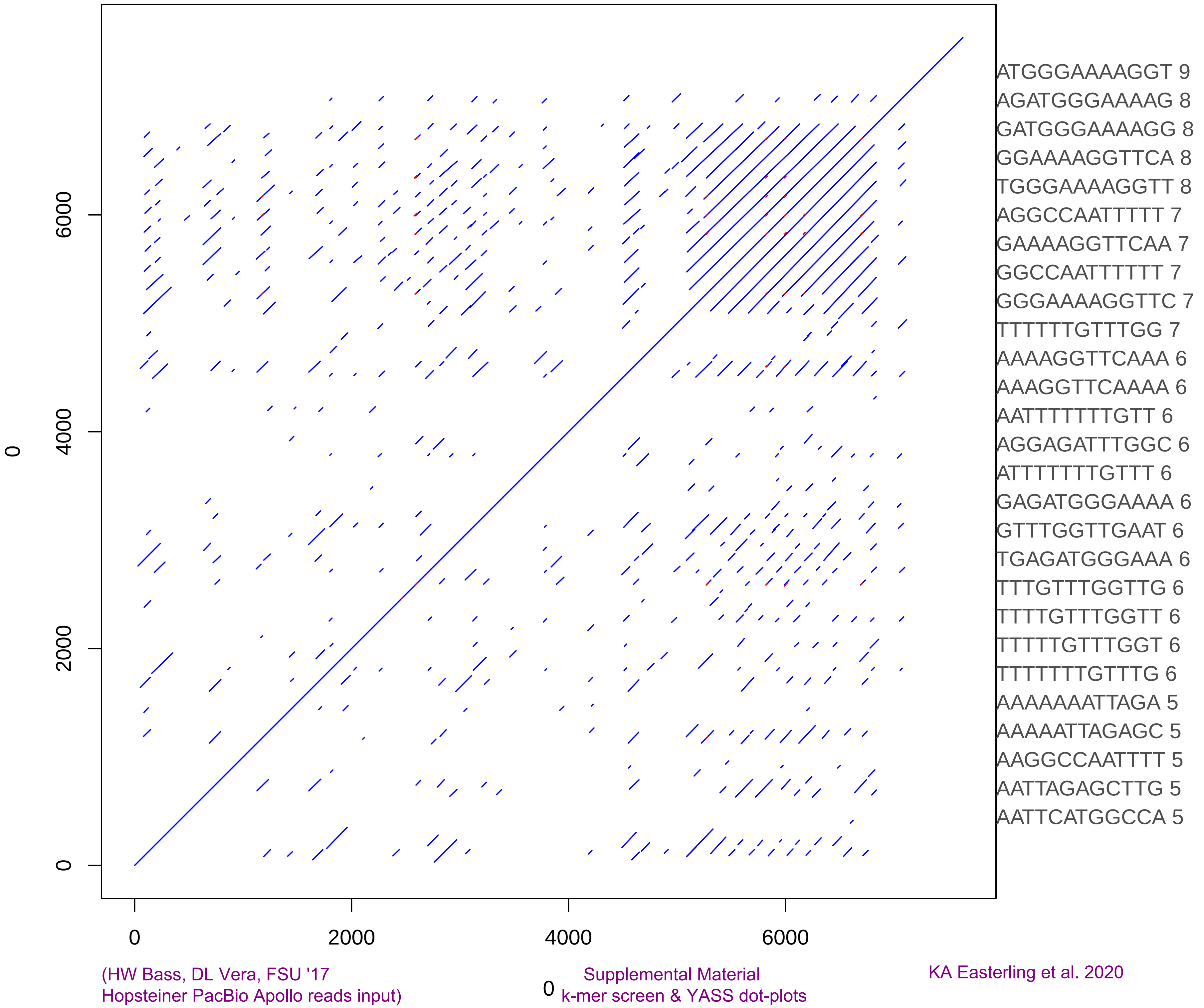

>m141126_060447_42134_c100732262550000001823141405141500_s1_p0/100344/8907_14281

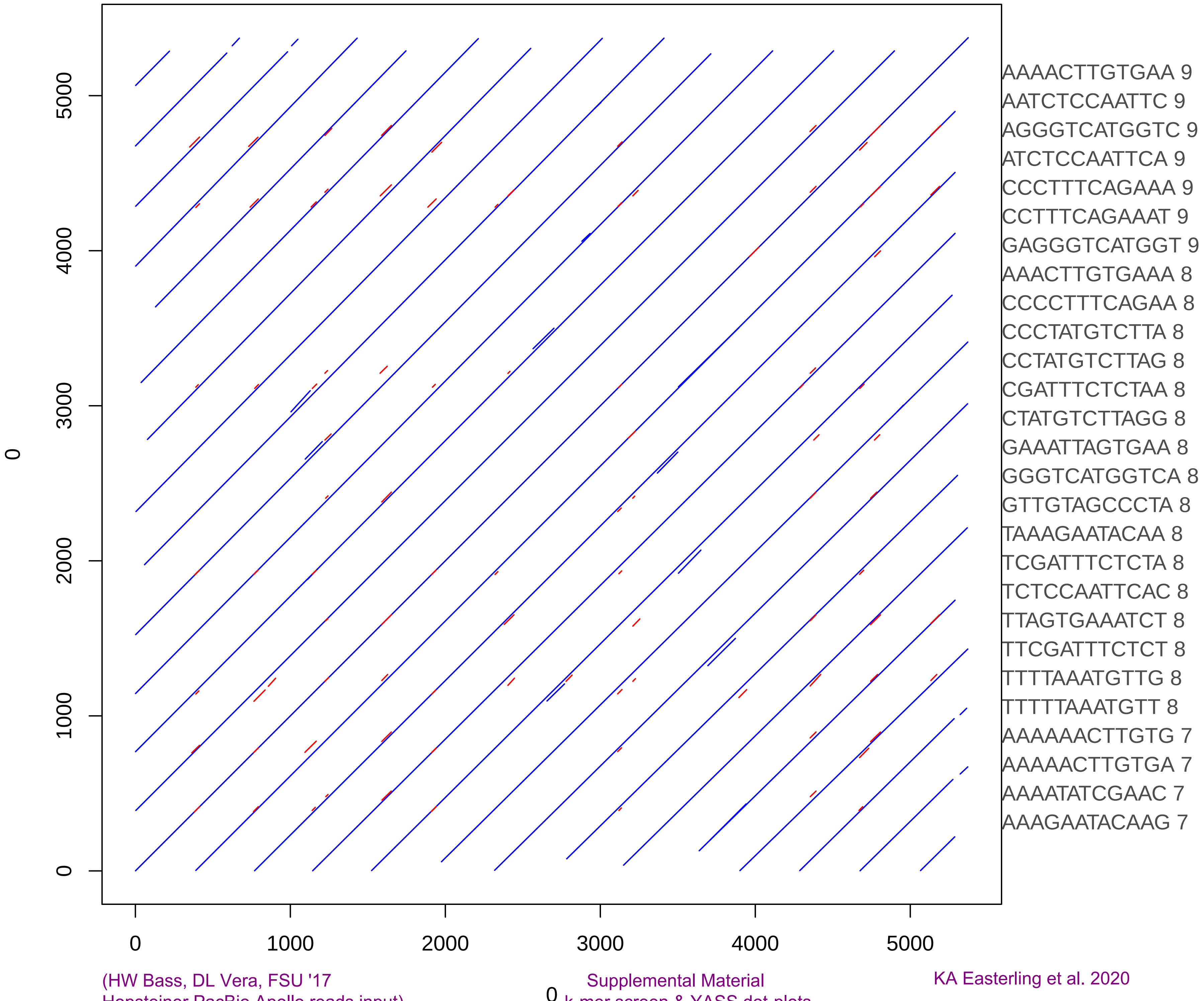

>m141126_060447_42134_c100732262550000001823141405141500_s1_p0/10726/1764_9077

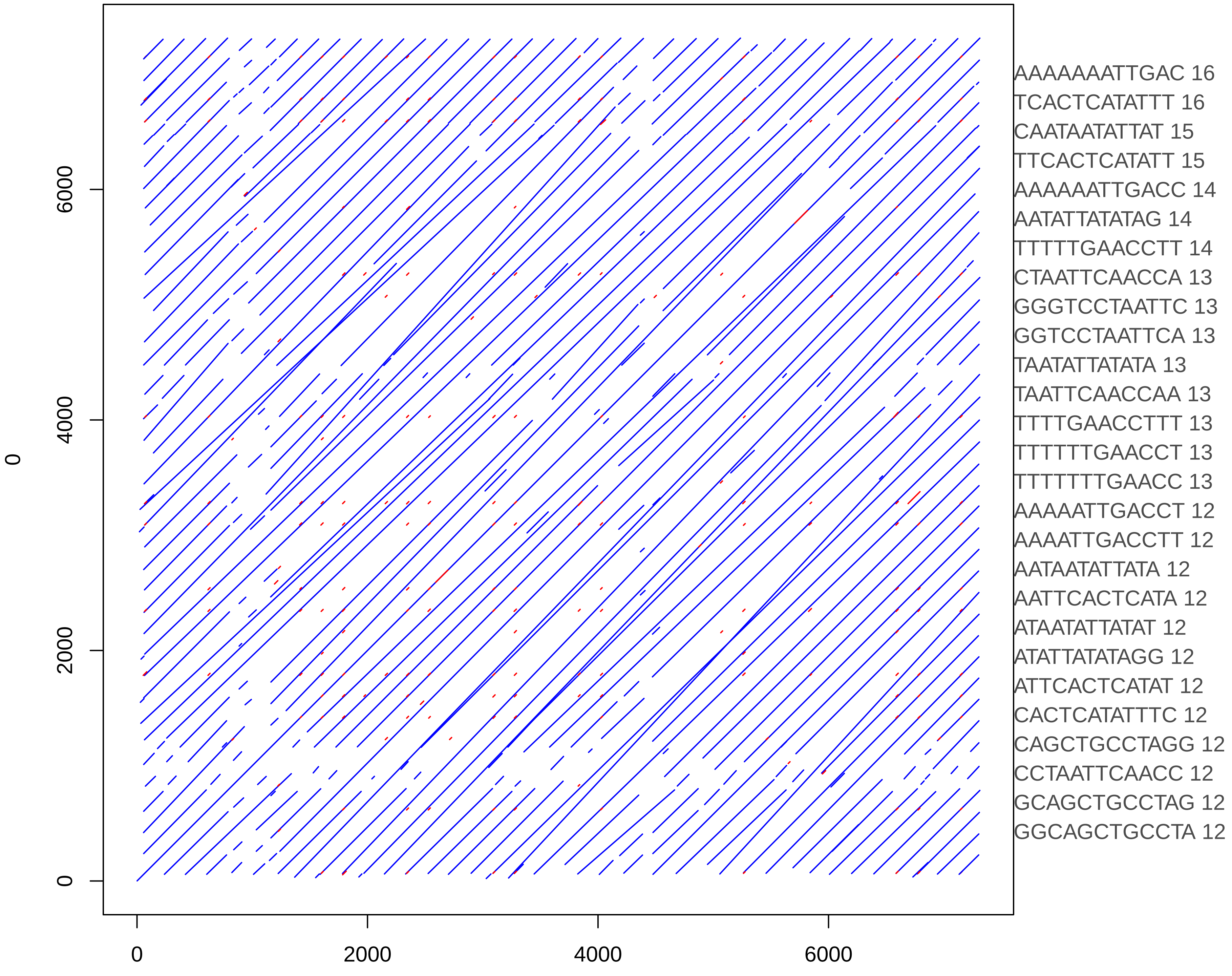

>m141126_060447_42134_c100732262550000001823141405141500_s1_p0/108942/7757_16544

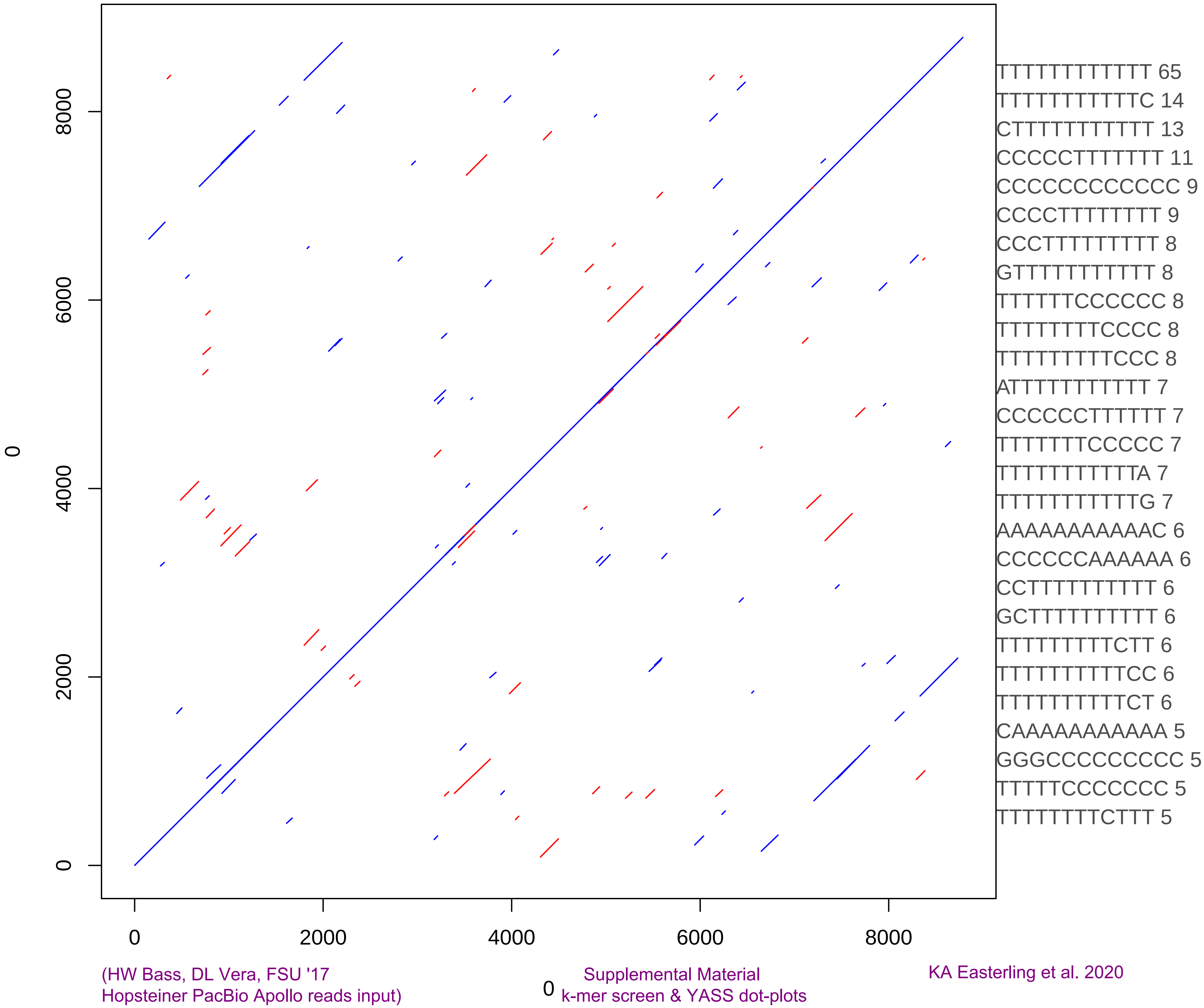

>m141126_060447_42134_c100732262550000001823141405141500_s1_p0/117603/6437_13726

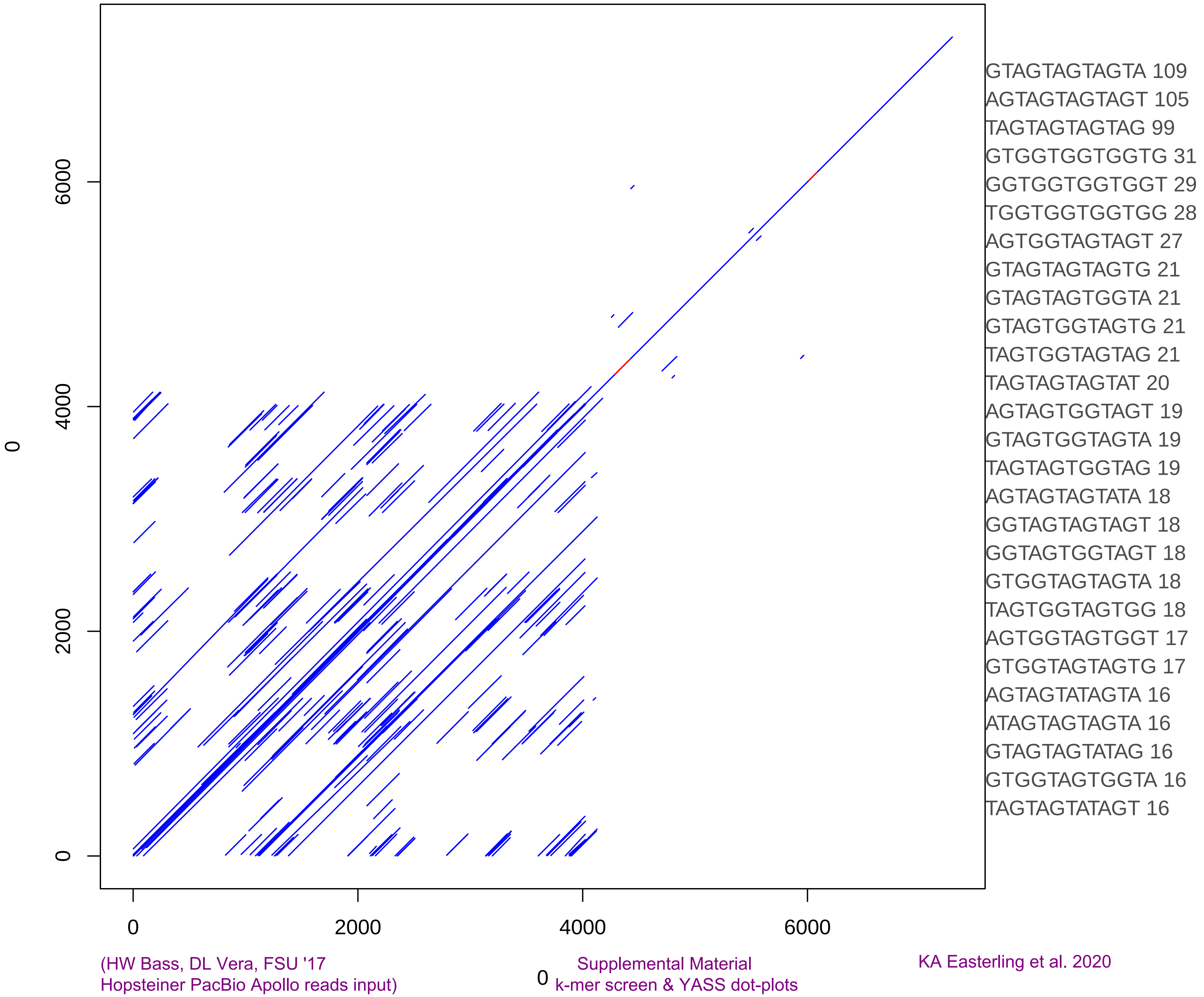

>m141126_060447_42134_c100732262550000001823141405141500_s1_p0/120397/7576_14316

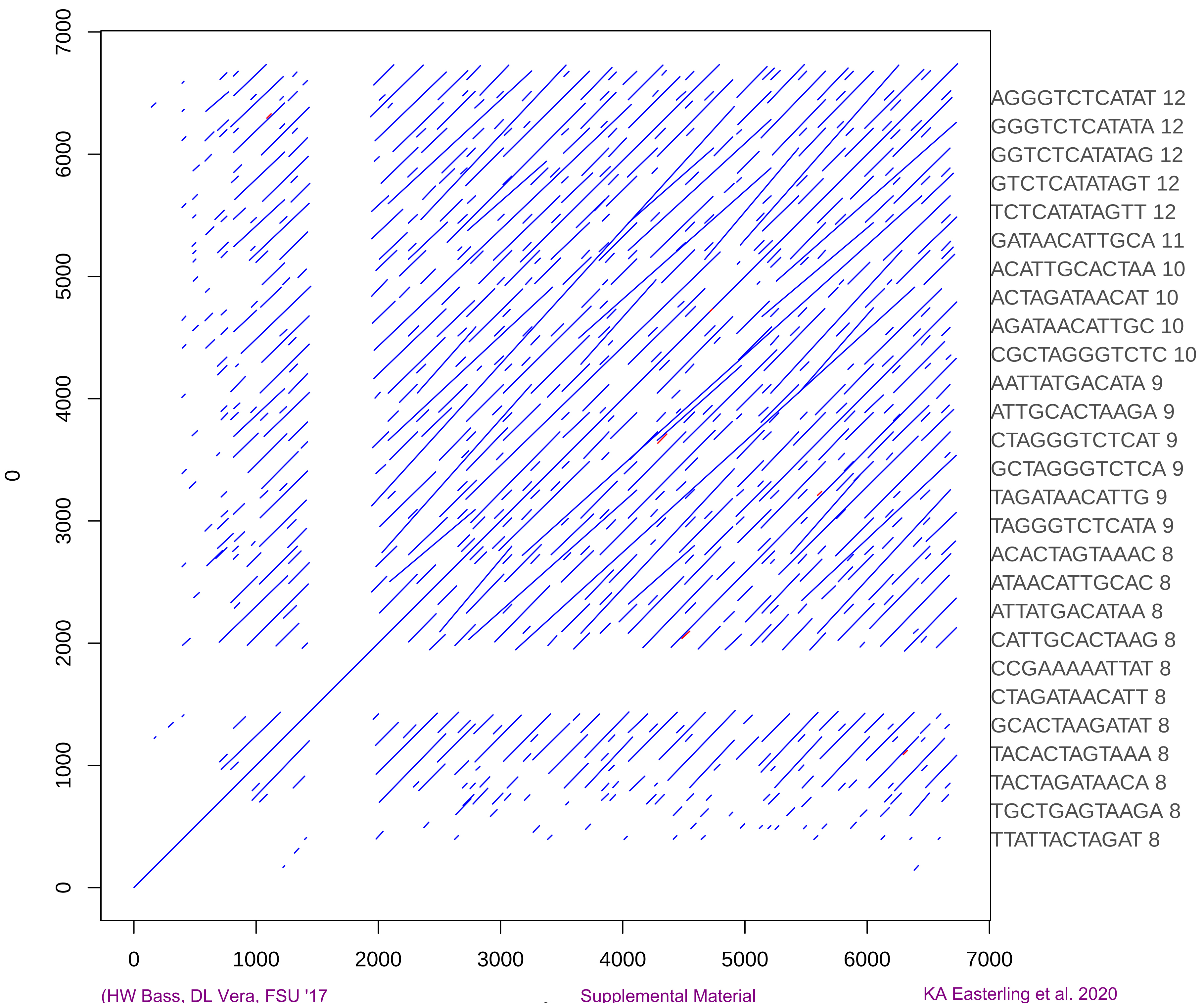

>m141126_060447_42134_c100732262550000001823141405141500_s1_p0/124043/0_8287

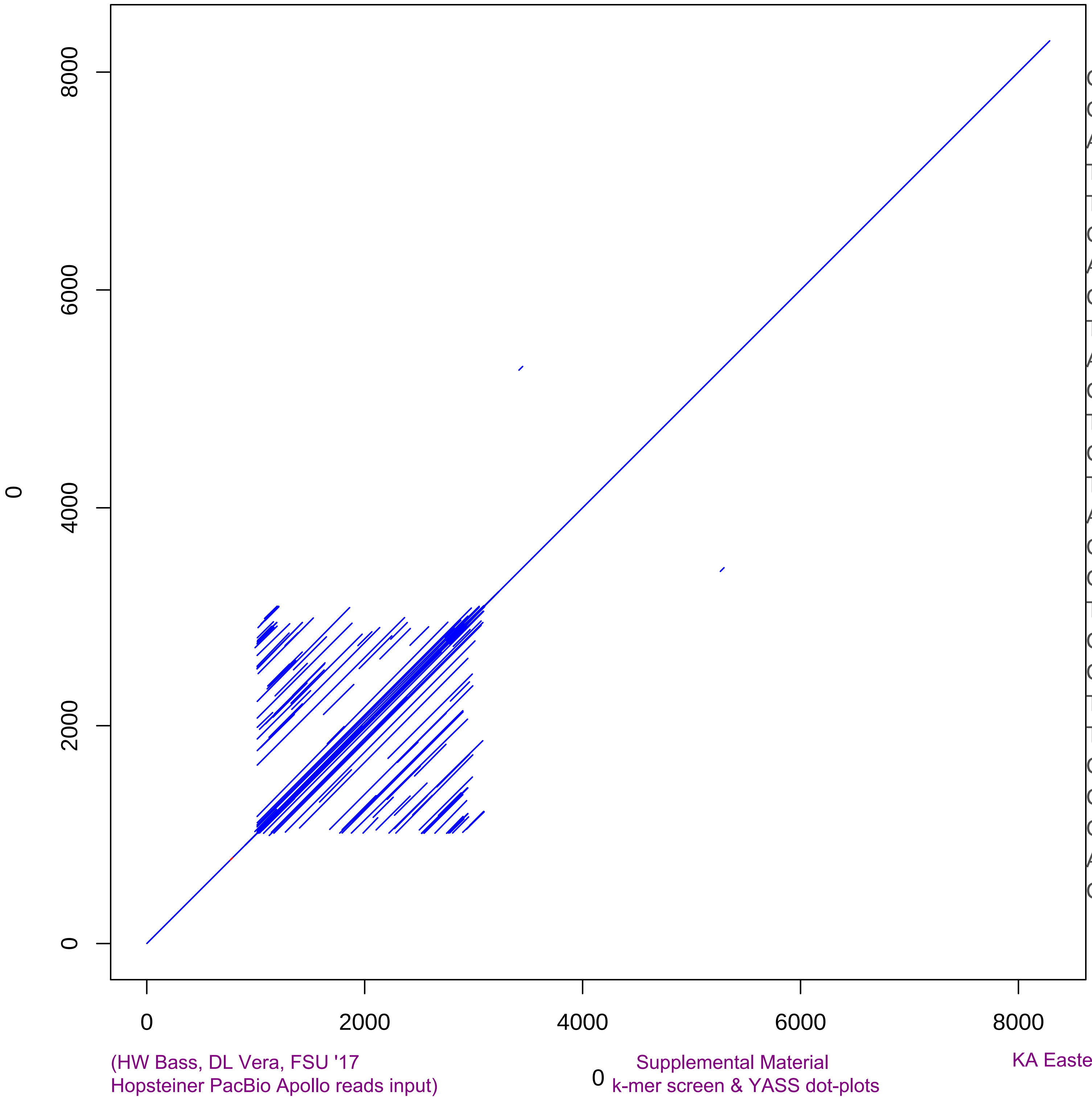

CTGCTACTGCTA 36
GCTACTGCTACT 35
ACTGCTACTGCT 33
TGCTACTGCTAC 32
TACTGCTACTGC 31
CTACTGCTACTG 30
ACTACTACTACT 27
CTACTACTACTA 25
TACTACTACTAC 24
ACTGCTACTACT 22
CTACTGCTACTA 22
TACTGCTACTAC 21
CTGCTACTACTG 19
TGCTACTACTGC 19
ACTACTGCTACT 17
GCTACTACTGCT 17
GCTGCTGCTGCT 15
TACTACTGCTAC 14
CTGCTGCTACTG 13
CTGCTGCTGCTG 13
TGCTGCTACTGC 13
TGCTGCTGCTGC 13
CTACTACTGCTA 12
CTACTGCTGCTA 12
GCTGCTACTGCT 12
ACTGCTGCTACT 10
CTACTACTGCTG 10

1,121 hits for repetitive sequences
passing 12mer abundance criterion

For Tandem Repeat families of ### bp:
PacBio Read named "HuluTR###-r564"

>m141126_060447_42134_c100732262550000001823141405141500_s1_p0/128576/19697_24911

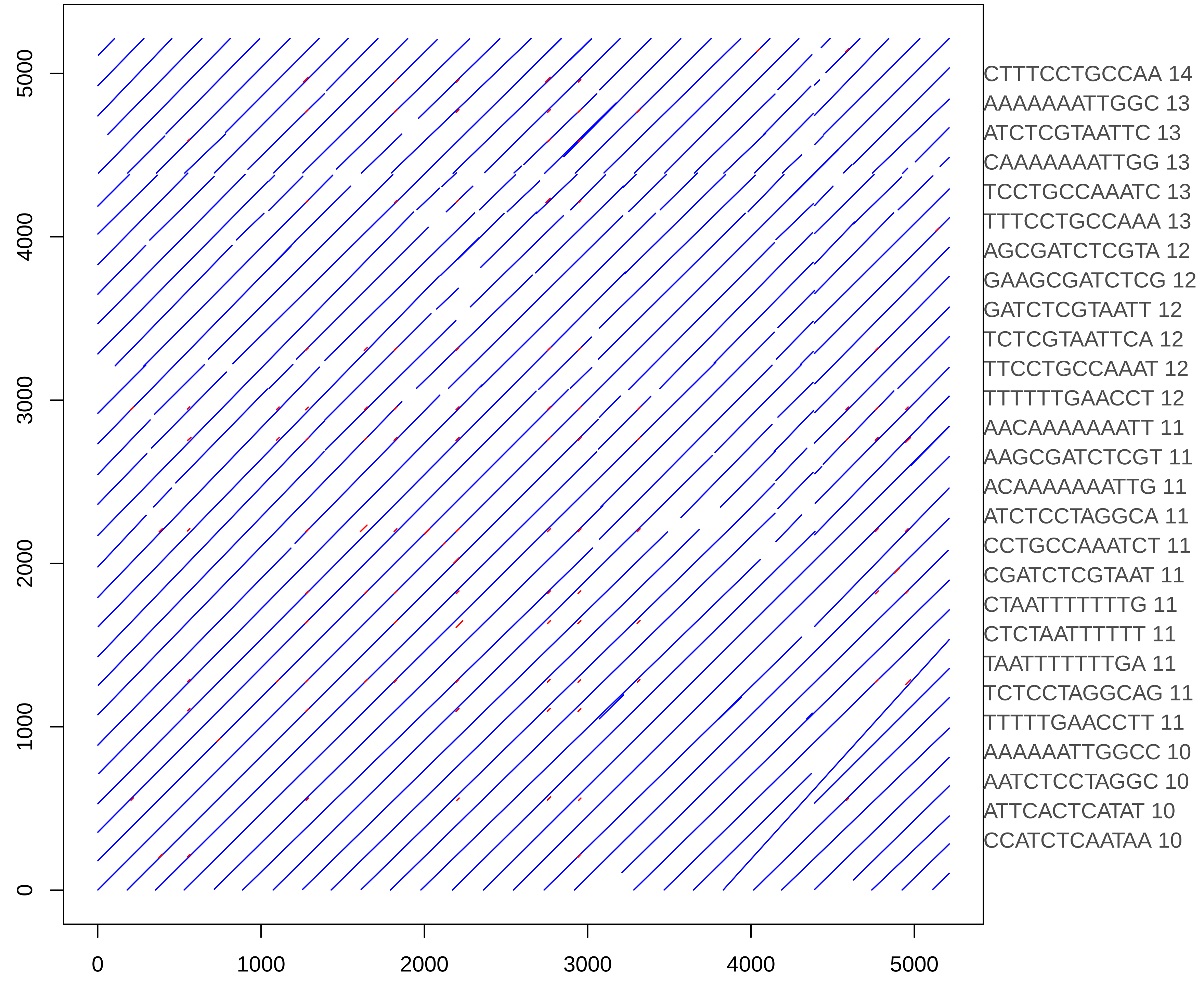

0

(HW Bass, DL Vera, FSU '17
Hopsteiner PacBio Apollo reads input)

Supplemental Material
k-mer screen & YASS dot-plots

KA Easterling et al. 2020

>m141126_060447_42134_c100732262550000001823141405141500_s1_p0/129186/0_8219

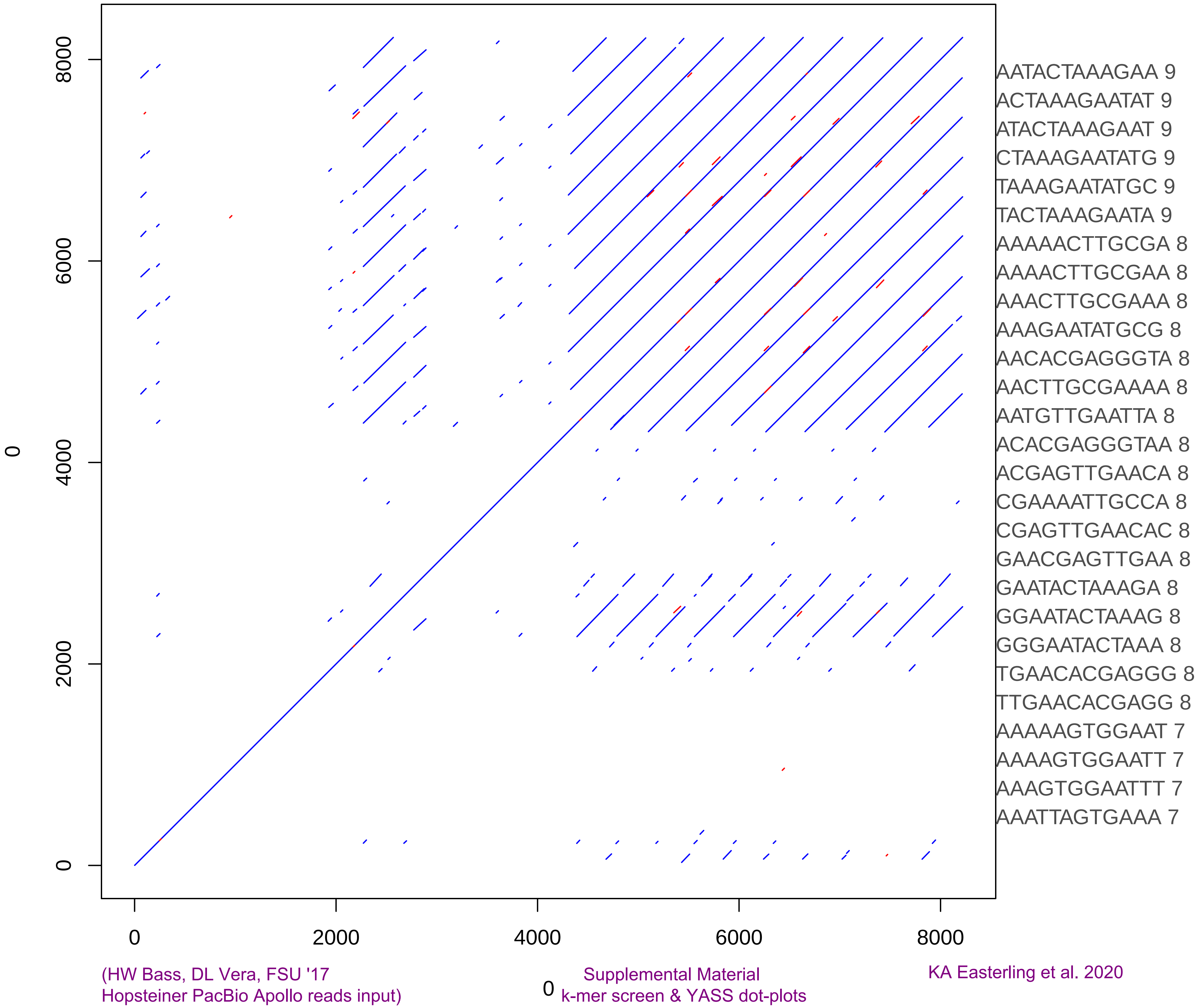

>m141126_060447_42134_c100732262550000001823141405141500_s1_p0/136663/9591_17184

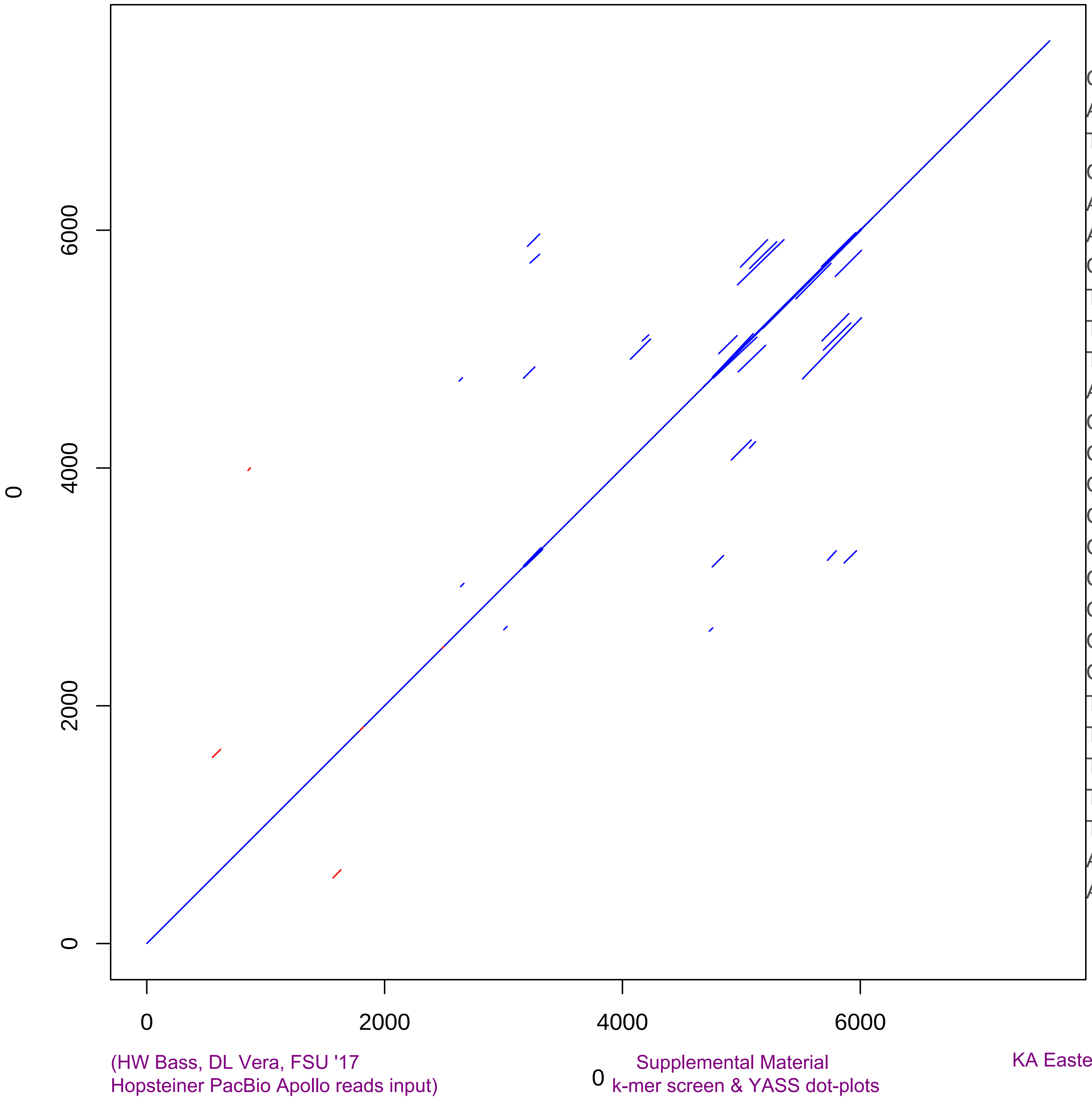

GTAGTAGTAGTA 16
AGTAGTAGTAGT 14
TAGTAGTAGTAG 13
GTGGTAGTAGTA 9
AGTAGTGGTAGT 8
AGTGGTAGTAGT 7
GTAGTGGTAGTA 6
TAGTAGTGGTAG 6
TAGTGGTAGTAG 6
TGGTAGTAGTAG 5
AGTGTGGTAGTA 4
GGTAGTAGTAGT 4
GGTAGTGTGGTA 3
GTAGTAGTAAGT 3
GTAGTAGTAATA 3
GTAGTAGTAGTG 3
GTAGTAGTGGTA 3
GTAGTGTGGTAG 3
GTGGTAGTAGTG 3
GTGTGGTAGTAG 3
TAGTAGTAGTAA 3
TAGTGTGGTAGT 3
TGGTAGTAGTAA 3
TGGTAGTGTGGT 3
TGTGGTAGTAGT 3
AAGTAGGTGTAG 2
AGAGCTAGAGTG 2

>m141126_060447_42134_c100732262550000001823141405141500_s1_p0/13935/373_16162

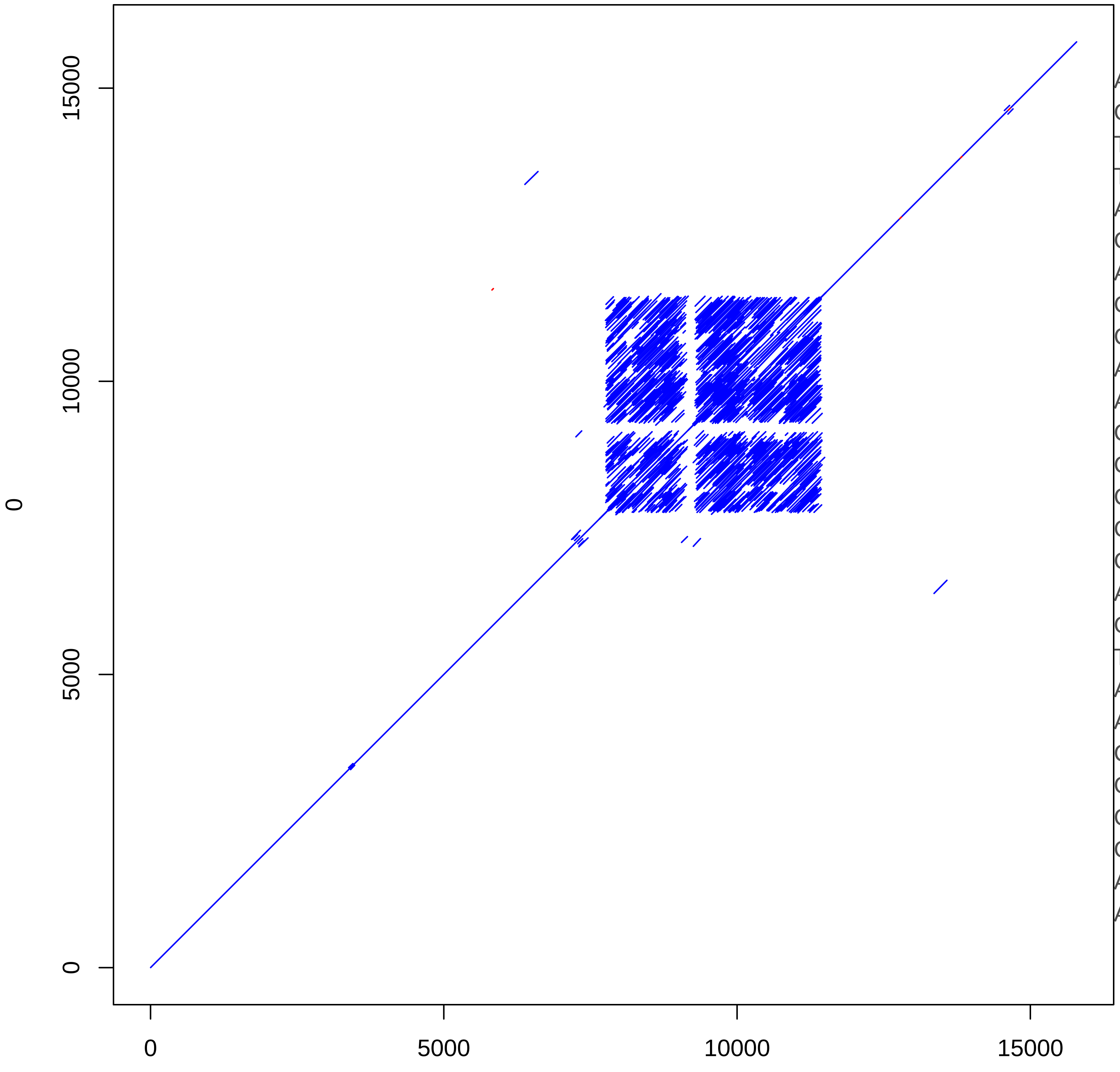

AGTAGTAGTAGT 29
GTAGTAGTAGTA 29
TAGTAGTAGTAG 29
TAGCAGTAGTAG 15
AGCAGTAGTAGC 14
GTAGCAGTAGTA 13
AGTAGCAGTAGT 12
CAGCAGTAGCAG 12
CAGTAGCAGTAG 11
AGCAGCAGCAGC 10
AGCAGCAGTAGC 10
CAGCAGCAGCAG 10
CAGTAGTAGCAG 10
CAGTAGTAGTAG 10
GCAGCAGTAGCA 10
GCAGTAGCAGTA 10
AGTAGTAGCAGT 9
GCAGCAGCAGTA 9
TAGCAGCAGTAG 9
AGCAGTAGCAGC 8
AGCAGTAGCAGT 8
CAGTAGCAGCAG 8
GCAGCAGCAGCA 8
GCAGTAGTAGCA 8
GTAGCAGCAGTA 8
AGCAGTAGTAGT 7
AGTAGTAGTAGC 7

>m141126_060447_42134_c100732262550000001823141405141500_s1_p0/141175/0_5913

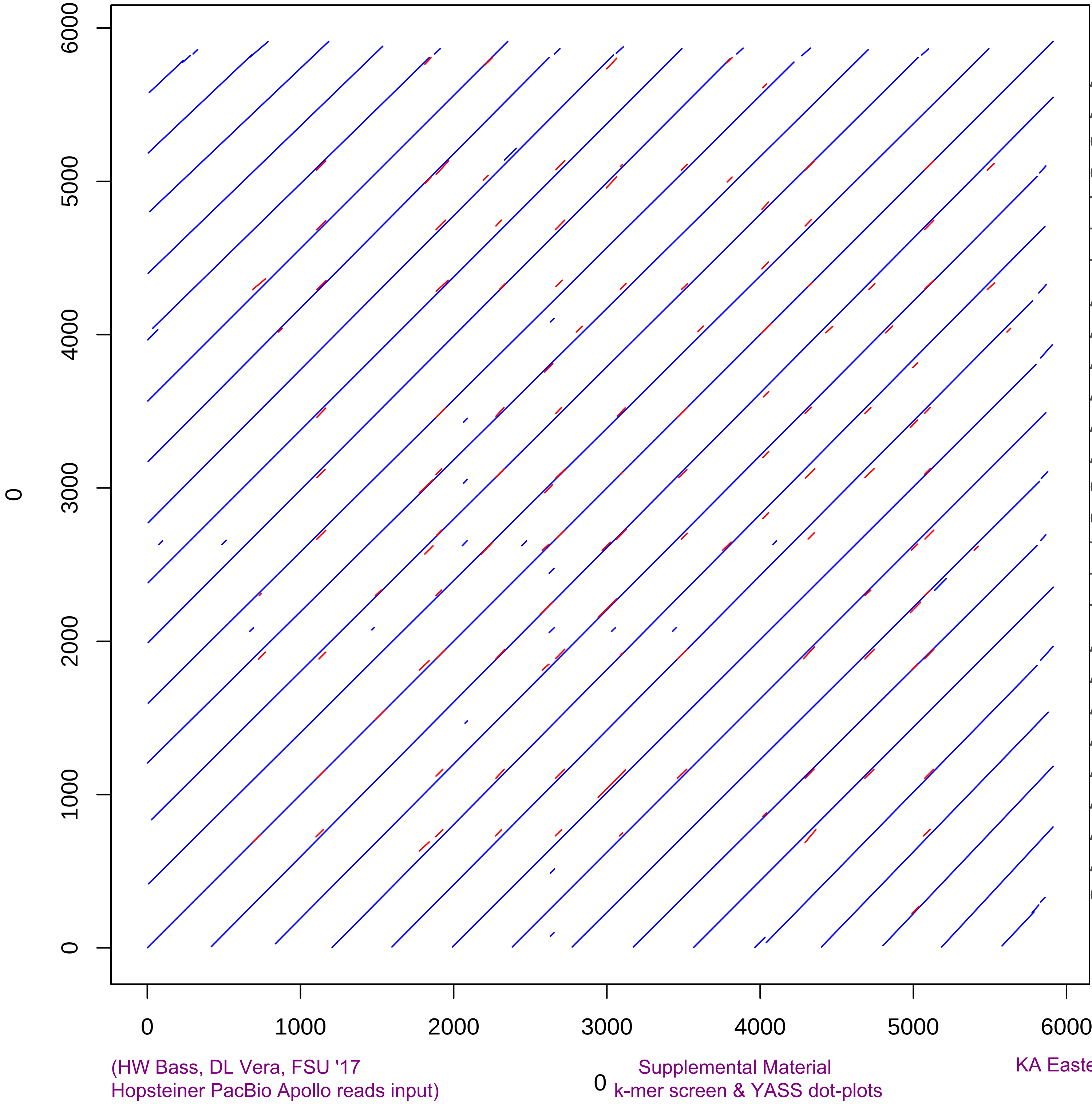

>m141126_060447_42134_c100732262550000001823141405141500_s1_p0/141387/0_6309

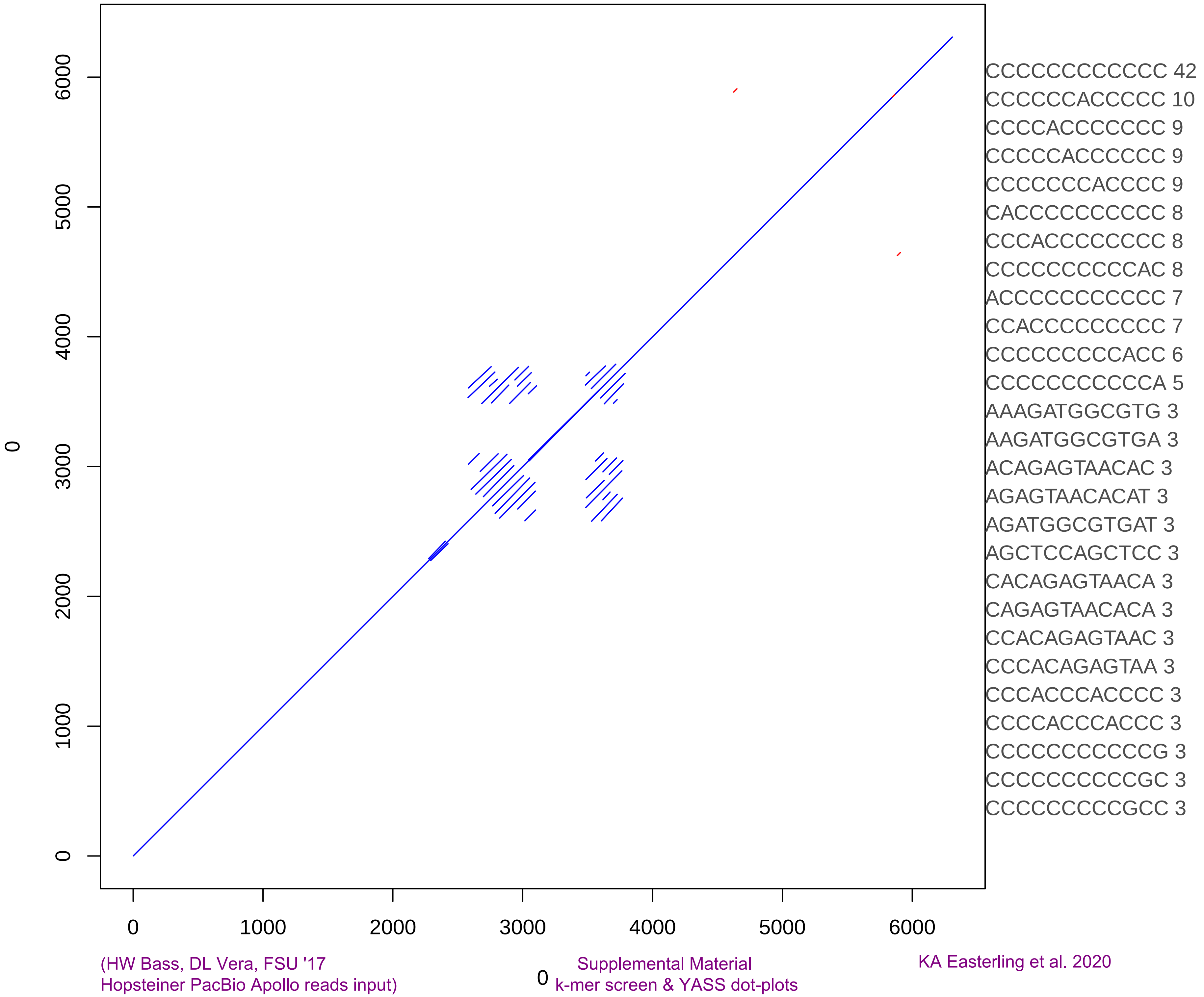

>m141126_060447_42134_c100732262550000001823141405141500_s1_p0/144025/2472_8390

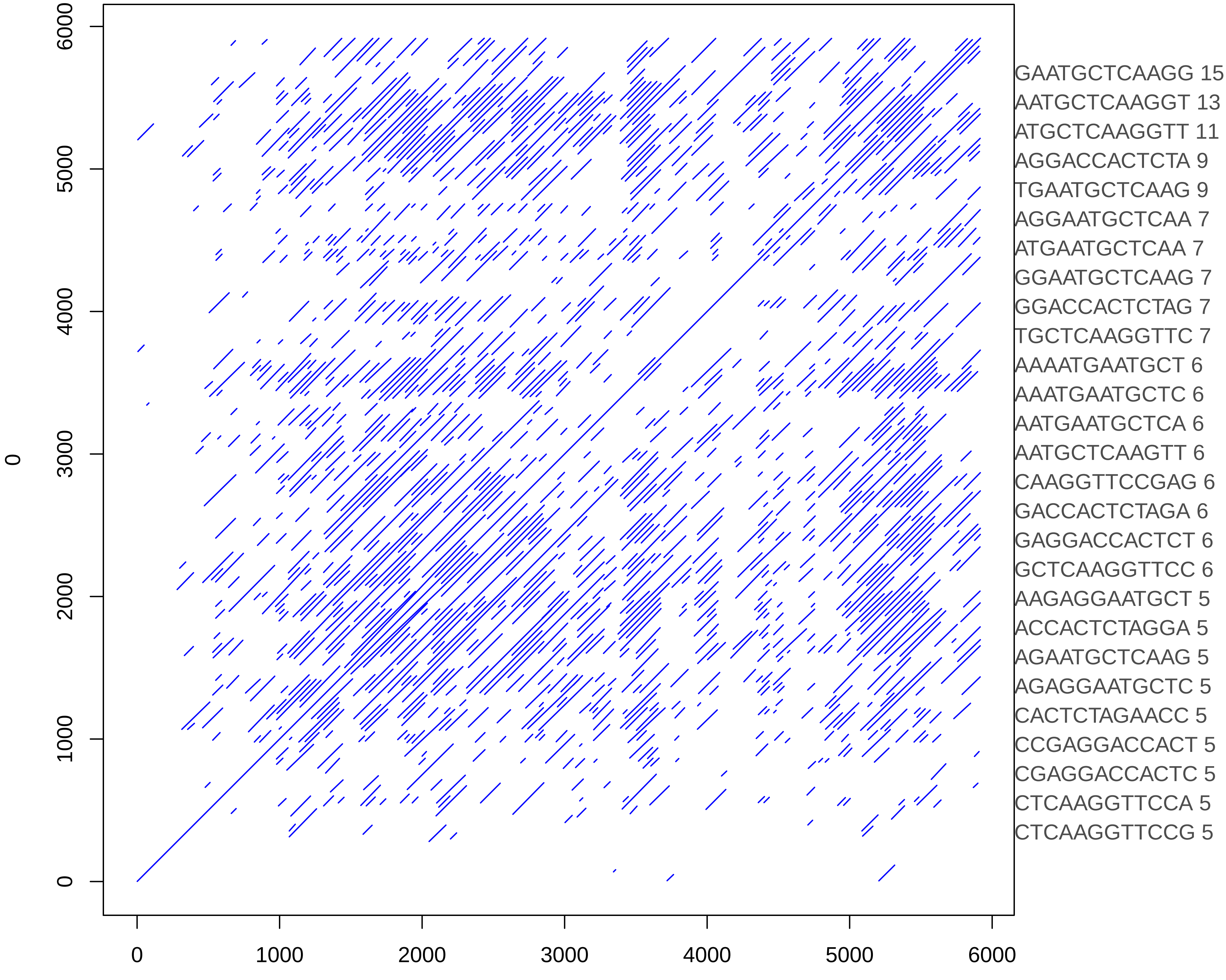

>m141126_060447_42134_c100732262550000001823141405141500_s1_p0/150712/0_5483

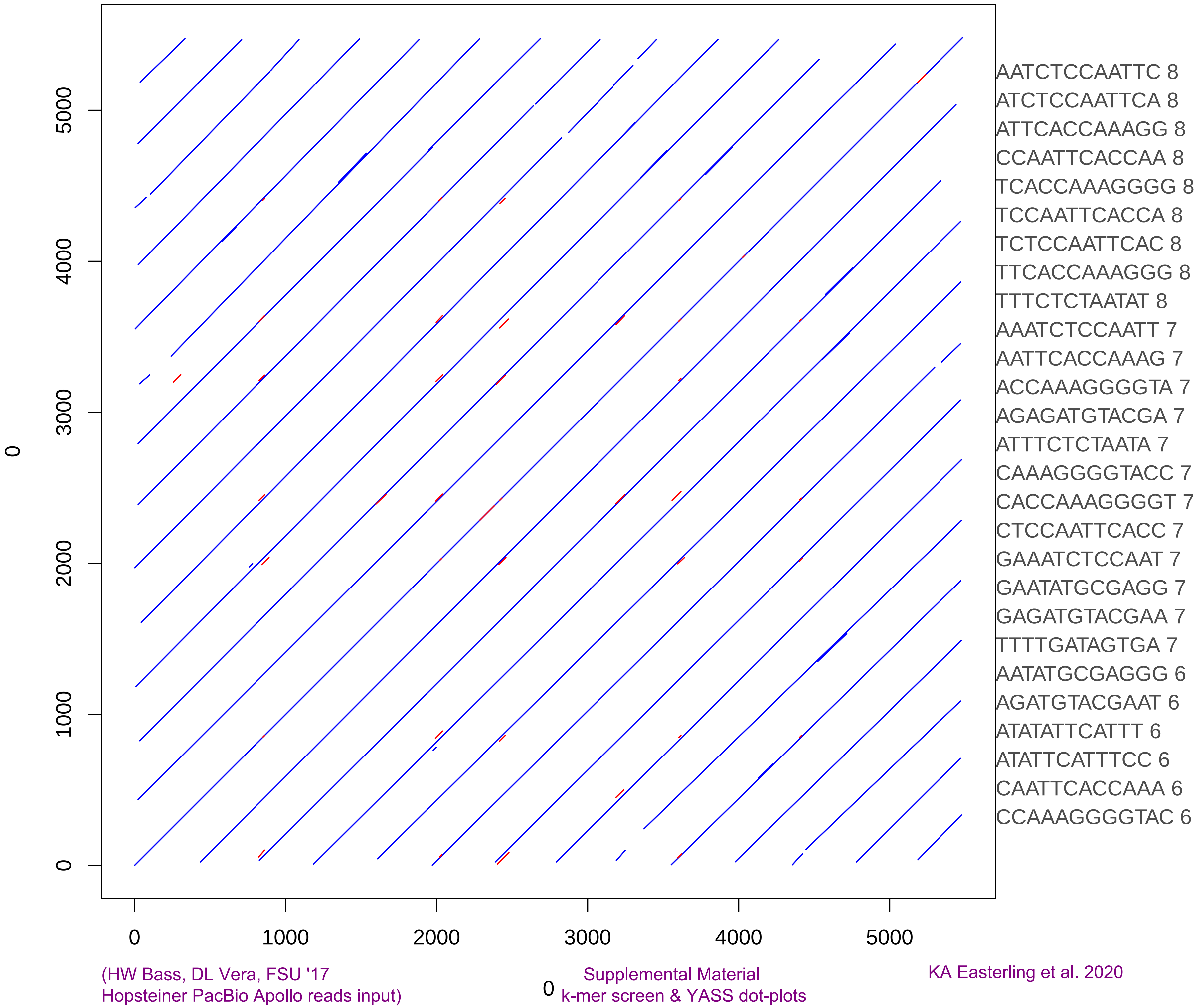

>m141126_060447_42134_c100732262550000001823141405141500_s1_p0/1526/0_8245

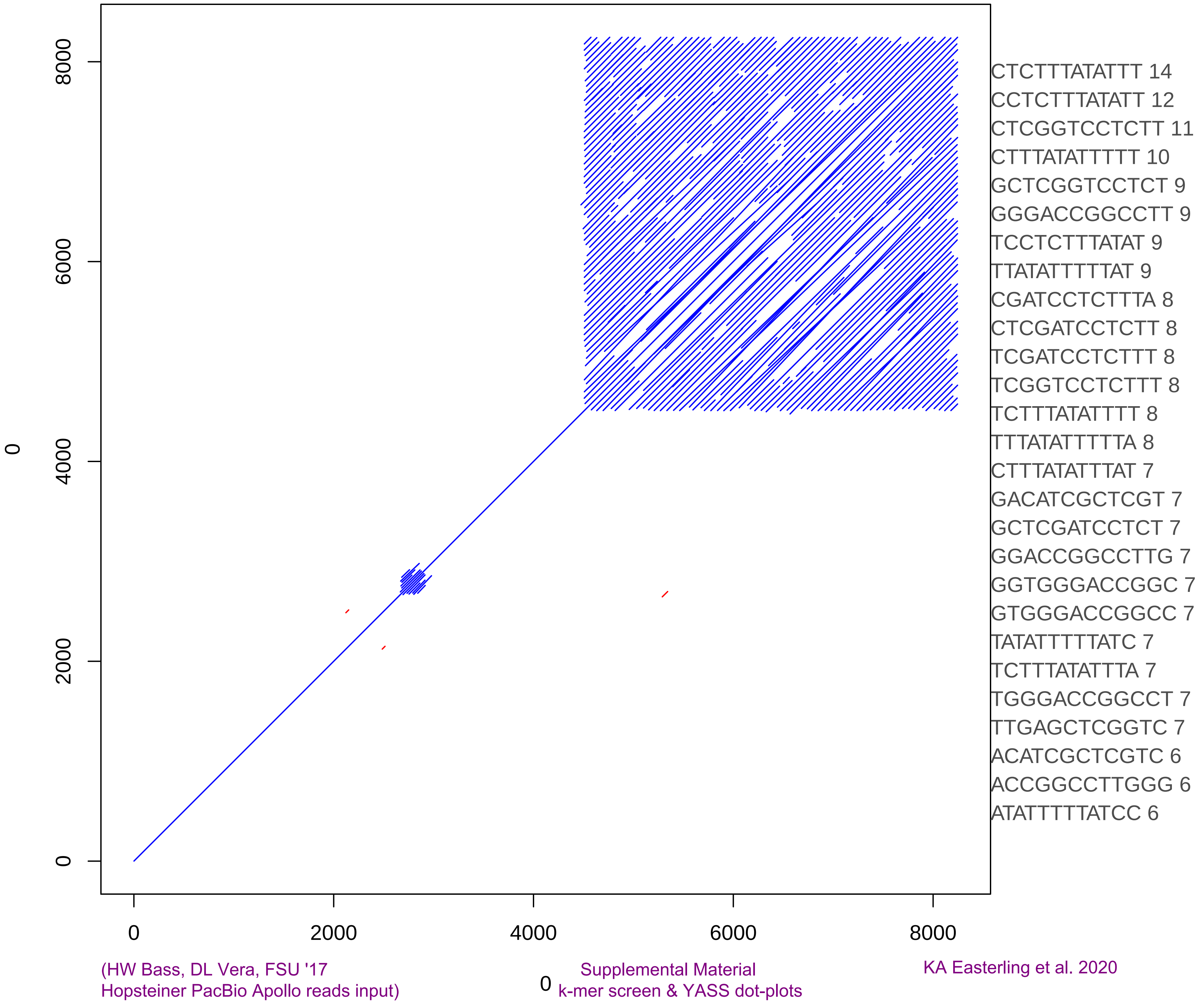

>m141126_060447_42134_c100732262550000001823141405141500_s1_p0/155825/0_13654

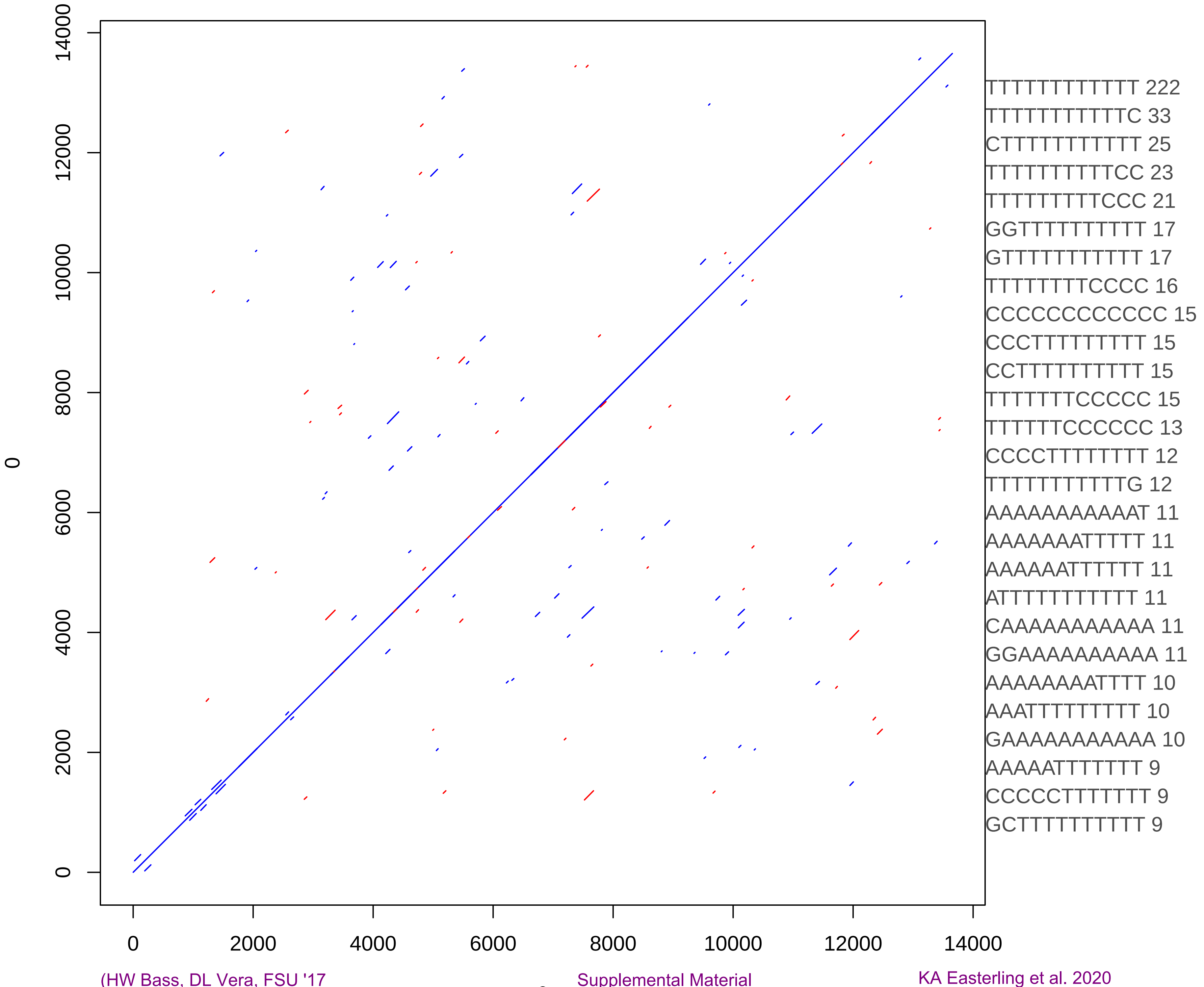

>m141126_060447_42134_c100732262550000001823141405141500_s1_p0/156511/0_7236

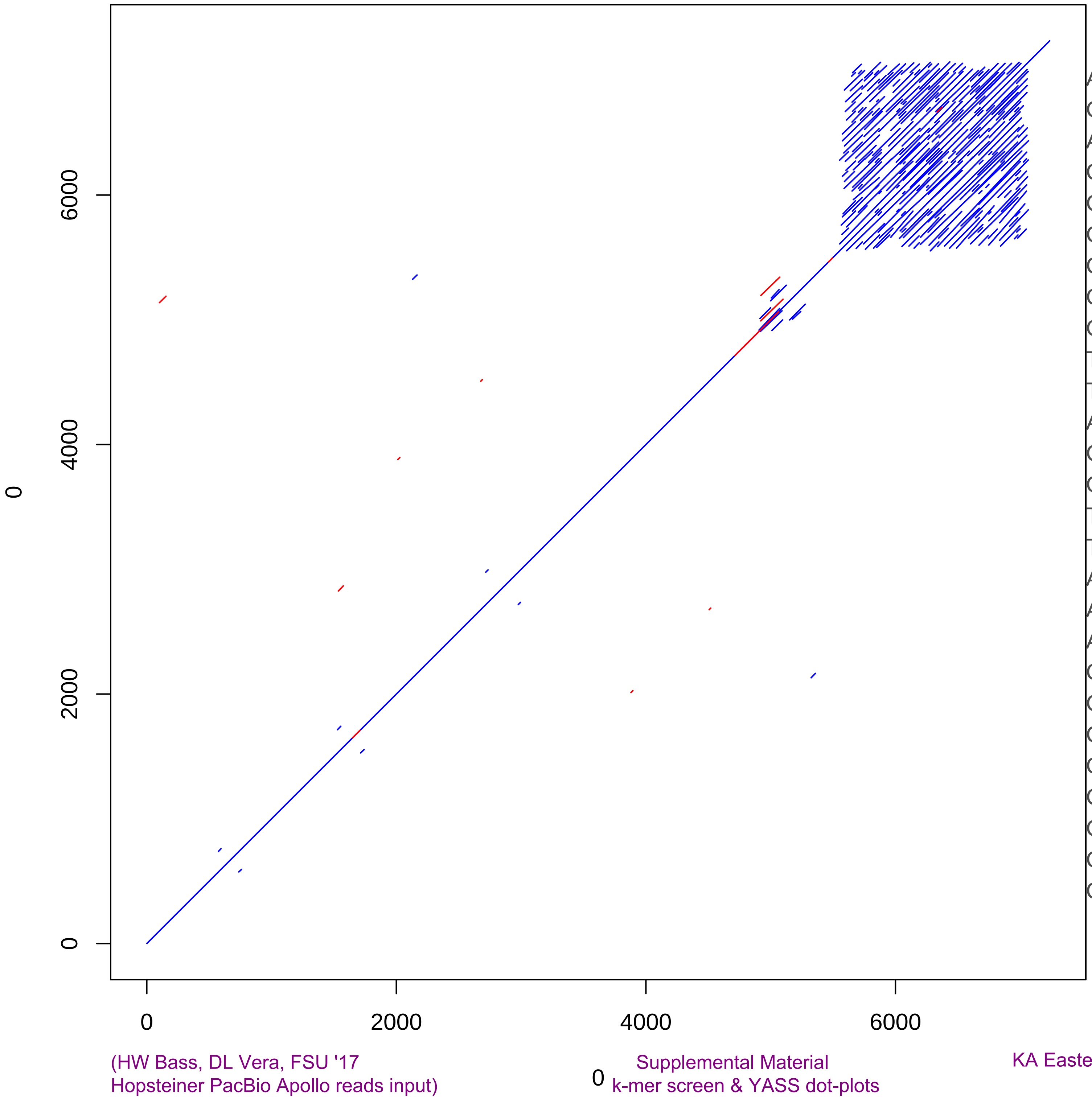

AAGGGTCTCACT 11
CAAGGGTCTCAC 11
AGGGTCTCACTA 9
CCCCCGCATGCG 8
CCTTCCCCCGCA 8
CGAGGCTACCCT 8
CTTCCCCCGCAT 8
GAGGCTACCCTT 8
GGGTCTCACTAT 8
TCCCCCGCATGC 8
TTCCCCCGCATG 8
ATGCGAGGCTAC 7
CCGCATGCGAGG 7
GCGAGGCTACCC 7
TGCCAAGGCCAC 7
TGCGAGGCTACC 7
AAGGCCACAAGG 6
ACAAGGGTCTCA 6
AGGCCACAAGGG 6
CAAGGCCACAAG 6
CACAAGGGTCTC 6
CACTATGCGAGG 6
CCAAGGCCACAA 6
CCCCGCATGCGA 6
CCCGCATGCGAG 6
CGCCTGCCAAGG 6
CTGCCAAGGCCA 6

>m141126_060447_42134_c100732262550000001823141405141500_s1_p0/157526/2733_8142

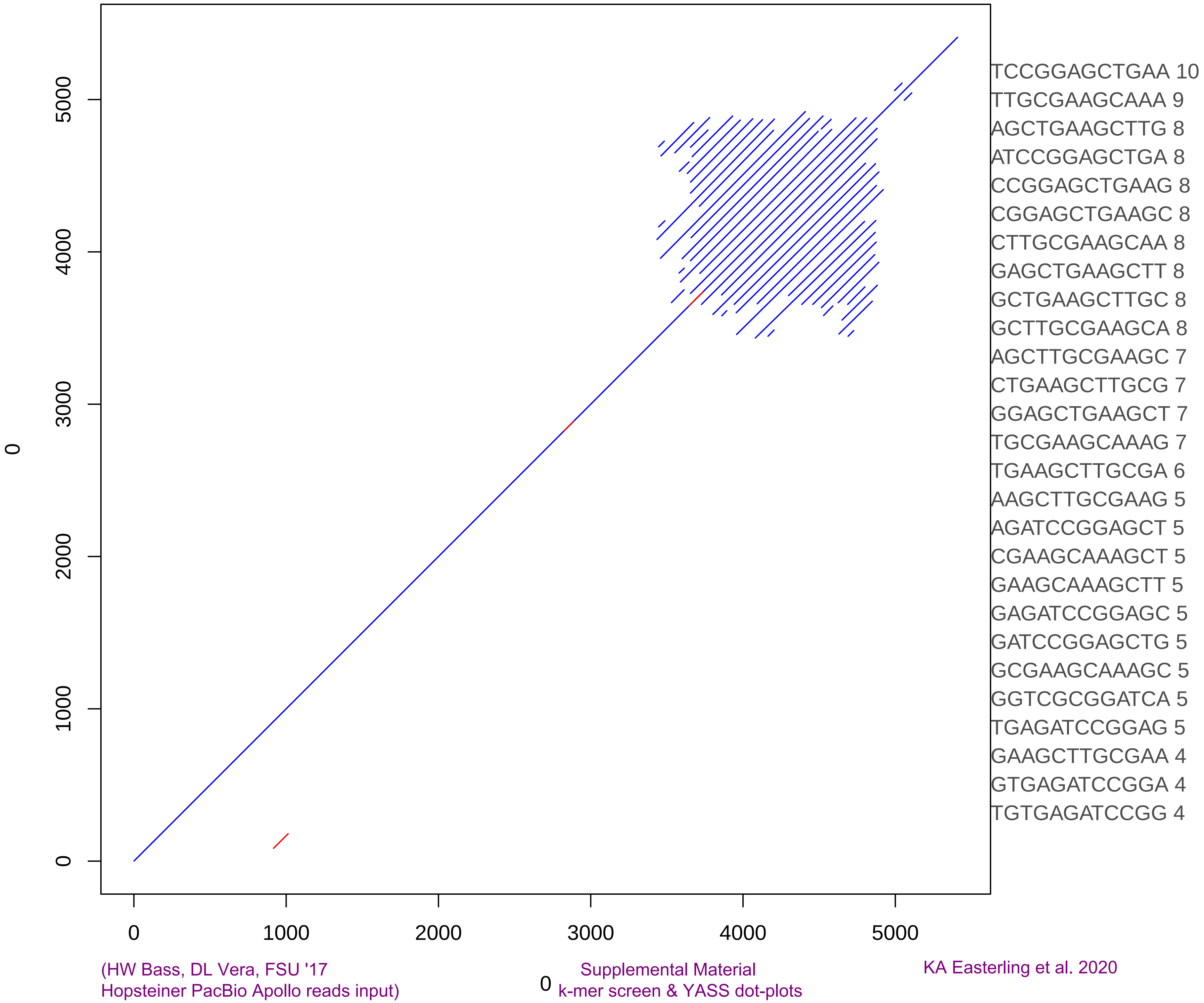

1,121 hits for repetitive sequences passing 12mer abundance criterion

(Read-Number)
PAGE 576

For Tandem Repeat families of ### bp:
PacBio Read named "HuluTR###-r576"

>m141126_060447_42134_c100732262550000001823141405141500_s1_p0/157630/0_8380

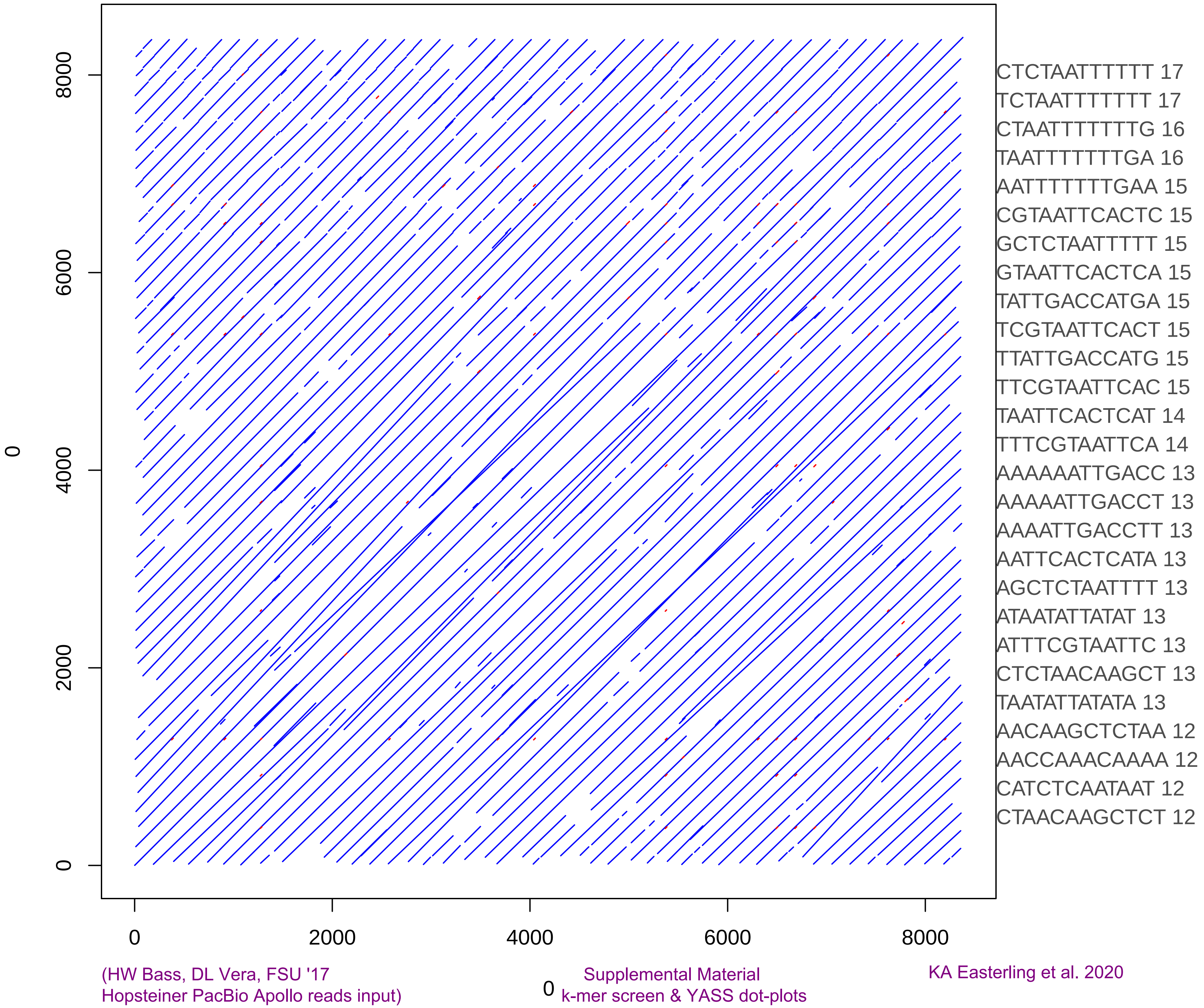

(HW Bass, DL Vera, FSU '17
Hopsteiner PacBio Apollo reads input)

Supplemental Material
k-mer screen & YASS dot-plots

KA Easterling et al. 2020

>m141126_060447_42134_c100732262550000001823141405141500_s1_p0/159662/0_5117

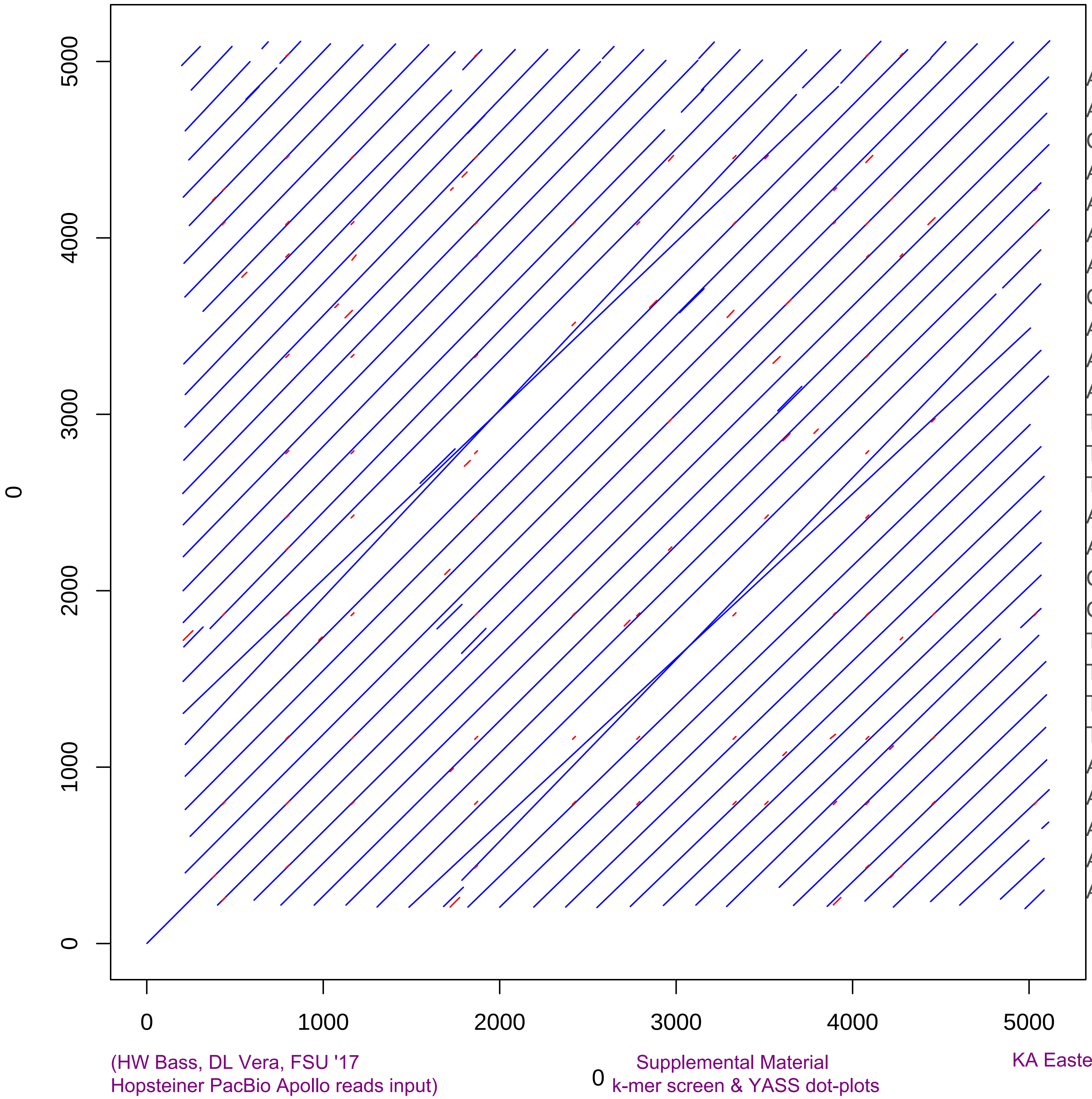

>m141126_060447_42134_c100732262550000001823141405141500_s1_p0/161581/0_5104

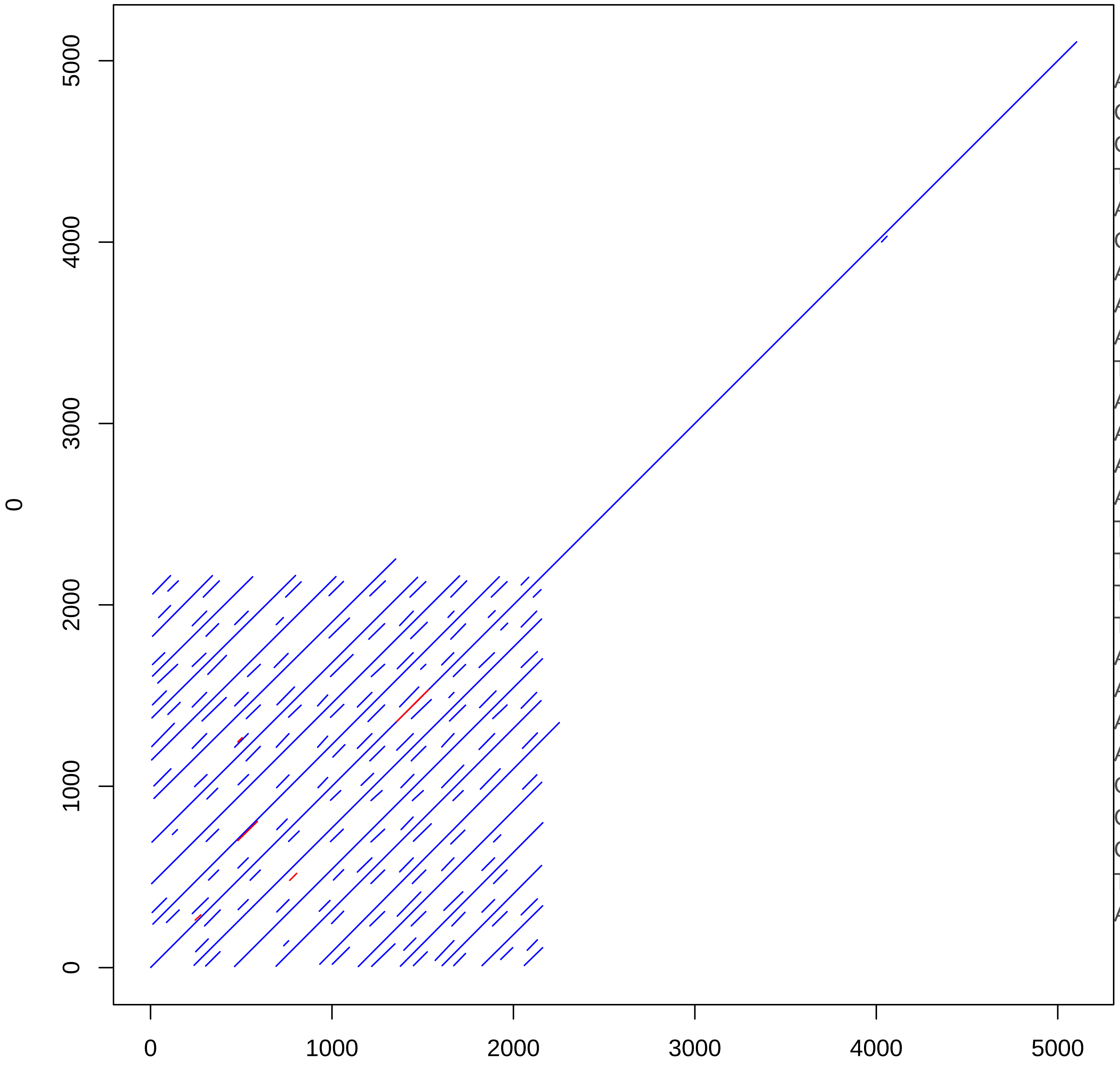

ACATTGCACTAA 10
CATTGCACTAAG 10
CTAGATAACATT 10
TAGATAACATTG 10
AGATAACATTGC 9
GATAACATTGCA 9
AACATTGCACTA 8
ACTAGATAACAT 8
ATAACATTGCAC 8
TACTAGATAACA 8
AAAAATACTACT 6
AAAATACTACTA 6
AGTAGTGAATTT 6
ATTGCACTAAGA 6
TAACATTGCACT 6
TAGAAGGGGATC 6
TTAGAAGGGGAT 6
TTTAGAAGGGGA 6
AAATACTACTAG 5
AATTTAGAAGGG 5
AGAAGGGGATCC 5
AGTGAATTTAGA 5
GAAAAATACTAC 5
GAATTTAGAAGG 5
GTGAATTTAGAA 5
TGAATTTAGAAG 5
AAATTATTACTA 4

>m141126_060447_42134_c100732262550000001823141405141500_s1_p0/162489/0_12391

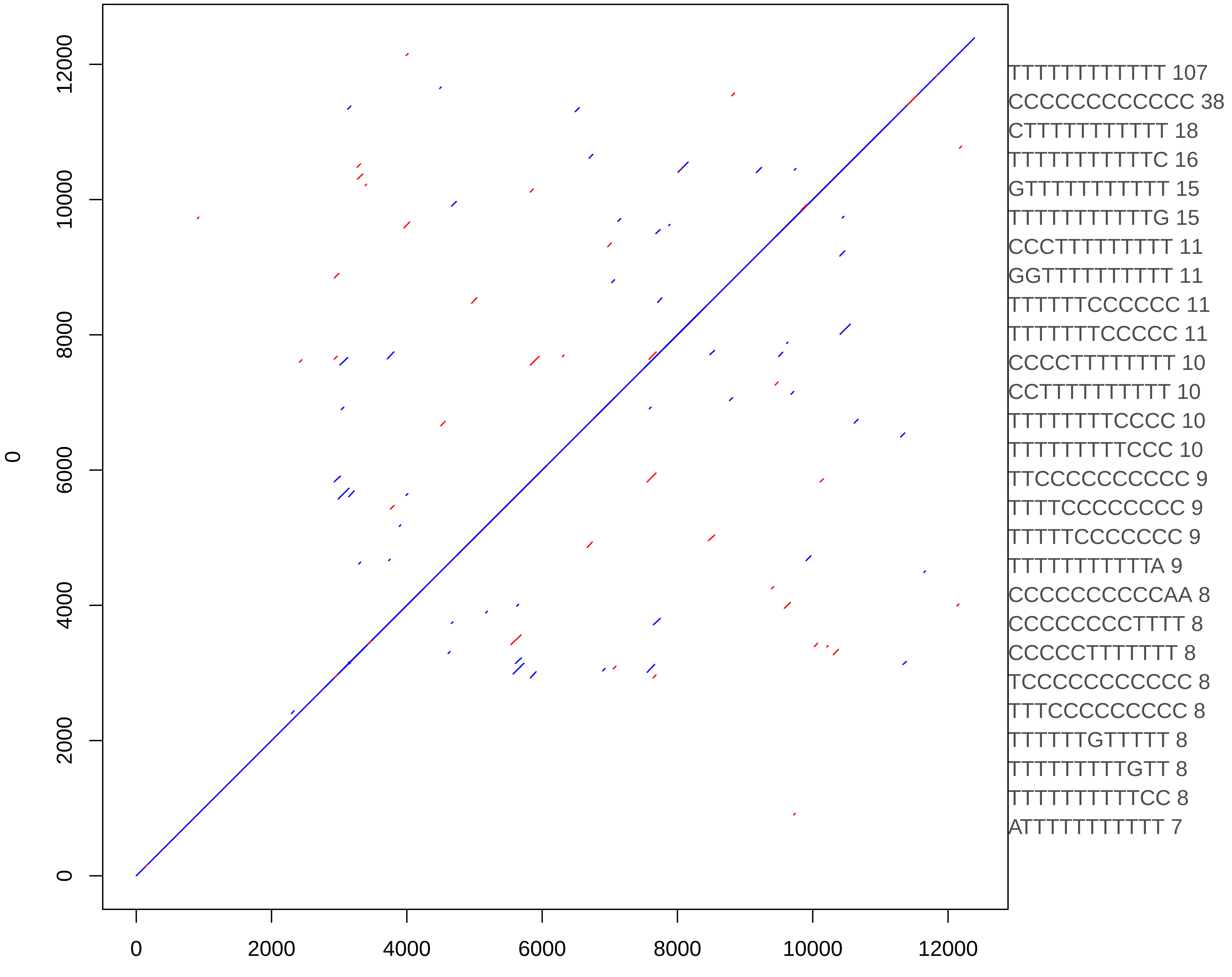

0

>m141126_060447_42134_c100732262550000001823141405141500_s1_p0/16856/2450_10323

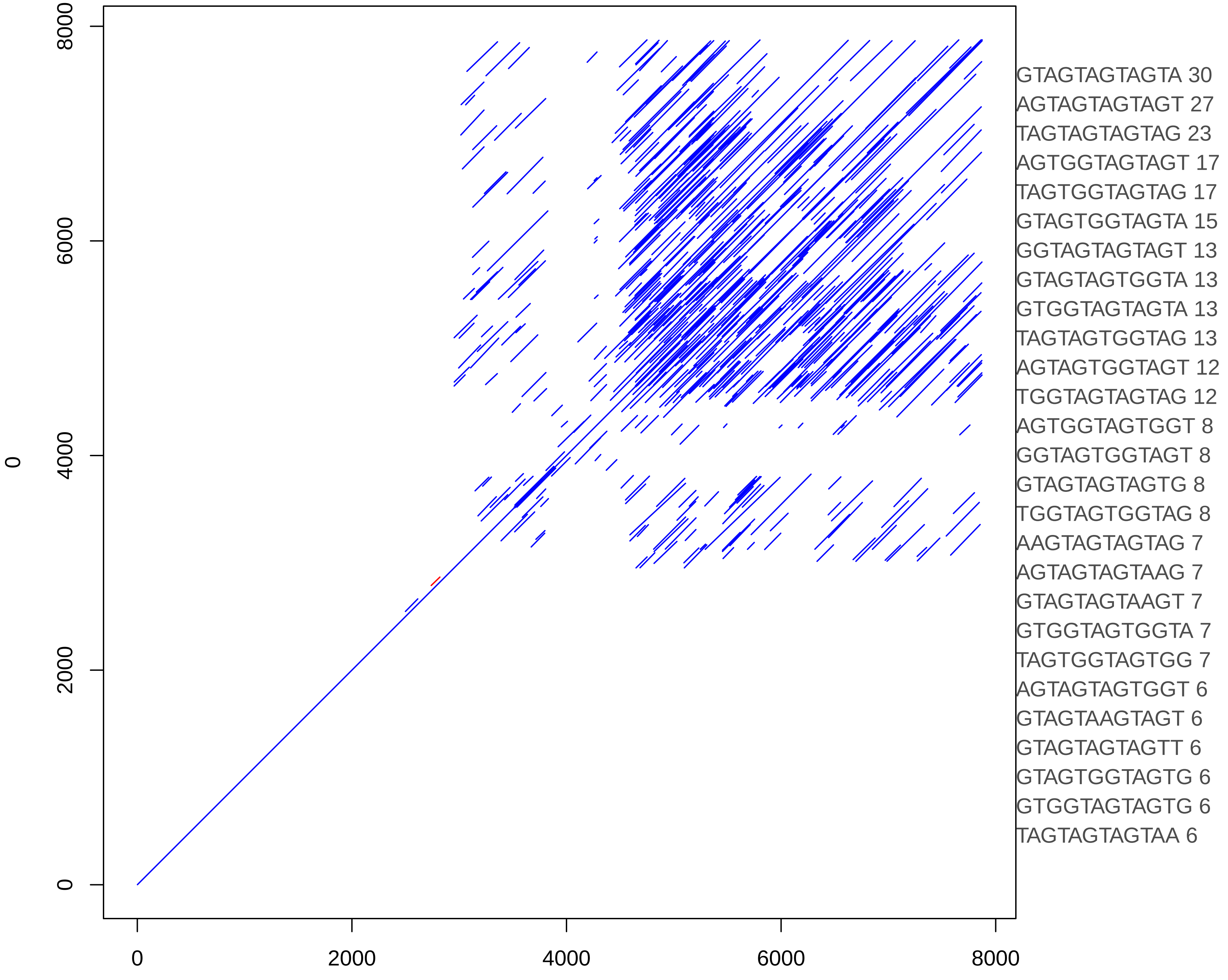

>m141126_060447_42134_c100732262550000001823141405141500_s1_p0/1814/0_6842

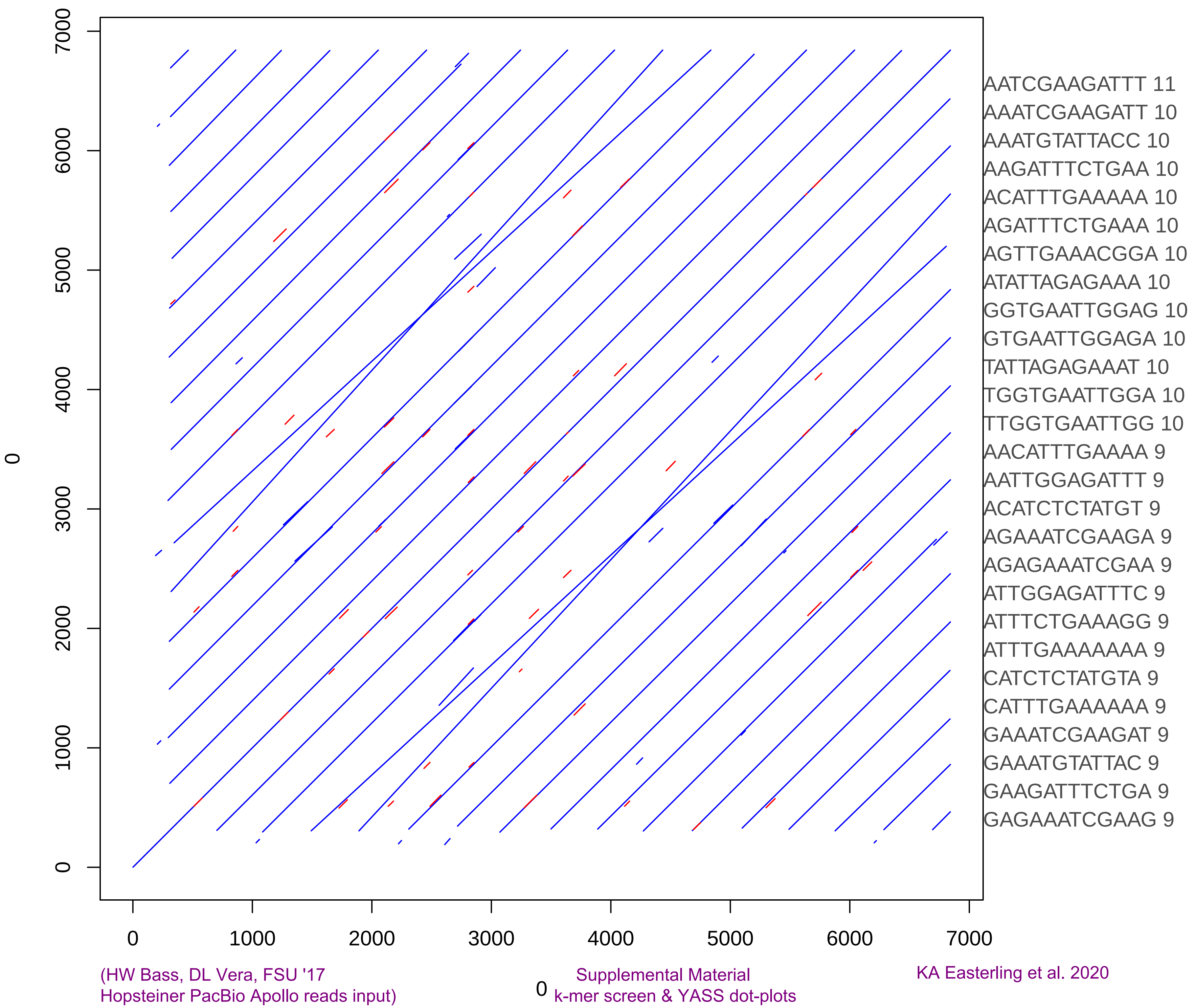

>m141126_060447_42134_c100732262550000001823141405141500_s1_p0/19091/0_10797

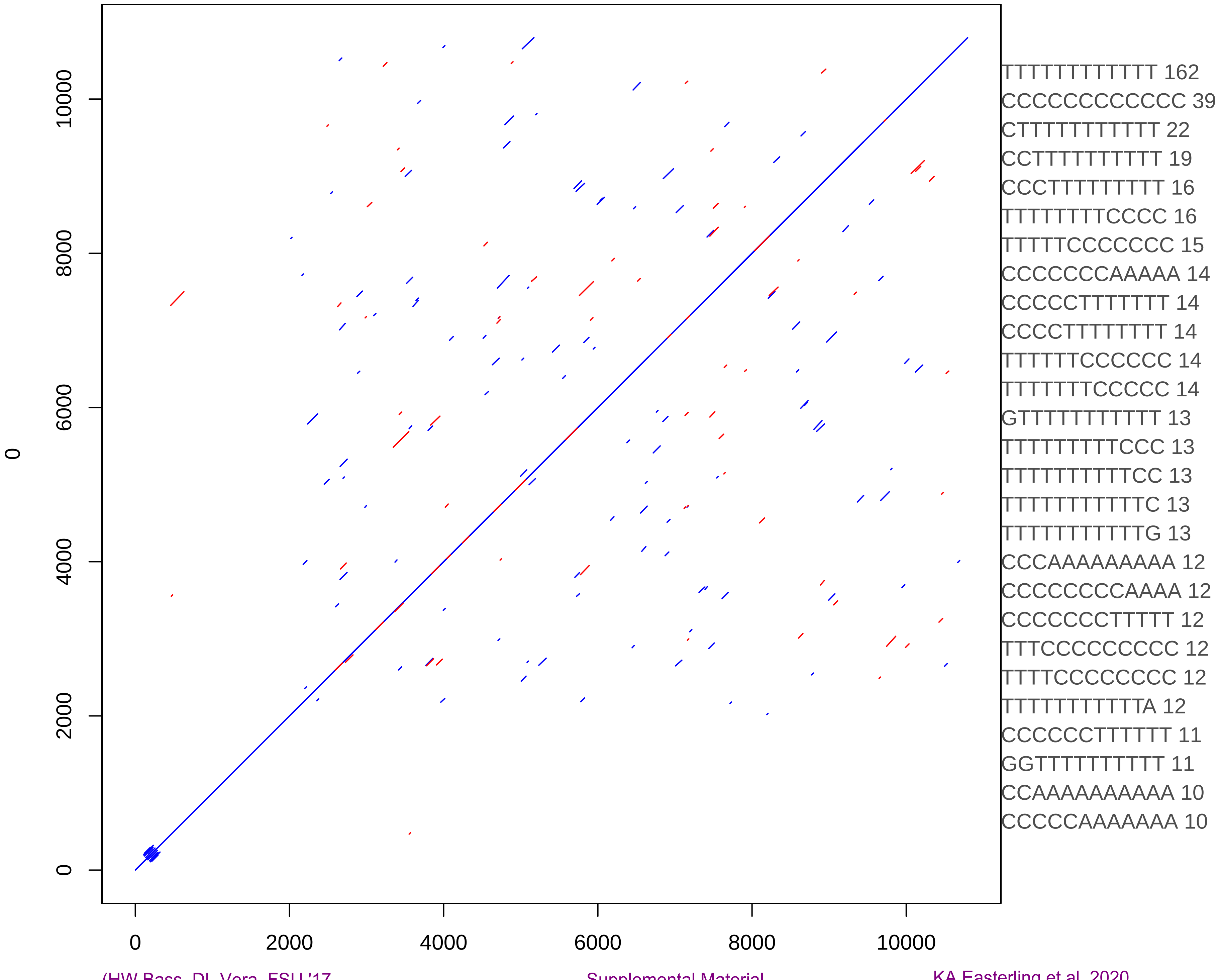

>m141126_060447_42134_c100732262550000001823141405141500_s1_p0/19751/12978_20690

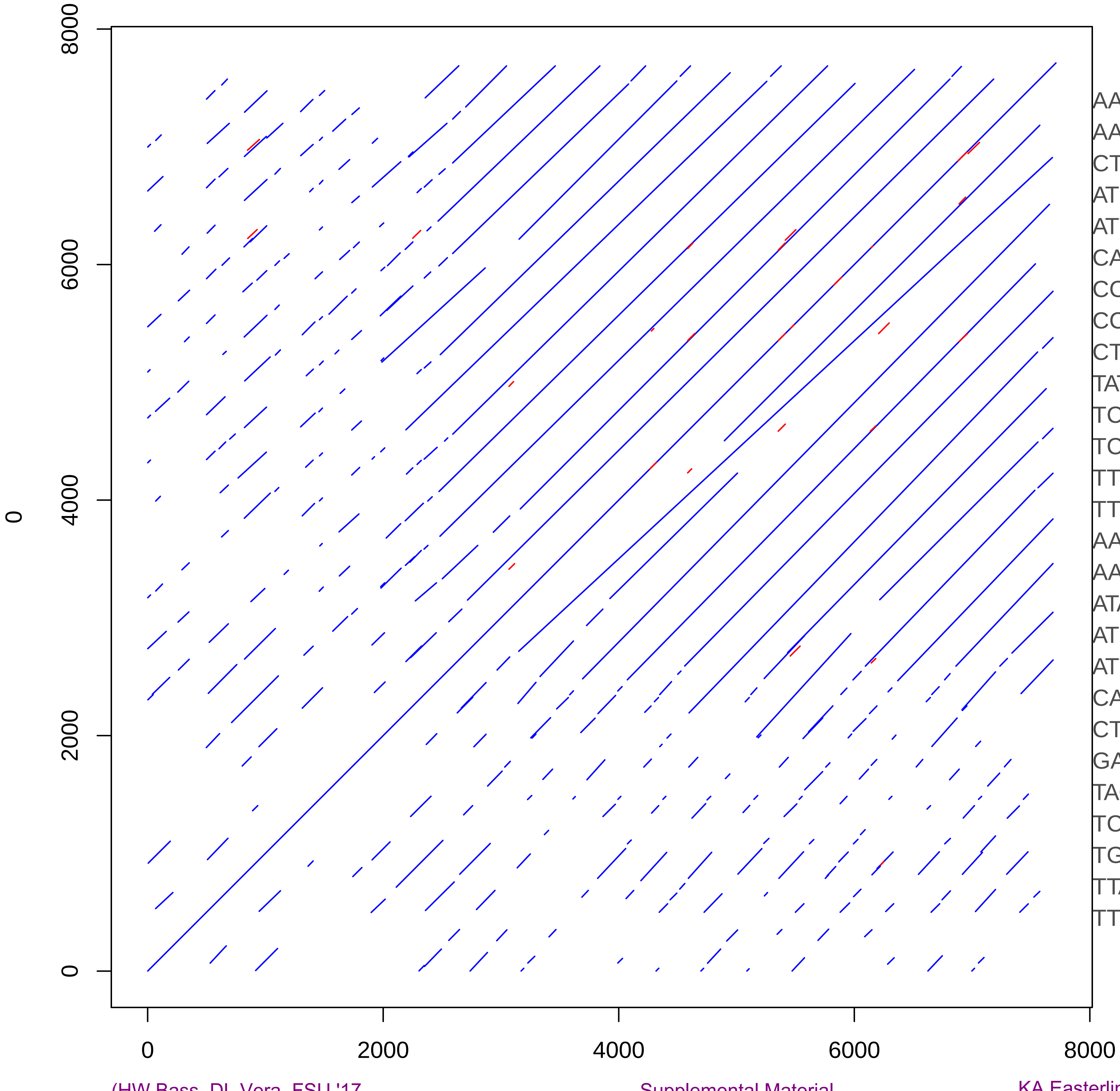

AAAAAAATTCCC 12
AAAAAAAATTCC 10
CTCATGGGGAAA 9
ATCTCATGGGGA 8
ATTTCAAAAAAA 8
CAAAAAAAATTC 8
CCCTTTTTGATG 8
CCTTTTTGATGG 8
CTTTTTGATGGC 8
TATCTCATGGGG 8
TCAAAAAAAATT 8
TCTCATGGGGAA 8
TTTCAAAAAAAA 8
TTTTTGATGGCA 8
AAATCGAAGATT 7
AATGAATATATT 7
ATATCTCATGGG 7
ATGGGGAAATGA 7
ATTGGAGATTTC 7
CATGGGGAAATG 7
CTCGGATTTGCA 7
GAAATCGAAGAT 7
TAGAGAAATCGA 7
TCATGGGGAAAT 7
TGGGGAAATGAA 7
TTAGAGAAATCG 7
TTTGATGGCAAT 7

>m141126_060447_42134_c100732262550000001823141405141500_s1_p0/2238/0_6520

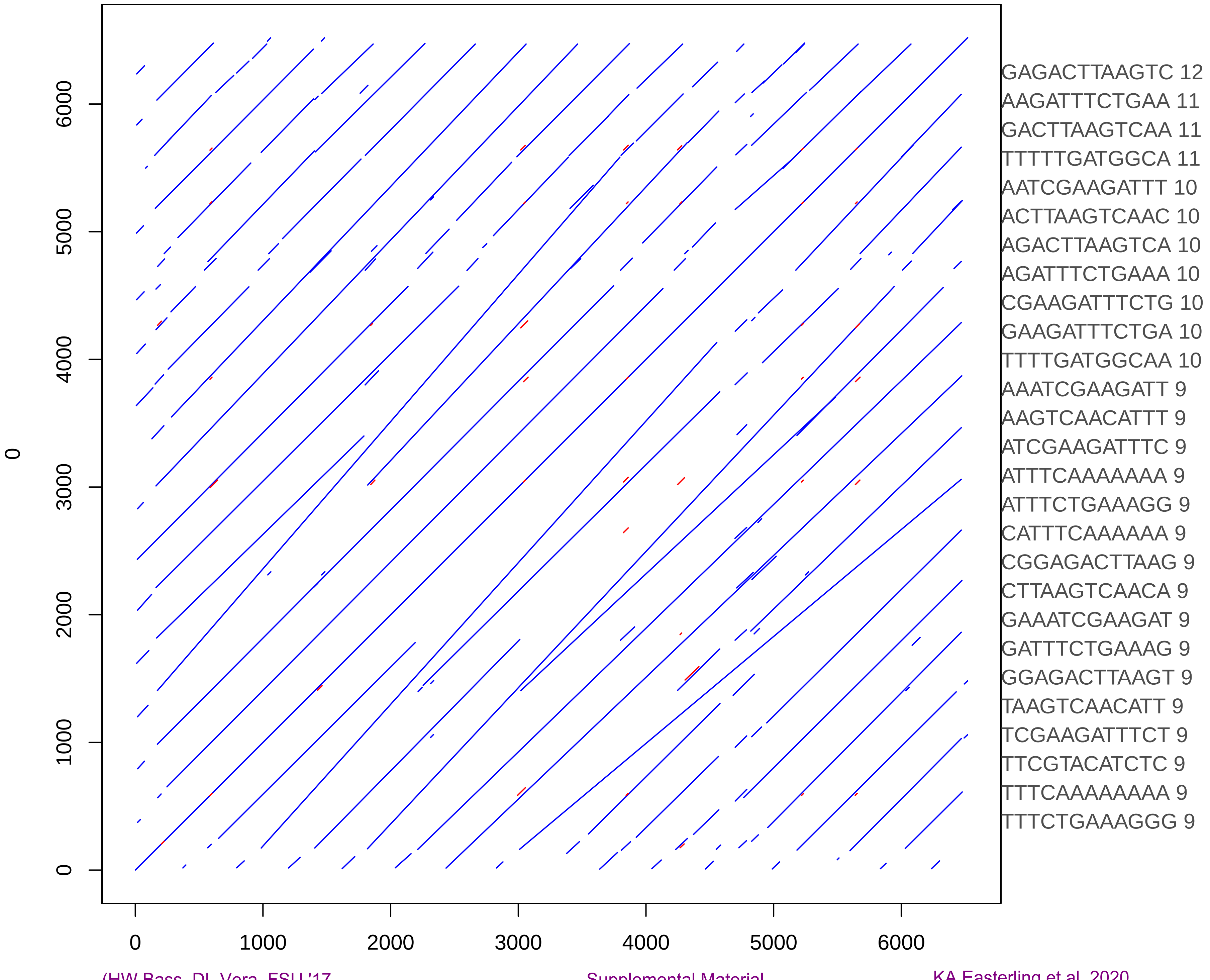

>m141126_060447_42134_c100732262550000001823141405141500_s1_p0/24549/9117_15526

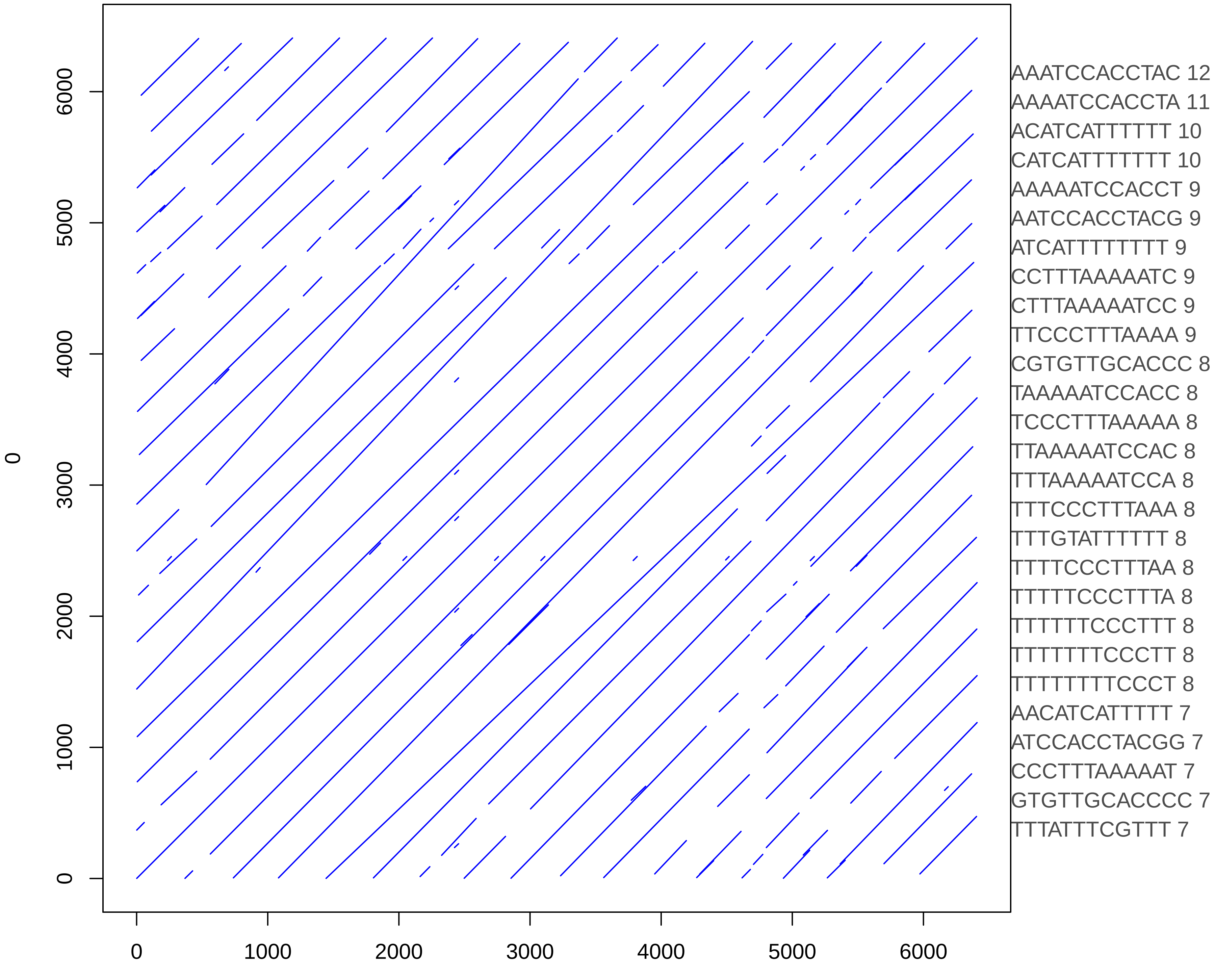

>m141126_060447_42134_c100732262550000001823141405141500_s1_p0/24865/4263_12341

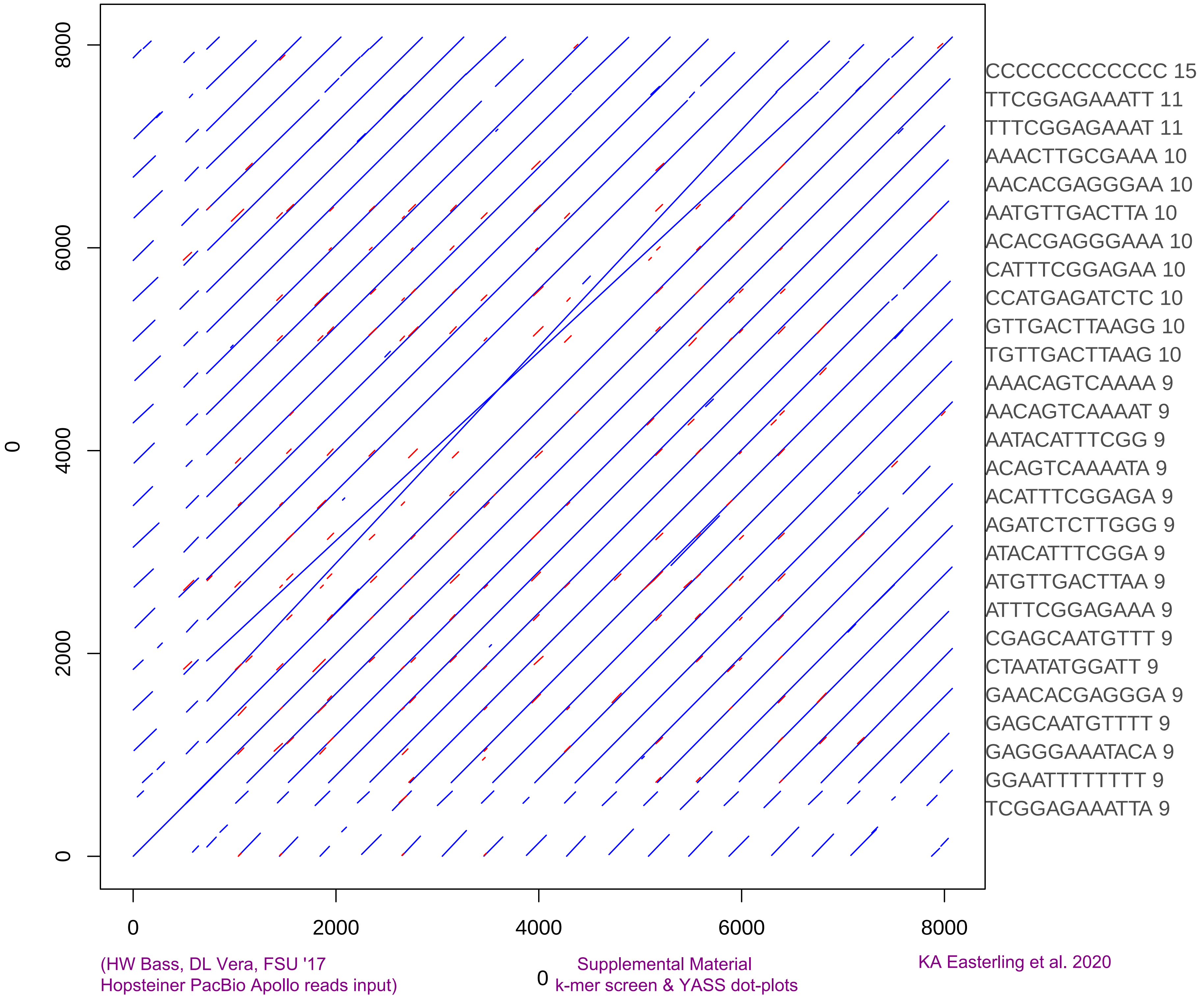

>m141126_060447_42134_c100732262550000001823141405141500_s1_p0/2617/8306_16914

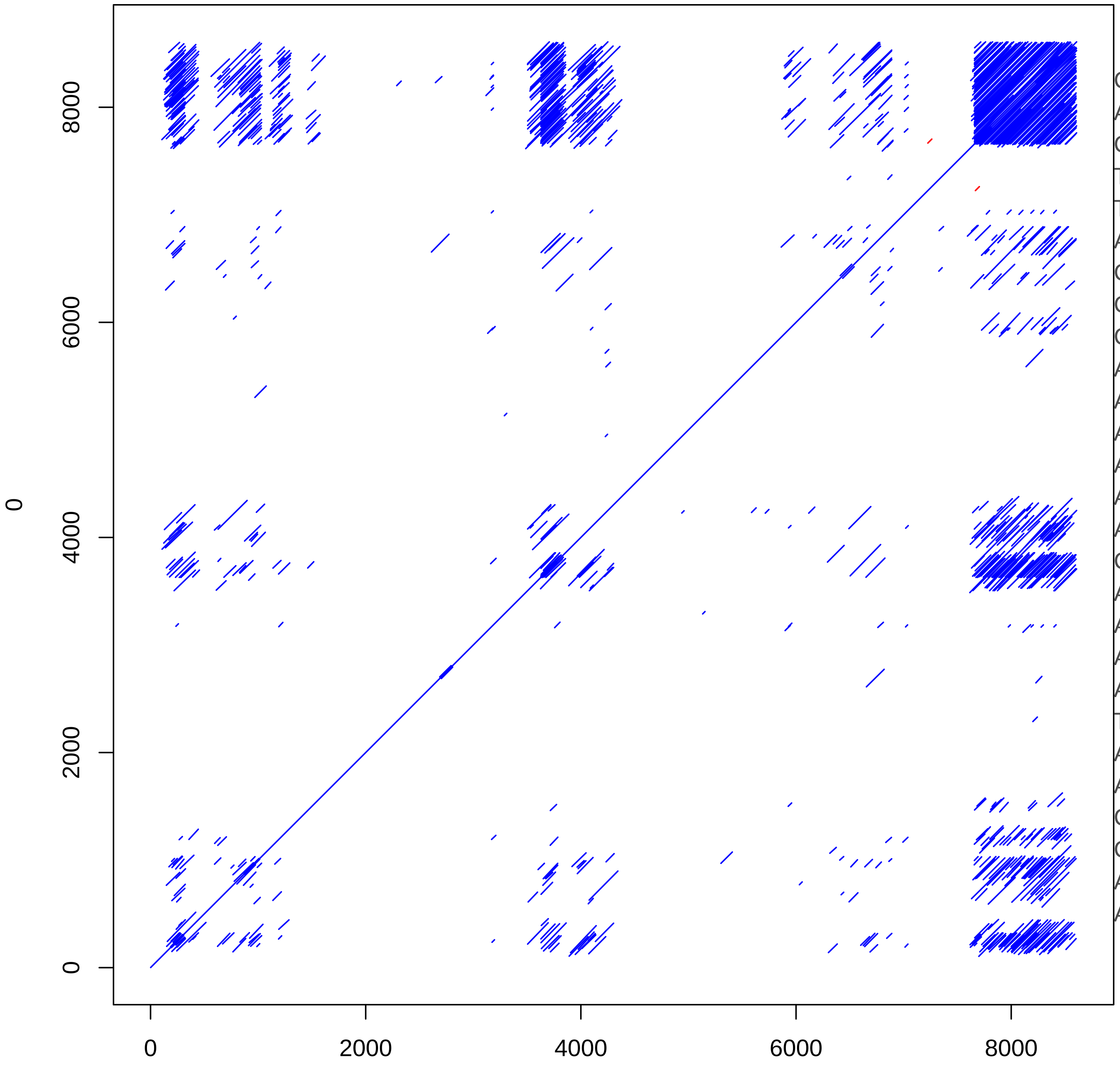

>m141126_060447_42134_c100732262550000001823141405141500_s1_p0/34940/4242_11465

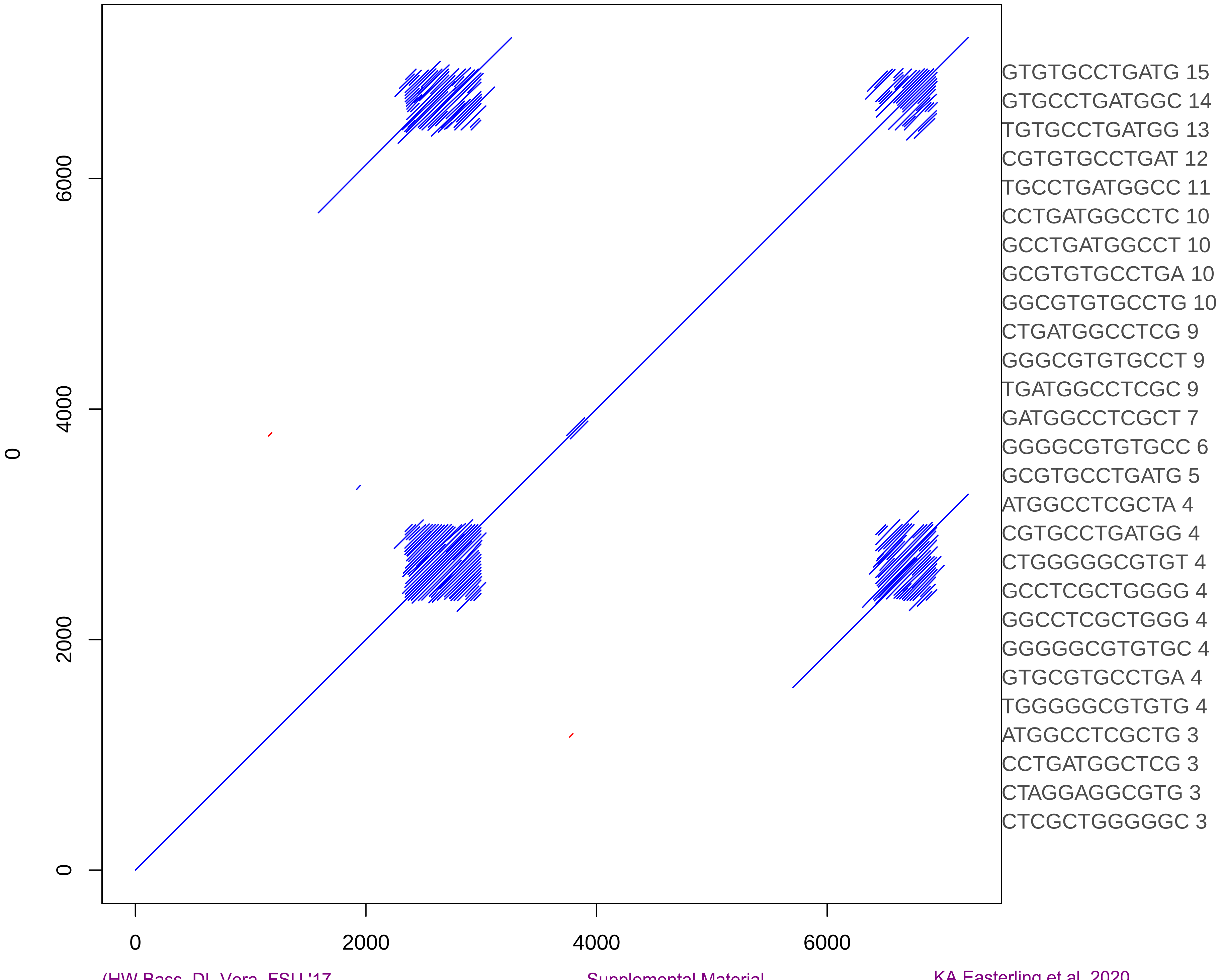

>m141126_060447_42134_c100732262550000001823141405141500_s1_p0/3924/0_8907

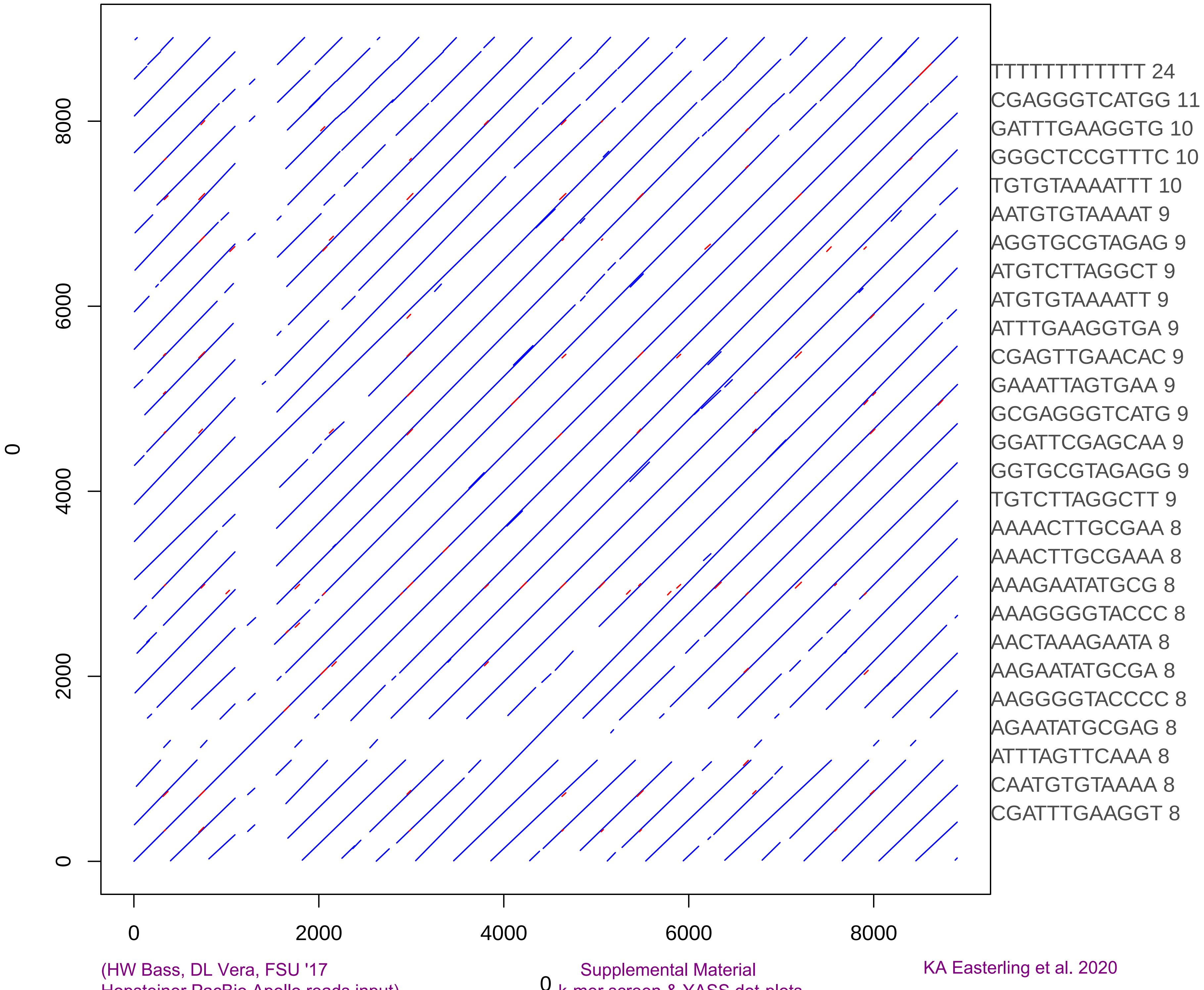

>m141126_060447_42134_c100732262550000001823141405141500_s1_p0/40644/691_14124

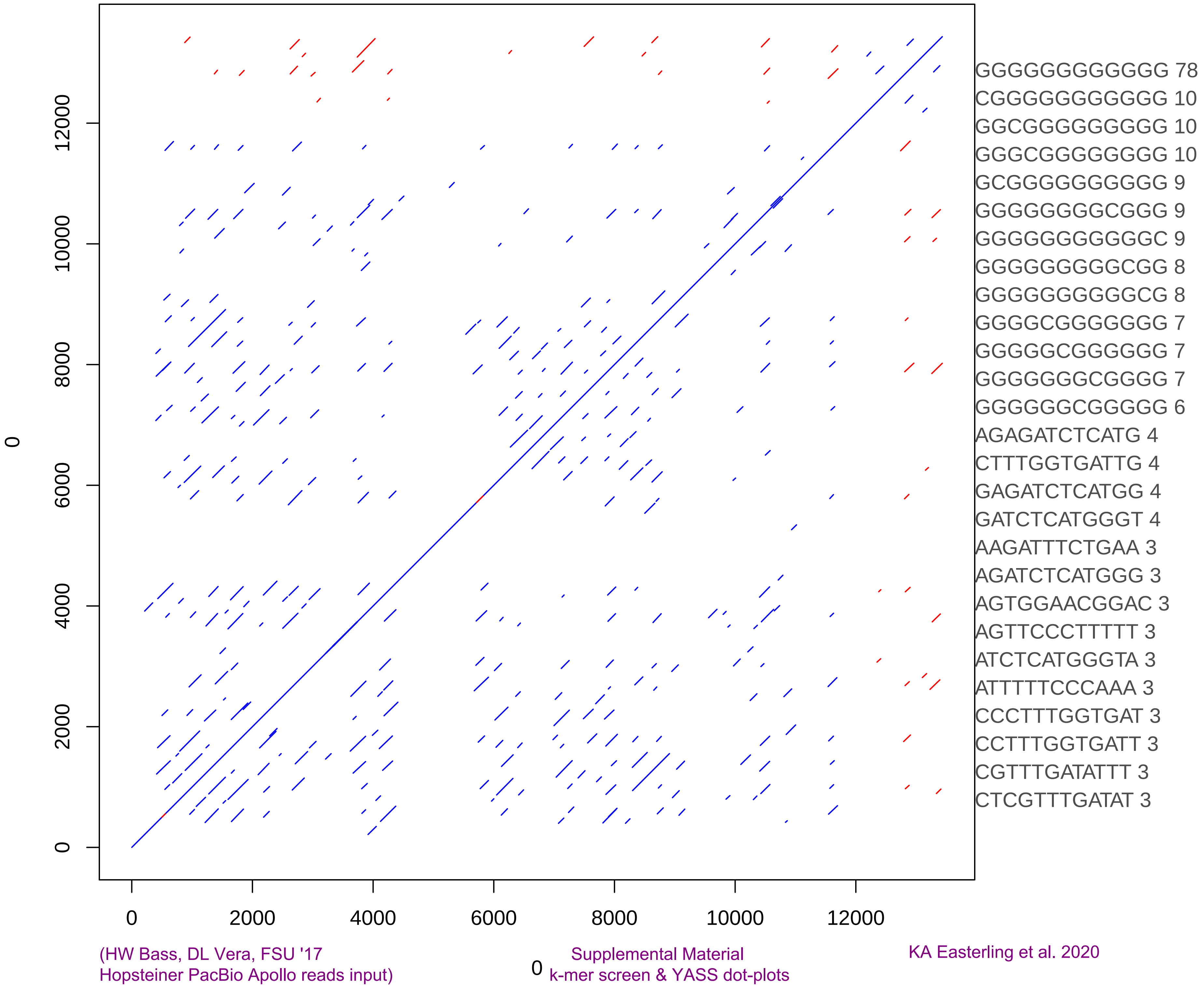

>m141126_060447_42134_c100732262550000001823141405141500_s1_p0/41951/334_6948

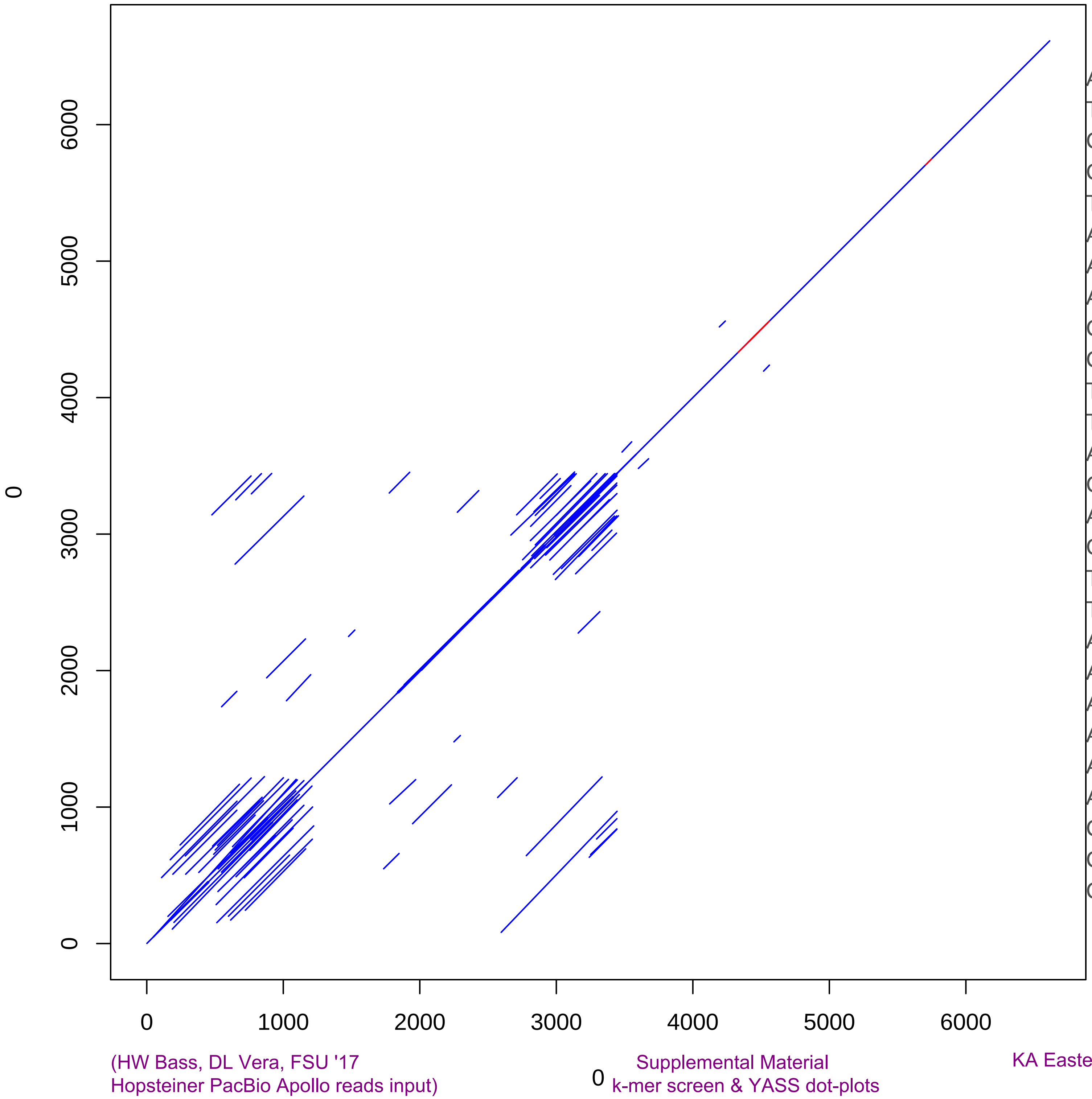

ACTACTACTACT 66
TACTACTACTAC 61
CTACTACTACTA 58
CTACTAACTACT 14
TACTAACTACTA 14
ACTAACTACTAC 13
ACTACTAACTAC 12
ACTACTACGACT 11
CTACTACGACTA 9
CTACTACTACTT 9
TACTACTAACTA 9
TACTACTACTTA 9
ACTACTACTTAC 8
CTAACTACTACT 8
ACTACTGCTACT 7
GACTACTACTAC 7
TAACTACTACTA 7
TGCTACTGCTAC 7
AACTACTACTAC 6
ACGACGACGACG 6
ACTAATACTACT 6
ACTACTACTAAC 6
ACTACTACTACG 6
ACTGCTACTGCT 6
CGACGACGACGA 6
CTACTGCTACTA 6
CTGCTACTGCTA 6

>m141126_060447_42134_c100732262550000001823141405141500_s1_p0/42246/0_8678

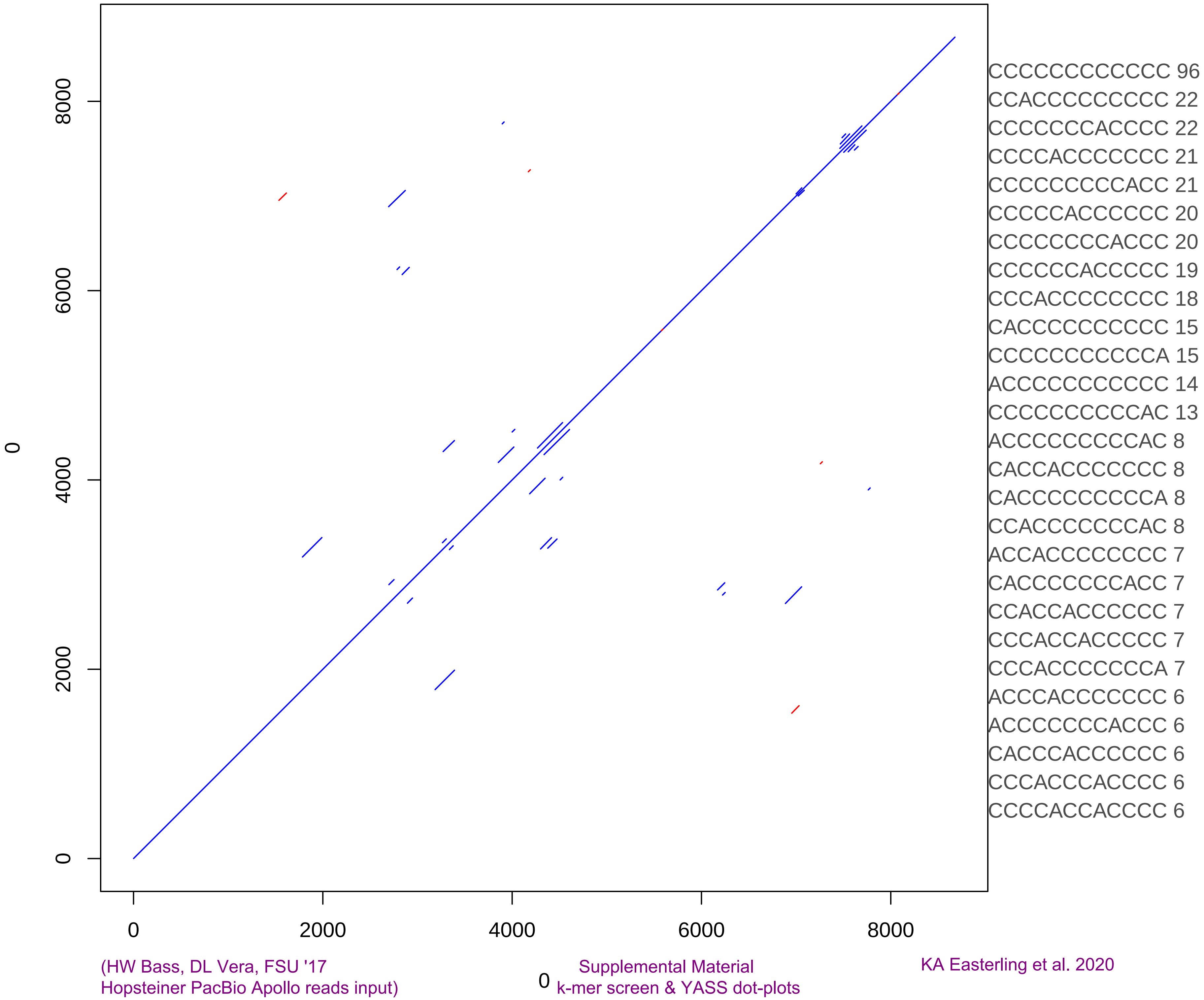

>m141126_060447_42134_c100732262550000001823141405141500_s1_p0/44383/3039_10119

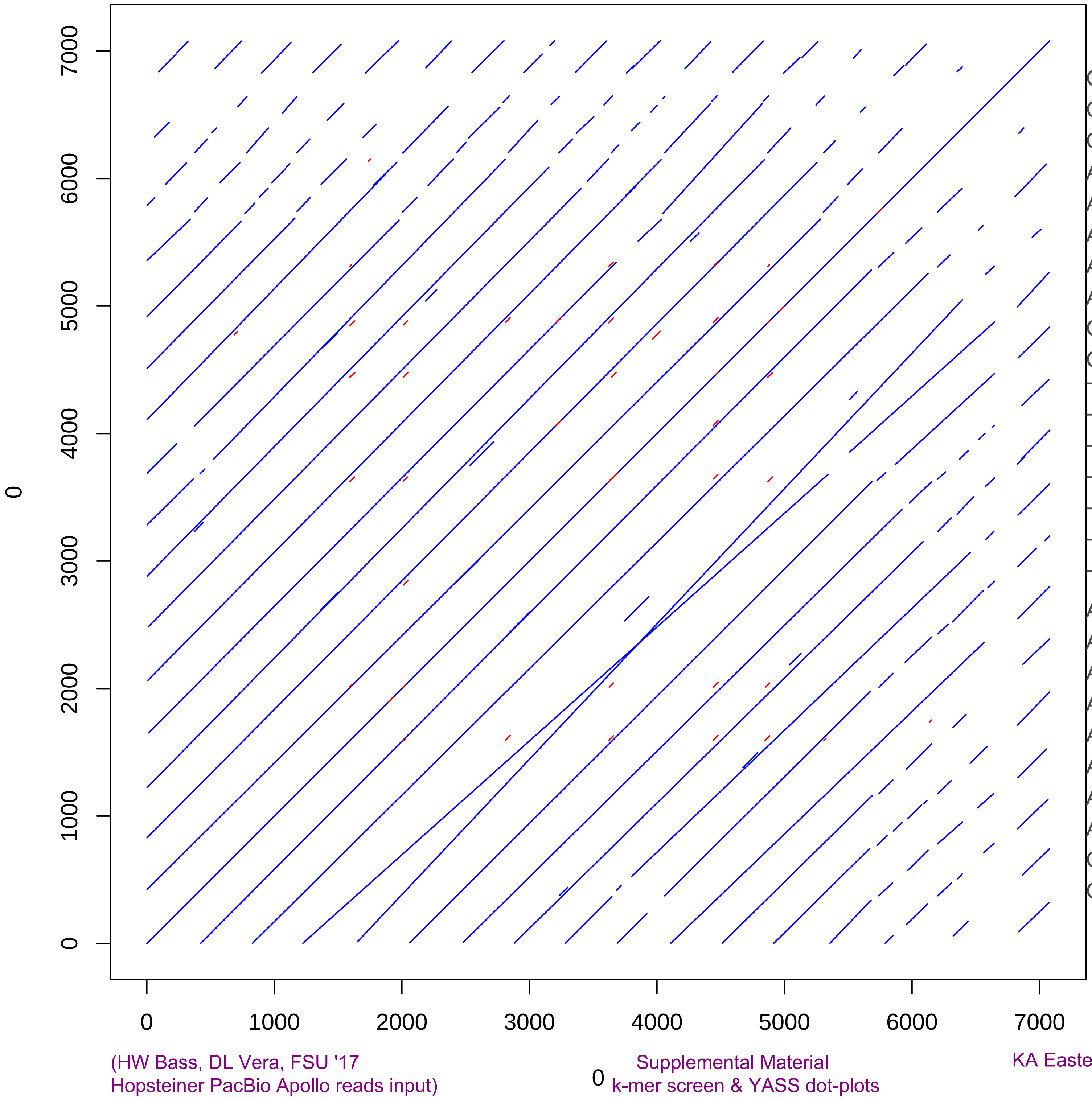

GAATTTTTTTTT 11
GGAATTTTTTTT 11
GGGAATTTTTTT 11
AAAAACTTGTGA 10
AAAACTTGTGAA 10
AAACTTGTGAAA 10
AACTTGTGAAAA 9
ACTTGTGAAAAT 9
CTTGTGAAAATT 9
GTGAAAATTGCC 9
TGTGAAAATTGC 9
TTGTGAAAATTG 9
TTTAAATGTTGA 9
TTTTAAATGTTG 9
TTTTTAAATGTT 9
TTTTTTAAATGT 9
TTTTTTTTAAAT 9
AAATCTTCGATT 8
AAGGGGAATTTT 8
AATCTTCGATTT 8
AATTTTTTTTTA 8
AGGGGAATTTTT 8
AGTTGAACACGA 8
ATCTTCGATTTC 8
ATTTTTTTTTAA 8
GGGGAATTTTTT 8
GTGTGTATAGGG 8

>m141126_060447_42134_c100732262550000001823141405141500_s1_p0/45936/2774_9356

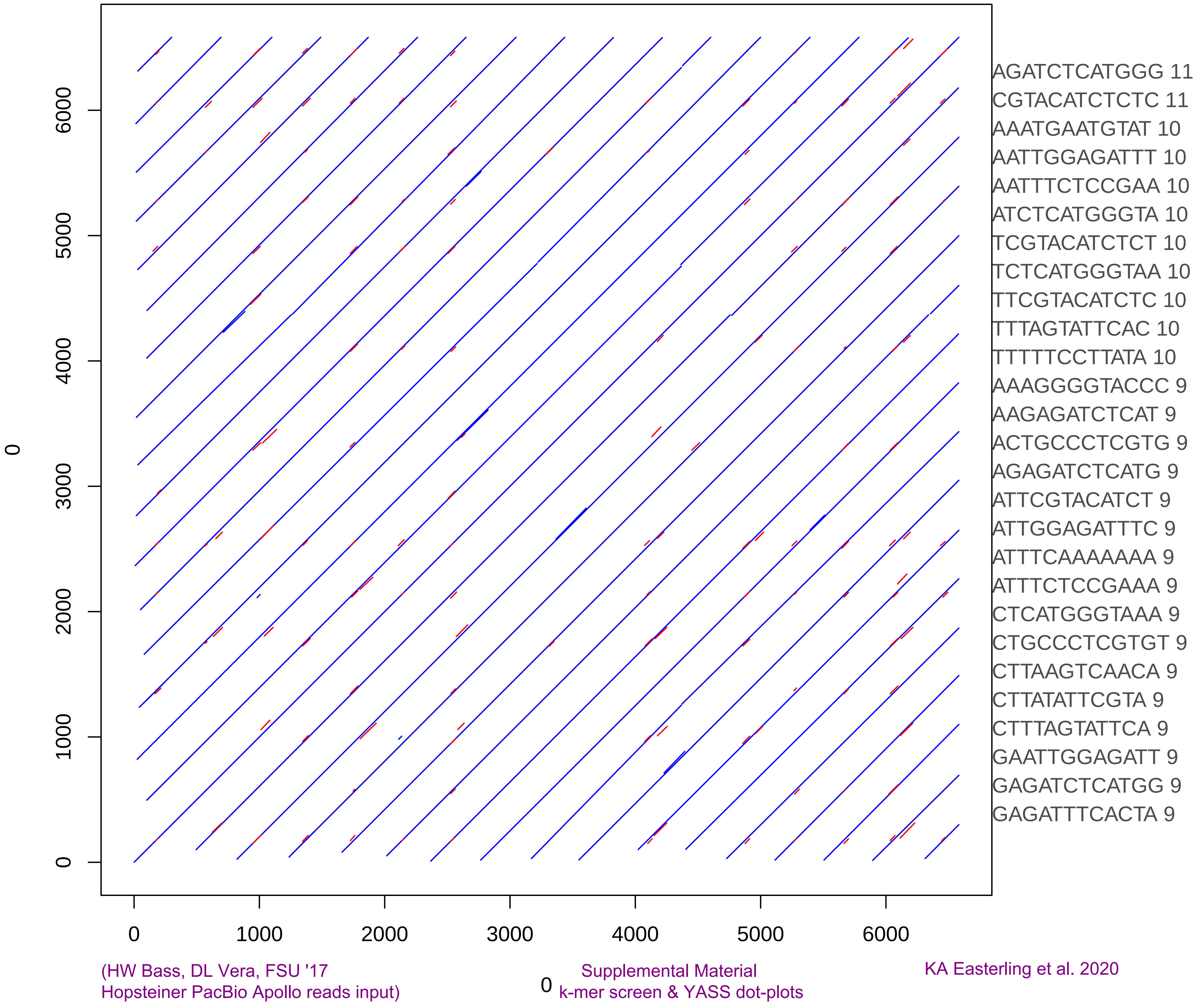

>m141126_060447_42134_c100732262550000001823141405141500_s1_p0/47020/0_11954

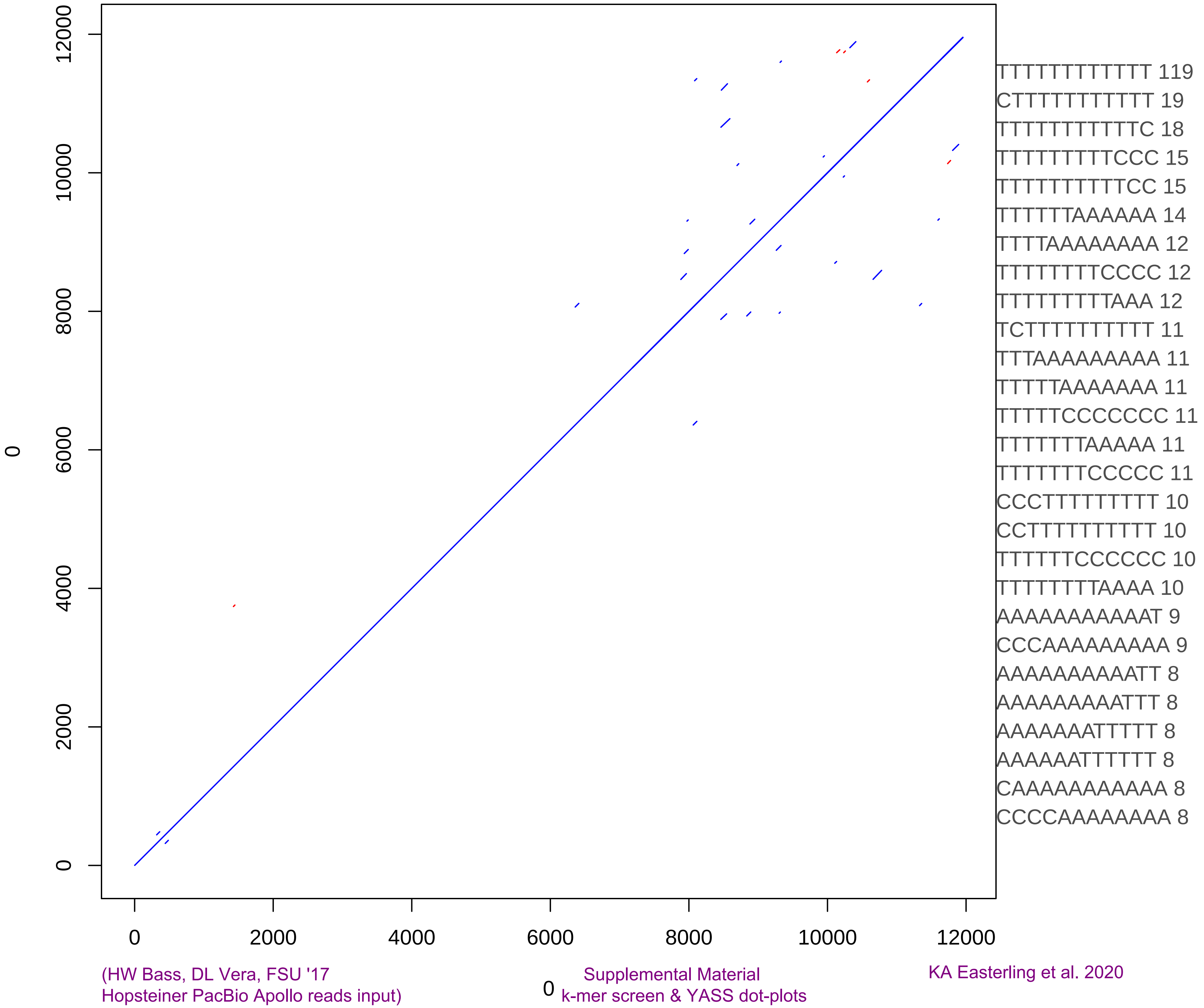

>m141126_060447_42134_c100732262550000001823141405141500_s1_p0/47153/5535_13649

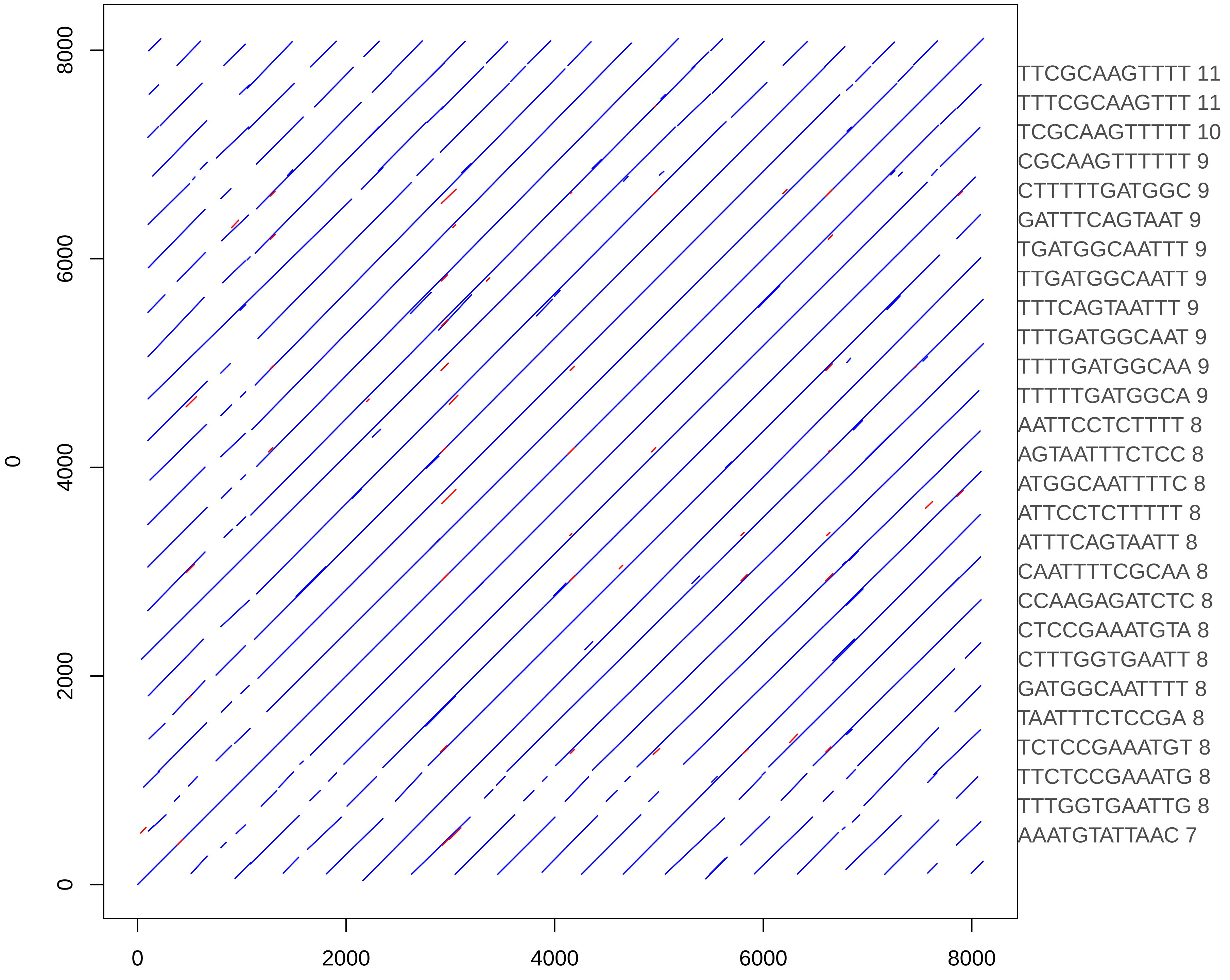

(HW Bass, DL Vera, FSU '17
Hopsteiner PacBio Apollo reads input)

0

Supplemental Material
k-mer screen & YASS dot-plots

KA Easterling et al. 2020

>m141126_060447_42134_c100732262550000001823141405141500_s1_p0/48756/668_11761

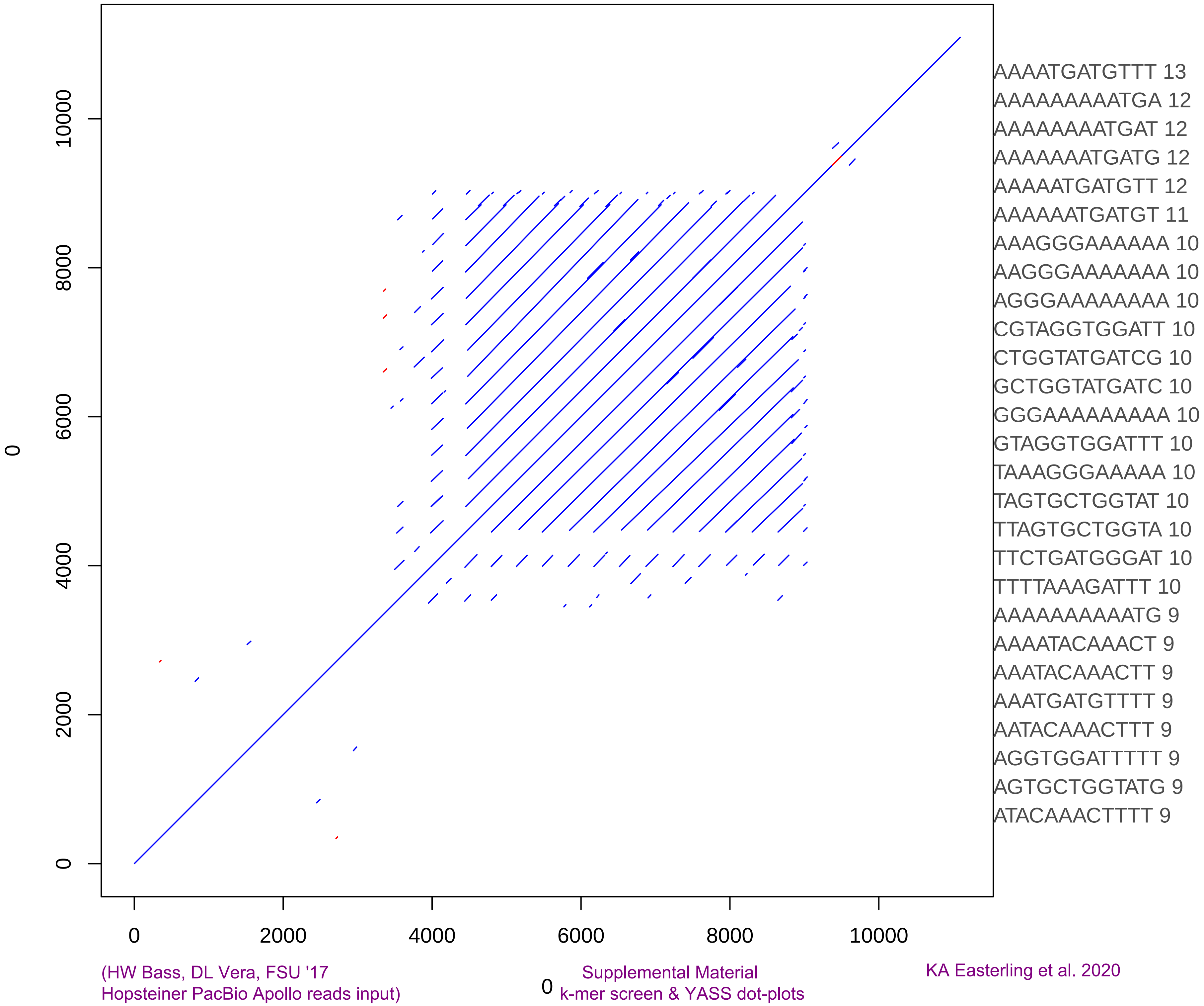

>m141126_060447_42134_c100732262550000001823141405141500_s1_p0/50878/4215_14777

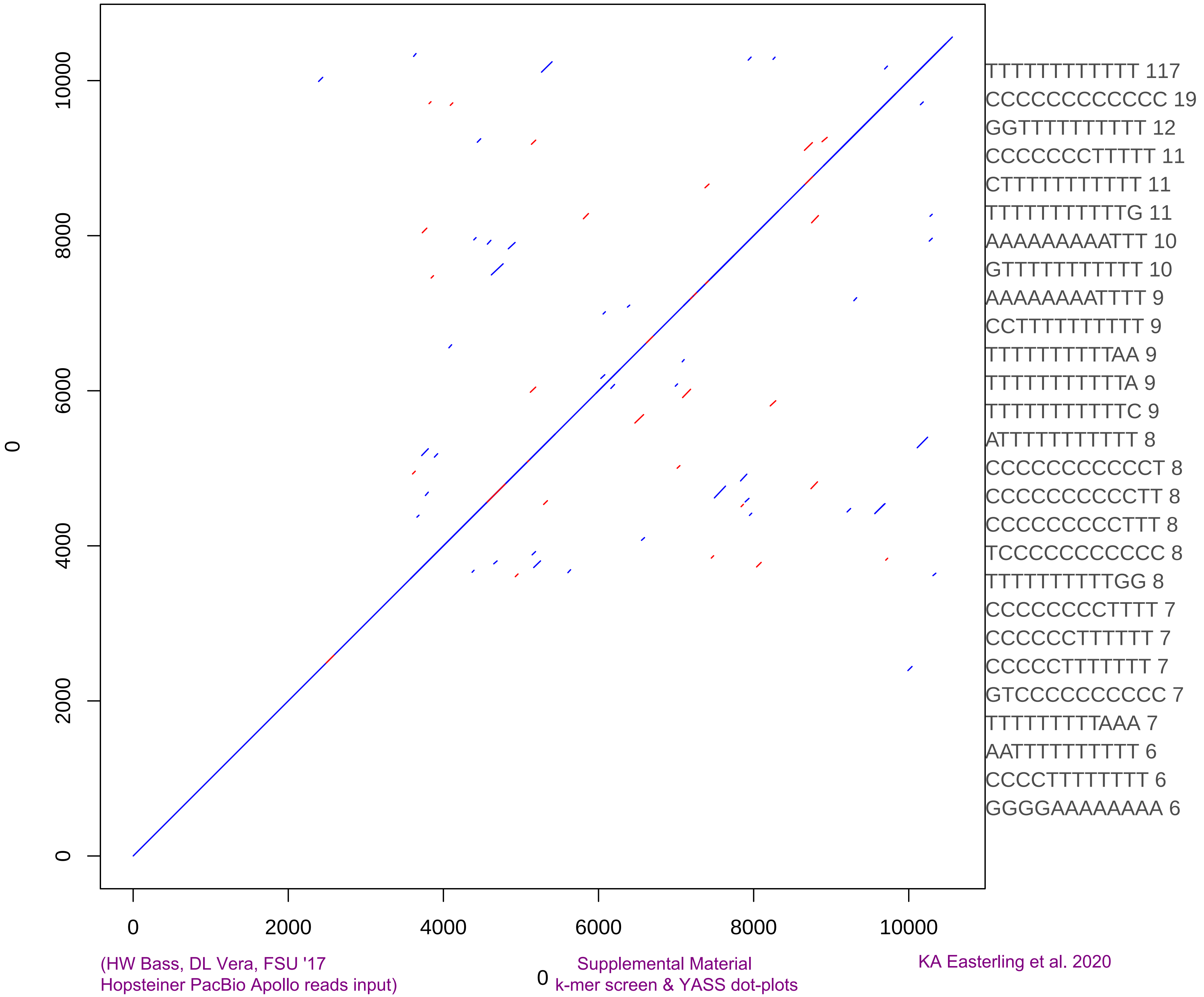

>m141126_060447_42134_c100732262550000001823141405141500_s1_p0/51831/0_10869

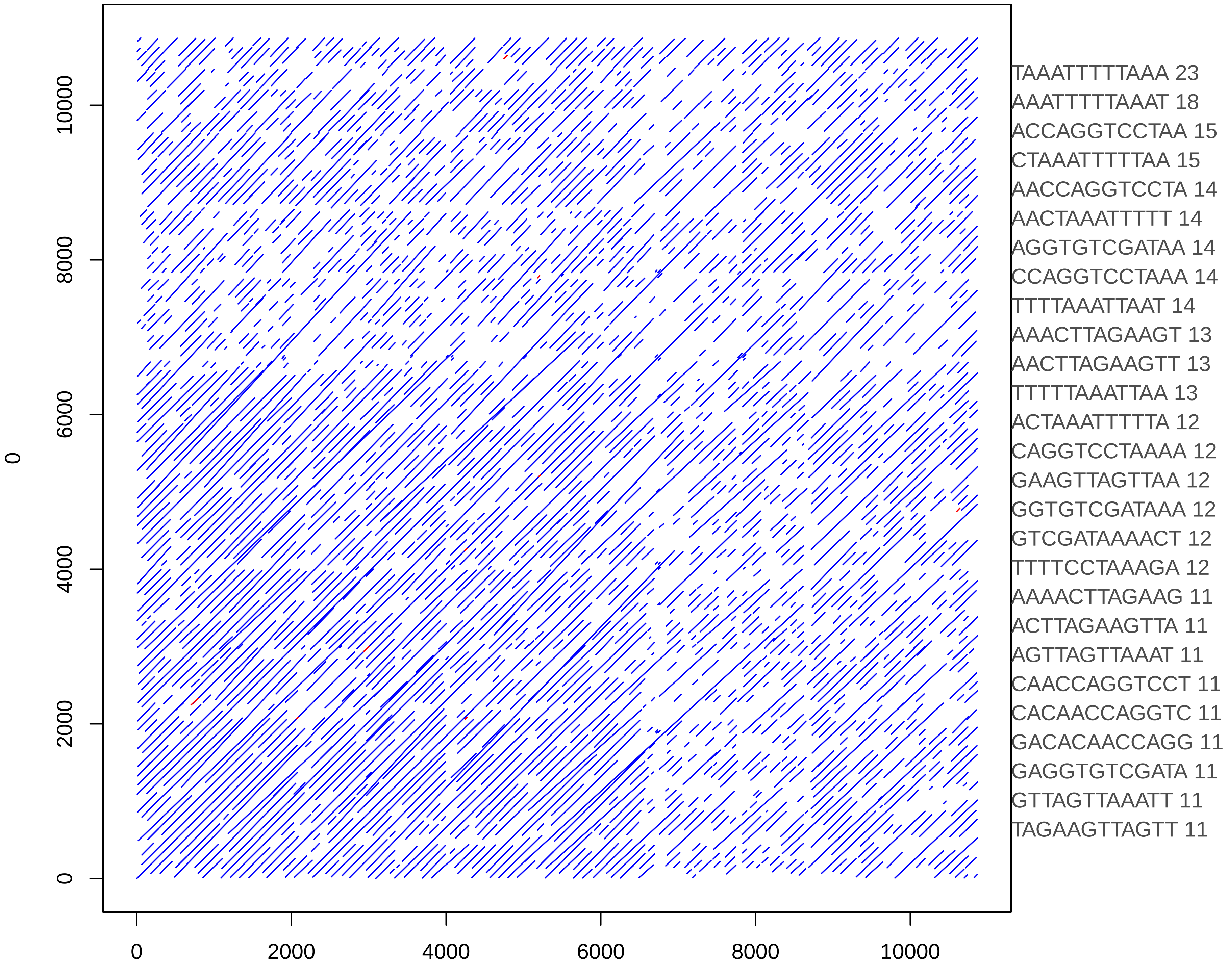

0

>m141126_060447_42134_c100732262550000001823141405141500_s1_p0/54347/0_8775

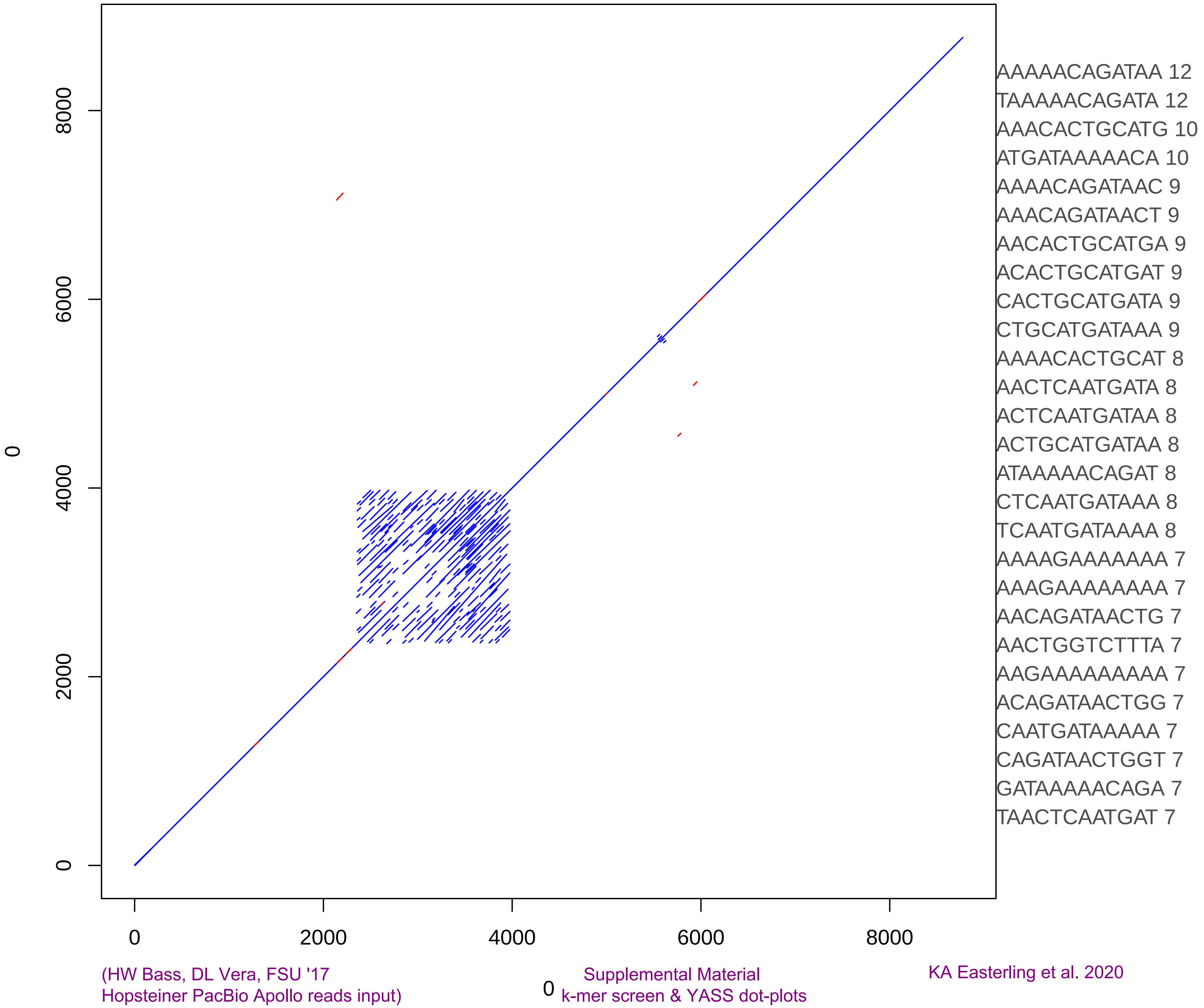

>m141126_060447_42134_c100732262550000001823141405141500_s1_p0/6296/4814_19431

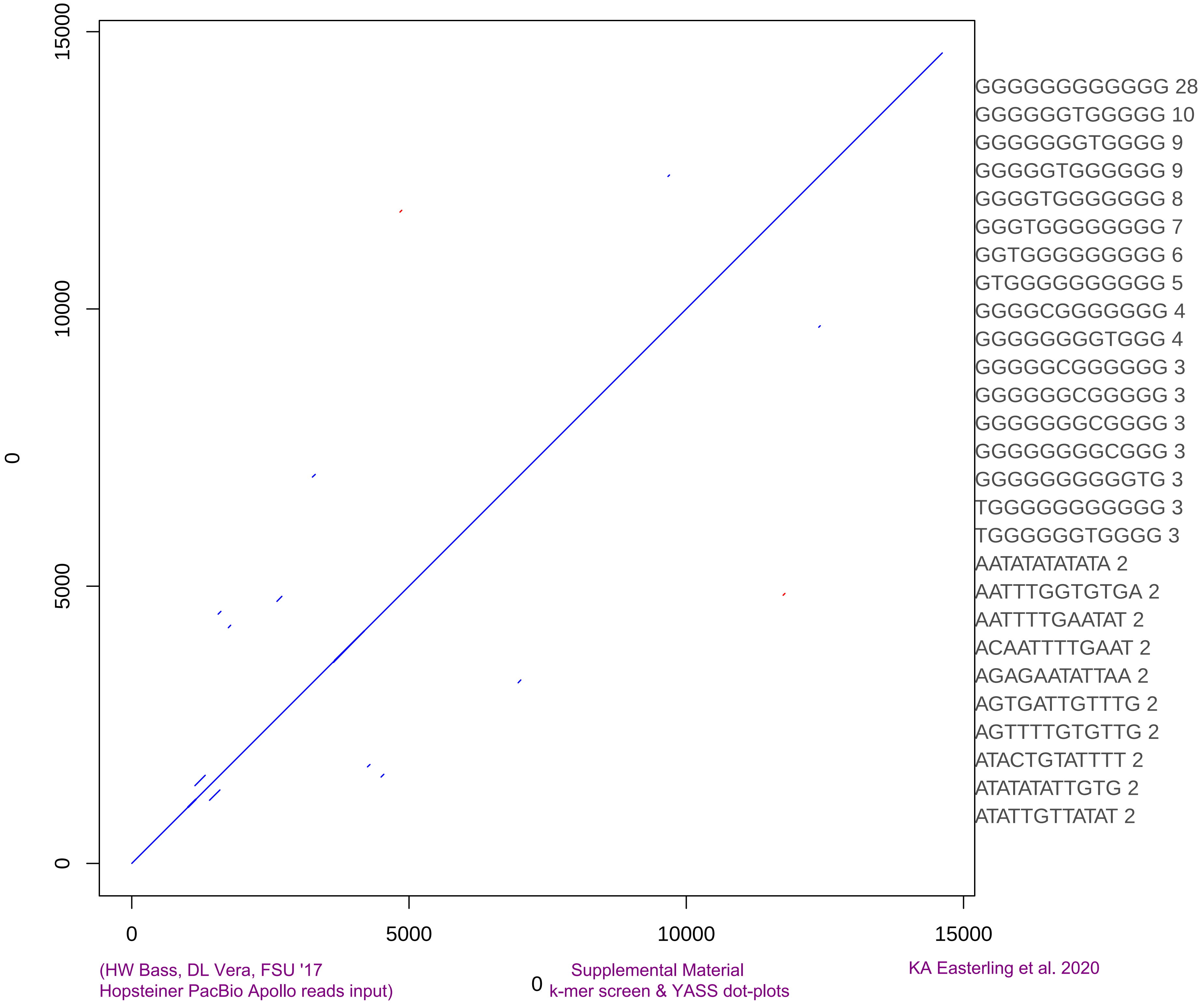

>m141126_060447_42134_c100732262550000001823141405141500_s1_p0/63688/0_5568

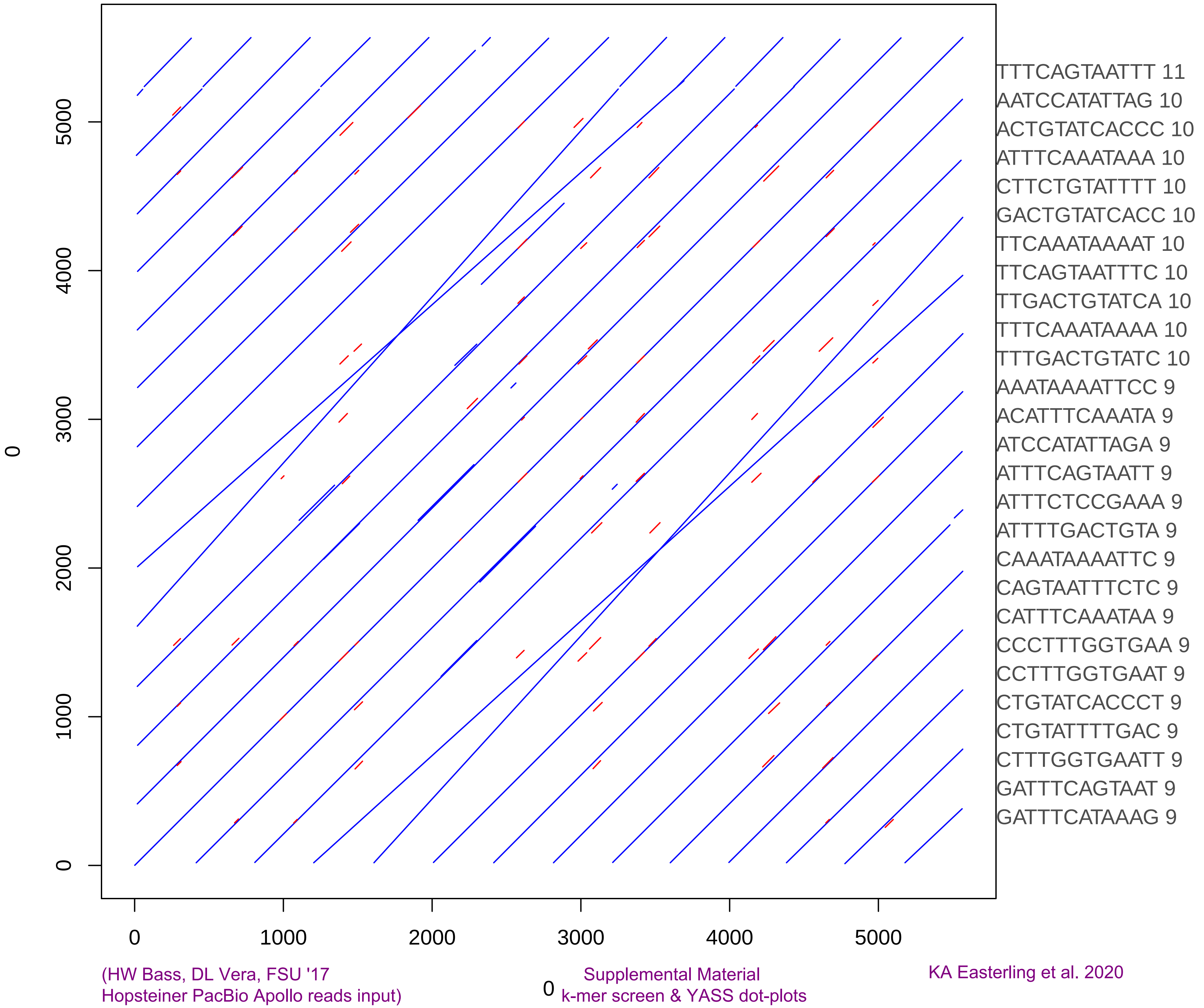

>m141126_060447_42134_c100732262550000001823141405141500_s1_p0/63805/0_9527

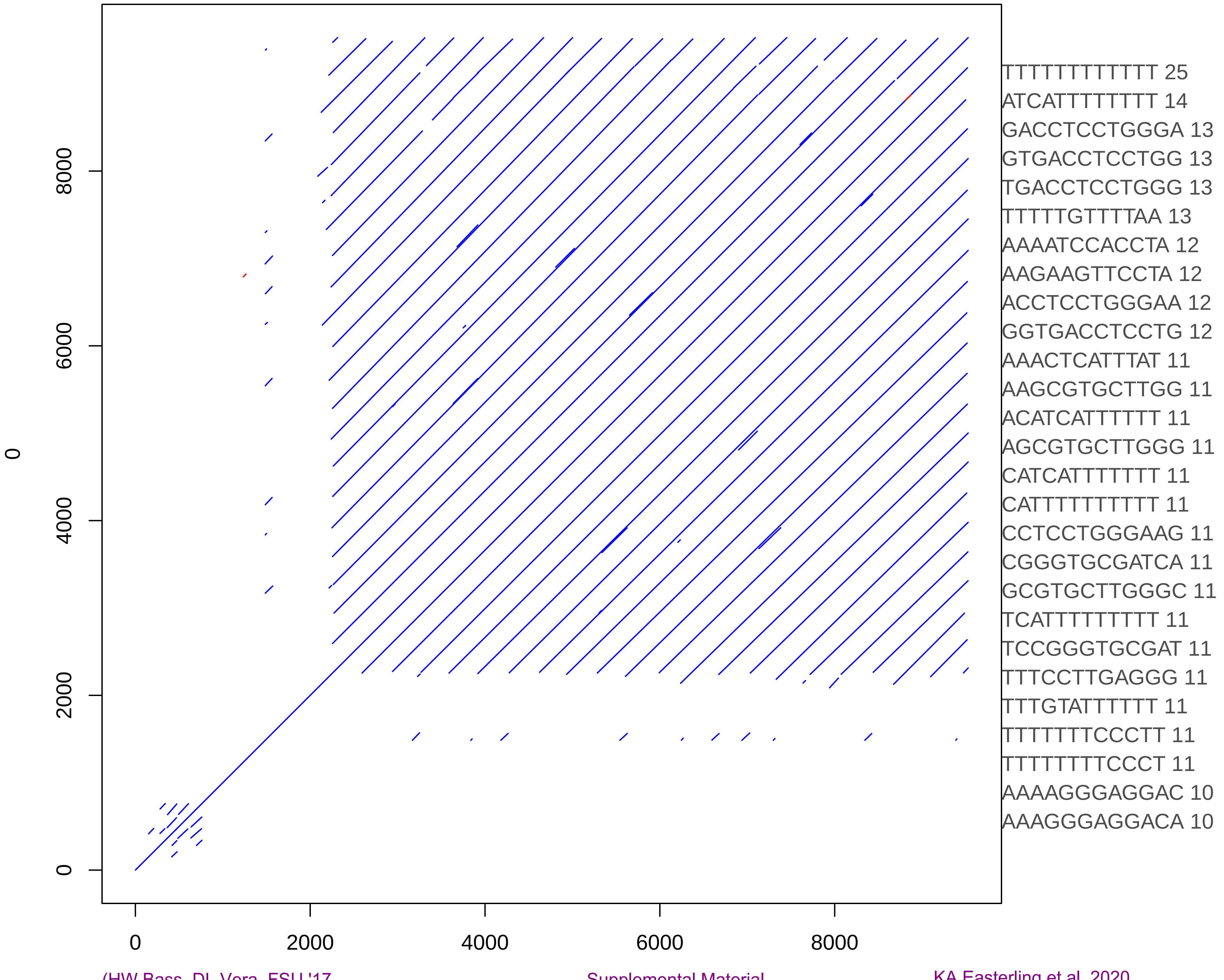

>m141126_060447_42134_c100732262550000001823141405141500_s1_p0/64071/0_5113

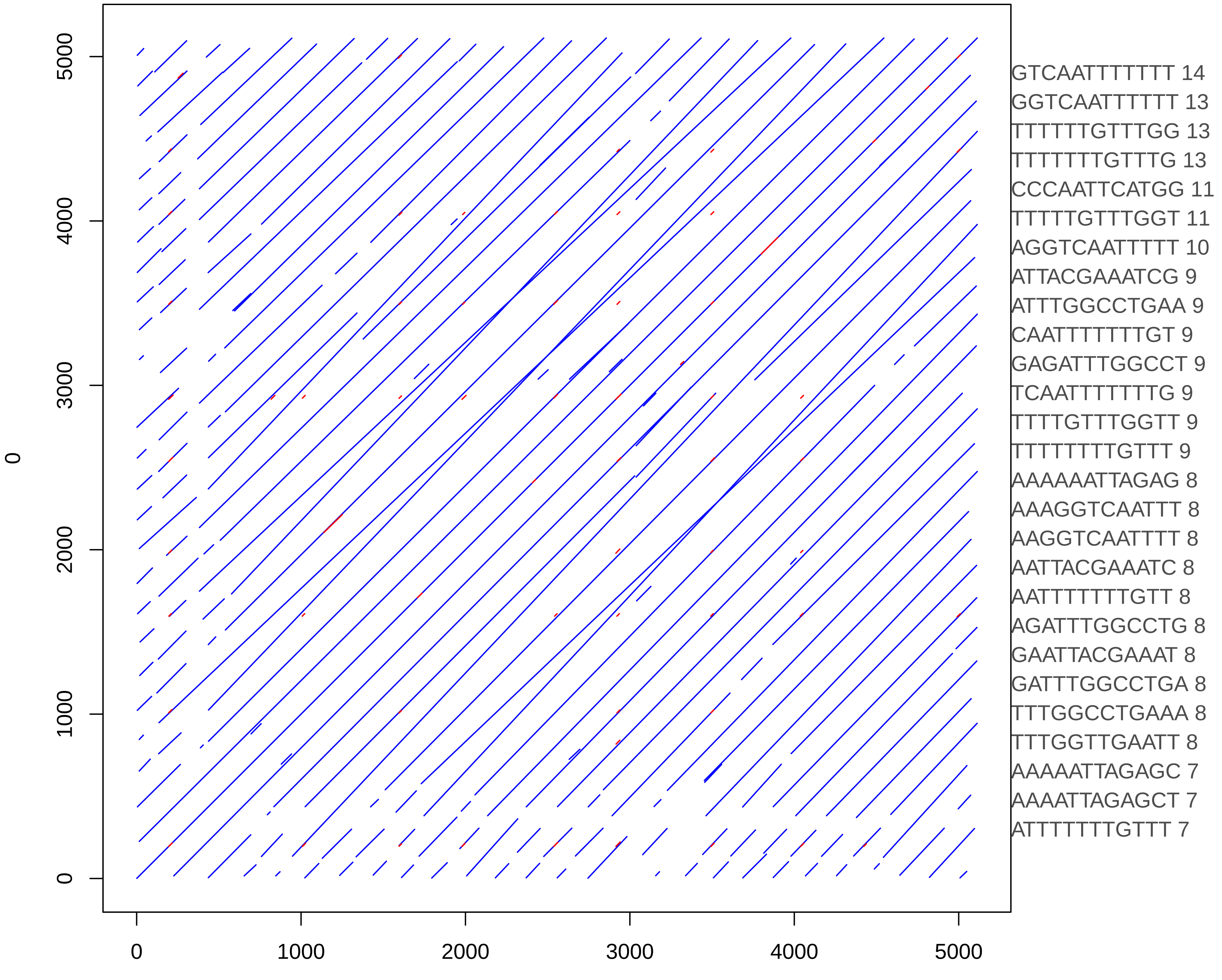

>m141126_060447_42134_c100732262550000001823141405141500_s1_p0/64263/4527_14321

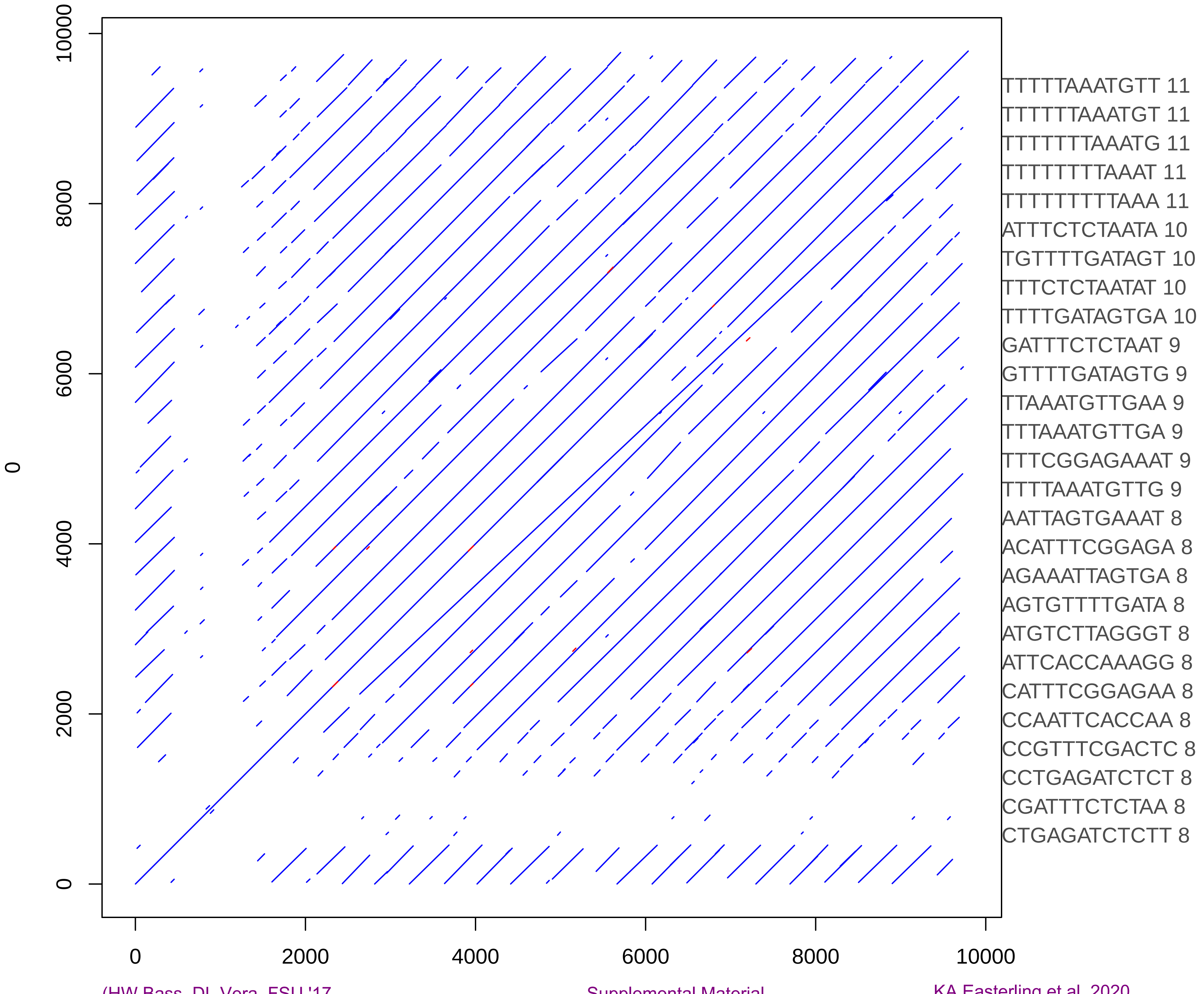

1,121 hits for repetitive sequences
passing 12mer abundance criterion

(Read-Number)
PAGE 606

For Tandem Repeat families of ### bp:
PacBio Read named "HuluTR###-r606"

>m141126_060447_42134_c100732262550000001823141405141500_s1_p0/64394/0_7480

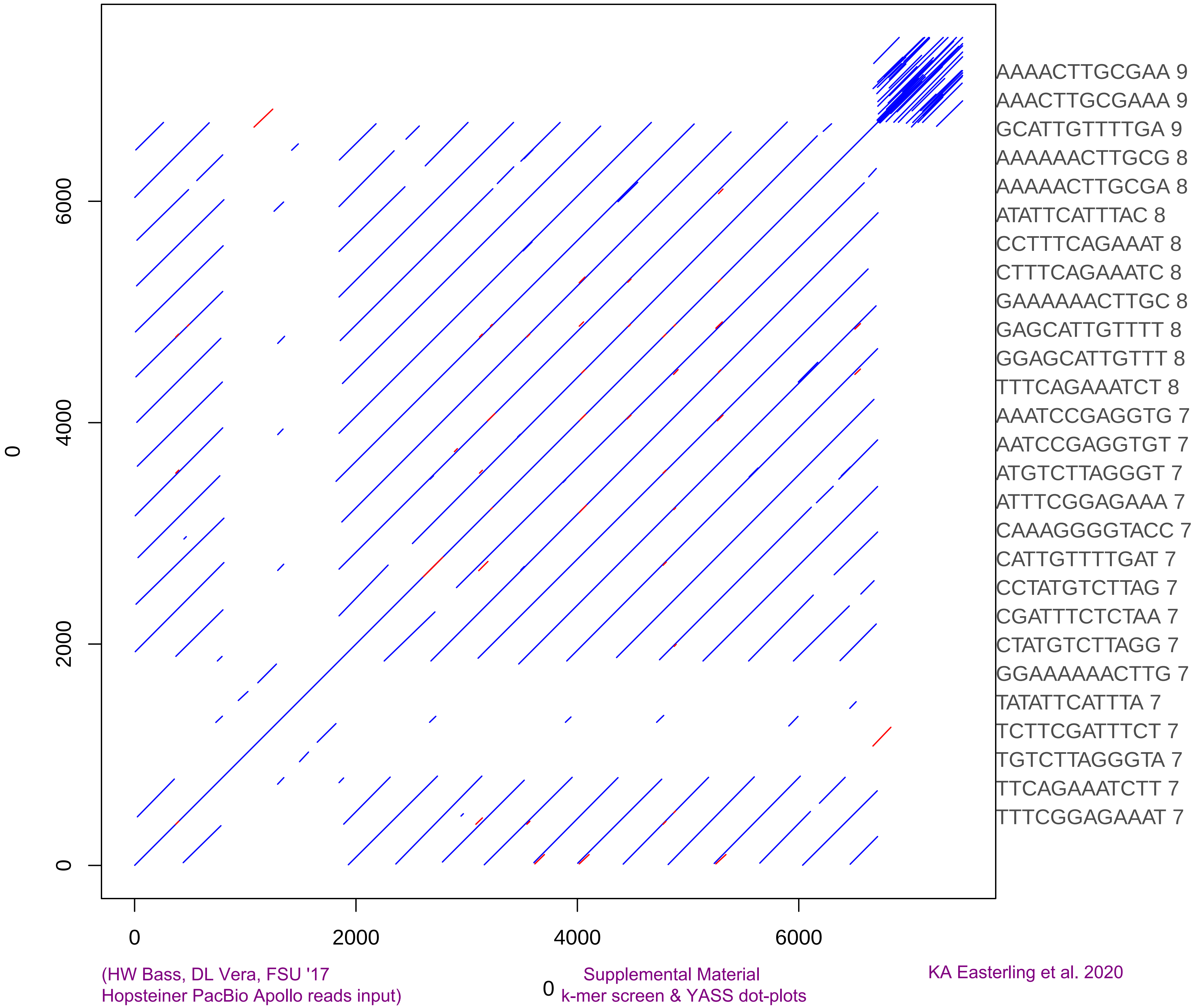

(HW Bass, DL Vera, FSU '17
Hopsteiner PacBio Apollo reads input)

Supplemental Material
k-mer screen & YASS dot-plots

KA Easterling et al. 2020

>m141126_060447_42134_c100732262550000001823141405141500_s1_p0/71587/6545_12608

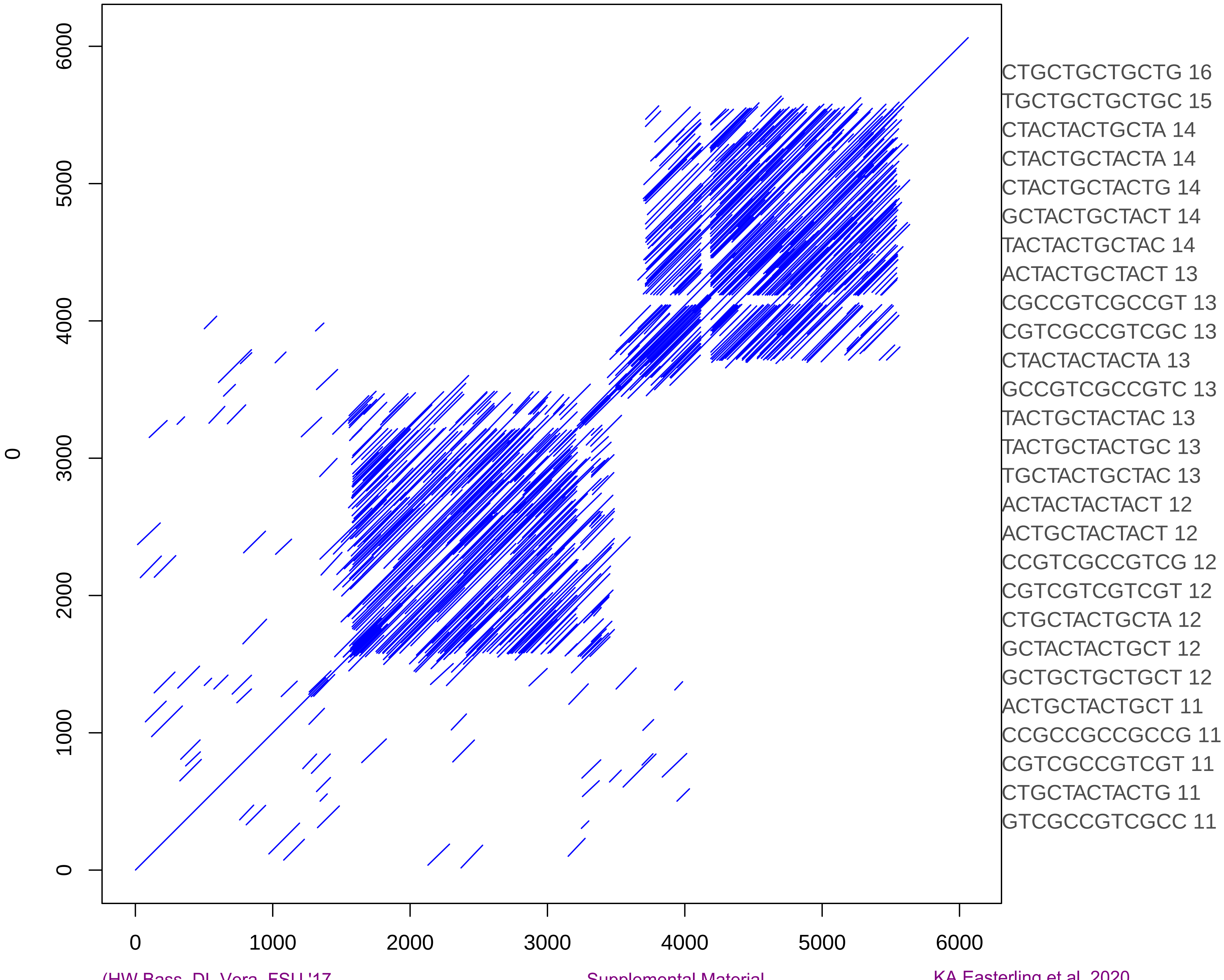

>m141126_060447_42134_c100732262550000001823141405141500_s1_p0/71820/264_9861

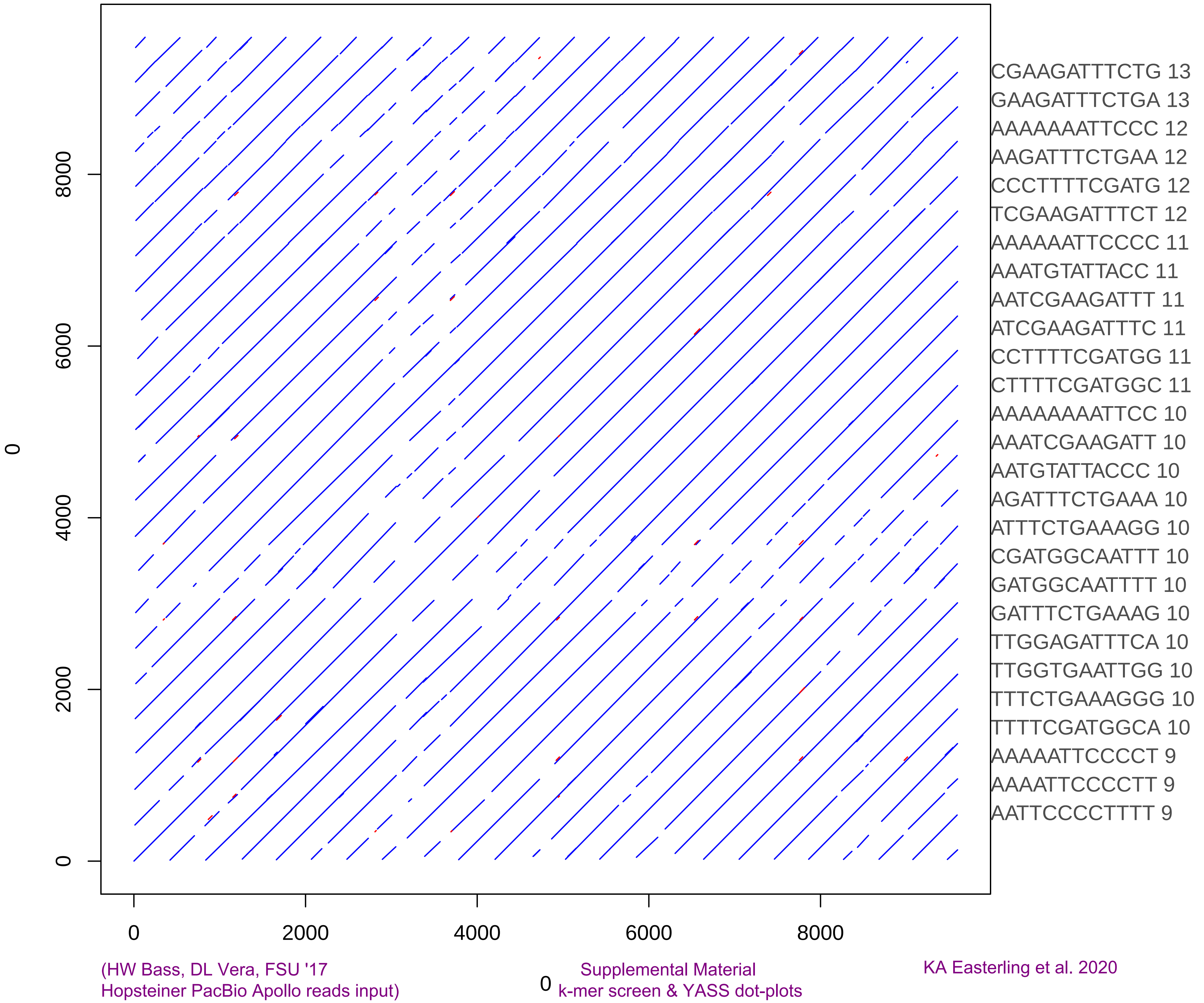

>m141126_060447_42134_c100732262550000001823141405141500_s1_p0/76452/252_6548

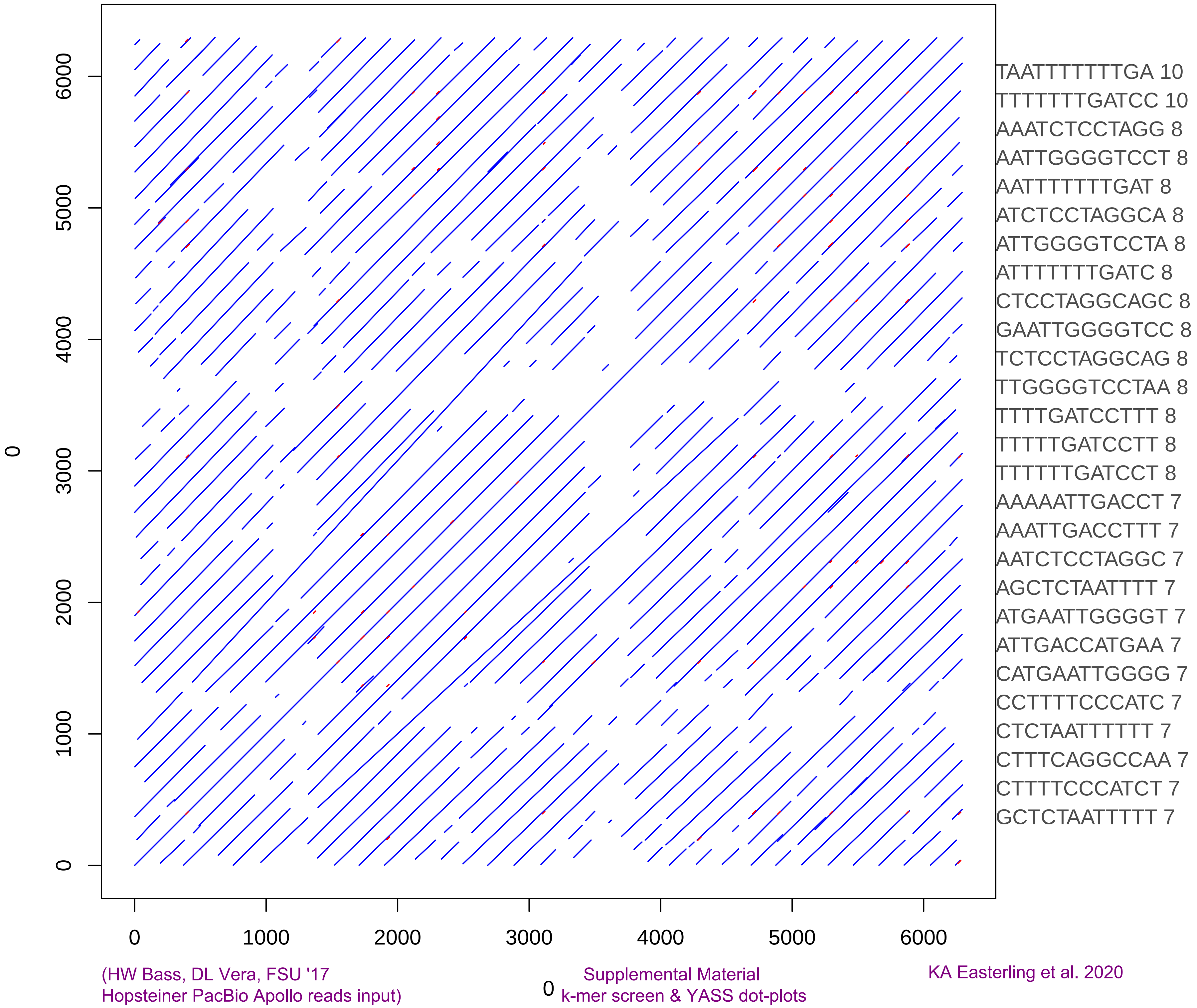

>m141126_060447_42134_c100732262550000001823141405141500_s1_p0/7952/0_7598

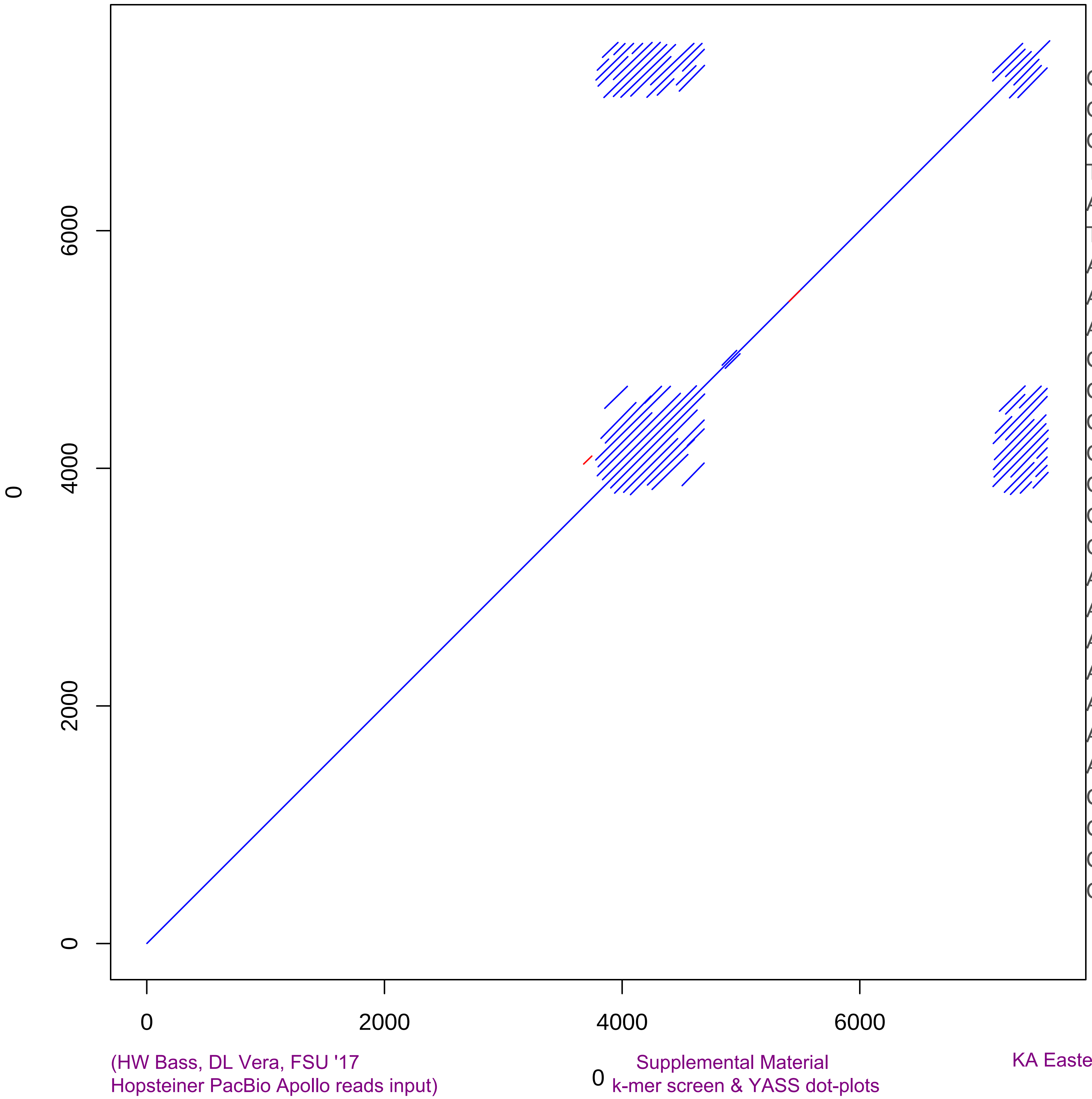

CTTGCGAAGCAA 10
GCTTGCGAAGCA 10
GCGAAGCAAAGC 9
TTGCGAAGCAAA 9
AGCTTGCGAAGC 8
TGCGAAGCAAAG 8
AAGCTTGCGAAG 5
AAAGCTAACAGC 4
AAGCAAAGCTAA 4
CAAAGCTAACAG 4
CAGAGGTCGCGG 4
CCAGAGGTCGCG 4
CGAAGCAAAGCA 4
CGAAGCAAAGCT 4
GAAGCAAAGCTA 4
GAAGCTTGCGAA 4
AAAGCACCCAGA 3
AAGCACCCAGAG 3
AAGCTAACAGCG 3
ACCCAGAGGTCG 3
AGCAAAGCTAAC 3
AGCACCCAGAGG 3
AGCGGTCGCGGA 3
CACCCAGAGGTC 3
CAGCGGTCGCGG 3
CCCAGAGGTCGC 3
GAGCTTGCGAAG 3

>m141126_060447_42134_c100732262550000001823141405141500_s1_p0/80295/2713_9960

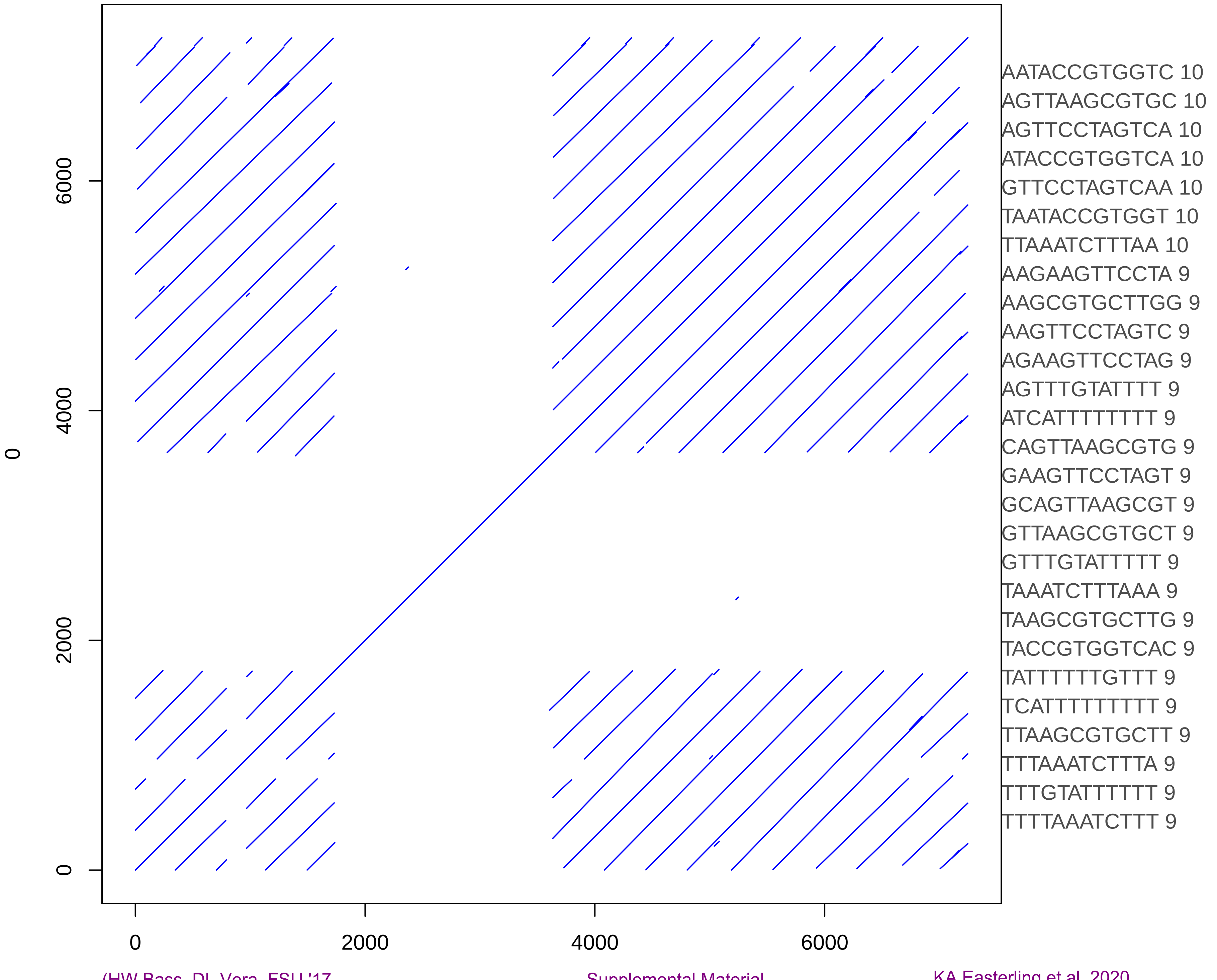

>m141126_060447_42134_c100732262550000001823141405141500_s1_p0/83119/0_6637

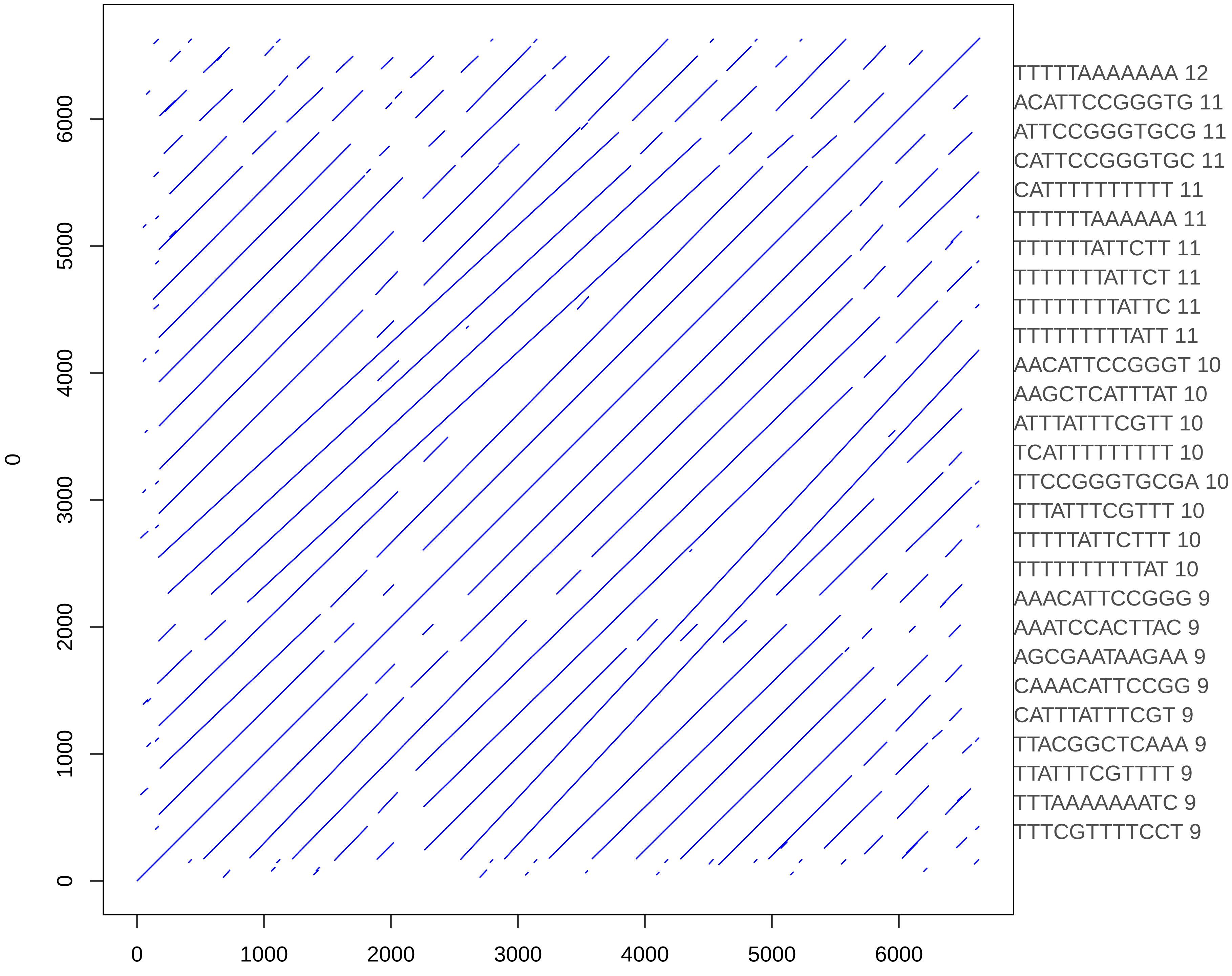

>m141126_060447_42134_c100732262550000001823141405141500_s1_p0/89680/6595_20680

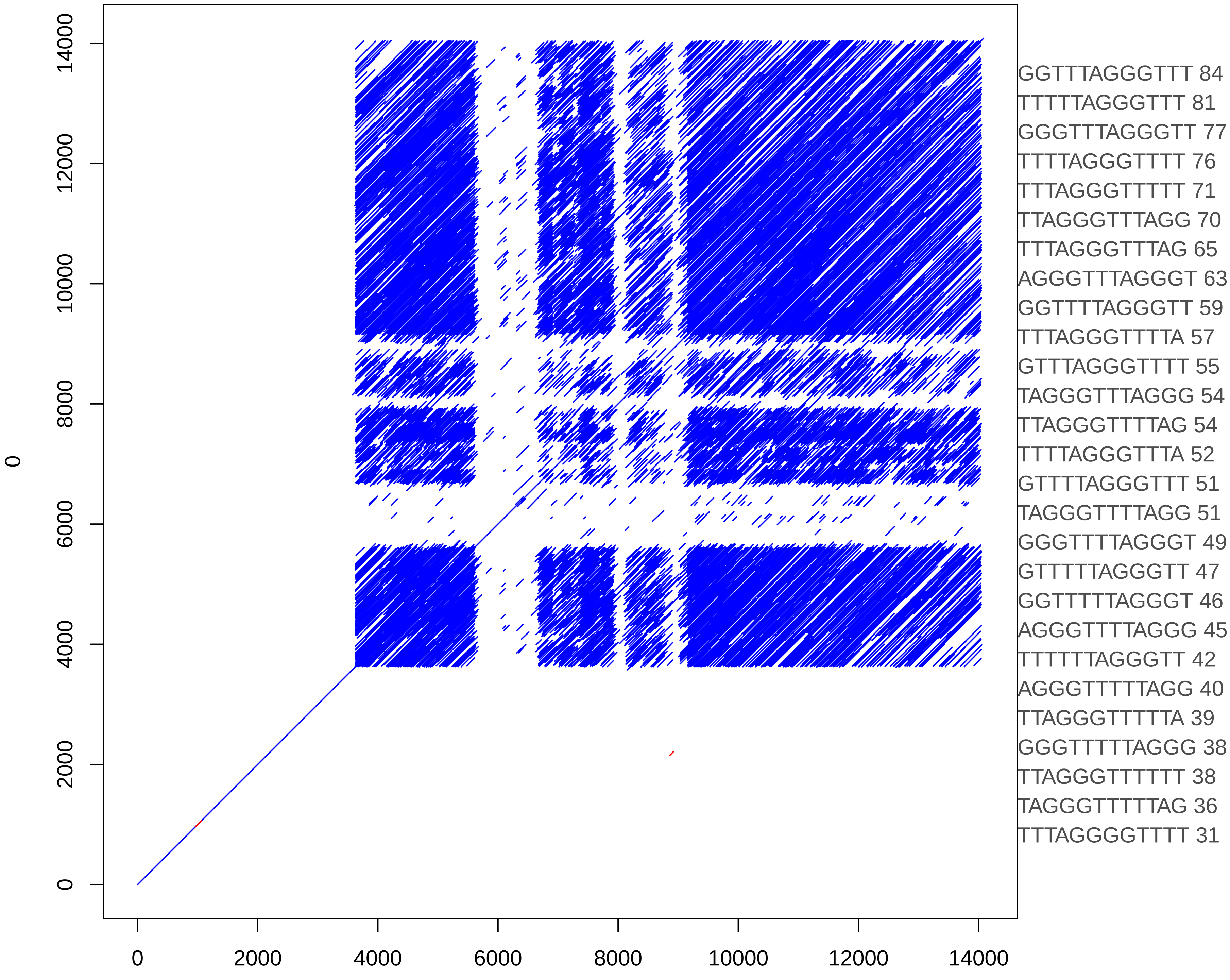

>m141126_060447_42134_c100732262550000001823141405141500_s1_p0/94289/0_6334

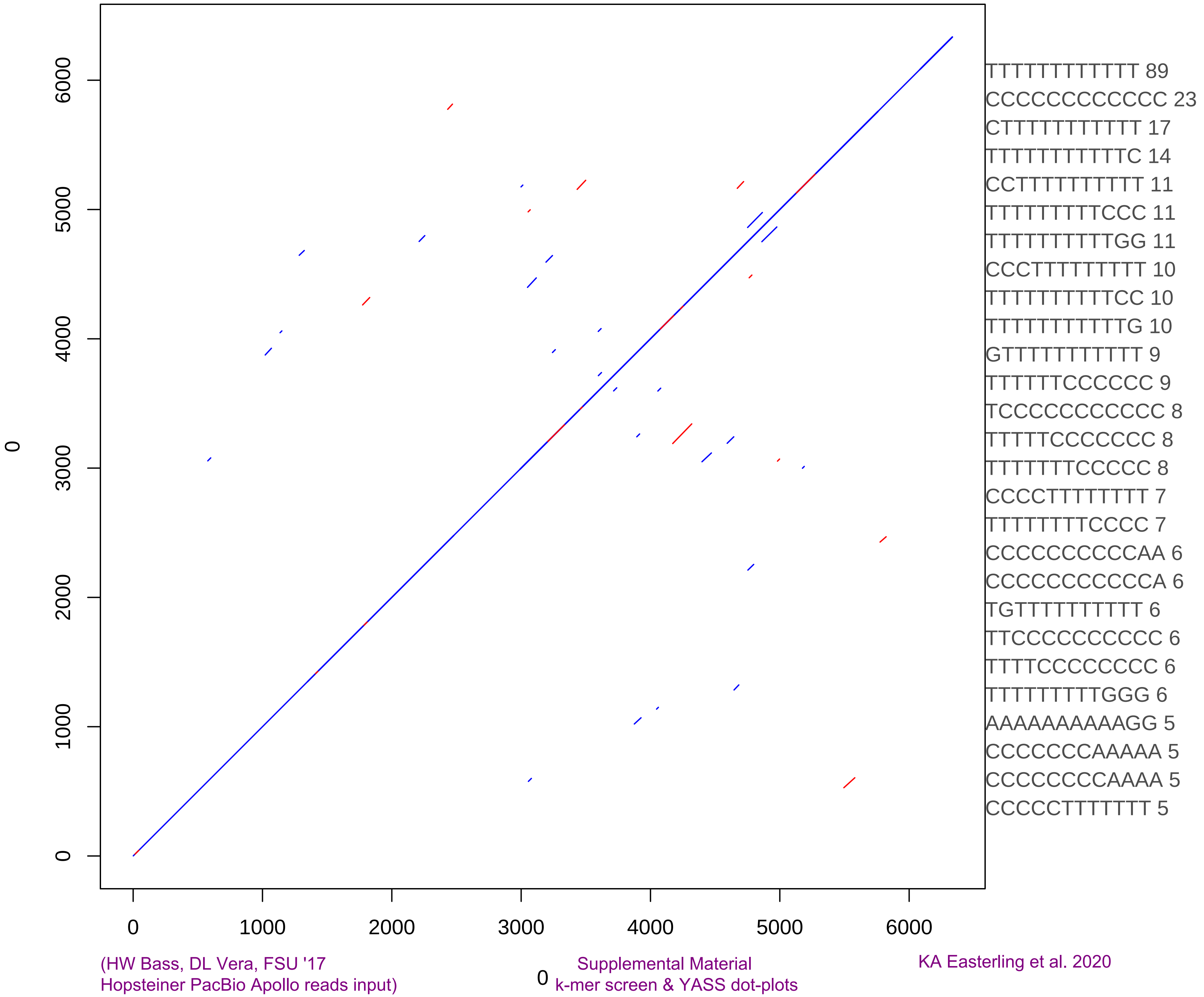

>m141126_060447_42134_c100732262550000001823141405141500_s1_p0/94993/6007_15761

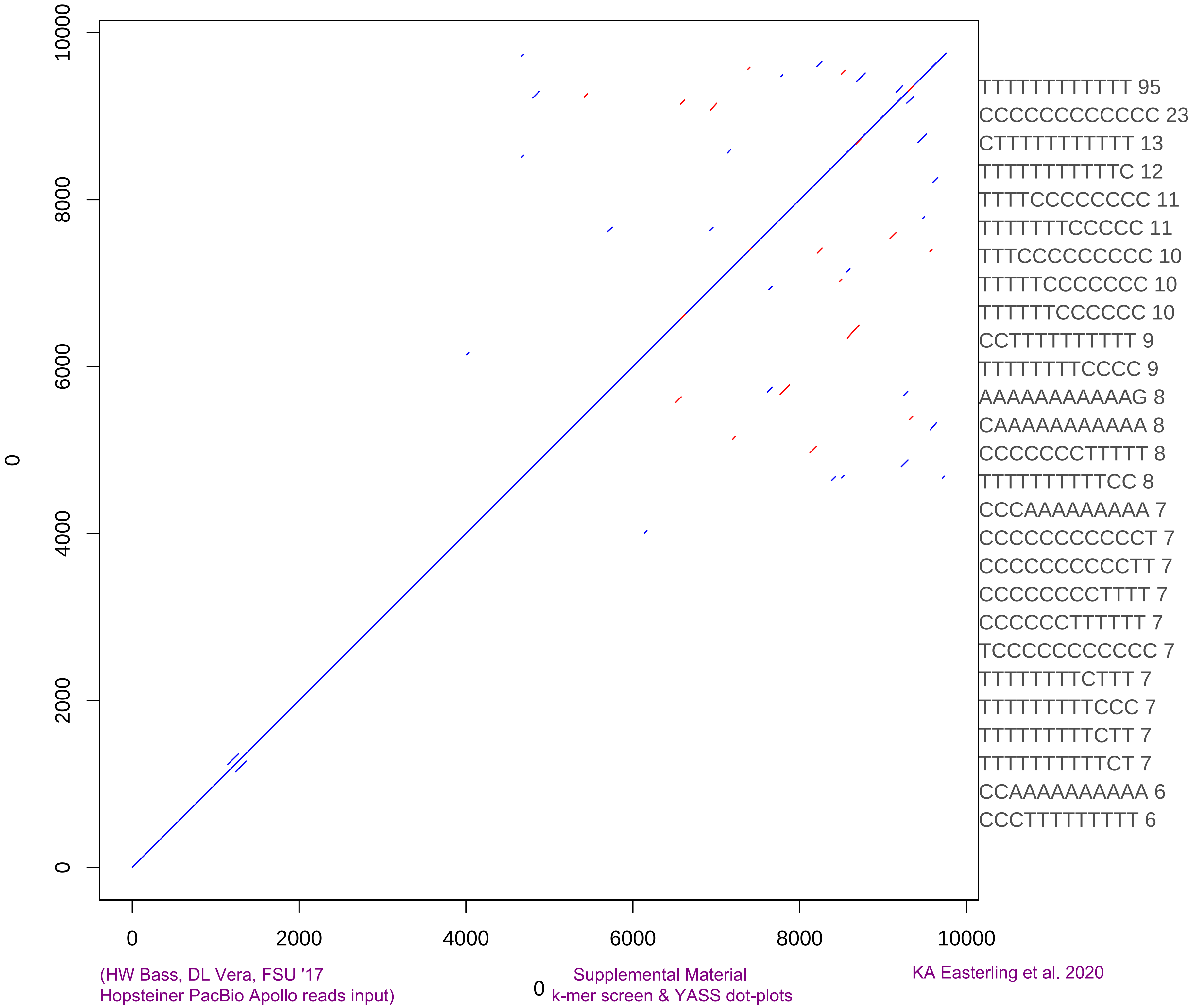

>m141126_060447_42134_c100732262550000001823141405141500_s1_p0/94997/0_8387

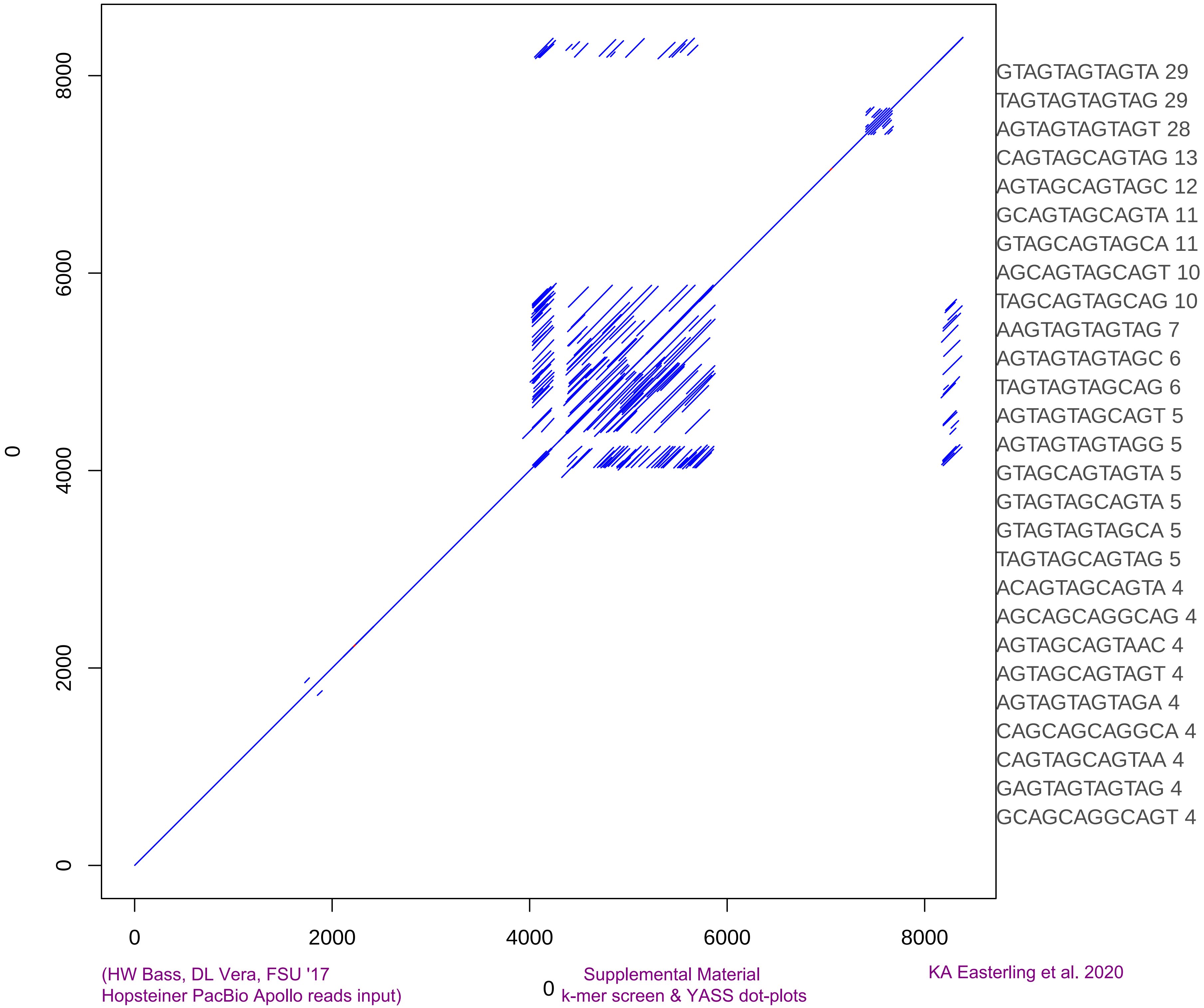

>m141126_060447_42134_c100732262550000001823141405141500_s1_p0/96149/4022_12553

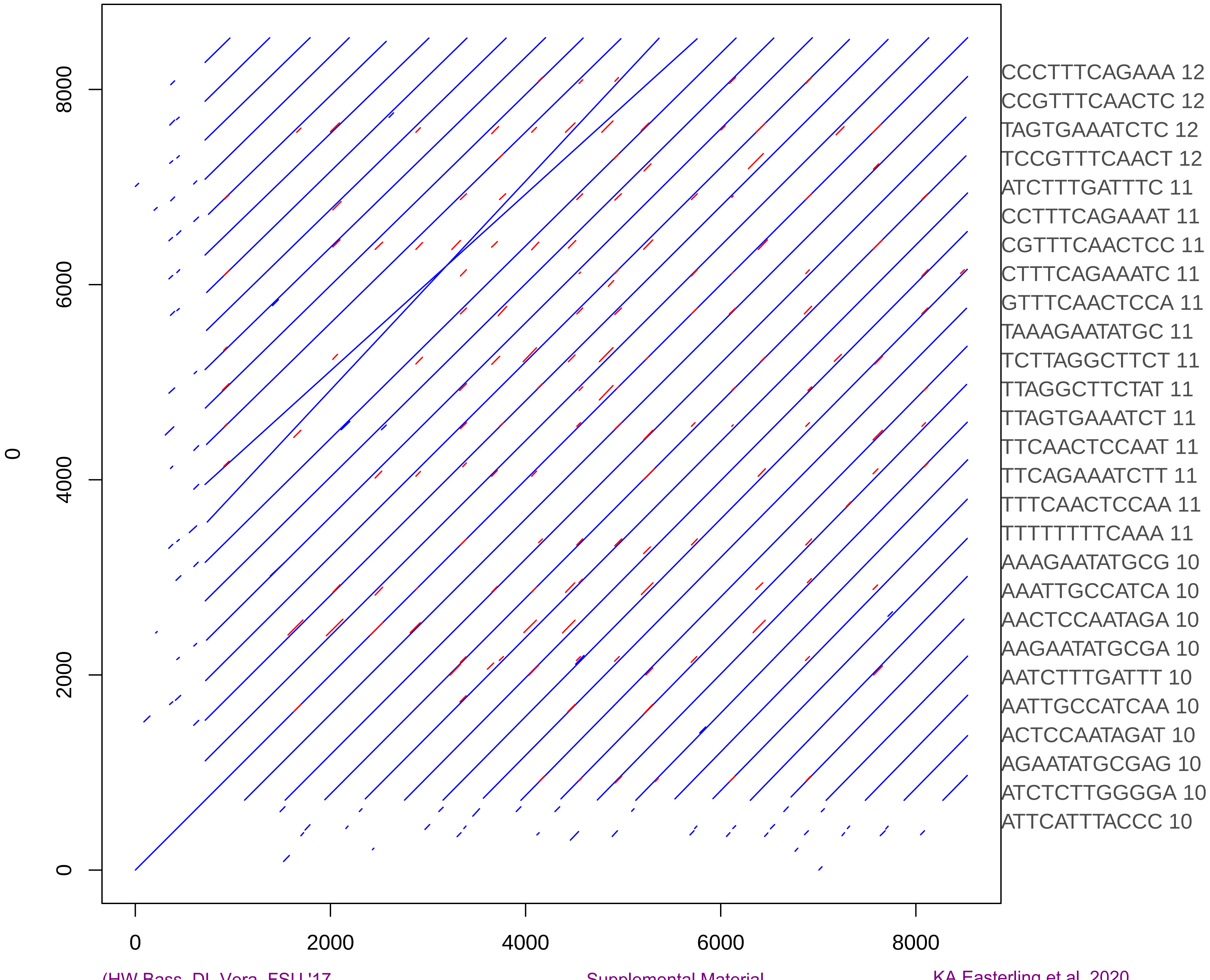

>m141126_082622_42134_c100732262550000001823141405141501_s1_p0/107110/353_8647

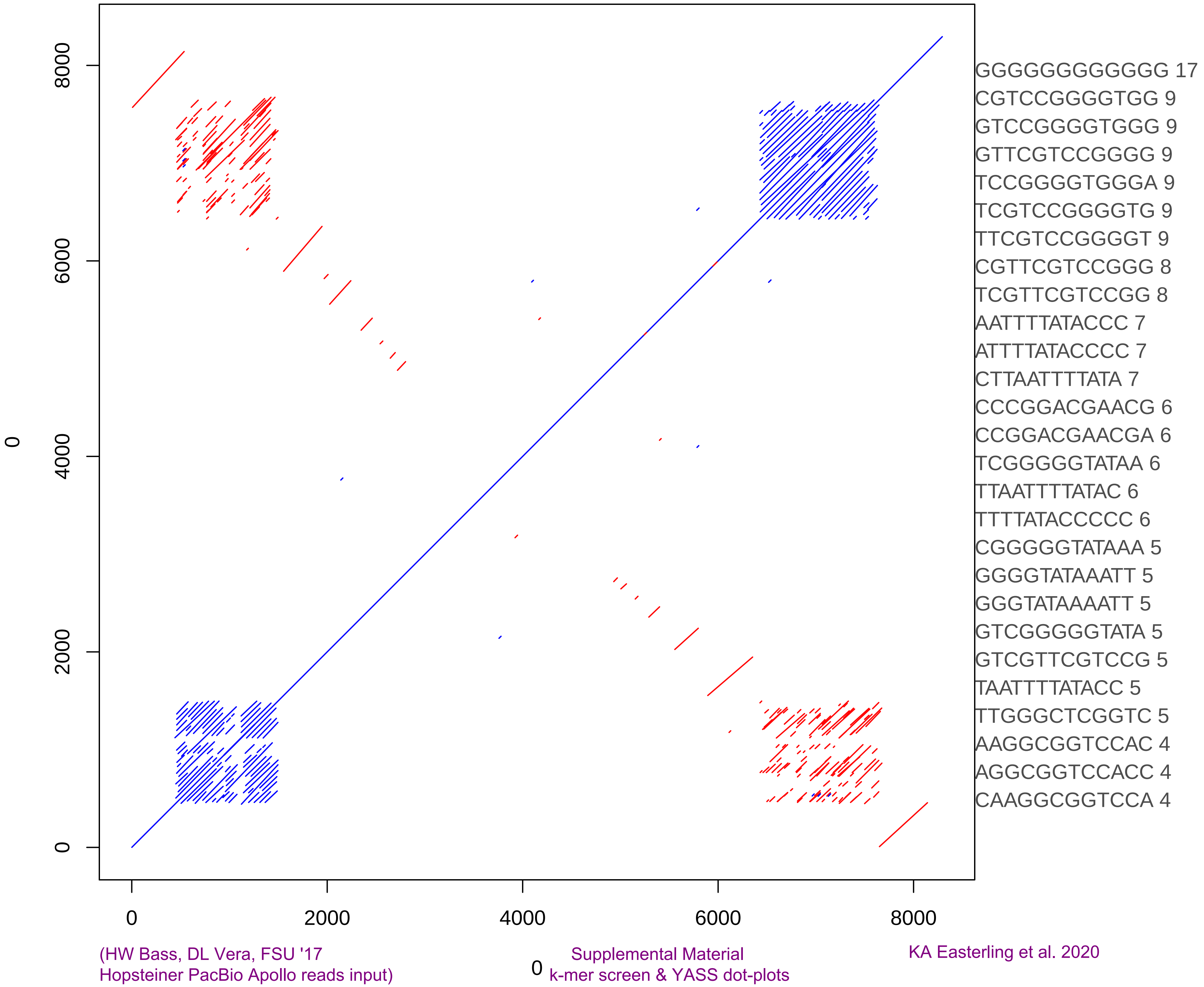

>m141126_082622_42134_c100732262550000001823141405141501_s1_p0/107260/1644_12501

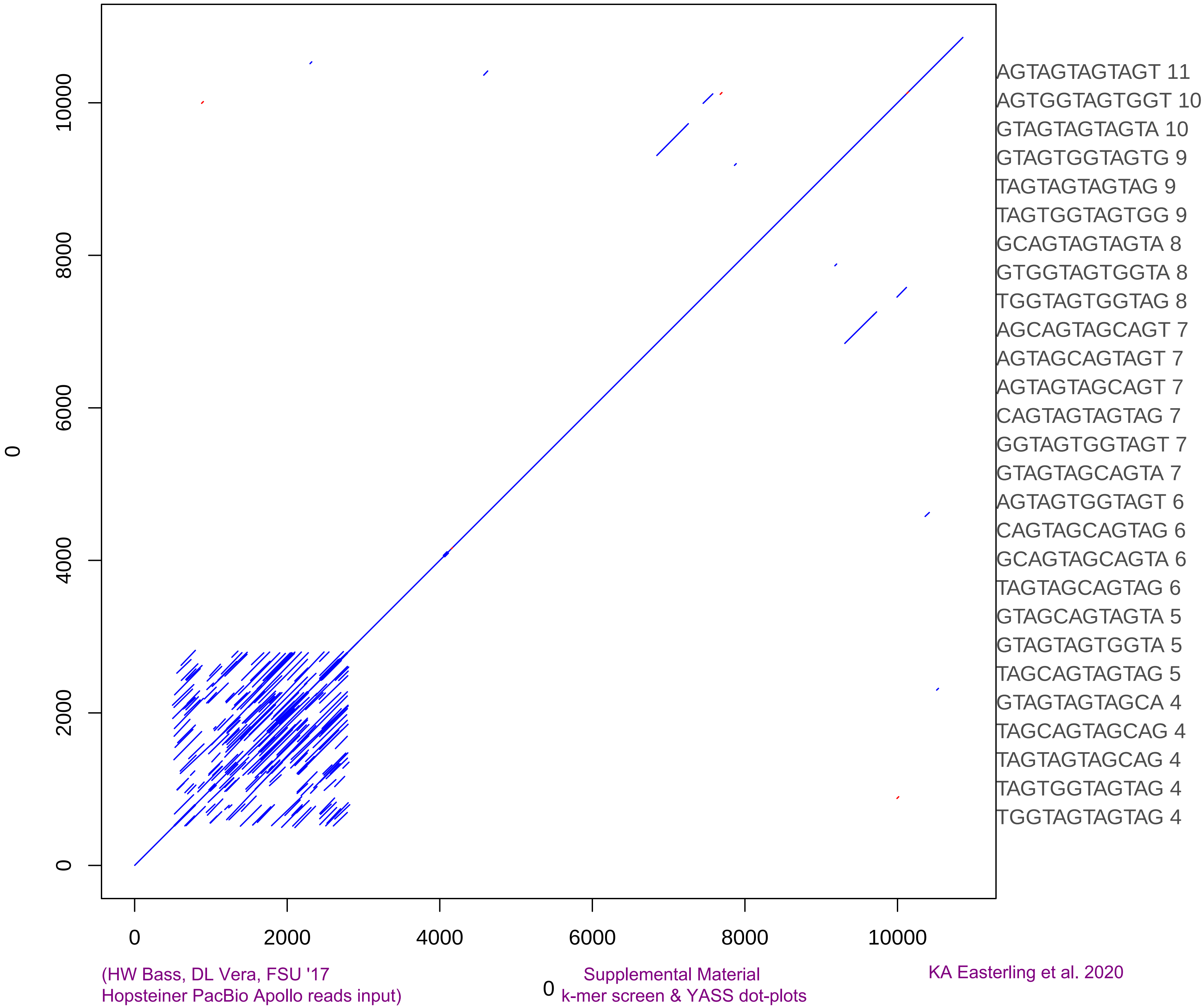

>m141126_082622_42134_c100732262550000001823141405141501_s1_p0/10807/11291_16755

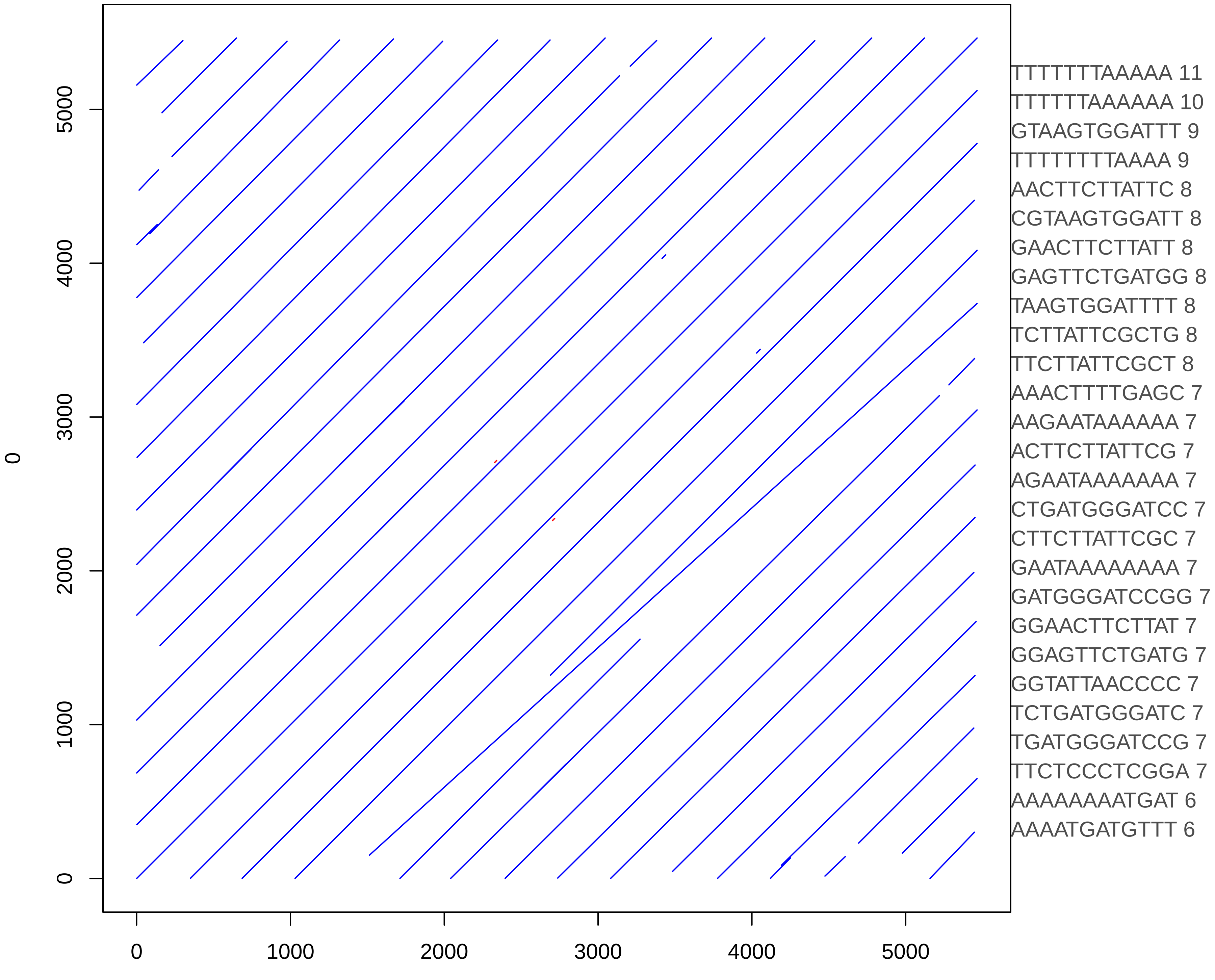

>m141126_082622_42134_c100732262550000001823141405141501_s1_p0/110394/3753_13721

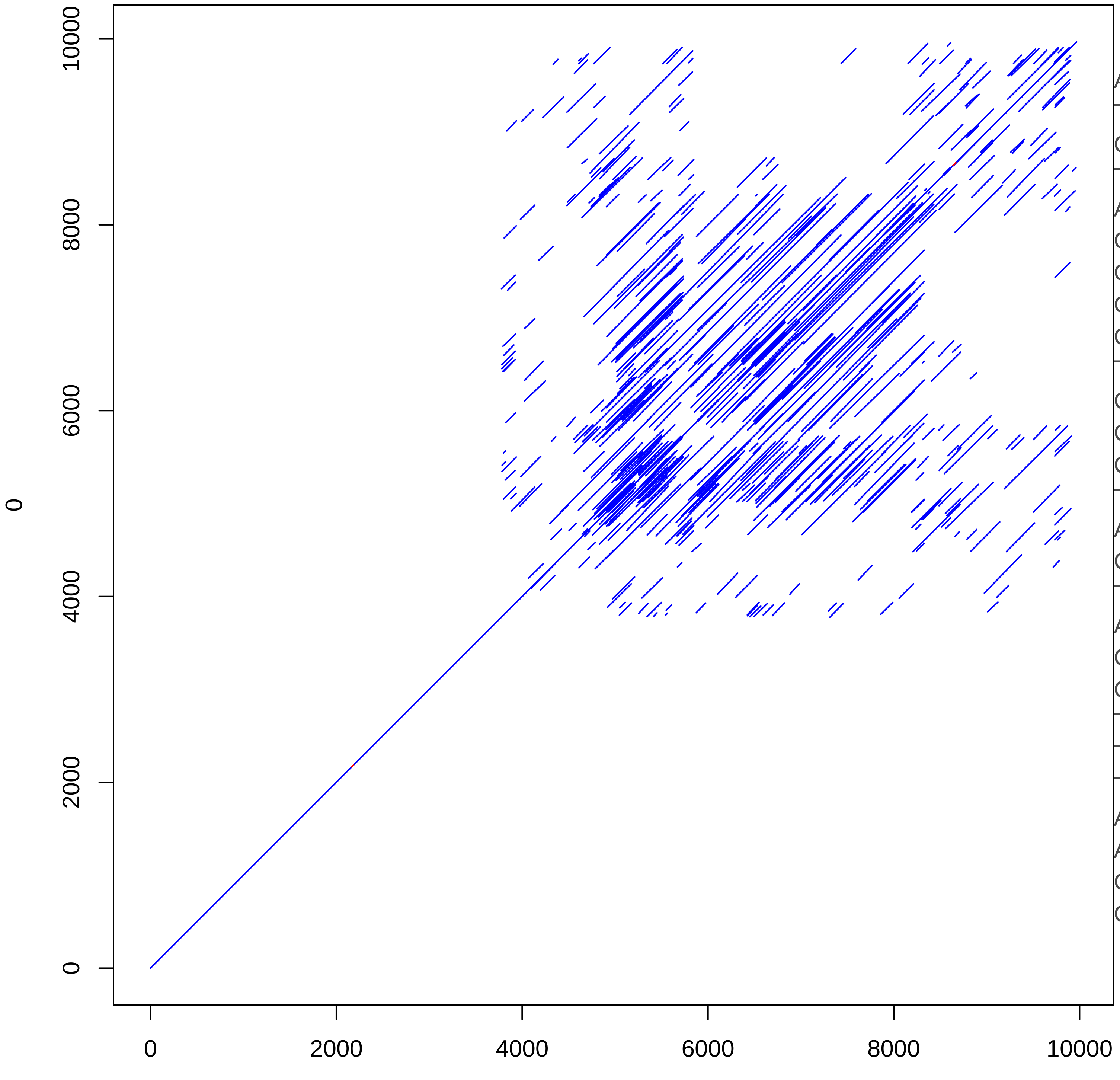

AGTAGTAGTAGT 50
TAGTAGTAGTAG 50
GTAGTAGTAGTA 47
TGGTGGTAGTAG 23
AGTAGTGGTAGT 19
GTAGTAGTGGTA 19
GTGGTGGTAGTA 19
GGTAGTAGTAGT 18
GTAGTAGTAGTG 18
TGGTAGTAGTGG 18
GGTAGTAGTGGT 17
GGTGGTAGTAGT 17
GTGGTAGTAGTG 17
TAGTAGTGGTAG 17
AGTGGTGGTAGT 16
GTAGTGGTGGTA 16
TAGTGGTGGTAG 15
AGTGGTAGTAGT 14
GTGGTAGTAGTA 14
GTAGTGGTAGTA 13
TAGTAGTAGTGG 12
TAGTGGTAGTAG 11
TGGTAGTAGTAG 11
AGTAGTAGTGGT 10
AGTAGTGGTGGT 9
GTAGTAGTAGCT 9
GTAGTGGTAGTG 9

>m141126_082622_42134_c100732262550000001823141405141501_s1_p0/111468/0_6759

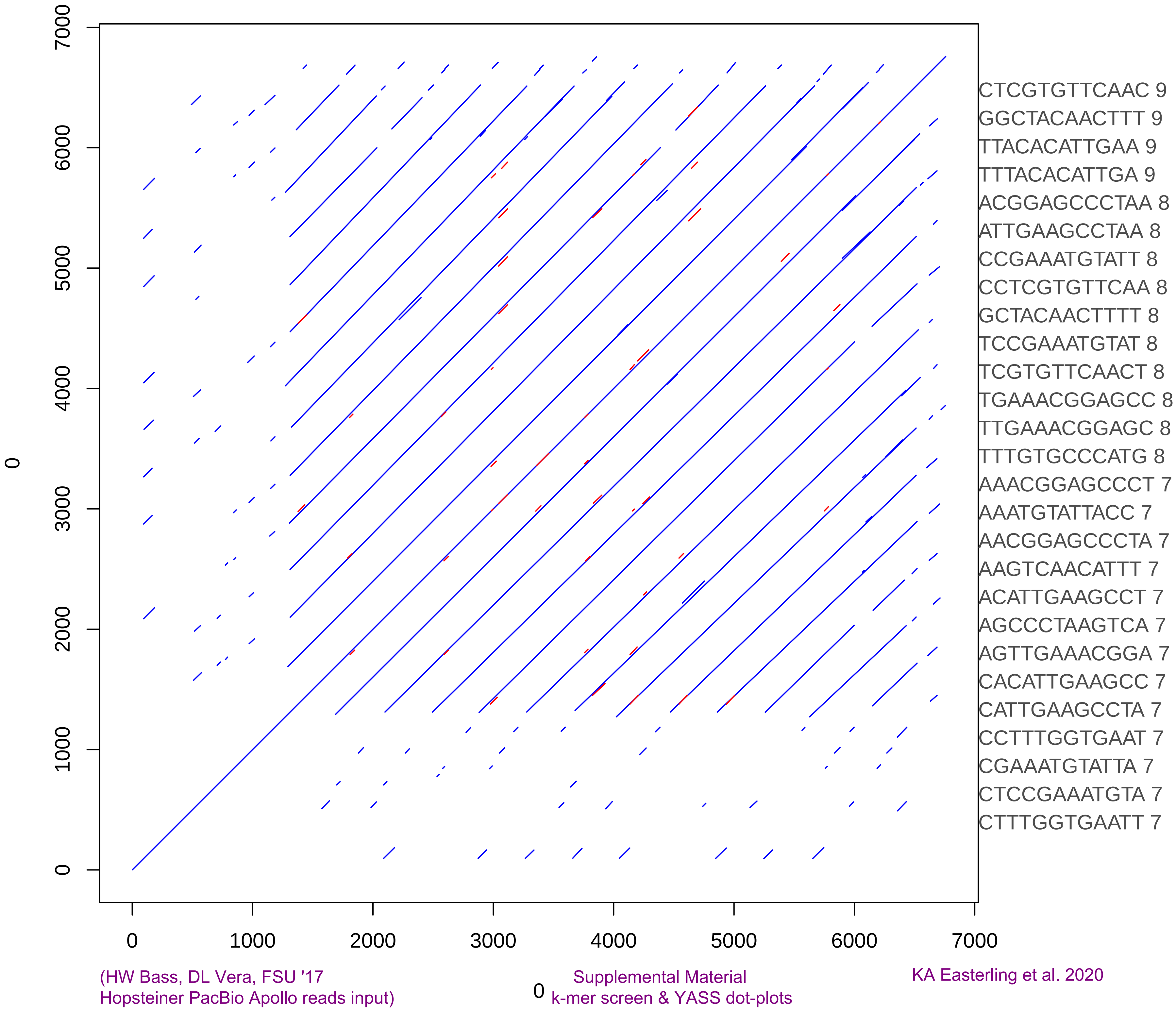

>m141126_082622_42134_c100732262550000001823141405141501_s1_p0/112175/1036_7360

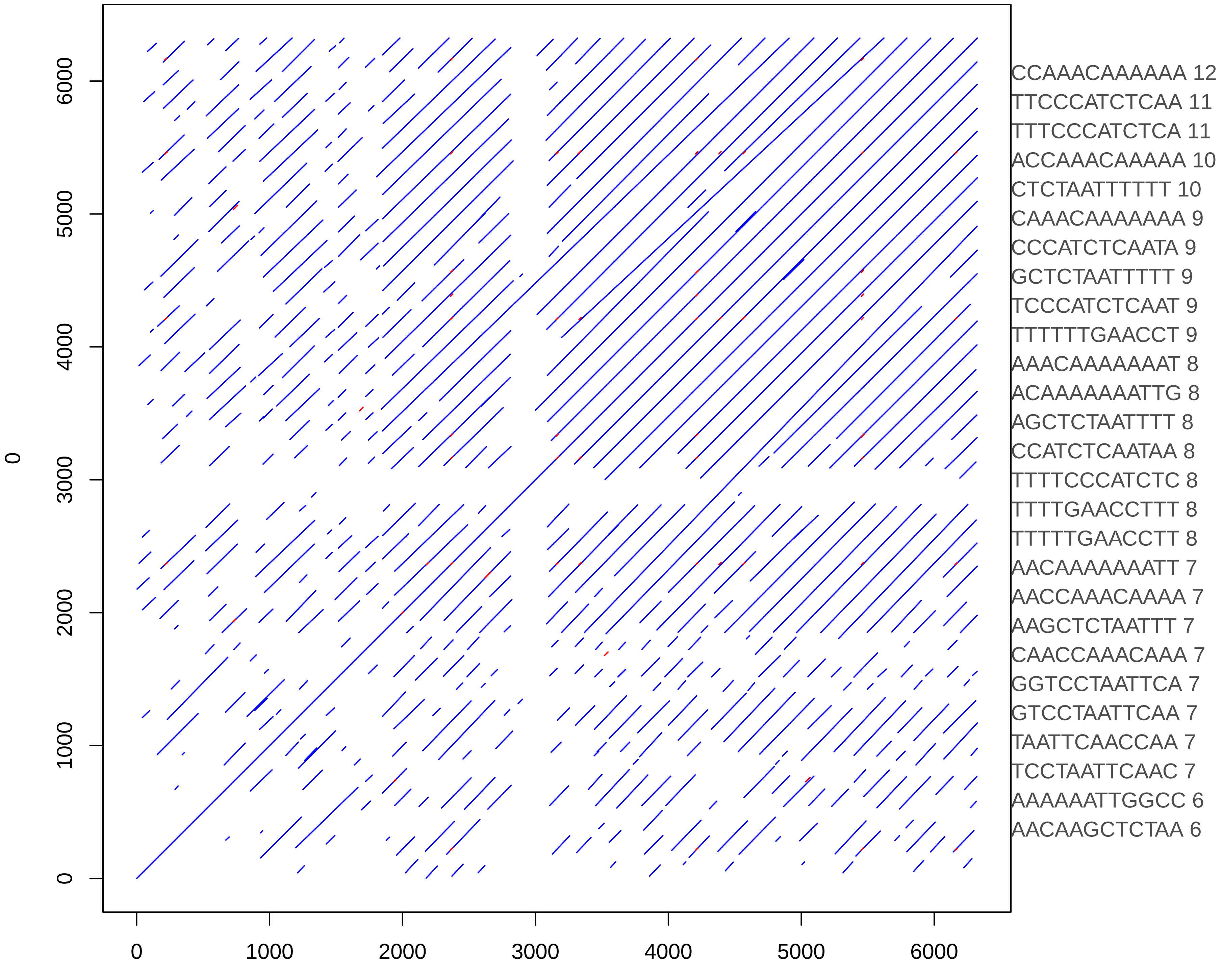

>m141126_082622_42134_c100732262550000001823141405141501_s1_p0/12282/1533_8559

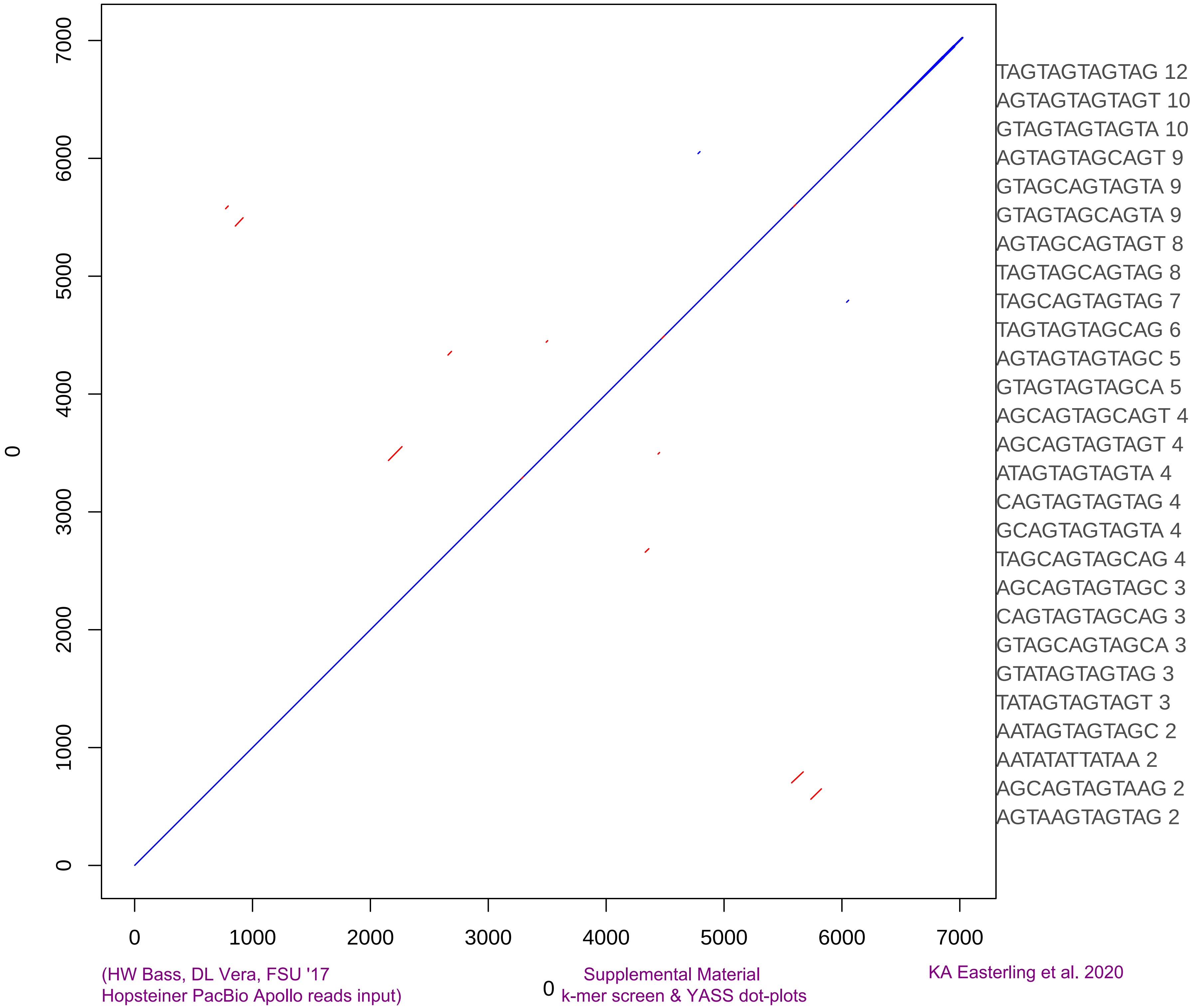

>m141126_082622_42134_c100732262550000001823141405141501_s1_p0/125391/5975_13618

Representative (only one) Read (Table 1): HuluTR350-r625, 1 ppm, 67% A+T, GenBank Acc. MN537577

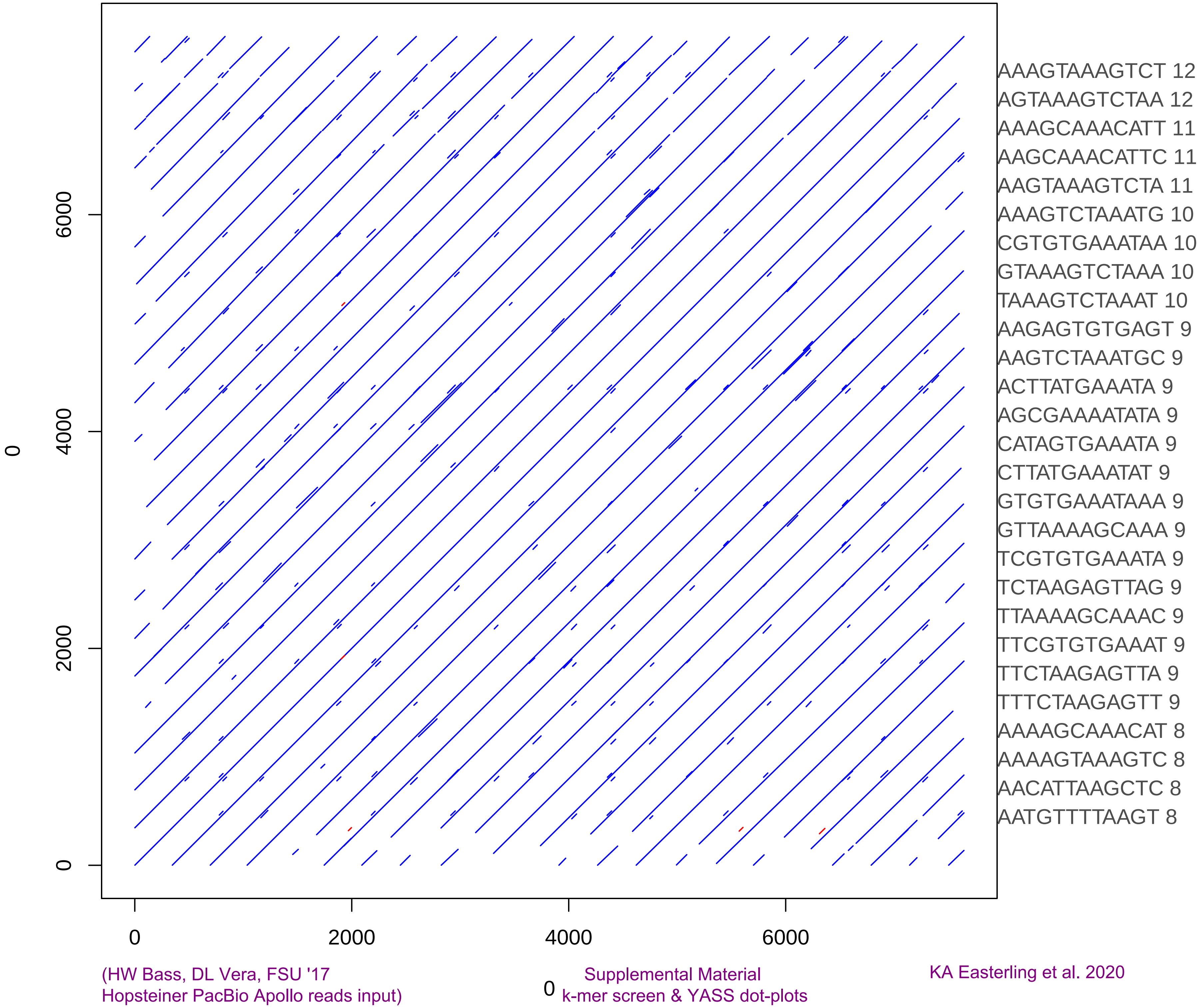

1,121 hits for repetitive sequences
passing 12mer abundance criterion

(Read-Number)
PAGE 626

For Tandem Repeat families of ### bp:
PacBio Read named "HuluTR###-r626"

>m141126_082622_42134_c100732262550000001823141405141501_s1_p0/129589/4138_15238

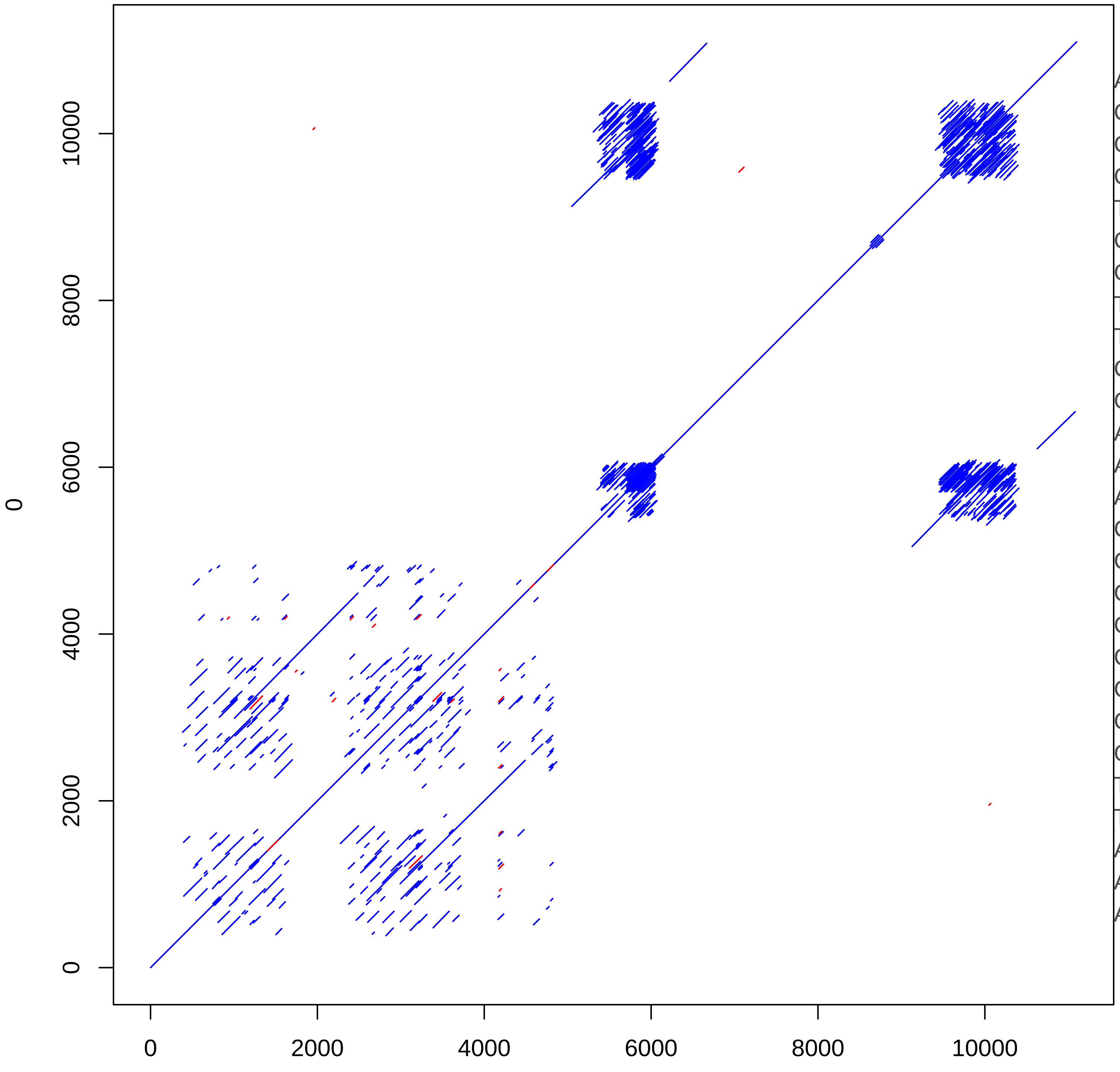

ATGGCCTCCTCC 12
CATGGCCTCCTC 12
CCATGGCCTCCT 12
CCCATGGCCTCC 12
TGGCCTCCTCCA 8
CCCCATGGCCTC 7
GCCCATGGCCTC 7
TAGGGGTTTAAA 7
TTAGGGGTTTAA 7
CGCCCATGGCCT 6
GGCCTCCTCCAT 6
AGACTTGGATTA 5
AGAGACTTGGAT 5
AGGGGTTTAAAT 5
CCTCCCATGGCC 5
CCTCCTCCATGG 5
CTCCATGGCCTC 5
CTCCCATGGCCT 5
CTCCTCCATGGC 5
CTTAGGGGTTTA 5
GAGACTTGGATT 5
GCCTCCTCCATG 5
TCCCATGGCCTC 5
TGGATAAAAATT 5
AAAGAGACTTGG 4
AAGAGACTTGGA 4
AATGGATAAAAA 4

(HW Bass, DL Vera, FSU '17
Hopsteiner PacBio Apollo reads input)

Supplemental Material
k-mer screen & YASS dot-plots

KA Easterling et al. 2020

>m141126_082622_42134_c100732262550000001823141405141501_s1_p0/131442/9353_16828

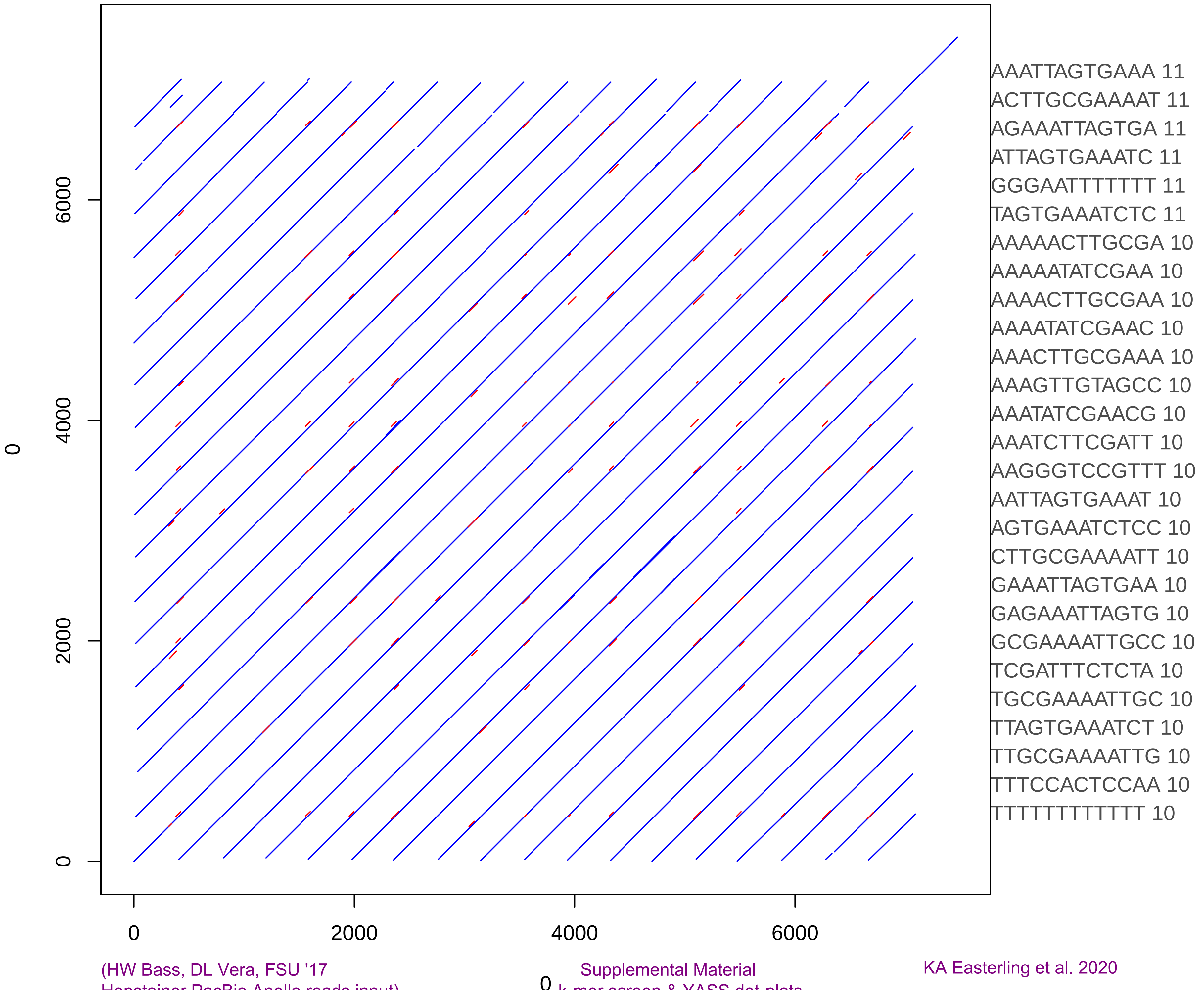

>m141126_082622_42134_c100732262550000001823141405141501_s1_p0/134643/0_14072

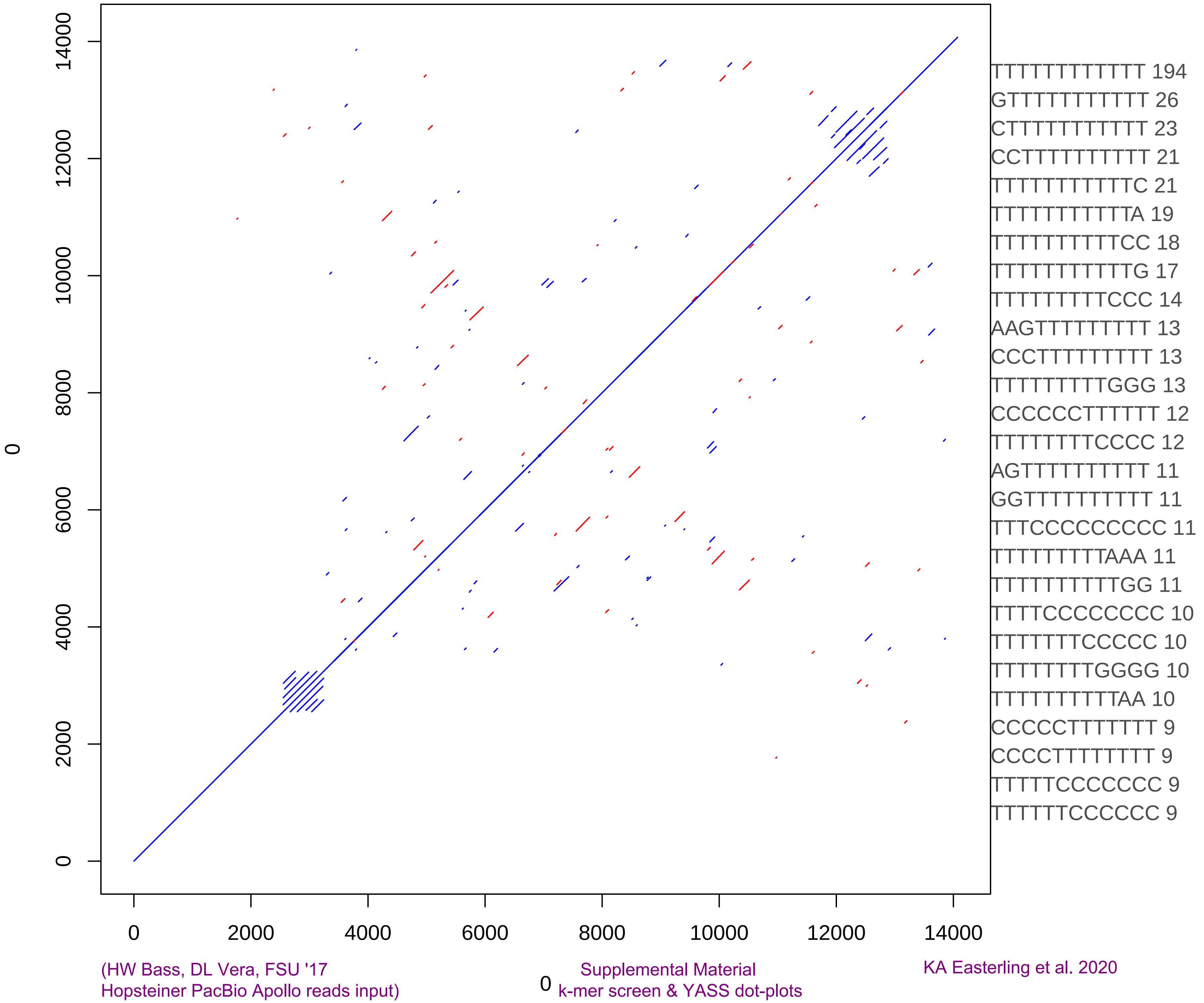

>m141126_082622_42134_c100732262550000001823141405141501_s1_p0/139358/0_6351

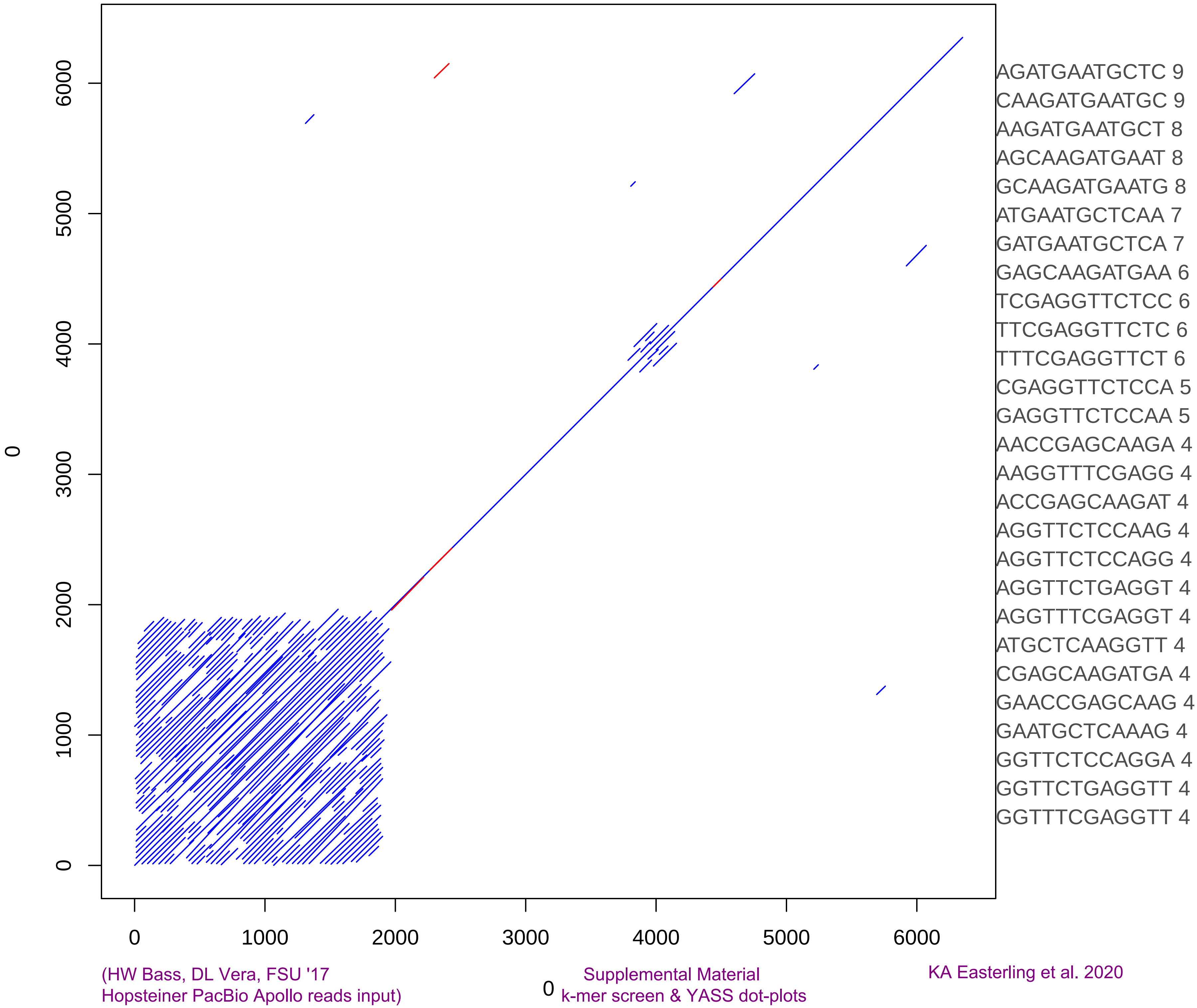

>m141126_082622_42134_c100732262550000001823141405141501_s1_p0/141239/8033_17955

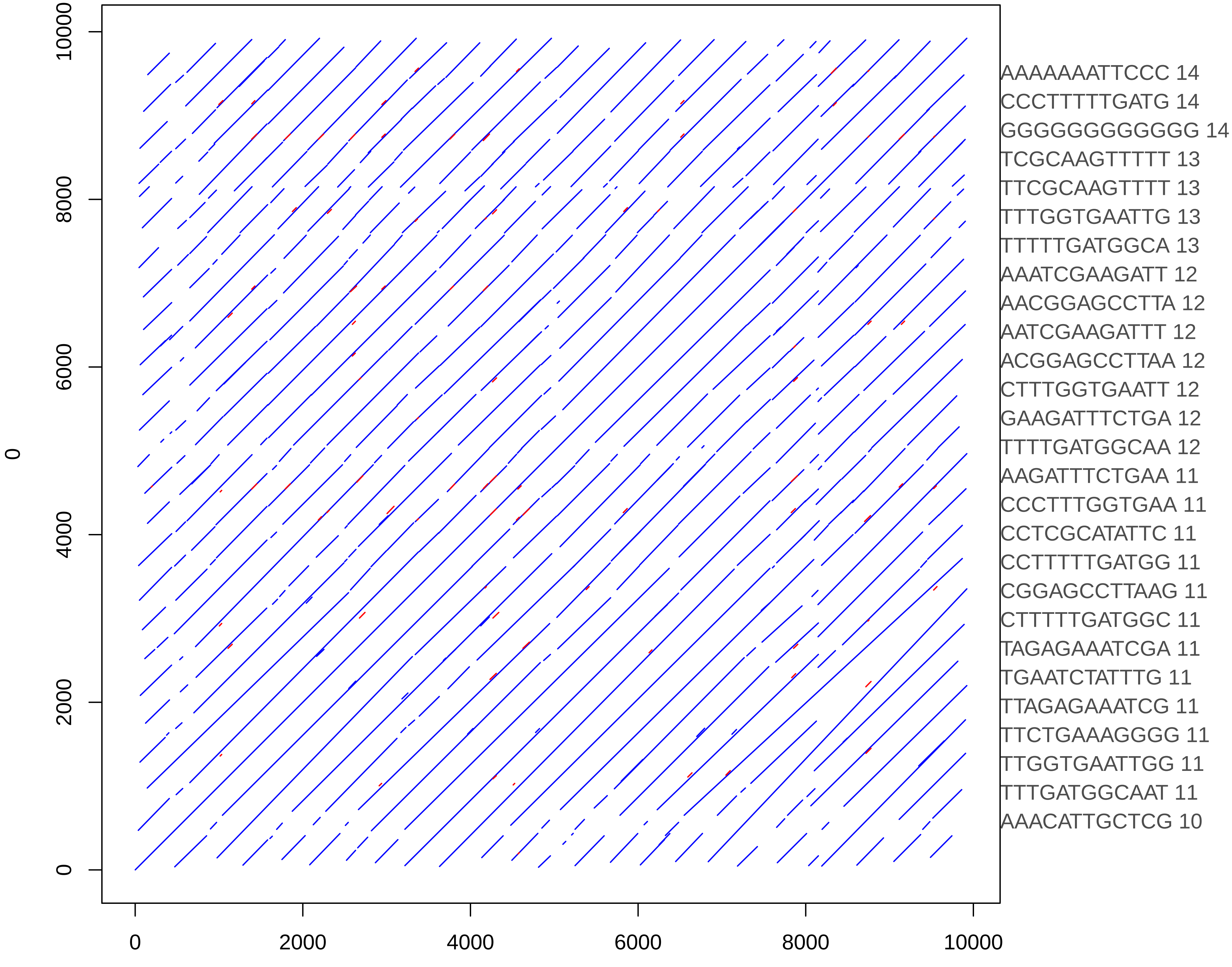

>m141126_082622_42134_c100732262550000001823141405141501_s1_p0/155050/0_13539

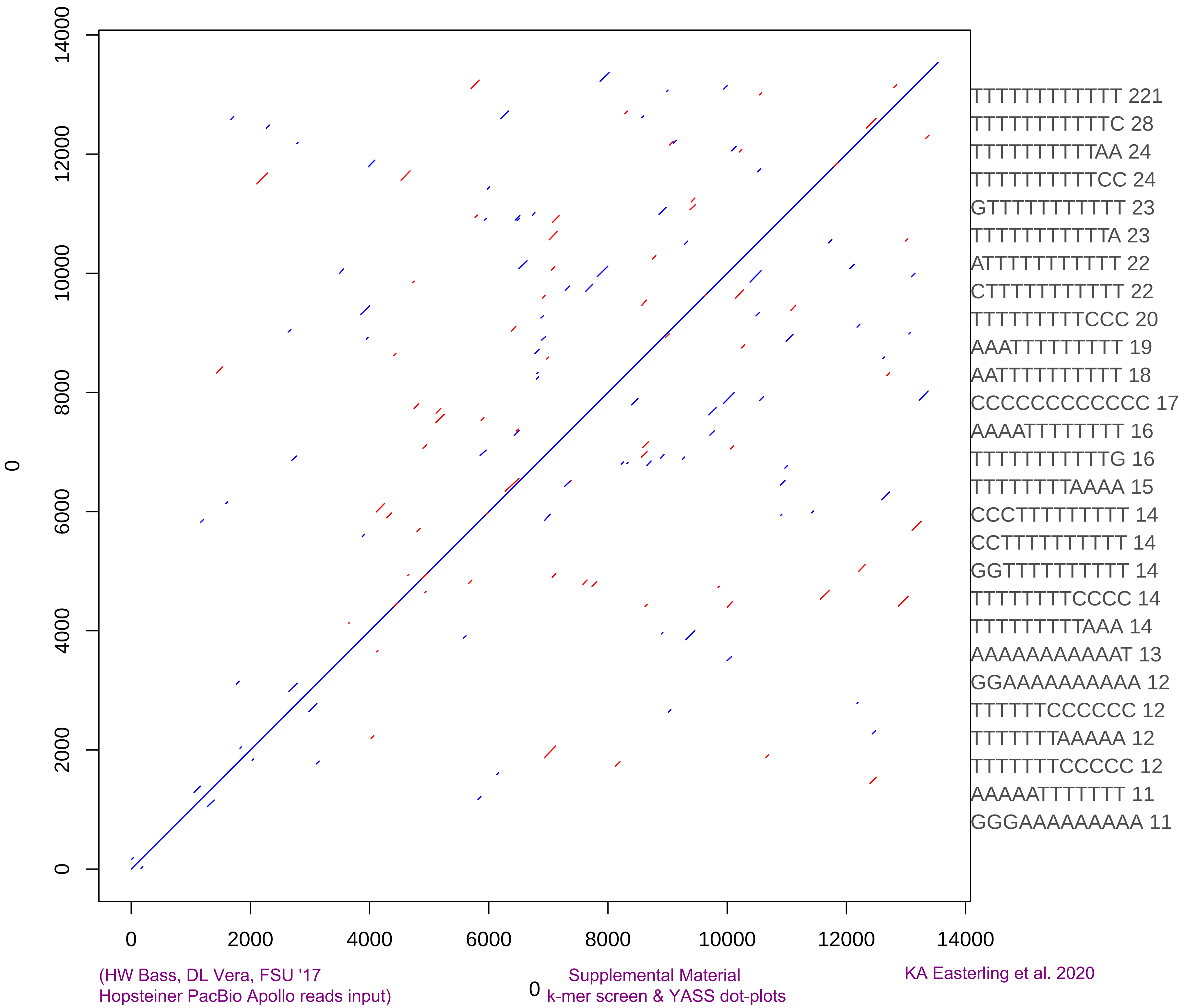

>m141126_082622_42134_c100732262550000001823141405141501_s1_p0/155475/5514_15124

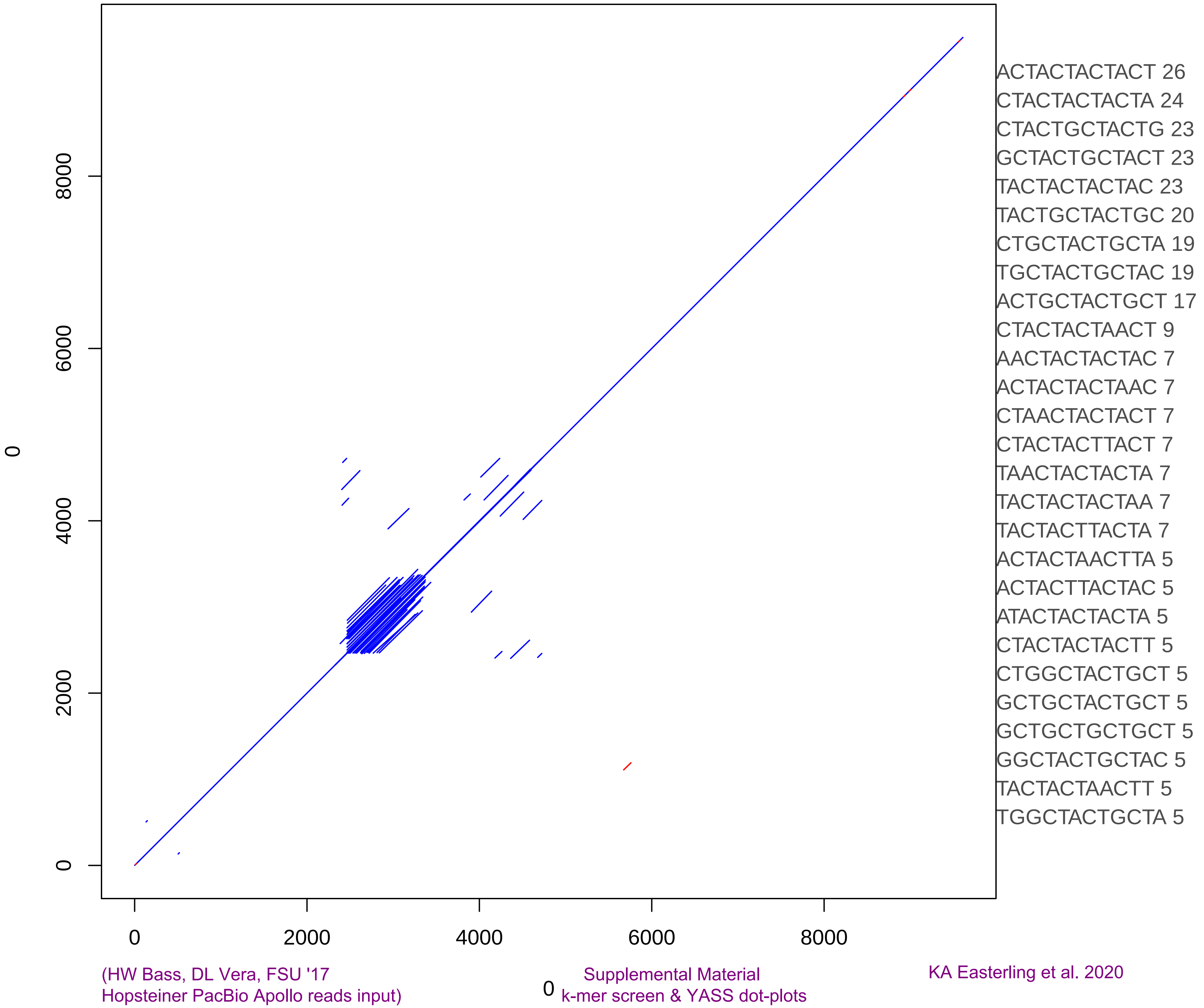

>m141126_082622_42134_c100732262550000001823141405141501_s1_p0/15894/10274_15816

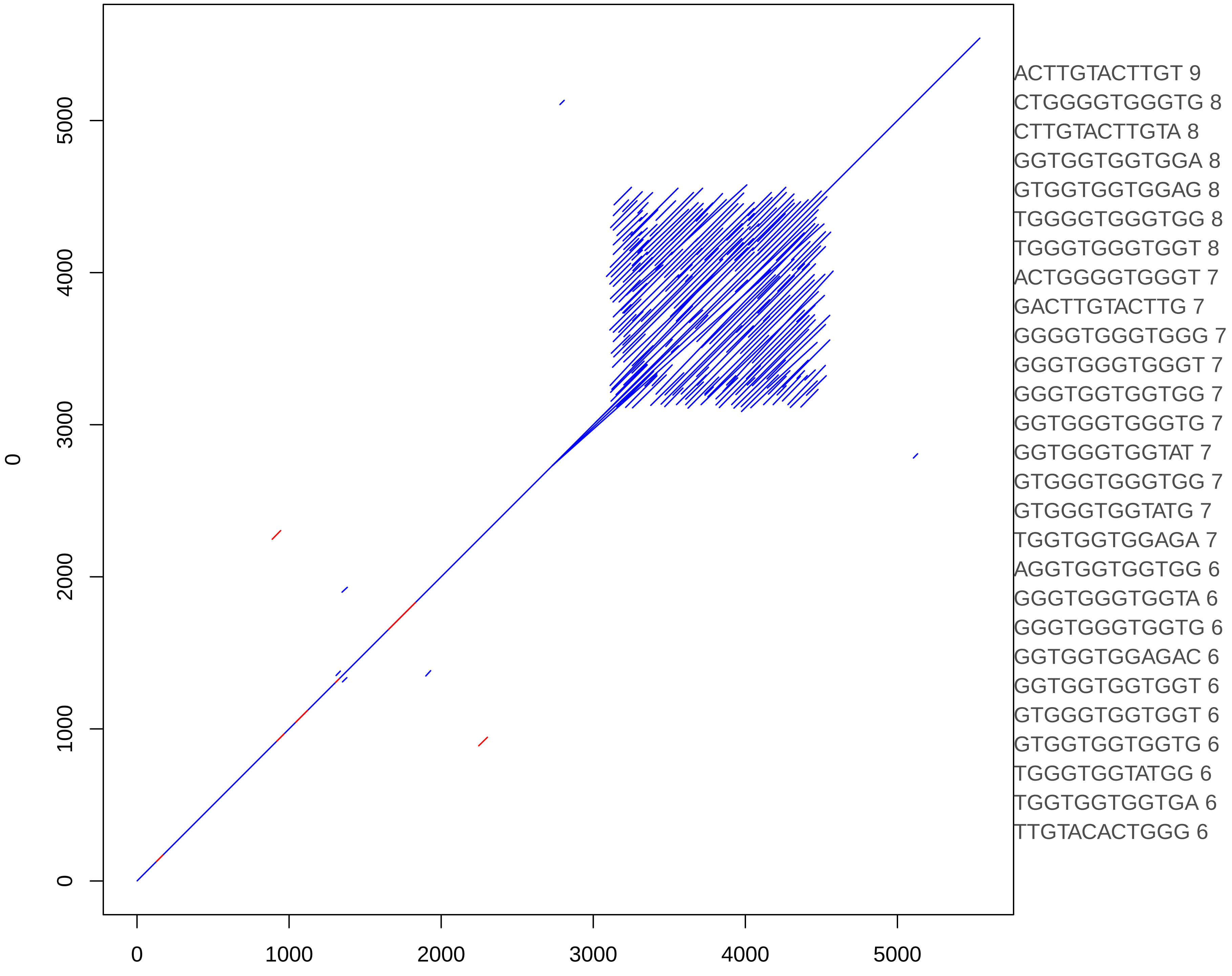

>m141126_082622_42134_c100732262550000001823141405141501_s1_p0/160267/383_5466

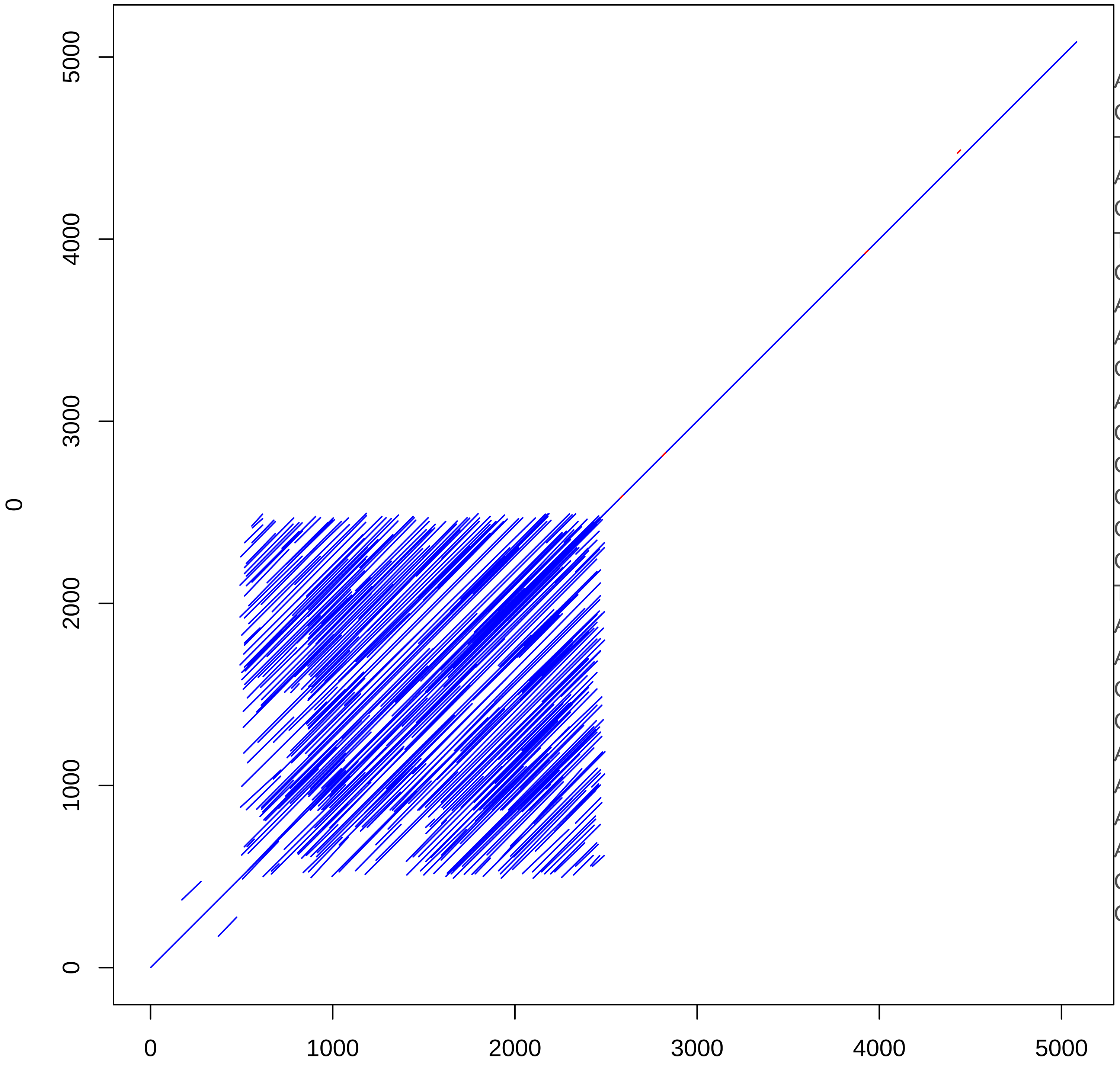

AGTAGTAGTAGT 20
GTAGTAGTAGTA 19
TAGTAGTAGTAG 19
AGTAGCAGTAGC 17
GTAGCAGTAGCA 17
TAGCAGTAGCAG 16
CAGTAGCAGTAG 15
AGCAGCAGCAGC 14
AGCAGTAGCAGT 14
GCAGTAGCAGTA 14
AGGAGTAGGAGT 11
GCAGCAGCAGCA 11
CAGCAGCAGCAG 10
GAGTAGCAGTAG 9
GAGTAGGAGTAG 9
GGAGTAGGAGTA 9
TAGGAGTAGGAG 9
AGTAGCAGCAGC 8
AGTAGGAGTAGG 8
GTAGCAGCAGCA 8
GTAGGAGTAGGA 8
AGCAGTAGCAGC 6
AGCAGTAGGAGT 6
AGGAGTAGCAGT 6
AGTAGCAGTAGG 6
CAGTAGCAGCAG 6
GCAGTAGCAGCA 6

>m141126_082622_42134_c100732262550000001823141405141501_s1_p0/160267/5512_11022

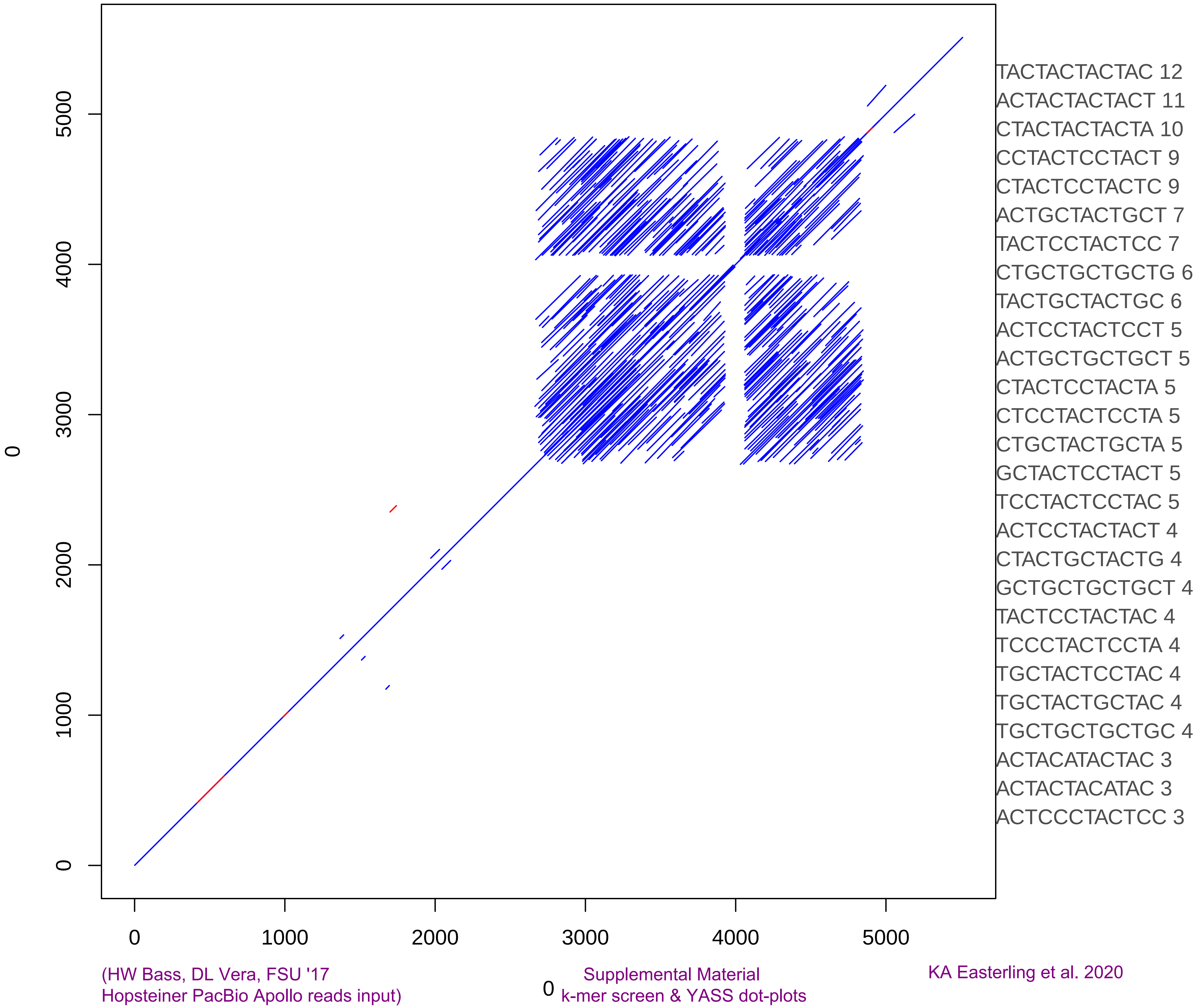

>m141126_082622_42134_c100732262550000001823141405141501_s1_p0/160589/0_9994

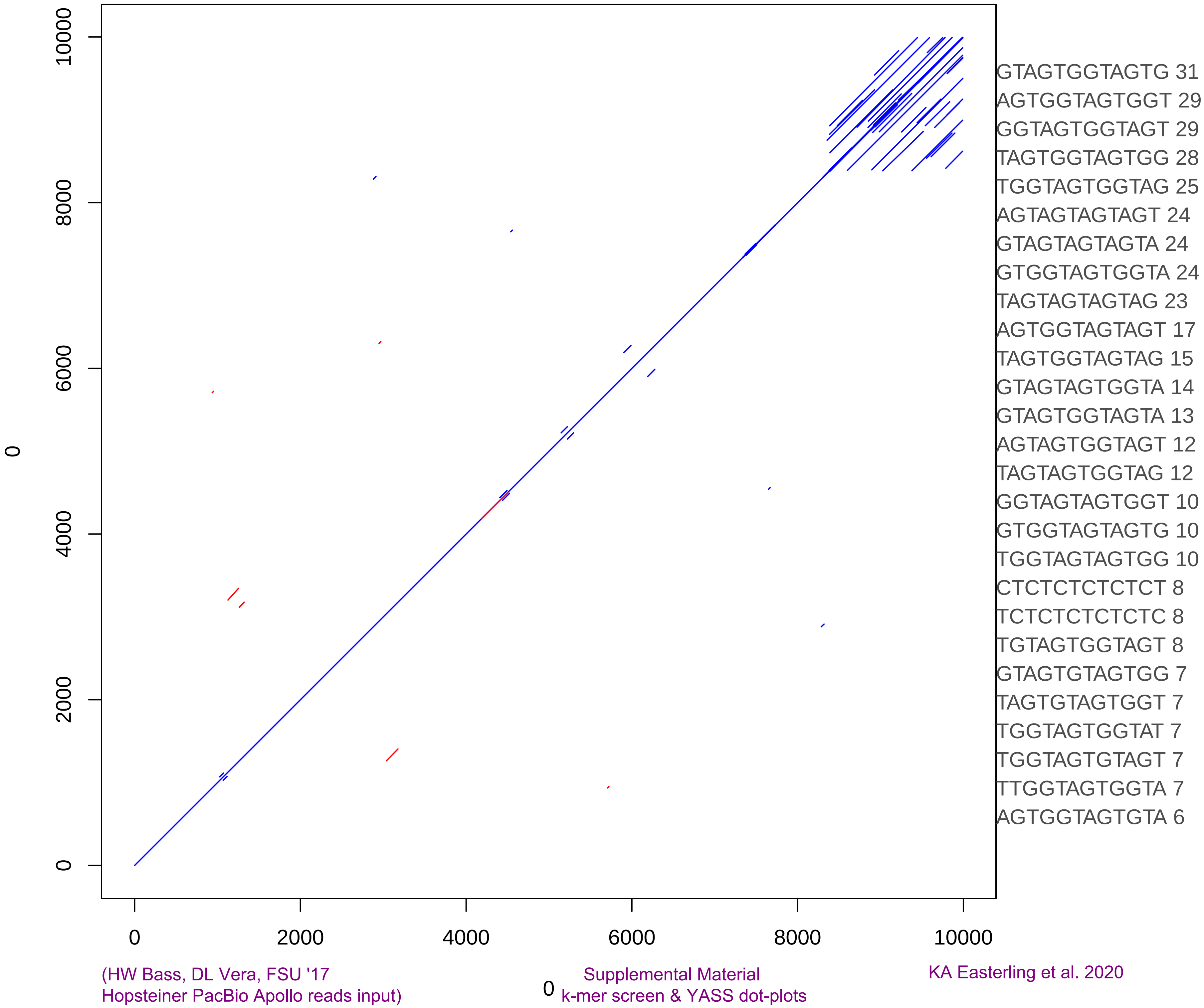

>m141126_082622_42134_c100732262550000001823141405141501_s1_p0/160712/0_14264

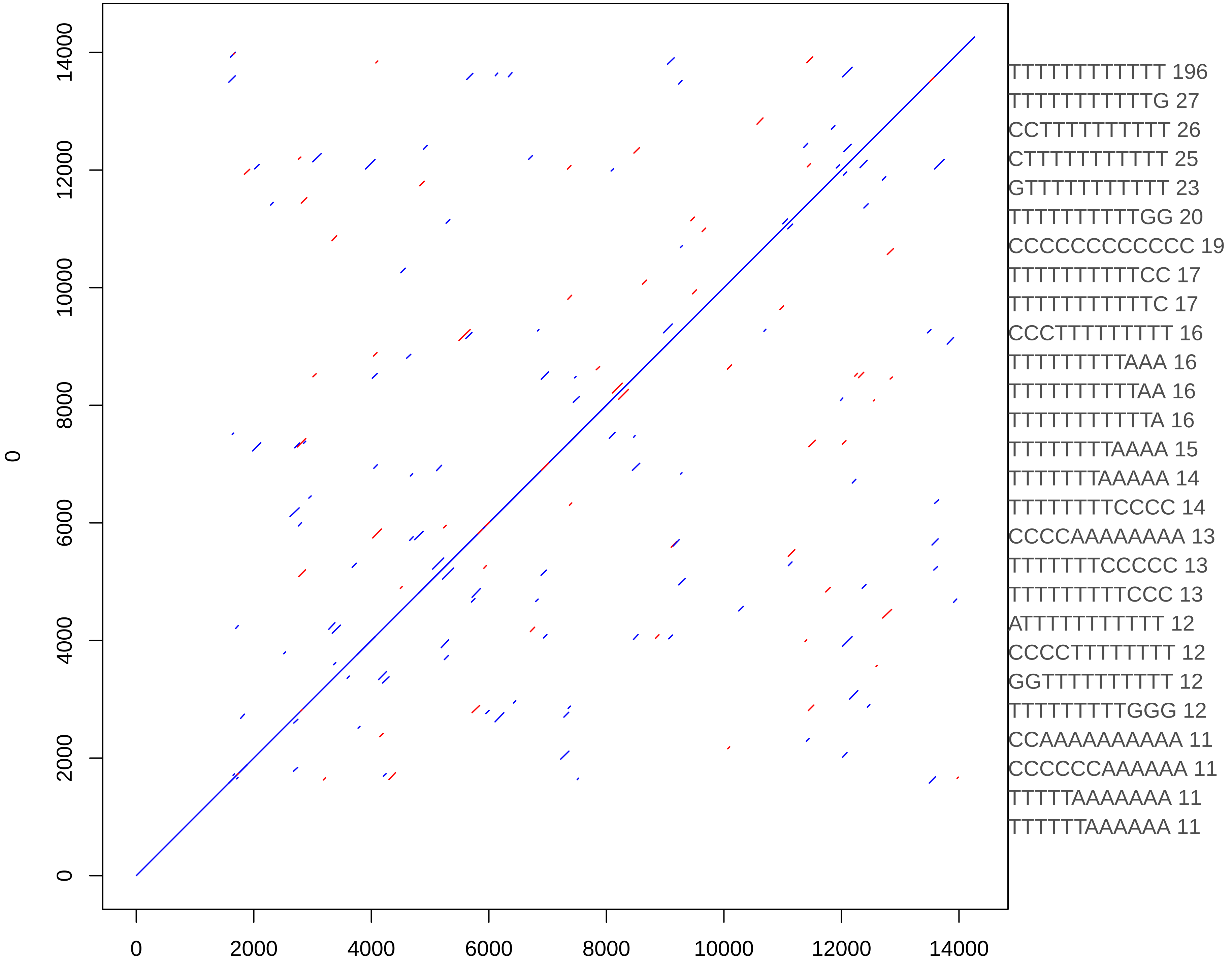

>m141126_082622_42134_c100732262550000001823141405141501_s1_p0/161171/252_10527

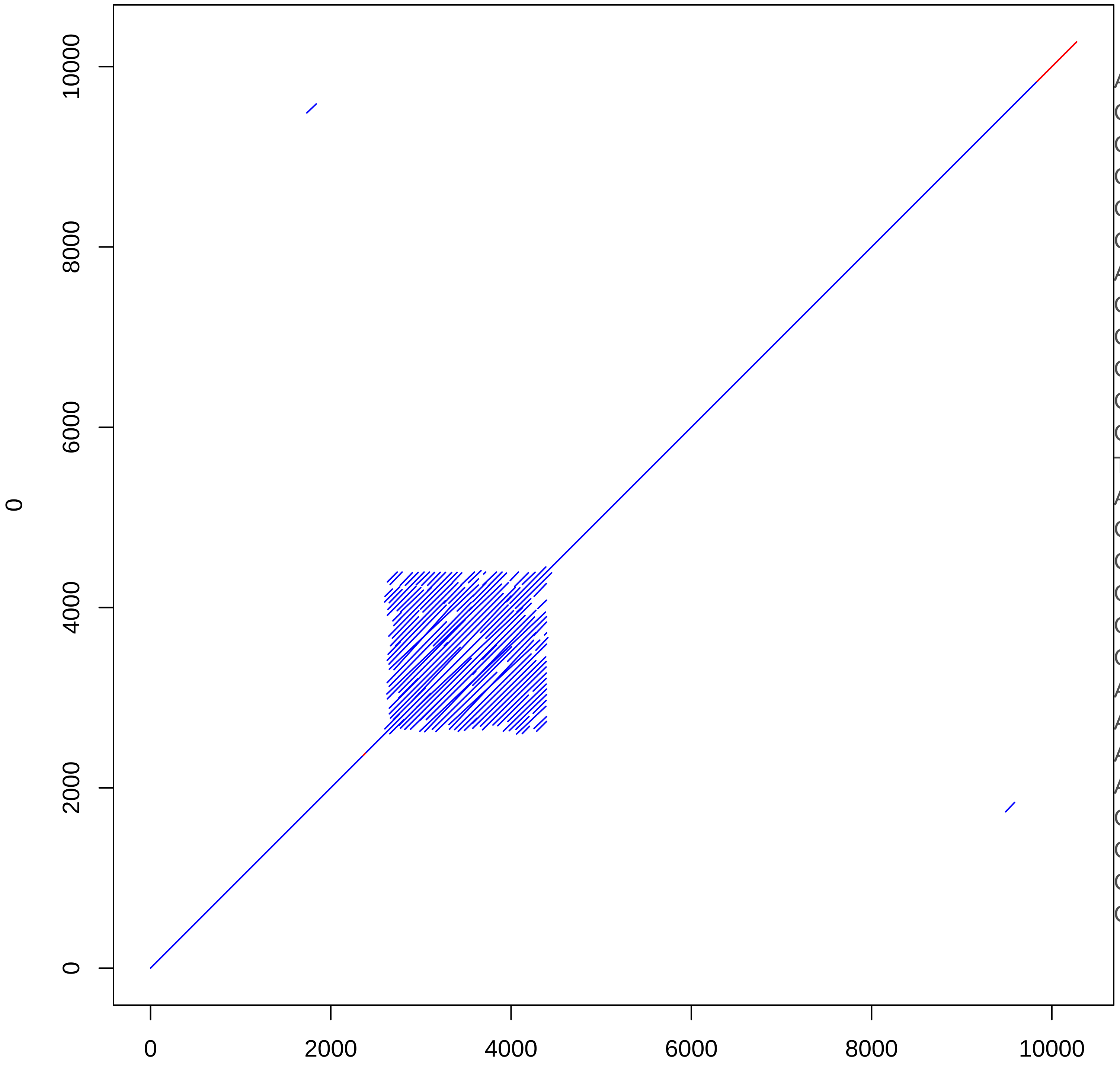

ACCCCGGACGAA 10
CACCCCGGACGA 10
CCCCGGACGAAC 10
CCACCCCGGACG 9
CCCACCCCGGAC 8
CCGGACGAACGA 8
AAGGCCGGTCCC 7
CAAGGCCGGTCC 7
CCAAGGCCGGTC 7
CCCGGACGAACG 7
CGGACGAACGAC 7
GACGAACGACGT 6
TCCCACCCCGGA 6
ACGAACGACGTC 5
CCCAAGGCCGGT 5
CCGGTCCCACCC 5
GGACCGAGCCCA 5
GGACGAACGACG 5
GTCCCACCCCGG 5
AACGACGTCCGG 4
ACGACGTCCGGG 4
ACGTCCGGGGTA 4
AGCCCAAGGCCG 4
CGAACGACGTCC 4
CGACGTCCGGGG 4
CGAGCCCAAGGC 4
CGGTCCCACCCC 4

>m141126_082622_42134_c100732262550000001823141405141501_s1_p0/16461/347_6465

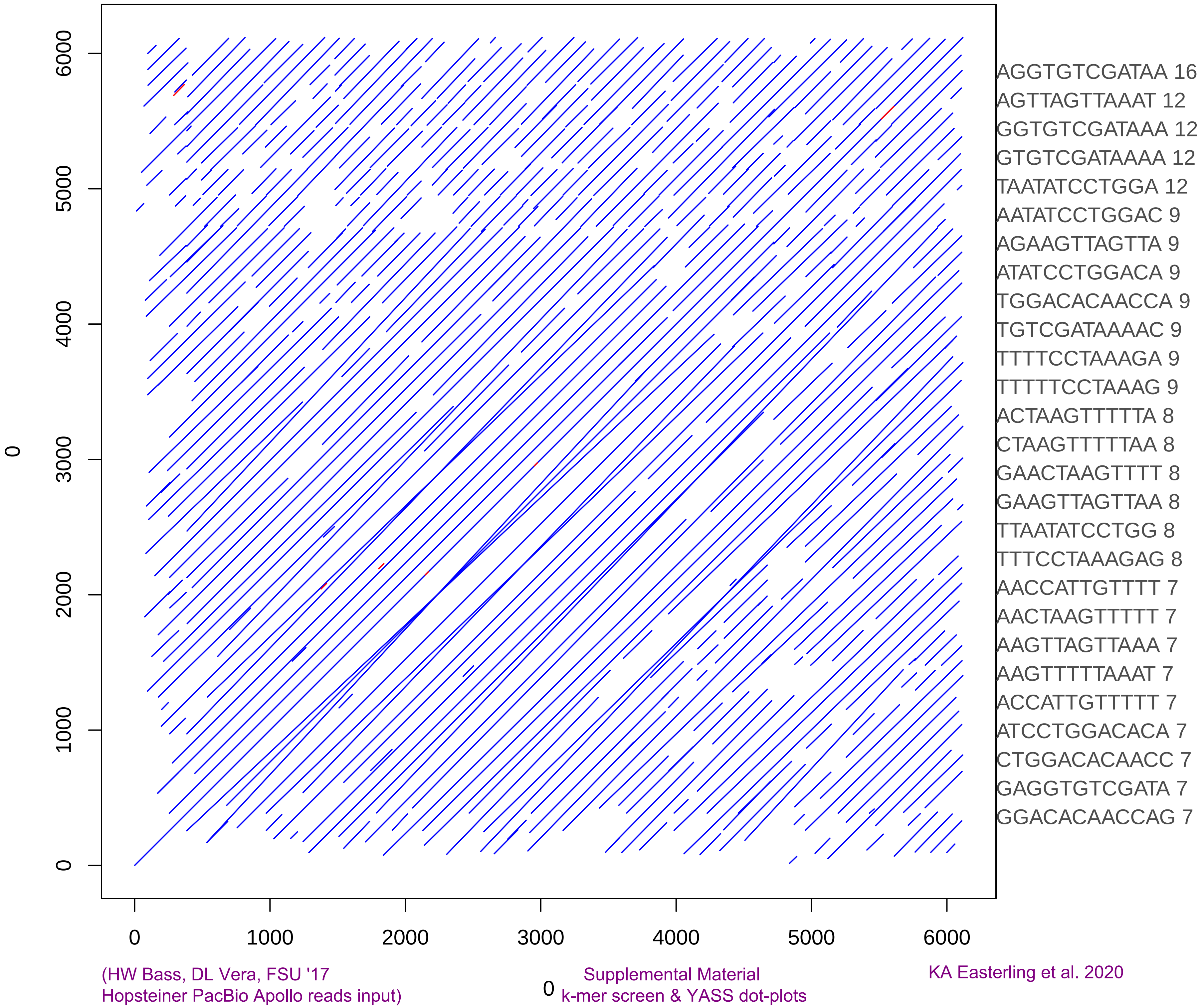

>m141126_082622_42134_c100732262550000001823141405141501_s1_p0/17727/8616_16416

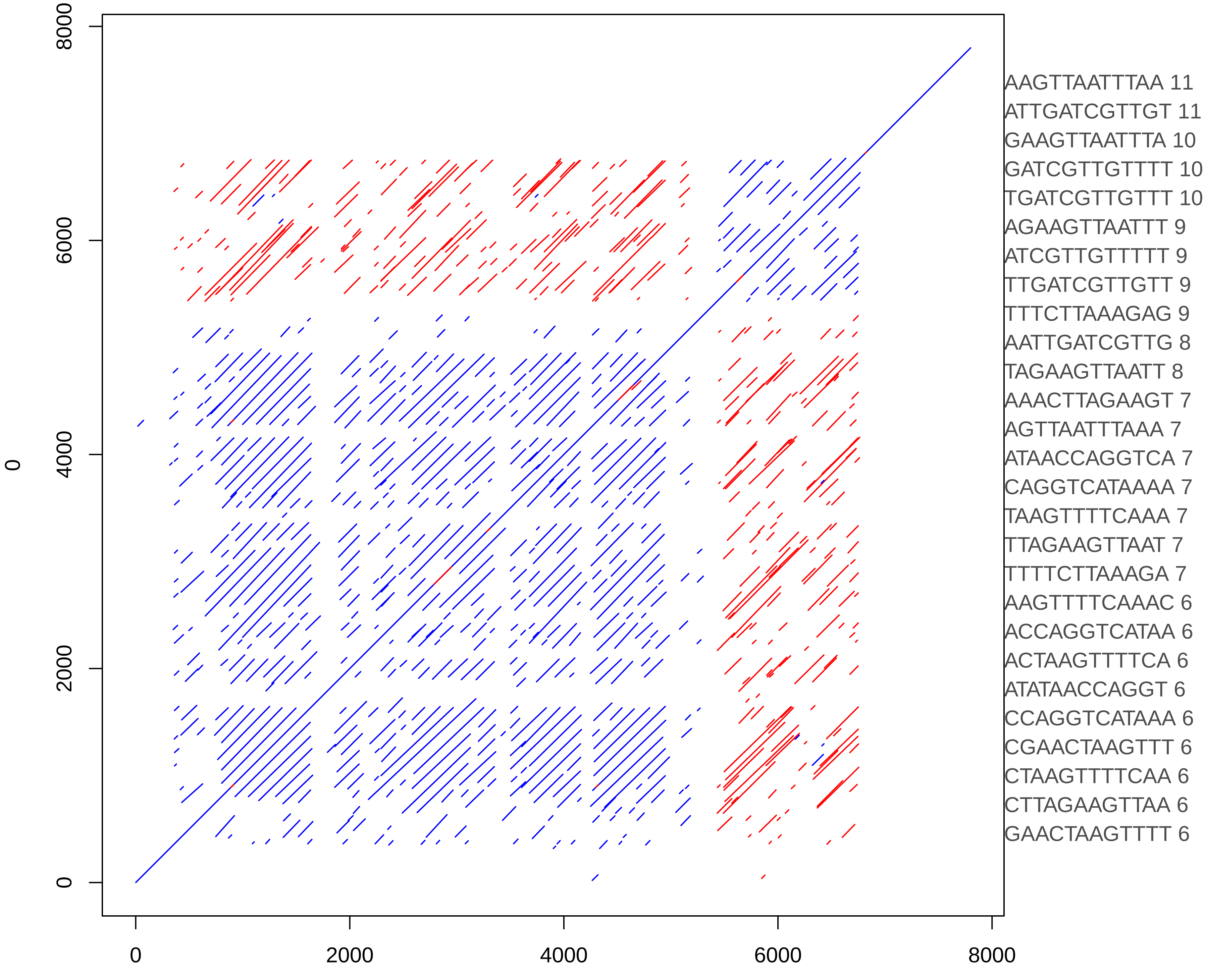

>m141126_082622_42134_c100732262550000001823141405141501_s1_p0/2368/0_7694

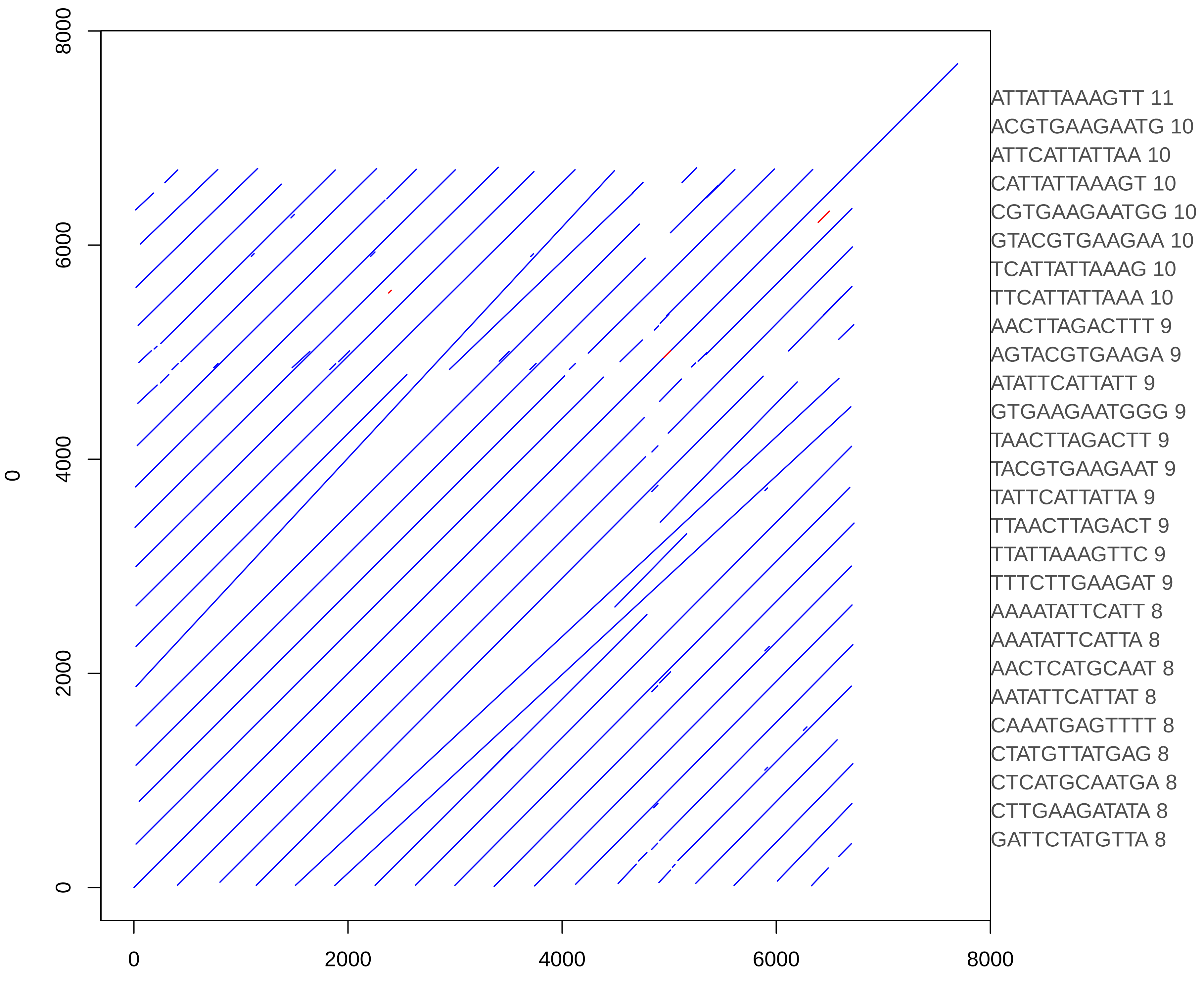

>m141126_082622_42134_c100732262550000001823141405141501_s1_p0/2368/7738_14518

Representative Read (Table 1): HuluTR360-r642, 2 ppm, 66% A+T,
GenBank Acc. MN537578

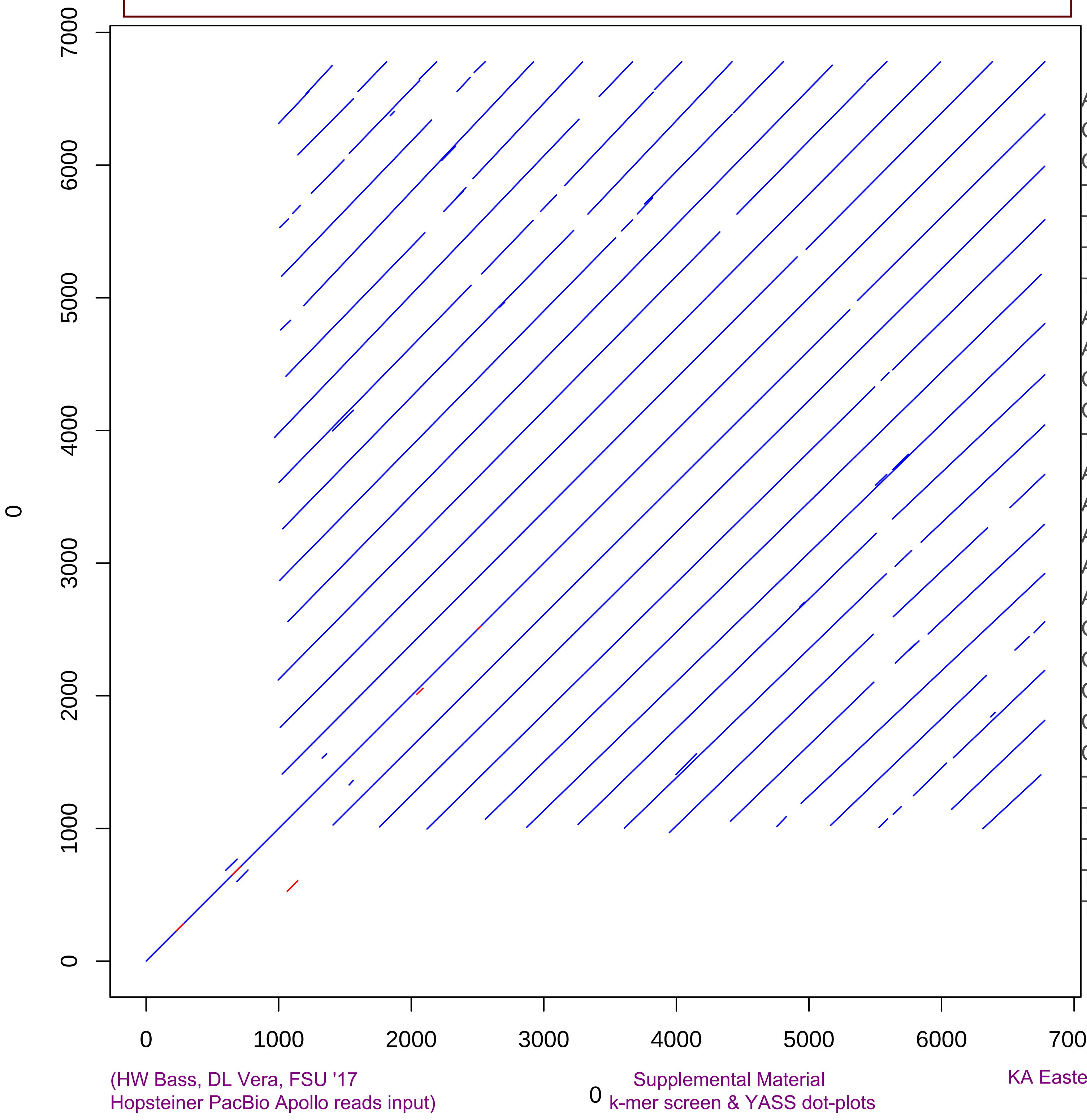

ATGAGTTGAATT 8
CATGAGTTGAAT 8
GCATGAGTTGAA 8
TATGTAGTACTC 8
TGAGTTGAATTA 8
TGCATGAGTTGA 8
TTGCATGAGTTG 8
AGTTGAATTAAT 7
ATTGCATGAGTT 7
GAGTTGAATTAA 7
GTTGAATTAATA 7
TCATTGCATGAG 7
AAAGTCTAAGTT 6
AGAAAGTCTAAG 6
ATCGCATTGAAA 6
ATTCTTCACGTA 6
ATTGTAAGTATA 6
CATTCTTCACGT 6
CATTGCATGAGT 6
CCATTCTTCACG 6
GAAAGTCTAAGT 6
GAACTTTAATAA 6
TGAATTAATATA 6
TGTAAGTATATC 6
TTGAATTAATAT 6
TTGTAAGTATAT 6
TTTAATAATGAA 6

>m141126_082622_42134_c100732262550000001823141405141501_s1_p0/23881/2162_11342

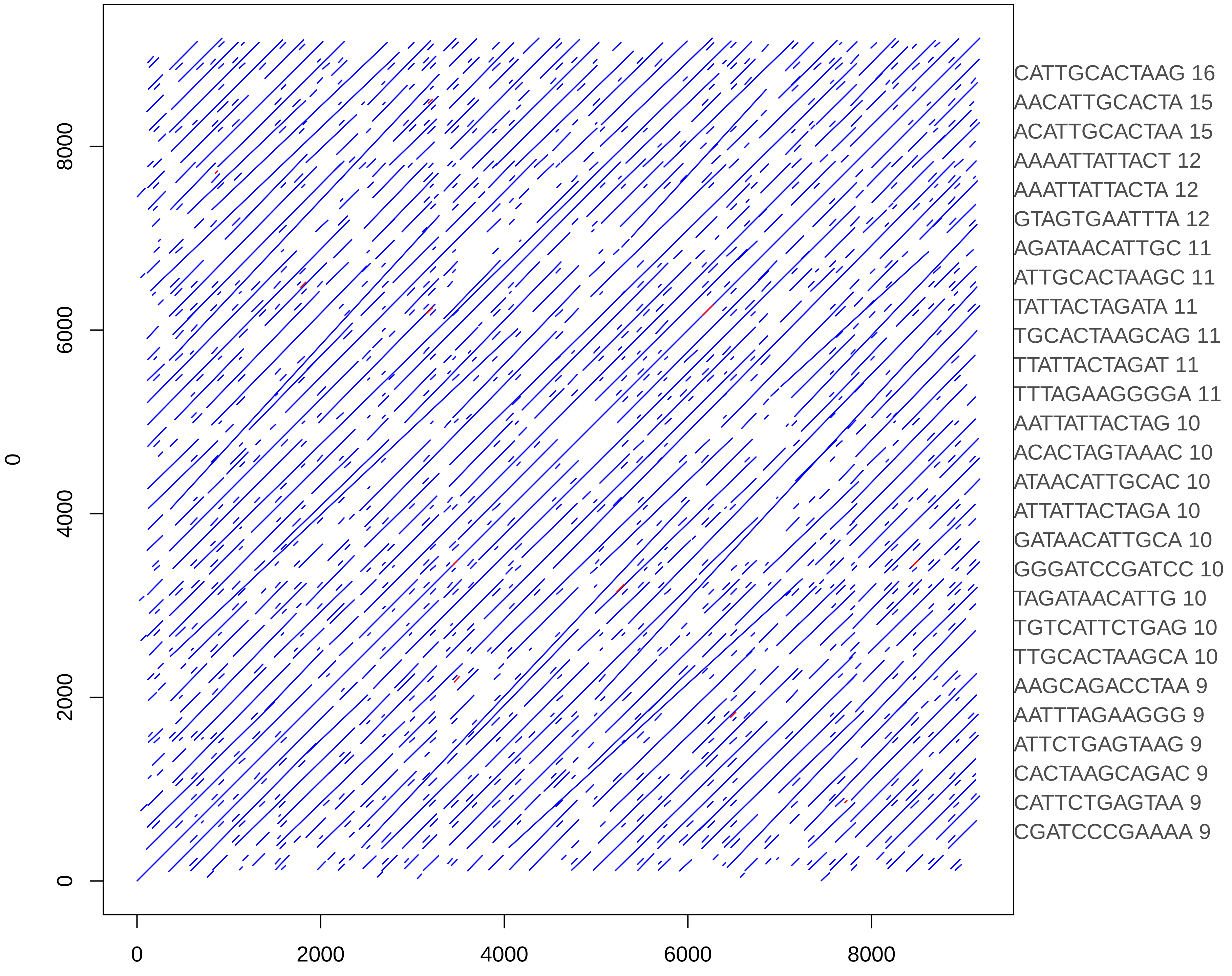

1,121 hits for repetitive sequences
passing 12mer abundance criterion

(Read-Number)
PAGE 644

For Tandem Repeat families of ### bp:
PacBio Read named "HuluTR###-r644"

>m141126_082622_42134_c100732262550000001823141405141501_s1_p0/27521/11592_17889

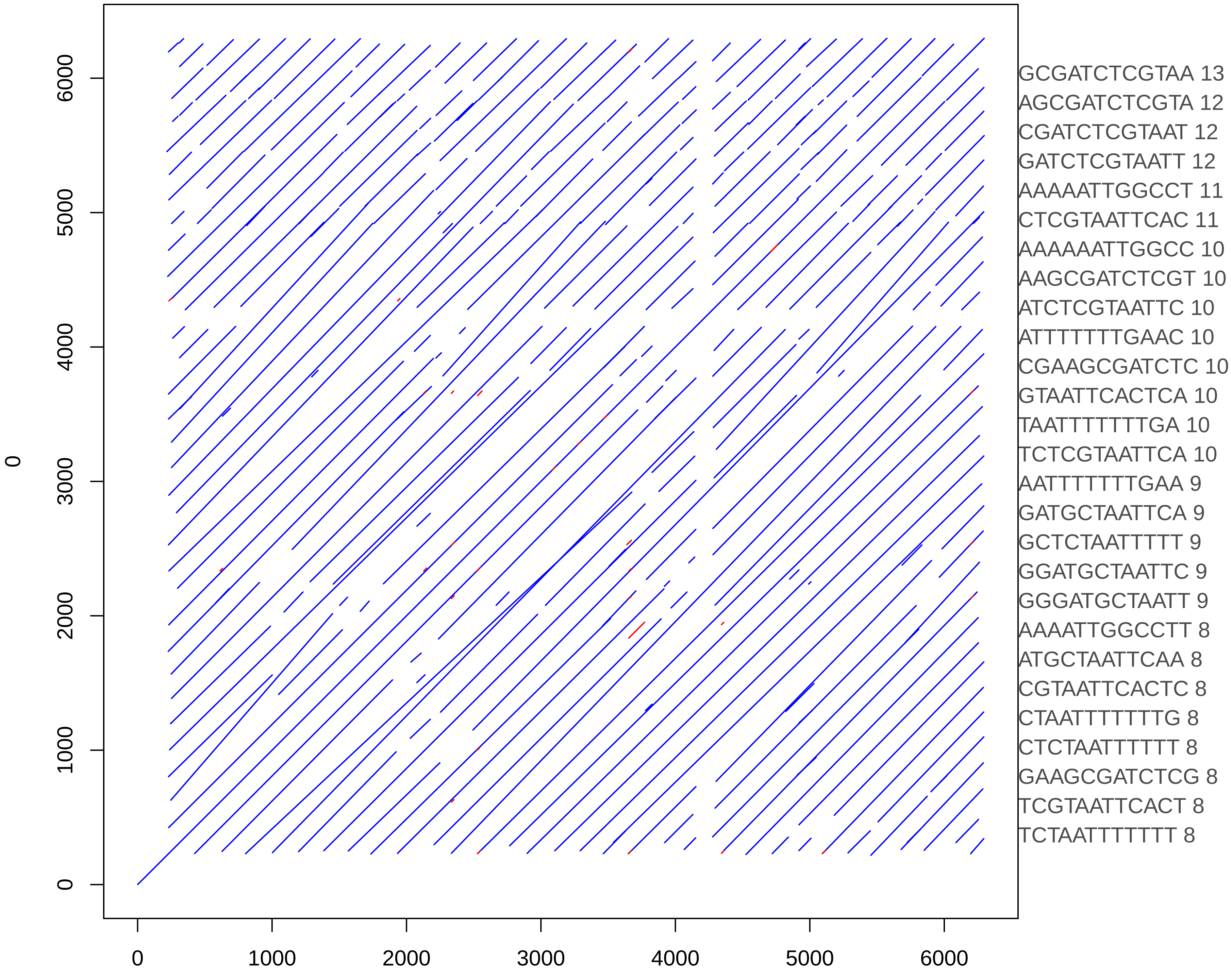

(HW Bass, DL Vera, FSU '17
Hopsteiner PacBio Apollo reads input)

0

Supplemental Material
k-mer screen & YASS dot-plots

KA Easterling et al. 2020

>m141126_082622_42134_c100732262550000001823141405141501_s1_p0/28460/302_14518

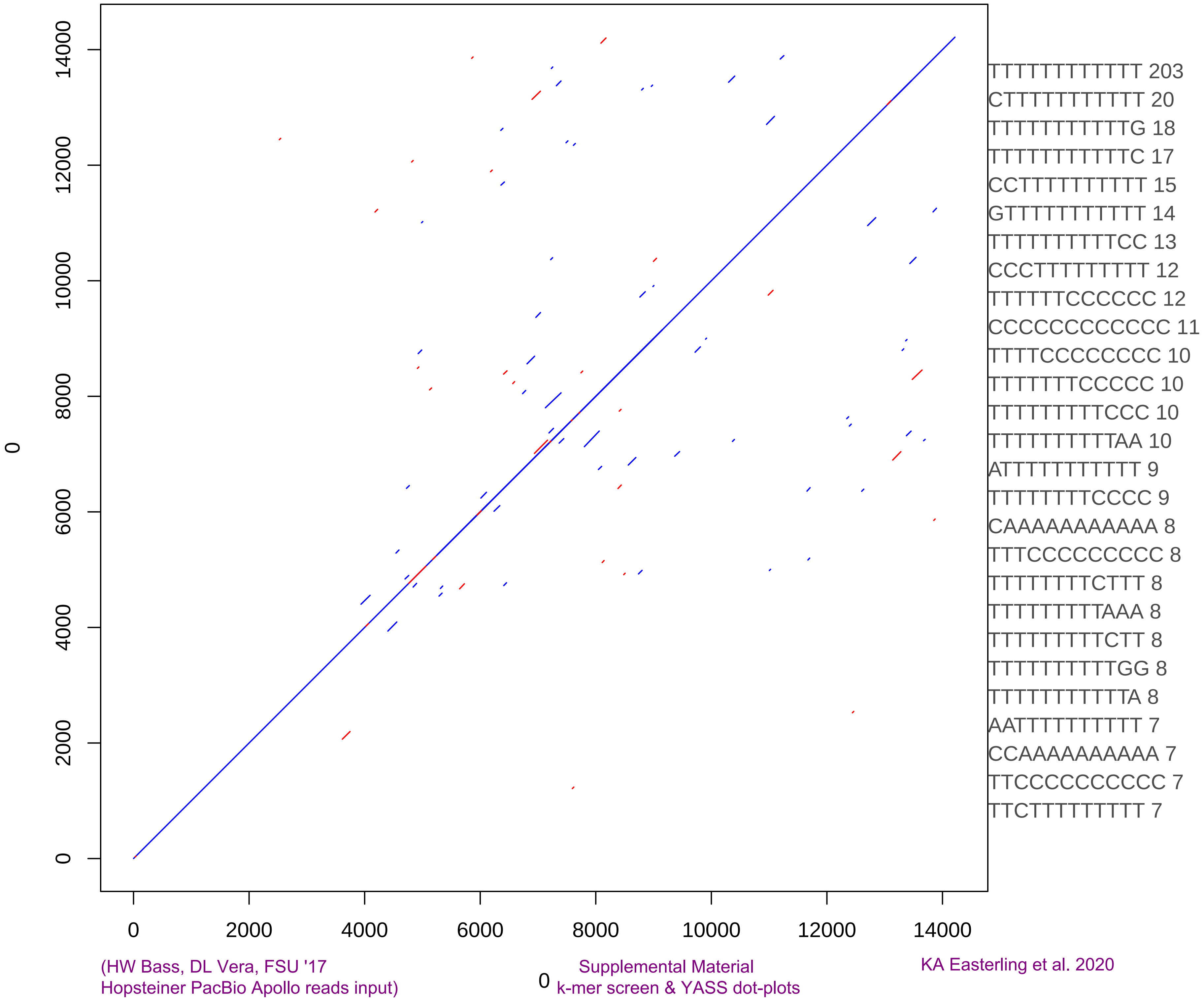

>m141126_082622_42134_c100732262550000001823141405141501_s1_p0/28823/451_9022

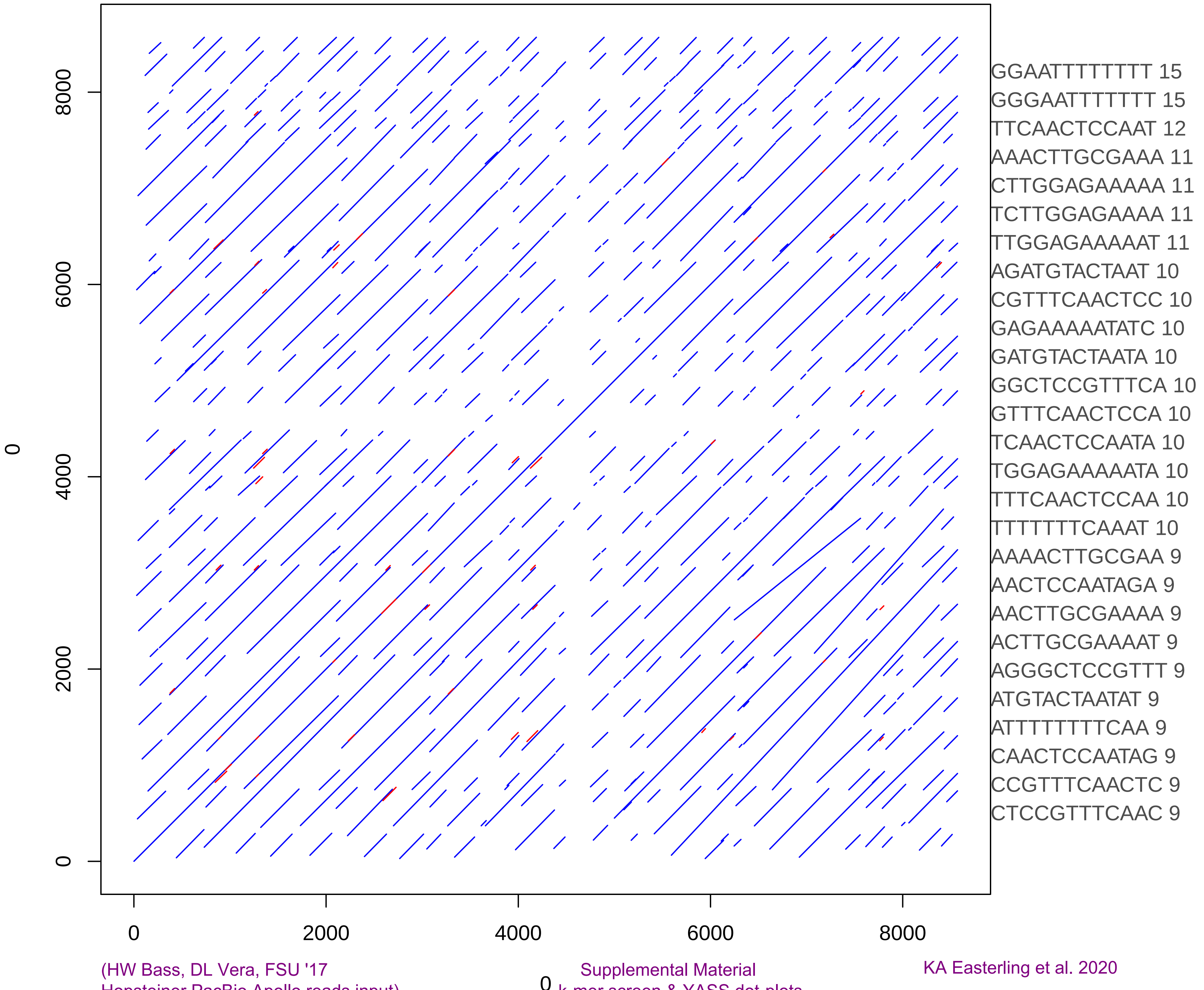

>m141126_082622_42134_c100732262550000001823141405141501_s1_p0/30732/2570_7969

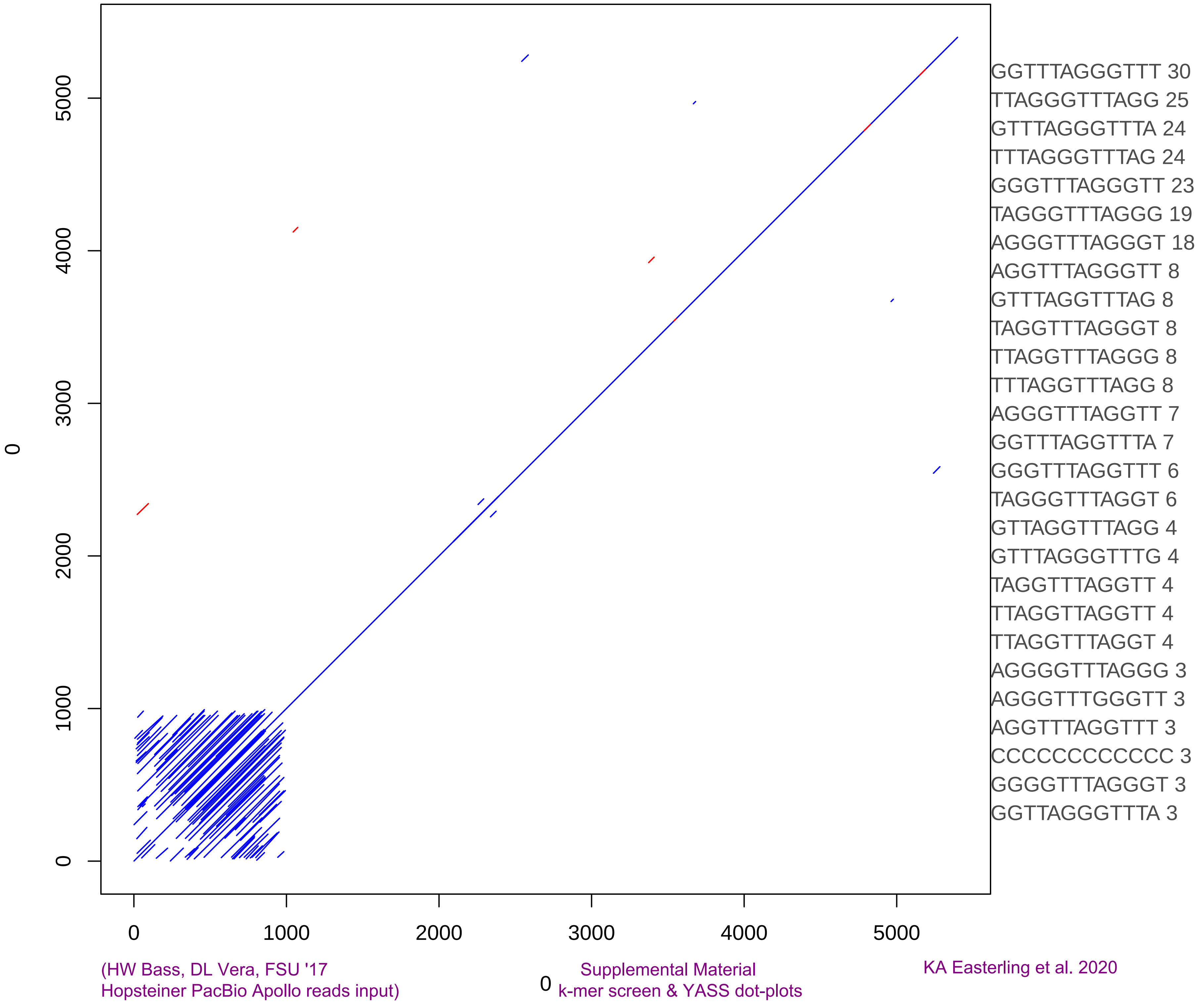

>m141126_082622_42134_c100732262550000001823141405141501_s1_p0/32846/4452_15376

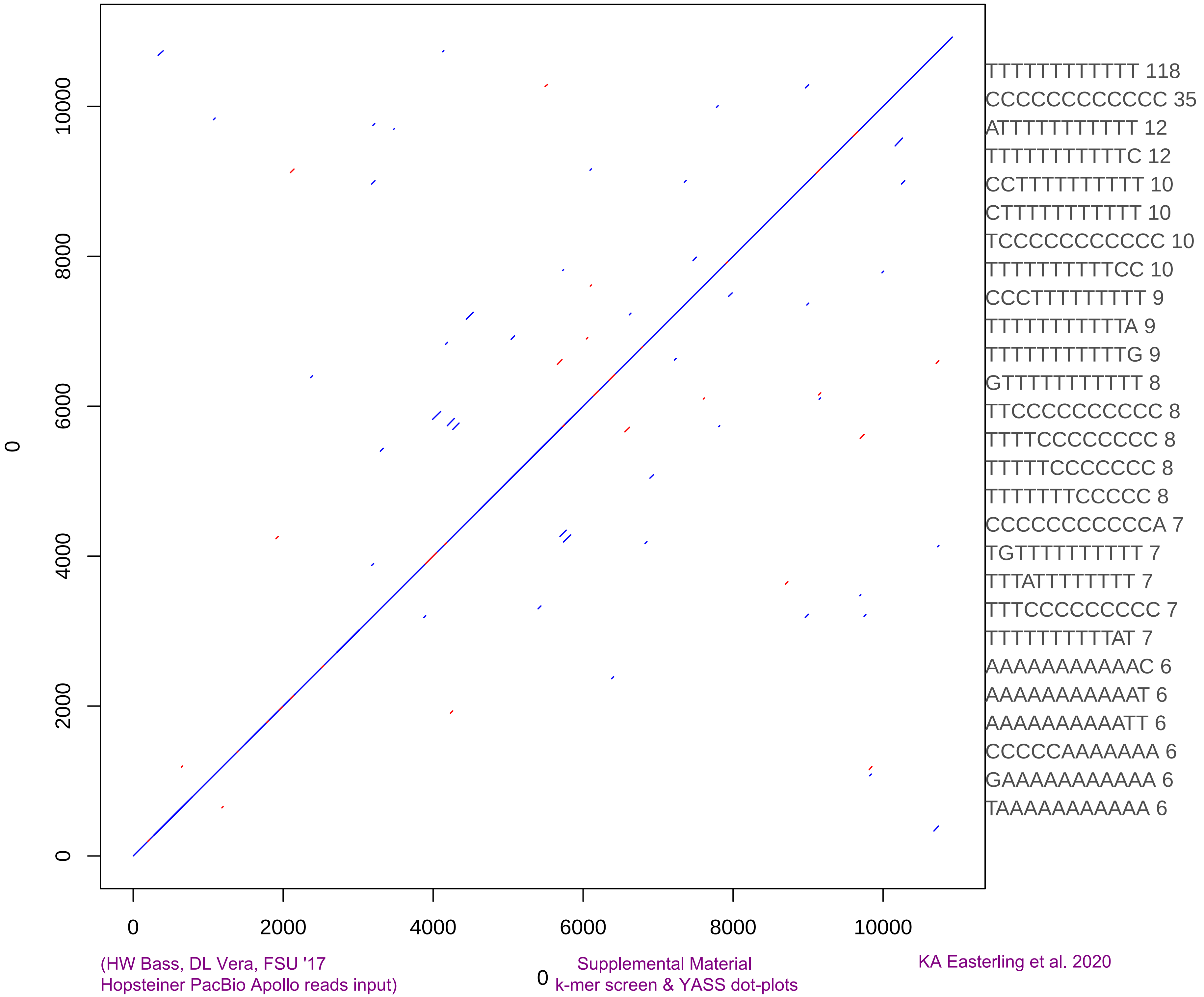

>m141126_082622_42134_c100732262550000001823141405141501_s1_p0/36614/0_5182

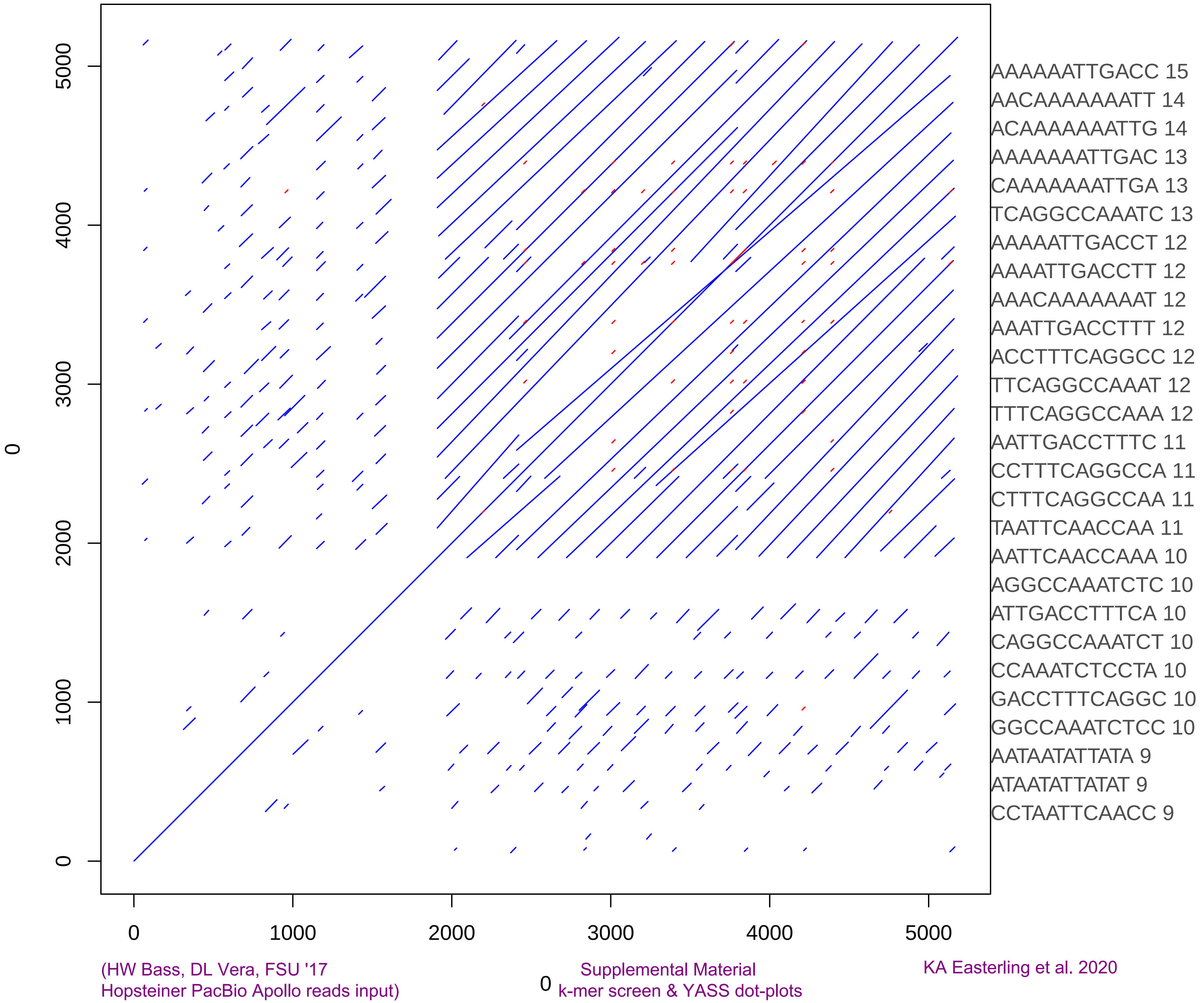

>m141126_082622_42134_c100732262550000001823141405141501_s1_p0/37397/8497_18142

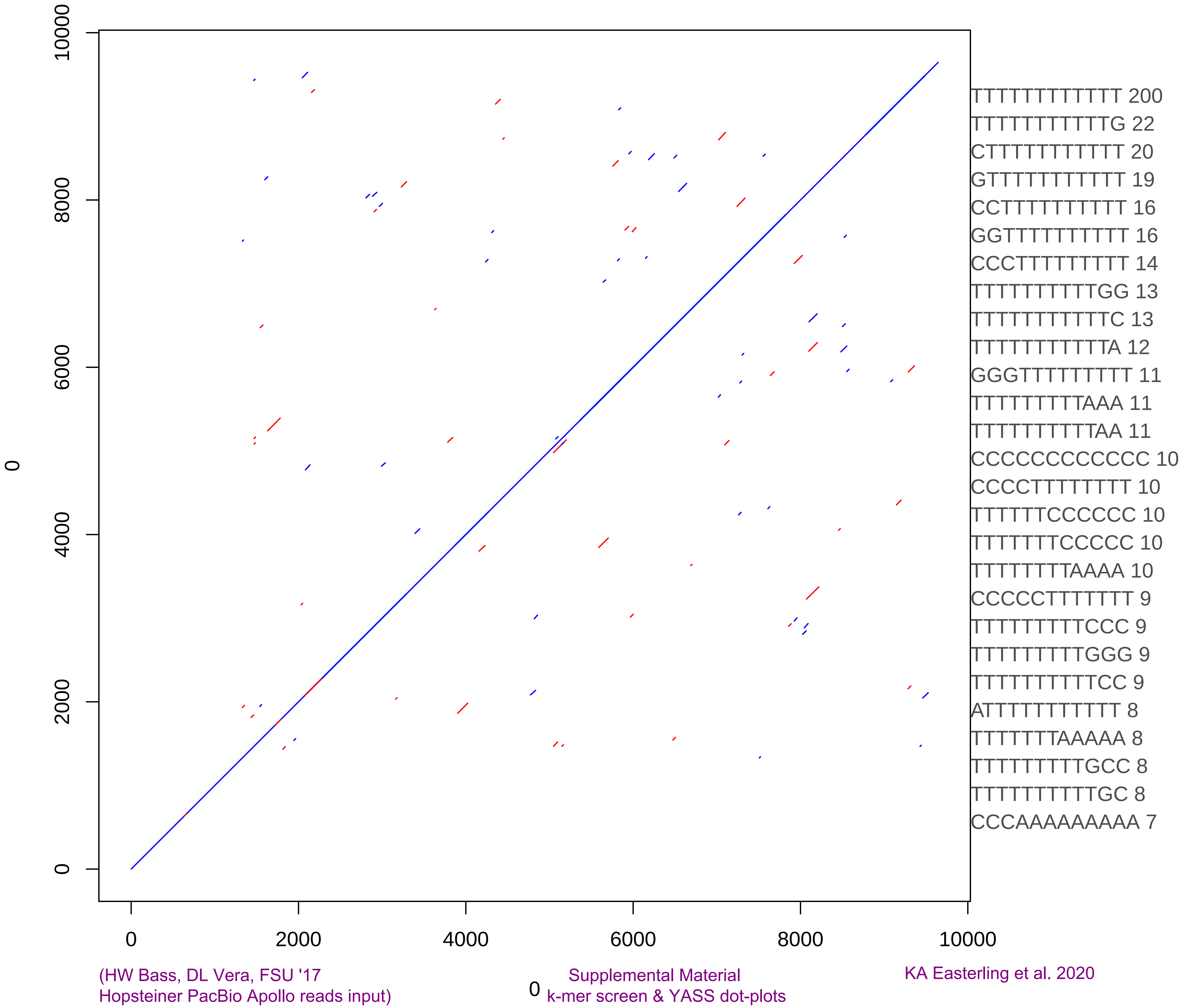

>m141126_082622_42134_c100732262550000001823141405141501_s1_p0/38687/3146_9210

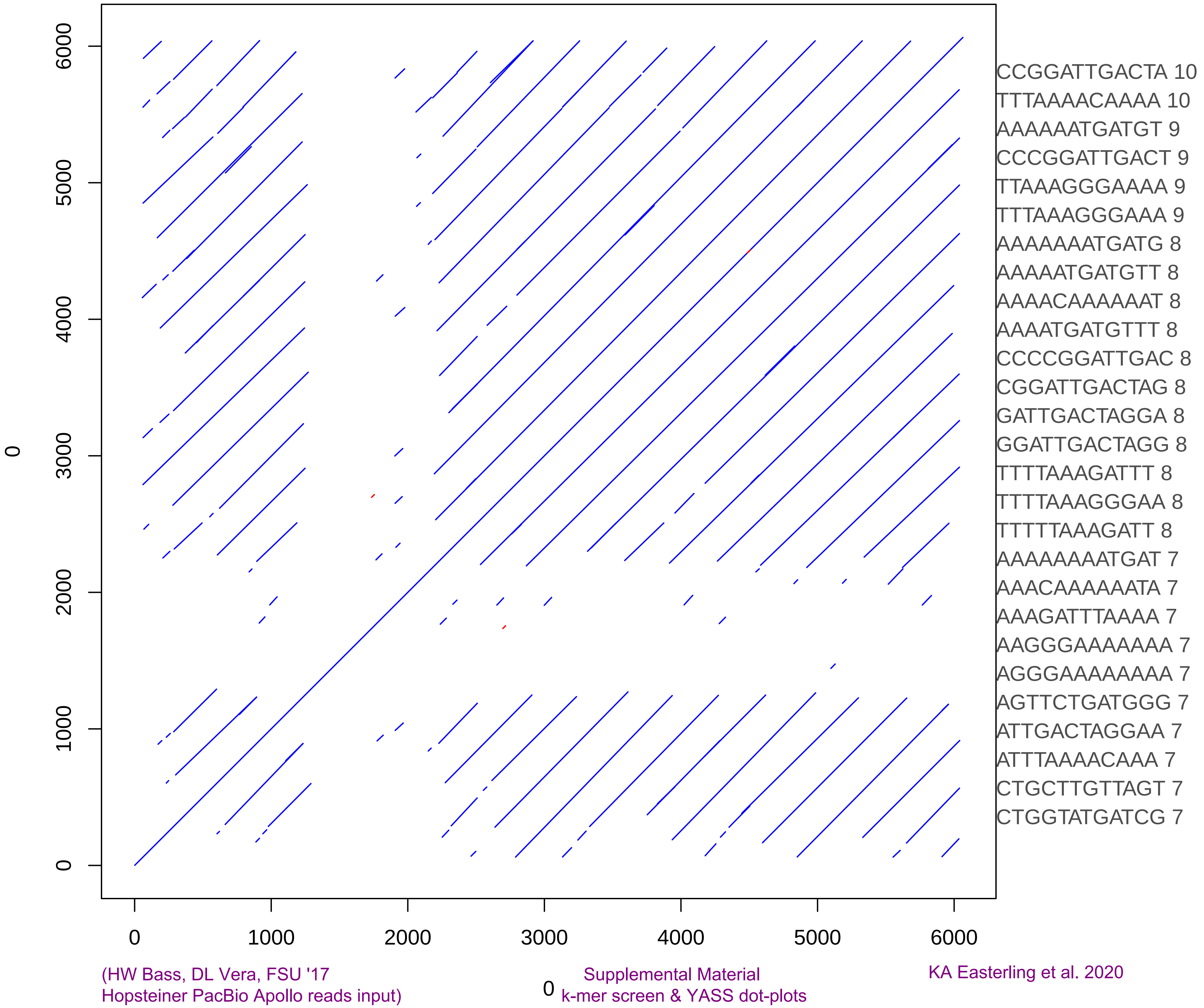

>m141126_082622_42134_c100732262550000001823141405141501_s1_p0/45438/9815_15445

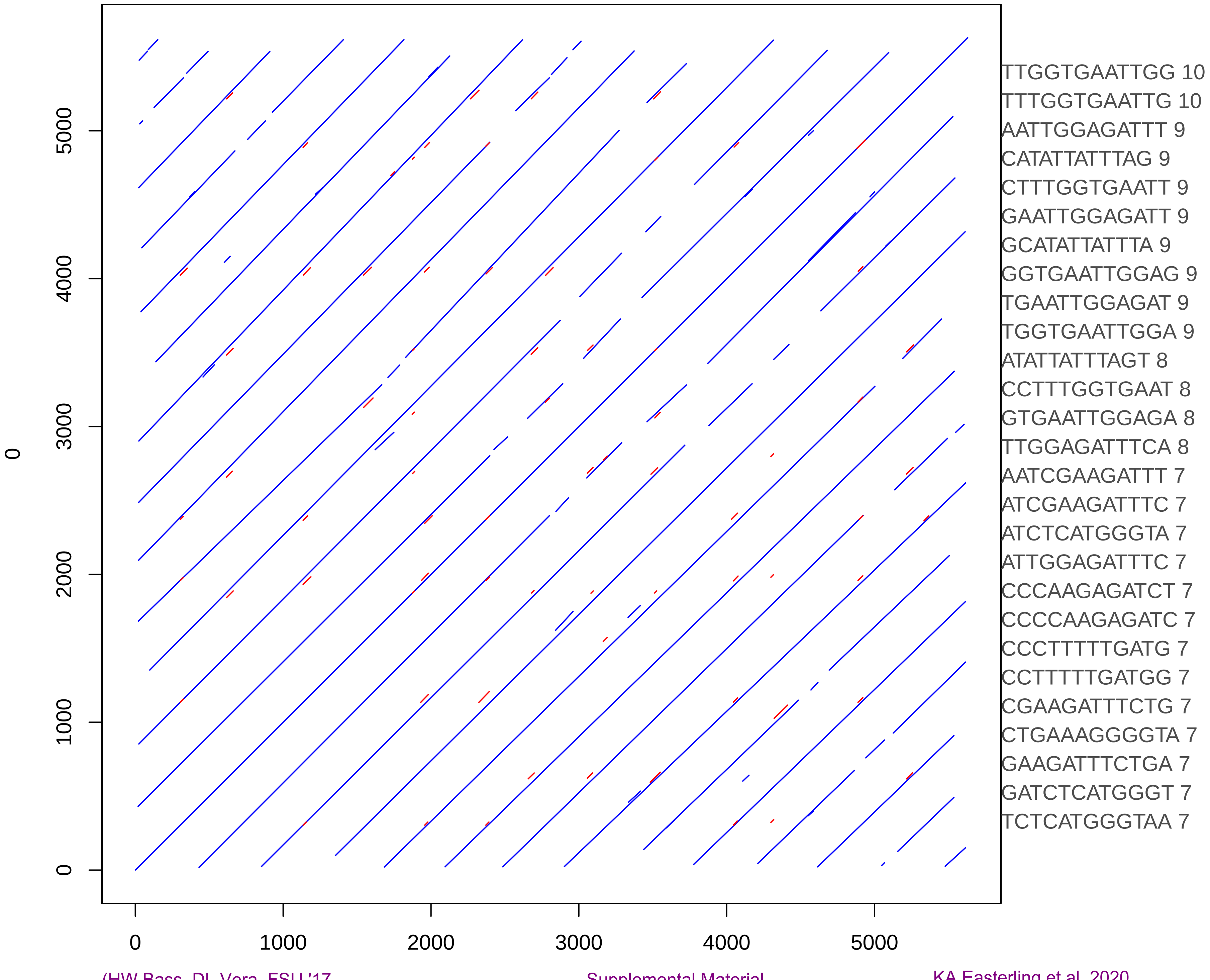

>m141126_082622_42134_c100732262550000001823141405141501_s1_p0/51844/7419_13938

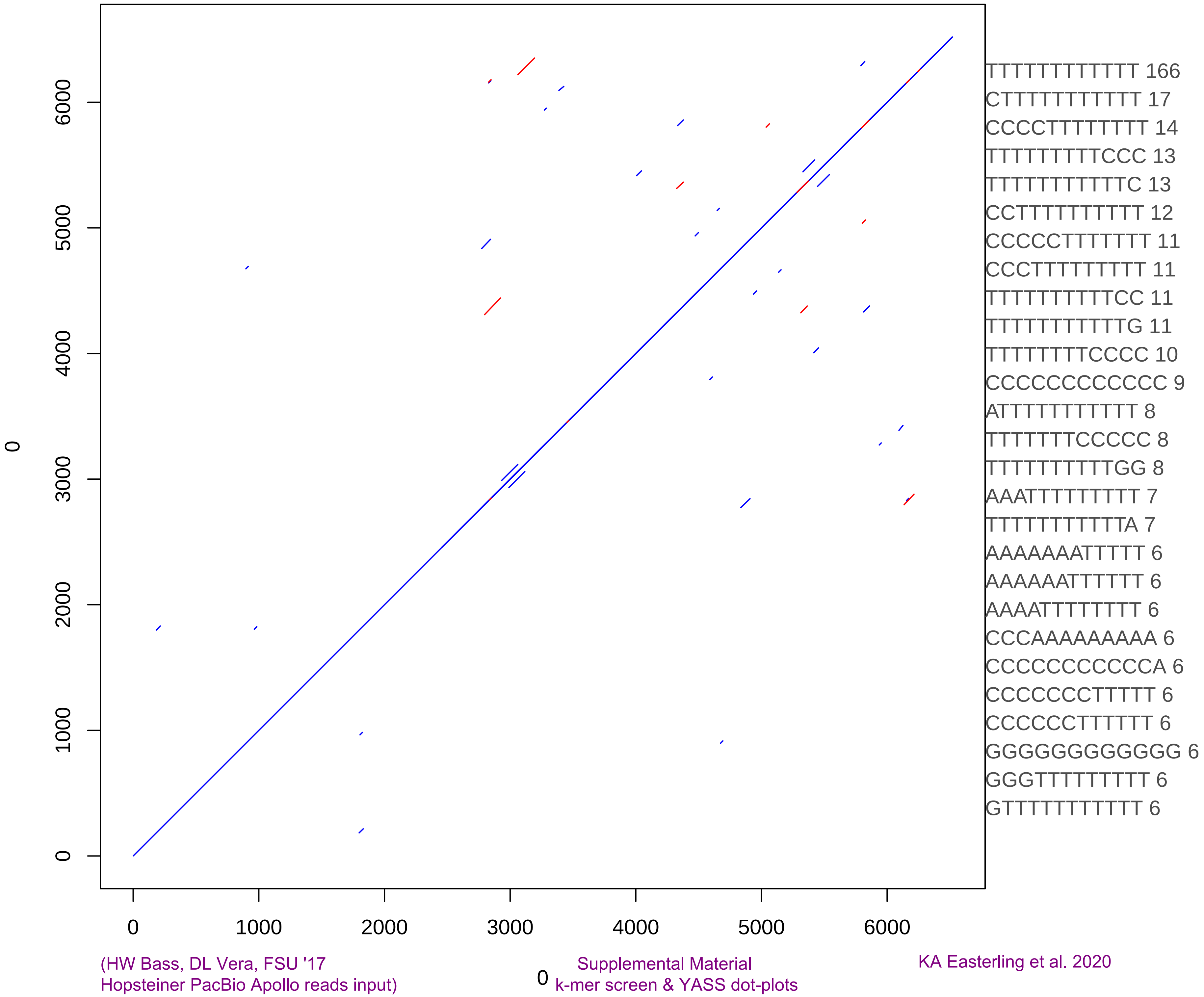

>m141126_082622_42134_c100732262550000001823141405141501_s1_p0/53279/13516_19066

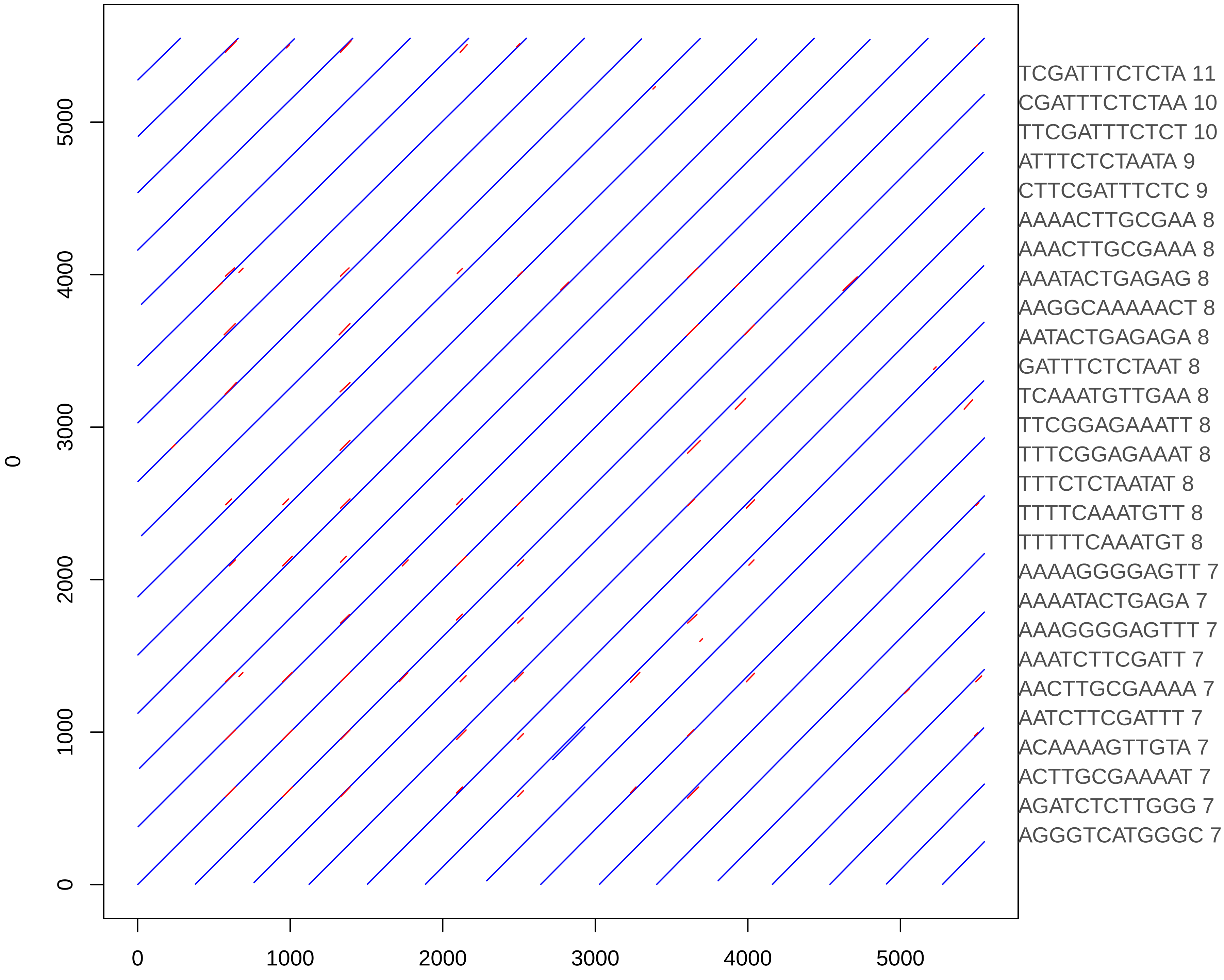

>m141126_082622_42134_c100732262550000001823141405141501_s1_p0/60172/247_9359

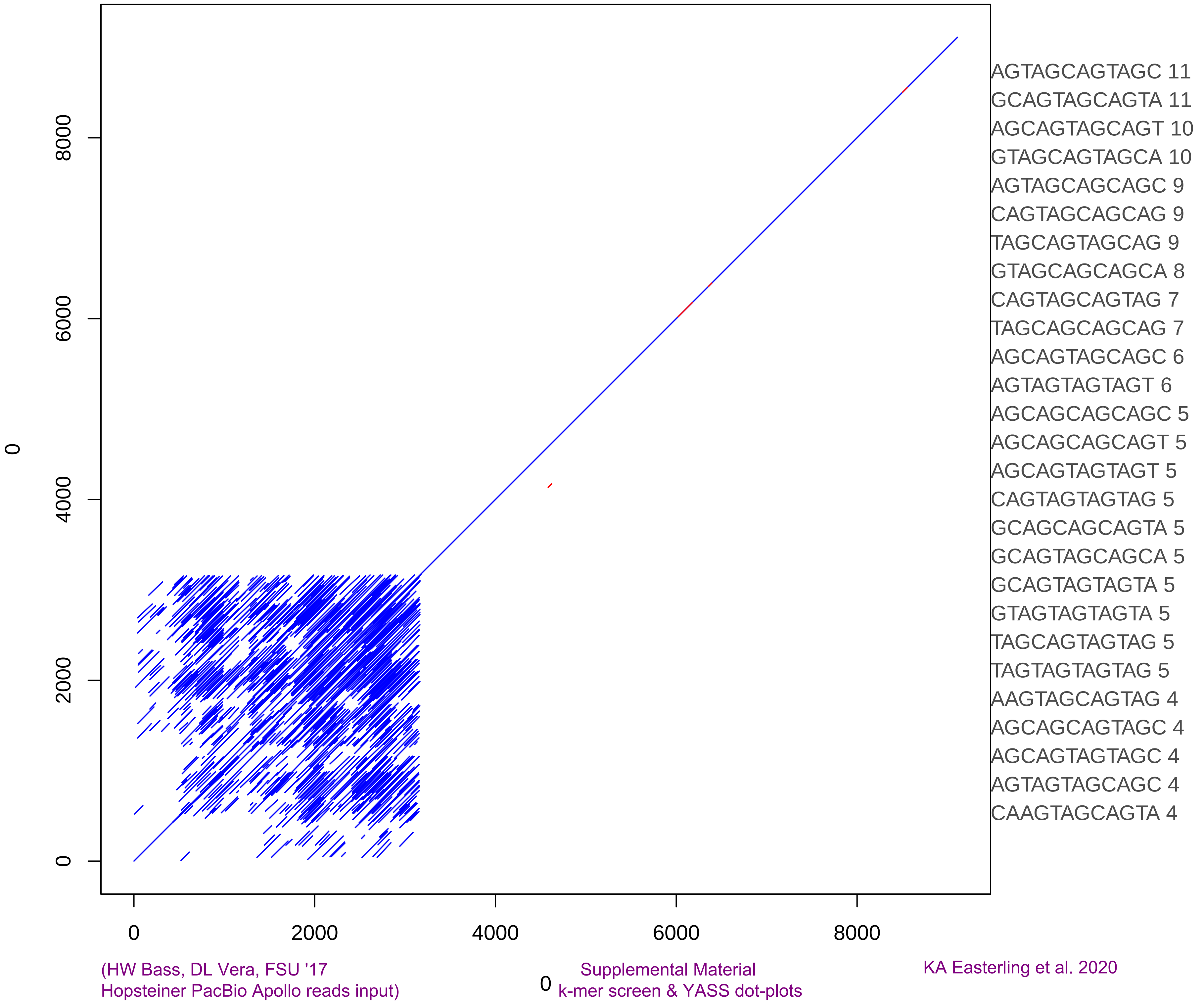

>m141126_082622_42134_c100732262550000001823141405141501_s1_p0/65343/0_13393

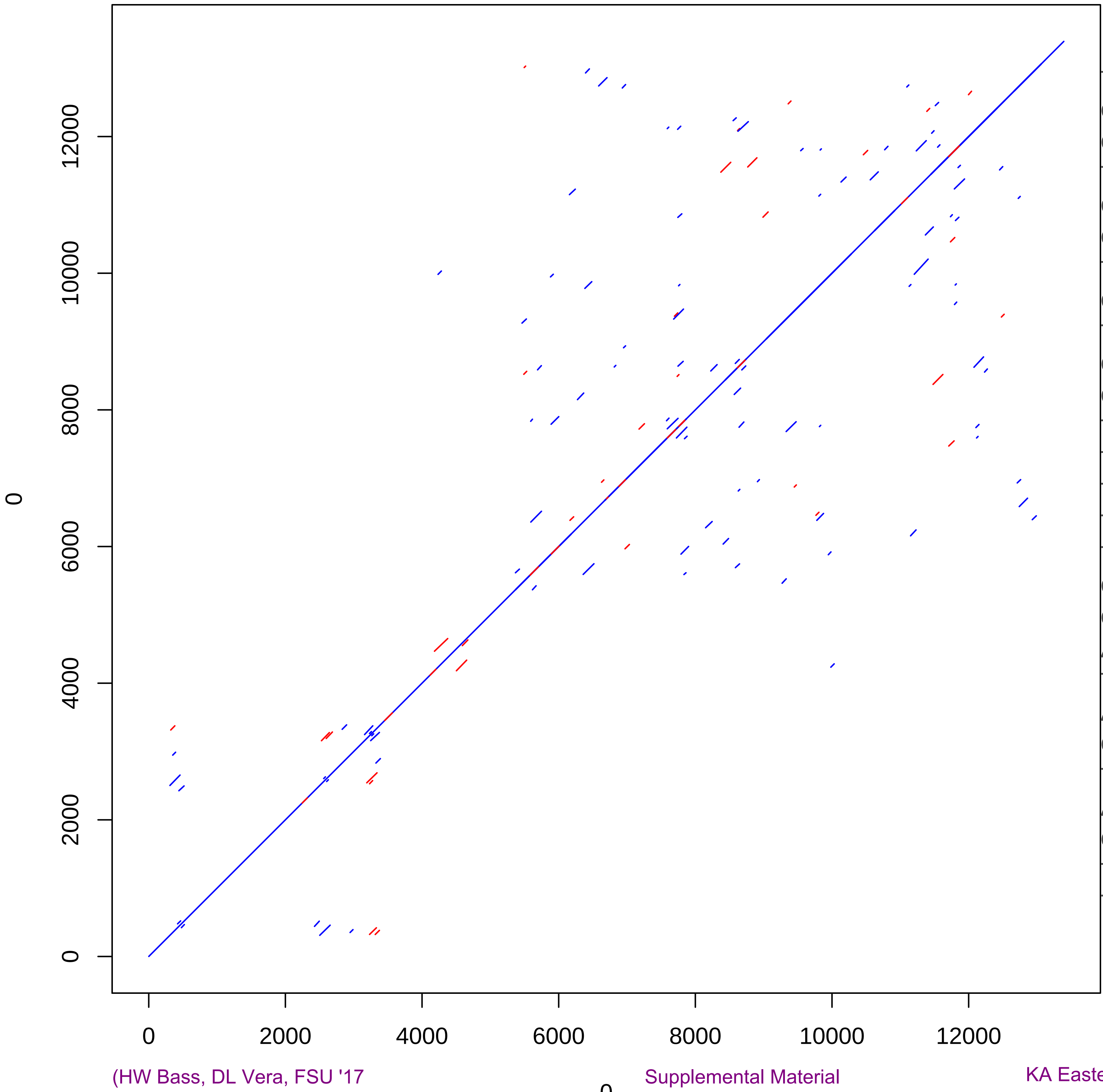

>m141126_082622_42134_c100732262550000001823141405141501_s1_p0/690/2600_12013

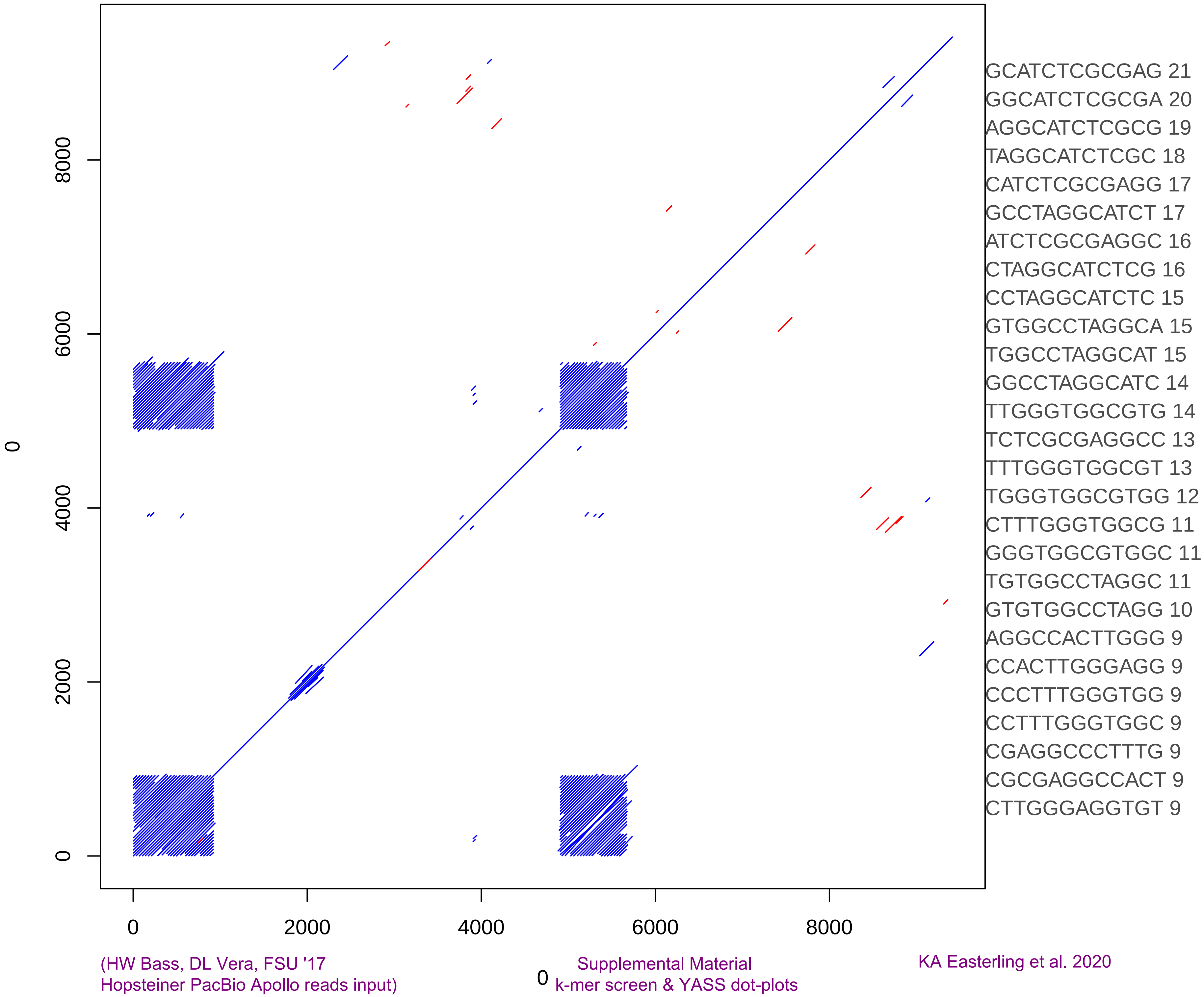

>m141126_082622_42134_c100732262550000001823141405141501_s1_p0/71108/0_6889

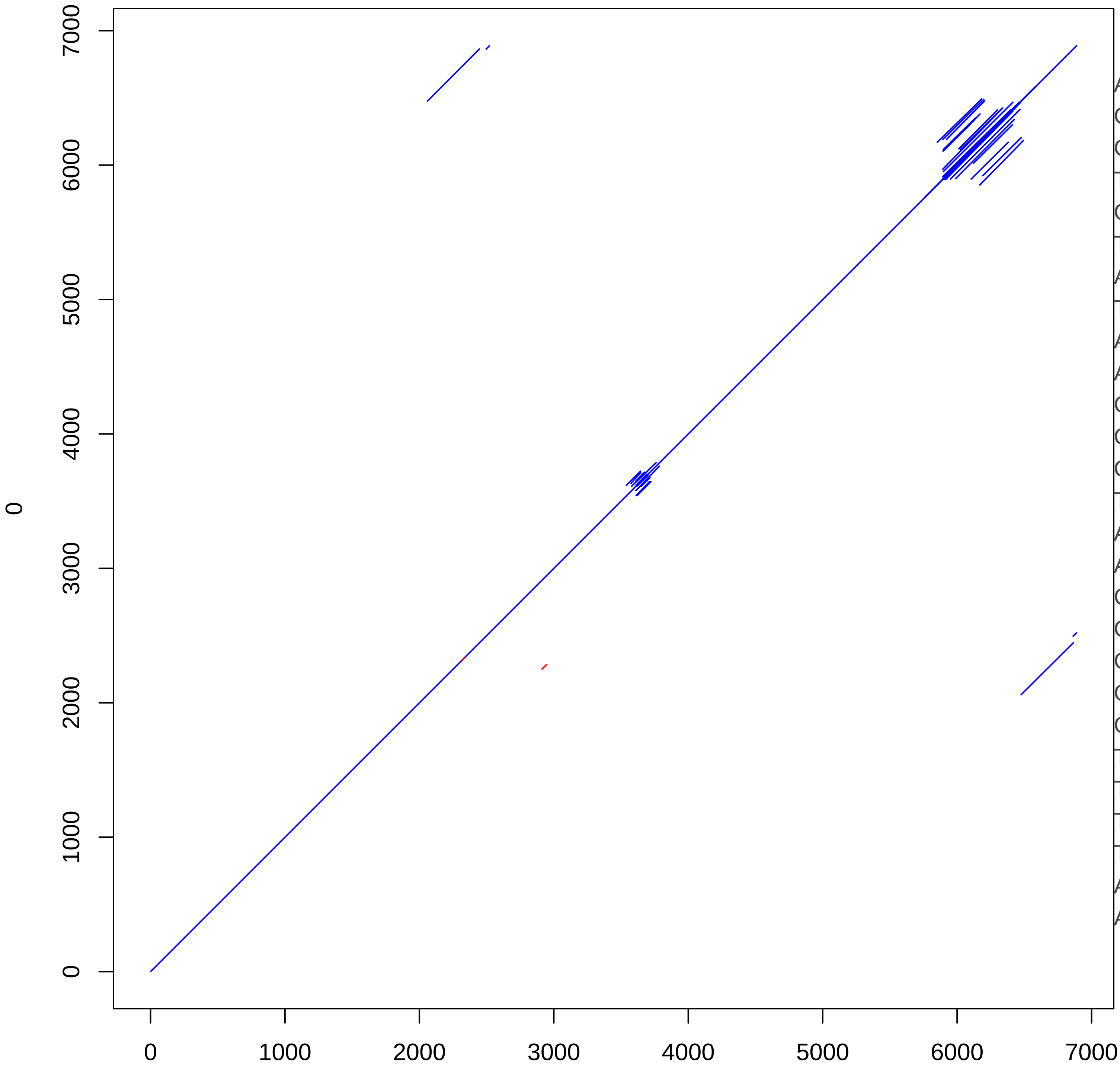

>m141126_082622_42134_c100732262550000001823141405141501_s1_p0/72505/5577_17133

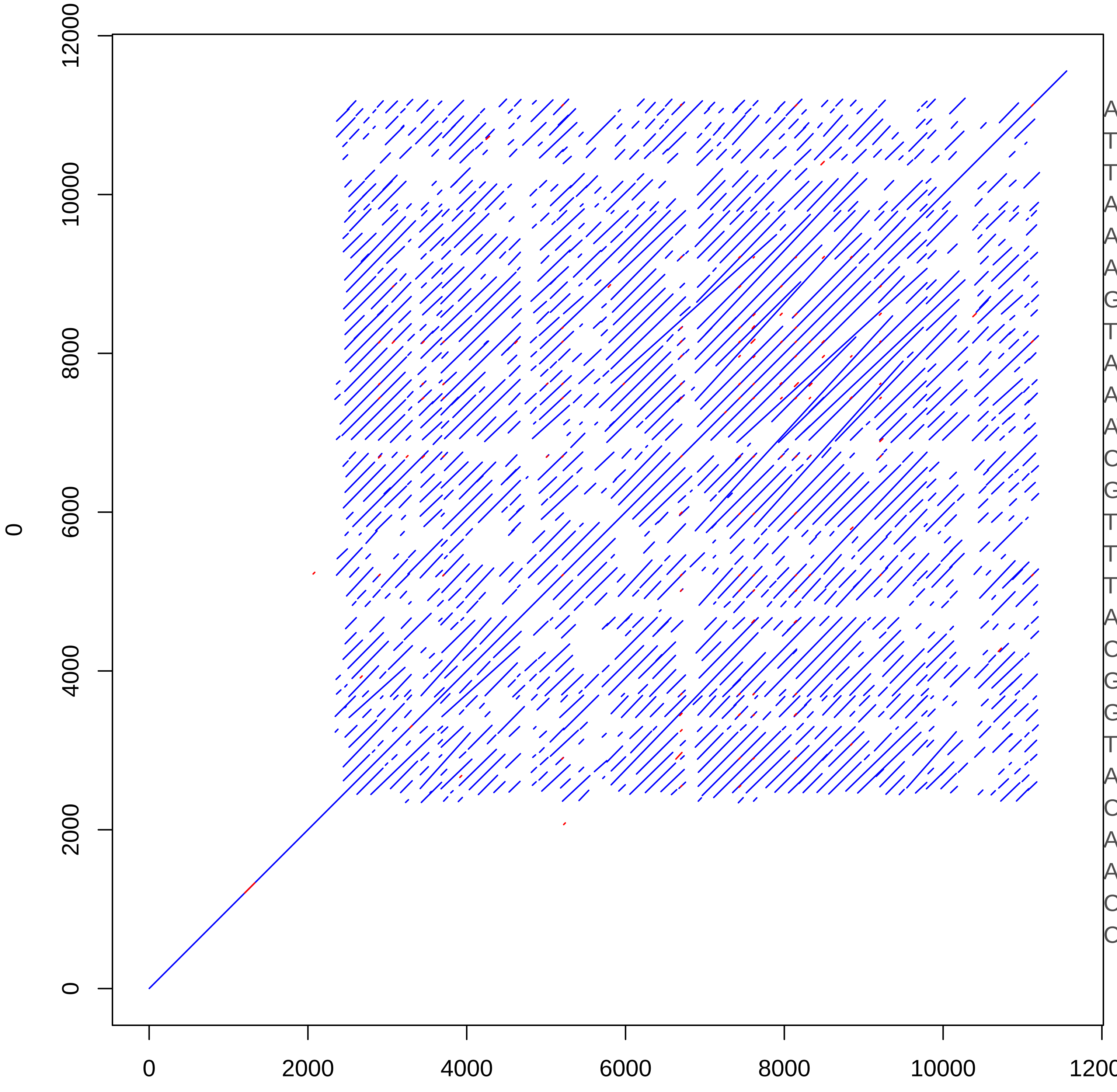

AAAAAAATTAGA 15
TCAAAAAAATTA 14
TTGGCCTGAAAG 13
AAAAATTAGAGC 12
AAAATTAGAGCT 12
AGGTCAATTTTT 12
GGTCAATTTTTT 12
TTTTTGTTTGGT 12
AAAAAATTAGAG 11
AAGGTCAATTTT 11
ATTTGGCCTGAA 11
CCCAATTCATGG 11
GTTCAAAAAAAT 11
TTCAAAAAAATT 11
TTTGGCCTGAAA 11
TTTTTTGTTTGG 11
AAATTAGAGCTT 10
CAAAAAAATTAG 10
GAGCCCTAGGCA 10
GATTTGGCCTGA 10
TTTTGTTTGGTT 10
AAAGGTCAATTT 9
CCAATTCATGGT 9
AATTCATGGTCA 8
AGAAATATGAGT 8
CAATTCATGGTC 8
CCCCAATTCATG 8

>m141126_082622_42134_c100732262550000001823141405141501_s1_p0/74245/6562_12092

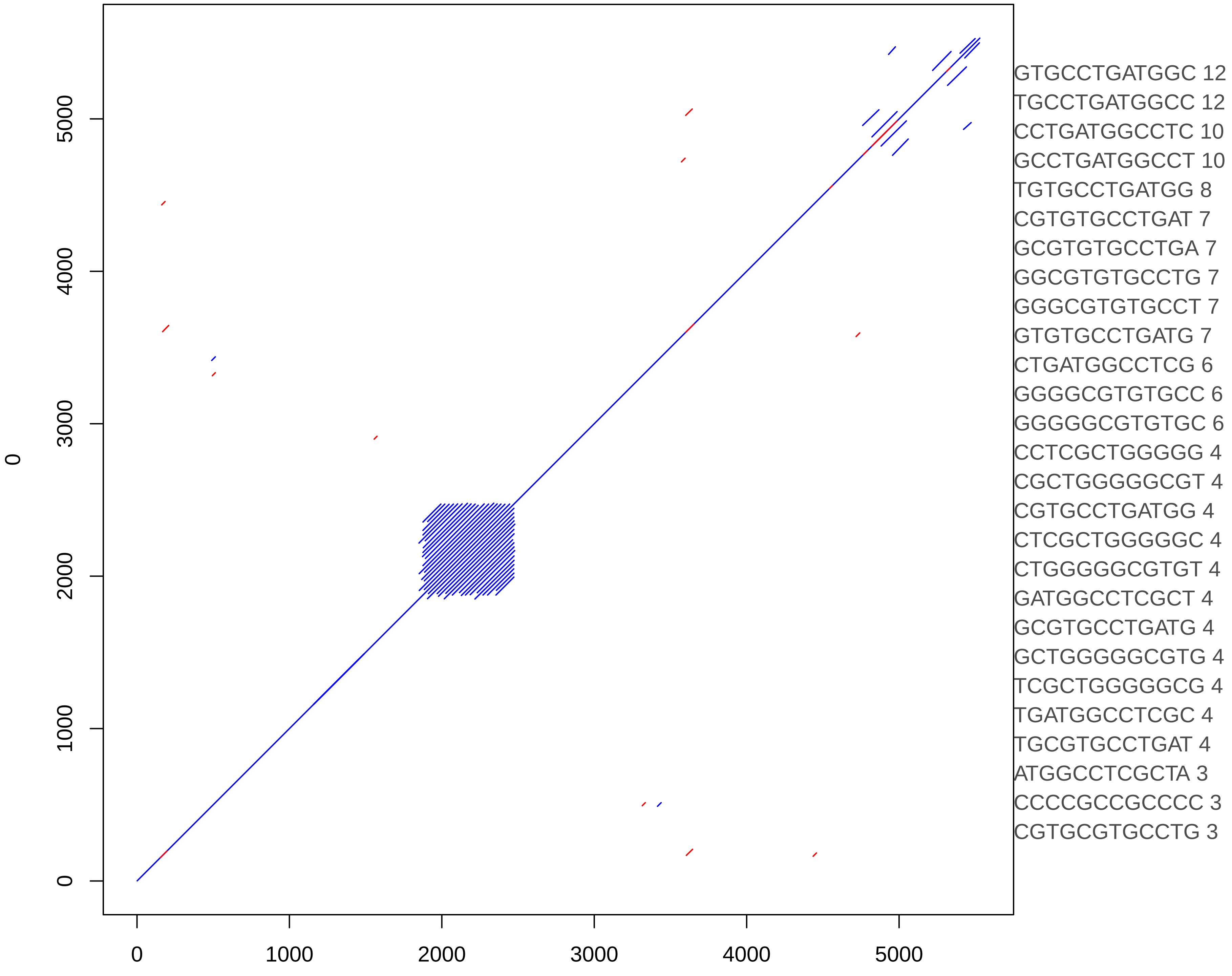

>m141126_082622_42134_c100732262550000001823141405141501_s1_p0/7529/0_10943

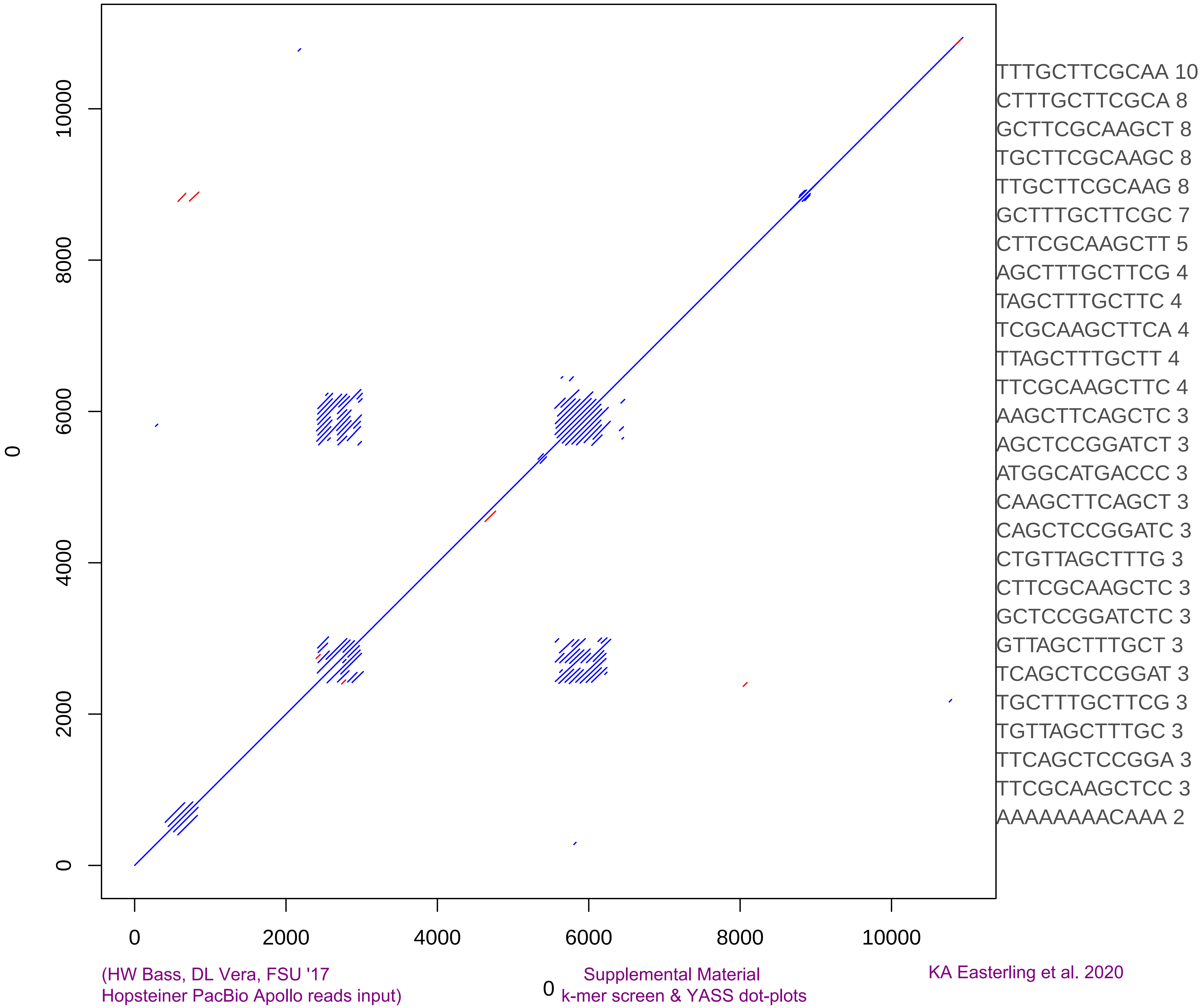

>m141126_082622_42134_c100732262550000001823141405141501_s1_p0/76440/3614_8971

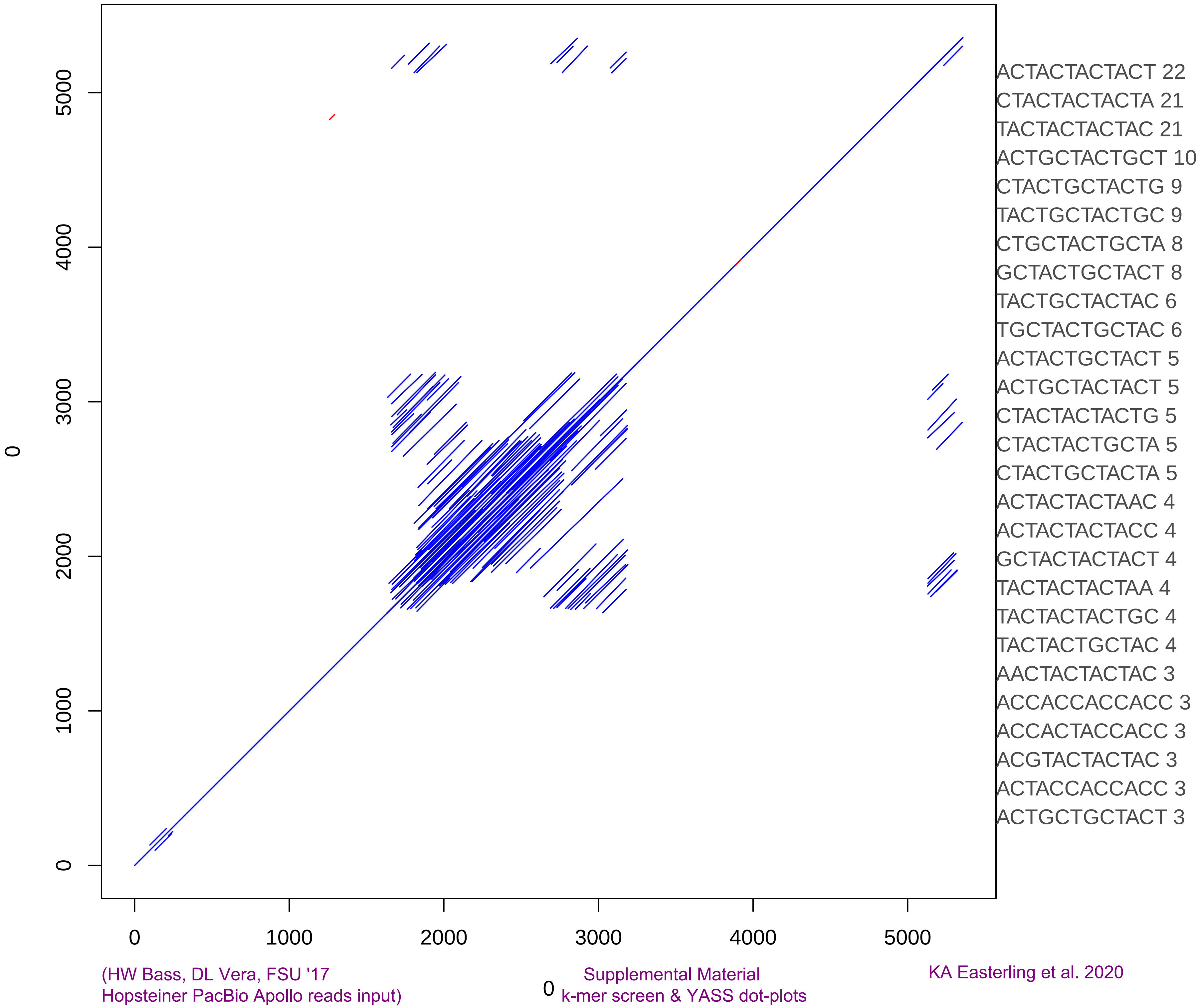

>m141126_082622_42134_c100732262550000001823141405141501_s1_p0/79174/0_11623

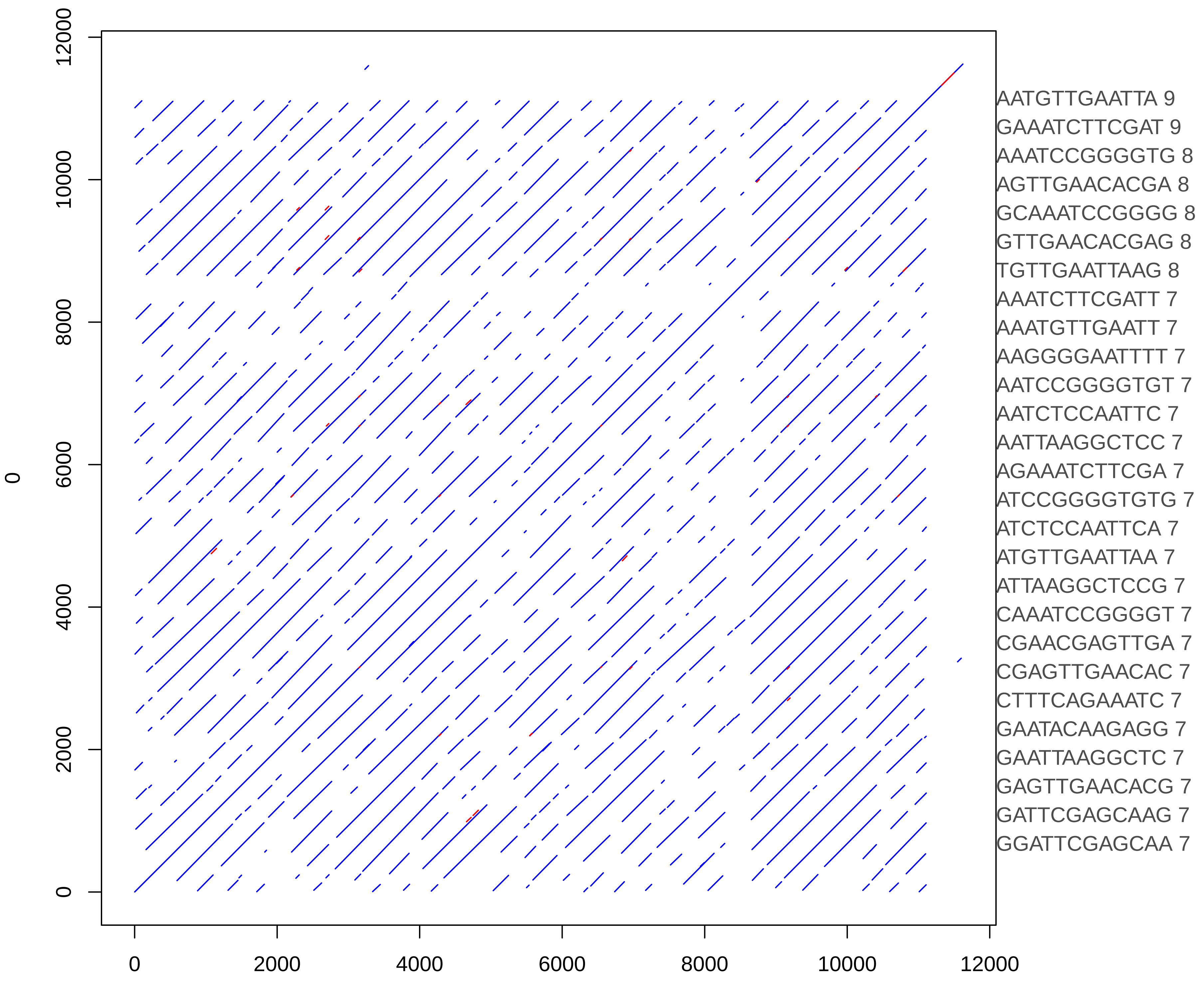

>m141126_082622_42134_c100732262550000001823141405141501_s1_p0/86341/9136_14966

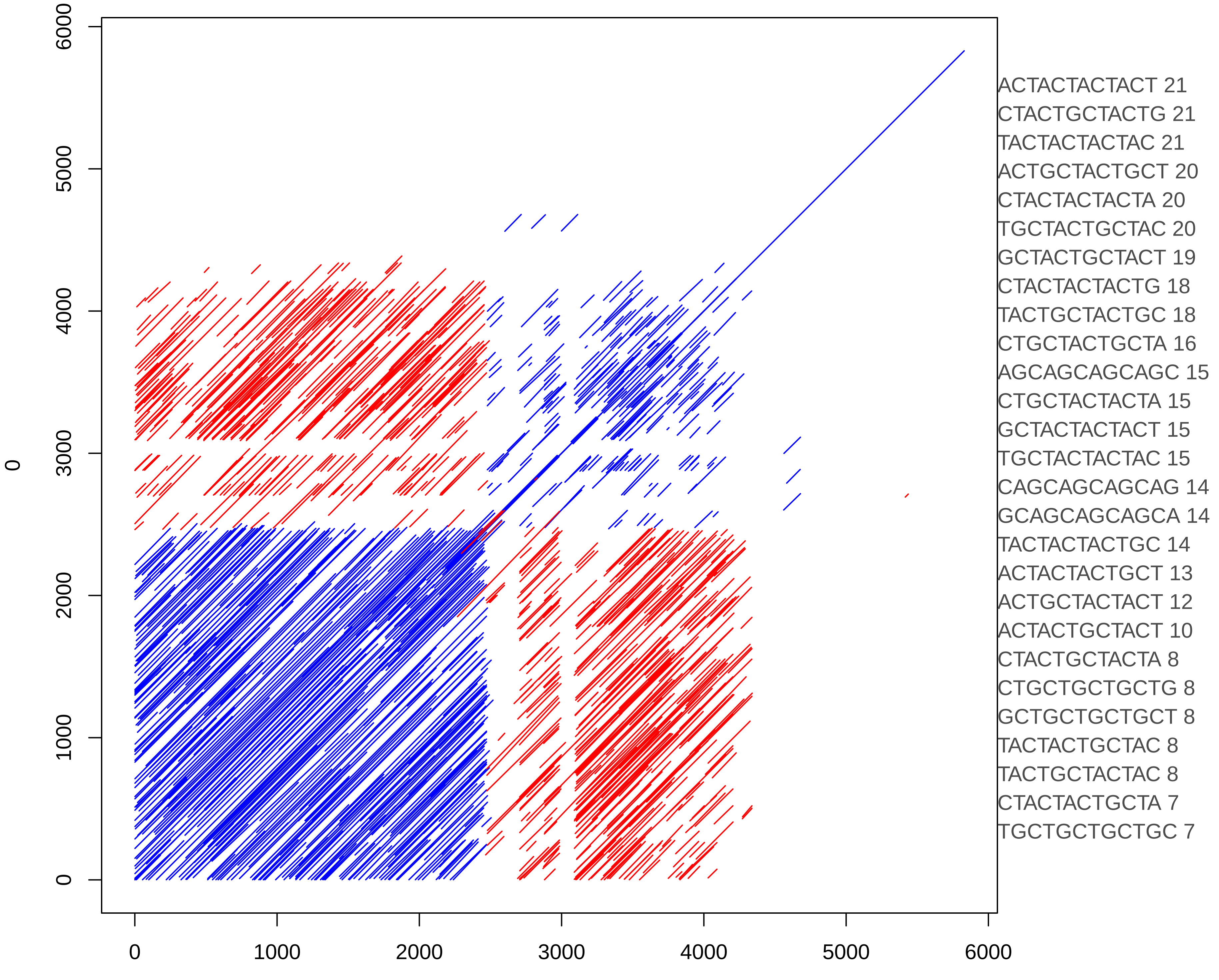

1,121 hits for repetitive sequences
passing 12mer abundance criterion

(Read-Number)
PAGE 665

For Tandem Repeat families of ### bp:
PacBio Read named "HuluTR###-r665"

>m141126_082622_42134_c100732262550000001823141405141501_s1_p0/8967/4255_14097

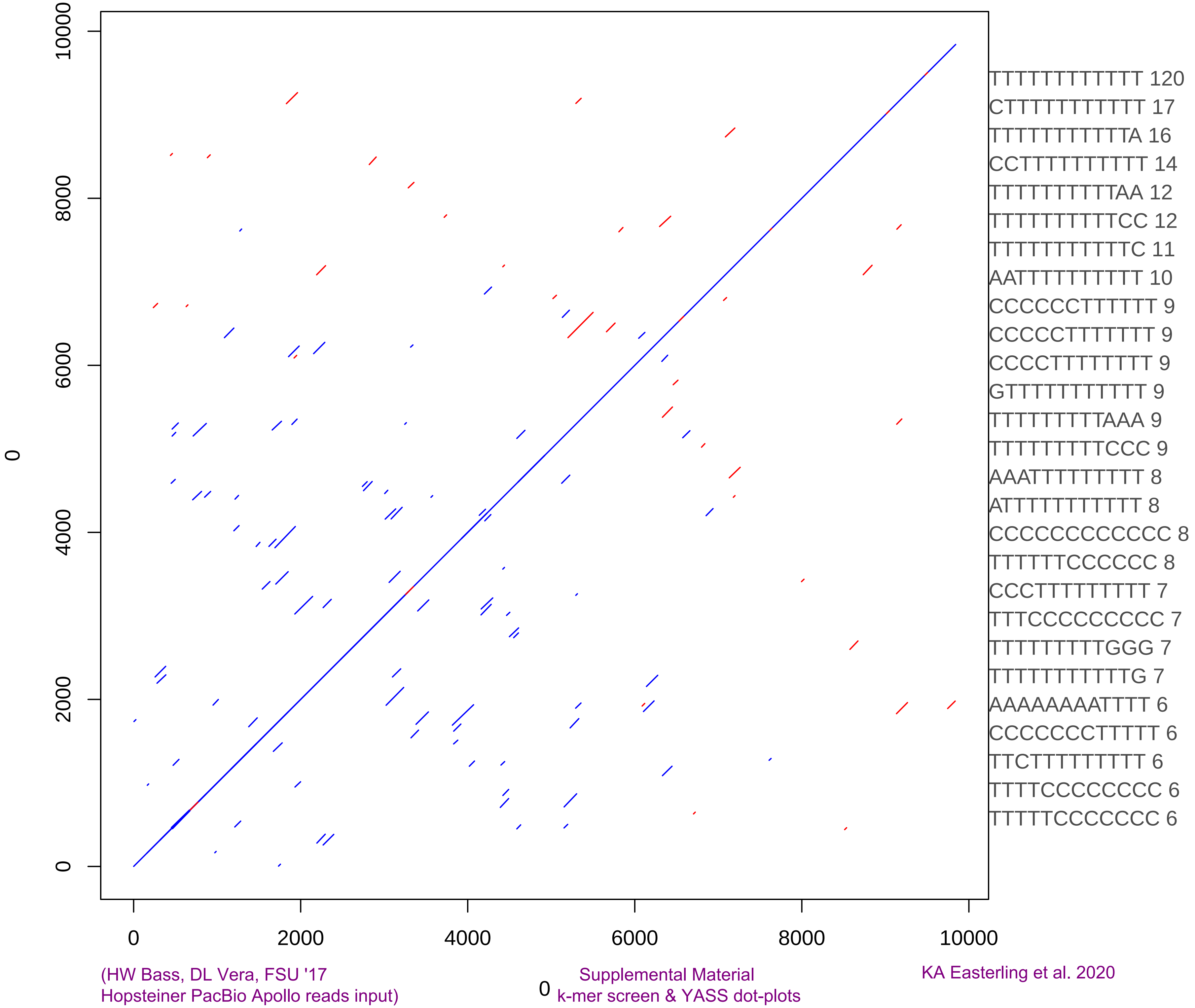

(HW Bass, DL Vera, FSU '17
Hopsteiner PacBio Apollo reads input)

Supplemental Material
k-mer screen & YASS dot-plots

KA Easterling et al. 2020

1,121 hits for repetitive sequences
passing 12mer abundance criterion

(Read-Number)
PAGE 666

For Tandem Repeat families of ### bp:
PacBio Read named "HuluTR###-r666"

>m141126_082622_42134_c100732262550000001823141405141501_s1_p0/9196/3353_12390

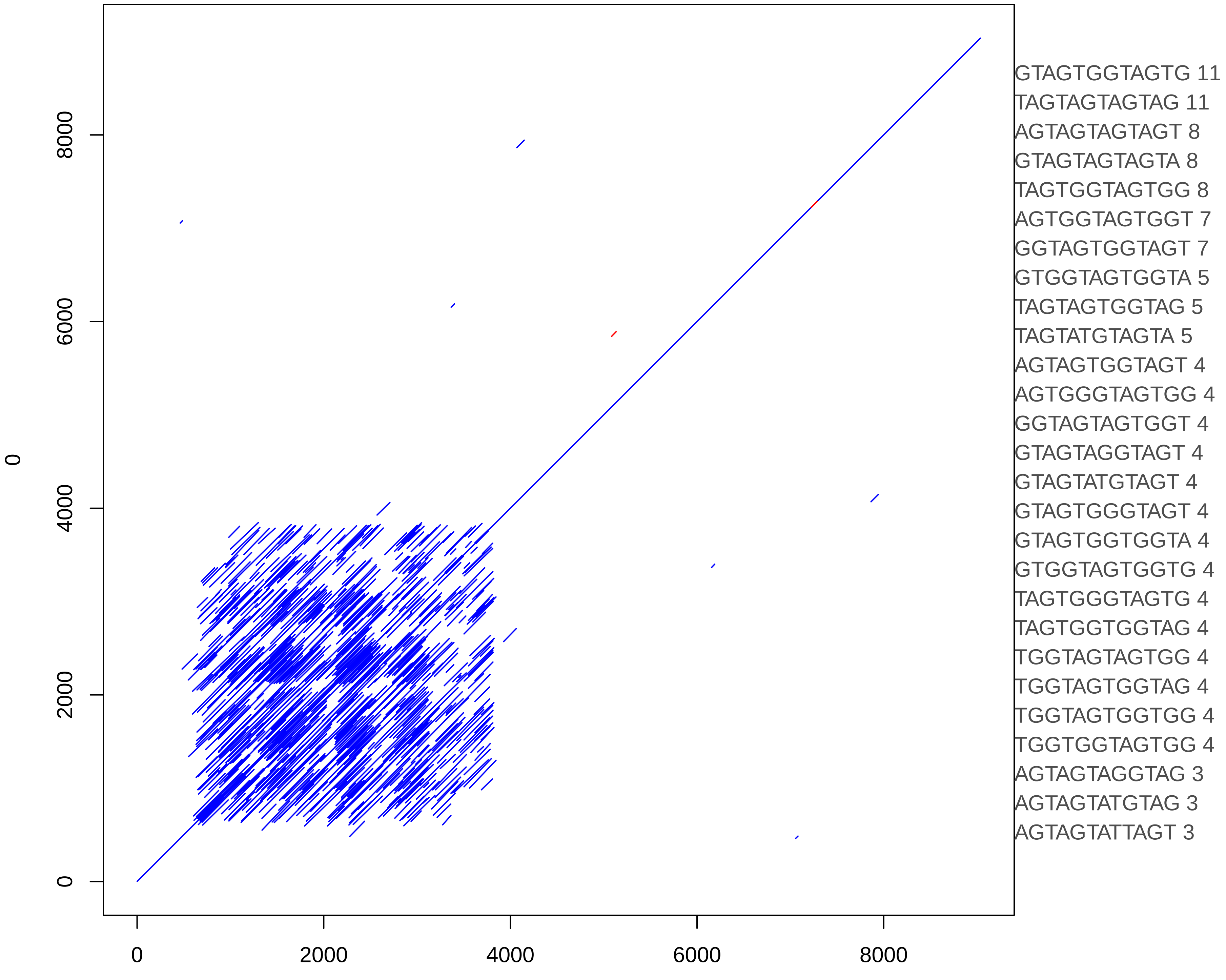

0

(HW Bass, DL Vera, FSU '17
Hopsteiner PacBio Apollo reads input)

Supplemental Material
k-mer screen & YASS dot-plots

KA Easterling et al. 2020

>m141126_082622_42134_c100732262550000001823141405141501_s1_p0/92964/391_8454

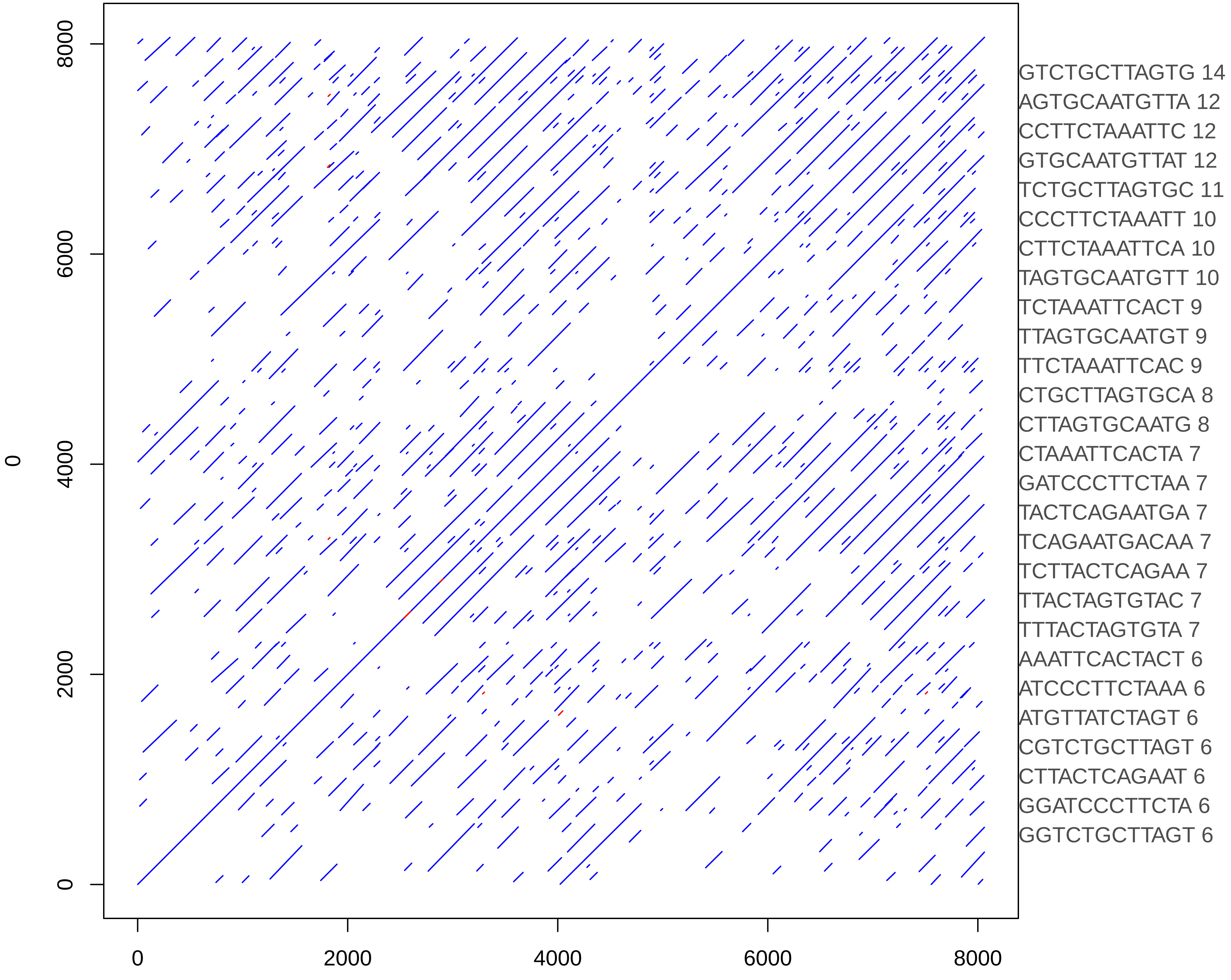

>m141126_082622_42134_c100732262550000001823141405141501_s1_p0/96642/7963_13069

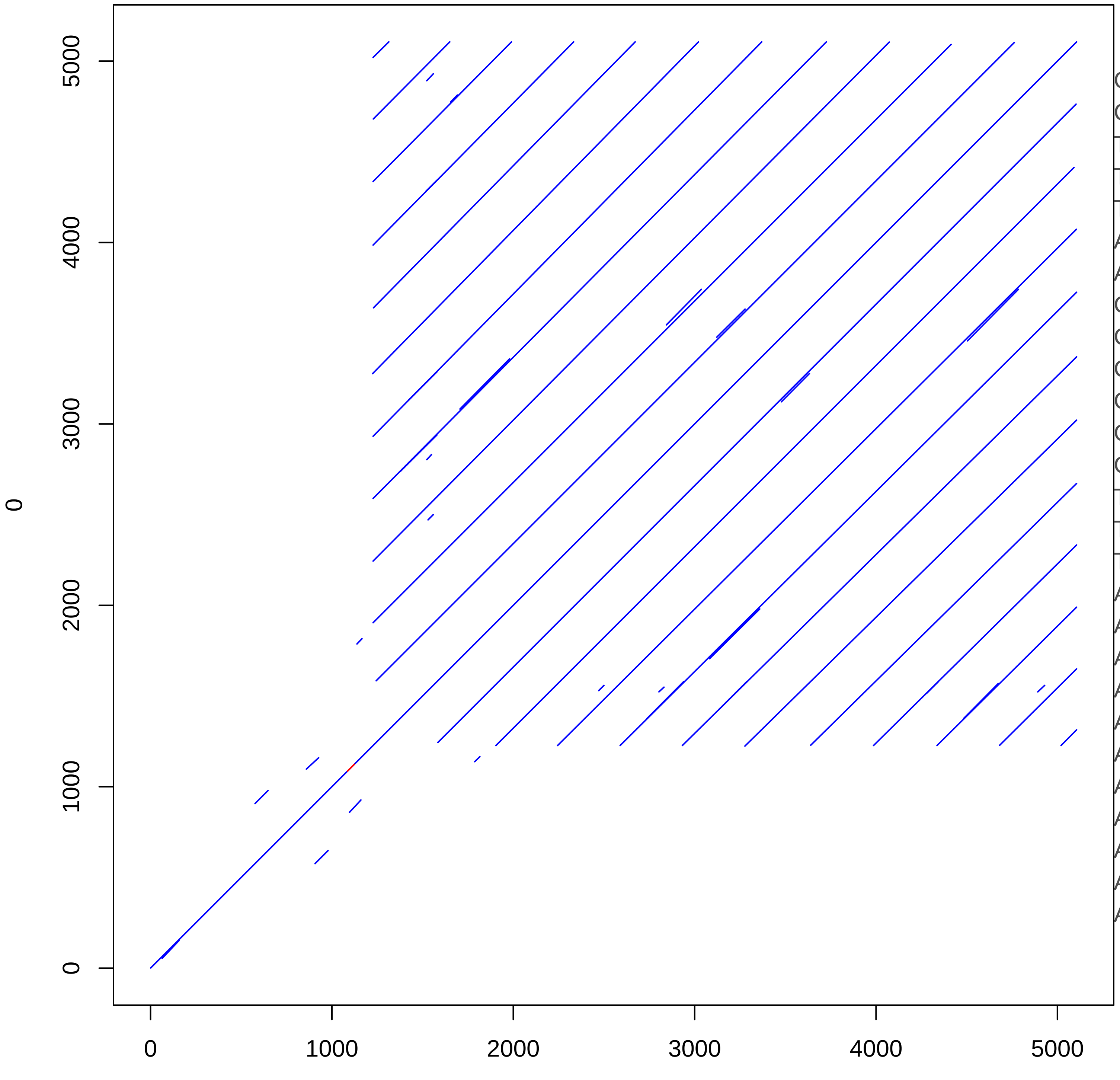

GCTTAACTGCGG 10
CTTAACTGCGGA 9
TCTTATTCGCTG 9
TTAACTGCGGAG 9
TTCTTATTCGCT 9
AAAAAAAAATGA 8
ACTTCTTATTCG 8
CCCCTCAAGGAA 8
CCCTCAAGGAAA 8
CTTCTTATTCGC 8
GGATTTTTAAAG 8
GGTGGATTTTTA 8
GTGGATTTTTAA 8
TAACTGCGGAGT 8
TGGATTTTTAAA 8
TTAAAACAAAAA 8
AAAAATACAAAC 7
AAAATACAAACT 7
AAACGAAATAAA 7
AAATACAAACTT 7
AACCCCTCAAGG 7
AACTGCGGAGTT 7
AAGGGAAAAAAA 7
AATACAAACTTT 7
ACCCCTCAAGGA 7
ACGCTTAACTGC 7
AGGGAAAAAAAA 7

>m141126_104651_42134_c100732262550000001823141405141502_s1_p0/101289/3334_11883

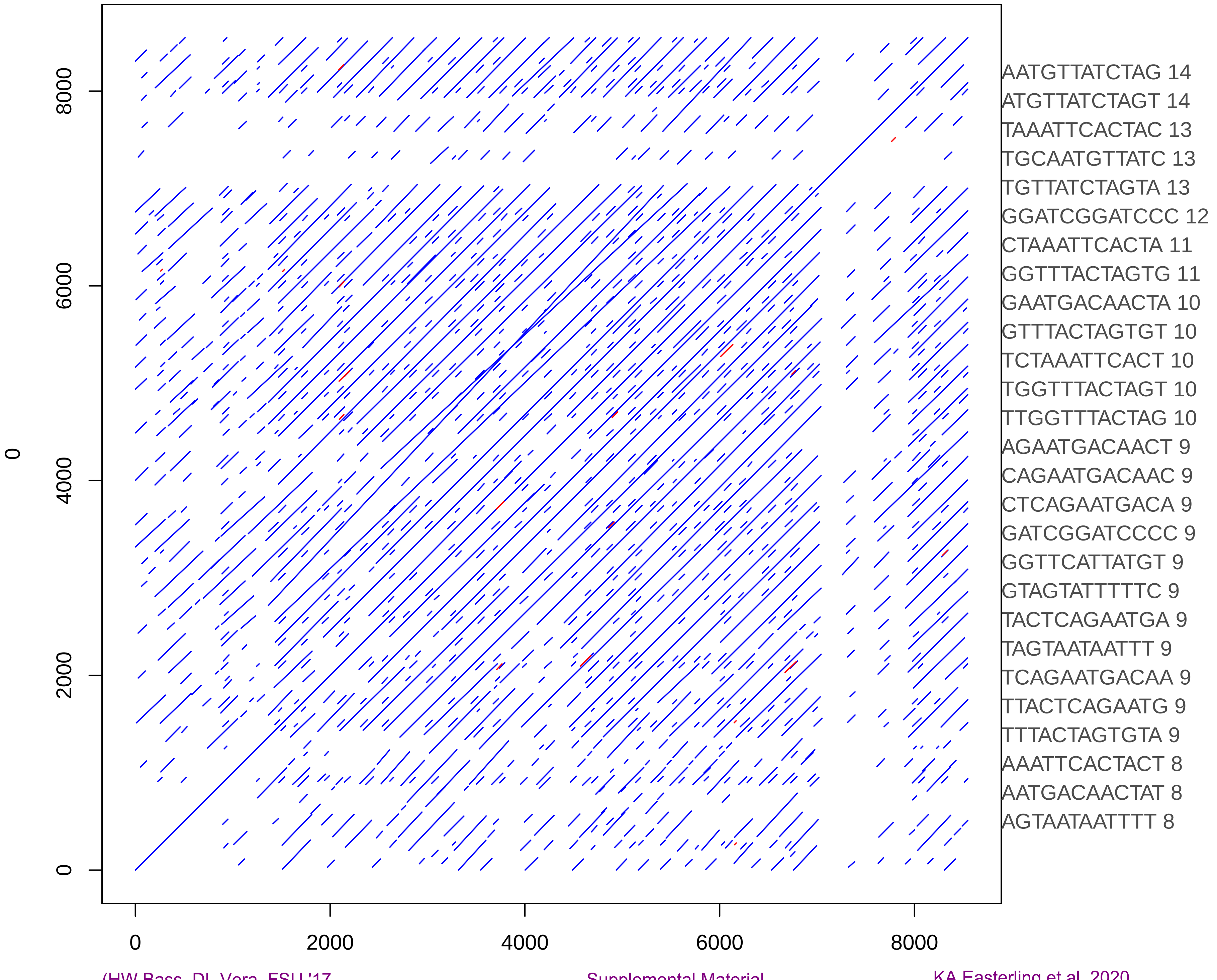

>m141126_104651_42134_c100732262550000001823141405141502_s1_p0/102689/0_5898

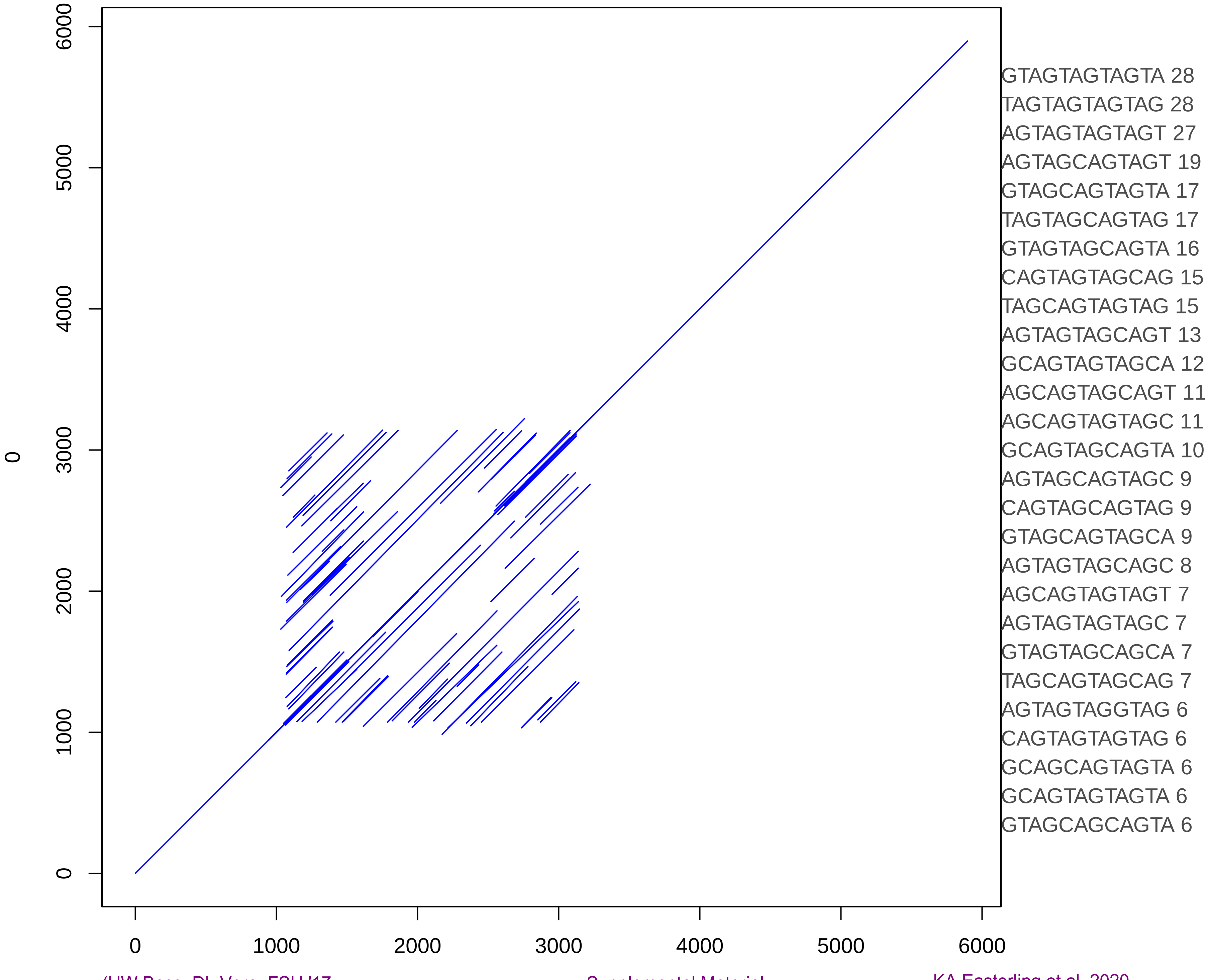

>m141126_104651_42134_c100732262550000001823141405141502_s1_p0/102689/5947_11874

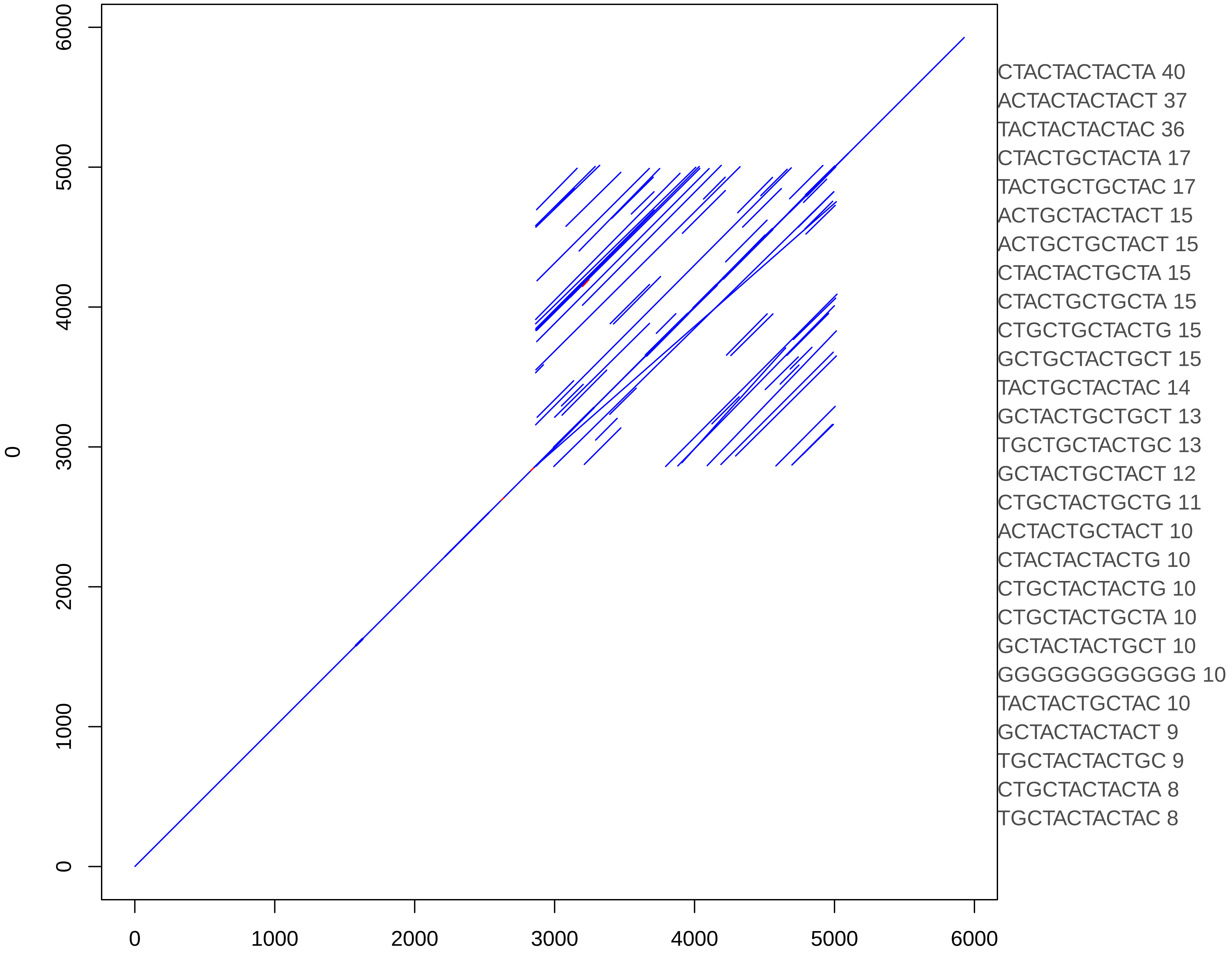

>m141126_104651_42134_c100732262550000001823141405141502_s1_p0/10502/182_7780

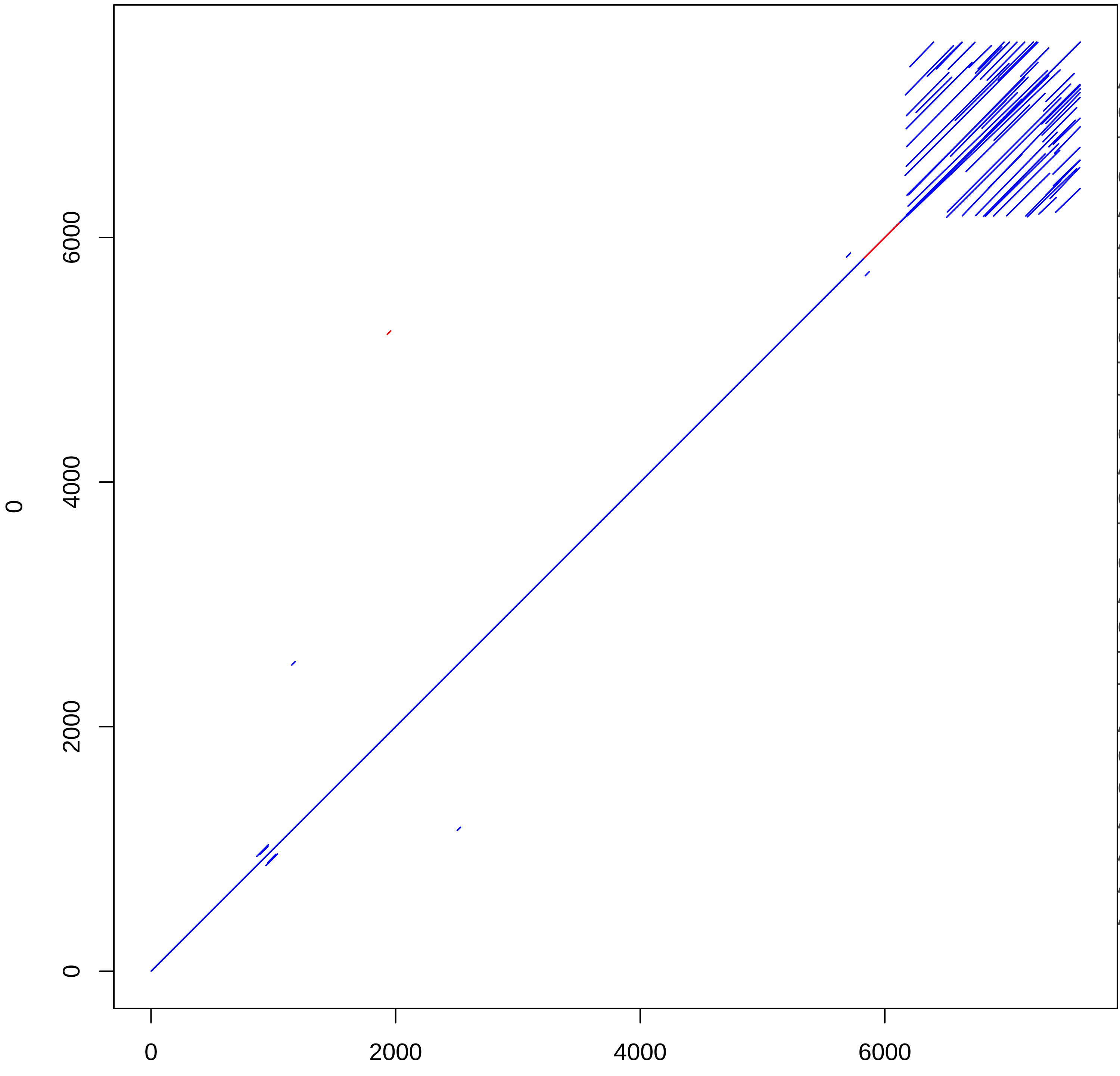

>m141126_104651_42134_c100732262550000001823141405141502_s1_p0/106739/2296_9508

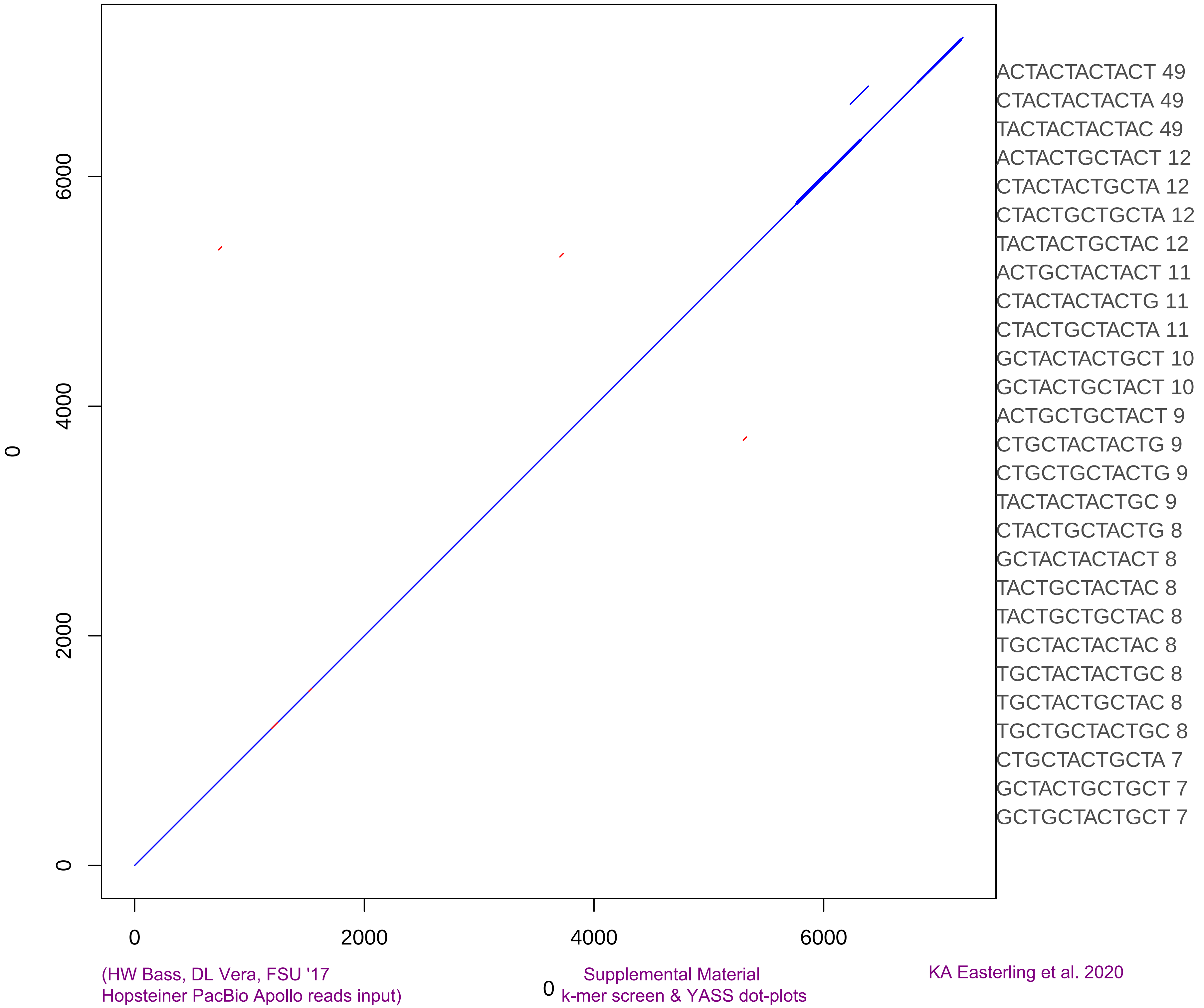

>m141126_104651_42134_c100732262550000001823141405141502_s1_p0/106739/9553_16506

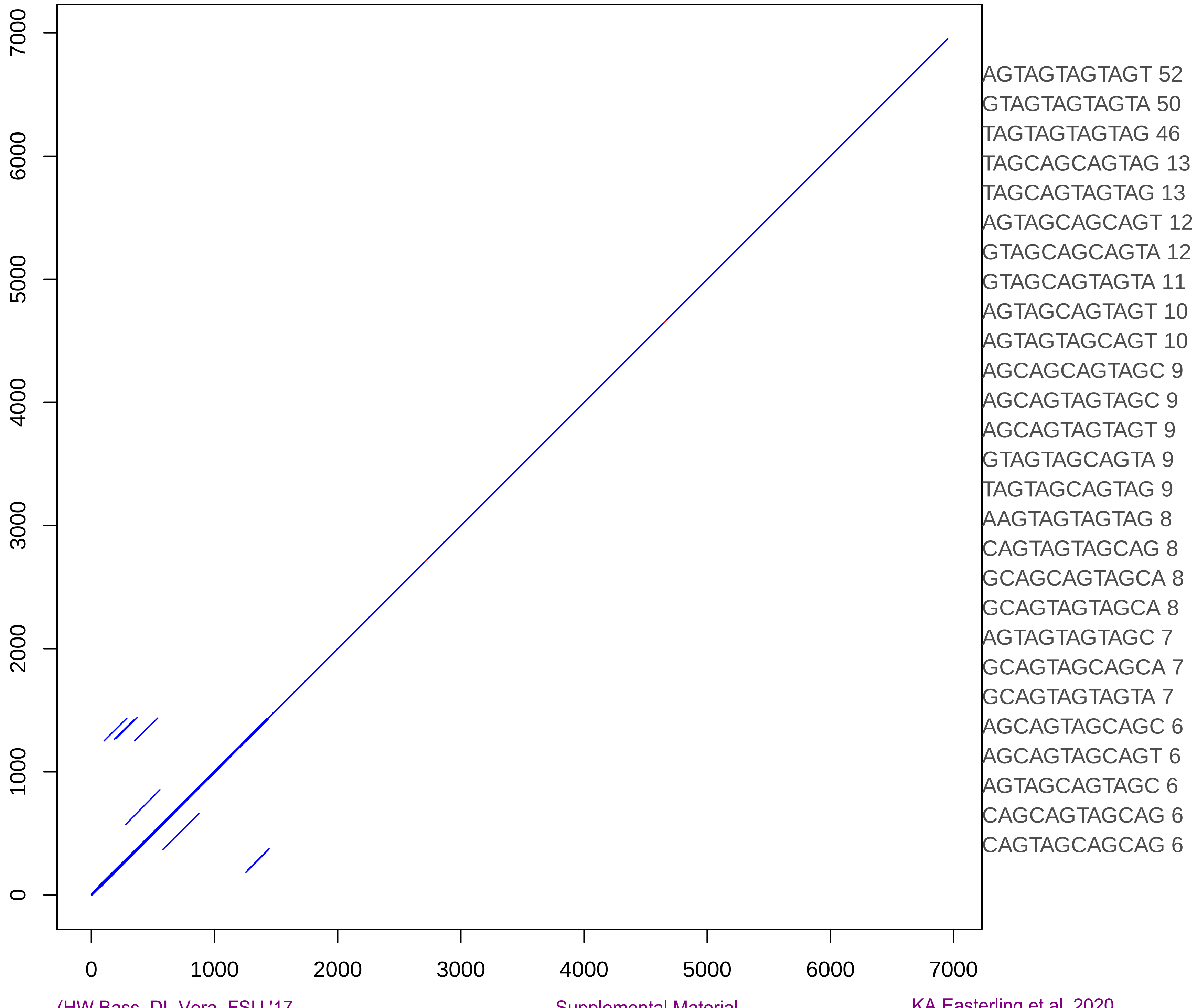

>m141126_104651_42134_c100732262550000001823141405141502_s1_p0/106765/6901_17256

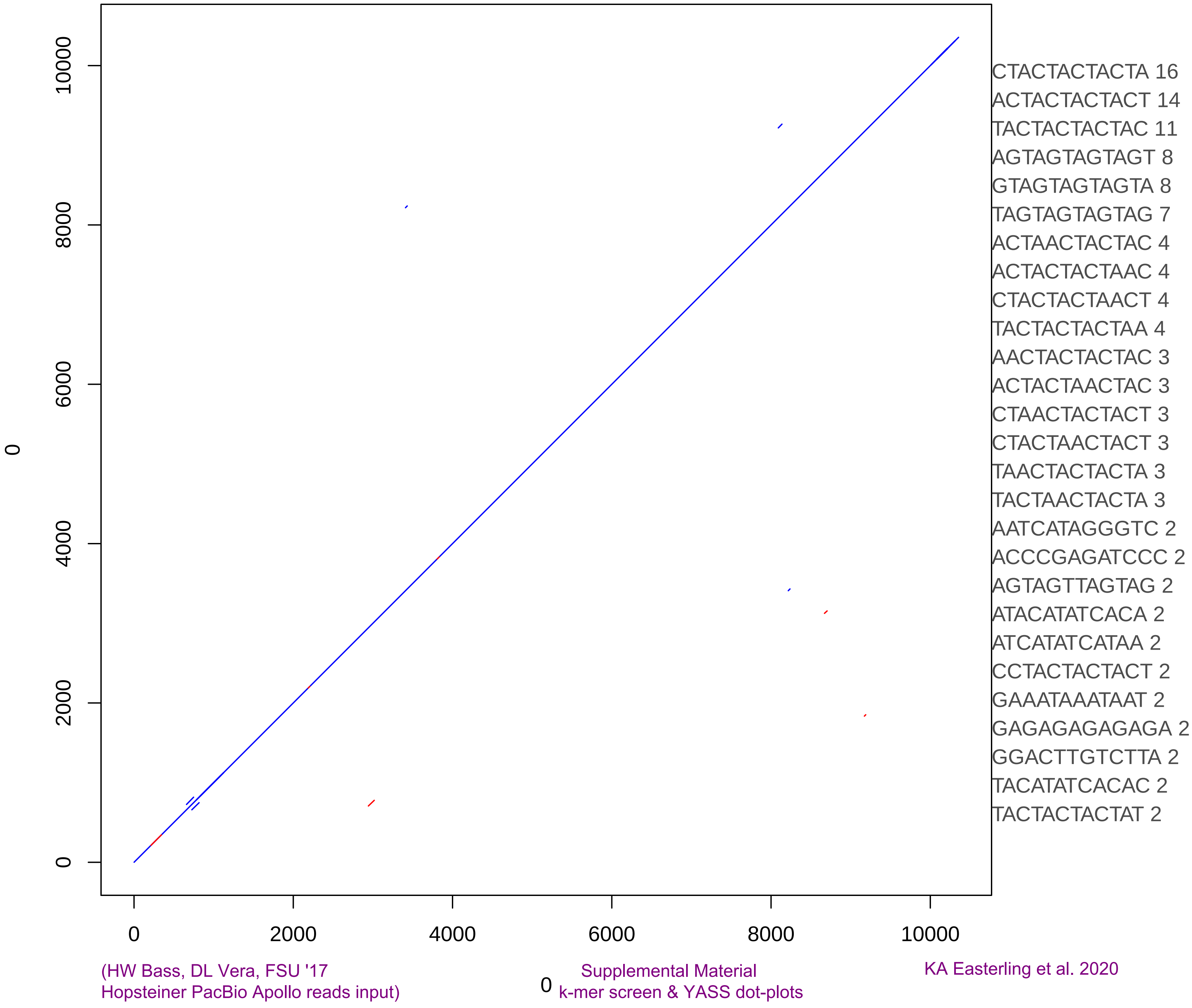

>m141126_104651_42134_c100732262550000001823141405141502_s1_p0/109278/0_7974

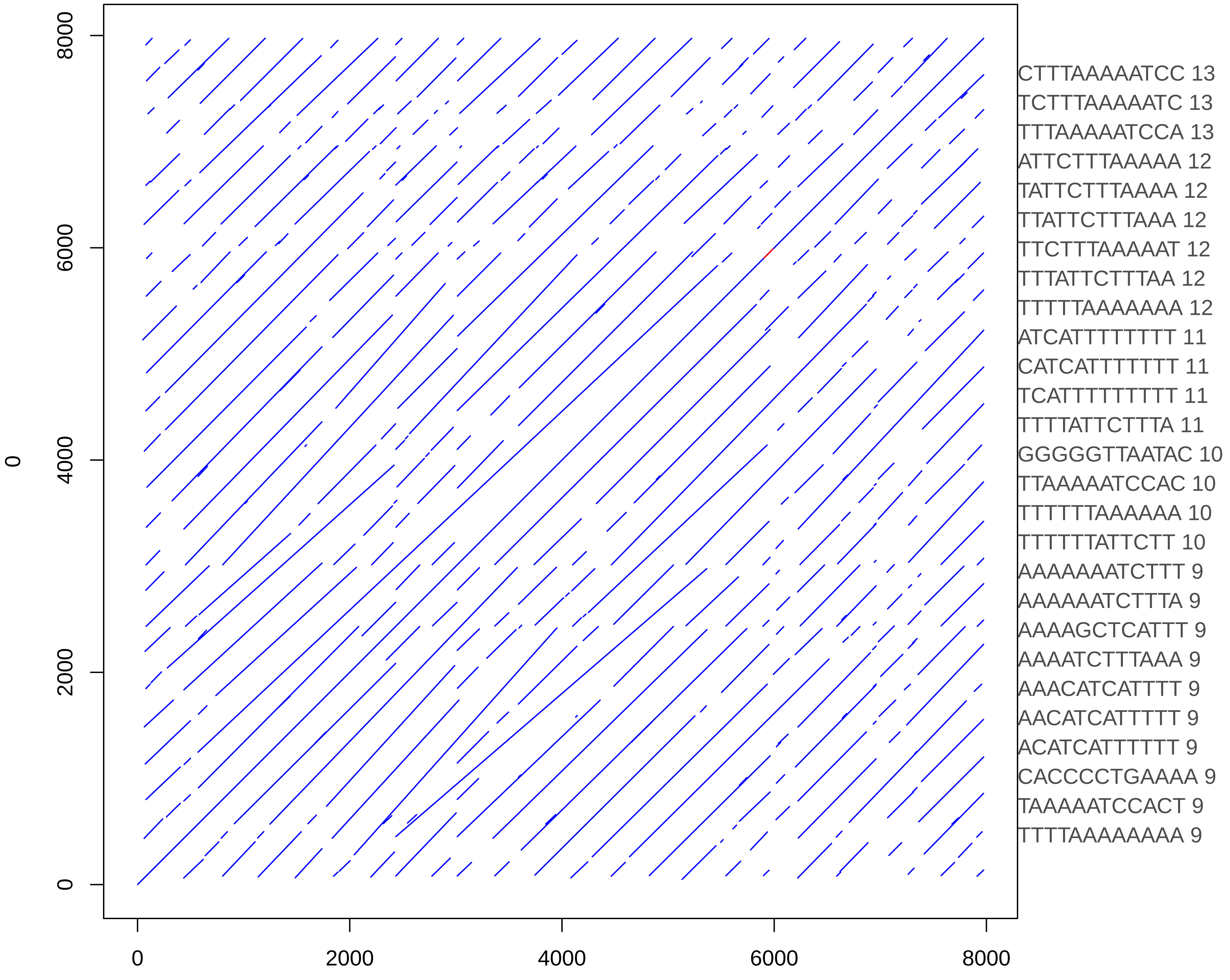

>m141126_104651_42134_c100732262550000001823141405141502_s1_p0/109960/0_7016

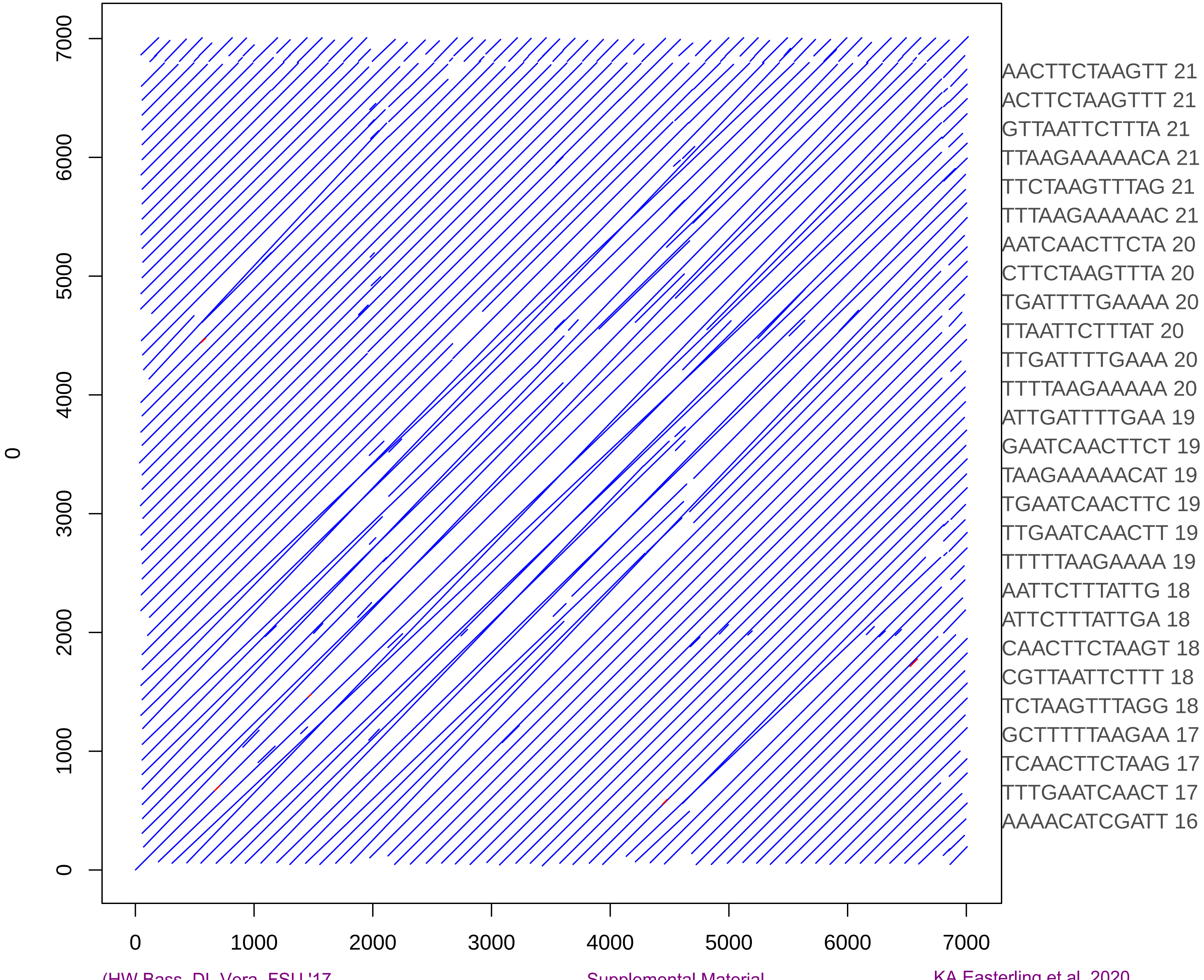

>m141126_104651_42134_c100732262550000001823141405141502_s1_p0/11065/6074_11424

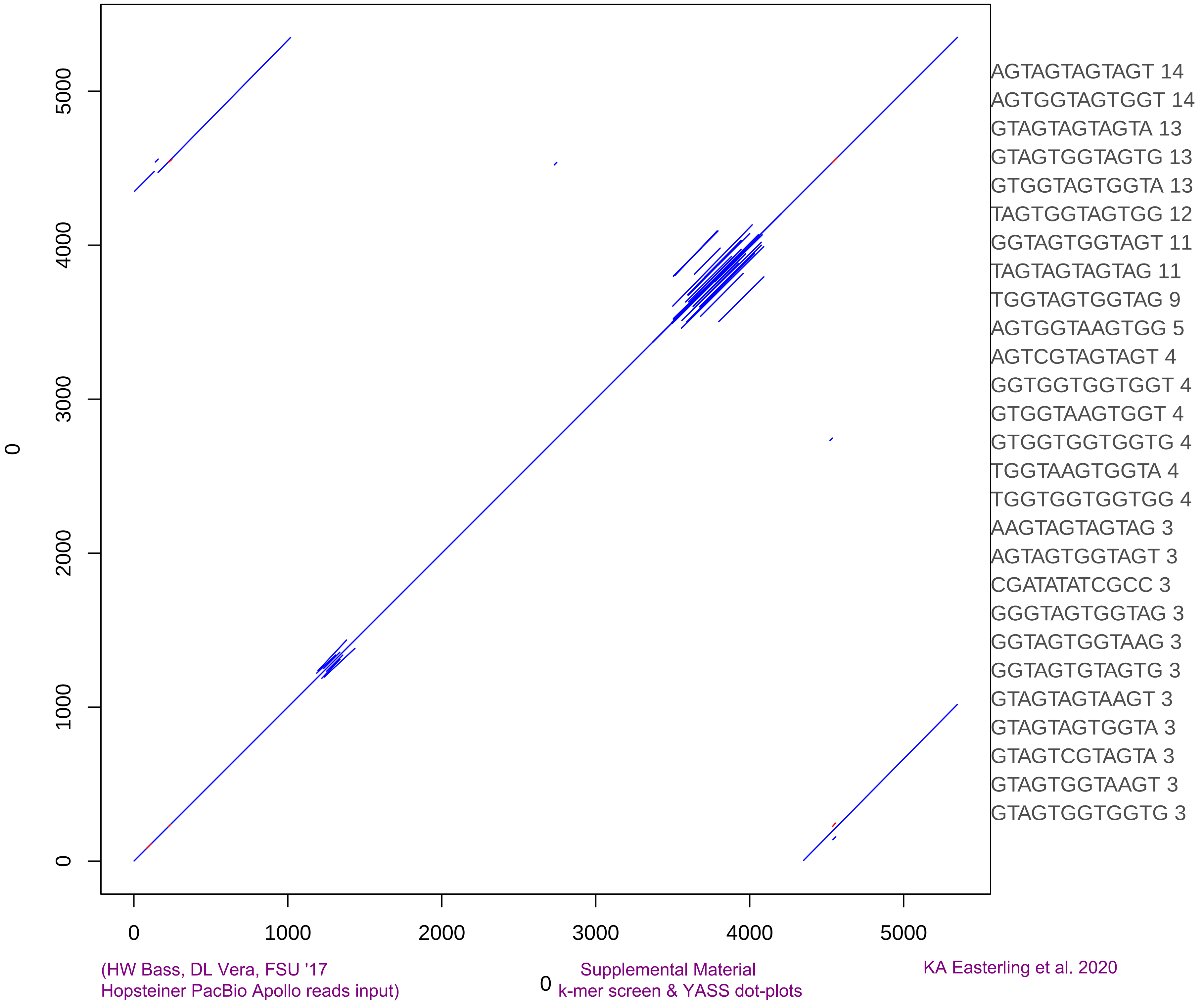

>m141126_104651_42134_c100732262550000001823141405141502_s1_p0/113130/0_7008

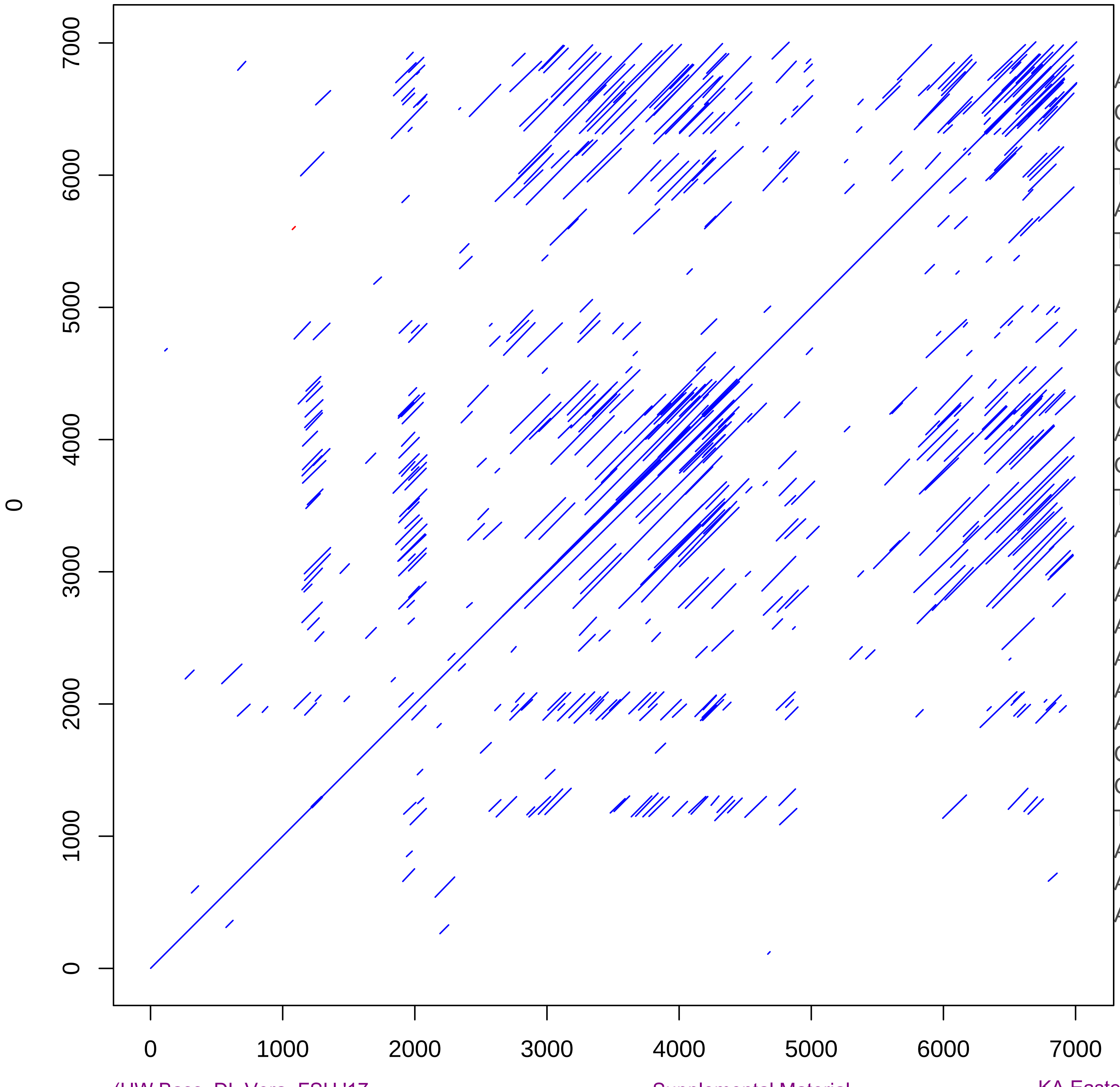

AGTAGTAGTAGT 23
GTAGTAGTAGTA 23
GTAGTAGAAGTA 22
TAGTAGTAGTAG 22
AGTAGTAGAAGT 21
TAGTAGAAGTAG 19
TAGTAGTAGAAG 15
AGTAGAAGTAGT 12
AGTAGTAGTAGA 12
GAAGTAGTAGTA 12
GTAGTAGTAGAA 12
AAGTAGTAGTAG 11
GTAGAAGTAGTA 11
TAGAAGTAGTAG 11
AAGTAGTAGAAG 10
AGAAGTAGTAGT 10
AGAAGTAGTAGA 9
AGTAGAAGTAGA 9
ATAGTAGTAGTA 8
AGTAAGTAGTAG 7
AGTAGTAGTACG 7
GAAGTAGTAGAA 7
GTAGAAGTAGAA 7
TAGTAGTAGTAA 7
AAGAAGTAGTAG 6
AGTAGTAGTAAA 6
AGAGTAGTAGTA 5

>m141126_104651_42134_c100732262550000001823141405141502_s1_p0/117618/0_11698

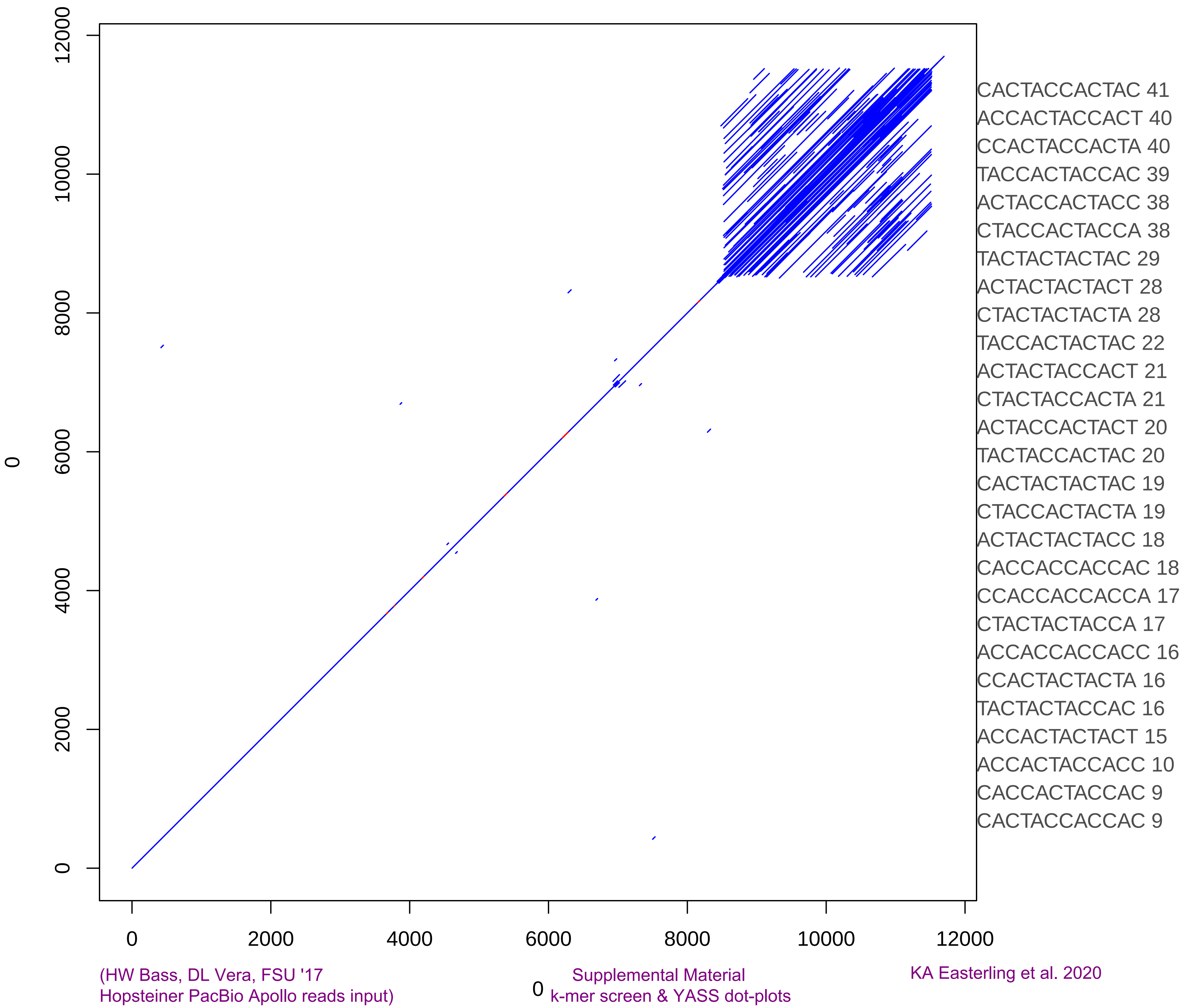

>m141126_104651_42134_c100732262550000001823141405141502_s1_p0/118157/6634_13817

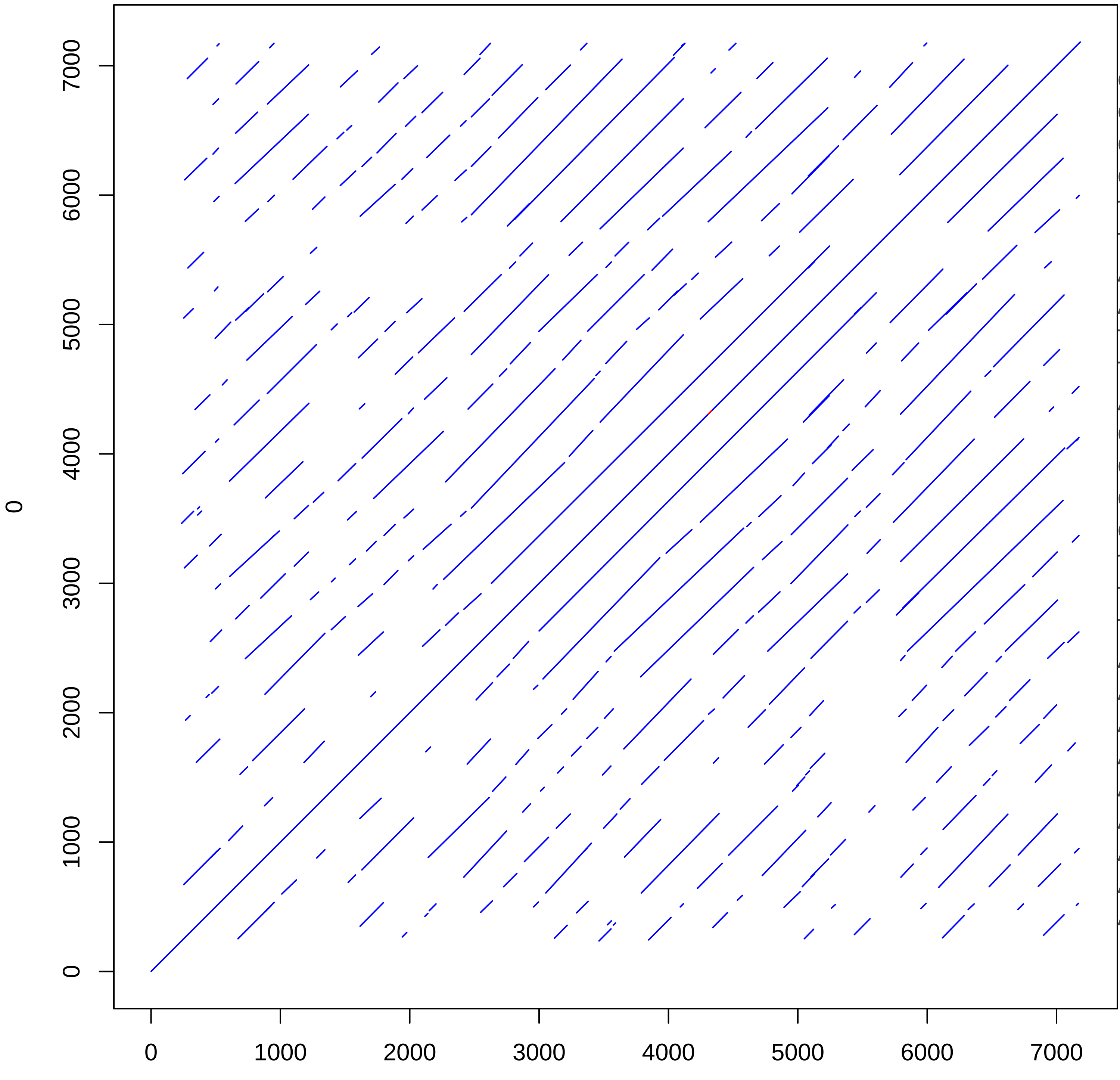

>m141126_104651_42134_c100732262550000001823141405141502_s1_p0/119956/1779_13137

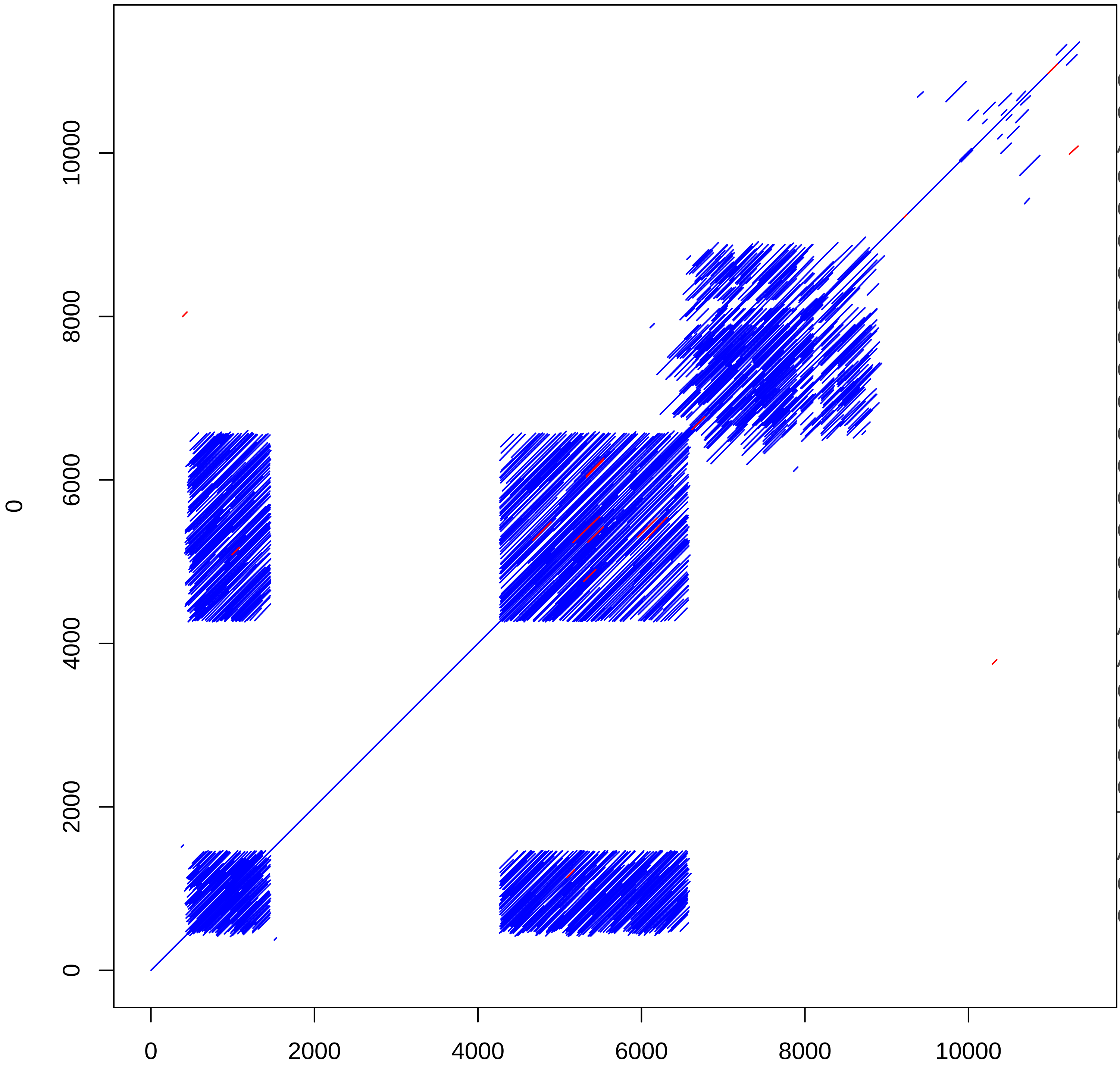

>m141126_104651_42134_c100732262550000001823141405141502_s1_p0/12010/0_7943

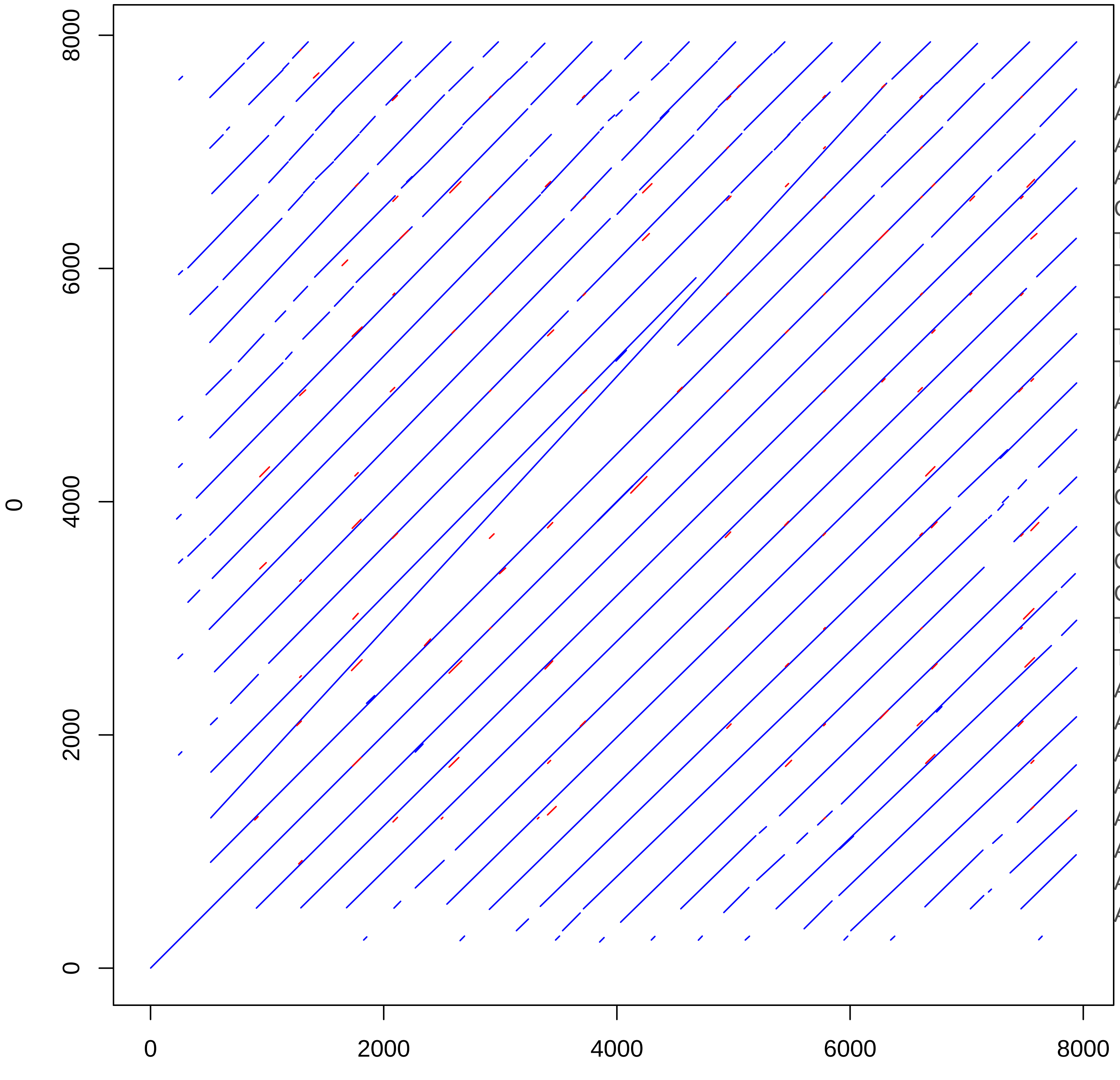

AAAAAAATTCCC 9
AAAAAATTCCCC 9
AACATTGCTCGA 9
ATGGAAGACTTA 9
GGGGGGGGGGGG 9
TGGAAGACTTAG 9
TTCCCCAAGAGA 9
TTTCCCCAAGAG 9
TTTTCCCCAAGA 9
TTTTTCCCCAAG 9
AAAAAAAATTCC 8
AAAAATTCCCCT 8
ATTTTTCCCCAA 8
CCAAGAGATCTC 8
CGCATATTCCTT 8
GCATATTCCTTA 8
GGGTACCCCTTT 8
TCCCCAAGAGAT 8
TTTGAAAAAAAA 8
AAAACATTGCTC 7
AAAATTCCCCTT 7
AAACATTGCTCG 7
AAATGTATTACC 7
AAATTCCCCTTT 7
AAGATTTCTGAA 7
AATTCCCCTTTT 7
ACATTGCTCGAA 7

>m141126_104651_42134_c100732262550000001823141405141502_s1_p0/121189/0_9622

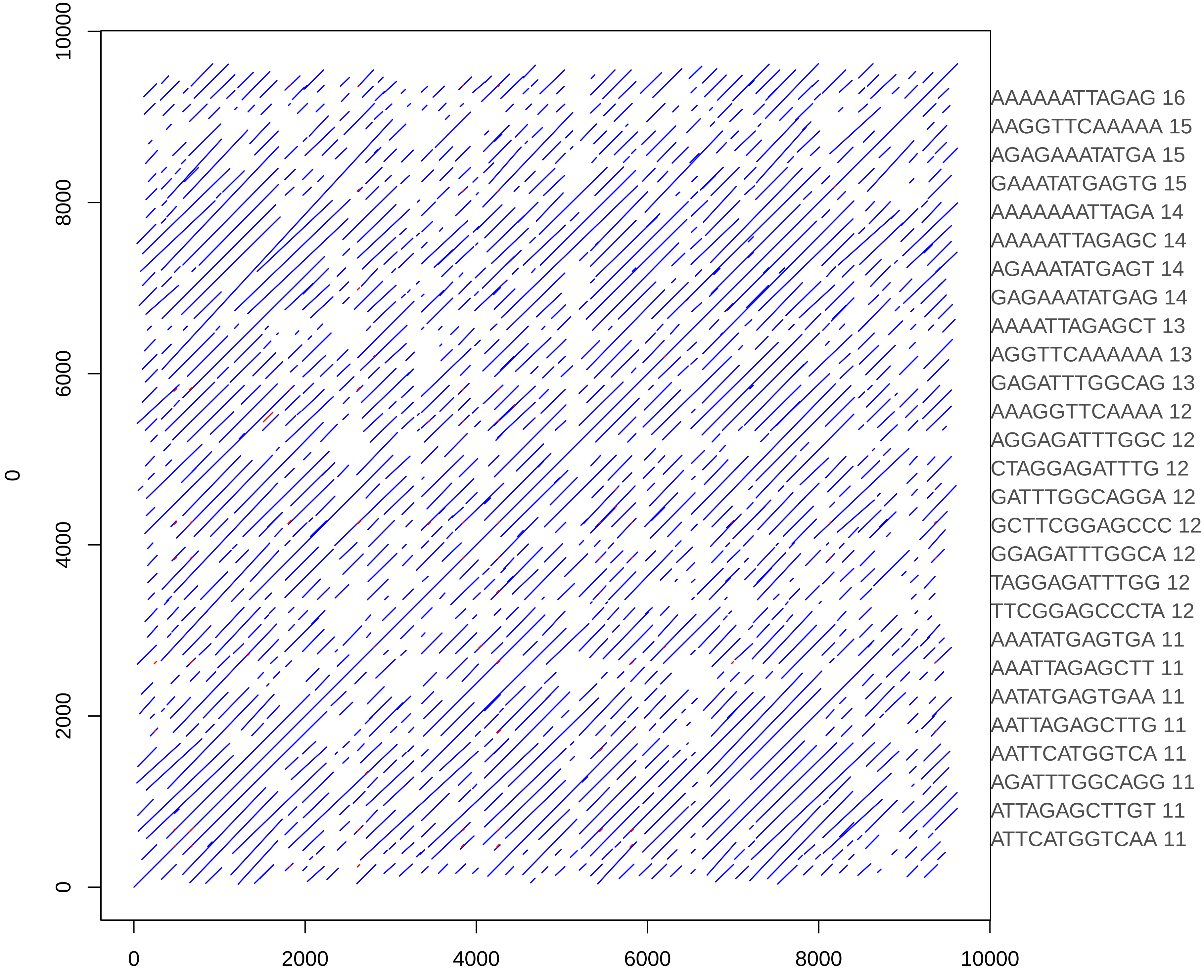

>m141126_104651_42134_c100732262550000001823141405141502_s1_p0/128472/0_5743

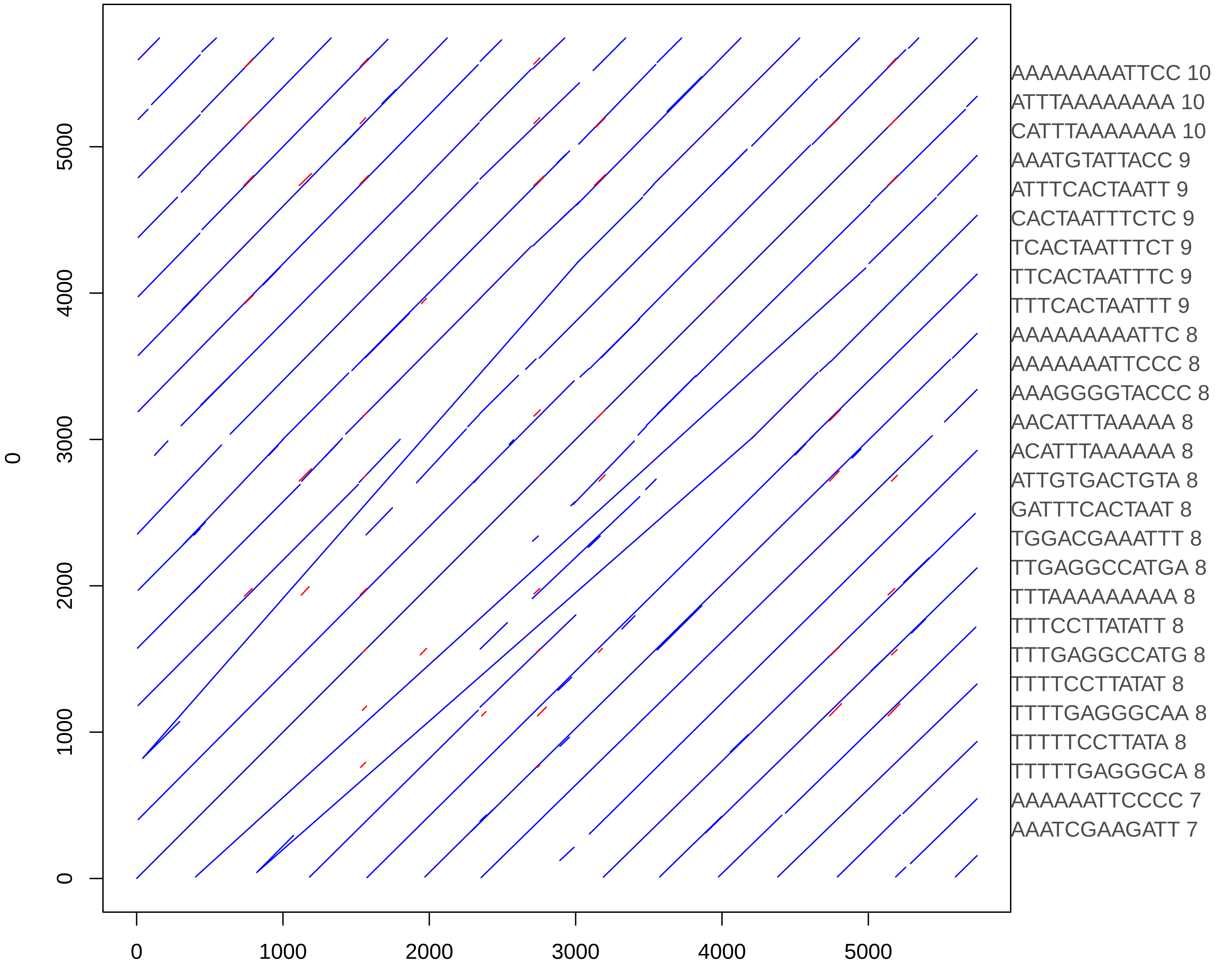

0

>m141126_104651_42134_c100732262550000001823141405141502_s1_p0/131362/5239_17473

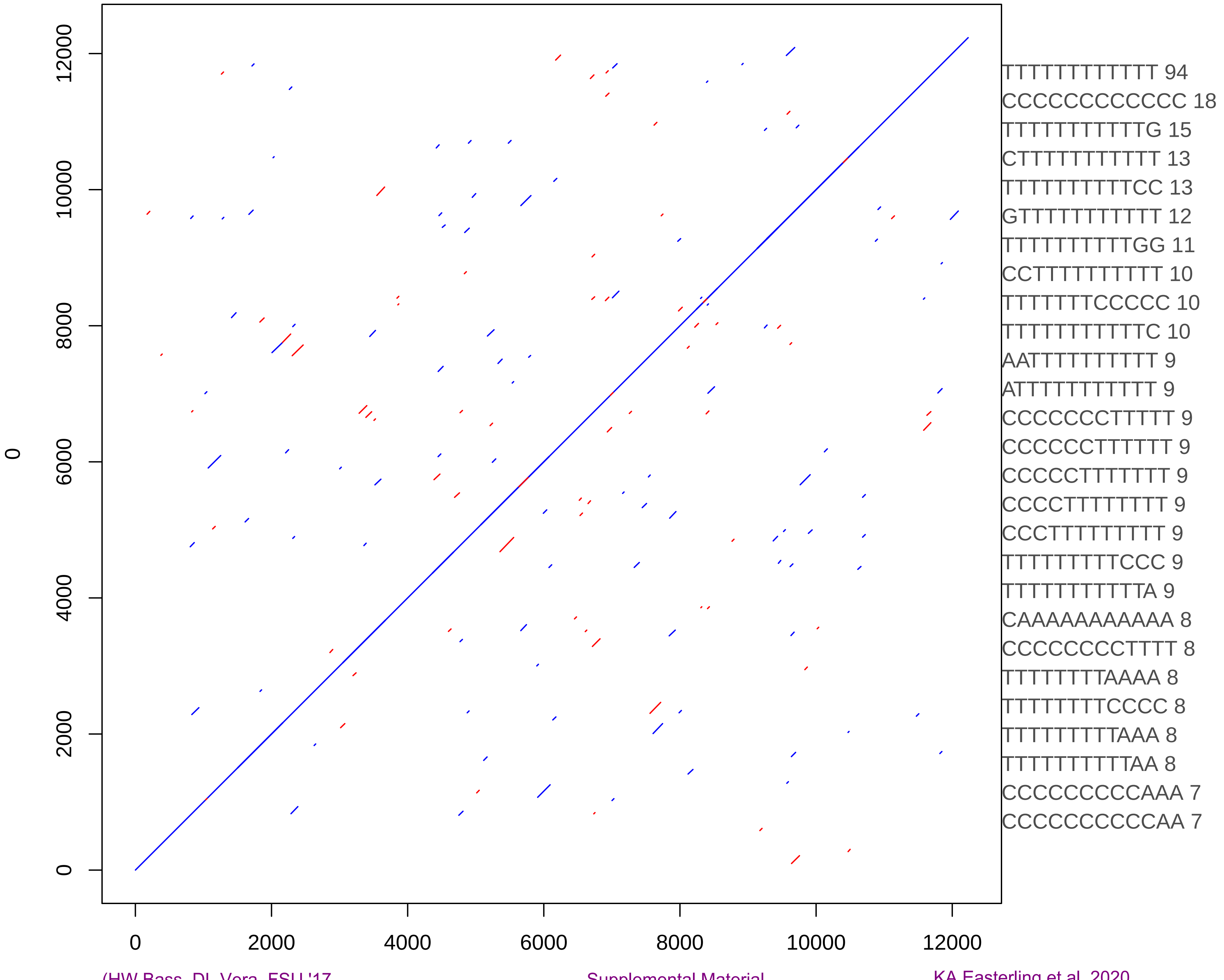

1,121 hits for repetitive sequences
passing 12mer abundance criterion

(Read-Number)
PAGE 687

For Tandem Repeat families of ### bp:
PacBio Read named "HuluTR###-r687"

>m141126_104651_42134_c100732262550000001823141405141502_s1_p0/136049/7637_13779

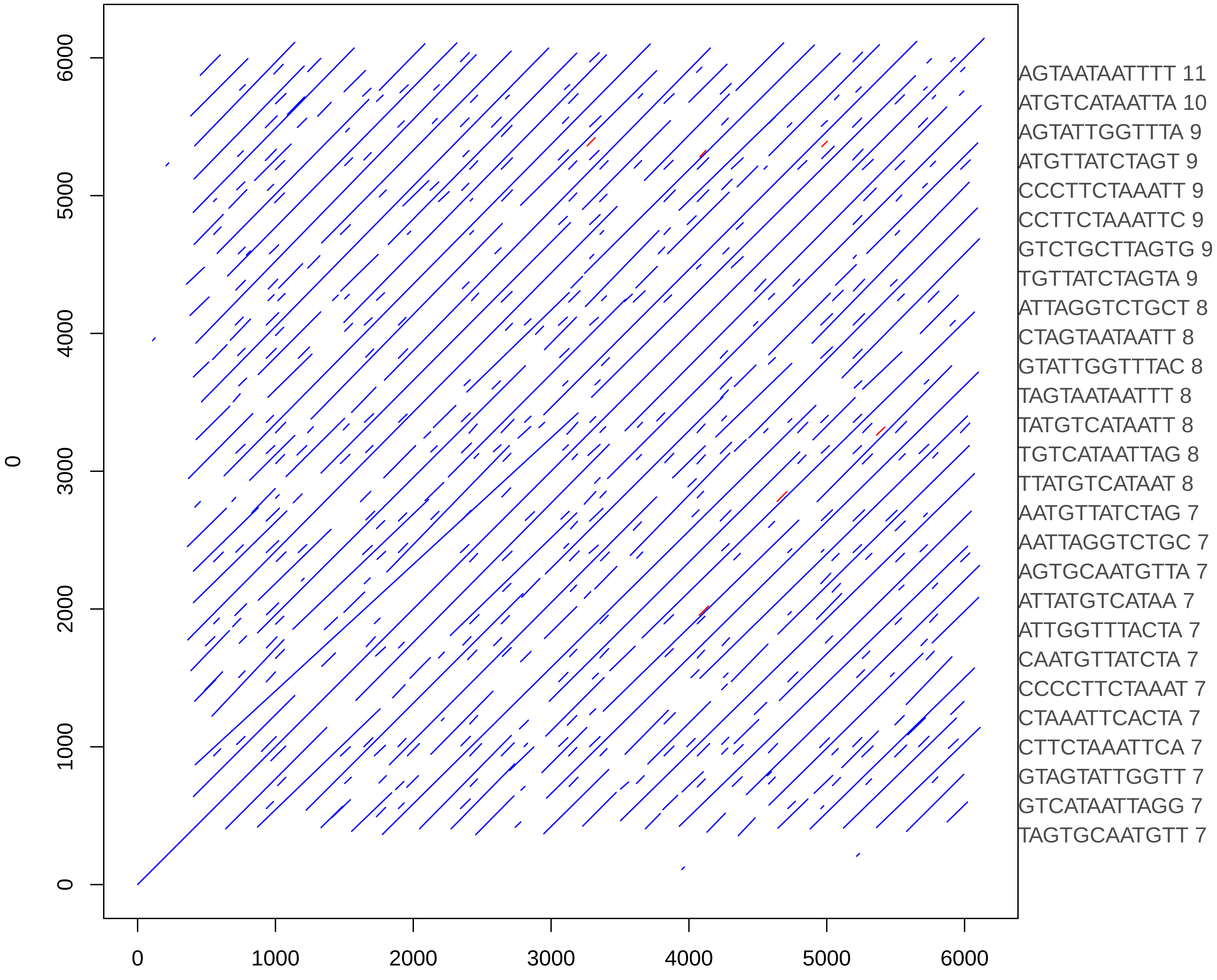

(HW Bass, DL Vera, FSU '17
Hopsteiner PacBio Apollo reads input)

0

Supplemental Material
k-mer screen & YASS dot-plots

KA Easterling et al. 2020

>m141126_104651_42134_c100732262550000001823141405141502_s1_p0/136730/17506_23532

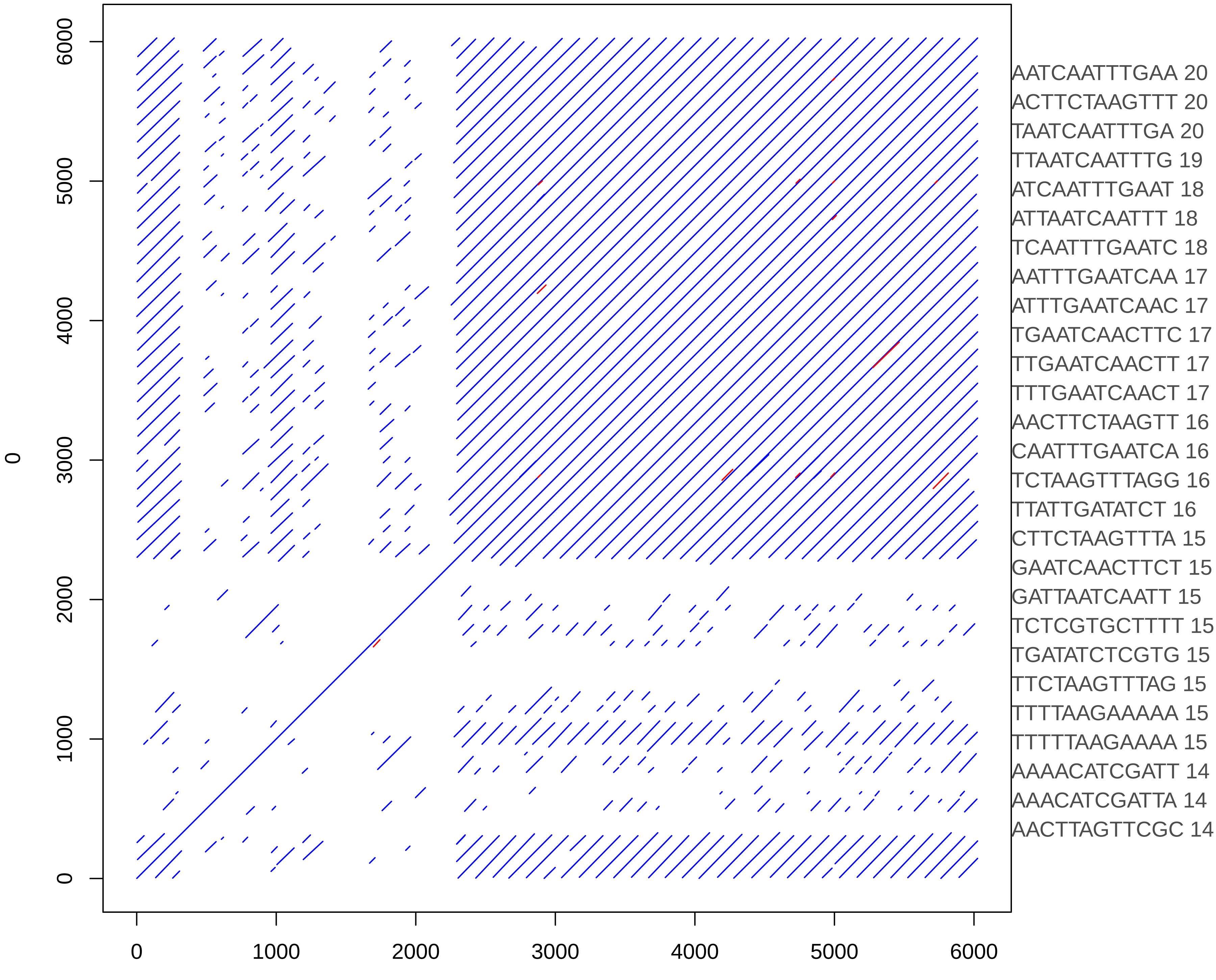

>m141126_104651_42134_c100732262550000001823141405141502_s1_p0/137445/1414_9508

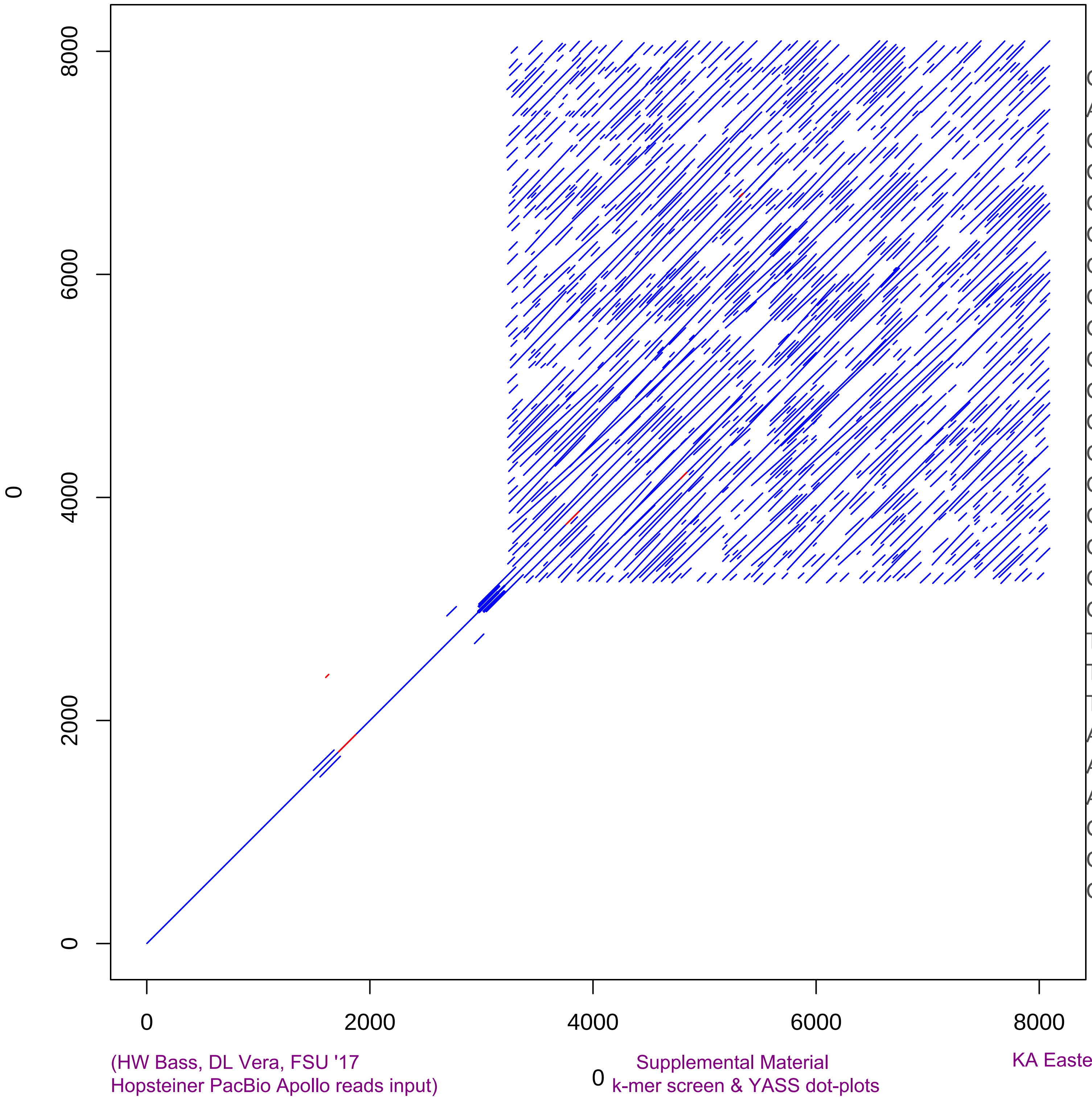

CGGACCATTTAG 12
ACCATTTGGTGT 11
CATTTGGTGTCC 11
CCCGGACCATTT 11
CCGCCCGGACCA 11
CGCCCGGACCAT 11
GCCCGGACCATT 11
GGACCATTTAGG 11
CCGGACCATTTA 10
GGATGGGTTTGT 10
CCATTTGGTGTC 9
GACCATTTAGGT 9
GCGTCCCATTTG 9
CGGGGATGGGTT 8
CGTCCCATTTGG 8
GGATGGGACTAG 8
GGGGATGGGTTT 8
GGGTTTGTACCC 8
TGTCCGGGATGG 8
TTAGGTCGGGGA 8
TTTGGTGTCCGG 8
ACCATTTAGGTC 7
ATGGGACTAGGC 7
ATTTGGTGTCCG 7
CATTTAGGTCGG 7
GATGGGACTAGG 7
GGGATGGGTTTG 7

1,121 hits for repetitive sequences passing 12mer abundance criterion

(Read-Number)
PAGE 690

For Tandem Repeat families of ### bp:
PacBio Read named "HuluTR###-r690"

>m141126_104651_42134_c100732262550000001823141405141502_s1_p0/138210/3434_13023

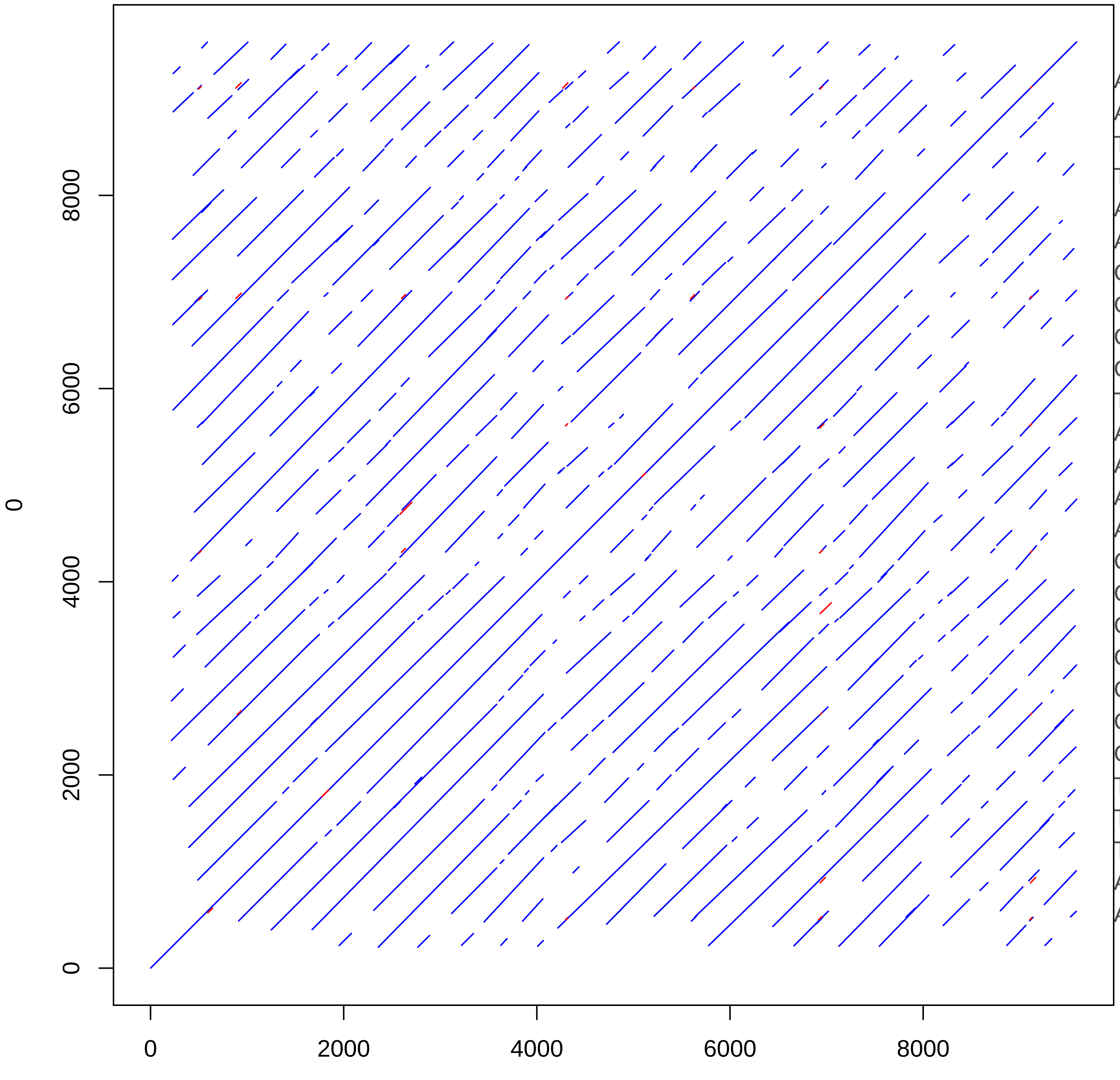

ATCTCATGGGTA 9
ATTTCACTAATT 9
TTCACTAATTTC 9
TTTCACTAATTT 9
AACATTTAAAAA 8
AGATTTCACTAA 8
CCCTTTTTGAGG 8
GAGATTTCACTA 8
GATTTCACTAAT 8
GTCGAAACGGAG 8
TCTCATGGGTAA 8
AAAAATAATTCC 7
AAAATAATTCCC 7
AGATCTCATGGG 7
ATTGGAGTCGAA 7
CAACTTTTGTGC 7
CCCTTTGGTGAA 7
CCTTTGGTGAAT 7
CCTTTTTGAGGG 7
GAATTGGAGATT 7
GATCTCATGGGT 7
GTGAATTGGAGA 7
TCACTAATTTCA 7
TCGAAACGGAGC 7
TGAATTGGAGAT 7
AATTGGAGATTT 6
ACATTTAAAAAA 6

(HW Bass, DL Vera, FSU '17
Hopsteiner PacBio Apollo reads input)

0

Supplemental Material
k-mer screen & YASS dot-plots

KA Easterling et al. 2020

>m141126_104651_42134_c100732262550000001823141405141502_s1_p0/143360/1823_10918

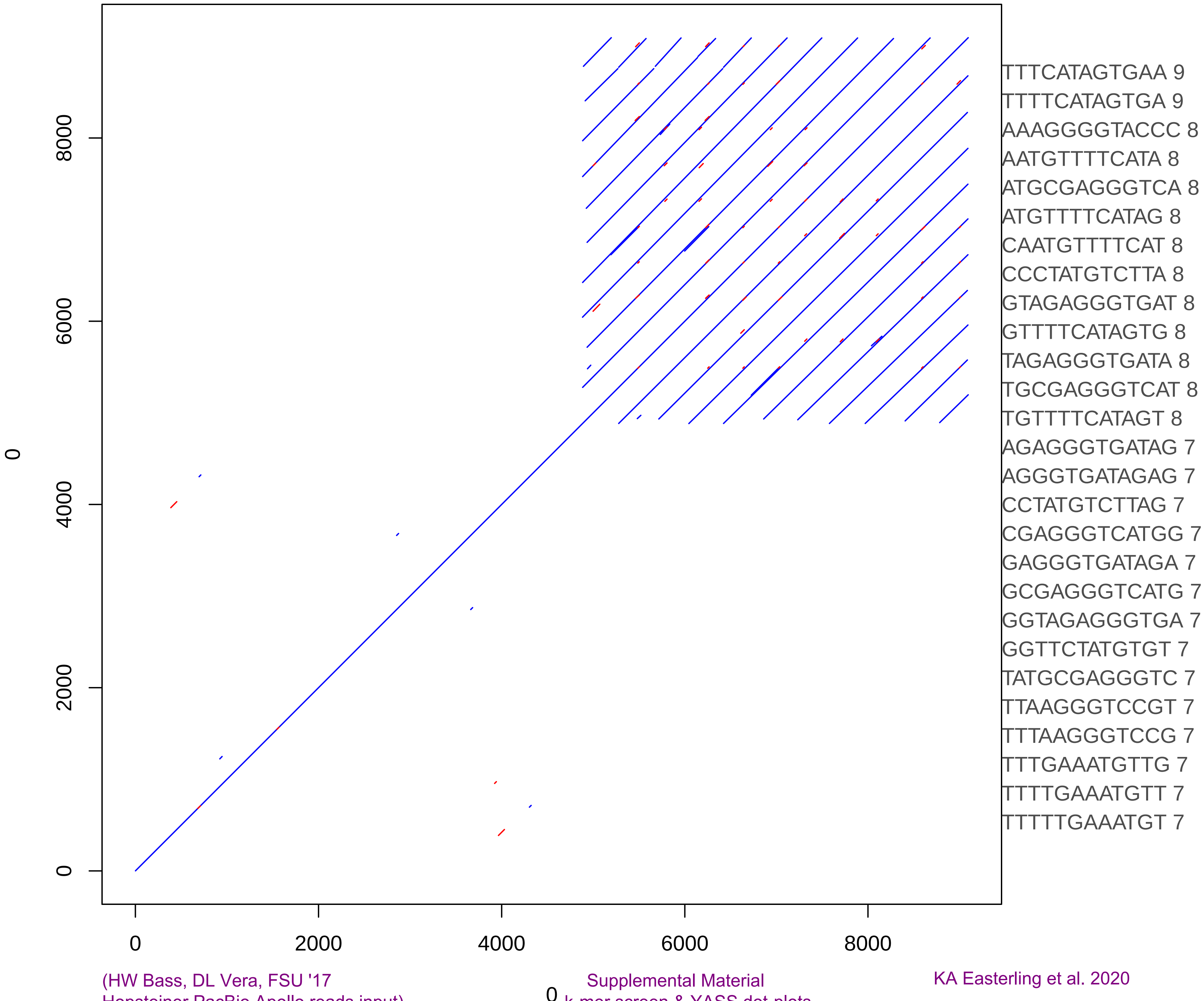

>m141126_104651_42134_c100732262550000001823141405141502_s1_p0/144778/2285_16117

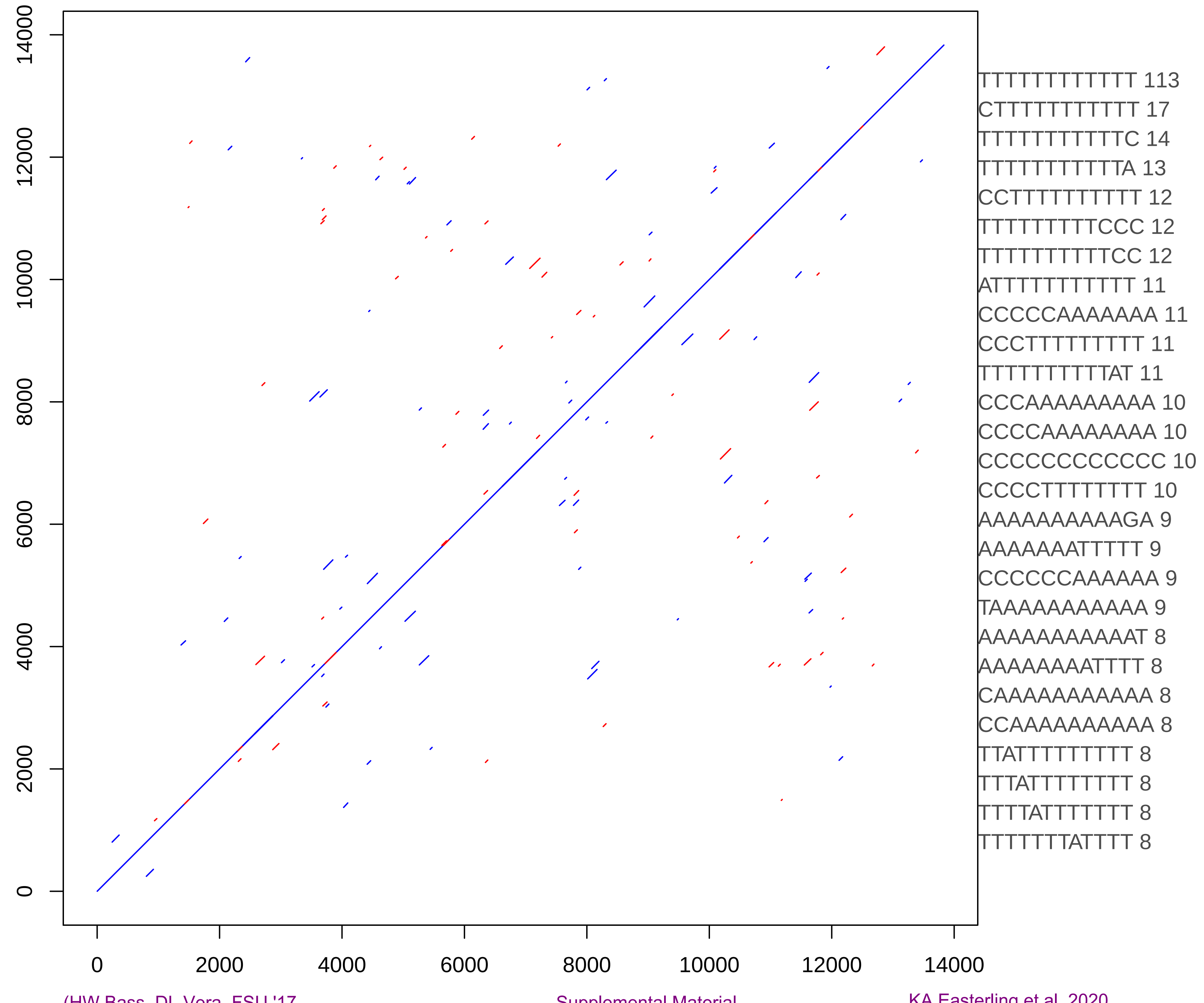

>m141126_104651_42134_c100732262550000001823141405141502_s1_p0/144984/5044_14842

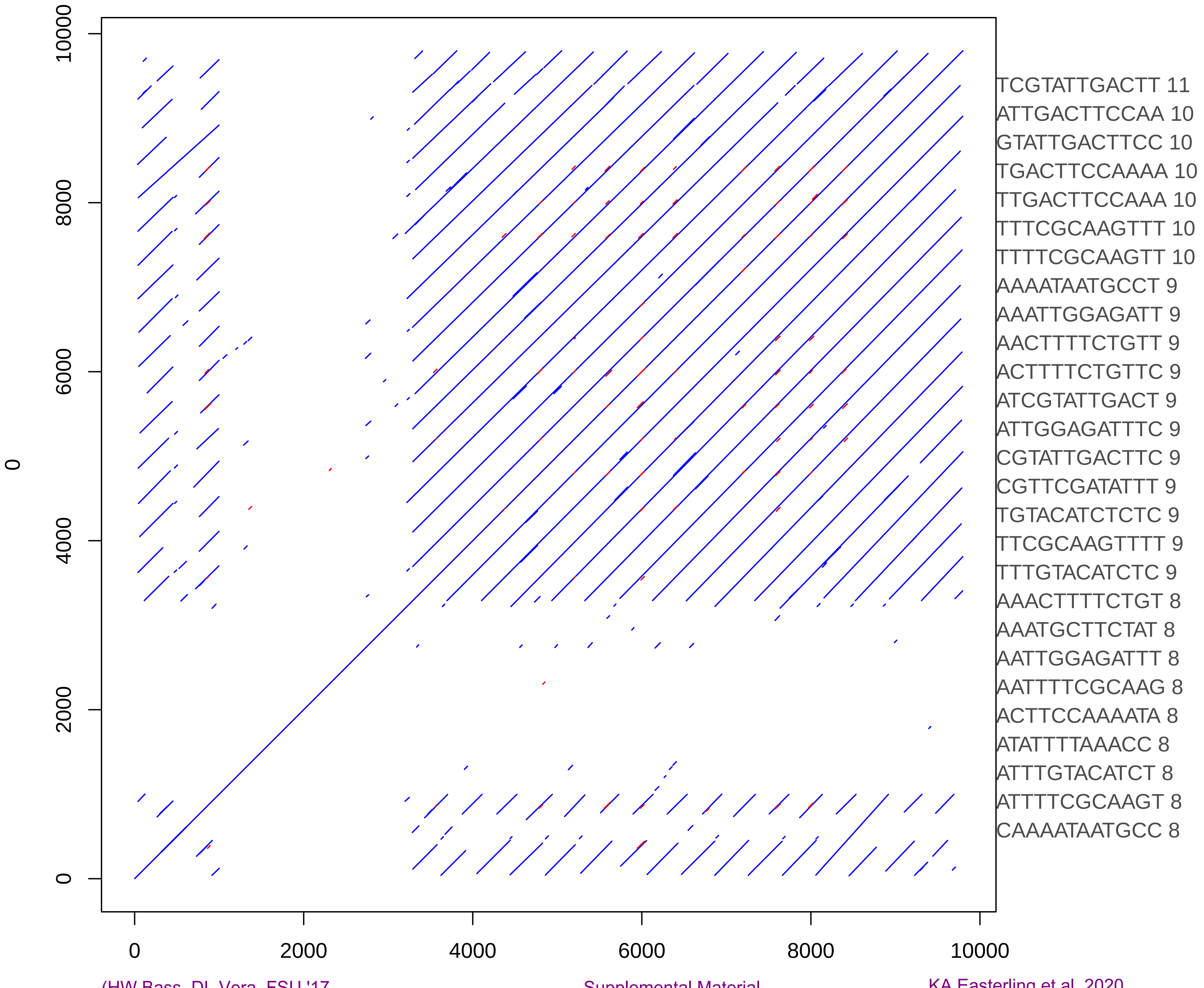

>m141126_104651_42134_c100732262550000001823141405141502_s1_p0/145652/6527_18333

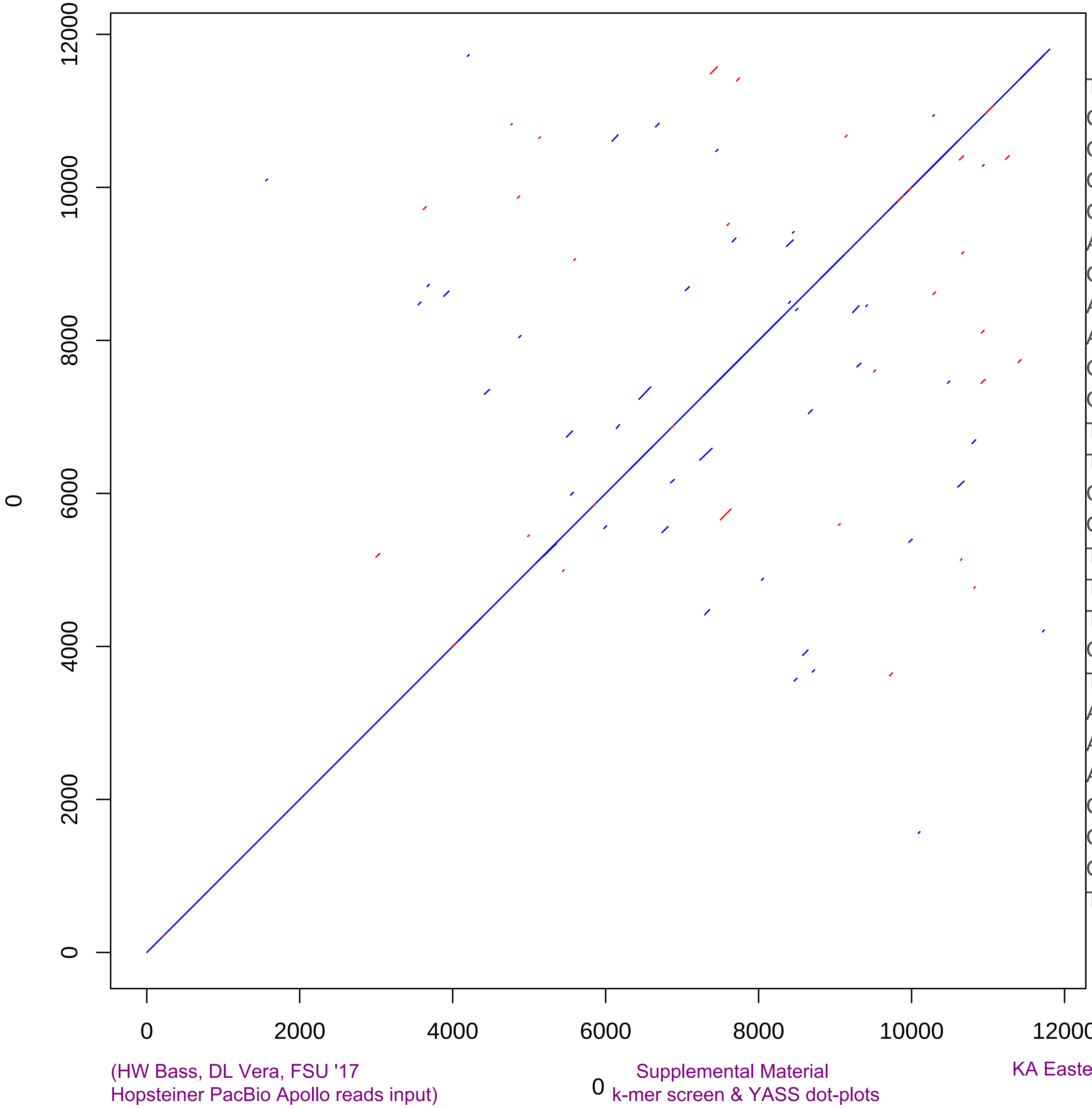

TTTTTTTTTTTT 113
CAAAAAAAAAAA 19
CCCCCCCCCCCC 16
CCCAAAAAAAAA 15
CCAAAAAAAAAA 14
AAAAAAAAAAAT 13
CTTTTTTTTTTT 11
AAAAAAAAAAAC 10
AAAAAAAAATTT 10
CCCCAAAAAAAA 10
CCTTTTTTTTTT 10
TTTTTTTCCCCC 10
TTTTTTTTTTTC 10
CCCCCCAAAAAA 9
GTTTTTTTTTTT 9
TTTTTTCCCCCC 9
TTTTTTTTTCCC 9
TTTTTTTTTTTA 9
CCCCCCTTTTTT 8
TTTTTTTTAAAA 8
AAAAAAAAAATT 7
AACAAAAAAAAA 7
ACAAAAAAAAAA 7
CCCCCAAAAAAA 7
CCCCCTTTTTTT 7
CCCTTTTTTTTT 7
TTTTTCCCCCCC 7

>m141126_104651_42134_c100732262550000001823141405141502_s1_p0/147517/3689_14178

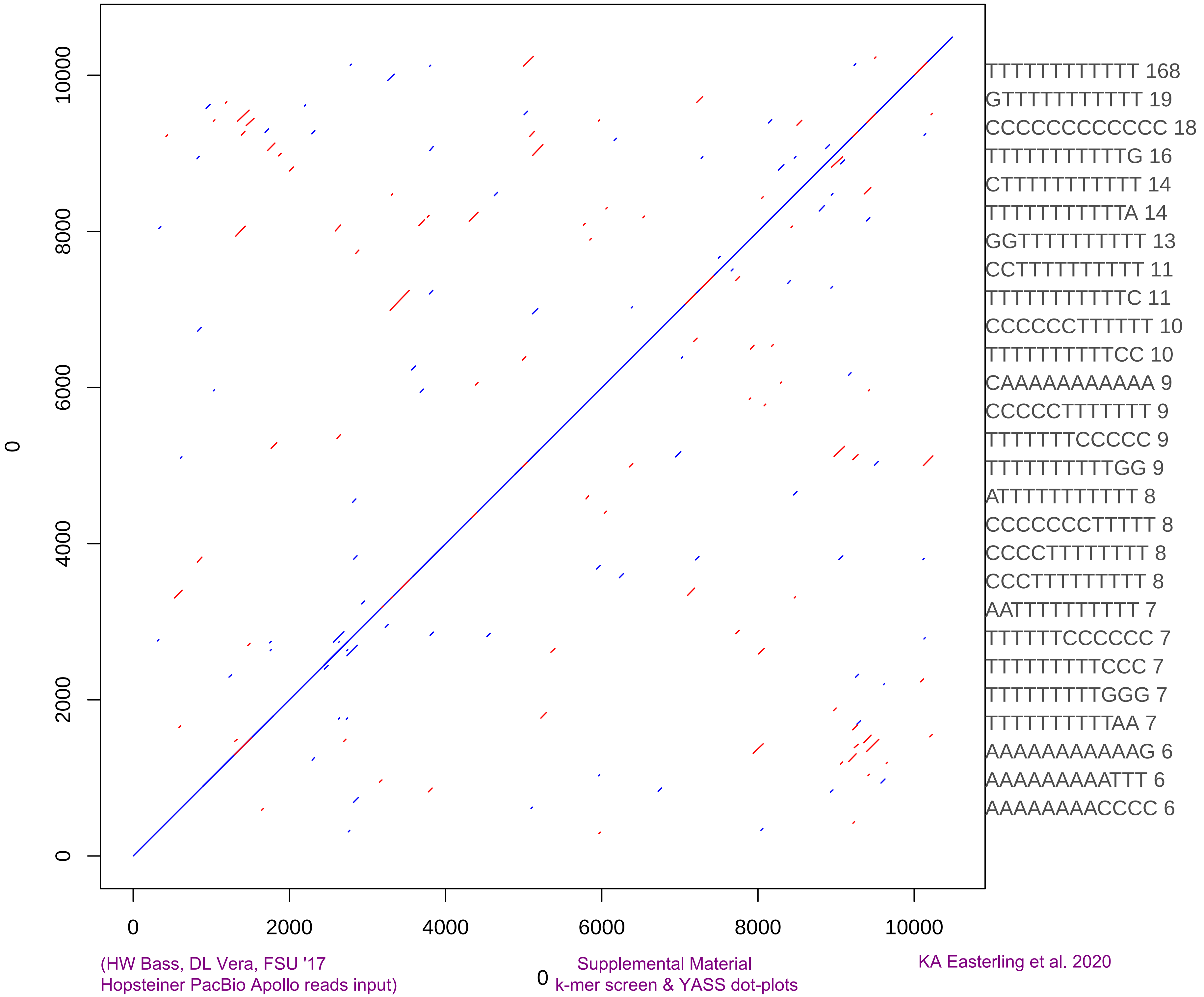

>m141126_104651_42134_c100732262550000001823141405141502_s1_p0/148693/6989_12396

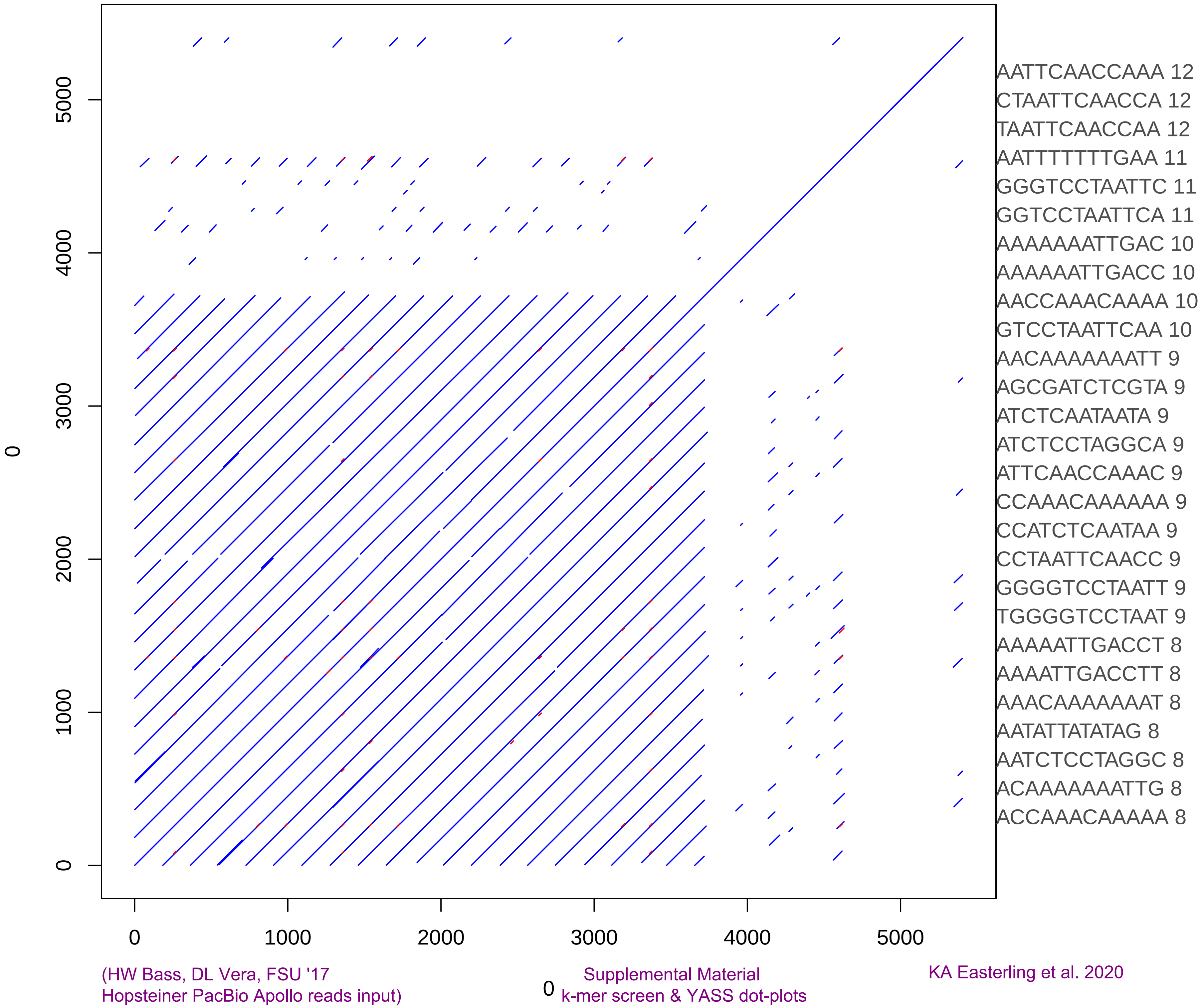

>m141126_104651_42134_c100732262550000001823141405141502_s1_p0/152262/0_7229

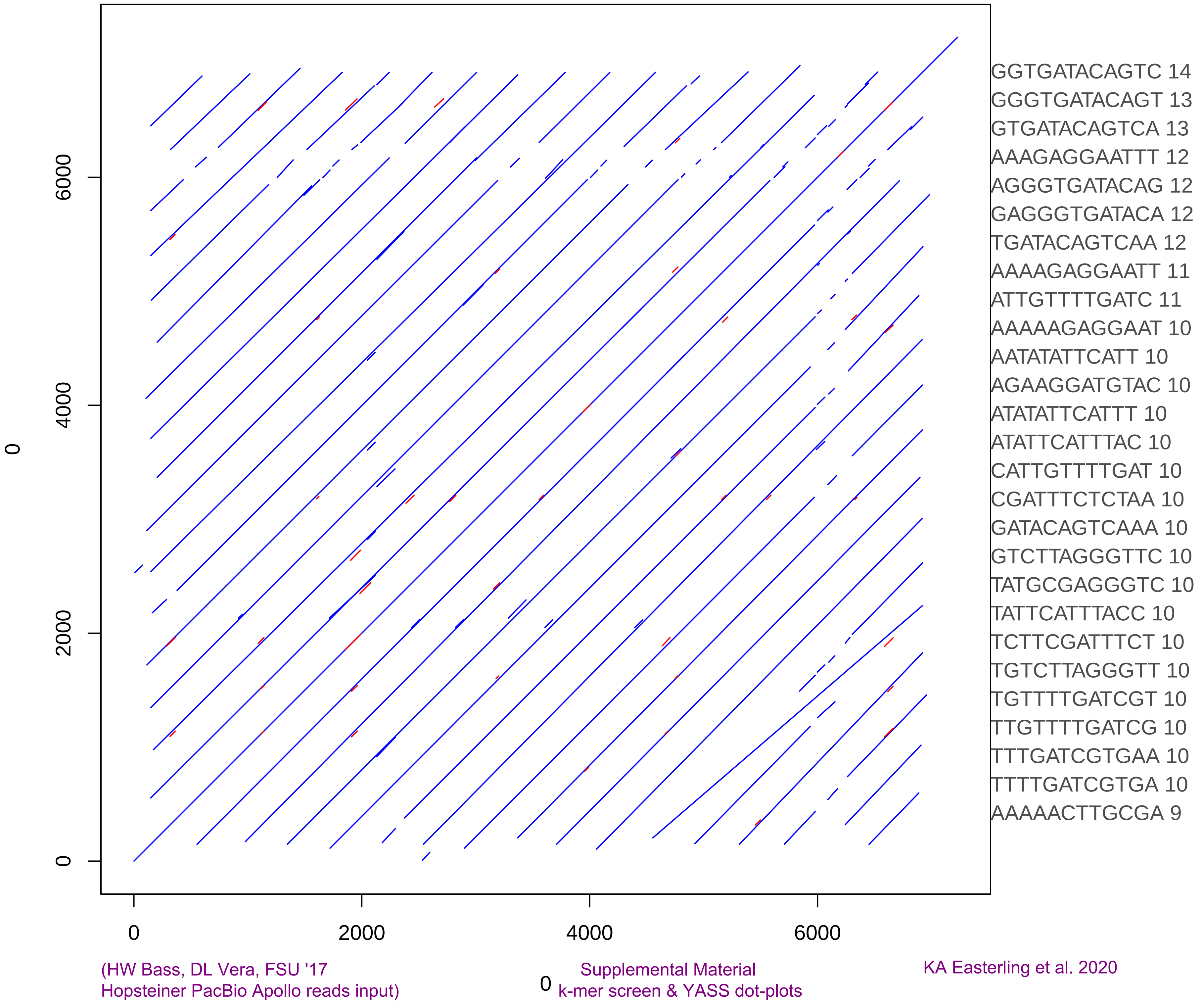

>m141126_104651_42134_c100732262550000001823141405141502_s1_p0/160832/2590_15855

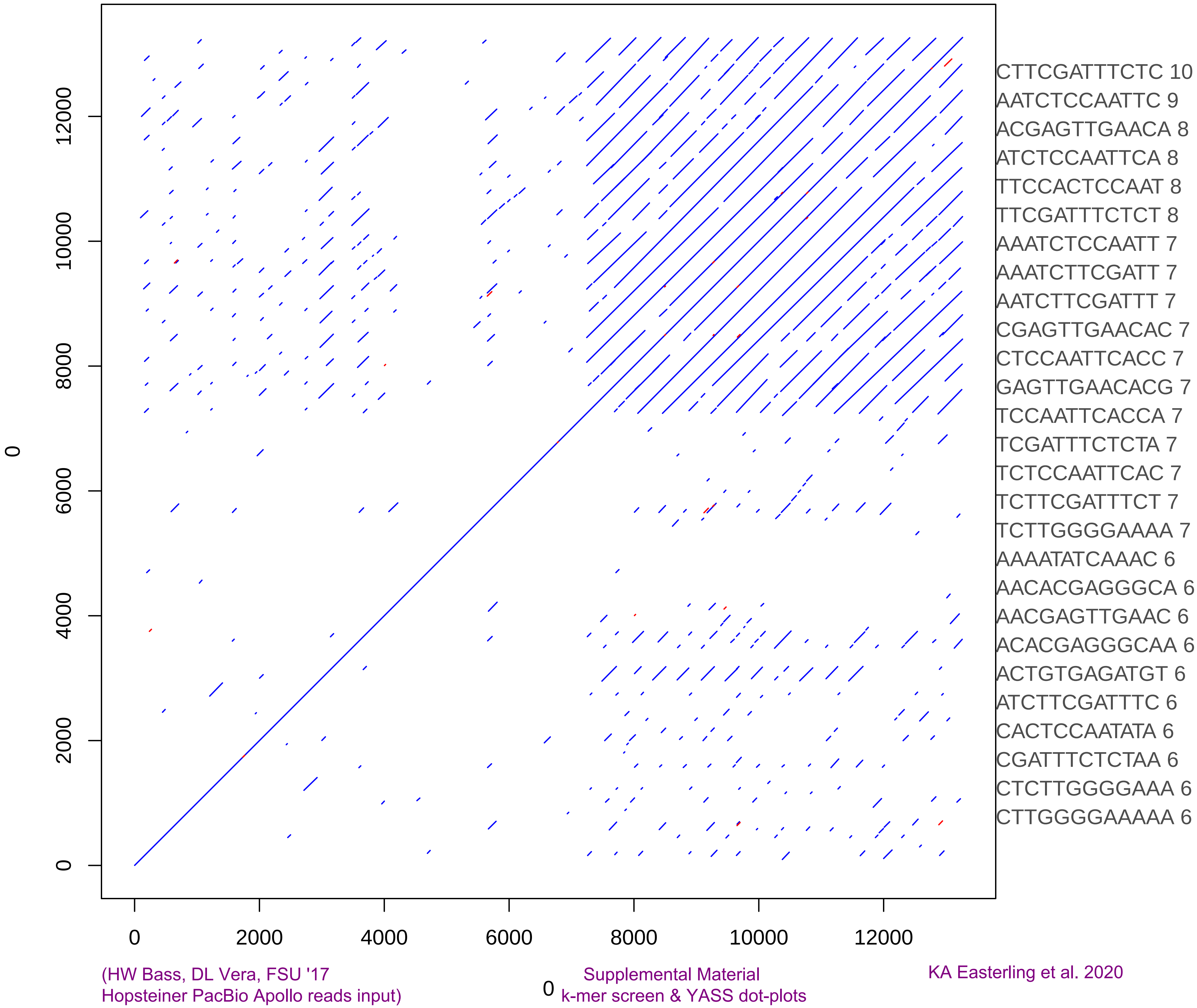

>m141126_104651_42134_c100732262550000001823141405141502_s1_p0/161810/2596_10565

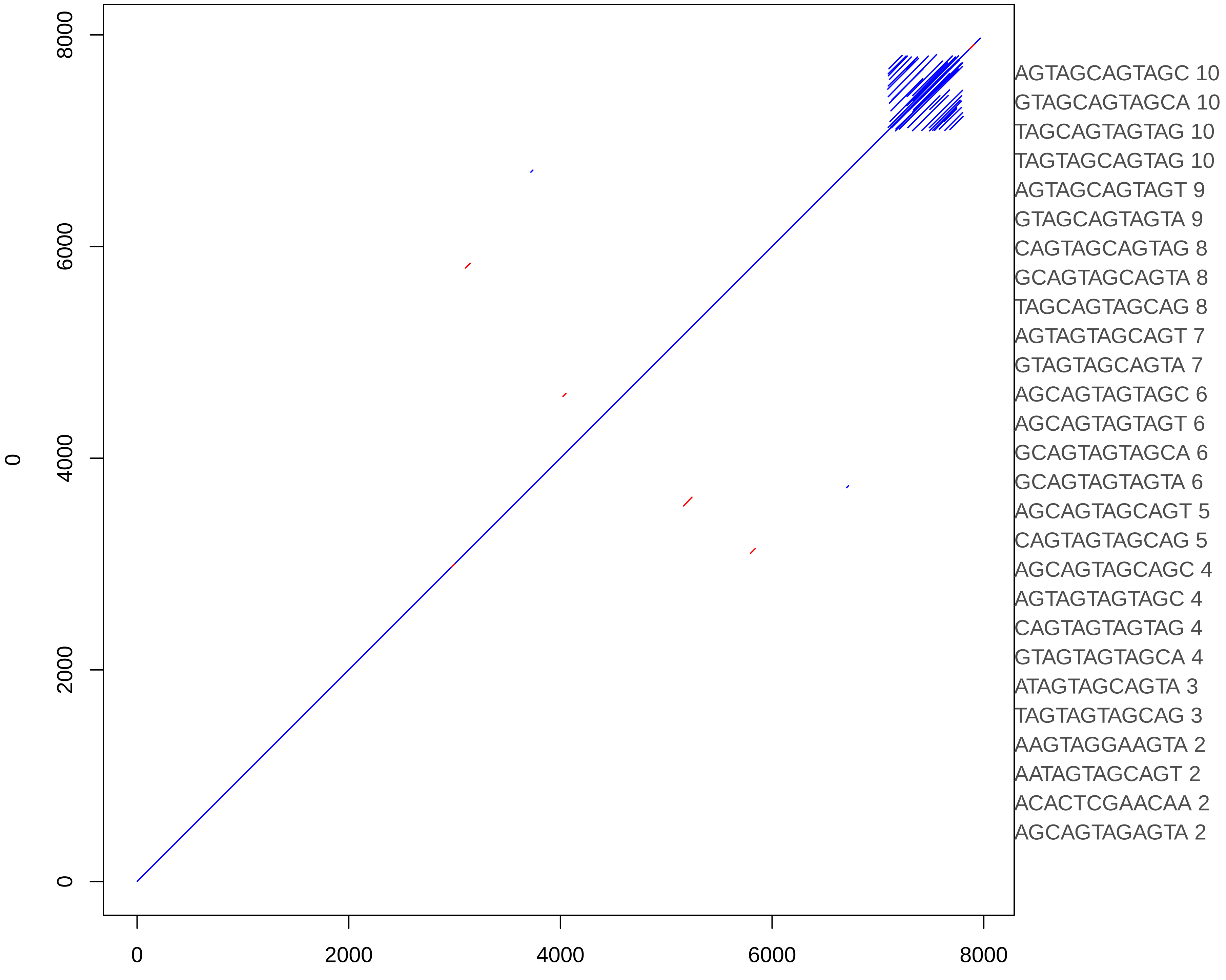

>m141126_104651_42134_c100732262550000001823141405141502_s1_p0/162448/1871_11251

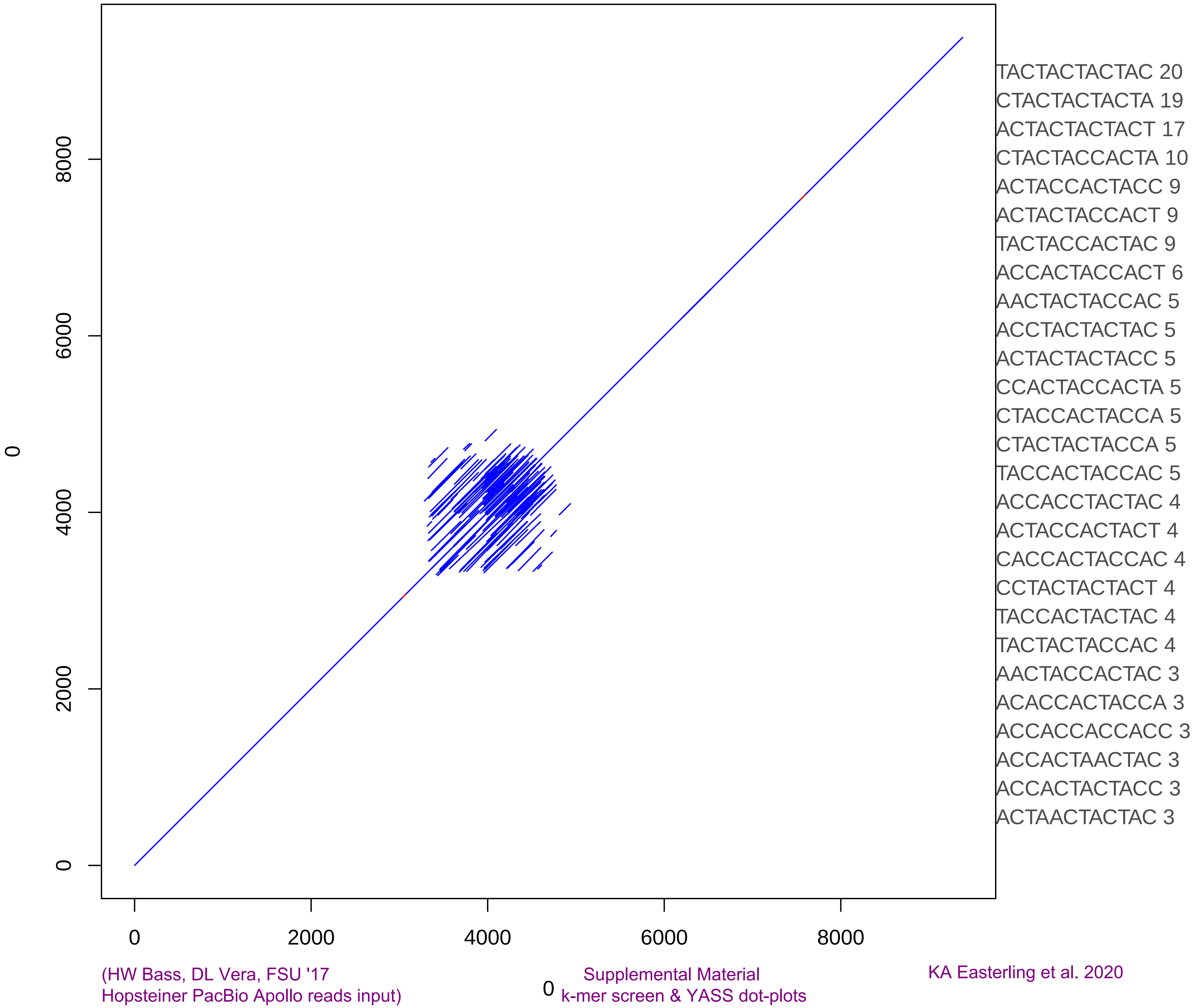

>m141126_104651_42134_c100732262550000001823141405141502_s1_p0/19232/179_7024

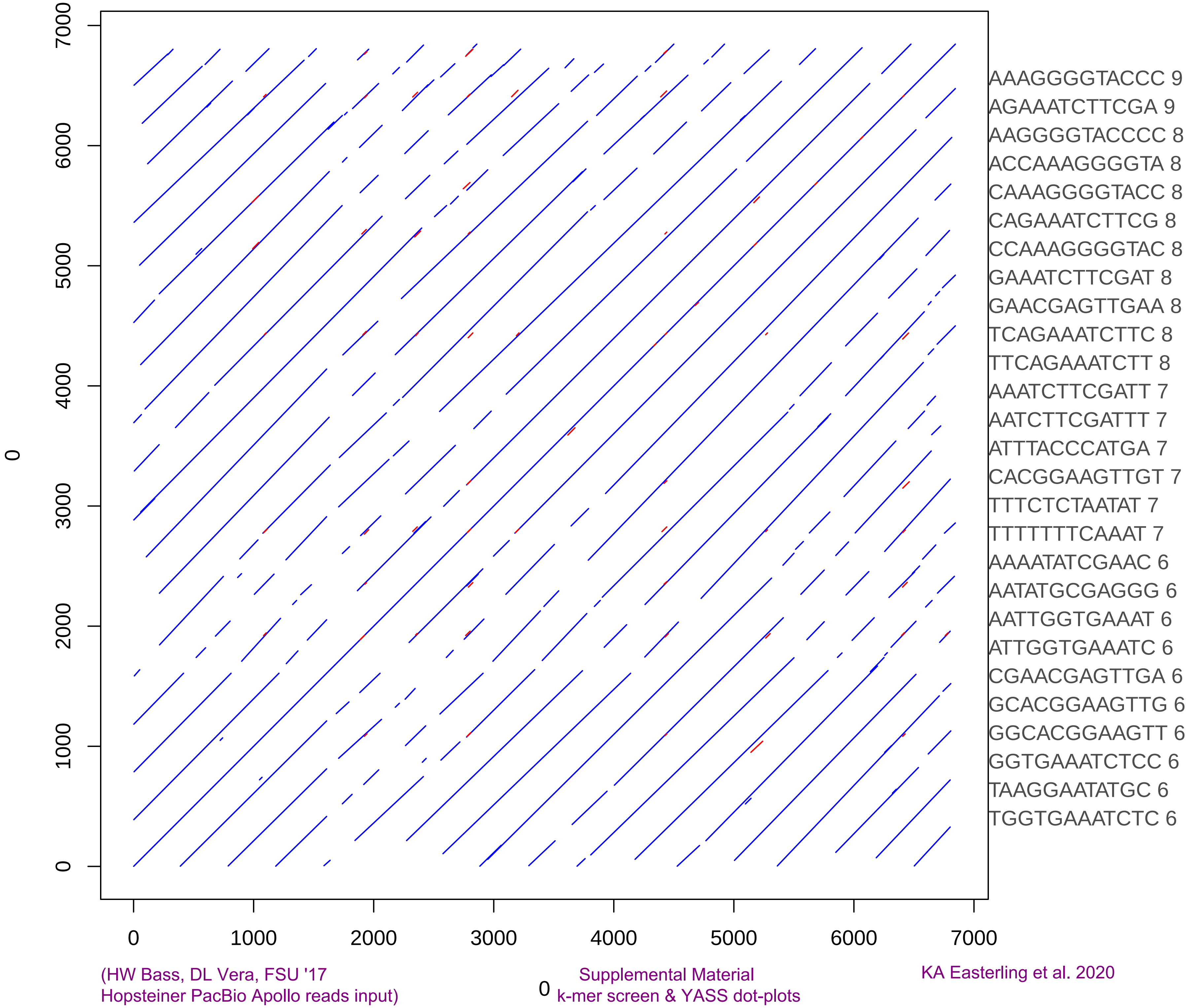

>m141126_104651_42134_c100732262550000001823141405141502_s1_p0/22104/538_12592

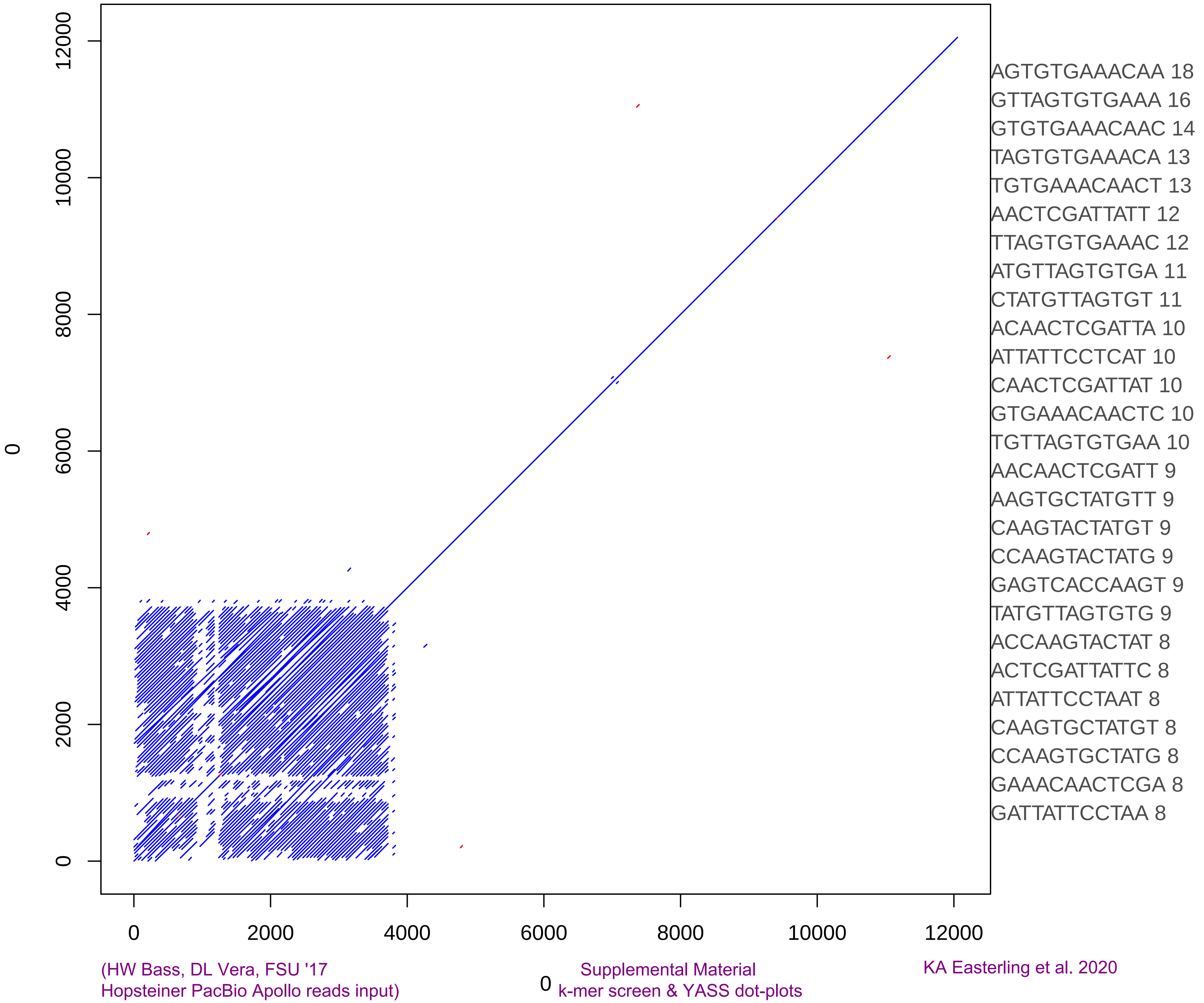

>m141126_104651_42134_c100732262550000001823141405141502_s1_p0/22410/0_9929

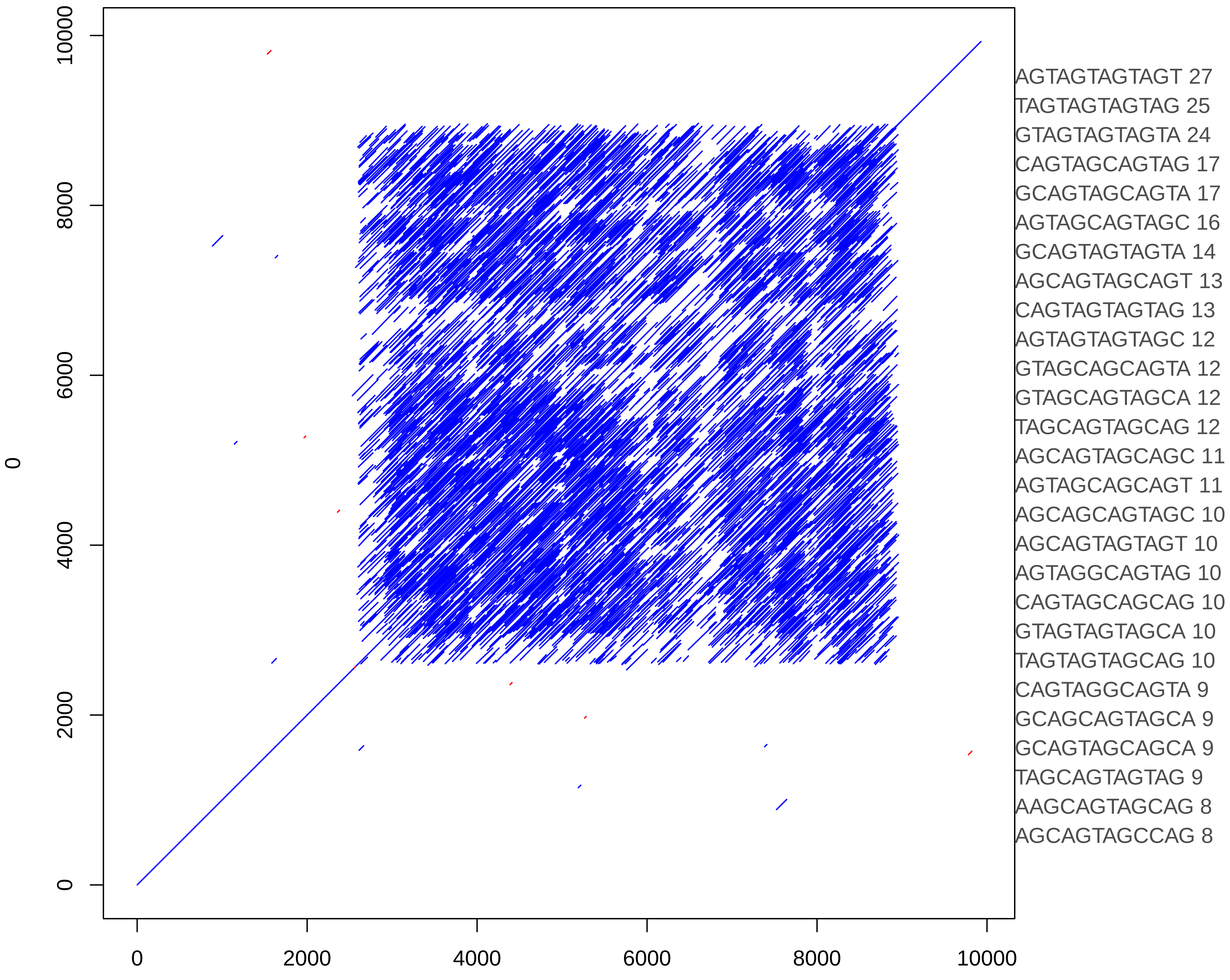

>m141126_104651_42134_c100732262550000001823141405141502_s1_p0/25272/240_10607

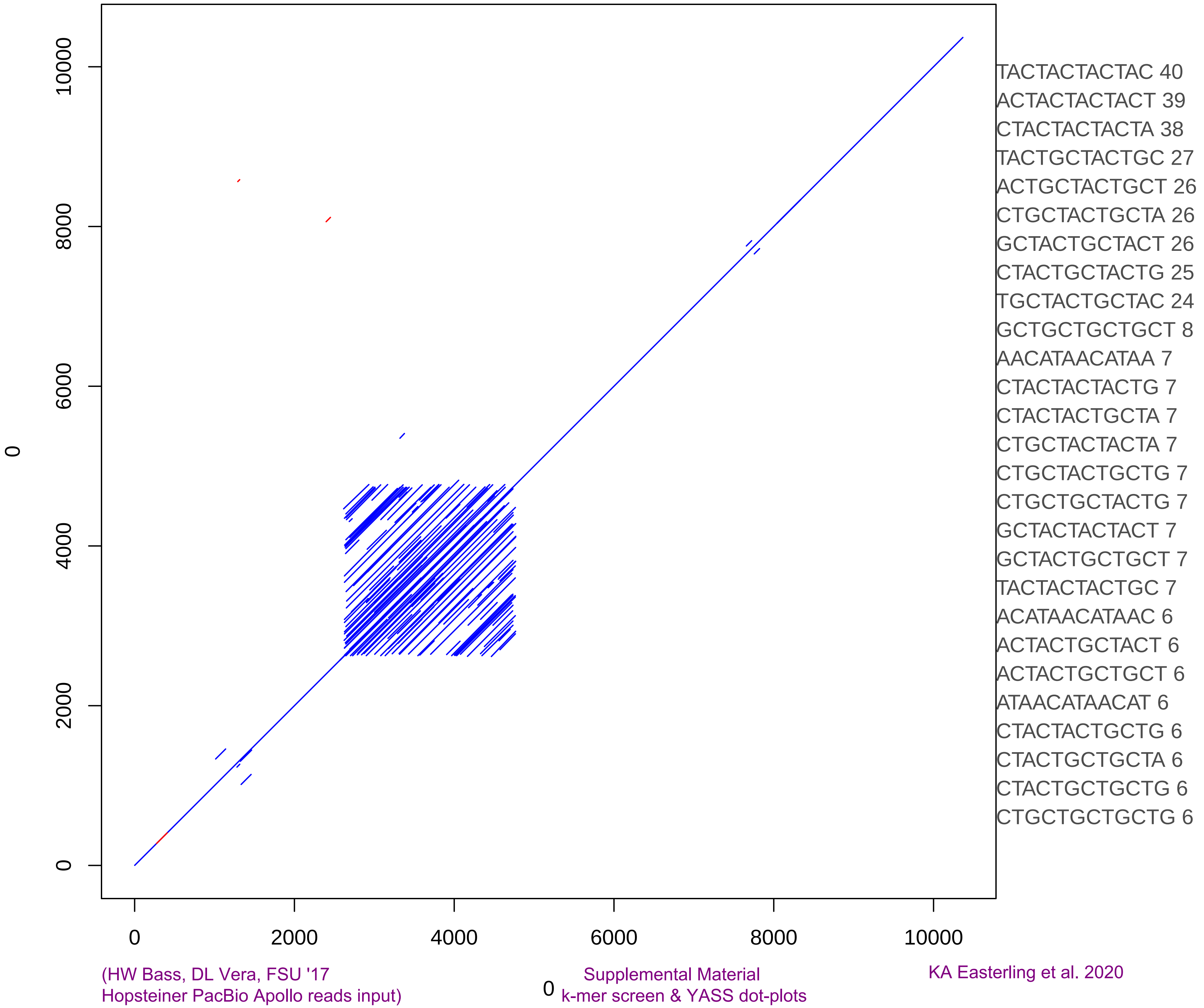

>m141126_104651_42134_c100732262550000001823141405141502_s1_p0/25743/3368_14749

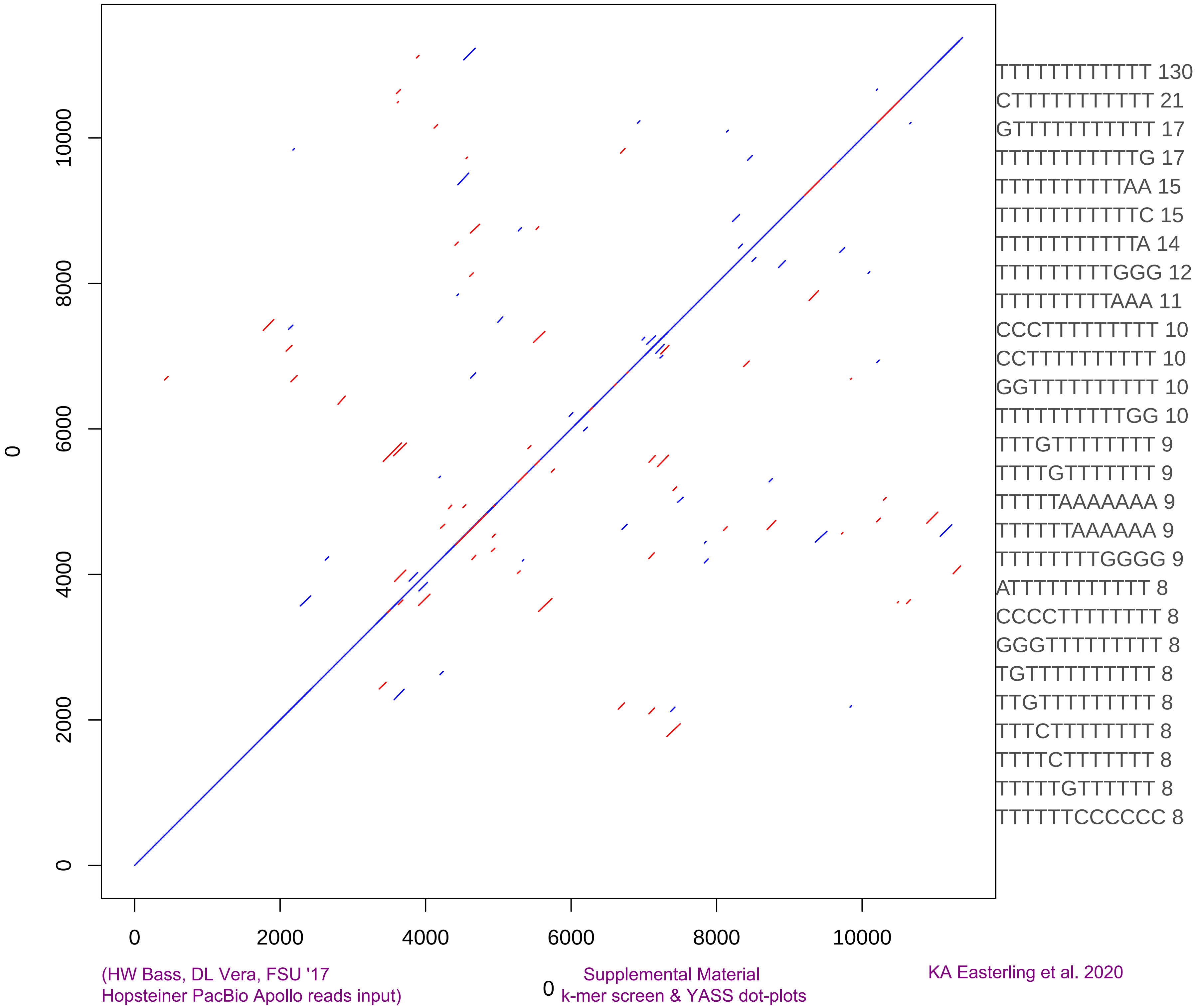

>m141126_104651_42134_c100732262550000001823141405141502_s1_p0/26720/7486_14253

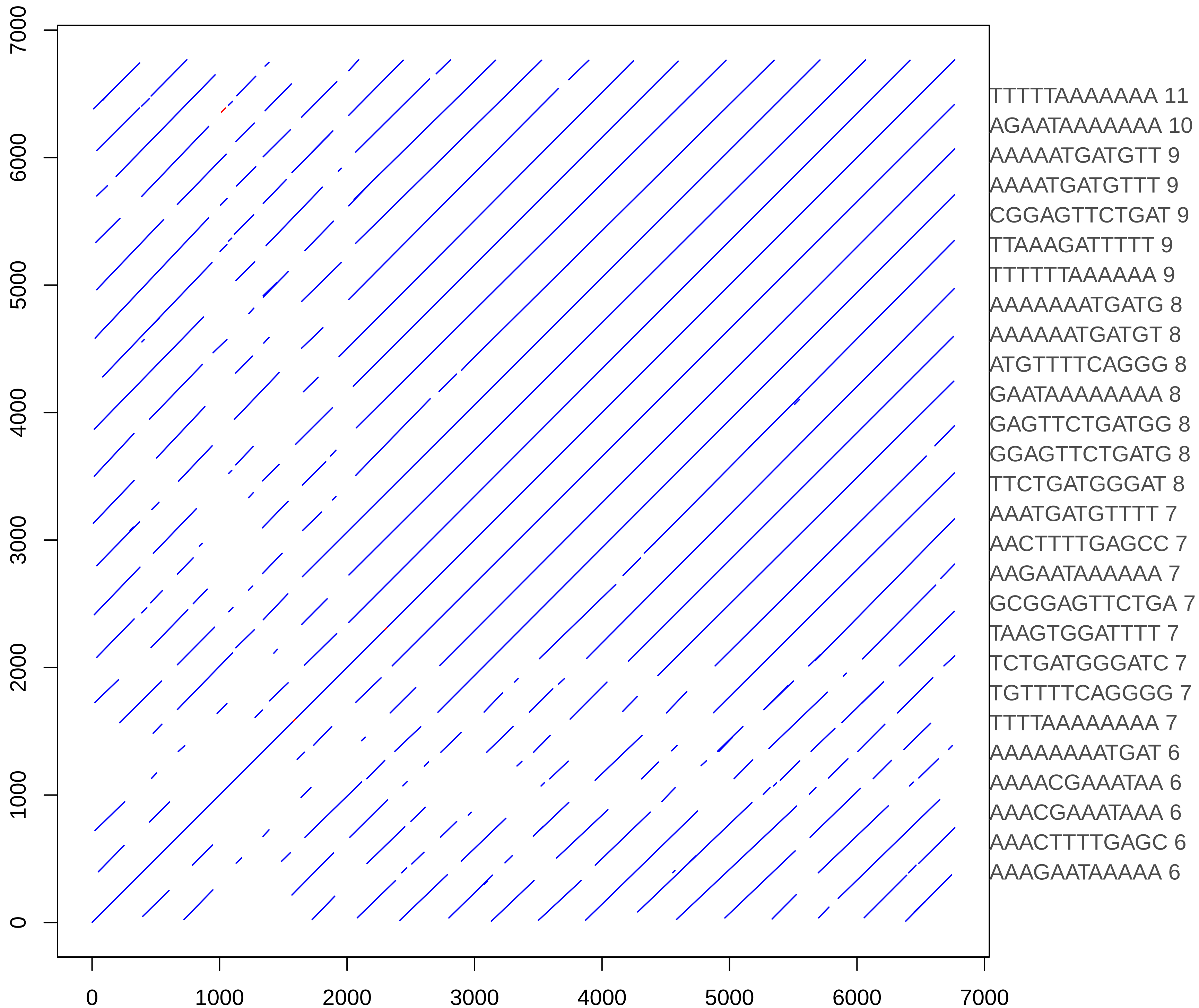

0

>m141126_104651_42134_c100732262550000001823141405141502_s1_p0/28165/0_5342

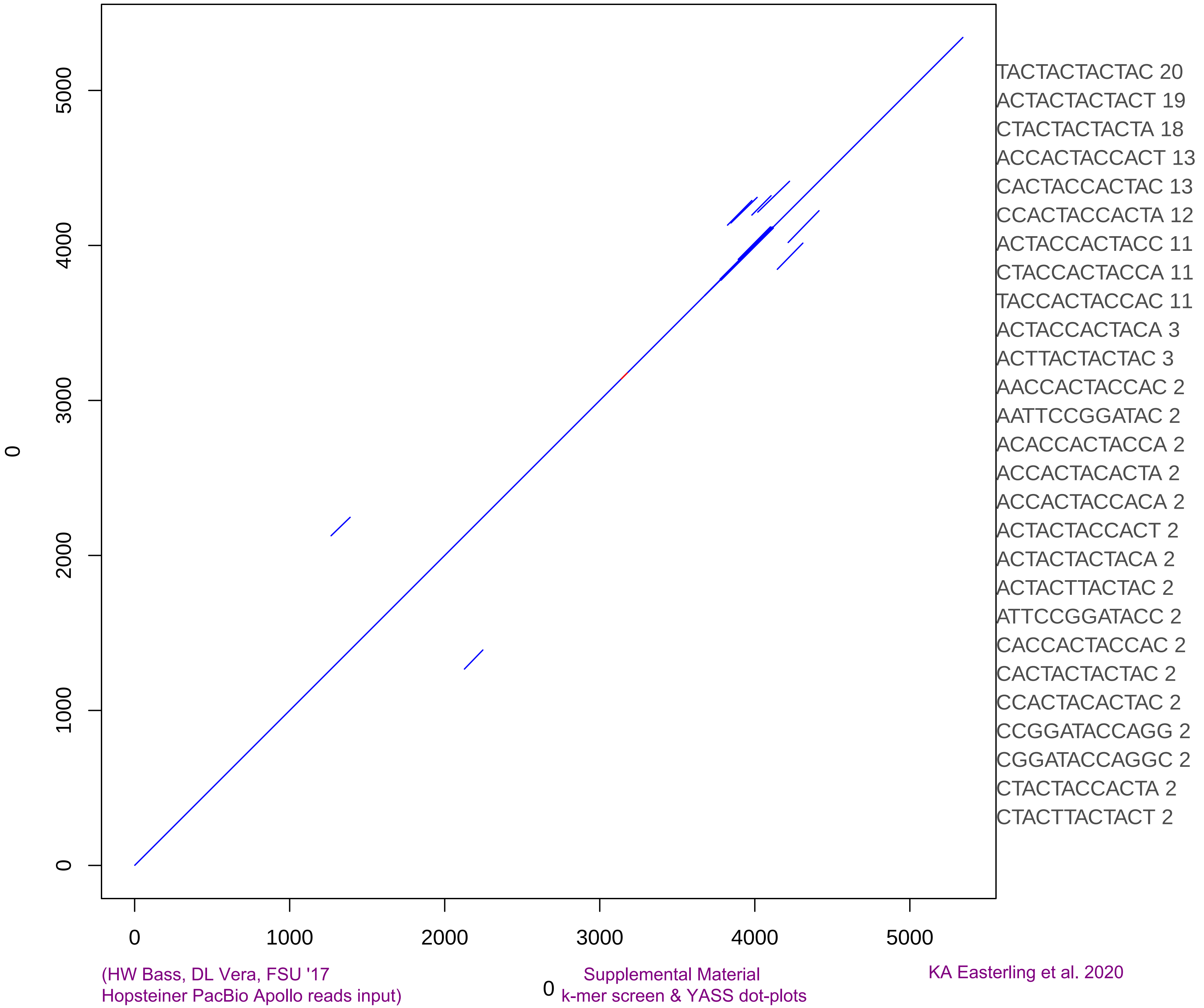

>m141126_104651_42134_c100732262550000001823141405141502_s1_p0/28342/9849_15439

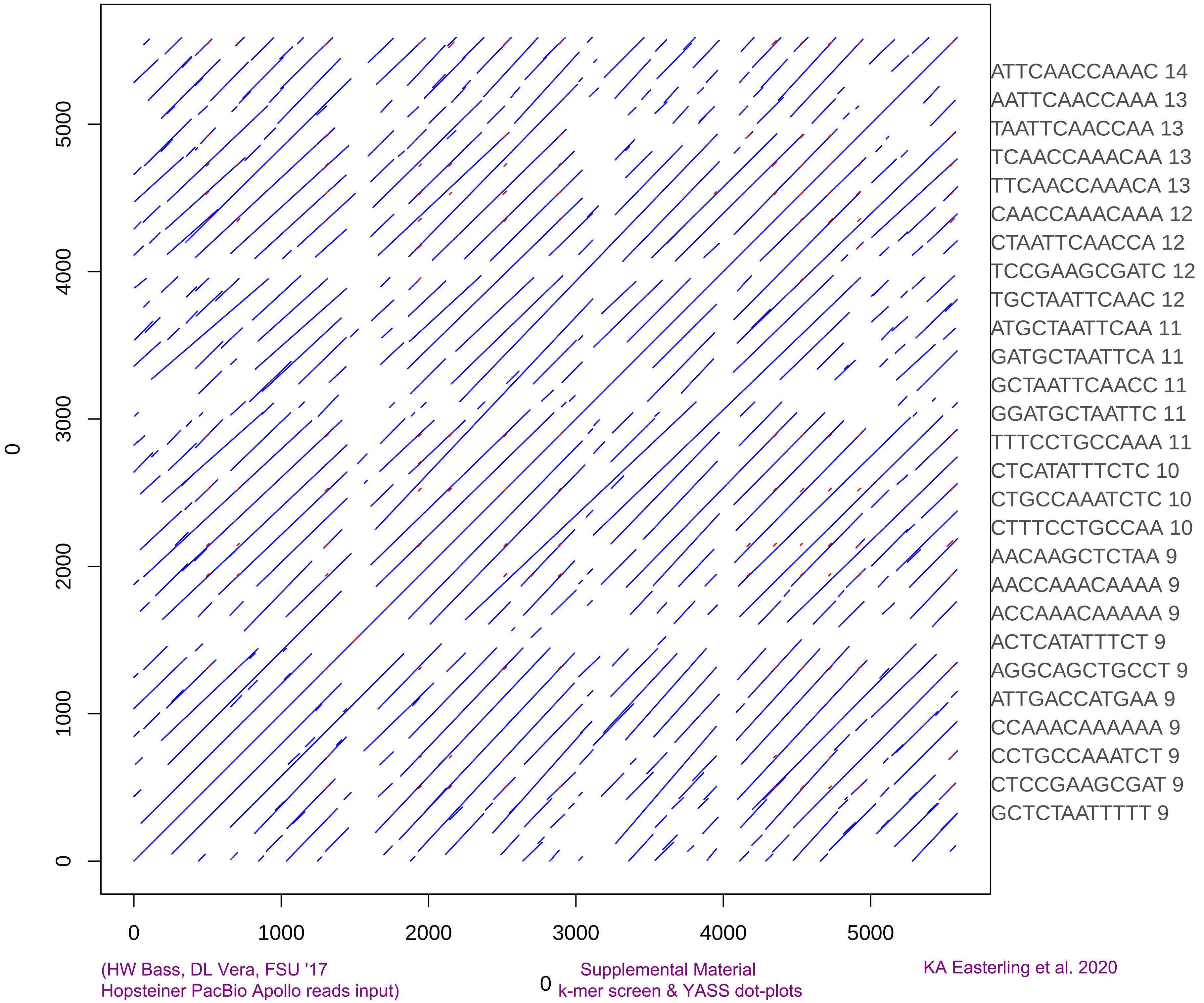

>m141126_104651_42134_c100732262550000001823141405141502_s1_p0/2967/7278_12936

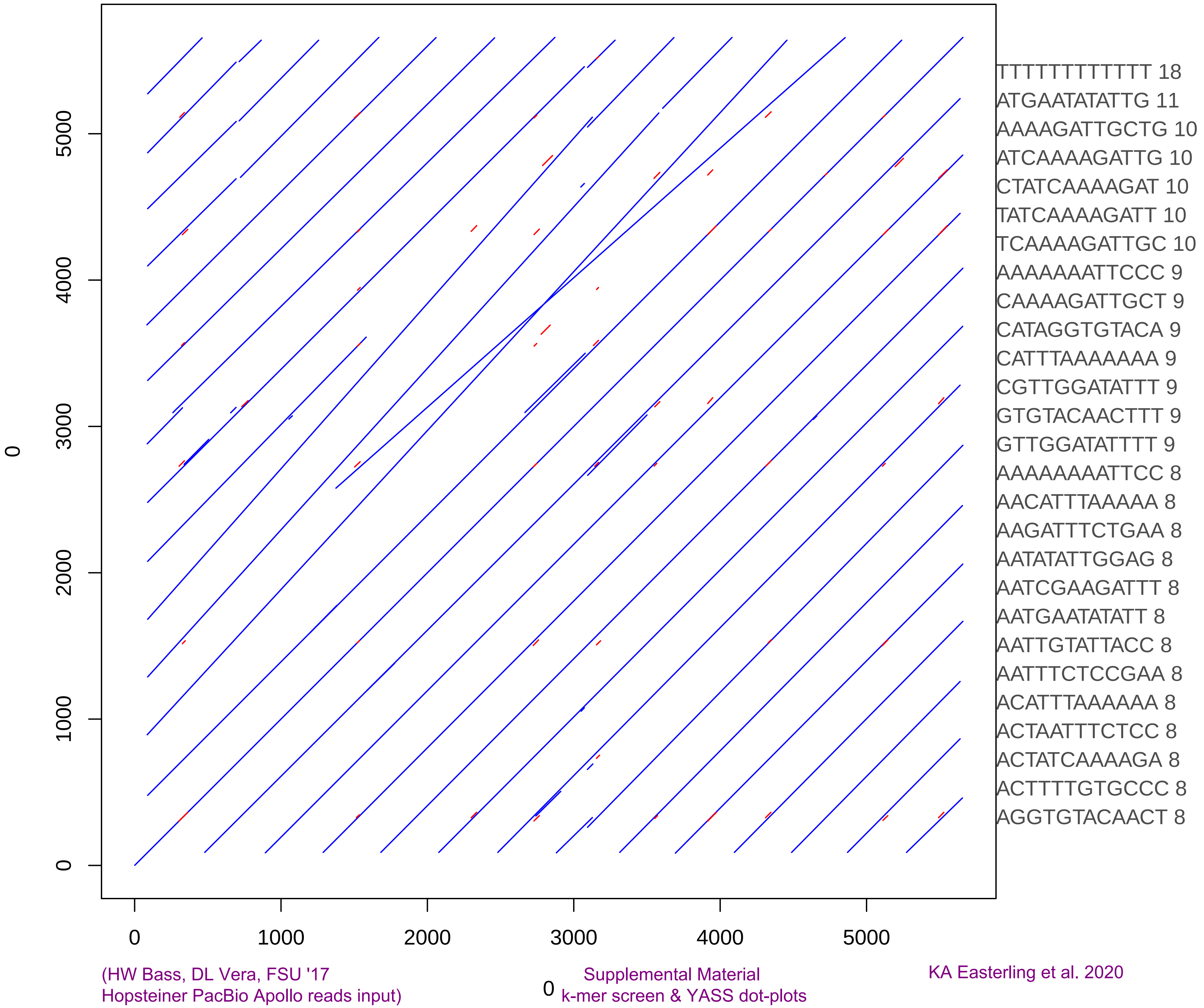

>m141126_104651_42134_c100732262550000001823141405141502_s1_p0/31502/0_6236

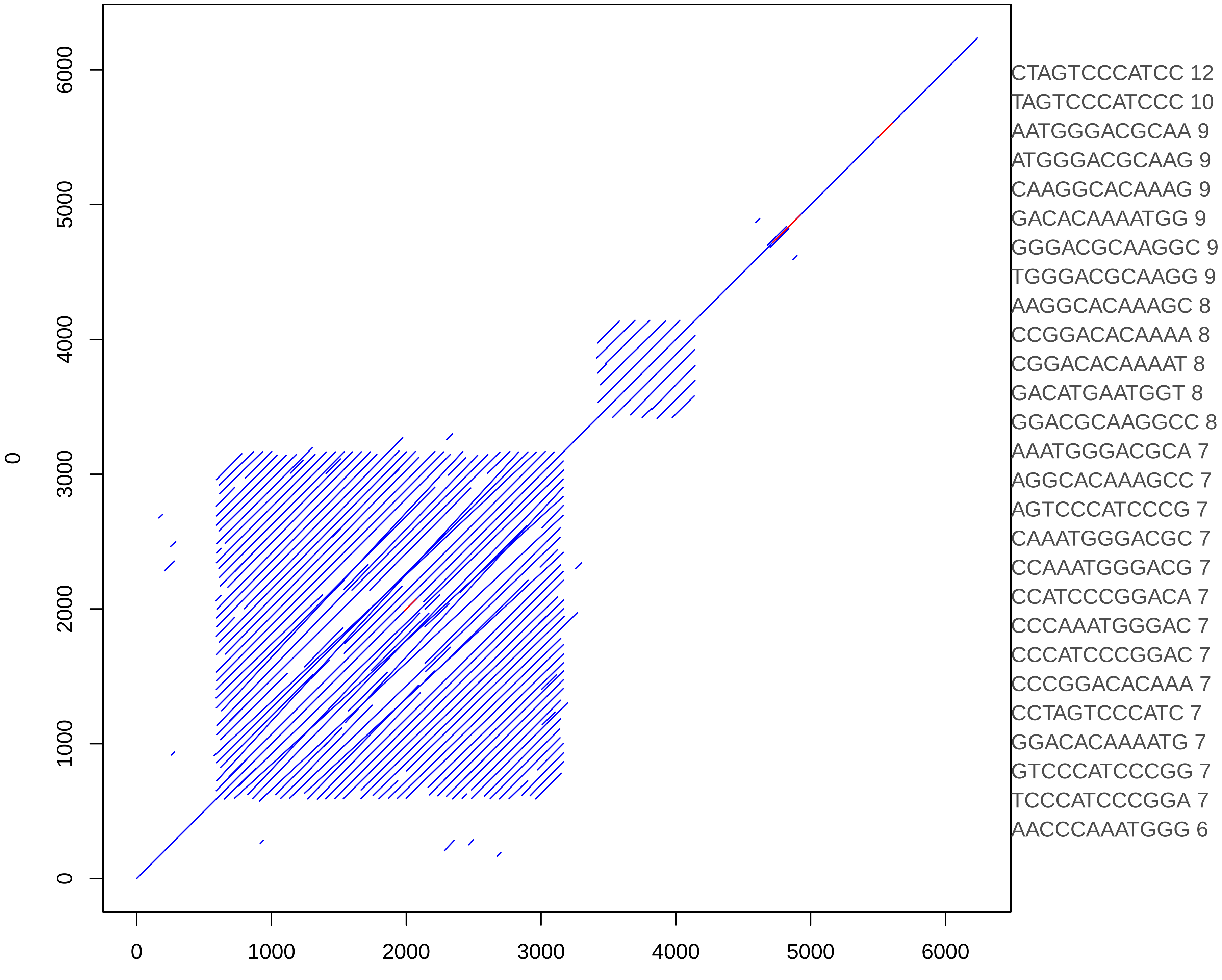

0

>m141126_104651_42134_c100732262550000001823141405141502_s1_p0/31502/6281_12262

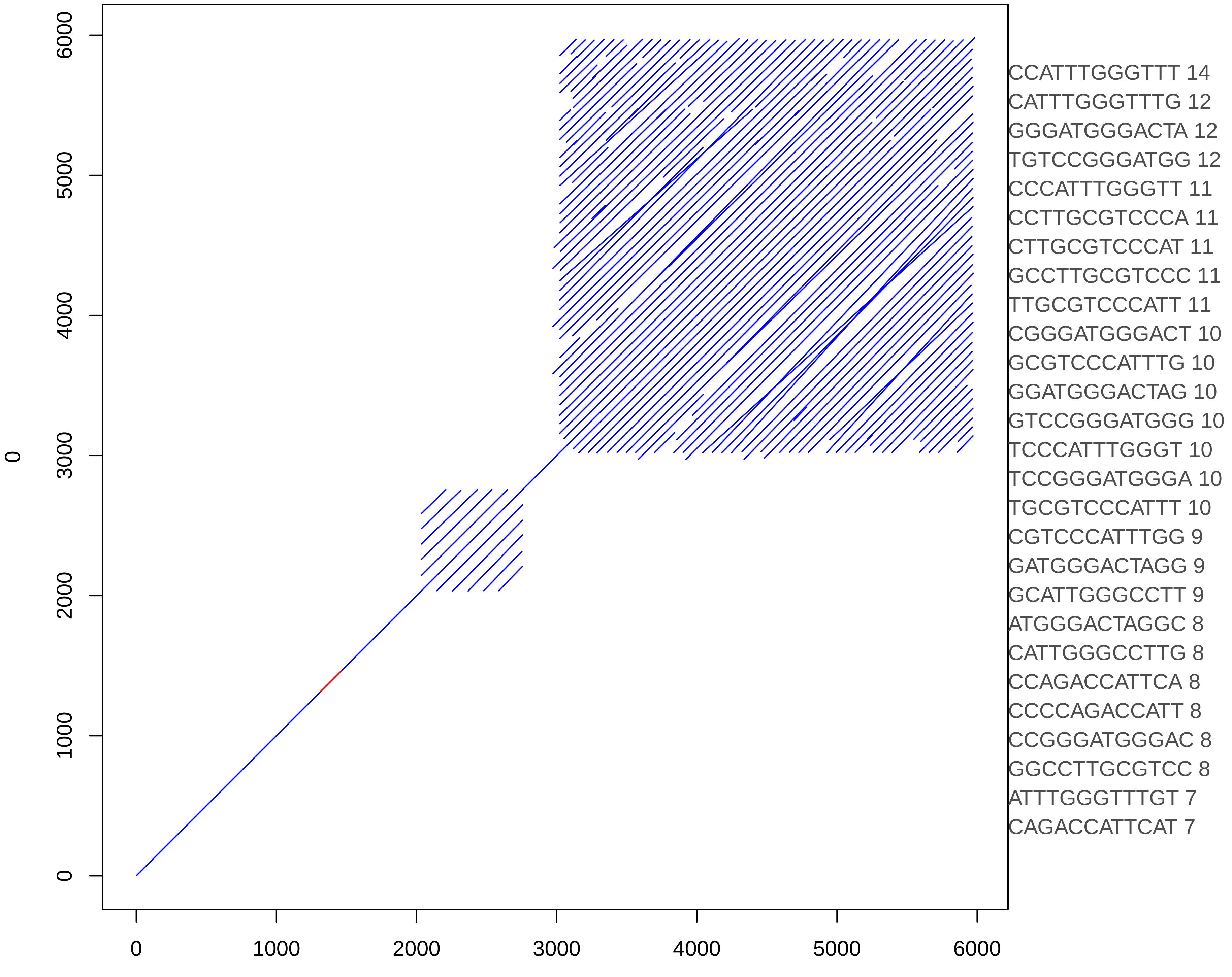

>m141126_104651_42134_c100732262550000001823141405141502_s1_p0/35030/0_6289

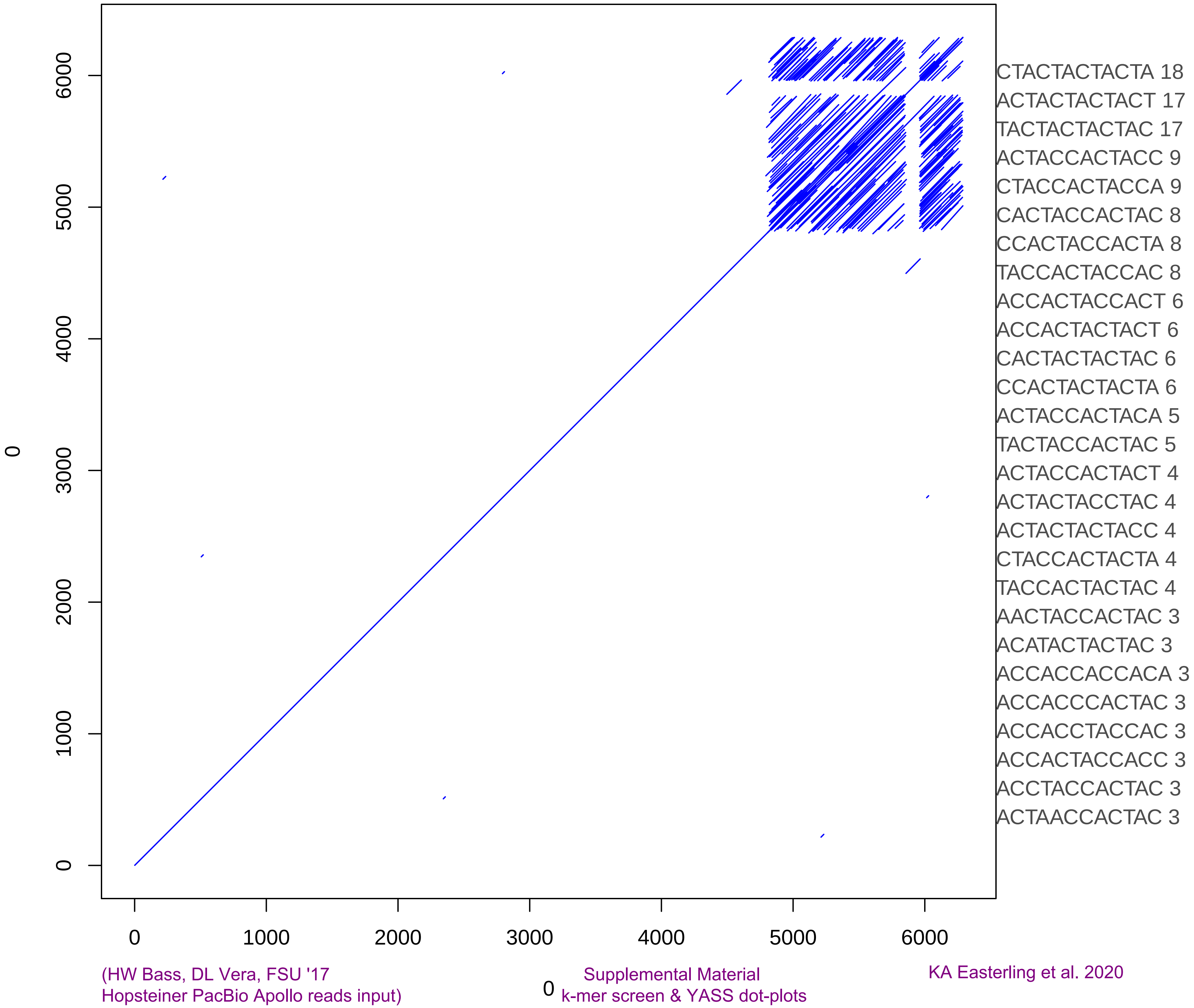

>m141126_104651_42134_c100732262550000001823141405141502_s1_p0/37047/3234_9049

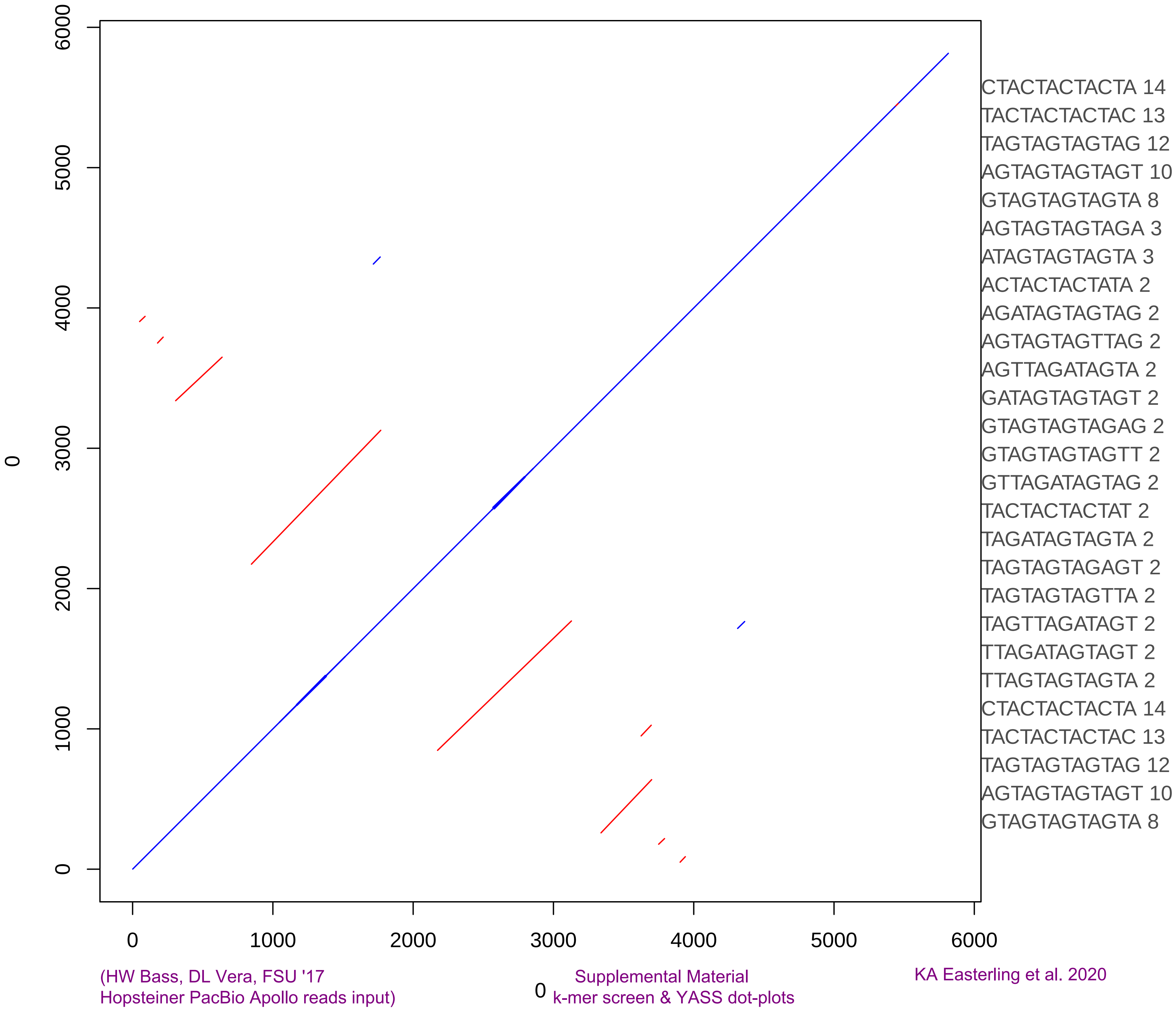

>m141126_104651_42134_c100732262550000001823141405141502_s1_p0/37932/717_13563

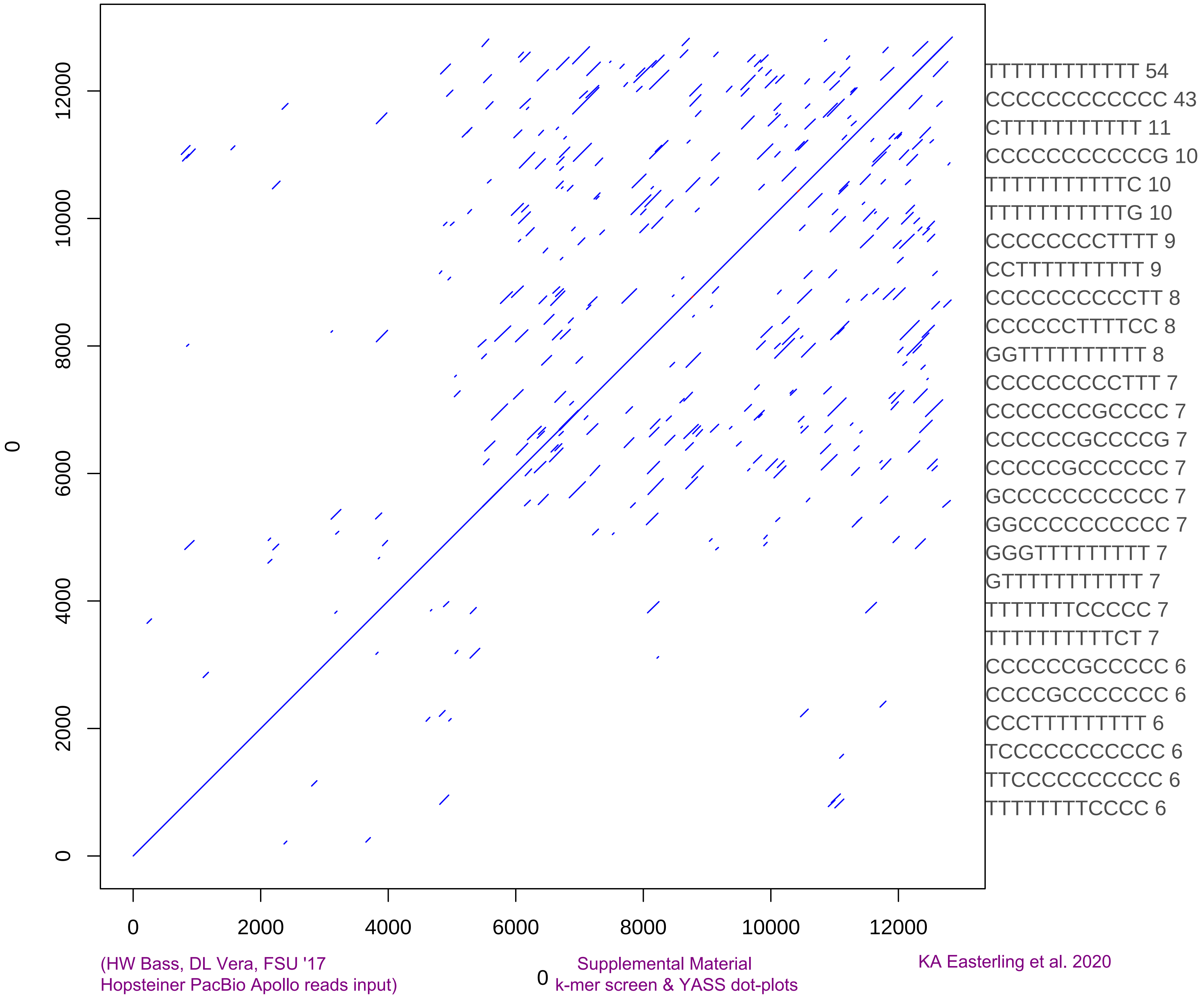

>m141126_104651_42134_c100732262550000001823141405141502_s1_p0/4125/0_5931

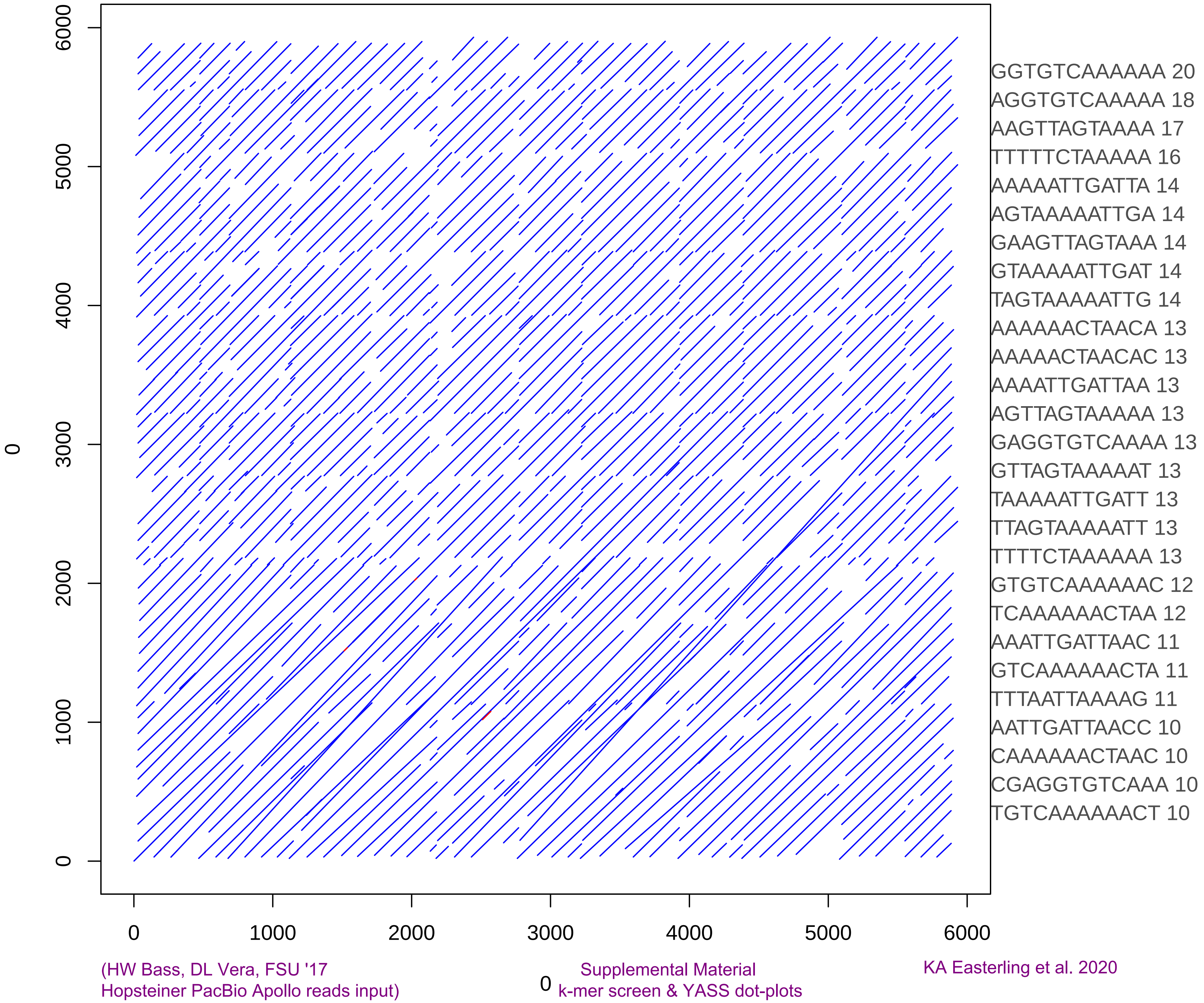

>m141126_104651_42134_c100732262550000001823141405141502_s1_p0/41997/3022_15457

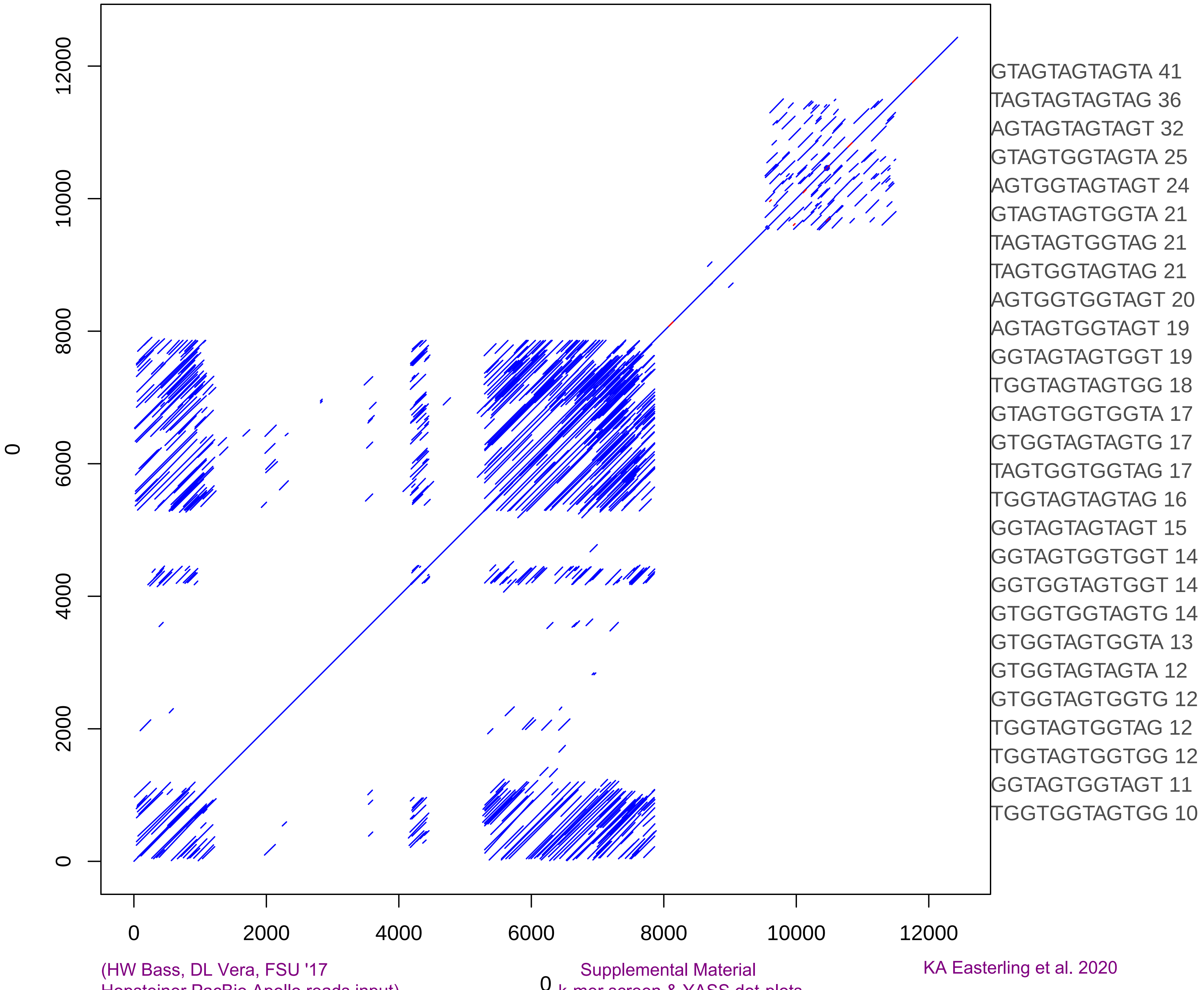

>m141126_104651_42134_c100732262550000001823141405141502_s1_p0/43126/7594_13117

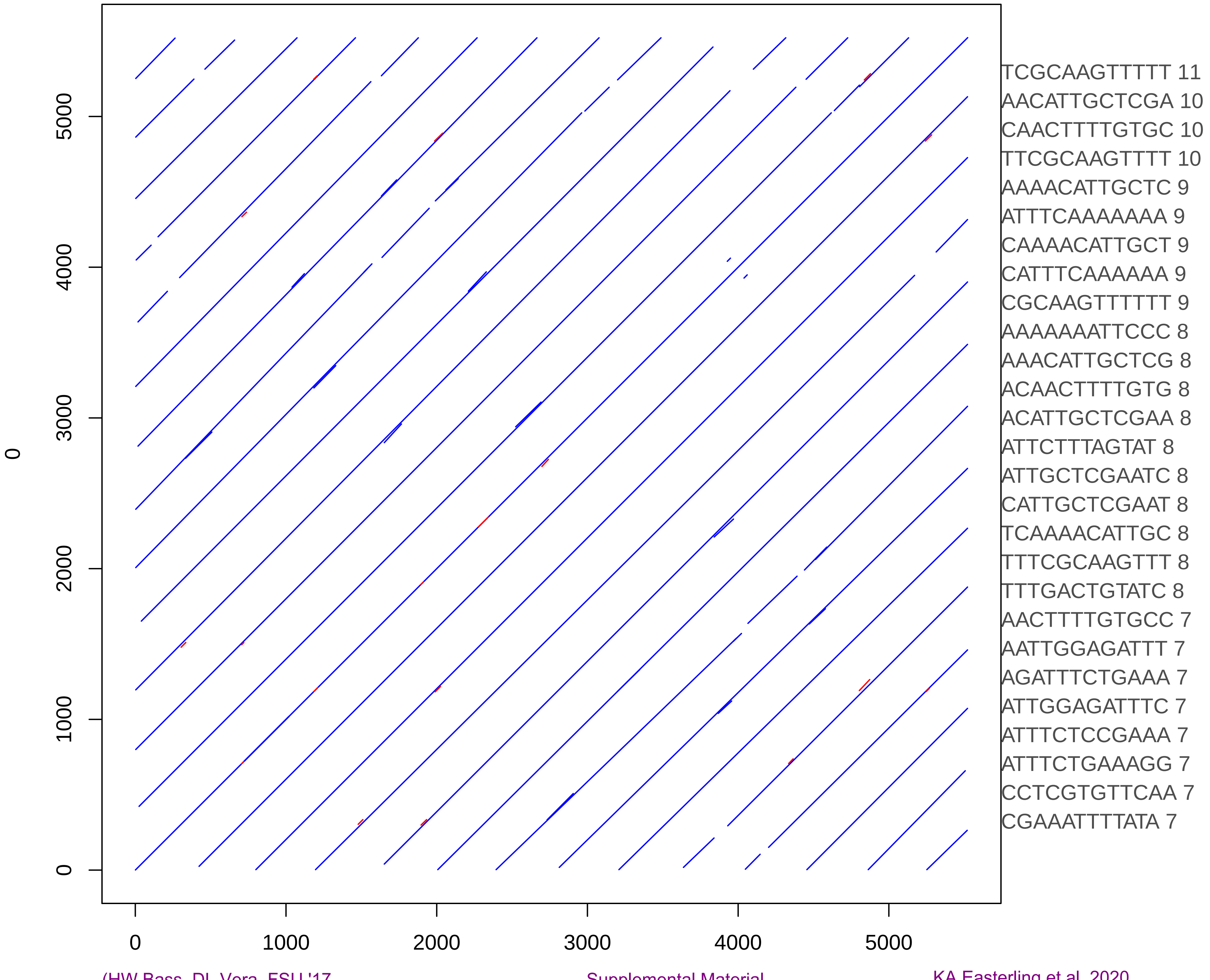

>m141126_104651_42134_c100732262550000001823141405141502_s1_p0/49348/0_10676

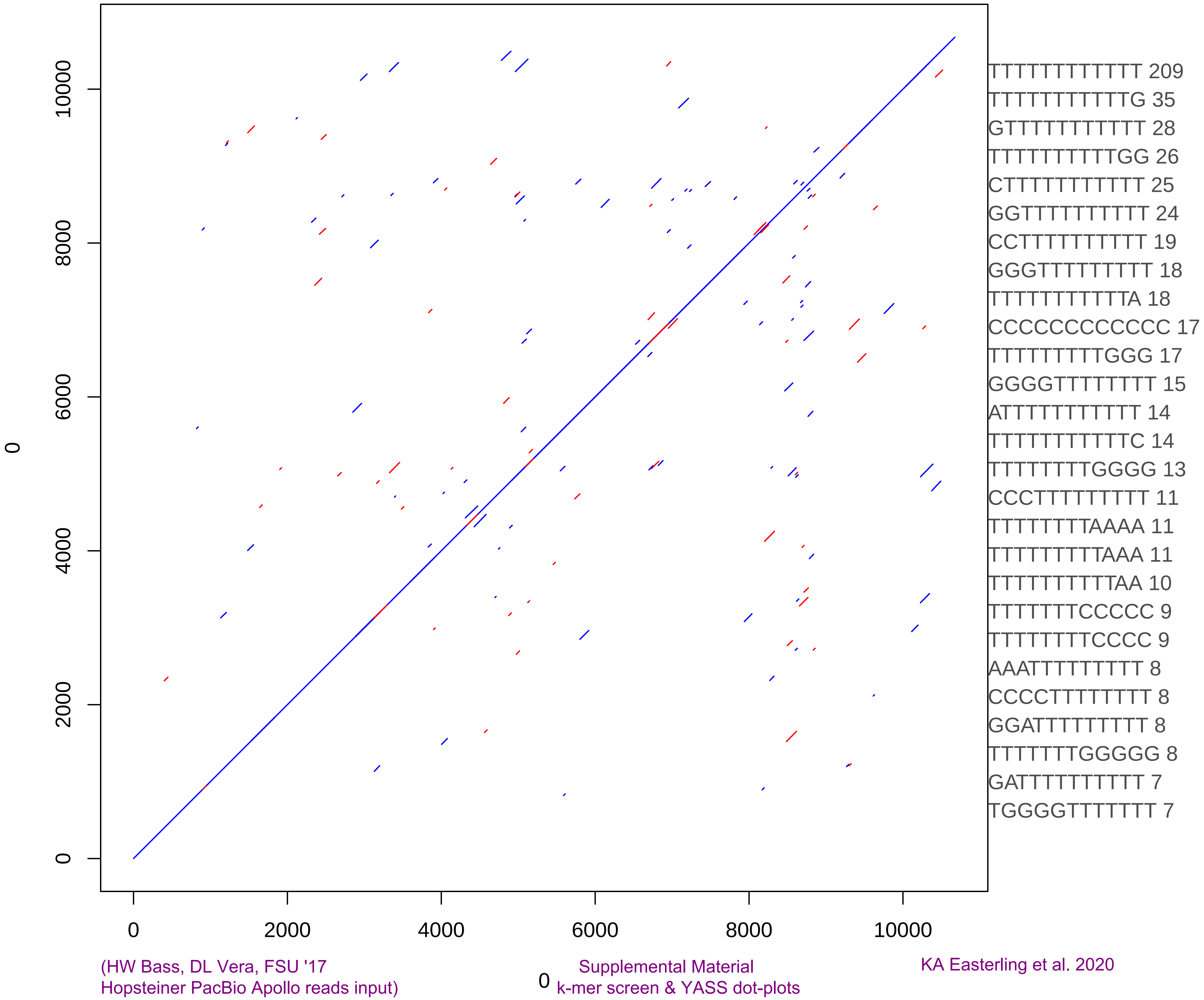

>m141126_104651_42134_c100732262550000001823141405141502_s1_p0/5066/11632_17139

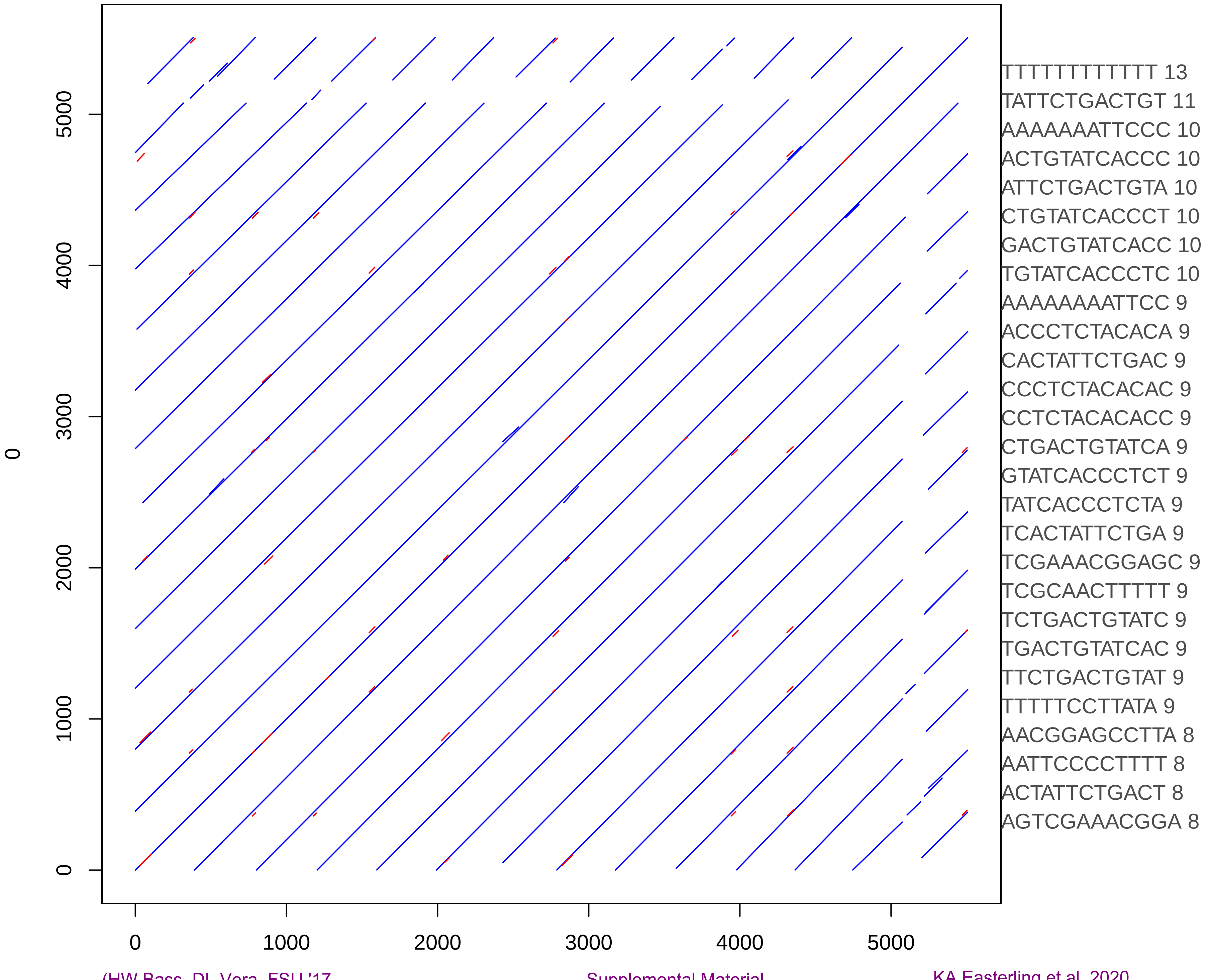

>m141126_104651_42134_c100732262550000001823141405141502_s1_p0/5066/2738_11587

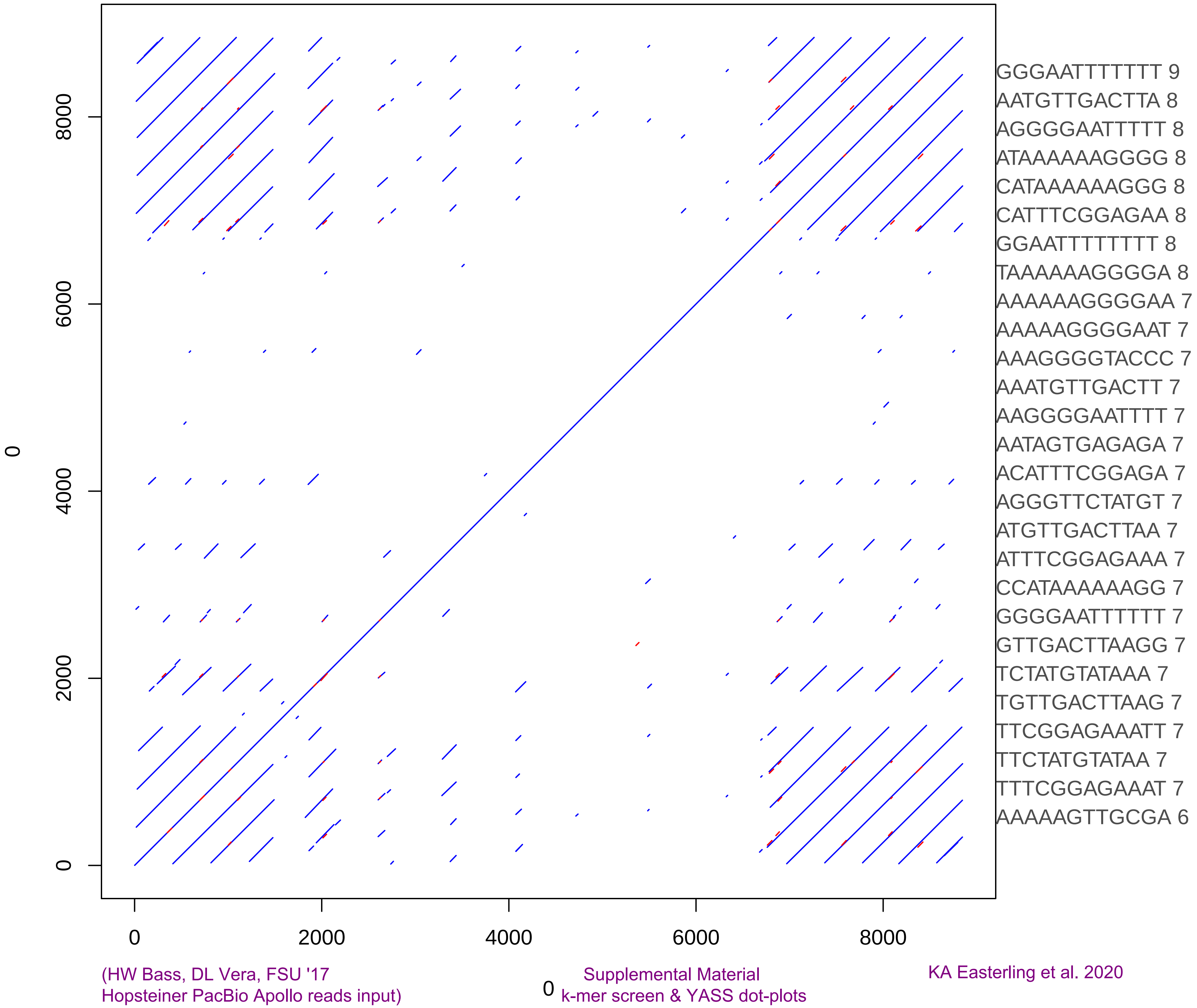

>m141126_104651_42134_c100732262550000001823141405141502_s1_p0/55757/10031_18632

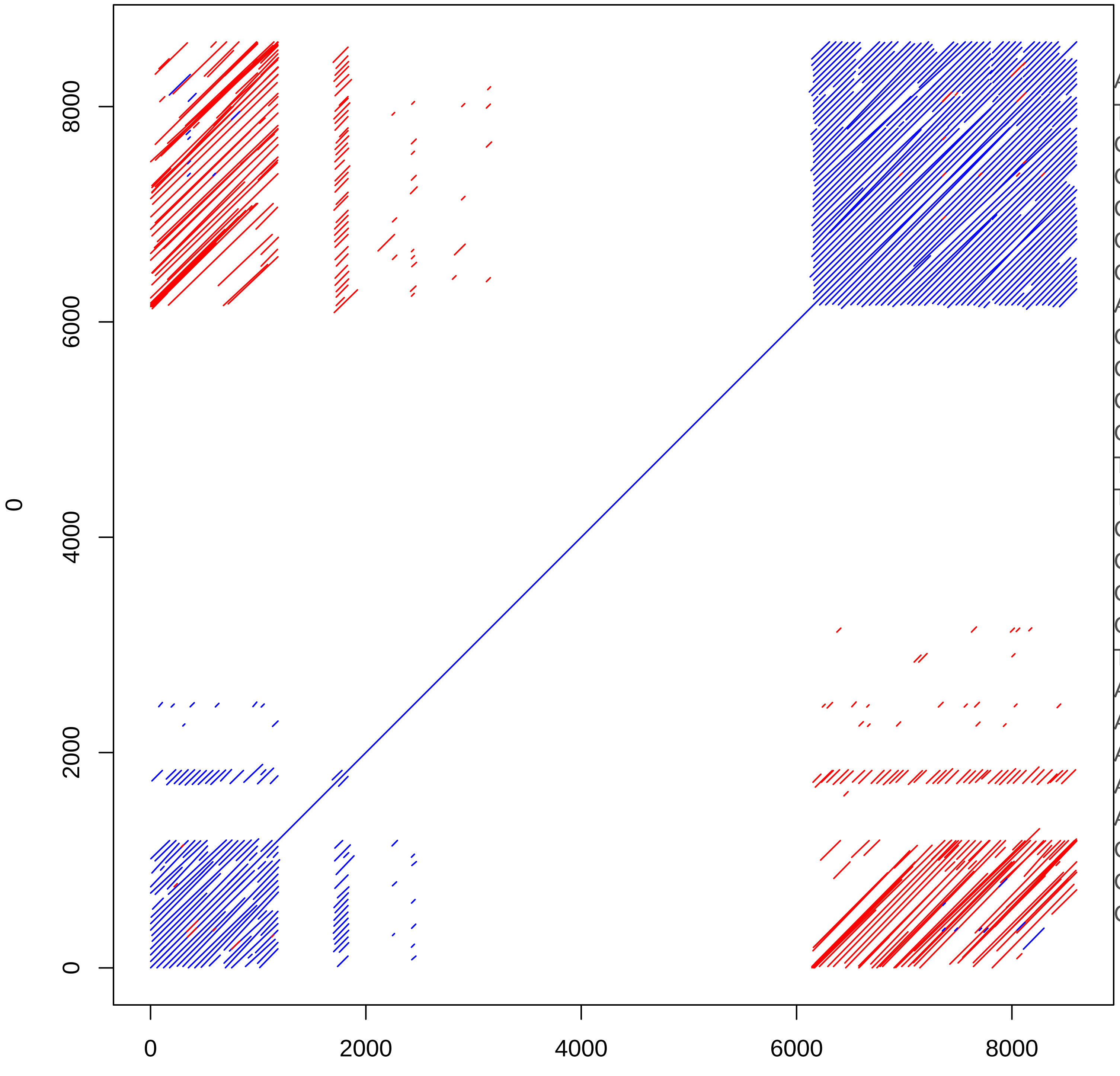

AGGCTTGGTCCT 12
TGAGGCTTGGTC 11
CCTGAGGCTTGG 10
CTCTTTATTATA 10
CTGAGGCTTGGT 10
GAGGCTTGGTCC 10
GCCTGAGGCTTG 10
AGCCTGAGGCTT 9
CCCCGGACATCG 9
CCCGGACATCGT 9
CTTGGTCCTCTT 9
GCTTGGTCCTCT 9
TCCTCTTTATTA 8
TTTATTATACCC 8
CCGGACATCGTT 7
CCTCTTTATTAT 7
GCTTGGTCCTTT 7
GGTCCTCTTTAT 7
TTGGTCCTCTTT 7
AAGAGGACCAAG 6
ACCAAGCCTCAG 6
ACCCCCGGACAT 6
AGAGGACCAAGC 6
AGGACCAAGCCT 6
CAGCCTGAGGCT 6
CCCCCGGACATC 6
CTTGGTCCTTTT 6

>m141126_104651_42134_c100732262550000001823141405141502_s1_p0/5785/1635_11447

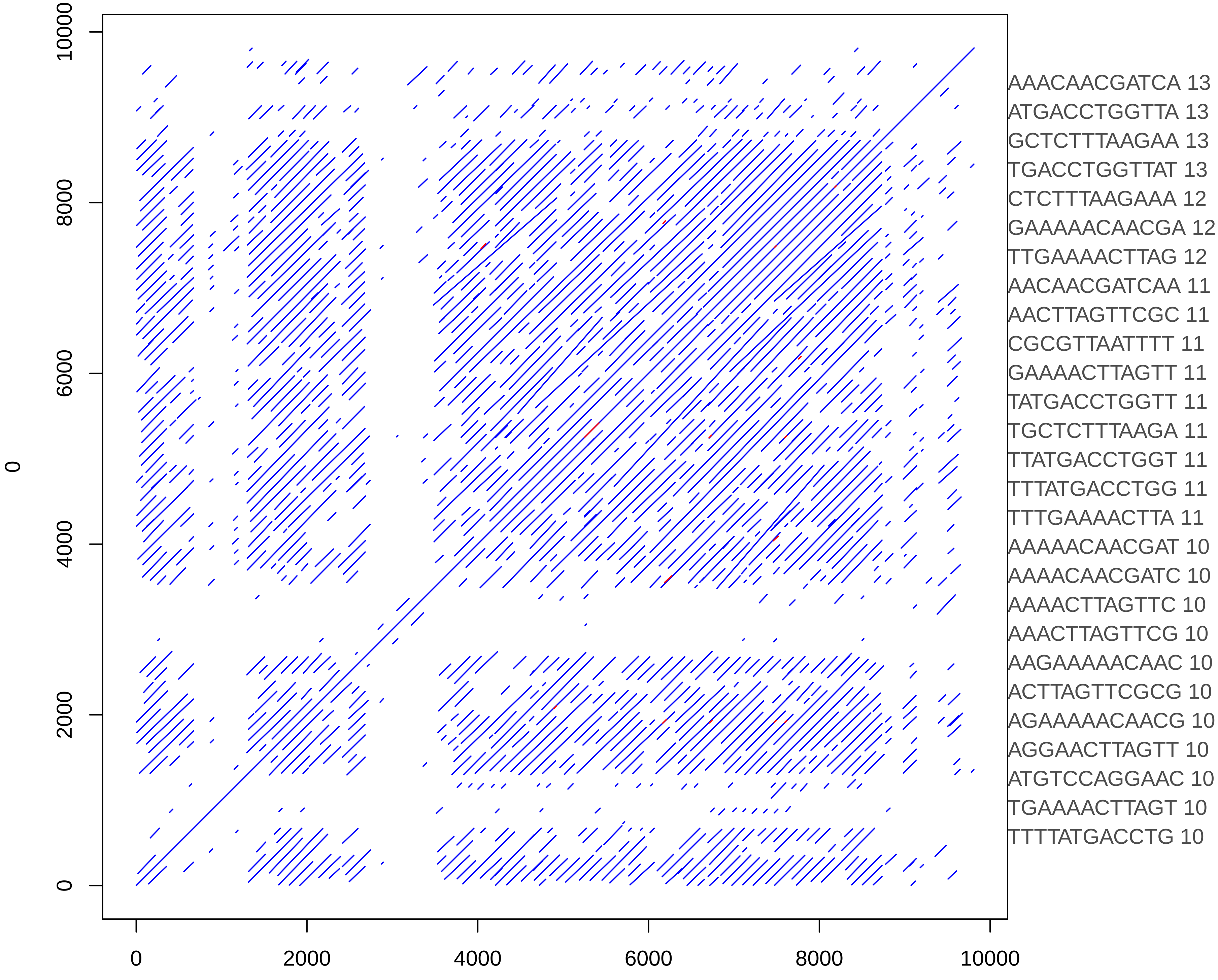

>m141126_104651_42134_c100732262550000001823141405141502_s1_p0/66764/1402_8305

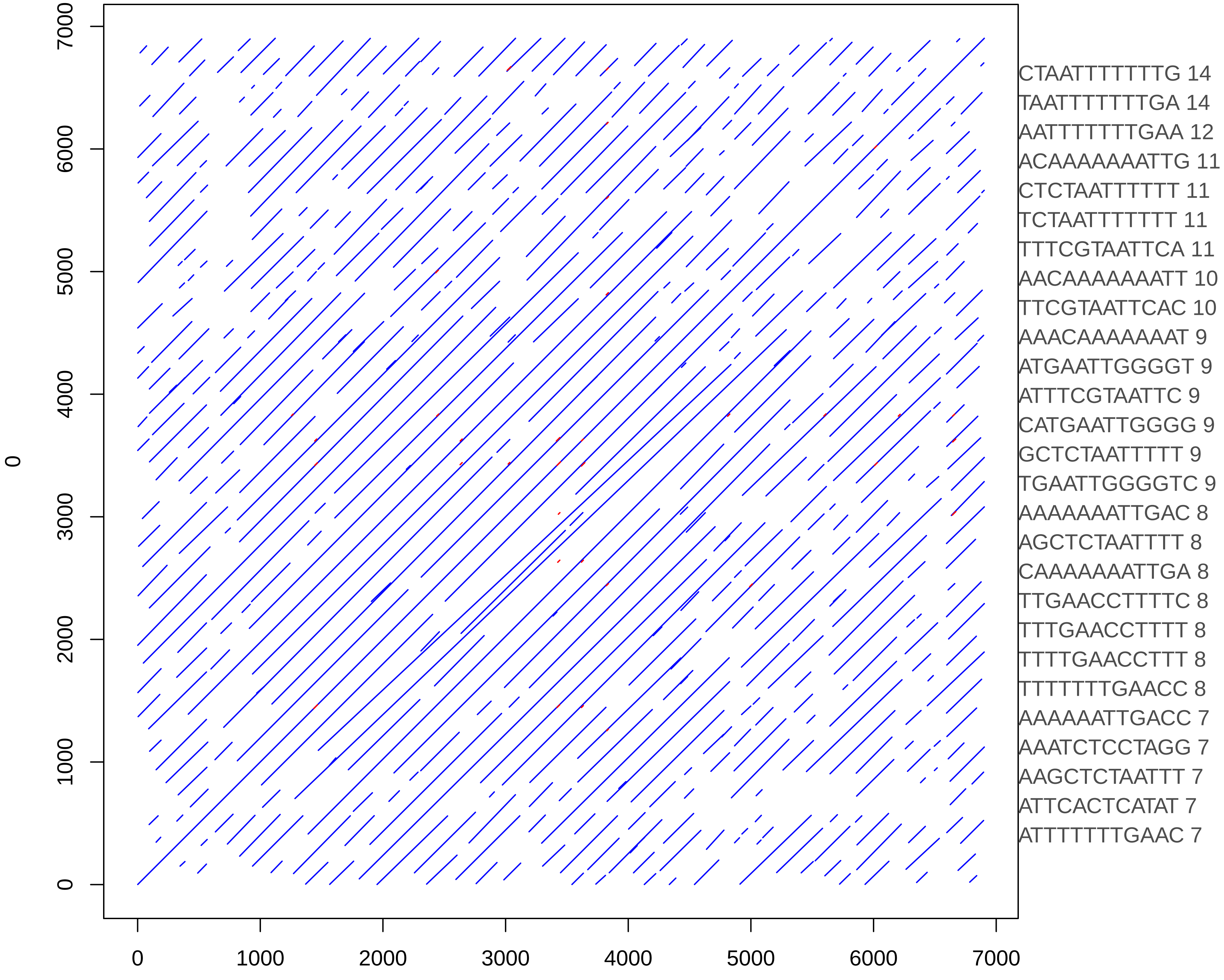

>m141126_104651_42134_c100732262550000001823141405141502_s1_p0/66775/3845_14898

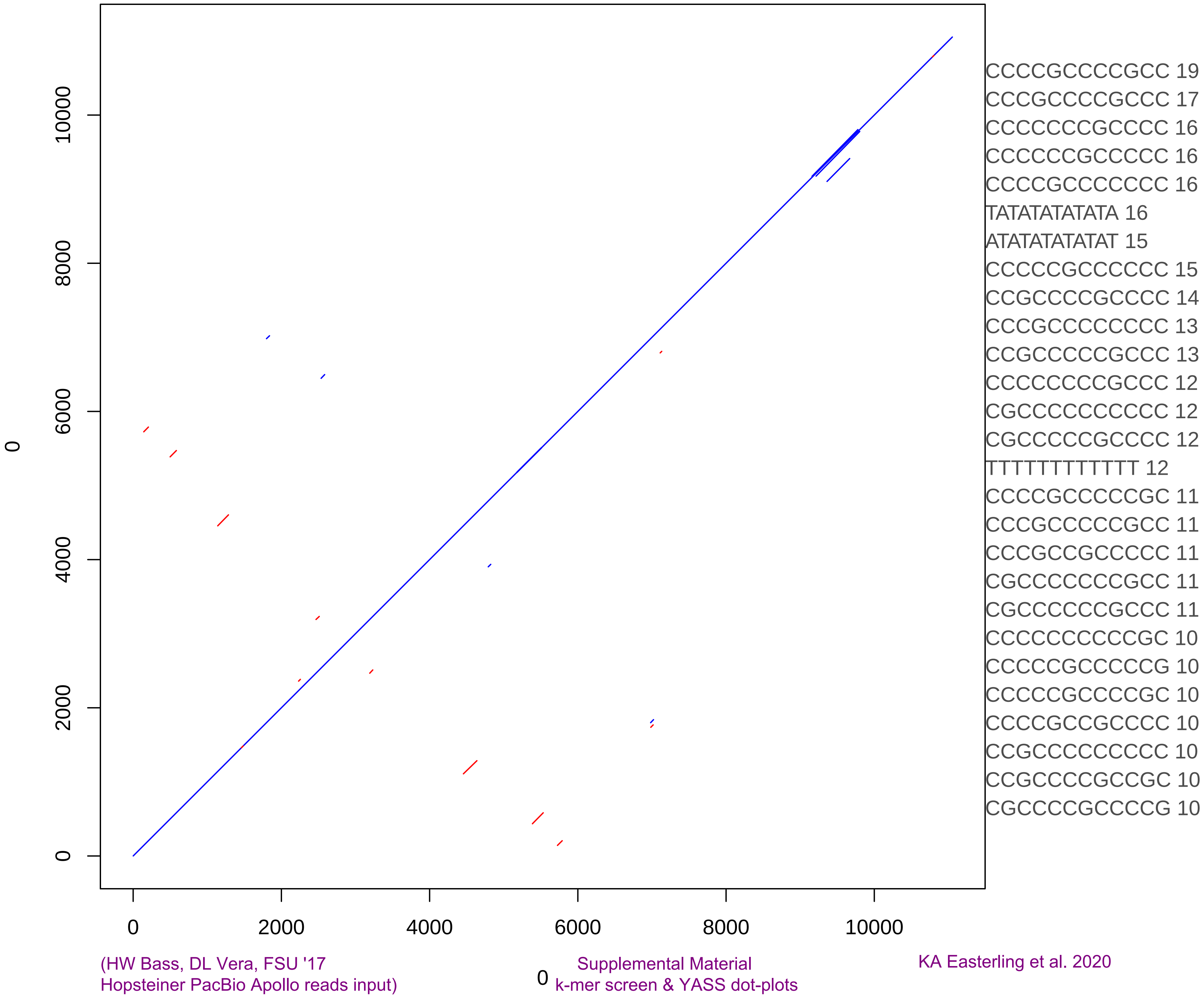

>m141126_104651_42134_c100732262550000001823141405141502_s1_p0/72543/8507_15314

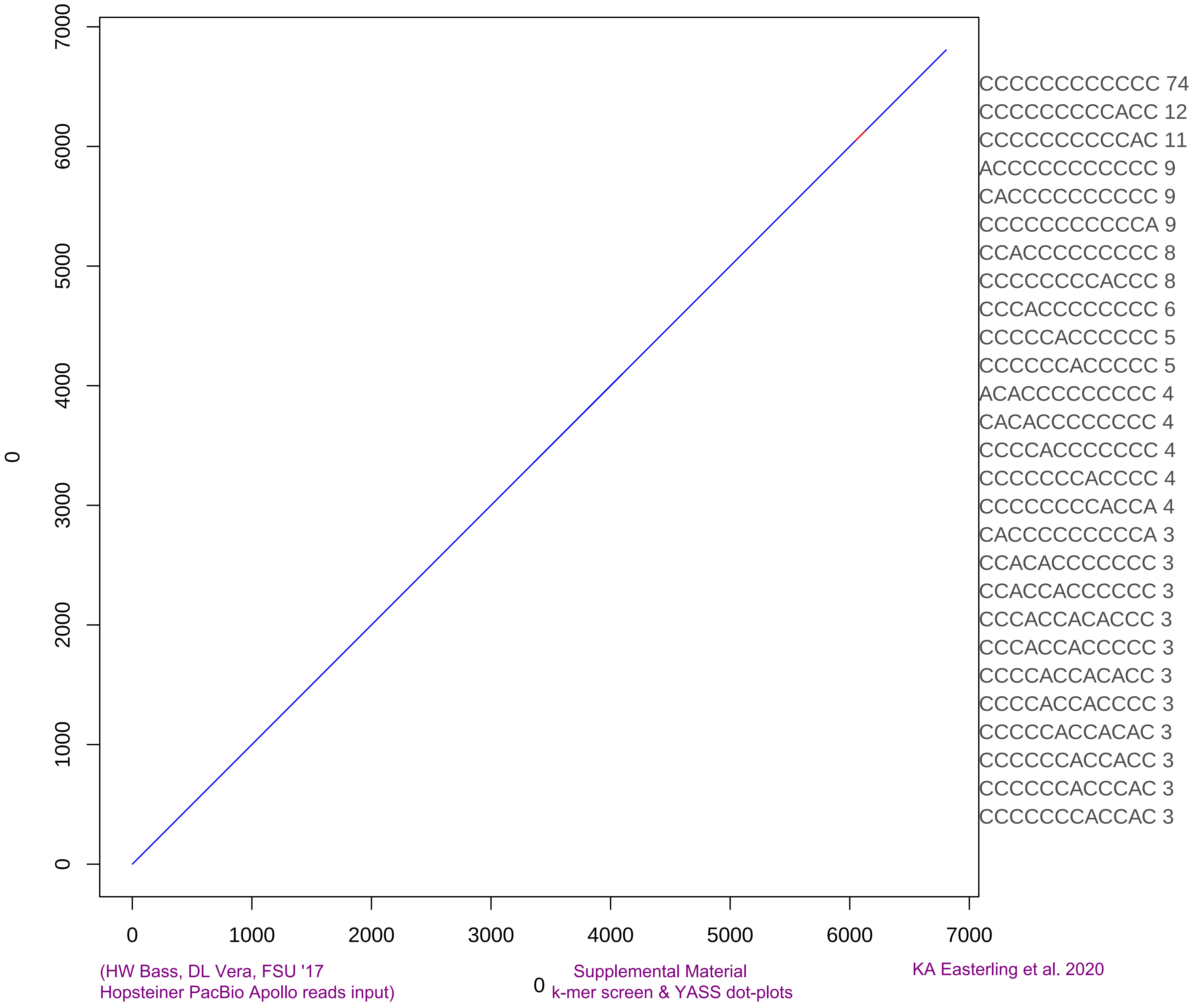

>m141126_104651_42134_c100732262550000001823141405141502_s1_p0/72689/719_7598

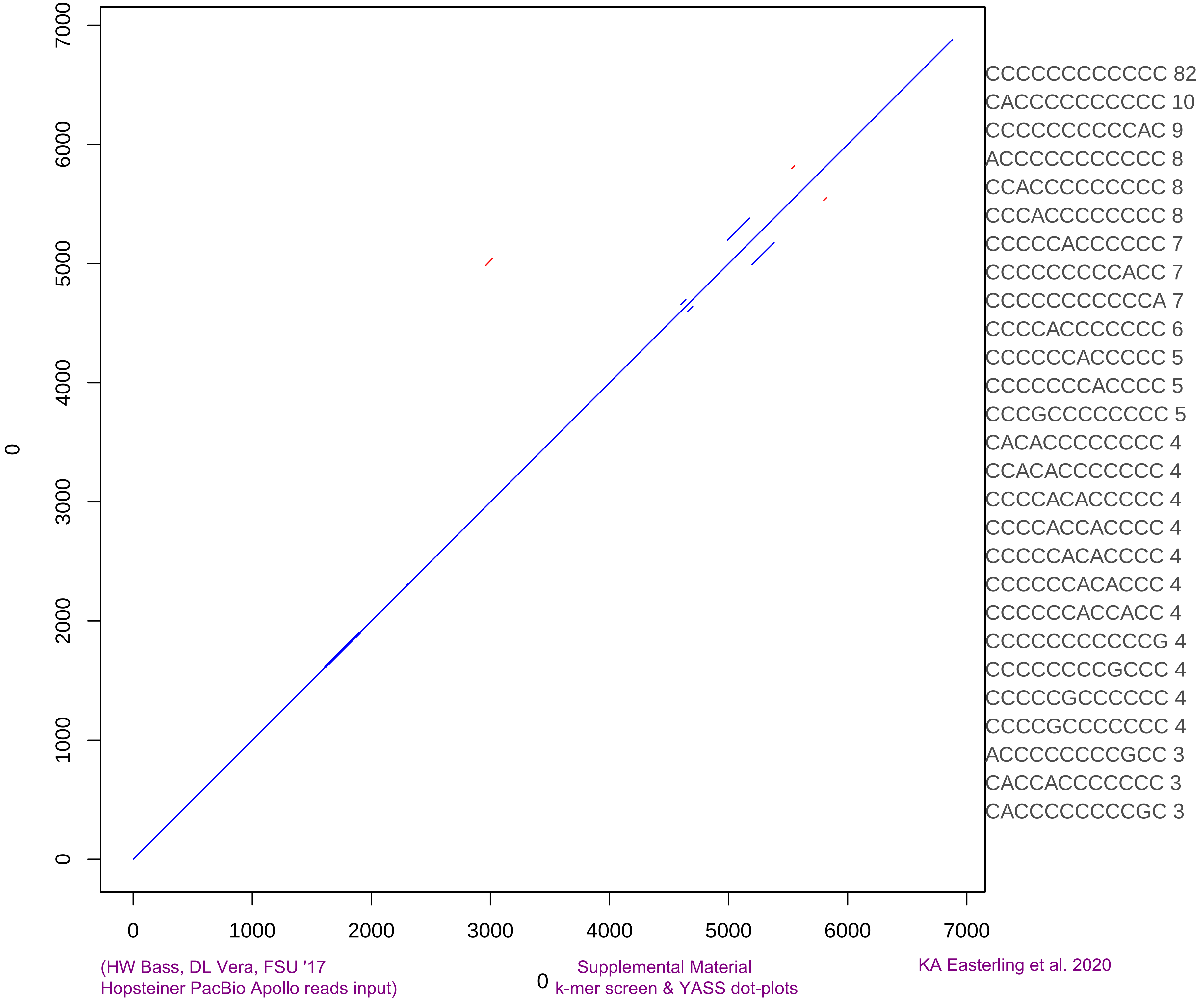

>m141126_104651_42134_c100732262550000001823141405141502_s1_p0/81441/0_6232

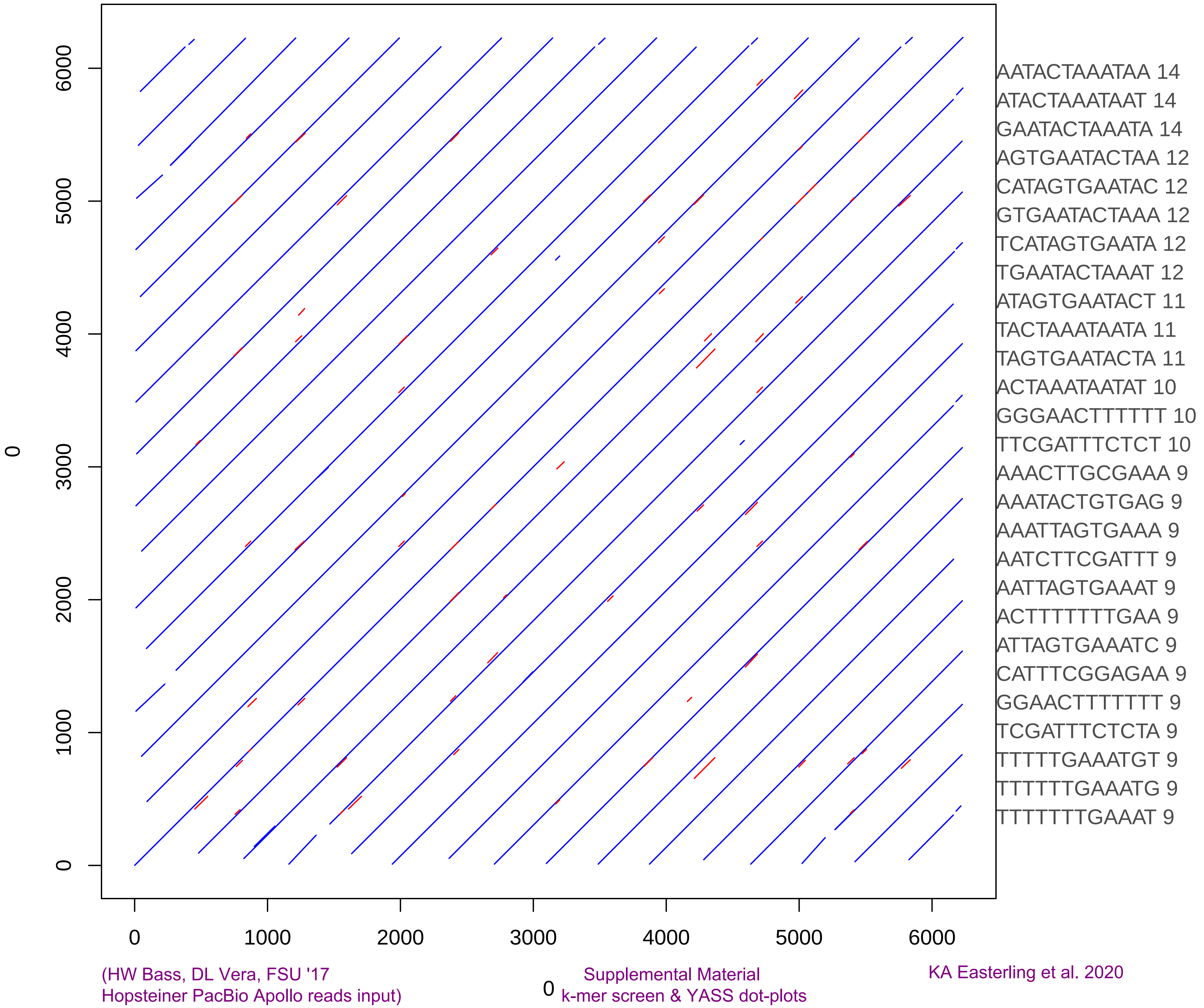

>m141126_104651_42134_c100732262550000001823141405141502_s1_p0/8244/0_5115

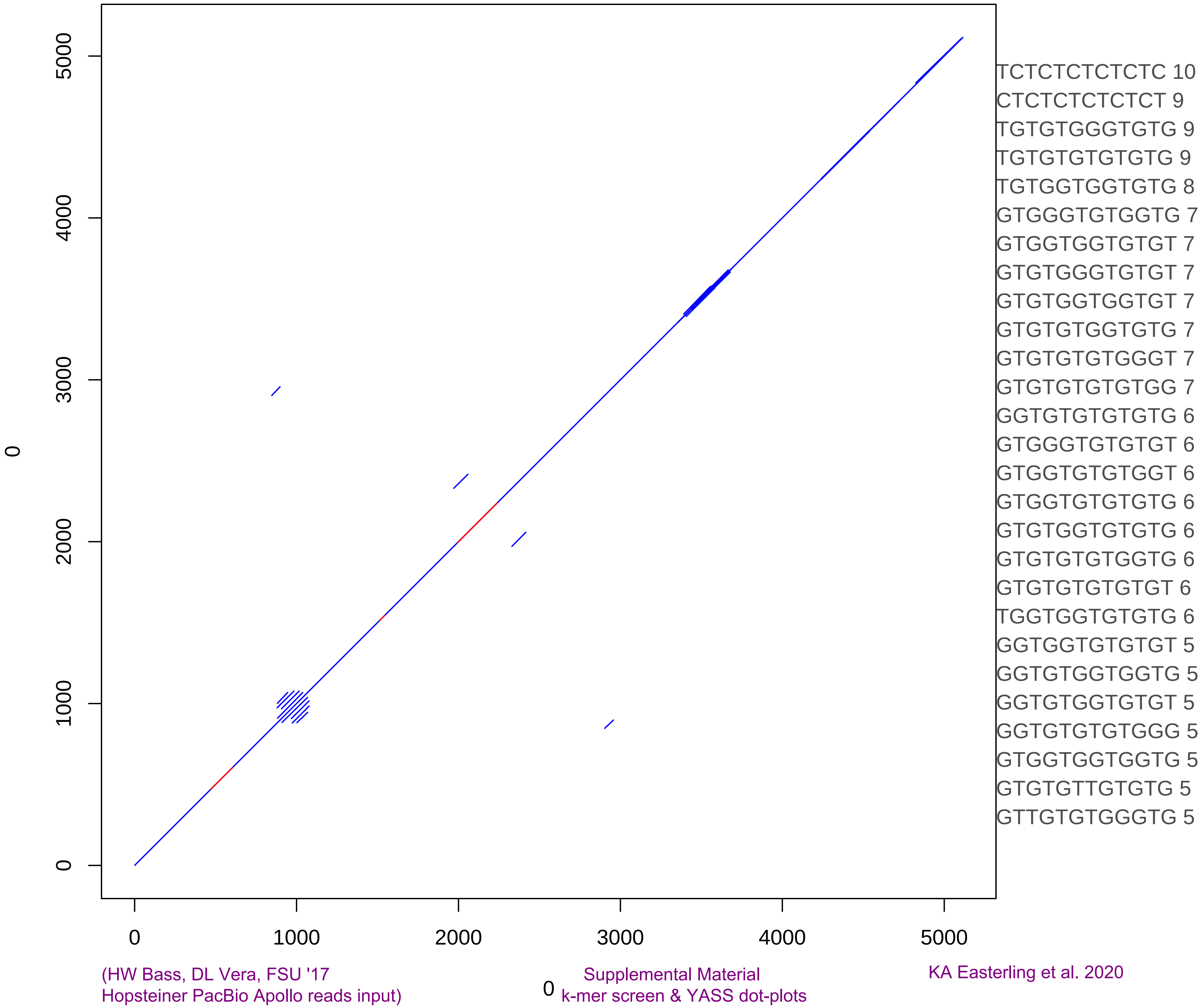

>m141126_104651_42134_c100732262550000001823141405141502_s1_p0/86027/4921_12032

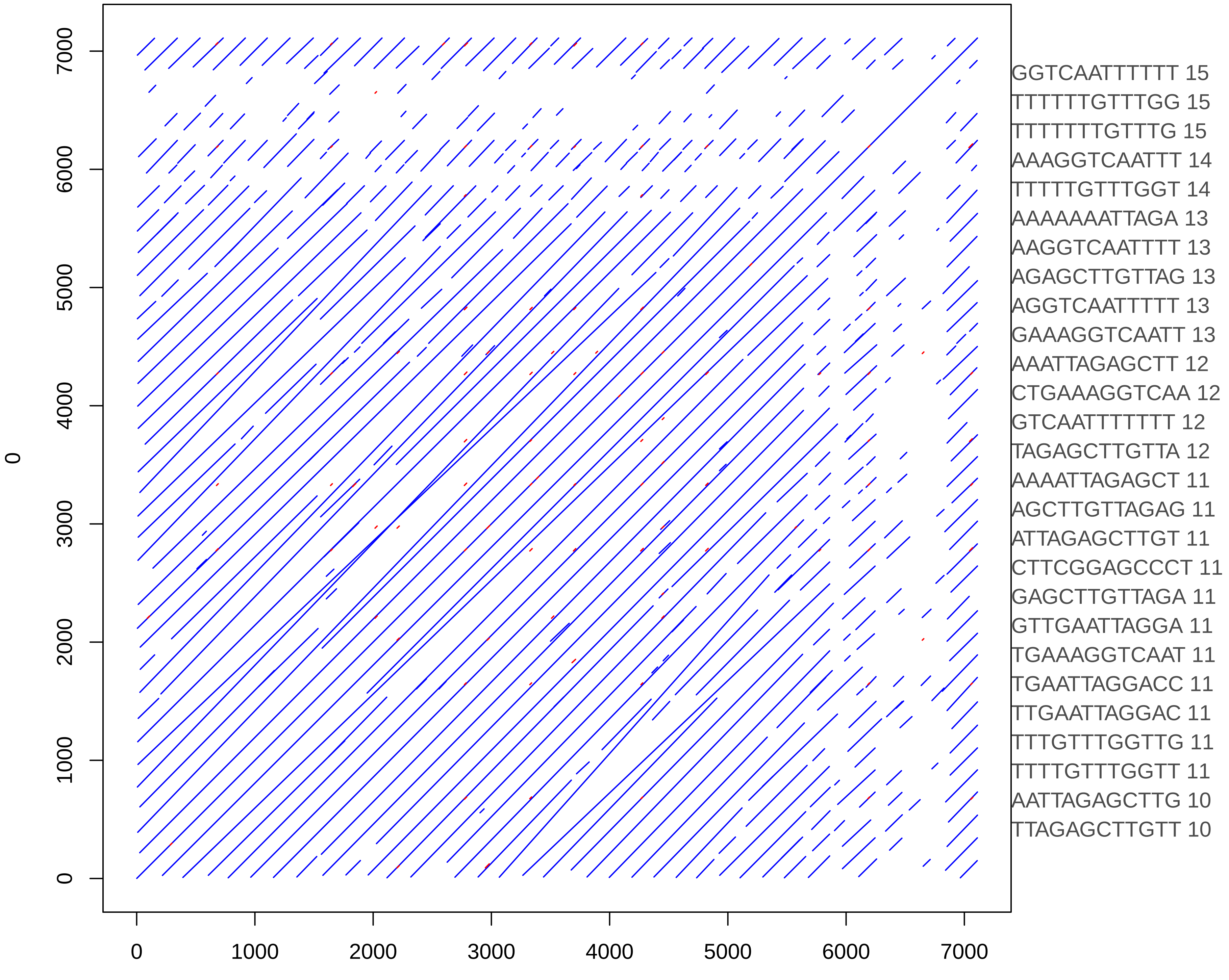

>m141126_104651_42134_c100732262550000001823141405141502_s1_p0/86682/3238_18030

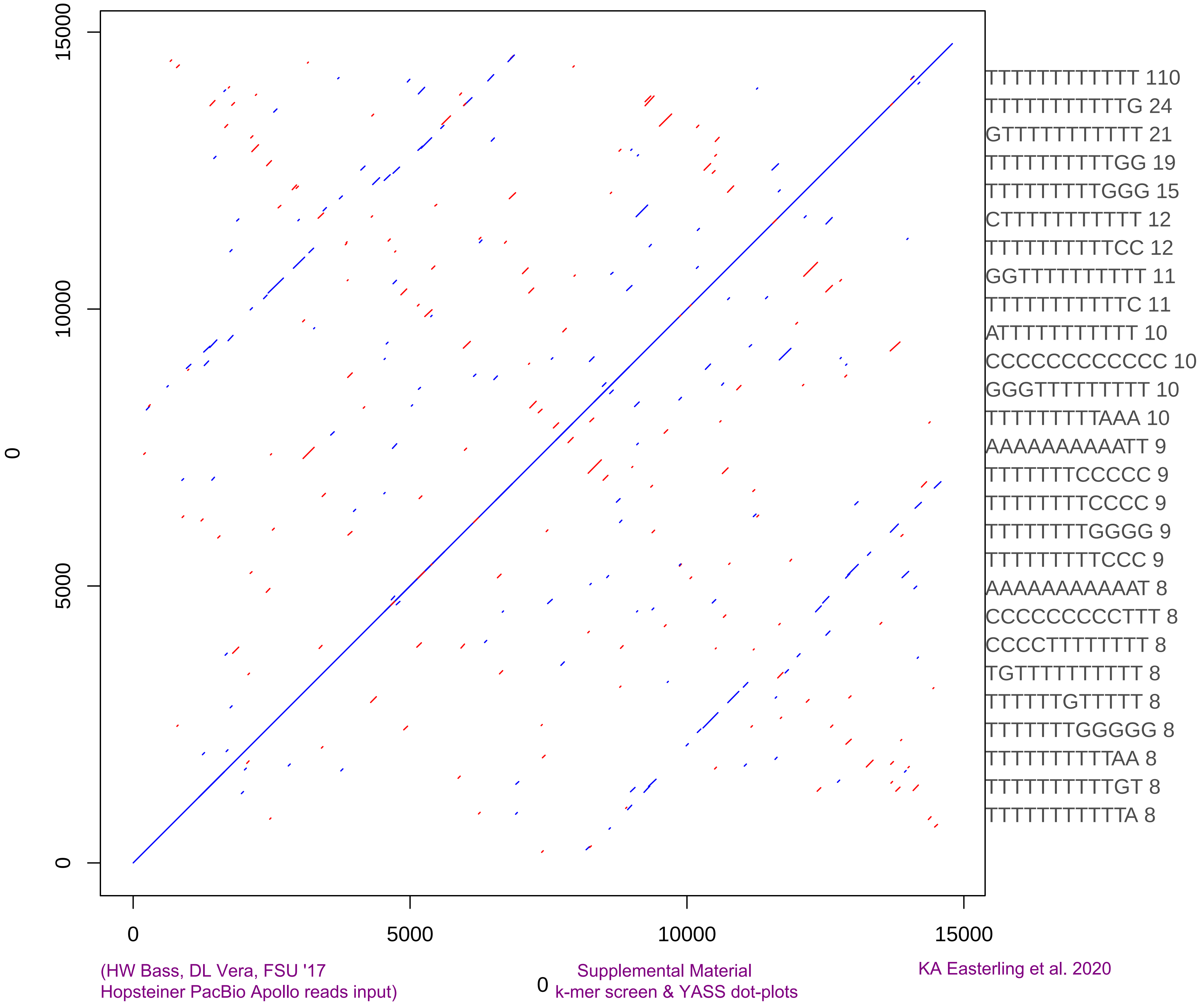

>m141126_104651_42134_c100732262550000001823141405141502_s1_p0/88261/7313_18153

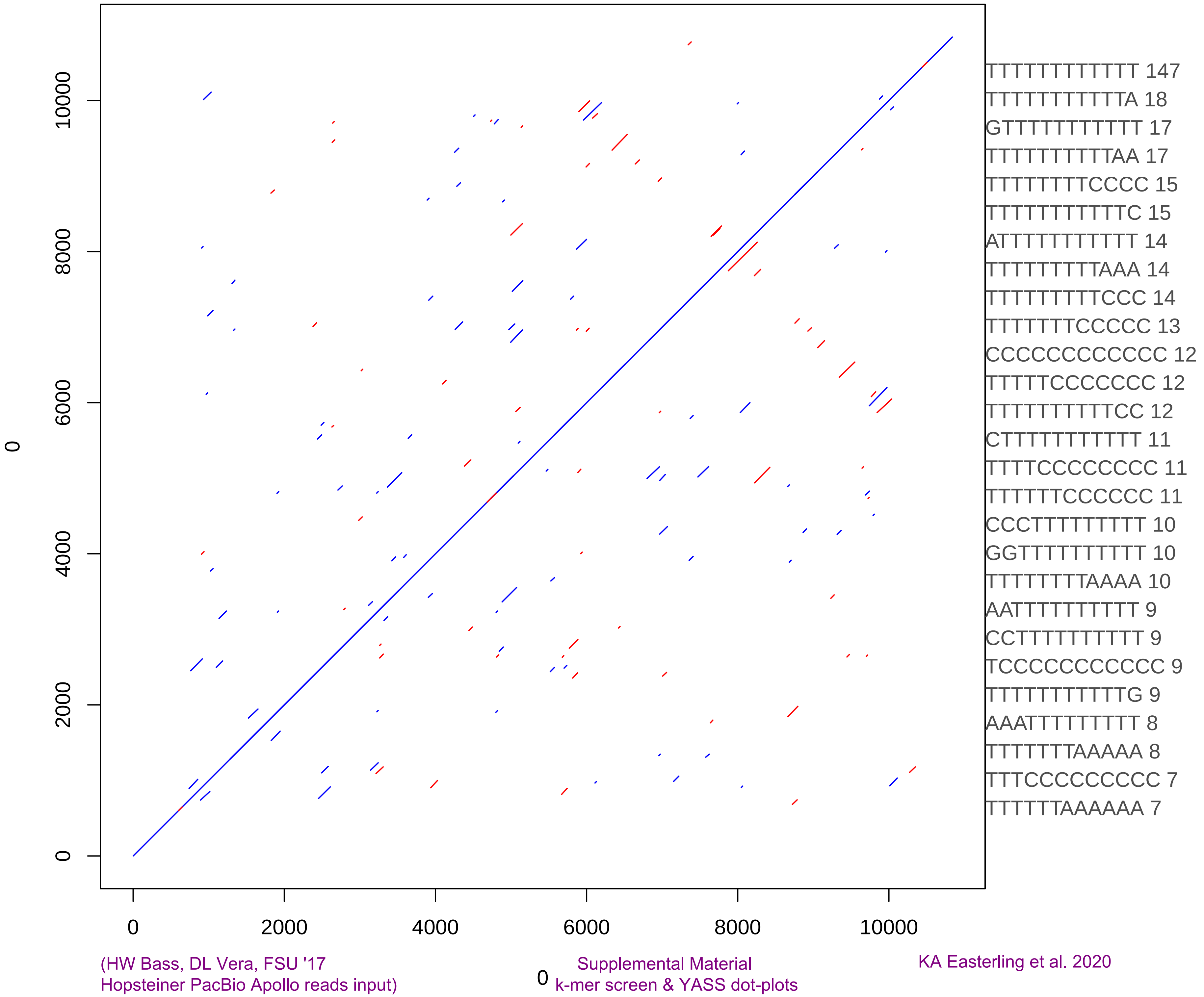

>m141126_104651_42134_c100732262550000001823141405141502_s1_p0/8883/203_5867

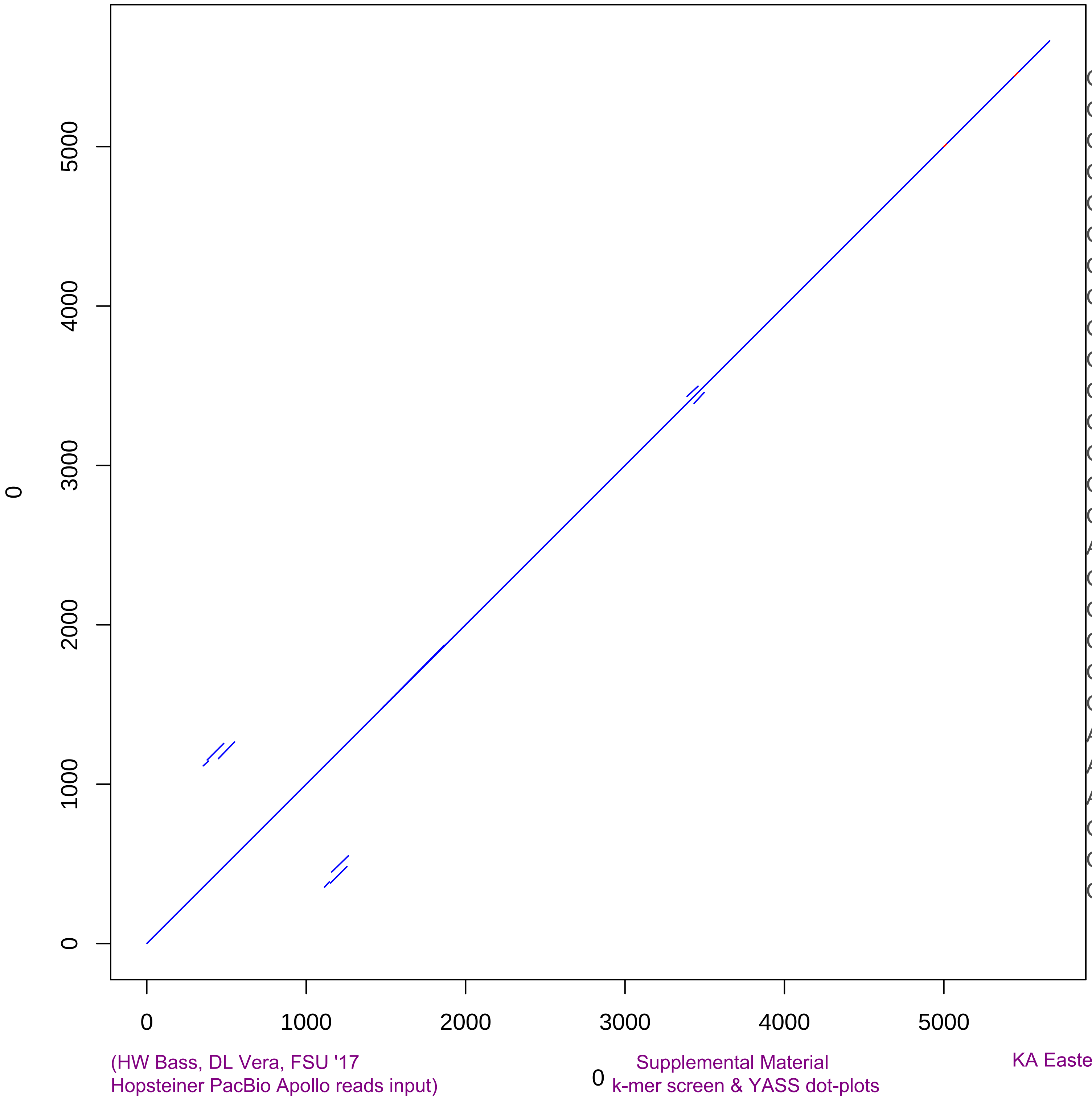

CCCCCCCCCCCC 113
CCCCCCCCCCGC 12
CCCCCCCCCGCC 12
CCGCCCCCCCCC 11
CCCCCCCCCCCG 10
CCCCCCCCGCCC 10
CCCGCCCCCCCC 10
CGCCCCCCCCCC 10
CCCCCGCCCCCC 9
CCCCCCCGCCCC 8
CCCCCCGCCCCC 8
CCCCGCCCCCCC 8
GCCCCCCCCCCC 8
CCCGCCCGCCCC 4
CCGCCCGCCCCC 4
ACCCCCCCCCCC 3
CACCCCCCCCCC 3
CCCCCGCCCGCC 3
CCCCGCCCGCCC 3
CGCCCCCGCCCC 3
GCCCCCGCCCCC 3
ACATAAACATAA 2
ATAAAATTCTAT 2
ATAATGCAATAA 2
CATAAACATAAA 2
CCACCCCCCCCC 2
CCCACCCCCCCC 2

>m141126_104651_42134_c100732262550000001823141405141502_s1_p0/89525/3680_11800

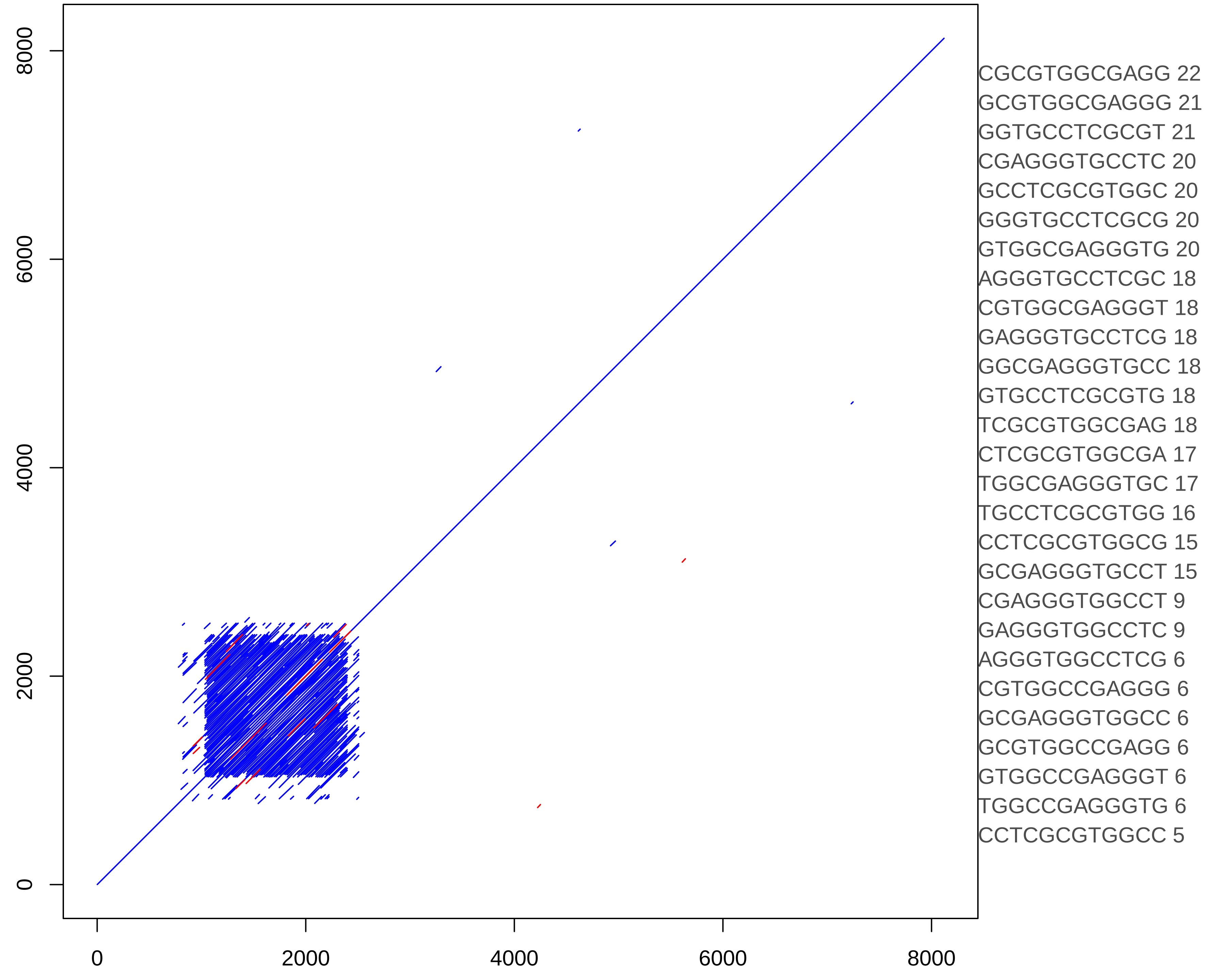

>m141126_104651_42134_c100732262550000001823141405141502_s1_p0/92165/4684_14743

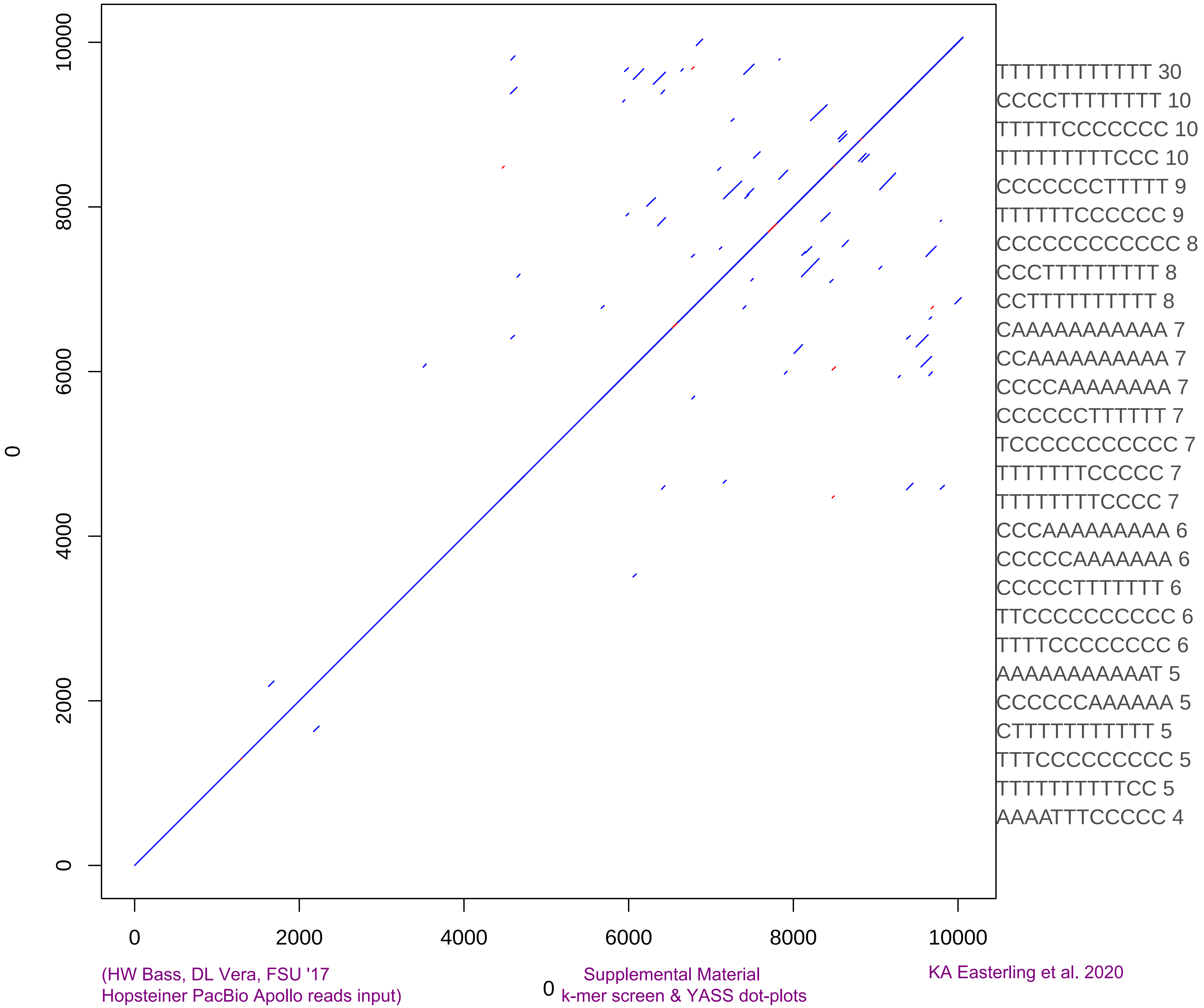

>m141126_104651_42134_c100732262550000001823141405141502_s1_p0/95911/7059_13349

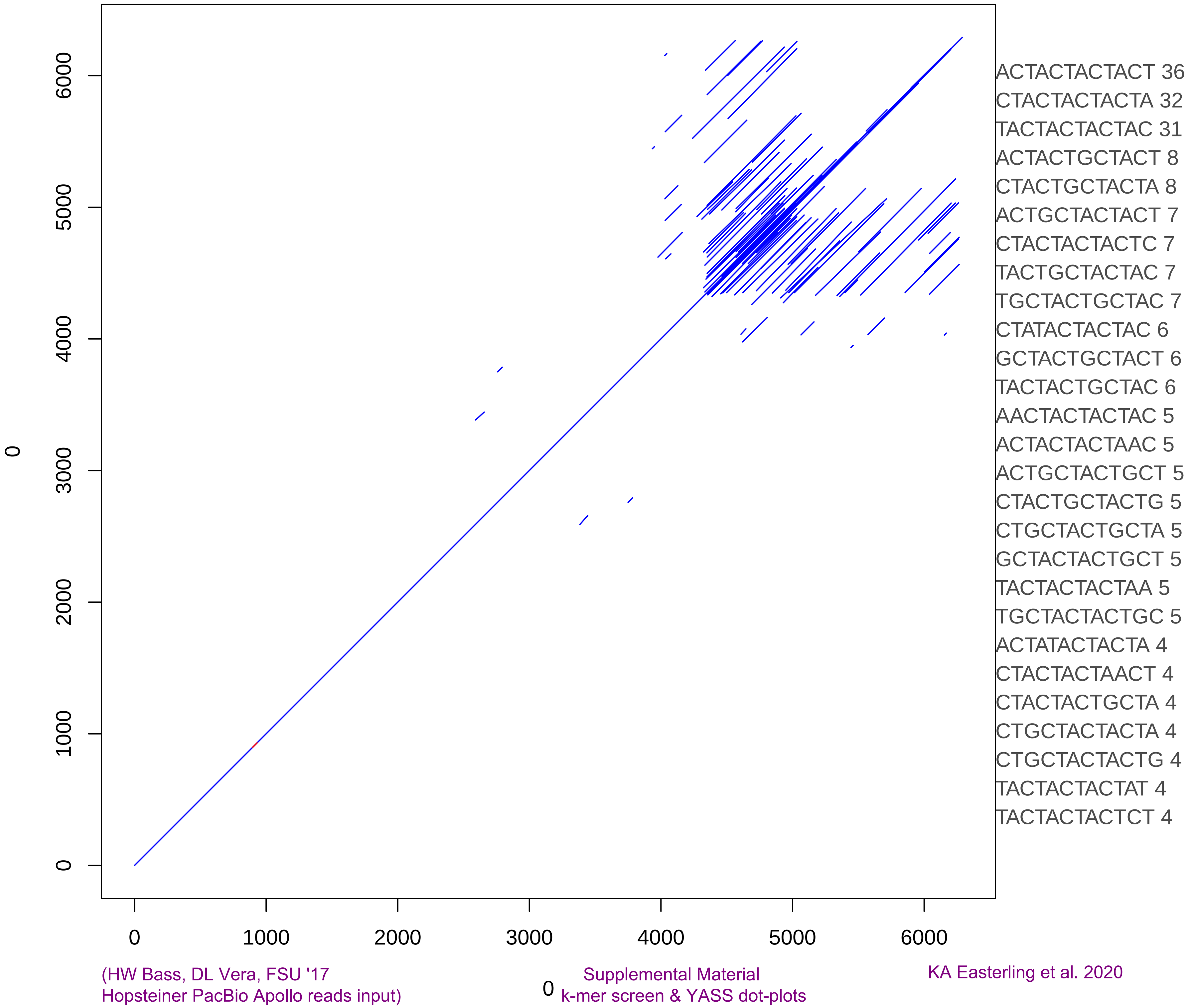

>m141126_104651_42134_c100732262550000001823141405141502_s1_p0/95941/8028_14582

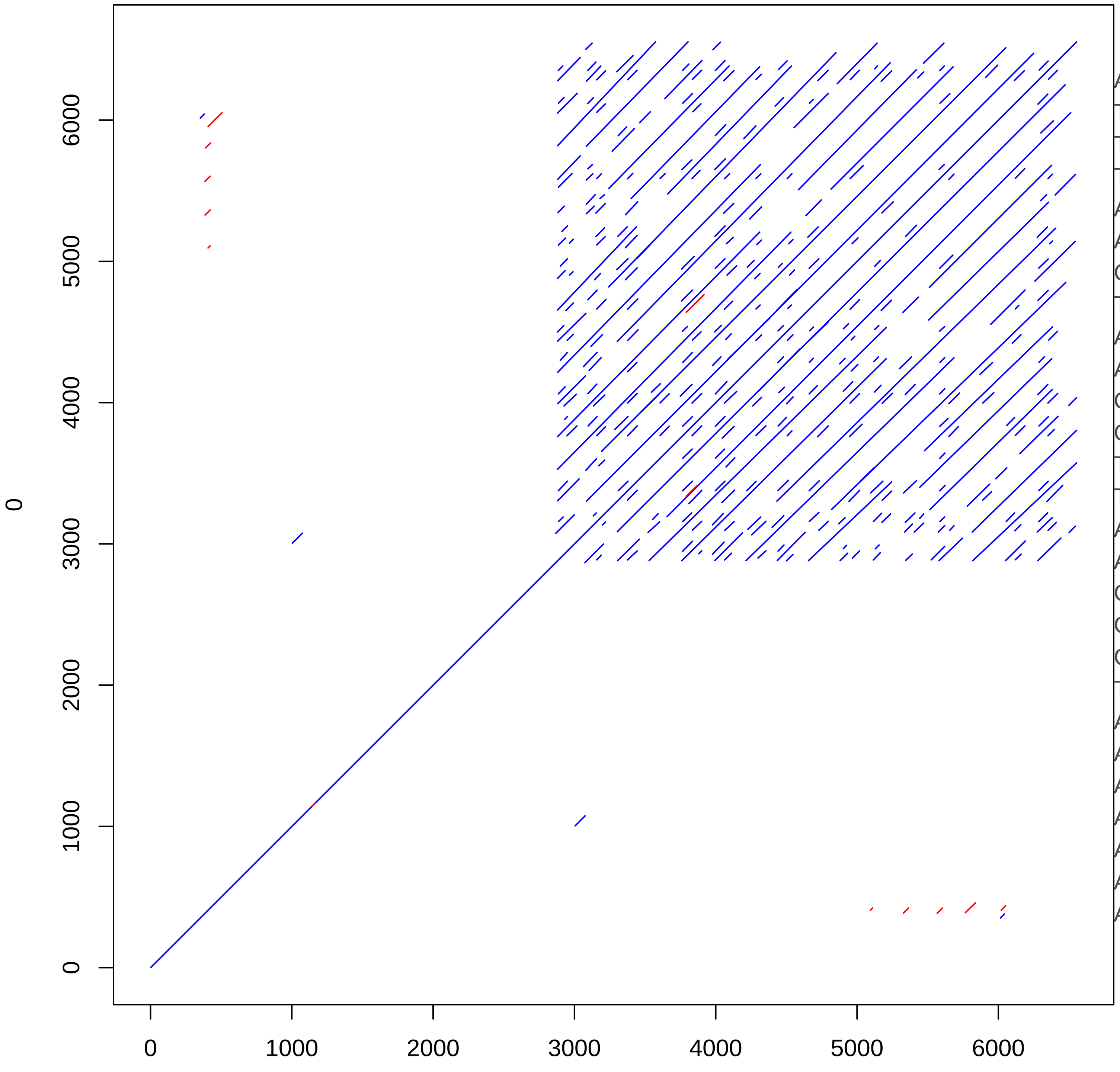

>m141126_130806_42134_c100732262550000001823141405141503_s1_p0/102620/461_10383

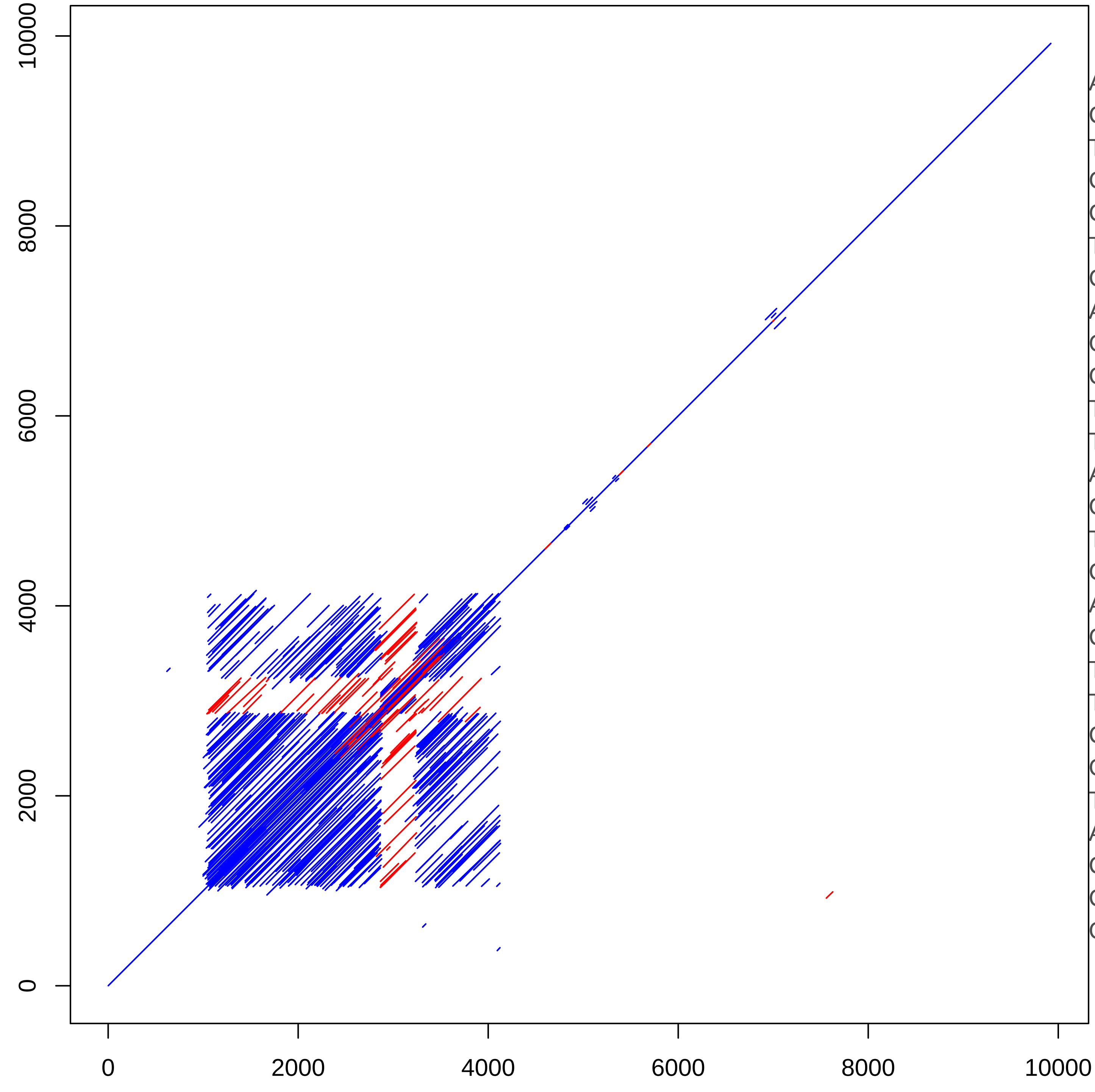

ACTACTACTACT 60
CTACTACTACTA 58
TACTACTACTAC 58
CTACTACTACTG 21
CTGCTACTGCTA 21
TACTGCTACTGC 20
CTACTGCTACTG 18
ACTGCTACTGCT 17
GCTACTGCTACT 17
GCTACTACTACT 16
TACTACTACTGC 16
TGCTACTGCTAC 16
ACTACTACTGCT 15
GCTGCTGCTGCT 14
TGCTGCTGCTGC 14
CTACTACTGCTG 13
ACTGCTACTACT 12
CTGCTGCTGCTG 12
TACTGCTACTAC 12
TGCTACTACTAC 12
CTGCTACTACTA 11
GCTGCTACTGCT 11
TGCTGCTACTGC 11
AGTAGTAGTAGT 10
CTACTGCTACTA 10
CTGCTGCTACTG 10
GCTGCTGCTACT 10

>m141126_130806_42134_c100732262550000001823141405141503_s1_p0/103884/2872_9234

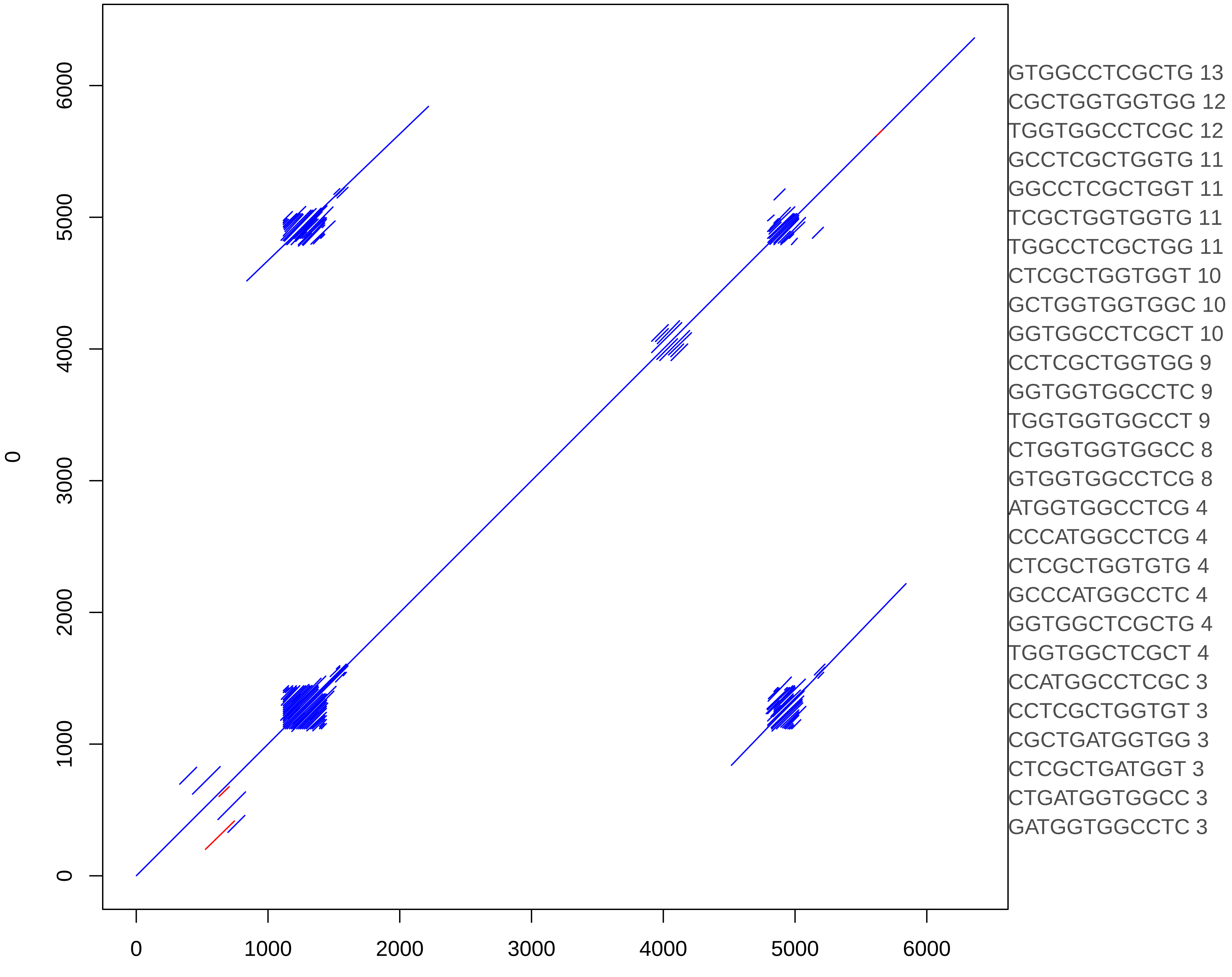

>m141126_130806_42134_c100732262550000001823141405141503_s1_p0/11670/7590_14159

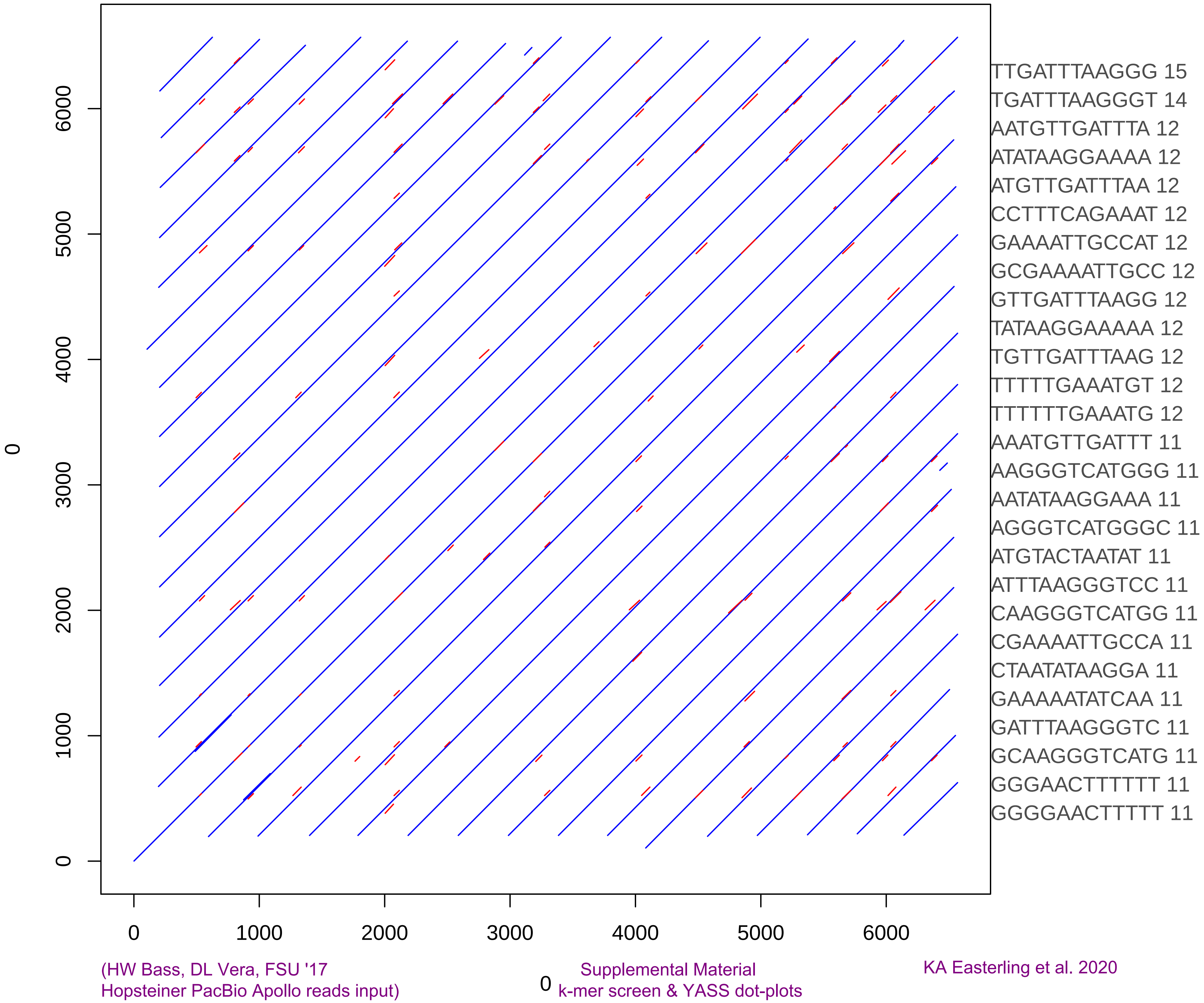

1,121 hits for repetitive sequences
passing 12mer abundance criterion

(Read-Number)
PAGE 740

For Tandem Repeat families of ### bp:
PacBio Read named "HuluTR###-r740"

>m141126_130806_42134_c100732262550000001823141405141503_s1_p0/117470/6446_15759

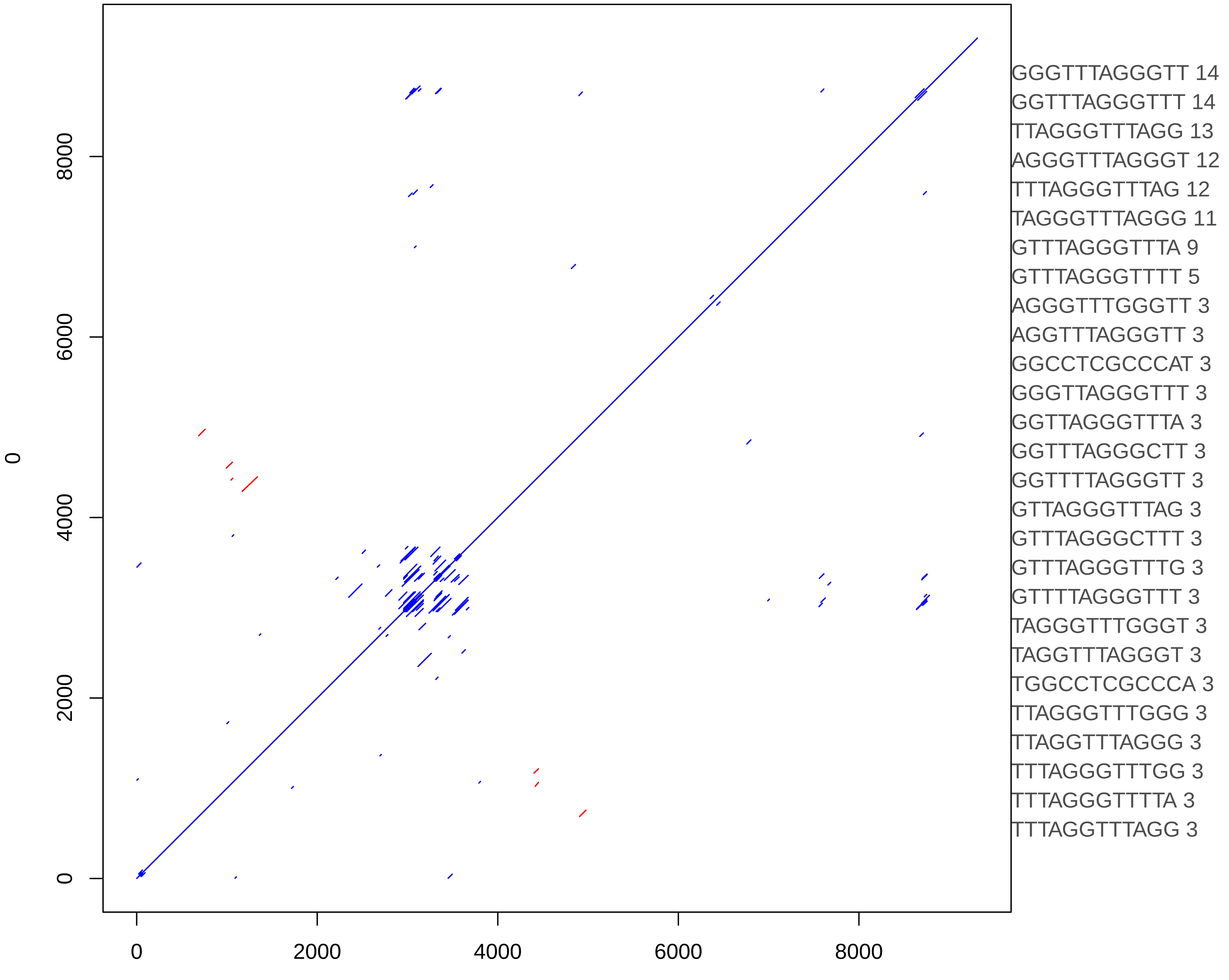

(HW Bass, DL Vera, FSU '17
Hopsteiner PacBio Apollo reads input)

0

Supplemental Material
k-mer screen & YASS dot-plots

KA Easterling et al. 2020

>m141126_130806_42134_c100732262550000001823141405141503_s1_p0/119301/424_9430

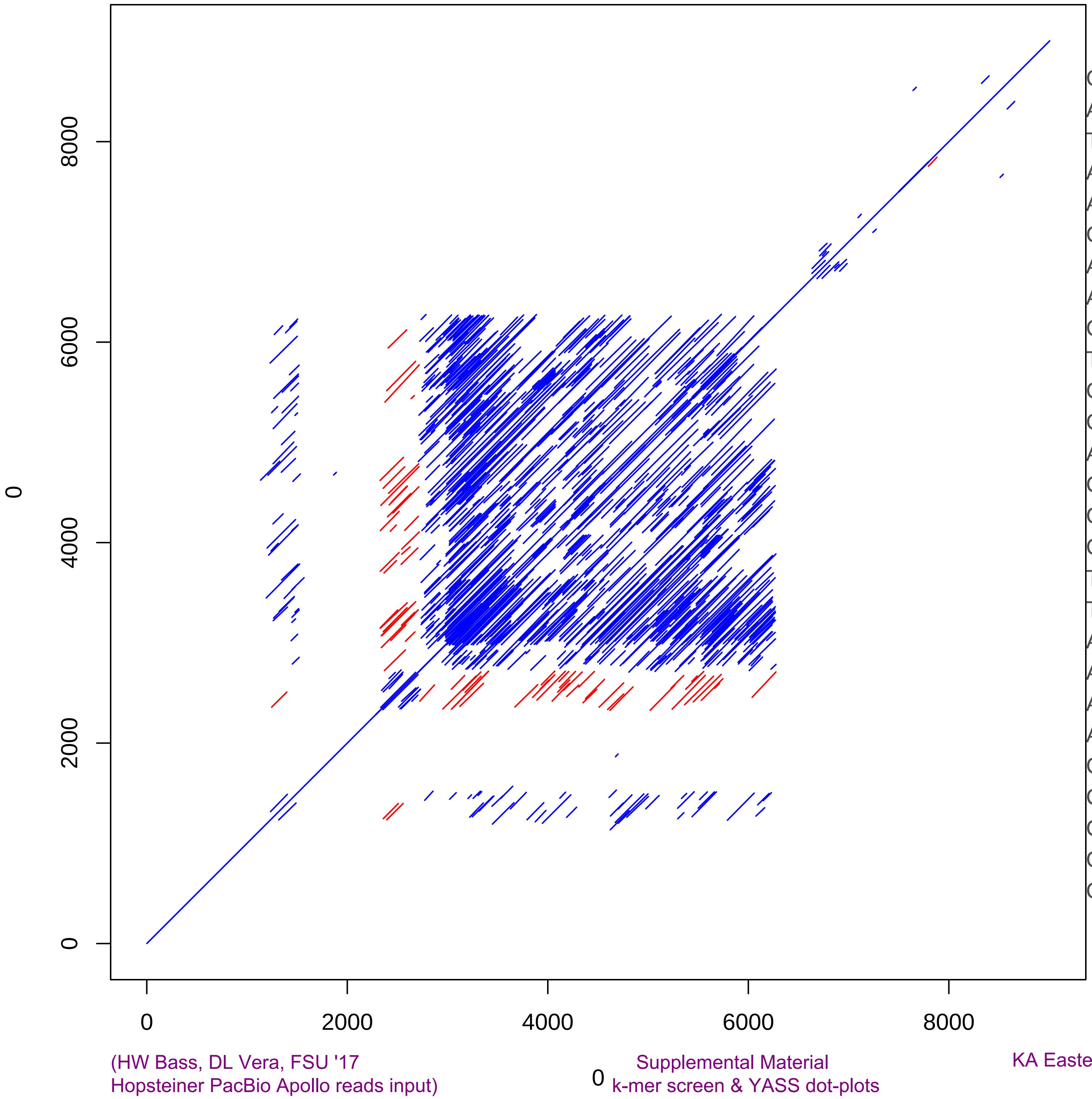

GTAGTAGTAGTA 38
AGTAGTAGTAGT 36
TAGTAGTAGTAG 32
AGTAGTATAGTA 11
AGTAGTAAGTAG 10
CAGTAGTAGTAG 10
AGTAGTAGTAGC 9
AGTATAGTAGTA 9
GTAGTAGTAGCA 9
TAGTAGTAAGTA 9
GTAGTAGCAGTA 8
GTAGTAGTAAGT 8
AGTAAGTAGTAG 7
CAGTAGCAGCAG 7
GCAGTAGCAGCA 7
GTAGCAGCAGCA 7
TAGTAAGTAGTA 7
TAGTAGTAGTAA 7
AAGTAGTAGTAG 6
AGTAGCAGCAGC 6
AGTAGTAGTAAG 6
ATAGTAGTAGTA 6
CAGCAGCAGCAG 6
CCAGTAGTAGTA 6
CCCCCCCCCCCC 6
GTAAGTAGTAGT 6
GTAGCAGTAGCA 6

1,121 hits for repetitive sequences
passing 12mer abundance criterion

For Tandem Repeat families of ### bp:
PacBio Read named "HuluTR###-r742"

>m141126_130806_42134_c100732262550000001823141405141503_s1_p0/119301/9475_16193

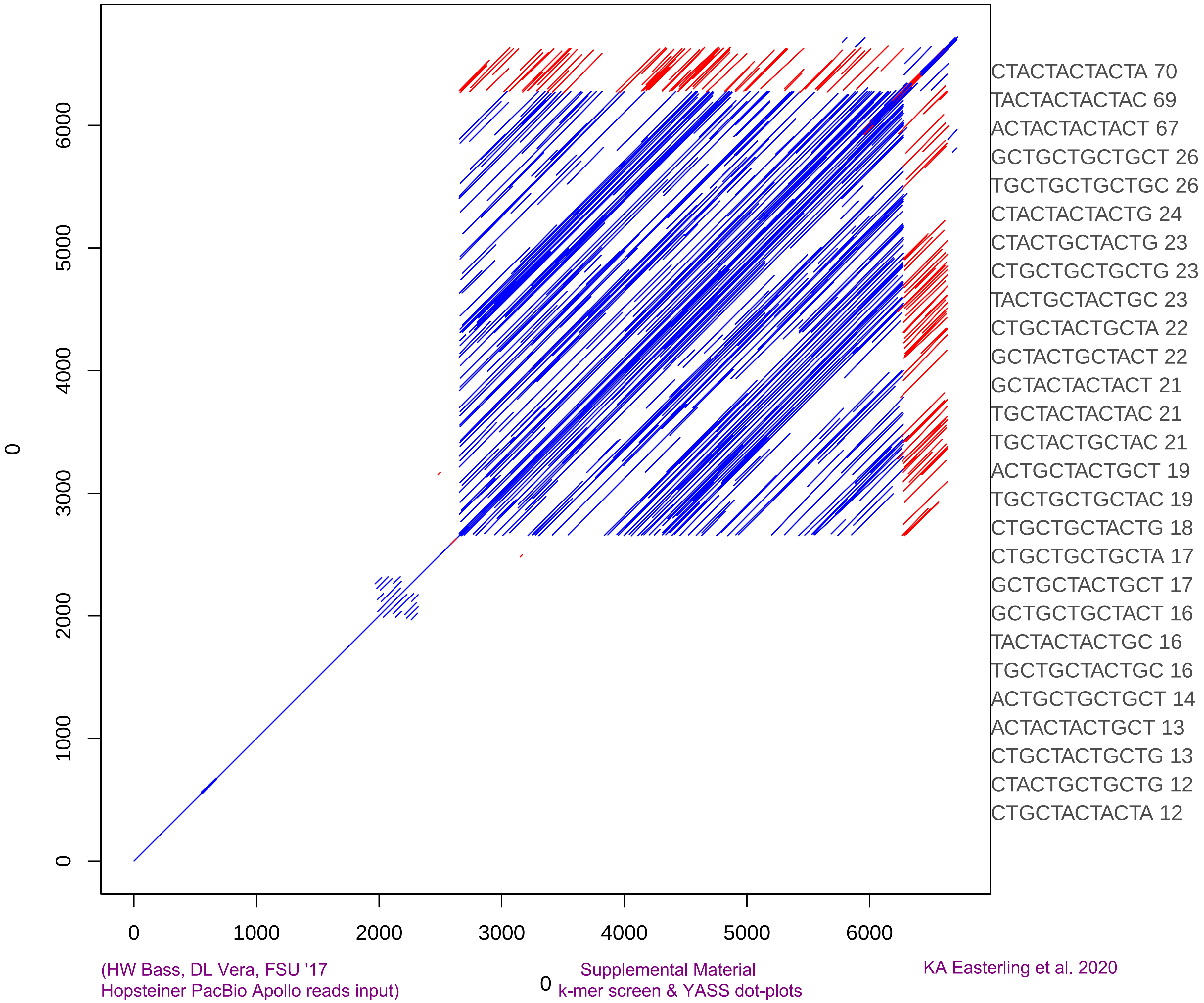

(HW Bass, DL Vera, FSU '17
Hopsteiner PacBio Apollo reads input)

Supplemental Material
k-mer screen & YASS dot-plots

KA Easterling et al. 2020

>m141126_130806_42134_c100732262550000001823141405141503_s1_p0/126060/0_8229

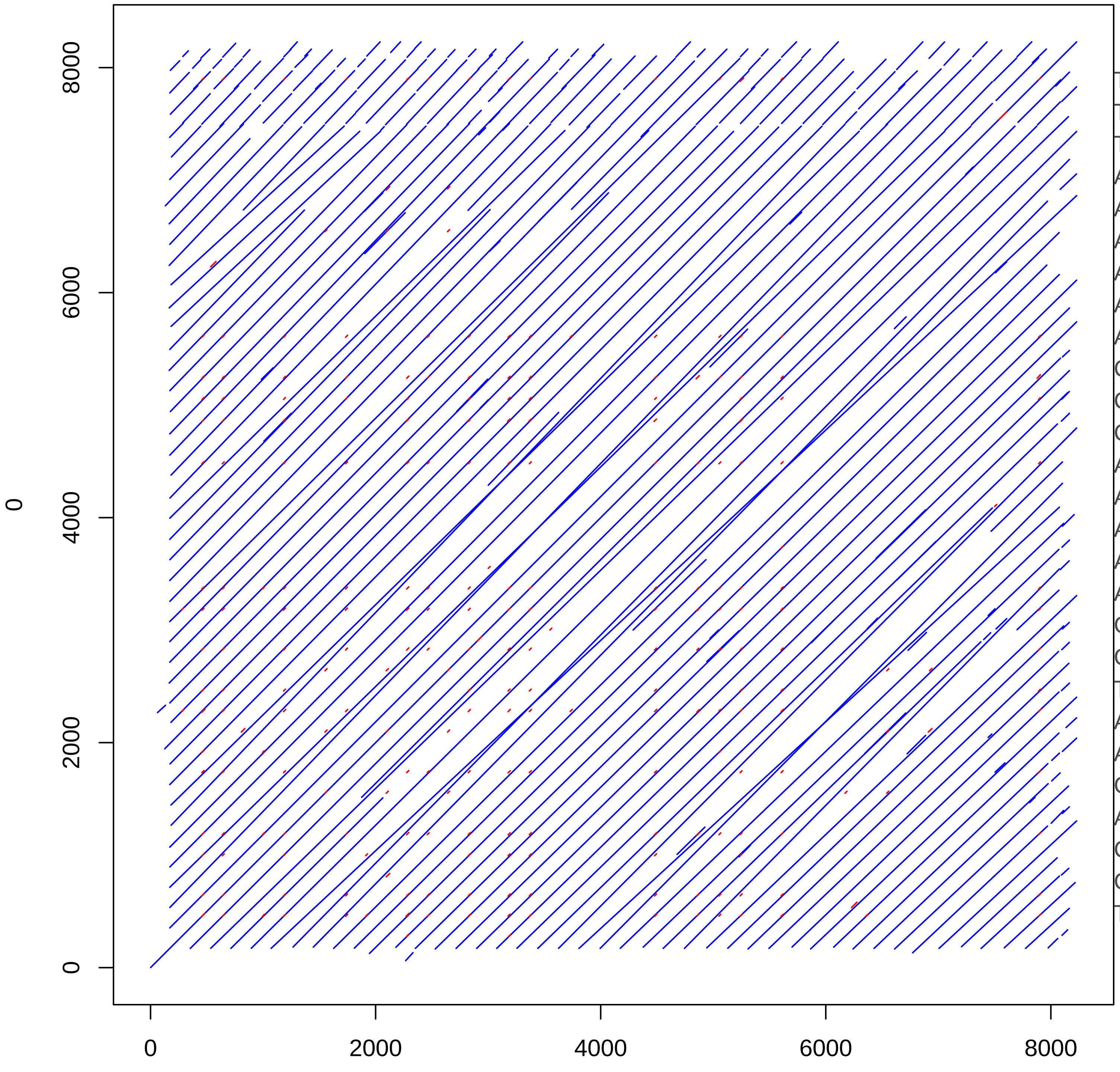

>m141126_130806_42134_c100732262550000001823141405141503_s1_p0/127225/6888_13089

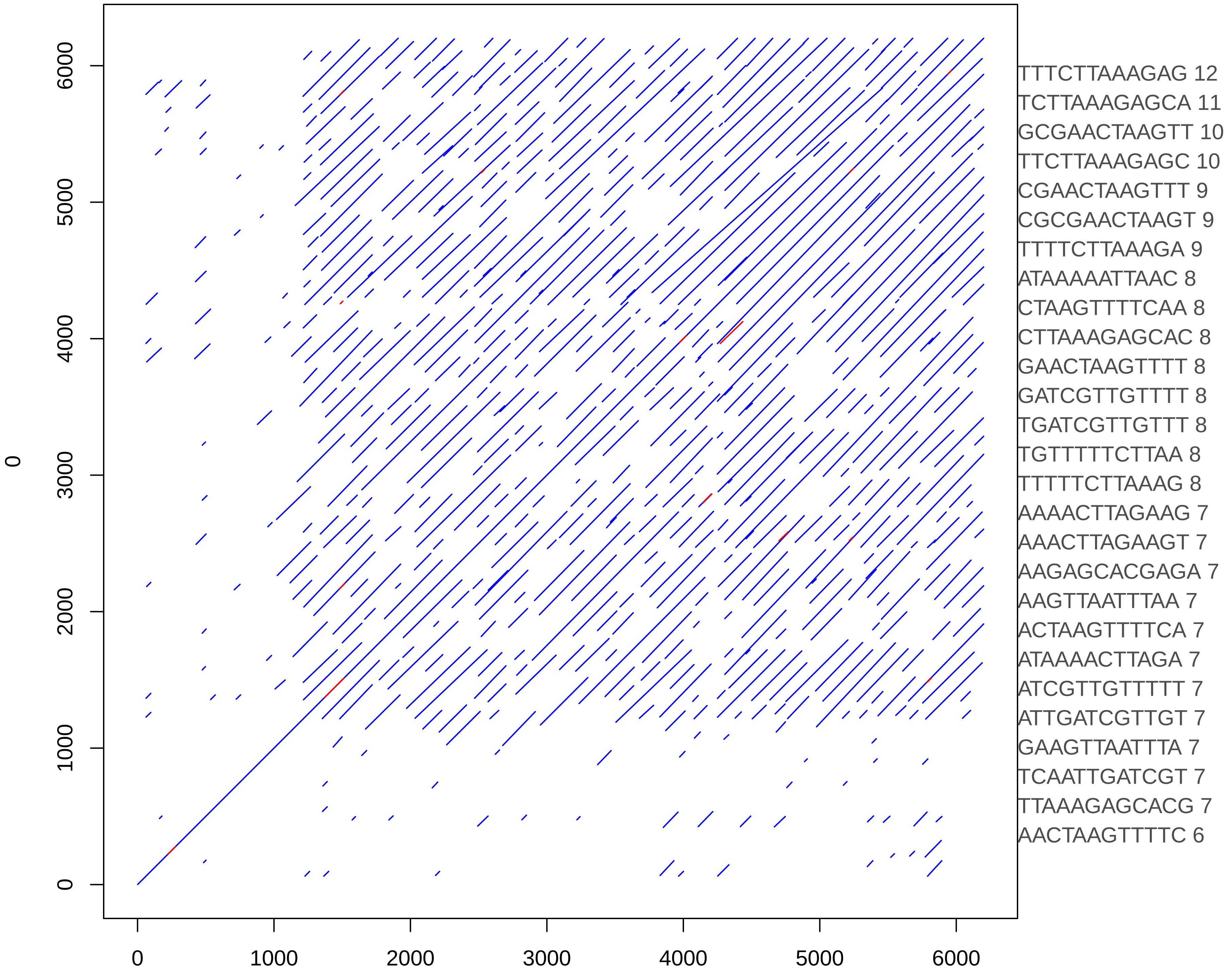

>m141126_130806_42134_c100732262550000001823141405141503_s1_p0/132914/3363_10190

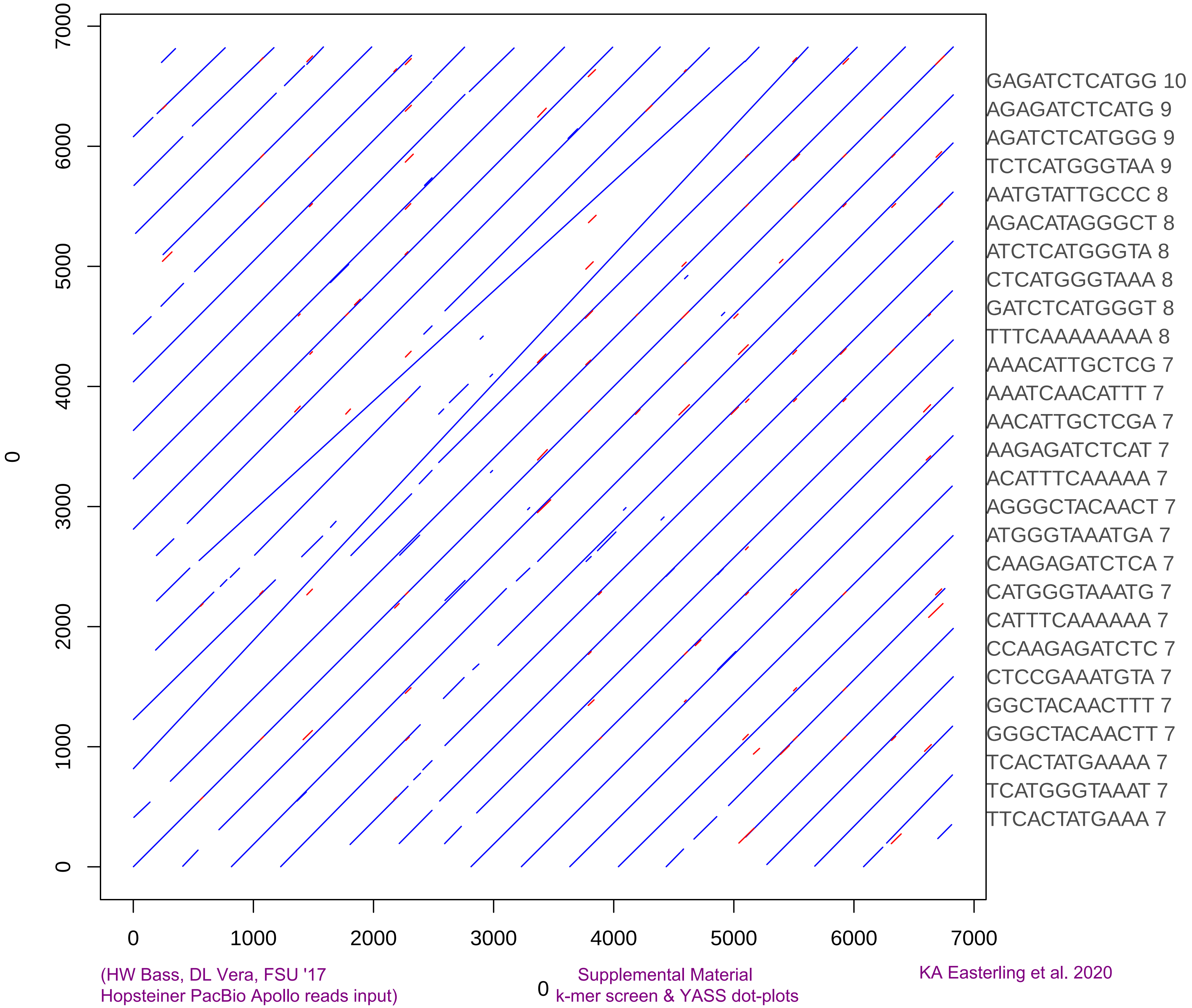

1,121 hits for repetitive sequences
passing 12mer abundance criterion

(Read-Number)
PAGE 746

For Tandem Repeat families of ### bp:
PacBio Read named "HuluTR###-r746"

>m141126_130806_42134_c100732262550000001823141405141503_s1_p0/134801/3264_13068

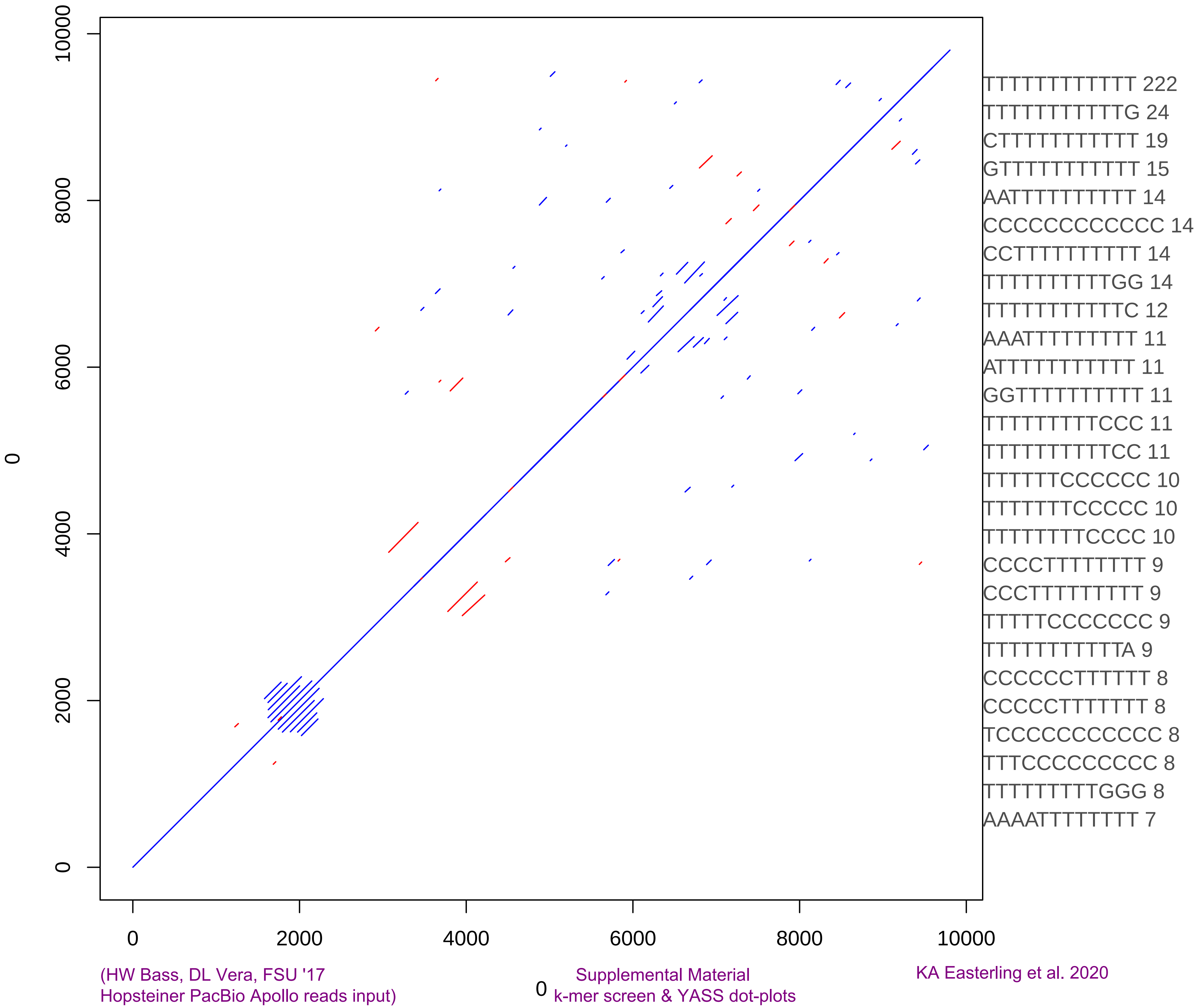

(HW Bass, DL Vera, FSU '17
Hopsteiner PacBio Apollo reads input)

Supplemental Material
k-mer screen & YASS dot-plots

KA Easterling et al. 2020

>m141126_130806_42134_c100732262550000001823141405141503_s1_p0/143489/5925_19961

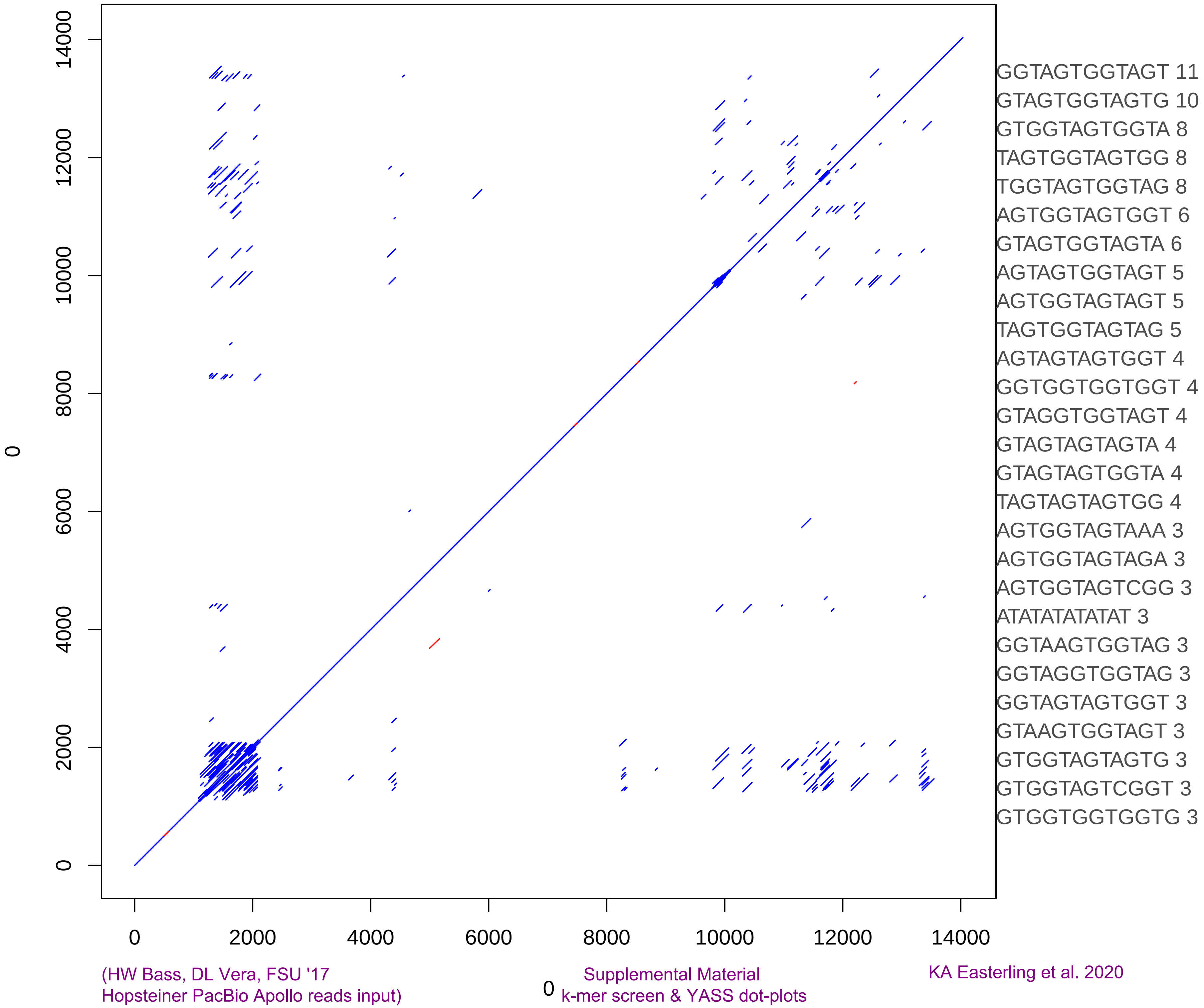

>m141126_130806_42134_c100732262550000001823141405141503_s1_p0/14823/0_8021

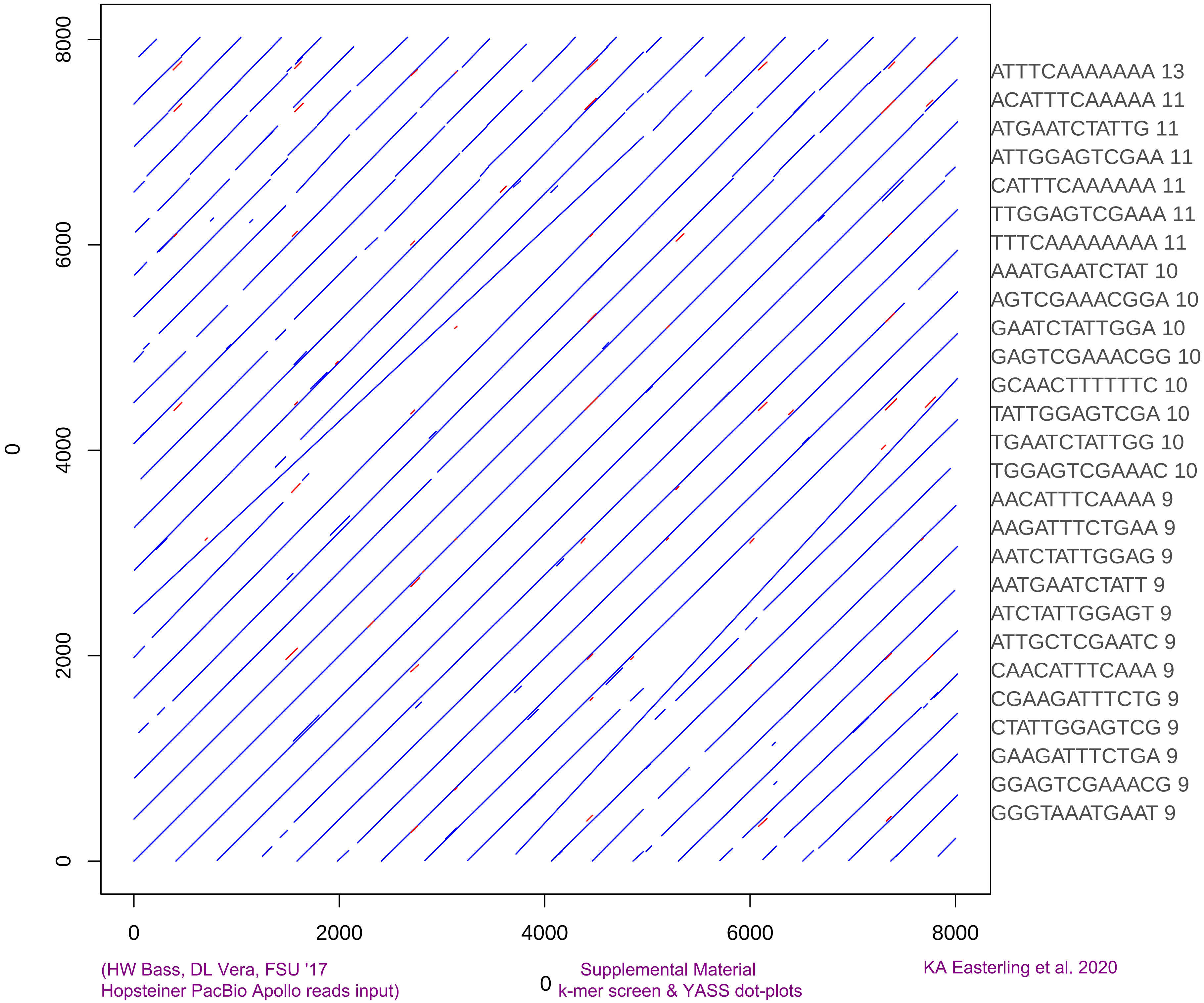

>m141126_130806_42134_c100732262550000001823141405141503_s1_p0/149544/9716_15198

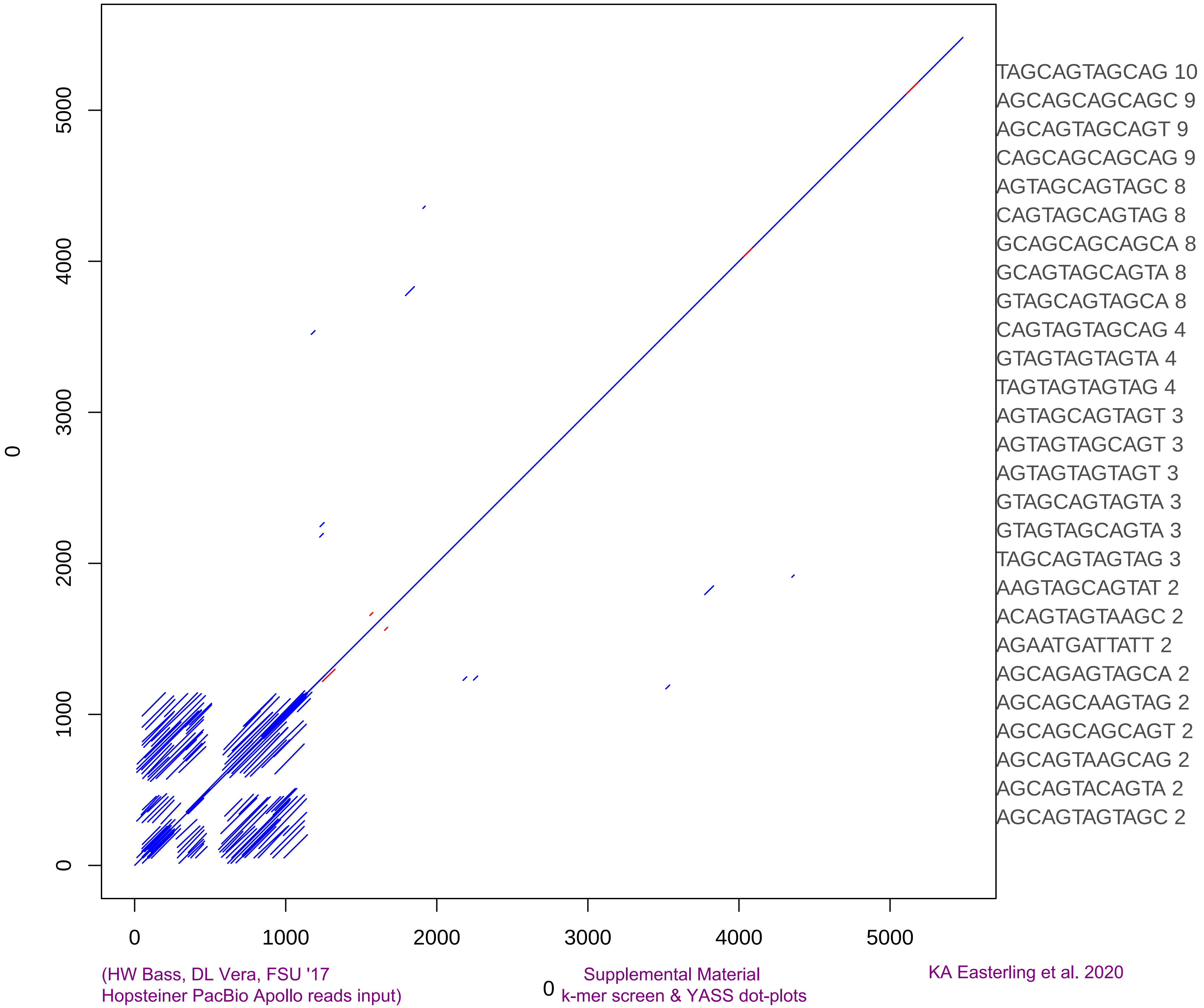

>m141126_130806_42134_c100732262550000001823141405141503_s1_p0/14965/0_7179

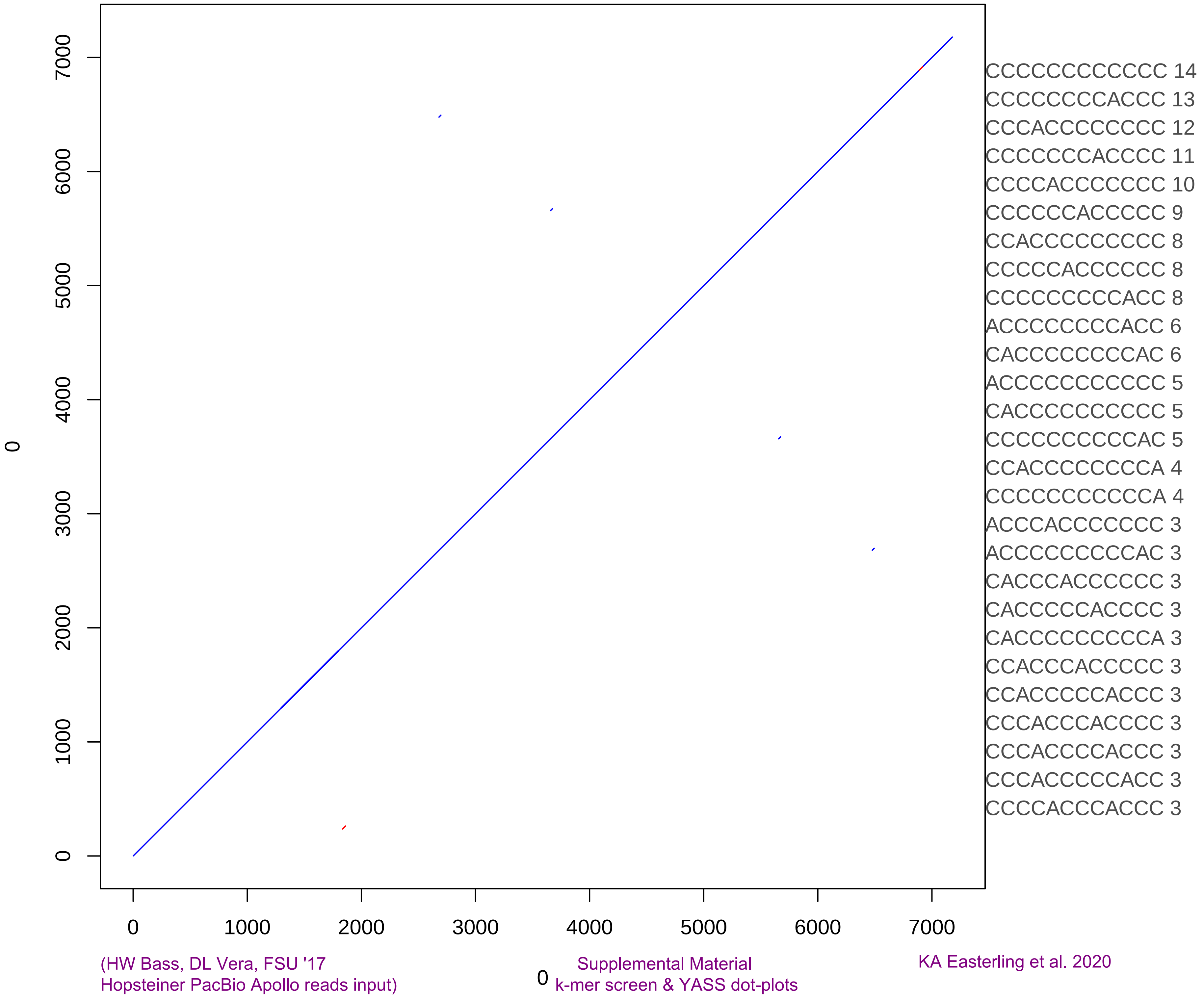

>m141126_130806_42134_c100732262550000001823141405141503_s1_p0/150054/1423_7056

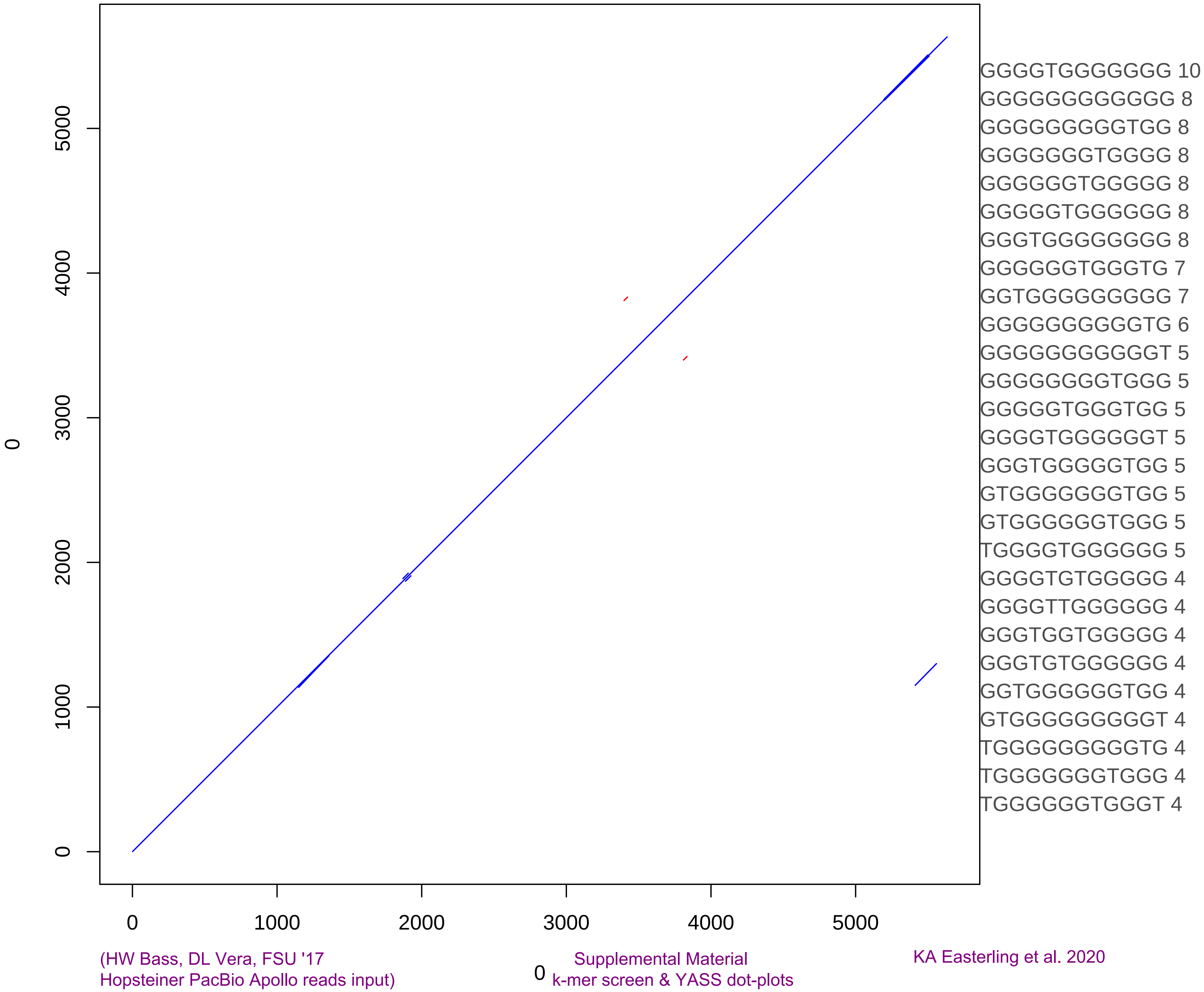

>m141126_130806_42134_c100732262550000001823141405141503_s1_p0/154223/2872_12445

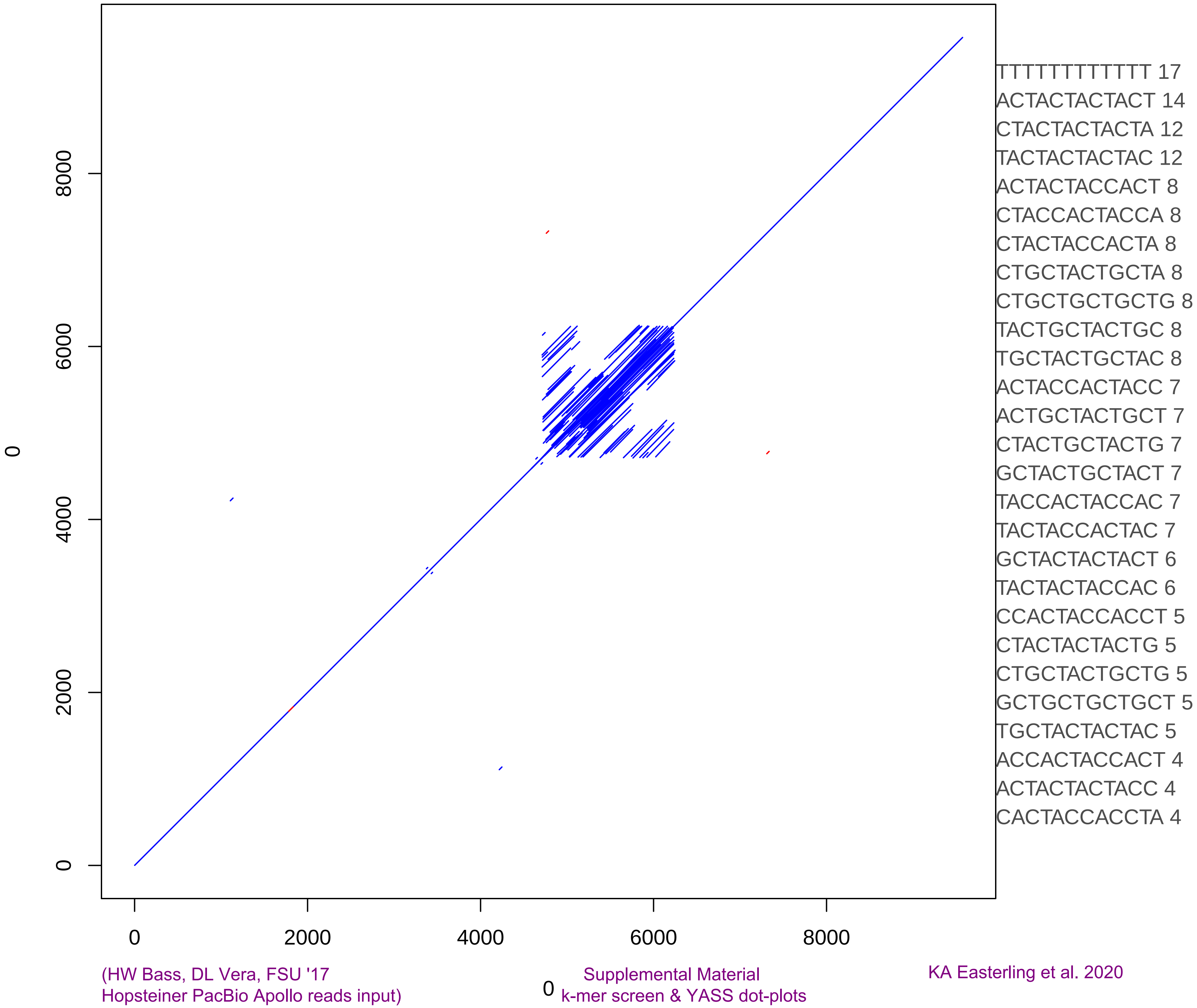

>m141126_130806_42134_c100732262550000001823141405141503_s1_p0/157448/1012_6151

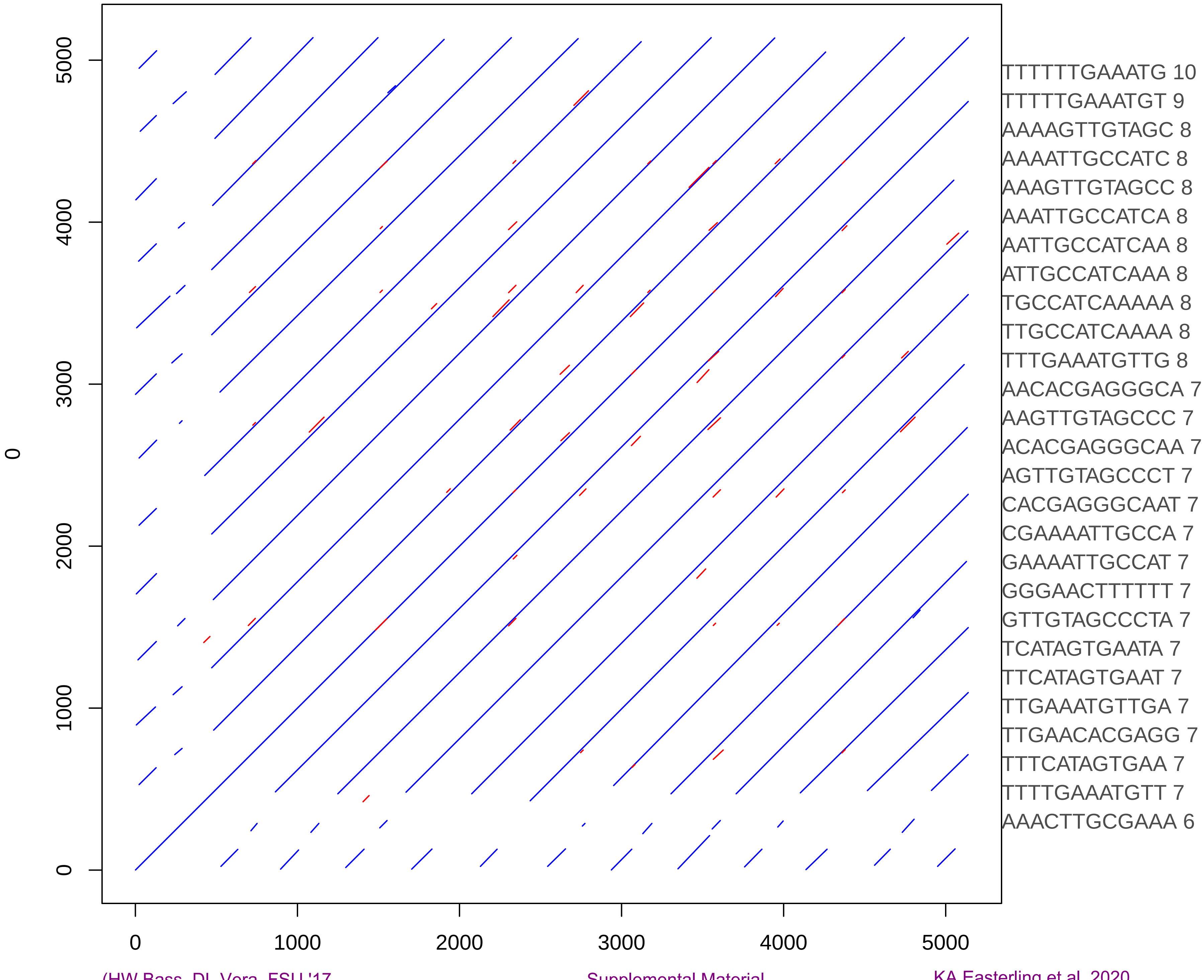

0

>m141126_130806_42134_c100732262550000001823141405141503_s1_p0/161444/7453_14769

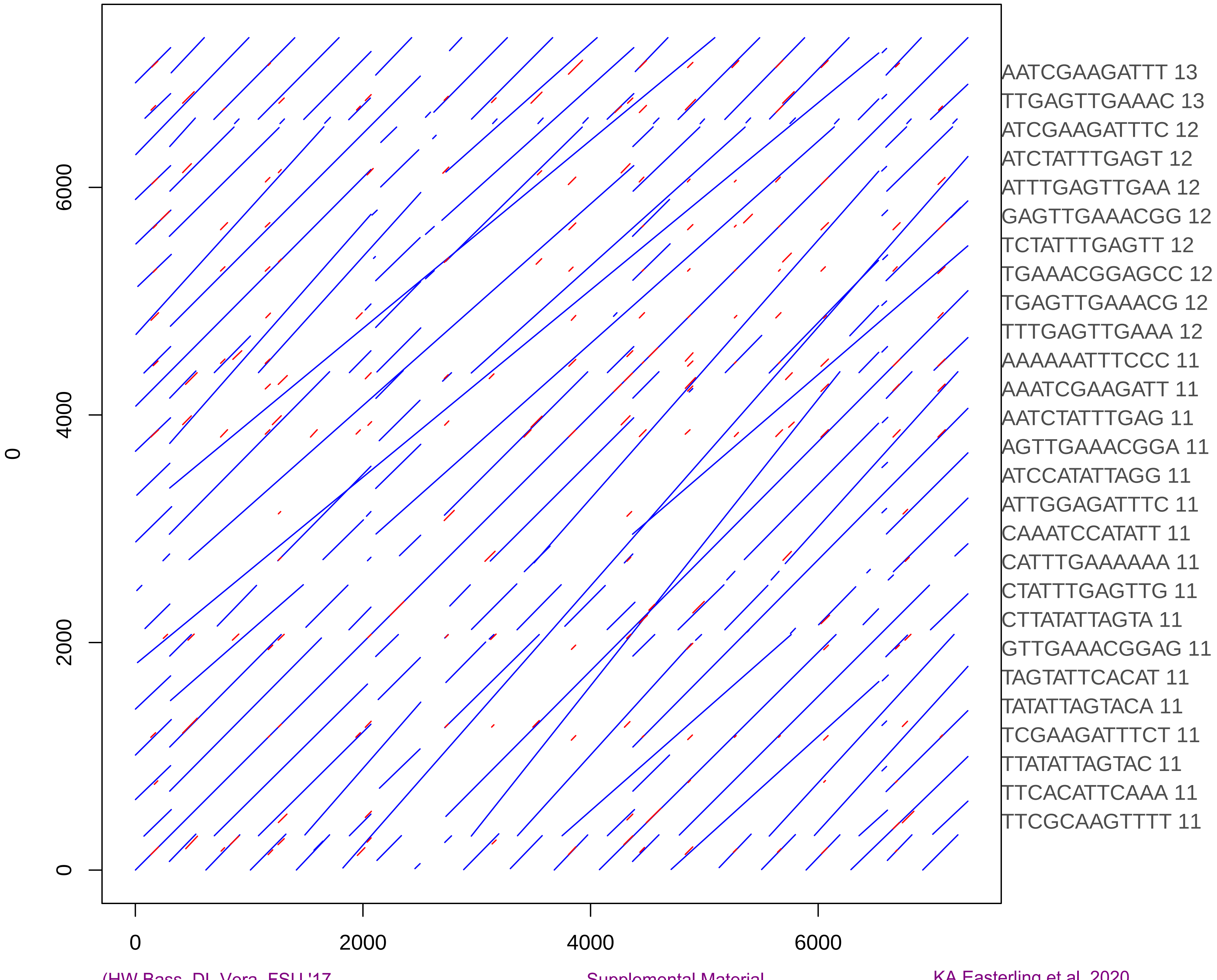

>m141126_130806_42134_c100732262550000001823141405141503_s1_p0/20101/5442_12969

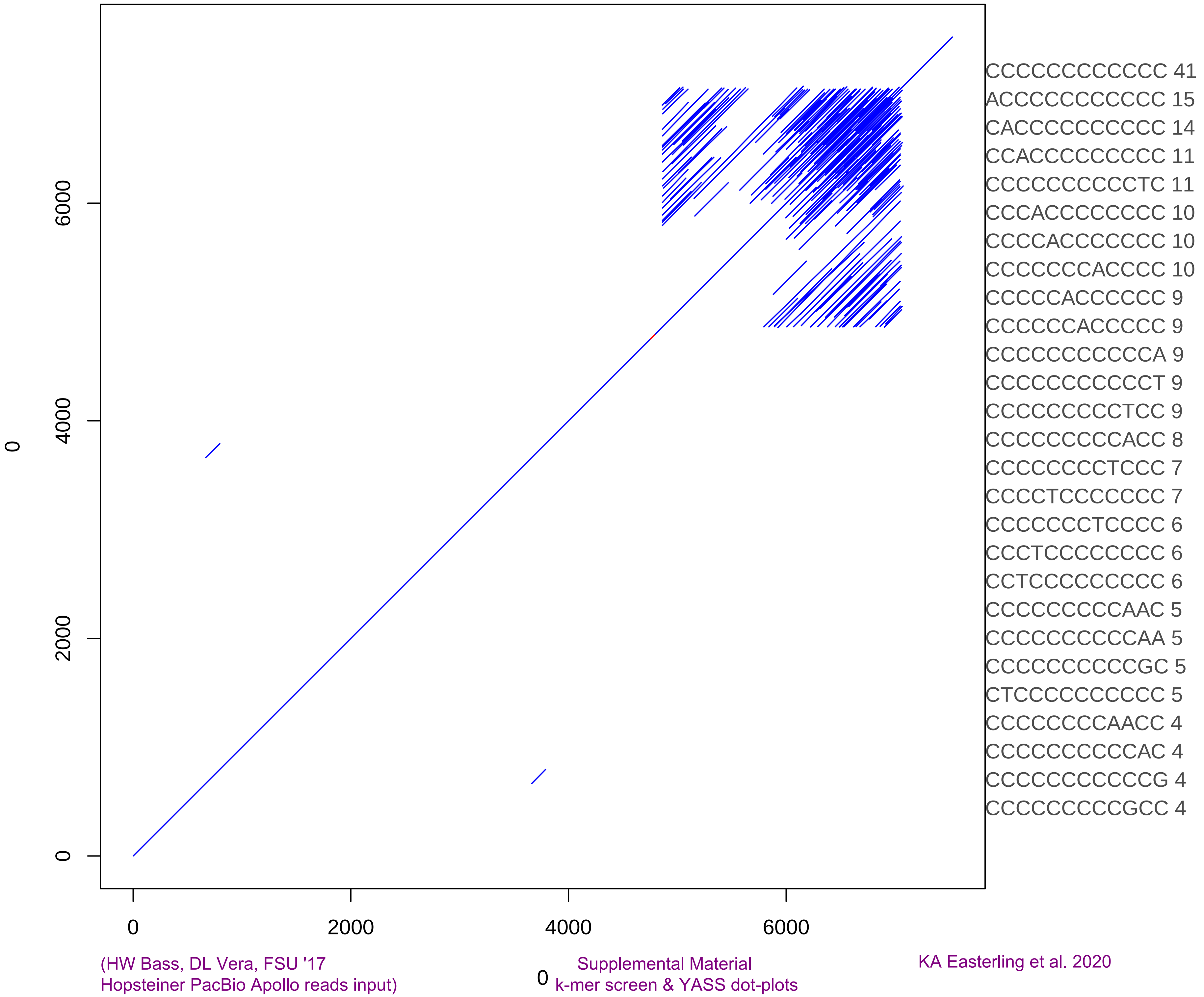

>m141126_130806_42134_c100732262550000001823141405141503_s1_p0/21270/3504_9911

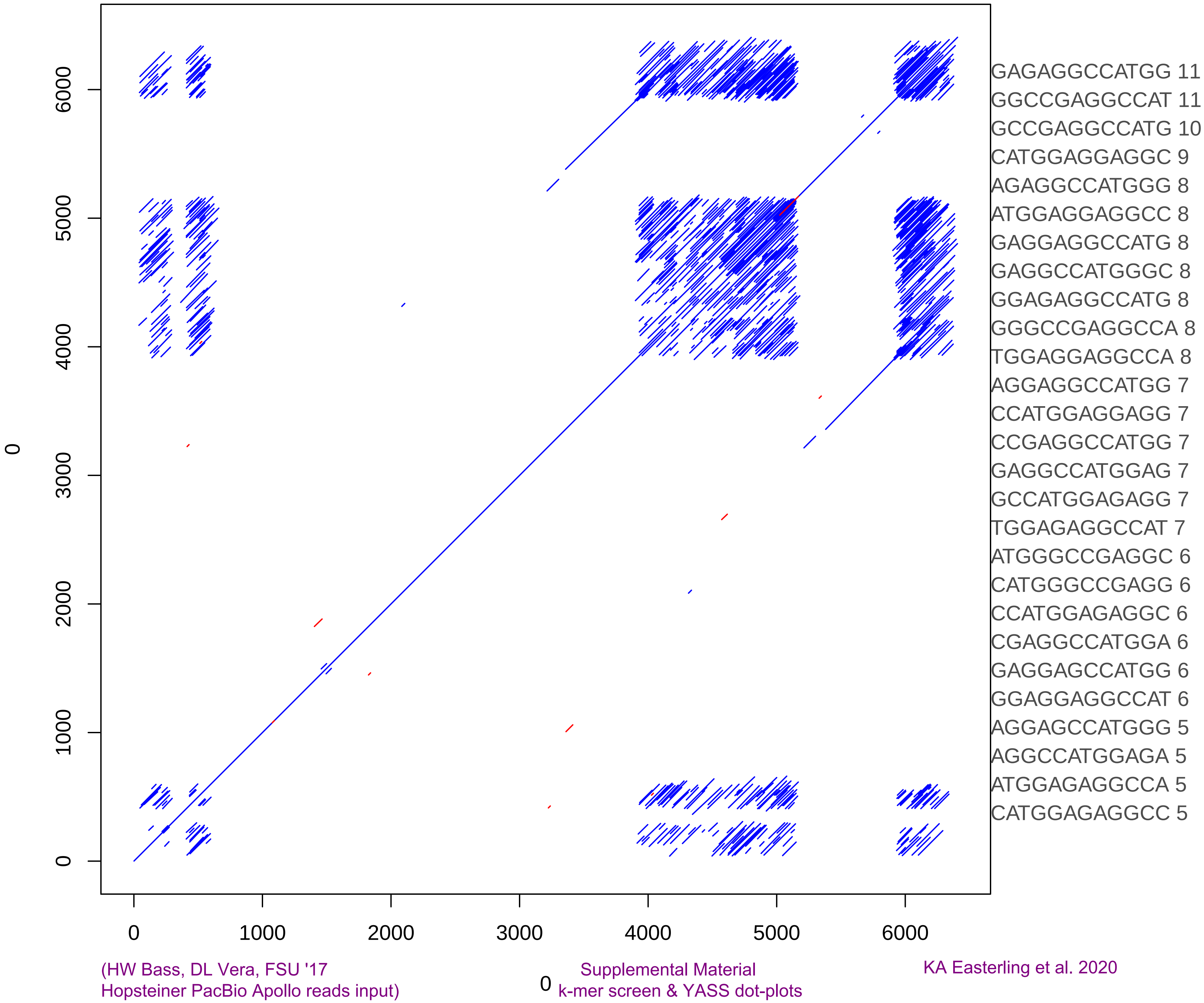

>m141126_130806_42134_c100732262550000001823141405141503_s1_p0/21270/9962_15667

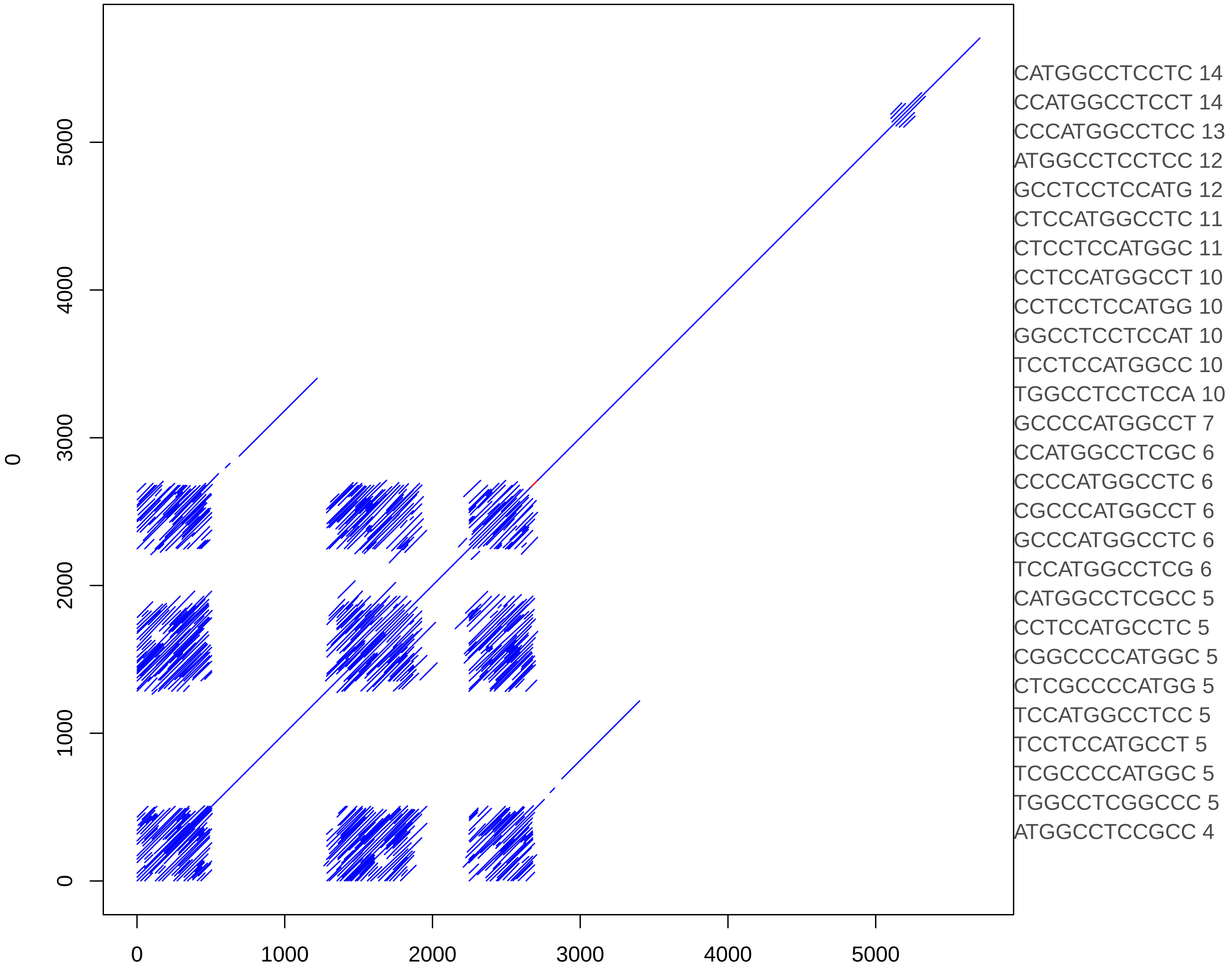

>m141126_130806_42134_c100732262550000001823141405141503_s1_p0/22551/0_8578

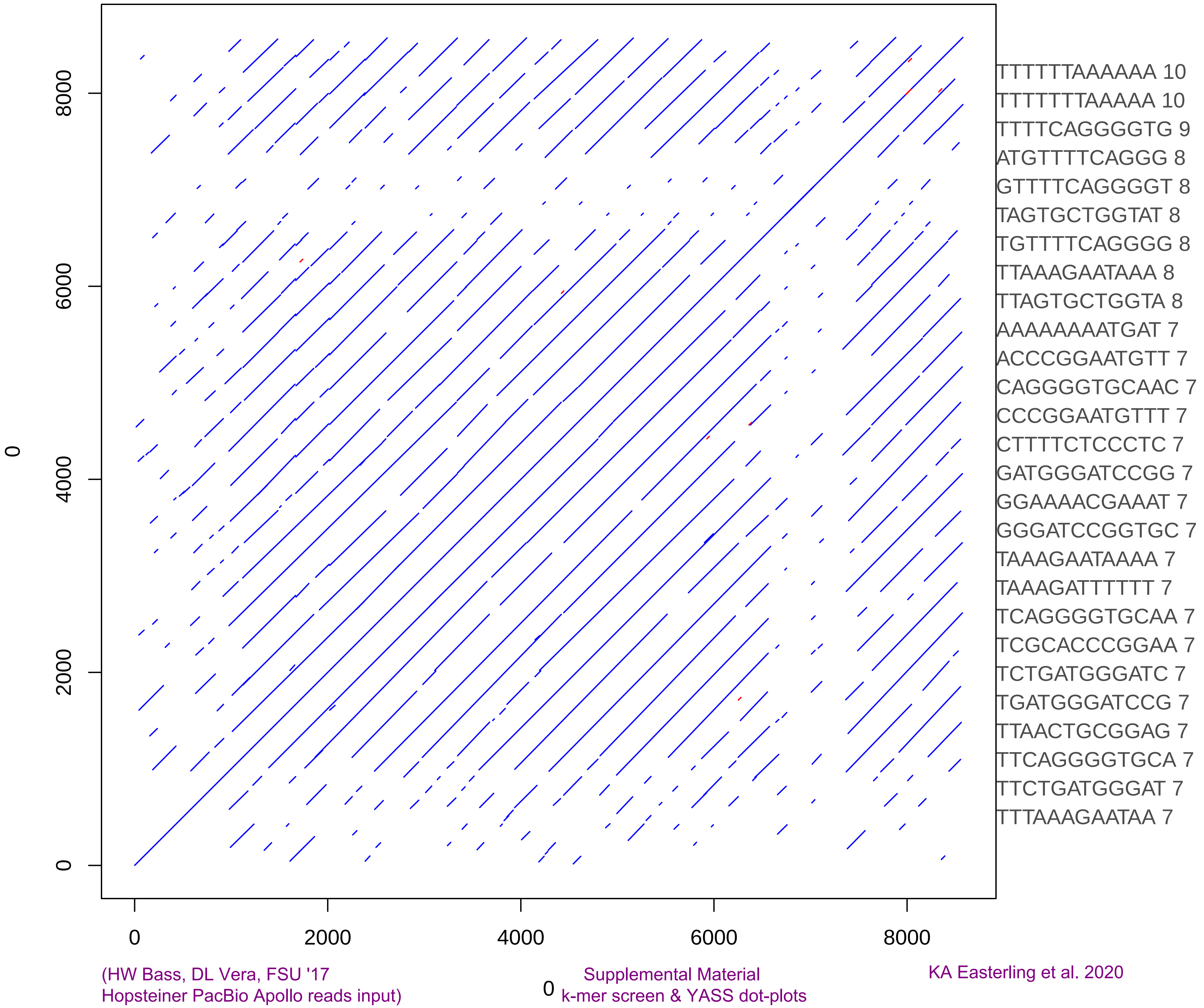

>m141126_130806_42134_c100732262550000001823141405141503_s1_p0/25215/0_5203

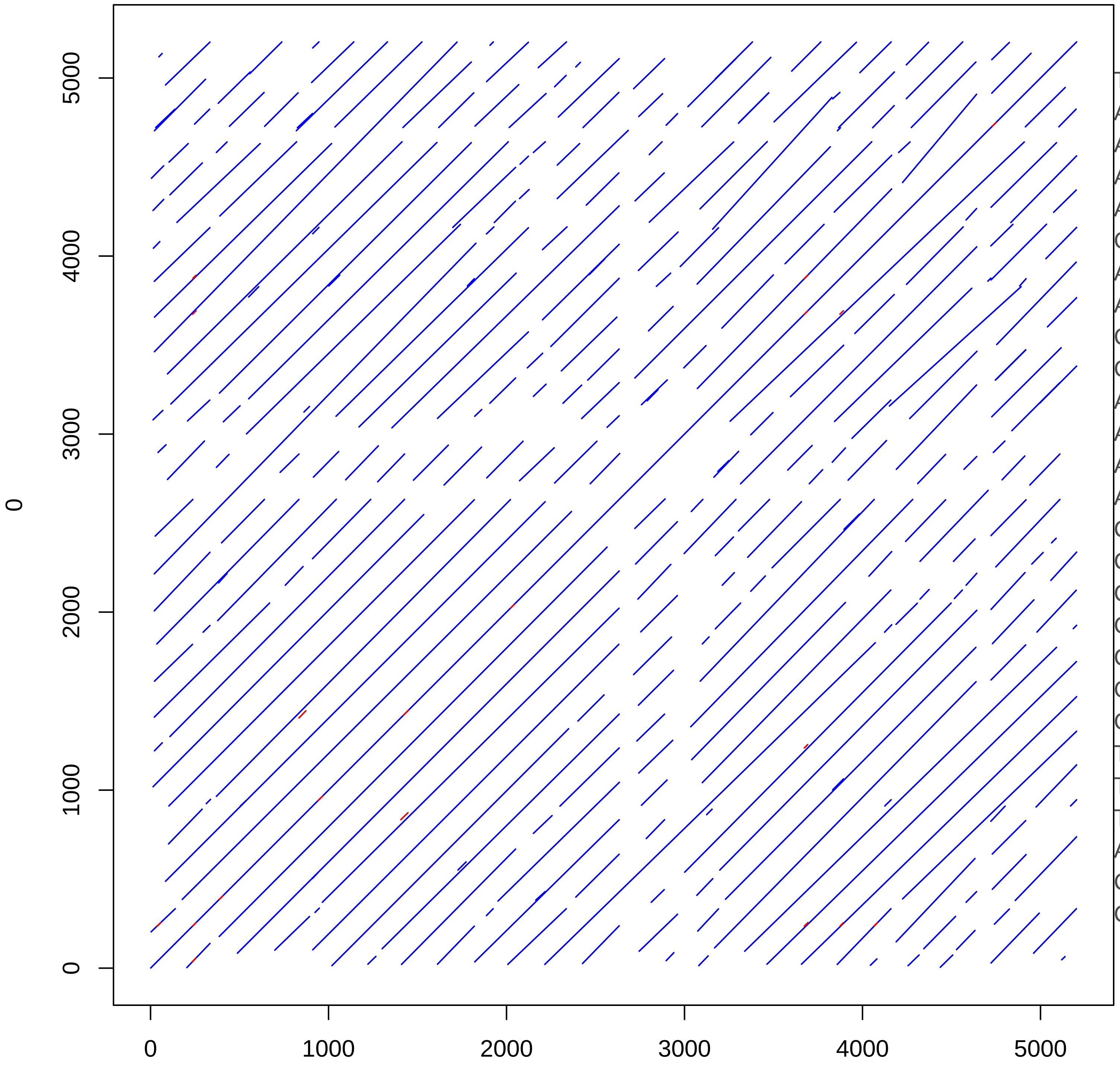

TTTTTTTTTTTT 84
AAACAAAAAAAT 10
AACAAAAAAATT 10
ACAAAAAAATTG 10
AGGGCTCCGAAG 10
GCTCTAATTTTT 10
AAAAAAATTGAC 9
AGCTCTAATTTT 9
CTCTAATTTTTT 9
GAATTGGGGTCC 9
AAAAAATTGACC 8
AAGCTCTAATTT 8
AATTGGGGTCCT 8
ATTGGGGTCCTA 8
CAAAAAAATTGA 8
CAAACAAAAAAA 8
CCCATCTCAATA 8
CCTTTTCCCATC 8
CTTTTCCCATCT 8
GATCCTTTTCCC 8
GGGCTCCGAAGC 8
TCTAATTTTTTT 8
TTCCCATCTCAA 8
TTTTCCCATCTC 8
ATCCTTTTCCCA 7
CCATCTCAATAA 7
CTAATTTTTTTG 7

>m141126_130806_42134_c100732262550000001823141405141503_s1_p0/26635/1501_9764

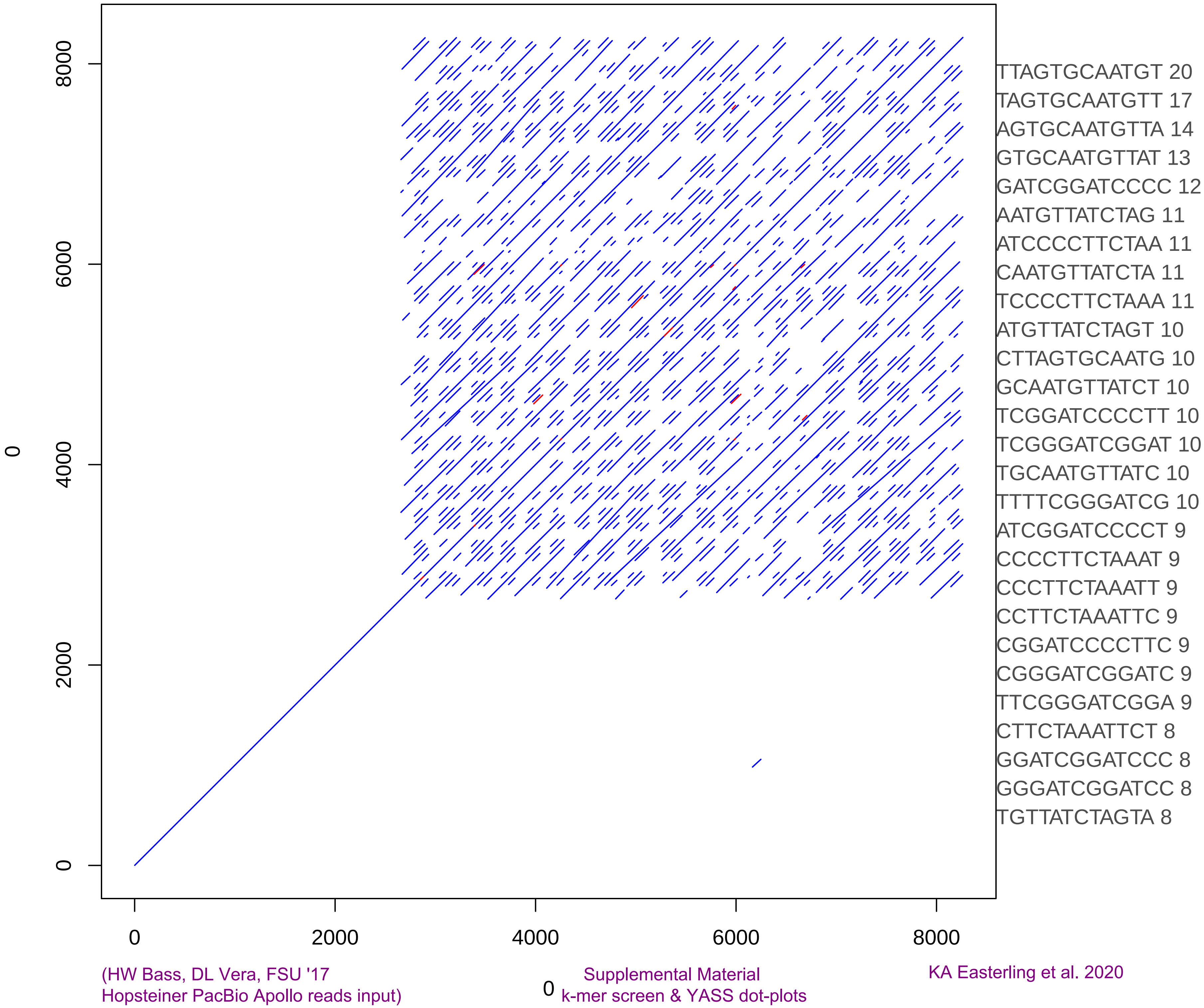

>m141126_130806_42134_c100732262550000001823141405141503_s1_p0/27629/6240_13346

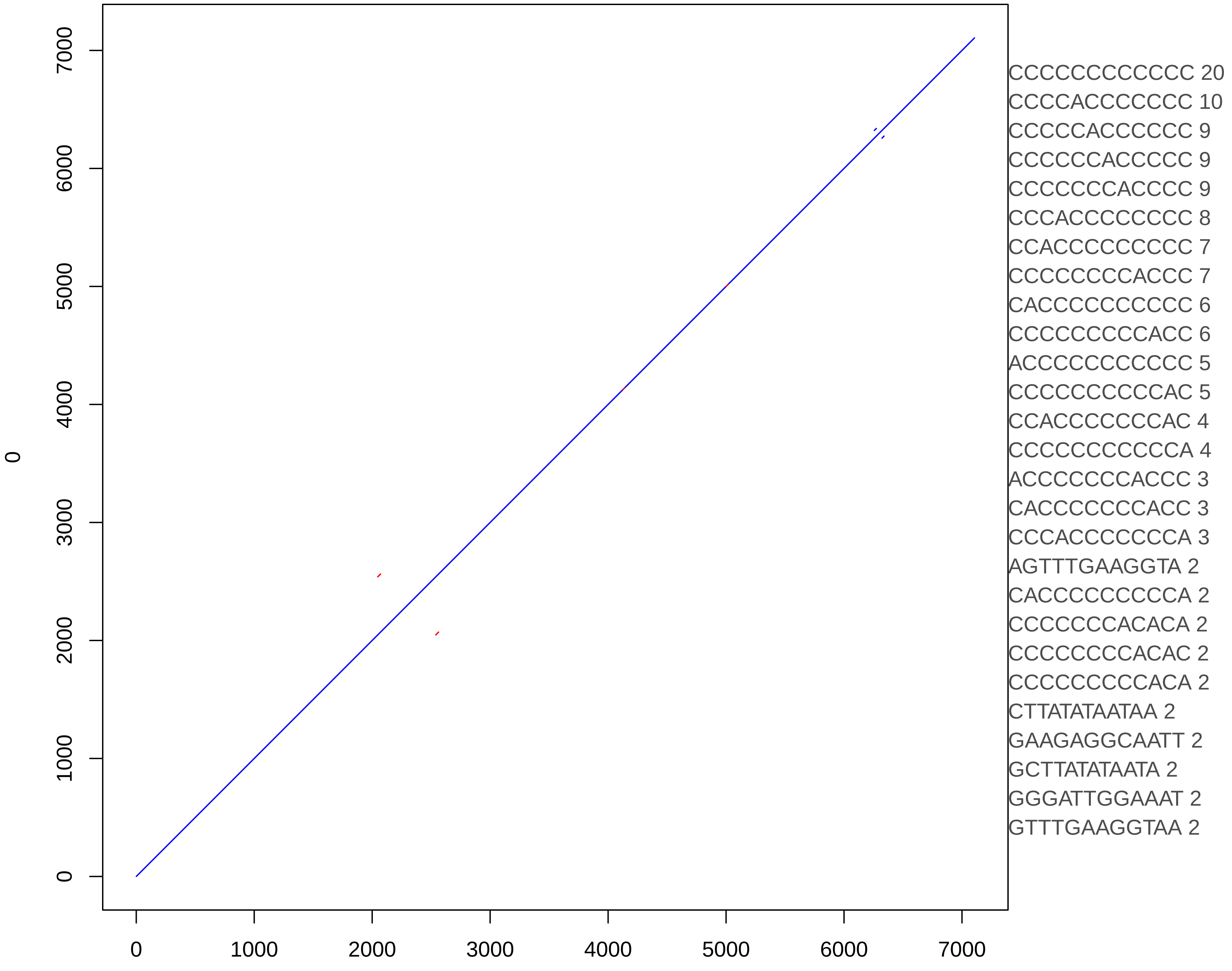

>m141126_130806_42134_c100732262550000001823141405141503_s1_p0/32098/5256_11362

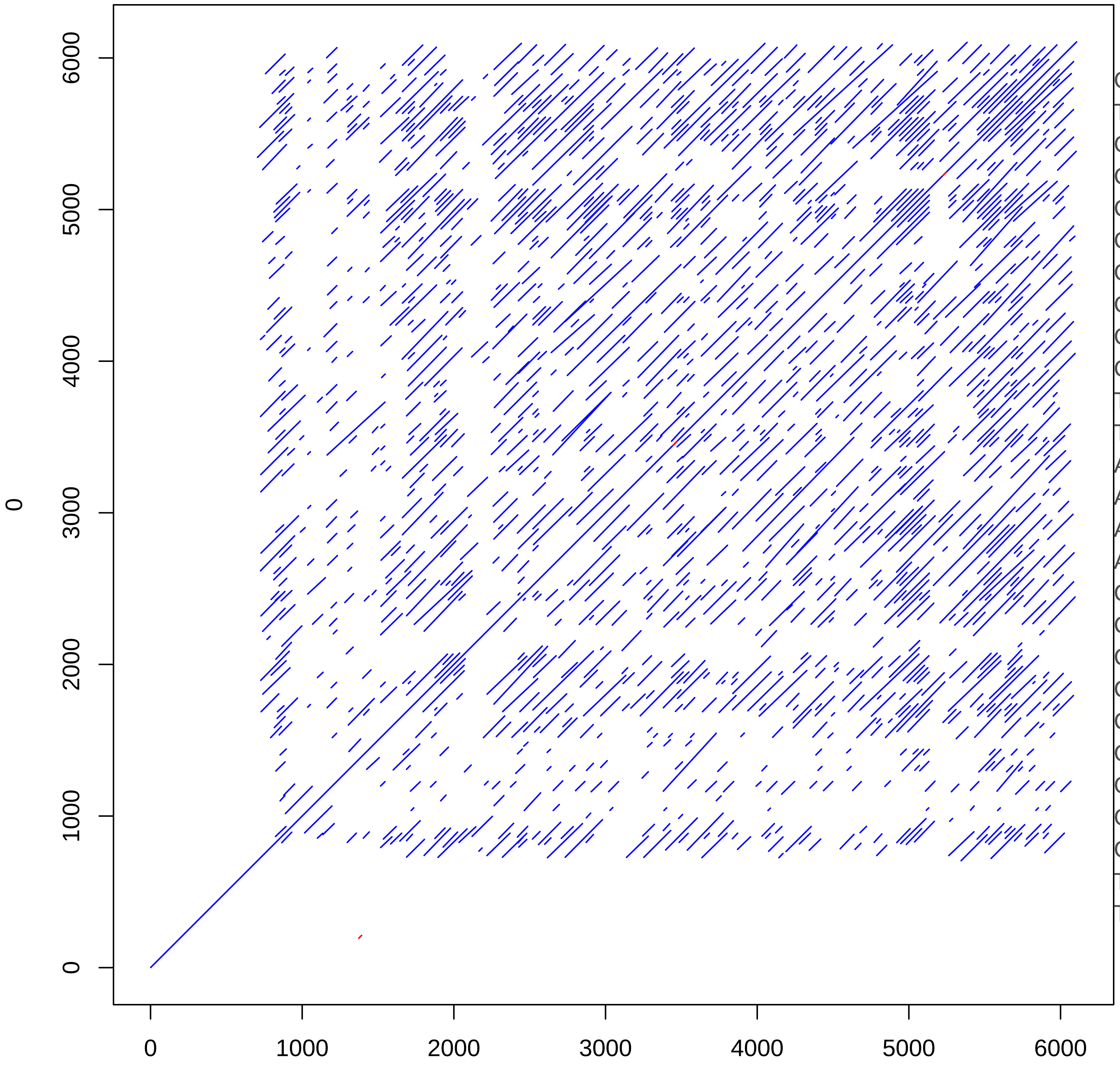

GGGTTTGTACCC 14
TGGGTTTGTACC 13
GGTTTGTACCCG 12
CCATTTGGTGTC 10
CCCGGACCATTT 10
GCGTCCCATTTG 9
GGCCTTGCGTCC 9
GTTTGTACCCGC 9
GATGGGTTTGTA 8
GCCCGGACCATT 8
TTGCGTCCCATT 8
TTTGTACCCGCC 8
ACCATTTAGGTC 7
ACCATTTGGTGT 7
ATGGGTTTGTAC 7
ATTTGGTGTCCG 7
CCATTTGGGTTT 7
CCCATTTGGGTT 7
CCTTGCGTCCCA 7
CGCCCGGACCAT 7
CGTCCCATTTGG 7
GACCATTTAGGT 7
GGGCCTTGCGTC 7
GTACCCGCCCGG 7
GTGTCCGGGATG 7
TGCGTCCCATTT 7
TGTACCCGCCCG 7

>m141126_130806_42134_c100732262550000001823141405141503_s1_p0/34540/487_12558

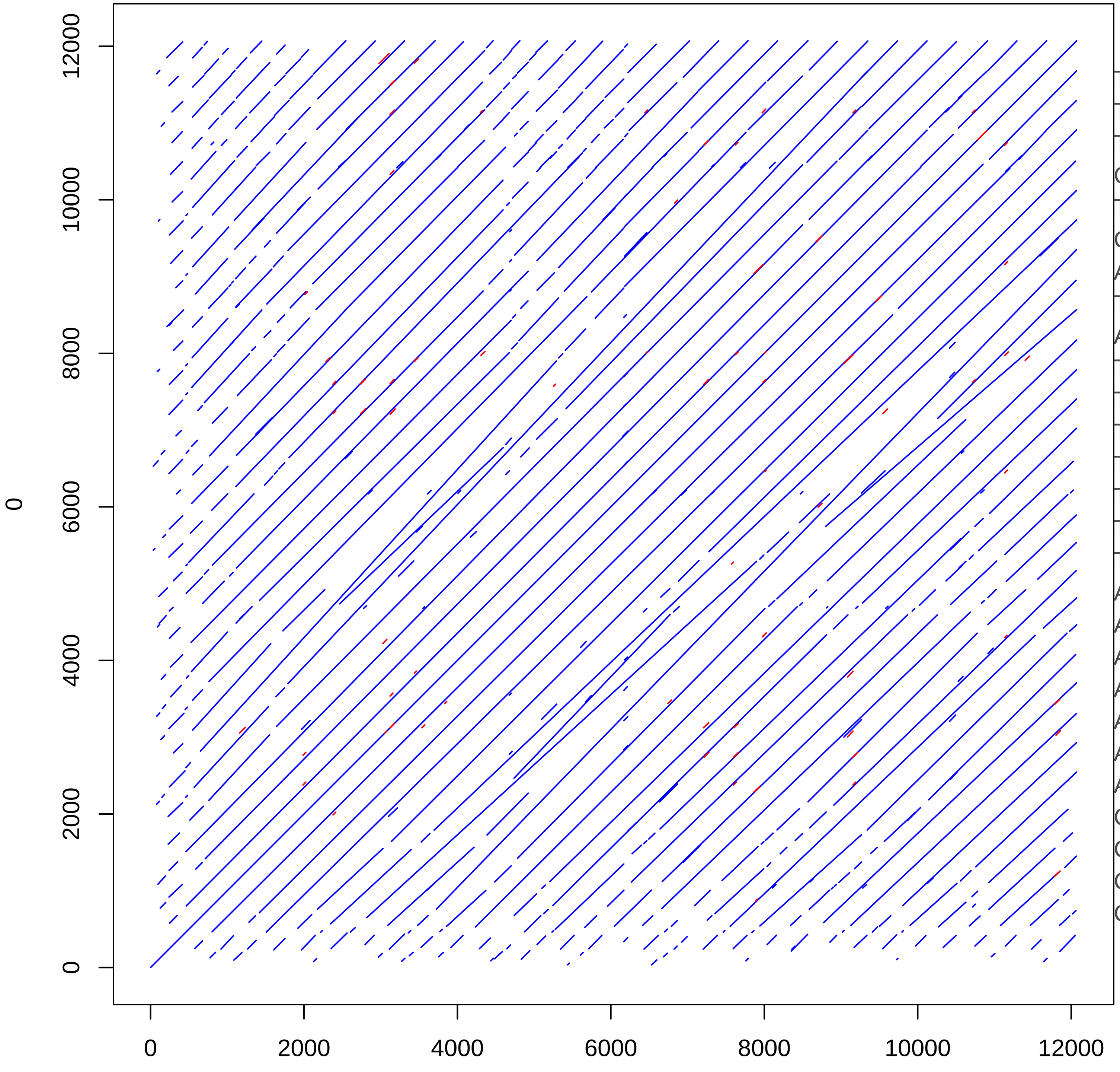

TTTTTCAAATGT 19
TTTTTTCAAATG 19
TTTTTTTCAAAT 19
GAAAACTAAAGA 17
TTTTCAAATGTT 17
GGGAATTTTTTT 16
AAAACTAAAGAA 15
TTCTCTAATATG 15
AAAACTTGCGAA 14
TCTCTAATATGG 14
TGAAAACTAAAG 14
TTCAAATGTTGA 14
TTCGATTTCTCT 14
TTTCAAATGTTG 14
TTTCTCTAATAT 14
TTTTTTTTCAAA 14
AAAAACTTGCGA 13
AAACTAAAGAAT 13
AAACTTGCGAAA 13
AAATCTTCGATT 13
AAATGTTGACTT 13
AATGTTGACTTA 13
AATATGGATTCG 12
CAAATGTTGACT 12
CATTTCGGAGAA 12
CGATTTCTCTAA 12
CTCCGTTTCAAC 12

>m141126_130806_42134_c100732262550000001823141405141503_s1_p0/34947/12340_21021

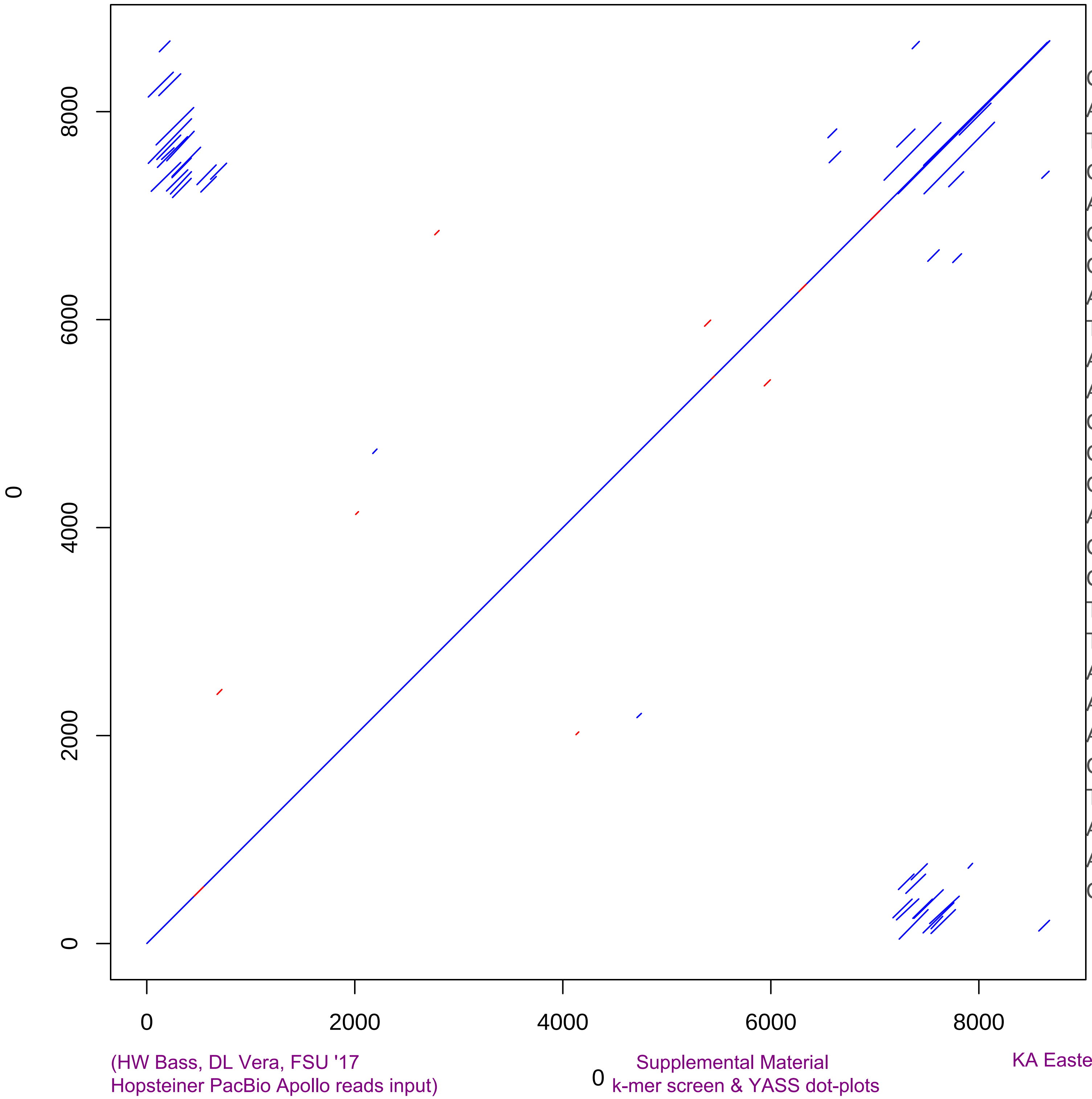

CTACTACTACTA 38
ACTACTACTACT 36
TACTACTACTAC 36
CACTACCACTAC 17
ACTACCACTACC 16
CCACTACCACTA 16
CTACCACTACCA 15
ACCACTACCACT 14
TACCACTACCAC 14
ACTACCACTACT 10
ACTACTACTACC 9
CCACTACTACTA 8
CTACCACTACTA 8
CTACTACTACCA 8
ACTACTACCACT 7
CTACTACCACTA 7
CACTACTACTAC 6
TACCACTACTAC 6
TACTACCACTAC 6
ACATACTACTAC 5
ACCACTACTACT 5
ACTACTAACTAC 5
CTACTAACTACT 5
TACTACTACCAC 5
AACTACTACCAC 4
ACTAACTACTAC 4
CTACCACTAACT 4

>m141126_130806_42134_c100732262550000001823141405141503_s1_p0/35739/6151_15569

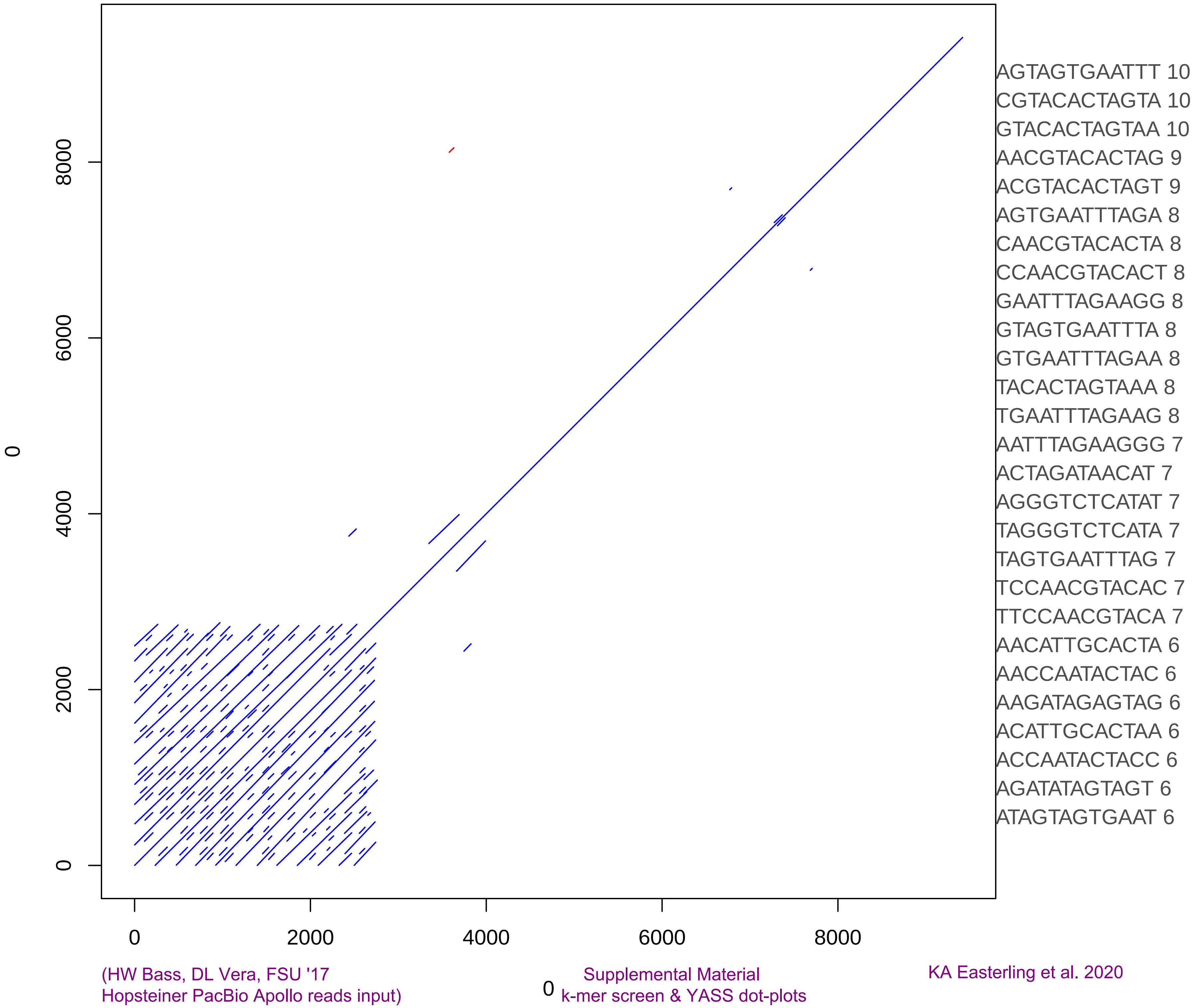

>m141126_130806_42134_c100732262550000001823141405141503_s1_p0/37414/403_7248

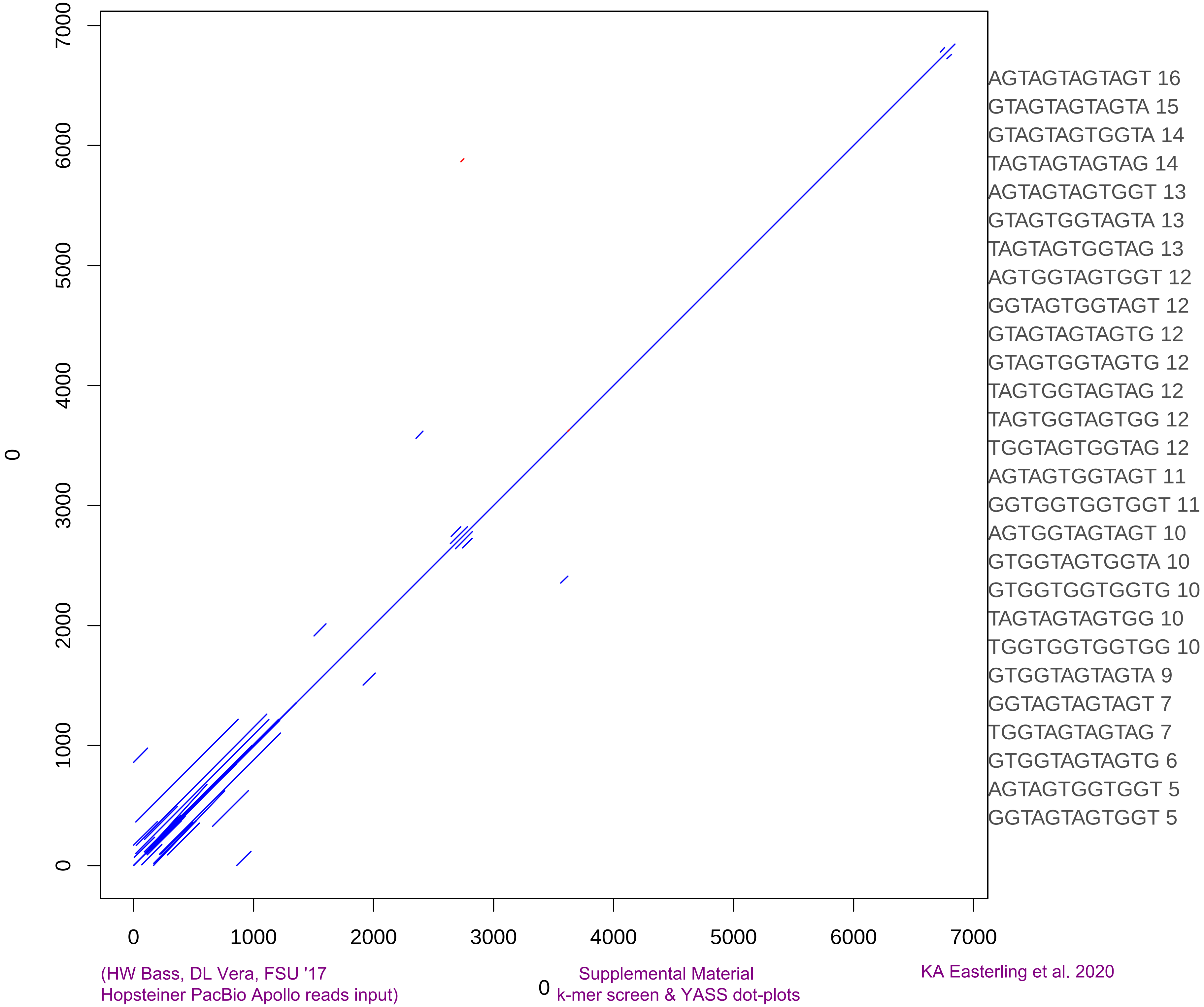

>m141126_130806_42134_c100732262550000001823141405141503_s1_p0/38425/3464_14642

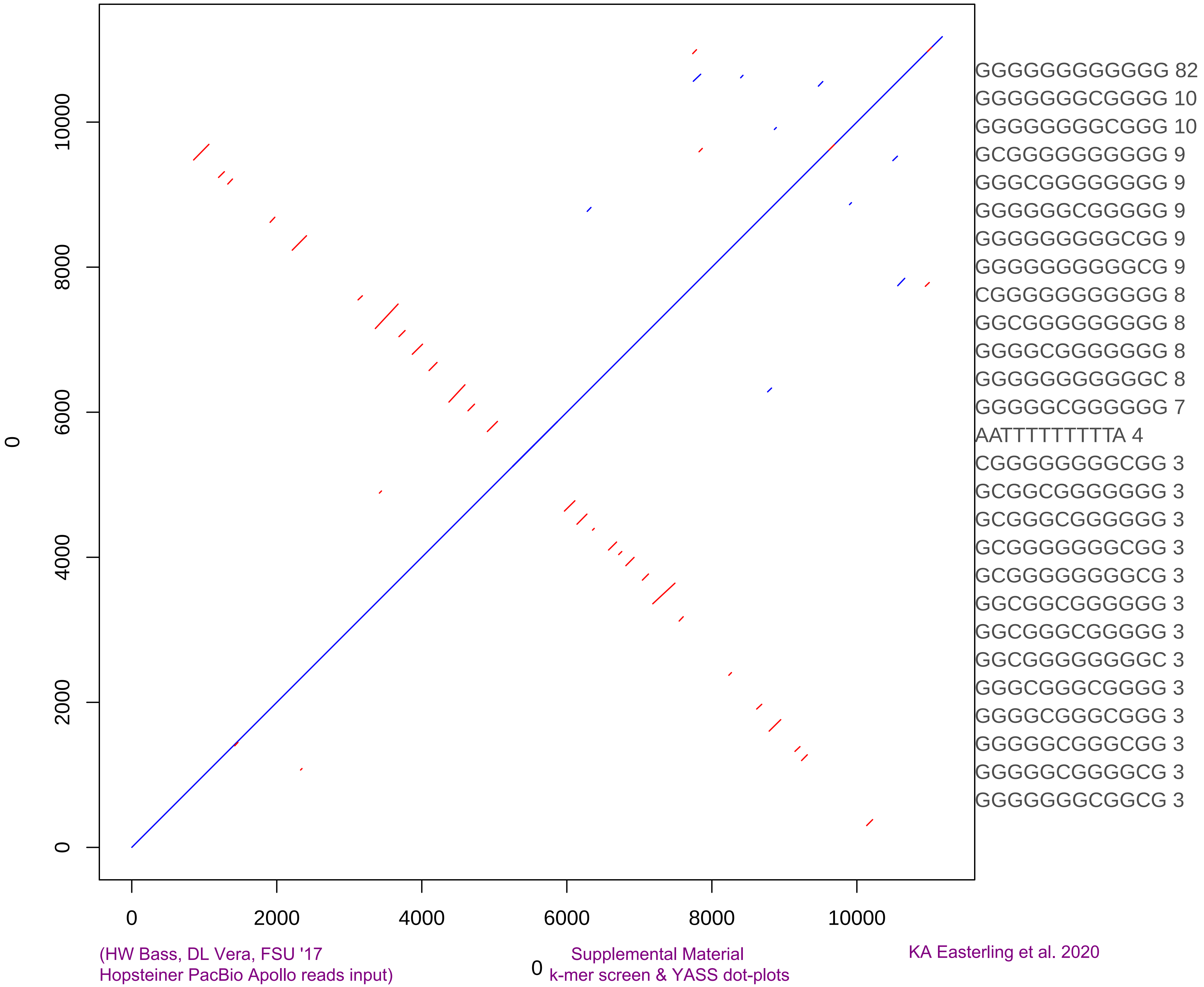

>m141126_130806_42134_c100732262550000001823141405141503_s1_p0/39571/3077_14085

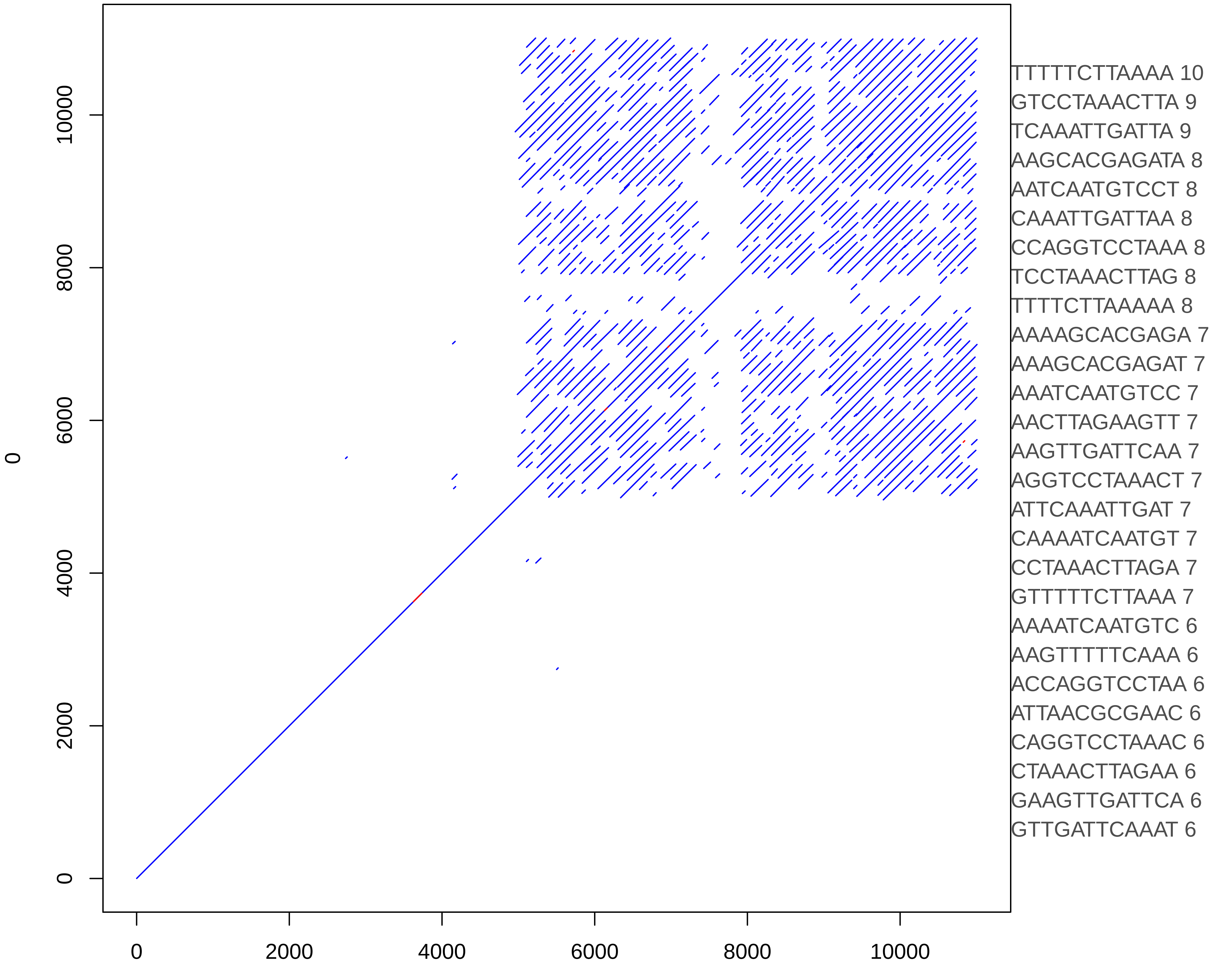

>m141126_130806_42134_c100732262550000001823141405141503_s1_p0/40310/8409_16802

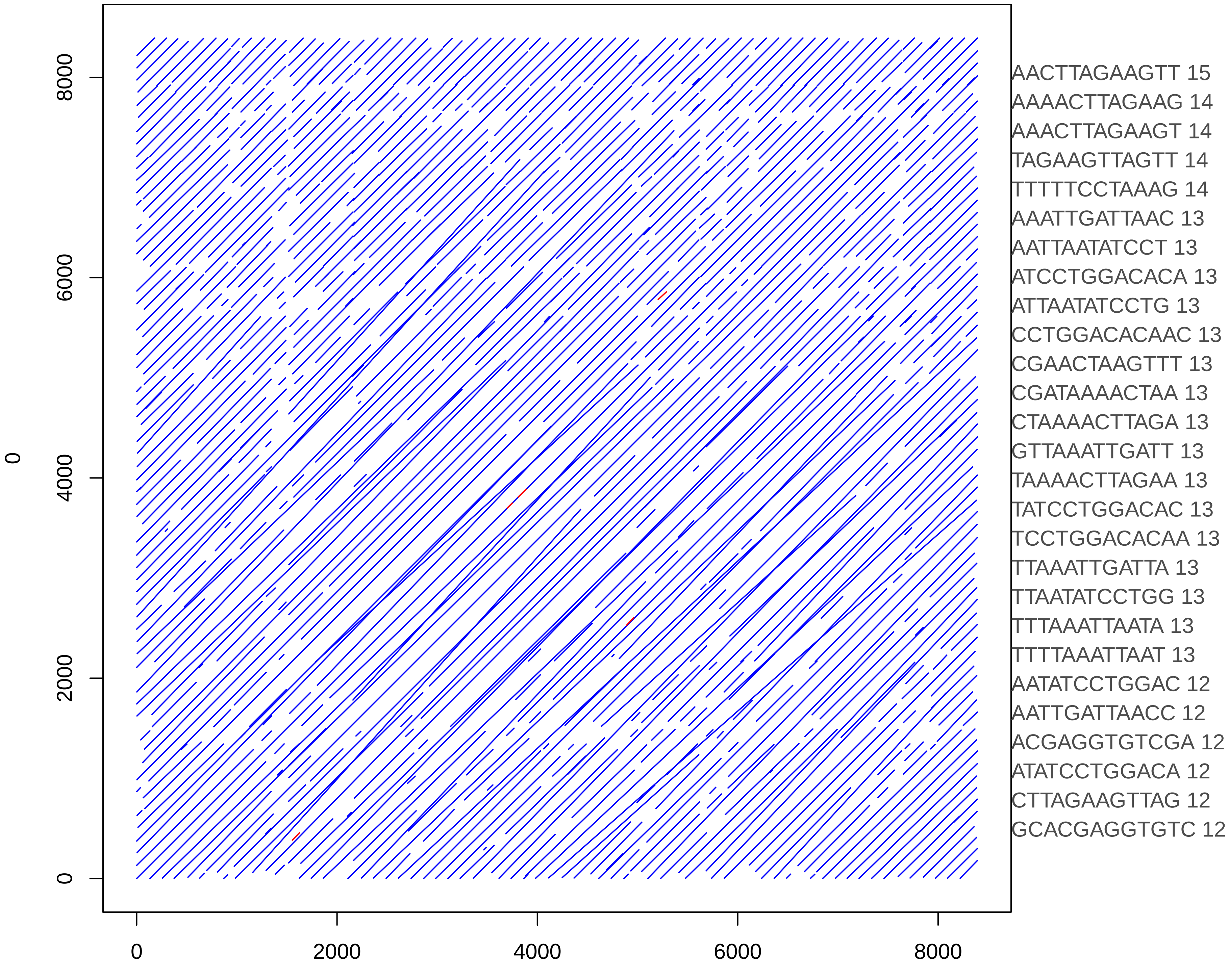

>m141126_130806_42134_c100732262550000001823141405141503_s1_p0/51239/3819_11591

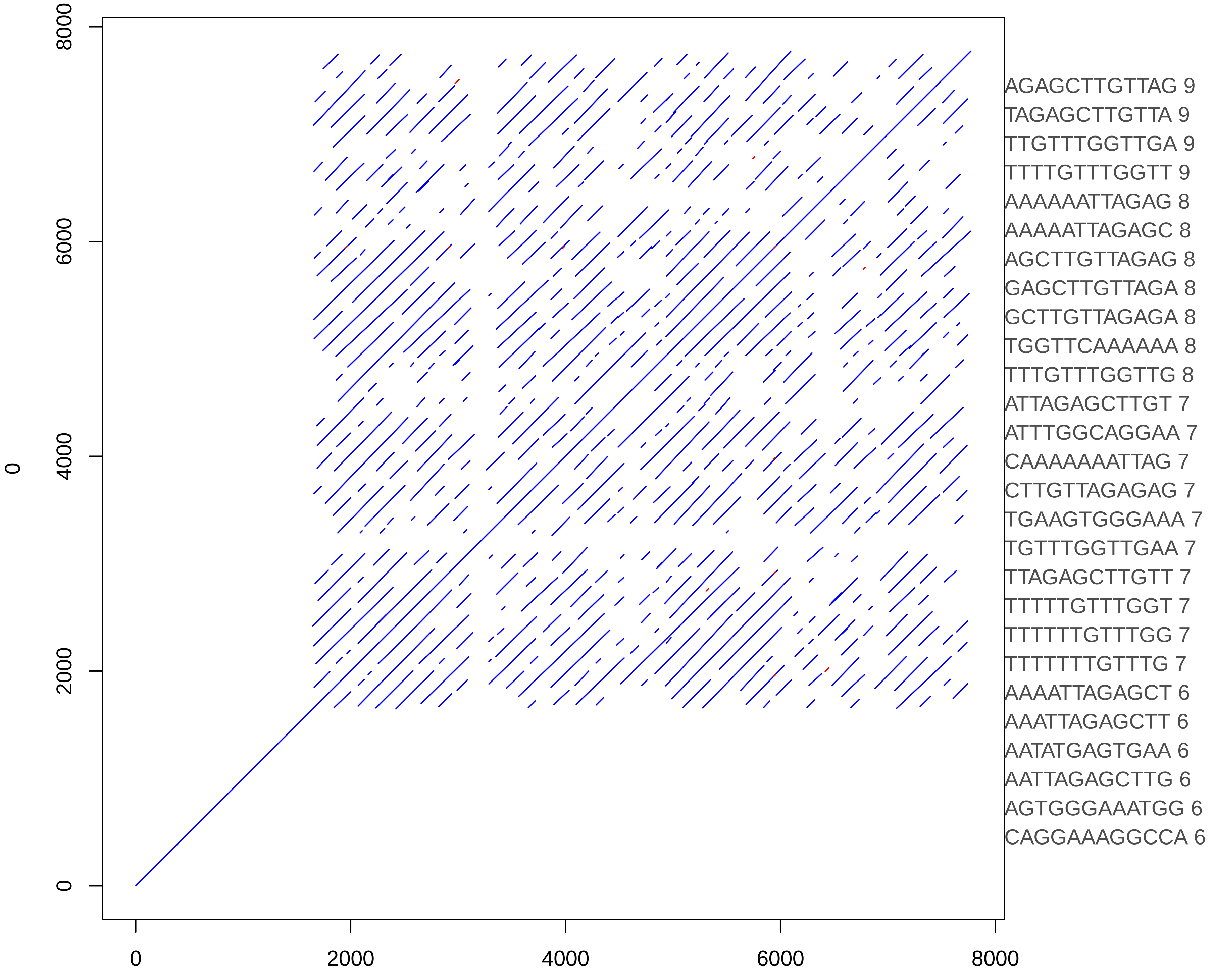

>m141126_130806_42134_c100732262550000001823141405141503_s1_p0/5124/8577_13959

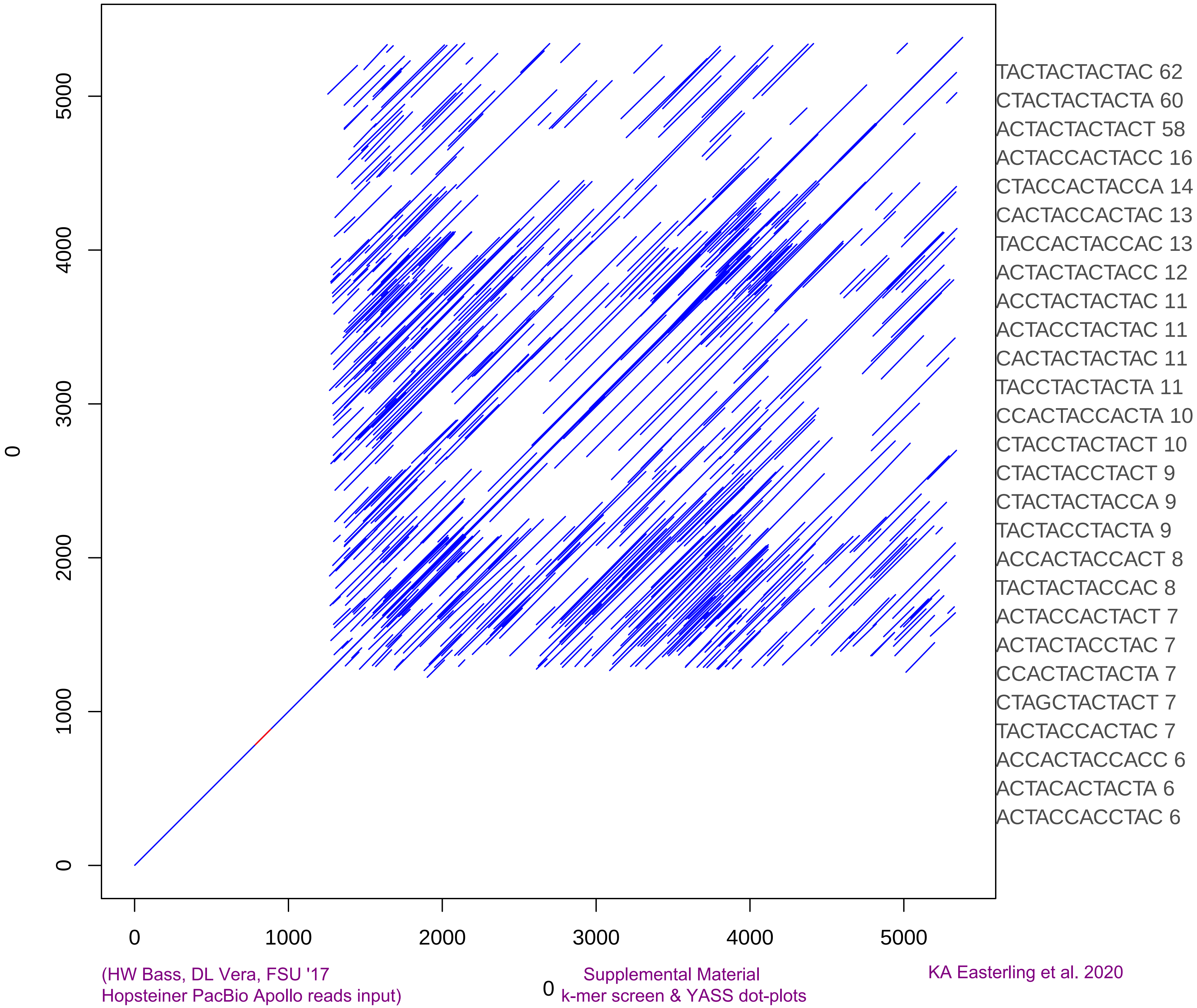

>m141126_130806_42134_c100732262550000001823141405141503_s1_p0/54042/4147_11066

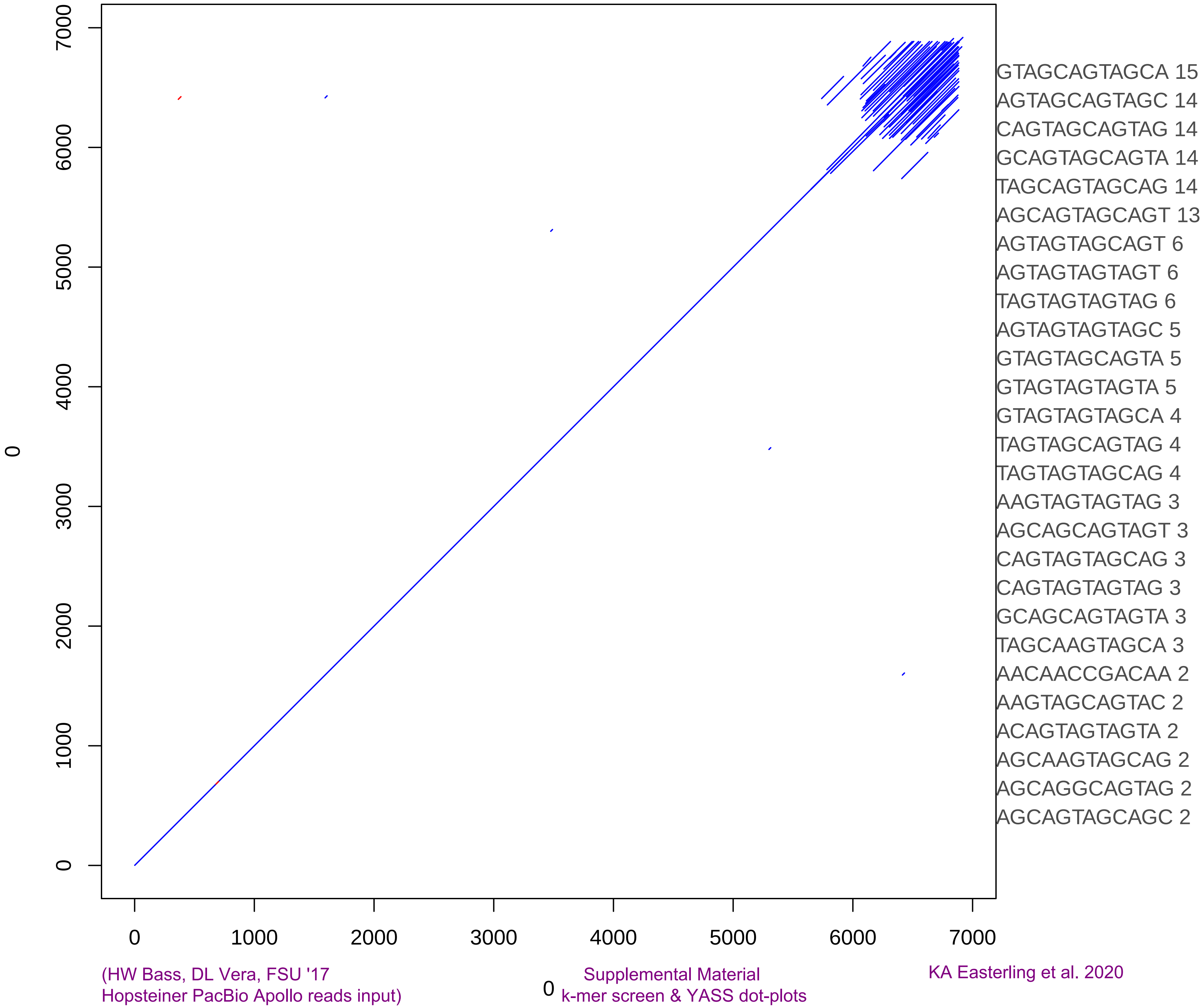

>m141126_130806_42134_c100732262550000001823141405141503_s1_p0/57054/2688_10283

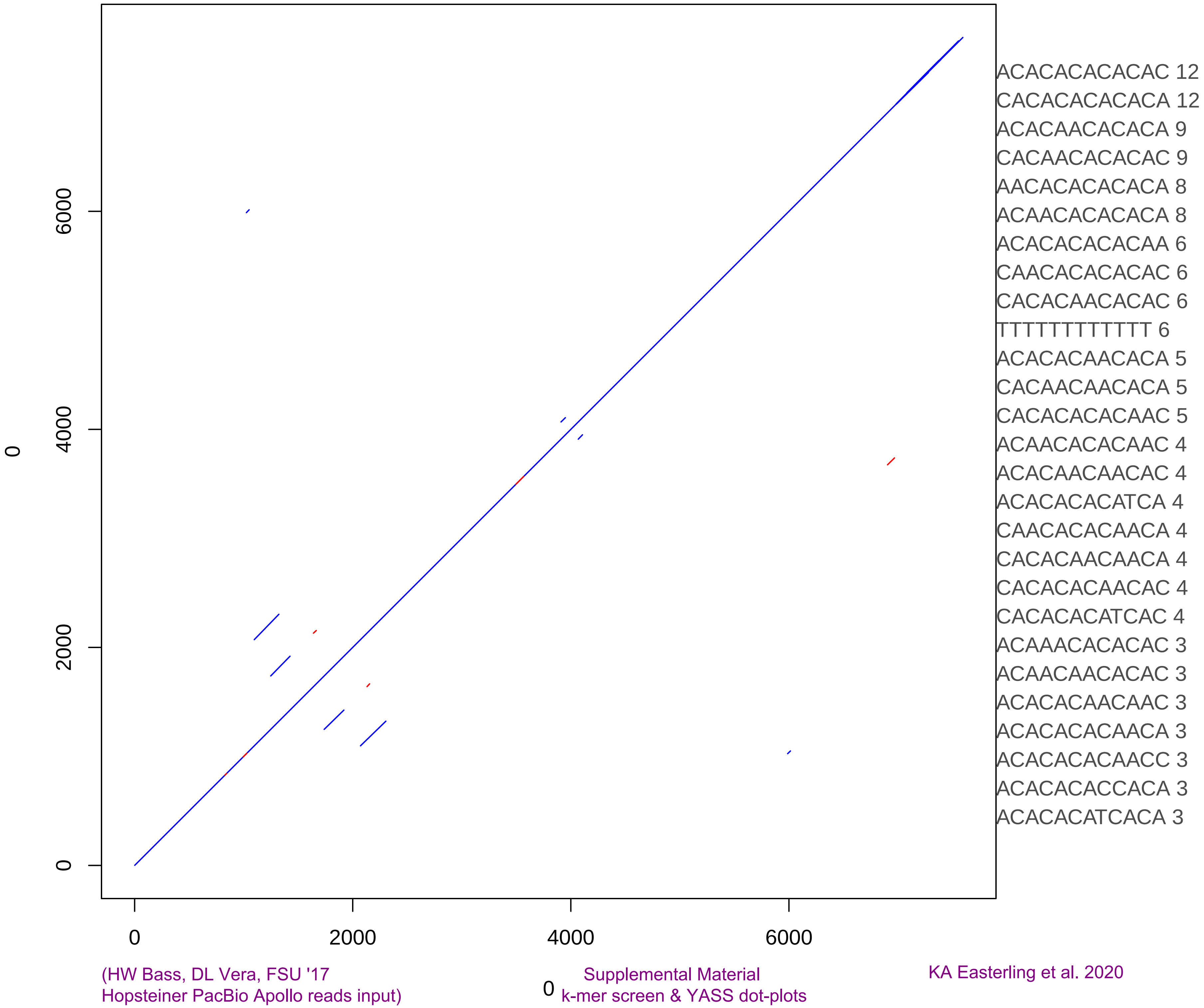

>m141126_130806_42134_c100732262550000001823141405141503_s1_p0/61221/373_9048

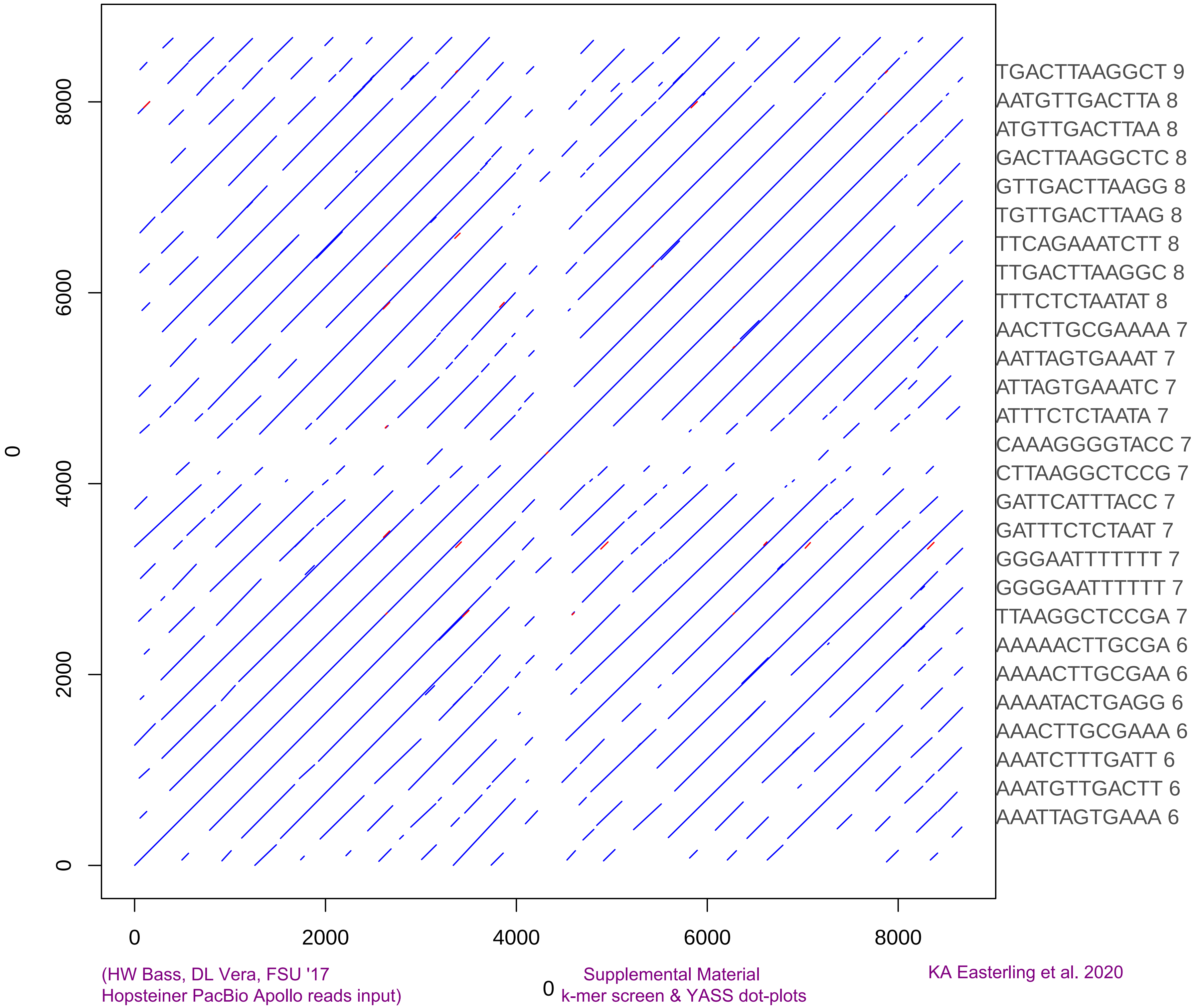

>m141126_130806_42134_c100732262550000001823141405141503_s1_p0/67131/0_13043

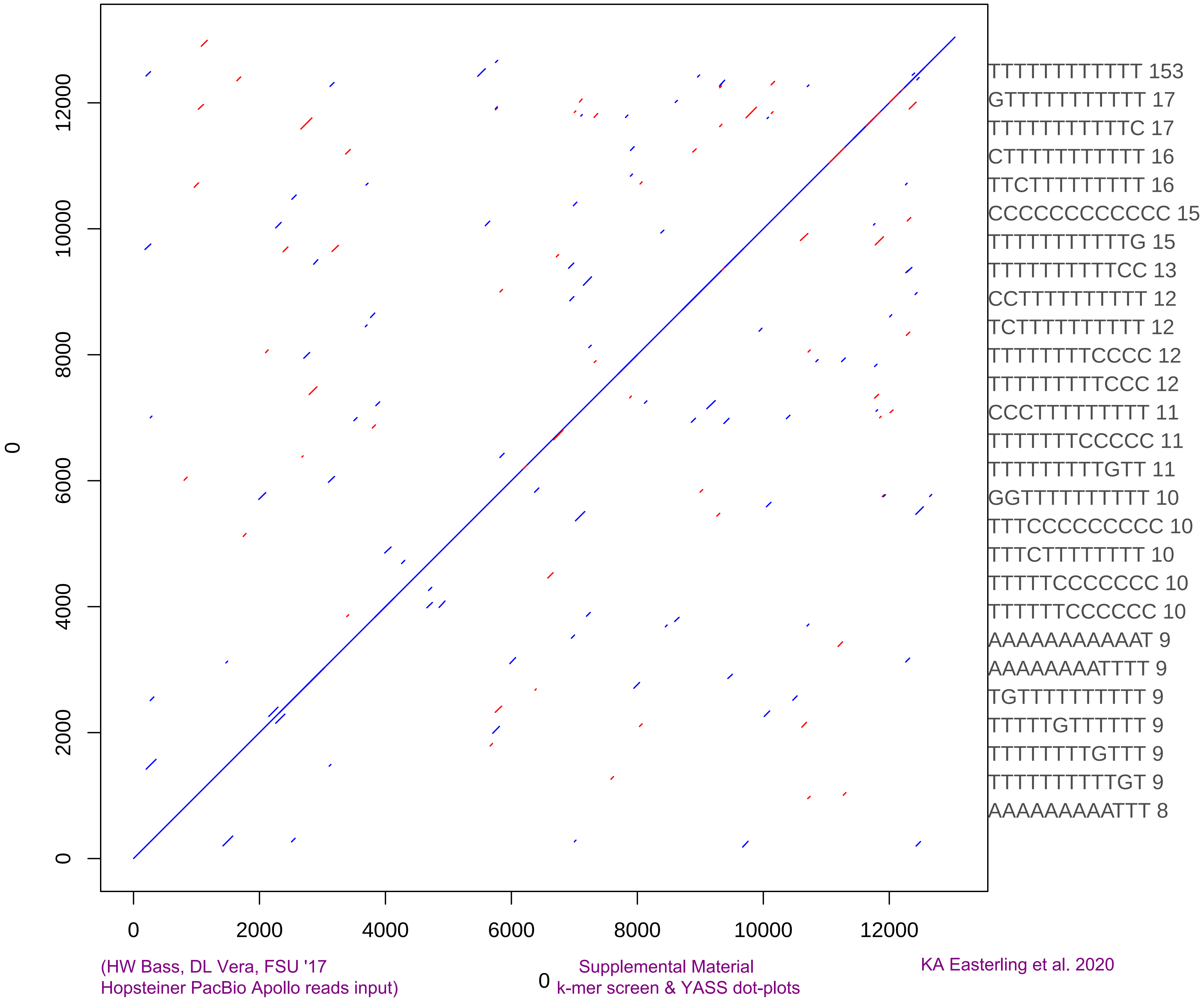

>m141126_130806_42134_c100732262550000001823141405141503_s1_p0/72604/3685_9165

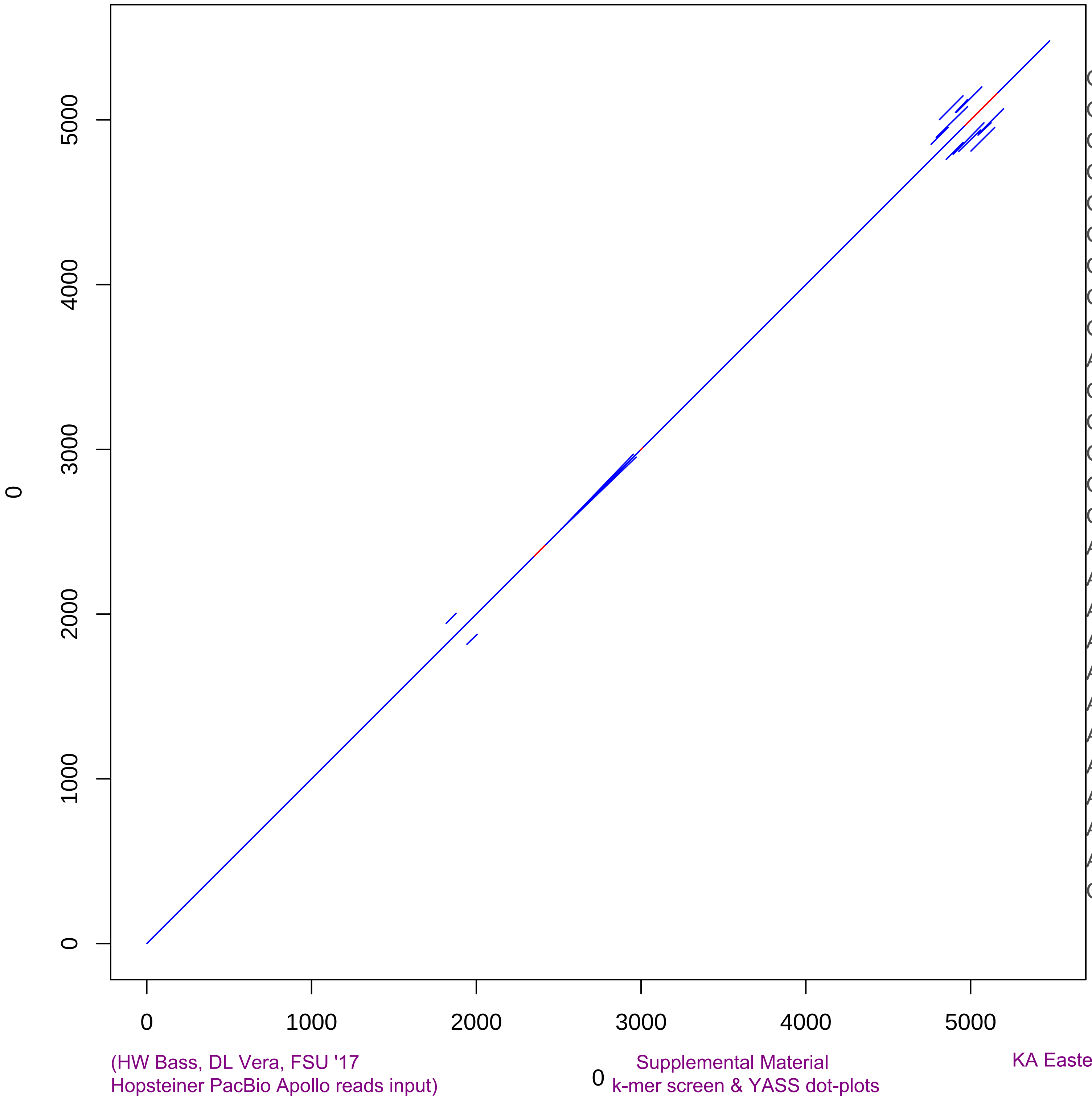

CCCCCCCCCCCC 25
CCCCCCCCCACC 9
CCCCCCACCCCC 8
CCCCCCCACCCC 8
CCCCCCCCACCC 8
CCACCCCCCCCC 7
CCCCCACCCCCC 7
CCCCCCCCCCAC 7
CCCCCCCCCCCA 7
ACCCCCCCCCCC 6
CACCCCCCCCCC 6
CCCACCCCCCCC 6
CCCCACCCCCCC 5
CACCACCCCCCC 3
CACCCCCCCCCA 3
AATATATTATAC 2
AATATTATATTG 2
ACCACCCCCCCC 2
ACCCACCACCCC 2
ATAAGTTATTTA 2
ATATTATAAGTT 2
ATATTATATTGT 2
ATATTGTTATAT 2
ATTATAAGTTAT 2
ATTATATTGTTA 2
ATTGTTATTTAT 2
CACACCCCCCCC 2

>m141126_130806_42134_c100732262550000001823141405141503_s1_p0/77489/203_11648

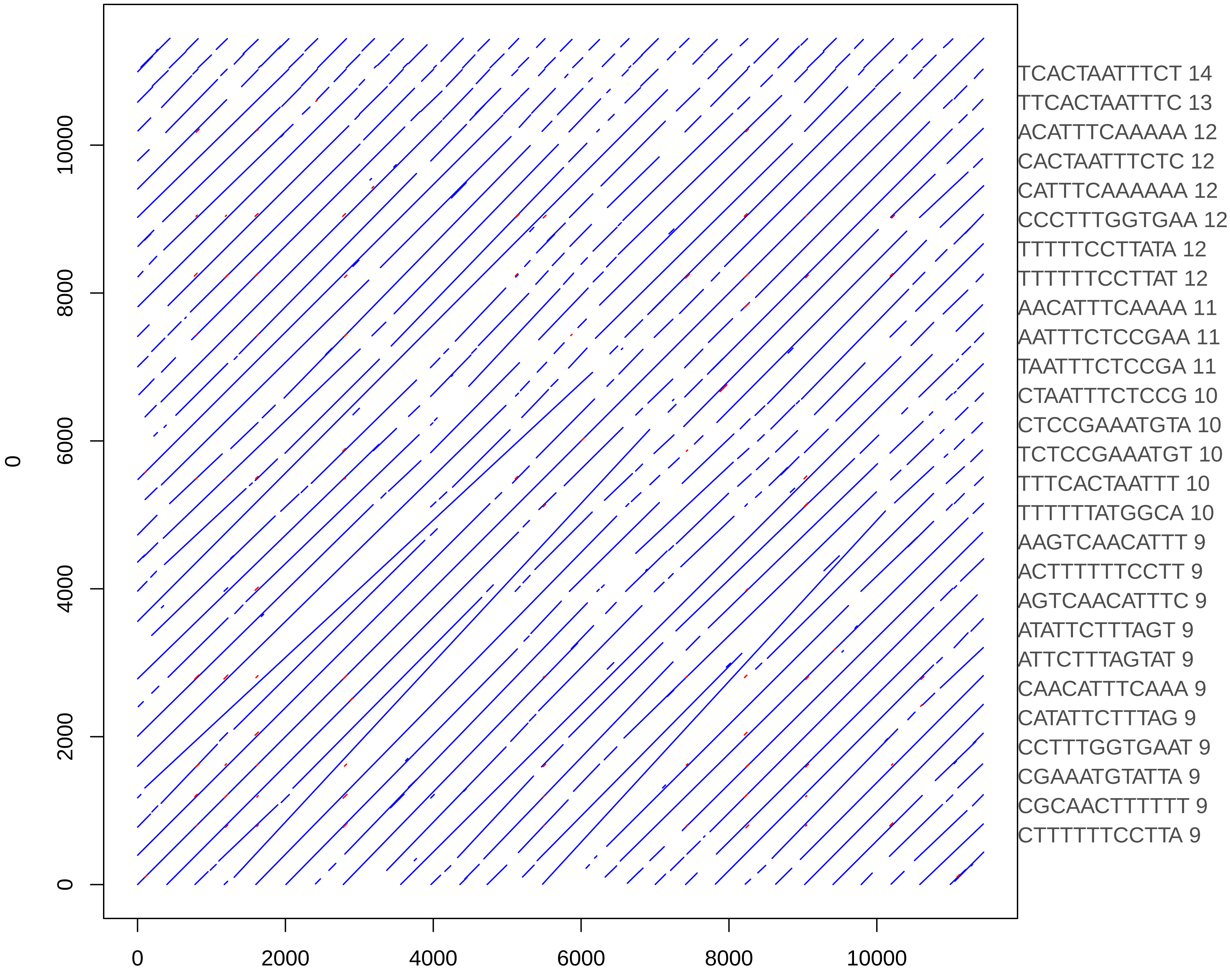

>m141126_130806_42134_c100732262550000001823141405141503_s1_p0/77607/8072_17965

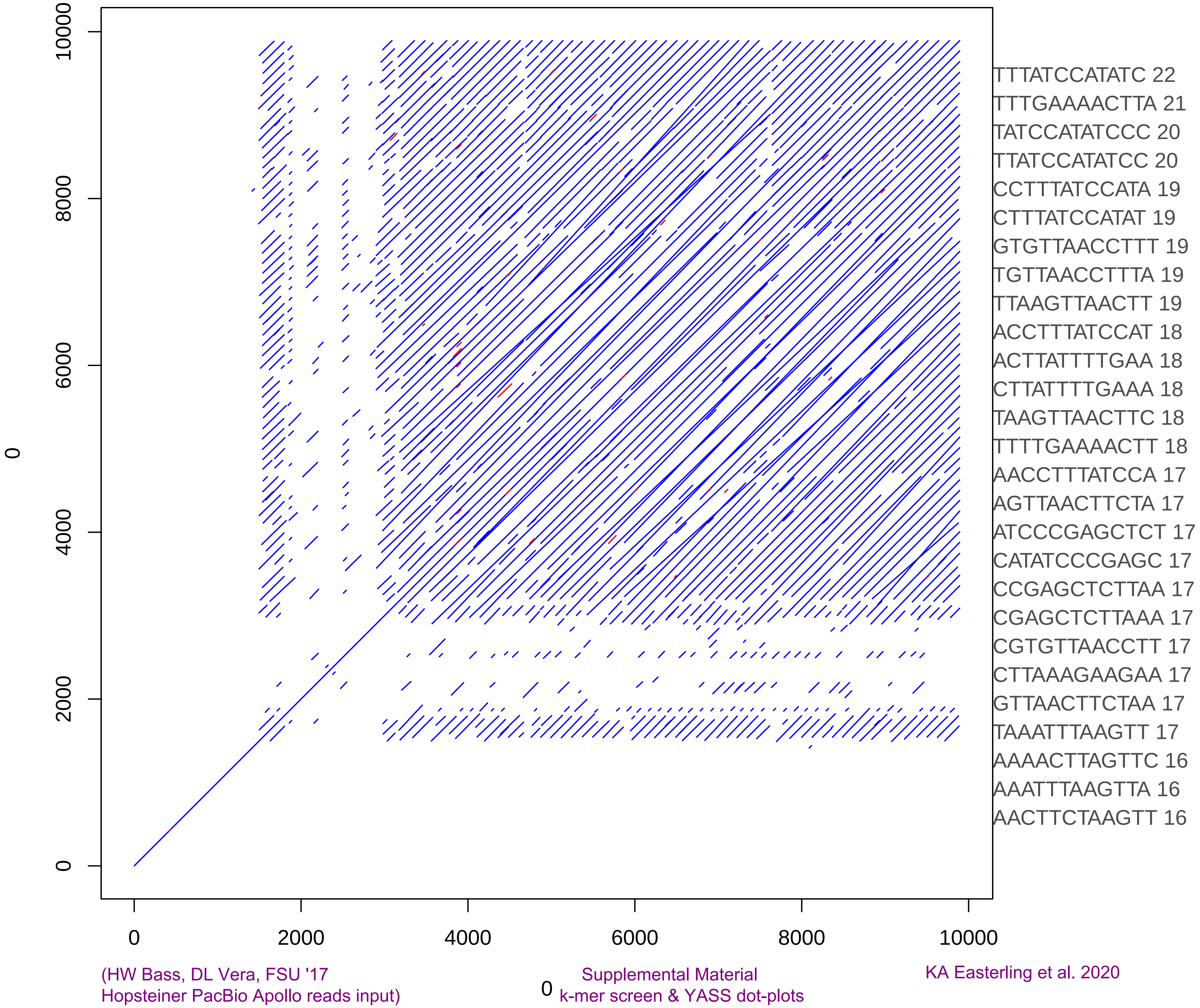

>m141126_130806_42134_c100732262550000001823141405141503_s1_p0/79457/2501_9531

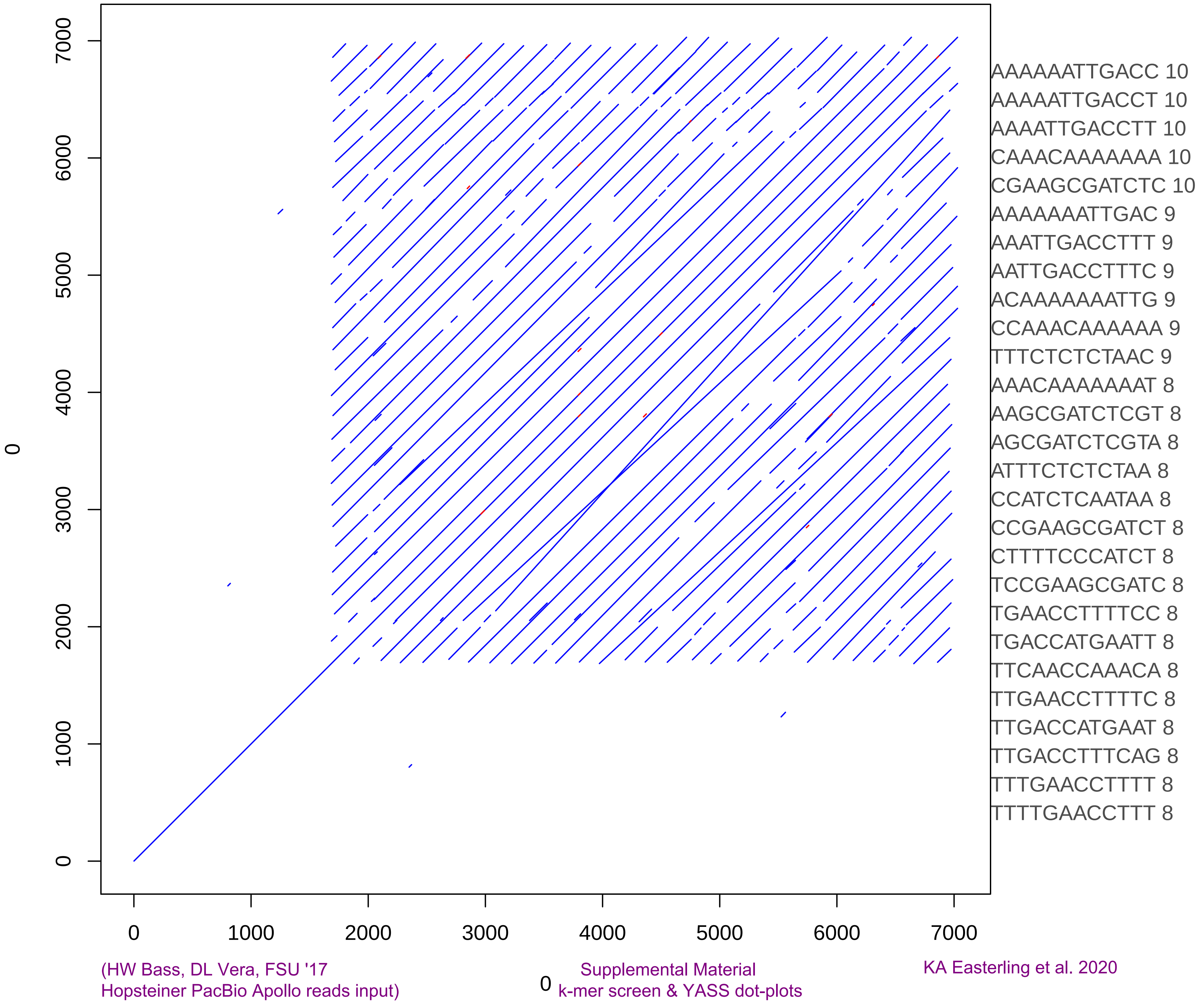

>m141126_130806_42134_c100732262550000001823141405141503_s1_p0/89934/0_6235

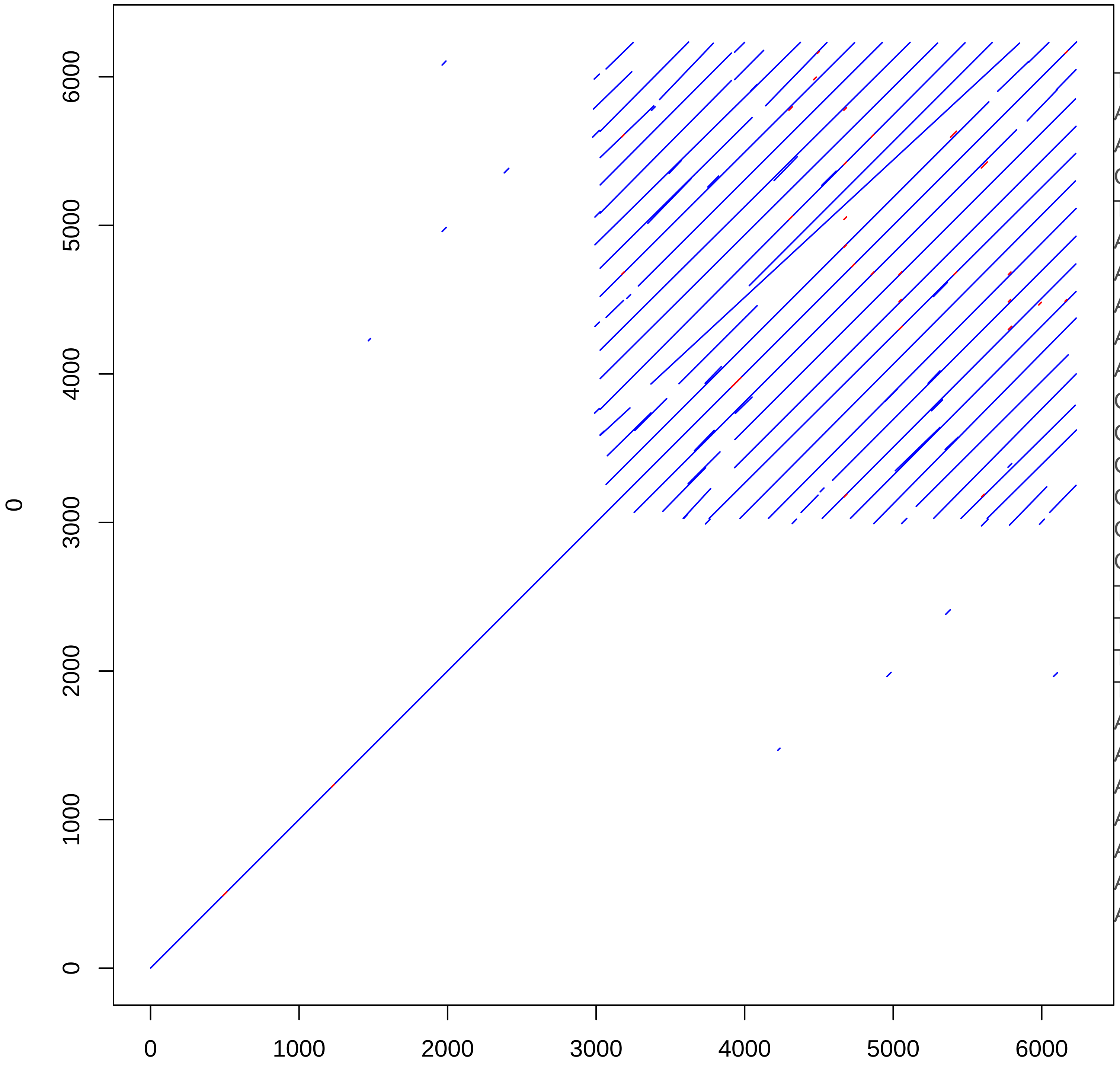

TATGAGTGAATT 11
AATTTTTTTGTT 10
ATATGAGTGAAT 10
GGCCTGAAAGGT 10
TGGCCTGAAAGG 10
AAATATGAGTGA 9
AATATGAGTGAA 9
AGAAATATGAGT 9
ATTTGGCCTGAA 9
ATTTTTTTGTTT 9
CAATTTTTTTGT 9
CCTGAAAGGTCA 9
CTGAAAGGTCAA 9
GAAATATGAGTG 9
GAGAAATATGAG 9
GCCTGAAAGGTC 9
TAGGAGATTTGG 9
TTGGCCTGAAAG 9
TTTGGCCTGAAA 9
TTTTTTTGTTTG 9
AAAAAATTAGAG 8
AAAAATTAGAGC 8
AAAGGTCAATTT 8
AAGGTCAATTTT 8
AGGAGATTTGGC 8
AGGTCAATTTTT 8
ATGAGTGAATTA 8

>m141126_130806_42134_c100732262550000001823141405141503_s1_p0/92446/3111_10628

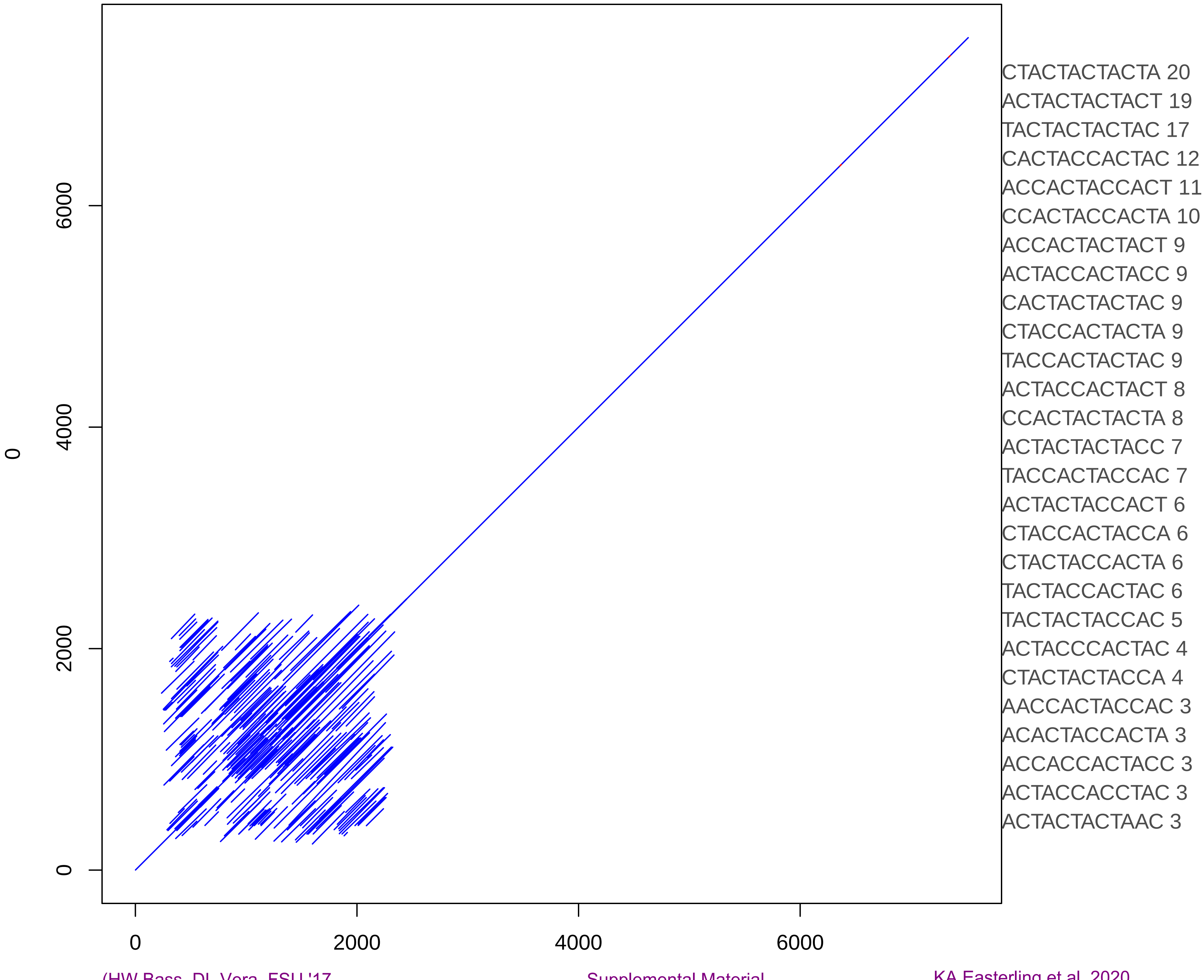

>m141126_130806_42134_c100732262550000001823141405141503_s1_p0/93321/9099_15093

Representative Read (Table 1): HuluTR120-r782, 69 ppm, 67% A+T, GenBank Acc. MN537570

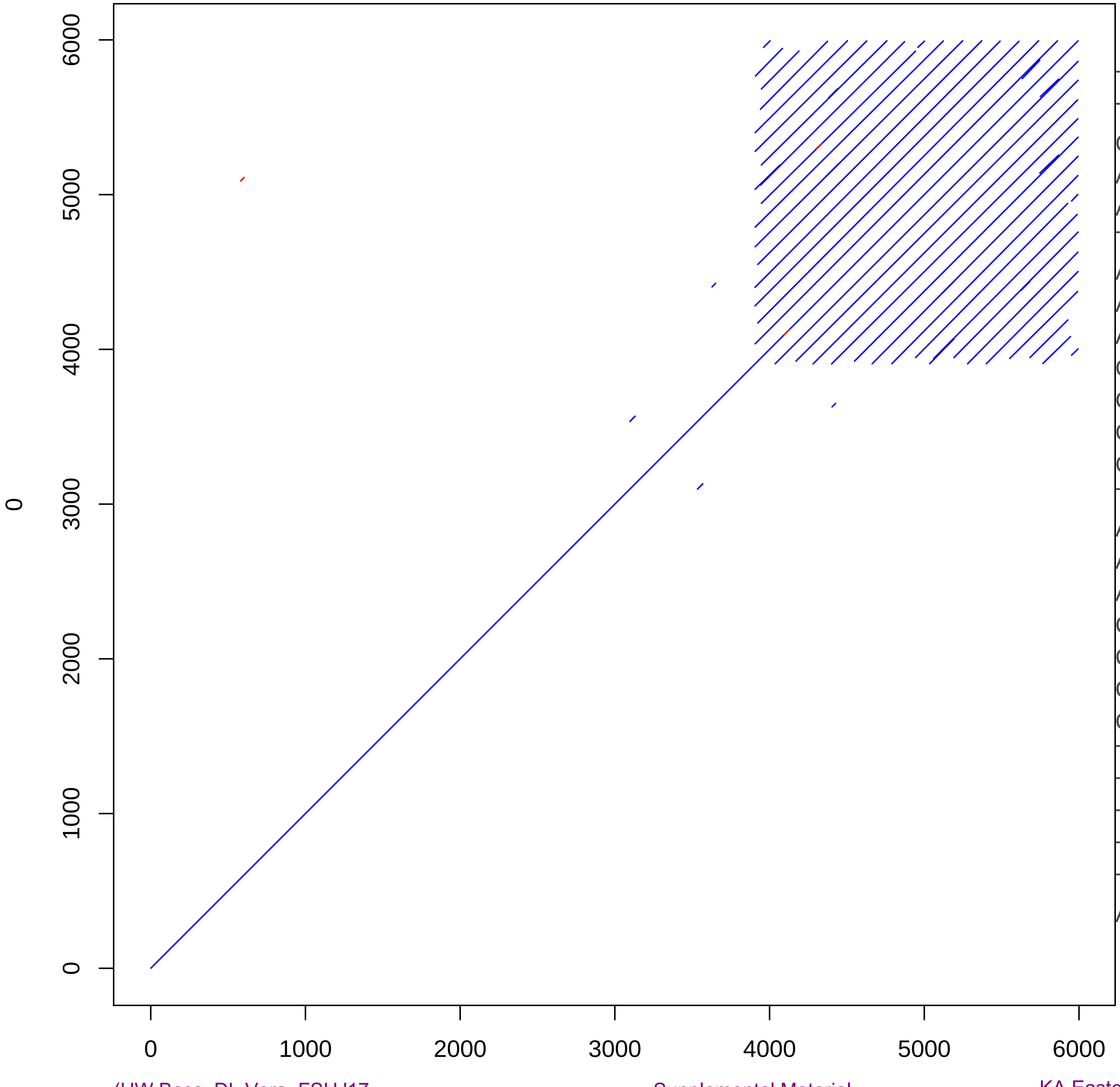

TTTAAGAGCTCG 14
TTAAGAGCTCGG 13
CTTTAAGAGCTC 11
AAGAGCTCGGGA 10
AGAGCTCGGGAT 10
TAAGAGCTCGGG 10
AAGTTAACTTAA 9
AGAAGTTAACTT 9
ATAAAACTTAGA 9
GAAGTTAACTTA 9
GAGCTCGGGATA 9
GATATGGATAAA 9
GGATATGGATAA 9
TAGAAGTTAACT 9
AAAACTTAGAAG 8
AGCTCGGGATAT 8
AGTTAACTTAAA 8
CATAAAACTTAG 8
CTCGGGATATGG 8
GCTCGGGATATG 8
GTCATAAAACTT 8
TAAAACTTAGAA 8
TCGGGATATGGA 8
TTAGAAGTTAAC 8
TTTAACCAGGTC 8
TTTTAACCAGGT 8
AAACTTAGAAGT 7

>m141126_130806_42134_c100732262550000001823141405141503_s1_p0/93723/0_7973

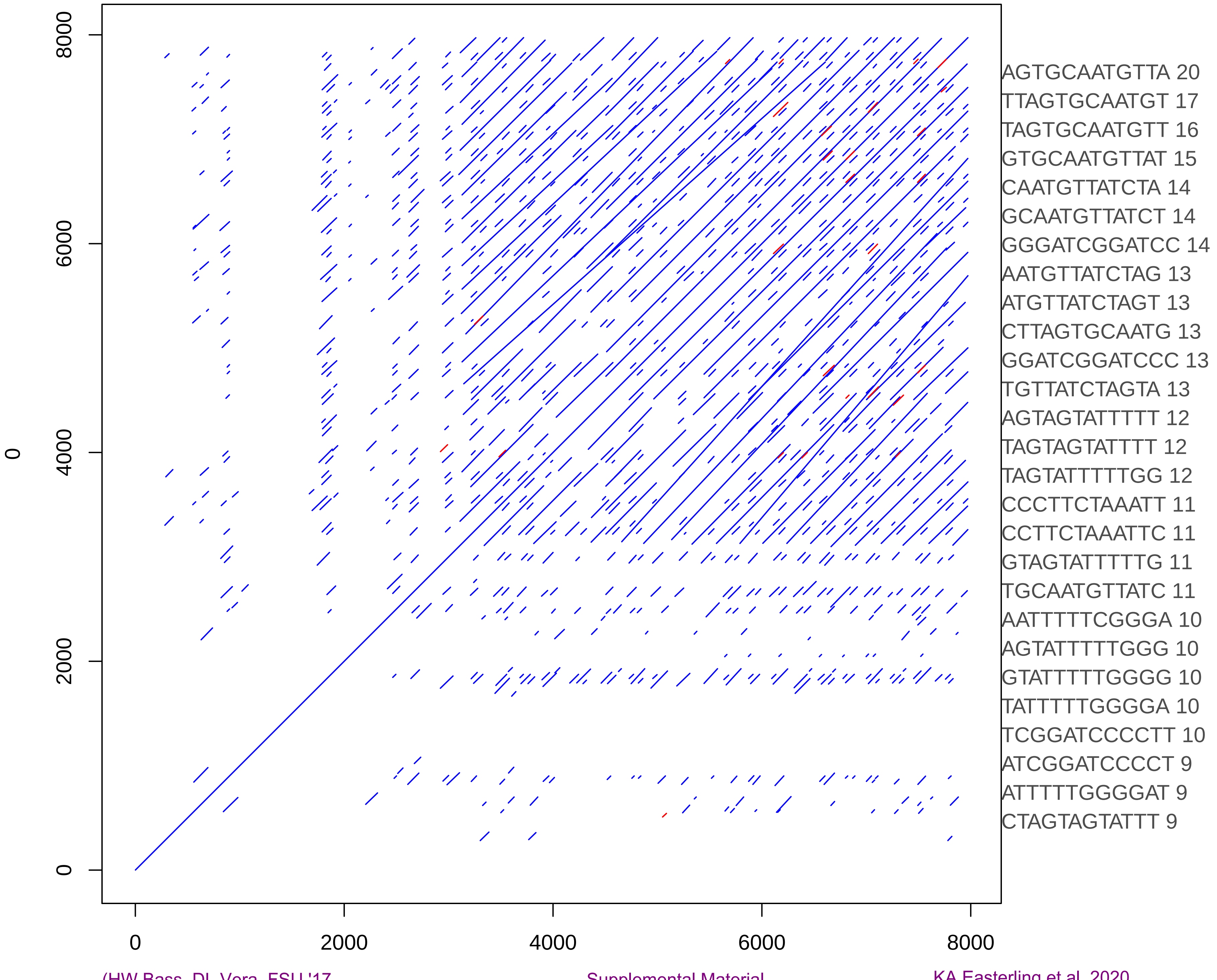

>m141230_233107_42134_c100756552550000001823146807221541_s1_p0/104255/5409_12136

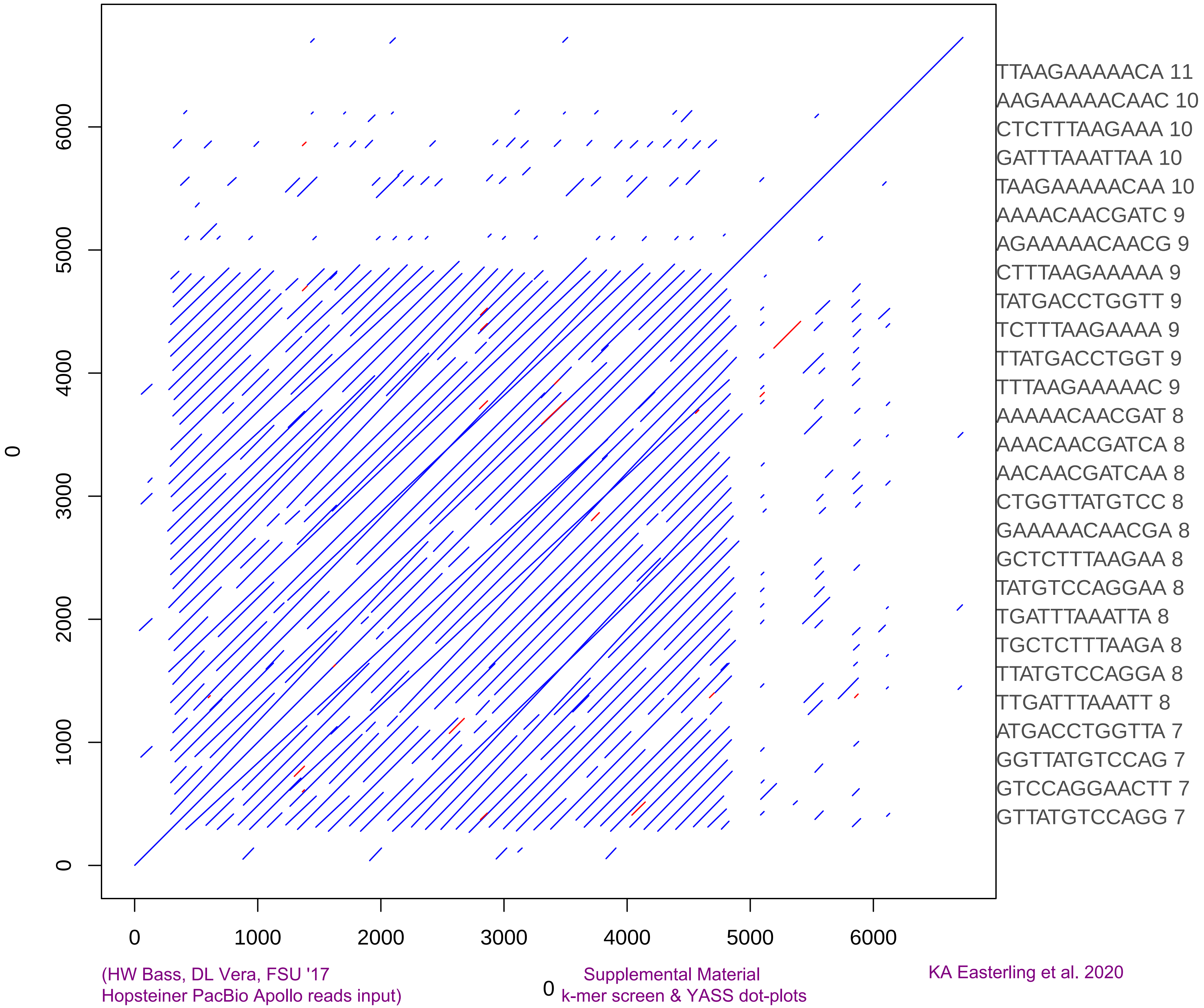

>m141230_233107_42134_c100756552550000001823146807221541_s1_p0/107422/10274_15519

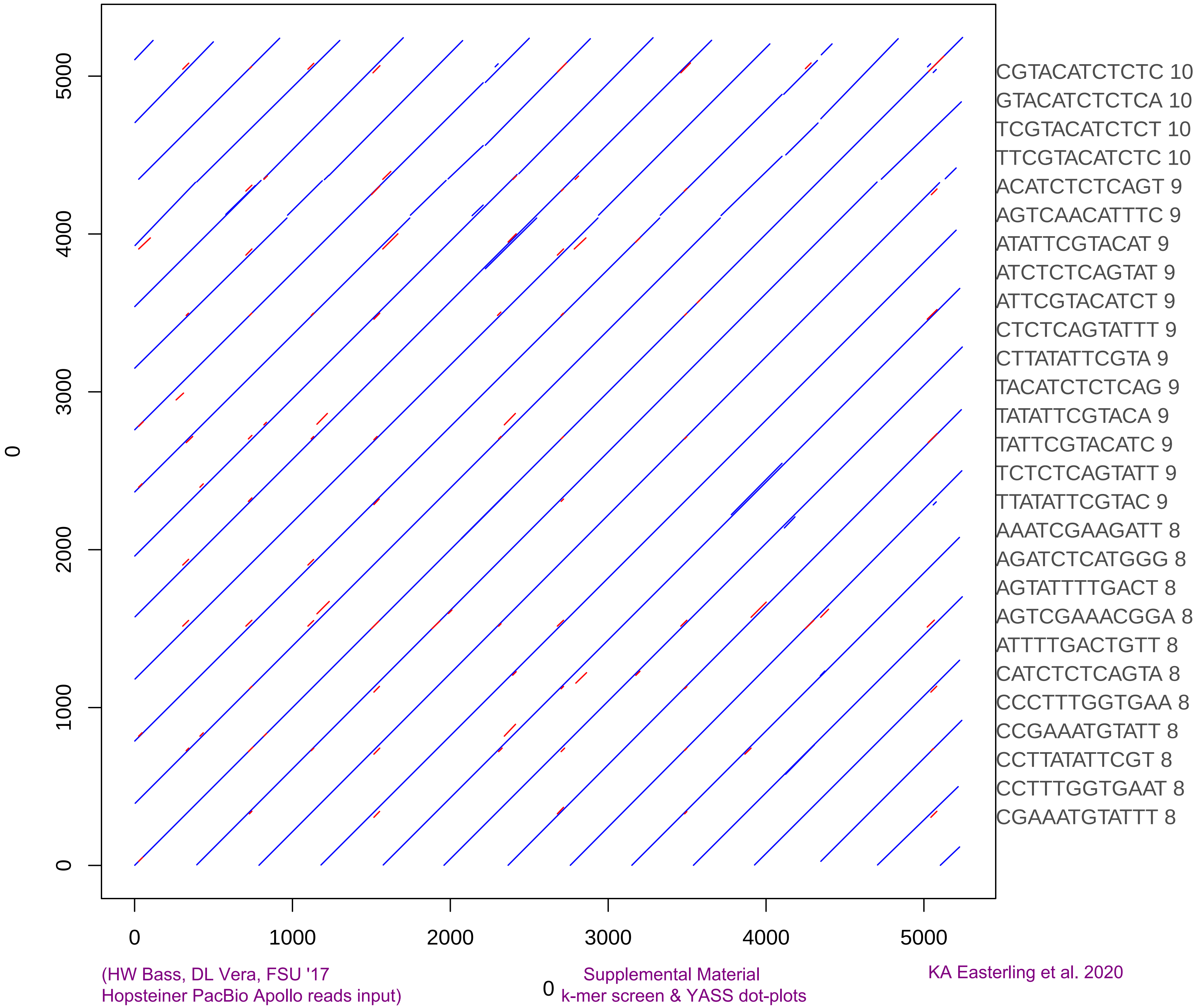

>m141230_233107_42134_c100756552550000001823146807221541_s1_p0/107537/8985_14590

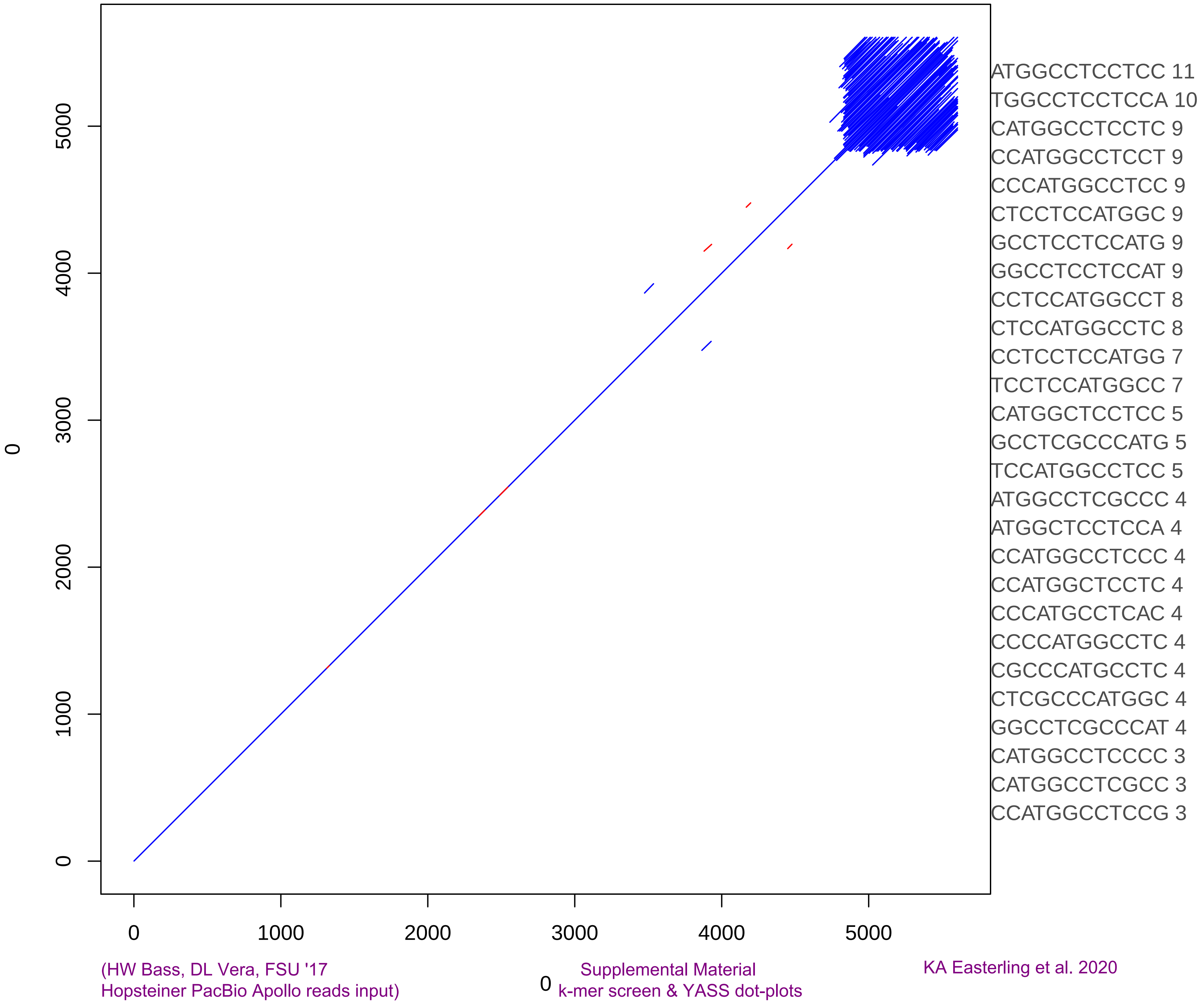

>m141230_233107_42134_c100756552550000001823146807221541_s1_p0/107741/0_13734

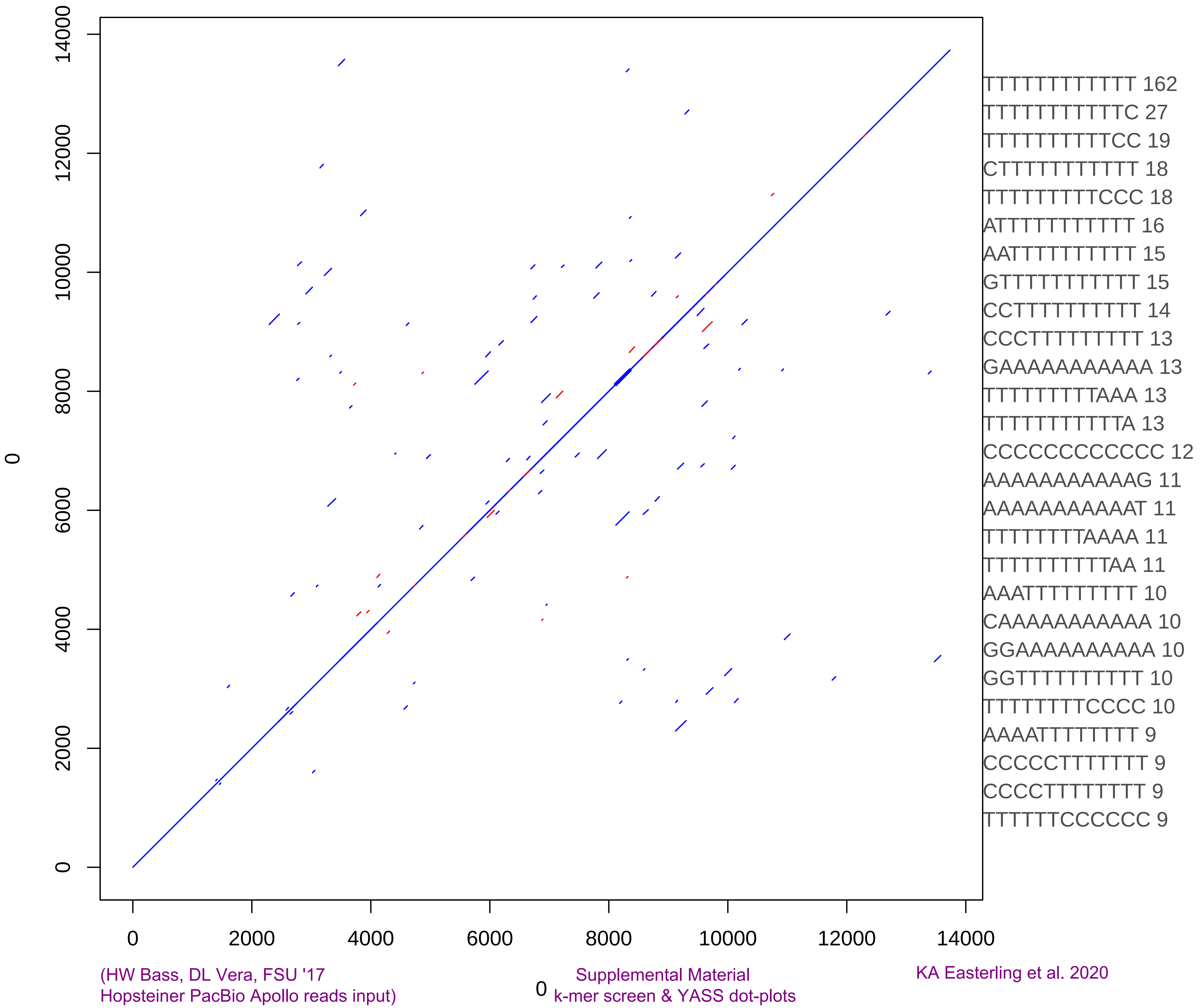

>m141230_233107_42134_c100756552550000001823146807221541_s1_p0/107769/296_8374

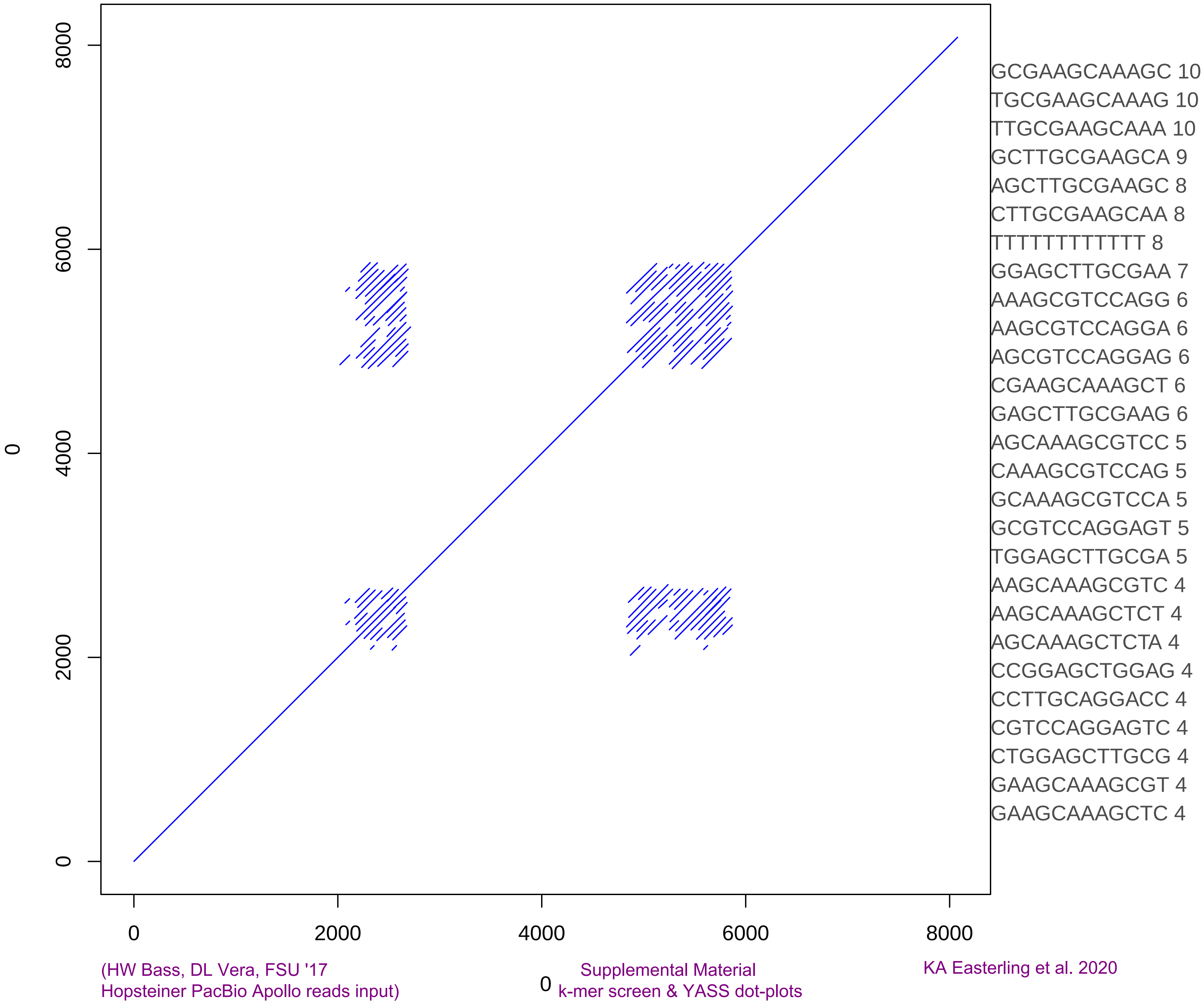

>m141230_233107_42134_c100756552550000001823146807221541_s1_p0/108312/463_12022

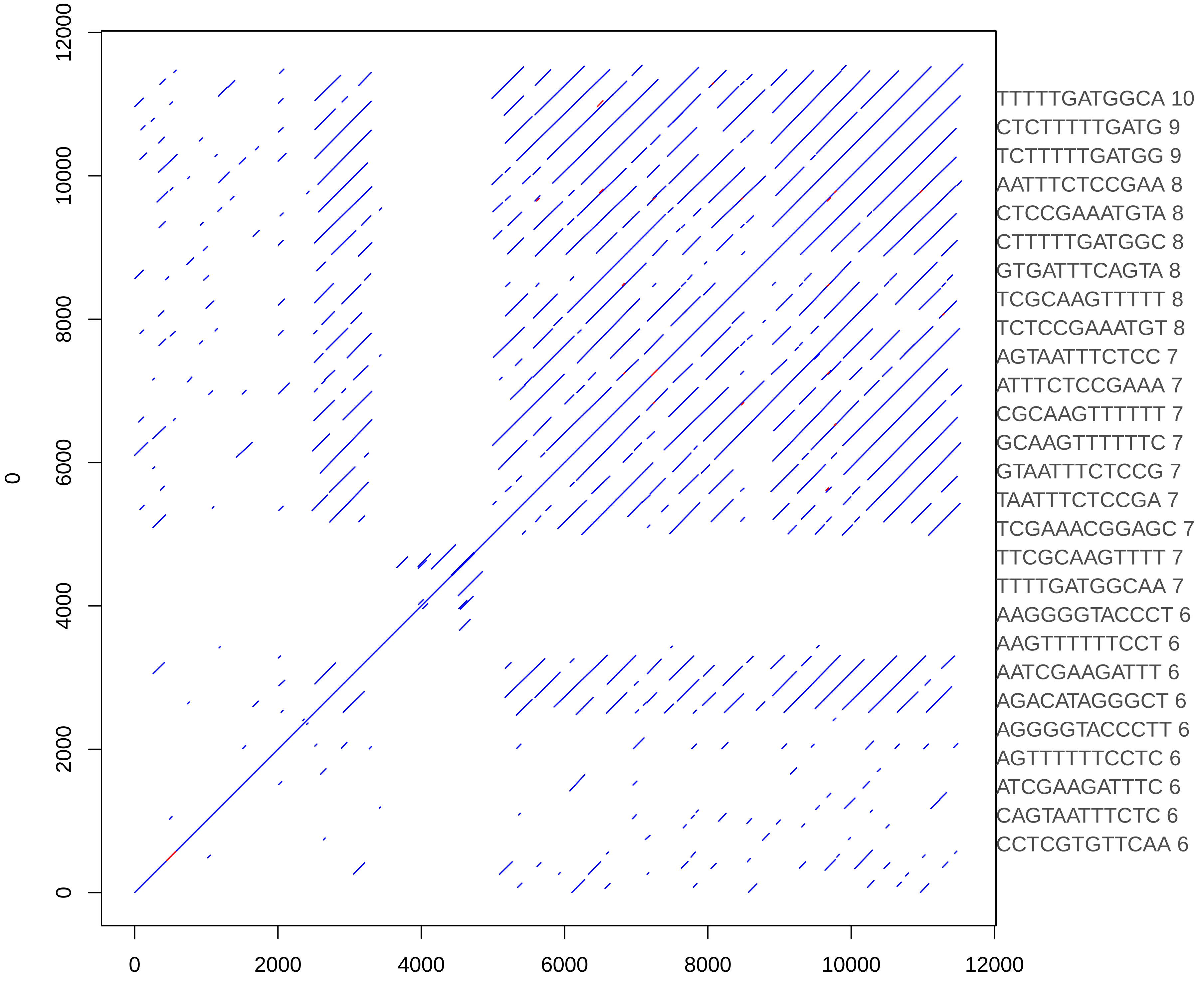

>m141230_233107_42134_c100756552550000001823146807221541_s1_p0/109885/3208_13862

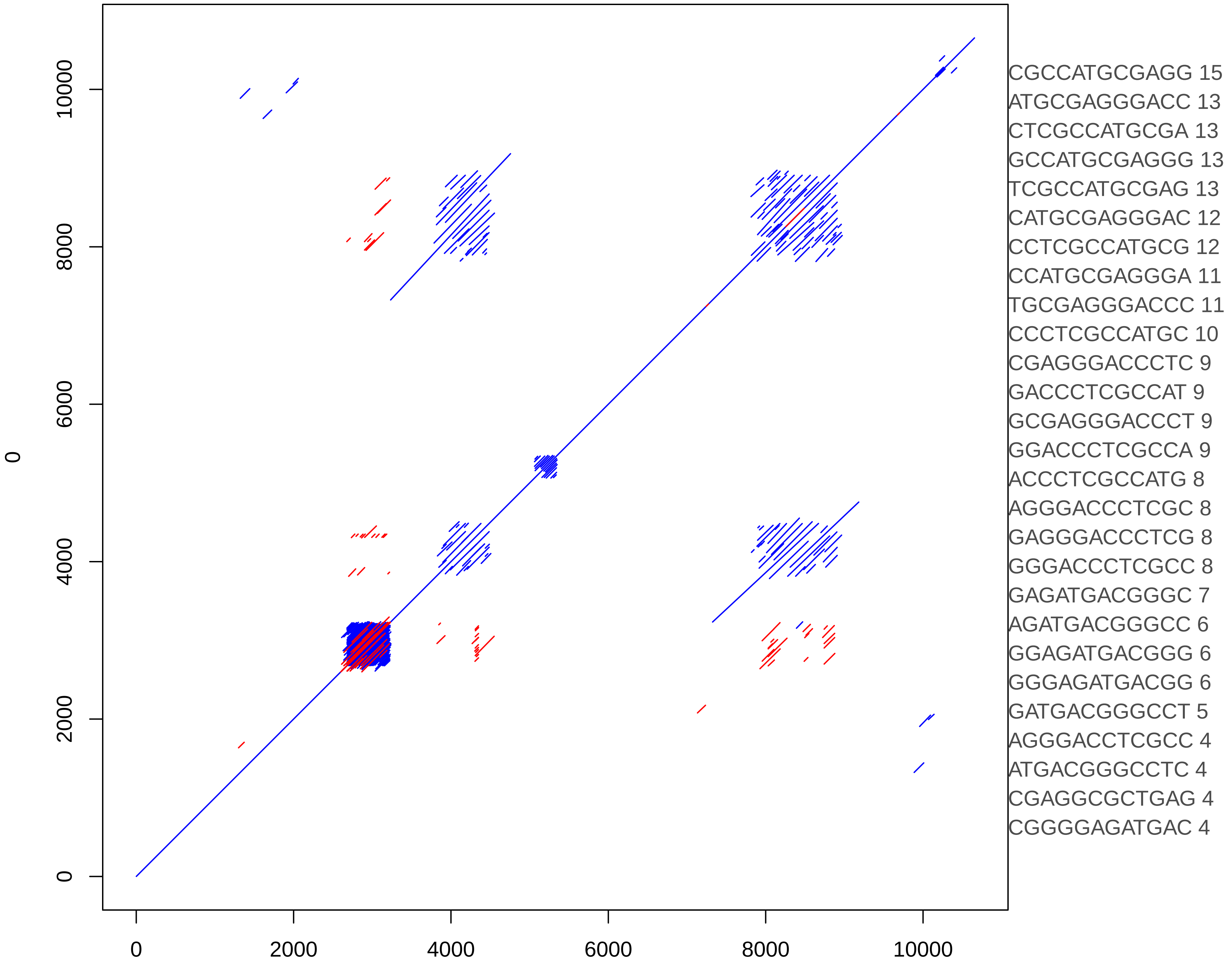

>m141230_233107_42134_c100756552550000001823146807221541_s1_p0/110454/708_14756

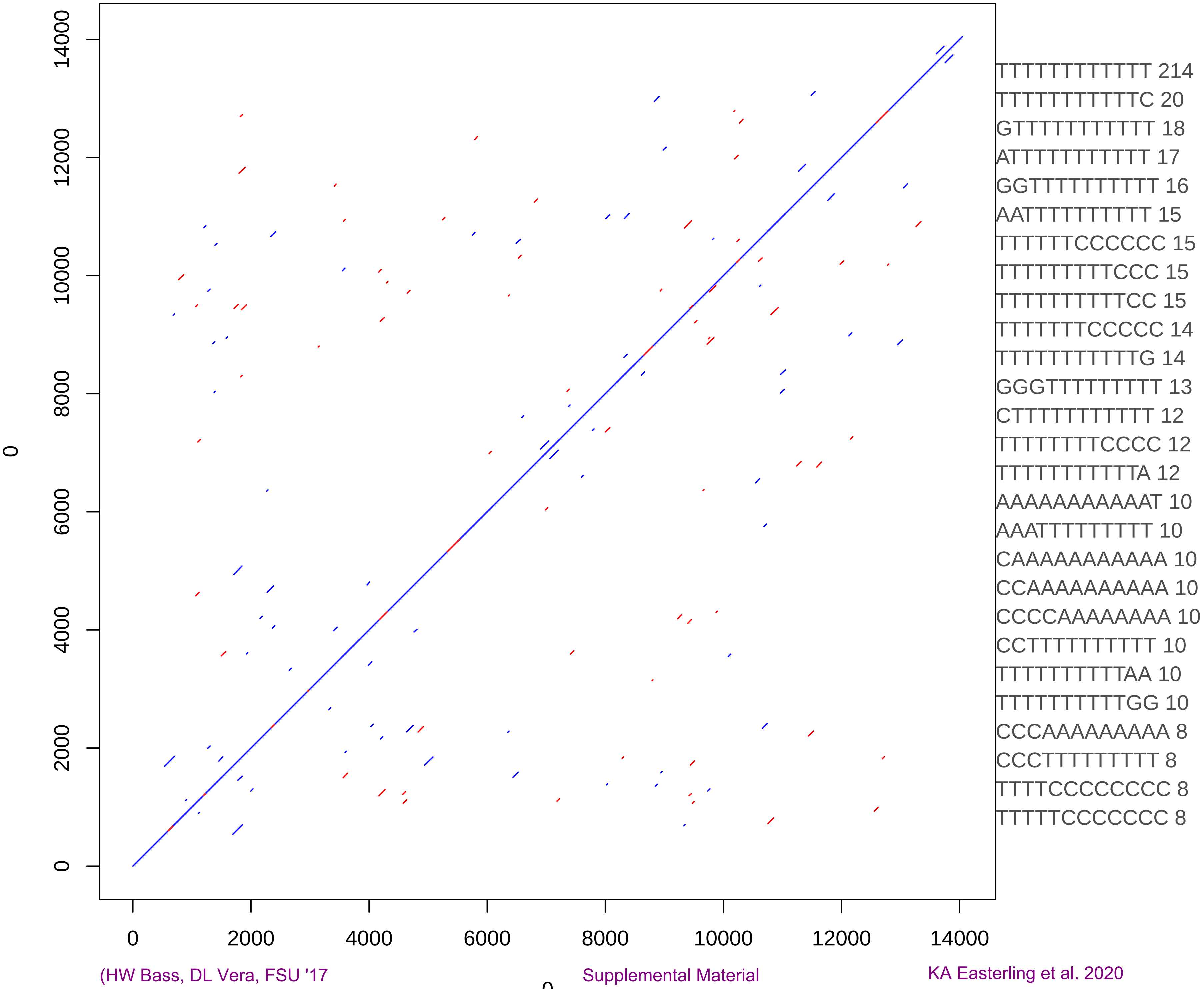

>m141230_233107_42134_c100756552550000001823146807221541_s1_p0/11198/2197_9715

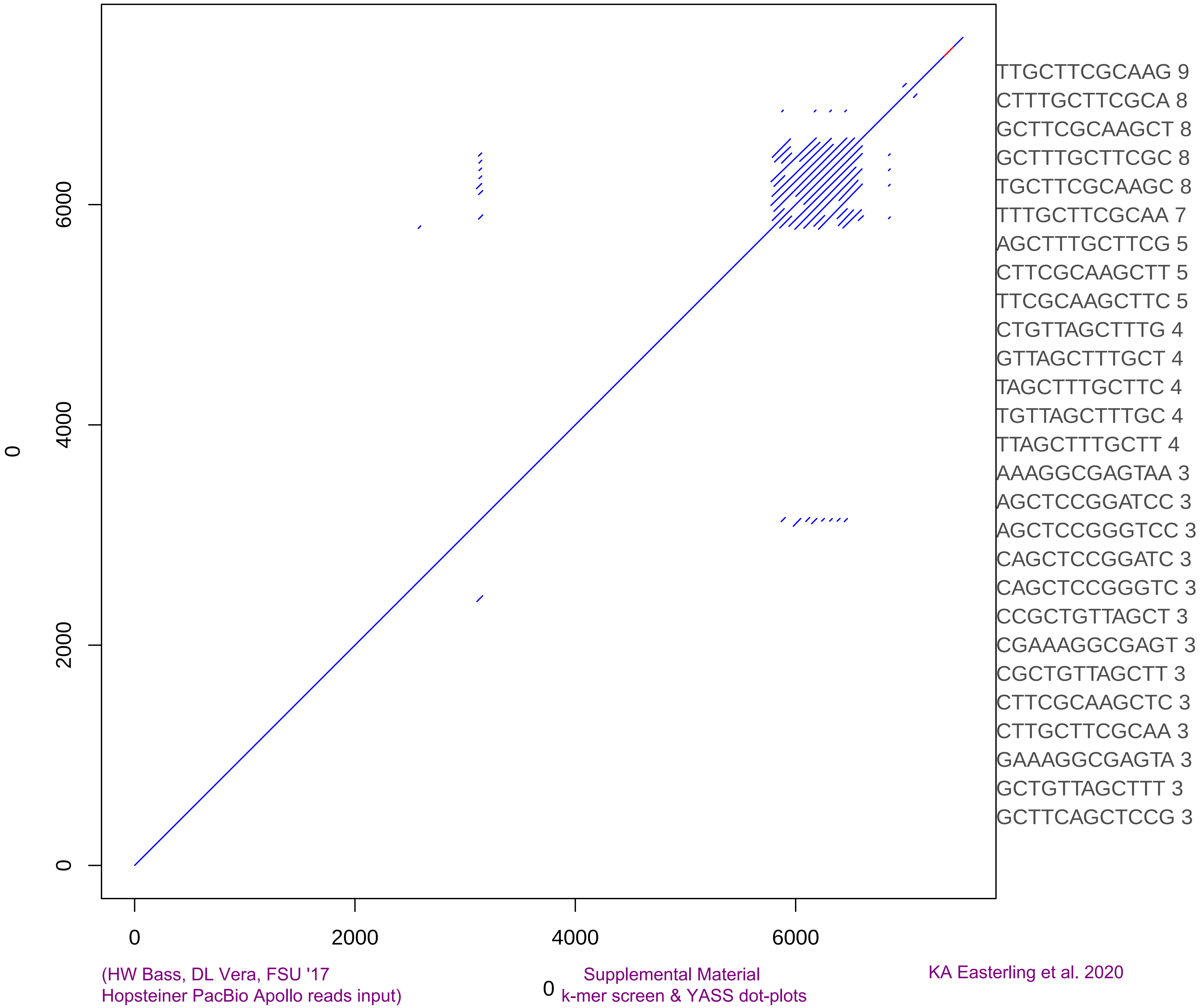

>m141230_233107_42134_c100756552550000001823146807221541_s1_p0/11336/291_6561

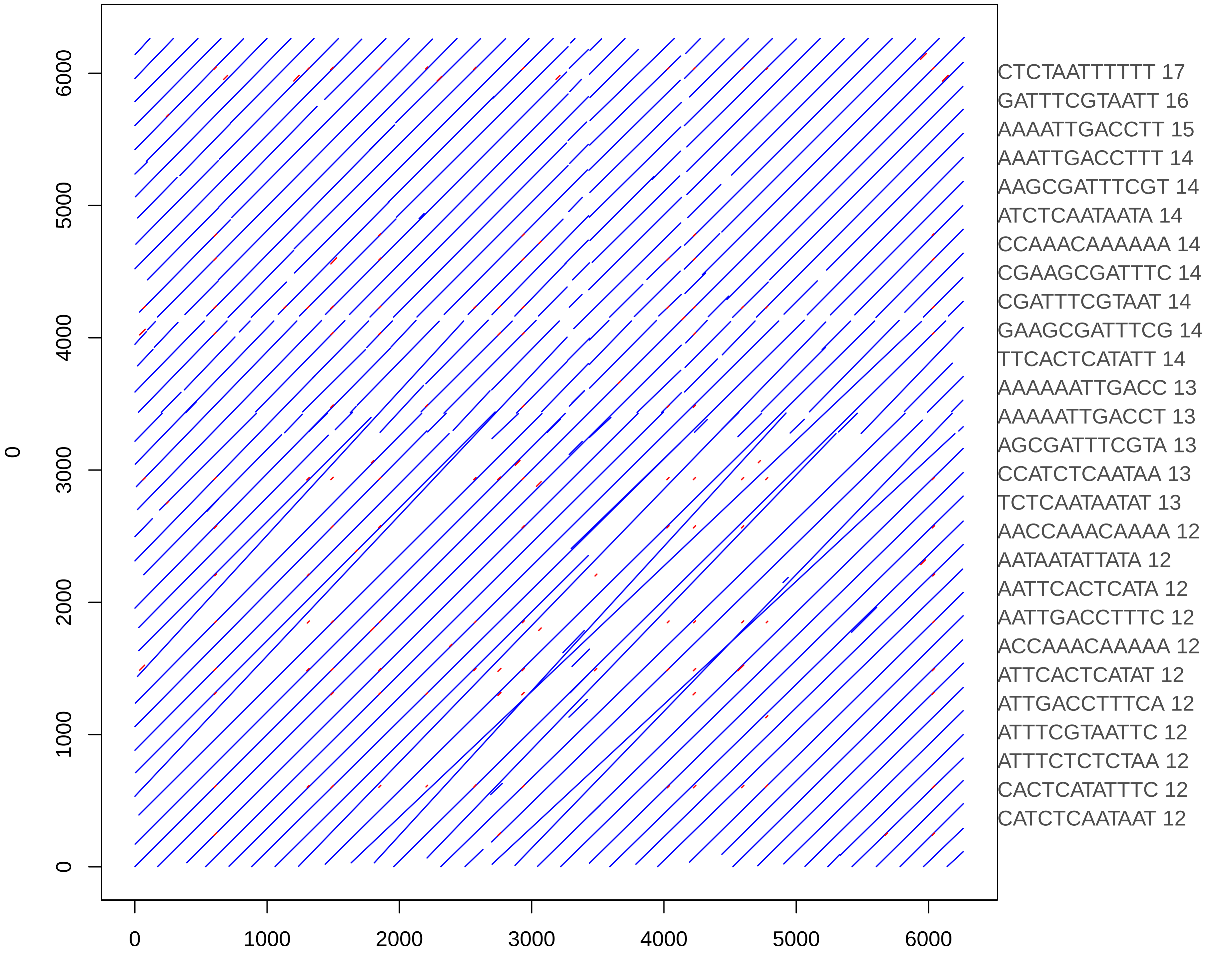

>m141230_233107_42134_c100756552550000001823146807221541_s1_p0/114022/0_9085

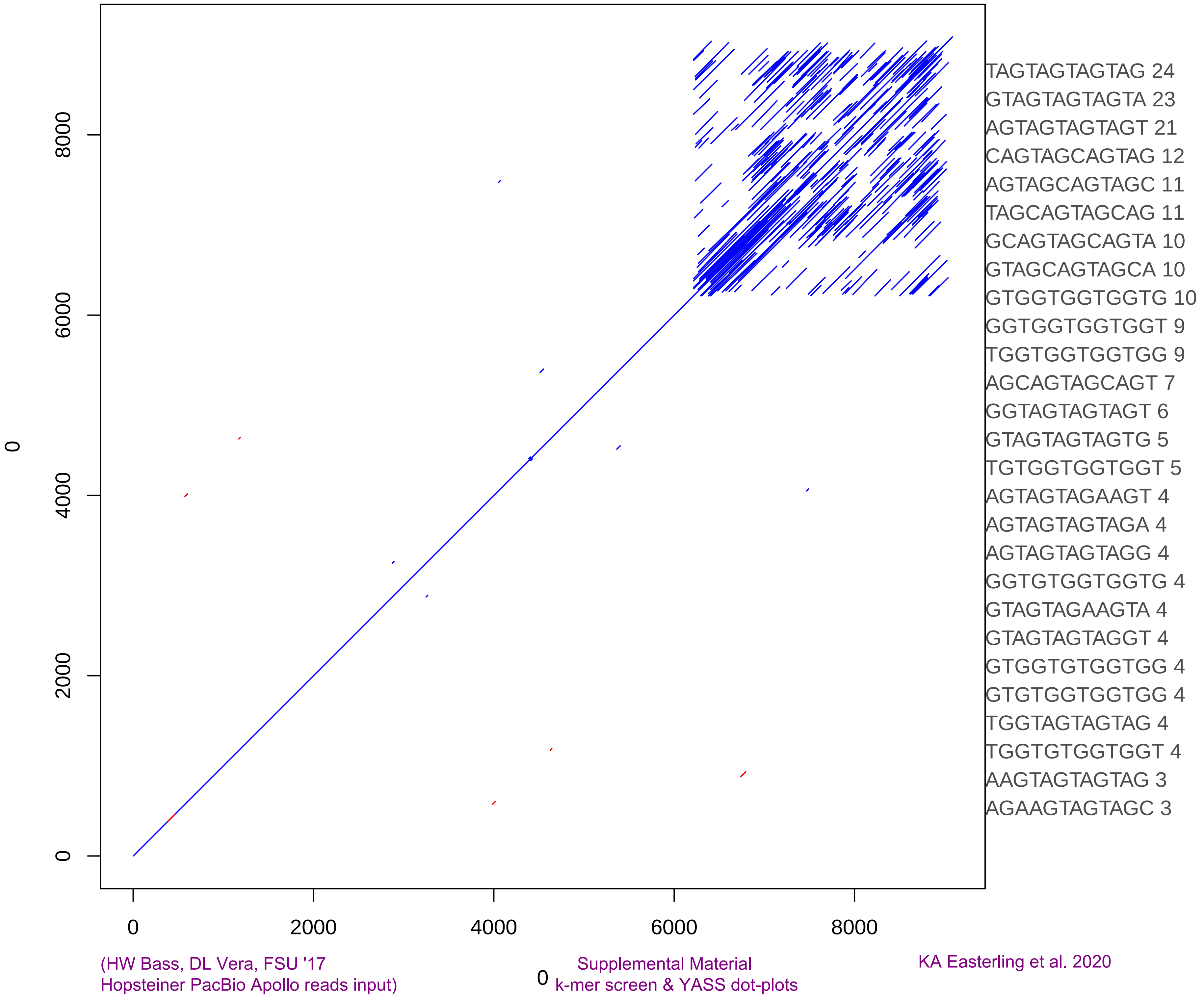

>m141230_233107_42134_c100756552550000001823146807221541_s1_p0/115274/0_5931

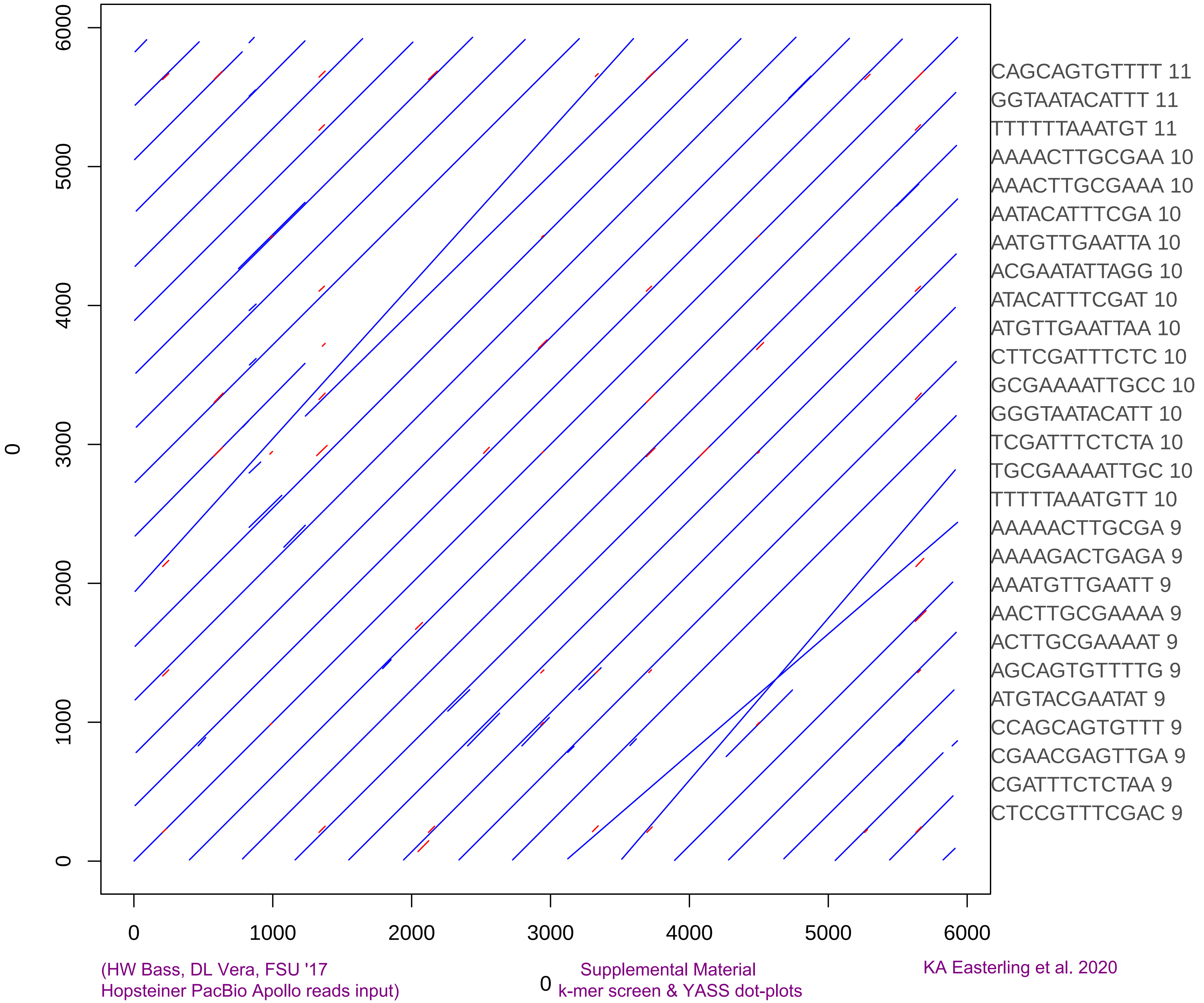

>m141230_233107_42134_c100756552550000001823146807221541_s1_p0/116481/585_6979

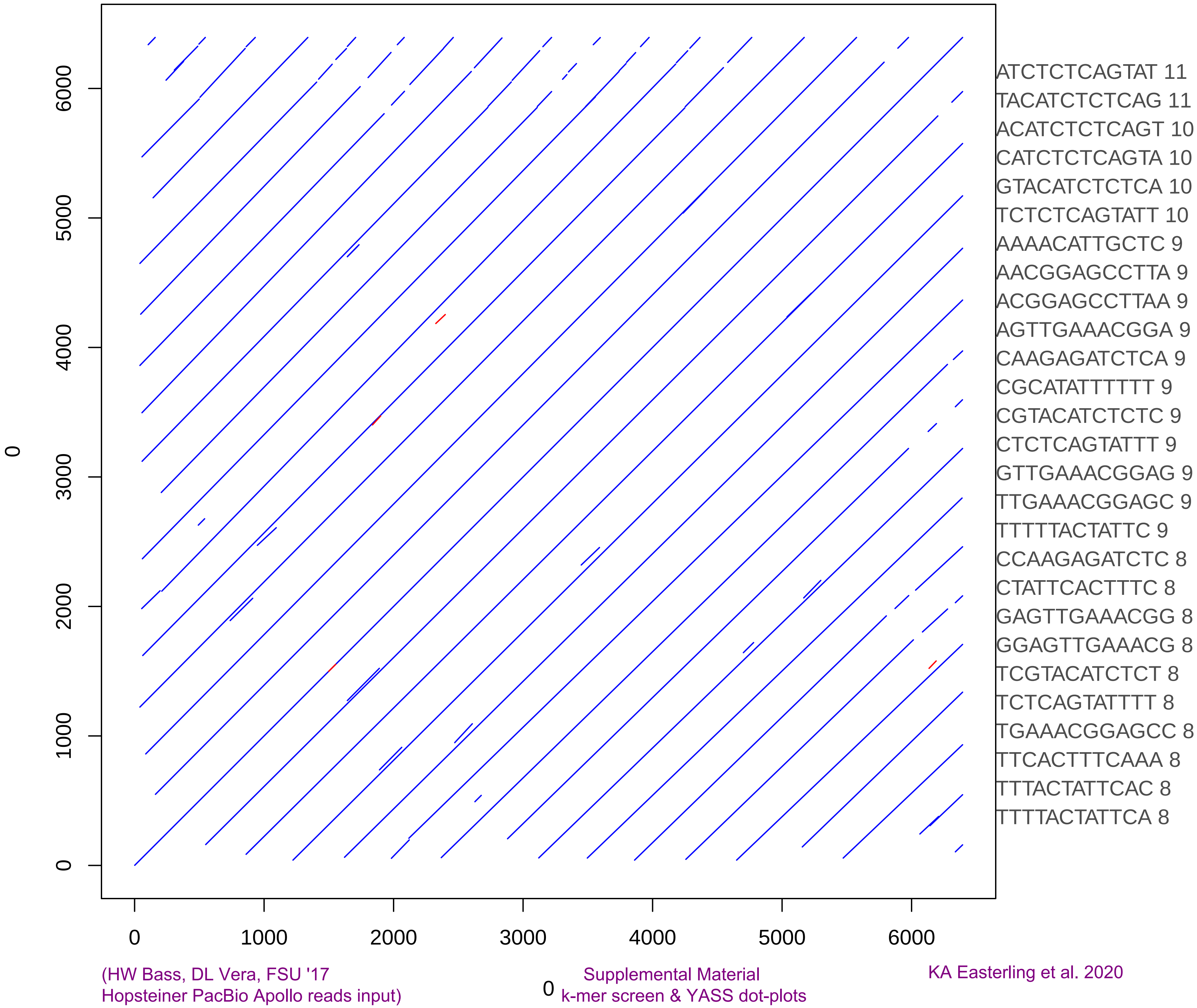

>m141230_233107_42134_c100756552550000001823146807221541_s1_p0/116787/1989_12684

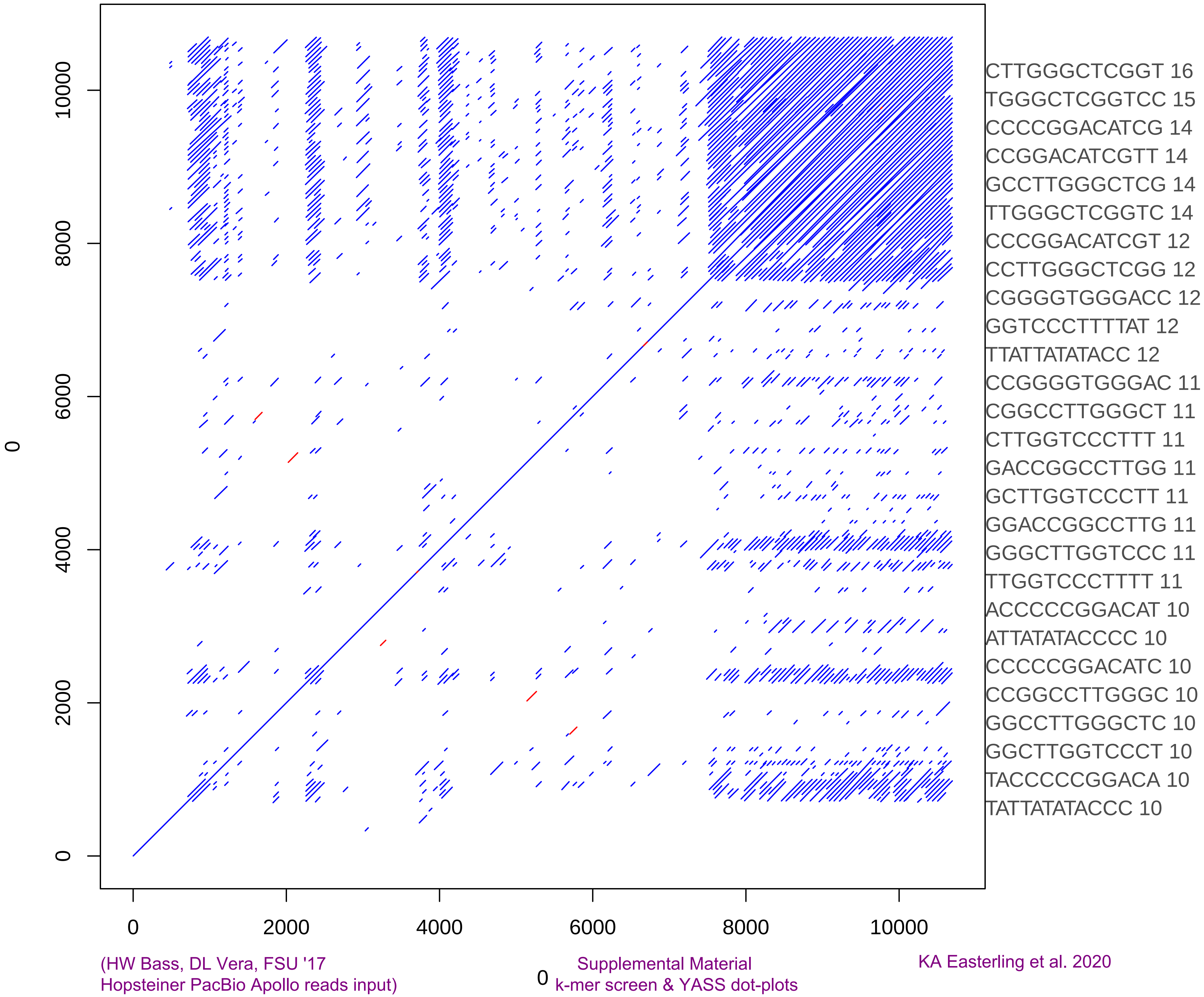

>m141230_233107_42134_c100756552550000001823146807221541_s1_p0/117912/721_5875

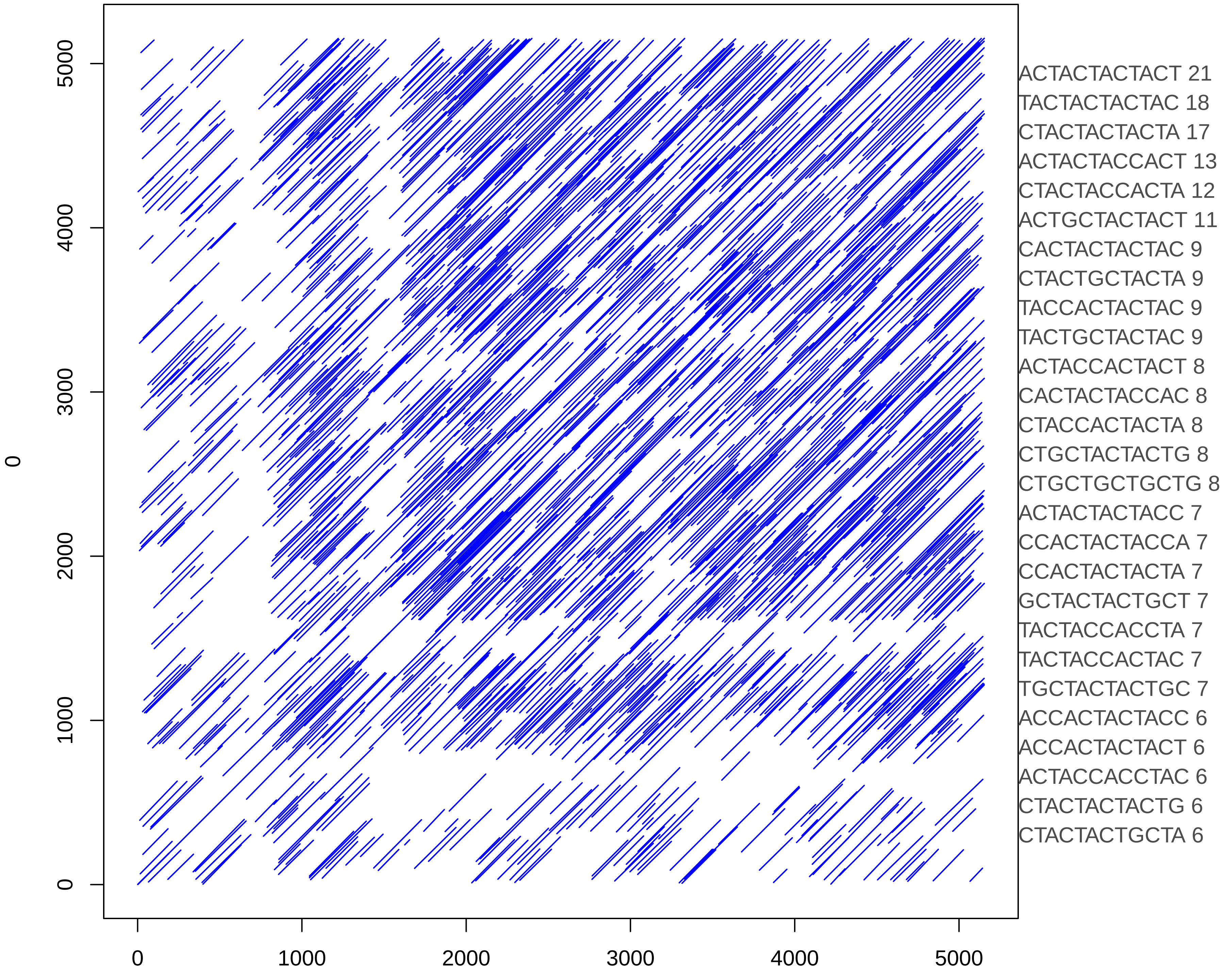

>m141230_233107_42134_c100756552550000001823146807221541_s1_p0/118639/553_10462

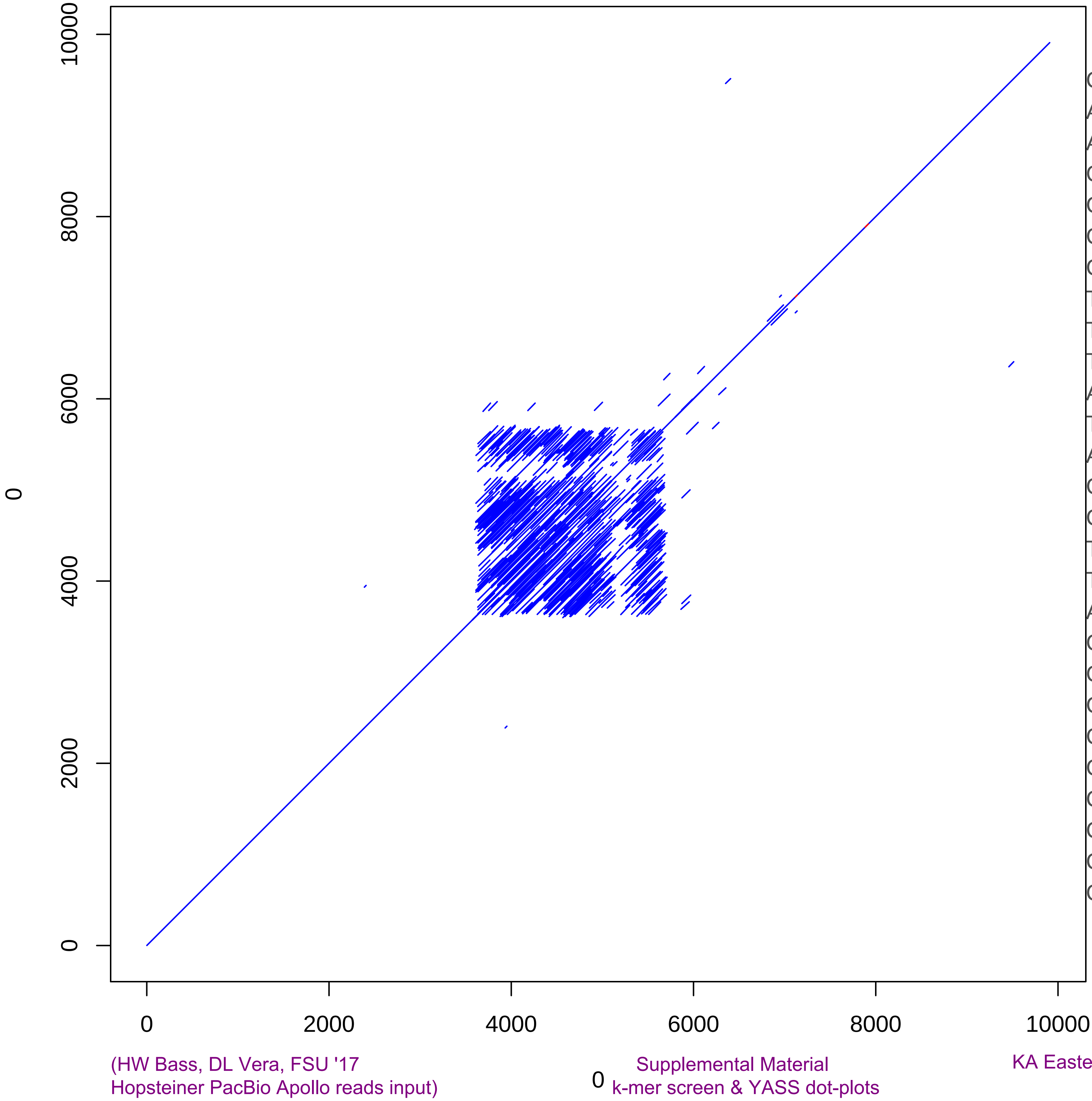

GGGGGGGGGGGG 18
AGTGGTAGTGGT 13
AGTAGTAGTAGT 11
GGTAGTGGTAGT 11
GTAGTAGTAGTA 11
GTAGTGGTAGTG 11
GTGGTAGTGGTA 11
TAGTAGTAGTAG 11
TAGTGGTAGTGG 11
TGGTAGTGGTAG 10
AGTAGTAGTGGT 7
TAGTAGTAGTGG 7
AGTAGTGGTAGT 6
GTAGTAGTAGTG 6
GTAGTGGTAGTA 6
TAGTGGTAGTAG 6
TGGTGGTGGTGG 6
AGTGGTAGTAGT 5
GGTAGTGGTAGC 5
GGTAGTAGTAGT 4
GGTAGTGGTAAG 4
GGTGGTGGTGGT 4
GTACGTGGTAGT 4
GTAGTAGTGGTA 4
GTAGTGGTAGCT 4
GTAGTGGTAGTC 4
GTGGTAGTAGTA 4

>m141230_233107_42134_c100756552550000001823146807221541_s1_p0/119988/584_15642

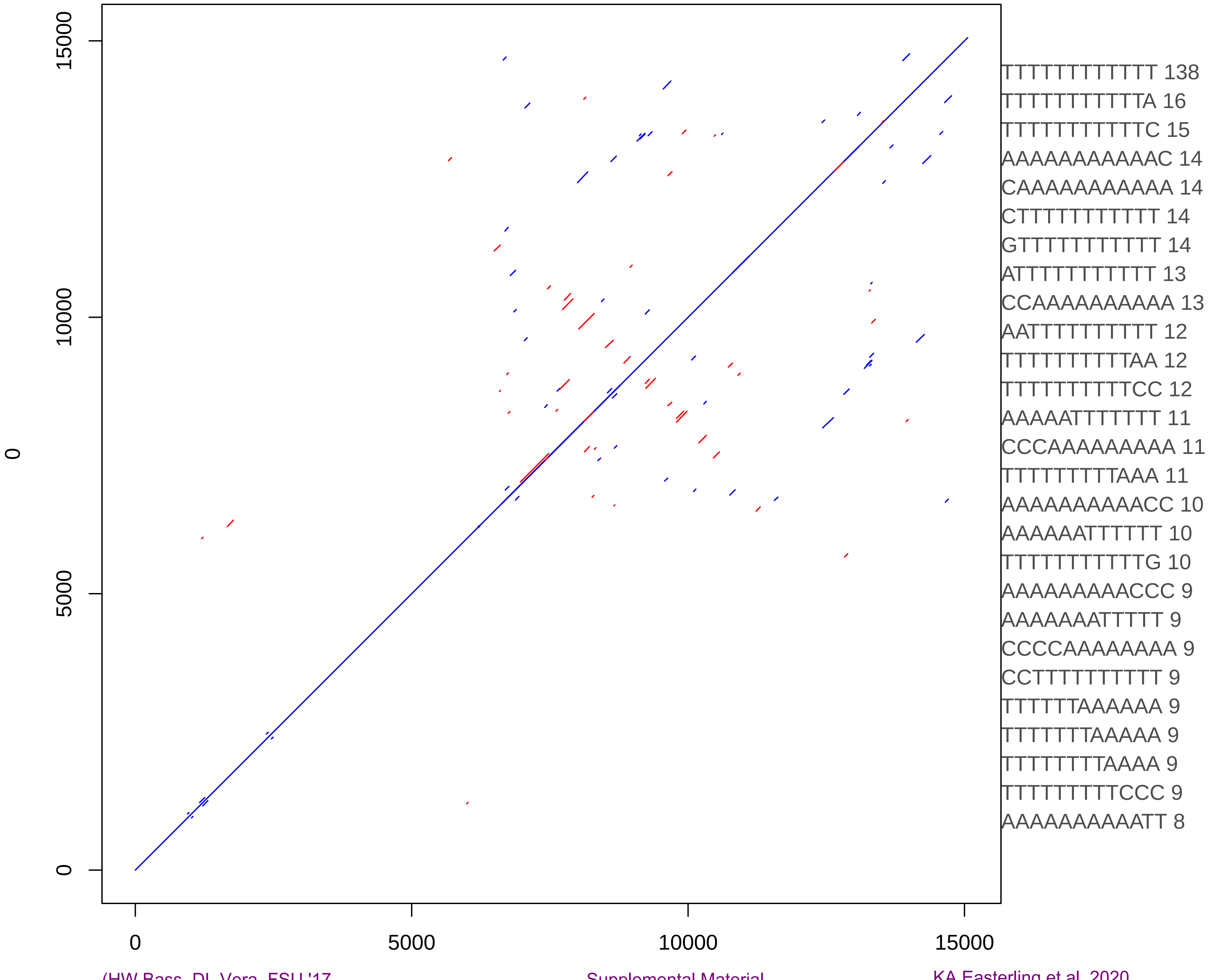

>m141230_233107_42134_c100756552550000001823146807221541_s1_p0/121589/3482_9098

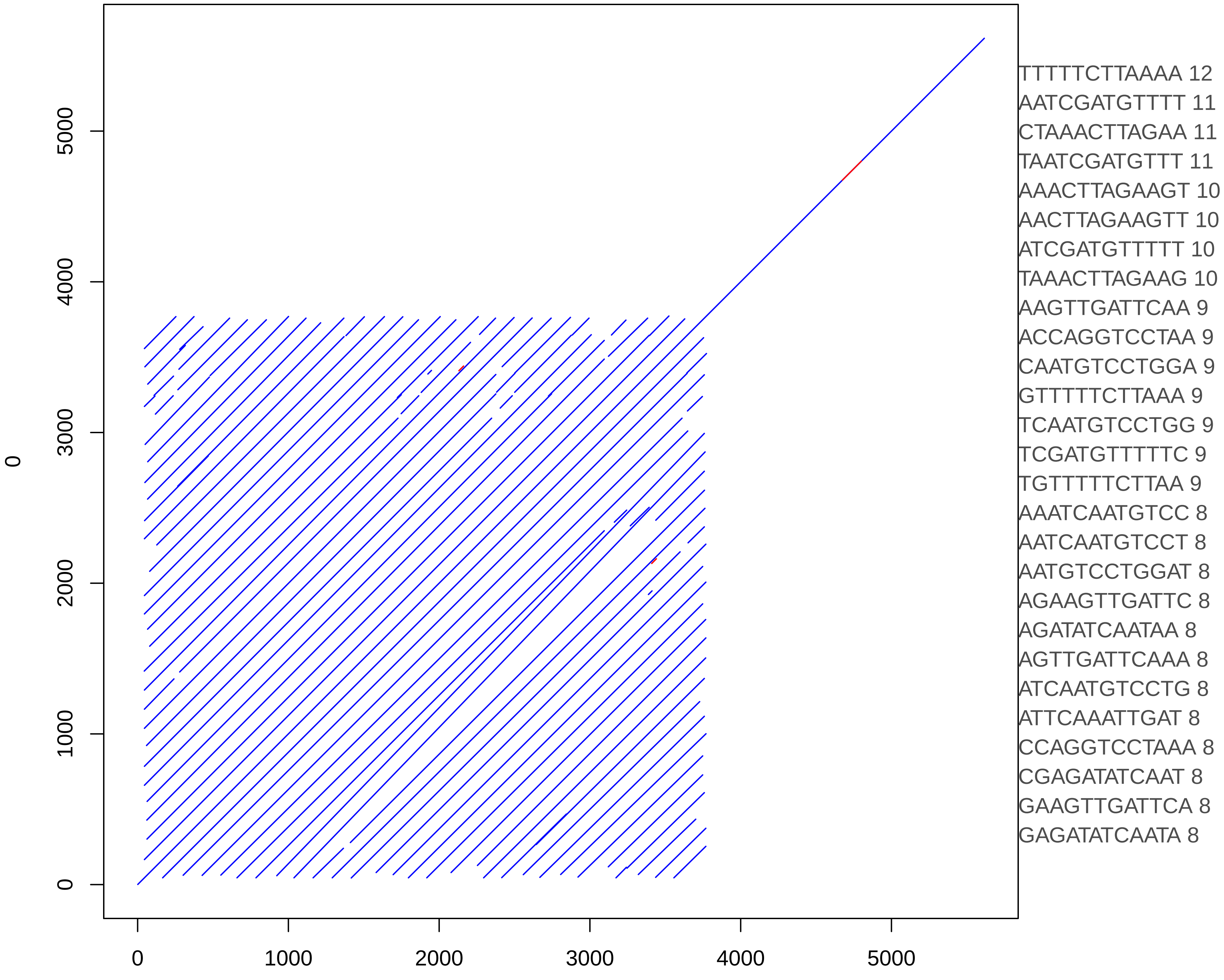

>m141230_233107_42134_c100756552550000001823146807221541_s1_p0/121641/8374_16279

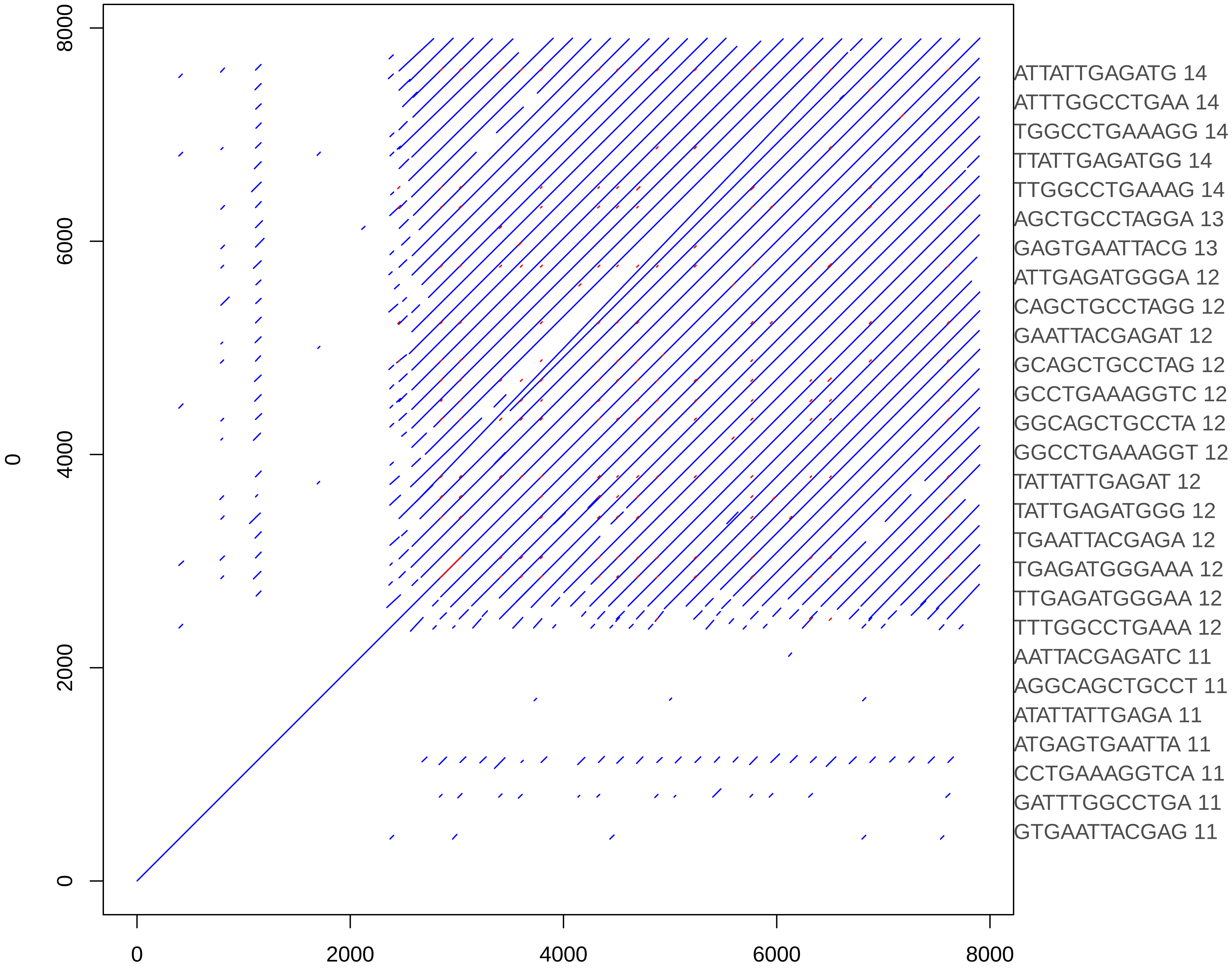

>m141230_233107_42134_c100756552550000001823146807221541_s1_p0/121793/450_5996

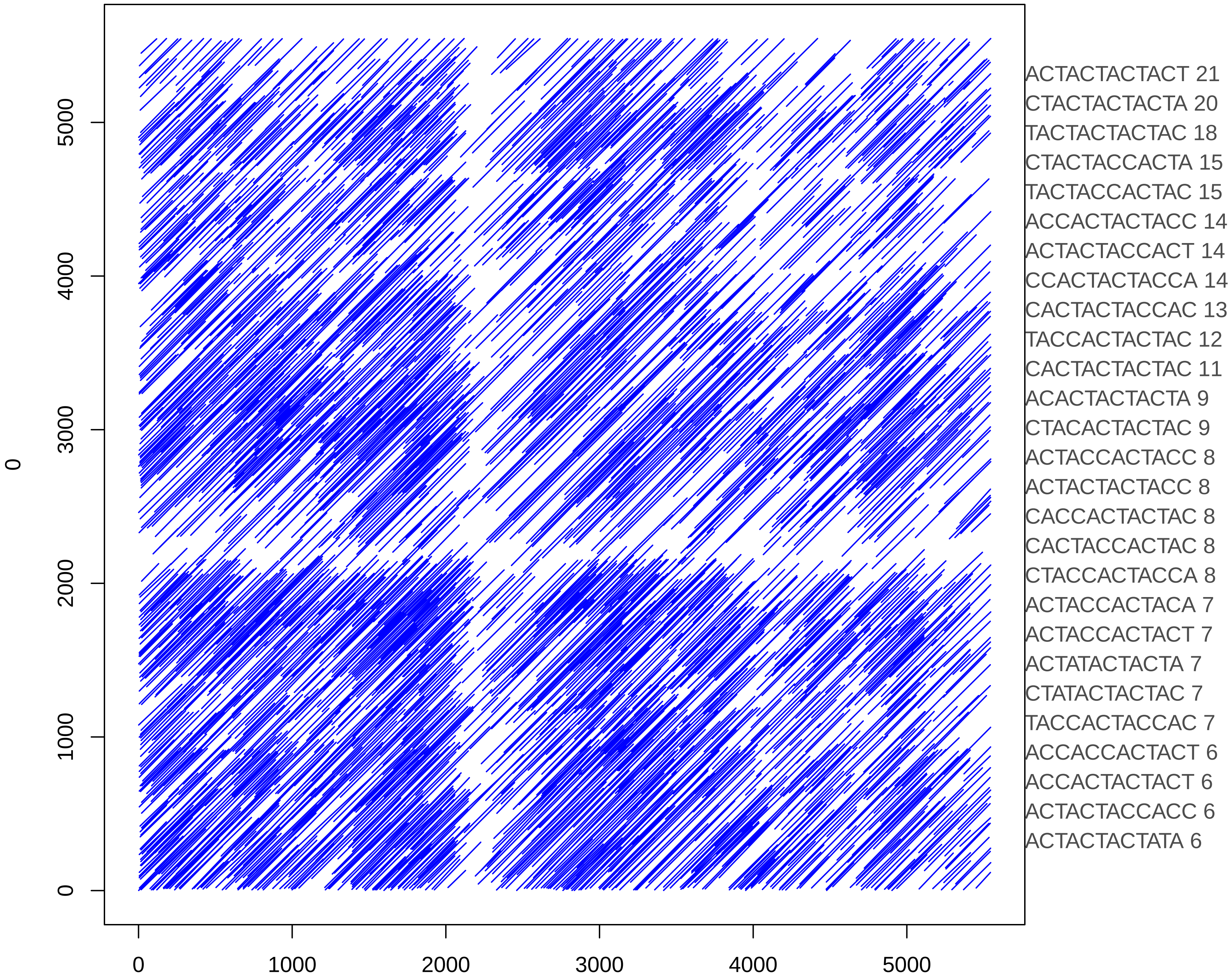

>m141230_233107_42134_c100756552550000001823146807221541_s1_p0/124577/7447_17206

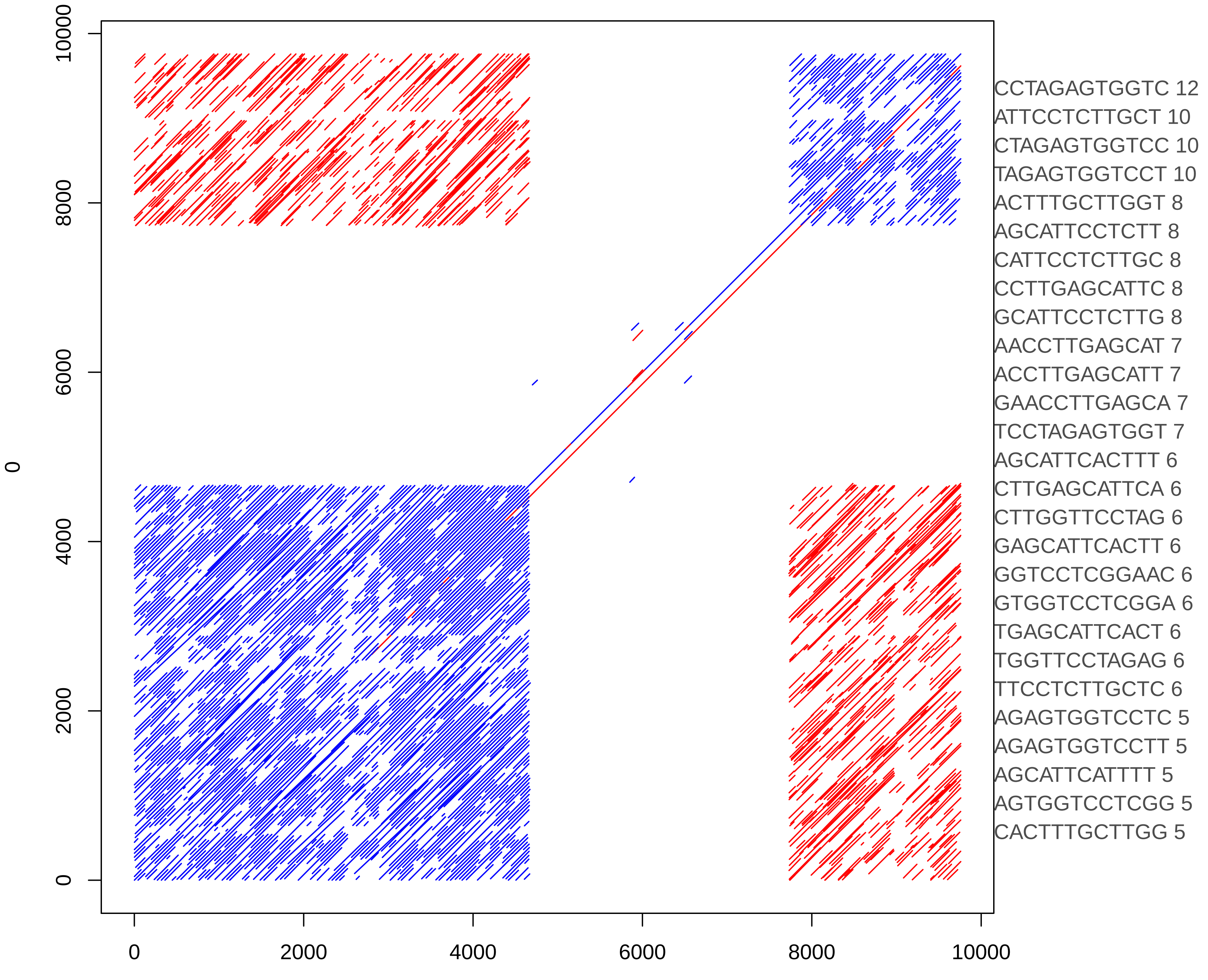

>m141230_233107_42134_c100756552550000001823146807221541_s1_p0/126603/787_6746

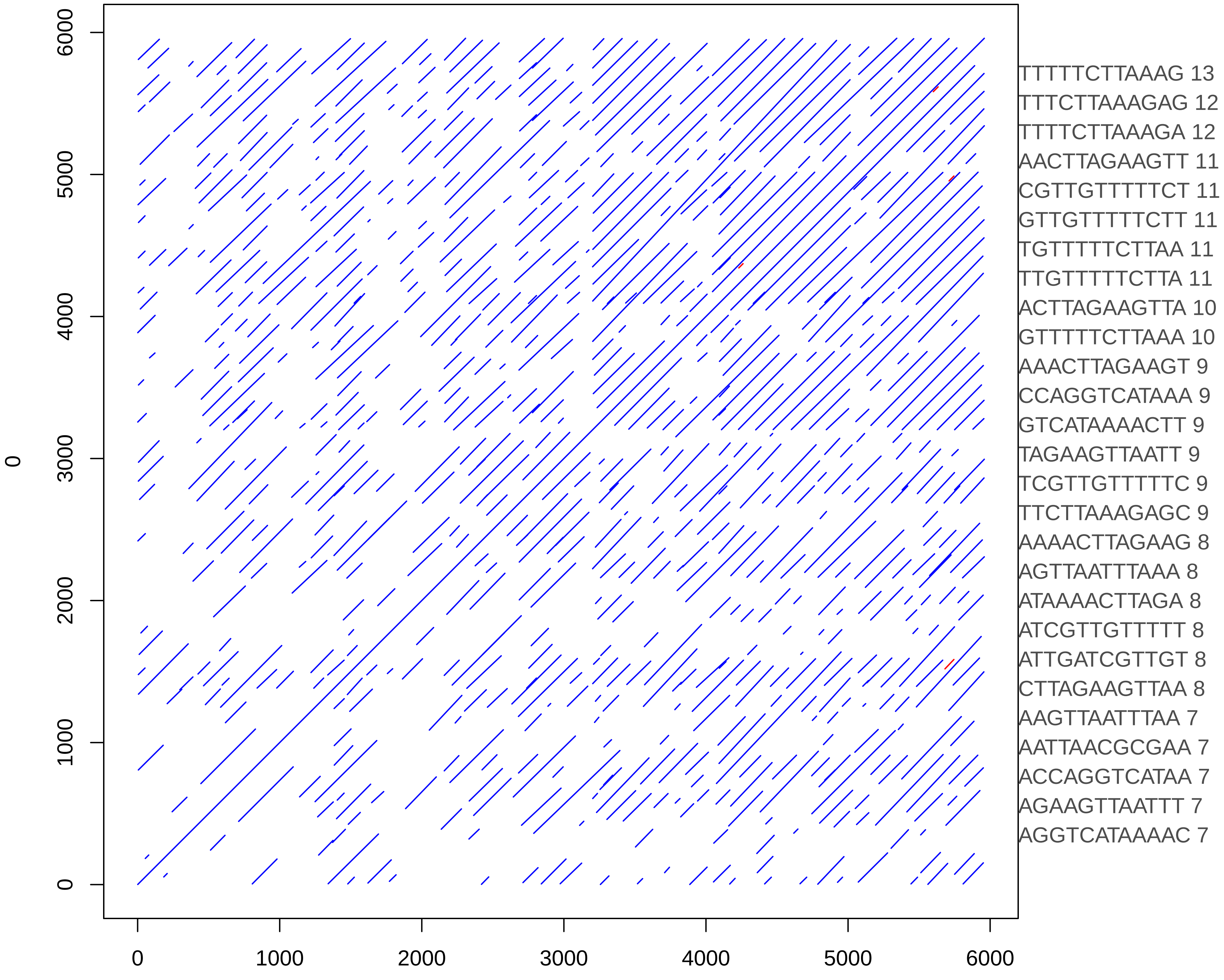

>m141230_233107_42134_c100756552550000001823146807221541_s1_p0/128422/616_11867

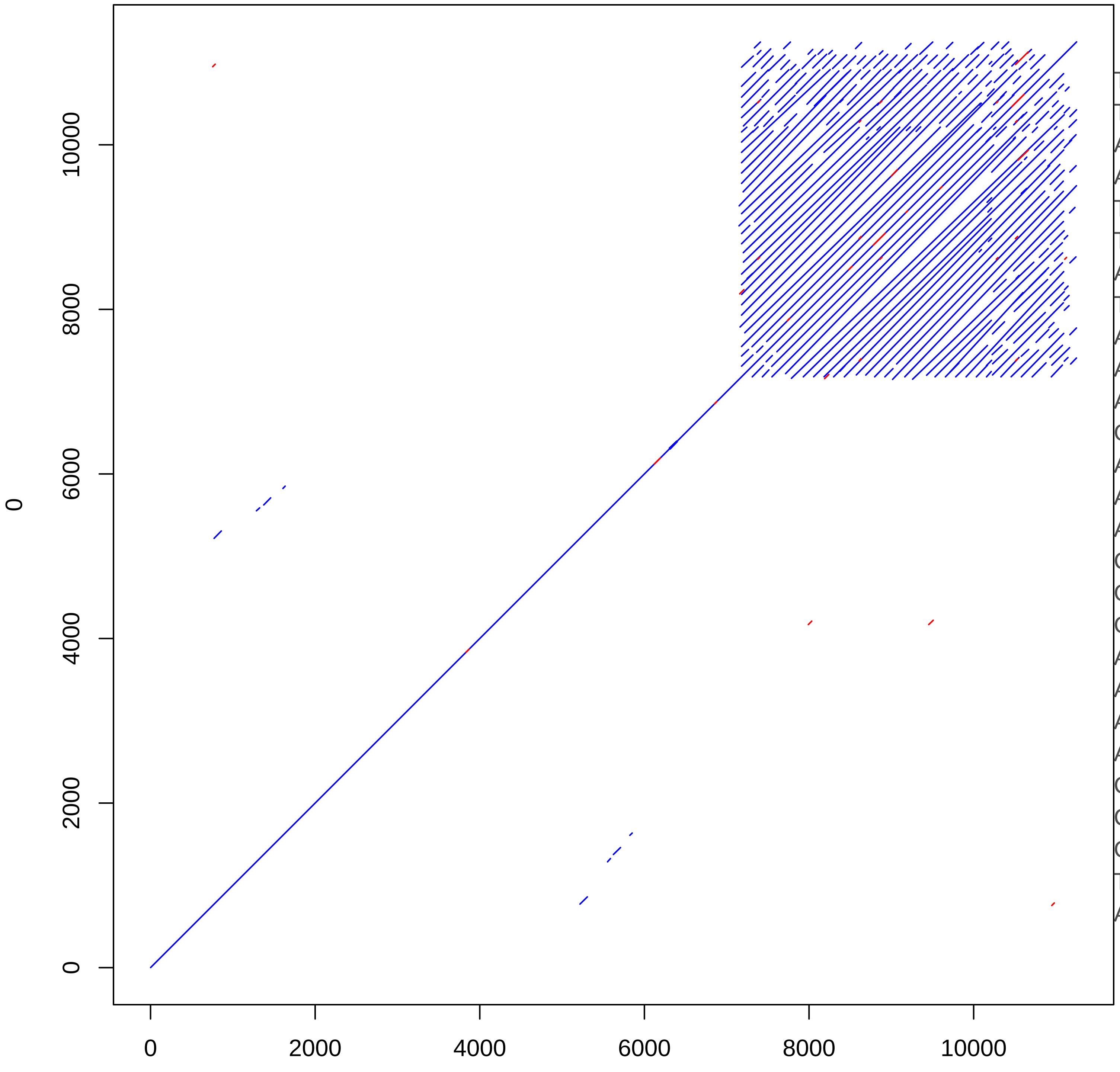

TATGACCTGGTT 15
TGACCTGGTTAT 15
AAAACTTAGTTC 14
ATGACCTGGTTA 14
TTATGACCTGGT 14
TTTATGACCTGG 14
AAACTTAGTTCG 13
TTTTATGACCTG 13
AAACAACGATCA 12
AACAACGATCAA 12
AACTTAGTTCGC 12
GGTTATGTCCAG 11
AAAACAACGATC 10
AAGAAAAACAAC 10
AGAAAAACAACG 10
CTGGTTATGTCC 10
GAAAAACAACGA 10
GAAAACTTAGTT 10
AATTTTTATCAA 9
ACAACGATCAAT 9
ACCTGGTTATGT 9
ACTTAGTTCGCG 9
CCTGGTTATGTC 9
CTTAGTTCGCGT 9
GACCTGGTTATG 9
TGGTTATGTCCA 9
AAAAACAACGAT 8

>m141230_233107_42134_c100756552550000001823146807221541_s1_p0/131689/695_6776

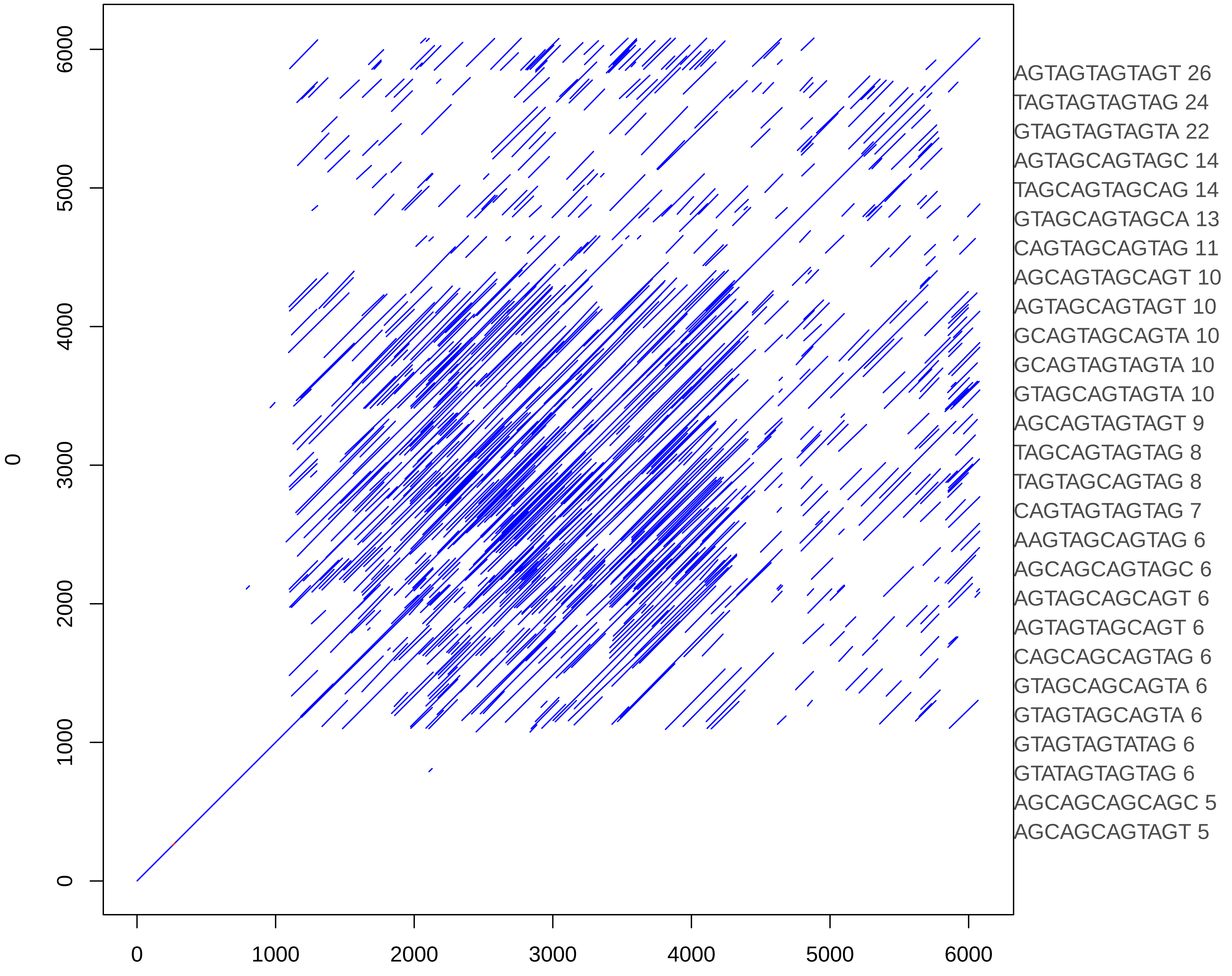

1,121 hits for repetitive sequences passing 12mer abundance criterion

(Read-Number)
PAGE 808

For Tandem Repeat families of ### bp:
PacBio Read named "HuluTR###-r808"

>m141230_233107_42134_c100756552550000001823146807221541_s1_p0/133229/0_9435

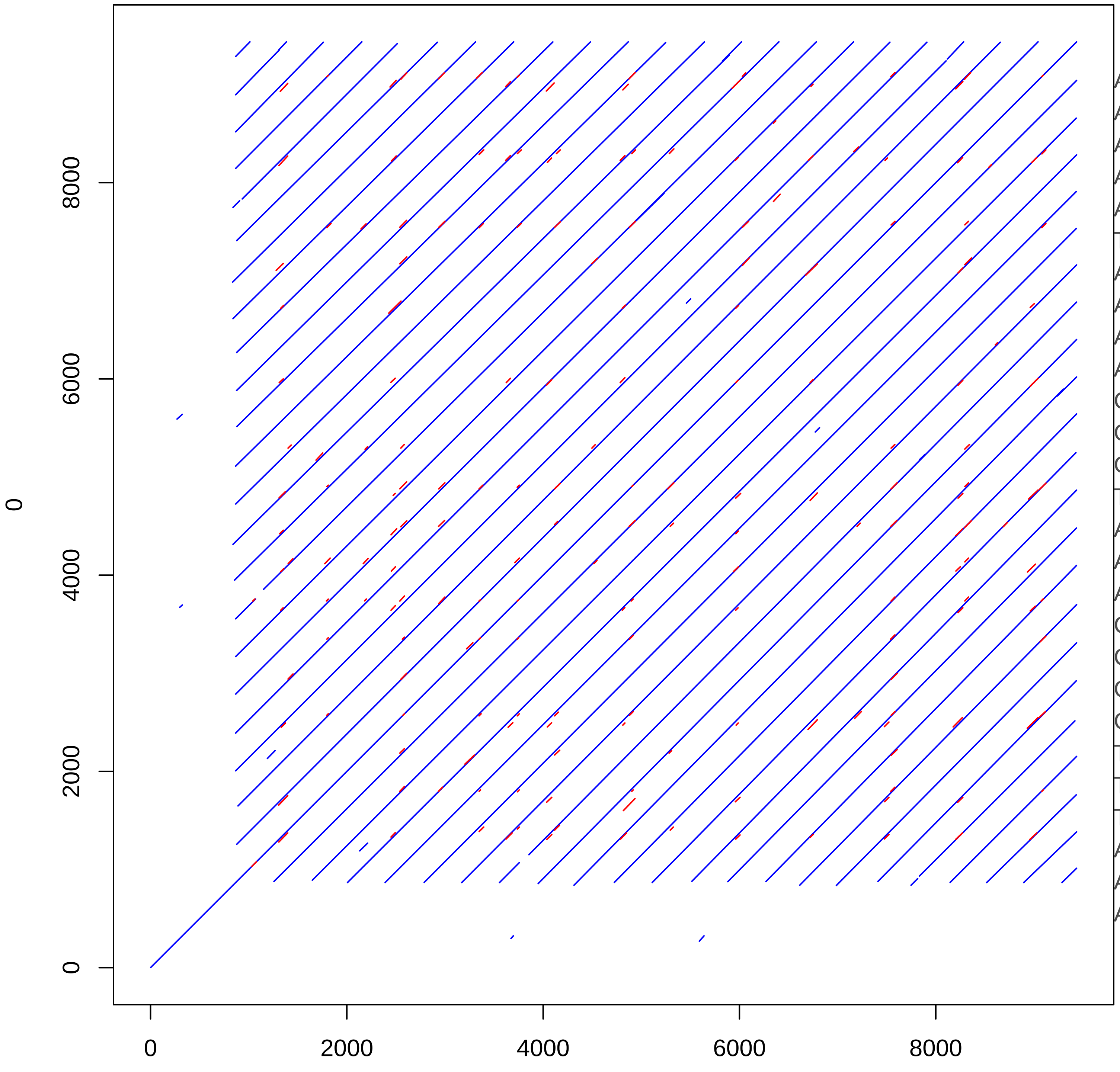

ACTTGCGAAAAT 16
AAAAAAGTGGAA 15
AAAAAGTGGAAT 15
AAAAGTGGAATT 15
AAAGTGGAATTT 15
TTTAAATCCGAG 15
AAACTTGCGAAA 14
AAATCCGAGGTG 14
AACTTGCGAAAA 14
AAGTGGAATTTT 14
CTTGCGAAAATT 14
GAAAAAAGTGGA 14
GTTTAAATCCGA 14
TAAATCCGAGGT 14
AAAATATCGAAC 13
AGTGGAATTTTT 13
ATTTCTCTAATA 13
CGATTTCTCTAA 13
CTGAGAGATGTA 13
GGGAATACTAAA 13
GGTTTAAATCCG 13
TGCGAAAATTGC 13
TTAAATCCGAGG 13
TTGCGAAAATTG 13
AATCCGAGGTGT 12
ACTGAGAGATGT 12
AGAAAAAAGTGG 12

(HW Bass, DL Vera, FSU '17
Hopsteiner PacBio Apollo reads input)

Supplemental Material
k-mer screen & YASS dot-plots

KA Easterling et al. 2020

>m141230_233107_42134_c100756552550000001823146807221541_s1_p0/134793/0_6086

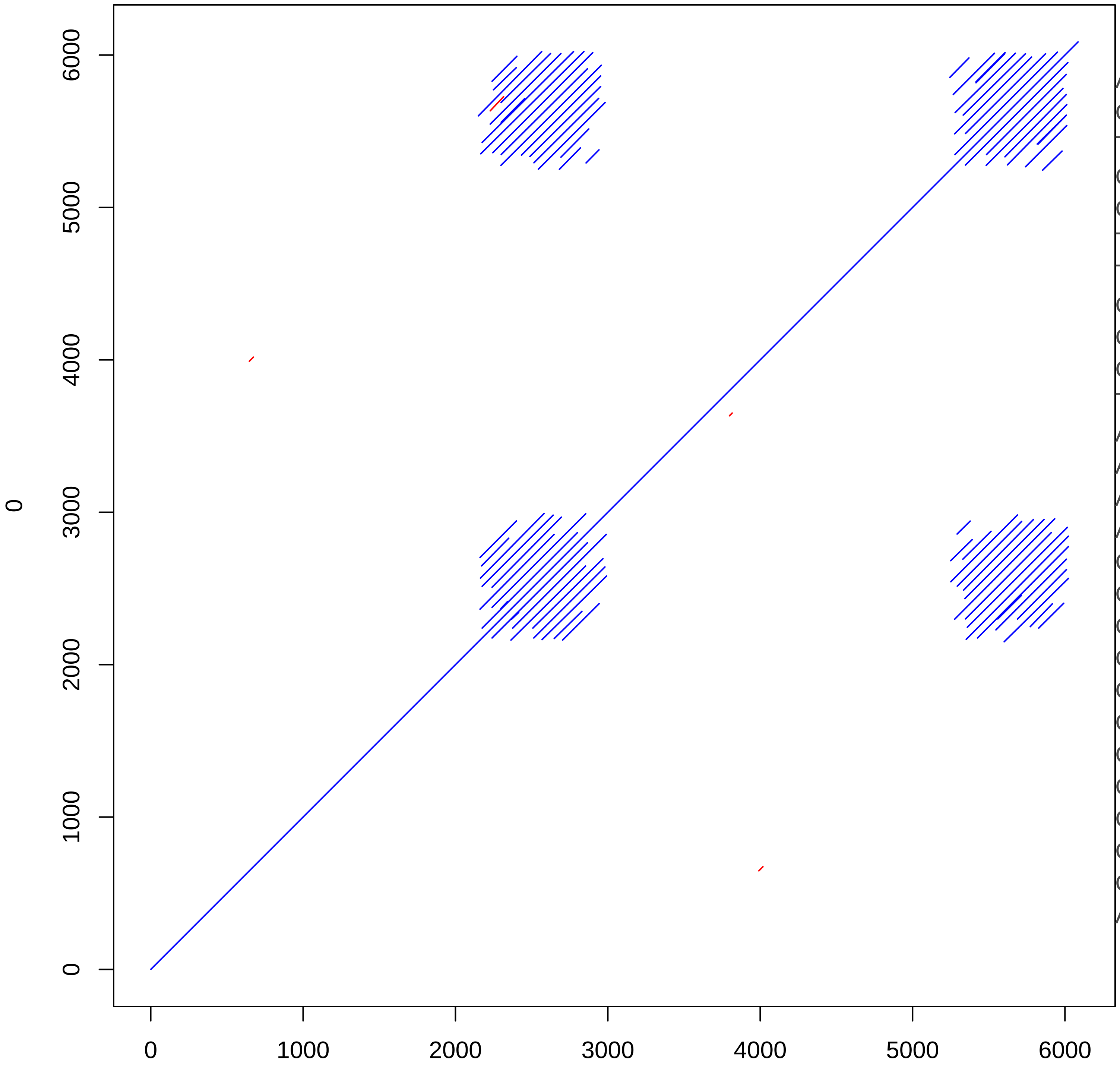

>m141230_233107_42134_c100756552550000001823146807221541_s1_p0/1362/2572_10145

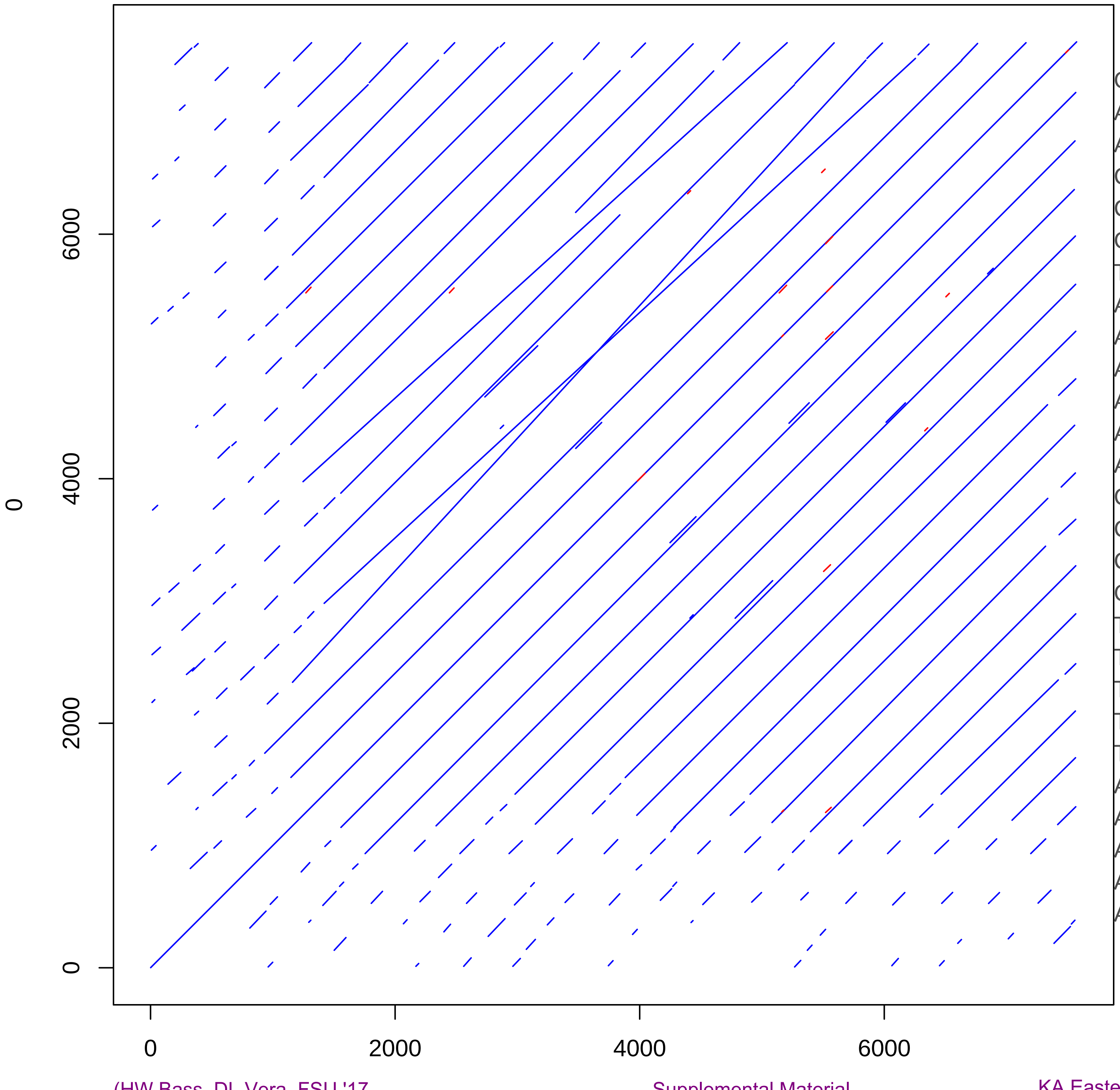

>m141230_233107_42134_c100756552550000001823146807221541_s1_p0/139697/4857_13343

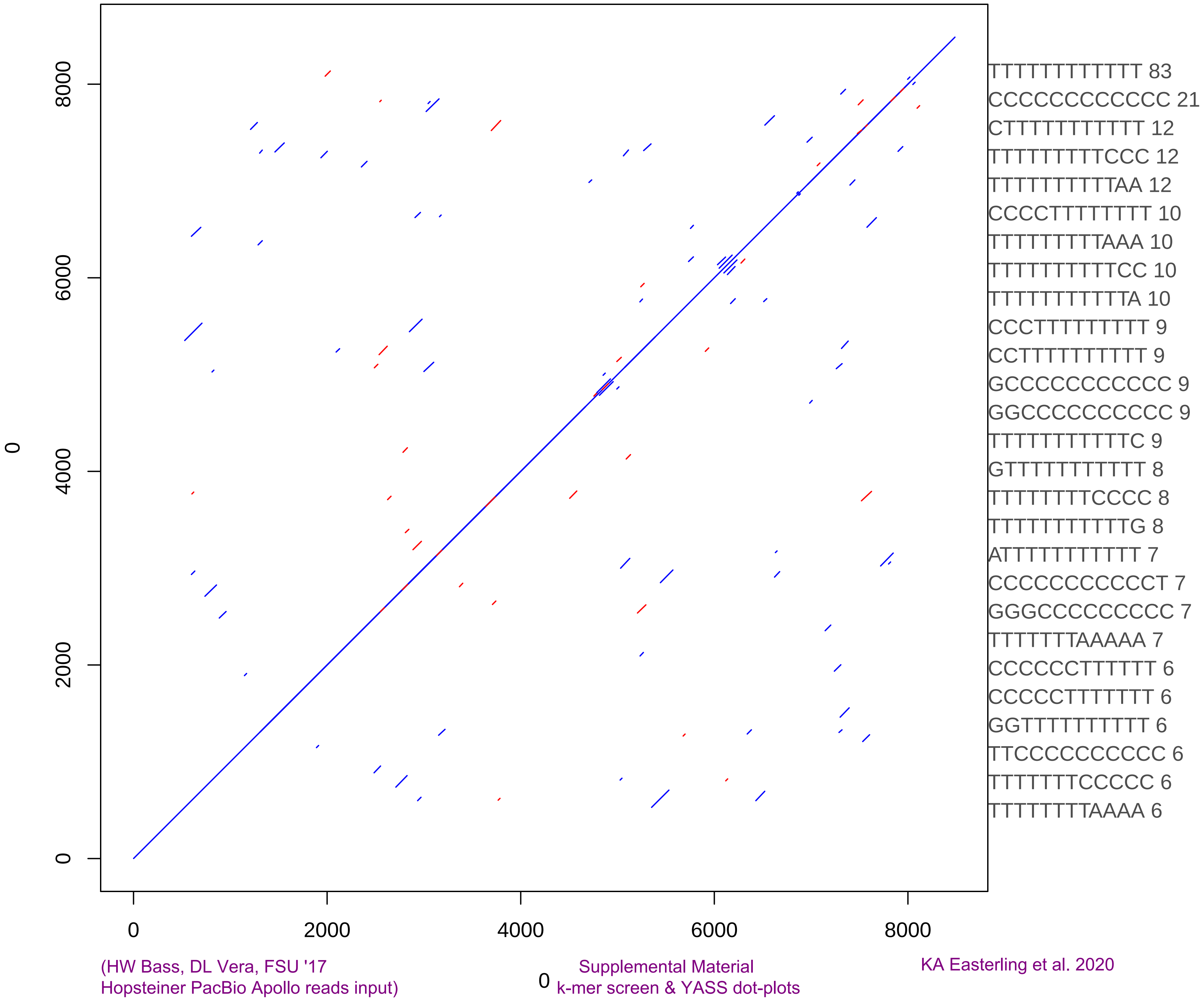

>m141230_233107_42134_c100756552550000001823146807221541_s1_p0/140709/989_9453

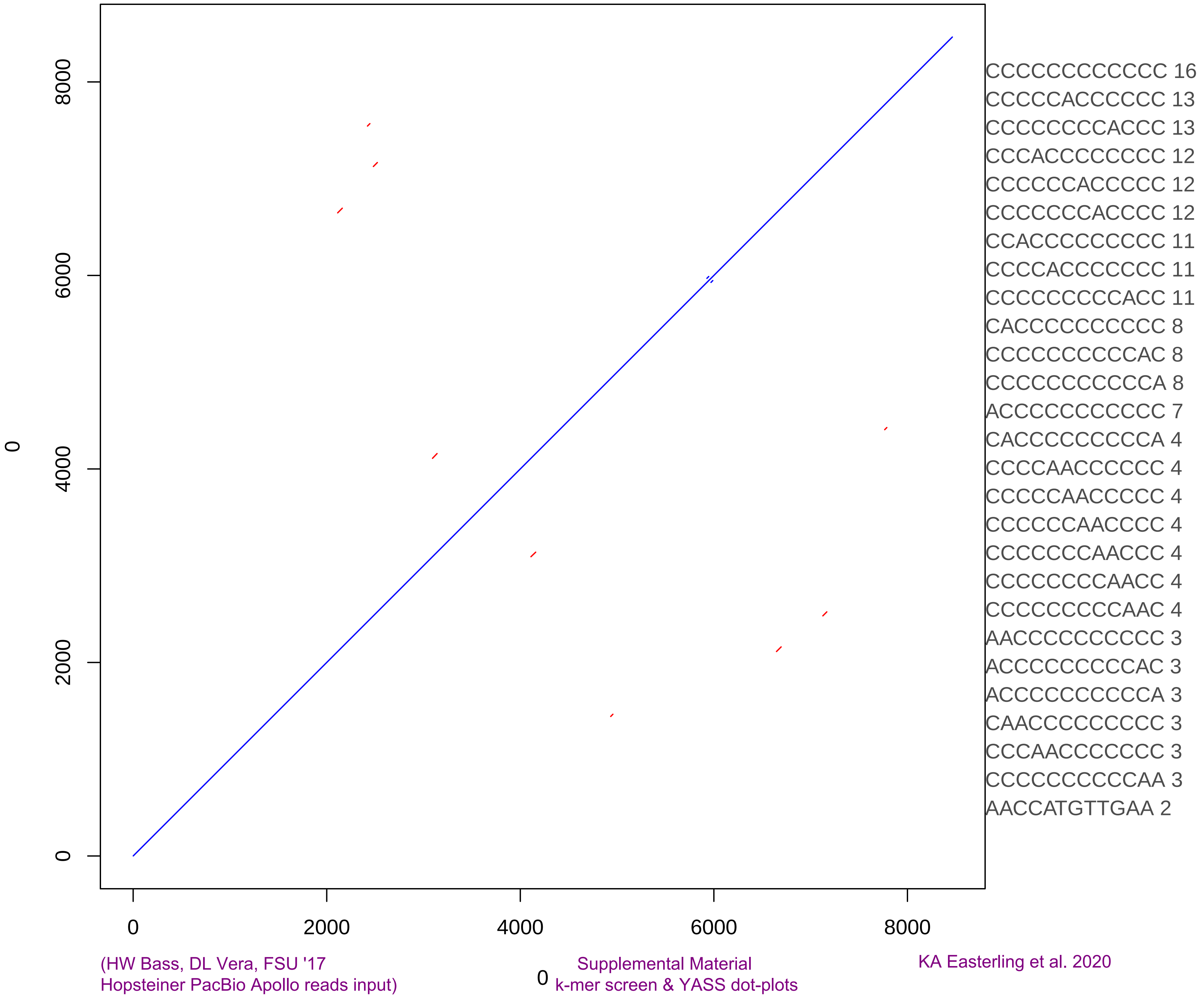

>m141230_233107_42134_c100756552550000001823146807221541_s1_p0/140837/460_11361

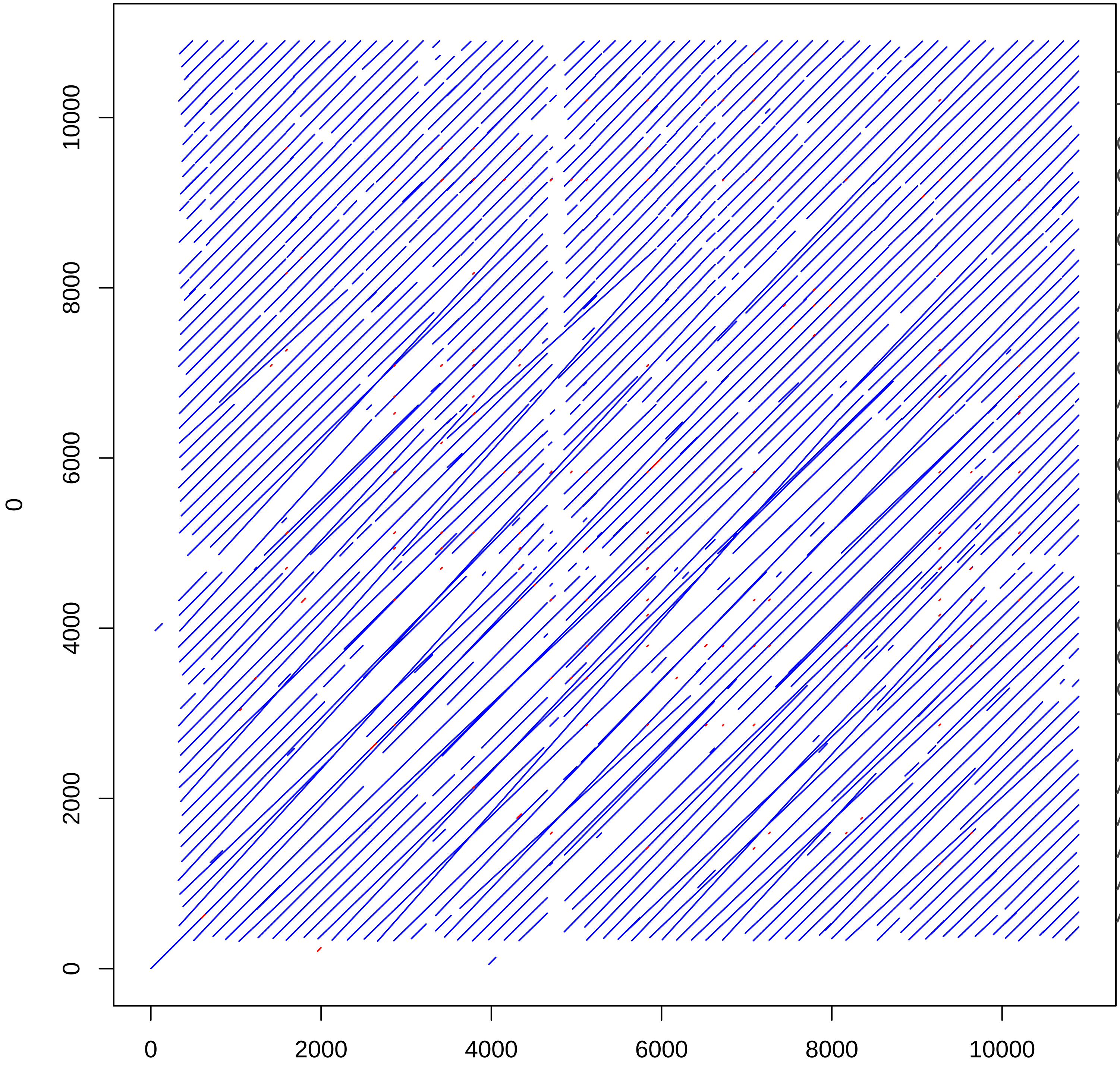

TTTCCCATCTCA 24
TTCCCATCTCAA 23
CCATCTCAATAA 21
CCCATCTCAATA 21
ATCTCAATAATA 19
CATCTCAATAAT 19
TCCCATCTCAAT 19
AGCGATTTCGTA 18
CGATTTCGTAAT 18
GATTTCGTAATT 18
AAGCGATTTCGT 17
ATTTCGTAATTC 17
GCGATTTCGTAA 17
GCTATTATTGAC 17
TTCGTAATTCAC 17
TTTCGTAATTCA 17
TTTTCCCATCTC 17
CTAACAAGCTCT 16
CTCAATAATATT 16
GACCTTTCAGGC 16
TCTCAATAATAT 16
AAAAAAATTGAC 15
AAAAAATTGACC 15
AAAAATTGACCT 15
AAAATTGACCTT 15
ACCTTTCAGGCC 15
AGCTCTAATTTT 15

>m141230_233107_42134_c100756552550000001823146807221541_s1_p0/143398/1840_9702

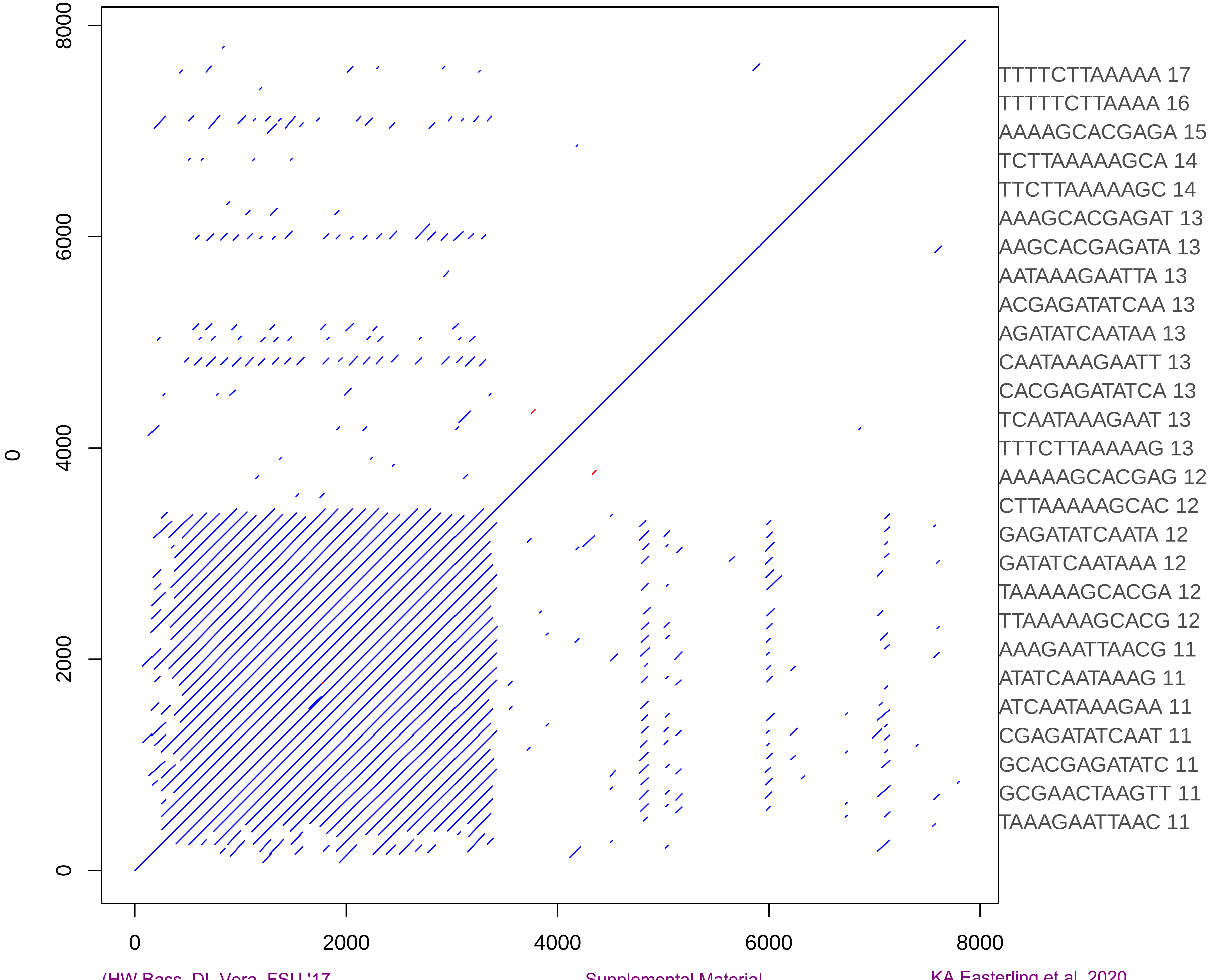

>m141230_233107_42134_c100756552550000001823146807221541_s1_p0/146066/470_8763

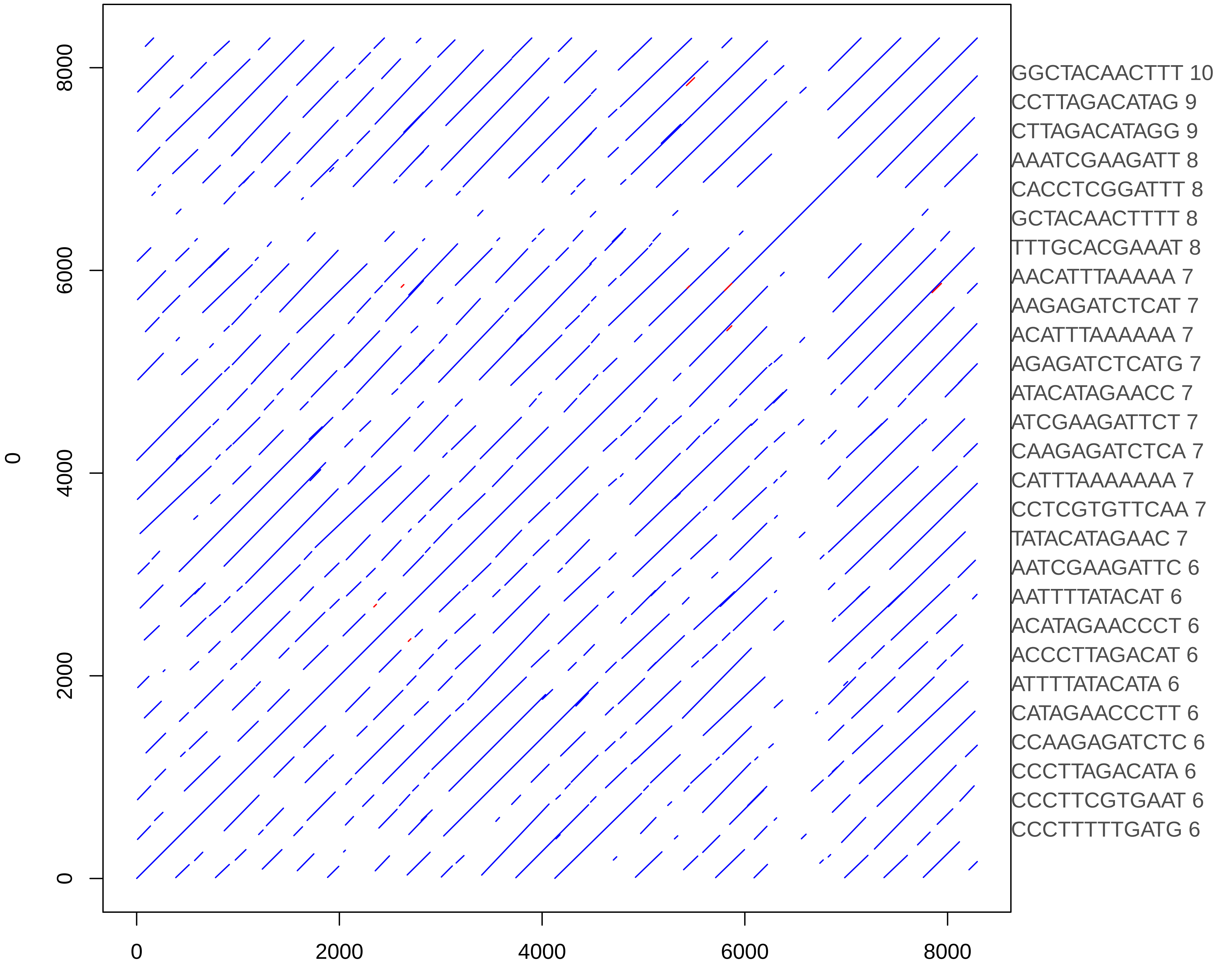

>m141230_233107_42134_c100756552550000001823146807221541_s1_p0/14681/0_7233

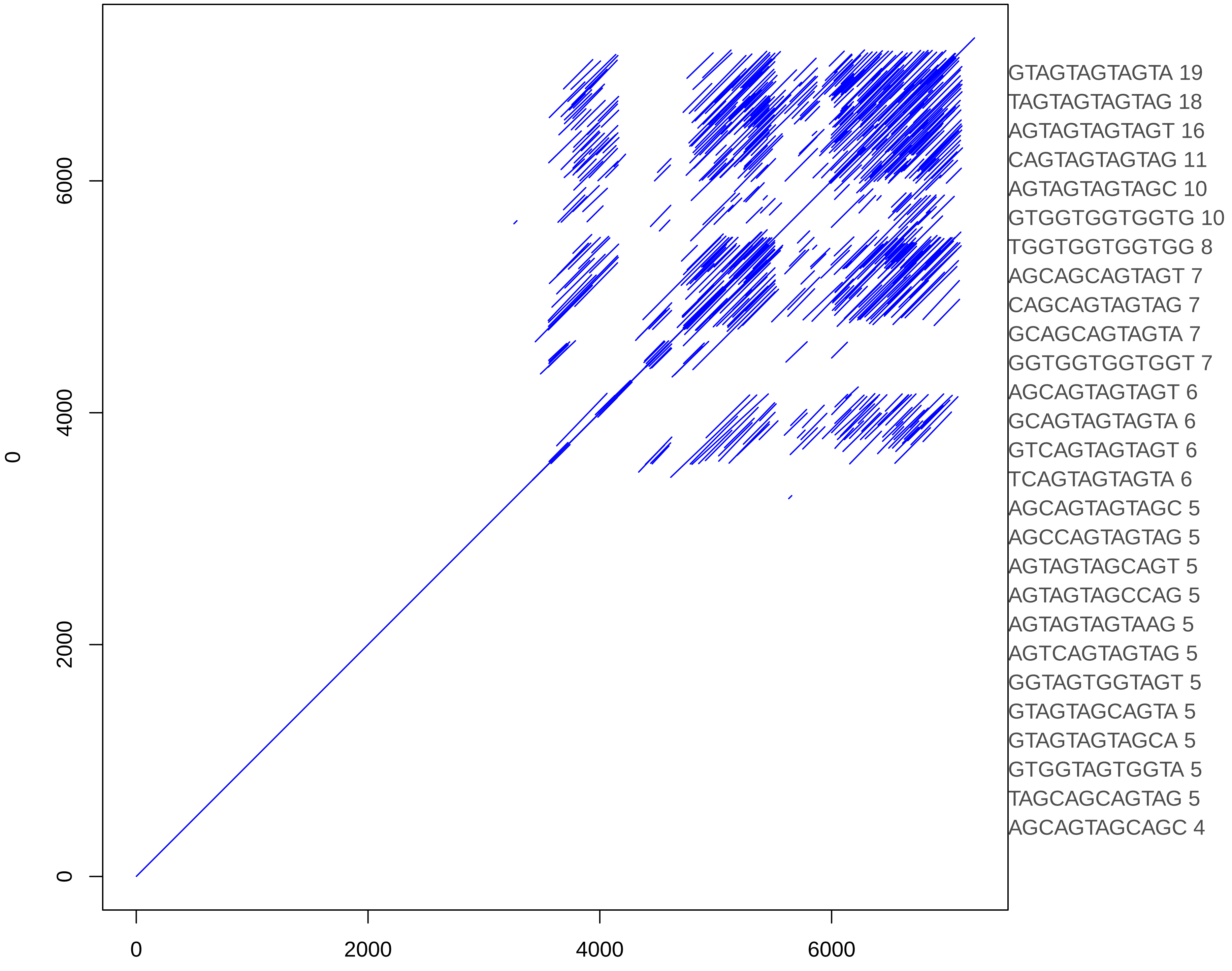

>m141230_233107_42134_c100756552550000001823146807221541_s1_p0/148750/4595_12468

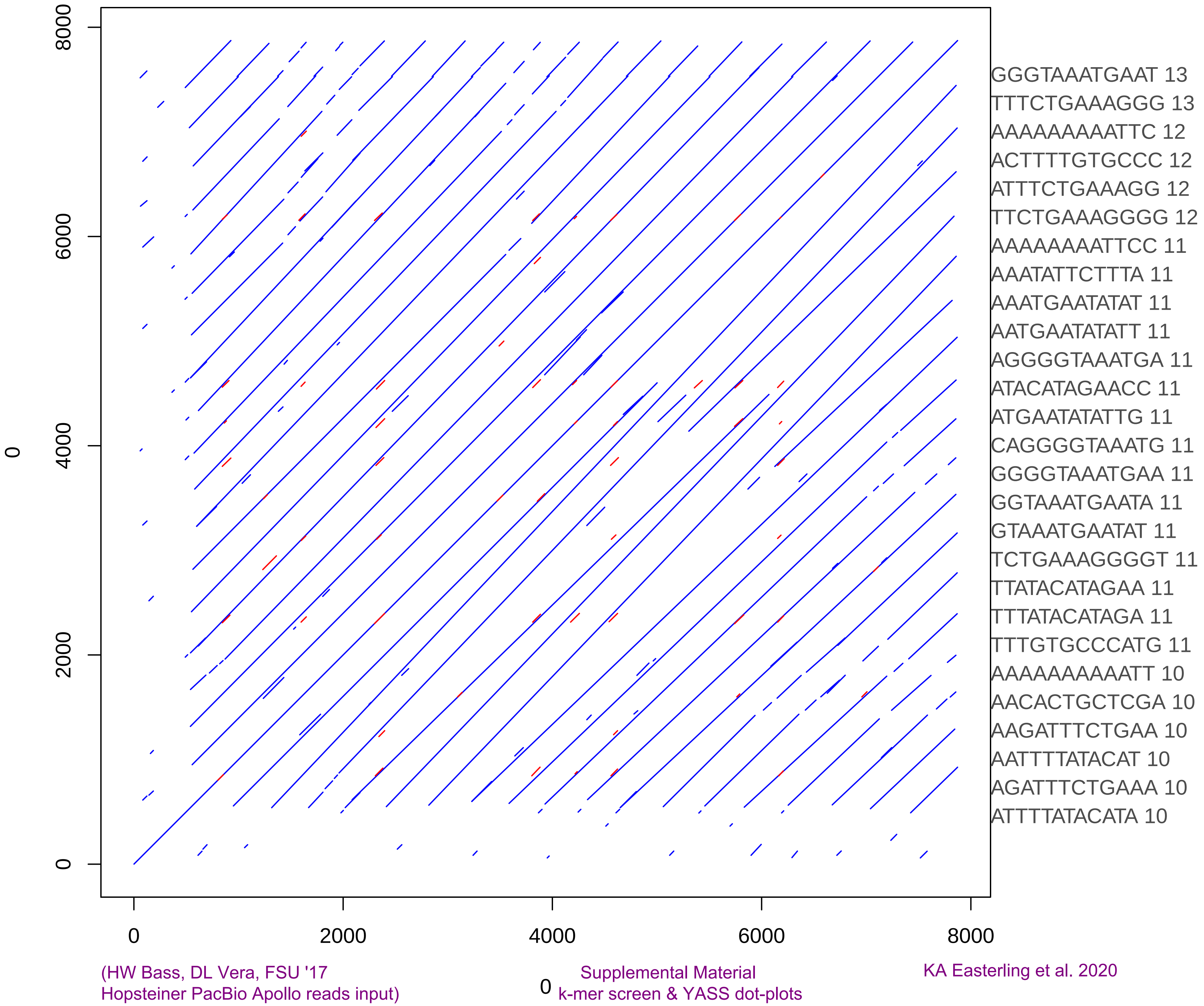

>m141230_233107_42134_c100756552550000001823146807221541_s1_p0/149196/0_11429

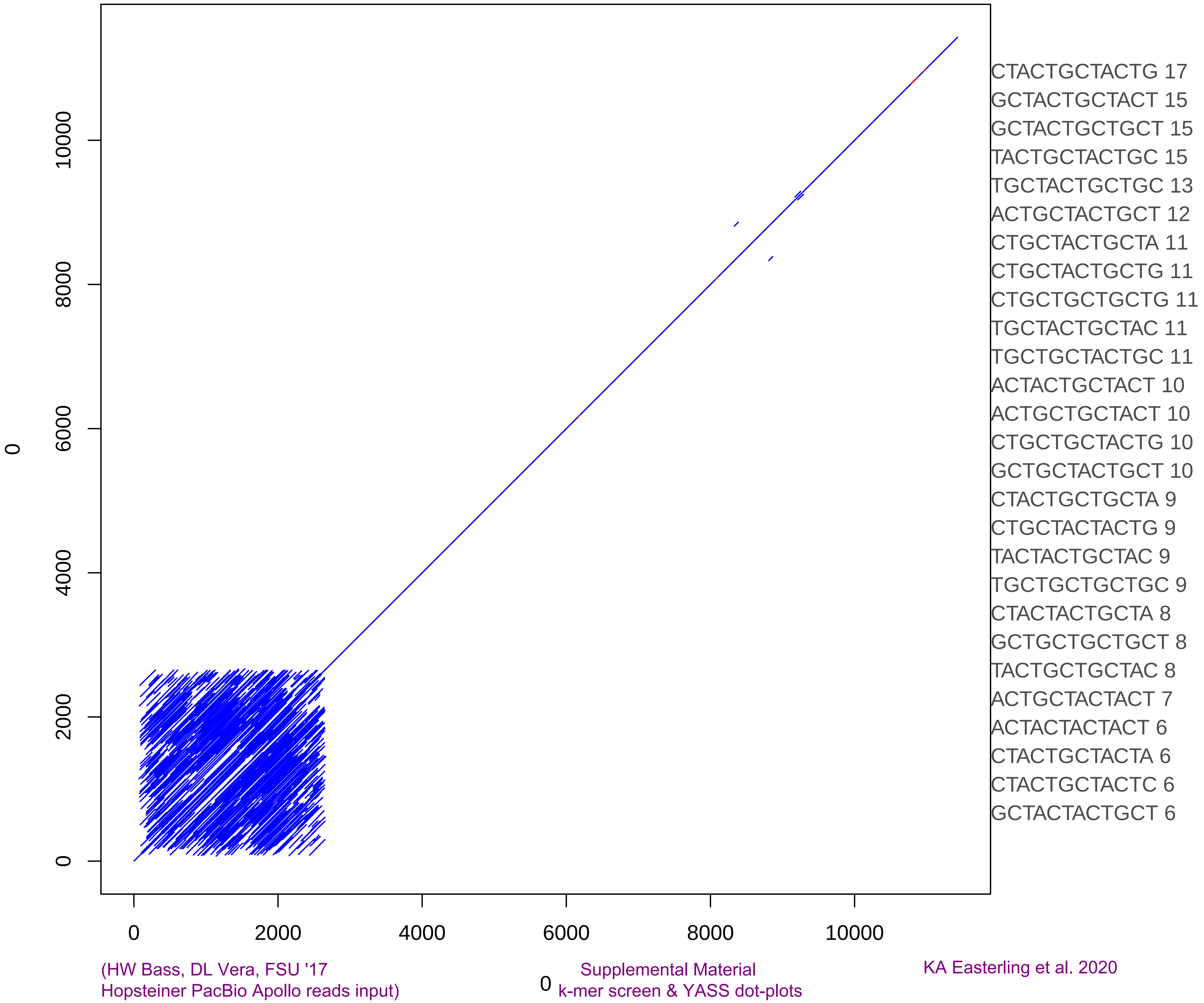

>m141230_233107_42134_c100756552550000001823146807221541_s1_p0/149589/0_12586

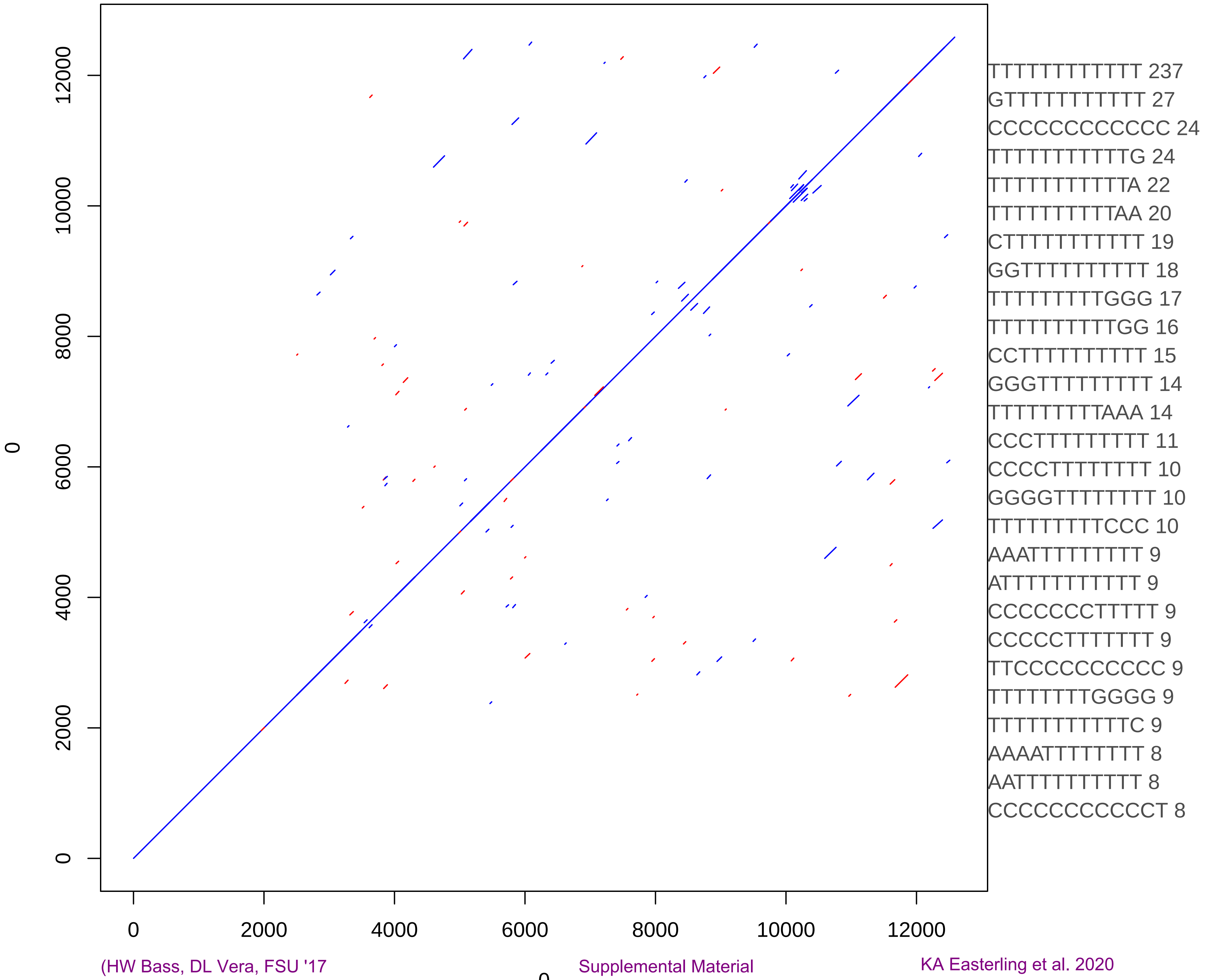

1,121 hits for repetitive sequences
passing 12mer abundance criterion

(Read-Number)
PAGE 820

For Tandem Repeat families of ### bp:
PacBio Read named "HuluTR###-r820"

>m141230_233107_42134_c100756552550000001823146807221541_s1_p0/150314/1078_13912

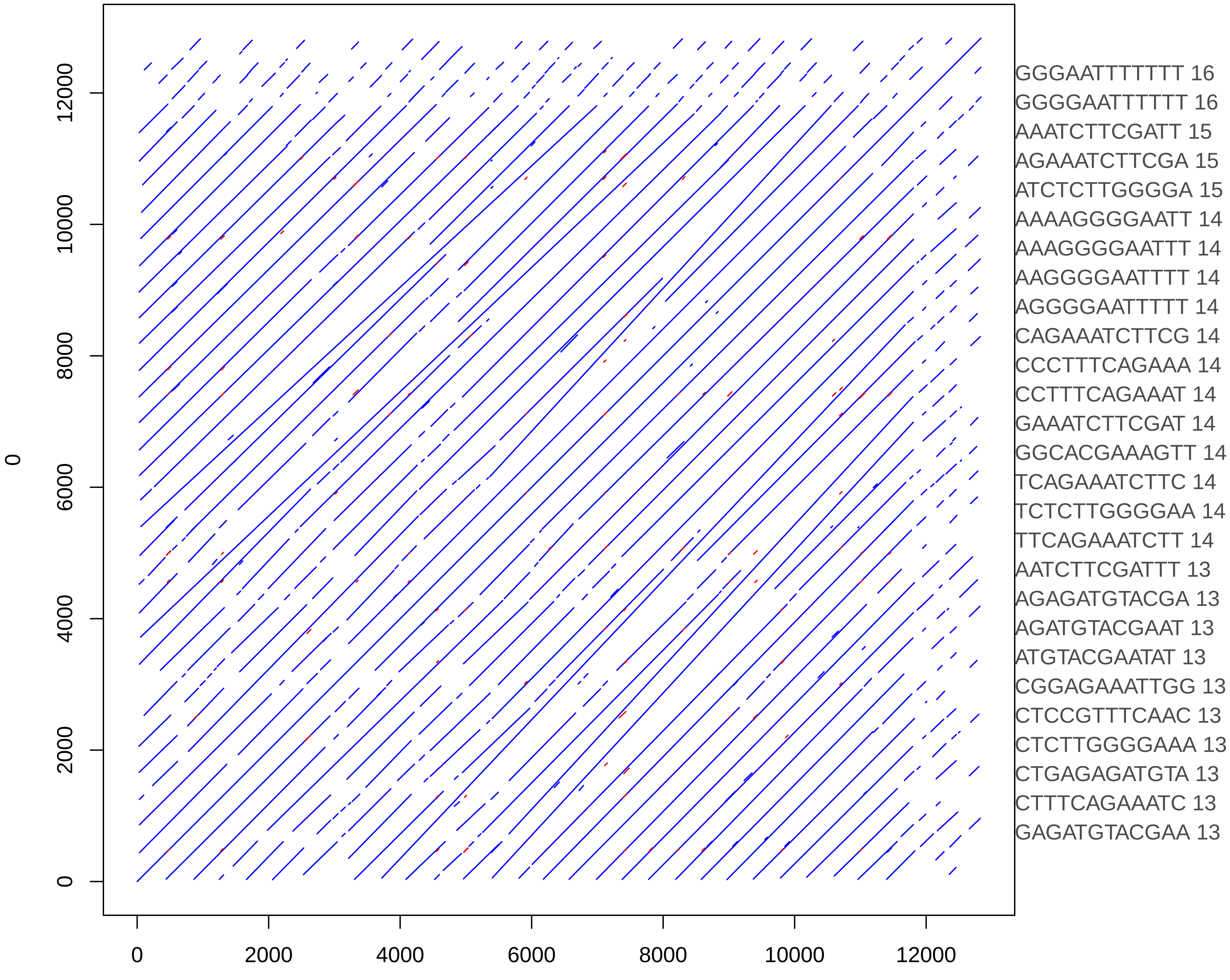

(HW Bass, DL Vera, FSU '17
Hopsteiner PacBio Apollo reads input)

0

Supplemental Material
k-mer screen & YASS dot-plots

KA Easterling et al. 2020

>m141230_233107_42134_c100756552550000001823146807221541_s1_p0/150543/6966_13758

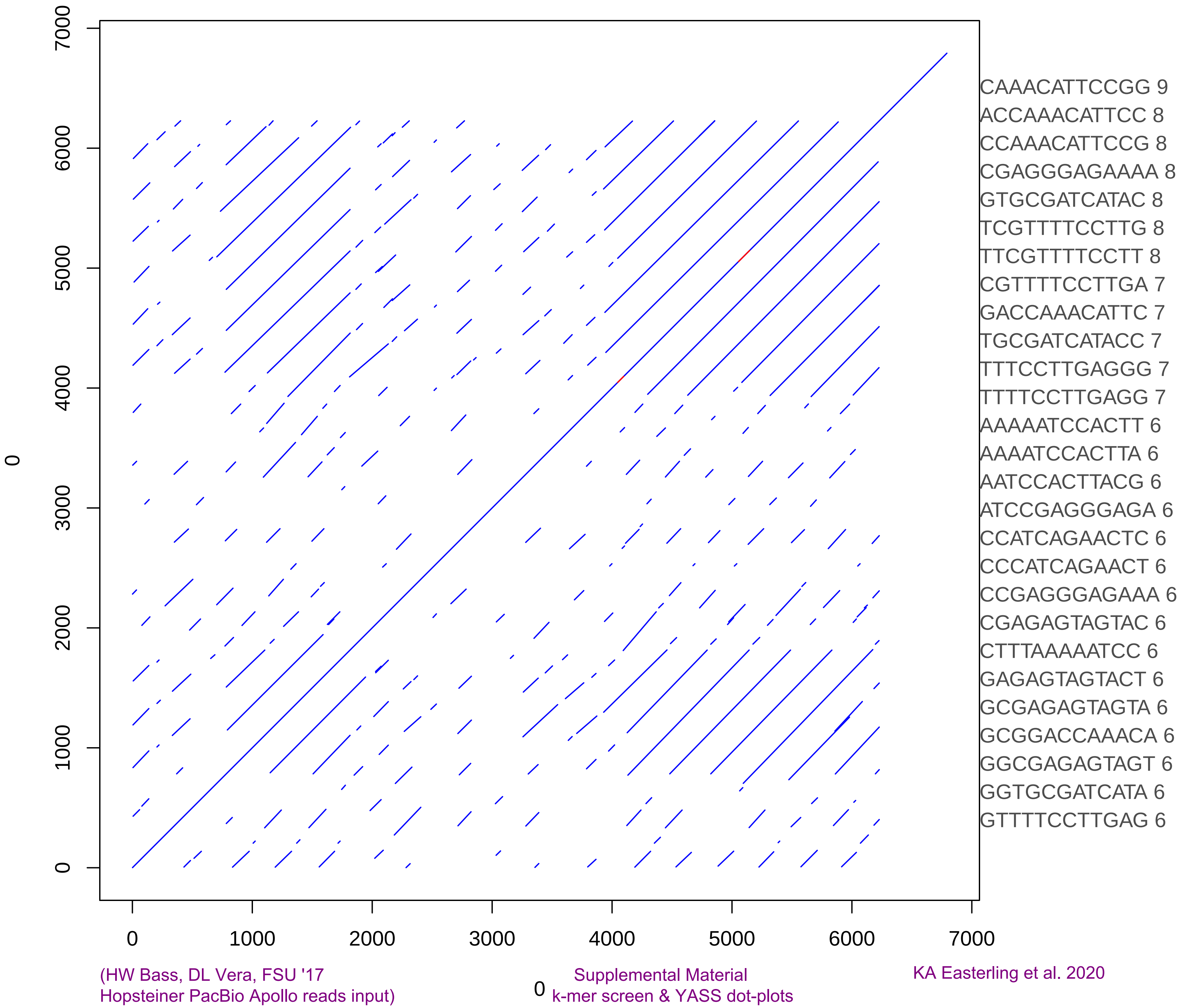

>m141230_233107_42134_c100756552550000001823146807221541_s1_p0/154463/0_9188

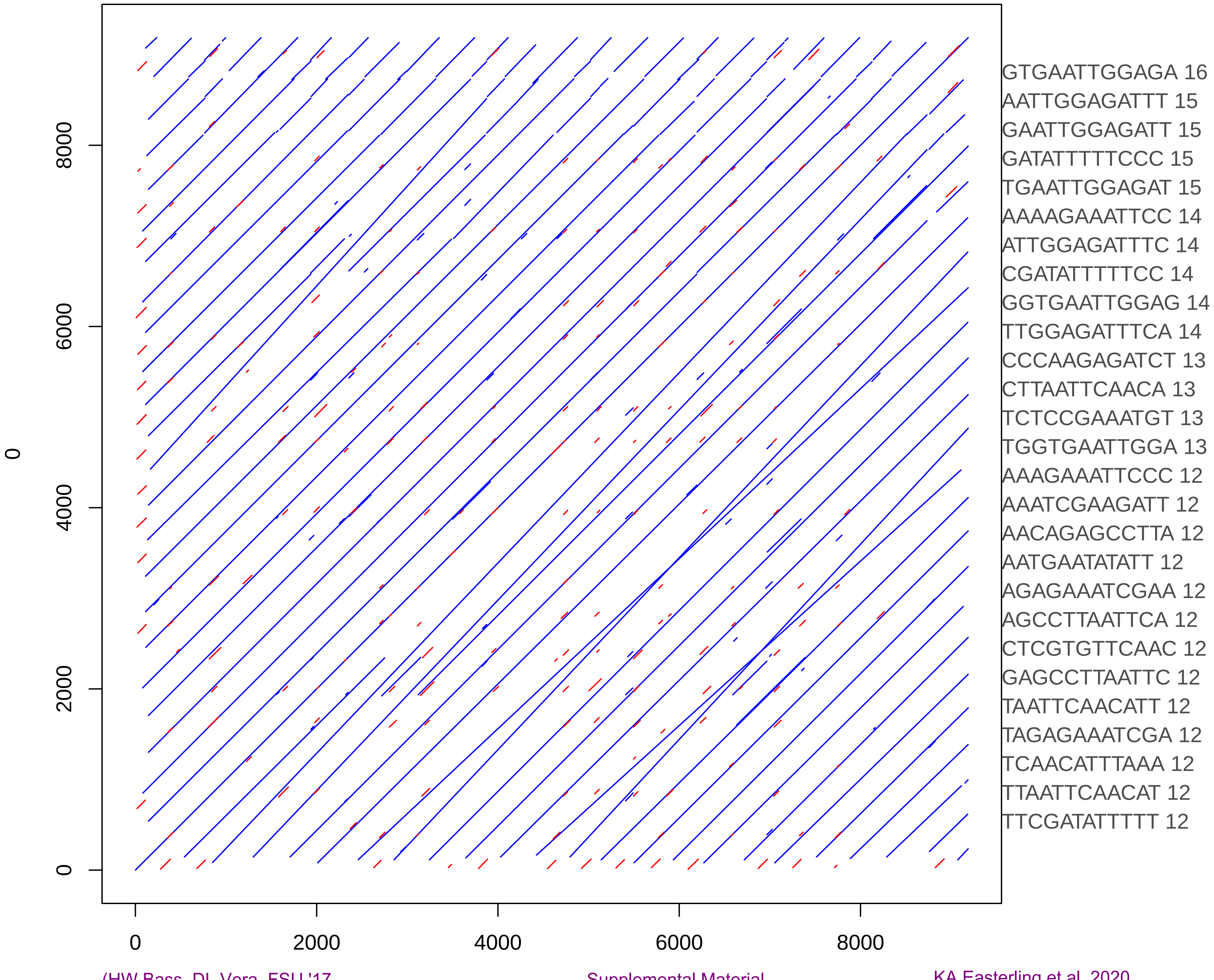

>m141230_233107_42134_c100756552550000001823146807221541_s1_p0/156096/2789_10990

Representative Read (Table 1): HuluTR625-r823, 4 ppm, 69% A+T,
GenBank Acc. MN537582

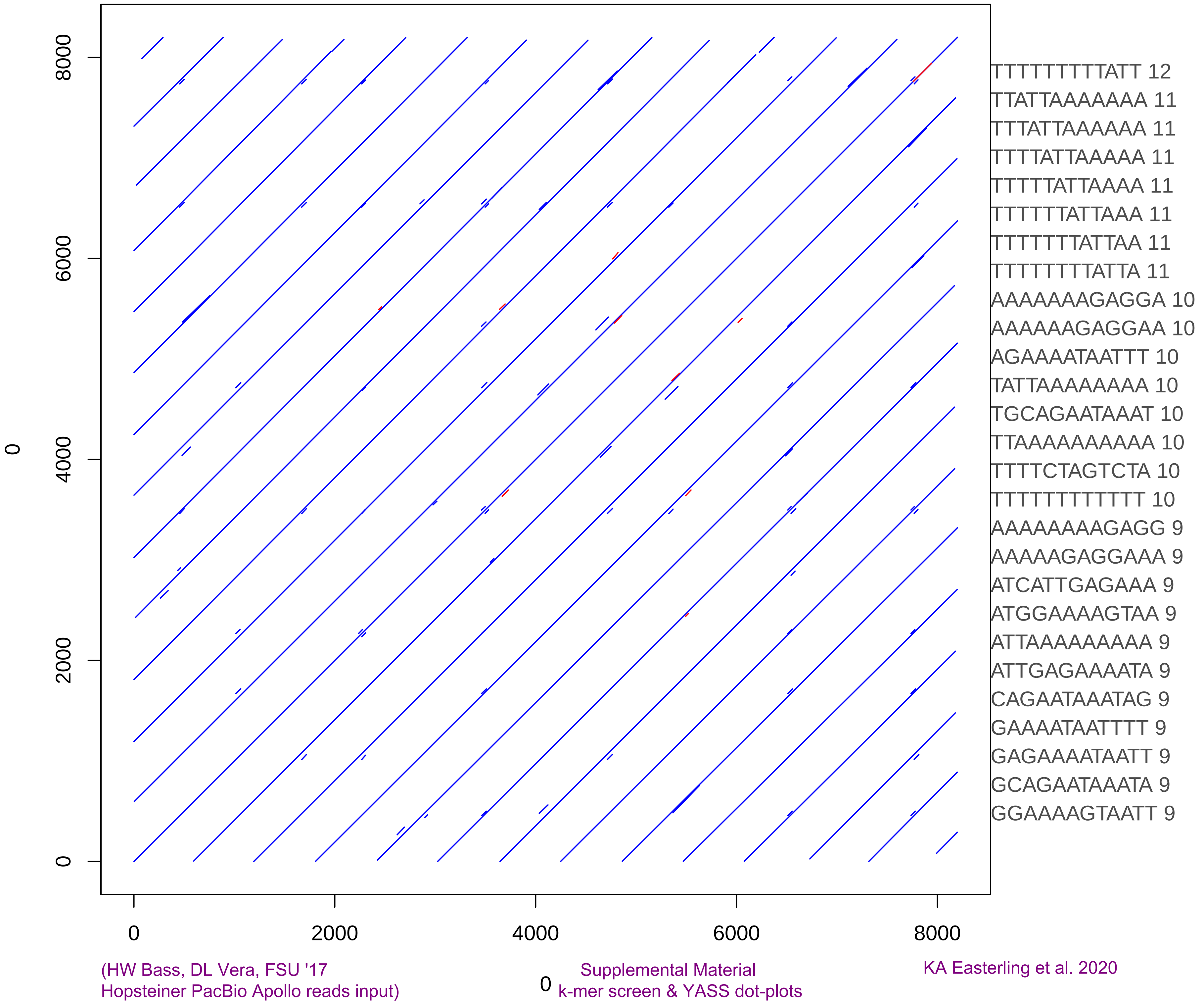

>m141230_233107_42134_c100756552550000001823146807221541_s1_p0/158270/0_8230

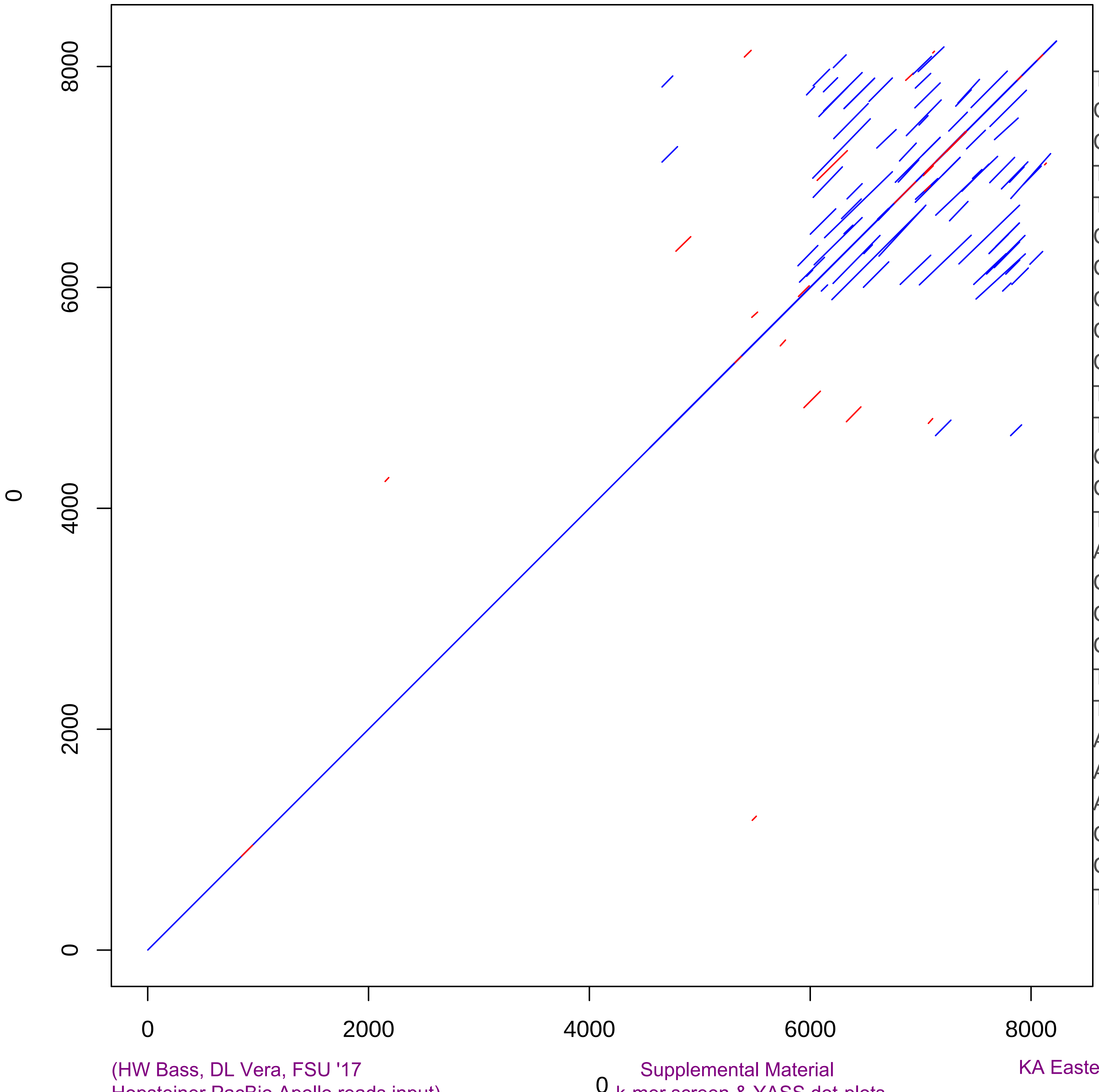

TTTTTTTTTTTT 86
GGGGGGGGGGGG 54
CCCCCCCCCCCC 22
TTTTTTTTTTAA 15
TTTTTTTTTTTA 15
GGAAAAAAAAAA 12
CCCTTTTTTTTT 11
GAAAAAAAAAAA 11
GGGAAAAAAAAA 10
GTTTTTTTTTTT 10
TGGGGGGGGGGG 10
TTTTTTTTTAAA 10
CCTTTTTTTTTT 9
CTTTTTTTTTTT 9
TTTGGGGGGGGG 9
AAAAAAAAAAAC 8
GGGGAAAAAAAA 8
GGGGGAAAAAAA 8
GGGGGGAAAAAA 8
TTTTGGGGGGGG 8
TTTTTTTTTGGG 8
AAAAAAAAAACC 7
AATTTTTTTTTT 7
ATTTTTTTTTTT 7
GGGGGGGGGGGA 7
GGGGGGGGGGGT 7
TTGGGGGGGGGG 7

>m141230_233107_42134_c100756552550000001823146807221541_s1_p0/158722/0_5782

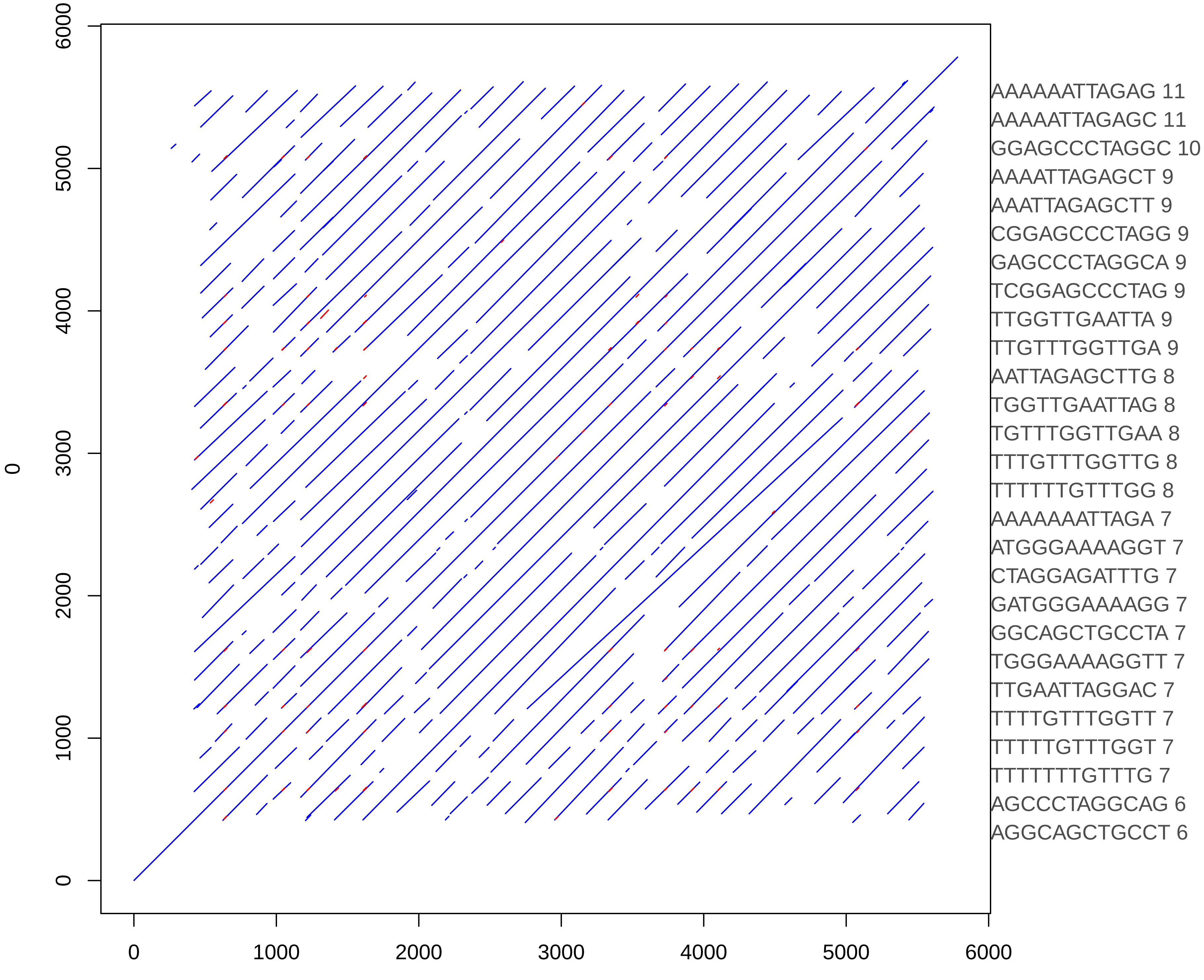

>m141230_233107_42134_c100756552550000001823146807221541_s1_p0/159189/2077_10748

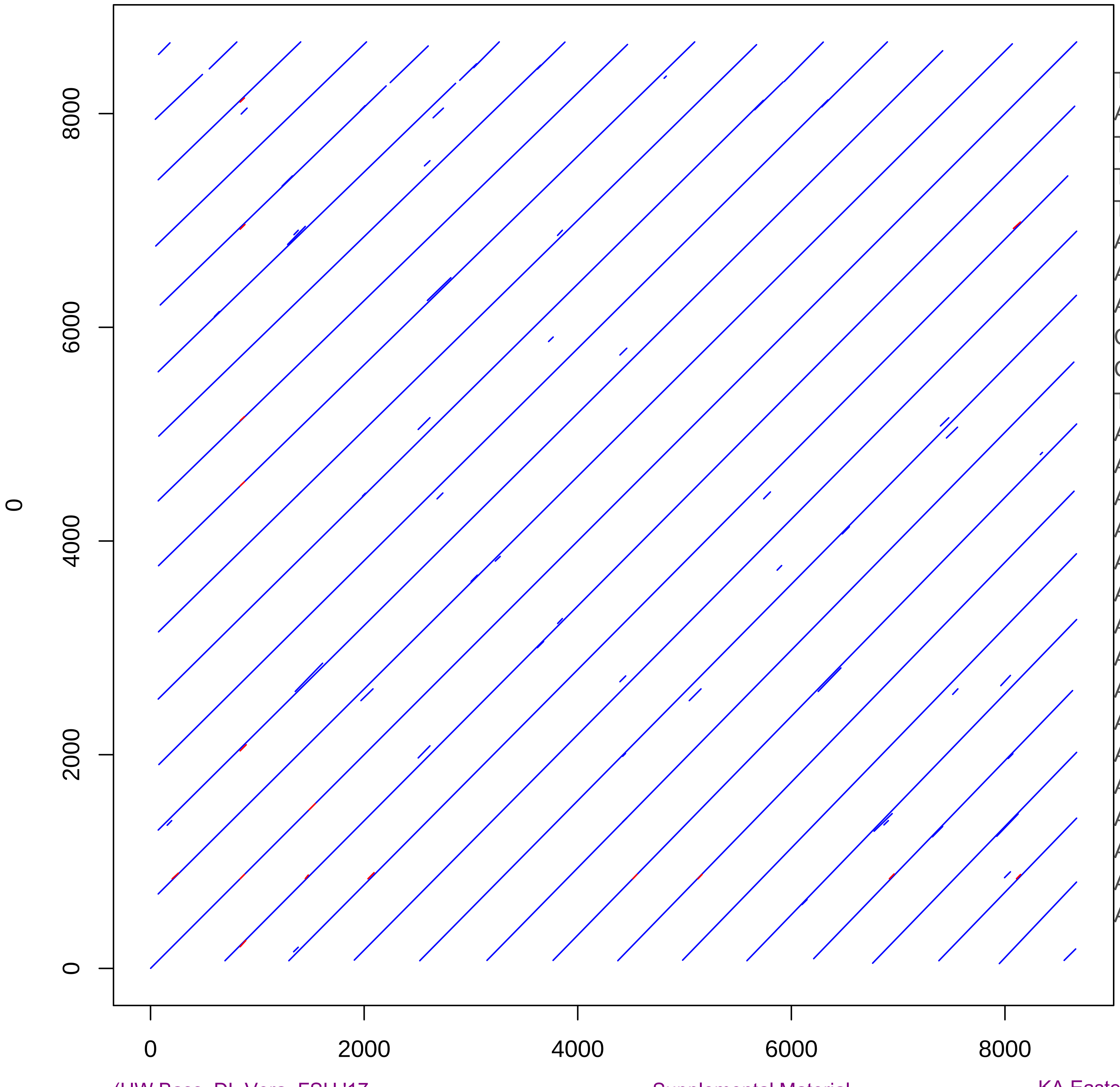

TTTTTTTTTTTT 24
AAATTATGGCAA 12
TGAAAGCTAAGC 11
TTGAAAGCTAAG 11
TTTGAAAGCTAA 11
AAAGCTAAGCTT 10
AAGCTAAGCTTA 10
AGCTAAGCTTAT 10
GAAAGCTAAGCT 10
GATGACTTATGA 10
TTTGTTATTGTT 10
AAAAAAAAGAGG 9
AAAAAAAGAGGA 9
AAAAAAGAGGAA 9
AAAAAGAGGAAA 9
AAAAGAGGAAAA 9
AAAGAGGAAAAC 9
AAGAGGAAAACC 9
AATCATTGAGAA 9
AATTATGGCAAC 9
AATTTAATCATT 9
ATATCTAATTTT 9
ATCATTGAGAAA 9
ATCTAATTTTTG 9
ATGACTTATGAA 9
ATGGAAAAGTAA 9
ATTATGGCAACT 9

>m141230_233107_42134_c100756552550000001823146807221541_s1_p0/161844/263_10390

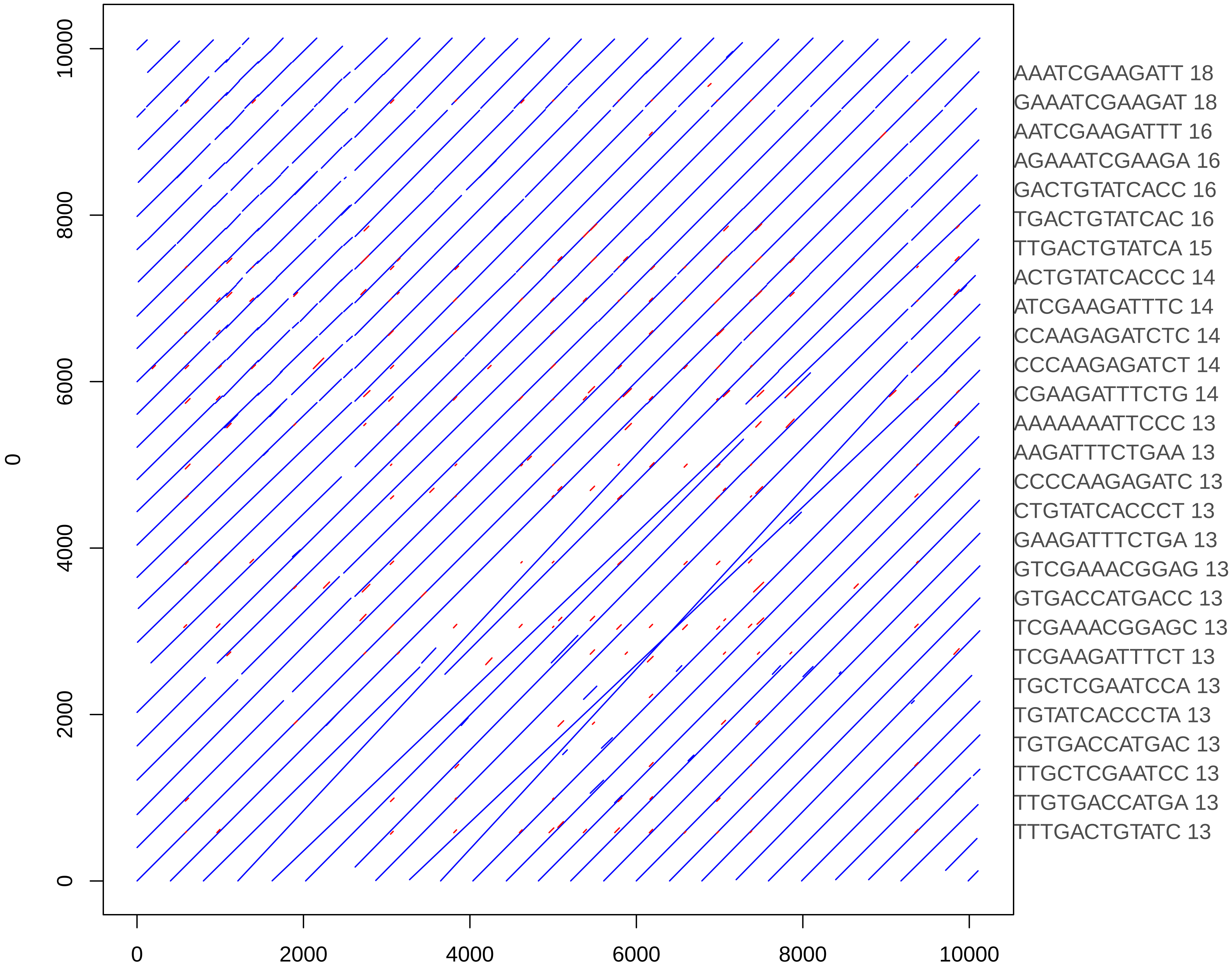

>m141230_233107_42134_c100756552550000001823146807221541_s1_p0/16737/6930_16934

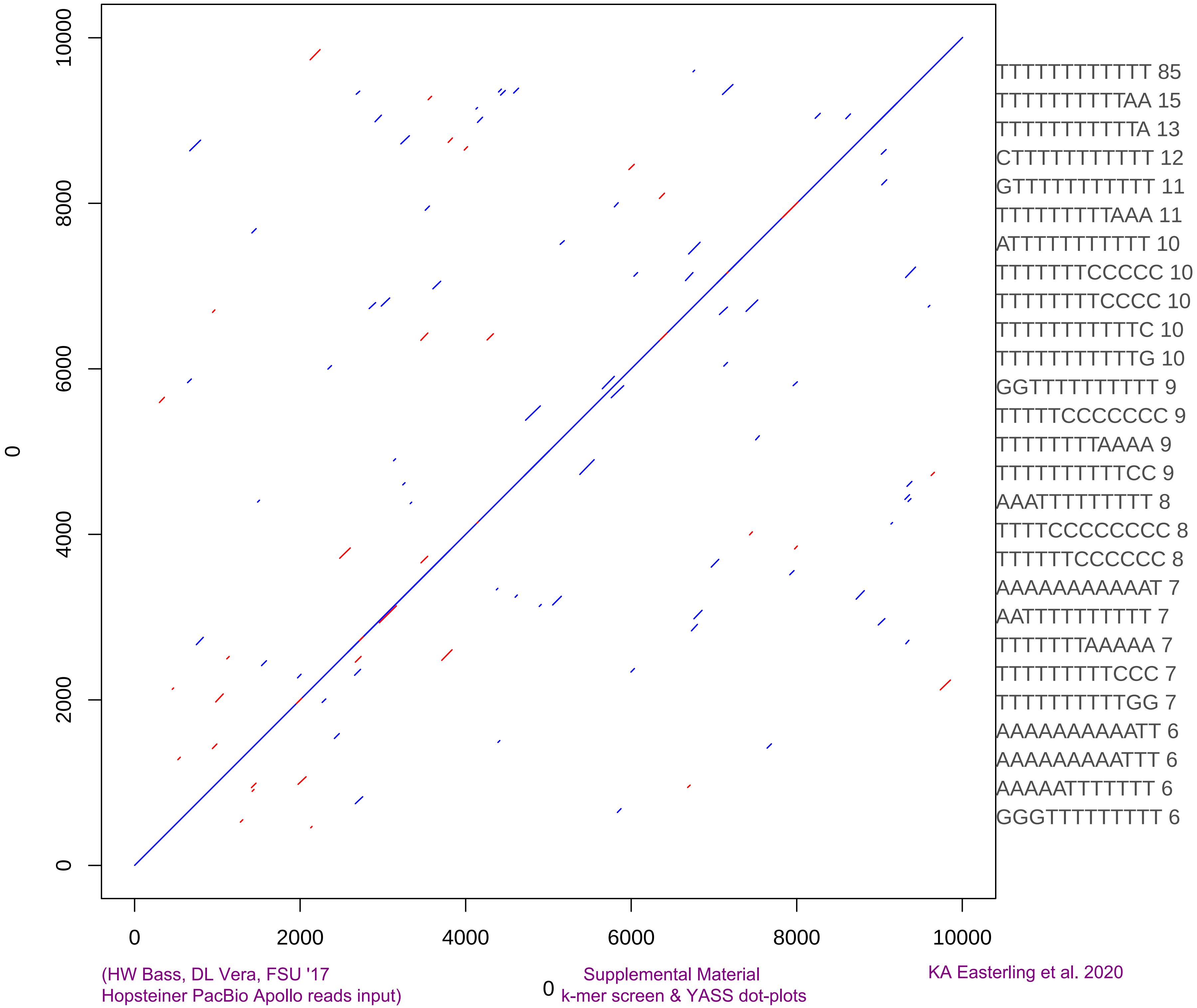

>m141230_233107_42134_c100756552550000001823146807221541_s1_p0/20206/341_8968

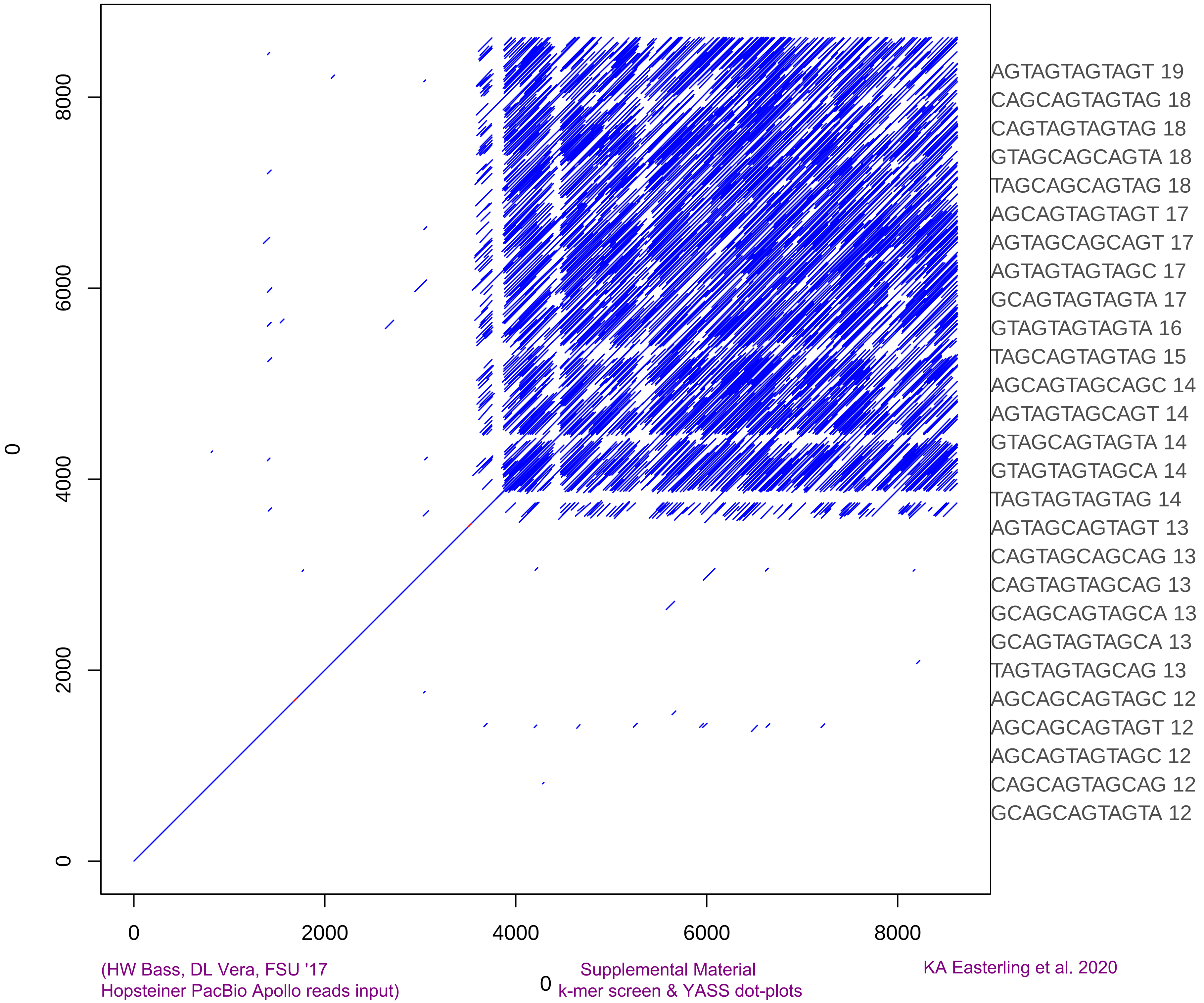

>m141230_233107_42134_c100756552550000001823146807221541_s1_p0/21970/2172_14357

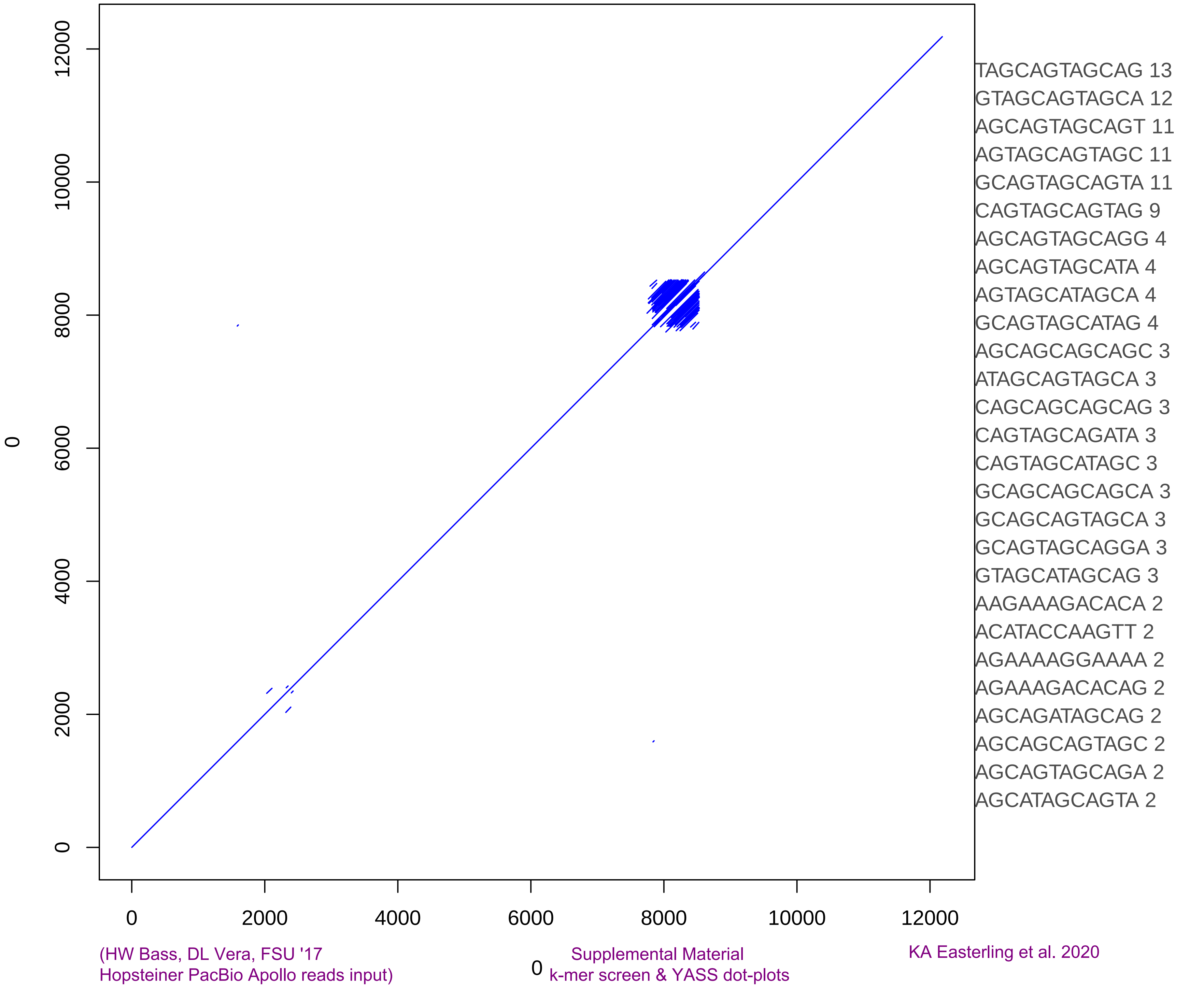

>m141230_233107_42134_c100756552550000001823146807221541_s1_p0/22633/6241_15548

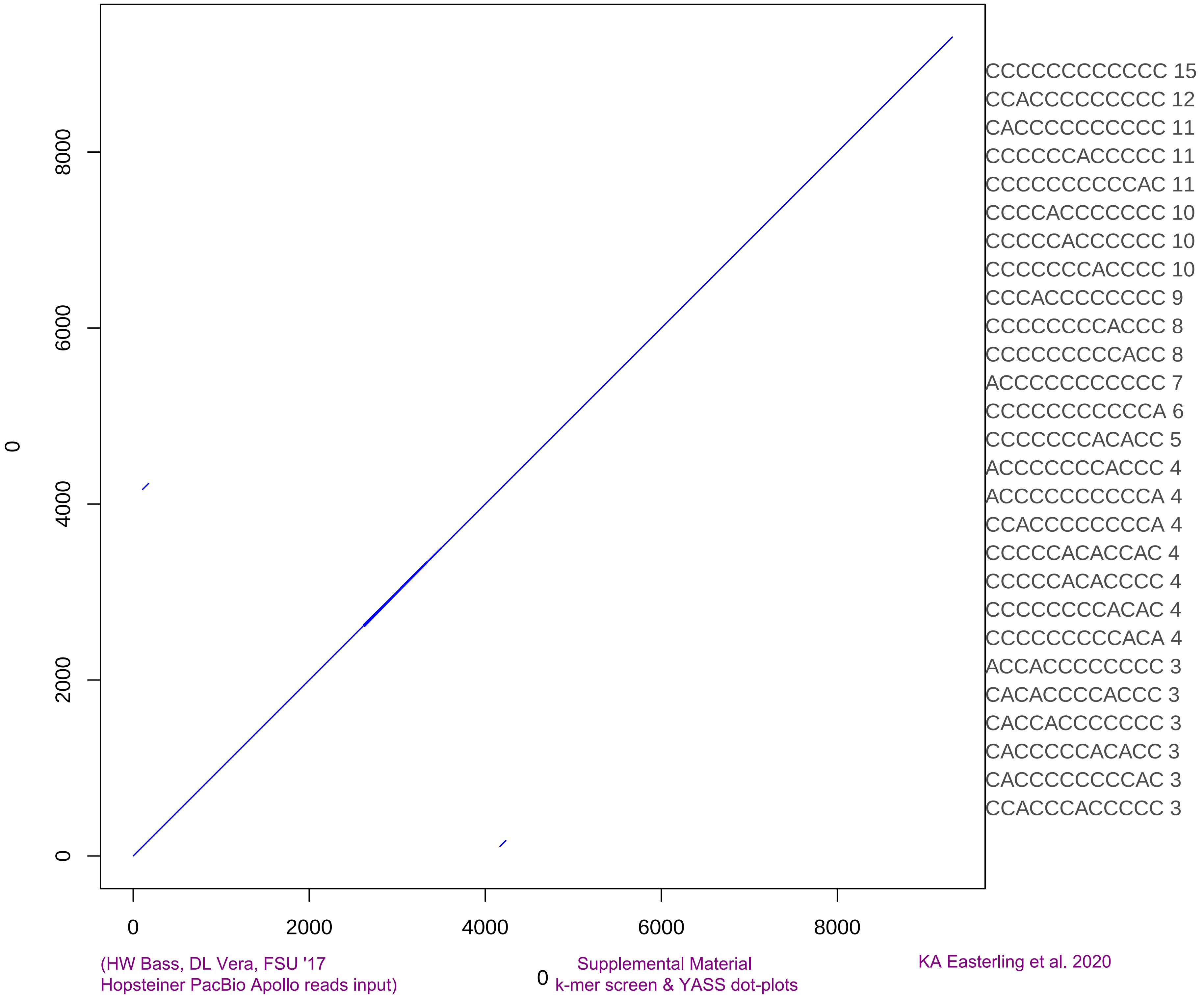

>m141230_233107_42134_c100756552550000001823146807221541_s1_p0/2321/0_6681

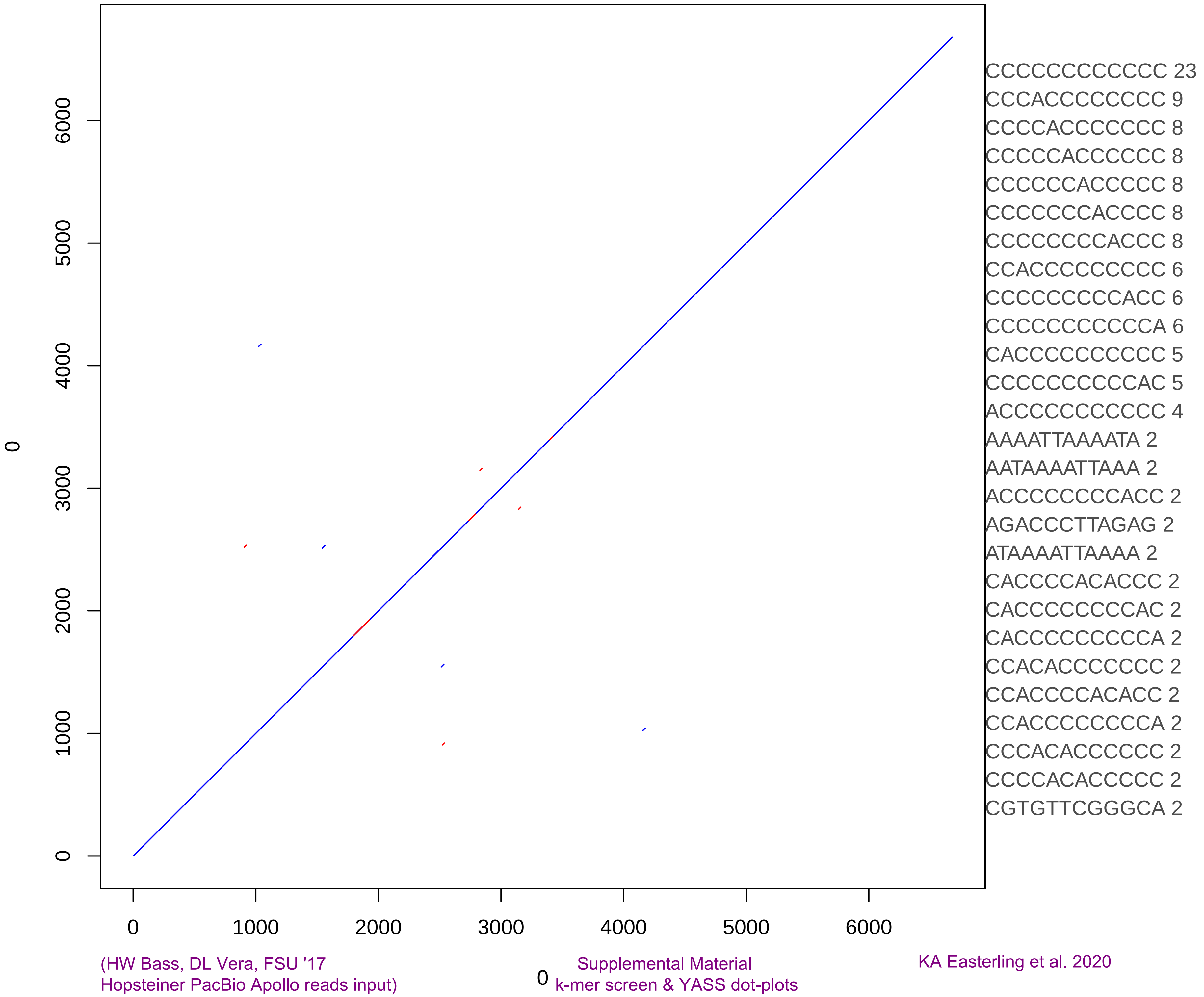

>m141230_233107_42134_c100756552550000001823146807221541_s1_p0/24811/6808_12765

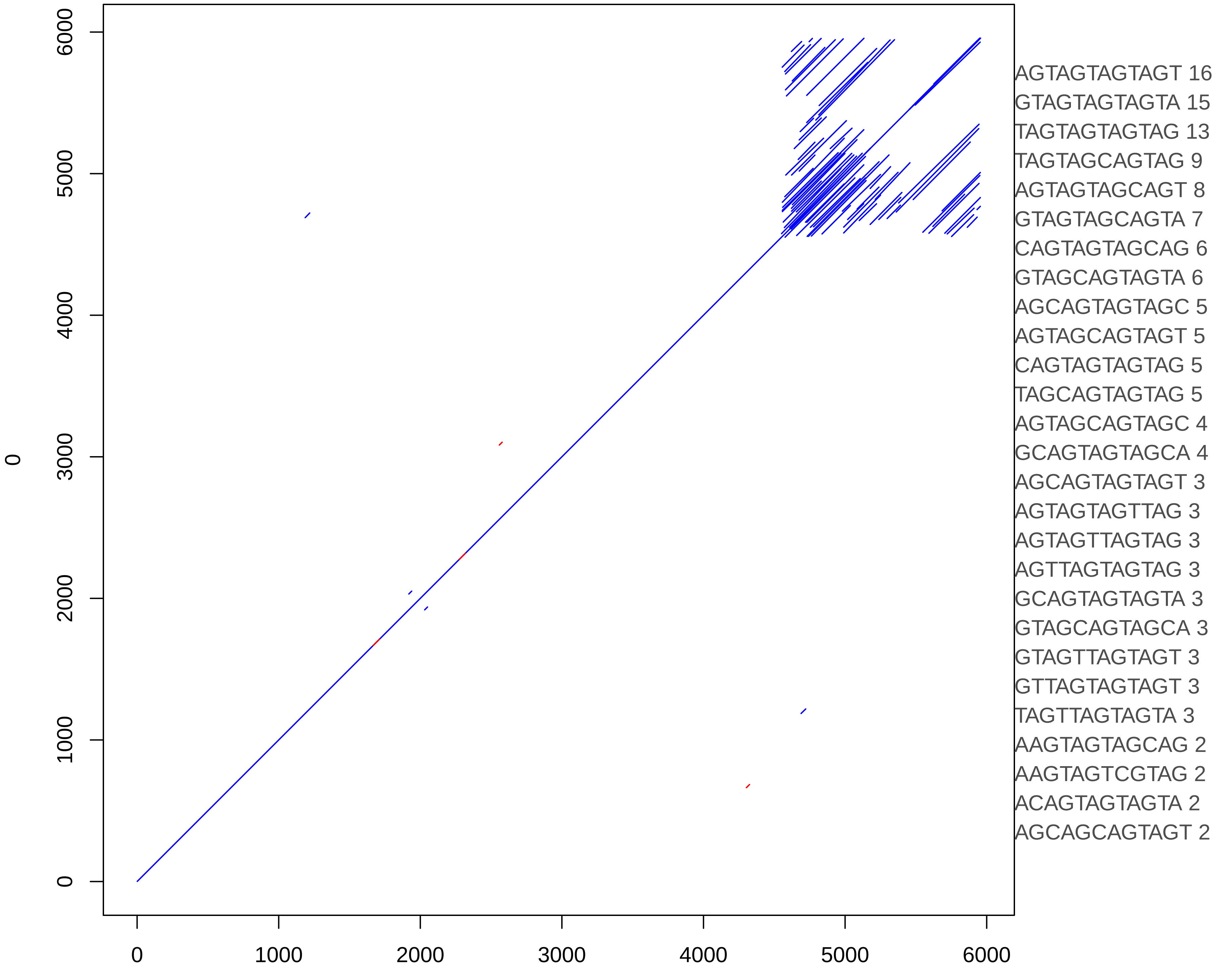

>m141230_233107_42134_c100756552550000001823146807221541_s1_p0/25040/8914_15997

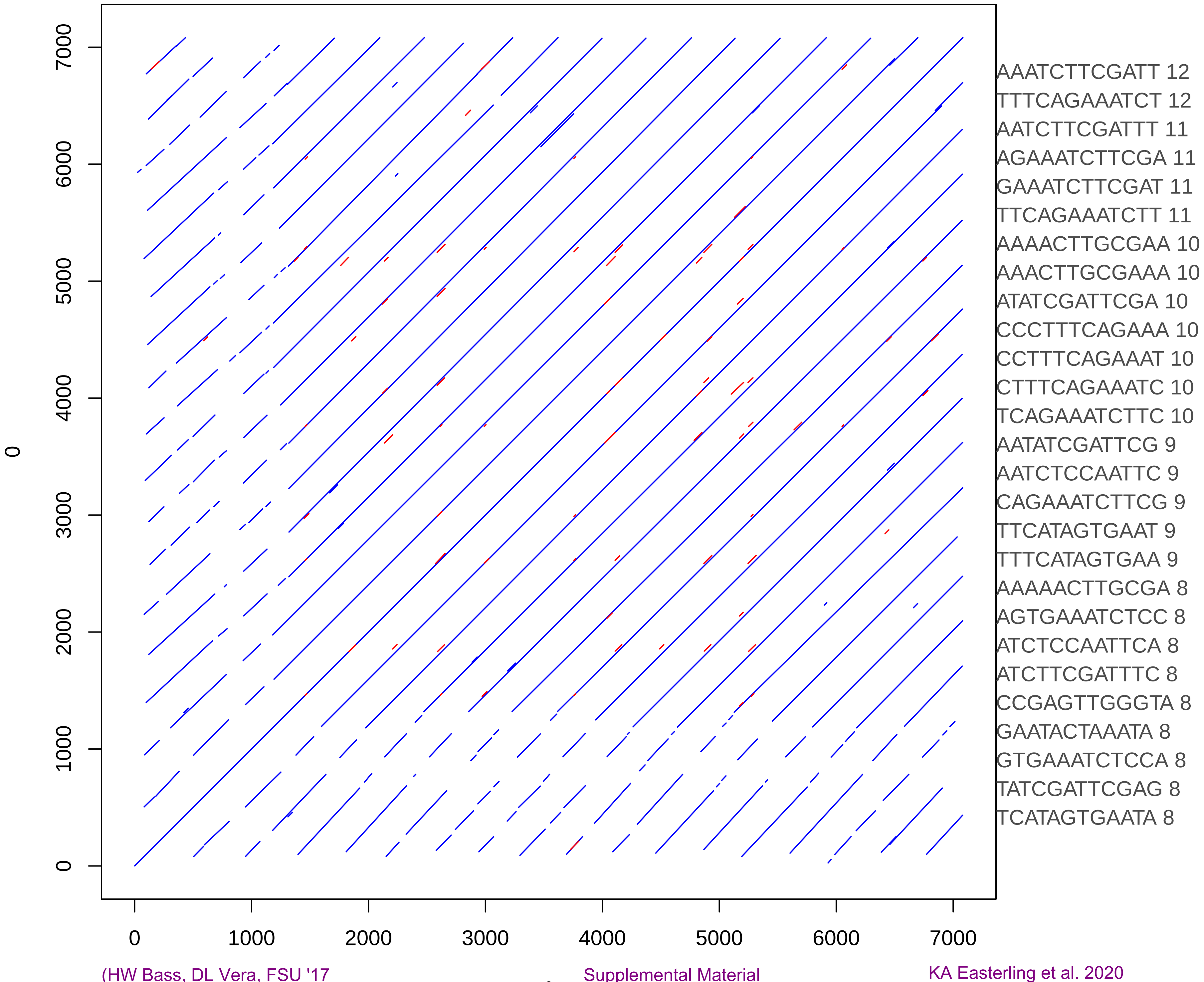

0

>m141230_233107_42134_c100756552550000001823146807221541_s1_p0/25931/3030_15084

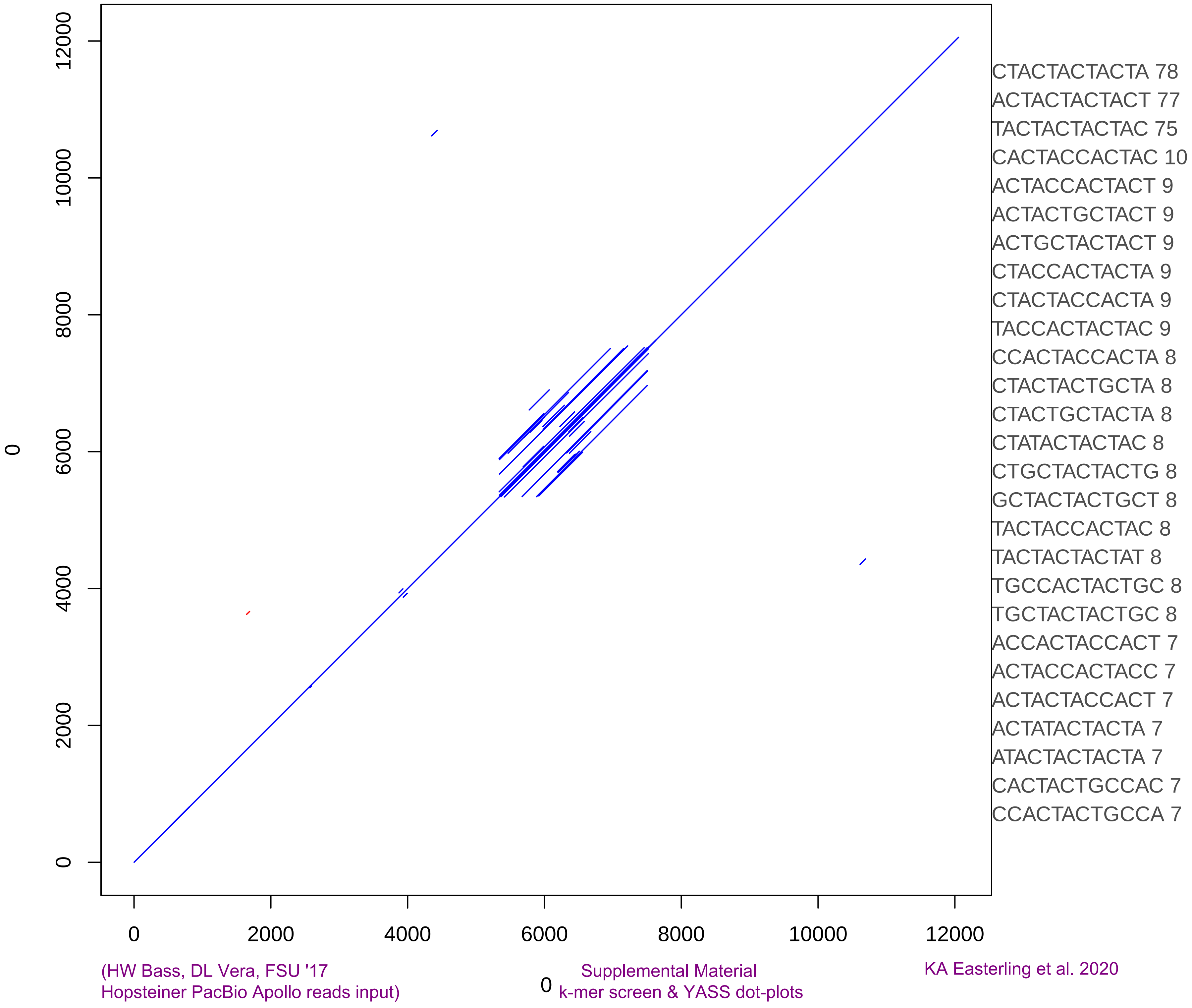

>m141230_233107_42134_c100756552550000001823146807221541_s1_p0/28216/2110_9537

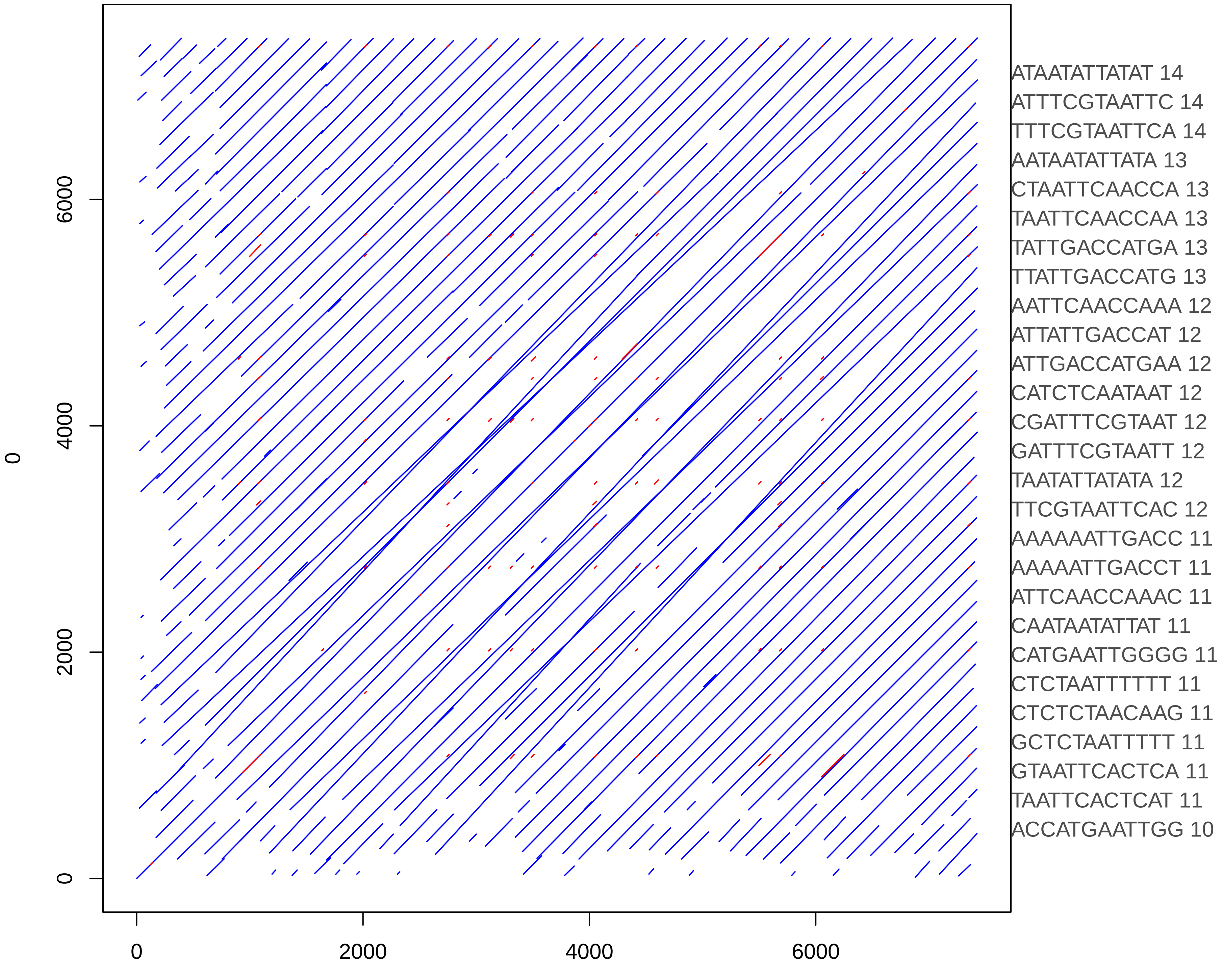

0

>m141230_233107_42134_c100756552550000001823146807221541_s1_p0/28966/0_13007

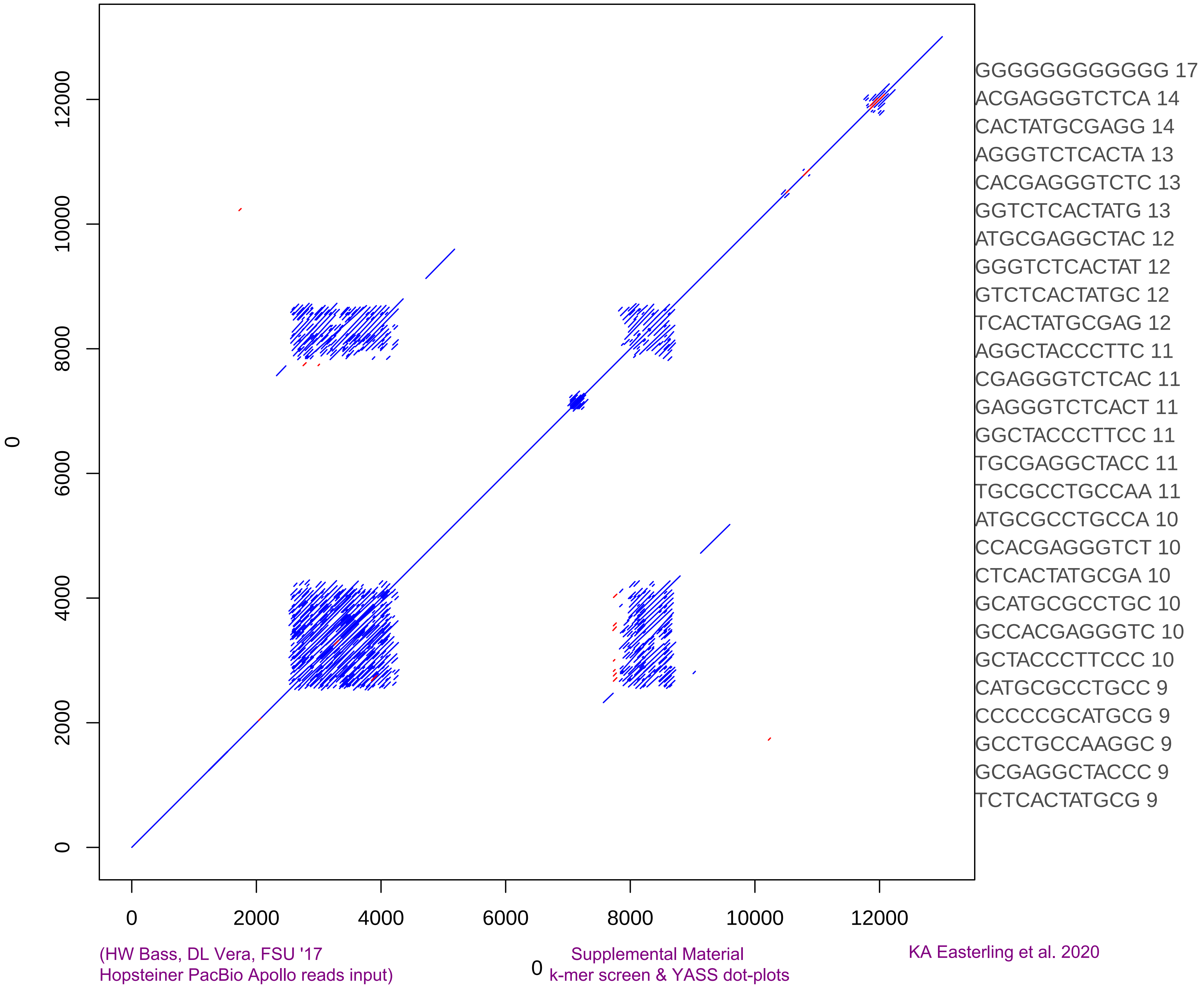

>m141230_233107_42134_c100756552550000001823146807221541_s1_p0/30028/2977_10959

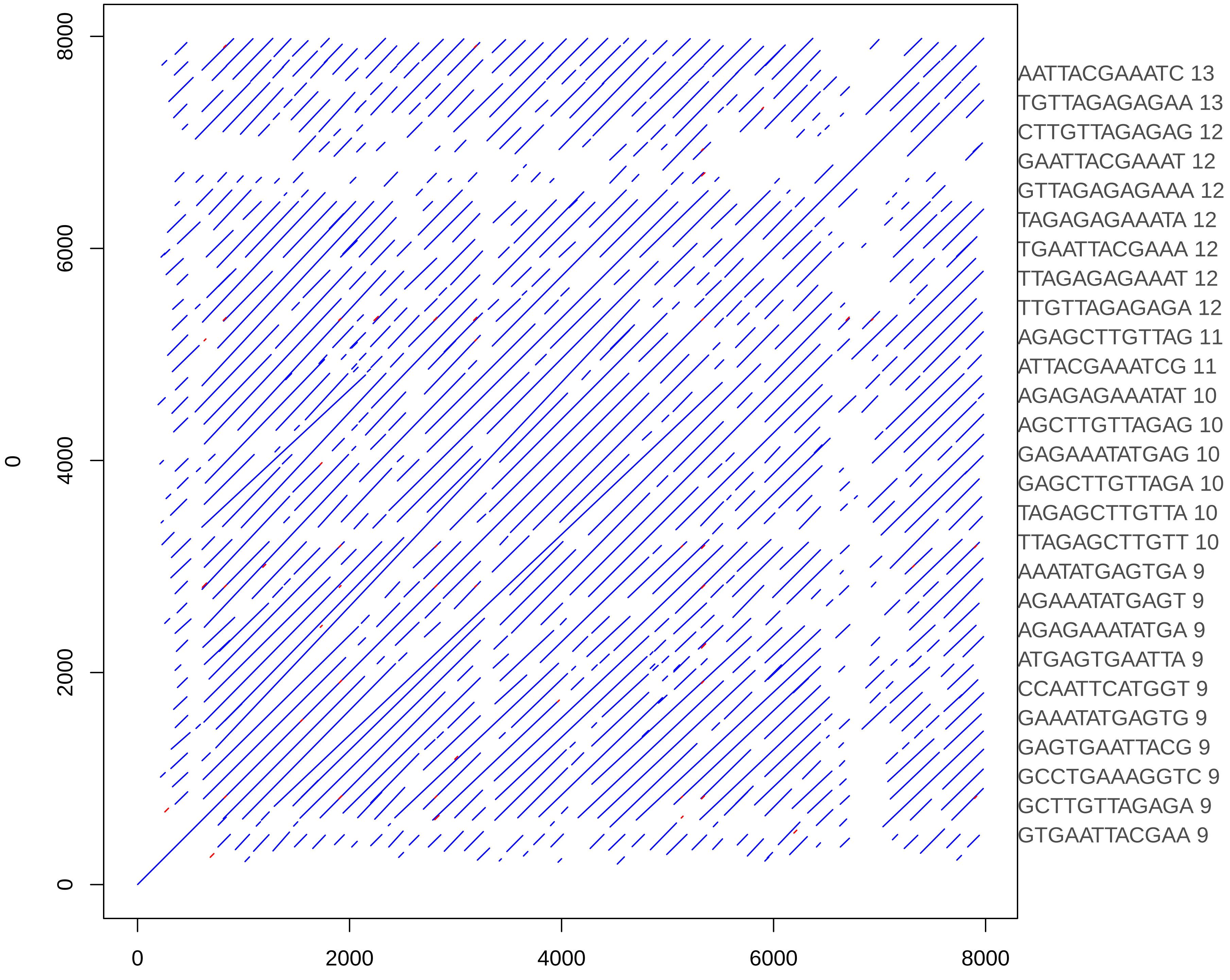

>m141230_233107_42134_c100756552550000001823146807221541_s1_p0/30618/161_6394

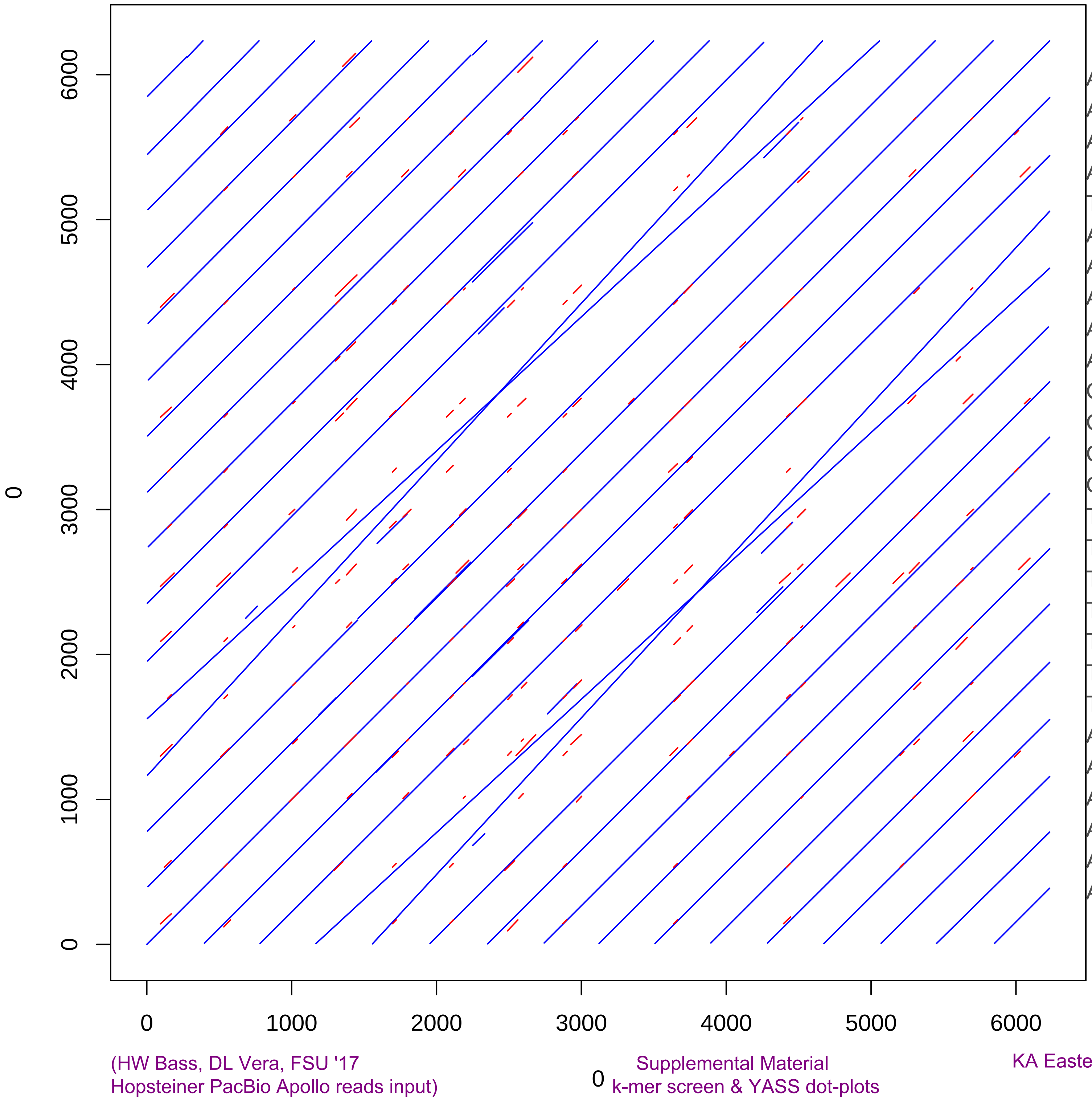

AATAATATGCGA 13
AATGTTGATTTA 13
ATAATATGCGAG 13
ATGTTGATTTAA 13
TAATATGCGAGG 13
AAATAATATGCG 12
AAGGGTCCGTTT 12
AATATGCGAGGG 12
ACTTGCGAAAAT 12
ATATGCGAGGGT 12
CCCTTTCAGAAA 12
CCTTTCAGAAAT 12
CTTGCGAAAATT 12
GATAGAGTCAAA 12
TAAATAATATGC 12
TGCGAAAATTGC 12
TGTTTTCATAGT 12
TTAGTGAAATCT 12
TTCAGAAATCTT 12
TTTCAGAAATCT 12
TTTTCATAGTGA 12
AAAATTGCCATC 11
AAAGTTGTAGCC 11
AAATGTTGATTT 11
ACACGAGGGCAA 11
ACATTTCGGAGA 11
AGTGAAATCTCC 11

>m141230_233107_42134_c100756552550000001823146807221541_s1_p0/31184/354_6356

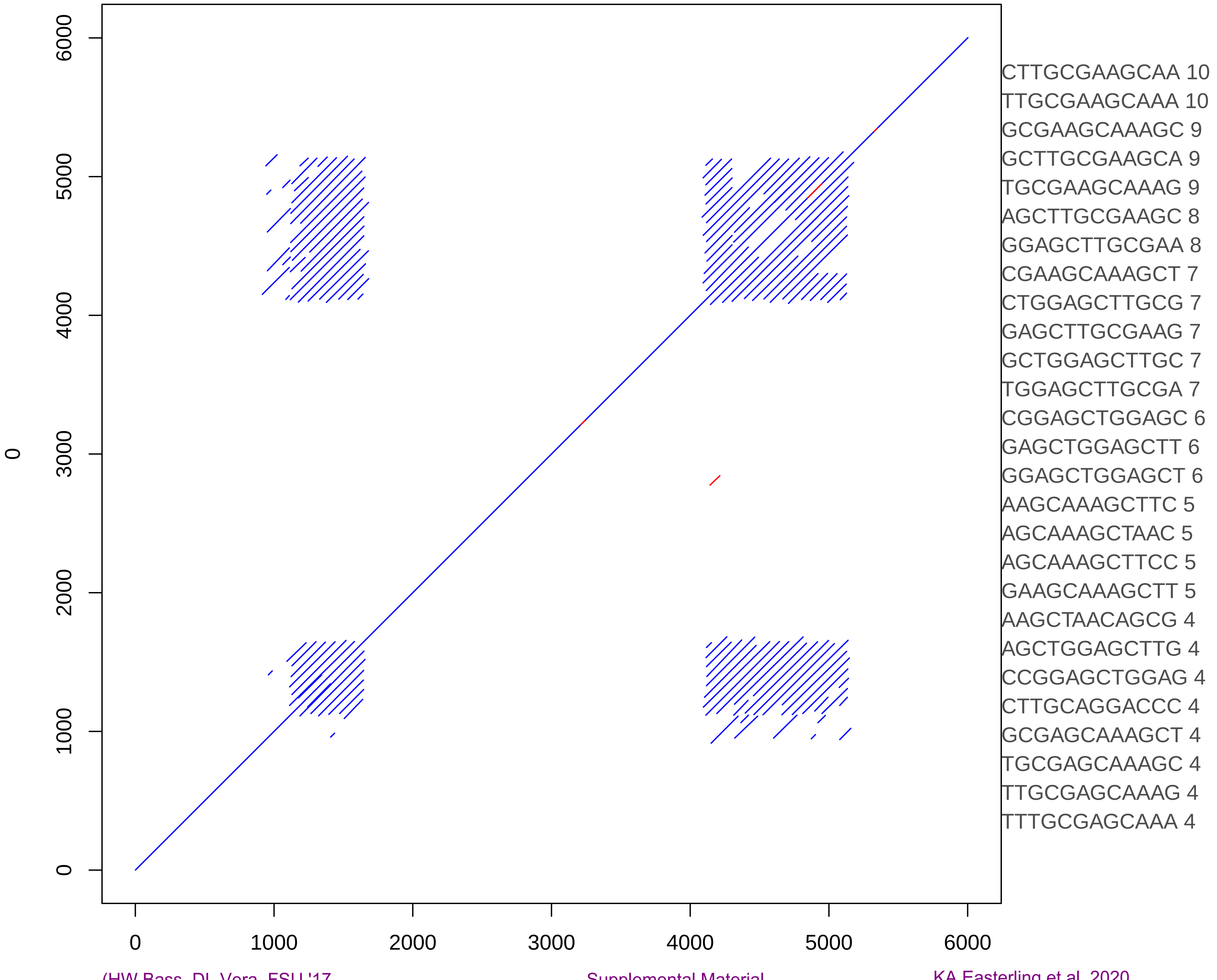

>m141230_233107_42134_c100756552550000001823146807221541_s1_p0/31785/727_7094

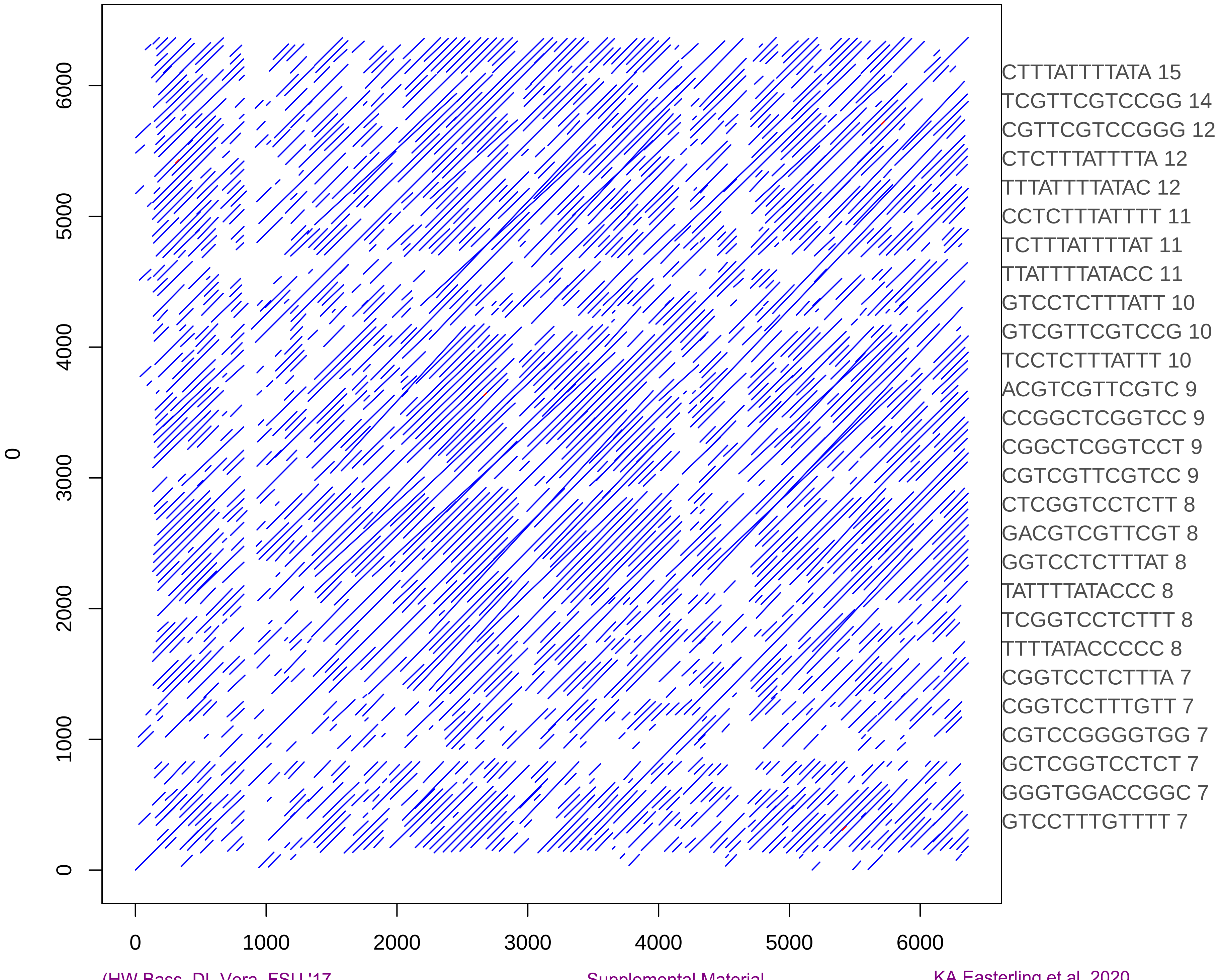

>m141230_233107_42134_c100756552550000001823146807221541_s1_p0/32389/508_6239

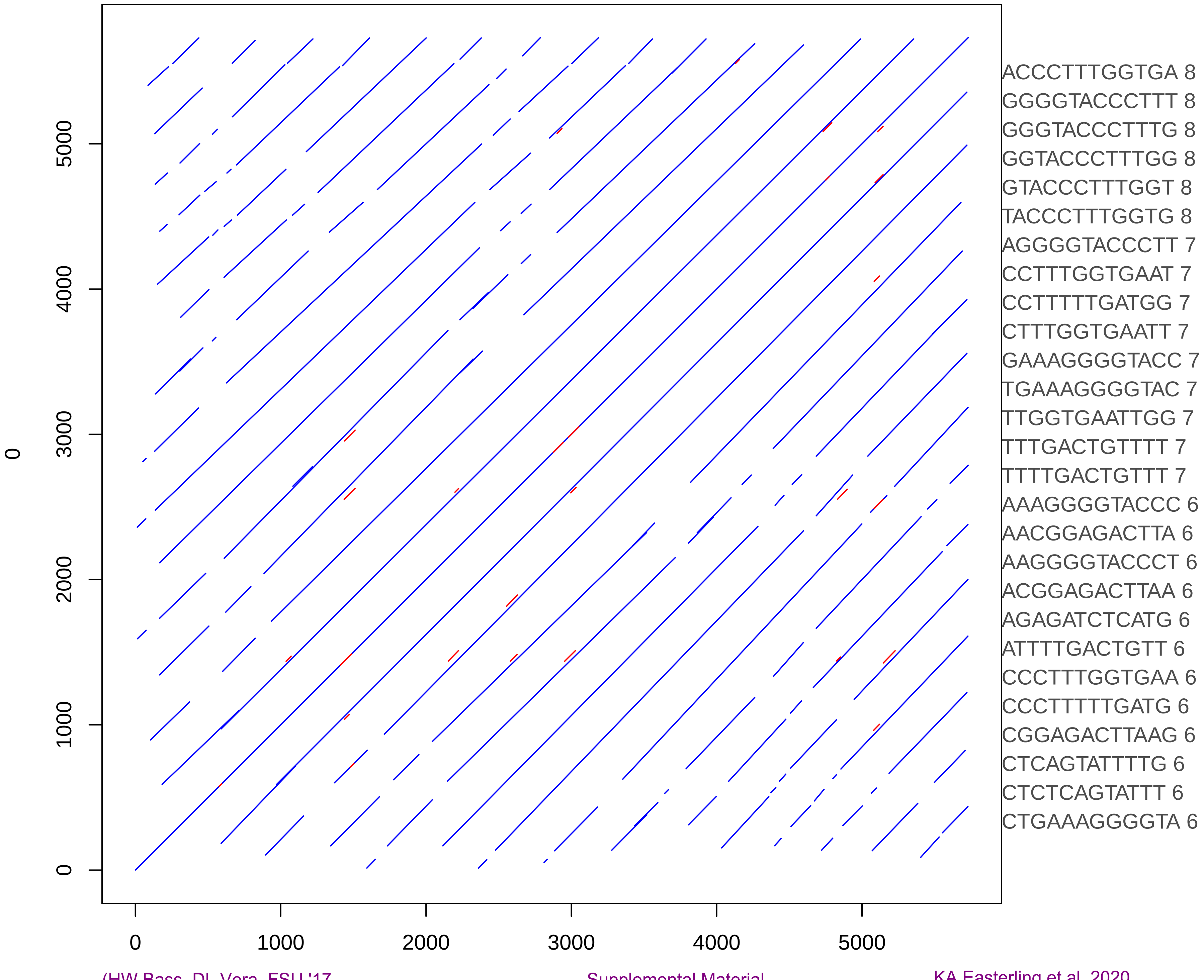

>m141230_233107_42134_c100756552550000001823146807221541_s1_p0/32498/8892_15563

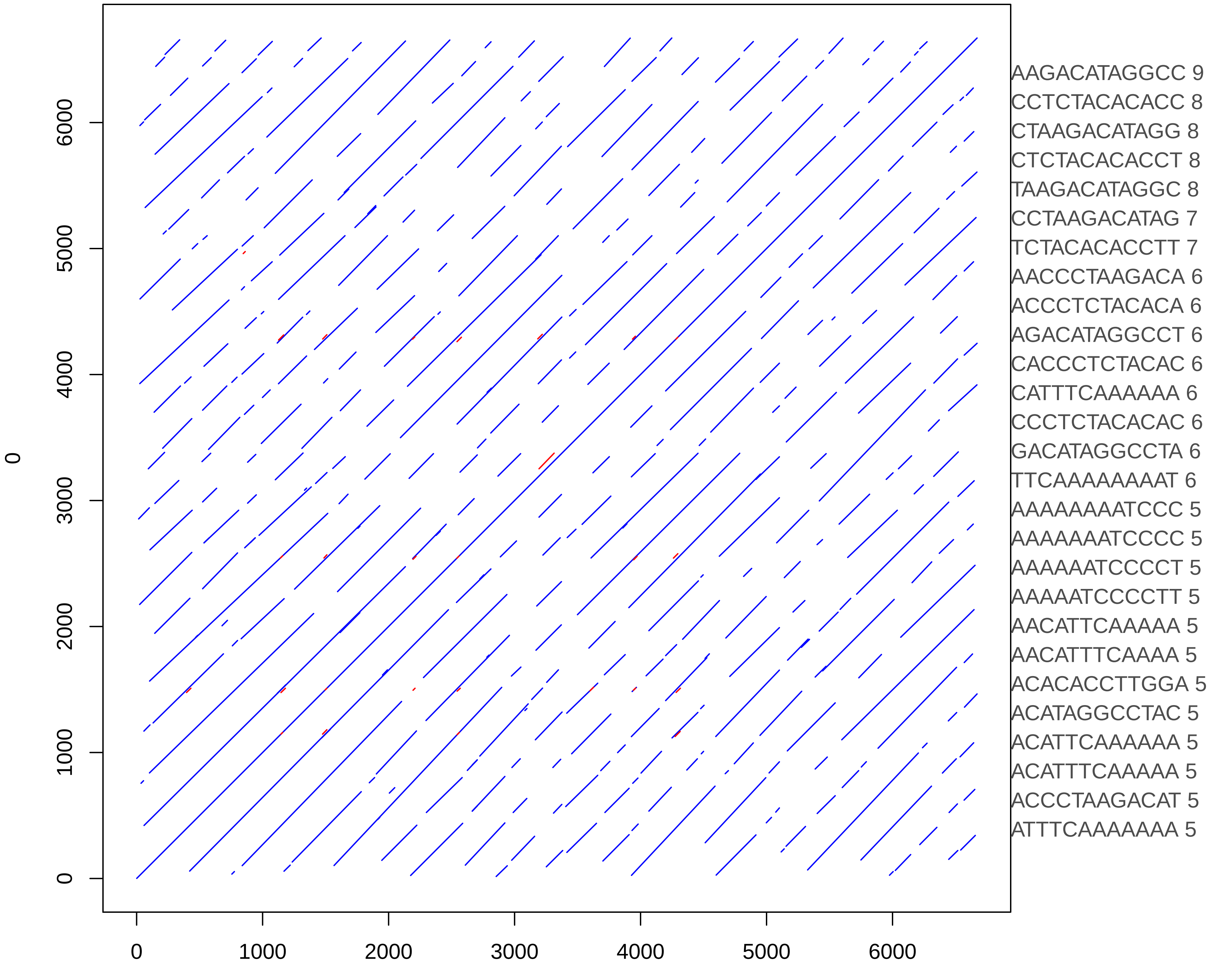

>m141230_233107_42134_c100756552550000001823146807221541_s1_p0/3358/0_5211

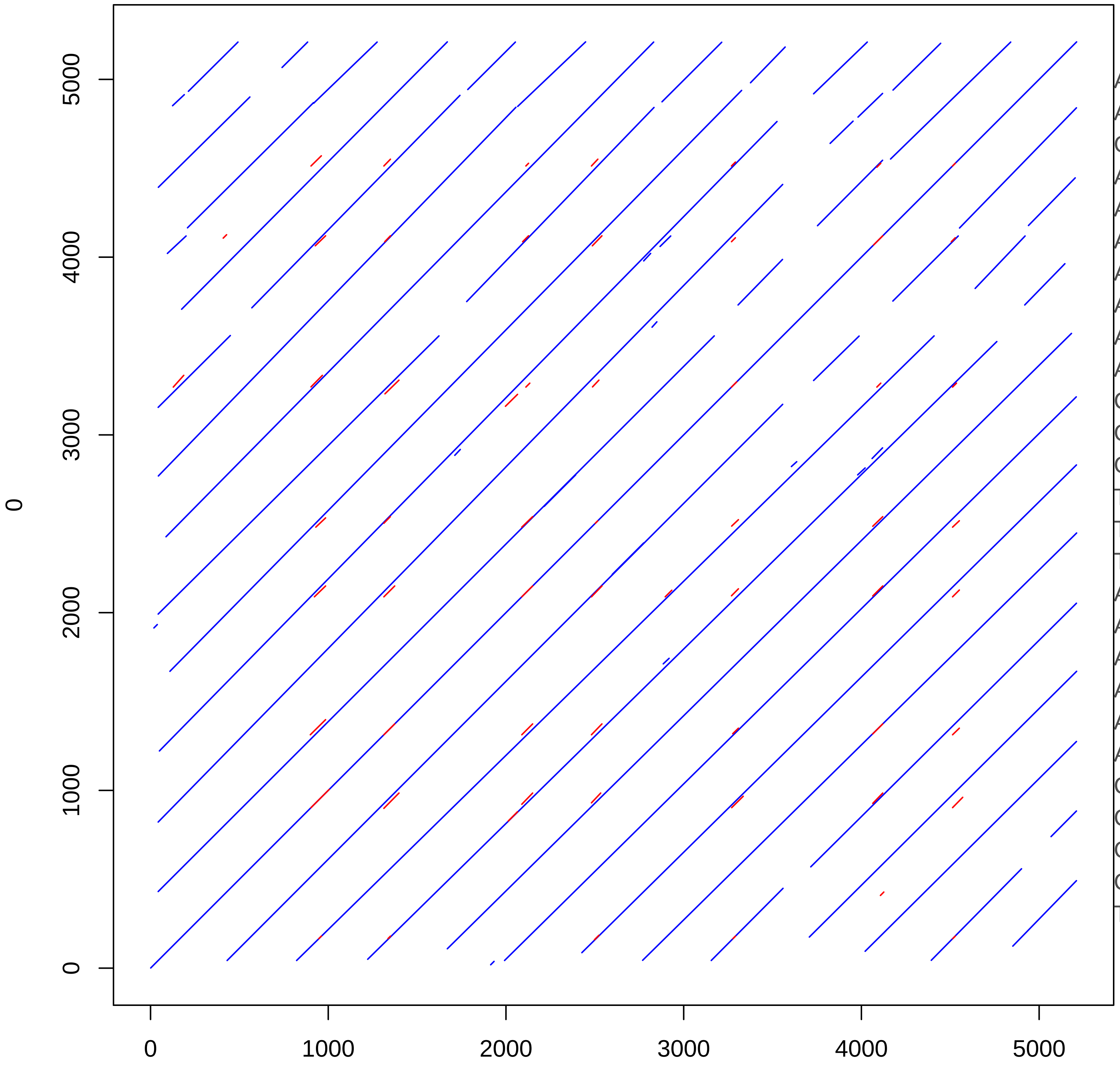

AACTTGCGAAAA 9
ACTTGCGAAAAT 9
GGGAATTTTTTT 9
AAACTTGCGAAA 8
AAAGGGGAATTT 8
AAATTGCCATCG 8
AAGGGGAATTTT 8
AATTGCCATCGA 8
AGGGGAATTTTT 8
ATTGCCATCGAA 8
CTTGCGAAAATT 8
GGAATTTTTTTT 8
GGGGAATTTTTT 8
TGCGAAAATTGC 8
TTGCCATCGAAA 8
TTGCGAAAATTG 8
AAAACTTGCGAA 7
AAAATTGCCATC 7
AATGTCGACTTA 7
AATTTTTTTTGA 7
ATGTCGACTTAA 7
ATTTTTTTTGAA 7
CGAAAATTGCCA 7
GAAAATTGCCAT 7
GAATTTTTTTTG 7
GCGAAAATTGCC 7
TGTCGACTTAAG 7

>m141230_233107_42134_c100756552550000001823146807221541_s1_p0/35046/5489_10823

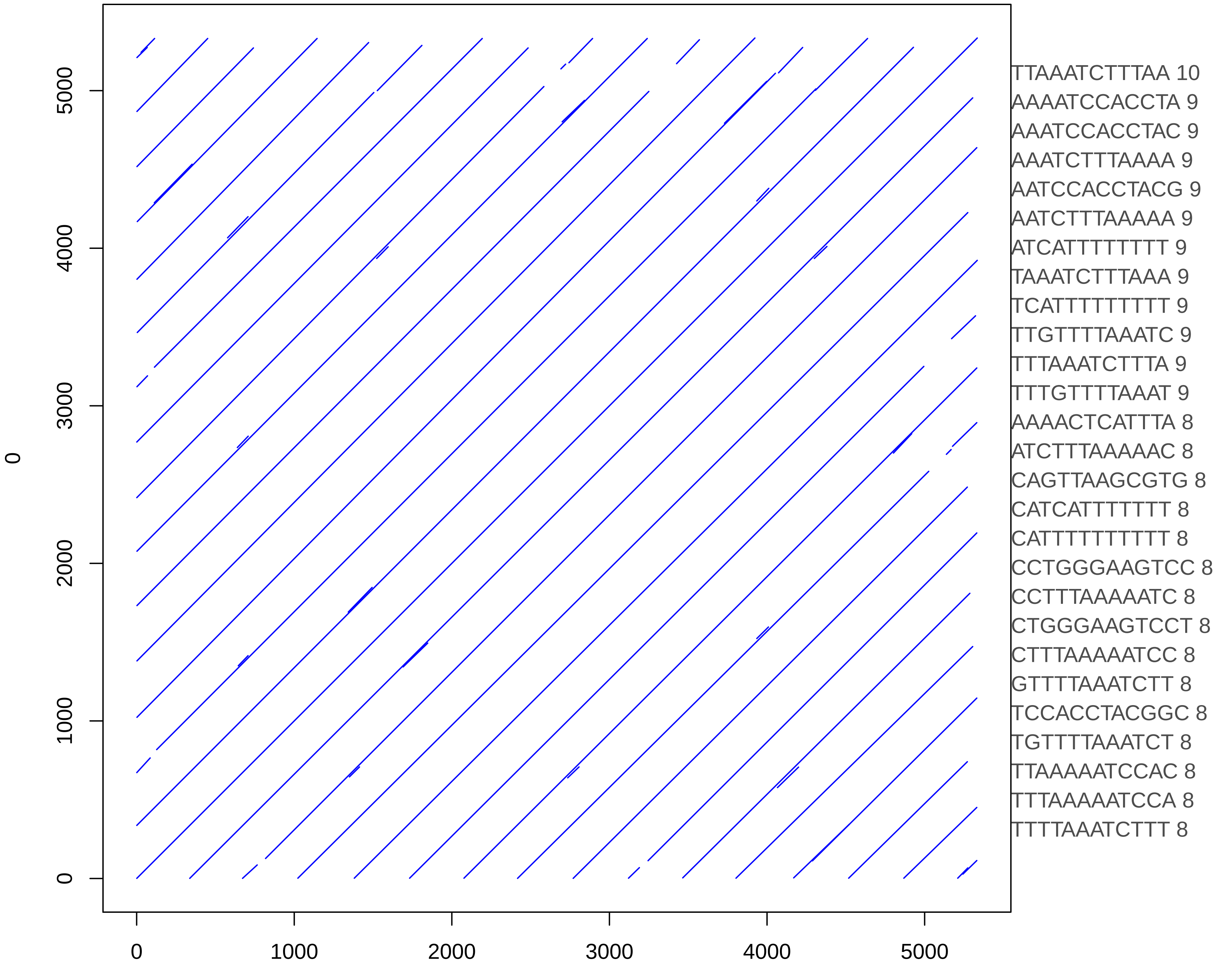

>m141230_233107_42134_c100756552550000001823146807221541_s1_p0/41563/10245_16148

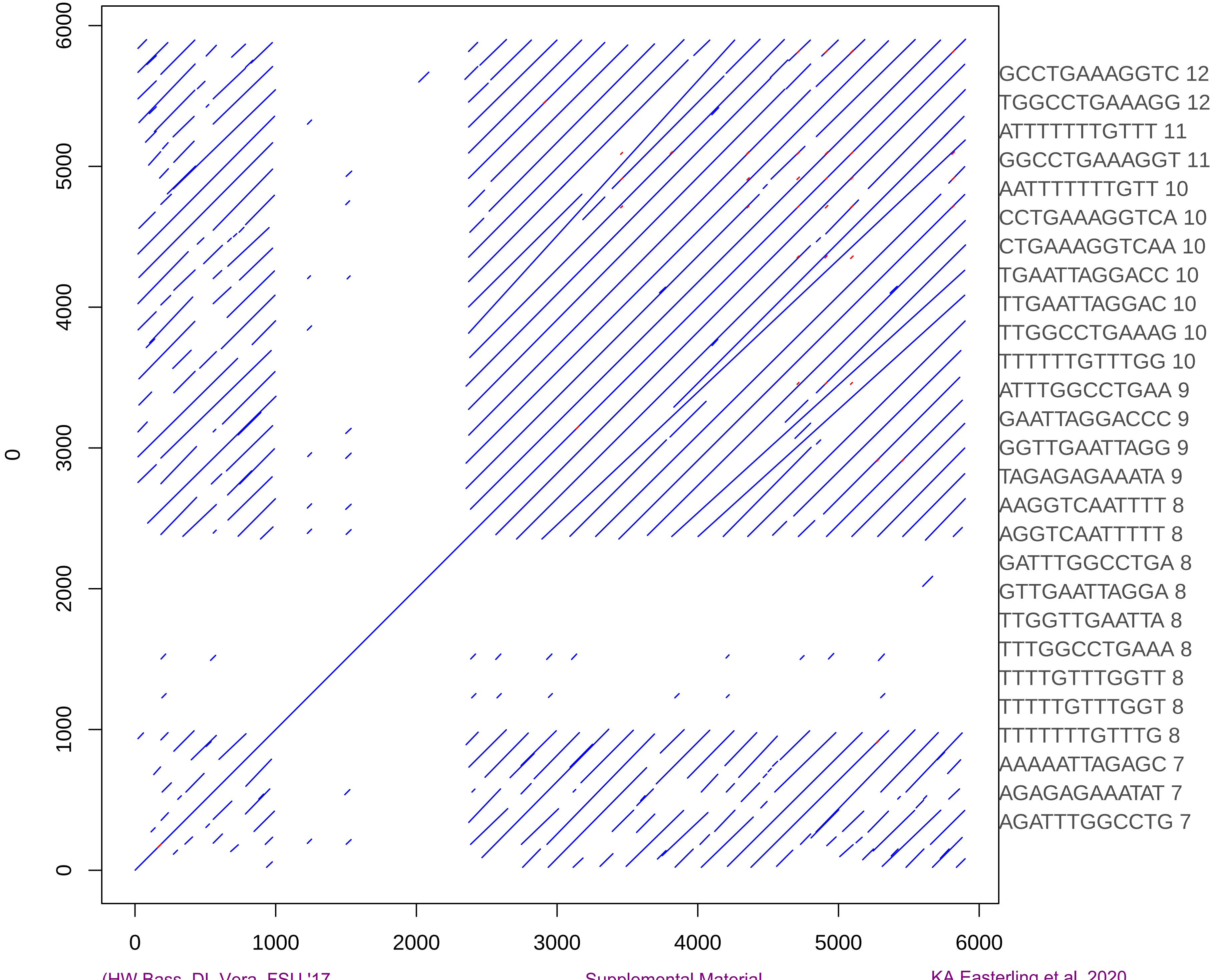

>m141230_233107_42134_c100756552550000001823146807221541_s1_p0/43114/4864_11538

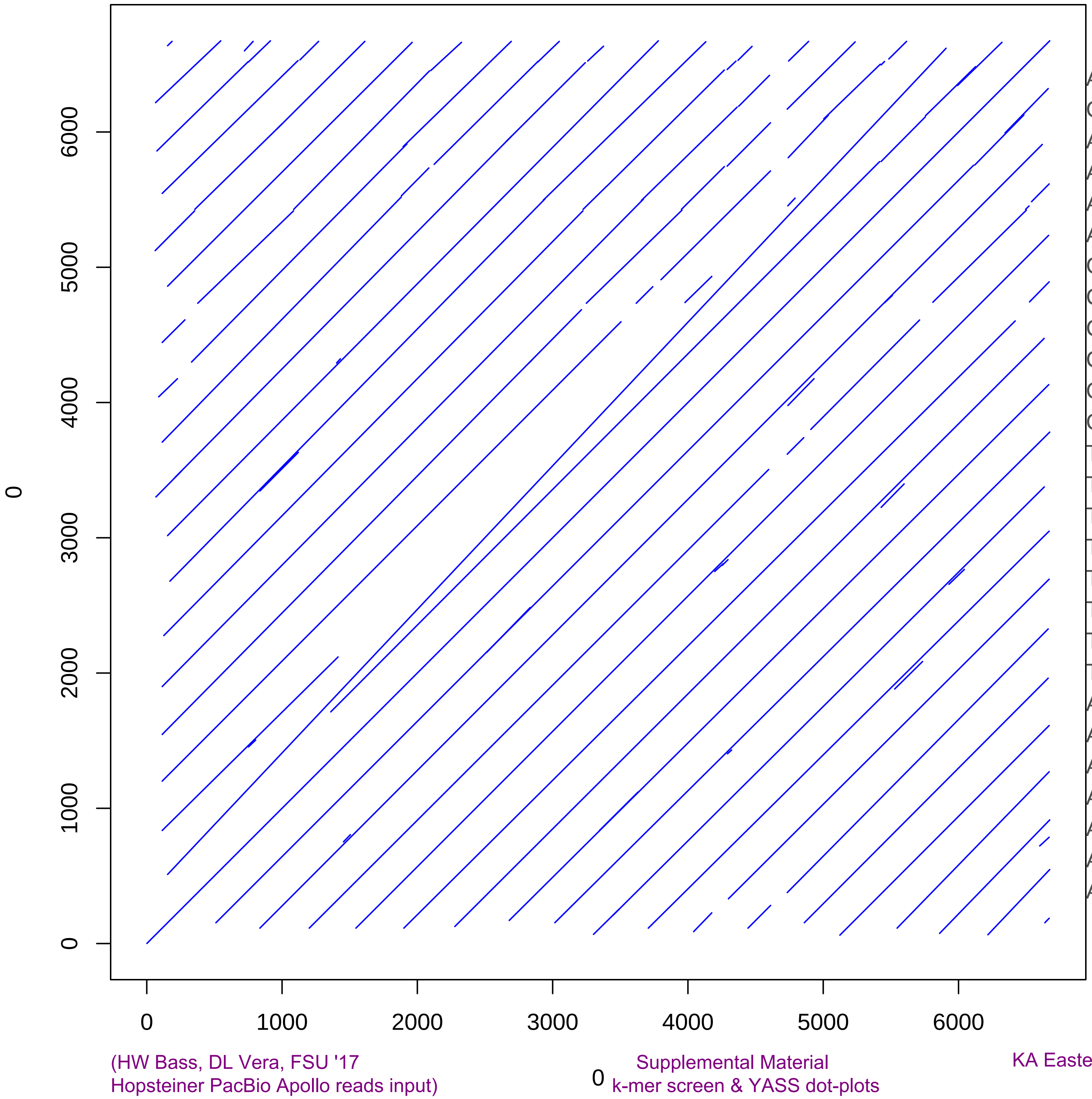

AGTCAATCCGGG 9
GTCAATCCGGGG 9
AATCTTTAAAAA 8
AGCACTAATGCA 8
AGCAGCGAATAA 8
ATACCAGCACTA 8
CAGCACTAATGC 8
CCGGGGGAGAAA 8
CCTAGTCAATCC 8
CTAGTCAATCCG 8
GCAGCGAATAAG 8
GGGAGAAAAGGG 8
TACCAGCACTAA 8
TAGTCAATCCGG 8
TCAATCCGGGGG 8
TCCGGGGGAGAA 8
TCCTAGTCAATC 8
TGCGATCATACC 8
TTTTGTTTTAAA 8
TTTTTTTTTTCC 8
AAAAACTCATTT 7
AAAAGTTTGTAT 7
AAAGTTTGTATT 7
AAGTTTGTATTT 7
ACCAGCACTAAT 7
ACTAATGCACCG 7
AGTTTGTATTTT 7

>m141230_233107_42134_c100756552550000001823146807221541_s1_p0/43771/451_8936

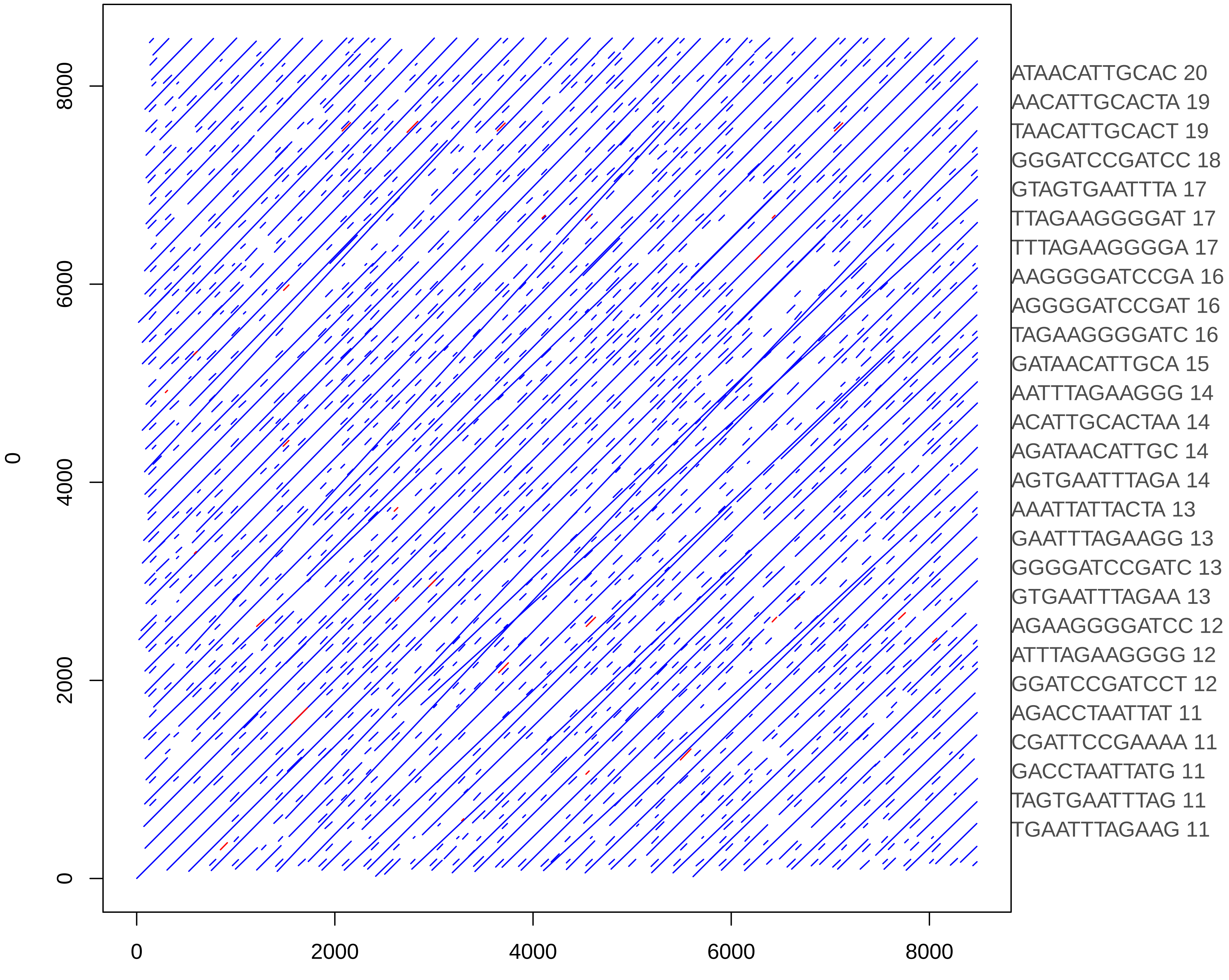

>m141230_233107_42134_c100756552550000001823146807221541_s1_p0/44359/6227_15339

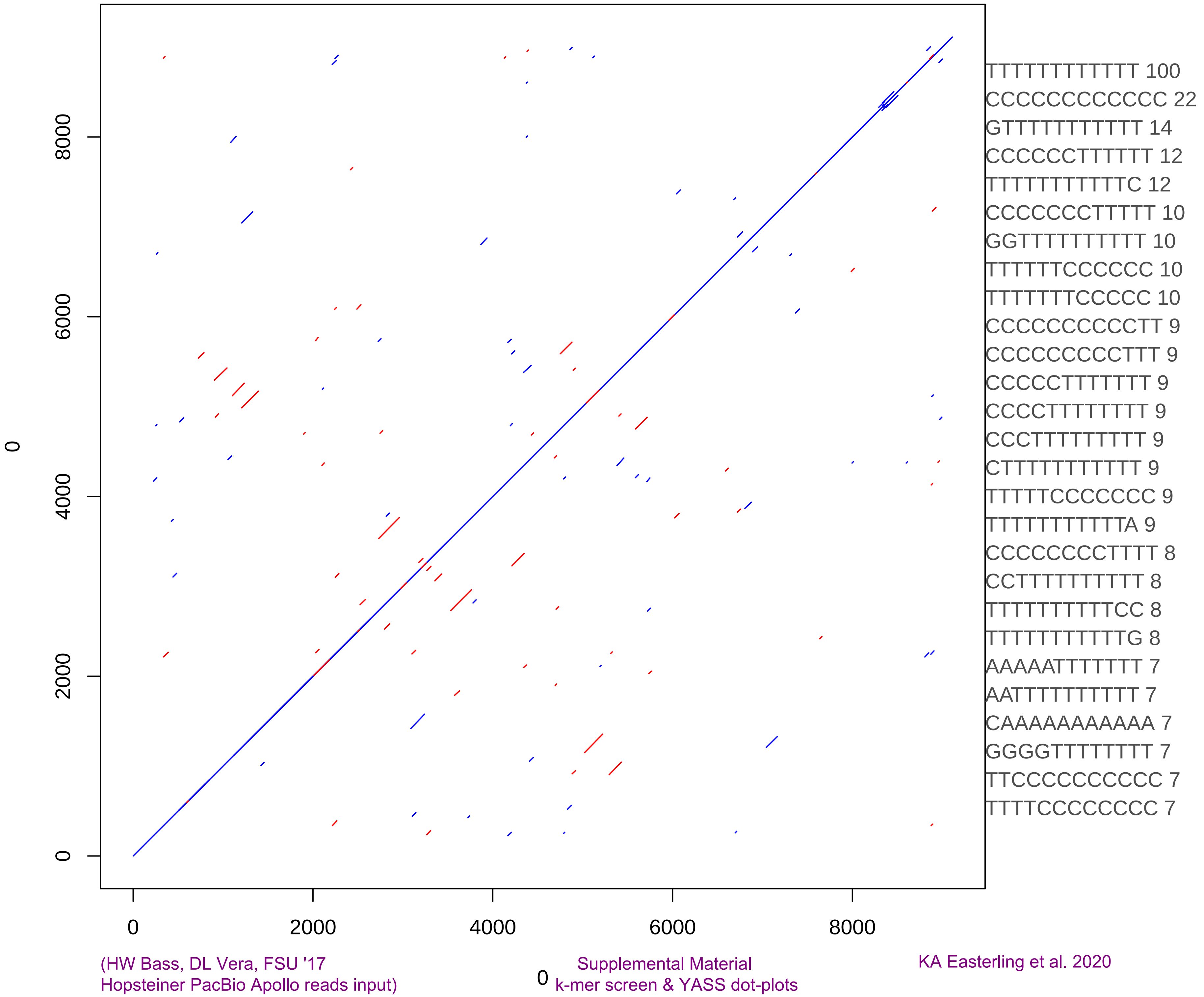

>m141230_233107_42134_c100756552550000001823146807221541_s1_p0/45060/474_6782

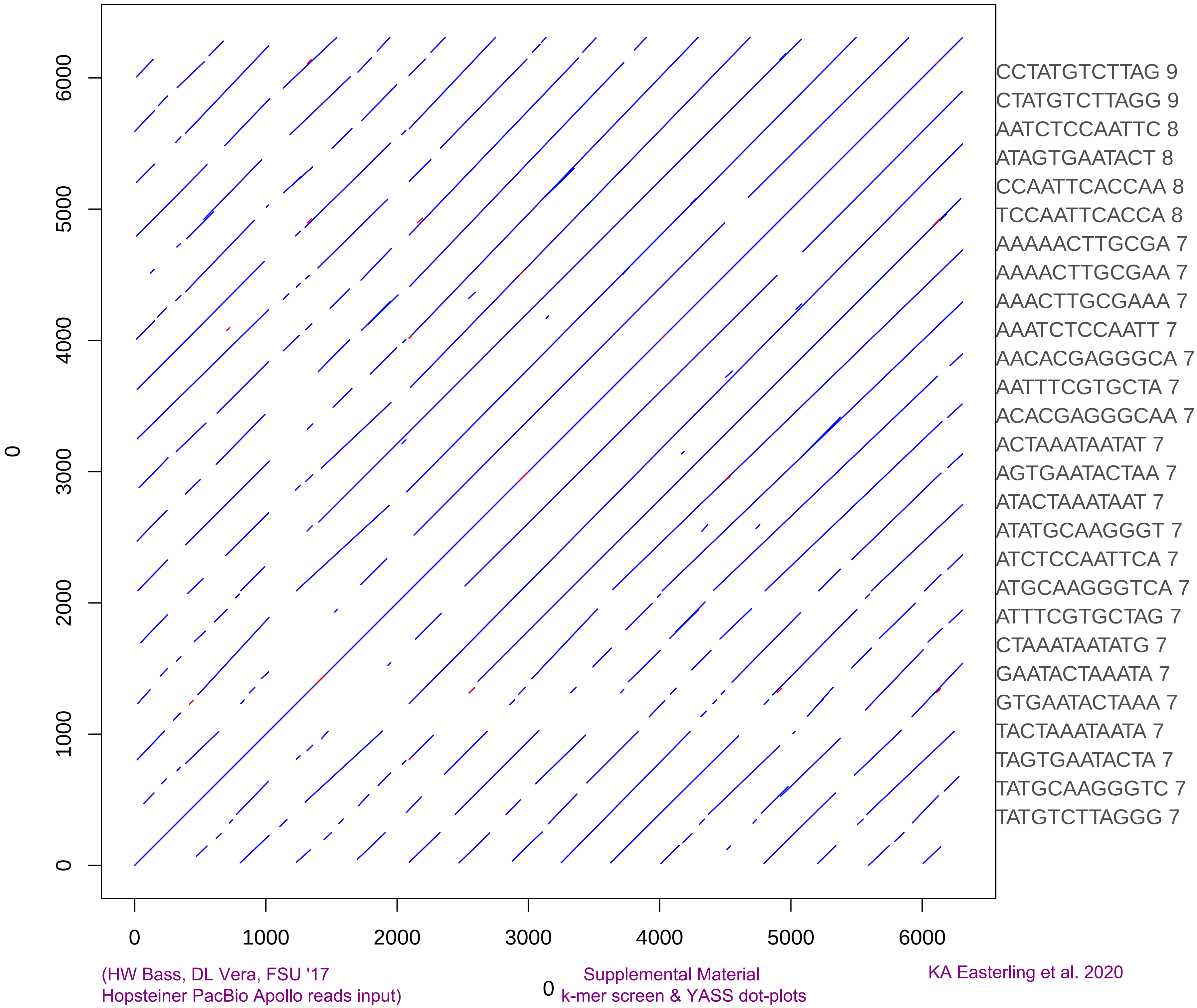

>m141230_233107_42134_c100756552550000001823146807221541_s1_p0/4581/0_6299

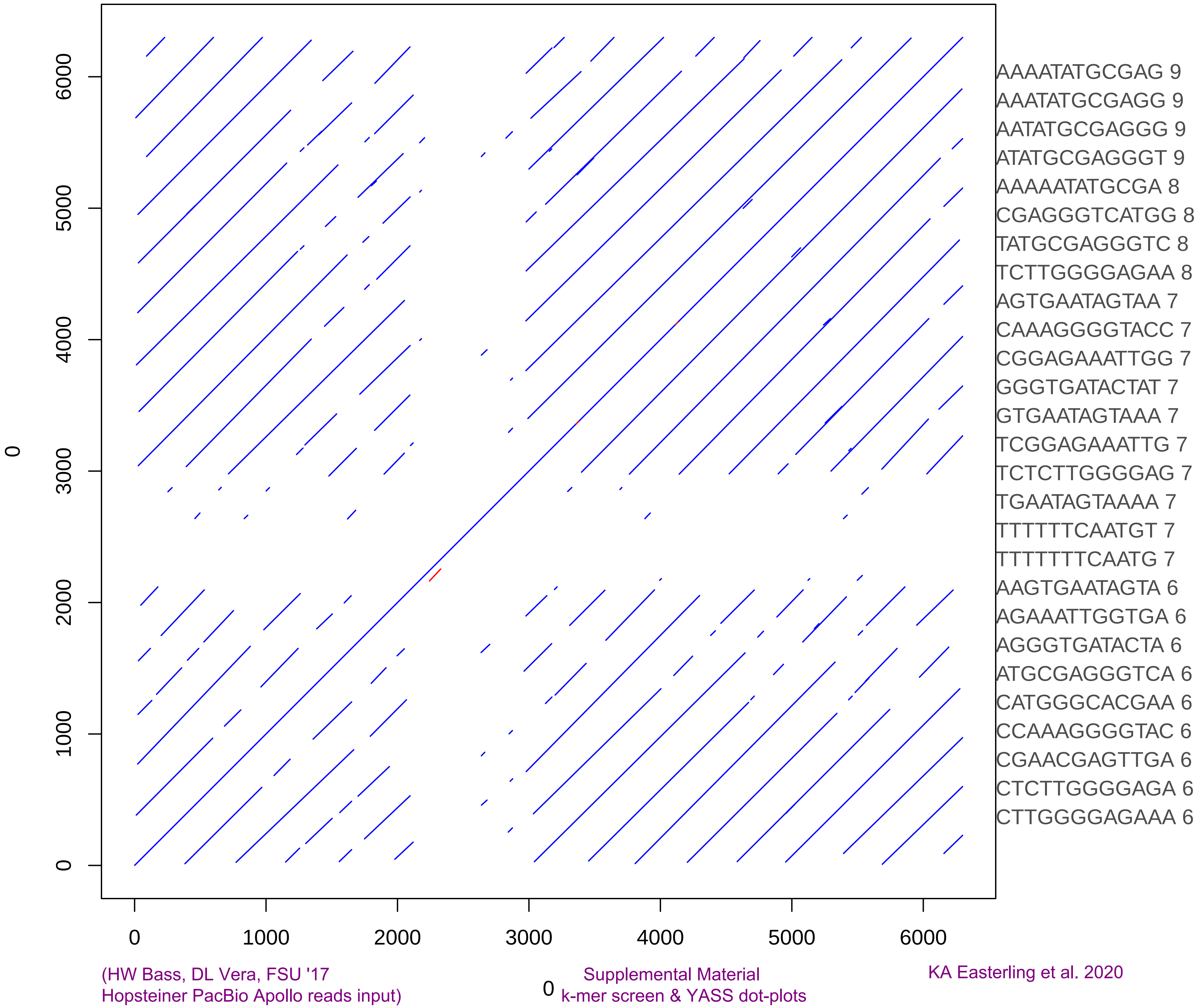

>m141230_233107_42134_c100756552550000001823146807221541_s1_p0/45942/365_5609

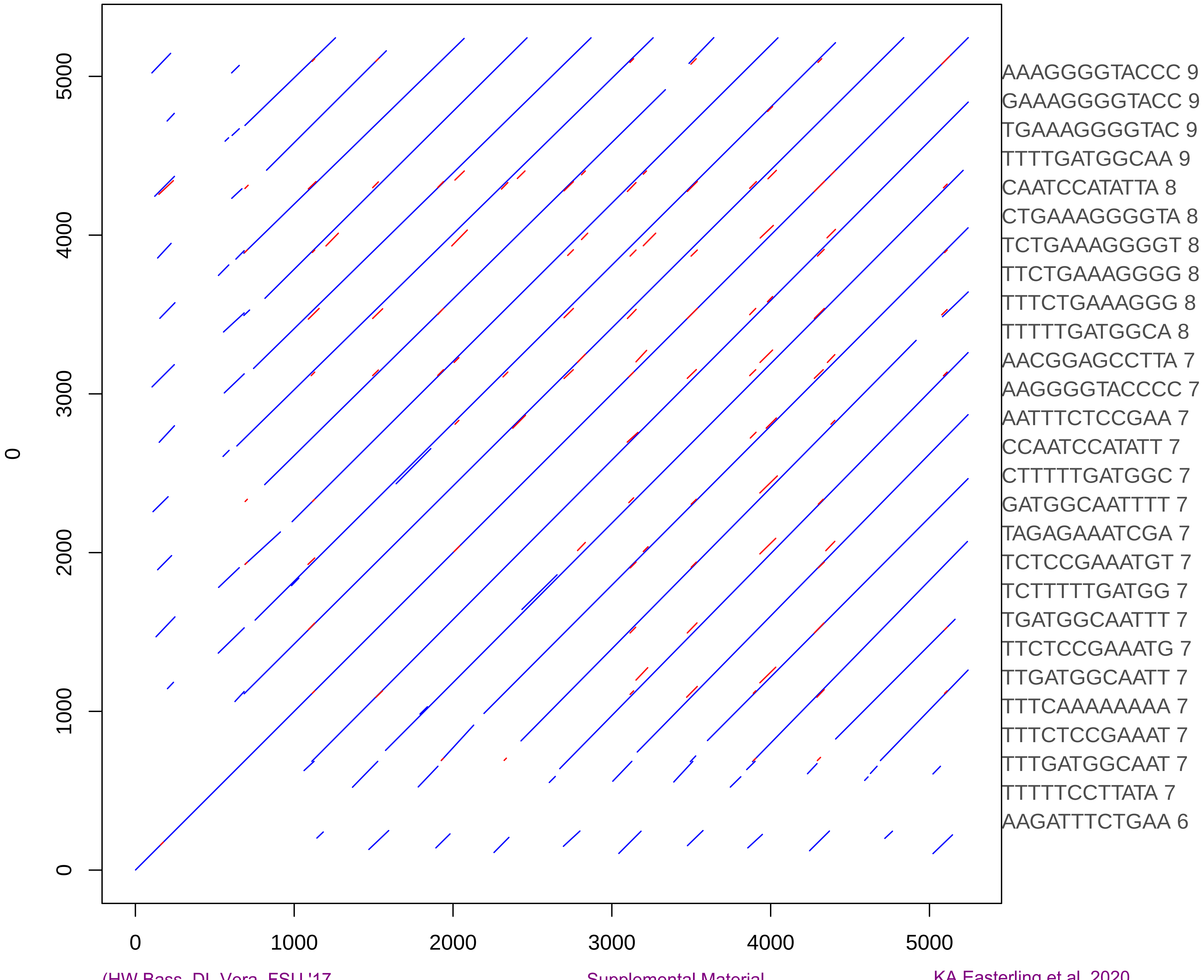

>m141230_233107_42134_c100756552550000001823146807221541_s1_p0/47360/3135_10378

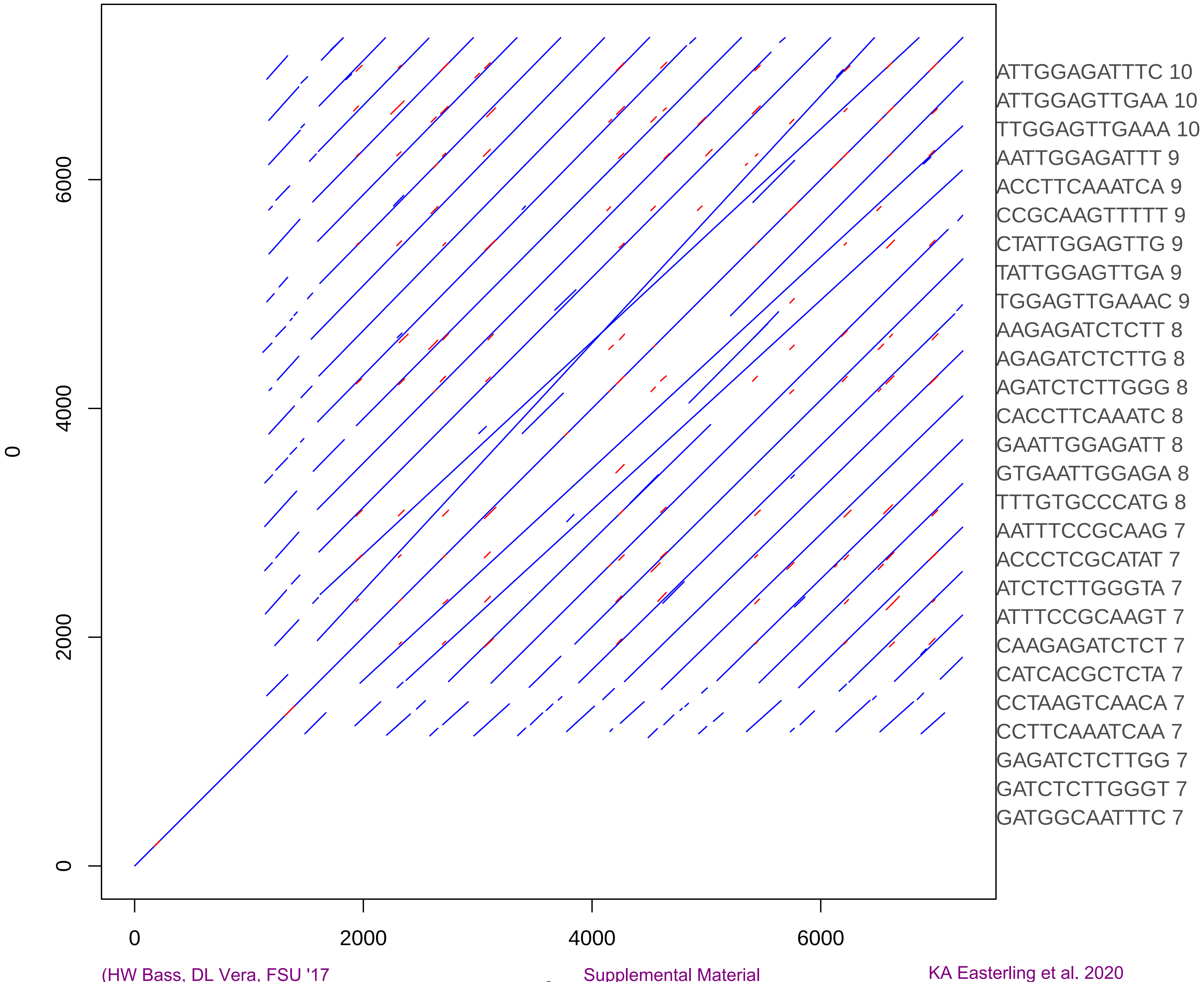

>m141230_233107_42134_c100756552550000001823146807221541_s1_p0/47683/4558_15459

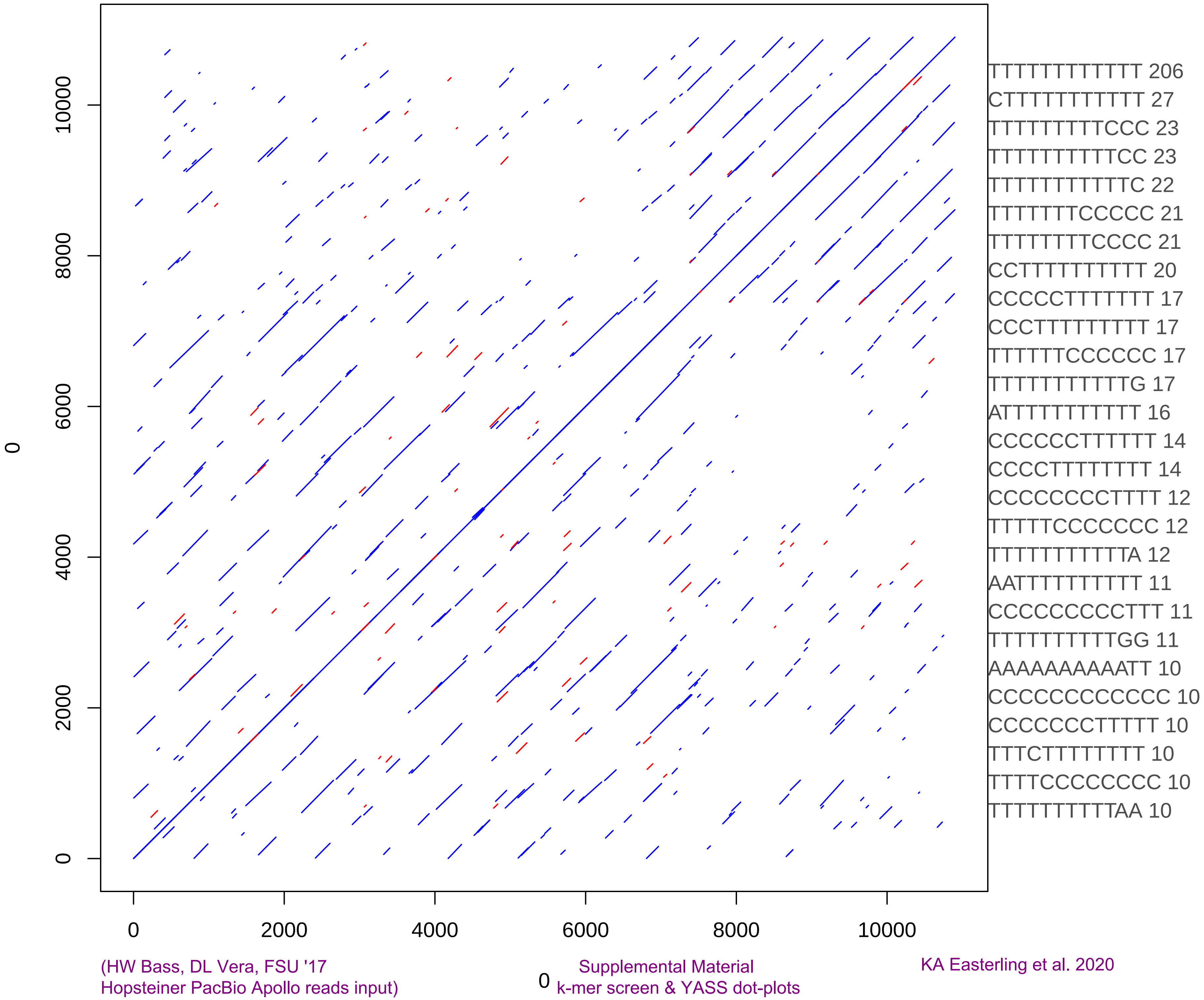

>m141230_233107_42134_c100756552550000001823146807221541_s1_p0/48312/233_7619

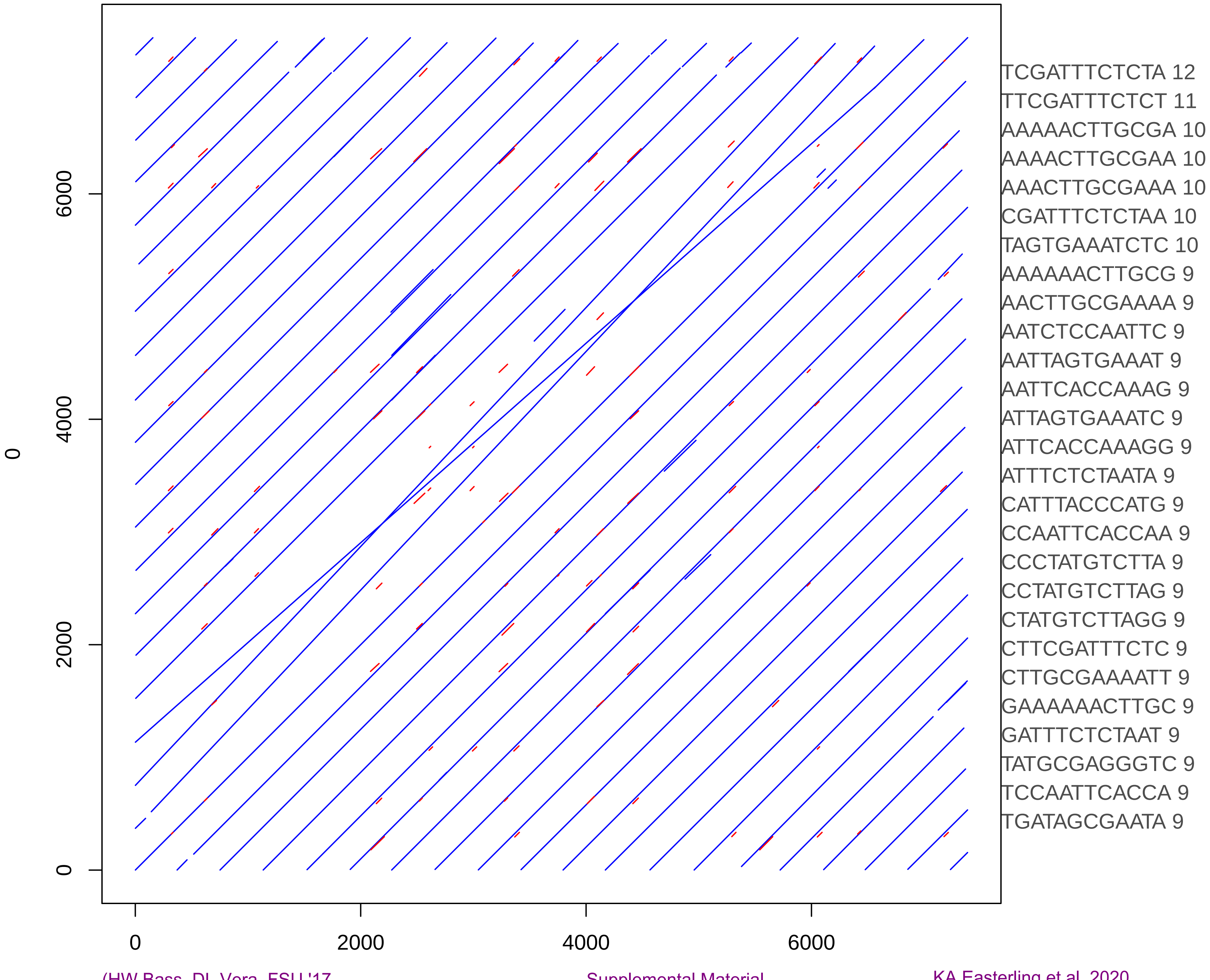

>m141230_233107_42134_c100756552550000001823146807221541_s1_p0/49150/847_5947

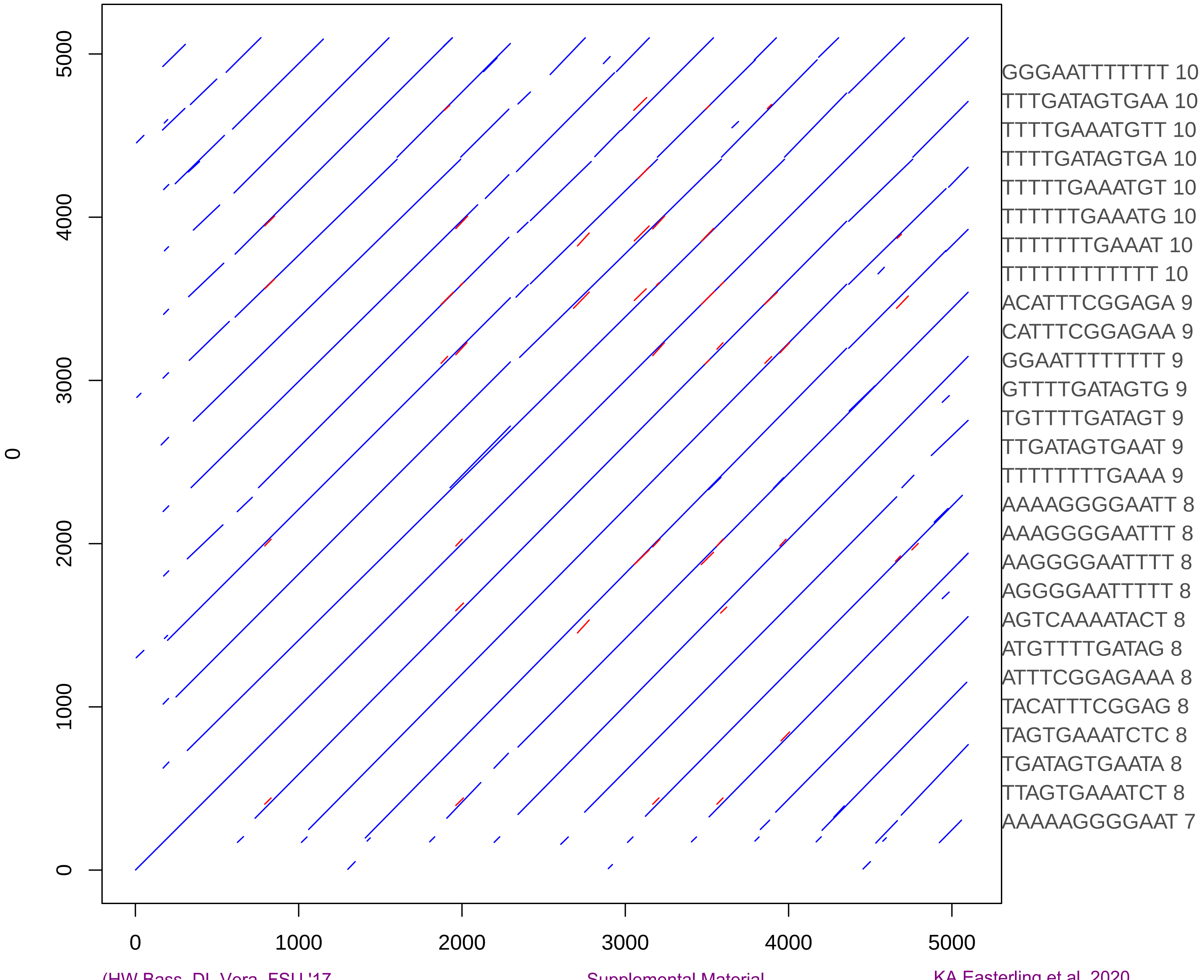

>m141230_233107_42134_c100756552550000001823146807221541_s1_p0/53127/3656_12730

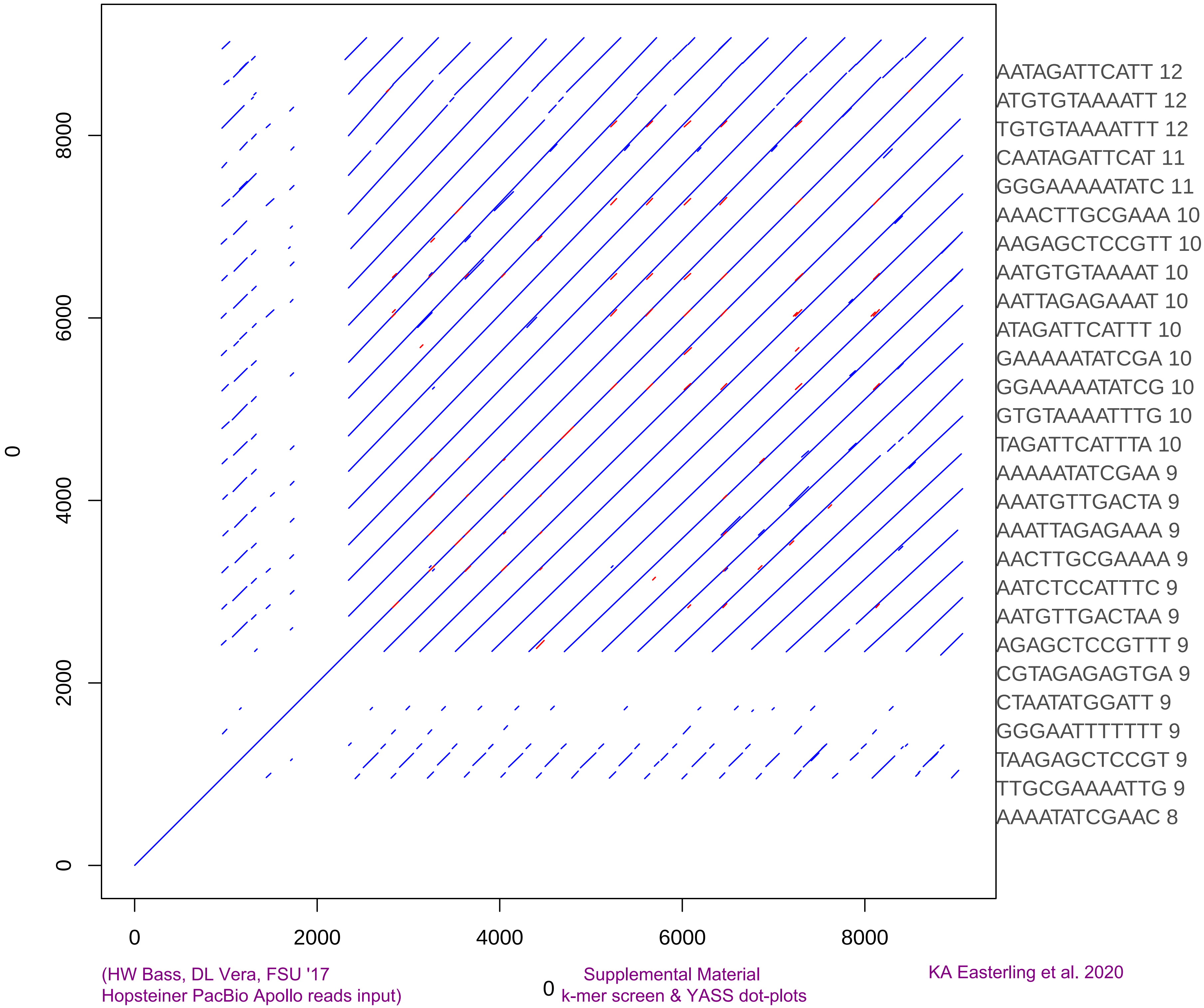

>m141230_233107_42134_c100756552550000001823146807221541_s1_p0/55371/7855_13808

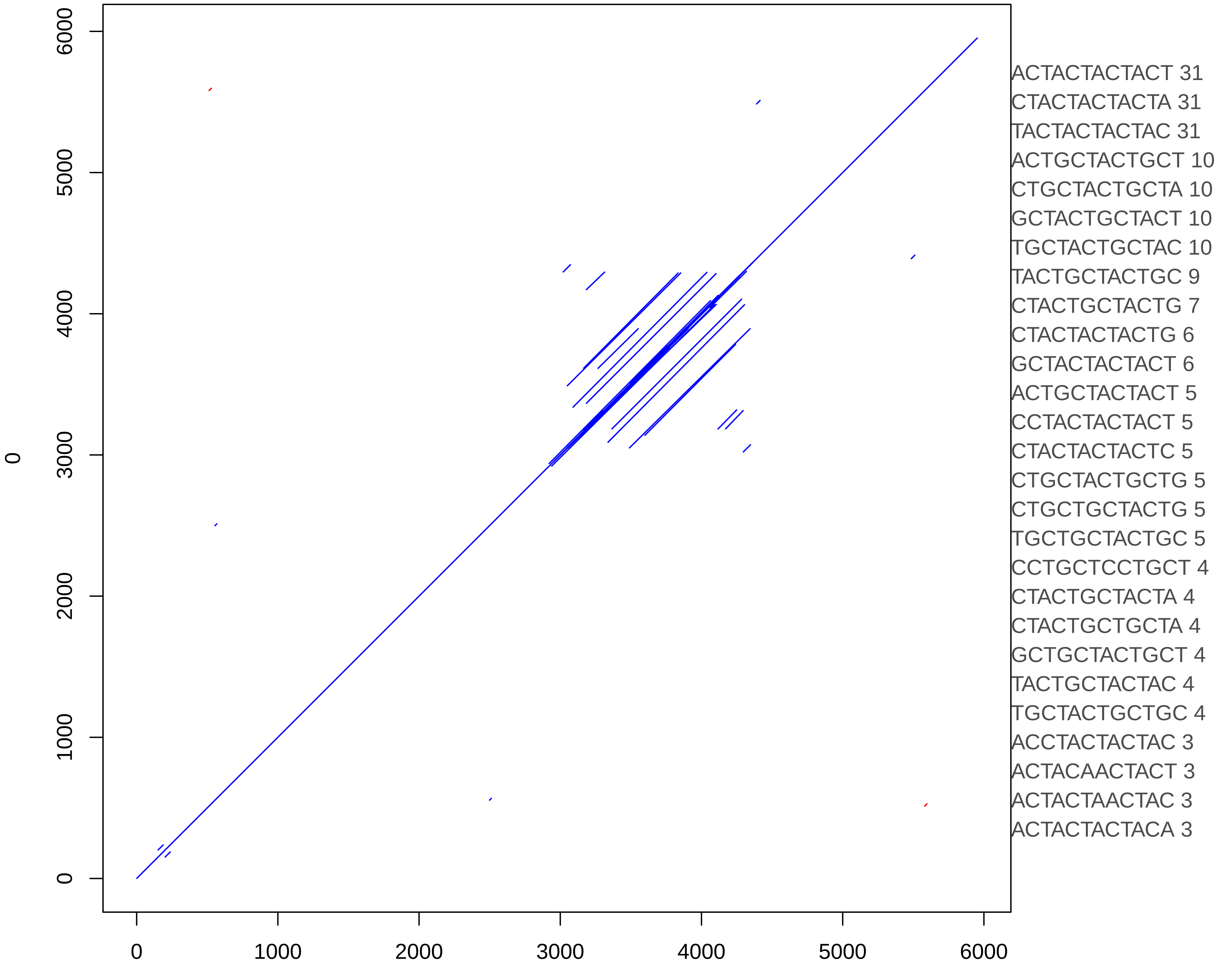

>m141230_233107_42134_c100756552550000001823146807221541_s1_p0/58330/571_7037

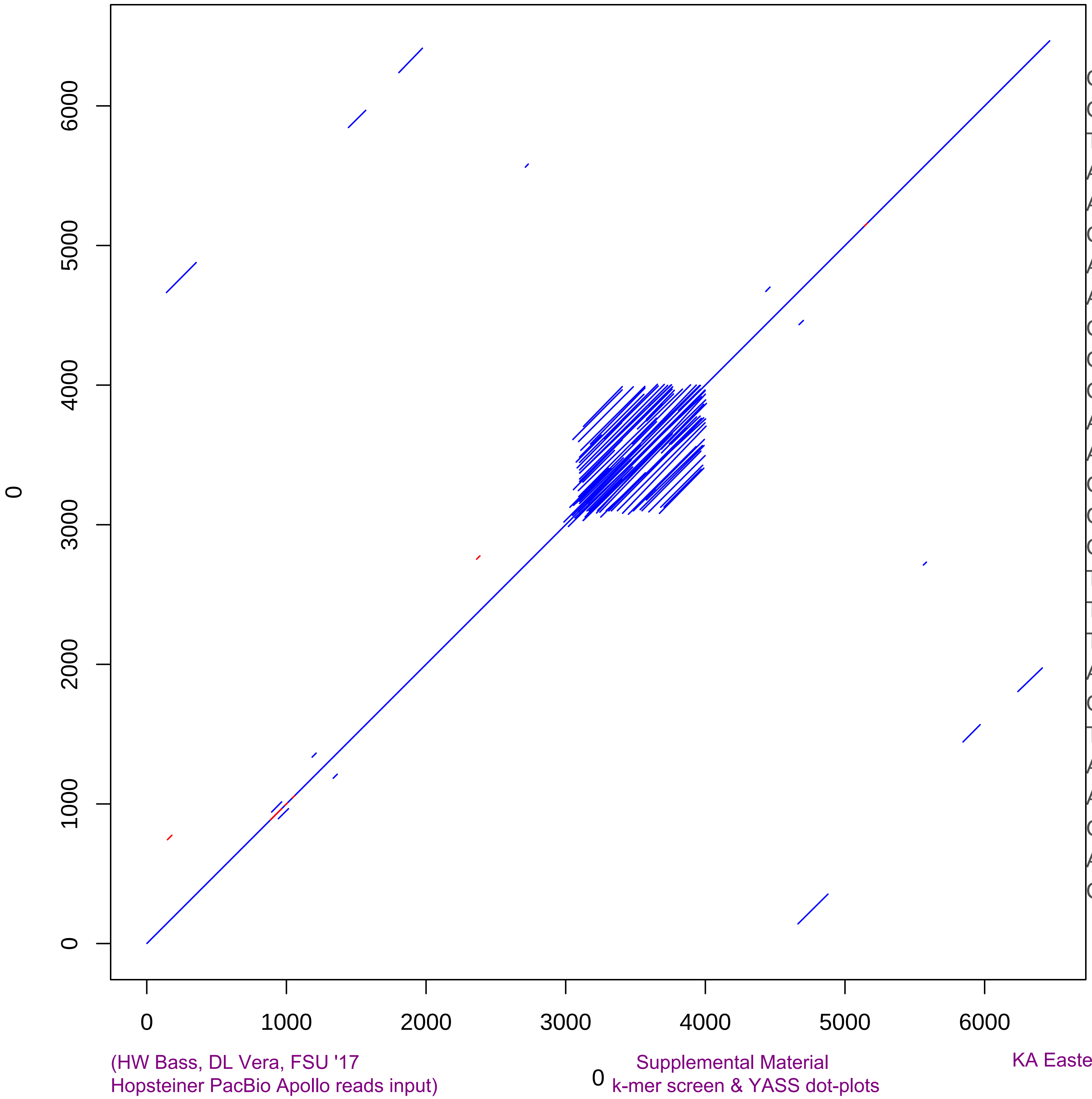

GTAGTAGTAGTA 10
GCAGTAGTAGTA 9
TAGTAGTAGTAG 9
AGTAGTAGTAGC 8
AGTAGTAGTAGT 8
CAGTAGTAGTAG 8
AGCAGTAGTAGT 7
AGTAGCAGTAGC 7
GCAGCAGTAGTA 7
GTAGCAGTAGCA 7
GTAGTAGTAGCA 7
AGCAGTAGCAGT 6
AGTAGTAGCAGT 6
CAGTAGCAGTAG 6
GTAGCAGTAGTA 6
GTAGTAGCAGTA 6
TAGCAGTAGCAG 6
TAGTAGCAGTAG 6
TAGTAGTAGCAG 6
AGCAGCAGTAGT 5
GCAGTAGCAGTA 5
TAGCAGTAGTAG 5
AGCAGTAGTAGC 4
AGTAGCAGTAGT 4
GAGTAGTAGTAG 4
AGTAGTAGTAGG 3
CAGTAGTAGCAG 3

>m141230_233107_42134_c100756552550000001823146807221541_s1_p0/59928/0_8243

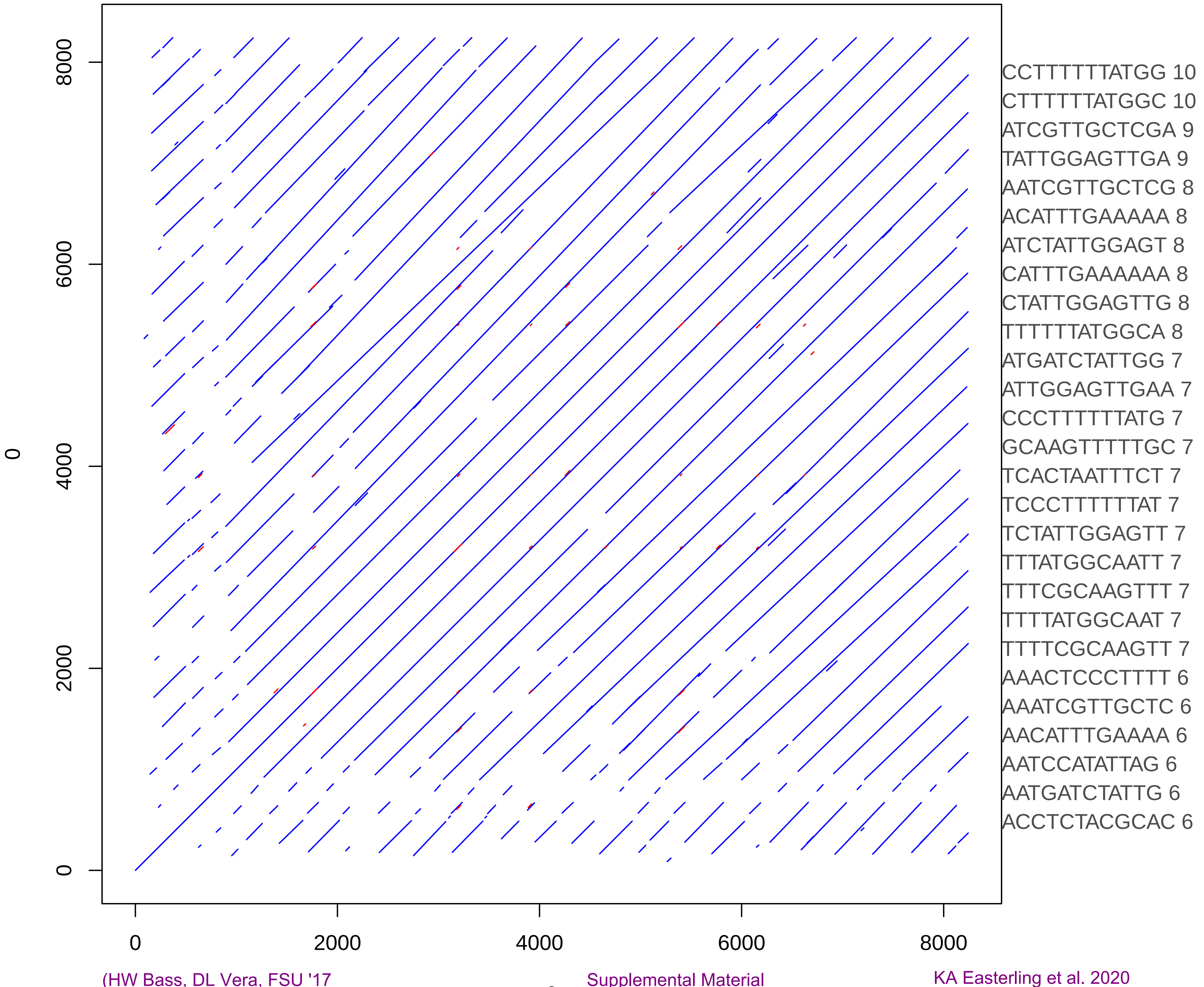

>m141230_233107_42134_c100756552550000001823146807221541_s1_p0/60800/0_10197

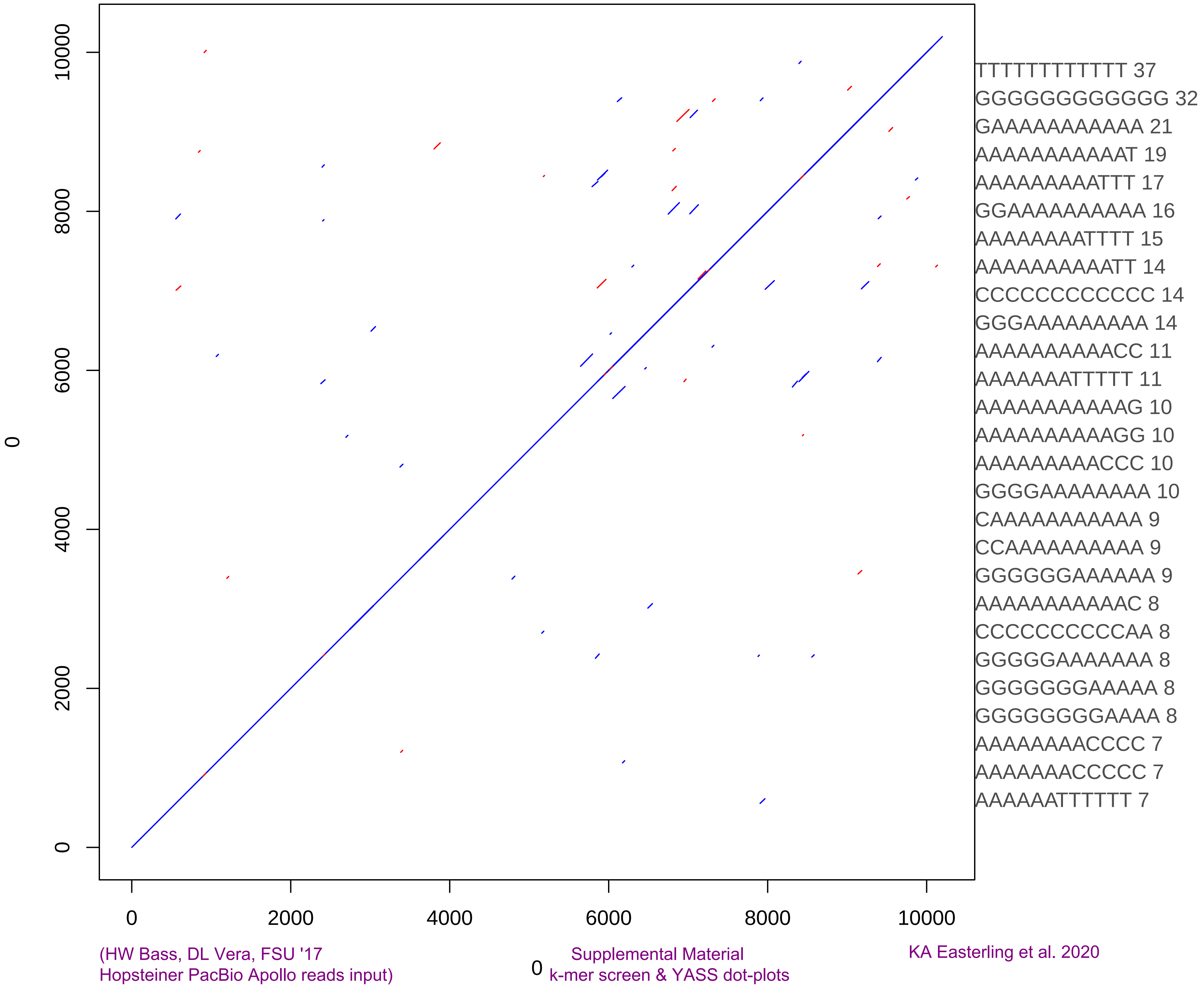

>m141230_233107_42134_c100756552550000001823146807221541_s1_p0/62243/7284_16724

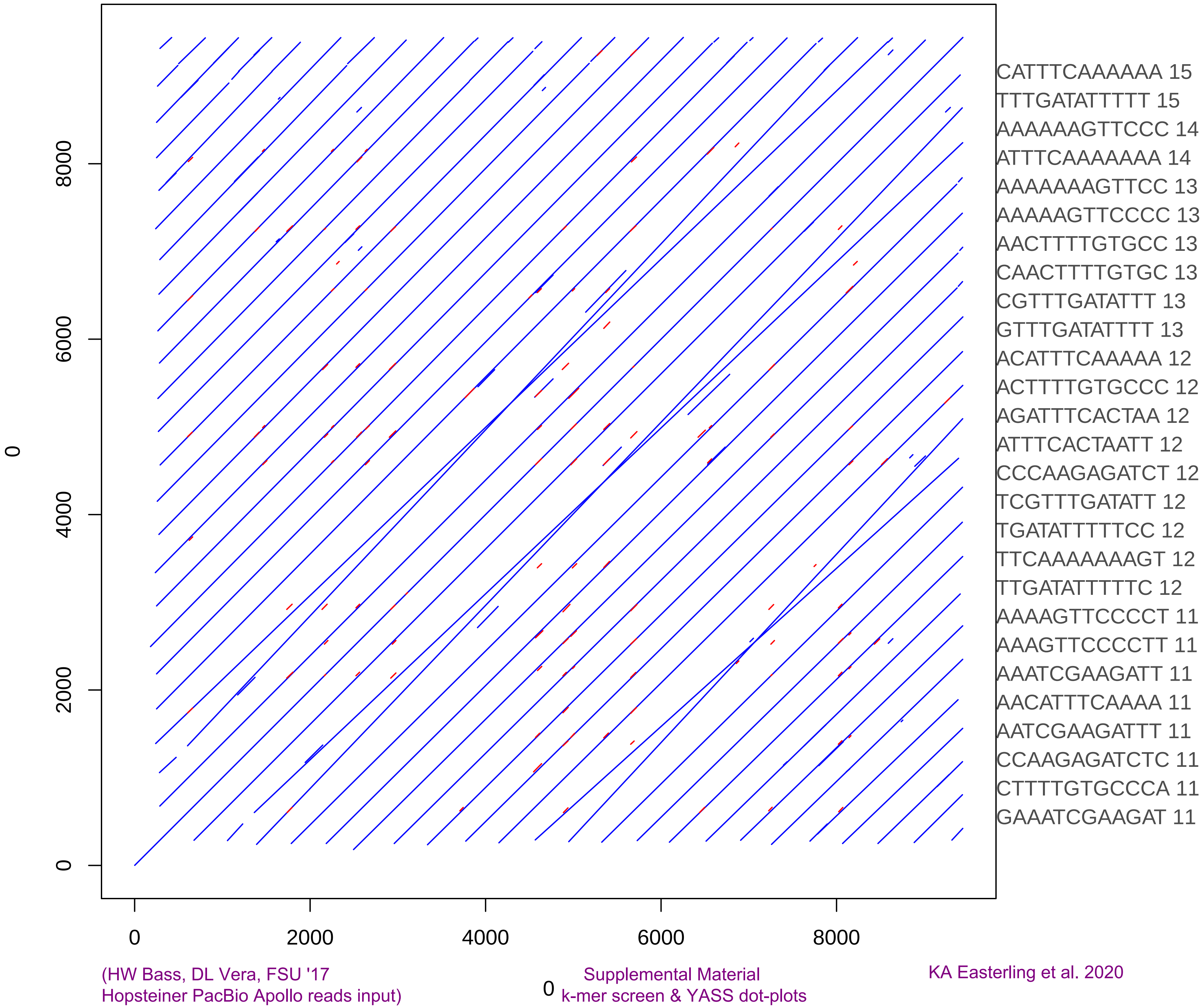

>m141230_233107_42134_c100756552550000001823146807221541_s1_p0/67295/1172_13482

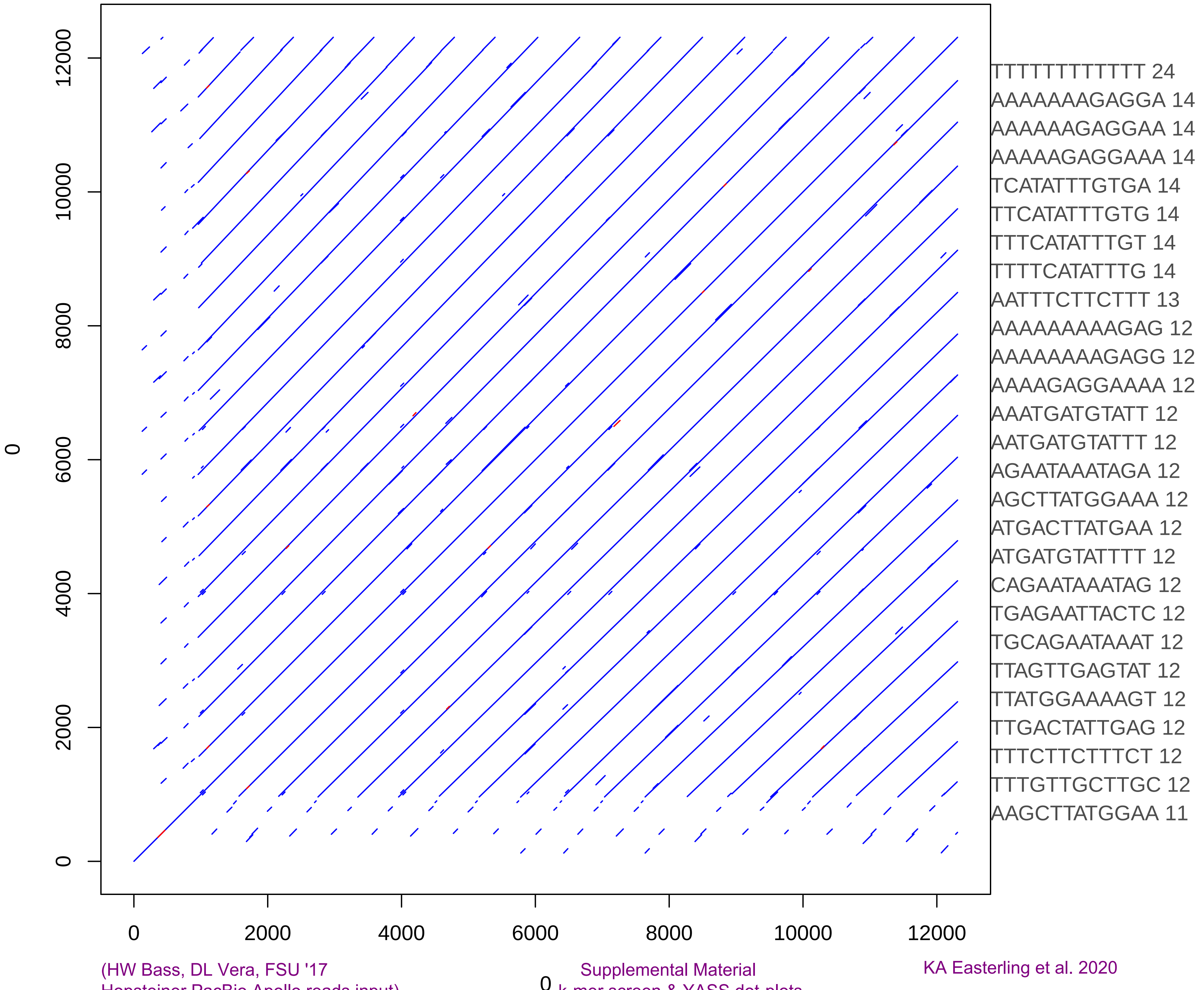

>m141230_233107_42134_c100756552550000001823146807221541_s1_p0/67314/0_5079

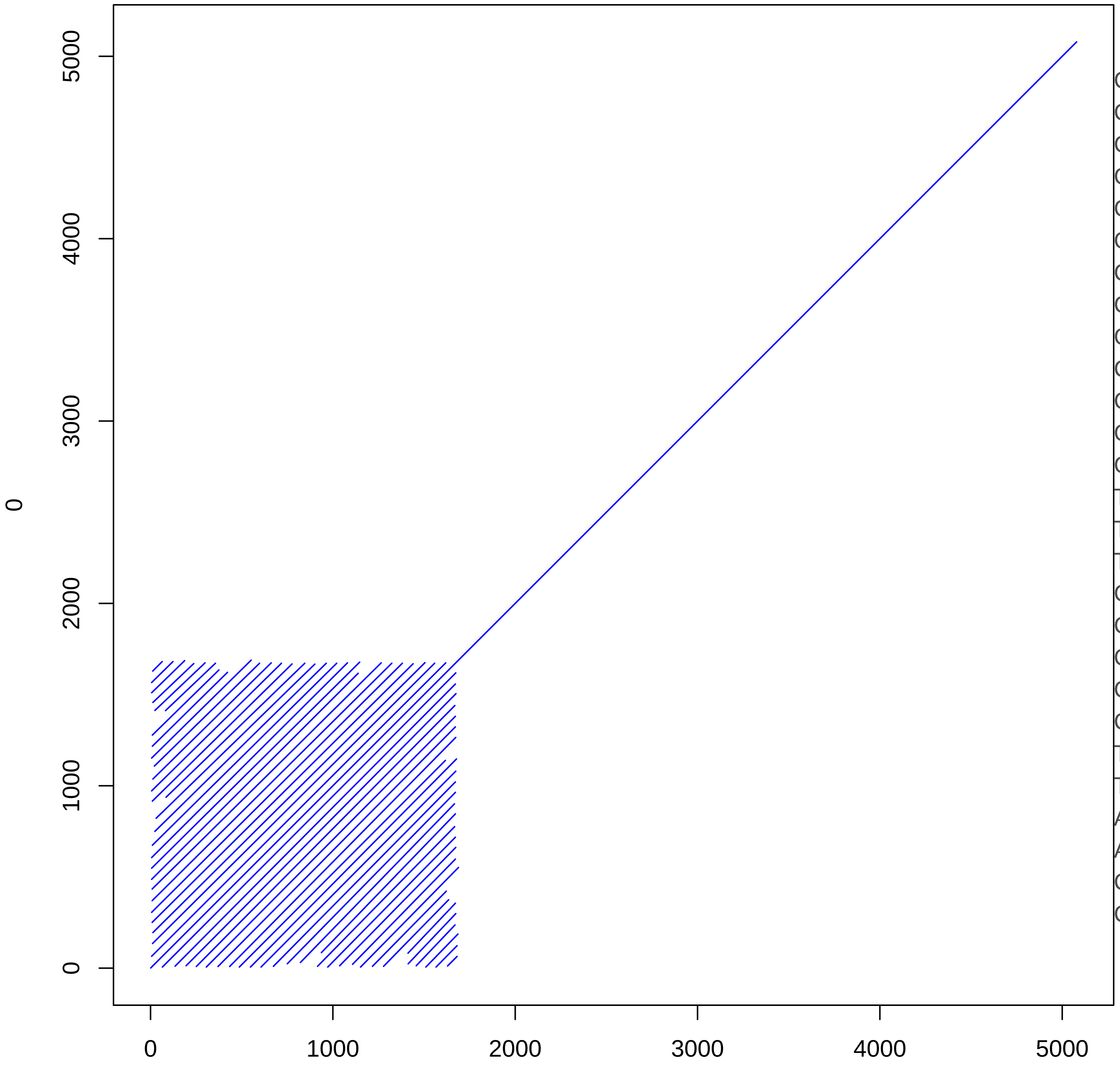

CTTGGGCTCGGT 12
CCGGACATCGTT 11
CCTTGGGCTCGG 10
GACCGGCCTTGG 10
GCCTTGGGCTCG 10
GGACCGGCCTTG 10
CCGGCCTTGGGC 9
CGGACATCGTTC 9
GGGCTCGGTCCT 9
CCCGGACATCGT 8
GACATCGTTCGT 8
GGACATCGTTCG 8
GGCTCGGTCCTC 8
TGGGCTCGGTCC 8
TTCCCTTTATTT 8
TTGGGCTCGGTC 8
CCCTTTATTTAT 7
CGGCCTTGGGCT 7
GCTCGGTCCTCT 7
GGCCTTGGGCTC 7
GGGACCGGCCTT 7
TCCCTTTATTTA 7
TGGGACCGGCCT 7
ACATCGTTCGTC 6
ACCGGCCTTGGG 6
CCTTTATTTATA 6
CGGTTCCCTTTA 6

>m141230_233107_42134_c100756552550000001823146807221541_s1_p0/67581/0_6133

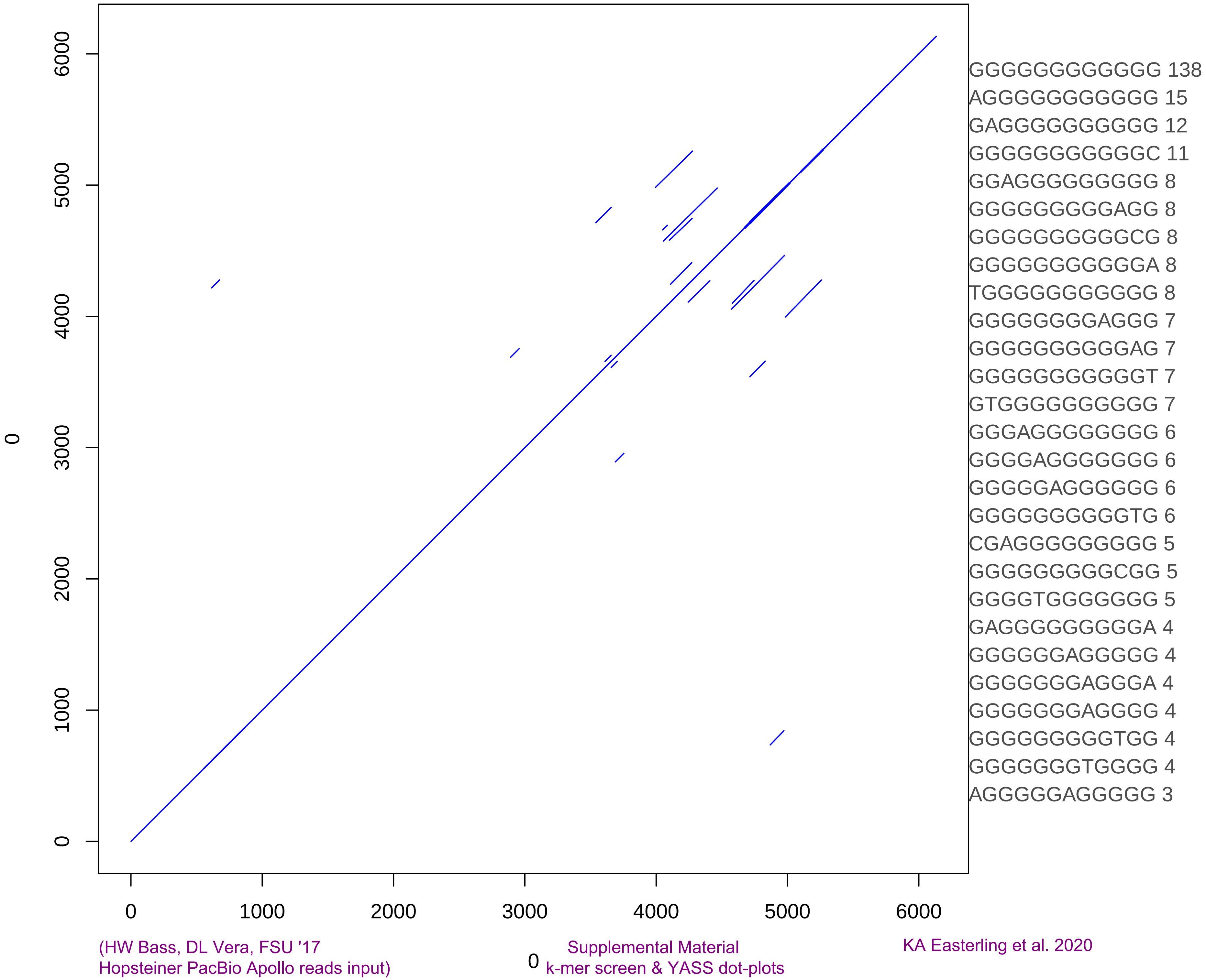

>m141230_233107_42134_c100756552550000001823146807221541_s1_p0/69014/2558_10496

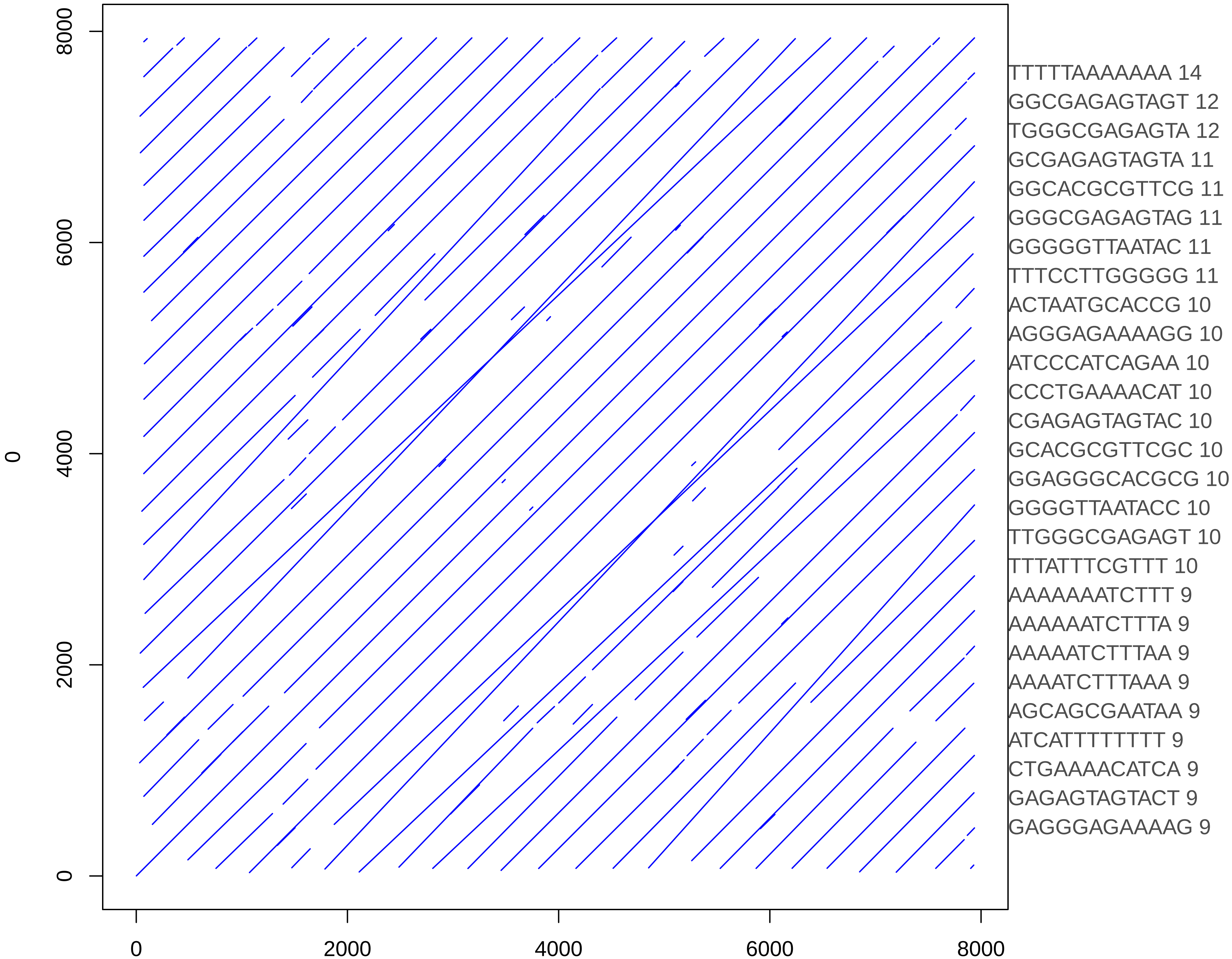

0

>m141230_233107_42134_c100756552550000001823146807221541_s1_p0/70476/0_10196

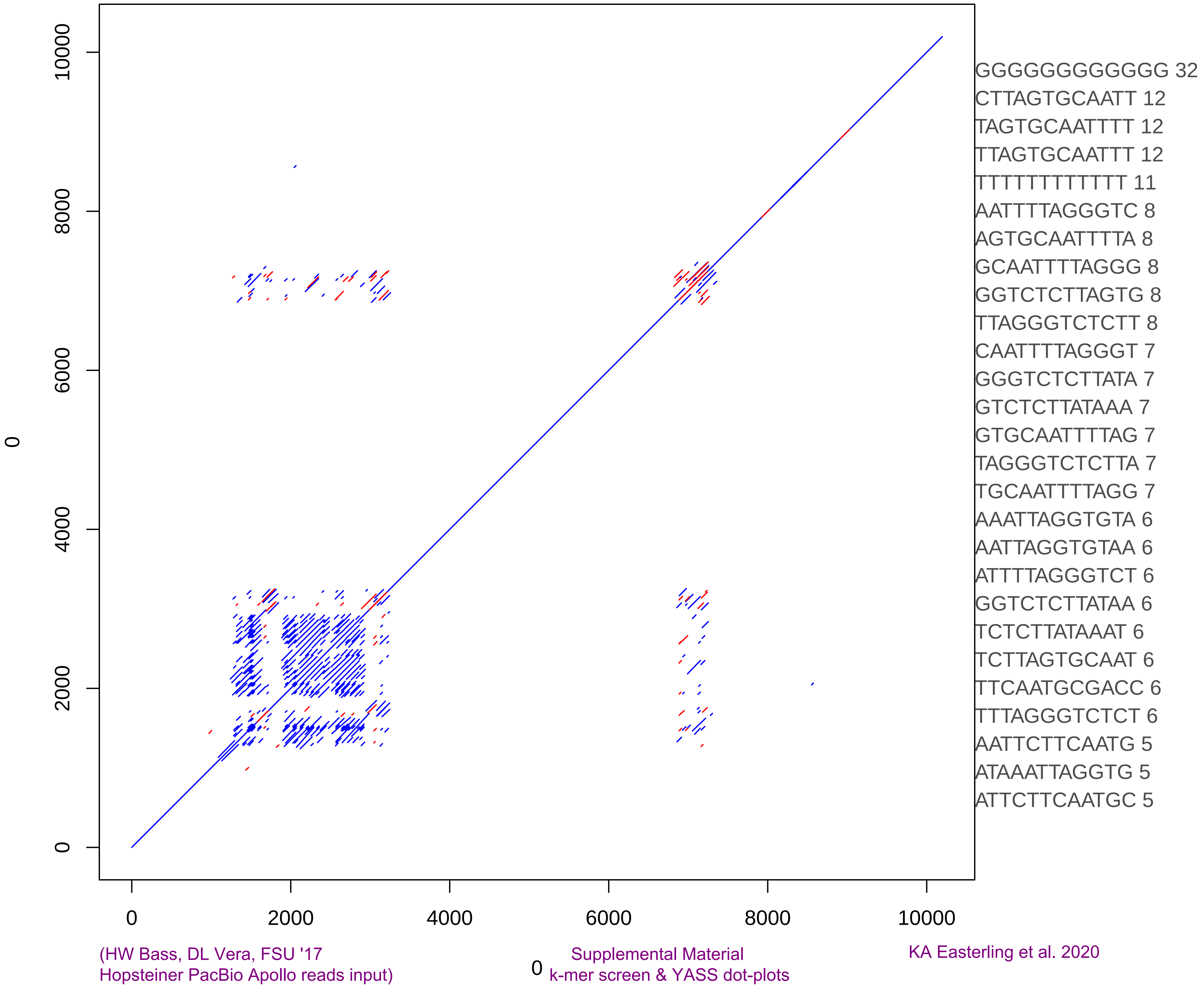

>m141230_233107_42134_c100756552550000001823146807221541_s1_p0/74407/277_9954

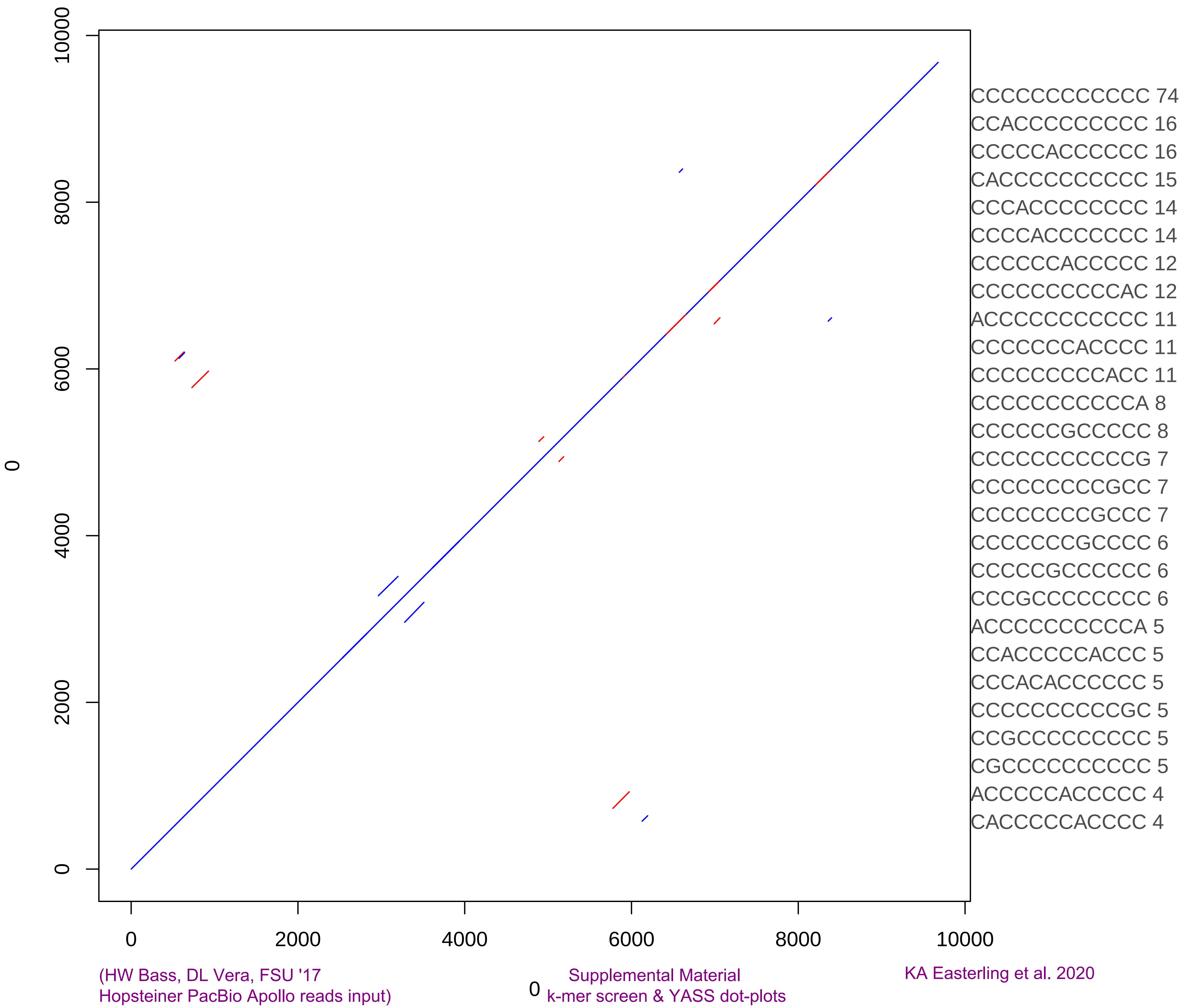

>m141230_233107_42134_c100756552550000001823146807221541_s1_p0/76115/0_11627

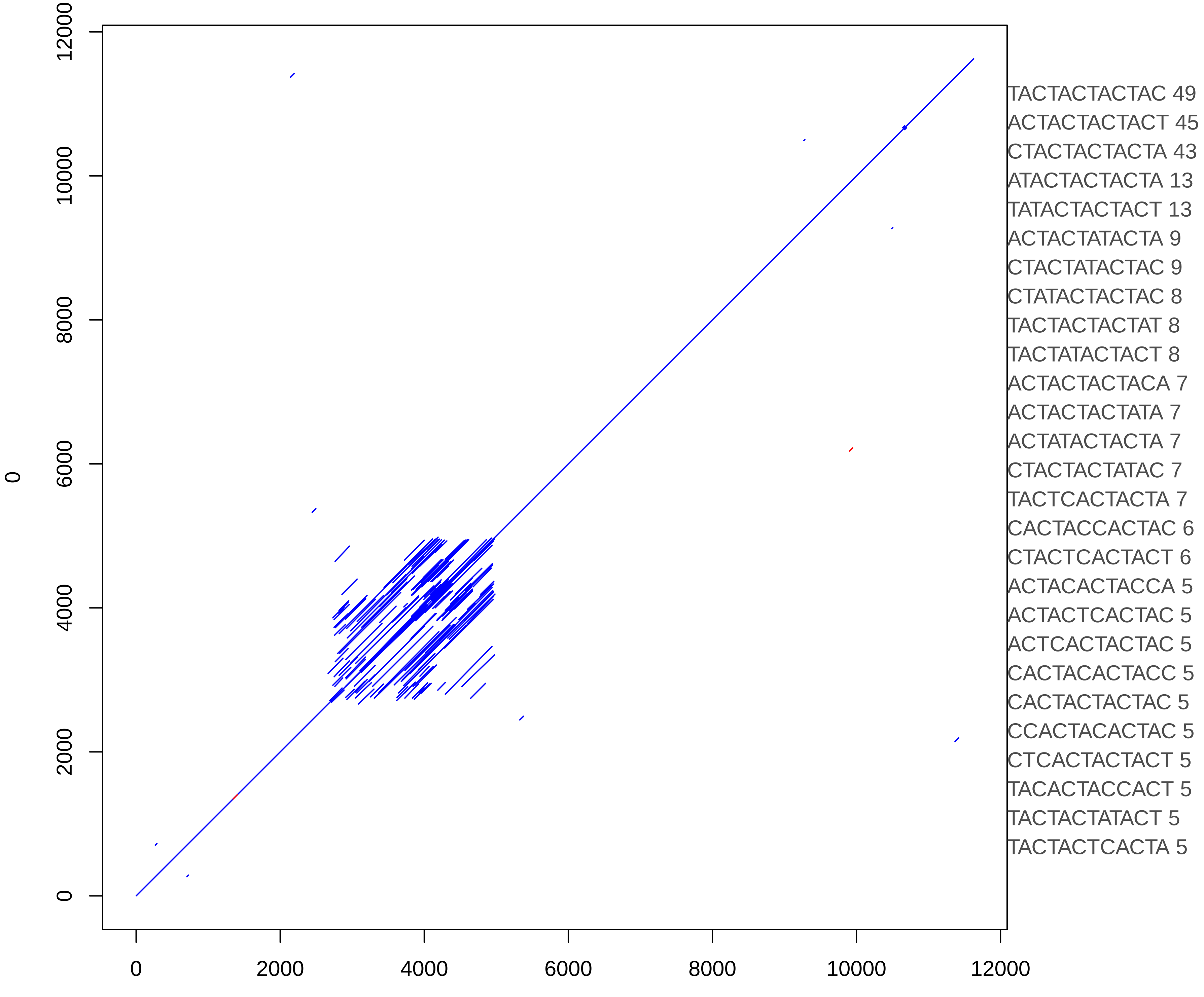

>m141230_233107_42134_c100756552550000001823146807221541_s1_p0/78757/2251_12150

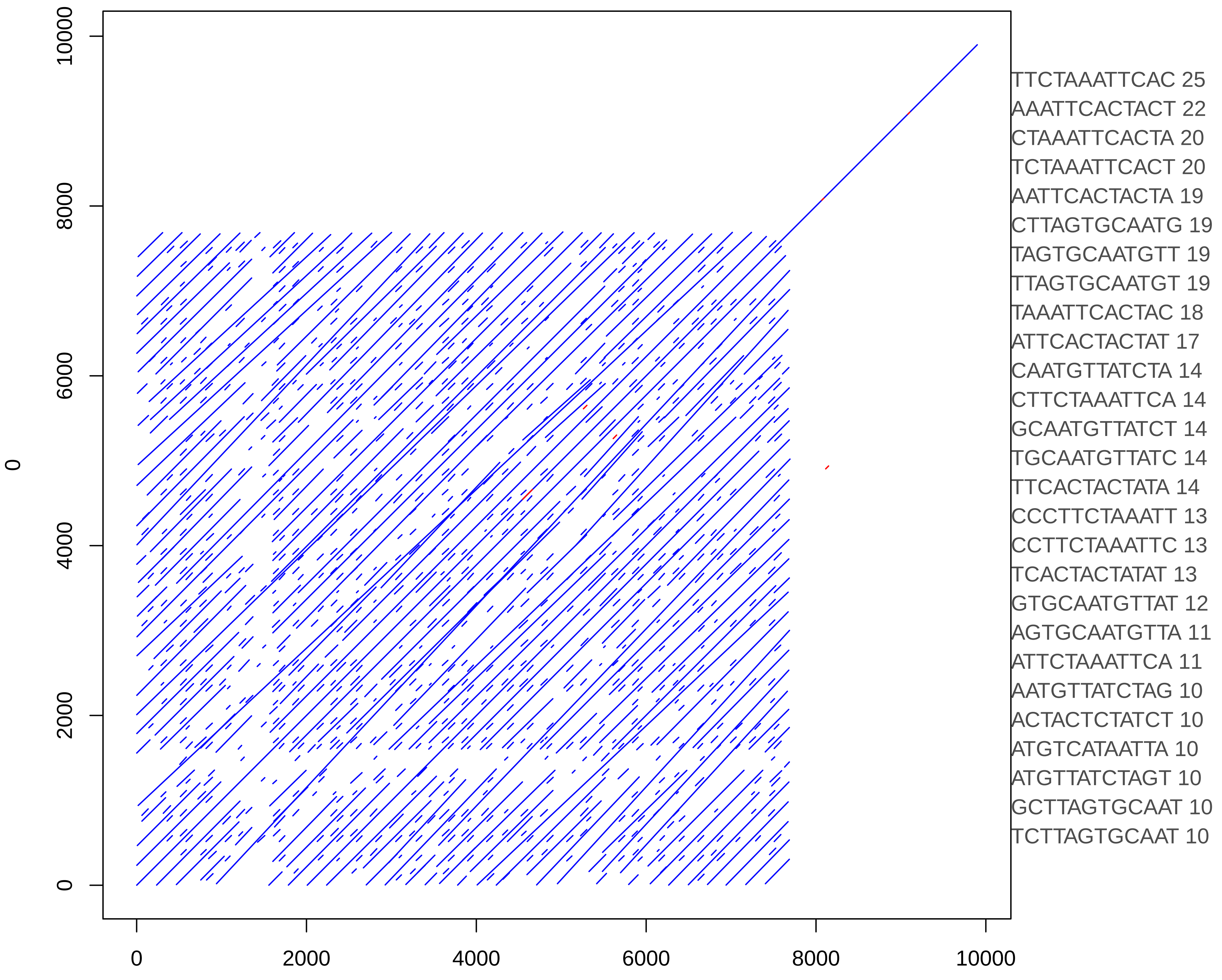

>m141230_233107_42134_c100756552550000001823146807221541_s1_p0/80432/990_6659

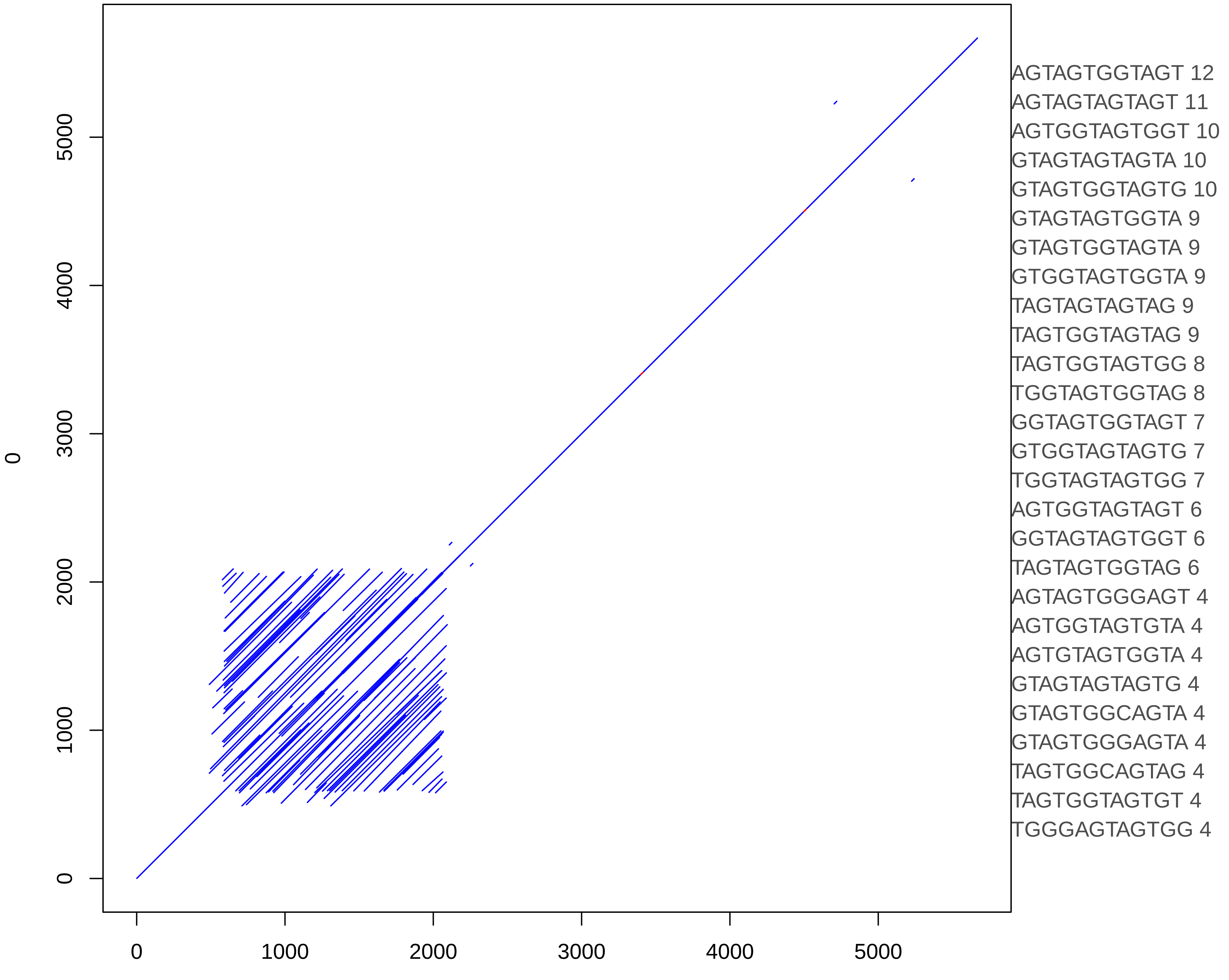

>m141230_233107_42134_c100756552550000001823146807221541_s1_p0/8124/4029_12612

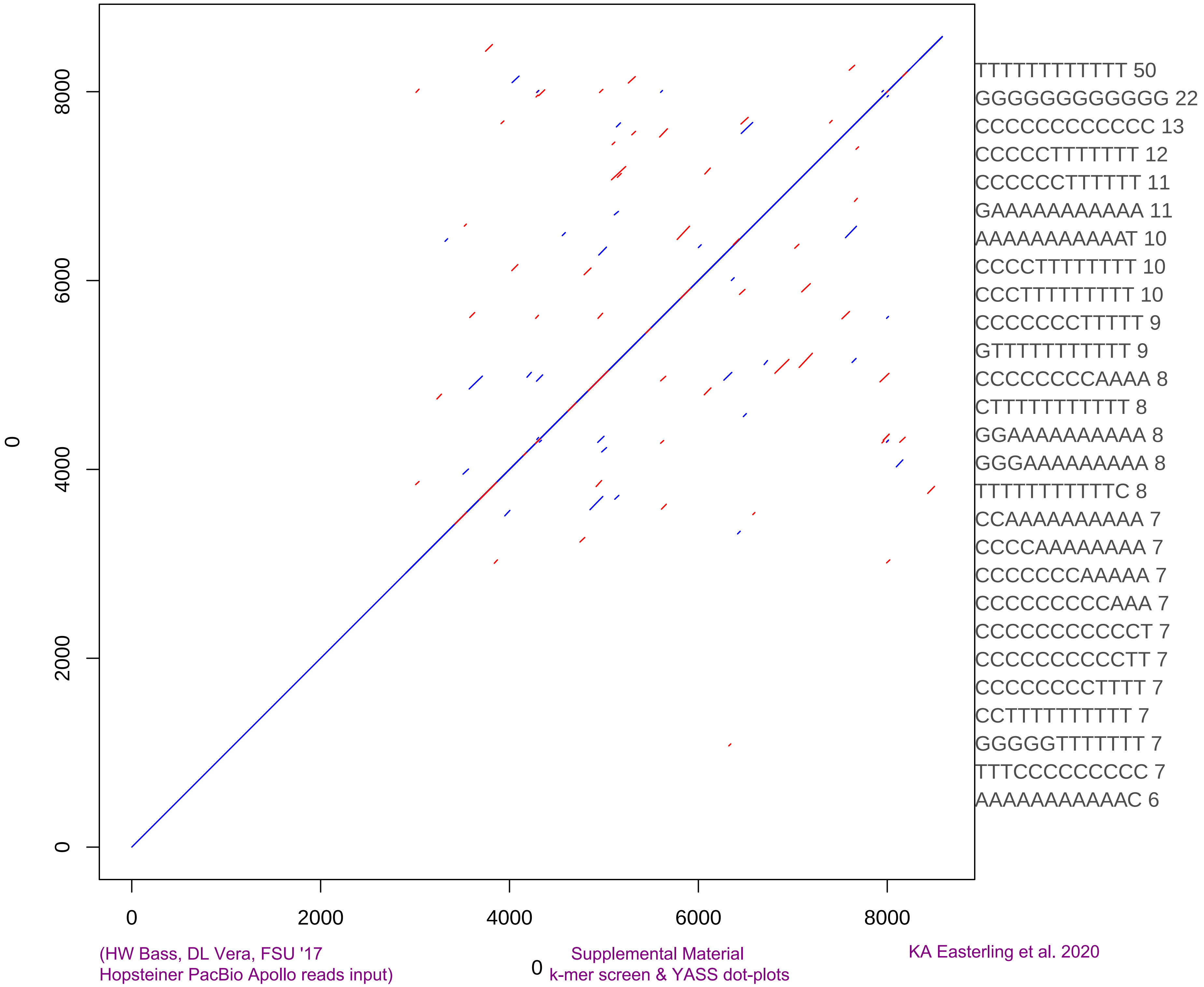

>m141230_233107_42134_c100756552550000001823146807221541_s1_p0/82275/0_8879

Representative Read (Table 1): HuluTR450-r873, 8 ppm, 77% A+T,
GenBank Acc. MN537581

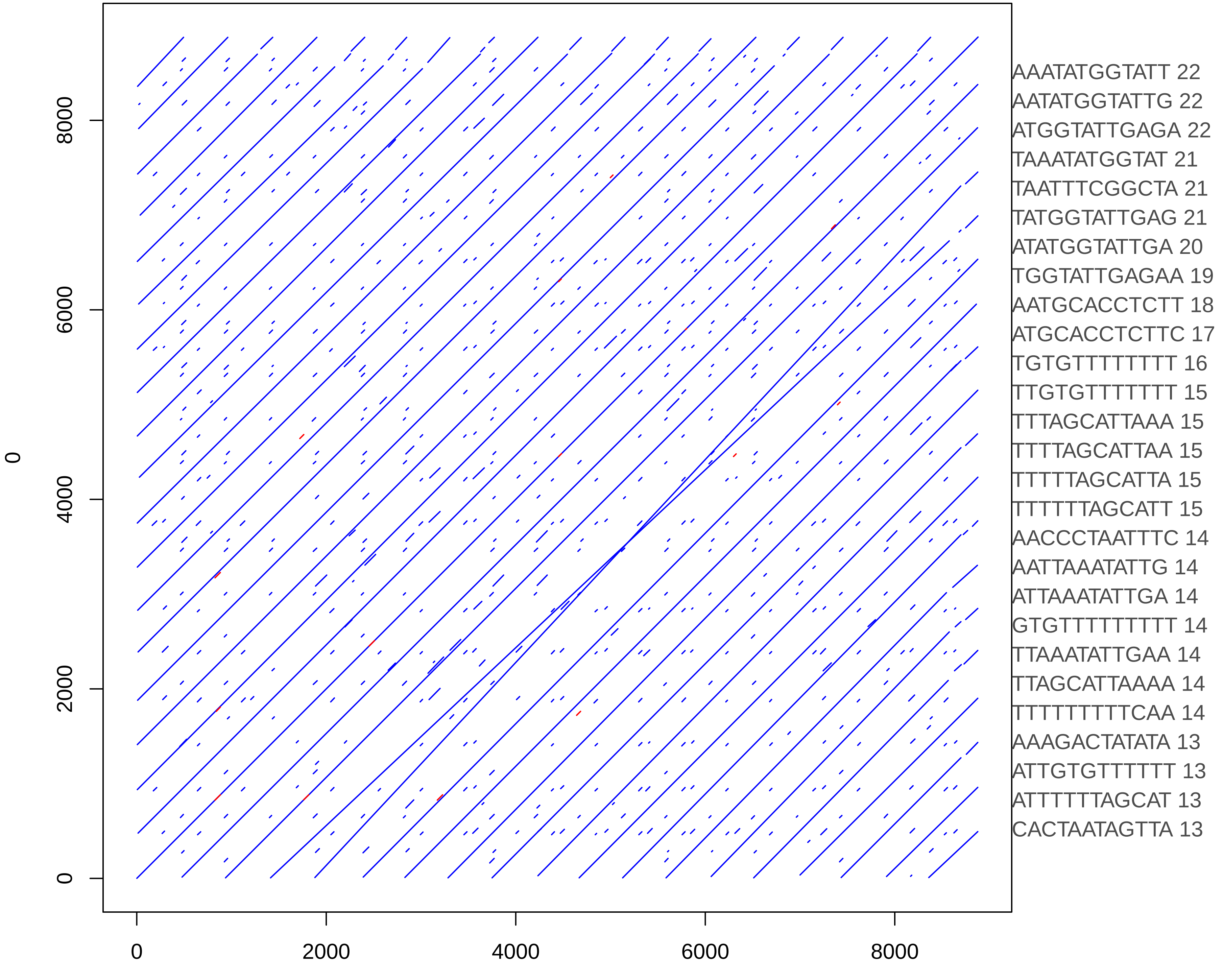

>m141230_233107_42134_c100756552550000001823146807221541_s1_p0/87464/5050_13947

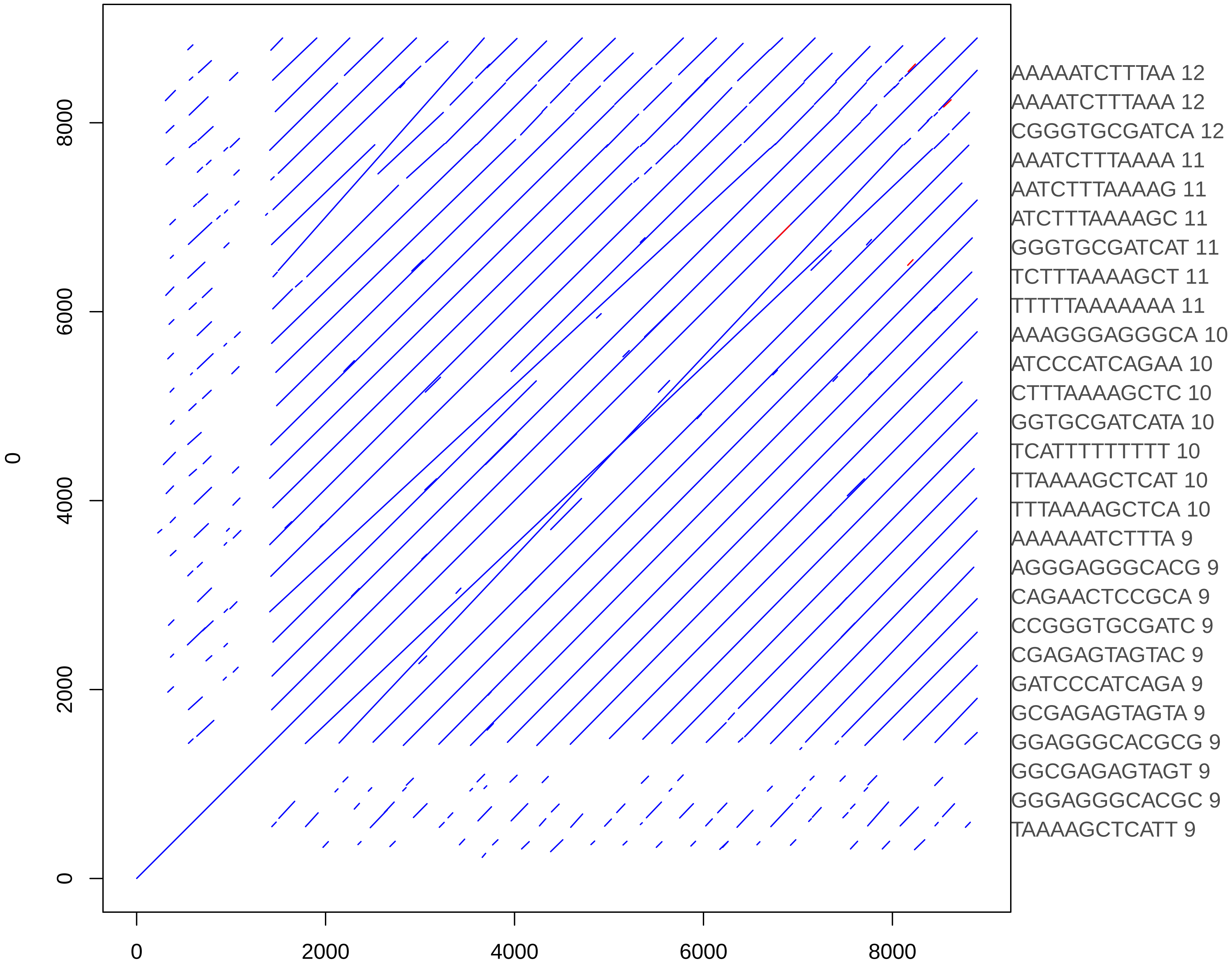

>m141230_233107_42134_c100756552550000001823146807221541_s1_p0/90982/3868_13909

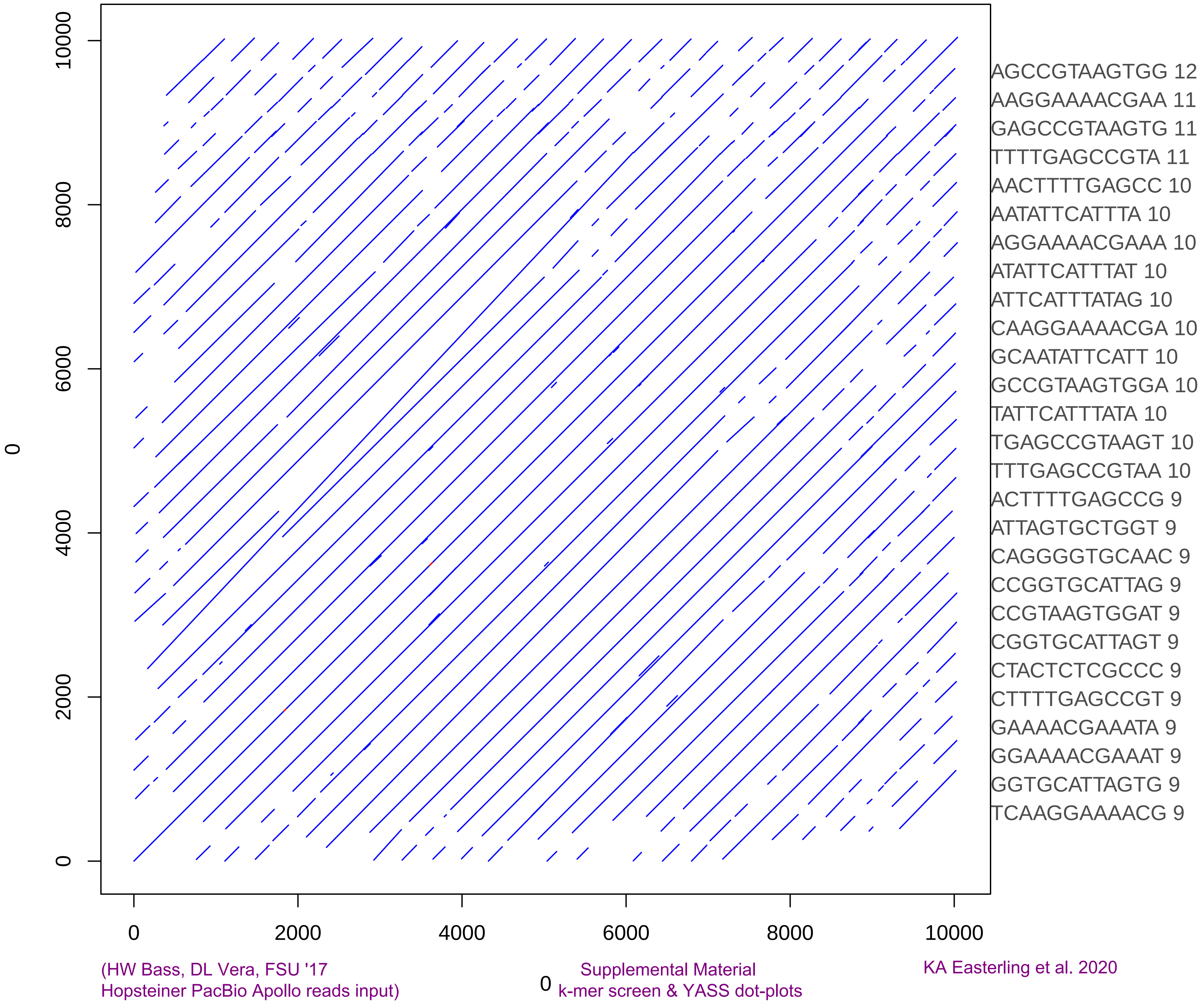

>m141230_233107_42134_c100756552550000001823146807221541_s1_p0/94957/0_6029

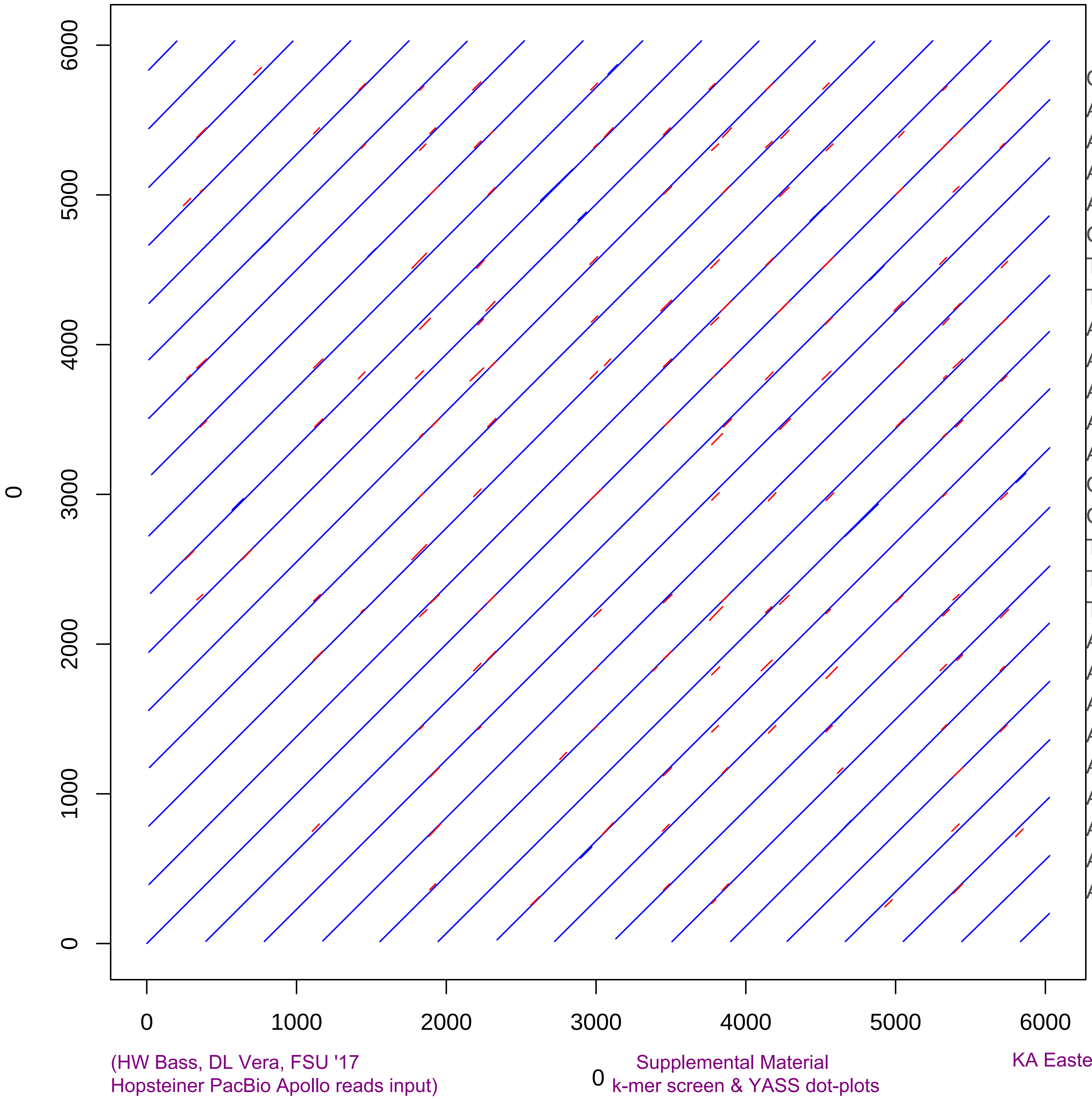

GGGAATTTTTTT 11
AAAACTTGCGGA 10
AAACTTGCGGAA 10
AACTTGCGGAAA 10
ATACATTTCGGA 10
CATGGGCACAAA 10
TCATGGGCACAA 10
TTGAAGGTGAAA 10
AAAAACTTGCGG 9
AATACATTTCGG 9
ACATTTCGGAGA 9
ACTTGCGGAAAT 9
ATGGGCACAAAA 9
GGAATTTTTTTT 9
GGATTCGAGCAT 9
TACATTTCGGAG 9
TGGATTCGAGCA 9
TTTGAAGGTGAA 9
AAAACTAAAGAA 8
AAACTAAAGAAT 8
AAATTTTGTGCA 8
AAGGGGAATTTT 8
AAGGTGAAAACT 8
AATTAGTGAAAT 8
AATTTTGTGCAA 8
ACTCCAATAGAT 8
AGGGGAATTTTT 8

>m141230_233107_42134_c100756552550000001823146807221541_s1_p0/96882/0_7335

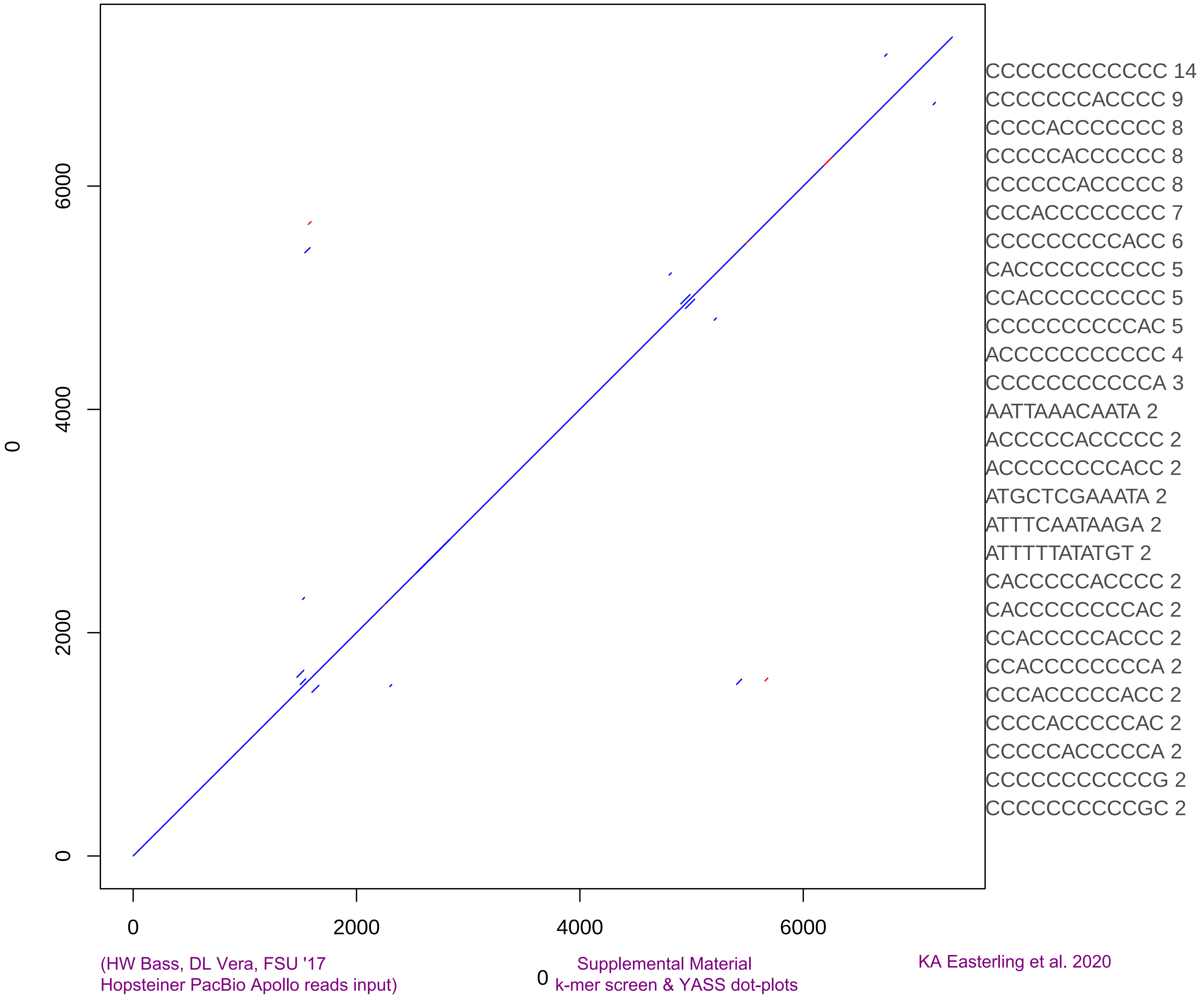

>m141231_015334_42134_c100756552550000001823146807221542_s1_p0/101361/1158_8367

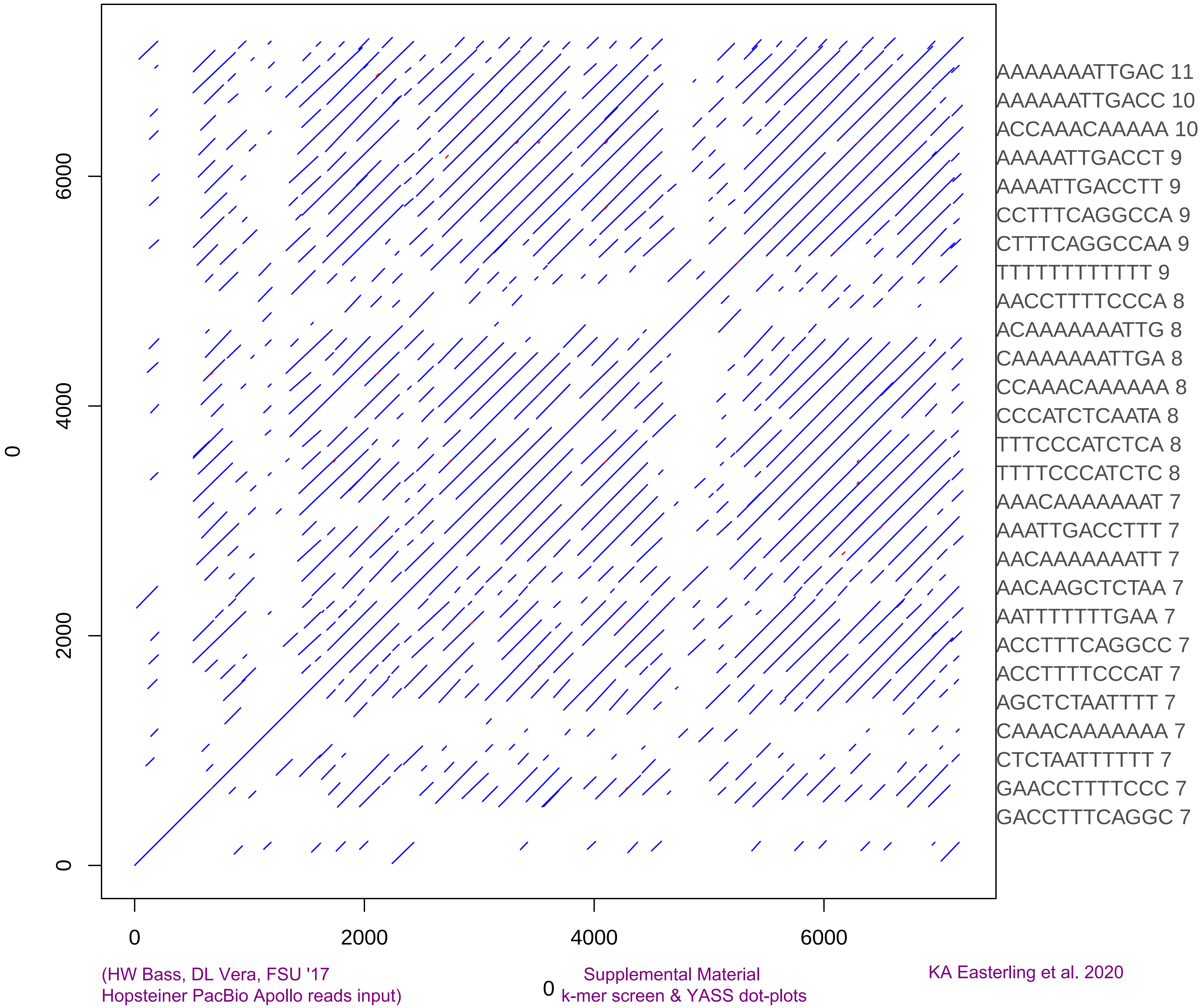

>m141231_015334_42134_c100756552550000001823146807221542_s1_p0/101633/621_6713

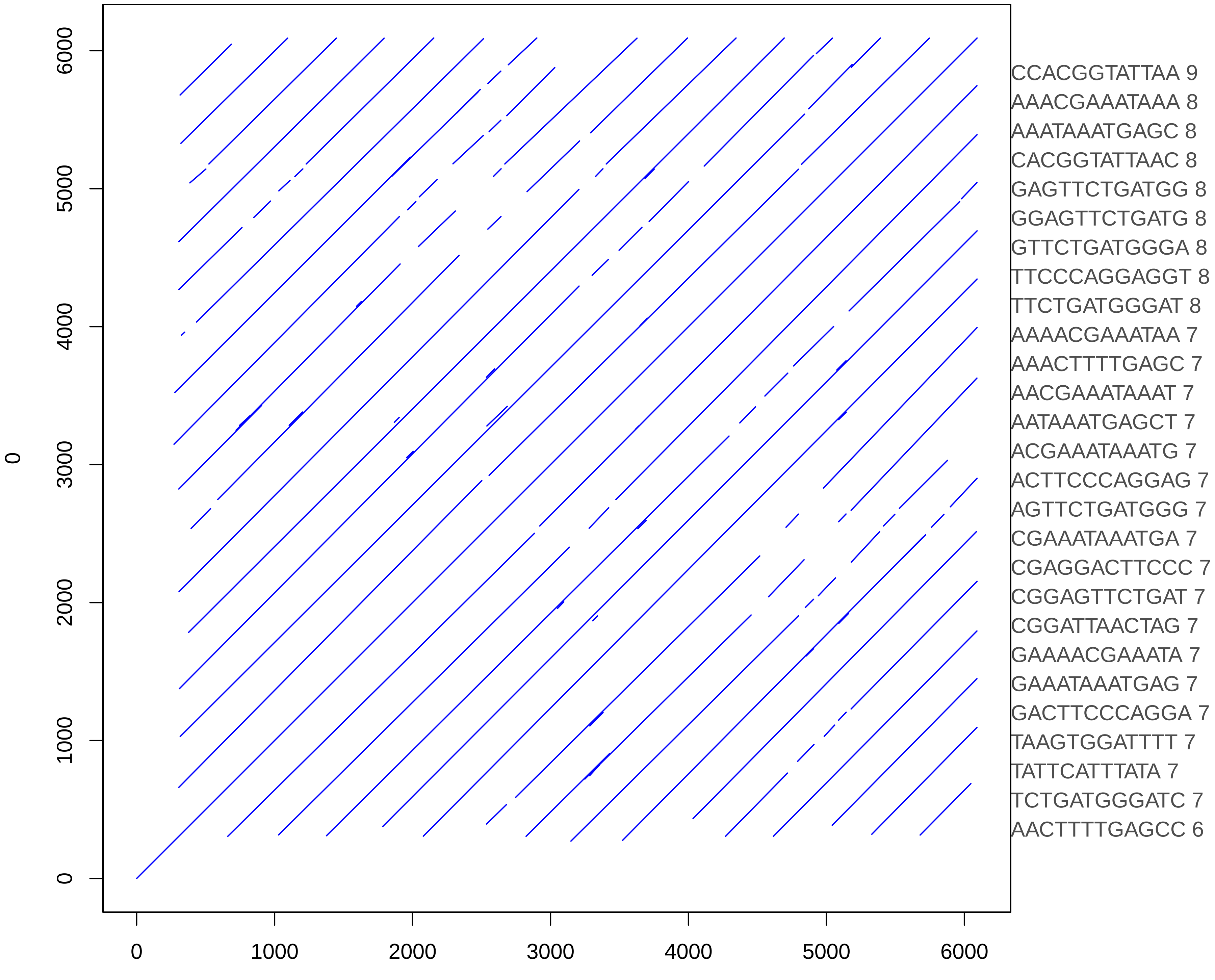

0

>m141231_015334_42134_c100756552550000001823146807221542_s1_p0/101676/1941_11435

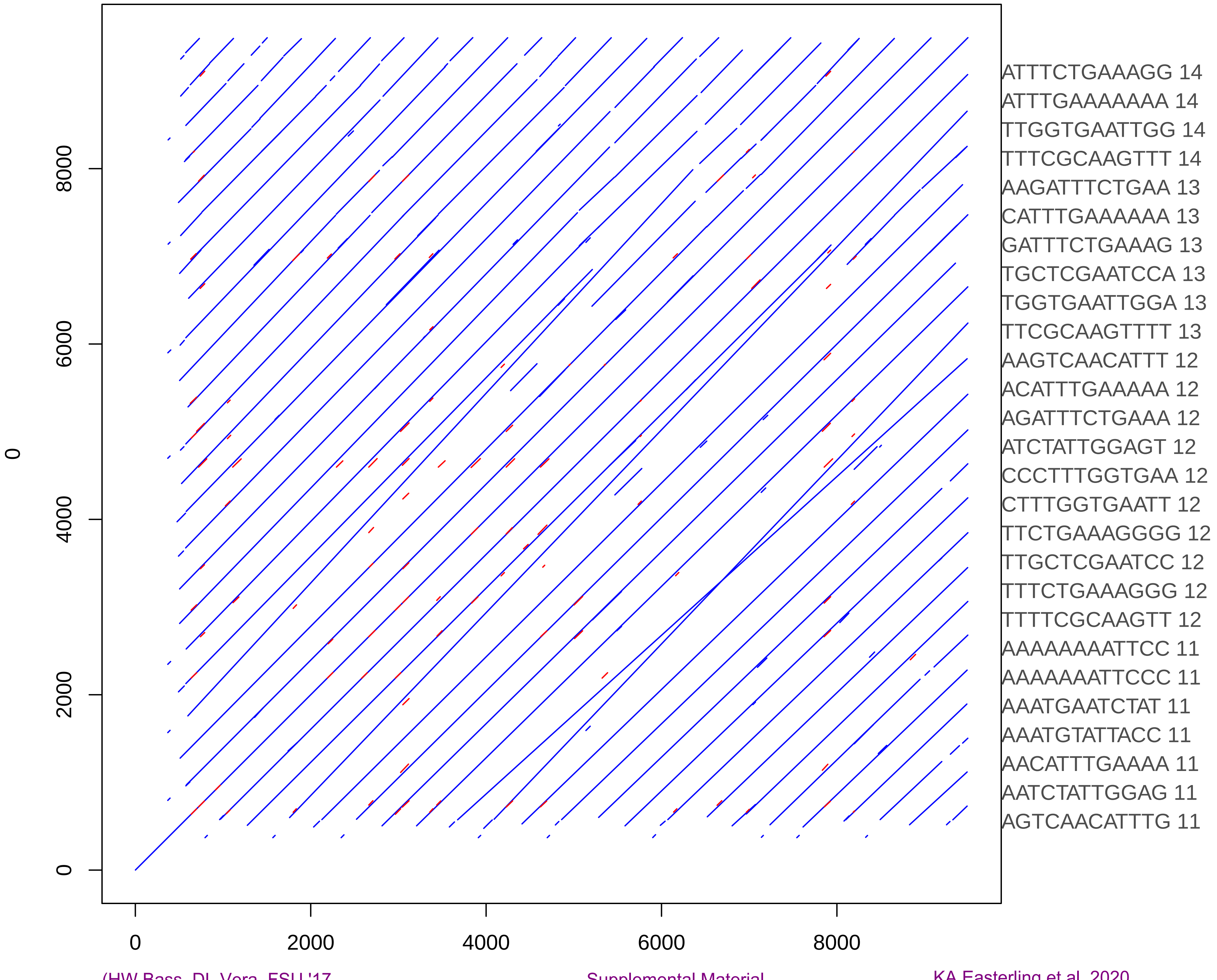

0

>m141231_015334_42134_c100756552550000001823146807221542_s1_p0/102945/9364_14436

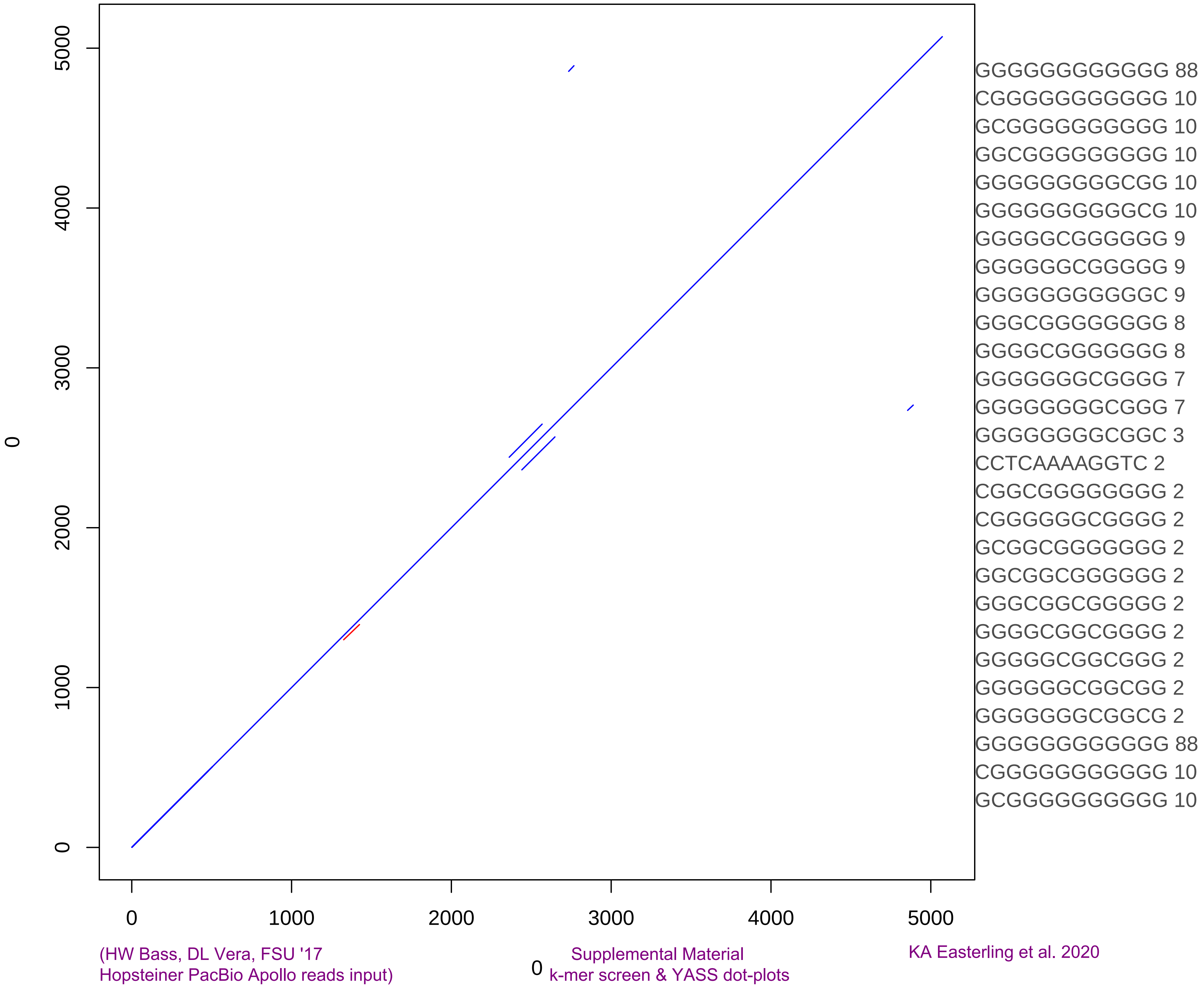

>m141231_015334_42134_c100756552550000001823146807221542_s1_p0/105335/3655_13646

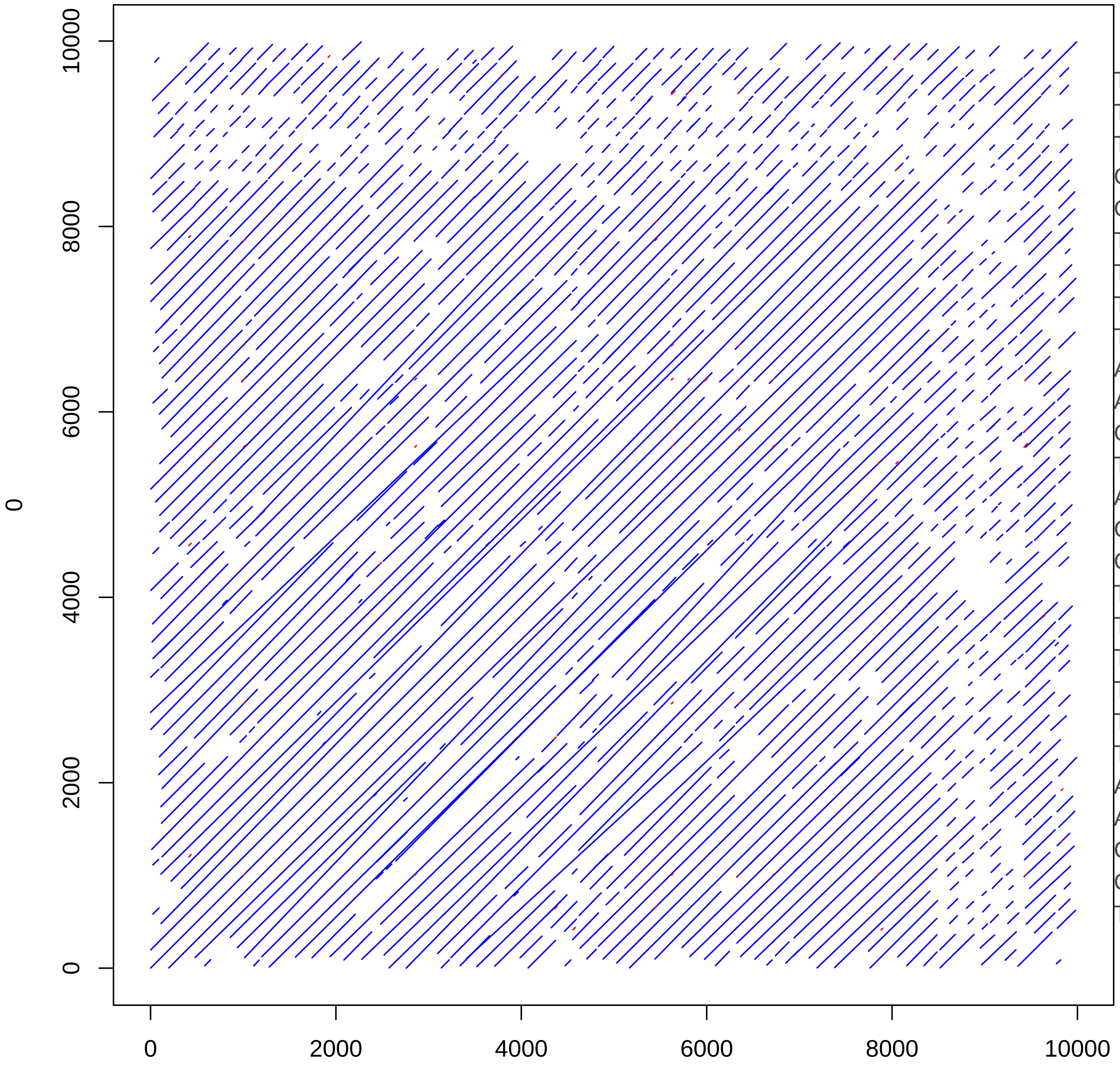

TGTTTGGTTGAA 18
TTGTTTGGTTGA 18
TTTGGTTGAATT 18
GTTTGGTTGAAT 17
GGTTGAATTAGG 15
TTGAATTAGGAC 15
TTGGTTGAATTA 15
TTTGTTTGGTTG 15
TTTTTTGTTTGG 15
AATTCATGGTCA 14
ATTCATGGTCAA 14
GTTGAATTAGGA 14
TGGTTGAATTAG 14
ATGGTCAATAAT 13
CATGGTCAATAA 13
GCCTGAAAGGTC 13
TCATGGTCAATA 13
TGAATTAGGACC 13
TGGCCTGAAAGG 13
TTCATGGTCAAT 13
TTTTGTTTGGTT 13
TTTTTGTTTGGT 13
AGTGAATTACGA 12
ATTTTTTTGTTT 12
CGCTTCGGAGCC 12
GAATTAGGACCC 12
TTGGCCTGAAAG 12

1,121 hits for repetitive sequences
passing 12mer abundance criterion

(Read-Number)
PAGE 883

For Tandem Repeat families of ### bp:
PacBio Read named "HuluTR###-r883"

>m141231_015334_42134_c100756552550000001823146807221542_s1_p0/106235/0_8612

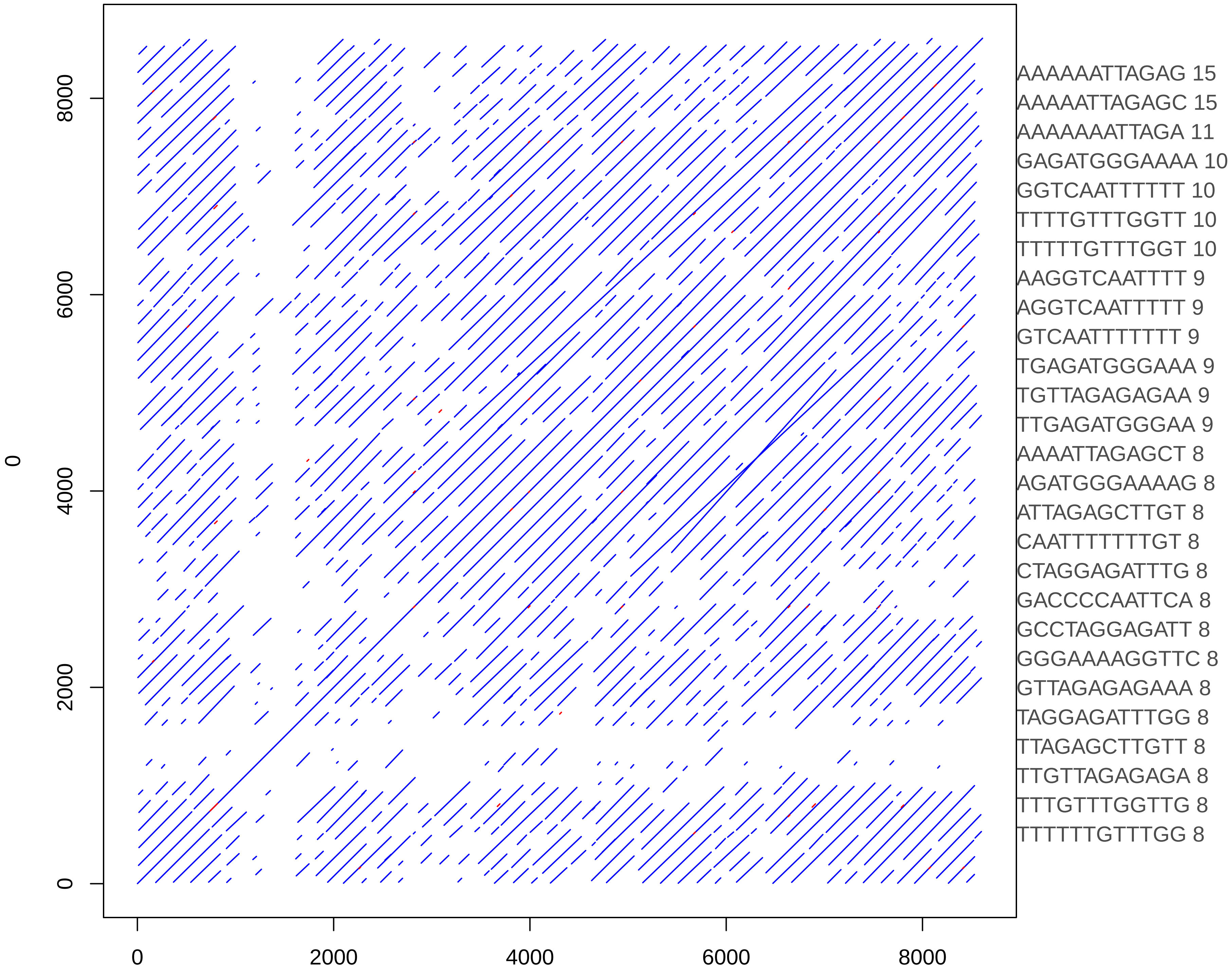

(HW Bass, DL Vera, FSU '17
Hopsteiner PacBio Apollo reads input)

0

Supplemental Material
k-mer screen & YASS dot-plots

KA Easterling et al. 2020

>m141231_015334_42134_c100756552550000001823146807221542_s1_p0/107068/0_10056

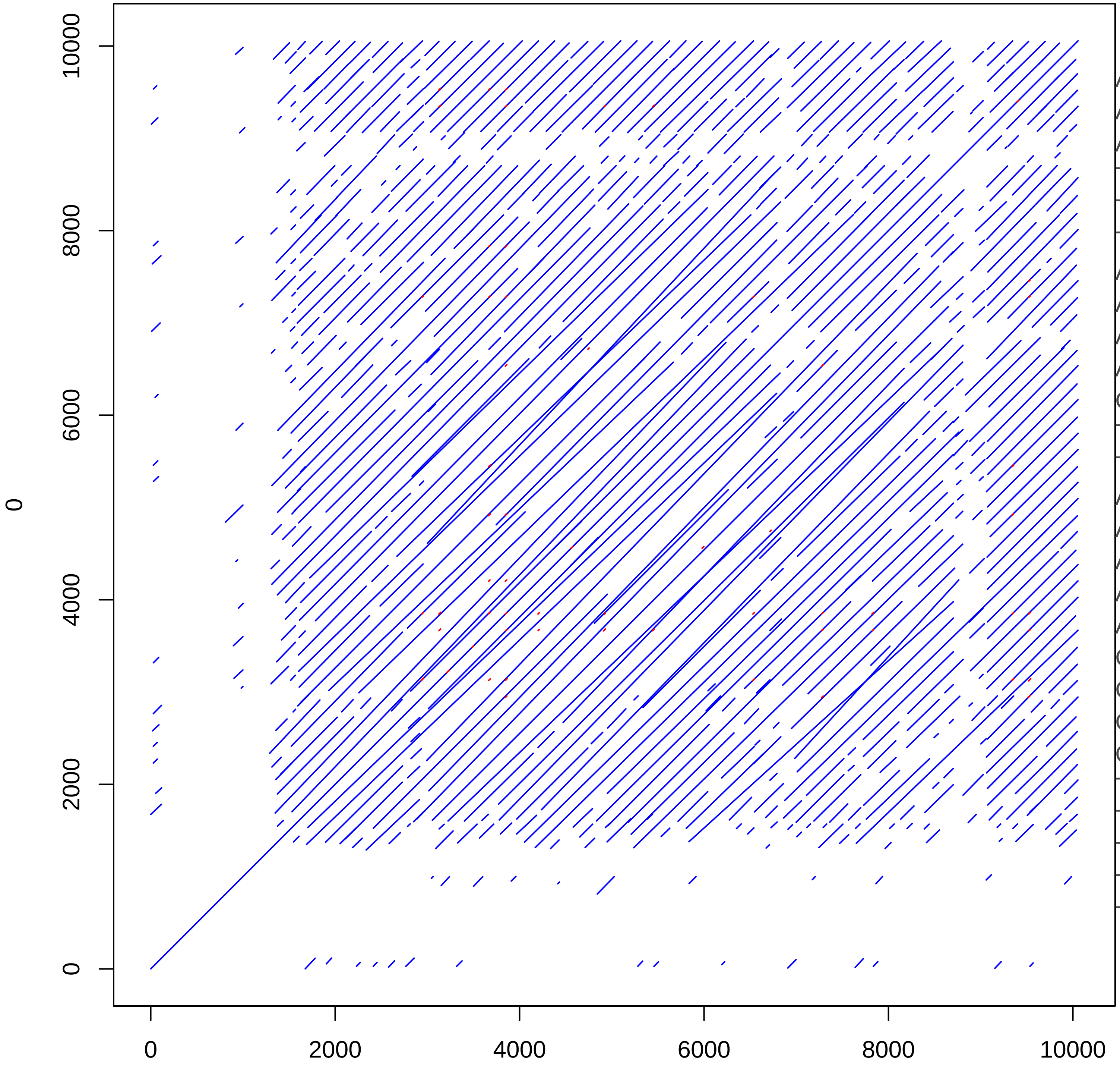

AATTCATGGTCA 14
ATGAGTGAATTA 13
ATTCATGGTCAA 13
TATGAGTGAATT 13
TTTTTGTTTGGT 13
TTTTTTGTTTGG 13
AAATATGAGTGA 12
AGAAATATGAGT 12
ATATTATTGAGA 12
ATTATTGAGATG 12
GAAATATGAGTG 12
TATTATTGAGAT 12
TTATTGAGATGG 12
AAGGTCAATTTT 11
AATATGAGTGAA 11
AGGTCAATTTTT 11
ATATGAGTGAAT 11
ATTGAGATGGGA 11
CAATTCATGGTC 11
CCAATTCATGGT 11
GAGAAATATGAG 11
GGTCAATTTTTT 11
TAGGAGATTTGG 11
TCATGGTCAATA 11
TTCATGGTCAAT 11
TTGAGATGGGAA 11
TTTTGTTTGGTT 11

>m141231_015334_42134_c100756552550000001823146807221542_s1_p0/107100/637_9443

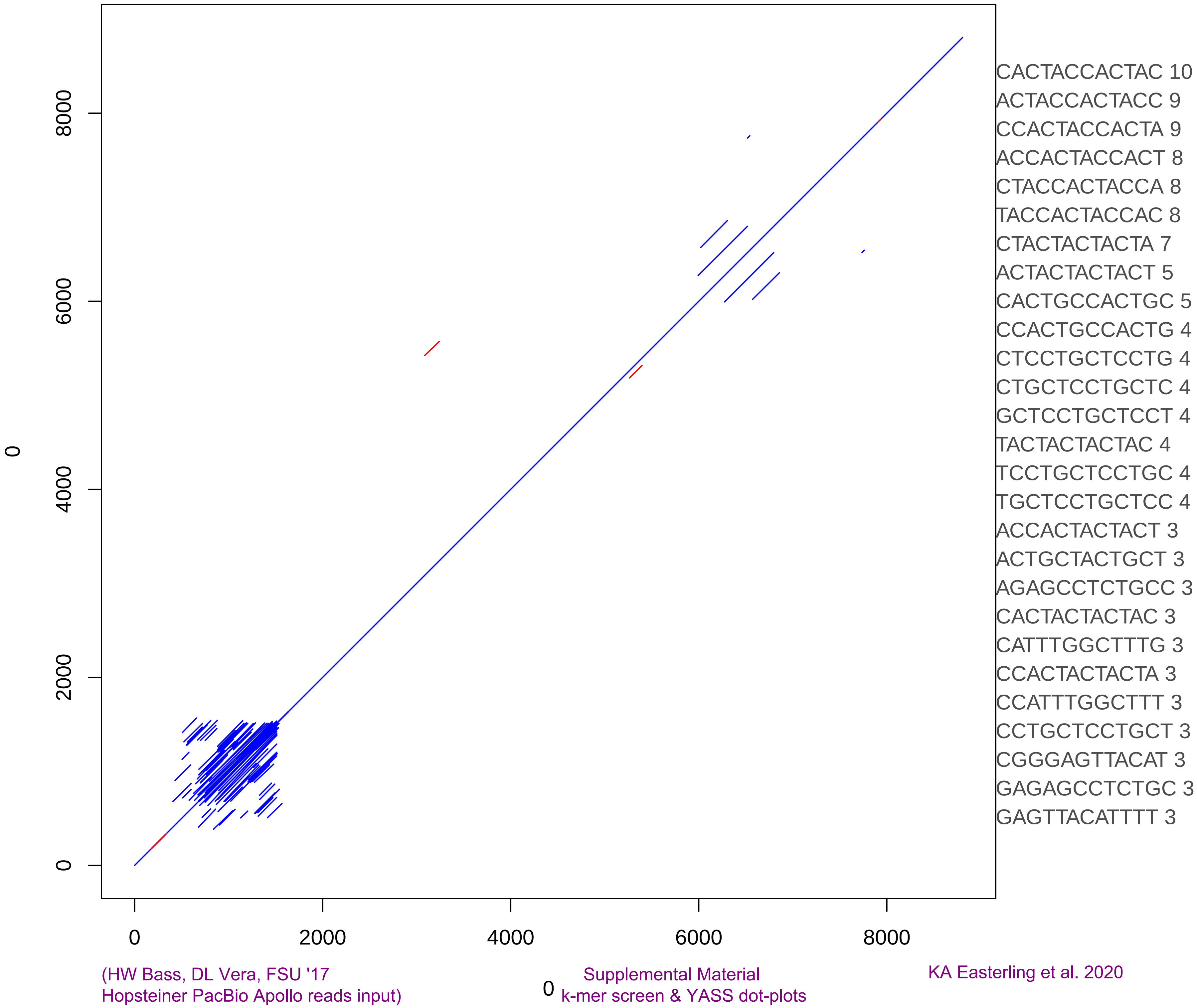

>m141231_015334_42134_c100756552550000001823146807221542_s1_p0/107346/0_11952

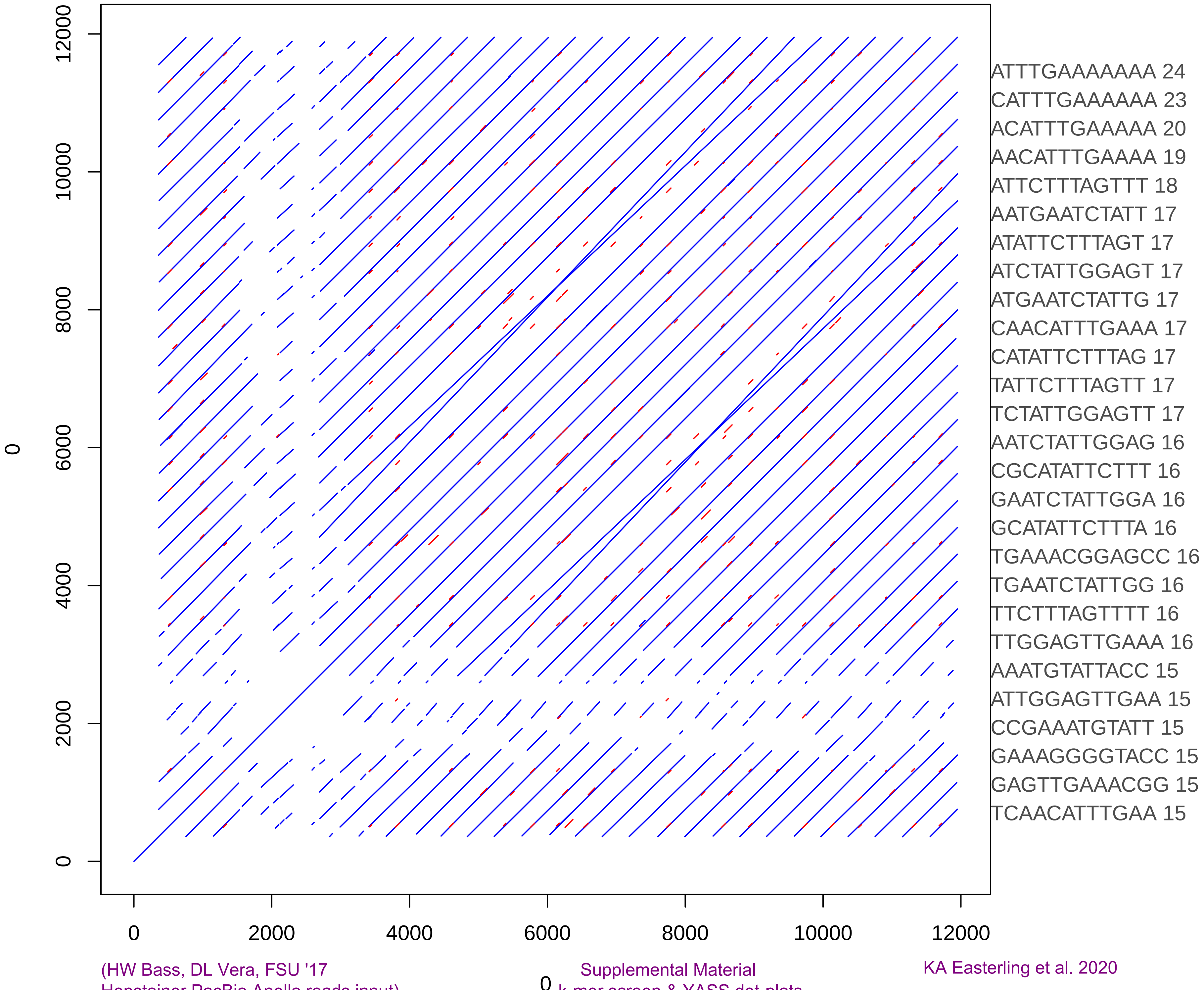

>m141231_015334_42134_c100756552550000001823146807221542_s1_p0/109600/12883_20073

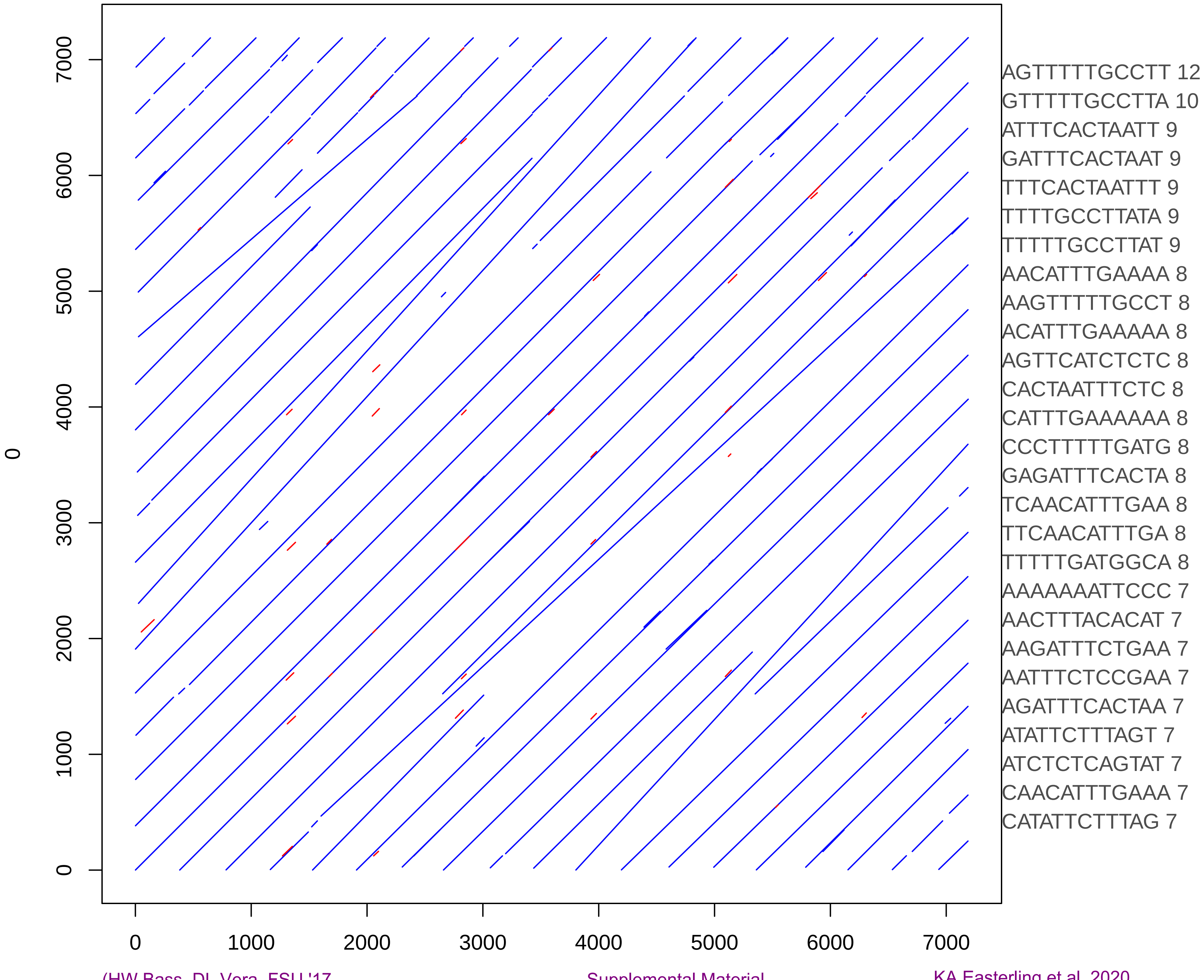

>m141231_015334_42134_c100756552550000001823146807221542_s1_p0/110736/13967_19471

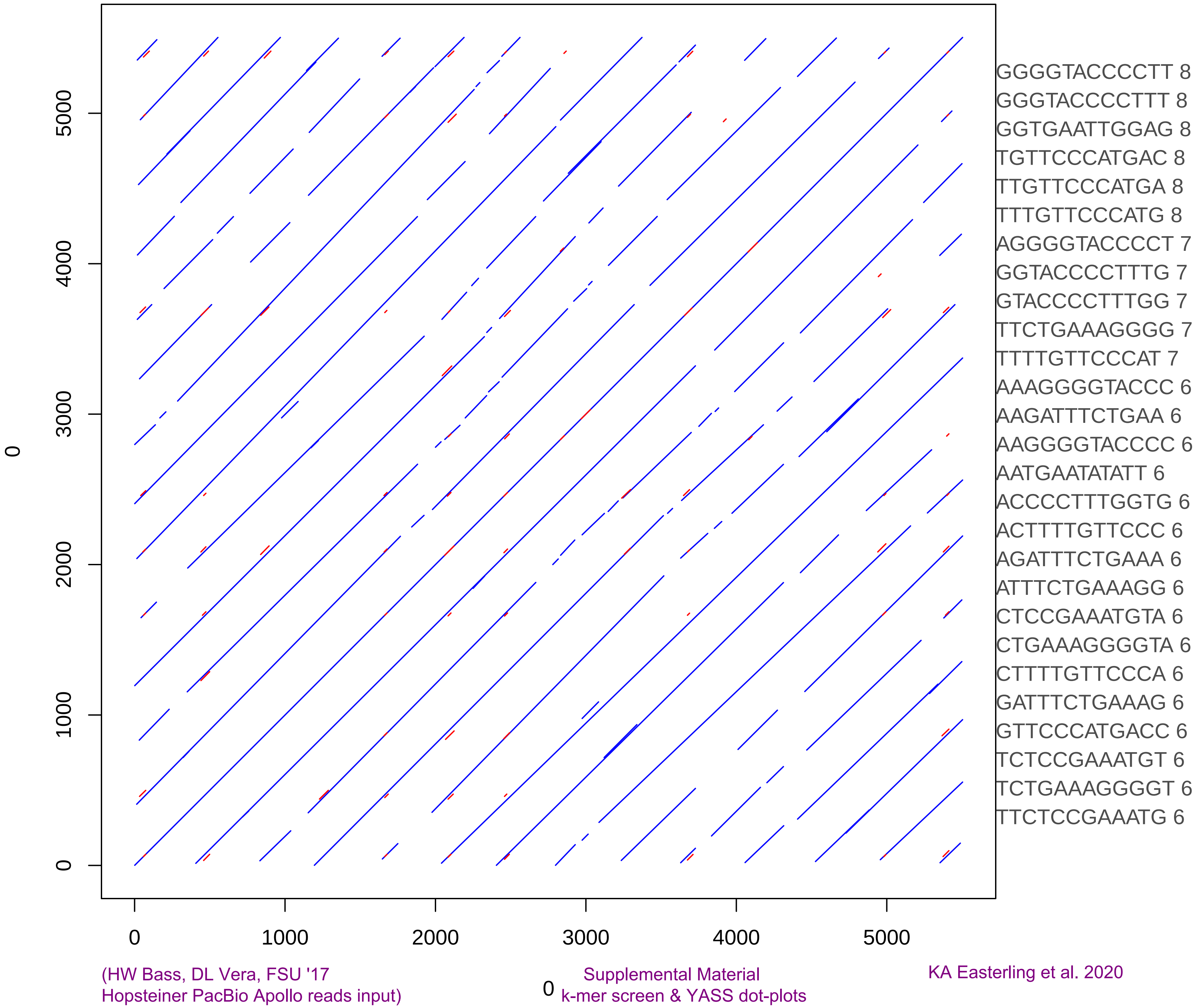

>m141231_015334_42134_c100756552550000001823146807221542_s1_p0/111096/2774_8500

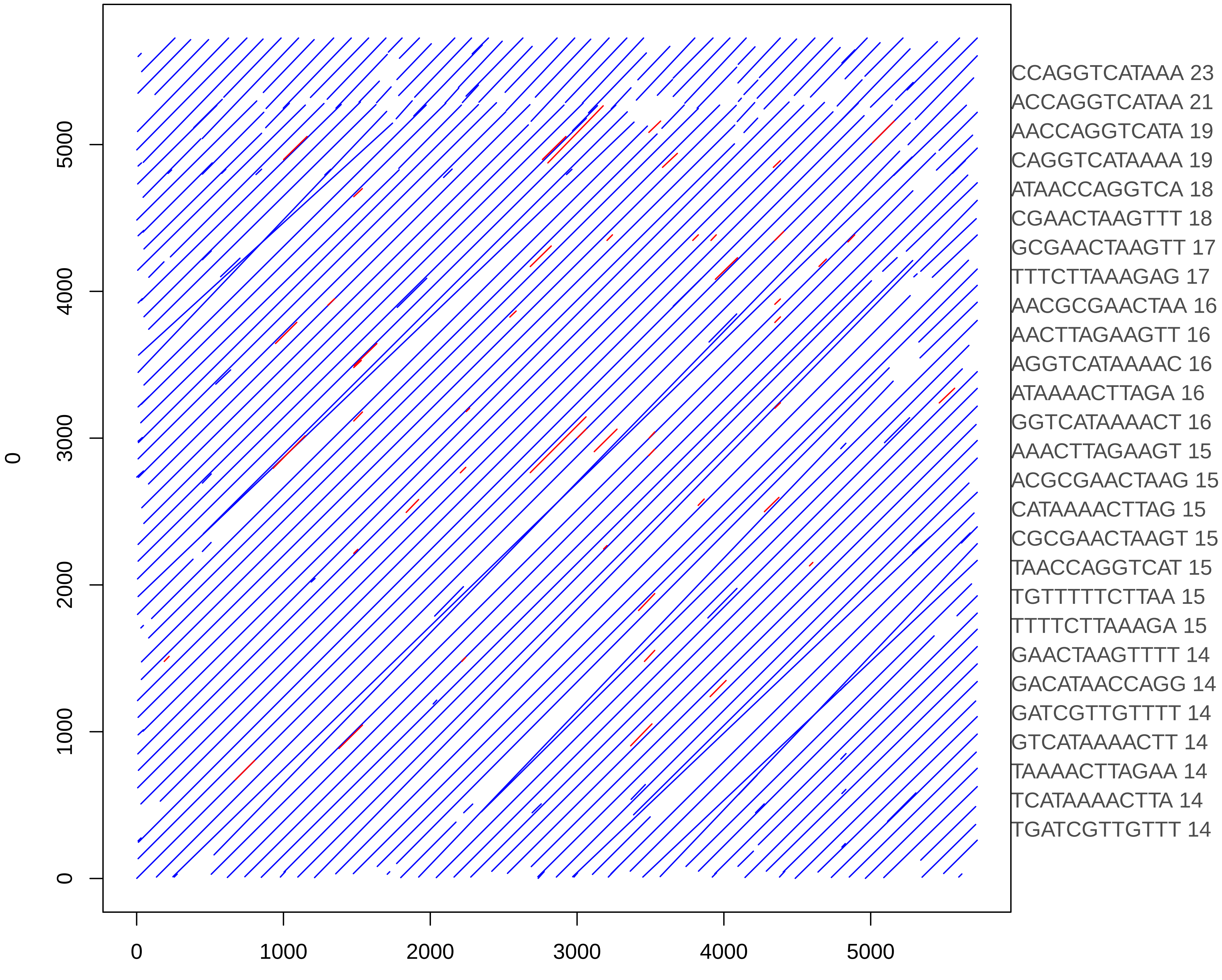

1,121 hits for repetitive sequences passing 12mer abundance criterion

(Read-Number)
PAGE 890

For Tandem Repeat families of ### bp:
PacBio Read named "HuluTR###-r890"

>m141231_015334_42134_c100756552550000001823146807221542_s1_p0/111724/336_9261

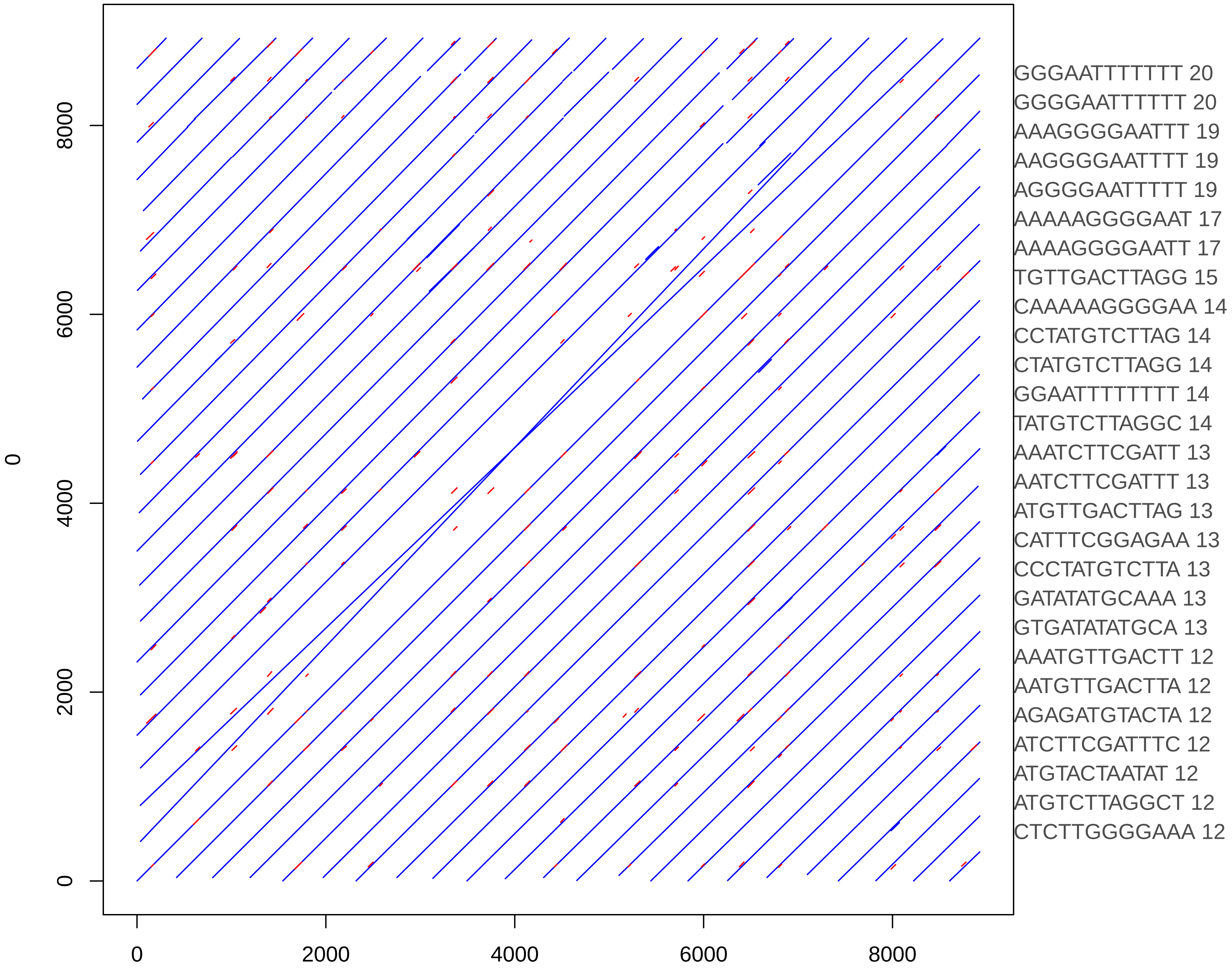

(HW Bass, DL Vera, FSU '17
Hopsteiner PacBio Apollo reads input)

0

Supplemental Material
k-mer screen & YASS dot-plots

KA Easterling et al. 2020

>m141231_015334_42134_c100756552550000001823146807221542_s1_p0/111838/762_9185

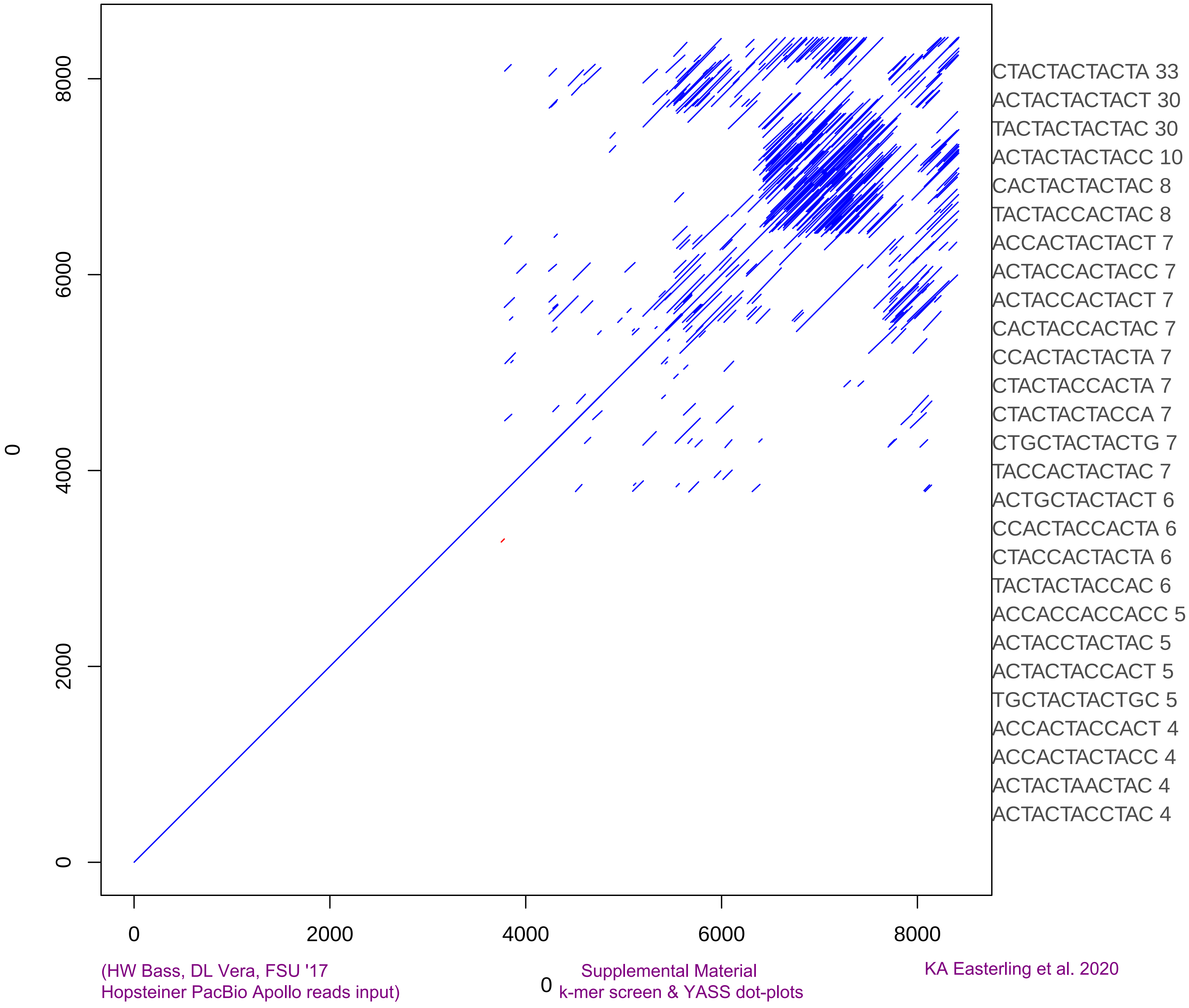

>m141231_015334_42134_c100756552550000001823146807221542_s1_p0/114166/0_8627

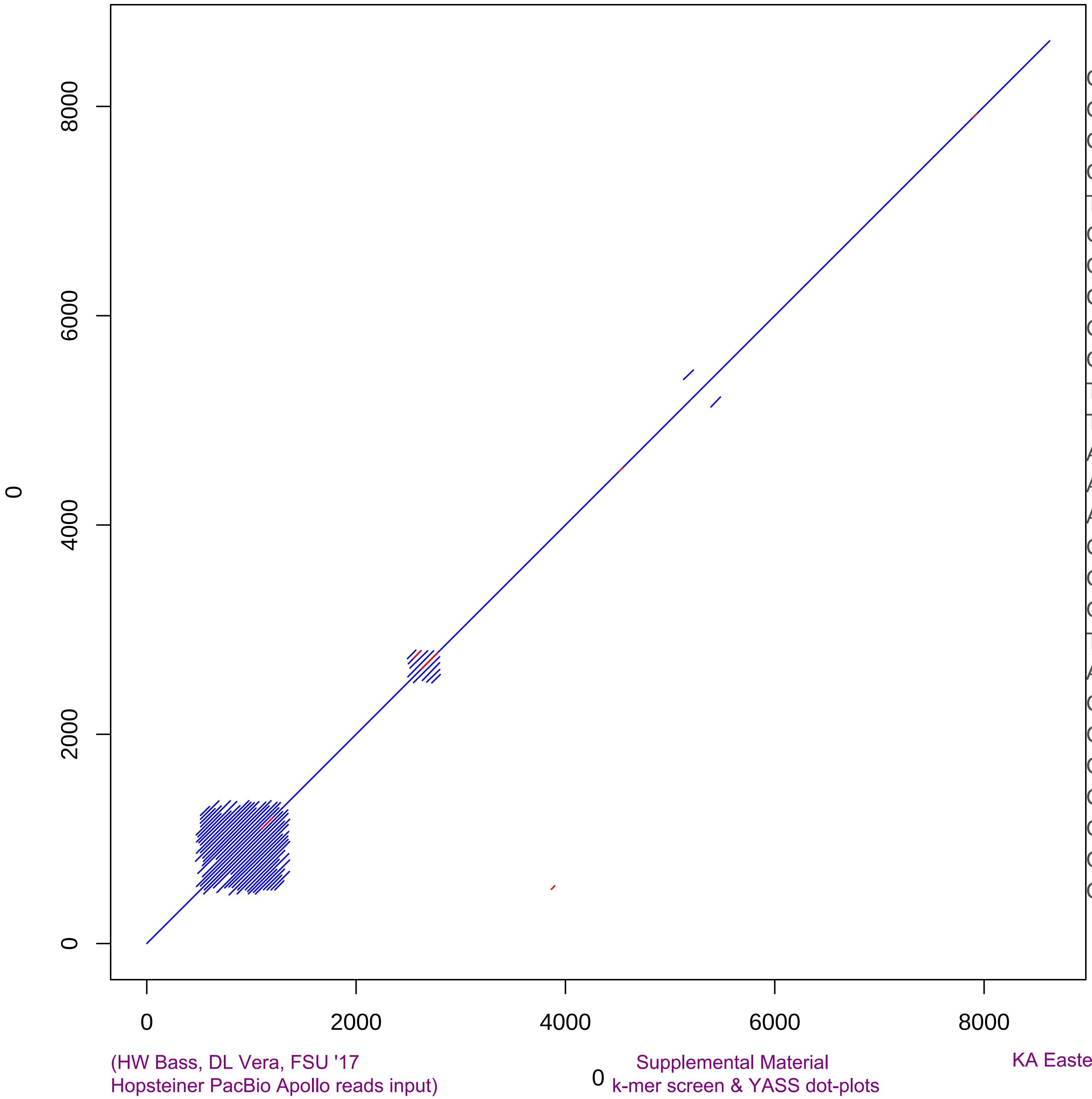

GTGGCCTAGGCA 10
CCTAGGCATCTC 9
GCCTAGGCATCT 9
GGCCTAGGCATC 8
TGGCCTAGGCAT 8
GCATCTCGCGAG 7
GTGTGGCCTAGG 7
CATCTCGCGAGG 6
GGCATCTCGCGA 6
GGTGTGGCCTAG 6
TCTCGCGAGGCC 6
TGTGGCCTAGGC 6
AGGCATCTCGCG 5
AGGTGTGGCCTA 5
ATCTCGCGAGGC 5
CTAGGCATCTCG 5
GAGGTGTGGCCT 5
GGAGGTGTGGCC 5
TAGGCATCTCGC 5
AGGCATCTCCGC 4
CCCTTTGGGTGG 4
CGCGAGGCCCTT 4
CTAGGCATCTCC 4
CTCGCGAGGCCC 4
GAGGCCCTTTGG 4
GCGAGGCCCTTT 4
GGGAGGTGTGGC 4

>m141231_015334_42134_c100756552550000001823146807221542_s1_p0/118340/2266_8852

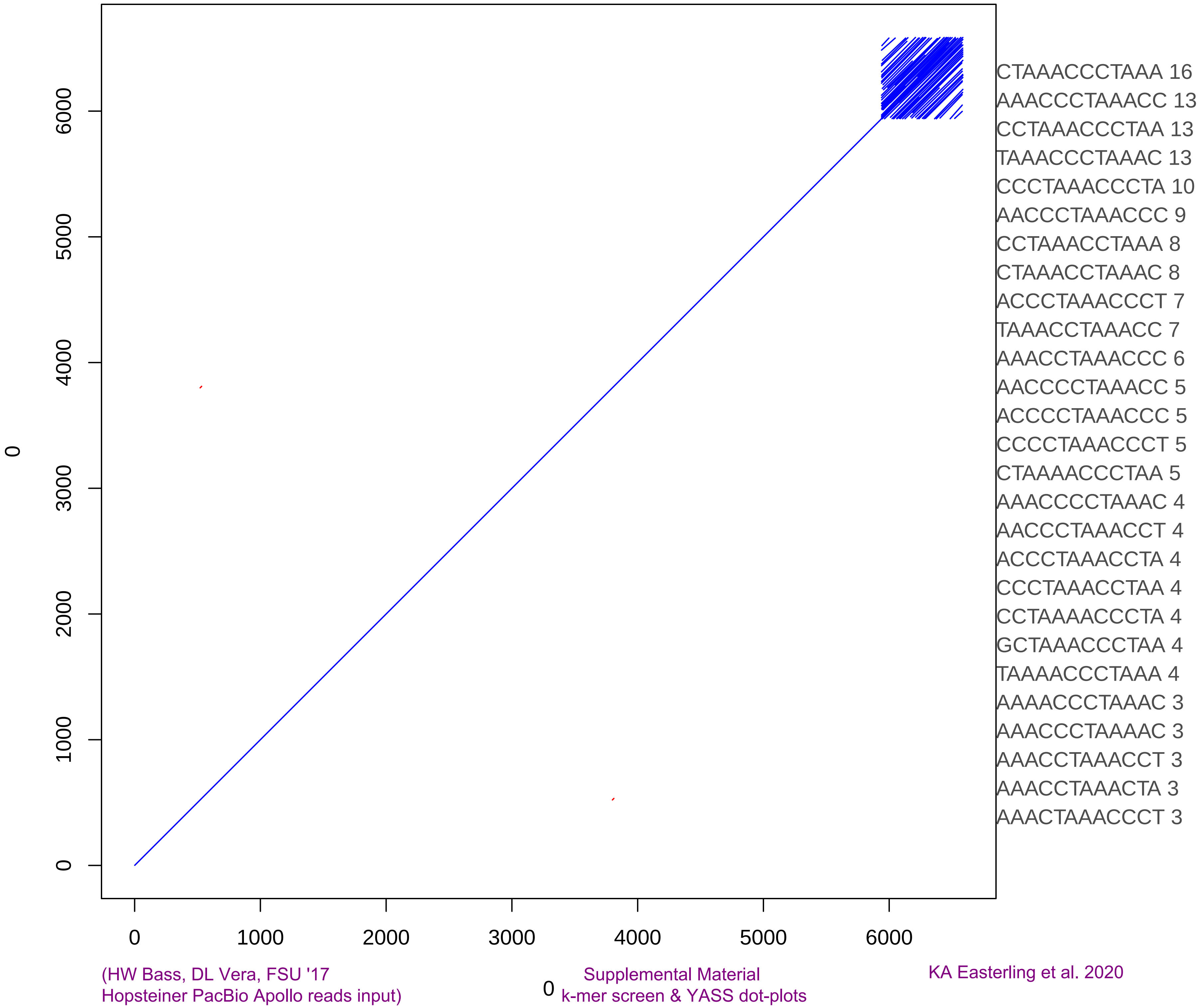

>m141231_015334_42134_c100756552550000001823146807221542_s1_p0/119555/1493_8125

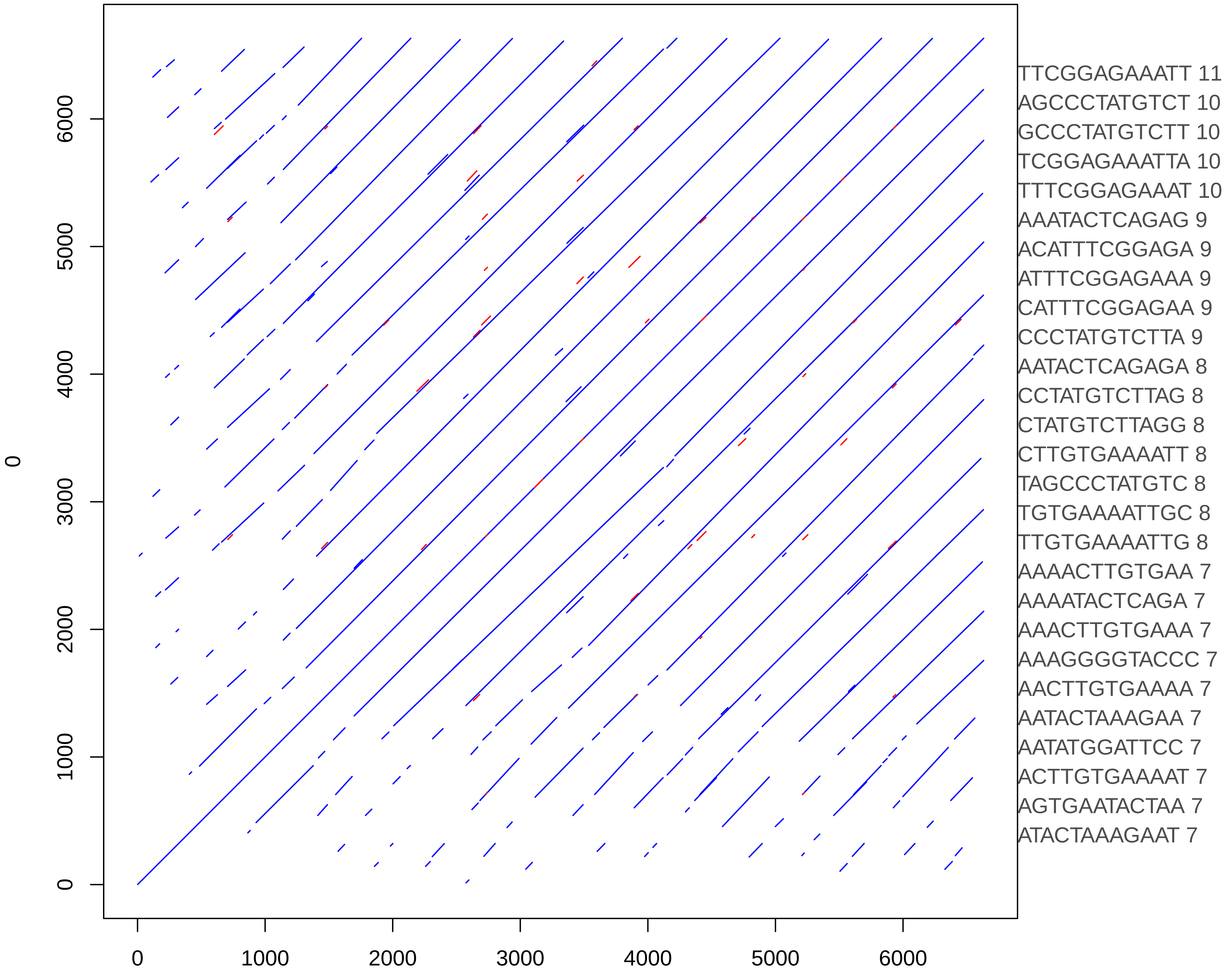

>m141231_015334_42134_c100756552550000001823146807221542_s1_p0/119555/8171_13386

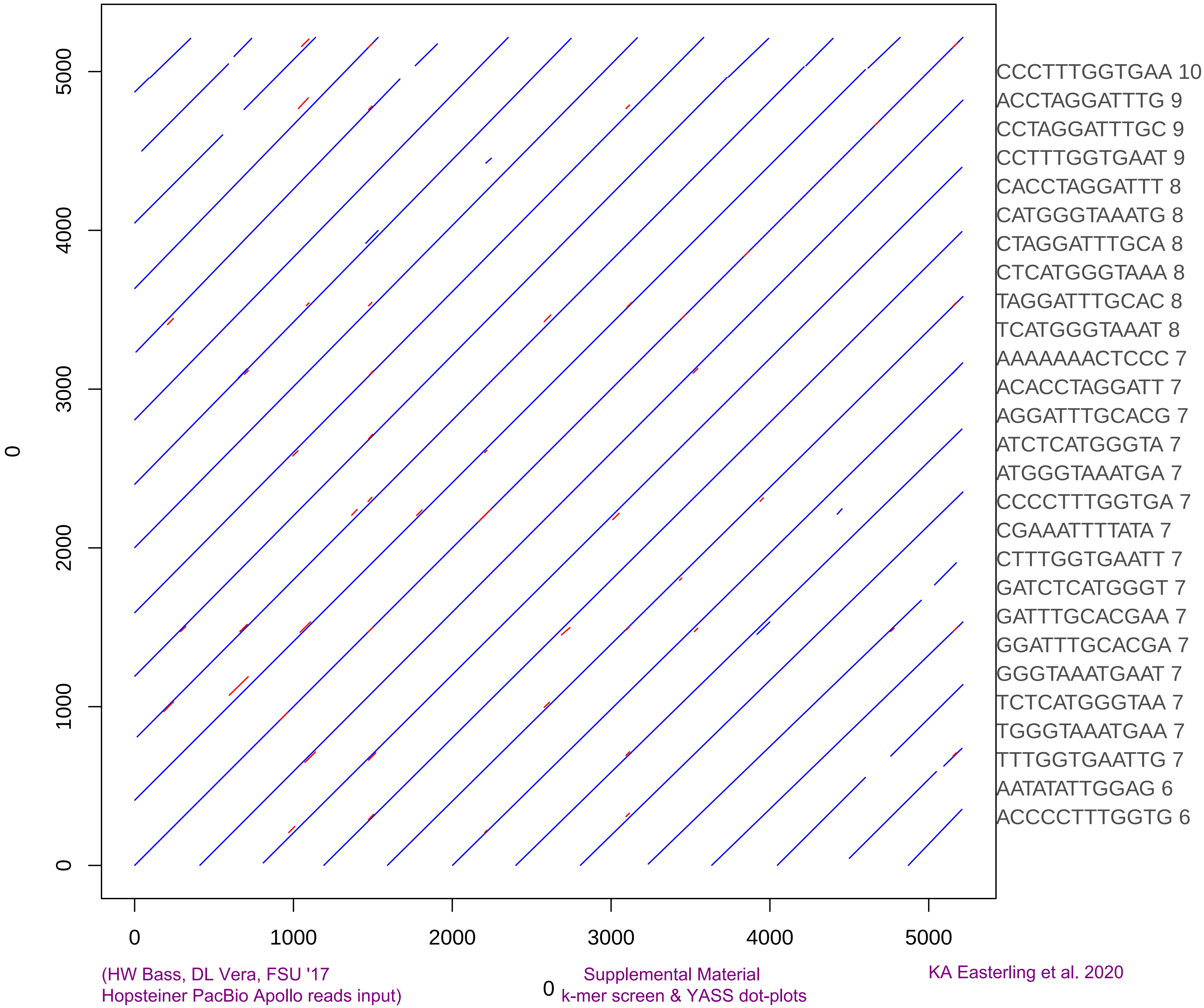

>m141231_015334_42134_c100756552550000001823146807221542_s1_p0/119919/964_6997

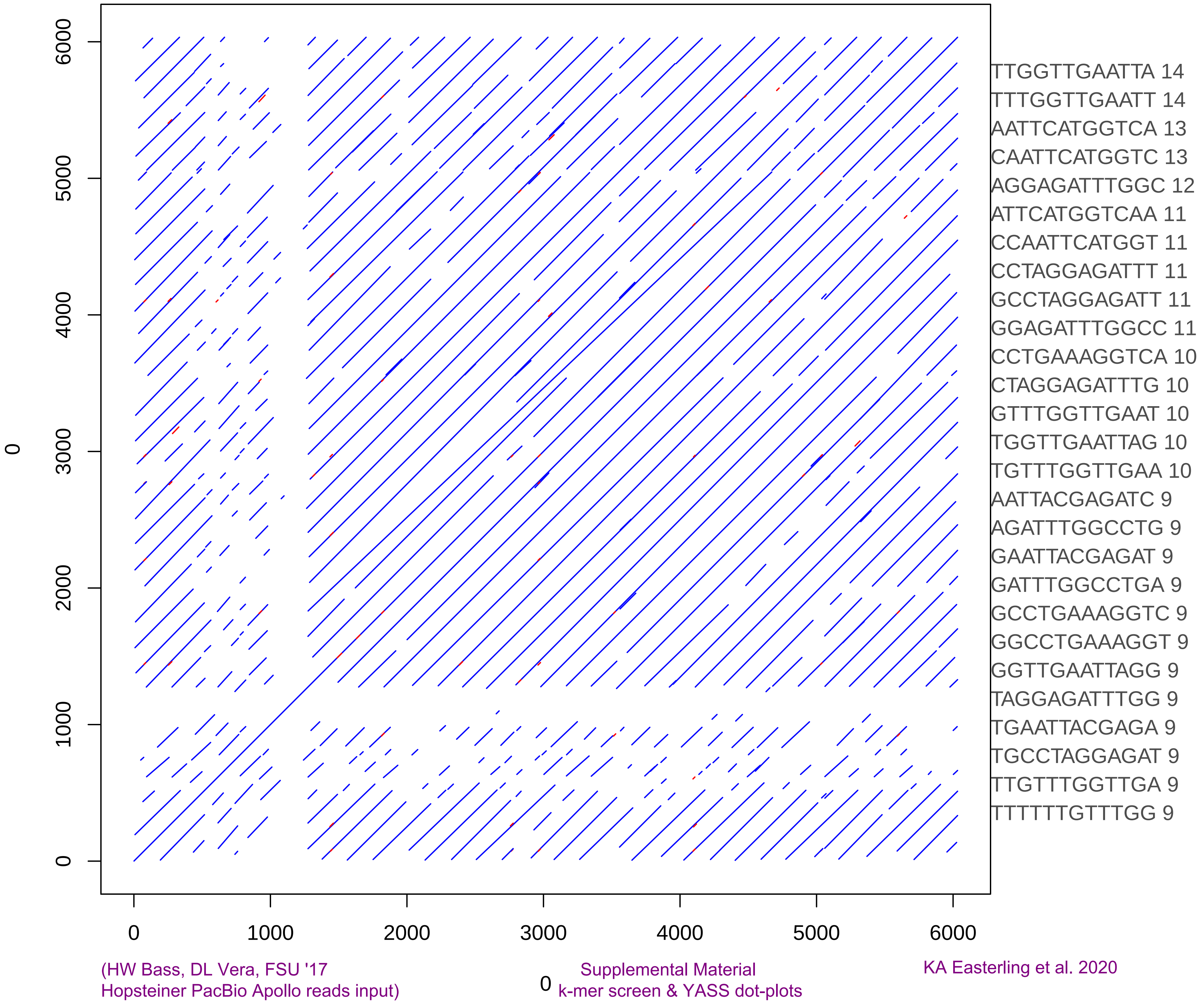

>m141231_015334_42134_c100756552550000001823146807221542_s1_p0/122663/2020_12156

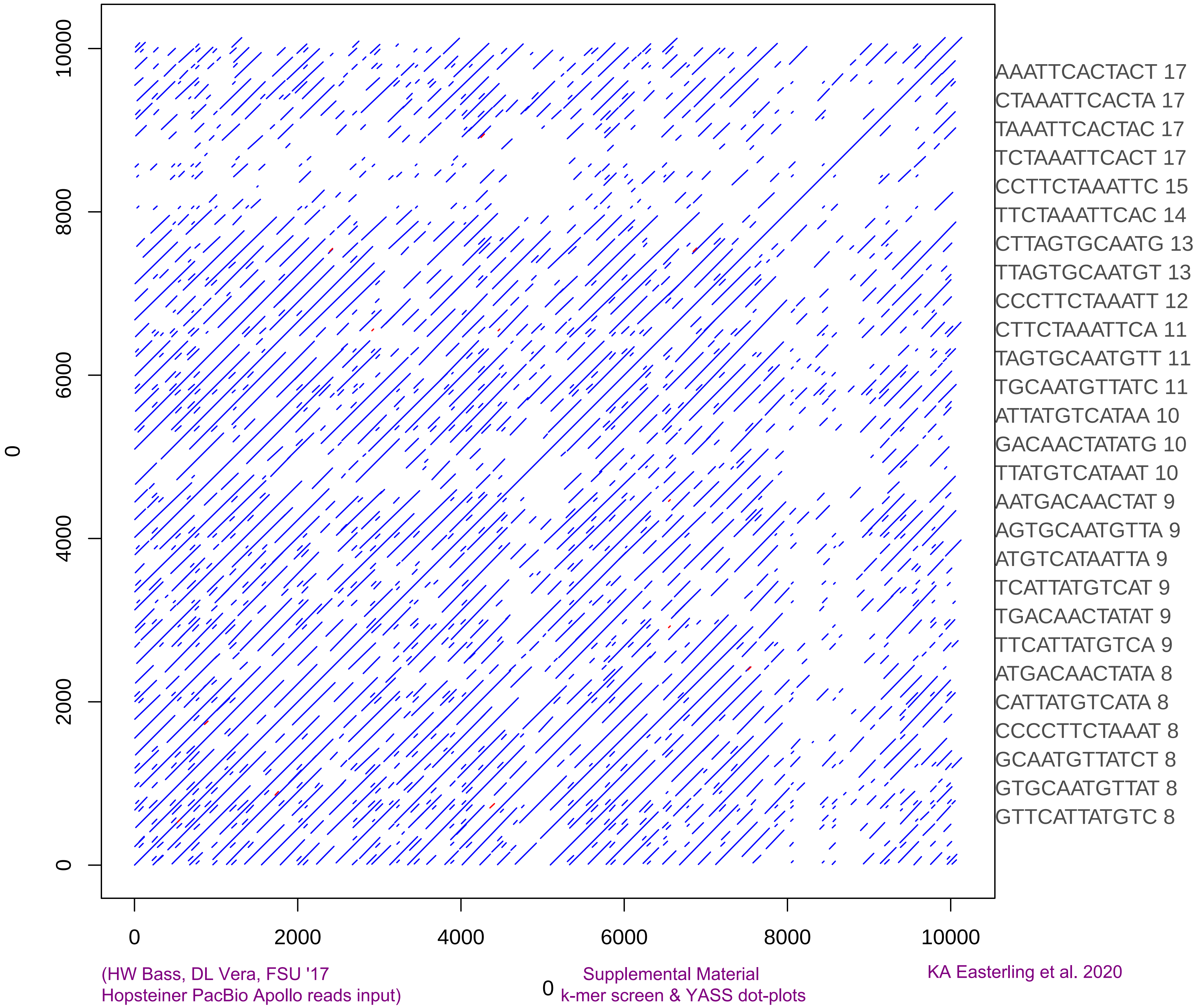

>m141231_015334_42134_c100756552550000001823146807221542_s1_p0/125461/537_11186

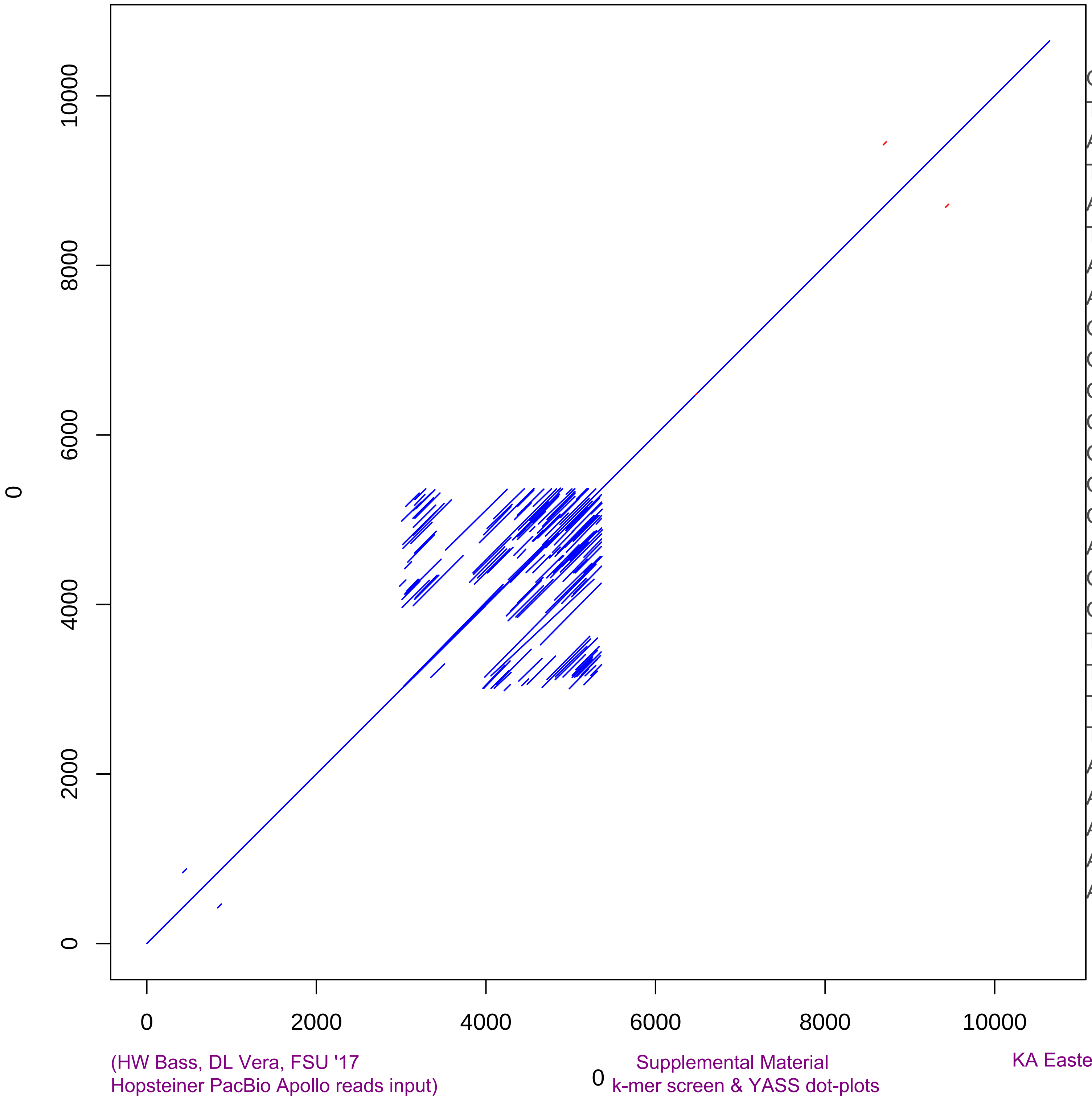

GTAGTAGTAGTA 71
TAGTAGTAGTAG 70
AGTAGTAGTAGT 64
TGGTGGTAGTAG 9
AGTAGTAGTAGC 8
TAGTAAGTAGTA 8
AGTAGTATAGTA 7
ATAGTAGTAGTA 7
GGTAGTAGTAGT 7
GGTGGTAGTAGT 7
GGTGGTGGTGGT 7
GTAGTAGTAGCT 7
GTGGTAGTAGTA 7
GTGGTGGTAGTA 7
GTGGTGGTGGTG 7
AGTAAGTAGTAG 6
GTAGTAAGTAGT 6
GTAGTAGTATAG 6
TAGTAGTAGCTA 6
TAGTAGTAGTAT 6
TGGTAGTAGTAG 6
TGGTGGTGGTGG 6
AGATAGTAGTAG 5
AGTAGTAAGTAG 5
AGTAGTAGTAGA 5
AGTAGTAGTATA 5
AGTGGTAGTGGT 5

>m141231_015334_42134_c100756552550000001823146807221542_s1_p0/125904/0_5494

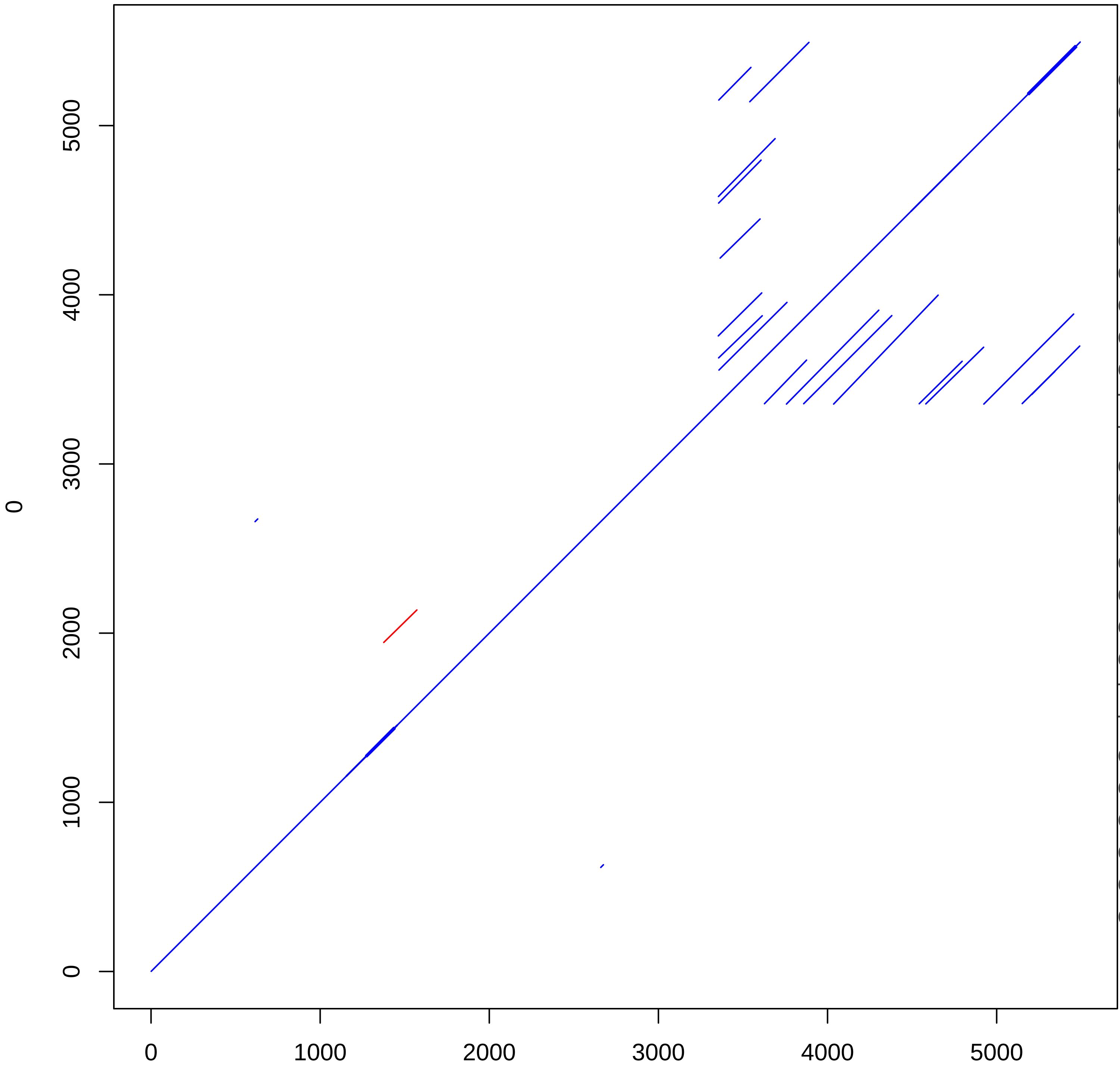

>m141231_015334_42134_c100756552550000001823146807221542_s1_p0/125979/1601_10212

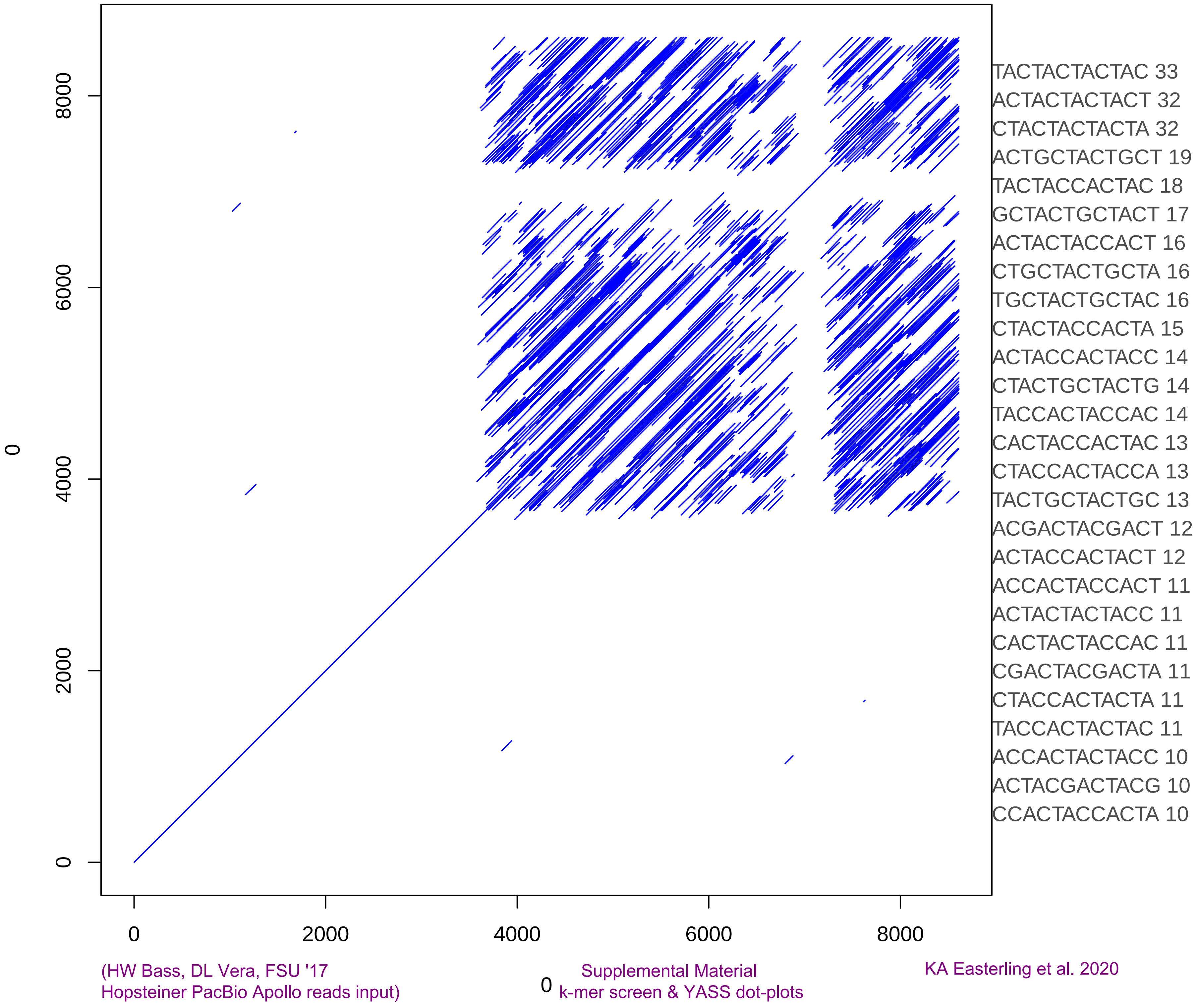

>m141231_015334_42134_c100756552550000001823146807221542_s1_p0/126099/593_7611

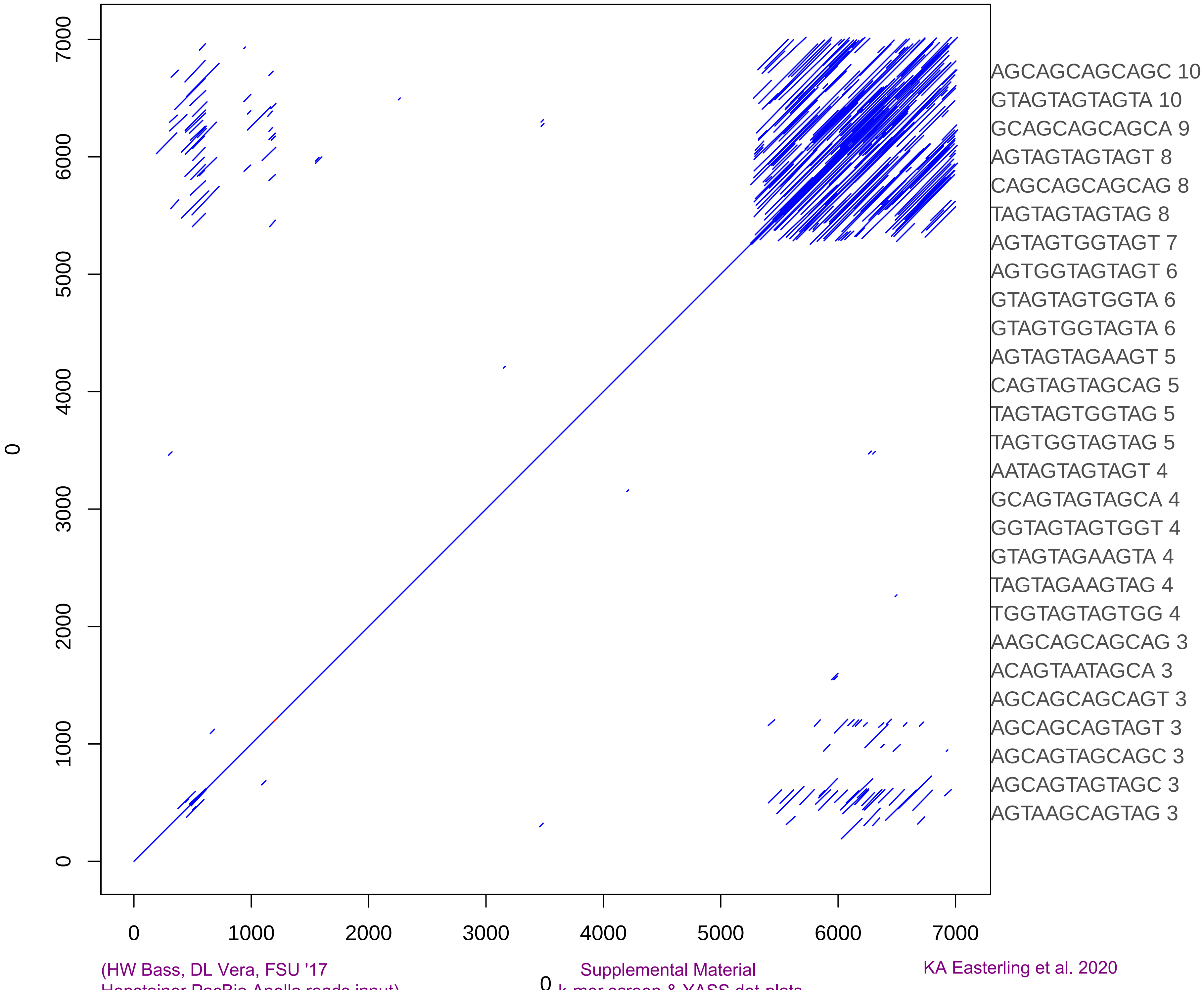

>m141231_015334_42134_c100756552550000001823146807221542_s1_p0/126190/0_5637

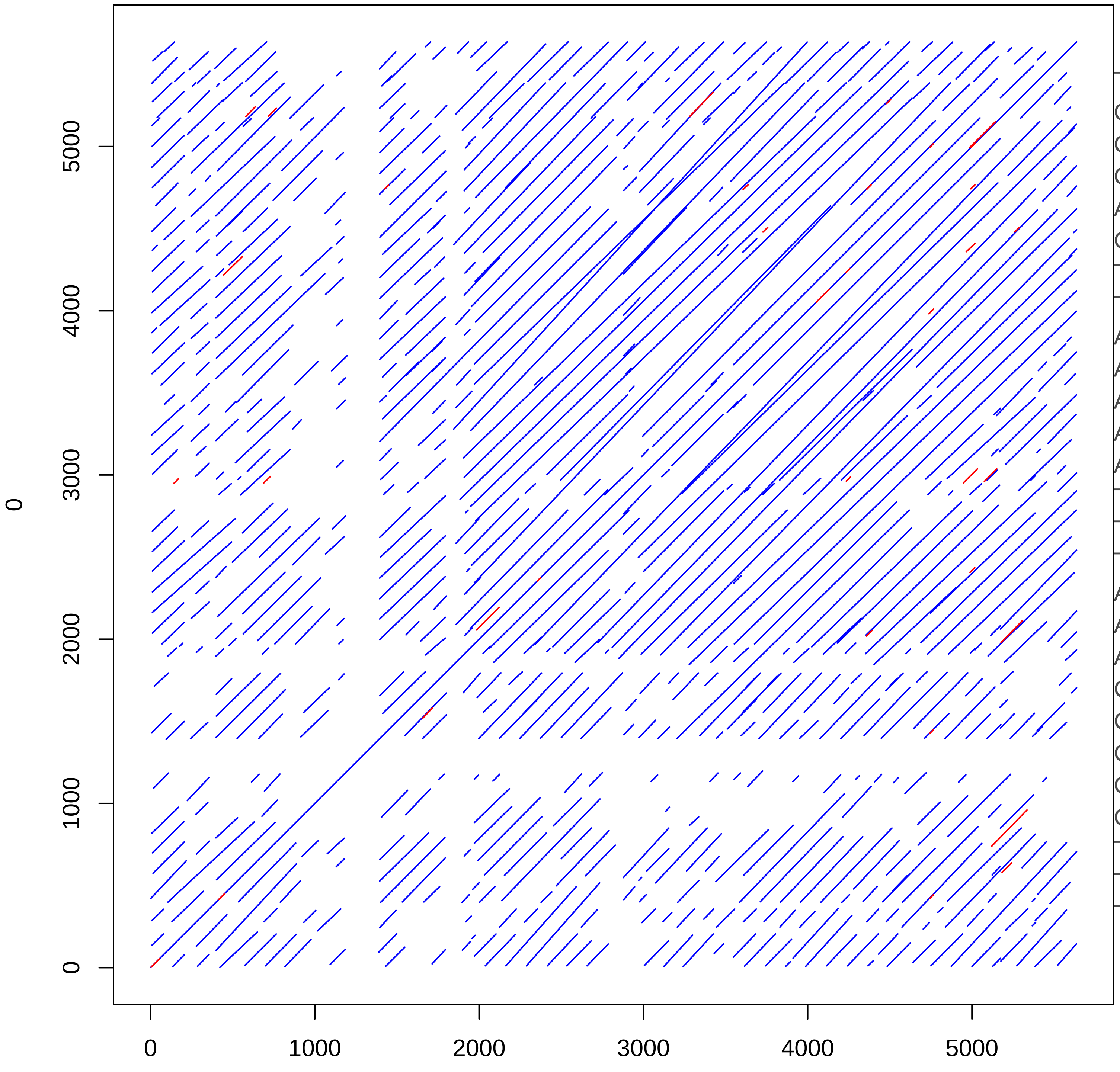

>m141231_015334_42134_c100756552550000001823146807221542_s1_p0/127932/251_10610

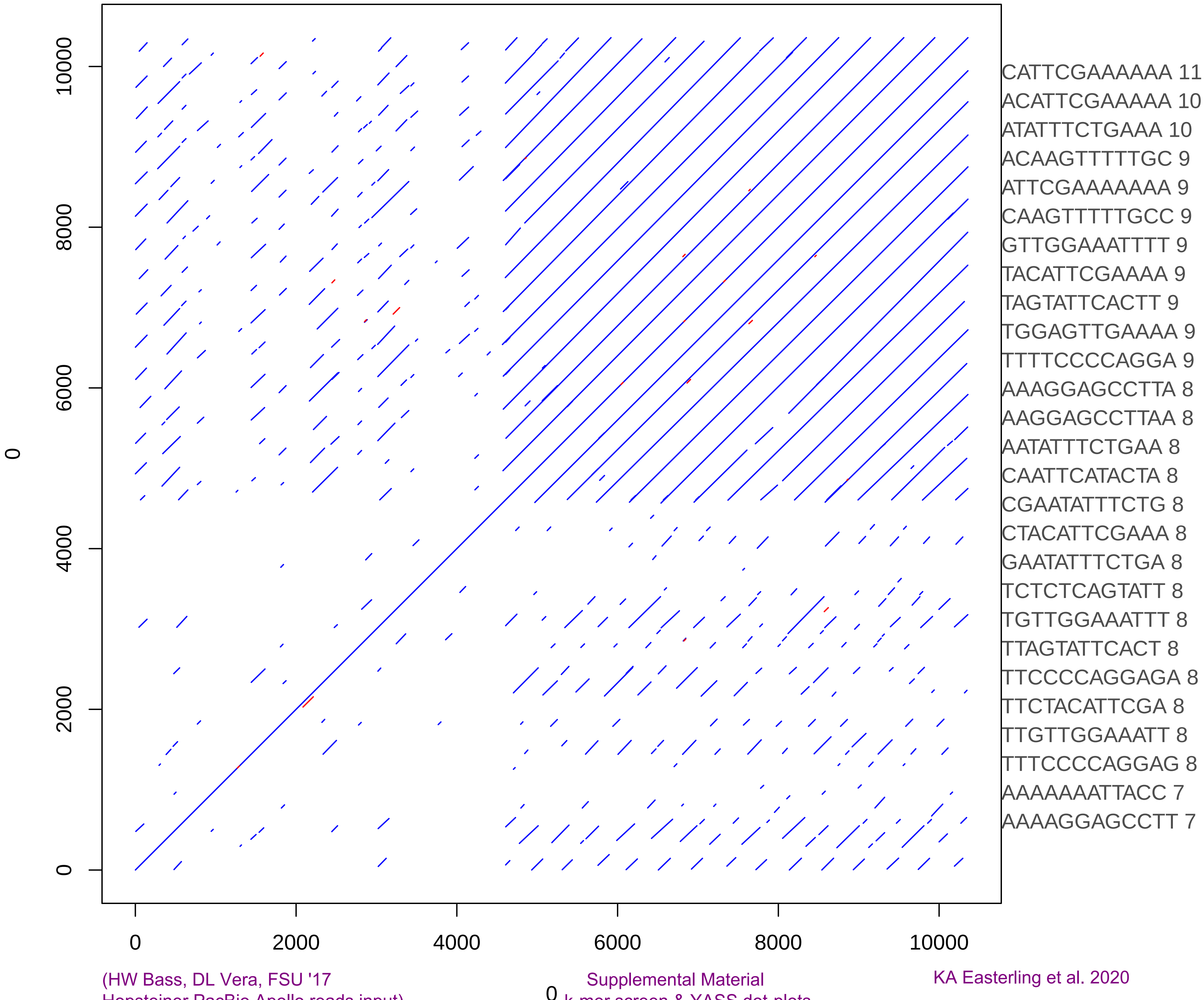

>m141231_015334_42134_c100756552550000001823146807221542_s1_p0/136075/8962_15018

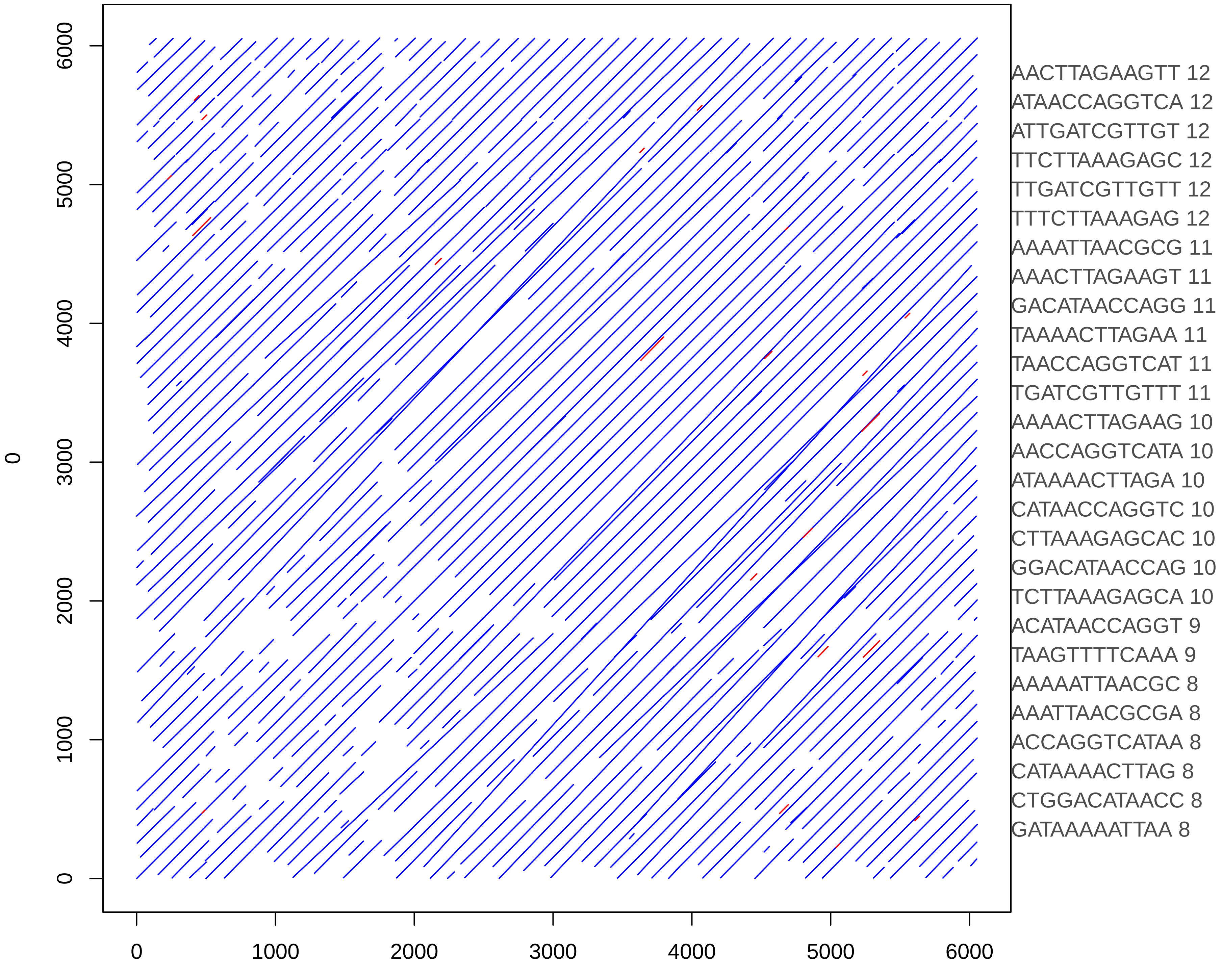

>m141231_015334_42134_c100756552550000001823146807221542_s1_p0/138253/0_10757

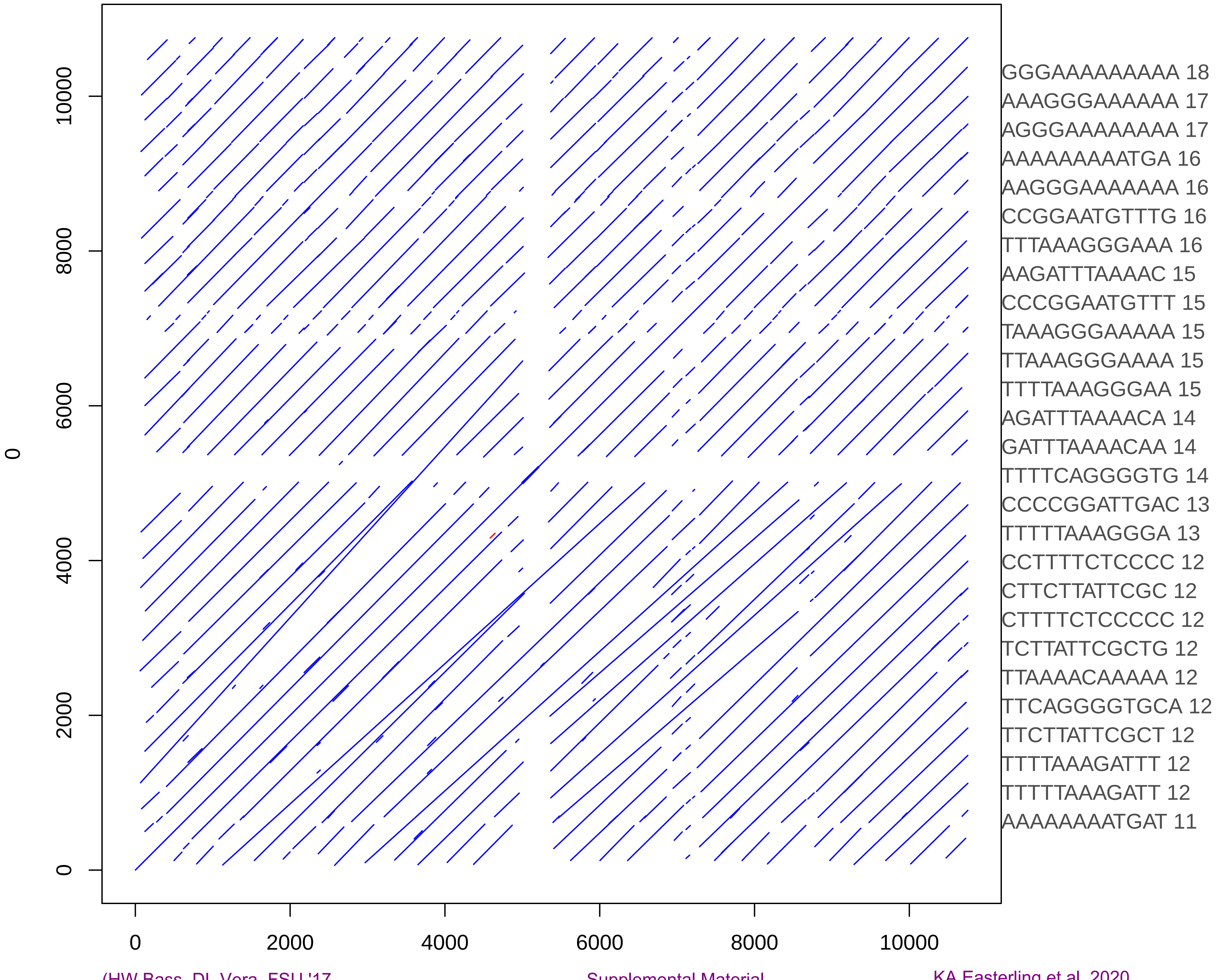

>m141231_015334_42134_c100756552550000001823146807221542_s1_p0/138747/6369_18250

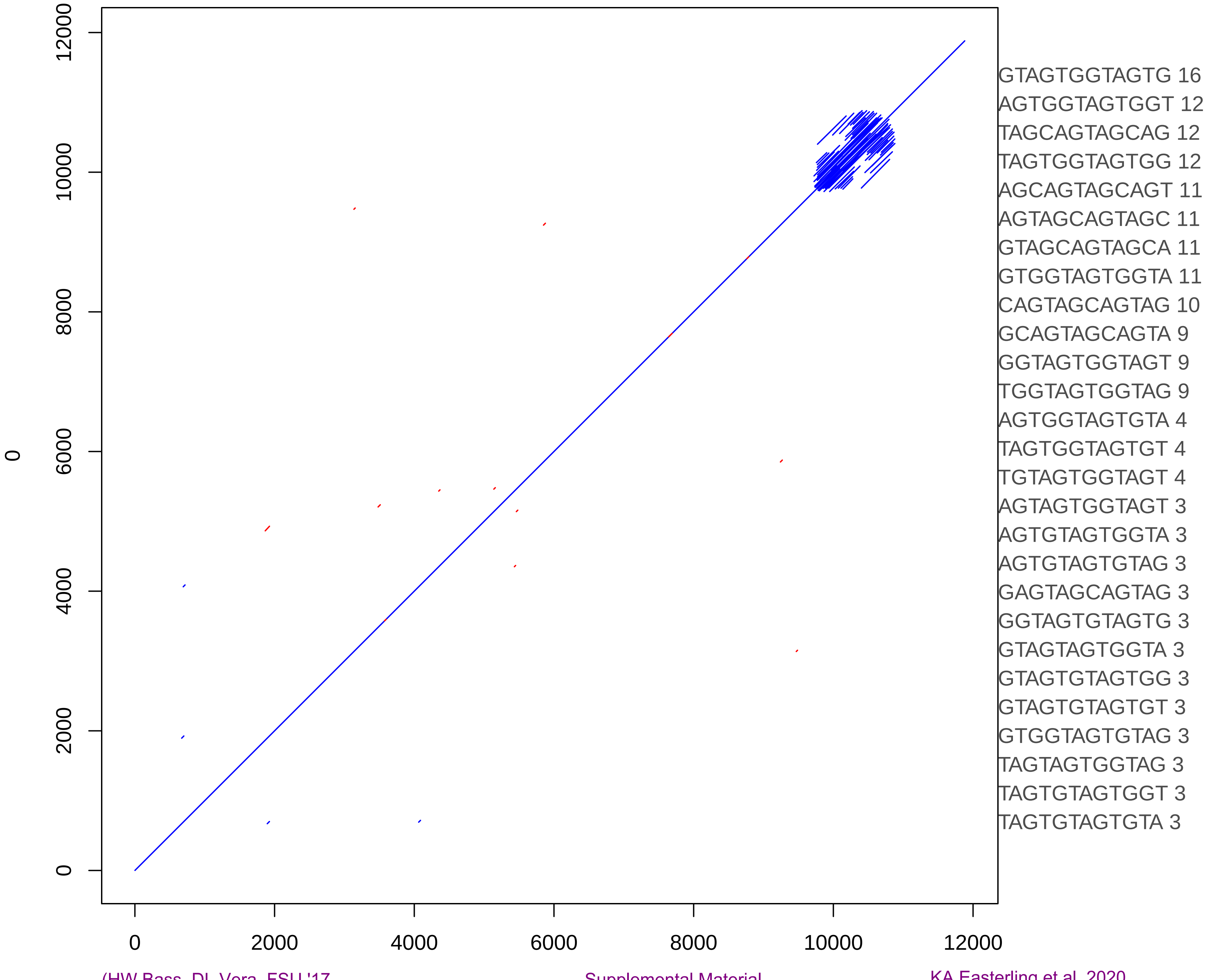

>m141231_015334_42134_c100756552550000001823146807221542_s1_p0/13876/561_12091

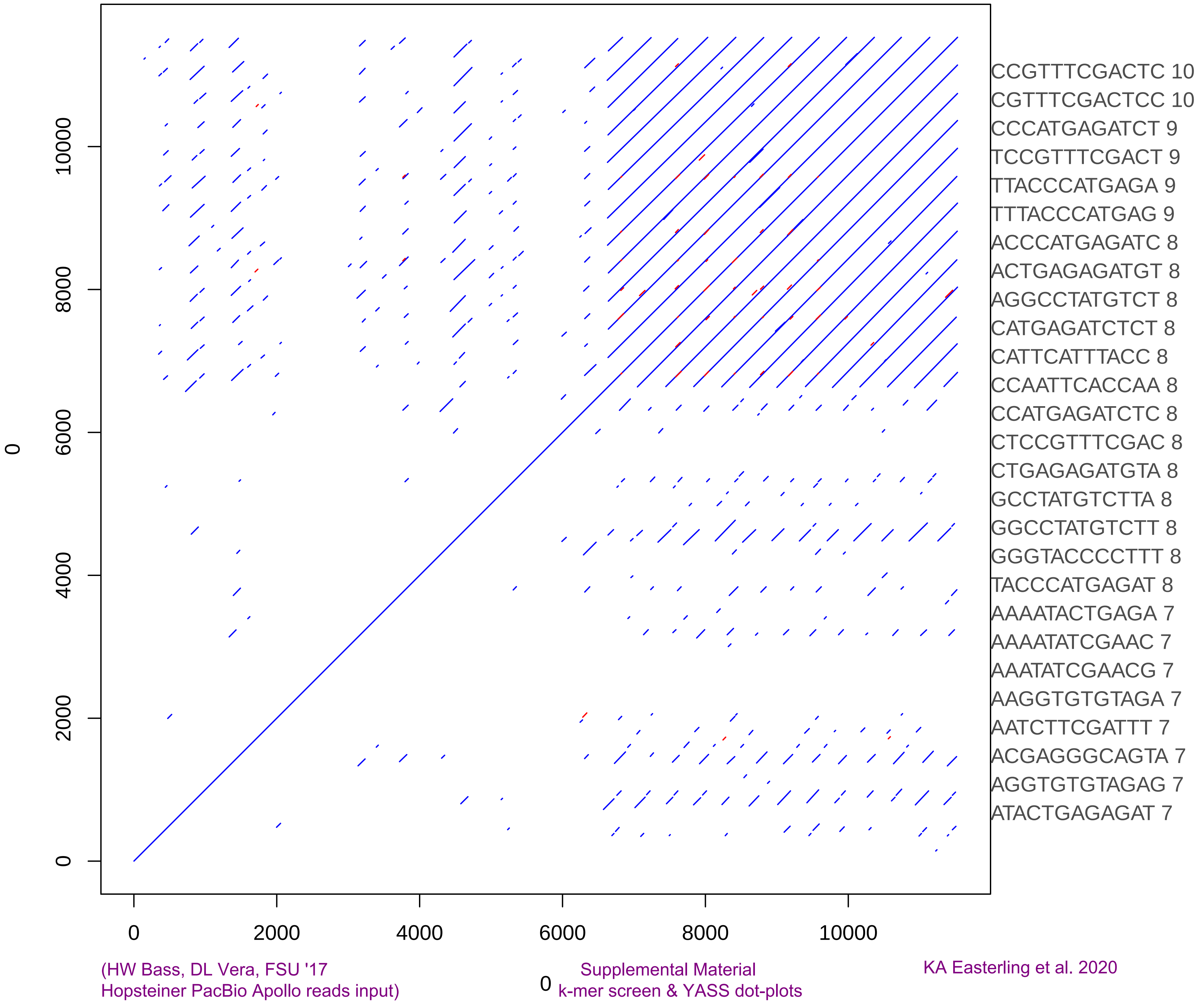

>m141231_015334_42134_c100756552550000001823146807221542_s1_p0/139618/0_5026

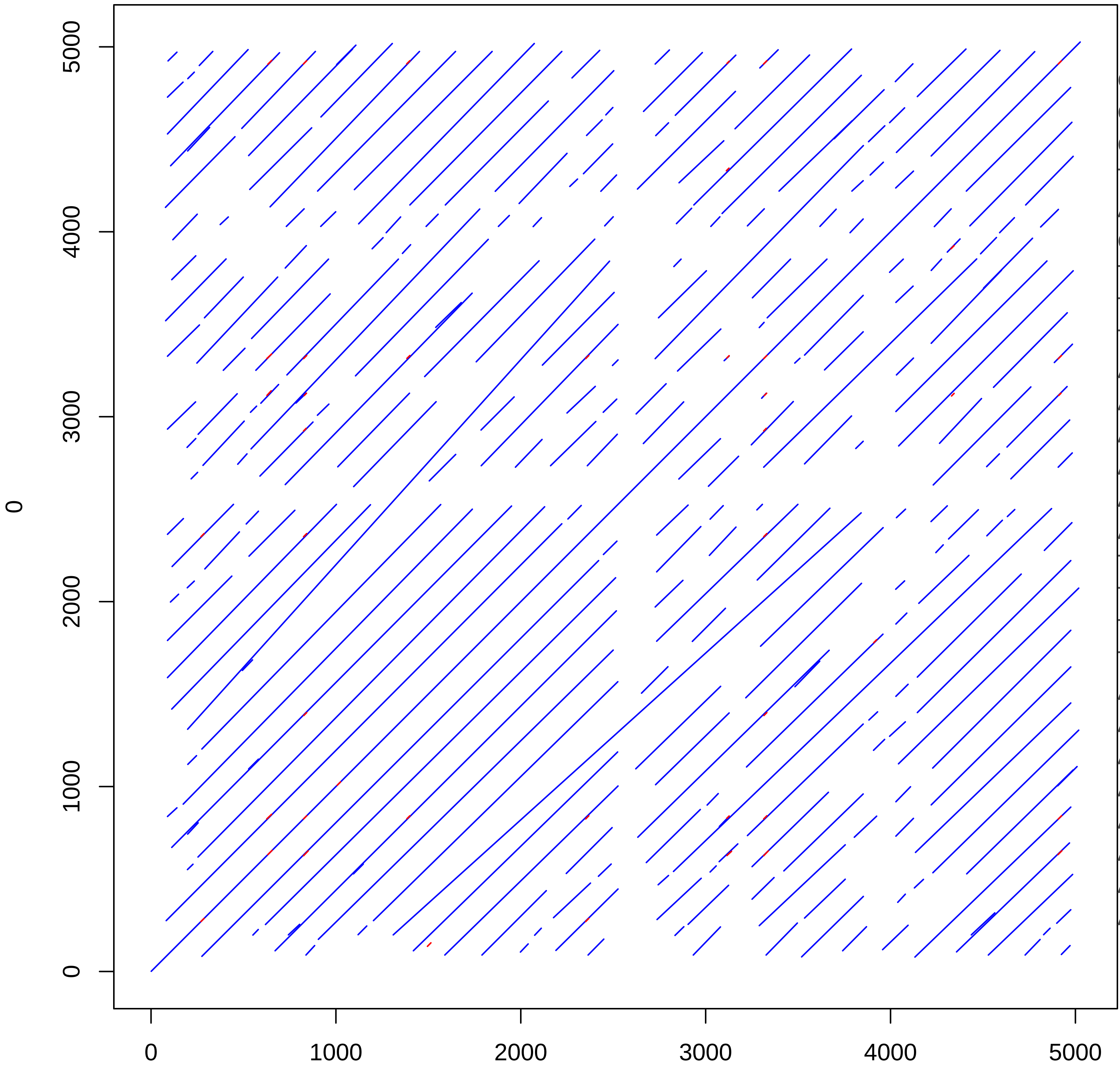

>m141231_015334_42134_c100756552550000001823146807221542_s1_p0/141107/8011_18504

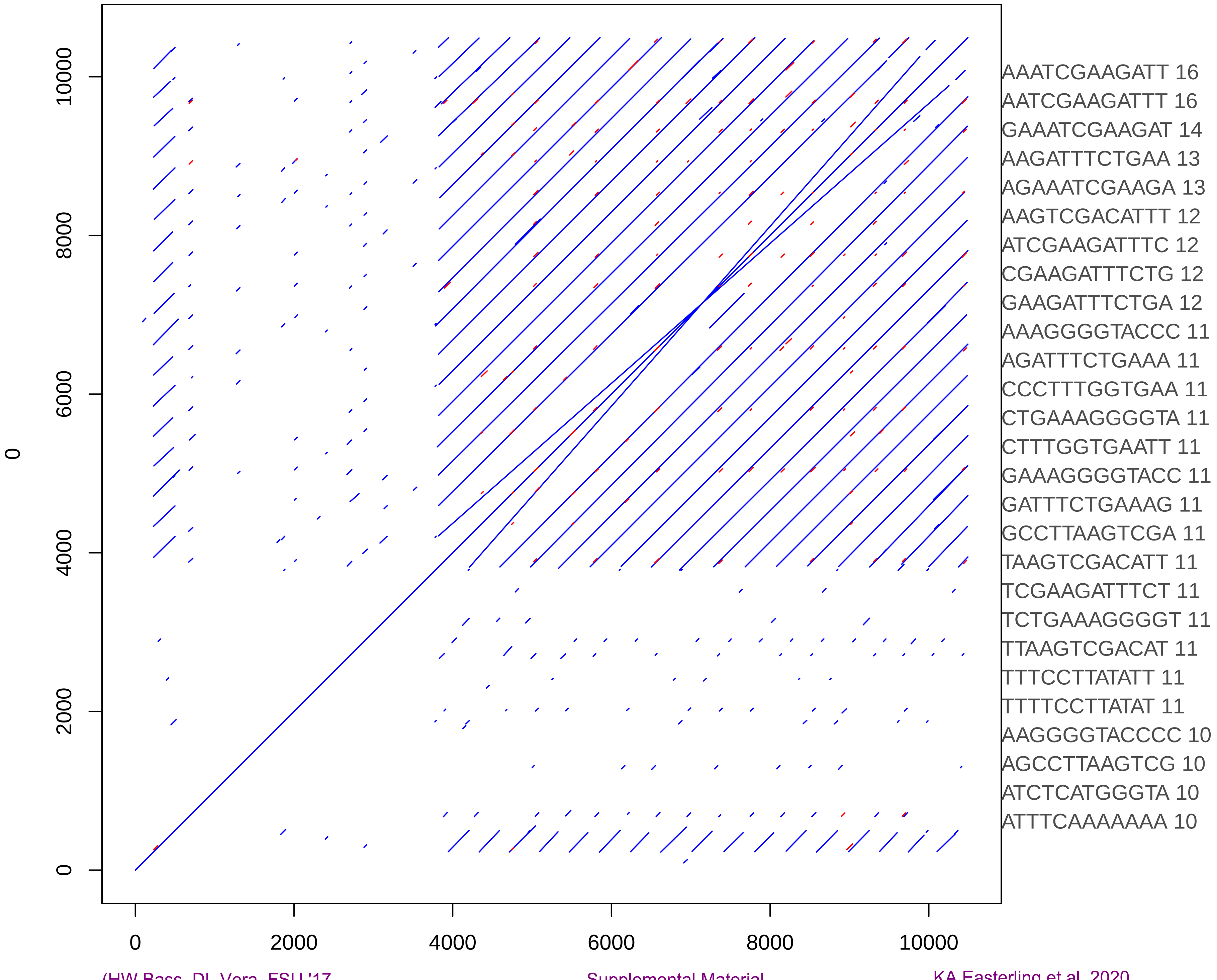

>m141231_015334_42134_c100756552550000001823146807221542_s1_p0/14401/0_11069

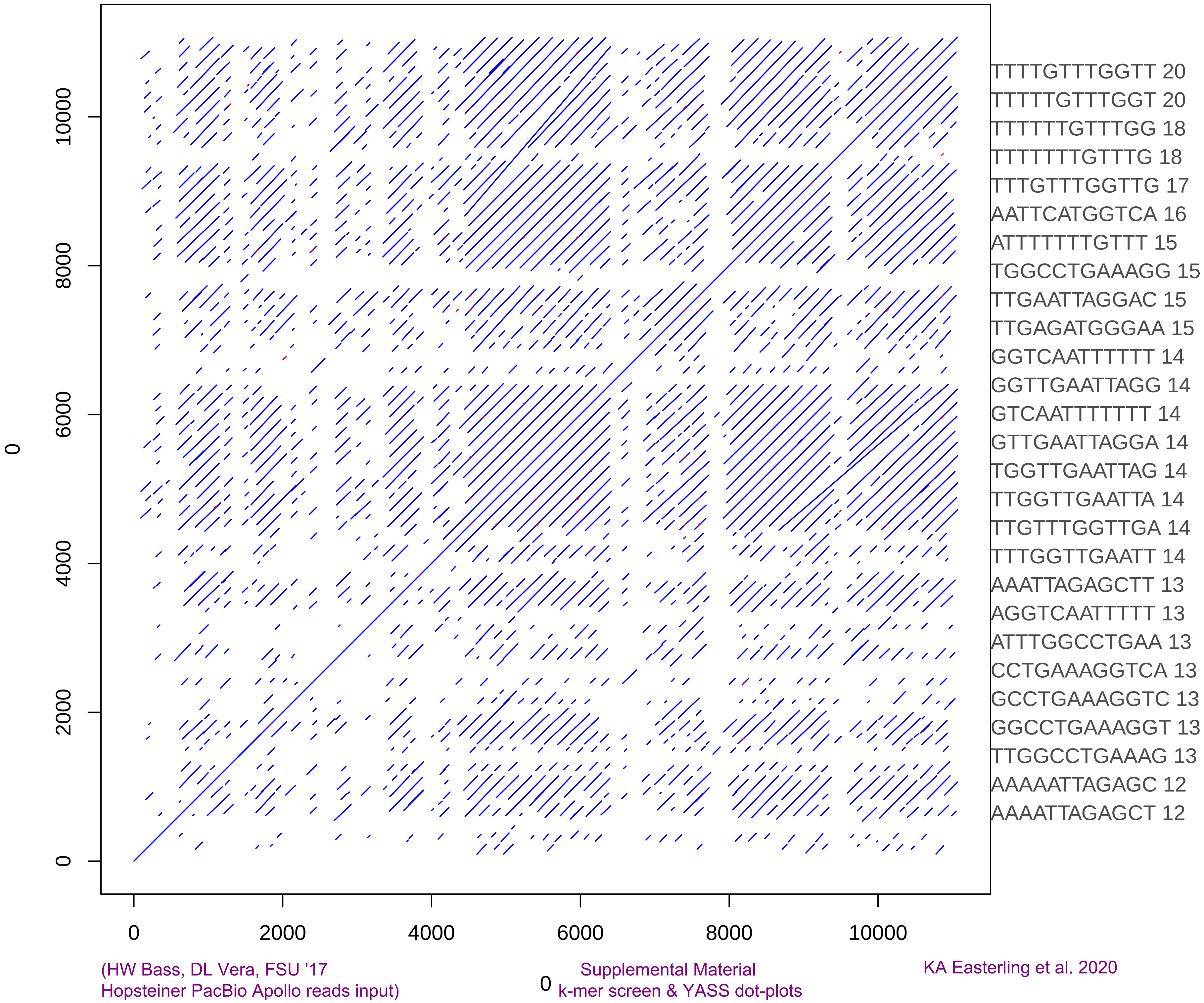

>m141231_015334_42134_c100756552550000001823146807221542_s1_p0/144226/0_10390

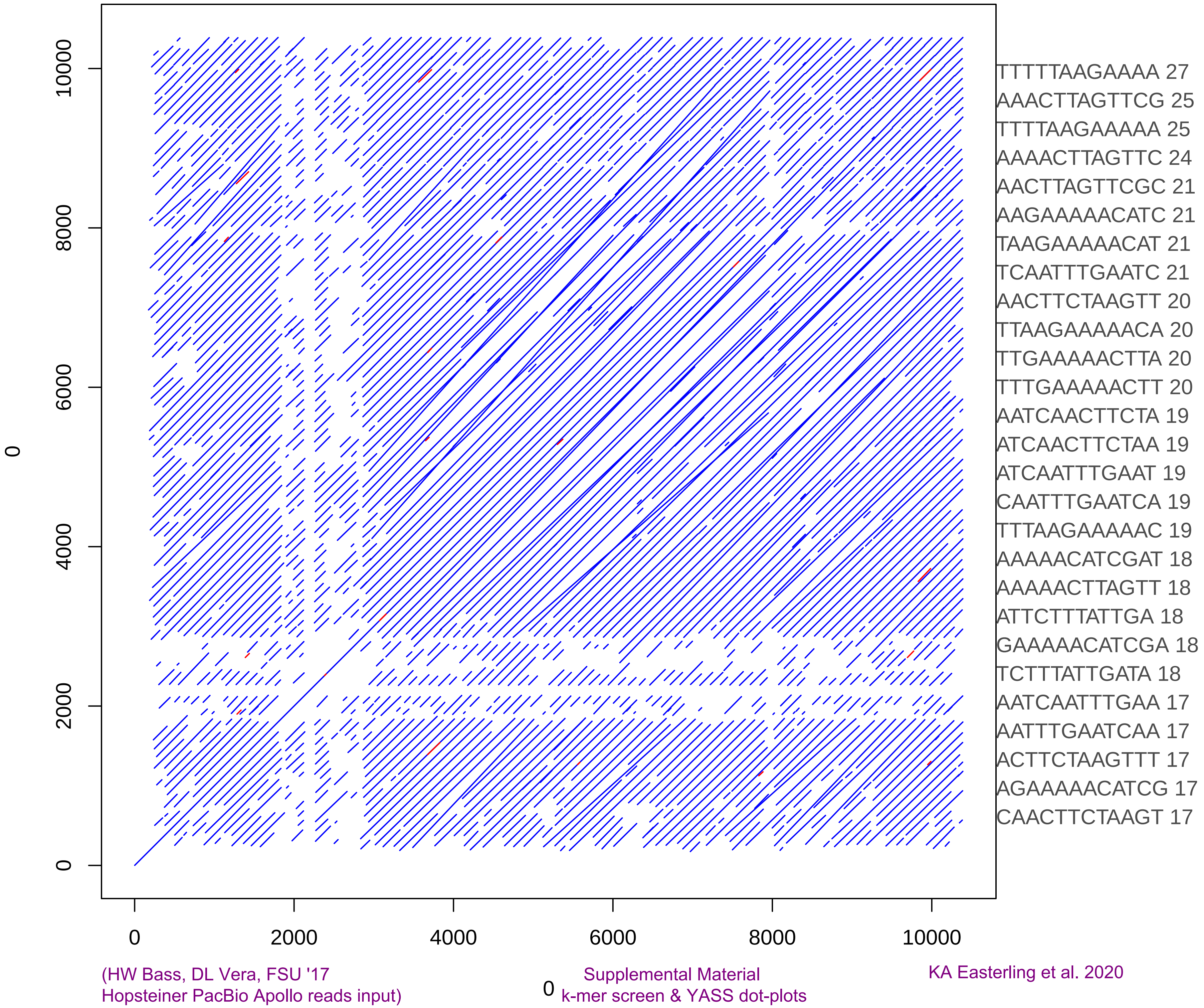

>m141231_015334_42134_c100756552550000001823146807221542_s1_p0/144488/3197_12020

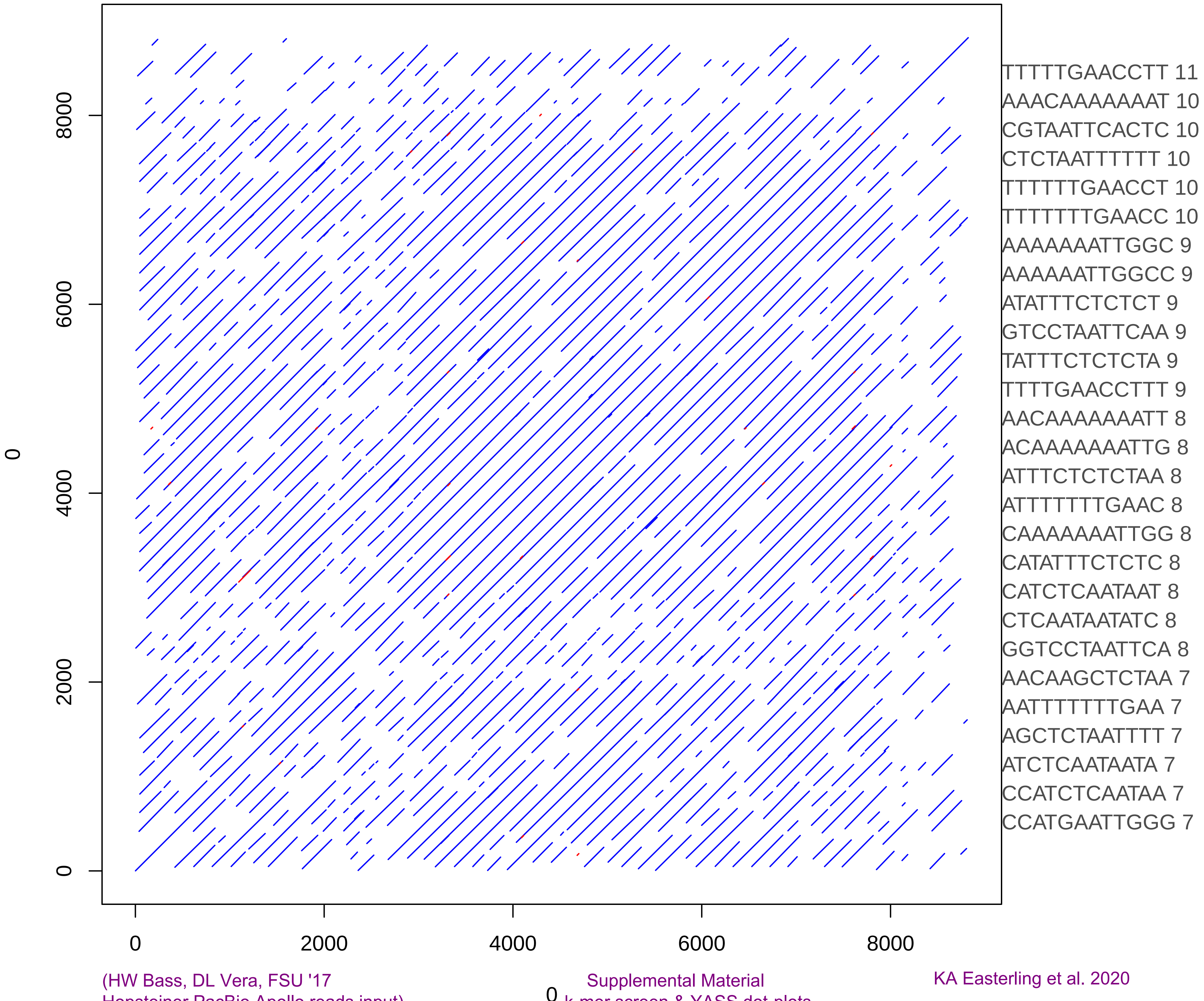

>m141231_015334_42134_c100756552550000001823146807221542_s1_p0/149672/0_10535

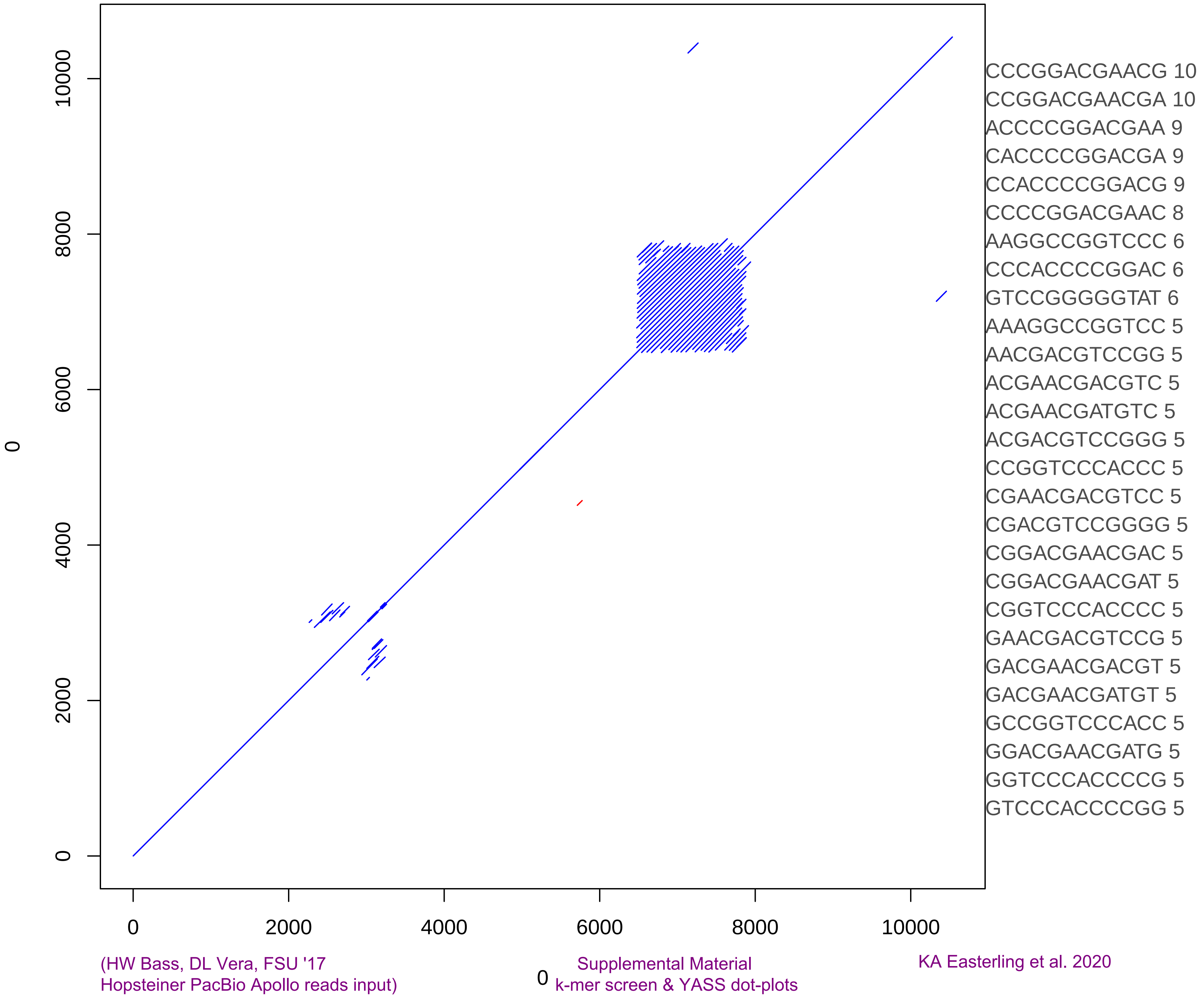

>m141231_015334_42134_c100756552550000001823146807221542_s1_p0/151360/2380_12946

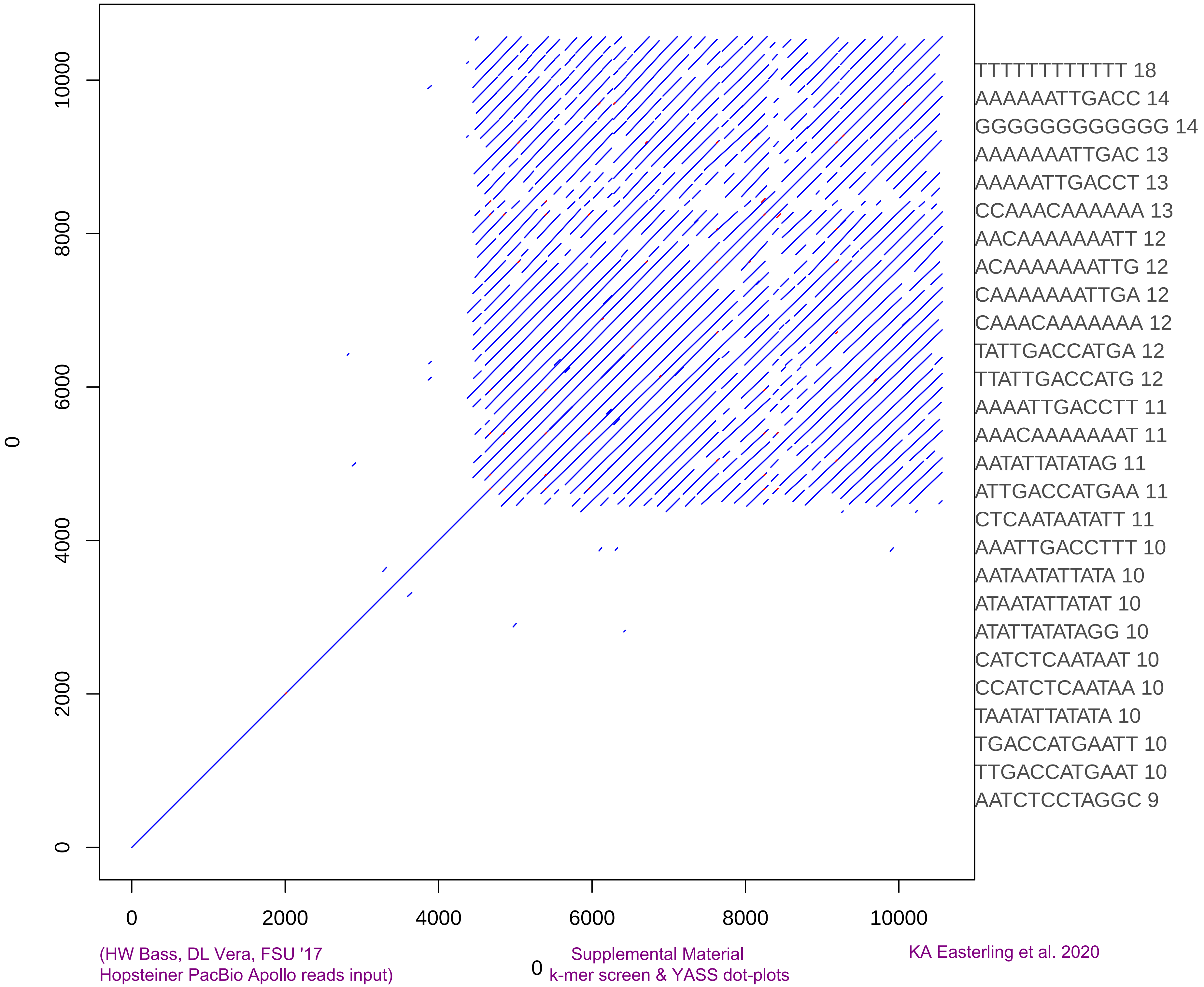

>m141231_015334_42134_c100756552550000001823146807221542_s1_p0/153412/9247_15477

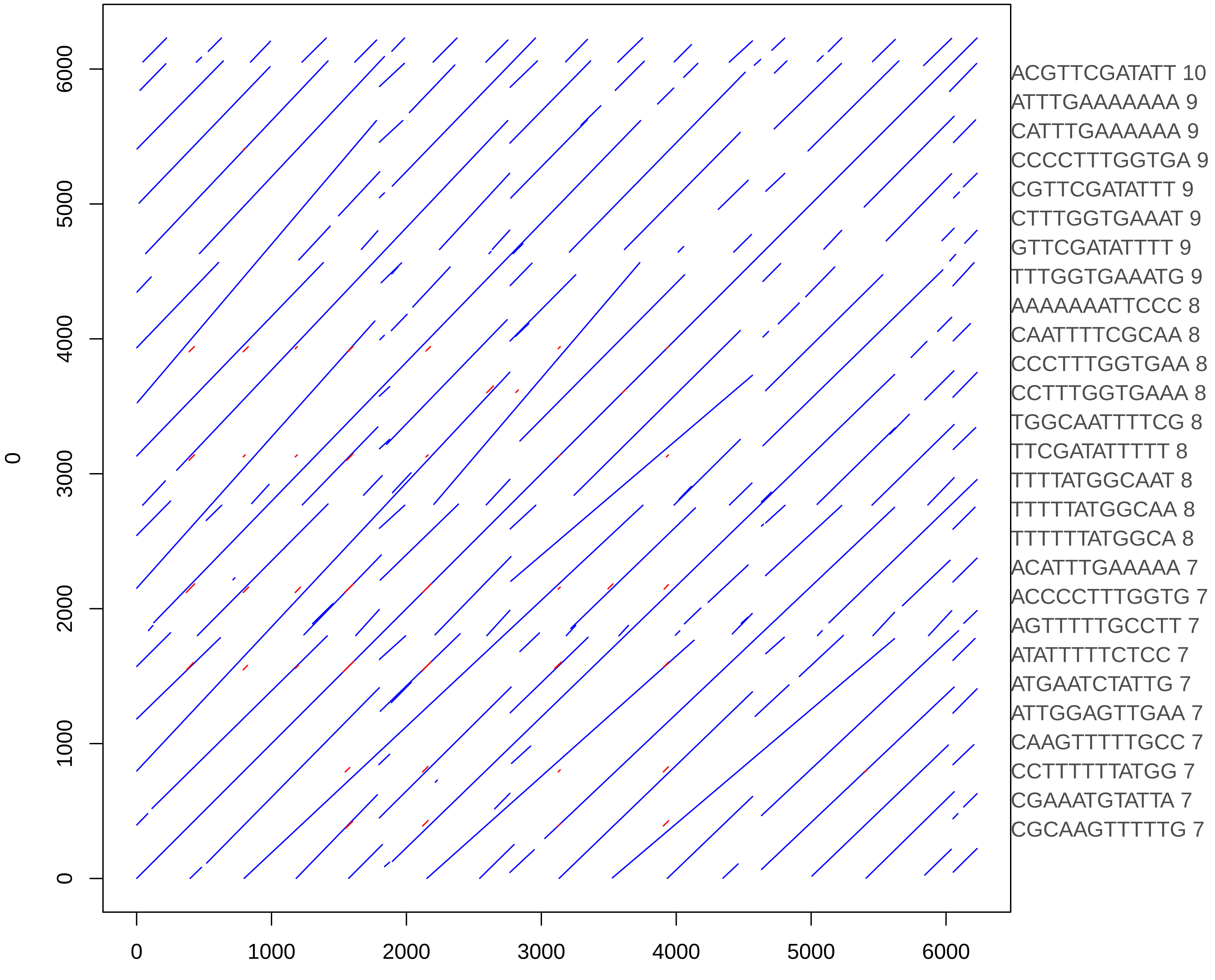

>m141231_015334_42134_c100756552550000001823146807221542_s1_p0/157181/0_6793

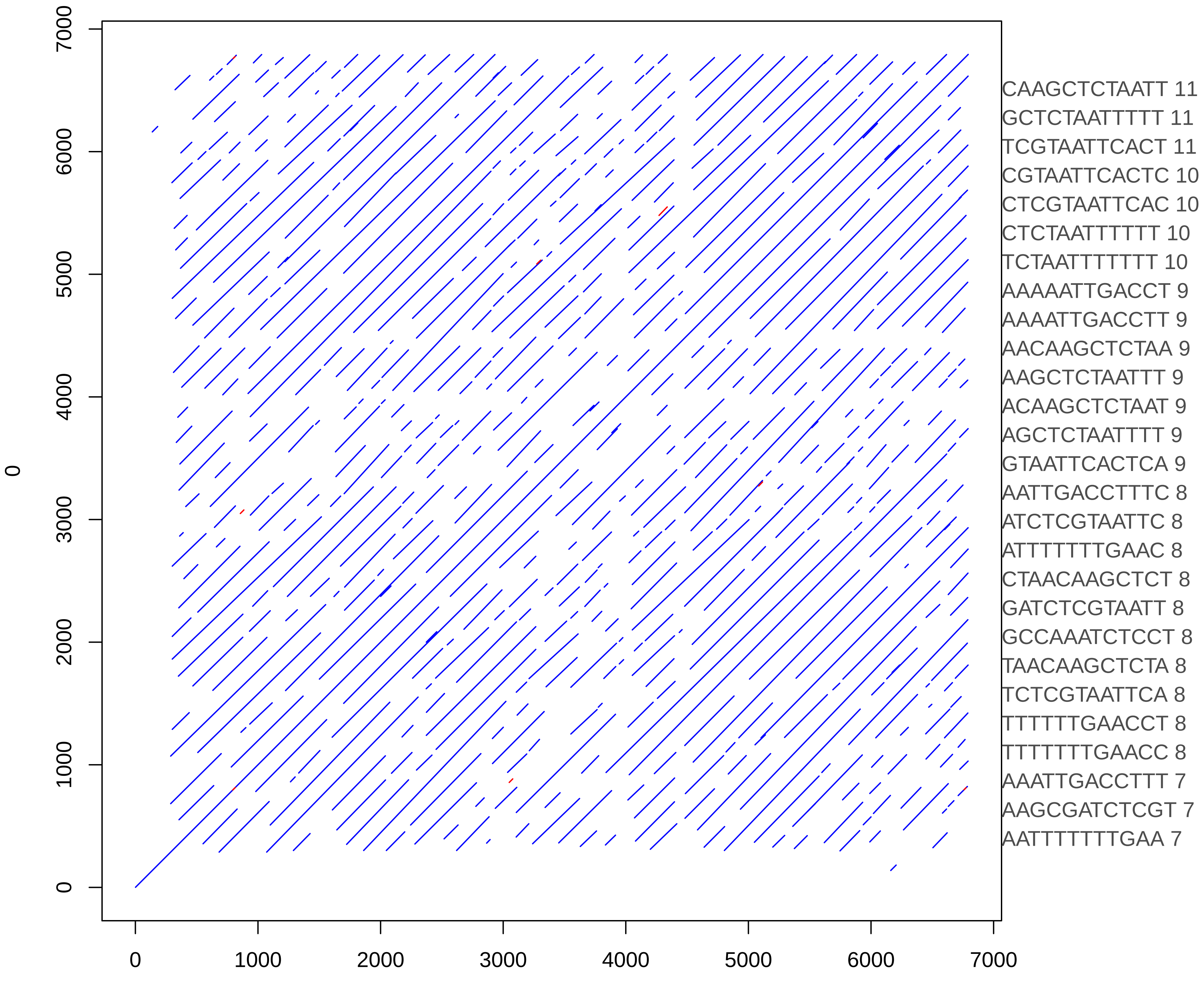

>m141231_015334_42134_c100756552550000001823146807221542_s1_p0/15751/0_6085

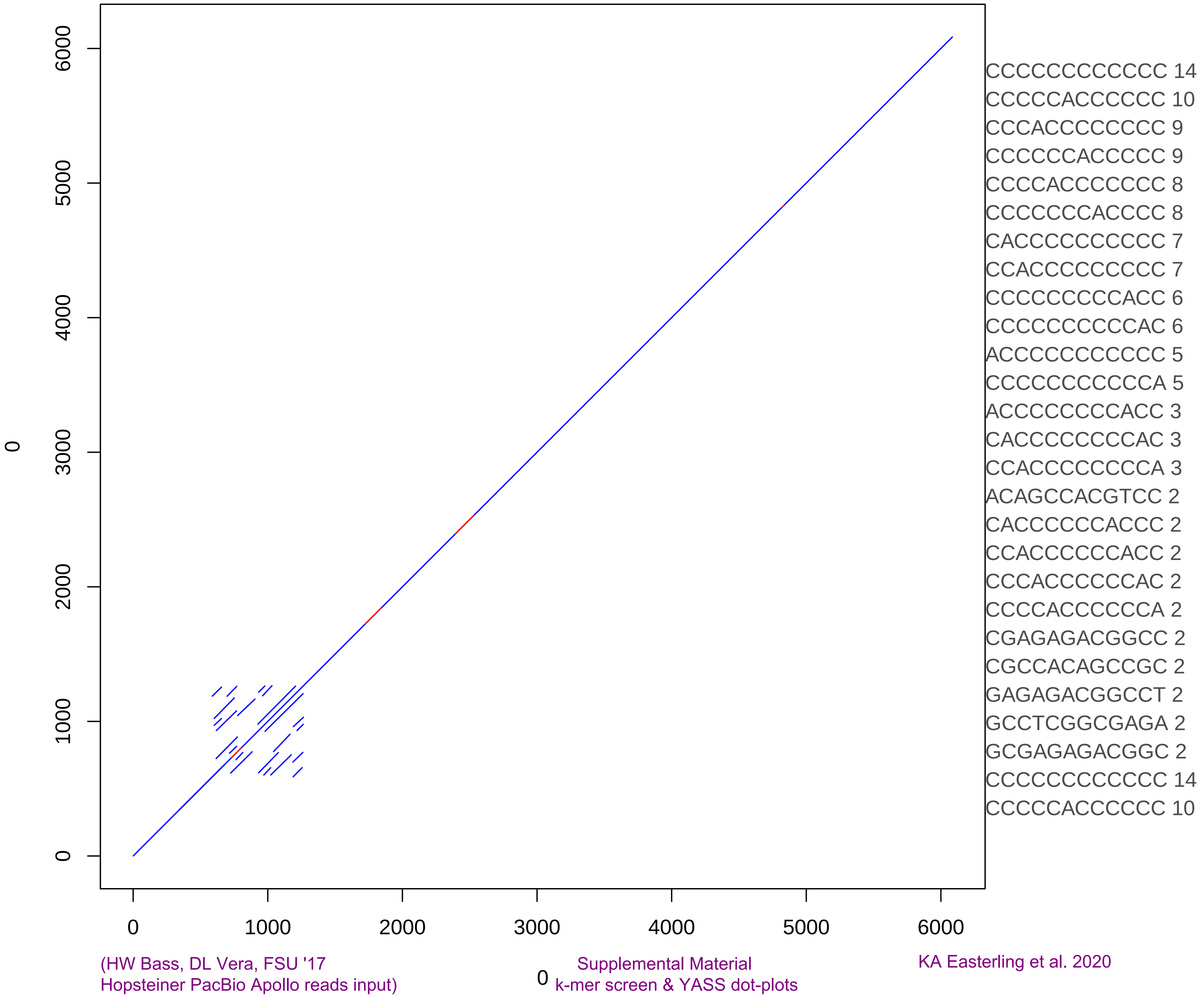

>m141231_015334_42134_c100756552550000001823146807221542_s1_p0/157834/4839_13142

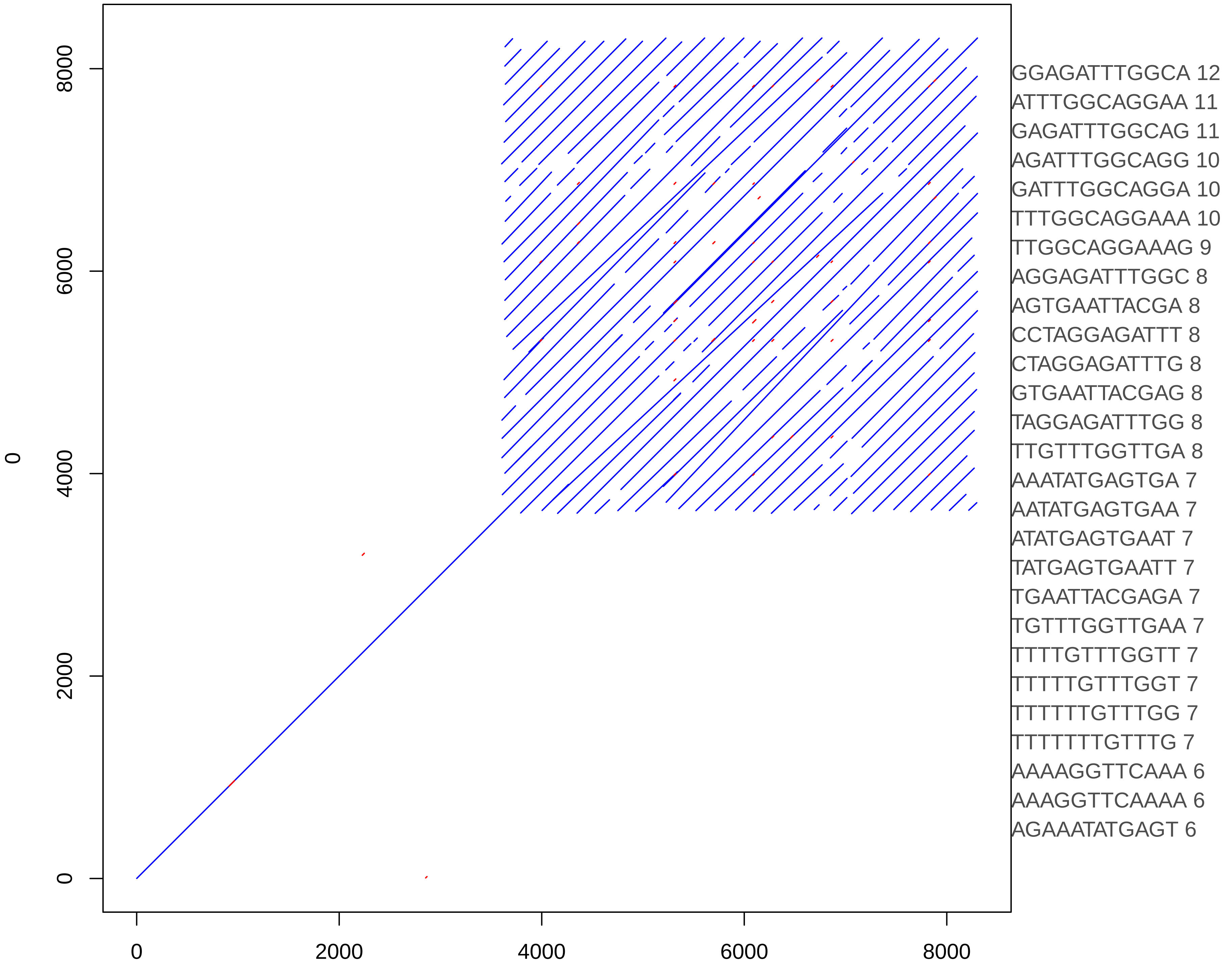

>m141231_015334_42134_c100756552550000001823146807221542_s1_p0/160063/668_10035

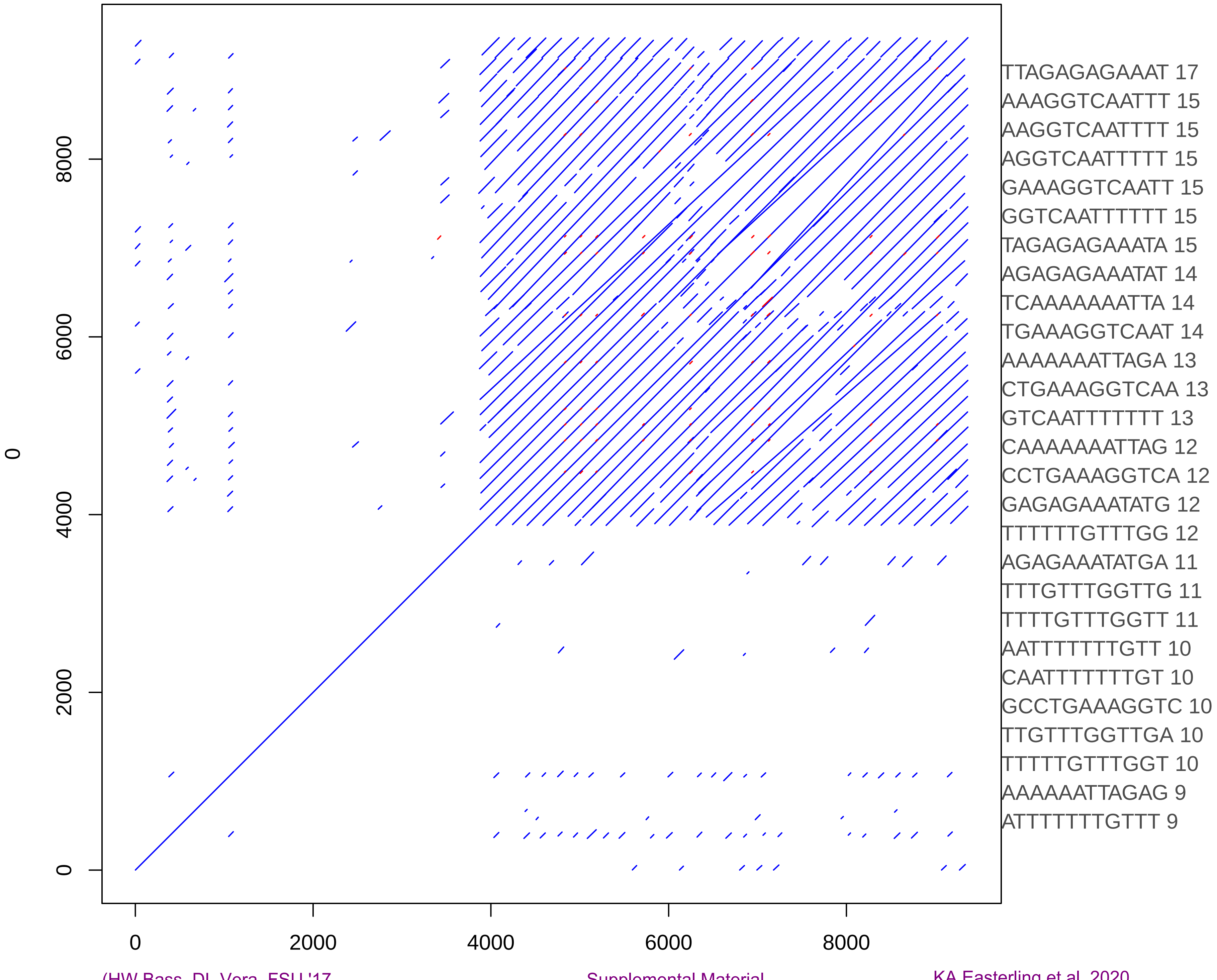

>m141231_015334_42134_c100756552550000001823146807221542_s1_p0/160525/0_5374

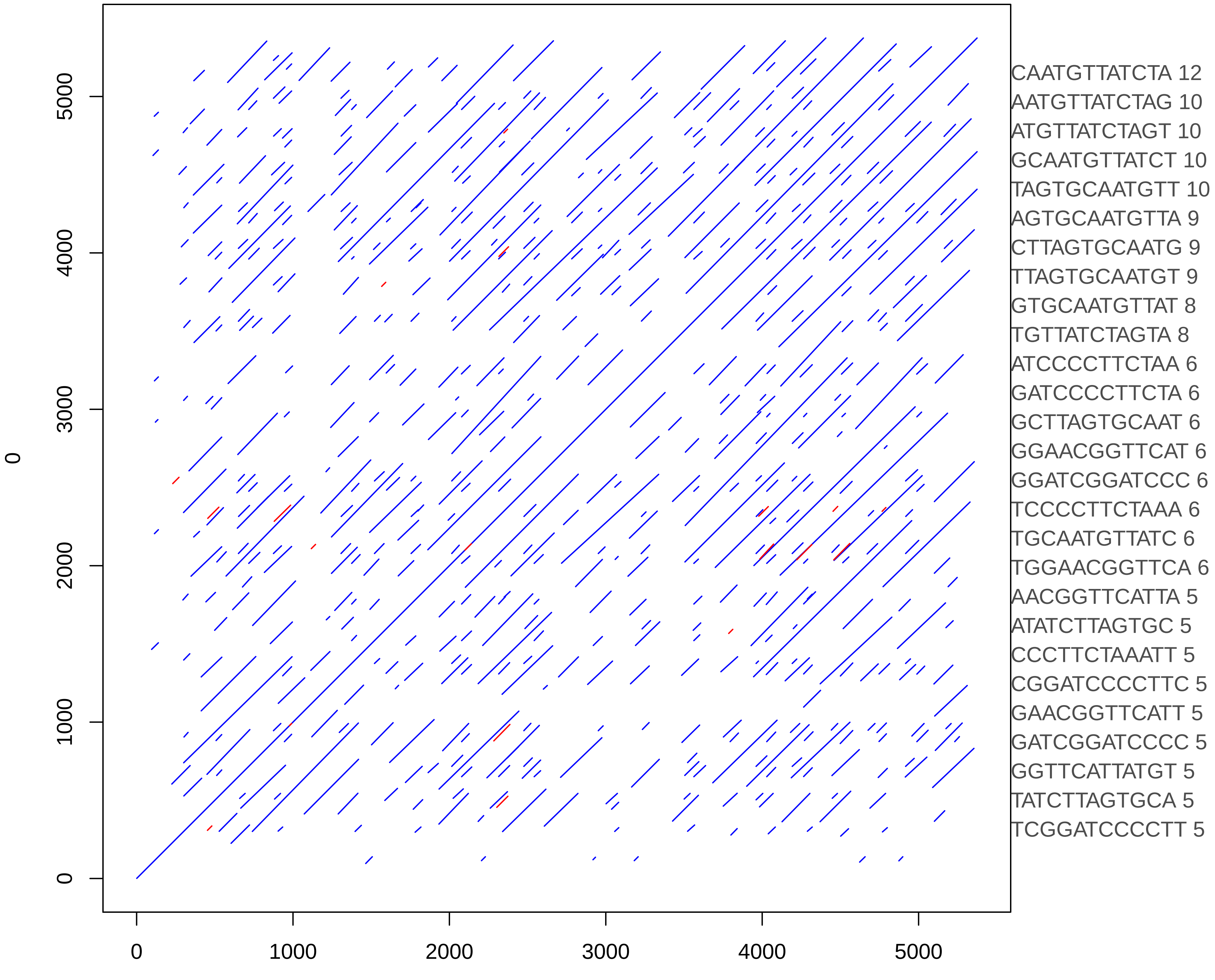

>m141231_015334_42134_c100756552550000001823146807221542_s1_p0/161963/266_12888

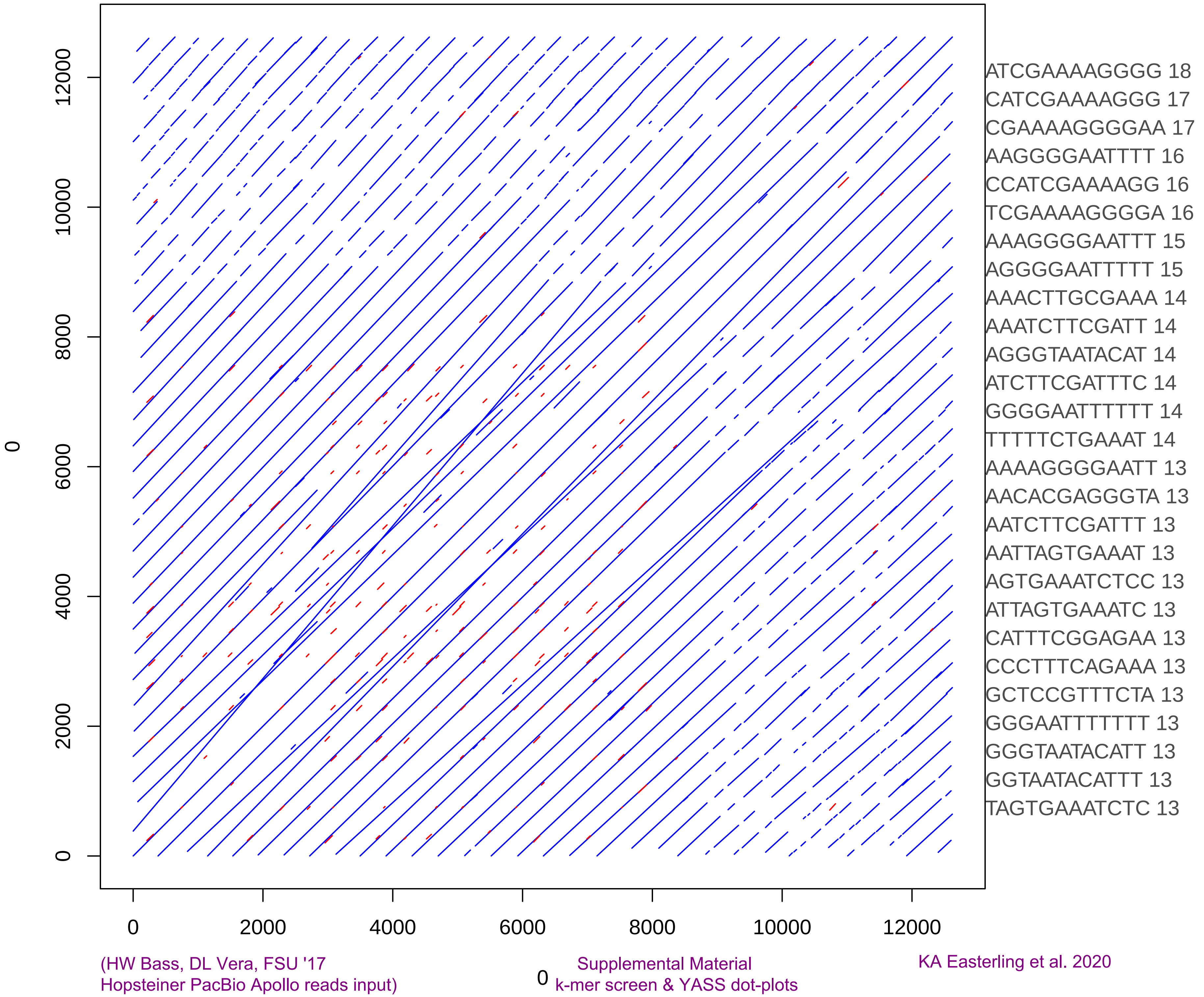

>m141231_015334_42134_c100756552550000001823146807221542_s1_p0/162838/0_6383

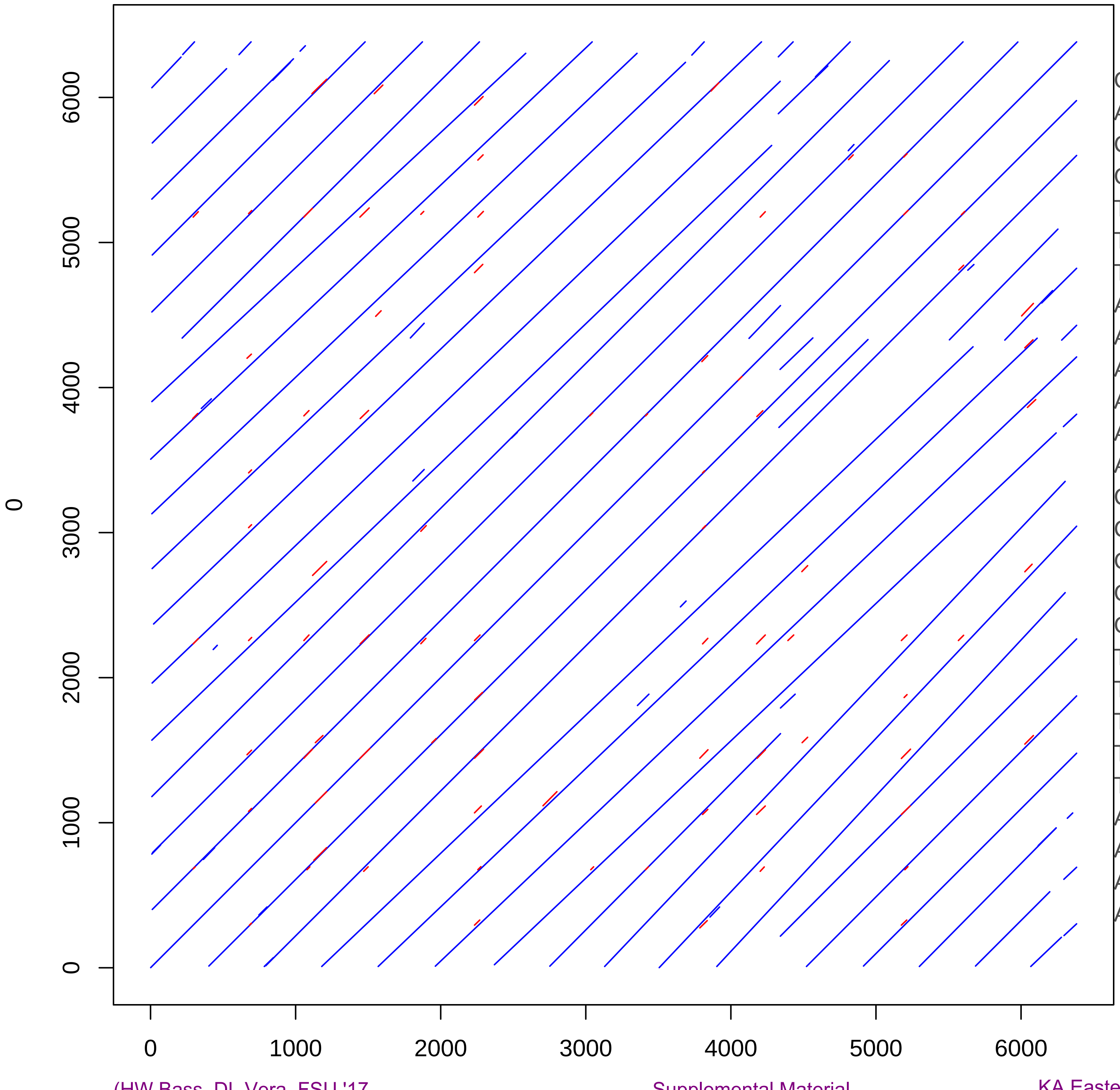

GCTCAAATCCAT 10
AAACTTGCTCAA 9
CTCAAATCCATA 9
GGGTACCCCTTT 9
TCAAATCCATAT 9
TGCTCAAATCCA 9
TTGTGCCCATGA 9
AAATCGAAGATT 8
AACTTGCTCAAA 8
AATCGAAGATTT 8
AATGTATTACCC 8
ATCGAAGATTTC 8
ATGTATTACCCT 8
CCGAAATGTATT 8
CTCGCATATTCC 8
GGAAATCGAAGA 8
GGGAAATCGAAG 8
GGTACCCCTTTG 8
TCTCATGGGTAA 8
TGTGCCCATGAC 8
TTTCGCAAGTTT 8
TTTGTGCCCATG 8
TTTTCGCAAGTT 8
AAAAACTTGCTC 7
AAAACTTGCTCA 7
AAATGTATTACC 7
ACATTTGAAAAA 7

>m141231_015334_42134_c100756552550000001823146807221542_s1_p0/16746/4294_16351

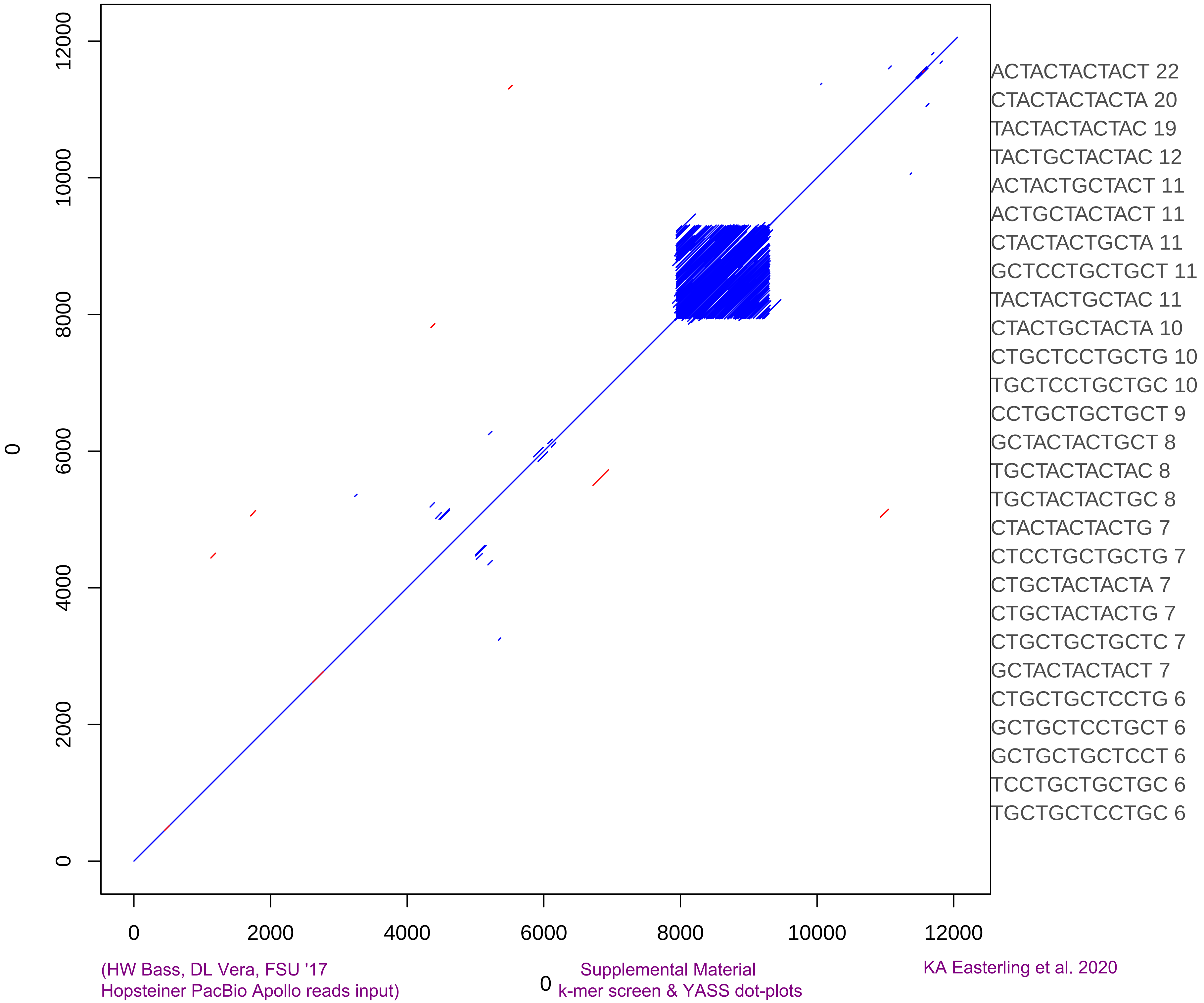

>m141231_015334_42134_c100756552550000001823146807221542_s1_p0/1813/5417_13511

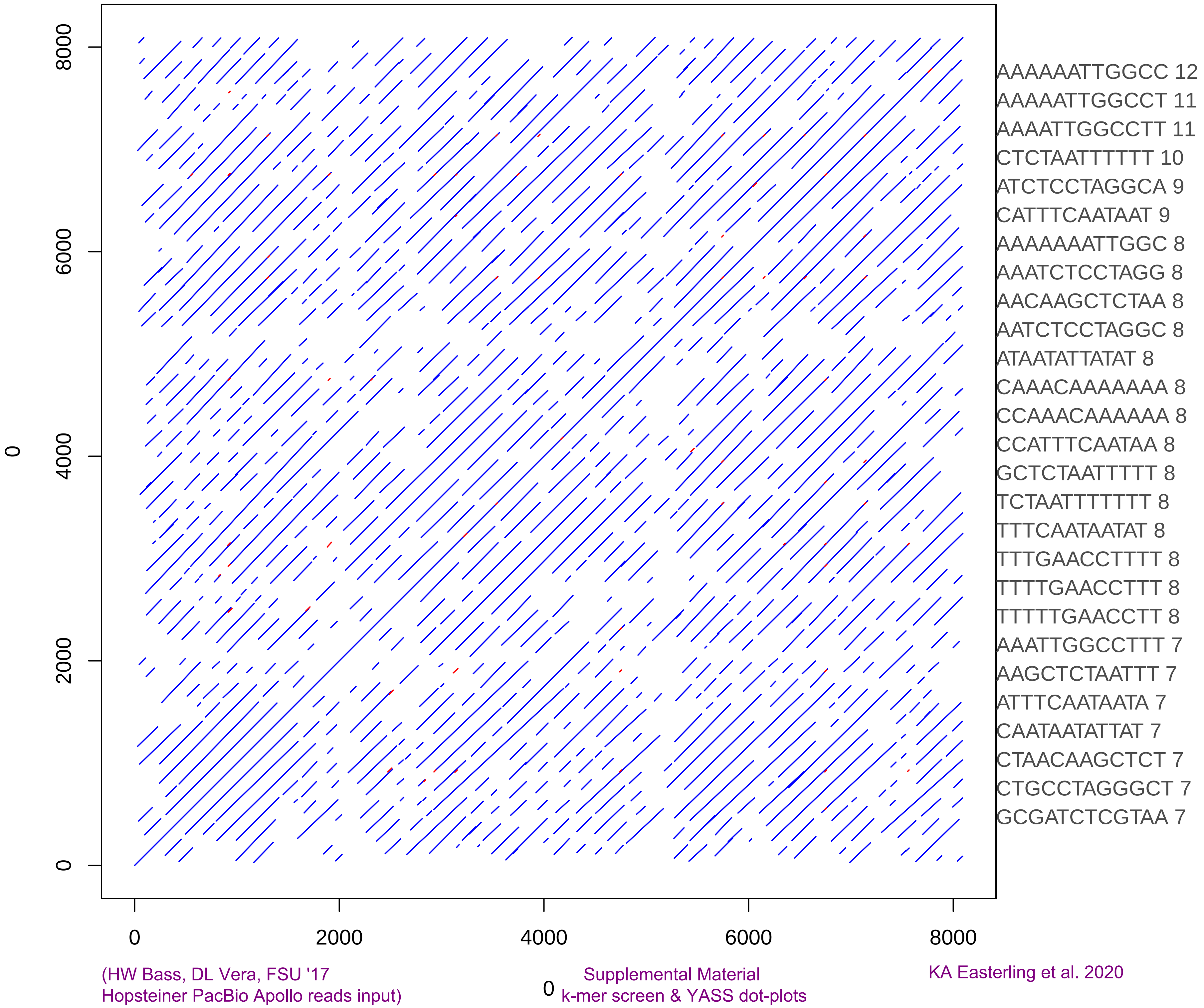

>m141231_015334_42134_c100756552550000001823146807221542_s1_p0/18375/0_9638

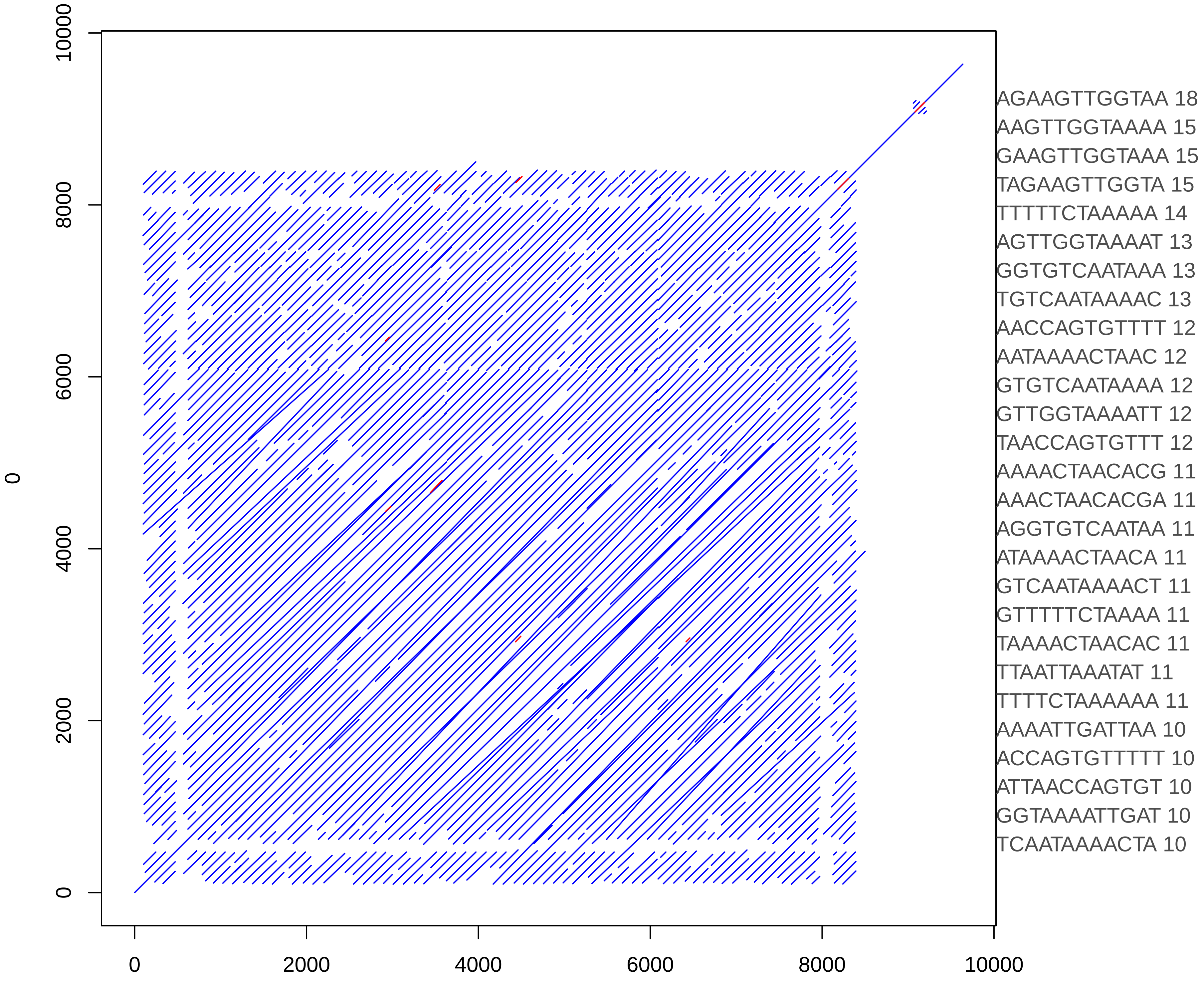

>m141231_015334_42134_c100756552550000001823146807221542_s1_p0/18472/5161_12723

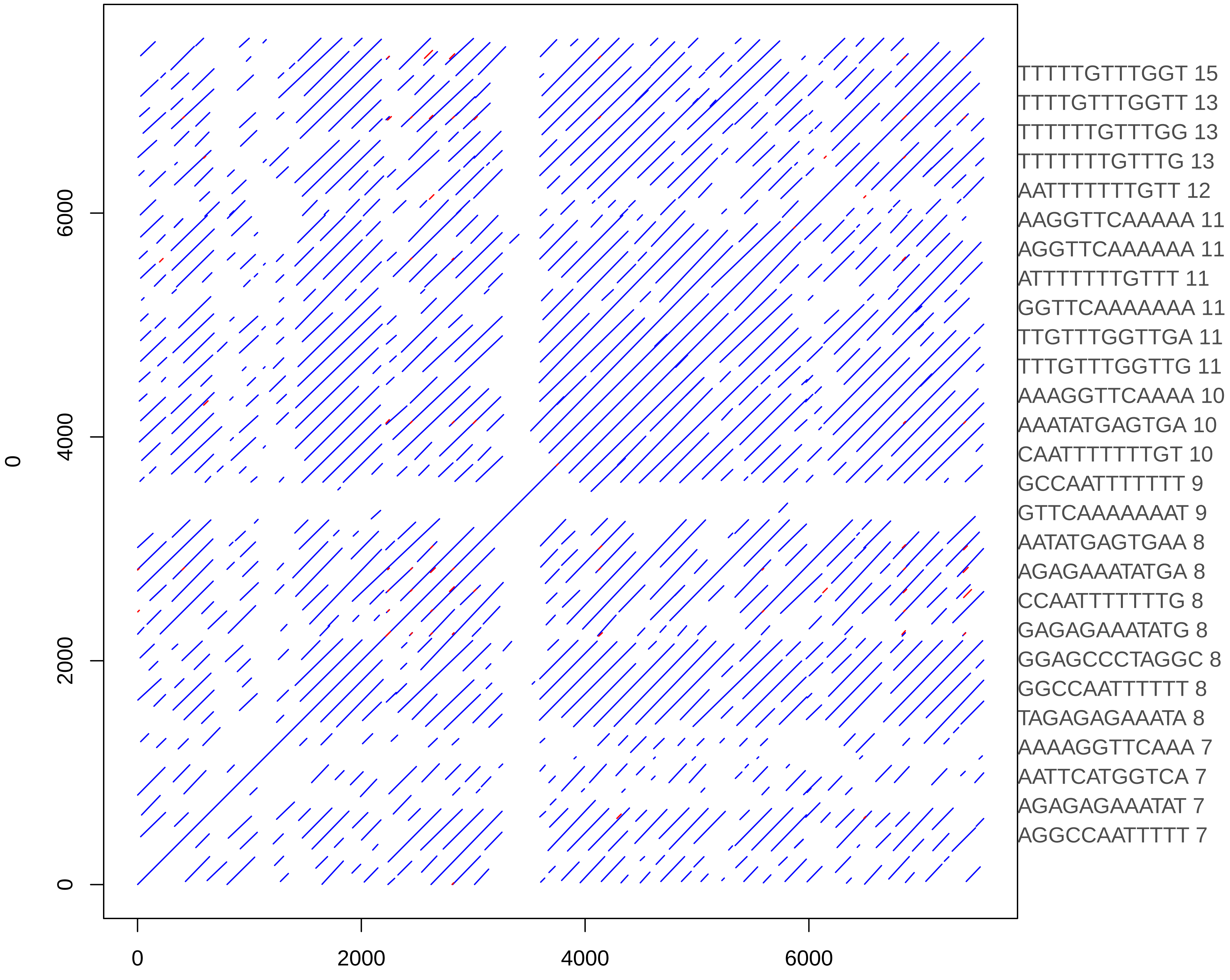

>m141231_015334_42134_c100756552550000001823146807221542_s1_p0/19011/2760_11314

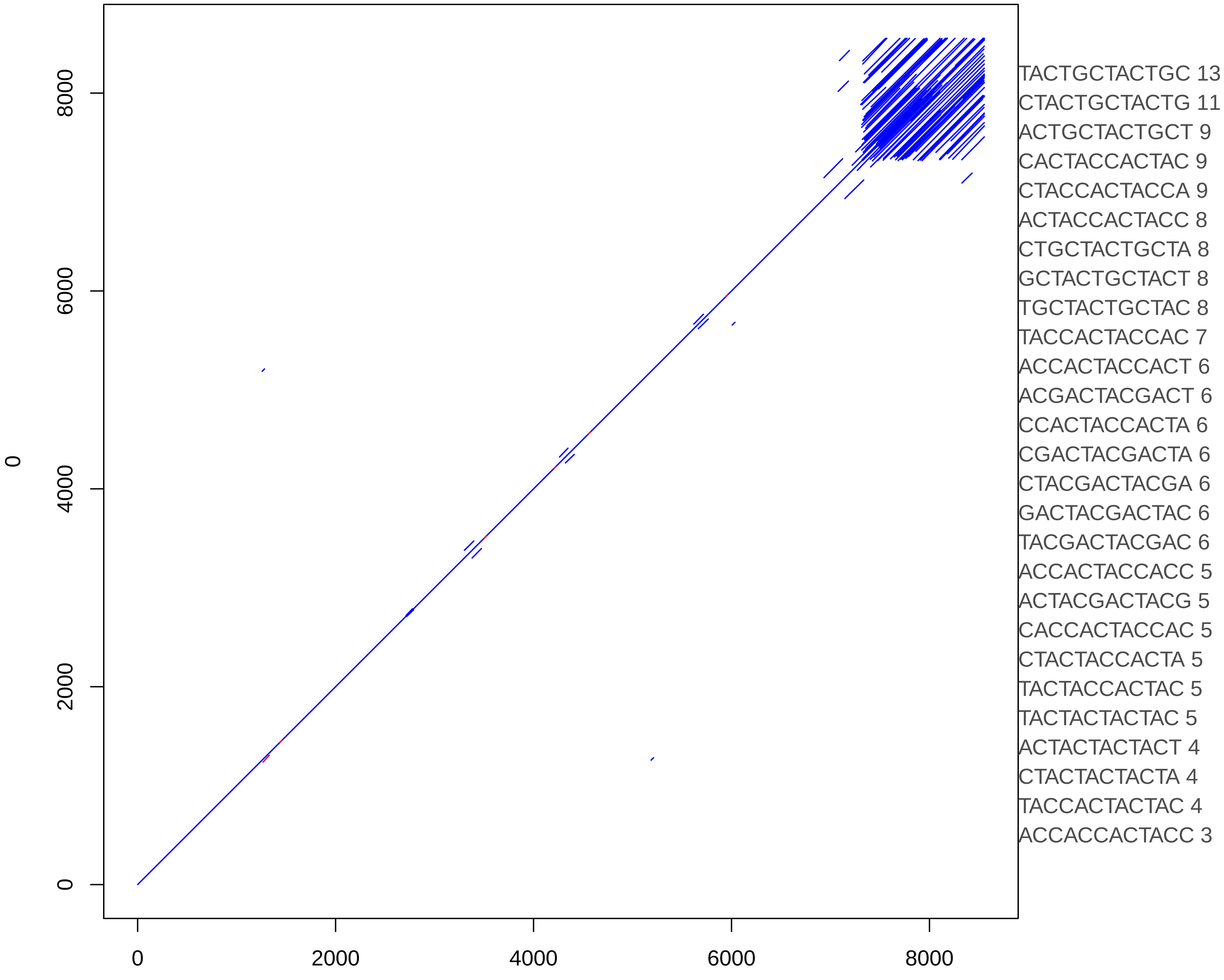

>m141231_015334_42134_c100756552550000001823146807221542_s1_p0/1918/764_6395

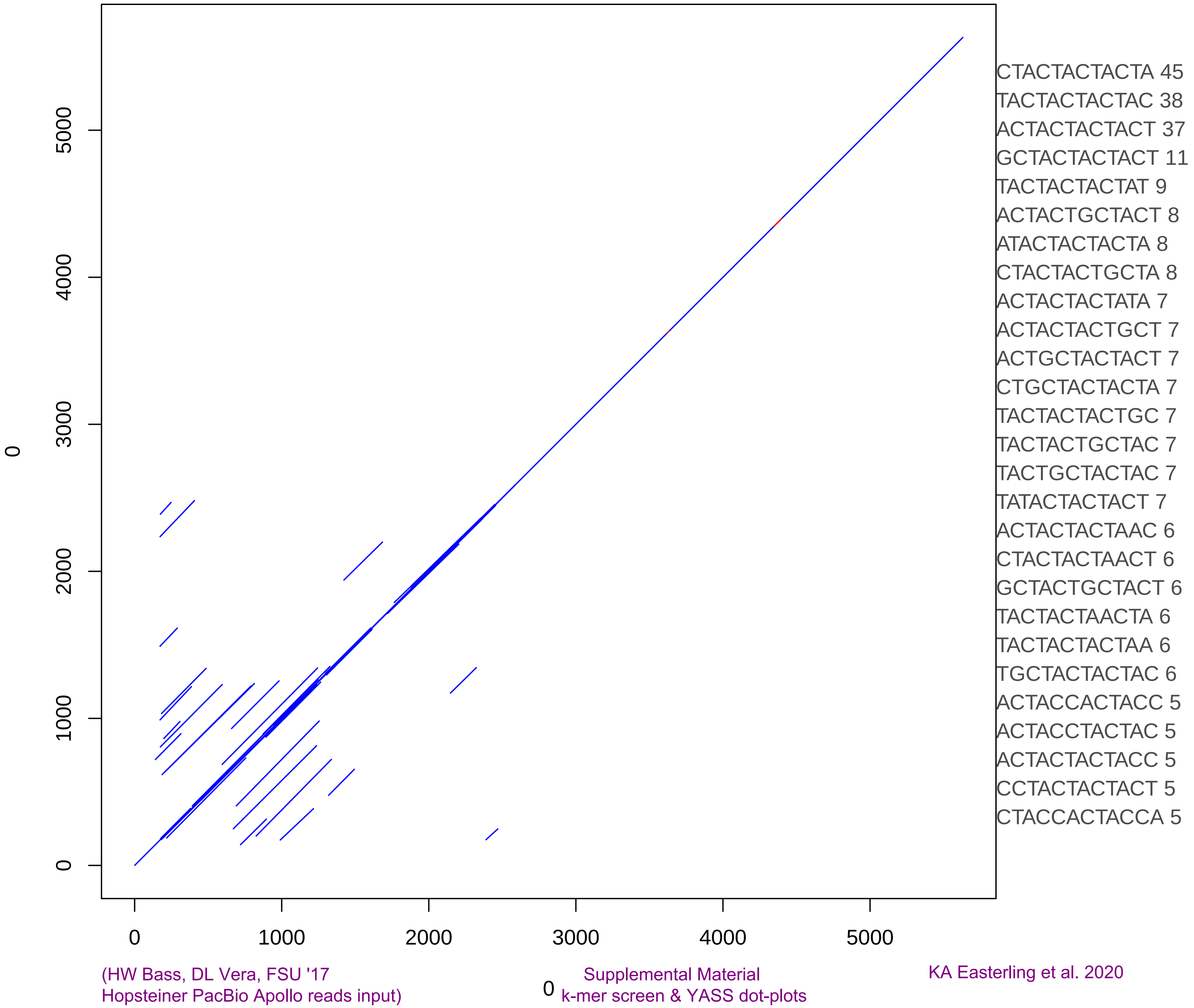

1,121 hits for repetitive sequences passing 12mer abundance criterion

For Tandem Repeat families of ### bp:
PacBio Read named "HuluTR###-r929"

>m141231_015334_42134_c100756552550000001823146807221542_s1_p0/19623/2430_12452

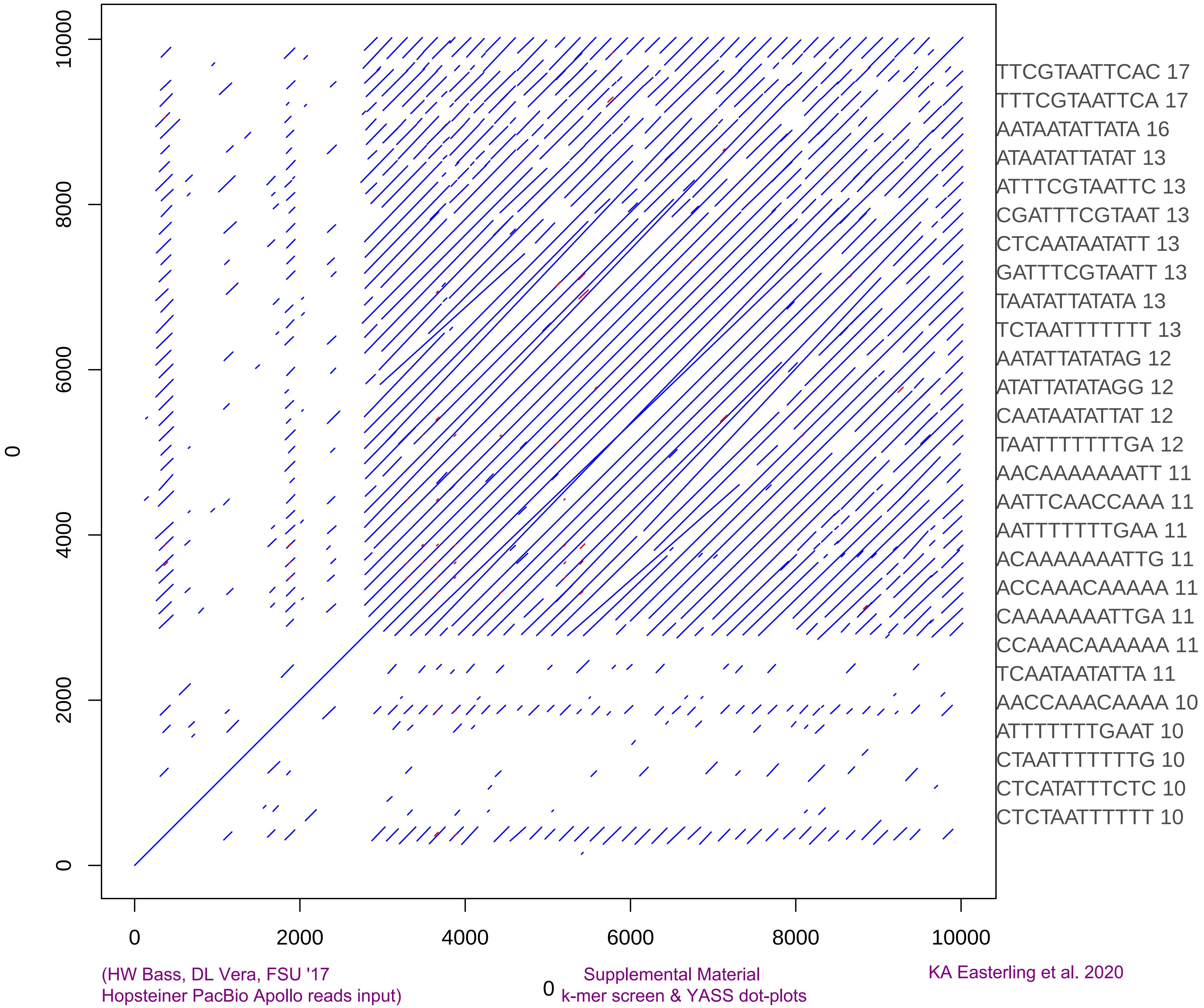

(HW Bass, DL Vera, FSU '17
Hopsteiner PacBio Apollo reads input)

Supplemental Material
k-mer screen & YASS dot-plots

KA Easterling et al. 2020

>m141231_015334_42134_c100756552550000001823146807221542_s1_p0/19905/677_5896

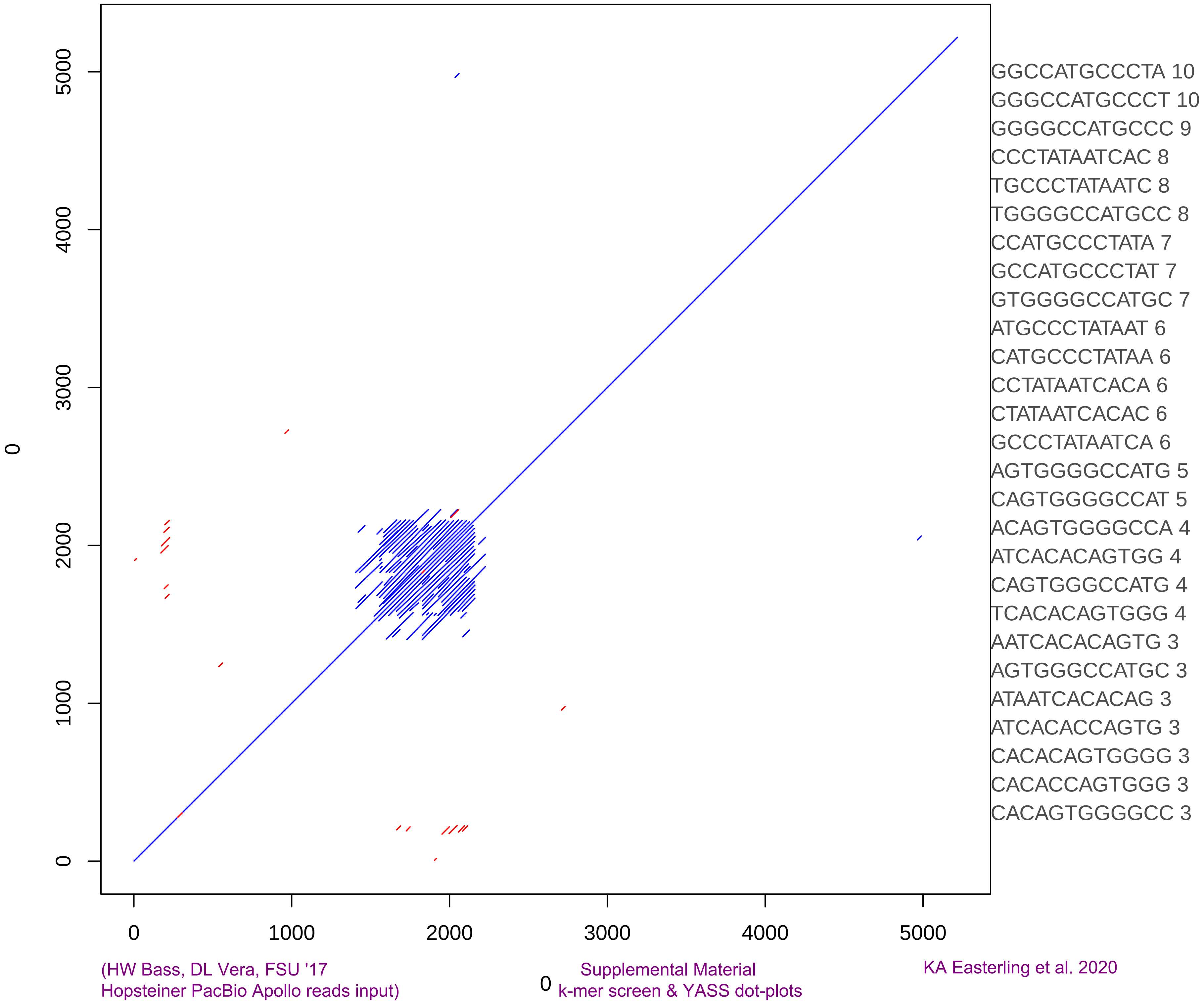

>m141231_015334_42134_c100756552550000001823146807221542_s1_p0/24009/783_7346

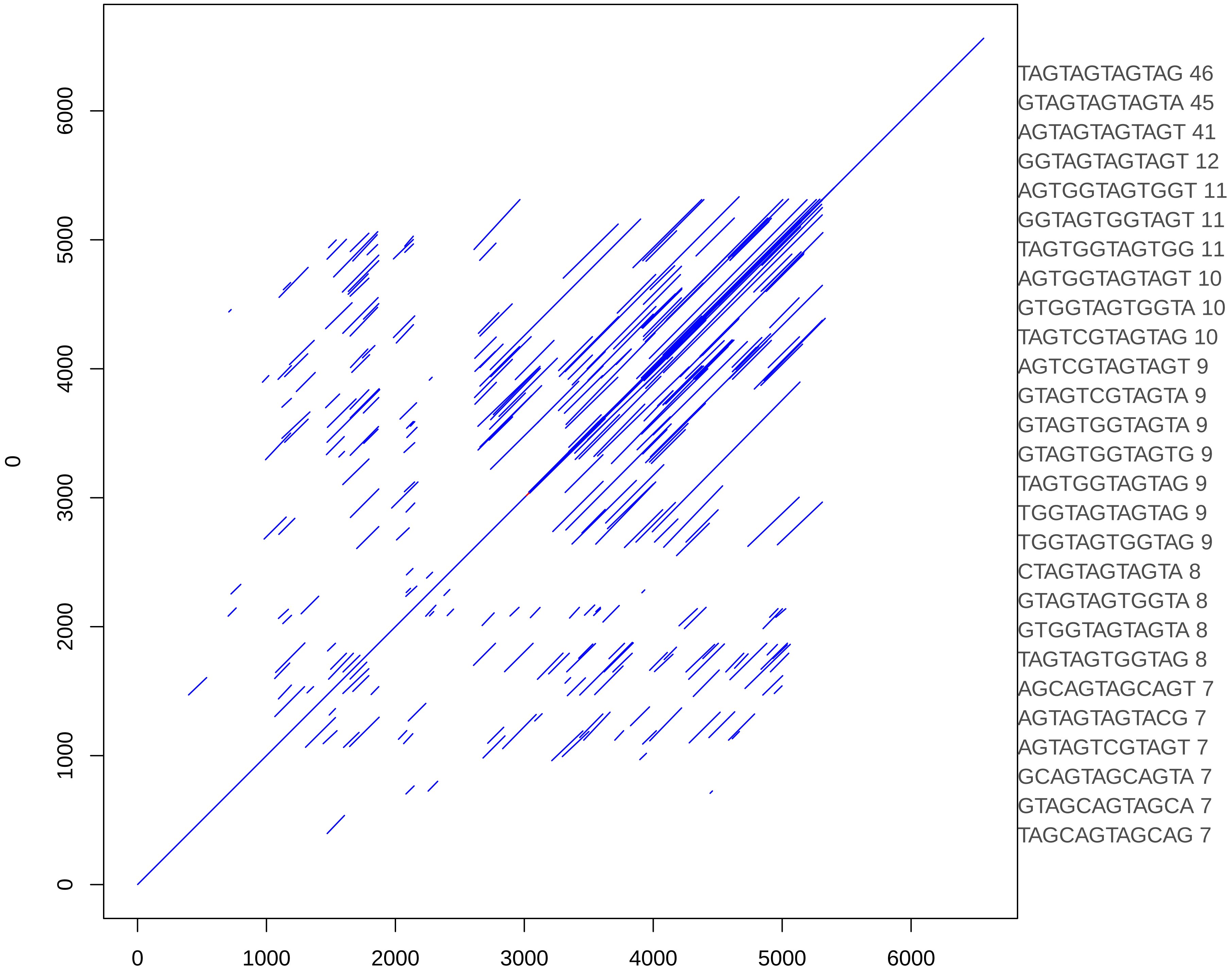

>m141231_015334_42134_c100756552550000001823146807221542_s1_p0/25862/0_11380

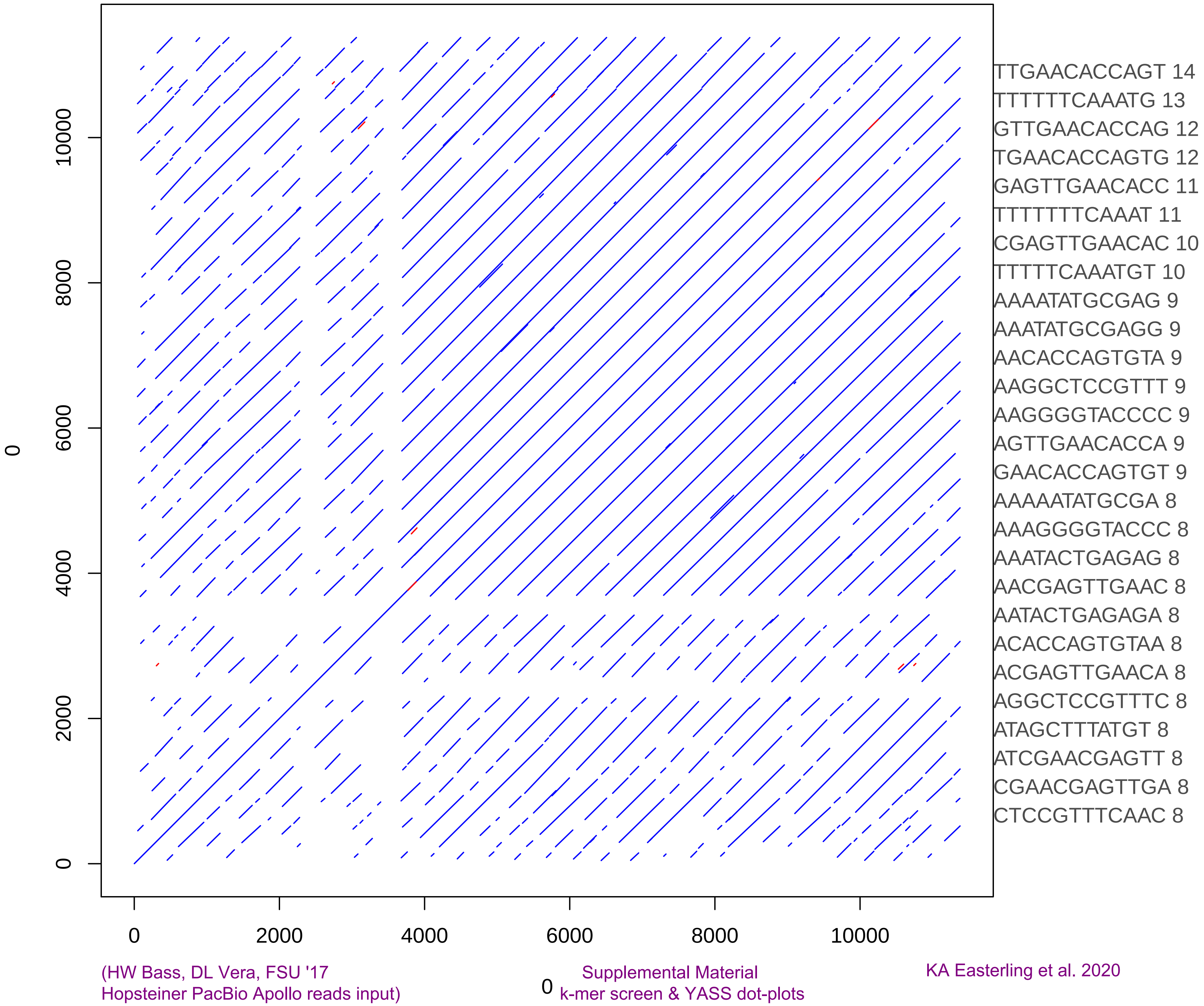

>m141231_015334_42134_c100756552550000001823146807221542_s1_p0/3008/684_7401

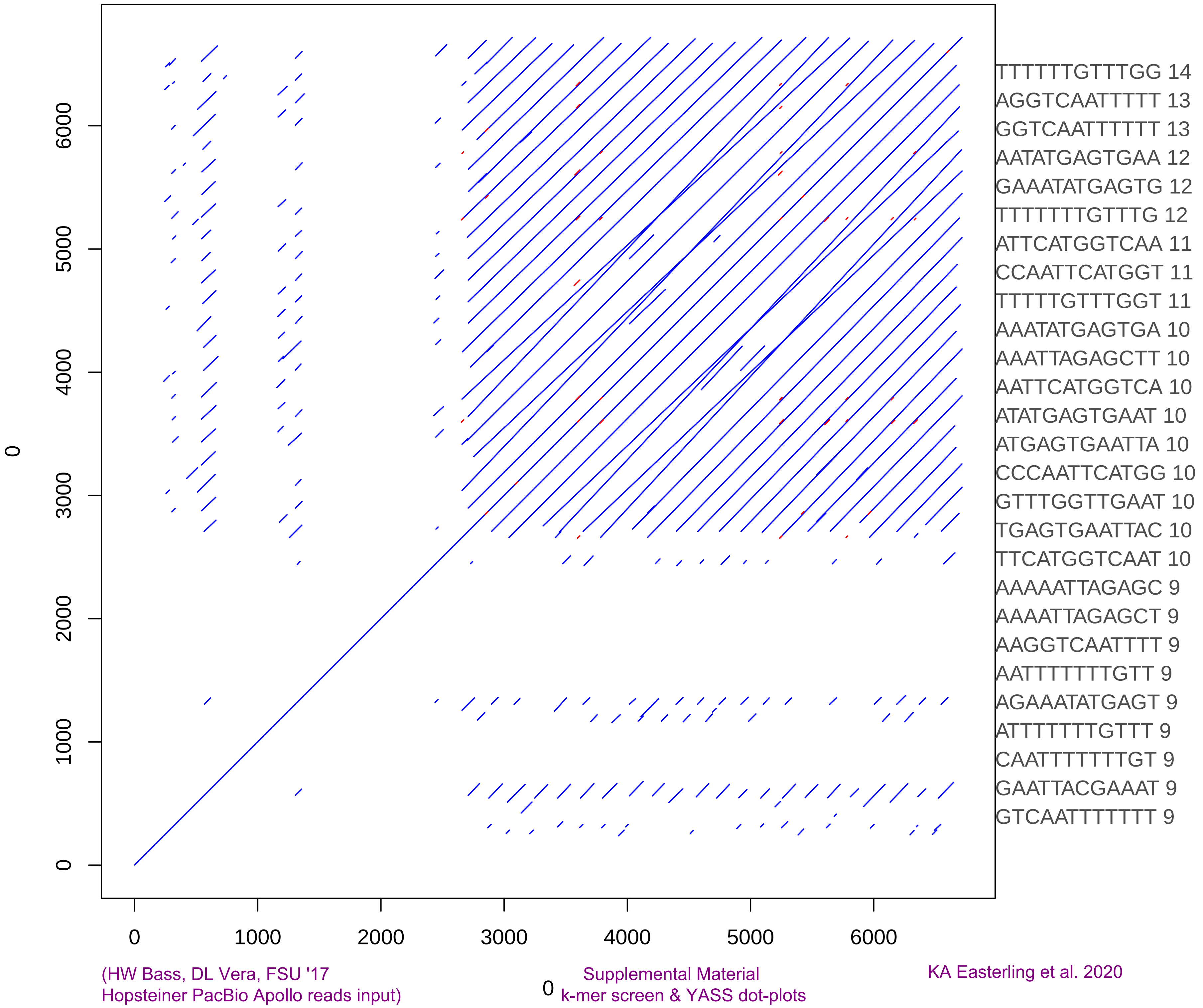

>m141231_015334_42134_c100756552550000001823146807221542_s1_p0/32370/2397_14113

Representative (only one) Read (Table 1): HuluTR285-r934, 1 ppm, 69% A+T, GenBank Acc. MN537576

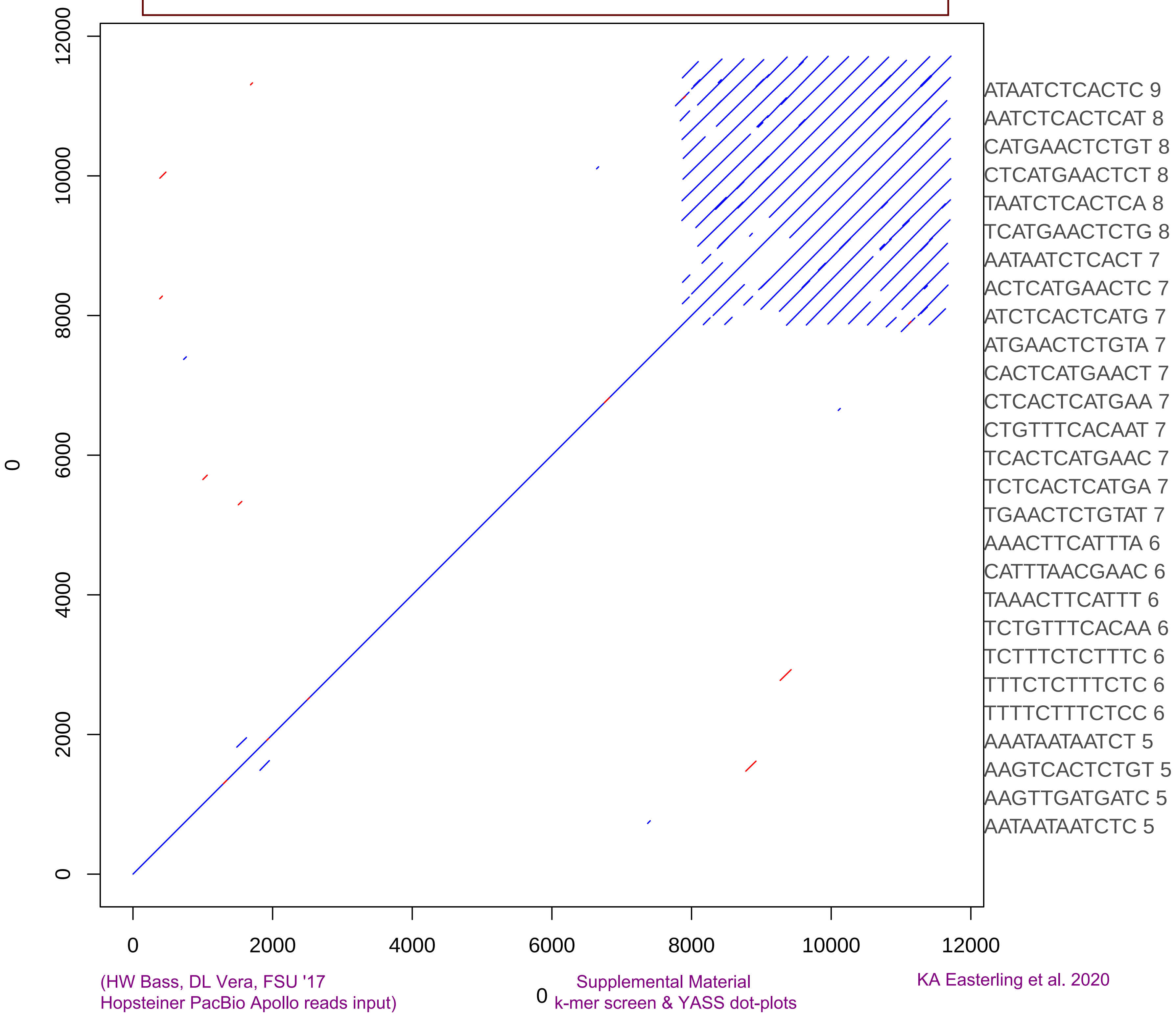

>m141231_015334_42134_c100765552550000001823146807221542_s1_p0/3272/1878_10233

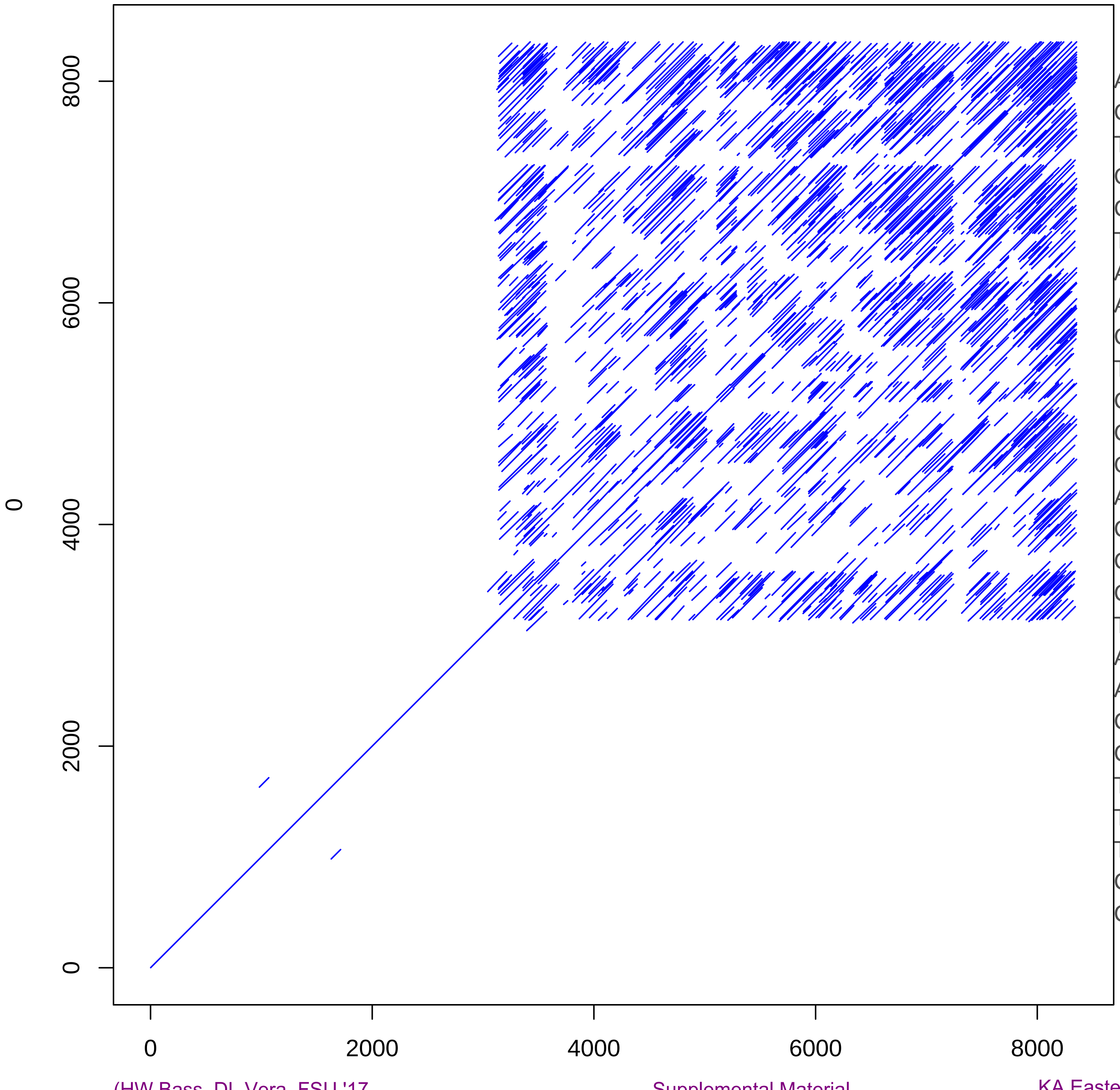

>m141231_015334_42134_c100756552550000001823146807221542_s1_p0/34414/13726_20768

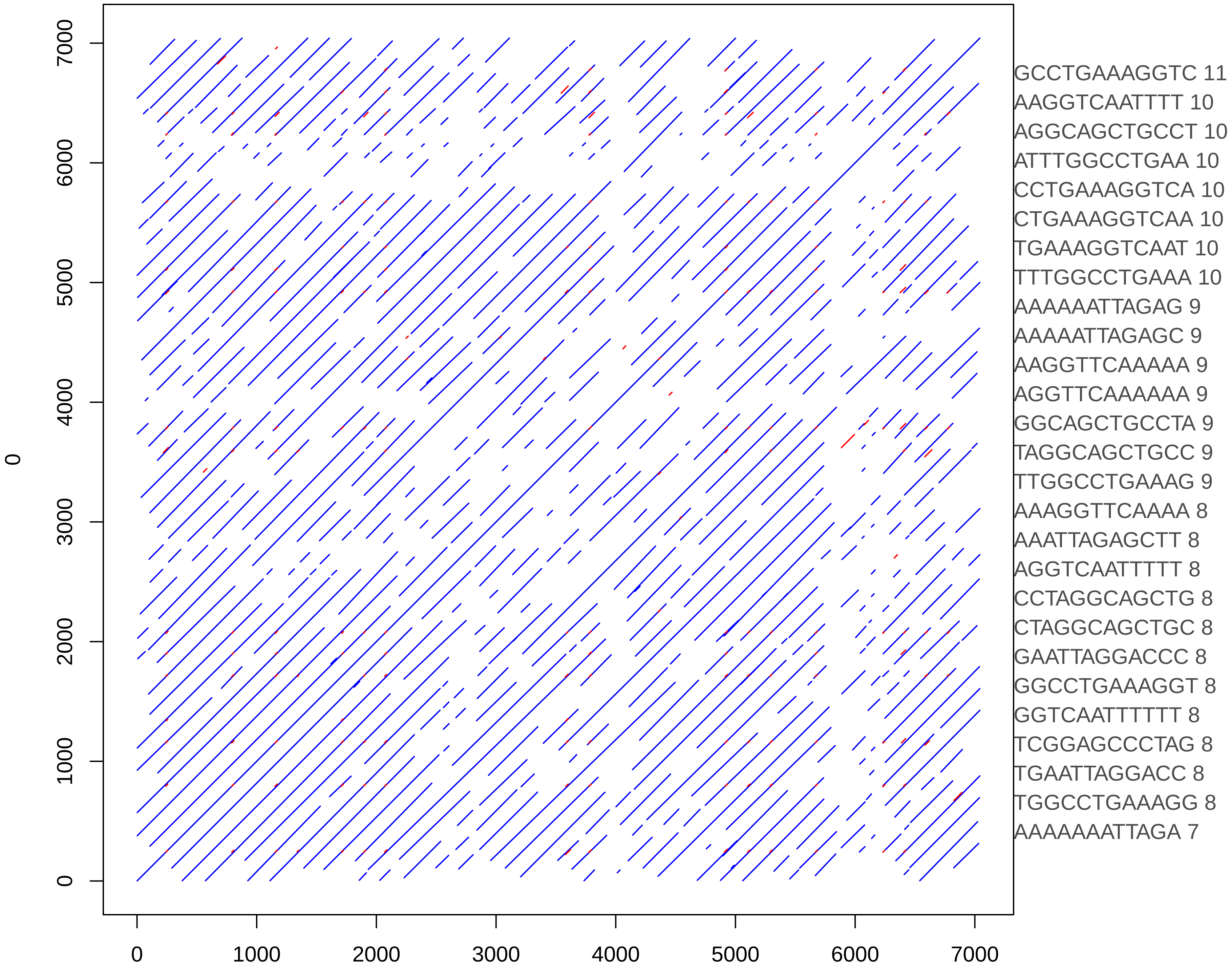

>m141231_015334_42134_c100756552550000001823146807221542_s1_p0/3705/3377_8561

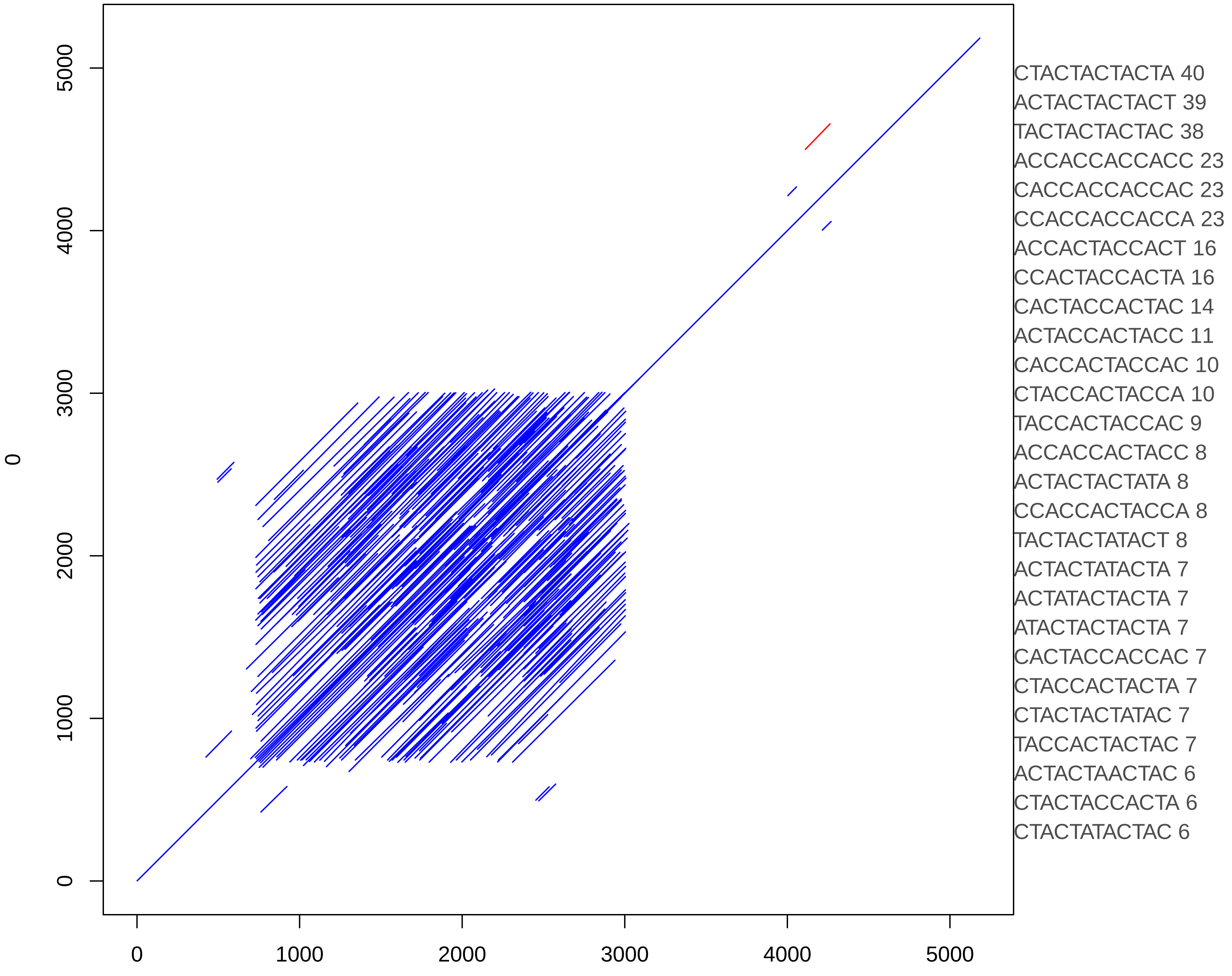

>m141231_015334_42134_c100756552550000001823146807221542_s1_p0/41906/0_6917

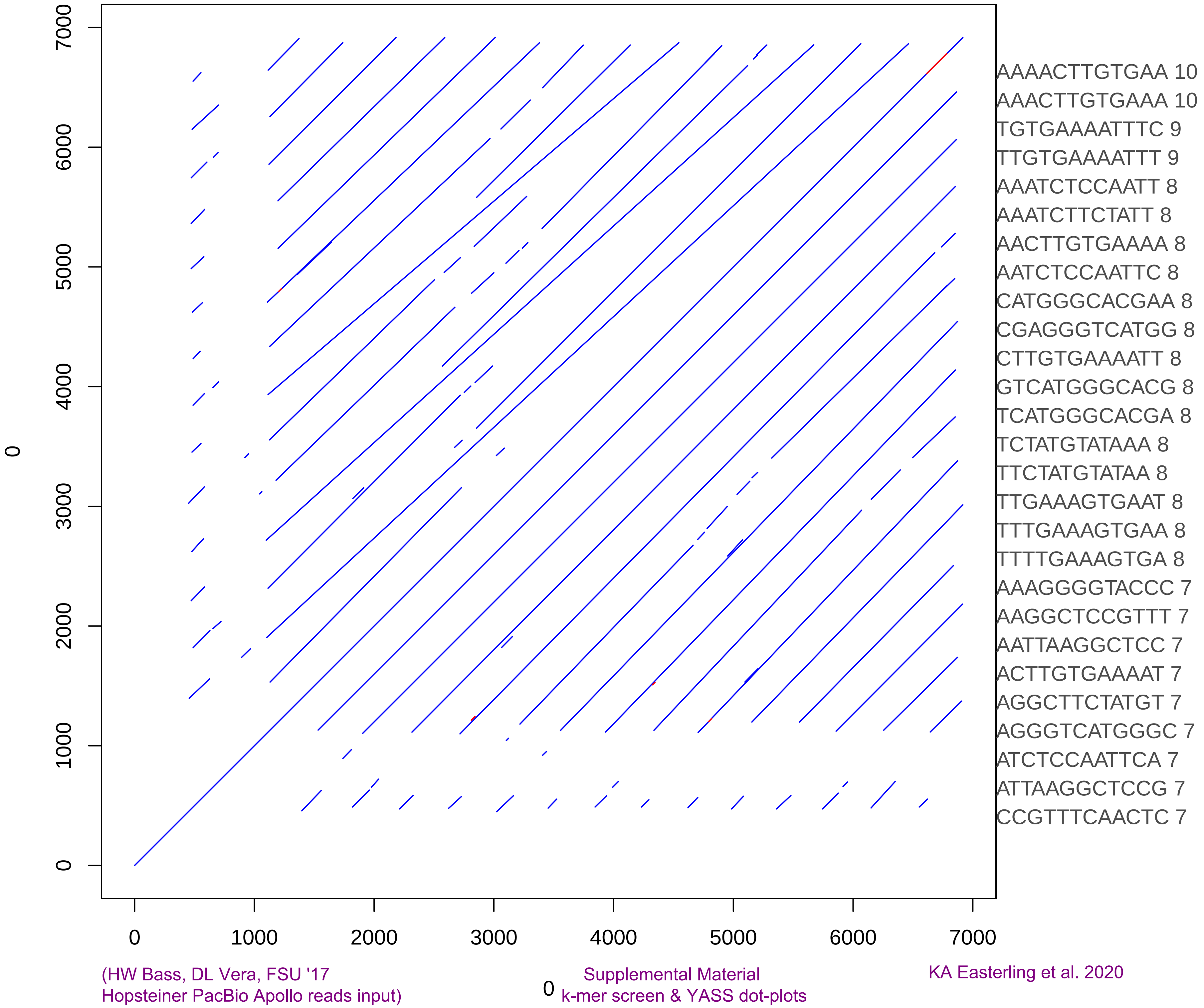

>m141231_015334_42134_c100756552550000001823146807221542_s1_p0/42717/0_15820

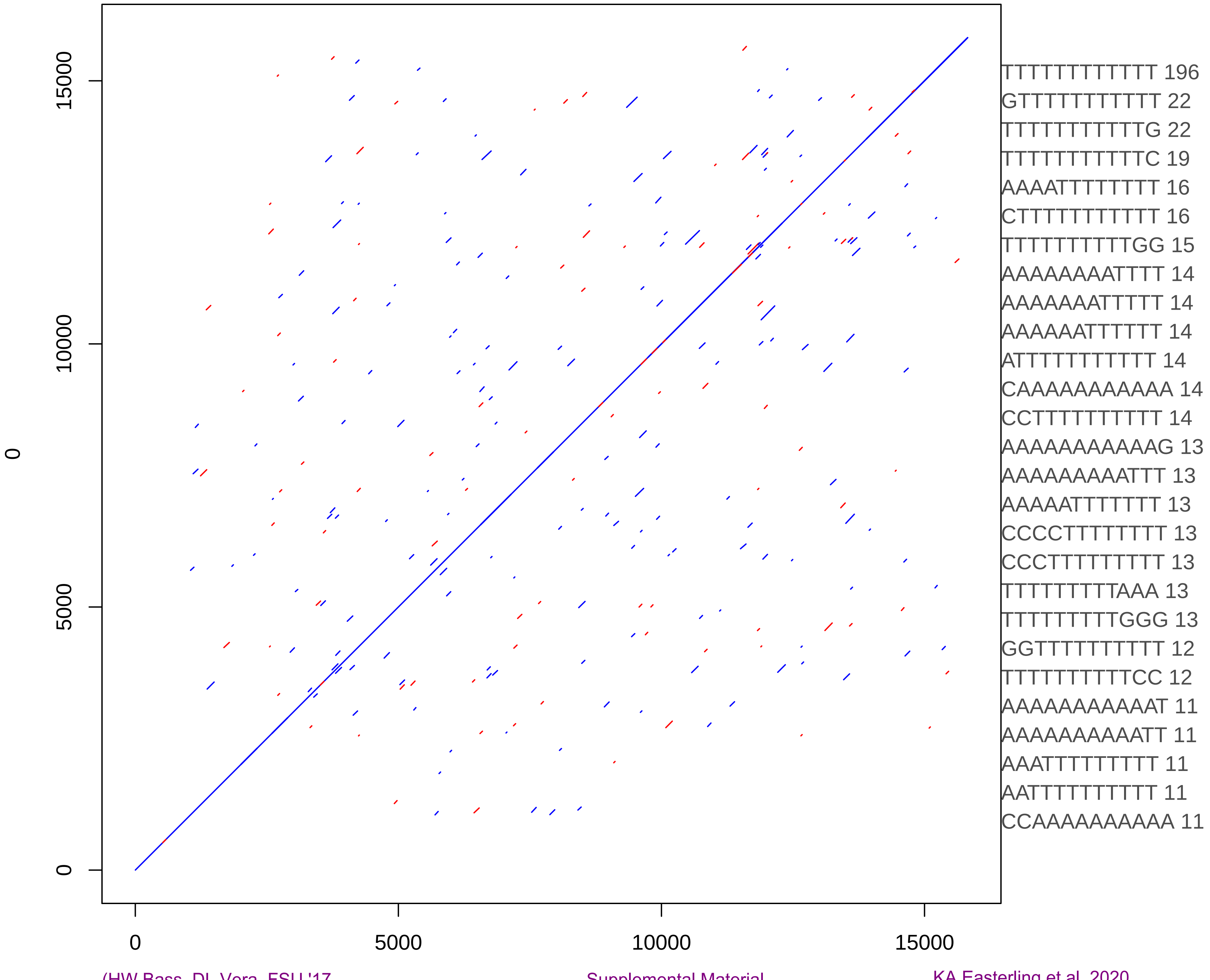

0

>m141231_015334_42134_c100756552550000001823146807221542_s1_p0/44381/0_10139

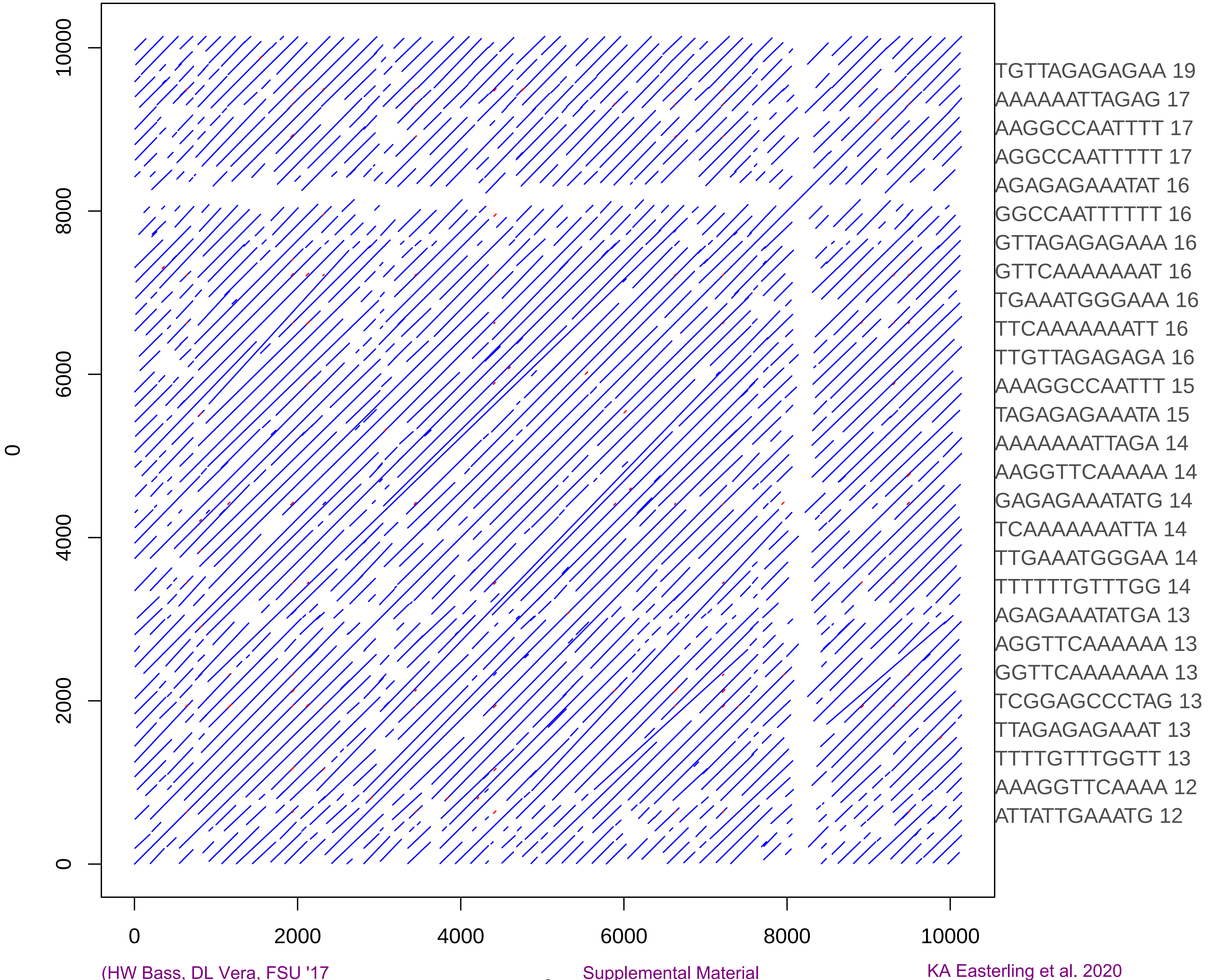

0

>m141231_015334_42134_c100756552550000001823146807221542_s1_p0/47265/179_8656

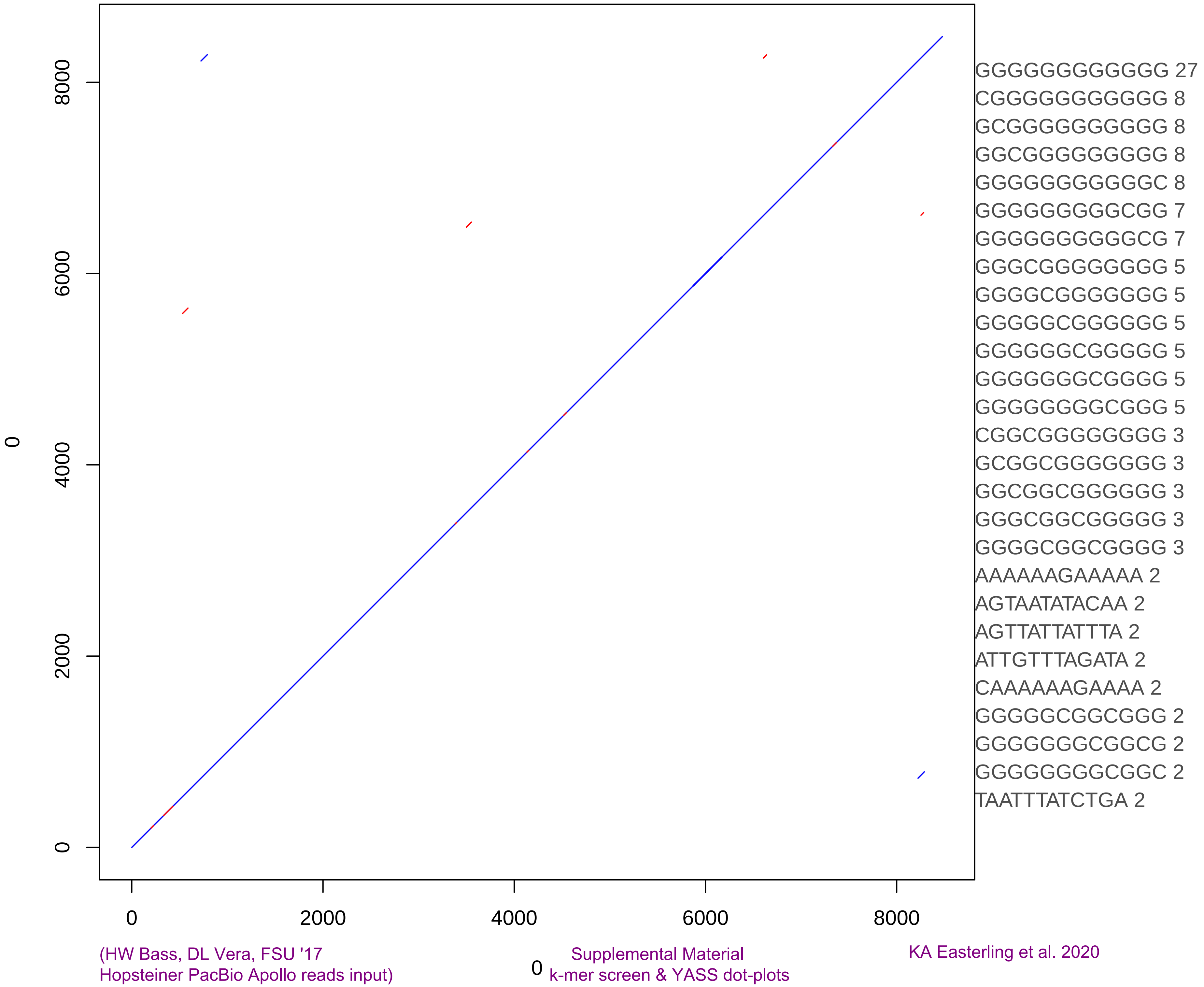

>m141231_015334_42134_c100756552550000001823146807221542_s1_p0/48240/927_6214

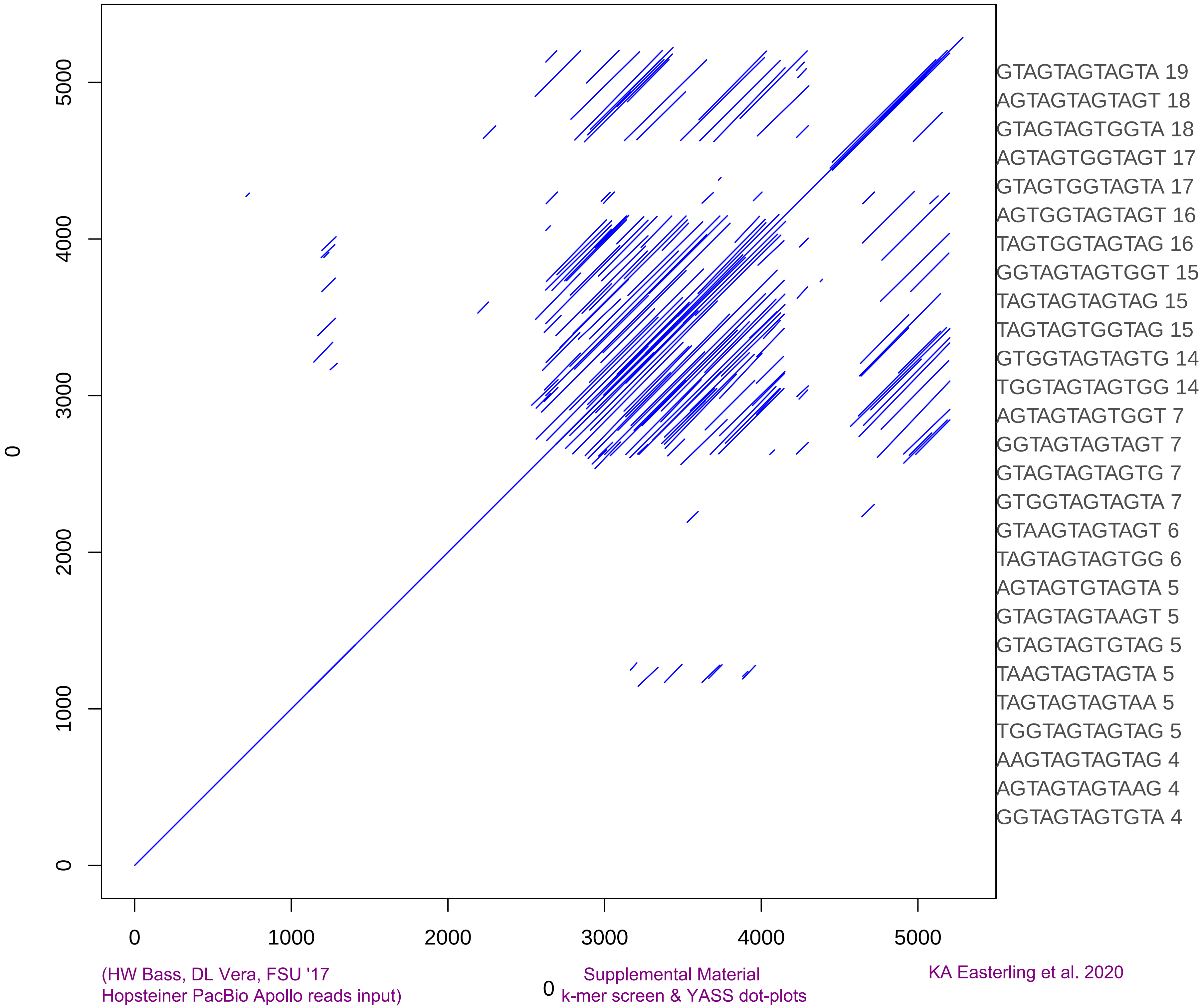

>m141231_015334_42134_c100756552550000001823146807221542_s1_p0/48804/2798_10862

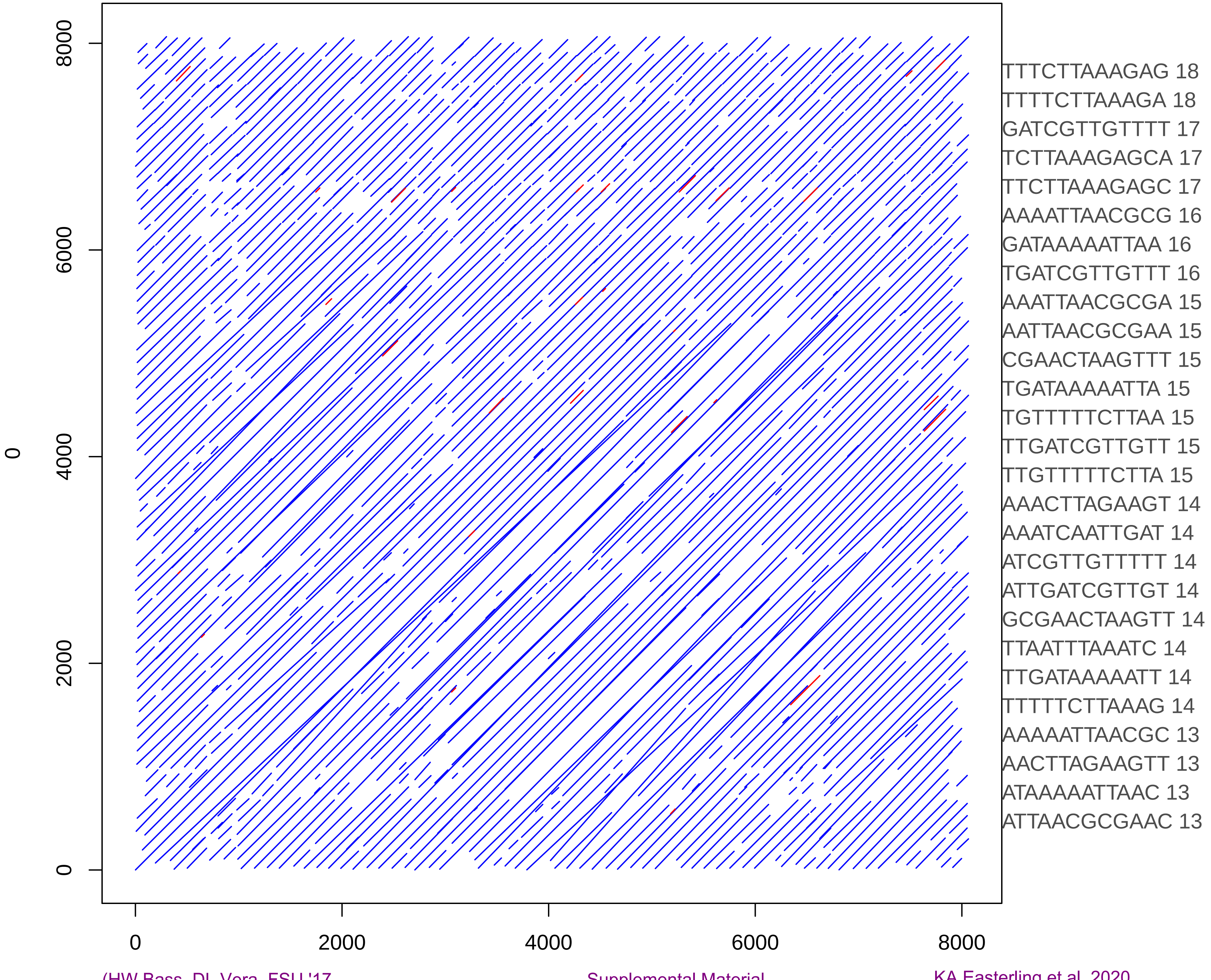

>m141231_015334_42134_c100756552550000001823146807221542_s1_p0/49745/3900_11334

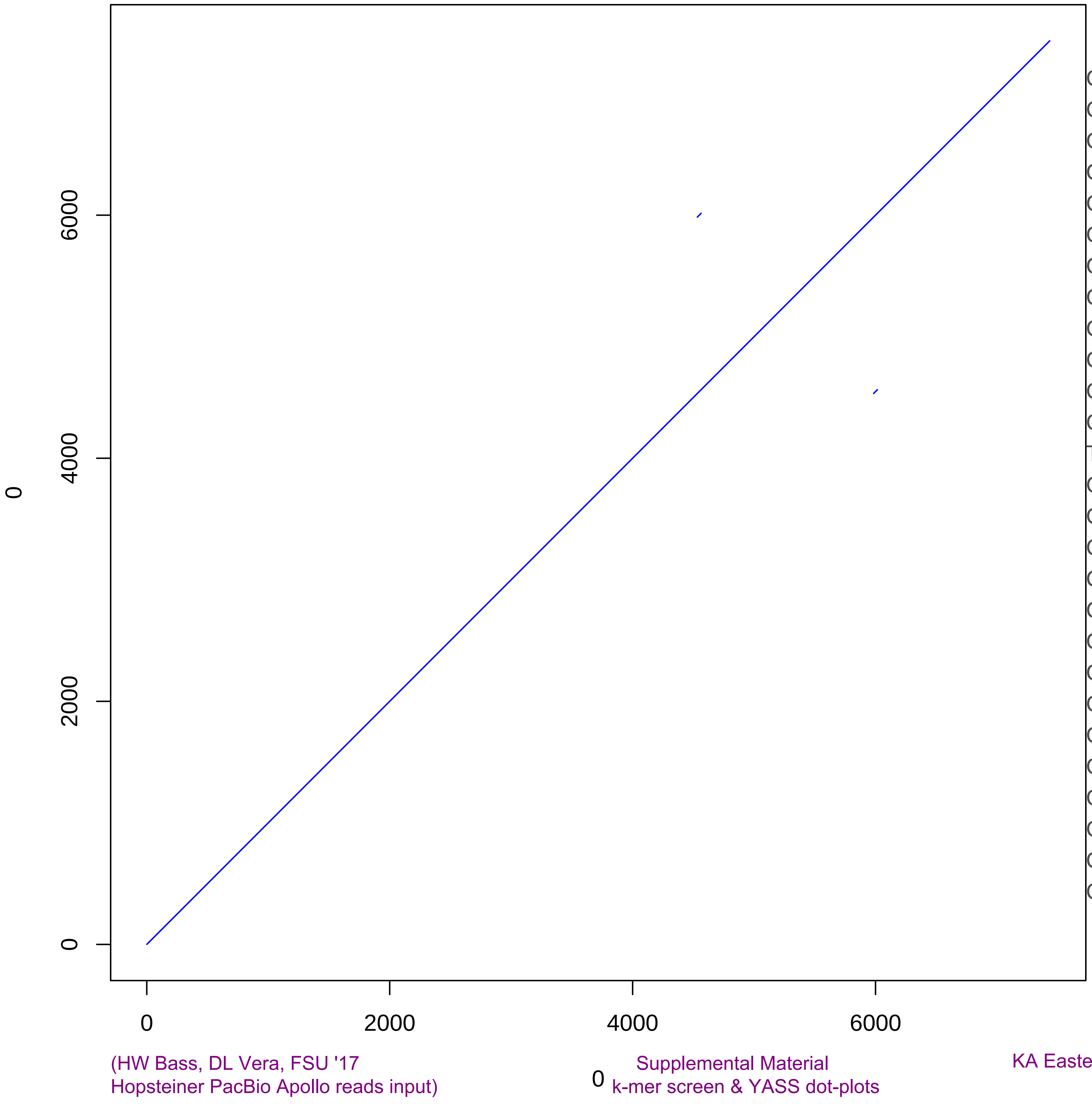

GGGGGGGGGGGG 18
GGGGTGGGGGGG 13
GGGGGTGGGGGG 12
GGGGGGGTGGGG 10
GGGGGGTGGGGG 10
GGGGGGGGGTGG 9
GGGGGGGGTGGG 9
GGGTGGGGGGGG 9
GGTGGGGGGGGG 9
GGGGGGGGGGTG 8
GGGGGGGGGGGT 7
GTGGGGGGGGGG 7
TGGGGGGGGGGG 7
GGGTGGGGGGGT 4
GGTGGGGGGGTG 3
GTGGGGTGGGGG 3
CCCGAGTCCTTC 2
CGGGGGGGGGGT 2
GCCCGAGTCCTT 2
GGGGGGGGTGGT 2
GGGGGGGTGGTG 2
GGGGTGGGGGTG 2
GGGGTGGGGTGG 2
GGGTGGGGGTGG 2
GGGTGGGGTGGG 2
GGGTGGTGTGGG 2
GGTGGGGGTGGG 2

>m141231_015334_42134_c100756552550000001823146807221542_s1_p0/49801/1456_10114

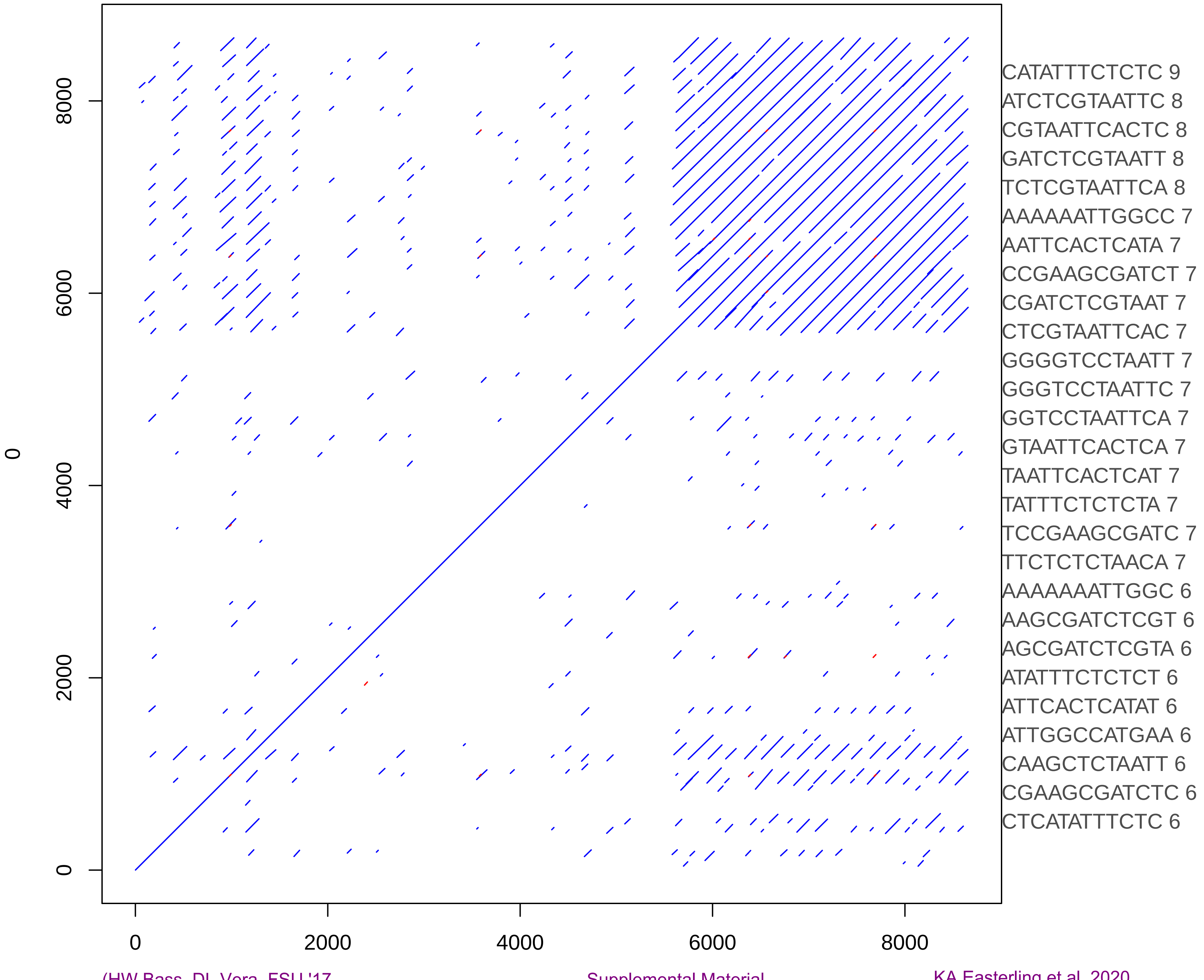

>m141231_015334_42134_c100756552550000001823146807221542_s1_p0/49827/0_5340

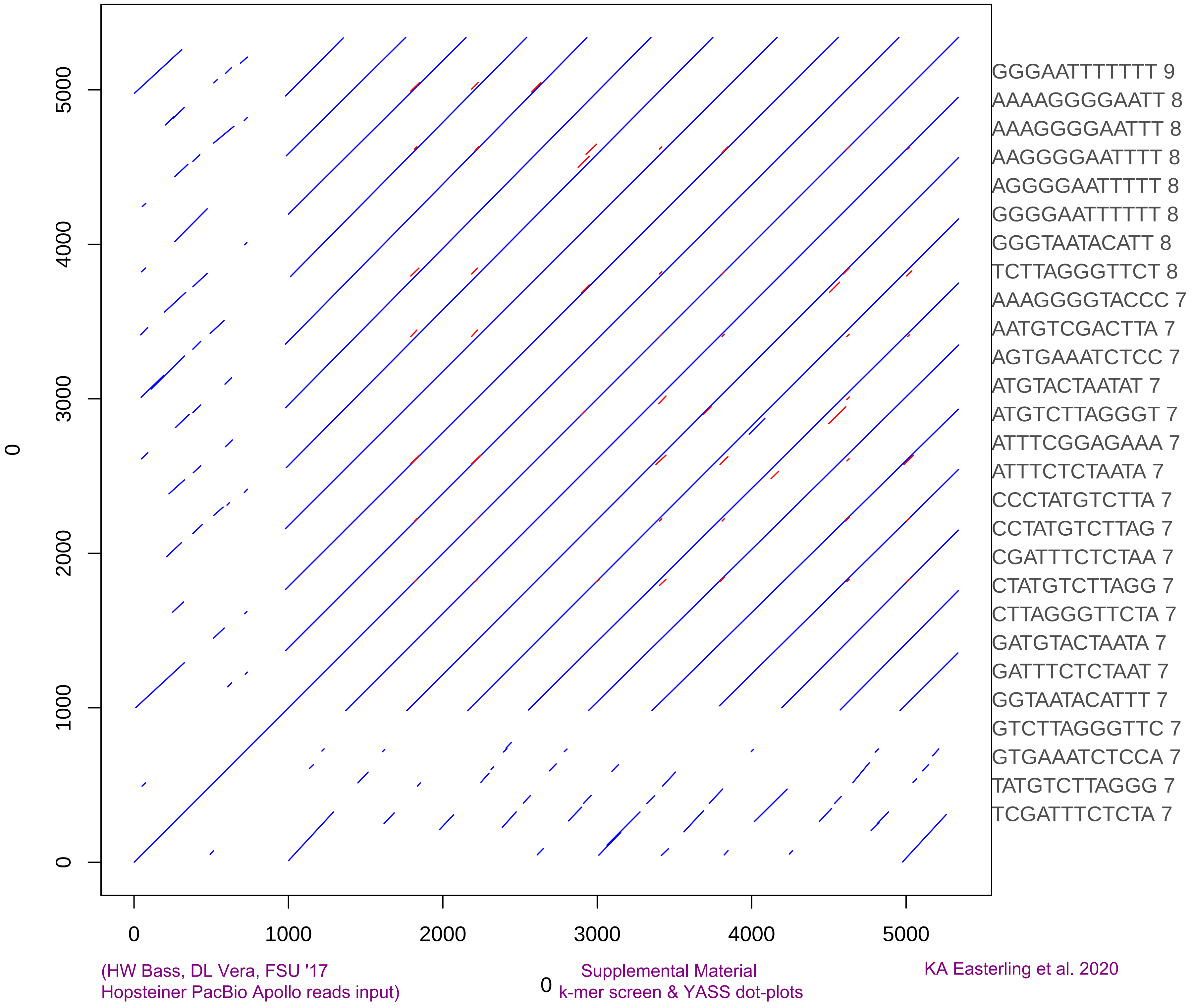

>m141231_015334_42134_c100756552550000001823146807221542_s1_p0/50078/3535_15420

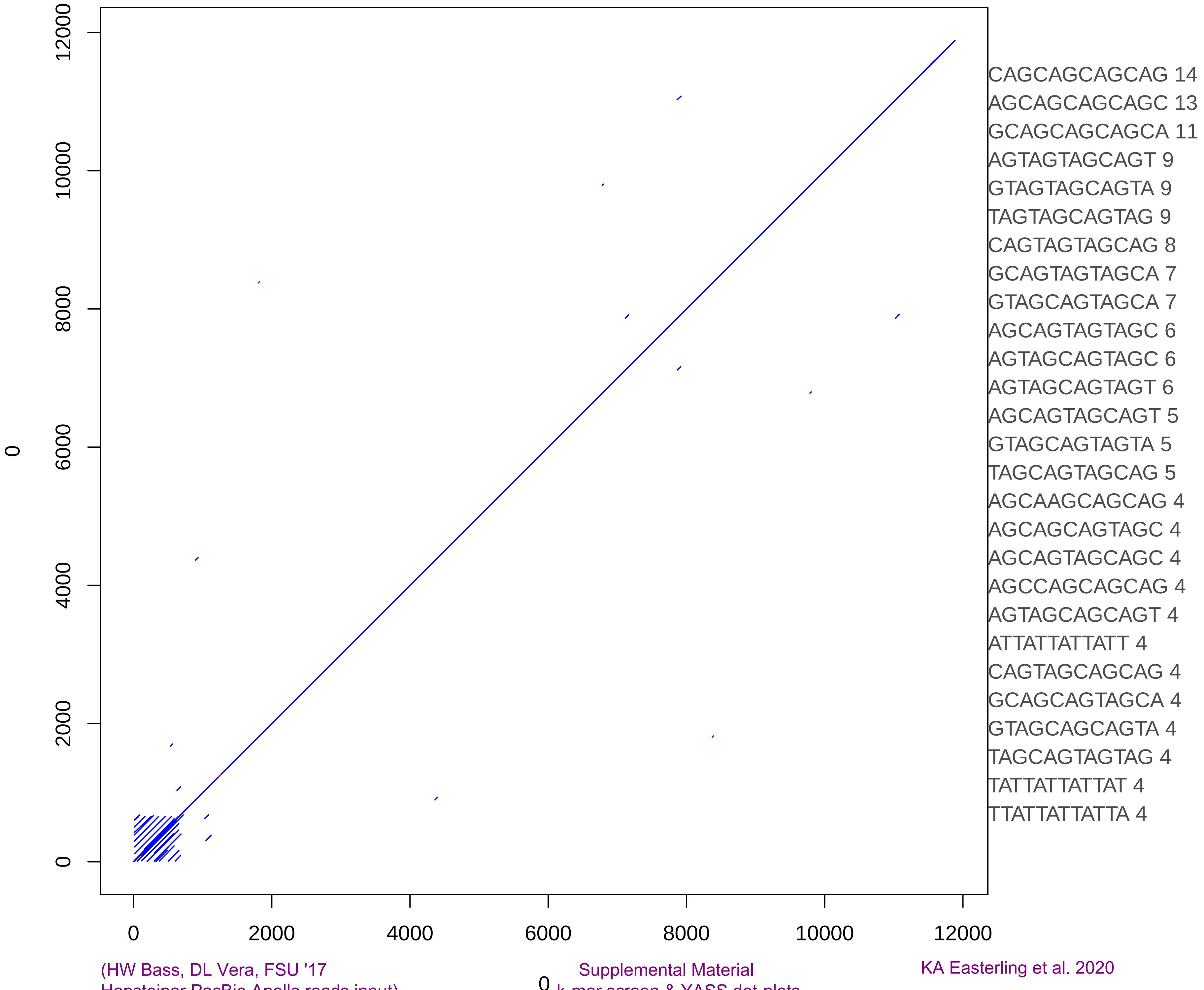

>m141231_015334_42134_c100756552550000001823146807221542_s1_p0/54282/8614_16454

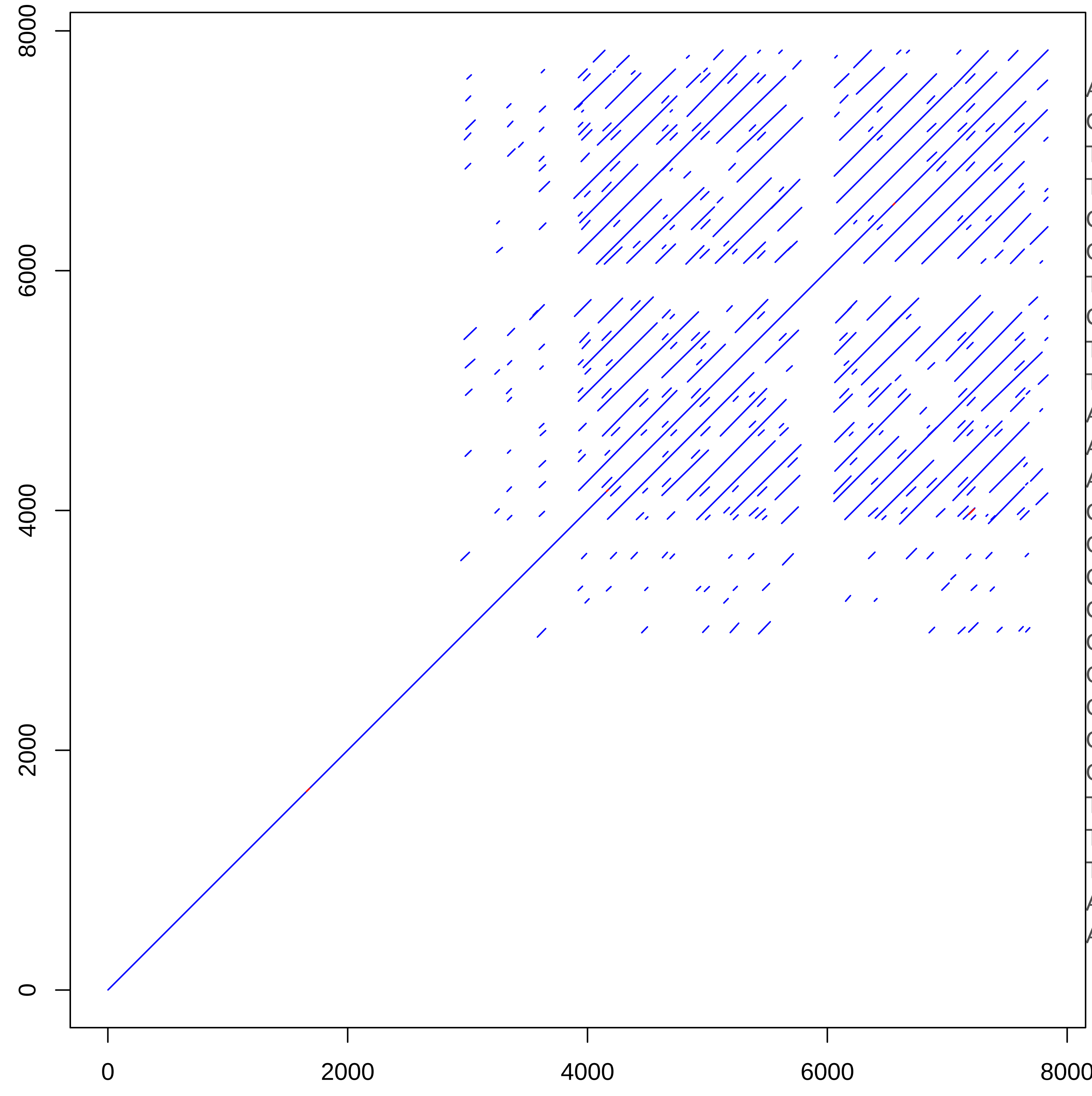

AGTGCAATGTTA 9
GTGCAATGTTAT 9
TAGTGCAATGTT 9
TTAGTGCAATGT 9
CTTAGTGCAATG 8
GCAATGTTATCT 7
TGCAATGTTATC 7
CTAAATTCACTA 6
TATTTTTCGGGA 6
TCTAAATTCACT 6
AAATTCACTACT 5
AACGGTTCATTA 5
AATGTTATCTAG 5
CAATGTTATCTA 5
CCTTCTAAATTC 5
CGGTTCATTATG 5
CTTCTAAATTCA 5
GAACGGTTCATT 5
GGAACGGTTCAT 5
GTAGTATTTTTC 5
GTATTTTTCGGG 5
GTCTGCTTAGTG 5
TAAATTCACTAC 5
TGGAACGGTTCA 5
TTCTAAATTCAC 5
ACGGTTCATTAT 4
AGGTCTGCTTAG 4

>m141231_015334_42134_c100756552550000001823146807221542_s1_p0/54634/3573_14992

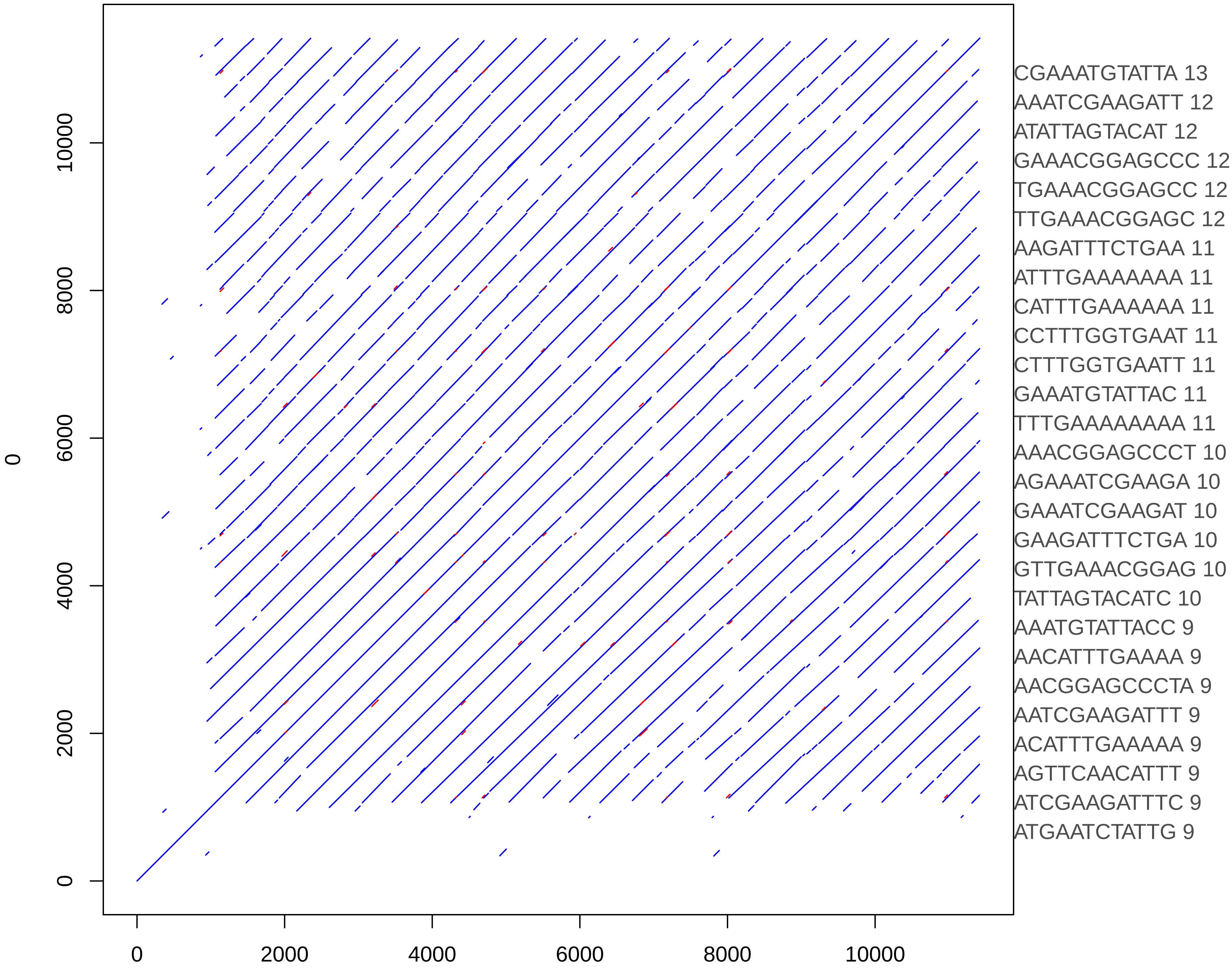

1,121 hits for repetitive sequences passing 12mer abundance criterion

(Read-Number)
PAGE 950

For Tandem Repeat families of ### bp:
PacBio Read named "HuluTR###-r950"

>m141231_015334_42134_c100756552550000001823146807221542_s1_p0/55310/0_9403

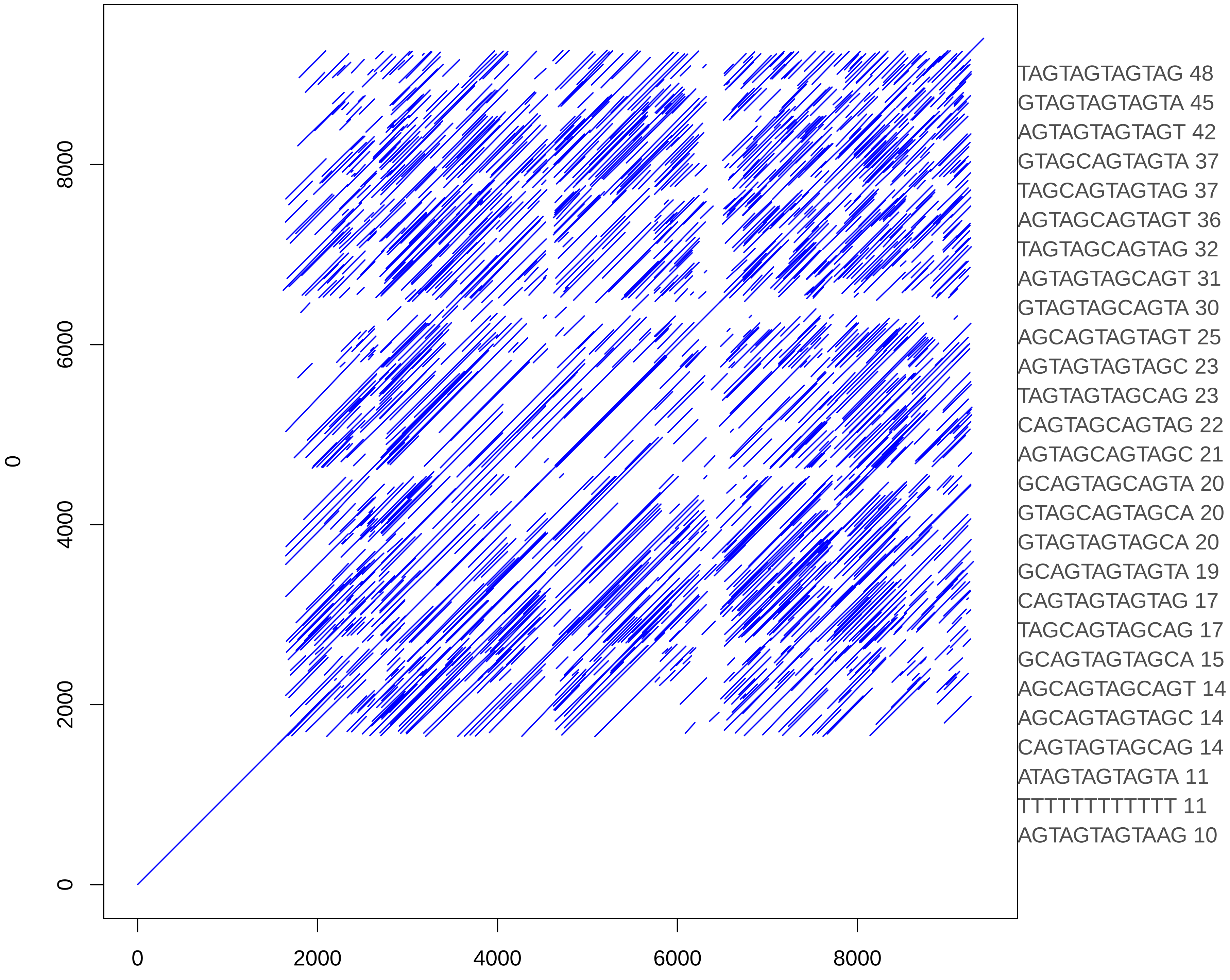

(HW Bass, DL Vera, FSU '17
Hopsteiner PacBio Apollo reads input)

0

Supplemental Material
k-mer screen & YASS dot-plots

KA Easterling et al. 2020

1,121 hits for repetitive sequences
passing 12mer abundance criterion

(Read-Number)
PAGE 951

For Tandem Repeat families of ### bp:
PacBio Read named "HuluTR###-r951"

>m141231_015334_42134_c100756552550000001823146807221542_s1_p0/57353/8475_20059

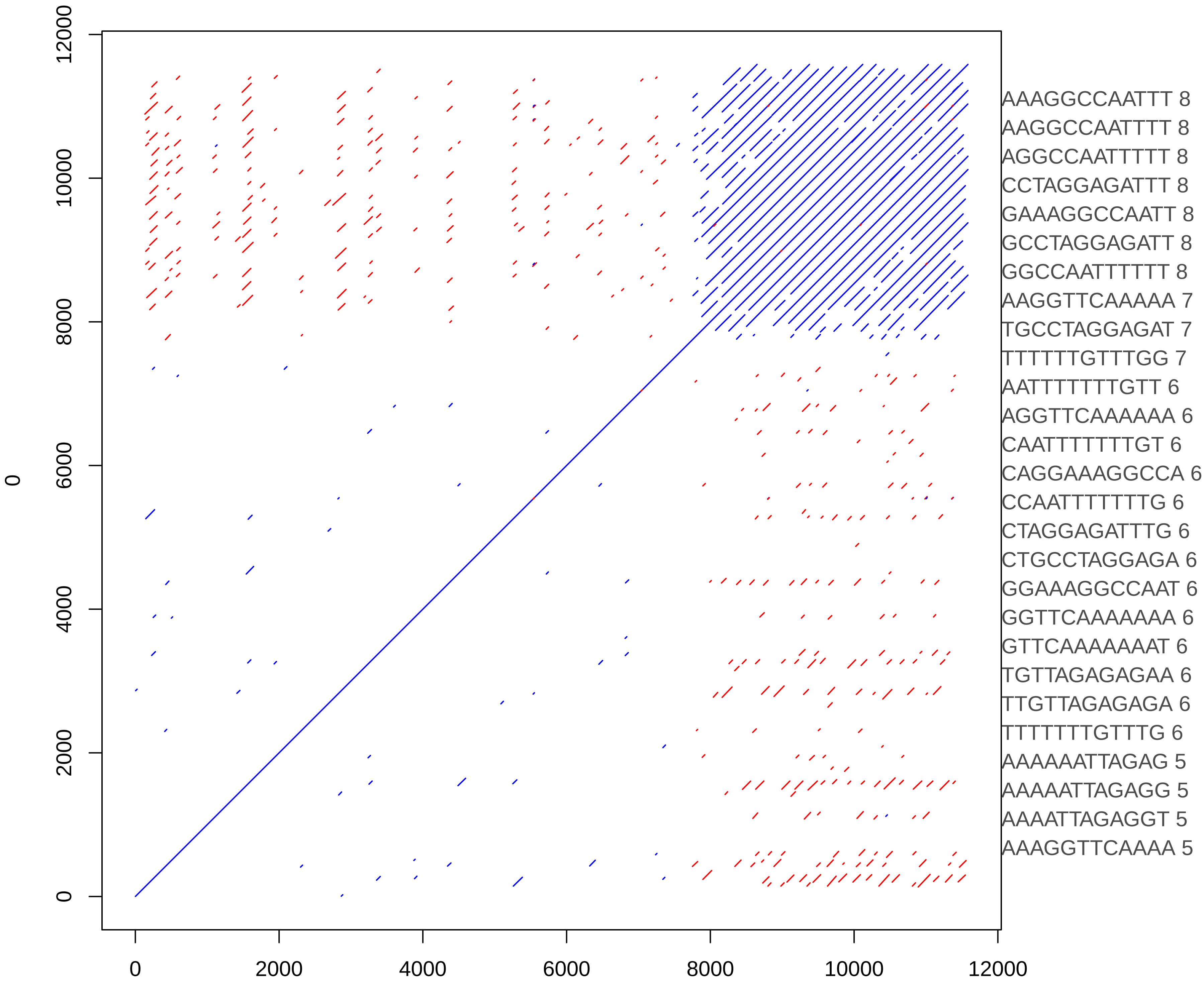

(HW Bass, DL Vera, FSU '17
Hopsteiner PacBio Apollo reads input)

0

Supplemental Material
k-mer screen & YASS dot-plots

KA Easterling et al. 2020

>m141231_015334_42134_c100756552550000001823146807221542_s1_p0/57564/1624_7576

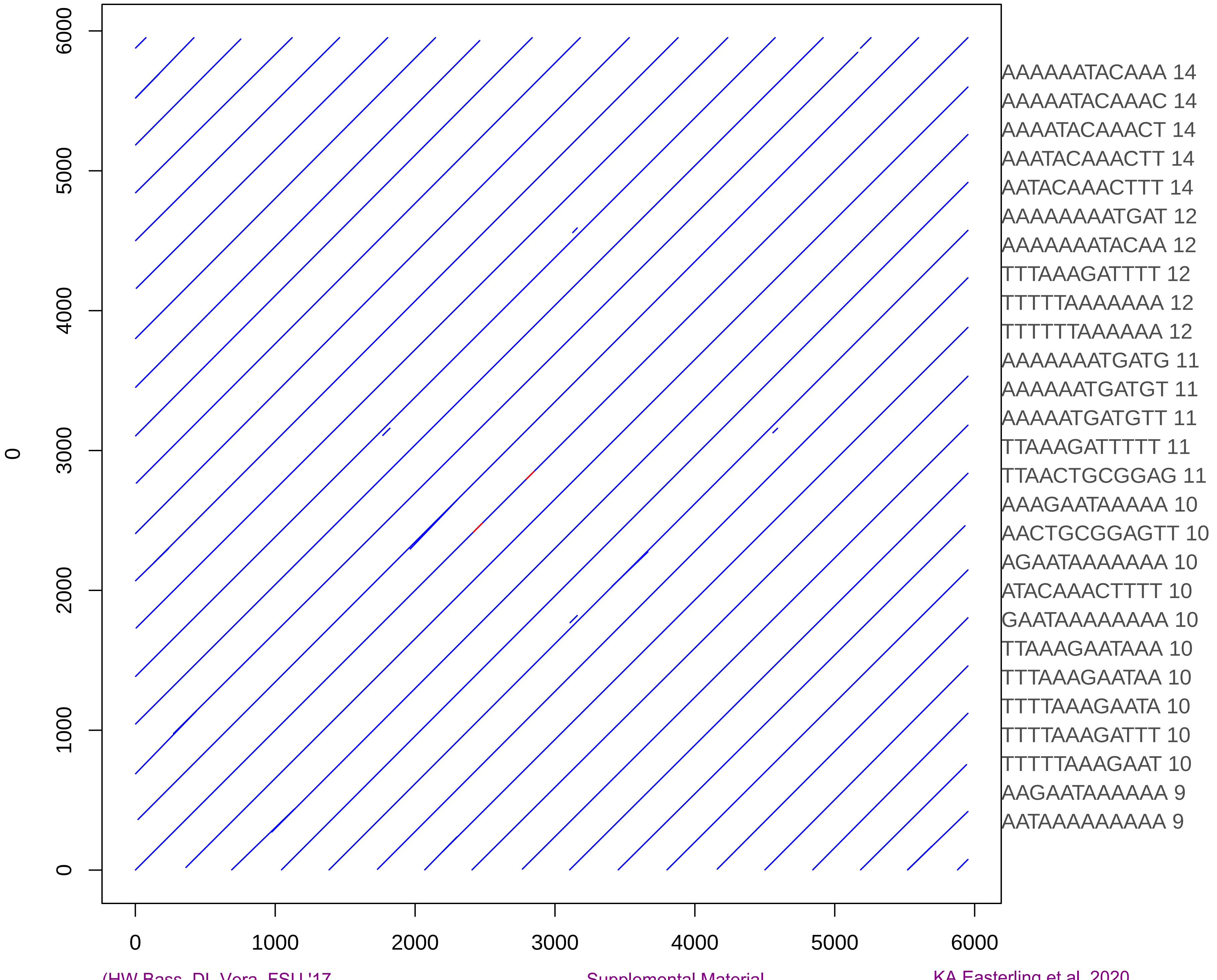

>m141231_015334_42134_c100756552550000001823146807221542_s1_p0/59603/436_8828

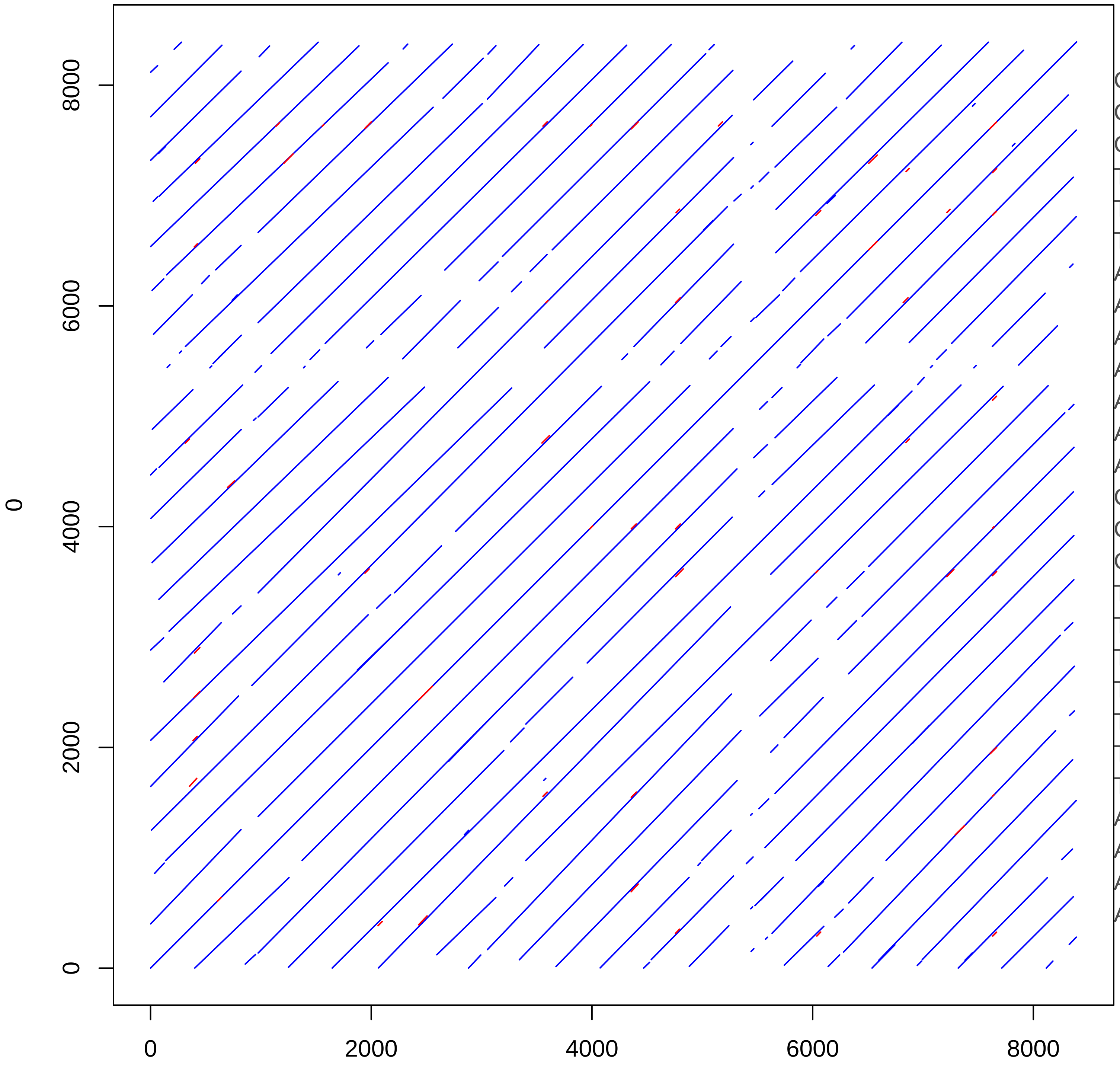

>m141231_015334_42134_c100756552550000001823146807221542_s1_p0/61757/0_10443

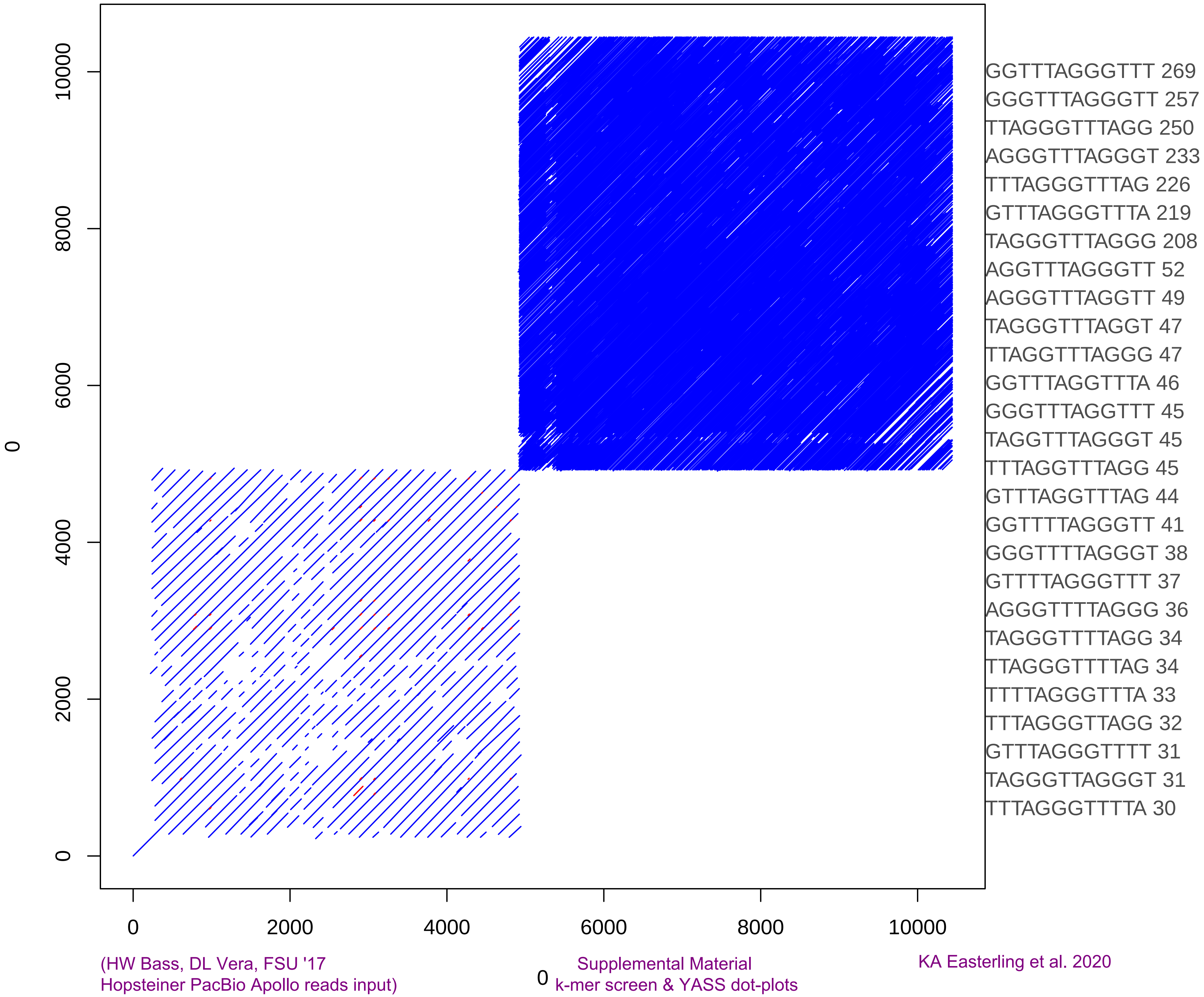

1,121 hits for repetitive sequences
passing 12mer abundance criterion

(Read-Number)
PAGE 955

For Tandem Repeat families of ### bp:
PacBio Read named "HuluTR###-r955"

>m141231_015334_42134_c100756552550000001823146807221542_s1_p0/62987/4042_11031

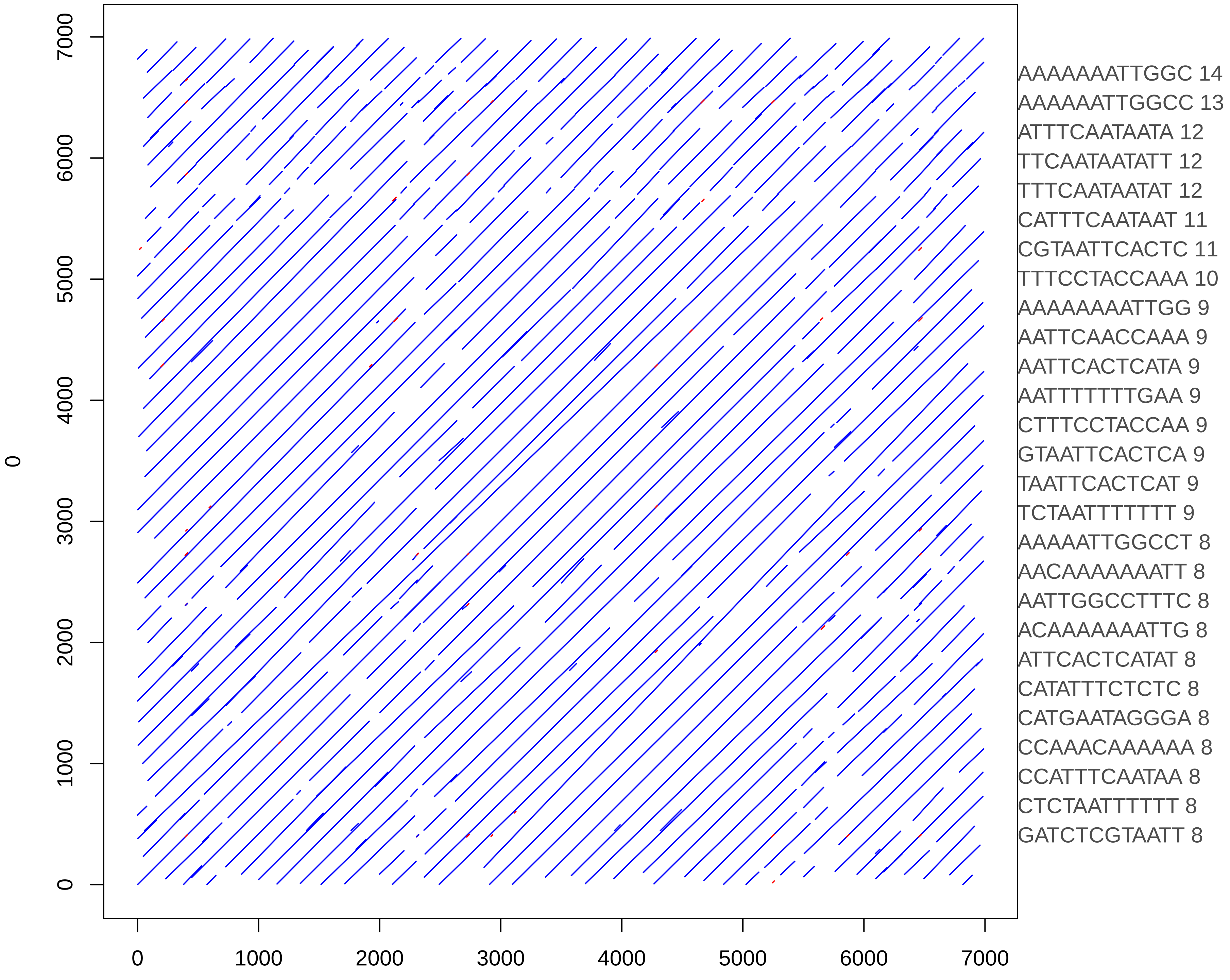

(HW Bass, DL Vera, FSU '17
Hopsteiner PacBio Apollo reads input)

0

Supplemental Material
k-mer screen & YASS dot-plots

KA Easterling et al. 2020

>m141231_015334_42134_c100756552550000001823146807221542_s1_p0/63025/2980_12169

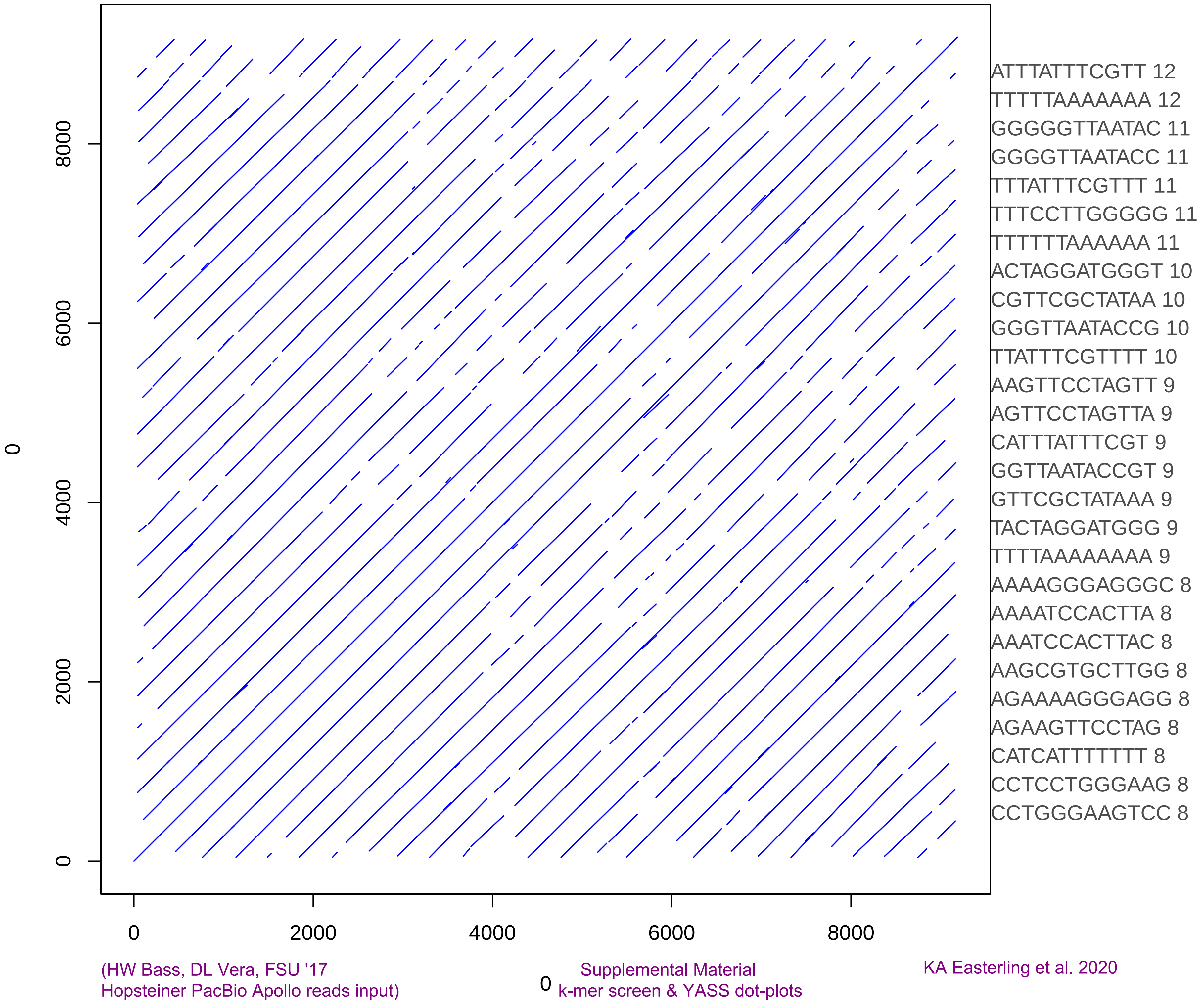

>m141231_015334_42134_c100756552550000001823146807221542_s1_p0/63130/2904_8726

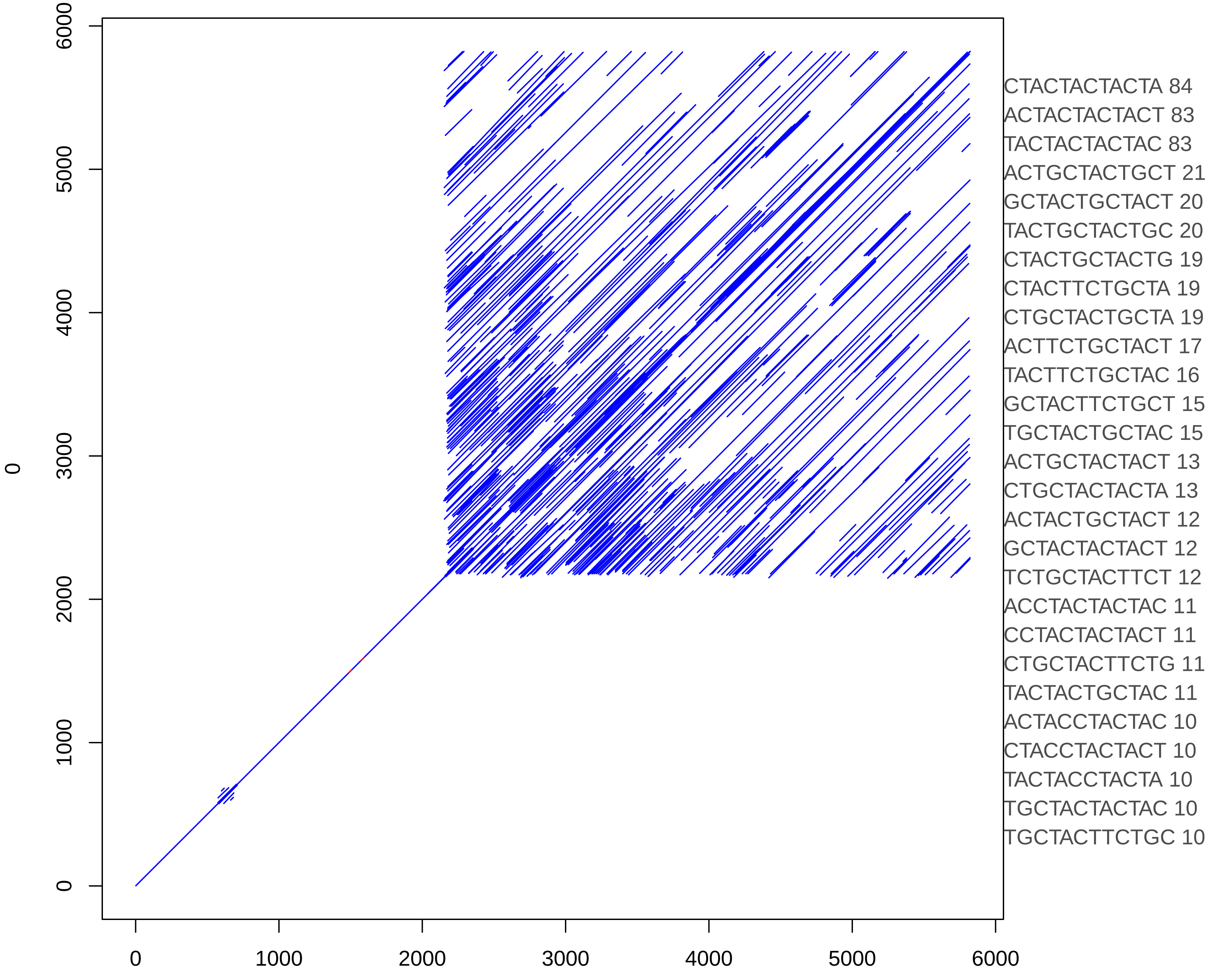

>m141231_015334_42134_c100756552550000001823146807221542_s1_p0/66403/3942_13320

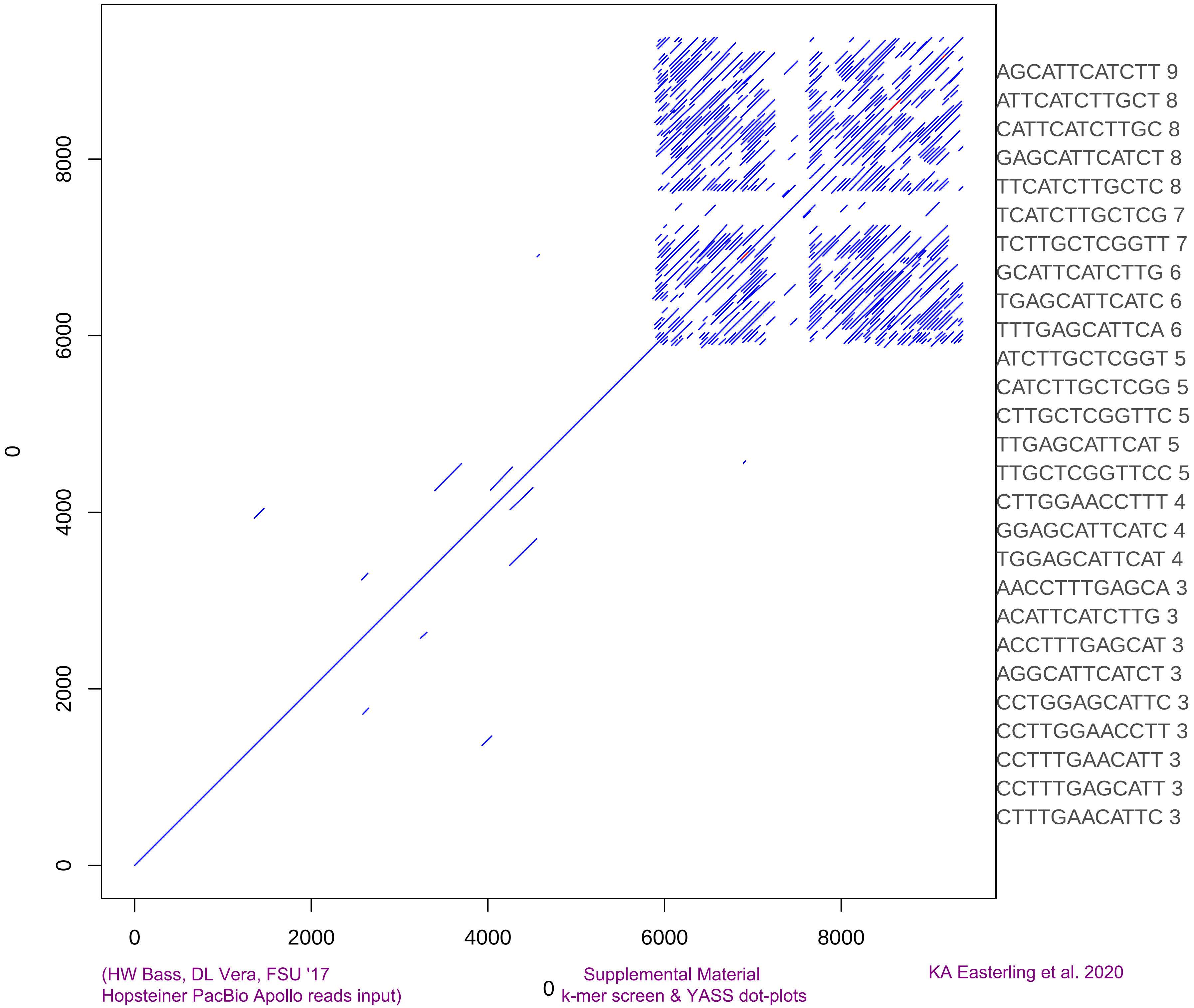

>m141231_015334_42134_c100756552550000001823146807221542_s1_p0/66650/0_7437

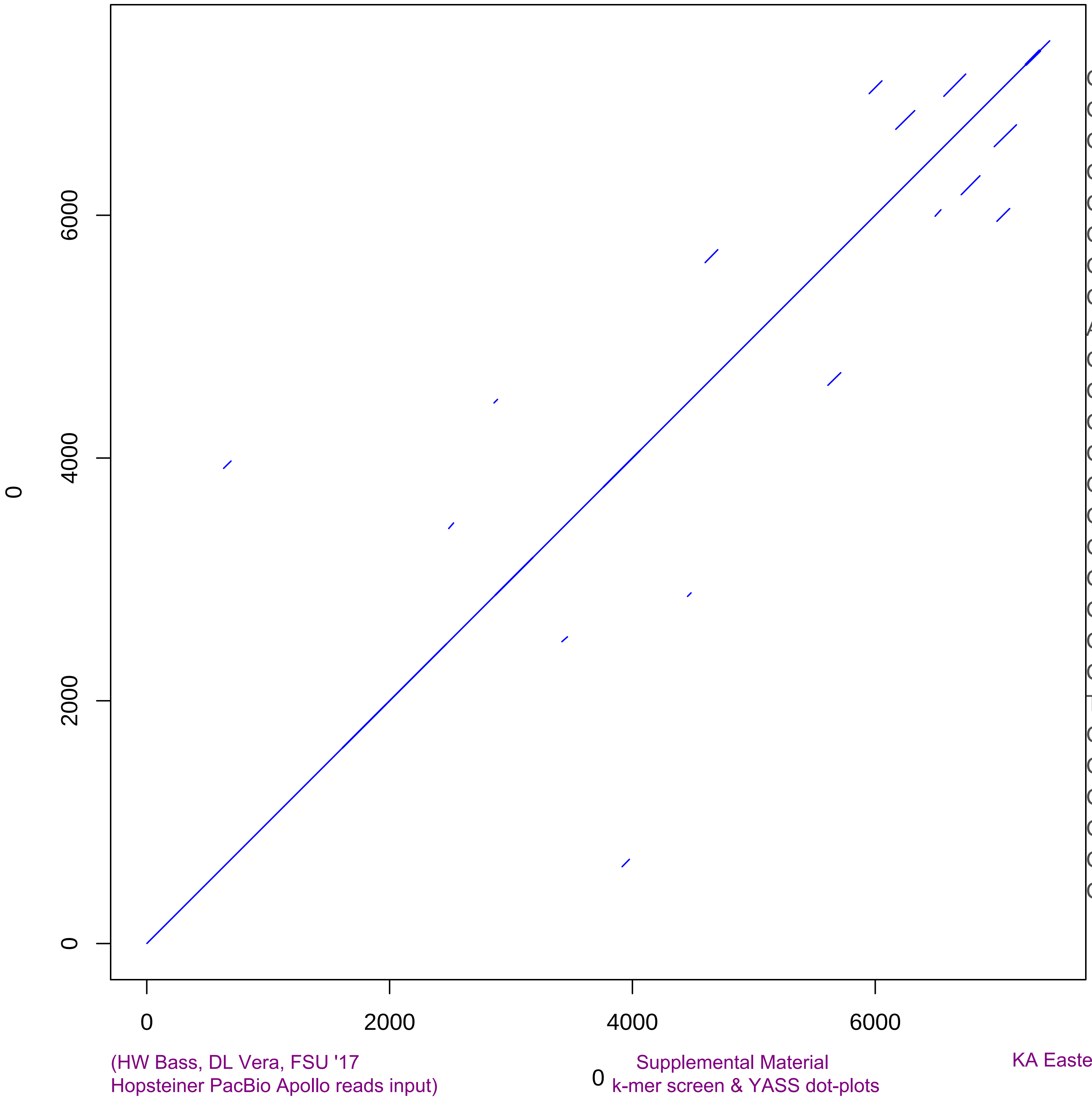

CCCCCCCCCCCC 155
CCCCCCCCCCTC 13
CCCCCCCCCTCC 11
CCCCCCCCTCCC 11
CACCCCCCCCCC 10
CCCACCCCCCCC 10
CCCCCCCCCCCT 10
CCCCCCTCCCCC 10
ACCCCCCCCCCC 9
CCACCCCCCCCC 9
CCCCACCCCCCC 9
CCCCCCACCCCC 9
CCCCCCCTCCCC 9
CCCCCTCCCCCC 9
CCTCCCCCCCCC 9
CCCCCACCCCCC 8
CCCTCCCCCCCC 8
CTCCCCCCCCCC 8
CCCCCCCCACCC 7
CCCCTCCCCCCC 7
TCCCCCCCCCCC 7
CCCCCCCACCCC 6
CCCCCCCCCACC 6
CCCCCCCCCCCA 6
CCCCCCCCCCAC 5
CCCCCGCCCCCC 4
CCCCGCCCCCCC 4

>m141231_015334_42134_c100756552550000001823146807221542_s1_p0/70407/907_6013

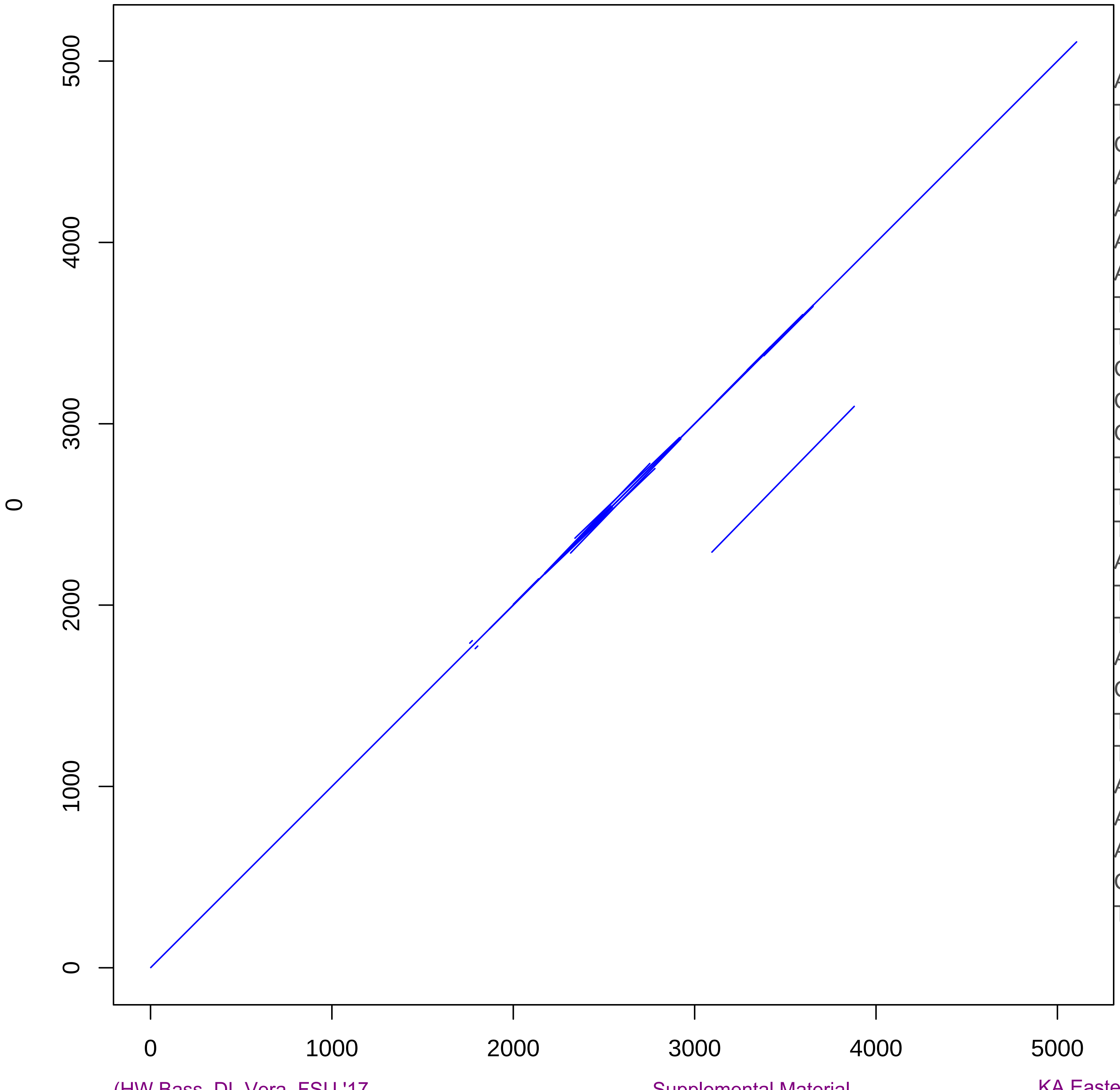

AGTAGTAGTAGT 56
TAGTAGTAGTAG 55
GTAGTAGTAGTA 54
AGTATAGTAGTA 14
AGTAGTATAGTA 13
AAGTAGTAGTAG 12
ATAGTAGTAGTA 12
TAGTAGTATAGT 12
TAGTATAGTAGT 12
GTAGTAGTATAG 10
GTAGTATAGTAG 10
GTATAGTAGTAG 10
TAAGTAGTAGTA 9
TAGTAGTAGTAA 9
TATAGTAGTAGT 9
AGTAGTAGTATA 8
TAGTAGTAAGTA 8
TAGTAGTAGTAT 8
AGTAGTAGTAAG 7
GGTAGTAGTAGT 7
TAGTAGTAGCTA 7
TAGTAGTAGTTA 7
AGTAGCTAGTAG 6
AGTAGTAGTAGC 6
AGTAGTAGTTAG 6
GTAGTAGTAAGT 6
TAGTAAGTAGTA 6

>m141231_015334_42134_c100756552550000001823146807221542_s1_p0/728/4505_11389

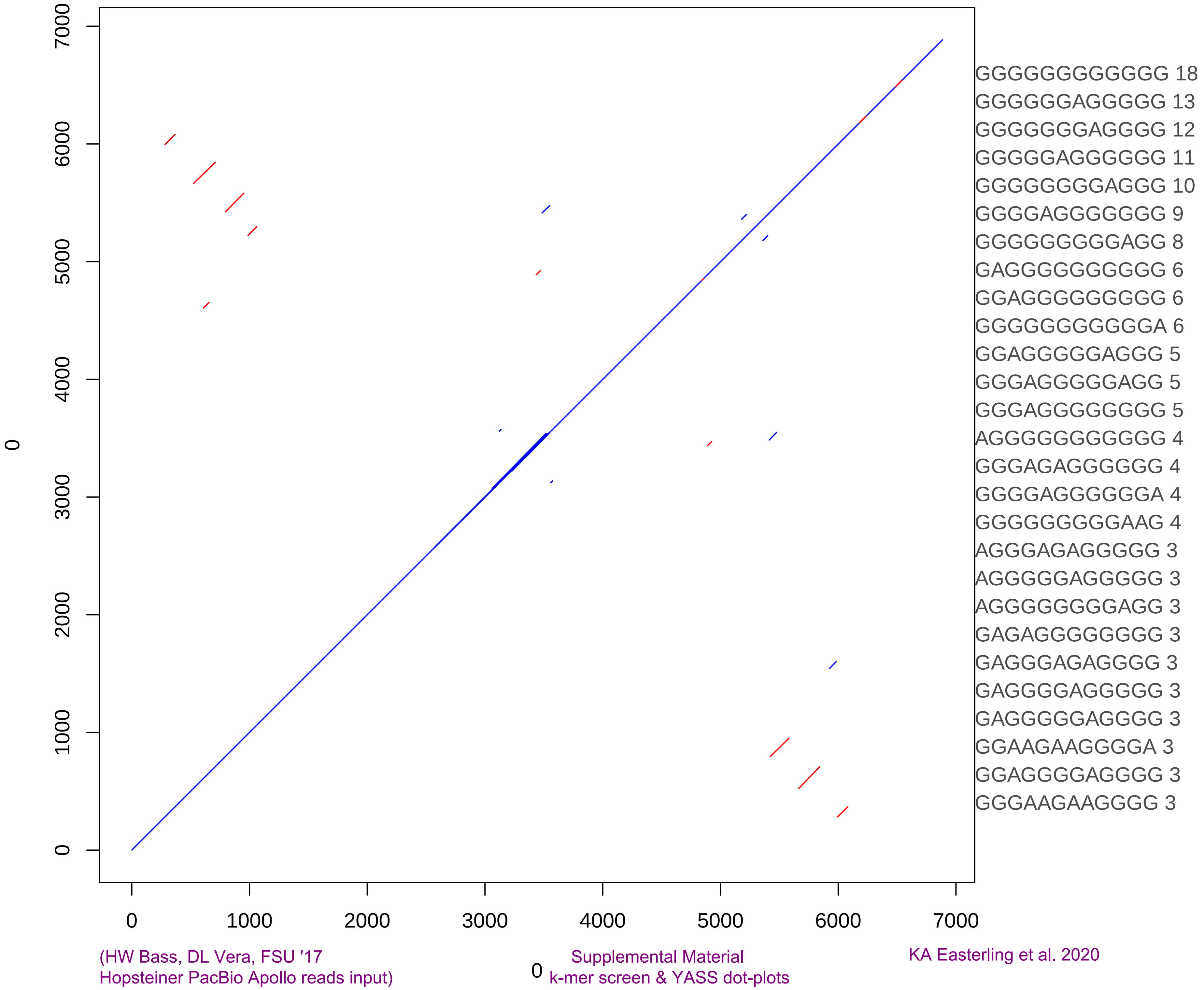

>m141231_015334_42134_c100756552550000001823146807221542_s1_p0/76778/3886_9694

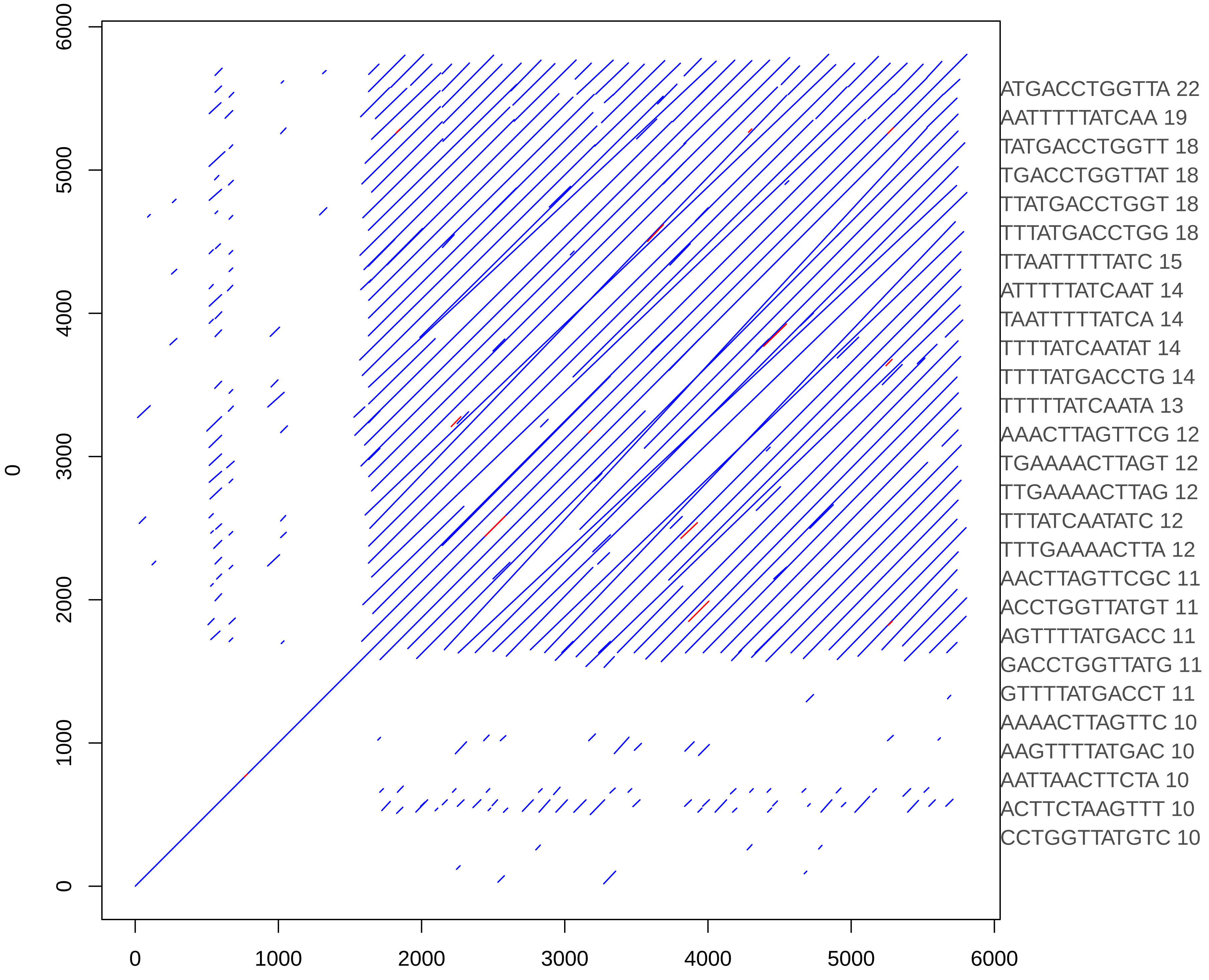

>m141231_015334_42134_c100756552550000001823146807221542_s1_p0/78578/476_10778

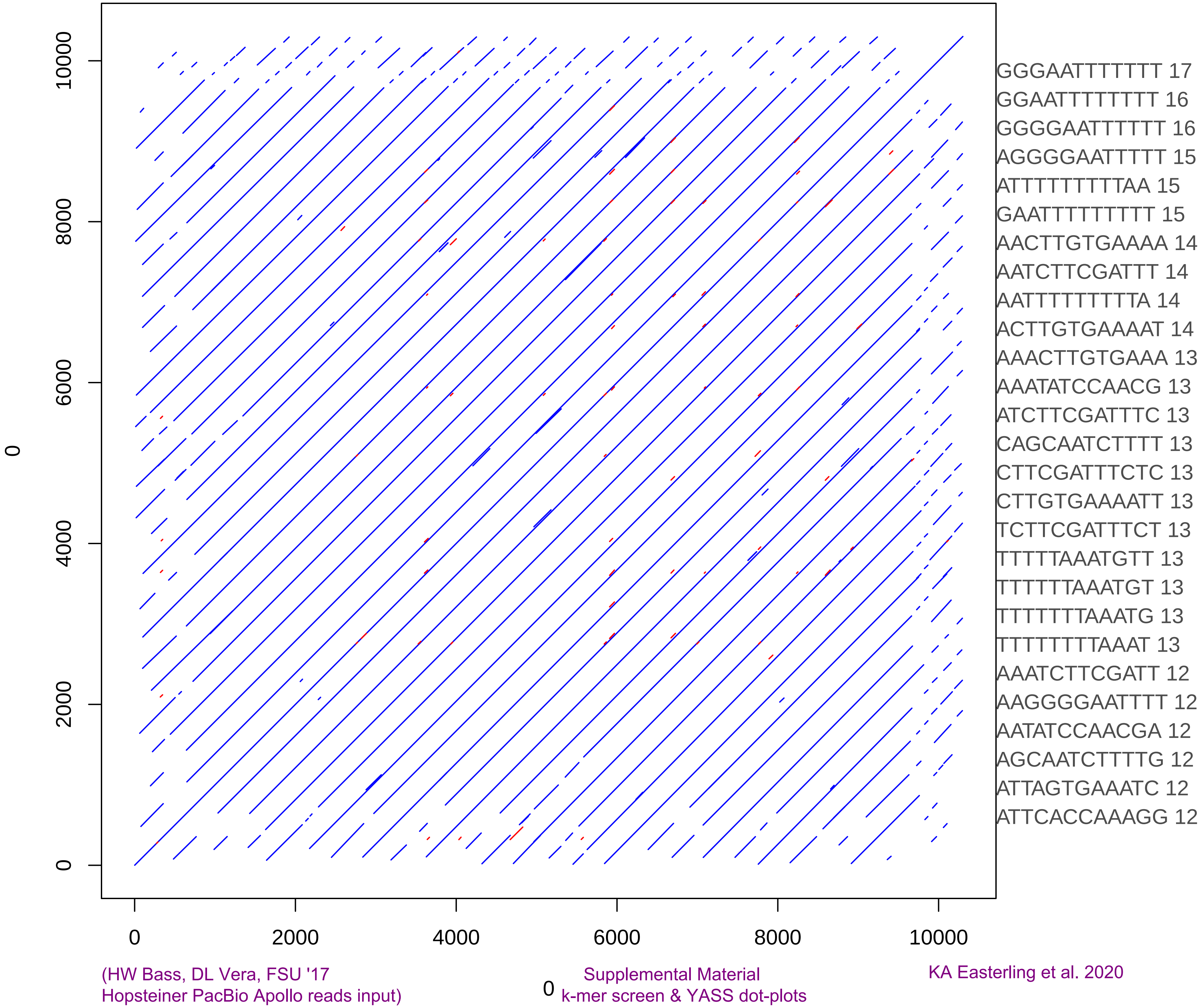

>m141231_015334_42134_c100756552550000001823146807221542_s1_p0/80804/4219_13170

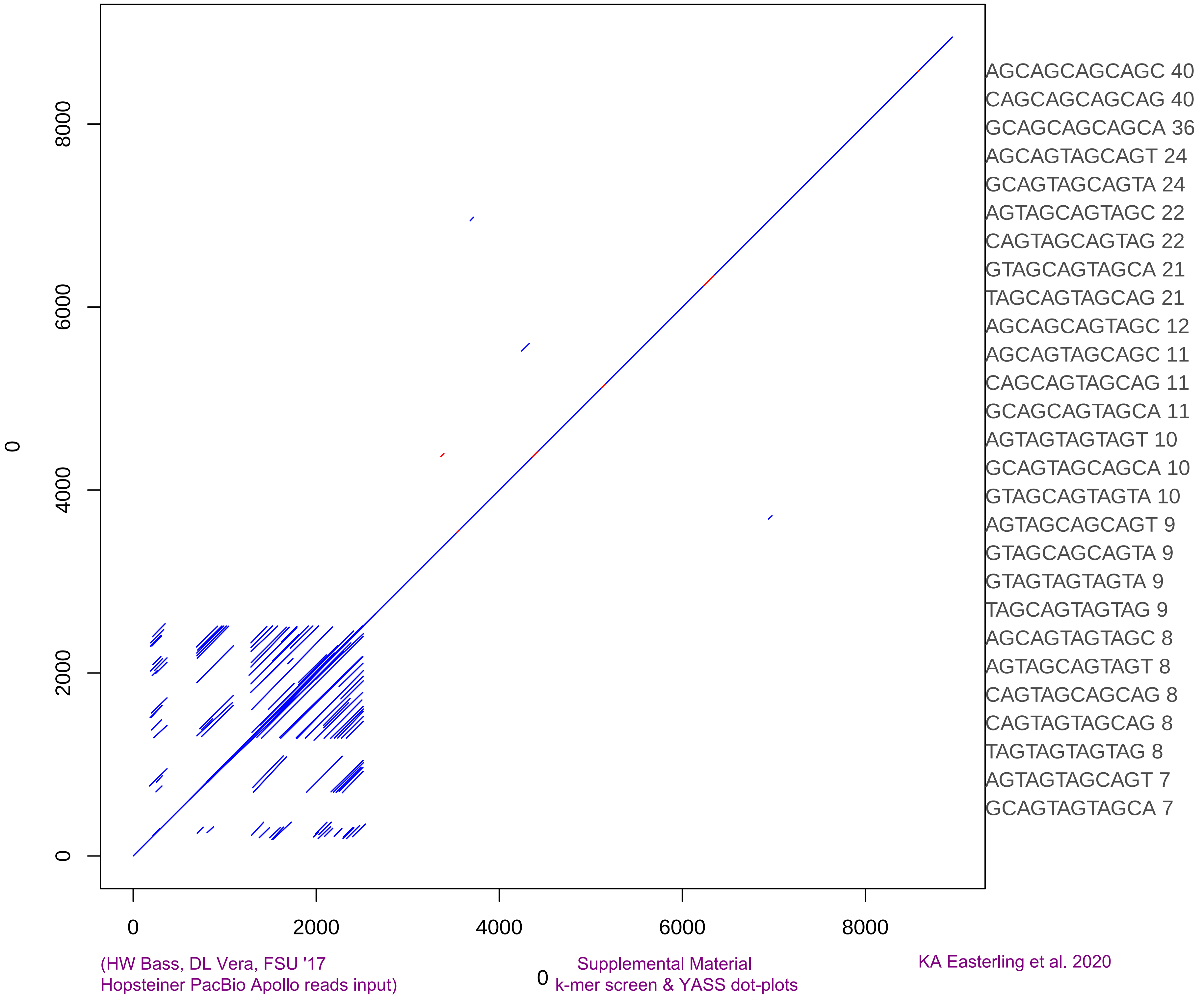

>m141231_015334_42134_c100756552550000001823146807221542_s1_p0/84065/1653_11072

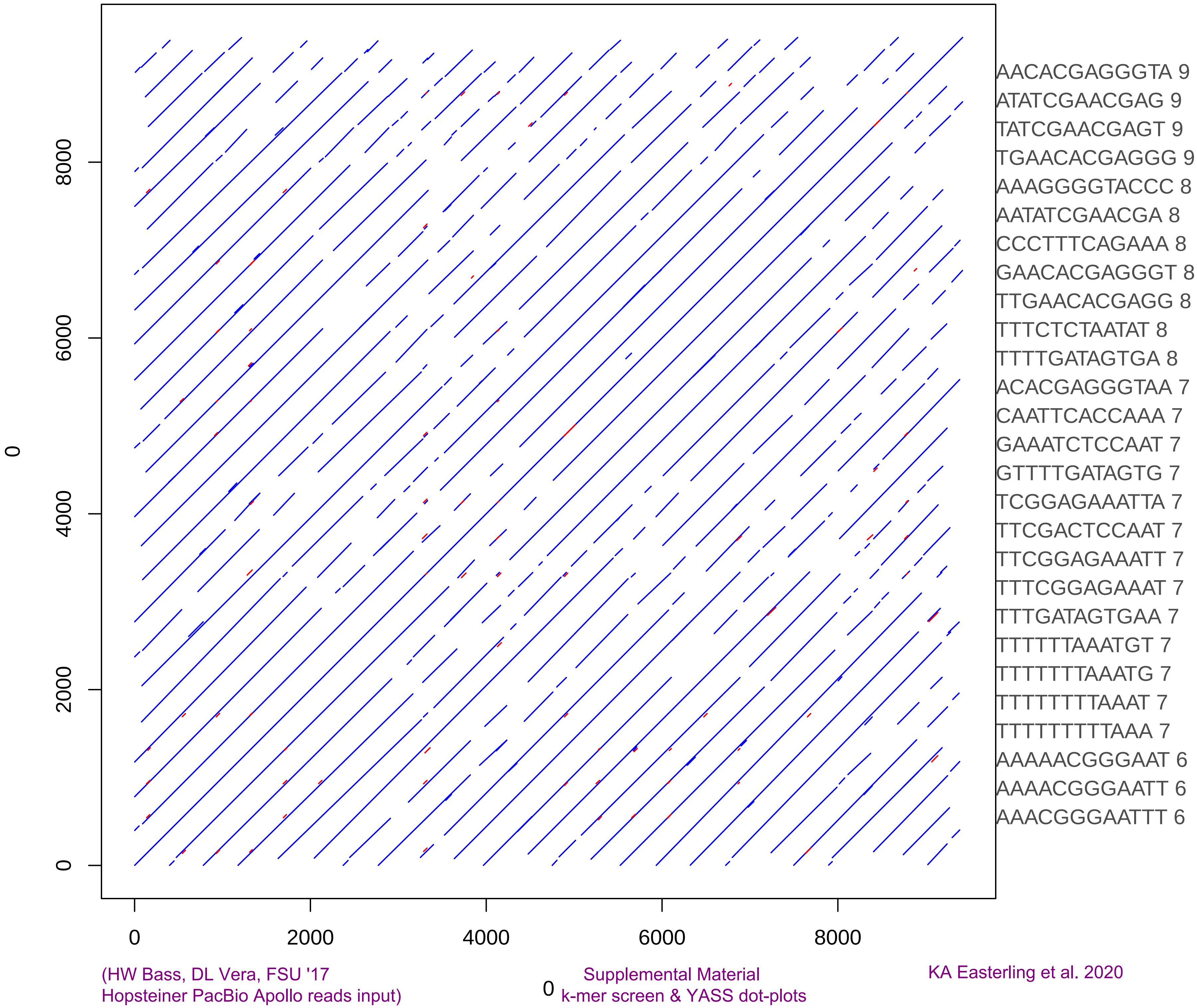

>m141231_015334_42134_c100756552550000001823146807221542_s1_p0/84760/6085_18140

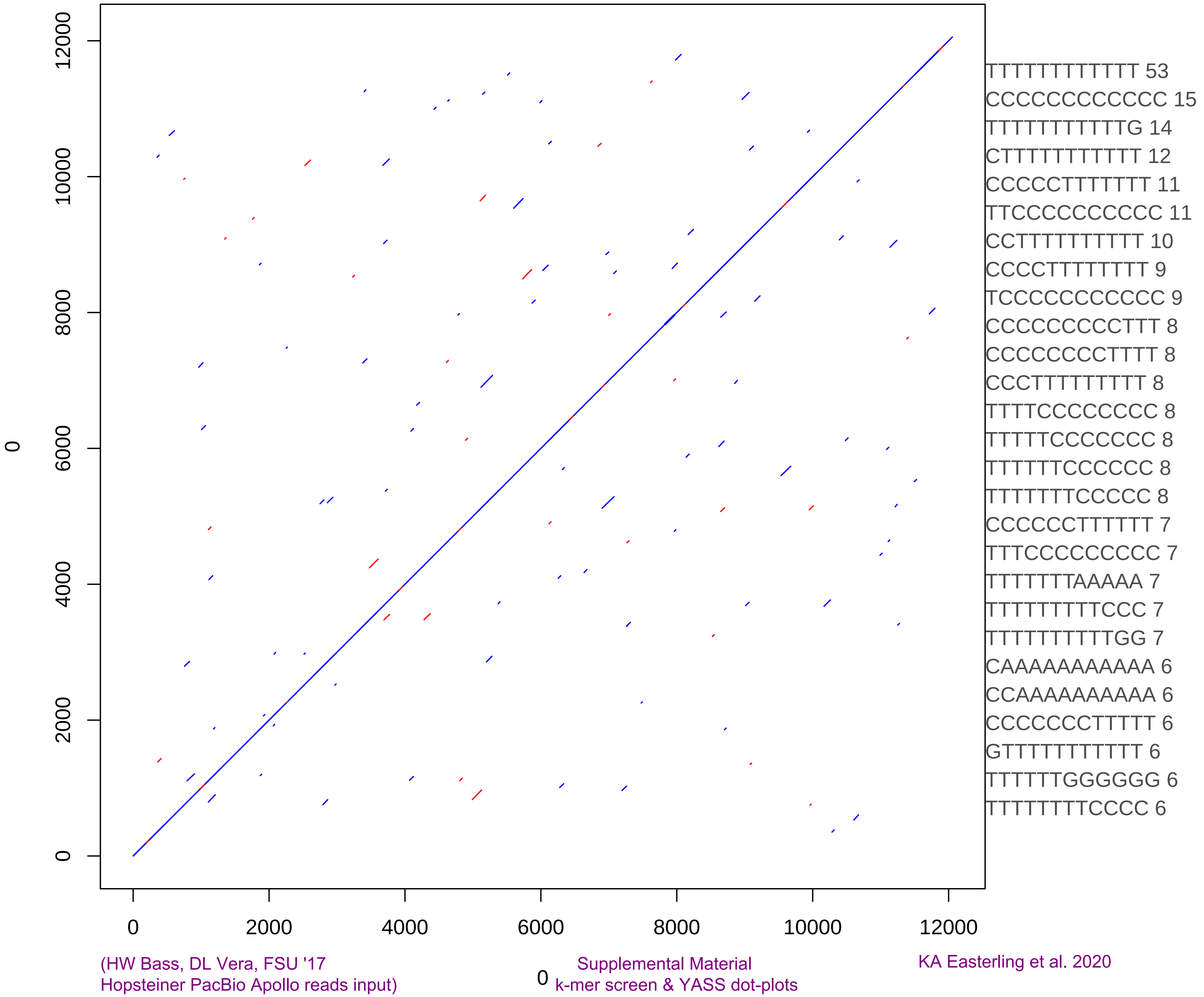

>m141231_015334_42134_c100756552550000001823146807221542_s1_p0/85435/416_5662

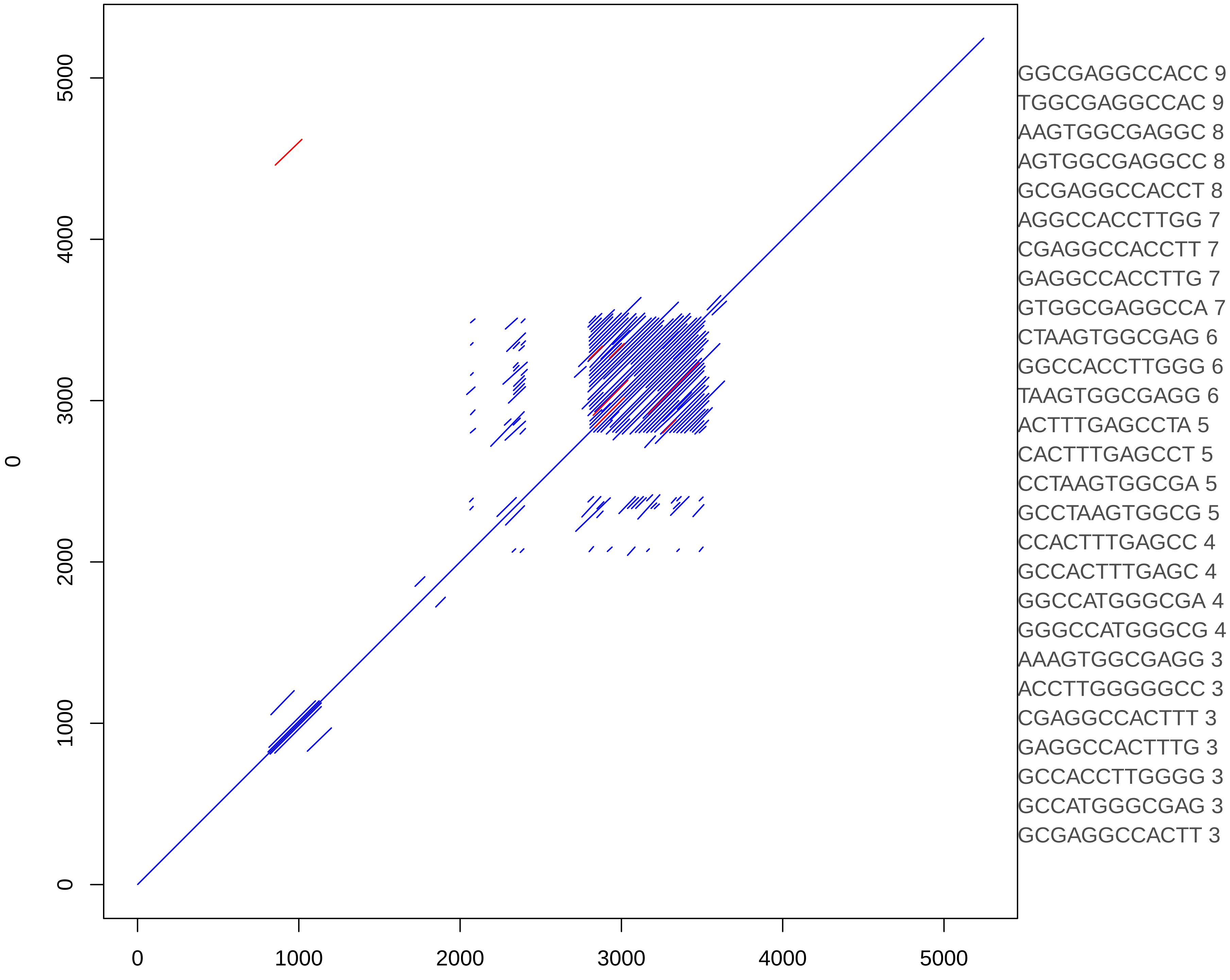

>m141231_015334_42134_c100756552550000001823146807221542_s1_p0/85713/8061_18664

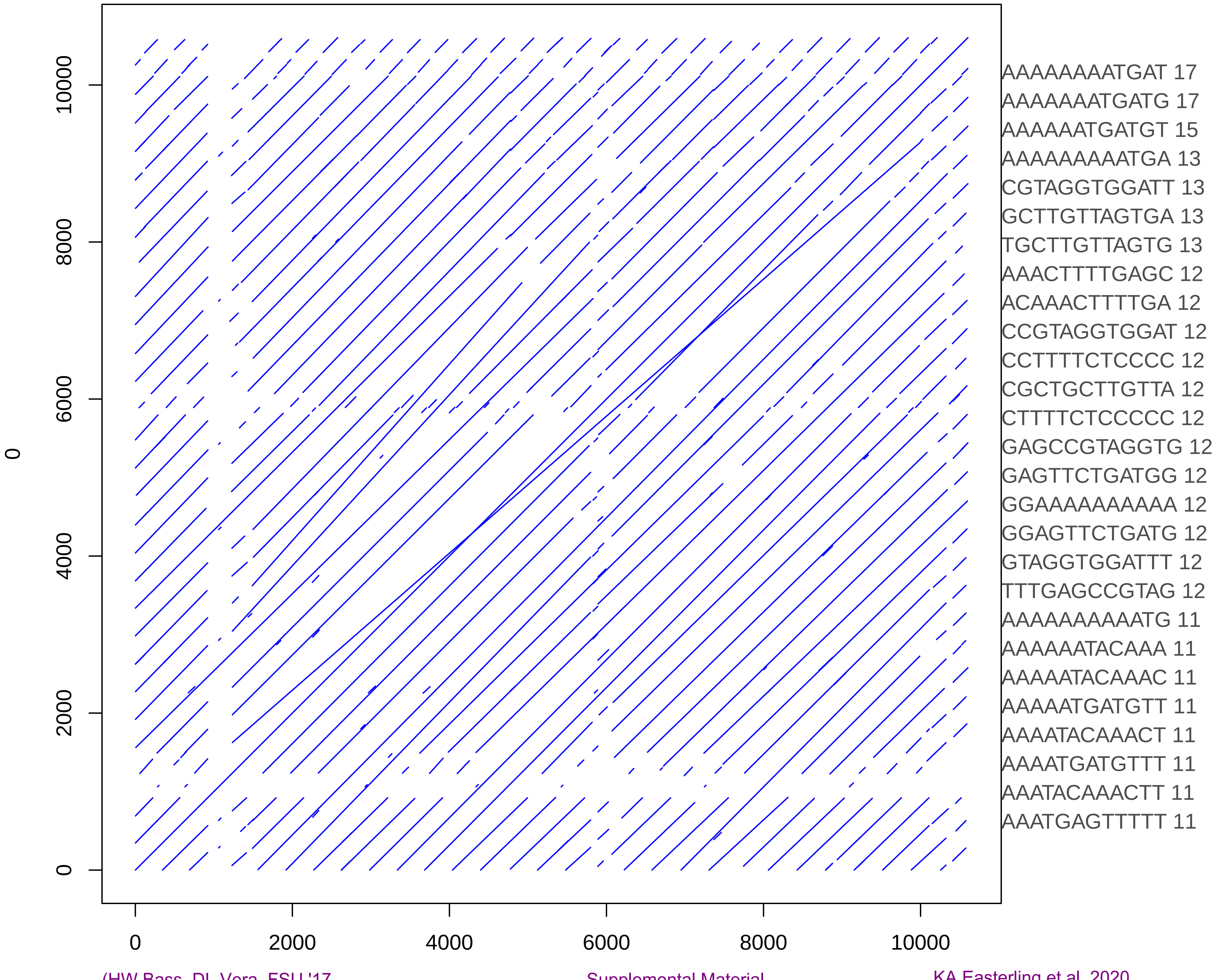

1,121 hits for repetitive sequences passing 12mer abundance criterion

(Read-Number)
PAGE 969

For Tandem Repeat families of ### bp:
PacBio Read named "HuluTR###-r969"

>m141231_015334_42134_c100756552550000001823146807221542_s1_p0/88002/0_14282

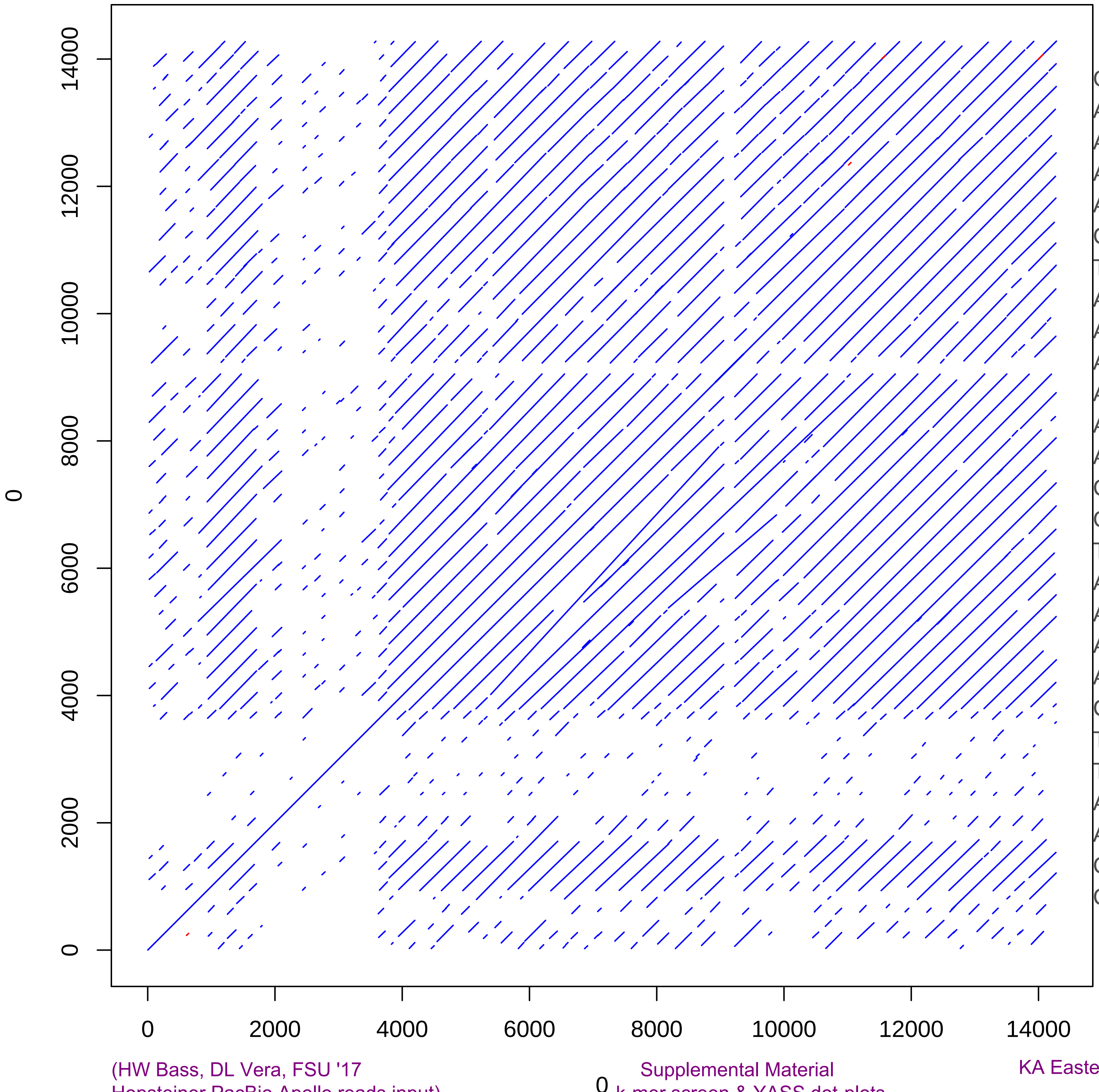

GGGGGGGGGGGG 58
AAAAATGATGTT 17
AAAATGATGTTT 17
AAAAAATGATGT 16
AAACGAAATAAA 15
CGAAATAAATGA 15
TTTTAAAGAATA 15
AAAAAAATGATG 14
AAAGAATAAAAA 14
AACGAAATAAAT 14
AAGAATAAAAAA 14
AATAAATGAGCT 14
ACGAAATAAATG 14
CTTATTCGCTGC 14
GAAATAAATGAG 14
TTTTTTAAAAAA 14
AAATAAATGAGC 13
ACGAGGACTTCC 13
AGAATAAAAAAA 13
ATAAATGAGCTT 13
CCCTTTTCTCCC 13
TCCCTTTTCTCC 13
TTTTTAAAGAAT 13
AAATGATGTTTT 12
ATTTTTAAAGAA 12
CGAGGACTTCCC 12
CTCCCTTTTCTC 12

(HW Bass, DL Vera, FSU '17
Hopsteiner PacBio Apollo reads input)

Supplemental Material
k-mer screen & YASS dot-plots

KA Easterling et al. 2020

>m141231_015334_42134_c100756552550000001823146807221542_s1_p0/91576/4323_16464

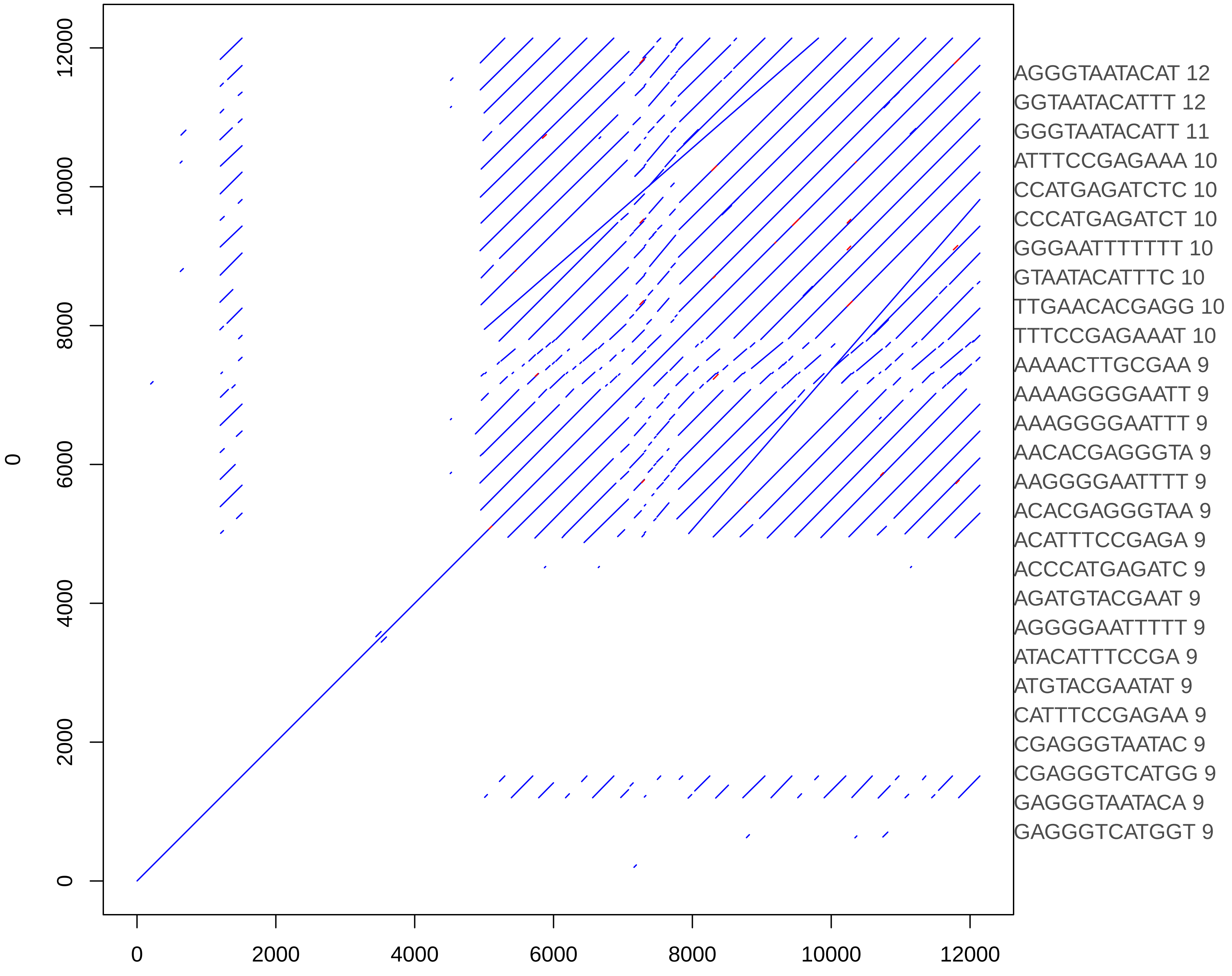

>m141231_015334_42134_c100756552550000001823146807221542_s1_p0/91998/410_9182

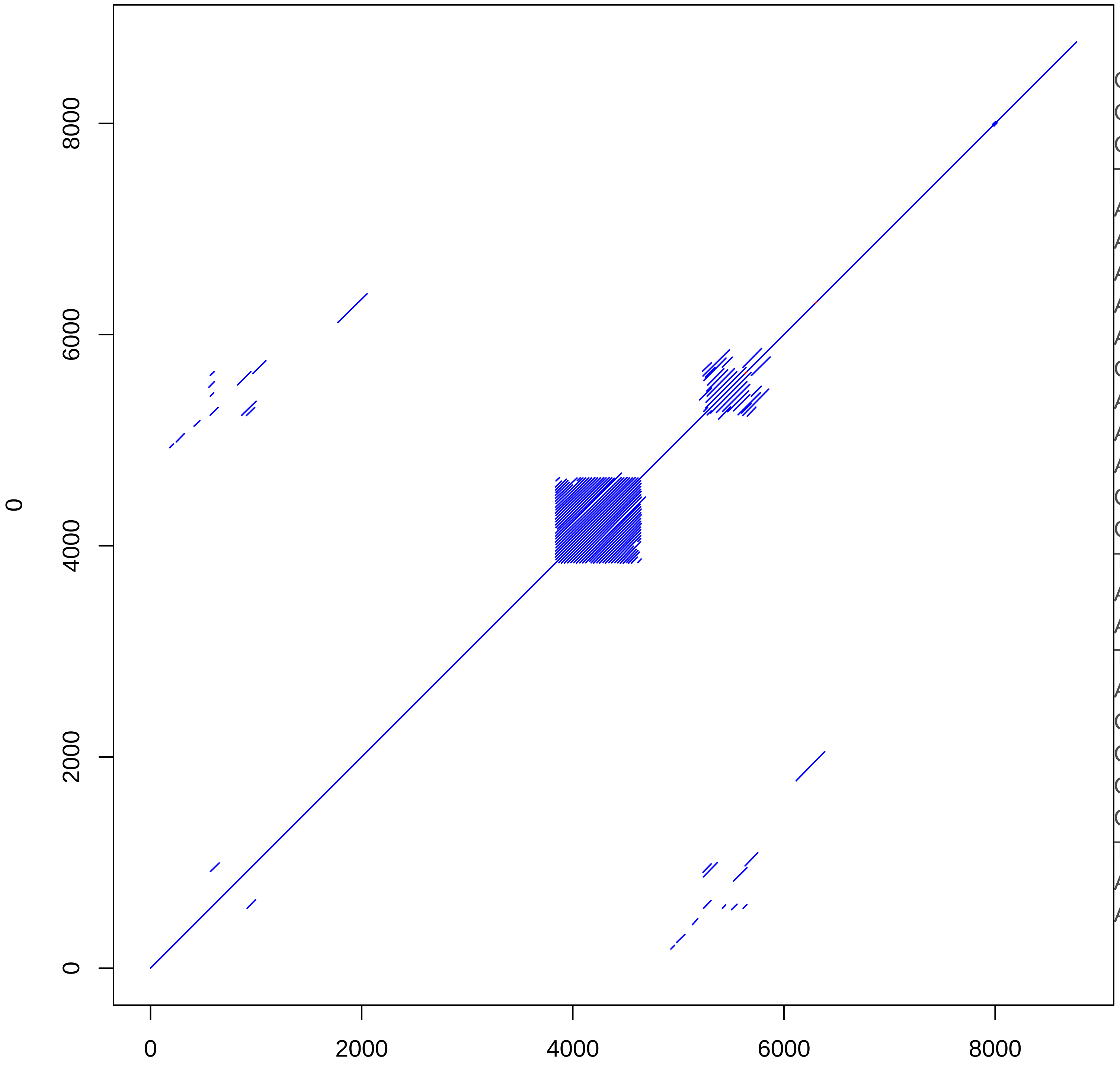

GGTCTCAAAAGT 12
GTCTCAAAAGTA 12
CTCAAAAGTAGA 9
TCTCAAAAGTAG 9
AAAAGGGTCTCA 8
AAAGGGTCTCAA 8
AAAAGTAGACGC 7
AAGGGTCTCAAA 7
ACGCTTGCAAAA 7
GACGCTTGCAAA 7
AAAGTAGACGCT 6
AAGTAGACGCTT 6
AGACGCTTGCAA 6
CAAAAGTAGACG 6
GGGTCTCAAAAG 6
TCAAAAGTAGAC 6
AAAAAGGGTCTC 5
AGTAGACGCTTG 5
TGCAAAAAGGGT 5
AGGGTCTCAAAA 4
CAAAAAGGGTCT 4
CGCTTGCAAAAA 4
GCAAAAAGGGTC 4
GTAGACGCTTGC 4
TAGACGCTTGCA 4
AAAAGAGTCTCA 3
AAAAGGGGTCTC 3

>m141231_015334_42134_c100756552550000001823146807221542_s1_p0/93968/6514_14421

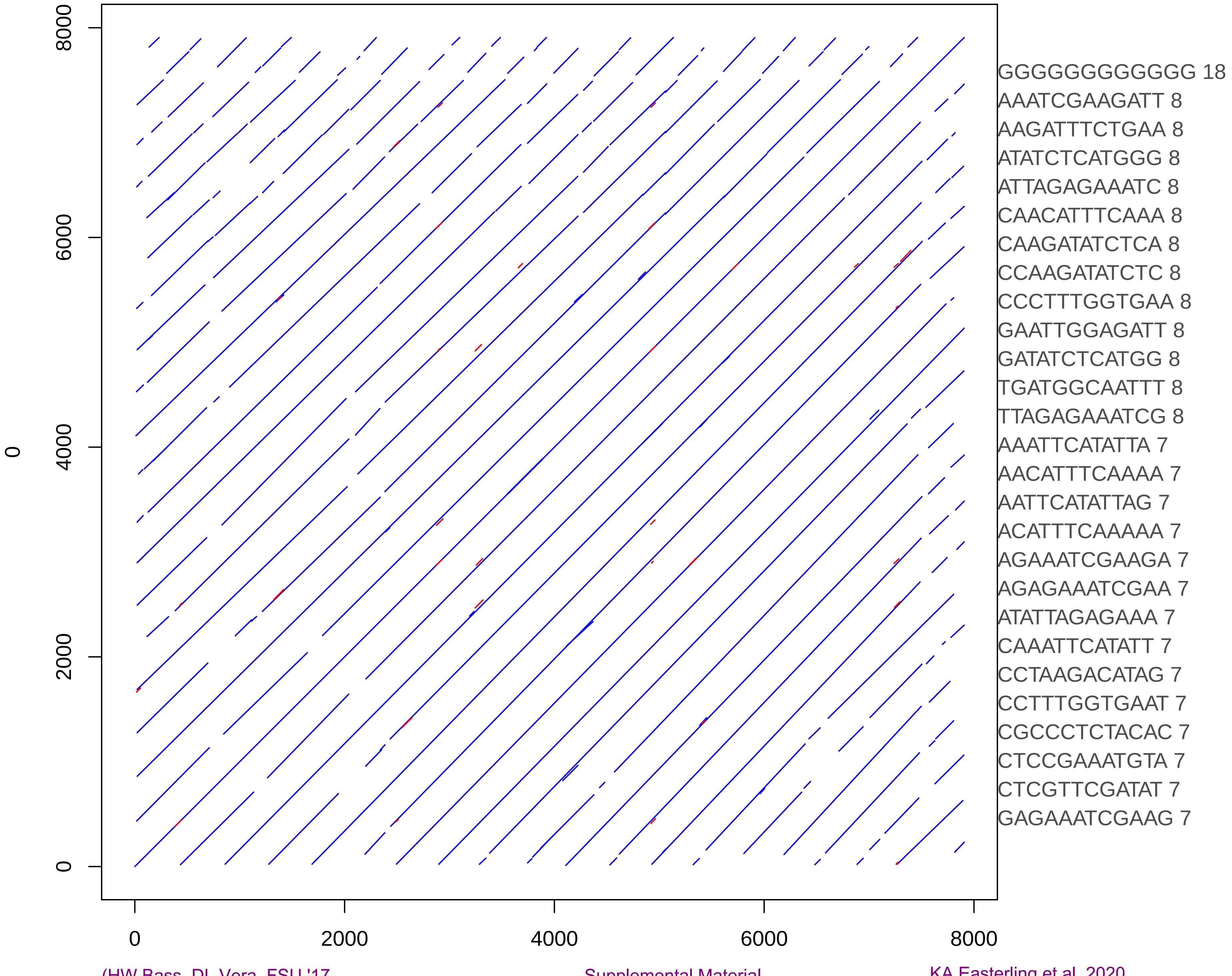

>m141231_015334_42134_c100756552550000001823146807221542_s1_p0/95947/2936_8663

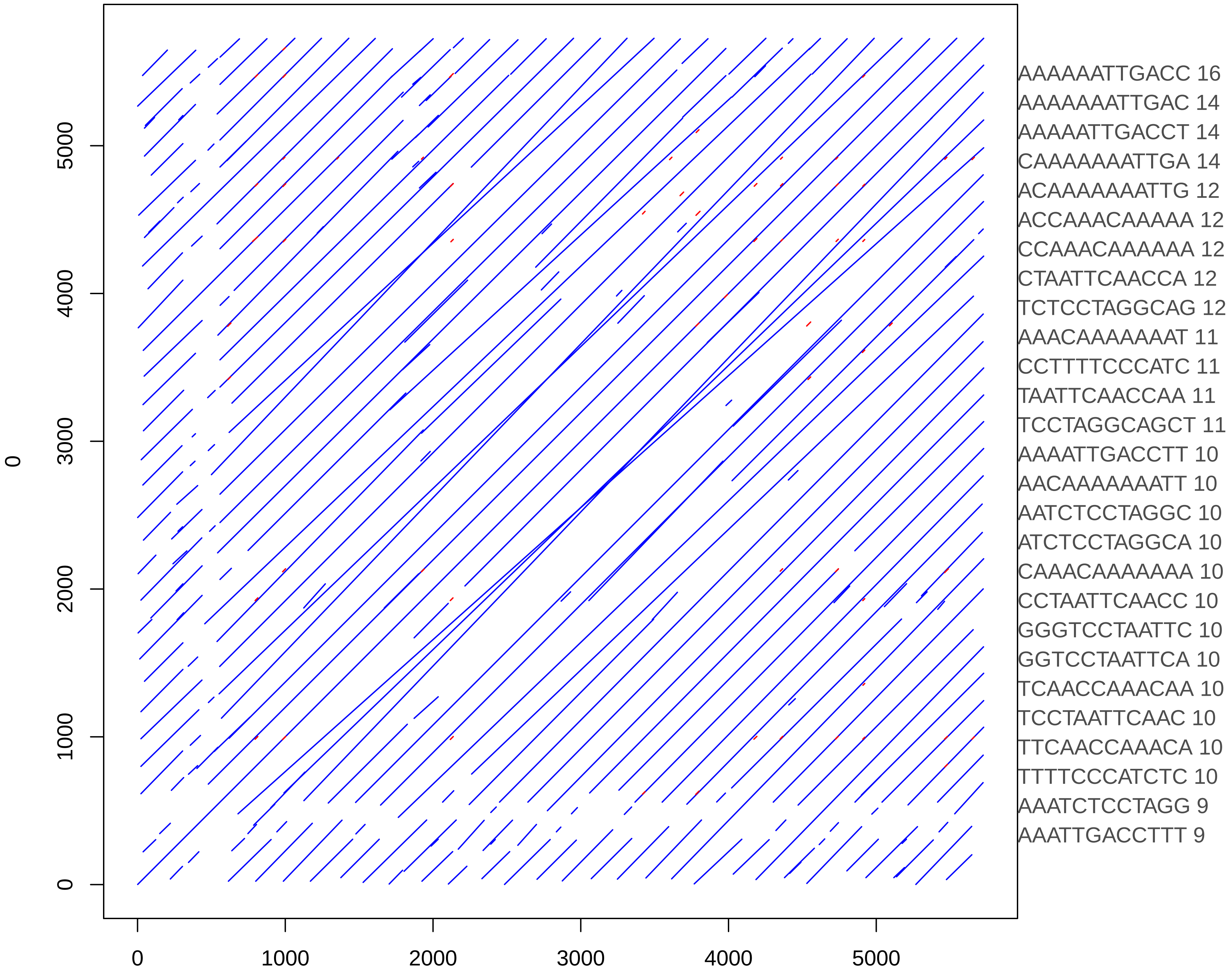

>m141231_015334_42134_c100756552550000001823146807221542_s1_p0/97552/2603_10572

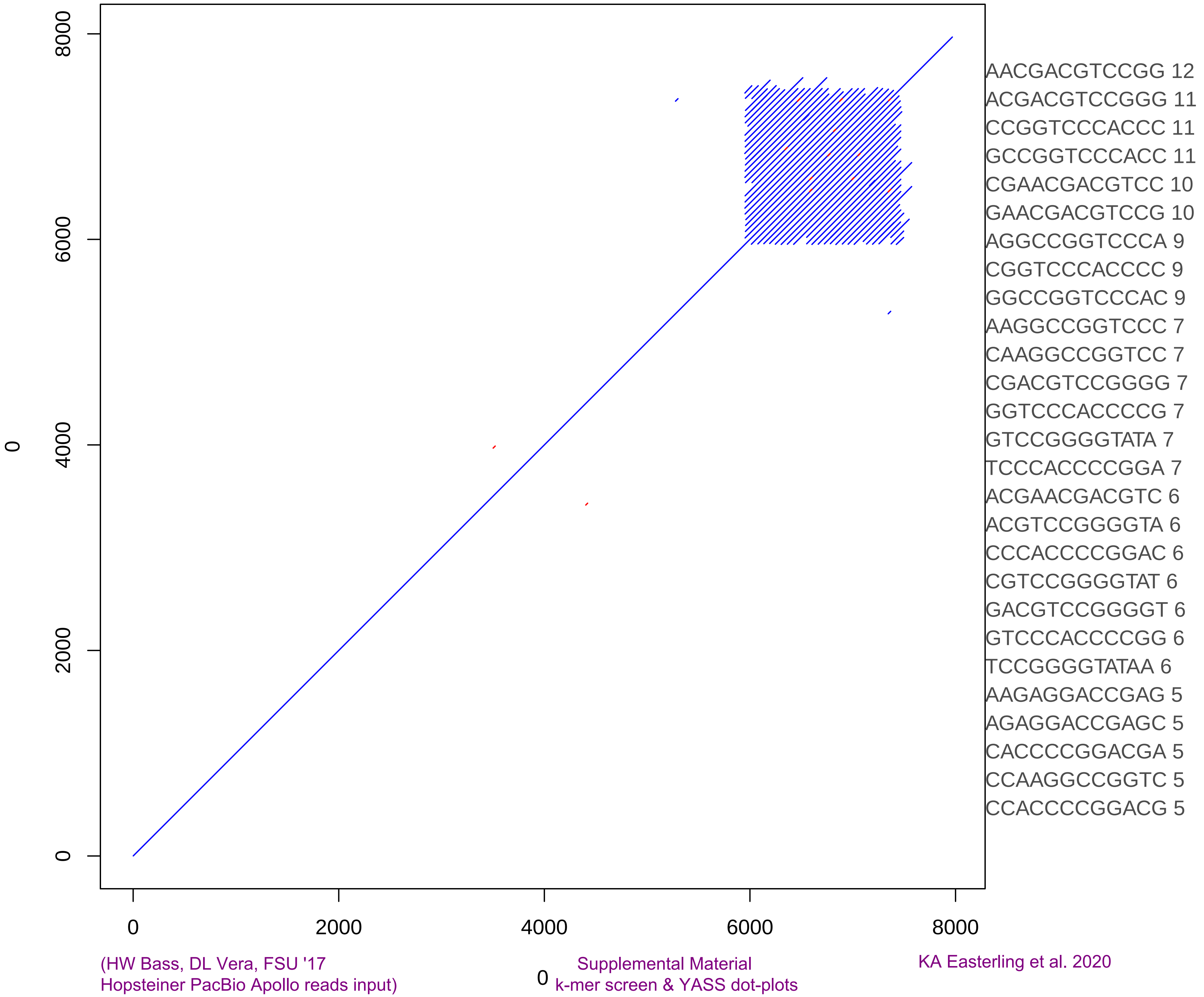

>m141231_015334_42134_c100756552550000001823146807221542_s1_p0/98162/0_5614

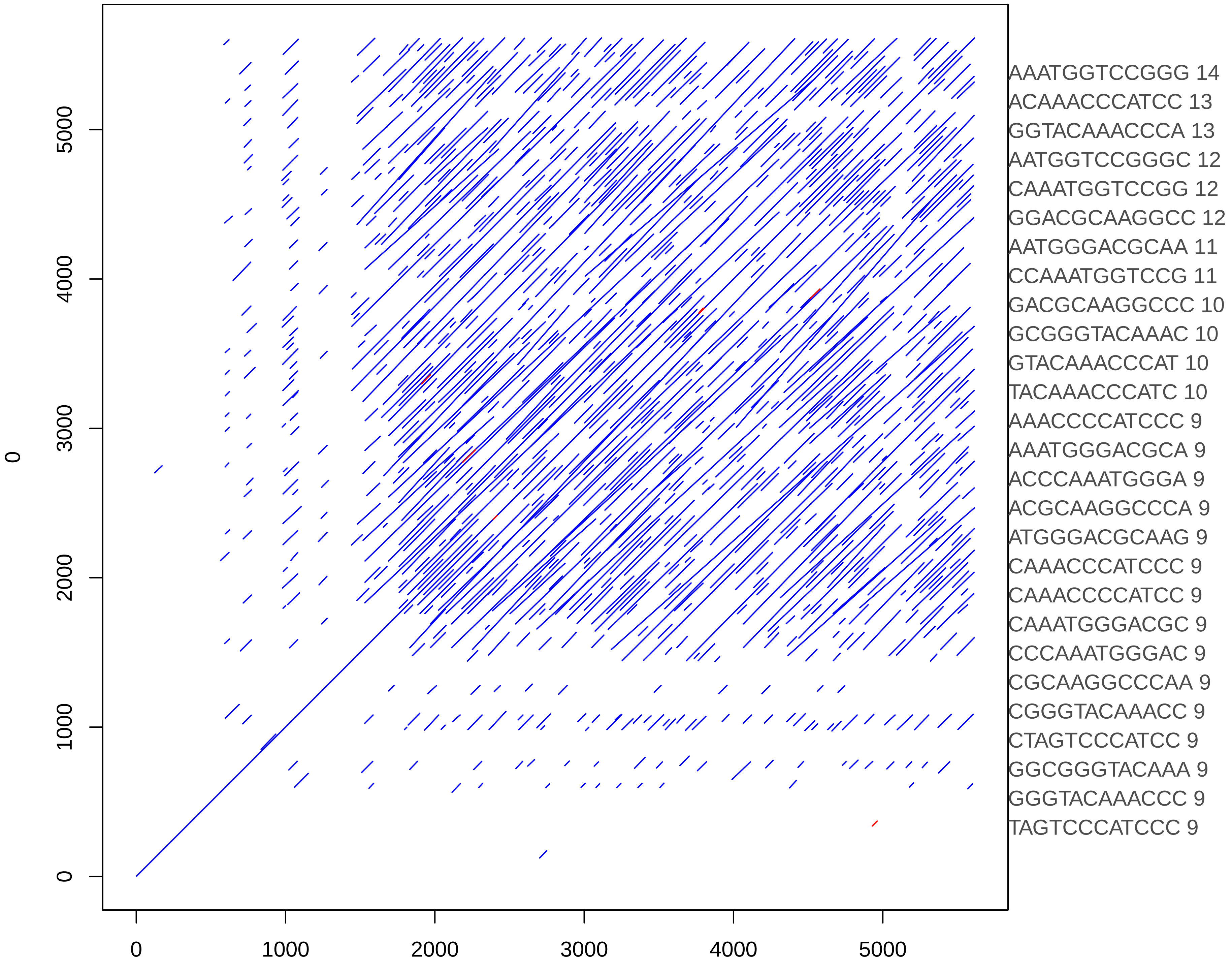

>m141231_015334_42134_c100756552550000001823146807221542_s1_p0/984/2521_9755

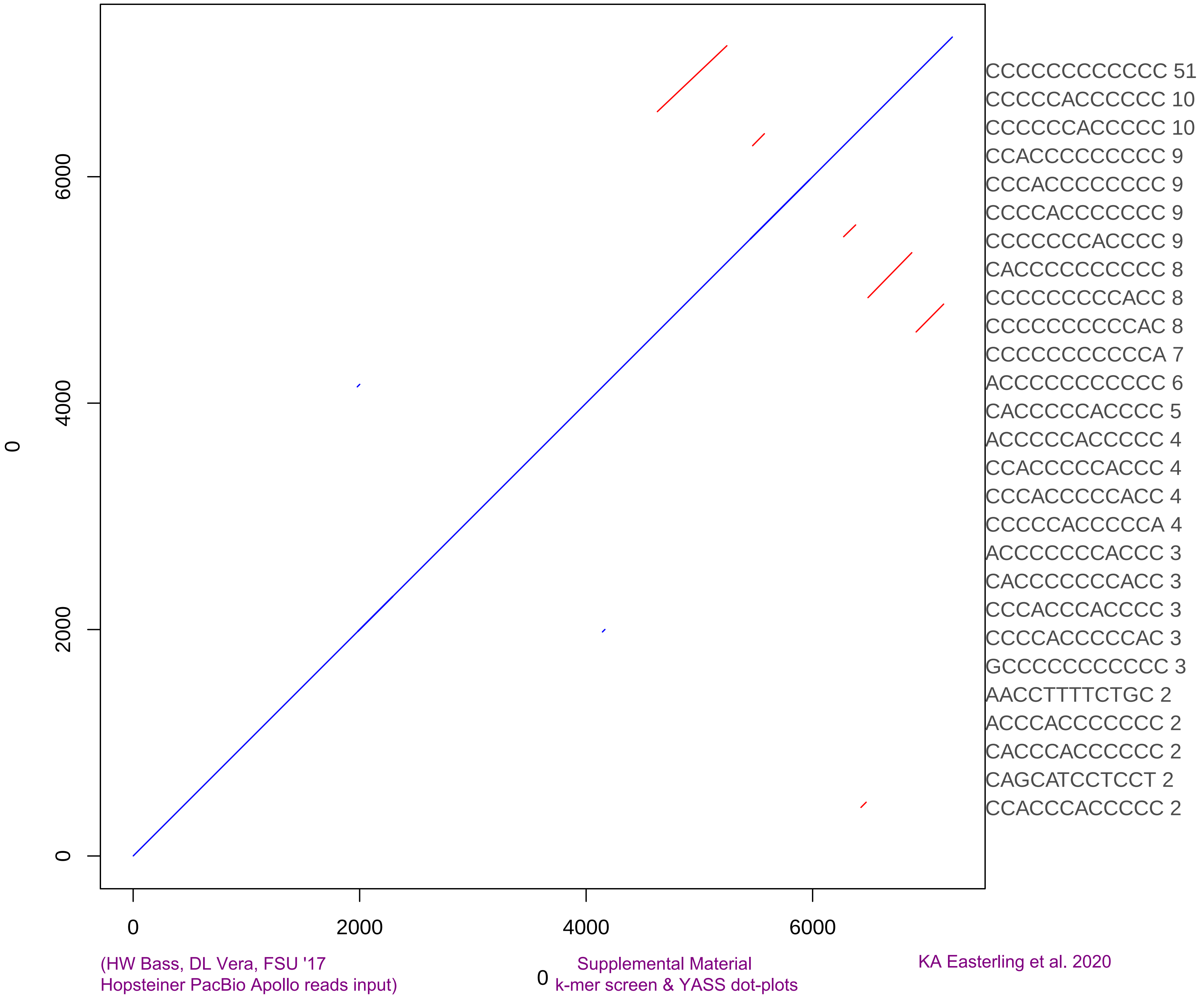

>m150106_022046_42134_c100761282550000001823158307221520_s1_p0/10233/0_6988

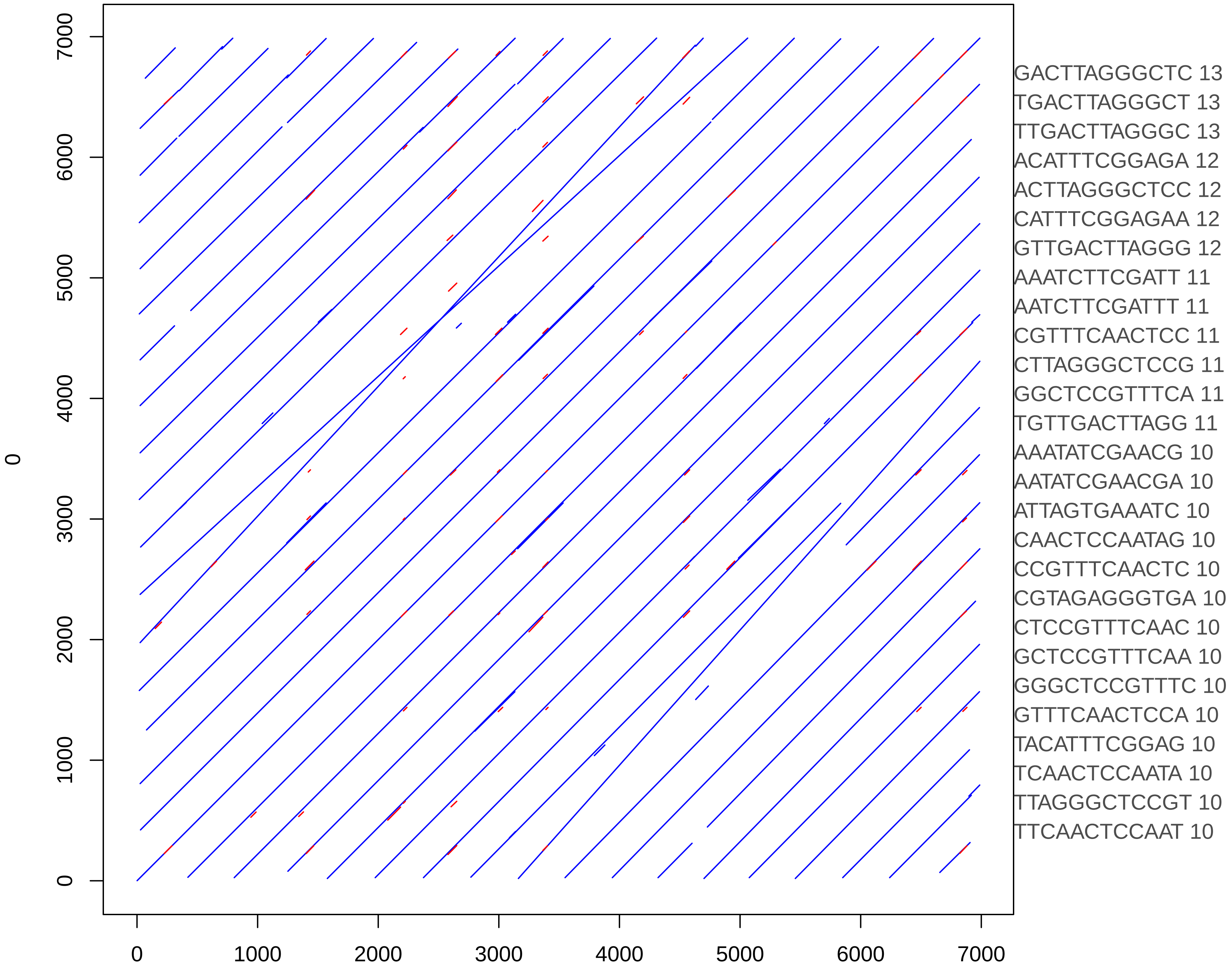

>m150106_022046_42134_c100761282550000001823158307221520_s1_p0/10233/7030_12643

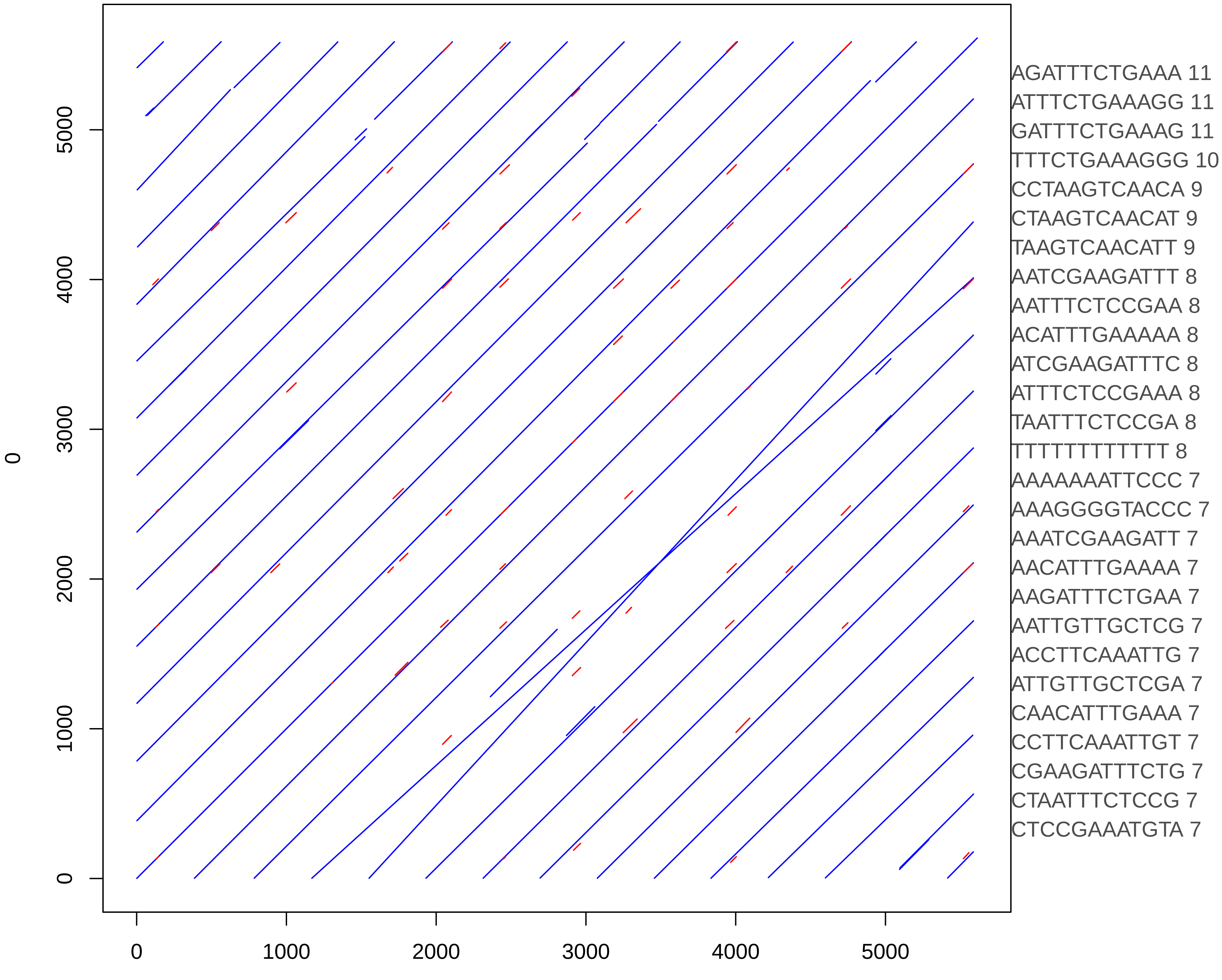

>m150106_022046_42134_c100761282550000001823158307221520_s1_p0/108628/215_7632

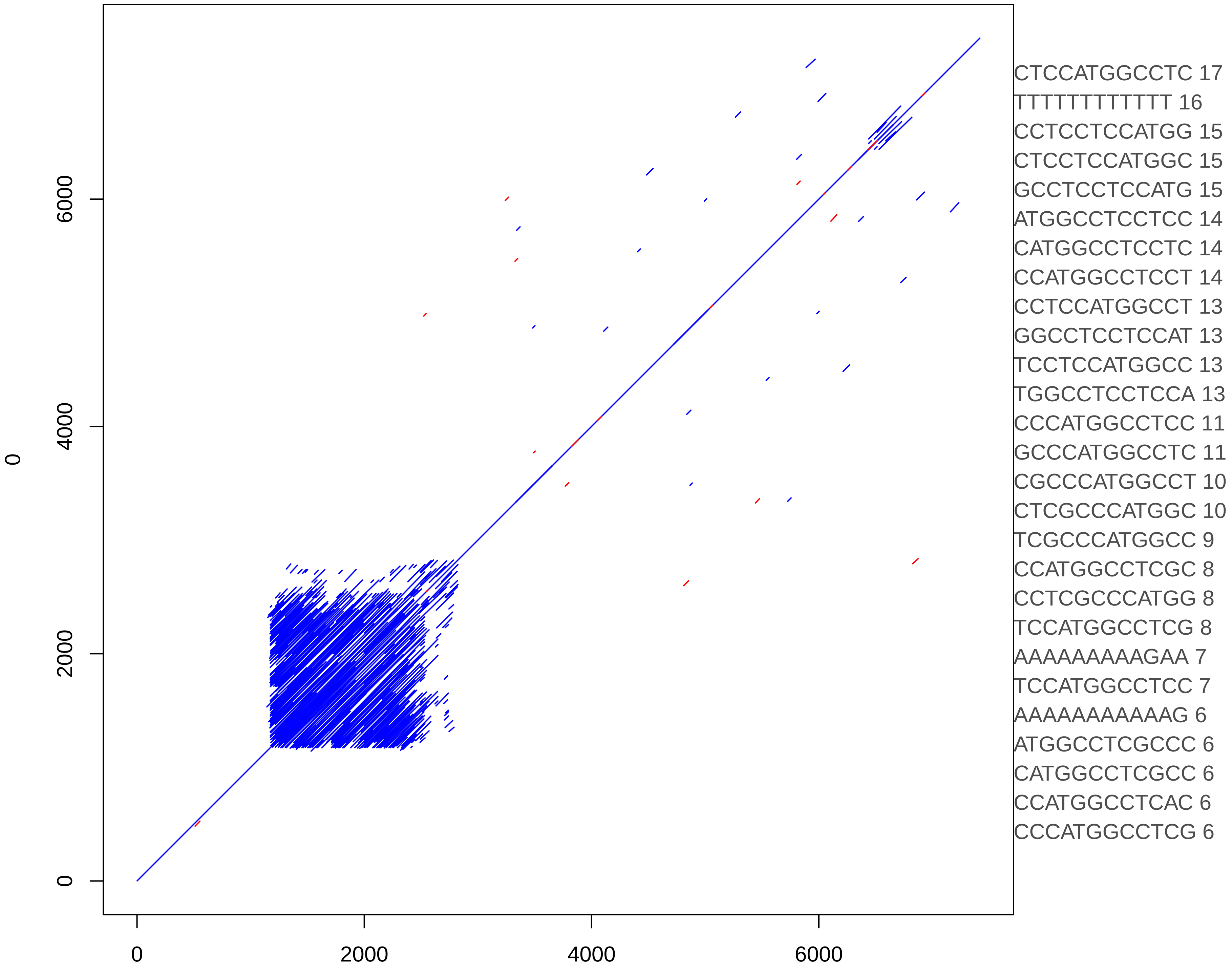

1,121 hits for repetitive sequences passing 12mer abundance criterion

(Read-Number)
PAGE 980

For Tandem Repeat families of ### bp:
PacBio Read named "HuluTR###-r980"

>m150106_022046_42134_c100761282550000001823158307221520_s1_p0/115652/2179_9481

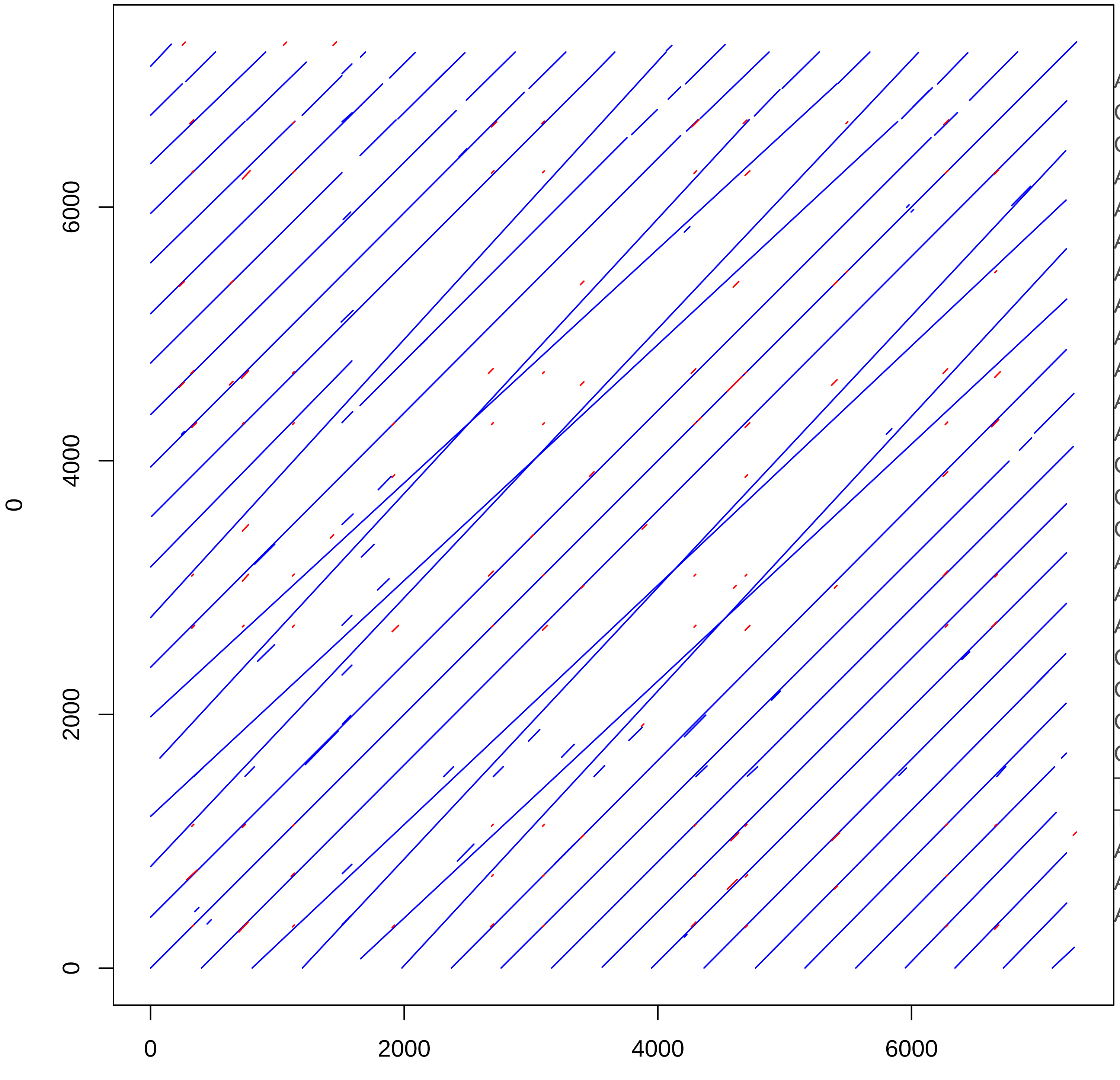

AAAAGTTGCGAA 13
GGGAATTTTTTT 13
GGGGAATTTTTT 12
AAAAAAGTTGCG 11
AAAAAGTTGCGA 11
AAAGTTGCGAAA 11
AAGTTGCGAAAA 11
AATCCGAGGTGT 11
AATGTTGACTTA 11
AGAATATGCGAG 11
ATCCGAGGTGTG 11
ATGTTGACTTAA 11
CCGAGGTGTGTA 11
GAATATGCGAGG 11
GGAATTTTTTTT 11
AAGAATATGCGA 10
AGAGGGTGATAC 10
AGGGGAATTTTT 10
CAAATCCGAGGT 10
GAAAAAAGTTGC 10
GCAAATCCGAGG 10
GGAAAAAAGTTG 10
TAGAGGGTGATA 10
TCCGAGGTGTGT 10
AAATCCGAGGTG 9
AATACATTTCGG 9
AATATGCGAGGG 9

(HW Bass, DL Vera, FSU '17
Hopsteiner PacBio Apollo reads input)

0

Supplemental Material
k-mer screen & YASS dot-plots

KA Easterling et al. 2020

>m150106_022046_42134_c100761282550000001823158307221520_s1_p0/116142/0_5105

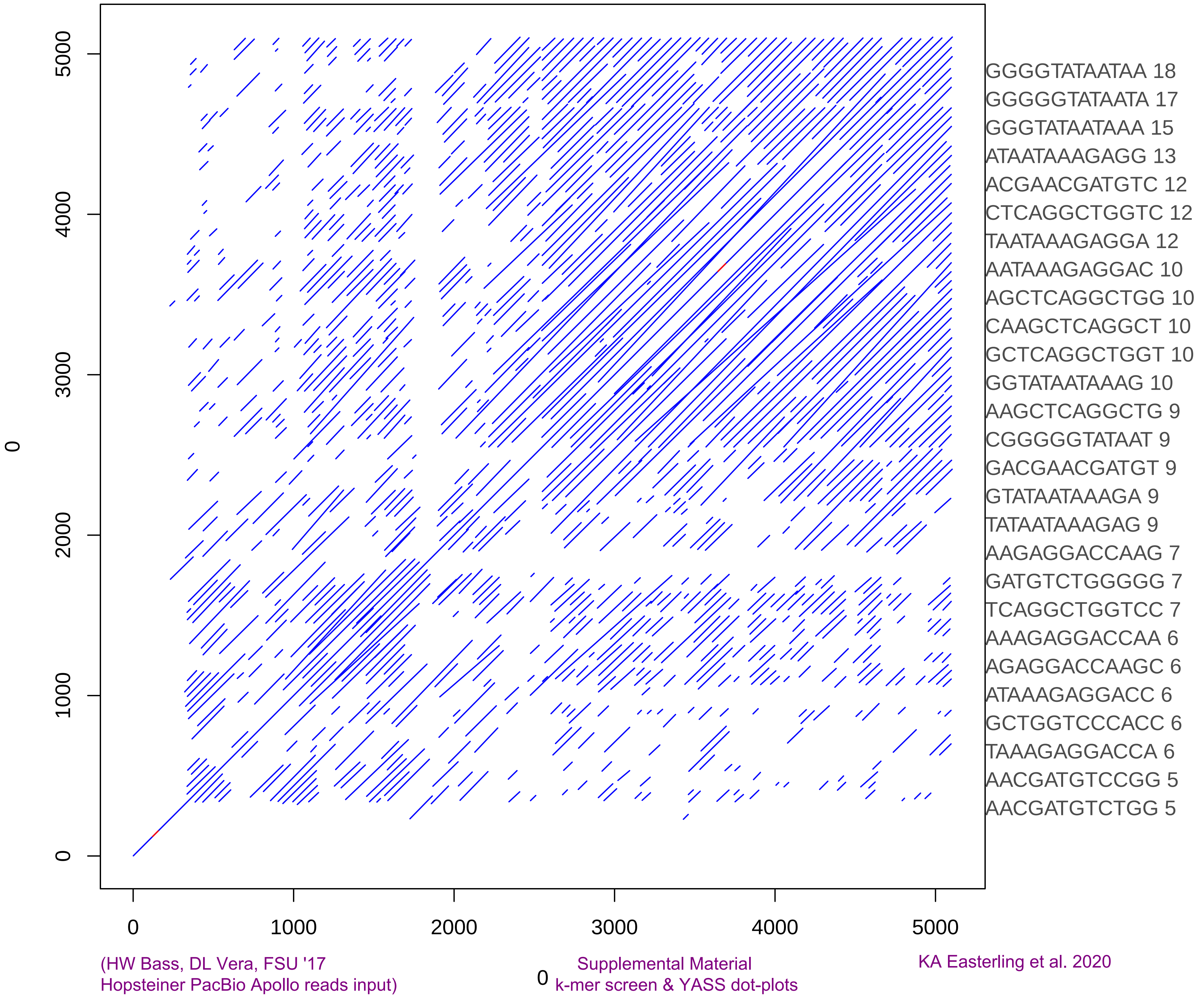

>m150106_022046_42134_c100761282550000001823158307221520_s1_p0/118459/139_6465

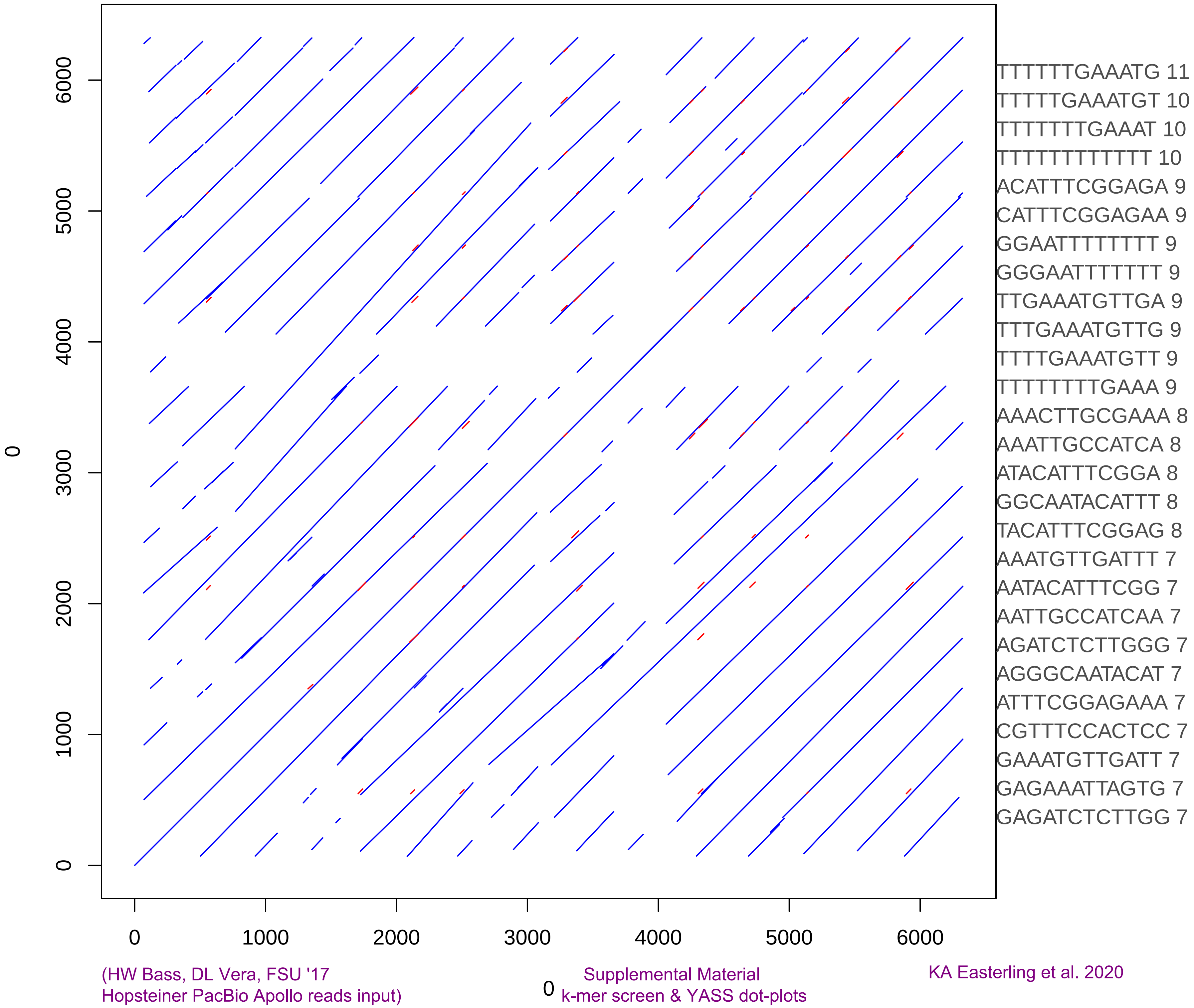

>m150106_022046_42134_c100761282550000001823158307221520_s1_p0/120507/0_8443

Representative Read (Table 1): HuluTR100-r983, 2 ppm, 62% A+T,
GenBank Acc. MN537569

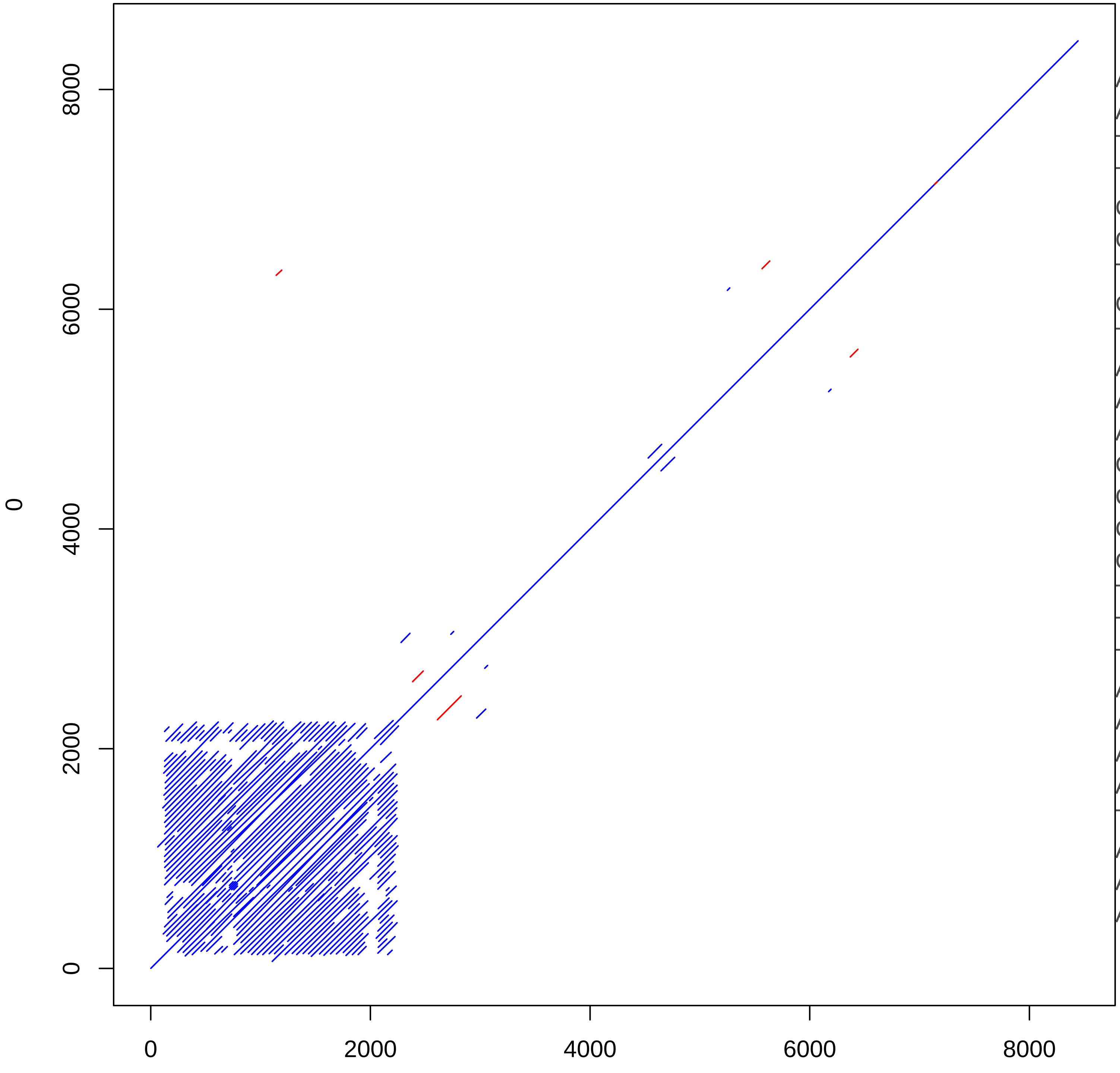

AACTTGGTACTT 10
ACTTGGTACTTG 10
TAACTTGGTACT 10
TTGGTACTTGGT 10
CTTGGTACTTGG 9
GTACTTGGTGAC 9
TACTTGGTGACT 9
GGTACTTGGTGA 7
TGGTACTTGGTG 7
ACACTAACTTGG 6
ACTAACTTGGTA 6
AGTTGTTTCACA 6
CACACTAACTTG 6
CACTAACTTGGT 6
CTAACTTGGTAC 6
GAATGAGGAATA 6
TCACACTAACTT 6
TTCACACTAACT 6
TTTCACACTAAC 6
AATGAGGAATAA 5
ACTTGGTGACTC 5
ATGAGGAATAAT 5
ATTATTTCACAC 5
TGAGGAATAATC 5
AATCGAGTTGTT 4
ATCGAGTTGTTT 4
ATTTCACACTAA 4

>m150106_022046_42134_c100761282550000001823158307221520_s1_p0/121311/921_6858

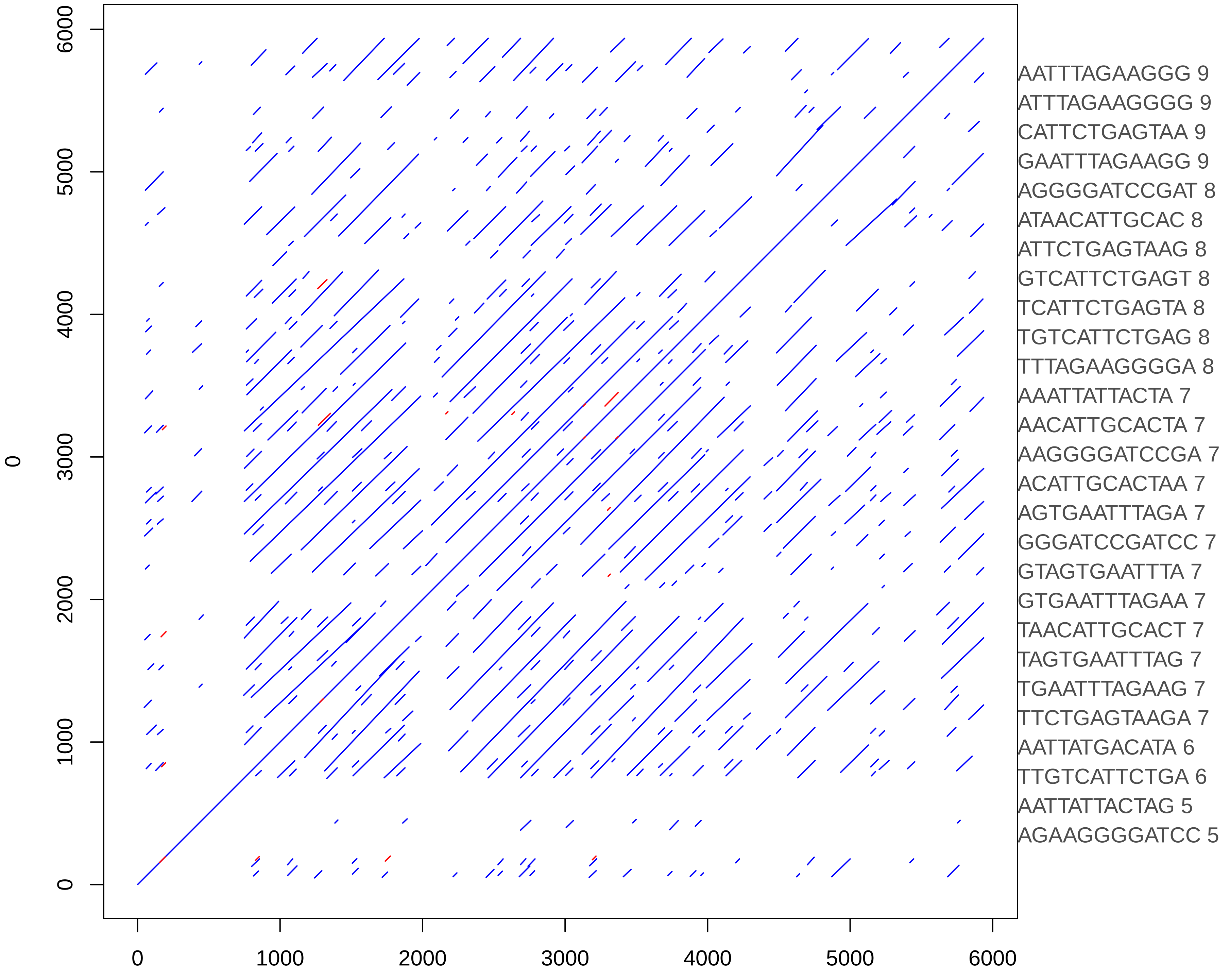

0

>m150106_022046_42134_c100761282550000001823158307221520_s1_p0/121993/2013_8755

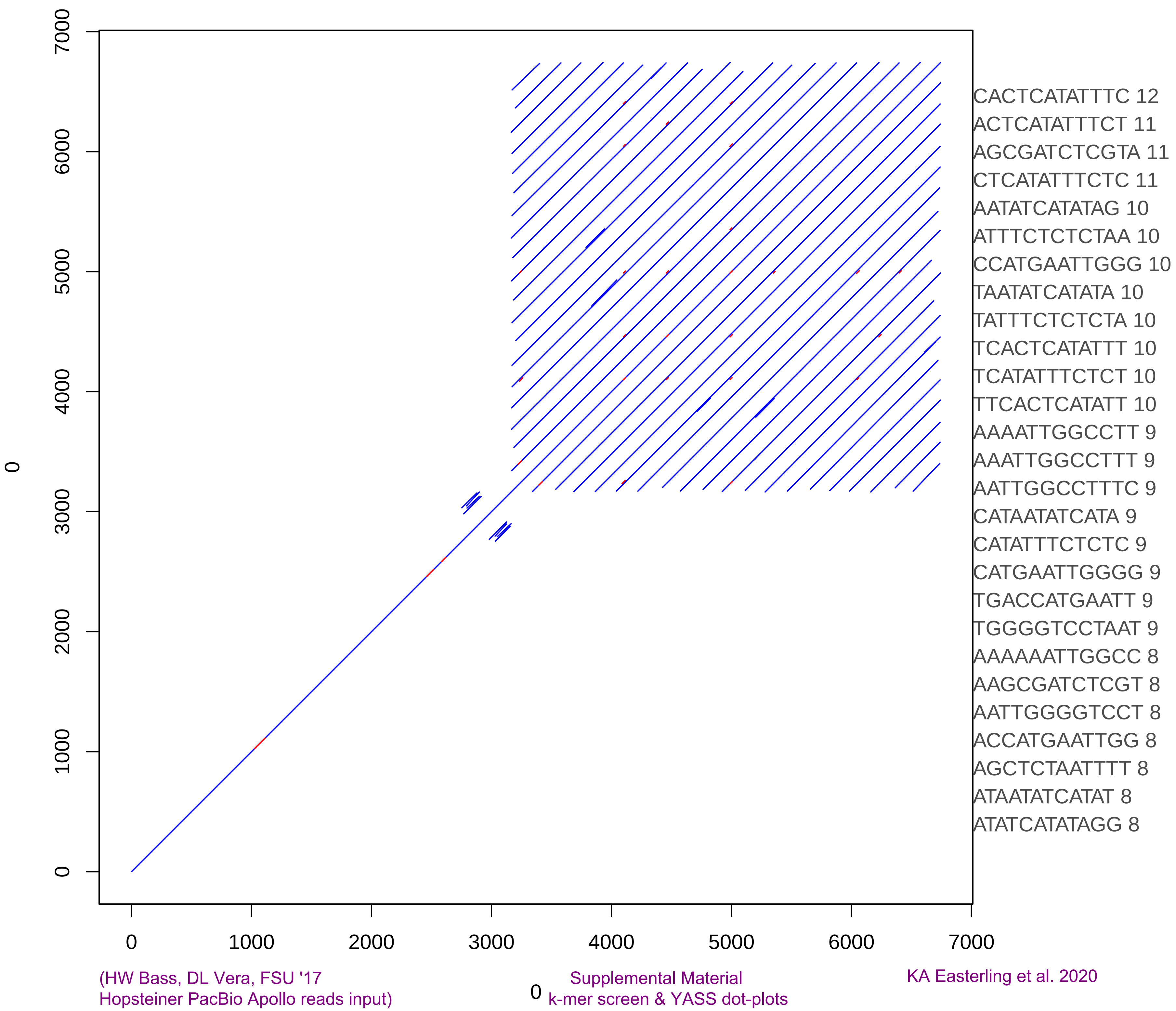

>m150106_022046_42134_c100761282550000001823158307221520_s1_p0/12230/341_10591

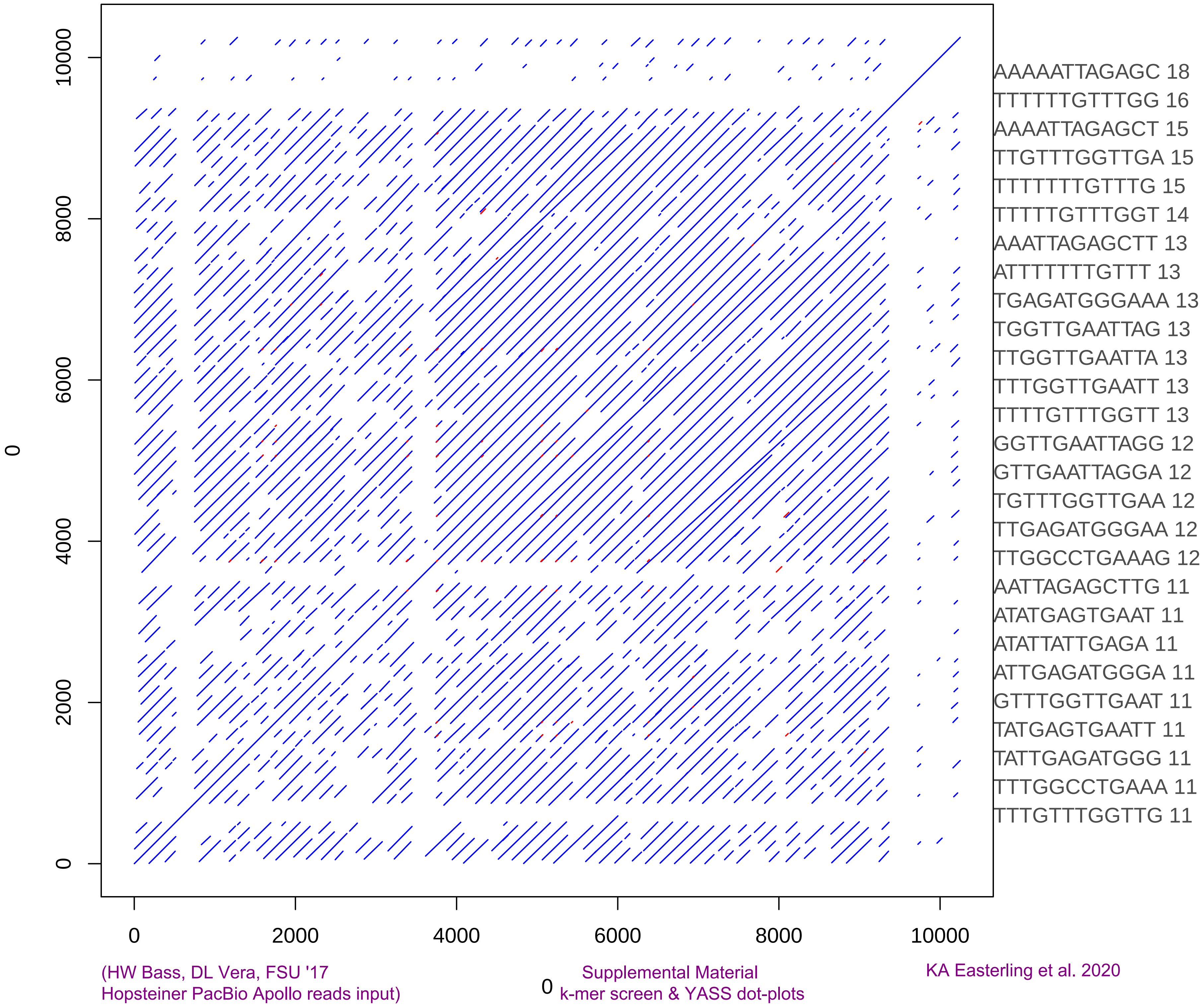

>m150106_022046_42134_c100761282550000001823158307221520_s1_p0/122736/684_14233

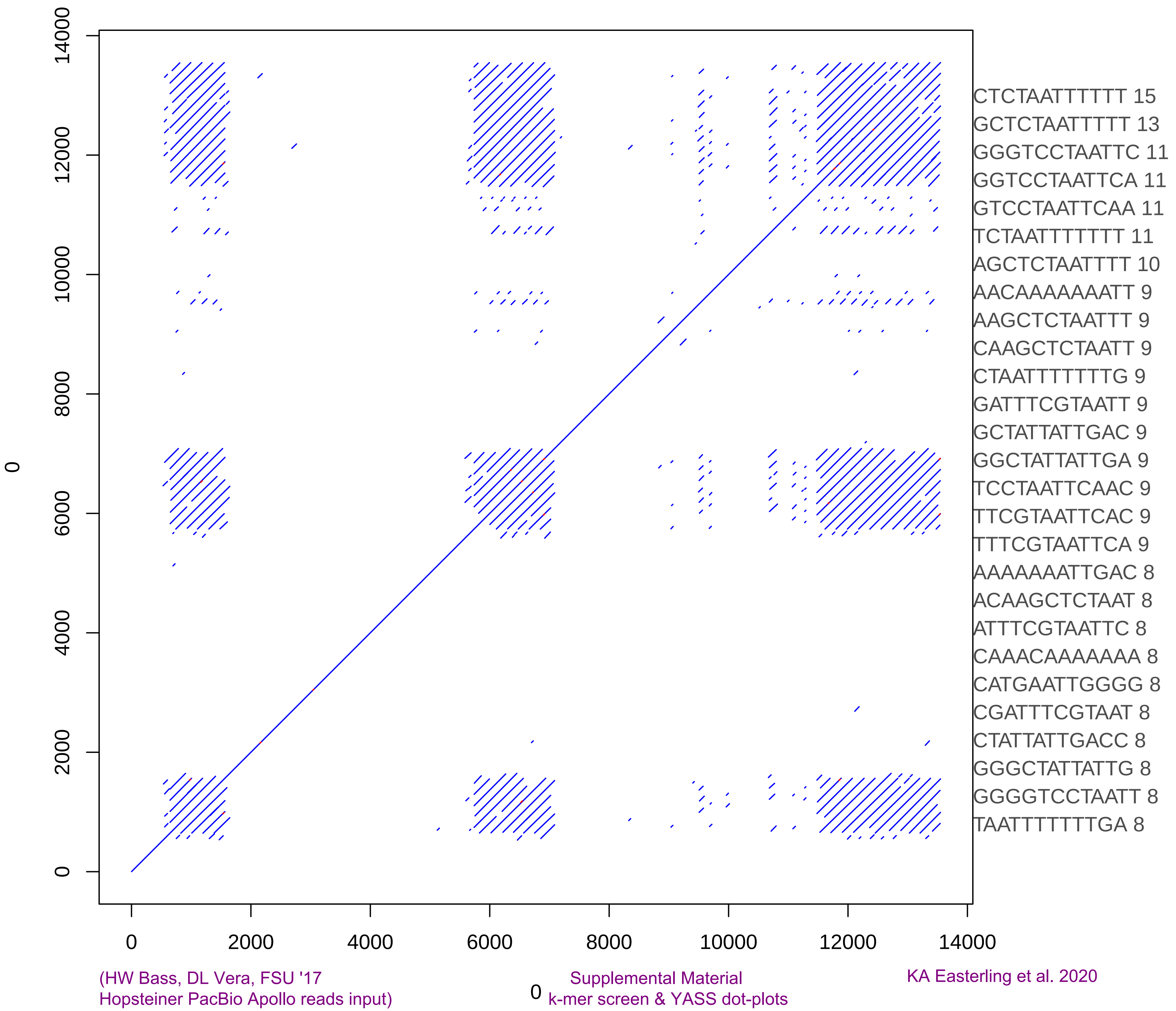

>m150106_022046_42134_c100761282550000001823158307221520_s1_p0/128999/726_9448

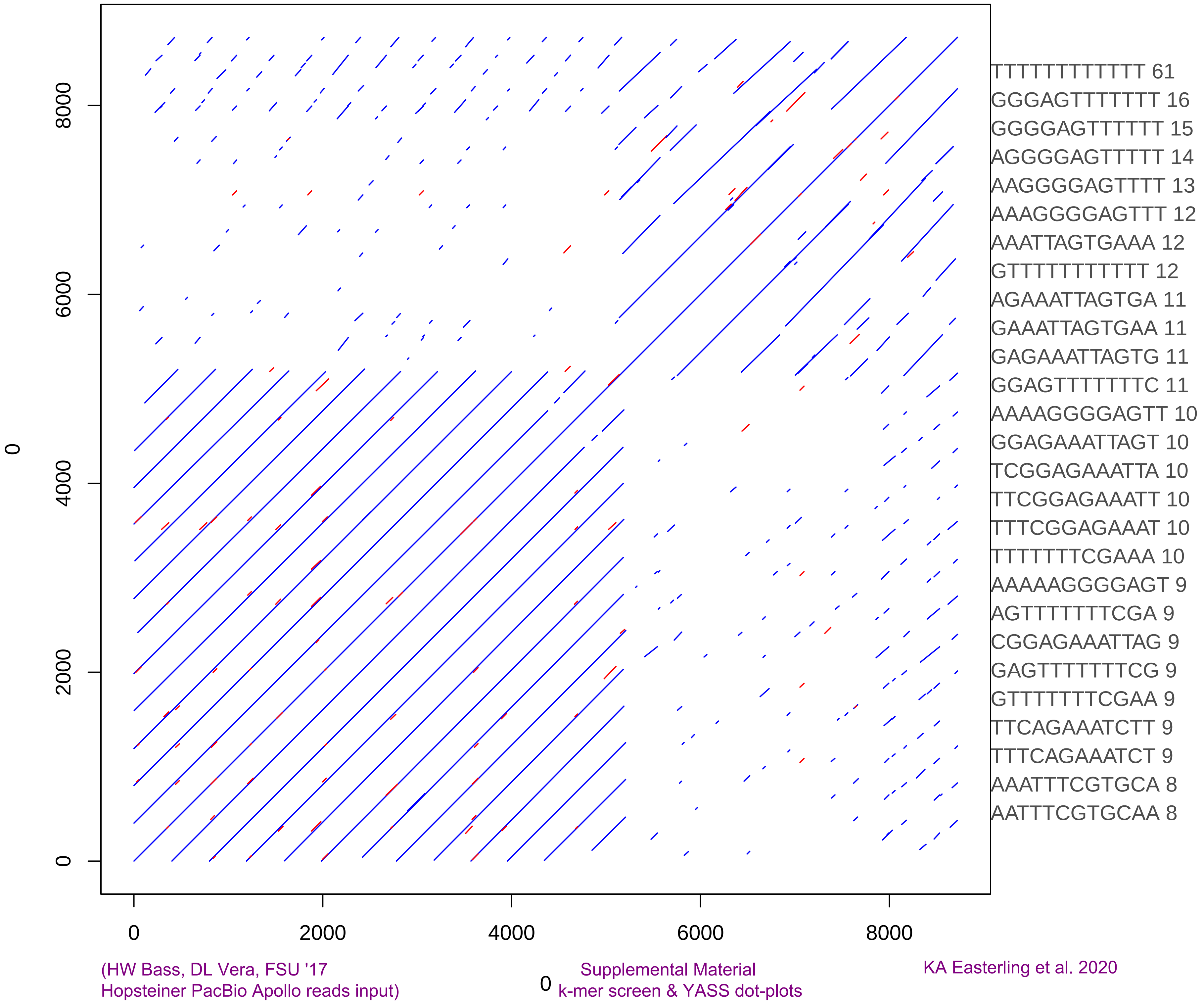

>m150106_022046_42134_c100761282550000001823158307221520_s1_p0/130600/0_5923

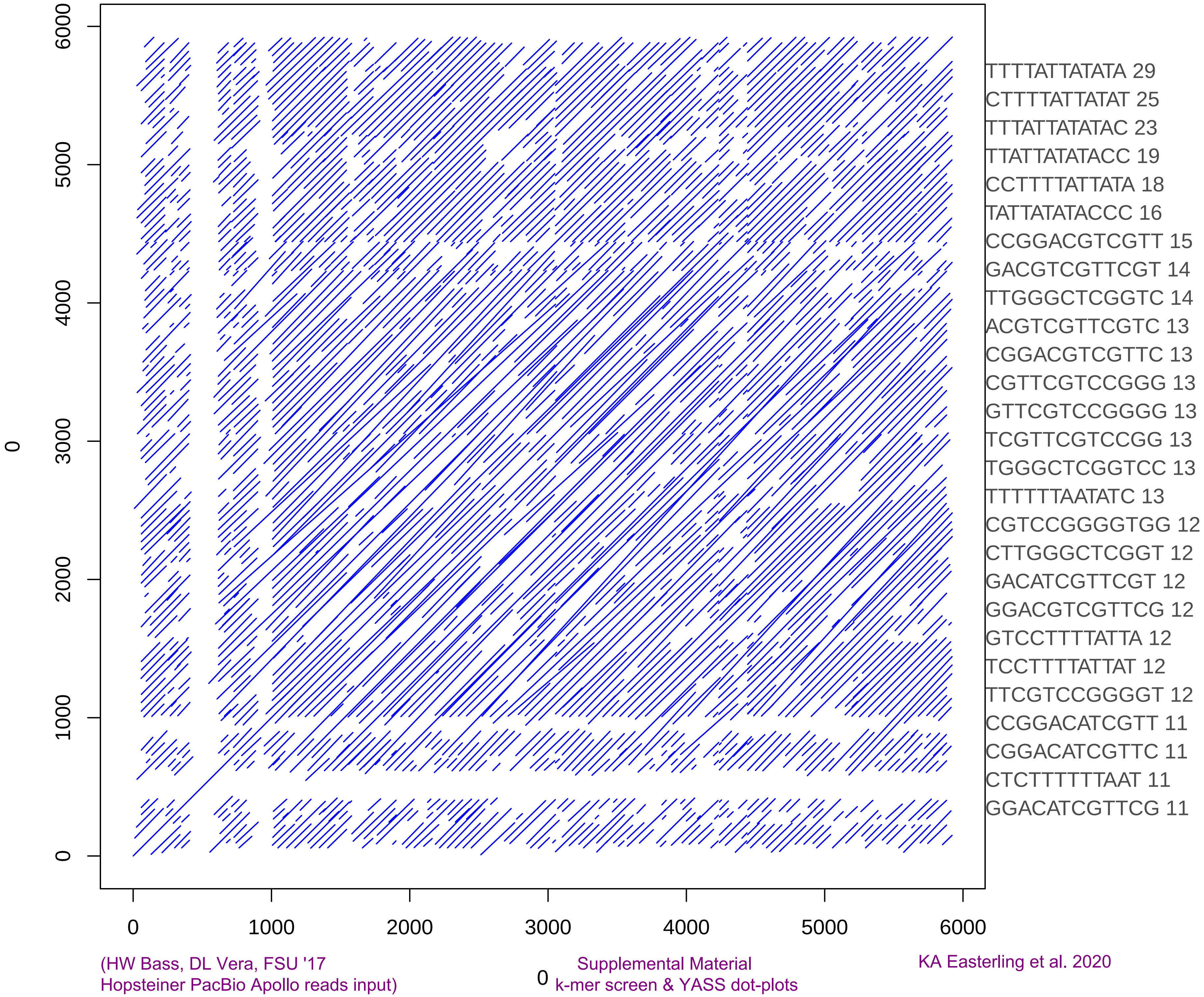

>m150106_022046_42134_c100761282550000001823158307221520_s1_p0/132248/342_5991

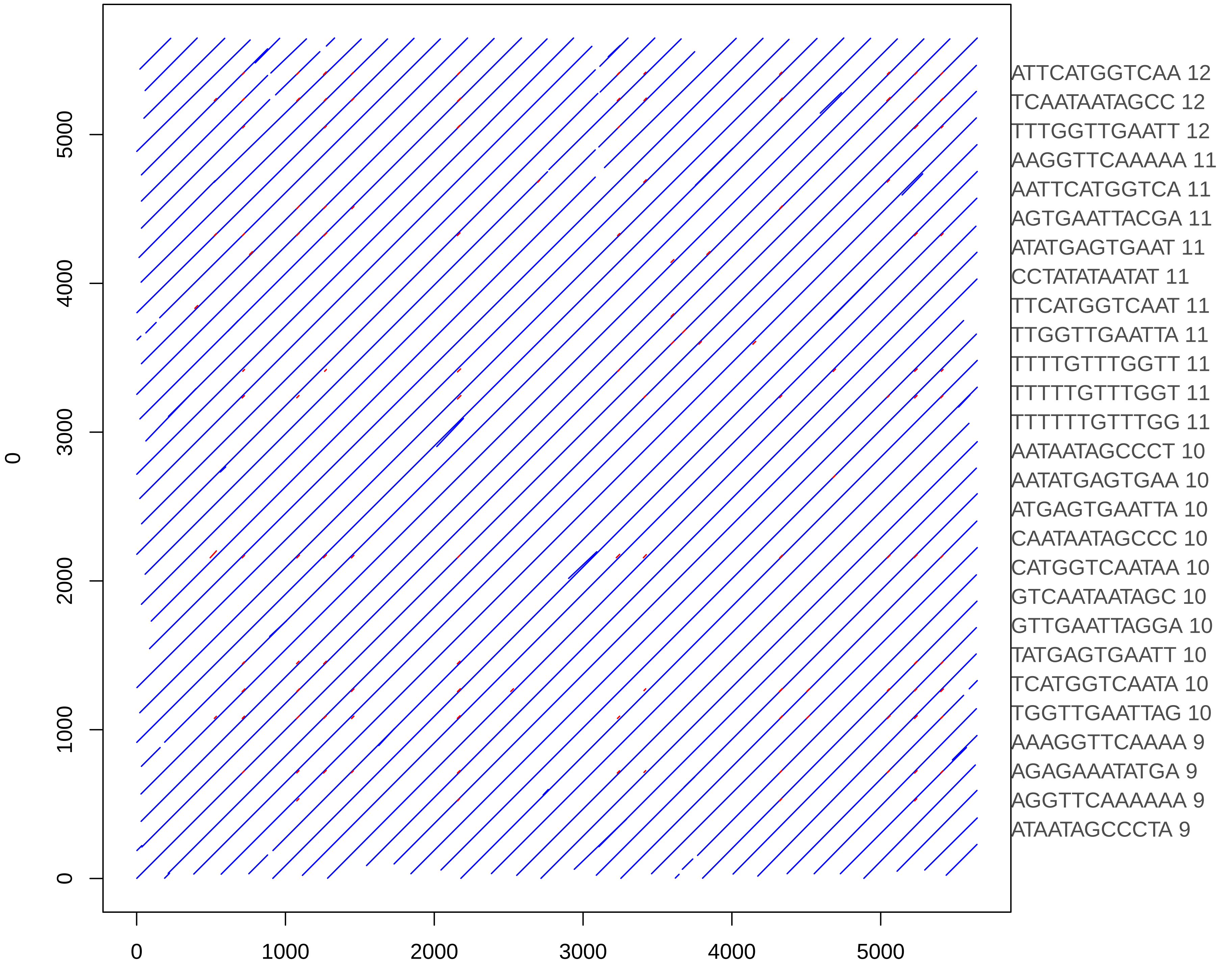

>m150106_022046_42134_c100761282550000001823158307221520_s1_p0/13714/0_5087

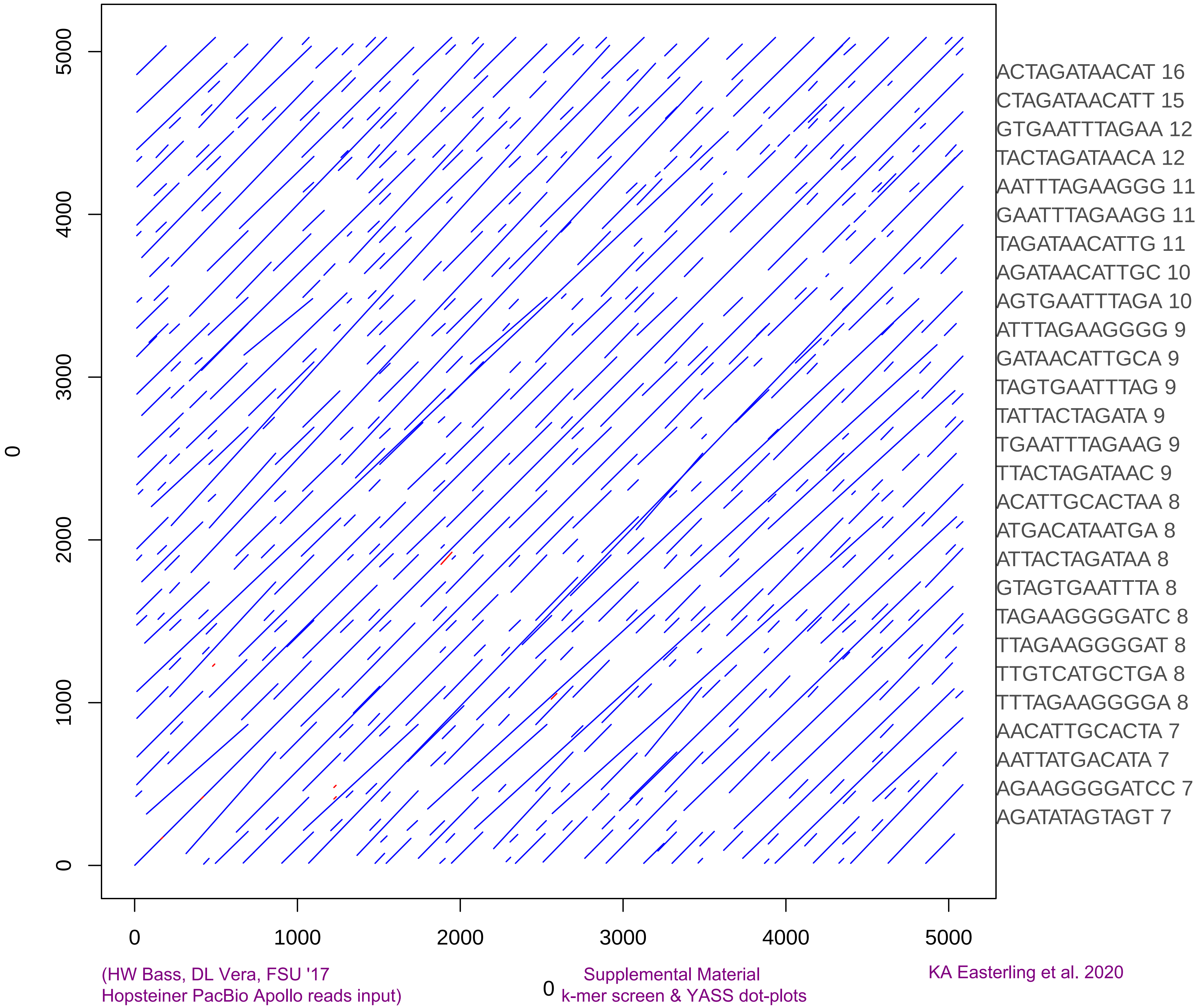

>m150106_022046_42134_c100761282550000001823158307221520_s1_p0/139299/4392_12389

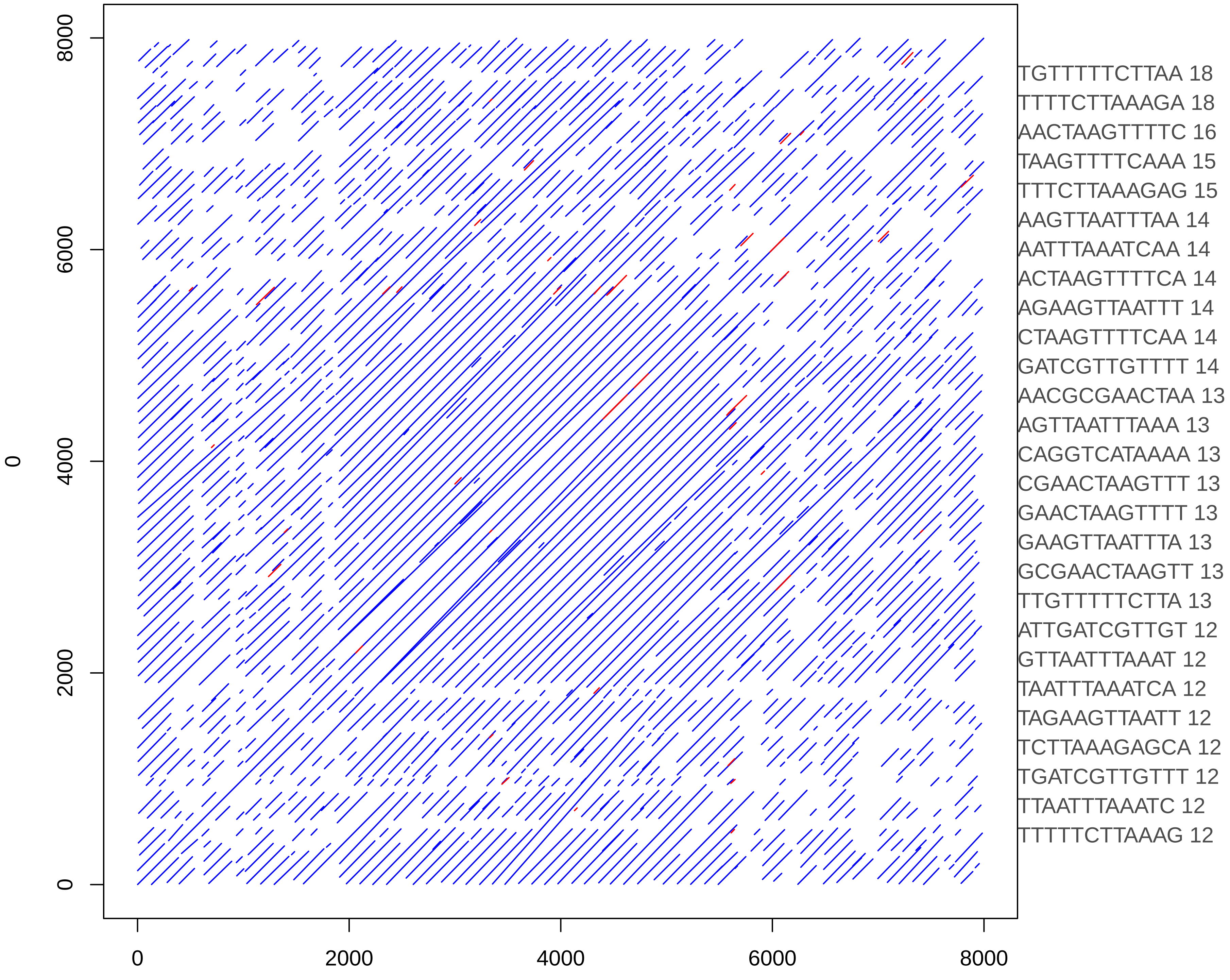

>m150106_022046_42134_c100761282550000001823158307221520_s1_p0/143938/338_6783

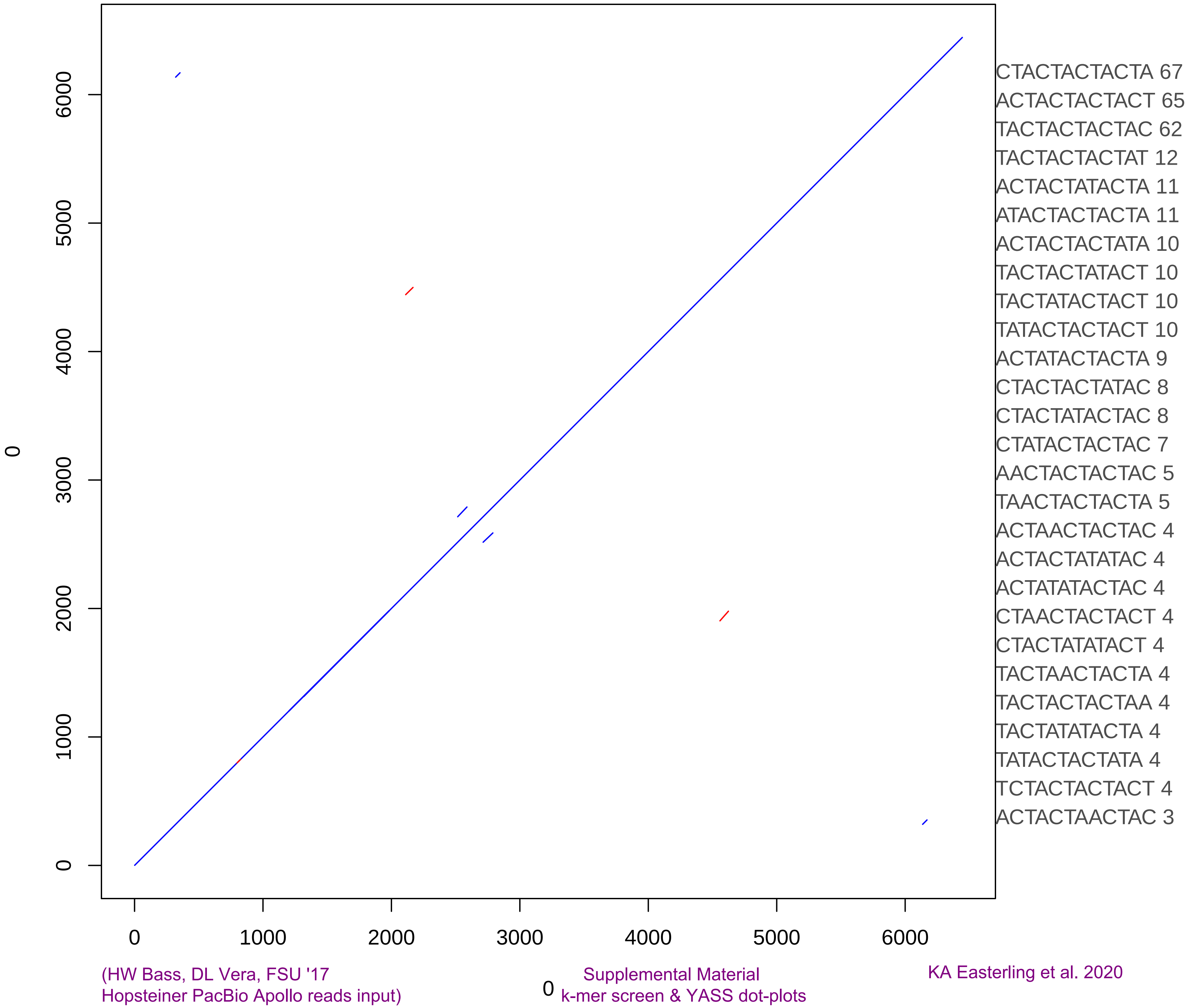

>m150106_022046_42134_c100761282550000001823158307221520_s1_p0/144589/0_6972

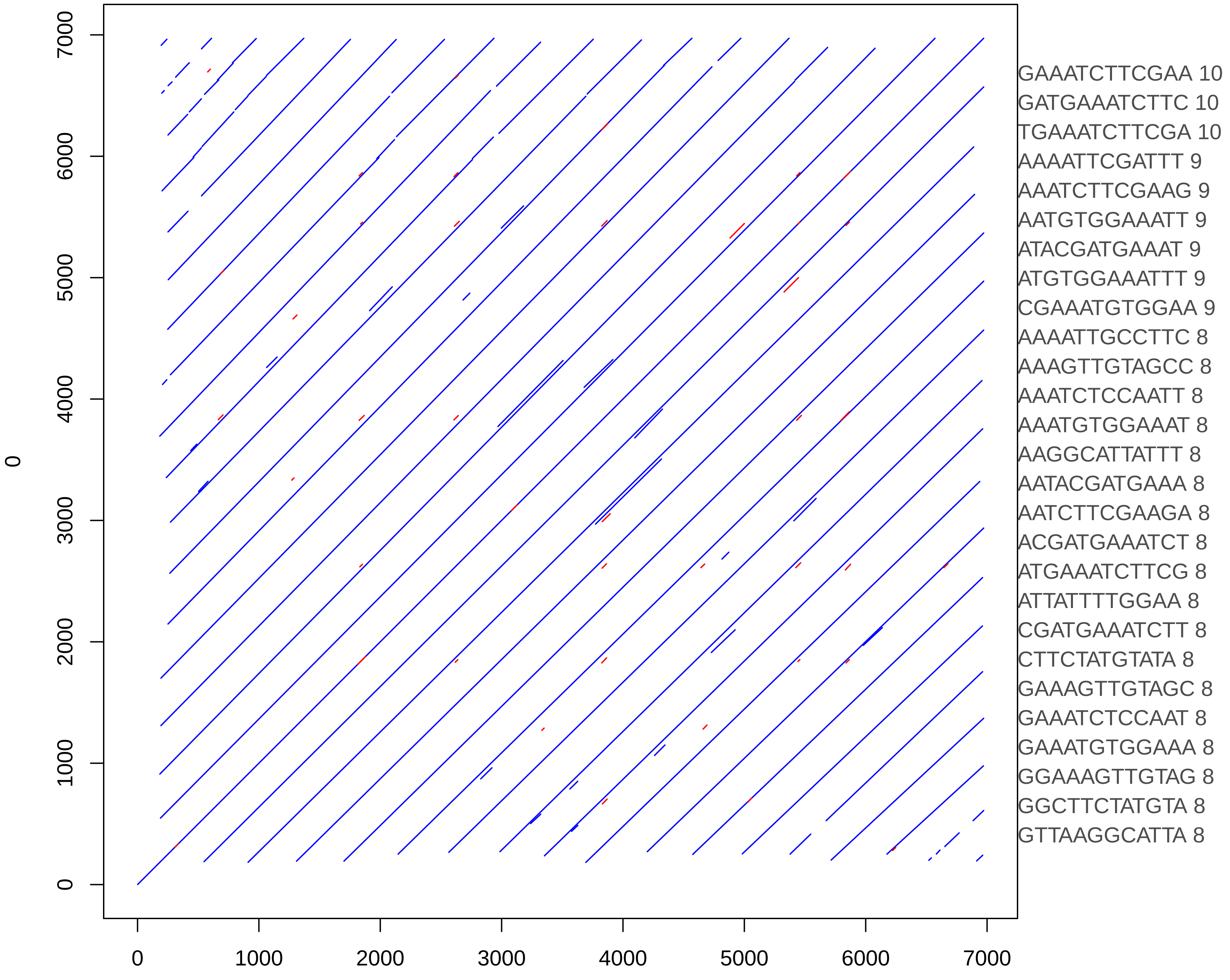

>m150106_022046_42134_c100761282550000001823158307221520_s1_p0/147554/8998_14851

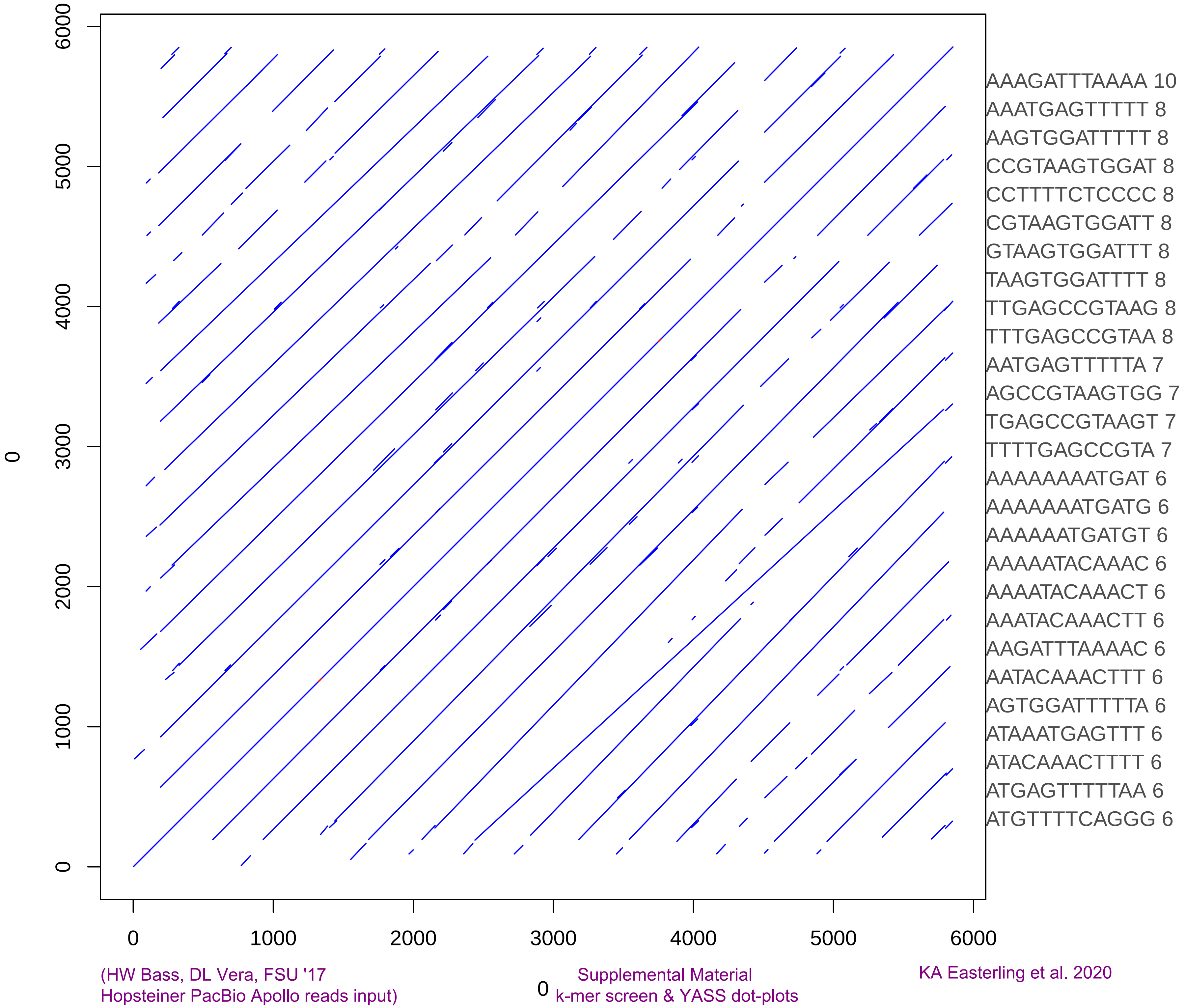

1,121 hits for repetitive sequences passing 12mer abundance criterion

(Read-Number) PAGE 996

For Tandem Repeat families of ### bp: PacBio Read named "HuluTR###-r996"

>m150106_022046_42134_c100761282550000001823158307221520_s1_p0/148102/861_6991

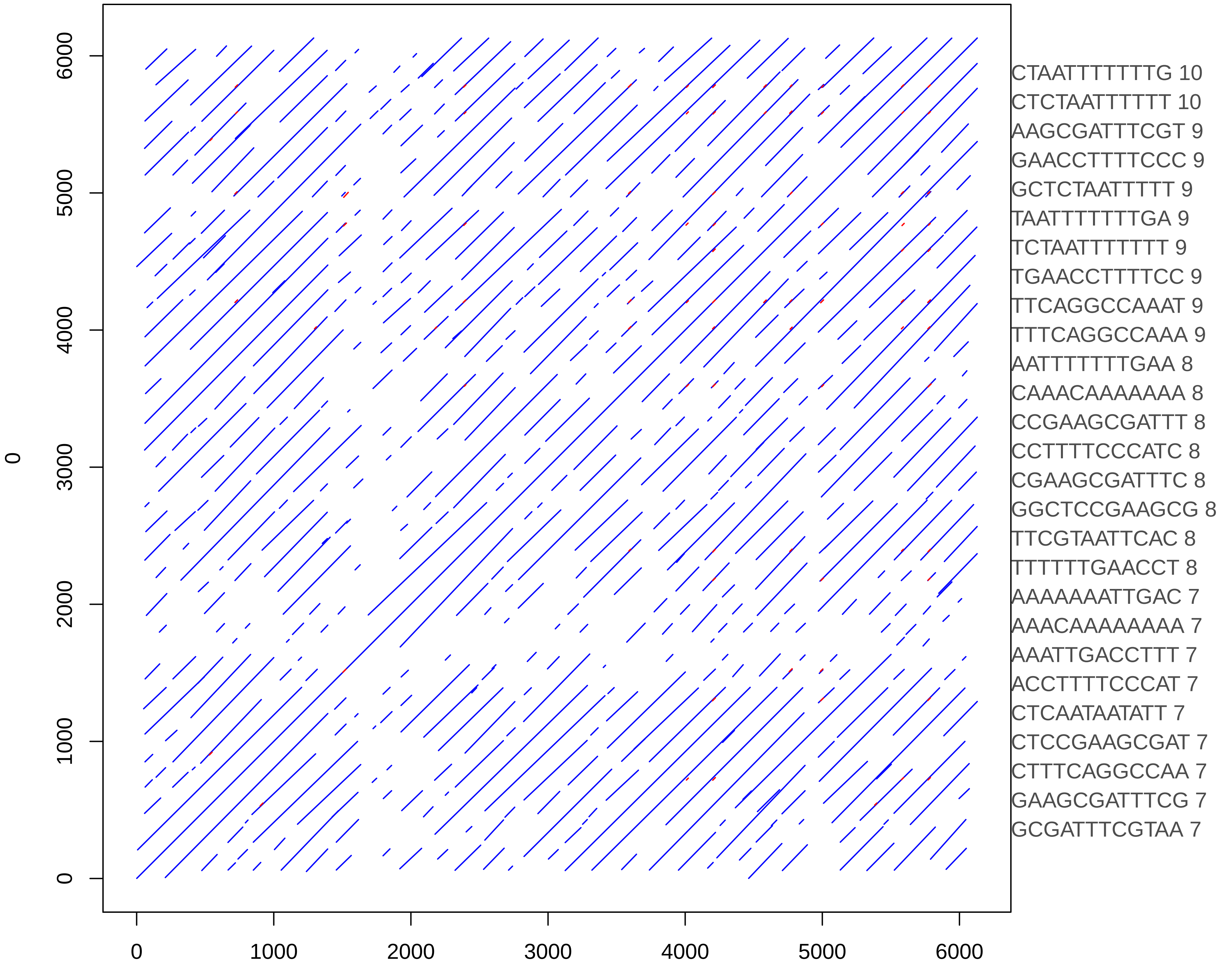

(HW Bass, DL Vera, FSU '17 Hopsteiner PacBio Apollo reads input)

0

Supplemental Material k-mer screen & YASS dot-plots

KA Easterling et al. 2020

>m150106_022046_42134_c100761282550000001823158307221520_s1_p0/149333/0_6614

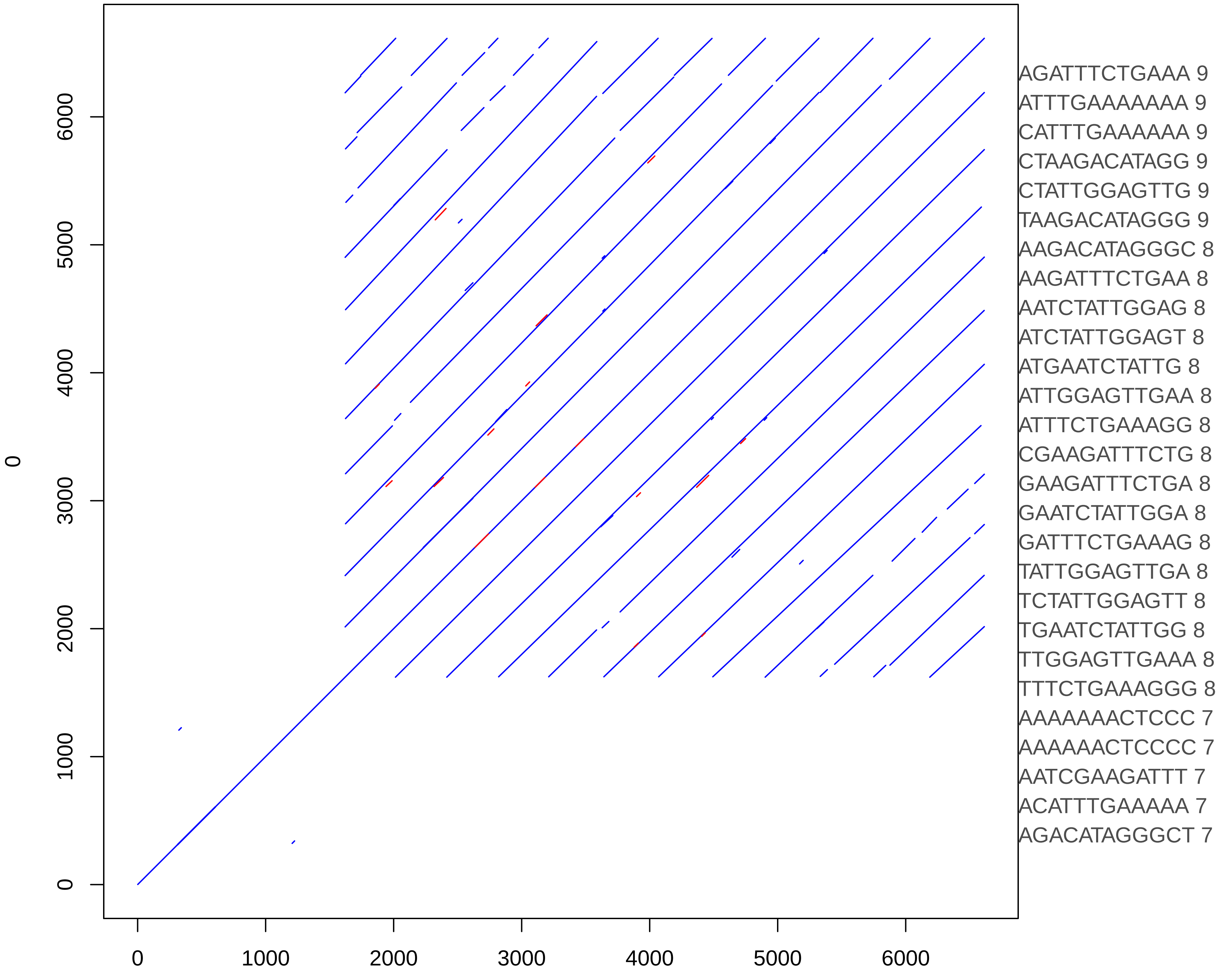

0

1,121 hits for repetitive sequences
passing 12mer abundance criterion

(Read-Number)
PAGE 998

For Tandem Repeat families of ### bp:
PacBio Read named "HuluTR###-r998"

>m150106_022046_42134_c100761282550000001823158307221520_s1_p0/151329/0_5273

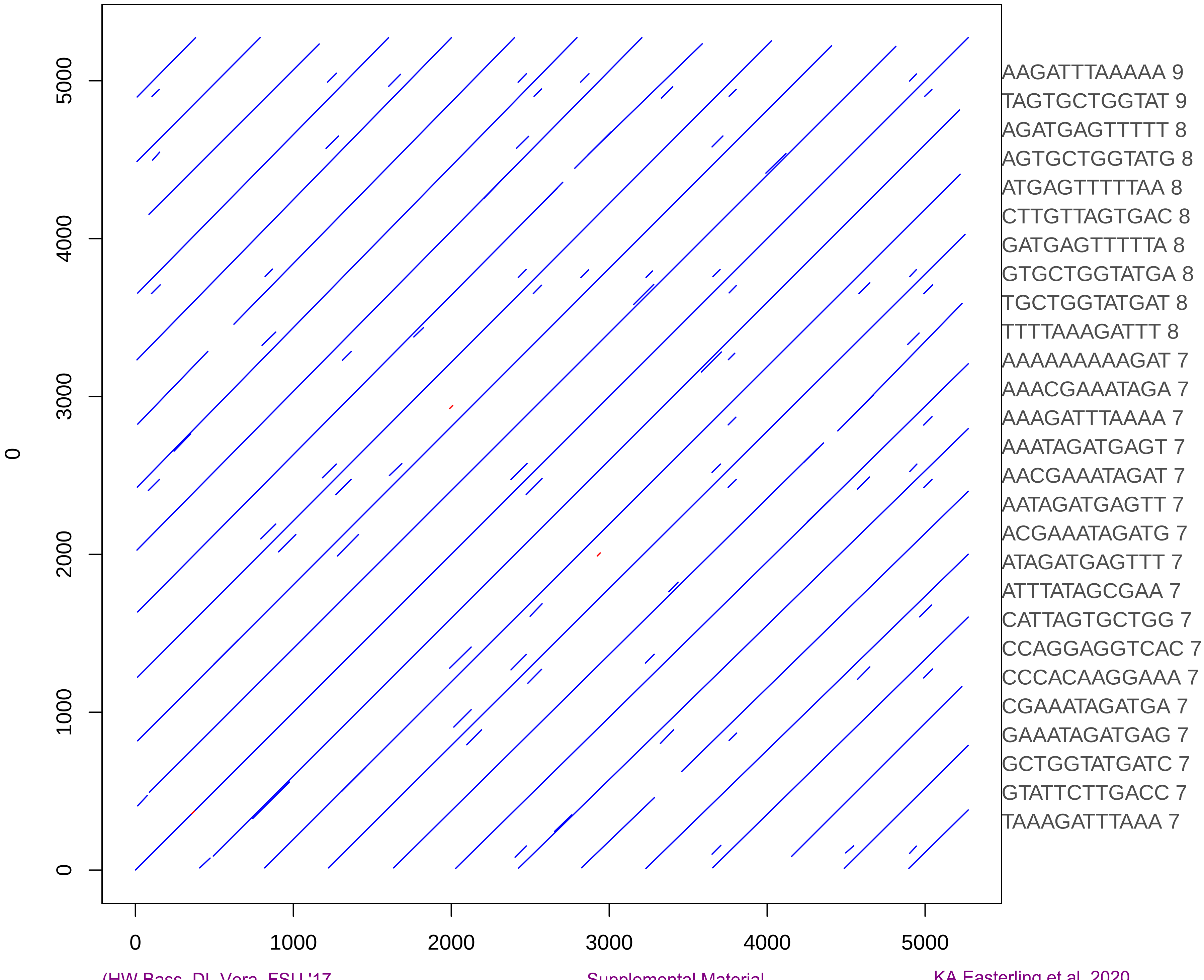

(HW Bass, DL Vera, FSU '17
Hopsteiner PacBio Apollo reads input)

0

Supplemental Material
k-mer screen & YASS dot-plots

KA Easterling et al. 2020

>m150106_022046_42134_c100761282550000001823158307221520_s1_p0/151371/0_6485

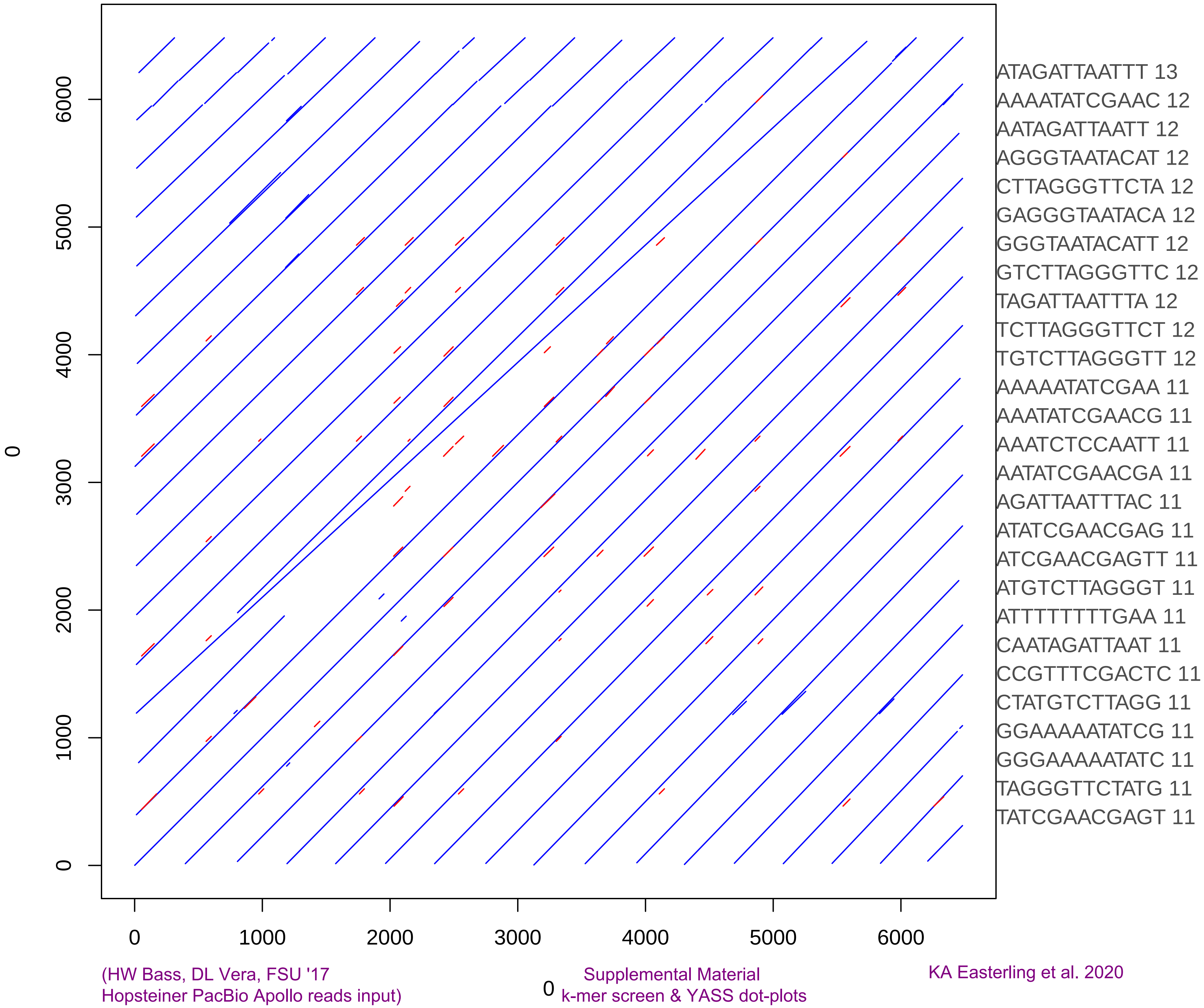

>m150106_022046_42134_c100761282550000001823158307221520_s1_p0/15184/407_10180

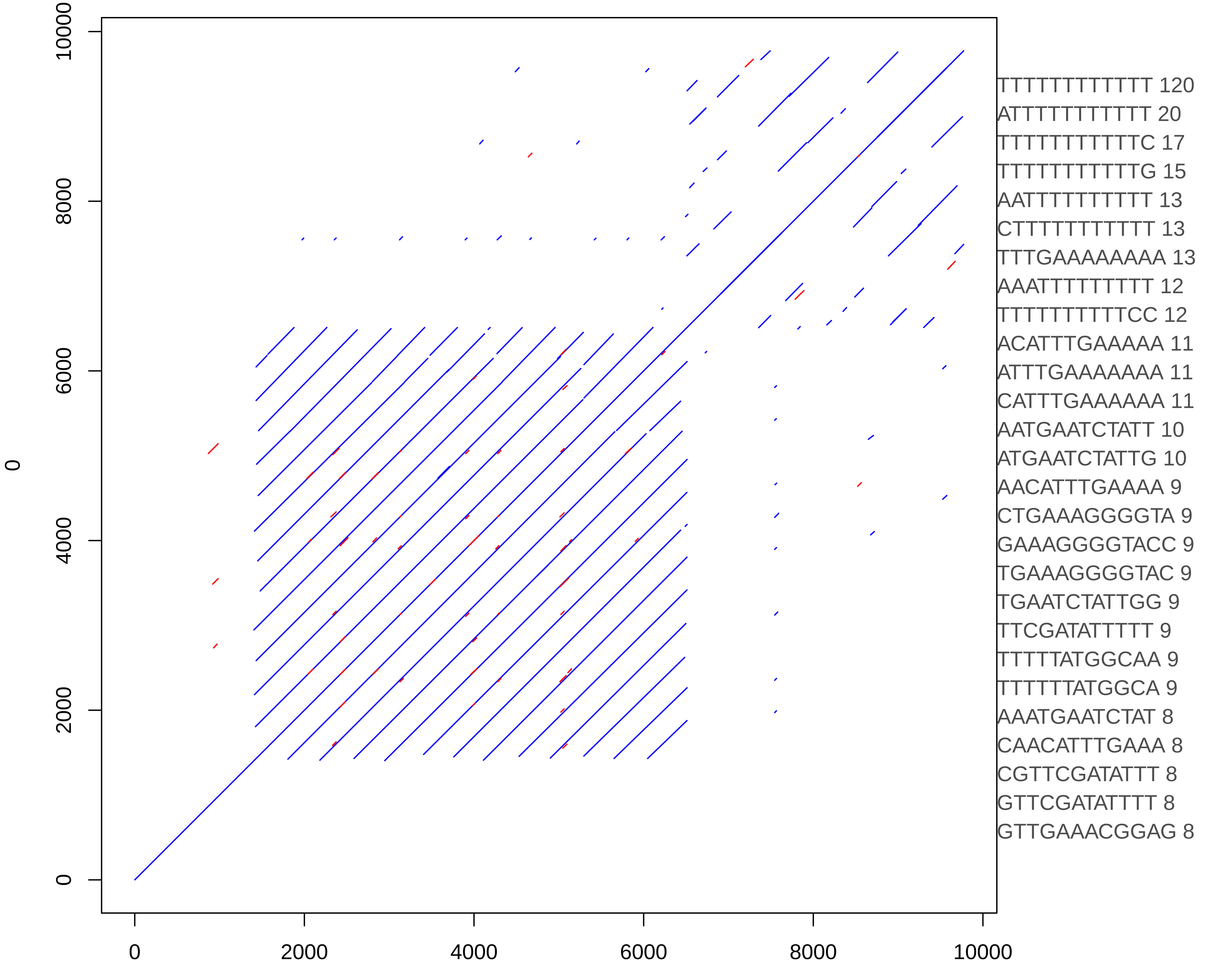

1,121 hits for repetitive sequences
passing 12mer abundance criterion

(Read-Number)
PAGE 1001

For Tandem Repeat families of ### bp:
PacBio Read named "HuluTR###-r1001"

>m150106_022046_42134_c100761282550000001823158307221520_s1_p0/153098/0_5991

Representative Read (Table 1): HuluTR240-r1001, 2 ppm, 71% A+T,
GenBank Acc. MN537575

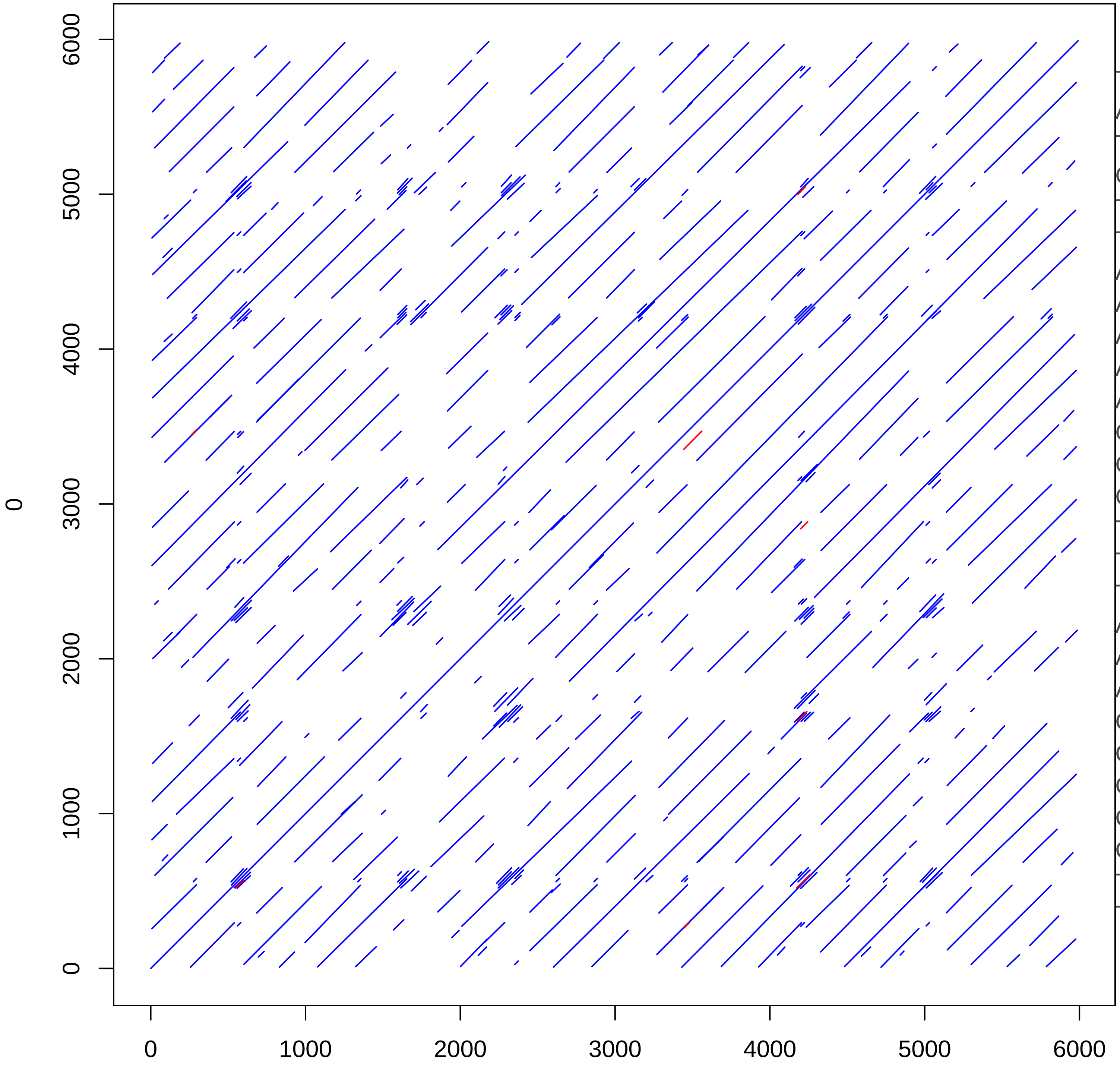

TTTATAAAGTTA 11
ATTATGATGATT 10
TTATAAAGTTAA 10
GATGATTGTGTT 9
TGATGATTGTGT 9
TTTTATAAAGTT 9
ACAACTTGTCTA 8
ATCTATTGAGGT 8
ATGATGATTGTG 8
ATGATTGTGTTT 8
ATTTTATAAAGT 8
GACAACTTGTCT 8
GAGATTATGATG 8
GATTGTGTTTAT 8
TATAAAGTTAAA 8
TATGATGATTGT 8
TTTATGAGATTA 8
AGATTATGATGA 7
ATATTTCAATCA 7
ATTGTGTTTATA 7
CAACTTGTCTAA 7
CGACAACTTGTC 7
CTATGATGATCA 7
CTATTGAGGTAC 7
CTTAATTCATTA 7
TATGATGATCAA 7
TATTGAGGTACT 7

(HW Bass, DL Vera, FSU '17
Hopsteiner PacBio Apollo reads input)

0

Supplemental Material
k-mer screen & YASS dot-plots

KA Easterling et al. 2020

>m150106_022046_42134_c100761282550000001823158307221520_s1_p0/154309/1055_9146

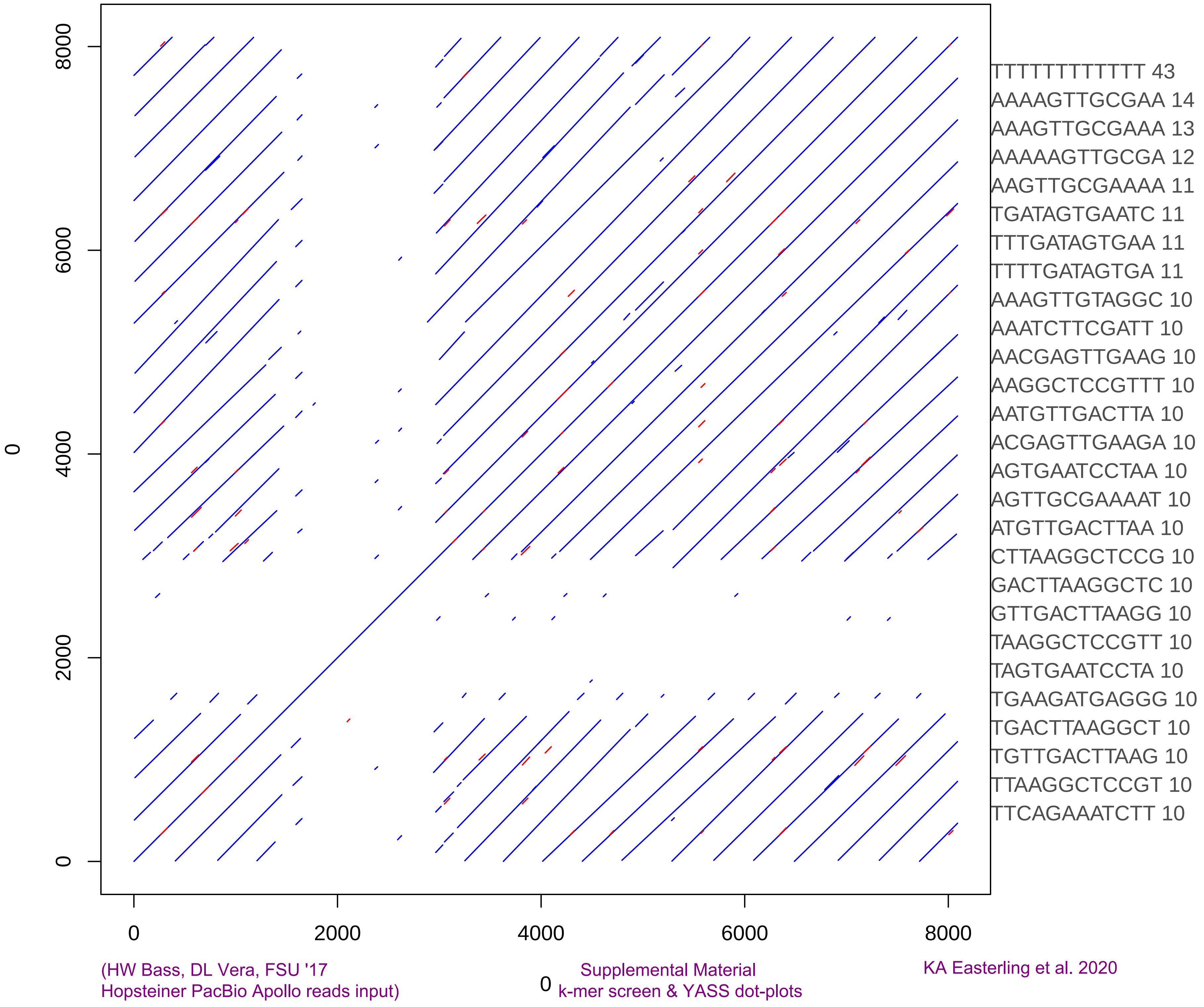

>m150106_022046_42134_c100761282550000001823158307221520_s1_p0/15548/0_6451

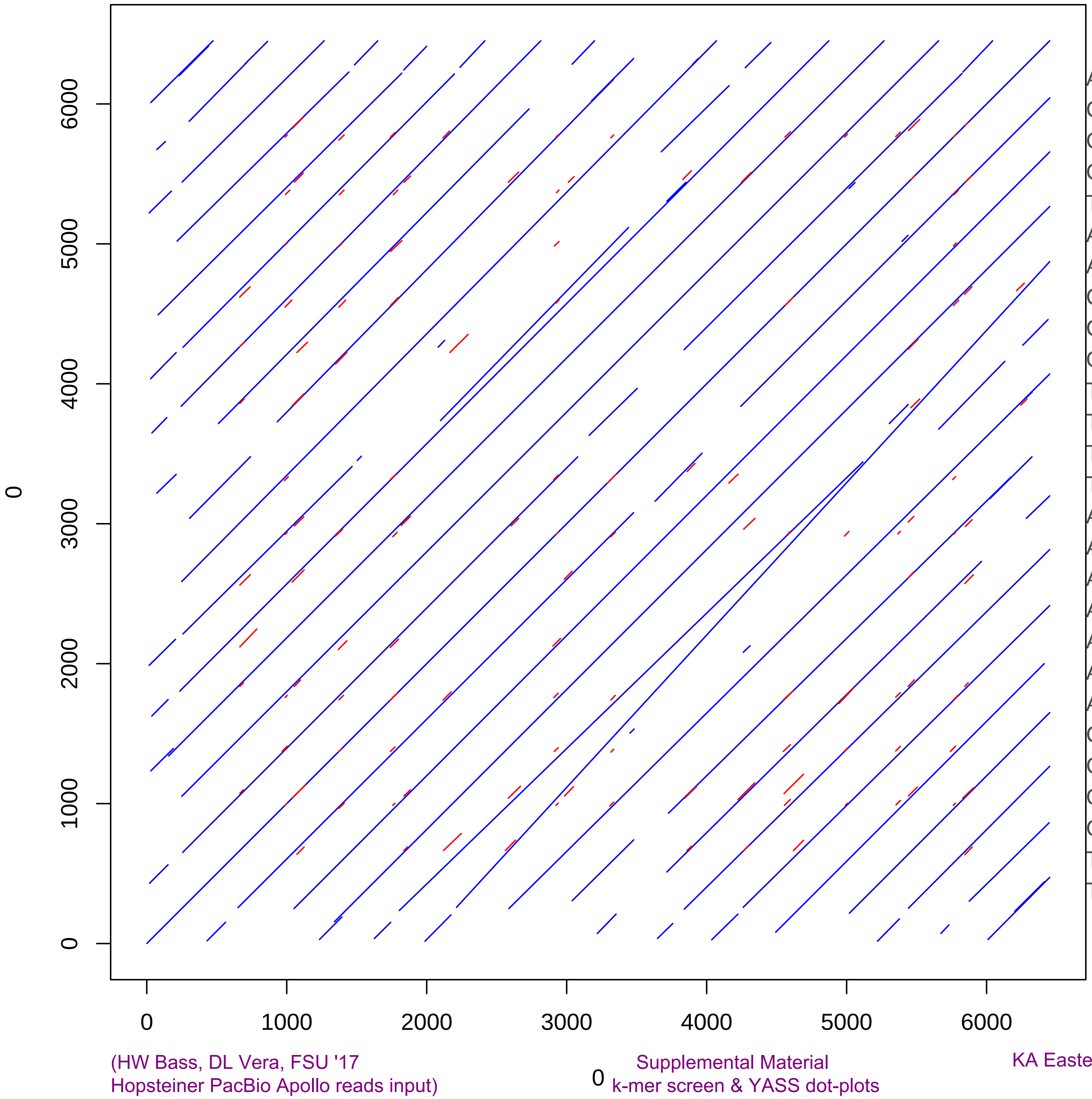

>m150106_022046_42134_c100761282550000001823158307221520_s1_p0/15548/6501_12965

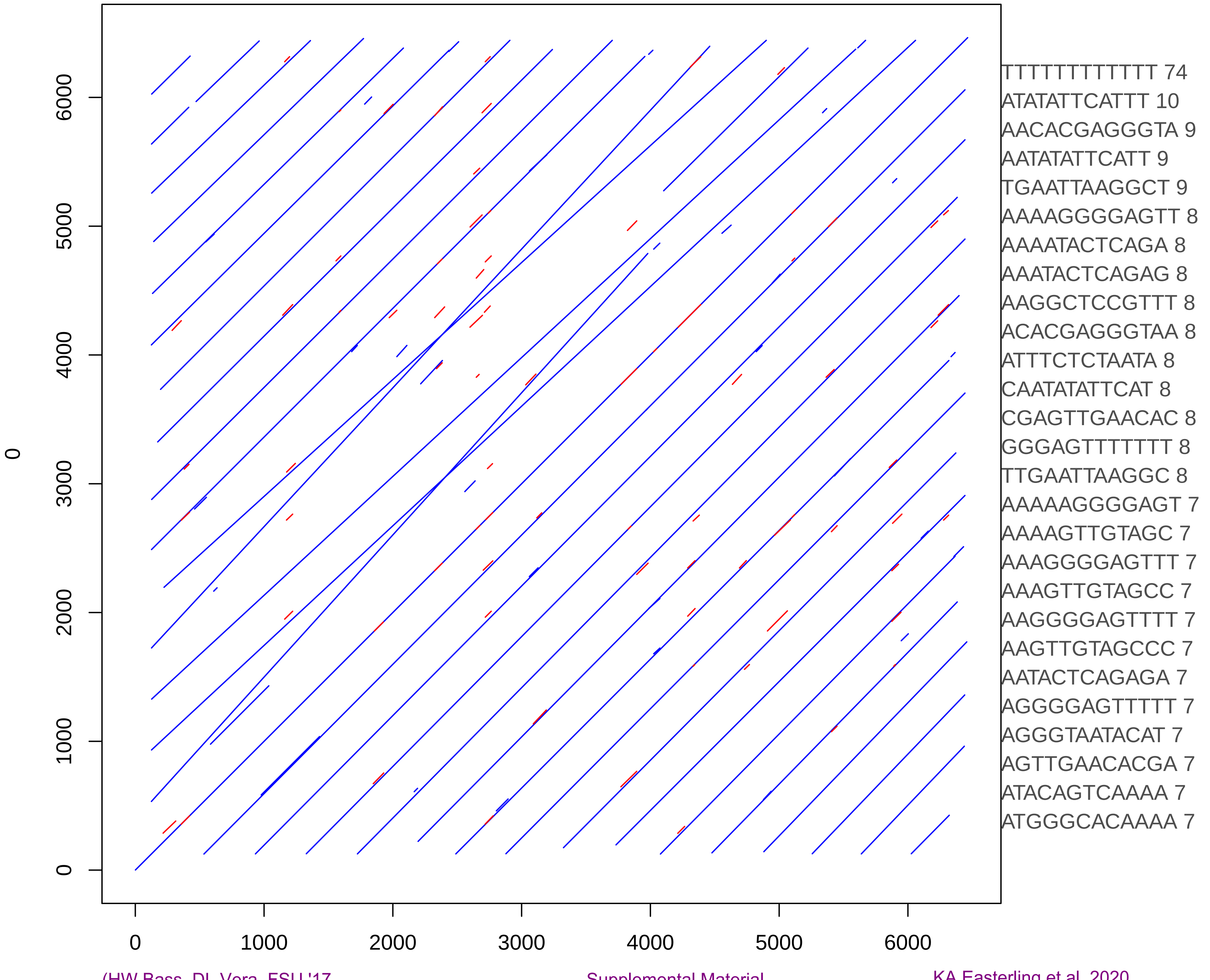

0

>m150106_022046_42134_c100761282550000001823158307221520_s1_p0/155746/3687_8869

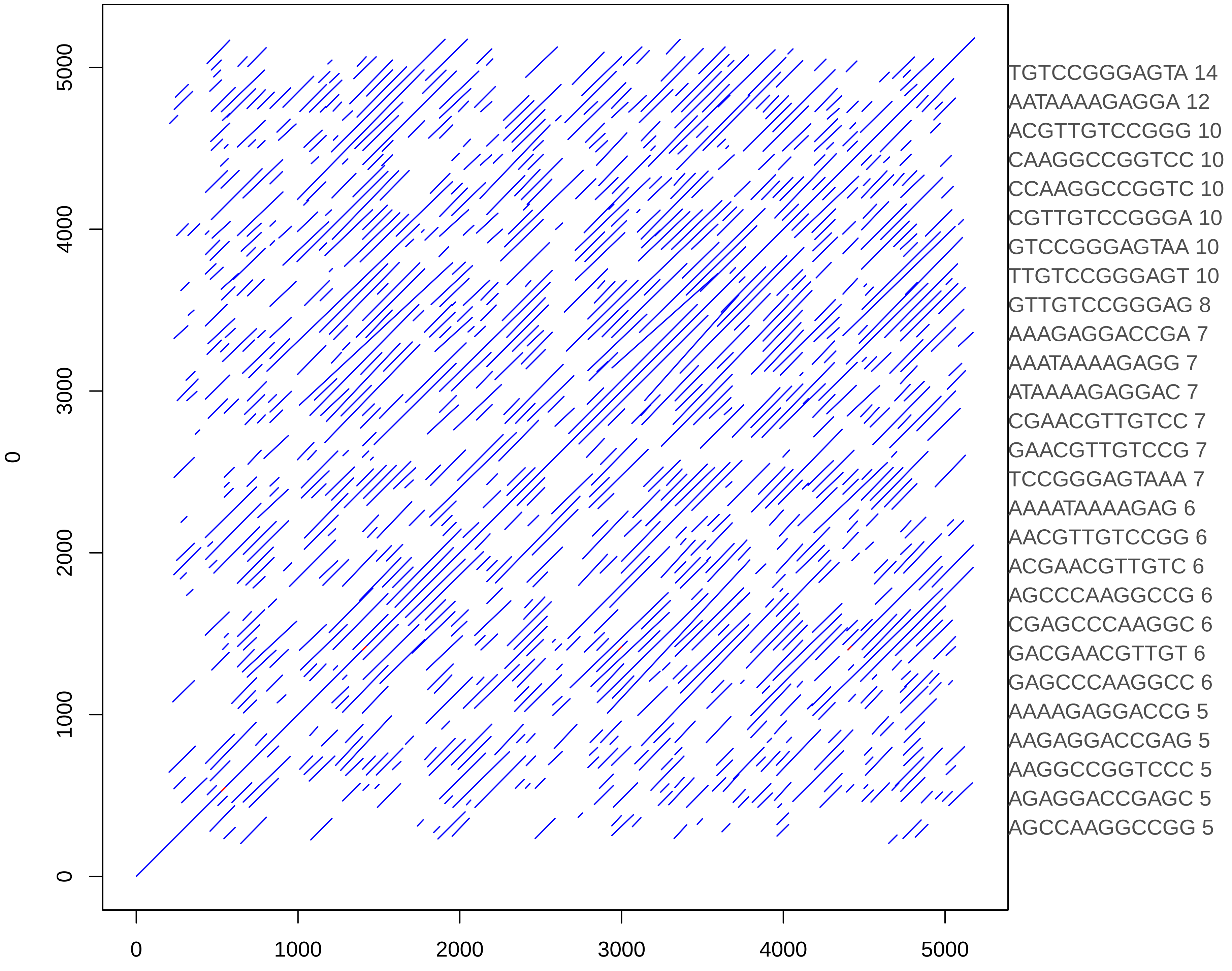

>m150106_022046_42134_c100761282550000001823158307221520_s1_p0/157379/0_6627

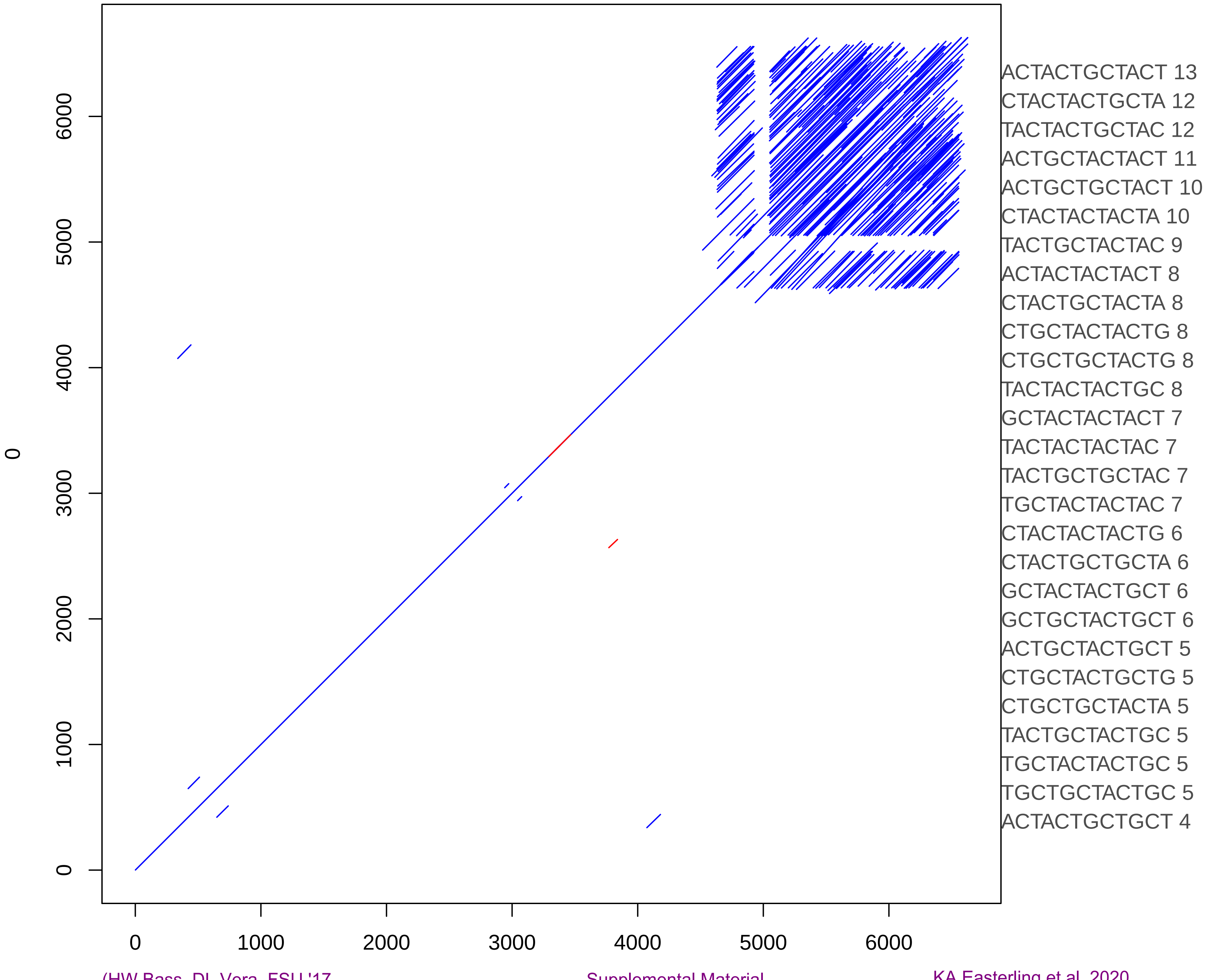

>m150106_022046_42134_c100761282550000001823158307221520_s1_p0/15966/0_5398

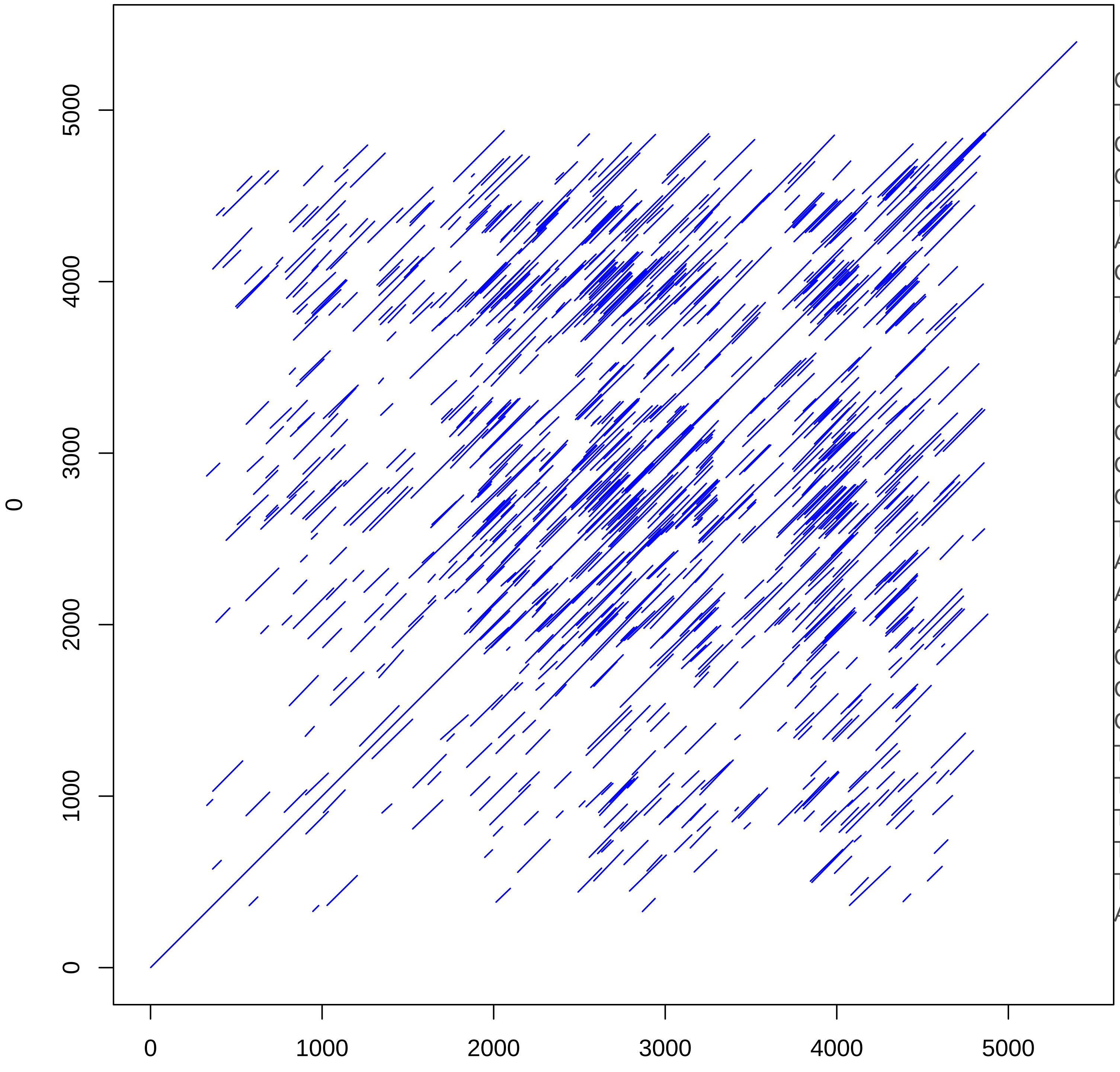

GTAGTGGTAGTG 14
TAGTGGTAGTGG 12
CAGCAGCAGCAG 11
GCAGCAGCAGCA 11
TAGTAGTAGTAG 11
AGCAGCAGCAGC 10
GTAGTAGTAGTA 10
TGGTAGTGGTAG 10
AGTGGTAGTGGT 9
AGTAGTAGTAGT 8
GGTAGTGGTAGT 8
GTGGTAGTGGTA 8
CGTAGTGGTAGT 5
GTAGTCGTAGTG 5
TGTAGTAGTAGT 5
AGTAGTCGTAGT 4
AGTCGTAGTGGT 4
ATGGTAGTGGTA 4
GTAGTAGTAGTG 4
GTAGTAGTGTAG 4
GTCGTAGTGGTA 4
TAGTAGTGTAGT 4
TAGTCGTAGATC 4
TCGTAGTCGTAG 4
TCGTAGTGGTAG 4
TGGTATGGTAGT 4
AGCAGCAGCAGG 3

>m150106_022046_42134_c100761282550000001823158307221520_s1_p0/161225/0_6371

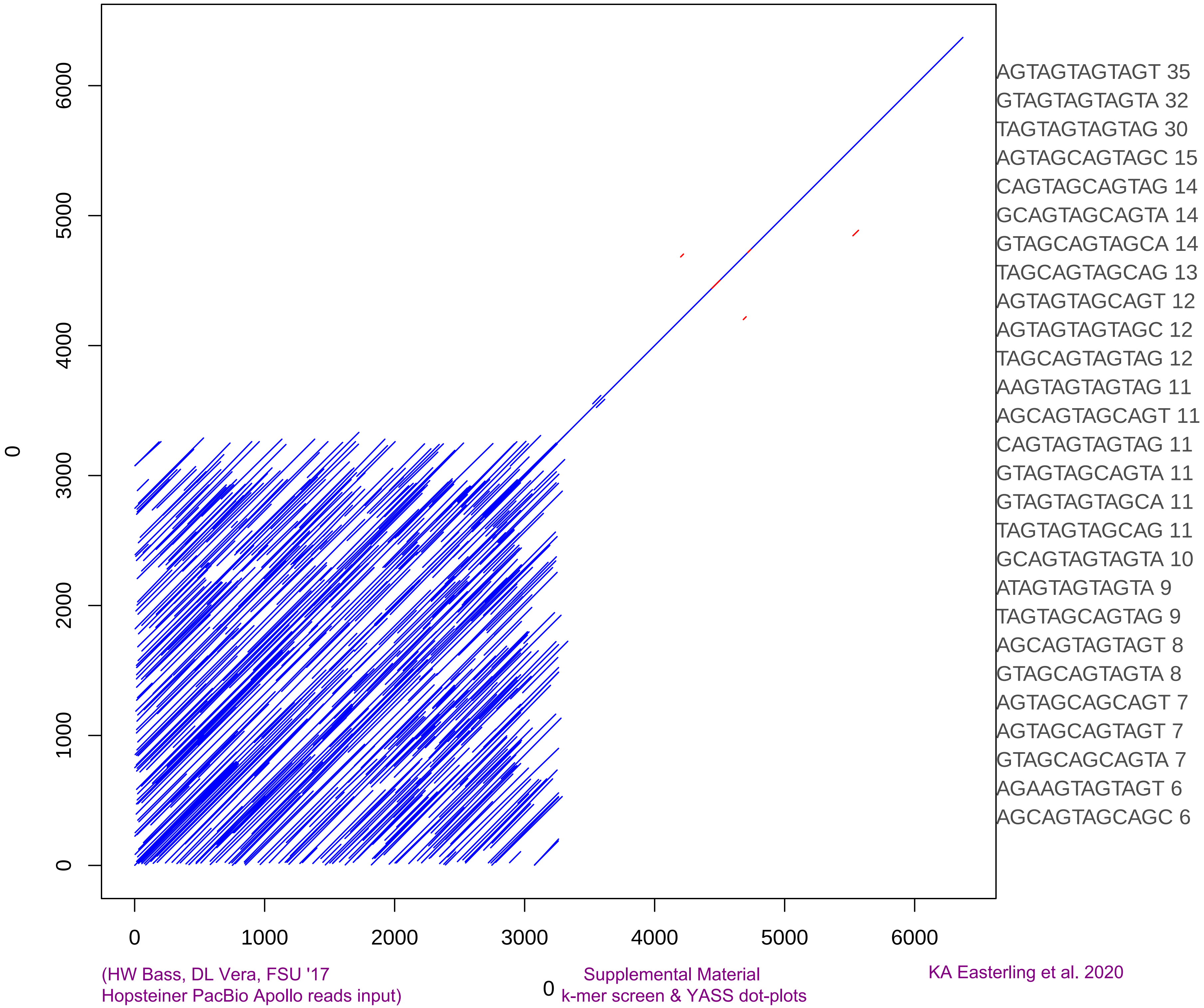

>m150106_022046_42134_c100761282550000001823158307221520_s1_p0/161912/533_6665

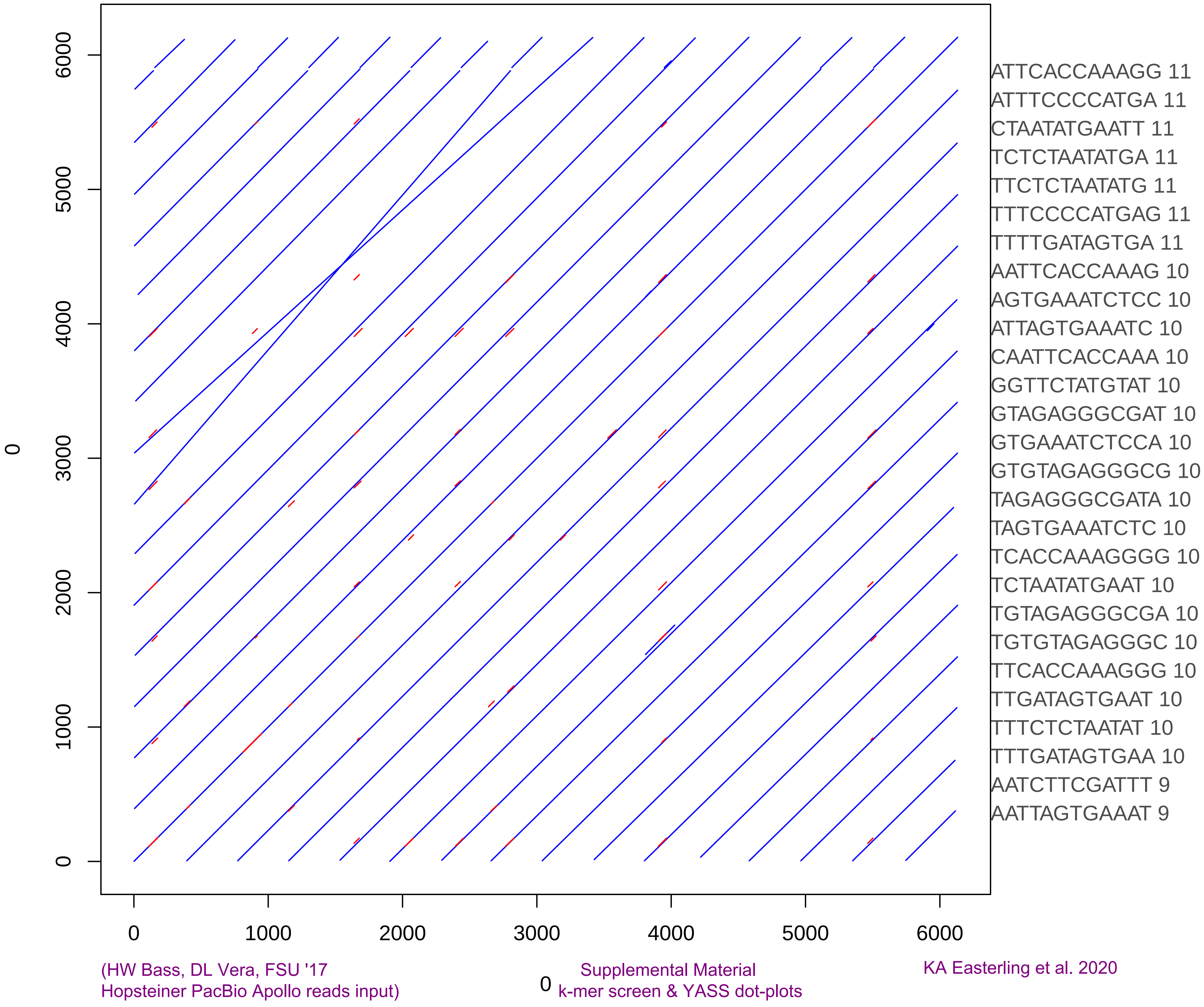

1,121 hits for repetitive sequences
passing 12mer abundance criterion

For Tandem Repeat families of ### bp:
PacBio Read named "HuluTR###-r1010"

>m150106_022046_42134_c100761282550000001823158307221520_s1_p0/16608/629_5846

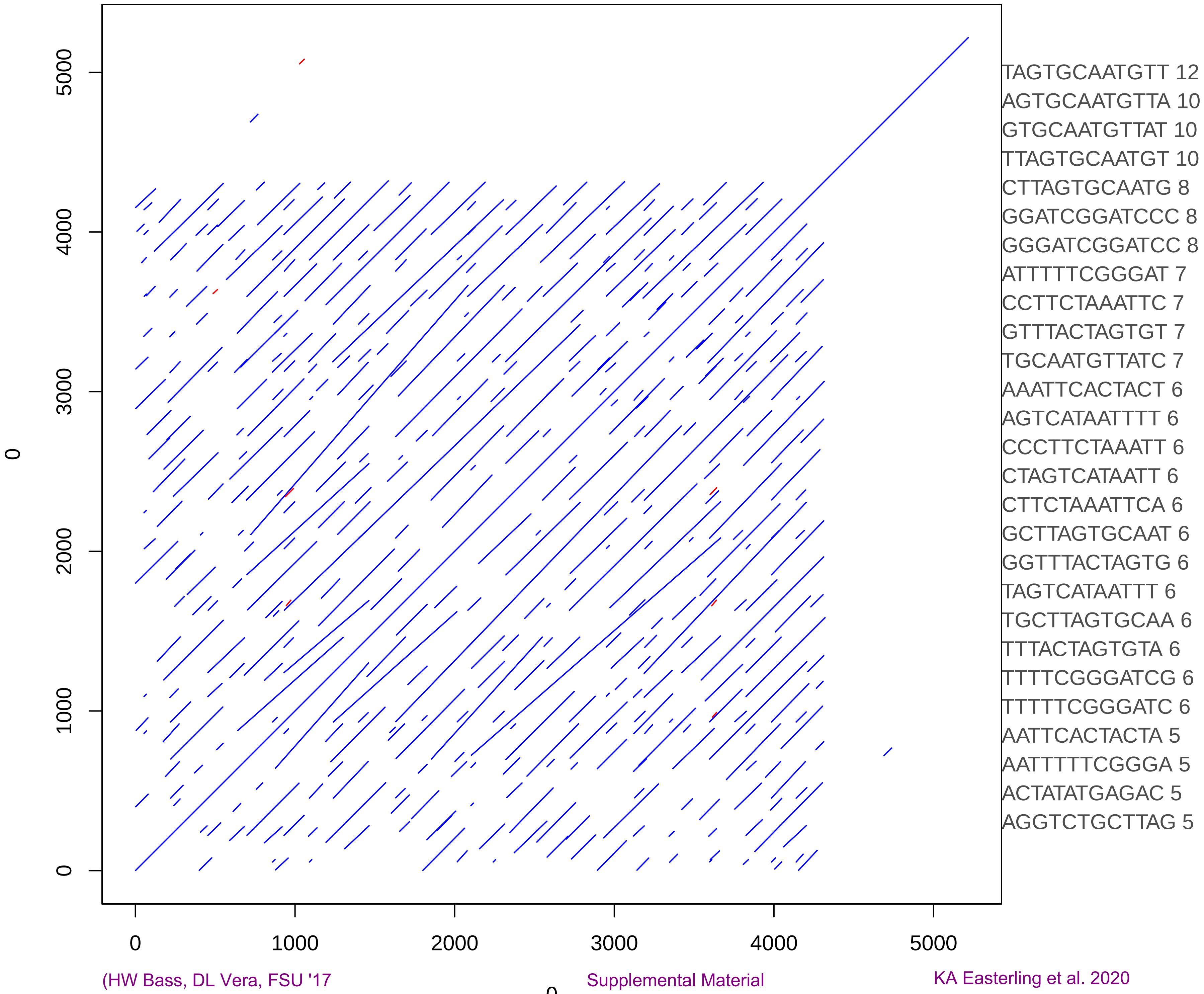

(HW Bass, DL Vera, FSU '17
Hopsteiner PacBio Apollo reads input)

Supplemental Material
k-mer screen & YASS dot-plots

KA Easterling et al. 2020

1,121 hits for repetitive sequences
passing 12mer abundance criterion

(Read-Number)
PAGE 1011

For Tandem Repeat families of ### bp:
PacBio Read named "HuluTR###-r1011"

>m150106_022046_42134_c100761282550000001823158307221520_s1_p0/20221/329_6296

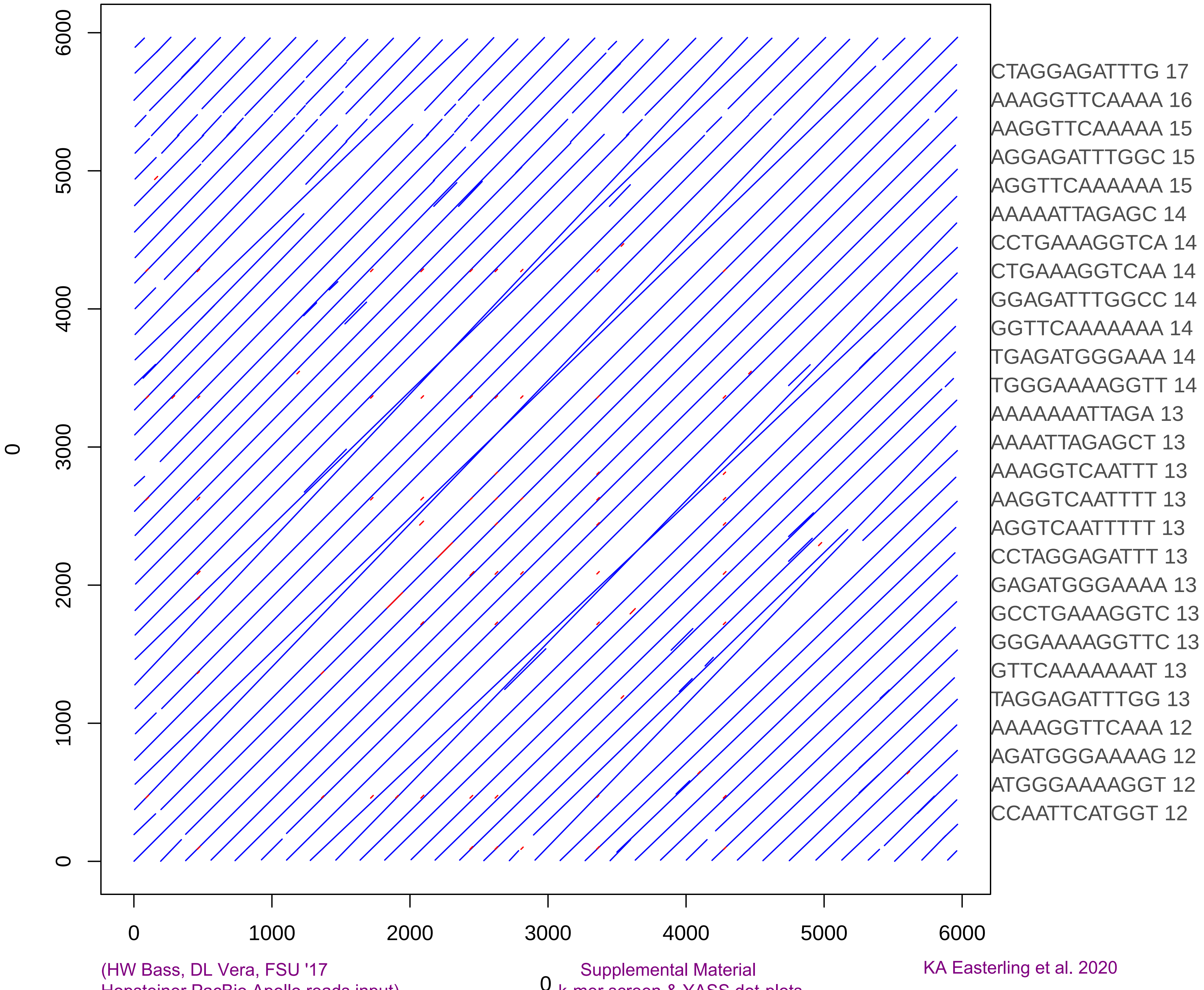

(HW Bass, DL Vera, FSU '17
Hopsteiner PacBio Apollo reads input)

Supplemental Material
k-mer screen & YASS dot-plots

KA Easterling et al. 2020

>m150106_022046_42134_c100761282550000001823158307221520_s1_p0/22332/0_5214

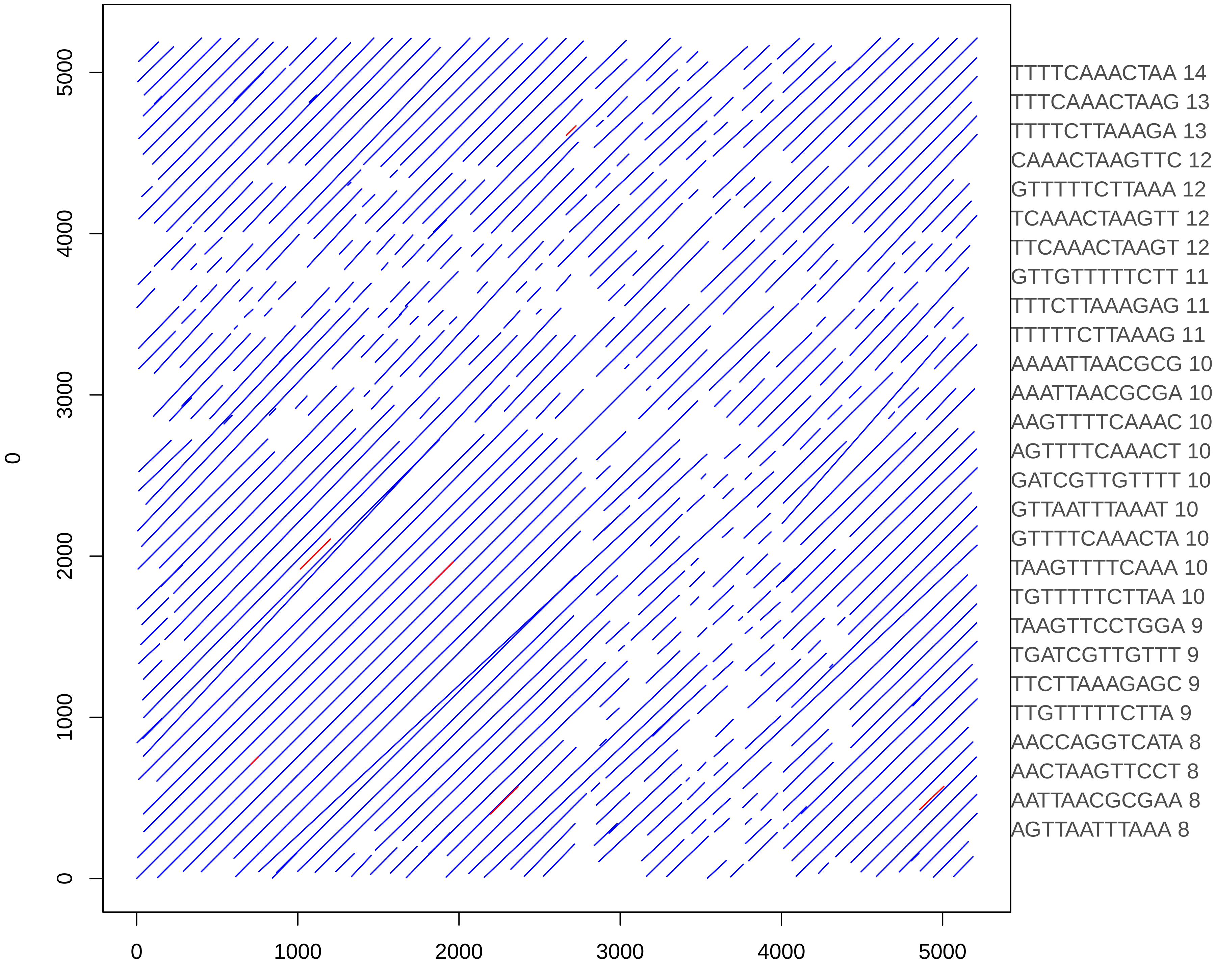

>m150106_022046_42134_c100761282550000001823158307221520_s1_p0/23037/285_7900

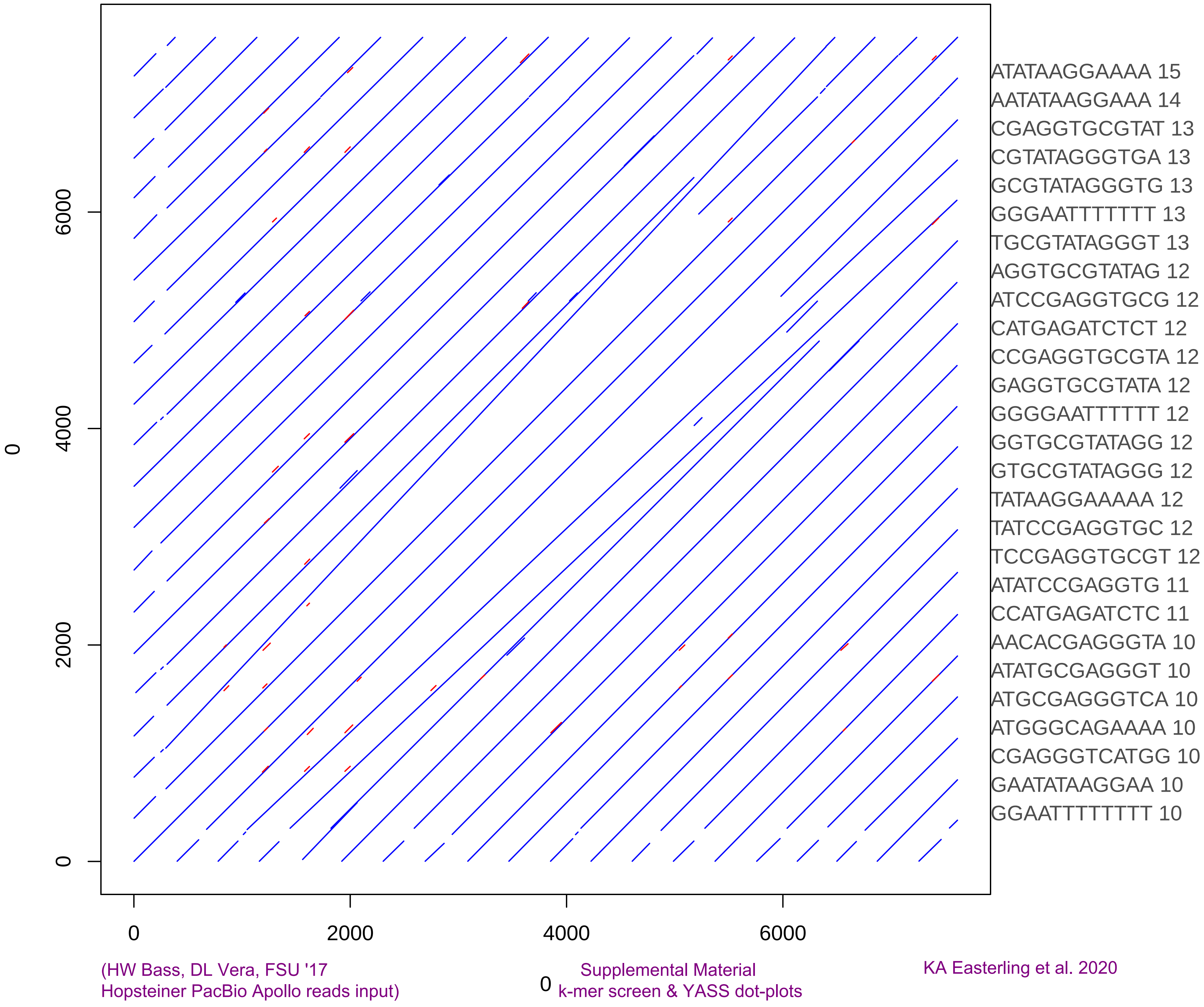

>m150106_022046_42134_c100761282550000001823158307221520_s1_p0/24547/3818_9011

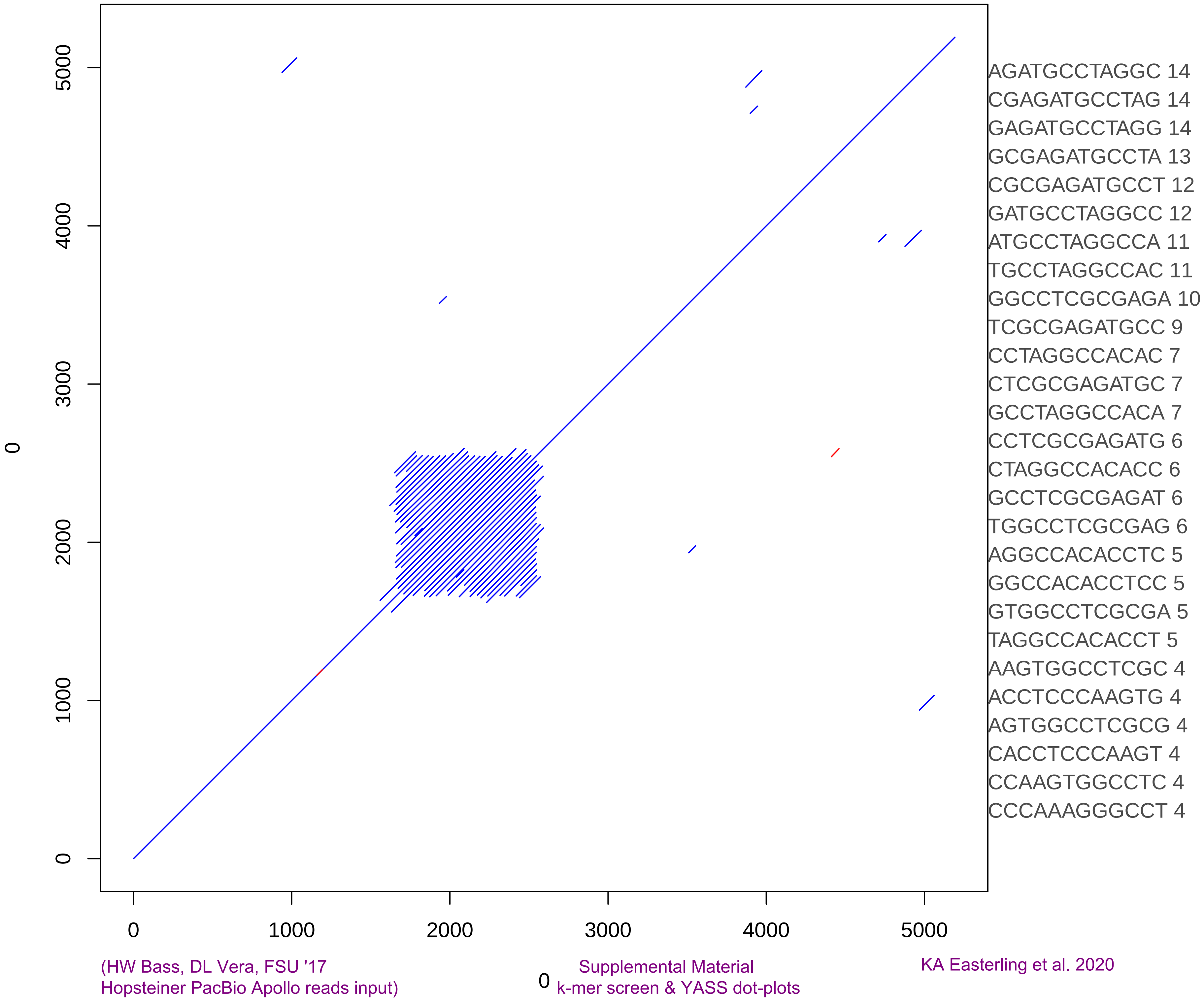

>m150106_022046_42134_c100761282550000001823158307221520_s1_p0/26030/0_5820

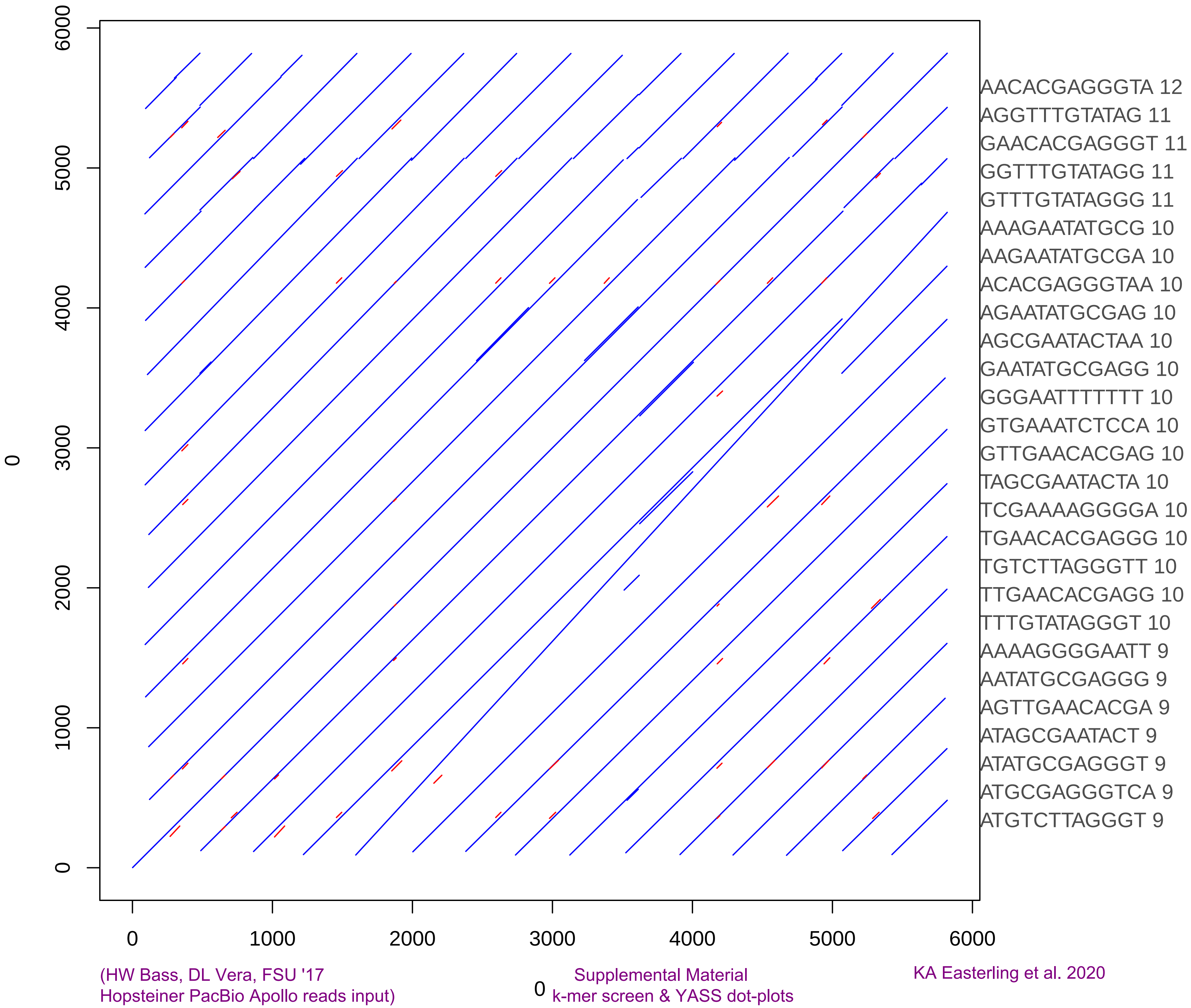

>m150106_022046_42134_c100761282550000001823158307221520_s1_p0/26359/0_10125

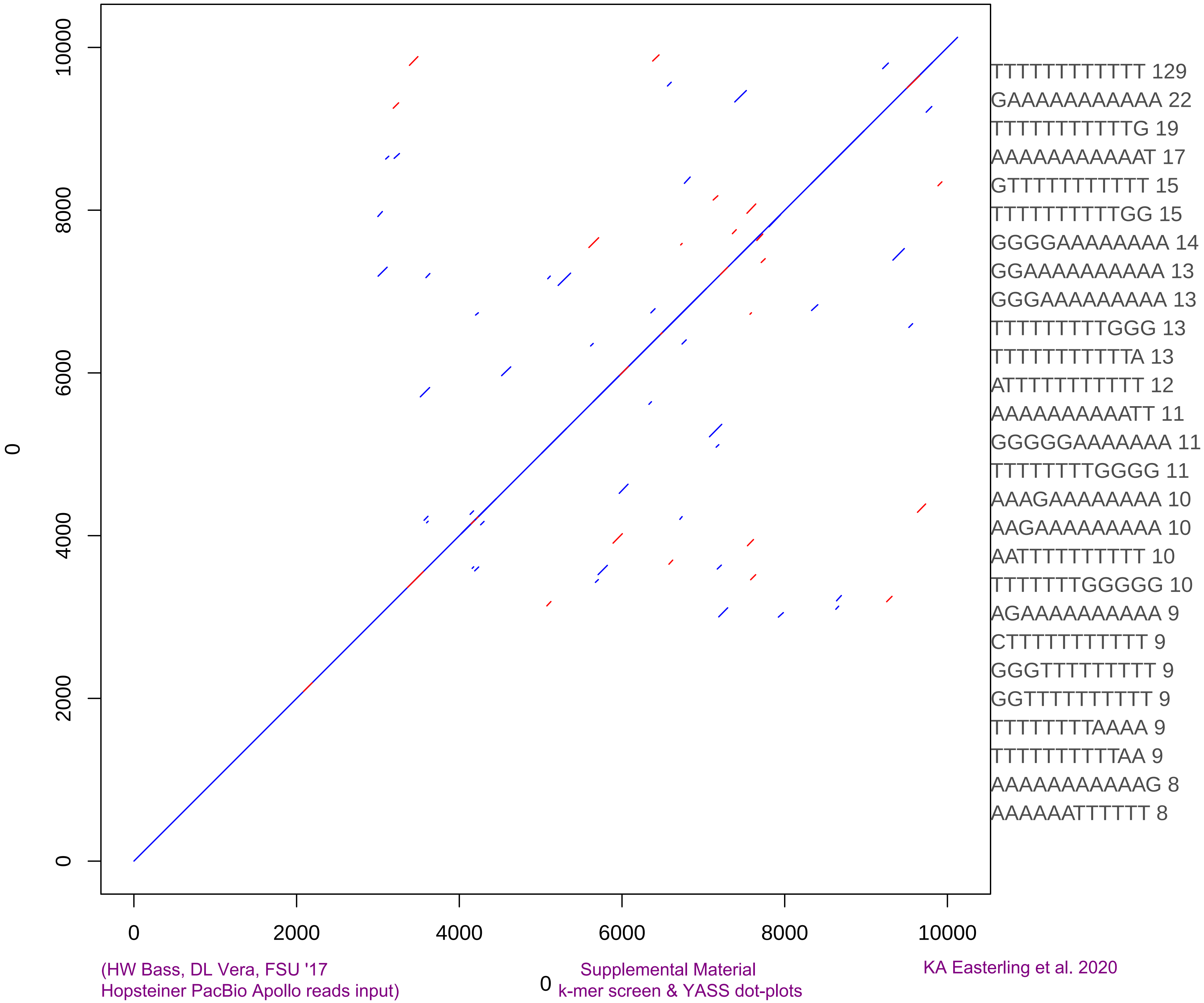

>m150106_022046_42134_c100761282550000001823158307221520_s1_p0/28239/237_9090

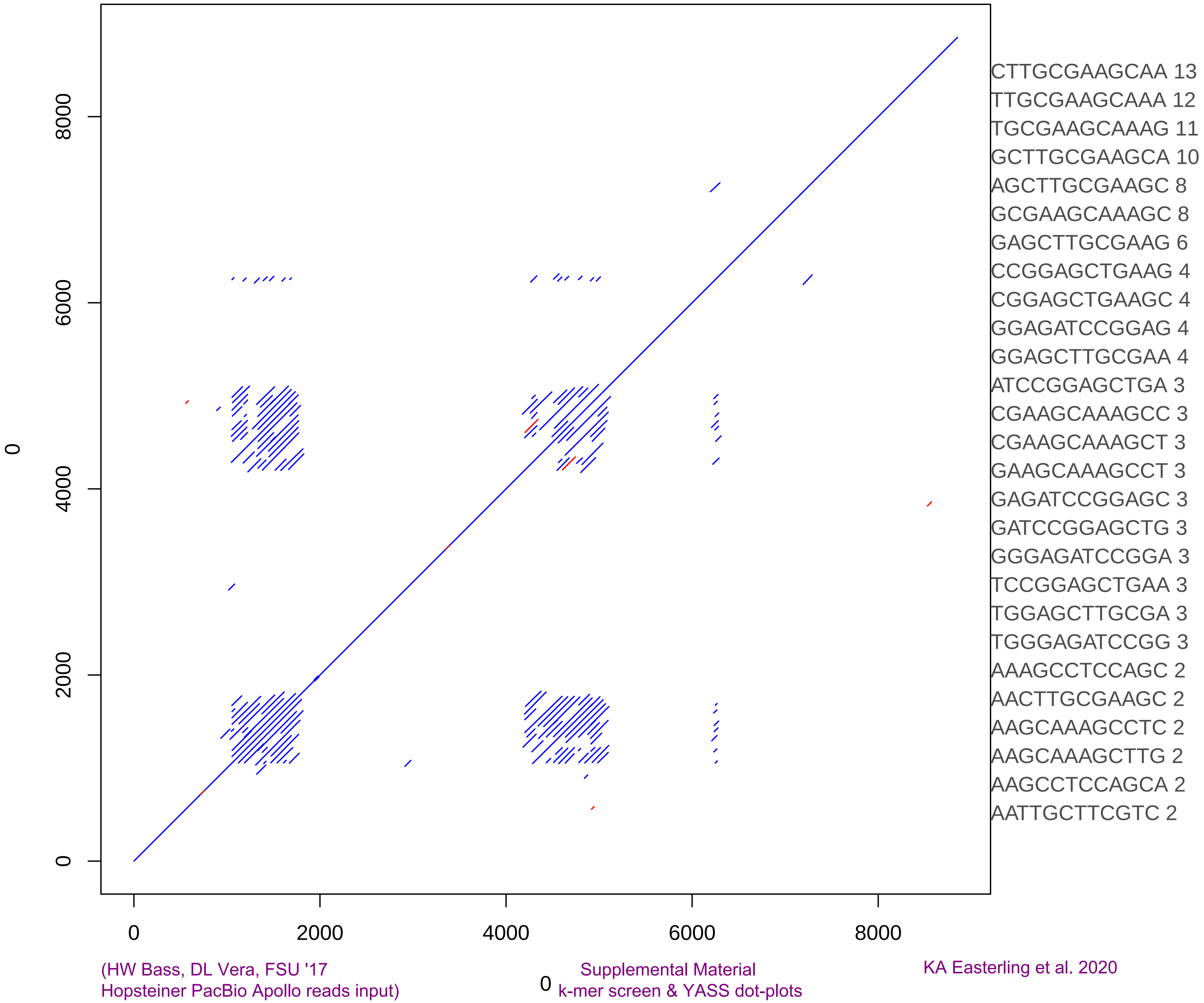

>m150106_022046_42134_c100761282550000001823158307221520_s1_p0/28456/0_13186

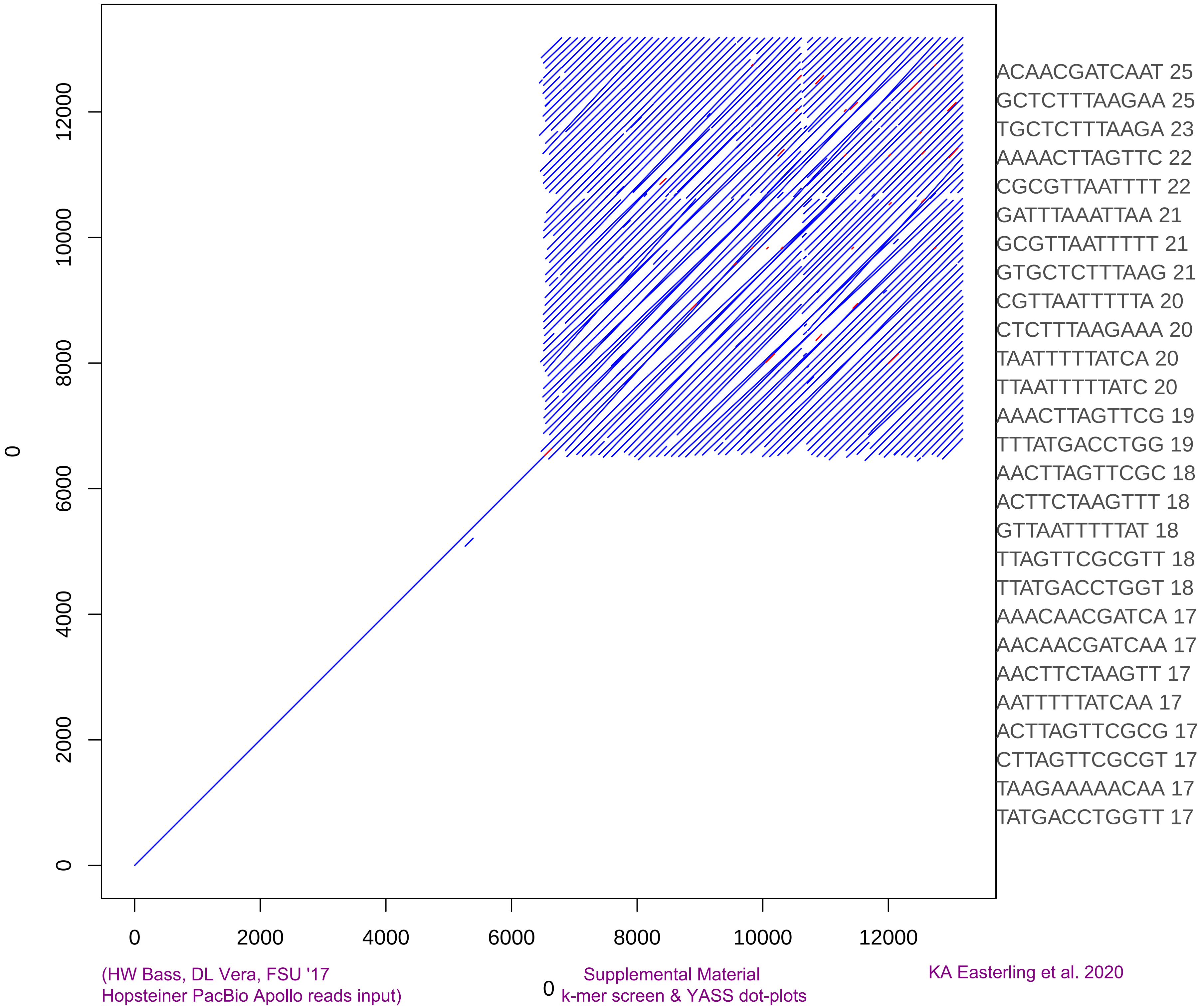

>m150106_022046_42134_c100761282550000001823158307221520_s1_p0/28943/194_7988

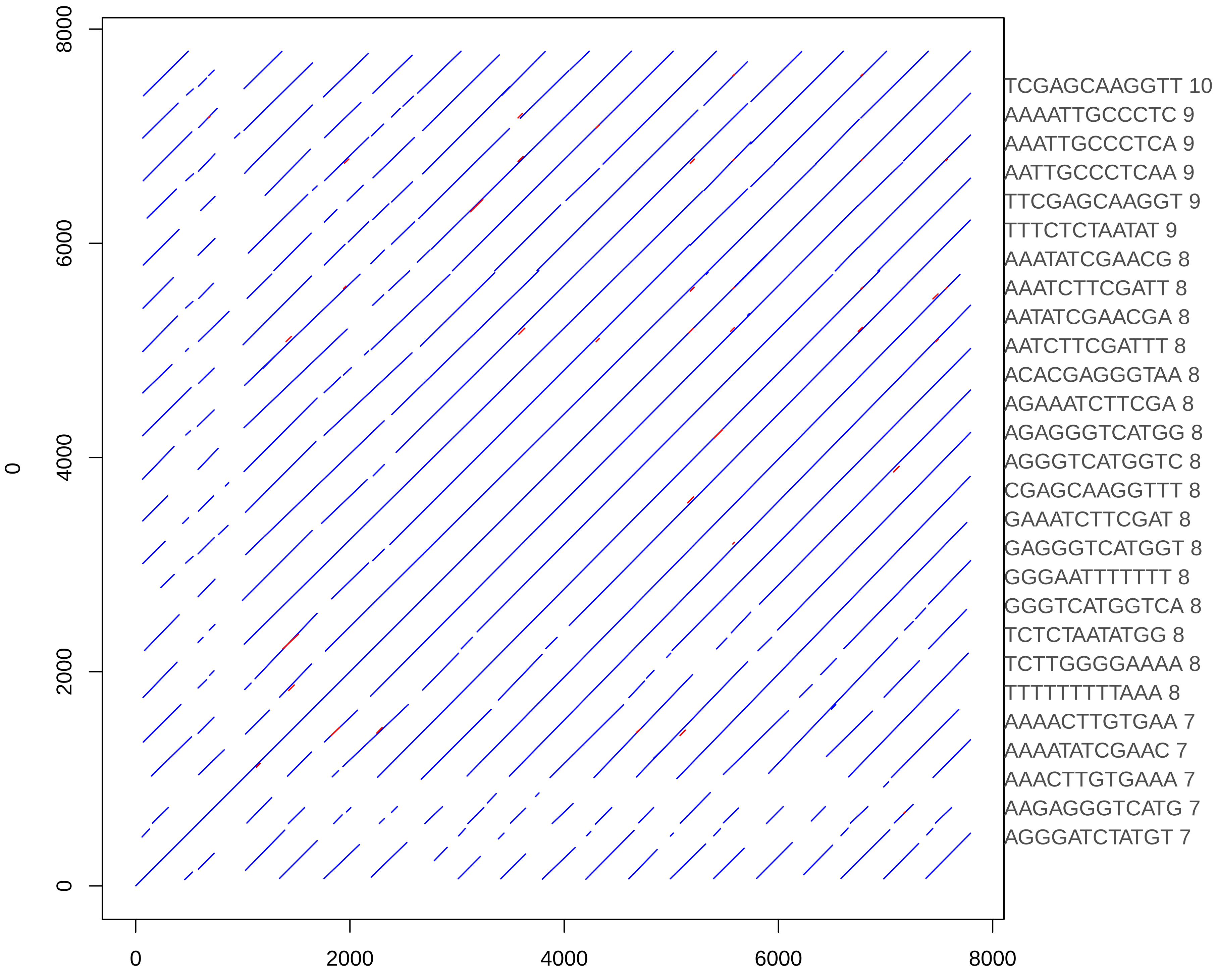

0

1,121 hits for repetitive sequences
passing 12mer abundance criterion

(Read-Number)
PAGE 1020

For Tandem Repeat families of ### bp:
PacBio Read named "HuluTR###-r1020"

>m150106_022046_42134_c100761282550000001823158307221520_s1_p0/29670/0_5573

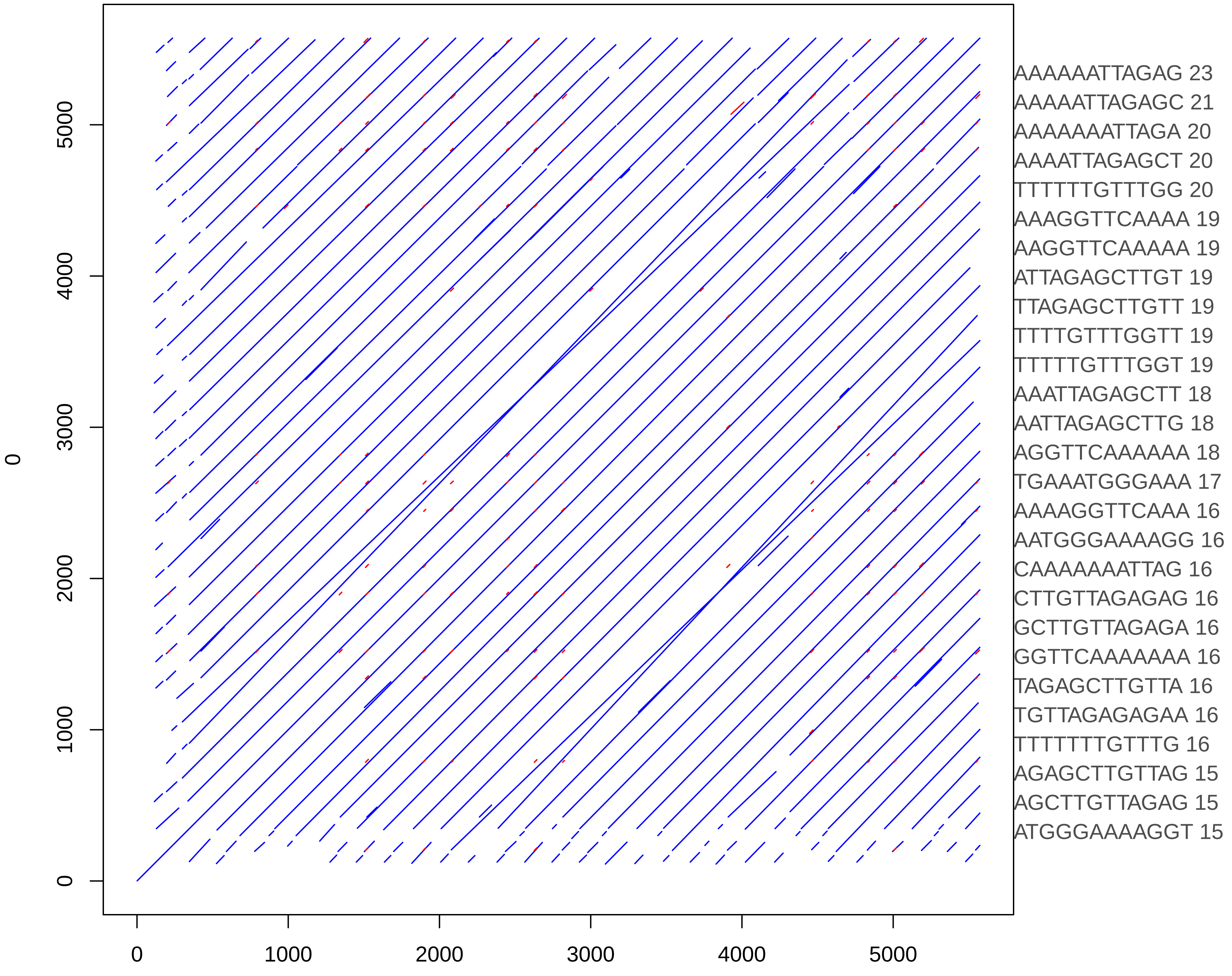

(HW Bass, DL Vera, FSU '17
Hopsteiner PacBio Apollo reads input)

Supplemental Material
k-mer screen & YASS dot-plots

KA Easterling et al. 2020

>m150106_022046_42134_c100761282550000001823158307221520_s1_p0/31776/788_8033

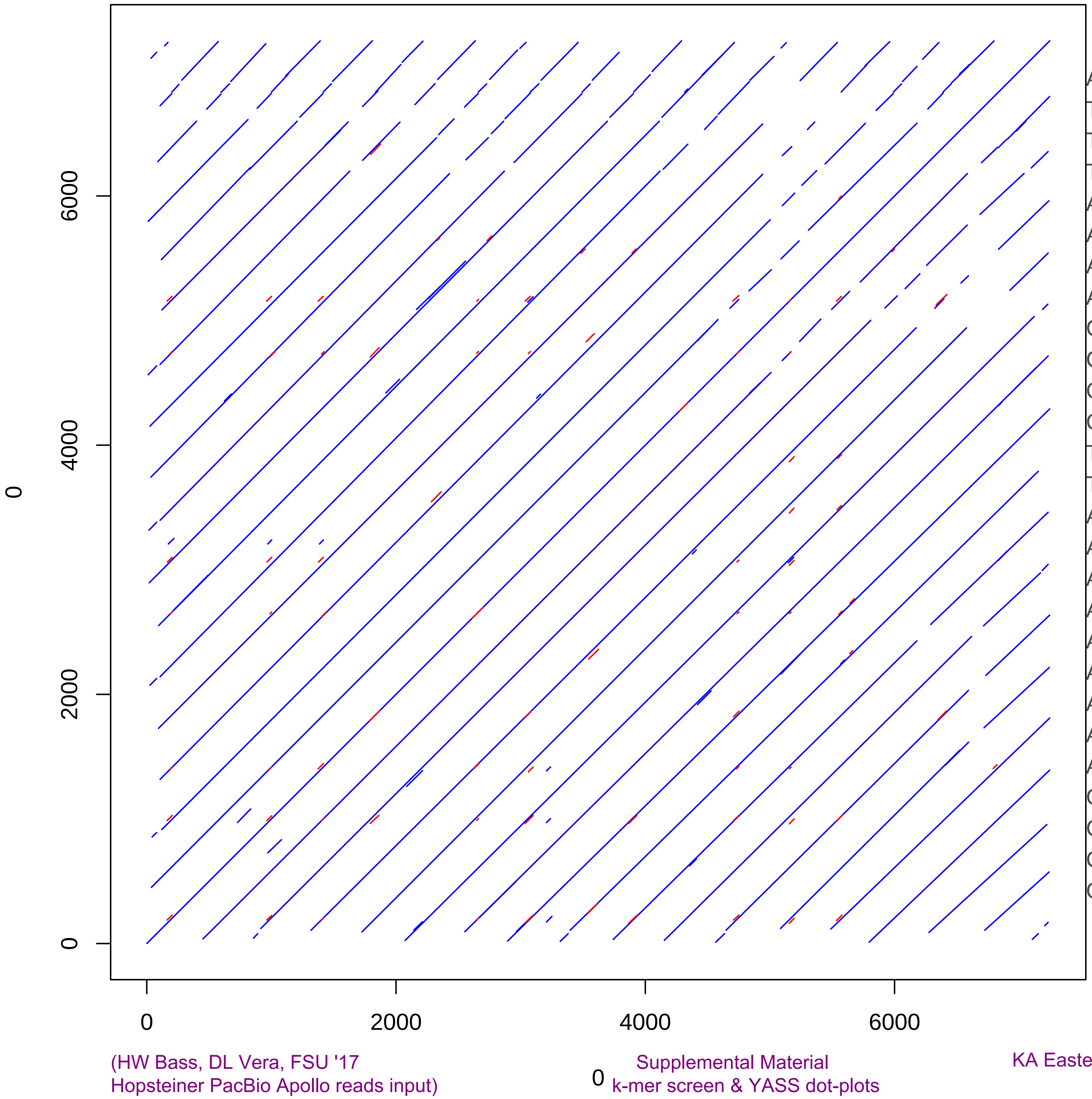

ATTGGAGTGGAA 11
TGGTGAATTGGA 11
TTGGAGTGGAAA 11
TTGGTGAATTGG 11
AAAGGGGTACCC 10
ACATTGCTCGAA 10
ATATTAGAGAAA 10
ATATTGGAGTGG 10
CATTGCTCGAAT 10
CTTTGGTGAATT 10
GAAAGGGGTACC 10
GTTTGATATTTT 10
TATTGGAGTGGA 10
TTTGGTGAATTG 10
AAACATTGCTCG 9
AAATCGAAGATT 9
AACATTGCTCGA 9
AAGGGGTACCCC 9
AGTGGAAACGGA 9
ATATTTTTCCCC 9
ATTAGAGAAATC 9
ATTGCTCGAATC 9
ATTTCAAAAAAA 9
CATTTCAAAAAA 9
CGATATTAGAGA 9
CTTATATTAGTA 9
GAGTGGAAACGG 9

1,121 hits for repetitive sequences passing 12mer abundance criterion

(Read-Number) PAGE 1022

For Tandem Repeat families of ### bp: PacBio Read named "HuluTR###-r1022"

>m150106_022046_42134_c100761282550000001823158307221520_s1_p0/35397/2414_12016

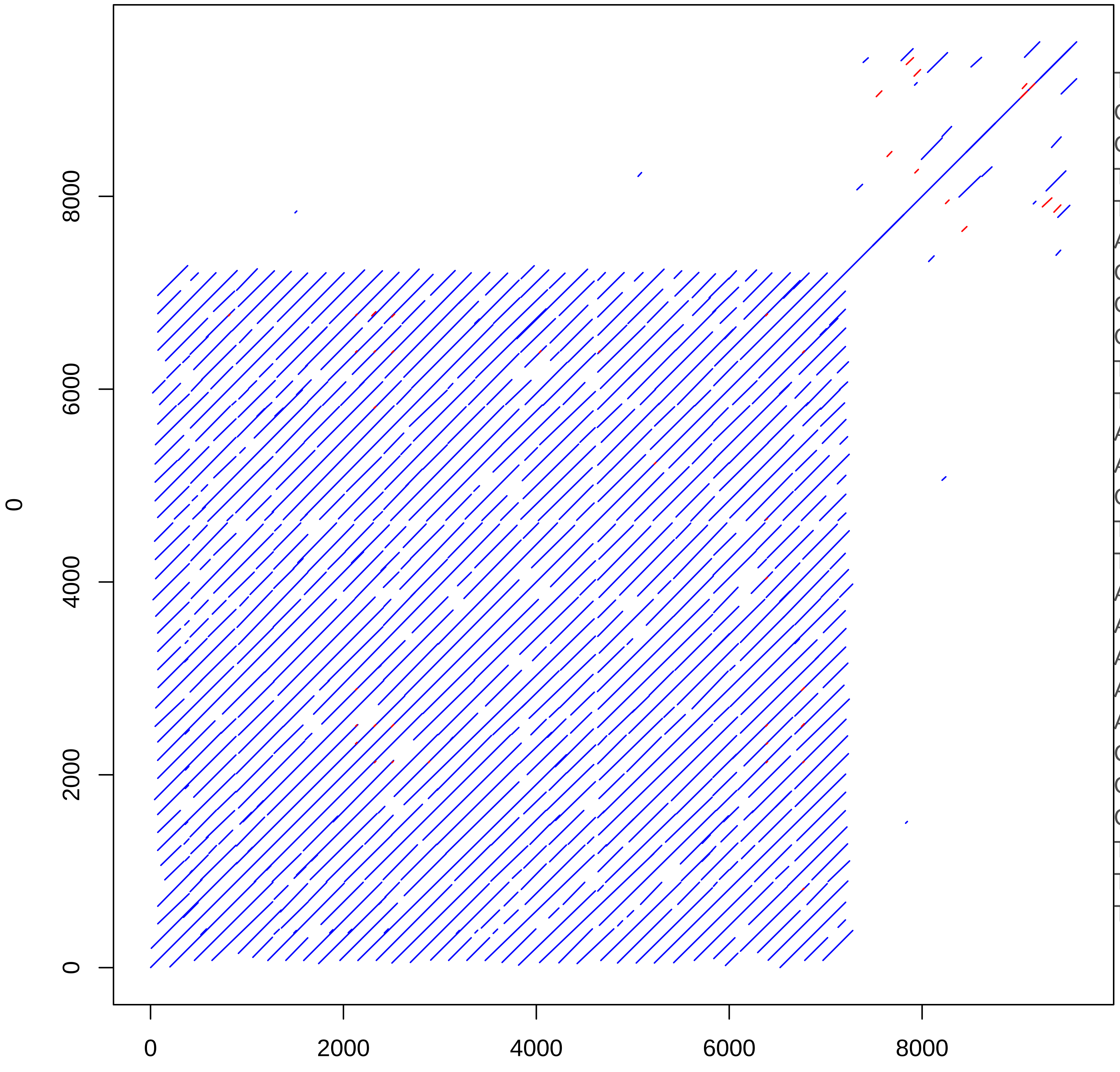

TTTTTTTTTTTT 44
GGTCAATTTTTT 15
GTCAATTTTTTT 15
TGGTTGAATTAG 15
TTGGTTGAATTA 14
AGATGGGAAAAG 13
GAGAAATATGAG 13
GAGAGAAATATG 13
GATGGGAAAAGG 13
TCAATTTTTTTG 13
TTTTGTTTGGTT 13
AGAGAAATATGA 12
ATTCATGGTCAA 12
GAGATGGGAAAA 12
TGAGATGGGAAA 12
TTTGTTTGGTTG 12
AAAAAATTTTTT 11
AAATATGAGTGA 11
AATTTTTTTGTT 11
AGGTCAATTTTT 11
ATATGAGTGAAT 11
CAATTTTTTTGT 11
GAGTGAATTACG 11
GGTTGAATTAGG 11
TATGAGTGAATT 11
TTGTTTGGTTGA 11
TTTTTTGTTTGG 11

(HW Bass, DL Vera, FSU '17 Hopsteiner PacBio Apollo reads input)

Supplemental Material k-mer screen & YASS dot-plots

KA Easterling et al. 2020

>m150106_022046_42134_c100761282550000001823158307221520_s1_p0/38094/540_7164

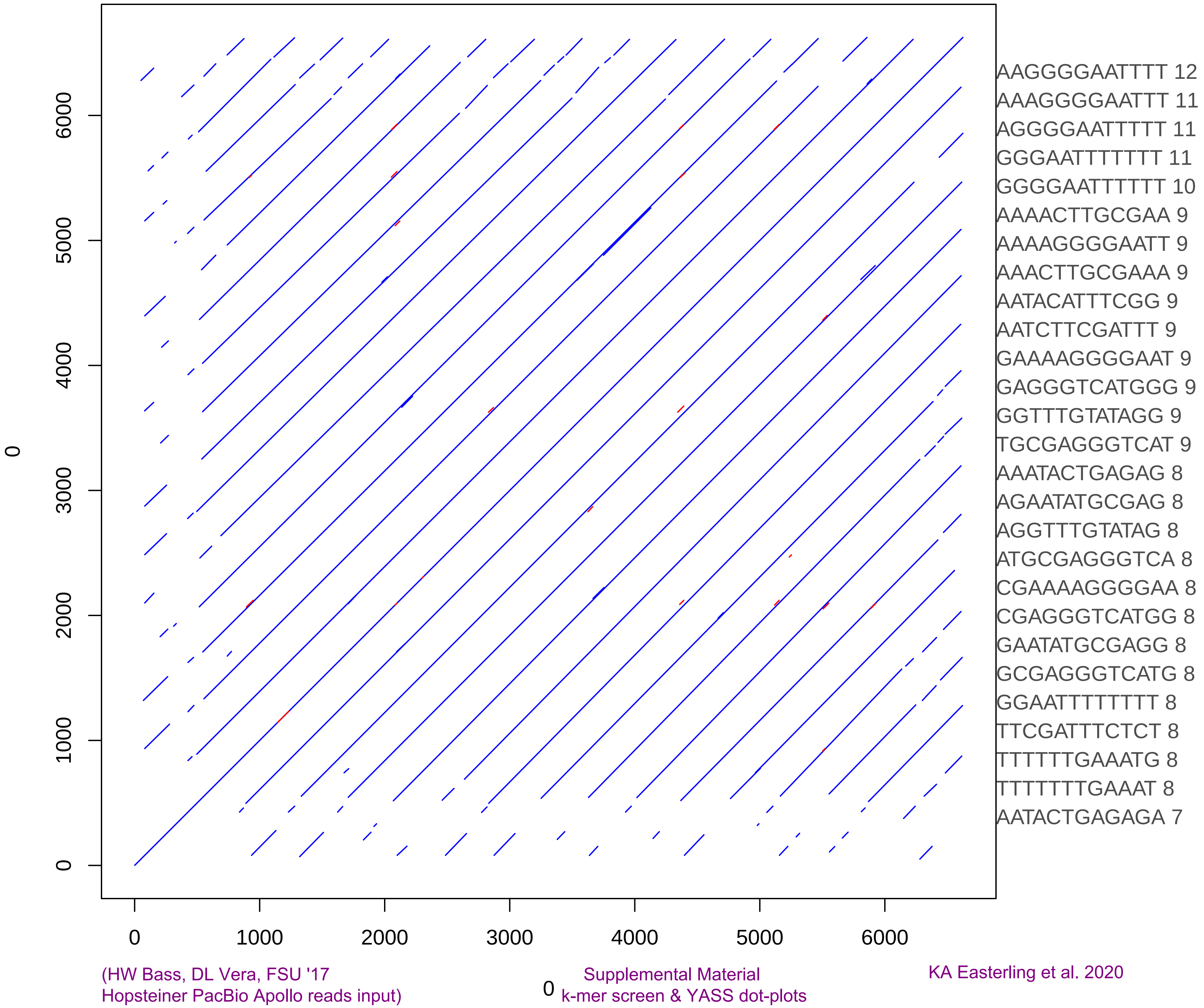

>m150106_022046_42134_c100761282550000001823158307221520_s1_p0/39024/0_7582

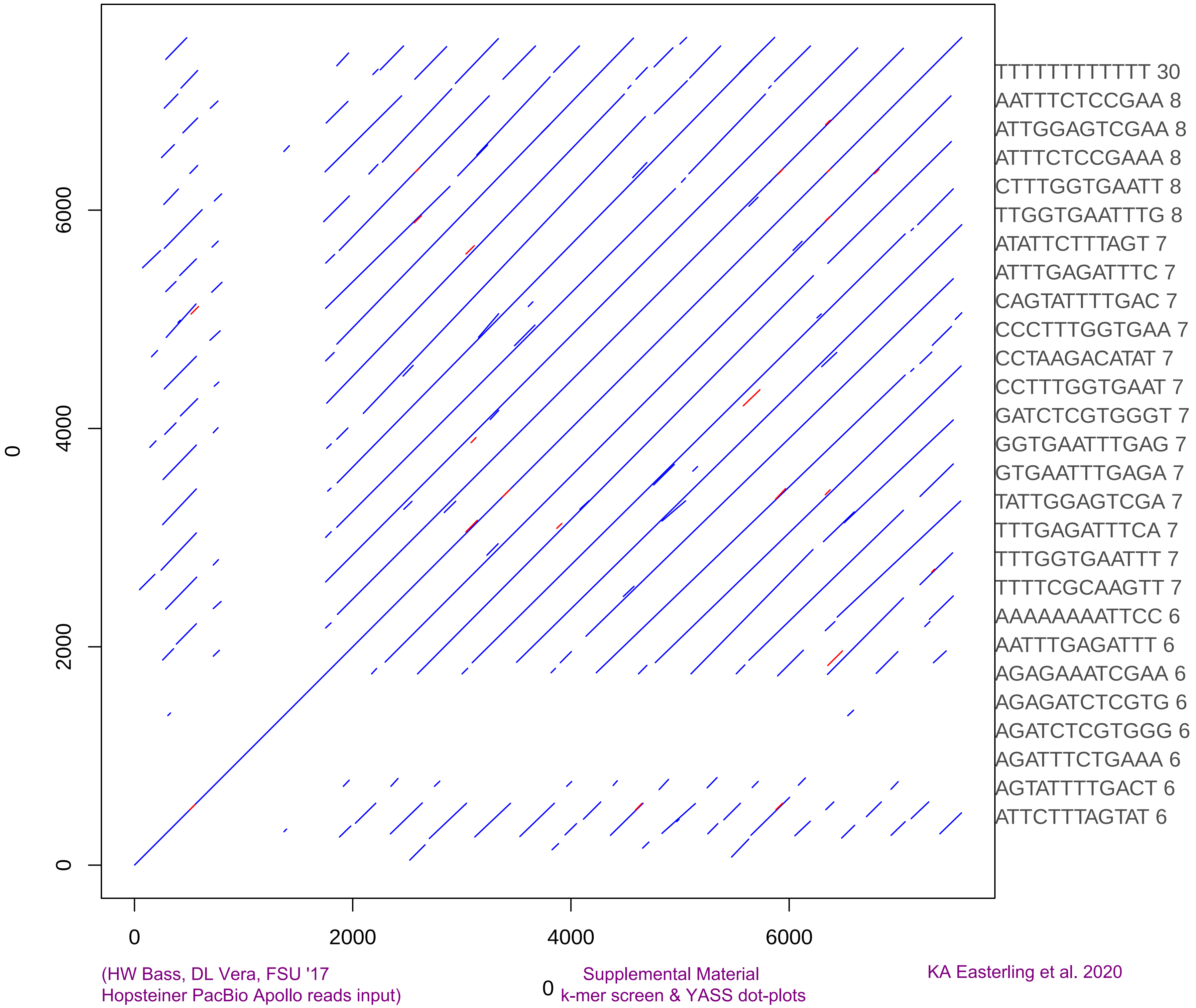

>m150106_022046_42134_c100761282550000001823158307221520_s1_p0/42244/0_5679

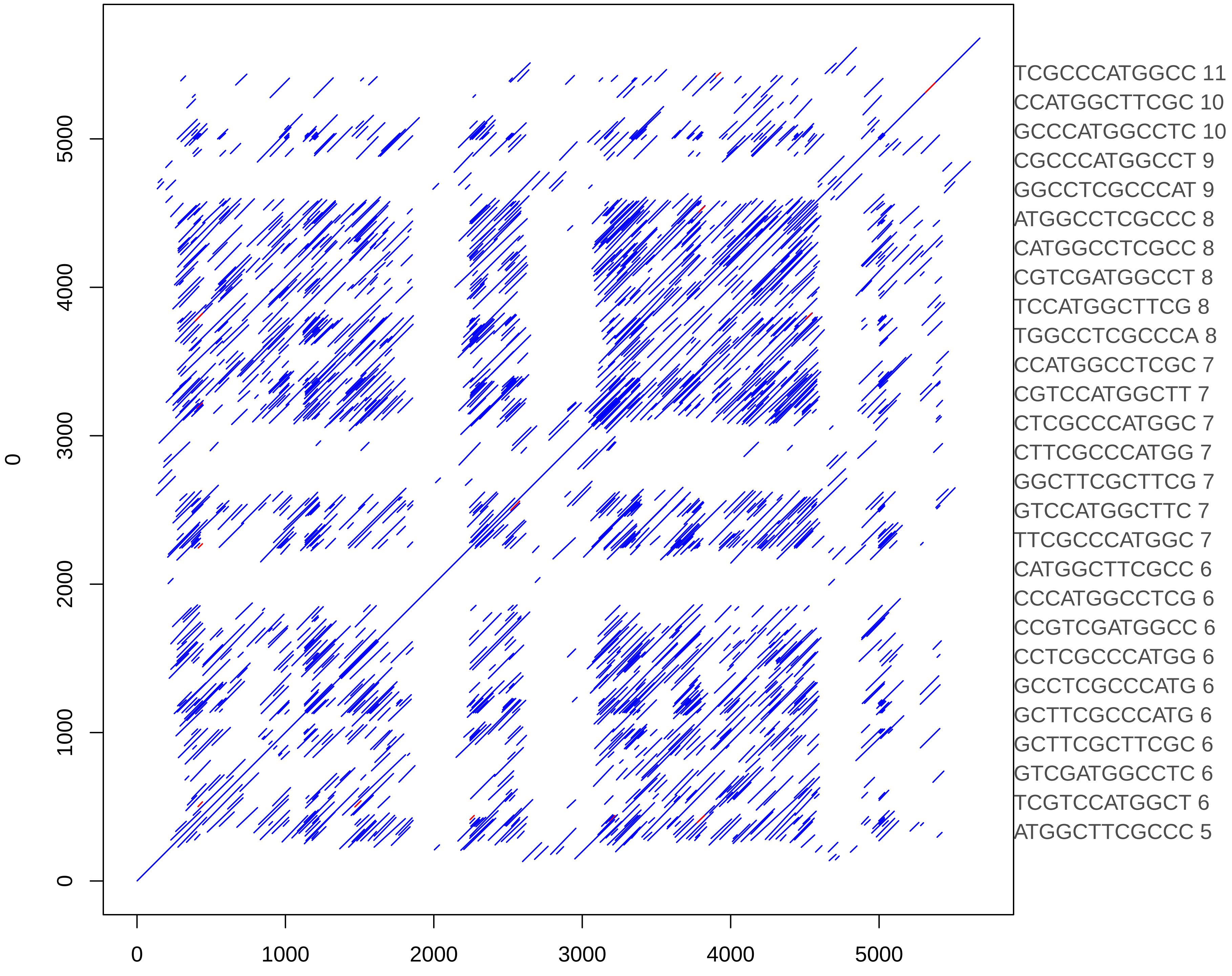

>m150106_022046_42134_c100761282550000001823158307221520_s1_p0/45088/1206_6863

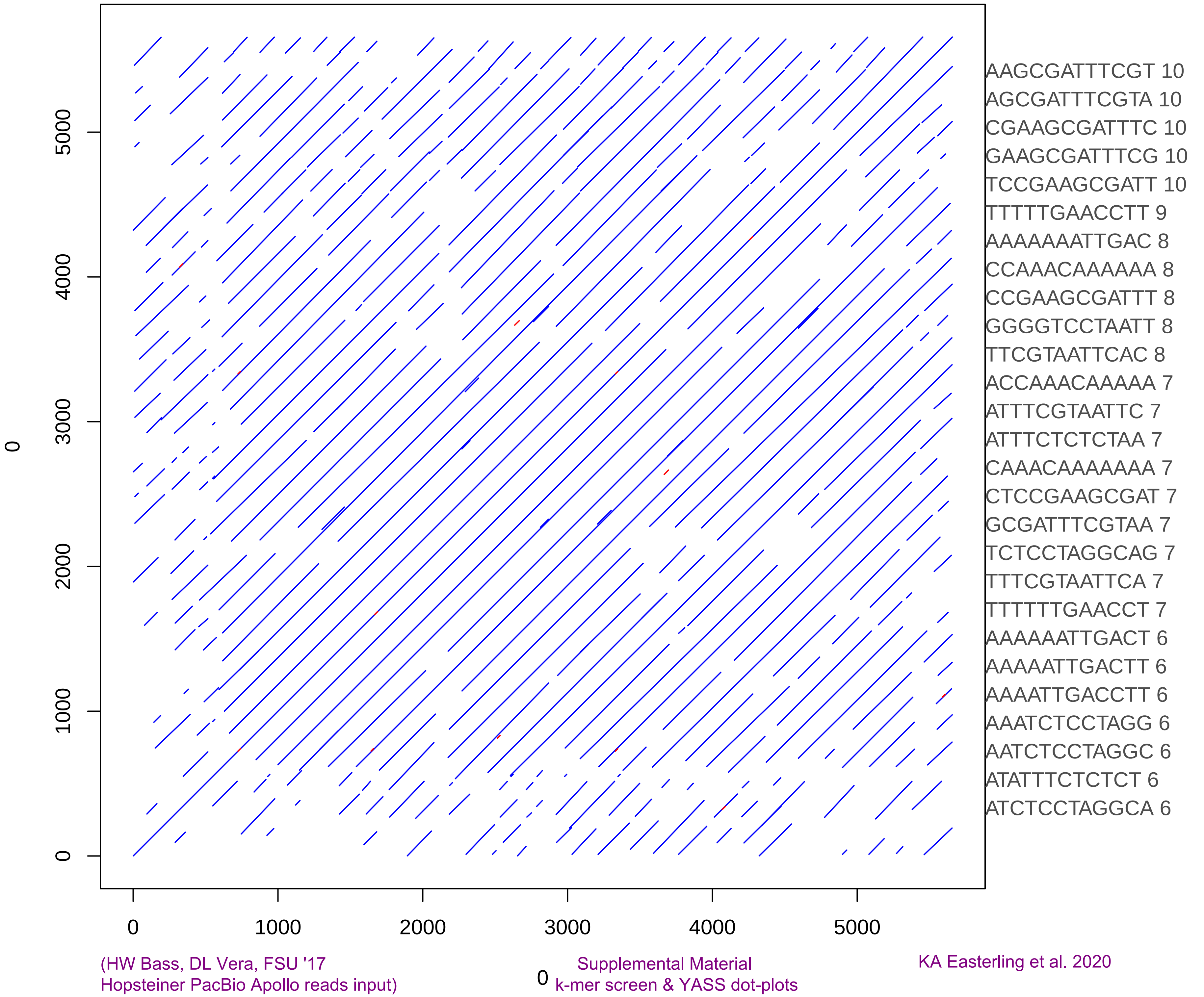

>m150106_022046_42134_c100761282550000001823158307221520_s1_p0/50139/255_6609

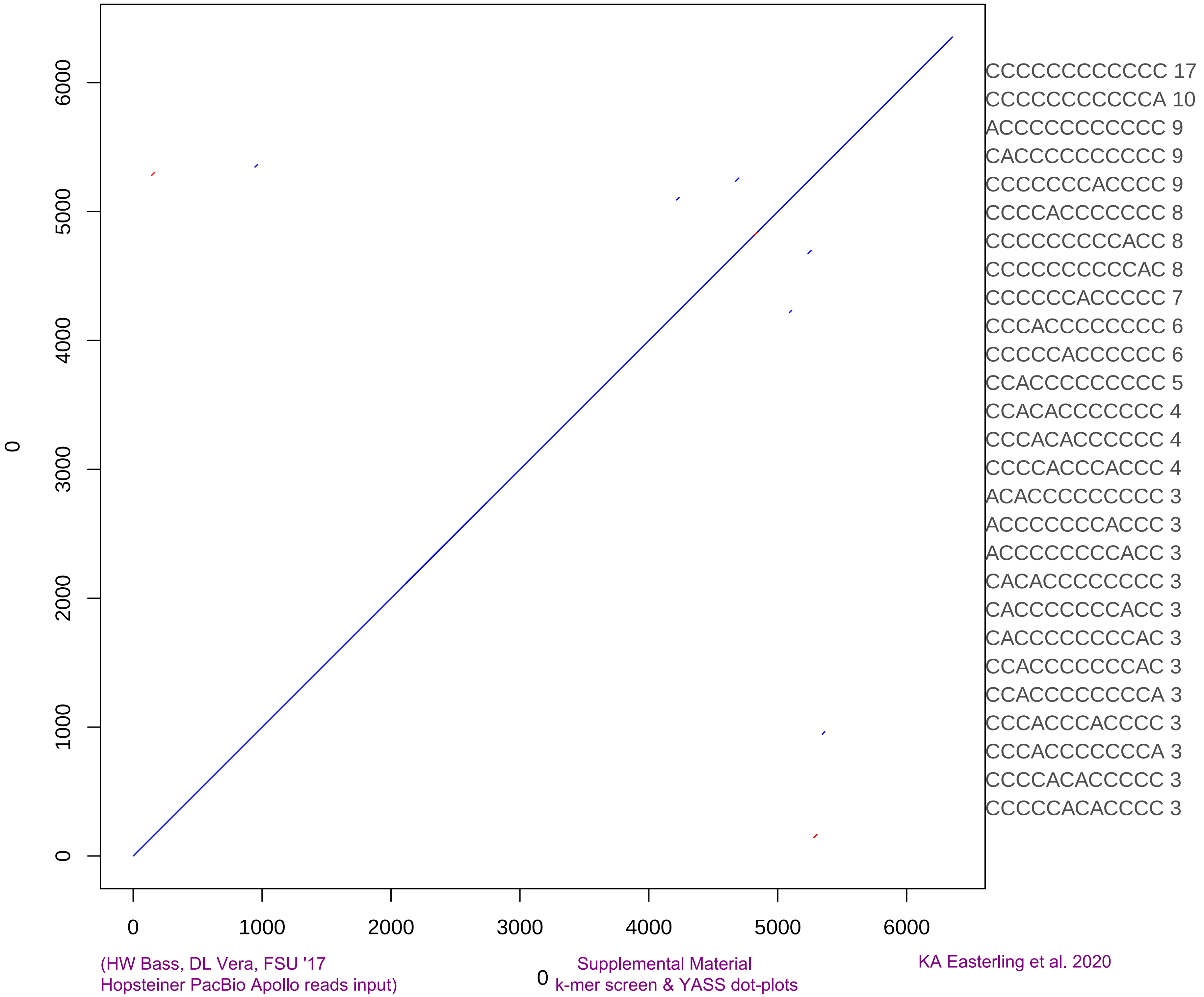

>m150106_022046_42134_c100761282550000001823158307221520_s1_p0/51898/301_8944

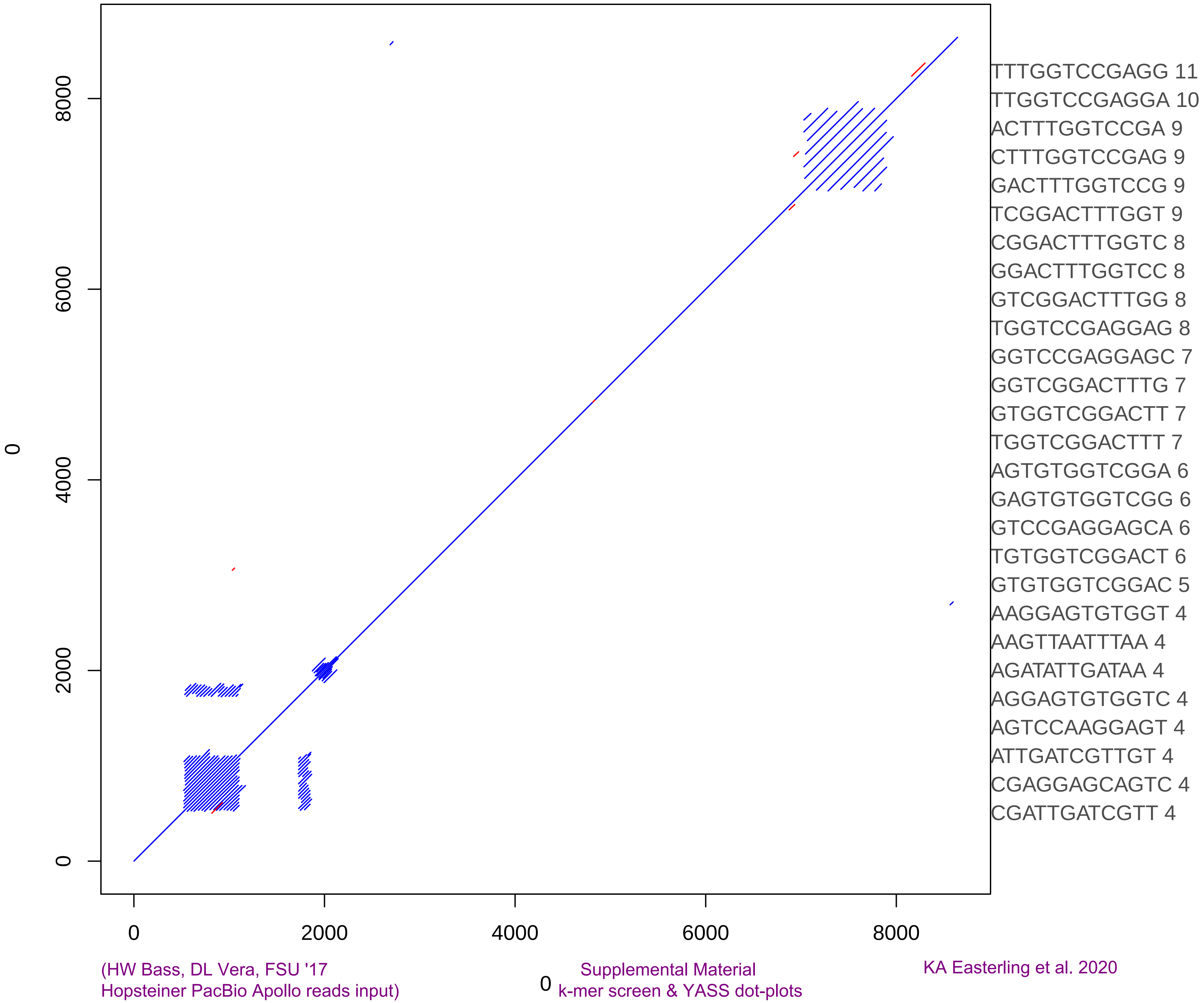

1,121 hits for repetitive sequences
passing 12mer abundance criterion

(Read-Number)
PAGE 1029

For Tandem Repeat families of ### bp:
PacBio Read named "HuluTR###-r1029"

>m150106_022046_42134_c100761282550000001823158307221520_s1_p0/5438/576_7054

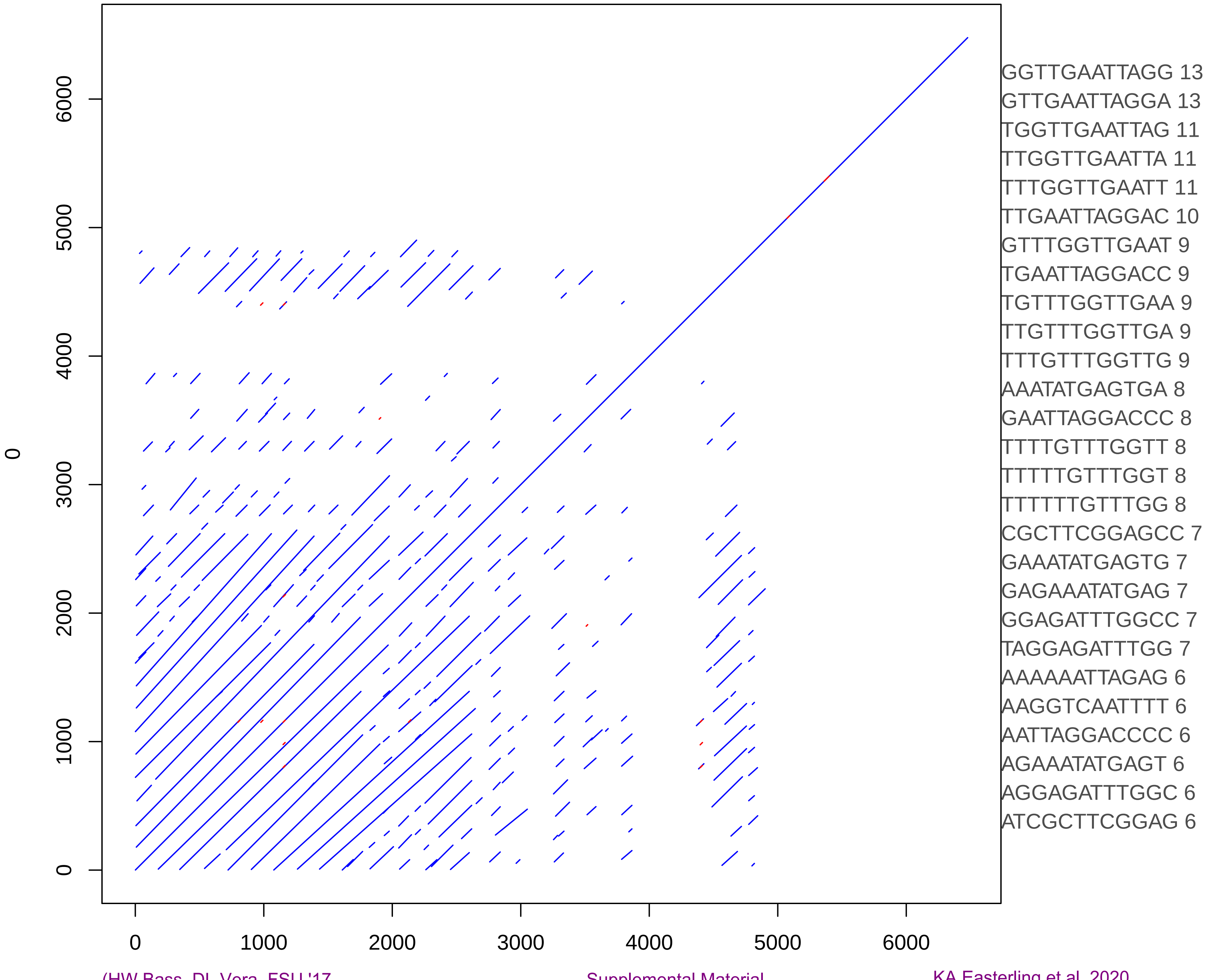

(HW Bass, DL Vera, FSU '17
Hopsteiner PacBio Apollo reads input)

0

Supplemental Material
k-mer screen & YASS dot-plots

KA Easterling et al. 2020

>m150106_022046_42134_c100761282550000001823158307221520_s1_p0/55844/249_14340

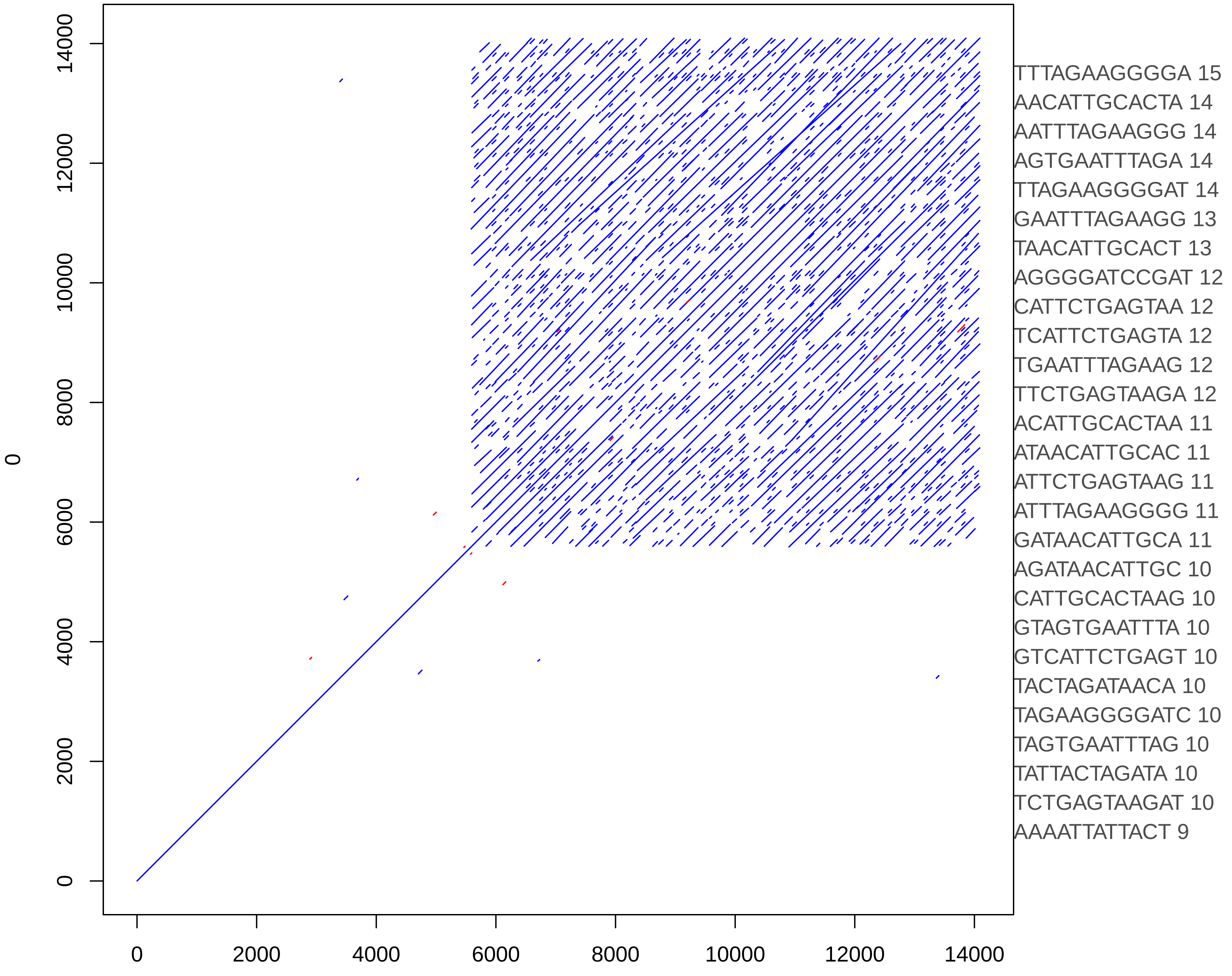

>m150106_022046_42134_c100761282550000001823158307221520_s1_p0/57689/0_7253

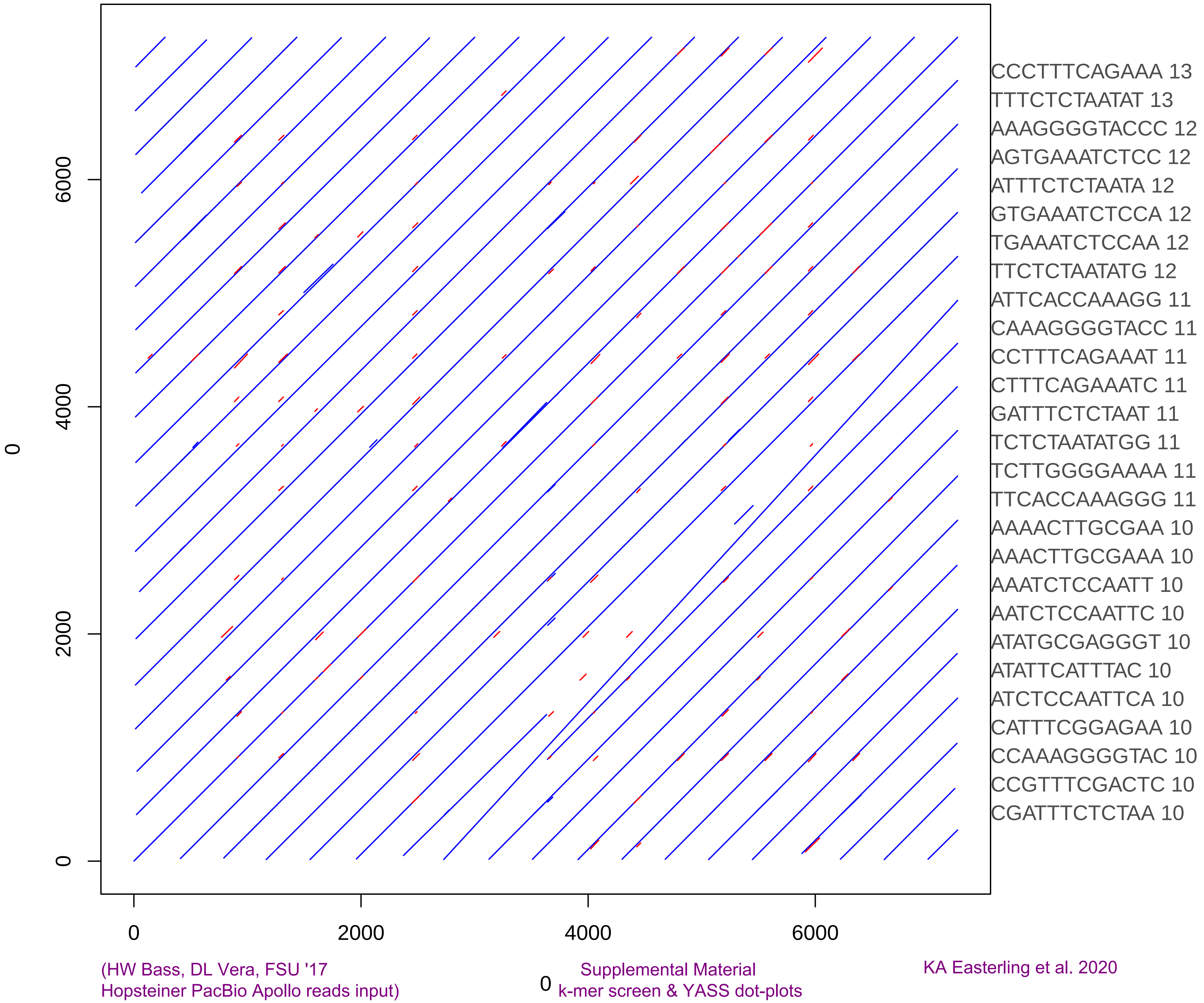

1,121 hits for repetitive sequences passing 12mer abundance criterion

(Read-Number) PAGE 1032

For Tandem Repeat families of ### bp: PacBio Read named "HuluTR###-r1032"

>m150106_022046_42134_c100761282550000001823158307221520_s1_p0/59023/0_5148

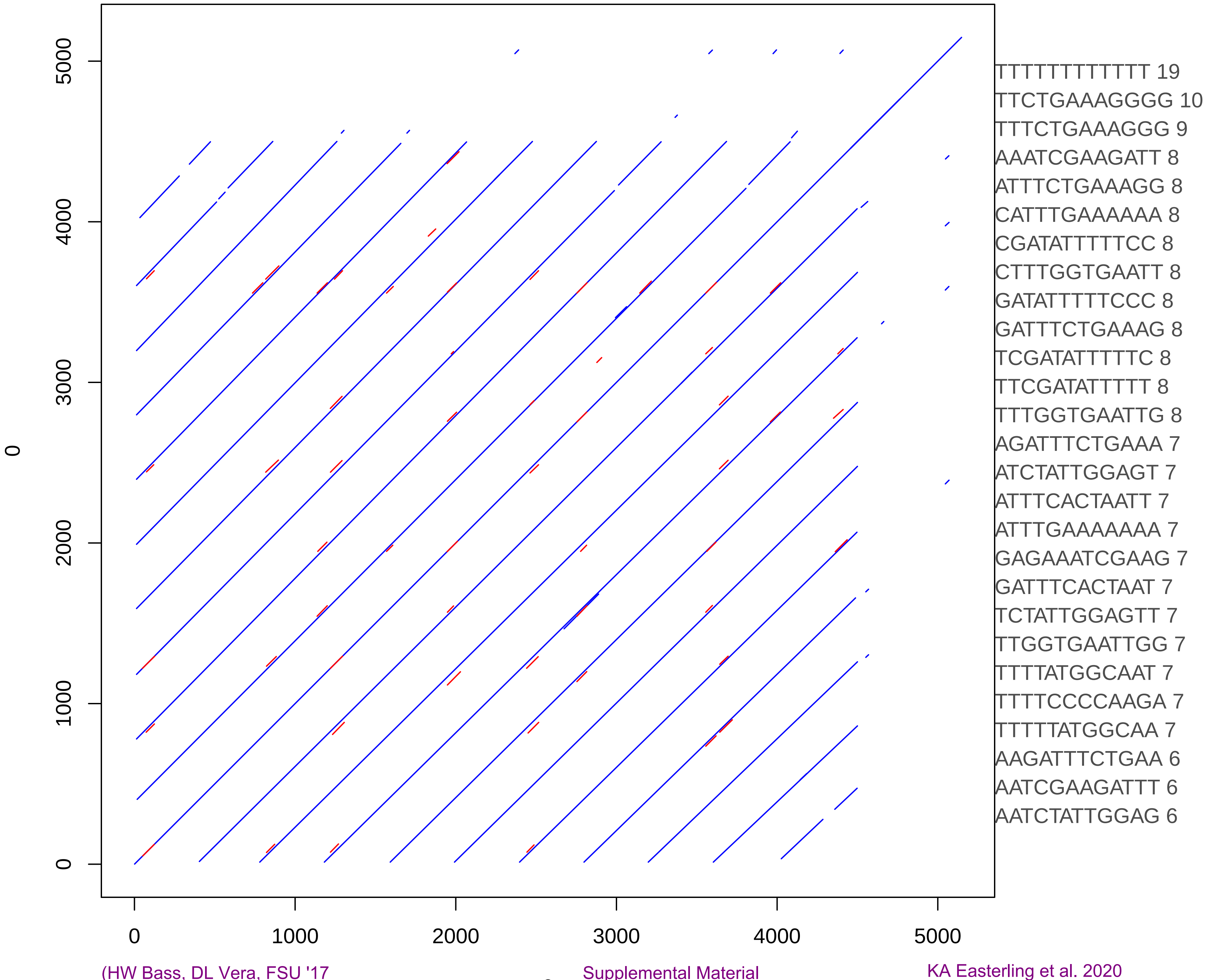

0

(HW Bass, DL Vera, FSU '17 Hopsteiner PacBio Apollo reads input)

Supplemental Material k-mer screen & YASS dot-plots

KA Easterling et al. 2020

>m150106_022046_42134_c100761282550000001823158307221520_s1_p0/59836/1764_7312

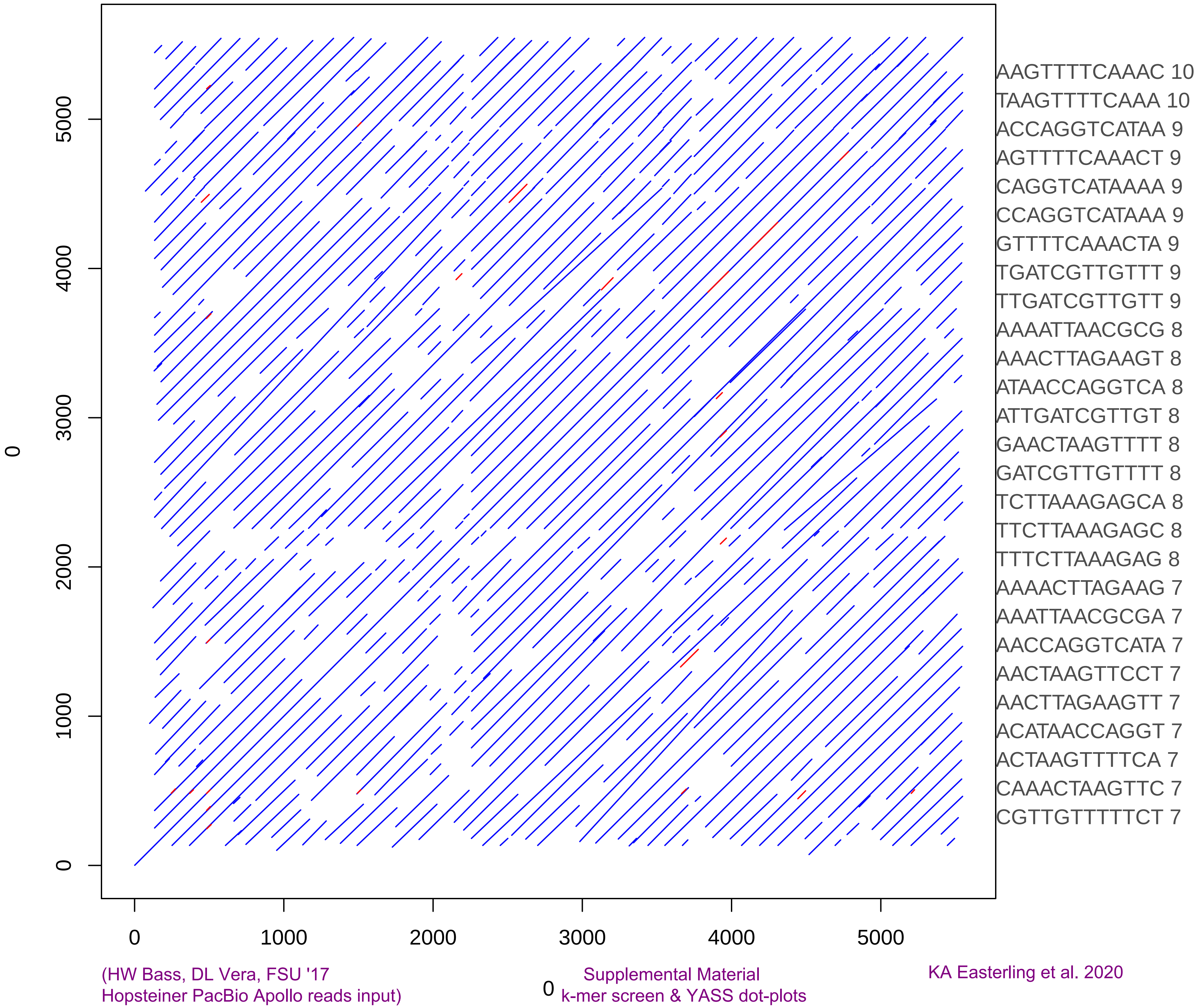

>m150106_022046_42134_c100761282550000001823158307221520_s1_p0/59852/8629_14402

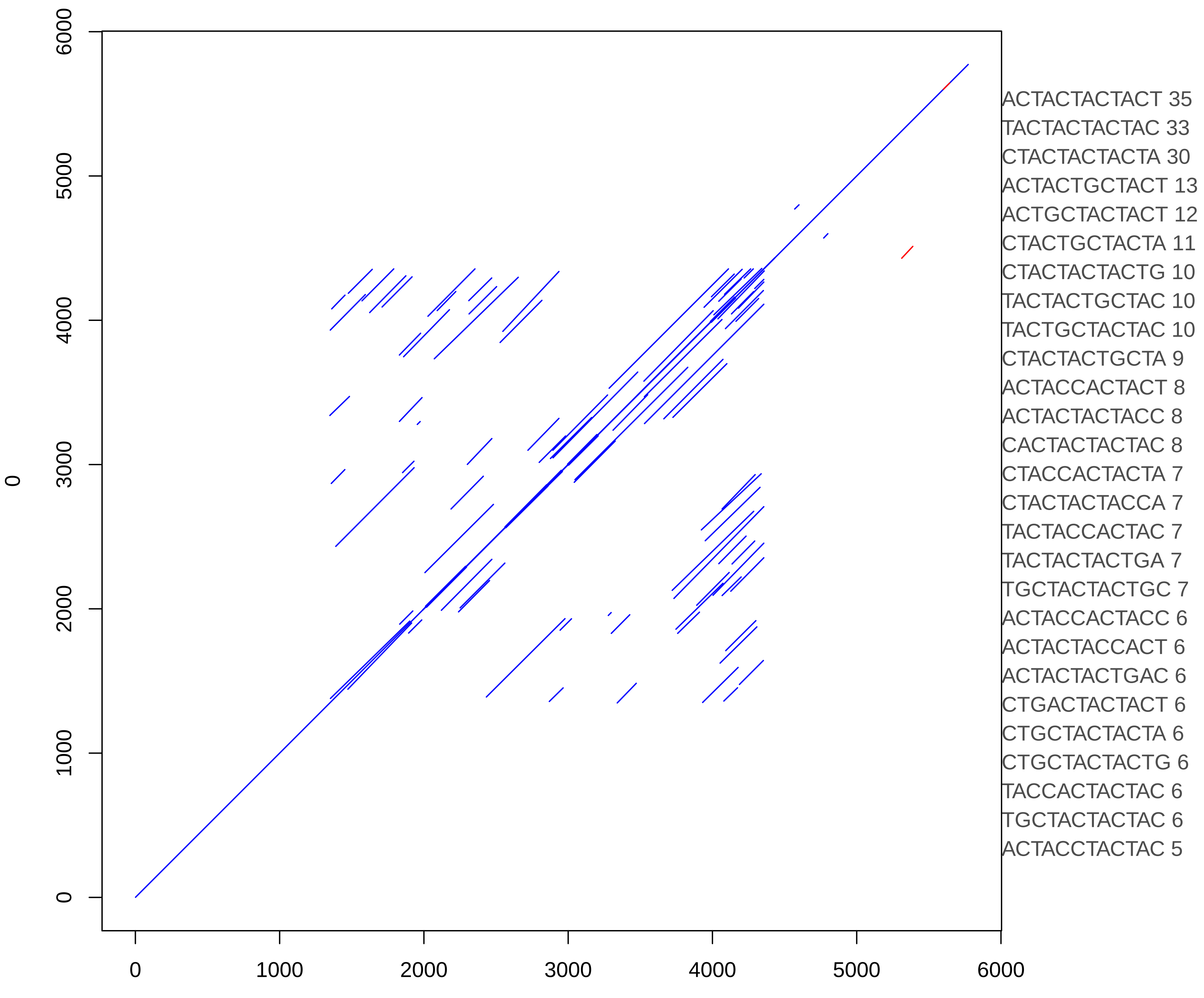

>m150106_022046_42134_c100761282550000001823158307221520_s1_p0/60815/279_8098

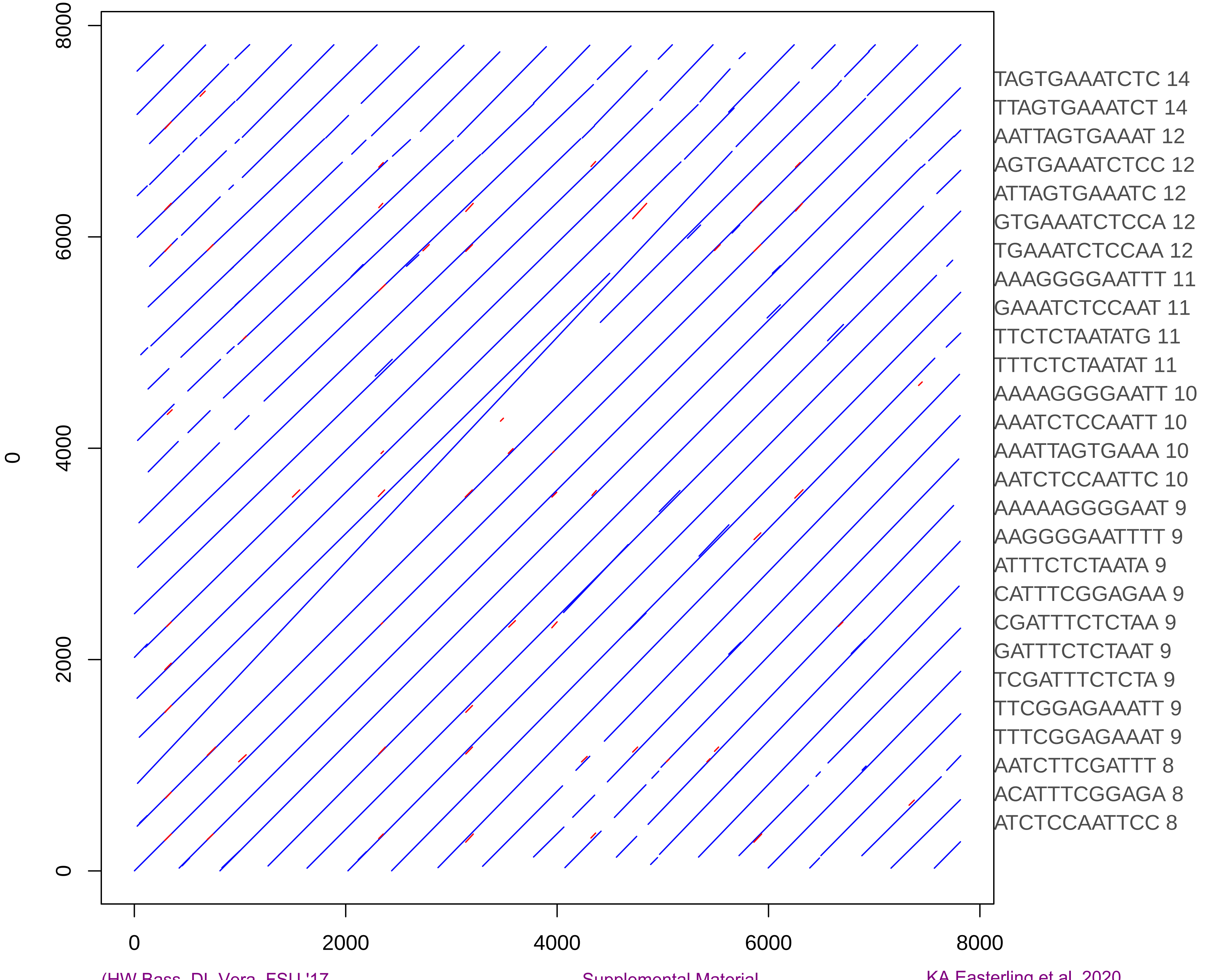

>m150106_022046_42134_c100761282550000001823158307221520_s1_p0/615/0_7653

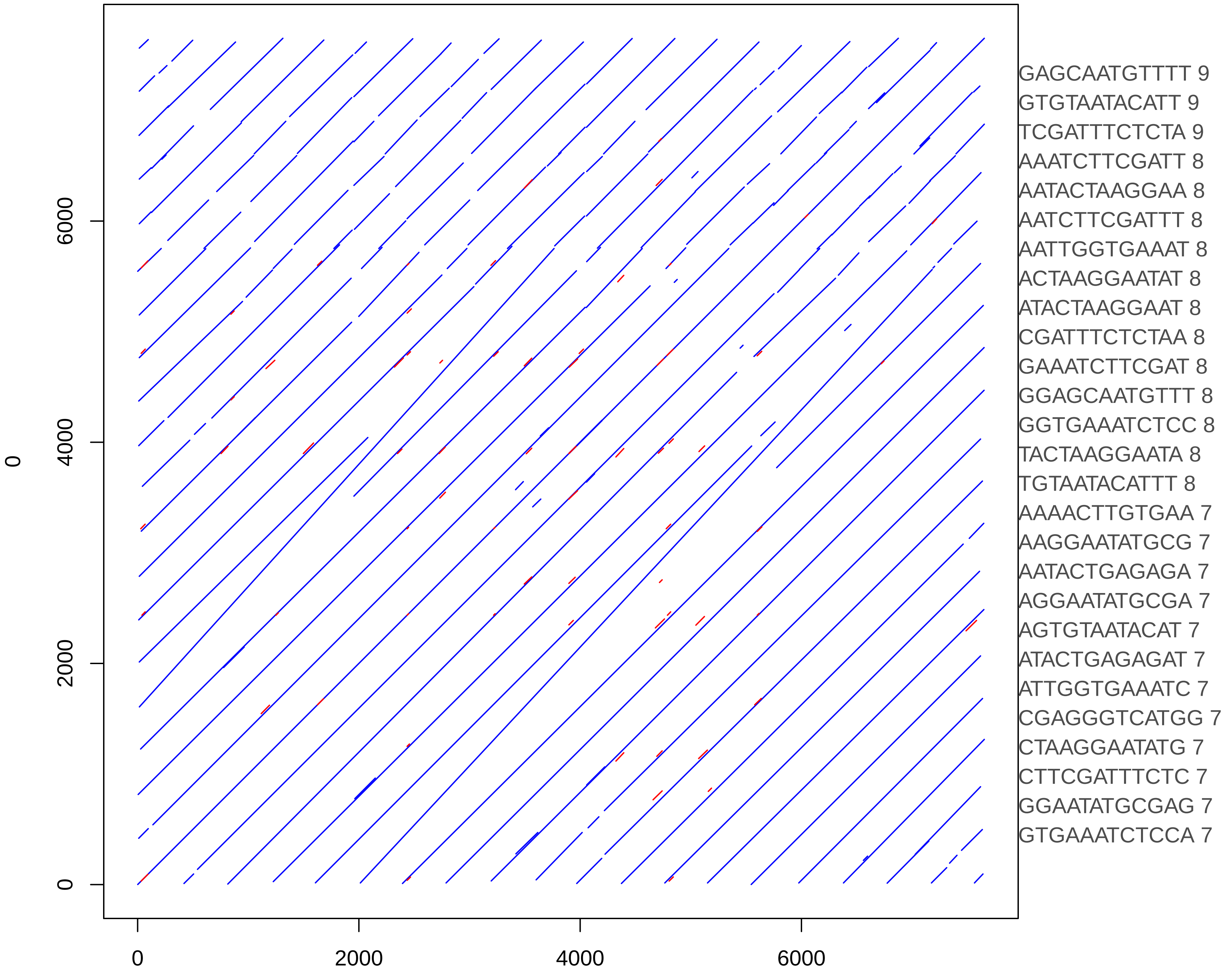

1,121 hits for repetitive sequences
passing 12mer abundance criterion

(Read-Number)
PAGE 1037

For Tandem Repeat families of ### bp:
PacBio Read named "HuluTR###-r1037"

>m150106_022046_42134_c100761282550000001823158307221520_s1_p0/6477/0_6149

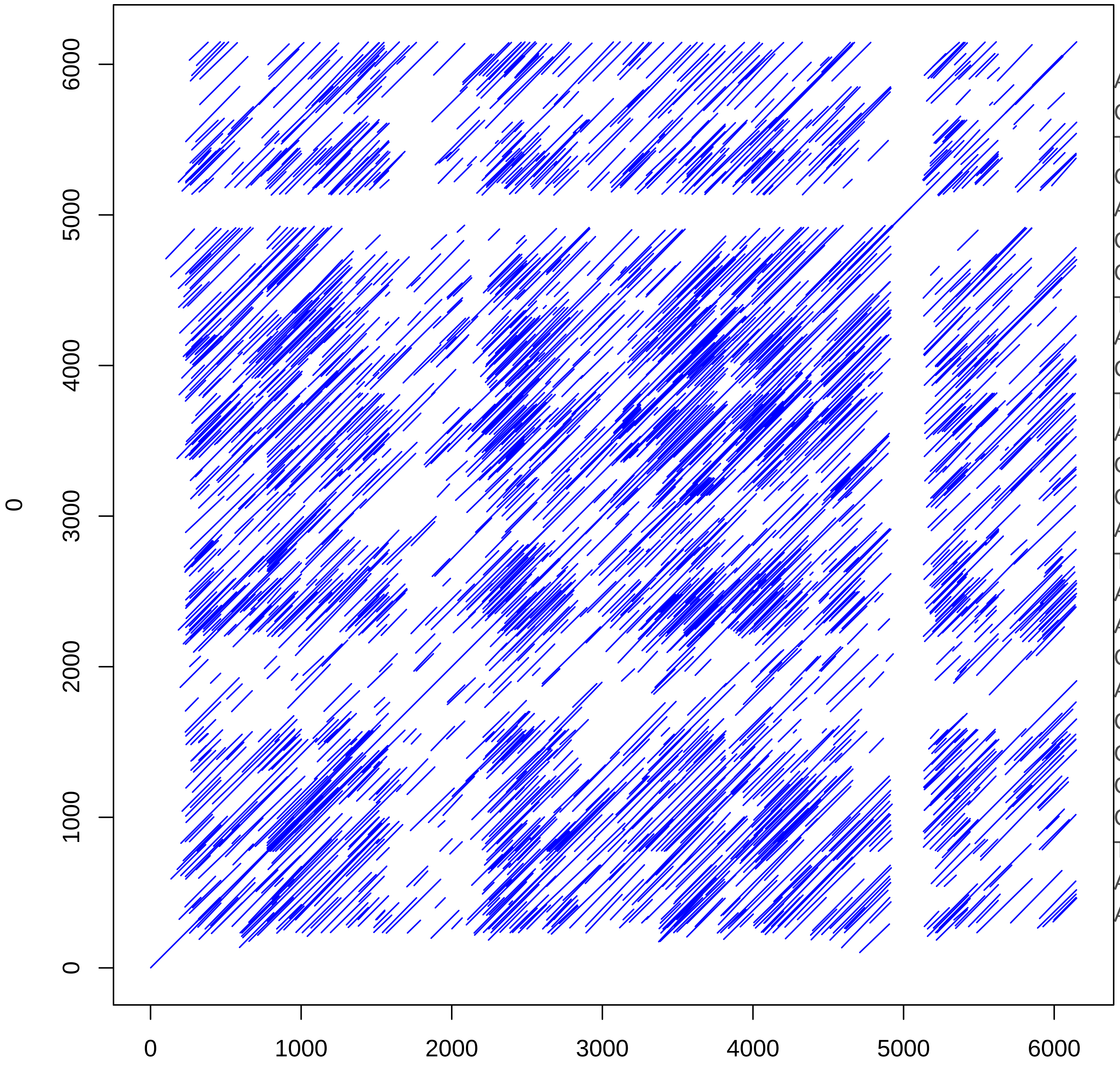

AGTAGTAGTAGT 36
GTAGTAGTAGTA 33
TAGTAGTAGTAG 32
CAGTAGCAGTAG 17
AGTAGCAGTAGC 15
GCAGTAGTAGTA 14
GTAGCAGTAGCA 14
TAGCAGTAGCAG 14
AGCAGTAGTAGT 13
CAGTAGTAGTAG 13
TAGCAGTAGTAG 13
AGTAGCAGTAGT 12
GCAGTAGCAGTA 12
GTAGCAGTAGTA 12
AGCAGTAGCAGT 11
TAGTAGCAGTAG 9
AGTAGTAGCAGT 8
AGTAGTAGTAGC 8
GTAGCAGCAGTA 8
AGTAGTAGCAGC 7
CAGTAGTAGCAG 7
GCAGTAGTAGCA 7
GTAGTAGCAGTA 7
GTAGTAGTAGTG 7
TAGCAGCAGTAG 7
AGCAGTAGCAGC 6
AGCAGTAGTAGC 6

(HW Bass, DL Vera, FSU '17
Hopsteiner PacBio Apollo reads input)

0

Supplemental Material
k-mer screen & YASS dot-plots

KA Easterling et al. 2020

>m150106_022046_42134_c100761282550000001823158307221520_s1_p0/67956/208_9075

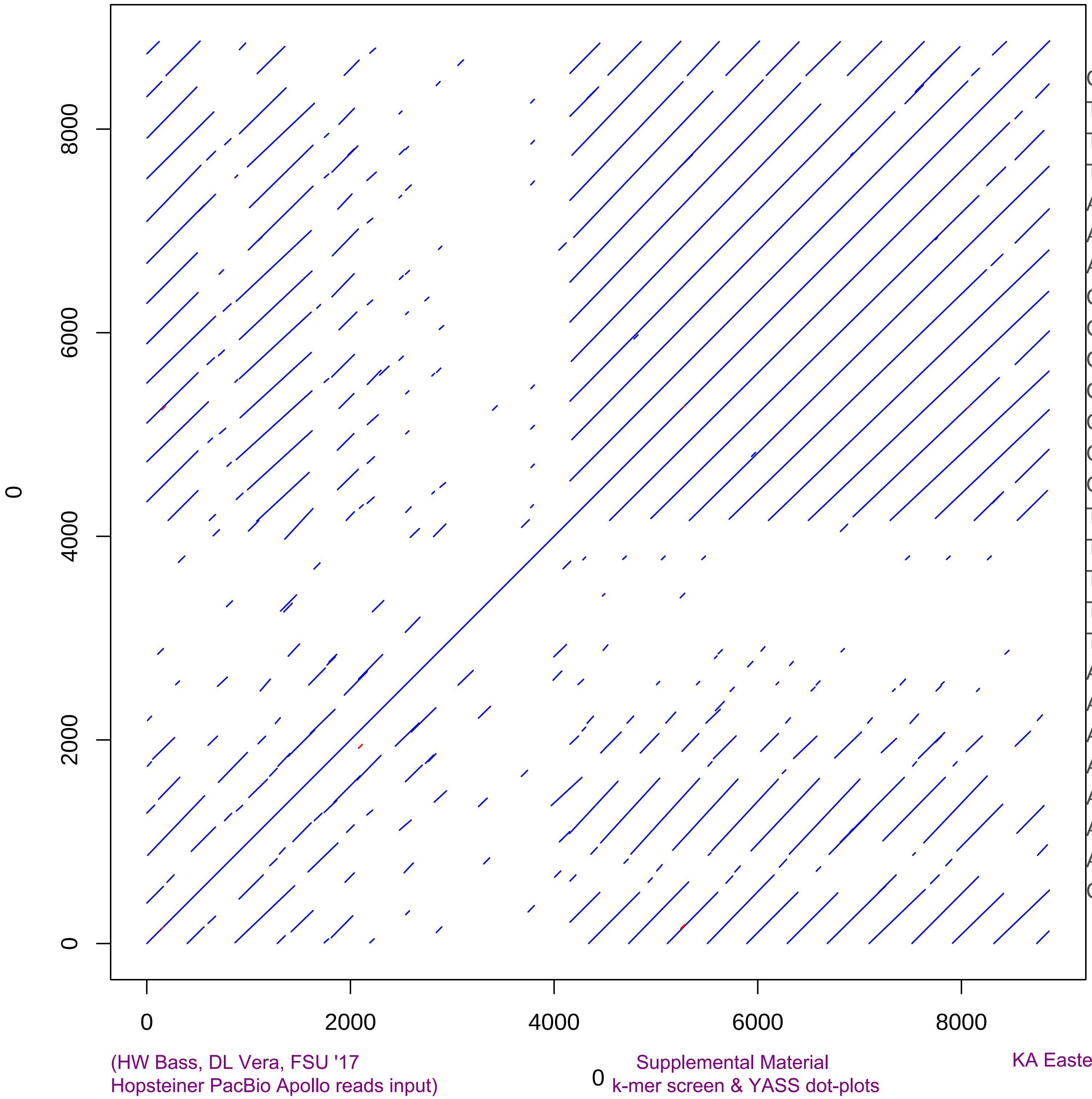

>m150106_022046_42134_c100761282550000001823158307221520_s1_p0/68507/1272_7307

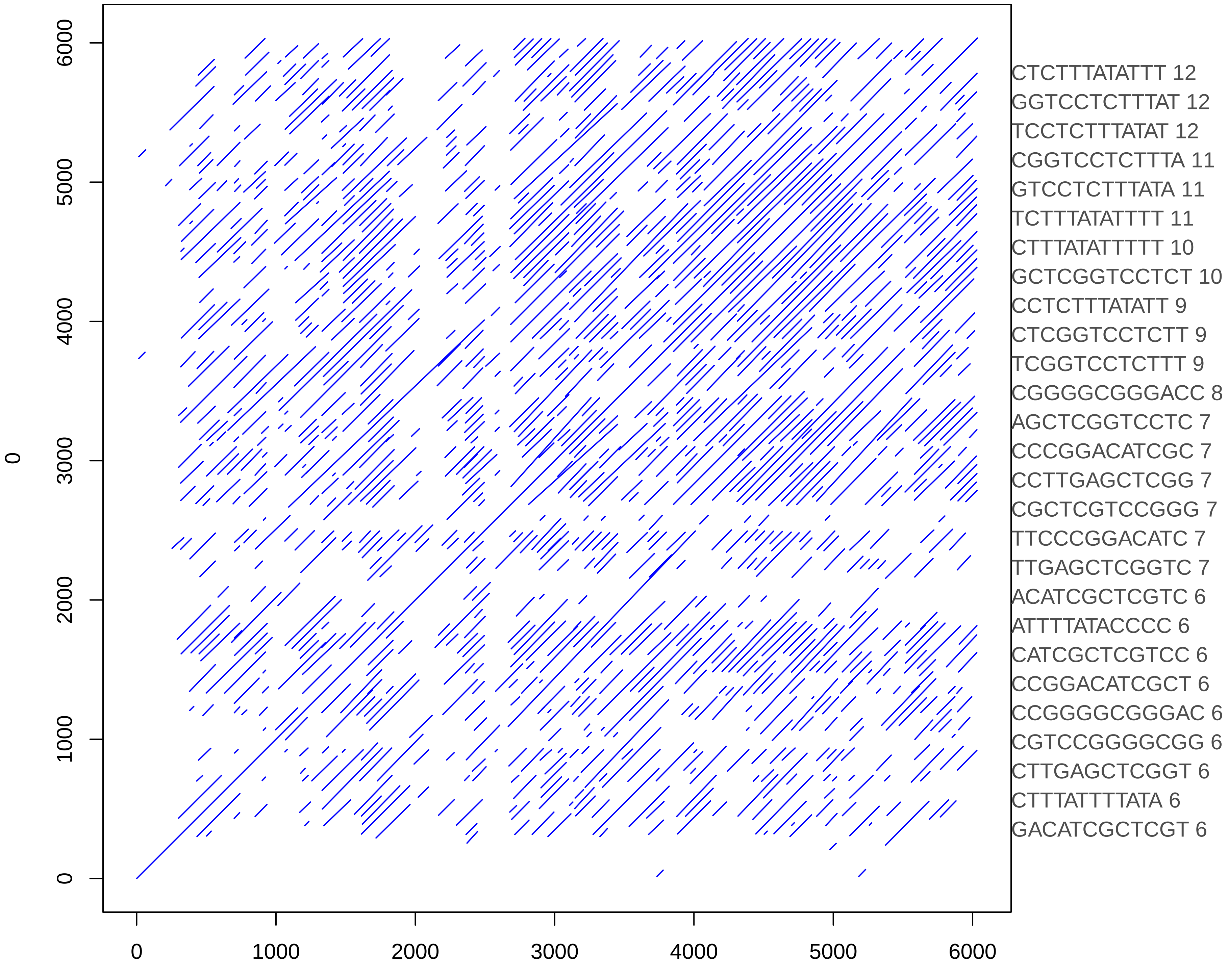

>m150106_022046_42134_c100761282550000001823158307221520_s1_p0/69276/0_5191

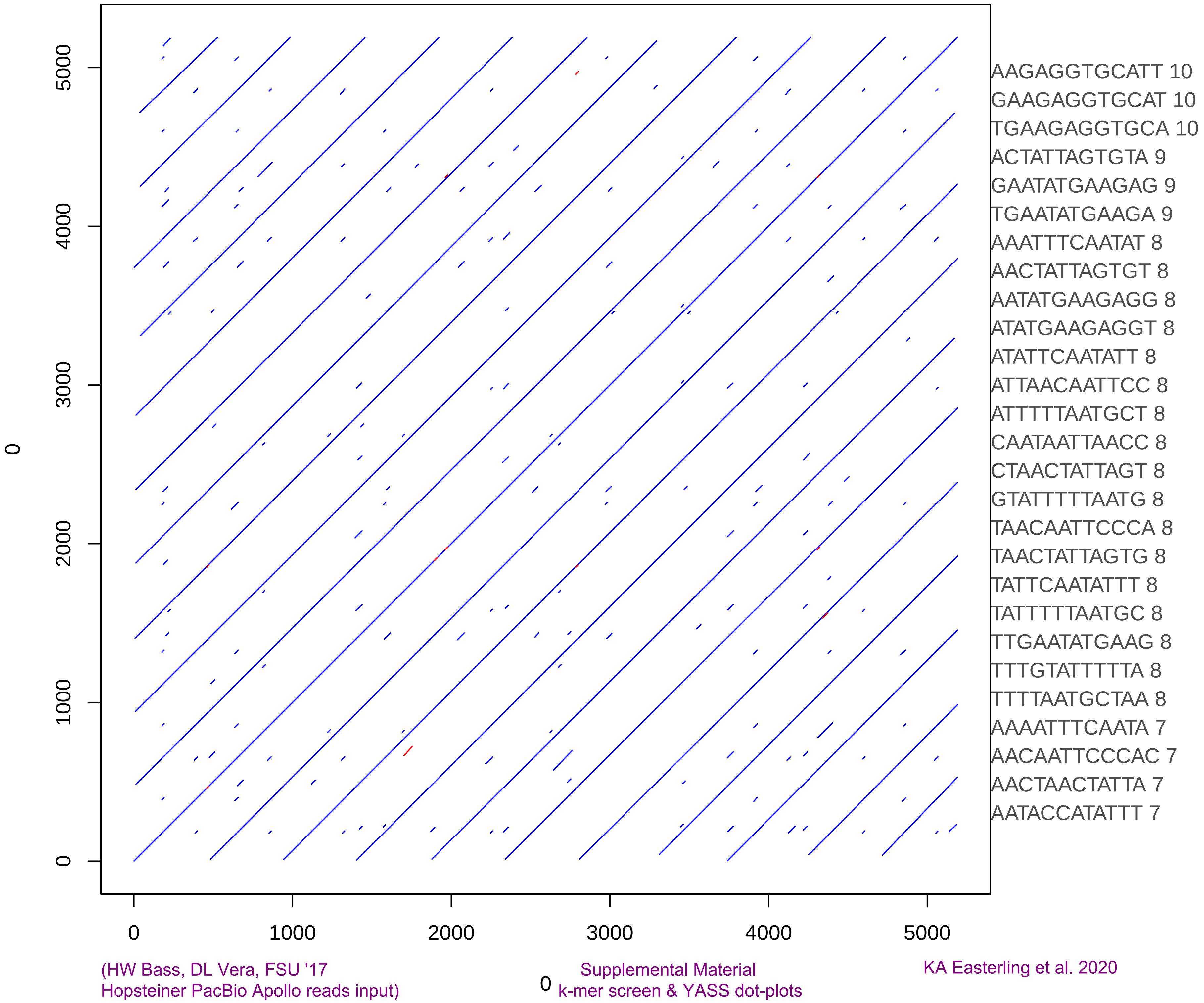

>m150106_022046_42134_c100761282550000001823158307221520_s1_p0/72142/0_9196

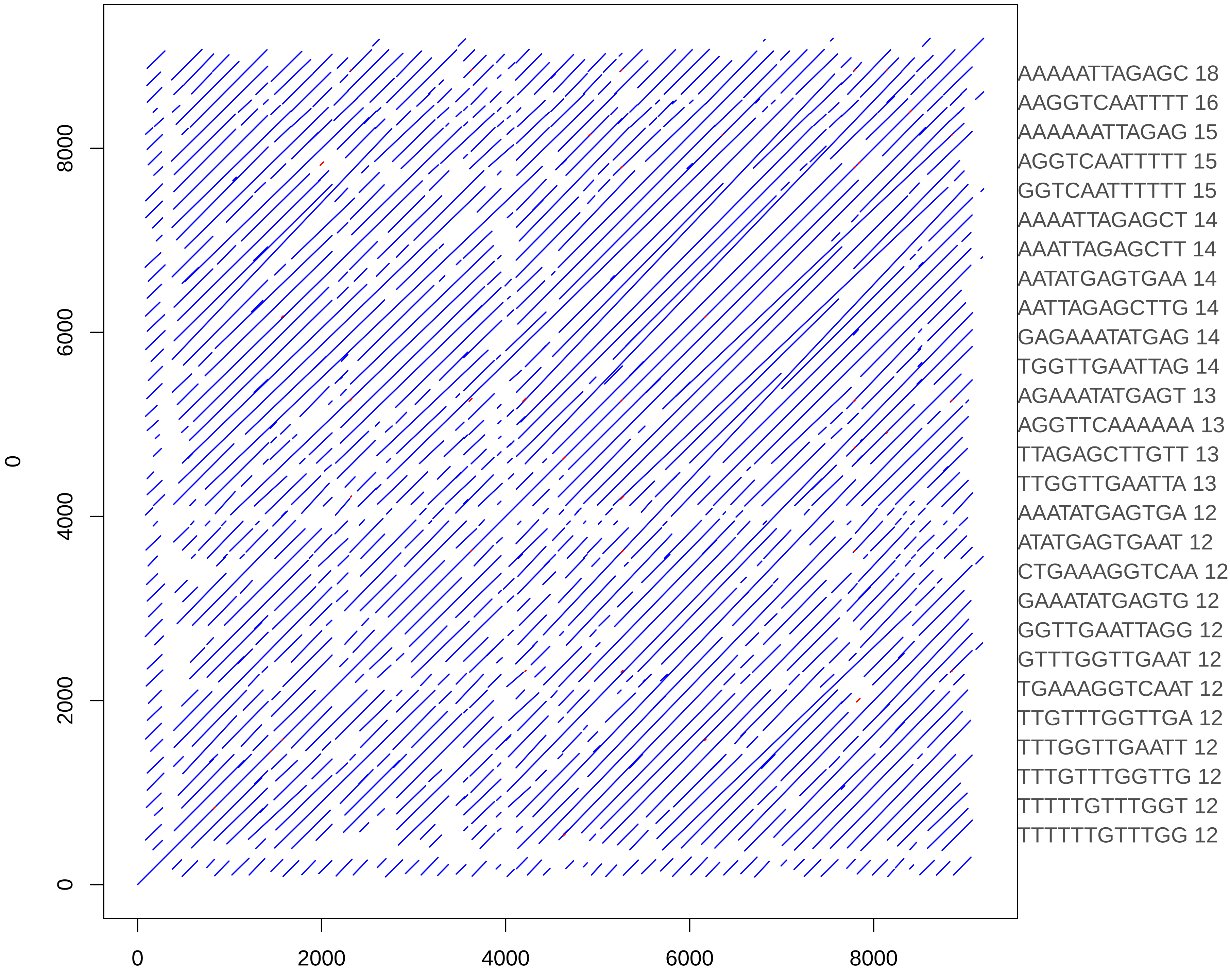

0

1,121 hits for repetitive sequences
passing 12mer abundance criterion

For Tandem Repeat families of ### bp:
PacBio Read named "HuluTR###-r1042"

>m150106_022046_42134_c100761282550000001823158307221520_s1_p0/73126/0_7035

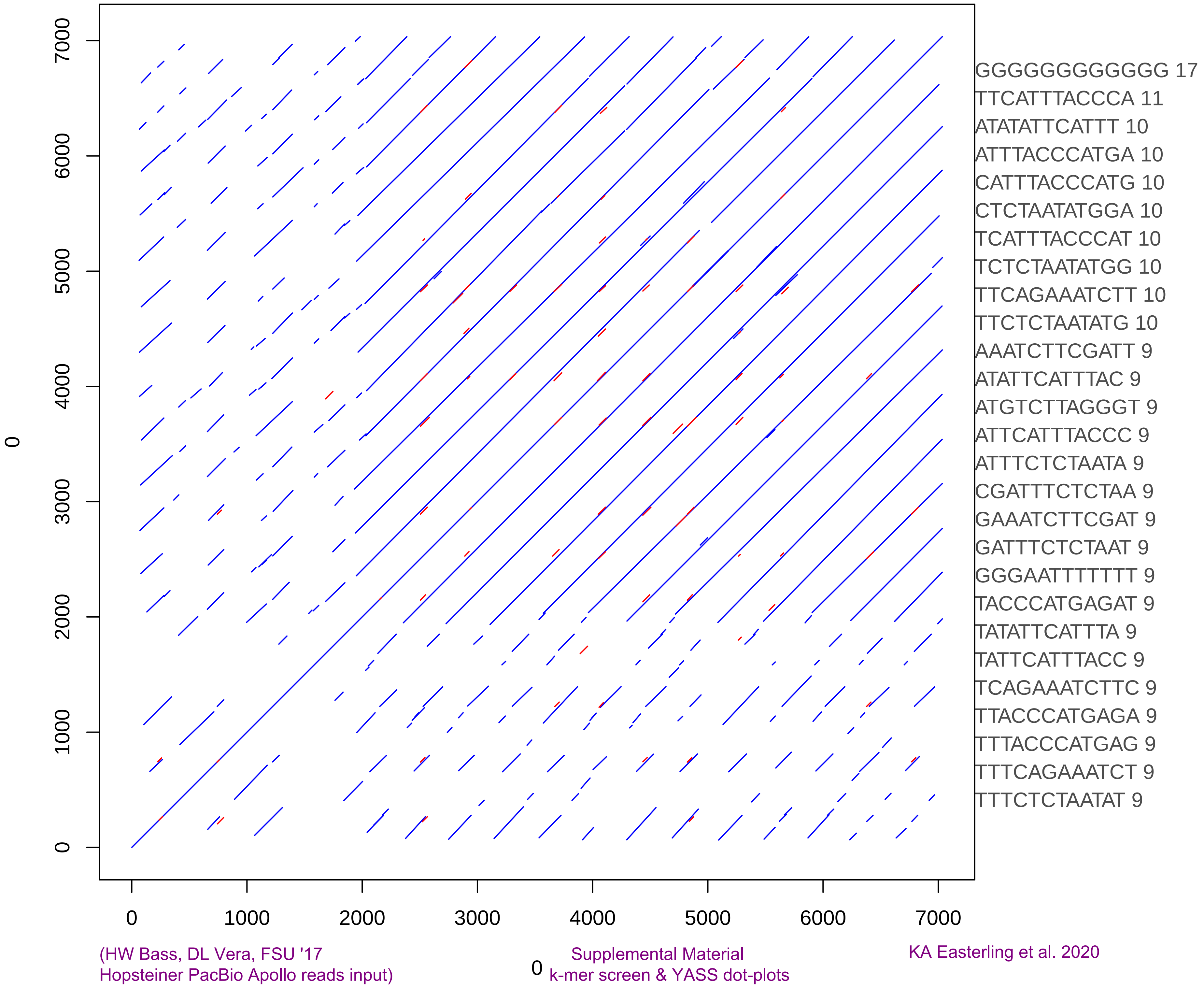

(HW Bass, DL Vera, FSU '17
Hopsteiner PacBio Apollo reads input)

0

Supplemental Material
k-mer screen & YASS dot-plots

KA Easterling et al. 2020

>m150106_022046_42134_c100761282550000001823158307221520_s1_p0/74159/4149_10929

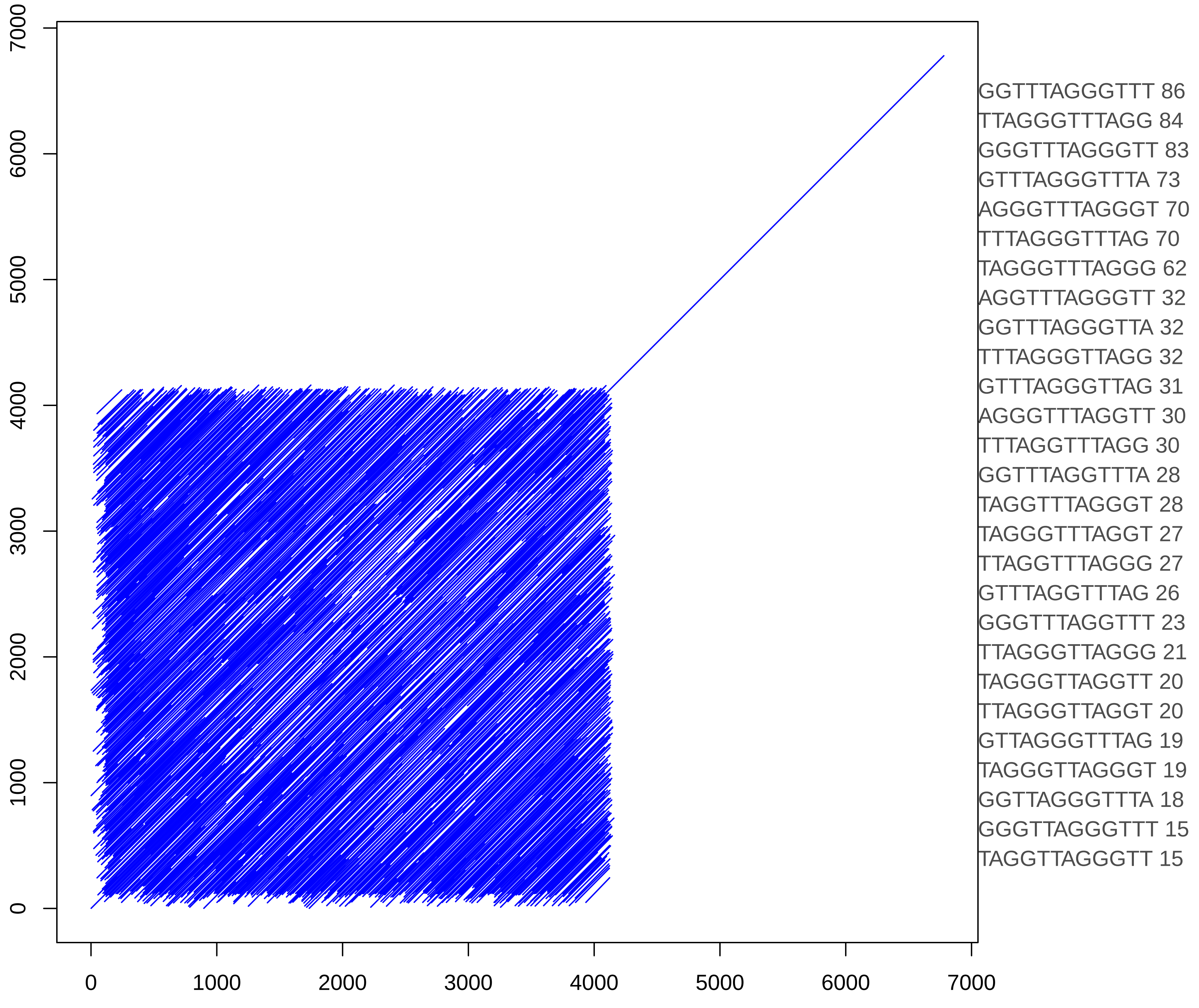

>m150106_022046_42134_c100761282550000001823158307221520_s1_p0/7564/404_7008

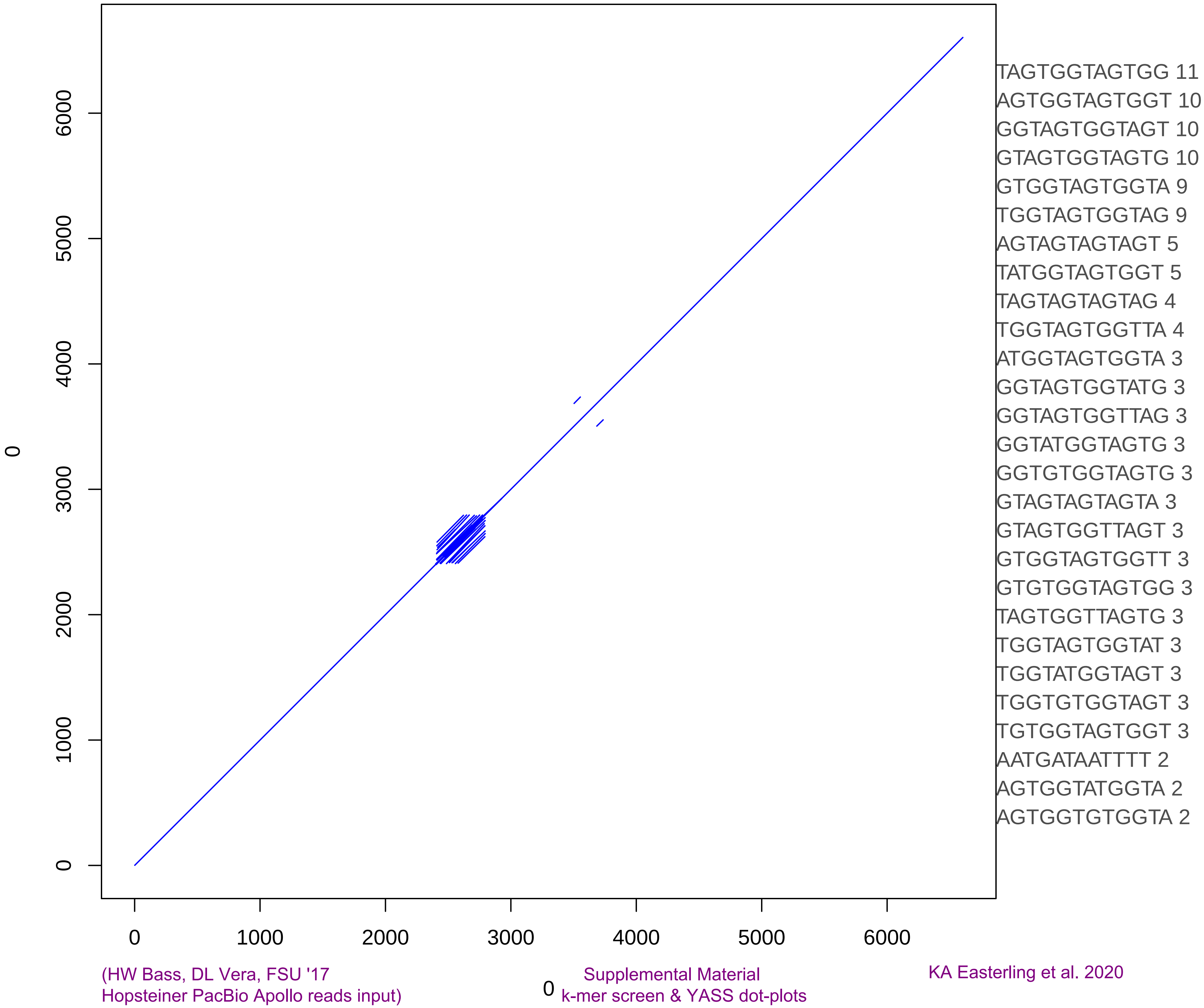

>m150106_022046_42134_c100761282550000001823158307221520_s1_p0/77130/169_9843

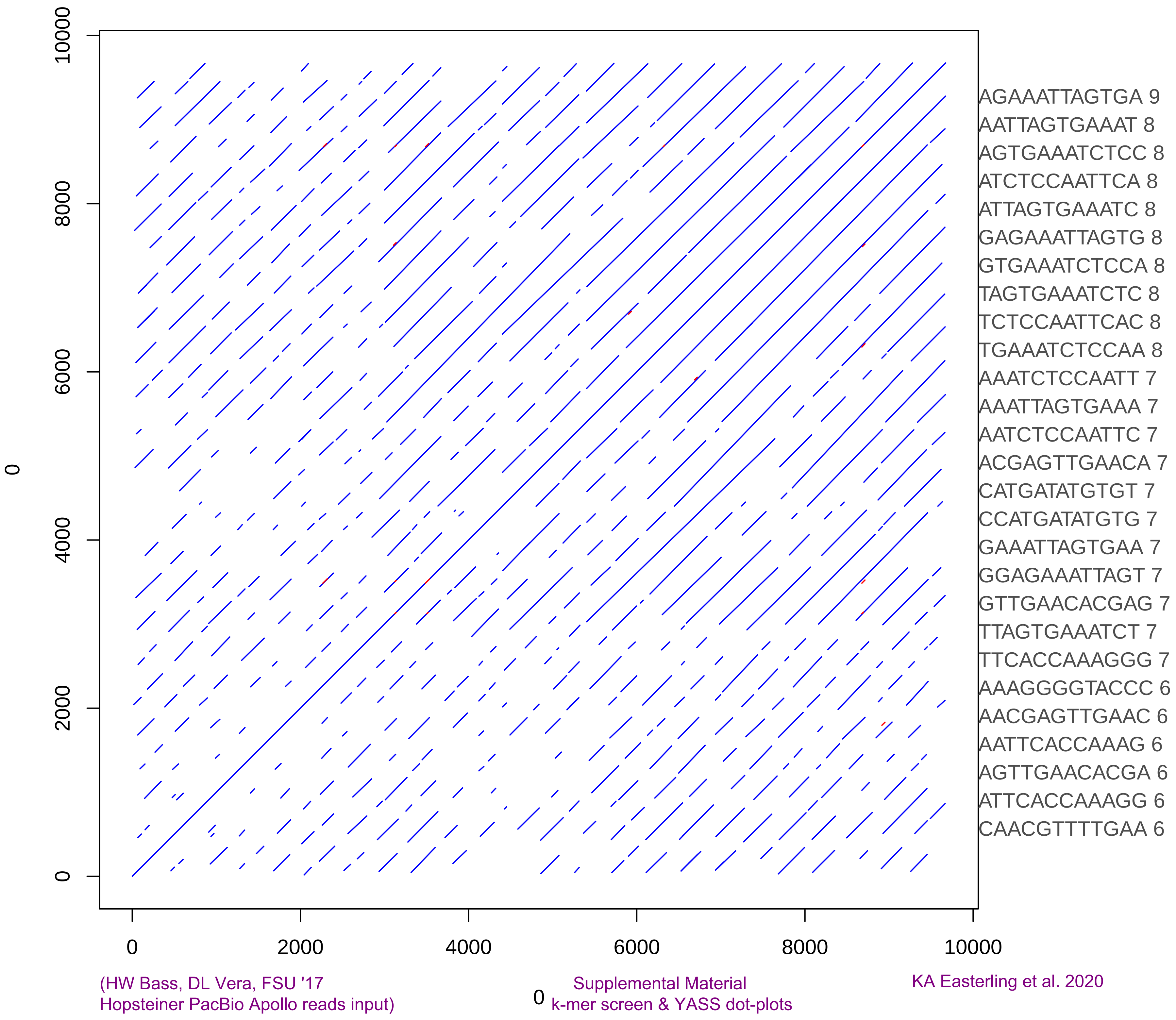

>m150106_022046_42134_c100761282550000001823158307221520_s1_p0/80118/504_12510

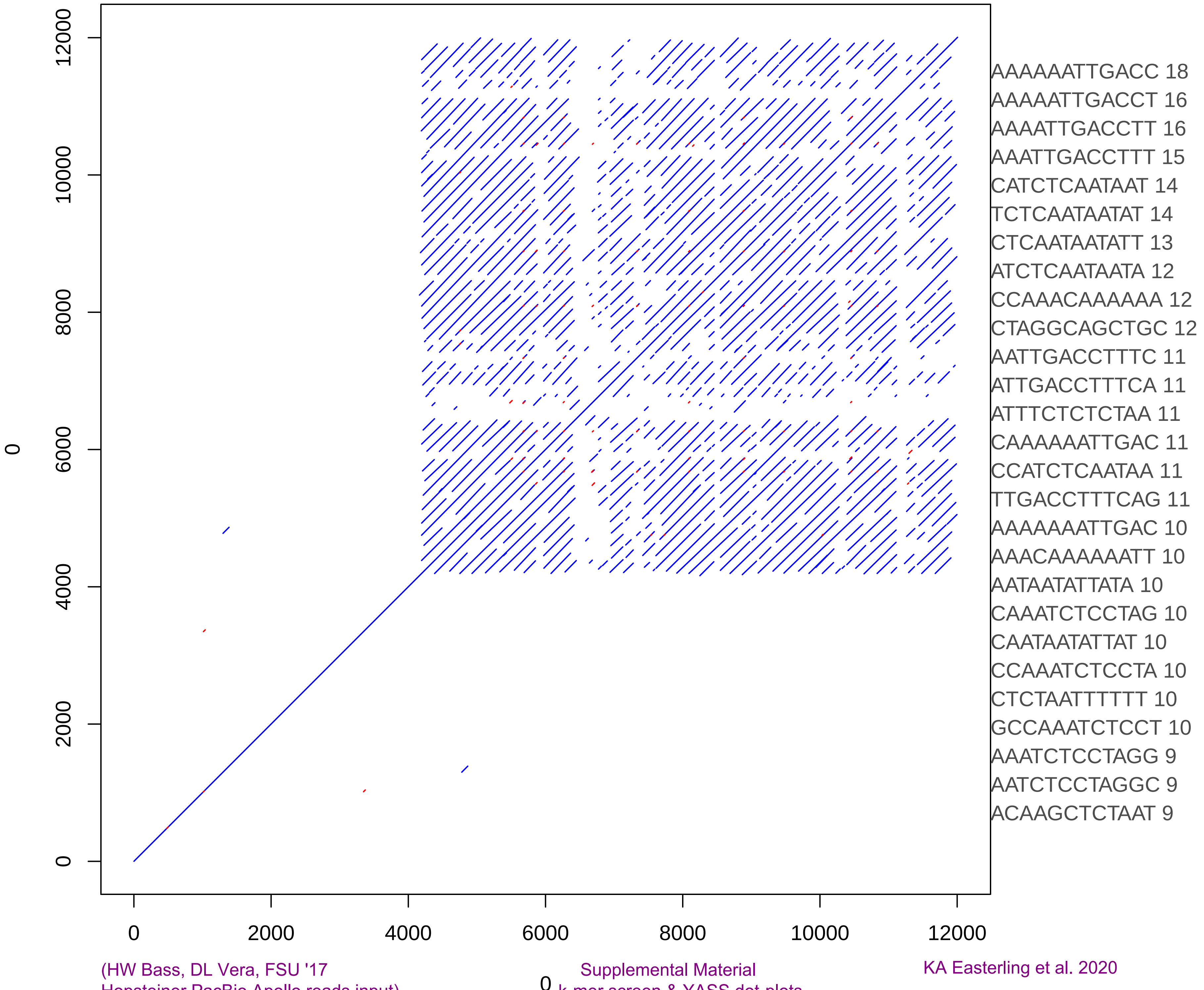

>m150106_022046_42134_c100761282550000001823158307221520_s1_p0/81807/399_5989

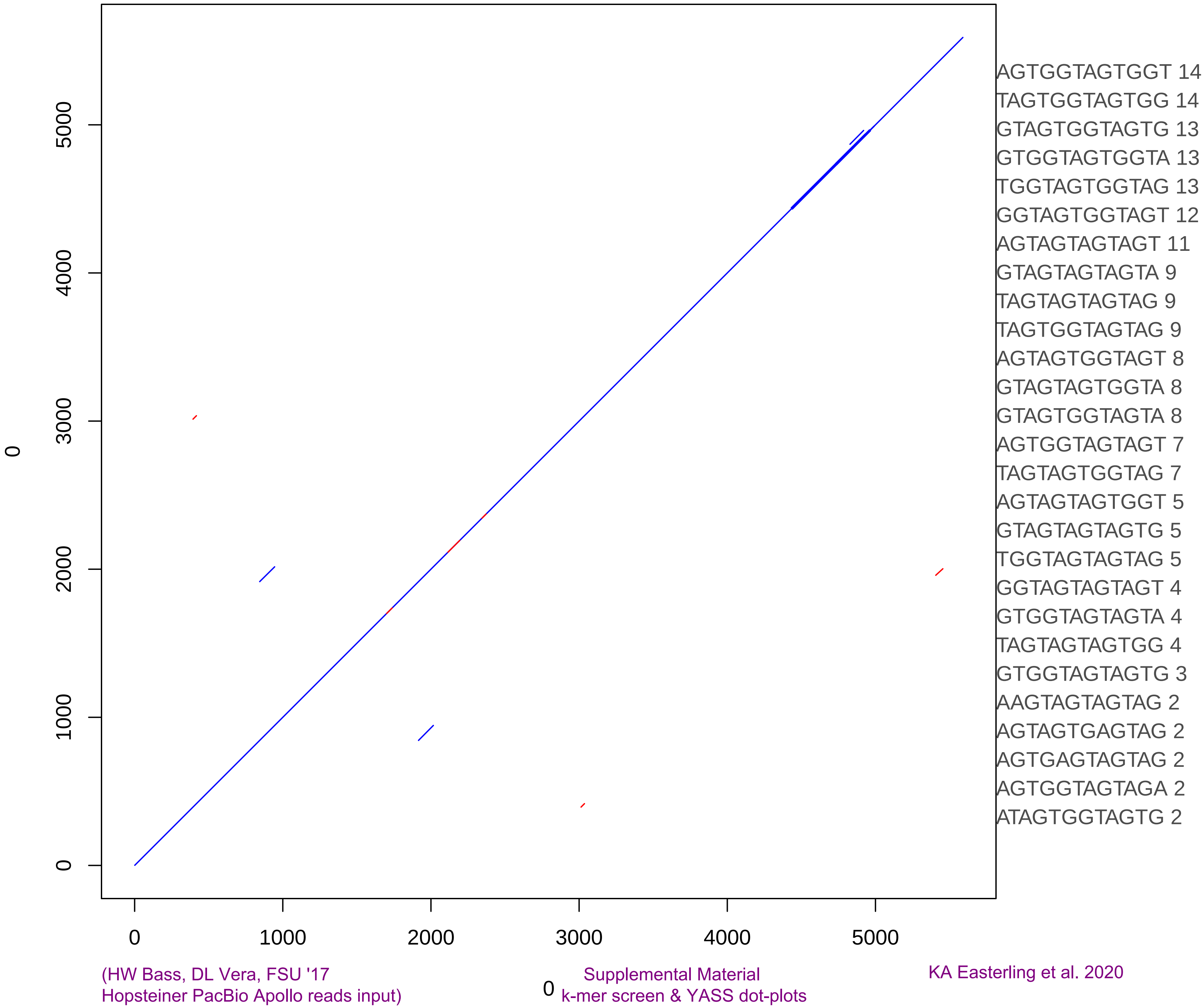

>m150106_022046_42134_c100761282550000001823158307221520_s1_p0/82041/307_11730

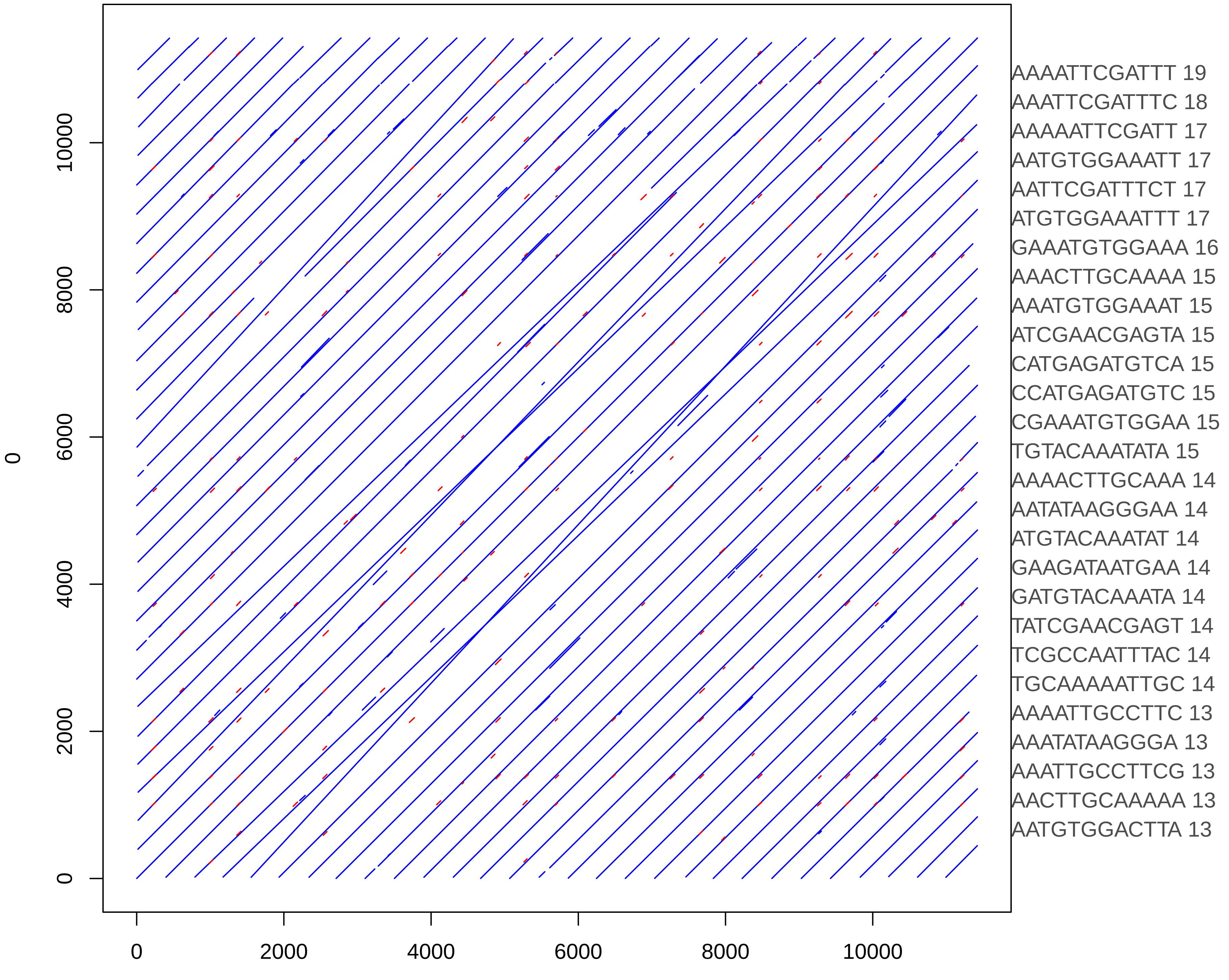

>m150106_022046_42134_c100761282550000001823158307221520_s1_p0/8360/0_6344

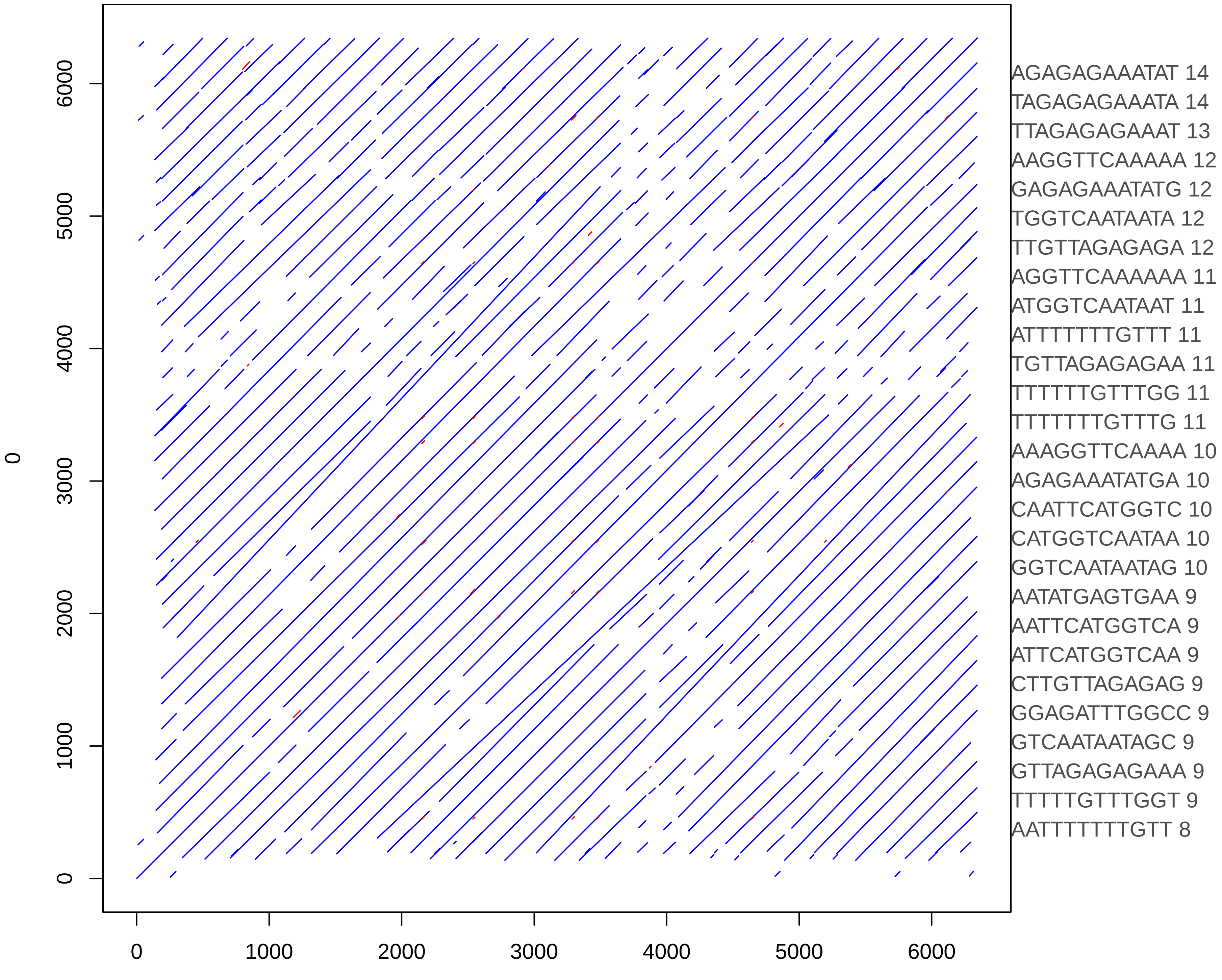

>m150106_022046_42134_c100761282550000001823158307221520_s1_p0/85611/0_5479

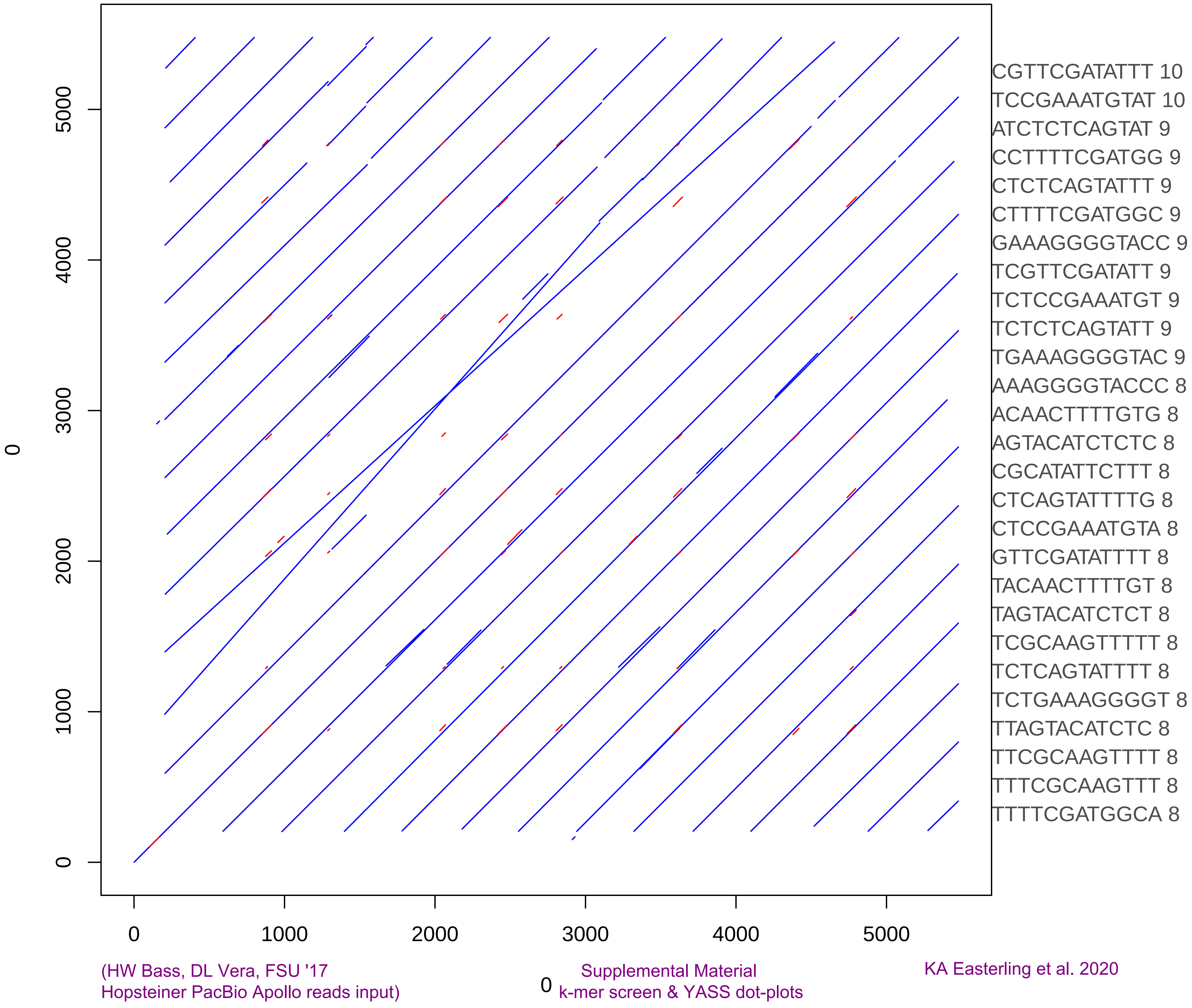

>m150106_022046_42134_c100761282550000001823158307221520_s1_p0/86900/5321_10649

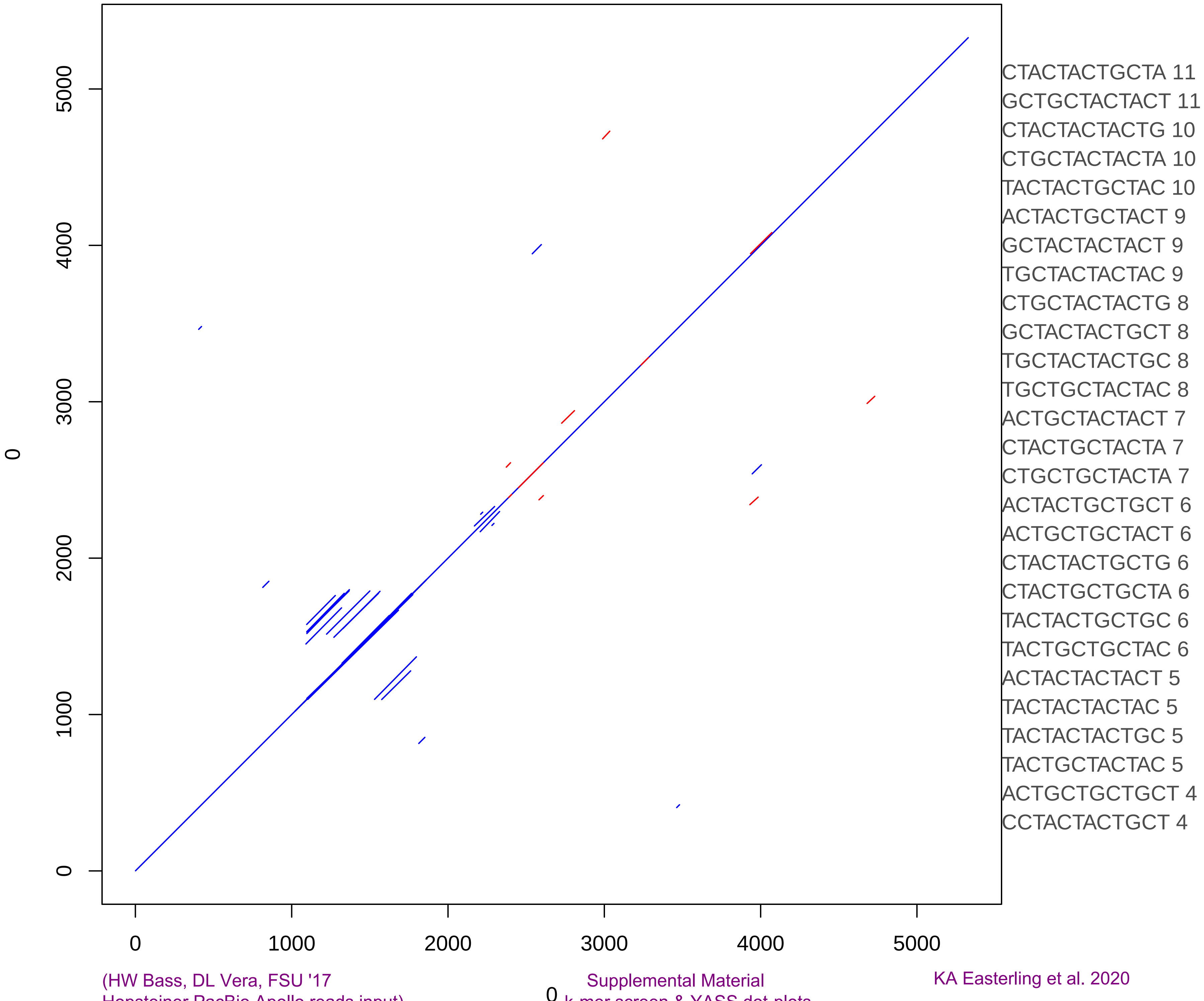

>m150106_022046_42134_c100761282550000001823158307221520_s1_p0/8910/0_5859

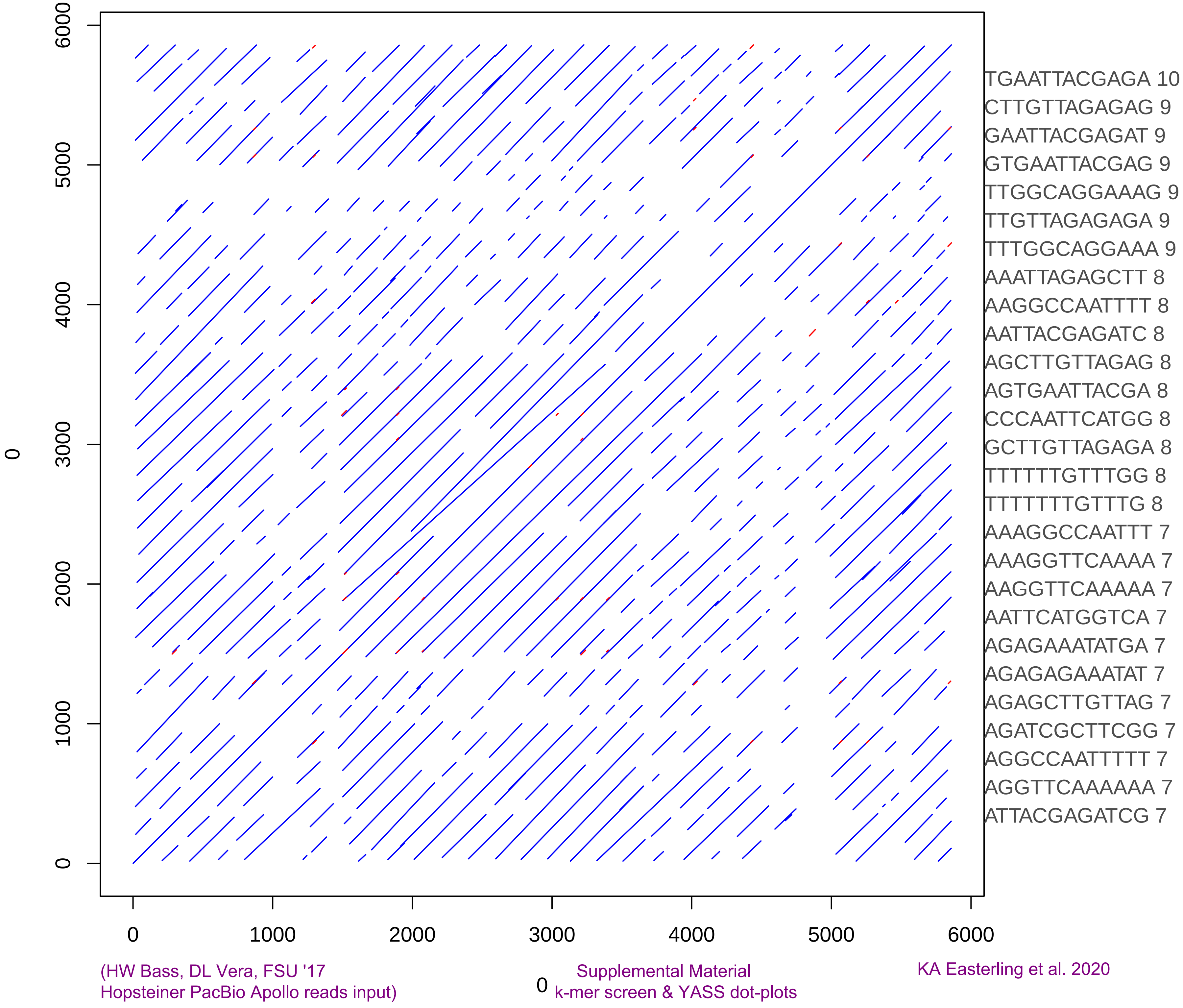

>m150106_022046_42134_c100761282550000001823158307221520_s1_p0/89672/293_6627

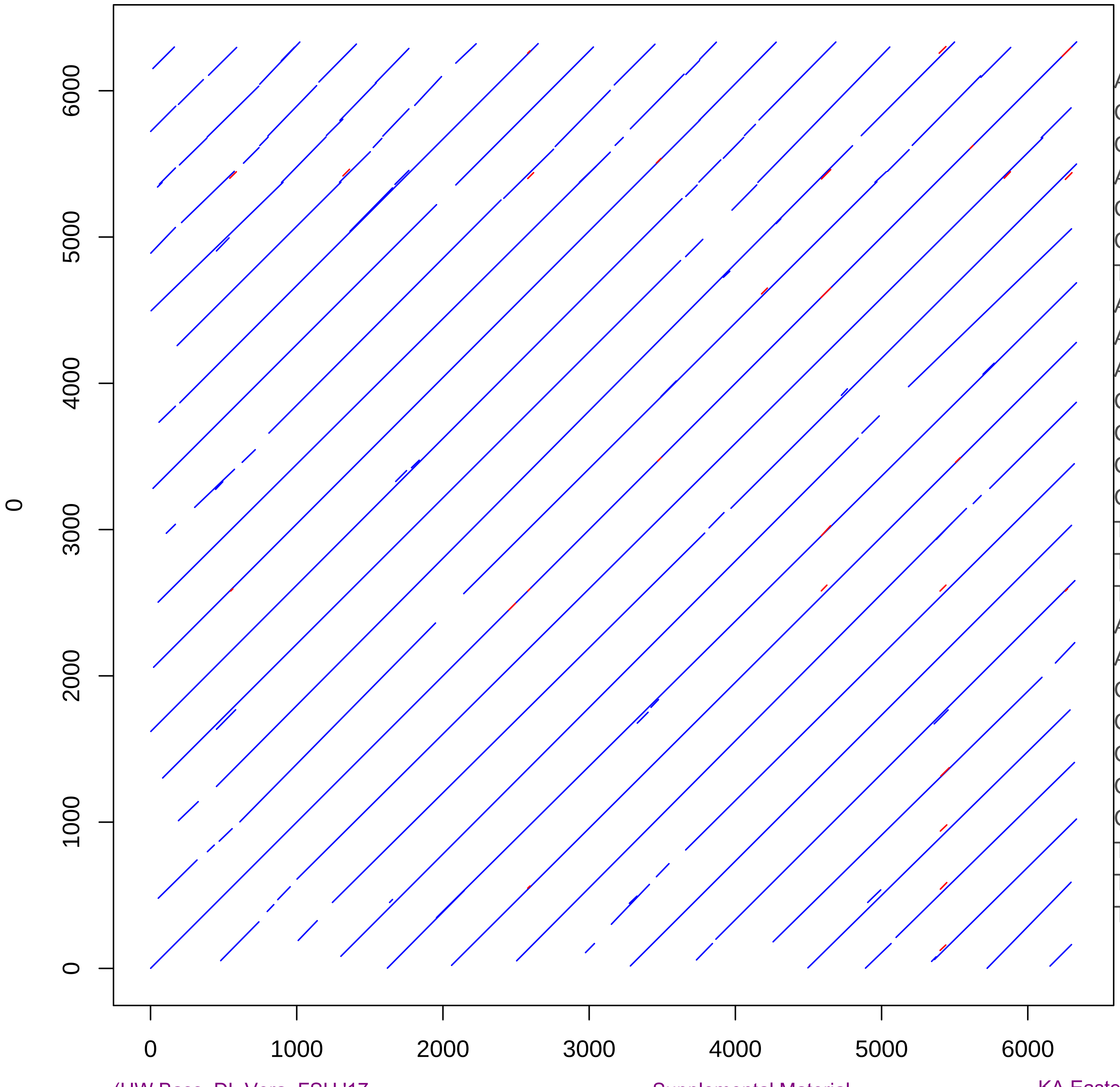

>m150106_022046_42134_c100761282550000001823158307221520_s1_p0/90089/798_6760

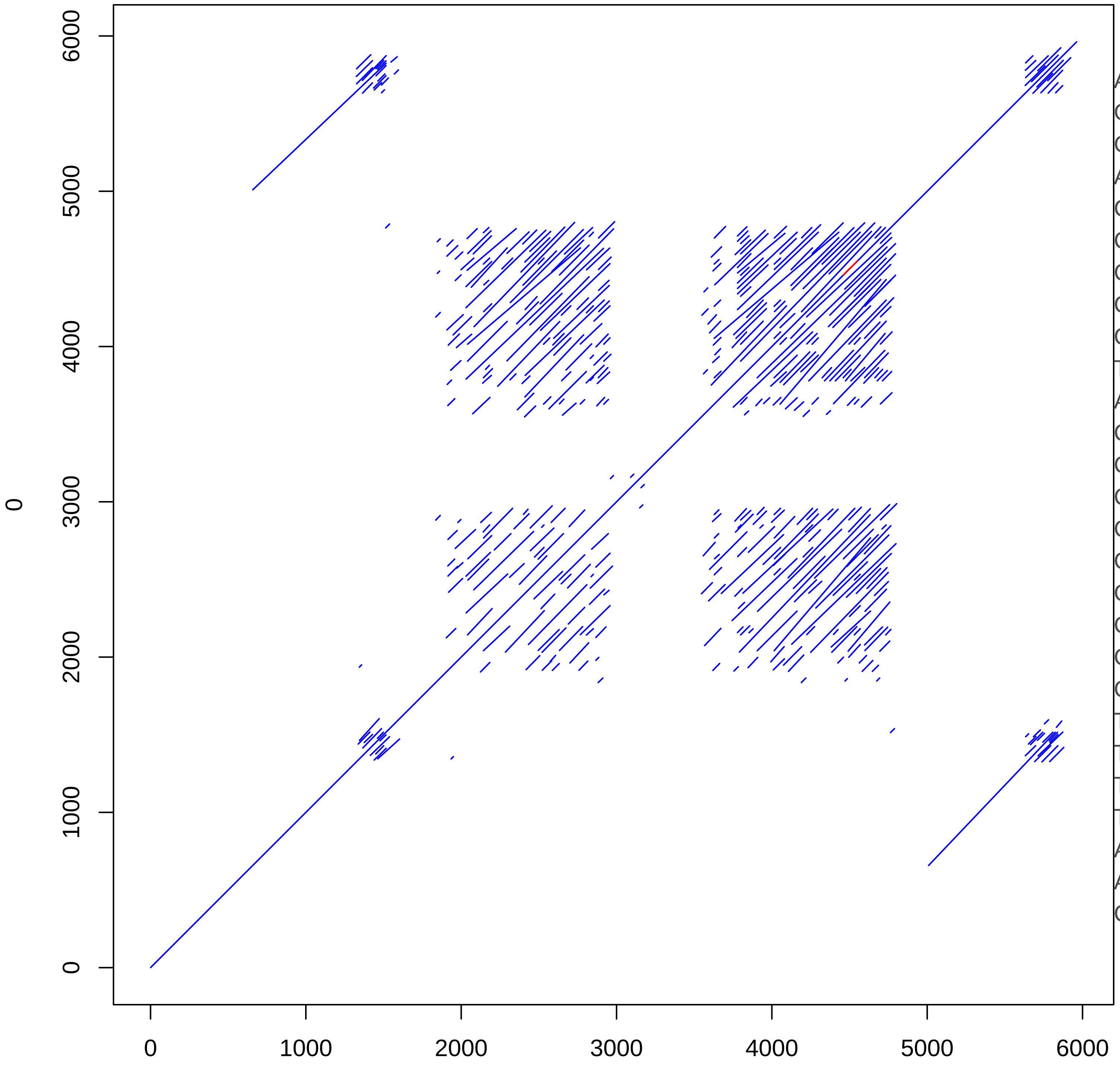

>m150106_022046_42134_c100761282550000001823158307221520_s1_p0/93585/6903_14786

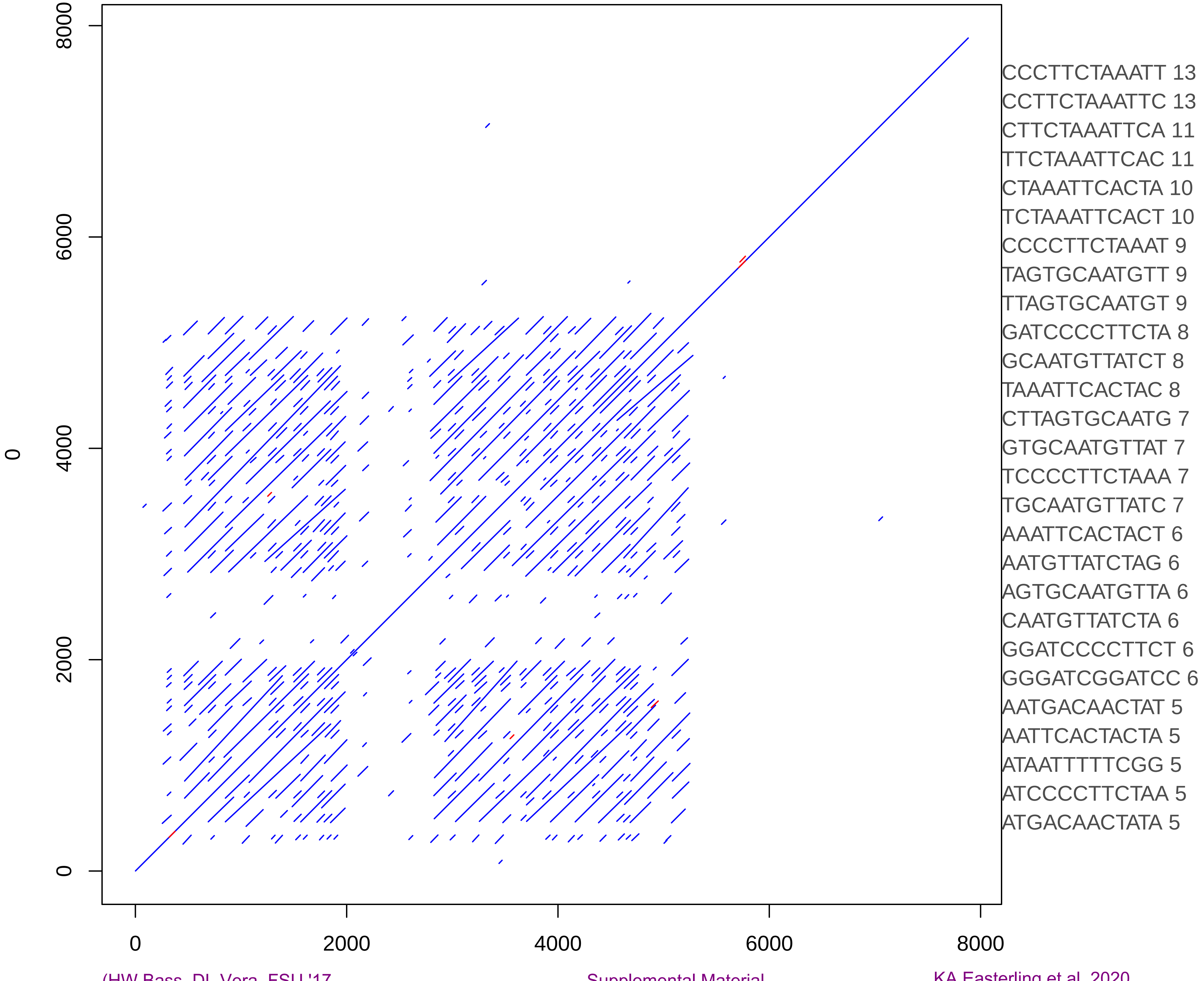

1,121 hits for repetitive sequences passing 12mer abundance criterion

For Tandem Repeat families of ### bp:
PacBio Read named "HuluTR###-r1056"

>m150106_022046_42134_c100761282550000001823158307221520_s1_p0/96259/304_9144

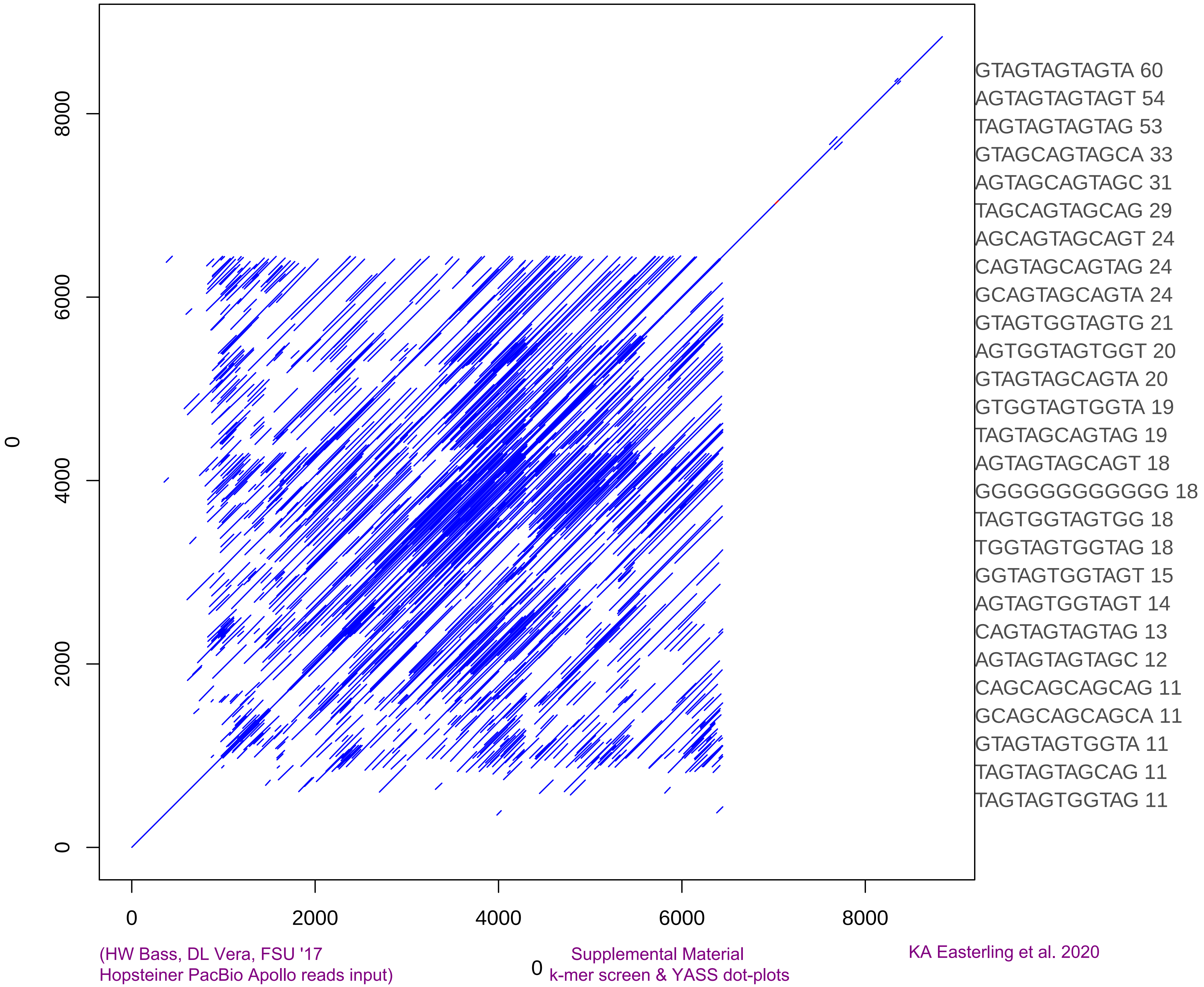

(HW Bass, DL Vera, FSU '17
Hopsteiner PacBio Apollo reads input)

Supplemental Material
k-mer screen & YASS dot-plots

KA Easterling et al. 2020

>m150106_022046_42134_c100761282550000001823158307221520_s1_p0/99569/826_8458

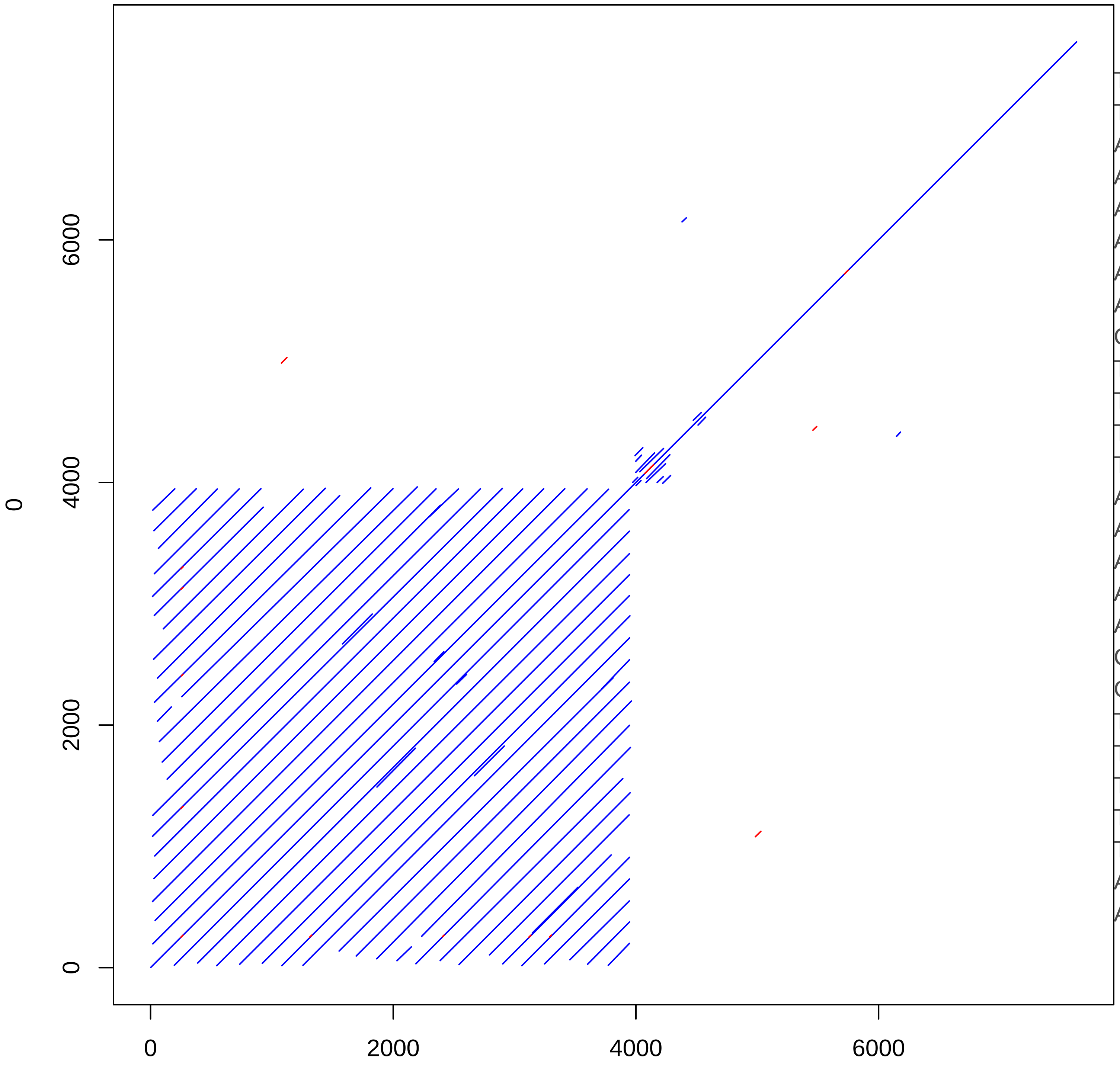

>m150106_022046_42134_c100761282550000001823158307221520_s1_p0/99785/382_5755

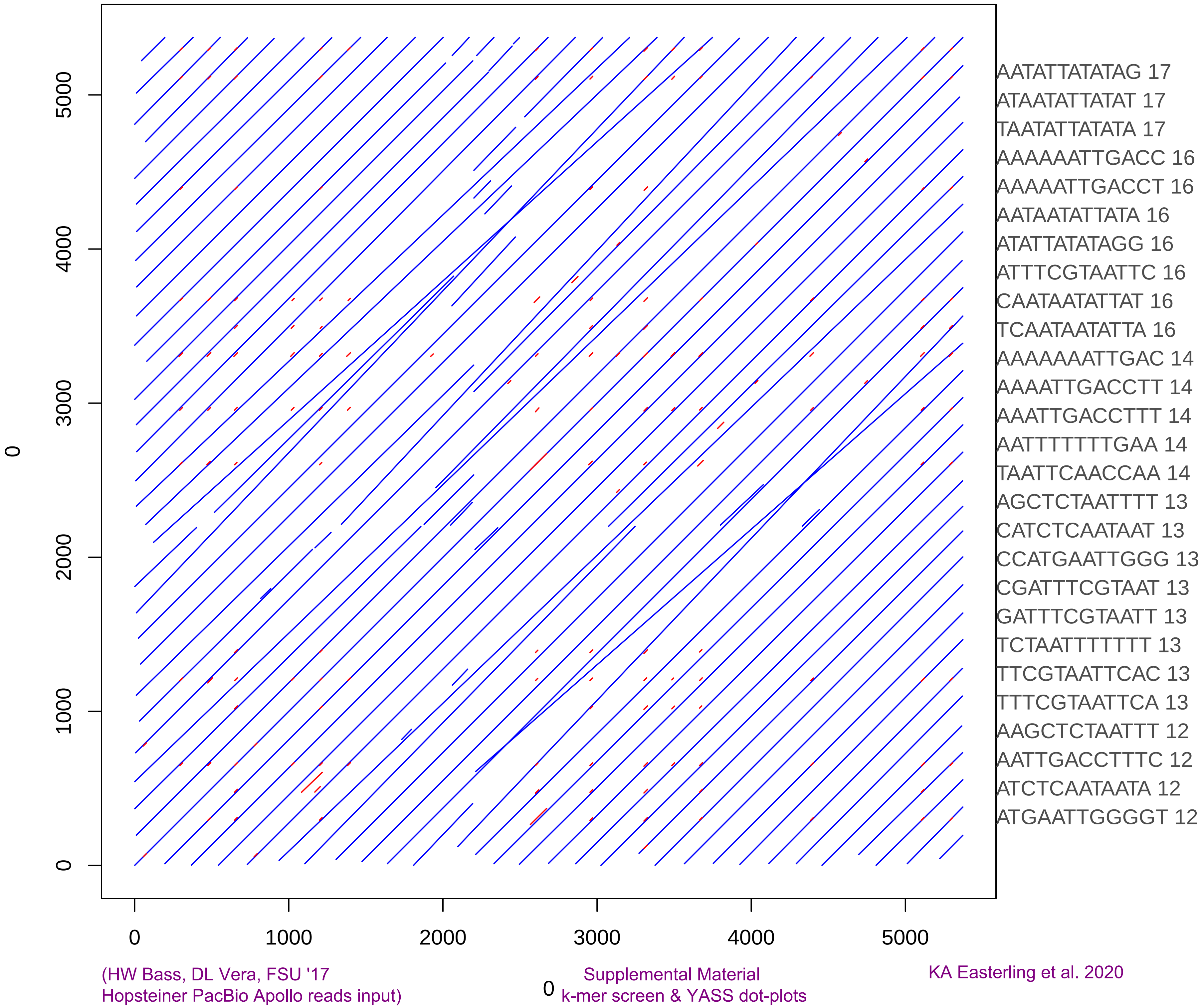

>m150106_044313_42134_c100761282550000001823158307221521_s1_p0/100527/0_6934

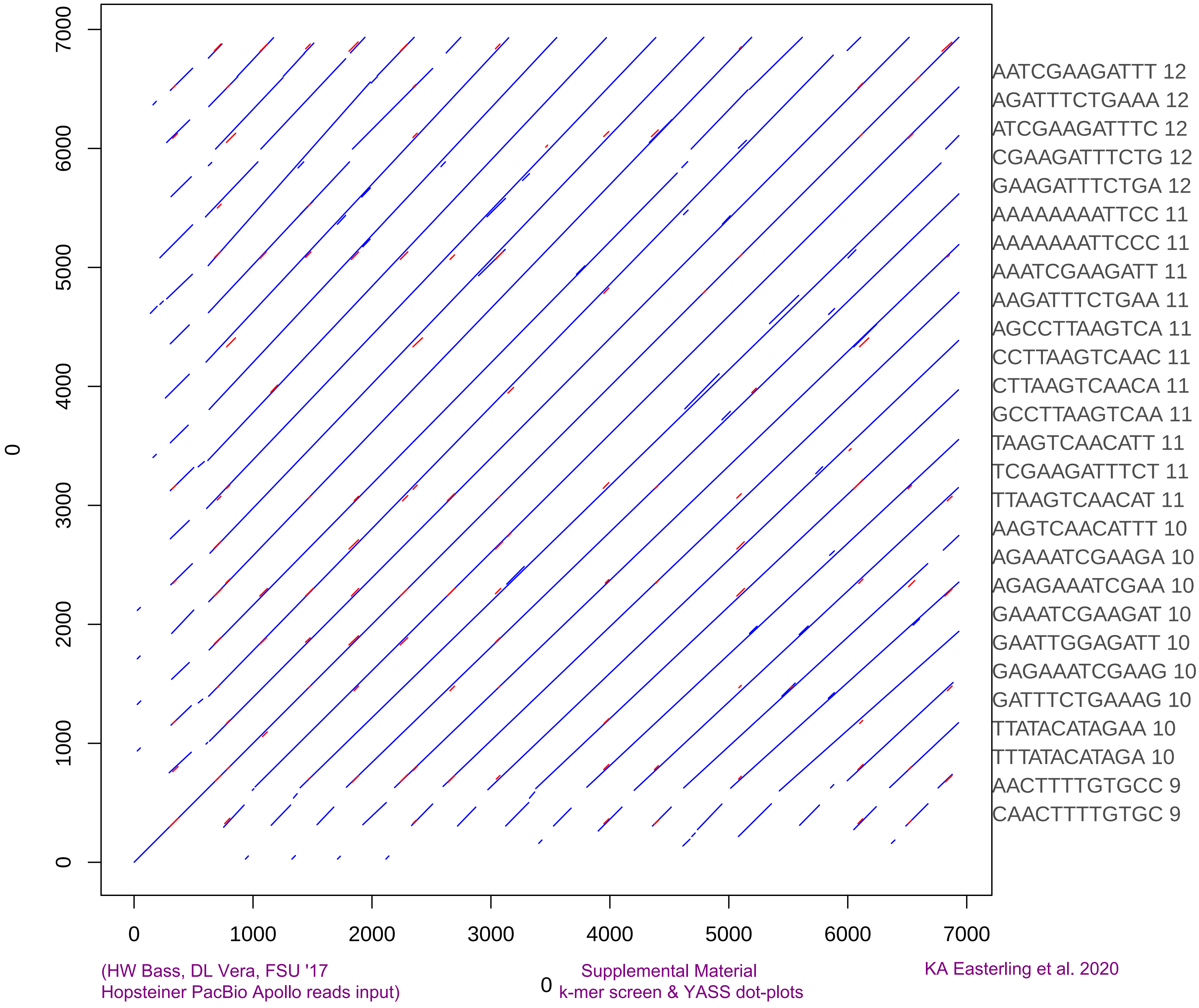

>m150106_044313_42134_c100761282550000001823158307221521_s1_p0/102123/0_7249

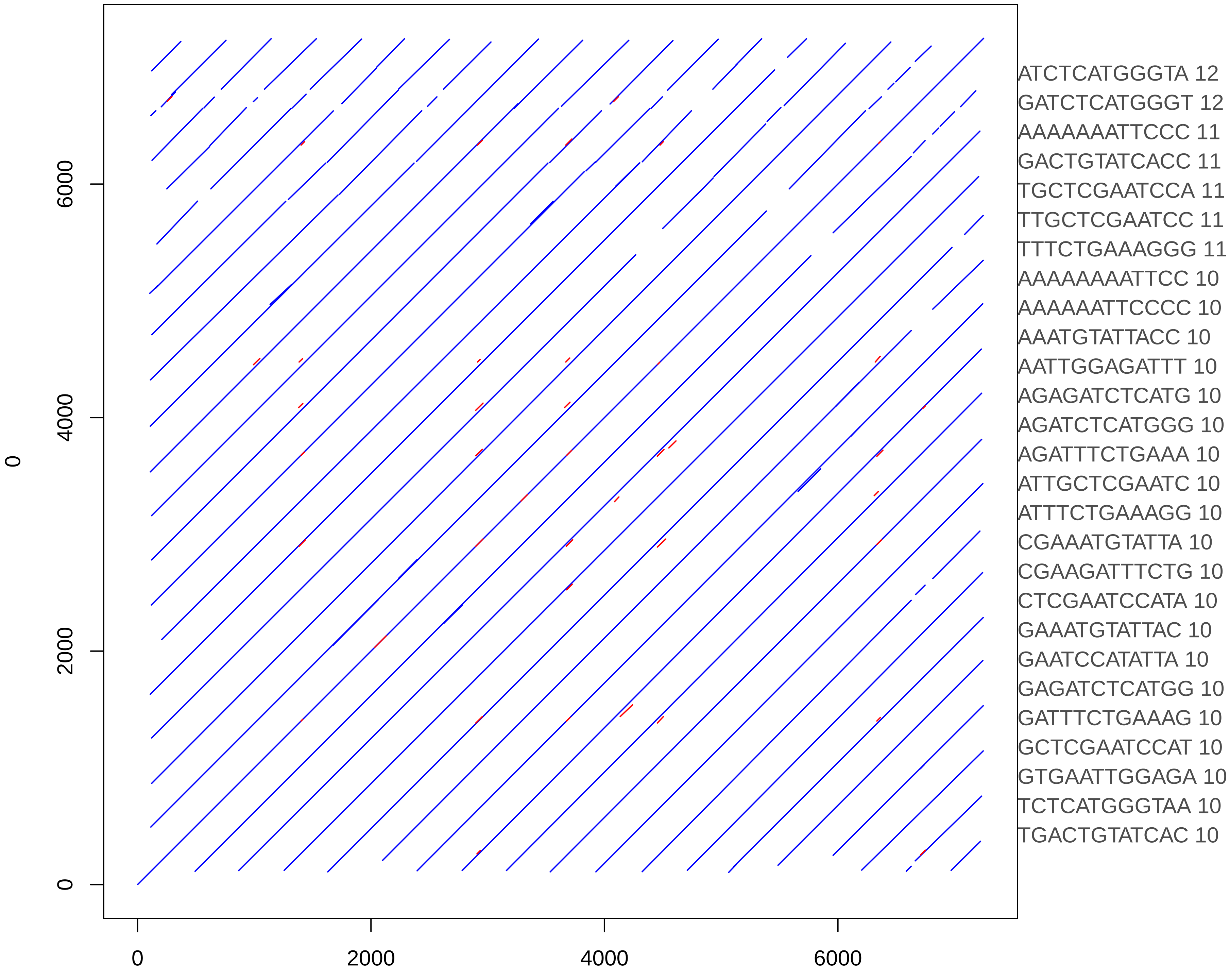

1,121 hits for repetitive sequences
passing 12mer abundance criterion

(Read-Number)
PAGE 1061

For Tandem Repeat families of ### bp:
PacBio Read named "HuluTR###-r1061"

>m150106_044313_42134_c100761282550000001823158307221521_s1_p0/105195/295_5992

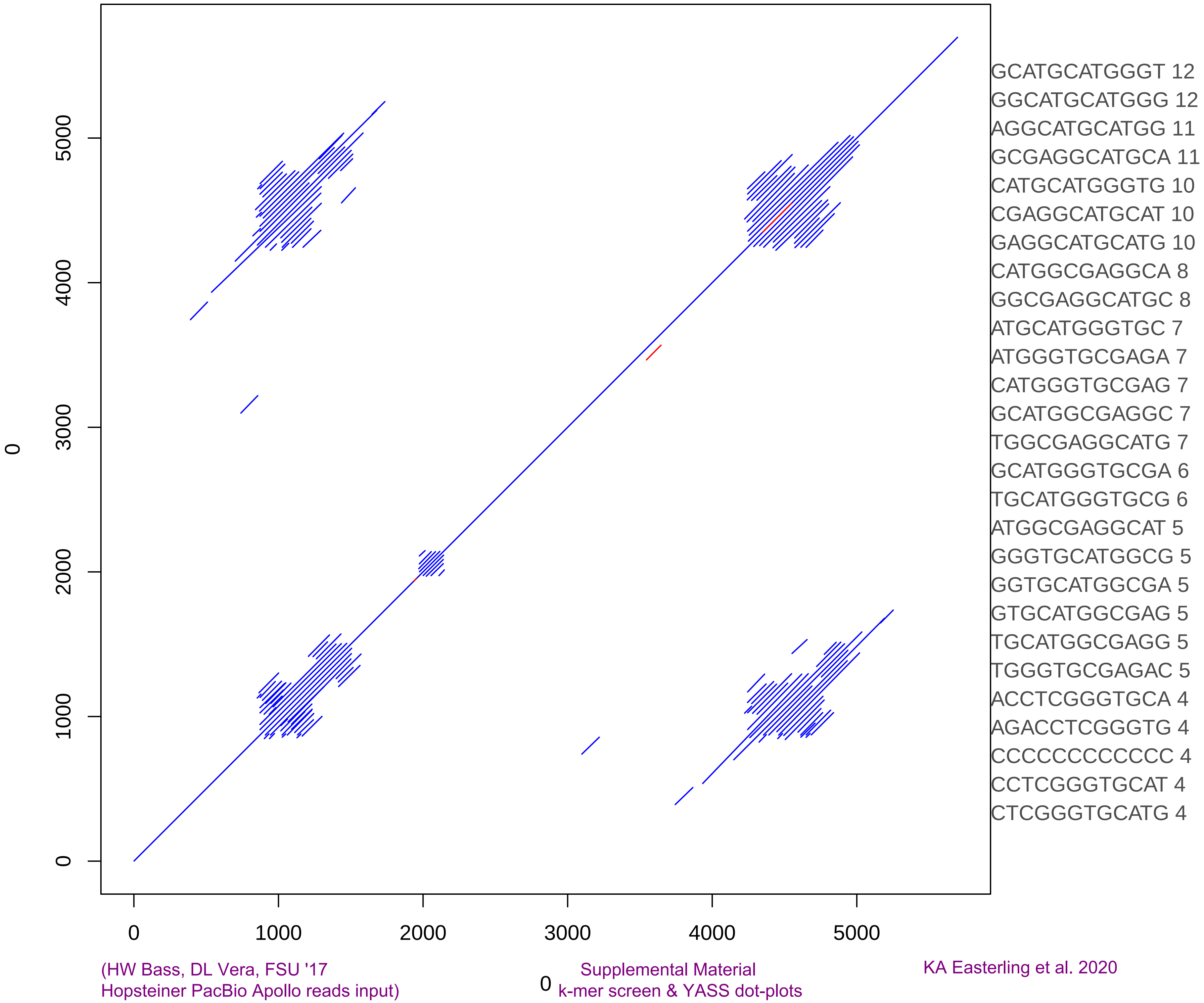

(HW Bass, DL Vera, FSU '17
Hopsteiner PacBio Apollo reads input)

Supplemental Material
k-mer screen & YASS dot-plots

KA Easterling et al. 2020

>m150106_044313_42134_c100761282550000001823158307221521_s1_p0/106104/893_7020

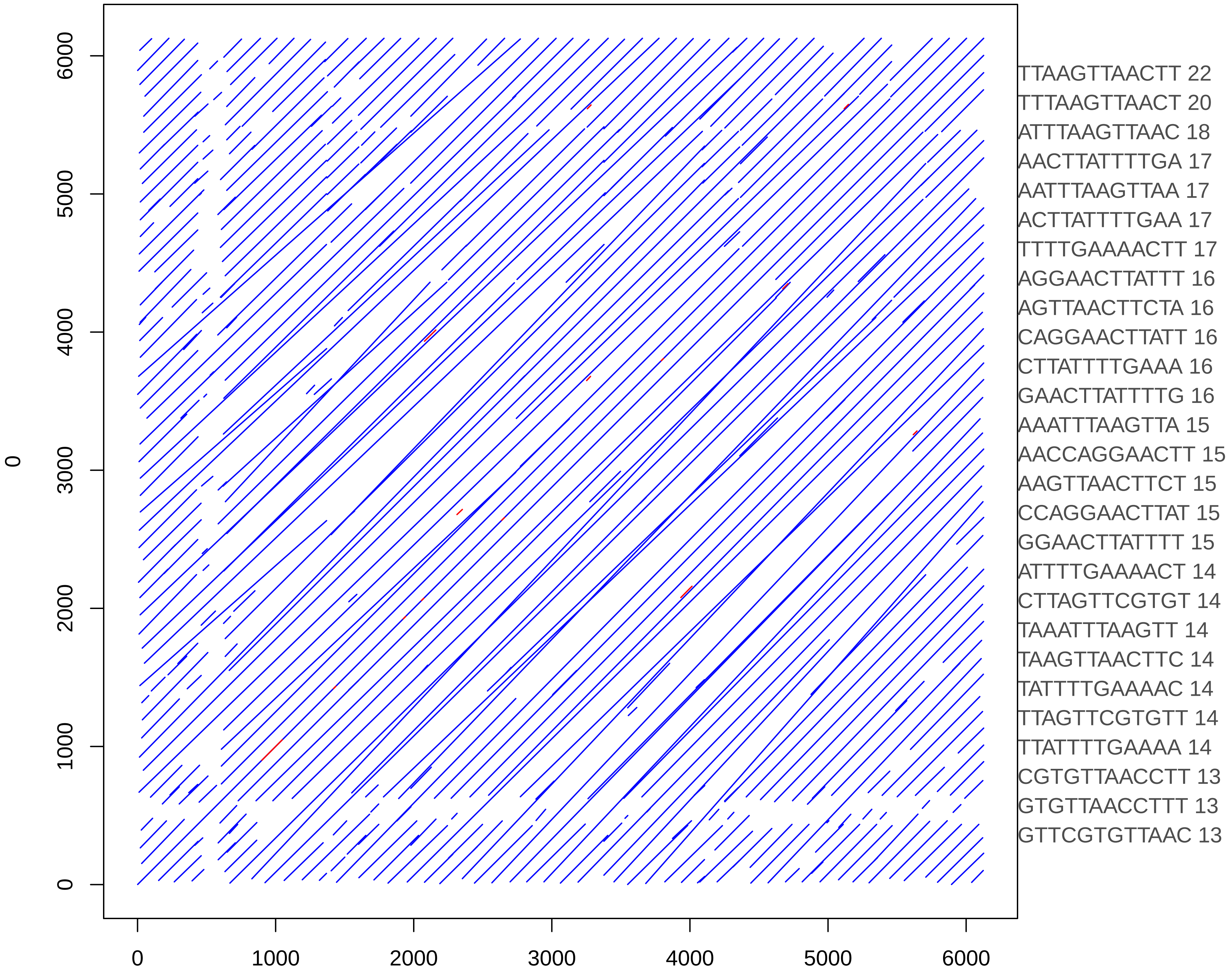

>m150106_044313_42134_c100761282550000001823158307221521_s1_p0/10819/644_7793

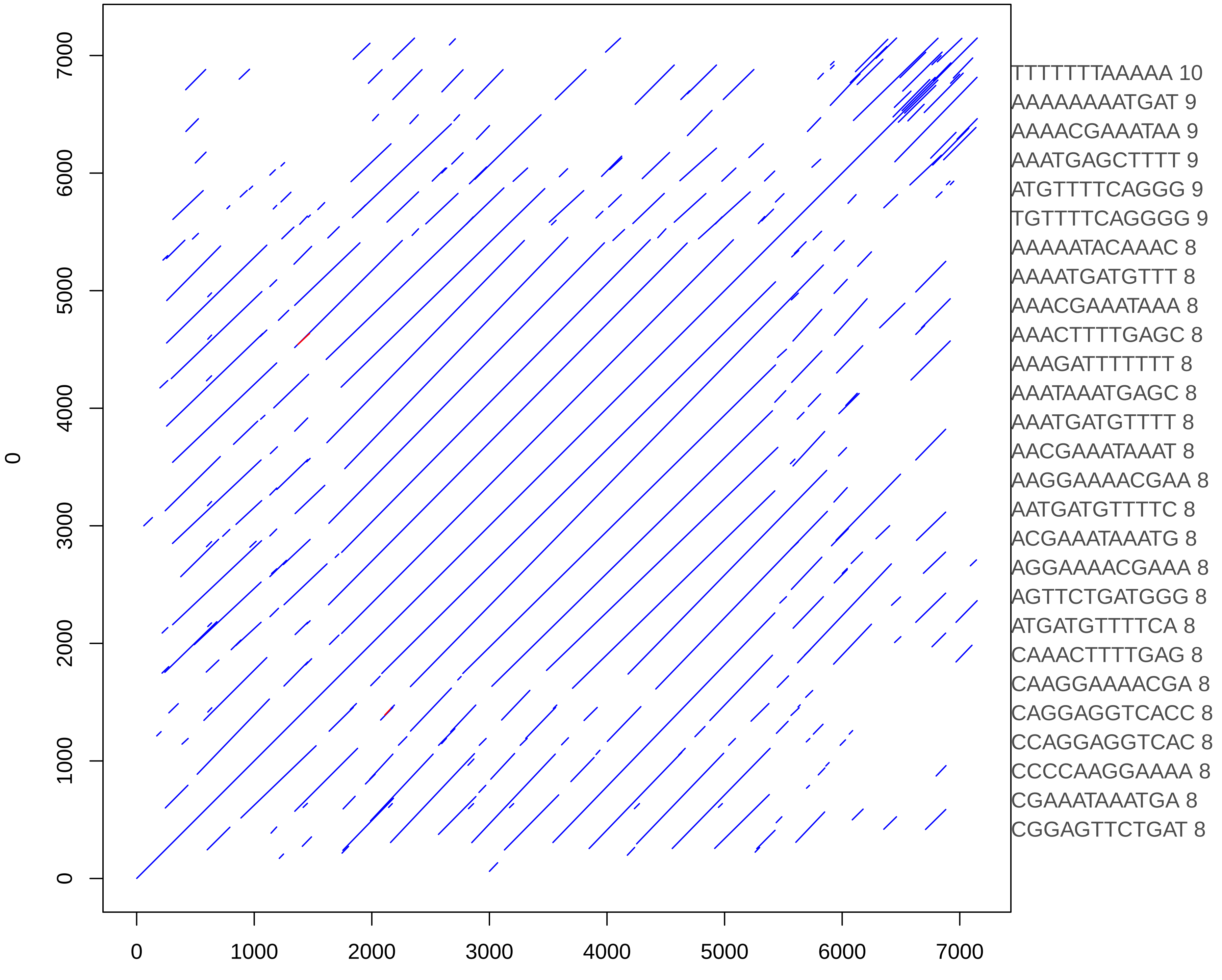

1,121 hits for repetitive sequences
passing 12mer abundance criterion

(Read-Number)
PAGE 1064

For Tandem Repeat families of ### bp:
PacBio Read named "HuluTR###-r1064"

>m150106_044313_42134_c100761282550000001823158307221521_s1_p0/110109/312_7245

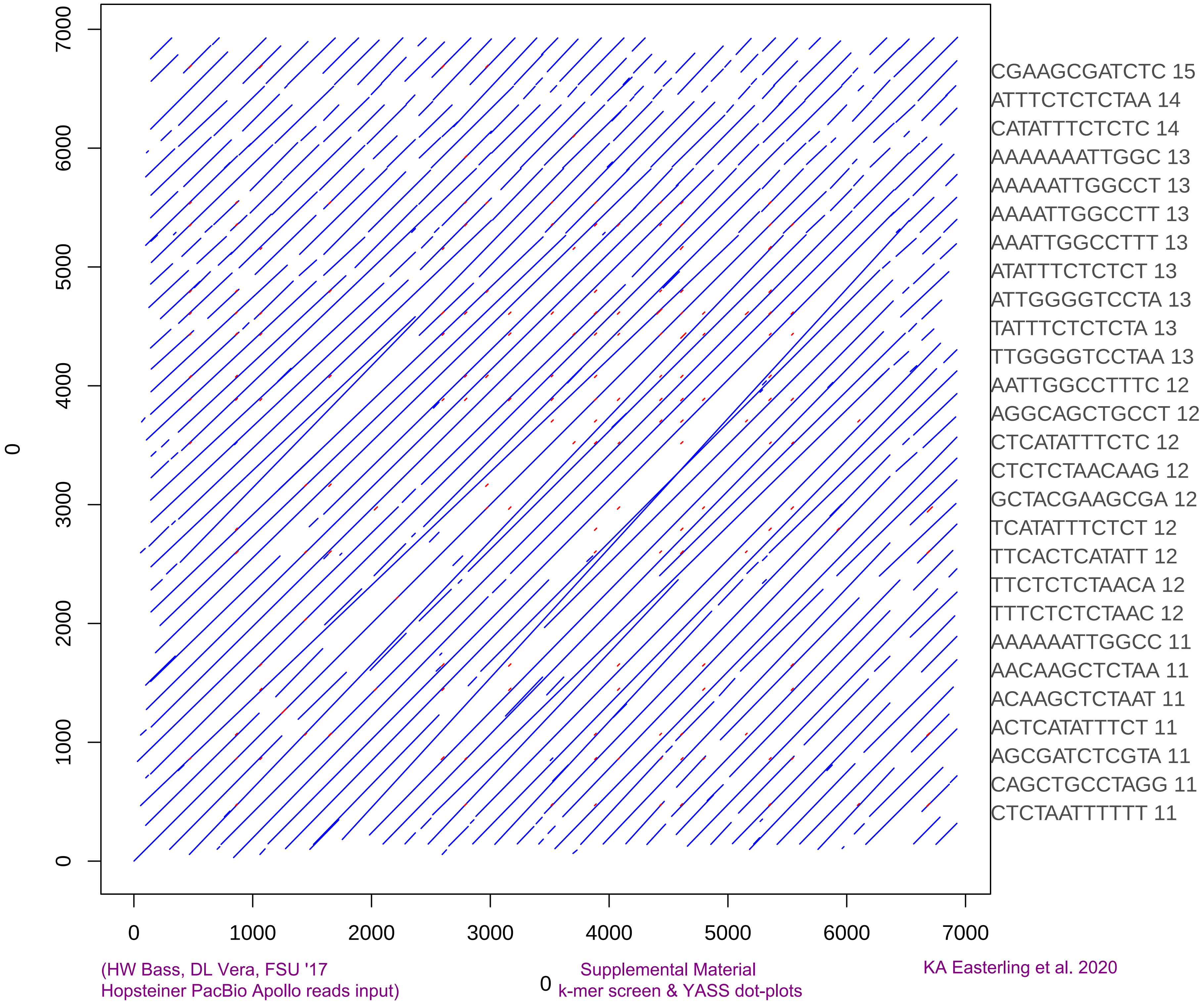

(HW Bass, DL Vera, FSU '17
Hopsteiner PacBio Apollo reads input)

Supplemental Material
k-mer screen & YASS dot-plots

KA Easterling et al. 2020

>m150106_044313_42134_c100761282550000001823158307221521_s1_p0/119007/0_10702

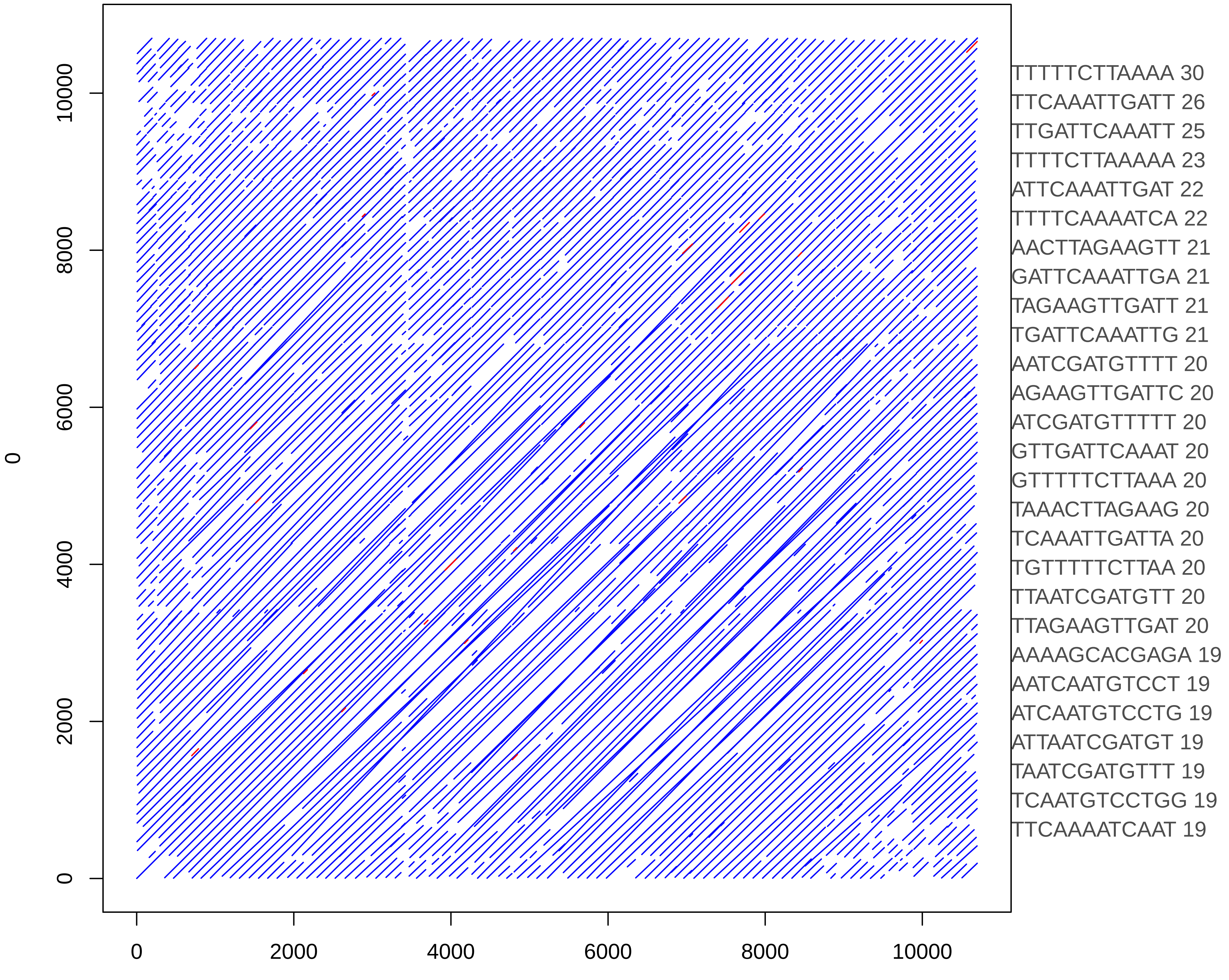

>m150106_044313_42134_c100761282550000001823158307221521_s1_p0/119305/0_9118

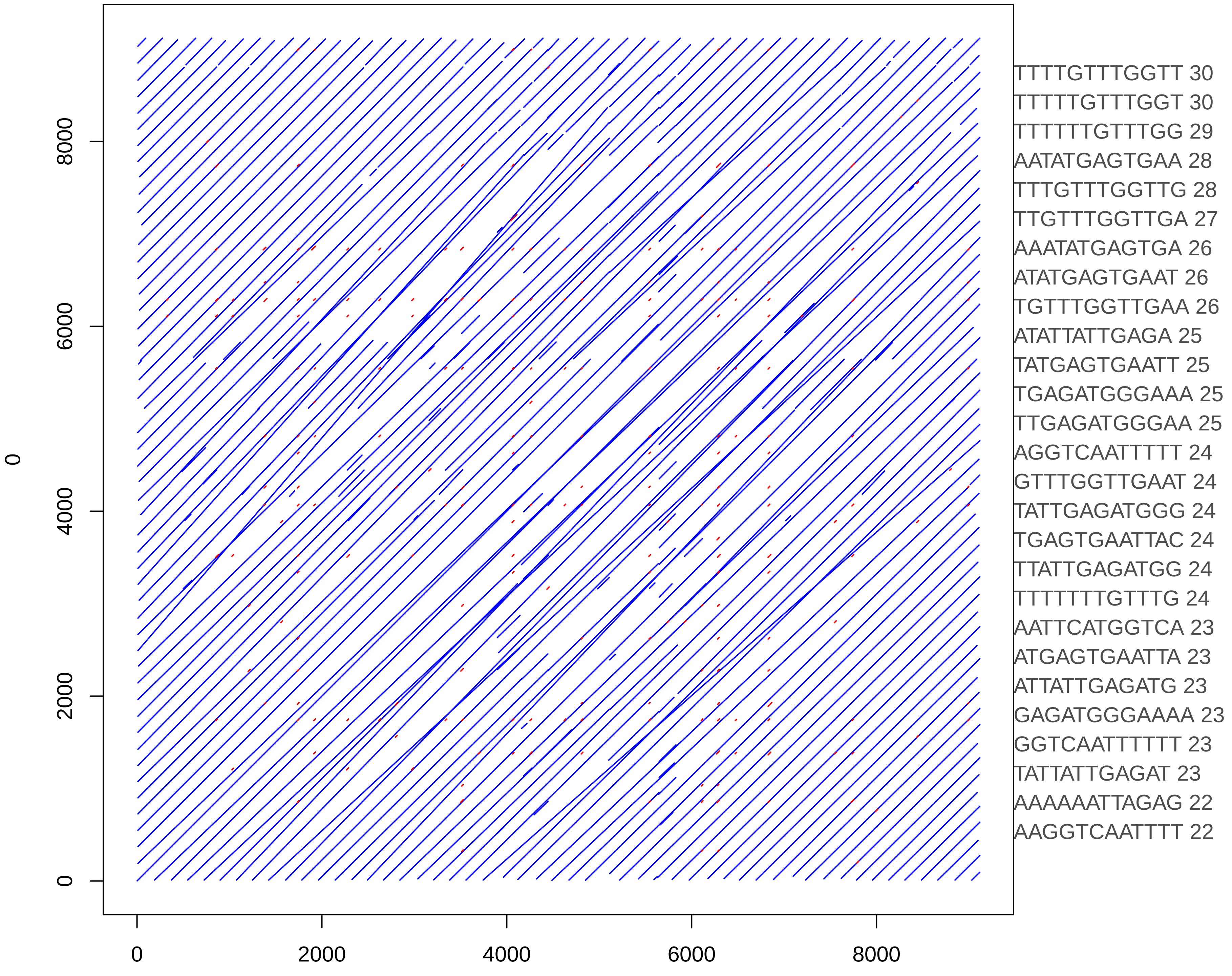

>m150106_044313_42134_c100761282550000001823158307221521_s1_p0/132980/1113_11246

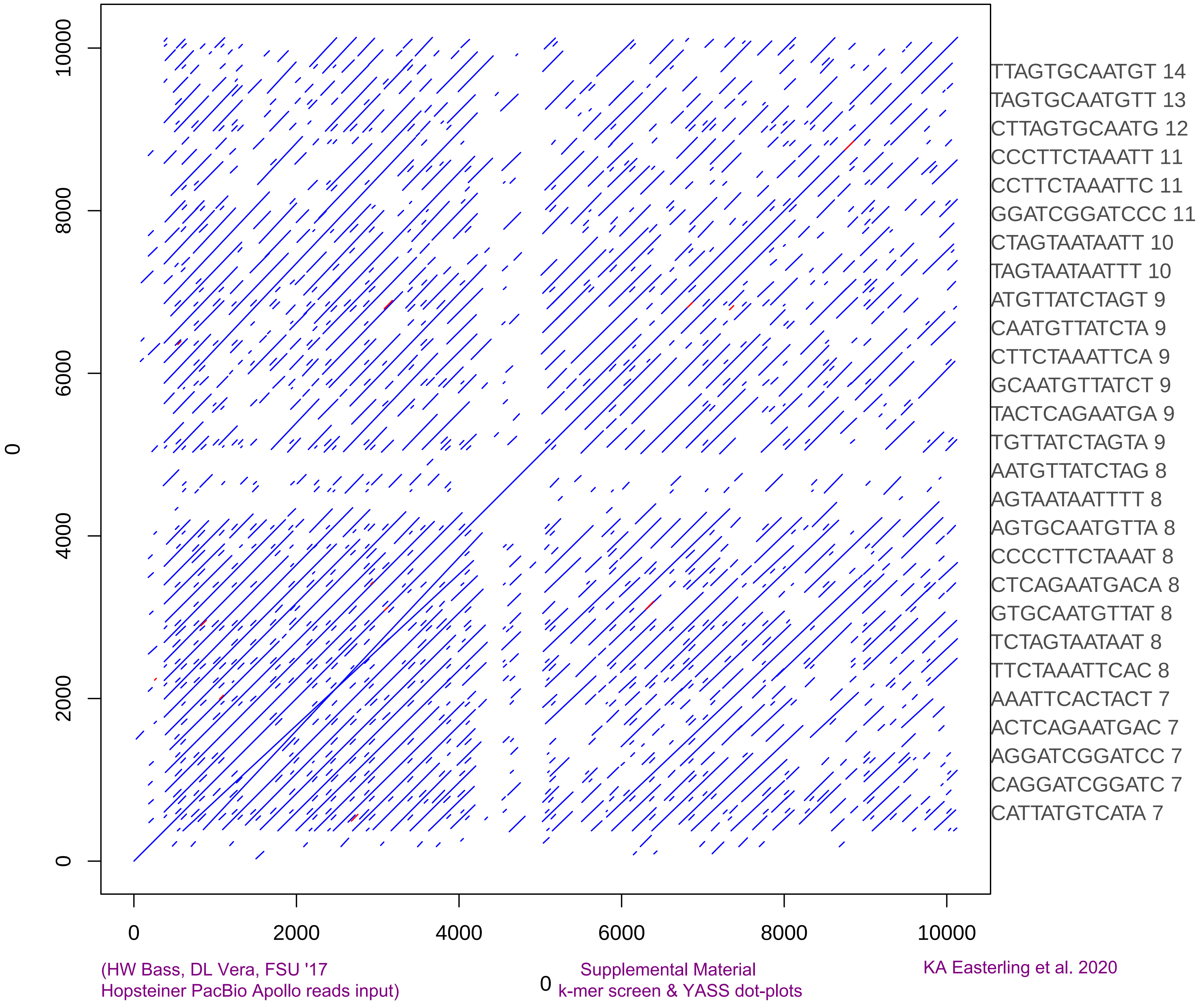

>m150106_044313_42134_c100761282550000001823158307221521_s1_p0/133471/0_7967

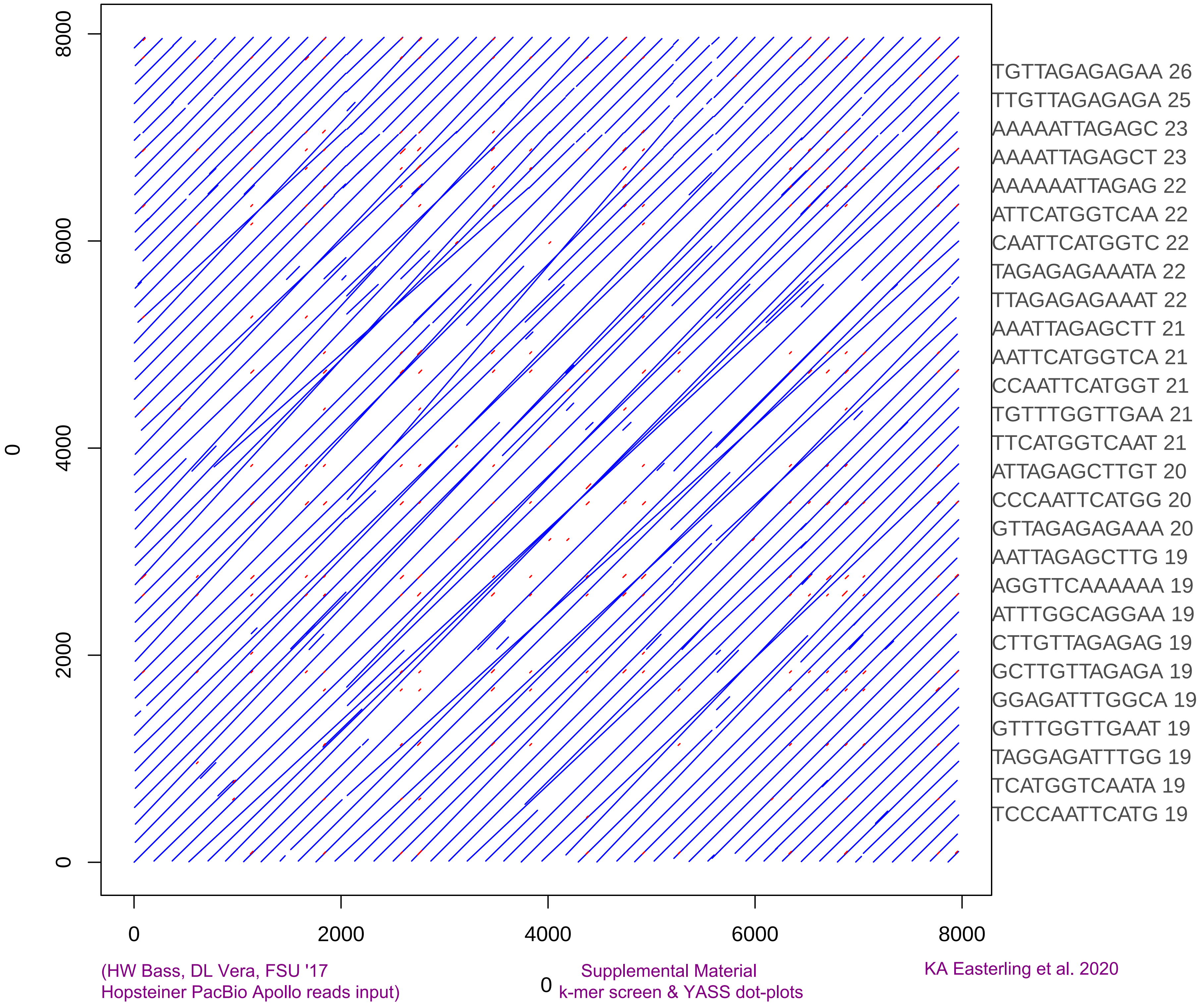

>m150106_044313_42134_c100761282550000001823158307221521_s1_p0/13539/0_10531

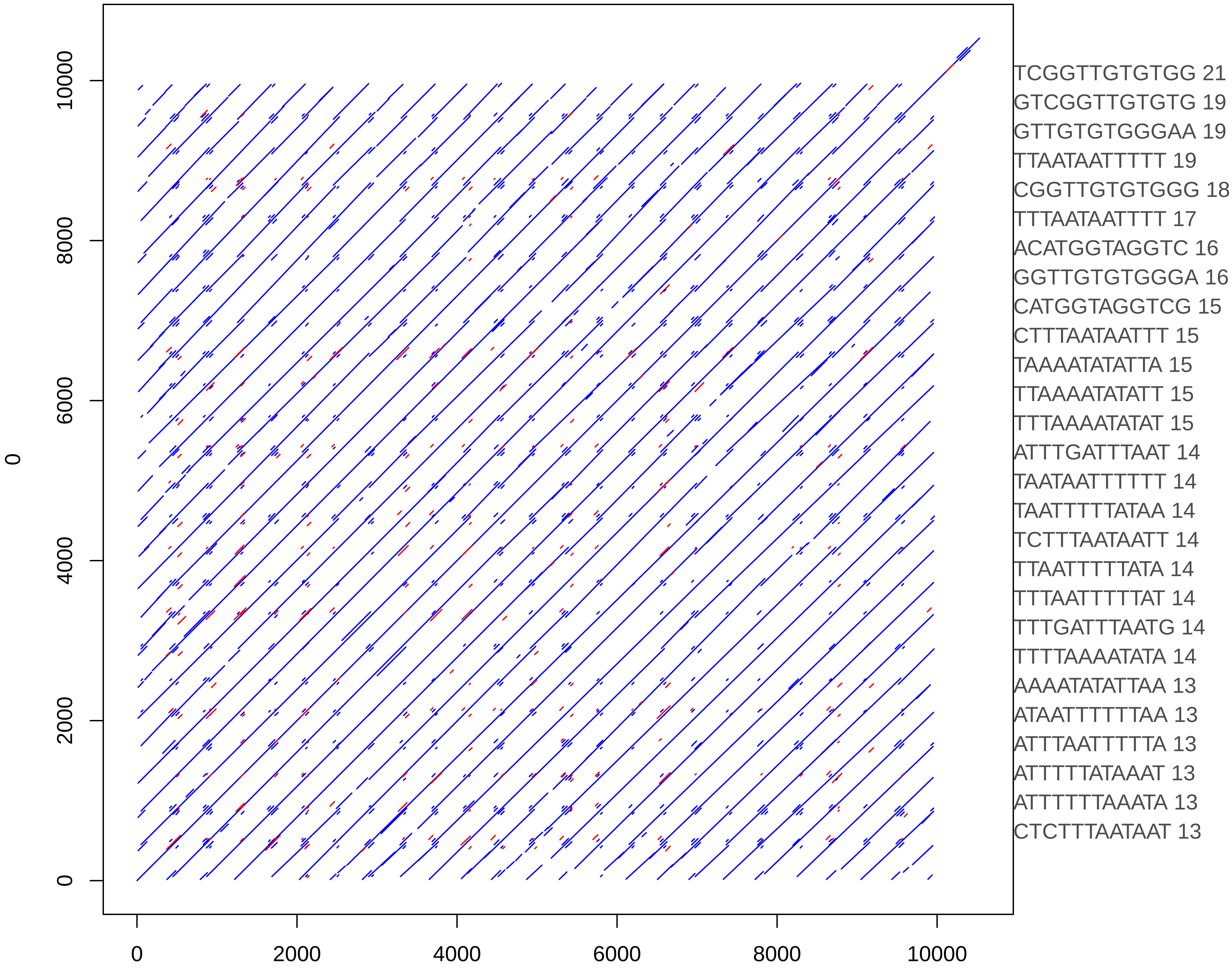

>m150106_044313_42134_c100761282550000001823158307221521_s1_p0/1412/494_9308

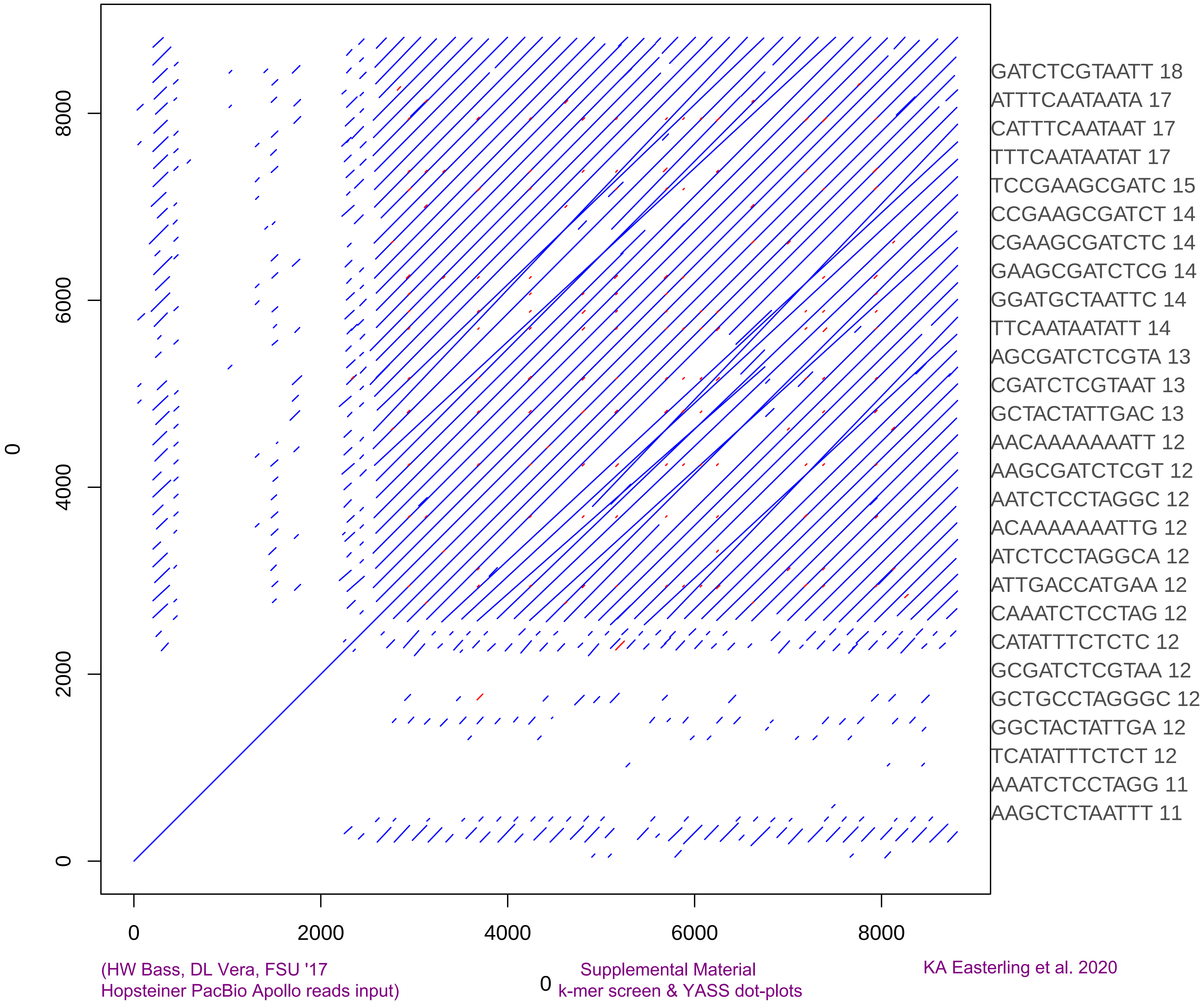

>m150106_044313_42134_c100761282550000001823158307221521_s1_p0/143626/774_6170

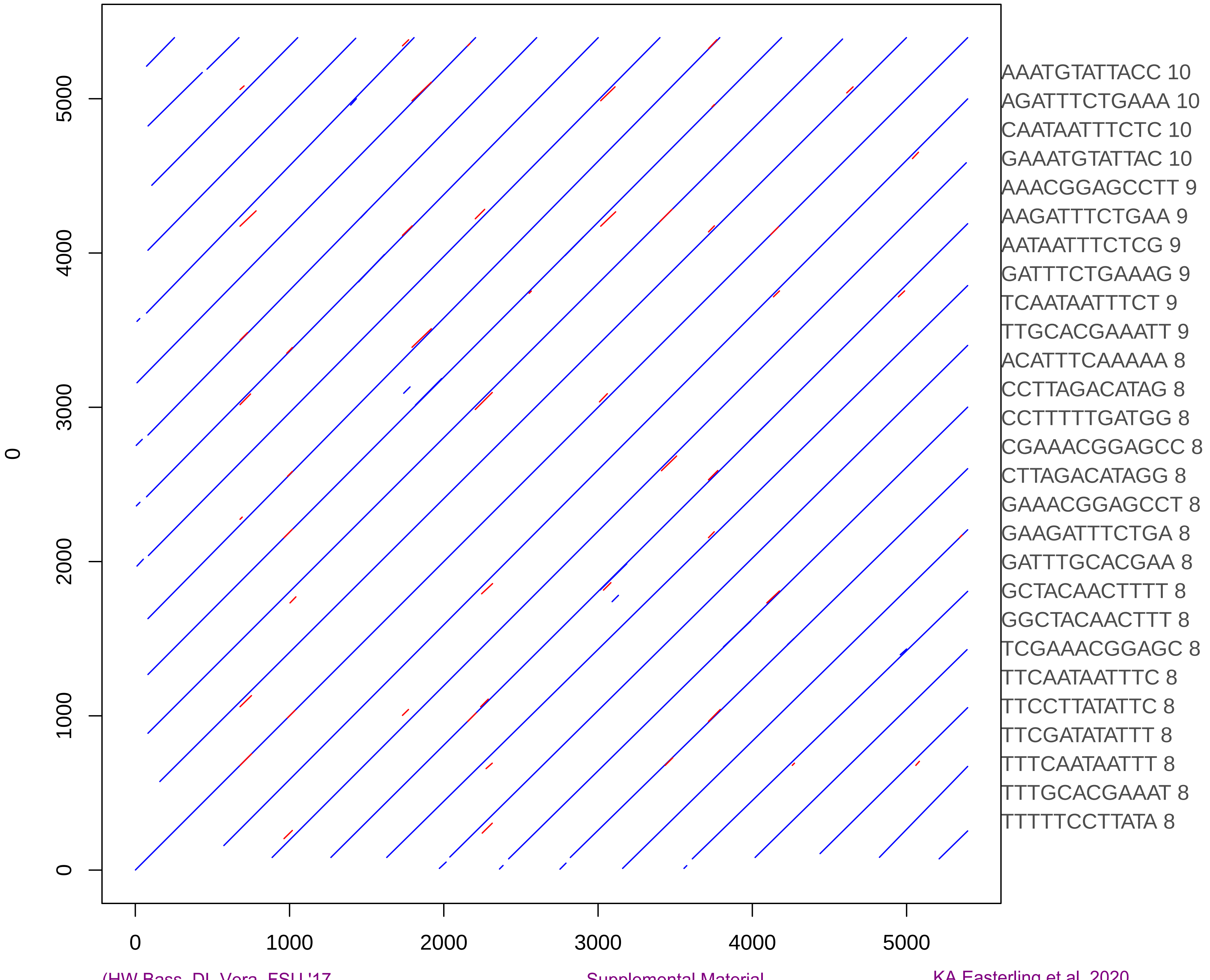

0

>m150106_044313_42134_c100761282550000001823158307221521_s1_p0/14421/0_5855

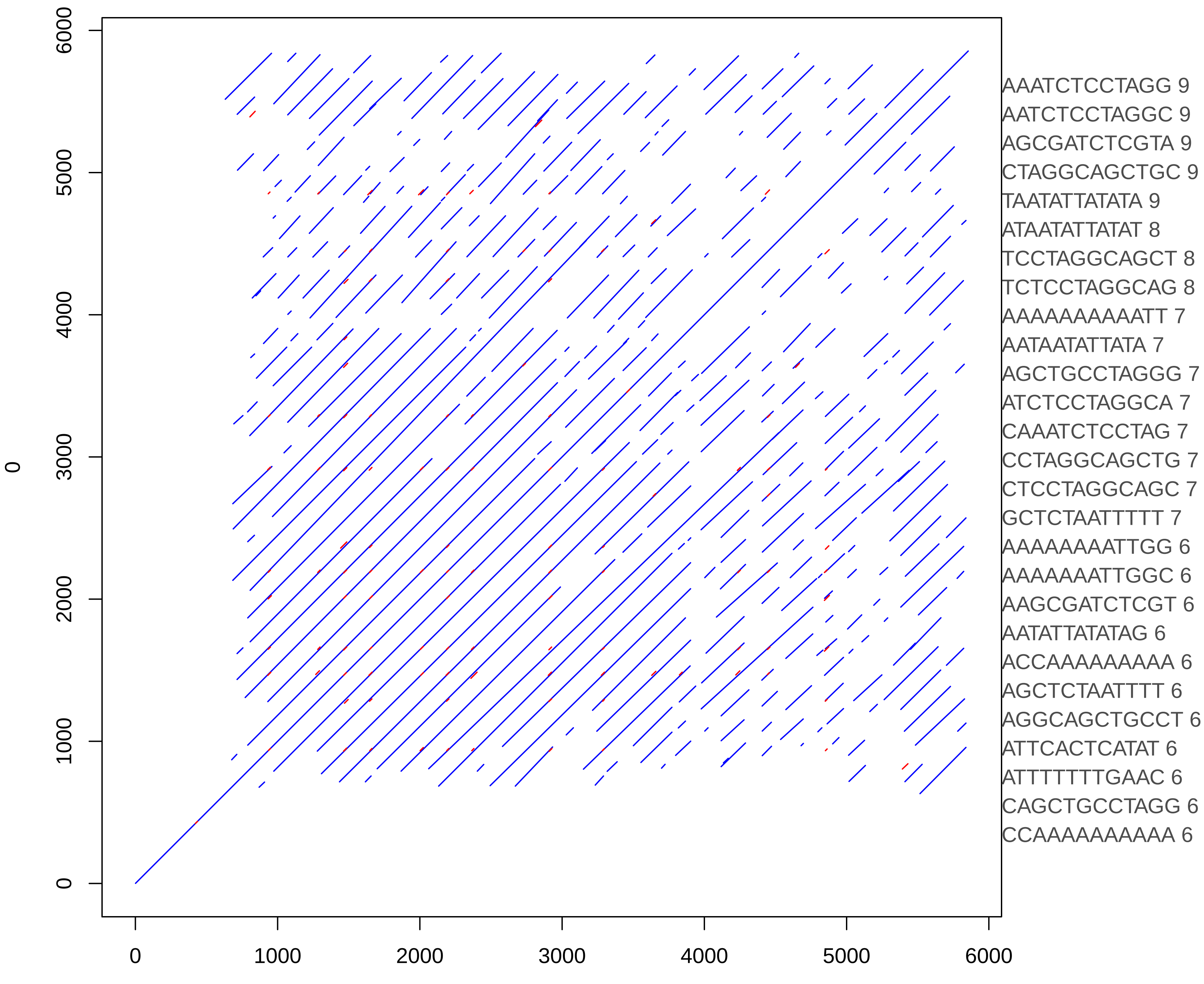

>m150106_044313_42134_c100761282550000001823158307221521_s1_p0/147268/888_12989

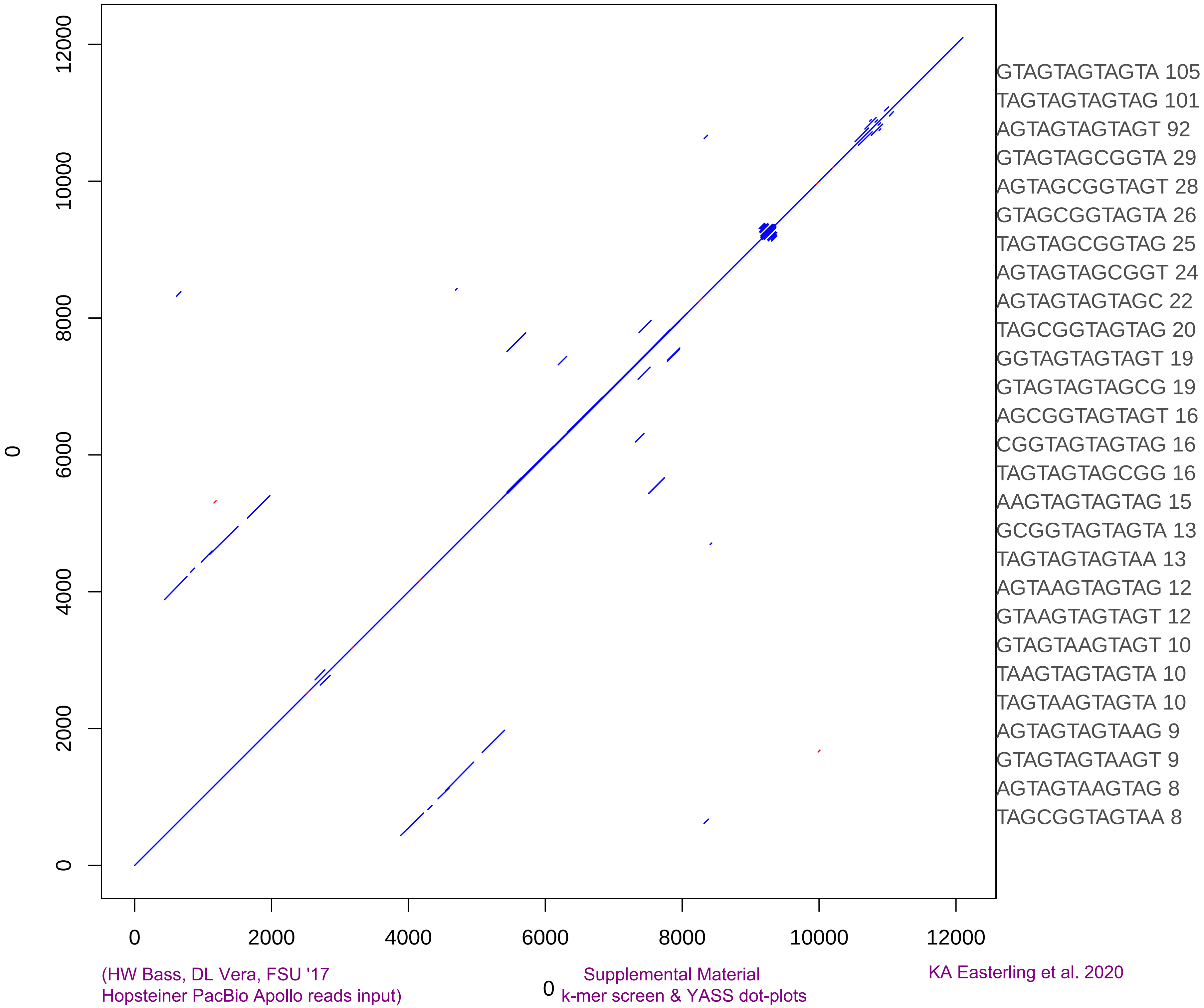

>m150106_044313_42134_c100761282550000001823158307221521_s1_p0/148822/1619_11245

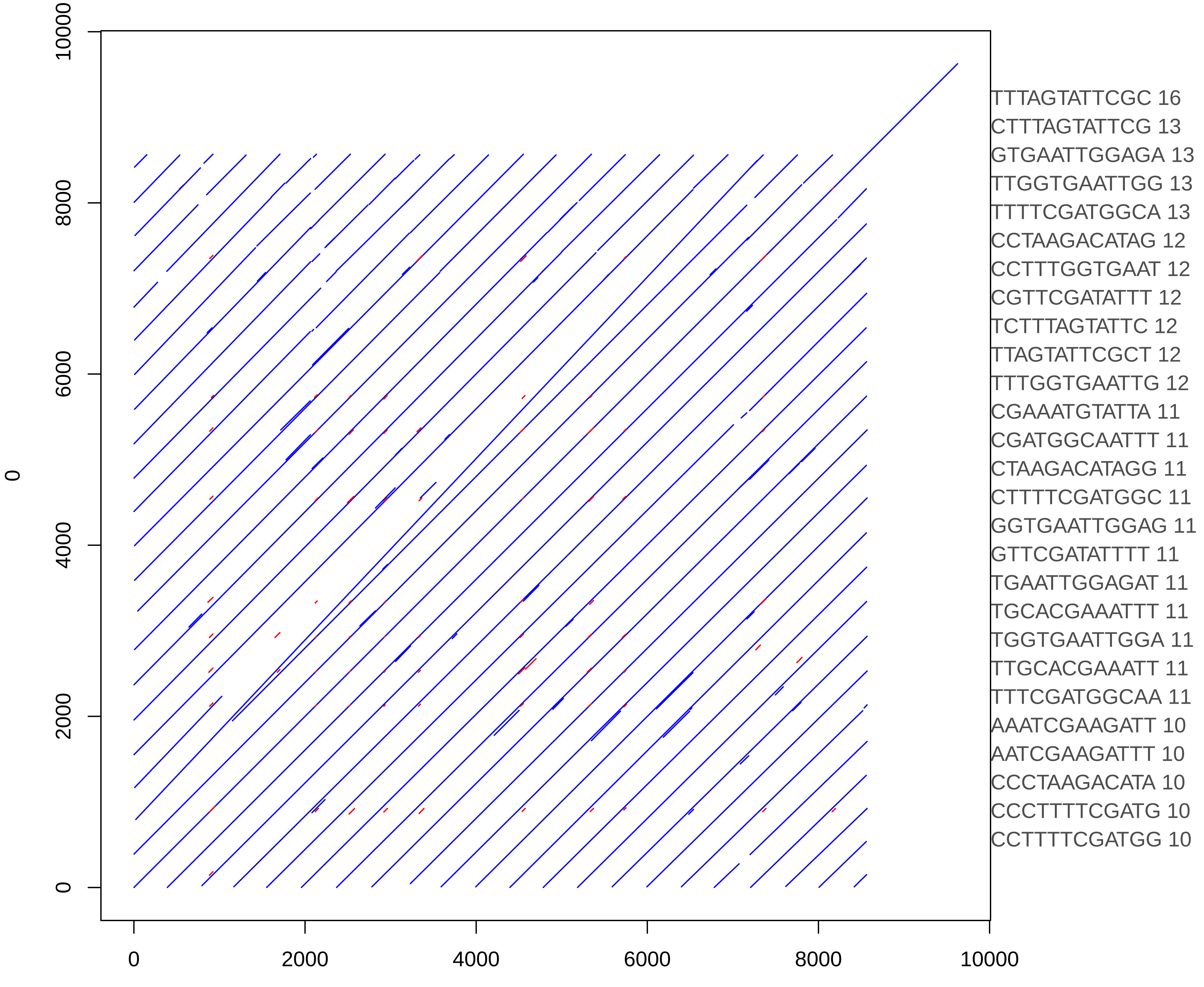

>m150106_044313_42134_c100761282550000001823158307221521_s1_p0/149651/428_6420

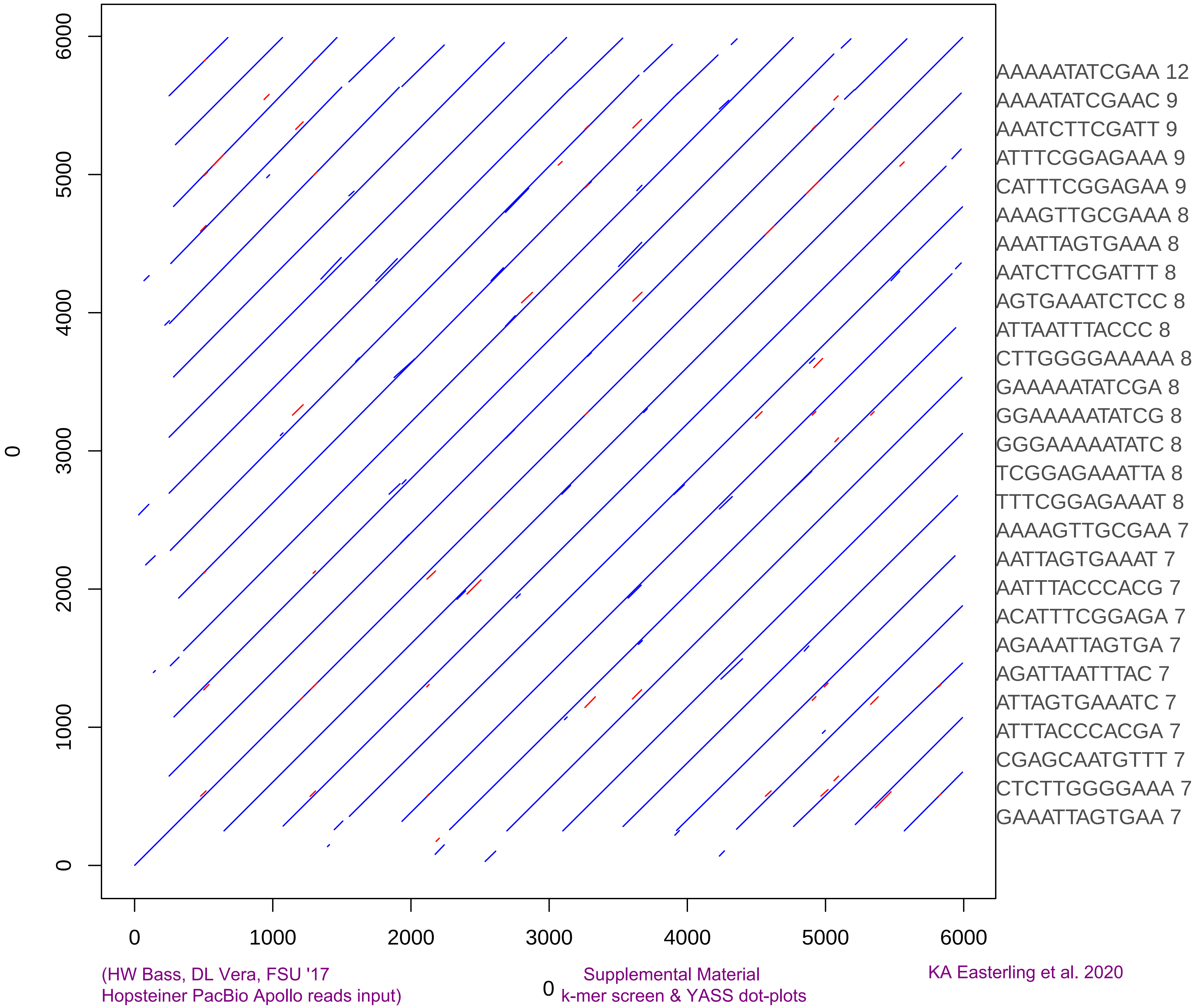

>m150106_044313_42134_c100761282550000001823158307221521_s1_p0/152838/0_5436

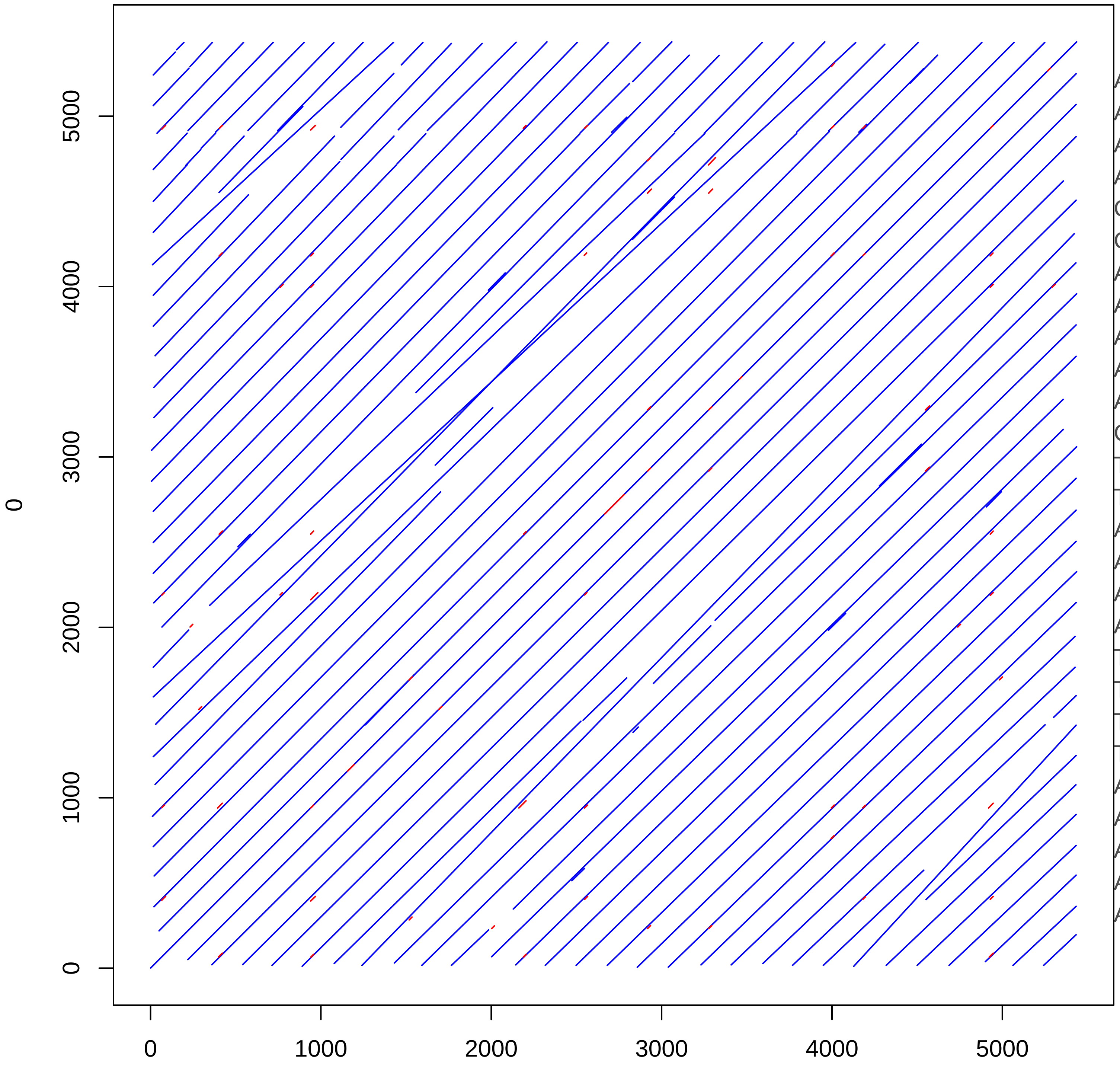

>m150106_044313_42134_c100761282550000001823158307221521_s1_p0/153206/6591_12372

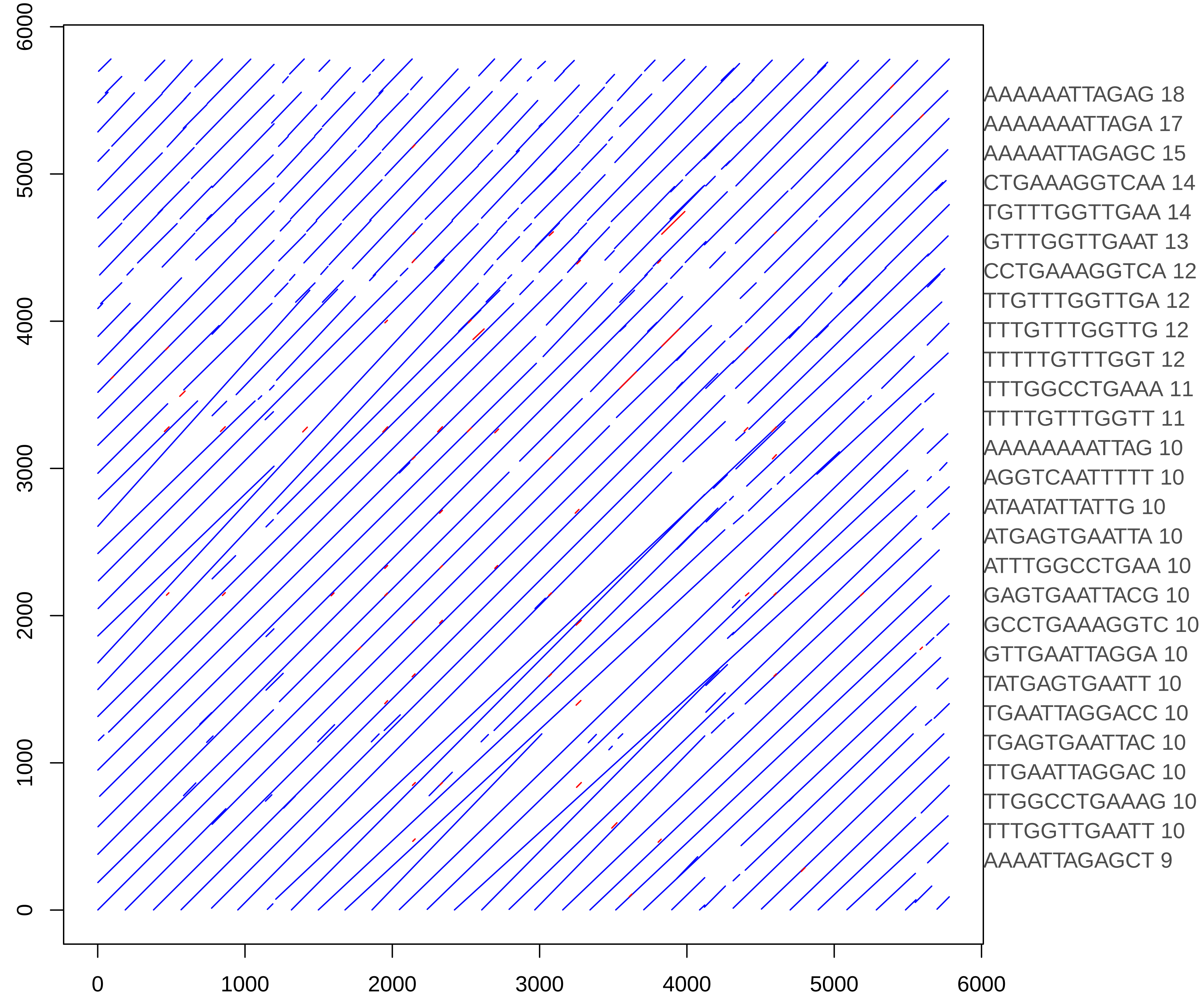

>m150106_044313_42134_c100761282550000001823158307221521_s1_p0/1558/0_5041

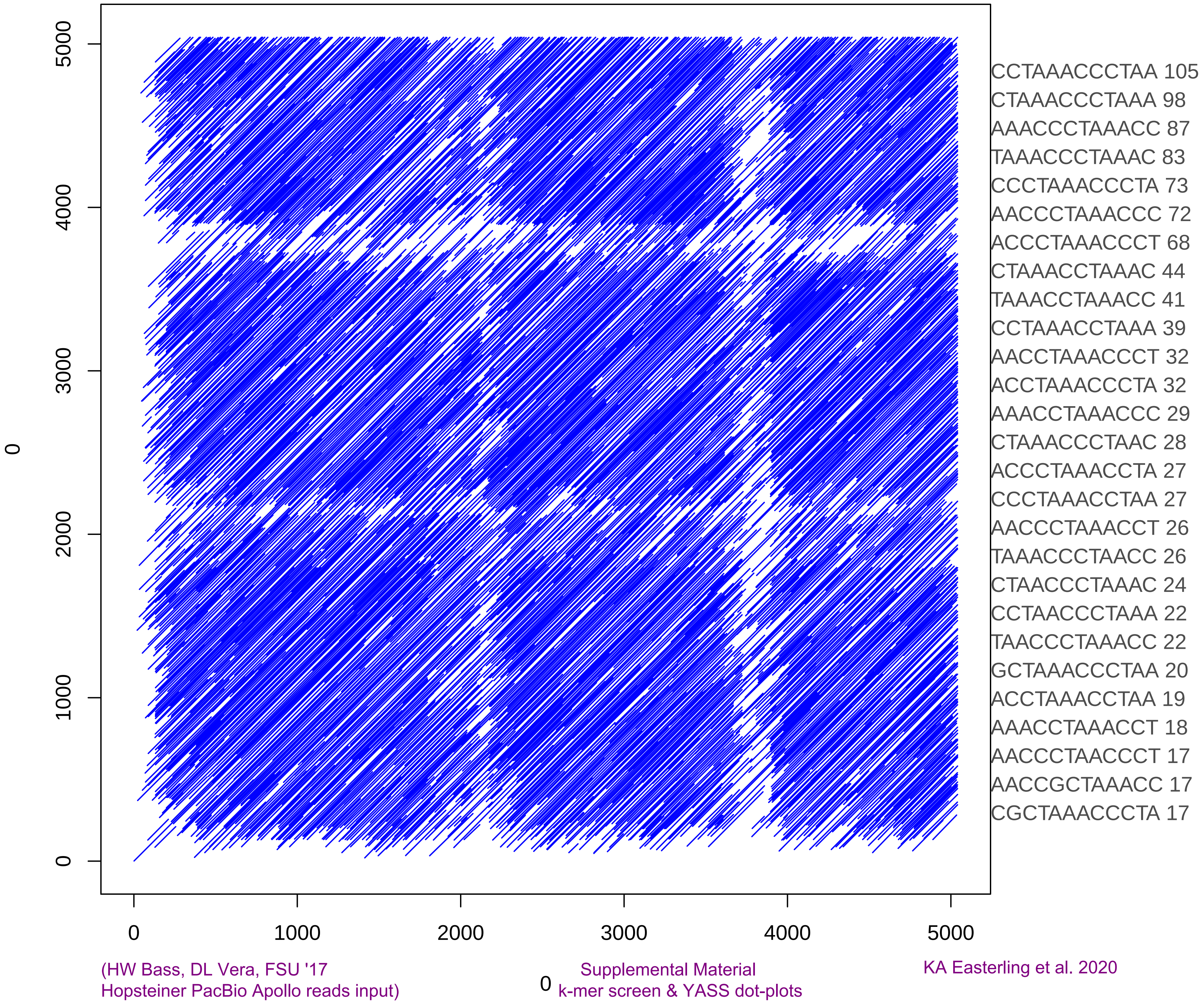

>m150106_044313_42134_c100761282550000001823158307221521_s1_p0/157398/0_5576

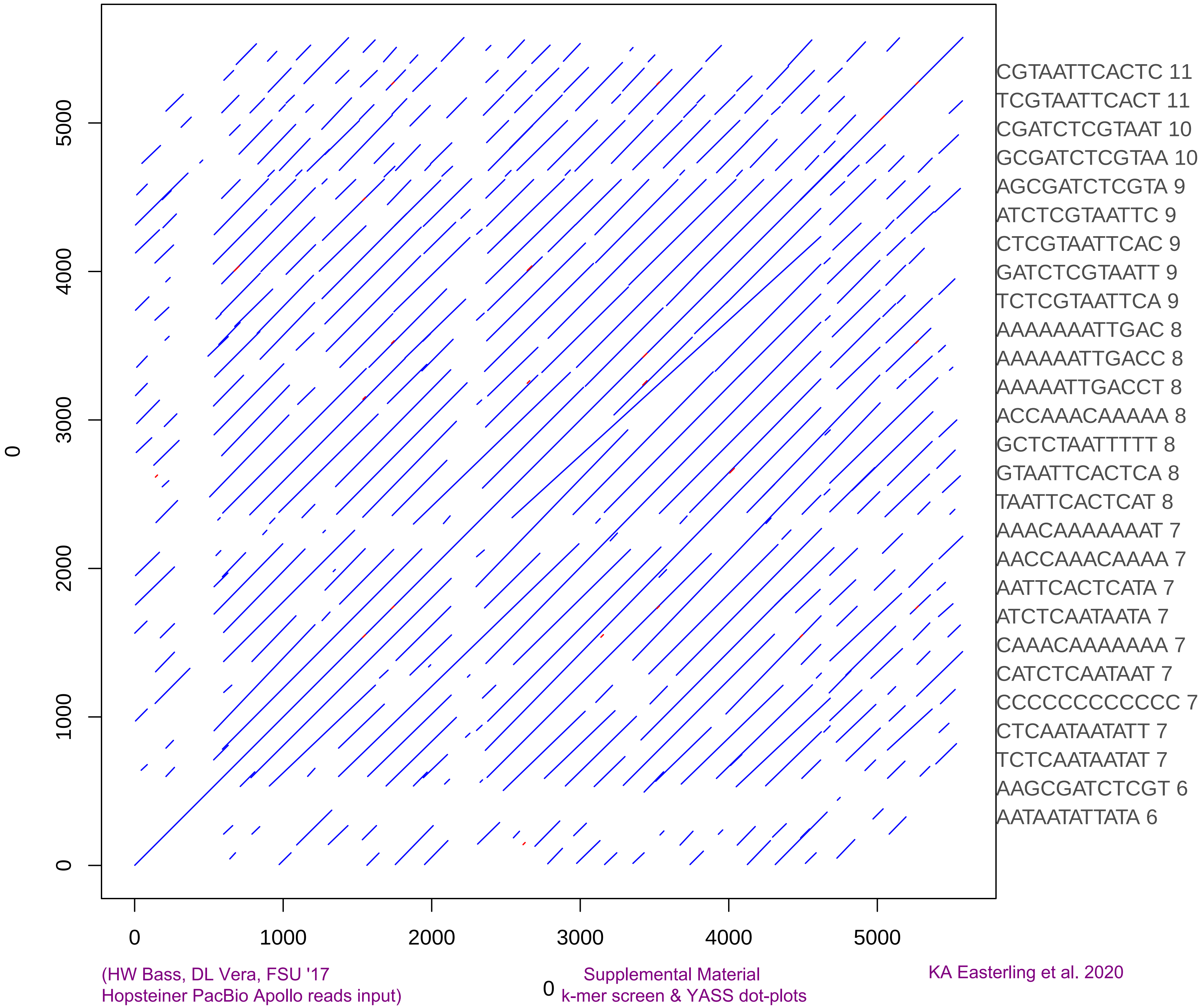

>m150106_044313_42134_c100761282550000001823158307221521_s1_p0/157558/2142_7505

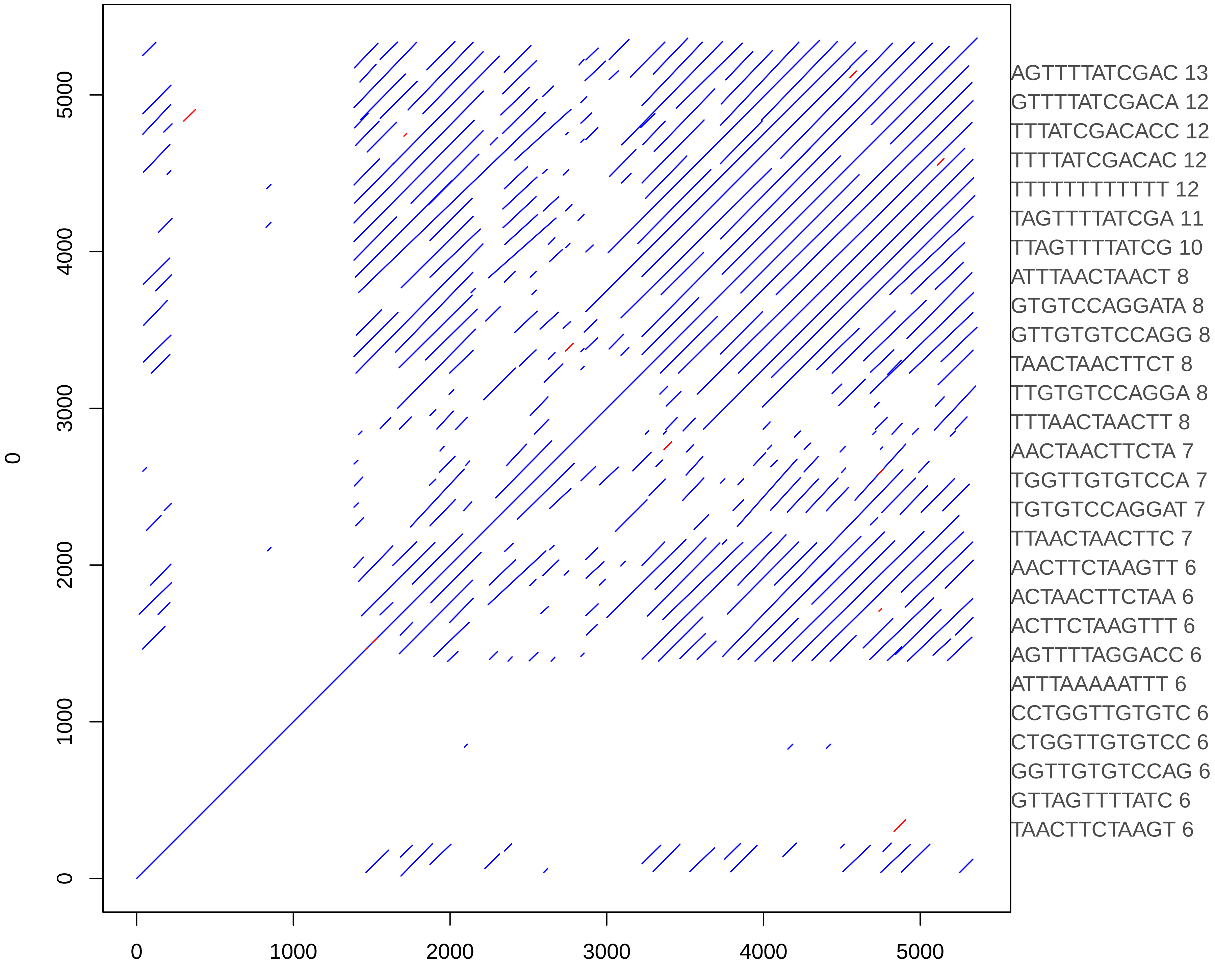

>m150106_044313_42134_c100761282550000001823158307221521_s1_p0/1583/0_11994

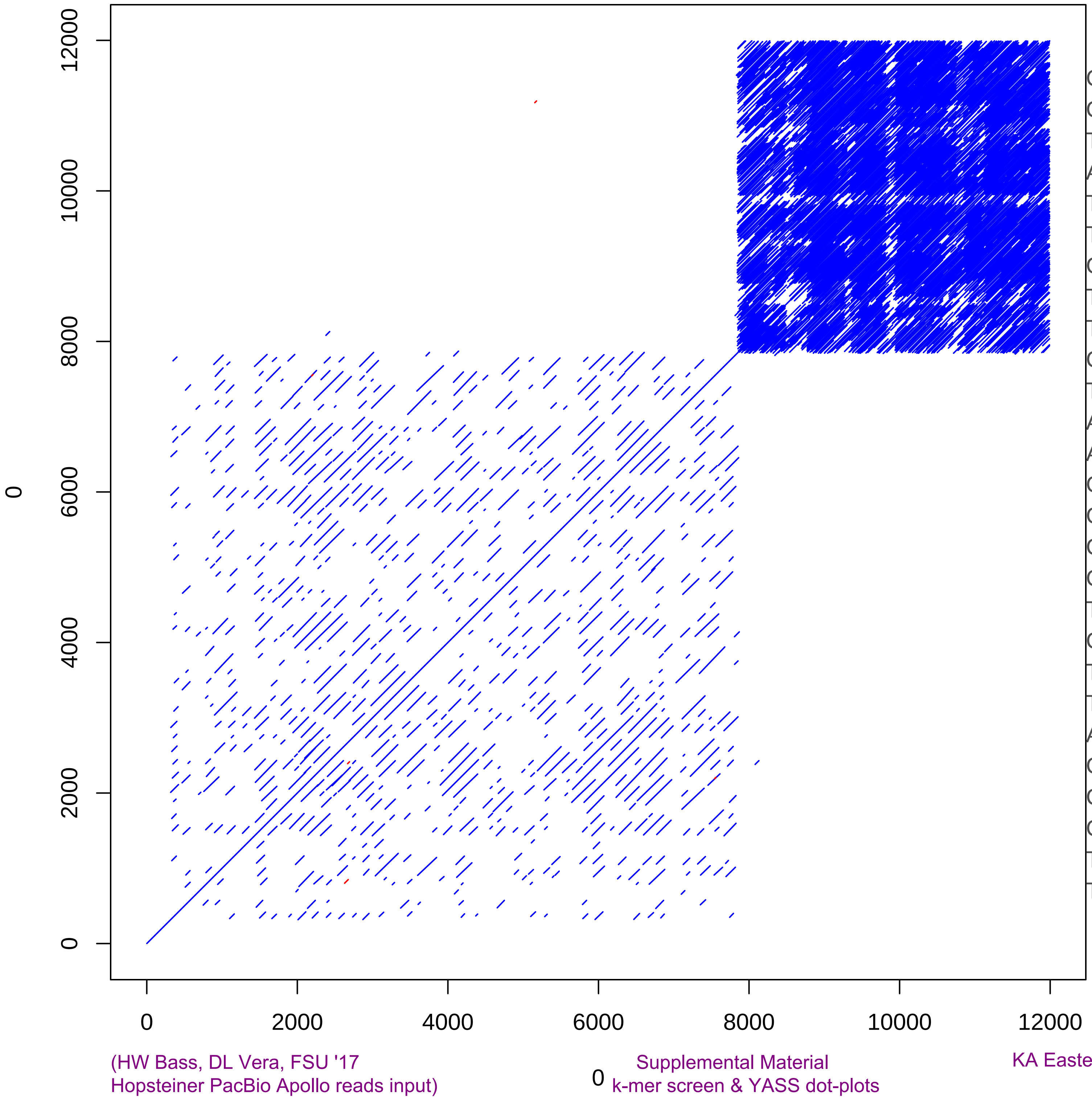

GGGTTTAGGGTT 58
GGTTTAGGGTTT 53
TTAGGGTTTAGG 53
AGGGTTTAGGGT 45
TAGGGTTTAGGG 45
TTTAGGGTTTAG 42
GTTTAGGGTTTA 37
TTTAGGTTTAGG 21
TAGGTTTAGGGT 19
GTTTAGGTTTAG 18
TTAGGTTTAGGG 18
AGGGTTAGGGTT 16
AGGTTTAGGGTT 16
GGGTTTTAGGGT 16
GGTTTAGGGTTA 15
GGTTTAGGTTTA 15
GGTTTTAGGGTT 15
TAGGGTTTAGGT 15
GTTTTAGGGTTT 14
TAGGGTTAGGGT 13
TTAGGGTTAGGG 13
AGGGTTTAGGTT 12
GGGTTAGGGTTT 12
GGGTTTAGGTTT 12
GTTTAGGGTTAG 12
TAGGGTTTTAGG 12
TTTAGGGTTAGG 12

>m150106_044313_42134_c100761282550000001823158307221521_s1_p0/158571/442_6851

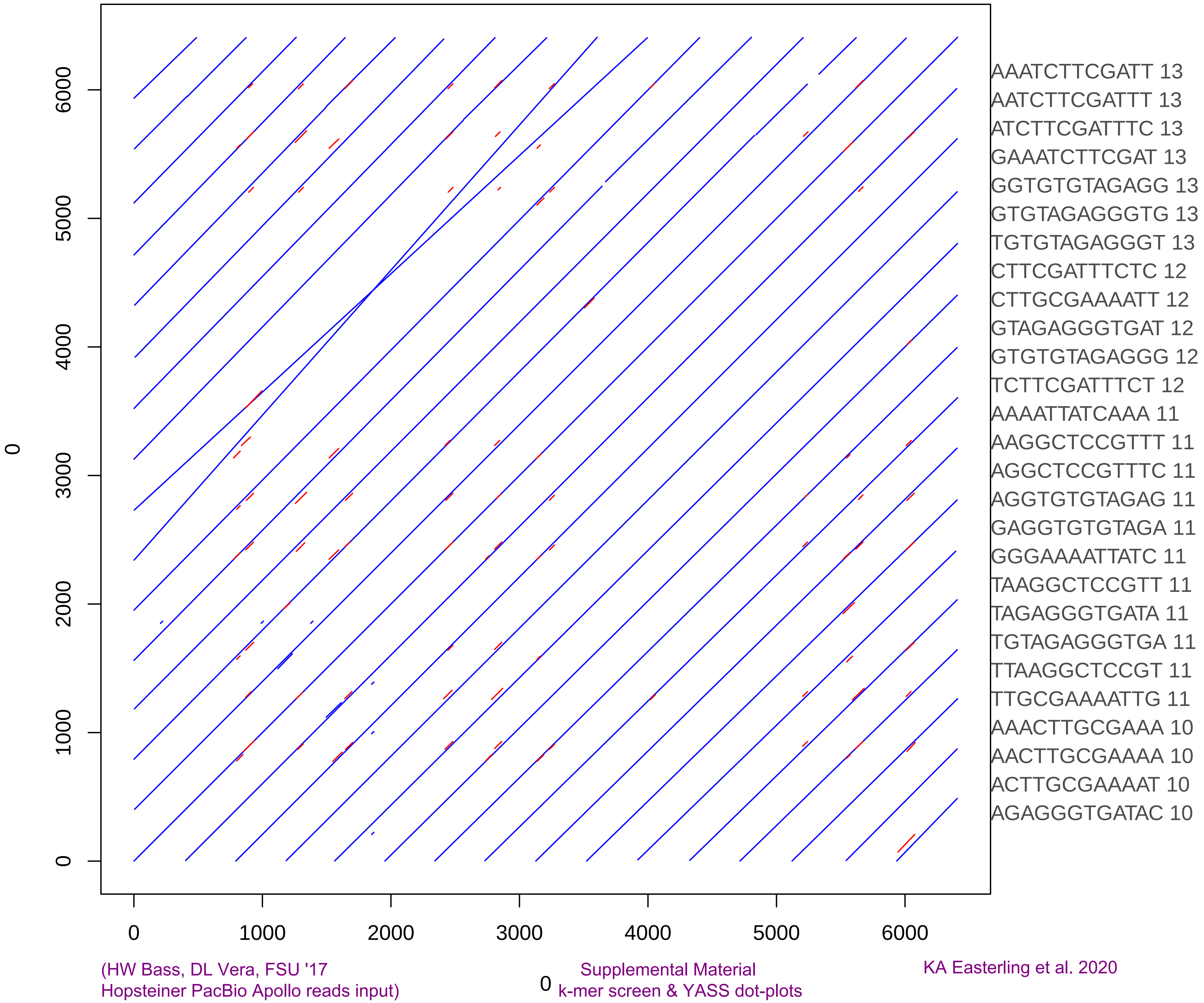

>m150106_044313_42134_c100761282550000001823158307221521_s1_p0/160814/0_8430

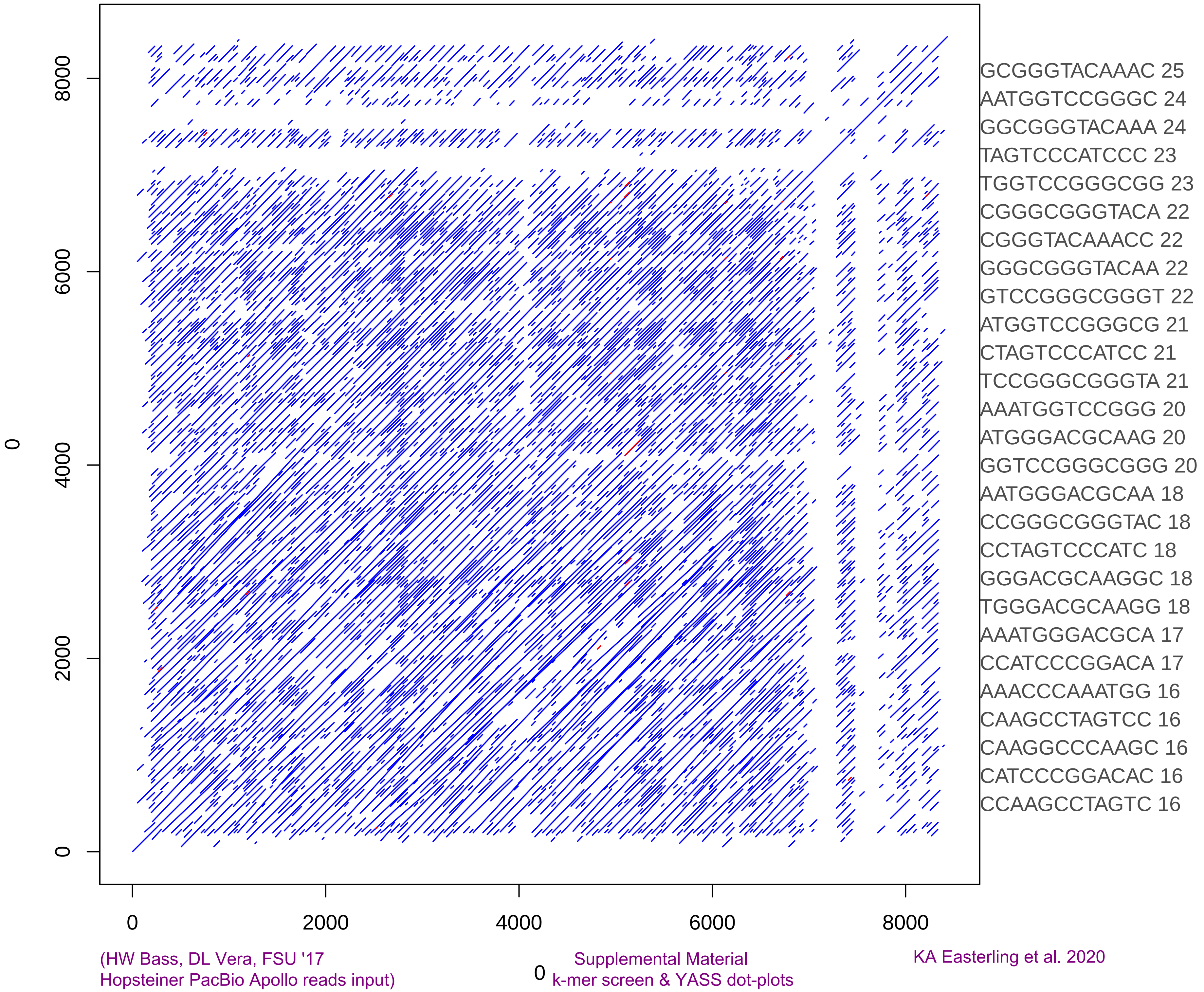

>m150106_044313_42134_c100761282550000001823158307221521_s1_p0/1689/1002_7054

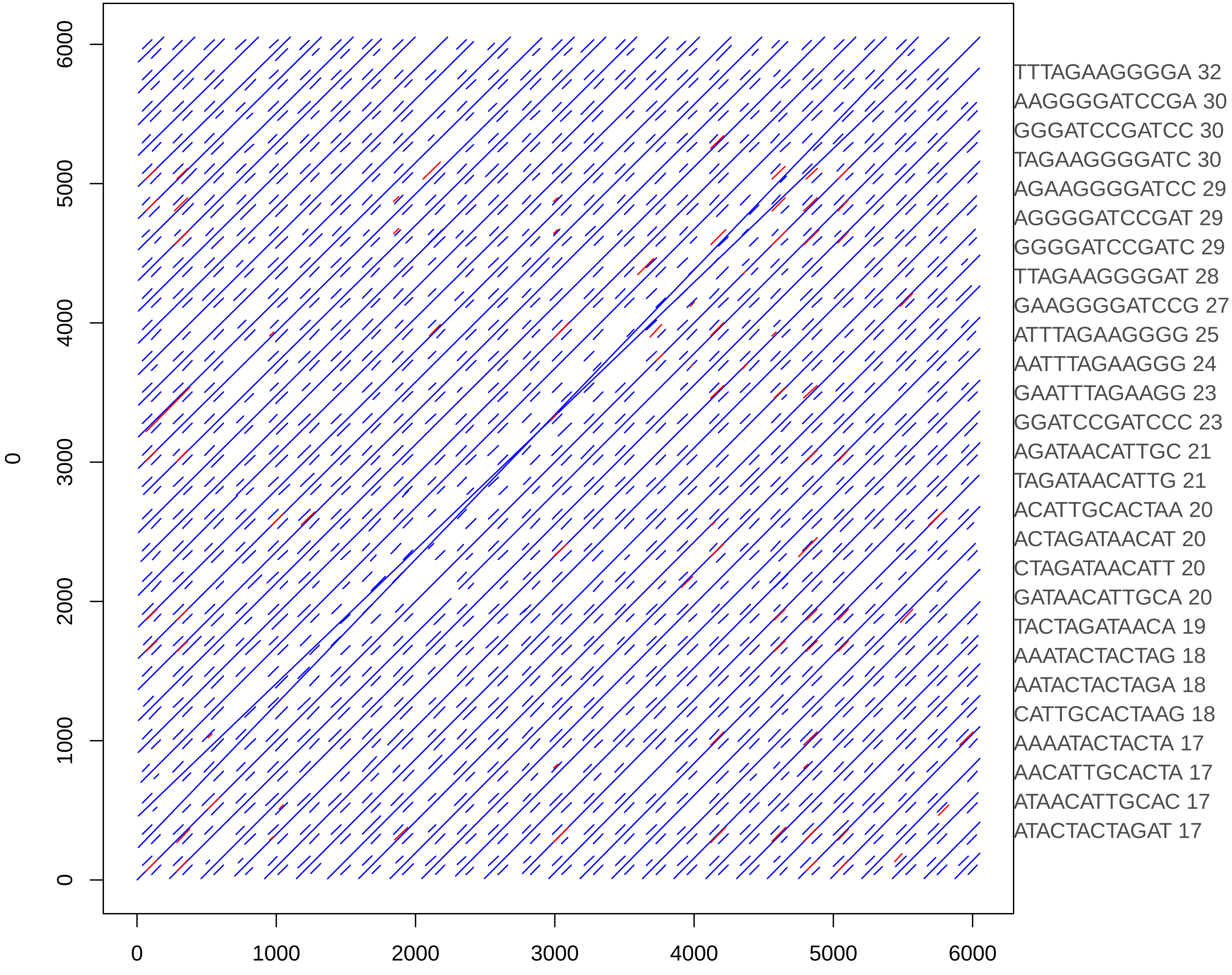

>m150106_044313_42134_c100761282550000001823158307221521_s1_p0/17034/0_7106

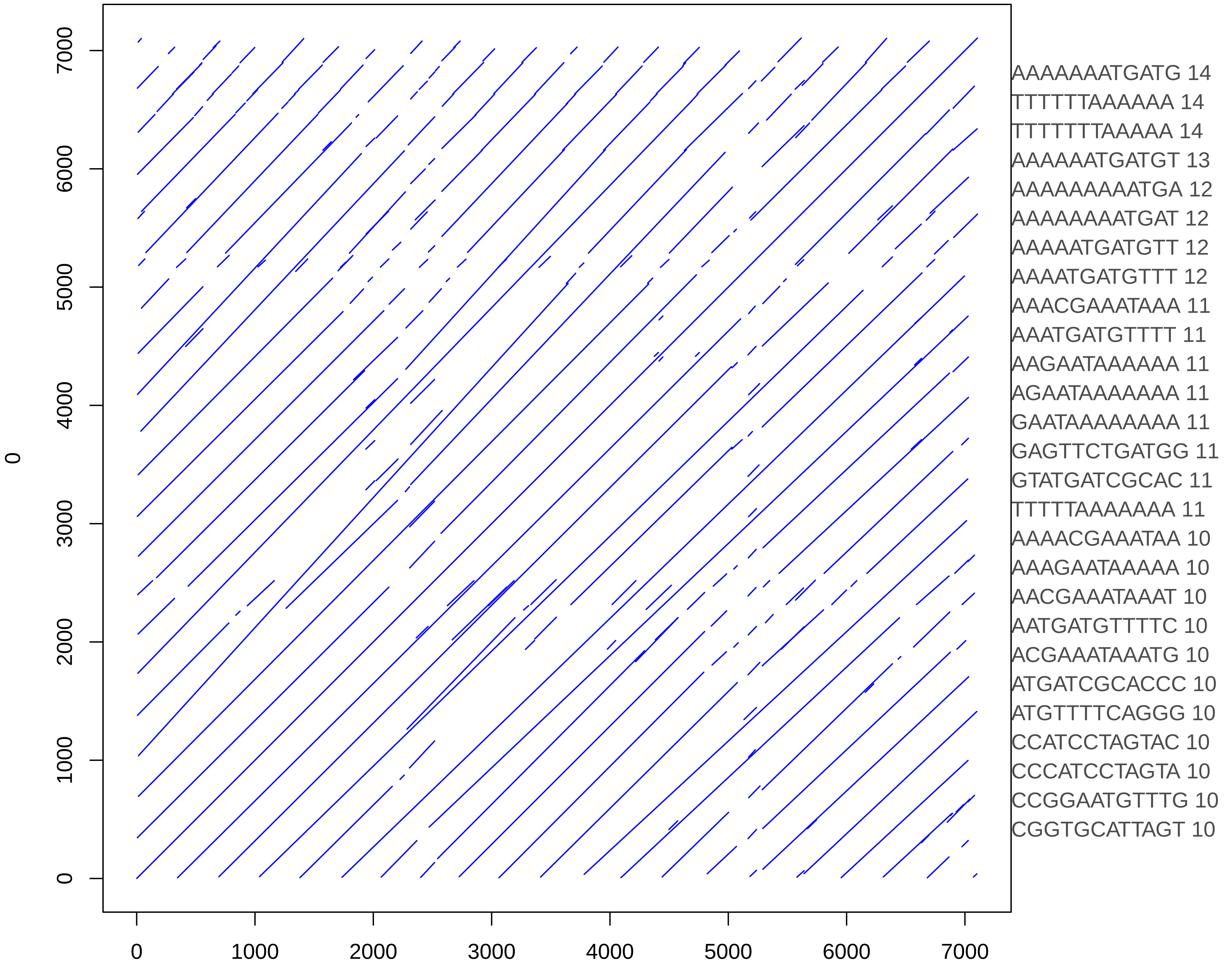

>m150106_044313_42134_c100761282550000001823158307221521_s1_p0/2044/691_5909

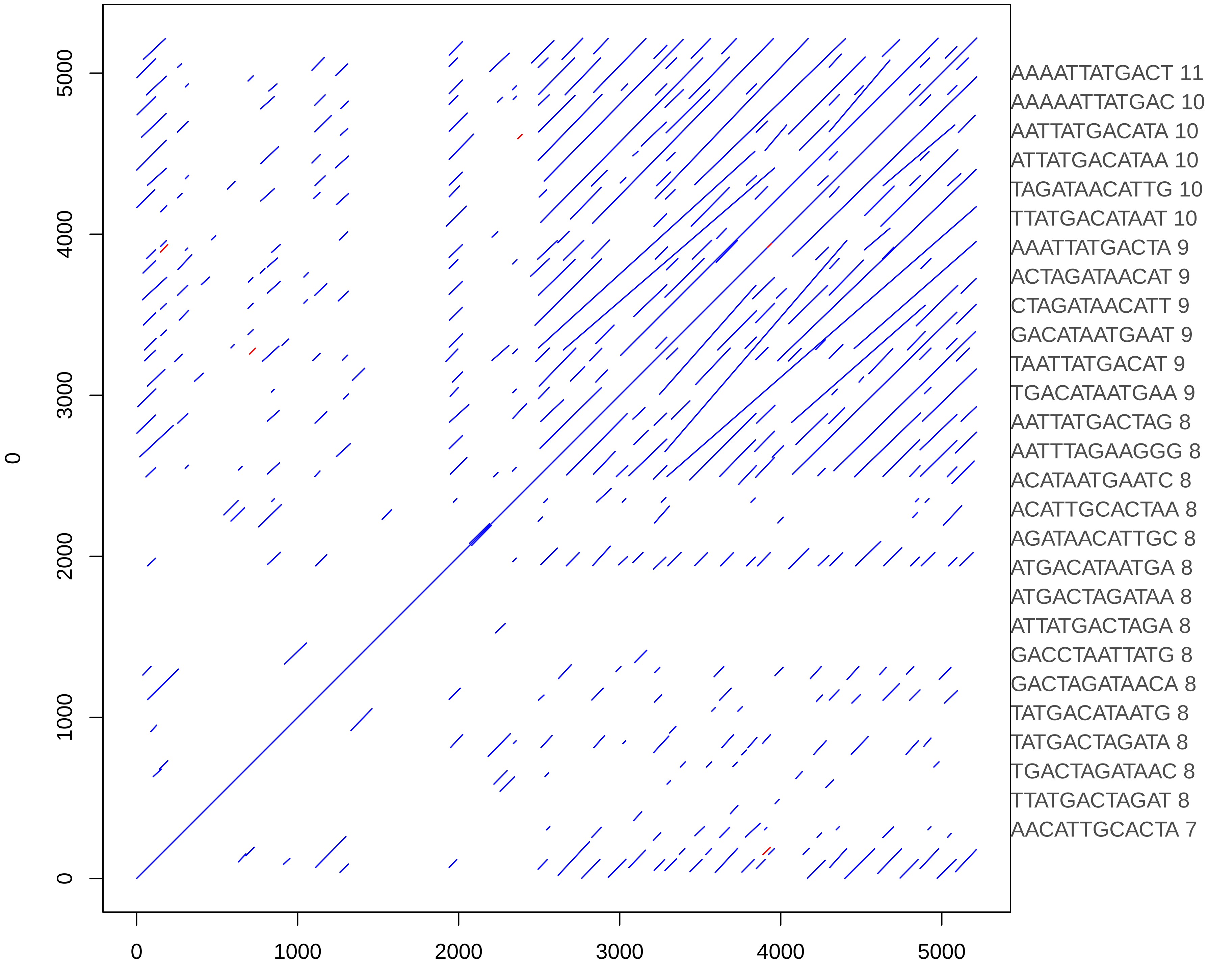

0

>m150106_044313_42134_c100761282550000001823158307221521_s1_p0/25677/0_10215

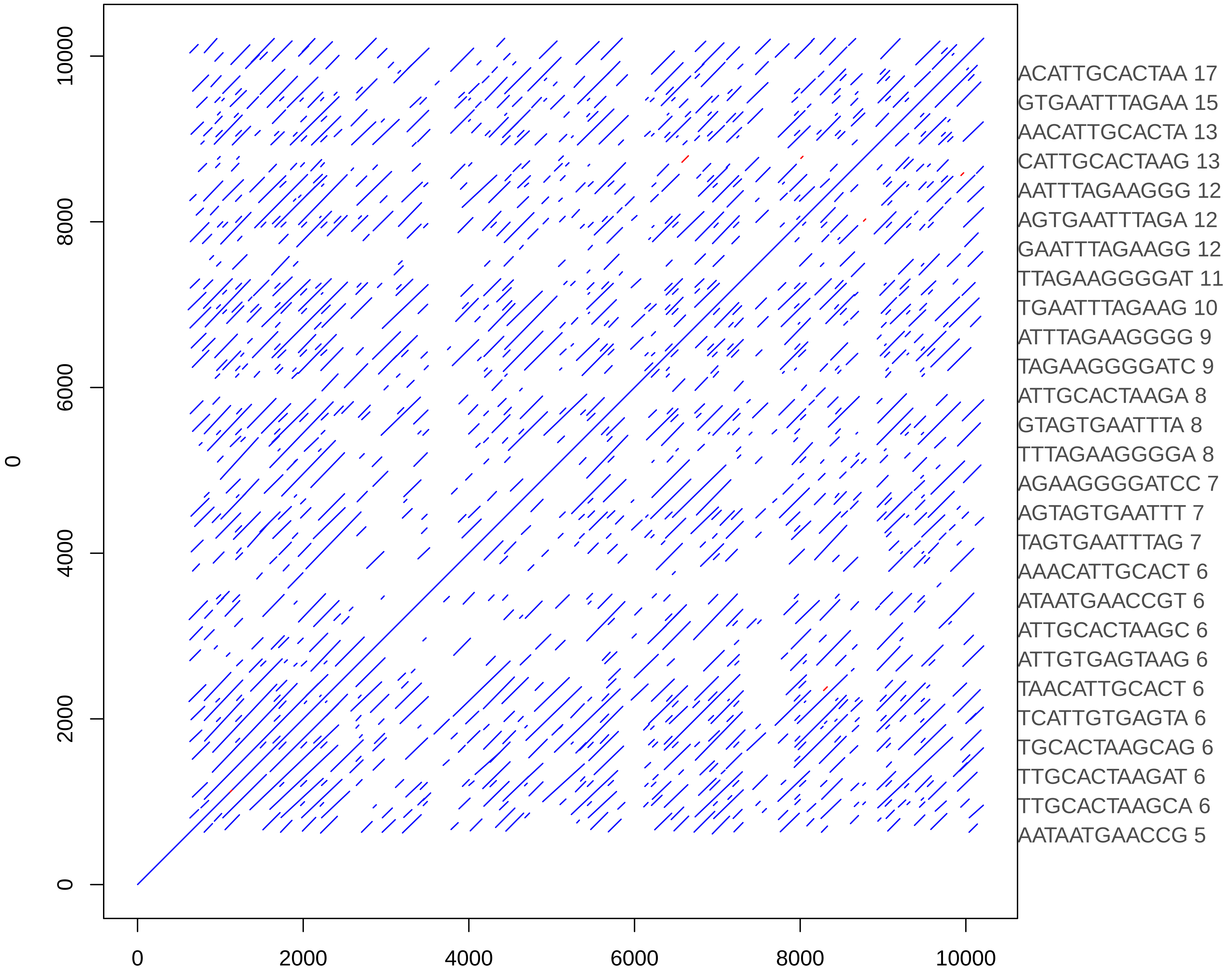

0

>m150106_044313_42134_c100761282550000001823158307221521_s1_p0/27961/0_7987

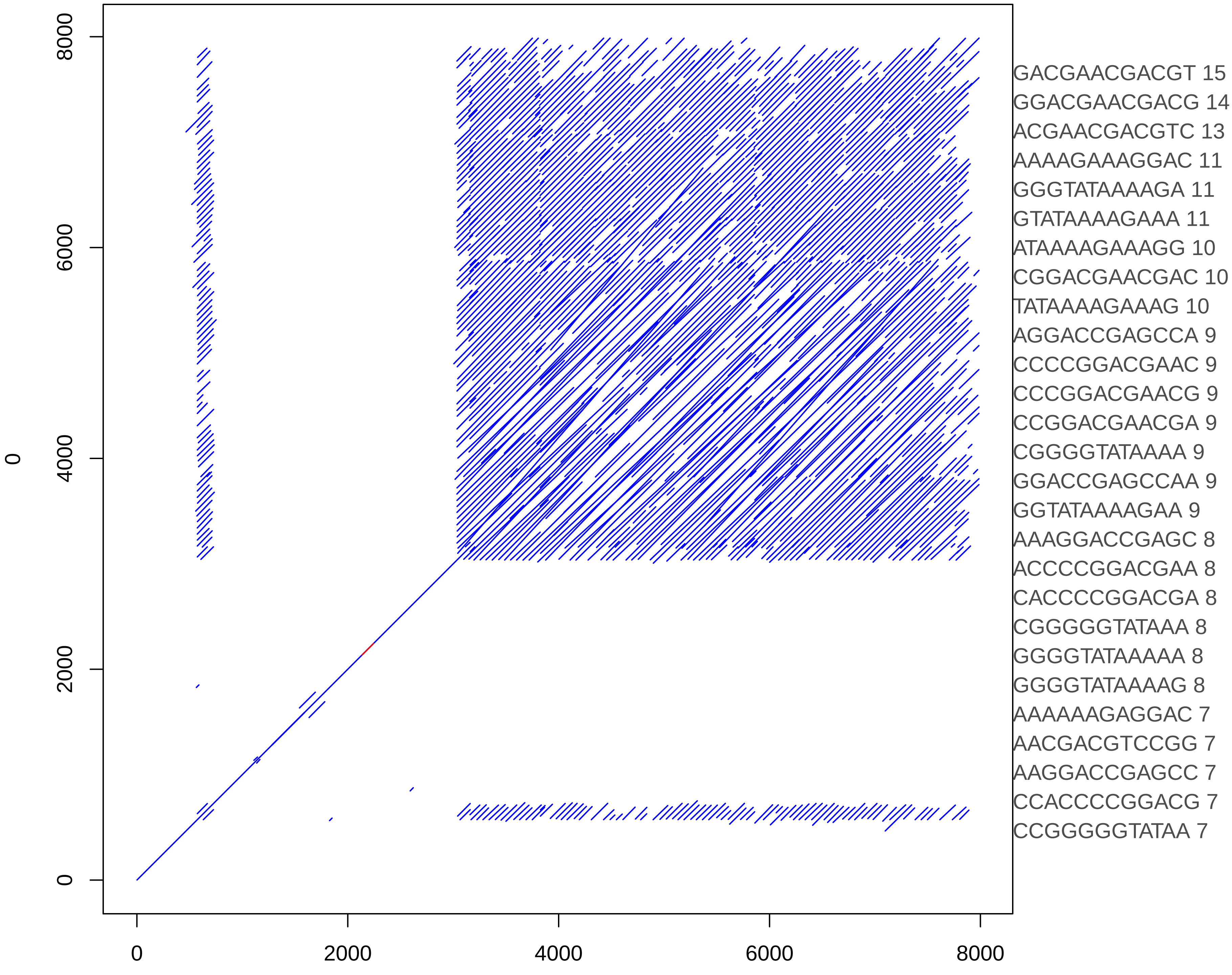

>m150106_044313_42134_c100761282550000001823158307221521_s1_p0/28154/0_10097

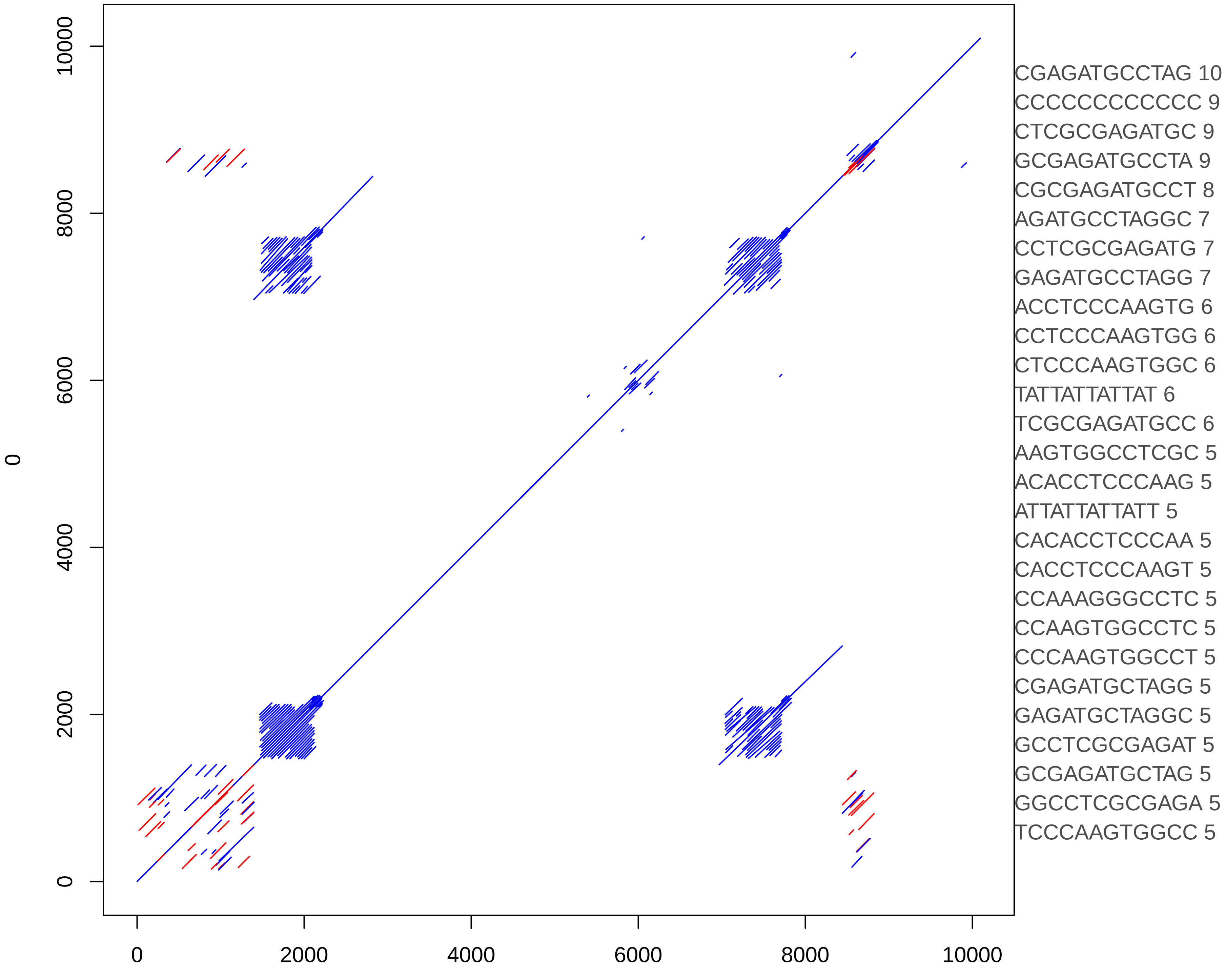

0

>m150106_044313_42134_c100761282550000001823158307221521_s1_p0/28465/0_5390

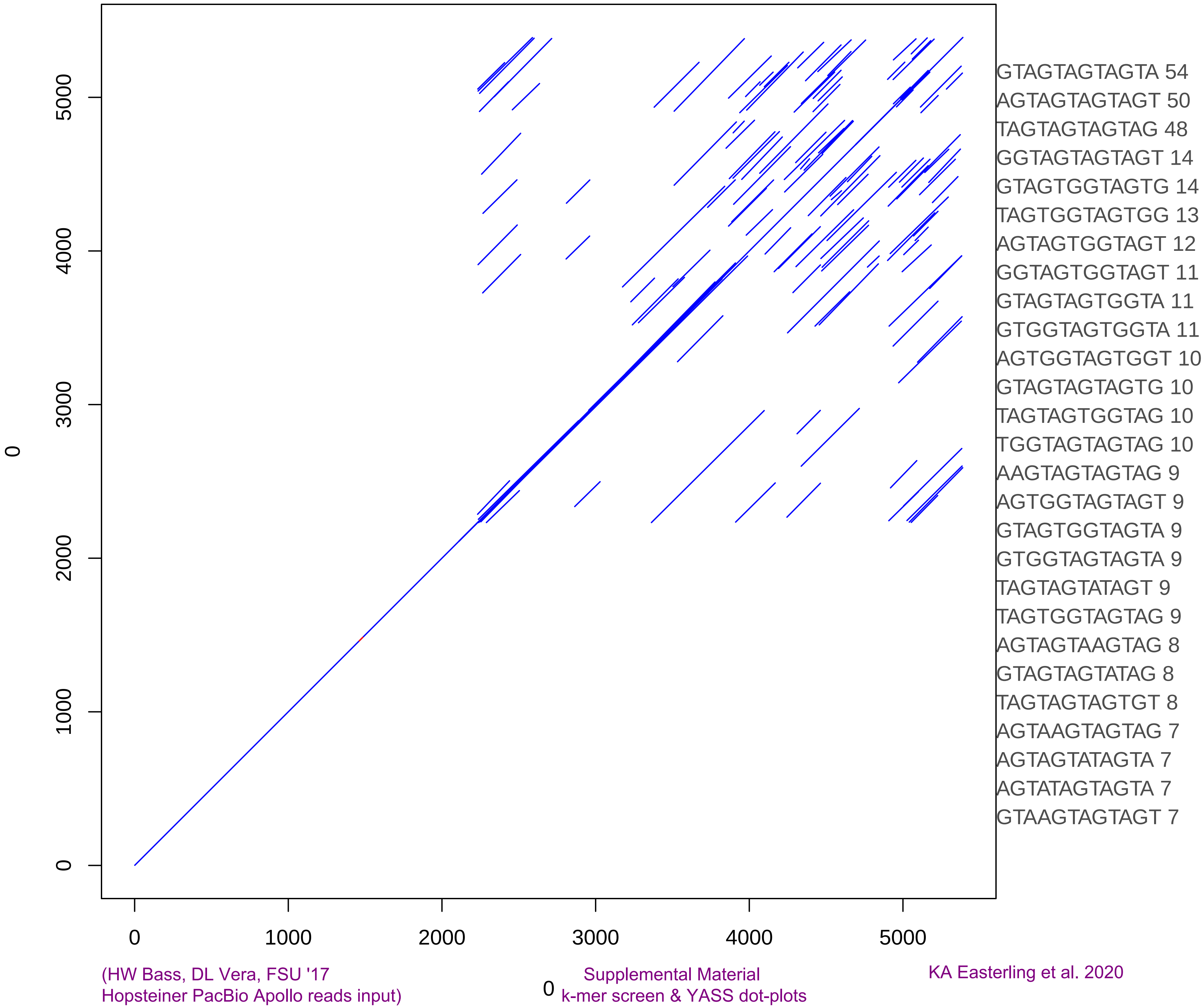

>m150106_044313_42134_c100761282550000001823158307221521_s1_p0/29138/788_8456

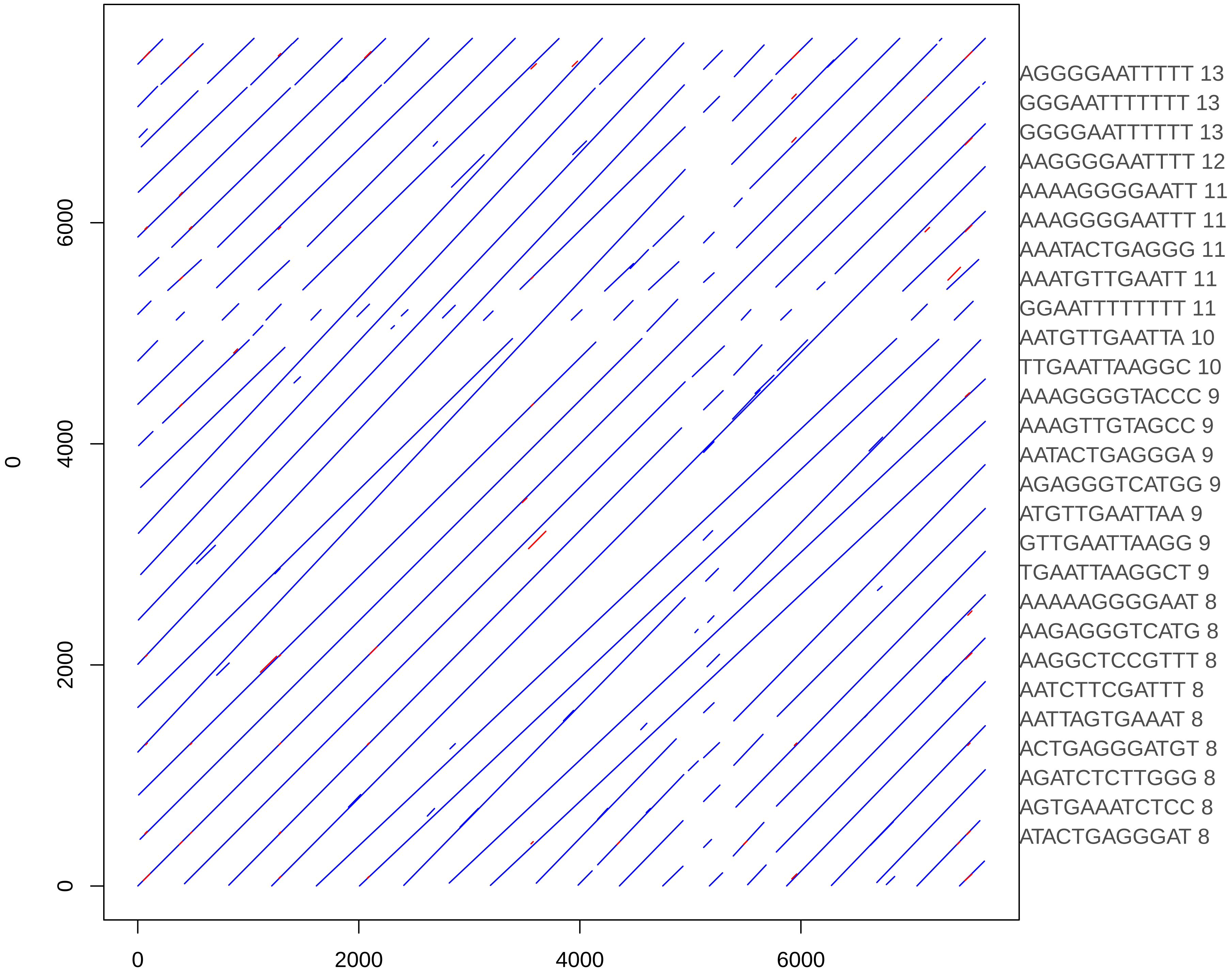

1,121 hits for repetitive sequences passing 12mer abundance criterion

(Read-Number) PAGE 1092

For Tandem Repeat families of ### bp: PacBio Read named "HuluTR###-r1092"

>m150106_044313_42134_c100761282550000001823158307221521_s1_p0/30331/0_10802

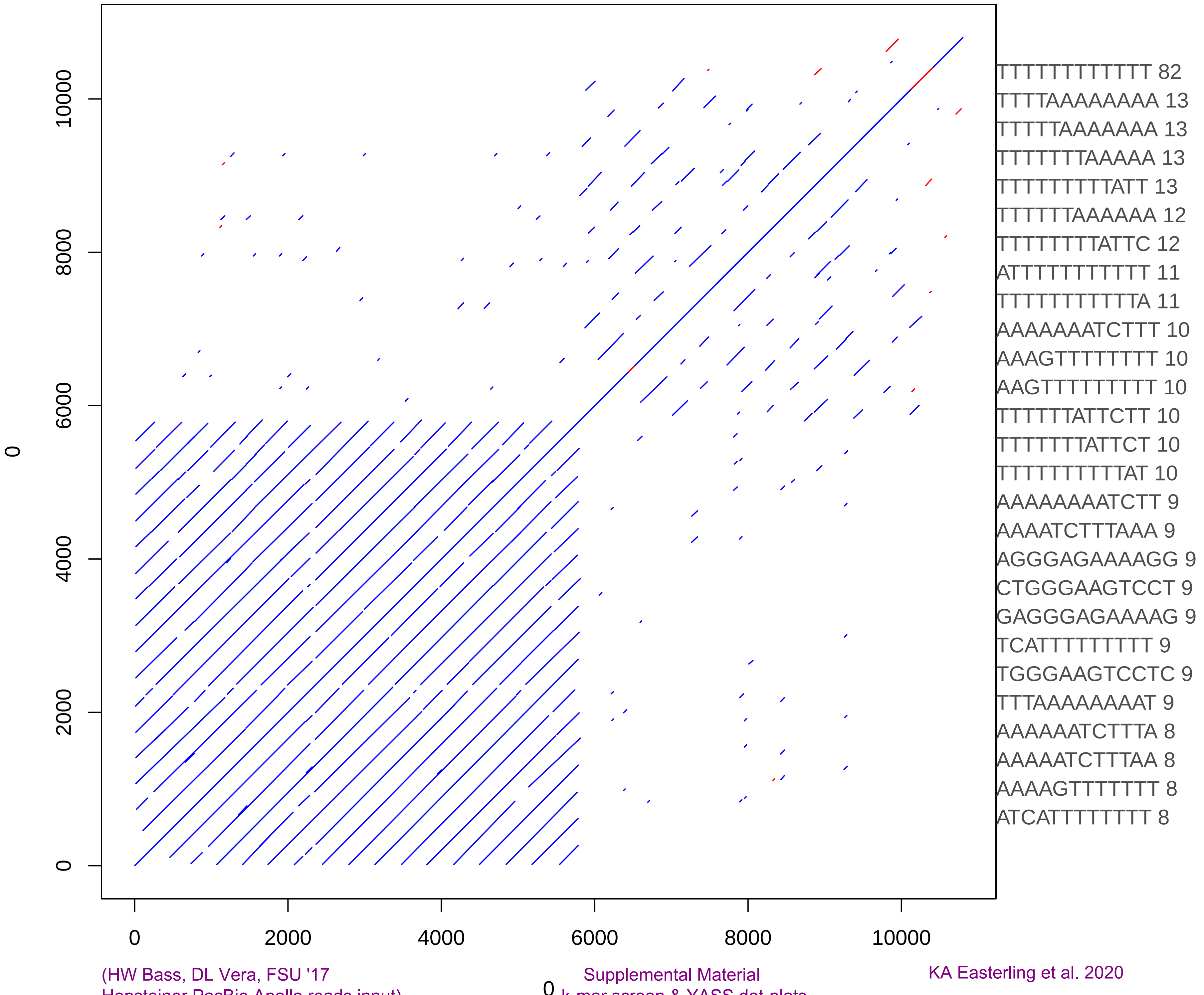

(HW Bass, DL Vera, FSU '17 Hopsteiner PacBio Apollo reads input)

0

Supplemental Material k-mer screen & YASS dot-plots

KA Easterling et al. 2020

>m150106_044313_42134_c100761282550000001823158307221521_s1_p0/30597/0_5849

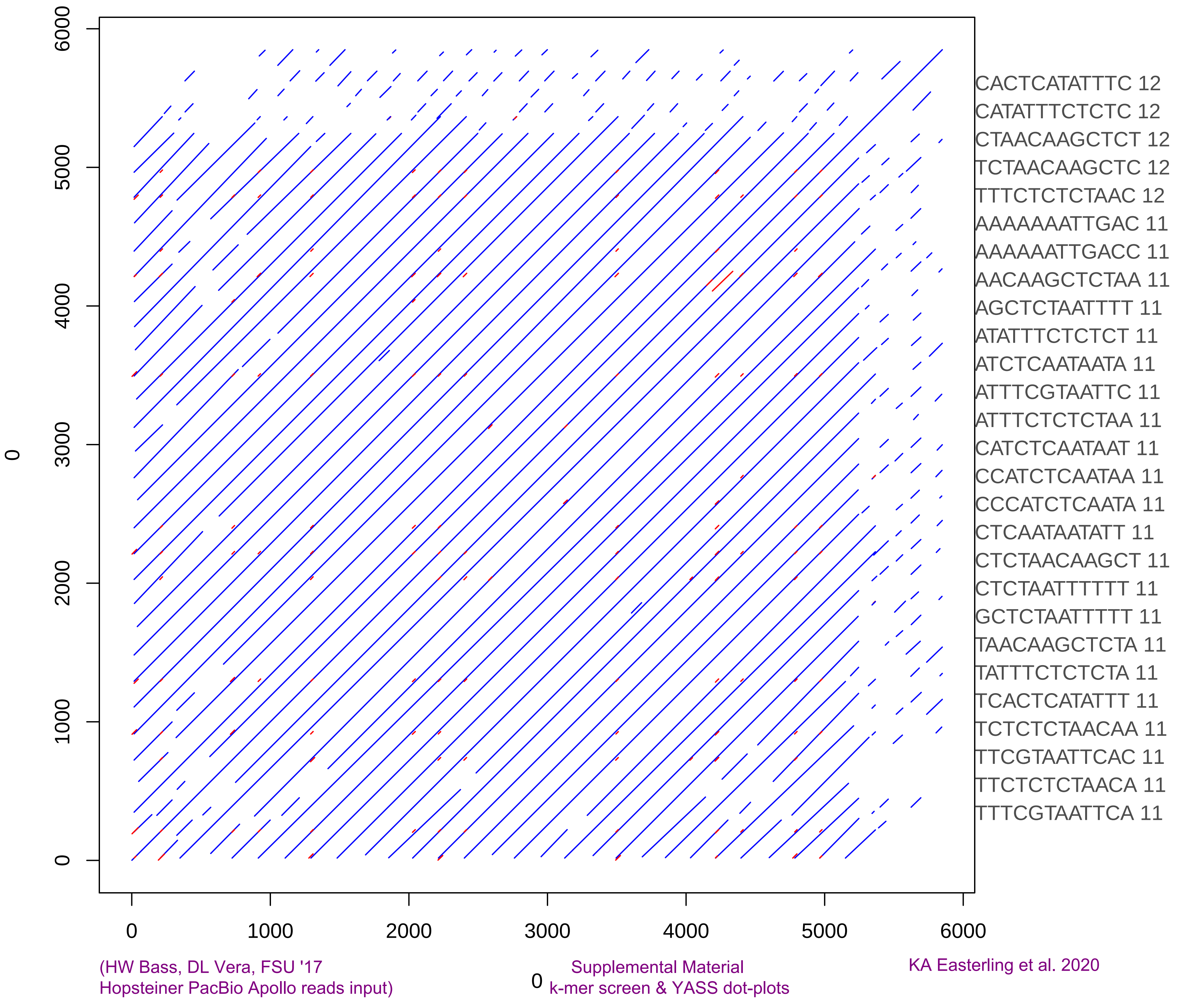

>m150106_044313_42134_c100761282550000001823158307221521_s1_p0/34230/1991_7794

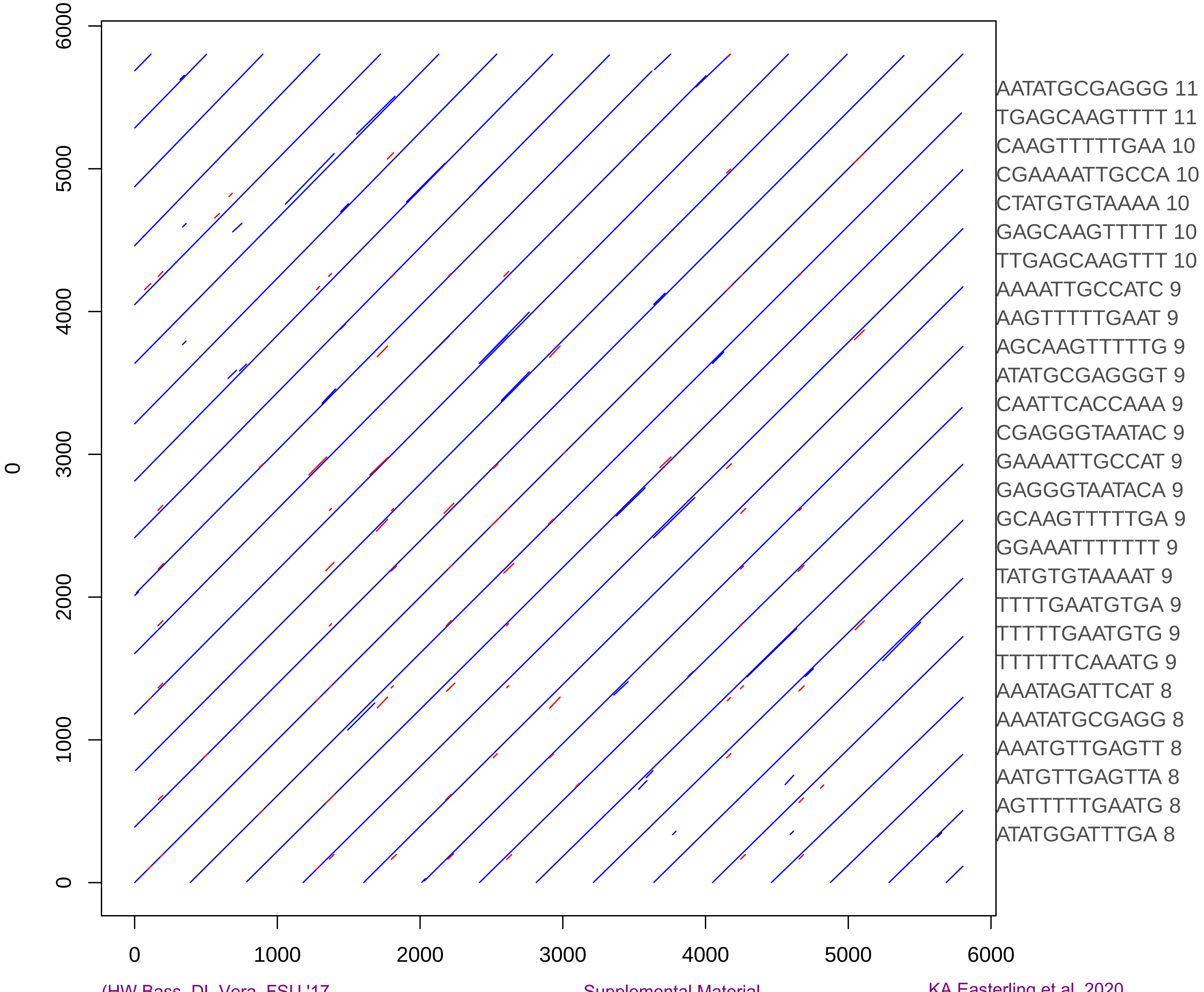

>m150106_044313_42134_c100761282550000001823158307221521_s1_p0/36116/5212_11982

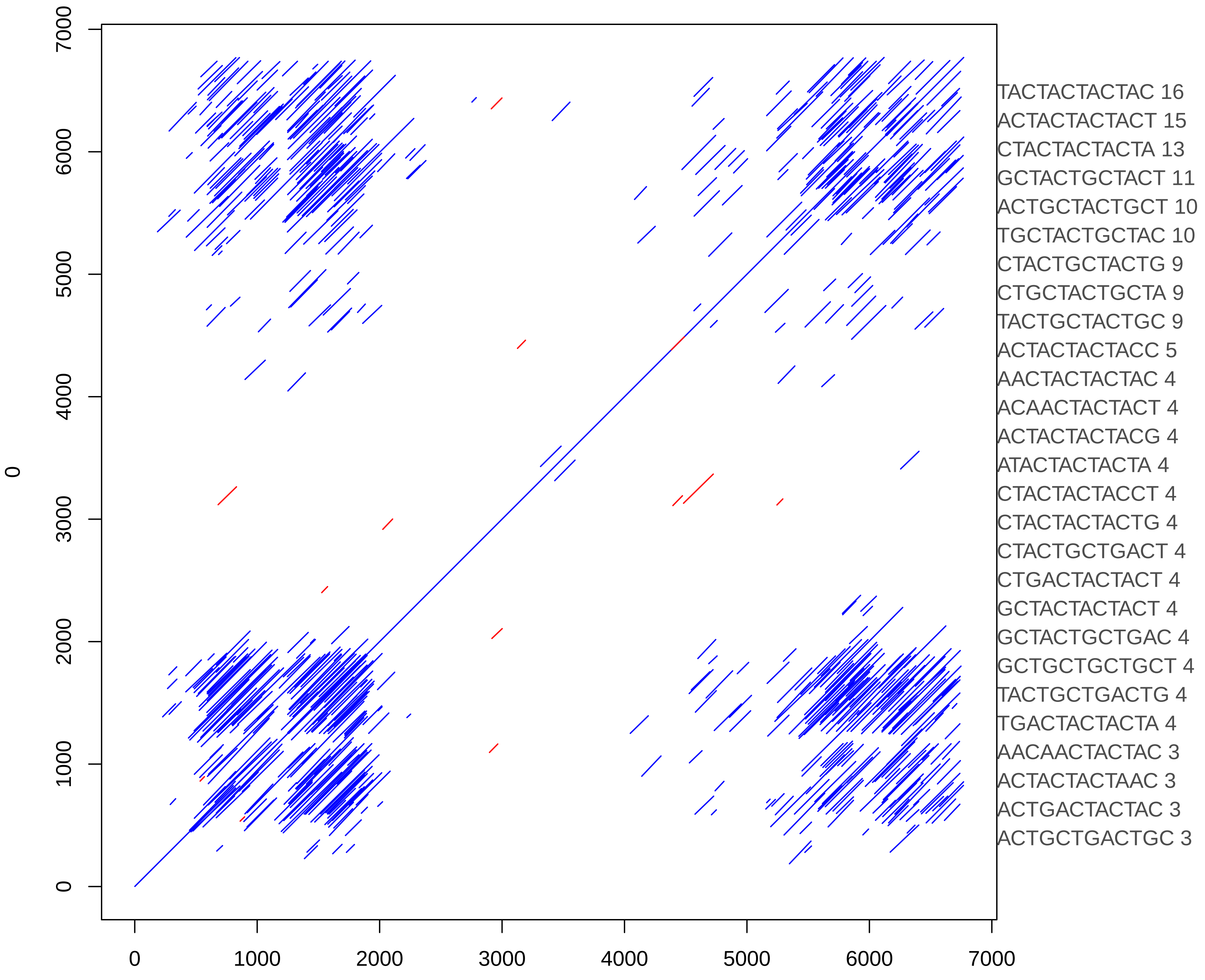

>m150106_044313_42134_c100761282550000001823158307221521_s1_p0/38718/0_7295

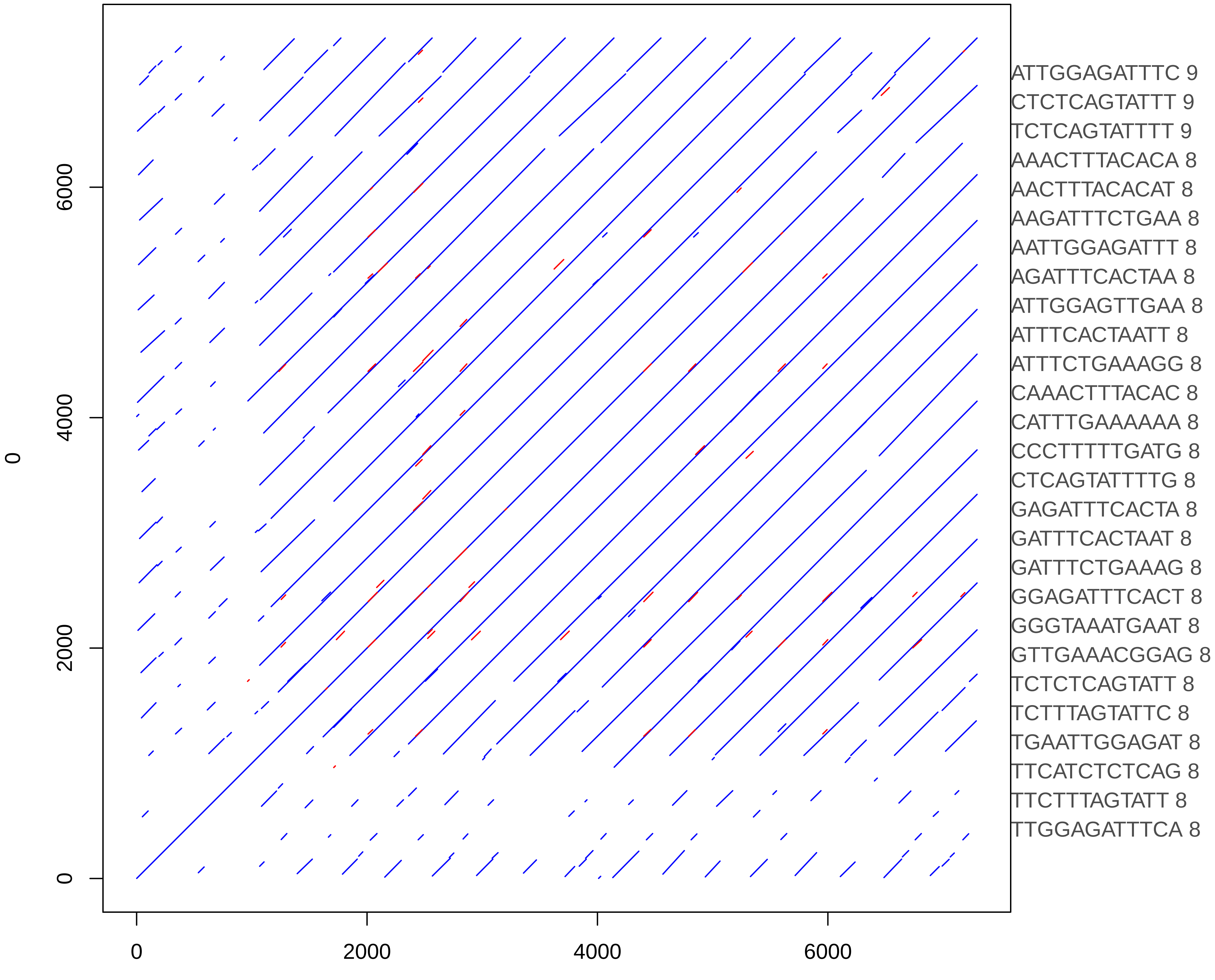

>m150106_044313_42134_c100761282550000001823158307221521_s1_p0/39106/0_9217

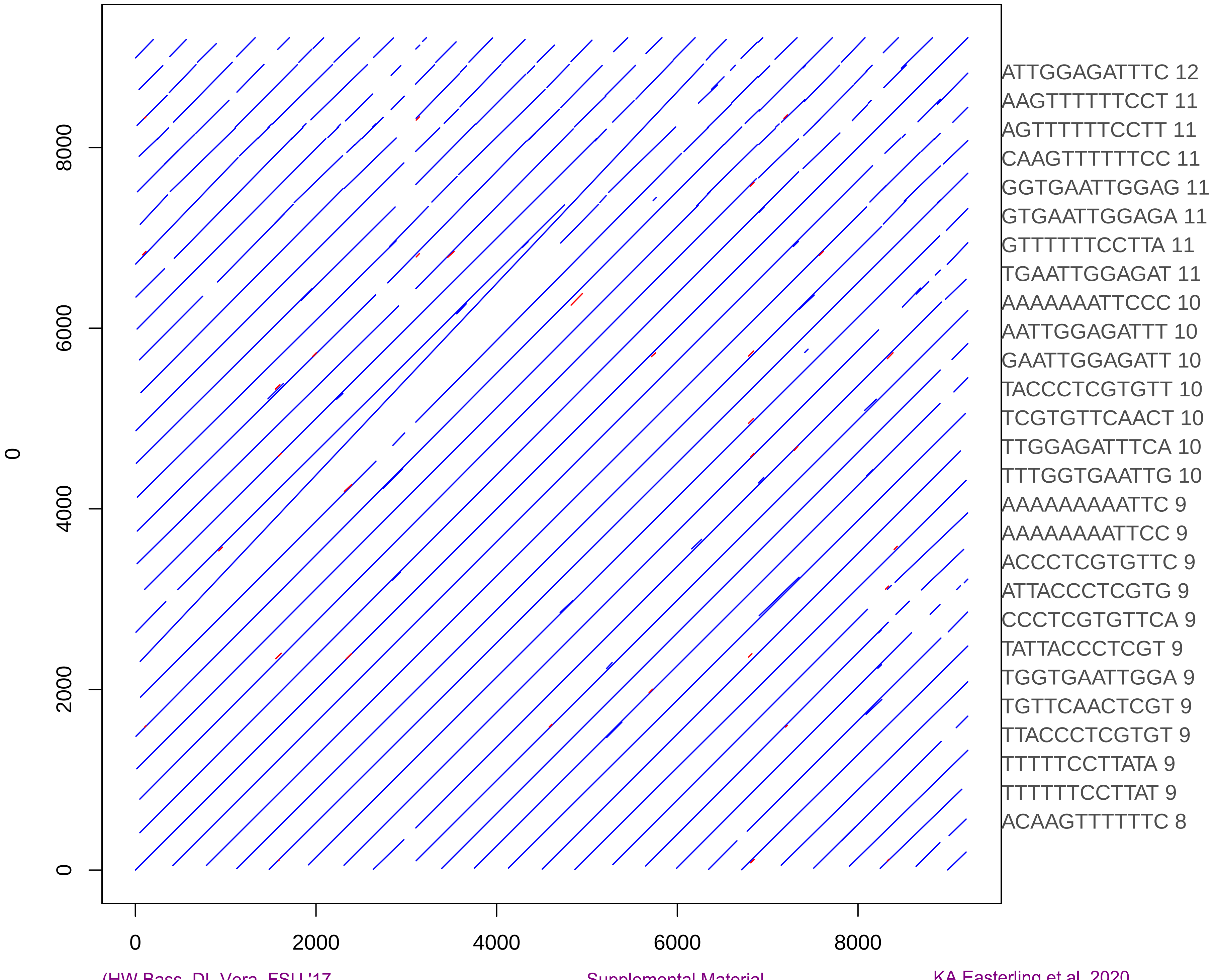

>m150106_044313_42134_c100761282550000001823158307221521_s1_p0/39247/184_7619

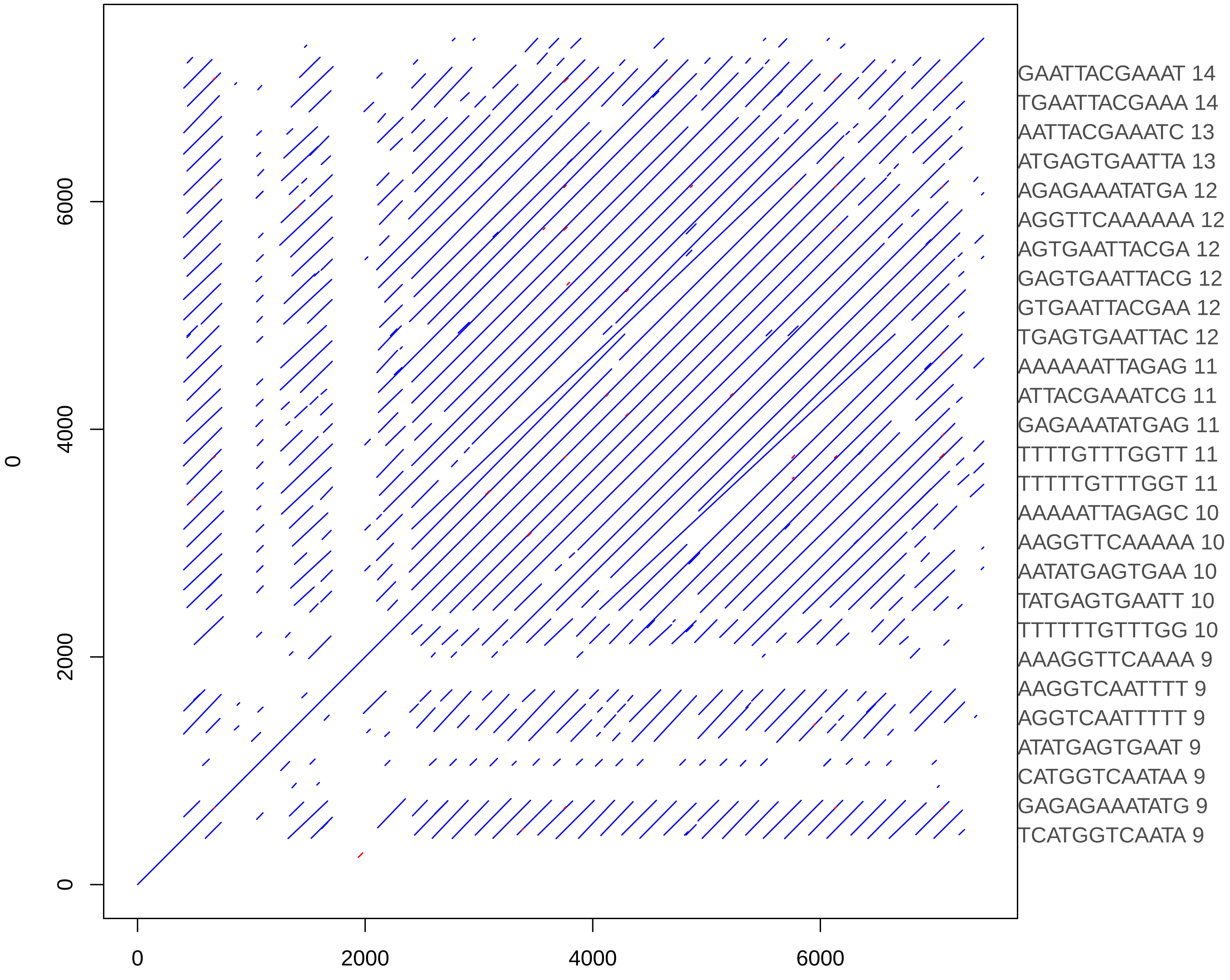

>m150106_044313_42134_c100761282550000001823158307221521_s1_p0/399/0_7966

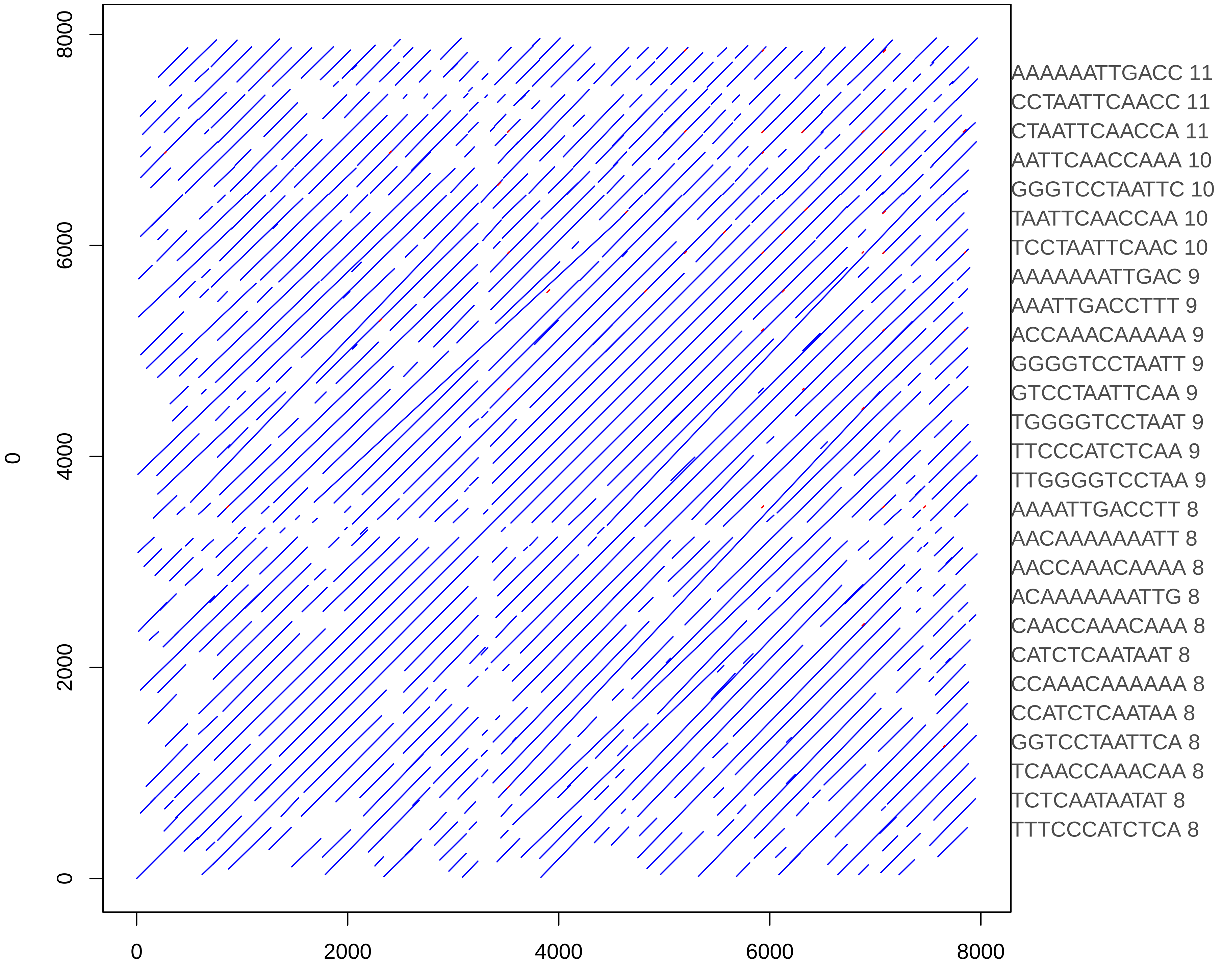

>m150106_044313_42134_c100761282550000001823158307221521_s1_p0/40756/0_7672

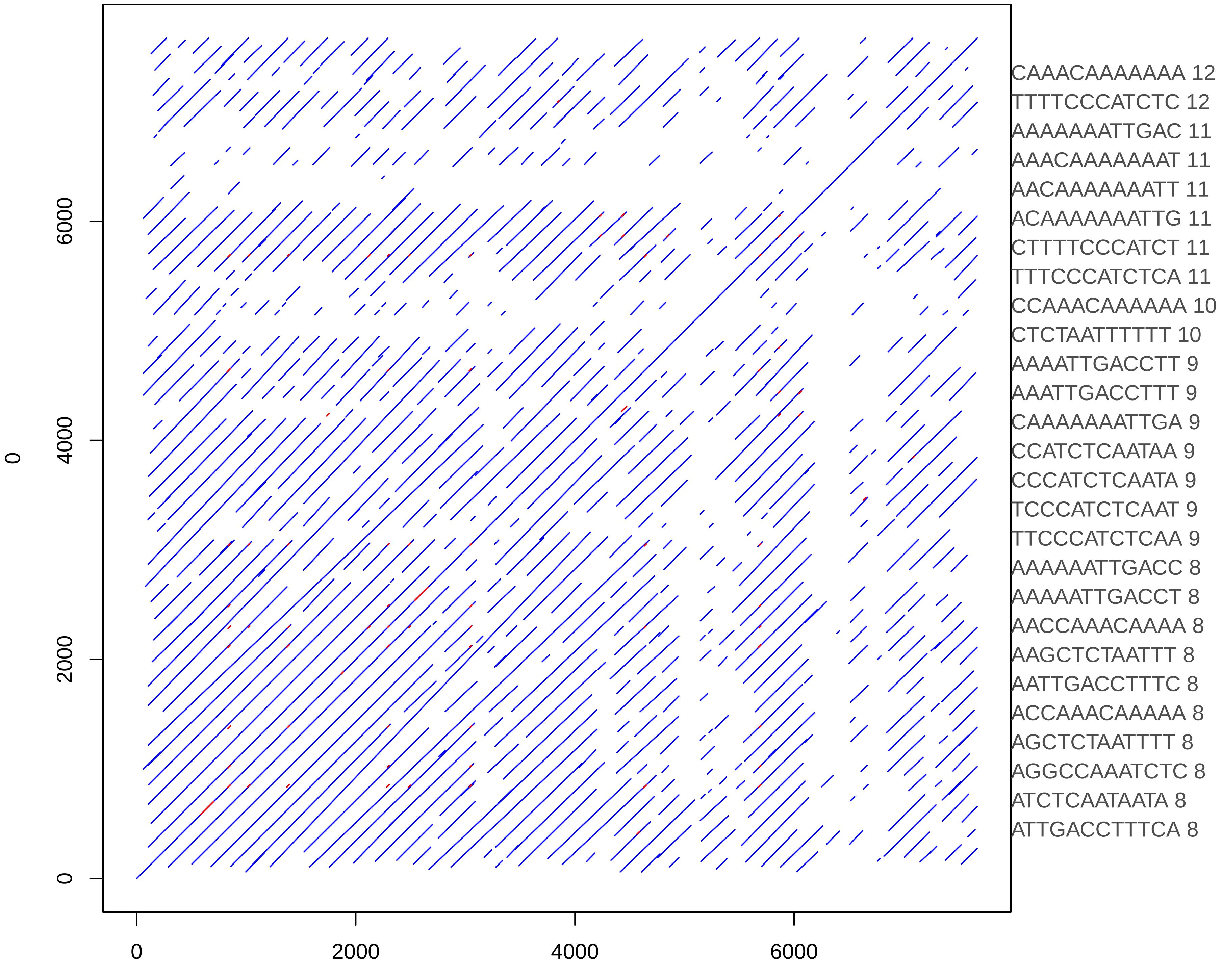

>m150106_044313_42134_c100761282550000001823158307221521_s1_p0/42433/0_6628

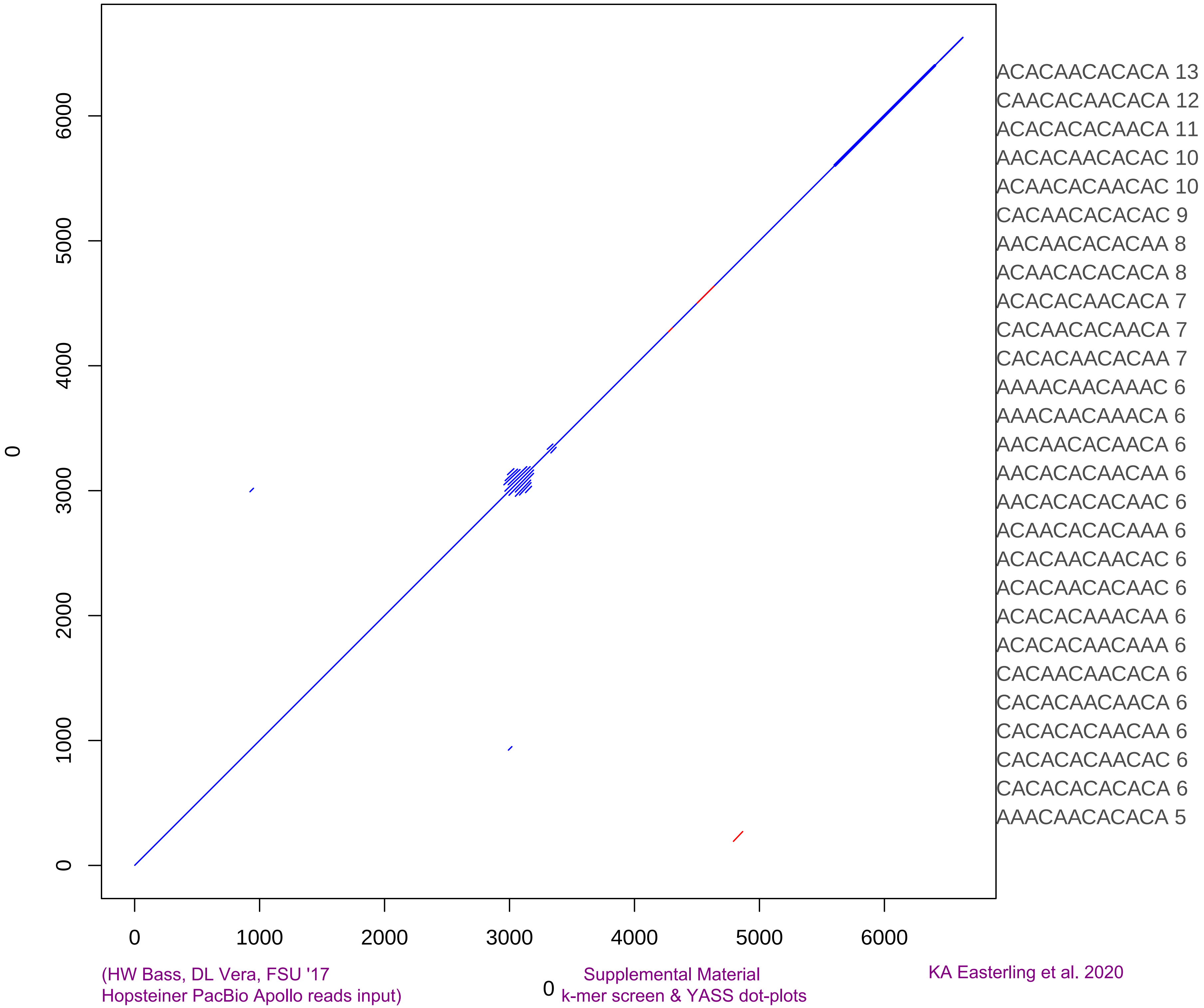

>m150106_044313_42134_c100761282550000001823158307221521_s1_p0/44418/1000_7819

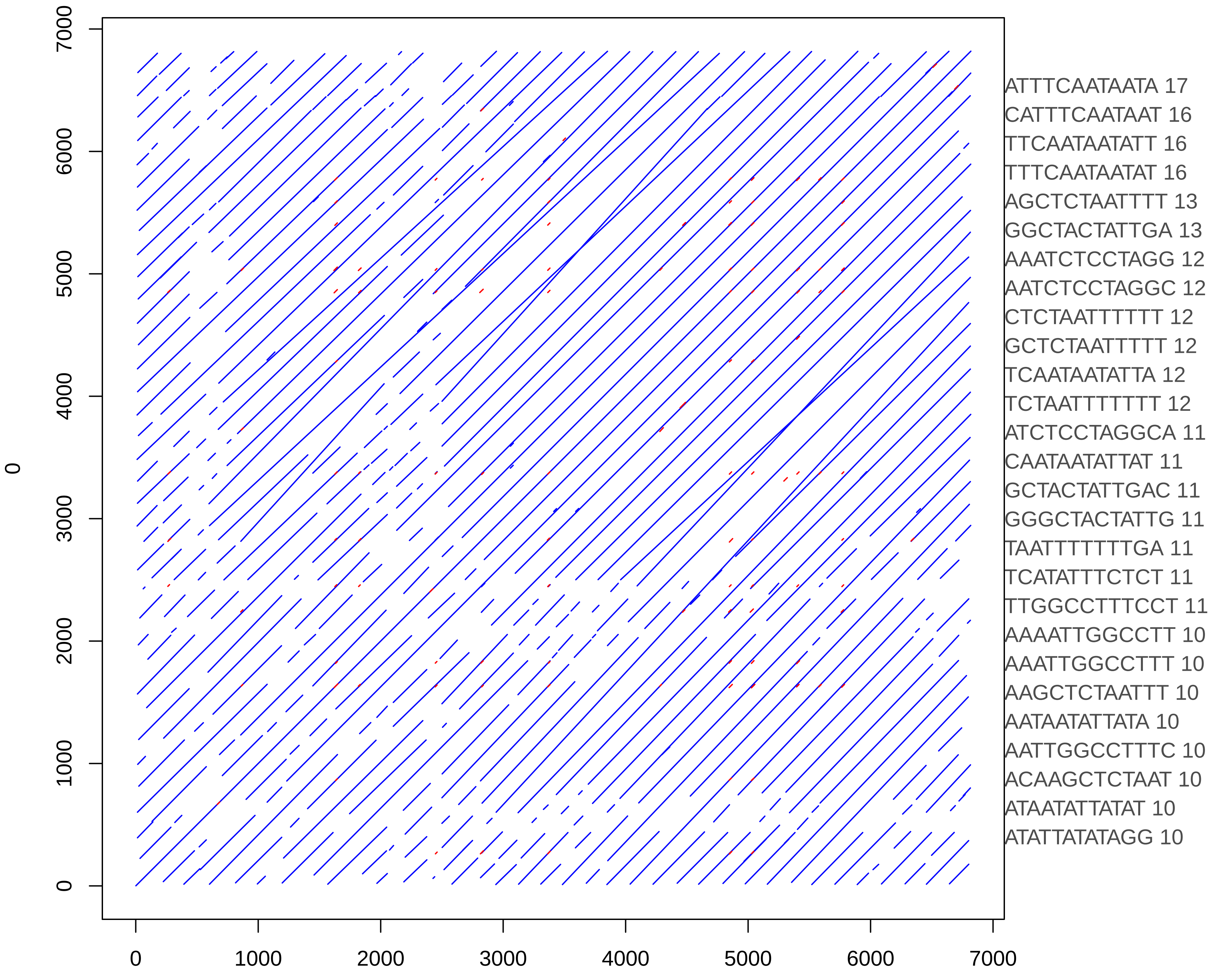

>m150106_044313_42134_c100761282550000001823158307221521_s1_p0/45080/894_10481

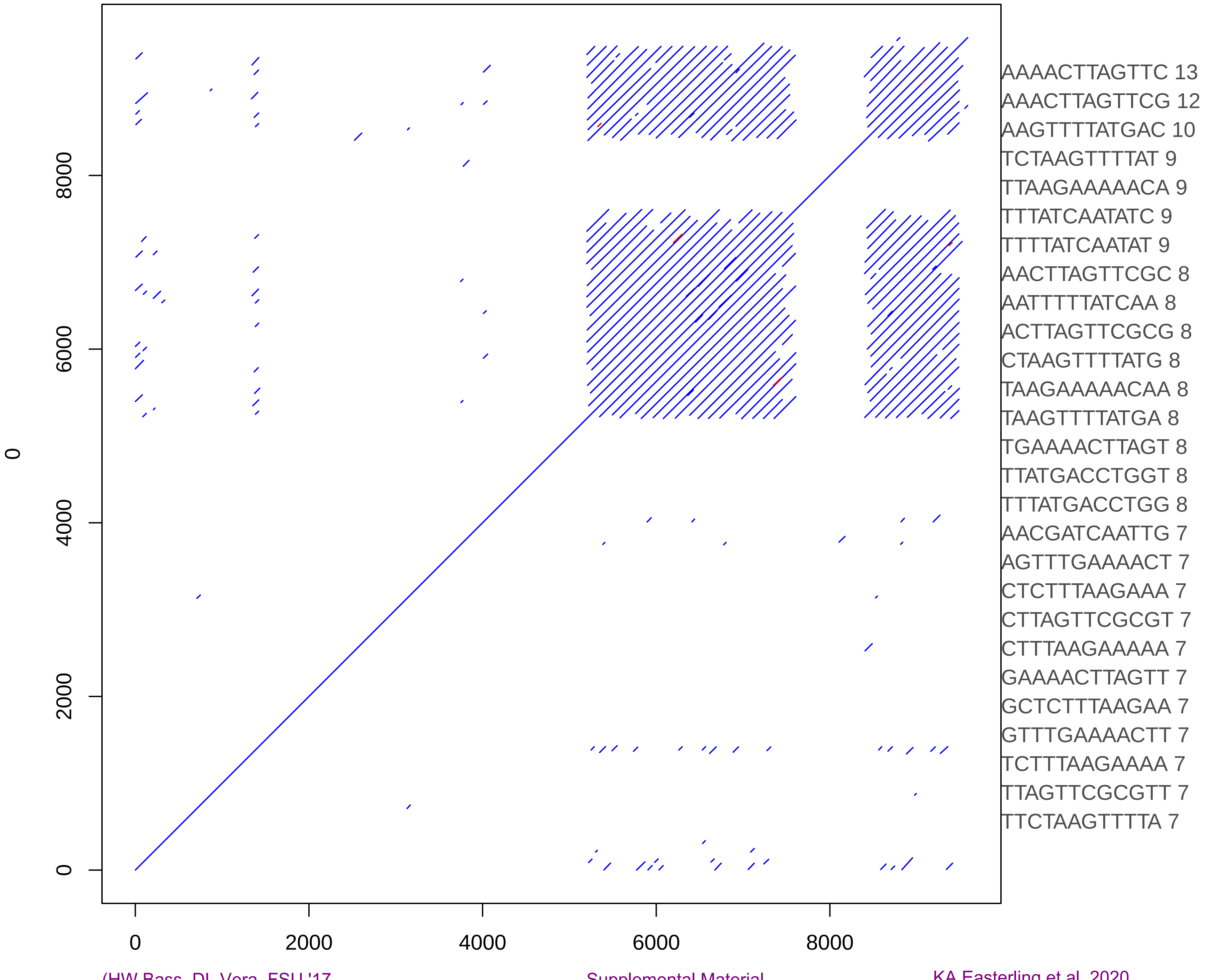

>m150106_044313_42134_c100761282550000001823158307221521_s1_p0/46340/0_13218

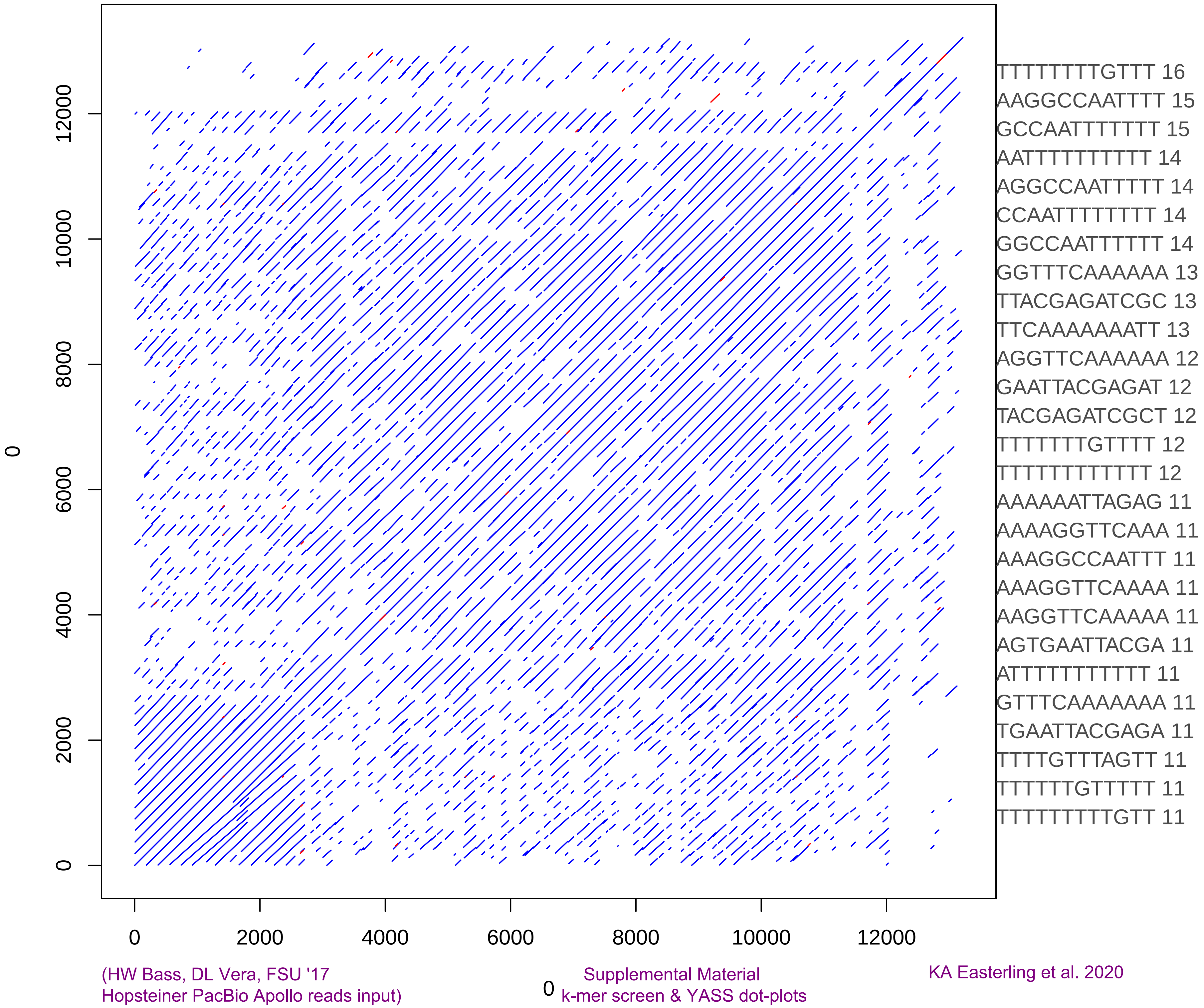

>m150106_044313_42134_c100761282550000001823158307221521_s1_p0/50922/0_5161

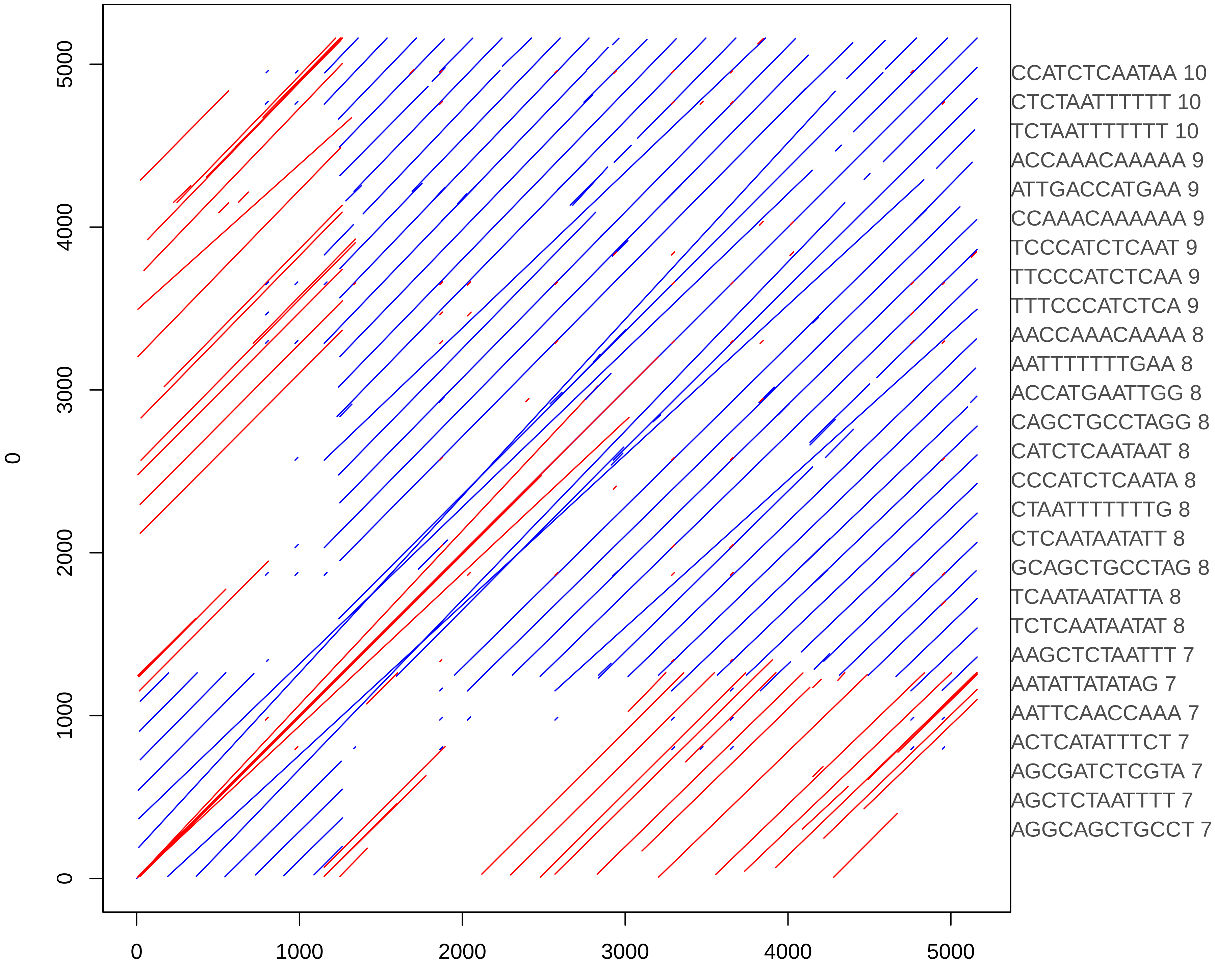

>m150106_044313_42134_c100761282550000001823158307221521_s1_p0/58962/0_12668

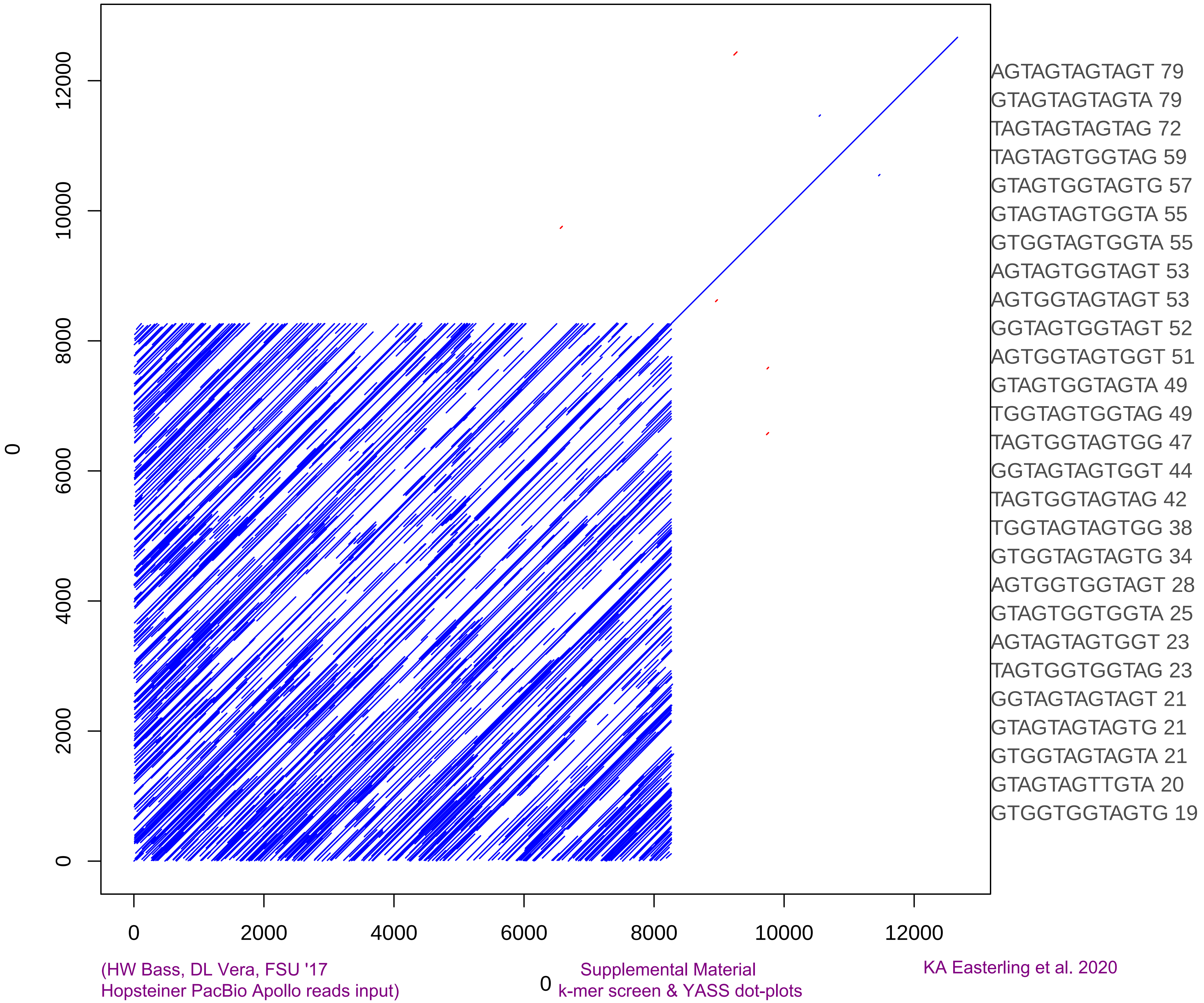

>m150106_044313_42134_c100761282550000001823158307221521_s1_p0/59665/535_10670

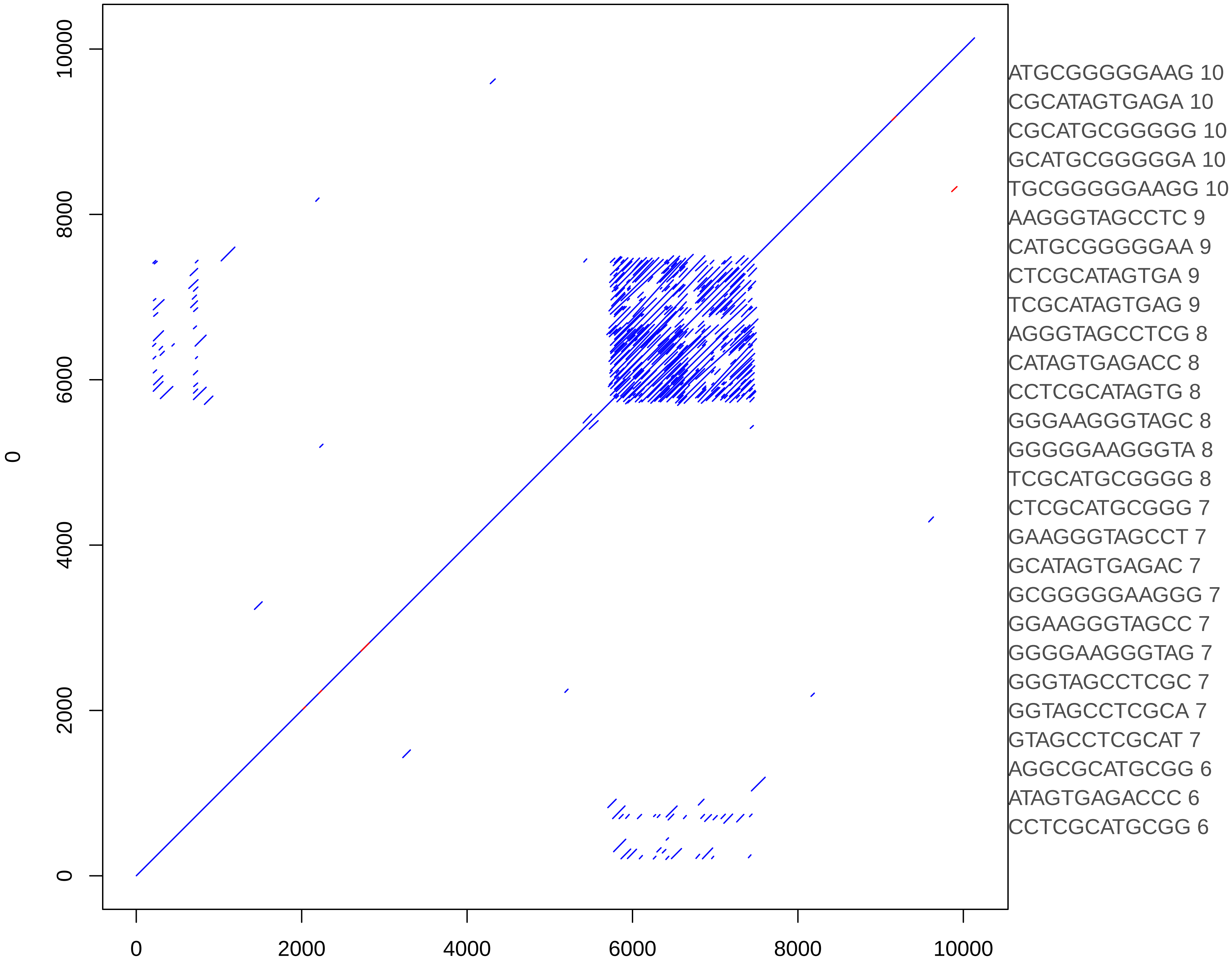

>m150106_044313_42134_c100761282550000001823158307221521_s1_p0/69392/1083_11334

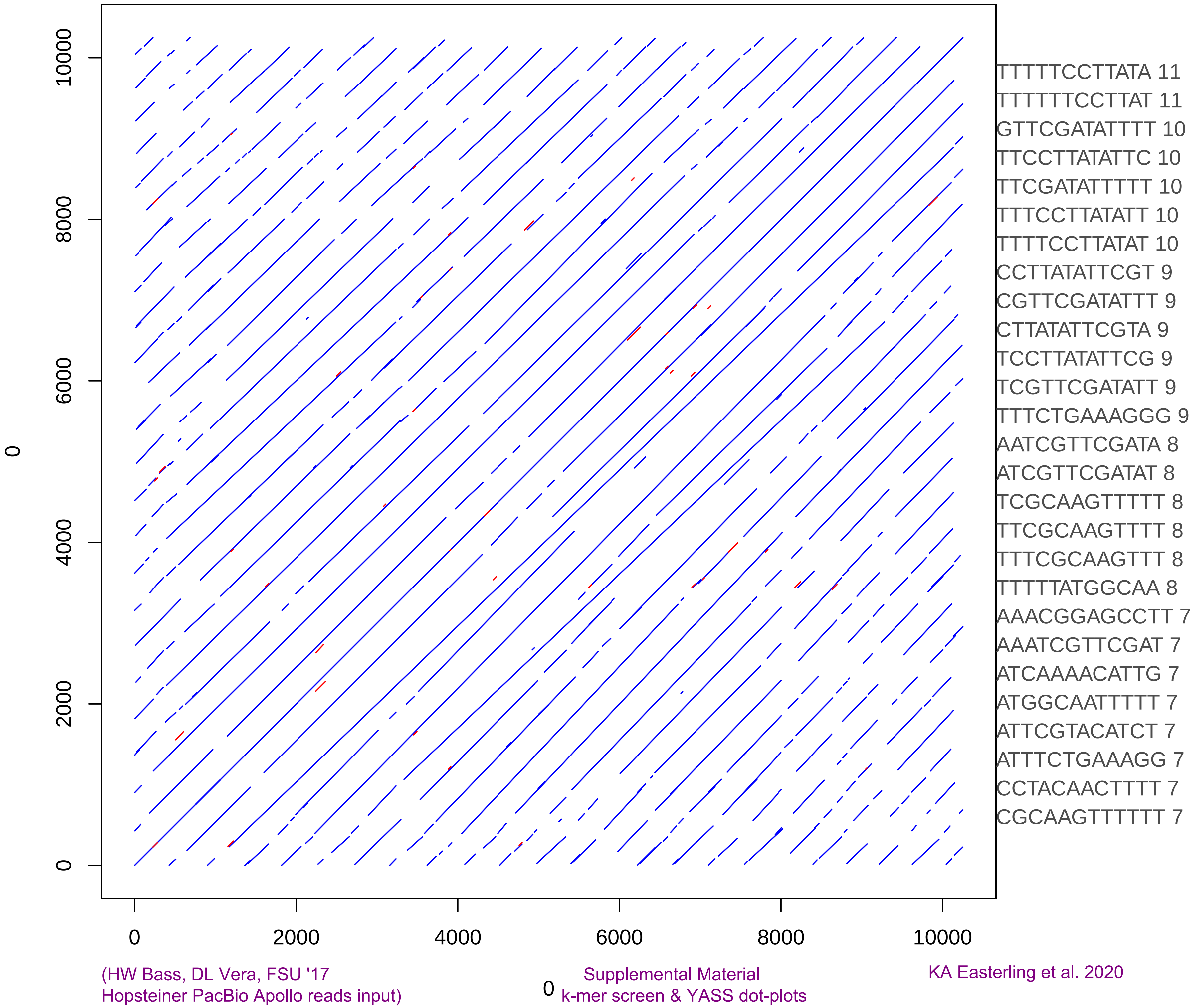

>m150106_044313_42134_c100761282550000001823158307221521_s1_p0/70157/0_5573

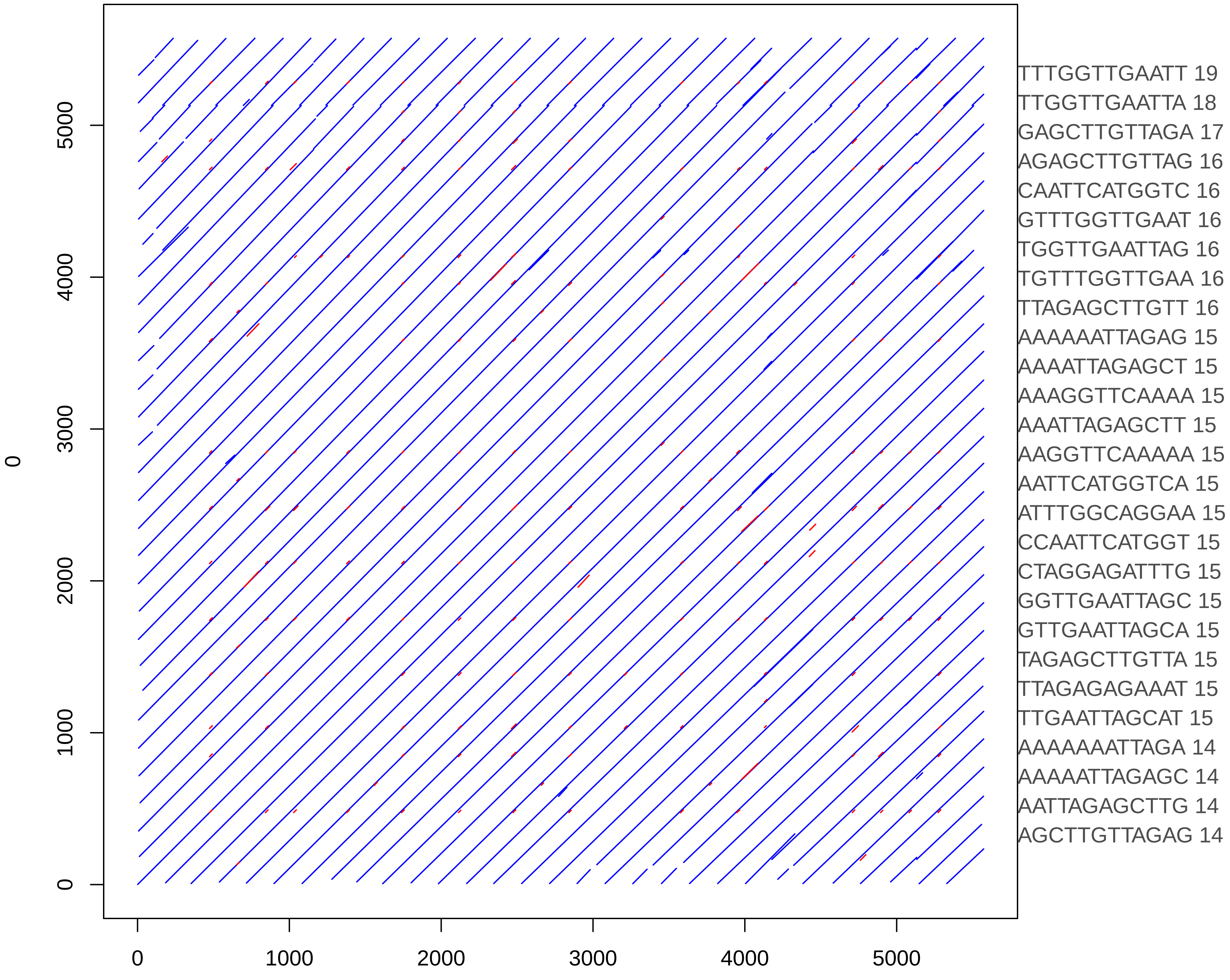

>m150106_044313_42134_c100761282550000001823158307221521_s1_p0/70678/286_5704

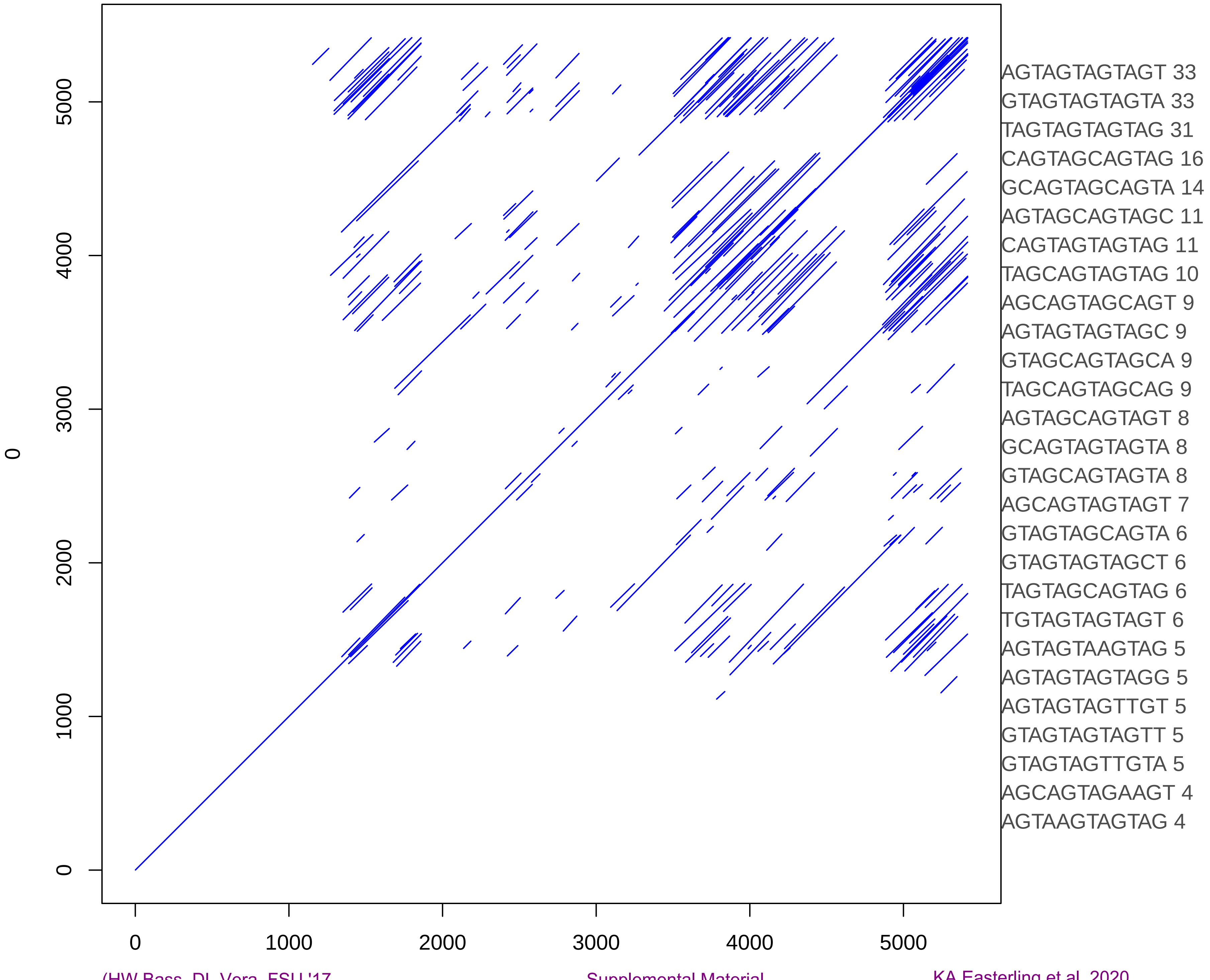

>m150106_044313_42134_c100761282550000001823158307221521_s1_p0/72516/0_5651

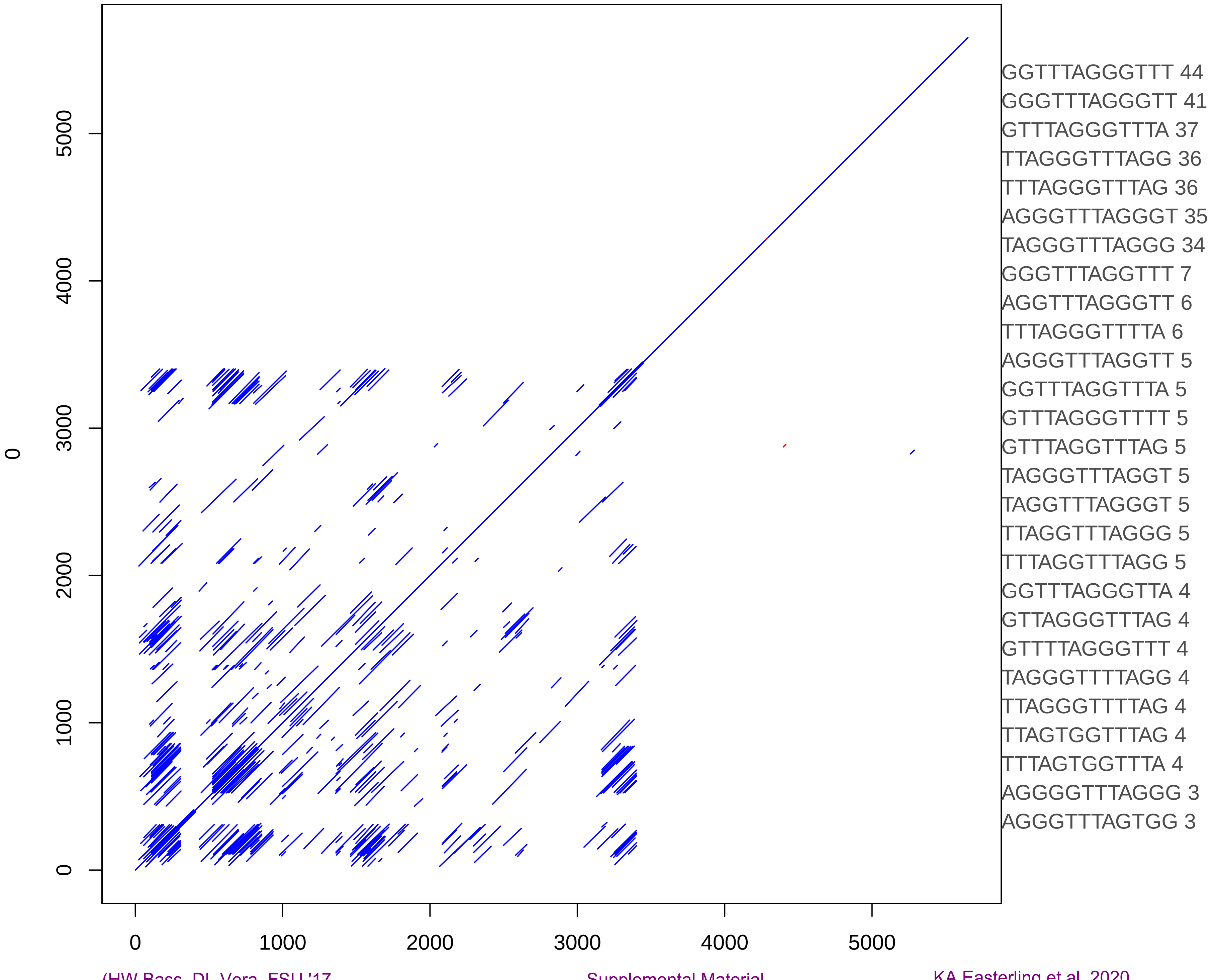

>m150106_044313_42134_c100761282550000001823158307221521_s1_p0/72721/245_6298

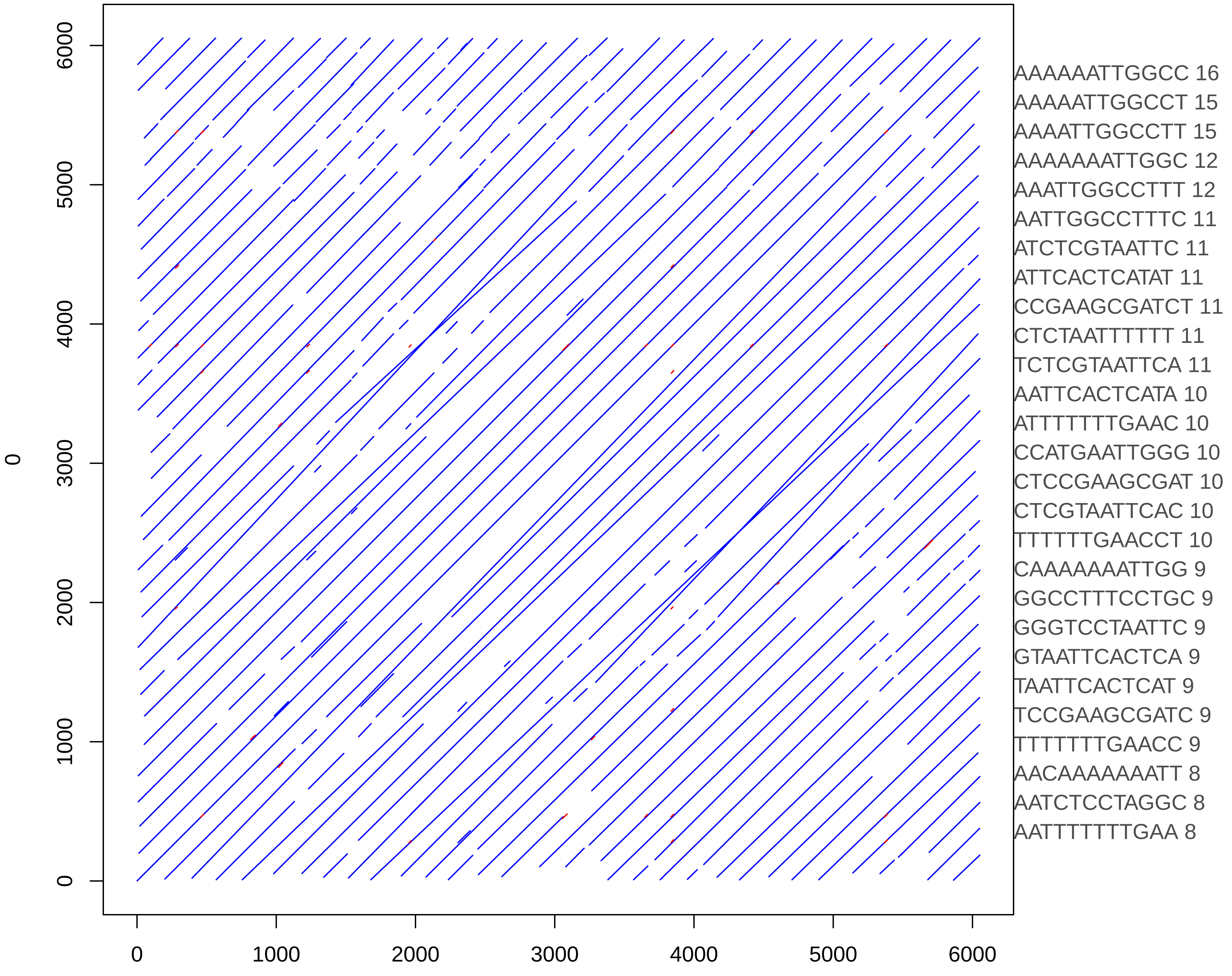

>m150106_044313_42134_c100761282550000001823158307221521_s1_p0/76484/0_9358

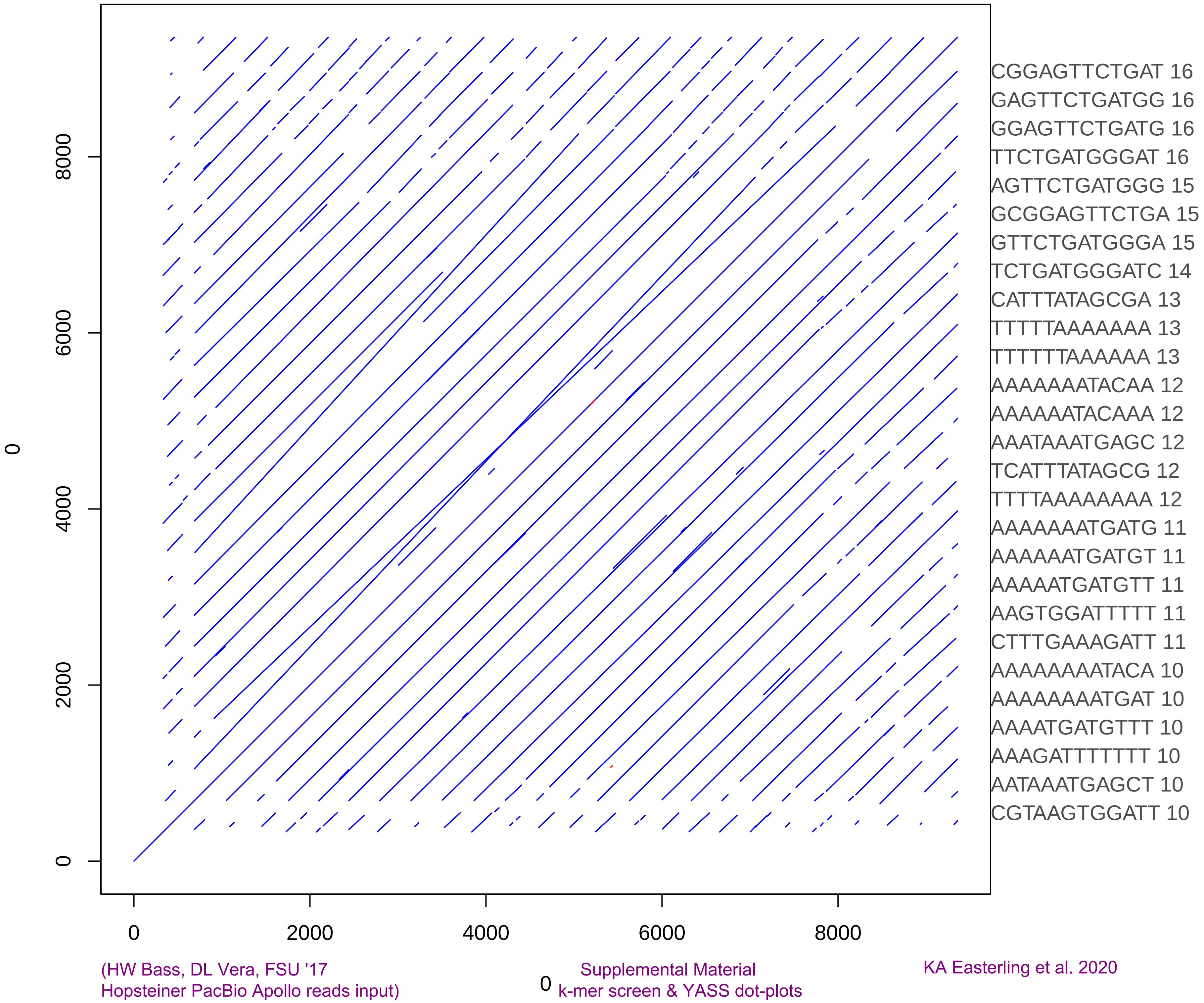

>m150106_044313_42134_c100761282550000001823158307221521_s1_p0/81085/956_10266

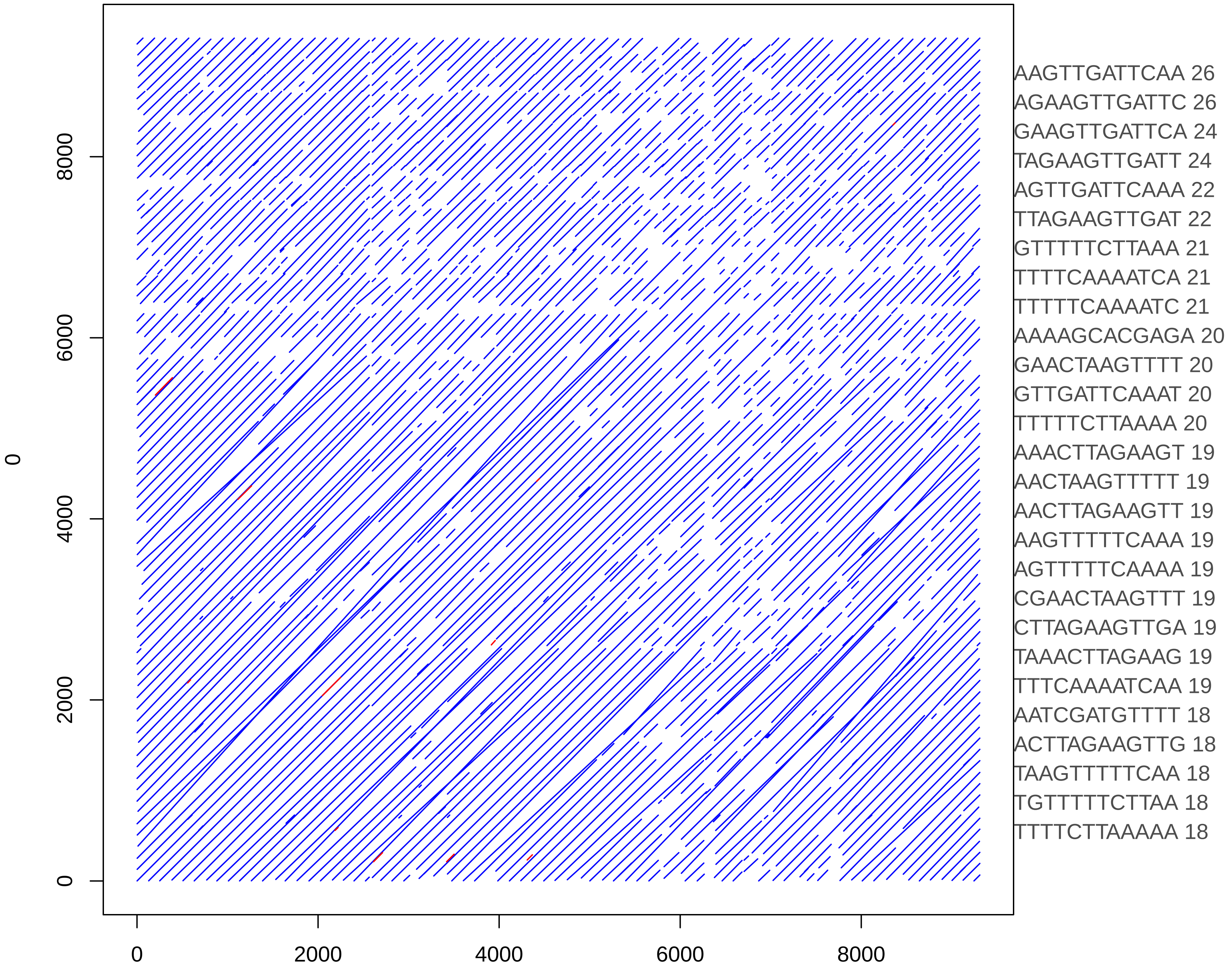

>m150106_044313_42134_c100761282550000001823158307221521_s1_p0/81942/189_9723

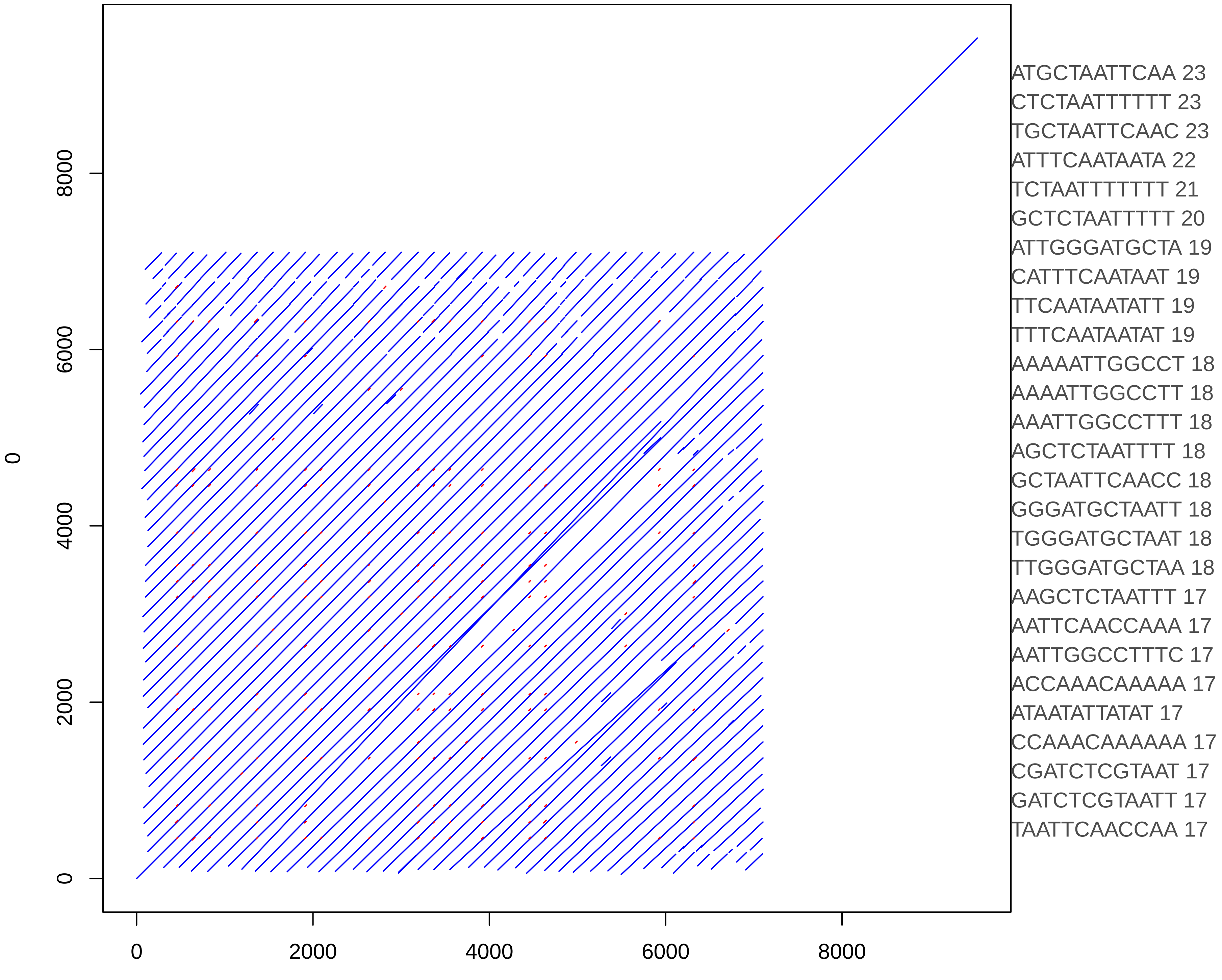

0

>m150106_044313_42134_c100761282550000001823158307221521_s1_p0/82787/0_5624

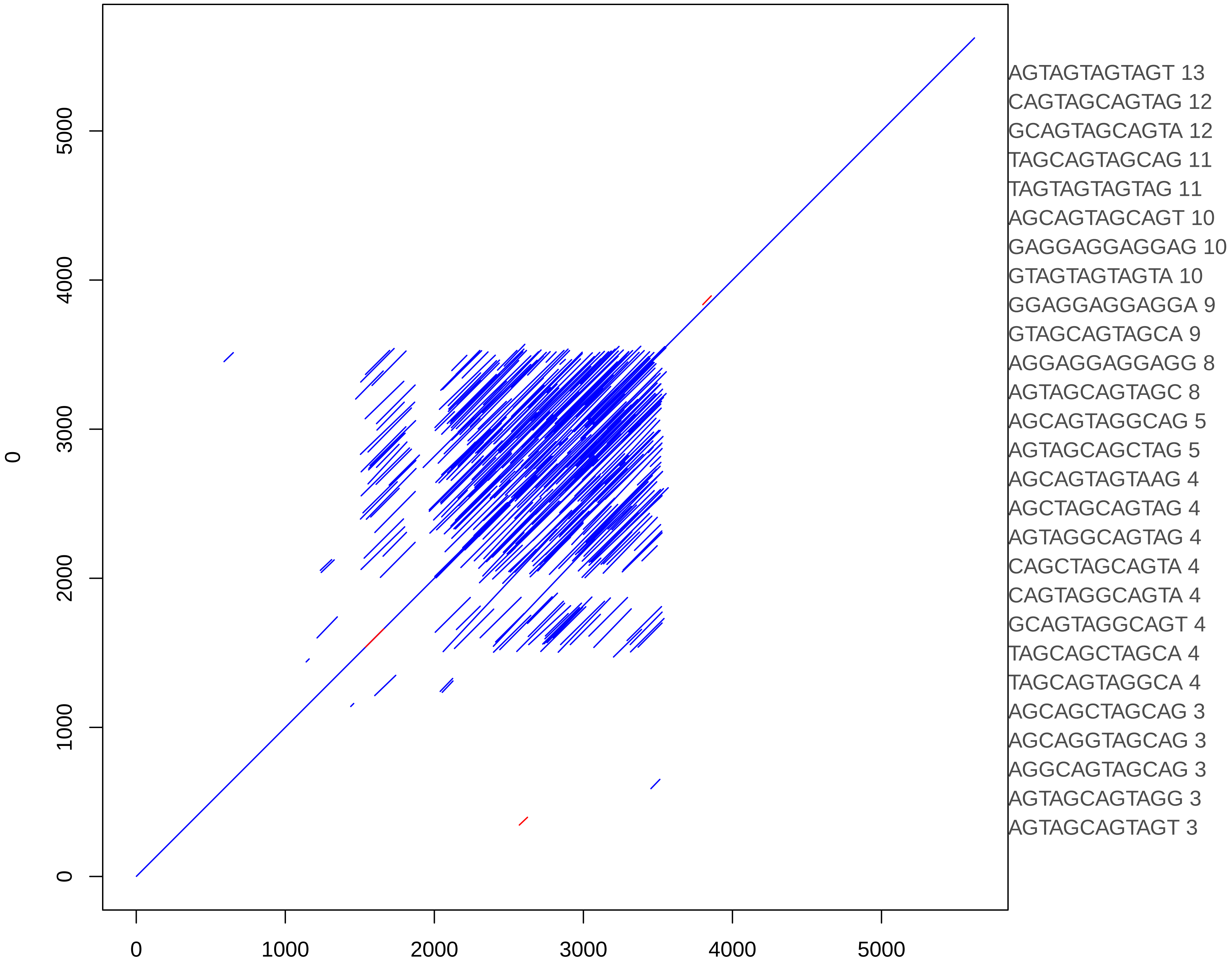

>m150106_044313_42134_c100761282550000001823158307221521_s1_p0/8548/0_7912

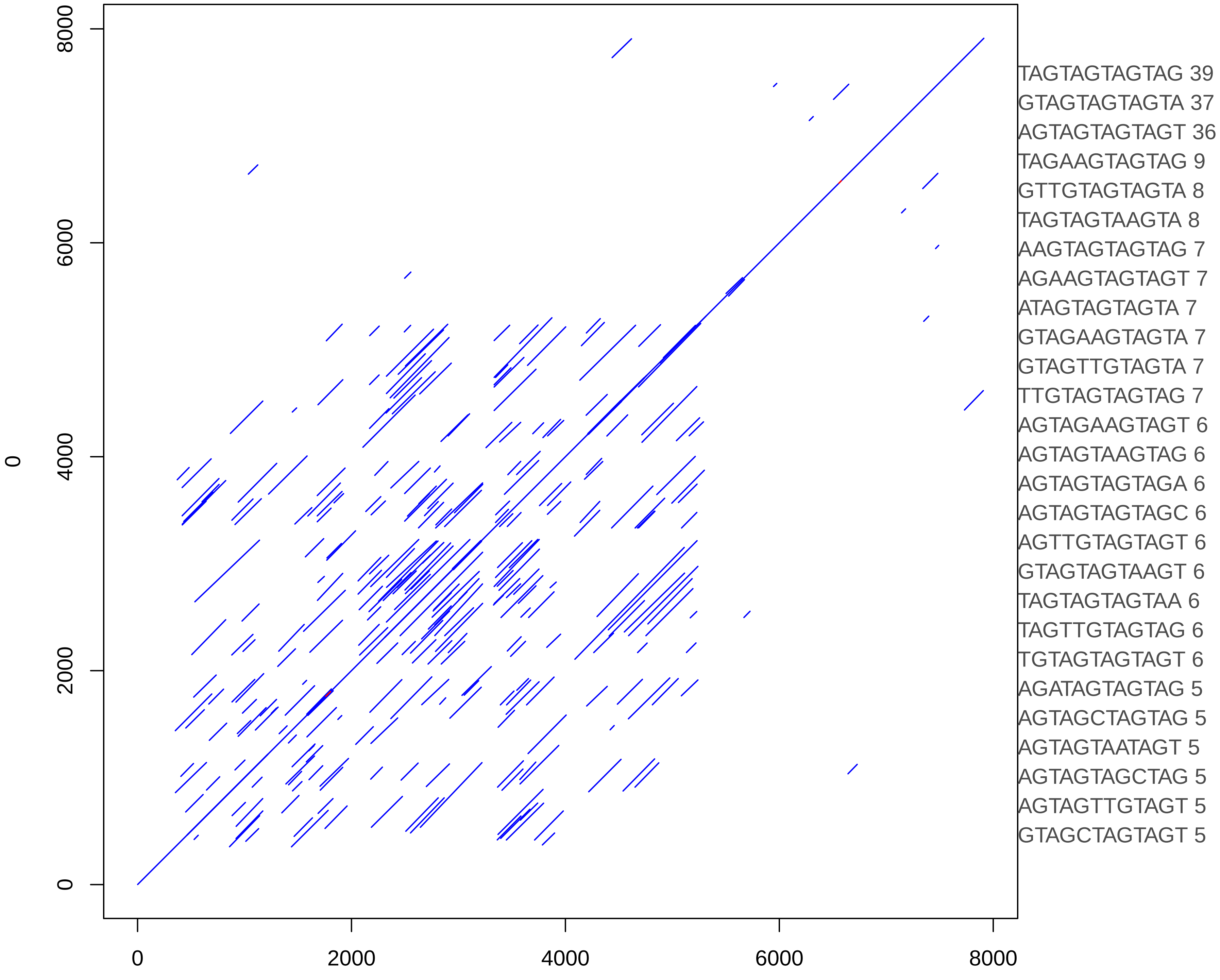

>m150106_044313_42134_c100761282550000001823158307221521_s1_p0/86681/0_6661

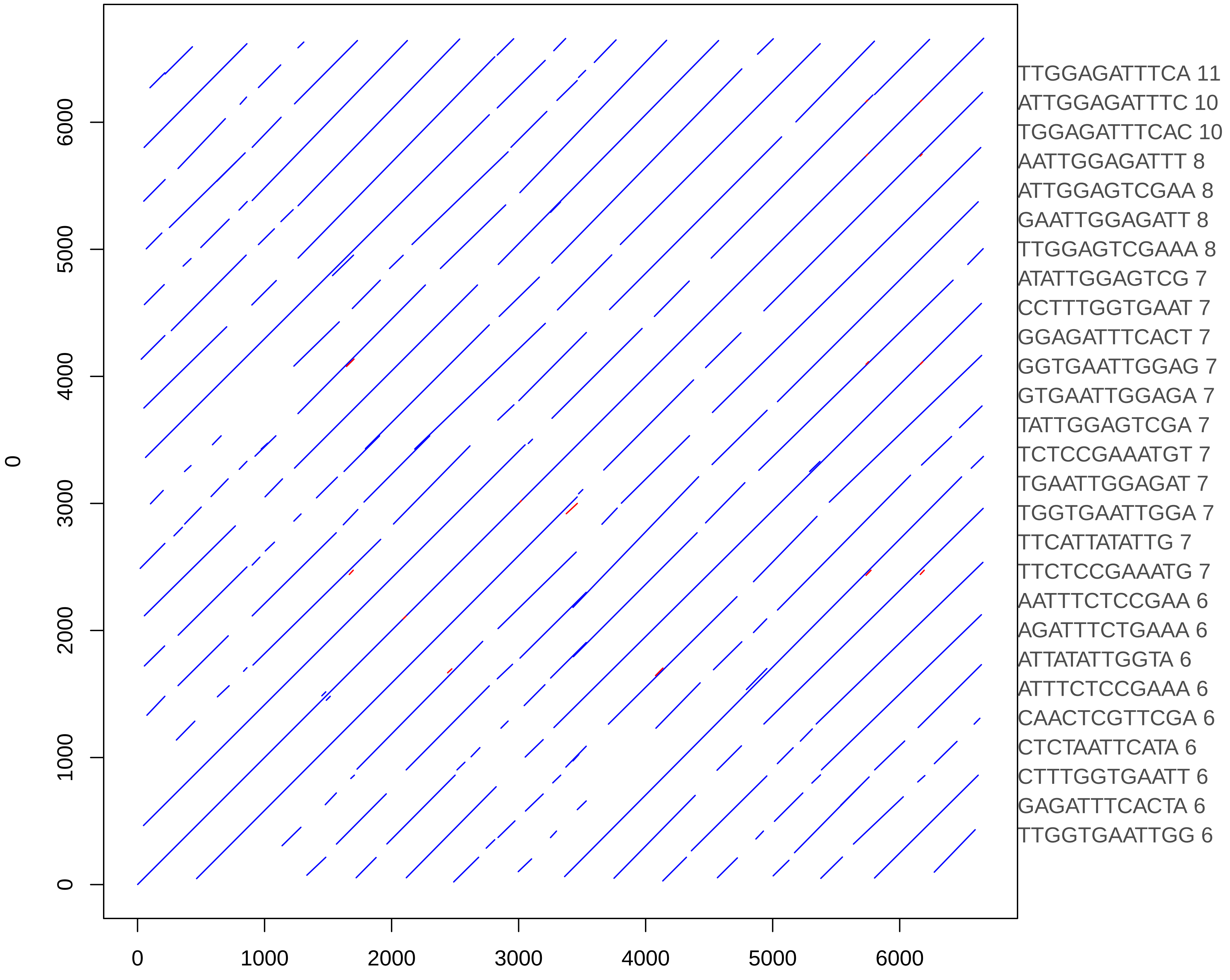

>m150106_044313_42134_c100761282550000001823158307221521_s1_p0/89148/0_5685

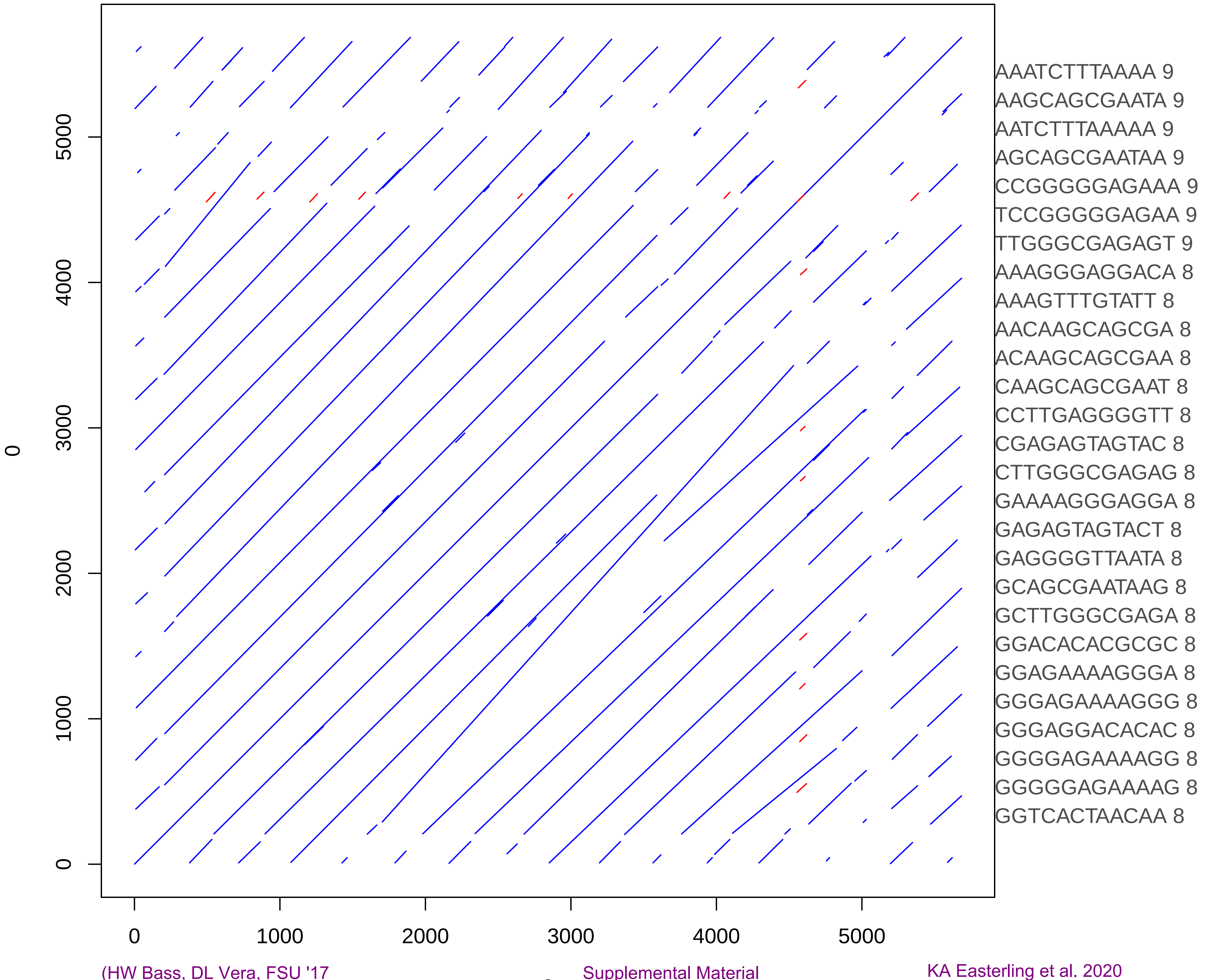

>m150106_044313_42134_c100761282550000001823158307221521_s1_p0/90245/0_6155

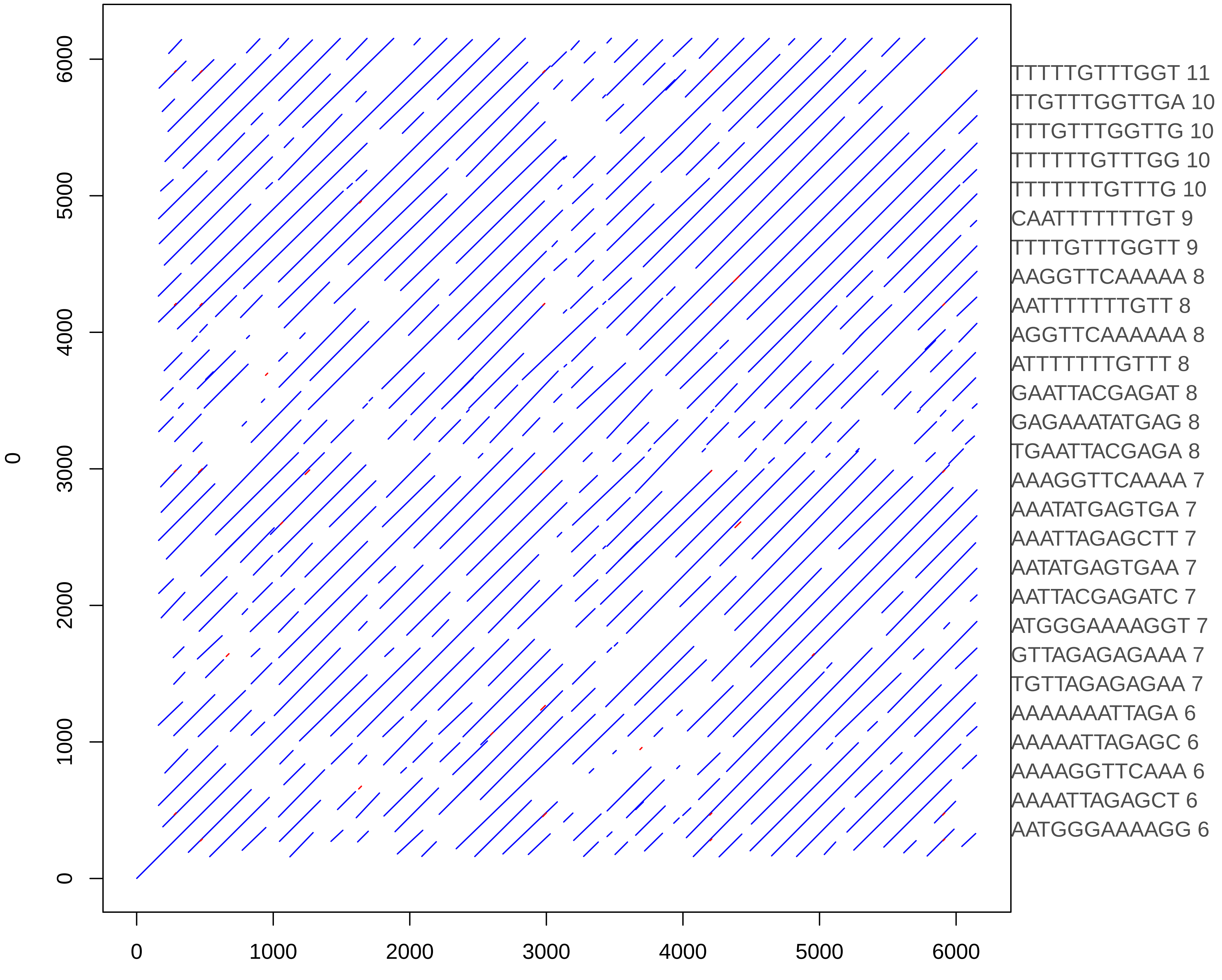

>m150106_044313_42134_c100761282550000001823158307221521_s1_p0/91848/0_6590

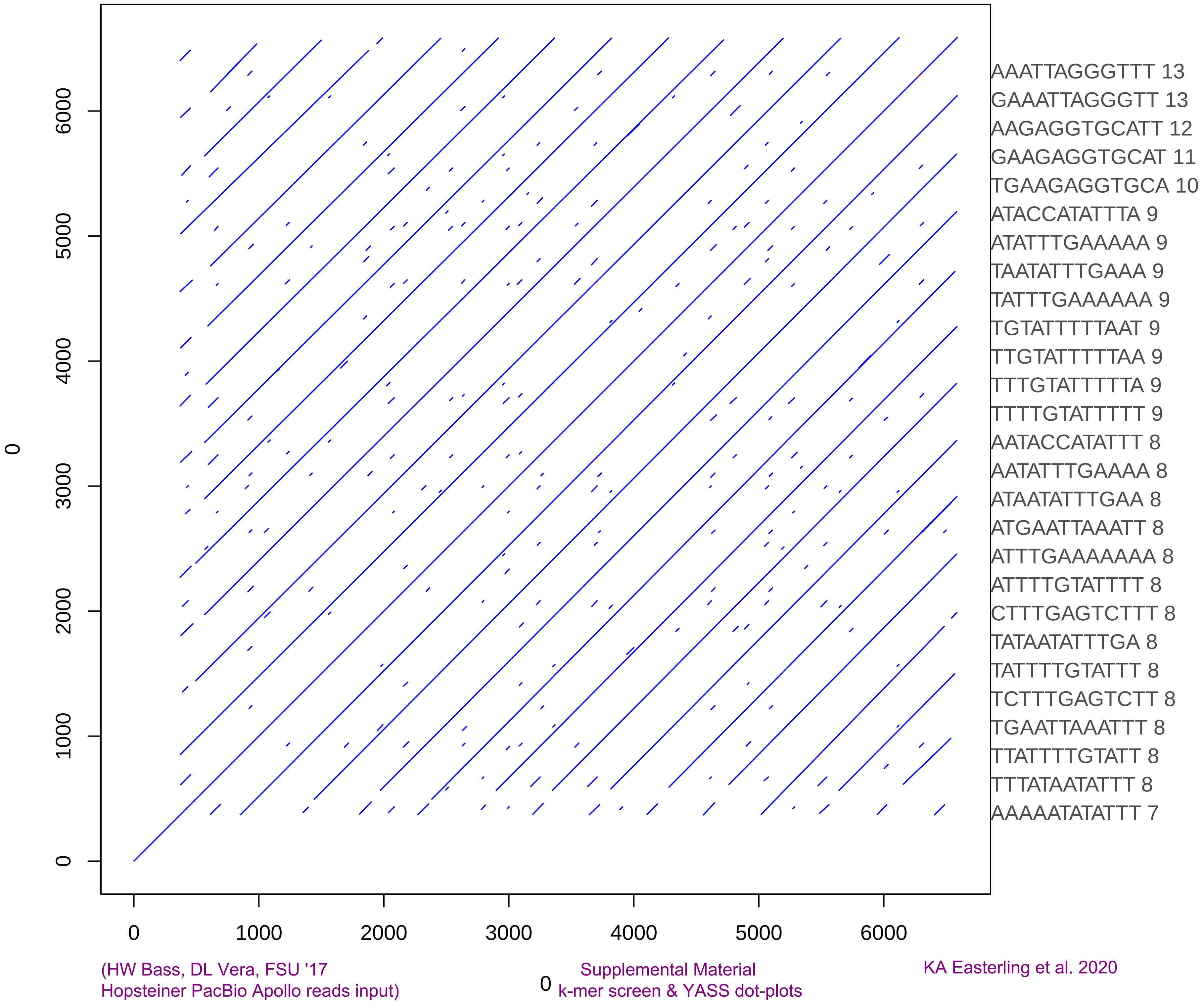

Supplement: S1 Data — (PDF) [file pone.0233971.s001.pdf]
