# Supplementary material for: Identification of tandem repeat families from long-read sequences of Humulus lupulus
Source: PLoS One. 2020 Jun 5;15(6):e0233971. doi: 10.1371/journal.pone.0233971 (PMC7274563; doi:10.1371/journal.pone.0233971)
Supplement: S2 Data — (PDF) [file pone.0233971.s002.pdf]

Supplemental Material - Data Sheet 2.

Original sequences from PacBio SMRT single-molecule reads for the 1,121 reads selected by the k-mer filtering. These sequences retain the original PacBio FASTA titles, which can also be found on the PDF Supplemental DataSheet-1 containing 1,121 pages of dot plots, file names, and most frequent 12bp k-mers. Note, these sequences are primary single-molecule sequences with an average base read quality of 82 and an estimated error rate of ~10%. From Easterling et al., 2020.

```
>m141122_014521_42134_c100732202550000001823141405141560_s1_p0/1189/0_9956
AAAAAAAAAGAGAGAGATAATCCTTGGGAGAAAAAATAGATCGACACGAGTTGATGGACGAGGGCTTAATAGCATTTCTGAGAA
ATTAGTGAATTCTCAAATTCCAAAGGGGTACCCCCCTTTATCAGAAATCTTCGATTTCTCTATATGGATCGAGAATGTTTTGAC
TCAAGCGTGCATAACTAAGAGAACTACCCTGCGAGGGTCATAGGGCACAAAAGTTTGTAGGCCTTTGTCTTAGGGTTCTATGTA
TAAAACTTACGTGCAAAGGGATCCGAAGGTGGTGTAGAGGGCATATACAGCTCAGAATAAGCTGAGAGATGTACGAATATTAAG
GAAAAAGTTGCAAAAATTGCATAGGAAAGGGGAATTTTTTGGAAAATGTTGACTTAAGGCTCCGTTTCGACTCCAATAGATTAT
TTACGCCACGAAGATCTCTTGGGGAAAAATATCGCAACGAGTTGATGAGGAGGGTATACATTTCGGGGGAAAAGAAATTAGGTG
AATTCTCCAATTCACCAAGGGGTACACCGTTTCCAGAAATCTTCGATTTCTCCTAATATGGATCTCGAGCAATGTTTTGATAGT
GGATCCTAAAGATATGCGAGGGTCATGGGACAAAAGGTGCGTCAGCGCCTCATGTCGCTTAGGGTTCTTATGTAGTAAAGCTTC
GTGCGAAATCCGAGGGTGTGATCGAGGGGTGAATACAGGTCAATAAGGTAGGTGAGAGATGTACGAATATACCGGGAAAAAAGT
TGCGAAATGTGCGATAAAGAAAATGGGAATTTATGTTTTGAAATGTTGACTTAAGCTCCGTGCTTCCGACTCACTAGATTAATC
TTAAACACGACGATCTCTTTGGGGAAAAATATCGAACGAGTCTGAAGATGGGGTATACATTTCGGCCAGAAATTAGTGAAAAGT
CTCCAATTCACCAAAGGGGTAACCATTTCAGAAATCTTCGATGTCCTACTATAATGGATTCCGAGCAATGTTTTTGATAGTGAA
TCTAAAGAATATGCGAGGGGATCATGGGCACAAAAGTTGTAGGCCTATGGACTCTTAGTTCATGCTATGAAAACTTGTGCAAAT
CCGAGGTGTGTAGGGGTGACACAGTCAAAATAGTGAGTGATGTACGAATTAAGGAAAAAGTTCGAAAATTGGCATAAAAAAGGG
TAATTTTTTTTTGAAAGTGATTAAGGATACGTTTCGGCTCCAATAGAAATAAATTTACCCACGAGATCTCTTGGGGAAAAATAT
CGACGAGTTGAAGACGAGGGTAATACATTTTGGAGAAATTAGTGAAATCTCCAAATTCACCAAGGGGTACTCTTCAGAAATCTT
CGATTTCTCTAATATGGATTCGAGCAATGTTTTGGATAGTGATCCTAAAAGATATGCGAGGATTCATGGGAAAAAAAGTTGTAG
GCCTATGTCTTTAGGGTTCTATGATGTTATAAAACTTGTGCAAATCCGAGTGTGTAAGAGGGTGATACAGTCAAAAAGTGAGAG
ATGTACGAATATAAGGAAAAAAGTTGAGAAAATTTGCCATAAAAAGGGTAATTTTATTTTGAAATGTGACTTTAAGGCTCGTTA
GACATCCAAGTAGATTGAATTTAACACGAGATCTTTGGGGAAAAATATCGAACGAGTTGAAGATGAGGGTAATACATTTTTCGG
AGAAATTAGTGAAATCTCCATTCACAAAGGGGATACCACCTTTCAGAAATCTTCGATTTCTCTAAATAATGGAATTCGAGCAAT
GATTTTTGGATAGTGAATCCTAAAGAATATGACGAGGGTCATGGGCAACAAAGTTGTAGGCCTATGTCTTAGGGTATCTATGTA
ATAAAGCTTCGTGCAAATCCGAGGTGTGGTAAGAGGGGTGATAAAGTCAAAATAGTGAGAGATGTACGAATATAGGGAAAAAGT
TGCGAAAATTGCCATAAAAAATTGGGAATTTTTTTTGAAATGTTGACTTAAGGCTCACGTTTCGACTCCAATAGATTCAATTTA
CCCACGAGAATCTCTTGGGGAAAAATATCGAACGAGTTGTACGATGAGGGTAATAAATTTCGGAGAAATTAGTGAAATATCCAA
TTCACCAAAGGGGTACCCTTTCAGAAATCTTCGATTATTCTAATATGGATTCGAGCAAGTGTATTTGAATAGTGATCCATAAAG
AATATGCGAGGTTCATGGGCACAAAAAGTTGTAGGGCCTAATGTCATATCTAGGGTTCTATGTAAAAAATTCGTGGCAAATCCG
AGGTGTGTAGAGGGATGATACACGTAAAATAGTGAGAGAGGTAACGTAATATAGAGGAAAAAGTTGCGAAATTGCCATAAAAAA
GGGTACATTTTTTTTTGAAAATGTTGACTTAGGCCTCCGTTTCGTATCAATAAGATTATTTACCCACGAGATCGTCTTGGGAAA
AATATCAGAACGAGTTGAAGACGAGGGTAATACATTTCGGGAATTAGTGAAATCTCAATTCACCAAAGGGCTACGACTTTCAGA
AATATTGATTTCTCTAATATGATTCGAGAATGTTTTGATAGATAGCATAATAAGGAATATGAGAGGGTCATGGGCACTAAAAGT
TGTAGGCCTTTGGTCTTAGGTTCTAATATGTATATAAAACTTCGTGCAAATCAGAGGTGTGTAGAGGGTGATACAGTCAGGGAA
TAGTGAGAGATGTAGCGAATATAAGGAAAAAAGTTGCGAAAATTGCCATAGAAAAGGGGAATTTTTTTTGAAATGTTGACTTAA
GGCTCGTTTCGACTCCAATAGGGAAAAAAAATTTTATTTACCACGAGATCCTCTTGGGGAAAATATCGAACGAGTTGATGACAG
GGTAATACATTATCGGAGAAATTAGTGAATTCTCCAATTCACCAACAGGGGTACCCTTTCAGAATCTTGATTTATCTAATCATG
GATTCGAGCAAATGTTTTGATAGTGAACCTAAAGAATAATGCCGAGGGTCAATAGGGCAGCAAAAGTTTGTGGCCTATGTCTTA
GGGTTATATGTAAAAAGCTTCGTGCAAATCCGAGGTGTGTAGAGGGTGATATCGTAAAAAAGTGAGAGATGTACGATATTAGGG
AAAAAGTTTGCGAAAATTGATAAAAATGGGATTTTGATTTGTTGACTTATGATTCCTTCGCTCAATAGATTAATATTAATCATG
AGAGTTCTCGTGTGTAAAAATATCGAACCGCGTTGAAGATCGAGGTAAATACATTTTCGGAGAAATTAGTTGGAAATCTCGCAA
TTCACCAAAGGGGTGGGTAACCTTTCAGAAATCTTCGATGTCTCTAAATATGGATTTCGAGCAAATGTTTTGATAGTGAATCAT
AAGAATATATATTGCGAGGTCATGGGCAACAAAAGTTGTAGCGCCTATGTCTTAGGGTTCTATGTATAATAAACTCGGCAAATC
CGAGGTGTGGTAGAGGGTGACACGAGTCAAAATAGCGATGAGTGATGTACGAATTAGAAAGGAAAAAAGTTGGCGAAAATTGCA
TAAAAAAGGGTAATTTTTTTTGAATGTGACTTAAGGCTCGTTTCGGATCCATAGATAAATTTTATACCACGAGAATCTCTTGGG
GAAAAATATCGAACGAGTTGAAGACGAGGGTAATGACATTTTTCGGAGAACTTAGTGAAATCTCCAATTAACAAAAGAGGTAAA
CCTTTCAGAATCTTCGATTTCTCTATATGGATTCGGAGCAATGTTTTGATAGTTGAATTCTAAAGAATAATGCAGAGGTTCATG
GGCACAAAAGTTGTAGGCCTATGTCTTAGGGTTCTATGTATACAAAATTCGTGCAAATCCGAAGGTGTGTAGAGGGTGATAAGT
AAAATACGTGAGAGATGTACGAATATAAGGAAAAAAGTTGAGAAATTGCATAAAAGGGTAATTTTTTTTGAAATGTTGACTTAA
GCTCCCGTTTCGACTCCAATAGATTAATTTACCCACGGATCTCTTGGGGAAAAAAGTATCGAAGAGTTGGAAGATGAGGTAAAT
```

ACATTTCGGGAAATTAGTGAAATCTACAATTCACCAAAAGGGGTACCCTTTCAGAAATCTTCGATTTCTCTAATATGATTCGAG
CAATGTTTTTGATAGTGAATCCTAAACGAATATGCGAGGGTCATGGGCCAAAAGTTGTAGGCCTATGTTTAGGGTTCTATGTAT
ATAAGGCTTTCGGTGAAATCCGAGGTGTGTAGAGGGTGGATACAGTCAAATAGTGAGAGATGTCGAATATAGGAAAAAGTTGCG
AAAATTGTCCATAAAAATGGGAATTTTTTTGAAATGTTGAATTTAAGGATCCGTTTGATCAATAGATTAATGTTACACACGAGA
TCTCTGTGGGGAAAAACTATCGAACGAGTTGTAGATGAGGGTAATACATGTTCCGGAGAAATTAGTGAAACTCTCAACTTCCCA
AAGGGGTAACCTTTCAGAAATCGTTCGATTCTCTCTAATGATGGATTCGAGCAATGTTTTGAATAGTGAAGTCCTGAGAAGAAT
ATGCGAGGTTCATGGGCACAAAAGTTGTAGGCCTATGTCTTAGGGTTTCCTATGTATAAACTTCGTGCAAATAGAGGTGTGTAG
AGGGTGATAGTCAAAATAAGTGAGAGATGTACGAATATAAGGAAACAAAGTTGCGAAAATTGCCATAAAAAAGGTAATTTTTTT
TGAAATGTTTGACTTAAGGCTCCGTTTCGACTCCAATAGATTAAATTTTAATCCACGAGATCTCTTGGGGAATAAATATCGATA
CGATTGAAGATGAGGGTAAATAATTTCCGGAGAAATTAGTGGAAATCTCCAATGTCACCAAAGGGGTACCCCTTTCAGAAATAT
TCGATTCCGTAATATGGATTCGAGCAATGTTTTGATAGTGATCCTACAAGAATATGCGAGGGTCATGGGGCAAAAAGTCTGTAG
GCCTATGTATTAGGGTTCTATGTAGTAAGCGTTCGTGCAAATCGAGGTGTGTAGAGGGTGATACAGTCAAAATAGTGAGAGAAT
GATACGATTATAGGGGAAAAGTTCGAAATATGCCATGAAAAATGGGAATTTTTTTGAAATGTTGACTTAAGGCTCCGTTTCGA
CTCCAATAGATTTAATTTACCACGAGATCTCTTGGGGAAAAATATCGAGACCGAGTTGTAGATGAGGGTAATACATTTCGGAGA
AATTAGTGAAATCTCCATTACCAAGGGGTACCGCTTTCAGAATTCTTCGATTTCTCTAATTATGGATCTCGAGGCAATGTTTTG
AGAGTGAATCCTAAATGAATATGCGAGGTTCATGGGCCAAAAAGTTTGTAGGCCTATGTCTTAGGGATTCTATTGTAATAAAAC
TTCGTGCAATCCGAGGTGTAGTAGAGGGTGATACAGTCAAAATAGATGAGAGATGTAACGAATATAGGAAAAAGTTGCGAAAAT
TGCCATAAAACAGGGTAAATTTTTTTTTGAAATGTTGACTTAAGGCTCCGTTATCGACTCCCAATAGATTATTATACCACACGA
GATCTTCTTGGGAAAAATATCGAATCGAGTTGAAGATGAGGGTAATACAATTTCGGAGAATTAGTGAAATCTCCAATTCAAAAG
GGTAACCCCTTTTCAGGAATATCGATTTCTCTAATATGGGATCGGCAATGTTTGATAAGTGAATCACTAAGAATATGAGGGTCA
TGGGCACAAAAGTGTAGGCTATGTCTTAGAGGTTCTATGTAATAAAGTTCGTGCAAATCGAGGTGTGTAAGAAGGGTGATACAC
AGTCAAAATATGTGAGAGATGTACGAATTATAGGGGAAAAAAGTTTGCGAAATTGCCATAAAAAATGGGAATTTTTTTGAAAT
GTTGACTTAAGGCTCCGTTTCGATCCAATAGATTAATTTTACCCACGAGAATCTCATTGGGGAAAAATATCGGACGAGTTGTAG
ATGAGGGTAAATACATTTCGGCAGAAATTAGGTTGAAATCTCCAATTCACCAAAAGGGGTACCCTTTCAGAAATCTTCGATTTC
TCTAATATGGATTCGAGCAATGTTTTTGGATAGTGAATCCTAAAGATATGCGAGGTTCATAGGGACGAAAAGTTGTAGGCCTAT
GCTCTGTAGGAGGTTAATATGTATAACTTTCGTGCAAATACAGAGGTGTGTAGAAGGTGATACAGTCAAATAGTGAAGAGATGT
AACGAATATAAAAGGAAAAAGTTGCGAAAATTGCCATAAAAAAGGGGTAATTTTTTGAAATGTTGAATTAAGGCTCGTTTGACA
TCCAATAGATTAATTTACCCACGAGATCTCTTGGGGGAAACAATAATCGAAAGAGTTGAAGATGAGGCGTAATACATTTCGGAG
AATTAGTGAAAATGCTCCAATTCACAAGGGTACCATTTCAGATATTCGATTTCTCTAATATGGATTCGAGAAATGTTTTGATAG
TGAATCCTAAAGAATATGCGAGGGTCATGGGCACAAAAGTTGTAGGCCTATGTCGTTAGGGTTCTATGTATATAAGCTTCGTGC
GAAAATCGAGGTGTGTAGAGGGTGGAATACAGTCAAAAGTATGAGAGATGTAAGAATATAGGAAAAAAGTTGGCGAAATTGCCA
TAAAAATGGGAATATTTTTTTTGACATGTTGAATAAGGATCCGTTTCGACTACCGAATAGATTAATTTGACCCACGAGATTCTT
GGGGAAAAATATCGAACGAGTTGAAGATGAGGGTGACATACATTTCGGAGAAATTGAGTGAATCTCCAATTCCACGCAAAGGGG
TAGCCCCTTTCAGAAATCTTCGATGTCTCTAATATGGAGTTCGAGCGATGTTTTGATTAGTGAAATATCCTAAAGAATCATGAG
AGGTCAATGGGAAAAAAGTTGTAGGCCTATGGTCTATAGGGTTCTATGTTAAAATGTTGCAAATCCGAGGTGTGGTAAGAGGGT
TGACACAGTCAATAATAGTGAGTGATGTACGAGATATAAGGAAAAAGTTGCGAAAATTGACATAAAAAGAGGGTTAATTTTTTT
TGAAAATGTTACTTAAGGCTCCCGTTTCGGTCCATACGATAACATTACCCACGAGATCTCTTGGGGAAAAATATCGAACGAGTT
GAGACGAGGGTAATACAATTTCAAGGAGAAATTAGTGAAATCTCCAATTCACAAAGGCGGTTACCCTTTCAGAAATCTTCGATT
TTCTCTAATATGGATTCGATGCAAATGTTTTGAATAGTGTAATTCCTAAAAGAATATGCTACCTGGGCACAAAAGTTGTAGGCC
TACTGTCTTAGGGTTCTATGTATACAAACTTCGTGAAATCCGAGGTGTGTAGAGGGTGATACAGTCTAAATAGTGAGAGATGTA
GAATTATAAGAAAAAAGTGCGAAAAATTGCCATAAAAAGGGTAATTTTTTTTTGAAATGTTTGACTTACAGGGCTCCGTTTCGA
CTCCAATAGATTCAATTTACCCACGAGATCTATTGGGGAAAAATCGAACGGTTGAAGATGAGGGTAATACGATTTCGGAGAAAT
TAGTGAAATCTCCAATTCAACAAAGGGGTACCCCTTTTCAGAAAATCTTCGATTTCTCTAATATGGATTTTAGAGCAATGTTTT
GATAGTGAATCTAAAGATGCGAGGGTCTGGGCAAAAGAAGTTTGTAAGGCTCATGTCCTTAGGGTTCTAATGTATAAAGCTTCG
TGCAAATCCGAGGTGTGTAGAGGGTGATACAGATCAAAATAGTGAGAGATGTAGATATAGGGAAAAAGTTGCGAATTGCAATAA
AAAATGGGAATTTTTTTGCTGAAATGGTTGACTTAAGGCTCCGTTTCGACTCCAAATAGAGTTATTTACCCACGAGATTCTTGG
GGAAAATATCGAACGAGTTGTAGATGAGGTAATATTTCGGAGAAATTAGTTGAAATCATCCAATTCACAAAGGGGTACCATTTC
AGAAATCGTTCGATTTCTCTAATTGGATTCGAGAATGTTTTGATAGTGTCTAAAGAAGTATGCGAGGTTATGGCAAAAAAGTTG
GTAGGCCTATGATTTAGGGTTATATGTATATAAACTTCGTTGCAAATCGAGTGTTAGAGGTGATACAGTCAAATAGTGAGATGT
ACGAATATAAGCGAAAAAAGTTGCGAAGTTGCCATAAAAAGGGTAAAGTTTGTTTATTGAAATGTTTGACTTAGGCTCAGTTTC
GATCACAATAGATTAATTTACCACGAGAATCTCTTGGGGAAAAATATCGAACGACGTTGAAGATGAGGGAATACGATTTCGAGA
AAGTTAGTGAAAATATAAATTCACAAGGGGTAGGACCTTTCAGAATCTTCGATTTATCTAATATCAGGAATTTCGAGCATGTTT
TGATAGTGACCTAAAGATAATGCGAGGGTACATGGGACAAAAGTTGTAGGCCTATGTCTTAGGGTTCATATGTATAAAGCTTCG
TGAAATCCGAGGTGTGGTAGAGGGTGATACAGTCAAATAGTGAGAGATGTAAGCGAATATACGGGAAAAAAGTTGAGAAAATTG
CCATAAAAAAATGGAATTATTTTTGCATGTTGACTTAAGGCCGTTTCGACTCAAATAGATTAATTTACCCACGAGATCTCTTGG
GGAAAATATCGACACGAGTTGAAGGATGAGGGTAATAAATTTCGGAGAAATTAGTGAATCTCACAATTCACAAAGGGGTACCCC
TTTCAGAAATCTTCGATGTCTTCTAAAATGGTGGTTCGGAAGCAAAATGTTTTGATAGTGAACCTAAAGATATGGAGGGTCAT
GGCACAAAAGTTGTTGGCATATGTTAGGGGTTTATGTATAAAACTTGCAAATCCCGAGGTGTGAGAAGGTGGGAACAGTCAAA

ATAAGTGAGTGATGTACGAATATACAGGAAAAAAAGTTGCGAAAATTGGGGCCCATAAAAAAAGGGTAATTTGTTTTTGAAATG
TTGATTAAGGCTACGTTCGACTCCAATAGATTATTTACACACGAGATCTCTTGGGAAAATATCGAACGAGTTGAGATGGAGGGT
AAATACATTTCGGGAATTAGTGAAATCTCCAATTCACCAAAGGGGTACCCCTTTTCAGAAATCTTCGATTTCTCTAATATGGTT
CGAGCAATGTTTTGATAAGTGAATCCTAAAGAATATGCGAGGTTAATGGGACAAAGTTGTATGCCTATGATCTTAGTTTCTATG
ATATAAAACTTCGTGCAAATACGAGGTGTAGTGAGTGTGATAGCAGTCAAGAATAGTGAAGATTTACAATATAACAGGAAAAAG
TTGCGAAATTGCTTAAAGAAGGGAATTTTTTTTGAATGTTGACTTAGGATCCGTTTCGACTCCAATAGATTAATTACCCACGAC
GACATATCTTGGGGAAAATATCGAAGCGAGTTGAAGATGAGGGTAATAACATTATCGCAGAAATTAGTGAAATATCCAATTCAC
CAAAGGGCGTACCCCTTCAGAATCTTAGATTTATCTAATATGGATTCGAGCATGTTTTGATAAGTGGAATCCTAAAGAATTGTG
AGGTCATGGGCACAAAGTTGTAGGCGATATGTCTTTAGGGATTCTATGTATGAAACTTCAGTGCAAAATCCGATGGTGTGATAG
AGGGTGATCAGTCAAATAGTGAGAGATGTACGATATAAAGGAAAGTTGGCGAAAATTGCTTAAAAAGGGGAATTTTTTTAGAAT
GTATGACTTAAGGCTCCGTTTAGATCAATAGAGTTAATTTATACCCCACGAGATTCCTTGGGGAAAAATATCGAACGAGTTGAA
GATGAGGGTAATACATTTCGGAGAAATTAGTTGAAATCTCCATTCCCAAAGGGGTACCCCTTTCAGAAATCTTCGATTTCTCTA
ATATGGATTCGAGAATGTTTTGATAGTGATACTAAAAGAATATG
>m141122_014521_42134_c100732202550000001823141405141560_s1_p0/12096/0_6154
AATAATATTGAAGATGATAGATTTAACTTCCAGTTGTTAGATTGTTTCTTTTCGCATACTTTTCTTTAATATTGGATTTCCAGC
GATGTTTTGGTAGTGCATACTAAGAATAAGCGGGGTCAGTGGGCCATAAAAGATTGTAGCCATATGTCTTAGGGTTTATATATA
AAGATTTCTGCAAGCATACTAGGTGTGTATAGGGTGATACAGTCAAGAATACCTCAGAGATGTACGAATGATAAGGAAGAAAAC
TGTGAAAATTGCCTCAAAAAGGGGAGTTTTTTTTTTGAAATGTTGAATTAAGGCTCCGTTTCGCACTCCAATATATCATTTCCC
ATGAGACGTCTTGGGTAAAAATTATGCAAACGAGTTGAACAGCGAAGGGTAATACATTTCGGAGAATTAAGTTGAAATCCCCAT
TCAGCCAAAGGGTAACCCTTTCAGAAATCTTCGATTTCTCTAAATATGGATTCACGCGATGTTTTGGTAAGTGAATACTAAAGA
ATAGGCCAGGGTCAGGGCACAAAAGTTGTAGCCCTAATAGTCTTGGGGTTCGCATATATAAAATTTTCGTGCAAATCCTAGGTG
TGTAATTAGGGTGATACAGTCAAAATACTCAGAGATGTACGAATATAAGGAAAAAACTTGTGAAAATTGCCCTCAAAAAAGGGG
AGCTTTTTTTTCGAAGTGTTGACTTAAGGCTCCGTTTCCACTCCAATATATCATTTACCATGAGATCTCTTGGGTAAAAATATC
AAACGAGTTGAACACGAGGGTAATACATTTCGGAGAAATTTTAGTTGAAACCTCCGAATTCAACCCCAAAGGGGTACCCCTTTC
AGAAATCTTCCATTTCTGCTAATATGGATTCGCAGCGATGTTTTGGTAAGTGAATACTAAAAGAATATGCGAGGTCATGGGCC
CATCAAAGTTGTAGCCCTATGTTTATAGGGTTCTATGTATAAAATTCGTGCAAAGCTAGGTGTGTATCAATGGGTGATACAGTC
AAATCACTCCAGAGATGTACGAATATAGGAAAAAACTTGTGAAAGATTGCCACAAAAAGAGTTTTTTTCGAAATATTGAATTAA
GCGCTTCCGTTTCCACTCCAATATATTCATTTACCGCATGGCATCTCTTGGGTAAAAAATATCAAACGAGTTGAACACGAGGGT
AATAACATTTCGGAGAAATTTAGTGAAATCCCCAATTCACCAGGGTAGCCCACTTCAAAATCTTCCATTTCTCCTAATATGGAT
TCCAGTGATTTTTGGTAGTGAATACTAAAGAATATGCAAAAGGGTCATGGGCAGAAAAGTTGAGCCCTATGTCTTAGGGTTCTA
TAACTATAAAATTCGTGCAAATCCTAGGTGTGTATATGGGTGAATACGTCAAAAATACTCAGAGATGTACGAATATAAGGAAAA
AACTTGTGAAATGCCTAAAAAAGGGGAGTTTTTTTTTGAAATGTTGAATTAAGGCTCGTTCAACTCAATGATATTATTTACCC
ATGAGATCTCTTGGGTAAAATATCAAACGAGTTGAAGCACGAGGGTAATACATTTCGGAGAATTAGTGATAATCCCAAAATTCA
CAAAGGGGTCCCCTTTCAGAAATCTTCGATTTCTTTAATATGATTCCAGCGATGTTTTGGTAGTGAATACTAAAGAATATGCGA
GTGTCATGGGGCACAAAAGTTGGTAGCCCCTATGTCTTAGGGTTCTATATATATATTTCGTGCAAATCCTAGGTGTCGTTAATG
GGTGATCACAGTCAAAATACTCAGAGATGTACGATATAAGCGAAAAACTTGTGAAAATTGACCTCCCAAAAAGGGGAGTTTTTT
TTTTGAAATGTTGAATTAAGGCTCCATTTTCACTCCCAATATATTCATTTACCTCAATGAGATCTCTTGGGTAAAAAATAGCAA
CGAGTTGAACACAGGAAATAACATTTCGGAGAAATAGTGAAATCCCCAAATTCCCAAAGGGGTACCCCCTTTCAGAAATCTTCC
ATTTCTCCTAATATGTGTTCCCAGCGATGTTTTTGGTAGGAAATACTAAAGAATTAGCGAGGGTTCATGGGCCCACAAAAGTTG
TGCCTATGTCTTAGGGGTTCTATGATAAATTTCGTGGCAAATCTCTAGGTGTGTATAGGGTGATACAGTCAAAATACTCAGGAT
GTACGATATAGGAAAAACTTGTAAAATTGCCACAAAAAGGGGAGTTTTTTCGAATATTGAATTAAGGTCCGTTTCACTCGCAAT
ATATTCATTTACCATGGAGATATCTTGGGTAAATATCAAACGAGTTGAAAACGAGGTCATACATGTTTCGGCAGAATTAGTGAA
ATCCCCCAATCTCACCAAAAGGGTACCCCATTTCAGAAATCTTCCATTTCTCTAATATGGATTCCGTGAATGTTTTGGTAGTGG
AATACTAAAGATAATATGCGAGGGTCATGGGAGAAAAGTGTCAGCCTATGTCTTAGGGGTTCTATATATTAAAATTTCGTGCAA
ATCCATAGGTGTGTATCAGGGTGATACAGTCAAAATACCCTCAAGAGATGTACGAGAGATAAGGAAAAAATTGTGAAAATGCCT
AAAAAAAAGGGGAGTTTATTTTTTGAAATGTTGAATTAAAGAGCTCCGTTTCCAATCCAATATATTCATTTACCCGCATGAGAT
CCTTGGGTAAAAATACAAACGAGTTGTAACACGAGGGTAATACATTTCGGAGAAATTAAGTGAAATCCCAATTCACGCAAGGGG
TCGCCCTTTCAGAAATCTTCGATTTCTTTAAATATGGATTCCAGCGATGTTTTGGTATGAATACTAAAGAATATGCGAGGGTCA
TGGGCCACAAAAGTTGTAGCCCTATGTCTTAGGGTTCTAATATATGAAAATTTCGTGCAAATCCTAGGTGTGTATAGGGGTGAT
ACAGTCAAAATACTCAGAGATGTAGGATATAAGGAAAAAACTTGTGAAAATTGCCTCCAAAAGGGGAGTTGTTTTTTTGAAATA
TTGATTAATGGCTCCATTTCCACTCCAATATAATTTCATTTACCCATTAGAGATCTCTTGGGTAAAAATACAAACGAGTTGAAC
ACACGAGGAAATACATTTCGGAGAATTAGTGAAATCGCCAACCCTTCACCAAAGGGGTACCCTTTCAGAAATTACTTCATTTCT
CTATATGGATTCCAAGAAGATGTTTTTGGTAGTGAATACTAACGAATATGGCGAGGGTCATGGGCAGCAAAAGTTGTAGCCCTA
ATGTCTTAGGGTTCTATAGTATAAAATTTCGTAGCAAATCCTAGGTGTGTATAGGGTGATATCAGTCAAAATTAAACTCGAGAT
GTACGAATATAAAGGAAAAGCTTGTGAAATTGGCCTAAAAAAGGGATTTTTTTTTGAAATGGTTGATAAGGCTCCGTTTGCCAC
CAAAAATCCCAATTATTCCAATTTACCCATGAGATCTCCCTTGGGTAAAAATTCAACGAGTTGAACACATAGGTAATACATTTC
GGAGAAATTTAGTGAAATCCCCAATTCACCAAAGGGTACCCCTTTCAGAAATCTTCGATTTCTCTAATATGGAATTCAGCGAT
GTTTTGGTAGTGAATACTAAGAATATGCGAGGGTCATGGGCACAAAAGTTGTAGCCCTATGTCCTTGGGTTCTATATATAAAAT

TTCGTGGCAAATCCTAGGGTGTATAGGGTGATACAGTCAAAATACTCAGAGATGATAGCGAATTAAGGGAAAAAAACTTGTGAA
AATTGCCCTCCAAAAGGGAGACTTTTTTTCGATGTTGAATTAAGGGCTCGTTCCACTCCCCAATATATTTCATTTCCGCAATGA
GATGCTCTTGGGTAAAAATATCAAACGAGTTGAACACCGAGGGTATATACATTTCGGAGAAATTAGTGAAACCTCGAATTCACC
AAAGGGGTACCCTTTCAGAAATCTTCCATTTTTCTAATATGGATTCCGCAGCGATGTTTTGGTAGTGAATACTAAAAGAATAAG
CGACGGTCATGGGCACGAAAAGTTGTGCCGCTGATGTCTTAGGGTTCTATGTATAAATTTCGTGCAAATCCTAGGTGTGTATAG
GGTGATACAGTCAAAATAGATCAGAGATGACGATATAAGAAAAAACTTGTGAAAATTGCCCTCAACAATAGGGGGAGTTTTTTT
CGAAATTGAATAAAGGCTCCGTTTCCCTCCAATGATATTCTATACCATGAGATCTCTTGGTAAAAATATCAAACGAGTTGAACA
CGAGGGTAATAACATTTCGGAGAAATTAGTGAAAATCCCCCAATTCACCAAAGGGGTACCCCTTTCAGAAATCTTGCCATTTTC
TAATATGGATTACCAGTGAATGTTTTGTAGTTGAATACTAAAGAATATCGCGAGGGTCATGGGGCACAAAGTTGAGCCCCTATG
TCTTAGGGTTCTATATAATGAAAATTTCGTGGCAAATCCTAGGTGTGTATAGGGTGATACAGCAAAATATCAGAGATGTAGAAT
ACCTAAGAGAAAAAACTTGTGAAAATTACCCTCAAAAAGGGAGTTTTTTTTGAAATGTTGAAATTAAGGCTCCGTTTCTCACTC
CAATATATTCATTTACCCATAGATCTGCTTGGTAAAAATTCAAAAACGAGTTGAACACGAGGGTAATACATTTCGGAGAAATTA
GTAAATCCCCAATTCACCAAAAGGGGTACCCCTTTCAGAAATCTTCGATTTCTCTAAATTGGATCCCAGGCGATGTTTTTGGTA
GTGAATACTATAAGAATATGCGAGGGTCCATCGGGCACAAAAAGTTGTGCCCTAATGTCTTAGGGATCTATGTATAAAATTTCG
TGCAATCCTAAGGTGTGTAATAGGGTGATACAGTCAAAATACTTCAGAGATGTACGAATATAGGAAAAAACTTGGTGAATAATT
ACCCTCAAAAGGGGGAGTTTATTTTTGAAAATGTTGAATTAAGGGCTCCGTTTCCAGCCTCCCAATCATATTCATATTACCAT
GAGATCTCTTGGGTAAAAATAGCAAACAGAGTTGAAACACGAGGGTAATACATTTCGGTAGAAATTTAGTGAATATCCGCAATT
CACCAAAGGGTACCCTTTCAGAATCTTCCATTTCTCTAATAATGGATTCCAGTGATGTTTTGGTAGTGTAATAACTAAAGAATA
TGGCGAGGGTCATTGGGGGGAGCAAAAGTTTGTAGCCCTATGTCTTAGGGTCTATATATTGAAAATTTCGTGCCAAATCCTAGG
TGTGTATAGGGTGATACAGTCAAAATACTCAGTAGATGTACGAATATAAGGAAATAAACTTGTGAAATTTACCCTCCAAAAAGG
GGAGTTTATTTTTTTGAAATGTGAATTAAGGCTCCGTTTTCCACTCCAATATATTCATTTACCATGAATTCTTGGGTAAAAATA
TCAAACGAGTTGCAACACGAGGGAAATACATTTCGGAGAATTAGTGGAAATCCCAATTCACGAAAGGGGTACCGCCTTTCAGAA
ATCTTCCATTTCTCTAATATGGATTCCAAGCGATGTTTTGGTAGTGAATCCTAAAGAATATAGCGAGGGTCATGGGCACCAAAA
GTTGTAGCCCTATGTCTTAGGGTTCTATATATAAAATTTCGTGCAAATCCTAGGTTGTGATATAGGGTGATTACAGGTCAAAAA
AACTCAGAGATGTAGCGGAATATCAAGTGAAAAAACATGTGAAATTGCGCCTAAAAAAAGGGGAGTTTTTTTTGAAATGTTGA
ATTAAGGCTTCAGTTTCCACTCCAATATATCATGTTACTCGCATGAGATCTCTTGGGTAAAAATAAATCAAACGAGTTTGAAAA
CGAGGTAATACATTTCGGAGAAATTAGTGAAATCCCAATTCACCAAAAGGGGTAGCGCCTTTCAGAAATCTTACCATGTTCTCC
TAATAATGGATTCCAGGGGATGTTTTGGTAGTGAGATCTAACAGAATTATATGCAGGGTCAATGGGGCCCACAAAAGTTGTAGC
CCTAATGTCTTAGGGTTCTATATATAAAATTCGTGCAAATCCCTAGGTGTGTATAGGGTGATACAGTCAAAATACTCAGAGATG
TAGCGAATATAAGGAAATAAAA
>m141122_014521_42134_c100732202550000001823141405141560_s1_p0/109291/1969_8215
TAAATGGAATACATGCCCGCGCCCTAACCTAAGAGCTTATTGAAAAAAGCTAATTCCCATAATGTGCGACATCTCCCCCAACAT
TAAACCATAAAACTAACAACTATATGTCTAATACCCTGGACTCTAACCTGTCAATGCTAAACTGATATCACTGGGCGTGCGAGT
TGCTCCGCTCGCAGTGGGATTAAGTACTTGCAGCAAATTAGACTCTGAGCCCATGAAGCGCCTATACAACGTTCAGGAAAACGT
TATATTTTGCCTTACTATTACTCGAGTACAGCCTAACTTTTCTACATCACACGCATCACTTACCATTACACTCCGCACTAGCCA
CCCGCATACTACAGGTAACTGCCTGATACCTTATCTCATCCTGATGATCACTAAACTGAAACTAGTAAGTACACTTGTGCCATG
ACAGCACATGCATAATGCATTCGTGGCTGTAACTAGCATATTAACTGGCTAACTAGTTGCATTCTTAGAACTCAACTCGTCGCA
AACCTAAAAAAAAACCTCGCATAACTTCAACATGCGTAAACTGCTAACAGCCATAATCTCGACCATTATCTGTGCATAATAGAAC
ACCTGACACACATTGAAATAAATCCCAACTTAAGCAGCTCTGCTACCTATAACATACTAGTCCCAGAACTACGCTAACTCAGCC
ATGGCTACCGTACTCAACTGTTCTTGACCCGTAACTACTATCTTCTATTAACCACAATTTGCCTTTAACCTTCAATACTGGCAT
CACTCCTAATCTTAAGTCTCAACATTCGTTAAGTACCACTAGTGCTAAAAACTAGCCTGCTGCCTGACGCAGGGTAGCATGCTG
CTGTGGGCAACTGCCCCCCAACGCTTCACTAATTCAATCCCTCGCTCACCTGCACCCCTCTGCATGCTGTCCGCTACCACGCTC
CCACGCTCACTGCTCTCTCTACTCGCTCGCTGCCCCGCTACACCACCGCTCTAGCACAAACCCGCTGAACTCCCGCAGCTACTG
CTCCCAATCAACGTTGCTAACTGTCACTGTACTAACTATCGCTAAAACCTGCTACTAAACAGACTAGCCTACACACTGCTAATC
TCTCCTGCTAAACTGTCTGCTTCCCTGCTACGACACCTAATCTACTAACTAATCAGTTACTGTACTCCTGCCTATCTGCCTCAC
TTGCTGCAACTGGGAACCTCTGCTAATAAAAACGCCAAACTCCCGAAACTTTATGGTAGCATACACTGAGTCAACACCCAAACA
AAAATTTGCCTGAGGCTTTACCTGCTTGGGCTATGAATAGAAAAGCTTACGCGCAGACGATGCTGCGCTCAGTCTTACCACAAA
TTGCCCAACGCACCGCCTCTGGTCCGCCAATCTATTTCAAATCGCTTTATGTGTTGCTATAATTTACAGCTTACATGCCCTAGA
AGGTCTTACTTGAGCCTCTTAATATTTTAGCTCTGGCAACTAGTATCTACTCGACAACTTCTCCCCTAACAGATCTACATCTAC
TGCTATGCTATGACGGCTAACTGCATTCTACTCGTACTGCCTATGCGCTACTTCTCAATTCATCAACTACGTCTCACTCCCAGC
CTAACAACATGCTATCACTGCCTTGCTGCCATGCTCAGTCCAAATTCATTTAGTTGGAGCACCTCGCTTGCTCAATGAATCCCT
GCCGCTTACATCTGGCCATTGAGCTAACTCAGCCTGGGCGAAAAGCTCTCGACTTCCTGATCTCTCGCTGCCCCTACCGCTACG
TGCCTGAAAAGTATCTACACAACCTACTCTATGCTTCACTACTATCTGCCAGTAGCCTCGAGTCATTAAATTCTACAAATGACT
CTATTCTACTTCTGCACATTAGTTATATGATACCTTCAATCTACGAGCGCTACTTAATGCAATGCGTACTGTCTGCTAATACCG
GAAGTGCTATTTGCCAGCCTACTCTACATCTTGCATTACCGGATCCGATTTCTGCCGTTACCTGCCCTGAAGCGCTTTCTGCCC
TGTGTGCCATTGACATACCCGTGCTGATGCCATCGCAACTGAGGTCAGCTGCTAACTTCATGCCCTTTGCCTGCCCTGGAGAAT
CCTTCTACAAATCCCTGTCCTGTAATGCTGCAAAAAACTCCTGCTAAACCAGCTAACCCCCTGCCAACCAGAAGCCAAATCCTC
TGAGGGCATCCTTGGCCTGGCTAAATTATTCACAAAGCCTCAAAACCGTTTAGAGCTGAAACCTGCTAAACCCTCACCGCTATC

TTGCTACTGAAAGTCAACTACCTCCGCTTACAAAATAGACAAAATTCATGATGTTAGCGCGCTACAACCATAACTCACCTAATC
GGCAATCGCTACTAATAACTCCAGCCATCTCCGTTAACTACTACGCCAAAGCTAATCTGCCTATAGCCTAAACTACTCTCAAGA
GCAAAACTAAATTATATACAAATATTACTACTAATCTACTAAAACACTAAAAACGTGCAATACTCCTGCTGCTAACTGCTGCTA
CCTAATGTATACCTATTAACAAAAACTGCTGCTACTACTGCGTACTGTGCAACTACATACTATTGCTAAACTACTTACTTAGAC
TGTCAGCTCATACGTAAACTGCTACTACCTGGCTCGCCATCGCTGCTCATCTTCCGCCACTGGCTACGTGAGCCTACTACTCAC
TATCAACTACCTGCGCAGCCAACTAAGCGATCGATCGAACTTTTTCATTGAAACTGCTACCTGCCTACCTGCTACTACTACTAT
TGCTACTACGTACTTATGCTCCTACTATCTGCTACTACTTGTTGACTATTGCTGTTACTTACTGCTACTAGCCTGTTCCTGCTC
TGCTGCATGGCTTCTGCGCTAATCCCTGCTGCTGCGATCCTGCTGCTGATCCTGCTCGCTCGCCTCTGCTGGCTGCTTCGTGCT
CAACGACCGTTACTGGCTACCGCTACTGCTACAGCTACTGCTCTGCGCTGACTTGCTAACTGCATATATATTGCTACTCTAACT
CCTACTGCTACTGCTACTACTACTGCTACTTCTACTCCTACTCCTACTAGATATTGCATACTGCTACTCTACTGCTAGCTACTA
TTACTATCTACTACTACTACTCTATACTAACTACTAGCCTATCTGCTATACTGCTGCTACGCTGCTCCTAACCTGCTACTACTA
CTAACAACTGCCCGGGCTACTACTGCTCTAAGACATAACTACTACTGTTGCGACAAACCTACTGACTACTGGCTCCTGACTACT
GCTACTACATACTACGCTACCTGCTACTACTACTAGCTACTCTACTGCTGCTATAACTGATGGCTACTACTACGGCTCACTCTA
TCTACTCCTACTGGATACTACGTACTGTTCTTACAATATACTACATGCTACCTACCTACTGCTGACATACTTGACTACTACTGC
TATACTACTGCTACATTCTACTGCGTGCTACTACTGCCTGCTACTACTATGCTACTTCCACTGCTACTCGACTACTACCTGTAT
ACTACTGCTACTGTATACTACTGCTACTGCTACTGCTACTGCTACTCCTACGTCCCTACTGCTACTACTACTCCTAAACTGCTA
CTACTACTGGCAAGTAGCTGTCCTAGCAAAGTGCTGCTGCTGGCACTAGCTACTGCTATAGTCTATTGCATGACTAGCTGCTGT
CTGTGACTGGCTACTGCTACTGCTGCATGGCTACTACTTGCTACGCTGCTACTACTTCTACTGCAGCTACATACTAACACTGCT
ACTATGCTACTGCTATGCTACTACTTGCTACTGCTACTACTACGCTAAAACCAGCTACTTGTACTACTACTGCTGCTTTACTAC
ACTGGCTACTATACTGTTGCCTGACAACTACTACTACTGCTCCTACTATGTACGTACTACTAGCCGCGTATGCTACTACTACTG
CTACTGATCTACTGTGCTACTAACTACTGCTACTGACTACTGGCTTACTATCTATCTGATGTACTATACTGCTCTAACTGACTG
CTATGCTACTACTACATGGCTACTGGCTTGACTGCTACTACTACTAGCTATCTTCCTACTGCTGCTCTCACTACTGCTTACTAC
TACTGTTGCTACAATACTACTACTGCTCTTCTACTGCTACTATACTACCGCTATTGCTACTACTACTGGCTACTTCTACTTGCT
GCTACCACTACGTGCTACTGAAATACTGCTACTATCTTACTGCTGGCTACTGACTCACTTGCTGACTACTACTCGCTAAGTGCT
ACTACTCATACTACTGCTACTGCTACGTGCTACTCCTACTCCTACTGCTAACTTATACTCCTACTGCTACTATACTGCAGTACT
GTCATCAAGTGCTGCTGCTGCATGCTACTGCTACTTCTAATTGCTACTGCAGCTAATCTGTCTAGCTACTACTGCTACTGCTGT
GCTACTTACTGCTCACTGCTCGATACTACTTCTACATAGCAGCTGACTACTACTAACTGGCTACTCACTGCTACTGTATGCTGA
CTATGCTACTCTGCTACTATGCTACTGCCTACTAATCCTACTACTACTGCTACGTACTTCTACTACTCCTACTCCTCCTACTGC
TACTGGGCAACTACACTGCTACTTCTACTCTACTCCTACTGCTATTGCTACTCGCTGACTACGCTACTACTATGTACTGACTAC
TACTAACTCCTACTACACTCTCCTGACTGCTACTACCTCTGCTGCTATGCTGCTGACTAACTGCTACTTACTACTACAACTGCC
CTACTACTGCTACTAGCTACTAACTACTGCATTGCCCTACTACTACTACTGCTCCTTACTACTGGCTACTACTGCTGCGCTAGC
TGGCTCACGCTACTGCTCCTGCTATGCTACTGCTGCTGCTGCTACTGCTGCTACTGTAAGCTGCTACTGCTCTCCTAACCTACT
GCTACTACTTACTCTACTGGCACTACTACTACTGCAATACTTCCTACAATGCTGTCACTGCTTACTGCACTGCGTATTGCTATC
TGCTGCTTCTGTATAGATACTACTGCTGACTGCTCAGCGTGCTACTACGTGCTAACTGCAGCTACTACTCTCTACTTGCAGCTA
CACTAATACTAGCTATGACTGCTACTTGAGCTACTGAAGCTACTACTAGCTACCTACGCTACTGCTACACCCTAACTAACTACT
GCTACTGCTACTCTCAACTGCCTACTGCTACTGCTACTGCTACTCACTGACTAGCGTACTCAACTCCTCCTACTACTGCTACTG
CTGCGCTACTGCTGCTAGCTGCGCTGGCTCCGCTGCTGCTGCTGTCGAGTCGCTGCTAAGCCGCAGCTGCTGCCCAGCTGCTTG
GCAGCCGCTGCTAGCCGGCTACTGCTGACTTTTACTGCTCCGCTGCCGGCTACCGGCATACTGCTACTGCTATTCTACTGCTTC
GCCATGCCGCTACCGCTACTGCAACTCCTGCTGCGCGTCACTAATGCTACATACTGCTACTTCGCCATACTGTACTGCCTACGT
GACTGCTACTGCTAATTCTGCTAGCTACTGCTGTTACATGCTGCGTACTACTACTTACTACTGCTACTATACTGCGTCTGCTAT
TGCCTACTGCTGCTACTAAAAAATAGCTAACTAAAAAACTGTTACTACAACAGCTGAGATGATTATGCAACGCAGGCTACTACA
AAATATACTAAGCTACTAGCTAACACACTACCTAGAGTACTAGCTATACTAGACATCTACCACTCATGAAAAATACCATACTGC
ATAAATAATTGCTACTACTGCTATGGCTGCTACATGCTGCGTGCTGCTGCTAGCTATCTGTGCTGCTGCTACTGCTGCGCGCTA
TCCGCTCGCAACTGCTCCTCCACGCTACTTACTGCTACTCCTGCTACTACTACGGTACTCCTGCTACTACGACTGACCTGCTAC
TTATGCTAAACTGCTAAACTGTACTGTCTACTACTGACTATCTGCTACTACTACTACATGGTCCTACTTCTGCTACTACGTTTA
TTCTGCTACTCACTGAGGCACTGGCTGTTGTTGCCTCCTGCATGCTGGCTTCGGCATGTAGCTCCTGCTGGCTGCCTCTATCAA
GTATAGTCTGCCGCTGCTACATCTGCTACGCTACCGTACTGCTACTGCTACTACTACTGCTACTCGCTATTGTGTACTTCCTAC
TCCATACTGCGGTCACTGCTATCTACTAAC
>m141122_014521_42134_c100732202550000001823141405141560_s1_p0/111276/2544_15960
AAACAAAGTTGAAAGGAAAAAAAAAAAAAGTTGGCACAATGGGAAATTAGAGTTAACAAGGAAATAAACCCGCTTGGCTTTCCAT
CAAAGACCATACAAAGGGCCCCAAAGCCCCCAAAAAAAGGCAAATTCCCCACAAAAAAAAACAAAATCCGGCAAATCTTTGGAA
AATGGTTAATCTATTTATCTTCTTGTTTTTAAGGAACCTGGTTGTGGTTCAGTTAATCCAATTTAAAAACTTAGTTCACGTTAG
TTTTATCGCATTCGTGATCTTTAGAATTATTAGTTATGTTTTAACTAACCTTCATAAAGTTTAGAACCTGGTTGTTCCAGGGAT
ATTAATTTATAAAAATTATTCCAGTTAGGTTTATCGAACCACTTGTGCCTTTAGGTAAATTATTAGCTACCGAAATGCATTTAC
CTACTTACTAAGTTTGGACTGGTTGTGTCAGGACTATTAACCATTAAAATTTATTGCAGTAGTTTTTATCGACACCTAAAGCTC
CTTTAGGAAAACACTGGTGAATTCAAATTTTAACTAAACTACTAAATAGGTCCTGGTTTGTGTATAGGATTTAATTAAAAATTT
AGTATCGTTTTATTTTTATCGACAACCTCATGCCCTTAGGAAAACACTAGCTAAAATTTAATAACTTCTAAGTTTTTAGGACCT
GGTTGTGTCAGGTAATTAAATTAAACAATTTCTTAAAATTTGTCGATCAGTTATTATCGACCCGTGAAGCCTTTAGGAAAAAAT

AGCTTGTTAATCTTTAATATAACTCCTTTTTTAGTTTTCTAGGCGTGGTTGTGTCCAGGATAATTTTATTTAAAACCTTAGTTC
ACATTAGTTTTTTTCGACCCACCCAATCGTGCTCTTTAAGGTAATAACAATGGTTAAACCTATTTTACTAACTTCTAAAGTATT
AGACATGGTTGTGTAACAAGATAATTAAAATTTAAAAACTTAGTTCACTTACATTATATCGACACAAAAAAAAAATCGGGCTCAT
TAAGGGAAAAACAATGGTTTAATCTATTTAACTAACTTCTAAGTTTTAGGCCTGGTTTGTCGCAGCCTATATTAATTAAAAACT
TAGTTCACGTTAGTGTTTTCGACACCAATTTGTGCTCCTTTTAGAAATTATTAGTTATGAATTTAACTACTTCTACAGTTTTAG
AACCTGGGTTGTTGCAGGAATATTAATTATAAAACTTAGTTCACGTTAGTAAATTTATGCGAACCCTTTTGTGGCTCTTTTAGG
TAAAATAATAATAGTGAATGAATTTAATAACTACTATGTACAAACTTAGGAACCTGGTTGTGTCGCGGATATTACTTTAACTAT
TAAATAGCGCAGTAAGTTTTATCGACACCTCAAGCTCTTTTAGGAAAACATGGTGATCAATTTAACTAACTAACTAAATTTAAG
GTCTAGTTGTGTCCCAGCCGCCTTTAAAATTAATGAAATTTAAGTTCGCGTTGTTTTATCGACACTCATGCTTATCAGGAAAAA
CACTTGGCAATCAATTTAACTAACTTCTTGTTTTAGGACTGGTTGTGTCCAGGTTATCATTATAAAAATTTGTTCATCAGTTTA
TGCGCCGTGAAGCCGCTTTATGAAATGCTGTTAATCTATATACTCACTTTTAGTTTTAGAGACGTGGTTTGTGTCAGGATTTCC
TATTTAAACTTAGTTCACATTAGTATTTCGACACCGTCGTGCCCCATTAATAGGAATACAAGGTTAGCCTATTTAACTAACTTC
TAAGTTTTAGGACGCTGGTTGTGTACAAGATATTATTGTAAAAACTTACTTCACGTTAGGCATTATCGACACCCAGGCATCAAT
TAGAAAAAGATGGTTAATCTTTTCAAACTAAACTTGCTAAATTTTAGGACATGGTTGTGTCCAGGATATTAATTAAAACTTAGT
TCAAGTTACTTTTATCGCAACTTGTCTCTTTAGGAATTCTTAGTTAATGAATTTAACTAACTTCTAAGTCTTTAAGGGCCTGGT
GTTTCAGAATATTAATAAGTATAAAAAACTTTGTTTCACGTTAGTTTTATCGACACTTTGTGCTTCTTTAGGAAAATTATAGTT
TAATGAATATAATAACTTAACTTCTATGTATTAGGACTGGTTGGTCAGGATATTAAATTTAAATTTTTAATGTTCGCAGTAGTT
TTTTATCGACGACCTCAAGCTCTTTAGGAAAGAACCACTGGTGAATCCAATTTAACTAACTAGCTAAATTCCCCAAAACAAGGT
CCTATGTTGTGTCCAGGATTTAAATTAAAATTTAGTTTCGCGTTTGTTTATCGACACCTCATGCCCTTATCGGAACAGCTGCAA
CCCCCCAATCAATTTAAAACACTTCTTAGTTTTTAGGCCTGGTTGTGTCGCGGTTTAATCATTTTAAAAATTTGTTCGGATCAG
TTTTATCGACCGTGAAGCCCTTTATGAAAAATGCTGTGTTATCTATATAACTCCTTTTTTAGTTTTAGGACGTGGTTTTGTGTC
CAGGATATTCCCCTAAAAATATTAAAAACTTAGTTTCACATTAGTATTTTTCGACACCAAAAAATCCGGTGCTCTTAGGAAATA
CAATGGTTACCTATTTAACTAATTCTAAGTTTTAGATGGTTGTGTACAAAGATATTAATTTAAAAACTTAGTTGCCGCGTTAGC
TTATCGACACGCAAAAAACTCGGCTCATTAGGAAAACAAGGTTTATCTATTTAACTAACTTCTAAGTTTAAGGACCTGGTTGTG
TCCCAGGATATAAATTAAACAAAACTTAGTTCAACGTTAGTTTTATCCGACCCGTTGTGCTCTTTAGGAAATTATTAGTTATGA
ATTTTAGACATTCTAAGTTTTAGAACTGGTTGTGTCTATGACCAGGATATTATTTAACAACTTAGTTCACGTTAGTGTTTCGCA
ACACCAAATTGTGCTCTTTAGGTAAATTAATTAGTGATGAATAACTAACTTCTATGTATTGGACCGTGGTTTGTGTCCAGTGAT
ATTAAATTTAAAATTTAATTCGCAGTAGTTTATCGACACCCCACCAAGTCCAAGGCTGCTTTAGGAAAACATGGTGAATCAATT
TAACTAACTCACTAAATTTTTAGGTCTAGTTTGTCCCAGGATATTAAATTTAATAAAAAAAATTTACGTTGCGCGTTTGTTTTT
TATCGACACCTCCATGGCCCTTGTCGGAAAACACGCAAATCAATTAACTAACTTCTTAGTTTTAGGAACCTGGTTGTGTTCCAG
GTTAATCATTAAAATTTTGTGCGGATCAGTTTTATCGACACGTGAAGCCCTTTTATGAAAAATGCTTTGTTAATCTATAATACA
AAAGAACTCACTTTTTAAGTTTAGGACATGGTTGTGTCAGGAATATTTATTTTAAAAACTTAGTTTCGCATTGTATTTTCGACA
CCCAATCCGTGCTCTTTAGGAAAATACAATGGGTTACCATATTTAACCTAACTTCTACTGTTTTAGGACTGGTTGTGTACTAAG
ATAATTAATTTAAACATTAGATATACACGTTAGCATTAATGACACTCGGGCTTCATTAGGAAAACAATGGTTAAAAAATACATA
AATTTAACTAACTTCTAAGTTTTAGGCCTGGTTGTGTCCAGGATATTAAATATTTACACACACATTAAGTTACAAGGGGAAGGA
AGGGACAGGAGGGCAGCGTTAGTTTTATCGACCACGTTGTGCTCTTTAGGAAAAAATTATTAGTTAATGAATTTTTAACTAACTT
CTAAGTTATATATAAGGGCCTGGTTGATTTTCCAGGATATCTAATTTAAAAACTTTGATTCACGTAATGAGTTTTATCGACTCC
TTTGTGCTGCTTTAGGGTATTATGTAGTGAATGAATTTAACTAACTTCTAAGTTTTAGGACCTGGTTGTGTCTAGGAGATAATT
AAATTAAAATTTTAATTCGCCAGTAGTATTATCGATCACCCTTTCAACTCCTTTAGGAAAACACACCTCAAGCTAATCAATTTA
ACTAAAGCTTACTAATTTAGGCCCTGGTTTGTGTCCAGTATATTAAGATTCAAAAATTTTAGTTCGCGTTTGTTTTATCGACAC
CATCAATGCCTTTAGGAAAAACAGCTGGCTTAATCCAATTTAACTAACTTCTAAGTATTCCCAAAAGAGAACCTGGTTGTGTCC
AGGTTAATCATTTAAATTTTTGTCGCATCAGTTTTATCGCCCGTGAAGCCCTTTAGCGAAAATGCTTGTTATCTATCTAACTCA
CTTTTTGAGTTTTAGGAAGATGGTATAGTAGATACCCAGGAAATTAAAGATTTAAAAACTTAGTCACGATTAGTTTTTTCGACA
CTCGTGGCCTCTTTAGAAATACAAAAGTGGTTAACCTATTTAACTAACTTCTAGTTTTAGGAAGCTGGTTGTGTACAAGATATT
CCCCAAAATAATTGATAAAAAAACTTACTGATCAACGTTGAGCATTATTCGCACTCGGGCTTCAATTAGAAACATGGTTAAATA
ATCTATTTAACTACTTCCTAAATTTTAGGACATGGTTGTGTCGCAAGGATATTGAAATTAAACTTAGTTCAAGGATTACTTTTA
TCGACCACCGTTGTGCTCTTTAGGAAAATTATTAGTTAATGAATTTAAGCTAACTCTAAGTTTTTAGGGCCTGGTTGTTTCCAG
GATATTAATGGTTAAAAAGCTTTGTTCACGTTTAGTTTTTATCGACACCCTTCGTGCTGCTTTAGGGGGTAATTATTGAAGTGA
ATGAATGAATAAAACACTAACTTCTACAGTTTTAGGACCGTGGTTGTGTCCAGGATATTAAATTATAAAATTTTAATTCGCCAG
TAAGTATTTTTATCGACACCTCAAGCTGCTTTAGGAAAACATGGCGAAATTAATTTAACTAAACTATTAACATTTTTAGGCCCT
TGTTGTGTCCAGTATATTAAATTAAAGTTTAGTTCGCGTTTGTTTTATGACACCTCATGCCCTTTAGGAAAACAGCTGGCTAAT
CAATTTAACTAACTTCTAGGTGTAGGACCTGGTTGTGTCCAGGTTAATCCATCTCTAAGAAAAAAATTAAGAATAGACCGGCAA
AAAAGTTTTATCGACCCGTGAGCCCTTAGGAAAATGCTTGTTAATCTATATAACAATCACTTTTTAGTTTTTGGACGTGTTGTT
GTCCAGGATATTTATTTACAAAACTTAGTGCGGGATTAGTTTTTTCGATACCCCAAAAAAAAAAAAACTCCGTGCATCAATTAG
GAAATACAATGGGTTGTAACCTATTAACTAACTTCTAAGTTTGGAGCCTGGTTTGTGTACCAAGATATTTATTTAAAAACTTAG
TTTCACGTTAGCATTATCGACACCCAAAATTCGGGGCATCATTACGGACAAACATGGTTAACTATTTTTAATAACTTTCTAGTT
TTAGGAACCTTGGTTTGTGTCCAGGATATTAAATTAAAAAACTTAGTTCAACGTTAGTTTTATCGACACCCCAATGAGCATGCT
ACTAATTAGGAAAAATTATATAAAAAGTTAATGAATTTAACAAACTCTAAGTTTTCAAGGGCTGGTGTTTCCAGGATATTAATT

AAAAACTTTGTTCACGTTAGTTATAATCGACACCTTTGTGCTCCTTGTAGGTATTAGTATGGAATGAATTGAACTAAAACTTCT
ATGTTATTAGGACTGGTTGTGTCCAGGATATAGTTTAAATTAAAAATTTAATTCGGCAGTGTTTTATCGACAGCCTCAAGCTCT
TTAGGAAAACACTGCGAATCAATTTAATAACTATACTAGCGCTTACTAAATTTAGGCCCTGGTTGTGTCCAGTATAATTAAATT
AAAAATTTAAGTCACGTTTGTTTTATCGACACCGTCATGCCTTTAGGAAAACACTGGCTTTAATCAATTTAAGCTAACTTCTAA
GTATTAGGACCTGGTTGTGTCCAGGTTAATCATTTAAATATTTTTGTTCGCCATCAGTTTTTTAAGACGAAAACCCGATGAAGC
CCATTTAAGGAAAATATGCTTGTATCTTCTTAGACTCACTTTTGAGTTTTAGGCACGTGGTTGTGTCCCAGGATATTTACCTCC
CCCCCCATTAAAAACTTTAGTTCACATTAGTATTTTTTCCGACACTCGTGCTGCCTTTTAGGATAATGGGTTTAACCTATTTGT
AACTAACTTCCTAAAAGTTTTAAGGACCCCTGGTTTGTACAGATACCCCCTATTAAAAAACTTACTTCACGTTAAGCCATTATC
GACACCACTGTCGGGCCTCATTTGAAACAATGGTAATAACTATTTACTAAGCTTCTAAAATTTTAGGACCTGGTTGTGTCCAGG
ATATTAAATTAAAACTGTAGTGTTCAAGTTGTTTTATCGACACGCGTTGTGTCTTAGGAAATTATTAGTTAATGAATTTAAACT
AACTGTCCTAAGTTTTAGGGCCTGGTTGTTTCCAGGATATAATATTAAAACTATATAGTTCACAAAGTTAGTTTATCTGACAGT
TGTGCTCTGTTAGGTAAGATTAATTAGTGAATGAATTTGAACTACTTCTAAGTTTTAGGTCCTGGTCTGATAGATTCACAGGAT
CATTAAAATAAAAAATTTAATCGGCAGTAGTTTTATGCGACACCTCAAGCTCTTTAGGAAAAACACTGGGCGAATCAATTTAAC
TAACTAGCTAAGTTTTAGGCCCTGTTGTGTCGCAGTATACCAAAAAAAAAAAAATTAAATTTAAAAAAATTTAGTTCCTCCGTT
TTGTTTTTATCACACCTGCAATGCCCCCTTATGGGAAAACACTGGCTAATCAATTTAACTACTTCTAAGTATAGGACCTGGTTG
TGTCCAGGTTAATCATTTAAAAATTTAGTTCGCATCAGTTTTATCGACCGCGTGGAAAGCCACTTAAGGGAAAAATGCTTGTTA
ATCTATATAACTCACTTTTTAGTTTTTGGACCGTGTTTGGTGTCCAGGATATTTATTTAAAAACTATTTGGTCATTTTTTTTAA
AAAAAAAAAAAAGTTTTCACATTAGTTTTTTCGATACAAAATCCGTGCTCGTTTAAGGAATACAAGGTTACCTAATTTACTAAC
TTCTCGTTTTTAGGACGCCTGGTTGTGTACAGATATCCCATAATTTAAAACTTAGTTCACGTTAGCAATTATCGACACCAAAAA
GTCCGGGGTCATTAGGAAAAAACAATGGTTTTAATTATTTAACTAATTCTTAAGTTTAAGGACCATGGTTGTGTCCAGGATATT
ATATTAAAACTTAGTTCACGTTTAGTTTTATCGACGACCTTGTGCTCTTAGGAAAATTATGTAGTTAATGAATTTAACTAACTT
CCTAAGTTTTAGGGCCTGGTTGTTTCCAGGATTTACATTTAAAAACTTGTTCACGTTAGTTTTTATCGACCTCCCTTGTGCTCT
TTAGGTAAATTATTAGTAGGAAATGAATTTAACTAACTTCTAAAGTTTTTAGGACCTGGTTGTGTCTAGGATCATTAAAATTAA
ACATAGTAATTCGGAGTAGTTTATCGAACCTCAAGCTCCTTTAGGAAAAACCACTGGCTAATCAATTTAACTAACGTACCGCCG
CCACTAAAATATCACAAGATAGAGCCCTGGTTGTGTCCAGTATGTTAAATTAAAATTTAGTTCGCGTTTGTTATTTATCCGACA
CCTCATGCCTTTAGGAAACACTGGCTTAATCAATTTAACTAACTTTATAGTATTAGCCTGGTTTGTGTCAGGTAATCATTTAAA
TATTTTTGTTGCGCATCAGGTTTTATCGACAGCCGTGAGCCGCTTTAGGAAAATGCTTGTTATCTATCTAACTCACTTTTGAGT
TTTAGGACGTTGGTTCGTGTCCAGGATATTTATTTAAAACTTAGTTCACATTAGTTTTTCGACAACGTTCGTGCTCCTTTAAGG
AAATACAATGTTAATATTTAACTAACTTCTAAGTTTTAGGACTGGTGTGTACAAGATACTAATTTAAAACTTACTTCACGTTAG
CATTATAGACACCCAATCTGGCATCAATTAGGAAAAACAATGTAATCTATTTAACTAACTTCTAAATTTTAAGGACATGGTTTC
GTGTGCAGGATATTAAGATTAAAACCTTTAGTTCAGTTATTTTTTTATGCGACCTTGTGTCTTATAGGGAAAATTATTAAGTTA
ATGATTAGACTAACCATTCTCAGTTTTTAGCGGCCCTGGTTGTTTCCAGGAATATTTAATTTAAACCTATTGTTCACGTTAGTT
TTATCGACCCGTTGTGCTCTTGTATAGAGTAAATTATTAGTGAAATGGAATTTTGCTTACTTACTAAGTTTTTAGGACCTGGTT
GTGTTCCAGATAATTAAATAAAAATTTAATTCGCGCAGTAGTTTTTATCGAACACCTCAAGCTCTTTTTAGGGAAAACACTGGC
GAATTAATTTAACTAACTATTAACTTATAGGCCTTGTTGTGTCAGTATATTAAATTTAAAAAAAAGTTTAGTTGCGCGTTTGTT
TTATCGACACGCTCATGCCTTTAGGAAAGAACTGGCCTTAATCATTTTAAAACTACTTCTAGGTTTTAGGACGCTGCGTTGTCC
GTCCAAGCGTCCCCCTCCACTCCCGATATAAAAATTTGTTCGCAAAAGTTTTATCGACCCGTGAAGCCAGTTTAGGAAAATGCT
TGTTAAATATATACACTCAGCTTTTTAGTTTTTGGCGTGGTTGTGTCCAGGATATTTATTTAAAAAACTTAAGTGGCGCATTAG
TTTTTTTCGATACCTCCGTGCTCTTTAGGAAATACAAGGTTTTAACCTATTTACACTAAACTTCTTAAGTCCTCTCTACGGCAC
TGAGATTGTTGGAAAATATTTATTTAAAAACCTTAGTTCTCACGTTAGCATTATCGCACCTCGGGCGTTCATTAGGAAAACAAT
GGTTTATCATTTAACTACATCTAAGTTTTTAGGACCTGGTTGTGGTCCAGGATATTAAATTAAAACTAGTCAAACGTTAGTATT
TTCGACCCCGTTGATATGCTCTTAGGGAAAATTATTAGTTAATGAATTTAACTAACTCTAAGTTTTTAGGGCCTGGTTGTTCCA
GGAACATATTACATTTAAAACTTTGTTCACGTTAGTTTTTATGCGACACACATGTGGGCTACTTAAATGTGAGGAAAAATAGTG
TGAATGAATTGAATAACTTCTATGTTTTAGGCCCTGGTTGTGTCCAGGATATTGAATTAAAAATTTAATTGCAGTAGTTCTTAT
ACGACCCATTCAAGCTCTTTTAGAAAGACACTGGCGAATCAATTTATAACTAATACTAAATTTTAAGGCCCTAAGGATTGATGA
TCCAAGTATATTAAATTAAAAAAATTTAGTTCAGCAGTTTGTTTTATCGACAACCATCATGCCCTTTAGGAAAACACTGGCTGT
TTTATATCAAATTTACTAACTTCTAAGTATTGCAGGACTGGTTCGTGTACAGGTTATCATCATTTAATATTGTTCGCATCACGT
CTTTATGCGACCGTGAAGCGCCCTTTAGGAAAATGCTTGTTAATTATCTAACTCACTTTTGAGTTTTAGGACGTGGTTGTGTCA
GGAATATTTATATTTAAAAACTTAGTTACACATTAGTTTTTTCCCGACAACTCGTGCTCTTTAGGTACATGGTTTAACTATTTA
AACTAACTTCTAAGTTTAGGACCTGGTTGTGTACAGATATTTATTTAAAAACGTTACCTTCACGTTAGCATTATGACACCCCCC
CCCCACAATCCGGGCTTCAATCTCTGGCAAAAACAAATCAGGTTATCTATTTACTAACCTTATAAATTTTAGGACGCTGGTTTT
GTGTGCCAGGATATTATGAAAAACTTAGTTGCAAGTTAAGTTTATCGCCTTTGTGCTCTTAGGAAATTATTAGTTAATGAATTT
TAACTAATTCTAAGTTTTAGGGCCTGGTTTGTTTTCCAGGATATTATTTAAAACGTTTTGTTTCACGTTAGTTTTTATAGACAG
AAGCACAGAAAATTAGATAGACATATATATAGGTAATTATTAGTTGTGAATTTACTATTCTCAAAGATTTTAGGTCCTGGTTGT
GTCCAGGATATTAAAAGATTAAAAATTAATTCGCAGTATGTTTTATCGACACTCCTCAAGCTCATTTAGGAAAAAACTGGGATC
AATTTTGACTAACTGACTAACATTTTAGGCGCCTTGTTGTGTCCAGTATTTCACCAAATTAAATTTAACGTCTCAAGTTTGTTT
TTCGAAGCGTCAATGCCTTCTAGGGAAAACACTGGCTAATCAATTTAATAACTTCTAAGTATTAGAGACCTGGTTGTGTCCAGG
TTAATCATTTAAATTTTAGTTCGGCTATCAGTTTTATCGACGGCCGTGAAGCCGTTTGGAAAATGCTTTGGTTATCTACTATAA

AAAAAAAAAAAAAAGACTCACTTTTTTAGTTTTTGGACGTGGTTGTGTCCAGGATATTTACTTTTAAAAACTTAGTTCACATTAG
TTTTTTCGATACGCGCACAGCAAACAGATCGTGCATCTAATATAAGAATACAATGGTTAACATAATTTAATAACTTCTACGTTA
TTAGGGACTGGTTGTGTACAAGAGTTATTTATTTAAAAACTTGTGTCACGTTGCTTATACAAAGACAACCTCGGGCTTCATTAG
GAAAAAACAATGGTTAATCTATTTAAACTAATTCTAAGTTTTAGGACTCGCCGCTCTCGTCCCCAGGATATTAATTAAAAACTT
AATTCACGTTAGTTTTATCGAGCACCCTTGTGCCTTGTAGGAAAATTATCTAGTTTAATGAATTTTAAACTTAACCTTCTAAGT
TTTTAGGGCCGGTTTGTTTGCGCAGGATATTCCATTTACAAAATTTGTTCACCCACGTTAGTCTTATCGACGTTGTGCTCTTTA
GGTAAATTATGTGTGACATGAATTTAACTCAACCTTCTAAAGTTTTACGAGACCGTGGTTGTGTCCAGGATATTGAAATAAAAA
TTTAAGTTCGCAGTAGTTTTCCCTAATCGACACCTCAAAGCTCTTTAGGAAAACACTGAGCAGAATCAATTTAACTAACTACTA
AATTTTTGGGCCTGGTTTTGTGTCCAGTATAATTAAATTAAAAATTTAGTTTCGCGTTTGTTTTATCGATCACCTCATGCACCT
TTGGAAACACATGGCTAATCAATTTTAACCTAACTTCTAAGTATTAGGACCGAAAAAATGGTTGTGTCGGGTTAATCTTAAAAA
TTTAGCTCATCGCGTCAAGTTTTATGGACCCGTGATAGCCCTTTAGGAAAAATGCTTGTTAATTCTATTACTCACTTTTAGTTT
TAGGACGTGGTTGTGTCCAGGATTTTATTTAAAAACATAGTTCACATTTGTTTTTCGACACCCTAACGCACCGTCGTGCTCTTT
TAGGAAAATACAATGGTTATACCTATTTAAGCTAACTTGCTAAGTTTTAGGACGCGTGGTTGTGTACAAAGATATTAATTTAAA
AAACATATAGTTCACGTTAGCATCTATCGACACCTCGGGCTATTAGAAGAAAAAACAATGGTTAATCTATTATAACCTAACTTC
TAAAGTTTTAGGACTGGTTGCCTAGTCCAGGATACTATAAAAAAAAAAACTTTAAAAACTTAGTTCACGTTAGTTTATCGACAA
CCTTGTGCCTCTCTCCTCTCACCGCGAAAAATTATTAGTTTAATGATATTTAAGTAACTTCTAGATTTTACGAACTCTGGGTTG
TTTTCCAAGGATATTAACCTCCATTAAAAAACAGTTGTAGAGTTCACGTTAGTTTTATTGACACCTTGTGGCTCTTTAGAGTAA
AATTATTAGTGAATGAAAAAAAAAATATTAACTAACTTCTAGATTAGGACCCTGGTTGTGTCCAGCCCCCCCAAAGCATATTAAA
CTTAAAAAAATTTAATTCGGCAGTAGTTTATGCGACCGCCTCAAGCCTCTTATAGGAAAAACACTGGTGGAATCATTTAACTAA
CTACTAAAATTTTAGGTGCCTGGTTGATGTCCAGGATATTAAATTAAAAATTAGTTCGGCGTTTATTTTATCGACCCCCAAACA
ACCAAATCATGCCTTTAGAAAAACACTGGCTTATCAATTTACCTAACTTCTATGTTTTAAGGAGCGCTGGTTGTGTCCAGGTTA
ATCATTAAAAATTTCTGTTGGATCAGCAATTTATATCGACCCCGTAAAGGAACCTTTAGGAAAAATGCTTGTTAACCCCTCCAT
ACCATAACTCACTTTTTAGTTTTAGGCGTGGTTGTGTCCACCAAAAAGGATATTTATTTAAAAAGCTTAGTTTCACATTAGTAT
AAAAATTTTAGACACCCTCAGATAGATCTTTAGGAAATCAACTGGTTAATATTTAACTAACTTCTAAGTTTTAGGACGCTTGGT
TGTGTAGCAAGTATTTAATTTTAAAAACTTGAGATATCACGTTAGCATTATCGACACAAAATCGGGCTTCCTTTAAGGAAAAAC
AATGGTTAAATCAATAATTATAAACTAACTTCTTGTTTAGGACCTGGTTTTGATTCAGTATATCAAAATTAAAAACTTAGTTCA
CGTTAGTTTTAATCGACACCTTGTGCTCTTTAGGAAAATTATTAGTTACTCGAATTTAACTAACTTCTAAGTTTTAGAAGCCTG
GTTGTTCCAGGATAATTAATTTTAAAACATTAGGTTCACGTTACGTTTATCGACCCTGTTGTGCCCTTTAGGAGAGTGAAATAT
TATTTAGTGAATGAATTTAAGCTAACTTCTAAGTTATTATAGGACTGGTTGTGTCCAGGATATTTCCCACCAGATTAAAAATTT
AAATTCGTAGTAGTTTTACTCGATCACCTCCAAGCTCTTTAGGAAAAAACTGGTGAAATCAATTTAACTAACTACTAAATTTGA
GGTTCCTCGGTGTGTCCAGAAAGATTTAATTGAAAAATTTCAGTCCTCCCCTAGCGTTTATTTATATCGATCACCCTCATGCCC
ATTTAGGAAAACCACTAGACTAATCATTTTAACAACTTCTCCAGTTTTCGCGACCGGTTGTGTCCAGGTTAATCATCCCCCATT
AAAAATTTTTCGTATACGGAATGCAGTTCTCTATCGACGCCGTGAAAGCGCTTAAGGAAAAATGGCTTGTTATGCTATATAAAC
AATCAACTATGTTATAGTAGGACGTGGTTCGTGTCCAGCGATATTTATTTAAAAACTTAGTCACATTAGTTTTTTCGACACCTC
GTGCTCTTTAGGAAATACAATGGTTACCTATTAAAAACTAACTTCTAGTTTTAGGACGCT
>m141122_014521_42134_c100732202550000001823141405141560_s1_p0/118648/0_5885
AAAAGAGATGAGAGTGCCATAGGTTGGATATGATGCCGGCAAGGTTCAATTGTTTGTTTTGGTGTGTTTGGGTTTTGAATATTT
AGGCTGCGCGAGATTTCATATGATTTTTAGTCAATATTAGCCCTATTATAGATATTATATGAGATGGGTAAGTTTCAAAAAAAT
AGAGCCTTGGTTAGAAGAAATATGAGTGAAATTACGAAATCGCAATCGGAGACCTAGGCAGCTGCTCTAGGAGAGATTTCCTGA
ATGGTCAATTTTTTTGTTCCTGTGGGTTTGAATTAGGACCCAATTCATGGTCAAATAGCCTATTAATGGAGGGAGGTAGAGGTG
TATGGTGAGATGGAGGAGGAGTGTTTGAAGAAGAGGTAGTTGAAGGATGTGAGGGAGGGAAGAAGGGATGGGAGGGGGGAGATG
TGGCGGAGATCGGGGCAGGTGGGTCGGTGCCCTAGAGAGAGGGCCAGCTGCCTAGGGAGTGGGCCTGGAAGGTAAGGTGTGTGT
GGTGGGTGGGGTGAATGTGAAGGGGAGTGCGAGAAGTCGATGGGGGTCGCGCAAGTAATCCTATATAAATAATGTATTGAAGAT
GAGGACAAAGGCTCAAAAAAATTAGAGCTTGTTAGGAGAGAAATATGAGCGGAATTGGAGACGGGAGGACAGCTACGGAGCCAG
TAGGCAGCTGCGCTAGGAGATTTGCCTGAAAGGTCAATTTATTTAGTTTGGTTGAATTAGGACGCCCAATGTCTATGGTAAATA
ATCGCCTATATAATATTTATGGAATGTGAAAAGGTTCCAAAAAATAAGAGCGCTTGTTAGGGAGAAATATGAGTGGAATTTGGC
CGAGATCGCTTCGAGCCTGGGCTTGCTGCCTACGGAGATTTGGCCTGAAAGGTCAATTTTTTGTTGGTTGAATTAGGATTCCAA
TTCATGGTCTCAATAATAGACCTATATAATATATATTGAGAATGTGGAAAATGTTTAAAAAAAATTAAGAGCTTATTAGGGAGA
AATAGAGGTGAATGTGCAGAGATCGCTTCGGAGCCCTAGGTCAGCTGCCTAGAATTGCCTGAAAGTCCAATTTTTTTGTTTGGT
TGAATTAGGACCCAATAATTCATAGTCCAAGTTAATAGCCCTATATAATAATTCATTTGGAGGGAATGGTAAGGAGTGTTCAAG
AAAAAATAGAGCCTTGGTTGAGAGGGAGAAGAATATGTGAGGTGAATGTAACGAGAATCGCGTTGCGGAGCCTAGGCAGGCGTG
CCTTAGAGGGAGATGTGTGTGTCCCGTGGAAAAGTCATTGGTTGGTTTATGTTTGGTTGAATTAGACCCAGATGTCATGGTCAA
TAAATAGCCCTATAGATAATATTATTGAATGGGAAAATGTTTATAAAAAATAGAGCCTTGTTAGGGAAAATATGAAGCGAATTG
CGAGATCGCTCGGACACCTAGGCAGCTGCCTAGGAGATTTGGACTGAAAGGTCCAGGGTTTGGTTTTGTGTGTGGTTTGGGTTG
AGATGAGGACGCCAGTTCATGGAAGATCAAGTAGGAGCCTAGAGATGAGGATGGATTATGGTGTGGGAGGATGGAAAAGGTGT
TTGTAATAAAAATGGTAGGAGGCTGTGTTAGGAGGAGAAGTATGAGCGAAGTGGTGTGCGAGAGGTCGGCTGGTCCGGAGCCGC
AGAGGCAGGCTGGCCTAGGGAGAGTTGGTCCGTGAAGAGGTCAATTTGTGTTGTGTGGTTGGTGGTGGTTGTGATTAGGCCGGC
CCAATTCAGGGGGTGCAACGTAAGTAGGCGTAGATAGTGATGGGGATTATTGGGGGAGATGGGAAGGAAGAGGGTCAAGAAGAG

AAGGATGTGAGCGGGGGTTGGTTAGAGGGAGAAAGGTAGGATGGAGGTGAGAGTGTACGAGATGTCGGCTTTTCGGAGCCCTAG
GCCCAAGGCGTGTGCCTGAGGAGATGGTTGGGGCCTGGAAAGGGTCAATTTGTTTTTTTTTGGTTAACGATAGGACGCCAATTT
CATGGTCAATAATAGCCTAAATATAAAGTTACTTGAGATGGGAAAAGGTTCAAAAAAAATTTAGAGCTTTGTTTAGAGAGAAAT
ATGTAAGGGAATTACGATGATCGCTACGGAGCCCTAAGGCAGCGCCTAGGAGATTTGGCCTGAAAGGTCAATTTTTTAGTTTGG
TTGAATTAGGACCCCAATTCATGGTAAATATATCGCCCTAATATAATATTATTGGAGATGGGAAAAGGTTCAAAAAAATAAGAG
CTTTTTAAAGGAGAAATATCAGGTGAAAATGCGAGATCGCTTCAGAGCCCCTAGGCAGCTGCCTAGGAGATTTGGCTGAAAGGT
CAATTTTTTTGTTTTGGTTGTATTAGACCCAATTCCATGTGGTCCAATAGATAGCCCTATATAATATTATTAAGAATGGGAAAA
GTTCAAAAAATTAGAACTTGTTAGAGAGACATATGAAGTGAAGTTACTAGACGTTTTCGAGCTAGGCTGCTTCCTAGGAGATTT
GGCCCTGAAAGGTCAATTTTCTTTTTTTTGGTACCAAAAAATTAGAGACAGCCAATTCATGGTCACATAATAGCCCTATAAATA
TATATTTGATGATTGGGAAAAGTTGAAAAAAAATTTAGTAGCTTGTTAAGAGAAATATGAATGAAATTACGAAATCGCGTTCCG
GAGCCCTAGGCAGCTGCCTAGGAGATTTGGCCCTGAAAGGTCATTTTTTGTTTGGTTGATAGGAAACCCCAAATTCATAGTCAT
AATAGCCCTTATATAATATATTATTGAGAGGGTAAAGTTTTCAAAAAATTAGAACTTTTAAAGAGAAATATGAGTGAATTACGA
AATCGCTTCGGAACCCTAGGCAGCCTGGCCTATGGAGATTTGGCTGAAAAGGTCAATTTTTTGTTTGGATGAATTAGGACCCC
AATTCATGGTCAATACTTGCACCTATATATATATTATTGAGATGGGGAAAAGGTTCAAAAAAATTAGAGCTTTTTTTTTGTTAG
AGAGATAATATGACGGAATTACGAAATCTTGCTTCGGTAGCCCGTAGGCAGCTGCCTAGGGAGATTTGTCCTGGAAAGGTCAAT
TTTTTGTTTGGTTAAATTAAGGACCTCCAATTCATATGTCAGGAATAGCCCTATATATTATATTAATTGAGATGGGAAAAGGT
TCAAAAAAATTAGAGCTTGTTAGAGAAAAATGAAAGTGAATTAACGAAATCGCTTAGGAGACCTGGGCCTGCAGCCTAGGAGAT
TGTGGCCTGAAAGGTCAATTTTTTGTTTGGTTGAATAGATACCCAATTAATGCATAATAGCCCTAATATATTATATTATTGAGA
TGGGAAAAGGTTTAAAAAAATTAGGGCTCGTTAGGTGGGTAAATATGAGTGAAATTGCGAGATCGCTTCGGAGCCCTAAGGCAG
CTGCCAGGAGAATTTGGCCTGAAAGGTCAAATTTTTTTGGTTTGGTTGTATTTAGGACCCAATCTCATGGTTCATGGAGTAAGT
AGCCTATATATATTATTTTGAGATGGGAAAAGGATCAAAAAATTTAAGCTTGTTAGAAGAAATATGATGAATTACGAATCGCTC
GGGAGCCCTAGAGAGGGCGCAGCTGCCTTAGTGAGATTTGGACTTTGAAAGGTTCAATTTTTGTTTTGTTTGGTTGAATTAGGA
CCAATCCATGATCAAGTAATATCCCTATATAATTATTATTGAGATGGGTAATTTAGTTTTTCAAAATAAATTTAGAGCTTTTGT
TTTAGAGAGAATATATGAGTGAATTTACGAAATCGCTTTCCGGAGCCCTAGAGGCAGCTGGCCTAGGAGATTTTTGGCCTGAAT
AGGTCAATTTTTTTGTTTTGGTTGGAATTAGTGATCCCCTAATTCATGATCAGTAATTGCCCTATATTAATGATTTTGATGATG
GGAAAAGGTTCAAAAGATTAGAAGCTTGTTAGAGAGAAATATAGTGAGATTAACGAAATCGATTCGGAGCCCGTAGGCAGCTGC
CTAGAGATTTGCCTGAAAGGTCTAATTTTTTTGTTTGGTTTAATAGTACCCAATTTCATTGGTCAAAGATAATTTGGCCATATA
CATTGGTGTGAGGGCATGGGGGGAAAAGGGTTCAAAAAAATTAGAGCTTGTTAAGAGAAAATATGAGTGAATTACGAAATCTGC
TTCGGAGCCTAGGCAGCTGCCTAGGAGTATTTGGCCCTGAAAGGTCAATTTTTGTGTTGGTTGAATTAGGAACCCCATTCATAG
TCAATAATAATAGCCCTATAATAATAAATTATTGAGATGGAAAATGTTTAAAAAATTAGAGCTTGTTAGGGAGAAATATTGAGC
GAATTCGAGATCGCTTCCGGAGCCCTGAGGCAGCTTGCCAGGAGATTTGGCCTGAAAGGTCAATTTTTAGTTGGGTTAATTAG
CGACCCAAATTCATGGTAAATAATCGCCCTATATAATATTATTGAGATGGGAAAAGGTTCAAAAAAATAAGAGCTTTTTAAGGA
GAATATCAAGTGAATTGCGAGATCGCTTCAGAGCCCTAGGCAGCTGCCTAGGACATTTGGCCTGAAGGATCAATTTTTTTGTTT
GGTTGTATAGGACCCAAAAAAATTCATGGTCAATAATAGCCCTATATAATATATTAAGATGGGAAAAGGTTCAAAAAAATTAGA
ACTGTTAGAGAGACAATATGAGTGAATTACTAGATCGTTTTCGGAGCCCTAGTGCTTGCTTTCCTAGGAGATTTGGACTGAAAG
GTCAATTTTTTTTTTTATGGGTTAATTCGGACGCCAATTCAATGGTCAATAATAGCCCTATATAAATATTATGAGATGGGAAAA
GGTTGAAAAAAATTAAGAGCTTGTTAGAGAGAAATATGGAGTGAAAATACGAAATCGCTTCGAGCCACTAGGCAGCTGCCTGGA
GATTTGACCTGAAAGGTCCAATTTTTTTGTTTGGTTGAAATTAGGACCCCAATTCCAGGATGGAGTCAAGTAAGTGAAGGGCCT
ATAGTAAGGATATTATTGAGATGGGTAAGTTTCAAAAAAATTAGAACTTGTTAAAGAGAATAGGAGTGAATTACGAAATCGCTT
CGGAACCCTAGGCAGCTGCCTAGGAGGATTGTGGCGCGCTCGAAAGGTCAATTTTTTTGTTTGGTGGAATTAGGACCCCAATTC
ATGTCAATACTTGCCCTATAGACTAATATTATTGAGATGGAAAAGGTTCAAAAAAATTAGAGCTGTTAGAGAGAAATATGACCG
AATTACGAAATCGCTCGGAGCCCTAAGGCAGCTGCTAGGAGATTTGTCCCTGAAAGGTCAATTTTTTTGTTTGGTTAAATTAGA
ACCCAATTCATAGCGGAATACCCGTATATTAGATAAATTATTTGAGATGGAAAAGGTTCAAAAAAATTAGAGCTTGTGAGAGGA
AATATGAGTGAATTACGATAAGCGATTAGGATACTTGGCTGCAGCCTAGGAGATTTTGCCTGAAAGGTCAATTTTTTTGTTTTG
GATTGAATTAGGAACCCAATTAATGGTCAATAATACCCTATATAATAATTATTGAGATGGAAAATGTTTTAAAAAAATTAGAGC
TTGTTAAGGGGGAAAATGATGAGGTGAATGGTGCGAGAGTCGCTTTCGAGCCCTAGGGCAGCTGCCTAGGGAGATTTGGCCTGA
AAGGTCAATTTTTTTGTTTTGGTTGTATTAGGACCCCAATTCATGGTCAATTAATAGCCCCATATATATATTATTGAGATGTGG
AAAAGGTTCAAAAAAAATTATGAGCTTTTAGATGAGATAAGATGAGTGAATTACGAAATGCTTCGGAGATCTCTCGTCAACCCA
AACAC
>m141122_014521_42134_c100732202550000001823141405141560_s1_p0/120181/0_11846
AAGAGAGAGATAAAGCACGAGATAGTCAATAGAAATTAACCCGAACTAAAGTGTAATGTATCAAAAACAAGTATCCTTGTAAAT
ATGATACCAGGTCCCTTAACAGGAAGGTGATCAAATTGATTAAAGTCGAATGTGGGTTTCATAAAAAGCACGAGATATCACATA
AAGAATTAACGCGAACTAAGGTCTTTTCAAAATCAATATCGTCTTTTGGAATATTAAGTACGGGAGGCAACTACGGTAAATCTT
ACGAAGTTATTTAAAATGATCAGTCGAGGTTTTCTTACAAAAACACGAGATATCAATAAAGATATTAACCGTGAACTAAAGATT
CTCTCATACACTACAAAATCCTCTAGCGAAATAACCAGGTCCCAACTTAAGAAGTTTATTAAATTGATTCTAAGCTCCAATGAT
TTGGTTTCTTAACATATAGCCTCGCGATCCATATTGTCGTCAGTTATAAGCAGGTATCATTTATGCGAACTAGTTTTGTTCAAT
ACAACTTGTGGGTACATGGATGCAACAGGGTATCTCCTAAAACTTAGGAAGTTGATTCAAATTGATGTAAATATGATGTGTATG
TATCCTTCTCAAAAACATTTGCCCAAGACTAGCTCGATGCAATAAAGAAGTTAACGTGAACTAAGTTTTCAAAATCAGAGCTGA

TCGCTACACATATCTAACCCCAGTGTCTAAACGATATAGAGAATTGATATCAAAATTTGATTTATATTTCGATTCGCCTCTATC
TTAAAGCACTAGATAGTCTCAATCTAAAGAAGACCGTGCCTCTATCCACTTTTTTCAAATACAATATAGAGCAATAAACACAGA
GTACTTGAAACTTCTACAAGTTCGACTATCAAAACTAATGAGACTCTGAAATAGAGATGTGGGCGTAAAAAACATGACTACTTC
AAACTCTTCAGTGCGCAACGGTGAACAAAACCTCTTTTTCAACTAAGCCATTATCCTTGGATAATAACCAGGTCCTGCACACCA
CTTAGAAGTTTATCATTGATTAATTGATGGATTCCTCTTCTTAAAAAACAGATGAGATATCAATTCAAGAATTGACAATCGTTT
AGACCTAAGCCCATATCAAAAGTAGGAATACGCCCTGGAAAGATTGAGCAACCAAGCACATAAAACATATAGTGCTGATTGGCC
CTCAATTAATTAAGCTCGATTTCTCACCTTAAAAGCAAGAGATATCAATAAAGAATTAACGAGAACTAGTTTGCTCCAAATCAA
TATCCTGAAGTATAGTATACCTGAGGCTACCTAGAACTTAGAAGTGATTTTAAAATGATCAGTCCAATGTACTCTTCTTAAAAA
ACTGCGGAGCTCGTCAATAAGACGAAGATTCTGGTTAATCTGCCTGAACTAAGTCATATATACAAGAATCAATATGTACATCGG
AAATAACCAGGTCCTAAATTAGCAGGTTAATTCATAATTGATTCACAACACTCGTTATATTCATAAAACGCCACAAGATAAAGC
ACAATAGACAGAATTAATTTGAACTCATAGTATGCTGTGTTCTATAATCAGGACCGTAGTCAAACCATAATGCGTTGGATGCAA
CCAGGTCCTAAACTTAGAAGTTGATTCAAATTTATCAATTGATCGTTGCTTTGTCTTACACAAAACAAACAGATACTGACCAGG
TCAGGCTACATCAATACAGAATTAACGTGAACTAAGTTTTTTCCGACGACATCTATGTGTGACCGCTAGGAAATAACCAGGTCC
TAAACTTAGGGAGTTTTGATTCACCAATATAGACTAATATTAAGGCAAAAGGTGCCACACCTGCCTAATCGCATGTTTTTCTTA
TATATCTACTGATCACTCATTTTTGTCAGGGGGTGGCCAATTAAAGAATTTTAACGCTGAACTAAGTTTTGTTCTAAAATCAAA
TATCTCTGGAAATAACCAGGTACTACACACTCCCTCTTTATAGTTTTACTCAATTATGTTTCATATACATCGCGATATATTTCT
TAAAGAGAATATTATAAAGAGCTCAGAGCCTATAGCACTCATAATCTCAGTGAACAAAGCTTTTCAAATTAGTTATCCTGGAAC
TTATTAACCAAGGTCCTAAACTTAGAAGTATGATTCAAAGATTAGAGTTATACTTGAATTTCTTCTTAAAAAACGATCTGGAAT
ACAATGGCCAGTAGACCTCTACCACCCCTCATATTGTATATTAACGTAGAACGTAAGATGTTTCAATAATCAACTATCCTCTGT
ATGAACTCATCTTAGGATCCAATAAACCTTAGAACGCATATGCACTTTCCAATACTTCAATCGAATGTATATTCTTATAAAGCC
ACGAGATATCAATAAGAGAATTAACGGGGCGAGATACTGACTTCTCAACAACTCATATCTGTCCGCCAACAGCAGACCTAAGAA
CTTAGAAGTTGATTCAAATTGATTAATCGATGTTTCCTTTTCCTTAGAAAGACACACGAGATATCGACAGACAGTATTTAGTCC
GAACCGAACTCAAGTTTTTCAAATCAATGTACTGGATGCAACCAGCGCTCCTACCAACTTCTAGAATTGATTCAATCTAGTTGA
GGTGATATACAGAGATATATATCTTAGAAAAGCACTAGAGATACACTCAACAAAGAATTAACGCGAACGTAAGTTTCTCAAAAT
CAAATGTCCTGGATGCAACAGGTCCTAAACGATATAGTTAAGATTAAGTCAAATTGATTTAATCCGTCACCTCTCTACTCTCTA
AAAAGCCTGAGAATCACGTTGCTTCAATAAAGAGATTAACGCGAACTAAGTTTCTCAAAAATCAATGTCCTGGATCAACCAGTC
CTAAACTTAGAAGTTGATTCAAATTGATTAATCGATGTTTTTCTTAAAAGCACGAGATATCAATACAGAATTAACCGCGAATAT
GTTTTTCAAAATCAATATCTGGAAAAGACAGGCCAGAGCTAAATAGCAAACTGGAGAATTGATCAAATTGATTAATTGGATTGT
TCACTTTCTTAAAAAGCTAGCGATTAATGGAAAAAAAGTAATGCGAACATAAAGTTTTATCAAATTTTCCTTGCCTAGGGTACG
GAAACCCAGAGGTCCTTAACCATTTTTACATGTCTTGTTTGCAATTGGTAAATCAGACTGTTTTTTCTTAAAAGCACGCAGATT
ATTTCCTGTTCTTAAAGAATATAATTTGAACTAAGTTTTCTCAAATCGACATTCGTCGCTTAGGTTGCATCAAACCAGGTCCTA
AACTTAGAAGTTGTTCAAATTGATTACTCGATGTTTTTCTGTTCAACAAGCAGGAACACGACTGAGTCGAGACTTACAGGGAAA
TCCAAGCGAATTCCTACATAATTCTCTTCAAAATCACCAGTCCAGAGATAGACGGGACCAGGTCTCGTAGAGACTTAGCATATT
GGAGCAACATTGCTTAGCCAACACTTGTTTTCCTCTAAAAAGCACAGAAGATACATAAGATATGTCAACTGAGCAGAACTAAGT
TTTTCAAATCAATAACCTGAAGAGCCAGACCTAAACTTAAAAGTTGATTCAAATTGATTAATCCCGAAGTTGTCATTTTACATA
CAAAGAGACAGCGCCACTATAAGACTTCAGTCAGGAAGAATTAACCTGGACTAAGTTGGTTCGAGAGAGGAATTATCTCCGAAT
ATCCTAGGGCGATACAGAAAACGAACTTAAGCCCTACGGGTTGAGGCAATCTATGTGTCTCTATCTTGACAGATAACCGACAGA
AAGAATACCCGAACAGCATCGACAGCCGATGCAAGAGATGTACGTGACTAACCGTTCTCTCTCTACAAAGTCCACGACATCTCG
AGACTTATAGCGCAAGGCGCTACAAACTTCTGGGAGTGAGTGCGAATTAACTATTCATAGTAATCCATGTTTTTTTCTTAAAAA
GCCAGAGTAGAACCTGGCCATCAATAAGAGAATTAATGCGACCACCCGCAAGCAGAGGACCTTCAGAAGTTTTTCACACTCATG
TCCGTGCTAAGCGAGCGGGCGACAACTATAGGACAATGGGACATTCAAATATAGAAGTTACCTGAATATTCCTCTAAAAAGAAA
ACACGAATATCAAGAAAGAATCATTAACGTGAACTAAGTTTTTCAAATCATATCCTAGAAATAAACCAGGTTCCCTAAACATAG
AAGATCTTGATTCATCTATTGAGTTAACGAGATGTATTTTTCTCGTAAAAGCGCGATGATAAACGGTACAAAATTGCTTTTTAA
ATACAATAATTTTCGCAAATCCCCCTATCATCACAGACTAAGTTTTCAACTTCATAGTATCCCTGGAAATAACCAGGTAACTAA
ACTTAGAATTGATTCAAATTGATTATATTGATGTTTTTCTAAAAAACATGAGATATCAATAAAGAATTAACGTGAAAAGCTTTT
CAAATCAGTGAATCCTGGAAATAACCAGGTCCTAACTTAGAAGTTGAAGTCAAATTGATTAATTTGAATGTTTTCTTAAAAAAC
AAGTGAGATAGCTCAATAATGAATTACGTGAACTAAATTTTCAAAATAACTATCCTGGAAATTAGCAGGTCCGTAAACTATACA
AGGTTGAGTTCAAATTAATTAATCGTGTTTTTCTTAAAGCAAGTAGATATCAATAAGAATTAACGAGAACTATGTTTTTTCAAA
ATTCAATATCCTGGAAATAACTAGGTCTCTAAACTTAGAAAGTTGCTTTTAAAATGATCAGTCGATGCTCTTTCTTAAAAAACA
GCGAGATATCCAAAAAAGAATTAACGTGAACTAATTTTCCAAATCAATATCCTGGAAATAACCAGGTCTAAACTTAGAAGTTTT
ATTCAAATTGATTAATCCATGTTTTTCGATAAAAAGCACGAGATATCATAAAAGAATTAAATGCGAATAGCTAATTTGATTGGG
TTCAAACAAGGCTGTACCTGTTAGGATTCGTCCCCCAACCAGTCTTCTTAATAAACTTAGAAGTCAGGAATTCAATCTTACCAT
AGAATAGCATCGGAGTGAGTGTTTCATGTACACAAGATAATTCGACACGACCAGTAGTTAGATAGAGACTCTGTGGGAGCGCGG
AGGAGCTTACAAGTTTTCTTTTGTATCAAATATCCCTTTTACTCTTTGAAATCTCTAGGTCCACCCTACTTAAGCACTAGCTCA
ATGTAATTTCGTACACAACTCGGTGTTGTTTGTGATTCGCTTTTTCTTGTGCAAATATAAAACAGTGAGATTAGCTCTGGAGAC
GTCTCTTATATACGCAGACATTTAACGTCGTAACAGGCGCTTTTCAAAATTAGTATGCCGCGCCTGGAAGACTTAACTAGGTTC
CTAAACTTAGAAGTTGATTCAATATGCTTCGCGACAGATTGGAAGATATAGGTTCTATAAAAACAGCTAAGAGAAAGGGCCCCG
AAGGGGACACACAAACCCTTCCATTAAGAAGTGAACTAAAAGGCGATTGTGTCACAAAATCATATCTATCATACATCCGGAAAT

CGTCACGCCAGTGTCTCTAAAACGTAGAGATTACTATTCCACCATTAATCCAATGCCCCCACCGAGTTTGCGAAAAACCAGGCT
ACGAGCAGGCATCAATAAAGAATTAACTGCGAACTAAGTCTCTCAAACATCTCAATGTCCTGGATGCAACCAAGTCCTAAATGT
AAGAAGTTGATTCAAAATGATTAAATCAGATGTTGTGTGCTCTTGAGAAACCAGACCCACAGAGACTTATCCCTTAATAGGAGA
GCTCCTGACTGGACCGACGAGAGAGTAGTATCTTTCAAGAGATCCAATGATCCTGGTCCGGAGGTAGCGGCATACTAGGCGATC
CAAAACTCCTCTCAGTAAGGGTTCTGTGGAATTCGAACTTGGGATGAGAGACGATTAGATTTTTCTTAAACAAAGCATCACTCA
AGAGAGTCCAGAAATACAGCACGATAGAGACCCCGCGAACGTAGTTTCTCAAAATCAAGCCATCTGATATCGGAAACCAACCCA
GAGTCCTAAACTTCAATAGTATGTGCGAAATCTGATTCCTCGATGGTTTATTTATCTTACAGCACAGATGGAGAAATGAGTACT
CGTCAATGACAGTAAGTCATCTTTTGAGCAGATTCAACACTTGCCGACTATCAGTGATTCTCTATACATTTCCTTTAATCCCTG
GTATAATACATTATCTCGAGTCTTGCGCAGGTAAACTACGAAGTGGAGTTCAATTGATTAGTGAGGTTTTTTAAAAAGCTCGGG
GATACTTAAGGTCTTTACCATACTAGCTTAAGTTCGCATGGTCGACTGAGGAGGGTCTGCAAGGTATATCAAATCGTCCGTGGT
ACGATGAAACCAATGCGACTCACGCTTATGTGCAGATAGGCATAATCGAAAATAGAGCTAATAATCGTATGGGACGAGAACAAA
AATAGTTTCTTACAAGAGGACAGAACAATAGGAGCACAACACAATAAAGCTTGAATGAACGAGCTGATTATGCGTAGGACGAGA
CAGGTTCTTCTAAATAAACCTGCGAAAACTTACCACGCGCATCGCATGCTTCAGGACTTAAGCAAGGGGGCGCGACCCTAGTAG
AGTTAAGTCCGATATCATATATACATATAAAGAAGCGACCGAGGACTAGGGCTAAAAGGAGGAGCGATAGATTCTGAGTATCTT
AACTACCAAGACGAAAACATATCTTCACACAATCTACTCGATGACAAATCTAGGTCTCACACACAACTCTAGGGGTATGTAGAT
GCGAGACATTGAATATAGATCCGTAATAGGGTCGTGAGAGGAGACGGGAGAGTAAGAGGAGAGAGGGGATTTAACGCGAACTAA
GTTTCTCAAGGAGTCGAGTCCTGGATGCAACCAGCAATCCATACTATAAGAGTTAGTTCAAATTGATTAATTGCGAGGATGTCT
CTTAAAAGAGCAACGAGAGAGCGAGAGGGCTCAACGTGGCAGGACAGAACTAAGTTTTTTCAAGAGCGAAAGTATCCTGGAAAT
ACCAGGGTCCTGAAACAGTAGAAGGTTTGATTCAAATTGTTTAAGTCGAATTTTCATAAAAAGGCACGAGAATGGTCGCTAAGA
AATTTAACGCGAGACGGAAGAGAATATGTTTCAAATCAAAATAGCGAGGAAATAACTAGGTCTAAACTTAGAAGTTGATTCACA
CCTGCGCCCTCTCTGGGCTTGTCTTTTGAACTCTTTAATAAACAGACGAGACAACCAGGAGGGCCTCAACTATTATACCGAAAT
CTAATACGTGACCGAACTTAAGTTTTTCTACAGACAGATCAATTATACTGAGAAGGAAATAACCAGCGTCCTAAATTAGAGTTT
ATTTGCATGAGATTCGATTATATTGCCGGGCCACTCCTTTCGAAAAGCACAGATTAGGAAGGGCATCAGGAATAAAGAGGTGAC
ATATAGCGGAGCTACAACAGGTGTTTTCAAAATCAATGTCCGTGATGGCAACCAGTCGCTAAACTTAGAAGTTGATTCAAATTG
ATTAATTGATGATTTTTCTTAAAACGACGAGATATCGAGGAGAAAGAATGTAAGCGTGAACTTAGTTTTTTCAAAATAATATCT
AGAGAAGTGTAACCAGGTCCTAAACATAGAAGTTGATTCAAATGATTATATCGATTTTTTTCTTAAAAAACAGCGAGATAATCA
ATAAGAATTAACGCGAACTAAGTTTTCAAAATCAATATCCTGGAAGATAACCAGGATACTCAAACTTTAGAAGTTGATCTCAAA
TTGAATTAATTGATGTTTTTGCTTAAAAAACAGTGAGATATCAATAAAGATTAACGTGAACAAAGCCTTTTCAAATCAGTCTAT
CCTCGGAAATAACCCAGGTCCTAAACTTAGAAGTTGATTCAAATTGATTATATTGATGTTTTTCGTAAAAACATGAGATATCAA
GTAATGAATTAACGTGAACTAGATTTTCAAATAAATATCCTGGAAATTAGCAGGTCCTAAACTTAGAAGTTGATCAAATTAATT
TAACGATGTTTTTCTTAAAAGGCAAGAAGATATCAATAAGAATGTAACGAGAACTAAGTTCTTTCCAATCAATATCCTGGAAAT
AACTATGGTCTGAAAGCTTAGAAGTTGATTAAAATGATACAGTCGATGTCTTTTAAAAACACGAGATATCAAATAAAGTAACGT
GAACTAAAGTTTTCAAAATCAATTATCTGGAAATAACCAGGTCCCTAGAACTTAGAAGTTTAATGTCAAATTGGATTAATCCAT
GTTTTTCATAAAAAGGCACGAGAGTATCAATAAGAATTATGCTGAACTAAAGTTTTTCAATAATCAATGTCCCTGGATTGGCAA
CCAGGGTCCTAAACATTAGAAGTTGATTCAAATGGTATGTAATCGATGTTTTTTTCTTAAAGCAGCCGAGATACGTCATAAGAT
TAACGCGAACTAAGTTTTCAAAAATCAATATCCTGGAAATAACTAGGTTACTAAACTTAGAAGTTTATTCCAGATTGATTATTG
ATGTTTCCCTTAAAAACATGAAGTATAAAAAATTAACCGTGAACAAAGCTTTTCAAATTAGTAATCCTGGAAATAACCAGGTCC
CCTAAACTTAGGAAGTTGATTCAAATTGATTAATTGATTGTTTTTCTTAAAAAACATGTATCTAATAAAGAATTAACGTGAAGT
AAGATTTTCAAAATCAATATCCTGGAATATTAGCAGGTCTAAACTTAGAAAGTTGAGTTCAAATTTAATTATCGAGTGTTTTTC
CCTTAAAAGCAAACGGATATCAAATAAAGAATTACGCAAACAAGTCTCTCCAATAATCAATGTCCTGGAGCAACCAGGTCCCTA
CTTAGAAGGTTGAATTCCAAATTTGGATTAATCGTGTTTCTAAAAAGCAACGAGATATCAAAAAGGAATTAACGCGAAACTAAG
TTTTTCAAAACAAGTCTGGATGCAACCGGTCCTAGAACTTTAGAATTGAATTCCAGAAATTGATTTAATCGTATGTTTTTCTTA
AAAGCACGAGATATTCAATAAAGAATTAAAAAACGCGAACTAAGTTTCTCCAAAATCAATGTCCCCTGGATGGCAACCAGGGTC
CTACAACTGTAAGAAGTTGATTCAAATTGATTATCGATGTTTTTCTTAAAAAGCACGAGATATCTATAAAGAATTAACGCGAAC
TAAGTTTTTCCAAAGATCAATAATCCTGGAAATATACCAGTAACTGAAACTTATGAAGTTTGATTCAATTGATTAATTGAGTTT
TTTCTGTAAAAAGGCTCGAGATAGATATAAAAAATTAAGCGAACATAAGTTTTTCAAATCAATGTCTTGGATGAAACCAGGTCC
TAAATTTAGAAGTTGATTCAAATTGATAATCGATTCTTTTCTTAAAAGCAAAAATATCTATAAAAATAACGTGAAACTAGGTTT
TTCAAAATTAAATATCTGGACAATTACGGGTCCTGAACTTAGAAAGTCGATTCAAATTGATTTAATCCGATGTTTTTTTCTTAA
AAGCACGAGAATAATCAAAAATTAAAGAATTATGCGAACTAAGTTTTCAACATCACTGTCCTGGAATCGCAACGAGGTTAAACT
TAGAAGTTGATTCAATTGTTAATGGAAGTTTTTTCTTAAAAAGCAAGAGATATCACTAAAAATTAACGCGAACTAAGTTGCTTC
CAAAATCAACTGTCTGGATGCAACCAGGTCCTACCACTAGATAGTTGATTCAAATTGATGTAATCGATGTTTTTTCCTTAAAAG
CACGAGATATCAATAAGATGTAACGCGAAACTAAAGTTTTTCAATCAATATCCTTGGAAAATAACAGGTCTAAACCTGTAGAGT
TGATCAAGATTGTTAATTGATGTTTTCTTAAAAACACGAGAATATCAATAAAAGATTTAAGTAAATAGTTTTTTCAAAAATCAA
TATCCTGGAATATACTAGGTCCTAAACTTAAAGTTGATTTAAAAATGTTCAAAGAAAGTCGATGTTCTTTCTTAAAAAAAGGAG
ATTACAATAAACCAAGTAACGCGAACAAGTTTTTCAAAATCAATACCTGGAATAAACCAGGTTATAAACTTAGAAGTTGAATTC
ACAAGTGGATATGATGTTTTTTCTTAATAAAAACATGATGATAATCAATATAGAATAACGTGAACAAAGCTTTTCAAATCAGTA
TTCCTGGAGAATAACCAGGTCCTACCTTGTAGAAGTTATTAAATGGATTAATTGAATGTTTTTCTGTAAAAACATGAGATATCA
ATTGAAGATTAAACAGTGCACTAAGATTTCAAGATATAATATCCTGGAAATTAAGCAGGTCCTAAACTGTTAAAGTTGAATTCA

AAGAATTAATTAATCGATGTGTTTTTTCCTGAAAAGCAAGGATATAATAAAATTAACGAGAACTAAGTTTTCCAAATCAATATC
CTGGAAATAGACTAAGGGTTCCTGAAACCATAGATGTTGGAGTTTTAAAGTGAGTCAGGCTCGATCCGTTTTGCTTCAATAAAA
ACACGAGATAGCGAAGCTTCAATAAAGGATATTCACGTGAACTCCATTTTTTCAAATCAACTGCATCCCTGAGACGACAGATAA
CCAGGGGTCCGAAACTTAGAGGTTTATTCAGACTTCGCATTTAACCCCTCTTTTCACACCAAGACACGAGATACCTACAATAGA
AAACTAACGTATCTCTCCAAGTTTTTCGAAAGAGCAATGTCCTGGATGCAACGGTCCTAAACTTAGAAGTTGAATTCAAATGGA
TTAATGATGTTTCTTAAACACGAGATATCAATAAGAATTAACGCGAACTAAGTTTTCAAAATCAATATCGCCTCTCGCTCACTA
GGGTCTCTTGACCACTAAGAAGTGATTCATAAGCTCGATGAATGAATAGTTTTTCTCTAAAAAACATGAGATTATCAATAAATT
GAATTAACGTGAAAAGCTTGTTTCAAAATTAGTTATTCCTCGTGTAACTATTATCCAGGTCGACTACAACGTAAGAAGTTGATT
CAAAATGGATTTAATCGATTGTTTCTTAACAGCACGAGATATCATAAAGAATTAACGCGAACTCTAAGGTTTTCAAAATAATA
TCCTGGAAATAAACTAGGTACTAAACTTAGAAGTTTTTTTGATTCAAAATGAATTTTAAATTGATGTTTTTTAAAAAACATGAG
TATCAATATAGAATTAACGTGACTAGATTTTCAAAATCAATAATCCTGGATATAGAGCAAAGGTTTGCTAAACTTAGAAGTTGA
TCCAATTAATATTCGTATGGATTTTCTTAAAAGCACGAGATATCAATAAAGAATTACGCGAACTAAGTTTCTCAAAATCAATGT
CCTGGACTGCAACCAGGTCCTAAACTTAGAAGTGATTTCAAATTGATTATCGATGTTTTCTTAAAAAGCACGAGATATCAATAA
AGAATTAAACGCGAACTTAAGTTTCTCCAAAATCAATGTTCCTGGATGGCACAAGATGTTCCTAAACTTAGAAGTTGATTCAAT
TGATTAATCGATGTTTTCTTAAAAAGCACCGAGAATAGTCAATAAAGAATTAACGCGAATAAGTTTTCTCCCAAATCAATGTCC
TGGAGCAACCAAGGATCCTAATAACTAGAAGTTGAATTCAATTGAATTTTATCGATGTTTTTTAAAAAGCACGAGATATCAATA
AAGACATTAACGTGAACTAAGTTTTTCAAAATCAATATCCTGGAAATAACGCAGGCTCCCTAAACTTAGAAGTTGATTCGAAAT
TG
>m141122_014521_42134_c100732202550000001823141405141560_s1_p0/121672/9680_16266
CGAGGTTAATCCATTTTCGGACGAAATTACTGAAATCACCAATTCACCAAAGGGGTTACCTCCTTTAGAAATCTTCGATTATCT
CTAAATCGGAGTGAGCATGTGTTTTGGATCGTCGAATACTAAAGAATATGCGAGGTCATGGGTACAGAAGTCGTAAAGCCCTAT
GTTTTAGGGTTCTATGTATAAAATTGCGTGCAAATCCGAGGGTGTGCGGTGATACAGTAAAAATACAGAAGGATCGTAACTAAT
AATGAGGACAAAAACTTGCGAAAATTGCCATCAAAAAGAGGATTTTATTTGAACTGTTGACTGTAAGGCTCGTTCGACTCCAAT
ATAGTTCATTGTACCCATGGAAATCTCTTGGGGAAAATTATCAAAACGAGTGTGAACACAAGGTTTAATACATTTCGGAGAAAT
TACTGAAATCACCCAAAATTCACCAAAGGGACTCCTTATCAGAAATCTTCGATTTCTCTAATATGGATGCGAGCATTGTTTTTG
TCATGAATAACAAAGAATAATGCCGAGGGTCATGGCTCAGAATCGGTAGCCCTATGTCTTCGGGTTCTATGTACGTGAAAATTG
CGTGCCAAATCCGAGGTGTGTAGAGGGTGATACATCAACATTCATGGAAGGATGGTACTAATATGAGGAAAAAACTTGCGAAAA
TTGGCCATCAAAAAGAGAATTTTATTTGAAATGTTGACTTAAGGCTCCGTTCGACTCCAATATATTTATTTTAACCATGAAGAT
CTCTTGGGAAAAATTATCAAACGAGTTGAAACGACGTTTAATACATTTCGGAGAAATTACTGAAATCTCCAATTCACAAGGGGAT
ACCCCTTTATGAAATTTCGATTTCTCTAATATGGTTCGAGCATATTTTGATCGTGAATGGAACAAAGAATCCTCATGGGCATCA
GAATTGTAGCCCTTGTCTTAGGGTTCTATGCTATCAATTGCGTGCAAATCGAAGGTGTGTAGAGGGTGATACGTCAAAATACAG
AAGGATGTACCTAATATTGAGGAAAAACATTGCGAAAATTTGCCATCAACAGAGAATTTGTATATTGAAATGTTGACTTAAGGC
TCCGTTTCGACTCCAATATATTCCACTTACCCATTGGATCTCTTTGGGGAAAATTATCAACGAGTGAACACGAGGTTAATACAT
TTCGAAGAAAATTACTGAAATACCATTCACCAAAGGGGTACTCTTTCTAAAATCTTCGATTTCTTCTAAAGTATGGATGGAGCA
TTGTTTTGATCCGTGACATAACAAAGAATTTGCGAGGGTCCATGGCACAGAAGTTGTAGCCCTATGTCTTAGGGTTCTATGTAT
AAAATTGCGTGCAAATCCGAGTGTGTAGAGGGTGATACAGTCAAATACCGAAGATGTAATAATATGAGGAAAAATTGCGGAAAA
TTGCCATCAAAGGGAATTTTTTTTATTTGAAAATGTTGACTTATGGCTCCGTTTCGACTCAATATATCATTTTACCCCATGA
GATCTCTTGGGGAAAATATCAAAAACGATTGAACACCGAGGTAATACATTTGCGAGCAGATTCTGAATCACCATTCAGCCAAAG
GGTGGTACTCCTTCAGAAATCTTCCGATTTCTCTAATATTGATTGGCAGCATTGTTTTGATGCCGTGAATAATAAAGAATATGC
GAGGGCATGGGCACAGAAGTCCGTAGCCCCTAATGTCCCTAGCGTTCTATGATATAAATTGCGTGCATCCGAGGTGTGTAGAGG
GTGAATACGTTCAAAATAAGAAGGATGTTAACTAAATGAGGAAAAAACTTTGCGAAAATTGCCATCAAAAAGAGGAATTTTATT
TGAATGTTGACTTAAGGCTCCGTTTTCGACTCAATGATATTCATTTACCTATCGAGATCTCCTTGGGGAAAATTTCAACGAGTG
AACACGAGGTTATACATTTCGGAGAAATTTACCTGAAATCAGCCAATTCACCAAGGGTATCTCCCTTTCAGAATCTTCGATTTC
TCTAATTAGGATGGAGCATCTGTTTTGATCGTGATACTAAAGAATGCCGAGGGTCATGGTACGAAGAAGTCGTAGCCCTATGTC
TTAGGGTATCTATGTTAATGAAAATTGCGTGCAAATCCGAGGTGTGTCAGAGGCGGTGATCAGCAGTCAAAATACAGAGGAATG
TACTAATATTTGAGGAAAACACTTGCGAAAATTGCCATCAAAAAGAGGAATGTTATTTGAAATGTTGACTTACAGGCTCCGTTT
CGACTCCCAATATATTCATTTACCCACTGGAGATCTTCTTGGGGAAAATTATCAACGAGTTGAACACGAGGTTAATACATTTCG
GAGAAATTCTGAAGTCACCAACTTCACGCAAAGGGGTAGCTCTTTCAGAAAATCTTCGATTCTCTAATGATGGACATGGAGGCA
TGTTTTGATCATGAATAAGCAAAGAATATTGCGAGGGTCATGGCACCAGAAGTCGTAGCCCTATGTCTTCGGGGTTCTATGTAT
AAAGATTGCGTGCAAATCCGAGTGTGTTGTAAGGGTGATAGCAGTCAAAATACAGAAGGATGTACCTATATGAAGGAAAAACTT
GCGAAAATTGCCATCAAAAAGAGGAATTTATTTTAAATGTTGACTTAAGGCGTCCGTTTCGAACCCAAAATAATCATTTACCCA
TGAGATCTCTTGGGGAAAATTATTCAAACGAGTTTGAACACGAGTTAATACATTTTCGGAGACAATTACTCGAAATCTTCCATT
CACGCAAAGGGGTCCCTTTATGAAATCTTCGATTTCTCTAATATGGATTCGAGCCAATATTTGATCGTGAAATAACAAAGAAAT
TTGCGTAAAGGTCCATGGGCCACAGGAAGTTGTAGCCGTATGTCTTAGGTTCTATGTATAAAATTGCGTGCAAAATCCGAGGTG
TGTAGAGGGTGATACTCAAAGCAATGACAGAAGCATGTACTAATATGAGAAAACATTGCGAAAATTGCCAATCAAAAGAGGAAT
TTTATTCAAAAAATAAAAAAATGTTGACTTAGGCTCGTTTCGACTCCAATATATTCATTACGCCATTGGATCTCTTGGGGAAAA
TTATTCAAACGAGTGAACCCGAGGTTAATACATTTCGGACGAAATTACTGAAATCTCCAATTCACGCAAAGGGGTAGCCCCTTT
ATGAATCTTCGATTTGCCCAATATGGATTCCGGCATCTTTTGTATCTGAATACAAAGAATAATGCGAGGGTCAGGGCACAGAAG

TCGTAGCCCCTATGCTCTTAGGGTTCTATGTAGTAAAATTGCGTGCAAATCGAGGTGTTGTAGACGGGTGATAGCAGTCAAAAT
ACAGCAAGGATTGTACTCAATATGAGGAAAAAACTTGCGAAAATGCCATCAAAAAGAGGAATTTTATTTGAAATGTTGAACTTA
AGGCTCCGTTTCGACTCCAAATATTCATTTATCCATGAATCTCTTGGCGGAAAATTATCAAATGAGTTGAAGCACGAGGTTATA
TAAACATTTCGAAGAAAGTTAGCGAATCCTCCAATTCCACCAAGGGGTAGGCCCTAATATGATCTTCGATTTCTCTAATATGGA
TTCGAGTAAAATATTTTGATCGTGAATACAATAGAATAGCGAGGGTCATGGGCACAGACAGTCGTAGCCCTATGTCTGTAGGGT
TTTATGTATAAAATTGCGTGGCAAATCCGAGTGTGTTAGAGGGTGATACAGTCAAAAATACCATAAGGATGTATAATATGAGGA
AAAAACTTGGCGAAAATTTGCCATCAAAAAGAGGAATTCTTATTTGAAATGTTGACTTAAGGCTCCGTTTCGCTCATATATTTC
ATTTACCATGAGATCTCTTGGGGAAAGATTATCAAACGAGTATGAACACGAGGTGTCAATCACATTTCGGAGATAATTACTGAA
ACTTCACGCAAAATTCACGCAAAGGGTACCTCCTTTCAGAAATCTGTCGATTTTCTAAATATGGATTGGAGCATTGTTTTGATC
ATGAAATATACAAAGAATATGCTGGGGCGGTCATGGGCACAGAAGTCGTAGCCCTATGTCTTCGGGTTCTCCCATGTATAAATT
GCGTGCAAACATCCGAGTGTGTAGAGGTGATAGCAGTCAAAATACAGAAGGATGTACGAATATGAGGAAAACTTGCGAAAATTG
CCATCAAAAAGAGGGAATTTTATTGAAATGTTGTCTTAAGGCTCCGTTTCGAACCTCCAATAATTCATTTAGCCCATGAGACTC
TTGGGAAAATTAACAAACGAGTTGAACACGAGGTTATACATTTCCGGAGAAAATTACTGAAATCACAATTCACCAAGGGGTACT
CCTTTCAGAAATCTTCGATTTCTCTAATATGGATTTGGAGCATTGTTCTTGATCCGTGAATAAAAGATATGCGAGGGTGATGGG
CACAGAAGTCGTAGCCCTATGCTCTTAGGGTTCTATTAACTAAAATTGCGTGCAAATCCGACGGTGGTAAGAGGGTGAAATACA
GTCAAATACATGAAGGATGTACTAATATGAGGAAAACTTGCCGAACATTGGCCATCAAAAAGAGGAATTTTAGTTGAAATGTTG
ACTTAAGGCTCCGTTTCGACTCAATATATTTCATTTACGCATGGATCGCTTGGGGAAAATATCAAAACGAGTTGAAGCCACGAG
GATAATACATTTCGGAGAATTATGAAATCACCCGCAATTCACGCCAAGGGGTACTCCCTTTCAGAAATATTCGATTTCTCTATA
GGATTGGAGCATTGTTTTGATCATGAATACTTAAAGAAATATGGCGAGGGTCATGGCACAGGCAATGTTCGTAGCCCCTATGTC
TTAGGGTTCATGTTATAAAATTGTGTGCAAATCGAGGTGTGTAGAGGGGATACAGTCAAAATACAGAAGACTGTACTAATATGG
GAAAAATTGCGAAAATTGCCCATCAAAGCAAGAGGAAATTTTAATTGAAAATGTTGACTTAAGGCTCCGTTTCGACTCAATATA
ATCATTTACCCATGAGATCTCTTGGGGAAAAATTATCGAAACGATTGAAGCACGAGGTTATATACATTTCGGAGAATTACTGAAA
TCACCATCTTCACCAAAGGGGTAGGCTCGCTTTCAGAATCTTCGAATTTCTCTAATATGGATTGGAGCATTGTTTTTGATCGTG
AATACTAAAGAATATGCGAGGGTCATGGGTACAGGAAGTCGTAGCCCTATGTCGTTAGGTTTTATGTATAAATTGCGGCAAACT
CCGAGGTGTGAGAGGGTTACAGTCAAATACAGAAGGATGGTACTACATATGAGGGAAAAAAACTTGCGAAAATGCCATCAAAAA
GAGGAATTTTATTTGAAGATGTTGACTTAAGGCTCCGTTTCGACTCCAATATATTCATTTACCGCATGAGATCTCTTGGGGAAA
ATTATCAAACAGTTGAAACACGAGGTTATACATTTCCGGCAGAAATTACTGAAATCACCAAATTCACACTAAAGGGGTATCCCT
TTCAGAAAATCTTCGATTTCTCTAATATGGATTCGGAGGCTTGTTTTGATCGTGAATAATAAAAGACATATGCGAGGTCAATGG
CCCCCCCACAGAAGGTCGTAGCCTATGTCTTAGGGTTCTTATGTATAAAAAATTGACGTGCAAAATCCGAGGTGTGTAGAGGGTG
ATAGCAGTCAAATGACAGTCAAGGATGTACTAATATAGGAAAAAACTTGCGAAATTGCGCATCAAAGAAGAGGAATGTTTATTT
GAAAGTTGGACTTAGGCTCCGTTTGACTCCAATCATATTCTTTACCCATGAGATCTCTTGGGGGGAAAATTATCAAACGAGTTG
AACACGAGGTTAACTACATTTCGGAGGGAAAATTACTGAAATCAGCCAATTCACCAGAAGGGGTACCTCCTTTCAGAAATCTTC
GAATTTCTCTAATATGGATTGGAGCATTGTTTTGATCGTGAATACTAAAGAATGCCGAGGGTCATGGCCCAAGCTAGAAGTCG
TAGCCCTAATGTCCTTACGGGTTCCTATTTAAAATTGCGTGGCAAATCCGAGCGTGGTGTAGAGGGTGATAGCAGTCAAATAAG
AGAAGGATGTACTAATTAAGGAAAAACTTGCGAAAATTGCAATCAAAAAGAGGATTTTATTTGAAATGTTGACTTAAGGCTCGT
GTTCGACTCGCAATATATTCATTTACTACATGCGAGATCTCTTGGGGAAATTTATCATAATGATTGAACAACGAGGTTATACAT
TTCGAAGAATTACTGAAATCTCCAATTCACCAAAGGGGTACCCCTATCATGAAATCTTCCGAATATTCTTCTATATATGGATTG
CGAGTAATATTTTGATCGTTAATTAACAAAGAAGGATGCGAGGTTCATTCGGGCAGCAGAAGTCGTAGCCCTTGTCTAGGGGGT
TCTATTTAAAATTGCGTGCAAATTCGAGGTGTGTAGAGGGTGATAGCAGTCAACAATACATGAAGGATGTATAATATGAGGAAA
AAACTTGCGAAAATCTGTCCATCCAAAAAGCGAT
>m141122_014521_42134_c100732202550000001823141405141560_s1_p0/122288/4893_12286
GCAATTATTTTTGAGTAGGAGAGATGAGGTAGAATTTAAATGAGCTCATCAATTTAGTGATTGCTGTAACGACCTGATTGCTCC
TTAAGAATGAGCATTGCGATTTGTATGATCTGTGCATTGAGTGGCATAAGCTTGATAATGGTACTATGTTGAATTTGTGCCTAT
GTGTGTGGTGGTGTTTAGTTATTGAGTCTGCACGGGCATCGATTGACGGTTCCTAGTTGCAATTATGTCTAATGAAGGTCACAA
CTGGGATATGTTGTACACTTGTTCGGCCTCTGGGTGTAGGAGCCTAGGGTAGCTTGTTTCGGTGTATTGCTTATTAAGGGGTTT
TGAGGTTGAGAGGATTTGGAGTTGGTTTAGTATTTTGGTATATCTGGGTATTATCCTGGAAATTACCGTTAGGGGGGGGTTGGG
GGGGGGGGTGGGTGGGGTGTGTGGGGTGGGGGGGGTTGGAGGTGGGGGGGGGTGGGGGGGTTGGGGTGGGGGTTGGGGCGGGT
GGGGGGGGTGGGGGGTGGGGGGGGGGGGGGGTGTGGGGGGTGGGGGGCGGGGTGGGGGTGGGGGGGGTGGGGTGGGGTGGGGGG
TGGGGTGGGGGGGGTGGGGGGGGGGGGGGGTGGGGTGTGGGGTGGTGGTGGGGGTTGTGTGGGGGTGGGTTGGGTGGGGGGGGG
GGGGGGGGGGGTGGGTGGTGGGTGGGGGTGGGGGGTGGTGGGGGGGGGTGGGGGTGGGTGGTGGGGGGGGGTGTGGTGGGGTGT
GGGGGGTGGGGGGGGGGTGGGGGGGTGGGGTGGGGTTGGGGGGGTGGGGGGGGGTGGGGGGGGGGTGGTGGGGGGGGGGGGGGG
GGGGGGGGGGGGGGGGGTGGGGTGGGGGGTGGGTGGGGGGGTGTGGGGTGGGGGGGGGGGGGGTGGGGGTGTTAATGTTTGGGTT
AATTGCAATGTTTTAGAGAGTAGAAATTGTGTATAGAAGTTCGTGAAGAGTAGAATTGTTAAAGACTGGTAAGTGTCCTTGATG
GTTGTAGTGTTAGGGAGGATTATTAGGAAGGATAATTCTGGTTTTCTTTTGGCAAGGGAGTTATTAAATTTCAGCTGTAGATAT
AATTGTTGCTTACTGGTTTGCTATTTGGGGTCACTTGTAAGCTTTGGATAGAAGAGTTGAAATGAGTAAAAAGAGAAAGTTGAG
AAAGTAATGTAGCTATGGCAAGTAAATAAGAGAATGAAGTACGGAAGGTAGTGAAGTGGCTGAAATTGAGGAGCTTAAGATGGA
GATTCATTTGAAGCTGTTGGAGGATTTTCTTACTTGAGGTATGGATATTTAATACATCATCTTAAGTTGTTGTTTTTCTATTGT
TGATTTGAGGATTTTATTATGCTTGTAGTTATTAGCTTTTTCTAAGGTTTTTGGTTTGAGTGGCCTGAAATTAGAAGATCAAAG

TATGGATTCTTTGGTGTGTTTGATTGTTTTGATCTTGTTCTAGTGTCCTATGGTTGTTAGAGTTGTTCATATAAGTTTTGGTAA
ATTTTTAGTTTTTTTGTGGGTTGTACTGTTGTGCTGGTTTGGGGTTATTTTGGTATTGAAGGAATTTTGAAGAGAATAAAACCC
AAAAATTCGGGTTTCGTATAGGCGCGCCGCGGCGCGATGGCTTGGTTCCTAGGCCGGTGTGGCTCTCTTTGACTTGTTTTTAAG
CGCCTGTAGGCCTTCTGTAGGGCTGAGCCTGCGCGCTAGTGTAGCATTTTTTGGGGCACAAAATTTTTTTTTCCTCCCAAAAAT
TTTGTGGAGCTTTTGTCCAGGTGTTCCGGGGAATGTTTCCACATGAACTTATTTTTAAGATTTTTGGGAGTCCCAAATGAGTAT
GCTGGATTGGTCCCGGAAGTGGTTTTTGGGTTTTGTATAGTATTATAAAGAGTGTTTTATGTTGTGTTGTGACTAGATTTTCTG
GAGAGGTCGTGCTAGAGGTGACCTGGCTCGAGTGACTCGGTGTGCATCGAAAGCTGGGTATGCGGGTATAGAAATACCGTAACA
AACCCAGTACTGAGGTGCATTGACCCCACTTTTGTGTGTGATTCGCAGGGCATTGGCCTAGATTTTGTATTATGTTGCAAATGT
TTAACCCTTAAATGAAGCATGATGTGTGGTGAATTGATTGATTATCGAGATGTATCTATATTGTTCTGTGATTATAATGAATAA
CGGCAGTGCCGATGTGCGCTGTAAGCCGGTACGAGTCGTGGGGCCGATAAGTAACAACTCAGGCACATGGATGGCTTAGTGAGG
TTCAGGTGGGACCCAATGGGATGAAGCATTAGTTATATTCCTTTTCGCGGTGAGTCATACCGATATCACCAGGCTTTGTTGATA
AGGCCTCTGGGGCGGCATGTTGGCCGTTACTTGTTTGATTTATGATGTTTGTTTCCTATTTATCAGTGATATTAGTGCCAAGTA
TAATGATTGCCATAATGTTATTGTAGGTAATGTTGGTTTTGTCTTGGGTTTGCCTTTCGCGCTTGCACTTGTGCTCCGTGTGGC
AGGTAAGAACAGGGAGTTTCAGTCAACCGGCCATGAGTATGGAGAGCGTGGGGACGGCGCGTACTATGTTCTGATTCTTGCCCG
ATCTTGTGTTGGTGGGGCATTTGTGTATATTTGCGGTATTAACCGATTCTTTTGATAGTTGATCAAGTGTTAATTATTTGAGTT
GTAATATATTTTGTAAACCTTATTTTTTGGGGTTGTGTGGTGGTTGGGATTCCCTATGATGTTTGTATTACTTAAGTTTTCAAA
AATAAACATTTTCGAAGAGTACAGGCCTTAAATTGTGTCTGTTTATTCAACACGTGTTGGGTTTTATGAAACCACTTAGGTTTT
GACTAAATGCAGGTCTAATTTCCTGTTTTAAACCACCTAAAGTATTGGCTCTAGGAGGTAGTGGAAGGGTGGCGTTACAACTTT
ATTCCTTTTTTTATCGTGATTACAGGGCTTTTACTGACCATTGACAGCTGTGCTGGTCCCGTCGCTGAGTGATTGGTGTCCTCG
GGCCAAAAATTTTGGACACCTAGTCGTCGTGAGGCTAAGAAAGCACCCTCGGAGAGAATACAACTTAGTGCTCGAGTAGAACAA
TCGACTAACTATGCATGAGATTGGTCAGTTCTTGAGAAGGAGTACAAAGCTGCTAAGTTAAAAAAGGAACTGCTCCGAGCAAAA
ATCTCACTTCTGACTGAGGAGCTGCTGGGTTTGGAATGGCCTGAAGAAATCCAACGAGAAAAGAAAGTTCAGGAATGCGCCAAA
CTCAACTACCAGCAGGCCGTTAATGCTCAGATGCTTGATTTGATTGCGTGCAGGCCAAGAGACGGAGGACTGAGAAAGCGCATG
GGAGCTTGAGAAGCTGAGCCAAGACTTTGGAAAAATGCTACAAGGAGCCACTCGATCTTTGCTTCTACGAGTTTGCGAAGCACA
ACCGGGAGGCTATGAAATTCAACTATCTGTCGAACGGCCTGAGGCGGACGTCTAAATGTCTCTTAGGTGCCATCTTGCTGGAGA
AAGAAAAGGCTAAAAGTACCTGCTTCCCCAAAGATTTCTCTGGCAACAGGGATAGATGTGTGCATGACAATGAAGTGTAAGCTG
CAAGCTGTCGACTAGGTATTTCCTCGAAGATCCGTCCAGCGCCCTTTATGTGTGTCTTTTTCCTTTTTTTTTCTTTCTCTTTTGA
TAGTTAATTACATGTGGACTGTTTTACTTAGTATTTGATGTAAAGATAGATTATTTTATTGCTCGGCGGGCAACCTATTTTCCT
TGTTAAGAGTCAAGACTTTTACATCCGAAGCAGGTGGTTCTTGGCTCGTTGGATGTTATTATAGAATTTATTTTGTGATATTAT
ATTACAACATTTTTAATATTTAATATTCTGTTCCACTAGGTTGCCATAACTTAGCATAGCACTTTGTTTGATGTTTAACAAAAT
ACATTATATTTTTTAAAAAATACTCTTAAGTACCTAGCATGCTTCACCTTTCTATTGATTGATAAATTGTTTTTACCATTACTT
TTTTAATATATGCTTGCTGTACTTGTACCTTATTAGTGTCCCGCCCGGCCAGAGTGATTCGATTGGAGTTCTTTAGGTTCTCTT
GTTCACATGCCTTGACCCGAAAACCTGTTGATTTAAATGCTGCTGGTGTAGAGCAAAAATAATTTTGGAATATTTGTTAGTAGG
CATATATACAACACACGTAATGAGCTAATTGTACTTTAAATAACAAAATTAATGTGGCAAGAAAGGTGACTGGTCTGCGCACAT
CCTTATATTCTCGATAATAAATGGGACATAAAACATGATTTGTTACAGTTAGTTATCAAGTGATAAGATCTTACACTTATATAC
GACGATCACGTAAAAATGACCAATCCCTGTTTTCTATACTTTGTCAGAGAATGTAAAATTATAGGTACAAGCCAATTGTCTTAT
GTAAAGATGAATTTGGTTTATGTTGGTGTAGATTTACTTTGCGTAGGTGTTCTCGCATTGTTCAATAGTGAGGAACAGATCTCC
CTTCAAGCCGTAGAAATTTTGATAAGTGCTATGGGTTGAGGCTTTCTCAATCTGGTATATGGGCTGTTCTCAATTAGGGGGGCC
GAGGTAACTGTTCCAGCTTATCTTGTGTTGGCTGGTGTTTGTTATAAAAATCTCTGGTCGGAGTTAAACAATATTCCGTCCAGA
CGCTGAACTTTCCTCTCTCGAACTTTAGAGAGTTAAAATAATAACTTGGGCTGACTTGTTCGGGCTGGTACGGCTGCTACTCGA
CGTTTGGCCTGTTCATGTTTTTTCTTCTATGCGATTGAGTCCTAGGGATTCCATCAATTTATGCTTGGCGTTCGATATGTTTGA
TCGTTTTATCGTGTATTTTCTACGATGTGCGTATAGGCAGAGGTCTATACTTCTAACAGGCAATCATATAGTGTGCTTTTCATT
ATTGGTTTGATCCATTGGAATCCAAGAGAATGGATTGTATGTACCCGTTGCTGTTCGGTTTGGATAGTTCTTTTCGGGCCATTT
GTGGGCCCCCTTAGCTTCTTCGTTAGCCTTTGTCTTTCCTGTATTCCCTCAGTGTGATTGTTGACTGCTTCGACTTTCCATTTG
TTTCTTTGTGGTGGATGGGTAGCTGTAGTAGAGAGAGCTTCTTGAGTGATTATACCTTTTGGTCGGTTTCGTGTAGAAATCTGA
ATAAATTTCACTGTCAAATCTGGGGTTCTCATTATTCGCGGACATAGTGTCTTCCTTTTGCAATTCCATAGCTGTACATATTCT
ATTTGTGTTTTTGAGTCAAAAGATCCAACAACCTTTTCGTTGTCGTTATTGGTTGCAAGGTGTTCAGCCGGTCGACCATGTTGT
TGAAGTTATGGTCGTGGTGGCATATAAAAATAAATACCGCCACAGCATATTTGACACTGCTCTTTGGTGTTTTTTCCTCTGTGG
TTAAGAGACTCCCGATTAGGGTCTGTATATTCCCCATTGTGAACTTTTTGTTTGTTTTACGTGGTTTGGCATAATGTGCCATTT
GGGTTCTTCATCGGTTATTTAATGCGTAATTATTTACGGGGCGTTTGTCTTTGGGAGTTTGGAGAATCTCTGGCCATTTATTTC
TACTCCTGTCGCCATCGAAATCCTATCTGAGTCTTCGATTGTCATTCGTTGGTCAAGTAAATATTCACTTAGCCTTAGGATTCT
TTTTACAACTCTGCCGGCCCCAACGCTGTAATATCAACTTCTAATGCAAATATTAAATAATAATGATTGATCTAAAAAGCGAGT
GTGTAGTAACTGCTATATTGACTGAAGATTAGTGGAATGAAACGAGACAAGAAAGGAAGTGGATAGAAAGAGAAAAGAGAACTT
ACGGTGATAAGATTAGCGGAACTTCTATGGAACAAAAGATTTACTAATTTTATACAGGTAAGGTTTTGAGAACAAAAGTATTAA
AGAGTAAAGAAGATTAAAGGACGGTTCTAAAAAGTTACAAGTAAGTATGTGATGAGAAATGAAGAATAGATAAGAAAAGCAATT
AGGAGATATAAAATGTAAGGAACTATAAACAATTATTATAATTTGGTATCTCTCACTTACACAACCTTAAGTAGTGTATAGGTT
TTTGGGATTCACGAACTTTGAAATCTTATTTGAGTGTTGTGTGAGAGATACCTTGTGTCCAATATGAGCTGTATTTCCGCCCTG
AGACGTCAAGCAGCTAACGGGATCTTCCTCTATAGATTTTGGAAATGCTTTCTGGAATATTTATCAAGTACCTGAAGGTGTAG

TGTTTCAGCCAATTGTGTTTTGTTGGATGGAATATATGTGTGTCTTTACAAGTGAGCATTAGGCTCCTATATTATTATTTATGT
GTTTGATGTATACCTTTGCATTGAATTTTAATTCCACCAACACCCCATTTGACTTATTACCTTTTAGATGTTTAATTGGATGTT
TTATTGGAATAAAATGAAGATTTGGAGTTATTTGGAATTGCTTAGAACCGTTCATATTTAGAAAAACGAAAAACCATATAGCTT
GGTTCAGCCTTACTCTGCCGGGCCAGGATTATTCCCAGTGCCTCGCGGCCACTAGCTCTGTCCACCAGGTTGTTTGTTGGTTCG
CGGCCAGTGGAAAGACAGTGGCCGCGGCCAGAAATACAGACATGTGGCCGCCGGCCACAGCCTATGTTTCAGCACAAAAATTGT
CTTTTCCAAATCATGTCCAATAACGGTCACAACATGAATTTTGTAACCTCAAACAAACACCCTATTGGGATTTAAACCAACATG
TCTCCCCAACTTCCATATTTCATTATGGCTTTATGAAATCCAATCTTAAATATGTAACATATATATAATACACATTATTGGGTA
AATTTGGGAGTTACAATTTGTAACTGATTTTTGTTAACTCCAAAATAATTCATAACTTTGAGGCACACACATGTGTGCCAATTT
TGTGATCTCTCAATAATATTTTACAGAGGTGTGATAAATTCACATTTTTGGTGTGACAAATCTAATTTTTTCACAGTTATTATG
ATCTAGGATTATTTTATATTATATGAAATAATAATATTATCTTTGTGTAATCACCTGAGGAGTGCCATTGAGTATCCTTCGGCT
TTTTAAATAAATGCCGAGTCGACCAGTGATTATTACCGGTGTAGCAGCCTGTCGCTTGAATTGTGTCGTTGGCTTGTGTTCCTG
TCCGTTGTGTAACTTTCACCATTTTTGTGTCATATGACTCGGTGACGATATTGTTGACACCATCCACTCTGTATCAGCCATCTA
GATCCACCAAGGTGATTCTCCTTCTGCTTGGATGCTTTCAGATATTCGTTCGATATGGGCACTATGGTACGAGAGTGTCAGCAT
C
>m141122_014521_42134_c100732202550000001823141405141560_s1_p0/123417/14610_20842
GGTTTCTCAACATATTGGACGTCTTTTATAGTTCAGGTATGCTTTGAATTAAATAAGAAAAAAAATAATAATTTGTTACTCTAG
TGAACTTATTTACTTTAAACTAACTTATGGATTATATTCTTATCTATGTTTTTCCTTCTAAAGATAATATTGCATAATTAGATA
TATCTTTTTCCTATAACTAAATAAAAAATTTACATTTATCCCACTTGAGTCGAACAGATGTAGGAGAGATCCGTTTTTTTGGTT
TGACAGTGTTTTGGAGAGAAACTATCGGAAAGAAAGATATAGGTAATAGCACCTTCTACAGTAGTAGGATCATGAATAAGACAG
TAAAGAAAAAGATACTATTATAGGCACGTTTTGGAGTTTTTCTGTTTTTTTCACTGGTGGTTTATATTTTTTTCCCACCAATA
CACTCCTGAGATCTCCCAGTTTAATTCTTACTTAATACTGTAAGATAGAAAGCAGATATAATATATTACTACTATTTTACCGAT
CAATAGACTGTAAAAATATTAAAAATATAATTGTTAAATAATATTAAAATAAGATATGTAAAGAGCTAGACTTTTCGGAAGAAA
AGAACGAGAGGTGAAGGCTTTTGATTTTAAGTTTTCGTGGCGTTAGGTAGGTGGGGGGTGAGGTTGTTTTTGTTATAACCGTGA
TGGAAGGTAGACGAGGTGAGGAGTCTACTGGCTGGAGACACGTTCAAGAGAGGTGCAGCGTGGGAACCTCAGTTGTCCGCTTTT
CATTTAGTCCCCACTATTATGACCCACACACCCTTGTTGATCTATGATCTACCCCACACCTGTTTTTTGGTTTTGGTGTGGGTT
TGGGTGATGGTTGGTGTTGATTTGTTTTGTTTATTTTTGTGTTTGGGTTGTTGGGCTTATCTAAACTCTATATTCAGAATGTTT
TTCACATTACTGACATTCCTTTTTATTGAGTTTGTGGTACGTTTTGTATTCTCTTTTATTGTACATCGGTATAGCCAAAAGGGT
ATCCGTGATTCATATTTGTTGCTTCGAAGAGTGTAAATTAAACATATTATCTCTAGCTTAAGCTAGAATAAAAAGGACCCAAAA
TGAAGATATTACCAGTCTGGAGAATTCTTATACGCTTTTTAATGCGATTTTTGTTTTGTACTCTCTCACTTCTCCTCGTTGCGA
ACCACTGTTCTATTTTTGTATGGTTATTAGTTTCATCCTCACACACTGTCTCGTAAACATTTTTCTTCTGTGATTCTTGCACAA
GGTTTTGTTTTGTTTTGGGTTTAAGCCCTCGACATCTGTGAGTTTTGAGTTGAGTTGAGTCCAGTACTTTCAACCAAACATGCC
CGTTTGCACTCTATTTATTAAGCTCTATTCCCTCTATTAATTTATGGTGAAACAATTAGGCAGTTTTTAAGTCATTTTTTTACT
CTGACATATTCAGCTTTGGCTCGGTCTTTTCTTTATTTTCAATGCTCTTTATACATATATCCTATAAAGGAATCAGTGATGATG
GTGGATCATCACTGCTACAAACAACCAAAGTTTCTTAGATCCATCGGTTAAGTTCACGAGCACAAATCACACACAACCCGTAAG
CTCTTTCATCAAGCCCGAAACGAAACCGAACCCACCATGAACCCGCTCAGAAAAAGAATCGTTCGGATCATAATCACTTGGACC
GCCCAGATGCCTACCGATTTTTTCATCCTAGCGACGAAGACGACTCGATTGCCGTTACCTCACGGAGAGTCAAAGAGGCAATGT
GAGGGAGATCAGTTTACAAAACGGTCTTCTCCTCCTCCCGCCTTCACCGACTTAGTTACTAGCAAGAGCGAGCTCCCAAGAGAG
TGATAAAATCGCCGGAACCGATTCCGCTTTCGCCGGAAAAAGTTTAAGAGGCGTTTTTACGGCCGAAGACCATGGGGTCGCTGG
GCGCCGAGATCCGAGACCGACTCAGAAGAAAACGGGTTTGCTGGGGACTTTCGAGTACGGCGGAGGAAGCCGCCACCCGTCTAC
GACCAAGCCTGCGCTGAAGCTTAGCGGCTAACGCCCGTTCCAACTTTCCTAAACGTCGCTAACGAGCGAGTCGACGGAGCGCCG
TTACGGGAAGTGAATGGAGAAGCTCGTCCCGCGAGGTTTCGTCCCGTCGTTCCAGGGGGCGCTGTGGCATTGGCCCTCGTCGCC
CACGTCGGTTCCTCCGTTACGAAGAAATGACGCCGTTTGAAGATTTCGGTTTCTTCGGCCCTGAAAATTATGGGTTCTGATTTT
TGCCGAGCCCCCGGTTGCCAGTTGGTACGATTATTATGCTGCGCGGAAACAGTATTGGCTTAGCTGAAGGTCGAGTTCCAGCGA
GTTCGACTTTGACTGGACGTCGTTTTTTCTAAATTGGTAATATCTTCGCCACGTGCTTCAGAAGCTTGCCCACTTCGGTCTTCC
TTGATTGCTAGGGGCAGTTTAATAATTTCACATCAACTGTTCCATAGATTGTGGGGTACGTGGCACATTGTGATTAGCCATTCC
TATAACGGACAGTGTTTTCGGGGAAACAAAAAATTGTTTCCGAGTTTAATGTTAACAGCGGTCAAGTTGTCCAACGTGCTACTA
CTTAGTCAGCCGCCTTTTACCCCAAAATATACGGCGATTTTGTTGTGTGTCGTAGAGAAAAAAAGGTGAATAATCTGTAAGTTA
TGATGTTTTATAATTGTATTTACGTGAATTAATTATTTAATTATTTAATTAGGTTGGCTAAGAAAGAGTGAGCGTGGATGGTTG
AGAGAGAGTGTGAGCTTGGAGGAGGAGAAGATATTATATTGTATTTGGAATTTGGATAGAATCGAAACATTATGTAGAATTGAA
TTTTTGGGATCTATTTAATTTATTTTTATTTTTAATATGTTTAGTGGCTTTTGGGTGGCGTGCTTACTAAATCGCCTACTTGGA
ATGTTCAATATTAGAAGCACGTGGTGTACTAATCACGCCAATTTTATTTTTTATCTTAAATTAGAGAAATCCAATTTACAAATT
TCTATGCTTTTCTTTTTTGTTGGCACTGGGTGAAACTTGTGTACAGACTATATCAATCTATTCATGACGTCGTTTCCTATTGAT
GAGGATATTTATCTGGTTACAATAATCTCTCCTGCATAGAGTTACAGACACTATTAAATAATTCTTTGCTACTTCCCTTTTTCG
AAGATGAGGTGCGACCAAGCGTGGGGGGGTATTTTCATGTTTAAAGAGGTCCACGCAAAGTGATGGAGATGAGCAATGTTTACT
AAAATTATTGGGGCTACTATGGGGTCTCTTCTCGTTACTAAAGCAGTGTTGGAATGTCTTAAGTGGATCAGTTTTTCTTTCTCT
TATTAACTCACGGTGGTTACTTTAATAACCAAAGTTAAATTGACTACTAGCTGGTAAAGATTTTTCCATAACAAGGTTAGCTTA
TGTAAGTGGTTACAAAATAGTCCCCCTCCAAAGTTTTGGTACTTCGTTGGCATCATGGTTTGGATGGTATAATTTCATATTTAG
GAGAGTGCTTTTATTGAGGGACCGTCGGATTTATTGATAATGTTGATGTTGCATATGAATTACTACATTCCTTACCGCACTTAA

ACCTAGGGGAGAAAGTGGTTATTGTCATAATTGAATTAGAATGAAAGGCATATGATCAGGTTTGAAATGATCTTTTTAGAATTG
GTCATGGCTCAAACCTTGGTTTTTTGCCTCACCCTGAGGTTTAATTTGTTTGATGAATTGGTTTGTTCGTTGTCTGTTCCACGT
ATTCATTTAATATTAATGGTGATGTAAAATGAAAAGTGTTTCCTTTGCGAGGATTTGCGTCAAGGGGATCCACCTTTCAACTAT
ATCTCTTTCTGATTCGGCGTGGAAGGGTTTTTCATCCCTTCCTACTCAAAGCCGAAAGAAATGGAGATCTTGTATGATATCAAG
TGCACATGCAGCTCCTTCTATTACTCAACATTTCTTCGTGGATGGATAGTCTTCTTTTTTGTAAGGCCTGAGAATCATGTACAA
CATTGGTCAATATTGAACAATTTATATTCTAATAGCTTCGGAATAGGTAATATTTGAGTCCTACCAAGGTTTCTTTTGCTATAG
TCCTCATTGTGAACGTTCCCAATGGTAATAAGGCTTAGGCGGGGAGGTGGCGGAGGATGATAATTTAGAAATTATGCGATTATG
TGAATATTATATGTTTCAAATATGTATTATTGTAGTTAATATTATAATATGACTTGGATGAATGCATGTTTTGGTATATTATAA
TATGCAGTGATAGGGCCATTTCTGATTAAAAAGGGCCAATTTTTGTAATGTTGGCATTTGTTTATGAGGTATATTTGCTATATG
TGATATATGTGTGAGATCACATTATTTTATTTGGGGATTATTATTTTGTGTGCTTTTACTTTCAGCACTGAGACGATCCTAGGA
TGAGGCAAGCTAGTGGGATAAGTCACAGCGGGCCCATATTCGACTCGGTGTGAGTCCAAGGAGTACTTTCGATATTTAGTACAT
TACCGAATATTGGGTAATGAGAATGATTTATTCTTAGGGTGATATATCTTGGGGTGGGAGTGGTGAGATCATGGGAGTGGAATT
TTGGGTGGCTTAGACTTTTTTGACGTTCTTTCGTGGGGCGTTATTTGGACCCGAGCAATTGGGATTTGCTTGAGTGTTACTTAA
GCTTGAAGTAACCTGTCCAGTTTGGGGAAATAAAAAGAAACGTTTCAAGGTGGTTATTTTCGTCTCTTCTCTCCTCTCTCTTTT
GTTTCGTTTCATTTTTTTTTTTTGCGCTCGGTGGGGGGGAGCTTTTTCAAAAGAGACTTCGAGTTTTTGTAAGGGACTCGGATT
TAAGCAAGTGTTTTTAGAGTCCAGTAAACGATTCTATGTAATTATTATTTAGGAAGCTTGTTAGCTGAAGGATTTAACTTAGGA
TTAAACGACTAAATCAGAGGTAATCCAAGCTTTAAGTTTTGAAGTTTTCGAGTTTTTAAGGTCTTTGATGTGGATTTTACATTT
TGATGAGTTTTTGAATGGTTTGGGACTTGGTTTTGATGGTTTTGGGCCATTGAAATGTTTGGAAGTTTACAGTACCCTAAAGTT
ACAAAAAAATTATTTAATTATCTAAATCACAAATAAATAAAATATCTAAATCACAAATAATAAAAATTGTGACAAATGTCATGC
TTCCTAACAAATTCTGGTGAAGACAAAGTCTTATATTTCCCTTATTTTATCTTAAAATAATATATTCCTTAATAATCATACATA
TGACAAGTGTCATATTCTCTATTGGCTCATCTAACCTTAGGAATAATCCTCTTGTCTCATGACAAATGTCATTTCTATTAGCTC
TTTCTAAACTTAGATTATAATAATTAATCATATTTAATAACCATGCAAATATTAATCAAACCTTATGTTTTTTATAATTAATAT
TCTTAAACTATAGGTTAAACTTATAAATCCATAATAATTGCTAGGAATTTCCAACTAAGTTCCCGGCTTGAACCTAACTACCAC
TACCCACTACCACCACCACCACACCACCACACACCAACTACCACCACCACCACCACCACCACCCTCGACCACCACCACTACCAC
CACCACCACGCACCCCACCACCACTACTACCACATAACCACCACTACGACGACCACGCACCACTACCACGACACTACCACCGGG
ACCCCACTTACTACTACTACTACTACTACTAACTACTACTACTATTGCTAGGAATTCACTATAGTCCCGGCTTGACTAACTACA
CTACCACTCTACCACGTTTAACCACCTACCACCGCACCACCAACACCAACCACCACACCCGACGACCACCACCACCACCACTAC
GCACCACCACCACTACCACCACATACCTACCACTACCACCACCATCACTACCACCACGCACTACACCAACCTACCACGGGACTA
CTACTACTACTATACTAAACTACTAACTACTACTAACTACTACTACTACTACTACTACTACCACTACTACTACTACTACGACCC
AACCACCACCACCACCACCACCACCACTATACTGGCTACTACTGCTACCTTTATTCCACTACTACGAAACTAATTACTACTGAC
TACTACTACTTACTTACTATTGCTAGGAATTTCCAAACTAAGTCCTGGCTGATACCTAACTACCTACTACCACTTCTACCACTA
CCACTACACTAACACT
>m141122_014521_42134_c100732202550000001823141405141560_s1_p0/124459/0_10373
AGAGAGAAAATGTAAGGGAGGTGTAGATTATATTCTTCTTAAGGTTTGGCGAATACCTTTATGCCAGAGGTTCTAATATGCTCC
AGTCGTAAGTAAGAGTAGCATGATGTGCTTTACAACCCTATATAATGATTCCGTGTCAGCATTCGAACCACTTGCGAAAATTGC
CATCGATATAGGGTGAAATTATACTTTTTTTGAAAAAGGTAGAACAGGACGTTAACTTCACTGAATAGGGCCGTTGATCTCACA
CTAACCGAACTGAAATTGTCAGAAAGGATTAATTCTTTCACACTTCTACTACCTTGGTATGAGATCTGTTATGTGGGAAAATAC
TATCACGAGGCACATACGAAGGTTGAACACGAGGTTTAATACAGATTTCGGCGAGAATGAGTGAAACGCCAAATTCACCCAGAA
TGCTACCTCTTTTCAGAATTCAGTTTGCGATTAATCGTCTCCAACTATTGCGAATAGTGGCCGATCAAGACACATGAAAGTAGT
TTTGTAATAGCGAATACTAAATAATATGCGAGGGTTCCATCTGGTCACTAAAAGTTGTAGCCCTTCAGAGTATCTTTTAGGGTT
CTGTCATTGTCAATTGCAAAATTTGAGCAATATTATCGAGGTGTGCTATCCAGGGTGAATACAGTCAAAATACTGAGACATGTA
CTAATATCAAGGAAAAAAATCTTGCGAAAAATTGCCATCGAAAAGGGGAATTTTTTTTGAAAATGTCCGACTTAAGGCTCCGTTT
CGACGCAATATATTCATTTACCATGAGATCTCTTGGGGAGAAACATATCGAACGAGTTGAATACACGACGGGTAATACATTTAG
GAGGAAATGAGTGATAATCTCCATTCAACAAAGGGGTACCCTTTCGAAATCTTCGATTTCTCTAATATGGATTCGAGCAATGTT
TTGAATAGGGAATACTAAAGAATATGCGAGGGTCATGCGCCAAAAGTTGTACCCTATGTCTTAAGGGGTTCTATGTATAAAATT
TCGAGGCAAATCTAGGTGTATAGGGTGATACAGTCAAAATAACTGAGAGATGGTACTAATATAAGGAAAAAACTTCGAAAATTG
CCATCGAAAAGCCGGTGAAATTTTTTTTGAAATGTCGCTAATGGCCTCCCGTTTCTATCCCAATATATTTCATTTAACCATGAA
ATCTCTTGGGAAAAAAAATATCGCGAACGAGTTGAACACGAGGGTCAATACATTTCGGAGAAATGAGTGAAATCTCCAATTCAAC
CATAAGGCGTACCCCTTTCAGAAATCTTCGATTCTCTAATATCTGATTCGAGCAATGTTTTGACAGCGAATACCTAAAGATTAT
GCGAGGGTCATTTGGGCACATTCAAAAGTGTAGCCTATGATCTTAGGGTTCTATTTATAAAATTTCGTGCACAATCCGAGGTTT
GGTATAGGGTGATAACAGTCAAAATACTGAGAGATGTACTAATATAAGGAAAAAACTTGCGAAAATTGCCATCCGAAAAAGGGG
AATTTTTTTTGAAATGCCGCTAAGGGTCCGTTTCTACTCCCAATTATATCTTTACCGATGAGATCTGTTGGGGAAAAAAATATCA
AACTGAGTTGAACACGAGGGTAATACATTTTCGGAGAAATTAGTGAAATCTCCAATTCACCAAAAGGGGTACCCTTTCAGAAAT
CTTCGATTTCTGTATATGGATTCGACAAATTTTTGATAGCGAATACTAGAATATGCGAGGGTCATGGCAACAAAAGTTGTAGCC
CTATGTCTTAGGGTTCTATGTATTAAAATTTGAGCAATAGCCGACGTGTGTGTAGGGTGATATCTTCAAAATACGGAGAGATGT
ACTAATATAAGGAAAAAACATGCGAAAATTGACACATCCGAAAGGGAATTTTTTTTGAAATGCCGACTTAAGGCTCCGTTCTAC
TCCATATATTCATTTAACCCCCATGAGTCTGTTGGGGAAAAAATATCGAACGAGTTTGAACACGAGGGTAATACATTTCGGAGAA
ATGAGTGAAATCTCCAATTCACCAAAGGGGTACCCCTTTCCAGAAATCTTCGATTCTCTTAATATCGATTCGAGCAACTGTTTT

GATAGCGAATTACTAAAGAATATGCGAGGGGTCATGCGGCAACAAAAGTTGTAGCCCTAATGTCTTAGGCGTTCTATGTATAAA
ATTTCGGCCAAATCCTAGGTTTGTATAGGGTGGTACCATGTCAAAATACTGAGACATTGTACTAATATAAGGAAAAAATTTGCG
AAAATTGCCATCGAAAAGGGGAATTTTTTTGAAAGTCGACTAAAGGCTCCGTTTCTACTCCAATATATTAATTTAAGCCATGA
GATCTCTTGGGGAAATATATCGAACGAGTTGAACACGAGGGTAATACATTCGGAGAAATTGAGGAATCTCCAATTCACCAAAGG
CCGTACCCCCTTTCAGAAATCTTCAGATTTCTCTAATAATGGATTCGAGCAATGTTTTTGATAGTGAATACTAAGAATATGCGA
GGGTCATGGAACATAAAGTTGTAGCCCTATGCTTTTTTTAGGGGGGTTCTTTTAAAGTAATAAAAATTTTTGGAGCAAATCCCA
GGTGTGTATAGGGTGATACAGTCAAAATACTGAGACATGTACTAATATAGGAAAAAACTGCGAAAATTGCCCATCGGAATAGGG
AATTTTTTTTGAAATGTCGACTTAAGGCTCCCGTTTCTGACGCCATATATTCATTTACCCATGGAGATCTCTTGGGGAAAAATT
ATCGAAACGAGTTTTACACGAGGGTAATACATTTCGGAGAAATGAAGTGAAATCTCCAATTCACCAAAGTGGTCCCTTTCAGAA
ATCTTCGATTTCCTTAATATGGATTCGAGCAAGTTTTGATAGGAAATACTAAAGAAATATGCGAGGGTCATGGGCAACAAAAGT
TGGTAGCCCTATGTCTTAGGGTTCTATGTATAAAAATTTCGTGCAAATCCGAGGTTTGTATAGGGTGATACAGTCAAAATACTG
AGGGATGTAACTAAATAAAGGAAAAAAACTTGCGAAAATGCCATCAAGAAAAGGGGAAATTTTTTTTTGAAATGTCGACTTAA
GGCTCCGTTTCGGACGCCAATATATTCATTACCCTATGAATCTGTATGGGAAAATATCGAACGAGTTGAACACGAGGGTAATAC
ATTTCAAGAAGAAATGAGTGAAATCTCCAATTCACCAAGGGGTACTCCTTTCAGAAAATCTTCGATTTCTCTAATATTGATCGA
GCAATGTTTTGATAGCGAATATAAAGAATATGCGAGGTCATGGGCAACAAAAAGTTGTAACCCTAATGTTCTTTAGGGTTCTA
TTGTATAAAATTTCGGCAAATCCTAGGTTTGTATAGGTGATACCAGTCAAAATAACTGAGACATGTTGTAATATAAGGAAAAAA
CTTGCGAAAATTGCCATCGAAAGGGATTTTTTGAAATGTCGACTAAGGCACGTTTCGACGCCAAATATATTCATTTACCACCAT
TGAGATCCTCTTGGGAAAAAATATCGAACGAGTTGAACACGAGGGTAATACATTTCGGAGAAATGAGTGAAATCTCCAATTCACA
CAAAGGGTACCCCTTTCAGAAATCTTCGATTTCTCTAATATGGATTCGAGCAAATGTATTGGATAGGGAATCACTAAAGAATAT
GCCGAGGGTCATGGGCACAAAAGTTGTAGCCCCTATGTCTTCTAAGGGTTCTATGTAATAAATTTTCGTGCAATATCCGAGGGT
TTAGGTATAGGGTGATACAAGTCAAAATACTGAGGGGATGATACTAATATAAGAAAAAAACTTGCGAAAATTGCCATCAAAAGG
GAATTTTAATTTGAAAATGTCGACTTAAGGCTCCGTTTCGACGCAATATATTCATTTACCCAGTGAGATCTGTTGGGGAAAAAT
ATCGAACGAGTTGAACACGAGGGGTAATACATTTCTGGAGAAATGAGTGAAATCTCCATTCACCAAAGGGGTACCCTTTCAGAA
ATCTTCGATTCTCTAATATTGAATTCGAGCAATGTTTTGATAGGCGAATACCTAAAGATATGCGAGGGCGTCTGCACAATAAGT
TGTAACCCTATGTCTTAGGGTTCTATGTATACATAATTCGAAGCAAATCCTAGTTTGTATATAGGGTGATACAGTCAAAAATAC
GTGAGACATGTACTTAATATGAAGGAAAAAACTTGGAAAATTGCCCATCGAAAAGGGGAAATTTTTTTGAATGTCGCTTAAGC
TCCGTTTTCGACGCAATATATTCATTTACCCATGAGATCTCTTGGGGAAAAAATATCGAACGAGTTGAACACGAGGGTAAAATAC
ATTTCGGAGAAATGAGTGATCTCCAATTCACCCAAAAGGGGTACCTTGTCAGAAATCCTTCGATTTCTCTCCTAATATCTGGAT
CGAAAGCAATGTTTTGATAGGGAATACTAGAAGAATATGCGAGGGTCATGGGCAGCCATAAAGTTGTAGCCTATGTCTTAGGGG
TTCTAATGTAATAAAATTTCGTGCAAATCCGAGGTTTGTATATGTGATACAGTCAAAAATACTGAGGGATGTACTAATATATAG
GAAAAAAACTTGCGAAAATTGCCATCGAAAAGGGGAATTTTTTTGAAATGTTCGACTTAAGGCTCCGTTTCGACGCCAATATAT
TCTTTTACCCAAATGAGATCTGTTGGGGAAAATATCGAACGAGTTGAACAACGAGGGTAATCATTTCGGAGAAATGAGTGAAAT
CTCCAATTCACCAAAAGGGGGTGGGTACCCTTTCAAGAAATCTTCGATTCTCTAATATTGATTCGAGCCATGTTTGATAGCTAA
TACTAAAGAATATGCGAGGGTCATGGGCACAAAAGTTGTAACCCTATGTCTTAGGGATTCTAATGTATATAAATTTCGAGCCAA
TCCTAGGTTTGTATAGGGTGATACAGTCAAAATACTGGAGATGTACTAATATAAGGAAAAAACTTGCGAAAAATTGCCATCGAA
AAGGGAATTTTTTTTTGAAATAGACCGACTTAAGGCTCCGTTTCTACTCCAATATATTCAATTTAACCGATGAGATCTGTTGG
GGAAAAATATCAAACGAGTTGAACACAGGGTAATACATTTCGGAGAAATTAGTGAAATCTACAATTCACCAAAGGGGTAGCCCT
TTCAGAAATCTTTCGATTTCTGTAATATGGATCGAGCAATGTTTTTGAATAGCGAATACTAAAGAATATGCGAGGGTCATGCAC
ACAAAAGTGTAGCCCTATGTCTTAGGGTTCGTATGTATAAAATTCCGAGCAAACCTAGGTTTGTAATAGGGTTGATACAGTCGA
AATACGGAGATTGTACTAAATATAAGAAAAACTTGCGAAAAATTGCCATCGATAAAGGGGAATTTTTTTTGAATGCCGACTTAA
GGCTCCGTTTCTACTCCAATTATTCATTTACCTGATGATATCTGTTGGGGAAAAAAATATCAAACGAGTATGACACGAGGGTAAT
ACATTCCGCCAGAAATTAGTGAAATCTCCAATTCACCAATGGGGTACCCCTTTCAGAAATCTCGATTTCTGTAATATGGATTCG
AGAAATGTTTGATAGCGAATACCTAAAGAAATATGCGAGGTCATGGGCACAAAAGTTTGTAGCCTAGTCTTAGGGTTCTATGTA
ATAAAATTTCGAGCAAAGCCGACGTGTGTGTAGGGGTGATAACTGTCAAATACGGAGTAGATGTACTAAATATAAGGAAAAAAC
ATGCGAAAATTGCCATCGAAAAGGGGACCTTTTTTTTGAAATCCGACTTAAGCTCGTTTCTACTCCAATATAATTCATTTACCC
ATGAGATCTGTTGGGGAAGAAATAATCGAACGAGTGAAACACGAGGGTATACATTTCGAGAAATGAGTGGAAATCTCCAATTCA
CCAAAAAAGAGGTAACCTTTCAGAAATCTTCGATTTCCTCCTAATATGGAATTCGAGCATGTTTTGATAGGGAATACTAAAGAA
TATGCGAGGGTCATGGGCACAAAAGTGTAGCCCTTGTCTTAGGGTTCTATGTATATAAATTTCGTGCAAATCCGAGGTTTGTAT
AGGGTATACAGTCAAAATATCTGAGGGATGTAACTAATATAAGGAAAAAAACATTTGCCGAAAATTGCCATTCCGAAAAGGGGA
ATTTTTTTTGAAATGTCGACTTAATGGCTCCGTTTCGACGCCATATATTCATTTACCCATGAGATCTGTTTGGGGAAAATATCG
AACCGAGTTAACACGAGCGGTTAATACATTTCGGAGAATGAGTGAAATTCTCCAATTCACCAAAGGGTACCCCTTTCAGAAATT
CTTCGATTTCTCTAATATTGATTCGAGCAATGTTTTGATAGCGAATACTAAAAGAATAATGCGAGGGTCATGGGCACAAAAGTT
GTAAACCCTATGCTTAAGGGTTCTATGTATAAATTTTCGAGCAACATCCTAGGTTTGTATACGGGTGATACAGTCAGAAATACT
GAGACATGTACTAATATAAGGAAAAACTTTGCGAAAATTGGCCATCGAAAGAGGGGAATTTTTTTTGAAATGTCGAAATAAGGC
TCCCGTTTCCGACGCCAATGATATTCATTTACCCATGAGATCTCTTGGGGATATAAAATATCGACACGAGTTGAAACACTGGGG
TAATACATTTCCGGAGAAATGAGTGAAATCTCCAATTCCACCAAAAGGGGTACCCCTTTCAAGAAATCTTCGATTTCTCTAAAT
ATGGATTCGGCCAATGTTTTGATAGGGAATACTAGAAATATCGAAGGGTCATGGGCACAAAAGTTGTAGCCCTAATGTCTTAGG
GTTTCTATGTAATAATAATTTCGTGCTAATCCGAGGTTTGTATACAAGGGTGATATAGTCAAAATACTGAGGGATTACTAATAT

AAGGAAAAAAAATTGCGAAAATTGCCATCGAAAAAGGGGAATTTTTTTTGAAATGTCACTTAAGGCTCCGTTTCGACGCAATCA
TATTCATTTACCAAATGAGATCTGTTGGGGAAAAAAAATATCGAACCGAGTTGAACACGAGGGTTAATACATTTCGGAGAAATGA
TGAAATCTCAAATTCAACCAAGGGTACCCCTTTCCCAGAAATCTTCGAATTTCTCCTAATATTTGAATCGACAATTTTTGATAG
CGAATACCTAAAGAATATGCGAGGGTCATTGGGCAACTAAAGTTGTACCTATGTTTAGGGTTGCTAATGTAATAAAATTATCGT
GCAAATCCGAGGTTTGTATAGGGTGATATCAGTCAAAATACTGAGGGATGTACTAATATTCATTACCAAGGAAAAAAACTGCGA
AATTGCCCATCGAAAAGGGTAATTTTTTTTGAAATGTCGACCTTAAGGCTCCGTTCGACGGCCAATTATTCATATACCCATGAA
TCTGTCTATAGGGGAACAATATCCGAACGAGGTTTGACATCCCGAGGGTATACATTTCCGGAGAAATGAGCTGAAAATCCTCCA
ATTCACCCAAAGGGGTACCCCCTTTCCAGAATCTCCGATTTCACCTAATCCATTTGATTCGAGCATGTTTTGATAGGGCGAATC
ACTAAAGAAATAGTGCGAGGGTCATGGGCACAAAAGTTGTAGCCCCTATGTCTTAGGTTGCTATGTATATAAATTTTTCGAGCA
AATCCTAGGTTTGGTAATAGGGTGATAACAGTCAAATACTGAAGAGATGTACTAATATAAGGCAAAAAAACTTGCGAAAATTGC
CAATCGAAAAGGGGAATTTTTTTTGAATGCCGACATATTAAAAAAGTTAATAATTTTTTTTTTTTTTTTTTTTTAAAAAAAAA
TATTTTATAAATTTTTAAAAGTTCTTAAGGCTCCGTTTCAACTCCAATATAATTCATTTACCGATGAGATTGTTGGGAAAATAT
CAAACGAGTTGGAAACACCGAGGGTAGAAATACATTTCGAGAATTAGTGAAACTCCCAATTCACCAAAGGGGTACGCCCTTTGA
ATCTTCCGATTTCTGTAATAATCAGGATTCGAGCAATGTTTTGATAGCAATAATAAGAGAAATATGCGAGGGGTCATGGCAGC
AAAGTTGTAGGCCCTATGTCTGTAGGGTTCTAATGTATAAAATTTACGAGCAACCAGCCGACGTGTGTAAGTAGGGTGATCTGT
ACAAAATGACGGAGAGAATGTACTAATATAAGGAAAAAACTGCTGAAAATTGCCATCGAAAAGGGGATATTTTTTTTGAAATAA
GCGACCCTTAAGGGTCCGTTTCTACTCCATATATTCATTTACCGAATGAGATCTGTTGGGGAAAGAATATCCGAACGAGTTGAA
ACACGTAGGGTAATACATTTCCGGCAGAAATGAGTGAAATCTCAATTCACCAAAGGGGTAACCCCCTTTCCAGAAATCTTGCGA
TTTCTCTAAATAAGGATTCGAGCATGTTTGATAGGCGATACTAAAGAATATGCGAGGTCCAATGGGCAGCAAAAGTTGTAGCCC
TATGTCTTAGGGTTCTAATGTATAATCTTCGAGCAATATCCTAGGTTTGTATAGGTGATACCAGTCAAAATACTGAGAGATGTA
TCTCAATATAAGGAAAAACTTTTGGCGAAAATTTGCCAAATCCGAAAAGGGAAATTTTTTTTGAAATGCCCCGACTTAAGGGCT
CCCGTTTCTACTCCAATATAATTCATTTACCGATGGATCTGTTGGGGAAAAATAATCAAACGAGTTTTGAAACACGAGGGTATA
CCATTTCGGAGAAATAAGTGCAATATCTCCCAATTCACCAAAGGGGTACCCTTTCAAGAAATCTTCCGATTTCTGTAATATGGA
TTCGGAAATGTTTATGATAAGCGAATACTAAAGAATACTGCGAGGGTTCTGGGCCAGCAAAAGTTGTATAAGCCCTATGTCTTA
GGGTTCTAATGGTATAAATTTGCGAGCAAAGCCGACGTGTGTGTAGGGTGATACTGTCAAAATACGGAGAGATGTAGGCTAATA
TAAGGAAAAAACGATGCGAAAATTGGCCATCGAAAAAGGGGAATTTTTTTTGAAATGCGCGACTTTAAGGGGTCCGCGTTTTCT
ACTCCCCCCAGATATATTCATTTACCCGATAGATCCTGTTTGGGGAAAAAATATCGAACGAGTTGACAGCGAGGGTAAATACATT
TCGGCCAGAAATGAGTGAAATCTCCCAATTCAGCCCCAAAAGGGGGGGTACCCCGTTTTCCAGAAAATTCTTAGATTTCTCTAA
TATGGATTCCGAGCCAAAATGTTTTGATAGCGAATTAGCTAAAGAATAATGCGAGGGTCAATGGCACCAAAAGTTGTAGCCGCT
ATGTCTAGGTTGCTATGTATAAATTTCGAGCAAATCCCTAGGTTTGTATAGGGTGATGCAGTCAAAATACTGAGAGATGTACTA
AATAAGGAAAAAACTTGCGAAAAATGCCATCGAAAAGGGGGAATTTTTTTGAATGCCGACTTACAGGCTCCGTTTCGCTCTCCA
ATATATCATTTACCGATGAGATCGTTGGGGAAAAATATCAAACGAGTGAACACGAGGGTAATACATTTCGGAGAAATAGTGAAA
TCTCCCAATTCACCAAAGGGGTTTACGCCCTTTCAGAAATCTTGCCGATTTTCTCTAATATGGATTCGAGCCAATGTTTTTGAT
AGGAATCTACCTAAAGAAATATGGCGAGGGTCATGGCCACAAAAGTTGTCATGCGCTATGTCTTACCGGGTTCTATGTAATAAA
ATTTCGAAGCAGCCCGACGTGTGTGTAGGAAGTGAAGCTGTCCAAATACGGAGAGATGTAACTAATAAAGGAATAAACATGCAA
ATTTGCCATCGAAAAGGGGGGAATATTTTCTTTGAAATGCCCGACTTAAGGGTCCGTTTCCTACTTAGCAATATATTCATTATC
CGATGAGATCTGTGGGAAAAAATATCGAACAAGTTGAACACGAGGTAATACATTTCGAGAAATGAGTGAAATCTCCAATCACCAA
AGGGTACCCCTTTCAGAAATCTTTCGATTTCTCCAATATGGATTCGAGCAATGGTTTTGATACGGGAAATAGCTAAAAGAATAT
GCGAGGGCATCCTGGGCACAAACAGTTGTAGCCTATGTCTTAGGGTTCCTATGTATAAAATTTCGTGCAAATCCGAGGTTTTGT
ATAGGGTGATACAGTCAAATACTGAGGGATGTACTAATATAAGTGAAAAAACTTGCAAAATTGGCCCATCCGAAAAGGGGAATT
TTTTTTGAAAGTCGACTTAAGGCATCCGTTTCCGACGGCCC
>m141122_014521_42134_c100732202550000001823141405141560_s1_p0/126506/1802_8211
ATTAAGGATGAAGCAAAGTTGTCACACAGCAGAAAATATACGTGGCTCATTGCCTCCTACGAATGTATTGCAAACGACACACCG
AAGCGGAGCACCTCTAACCGTAGCTGGGTGTTACTCGCATACCTTCGAGCTAAAAGTTCCAGAAATGGAAGTATGAGTATGATG
ATCAGCATATGGACTTTGCCGACTTTGGACTTCCGAAGATATCCCTAAAGGTCATCACGAGATATCAAGTGTAAGGGTTATTGT
TGATAGATTGACCAAGTCGCACATTATTGAACCCCTCTATACCTCTAGGTGATGTATTATGAAGACATGAGAGACATTATGTCC
CTAAGTCAAGCATTTGAAAAAAAAATTCCCTTTTTGATGGCAATTTTTCGCAAGTTTTTTGCGCTTATATAAATAGTACCATCTC
TCAGTATAATGACTGTACTCAGGCTTCTACGCACCTCGGTTTGGAAGTATTTCACACACATTGAAGGCCTAAGACATAGGGCTA
CAACTTTTGTGGCCATGATCCTCGCATACTTTTTAGTCTTACAGTGCTTCCCCCAAATCGTTGCTCCGAATGCCATATTCAGGA
AAATCGAAGATTTCTGAAAGGGGTACCCCTTTGGTGAATTGGAGATTTCACTAATTTCTCGCGAAATGTATTAGCCCTCGTGTT
CAACTCGTTCGATATTTTTCCCCCAAGAGTCTCATGGGTAAATGAATCTATTGGAGTTGAAACGGAGGCCCTAAAGTCAGGCAT
TGAGGGAAAAAAATTCCCTTTTTGATGGCAATTTTCGGCAAGTTTTTGGCCTTATATTAGTTCATCCTCTCCCAGTTATATACTG
TATCAACCTTCTACGCAGCCTCGGATTTGGAGAGTAATATTACACATTGAAGGCCTAAGAGCTCATAGGGCTACAACTTTTGTG
GCACCATGACGCCTCGCATAATCTCTTTAGTTTTGCACCTTCAAAATCGTTCCTCGAATCCATATTAGAGAAATCGAAGATTTA
TGAAAGGGGTAGCGCCCTTTGGTGAGATTGGAGATTCGCACTAAATTTCTCCGAAATTGTATTACCCCTCGTGTTCAACTCGTT
CGATATTTTTCCCCAAGAGATCTCATGGGTAACATGAATCTATTGGAGTTGAAAACGGAGCCCTAAGTCACACCATTTGAAAAA
AATTCCCTTTTGATGGCAATTTTCGCAAGTTTTTGGCCTTATATTAGTACATCTCTCAGTATAATGACTGTATCACCTCTACCA
CCTCGGATTTGGAAGATATTTAACCAATTGAAGCCTAAGACATAGGGCTAAAACTTTGCTGGCGCATGACCCTCGCATATTTCT

TTAGTTCTTCACCTTCAAATCGTTGTCGAATCCATATTAGAGAATCGAAGATTTCTGAAAGGGGTACCCTTTTGGTGAATTGGA
GATTCATAATTTCTCCGAAATGTAATTTACCGCTCAGTGTTGCAACTCGTTCGACTATTTTTCCCCAAGAGATCTCATTGGTAA
ATGAATCTATTGGAGTTGAAACGGTGCCTAAAGTCAACATTTGAAAAAAAAATTCCCCTTTTTATGGGAAAAATTTCGGCAAG
TTTTTGGCCCTTATATTAGTACATCTCCTCAGTATATTGACTATATCACCTTATACGCACCTCAGATTTGGAAGATATTTTACA
CATTGAAGCCTAAGACATAGGGCTACAACTTTGTTGGCCCATGACGCCTCGCATATTCTTTAGTTTTCACCTTCAAAATCGTTG
CTCGAACCCATATTAAGAAATCGAAGATTTCTGTAAAGGGGAAGCCCCTTTGGTGAATTGGAGATTTCACTAATTTCTCCGAAA
TGTCATTACCCTCGATAGTTCAACTCGTTCTATTTTTCCCAAGGATCTCATGGGTAAATGATCTTATTGGAGTTGAAACGGAGC
CCTAAGTCAACATTTGAAAAAAAATTCGCCCTTTTTGATGCCAATTTTCGCAAGTTTTGCCTTATATTAGTACATCTCTCAGTA
ATACATGACTATCAACCTTCTACGCACCTCCGGATTTGGAAGATATATTACACATTGAAGCTAAGTACATAGGCTAACACCTTT
TGTGGCCATTGACGCCTCCCCATATTCTTTAGTTTTCACGCTTCAAAATCGTTGCTCGAATCCATATTGAGAAATCGAAGATTT
ATGAAAGGGAGAACCCCTCTTGGTGAATTGGAGCTTCACTAATTTCTCCCGAAATGTATTACCCTCGTGTTCAACTCCGTTATA
TTTTTCCAACCCAAGAGATCTCATGGGTAAATGAATACTTATTGAGTTGAAACGGAGGGCCCTAAGTCAACATTTGAAAAAATT
CCCCTTTTTTGATGGCAATTTTCGCAAGTTTTTGCCTTATATTAGTAGATACTCTCAGTAGTAATGACTGTATCACCTTTCTAC
GCACCTCGGATTTGGAAATATTTAAGACATTGAAGGCGCTAAGACAATAGGGCTATCAACTTTTGTGGCCATGATCCTCGCATA
TTCTTTAGTCTTCACCTTCAAATCGTGCTCGAATTCACATATTAGAAGAACTCGAAAGATTTCTGAAAGGGGTACGCCCCCTTTG
GTGAATTGGAGTTTCACTAATTTCTCCCGAAATGTATTACCCTCGGTGTTCAACCTCGTTCGATATTTTTCCCCAAGAGATCTC
ATGGGTAATATGAATCTATTGGAGTGAAACGGAGCCCTAAGTAACATTTGAAAAAAATTCCCCTTTTTGATGGCAATTTTCGCA
AGTTTTTGCGCTTTATATTAGTACTTCATCTCTCAGTATAATGCTGTATCCACCTCTACGCCACTCGGATTTGGAAAGATATAC
TTGTCTGCTAATAAGCACATTGAAGAGCGCCTAAGAAAGCTTATAGCAGTGGTGCTACAACCTTTTGTGGCCCATGACCGCTTC
GCATATGATCGTACTTAGTTTTCACCACTATATTGCTCAAAATCGTGTGCACTTTTGCGCGACATTAACGAAACTATATACGCT
AATTAAATGATCAGCTTATGTAAATAGATCGAAGATTTTACACTTTGAAAAGTGGCGAAAGAATACATAATTTTATTAAACTGT
TACGCGTAAAAATTAAAAGTTACCTTCTTGGTACATTGAGAGAATAACCTTGGAGAATTCCCACTAATAATTAAAGGATCATTC
TCCGCGGAAATAATCCATTGATAACCTTCTAAAAACAAAACATCGATTTAAAATAAGACTCATAAAGTTAGCACATTACATAAC
CATGCGCTTGCTGAGCGGAAAATATCAGTGTAAAAAAAAATATAATAAACAGAAACTTATATCCTTCTTCCCCAAGGAGATCTG
CAAATCGAATTAAGCGGCTACGGCTCACCTTGATCCATTCACGCATCCAGATACACATCCTCTACATGACGTTTCACGCGGAGT
TGAAACGGAGCCTAAGTCAAGCCAGATATATCTTGACAAAAAAAAAGCCTTCTCTCTTAATATATTAGCTTTAGAGTATTGGCA
CACTTTGCACATGAAAACGTAATATGTTGTAATGTGCTTATAAGCCTAATTTGCCCAGCAAAGAAAGTATCACTAGACACTACA
TAGTCATTATCAGAGAATCAGATATAATTTCATAAGAAAATATGATGATTATCACCTTCTACGCACCTCGGATTTGGGAAGAAT
ATCTAACACATTGAACCTAAGACATAGGGCTACAACTTTTGTGGCCATGACCCTCGCATATTCTTTAGTCTTCACCTTCAAATC
GTTGGCTCGAATCCATATTAGAGGAAATCGAAGATTTCTGAAAGAGGTACGGGCCTTTGTGAATTTGGAATTTCACTAATTTCT
CCGAAATGTATTACGCTTCCGTGTTCAAAAACTCCGTTGCGATATTTTTCCCCCAAGAGTCTCATTGGTAAAATGAATCTATTG
GAGTTGAAACGGTGCCCTAAGTCAACATTTGAAAAAAAATTCCGCTTTTTGATGGGCAATTTTCGCAAGTTTGCATTATATTAG
TACTCCTCTCCAGTATTTGACTATATCCACCTTCTACGCACCTCAGATTTTTGGAAGATATTTTACAGCATTGAAGCCTAATAC
ATAGGGGCTACAACTTTGCTGCCCATGACCCTTCGCATATTCTTTAGTTTTCACCTTCAAAGTCATTGCTCAACCCATATTCAG
AAATCGAAGATTTTCTGAAAGGGAACGCCTTTGGTGAATTGGAGATTTCACTAATTCTCCGAAATGTATTACCTCGTTTCAACT
CCGTTCGATATTTTTCCCCAAGAGATCTCATCGGGTAAATGAATCTATTGGAGTTGAAACGGGAGGCCCTAAGTCAACATTTGA
AAAAAAAATTCCCTTTTTGATGGCAATTTTCGGCAAGTTTTTCCTTCATAGATTAACCACTCTCATCAGGTACTTAATGACGTG
TATCACTTCTACGCAGCTCCGGATTTGGAAGATATATTAGCAGGCATTGAACCTAAGAATAGGCTAGCAACTTTTGTGCATCGA
CCCTCGCATATTCTTTAGTTTTCACTTCCAAATCGTTGCTGAATCCCATATTAGAGAATCGAGATTTCTGAAAGTGGTACCCCT
TTGGTGAATTGGAGAATTCGCACTAATTTCTCCGAAATGTATTACCCCTCGTGTGTCAACTCGTTCGACTATTTTTCCGCCAAG
AGATCTCATGGGTAAATGAATCTAGTTGAGTGAAAACGGGAGGCCCTAAGTCAAATTTTGAAAAAAATTCCCCTTTTTGATGCA
ATTTTCGCAGTTTTTGCTATATTTAGTATCTCTCAGATTATTAGATGAGCTGTATCAGCCTTCTAGCACCCCTCAGATTTGGAA
GATATTGTTACACATTGAAGCCTAAGACATAGGGCTACAACTTTTGTGGCCCATACCCTCGGCATATTCTGTGTTTTCACCTCC
AAATCATTGGGCTCGAACTATATTAGAGAAATCGAAGATTTCTGAAAGGGGAAGCCTTTGGTGAATTGAGATTTCACTAATTTG
CTCGAAAGTATTACCTCCTTTCCCGTGTTCAACTCGTTCCGATATGTTTTCCGCCAAGAGATCTCATGGGTAAATGAATCGTAT
TGGAGTTGAAACGGAGCCTAAGTCAACATGTTGATTAAAAATAATTCCCCTTTTTGATGGCAATTTTCGCAAGTTTTTGCCTTA
TATTAGTCATTTCTCTCAGTATAATGATGTATCACCTTCCTACGGGCACCTCGGATTTGGAAGATATATTTACACATTGAAGGC
CTATAAGAACATAGGGCTTAGCAACTTTTGTGCCCATGACTCGCATATTCTTTAGTTTAATCAGCTCAAATCGTTGCTCGAATC
CATAATTAGAGAAATCGAAGTTTCTGAAAGGGTACCGCCCTTTGGGAATTGGAGAATTTCAGCTAATTTCTCGCGAAATGTTAT
TAGCCCTACGTGTTGCAAGGCTCCCCGTTCGATTTTTTTCCCGCGCAAAGAAGTCCGTCATGGGTAAATGATCTTGGAGTTGAA
ACGGCACCGCTAAGTCCAACATTTGAAAAAAAATTCCCCTTTTTGATGGGGCATTTTGCCGGCCAAGTTTTTTGCAAATTATAT
TAGTACATCCTCCTCAGTATAGGATGACTTATCGAACGCTGTTACGGGCACCTCGGATATTGGAAGATATACATTACCCATTG
AACGCGTTCCTAGAGACATAGGGCTACACGCTTTTGTGGCGCATGAGCCTCTCCCCATATTCTTTAAGTTTTTTCTAGCGGCTT
CCAATCGTTGGCTCCGAATCCATATTAGGACAAATCGAAGATTTCTGACAAGGGGAAAGCCGCTTTGGTGAATTGGAGCTTTCA
CTAAATGTCTGCGCGAAATGTATTAGCCTGCCGGTGTTCAACTCCCGTTCGACCTATAATTTTCCCCCAAGAGATCTGCTGGGT
AAAATGAATCTATTGGAGTTGAAACCCGCAGCACTAAGTCAACATTTGGAAAGAAAAAATTCGCCCTTGTTTGATGGCAATTTT
CGCAAAGTTTTTGCGCTATATTAGTCAACACTCCTCTCAGTATAGGATGACTGTGATCACCTTCTACTCGCAGCCCTCCGGATT
GGAAGATAGTTTAAGCACATTTGAAGCGGCTAAGAGCATAGGGCCTAGGGCAACTTTTGTGGCCATCGATCCTCGGCATATTCT

TTAGTCTTCCACGCTTGCATGCGTTTGCTGCGAATGCCATACTTAGAGAAATCGAGATTGCTGCTGAAAGGGGGGTACCGCTTC
GGGTTTGGTGAATTGGAGATTTCACCTAATTTCCTCCGAAATGTATCTCGAACCGTCTCGGTGTTAACTCCCGTTCGATATTTC
CGCCAAGGAAGATCTGCATGGGTAA
>m141122_014521_42134_c100732202550000001823141405141560_s1_p0/131333/0_6132
ATAGAGTGAGTAAGAGAGTATTAGGATCCCGTACGCATTTTTTATTTATGTCTTCTGTTCTCATCTTTATATTGAATAGATTCA
CGGATGCTGCAGCGCGCGATCGGATATTACGAGAAATCGAAGGATCTTTTGAAAGGGTACCCCTTTCGGTGAATTGCGAGATTC
TCACTACATTTCTCCGATTATGTATTGTCCCTGCCCTCTGTGTTCAACTCGTTCGATATTTTTCCCAAGAATCTCATGGGTAGA
GAATATATTGGAGTGGAAATCGGACTCCTTAAAATAACATTTCAAAAAAAATATCCCCTTTTGGATGGCAATTTTCCGCAAG
TATTTTCTTATATTAGTACATCTAAACAGTATTGTTAACGTATCTACCCTCTACCGACAACTCGGATTAGCACAAAATTTTACC
AATAGAACCCTAAGACATAGGGCACAACTTTTGTGCCCATGACCCCTCGCATCTTTATTTAGTATTCACTATACAACGTTGCTC
GAATCGATATAATTATAAATTAAGAATCGAAGATTTTATAGAAAGGGGTAACCCCATATATATAGGTGAAAATTGGAAAGAATA
TTCAATATATTTAACTCCCGAAATGTAATGCACTCAAAGTGTTCAAACACATAAAAATCGATATTTTAACCCAAGAGAATTCAT
GAGAAATAGAAATATTATTAAAAAGGAGTAGAGAAACGAACCATAATATATATATAATTATCAACATTAAATCATATTTTTGAT
GGCAAAATATAGAGTATTTTCCTATATAAATAGTAACATATATACTGAGAATCAAACCAATCCATAACCAACCAAAACAACTCG
GACTAGCCCGAAATTTTACACACAGAACCCTAAGAGAGAAAATAGGCTACAACTTTTGTGCCATGACCCTCGCATTTTATTTAC
GTATTCACTATGAAAAACGTTAGCTCGAATATTAGAAGAAATACGAAGATTATTGAAGGGAGGATTAAAAAAAACAATAATTAT
AGAATAGAGTTTCACTAATTTCTCCGAAATGGTATTGCCCTCGTGTTCAACGTCGTTCGATATTGTTTCCCGCAAGAGTCTCAT
GGGTACATGAATATAATGGAGTGAAACAGACCCTTAAATCAACATTCCAAAAAAAAAATTCCCCTTTTTGGATGGCAATTTTCG
CAAGTTTTTTCTTAATTAGTACATCTCTCATAAAAATTATGACTGTATCACCCCACCACACACCTCGGCTTTCCCGCCGGCGCG
CCAGGAAAAATAATAATAAATTTAATAATTACATGAGAGGAGAAGAAAGAGAGAGAGAGAAGAGAAGGAGAAGAGAGAGAGGAG
AGAATAAACAAAACATAATATATCACATAAGGGGCAACAAAAAAACAAAAAAATATTAATATATATTATTACAATATTAGACTA
TATTTAAAAGAATAACCGAATGATATTAGAGTAAATCGAAGATTTCTGAAAAAGGGGTACCCCTTTGGTGATATTGGAGATTTC
ACTAATTTCTCCAAATGTATTGCCCCTCGTGTTCAAGTCGTTCGATATTTTTCCCCAGAGATCATCATGGGTAAATGAATATAT
TGGAGTGGAAACGGACCCTTAAAGCTCAACATTTTCAAAAAAAAATTCCCCTTTTTGATGGAAATTTTCCGCAAGTTTTTCCTT
AAATATTAGTACCATCTCTCAGTATTATGATGACTCACCCACCACACACCTCGCTTTGCAGGAAATTTTACACATAAAACAAAT
AAGATATAGGGCTACAACTTTTTGTGCCCATGACCCTCGCATATTATTTATTATTCACTAATGAAAACATTGCTCGGAAATCCA
TATTAAAGAGAAAATCGAGAGATTTCTGAACAGGGTACCCTTTGTGAATCGGAGATTCACTAATTTCTCCGAAATGTATTGCCC
TCGTGTTCAACTCGTATCGAGTAATTTTTCCCCAAGAGATCTCATGGTAAATGAATATATTGGAGTGGAAACGGACCCTTAAAT
CAAACATTTTCAATAAAAAATGCCCCTTTTTGATGGCAATTTTCGCAAAGTTTTTTCCTTATACTCTAGTAATCTCTCAGTATTA
TGACTGACTCACCCACCACACACTCGGCTTTGCAGGAAATTTGTACACTATAAAACAAATAAGATATCAGGGCTACAACTTTTG
TGCCATGACCCTCGCATATTAACTGTTATTATTCACATATGAAAACATTTGCTCGAAATCCATAATTAGAGAAATCGAAGATTT
CTGAAAGGGTACCCCTTGTGAATCGGAGATTTCACTAATTTTCGCGAATGTATTGCCCTCGTGTGTCAACTCGTTCGATATTTT
TCCCCAAGAGATCTCCATGGGGTAACATGAAATCATATTGGAGTGGGAAACGGACCCCTTAAAAGTCAACATTTCCAATAAAGA
TCCCTTTTTGATGGCAATTTTCGCAAGTTTTTCCTTATATTAGTAAATCTCTCAGTACTTATGACTGACTCCACCCACCCCACAA
CACCTCGCTTTGCAGGAAATTTTACAACATAAAAACAATAAGATATAGGGGCTACACTTTTGTGCCCATGACCCTCGCATATAT
TATGTTATTATTCACTATGAAAACATTGCTCGAATCCATACTTAGAGATAATCGAAGGATTTCTGAAAGGGGTACCCTTTGGTG
AATCGAGATTTCACTAATTTCTCGAAATGTATTGCGCCTCGTGTTCCAACTTCGTTCAGATATTTTTCCCAAGAGTCTCATGGG
TAAATGAATATATTGGAGTGGAAACGGACCCTAATCACCATTTCAAAAAAATTCCCTTTTTATGCAATTTCGCAAGTTTTTTCC
TAATATTAAGTACATCTCACAGTATTTTAACCCTGTATCACCCTCAATACCCAACTCGGACTAGCACGAAATTTTACACATAGA
ACCTAAGCATAGGGCTAAAACTTTTGTCCATGACCCTCGCATATTACTTAGTATTCACTATGAAAACATTGCTCGAATCGATAT
TAGAGAAATCGAAGATTTGAAAAGGGGTAACCCTTTGGTGAATTGGGAGACTTTCACTAATTTCTCTCCGAATGTATTGCCCTC
GTGTTCAACCTCGTTTCGATATTTTCCCAAGAGGATCTCAATGGGTAATTGAATATATTGGAGTGGAACGGACCCTTAAATCAA
CATTTTCAAAAAAATTCCCTTTTTTGATGCAATTTTCGCAAGTTTTTTCTTATATTAGTTACATCTCACAGTATTTTAACTGTA
TACCCTCTACCCAACTCCGACTAGCACGGAAATTTTACACATAGAGACCCTAAGACATAGGGGCTAACAACTTTTGTCCCATGA
ACCCTCCGCATATTATTTAGTATTCACTATGAAAACCGTTGCTCGAATCGATATAGAAGAAACTCGAAAGATTTTGAAGGGGGT
ACCCCTTTGGTGAATTGAGATTTTCACTAATTTCTCCGAAATGTATTGCCCTCGTGTTCAACCCGTTCGATATTTCTTCCCCAA
GACGATCTCATGGGTAAATGAATTAAATATTGGAGTGGAAACGGACCCTTAAATCAACATGTTCAAAAAAAATTCCCCCTTTTT
GATGGCAATTTTCGCAAGTTTTTCCTTATATAGTACAATCTCTCAGCTATTATACTGTATCACCCACCACACACCTCGGCTTC
AGCACGAAATTGTTACACATAAAACCCATAAGATATAGGGCTACAACTTTTATGCCCATGACCCTCGCATATTATTTATTATTC
ACTAATGAAACATTGCTCGATCGATATTAGAGAAATCGAAGATTTTTTGAAAGGGGTACCTTTGGTGAATTGGAGATTCACTAA
TTTCCTCCAAAATGTATTGCCTCGTGTTCAACGCGTTCGATATTTTTCCCCAAGAAGATCTCATGGTAAATGAATATAAATTGG
AGTGGATAACGGACCCTTAAATCAACATTTCAAAAAAAATTCCCCCTTTTTGTATGGAAATTTTCGCAAGTTTTTTCCTTATAT
TAGTACATCTCTCAGTATTATGACTCGACTCACCCACCACACACCTCGGCTTTGCAGGAAATTTTACACAATAAAACAATAAGA
TATAGGCTAACAACTTTTGTGCCCCATGACCCTCGCATATTATTTTTATCACGGTATGAAACATTGCTCGAATCCCATATTAGA
GAATCGAAGATTTCTGAAAGGGGTACCCCTTTGGTGATCGGAGATTTCACTAATTTCTCCGAAATGTATTGCCCTCGTGTTCAA
CTCGTTCGATATTTTTCCCCCAAGAGATCTCATGGGTAAATGAATATATATTGGAGTGGAAACGGACCCTTAAATCAACATTTC
AATAAAAATGCCCCTTTTGATGGCAATTTTCGCAAGTTTATTTCCTTATATTAGTAAATCTCTCCAGTATTAATGACTGACTCA
CCACCACACACCTCGGCTTTGCAGGAAATTTTACACATAAAAAATAACGATTAAGGGCTACAACTTTTTGTGCCATGACCCTCG

CATATTATTTATTATTCACTAGTGAAAACAATTGCTCCGAATCCATATTAGAGAAATCGAAGATTTCTGAAAATGGGGTACCTT
TTGGTGAATCGGAGATTTCACTAATTTCTCCGAAATGTATTGCCCTCGTGTTCAACTCGTTCGTACTTTTTCCCCAAGAGAATC
TCATGGGTAAATGAATATATTGGAGTGGAAAACGGACCCTTAAATCAACATTTCAATAAATGCCCTTTTTGATGCATTTTCGCA
AGTTTTTTCCCTTATACTTTCAGTAATCCTCTCAGTATTATGACTGACTCACCACCACACAACCTCGGCTTTGCAGGAAATTTA
CACATAAAACAATAGAATTATGCTACAAACTTTTGTGCCCATGACCCTGCGCATATTATTTATTATTCACCTATGGAATAACAT
TGCTCGAATCACATATTAGAAGGAAATCGAAGATTTCTGAAAGGGGTACCCCTTTGGGTGAATCGGAGATTTCACTAATTTCCT
CCGAAATGTATTGCCCTCTGTGTTCAACTCCGTTCGATATTTTTCCCCAAGAGTTCATGGTAAAAATTTTTTGGAAAAAAAAAT
TATATTGGATGGAAACCGGAACCCTTAAATCACATTTCAAAAAAAATTCCCCTTTTTGAATGTCAATATTTCGCAGTTTTTTCC
TTATATTAGTACATCTACAGTATTTTAACTGTATCAACCCTCTACCCACTCGGACTACACGAAATTTACACATAGAACCCTAAG
ACATAGGGCTACAAGTTTTGTGCCCATGACCAATCCGCATTTTATTTAGTATTCACTATGAAAACGTGCTCGAATCGTTATAGA
GAAATCGAAGATTTTTTGAAAGGGGTACCCCTTTGGTGAATTGGAGATTTCACTAATTTCTCCGAAATGTAATTGCCCTCCGTG
TATCAACTACGTTTCGATATTTTTCCCCAAGAGATCTCATGGGTAAATGAATATATTGGAGTGAAACGGAACCCTTAAATCACA
TTTCAAAAAAAATTCCCTTTTTGATGGGCAATTTTCGTAAGTTTTTTCCTTATATTCAGTACATCTCACAGCTATTTTAACTTG
TATCACCCCTCTATCCCAACTCGGAGCTAGGCAACGAAATTTTTTACACATAGAACCCTAAGACTATAGGGCTACGCAAACTTT
TGTGCCCATGACCTCGCGATTTTATTTAGTATGTCACATGAGAAACGTTGCTCCGAATGCGAATTAGAGAAAATCGAAGATTTT
TGAAAAGGGGTACCCCTTTTGGTGAATTGAGATTTCAGCTAATTTCTCCGAAATGTATTGCCCTCGCGTGTTCAACCTCGTTCG
ATATTTTTCCCCAAGGATCTCATGGGTAAATGAACATATATTGGAGTGGAAACGAGACGCCTTAAATCAACATTTCAAAAAAAT
TCCCCTTTTTGATGGCAATTTTCGTAAGTTTTTTCCTTTATATTCAGTACATCTCACAGTATTTTAACTGTATCCACGCCTCCT
AACCAAACTCGGAACTACCGCACGGAAATTTTAACACATAGAACCCTAAGACATAGGGCTAGCCAACTTTTGTGCCCATGACCT
>m141122_014521_42134_c100732202550000001823141405141560_s1_p0/136676/13135_18627
ATTCACTTTCAAAACCACGGCTCCAAGTTCATATTGGAAGAATCGAATATTTCTGAAAGGGGTACCCCCTTTTGGGTGAAATTG
GAGATTTTTCACCAATTTCTCGAAGGTATTACAGCATGGGTTCAACTCGGGATATTTTTTCCCCAAGAGATCCATGGAAAGTG
CATCTTGTGGAGTTGAAACGGAGCCGTTAATTCAACATTTGAAAAAAAATTTCCCCTTTTTGTTCGCAAATTTCCAAGTTTTTG
ACTATATTGGTAATCTCTCAGTAATTTTCAAGCCGTTCACCCCTCGTACAGCACGTCGGATATAGCACGTATTTTATAAATAGA
AGGCCTAAGACATAGGACCTGACCAACTTTTCGTTCCGCATGACCCACCGCATATTCCATAGTATTAACTTTCATACAGCTGCT
CCAATTCATATTAAGAGATATTGAAGATTTCTGAAGGGGTACACCCTTTGGTGATATTGGAGATTTCACCATTTCTCCCGAAAT
GTATTACAGAGGGTTCAAACTCCGGTCGATATTGTTTCCCCAAGAGATCTCATGGGAAATGCATCTTGTGAGTTGAAACGGAGC
CTTAAATGCCAACATTATGAAAAAAAAACTTCGCCCTTTTTGTTGGCATTTTCACAAGTTTTTGACTTATGTTCGTACATCTCT
CAGTATCCTTCAGCCGTACTCACCCTCTAAAACCGTCGGATATGCCACCGATATTTTAATACAATAGAAGGGGCTATGACATAG
ATCTACAACTTTCGTGGCCCATGACCCACGCCATTATTCCATAGTAGTTCACTTTCAAAACACTGGGCTCCAATTCATATTACG
AGGAATCGAAGATTTCTGAAGGGGCACCCCCTTTGGTGAATTGGAGATTATAAACCATTCTCCGAAAGGTATCTACACAGGGTT
CAACCCTCGGTCGAGTATTTTTCCCCAAGAGATCTCAATGGGAATGCATCTTGTGGATTTGAAAACGGAGCCTTATATTCAACA
TTTGAAAAAAAATTCCCTTTTTGTTGCAATTTTCGACAAGTTTGTTGAGCTTATATTCCGTACATCTCTCAGTATCTTCACCCG
TATCACCCTCTAAACCCGTCGGATATGCAGGATATTGCCTTATACATAGAAGGCTATGACATAGGGCTATAAGCTTTCGTGGCC
CATAGAGCCTCGCATATTCGATTTCAGTATTCACTTTCAAACACTGGCTCCAATTCATTATTAAGAAGGAAATCGAAGATTGCT
GAAAGGGGGTACCCCTTTGGTGAATTGGAGATTCACCATTTTCTCCGAAAGGTATAAACCAGGGTTCAACTCGGTCGAATTTTT
CGCAGAGAGATCTGCATGGGAAATGCATCTTGTGGAGTTGAAACGGAGCCTTAAATTCACATTTGAAAAAAAAGATTCCCCTTT
TTGTTGGGGCAATTTTCACAAGTTTTTGACTTATAATTCGTACATCTCTCAGTATACTTCACCGTATGCAGCCCTCTAACCGTC
GGTATATTGCAGCGATTTCTTATACAACCGAAGGCTATGACATAGGGCGTATAACTTTTCGTGGCCATGAGCCTGCGCATATTC
GATAGTATTCCACTTTTCACAAAAACATGGCTCCAATTCATATTAGACGCAAATCGAAGATTTTCTGAAGGGGTGCCCCTTTGG
TGAATTGGAGTTTTCACCATTTCTCCGAAAGGTATTACACAGGGGGGGTTCAACTCGGTCCGTATTTTTCCCGCCAAAGATCTC
CATTGGGAAATCTCTTGTGGAGTGAAACGGAGCCTTAATTCAACATTTGAACAAAAATTCCCCTTTTTGTTGGCAATTTTCACC
AGTTTTTGACTTATATTTCGTAAATCTCTCAGTACTTCCCACGTACCACCCCTCTACACCCGTGCGGATATGCACGTATTTTAG
ATACATAGAAGGCTATGACTAGGGCCTAGCAACTTTTTGTGCCATGAGCCTCGCATAATCCATAGTTATTCACTTTCAAAAACA
CTGCTCCAATTCATATTAGAGAAATCGAAAGATTTCTGAAAGGGGTAGCGCCGTTTGGTGAATGGAGATTTCACCAATTTCTCC
CGAAGGTATAACACAGGGGTTCAACTGCGGTACGATATTTTTCCCCAAGACGATCTCCATGGGAATGCATCTTGTGAGGTTGAA
AGCGGAGCTTAATTCAACATTTGAAAAAAAACTTCCCCTTTTTGTTTGGCCAATTTTCAGCCAAGTTTTTGACTTATATTCTAC
ATGCTCTCAGTATCTTTCACCGTATCACCCTCTATACCCGTCGGATATGGACGATATTTATACATAGAAGGCTATGACATAGGG
CCTATAACTTTTCGTGCCATGACGGCCTCGGCATATTCGATAGTATATCACTTTCGCAAGAACGACTGCTCAATCCATATTAGA
GAAATCGAAGATTTCTGAAAGGGGTAGCCCCTTTGCGTGAATGTGGAAGATTTCACCAATTTCTCAAACGAAGGTATTACAGCC
CGGGGGGTTCAACTCCGGGTCGATATTTTCCAAGAGATCTCGCCTGGGAAATGCATCTTGTGGAGTTGAACGGCGCGCTTAATT
CAACAGTTTGAAAAAAAATTCCCCTTTTTGTGGCAATTTTCAACAAGTTTCCTTGACTTATATTGCGTAAAATCTCTCGTATCT
TCAGCCCGTATCAGCCGCTCTAAACCCGTCGGAGTATGCACTATTTTGTATACAGTACCGGAAGCTAGACCTAGGGCTACAACT
TTTGTGCCATGACCTCGCATTATTCCATAGTATGTCAGCTTTCAAAACAACTGCTCCCAATTCATTAGCGAAGACAATCGAAGA
TTTCTGAAAGGGGTAGCGCTTTGGTGAATTGGAGATTTCACCAATTTCTCGAAAGGTATAACACAGGGGTTCAACTCGGTCGAC
TATTTTTCTCCACCAAGCAATTCTCATGGGAATGCATCTGTGGAGTTGAAACGGAGAACACATTATTCAACGATTTTTAAAAAT
TCCCTTTTTGTTGGCAATTTTCACAAGTTTTTGACTTATATTCGTACATCTCTCAGTTCTTCCCGTATCACCCCTCTAAACCCG
GTCGGATATGCACCCGATATTTTATACATAGAAGGCTATGACATAGGGCTCAAGCGTTCGTGGCCCATGAGCGCCCGTCGCATA

TTCGATAGTATTCACTTTCAAAACACTGCTCGCAATTCAATATTGAGAAATCGAAGATTCTGAAAGGCGGGGTACCCCTTTGGT
GAATTGGAGATTTCACCAATTCTCTCGAAATGTATTACACAGGGGTTCAGCTCGGTCGATCTATGTTTCCCAAGAGATCTCCCA
TGGGAATGCATCTTGTGGAAGTTGAACACGGGGCCTTAAATTAAACATTTGAAAAAAAATTACCCTTTTGTTGGCAATTTTCAC
AAGGTTTATATGAGCTATATTCGGTACATCTCTCAGTATACTTCCACCGTATCACCTCTAAACCGGGGGGTCGGATATGCACGA
TTTTTTATACATAGAGGCTATGACATAGGGCTATCACACTTTCCGTGCCCATGAGCCTCGCCCATATTCGATAGTATTCACTTT
CCAAAACCCTGCTCCCAATTCATATTAGAGAAATCGAAGATTTCTGAAATGGGTTGGTACCCCTTTGGTGAATTGGAGATTTCA
CCATTCTCGCGAAAGGTGTATTAGCACAGGGGTTCAACTCCCCGGTCGATAATTTTTCCCAAGAGATCTCCACGGGAAATGCAT
CTTGTGGAGTTGAAACGGAGCCCTAATTCAACATTTGAAAAAATTCCCCTTTTTGTGGCAATTTTCGACAAGTTTTTTGACTTA
TATGACGTAAATCTCTCAGTATCTTCCGCACGTAATCAGCCCTCTAACCCGTCGGATATGCACGATACTTTTATACATAGAAGG
CTATGACATAGGGTACAACTTTGTGCGCCCATGAGCGCTCGCATATTCCCAAGTATTCACTTTCAAAACACTGCTCCACCATAT
TAAGAGAAATCGAAGATTTTGAAAGGGGTCCCCTTTGGTGAATTGGAGAAAGCACCCAATTTCTCGCGAAGGTATAAACACAGG
GGTTCACTCGGTCGATATTTTTCCCAAGAGATCTTCCATGGGACAATGCATCTTGTGGAGTTGAAACGGAGCCTTAATTCAACA
TTTTTAAGAAAAATTCCCTTTTTCGTTGGCATTTTCACAAGTTTTTGACTATATTTCGTACATCTCCCCAGTATCTTCACCGT
TCAGCCCCGTCCCTACCCGTCCGATATGCACATATTTTAATACATAGAAAGGTATGCGATAGGGCTAGCAACTTTCGTGCCCCA
TGACCCTCGCATTTCGATAGTTTCACCTTTCAAACCACTGCTCCCAATTCCATATTAGAGAAATCGAAGATTTCTGAAAGGGGT
ACGGCCCTTTTGGTGAATTTGACGCATTTCACCAATTCTCCCGAAAGGTAATTACACAGGGGGATTGCAACTCGTCCCGATATG
TTTTCCCCAGAGATCTGCATCGAGGAAATGCATGCTTGTGGAGTTGAACGGAGCCTTAATTCAACATGTTGAAAAAAAATTCCCC
TTTTTGTGTTTGGCATTTTCACAAGTTTTTGACTTATACTGCGTGACATCTCTCAGTATCTTTCACCGTATCACGCCTGCTAAA
CCGCGTCCCCGGATATGCCGATATTTTAATACATAGAAGGCTATGACATAGGGCCTAGCAATTTTTTGTGCCCATGAGCCTCCG
CCATATTCGATAGTATCTCACTTTCAACACTTGGCTCCCAATTCATATTAGAGAAATCGAGTAATTCTGAAAGGGTACCCCCCT
TGGTGAATTGAGATTTCACAATTTTCTCCGCGAAGGTATAACCACAGGGGTTCAAGCTCCCGGTCGATATTTCCCCTCAAGAGA
TTGCATGGGAATATGCATCTTTGTGGAGTTTGAAACGGAGCTTAATTCAACTTTGAAAAAAATTCCCCTTTTTGTTGCATTGTT
GCACAAAGTTTTTGACTTATATTCGTACATCTCCCTCAGTATCTTCACCGTATCACCTCCCCTAAACCGTCGGATATGGCAGA
TATTTTATACATAGAATCTATGAATAGGGCTATTAACTTTGCGTGCCCATGAGGCCTGCGCATATTCGAAGTATTCAGCTTTCA
AACATGCTCCAATTCATGATTAGAGAAATCGACCAGATTTCTGAAAGGGGTGACCCCTTATGGTGAATTGGAGATTTCCACCAA
ATTGTCTCCGAAAGGTATTACACAGGGGGTTCAAGCTCGGTCGATATTTTTCCCCAAGAGATCCCATGGAATGCCATCTTGTGG
AGTTGAAACGGAGCCTTAATTCAAACATGTTGAAAAAAAAAATTCCGCTTTTTGTTGGCAATTTCACAAGTTTTTGACTTATATT
CGTAAATCTCTCAAGTATTCTTCACCGTATCA
>m141122_014521_42134_c100732202550000001823141405141560_s1_p0/137302/0_5363
AGAGAGAGGTATAATAATCAGAGATTGTACAATATAGAAAACTTGTGAAATTGCCCACAAAAAAGGGGATTTTTTTCGAATATT
GGAATTAAGGCTCCGTTTTCCACTCCATAATTCATTTACCCATGAGATTCCTCTTGGGTAAAAAATATCCGAACGAGTTGAACG
AGGGTAAATACATTTCGGAGAAATTAGTGAAATCCCCAATTCACCAAAGGGGTACCCTTTCAGAAATCTTCCATTCTTCTCTAA
TTATGGATCCAAGCGATGTTTGTAGTGAATAACAAAAATAGCGAGGGTCATGGCACAAAAGTTGAGCCCTATGTCTTAGGGTTC
TATGTATAAATTTCGTGCAAACCTAGGTGTGTACTAAGGGTGATACAGTCAAAATACTCAAGAGTACGAATATAAGGAAAAAAT
TGTGGAAATTGCCCACCAAAAAGGGGAGTTTTTTTCGAAATATTAATTAAGGCTCCGTTTCCCACTCAATATATTCATTTACCC
ATGAATCTCTTGGGTAAAAATATCCGAACGAGTTGAACACGAGGGTATACATTCGGGAAATTAGTGGAAAATCCCCAATTCACC
AAAGGGGTAGCCCCTTTCAGAAATCTTCCATTCTCTAATATGGATTCCAGCGATGTTTTGGTAGTGAATATCTAAATGAATATG
CGAGGGTCATGGGCAAAAAGATGTAGCCCTATGACTTAGGGTTTCTAATATAATTCTGCAAATCTAGGTGTGTATAGGGTGATA
CAGTCAAAATATCTCGGTGTACGATATAGAAAACTTGTAAAATTGCCTCAAAAAGGGAGGAGTTTTTTTTGAAATGTTGAATAA
GGCTCGTTTCCATCCATATATTCATTTACCCATGAGATTCTTGGGTAAAAATATCGAACGGTGAACACGAGGGTATACTTTCGG
GTTGTGAATCCCAATTCACCCAAAGGGGTACCCCTTTCAGAAATCTTCCATTTCTCTAAGTATGGATTCCAGCGATGTTTTGGT
AGTGAATACTAAAGAATAGCGAGGGCAAATGGGCACATGTAGCCTATGTCTTAGGGTTCTATGTTTTTTCGTAGCAAATCCTAG
TGTGTATAGGGTGTACATCAAAATACTCAGAGATGTACGAATATAAGGAAAAACTTGTGGAAATTGCCCACAAAAAGGGGGAGT
TTTTTTCGAATTATTGAATTAAGGCTCCGTTTCCACTCCAATATATTCCATGTTACCCATGGAATCTCCTTTGGGTAAAATATC
GAATCGAGTTGAACACGAGGGGGTAATTACATTTCGGAGAAATAGTGAAATCCCCAATTCACCAAAGGGGACCCTTCAAATCTT
CGATTTCTCTAATATGGATTCCAGCGATGTTTTGGTATTGGAATTACCTAAAGTATTCGAGGTCATGGGCACAAGTTGTGCCCT
ATGTCTTAAGGGTTCTAGTATATAAAATTTTCGTGCAACTATCCTAGGTGTGTATAGGGTGATACAGTCAAAATACTCAGAGAT
GTACGAATATAAAGGAAAAAACTGTGAAAATTGTACCTCAAAATGGGGAGTTTTTTTTTGAAATGTTGAATAAGGCTCCGTTT
CCACTCCAATATATTCATTTTTACCCATGAGATCTCTTGGGTAAAATAGCAAACGAGTGAACACGAGTAATACATTTCGGAGAA
ATTAGTGAAATCCCCAATTCAACCAAAGGGTACCCCCTTTCAGAAATCTTCGATTTCTCTAATATATATTCCAGGCGATGTTTT
GGAAGTGAATACTAAAGAAATATGCGAGGTCATGGGCATTAAAAGTTGGTACCTATGTTTAGGTTCTATGTATAAAATTTCGTG
CAAATCCTAGTGTGTTATAGGGTGATACACGTCAAAATACTCCAGAGATGTACGAATAATAAGGAAAAAATTGTGAAAATTGCC
TCTCAAAAAGGGGAGTTTTTTTTTGAAATGTGAATTAAGGCTCCGTTTCCACTCCAATATATTCCATTTACCCATGAGAATCTC
TTGGGTAAAAATACAAACGAGTTGAACACGAGGGTAATACATTTCGGAGAATTAAGTGAAATCCCCAATTTCACCAAAAGGGGT
ACCCACCTTCAGAAATCTTCCATTTCTCTAATATGGATTCCAGCGATGTTTGGTAGTGAATATAAAGAATATGCGAGGGTCATG
GGCACAAAAGTTTGTAGCCCTATGTCTTAAGGGTTCTAATATATATAATTTCGTTGCAAATCCTAGGTGTGTATAGGGTGATAC
ATGTCAAATACTGAGATGTACGGAAATATAAGGAAAAAACTTGTGAAAATGCCCTCAAAAAGGGGAGTTTTTTTTGGAAATGT
TGAATTAAGCTCCGTTTCCACTCCAATATAATTCATTTACCCATGAGGATTCTCCTTGGGTACATATCTGAACGATTGAACACG

AGAGGTAATCATTTCGGGAAATTAGTGGAACCCCAATTCCCAAAGGGGTAACCATTTTTCAGAAATCTTCCATTTCCTCTAAAA
TATGGATTCCAGCATGTTTTGGTAGTGAATACTAAAGAATATGCGAGGGTCATGGGCAAAAAAGTTGTAAGCCCTATGTCTTAA
GGGTTTATGGATAAATTTCGTGCAAATCCAGGTGTTGTAAGGGTGATACAGTCAAAATACTCAGAGATGTACGAATATAAAGGA
AAAACTTGTGGAAATTGCCCACAAAAAGGGCGAGTTTTTTTCGAAATAAAAATTGAAATTAAGGCTCCGTTTCCACTCCAATAT
ATATTTACCCATGAGTCTCTTGGGTAAAAATATCGAACGAGTTGAACACGAGGGTAATACATTTCGGAGAATTTAGTGAAATCC
CCAATTCACCAAAGGGTACCCCTTTCCAGAAATCTTCGATTTCTCTAATTATGGATCCCGATGTTTTGGTATTGAATACTAAAG
AATATGCGAGGGCATGGGCAGAAAAGTTGTAGCCTATGTCTTAGGGTTCTATATATAAATTTCGTGCAAATCCTAGTGTGTATA
GGGTGATACAGTCAAAATACTCAGAGATGTACGAATAATAAGGAAAAAACTTGTGAAAATTGCGCCCTCAAAATGGGGAGTTTT
TTTTGAAATGTTGAATTCAAGGCTCCGTTTCCACCTCCAATATTTCATTTAACCCATGAGATCTCTTGGGTAAAAATAGCAAAC
GAGTTGAACACGAGGTAATAATTTCGAGAATTAGTGAAATCCCCAATTCACCAAAGGGGTACCCCTTTCAGAATCTTCGATTTC
TCTATATATATCCAGCGATGTTTGGAAGTTGAAGTACCTAAAATATGCGAGGGTCATGGCATAAAATTGTAAGCCCTATGTCTT
AGGTTCTATGTATAAATTTTCGTGCAAATCCTAGGTGTGTATAGGGTGATATCAGTCAAAATACTCCAGAGATGTACGATATAA
GAGGAAAAACTGTGAAAATTGCCTCAAAAAGGGAGTTTTTTTTGAAATGTTGAATTAAGGCTCCGTTTCCACTCCACGAATAT
ATTCATTACTCCATGAGATCTCTTGGGTAAAAATATCAAACGAGTTGAACATCGAGGGTAATACATTTCGGAGAAATTAGTGAA
ATCCCCAATTCACCAAAGGGGTACCCGAATTTCAGAAATCTTCGATTTTCTAATATATATTCCACCCAGCGATGTTTTTTGGAA
GTGAATTACTACCAATAATATGCGAGGGTATGGGCACAAAAGTTGTACCCTATGTCTTAGGGTTTATAACTAAATTTCGTGCAA
ATCCTAGGTGTGTATAGGGTGATACAGTCAAAATACTTCAGAGATGTCGAATATATAAGGAAAAAACTTGTGAAAAATGCCCAC
AAAAAGGGGAGTTTTTTCGAAATGTTAATTAGCTCCGTTTCAACTCCCAATATATTCATTTACCCATGAGGATCTCTGGGTAAA
GATATCGAACGACTTGAAACACGAGGGTAATACATTTCGAAGAAATTAGTGGAAATCCCAATTCACCAAAGGGGTACCCCTTTC
AGAAATCTTTCGATTTCTCCTAATATATGGATTCCAGCGATGTTTTGGGTATTGAATACTAAATAAATGCGAGGGTCCATGGGC
ACAAAAGTTGTAAGCTATGTCTTAGGGTTCATGTATAAATTTCGTGCAAATCCTAGGTGTGTTTTAGGGTGATACAGTCAAAAT
ACTCAGAAGAGGTACGAATTATAAGGAAAAAACTTGTGAAAATTGCCCTCAAAATGGAGTTTTTTTTGTGAAATGTTGATTAAG
GCTCCGTTTCCAACTCCAATATATTCATTTACCCATGAGATCTCTTGGTAAAAATAGCAACGAGTTTGAACACGAGGGTAATAC
ATTTCGGAGAAATTAGTGAAATCCCAATTCACCCAAGGGTACCCTTTCAGAAATCTTTCGATTTCTCTAATATGAATATACAGC
GACTGTTTTGGAAAGGAGATACAATAGAATATGCGAGGGTCATGGGCATAAAAGTTGTAGCCCTATGTCTAAGGGTTCCTATGT
AATAATAATTTCCGTGCAAATCCTAGGTGTGTATAGGGTGATACAGTCAAATACTCAGAGATGTACGATATAAGGAAATAAACT
TGGAAAATTGCCTCCCAAAAAGGGGGTTTTTTTGAAATGTTGATAAAGGCTCCGTTTTCCCCCACTCCAATATATTCATTTACC
CATGAGATCTCTTGGTAAAAATCAACGAGTTAACACTGAGGTAAATACATTTCGGAGAAAATTAGTGAATATCTCCCAATTCAC
CAAAGGTTACCTTTCCAGAAATCTTCGATTTCTCTAATTATATACATTCCCAGCGATGTTTTGGAAATGAATACTAAATAATAT
CGAGGTCAGGGGCCAAAGTGTAGCCCTATGTTCTTAGGGTTCCTATATCTATGACAATTTCGTGCCAATGCCTAGGTGTGTATA
GGTGATAACAGTCAAAATACCTCCCCCAGAGATGTACGAATATAAGAAAAAACTTGTGAAAACTTGCCCACAAAAAGGGGAGTT
TTTTTTCGGAAATGTTGAATTAAGGCTCGCGTTTCCACTATCCACCAACCCCCCCCATATATCATTACCGCCCATGAGATTCTG
GGTAATAGATTGAACGACCTTGAACACGAGGGTAATACAATTTCGAAGAAATTAGTGAAATCCGCCAATTCACCAAAGGGGTAC
CCTTCAGAAATCTCGATTTTCTAATATGCGATTCCCAGCGATGTTTTGGTAGTGAATAGCTAAATAATATGCGAGGGTCATGGG
GCCCAGCAAAAGTTGTAGCCCTCAGCTTAGGGTTCTATGTAGAAAAAATTTCGTGGCAAATACCTTAGGTGT
>m141122_014521_42134_c100732202550000001823141405141560_s1_p0/101479/8146_17057
CCTGACTTCCAATAGATTAATTTACCTCTCACACCACGAATTCCCTGCAGGAGAAATATCGGTACACACCTGCAGTCGCACGAG
GATAACTACGATTGATCGGAGAAATTTATGAGCCATCGTCTATACTAGCCCAAGCCATACCTACCCACGGTGTACCTCACTTCG
AGTCAGTTCCAACCATGCTACAGATTGATCGTCAATAGAGCCGAGTTTTAACGAGCCAACCACCTCATGATTATCAAGAATGAG
AATAGAGGCGCAGAGAGGGTGCATCGCCGGCACCCAAGGTTTTGAAAGCCTTATGGTTTCCCCCAAACTGTGCGAACGGCCATT
AGGGTGGAGAGAGAACGAAGATTCACTATCCTTCAGCTCGATTGAGGATCGCCGTTCCAGAACACGATGCGTGGATAGAGGTGC
AGCGTTTATGGGCATTTGTCGGCATTTCTTCCAGTGATTCGAGAAAAATAATCGGAGTTACATGTATTGGAACAGGAGATATAG
GTATCCAGGTAAATCCTGTAAAGCCATCGAGGGCAGACATATTTCTAATAAAGAGCAGAGGGCTAACTTGCTCTTTTGAACGCC
CTTCCAGGGCCGATGTTCGACTGCTAAGAGTCTCCTCTAAAATAAACTCTCATCGCGCCACCAACCTACATCGGAAGGGGGCCG
TGTAATGCATGGATCGTAGCCTGGACAGAGAACGCACGCGCGCAAGGATGATGAGGATAATTTTGCTGAGGAAATGTAGTGAAA
TCTCCAATTCACCGAGAGAGGGTTACCCCTTCACTCGGTCCTGCGTTCGAGATATTAACCCATCGTACACATCGTGATTCCGAC
TGACGCATAATGTTATCTTGGTGAGCTTTGATAAATAGGCTAAACAGATACGCCAGGCGCACTATTCATCACTACGTCCTATAG
CGTTGTAGGCTTTGTCTTAGGGTTCGTTATCGAGAGCTAGACTTTCCCCAAACTCCAGAAGCTGTGGACAGAGACAGTAGTGGA
ATCCATCCATCCGGAGAATGTAGGGTGGGAGAGATTACGATATAAGGAGAGAGACATAGCTGCAGACGGCCACCATGAAGCCAT
CAAAGGAATTTGATCATGAACATACTAGATGACCTTAAGGCTCCGTATCCGATCGCGAACTTTCATTGCATAATAATTACCACC
GTACGATCCTTCTAGTGGTATACATCACAATACAGAATGACTACTGTGTTCGACTAAACCCGCCAGAGAGGTCATCCGATCTCT
CAGAGAGGTGGAAATTAGCTTGGCAATCCAGATGCACTCCTGGACATAACGGTGGGGTGGTACCTCCATGACAGAGATTATTCG
ATTTTCTCCATATCTTGAGGCCTTCGCCGCTCCATACGTTTGCGGTGTACATACACCTCACTATTATACCTGCTGGTGTGCCTA
TCATGGCACAATAAGTTGTGGCTAACAGCTGCATCTTTACTTCTTTGGGATTTCCAAGTAGCGTCTTCGGAATAATTCGCACAG
CGAGGTGTGTAGAGGGGTGATAACAGTCTAACACTGAGGTGATTTAGAGATGTCCACGAATAAGCCGAGCATGGGACAGGTAAA
ACATAGCGAACACGCATAGAACACGACGTGCGACACTTTCAACTCATTCGTGTACTAATTACCAGCCCTGCAACCAGCACCGTT
ACGCTTTCTCAGATACATTACGTGAATCTATCAATAAATACATAGATGGAGGGCTCCTTTGACGCCGAACCTATTAGACAGCCT
CGAGAGTGACAGAAGTTTAGTATGCACACCACGAAGGGATAATAACAAATTTCGGAGAAAATAGTGAACATCTCCAATTCCACC

AAAGGGTACCACTTTCAAATACTTCGATATTCTCTAACATATGAGATTAGAGAAATGTTTTGATATGTGAATACTAAAGAATAT
GCCGTAGGTCCATATGGGCACAAAAGTTGTTAGGCCCTTTGCTTAGGGTTCTAATGTATAAATACTTTCGTCCAAGATCCGAGT
GTTAGAGGTGATACAGTCAGAATAGTCAGATGAATGCAACAATCTCCAAGGAAAAATAGTTGCGAAAATTGCCATAAAAAAGGG
AATTTTTTTTTTTAAATGTCTGACTTAAGGTCGTTTCGACTCCAAATAGAAGGTTAATTTACCCACGAGATCTCTAGGGGAAAA
ATATCGAACGAGTGATGACAGGATAAATTTCGCAGAAATTATGAAATCTCCAAAATCACCAAAAGGGACCCCCTTCAGAAATCT
TTCGATTTCTCTAATATGATCGAGCAATGTTGGTTGATATGAATACTAAAGAATATCGAGGGTCATGGCAACAAAAGTTGTAGG
CCTAGGTGTCTTAGGGTTTCTATTGTATAGAAACTCGTGCAATCCGAGGTTGTAGAGGGTGATAACAGTCAGAATAGTGAGGAG
ATGTACGAAGATAAGGGAAAAAAGTTTTCGAAATGCCATAAAAAAGGGAAATTTTTTTAAATGTTTGACTTAAGCTCCGTTTCG
ACTCCAATTGAGATTAATTTACCACACAGAGATCTCTTGGGGAAAAAGATCGAACCAGTTGATGACGAGGGTAATACTTTCGGA
GAAATTGTGAAAATCTCAATTCACACAAAGGGTACCCCTTTCAGAAATCTTCGATTCTCTAGATATGGATTCGGATGCATGTTT
TATAGTGAAATACTAAAGAATAATGCGAGGGTCATGGGCACAAAGTTGGTAGGCCATGTCTTAGGGTCTATGCTATAAAACTTC
TGCAAATCCGAGGTGTGTAGAGGGTGATACACGTGCAGAATAGTGAGAGATGTACGAAATAAGGAAAAAGTTCGAAAATTGCCC
ATTAAAAAAGGGAATTTTTTTTGAAATGTTACTAAGCCCGTTTCGACTCCAATAGATTTAATTTTACCCACGAGATCTCTAGGG
AAAAATACAGAACGAGTGTGACGAGGGTAAATCACATTCGCAGAAATTAGTGAAATCCCAAAATTCACCAATGGGTATACCCCT
TCTCAGAAATCTCGATTTCTCTAATATTGGACCTTCGATGCAAGTTTTGATATAATACTAAAGAATAATGCGAGGGTCATTGGG
CCCCACAAAGTTGTAGGCCTTTGTCTTAGGTTCTATGTTATATAACTTCGTGCAAATCCGAGGTTAGTAGAGTGTGATACCAGT
CAGAATAGTGAGAATGTAGCGAATATAAGGAAATAAAGTTGGCGAAAAATTGCCCCATAAAAAAGGGAATTTTTTAAAATGTTG
ACTTAAGGCTCCGTTCTACCTCCAATAGATAATTTACCCACAGAGATCTCTAGGGAAAAATATCGAACGAGTTGATACGAGGGT
AATCATTTCGGAGAAATTCGTGAAATCTCCATTCACCAAAAGGGTACCCTTTCAGAAATCTTCGATATCTAATTGGATTCGAGC
CAATGTTTTTCGATAGTGAAATACCAAAGAATACTGCGACGGTCATAAGGCACTAAAGTTGTAGGCCTATGTCTTAGGGATTCT
ATGTAATACAAACTTCGTGCAAATCCCGAGGTGTGTAGAGGGTGATACAGTCAGAAAGTGAAGATGTACGAATATAGGAAAAAG
TTGCCGAAAATTGCCATAAAAAAGGGAATTTTTTTTTAAATGTTGACTTATAGGCTCCCGTTTCGACCTCCAAATAGATTAAA
TTTAGCCCACGAGATCCTCTGGGGAAAAAGATCGAATCGAGTTGATGACGAGGGTAAATACATTTCGAGAAAATTACGCTGAAA
CTCCAAATTCACGCAAGGTAACCCCTTTTCAGAAAATCTTCGATTCTCTAATATGGATTCGAGCCTGTGTTTGATAGGAATACT
AAGAATATGCGAGGTCATGGCAGTCAAAGTTGTAGCCTATGTCTTAGGGTCTATGACTAAAAGTTCGTGCATATCGCCGAGTTG
TGAAGGTGGATAACATCAGAATAGTGAGATATGTACGAATATAAGGAAAAAAAGTGCGAAATTGCCCTAAAGAAAAGGGGAATT
TTTGAATGTTGACTTAAGGCTCCTTTCGACCCAAATTTATTCCATTACCATGAATCTTGGGGAAAAATATCGAACGAGTTGAAG
AGAGGGGTAATACATTTCGGAGGAAATTAGATAATCGCTCCAATTCACCAAAAGGTACCCCTTTCAGAATCTTCGATTCTCTAA
TATGGATTGCGAGCAATTGTTTTGATAGTGAATACTAAAGAATATGCGAGGTCATGGCACAAAAGTGTAGGTCCTTTGTCTTAG
GGTATCTATGTAACAATCGTGCAAATCCGAGGTTGAGAGGGTGATACAGTCAGAAAGTGAGAGATGTAGCGAATATAAGGAAAA
AAGTTGCCGAAAATTGCCATAAAAAAGGGAATTTTTTAAATGTTGACTTAAGGCTCCGTTCGACCAATAGATTAATTTACGCAA
CGAGATCTCTTAGGGGAAAATATCGAACGAATGTTGATGACAGGGTAATACATTTCGGGAAAATTCAGTGAAATCTCCCAATTC
ACCCCAAAGGGGTACCCTTCAAAATCTTCGATTTCTTAATATGGATTATGGATTCGATCAATGTTTGATAGTGAAACTAAGAAT
ATGAACGAGGGCATGGCACAAAAGTGTGTAAGGCTATGTCTTAGGGTTCTATGTATATAAGACTTCGTGCAAATCCGAGTGTGA
GAGGGTGATACAGTCGAATAGTAGAGATGTACGAATAAAGGAAAAAAGTTGCGAAAATTGCCCATAAAAAAGGGAATTATTTTT
AAATGTCTTGACTTAAAGCTCCGTTTCGACTCCCCAATAGATTTAATTACCCACAGATCTCCTTGGGAAAAAGATCGAACGAGT
TGATGACGAGGGAATACATTCGGAGAAATTAGTGAAATCTCCAAATTCACCCAAAGGGTACCCCTTTCAGAATCTCGATTCTCC
TAATTGGATTCCGAGCAATGTTTTTTGATGTGAATAGCTCAGATATAGTTGCGAGGGTCATGGGAGCAAAGTTTAGGCCTATGT
CTTAGTTCTATGGTATAAAACTTCGTGCAAATCCGAGGTGTGAGGGGTGATAACAGTCAGTATAGTGAGAATGTACGAATAATA
GGAAAAAAGTTTGCGAAAAATTGGCCAAAAAAAGGGAAATTTTTTTTGAAATGTTTGACTCTAAGGCTCCGTTTCGACTCCAAT
AGATTAATTTACCCACGAGATCTATAGGGGAAAATATCGAACCGAGTTTGATGACGATGGAATACATTTTCGTAGAATTATAAG
ATCTCCAATTCACCCAAAGGGGTACCCCTTTCAGAATCTTCGATTTTCTCTAATATGGATTCGAGCAAATGTTTTGATAGTGAA
AGTACTAAAGAATCATGCATGGGCATGGGCCACAAAAGTTGTAGGCCTTTGTCTTAGGGTCTATGTATATTTTAAACTTCGTGC
AAATCCGATGGTGTCGTAAGATTGGTGATACAGTCAGAATTAGTGAGAATGTAACGAATATAAGGAAAAAAAATGCGAAATTGC
AATTAAAAAATAGGGGAATTCTTTTTTAAATGTGACTTAAGGCCCGTTCGACTGCCCATCATGATTTAATTTACCACACGAGATC
CTCTAGGGAAAAAGAGTCGACACGAGTGATGACGAGGGTAATTACATTTCGGCAGAATTAGGAATCTCCAATCCTCACCAAAAG
GGGTACCCCTTCAGAATCTTCGATTTCTCTACAAAATGGACGAGGCAAATGTTTGATAGTGAAATACTAAAGATAGGCAGGGTC
ATTGGGCCCCAGCAATAAGTTGTAGGCCTATGCTTAGGGTCCTAATATAAAACTCGTGCAAAGCCGAGTGTGTAGAGGGTGAAC
AGTCAGAATAGTGAGAGATGTACGAATATAAGGAAAAGTGCAAAATGCCCCATAAAAAGGGGATTTTTTAAATGTTGACTAGAG
GCCTCCGTTTCGACTCCAATAGATTAATTTCCCACGAGATCTCTTGGGGAAAAAAGATCGAACGGAGTTGATACGAGGGTAATG
ACATCTTCGGAGATTAAGTGAAATCTCAATTCACCAAGGGGTACCACACTTTCAGAAATCTTCGATTTCTCTAATATAGATCGA
GCATGTTTTGATAGTGAATTTCCCCCTAAAGAATAGGTGCGAGGGTCATGGCCACAAAGTTGTAGGCCTATGTCTTAGGGTTCT
ATGTACGTATAAAGTTCGTGCAAATCGAGGTGTGTAGAGGTGTGATAGCAGTCAGAATAGTGAGAGATGTACGAATAATAGGAA
AACAAAGTTGCGAAGATGTGGCCATAAACAAAGGGGAATTTTTTTGAAATGTTGACTTCAGGCCTCCGTTTAGACCTCCAAATT
TATCATTAAACCCATGAGGATCTCTTGGGGAAAATATCGACACGAGTTGAAACGAGGTAATACATTTCGGAGAATTATGTGAAA
TGCTCCCAATTCACAAAGGGTACCCTTCAAGAAATATTCGATTTCTCTAATATGGATTCGAGCAATGTTTTGATAGTGAATACT
AAAGAATTCATGCGAGGGTTCCTGGCAGCAAAAGTTTTTAGAGCCTAATGTCTTAGGGTTCTATTATAGAATTGTTCGTGCAAA
TCCAAGGTGGTAGAGGGTGATACAGTCAGAATAGGAGAGACTGTACGAAGTATAAGGAAAAGTTGCCGAAAATTGCCATAAAAA

ATGGGGAATTTTTTTGAAATGTTGACTGTAAGCTCGCGTTTCGACTCCAAATAGATTAATTTACCGACACGAGATCTCAAAGGG
GAAAAAAATATCGAATGAGTTTGATGACGAGGGTAATCATTTCGGAGAAATTAGTGAATCTCCAATTCACCAAAGGGACCCCTTT
CAGAAAACTTCGATTATCTCCTAAATATGGATTGAGGCAACTGTTTTGATAGTGATACTAAAGAATAATGCCGGAGGGTCATGG
CACAAGTTGTAGCCCTATATCTTTAGGGTTCTATGATCAAAAGTTCGTCTAATCCGAGTGTGTAGAGGTGATACAAGTCAGGCA
ATAGTGATGAGATGTACGAATTCCAAAGGAAAAAAGTTGCGAAAATTGCCCATAAAAAAGGGGAATTTTTGAAATGTGTACTTA
AGGCTCCGTTCGACTCCAATTAATTCATTACCCATTGAGAGTCTCTTGGGGAAAAAAATATCGAACGAGTTGAAGACGAGGGTAC
TATACATTTCGGAGAAATTAGTGAAATCTCCAATTCCACCGCAAAGGGTCCCTTTCAGCAAATCTTCGATCTCTAATTGATCGA
GCAAAGTGTTTTGATAGTGAATACTAAAAGATATCGAGGTCATCCGCAACAAAAGTTGTAGGCCTTTGTCTTAGGTTCATGTAT
ATAACTTCGTGCAACCGAAGGTGTGTGAGGGTGATACAGTCAGAATAAGTAGAGGATGTACGAAATAAGGAAAAATAGTTGGCG
AAAATTGCCATAAAAAAGGAATTTTTTGAATGTGACTAAGCTCCGTTTCGACTCCAGTAGTAGTTAATTTACCCACGAGATCTC
TTGGGGAAACAGCGAAATATCGAACGAGTTGATGACAGAGGTAAATACCATTTCGGAGAAATTAGTGAGAATCTCCATCTCACC
AAAGGGGCGTACGCCCTTTCAGAATCTTCGATTTCTCTAATAAGGATTCGAGGCAATGTTTGAATGAATAACAAAGAATATGCG
AGGGTCCATCCGGGCACAAAGTGTAGGCTATGATCTTGAGGGTTCTAATGTATAGGAATGTTGTGCAAATCCGAGGTGTGTGAG
GGTGGATATGCAGTCAGAATATGAGAGATGTGCCGAATCATAGGAAAAATTGCGAAATTGCCATAAAATAAGGGGAAGTTTTTT
GAAGATGTTGTACTTAAGCTCCGTTTCGACTCCAATTTATCATTTACCCACGATATCTCTTGGGAAAAAATATCGAACGAGTTGA
AGACGAGGGTAGCATACATTTCGGAGAAATAGGCAATCTCCAATCACCAAAAGGGGTACCACCTTCAAGAAATCTTCGATTTCC
TCTAATATGGATTCGAGCAATGTTTTGATAGTGAATACTAAAGAATATGCGAGGGTCATTGGGGCATCCAAAAGTTGTAGGCTT
TGTTCTTAGGTTCAGTGTATTAGAAACTTCGTGCAAATCCAGGTGTGGTGAAGGTGATAACAGTCAGAATGTGACGAGATGTAC
GAATATAATTGAAAAAAAGTTGCGAAAATTGCATAAAAAGAGGGGAATTTCTTTTTAAATGTTGACTTAAGGCCTTCGTTTCGA
CTCCAATAGATAATTTACCCGCAGAGATCTCTTGGGAAAAGACCGAACGAGTTGATGACGAGGGTAATGACATTCGGAAGAAAT
TAGTAAATCTCCAATTCACCAAGGGGTACCCCTTCAGAATCTTCGATTCTGCTCTAATATGGATTCGAGCAAATGTTTTGATAG
TGAATACTAAAGAATAATGCGAGGGTCATCGGCAGCGAAAAGTTGTAGGGCCTATGTCTTAGGGTTCTATGTATAAAACCTTTC
AGTGCAAATCCGATCAGTGTGTAGAGTGTTGATACGCAGTCAGGGAATAGTGAAGATCTACGAATATAAGGAAAAAAGTTGCGA
AAATTGCCATAAAAGAAGGAATTTTTTTTTGAAATGTTAGCTTAAAGGCTGCCGTTTCGACTCCAATAGATTAATTTACCCCCA
CCGAGATCTCTAGGGAAAAATATCGAACGAAGTTGATGACGAGGGTAATACATTTCCGAGAAATTAAGTGAAATCTCCGCATTC
AAAGGGGTACCCCCTTTCAAGAAATCTCGATTTCTCTAATATGGATTCGAGCAATGTTTTGATAGTGAATCACTAAAGAAATGC
GGGGTCA
>m141122_014521_42134_c100732202550000001823141405141560_s1_p0/105534/0_6028
AAAGAAGAGAGATTCATAGAGAGAAGAGGAGAGCACATGGTACAATTGTTGCGAATTTTAAGGGCTCAAGAAAGTGTTGCCCTC
GGCAGTGACCCCAGTTTGAGGCGGCTGGGGGTATTAGACTACCGTGTTCAGGGCATCGATTGAGCCTGAAGCGGGCTCCTGATT
GGGGGCCACACGAGCCGAATGGTGGGGAAGAAGTGCAGGTAGAGGCGCCTCAAACACATGGTGAGGGCAGAAGTATCGTACATG
CTGAAGCAGATGCTGTCGTGTGAAGACATTCACCGTTTCCTAAACAGCGTGCCTCCTTGTCAACCGCTGGGCGCGGTTGTCACG
CTGTGCCGTACGCATGGAGCCGGGCTACTTGAAAATGGCAATATAGTTGAATTGATTGCTGCTGCTAGAATGTATGCCCACAGA
CAAAGGTCCATTGGACATCCTGAGTGTGGGTCGCGTTTGGGTCTCGGAAGGTGGGAGAGTCAGACCACCGCTGGCTGTTGTGAT
TGATGCATGGCTAATATGGAGATCGAATGAATCGCGCTGGGAGCATGTTGTGTTCCCTACTTTCCACGAGCGAGGCATGCCTGC
TGAAGATGGCCGGCTTATGAAGTTGGCGCTTCCTTTGATAATACTGACCACTTGTAGTTGTTCCCATTCTTTTGCCTTGATAAT
TATTATGTTTTAATTTTTCTCCCTCCTATTTGCTTTCCAATTCGATAATACAAACTTTGAACATGGGCTATGCATGACAGGAGG
AACCGACGGTTTTCCATATACAAATCTATGTTGCCAGTCCCTATGGGGAAATTCAACCCTGGAATTAACTAACTCCGGAAAATA
CTTTATTCAACCATATAAAAATAAGGGAGTCAGAAGCTTTATAGGTCGTTGGTTCTCTTTGTATAAATAGGTTTAGATTATATC
CCCGTGCATTTCAATTTAATCTTATGGTGAGTGAGAGAGTTTAAGTTTAACATTGCACCCTTCCTTGAAGTGCTACCAAGCACA
TCGTTCCAAGATACAGAAGTCAACAAAAATACTTGGCAAAGAACGCGCCAAATATCCCCTGCTTGGTGGAAATCGATCCCTTTT
ATACACTAACCCATAGGCCATCACTTAGCCACTATTTTTTATTGCCCCGAACCCTAATACAACTAAATTATAGCCAAAAATACA
CAAAAATATATATCAGAGATGCTAAGAATAGAGAGACCCCCGACAATCCATGAAGACGTTCAAAAGATGGTGAGCGCTCTGCTT
CTCAAGACCCTTATGGGAAACTGGTTATATAAATTTCATCAATATGAGACATTGAAAACTCAAGAACTAACAAACATTAATATT
CGTGCTGTTATAGTCCGACCATATTTTATTTGGTGGCGTTTTGAATTGGATGGAAGCGGAAGGGCTAGAGATTAACTTCTAGTG
AACCTTGTAAAGTTATCTTTTTGTGCAAGAATTGCCTGCTTTGGGGTGAATATTGATACTCAGAGCGCAGGAGAGGGTTACCCT
CGCAATCGTATGATCATGAGCCACCAATACTTGATTCCCGTGTAGCCAACTGGAAGAGGAGGACTATCCTGTCTATCAAAGGGG
TAGCATGTAAGGCAATCCCTGAGCTGACTTAAATGTTCCCAATATCAATTACGCCTGTCCTATCTGGATCTGAGGTGGTTCAGG
GGACTTATTGATCCGCGATTATTATCAGAGGCGTTCGATTGGCGCTAGGCTTCGGATGCGCCTACTCTTATCTCCCTCTCTATT
GTACGCCATTTACTCTCATAATAAAGTGTGGGAAAAGCTTTGTTGAATTGAAAAGTAGGAACATAAGTTCGCAGGTAGAGATTG
TATCTTCCTTAGAAGTATCATCACCGTGGAAAGGGGGTGATGAGTTAATGGACAGTTGAGCTCTAGAGTAGGAGTAACAATTGG
GGGAGTCCTGATAGGACTGGTTAGGTTGATTTTGGTTTCGACCTTATCGTTTGGCACCACGAACACTTCTTCTCTGAGTCCTTG
CATAGGTAATCCAACCATTCTTGAAACACCCCCTATATCGATTTGTCTTCAGGGCCCCGTCAGTGATGATTCCCACTTTTCTCC
TCCATCGAACTCCAAAATTGTCACGAGTGGCGTTTACGTCTGTCGACCACTTTTTGTGGGGTGGGGAGAGAGACAATCGGTCGG
TTGTGACAGTAGTGATGCAGCGTTATCTGCCAAGAATTCCTTTTTTGGGAGTCTTTGCATTTTAAGAAGTTCTTGAAGTTCACA
AGTATGCTCTCAATGATATAAAGTCCCAAAATATAACCCTAATATTATGGTCCAAACTGACAACGGAGAGGCTCAATAGAAAAT
CAGACAGGAAGTAATAGACCAGATATAGAGCCAGAAAGAATAAGTGAAGTAAAATATCTGAGGAACATAAGAAAAATAAAGAGA
TATATCACGCGAGATTATGGAGAAGTTAGGTTCAAGCCCCAGTGAAGTTTAGTGGTTAATAGCACCATATCTCCATGTAAAAAA

ATACTGAGCCTAGTCGAAGGAATGACCTAGAAAACCCAGGGGACCGTTAGTAGTTTACCGGGAAATACAAATGTGGGTTAGATG
AGACATATTTGTCCATTAAATCGATCTTGGCCCCGCGAAATCGCAGGTGTATACAGCTTAAAAGTTATTGTATCAGTAGCGGAC
AAAAGGTTTTACTGGGAGATTAGGAGAAGGGGATAGTTGTAATTTTGTGCGCGTTCCAAAAATCTTGTCTCTTATATTGTTTAG
GGATTTTACAAGACCTTTTGAATTAGTATGAGGCCAAGGCAGAGTGTTAGGGCCATATTCACAATAAAAAAATATTATAGAATT
TTAATTTTTAGAGTGTCATTATAAAAAGTTAATATTTATAACAACAGACCTGAGTCTAAAAAGATACGGGCCAACAACATGTTT
TAGGTCTATAGGGTAAAAATCCAATTAGTGCATCGCTCTGTACAAACCATCATTTCTCTTGGCACGTAAGTTGTGGGTGTGGTG
TGTCGATGAGCTCATAGTACAGCATCGCCACCTAGAGCCTCTCTCAACTCAAGGATGGTCCAGCTGTTTCTTTTGCTTTTACCT
GCACGTACAGTAGCCTGCCCGTGCCGAAGACTGTTGCAGCAAGAAAAATCGTCCAATATGCTTCATGAATATGTAATAGACATG
TCATCAAATCAATAGTGGTGGCAGCACCCTAACAGATATACCTAGGGCCAACAAAGTGCATATCTATATATATCCCTGTGAATG
ATGGGGTGTATCCAAGGATATGAGAAATCATTTGGTCCGCTCCGCTGATGGTGATGGACTATTCAAATCAATTTCACGAAATCA
GAGTAGAGTGAATTATCACTGGTGGTCCGGTAACCATCGGGCTTAGATAGCCTCCGTGGAATAGAAAGGTGACAGTTGGGACAA
GTTCACTAAGGGGTCATTCCTTTTATGAATAAGTGAGTCCTTTCTACGTAGCGTTAATATACAGGATAGGGGTTTCGATGATTT
AGTACGCATAACCCATAACCTTTCTTGTCATGACTTAGGAGTCCAAGTAACTATGGAACCTGTTCCTCCGATCCTAGTAGCAGG
TGCAGTTGGTGATCTCTGTTGTAGGCTGTTAGGTCAGTGGGCCTCTTGAGAACTTAGTCCGTAGATCTAGACAAGGTGCTTAAT
TTTCAATCGAGCCTTGGTGCGTCGTCCAGCGCAAGTTAATACCCCATAGTGAGTCATTCTAGTGCTGATATTCGATGTTAGATC
TAATCTGTCATTGTTGGCTGTGGCGTTCTCGAACGCTATGCGTTTCTTGACTCTTAAGGCAGTAAAACAGACCAAGTGGTGTTT
CAAACCCGTGTGGAAATTGAACTAGTAATGTCATAGCTGTCACAAACGATTTACACCATTGCCGGATTCTGACAGTTAAGGGGT
GTACGAGGTGCCACACAACACGAATGTAAGCAATGCATCTACATATAGCTTATATGGTTAAGAGTATCCAAGGGTACAGTGGTT
GCAATTTTGACATGTACTTAAAAGATTGCCTTTAGCATCATTAGGACCGTGATGCATTGAAACACAGAGGCCTCAAGCTCTGAA
CAATCTCAAATACTCAATGTATACGATTAGTCAGCAGTGGCTCTCTACTCGAGCTATGTTTTCTCGGTTGTGCAACATCATTGG
CATACAGCATTAGAGGTCCTTGACTATGTGCATTGCAATGAAAACCTGATGCAATGTTCACATATAGCCACAGGGTGCCACAGT
TTTGTTCTTACCTTTGGGTCCATATGCAAACAGGTTACCATAAGATGAGTCCCCAACACTACGTGATCCTGGATTTTGTTCCAA
GGCCCTAGGTGAGTAACGCTTTGTAGTCACAACCAGTTGAATATGTTAGAAACCTCATCAAAAATGAGTAAATAAAATACTTCC
AAACCCAACCAAAGCCTCCGGGACATCGAATTCCCAACTGCAGCTAGGTAGGAGGAATCGTAGCCAGGCCCTTAGGAGTTTAGA
TTCCCTCAGACTGAAAAAAACTCATGTTCTTAGTGAATTTTTTTCCGCTTAAGGGTGGTGGCCCGACCCTGGTGCCAGGATCTC
ATCGGCCCGTGTGGCGGACCTGGTCGCCCCTAGCTAAGCCTATCACTCGACCGCTTCATCAAGCTAGGGTTCCCCAGCGCCCTA
GAACAAGGCATGGCGGCAACCGTGGTGTGTGTTGTCGCACTCGGAGCCTAGAAAAGCCTTTCGTTTTTTGCCACTTTTTTTAAA
AACTTTCCAAGAAACCTAAGTGATCCATGACCAAATGACATCTCCTTGAAATCCCAAATCAAAGTTTCCCAAACATCCCGATGA
CGCCAAAACCATAAAATCAAAGCTCGAATTCAATCAAATAAAGCTCATCAAGAGACACAAAATCCAATTCAAGCTTAAAAAATT
TAAACTTAAAACTGGAATACCTCCAGATTGATTTGTTTCCGCAATTTGTGGGTGTGTGTGGTGTTGTGGTGTGTGTGGTGTGTG
TGTGCTGTGTGTGTGTGTGTGTGTGTGTGTTGTGGTTGGTGTGTGTGATGGTGGTGTGTGTGTGTTGTGGTGGTTGTGGGTGTGTG
GGTTTGTTGTGGTTGTGGTGGGTGGTGTGTGTGTGTGGTGTGTGCTGTGGGTGTGTGGGTGGGTGGTGTGGTGTGGTGGTGTGT
GTGGTGTGGTTGTGTGGTGTGAGTTGTGTGTGTTGTGTGGTGTTGTGTGTGGGTGGTGGTGTGTGTGTGTGTGCTGTGTGGTTG
TGGTGTGTGTGGTGTGTGTGTGTGGTGGTGATGTTGTTGTGGTGTGGTGTGTGTGTGGTGGTGGGTGTGTTTGTGTGTGTGGTG
GTGTGTGTGTGGTGTTTGTGTGCCTGCGTGGGTGGTGTGGTCGTTGTGTGTGTGTTGGCTGTGTGGTGTGGTGTGTGTGTGTGG
GTGTGTGTGGGTGTGTTGTGTGGTGTGGTGGGGATTGGTGTGGTGGTGTGGTGGTGGTGTGTGTGTTGTGGTTGTGTGTGATT
GGTGGTGTGTTGTGTTGGTGTTTGTGTGTGATGTTGGTGTGGGTGGTGTGGTGTAGCCTGTGGGTGTGTGTGTGGGTGGTGTGT
GTTGGTGTGTGTGTGGTGTGGTGGTGTGTGTGGTGGTTGTGTTGGTGTGCGTGGTGTGGTGGTGTGTTGTGTGTGGTGTGTGTG
TTGTGGTGTGTGTGTTTGTGTAGTGTGTGATGTGGTTGGGTTGTGTTGGGCGTGTGTGTGTTGTGGTGGTGTGTGGTGGGGTGA
TGGTTGTGTGTGTGGGTGGGTTGTGTGTGTTGTTGATGTGTGTGGTGTGGTGGTGTCGTTGTGTGGGGTGGTTGGGTGGTGGTG
TGGGAGTGTGGTGTGTGTTTTGGTGGTGTGTGTGTGATGGTGGTGTGTGTGTGTTGGGTGTGTGTGTGATGTGGTTGTGGTGTT
TGTGGTCGTGGGGGGTGTGGGGGTGTGTGTGTGCTGTGGTGTGTGTGTGTGTGTGGTGGTGTGG
>m141122_014521_42134_c100732202550000001823141405141560_s1_p0/106662/2616_10574
ATATAAAGAAAATACATGCCGATTAATTCAATTTGAATCTAACTTCTAAGTTAGGAACCTGTTATTTTTTCATGATATTTGAAT
CTTGAAAACATAGTCTTCAACGTTAATATTCTTATCTTTATTGCATATCTCGTGCTTTTTAAGGCAAAACAATCGCACTTAATC
AATTGAATCACTTTCTAAGTTTAGGAGCCTGGTTCGCATATTACCAGGACATTTGCTATTTGGAGAAACATATAGTTCGGCCGT
TAATCTTTATTCATTATACATCCGTGCTTTTAGAAAAACATCGATTAAATCAATTCGAATCTACTTATCTTATGTTTAGGACCT
GGTTGCATCCCAGGACATTGATCTGAGTATACTAGTTTCGCGTGTAATTCTTTATTGATATCTCGTGCTTTTTAAACATGAAAC
ATCGAAAATCTCAATTTTCTGAATCAACTTCTAAGTATAAGACCCACTGATTGCAAGCAGTCTCAACACAGACATTGATTTTAA
GAGAAACTTATCGGCCGTTAATTCTTTATTGATATCTGTGCTTCTTAAGAAAAATTCATCGATTAAATTAATTTGAATCCTACT
TCTAAATGTTATGGACCTATGCTATATCCAATGATAAATTATATCTTGAAAATCATAGTTCACGTTAATTCTATATTGAGTATC
TCATGTTTTTAAGAAAACATCCATTAATCCAGATTCCTGATATCAACACAAGTTTAGGACCTGGTTATTTTCCAGCTATACCCA
GATCTTTGAAAAGTATTTGTTCACATATAATCTTCTTATTGATATCTCGTGTTGTTAAGATTAAAACATCGACTTTAATTTACA
TGAATTCAACTTCTAAACTAGTAAAAGGACCTGGTTGGTATTCCAGTGCACATTTGATTTTTGAGAACTACACTATCATATCCT
TTATAGTTCTGCGCATTAAGGGGTTTATATTTCTTTATTGATTATCATCGGGCTTTTTCTTAAGAAAACATTCGATTAATTTCA
AGTATTGAGGAGATCACACTTCTAATTTTTAGTGACCTGGTTCATCCAGGACTTAGAATTGACCTTAGTTCAGCGTAATCTTTA
TTGAATATCCTCTATGACATTTTACAAGAATAAACATCTGCTTAATTTCTCCATATTAGAACTTCTTAAGTATTAGTGACCACT
GGTTCTGCATCCCGGACATTGTTTTGAAAACTTATAGTTGCGTTAATTCTTATTGCAGTACCTTCAGTGCTTTTTAGAAAACCT

CTGAGATTAATCAATTTGAATCAACTTCTAACAGTATTTAGGGCTACATGGTTGGCATCCAGGACATTGAATTTTGTAAACTAT
TAGTAATTCCCAATGATAATAAATCTTTTTTGAAATCTCCGTCGCTATAAGACCAAACATCGATTAATCTCTATCAATTTGAAT
CAACTTCTAAAGTTTAGGACCCTTGGTTATTTGCCAGTGATACTGATTTTTGAAAAGATTTGTTCAGCGTTAATATTCTTATCT
TGTACATCATCAGTGTTTTTTAACAAAAAAGCATCAATTTAACCAATATTGGAATACACTCTAAGTCTAGGTACCATCCCAGAA
GCATTGAATTTGAAAAACTAGTTCGCAATAAATAACACAGAACATCAAAATGAAGTAACAAACTATTATACATTCTAATGGCCT
ATGCCAAATCTAAAACTATATCATAATGCCGTGCTTTTTAAGAAAAACATCGTATTAAATTGCATTAATACAACTTTATCAAGT
TTATCGGACCATCCGTTATTCCAGGATATTTGATTTTGAAAATACTTAGTTCACGTTAATTATCTCAACTCAAATATCTCGTGT
TTTTTAAGACAAAGACATCGATTTATCACTCTTCTTTAAAAAACCATCTAAGTTTTACGGAACCTAACGTTAAGTTAATCCAGG
GATCCATCTCCACATTTTGTAAAACTTAGTTCAAGTATAATTCTTTACTTGATATTCTCTGTAGCGTTCTTCTCTAAGAAAACA
TCGATGTAATCAAATTGAATCAACTTCTAAGTTTAAGTGACTTGGTTATTGTCCAAGGTATAGATAATTGAAAAACTATAGTAT
CTGCGTTAATTCTTTATACGCAATATTCTCCGTGCCTTAAAGAAAATACATCGGATTAATCTATTTGTAATCAACTCTAAGGTT
TAGGACCAGGTTGCAATCCAGAGACATTGCCTCCAATTAATGAGAAACTTAGTTCGCGTTAATTCTTTATTGAATATCTCGTGC
TTTTTAAGAAAACATCCGATTAATCAATTGGAATTAAGCTTCAATTTTAGCAGCGCACCTGATTGCATCCAGGCACATTCTGAT
ATCTATGAAAAACTTAGTTTCGCATTAATTCCTTTATTGATATCCGTACTCTGTGCTTTTAAGAAAACACTCGATTAAGATCTA
TTTGAATCGACCTTTAAGTTTATGGCCCTGGTTTTCATTTGCAAAACATATGGATTTTGAAAAACTTCAAGTCGGCATTCAATA
ATTTTTCTTATAAAATTATACCTTCCGTCTTTTTAAGAAAAACATCAATTATCAATTTGAATCAACTTCTAAGTTTATGTACAC
TTTAGTTATTTTTTTTTTTTTTTTTTTCCCAGGATATTGGGATTGTTTGAGGCAAAAACTTAGTCCGCGTTAAATTCTCTTTATTG
ATTAAGTGCATTTAAGGAAAAACATCGATTTAAATGCAAGTTGAATCTAACTTCATAGTTTAGCCTAAGCGTTGCATCCCAGAT
CGAATTGATTTTTTGAGAAACCTTAGTTATCGCGTTAATTCTTTATTGAATATCTCGAGCTACTTTAGAAAAACATCGATTAAC
ATAAATTTGAATCAATCTTCTATAGTCTTAGGACCCACCCATGGTTGCTATCGCAGAGAATTGATTTGAAAAACTTAGTTTCTT
GCATAATTTTCTCTTTTTGAATATCTCGTGCTTTTAAGAAAAACATCGATTTAAATAATCAATATTAGAACTCATACCTTCTAA
GTTTAGCGAACTTGTGTTGCATCCAAGGACATTGATATTTGAGAAACTCTAGTTCGCTTTGTTAGAGTTCTTTATTGATATTCC
GTGCTTTATTAAGCAAAAGGCATCGATTAATTAACACTTGCAAACAACTTCTAAAGTTTATCGGACCGAGCCTATTATGTTCCA
GGAGATATTGATTTGAAATACTTATTTCACGATTAATATCTCTTTATTGTATCTTCATGTTTTTAAGAAAACAATCTCATTAAT
TACCCAACAATTTGATATTGCAACTTCTAAGTTTAGGACTCTGAAATAATTTCCAGGAATCTCGACATTTGAAAACGATTATGT
TCAACCCAGTCTTCAATCCTCTATTATTGATATCCTCCTAATGGTATACTTCTTAAAAAATAACAATAACACATCAATAATCAA
ATGCATCAACTTCTAAAGTTTAACCTGCATTATTCCGAGGATAAATTATTATAACACCATATTTCATCATATCTTTATAATTAC
CTAAATGTTCTATCATTATTTTTCCATGCATCTTTAAAATTTTATTCATTCATATATCACATCATCCTATCAAATTTCATGGGA
TCCCAAATAGCCAAGATTTTCAATTTCATTTTACCCAAATTTACATGGTCACCTACATGGATCCTAATATTTGGGAAAATCATA
CACTCAAAAGGACAAAAATATCAAGATTATTCCTTAGGGTAAATGTGCCTAATGGTCCCGAATTACCAGATATTTTGCTATTCA
TTGATTTGAAAGACTTAGTTTCGCGTTAATTCTTTATTGATATCCTCGTGCTTTAAAAAAAACATCGATTAATCTAATTTCACA
TCAACTTATCCTAAGTTTAGGACTGGTTGCAATCCCAGGACAGCGATTTTGAGAAACTTAGTTCGCGTTATAAAATTCTTTGTT
GATATCTCCGTGCTTCTTTAAGAAAAATCGAGTTAAATCAATTTTGAATCAACTTCTAACAGTTTAGGGACCTGCGTAATATTC
TCAAGCATACTGAATTTTGAAAAGCTTTTGTTCACAGTTAGAGATTCATATATGTGTATCACCATTGCGTTTTATAAGAAAACA
TCAATATAAACCTCAATTTGAATCAACTTCAAAGTTTAGGACCTAGTGTTCCAAGGATATTTGATTTTGAAAAACTTAGTTTCA
GACGTTAATTCTTTAGTTGAACCTATCTCGTGCTTTTAAGTACAAACATACAATAACTCAATTTGATATACAGACTTTCTAAGG
TTAGAAGCCTGTGTTATTTCCAGGATATCCGATTTTGAAAAACTTAGTTCAACTGCTAAATGCCCAAATTCTTATATATAATCA
TCTCGTGGCTTTTTAAACATAAACACGATTAATTACAATTTGTATTCAACTTTCCTTAAGTTATATGGTACCGCTGGATTGCAT
CTCAGGGAACACTTGATTTATGGAGAAACTTATTTTCAGACAGTATAGATTCGTATTGTATACATCCCGTCAGGCTTTTTGAAA
AACATCGATTAATCATTTGAATATCAACTTCCTACAGTTAGGAACAAGTGGTTGCATCCCAGGACAACTACAGATATATGTGGA
GAACTTAGTTCGGCAGTCTCAATCTCTTATTGATATCTCGTGCTTTTATAAAGAAAATACATTTGTTAATCATATTTCAATCAA
CTTCTAAGTGTAGGACCTTGGTTGCATCCCCAGGACATTGAAATTTTGAGAAACTTATAGTTCCGCCGCTTAACAATTCTCTCT
TATCGATATAAACACGCTGTTTTTTAAGAAATCATCTGATTTATAATATGAAATCAACTTCTAAGTTTACACCTGCTTAACGTT
GCCAGGATATCCCCGATTTTGAAAATCTTATAGTTTCACGTTACTCCATTCTATATTAGAATATTCTCCCATTGTTTTTAAGAA
AACACATCATTAATAATCAATTTGACATCAACTTCTACAGCTATTAGGAACACCCATGGTTTCCTAAATTCCAGGATACAACTG
AATCTTCCTTTGAAGATTTGTATCACGTATTAATCCTATATTGATATCGTAAGTGTATTTACTTTACAAAAACATCAAATTAAT
CAATTTTGAACTCAACTTTCTAAGTTTAAGACCTGGTTTGCATGCCAGGACAAAGGATTTGATTTTCTCGACAAAAACTTAGTT
CGCAAATACTGCCTATTCGATATCTCGTGCTTTTAAGAAAAAACATTGGATTGAAACAATTCTGAATTACAACTTCTAAGTTCA
GGAAACTGTGTTATTTCCAGGAATATATTGGAGATTTTGAAAACTTAGTTCACGTTAATAGAAATCTTTATTGATATCATCGTG
TTATTTTAAGAAGACAATCGACTGAATGACTCATATTAATAACCTTCTCTAAGTCTAGGAACCAGAGTTTAATCTCCATTCCCA
GGACCTAAATTGATTTTGAAAAAACTTAGTTCGCGTTAGATAGTCTTTATTGATATCTCCGTCTTTTAAGAAAAACATCGATTA
AGTCAGATTTGAAATCAACTCTACAAGTTATAGTAGGACCTAGGTTATTATCCAGGATCCCCAATTGATTTGGAAAAACTTACG
CTTCGCGTTAATTCTTTAAGACGTACTAACTCGTGCTTTTTAAGAAAAACTTCCGATTAATCTAGTGTGACATTCAACTTAATA
AGATTAAGAGACACCCTGGTTGCAATCCAGTGACATTGATTTTTGCAAACTTACGCGTTAGCGGTTTTGATTCTTTATTGATAT
ATCGTTGCGTTTTTTAAGAAAAACAGTCAGATTAATTCAATTGAATTAAACTTACCTAAGTTTAGAGAACCTTGGTTGCATCCA
GGGACATTGATTTTGAACAAACTAGTTCGGCTATTAATATTTCTTTTATTGATATCTCGTAGCCTTTGTAAGAAAAACATCGAT
TAATCTATTTGAATCGACTTTAAGTTTTGTAGGGACCTGGTATCATGCCATAATCAGATGAGGGGAGAGGAAACTGATTTTGAA
AGAAACTTTAGTCGCTAATTAATTTTTTTATTAATGTGATGGTCATCGGCTTTTAAGAAAAACATCAATATAACTTCAATTGGA

CCATCCAACTTCTATATGTGTAGGGCTACTCTTGGTAATTCGCAGGATCACTTCGCACTTTTGAAAAACTTAGTTCGCGTTAAA
TTCAGTATATTGATAACTCGTGCTATCTATGTAAGAAAATAGCATCGATTAATCCAATTTGATCAACGTTCTAAGTTATGGACG
CTGCGGGTTTGGCAATCCAGAGAGACATCTGATTTTGAGAAACTAGTTCACGTTATTCTTTATTGATATTCGCGTGCTTTATTA
AGAAAAACATCGATCTTATCAATTTGAATCAGAGCTGTCTAAATTTCAAGGACTGTTTGGCATCCCAAGGACAGTTGATTTTGA
AAAAACTTAGTTTCGCATTAATTCTTTATTGACTATCTCGTGCTTTTTAAGAAAATAAATCATCGATGTAATCTATTTGGAAAT
CGAGCTTCCGTAAGTTAGGACCTGGTTGCATGCCAGCGACATTGATTTTGTTGGACAAAACTCTCAGTTCATTAGCTCTTATTG
ATATCTTCGTGCTTTTTAAGAAAATACACTCCGTGGAATCAACCAAGTCTATGTAAGTCTGCGACTTCTAAGTTAAGTGACCTG
GTTTCTATGCAGACATTTTATATTTGAAAACTTAGTTCGCATTATTTTTTTTATTAATATCATCGGCTTTTTAGAAAAACATCA
ATTAATCAACCTTTGAATCAACTTCTAAGTTTAGTAACCTGGTTTATTTCCCAGGTAATTCGATTTTGAAAAACTTAGTTCGCG
TTAATTCTTTATTGATATTCCTCGTGTTTGTTTAAGAAAAAACATTCGATTAATCAATTTGAATCAACTTCTAAACAGTTTAGG
ACCTGGTTGCCATCCCAGGACAGTTGATCTTTTGAGAAACTAGTCTAGCGTTAATTCTTTATTGATACCTCCGTGCTTTAAGAA
AAAACATCGATTAATCCGATTTGAATCAACTCTAAGTTTAGGACTGTGGGCATCCCGGACATTGATTTTGAAAACTTAGTTATT
CCATTAATTCAGCTTTTTTGAAATCTTCGTGCGTTTTAAGAATAAAACACGATTAATCAATTTGAATCAACTTTCTTAAGTTTA
GGACCGTGGATTGCATCCCAGGACATTGATATTGAGAAACTTAGTTCGGCGTTAATGTCTTTATTGATATCCCGTGCTTTTTAA
GAAAAGCATCCGTTAACGTTAATTTGATCAACTTCCAAAGTTTAGGACCAGCTAATTTCCAGGGATAAGTTGATTTTGAAAATC
TTAGTTCACGTTAATTCTTTATTGATATCTTCATGTTTTTATAAGAAAACAAGCTCAATTAAGGTCAAATTTGATACACTCCAA
CTTCAAGTTTAGATGACCTGGTTTATTTTGGCCACGGCCACATAGCTTTGATTTTGAAAGCTTTGTTCACGTTAATTTCTATTT
ATTTAGACTAGGTTCCCATGGTTTTTAAAAAAAACATCATTAAGTCAATTTTGAATCAAGCTTCTAATGTTGGTTAAAATATCC
TGAGCCATATATTTTCCAGATAATTGATTTTGAAAGAACTTAGTTCGCAGGGTTAATTCATTTTTATTTGAAAGTAGTCGCGTG
CTTTTAATGAAAAACCATTACGATTAACTCAATTTAACAATCCAACTTCTAATATGTATGGAACTCTTATGGTTGCATCTCCAG
AAAGACACCATTGATTTATGAGAAATGCTAATAGTTCACATGTTTAATTCCTTTGTTGATTCTGCGTGTTTTTAGAGAAAAACC
TCGTTAATCGAATTTGTCAACTTCTAAGTTTAGGACCTGGTAATGAGCTATGCTTCCTCCTC
>m141121_232159_42134_c100732402550000001823141405141507_s1_p0/425/0_6342
TAAATACAGTTAGAGACGAATGTTTGTTCTGATGGTTTTACTGGCCCTGCGAGCCTACATGCGTGGGTGTTGTGAGCGGCGCTG
GGTCCATCTTGCAGCTCCATTGCGTAAAAAGAACCAAACTTTCGGCAGAAGCGGCACATCCGGAAGAGCTGAGGAGCTTTCCGA
AGACGCCCTACCAAATGCCGGGACACCGGGGGCATAGCGTTATCGGGGTGAGGTAACTCCCCACCTAATCTTAATTATGCTCCC
CAAAATCAGGGACTTAGCTAGGGATAGGCTAGGACCGCGCGAACCCCGCGTTTGGGCTGACGGGTGAATGAGGCCTGCATCTCC
TGTCCCTGTGGGATCAGAGGTCCTAAGGTTCTTGTCGGTTCCCTCCAGTACGGGGGTCCCTGACCCCGGACAACGCAGGGAGAC
CGATCCACTCGTTCGCTTTAACGGGAACTGAGGTCTCGCCTGGGGCTTAGAAGAGGGCTCGTCCCGGCCATTTTTTATTGTGTA
TTTTCATCTGTTTTGCTTATCGGTCCTAACTCCTTTCCTCCTGGTACTTTTTGCAGAGGATTTCCGACATGTCTAACCCGGCCA
GCGAGATTGAGGAGGCTCATCAAGAACGGTCGACCAGAATGGCGGCCAGGAGGTCCAAACGTGTGTGGCGTGCACGGGAGCCCT
CCCCACCAGTGAACACCCGGGATAGTGTCCCTGTCCCAGGCGGGCTCTGGCCGTGGTTCCTTTTTCCAGGCGTGTGAGCCTGGC
CGCCGACTTCTCTTCGGTACCAGCCTCCGGTGATTGAGTTTGGAAGCGGGCGAGGCCGTCAGCGCGGAGCCAGGTAAAGAGGGG
CCCCGGAAGTCCGATGCCGGGGAAGGCAACCGAAGTAGGCCCCCGGATGACGCGGTCAGAAACAGTGAGGAGTCGCATGGGGAG
AGTCGGTAACTGCGAAGGAGATCCCTGCTCCGCCTGTCCCTTCCCCTCCGCCCAACTCTTTCTCGAGAGGGGAGAAGTCCCGCC
TTGCGGGACGAACAGTCCGGCTGTATCAACCGGAACCTGGGGAAAACGGTCCCGGTTGCTGGAGGGACCCCTTACAAGGCCCTG
ACGAATCTGTCGTCAGTCGAGTTCTCCGGAGCGTCCTGTCCGAGTTCAGCGGCTCCCCAGCCAGGCCGATCGTGGACCGGCTAC
TGCCAGGCGGAGCTTTTCACCCCAAGCTGGGCCAGAAACATCGCCCCATGCTGCTGATCTGCTGGTCAGGTTGGGAGCACTATT
CCAATCCTTCAGCTGAAGAGGTACGCCTACGATAGCCAACCCCGGACTGCTGTTCATCTCATCAGGCTCTCCGCCCCAGGCCAC
CCGCGGTAATGTTCCACCCTCACTCATTCCTTCTGTCATTTGTTTGTGGTGGTTTATCTTCTAACTTTTTTGCTCCAGGTTGA
TTTAACTGTTCCAGAGGAGTGCCGATCTAGTAAGCCGAGCTTTTGCCACTAAAGATGGAGACGGCCAAGCTGGAGCTGGAGGCA
GCCGTGAACCAGAGGGAGGCCATCAAGGAACCCTCAGCGGGAAAATGGCCCCATGTCCAGGAGTAAGTGGGCCCACCTGAAGGC
GGAGCGGCGAGGAAAGTTGGACGCTGCGCAAGGTTGGGCTGGAAGAGGAGCTGTCCGAAGACAAGAGTCACCGATGAAAAGGGC
GACGCTCTTGGAGACGGCGCGGCAACAGTCTCCTCTGGACATTAGAGGGATCCTGGCCTGAAAGCCGAGTCAGGGAACTGGTCG
ACTCCCTGCTCAGGAGAAGGCGGCGCAATGAGACCGACTCGTCAAGAGAAGGAGGAGGCCGATTACCTTTAGTGGATGTTACCT
CGACGAGGCCACTATATGGCCTGGTGCAAGGATAAGAACATGAACCTCCCTCGTCTTCCCTGATCCACAATCGGAAAGCGTGCC
GAAATATGAGCCAAGGAGAGGAGGATGACGGACCTCCGGGCCGATGCGGTTGTAAGGCCCGTGTCCTGGCCTTGCAACTTTTGT
TACTTTTTGGCTTGTAATTAAATTCTTAAGCTTGTGCTTTCTTTAGTTTGGTAAGGAAACTTCCTTCCATGGCTCAGTAAGAGA
TTCGATTTTATATTGCCGGCGTCTTAAGTGAATGTAGGTGGTTTGGTTTCTGGTTCCAACGGCCGGGACGAGACCACAACTAAT
TTTCCAAGTCTCTAGCCTGGCACTCAGTCCAGGGCACCGGGGCTTTGGCTTGAACTGGGCGTTGTCGGTCGCCTTGTTCAGTTT
TAAGCCCCGGCCCACCTCCGGGGCAAAACCGATCCTCCTTTACATTCCCCGGTCAATGGTCCGGCGGAACAAGCTTGGCTCGGT
CCCCTTTTTTTTTTAATATACCCGGTACGTCGTTTCGTCCGGGGCGGGACGAGCCTTGGGCCTTTCCCTTTTTATTAAATATAC
CCCAAAATATTGTTCGTCCGGGCAGGACCGGCCTTGGCCTGGTCCTTTTTTAATATCCCCGGACATCGGTTTCGTTCCGGTTGG
CGGACGATGCCTTGGGCTTGGCCCCTTTTATTAATATATCCGAACATCCATTCGTCCGGCGGGACTGGCCTTGGGCTATGTTCT
TTTTTAATATCCCCCAGACCGTCGTTTCGTCCGGCGGGATCGAGCCTTGGCTTGGTCCCCTTTATTATCATACCCAGACATCCG
TTCTCCGGGCGGTAGACCGGCCTTGGGCTCGTCGTTTTTTTAATATCCCCGGACGTCGTTTTGTCCGGGGCGGGACGACCTGGC
TTGGTCCCTTTTTAATATACCTCCCGGACATCCGTATCGTCCGGCGGGAACTGGCCTTGGGCTCGGTCCCTTTTTTAATATCTC
CGGACGTCGTTTCGTCCGGGCCGGGACGAGAATTTGGGCTTGTCCTTTTTTTAATATCCCCGGACCGTCGTTTGTCCGGGCGGA

CGAGCCTTGGCTTGGTCCTTTTTAATTATATACCCCGTACATCCGTTCGGTCCGGGCGGGACCGCCTTGGGCTCGATCCTTTTT
TAATATCCCCCGTACGTCGTTTCGTCCGGGGGGGCGGGACGAGCCTTGGCCCCTTTTTTTAATATACCCCCGGACATCGTTCGT
GACGACCGGCCTTGGGCTCAGTCCTTTTTTAATATCCCGGACGTCGTTTCGTCCGGGGGGAACGAGCCTGTGGGCTTGGTCCCT
TTTATTATATACCCGAACATCGTTCCGTCAGGCGGACCAGCCTTGGGCTCGGTCCTATTTATATTACCCGGACGTCGTTTCGTC
CCGTGGGCGACGAGCCTTGGGGCCCTTGGTCCATTTTATTATATACCCGGACATCGTCGTCCGGGCAGGACGGGCCTTGGCTCA
GGTCCTTTTTTAATATCCCCGAACGTTCGTCCCGGGGCGGGGATGGAGCCTTGGTGCTTGGTCCCTTTTTTAATATACCCCCGA
AACATCGTTTGTCCGGGTGGGACCGGCCTTTGGCTCGGTCTTTTTTAATATCCCCCGACGTCCGTTTCTCCGGGCGGGACGAGC
TTGGGTTGGTCCCTTTTATTTAATATCAACCGAACGTCGTTCGTCCGGCGGCCGGGACGAGCCTTGGGCTTTGGTAACTTTTAT
TATATACCCCGGACATCGTTCTCCTGGGCGGGAACCGGCCTTGGTTTCGGTCCTTTTAATATCCCCCGGACGTCGTTTCGTGCG
GGGCGGGACAGCCCTTTGGGCTTGGTCCCTTTATTATATACCCGGACATCGTTCGTCCGGCGGGACTGGCCATTTTTTTTTTT
TTTTTGCCTGGGCTCAGTCCTTTTTTTAATATCCCCCGGACGTCGTTTCGTCCGGGGCGGGACGAGCTTAGGCCAATTGGTCCT
TTTTTTAATATAACCCGGCATCGTTCTTCGGGCTGGACGGCCCTTGGTTCGGTCCTTTTTAATATCCCCGGACGTCGTTTCGAT
CCAGGGCGGGACGAGCCTTGGGCATTGGTCCCTTTTTTAATATCCCCGGACGTCGTTTCGTCCGAGGGCGAGACGAGCCTTGGG
CTTGGTCCTTTTTATTATATACCGTACATCGTTCGTCCGGCGAGACCGGCCTTGGATCCGATCCCTTTTTTAATATCCTCGGAC
GTCATTTCGTCCGGGCGGGGACGAGCCCTTAGGCCCTTTTTTAATATATCCCGACATCGCTTCGTCGGGCGGGACCAGGCCATG
GACTCAGTCCTTTTTTTTAATAATCCCCCGGACGTCGTTTCCGTCCCCGGGCGGGACGAGCCTTGGGCTGGTCCTTTTATTATA
TACCCCCGGACTATCGTTTGTCCGGGCCAGGGACCGGCCTTAGGTTCAAGTCCTTTTTTAATATCCCCGGACGTCGTTTCGTCC
GGGGCAGGACGAGCCTTGGGCTTGGTAACCTTTTATTATGATACCCCCGGAAATTCCGTTCGTCCAGGCAGGACCGCCTTGGGC
TCGGTCCTTTTTTTTATATCTCCCGGACGTCGTTTTCGTCCGGGCATGACGAGCATTGGCTTGATCCCTTTTTTAAATATACCC
CCGGACAGTCGTTCGTCGGGCGGACCGCCGTTATGAGCTTCGGTCCTTTTTTAATATCCCCGACGTCGTTTCGTCCGGGGCCGG
AGGAGCCTTGGGCTTGGTACCTTTTATTTATATACCTCCGGACATCGTTCGTCCGGGCGGGCCGGCTTACGGGGCTCGGTCCTT
TTTTAATATCCCCCGACGTCGTTTCGTCCGGGGTCGGGACGAGCCTGTGGGCTTGGTCCCTTGTTTTTATATACCCGGACATCG
TTCATCCCGGGCGGGAGCGGAATTGGGCTCGGTCTTTTTTTAATTGTCCCCGGAACATCGTATTCGGTTCGGGCGGGACGAGCC
TTGGCTTGGTCCCTTTTAGTTATATATCCCCCGGACATCATTCATCCGGCGGGACCGGCCTGTGGGCCTCGGTCCTTTTTTTAA
TATCCCGGACGTCGTTTCGTCCGGGGCGGGACGAGCCTTCTGGGGGTTGGTACCTTTTATTATAATACCCGGACATCGTTCGTC
CGGGCGGGACCGCCTTGGTCTCGGTCCTTTTTTAAATATCCCCAGACGTCAATTTCGGACCGTGCCGGGATCGCAGCCTTGGCT
TGTCCCTTTTATTATTTACCCCGGACATTCGTTCGTCCGGCGGGAACCCGGCCTTGGGCTCGATCCTTTTTTATATCCCCGGAC
GTCGTTTTGTCTGGGCGGGACCGGAGCCCTTGGGCCCTTTTTAATATACCCTGGACATTCGTTCGTCGGGCGGGACGCTTAAGG
CTCGTTTTTGTTTAATATCCCGGACGTCGTTTCGTCCGGGCCGAGACGAGCCTTGGGCTTGGTTCACTTTTTTTTTAATATCCC
CCGACGATCGTTCGTCCCGGGGTGGGACTGGGCCTTGGGCTCAGCCCTTTTCGCGATGTGCCTGGAATGAACACCCCCGGAAGT
GAGCGGAGACGGTCTTGGGGTCATTGAAAGAAAATTCGGAAAGGAATTCCGCCCTGAGGCGTACATTTTTCTTTGGTGACAAAT
AGCTTTTTACGAACGTTTTAGACACGCCGTTCATTGTTCAAAAGGAATTGCCTGAGGCTACATTGCTTACACCGTAAACCACTT
AAACGGGAACGAGTTTCTAGGACTTACTGGTAGTTACGAGATGGTGCGCGTTCCAGGCTCGCGAAAACAGAACCATCGAGCCGC
TTCAAGCGTGTACCTTCCGGGCCAGACCTCGTGCCTGGATCTCTTATGGCACCTTCCCAAATTAGGGCCCAGAACCCCTACTCC
CACATCTGAGTGGCAGGGAAGACCCTTCCGGAGGACGTAGATTGCCGGTTTTTCAACTACGGCTTTTGACTCAGAGTTGAAATG
TTCGCGCCGATCTTGCCCCTGGTACATGTCAACCTTCACTTGCAATTCCTTCCCGATCCCTCGATAAGATCCAGCGATTCCTGG
AGTAAGGGGGGGGGGGGGGGTGTGGTTGGGGTGGATCCATGGTCGCGTCGATTGGGAAACGGACGTCGCTTCAATTGGGGATG
ACGGCTTCGCATCCGTAACCATTGAGTAGGGGGTGTGCCCCAGTTATGTCCTTTCAGTTGTCTTGGTAGGCCCACAGATAACGC
CGGTGAGTTTCCTCGGCCATTTGCTTTTACAGGCCGGAGCTTTTTCCTTCAGCGTTCCCTTTTAGGGAATTTTTTCACAGCTTC
GGCCCTGCCGTTAGCCTGGGTCCTGGGCGACTGCCGAAGAAACTCTTCACTATACCGTGCTTTCGCAAAAAAATCCGTGAAGTG
GAGCTGTCAAACCTGCTTCCCGTTGGCCGGACACGATTTCCG
>m141121_232159_42134_c100732402550000001823141405141507_s1_p0/4972/5876_12915
AACGCAATCATCAGTCGAGCGTTTCTATTTAAGAGCACATGTTGTGCTTGGGCATTCTAGTTTTTTGCATGAATTAAAAGTTGT
AGGCCTATTGTCTTAGGGTTCTTATGATATACATTTCGTGCAAATCCTAGGTGTGTAAGGTGATACAGTCTATAAATACTAGAG
AGATGTACGATATAAGGAAAACTGCGAAAATTGCCAAAAAAAAGGGGAATTTTTTTTGAAATGTTGACCTATTAAGGCTCCGTT
TCTACTTTCCAATTAGATTTCATTTACCCACGAGATCTTCTTGGGGAAAAAATATCGAACGATTTTGAAGACGAGGGTATTACAT
TTCGGAGAATTAGTGAAATCCCAATTCACCAAAGGGTAAGCCTCCTTTCAGAAATCTTCGATTTCTCTAATAAATTGGATTCGA
GCAAATGTTTGATAGGAATACCAAAAGAATAGTTGCGAGGTTTTCATCCGGCCAAAAAGTTGTAGGCCTATGCTTAGGGTTCTA
TGTATTAAAAATTCGTGCAAATCCGAGATGTGTAGAGGGGGTGATAACAGACCAAAAATACTGAGAGATGTACGAATATAAGGA
AAAAAACTTGCGAAAATTGGCCATATTAAAAGGGGAATTTTTTTTGAATGTGTGACTTTAAAGCTCCCGTTTCGACTCGCAATA
ATTCCATTTACCCACGAGATCTCCTTGGGGGAAAAAATATCGTAACGATTGAAAAGACCGAGTAATACATTTCGGTAGAATTAGT
TGAAATCTCCAATTCACCAAAAGGGGTAACCCCTTTTCAGATTCTTGTTCGATTTCTCTATATTATCAACATTCGAGCAATTTG
TTTTGATAGTGAATACAAGAATATGCGAGGGGTCATGGGTGCACAAAAGTTGTAGGCCTAATGTCTTAGGGTATGCTTATGTAT
TAAAATTCGTGCAATCCGACGATTGGTAGAGGTGAATAACAGTCAAAATACTGTAGAGATGTACGAATATAAGGAAAAAACTTG
CCGAAAATTGCCATTATAAAAGGGGAATTTTTTTGAAAAATGTTTGACTTAAAGCTCCGTTTCGAACCTCCCCAATAGATTTC
AATTACATATTTCACCGAGATCTCTTTGGGGAAAATATCGACGATTTTGAAGATTGAGGGTAATACATTTTTCCGGAGAAAAT
TAAGTGAATTCTCCCAATTCAGCGCAAAGGTGGTACGCCCTTTCAAAATCTTCCGATTTCCCTCCTTATATGATTCGGACAATT
GTTTGATGATAATGAATACCTAAAGAAATATTGCGAAGGGTGCATGGGCCCACTTAAAGTTTGTAGGCCCTATGTCATTTAGGG

TTCTATGTATAAAAATTCCGTGCAAATCCGAGTGTGTATTAGGGTGATTACAGTCAAAATACTTGAGAGATGTACGAATATAAG
GGAAAAAACTTGCGAAAAAATTGGGCCATATATAAAGGGAATTTTTTTGAAATGTTGACTATAAGCTCCGTTTCTGACTCCAA
TAGGATTCCATTTACCCACGAAATATTTTTAATTGATCTCTTGGGGGAAAATATCGATACGATTTGAAGATGAGGTAATACGA
TTCGGGAGAAATTTAGTGAAATCTCCAATTCACCCAAAGGTGGGTACCCCTTTTCAGAAATATCTTCGATTTCTCCTTATAGG
ATTCCGAGCCAATGTTCTCTGATAGTCGATATACAATAGAAATAATGGCGAGGGTCAATAGGGCCAAAAGTTGATAGGCTTATG
TTCTTAGGGTTCTATGTATACACATTTCGTTGCAAAATACTAGGTGTTTGTAGAGGGTGATAACAGTCAAAATACTGAGTGTGT
CGAATTATAAGAAAAAAGTTGCGAATTCCATAAAATAGGGGAATTTTTTTTGAAATGTGACTTAAGGCTCCGTTTCGACTCCAA
TGAGATCATGTTACCCACGAGATCGTCTTGTGGGGTGAAAAAATATAAAAAAATAATAAAAAAAAAAAAAAATAATCGAACGAT
TTGAAAGACGAGGGTAATACAATTTCCGGAGAATTAGTGAAATCTCCCAATTCACAAAAGGGGGTTTACCTCCTTTTCGAGAAT
CTTCGATTTCTCTAAATGGATTCGAGGCCAATGTTTTTGTATAGTGAATTACTAAAGAACATATGGCGAGGGTGTCTATGGCAC
ATGAAAAGTTGTAGGCCTATGTCTTATGGATTCTATGTGATTAAACATTTCGTGCAATATCCTAAGGGTGGTGTAGATGGTGAT
ACAGTCAAAATAACTGGAGATGTACGAATATAAGGAAAAACTGCCGAAAATTGCCCCAAAAAAAAAGGGAATTTTTTTTGAAA
TGTTGATCTTTAAGGCTCCGTTTCGACTCCCAATAGATTCATTTACCCAACGAGATCTCTTGGTGGAAAAATAATCGACGATTT
GAAGCGGAGGGTAATTAATTTCGGTAGAAAATTAGTGAAATCTCCCATTCATCCCAAAAAAAGGGTACCCCTTTCAGAAATCCT
TCGTTCTCTAATAATTGGACGAGCCCAAATGTTTGATAGTGAATACTAAAGAATTTGCAGGGTCATGGCACAAAAGTTGTTAGG
CCCTTTTAAAATTTTTGTTTTTTTCTTTAGAGGTTCTATGTATAATTAAAATTAAGTGGGCAAAATCCTAGGGTGTAGAGGGTG
ATACAGTCAAAATACTGAGAGATTAACGAATATAAGAAGGAAAAAACTTGCGAAAACTTGCCAAATATATTAGGGGAATTTATT
TTGAAAATGTTTGACTTTAAAGCTCCGTTTCGACTCCCCAATTAGATCCATTTACCTCGAGATCTCTTGGGGAAAATTATCGAA
ACGGATTGAAGACAGGGTAATACATTTCGAGAAATTAGTGAAATCTCCAATTCCCCAAAGGTGATACGTCCCTTTCGAAATCTT
CGATGTTCTCCTAATATTGGGATTCAGCAAATTGTTTTTGATAGTGAATAACTAAAGAATATGCGAGGGTCATGGGCATTAAAA
GTTGTAAGGACCTATGTCTTAGGGTTTCCTATGTATAAAATTGTGCAAATCCGAAGATGTGTAGAGGGTGATACAGTCAAATAT
ACTGAGGTGTCATTAATGGAAAAACTTGTCGAAAATTGCCAATAAATAAAAGGGGAATTTTTTTGAAATGTTGACTTAAAGCTC
CGTTTCGACCTCCCTCCCACATAGATTCATTTACCTCACGAGATCTCTTTGGGGGATAAATAATCGAACGATTTAAGATGAGGA
GTAGGATTATCTATTTCGGAGAAAAATTAGTGAAATCTTTCAATTCACCAAAGGGGTCCCCGTTTCAGAAATCTTCGTTTCTCT
TTATGGATTCGAGCAATGTTTGATTAGTGAATACTAAAGATATGGAGTGGGTCATGGGCAGCTAAAAGTGCTATAGGCCTATGT
CTGTAGGGTTTCTATGTATTAGAAATTCCGTGCAAATCCGAGTTGTGTAGAGGGTGATACACGTCAAGAATAACTGGAGGATGT
ACGAATATAAGGATAAAAACTTGCGAAAAAAATTGCATATAATAAGGGGAATTTTTTTTTGAATGTTGAACTTAAAGCTCCGTT
TCGACCTCAATAGATTTTCATAATTAGATCCCCACCGGATCTCTTGGGGAAGAATATCGAATCGATTTGAGATGAGGGTAATTA
CATCTCGGAGAAAATTATGTGAAATCTCAATTCACCAAAAGGGGTTTACCTTTTCAGAAATCTTCGATTCTCTTATATGGATCG
AGCAATGGTTTTTGATAGTGAATATTAAAGAAATACGAGGGTTCATGGACAAAAAGTGTAGCGCCTATGTCTTAGGGTTCTATG
TATTATGACATTTCGTGCAAATCCTAAGGTGTGTAGAGGGTGATACAGTCAAAATACTGAGTGATGTACGAATAAAGGAATAAA
AGTTTGCCGAGAAAATTTCCATAGAAAAAGGGGAATCTTTTGATTGAAATGTTGACTAGGCTCCGTTTGCGGACTCCCACCAAT
AGATTTCATTTTACCCAACGAAGATCCCTCTTTGGGGGAAATATCGATACCGATTTGAAGACGAGGGTAATAACATTATCGGAG
AAATTAGTGAAATCTTCCAATTCCTTCACCAAGGGTACCCCTTTTCAAGAAATCTTGCCCGATTTCTCCTAAATATGGATTACG
AGCAATGTTTTTGATAAGTGAATACTTAAAAGAATAATGCGAGGGTCATGGGTCACTTAAAAGTGTAGGCCTATGTTCTTAAGG
GTTCTATGTAGGCTTATTAACATATTCGTGCAAATCCAGGGTGGTAGAGGGTGATTTACAGTCAAAATACTGAGAGAATGTACG
AATTAATAAAGTGAAATAAACTTGCGAAAATTGCCAAAAAAAAAAGGGGAATTTTTTTTGAATGTGACTGGCTCCGTTTCGAC
TCCCCCAAATAGAAGTTCATTTACCACCGAGAATCTGTCCTGGGGATAAAATATCGAACGATTTTGAGACGAGGGTAATACATT
TCGGAGTAATTAGTGAGAATCTCAAATTCACCCCAAGGGTACCCCTTTCAGAAATCTTCGTTCTCCTAATATGGATTCTGAGCA
AATGTTTTGATAGTGAATACCTAAAGAATATGCGAGGTCATGGCACGAAAAGTTGTAAGCCTATCGTCCATTAGGTTCTATGTA
ATTAAAAATTCGTGGCAATATCTAGGTGTGTCAAGGGGTGATCAAGTCAAAATACTGAGAAGATGTACGAATTATAAGGAAAAT
AAACTTGCGAAATTGCATAATAATACAGGGTGAATTTTTTTTTGAAATGTTGAGCTATAAGCTCGCGTTTTCGAGCTCCAAAT
AGATCATTTACCGGCCACGAGATCCTTGGGGGAAAATATCGAACGATTTGAAGAATGAGGGTAATACATTTCGGAGAAATTAGT
GAAATATCCAATTCCCAAGGGGTGACCCCTTTTCAGAAATCTTCGATTTCTCTATATATGATTCGACAATTGTTTGATTAGTGA
ATACATAAAGAATATGCGAGGGTCATTGGGCACAAAAAGTTGTAGACTATGTCTTAGGGTTCTATGTATTAAAAAATTCCGTAC
AAATCCTATGGTGTGATAGAGGGGTGATAAGTTCAAAAATACTGAAGAAGAGTGATATAAGGAAAAAACTGCGAAATATTGCCA
TAAAAAGGGGAATTTTTTTGAATGTATTGACTATAAGGCTCCGTTTCGGACTCAATAATTCATTTACCAACGGATCTCTTGGG
TGGAGAAATAATTGAATATTGATAGACCGAGGGTAATACATTTCGAGAAATAGAAATTCTCCAATTCACCAAAGGGTACCCTCC
TATTCAGAAATATTCGATTCTACTAACATATGTTGATTCATGCAACTTGTTTATGAATAGTGAATACTAAAGAATATGCGGAAG
GGTTCATGGCCCAACTAAAGTTGTAGGCCTATGTTTTAGGGTTCTATGTATAAGAAAATTCGTGCAAATCCGAGTTGTGTTAGA
GGGTGATACCGTCAAAATACTGAGAGATTATGGAAAATATAAGGAAAAAACTTGGCGAAAAGTCCATATCAAAGGGGAATTTTT
TTGAAATGCTTGCTTATATTAGCTCCTCGTTTCGACTCCAATAGATTGCATTTACGCCGCACGAGATCTCCCTTGGGTGGAAAA
TATCGAACGATTTGAAGACCGAGGGTATATACATTTCGGAGAAATTAGTGAAATCTCCAATTCAGCTCTAAAGGTAGCCCCTTT
CAGAAATCTTCGATTTCTCTAATATGGATTCACAACATGTTTTGATAGTGAATACTAAGAATAGTGGCGAGGGTCCATGGGCCA
CAAAAAGTTGTAGGCCCTATGTCTTGGGTTCTAATGTATAACAATTTCGTGCAACCATCCTAGTGTGTATGAGGGTGAGTACAG
TCAATAATGACTGAGAGATGTACTAATATAAGGAAAAAACTTGCGAAAGTTGCCAAAAAAAAAGGGGAATTTTTTTTGAAATAG
TTGACTTAAGGCTTCCGTCTTCCGACTCCAAATTAGATTCATTTAGCCACTGAGATTCTCTGGGAAAAAAATATCCGAACCGAA
TTGAAGACGAGGGTATTACATTTCGGAGAAATTAGTGAAAATCTCCAATTCACCCAAAGGGTACCCCTTTCAGAAATCTTTCCG

ATTTCTCTAATATCGGATTCGAGCAAAATTGTTTTTGATAGTGAATCACTAAGAAATTGCGAGGGTCATGGGCAAGCAAAAGTT
GTAGGCCTATGTTAAGGGTTCTATGTATTACTAAATTGCGTGCCAAACCGAGATGTGTAGAGGGTGATTCAGTCAAAATACTGA
GATGAATGTACGAATATATAGGAAAAACTTGCGAAAATTGCATATAAAATTAGGGAATTTTTTTTGAAATGTTTGACTTAAAG
GGCTCCGTTTCGACTCGCAATAGATTCATATTGCCCAAGCGAGATCCTCTTGGGGAAAATATCCGAACCCGATTTGAAGACGAG
GGTAATACATTTCGGAGAAATTGAGTGAAATCACCAATTCACCCAAAGGGGTCCCTTTCCAGAAAATCTCTTGATTTCTCTATA
TGGATTCGAGCAACTGTTACCGATAGTGAATACTAAAGAATATCGAGGTCATTATGGCACAAAAGTTGTAGGCCCTTATGTCTA
GGTCCTATGTATTATAAAAGTTCGTGCAAAATCGCGAGATGTGTAGAGGGTGATACAGTCAATAATAACTGAGTAATGTACAGA
TATATAGGAAAAAACTCTGGCGAAAATTGCCCCATATAAAGGGGAATTTTTTGAATGTGAAACTATAAAAGCTCCGTTTGCGA
CTCCCAATTAGATTCATTTACCTTGCACGAGTATCCTCATTGAGGGATAAATATCGCTGTAACATTTGAAAGATGAAGGGTAAT
TACATTTCGGAGAAATTAGTGAAAATCTCCAATTCAATCAGCCAAAGGGGTCCCCTTTTCAGAATCT
>m141121_232159_42134_c100732402550000001823141405141507_s1_p0/5531/4265_10239
GCCATTATTCCTGGCATATAATCTTAAAGGTTCGAGTGATCGCAAGTGCAGGAGATTGAGAGTATACGACCTATGGTTATTGGA
AAATCATATAGTACAGTAATAGCGAGCGAAAAGCTTATGCATAGCGCTTTGGAAGTAATAAGGTTCCTTTTAGTATTGTAGGTT
ATTGTGGTAGATTTAGGTTTAATATCCCTTGAGTAGAATCCAAAAACTCTAGTTCATTTGGTTTTAGATGTATTTTCTTCTCCG
ATCTTCGTACTATACATCCCCCTAATGCAATTATATTTTGGTAGGGGAATCGTATAGTCTGAGCACATTAATTTAGTAAGTTAA
CCGTTAAATGTTAGGTATTTCCAGTGTCCTTTCATTCAGCTCCTTTTCTCATTAGAAACCTCCCTGTTATTAGTATGTTTAGGG
TGGGTAGGGTAGATGTTCCACAAAAGTCAAAACGGCTGTCTCGGTCTATGATCCCAGTGAACAATTAGTGGAGTGAGAAAAGGA
TAGAATTCATATGTTAGTTATCTATTGTTATCTATAGGTGTTGAGTTGAGTGTATATTTAAAATAATGTGTGATGATTGATATA
GTTTAGTAGATTATGAGGTTGAGTAAGATCGTTGACATAAGACCCATATGATAAACAATGACTCCAAATGGGTGTAGGCATAGA
GCTTAGCATTAGAGCTAGGACTAGACTAAATGATTGTTAGGTCCTATGGGGAACCCCAAAGGAATAATGCAAATATATTTGTAT
TAAATAAGATGTTGTTAGTCTTTGATGTTGGCTTATGAAAGTTATGTTTTATGGACTATGTGATTGAGATTTATTCCTATGCAT
GTAGGCATTAGGCTTCATAGTCCTTTTTTGTTTTAGGTGTGCAGAGAAAAGAGCTTTTTTGTGATAAGGCGTATTGGTAATGCT
TTGAGGTATGGGATTATCGATGTGAATGAAGTCATGGAGCTACGAAGTTCATGTTCTCGATATTAAGTTATCACTTTGTAGTGG
TTTTTAAATCGTATTATGATCTGCACTTCTTAATGTAAATTCTTACGTCATTTTAAATCATGTTTTTGTTTATTAAAGACGATC
TTGATGTGTAAAGGTTATAAGCTCTTATCATTACAAGGTTTTTCAATAAAATTAATGTACTGTTTCCAAATGTAGGTTTTATAG
TAAGAATTATTATGAGTATATGGGTTCCGTCGTTATACGAAAAGTCTAGAGTAGAGGTCATATACACCGAGCGTTTAGGACTTT
GCTTTGATGGCGCTACTTATAAACTTGAAGGTAACCTTTTAAGAAAAAAAGAATACAGTTCAATGTGTAGGGTTAGTGTAGTAG
TAGGTGAATAAGCGTCCCATCCCTCGTAAGTAGAAGTTCTATTCGTTATTCCGATCACATTTTCGGAAAGGTAAACTTGAGGTA
TCTGAGGACTGACTGAGTTCGAACGAATGTCAAGAGCAGTATAGGCGTGATGACTAAGAGAGATAGAAGGCTGTATTAGCCGAA
AATTATTTGAAAAAACTTCAGTGGAGTATATATGCAGAGTCTGTTTAGAGTTCTAAGTGTTAAATATATATGTTAAGAAAATTT
TTAATATGTTTGATGGAGTATTTTGTTAGATTGGAGACTGTATGGGTTTTAGGCTTGGCCTGATGCCGCACTATGTTTTGCTAG
GTGTATATAAGTATGAAGAGTGAGGAGCCAGCTACGATAAGGAATATTTGAAGCCATTAAAGTTATGTTATAACGTAGGTACAA
AGCCACGCGTAATATCGTCCGGGATCATTTCCTACACCCTGATTCTTAAGTTGATGTTATGTCTTGAGGCAGTGTTTATGTTCG
GATGTTTTGAAGACTGTAAGACTTATTGGATCAACGTATGTGGATTATTTGACTGAGTAGTTATCGACAAGGCTTGGAGTATAG
ATCGTGCTGGTGTCTCGTTTATTGGTAACCTTGTGCTTGGGTACAAAGGTAAGAAACTGCACCCTAAGTGGTATAATACATCTG
GCAGTGGTTATATCTTGATGCATGGTTGGATATGTTTAATATTGAGAGCATGGTGATTTGTATTAGTGCATATCAGTGATTAAA
TGTAATCAATTGTAGAGTAGAAAACATTGAATTAAGGGGCAGTCGATATGTAATAATGAGGCGAGATAGATTCAGAGCTTGAGT
CTCATGATGTATCGATTAGCGTGAAATTATGCTAGAAATTTATTAATCCATAGCATTCGAGCTACTGTATAGTAGTATGGATAT
AAGATTACTGATCTAGGTTGTGGGTCCGTATCGGTAGTAGTAGTATAGTGGTAGTATTAGTAGTAAGTAGTAGCTGTAGTAGAG
AGTATAGTTATAAAGTGCTAGTAGTGGTAGTATGTAGTAGTGGTGTAGTAGTATAGTATGGTAAGTAGTTGGTAGTAGTTATAG
TATGGTAAGCTAAGTGGAGGTAGTGTGAGCTGTAGTATGTAGTAATTAGTATATAAGAAGTGTATAGTAACGACGTAGTAATAA
GTAGTATAGTAGGATGAGTGCCGTAGTCGGTGGTAGTGGTAGTATAGTAGTGTAGGTAGTGGTAGTAGTGCTAGTACTGCAGTG
GAGTTTAGTATAGGTATCAGGTAGTAGTGGAGGAGTGTAGAGCGTGGTCATGGCTAATGCTAGGATAGTAGTAATGTAGGTCGT
AGTGAAGGGGTAAGTAGTGGTAGTAGTAGTAGTAGTGGGTAGTTGGGTAGTATAGTATGTAGTAAGTGTAGGGTGAGTATGTGT
AGTGTAGTAAGTGGGATCTAGTGAGTAGTGGTAGTAGTGGTAGTAGTGTAGTAGTGTGGTAGTCGTTTATATATTAGTAGATAT
GTAGTGGTATAGTGGTAGTAGGTAGTAGCTAGTGGTATTTAGTAGTAGGTAGTTGTGGTAGTAGAAGTATAGTAGTGGTATAGT
GGTATATGTCAGTATATAGTCTAGTGTAGGTAGTGGTAGTAGTACAGTAGTAGAGTAGTATAGTAGAGTGTTTAGAAGTAGTAG
TGATAGTATTTTGGTGTGGTGGTAAGTTGGTAGTCTAGTAGTACTGAGTAGTATAGTAAGTGGTAGGTAAGCTGGTTAGTGACT
CGGGGTACGTTGGTAAAAGTGGTAGTTATAGTATAGTCGTACTGTTTAGTGGAGTAGTGTATAAGTGGTGTATTATAGTAGGC
TGGTAGTTTGTAGAGTAGTAAGGTAGTATGTATAGTGGTCAGTAAGTGGTATGGGAGCTAAAGTCGGTGTAGTCGGTGGTATGG
TAAGTAGTGTATTAGTGTAATCCGGCCTATAGTAGTAGTAGCTACGCTAGAGTGGTAGTAGTGGTAGTTGGTAGTAGAGTAGCT
AGTGGTAGTATGTATAGTCGGTAGTCGGGGTAGCCTGGTAGATAGTAGTAGTAGTAGTAAGTAGTAGTCGTATTAAAATAGCAG
TAAGCACGCAGTCAGTAAGTACGGTCAGTGGTAGTAGTAGCTGAACTAGTAGTGGTGGTAGTGGTGGTAGTGTGTAGTATTGTT
AAGTATGGTTAGTATAAGTAGTGGTACTGGTAGTAGTAGTGGTGTAGTGGTAGAGGGTAGTGTTAGTTAGTGTCGTATGCTAGT
TACTGGATAGTTGGTAGCGGATAGTAGTAGTAGTAGTAGAGTGGAGTATGGATAGTGTTGGCGTAGTAGTAGTAGTATAGGAGC
ATAGCAGTAGCAGTGCATAGTAGGCGGCATGTGGTAGTGGTGGTAGTAGTGGTATATGCAGTAGTAATTAGTAGTATAAGTTAG
TAGTGTAGTAGTGGAGTGGTAGATAGTAGATGGTAGTAGTGGTAGTAGGTATGTGTAGTGTCGTAGTGGTAGTAGTGTCTAGTG
GAGTAATGTAGTAGTGTGGTAGTCGTGTGTAGTACTGTAGTAGAATAGTAGTAGTAAACTATAGTGGTATGTGGTAGTAAGGTA

TCCATAATAAGCGGAGTAGTGGGTAGTAGTTTAGGGTATGTTGTGGTAGTAGGTAGTAAGTGAGTAGTTAGAAATAGATAGTAA
GCAGTAGCAGCAGAGGTAGTAGCGGCATTTGGTAGTGGTGGAGGTAAAGTGTAGACGTGTAGAGTGTAAGTGGGGTAGTGTGGT
ATGGTATTGGGTAAGAGTGGGTACTAGTTTAAAGTAGGTCAGTACGGGTATAGTGGAGTAGTGGTAGTGGTAGTCGCTAAAGTA
TTATAGTTAGTAAGGTAGTTTTGTAAGTGGTAGGTGGTGGTATGGTAGTTAGGGTAACTGAAGTAAGTATAGAGTAGTAGAGTG
GTCAGTGTAGGGTACGTGTGTAGGCTTGGGTCGTCTCGTACGTAGGAGTAGTAGTAGGAGTAGCTGTATGTGGTAGTATGGTTA
GTCCTGGAAGTGGTTACCGTTAGCCTAGAGTACGTGTAAGCAGGCTCCTTAGTGGTGAGTAGTTGGAAGTATGCTGGTATTCCC
AGTGAGTAAGTAGGTGTCAGTAGTGTAAGTAGTAGTAGTAGGTGTAGTAGGTCGTGGTAGTAGGGTAGTAGTACGTAGGTAGCT
AAGTAGGAGTGGAGTATGGTTGATAAATGTACGTTGGAGTAGTCTGTAGGGGATAGTGGATAAAGTAGTGGTAGCTAGTGGTGT
AGCGTTGTAGGATAGAGTAAAGTAGTATTAAGTGGTAGTAGTGGTTATAGTGGAATGTGGTAATAGTATAATAAGTCGTGTACG
TGGTAGTGTAAGTATATAGTAAGACGAGTAGTAGTATATAGTAGTGATGTAACAGTAGTATAGTAGTAGTTATGGTATACGTGG
TATGTGGGCCCTACTGGTGCGTAGTCGGTAGTGCGCTATAGTAACGTAGCGTAAGTGGTAGTAGTGGTAGTAGTGGTAGTGGTA
AGTGTATGTAGATAGTAGTCAAGTGATAGTAAGTAGTAGTGGTAAGTGGTAGTATAGTGATATAGTTAGTAGAAAGTATGTAGT
AAGCTGGTAGTATGGAGTGTAGTGGTAGTGGTACGCTAAAAGTAGTAATAGTAGTAGGTATAGTAGTGGTAGTAGGTGGTAGAC
TGGTAGTCGTGGTAGGGTAGTAGTATAGCTATAGAGGTCTAGTGGTAGTATGTAGAGTAGCTGGTAGTCAGTAGTACCGTATGT
ACCTGGGTAGTAGTAGTAGTAGTAGTAGTAGTTAGTAGTGTTATATGGTATAAGTGGGTTGTGGTAGTATGGTCGTAGGTGGCTATG
GTAGGTAGTAGTGTATAGTGGTATATATGTATGTGTGGTGGTAGTGGTGGTTCAGGTAGTGGTCAGTTGGTAATGTAGTAGTAA
AGTAAATATGGAGTGTATCCGTAGTCACGTAAGTAGTAGTAGTAGTAGTTTGTAAGTGGTAGTATTGGTATCCTGAAGGTGTAGTGG
TAGTTAGTTTGGGTCGTATGGATTTGTGGTAGTAGTGGTTAATGGTAGTGATACGTAAGTAGTGTAAGTGTAGTAGTGGTAGTA
TAGGGTTATAAAAAAAAAAAAAGTACGTAGTGGAGTATGGTAGTGGTAGTATGGTAGTATAGAGAAGTAGTAGTAGTAGCATAC
CAGCAGTAGTAGTAGCGGCAGTGTAGTAGTGTGTAGTAAGTGGTATAGTGGTAGTAGTGTAGTAGTGGTATAGTATTAGTAGTA
GTGAGGTAGTATGGTAGTGGTTTGTAGTAAGTAGTAGTGGTAGTCAGTGGTATAGTGGTACGCTCCCGGTAGTAGTGGTCGTTA
GTGGTAGTAGTGGTTCGATAGAGGTAGTAGTGGTCATAGTCTTAGTAGTGTGTAGTCGTTGTAGTATATAGGAGTAGTAGTAGT
AGTTAGTGGTAGTAGTCGAGTAGTAGTAGTATGGTAGTAGTGGTAGTAGTATAGTATAGTAGTGGTAGTAAGAAAAGTAGATAT
GGTAGTAGTGGGTAGTAGCAGTGCAGCACGTAAGTAGCCGGCAGTGGTAGTGGTAGTAAGTGGTAGGTAGTTATAGTAGTGGGC
TAGTAGTGGT
>m141121_232159_42134_c100732402550000001823141405141507_s1_p0/5811/3836_14090
GCCACAATGTATTACCCCTCGGGCTCGCTCAACTCTCGTTCCGACCGTTATTCTTTCCCCCGCAAGAGAATCTCATGGGTAAAA
TGAAATACTAATTGGAGTCGAAACGCGCGGCCTTAAGTTCAACATTTCAAAAAAAATTCCTCTTTTTTGATGGCGAATTTTCGC
AAGTTTTTTTTCTTTATATTAGTAGTACGTCCTTTCTATAATTTTGGACTCTATCACCCTCTACACACCTCGGATTTGCACGTA
AATTTTATAACATAGTACACCTAAAGTACATAGGGCTACAACTTTTGTGCCCAATGACTCGCATATTCTTTATTATTCACGATC
AACAACAATGCTCCAATCCATATTTCGAGAAATCGAAGATTTCTGGAAAAGGGTACCCTTTGGTGAATTGGAGATTTCACATAA
TTTCTCGAAAATGTATTAACCCTCGTGTTCAACCTCGTTCGATATTTTTCCCCAAGAGAATCTCATGGGTAAATGAATATTATT
GGAAGTCGAAACGGAGCCTTGAAGTCAACTTTCCAAAAAAAAATTCCTTCTTTTTGATGGCAATTTCGCAAGTTTTTTCCTATA
TATTAGTGTCCTTCTATATTTTCGACTGTATTCACACCGTCTACACACCTCACCGGATTTGCAACGTACTTTTATACATAGTAC
CCTAAGACATAGGCTACAACTTTTGTGCCCATGACCTCCTCGCATATTCTTTAAGTATTACAGTCAAAAGAACAATGCTCCAAT
CCATATTAGAGAAATCGAAGATTCTGAAAGGGGTACCCCTTTTGGTGAATTTGGAATTTCACTAATTTCCTCCGAAATGTATTT
ACCCTCGGTGTTCAACTCGTTCGATATTTTTTCCCCAAGAGATCTCCATGGGTAAATGAATATATTGGAGTCGAAACGGAGCTT
AATCAACATTTAAAAAAAAAAATTCCCCTTTTTGATGGCAATCTTTCGCAGTTTTTTTCCTTATATTAGGTACGTCCTTCTATA
TTTTGACTCTATCACCCTCTATCACACCTCCGGATTTTCACGTAATCTTATACATAGTACCCTAAGACATAGGCTACAACTTTT
GTGCCCATGACCCTCGCATATTCTTTATTATTCACGAATCAAAACATATGCTCATCCATATTAAGAAATCGAGATTTCTGAAAG
GGGTACCCTTTGGTGAATTGGAGATTTTCACTAATTTCTCCGAAATGTATTACCCTCGGTGTTCAACCGTTCGATATTTTTCCC
AAGAGATCTCATGGGTAAATGATATATTGGAGTCCGAACGGAGCCTTAATCAACATTTCAAAAAAAATTCCCCTTTTTTGATGG
CAATTTTCGCAAAGTTTTGGTCCTTATCATTGTACATCTCTCCAGTGATTTGACTGTATCACCCCTACATCACCTCGATTTGC
ACGTACTTTTATACATAGTACCCTAAGACATAGGGCTACAACTTTTTTGCCCATGACCCTCGCATAATTCTTTAGGTATTCACT
GATCAAACATGCTCCAATCCATAATTAGAAGAAATAGAAGATTTCTGAGGGGTCCTTTGGTTGAATTGGAGATTTCACTAATTT
CTCAGAAATGTATTAACTCAGTGTTCAAACTCGTTCGATTTTTTCCCCAAGAGATCTTCATGGGTAAATGAATATATTGGAGTC
GAAACGGAGCTTAAGTCAAATTTAAAAAAAAATTCCCTTTTTGTATGGCATTTTCGCAAGTATTTTCCTTATATTTAGTCGTCC
TTCTATATTTGACTCTAATCACACTCTACCACTCTCGGATTTCACGTAATTTTATAAATAGTAGTACCCTAAGACATAGGGCTA
CAACTTTTGTGCCCATGACCCTCGCATATTCTTTTATTATTCACGATCAAACAATGCCCAATCCATTATAACGAAATCGAGATT
TCTGAAAGGGGTACCCCTTTGGTGAATTGGAGATTCACTAATCTTCTATCCGAAATGTAATTACCCTCATGTTTCAACTCGTTC
GATATTTTTCCCCAAGGATCTCATGGGTAATGAATAATTCCCGGAGTCGAAACGGAGCCTTAAGTCAACCATTTCAATAAAAAA
TTCCCTTTTTGGATGGCAATTTTCGCAAGTTTTTTCCTTATATCTCCGTACATACTCTCAGTATTTTTGAACTGTATCACCTCT
ACACACCTCGGATTTTCACGTAATTTTATACATAGTACCAAGACATAGGCTACAACTTTTGTGCTCCATTGATACTCGCCATAT
TCTTTATTATTCACGATAAAAACAATTGCTCTCAATCCATATTAGAGAAAATCGAAAGATTCTGAAAGGGGTAACCCTTTGTGA
ATTGGAGATTTCACTAATTTCTCCCGAAATGTACTTACCTCGTGTTCAACTCGTTCGATATTTTTCCCAAGGATCTCCATGGTA
ATATGAATATATTGGAGTCCGAAACAGGAGCTTAAGTCAACATTTCAAAAAAAATTCCTCTTTTTGATGGCAATTTTTTGCAAG
TTACTTTTCCTTATATTAGTACGTCTTCTATATTTGACTCTATAACCCTCTACACCGACGCCCTCGGCCTCTTTCGCCACCTA
ATTCTCTCCATACATAGCTCCACACTCAAGACATAGGGCTACAACTTTTGTGCCATGACCCTCGCATATTCTTTAGTATTCACA

GATCAAAACAATTGCTCCAATCATATTACCGAGAAATCGAAGATTTCTGAAGGGGTACCCTTTGGTGAATTGGAGATTTCACTA
ATTTCTCCGAAATGTATTACCCCTCGTCGTTCATCGTTCGACATAATTTTTACCCAAGAGATCTCATGGGTAACAATATATTGA
AGTCGAAAAGGCCTTAAGTAACATTTAAAAAAAATTCATCTTTTTGATGGCAATTTTTTGCAAGTTTTTTTCCTTATATTAGTA
AGTCCTTCTATATTTTATTGTTATCACCCTCTACACACCTCCGGATTTGCACGTACTTTTAACATAGTACCCTAAGAATAGGGC
TACTAACTTTTTGTGCCCCATGACCCTCCGCAATATTCTTTAGTATTTCACGATCACCAACAATGCTCCAATCCATATTACAGA
GAAATCCCGAAGATTTCTGAAAAGGGGTAACCCCTTTTGGTGAATGTGGAGATTATCACTAATTTCTCCGAAATGTATTACCCT
CGTGTTCAACTCGTTTCGATAATTTTCCAAGAGATCTCAATGGGTAATGATATATTCGGATTCGAAACGGAGCCTTAAGTCAAC
ATTTAAAAAAAAATTCCCTTTTTGATGGCAATTTTCGCAGTTTTTTCCTTATATTAGTAGTCCTTTCTATATTTTTTGACTCC
TATCACTCTTAACACACCTCGGATTTTCCACGTAATTTTATACATAGTACCCTAAGACATAGGGCTACAACTTTTGTTGCCATG
ACCCCTCGCATAATTCCTTATTACGATCAAGCAACAATGCTCCAATCCATATTAGAGAAATCGAAGATTTCATGAAAGGGGTAC
CTTTGGTGAATTAGGAGGATTTCACTAATTTTCTCCGAAATGTATTACCTCGTGTTAATCGTTCGATATTTTTCCCCAAGAGAT
CTCATGGGTAAATGAATATAATTGGATCGAAACGGAGCTTAAGTCAACATTTCAAAAAAATTACTCTTTTTGTGGCAATTTTCG
CAAGTTTTTTTCATTATATTAGTACGTCCTTATATATTTTGACTCTATCACCCTCTACACACCTCGATTCTGCACGTAATTTTA
TAAACATAGTACCCTAAGAATAGGGCTACACACTTTTGTGCCCATGACCTCGCATATTCTTTATGTGATTCACGATCAAAATAA
TGCTCCAATCCATATTAGAGAAATCGAAGATTTCTGAAAGGGGTACCCTTTGTGAATTGGAGATTTCCTAATTCTCCGAATGTA
TTACCCTCGTGTTCAAGCTCGTTTCGGCCCCGCTACTCTTTCCCACAGAGATCCGTCCATCCGGTCACACATGCCACATATACT
CCTGGAGCTCGAAAACGCGGGCCTTAAGTCAACATTCCTCAGCACACAGAAACAACTTCTCTTATTCTTGATGGAATCGTTTCG
CCAAGTTTTGCTTCCCTATCACCACCCTCCCCTCAGCTACGTCCTTCCCATATATCGCTCTGACTCTATCCCCGCCGCTCCGAA
CACCACCATTCCGGATGTCTGCCGTCAATTCGGTTAGTCCACGTCACCGGCCACTACCCTCAAGGAAGCTAGGGCCTTACCACA
TTTCTTGTGCCCACGATGACCTCCCGCGCACTATGACTTCTCTTACTTACTCCACCCCGATCAGGAAACCACTGCCCTCCAATC
CATATGACCAGACCACTCGACACGACGTTCCCGTTTCTGGAAACGCCGGGCTACCCCTTCTTACGCATGGAATTGCCGAGCATT
CTCACTAAATTCTCCCGAAATGTATTACACTCGTGCTTCAACTCGTGTCGATATCTTTTCCCCAAAGAGATCTCATGGGTAAAT
ATATATTGGCAGTCGAAACGGAGACTTAACATCAACATTTTCAAAAAAATTCCTCTTTTTGATGGCAATTTCGCAAGTTTTTT
CCTTATATTAGTACGCTTCTATATTTTGACTCTATCACCCTCCCTACACAACCTCGGATTTGCACGTAATTTTATACATAGTAC
CTAAAGACATAGGGCTCAACTTTTGGTGCCCATTTGACCCTACGCATATTCTTTCTTATCACAGATCAATAATGCTCCAATCCA
TATTAGCAGAAATCGAAGATTTCTGAAAGGGGGTACCCCTTTGGTGAAGTGGAAGATTTACTAAATTTTCTTCCGAAATGTAAT
TACCCTCAGTGTTCAACTCGTTCGATAATTTTTCCCCAAGAGATTCATGGTAATATGAATATATTCGGGTCGAAACGGCCTTAA
GTCAACATTTCAAAAAAAATTCCTCTTTGATGGCATTTTTCCGCAAGTTTTTTCTTATATTAGTACGTCCTTCTATATTTGTGA
CTTATCACCCTCTACACACCTCGATTTGCACGTACATTTATACATAGTACCCTAAGACATAGGGCTACAACTTTTGTGCCCCAT
GAACCCTCGCATATTCTTTATTATTCACGAAGTGAGAGAACAAACAATGCTCCAATCATATTAGAGAAATCGAGATTTCTAAAA
GGGGTACCCCTTTGTGAATTGGAGAATTTCACTAATTTCTCACCGCGAAATCGTCACCTTCCCCCTCCGTGTGCCTCAACTCGT
CGCCCACTACCTCTCTCTTCCACAGAGCTCCTCCATCGCGTAACGCACCAACCCAGACTCCCTCATTGCCAGCCGCGACCGCGT
CGAAACCGGACGCGCGCTCTCTGTACACAACAAGCTCAACATTTCACAAAAACCTCGTCCTCTCTTCTCTGATGGCACAATTTC
TCGCAAGTTTCTTTCCTTATGTTAGTCACGCCTCCTTCATCCATAGTTCTTGACTTCCTATCCCCACCCCTCTCACACGCCTCG
GAATTTGCACACGTAATTTTATACATGTACCCTAAGACAGTAGGGCTACAAATTTTGTGCCCATCGACCCTCCGCATATTCTTT
AGTATTCACGATCAAAACAATGCTCCACACTCCATATGGATCAAACTCCGCAACCATTTCTGAAACGCCCGGTACCCTCTTGG
TGACCATTGGAGACTTTCCACCAATTCTCCTCTTCTGACCAACGCTAGCTGCTCCACCTCCCGCCTGTCTCACTCGTTCGATAT
TTCCCAAGAGATTTTTTTTTTTCTCATGGGTAAATGATATATTGGAGTCGAAACGGAGCCTTAAGTCAACATTTAAAAAAATTC
CCCTTTTTGATGGCAATCTTTCGCCAAGTTTTTCCCTTATATTAGTACGTCTTCCTATATTTTGACTCTATCACCTCTACACC
ACCTCGGATTTTCACCGTAATTTTATACATAGTACACCAAGACATAGGGCTACAACTTTTTGTGCCCATGACCCTCGCATATTC
TTTAATTTTCACGATCAAAACAATGTCCAATCCATATTAGATGATAATCGAAGATTTCTGAAAGGGGTAACCCTTTGGTGAAAT
TGGACGATTTCACTAATTTCTCCCGAAATGTATTACGGCCTCCGTGGTTCAACTCGTTTCGACTATTTTTCCAAGAGATCTCAT
GGGTAGAATGAATATTATTGGAGGTCGAAACGGTAGCCTTAAGTCAACATTTCAAAAAAATCCCTTTTTTGATGGCAATGTTCG
CAAGTTTTTTCCTTATATTTAGTACGTCTTTCTATATTATTGCCTGTATACACCCTCTAACACACCTCGGTTTTACGTACTTTT
TATACTAGTAACCTAAGCATAGGGCTACAACTTTTGTGCCCATGATCCTCGCATATTCTTTAGTATTCACGATCAAAACAATGT
CCAAATCATATTAGACAATCGAAGATTTCTGAAAGGGGTACCCCTTTGGTGATTGGAGACTTCTCACCGTAATGGTTCTCTCGC
CGGCCAATGCGTATCTCAGCCGGACGCTCGCGCCGTGTTCAACCGCTCTCTCCCGTCCCGACCCGCGCACCCGCCCACCCTCAC
GCCGCTTTTTCCCAGCAGCCGCACCCTCTCATGGGTCTGCCAATACGTACCTTGCGAGTCGAAACGGACACGCCTATTAAGCTC
AACATTCACTCACAAATCCCTTTCTTTCCTTGATGGGCGCACATGTTTTCGCCCACCTTCTCTTTCCTTATGACCCCTTCCGAG
GGATACGTCCCTCTTGCGCGCCCCTTCCGACATTGCCTCTGGCGACTGCTCACTCCTCACCCCACCCCCTCTACACCACCACCC
ATCCGGATTTCTGCACGTGACTCCACCTACCGCTACACTACGACGACTAGGGGCCTCGACAACTTTTCTGCTGCGCCCAATCGC
GACCGCTCCGCCAGCATGATTCGCTTTCAGGTATCCACCTCACCGATCCCGAGCACTCTCCGCTCACCGCAACACGCTCGCTCC
AATCCATATTCCCCAACGAAGGAACTCGCACGAACCTTCTCTCGCCAAGCCGCTACCCTTCTGCGTCGAACGTCTGGAGCTCTT
CCTCAATTCTCCGACAATGCAATCCTAACGACCGCCTTCGGCGCCTTTCTTCCCACCACACCTCGTTCCGAGTATTCTTCTTCC
CCAAGAGATCTCCATGGGTAAGCCCAGTGACCGTCACCTATTGGCGTCGCAAACGGCGCCTTAAGTCCATATCTCAAAACACAC
GTTCTCTTTCCCTGATCGCGCAAGTTTTCGCAAGCTATCTTTTTCCCTTATACGACCGCCATTCAGTCGACGCTCCCTCTCACC
CTACCTACCCCCCTCTCTGCCATGACTCTATCCACCGCCTCCCTACCCACCCGACCTCCGCGACGTTCCGTGCACGCGTATTTT
CATACAGTAGCTCACCCGCTAAGCACCAATAGCGGCTACAACCTTTCCCTGTGTGCCCATCGACCCTCTCGGCATAGATTCAGC

TTTAGCATCTAGCTTCCACGACCTCCCCCACCGAAAACTGCTCCAATCCTCATATTACGGAGCACAGCCAACTCGACAGATTTC
CTCAAAGGGGTACCCTATTGGTGCAATTGGAGATTTCACCTACTTTCTACCCGAAATGCATATTACCCTCGGCTGTTCAACCTT
CCGTCTCGAGTATCTTTTTCCCGCCTAAGAACCCGTCCGTCTGGTCACCCAGCATGAATATATTGGAGTCGAAACGGAGCCTTA
AGTCAACATTTTCAAAAACAATCCCCCTTTTTGAATGGCAATTTTAAGCAAGTTTTTCCTTATATTAGTAGTTCCCTTCTCATA
TTTTGACTGTACTCCCGGCGCCCGACGCCTCCCCGCCGCCTCAGCCCGCCACAGCGGCCCGAGCCTCGCTCTTCGCGTCACTGC
CTTCCTCCGGCTTACGTACGCGACCCACCTCAAGCGCCTCGCGGGCTCCAGAGCCAACTTCTTGTTCGACTCGCCCATGACCCG
CTGCGCATATTCTTTAGTATTCACGATCAAAACAATGCTCAATCCACTATTAGAGAAATCGAAGATTTCTGAAAGGGGTACCTC
CTTCTCTGATTGGAGTTTCACTAAATTTCTCCGAAATGTATTACCTCGTGTTCAACTCGTTCGACTATTTTTCCCCAAAGAGAT
CTCATGGGTAAATGAATAATATTGGAGTCGAACCGGAGCTTAAGCCCCCAAACATTTCAAAAAAAATTCCTCTTTTTGATGGAA
TTTTCGCAGTTATTCCTTATATTCATACTGTCCTTCTATATTTGACTCTATCACCATCTACCACCTCGGATTTGCACAGTAAT
TTTTTATACATAAGTACCTAAGAACATAGGCCACAACCTTTTGTGGCCATGACCCCCCATATTATTTATTATCTTCACGATTCA
AAACAATCGCTCCAAATCCAATATTAGAGAAATCGAAGGATTTCTGAAAGGGGTACCCCTTTGGTGAATTGGGATTTCACTAAT
TTCTCCGAAATGTATTACCCTCGTGTTCAACTCGTTCGATATTTTTTCCCCAAGAGATCTCAATGGGTAAATGAATATATTGG
AGTCGAACGGAGCCTTAGTCATCATTTTCAAAAAAAAATCACCCTTTTTGGATGGCAATTTTCCAAGTTTTTCATTATAGTTAT
AGTCTTCTATATTTTTGACTCTATCACCCTCTACCACTCGATTTTGCACGTATTTTTATACATAGTAACCCTAAGACATAGGGC
TACCAATTTCTGTGGCCACTGACCCTCGCATAATTCTTTATTATCACGATCAAAAACAATGCTCCAATCCATATTAGAAAAATA
AGAAAGATTTCTGAAAGGGGTACCCTTTGGGTGAATGGAGATTTCACTAAATTTCTCCAGAAATTATCACTCGGTGTTACCCGT
TCGATATTTTCCCCAAGAGACTCTCATGGGTAAATGATCATATTGGGAGTCAAACGGAGCCTTAAGTCAACATTTCAAAAAAAT
CCCCCTTTTTGATGGCAATTTTCGCAAGTTTTTCTTATATTATACGTCCTTCTATATTTGACTCTAGTCACCTCTTACACAC
CTCGGATTTGACGTATTTTATACATAGTACTCCTAAGCATAGGGCTACAAACTTTTGTGCCATGACCCTCGCAATATTTTTATT
AATTCACGATCAAAACAATGGCTCCAATCCCATATTAGAGGAAATCGAAGATTTCTGAAAGGGGTACCCCTTTGGTGAATTGGA
AGATTTCACTAATTTCTCCGAAATGTATACCCATAGGTGTTAAAACTCGTTCGATATTTTTTCCCCAAGAGATCTTCATGGTAA
ATGAATATATTGGAGTCGAACCGGGCCTTAAGTCACATTTCAAAAAAATCCCTTTTTTTGATGGCAATTTTTCGCAAGTTTTAT
TTTCCTTATATTAGTACGTCTTCTATATTTTGACTGTATCCCTCTACACAACATCGGATTTTAACGTCTTATTGATAATAGTAC
CTAAGACATAGGGCTACAACTTTTGTGCCCATGGACCCTCAGCATTTTTTAGTATTCACGATCAAAACAATGCCTCCAATCCAT
ATTAGAGAAATGAAGATTTCTGAAAGGGGTACCCCTTTGGTGAATTGGGGATTTCACTAATTCTCCGAAATGTATTACACTCGT
GTTCAACTCGTTCGATATTTTTCCCCAAGAGATCCTCATGGGTCAAATAATATATTGGAGTCGAAAACGGAGCCCTTAAGTCAC
ATTTCAAAAAAAATCCCCCTATTTTGATGCAATTTTCGCAAGTTTTTTCCTTAATATTAGTACGTCCTTCTATATTTTGACTCT
ATCATCACCCTCTACACACTCGGATTTGCAACGTAATTTTATACATAGTACCCTAAGACATAGGGCTACAACTTTTGTGCCCAT
GACACTCCGCATAATTCTTTATTATTCAACGATCAAAACAATGCTCCAATCCATATTAGAAAATCGAAGATTTCTCTGAAAGGG
GACTTTTGTGATTGGAGATTTCACTAAATTACTACGAAATCGTATTACCCTCGTGTTCAACTCGTTCGATATTTTTCCCCAAGA
GATCTCATGGGTAAAATGAATATAAGGAGTCGAAACGAGCCTTAAGTCAAACATTTCAAAAAAAATCCCCCTTTTTGATGGCAA
ATTTTC
>m141121_232159_42134_c100732402550000001823141405141507_s1_p0/8659/1581_7400
CCTTGATGTTAATATAGTGAACTATCTCAGATGAATCGTAAGAAATCAAATCTTGGGAAGAATAAAAAGCGCGAGGTTGAGATG
GTCGCATACCTTCAACAAAGAAAAGGAATCTATTAGCCCATTCGACAGAGTACACTCATCATAGCAGAGAGATTTCGCATGCGA
ATGAGTCGCCAGATTTTATATTCCCATGACGTCTGGTTGAATTTAGGACCTTATTCATAGGTCAAAAATAACCTTATATAATGA
TTTCATTGAGATGGAAAAGGTTCAAAAAAATTAGAGCTTGTTAGAGGACGAAATATGAGTGAATTACGAGATCGCTTCGGAGAC
CTAGGGGCTGCCTAGCGAGATTTGGGCAGGAAAGGTCCAATTTTTTTTTGTTTGGATTTGAATTAGGGTACCCCACATTCATGG
TCCCCCAATTAATATACTATATGAATTATTTGAGATTGGATAAATGGTTCAAAAAATAGAGGCTTGTTAGACGGAGAAATGAAT
GAGTGATCATGTAGGAGATCAGCTTCGGAGCCTAAGGCAGCTGCCTAAGGAGATTTGGCAAAGGAAAGGCCATTTTTTTTGTTT
GGTTGAATTCGGACCCCCAATTCATGGTCTATAATATACACTATATTCATATTAATTGAGATGGGAAAAGGTTGCAAAAAATTA
GAGCCTTTGTTAGAGAGAAATATGAGCTGACGATTACGAGATCGCTTTCGGAGCCCTAAGGCAATTGCCTAGAGATTTGGCAGG
AGAAGGCCATTTGTTTTTGTTTGGCTTGAATTAGGACCACAAATTCATGTGGCAAATAGTATACCTAAATGACAATATTGAGAT
GAGAGAAAGGTTCAAAAAAATTAGAGGTTGTAAGACAAAATAATTGAGTGAATTACGAGATCGCTTCGGAGCCTAGGGCACGCG
TCCTAGAGATTGTGGTCAGCGAAAAGGCCTATTTTTTTTTTGTTTGGGTTGAATTAGGACCCTTATTCAATGGTTAAAATATAC
CTAAATATCAATATATTATTTGAGATGGGAATAGGTTCCAAAAAATTAGAGCTTGTTTTAGAGAGAATATGAGTGAATTACGAG
ATCGCTTGTCGAGACGCTAAGGCTGCCTAGGAGGATTTGGCAAGAAAGGCCAATTTGTTTGGTTTGGTTGATATTGAACGGACC
CCAATTCAATGGTAATAATAAAACTCTAATATGATATTATTTGAGATGGAGAATAGGTTCAAAAAAATTAGATCTTGTTAGAGA
GAATACTGAGCTGAATTACGAGATCGCTCTCGCAGCCCTAGGCAGGCTGCCTAACCGAGATTTTGGCCAGGAAGGCCAATTCTT
CTTGTTTGGGTTGGATTAGGACCCCAATTCATGGGTCAATAATATACTATATGCTATTTAGCTTTGAGATGGGACAAATTGTTC
AAAAAAATTAGAGCTTGTTAACGAGAAAGTATGAGTGTAATTTACGAGATCCGCTTCGGAGCCTAGGCTGCTGCCTTAGAGATT
GGCAGAAGGGCCAATTTTTTGTTTGGATTGAATTAGGACCTTATTTACTGTCAAAAATATACCTATATAAATATTACTTGAGTA
TGGGAAATGTTCAAAAATATTATGAGCTTGTAATAGAGGTGATATGACGTGGAATTGACGAGATCGCTTCGAGACTAGGCGGCC
GTAGGAGATCTTGGCAGCGAAAGGACAATTTTCTTTTGTTTGGCCGCTTTGAAATTAGGAACCCCAATTTCATGGTCAATAATA
TACCCTATATGATGATTATTGAGATTGGGAAATGGTTCAAAAAAGATTCAGAGCTTTGTTTAGAGAGAAATATGAAGTGAACAT
TACGAGATCGCTTCGGAGCCCCTAGGCAGCCTGCCTAGGAGATTTGGCAGGGAAAGGCAAATTTTCTTGTTTGGTTGGATTAGG
ACCCCAATTCATGGTCAATTAATTACCTATTATGAAAATTATTTAGATGGGAAATGGTTCAAAAAAATAGTCTTATTTAGAGAG

AAATATGGAGTGAATTACGAGATCTGCCATTTGGAGCCCTAAAGGGCAGCTGCCTAATAGATTTGGCATGAAGCAATTTTTTTG
TTTGGTTGATTAAGGACCCCCTATTCATGGTCAAATAATAGTACCTATATGTATATTTATTTAGATGGGAAATGGTTCAAAAAA
ATTAGAGCATTTTTTAGAGTGGAAATAATATGAAATTACGAGATCCGTTTCGAGCCCTAAGGCAGCGGCCTAAGGGAGTTGGCA
GGAAAGGCCAATTCTTTTTGTTTGGTTTGCAATCTTAGGACACCCAATTCATGGCCAATAGTGATACTATAATGACGACTATTG
AGAATGAGAAAAGGTTCCAAAAAAATTAGAGTTGTTAGAGAAAATAATGAGTGAATTACGAGATCGCTTCGGAGCCCTATGACG
TCCGAGGAGATTGTGGCAGGAAGGCAAGTTTTTTTGTTTGGTTGAATTAGGACCTTTATTTTATGGTCAACAAATATACCTATA
TATATATGTTATTGAAGATGGGAAATGGTTCAAAAAATATTTAGAAGTTTGTTAGAGACGAATATGAGTGTATATTCGAGATCA
CTTCGGAGGGACCCTAGGCTGCCTACGGACGATTTGGCAGGAAAGGCGCAATTTTTTGTTTGTTTGAATTAGGAACCCAATTCA
TGGTCAATAATAATACCTATATGGATATTCAAGTTGGAGATGGGAAATGGTTCAAAAAAGTTAGAGCTTGTTTAGAGAAGAAGA
TATGAGTTGAATACCGAGATCGCTTCGAGCCCCTAGGCTGCTGCCTAGGAGAATTTGGACAGGAAAGCAATTTTTTGTTTTGGT
TGGAATTAGGACCCTTATTTAGGTCAAAAATTACTATATAATATTATTCCGAGATGGGAAATTGGTTAAAAATAATTAAGCTTG
TTAGAGTGAAATATGAGTGTATTACGAGATCCGCTTCGAAGACGCTAGGCTGCCTAAGGAGATTTGGCAGGGAAAGGCCAATTT
TTTTTATTTTGGTTGAATTAGGAACCCAATTCATGGTCATAAATACCTATATGGATATTCCATGGTTGGGAAATGGTTTCAATA
AAGAAATTAGAGCTTGTTAGAGAGAAATGATGTAAGTGAGTTAGCGAGTCGCTTCGGAGCCTAGGCCAGCTGCCTAGGAGAATT
TGGCAGGAAAGGCCAATTTTCTTGTTTGTTTGGATTTAGGACCCCAATTCATGGTCCAATAATATACCATATTATGATTATTA
TTGGTGAGATGGGAAAAATGGTTCAAGAAAATTAGTGCTTTTATAGAGAGAAAATATGAGTGGAGATTACGAGATCTTGAGCTT
CGGATAGCCCTAGGCAGCTGCCCTAATAGCATTTTGGCCACTGAAAGGGCCATTTTTTTTGTTTGGTGAAGTTAGGACCCATAT
TCAATTGGTGCAATAATATGCCTATATGATATTATTGAGAATGGGATAATGGTCAATGAAAAATTAAGAGCTTTTATTAGAGTG
TAATATGAGTGAGACTTACGGATCGATTCGGAGCCTAGGCAGCTGCCTAGGAGATGTTGGCGCAGGAAAGGCCATTATTTTTGT
TTTGGTTGAATTAGGACCCCATTCATGGCAATAGTATATCCTAAATGACACTATTGAATGAGTGAAGAAAAGGTTCAAAAAATC
TAGAGGTTTTAGAGAGCAAATATGAGTTGAAGTTACGAGATGCCGCTTAGGAAGCCCTATCCCACCGCGTGCCTAAGGAGATTT
TGGTCCTAGAAAGGCCAATTTTTTTGTTTGGTTGAATTAGGATCCTTATTTACTGGTCAAAAATATACCTATAATAAATATTAT
TGAGATGGCTGAAAGTGGGTTCAAAAAATAGTTAGAGTTTGTTAGAGAGAAATATGATGTGAAATTACGAGATCACTTCGAGAC
CTAGGCTGCCTTACGGGAGATTTTGGCAGGAAATGCCAATTGTTTTTGTTTTGAATTAGGTACCTCCAAATTCTGGGTCAATGT
AATATACCTGATATGATATATGAGAATGGAATGGTTCAAAAAAAATTAGAGCTTGTTAGAGAGAAATAGAAGTGAATTACGAGA
ATCGCTTCGGAGCCCTAGGCAGCGTGCCTAGGAGCATTTGGCAGGAAAGGCCATTTTCTTGTTTGGTTTGGATTAGGACCCGCA
GATTCATGGTCAATAATATAACTATATGATATAATCTGATGACGGAAATGGTTCAAAAAATAGAGGCTTTTTAGACGAGAATAT
GAGTGAATTCAGAGATCGCTAGGGAGCCCTAGGCAGCGCCTAATAGCCATTGTGCATGAAAGGCCAATTTTTTTGTTTGGTTGA
ATTAGGACACCTATTCAATGGTCCAATAATATCACCCTATATGATAATTATGAGATGCGGGTAATGGTTCAAAAAAAGTTAGAG
CTTTTTAGAGTGATAATGAAATGAGTGAATTACGAAATCGCTTCGGAGCCCTAGGCAGCATGCCCTGGAGATGTTGGCAGGAAA
AAGGCCAATTTGTTTTGGTTTGGATAGAATTAGGACCCCAATTCATGGTCAATTTAATGATACCTATTATGGATATTATTGAGA
TCGGGAAATGGTTCAAAAAATTAGAGCTTGTGCTAGTGAGAAATGATAGTGAAATTACGAGATCGGCTTCGGAGACCCTTGGCA
GCTGCCTAGGGTATTAGGCAGGAAAGGCAATTTTTTGTTTGGTTGAATTCGGACCCCAATTCATGGCAAATAATATACCTATAT
GATATTACTTGAGATGGGAAATAGGTTGCAAAAAAATTAGAGCTTGTTAGAGAGAAATAATGAGTTGAATTAACGAGATCGCCT
TCGGAGCCCTTAAGGCAAATTGCCTAGGAGATTTGGCAGAGAAAGGCCAATTTTTTTGTTAGGTTGAATTAGGACGCCCAAAAA
TTCAATGGCCAAATAATACGTACTAAATGGACACTATTGAGATGAGACAAAGGTTCAAAAACAATTAAGGTTGTTAGAGACAAA
TATGAGTGAAGTTACGAGATCAGGCTTCGGAGCCCTATTGCACGCGTCCTAACGGCACGATTTTGGCTAGGAAAGCAATTTTTA
TTGTGTTGGTTTGAATTAGACCCTTATTTACGGTCTAAAATACACCTAGTATAAATATTATTGAGATGGGAAATGGTTCAAAAA
TTAGAGTTGTTAAGAGAGAAAATATGAGTGACTTACGAGATCGCTTCCGGAAGACCTAGGCTGGCCTAGGAGATTTGGCAGGAA
GGCCAATTTTATTTTTGTTTGGTTGAATTAGGACGCCCAATAGTGCATGGTCAATAATATACCTATAATGATATATTGAGATGG
GAAATGGTTCAAAAAATTAGAGCTTGTTAGAGAGAAATATGAGTGAATTACGAGATCGCTTCGGAGCCCTAGGCAGCTGCCGTA
GGAGATTGGCAGGAAAGGCCCAATTTTCTTGTTTGGTTGGATTAGACCCCTATTCATGCGTCAAATTAAGTATACCTATATCGC
ATATTTATTCGAGATGGGAAATGGTTCAAAAAAAATTAGAGCTTTTTTTAGAGTGAAGAATATGGCAGTAATTACGAGATCGCT
TCGGAGCCCTGATGCCAGCCGTCCTATGGAGGATTTGGCAGTAAAGGCCAATTTTGTTTGTTTGGTTTGTAATTAAGGACCCTT
ATTTATGGCTAAAATAACCTATTAATATTTATTGAGATGGGAATGGTTCAAATAAATTAGAGCCTTGTTTTAGAGAGAAATATG
AGTGAATTACGAGATCGCTTCCA
>m141121_232159_42134_c100732402550000001823141405141507_s1_p0/10893/3398_9324
TGCACGTGAGAACCATACCCCAGGAGGGTGGCACCGTTCCATTATCGCTATTCTCAATTTCAAAACTGGTCCGAAAATGAATAA
GATGCGGAACAAAACATATCCGGGTGCGATCATACAGCACCTACATAACACCGATCACCCTTTTCTTAAACTGGGCTTATCAGA
GACCACCGCAGTCTCACGCGTTCTTGAGCCGACGAGTTGTACTAGGATGGGGTGACCTCTCTGGGAAGTGGGCATCTCGTGTCT
GCATCCTGTTTTGACTGAAAACACTCATTTTTTTTTTAATTCTTTTAAAAATCCACATTACGGCTCCAAAAGTTTGTAGCTTTT
TAAAGAGAGGAGATCTCTCACCAACCTTCATCATTTTAATGAATCTTCGTTTTCCGTTCTGGGGGGGTTAATGACCGTGGCTCA
CTGACGAGCAGCGAATAAGAAGTTCCTAGTTATCTATCCGAGGGAGAAAAGGGAGGCGCCTAACCCCGGACTGCGTTTCGCTAT
ATGAGAGACATGTGCGGACCAAACATTCCGGGTGGCGAACCCCATTGCACCAGTGCACGTTGGTGGAATGCCAATCGAGATACA
GACCAATCCAGACACTTCCTGCGAGCGTGTAGCAACGCGATATGCTGCCACGGCGAGTTAGGAGTCTACTGAGGGATTGTCGGT
GGTGACCTCCCCTGGGAGTCCTCGTGTTTGCAGCCCCTGAAAAACATCATTTTTTATTATTTAACTCTTTAAAAATCCACTTTT
ACGGCTAAATAGTTTGTGGGATTTCTTTTGAAAAAAATCTTTAAAAGCTCATTTATGGGGATTCGTTTTCCTTGGGGGGTTAAC
ACCGTGGCCACTGACAAGCAGCGAATCAATCGAAGGTTCCCTAGTTCCAAATCGAGGGGCGACAGAAAGGGAGGGCCACTGATG

CGTGTCGCGTATAGAATAAATGATCCATTGCGGAAACACTGGTCAGGACATTCCCGCGAAGAGATGCAGTCTATATACCGCCTA
GCGACCTCAAAATGCAGCTCGGAATCCATAACAAGAACTCCGTAGTTAAGCGTGCTTTGGGCGAGAGTAGTACTAGGATTGGGT
GACCTCCTGGGAAGTCCTCCCGTGTTGCCACCCCTAAACAATCATTTTTTTTTATTCTGTTAAAAATCACCACTTACGGCTCC
AAAAGTTTGTATTGACTTTTTAAAAAATTCTTTAAAGCTCATTTATTTCGTTTTCGCTTGGGGGGTTAATACCTGGTGGCCCA
CTGACAAAGACAGCGAATAAGAGTTCCTAGTTAATCCGAGGACGAAAAGGGAGGGCACCGCGTTCGCTATAAAGATAATGCGGA
ACAAACATTCCGGTGCGATCCATACAGCACTAATGCACCGGTTCCCATCAGAGACTCCGCAGTTAAGCGTGCATGGGCGAGAGT
AGTACTAGGATAGGGTGACCTCCTGGGAAGTCCTCGTGTTGCACCCCTGAAAACATCATTTTTTTTTTTAAAATTTTATTTAAA
AATCGCACCTTACGGCTCAAAGGTTTGTATTTTTTTAAAAAAATCTTTAAAACCTCATTTATTATCGTTTTCCTTGGGGGGGT
TAACACCGTGGCCACTGAAAACGCAGCGAATAAGAAGTTCCTAGTGAATCCGAAGGGAGAAAAGGGAGGGCACGGCGTTCGCTA
TACATGAATAATGCGGAACAAACATTCCCGGGTCGCGATCATACCAGCACTAATGCACCGGATCCCATCAGAATCCCGCAGTTA
AGCGTGCTTTCGGCTAGAGTAAGTACTAGGATTGGGTGACCTCCCTGGGAAGTCCCTCGTGTTGCACCCTGAAAACATCATTTT
TTTTTTATTCTTCTAAAATCCACTTACGCCTCAAAAGTTTGTATTTTTTTAAAAAAATCTTTAAAAGCTCATTTTATTCGTTTT
CTTGGGGGGTAATAACCGTTGGCCACCTGACAGAGCAGCGAATAGAAGTTTCCTAGTTAATCCGAGGAGAAAAGGGAAGGGCAC
GCGTTCGCTATAAATGAATAATGCGGAACACAATTCCGGGTGCCGATCATACAGCACCTAAGCACCGGATCCCATTCAGAACTC
GCAGTTAAGCGTGGCTTGGGCGAGAGTAGTAACTAGGATGGGTGACATCACTGGGAAGTCCTCCGTGTTTGCACCCCTGAAAAC
ATTCCATTTTTTTTTATTCTTTAAAAAATCAACTTACGGCTCCAAAATTTGTATTTTTTAAAAAAAATCTTTAAAAGCTCAATT
TATTTCGTTTTCCTTGGGGGGTTAACACCGTCGGACCACTGACAAGCAGCGAATGAAGAAGTTCCTAGTGAATCGAGGGAGAAA
TGGAGGGCACGCGTTCGCTATAAATGAATAATGCGGAACAAGACATTCGCGGGTGCGATCATCACCAGCAATAATCACCGGTTC
CCATCAGAACTCCCGCAGTTAAGCGTGCATGGGCCGAGGAGTAGTACGTAGGATGGTGACCTTCTCCTGGGAAGTCCTCGTGTG
TGCCACCCCTGAAAACATCATTTTTTTTTTATTCTTTAAAATCCCACTTACGCTCAAAAAGTTTGTATTTTTTAAAAAAAACCC
CTTTCTTAAAAGCTCCAATTTAATTTCGTTTTCCTTGGGGGGTTAACACCGTGGCCACTGACCAAGCAGCGAATAAGAAGTTCC
TAGTGGAATCCCGAGGGAGAAAAGGGGGGCACGCTTCGCTAATAAATGAATAATGGCGGAACAACATTCCCGTGGTGCGATCAT
ACAACAGCACTAAATGGCACCGGATCCCATCAGAAACTCCCGCAGTTAAGCGTGCTTCGGCTAGAGAGTACTAGGATGGGTGAC
CTCCTGGGAAAGTCCTCGTGGTTGCACCCTTGAAAACATCATTTTTTTTTTATTCTTTAAAATCCACTACGGCTCCAAAGTTTG
TATTTTTTTAAAAAAAATCCTTTAAAAGCTCATTTAATTATCGTTTCCTTGGGGGGTTAATACCGTGGCCACTGACAGCAGCGA
ATAAGAAGTTCCTAGTTAATCCGAGGGAGAAAAGGGAAGGGCACCGCGTTCGCATATAAAAAATGAAATAAATGCGGAACAAAC
ATTCCGGGGGCGATCATACCAGCACCTAATGCACCGGAATCCCATCAGAACTCCGCAGTTAGCCGTGCTTGGGCGAGAGTAGGT
ACTAGGATGGGTGACATCCTGGGAAGCTCCTCGTGTTTGCACCCCTGAAAACATCATTTTTTTTTATTCCTTTAAAAATCAACA
ACTTCACGGCTCAAAAGTTTGTAAATTTTTTTAAAAAAAAATTCCTTTAAAGCTCATTTATTTCGTTTTCCTTGGGGGGTTAAC
ACCGTGGCCCTGACAAGCCAGCGAATAAGAAGTTCCTAGTGAATCCGAGGGAGAAAAATGGAGGGCAACGGTTCGCTATAAATG
AATAATGCGAAACAAACCATTCCGGGTGCTAATCATACCAGTCACTAATGCACCGGAATCCCATCAGAACTTCCCTACAGTTAG
CGTGCTTGGGCTGAGGGTAGTACTAAGGATGGGTGACCTCCCAGGGAAGTCCTCGTGTTGCACCCCCTGAAAACATCATTTTTT
TTTATTCTTTCAAAATCCCACTTACGGCTTCAAAAGTTTGCTATTTTGTTTAAAAAAATCTTTAAAAGCTCATTTATTTCGTTT
TCCTTGGGGGGGTTAATACCGTTGGCCACTGACAAGCAGCGAATCAAGAAGTTCCTGTGAATCCCCGAGGAGAAAAGGAGGGCA
CGCGTTCGCTATAAATGAATAATGCGGAAACAAACATTCCGGTGCGATCATACCCAGCACCTAATGCACAACGGATCCGCATCA
AGAACTCTGCAAGTTTAAGCGTGCTTGGGCGAGGGTAGTACTAGGATGGGTGACCTCCAGGGAAGTCCTCCGTGTTGCACCCCC
TGAAAAACATCCATTTTTTTTTATTCTTTAAAAAAATCACTTAGCGGCGTTCAAAAGTTTGTATCTTTTTTAAAAAAAAATCCTT
TAAAAGCTCATTTATTTCGTTTTCCTTGGGGTGTTAATACCGTGGCCACACTGACAAGCAGCGAATAAGAAGTTTCCTAGTTAA
TCCGCAGGGAGCAAAAGCAGGGCACGCGTTCGTATAAATGAATATTGCGGAACCAAACATTTCGGGTGAGATCTATACCAGCAC
TAATGCAGATCCCATCAGAACTCCGCAGTTAAGCGCGCTGGCGAGGAGTAGTACTAGGAAATGGTGACCTCTGCTGGGAAGCCT
CGTGTTGCACCTGAAAACATCCATTTTTTTTTTTTTCTTTAAAAAAATCCACTTACGGCGAAAAAGTTTGTATTTTTTAAAGAAA
AATCTAAAAGCTCAATTTTATTTCGTTTTCGCTTGGGGGTTAATACCGTGGAAACACTGACAAGCAGCGAATAAGAAAGTTCCT
AGTTTAATCGAGGAGAAAAGGGAGGGCACGCGTTCGCTATAAATGAATACAGCGGAACAATACATTTCCGGGTGCGGATCATAC
CAGCATAATGCCACAACGGATCCCCATCCAGAACTCCGCCAGTTAAGCGTGGCTTGGGCCGAGAAGTAGAACTAGGATGGGTGA
CCTCTTGGGAAAGTCCTCGTGTTGCACCCCTGAAAACTCATTCTTTTTTTATTCTTTAAAAATCCACTTACGGCCCTCAAAAGT
TGTATCTTTTTTAAAAAAAAATCTTTAAAGCCTCATTTATTTCGTTTTCCTTGGGGGGTTAATACCGTGGCCACCTGACAGCAGA
ACCGAATAGAAGTTCCTAGTTACATCCGAGGGATGAAAAGGGAGGGCTACCGTTCCTATAAAGGAATATTGCGGACCCAAACAT
TCCGGGTGCGATCATACCAGCCTAAATGCACCGGATCCCATCAGAACTCCGCAGTTAAGCGTGCTTCGGCGAGAGTAGTACTAG
GATGGGGTGACCTTCCTGGGAAGTCTCGTGTTGCACCCCCTGAAAACATCATTTTTTTTTAACCTCTTTAAAAATCCACTTACG
GTCAACAAGTTTTGTGATTTTTTTTAAAAAAATTCTTTAAACGCTCATTTATTTCGTTTTCCTTGGGGGGTTAACCACCGTGGC
TACTGACAAGCACGCGAATAAGAAGGTTCCTAGTTAACCGAGCGGGAGAAAAGGGAGGGCAGGGCGTTCGCTCATAAAATGAAT
AATGCGGAACAAACATTCCGGGTGCGATCATACCGCAGCACTAATGCACCGGATCCCCATCAGATATCCCGCAAGTTAACGTGC
TTGGGCGAGAGTAAGTACCTAGGATGGGTGACCTCGCCTGGGAAGTCCTCGTGTTGCACCCCCTCGAAAACATCATTTTTTTTT
TATATCTTTAAAAACCACTTACGGCTCAAAAGTTTGTATTTTTTTAAAAAATTCGTTTAAAGCTCATTTATTTCGTTTTCCTTG
GGGGGTTAATACCGTGGCCACTGAACACAGCGATAAAGAATGTTCCCTAAGTTAAATCCGAGGAGAAAAGGGAGGGCAACGCGT
TCGCGTATAAACTGAATGAATGCGGTAACAAACATTCCGGGTGCGATCATACCAGCACTAATGCACCGGTTCCCATCAGAACTC
CCGCAGTTAAGCGTGCTTGGGCGAGAGTAGTACTAGGATGGGTGCCTCCCTGGGAAGTCCTCCGTGTTGCACCCTGAAAACATC
ATTTTTTTTTATTCTTTAAAAATCCACTTAACGGCTCAAAAGTTTGTGATTTTTTTAAAAAAAAATCTTTAAAGCTCATTTTTT

ATCGTTTTCCCTTGGGGGGGTTAACACCGTGGCACTGACAAGCTAGCGAATAAGAAGTTTCCTAGTGAATCGAGGGAGAAAAGG
GAGGGCACCGCGTTCGCTATAAATAAAAAAAATGCGGAACAAACAT
>m141121_232159_42134_c100732402550000001823141405141507_s1_p0/14387/6265_11792
TCCTCGCTAGCGTAACTTAGCGTGCTGTTGCCTACGGGACCGCTCCCGAAGCGATGCAACTATCACAAATTGTAGTAATCTAAT
CATCTATCATATTGTCTTCTCCTACAAGCTTCTAATTTTTTGGTTTTAGAGTACCTTTTCATCTCATCTAGATAATTCTTCC
AAAAATTAAAGCTAAGAAAGAGGAAAACCTTTAATCGCTGTTATTGACCTATATGAAATTGGGGTTCCCTCAACTAACCAAGCC
ATTTGAACAAAAAAGGTTAGAACTTTCAGACAAGCCCACCAATCTTTCCTAGGCAGGCTGCCTATGTAGTGCTCCGAATGCTTG
ATTCGTAATTCACTCTCCATATTCTCCTCTATTAAAATTTGTGCTATAATTTTAATTTTGAACCCCTTTTCCCATCCCTCCAA
TAATATATAGAGCTTTAAGGGCTGCTGTATTTTTGAACCATTACAGAATTGGGGTTTCCAATAGTATCTTCTTAACACCCCAAT
AAACATAAAATTGCACAATTCAGGGCCAAATTCTCCTTTAGGGAGAAACATCCAGAGAAGCAAGATCAACGTAATTCATACTCA
TATTTTCCTTTCTAAGCAAGCTTTTAATATTTGATACCCTTTTCGCCCATCCAAATCAATAATCCATTATACTCTATGGACAGG
AGTCTCTATAAATTCCATGTAATTTGAGGGGTCTACTTTCAAACCAAAACAAAAAAAAATGACCTTCTTCAGCCAATATCTTTC
CAAGCGCAAGCATGCATAAGGGCTCCCGAATGCGATTTCGTAATTCTACTCAATATTTATACATCCTAAAAAAGTCTTAGTTTA
TTTATTTTAGACCCTTTTCATTTCTCAGATAATTATTAATATATGACGGTGCTGTTTATCTTGACCGGAGTATAACTTAGAATT
GGGTTGATCCTAAATATTCAATCTTAAACACAAAAAAATAAACACCTTACAGTTGCCAAATATCCTGGAGAGCCTGCCTAAAAA
TAAGAGCTTTCCGAGACAGCTGACTCTCTCGTATTCACTTCCATATTTCTCATCCTAACTAAGTACCACTTTTTTTGAATCCTT
TTTCCCATCTCATATAACCTTATTTGTTTATAGGGCAATTATTTGACCATAAATTGAGAATATTGGGTCCTAATTCCCAATAAA
AACAAAAAAATTAGACCTTTCCAAGGCTAAAATCGTTCCGTAAGCTGAGCTGGGCCAATTTATTCGTTGAGCTCCAGAAGCCCG
ATCATCGTTAGTAAATTCATCATCATATTCTCTCTAACAAGCTCTAATTTTTTTTGATCCCTTTTCAAACATCCTCCAATAATA
TTATCATAAGGCTGTTAGTTGACGTGAATTGGGTCCTAATTCAACAACCCCACAAAAAAAATTGAACCTTTCAGGCAAACAATC
TCCTAGGCCGCTGCCTAGGGCTCCCGATGCGATTTCGTAATTCACTCATATTTGTCTCTCTAAAAAAGCTCAATTTTTTTGAAA
CTTTTCCCATCTCAATATATTATATTAGGCCCTGTTATTGACCATGGAATTGGGGTCCTAAATTCAACGCAAACAAAAAAATTG
ACCTTATCAGCAAAAATCTCCTAGGCCAGCTTGCCTAGGGCCTCCGAAGTGATCTCGTATTCACTCTATATTCTCTCTCTAACA
AGCCTTCTAATTTGTTTTGATCTTTTCCGATCCACAATTAAGTATTATAATAGGGGCCTATTATTGACCATGAATTGGGGTCGC
TAATTCAAGCCAAACAAAAAAATTGACCTGTTCAGGGCCAAATCTCCCCTAGGCAGGCTGCCTAGGCTCCGAAGCCGATTCGTA
ATTCACTCAATATTTCTCTCTAAAAACTCTAAATTTTTTGGACCTTTTCCCATCTCCAATATTCTCTAATATTATCATAGGGCT
GTTATTGACCATGAATTGGGGTCCTAATTCAACTCCCCAAACCAAAAAAATTGACAACTTTCAGGCCCCAAAAAAATCTCCTAA
GGCAGCTTGCCCAACGGCTCCCCGAAACGATCTCGTAATTCCAGCTTCAATATTTCTCTCCCTAACAAGCTCCTAATTTTTTTG
ATCCTTTTCCATCTCATAATATTACTATAGGGCTGTTATTGACCATGAATTGGGGTCCTAATTCAACCCAAAAAAAAATTGACC
TTTCAGGCCAAATCTCCTAAGGCAGGCTGCCTAGGGCTCCGAAGCGGATTTCGTAATTCACTCATATCTTCTCTCTAAAAAGGG
CTCTAATTTTTTTGACCCCTTTTTCCCATCTCCAATAATATTATATAGGCTGTTATTGATCCCAATGAATGTGGGAGTCCCTA
ATTCAACCCAAGACAAAAAATGACCCTTTCAGGCCAAAAATCTCCCTAGGCCAGGGCGCCTAAGGGCTTCGAAGCGATTTCGTA
ATTCACTCCATATTTCTTCTCTAACAAGCCTCCCAATTTTTTTGAAACCTTTTCCCCATCTCAATAATTATTGTATAGGGCACT
TATTGACCATGAAATTGGGGGGTCCATAATTCCAACCAAACAACCAAAAAATTGACCTTTCAGGCCAAATCTCCTAGGCAGCCT
GCCTAGGGCTCCGAAGCGATTTCGTAATTCACTCATATTTCTCTCTAACAGAACGCTGCTAACTTTTTTTGAATCCTTTTTCC
CATCTCAATATATTATATAAGGGCTGTTATTGACCATCGATTGGGTCCTAATTCAACCCAAACAAAAAATTGACGCTTTCAGGC
CAAATCTGCCTAAAGGCAGCTGCTTGGGCTCCGCGATGCGATTTCGTAATTCACCTCATATTTCTCTCTAAAAAGCTCTAATTC
TTTTTGAACCCTTTCCGCATCTCCAATAATATTAATCATAGGCTGTGTTTTGACAATGAATTAGGGGTCGCTAATTCAAACCAA
ACAAAAAAATTGGACCCTTTCAGCCAAATCTCCTAGGCCCAGCCCCTGCCTGAGAGCTCCGAAAAGCGATTTCGTAAATTCACA
TCCTATTTCTCCTCCTGAAAAGTCTAAATTTTTTGGACCCCTTTTCCCCATCTCAATAATCCCGATTATCAAATGCACTACGGG
CCCTGTTATTGACGCATGATTGGGGTCCTAATTCAACCAAACAAAAAAATTGACCTTTCAGGCCACAATCCTCCTAGGCAGCTG
CCCAAGGCTCCGAAACCCCGATCTCCGTAATTCACTCATATTTTCTCTCTAAAGCAAGCTCCTAATTTTTTTGGATCCTTTCCA
TCTCAATTAATATTGTGTCATACAGGGCTGTTATTGACCAATGATTGGGTCCTAAATTCAACGCAAAAAAAAAAAAAAATTGACC
TTTCAGGTACAAAATCTCCTAGGAGACTGCCTAGGGCTACGAAGCGATTGTCGCTAATTCACTCATATTTTCCTCCCTCGCGCT
AAAAGCTCTAGTTTTTTTTAACCGCCTTTTTCCGCATCTCCAATAATATTATACTACGGGCTGTTATTGACCCATGAATTGGGG
TCCTAATTCAACAACTCACAAAAAATTGACCTTTCAGGCCCAAAAAATCTCCTAAGGGCAGCTGCCTAGGAGGCTTCGTAAGCG
ATTTCGTAATTCACTCCACTTAATTTCTCCCTCCTAACAAGGGCTCCAATTTTTTGAACCTTTTCCCATCTCAATAATAGTTGG
TATAGGGCAACTTATTGACCATGAATTGGGGTCGCTAATTCAAACAACATACAACAAATATTGACGCTTTCAGGCCAGAAATCT
CCCTAGGCTGCTGCCTACGAGGCTCCGAAGCGATCCTCGTAAAATTCACTCATATTCTCTCCTAACAAGCTCCTAATTTTTTTG
CGATCCCTTCTCTTTCCCATCTCAATAATATTAATATAGGCTGTTATTGACCGTGAGATTGGGGGGGTCCTACATTCAAAGCCA
AACAAAAAAAAATTGACCTTTCAGGCCCAAAATCTCCCTGGCAGTGCCCTAGGGCTCCGATGCGATTTCAGTAATTCACCCATAT
TTCTCTCTAAAAAGCTGCTAATTTTTTGACCTTTCCCCATCTCAATAATATGATATAGGGCCTGTTATTGACCAATTGGATTGG
GGTCCGTAATTCAACCAAACAAAAAAAATTGACCTTTCAGGGCCCCAAATCTCCGCTAGGCAGCGCTGCCCTAGGGCTCCGAAGT
GATCTCGTAATTCACCTCATATTTCTCTCCCTAAACAAGCTCTAAATTTTTTTGATCCTTTCCCATCCCAATAAATAGTGATAT
AGAGGGCTATTATTGACAATAGCCGAATCTGGGGTTCCTAATTCAACCAGACAAAAAATTGACCTTTTCAGGCCCAAATCTCCC
TAGGCAGCTGCCTAGGTCCGAAGTGATCTCGAATTCAGCCTCCCATCATTTCTCTCCTAAGCAAGCCTCTAATTTTTTTGATTC
CTTTTCCCAGTATCCACAATAATATTATATAGCCTATTATTGACATGAATTGGGGTCCCTAATTCAAACCAACACAAAAAATTG
ACCTTTCAGGCCAAAACTCCTCCAGGCAGCTGCCTAGGGCTCGCGAAGGCGATTTCGTAATCTCAGCTCCCATATTTCTCTCTA

AAAAGCCTAAATTTTTGGAACCGCTTTTCCCATCCCTCAATATATTCATATACGTGGGGCTGTTATTGCACCATCGAATTGGGT
CCTAAGTTGCAACCAACAAAAAAAATTGACCCTTTCAAGGCCAAATCCTCCTAGGCCAAAGCTGCCAAGGGCTCTTCCGAACGAT
CTCGTAATTCACCTCATATTTCTCTCTAAAAAGCTCCTAGATTTTTTTGATCCTTTTCCCATGCTCGAAAATAATATGTCCGGA
CGCCATATAGGCTGTTTTTGACATGAATTGGTTCCTACATTCAACCAAAAAAAAAATTGACCTTTCAGGCCAAGATCTCCTAAGG
CACGCTGGCCTAGGGCTCGAAGCGCGATTTCGTAATTCACTCCATGATTTCTCACTAGAGTAAAAAAAGCTCTATTTTCTTTGA
ACCTTTTCCATCTCAATAATATGCTATATACAGGGCTGTTATTGACCATGAATTGGGGGTCCCTAATTCAAACCAAACAACAAT
TGACCTATCTCAGGGCCAAATTCTCCCTTAAGGCGCAGCCTGCTTTGGGCTCCGAATGCGATTTCCGTACTTCACGATCATATC
TCTCTAAAAATGCTCCTAATTTTTTTGAACGCTTTTCCCATCCTCCCAATAATATTATATAAGGCTCGTTTTATTGACAATGAA
TTGGGGTCTTAATTCACGCAAACAAGAAAATTGACCTTTCAGGCAAAATCTCCATAAGCGCAAGCTGCCCTAGGGCTCCGAAGC
GATTTCGTAATTCACGATCCATATTTGTCTCCTCCTGACAAAGCTTAAATTTTTGGACCTTTTCCCATCCCTCAATGAAATCAT
TCATATTAGGCCTGGTTATGTGCGCATGAATTGGGCGTCCTAAATTCAACCCAAACAAAAAATTGAC
>m141121_232159_42134_c100732402550000001823141405141507_s1_p0/16464/7530_12898
CTTTCTCGTTCTCATTGGATATCCCTAGAATGATTTTTTGGGAAATACTTCAGAGAATTTTCCATTACTATCAGTACATCATTC
GCTTGCAGCTTTTTTTCATTCTCCTTTTACCACGATACTCTGTAAGGTATTTCAATGCATCCATGATCAATCATCGCTTCTCGC
CTTCAGTGCTGAGATCATTAGCCATTTCTTCTCTCGGGGCTGCCTTTGAATTCAAGGATGCTCACCACCGGAGTGAAATATCG
ACCTTTCTAAACTTGCAATTTATAATGTCCACGCATTTTGGTCAACCAATCCATATAGAAATATGAATCCTATATAGTATAGTA
ATATCCAAATCTAACAGAGTCGAGTTATCAGTTCGATCTTCTACCCGAGAACAGACATAGGGTAATAAGCTCTAACCCATGACC
GACAACCAGCCATGACATCCTTTTTTGCATACAACTTGTTTCTTGTTTTGTAGAGGAACCATGTAGAGAGAATACTGATGAGAA
AATAATACCTTGACTCAAATAGCTTATTAATATAACAATTAGGTACTCCAAACATATAACACATAACATCGAAATAACAGCTAG
AACAAATAATCAACACTTCTACGTACTACTGCTACGACCAGGTGCAATAATTTAATGACCACTACCAGTCAAATGTCACTATAC
TGTTTAAAGTCACAAGCGACCCACTTAGTCATTTACCTACAACAGAACCGACTGTTCCACATACCACAATTATACGCCTGAAGT
ATCGATTAACTAAGCATCTAGTTAGCCAAACAGACCACATAGCCATTCTTCAATAACACTTATCTATGTCCTAATTTATCAATA
CTAGCGACTATTCTAGCACTACTAACCTACATAGAACTCCATATAACTTAAGACAAGATTGTTCATTTAACAAAGACTACAAAC
ACTCTAACTAATACCACTTTAGCTTTCAGTCTATGTCTCACATCTTACATCTCATACGACTGATAAACTATCTGTAACTATAAC
TACTACTACTACTACTACATCTAACTACTACACCATCTTCAGCTCACTAGTCACTCTACACAGTAGAGATTATTCACTTAACGA
TATATCCTATATTACCTCCTCAGCCTCACGTACTTTCCATACACATAGTCCTCATTTAGGGTACCTTCTTCCCGGAATCCTATT
CCATCTCAGCAATTCACTTGCATCACACCACCTCACACGTGATCTCACCTGTCTCGACGCCTAACCCGGCTGCTGTTGCAGTCT
CCTCGGCTGCCTTCCTCTACGATCACAACCCGGCCACTTGAGCTGCCCTCCTCTCTATGGTCTCGCCTACTCCGATATGGGTTT
GATGACCCACTGCACCCCTAATTTGAAGTGTAGCCCTGGTTTGACGCAAGTTTTGCTATATGTACATAGGTGCCCGGCATCCAC
TATTCCTAATACCCAAATATACATCAACCCTCTAACTTAAATACAGCTCAGTAAAAACATTCGAAGTAACGAATGGCTAGTCCT
AAACTGTCTAAACGCTAGTACCATCGACTCTAGTACACGGCCTGTCAACTACCACCTGCAGCTGGTTAATCTTACCCTAGAAGC
TAGGGCGTACTAATCGAGTCAGAATAGTTTTCTGCTCTACAATAACTAGACTGACGACTAAAAAGGAACTTAATACAATGACTA
CTAAAACTCACTATGTCGAATAGAGCGATACAACGTGTTTTAGCTCTTTGTTGGAGAGGTAATCTAAATACCTGGGCGTAGACC
TAACTCACTATATAACAATTACGCGTTACCTCTCCATACATACACCTACTACCCTACAATTTGATAAACTATATACAGAAGTAA
TAACCACAACTATAAACCAACTAAAATAAATTTAAAAAGTAATATTTACAGACTTGTTCGTATACAGAATCCTCTATATACATA
GACCGGACATATACACTGCAGGGTTTCTAGTTAATCAGACTATATAAAGTAATATACCACAATACCAAGACTGCAATAACTGAT
CACCCACTTGGAACGACCCCAATAAATAATAACACTAAATCAATCTCTATAGAATACTTTAACACATAGTCTTATATACCCTAT
ATAAAACAATGACCCTCATTACCAACAAATACAGCGCTAACCACAAAGACCTATATCTTCAGCCCGTAATATAGATAGATAGCA
ACCACTCTAACACGCTACCATTAAACTTCAACATTAGCCTACAACCAAAACTACCTACTATTTTACACTATTAGTAAAACCTTG
ACATTATATACCACACAAGAGATACCAAATTCTCAAAACTACTCATAGGACCCCATAGTACCATTTCAAGAAGCACATAGACTA
CCTACTCACCTCTTCACTTTGATCAGACTACTATTAAGCTTACTACTTCATATCTAGCCTTACTACCACAACATGCAATATAAG
TAAGCGCAAAACTTTAGGTAAATCAATCTAGCAATACTAGTAAGGAACAACAATAGGAATCAATGACCTAGAAATTTTCCTCCA
CAACATATAAAATCCAAAGATAAATCTAGGCCTTAATCTAAATAATCAAGAACACCTAATTTTTCCCGGTGATATCACAGTACC
CTACCAGACTGAAATCAGCACAACCAAAATCCTGGTAGACTCCACTACCAGTACGCAGAACTACCCCATACCACGTATGGCTAA
TCCACAATTATCTTAACCATACTAACCACTAACCACTTACACCTACTTCAAACATACTAACGTACGAACACTACACTAATTCTA
CCACTAATACTACCTCTTAACTACTACCCTACTACTACCAACTATAACATACGCCAATACTACTACTGACTATTACTTACCACT
ACTCAACTACTACTTATACTTACATACTACTTCCACTCACTACCACTAACCACTACCACTACCGATTACCATTAATACTACCAA
ATACCGACTCACCAGTACGAACTACCCAGACACGTACCACAACTACCACAACTACCACCCCTTCCACCAATACCACTATCCACC
ACTTCCACTAACTACACAACCACTACAACCTACTAAATAACTACCAGCTACTACAACTACTACACAACAAACACTACTCCATAC
TACACTATACTTCTCCTTTTCTACGACTGCTACGACTACTTACGATCAACTTGCCGACTATCAACTTACGACTACTGCTACGAA
TTTACATACCACATATTACCACTTCTACCACTACCACTACCCAGTATAACAATGAACTCTACGATTACGACTACTAATACAACC
ACTACTACCACCACTACAACCACCACCACTCCCACCACAACCACCCACTACCAATCACTACCACCATTTACCACTCATTACCAA
CTACTACCACTACAACTACCCTTACCAACCAACTACTGACCAACTACCACCACTACCACCCACTACGTACTACCAACTTCTACT
ACCAACTACAAGCTAACAACTACTTCTACCACTACTACTCACCACTACTACCCAGCCACTACTACTACTACCCGACGACCACCT
ACCATAACCACTACCCAACGACCAAGACAAAACTTAACCGTTACACTATTCACGTTATCGAAAAGACCTCACCCTGACCACCAC
CCACTACAGGCGAGGAAAGAAAGACATTACGCTACACTATACTTGCTACTTCTACTACTACTATACATACAACAACATACTACA
TACCCACCACATAGCACTCCTTCAGCACCTTCCACCCACTTACACTAATGACCACTAACCTACCCACTAAATACACAACTAATC
TATTACTACAAACTACTACAACTGCACTACTACACTACTACTACTACTACAACTACTTACACTTCCACTACACTACCACATACC

ACTACCACTACCACTACCACACACCACTACCACTACAACTACCTCTACATTACCACCACTACCTACAACTACCACCACTATAAC
CACTACCACTACCCACCTCTAAACCACTACTACTACCAACTACTAAACCATTTACTAAATACCACCTGACCATACTTCACGATC
ACTACTACGCCACCGAACCACTTGCCACTACTAAGCAATTCATAACTTAACCAATACATAATTACTTATACTACTAACTAACTA
ACATACTAACTGCTAACTAGCTACTACCACATAACCACTAACAAACAAAACCACACCATCTTACTAAACCATAACTACCACAAC
CACCACTAACTACTAAAAACTACACATAACACAGTACTTCATAACACAATAATAACACAAACCACTACTATCAACCTACTACAT
AACTGCCGCAACACAACAAACATAGAACACATACACATCGACCACGACCCTACCGCTACCCACTATCCACGACCAAGAGCCGAC
CACTACCCATACCACTACCCTACCACTACTACTAACTAATACTACTACTACATACTACCATACTACTAACCTAACAACATACCA
CTACTAATACCACCATCCTACTACGTACCATACATACTACATATACAATCTACTAACTACATACTAATACTACTACTTCTACTA
CCTACTCCACTATACTAACTCATATTACATACTACATACCACTATTTACTAATTCTACTACTACTACATCTGAACTACAGGCTA
CCCACAAGACCCTGACACGACCCCAACCGGACCACCGGACCACCCTGCCAAATAAAAGTACCACTACTACCACACTACTATTAC
TAACATACTACTACTACTATACTACTACTACTATACTAACTACTATGCTACATACATACTTCTGATACTACTACTACTACTACT
AATAACTACTACTAGGAATATTACACTACTACCACCTTCCACCTCACCACTTACCACACTACCACTTGCCACTACCACTACCCA
CTACCATACCACTAACCCTGCACCTTCCACCATAACATTACACTGACCACCACCACTTACCACTACCACTACCGTTTCCACCAC
CACTACTGACTACTACATACTACGTACTTACACTATAATATATAACTACTACCACTACTACTCAGCCACTATTACGATTATACT
ACTCTACTACACTACTATTGCTGCATATATATTACATACACATACCACACTATTACCCACGACCACTTACAACGACAACCATGC
GCACTAATACTACTACGAATCACTTGCACGGCCACCTACCACGACCACTACCACGACCACGTACCCACGACCACCGACCACCTA
CCGAACATACCACCTACAATCTACACTAAAACTACACCACTACCACACTACGACAAATACCACTACTAACACTTAATCTATCAA
TACCTACTACTACCACCACAACCACTAACAACACAACCACTACCACTCACCTACATAGCCCACTACCAACACCACT
>m141121_232159_42134_c100732402550000001823141405141507_s1_p0/16933/1001_10367
CCCACCGGGGGCCGGAGTGTTCCAGCATACCGACACACACAATTTTTGCGCGGTCACCCTGACCAGTTCTTTTTCCACATCCCT
GGACATCCTTCCCCCCCCTCACGTTGCCGGCAGGCCCGCCCTTTCCCCTCTTGCCGCTCTTCTTCCATCCTTGCGCCCGTCCTT
TTGCGCGTTCCCTGCAGCCGGATTTTGCCCCCTGTTTCTCCGCTTCCTTTGCTCCCCCGGAGTTGGTTCGGCCCCCTTGTTCAA
CCACCTCTGGCTCTTGTCCGCACGACCCTCTGGTACGCCTCTTTGTTTCCCCGCTTTGCTTCGCGTCTTTTCGCTGCACACCGC
GTTCCCCCCCGGCCCCACTCCGCTGTTATTGATTTGCTCTTGTTCCTTTACCCAGGCCTTCAGCTCCTTCTGCCCGGCTCCTGT
CAAACCCGCTGTGGCACTCTTCCCTGTTCTGGCTTTCCCCACGTTGCCCCCCCTGGTCCCGATCACCCCTCCCAACCCTATAAC
GGAGGCTGCCATTCCTGCTTACTCTCCTGTTTCCTCCCCCGTTTGCTTTCACACCCCGCTGTCCTGCTTTCCATTCTTCGCCTT
CTCCGTCGATCGCCCCCCCTTCTCGTCCGGGCTCTCCCCCAGTCATTCCTCCTCGCCTCCGCCGTGGCTTGCTGCCCTTTTTGCA
GCGCCAGCCCAGCTCGCCCGCGGCCGAAACGTCGATCTTCTGTCTCCCTCTGCGTGATTGTTGGCCCCCATCGTCCAAGCCGCC
CCGGATGACGGTCAGTGGCTCCCAGACAAGTCCATTTCTTTGGACCGCCTCCCCTCTGCTGGTTCTGTTGCATGGTGCTACGCC
GGACTGGCGGTTCTCCCCAGACACTCCGCCTTGGTCCTCGCGGCCTCCGGTCCAGCAGCTTCTGCTGCCTTTCACTGCGCGCCT
TTGCCTCCCACTTTCGCATGCCCTGGCTGCCCCCGCCTTTCCTTTCACATCCCCCTCCCGGCGTTACCCATCCCCCCGTCACTT
CTCAGTGCAGACCTGGCCTCTCCTCGGCCACGTGCTGTTGCACGTTTGCCCGTCCTGTACTTTTCGCGGGTTCTTCACCCTCCT
TCGCCTGCTGTCCGGATCTGCTGCGGATTGGCTCCTGCCCGGCTTGCTGTCCCCACTCCTTGGAACCCGATACAGGCCGTATTC
TGCGTTGGCCCTCTACTTTCCACCTTAACACTCGTGCGGCCCGGTTCCCTCCCTGCCCGAAGCATCAAGGTCCCCGCCCCTCCT
CACCCTTCTTCCCTGGAAAATGAGTCCCGTGCCTCGCGCTGTTCCCCGTCCTTTCTTCGCCCGTCCCTGCGGCTTCTTTTCCGC
TTGGTCTCACTCTTGACTTCCTGGTTGGTCACGGACCTCTCTCTTATCCGTTCTCTGGTCCGCCCTCCTCTGGTGCTCGGCTCT
CCGCCGTCTCCCTTACCCTTGACACCACCCTCCCTTCCCATCTTGCCTCTCTGCTGCTCATCTCCTTCACTGTGTTCATCAGCT
TGCCCCTGCTCCGCGGACCCCCCTCGTGCCCGTCAGCATTAGGGCCCTTCAGCCGTTCAATCGCGGCCTTTCTGTGGCCCTGAC
ATCCCTCTCTTGGCTCACGGTTTGTCTTCTTCTTGCGTTCCCCAGCCAGCGGACTCTCTTCCCTCCGTTCTATTCCCGCCTTCT
CTGCTTTTCCTTTACGCCTTTCCTCTCTGTCTCTCCTGTCTCTGTCATGCCTTTTGTTCTCTCCATGCATTCTTTGGACTTTGG
GTTTTCCTCTGTTTGCCGCCTCTTCTGCCTCCTTCTGTTTTTTGTCTCCCTCCACTCAATCCATCATTTGGACCGGCTGTCTTT
CCTACTTTCCGTCGTTCCGTTCCTTTCACTCTTCCCTCCCTCCCTTGTGCTCTACACGTCTTCCTTGACTTTCTTGCCTTCTTT
CCGTCATTTGCTTCATTTCTCTTTGTTCTGTTCGTTTTTCCACCCCCTTTACCCTTTGGATAGTTTGGTGACAGACCTCTCGCC
CTCTTGTGGTTTCTCTGTTACTTTCTGCAGTCTTTTCCTCACGCTTTTACGGTTCTCCCATTTTTTTTTTTTTTTTTTTTTTTT
TTTTTTTTTTTTTTTTTTTTTTTTTTTTTTTTTTTTTTTTTTTGGAGGTTGCTTGGAGTCGTTCTTGCTTCCCCCTGGTGGGACC
TTTCCTTTCCCTGCTTGTTGACCCTGCTTCCCCCCGGTTTCGTTGTACTCCCTCTCGTCCATGGCCAGTCCTGTGTGTCTCATC
AAGTCCACTTTGCCCGCCCCTTGTCTGTCTCTTGTTTCCTCGTCCGGTCTTGTGTCCTTTTTCGTTCCCTCCACCTCTCTTTGT
CTTCCGCCGCCCCCTGGAGCTCTGTTGCGCTCGTCCTTGCGCCGCCTGCTTTTTTCTGTTTCCCCGCTCACGTTTCTCCCGGTC
TCTTCTTCTCTGTCCCTGTCCTCATTTTGTGTGCTCTCCTCCCTGTTTCCTCTTTGCCCCCTGGTCCGTTTTGCCACCGGTGTC
TACCTGCAAGCAGAGCACTGCTGTGACACCGCTTGTTTTTGTCTCCGTCTGGCTCCGTTCCGCTGTTTCCGTGTCGTCTTTTCG
CCCTTTTCGCTTTTCCCCTCCTCTTTCTCTCTCTCCCCCCCCCACCGGCGGAGGCGTGCCAACGACGACGCTCCGGCTGCTGCT
TGCCATCCCCATTTCTCGCCTCCTTCCCCTTCACATCTGCCCGGCTCCTCCTGCGACCCCACCGGGCCTCGTCCCGCCGTGTCT
CGTGCTTTCCCTTTGCCACCTGATTCCCGGAACCCCGTTCTTTCCCCTCTGACGCCCTTTCTCGCGGCCCCCCCACTGCTTCAC
GTTCACCGCTCCCATCCCTTCGCCCCTGTCCCTTTTCCCCTCAGCCGTCTTCTCACCCTTCCGATCTCCACCCAGCGGTCTTCC
CTGATGCGTCTTTCTGTATCCCCCTCCTGCCTTTTCTTCACCTCCACTCCCCCACCTCCGTCCTGGTAGTCCCCTTCCACCCCC
GGCCCCCCAACGGTTCTGTGTCCACCCTTGTACTTGCCTCCCACCCCCTTGACTGGCCTTTTCACACTATCGTGCGTCCCACCC
TGGTTTTTCCGCCCTTCCCCCCTCCGTGCCCTGGCCCGTTCCCGCCTGTTGCAACTCCCCTCCTCCCGCACCCTCACCGGCTCG
TCCTTCTGCTTCCAATCCTGCAGCCCATCACCTGCACCCCCTCCCTACTGGGCTCACTTTCTTCCCCCCCCCCCACTTCATTTT

CACCCCCTCACTCTCCCGCATCCTCCGCCCCTCCGCCCCATCCCCGCCCTTCAACGTCCTGCCTGTCTCTCCACCTCTTCCTTT
TCCCTTCACGGACTGAACCTCCTGACCTCTTCCCTACGCCCTTCCCACCTCTGGCTTCTGTTGCTTTCTCCCCCTCCCGCCGCG
CTGCCCTTTGCCCCCTCTTCCTCCCCCCTCCCGCTTACTGCTTCCCCATGCCTACATCTCCTGCTCGCCCTCTCTGCCCATCTC
ACCTGCCACTCATCTACGCAAGTCTTCCTCATCTACCCTTCTGGCCCGGTCTCTTTCTTTGTCCGCCCTCCGCCCACACCCTTC
GTCACCCTCCGTGCTCTTTGCCACACGCCGCCTCGACTTCCTGCTGCAGTGCCCCCCCACGAACTGCTTTACTTGCCACTCGGG
TTTTTCCTCGGCTTCCCTTGGCCATCTCTGCCAACCCATACCTCTCTATCTCCTCACTATCGTCATTCTTTCTTTTCTTTTTCC
ACTGTACTTTCCTCCTGCTTAGCCCTCCCCTCGTCCTTCTGCACCAGCTCACCTTCCAAGCTTATGCCTTCCCCCGTCATTCAC
TCTTCTCCATCCCACTCTCCCACCCTCCTCCAGTTCCCTGCTCGCCCAGCTACTATGGCCCCTCTCGCCGTCCCCGGTCTCCCC
CGGTTGGACGACATTTGCCGGGAATGCTGGGCTCTGCCTCTGGCACTTGCGTTCCCCTTCCGCTCGCTTTTCCAACGGTTTTTT
TTTTTCCCCTTTGGCCCTGTCCACCACCACCCACCTTCCCTCCCACCCAACCGGACCTAACCGTACGCCTTCCCACTTTTTTCA
TCCGTGTCCACCCCCCCTGGCCTTTTTTTTCCAGGCCCCCGGGCCCGGGCCCCCCTGACTCCAACCCCACCGCACCTTCCTCGC
TCCCTTTTTTCCCCTTCCCCCCCGGGGGCCGCGGAGTTTTGCCCGGACCCCCCTTCCTTTTTGACCCATTTTCACCCTCCTGGT
TCCCACCCTTTTTCCCCCCCCCTGGGTTTTCCCCCTGGCCCGGCCGGGGTTTTTTTCCCTTTTTTTGCCCCCCCTCTGCCCCT
TACCTTTTGGGGTTTTTTGGTCACTGTTCACCCCTCCCCTTTCCGTTTTTCCCAACCTTTCCCCCCCGAACTCCCGTTGGTTTT
TCCGCGCCCCGGGCCCACTCCCGCACAACTTTTTTTTTTTTTTTTTTGGGCTGGCCCGTTTTTTCCCCCCTCCCCCTGCCCAC
TTCCCCTCCTCCCTCCCCCCGCTGGCTTGGGCCGGGGGTCGCCCCCGCCTCCGTTTCCCCGGGCCCCCCCCTTTTTGGGCCCTT
TTTCCCTTTTTGGGGGGTCCGCCCCTCCCCCCCCCCTTTTTTTTTTCCCCTGGGGCCCTTTTTACTCCCCTCCCCCCCGGCCCA
CCCCAACCCCCGCCCCCCACGTACCCTACTTTTTTCCCTCCCCAAAAACCCAGCCATTGCACACCCCCTCCCCTTTTTTCCCCC
TTTTTGGACCGCCCCCCCCCACTTACCCCGTTTTCTCCCTTTCTTTTTTTTTTCCCCCCTTTGCCGCTGGTTTTTTTCCCCCCC
CCCCCACTTCCCCCCCGCCGTTGGGCCACCTTTCACATCCCCCCCCGCCTTTTCGGCCCGCCCTTTGGGCCCCCCCCCGGGGGGC
TTCTTTCCCCCCCCTTTTGCCGTTTCCCCCTTTTTGCACCCCCACCCCGTTTCCACCCTCCCGTTCCCCCCCCCCTTTTTCTTTC
AAATTTTGGCGCGGGCGTTTCACCAACAACCCTCCCCCTGGTTTTGTTTTTTTTTTACACAGTCGGCCCCCGTCCTTCCCCGCC
ACCCTTTTTTTGTTTTTTCCCTTCGCCCCTGATACCTCCACCACGCCCCTTTTTTCCGACCTGGCCCCCACCCCTTCCCCCGGC
CACCCCCCCGCCCTGGGTTGCCCCTCCCCCCCCCCCCCGTTGGGGAGGAGGTCCCCCCCACCCCCCCGCTACTCCGGCCCCCG
GGGTCCCTGGGCCACCACCACCGGCCCCCCTTTTTCCCCCTTTTTCACCACAGTTTCGTGGGGGTTTATTTTTTTTTTTCCTC
CGTTGGGGGCCCCCCCCCCCCCACCGTTGGGTGGTTCCCCCCCCCCCCCCCCTTGGACCCCCCGCCCACCCCCTCCCCCCTTTC
ACGCTGGCCCCCCCCCTTTGTTTTGGGCCCCCCACTCGCCTTCCCCCCCCCCCCCCCCGCCCTCTAAGCTTTTTCCCACTTTTGGG
CCCCTTGTTTTTTCCGGGACAACCCCCCTCCGGGGCCCCCCCCCTCCCCCCTTTTACCCCCACCCGGCCGGACAGTTTTTGGGAG
GGGGCCTTGGGGGGGGGTGGGCCCCGCCTGGGTTCCTTCCTCTCCGGCCGCCTCGGGGCCCCGCCTTTTGGTTCACCCCCCCGC
GACCGGGTTCCCCCCTGTTCCCCCTCCCCTTTTTTCCCCACCCTTTGGGCTTTTTTTTTCCACCCCCTCTCCCCCCGCGGTTTG
TTTTTTTTTTTTTGGCCCCCCCCACGGGTTTTTTTTTTGGCCCCTCACCCACCACTTTTCCCCCCCCCCCTTTTTGGCGGGG
GGCACTCCCTTTCCTCGTTGGGGCGGGCCCCCTTGGGGGGCCCCCCCCACTTCCCCTTTTTTTTTCCCCCCCACTGGTTTCCCT
TCTTTTTTTTCCCCACCCCACGGCCCCTTTTTTTCCGGCACCCGCCCACCCCTTGGGGGGGGCCCCCGACTTTCCTATTCTGCC
CCCCCGCCGTTTTTTTGCCCCCAGGTTTGGGGCCCCTGGCCCTTGCCCCCGCCCGCCACCCTCTTTTTTATTGGGTGGGGGCCA
CTCCCCCCCTCCACACCCGCATTTGGTTTGGGGGCCCCCCCACCATCCCCCTTTTCCTTTCCCTTTTCCTTCCCCCCCATTTTG
GGCCCTCTACCCCTCCCCTTTTGGCCCACCCCCCCCCTTTTGGGGGCCCCCCTTTTTGGCCTTTTTTTTGTCACTGCCACCCCA
TCGGACCCCCCCCCCCGGGCCAGCCAACCTTTCCTTGGGCCACTGCCCCCCCCCCCCCCCCTGGGGCCACCGCCAGTCCCCCCG
CCCAGGCACTGACCCCCTTTTTCCCCCCCCGGGGGCCCCCTTCCCCCCCTTTCCCGGGGGCCCCCCTTCCCCCTCCACCGGCCC
CGCTTTTGCCCACCGGGGAACACCCCCCCCCCCCCCCCCCCCCATCTCTTTTTCCCCCACCTTCCTTTTGACCCCCCTTTTTTCC
CCCCCGGGGGGGCTTTTTTCAGCCTTTTTTGGGGCCCTTTCCTCCCCCACAAACACTCCCCCCCCCCCCCCCCTTTCTTCCTCCT
GGGCCTTTTTGCCCCCGACAATCCCCCCTTCCCCCGTTTTTCCCCCCCCCCCGGCTTTTTCTTTTTTTTCACCACCCCCACTTT
GGCCCCTTTTTTTCCCTTTTTCCGGGCAGGTGGGGGGCCCCCCCCCCCTTCTTGGCCCTTTCCGGCCCCCCCCCCCCATTTTC
CCTTGTGGTTTGGTTTGCGCACTCCTTCCTTTTGCCGGTTTTTTCCCCCTCTTCCCTTCCCGGGGGTTGTTGTGGGGTCCCCGC
CCTCCTCTTTTTGCCCTTGGTGTTTTCAACACCTGGCACCCGCTTGGGGTTGCCCCCCCCCTTTTTTTTCCCCCCCCCCTTGACA
CCGCCCCCTTTTCCCTCCCCGTCCCCGTTGTTTTTCTTGGTTCACGGCCCCCCGAACCCCCCCTTCCACTCTTGGGAGGTTCCC
CCCCCCCCCCGCCTTTTTTCCCCGAACGCCCCCCTTTTTCCTTTTTTTTTGCTTTTGTCCATAATGCAACTTGCAACCACCACT
CCCCCGGGCCCCCCCCCCCCGTTTTTTCCTTGGCGGGGCCCCCACATTTGCCCCCTTTTTTTTTCGCTCCAGGCCCCCCACTTT
TTTTTCCCCCGGCTCCTTTTTTTGCTCTTTTCCCTTCCCTCCCCCCCCCCACCCCCCCCCGGGGGGGCCGGGCACAACGGGGCAC
GGGCCCCCGGACACGCGCGCCCCTTTTTTTCTACACCCCCAGCTTTTCGGGGTTTTTTCCCCCACCCTTTTTGGGCTTTTTTTT
TTTTTCCCATCGCCACGCCCCACACCCTTTGCCCCTCCCCACGGTTGGGGCCACTTTTTGGGCCCCCCCCCCTGGGGCACCCCTT
TTTTCCTCACTCCCTTCCCCCAACGCCACCCGGAACCGGGGCTTTTTTCCGGACGCGGGGGCCCCCCTTTCCCCCCCCGGCACTA
CAGGGGGTTCCCTTTTTGCGGCTTGGCCCCCCAAACCGCCCCCTGCCTTTTACTGGGGACGGGGTCTTTCCGCACACGCACTCC
CCACACCGCTGGGGGGGGGTGGCCTGAACCCGGCCAACGTATTCCGGCCCCCCCCACCCCTTTTCCCCAGCCGCCTGGGACACCC
CCTTTCCACCACCCACGCGCTTTTTTCCTTTTTCCCCCCCGGTCCACAACCTTTTGTTTTCCCCTAAACCACTTTTTTTCCCA
CCCCGCGGCCCCCCCCCTCCCCCTGCACCGGGGCAACCCCCCTGTCACTACACGGCCTTTTTTTGGCCCCCCCGGCCGGAGGGA
ATTGTCCCCCCTTTTATGCCCCCCCCCCCAGGCGGCGGGACCCAGACCCAACCGTTTTTTTTTTTTTTCTTTTTTGCTGACCAC
CCTTCGCCCCCCCCCACAGCCCTTTTTTTTCTCCCTTTTTTTTTGGGGGGGGGGGGGGGCTCACTCCCTGTCACACCTTAAAC
CTGGGGTTCTGCAACTTTGGGGTTCCCCCCTTGCAACTCTCTTCTTTTACCGTGGGGGGGGGCCATTGGCCCCTCCGTACCCG

GGTGGGCCCCCTTGGCCGTTTTTTTTCCTCCTTTTTGTCTGGACCCCCTGGGTGGGTTTGGCCCCCCCCCCCCCGGGGTGGGGG
TATTTTTGGCCCCCCCCCTTTTTTTTGCCTTTTCCCCCATTTTCCACTTCTTTCCCCCGGGGGTCCCCCTTTTTTTTTGGCCTCA
CCCGACCCCCCCCCCCCCCCAGGGCCCCCCCCCACCGGGGGGGGGGGGGGGTTTTCCCCCACACGGGTACGTCCCCCCCCCCCC
CACCACACTTTTTTTTTGCGCCCATCACCACGGGGTTTCCCACTGGGGTCAACACCACCCAGTTAACTTTTTTTCCCCCACCCC
TCTGGCTTTTGCTCCCCCCCCCCCCCCCTCCACCCCCGGTTTTTTTTTTTTTTGGGCGTGCACGGGGGCCCCCCCACACGCACCC
CTTTTCCCCACACCACCTTTCTTGGGGGGCCGCTTTCATTTAACCACTTTTTCCCCCCATTCTTGGCCCCGCCCCGCCCCCCCTT
TTTTCTGTTTTTGACGCAAGTTAACACCTGGCCCCGGTCTTTTTTGCCCCCCCAAAACAAAAATTGTTTTTTTTTTTTTCCATC
CACCCGGAAAACTTAAATTGGTTCCCCCCCCCCCAAATAAAAAAAAGGGGAAAAAGAGTTGTGGGGTTTTAAAAAAAAAAAGGC
CCCCAAAAAACCTTTGGTTTTTTTTCCAAAACACTTTCCCCCTTGGGGATTTTAATTTGGTAAAAAGCAAAAGAAACCCATACA
AATTGGGGTAAACCCCAGTAACTCCCCTTTCTTTTTTTTTCTTTTTGGGGGTTTTACCCAAAAGCCTTTTGCTTTCCCCCGGCCT
TTCTTTTTTTTTTTTGAAATGAACCTAAGCGGGTTTTTTTTTTTTTCCCACCCCAAAAGGCCCCACCCCCAAAACTTCGCTGTT
TTTTTTTTAAATTTGGAGTTTTTTTGGGGCTTTTAAATTGGTTTTCCCCCTTTGTTTTTAAAAACAAGGCCCTATTTTCCAGCT
TTAAATTTCCCCCCTCTGAGAAAAGGCCCCCCCGATTATGTTCAAAAAAAAAAACCAGTGGCCCCAAAAAAATTCCTTAAATGT
TTTTAATGGCCCTTTTTTTTTTTTTTTTTTTTTTTCCCAAAGTTGAACCAACCACTTTGGGTCACCCCCAAAAAAAAACGAATTT
TAAAAATAAAACCAAACCTTATATAATCTAAGGGAAGTGGCC
>m141121_232159_42134_c100732402550000001823141405141507_s1_p0/17363/7240_13436
CGAGGAATAGATAATATTTGTCGCAGCTCAATAGTGCTTAAGAGTGTGTGTGTGTGAGAATGGTAGAGGGAAGTACACAGGAGT
GGTTTATCCTGCAGAGAGGTATGTTTGTCCTTAACCGCAAGTGAGAAGTTATGCCTAGCTATTGTAAAGTGGTTCATTCCGAGG
ACTCAGGTTTTCGCTGAGCCGCGGCGAGATGAGAGGGTAGGGTGAGGGGTGTGGTGGCGTGAGGGTGAGGGGGTTGAGGTGGTG
GTGAGGTCGGGGGGGGGGAGGCGCGCGTGCATGTTGGTGAGTAGGGTCGTCGGGTGGTGAGGGTAGATTTGGATATGAGTTTAA
AACTTAGGGACAAATATCTTTGTTGATAGATGATTTTGAGATACCAGGTTTGTGAACAGAGTTGCTGTTGTGCTCGGTTGAGAG
TATTCTATTCAAATTCACGGAATAGGTATTTCGATGTGCGTTACCGAATCGAGAGAGGTTGAGTGAGGTGTCAAAACTGGGGAG
CGGAGAGCGTGAGTGAGGAGAACTTGGAGAATCAATGAACTTAGTCGTGATACGGAGGGCATTGGGGATCTGGTGTAGTGGCTA
AGGCAATGTCGCGAAGGCATGTGGTTAGCGATCATGCAGAACCGAAGATAATGACGGTTCGATGCGGTAATCGTGATTGGCCAG
ATGCGGGTTTGTTGTTTATGACTATTGCTTGCATTGGGTACATAGTATTCAGGATTTACACATTATGTTTTGTTGGGCACGTTG
TTTAATAGTTTTTTTATTGCTATACTTCTTAATAGCTTTCATAGGATGTTCATAGGCATTTTTTGACTTTATTGCGCGATGTCT
GTTACATTGTATTATTTGTTAGATTGTCTAGGAGAACTATCCACTGTATAAGGTGTTGATATGTACGGTCTTGGCATTTTTAAG
TTAGAGTGTCTCTATATATAATGCTGAGTGTTTCTCCTGGAACTGACTTGTCCACAATTGGGCCTGCGAACTTGGAAGAGCTGA
GCTTCACAAGTCGATATAGGTGCCGGCTAGCCTATATGGACGCTTATCTCCAATGGTGGCAACCAATAGTTGTGACTAGTGATA
GTGGCGTCGTTTGTATTCAATTATTTTAAGGCAGTTAAAGTACGCAGCTACAATGATATCCTATAGATCTCGGGTTTCATTCAA
ATCTAATGCTCTATTTCATCTCTCCATGTGGGGAGCTATTGTGGTTTCGTGCTATTTGATGATAGTAATAAGGACGTATTAGTG
CATGTCGCATGTGTTGCATATGTTGTGAACCTTACTGATGCTTGTAAAATAAGATGATGGATTTTTGTAATGATATATGAAGAT
ACCTCGCTATCCCGCCGGATTGATGATTTGTTTATCAATTCTGAGCGTACCGTGTATATCCAAAGATTGATCTATGGTCCAAGG
CATCACCCGTTGATGTATGGCTTTCTACGGTAGTCCTTGGTTGGGCGGGGGTGTGGTATGGGGTGTAGTTTTGGTTTGCCAGTG
AAGGAGACATGTTTCCTAAGGCTATGCCTGTGTTTTAGTAACTTCGTTAGTGGGTCATGGTTTACTGAGTTTGTTGTATGTATC
TTCCTTGTTGTCTTGTATCAAATTTTTTTGCTTTCAAGCCGTTTATTGAGGTGGAGTTTTTATATTGATAGGGTCTTGTACAGG
AATACTGTGGATAAATTCATTCGTATTAATGTGGTTGTTTCATTTCGACTGAACATCCTGTGGGTTATCTCAGAGGGACGATAG
TCGGCACAGTTGTGTTCTGTGGTTTGTGTGAGAGGTTTCTATTTTAGAGGGTTGTATGGTTTGTTACGAATCTTAATAGGATCA
TCGAGACTGCTATTTCCTGGATAAGTTCATAGAATATGTTGAGGTTGTTGTTGTGTTTTTAGTAAGTGGCATTGCCTTCATCAC
TGATTATACTTCCAGGACGGAGTTTTGCAGTTAGACCTCAGTGCAAGAGGTTATGTGAGGTGGTGATAGGTGTTCTGCTACAGG
GACGACAAAGAAGAATCAGAGTGTTTAAAGGGAGGATCTTTCTAGATTAGTGAAGGGTTTTTTTCAGATTTTGTAGAGGTATTT
GTTCTTAAGATTTTTGTAGGCCACTCGCTTCACCTAACCTAACCCAGGAGAGGCAGCAAAGGTTGCGAAGCTAGAACTGATAAG
AGTGGTGTTTAAGAGAGTCTTCTCTAGTTGCGTTAGGTGATATAGCTGTGCTCAGCAGCCAGTATTTAGTGTTATGTCTTGATT
ATCCCGAAATGACAGTTTGTGTTAGTTCTATTGTGATAAGTGGCGTCGAATCAGAGGGTTAGGGGGTGTTGTTGTTATGATTGC
ATAATGGACAAAGGTTATAAGAGTGCCTTATATTCTCTCTCATATAGTGGGGCGAGGCTGATAGGGTAGTACTGAGCTACAAGT
TTGCTATGAGCGAGACTCATGGGATGGTGTGAGGTTGTAGTGGTAGGTTTGTTTGCGTTAAGAGAAAGTTTGTTGGCGACCATT
ATGTGTATCGGAGAAGCTGGGTGTGAATTTAATAGCATGAGGACCGGATAAAAGTTTAATAATCACTTCTTCATTGGTAGGAAG
TTGTTTGTAGTGTGAATGCTTAAACATGAGGCAGCGCAGGTGGTTAGAGACTGAGAGATATAGATTTACGACTATTGTAGAGTT
GGGTATTTTGGTATCGCTGTTACCTGATCTGGGGATGTTTATGGTTGACCTTTTTCGGTAAGGCAGATGAGCGTAGTTGTATTT
TGATGTCTCTTACGATTGTGTGAGATTTGGTATGGAATTTATATTTTGGCTTTGGGCATCTAGCAGAATAGAAAAGCGCTACAG
CCAGGAGCTGATAAGTGTTGCCGGGAATAGAAGTAGGGGTCATCCGGTATATGGGAGTGTGCTGAATTTTCTCGTATTCAGTCA
TAAGTCCTGTTGAGAGGTTAGATGAGTTATCGTGATTTGGGAAATGGCGATGTTATTACAAATTAGTGCACCAGGGTTATGGAG
TGGGAGAGATTAGAGAGTTAGGCGCAAGCAGCAGAGTTTCTCAATAGTGTAGTGGTCAGAGAGGTATTGAGGAGAAGTGTTGGG
GGGGGTTGTGGGATTCGAGTATGTGTGCCAAATTGTATTCAAGGGAGAGAGTGAGAGAGAGGGAGCGATGTGCTAGATAGAAGC
AACACAGTACCTCCATAATCAGTTCACCAACGTCGATCCCAAATGATCACGACATCAAGGAATATATTCGGTGGCAGGAATGAA
GAAGGACATAGCATAATTTGTGTCTTAGATGCTTAGTATGCCAGCAAGTGAAAGCAGCAACATCAACGGCATGACAGGCTTATT
GCAACCAACTTAGCATACAGAATTGAAATAGGACGATATATAGCTATGGATTTCGTGACAAGTTTGCACGATAAGTAAATAGCA
TGATTCAGCTTAGGTAGTAAATAGATAAGAATAACCAAGTCCACTATTTCTACCTGTTAAGAACTTCATAAAAACATAGTGGAT

CAATAACAGACATCTATGTACAAGAGAAGTAACGTTGCATGGAAATCCCTAAGACAATAGTATCCGATAGAGGATCGGTGTTTA
TATCGATGATTTTGGGGGAAATGCTTACAACAAGCATGGGTACTAAGTTAAGTCCTTAGTACAACATTTCATCCTCAGACAGAC
GGTCAGTCCGAGCACTGACCATACAGGATTTTAGAAGATTATGTTGACCTTGTGTACTTGATTTCAGAGGATCATGGAAATAAG
TGCTTACCATTGATATGAGTTCTCCTAAAACAACAATAGCTCCAGTAATATCAGGATGGCACCTTATGAAGTTACTTTATGGAA
GAGGTGCTGAGCGCGTACATTAGGCACGACGTAGGAGAAAAGGCAGCTTCTTAGTCCCGAAAGCTGTTAGAAAAGCTCAGGAAG
CAAGTAGCGGCGTAAATTAGAAAATGTATGCTCACTTGCTCAAAGCCAATCCAGAATAGCTATGCAGATGCCAAGTCAACGTGA
GTGGAATTCAAAGTTGGAGATCAAGTCTTTCTGAAGAGTATCTCCTATGAGGTGTAAGCGGTTTTGGGAAGAAAGGCAACTTTA
TATAGGTCCTTTTGAGATATTGGACAGAGTGGGATTAAGTTGCAATATAGATTAGCCCTTCTGCAGCTAGCAGATAGCCACAAT
GTGTTCCACATCTAAATGCTAACGAAAATCGAAAGTATGTGTCAGACCATCTTCACATCCTCAACAGTACGATACAATAACACT
CCAGATAAGACTTGAGTTACGAGGAACGACCAGTTAGAATCCTGATAGAGGGATGAAGTACTATGTCCAAGAGTATTCCGATAG
TAAGTAGTAGTAGATAGTAGTTAGTAAGTAGTAGTAGTAGTAGTAGTAGTAGTAAGTAGTAGTAGTAAGTAGTAGTAGTAGTAG
TATATAGTAGTGGTAGTGTAGTGGTAGTAGTGGAGTGGGTGGTAGTGGTGGTGGTGGTGGTGTGTGGTACGTAGGGTGGTATGT
AGTAGTAGTAGTATAAATAGTGGTATAGTGGTAGTAGTGGTAGTAATAGTAAGTAGTAGTAGTAGTAGTAGTATAGTAGTAGTA
GTGTAGTAGTAGTGTAGTAGTAGTGGTAGTATAGTAGCTATTAGTAAGTAGTAAGTGGGTGTAGTAGTAGGTGTAGTGGTAGTA
TAGTGGTAGTAGTATGTAGTGGTATAGTAGTAGTAGTATAGGTAGTGGTTATATCAGTAGTAGTAGTAGTGGCGTGGGCAGTAG
TATAGTGGTATTGGTAGTAGTAGATAGTAGTAGTAAGTAGTAGTAGTAGTAGTAGTAGTGGTATAGGTAGTGTAGTGGTATGGT
AGTGGTAGTGTAGCAGATAAGTAGTAGTGTAGTAGTAGTAGAGTGGAAGTGGGTAATGGTAGTGGTAGTGTATGTGGGTAGTGG
TAGTGGTAGTGGTAGTGGTATGGGTAGTAGTAGTAGTGGTAGTGGTAGCGGTACAGTAGCAGTAGCAGTACATACAGTAGCAGA
TAGCAGTAGCAGTAGCAGTAGCAGTACAGTAGCAGTAGCAGTAGCAAGTACAGTAGCAGATAGCAGTAGCAGTAGCAGTAGCAG
TAGCAGTAGTCAGTAGCAGTAGCAGTAGCAGAGCAGTAGTAGCCAAACTTATAGATTTTATAGTTTAACTATATTTAAGAATAT
TAATTATAACATAAAGGTTTGATAATATAGCTGATTATAAAGATAATATTTAATTAAATTATAAGCAATAATAAGATCAGACAC
TTGTCAATAAGTGCATGTTTATTGAGAAATTAAGTATTTTTGGACAAATAGTTGTATAAGAAATATTTGAAAACCCCAGAATCT
GCCAATAGCTTTAAAATTGCTTTTATGACTAGTCAAAGTTGATTAATCAATAAAAATTAGGCTGAAAAAGTGCAATTACGGTAT
ACATAAATTTCAGCGTATCCGATATATCGCAGCTCTAGGGGCGATATATCGCAACAACGGGGATACGAAAAACACGTAACTGTC
GCAAACGAACGCATCGACAAGCCCTCGAGAATATAAGCCCCAGGTCGATATATTCGCCTACCATGGCGATATATCGGCTCTAAG
GAAAGATTTTTGATGGTTTTGAAACCGAGCTCAAATTCAACCATAACCTCTTTAACCAAGCCTCTGATCTTTTTACAAATCTTA
AGACCTCTGGCTAAAACGAATATTCAATGATTTTCAATTAAAATATTCATTATTTTTTATTCAAGATAAAAGAAAGATATTTTC
ATTCTGAAATAATATAATAAAGACCTGTACACCAGTCCATTTCTTTCATTCTTCAGCTGAGTTCAGAGCCTTCAAGGCTACTAA
GGTTTCTTTAGAGTGTTAAACACTTGGTTGGGTTATAACTTTATATTTAAGCTTTAAAAACCTTGGGAAGTAAGGTTAATTGTG
TTTTTTTGATATCAAGGTGTAGTTCGGTTCTAGTTCATTCAAAGGTATTCCTATTCCTAGTTCATGTTCTTATTTTCATTAGTT
TTTTATACTTTTTCTCTACTCAAATCCTAACTCGCTATTTTCTCATTATCATTTGGTAGGTATTTAGAGTTCTTTGAACTTAAG
ATTATCTTGTTGGAAGTTTCTCTCAGTGGATAGTTCATTCCTTTTCATCTCCTTTCTTTTAGAA
>m141121_232159_42134_c100732402550000001823141405141507_s1_p0/25564/8532_13927
AGCGCACTCACAAGCCTCATTCAATTTTTTTTAAAAAAGGTTAATGTGTGTTTTTTCTTAAGATTAAAATTGAAATTGTATTTA
AATAAGGTTTTTACTTCCGGATTTTGACAGAATTTTTTCCTTTTTTTTTACTTTGGAAGGGGGTCTTGTTATGAGGGTGATACA
GTCAAAATTTACCGATCTCGGATGTACGCAATCATGAAGGAATAAAACTTGCGAAAATTGCATCCGAAAAAGGGGAATAATTTT
TTTGAAATGTTTGGACCTTAAGGCTCCGTTTCGACTCCAATAATATTAGATGTTCGCCATGAGATCTCTTGGGGGAAGAAATAT
CCGACACGAGTTGAACGTGAGGGTAATACATTGGTCGGAAAATGTAGTGAAATCTCGCAAGTGTCACCCAAAGGGTACCCCTTT
CAGAAAGTCTTCGACTTTTCTCTAATGTGAATTAGGCAATGATTTTGGTAGTGAATACAAAGAATAATGCGAGGGTCATGGGCA
CAAAAGTTTGTAGCCCTATGTCTCTAAGGGTTCTATATTGTAAAAATTGTTCGTGCGAAGAACCGAGGGTGATAGAGGGTGTTA
AGTAAAAAAATACCGAGGAGATGTACGACTATGAAGGAAAAAAACTGCGAGAAATTGCTCATTAAAAAAGGGGATTTTTTATTTT
GAAATGTGACTTGAAGGTCTCCCGTTTCGACTCAGATATATTCATTTCGCCATGATAATCTCTTTGGGGAAAATATCAACGGTG
TTAACACGAAAAATAAAAAATCATTTCGGAGAAATTTAGTGAAATCTTCCAATTCACCATAGGGGTACCCCTTTTAGAAATCTT
CGATTTCTCTAATTTGAATTAGGCAGCAATGTTTTGCCTATGGAAATACTAAAGATAATGCGAGGTTTCATCGGCACAAAAGTT
GTACGCCCTATGTCTTTCAGGGTTTATTTGAAGCATTTTGTAGCAAAACTCCGAGGTGTGTAGAGGGTGTTACAGTCAAAATAG
ACCGAGAGAGTTACGATATAAGAAAAAAACATGGCGAAATTGCATCAAAAAAGGGAATTTTTTTTTGAAAGTGTTTGACTCAGG
CTCCGTGAACTCCAATTTATTCATTTCGCCATGAGAGTCTCTTTGGGAAAAATACGAACGATTGAACCGAGGGGTAATACAGTTT
CGGAGAAATTAGTAAATCTCCAATTACAAAAGGGGGTACCCCTTTCAGAAATCGTTCGATTTCTTTAATATGAATTAGAGCCA
AATTTATGATTGTGAGATACTAAAGAATAAGCGAGGGTCCATGGAGCACAAAAGTTGTAAGCGCTATGCTTAGGGTTCTATTTG
TAAAATTTCGTGCGATATCCGAGGTTGTAGAGCGGTTGATACAGTCAAAATAACCCTAGGATTACGAGTATAAGGAAAAAACCT
TGCGAAAATGCAATTAAAAAAGGAGGAAATATTTTTTTTGAAATGTTTGACTTAAGGTCCGTTTTCGACTCCAATATATCATTT
CGCCGATGAGATCTCTTGGGGAAAAAATAGTCGACGAGTTGAACACGCAGGGTAAATAATTTCGGAGAAATTTGTGAGATCTCCC
AATTACCAAAGGGGTACCCCTTTCCAGAATCTCCGATTTCTGCTAATATCGAATTAGAGCAATGGTTTTTGATGAGTGAATAAT
AAATAATACGTTGGCGAGGGGTCATCGGGCAAAAAAGTTGTACCTTGATGTATTAGTGTTCTATTTGTAAAATTTTATGCGAAT
CTCGAGGTTGTAGAGGGTGGATACATCAAAAGTACCGAGAGATGTACGAAATATAAGAAAAAACTTGCGAAATTGCCATCAAAA
AGGGGAATTATTTTTTTTGAAATTTTGACTAACGGCTCCGTTTCGACTTCCCAATATGATTCATTTCGCCGATGCGAGCATCTC
TTGGGGAAAAAATAATCGAACGAGTTGAACACCGAGGTAGAACATTTTTCCGGAGAATTAGTGAAATTCCCAATTCACCAAAGGG
GTACCCCTTTCAGAAATCTTCGATTTCTCTAATGTGAATTAGAGCATGATTTCTGGTAGTGAAAATACTAAAGAATAACGAGGG

TCATGGCAAAAATTTGGTAGCCCTATGGTCTTAGGTTCTATTTGATTAAAATGTTCGTGAATGATCCGATGGTGTGTAGCAGG
GTGTTCAGTCAAAATACCAGAGTGTACGAATATAGGAAAAGAACATGCGCACAAATTGCCATTAAAGGAACGGGAATTATTTTT
TTGAAATGATTGACTTAAGCTCCGTTTCGACTCCAAATATATTCATTTCACCTGAGATCTCTTGGGGAAAATATCGAACGAGTG
AACACGAGGGGTAAACATTTCGGAGAAATTAGTGAAATCTCCAATTACCCCAAAGGGGGTTACCCCTTTTCAGAAAATTCTTCG
GATCTTCTCTATCATGAATTAGAGAAAATTTTTGTAGTGATACTAAGATATGCGAGGGTCATGGCAAAATTGTCAGACTTATGA
TACTTAGTGTTCTATTTGTAAAATTTCATGCGAATCCCGAGGTCTCGTAGGAGGTGATCAACATCAAATACCGAGCAAGATGTA
CGAATAAAGAAAAAACTTGGCGAAAATTGCCAAACAAAAGGGGAATATTTTTTTGTGAATGTTGACTTAAGGCTCCGTTTCGAC
TCCAATATATTAATTTCGCCATGAGATCTCTTGGGAAAAAATATCGAACGATTGAAACGAGGGTACTACATTTCGGAAAAAGTAG
TGAATCCTCCATTCACCAAAGGGGTACCCCTTTCAGAAATCTTCGATTTCTCTAATGAGTCGCACATTCGAGAAACTGTTTTGG
TAGTGAATACTAAAGAAATAAGAGGCGTATGGCCATAAAAGTTGCTAGCCCTATGTCCTGGGGTTTATCTTTGTAAACCATTTC
GTGCAATCCGAGGTGTGTAGAGGGTGTTACAGTCACAAATAACGAGAGATTGAATATAAGGAAAAAACTGCGAAAATTGCCATT
AAAAAGGGAATTTTTTTTGGAATGCTTGACGTTTCTCTTATGTTTTGTTTTTTTTTTCTTTTTTTTTTTTTTTTTTTTTTTTT
TCTTTTAGGCTCGTTTCGACTCCAATATAATTCATTTTCGCCATGGAGATATCTTGGGGAAAAATATCAACGAGTTGAACACGA
GTAATACTTTGGAAAAAAAATTGTGAAATTCTCCAATTCAACCAAAGGGTACCCCTTTAAAAGATCTTCGATTTAAATCTAATAT
GAATTAGAGGCAATGTGTTTGATAGTGAATACTAAGAATATGCGAGGGTCATCGGGCACAAAAGTTTAGCCCTATGTCTTTAGG
GTTCTATTTGCTAAATTTTGTACAAAATCCGAGGTGTGTGAGGGTGTCTACAGTTTCAAAAATACCGAGAGATGTACGAATATA
AGGAAAAGAAACATGCGAAATTGCCATCAAAAAAAGGGGGAATTTTTTTTTGAATGTTGACCTCTAAGGTTCCCGTTTCGACTC
CAATATAATTCGCATGAGATTCTTGGGAAAATATCGAACGAAGTTTGAACACGAGGTATACATTTCGGAGAATTAGTGACAATC
TCCAATTCACCAAAGGGTACCCCTTTCAGAATTCTTCGATTTTCTAATATGAATTAAGAAAAGTTTCGATAGTGAGATACAAAG
AATATGCGACGGTCATGGCACAAAAGTTTAGCCTTATGTATTAGTGTTCTATTTGTAAATTTTCAAATGCGAATCCAGAGGTCT
GTAGAGGGTGATCAGTCAAAATACGAGGATGTACGTAATATAGGAAATACCTTTCGGCGAAAATTTGCCATCAAAAAAGGGCAA
ATATTGTTTTTGAAATGTTGACTTAAGTCTCCGTTCGACTCCAATCATATTAATTTCGCCATGAGATCTCTTGGGAAAAAATATC
GAATCGACGTTAACCGAGGGTAACATACATTTCGGAAAAAAATTAGTGAACATCTCCAATTCACCAAAAGGGGTACCCCTTTAAA
AAGTCTTCCGATTTATCTAATATGAATTAGAGCAATGTTTTGATTTGTCGAATACTAAAAGATTGCAGGGTCATGGGCAACAAA
AAGGTTTTTGTAGCCTTATGTATTAGGGGTTCTATTTGGGTAAATTTCATGCGAATCTGAGGTCTTAGATGGGTGATACATCAA
AAATACCGAGGAATGTACGAATATACAGGAAAACACTTGCGAAAATTGGCCATCAAAAAGGAGAAGTTTTTTTTGAAAATGTT
GACTTAAGGCTCCGTTTCGACTCCAATATATTCATTTCGCCATGAGATCTCTTGGGGAAAAAAATATCGAACAGTTGAAACACGA
GGGTATACATTCGGAGAAATTAGTGAACGAATCTCCAATTCCACCAAAGGGGTACCCTTTCAGAAATCTTCGATTTTCTAATAT
GAATTAGAGAAAAGTTTGTATGAATACTAAGAATATGCGAGGGTCATGGGCACAGAAGTTGTAGCCTTATGTATAGTGTTCTTT
TTAAAATTTCATTGCGATCCGAGGTCTGTAGAGGTGATACAGTCAATACCGAGAGATGTCGAATATAAGGAAACTTTGCGAAAA
TTGCCATCAAAAAGGGGAATATTTTTTTGAAAATGTTGACATTAAGGCTCCGTTTCGACTCCCAATATACTTAATTTCGCCATT
GAGATCTCTTGGGGAAAATATCGAACGAGTTGAACATGAGGGTAATACATTTCGAAAAAATTAGGTGAAATCTCCCAATTCACC
AAAGGGGTACCCCTTTCAGAAATTCTTCGATTTCTCTAATGTGAATTAGAGCAATGTTTTGGTAGTGAATACTAAAGAATAAGC
GAGGGTCCATGGGCAAAAAATTTGAGCCCTTGTCTTAGTGTTCTATTTGTAAATTTTATCGAATCCTGAGGTTTGTAGAGGGTG
TACAGTCAAAATACCCGAGAGATGTACGAATTATAACGGAAAAACTTGCCGAAAATTGCCATCAAAAAGGGGATATTTTTTTGA
AATGGTTGACTTAAGGCTCCGTTTTTCGAATCCAATATATTACATTTGCAATGAGATCTCTTGGGGAAAAATATCGAACGAGTG
AACACGAGGTAATCATACCATTTAGGAAAAATTAGTGAAATCTCAATTCACCAAAGGGGTACCCCTTTCAGAAATACTTCGATT
TCTCTAATGTGAATTCGAGAAATGTTTGGTAGTGAATACTAAAGAATAAGCGAGGGTCATGGGGCATAAAGTTGTAGCCGCTAT
GTTTAGGGTTCTATTTGTAAAATTTCGTGGCAATATCAGGAGGTGTGTAGGGGTGTTACAGTCGAAAATACCGAGAGATGTCGA
TAGTAGGAAAAAACATGCG
>m141121_232159_42134_c100732402550000001823141405141507_s1_p0/28297/6130_15512
AAAGAGTGTTACTTGAAGAGACGTTTGTCTTGATGTACAACACAATGGTGGGAAGGAAGAGCAGGGTTTTGAAGGGGAAGGAGG
AACGTCCCGGTATGCGCCATTTATGAAGAAGTAGTGGTGCACATACGTGAGAGGATTGGACACATGGTTCATGTGCTTGAGACA
GCCACAAGTTTTGGAATTGTATATTCCCATTGGTAGGAAACGTTTTAATGTTTTCACGCACACCCCTCACGGGAATCCCTTTAC
TTCCGTCGGTGCGTGGAAGACGTGTAGGTATACCTCGCCCGTCAGACGATCTCCGGTGTTAGTAAGTTGTACGCGGGGCAGGGT
TTGTTCACCGCCCCCGCCTAACTCAATAAATGTGTAAACGAACTCATGCCTCTCCAGAGAAGAATGATGCGTCCCTGAGGATAA
TTTGTACACTTGATCTTCACTCAACCGGTCAGTTAACTGGGTATTAACGGGGATGGTCTTGTCACCCGCAGGACCCTTTTTGTC
CCGGGCTGGAAGCTCACAGGATGGGCCTTGCGTTCCCTTCTAGGGTTTTACACACCACACCACAAGAGCACGACCTTCTTACGG
CCGGGGTGCGTTCTGAGAAGCAAGCATCCGATATAAGTAATTACAGGCAGTGGTATGATCCACAGCCCGGAAATGCCCGTTAAC
TGATAGTGATTGGATCGACTAGATAACCACCGGAAGGGAGCTGTATATAAATCTCTCAATCAAATTATTCGTATAAACACTGCC
AAAGCCCGTCTATTCAAAAATCGTGGGAGCTATTATCTAGTCGACGCTAACAACCATAAGAGGCCCTGAACATTTTGGTTCGGG
GCTGGTAGTCTGGAATACCGACCACCCACTATTTGCTGGACTCTCACTTGGGCCTTCCACACAACAACAACATATAGTGCCTTG
TGCACACGACACAAGATATGTGCGGTTGCCCATACCCAAAGAATTCCTAGTGATGATAAGTACAATGGTGAGAATTGGTATATG
GTTTCCCACACATACATCCGCGTGCGAAGAAACAGATCCGTGGAGCGCGAATTTCTTGTGACCAAAGAGACCCTTAAAAGCAAA
TGTGCGGAGATGTATAACTTAGCGAGTATCCCATTCCTTGGGTGTTAAGAAGAGCCTATCTAAGTCCAAAGCGCGGAATTCCCC
TCGCAAGGGTTACAGCGTATATTCTGCAAGCCCCGAACAACCATTTAGGTTCGGGGAATGGTTTGTAAAACCCAACCCGGTCTG
AACTTTCTTTGTGTTTCCCCACCTATATTCCTATCTCTGCGGTAACGCTCAGCATTTAGAAGCGCCCTTTATCGCAGGAACCAT
CCGCAGTTCGGCGCGATTCAATCTCCGTACCGCGTTACGCCTCGACTGTAACATATACTTGGGCCGCTCGAGTGGAACGGCCGG

TCGCCAGGCGACCCAATCTCTTCTTGTTGTGCCGAAGGGAAGACACCCCTGCACGGCATGGTGTGGTGCCCCAACTTGTCTTGG
CGCTTCTGGAAACCCGCCTAGGACTAATTTCACCTCTCCAGGGATTCTAGTTGGTCAGTCGGCTAACCCACGAACCAAGGGTTT
AATGCCGCTTCGATGTTTCAGTTGCGAGCAGAGCACCAAGAAGCAGATTGTCCTCACACGAAAGTCCGTGCCCTAGAAATCTCA
TCGACACGTGATCTCTATGGCGTGCACCTTGTTGTGTATTACTATATATCTTCCCATTTGCCAGCGTTAACCCGTTCATACACT
GACCGTGCCCACCTTGGATGCGGCCTTCTAACAACACGCATAGTCTGGAAAGTACTGACGCGGGTGTCGGGGTAGACCCGTGGA
TACCCACCTGCCCGGCCTACGAACAGACGGATGTACCGGGAATAGAGAGTATAAAGGCTTTGGGTTTGCAGTCCCAATTTGGGA
ATTTTTTAAACTCCACCGGACCTCCGAAATTAGTCAGACGATATGTGGTTTGGATACGCCGCCAGGAACTATCCTTCAATGATA
TAATAAGTATTGTCACGCTATCGCTCTGACTTTTGTGTCCCGGAATGGGAAACCAGACTTGGGCTTGCGTGCCATTTGGTTTTA
ATCCCCCGAACCCATTTAGGTCGAGGATGGTTTGTTCCAGCCCGGACCATTTGGGTCAGGATGGTTTGTTAAACCCGCCCGGCA
ACCATTTGGTTGACCAAGACTATAATAGGCCATTGATCAATCTACTACCGTTTTCAACTCAGCGCGTAGCCGATTAGCCAGGGT
TTGTATGCTGTATTTAGTGGGGGAGCGAGGCGCGGGAGGCGCGAGTGGGTGCGGGGGAGGGGGGGGGGAGAGGGGCGGGCGATG
GGTGGGCGCGGAGGGAGCGTGGGAGGGAGCGTGCGAGGTGGTGTGGAGAGAGCGGGCGCCGGGGGCGGAGGGGTGCGGGGTGGG
TAGGGGAGAGAGGCGCGGGGCGGCGCGAGGGAGCCGGGAGGTGGGCGGAGGGAGGGGGGGGGGAGCGGGGGCGGACCTTTGGTG
CGGGGGGGGGAGTGGGGGGAGGGGGGGGGGGAGCGCGGGGTGCGCGGGTGTGGGGGGGGCGCGGGTGTGGTTTGGGGAGGGGAG
GGCGCGGGCGAGGGGGCGAGTGTGGGGTGGGGGGTGTGGGCGCGCGGGTGGTGGGGGTCTAGACCGGGATGTTACACGGAAGTA
CTCCTTCAGCCAACAGCTGTATTATATTCTGGATTGTCTGGGATGGACCACACACGTATGGACTTATTGTGGTGCCTTAGGGTC
GTCACCCATCTTTTGGTATCTTCATGATCTCACTGCTCGGTTGGATCTGTTCGTCGCATGGTGACGGAGGGAAGAATTGGGTGT
AGATGATACCCGTCCCCTAGCTATTGTAACCTTGCTGTTGTGTTGCCGATTATTAGATGGGTCTTAAAGAGCAATTAACTTTGA
CCGTCGCTGGGGGTATAGGGACTTCTGGCAACATGGGGCTTTTGCTTCAACTATTGTGAAACCACCAGACCATGTTAATTATGT
CAGGCGAATAGGGGTGCGACCAGGTAACCAAGCATTAGCTCACACCGTCACCGCTTTATGGTAGAGATATATTGTACACCTCCG
GAACCTTTGGTGCCCGGTTGAATGGGACTGGAACGGTTCGACATCGACATATAGCGTCCAAAAAATTTGGGATTTAACAGCCAC
CCCGCACCCGATTAAGGATTAAAATTAGGCATCTGTAAGATGGGAACTGGAAAAGCTGCCCTTACGTCCACACACGCTCTTCCG
GTGTATGTGTATACTGAACCGCCGGAGAGATTTAAAGCGGAGCGATGGGTTTGTTCAGCCGAATTTGGGTCAGGGATCGGGTAG
CTGAACGCGATCCATACCCACGGACAATTTCCGTTTTACGAGGGGATGGCGAATATAAAGCCCTTGTGGTTCTGGCAAGTAGGT
CGAACATATTTTTATATTTAAAATCACCCTGGAACCCGCTATGTGTAGAGGTGGAGGAGGGGGATATTGTAACAAGCTCGACCA
TTTGCGCCGGGGATGGTCCTCGTAGAAACGCCCTCAGGTACTAATTTGGTTCCTGGATGGACAAAAGCGCATGGACTCCCAGAG
CGTCACATTGGAGTTCAACAATACTCCCCGGACCAAATTAGGTCTCGGGGTCGGATGGTTTTGTACCCGCTCAGATCGCATTTG
GTCAGGGACAGGTTTGTAACTAGCCGACCATTTGTGTCCGCATGGATTAGGCCTTTGGATTTGCGTCCCATTTGGTTTAAATCC
CCCAGACCATTGTAGGTCGGGGATGGTTTGTACTCTACCGGCCATTTGGTCAGGGATGGGTTTTACCCGCCCGGTAACTTTGGT
GTGGGAGGACTAGACTTGGGCCTTGCGTCCCATGTTTGTTTTAATGCCCACCGACATTTAGGTCGGGGATGGGTGGTACCTGCC
CTAACCACTTTGTGTCAGCGGATGGTTTGTACCAGGCCCGGACCATTTGTGTACGTTATGGGACTACAGGCTTGGGACTTGCGT
CCATTTGGGTTTGTAATCCCCCCCCAAGACCATTTATGTCGGGGATGGTGTGTACACAGCCGAACCATTTGGCAGGGAAGTGGG
TTTGTACCCGCCCGTACCTTGTAGAATACCCACTCCGACATCAGCAGATTCTGATGGCGAGGGCGGTGGCCTAGCTTGGATATT
AAGGTCAACGGAACAGGGATTGTCCTCCTGACCACCCGTCCGTTGCGCGGACTGGGACTAAAATGCTTGGGTCGATTTTGGACT
TGATGCGCCCTATGTGACACCTGGCGAACGTCGAGGCTCGGAGAGGCCAGTGATCCGGAAGAGAATGGGGCGAAATAATAACTA
TGGGCCCTTTTGAAACAGACCTCAGGTCGTGGGGGTTTTCTGTAATCTCTTCACAAAGACTTATTACAAGCGGGATGGTTTCCA
TGTTCCCTGAAAGAAGACAAGAGCCAGCCCGGAAATAGCACCAACGTTGTGGTGCGAGAGGGAGAGGATAGTGTTCGTACCGCG
ACAGGACAAAATAAAGTAATTCTGGCGTGGAACTTCTGGTCGGTATGGTGCGCGCTCCCTTTGGGGTTTTAAAAACCCCCCCCC
GAACCATGTTAACGGTCCGGGGGATGTTTTTAATCAGCCCGTTACTCTCATTTGCGGTCTTGATGAAGCAGCTGGGTGACTGTA
CGAAGAGGTCGCCGCGCCGCGTCCACCGATTGGGTCGGAATGGAACTAAAATAGGCTTTTCACCTCTCCACTAAAACCCCCACC
CCCGGACCATGTTAGTCGGCAAATCGGCATGCGTATTTGTAAAAACGCCGTCTGCATCAGACGAAATACAGTATGGGAGTACAT
AGGAGGAGAGAGGTGATATTGATTAAACATAAGCCCGGAATCAATTGTGTCTCGATGGAACCTAGCTTTTGTCCTGCTCCCAAT
ATAGTGCTGTTTTATATCCCTTACGAGGATATTAGATCGGGCTCGTTGGTTGTACCACACGGCCCAACTTTCTAGGTCAGGGAT
GGTGATGATAACGCGGAAAAAAGTCATTGGTTGCGTGGTTAATGGAACTCAGAATCGCACTGGCGTCCCAAGTGTCATTTTACT
ATTTAATGCCCCACACAGAAAAATGCTAAAAGTTCTGTTTCCGAGTGAGCATTACCGAAGTGTTTTAGCAGAAACGGACCATAA
AAACATTTGTGGTCCAGGAGGGTGAATAGGGTAAACCGAAAGGTGCTCTGTTGCAACATTTGGTTCCGTTATGCATACGCCTGG
GCTACAGTTGGATTTTAATCAACCAGACCATTTATTCGGAGGTAGGGTGGTAACAGCCCGAACGCATTTTGCCAGGGATGGTTG
TACCGCCGGACCATTTGCGTCCGTGATGGACTAAGCTTGGGCCTTGCGTCCCATTTGGTGTGTTTACCCGGCCGGACCATATGG
TGTCCGGGATGGGACTCAAGCCTTGGCCTTGGTCCCCATTTGGTTGTACCCGCCGGACCATTTAGGTCGGGATTGGTTTCTACC
CGACGGACCATTTGGTGTCCGTGATGGACTGATGCTTGGTACTTCGCGTCCCATTTGGGTTTTTAAACCCCCCGAACAATTTAG
GGCTCGGGATGGGTTAGACCATGTACCATGCCGACCATTTGTTCGGGGAGGGTTTGTACCCGCCAGACCATTTGTGCTGGGATG
GACTAGTTGGCCTTGGCGTCCCATTTGTTTAAAACCCCCGGACCATTTTAGGTGGGATGGGTTTGTACCAGCCCGACCATTGGT
CGGATGGTTTGTAACCCGCCGACCATTTGGTGTCCCGGAATGGGACTAGGCTGGGCCTTGGCGTACCATTTGGGTTTTAAACCC
CCGAACCATTTAGGTCGAGGATGGGTTTTACAGCCTGGCCATTTGGGTCGGATGGTTTGTACCAGCCCGGACCATGTTGGTGT
CCGGATGGGACTAGGCTTGGGATTGGGTCCATTTGGGTTTTAACCGCCCTGAACCATTTAGGTCGGGGATGGATTGTTTACCAC
CCCACAATTTTAACTTTCGGATGGTTTGTACCAGCCGGACCATTTGGTGTCCGGAGGGACTAGGCTTGGGCCATGCTCCCATTT
GGGTTTTAAACCCCGCGGACCATTTAGGTCACGGTGGTTTGTACCAGCCCGGACCATTTGGGTCAGGATGGTTTGTACCATCCC
CGGACCATTTTGGTGTACCCGGATGGGACCTGCAGCTTGCCTTGATCCCATTTGGGTTTAAACACACCGGACCATTACGGTCGG

GGATGGTTTGTACAGCCGGACCATTGGGTTAGGAGGGTTTGTACCCGCCCGACCATTTGGTGTCTGGATGGGACTAGGGCTGGC
CTTGAGTCCCAAATTGGGTTTGTACCGCCCGAACCATTTAGGTCGGACGGGGTTGTTACACGCCCGAACCTTCGGTCGGATGCG
GTTTGTACCGCCCGACCATTCGTGTCCGGGATGGGACTATAGGCTTGGCCTTGCGTCCCATTTGGGTTTTAAACCCCCCAGACC
ATTTAAGGTCGGAATATGTTTTACTAGTCAGTACATTTGGGATCGGATGGGTTTGTACCCGCCCGGAACATTTGGTGTCCGGGA
TGGCAGGCTTGGGCTTGCGTCACATTTGGGTTTTTAACCCGCGACCAATTTAGGTCGCGGAAGGTTTGTACCTTGCCCGACCAT
TTGGGCAGGGATGGGTTTGTACCCGCCCCAGACCATCTTGGTGTCCGGATTGGACTAGCTTAGGGCTTGCGTCCCATTGGGTTT
TAAACACCCGGACCAATTTAGTCGGGATGGGTTTTGTACCAGCCCGGACCATTTTGGGTTAGGGATGGGTTGTCCCGCCCGACC
ATTTTGGTTCTGGGATTGGGAACCTAGTCTTGGCCTTGCGTCCCATTGTGGGTTTGTACCTGCCCGGACCATTTAGGTCGGGGT
GACGGATGTGGTTGACACGCTTCGAACCATTTAGGTCGGATGGGTATTTACCCGCCCGGACCATTGGTGTCCGGATGGGACTAG
GCTTTGCCTTGCGTCCCATTTGTTTTAAACCCCGGACCATTAGGTCGGGAATGGGTTTGACTAGCCAGTACCATTTGGACGGTG
GTTTGGTATAGTGTTGTACCCCGCCGGCACAATTGGTGTCCGGGATGGGACTAGCTTGCCCTTGCGTCCGCATTTGGGTTGTAC
CCGCCGACCATTTAGGTCGGATGATGTACCCGTCCCCGGAAAATTTTTTTTTTTTGTTTGTGTGGCTGGATGGACTAGGCTTG
CCTTGCGCCCATTTGGTTTTAAACCTCCCGACCATTTAGGCAGGGATGGGTTTTGTACCCTGCCCGGACAACATTTGGGTCCGG
GGCAGTTTGTCCCGCCGGACCCATTTGTGTCGCGGATGGACTAGGCTGTCTTGCGTCCCATTTGGTTTTCGTACCCGCCCGAAC
CCATTTGGTGTCTGGATGGGACTAGGCTTGGCCTTGCGCCTAATTGGTTTTATAACCCCGAACCATTTGGTCGGTGGTTGACAC
ACGGACCACTTGGGTAGGGATGCGGTTTTACCCGCCCGGACCTTTTGGTTCTGGATGGACAGACTTGGCCCTTGCGTCCCATTG
GTTTAAAACCCCCCGGACCATTTAGGTTCGGGATGGGTTTGTACCAGCCCGGCAACCATTTGGTCGCGGATGGTTTGTACCCGC
CGCACCATTGGTGTCCGGATGAGACTAGGCGTGGTCTTGCGTCCATTTGGTTTTAAACCCCCCGAACCATTTAGGTCGAGGTGG
GTTTGTACAGCCCTGGACCATTTGGTCGTGGATGCGTTTGACCAAGCTCAGACGCATTTGTGTCGGATGGGACCTAGGCTTGGG
ACTTGGTTCCCTTTTGGTTTTAACCCCTGGACCATTTAGGTCGGGACTGGTTTTGTACCCGCCCGAAACCCATTAAGGTCGGGG
AAGGGTTGGTACAGCCCGGAACCAATTTGTGTTCCGGATGGACTTCAGCTTGGCCCCTTGCTCCATTTGGGTTAAACCCCGCGG
ACCCCATTTGGTCTCGATCGGTTTGTACCAGCCCGGCCATTTGTCGGGATGGGTTTCGTACGCCGCGCCGGACCATTCGGTGTC
CGGGATGGACACAGCCTTTGGGCCTTTGCCAATCCCATTTGGGTTTTAAACACCCGGACCAATTTAGGTCGGGGAATGTTTGTA
CCAGCGCCGGACCATTTGGGTTAGGGGATGGGTTTGTACGCCCCTGACCATTTGGTTCTGGATGGGACTCAGGCTTGGCCTTGC
GTCCTATTTGGGTTTGTACCCGCCTGGACCAATTTAGGTCGGGGTGACGGGTTTGTACACGCCCGAACCTTTAGGTCGGGAATG
GGTTGTACCCGCCCGGACCATTTGGTGTCCGGGATGGACAGCTTGCTTGCGTCCCATTTGTTTAAACCCCCAGACCCATTTTGG
TCGGGATAGGTTTGTACTATCCAGTCCATTTGGTCGGCGGATGGGTTTTACCTGCCCCGGACCATTTGTGTCGGGATGGAACTA
GGCTTGGCCTTCGCGTCAAATTTGGGTTAAACCGCCGCGGACCATTTAGGTCGCGGATGGATTGTACGCCAGCCGGACCATTTG
GGTCAGGGATCGGTTGTACGCCTCCCCGACCAATCTTGTGTCCGGGATGGGACTAGCTGGCTTGCGTTCCCATTTGGGTTTTAA
CACCCCGCCCATTTTAGTCGGATTGTTGTACCAGCGGACCATTTGGTACAGTGGATGGGTTAACCCGCGCCGTACCTATTTTGT
GTCCTTGGTATGGACGTAGGCTTGGGCCTTGCGTCCCATTTGGCTTGTACCCGCCGACCATTTAGTCCGGGCGGTGGTTGTAAA
CGCTAGACACCATTTAGTGTCGGGGAGGTGTTTGTACCCGCACGGACCATCTTGGTGTCCGGATGGGATGTAGCTTGGCCCTGC
GTCCATTTAGGTTAGAACCCCTCCGGACATTTAGGCCGTCGGTGAATGGTTTGTACTAAGCCAGTACCCATCTTGGTCGGGGCA
GGTTGTACGCCGCCCGACAATTGGCCGGGATGGACTCAGCTTCCCGGTCCTTGCGCCCATCTGGGTTTGTACCGCCCGGACCAT
TTAGGTCGGGGATGAGTTTGTACCCGCCGCCGGAAAATTTTGTGTCCGAGATGGGACTAGGCTTGGGCCTTGCGTCCGCATTTG
GGTTTAAACCCCCCGACCATTTAGGTCTGGATGGGTTGGTTGTACCTGCCCGGTCACCATTTGGATCCGGATGGGTTTGTTCC
CTGCGAACCATTGGGTCCCGGGTGGGACTCAGGATTGGGCCTTGCGTCCCATTTGGGT
>m141121_232159_42134_c100732402550000001823141405141507_s1_p0/34471/7814_13094
ACAAAACATAACGTACTCAATCCACCTCTTCATAAAAACTTACTACTATATCTTATACAATGTTATTTTCTTTTTATGTAATGT
TTTTTTGGATTTGGTATCCCTCATCTATATATTCACTATCATCCATCTTTACTCCAGTGAATGACAACATTTTATTGAGTATCC
CTAATGCGGTGTATTTGTATTACTTTGTATCGGTTGATTACGGTTTCATTATGTCATAATTATCTCTCTAGTGAATGTTATTCT
GATGTATATTTATATTTATGTTGTATCGGATCTCTCCTTCTAAATTTTCAATACTATAGTCATAGTGCAATGTTAGTCTAGTAG
TATTTTCCGGGATCGGATCCCTTGTAAATTTCCGCTACTACTAATCTTAACCTGCAGAATGAAAACTATATGAGACCCCCTGAG
CGGGCAGTATTGGGTTTACTAGTGTTAAACTTGGAGCCGGTTCCATTATGTCATAATATTGAGGTCTGCTTAGTGCAATGTTAT
CAAGGTAATAATTTGGGGCAGGATCCTGGAATCATCTTCGTAAACTCACTAATATATGCCTTAGTGGCAATGTTAATCTGGGAG
GGGTATGTTTTTTGGGATGGGATGCCCCCATTCCTAAATTCACCTTACACGTCTTACTCAAAATGACAACAAATAGAGACCCTG
AAGCGTTTGTATTGATTTACTAGTGACGTTGGAAAGGCTTCATTATGTAAATAATCACGTTGCTTAGGTGCAATGTTATCTAGT
AAATAATTTTTCCGGGAATTATGACCCCCATCGGCAATTCACACTAATATTTCTTTATTGCAATGTTAGTCCTAGTATATTTTT
CCAGGGATCGGAACCTTGTAAATTACTTTCATCTTACTCAGAATAAATCTAACTATGAAGCCTAAAAGAGGACGAATTGGTTTA
CTAGTGTACGTTGGAAAGGTTCAATATGTCATAATTAAGGTCTGCTTAGAGAAAATTGTTTATATAGAAATAATTTTGGCAGGA
TCCGGAATCATCTGTCTAAACTCACTACTATATCTAGCTGCAATGTTATCTAGTAGTATTTTTCGGGAATCGGATCCCATTGTA
AATTCACCTACTCGTATCTTACTCCAAACGACAACCCTATTATCAGACCGAGAGGTAGTGATTGGTTTACCTAGTGTACGTTGG
AAAGGTTCAATATGTCATAATTAGGTCTGCTTAGTGCCAATGTTATCCTAGTAAGTAATTTTGCCAGGATAGGATCCCCCTTCT
AAAAATTCACTACAATATCTTAGTGAATGTTATGTAAGTAAGTATTTTCGGAATCCGGAATCCCCCTTCTAAATTCACTACCCA
TTTTACTAAAAAGAAATGACAAGCTATATGAGACGCCTTGCTGTAGTAATTCACATTTACTACGTACATTGGCAAAGTTCATAT
ATGTCATAATTAGGGTCTGCTTGTGCATTATCTAGTAATAATTACAGATCCGGATCCCCTTCCTAAATTCACTAAAAATCTTAG
TGCAATGTTATGTTGTAAAGTAATTTTTCGGAATCGAATCCCTTAAAAATTCACTACTAATAAACTCGTTAGTAATATAATGCC
CAACTATATGAGACCTAGAGCAAGTAATTAGGTTTACTAGTGTACTTGGAAACGGTTGCATTATGTCCATAATTAGACTGGGGG

CTTAAGTGCATGTTAATCTAGTAAAAATTTGAAGGATCAGGATGCGCCATTCTAAACCCTCCCAGCTAACTAATCATGAAGTGC
AAATGTTATCAGTTAAAGATTTTTCGGGATTAGATCCCCTCTAAATTCACCTAAGTCTATTTTTACTCATAATGAAAACTATAT
GAGACCCTAAAGCGTAGTATAGTGGTAATACTAGTAACGTTGGAACCGGTTCATTATGTCATAATTAGGGCTTCTCCTTAGTGC
CATTGTATCGAGTAATAATTTTCAGGAATCGGATTCCCCTCTTCCTACAATTCACTACTAATATATAGTGCAATGTTATCTAGT
AGTATTTTTTCGGGATCGATCCCCTTGTAAATCCGCTAACTCTTATCTTACTCAGAATGAAAACTATAATAGAGAACCCTAGCG
GCAGTATTGGTTTACTAGTGTACGTGAACGGTTGTTATGTCCATATTAGGTCTGCTTGTTGCAATGTTATCTAGTAAATAATTT
TTGCAGGGATCGGAAAAATCATCTTCTAAACTTAACTAACCTATTATCTTATCAATGTTAGCTAGTAGAGTTTTTTGGATTGAT
CCATCCTAAAATTAACTACACAGTCTTACTCAGATGAACAACAATATGAAACCTAAAAGCGGTTTGTATTGATTTACTATGACG
TTGGAAAGGTTCAATGTATGTAAATATCACGTCTGCTTAAGTGCAATGTTATCTAGTAATAATTTTCGGGATCGGACCCCCCTT
TGCAAATTCACAATAAATATCTATTGCCCATGTACTAGTAGATATTTCGGGATCGGATCCCCTTGTAAATTCACAACTCTATCT
TAACCAGAATGAAAAACTAATATCAGACCCTAGAAGGTAGTATGGTTAAACTATGTACGTGGAAAGGTTCAATATGCTAATAAG
GTTCTGCTTAGTGATAATGTTATCTAGATAATTTTGCCAGGATCAGATCATTTCTAAACCCTCACTACTATTGTTAGTGCAATG
TTATCTAGTTAGTTGTTTTTGGAATTGGAATCCCCATCTAAATCACTAACAGTATTACTTAAAGACAAAATGAGACCCCCTTG
CGGTAGTATTGATACTGTGATAACGTGGAACGGTTATTTATTGTATAATCACGTCTGCTATTGGCAATGTTATCTAGTAATAAT
TTCGGATACGACACCCCTTGGCCAAATTCACTAATATTCTATTGTATTGTATCAAGTAGTTAAATTTTCGGGATCGGCTCCCCT
TGTAAATTTACTACTCTATCTCACTCAGAATGACTACTATATCAGACCCCTAGAGAGTATAGTTTATAGTGTACGTTGGAACGG
TCAATTATGTCATATTAGTGCCTTAGTGCCATGTTTATCTAGATAATTTGCCAGGATCGGATCATCTTCCTAAAAAACTCACTA
CTATATCTCAGTTGGCAATGTATCTAGTAAGTGTTTTTTTTGGCAAAGGATCCCAATCATAAATCAATACACGTCTTACCCCGG
ATGCAACAATATGAAGAACCCTAGCGGTTGTATTGATTTACTAGTGTACGTTGGAGAAGGTTCATTAATGTTAATAATACGTCT
GCTTAGTGCAATGTTATCTAGTAATAATTTCCGGGATCGACCCCCTTGCAAATTCACTAATATTTCTTATGCAATGTTATCTAG
TAGTTTTTTCCGGATGATCCCCTTGTAATTCACTACTCCTCTTACTCAGAAATGAAAATCTATATGACCCTAGAGGTAAAATGG
TTTACTAGTGTACCTGGAAAGGTTCAATATGCTAATTTAGGTCTGCTTAGAGAAATTTTATATAGAAATAATTTGCAGGATCGG
ATCATCTTCCTAAATTCACTACCTATTATCCTAGTGCAATGTTATCTAGTAGATTTTTCGGATCGGAATCCATTGTAAAATCAC
TACCTCTATATACCTTCAGATGACAACTATACAGACTCCGAGAGTATTATTGGTTTACTAGTGACGTTGGAAGGTTCAATATGT
CAATAATTAGTCTTTGCTTGTGCAATGTTATCTAGTAAAATTTGCAAGGATCGGACCCCTTCTAAATTCACTACAATATCGTAG
TGCAATGTTATGTAGTTAGTATTTTTTCGGAATCGACCCTTCTTAAAATCCTCTACTCCAAATTTTTAACTCCAGAAATGACA
ACTATTATGAAGTACCTTGCTGTAGTATCATTTTTACTGTTACCATTGGCAAAGTTCATTATTCATAATAGGTCTGCCCTTAGT
GCATTATCCTAGTAATAATTTTAACCAGGATCGATCCCCCTTCTAAAATTCACAATACAATATCTTATGCATGTTTAGTTTGTA
GTATATGTCGGAATCGATCCACTTCTAATTCACTACTATGATCTTAGTAAATGCCAACTATATGAGAACCCTAGAGCAGTATTG
GTTTACTAGTGGTAACGTTGGATACGGTTCATTAAATGTCTGAATTAGGTTCTTGCCTTAGTGCCAATGTACTAGTAATAAATT
TTAAGGATCGGAAATTCCCATTTCTAAACTCAAACTACTATATCATCAGGCAATGTTATCAAGTTAAGTATAGTAATTTTTCGG
GATTAAATCCCCCTTCTAATCCACATAGTCTATTCTTACTCCATAATGCAACATATTGGAGACCCAAGCGGTATATGGATACTA
TGTAACTTGGAACGGTCATTATGTCATAATTAGGTCTCTTAGTGCAAGTTAATCGAGTAATAATTTTTCAAGGAAGCGGAACCC
CCTTCTTTAAAATTCAACTACTATGGATCATAGTGCATGTTATCTAGTAATTTTCGGGATCGGCATCCCTTGATAAATTCGCTA
TCTATCTTACTTCAGAATGAAAAACTATAGAGACTAGCGGCAAAGTATTGGTTTACTAGTGGTACGTTTGGACGGTTCGTATGA
ATAATTAGGTCTGCGTTGTGCAATGTTATCTAGTAAATAATTTTGGCAGGATCGAGTCCTAATCTTTCCTAACTCTACTACTAT
ATCTTAGTGCAATGTTTATCTAGTAATTTTTTTGGGATGATCCCCATCTTAAATTCCACTACACAGTCTTATTCGATGACAACA
ATATAGACCGCTAGCGGTTGTATTGATTTACTAGTGACGTTGAAAGGTTCATTATGTAATAATACGTCCTGCTTAGTGCAATGT
TATCAGTATAATTTTCGGGATTCGGCCCCCTTGCAAATTCACTAATAAATTTATTGCAATTTATCTAGTAAGTATATTCGGGAT
CAGGATCCCCTTGTAAAATCACCAACTCTATCTTACTCCAGAATGACCAAACTATATCAACCTAGAGGTGTATTGGTTAACTAG
GTGACGTTGGAAAGGTTAATATCTCATATTAAGGTCTGTTAGTTGGAAATGTTTTAATCTAAAATAATTCCAGGAAACAGATTC
ATCTTTTCTAAACTGCACTACTATATCTTAGTGCCAATGTTTATCTAAGTGAGTGTTTTTTGGGATGGAGTCCCCCATCTAGGA
TTCACTACAACAGTCTTACCTAATAAAGAACCAAACAATATGAACCCGACCCTTGCGGTAGTATTGATTTA
>m141121_232159_42134_c100732402550000001823141405141507_s1_p0/36512/3233_9404
AGAGATGTTAAGTTTCCCGACTACATTTGTGGCTTATATGAGGGTCGAGCAAATAGTTGTGTAGGGTGGAGTTAGGTGTCCGTA
TTATGGTCTGTAGTGAGGAAGTGACGACTTATGAAGTAGTTAAAGTACTTGGTAATGGGTGGAGATGAGATTGAGTAGTTCAG
ATGCGGATGTCGATTACGATAGGGAGTGCGTTAGGAGGAATTAGTGAAATTTTGGTTATTAGTTGGTATGTGTTGGAGGTGGTG
GTGGGTGTAGTGTGGTGGGGTGGGTGGTAGTGTAGGGGTATGAGTGGTAGTTGTGTGGTAGTAGGGTGGTATGGTAGTGGAAGT
GGGTCCGTGTTGTGTGGGTGGTGGTGGTGGTGGTGGTAGTGGTAGCTGCTAGTGTAAGTGTAGTAGTAGGTAGTTAGTGGTATA
AATGGTATAGTGGTAGTAGTGTAAGTAGTAAGTATGTGTACTTAGTAAAGTAGTAGTAGTGGTAGAAGTAGTGTAGTTAGTAGT
TGTAGTTGTGGTAGTGGTGGTAGTGGTAGTAGCTGGCTAGTAGTCAGTTAGTATGTGTAGGTTGACGAAGACGTAGTAGTGTAG
TAATGGTGTAGTGGTAGTGGTAGTAGTGGTACTAAGAGTAATAGTAAGTGGAGAAGTAGTATGGTAGTGGTTTGTTGTATGGTA
GTGTAACTATATAGTAGTAGAGGAAGTTGTGTAGTGGTTGTTTTTAGTGGTAGTGGAATAGTAAGTAGAGTAGTAGTAGTAGTA
AGATAGTAGGTAGTAGTGGTATTAGTGGTATGGTAGAAGTGGGGTAGTGGTAGGTAGTGGAGTAGTGGGTAGAAGTGGTGATGT
AGTGGAGGAGTGGTAGTAATGGTAAGTAGTATAGTGGTTGGTGGCGGTGGTGGTAGGTATTGGTGGTTTGGTATAGATGTATGT
AGTGGTAGTAGGTGGTAGTAGTGGTAGTTGGAGTATAGTAGTAGTAGGGTAGTGGTAGAGGTAGTGTAGGGTAGTAGTGGTAGT
AGGGTAGTAGTGATAGTAGATAGTAGTGGCAGTAGTATAAGGGTAGTTGTTTGTAATAGTGGTAGTGGAAGTAGTATGTATTAG
TAGTAGTATGAGTAGTAAGTTTTGTGTTGTATTGGTAGGGTAGTTATAGTGGTGGTGGGGTGGGGTGGTGGTGGTGGGCGTAAT

GTTATAGTGTAGTAGTAGAAGAGTAGTATTATAGTAGAGAGTAGTAGTGAGTAGTGGGTAGTAGTGTGGTGGTAGTGGTAGGTG
TGTAGTGGTGGTGTTGATGGTAATAGTGGGTAGTGGTGTAGTGGTAGGTAGTAGTAGTAGTGTGGTAGTAGTGGTAGTGGTTGT
GGTGGTAGTGATGGTAGAAGTGGATGTAGTGGTAGTAGTAAGTAGTGGTAGTAGTGGTAATATTAGTATTAGTAGTGTAGTAAG
TGGGAGTAGAAGGAGTATAGTGGTAGTGGTAGTAGTAAGTAAAAGTAGTAGTAGTGGAAGTAAAAGAAGTAGTTGCTAGGTGGT
GGTAGGTGCAGTAAGTGTAGTAGTATAGGTGTAGTAGTGGATAGTGTAGTATGGTAGTAGTTGTAGTGGTTGGTGGCGGTGGGT
GGTAGTATTGGTGGTGTTGTGTAGTAGTAGTAGTGGTATGAGTAGTGTAGTAGTGGTAGTGAGGTGGTGTAAGATGTGGGTAGTAGT
GGATTGAAGTGGTAGGGAGAAGCAGTATGTAGTAAGGTAGTAGTGAGTTAAGTAGTAGTGGTAAGTTGTGGGTAGTATTAAGGA
GTAGTAGGACTAGTAGTGGTGTAGAGGTATATAAGTGGCTAGTAGTGTAGTAGGTGTAGTATAGTGGGGTTAGTAGTGGTAGTG
GTAGAAGTGGTAGTTAAAGAGTAGCGGTATTTGGTGGTGGAAGTGGTAATGGTAGTTGGTAGTAGTGGAGTAGTTGAAGTAAGT
AGTAGTAGTATTTCAGTGGTGGTGGTGTGTGGTGGTGGTAATGGTAGTGTAGTATGGTAAGCTGGTATATGGTATGTGAGTAGA
AGTGTAGTAGTAGGTGGAAGTAAGAAGAATGGGAAGCGGTGTAGTTAGGCGTAGATGGTAGTAGTTAGTGGAAGTAGTAGTAGT
GGTAGTAGAGGTAGAGGTAGTAGTGGTAGTAGGTAGTCTGGTTGTGGTGCGGTGGTGGTAGTAGTGGTGGTGGGGTGTAGAACG
GTATGGTAGTGGTAGTGGTAGTAGTGGTTAGTAGCGGTAGTAGGAGTAGATAAGTAGCAGTGGTAGCAGGTGGCGGTAGGTGGT
AGTGGTAGTGGTTAGGTGGATAGTAGGTAGTATTGGTACTTAGTGTCCGTGGATATAGTGGTAGTAGTAGTTGTGGGTATAGTA
GAAGTAAGTAATAGTTACGGGAGTGTTGTAATAGATGGTAATGGAAGTAGTAGTAGAGTAGCATATTAGTATAGTAGTAGAGAG
TAGTTGTTAACGCCTAGCTTCTTAGAGCCGGTTACTAGTGAGCTTTTCAGAGACAAATCAGTGTGCAAAGATTCCCGCTAACCT
GAGGTTTTGAAACGGAAAGTGCTCGGACTAATTAAAGCTTACAGGCTGTAGATCGATGTAGAAAAATCGTTGTTCAATAAAAAC
TCATAATATTCTAAACATTGGGATCCCAAAATAAGGTTTGAAAACTATTACTCTAAAAAAATTGGTTCACGAGTTGATAAAGTC
ATAAAAATCAAGATTTATTATGCAGCATTTGTTCAAATAAACCCCCAACCAAAGCAGTCCGAGGCAGGCCAACATGTTACGCGT
CGCTTTCACCGCATCTCCGTACTCATGGTTGTTTGACTCAGTCATTGCCCTTACCCTGCAACACAGACACGCAGCAGTCGTGAG
CCGAAGCCAGCAAGAAAACTCATCAGAACGATAACATATGCAATGTATAACAGTTTAATCATAATCAGTATAATACCACAATAA
TACAAGTCAATCATTAGACTAAGCAATACACGCCATAGCCGCCCCAAGGACCCCTTACCTAAAGCCCCGTGGGCGTATCGGTTC
CCTCACCGCTGAGGATAATCTCATGTATCCCTTGGGTCCCACCTGATATATAGCATCCCCATGTGCTAAGTGTTACTTTTCGGC
CCACGGCCGCCCCGGCCTACCGCCGTAACTACGGCCCACGGCCGCCGGCTACGCCGTACTCGGCCCCACGCTCGCCCGGCTGTA
CGCGTACATTTCATCATAGATCACATATATATGGTAAATTTCGAAATAAACATTCACAACAACATCACAGGTTCATTTCGGTTA
ACATTTACACATAATACAATCCGGCGGCCAACGCCTACAAATCATCTCAATCAGCAACATGCAATAAAGGCCATGCTCGACCAT
GCACACTACGGACCCAGCACTATCCTACGGGGTTATATGTTCTTACCTGTATTCCGAGCTTCCTGATGCACTAAAGCCGCGAGC
ACGGTCCTCAAGCACGACCCCACACAACCTATTGTCACAACAACAAGAAACCCATCCCTCAATACTATCAACCCAAAACCACTT
CCCGGGACCGAATCCCGCGCACTCACCTCGGAACTCACCCAAATCCTAAAACAATTCCACCCGAAACATCCCCCCCGAACCCCC
GGAAGCAAAAGCTCAAAATGCAACTTTTGTGCCCTGAAAATGGCCTTGTGCCCGCGGCACCAAGAAGTGTGCCGCGGCATCATC
CCCATCCAGATCAGGGCTCTTTGCCGCGGCAACGAAAGAAAGCTGTGTCGGGCGGCAACCGCCTTCGCTAAGACCCCCAGAATT
CTGGGTTTTTTCCCTGCGTTTTCCTCGCGATGCCTAAAACCCTTCCCAATTCATACCAAATCCAAACCAAACCCCCCAAGAGAA
TCAAACCAAACTTGGCATATGAAACGCGCATGCTAACAACCCCAATAAACCATCAGTCAAAGGCAATCACACACAATCGAAATC
CCCAAACGAATCCCAACATCCCTTGATTTATAATTTCAGAATTAAAAAACTCACGAAGTCTTCAACACTTAACACCCATGCCCC
CTTTCCCCAAGAAAACTCTTAACCCATCAACATAGAATCCCCACCGTCTTAAAGATTGTAATAAACTCCTTAACCCTCAAGTAG
TAGAATCCCTCTCCAACGCTCCCTCCGGCGACTACCAATTCACTCTTAAGTTCACTTCAGCCCATTTTCCTCCTTCCTTTTCTT
TTTCCTTGCCGCCTATGGACACCTGCCCTCCCGCCCTTTCCATTTTAGAGCAAATAGCCAACAATATACTCCTAAAATGCCAAA
ACCGTGACCCAACTAAACAACCCAGCTGAGAATTGCCTATAACCCTTGGCCAAAACTGACCATTTTACGCTCTTTCTACTAATC
CTCCTAGCTAAACCCGTCAAAGACAGCTAAGTCTTTTCATTCCCCATTTCAATTTCTACATGACCCTGTTTTACCTTAAAACCT
TGTTACCACAGCTAGTAACTAATGGTTACCGCCAGGTTTACTAACATCAAAATCCTAATAACCTTTATCCCGCTAAACCAACTT
AGCCCATAAAATTCCTGTAGATATCCCCTAGCTCCTCGCCGAGCCCGGGTATGCAGAATCCCGTTGTGACTCTTAAGTTAGACT
AGCTCTGTCGACGGACCCGTCTCCGGCACGATGCATCACCCACATAATACAACCACAGACCACCACGTGGTACCAAATGTCACA
GAATACAATTATCCACCATATCAGTACACGTTATGCCAAACATGGCCCAAATAGCAATTACGGCCCCGTTCTAAAAAACGATCC
GGGCTAGCATGCATACTAGTAAGCTCACATACGCCCATTCGCATCTCAGCGTTAAACAGCATCGCCAAAATAACTGCGCTTCTA
AATCATAACCATTGCCAATTTAACTCCATAAATCTACAAAGATATATTCCCATAACCGTGCCCCCGCTGGCACACTAATCAAGG
CCTTAAGCGACCTTATTTAGCGATAATTGGGCTCGTTACACACTATCCCCCTCCTCAATATAAAATTGTCGTGCCCCTCCGAAG
ATTTATCCAAACTACCATCAGGTCAGGCAAACCCCAACTCTGACCCTCCAAAAATCCCCAAGGCCACGCGACCTTACTTATAGT
TCCAACCAACTCTCTTGCCAGGCTGCCAACAATGCTTGCCCCACACAGATCTGTCTAAACGGCCATCCCTACACCCGAATACGC
CTATCGTTTACACCGCAACCCTGCTGAAGCCTCAAGGTCTGGCCTAGATTTACAATGACCAATCTTCATTGTCTTATTCCTGAT
TCATAGATACCTCCAAAACGTAATCCAGACCTTCTACAAGGTGGGATCAGCTTTAGGCTCCGCTGGCCACTAAGACACTTAGTG
ACCAACTTCGAAATCTTTATGCTGAATCAAGAGGTCCAGAACTCTCCCTTCCTTGTCCTCCTGAACACCTACGTAGGAATCCTC
GTTCTGTTATGTACGCAGCAGATTACAATTCTTTCGCTTCCCGGAGCTTTATTCCTCCTCGATAATTATACACAATTCAGCAGG
CTCTTTAATTCTTTCTCAGCACTAGCAGGACACTCCTGCGCTTAACGAATTCCAACAGCGCCTCCCTCCTGCTTCCTCTCTAGA
GCCAGTTGCCAATAGTAGTAGTCCCACCATCTCTTCACCCTTTCCACCATGTCCTAGGTCACCGTTACGCAGTTAACAGAGACA
GCCAAGCAACTGGCCCAAACCACGGCTAATTCCTCAGGGATTACTTCCCTTAATACCTCCAAGTATTCGCCCCTACTCAGCGCT
CAATCCCTTTAACGATATTCCTGTATAAAACTTTTCAGACGGCCATTATTAAAAAAAAAAAAGAAAATAAAATTAAAACAAATT
AGCTTGCAATCAACTGAAGCCCAGGTTCCCACTCTGTTCCTTATTCTCGTCCCTCCATGGCCCATTCCCCAAAATTTAACACCT
ACTAAAGGGCCTCAATTCAGTATTCACACTTTCCACAATCAAAATCCTCAATAAGGCTGAAACTAACACCAGACACACAGAGCC

ATCAGTTAAAAACCAATGGCTAACTAGTTTACCTCAAGTTTCAGGTTATGGTGCTCCTTCAACCCAGTGGAACACCCTCCAAAG
ATAATCGAAGGCCTACGCTCCCAAAGCTCAAAATGCTGC
>m141121_232159_42134_c100732402550000001823141405141507_s1_p0/53991/182_8901
ACACTTGCCGAATCCATTATTATAGGTTTGAATCGAAGATTTCTAAAGGGTACCCGTTTGGTGACGATTGGAGATTTCATTAAT
TTCTCCGAATGTATTAACCACCGATCAGATCTCGTTCGAATTGTTCCCATTGATATCTCATTGGGAACCCCAATGGAATCTATT
GGAGGTTGAAAACAAGGAAGCCTTAAGATCAGCATTTGAAGGAAAATTTCACTTTTTTTATGGTAATTTTCGCAGCTTTTGCCG
GGTTTCCGTACAATCGCGTCAGCATTTGCACTGTAATCACCCTCGTTACACACCTCGATTTCCAATCTGAAATTGTTATTCGTA
CAAGCTAAACCACATAGCGATACAACTTTTTGTGCCCATTTGACCATCGCATATTTCTTTAAGTATTCAGTGTCCCAACAATTG
CTCCGAAGCCCATATAGAGAAATACGAAAGACTTTCTGAAAGGGGACAAAAAAAAAACTTTACGCACTACCACACGCATACAAT
ACCCACCCCTCCCCCACCACAAACGCCAAAACACAAAACAAAAAACAACAAAATCACAAGACATAACAAACACAAAATGGACAC
ACTCGTGCGGGCTCTTTTCCCTGGCTCTCCTGCGGTCTGACTCTACTTCGCAGTGCCTGGCCGCCTTCGTCCTTTTGCGCACCT
TTCTCCTTTTTGCTGGCCACTCTCGCAGCCGTTTTTGCCTTCTTTCGACGCCTCTCTCACGTCTTTTGCTGTGTCCTCCTCTCC
CCACCTCGGCTTTCCATGCCCTTTTCTCCCGTCGCACAGCCGTCCCCATACGGGTCTCTTTGCCCCCATCCAGTCGGCACCCCG
TGCATCGTTCTCGCTATCGTGTCGCCCCCGTCGCGTTCTTCTCCGACGACCCTTCGGTTCGACCGCGCACTCCCCTCTGTCATT
GACAATGACAGTTTTCTTCACGGGAGTACGCCATTGGTCGAATTGAGGATCTTCACGTAATTTTTCTCCACGAAACTGTATTAC
CCGGCGTTCAACTGCGTTCGACGTATTTTCCCCATGAGCTATCAGCATGGGTAAATGAATCTATGTGGACGTGTGCGAAACCGG
AGCCTTAAGCAAACATTTGAGAAATATCTTTCTCACTTTTTTTATGGTATATCTTTCGCATGTTTCTTGCCCGTATTTCTTCGT
ACAACATCTCTCAGCTATTTGAGCTGTATCACCTCTACCCAGAGCCCTTTGTGATTTCAATGAAAAGTTTTATTGCCGTACAAC
AGCCAACACACCATATGGCGCCTACCAAACTTTTGTGCGCCCATGCACCGACCGCATATTTGATGTCAGGTGATTTTACACCCT
TCCAAAACCATCTGCTCGAAACTCCATGAATCACGCGGACCGAGCAACTCAACCCCAGATTTCTGAAAGGGGTACCCCTACATG
GAGACAATTGTAGATTTCACAAAAATTTCTCCTGGAAATTATTACCAATCTGTGTTGTTCAAAGTCCCGCCTCGATAGTATTTT
GCCCCATTTAGATAGTCATGGGTAAATTGAACGAATGGAGTTGAAACGGAGCCTTAAGTCGACATCTGAACAAAAAATTCAACT
TTTTGATGGCAATTTTGGCATGTTGTTGAACATATATTCCCGTCAATCTCTGAGTAATAGTTGACTGTATCGACCCTCCTACAC
ATCCTCGGTAATATCCCTTCAATATTTCATACGTAGAAGCTAACACATTGGGATAGAGCAACTTTTGTGCCCATGTGACCATCG
CATATTCTTAGTATTCGTGTACACAACAATGCTCGAATCCCATATTAGATGAAATCGAGTAGATTTCTGAAAGGGGTAAGCCAT
TGGTGAATTGGCGAGATCTCACTAATTTCTCCGAAATGTATTTACCTCCGTGTTCAACCTCTTCGAGTATTTTTTCCCAAGAGA
TCTCTCCTGGGGATATGAGATATTCGGAGTGAGAACGGATAAGCCAAGTCAAAAATTCTGAAAAAATTTCCCCTTCTTTGATG
CAATATTCTCGCCATGTTTTTCCTATATTTCGTACATCTTCCTCAAGTCTATTTGACCTGTATCAGCCCTCCTTACCACACGCT
TGGATTCAATGAATTTTATTCGTAAAAGCCAACACTATAAGCGCTAAAACTTTGTCCCATGTACCAAATCCGCGGCGTCAGTAT
GTCTTCTTAGTATTCACATTTTCAAACACATCTCGAATCAAATAGACGAAAATCAATAGATGTCTGAATAAGGGTCACCCCTTG
GCGAAATTGGAGATTTCACTAGATTTCTCCCGAAAATGTATTAACCCTCGTGTGCCAACTCCCGTCGACTATTTCCGCCAAAGA
GATCCCCCCATGGTAAACATGAATATATTGGAGTTGAAACGGAGCCTGTAGTCAAAATTGGAAAAAATCTCCCCTTTTTGATGC
AATATTCCGCAACAGTTTTTTCCTTATATCGTTACATGTCTCAGCTATTTCACGTATCGATACCCTCTACCACACCTCGAATTT
TGTGTCACGTAAACTTTTATACTTAGAACTCTACAGCACATTAAGGCTAAACAACTTTTGTTCCCATGGCCCTTGCATAGTTTT
TAGGTATTCAGTATCAATCATGCCCCGAATTCGGTATTCAGAGGAAATCCGAAGATTTCTGAAAGGGCGTACCCTTTGGTGAAT
TGGATATTTCACTAATTTACCTCCCCCGAGACGTATTCCCCTTTAGTTTTCAACCGTCCGAGTTTACTTTCTCCCCCGCAAAGA
GATCGTCATGGGTAACCATGAATTATATCGGACGGCGAAAACGTGAGGCCGATAAGTCAACCCATTTCAAAAAAAATTCCGCCC
CTGTTATATGTGCAATTCTTCCGCAAGTTTTTTCCCCTTCTATTCGTACATCTCCTCAGTATTATCAACCTGTAGCCAGCAGTC
AACTCCTCTACAATCAATGGTCGGAATTTGTACGAAAATTTACAATACATAGAACGCCTAAGGGACATAGGGCTACAACTTTGG
TTCCAATAGCCCACGCATGCATTCTTAGAATAATATTCGAAACATTGGCTCGATATTCGTATTACGCAGAAACCGAAGATTTCT
GAAAGGGGTACCCCTTTGTGAATGAGAGATTTCACTAATTCTCCGAACATGTCATCTACCGCTCGGGCGTTCAACTCGTTCCGA
CTATTTCTCGCCACCAGAGATCTTCATGTTTTAAGACTGAATAATATCTGGCGTCGAAACGGAGCCTAATAAGTCAATCATTTC
AAAAAAAATTCCCCTTTTATGGCAATTTTTCGCAATTTTTTTCCTTATATTCGTAGCATTCGTTAGGTATTCCTCACGTACCCA
CCCAATCCTACACCACCTCGGACTGCGGATTTCGTACGAAATTTAATTACATATGAACCGCTAAGACATACGGGCTAACGAGCC
TACAGATTATGTCCATGACCTATGCATCTGATTCTTAGTAATTCACTACATTCCAACCAACATTGTCGAAATTCGTGATTAGAG
AAATCGAGGAGATTGCTCTGAAAAGGGGTAGCCCCCTTTGGTGAATGGATAGTTCAATAATCTCTCTGAAATTGTATTTACCCC
TCAGAGTTCCAACTCGTTCGATGTTATTTCTCCGGCCCAAGAGATCTGCATGGGTAAATGAATATCATGGACGTCGACGAACGG
AGCATAAGTTCAACATTTCAAAAAAAATTCCCCTTTCTTATATGGCAATTCTTCGCCAGTTTGTTCCTTATATTTCGTACATTC
TCTCAGCGTATTTCACCTGTACGACCCTCTACCCAGCAGGTCGCATTTTGTACGAACAATTTTATACAGTTAGGAACCCAAACA
CAGAGCATATGCGCTACAACTTTTGTTCCCATGCACCCCCACGCATATTTTCGTAGATATCTCGACTATCGCAATGCAACAGTG
TGCTTCGAATGTCTGATTACGGCGACAATCGAAGAATTTCTGATAGAGGGGTAACCACGTTTGGAGAATTGGAGAAGTTCCACG
TAATTTCTCCCCGAAACTGGTATGTACCCTCCGCGTGTTCAAACCTCGTTTCGATCTATTTTCCCCCAAGAGATCTCATGTTTT
CAAATGAATTACTATTAGGGCGTGAGAACGGAGCCATGAAATCAACATTTCAAAAAAAATTTCCCTTTTGATGGCCAATTTTCG
GCAAGTTTATCATATACGTTCGCAGCAGCATCTCTTAGTATGCTCAAGACAGACACGCCTCTACAGCATCTTCGGAAATTTGTA
CGAATTGTTTACCAATAGAACCCTAAGACATTAGGGCTAACTGCACCGGTAATTATTGTTTCCCAATGCCCTAAGCACTCATTT
TTAGATTTCACTCAAGCATACATTGCTCGAAATTCGTATTAGACGGAAATCGAAGATTTCTGAAAAAGGGTACCGCCCTTTGTG
AATCGGGAGATTTCACCTAAATTTCCTGAAATGATTTAACCTTGCGGTGTTCAACCGTCGACCTATTATCCAAATGAGATCTCA
TGGGTAAATGAATTAGTAGCTTGGAGTGTCGAAAACAGAGCCATAATCAACATTAATAAAAAAATCCCCTTTTTTGATGGGCAA

ATTTTCGAAAAGTTTTTTCCTTATATTTCGTTAGCATGCCTCTCCAGTATTTCTTTCAACCTTGTGAACGGACCCTCAACAGAA
AAGCGGTTTTGTAACGAAAATTTATACATAAGAAACGACCAAGACATAGGCCTAAACAACTTTTGTGTCCCATGACTCGCACGC
ATTTCTTTAGATTAACCTGTCGAAACATTTGCTCGAAGTCGCGTATTAGAGGAATCGAAGATTTTCTGAAAGGGGTACCCCTTT
GGTGAATTGGAGATTTAACCAATTTCTCCCCGAAATGATACCCTCGTGTTCAATCCTCGTTCCGATATTTCTCCCCAAGAGATC
TCATGGGGTAAATGAATATTTGGAGTCGAAAGCAGAGCCATAAGTCAACATTTCAAGAAAAATAAATCCCCTTTTGATTCGCAA
TTTTCGAAAGTTCTTTTCCTTATGATTTCGTACATGCTCGTCAGTATTGTCAACGTAATCACCCCAACACACCTCGAATTTTGT
ATGAAATTTTATATCACTAGACACCCTAAGACAGTAGGAAGATCAACTTTTTTTCCATGACTCTCACCGCATATTTTTAGTATC
AGAATAACTATCCAGATACATTGCTTCGAATTCGTATAGAGAAATCGAAGATTCTGAAAGGGGTAGCCCCCCGTTTGGTGAATT
GGAGTTATATCAATCTAATTCTCCGAAGATGTATACCCTCGAGTTCAACTCCGTCGTATATTTTCCCCCCAAGAGACTCATGTA
AAATGAAATATTGGAGTCAAACCTAACGGAGCCCATAAGTCAACATTTTCAAAAAAATTCCCCTTTTATATGCAATTTTCGCAA
GTTTTTTCCTTATATCGTAATCATCATCGTATTTCAACTGGACCGGACCCTCAAAAAAAAAGTCGCGTTTGTACGAAGATTTAT
ACATAGAACCCTAAATGACATAGGGCTACAACGTTTGTTCCCATGTCCCACGAAATAATTCCCTTAGAATTAACTACGAAAACA
TTTGCTTCGATTATTCGTATTATAGATATATCGAGATTTCTGAAAGGGTACCCCTTGGGAATTGGAGATTTCACTAATTTCTCC
GAATGTATTACCGCTTCCGTGTTCAATCTAAGTTCCATATTTCTCCCCAAGAGATCTCAGGTAAGAGAAAGATGGTGTTCGAAA
CATGAGCCATAACGTTCAAACATTTTTCAAAAATAAATTCCCTTTTTGATGGCAAATTTCGAAAAGTTTTTTCCTTATATTACG
TAGCTCTCTCAGATTTTTTCACTGTACCACGCTCCCAACAAAGGATCGGTTTTGTACGAAAATTTATACATAGAACCCTACAGA
CATAGGGCTACAAGTTTTTTTTCCCATGACCCGACGGCGGATATTCTTTAGAATTAACTATCGAACCATTGATCGAATTCTATT
AAGAGAAATCGAAGAATTCTGAAAGGGGTACGTCCCGTTTTGTGATAGTGGATGATTTGCACTAAATTTCTCGAAAAGAAATGT
ATTACGCGCCTCGTGTTCTACTCCGTTCGATATTTCTCCCCAGAATTCCCCATGGTTAAATGATTATATTGGCCCGGAAACCGG
AGCCATAAGTCAACATTTCCAAAAAAAATTCCCCCTTATTTGATGGCATATTTTCGAAAGTTTTTTCCCCGTATATTCGACATC
TCGTCAGTATGTAGCTGTATCCGACACCTCCAACACACCCTCGAATTGTACAAATTTAAACCATAGATACCCCTCAAGACGTAG
AATATAAGCAACTTTGTTTCCAATGATTTCTAGCATATTTTTTAGTATTCAGGTATCCAATACATTGCTCCGAATGCGTATAGA
GGAAATCGTCGAAGATTTCTGAAAGGGGTAGCCCCTGTTAGGTGGATATTGGATAAATTCTCACTAATTTCTCTGAAAAGTATA
CCCTCCCCGAGTCAACTCGTTCGATATTTCTGTCTTCCCCAAGAGATCTCCAATGGGTAAATGAACATATATGGAGTCGAAAGC
GGAGCCATACAAGCTCAACACTTGTCAAAAAAAGATTCCCCTTTTATATGCAATTTTCGCCAGTTATGTTTCCTTATCTACATC
TCTTCCCAGTTATTTTCACTGTACCGCCACCCCTCTACACAGTCGGATTTGTACCGAAATTAGTACATTAGAACCGCATAAAGA
CATAGGGCCCTAGCCATTAACTTTTGTTCCGCCCATGACCACGCATAATTTCTAGTAATTCACTAATCAAAACATTGCTCGAAT
TCGCGTCATTAGCCGCCGAAATCCGAAGATTTCTGAAAGGGGTAACACTTGGAGAATTGGAGATTACTATTTCTCTCGAAATGT
ATTACCCTCGTGTCAGACTCGTTCGATTATTTTTCCCCAAGAGACTCTCATGTTACCAATGAATATATTGGCGTCGAATGGACC
ATAAATCAACATTTCAAAAAACAAAATTCCCCTTCTTTTGGATGGCAATTGTCGCAAGTTTTTTTCTTATATTTCGTAACATTC
TCTTAGTATTCTCACCATACCACCCCTCCCCGTAACACACCCCGTCGGACCTTTGTACGAAATTTTTATACATTAGAACGCCTA
TAGACATAGGGTCTCGAACTTATGTTCCATGACCCTAGCCAATAGCTTTCTTAGTATCACCGTATCAAAAACCATTGCTCCAAT
TCGTATTAGAGAAATTCGAAGATCTTCGGAAAAGGTACCCCTTTGGTGAAATTGCTGGAAGAATTTCACTAATTTCCCTCGAAC
ATGTAATTACCTTGTGTTCTAACTCGTTCGTATTATTTATCCGCACAAGGAGATCTCTATGGTAACATGAAATATATTTGGAGT
CGGAACAGACCATACAAGTTCAACATTTCCAAAAAAAATTCCCCTTTTTGAGTGGCAATTTTTTCGAAAAGTTTTTTCCTTATA
TTCGTAGCATCTCATCAGTATTTTCACCCAGTACCTACCCCTCCAACAAAAGTCGGCGTTTGCGCTTACGAAAAGTTATACCAT
AGAAACCCTAAGAGCATAGGGCGCTACAAGCTTTTGTTCCCATGGACGCTCACGCATATCTTTAGAATTAACTATCGAAACAAT
TGCTCGATTCGTAGTAGAGGAATATCGAGAGATTTCGAAAGGGGTACCCCTTTGGTGATATTGAGAATTTCACCAATTCTCCCT
GAAATGGTATTACCTGTGTTTCAATCTCGTTCGACTATTTCTCCCCAAGAGATTCTCAATGGTAATTAATGAATATTATTGGGA
TGTCCGAAACAGAGCCATATAGTCGAAACATTTCAAAAAAATTCCCTCTTTTGATGGGGCAATTTTCGAAAAGTTTTTGCCCTT
ATCATTCGTAATCTGTCAGTATTCGTATACTGTATCACCCTCCAACCACCTCGAATTGTACGAAATTCTAAGTACATAGAACAT
AAGACGTAGGATACCAATTTTGTTTCGCATGACACCTCTAGCAAATAGTTCATTTTAGTATTCAGTTCAAAATTACATGTGTCA
TCGATATTGATTAGATATGAAAATCGAAGATTTCTGAAAGCGGGTAAGCCCCTTTGGTGAATTTGGAGTAGTGTTCACCTAAAA
GATATTCTCCCCGAAATGTACATTACCTCCCCTGAGTTCAACTCCCCGTTCAGTTATGTTCCTCCCCGCAAGAGATCCATGGGT
AACAGTGATATACTCATTGGAGTCGAAAGCGGAGCCATTTAAGCAATCATTTCAAAAAAAATTCCCGCTTTTATGCTGTGGCAA
TTTTCGCAAGTTTTTTTCCTTATATTCGTACATTCTCTCGAGGTTATTGTTTGCACTGTACCAGCCCCTCTACAATGGTTCGGG
ATTTGTAGCGACAGTTATACCATAGAGACCCTGAAGACACTTAGGTGCCTACAACTTTTGTTCCCATGACCCACCGCATATTCT
TAGTATTCACTATCAAAACATTGCTCCCGAATTCGTATTAGGAAACCTGAAGACATTTCTGAAAGGGGTTAAACTTTGGAGAAT
TGGAGAAATTCACCCAGAATGTTCTCCGCATGTATTACCCTCGTGTCAGCCCCCGTTCGATATATTT
>m141121_232159_42134_c100732402550000001823141405141507_s1_p0/54455/2802_9817
AATTTTGAAAAGTTATCACTTGGTCATGACTTTCCATACCAAGGCTCAATATTTAAGTCCGTAGCGTCCTACCAATTTCGCCCA
TATCCCCCTTTTGGTTTGTGACTTCGGATCTAATTACGATACCAGTATTTACTTATATTTATACCTTTCATGACACAGGACACA
TACATAAAACTACTAGATTTACCCAAGAGGTAAATCATTAACTTCTTGATTCACCTCCCTAACGCTCACCTCTCAGCATTTCCT
CTTCCTTCTCTTCTTCCTCTCCCATCCCTACTCTCTTCCTCTTATTCTCTTCAAATTCTATCAGGCACTAAATGCATCCATGCC
ACCTGACATATAACATGAAAGCTATAAGACATGCCAAAAAGAGAACCATTTGTACCGTTTCCACTTATCTCCTAGTAGCTAAAC
CTCAAAGGCACCGTGTCGCTTCCGCCTACGAGATCGCTACGGAGGCCCTAGGCAGCTGCCTAGGAGATTTGGCCTGAAAGGTCA
AATTTTTTAGTTTGGGTTGAATTAGGACCCCAATTCATGGTAAAAATCCGCCCTATAGTAATATTATGTGAGAGGGAAAAGGTT
CAAAAAATAAGACTTTTTAAGGGAGAAATATGAGTGATTGCGAGATCGCTTCCAGAGCCCTAGGTCAGCTGCCTAGGAGATTTG

GCCTGAAAGGTCAATTTTTTTTGTTTGGTTGTCATTAGGACCCCAATTCATGGTCAATAATTGCCCTTATATAATATTATTAAG
ATGGGAAAAAGGTTCAAAAAATTAGAACTTGTGTAGAGAGACATATGAGGTGATTAGCTAGATCGTTTCGGACCCTAGCTGCTT
CCTAAGGAGAATTTGGCCCTGAAAGGTCAATTTTTTTCTTCTTTTTGTTAAATTAGGACGCCAATTCATGGTCAATAATAGCCC
TAATATAATATTATTGAGGATGGGAAAAGGTTTGAAAAAATTAGAGCCTTGTTAGAGAGAAATATGATGAATTACGAAATCGCT
TCGGAGCCCTAGGCAATGCTTGCCTAGAGAATTTGCCTGAAAGGTCAATTTTTTTGTTTGGTTGAATTAGGAACCCAAATTCAT
AGTCAATAATAGCCCTATATAAATTAATTTTTTTATGAGATGGGGTAACAGTTTCAAAAAAATTAGAACTTGTTAAAGAGAAAT
ATGAGTGATTACGAACACTCGCTTCGAACCCTAGGCAACTGCCTAGGAGATTTGGCATGGAAAGGATCATTTTTTTGTTTGGTG
GCATTAAGGGAGCGCCCAATTCATGGTCAATACTTGCCCTATATAAATATTATTTGAGATGGGAAAAGGTTCAACAAAATTAGA
GCTTGTTAGAGAGAAATATGAGGGAATTACGAAATCGCTTCGGAGCCCTAGGCAAGCTGCCTAGGAGATTTTGTCCTGAATATG
GTCAAATTTTTTTGCTTTGGTTAAATTGAAGGACCCAATTCATACGTCAGTCAATAGCCCTATATAATATCTATGAGATGGGAA
AGGGTTCAAGGGAAAATTAGAGCTTGTTAGAGAGAAATAGAGTGAATTACGAAATCGCCTTCGGAGACTTGGCTGGCAGCCTAG
GATTTGGCCTGAAAGGTCAATTTTTTTGTTTGGCTTGAAATTAGGACCCAAATTCAATGTCCACCTAATAGCCCTATATAATAT
ATTGAGATGGGAAAATGTTTAAAAAAATTAGAGCTTGTTAAGGAGAGAATTATGAGTGAAATTGGCGAGATCGCTTCGGAGCCC
TAGGCAGCTGCCTAGGAGACCTTTGCCTGAAAGGTCAATTTTTTTTGTTTGGTTGTATTAGGACCCCATTTCATGGTCAATAAT
AGCCCTATATATATTATTGAGATGGGAAACAGGTTCAAAAAATTGAGCTTGTTAGAGAGAAATATGAGTGATTACGAAAATCGC
TTCGGAGCCCTAGGCAGCCTGCCTAGGAAAAGGATTTGGACTGAAAGGTCAATCTTTTTTGTTTGGTTGAATTAGGACCCCAGA
GTCATGGTCAATAATAGCCCCTGTATACATATTATTGAATGGGAAAATGTTTAAAAAAATTAAGAGCTTGTTAGGGAGACATAT
GAGTTGAAATTGCGAGATCGCTTCGAGCCCCCTAGCTGCTGCCTAGGAGATTGGCTGAAAGGTAAAATTTTTTGTTTGGTTGAA
TTAGGACCCCAATTCCATAGTCCAATATAGGCCCTATATACGATGATTTATTGAGATTGCGGTTAAAGTTTCAAAAAAATTAAG
ATACTTGTTAAAGAAAATATGAGTGAATTAAGAAATCGCTTAAAGGAACCCTAGGCAACTGCCTACGGAGATTTGGGCCTGAAA
GTCAATCTTTTTTGTTGGTGGAAATTAGGACCCCAAATTCATGGTCAATACTTGGCCCTAATATAACTATTATTGCAGAATGG
GAAAAGGTCAAAAAAACTTAGAGCTTGTTTAGAGAGAAATATGAGGGGAATTACGCAAATCGCTTCGGAGCCCTCAGGCAGCTG
CCTAGGAGATTTTGCCTGAAAGGTCAAATTTTTTTGTTTGGTTAAAATTAGGACCCAATTTCATAGTAGCTAATCAGCCCTATA
TAATATTCATTGAGAATGGGAAAAGGTTCAAAAAAATTAGAGCTTGTTAGAAGAGAAATAATGAAGTGAATTACGAAATGCTTC
GGAGACCTTGGCTGGCAGCCCTAGGAGATTTGGCCTGAAAGGTCAATTTTTTTTGTTTGGTTGAAGTTAGGACCCCAATTAAAC
GTGGTCAATAATAGCCAACTATATAATATTGACTTGAGCATGGGAAAACAATGTTTAAAAAAATTAGAGCTTGTTAGGGAGAAA
TATGAGTGAATTGCGAGATCGCTTCGGAGCCCTAGGCATAGCTGCCTAGGAAGGATTGCCTGAAAGGTCAATTTTTCTGTTCGT
TTGGAGTTTGTATTGAGGAACCCCAATTCATCCGGTCAATAATAGCCCTATATAATATGTATTGGAGATGGGAAAAGGGTTCAA
CAAAAATTAGAGCTTGTTAGAGAGAAATATGAGTGAACCTCTAACGAAATCGCTCGGAGCCTTAAGGCAGCTGCCTAGGAGATT
TGGACCTGAAAGGTAATTTTTTTTTGTTTGGTTGAATTAGGACCCCAATTCATGTCAAGTAATAGCCCTGTACTATATTAGTTG
AGATGGGAAAATGTTTTAAAAAATTAGAGCTTGTTAGGGAGAAATAATGAGTGAATTGCGAGATCCGCTCTCGGAGCCTAGGCA
GCTTGGCCTAGGAGATTTGGCCTGAGGTCAATTTTTTTTGGTTGGTTGAATAGGACCCCAATTCATAGTCAATAATAAAGCCCT
ATATAAATATTATTGAGATGGGGTAATTTTCAAAAAAAATAGAGCTGTTAGAGAGAACATATAGTGAATTACGAAATCGCTTTC
GGAGCCCTAAGGCAGCTGCCTACGGAGATTTGTCCCTGAAGGTCAATTTTTTGTTTGGTTGAATTAGGACCCCCCAATCCATGG
TCAAAATAGCCCCTAATATAATATTATGAGATGGGAAAATGTTTAAAAAATTACGAGCTTGTTAGGGAGAAATATGAGTGAAAA
ATGCGAGATCGCTTCGAGCCCTAGGCCAAGCTGCCTAGGAGATTTGCTGAAAAGTTCCAATTTTTTGTTTGGTTGTATTGGACC
CAATTCAATGGTCAATAATACCCTATATTAAATATTAGTTGAGATGGGAAAAGGTTCAAAAAAATTCAGAGCTTGTTAGAGAAG
AAATATGAGTGAATTACGAAATCGCTTCGGAGCCCTAGGCAGCTTAGGAGATTTGGAGCTGAAAGTGTCTTTTTTTGCTTTGGT
TGAATTAGGACCCCAATTCATGGTCAATAATTAGCCCTGTTAATATTAATTGAGATGGGAATGTTTAAAAAATTAGAGCTTGTT
AGGCGAGAAATACTGAGTGAATTGCGAGATCGCTTCGGAGCCCTAGGCAGCTGCCTAGGAGATTTGGCCTGAAAGGTCAATTTT
TTTTTGTTTGGTTGGAAATTAAGGACCCAACTTCATAGTCAATAATAGCCCTAATATAATATTATTGAGACTGGGTAATATTTC
AAAAAAAATAGAGTTGTTAGAGAGAATATGAGTGAATTACGAAATCGCTTCGGAGCCCCTAGGCAGGCTTCCTAAGGTGATTTT
GGCCTGAAAGGTCAACTTTTTTGTTTGGTTTCAATTAGGACGCCAATTCATGGTCAATAATAGACCCCTAAATAATATTATTGG
ATGGAAAAGGTTCAAAAAAATTCAGAGCTTGTTAGAGAGGAAAATATAGGTGAATTACGCATGCTTCGAGCCCTAGGCAGCGCC
TAGGAGATTTTGGCCTGAAAGGTCCCAATTTTTTGTTTGGTAAATTAGGACCCCAATTCATCAGTCAGCTAATACGCCCTATAT
AATATTAATTGAGAATGGAAAAACCGTTTCGAAAAAAAATTAGAGCCTTGTGTAGAGAGAAATATGAGGGAATTACGAAGATCG
CTACGGACGACCCTAGGCAGCTGCCCTAGGAGGATTTGGCCTGAAAGGTCAATTTTTTTCAGTTTGGTTGAATTAGGAGGACCC
CAATTCATGGTAAATAATCGCCTCTATATTATAGTATGTGAGATGGGAAAAGGTTCAAAAAAATTAGAGCTTGTTATGGGAGAA
ATATGAGTGACATTGCGAGATCGCTTCGGAGCCCAGGCACTGCCTAAGGAATTTGCCTGAAGGTCAATTTTTTTGTTTGGTTGA
ATTAGGATTCCAATTCATGGTCAATTAATAGCCCTATATAATATTATTGAGATGGGAAAAGGTTCAAAAAAAATTAGAGCTTGT
TAGAGAGAAATATGAGGGGAATTACGAGATCGCTACGGAGGCCCTAGGCAGCTGCCTAGGAGATTTGGCCTGAAAGGTCAATTT
TTTAGTTTTGGTTGAATTAAGGACCCCCAATTCATGGTAAATAAGTCGCTCTATATTAAGATTCGCAGATGGGAAAAAGGTTCA
AAAAATAAGAGCTTTGTTAGGGAGAAAATATGAAGAGACATTACGAGATCGCTTCCGGCAGCCTTAGCGCCTGCTGCCTAGGAG
ATTTGGGCTGTAAAGGTCAATTTTTTTTGCTTTGGCTTTGTATTAGGACCCCAATTCAATGGTCAATAATAGCCCTATATAATA
ATATTAGATGGAAAGGTTCAAAAAAATTAGAGCTTGTAAAGAGATGAACATATGAGTGAATTACGAGATAAGCTTCGGAGCCCT
AGGCCTGCTTCCCCTAGAGATTTGGCCTGAAAAGTCAATTTTTTTTTTGTTAAATTAGGACGCCAATTCATGGTCAAATAATAC
CCTAAATAAGTATTATTGAGATGAAAGGTTCAAAAAATTAGAGCTTGTTAGAGAGAAATATGAGTTTAATTCGAAACCGCTTCG
GAGCCCTAGGCGGCTGCCTAGGAGATTTGGCCCTGCAAAGGTCCAATTTTTTTTGTTTGGTTGAATTAGGACCCCAATTCATTA

CGTCAATAAATAGCCCCTAATTAATATTATTGAGATGCGGTAAGAGTTTCAAAAAAATTAGAGCTTGTTAAAGAAAAATATGAG
TGAATTACGAAATCGTTCGGAACCACTAAGGCAGCGGCCTAGGTAAGATTTGGCCTGAAAGGTCACCACTTTTTTTGTTGGTGG
AATTAGGACCCCAATTCATGGTCAATAACTTGCCCTATATAATTTATTGAGATGGGAAAAGGTTCCAAAAAAATAGAGCTTGTT
AGAAGAGAAATATGAGTGAATTACGAAATCGCTTCGGAGCCTACGGCAGCTGCCTAGGAGATTTGGCTAAAGGTCTAATTTTTT
TGTTTGGTTAAATTGGACCCAATTCATAGTCGTAATAGCCCTATATAATCATTTTGAGATGGAAAAGGTTCAAAAACATCTAGA
GCTTGTTGAGAGAAAATATGAAGTGAATTTACGAAATCGCTTCGGAGACATTGCTGCAGCCCTAGGAGATTTGGCCCCTGAAAG
GTCCAATTTTTTTGTTTGGTTGAATTACGGCACCCCAATTAAATGGTCAAGTAATAGCCTTAAAAAAAACATAAATATTATTGA
GATCAGGAAAATGTTTAAAAAATTAGAGCGTTCGTGGTAGGGAGAAATATGAGTGAATTGCGAGGTCGCATTCGGAGCCCTAGG
GGCAGCTCCTAGGGAGATTTGCCTGAAAGGTCAATTTTTTTTTTTTTTGTTTGGTTTATAGGACAATTCCATGGTCAATAATAC
CCTATACCATAATATTATTGAGAGGAGGATGGGAAAAGGTTCAACAAAATAGAGCTTGTAGAAGAGAGAAATATGAAGTGAATA
CGAAATGCGCTCTGGATTCCTAAGGCAGGACGCAACCTAGGAGATCTTGGCTGAAAGGACATCTTTTTGTTTGGCGTGGAATTA
GGACCCAATTCATGATCACACTAATTGCCCTATACACCGAGTACATATTATTGAGGATGGGACGGGAAATCAGGTTCAAACAAT
AAGCTGCCGTTGTTAGAGAGAAATATGAGTGAATTACGAAATTCTTCAGAAGCCCCTAGGCCATACCGCTGCCTAGGAGGATTT
TTGGACTTGCACTGAAAGGTCAATCTTTTTTTGTTTGGTTGATGATTAGGCGCGGCCCAATTCCCATGAATCAATATAAGCCTA
TAATAGATATTATTGGAATGGGTAAAGTTTCAAAAAAAATAGAGCGTTGTTAGAGAGAAATATGAGTGAATTAGAATCGCAGCT
TCGGAGCCCCTACCGGGAGCTGCCTAGCGAGGATTTTGCCATCTTTTTTTGTTTGGTTAATAATTACTGACCCCCAATTATGGT
CAAGTAAATAGACGCCGCCTTGGTACTATATAATCTTTATTATTATGTAGATGTCGGAGGGAAAGGTCAAAAAATTAGAGCCTT
GTTAGAGAGAAATAGAAACGACCTAGGATAATAAAGTTATATC
>m141121_232159_42134_c100732402550000001823141405141507_s1_p0/122340/5808_12292
CTCATCAACACAGACAGGCTCAACCTTCTCGAATACGCCTATGAACATATTCGGCCAGCCTTTCCTTAGGCGAGTGCCGCAACG
GAGGCGACCTGAGCGACCGTTCCTCATCGCTCTCGGCTGTCAACAACTATGAGAATGAGACTCTCAGTTGCTCATAACTCCTCT
TACCCACTCACTTATTTTGAAAGTGAATTTTACAAAAAACAATATTTTTTAATTAAGCATTGTAAAAATAAGATTATTATTTTT
ACCGAAATGATTTCATTAACTACTAACTCAATTAGCAAGAAAAAAAAAAACCTACCTGCCTTCCGGACATTCTATTTCATTGTC
TGCTTCTAGTTAAAGAATGATCAATTTTCGCCGTGTGATAAGCACCGCACGTGCTTCTACAGCTACCCACACACAAATTAAACA
ACCTTTACATTTTGACTTCCTACATAGATTACGCTATTATGACTAACACGATCTGATCTATCCCCATGGAACTCACCATATCTC
ATACCACAACAGCTATAAAAAAACTGACGCTTCTCAAATAGCATGTCTACGCTCACGAAGTCGTACCTGTGCTACGCTTGCATA
TTCGTGTGGGTCAATCGATGATGGGATCGCCTCTTTGCATGAAGAACATCAGGTGTAACATTCTTAAATCATCATTCCGACGCC
ATCTTGTATGAAGTAACTAAACCAGCAGGGTATCGGTACACTTCTTGAGGGTATTTTCAGAAAAATTACACTTCAACTACTACC
ATGAAAAGTTCTAGCTATTCAGGCTTGCATTAGCGACTTTTGCATCTTGACTTGAGATGTTACTATCCCTTGGTTCGGTACTGC
ACCATTTCGTTTCATATCCATTTTGAATTAATACAATTTTTAAATGTATTATAAAAACTTCTTTAAATAATCCAGAATTTGGGA
TTTAACCTCCAAAGTCTCTCAAGCGCGCAATTTCTCTTGGGTATTTAATTCAAAATAAGATTACACGCACCAACTTTAATTTTA
CTCAAATGGAAATCTCCACAGAATCAATTAAACTAAAAATAAAAATACAATTAAATCAACTGTAAATAATCATTTATTATGGAA
CAACCATTGAACAACCATCTTTAATGTCAAATGTGTCTGACTTTTTTTATTACATAATTCACTGCGTCACACACATAGCAGTTT
CCCATTCTTTGCTGAATCTCTCCAAGACTTATCTTGTCTGGACCAAGGGGCTTGAGCGCGCGATGAGTCGGATCGCCAATGCGC
TCGCTCTGACTGTTCTGTTATACCTTGTAGGTATCGAGCATTGCGCGCACTCTAACCTCCAGACTGGGTAGCAGAGTTGCACAG
CGATGTACTTTGCGACTGTGTGTCGATGAGTTATGTCTCACCGTCTAGAAGGCCATTGGGCAACGGGACCATGGAGAGGCCAAT
GGAGGAGGGATGGGGTTCGAGGGACGCACATGGAGGCGTGAGGCGCGACTTGGTCCGCGGAGCCATGGGGTCTCGGGAGGCATG
CTAGGCGAGGCCGAGCCTGCCCACGAGTGCGCCATGGGGCATGAGCTAGGTCGCATCGCGGGGGCGAGCCCGAGGTCTCTTAAT
GAGACGGCAGGCTATGTAGTGACGAGCCCATGGGCCGAAAGGCAGATGGAGGAGATGGCCATATAGCGGACGTTACGCGCCATA
TCAGAGACGAGCCACTTGGAGCGAGCATGGGGGCGGAGGCCCATGGAGGAAGCCATGGGGCCGAGGCCATTGGAGGAGGCCCAT
GGGCCGAGGCCATGGAGGAGGCCAATGGGGCCGAGGCTCATGGAGCCGGAGGCCTTGGGCGGAGCCAATGCCAAAACGAGGGCG
ATGCACCGAGGCCAATAGGAGGAGGTCCATTGCGAGGGCCGACGGCATCAGGACGGAGGGCCATGGAGGAGGCGCCATGGCGAG
GAAAACACGGGCCCCCAGGCCATATGGAGGAGGCCACGAGGCCGAGCCATGGAGGAGGCCAGGCGAGGCCCATGGAGGAGGCCA
TGATGAGCGCCATGGCGAGGCCCATGGGACGGAGGCCCATGGCCGCCGAGCATGGAGGAGGCCCATGGGTGACGGCATGGGGAG
CCATGGCCAAAGCCATGGAGGAGGCCCACTGGGGCGAGGCCATGCGGGAGAGAGGCCATGGGGGCCGAGGCCATCGGAGAAAGC
GCCCATGGGGCCTAGGCCAATGAGGAGGCCCATCGGAGTGAGGCAATGGGTGACCAGGCCATGGAGGAGGCCCCAGGGCCCCGA
GGCCAATGGAGAGGCCATGGGCGGCAAAAAGAGGCCATGGATGAGGCCATCTGGGCCGAGGCATGGAGGAGGCCATGGGCAAGG
CCATGGAGGAGGCCATGGAGGAGGCCCCATGGAGGTAGGCCATCGGGGCCCGAGGCGTGGGCCTGAGTGCATGGGCGAGAGGGC
GGCCCTCGGCGAGCTACTGAGCCCTTGCACCTATGCCTTGCAACATGGGCCCACTTGGCATCGACTCCCCAACGAAAACGACCC
GACCTCGGTGTTGTGATGGCCCGATGCGGGCTCGGCGAGCACGCAGCGGTCGGTCTAAGGGCTTATGCTCAGACACATCTTTCT
CTTGATGAAAGAACAACACACCACGTCTGTGCCTCACAACAATATAAAAAACTGCTTTGCTTGAGAGACACAAGAAAAAAATTG
AAACTATGGCCTCTAGTCAATCTTTGGCAATTTCACCACTTGCCCCCGACCGTCTATGAGGAGTCTTTAGGCCTCTAGTAGCAC
TCATATTCAATGTACCACTCCCTACCTGCTCTGGTGGTACTTGCCTGGGTGATCTGTGTCCGTTGGTGCACTTTGATTACTTTT
GGTGAAGGATGATATTTGACCCCCTTGGGGTCTTTGCTATCCTTCATTTGTCGTGTTGTTTGCGCGCCCTTATTATTTGTGCGA
GAACGCCGCTTCTCTGGCCCTCGTGCGAGTGTGTGAGGCCATGAGTACTATCTTTTGCCAAGCGTTCTTCCTTTGAGGACCGCT
TATTTGGAAGCGTCTTCTTTGAGACCCTTTTTGCTGAAGCGTCTAACCTGCACCCGTTGTGTTTCCGCGGGCCCTACTTATCGG
CACCCTTGGTGGCAAGCGCGCGGCCGGGCGTCCTACCTTTGGAGACTCTTTGCGGGGCGCCTACTTATGAGCACTTTTTGCGCA
AGACACACTGTCTGGAGACCTTTTTGCCCAGCGCGTCTGACTTTGGACGACCCTTTTCGGCGGGCGCTATTATGAGACTTTTTT

GCAAGCGTCTACCTTTGAGACGCCTTTTCAGCGGGGCGCGCTACTTATGAGATACGCTCCCCCTCCGGGTGGGACTTCATGGCT
AGCAGTGGGTCTTCGTTGTAAAAAGACATCCTCCCAATTTGTGCGGCGACCCCTCCTAAGCCCGATGAGATGCCCCCTTCTACG
TAGCAGTGGGAACCCCAGACATAAATCGGCTAGTCATCTGCCCTGAGGAAGGGTTAGCCTCTCTTCGACCATGGAACGCGTTTC
TATGGCCTCCTTCCACACCGTATGTACTTTGTGAGTCATCCTCGATAGAAGGTCGTCTGAGGCAAGGATCCCGAAACTGTTCTC
TTTTGAGCATGAGTTATCGATTTTCTTCCTGGACGAACTCGGCCGGCCGTAATAGGTCCATCTTGAGCTCGCGCTAGTTTCGTT
AAATTCCCCACCATTTGCTGCTCTATCACTGAACCTCATGCGACTGTATCCTTCCAAATAACTTTCATTTCCGCGGAGCCGCGC
GCCGGGGGCCCGGGCGCGCACGACCGCGCAGAGCCGTCGCGGTTTTGAATGTAAGCGAGGCCGCTGGTAGTTGGACCAAATGAT
TGAGTGATCCTCGGCCCTCAACCATCCTGACACCACTTGTACGGCCCACATCTTTAGCGTAAACGACGATAGCAATTGACAACT
CGTTGCTCTGCTTATTGACACCTAAAATTACACCTCCATACAAGCTATTGAAGGAAGTCAACTAAGTGAACTATCTTGGGTCTG
ATAGTACCCCTCGTACGGAGTCACTCGTGAACCGGCTCTCCCTGAAAGCAACTGGAGCGCATTTGTTCAGCCTGCATCCTCCCT
TGTGAAACAAGGGGAATCACCGGTATAAACAATTTCCACCGGTCATCGCATCAGGCGGCCCCAAGGCGTGCTGTGGTGATCTTG
CCCGCAGGGGCTGCGACTTTATGTGCTCTAGCCCCCTCCTCTTTTCATTGCAAATCAAGGACGGTTTGGCGGTCTTGGGCTGAT
CCCTGTGCACTTGGTTGCTCGCTCCCTCCGGGTGGAACCCGCGGGCGGCACGATCTCCTTACTTCTGAGGCTCTCAAAACCCAT
GGATGCTCCTTTTCTCCGGCCACGAGGGGTGGCCTCTTCTGCAGGGGACTCGCTCCGACGCTCCTTCCACGCCATCCACGCTCT
TCCCTTCCACCTTGCTCCTGGGACGTCTCTTGTTGCCCTAGGCGCAACACCTGATACTTGTCTGGGGGGTTTTAAGCTGACGGA
AAGTTGGGTTCCCCCATCAAAAAAAACATCTACGGGGACAGTGCAACTCCCATGTTTTCAAAAACGCTCCTTACCCTTTACGCG
AGGAGGGCGTGTGCTTGACTTTCTTCCGCACGTAGGCGTCAATTTAAAACCTCTTGCATGTTCCTCTAATCCTGGGTCTTCCAA
GCTTTTCAAAGGTCAGAACACCTTTCTGTAACTCTTGAACTCATCCAAAAAAAAAAAAAAACCTATAACAGGTGAGTCGCTTGA
GCCTGAACTCCCAAAGCGCAGATCCCCCCGGGACTCTTGGCTTGGCCTGTGGCGTCTTTGATGGACCGGCCTGGTCCTGGTGGC
CTGAGCGTGGTGCTACCAGAGAAGCCGGTGAAGTGGAATGCGCCACCGGCCGGCTCAAAACTGCACCTCAAAAAACCGTCGGGG
GACCGGATGGCTCGGCAAGATGAGGCTTCTTTCTCCATCCCTTGGGAAGAAGAGAACTCCCCCCCGTCTGCCTCCCCAATAGTA
ATTAGGCGGAGGGGGGGTCTTTCCTGGTAGGGCCCGCTCCTACTTCATCCCGGAGAACCTCATCATCTTTGTACTTCTGGCTAT
CCAGGCACATTTTGAACCGTCTTAGCTCTTCGTTGCCTGGGAGTGAGTGGAGAACACCTGGCCTTGTGCTTGTTCTTCCCAAAA
GAGACGGCCGCCAAACTGTTGACGGTCAGAACTCGTCAACTAAGTTGAGTTGGAAGAAATTAATCAAGTTAGGAATCGTCAATA
AAAAACGCTATAGAAAGATCAAAGCCAAATATCCAAAAGAATTTATGTCAGAAGATCAACTGGAACCCGAAATAAATCACTCTT
TCATTAACTCCTCAGAACCTTGCCTACAGTCAGGAACTTCCCACCCCCCCTTTCAGGTGGTGTTAGAGTTTCCTTTTAGAGTAC
GGCTCTAGTGGCCCGCTTGTAATGCATGATGTCGGAGACCAAAGGGGTACATAAGTACTATCGTCAAGAGAGTGGCTTCAGAGG
TGTGGTCGTAGCCATCCCGGTAAGGAGGCGACGGTGTTAGGAGGACTGTCTCACCCAACCCTGTCTGTAGCCCCATCCAAAGTC
TGATGCATGGGGGGCAGGCGTAGTGGTGCAGGTGGTAGCGACATGTCCGACTCTCGACTCTGGCCAATAAGACGTACGGGCATG
AACTCCCTATCCTTGCATACCCACTCCCTTGCATCCCACGTAAGCTTGTCTGGTAGGCGGTAGCGCAGTATCCCATACTCTGAC
TTCTCTTTGGTTCTGTAGGTACATTCCCGCATACTCATGCCGTTGTGAGCTTGCCCTTGTGAGTATTCTCCACCTCTCAGGCCA
CTTGGGGCAGCGGGGACCCCATTGGAGCGAAAAAGCGCCACTGGGCGGCGAGCCATCGCGGAGGAGGCCCAGGGCGAGGCCTGG
CAGGAGGCCAACGAAAGCGACGGCCAAACAGGGCAGGCCAAACGGAGCGAGGCACATGAAAACAGGAGGAGGACAACGGCGACG
GGCCAATGGAGGAAAAGCCGCCGGGCACGGGGCGGCAGAGGCCCATGGAGGGAGGCCACGGGCCCCCCCAGGCCATGGAGGAGG
CCTATGGGATGAGGCCATGGGCGAGGCCATGGAGGAGCGCATGGCCGAGCATGGAGGAGGCCCCATGGGGGCCGAGGGCCATGG
ACGGAGGCTGGGGGTGCCGAGGGCGATAAATGGGCGAGGCCATGGACCCCGGAGGCCATGGGAGGAGGCCATGGGCACGGAGGC
CATGGGGCGAGAGCCCATCGGAGGGAGGCCAATGGGGGCCGAGGCCACAAATGGAGGAAGCATGGGGGCAGGCCAGGCATGGAG
GAGGCGCCATGGAGGAGCCGTGGCCCGAGGCCATGGATGGACCGGCCATCGGAGGGGCCATGGAGGAGGCCATGGGCCCGGCCA
TGGAGGCGAGGCCATG
>m141121_232159_42134_c100732402550000001823141405141507_s1_p0/123465/4790_13466
AAAAAAAAAATTGAAATTTAAGGAAAATTTAATAGGAAGCATGATAAGGGATAAAGGAGGAGATTTAGTAATAAATAATATTTT
ATATAAAAAAGATATAATTTTTTTTGAAAATTTTAAATATAAAAATATCTATATAGGGATATATATTGAAAATGAATTGGGTAA
TAATTAAAAAAAAATAAAAAAAATTGAATATAAGGAAAAATATACTGAATATGGATAAGAAGAGTTAATAAATTAATATAATTT
ATAATATAAAAAGATATAATTTTTTTGATAATTTTTAAATATAAATAATATTCATATAGGGATGTTATTGAAAATGAATTGGGT
CAATAATTAAAAACAAAAAAAATTGAAATTTAAGTTAAAAATATAATGGAAGATGAAATAGGGATCAGAGAGATTTAGGAATAA
TAATATTTATATATAAAATATAATTTTTTTGAAAAATTTAAATTAAATATATTACTATAGGGATGTTATTGGAATGATTGGGTA
ATAATTAAAGAATAAAAAAATTTAAATTTAAGGAAAAAATATAATAGGAAGATGAATAGGGATAGAAGAGATTTAGTAATTAAA
GTATATTTATATATAAAAAGAATATAATTTTTTTGAAAATCTTTAAATATAAATAATATTATAATAGGGATGTTGATTGAAATT
CGAATATGGGGTAATAATTAAACAAAAAAAAAAATGAAATTTAGGAAATATAATTAGGAAGATGAATAAGGGATAAAAAAAAAG
AGATTTAGTAATTAAATAATATTTATATATAAAAAGATATAAATTTTTTTTGAAAATTTTAATATAAATAATATTATATAGGGA
TATTATTGAAAATGAATTGGGGTAGATAATTATAAAAAAAAAAAAAAAATTGAATTTAAGGAAAAATATAAAGGAGATGAATAGG
GAAAGAAGAGATAATAGTATGATAAATAATATTTATATATAAATGAATATATATTTTTTGATAAATTAAAATAAAATAATATTA
ATAAATAAGGGATGTTATGAAAATTAGAATTGGGGTAATAATTAAAAAAAAAAAAAATTGAACATTTAAGGAAAAATAAATTGAG
GAAGATGAATAGGGAAGAAGCGATAAGTAATTAATAATATCTTATATATAAAAAATTTATTTTTTGATATTTTAAAATAAAAT
AATTTATATATAGGGATGTTCATTGAAAATAGAATTAGAGGGGTATAATTAAAAAAAAAAAAAAAAATTGGAAATAATTAAAAAAA
TATAAAAGGAAGGAAGAAAAGGGATAAAGTAGAGATATAGTAATTAATAAAATTTATATAAAAGATATAATTTTTTATTGATA
AATTTAAATATAAATAATATTATAATAGGAGAATGTTATTGAAAATGAATCTGGGTAATAATTAAAAAAAAAAAAAAAAATGAA
ATTTAAGGAAAAATATAATAGGAGCATGGAATAGGGGATAAGAAGAGATAATAAGTAATTAACTAATACTTTTCATATATAAAA

AGATTTAATTTGTTTGAAATTTTAAAATATAAATAATATTTAATCATACCGGCGATGTTAATTGAAAATGAAATTTGGGGGTAA
TAATTAAAACAAAAAAAAATTAAATTTAAGAAACAATATAATAGGAAGGACGAATGGGATAAGAAGAGATAACTAGATAATTAA
ATAATATGTATATATAAAAAGAATTAATTTTTTTGATAATTTTAAATATAAATAATATTATATAAAGGGATGTTATTGAAAATG
AATTGGGTAATACAGTAAAAAAAAAAAATAAAAATTGAAATTTAAGGAAAAATATAATAGGAAGAAATAGGGATAGTAGAGTTAG
TAATAAGTAATATTTATAAATAAAAGATATAATTTTTTGAGAATTCTAAAATATAAATAATATCTAGTATGGGATACTTATATG
AAATCGATTGGGGTAATAATAAGAAAAAAAAAATATTGAAATTTAAGGAAAATATAAAAGAAGATGGAATAGAGGATAGAGAGAT
ATAGTAATTAAATAATATTTAATAATAAATGATATAATTTTTTGATAAATTTAAATATAAAATAATATGTATATAGGGGATGTT
ACTTGAAAATGAATGTTGGGTAATAATTAAACAAAACAAAAAATAATTGAAATTATAATGAAAATTAAATAGGAATGATGAATA
GGGATAGAAGAGATTTAGTAATTAAATAATATTTAATACTACTAAAAAGATATAATTTTTTTGAAATTTTAAAATATAAATAAT
ATTATTAAGATGTTATTGAAATCGAATTGGGTATAAGTAAAAAAAAAAAAAAAAATTGAAATTTAAGGAAATAATATATAGGAAG
ATGAGAATAGGGATAAAGAAGAGATATAGTAATTTAAATATATTTATATATAAAAAGCTTTAAATTTTATATTTGATGAATTTT
AAAATATAAATTAATATTATATAAGGGATGTATTAGAAAATAATTGGGGTAATAATTAAAAAAAAAAAAAAAAATTGAAATTTAA
GGAAAAATATTAATAGGAAGGAGATTAGGAATAAGAAGAGATTACGTATTAAATAATATTTATATATAAAAAGATATAATTTTT
GAAAATTTTAAATAATAAATAATATTACTCATAAGGGATGTTATTAGAAAATGAGAATTGGGTGTAATTAAAAAAAAAAAAAAT
TGAATTTAAGGAAAATAAATACGGAAGCATGGAATACAGGATAAGAAGATATTTAATAATTAAATAATATTCTATATATAAAA
AGATATAATTTCTTTTTGATAATTTAAATAAAATAATACTATATACCGGGATGTTATTGAAAATGAATGGGGTAATAAAGTATA
AAAAAAAAAAAAAAAATTGAAATTTAAGGAAAAATATATAGGAAGATGAATAGGATAGAAGAGATTTCAAGTAATTAAATAATAT
TATATAATAAAAAGATATAATTTTTTTGAAAATTTTAAAATATAAAATAATATAATATACCTGAATGTTATGAAATGAATTGGG
GTAATAATATTCTAAAAAAAAAAAAAATTGAAATTTAAGGAAAAATATAATAGGAAGATAATAGGGATAAGAAGACCGTAATTAA
TAATATTTATATATAAAAAGATTTAATTTTTTTGAATAATTTTTAATATAATAAATTATCAATCAAGGGATGTTATTGAAAATG
AATTGGTAATAATAAAAAAAAACAAAAAATTGAAATTAAGGAAAAATAATAGGAAATGAATAGGGATAAGAAGAGATTTAGTTA
ATGTAAATAATATCTATATGTAAACGATATAAAGTTTTTTGAAAATTTTAAAATATAAATAATATTACAAGGGATGTTATTGAA
AATGAATTGGGGTAATAATTAAAAAAAAAAAAAATTGAAATTTAAAGGAAAAAATAATAATAGGAAGATGAATAGGGATAAGAAG
ATATTCTAATAATTAAATAATAAATCAATAGTATAACATATAATTTTTTGAAAATTTTAATTATAATTATAATCATATAGGATA
GTTAACTATATATAAAAAAAAAACAAGAGGCAGGAGGATAAAGAGGATTAAAAGAAATATATAAATCAATGACTGGGAAAACAG
GTTAAATAAAAATTAGAGATTAGAGATATACTATGAGTGAATTTATGGAAATACACTATTAGGAGAAATAGGAACGATGAATAG
GAGATTGGAATGAAAGGTAATATTTTTTATGTTTGTTAATTAGGAAAAAATTAATGGTAAACTAAACCGAATGTATACTAATAT
TATTGAGATGGGAAAAGGTTAACAAAAATTAGAGATTTTTAAAGAGAAATATGAAGTGATTAAGAATAGATTAGAGAAATAGGG
AAAGAATACGAGATTTGGAATGAAAGGTAAAATTTTTTTGTTTGGTTGAATTAGGAAAACAAAATTAATGGTAAATAAAAGAAC
TATATAATATTATTTGAGATGGGAAAAGGATAAAAAAATTAAAGATTGTAGAGAGATAATATGGGGAATTCAAGAATAGATTAA
GGAGAAATAGGAAGAGGAATAGGAGACTTTGAATGAAGAGGTAAATTTTATTGTTTGGTTGACAATTAGGAAACAAAAATCTAA
TGGTAAATAAAAGAAATATATAATATTATTGAGATGGAAAGGTTAAAAAAATTAGAGGATTTTAAGGAGAGAAATAGAGTGAAT
TAAGCAATATAATTTCAGGAGAATAGGAAGATTTGAGGAGATTGGAATGAGAAGGTAAACTTTTTTTGTTTGGTTGAATTAGGA
AAAAAATTAATGGTAAAATAAAAGGAACTATATAATATTATTGAGAGGGATACATGGATAAAAAAATTAGAGATTGGTTGAGAG
AAAATATGAGTGAATTATCGAAATATCATTGTAGGAGAATAAGGAAGATGAATAGGGAGATTTGGAAATGAAAAGGTAAATTTT
TTTGTTTGGATTGAATTAAGGAAACCAAAATTATAGGTAAATAAAAGAATTATATAATATATCGTCAGATGGACAAAGGTTAAA
AAAATTAGAGATTTTTAGAGAGAAATATGAAGTGATAAGAAATAGATTTAGGAAGAAATAAGAAGACGAATAGGAGATTTGGAA
TGAAACGTAAATTCTTTTTGTCTATTGGTTGAATTAGGCAAAAATTAATGGTAAATAAAAGAAATATATAATATTATTGAAGAT
GCGGAGAAAAGGATAAAAAAAATTTATAAGAGATTGTTAGAGAGAAATATGAGGGAAATTAAGAGATAGATTTAGGAGAATAGG
AAGGAGGATATAGGAGCATTTGGAAAATGAAAGGATAAATTTTTTTGTTTGGTTGAACTTAGGAAAAAAATTAATGGTAAATAA
AAGAAATATCATAATATTATTGAGATGTGGAAAGAGGTTAAAAAAATTAGAGATTTAGAGAGAAATATGAGTGAATTAAGAAAT
ATATTAGAGGAAATAGGAATGATGAATAAGGAGATTTGGAAATGAAAGGTAAATTTTATTTGTTTGGTGATAGGAAAAAAAATTA
TGGTAAATAAAAAAATATAATAATATTAATTGAGAATGGGACAATGGATAAGAAAAAATTAGAGAATGTTAGAGAGAAATATGA
AGCTGAATTAAGAGTAGCTTAGGAGCAATAGGAAGATGAATTGGAGATTTGGATGTAAGGTAAATTTTTTTGTTTGAATGAATT
AGGAAAAAAATTAAATCGGTAAATAATAGAACATATATATATCATTGAGAATGGGAAAAATTAAAAAAAATTAGAGAATTGTTA
GGGAGAAATCATGAATGACATTGTAAGTCAGAAGATAAGGACGAAACTAGGAATGATGAATAGGAGAATTTGGAATGTAAAGGT
AAATTTTTTTTTGGTTGAATTAGGAAAAAAATTAATGGTAAATAAAAGAAATATATAAATATTATAGGATGGAAACAGGATAA
AAAAAAAATTAAAGATTGTTAGAGAGAAATAATGATAGGGATTAAAGAGATACCGATTAGGAGAAATAGTGAAGATGAATAGGG
GATTTGGAATTGAAAGGTAAATATTTTTTTGTTTGGTTGAATTAGAAAGAAAATTAATGGTAAATAAAAGAAATAATATACATA
TTAATGAGGATGGGAAAGGATAAAAAAAATTAAAGATTGTTAGAGAGAAACTATTGAGCTGAATTAAAGAGATAGATTTAGGAG
AATAGAAGATGGATAGGAGATTTGGAATGAAAGGTAAAATGTTTTTTGTTTGGTTGAATTATGGGAAAAAAATTAATGGTAAAT
AAAAGAAATATATAAATATTATTCGAGAATGGGAAATGGATTAAAAAAAATAGAGATTGTAAGAGAATTTGAGTGAAATATAGA
GATAGATAAGGAGACATAGGAATGAGAATAAGAGATCTTGGAATGAAAGGTAAATATTTATTTTTGTTATGGTTGAATTAGGAA
AAAAATTAATGGTAAAATAAAGAAATAATTATAATATTATATGGAGATGGAAAAAGGATAAAAAAATTAAGAGATTGTTAGAGA
GAAATATGAGGGAATTAAGAGATAAGATTAGGAGAATAGGAAGATGAATGAGATTTGGATTGAAAGGTAAATATTTTGTTTGGT
TGAAATAGAAAAAAATTAAATGGAAAGATAAAAAGAATAATAGCATATTATTGAGATGGGAAATGGAAAAAAAAATTAGACGAA
TGTTAGAGAGACATATGAGTGGATTAACGAGATAAGTTAGGAGAATGGAAGATGAATTTTGGAGATTATGGAATAAAGGTAAAT
TTTTGTTTGGTTTGAAATAGGAAAAAAATTAATGTAACATATAGAAATATATAGATATTATTGAGATGGGAAAAGGTTAAAAAA

AATTATGAGATTGTTAGGAGAGAAATTGAAGTTGAATAGGACGAAATAGATTTGGAGAATAAGGCAGCATGAATAGGAGATTTG
GAATGAAAGATAAATTTTTTGTTTGGCTGAATTAGGAAAGAAAATTAAAGGTAACAAAAAGCATATACTTAGATATTATTGAGA
TCGGGAAAAGGTTAACAAAAAATTAGAGACTTCTCTAGAGAAGAAATATCGAGTGATTAAAGATAGATTAGGAGAATAAGGAAG
ATGGAATAGGAGATATTGGAATGAAAGGTAAACTTTTTTTGTTTGGTTGAATTAGAGAAAAAATTAATGTAAATAAAAGAATAA
TAATACATTATTATTGAGATTTGAATGGTTCAAAAAATTAGAGTTGTTAGAGAGAAATATGAGTGACATTAAGAATAGGATAAG
AAAACTAGAGAGGAATATGAAATTTGGAATGAAAGGTAAATTATTTTTATGTTTGGTTTGAATTAGGAAGAAAATTAATGGAAA
TAAAAGAAATAATAAATAATATTAATTGGAGAATGGGAAAAGGATAAAGAAAAATTAGAGATTGTTCGAAGAGAAATATGAGTG
AATTAAGAGATAAGATTAGGAGATAATAGAAGGAGATTATGGGAATGATAAGTAAACTTTTTTTGTTTGGTTGAATTAGGGAAA
AAATTATAGGTAACTAATTAAGAATATATAATACATTATTGAGATGGGAAAAGTTAAAAAAAATTAGAGACTTGTTAAGAGGAA
AAATACTAGTGAATTAAGAAATAGGATAAGGAGGAAATTAGGAAAGAGGAATAAGAATTTGTGAATGAAAGGTAAAATTTTTTT
GTTATTGATATAATATAAAAAAAAAAAAAAGGAGGAGAGAAAAGAGAGGAATAAAAAAAAAAATTTGAAAATTAAGGAAAAATTT
AATAGGAAGATGAATAAACGGGAGTAAGAGAGAGATTTAGTAATTAAATAATATTTTATAATAAAAAGATATAATTTTATATTG
AAAATTTTAAAATATAAATATATTATAATAGGGATATGACTGAACATGAATTCGGGGTAATAAAGTTAAAAAAAAAAAAAAAAA
TTGAAATATAAAGGAAAGATATAATGAAATGGATAAGAGAGTTATAGTAATTAAATAATACTTTATATATAAAAAGAATAATTT
TTTGATAATTTTAAAATCATAAAAATAATTACTTAATAATACGGGATTGTTATCTGAAAATAGAATTGGGTATAATTAAAAAAA
AAAAAAAATTGAAATTTAAGTAAAAATATAATAGAAGATGAAAGCTAGGGATAAGGGAGATTTAGTAATTAAATTAATATTATA
TAATAAAAATAATAATTTTTTTTGACAAAATTTAAAATATAATAATAATTAATATGGGATGTTATTGAAAATGAATTGGTGTAA
TAATTAAAAAAAAAAAAAAATTTAAATTAAGGAAAAAATATAATAGGAAGATGAAAGGGATAAGAAGAGATTGTAATTAAATAAT
ATTTATATATTAAAAGCATAATTTCTTTTGAAAAATTTTAAAATATAAAAATTTATATAGGGATGTTGTTGAAATTGAATTGGG
GGTAATAATTAAAAAAAAAAAAAAAATTGAATTTTAAGGACAAATATAATAGGAAGATGAATAGGGAAAAAAGAGATATTAGTAA
TTAATAATTTTATATATAAAAAGATATAATCTTTTTTGAAAATTTTTACAAATATAAATAATATTATAACGGGATAATTATTTG
AAAAGAATTGGGTAATAATTAAAAAAAAAAAAAAATGAAATTTAAGAGACAAAATATAAAAGGAGATGAATAGATAAGAAGAGAT
ATAGTAATTAATAATATTTATATATAAATGATATAATTTATTTTGGATAAATTTAAATATAAATAATAATTTATAATAGGGATG
TTGTTTATTGAAAATGAATTGGGTATAATTAAAAAACAAAAAAAAATGAAATTAAGGAAAAATCTCCACATACGGACCGCCACC
TGAACCTACGCCCGCGAAACTCAACGCACAGAGATATAGTAAATTAAATAATAATTTAATATATAAAAAGATTTTAATTTTTTT
GATAATTTTAAAATAAAATAATTATAATAGGGATGTTATTGCAAAAAATGATTGGGGTAATAATTATAAAAAAAAAAAAAAATTG
AAATTTAGGAAAATATAATAGTAAGAAGATAGGGATAAGTAGAGATATAGTAATTAAATAAAATTTATATATAAAAAGATATAA
TTTTTTGATTAAATTTAAAATATA
>m141121_232159_42134_c100732402550000001823141405141507_s1_p0/124964/1474_17123
CATAAAGTCAACAAGCCTAGGATACTCGAGTTTCTGATCGACTTATGATATCAGCAGGCGCAGATATTAGAAGTGAACTTACAG
ATAAAAACATACTCACAGCCCTTCACACTTCCCAAACAAGCGGCTTAAAACCGACTCAGATGATACACACACCGGCTCACTCGA
GAAGTTTCTTGCGCCTATCTGCCGCGTCGGTAGTGAATGAGAGAGGGATGGATTGGTAATTTCAGCATACTGGACAGAACACGC
TGACGTATGTTAATGAAGATGATAATACGAGAATGAACTCAACGGTATCGACACATCAAGAAGCAACGAGATAGAAGGTCTGAT
GACAGAACCTACAGCGGCAACTTCCCACCTAACTTTATAATGGATACATCCGATACGGCCCCGAAGTGGAAATAGTGAAACAAG
CACAGAGAATCAGGAACCACAAACCTTATTTTGAGTTCATTATGGCACACAAGAATCCAGAGCAGGAACAAGGGTCACTTCTAA
GCCTCCTGGAACACCCGTCCAAGGGCTCAGCTGCCCGGAGCAAACCCAATCACAAGGCAGTTCACTAAACCCACCCCACCCCCC
CCCCCCCACCAACCCCCCCAGTCCCCCCCCCCCCCCCCCCCCCCCCCCCCCAACACCCCCCCACCCCCCCCCCCCCTCCCCCCC
CCCCCCCCCCCACCATCCCCTCCTCCCACATCCCCCACCACCCCACCCGTCTACCCCCACCACCACCCCCCCCCCACCACCCCC
ACCCACGCCACACAACCACCCTCCCCCACCCCCACCCCCCCCCCCCCCCTACCCCCCCCCCCCCCAGCCCCCACACCCACTCCA
CGCCCCCCACACCTCACCCCCTCCCCCCCCCACTCCCCCCACCCCCTCCCCCACCCCCACCACCCCCCCACCCCACCACTCCAC
CACCCCCCCCCCCACCCCAGCCCCCCACCCCCCCCCCCACCCCCCCCCCCCCCTCCCCCCCCCCCTCCCCCCCCCCGCCCCCCC
ACCCCCACCCCCCCCACCCCATCCCTCCCCACCACCCCACCCCACCCCCCCACCCCCACCCCCCCCACCCCCCCAACCCCCACCC
CCCCCCCCCCCCCCCCCCCTCCCCCCTCACACTCCCTCCCCCCCACCCCCCCCCACCCCCCCAGCCCCCCTCCCCACCCCACCC
CACCCACCCCCCCCCAACCCCTCCCATACCAAACACGCACCGACACCACCACACCCGACACCCCGCCAGGCCCCCCCCCCCGCC
CCCCCCCTCCCCCCCACCATCCTCCCCCCCCCCCCCCACCCCCCCCCACCCAGCCCCCCGCCCCTCACCCACCCCCCCCCCCC
ACCTCCACCCCCCCCACGCCCCTCCCCCCCCAACCCCCCCCTCCCCCCCCCCACCCCGCCGCCCACTTCCCCGCCTATGCCCCC
CCCCCCCCTCCCCCCTAACACCCCCACCCACCCCGCCCCCCCGCCCTAACACTAACCTCGACACCGCACACCATGCAACGAAAT
ACAGCGCGCACCAGTGACACCAACCTTCACGAGACACTGATCCTCTCAAATTGCTGCCGCGCACGCTATTAACGACACTCCCAT
AACGGAAGCACGATTTTAAAAAAGAAAAGAAACGAAGAATAAGGAACCGCAGGCTGCAAAAACAACCTAAACTCAACAATAGCC
ACCGTACCCTCCCCACTACCCAATTCAACAAGAATCCATGCGACTAGCAGGCATAGCTCAAACTCAGGTACGCTCCCGGCAACC
CCGACCCCCAACAAACACCGAGTAAAATTCTGTCAGTGATCGGTTATTTCAATTTTATCCTGAATTCGCATCTCCGACCACCTC
TATCCTACGGATAATAATCCTCTGCTCACTCTTTCTATACTAATACGAGTCGATAGATTGGTGTCTGTCCGCTCACGCAAACCC
TCACGCCTCGCACCACCTCTTGGGTATGAAAGGGGGCACCTTCAAGGAACTCTCTGCTCCTCTAAACAATGGCAGCCGACAACT
GGATCCAAAATCCATACTAAGGAGACCCTTGGCTTACGCCACGCAAATTGATAGTAGAGCATCACCCCCGACAGTACACGCAGG
AGCTTGCCCATGCTGGACGACTTGATTCGAGTTACGCCGAGATCGGAGAGCACATTTCGAAATATGACTACATACCAGGGATCG
GAAATCGGTTATAAACCTCTCAGGGGTTCCCTCGAAACGAACCACAACACAAATTAATAAATCCCAAGGAACTAAGGCGCAATT
CAGACCTCTCAGTTGTGAAACGGCCGCGGCGAAAAAGATATGCTTACACTCCCTTGACCATTCGGCAAGTACGCCTCCTTAACC
TGAAATGATCCATAATGTTAAAACTCCCGATTGTCATCGCACTTATAGACATCATCACCCCCCTCGACCCACCACATCGAATGT

GGGAACCACAATTCTTCGGTTAAATCAGGGGACACTACCTTCCCGTAACTGATTCTAGAAAGATTCGATCCGGAGACTCACAAT
CTCGTTAGGGAGTGTCGCTAATAAAAGAATGTAAAAGAAATTAGTCCCCTTCTCCCTTTATGTGACATACCACACTCCACACAT
ACAAATGGACTTTATCATCTATTGTCACACTCATTCCTAGATTCGCTCTAAATTACTCTCGTTTGAACATCACATTCTCCCTC
CTGTACAATATAAGGCCAAGAATTAAATATTCAAGGTCCTACTTGCACCCGAGAATGTGATACCTGCTCACTTCAGTAACCGAG
ACGAATCTAACACTACAAATGACACCCTGAATAACCAAAACAACACCACAAAAAACATTAAAGTTATGGTAATTATTAATCTGG
CACAGCTTCGCCTAAGCCTAACGGTTTACACCATGATCATCCCCAATACATTCGCATGACCCCCAAAATAGATATAGAATTAGG
ATTATTGATGTCGCGGAGACAGCAAAATACCTAGGTAACAGAGTGATTTACTCACTACCCCCCCCCCTCATATCCCCGACATTT
ATTATCACTCCTCAACCATCGCGAACAGATATCGCAACCGTTAACAAGTATTTCACTACACAAAAAAATTTAAAAATCAAACGA
ATCCTCGTACAAGCTAATCCCGACTATCCACAATGACTCGATCAATCCTCTACTCGGACTACATACTATCACTACAATGATTTA
ACTCCTCCCCCGACACATAATGTTACACACTCGTTTGAATTTACACATATTTTGATCCCCTAATCAACCTCGATAGTTAACGCT
ACTACTCCCCCTCCCCGTAAAAAGAACTGCACGGACTAATCGGAAACTCCTCTTCGTTTTTCGGAAATCTCCTAACTATAATCC
CCCCGGCACTGGACTCGCGCGACAAACCTCACCTCACCCCTACCAACCAACACCCCTAAACCTCATTATCACAATATTTTAATA
AAGGATAACTCATACCTTCAACGCTCATTCACCATTAAGGGTTAATGCATATCGGAAATGACACCTGATTTCGCAGACTACACA
CACTATACCTCCACATTAACCGCACTCATAACGACTCTCCACCCCTTATCTAAATAGTCGCACCCCCACTCTAAATGGACCCAA
CTCCTACAGGACATCACAGCACACGTACCACCATTCACGCCCCACAACGGTTACAGATGGTCACAGCTTATAAAATCTATACAT
CTTATGAACCTAATTCTAATTCTCAAAGAGGACGTTTCGCAAATACCTCGTGTACAGCACATGCGTCTCACCCCCTGCACCCTA
AACTGGATCGCCCATACCTTCAAAACCAAGATTTATGTAAAAGCTCATACCTATATACCACCCATCACGACACGAGTCGACTAA
TTAATGACATTTATACCGCTCGACAGTCATACTCCGAAGCAATTGAATAACACTTCCCCCCCTAACCAATATCTCACACCCTTC
AACAGAGCTTACATTCACCACTTCCTACGCCTGTTCCCAAATTATTCGGAGCATCTCAACTCGCTTTCCACTCCCAGGCTAACA
TCGGTCATCACAAGAACACTGGACTCAAAAAAATCAAACCCCGTCAACACACCAGGAAAACATAAACAACAATCGGGCAAACAA
ATGAATTCATGTCGCCCATCACAGCTTACACACATCCCACTTCCTCTCATATGCTACACCAAACAATAACTTATTGTCCGCTCT
AACTTAACTCGTGTACCCTATTAACTTAAATTATTCTCGCCTCTCTAGTAGGACAAACAAATACCCTCTCCACAACCTCCTACC
ATTTTGAATTCAACCTTCTGCTTTTGCGGACTCTCTCTTGGTATTCTACAATAATCACCCCACACCACTACTAACACCCCTTGG
AGAAACATAAAACCCACCCCTCCCCCCCCCCCCCCCCCTCGAAATACAATCAAGAAATCCCGTGGCCTCAAAAGCTGTCCGCG
GGAAAAGTCGCCCAAAAAGGAAAATGCTCGACTAAACTGGAACCCAATAAGAGATGTAAATCCCGACGTTTTATTCGGCACCTC
TACGAAAATTTTAGCTCTACTCGTAACATACCCCTCTAACATAATAACCATCCCATGAGATAACCCTTCATTAATATGCATCCT
CAGAAATATTATGTATTTAAGAAAAAAATATTATAAACCCCTGATAAGCCCCCCCACCCTTATCATTTGACTCCTGTTTAAAA
TAAAACAACACACTCTCTTCACACTCATACTCTCCCGGCCTCCCGGCATCACCTCCACCCTTAAATAACCCTACGGCATATACA
CGCCCCCCCCCCTTCTTAATACGAAACACCTCCACAATTTAAGGATATGACACAACTTCATATACCCTGAATACTATATGACAG
CAGAAAGAGAAAACAGTCCACTGAACAACAATAAACCGGATACATCCTGCTTATTAAACAAAAAATCACGCGTCACACAAAATC
CTATGACTGGAGGATTTCCACAAGCAAACCACTGCGCACAGTGACAGCCATTATAAACTCGCACTCACGCATAATGTGCCTGA
CGTAGAGACTCATTGAGTTAAGTTTCAGGAATACAATCTCAGCGCAAAAGTTTGAAGAGGATATCACTAAATGTATGAGAATAC
GGCGTGTGGTAGGCTAGTATAAATAAAGCACATACCCGACCGGTCACCTTCACCAACAAACCGTACTTAATAACACCCCCACAT
TATACTCAGGATCACGACATTAACCTAATCCCGGTTCGGACGAAAGATGCCATTCCGCAACGTTATCGAACTCGATCGAACCAC
GGACAATTCCGTTAGCACACTTAACATCGCGGCGACAAGAGCGTACCGACTAGCTCCACTCTACTAAAGGCAGAAAACAAATCC
TCCCAATCCCCTAATCCGCTTCCCCTACCTACATTGCGGCCAGTCATGCCTAGCAACACGATATCGGGACACCCACGCACAGCT
CTCAAGGCCTTCCCTACGTCAGAAAGCCGACTCTCACTACACCTAACATAAGCACAGGATGGAAATAACTGAACAGGAAGAAAA
CGCCACCCTACCTCCTGGAGCGAAATGTTGAAAGTGAACTGTGCTCGACATGATTCCCTGCGGGATGACTTAATAAGCTCGAGT
AAGGCTACTATTATTGCTGTGAATGCAGAAATTGATCAGTATGCTTTTATGGATAGACTATGTTCCACTGATATAGTAGAGATG
AACTAAAGAATTCTTAGCAAATCACCTTAAAAAAACAAATAAAGAGAATATAGGAGTGGATGATACGACCAAGGACCTGAAGTA
TGAAGCTACACAATTCTACCCTTTGTACTATAATGCTGAAGGCTGATATATTCAGTAAAACTGAGACTCTATTAAAATATATGT
GAAGCAGCACCTGGCGCTCACCGAGACAACGACTTATTCACTGGCGCCTAACTTACCTACTGTGTAAGAATCGTGAGGAAGATC
GAACGCGTCAGAAACATATAGTGGCAATACCGAAACGACCTTAGATCACATTGCACGCACATGGCGAACTTTAGAATACATAAA
TCACAAGAATTCCTGAGAAAGTGTAGCTCGTTCTATCAGTCGATCGGTATGAATCTAACAAACGTAGCATAACTCAGAACTGAT
TGTGCACTAAAAACCTACACACAGGAATCACTATAAAGTAATAGTCAAGAATGCTAGTACAACGGACATAGAGTTAGAAAATTA
AGTAAAAAGGAGATAGTAACAAACAGCTCCTCATGGCTGATCTAGGGCGCAACCACATCGAGAAACACGAGCTTCCCTCGCTGT
AAAGTTTATGAGACCTACCTTCGAAAAACCTCGGGCTCTGGAGAGGTCACGACCTATAACTAGTATGGATTTAGCAACAGCTAT
CTATGTAATCTCCCTCATCTCTACTTTGTGACTGAATGCAATGGATGGATTATAGACTGAAAGATACACTCGAATTCATTGAGT
TGACGAAGACTCGCTTAATCCCGATGTATTAGTCTTAAAAATTTGTATTATGGATGAGATTGCACTTTTCACGGGGAGAGGTGT
CCTCACTGGGATCTCCATTGGAGTAATGATGACCAGACTAAGCAACCTACGTCTCTCATTGAGTCTTATGCTATGATCACGATA
AGCTAAGATACGCCCGCCTCTACTGCGGCACATTCAATAACCACTACTGGAGTCATCATGAAAGCGTATAAGGGTACCTTATTC
GAATTTTGTACACGCGCACTCACACACCGCCCCATCTAGAGACGAGACCACACCCGCAAACTATCCCACCACAACCGACCCATT
CACGTGAGAAAAAGTGATAACGGACGCGAAAAGAAAGGGCTTCACAGACCGCGCGCCGCGGAGAATGGGCCAATTGCAATAAGA
GATGAGGGAGAGGTAACACACGACACCCTTGTTATCTCAAGGTCTAATTCTAGAGGCACAAAACAATTCTATATAAGCGCACTC
GGCGGCATACTCGCTACCAACGGAAAAAACAACCCTCTCTTACCCTAATAATCGGCACATTCAAGGACACCCTTACTCCTAAAC
GAGCTGAATCGGAAGGAAACCCTAAGTCTGAATTTCAATTACAATTCTAAGTGGATACTTCCACATTCGTACCCTGTCATATAA
CTGTGGATCAGAGATCGCACATGTCTTCTCACCGTCTGCCAACTTACACAACTACCTGGAAACGTGGCGAAAACAAAGCTCTCT
CGGATACCCTTCCAGGGGATATCGGCTTCTGCTGACTCTTCAGAAGTTGAGCGGTATCCCCACAACTCCGAATCCACAACTGCA

CGCCATACCTCTACCCCTCCCCCCCGACCATACCCCTACCTCGTGTACCCCCCACCAAAATCTAGACCCGAAAACCCTGTAATA
CCGGGCAGAAGTTTACGAACCGGCACGAAGTAAACACAAGGGGTTGAGAAATCTACAACTACAAGGACGAAGGCGTACCCAGGG
TATTAAGGGTATCGGGATAACCCCTTCTCATGCGGGTTATTGGGTTTCATCCGAGAAATACACCTAACTTCTGGAGATATGTAT
TTGACTGTCACTCTCCACCCACCGAATGTTCACGGAGGACGCATACTATTGGTGAAATGTACACACCATCCCTATTGACAATAA
CCCTTTGCCGGAAAATTCTGGTATAACCGCACCCCCCCCCCCCCACCCCCAAGCACGGAACTTGACGCGTACAAAATCACTATCT
CACCCCCACTCTAAGCTCTCACCAATCACCACCCACAACAAAAACAATCACCGGACAAAACAACCCAAGAATCTCACTCTCACT
CACCACACAGCAAGATAAAAACAAATACCTCTAAATTCATATGGTTTCTCACCTTAAACAAAATTGCGCACACAACAATCAAAG
ATCAAAGACCTCAACCCTGAACTTCGCGGATCGCCCCTCGATGGTCATCTGAGACGAATTCTCAAGACTCTGGAAACTTAGAAA
CATACTCTAACGAAAACGAAAAATACCAATAATCAAACGCGCTCACACCCACTAACTACCCTACTCCCCCCCCGTCTCGAATGC
ACGTGTCACCATTCTCAACCGAATGGCTCTCCTTAAGGCTTCAACTACTTACGCAGCTCTTGAATCGTTGCCGAGGGATGCCAG
CGGACAAAATTCTGATTTCTTTATAAAGCCTTTCCCCCATGGCATTGACATTTTTTGTTGGCCCCCTGTCGTTATCGTATGACG
CCATCCCCCTCCCCCCCCCCCCGCTGACGCAGCGAACTGCAGCGATCAAATTTGCACTTTTATAAGGTTTTGGGACTCTCGATT
CTTCTACTTGTCTGGCCGTGTGTTATGCTTGACGATCACTCATGGTCATCCACCCACACAACTCCCCCCCTGATGACCTAATTT
AGCTATCACCGCCGGCTCTCCGCTGCGTCGCGCATTTTGGGGATAATTGGAATAGGATTTTTCCCCCCTAGCTGAAACATCGGG
CCGTTTCCTCTATGGAACGAACGGGCTTGGGCCCCCCTCCAAGACCGATCATGCTCCTCATCTCGAACGCTGACTCTCGAACTC
CCCACAGCACCTGAATCCCCCCCCCCCCCCCCCCCCCCCCGAGGAGAAAGGGACGCCATCATGGAATTCCCCTGCCGAGCCCGACA
CCCCAGGCTGACAAAGGGCAACGCGGACACGAAGACTACACACTGGCCACGTCACCCACAGAGAGGTCTAGAATCAACCTCGTC
CTACGGGGCGAGGGCAAGAGAAGATAGTCCCTCACTTCAGCGAACGTCCCCGCGATCAACAAGCACAACTCCTTGGACCTACTG
CCGAGGCTTTTCAAGAAAACGGGAAAACCCACTAAAATAGGGTTCGATACGGGACTATCCCCCCCGACTCCCCAACACAACAAA
AACCTCCCCCCTCGCTGTCCTCCCGCGGACATTCAGACTCTCCCAAAGGCCAAGCACTCAGAAATCTCACTAACTATCGCGAAC
AAAATCTAACTCAAGATATACCTTCACCCACTCCCCCGAACTCCACACTCTTTATTATCCCGACGTCCTGCTGAATAACGGCCT
TCTTTATTTCAACGGCCTCATACCGCACTACCGAGGAATCCCTGTGGCATACGTCGAATTAGAGGGCATTGGGCACACATCCTC
GGCACACCCACCCCCCCCAACCGCCGCGCAACCGCAAAAAAAATTGCTTCAAACACACTAGACCGAACCTTCTGTGTTAACGGG
ATGAAGAAACACTATGTTGACAGTGTGTCCCTGAAGCCACGGAACATCATGACACTTTCTGGTCTGAACACTCCTTGTCTACAG
TTCGTAGTACTTCTGATTCTATCTAATCAATGAATGTTGCAAATATGTCGCTACCAGACCGTCTAGCACAACCCCACAAGGACA
ACACACCCCTCCCCCCGAAGTTGAACGAAAACCACAGTGAGTCGAAAGGAACCGCACGCTCGACGCCACAAAGTGGGCACCTCG
GGAGTCAGAGTGGAGAGACACCCCCCGCTCGGAGTGAAAGTATGAAACAGGCAAGAAAAACCCATAAACAGGACTACCATGGCG
AATTACCCCCCCTCAATACCCCCCCCCCACCCCCACGGCTCGTGCGAGGAAAACCCCCACGATAAAAGGCGAATAGCTCAACCC
CCCCCAGCCAGAAACTGGTCAGACAGTGACTTGGAGTCACTGTCGTCTCACATAATTGATCGCATCTGTCTTCCAACTATCGCC
CATACACACGTGTTTCCCGGCCTGTGGAATCAATGCTCTCTCGTACGACTCCTCTGTCAGGTTCCCTTTTAATTGACTGGCCAC
TCAGGATAAAACGGGTCTGATCATGCCTGCGTCGAAGCGTAACGCGAAAAGGGGACACACTCAGACAACCCCAACCCAGAATCC
CGCACTCACACTCCCCAGGAGACGAACGTCGACAGAAGTCCTGAGTCAACACTCCTCATTGGAATGTAATAGCACTCCGAAACA
AACCGAAAACTGATCTTCGCAGCGCAGACATTGTATTACAAAACCAACACCTACCACCCTTTCCTTTAAAATAGTAGTAACAAT
TGGTTTCCCACTAACCTCCTAATCATGCTCCGTCAAATCACTCCTCTACTGAAAACCACATCATCATCCCTTGCACTAAACCCA
CAGCGCTCCAAACAAAATTTCTCATCACTCTTCAGTACAATGGACATTCACTCTCTAAAAGCTTACTTTATAATACTTCCTTGA
CCTGCACTCCTCTGAATTACACAATTTTGTTTAAAACACTTAGACTAAAGAATTACCCCCCAATCGCTACACCTTCACACCCAA
ACGTCACAACTTCGTTACAACCGCGACTCATAAGGCAAGTAGGCCGCAACGTCTACAAACTTGTATCCAAAGAGAGACGAAACA
GGTTCTCACTGGTTCTCAATGGAAGCATTTCGAAACGACCCAAATGTCACGATCCCCCCCAGGATGTAAAAGCCTGCACAAAGC
ATATGAATTAACCGGGTCTGCGTAGGCCCCACTGAACGCAACCACTGTACACTACCAAGTGACATTGAAAATGCTGCTCTTCCT
CAAATGAAAGTCGTTTCTCGAATGTTTCACAATCACCTTATTCAAAGACCCCGAATCCGAAATGAACCCCCTCCGACGTTAACA
ATATGACATGCATCGCCAAACGGACTCAGGAAAGAGCTTGCCCTACCCGATAGACTACTCATGCCCTCCCAATACTTACTCGTC
GTGTCTACCCTCGCTCTGCACACGATGTCTGCTCCGCCCCCTCGCATCGATGTGATCTTCGCCCGGCGACAGCTGGTTTCCTGC
ACAACTGTTGTCTTACAATCGATTAGACTCGACCAAAGCACGTCACATCTCGGCCGAACCACCGAACCCCCCCCCCCCTAATCT
TGTCACTGAGATGGAATAGCATCAACTCATATAGAATAACTGAAGGAATCACGCTACGCACGCACTCCGACAAAGAAAGCCCG
CTCGGAAGAAAATCCCACACAAAAGTAGACAAAGACACAAATTGGCAAGAGACTCACCTCTCGATGAATGGAAAGCAAAGAAAC
AAACAAGAAAAGCAGGAACGCAGAATCTTGGAAGTTGGAGAAGCCAAAGAACACAAACACCTAAGGTAACACTGGGATGAAAAG
ATAAAAGAAGGCCTCTGTGGAGAACAGTCGACAGGGCTAAGAATGTTTAACCCTGCGAGAATACGGTGTGGCAACAACATTCTT
TGTATTGAGCAGCGTTAAGGCAAAGAAACTCAATACAAAAAAATCGTGAAGGTGGATACCAGAAAAAAAAATGGAGTGAAGACAAG
CCTGGAGTAAATATGATTCAATAAAATTACTAGATCTTACTAGCGGTCCTAAGGGAAACCGTGGAGCCGTAACCACTTCGCTGA
CTAAGATAAATGTTAAACACGGAAAGACGAACGACATATGGTGGGACTGTGGTAAAGCTCTTTAAGAGAAGGAATCCCAGTGAC
AAATAAAATGTGAATCTGGCAAAGTTAAGAAAAATCTATTAAACCTGAGACACGCTACTAAAGCTCCGGTGGTCCCAGTCCTAG
AGATGTGAAGGATTGAAGAAGTTTAATACACACAACTCGAATCAAACAAACTCTCGAGTTGCAAACGTCAGCAAACTAACCGAT
ACTGATTGTAGCACTCTGAAAACACTTGGACGAAGTTCTGAGAAAATTTAGCTGAACGAATGATGAGGAACAGAACCACAAAGG
TATTATTAAGGGAGATTCGAATGTTATGAAAACCTGCTCAACAACTGATGTCGAACGAACTTAATCCCCCCCCTCTGGATTAAA
GACGAATAATAAATTATGATCGAGAGGTTGATAGAAAAGGAAATCTCCACACAGATAGGGCCGGAAAACAAACAAACAGATCAC
TGCGACCGACGCGACACGATGCAATCGTTACCCGAAGCGAATTCCGAAAACGGAGGATCACGTACGACATAATCTTTCTGTTGG
ATTCCAACTAAGGGAGGTGGTGGATACTAGCAGAGACAAAGACCGACAGGATAAACGATTAAATTTGTTTAAGTCAAGATTACA
AAGAATAGCAGGGAAAGGGGTGGACCTCCTAATAAAGGCAATCAAGGGAGACAAGCTGAAAAATGGACGCGCACTCTCCTTCCA

AGTCGTGTAAAAGGTGACAAGAAGCCAACGACAAACAAGGGTGCAGCTGAAAGATCTAGAGAATGATCCACTCTCTACTACTAC
AAAAAAATACACAAGGCAGGATGGAGAAAAAAGCAGAGAATGAACACAAGACACGCTATAATCAACTGAAATAAATATGGTGTA
AGCTAAAGTGATGACACAACGTGGACAAACAATGGCACACAAATAACTAAAGTCGTGTAAATTAACCGCATGGACCTGGACACT
GCCTAGAAGAACAGACAAGTCTGAAATAATCATACTGCACAATGGGAAGGAAAACACAAGCCGAATATGGGGAATTCAATATTA
TGATAAACCTACGAGAGAAAAACCACCGAAATGAATAACAACAAAAAGGAGCGAAAATGAAATAATGGAACTCAAAAGGATAAA
ATAAAGATAAATAACAAAAGATATCAGTATTTCTGGAAAAATTTCCAGAATGCTCAGAGGAATTGTATACAAAACTGCAGAACT
CAATACACGTAGACTACCTCGAATAAAAACAGCTACACTATCAAAATAATGAGCAAACTAATAAAACTCCTCAAACCCTAAAGG
CTCACCAAAATGCTCATACTCTATCTGCAGCATAAAAGCTAGGCAAAATCAACACACTACGTGGGAGACAAATTAGAAATCACT
TAAAATATAAAAGAATATTATAAAGGTCACAGAAAAACATTTTGAGTAAGGTTGATGAAAACTAAGTACAAAAATATCAAAAGG
ATTAGAAATAAAAAATGTAATAACTCATAATTGAAATCTCTACATGAAATACTTAACAACACGTAAAAATGGTAAATTAATTAA
TATTGACAATAAAAAATAAAAAAAAGATGAGGAATATCAATTGAAAACCTGTACACTCAGAAATACTAACAACTCAATACAATC
TTACATGAACACAACAATACAAACTATATATCCTCCGACGCGAACATTACATGACGAAATTCCTACTCCAAAATGATATGAATG
AAGTGGGTTATGATAATAAACCTTCACAAAAAGTGCCGCGAGGCACGTGACTCGCTCCCTTATGAAACCTATAAATAACACAAG
GCCATTAAAATACTTACTAACATAAACGGTTGAAAAAAGACAAATGGAGAAAAAAAAATGATGACGCACTGCGAAAGAAAGCTCG
AAAATGGTCTGACTTGATGGAATAAAAATGAAGAACCCAGCTATTAATATGTTAGGTCTGTACCGTCCACTTGACTTAATATCA
CAAACAATCATTCATATTGAAATTTCTGTGGCGGACAAAGCGTCGGCACGGCGTACATTGAAATGTCACCTCGATGAATAATAG
AACAAAATATCGCCGAAGAAGAGTACTAGACTGATTTAACCCCCCCGACGAATATAAAAAACACATCGACATGCAAAACCACCT
GAGAAGATACATCAAAAACACGAATCTACTCACCACACAACAACACTTCAAAAAAACAATTAAAACACTACAAATAAGAAAACA
AAAAATAAGTACAAACTAAACAGATCCAGACTATAACGAATCAAAACGGAAAACCTCGAACTCAGAATCCAATGAATATGTTAA
CATACATTTAATCCACCAACTCGGATATGTCACAAGCAAAGAGCCCACAAAGGAAACAAACGCGGACTCATTACAAAATTGTTC
CTATCAAGAAATCCTTACAGTAAACTTACAACACTAAAAACCCCAAATTAAAGTAGAGATTGCTAAAATGAAAGAGACGAGTCA
AATAGTTAGCCCTAAAACTACTAACGAAACACAATATTCACCAAACATGGATACGATAATGATAAAAAACTCGTTGCTACAAGA
GAACTAATTAATTACAGAAGCTAATCAAGCCCTAGCTTCTAACAAGTAGAACCTCTTAACGACTAGACACAGCTAGAGTCCTTA
CAATAACAAGTGTAATAAAGATAATGAAATCACAGACGAAACACTAAACATCTCTTGACAAATAAACTAAGCATCTCTAAATAA
AAAACCTAATAAACCCGTGTCCTTGAAACAATTGGTAAAAAAAATAAATCAGTTTCCAGAATATTCCCTAAAATACCAAGTAAA
AAAAAACGTTCGCAATACTGAAACAAACTAACTACGGTTTTACATAACAATTATGACCTATTACAATCAACACACTGGATTGTG
GGAAAGACTAGGAACGCGGAAAAAAAATCGTACAGAAAAGATTAAAACCGATATACACCAATCAGATAAATCGCGTTGCCTAGAC
ATTCTCAATTTGCAAACTCAACATGAAAACAACAGAGAAAACAAGTGAGACAGAAGAAAAGCAAACGCACACAAAATAAAAAGA
AGAACACAATATGAAAAAAAAAATTATAGAAATCCATACAAAAGGAATAACAATAGATCCCAACAAATACAGGAACCAACACCC
CGAAGCTAAAACGGAGATTTCAATCCAGGAAATGGTTAGCAGGCAAAAAAAAGTCAAGGACTACCTAACCCCCCCACACCCCCA
CCACAGACCCCCCCCCAAGGTACCAGAACTTCTCACGGAGTCATCAGTCAATGGAAAATTGCAATAAAGAGATAACAAAGCCC
CATCAAATAAACAAAGAAAACTCTCACGAACCCCGAATAGGGAACTTGAAACCTAAAAGTGGAGATAAAAAGCCAAAGTTAAAG
GAATCATTGTAGCCCAAATTCTAAAACACTATCGAAAATAAGGCCACGAACCCCACTACACCAACCGGACAATCCAAGTACCC
ACTTCCCAACAAAATTGAACACAACTCTCACACCAAAACCCCAGGGCACTCATGAACCTCGAACCAAAGAACCTCTCTCATCAA
CCACCATCAGCGCTCCATACAGAAGATGAACACAAACAGATTATACCCTGCAGCGACTAAAAGTCAGCGCCTCATTCTATCTAG
AATGGTATTACCCAAAAAGGATCTCAAAAACGAAAGAGAGATACAAGAGAACACATAAACTATTAACTCAAACGACACACTCAC
GCAACGCCACAACAAATATTAAACCTAAAGAAATACATAACCCACCGCACTCTAAAATTCAATACTCCTAGAGTAACATCGGTT
TCACTGACAGATATCCCCATTCATAGATTTCAAGGCGCAGCCCCCCCACACAAAAACAAAAAAAAACAAAAAAATACATAAAAA
AAAAAAAACACATAGAACCTCTAACAAGAAAAAAAGAAGGTGGCTCGGACACCACTAGATGAAACGAGAAACACATACGCTTCC
CCCCCCCAAGGCTTCAGACAGTGGATTAAGAATAAATCTCTAGAAAAAAATCTTAAACTTTTGAGCAAAGAGGAATGAACGAAC
ACCCTAGCAATCAAGAGGACAAAGCACAAAAGAACACAAAGAGGAGTACAAAAGACCTATACATGACGGAAACAATCCCGTCTG
AGATCGGAGGAAAATTGAACATAAA
>m141121_232159_42134_c100732402550000001823141405141507_s1_p0/127083/268_9991
GAAATATAGATCTAACAAACAATTTTAAGAGCTTTGTCCTCTCAGGGAAAAATAATGAGTGAATTTACCCGATCTGCTGGACAA
CGCCCTAGGCAACCTTTTATTGGAGGGAGTGTGTGTGAAAGGTAATTTTTTTGCTTGCTTGAATTAGTTACCTCCATTTATTG
CGTCAATTACATTACCGACCCCTATATATTATATTATAGAATGGAAATGTTCAAAAAAATTAGAGCCTGTCGAGAAGAAATATG
AGTGATTACGAATCGCTCCGTAATAGGATTCTACGGATGATGTCCTCGAGAACCAGGGCTATTTTCTTGTGTTATAGTTGAAGC
ATATGAGCGAATCCCATTATACGGTAACTCAATACCCGCATTATATATTATTGTTAGGAGTGGGAAACAGCCACCTTCTAAAAA
ATTTAGAAGGTTGTTATCCGGAGACCACTATGCATGAACCTTATCCGATTACCTTCGGAGCTATGCAGTTCTGCTAGGAGATTT
TTGGCCTGAAAGGTCATTTTTTGTTTGGGTGCGTCAAATTTAGGTCAATTCATGGTCAAAAATTAGCCTACCACACACAATATT
ATTACCCCGTTGGCCCCTGCGGACAAAAGGTTCCAAACAAAAACATTAGATCTTGTTAGAGGGAAATAGTGAGTTTAACTAGGA
CGAATCCCAGTAATGGAGACAATAGGCAACCATGCTACTAGATGATTGGACTGAAAGGTTCCAACTTTTTTTTTGGTTGAATTA
GGATAATCCCACATGGTCAAACATATATATACAACCATACATACTAATGATATTGATCATTTAGAATGGGATCAACGCCGCTTA
AGAAACAAATAGAGATACAAAAGGAGAATCACACTCTGATGATCTATCAGGCGGTCGTGCAGCCACATTAAGGCCCAGGTGCCC
TAACGGACAACCTTTGGAACATATCATAAGGTAATTTGGTGTGTTTGTGTTTGTTTGGGCTTGAATTTGTATCCACCAAATGTA
TTGGTACAATATATGCATCTTATAATATACAACAACCAATGGGGAACTGGTTCAAAAAAATTAGAGACTAGTTAGATAAATATG
AGTGAATCTACGAGATCGTTAGAGAATAGGTAGCTGCACTAGAGATTTAGACTGAAAGAAAAATTTTTTTGTTGGTTGAATTA
ATGACAAAATAATGGTCAATAATAGCTATATAAATCAACTTATTTTGAGGATGGGAAAAGGTACAAAAAATTATAGCTGTTTAG

AGAGAAATATGAGTGGAATTAACCCGAGATGCTTCGGAATATGCAATGCCGGGGTAACAGGCGGCAGATTTGGATTGAAGGTAC
TCTCTTTTGTTTGTTTGATTAAGGAAATAATTCATGGTCAAATCACTAATAGCTATACCAGAGCATATTATACATCGAGAGGGA
GAGTGGGCAGTTAAAACACACACCACAATAACCCCCATAACAATGAGAATAAAAATCATGTAATATATACCAGATACAAATAAG
ACTAGGCAGATGCATAGATACTTTGGCTGAACAGGTAATTTAAAAAATAAACCACAAACACCATATTTCATGGTCAAATGTAAT
TAGATATAAAATTATTAGTTGAGATGGAAAGGTAAAAAAAATTAGAGCTGTTAGGAGAAATATGTTAGTGAAATTCAAGATGCG
TGTAAGCGCTCGCTCCACACTTTCCATAGGCAAAGCGCATAAGGAATTTGGAATGAAAGGTAATTTTTTTTTTGCTTGACACTT
AGGAAAACAATCACCTGCGCTCAAACACATAAATATAATAAATATTAACATACTGAATGATGGGAAAAAACAAATAAAAAAATT
AGAGTTGTTACAGAGAAAATGAAGTGAAATTCAAAGAATGATTAGCAGCTAGCATGATGCAATTAGGAGATTTGGACTGAGAAA
GGTAATTTTTTGTTGTGGTTGAATTAAGGACAAATTAATGGTAAATAATAGACACTCACTATATTTTATGGACTCTTCTCACAG
TCTGCATATGGAGCGCACCTTGCGCAAAGGTTTAAAACAATATATTAGAGGTCATGTTAGAGAACGACAAATATGAGTGAATTA
CAGATTAACTCACAGAGGGACCCGCATCAGGGAAGACACTGCCATATGGAGACACTTTGGCACGTAATGGGACAATCCTTTTTG
TTTGGTTATCTAGTACCACACCACCGACAATTCCATCCGCTAAAATACTGATCACCACTACATCTATATTCATTTTATTTGTAG
GAAATACGACTTGACGAAAACAACAATTATTAGAGTTCGAAATACAGAAGAAATATGAAGATGACATTAAAGATCCGATTACAG
AACACATAACAGGCTGACTTGACACAAAACAACACACACAACAAGAGATTCTCCCGGCAGCATGAAAGGTGAAATCTTTTCATG
GTGTTGGGTTCTATATTAGGGAACAAACATTAATGGTCACAATAATAGATATAATAATGGATTCTATTCGAGCATCCGCCAACC
CCAGTACAAAAACACTTAGGAGGCATTGTGTGAGAGAAATATTAGTTTGATTAAGATCCAGTTCAGGAGAACAAAGGCAGCATG
CATTACCGGGAGCTTTGGTACTAAATACCGTAAATTTTTTGTCCTTTGGTTCAATTAGGACAATTCATGGTAAATAATTAGCAA
TATATAATATTATTATGGGAAAAACACACAAAAAATTAGAGCCACTAGTTAGAGAGAAAATATAGTGAATTAACGAGGATGCAA
TTAGGAGTACTAGGACTGCTGACTAGGAGATTTAGGAATGAAAGACAATTATTCATGTGTTCGGTTGAATTAGGAACCATTCAA
GGTCAATAATGAATATATAAATCAGGAACATTATCATAGATGGGAAACAAGGATTACATCAAAAACAATATAGCCGATTGTTAG
AGAAGGAAATTATGACGCTGATATGTTCGAGATATTCCACATAAGACTACGGAGCAATTACCAAGTGTTTAGGAATTTGGAATG
AAAGGTAATTTCTCTTTTTGCCTTGGTTGCAACCACAGGAAATAATTAATGGTAATAATAGAACTATATAAATATTATCTCGAA
GAATGGGAAATAAGGTTCAAGTAAAAGTTAGGCCTTGTTGAGAGAGGAATCAAAATGAAGAATTACCGAGATCTCGTTGGAGAC
ATAGGAACACTGCCATTAAGAGATTTTGTGATGAATAGGTCAATCTTTACTTTATGTTTGGTTGAATTCCAGGATAAAAATTAC
ATGGCTAAATACAGCCAATGAGCTAGTATAACGAGGTAGTTCATGTGAGCAGGGACCATAGGTTCGAAAAAAATTAAGATGTTG
TTAGGGAGCAAACTACGAATGAACCACTACACATTGCCCGTCCGGAGAAAACATACAGGCATGTCTGTAGGAGATTTGGACCAT
GAAAGGGTACAATTTTTTGTCTGTTGTGGTTAATTTAGGGACAAAGAATTAGATGGTTACACACACAGCTTCATAACCGCCATA
TATAGTTGCGGGGCACTGCTAAATGACATTCCAAGCCCACGGAACGGTTCAACACAAAAATTAAGGATAGTTAGAGAGAAATAT
GAGTGAATTAACTACGAGTCAGGGCCCATTCGAATTCGAGGAAGTACTGATAGTGCAAGATTCTCACTGTATAAGGTCAAATCT
CTATTTGCTTGGACTTGAATTAGGACAAACAATTCATGTAAAATAATAGCATATATAATATTATGAGATGGGAAAGTTCAAAAA
AATTAGCGCTTCGCTTAGAGAGAAATATGAGTGAATTAAGAACAAGTAACAGGACATAGGCAAGGTAAAAAAACACAACTGTAT
ATTAGTGTTAATTTTTTATGTTTTGGTTCAATTAGACAACAAATTATTGGAAATAAAGAACACTAGTATAATATTATTGAGATC
GAGGAAAAAGGGTTATTAAAAAAATTATGAGGTTGTTAGAGAGAAATATGACATGAATTCAGAGATCGCATTAGGAGCATAGGT
GCTGCATTACACATAGACTTGTTCGAAACGGTCAATTTATCTGTCTCTGGATTGAATTAGGAAAACAAATTTGAGTGTGGTAAT
AATAAAATAAGCCATATAAACATAATTAATCCGCTTTACACCACATAGGGTTACAAAAAAAAAATTAAGATAACCGTCAGAGAG
TAAAATATGAGTGAATTACGAGATACTTTACAGTTACATACGGAAGAACAATAAGGCAGTGATACACACAGGGAGAGTTGGAAT
TGAAAAGGTCAAGTTTTTCTTTCATGTTGTTGAAAATTAAGAAAAAAAATATAATGGATAAAATAATAGCATCTATACATATAT
ACATCTCTATGGGTGAGAAGAGGTGTCAATACAAAACATTAGAGCGACTTCCTAATAAGTTGCAAATATAATGACAGAGAACTC
ACGACACGATCGTCGCTTTAGGAAGACCATACGACCCAGCATGCTAAGAGGATTAGGATGAAAGGTTCAATTTTTTTGTTTGGA
TTAATTAGGTACCAAAATTAATTGAAGTGAAGTAACAGATAACCGTTGCGATCAAAAAAAAAATATGGCGGACCATAGGGAAAAG
GTTAAAAACAAATCATAGAGCTTGTTAGGGAGAAATATGAGTGAATTCAGAATAGCTTTAGGAAGACAATTTATTGAGCGAAAG
ATGAGACACAATACAAGGATTTTGGAATAAAAGTCAGTTTTTTTGTTTGGTTTGCAATTAGGAAAAAACATCTAGTTGGTAAGG
TAATAGCGCGAGAGTGAGGTGGTATTAGTTTATTGGATGCGGAAAACGTACACGCCCTAAACCAAAATTAGAGCATTGATAAGA
GGAGAAATATGAGCATGAATTAAAACAAGACGTGCCACTCGCATTCGTAGTTCCACATAGCATGCTGCACATAGGAGATTTGGA
ACTGAATACGTAAATTGTTTTTTGATTTCAGCTTGAATTAGGAAACAAAAATTCAAGTGGCTAAATAATAGAAATATTAAATAT
TATTGAGATGGGAAAAGGTTCAAAAAAAATTAGAGATTGTTAGGAGAATTATGAGTGAGATTAAAAGATCCGATTACAGAAGAC
TAGGAAAGCTGATAAGGAGATTTGGCATGAAAGGTCAATTTTTGTTTGGTTGAATTAGGAAAACAATTAATGGTTCAAATAATA
GACTATATAATATTTTGGAATGGAAGGTTAAAAAAATTGAGCATGTTAAAGAGAAATATGAGTGAATTACAGATCGCTTAGAAA
GAAATAGGCATGCGCTAGGAGATTTTGGATGAAAGGTAAAATTTTTTGTTTGGTTGAATTAGAAAAATTATGGTAAATATAGAT
ATATATTATTATTGGAATGGGAAAAGGTTAAAAAAAATTAGAATTGTTAAAGGGGGAAATATGAGTGAATTAAAAGATACGATT
CAGAAGAACATAGGAAGATGACTAGGAGATTTGGCTGAAAGGTCAATTTTTTGTTTGATTGAATTAGTAACAAAATGTATTGGT
ACAAAAATAGCTATAATAAAAGTATTATAGAAATGGGAAATGTTAAAAAAAATTAGAGATACTAAGAGGAAATATGAGTGAATT
AAGGATAGACTTAGGAGTACAATAGGCAGAGATAGGAAGATTTTGGAATGAAGGTGAATTTTTTTGTTTGTTTGAATTAGAAAC
AAAATTAATTGGTTAAATAATAGCTATATAATATTATTGAGTGGCGAAAAAGTTCAAAAAAATTAAGAGATTGTTAGAGAGAAT
ATGAGTGATATTAAGAGATCGATCAGAGAACTAGGAAGATGCAATAGGAGATTGATGAAAGGTAAAAGTTTTTTTGTTTGGTTG
AATTAGGAACACAATTAATGGTAAATAATAGACATATACAATAATATTATTGAGATGGGAAAAGGTTCAAAAAAAGTAGAGAAT
TTAGAGAGAAATATGGAGAAATTAAGAGATCGTTGAGTAGGCAGATGAATAAGACCAGATTTGGACTGAAAAGGTCATTTTTTT
GTTTTGGTATTGAATAGGATCAAAATTCATGGTAAATAATAGAACATAATATATTATTGAGATGGGAAAAGGTAAAAAAAATT

TAGAGCATTGTTAGGAGAAATATGAGTGAATTAAAGAGATAGCTGTCAGAGAACCTAGGATGATGTACGGAGATTTAGATTGAA
AGGTCAATTTTTTTGTTGGTTGAATTAGGAAAAACTAATTCATGGTCATAATAGCATATATAATAATATTGAAAAGAAAGGTTA
AAAAAAATTAGAGCTTGTTAGAGAGAAATATGAGTGAATAAAAGAGATAGATTAGAGACCAACCATAGGCTGATGCTAGGAAA
TTTGCATGAAAGTAAATTTTTTAGTTTGGATTGAATTAGGAAACAAATTATGTAATAATAGATATATATTATAGTTAAAGAAAG
GAAATGGTTCAAAAATTAGAGATAGTTATGAGAGAAATTTGAGTGAATTACGAAGATAGCTTAGGAGACATTAGGAAGATGACA
TAGGAGATTTGGTGAAAGGTACAATTTTTTTGTTGGTTGATTAGGACAACAAATTAATGTCAATAATAGCACATATATATTATT
ATTGAGATGGGAAGGTTAAAAAAATTAAGTTTAGGGGGAAATATGAGTTGATTAAAAATCGCTTCGGAGATAGGAAGCACTGAA
CATAGGAGATTTGGACTGAAAGGTCAATTTTTTTGTTTGATTGAATTATAAAACAAATTATTGGTTAATAATTGCATAATATA
AAATATTTGACCATGGGAAATGGTTAAAAAAATAGAGATATCAACAAGCAGAGAAAATATGAGTGGAATTACAGAGATAGATTG
AGGAGCCTAGGAAGCGGCCAACTAGGAAATTTGAATAAGCAATTTTTGTTTGTTTGAATTAGGACAAAATTAATGGTCAAATAA
TAGCACTATATAAATTATTGAGATGGGAAAAACATTAAAAAAAATTAGAGCTTGTTAAGAGAAATATGAGTGAATTACGAGATC
CTTACCGGAAGAATCATAGGAAGTGATTAGGAGATTTGGATTGAAGAGGAAATTTTTTTGTTTGGTTGAATTAGGACCACAAC
CAATTAATGGTAATAATAGATATATAATATATTGAGGATGGGAAAAGGTTCCAAAAAAGTTAGAGATTCGTTAGAGAGAAATAT
AGAGAATTACGAGATAGCTTCGGAAGACAAATAGAGATGTACTAAGAGATTTGGCTGAAAGGTCAATTTTTTTGTTTGGTTTGA
ATTAGGATAAAATATGTAAATAATACATATATAAATATTATTGAGATGGAAAGGTTAAAAAAATAGAGTTGTTAGGGAGAAATA
TGAGTGAATAGAGATAGATTATGGAGAAATAGGATGATGAATAGGAGATTTGGTTAAAGGTCAAAATTTTTTTGTTTGGTTGAA
TTAGGAACCAATAATGGCAATAAAGACATAATAATATAAATGAGAAGGAAACGGTTAAAAAAAATTAGAGCTAATGTTAAGAGA
AATATGGAGTGAATTATCAGATAAGCTTTGGGGACACGAGGACTGAACTAGGAGATTATGGAATGAAAGGTAAATTTTTTGTTG
GTTAATTAGGACAAAATTAGGTAACTAATAGTATATATAATATTATTGAGATGGGAAAAGGTTCAAAAAAAATAAAGATTGTTA
GAGAGAAATATGGTGAATTACCGAGATAGCCTTGGAGATAGGAAGAGGCATAGGAGTTTAAGACTGAAGGTAAAATTTTTTTGT
TTGGTTGAATTAAGGAACAAAATTCAATGGTAAATAATAGAAATATATATATTATTGAGAAGGGAAAAGGTAAATAAAATTAGA
GCTTGTTAGAGATGAAATATGAAAGTGAATTACGAGATCGTTGAACAGACACTAGGATGCTGCCTAAAAGGAGATTGGACCATG
AAAAGGTCAATTTTTTTGTTGTTGGATTAGACAAATTAATGGTAAATAATAAATATATATATAGTTAGAAAGGAATTGTTAAAA
AAAATAGAGATAGTTAGAGAGAAATTTGAGTGGAATTACGGATAGCTTTCGGAGAATAGGACAGCTGACTAGGAGATTTGGATG
AAAGGTAATTTTTTTTGTTTGGTTAATTATGGGCCTAAATTCCCTTGGTAAATAATATAACCTATATAAATATTATTTGATGGG
AAAAGATTAAATAATTAGAGATTGTTAGAGAGAAATAATGAGTGAATTAAGAGATAGCTTCACGGAGAATAGGCACAAGATGCC
TAGGAGATTTGTCACTGAAGGTAAATTTTTTTTTGTTTGGTTGAAATTAATGACACAAATTACATATGGTCAATAATAGGCACA
TATATAATATTATTGAGATGGGAAAGTTAAAAAAAATTAGAGATGTTAAGAGAAATTGAGTGAATTAACAGAGATATTTCGAAG
ATAGATGCTGCACAGTAGTCATTTGAAACTGGAAAGGTAAATTTTTGTTTGGTTGAAATTAGGAAAATTACATGGTAAATAATA
CAACATGTAATAAATTAATTAGATGGAATATGGTTAAAAAAATAGAAAGTTTAGAGAGAAAATATTTGTGAATTAAGAATAGCA
TACGGAGCACACTAGGAAGCTGCACTTTGGAGATTTGTGAAAAAGGTAAATTTTTTGTTTGGTTGAAATTAGGAAACAATTAA
TAGGTCAATACATAGAACTTATATATGATTATGTGAGATGGGAAATGGTTAAAAAATTAGAGATATAGAGAGATATATGTAGTG
TAATTAAAAATCGCTTCGGAGCCATAAGAGTTGCATTAAGGAAGATTTGGAATGAAAGGTAAATTTTTTTGTTTGTTTGAATAG
GAAAAAACAATTATGTAAATAATAGCATATATTAATATTATTGAATGGGAAAAGGTTAAAAAAAAATTAAGAGTATTGTTGGAG
AGAAAAATGGAATGAAGTTACGAAATCTACACTTTGGAGACAACTAAGGCAAATGTATGGAGAATATGGAACCTAAGGTCAATT
TTCTGTTTGGCTTGAAGTTGGACAAATTACTGGTCAAATAATAGATATATTATTTAATTTAGATGAAAAAGGTTAAAAAATAAT
TAAAAATGTTAGAGAGAAATTTAGTAATTGGAAAGTTCTTACGGAGTACTAGGAGCAAGCATAGGAATATCATGAAAGTACAAT
TTTTTTGTTTGGGTGTTAGGAACAAATTCATGGTAAATAATAGAACTATATATATTATTGAATGGGAAAAGGTTAAAAAATAGA
ATTGTTAAGAGAGAAATATGAGTGAGATTCGGAATCGTGAGCATTGGAGTGTAGGAGATAGGTGGCCAGAAAAGGTCAATTTTTTG
TTGTGGTTGAATTAGCACCAAATTCTTGGTTCAATAATTGCATAATATATTATTTAGATGAAAAAGGCAAAAATTTGAGCTTGT
ATTAGAGAGAAATATTAGTGAATTACAGAATCGATATAGGAGTAATAGGCAAGCAGGAATAGGAATAAGTATGGCAATGAAAGG
TATTTTTTTGTTTGGTGAATTAGGAAAAAATTATGGTAATAAAGTATGATAATATTATTGAGATGGGAAAGGTTAAAAAAAAAT
TAGAAAATTGTTAGAGAGATAAATGAGTGAATACGAGATCAGATTTAGGAGCATAGGTTGAGG
>m141121_232159_42134_c100732402550000001823141405141507_s1_p0/135006/4727_14468
TTAAAGGCTCTGAAACTTGCGAGCAGTCGAGAGCAAGATGTCTGATACCTAGGTCCTACTTTATCAGAGTTCAACGAGTGAAAA
ATCTCTTTAGCTTGAAATAAAATAACCTCGATGCTATTAAATGCAGAAATGAGTTCACCTGATATTCATTCAATCATCGGTGCT
TAAGAGCTCGTCAAAAACGTCTCTAGAGCCTAGTCAACGAGGTTAAGGGAATGAATCACCGCTCGGTTTCCTACCAACTTTTGA
AATTTATGGTCCTAAGGCCGACTATATCCGCCCACTCCTAGGTGAGTATGTCCCTGGGCCCTATGCTCGAGTGCTCACGATTGT
CGATCGTAGGCAAAGTGACGTGTTTTCGTATCCTGCTTGTGTGGACGATATAGTCGGCCCCTATGAGCTCGAGATATCCTCATC
AACATTTGTAAATGTATACGACACGTACCAATCGCACTTTTTCCAGCCTTACATTTCTATATCACTCGACCTCACCGCCCCAAC
CACCTTCTGACTAGTCATACATACAATTGTTAAAGCTCCACCTCGGAAGGTTCTAGACGACTTCTAAGACATCCATTTCTTTTA
ATAAAACGCGGGTTCATCAAAAATACTTAACTCTTAATAAGCTCCATGGCTTATGAAATGTCTCTCATGTTTATTGAGTGTCTC
ATTAAACTTATAGTGATAATAAATCGACATCTTTATAATCAGCTATATTCATAATCAACATTATTTTACATAACGAGTCTAATA
CTCATCTGAAACCTCATAGATCTTAAAATTGGTCCACAGAGAGATTCTAGAGTCTACCCCCAGGCCCTGCTCCCGTGCTCCACC
CCAGCTGTTCAAGCTCGCCAAGGTCCTCCCGGCCCTCGTGAACCAAAAATCCACAGTATTAAACATACTACTGCTACTATACTA
CTGCTATGTCACTGCTACCGCTGCGCTCCCGCTTCGCTCCCGGCAGCTCCCTGCTGACTCGGCTCGGCGCCGCGCCCGCTGCTG
CTCTCGCGCGCCGCTCGCCCCCGCCGGGCGCCCGCTCCTCCTCTGCTGCTTGCTTGCTGCTGCTGCTACACTGCCTCCTACTGC
TGCCCCCTCTCCGTACTGTGCTCCTCCCTCTACTGCTGCTTCTCTGCTAACTGCTACTGCTATGGCTACTGCTACTGCTACCTC

GCTATCGCTGCTGCTCTCTGCTGCTGACTCTGCTGCCTGAGTCAGCCTCCCTGCTGCTCACTACTACTGCGCTGCTGATGCTAT
CGCTACTCAGCCTCTGCTAGTACACTGCTACTGCTTACTGCTACTAACTACTACTCAATACCTACTACTACTACTACTAACTAC
TACTACTACTACTATACTACTCACCAACACTACCATACCACGACTACCCCAGCTACTACTCACCCACCACCTACTACTGACTAC
TTACTGAACTGACCACTCACACCTGACTACCCTCACGATGACTCAGCGTCACCCACGCCCGGCTACCGCTCGACCTACCCGAAC
CGCGCGCCCGCGCGGGCCCCTCACCCACATACCACTACCGCTACTACACTACTACCCTACCTACCACTCTAATCACGACTACTA
CTCACTACTACTACTCCTGCTCCCACTCCCGCCCCGCTTACTGCTCTGCTATGCTAACTCGCTACTGTCTTTACTGCCTACTGC
TCACTGCTACTGCTACCCTGCTGACTGCCCCGCTACTCGCTACTCCGCTCTGCTGAGCTGCTACTAGCTGTTGCTACGTGTGTG
CTCTGCTAGCTACTCTCCGGGCTGACTGAGCTACTTAGCTGCTAATTACCTGCTCGTAGTCGCTATGCTCAACTGCTATGGGTA
CTGCTACTGCTATTGCTACTCGCTACTGCTATTGCTACTGCTACCTCGCTTACTCGCTACTCGCTTACACCTCACACTATATAC
CACCCTACCCACTACACACCGCCACGTAACACCACACTACTACCCACTACTCATACCACCTATTACTACTATATACTACTACTG
CGTACTGCCTCTGCTACTGCTAACTGCTAGCTGGCTGCTGCTGCGTGCTACTGCTCTGCTGCTACTGCTATCTGCAATGCTGCT
ACTGCTACTCAGGCTACGTGCTTACTGCTATGCTACTGCTAATAGGCGTACTGCTACTGCTACCTGGCTACTGCTACTCGCTAT
GCACTCGCTAGCATCATGCTCATCTACTCGCTACTGACGCTACTGCTATGCTACTGCTTACTGCTACTGCTACTGCTACTCAAC
TAACTCCTACTAACTCTACTTACTTGACTACTATAAACTACTACTACTACTGTATACTAACTACTACTACTACTACTATCATAC
TACTTACTACTACTACATACTACTGATATACCTACTACTAACTACTACTACTACTACTAACCAATACCACCAACCACCACGCAA
CCACAACCAAACCGTATCCTAACACTAGCTAAGCGTACCAAGTTCTGGGACTCTACATGTTTGAAACGATCTAGTGCATGGGGC
GCTGCCACCTACATCAGAGAGCACCCTGCCGCGGAGCTCTCCTCTGCTAGTGTAGACGGCGCTGCATCCAAGTCAGACAGCACC
CCTAAGCTCTCCTGCTGCCTAGTGCTGGGGCACCAACTCGAAGACAGAAAATTCTAGTTTTTCTCCACTGCTTTTTCCCCTCTG
AAGTAACCAAGCTTCTAAACCAAACTAACAACCTCATCCAATTACCTCAACCTCATCAAGACCCATAACTCCTTGAACCATTTA
AAACCACCTCTCAAATAGCCACTTCTTAATAAAACTCTCAAGTTAGAACTAACTTAAAAACATAGGCAAACCAAAAACAACTCG
GCTGAAACCTCTACCTTTAATCCACAGGTTTTGAATGCCTTTCCATGATGCGCTTGAACACTAGTAAAACTCAACGTTGATGCC
AAAGTTGAACCTCAATCTTTAGTTCCAAAATTCAAGGAAAAAGAGGACGAAGTAGATGATCGTTGGAGGAGGAAAGAATATTAT
GGTTTTTACTTCAGCTTTATCCCTCAAATTAGCAAGGTTGGAGCACGCTGGCAGTGTCCCTCAATAAAGAGGCTTTTCTGCTCT
GCCTTGGACGCCCGCGGTCCTTAAGGGGCTTGGGTCGAAGGTCGCAACCCCTTTCAAACCGAGTCTGCTCCGTTGAGAGCGTTT
CTTAGCCTCGCAGACACCCACATCGTCTCACCCAGGCTCGGCGACATGGATTCTCTCCACCACCCCGGCCCTCTCTGCTCACAC
CGCGCTTCCGGCGCAGCGCTCACCTCACACACTCTCCCACACAGCTCTACTCTTGCTGCCGCATTTTGCACTTGCATCGCATGC
ATCATCTCACCCGAATAAGTTGTGAACAGGTTTTATGAGCCCAGCCTCCGAGTATAGAGGACTCAGGTAGCTCTGGCGATTCGG
CGGTATGCTACAGGAGCTCTCTGGGAGCACAGTAAGAAAACTGTTGACCCCCGCTCAGCCACGCAGCGGCACCTCAGGTCCCTA
GCTAGCTTTCGTATACGCAAGGGCATCCTGGCTGACATTGATTGATTGGCAGGGTGTTCAGCCTATCTATTTACCTGTTTACGC
CTCACCAACTGTTCATACATACCCTGCTCTGTCTAATTCACTTGTGCTCACCGTACCGTCCGCATACTTACTGTGACTGAACAC
GACACGGCCGGAAGCCCGACGAACGGCGGGCGAACGACGACGGCCAGCAAACCGCGGGCGCCCGCGACACCGCAGCCGAAAGCC
CACCAGGCGAGAACCCAGATCCACGGAGCCAAGCGCACGTACGCACAACCACGCGGAGGCGCTCCCGCCAGAAATGAAATAATG
AGCACACGCGAGCTGCATACCCAAAGTAATGGCAAGGGGCCAGGACACGACGTTCGAGAGCATTCCCGCGCCCATGCCTCAGCA
CACCCACCCCGGCGGAGTGGGAAGCGTGTAGGGGTGGACCCACTCCTAGGATGCGGGTCATCTCTGCGGTGAGACGCATACGAT
CCAGGGCCTGGTAAAGTGCCTGGACACCTGCGCCGCTATATGCTTTAGCCTGATTGGCTTGTATTTGTTAATATGCTGCTGCAC
CGATCCGGACGCGGGCCCCCACCCTCCTCGAAATTAGATATGCATGTTTTTCCGTTTTGTGTTGAGCTTTCCTGGCTGGGCTTC
CTGGCGATCATGGGTACTCTAACCTGCGTCGCAAGTCAGGGTCAGAGGGATGTCGAGCCCCCCAGACTCATGAGTACCGGAGAG
GCATCGGAGCAGATGGGTACAATATTCGGACCCCCCCACCTAACTGACCTCCATGGCTGGTGTGTATCTTTTGGGTAACCAATG
AAATGTACTCACAGTCCAATACAGTCTGTAATTTGTCGACACACGCCGACCCACTGTAATTTTTGACACTCATACTACTAGTCT
CTACTACAATAGAATTTCGGAGATCCCAACTATACGGACACTTTATGAGATTTCAATGAATGAACAAACTTTTTAATATTCATC
AGCAGCTGAATTATACTCATGGGTTTTTTCTCAGTTTAGTCACATACTTTTAACTAACCCCACGACCGGTCGATTAGTCCCGAG
ATTACATCGGCCGCAACAATTTTAAACGTCACTTAAGTAACGATCTAAGGAAAGTATGAGGCGATTAAAAGTATAGACCTGTTT
GATACAGGCTTGTATACACTTGTTCTTACAAAGCTTACAATCCTAACATAACTATTAGCCCCTATAATGTGAAGCAATATATCT
TGACCTGTCTCGAAACTCATAACTCTGAATACTTGTTTTTTGCATAGTTCGCTTCTCATCTGAACTCGGCTCCTGGCCTCACAG
GTGCGTGTATAGGATGGCACGGTAAACGCAAGGAAAATTAGAACCCAGAGTTGGAAGGCTCTAGGCAGCGCCAGGTGTGAACTA
TCAAAGCCCACCTCCCGGCTCCCACCCCCGCCCCACCCGCAGTCAAAGATACTCACTCTATCCTCCACACGGGACTCGCTACAG
CCTTTACAGATCCAACCTTTTTCTCCGATCCTAGTTCCGACTCACATCCTCTTCTGTTACCCGCACTGCGTTCATCGATCTGTA
CCTCTACCCTTGTTACCAACAAACTTTTTTTGGATTCGATCCTGTATACACCCTGCTTTCAAAGCTTTTCTCAATGAAACCAC
CGTTAACATATCCTGTTTGACACAAATTCTCATCCTCAGATACCCGTACCAACGCCATTGTTTACCAAATCTATCTCTACCGCA
CTTTTCGACCCTCTCACCATACCTCACTCTAATGAGCTTAACGCCTCATTCTCTCTCCAACATCGGCAGTACAGCTAACACAGC
TCTTGGATCTCACGCCTCAAGCCCGCTGCTCTCCACAACCACACTACCTTCCCCTCCTCATCACACCATTCATCTGCTGAAAAA
CAAGACACACGCTCAACTGATCCAATACTCAAACCAACCTTTCAAAATGGTCACGACAAACACTCCCCACATCAATCCAAAATC
CCAAACAGGCATCAGCCTAGGATCATCATTCAATGGTGTCCATCCCCCCAATACATCCTCCGTGCTTTCCTCACAATATTCTCG
ACTTACCTGGTTCGTTCCTGGTTTCACTTTTAAACACAGTCTTGGCTCCTGAACCTGCGCTCACAAGCGAGGGCTAGCTCCGCC
TCAGTCTGAGGTCAAGGTGACACTCTGGCAGGAGCGAGACTGTACACCCCACTGCTTTGGCTCCCTCATTTCCTCAGCGTTGTG
GGAAGTCCTTCTTTAATGATTAGGACTCCAACAGATAAAGGCCCCATAATACCTGCACTCTATCCTGATGTCGCGTCTGCACCG
ATGATCAACTAGGACAACCCTCCAGTTTTCACTCCACCCTGAACGGCTCACCTACAAGTCACGCCGTAGCCTCCCATATCCCGG
CTAGGAGAACAAAAGAATTGGGGTCTTTTTTCTTCCTGCTCAACGAGCCAGCTACCCCATCCGGACTGATACAATGAAACAGCA

GAGGCATAGCCTCTTGGCTCTGCGCCTGAAACAATGCTCTCTCTCCCATATTTTGATCTCACCGCCCGGTCAGGCTGTAAGAGG
CGTTGTCCACCAATGGCATCAAAGTCGTGTCTCTGGACCAAAGTATGCTTCCACATCGGCAGGATACATAAGATATCACATGCC
ATGCACAATCTATCGCTTCTTCGCTGCATCCATCGGACTAAATTCTACGGCAAACTTTGCCAACCGGGATCAAATCTCATGCGT
ACTCCCAGTACAAAGTTAAATGATTCGAGTTAGGTTGAGAACTTGTCGAACCTTATGCAGCTCGCGTACTGCTACTAGCTGAGA
AGTCACGATTTCTCATTAAAGATGTTTAGCTCTTCCAGTATACTGCCACATTCCTACTCTGAGTCACTAGCTCCACAGATGCGG
CGCCATCTATCTCTAAACATGTAGCTGCAAGAGCTACCTGCATCGTTCTCATATCCACCCTCATAGGAAGTGCCAAGATTGGAC
AGGAAAATCATATTGCATCCAACCATGCTCTAGCCCGCAGTGGGCTTGATCACCTGGAAGGAAGGAATGATGCTGCAACCTGAA
ACCTCCTCATACAAAACGGTTTCCAGCCCCAACACGACAGTGTTTCATAACAGGCTAACCGCCCGCCACCGCCGCCCCGCCGCC
GCGGCGCGCGGGCGCGGCGGCCCGCCGGGCGCCCGCGCGGCGCCACGCGCGCCCCGCGCGGCGCGCCACCGGGCGCGCGGCC
CAGCGCCGCGACGCGGGCACGCCCGCGCCGGCGCGCGCCGCGCGCGCGCGCCCGGCCGGCAGCGCCGCCGCCGCCGCGCGCC
CGCGGCGCCGGCGCCGCCGCCCCCCGCGCGCCGCCGCGACCCCGGCCGCGCCGCCGCCGCGCGCGCGGCGCCGCGCCGGCGCGG
CCACGCGCGCGCCGCAGCCCGCGCGCACCGCCGCCGCAGCGCCGCGCGGCGCGACCCGGGCGGCGCGGCGCCCGGGCGCGCCGG
CCGCCCGGCGCGGGCGGCCGCCGGCGCCGGGGCGCGCGCGGCCGCCCGAGCAGCGACGCGCGCGTCACGCACGCGGCGAGCGCG
CGCGCGCACGCGCCTGAGGGCGCACGTTGTTCGCGGGCGCGGCCGCCCGCACGCGCGGCGCTCCGCCGCTCGCCCGGCGTCGGC
GGCCAACGCGCGCAGCTCGCCCGCGACGCGCGCGCGGCGCGCCGCTCGCCCGCGCGCGGCTCCGCGCGAGCCGCCTGCTCGCCG
GCGCCGGCCGCTGCAGCCTCGGCGCTCCCGCGTCCGCTGCCGCGCAGCTCACGCGCGCTGCTCGGCGCGCATCTAAGAACACAC
ATCCCTTTGCCGACACCTTGCTGGGCACGTGGAGGGTCCTGGGTGCGGCGCGCGCGCGGCAGGGGGCGCGGGCGCCGCGCGGGC
GCGCCGGGGCGCGCGCGCGCGCCGCCCGCGTCGCCGCGGCGCAGCGCCGCGCGGCGGCGGCCTGAAACTGGGCGCGCGCACGCG
CGCCACCGGCGCCGCCGGGCCGCCGGCGGCCGCGCCGCCCGCCCCGAGCCCCGGCCAGCTCAATCTGCCCCCTCCCCCTGCATC
TCCCACGCTCCCGTGCCCCGCACAAGACAGTTGGCGCTGCCAATCAGGCAACGGATACACGAATCCAAACGCCCGCACCTCTCC
GAATTCATAATCAGTCAAGCCCAAACCATAACCACCCATAACAGTCACACCGCGAACCAAAGTGAACGGGACATCCATACACTC
CAGAATGCCGGCATGCCCCTGGGCATCGTACATGCATACGGTGTGATCCTTCATTCATGCATGCTCCATAACAGGCCGACACAC
AGGGGGCCGCAGCAACACCGCGGCACTCTTACACATAATTAAAAGCATCATACACTTCCAATCTTAATCTACCCAACCAACCCC
TCACTCGAGCTCTGGTCAACCCGACAGCTAGAGCGTCACACTGCTTCGGCCGCACTCCAATCTCCAGGAAGCCATCCAAAAAAC
GAAAACCCCACAACATCTGGGATCCGCGCACTCCTGAGCCGCTCTGCTAATCCCATAACCTTCCTCATCGACGAGTCCATCCTA
ACTCACCCAACGTGAGTCTTACAAATCGTCAGACTAACCCCCTCGCGTCCCCTCGCGAACCGTGTATCTTTAACAAGCTGAGCT
CAAACCCCAGCGATCAAACACAACACATATTACCAAAAATACTATACATTCCATCTCAACTCACTACAACGCGTTACTCTTGGC
ATCCAAAACACACTAGTATCCCTAGCCAAAAAGATATCTACAAATTCAAACACTGCAATCCACCCTCAAAATGTCAACAACCTA
CGGCTTGAATACCACATGTTCCACCAAAAATACCACTCGGCCCATCCCCGCACAGCCAGGCACGCACACCATGACGTCACGCGC
TCCACTACCCGCACCCTCTCGACTTCCGTCCGCACTCCAACCATGGTCCAACACTTCACTCCCTCACCTCCGCCCTCCTCACCC
CGCACCATCTGCCAACCAATCGAGGCCACGGCCGCAAGTCGAGCCGCAAGCCAGCAAAAACAACTCACACCAGCAAAACTAACA
GTATGCATGCACTCTTATGCCTCCCGCACCATCTAAACTCAACCGACCATAATCCCGAGTAAACACCGGCCCCCCGCCCCACAT
CACCCCGTCTCCCACCACGGTGGCTCTCACTAGCTGCTTCCGGTCTCACACCCCGCTGAAATACATCCTAGAAGGCTGATCTTT
AGGGTTCTCCGCCTGGCAACCCCACCCCGCACTGACCCCCACCACCGCCGTGCTACAACGTTGAAACCGCCACCCCGTTGCATA
AACTCGGACACATTTCTACCGCATATCCGGCCCTCGATCCCGCACCAGCTCTTGCGTAATTCACAATGATCATTGACATAACGC
TCATGAAATCTCAACCACCCCTACTCACAACTCATCTCCGCACCGGGGCCTCGCCTGCACACAGTCCACAACTAGGTCGTTTCG
CCTGCAACCACATGACTACTCGGGCCCTTGCGCCTACACTGACGACATATGAACAGCCTTTCATTGCCTGTGTCCCAAATGCCT
AATTCCGACGCACGTGGCGATCGAGCGAGCACCGTCTTCTAAAACCCCGAGCCTTGAAAGAGAAACATCCTAGTCCCACACGCA
TGAATCAAAATAATTACACCGAACACGTATCTACAACAATTATAATACCCGTATTCGAGAACTAATTCTACACCTCCGGACCGC
CCCTTGCTCTACCACGTCTTCTAGCAGCTTTTCCCGCGGCTTAACGTTTCAACAGAGTTCCTCCTAGCTAAAACCCTCATTTTG
CCAAAACATCAATTGGCAGGCAGCGTCCTTTAAAGCATCGCGGCATCGCACCCACCACCGCCCGAACCAGGTTAAGGACAATTG
AGTCATCACGTCTGATAGCCTACGGGGCCCGCGGCCCACCGACCCGCGCTTGCCGCGCGGCGCCTTGGCAAAGTCTCGAGCGAC
CACTCGAGCCTGTGACACTACCAAGCGGTGCTGCAGCGCTTGAAAGACAACAGAGGATGGCCCCCGGGCGCTGATCACCAGGGT
TCGCGACACAACCCTGAATCTAAGGAATCCTCATGGGAACTTTCACAATGCTAGACACTTCCCCTCGAACAACCCAATTGT
>m141121_232159_42134_c100732402550000001823141405141507_s1_p0/142171/1569_7124
GCATAATGTATTTTTCACTAATCTATATTCCTTACTACTATAGCAACTATAATGGACATGGTGCTCATGTGTCCATTTCAATTC
AGACGTTTCTTTGACTGAGCTGGAATAAGCGCATAGTTCGCCATCGGCCATCCCCCATATGGACCTTACTGTTGGGCGCTGCCT
CTGGAACCTTGCATTGAGCTGAGATGCGGCTGAGACACTGGGGCTGAGGCTAGAGTATGAAAATTAGTCTCAGTATAGTAGTAA
TGATGATCGATGCATATTGTCGATGCAGTTTCATTCCCCGACCGCAGTCCATACGTAGAAAATTGATGAAACCTTAATCGATCT
AAATCTTCAGAGTTGCTATGCTAAAACAACAAGGAGACAGAAATGTTAGAGACTGCAGATTAACGTTCCATATCTGGCGCTTTG
CAGCAGTAGCCAAGCGTCTCACCTTAAACGTCTCTATATATGATGCCGGACCGTGTTATCCTTGTAACAGTGACTTGAAGGCGA
GGAATCGTTATGCCAGTAGTTTTGACGCGGTGGCGCGGGACAACATTGGTCCGTTCGTGTACTCTAATTTATAAGAGTGAGACC
GCGTGCATACTTCGGAGCGACACTTCAAGGAACCCACTAGTCTAAACAGTCAGAGTCTGCATGAGAGACCACAAGCATTAGATG
AGACTTACACTATAATTTAGGCTCTAAGGAAAAACTTAGTCTAAGCAAACAGTAACTCGAACCTGATCAGTTTGTCTCTATCTC
AGTTATGTAAATAAATATAGGTGAGAAATGATTCCTTTATAATTGTAAGGGTGAAATCGGGTTCTCAACATACCAATCATCTAG
GAAAATTAATCCTAATCCATAATATGATTTATGTTTTTTTCTAACCATCTGTTCAAGTTAACAAGATATACCCCTTATTATCTA
AGTATAATATATATTCTCTAGAGACTACTGAACTTACTTATCGTTACGTTTTGTTCCAAAAGTTAAACCAGATTGGAGAGTGAG
TATGAGAGCCAAAGTGTAGCCATGTTATAAATTAATATTCTCCACAATACTATGGGCCCCAATGCTCAATCATGAGAGCCACAA

TACCTGGTCCATTGGATCTGTACCAACCCTCCACTGAACTTAGTCATTATTTATTGAATTTATTATTTATTATGATGTACAAAA
AAATCAAACTCATAGAACATGTCATTCAAAAAATAACAATTTAGTTCATTGGAAAAAGGTTTCACTAAGTACATCATAAATAAA
AATAAATAAATAAATAAGTTAACCAAACTAAAAAAATAACTAGGGTAATCTAAAAAACCCCCCCCCAGGATCTTCTACATCTGA
CCCTGTCATCTAGTCTATCATCGTCTATTTATCATAGTCCATCATTTCTTGAGCACTTCAAAAATACCTCAGGGAATTCAAATC
AAGATATATGCTTTGTTCCTTGTTGAATTGAAATTCAGAACTTAGAGGTGAACCTTGTATAAGAAAATAATATCTCATGGCTAC
CGGTAAATCATCCTCGTCGGTCTGTAGTCTCCATAATCTTCTAATGTTGAAATTACAAAAGGATTATCTTAAAACCTTAATTAT
AGGACATATTGTTCCATAGCTCTCATCATAATTTGCGTTAGTAGTTTGAAGTGAACTATCCCCTATGTGAAATAGGGATCAATC
CTTTGCGTGATTCTTTCTAACACGTCCTATTGTATTCCTTGCTCTCTAAATCCTTTTCAATGCCTTTAATGACTCGAATATTCA
TGATAGGTTAAGGCGCCAATCCATTTTAAACTGAAACATAATGGCTAAAACATTAGCTATTAACATAAACATAAAATCATAACA
TAAAAACACTTACATTGGGGTAAGATCGGAACTTGTCGTGTGTATCAACGAGAACGTTCATTCATATGAACTTGGCCTAATACC
AACTGTAATGCCCCAACTAATTCTAAGACTCGGACCATTAAAAACTACTAAAACATGAACTACTATTTGGAATACATAAAATAA
TGAACTTCATTTAAACCAAAATAATGTACAAATACAAATAAGTATAGGTAGCCCATTGTGTTAGTACATAAAACATAAAAACCA
TAAACGTTTAATCAATTGTTGTTTTAAATACTAAGTCGGAAATACATATAAACATAAATTGAAAAGAACTTTAAACAAATGCCA
TACTCAATCTTCCACTGTCCATTCAATCCATTCCATGTCTCAATACCAGTGCCAAGCTACCACTGAATCTGTTCCCGCTTCCAA
GTTCATTTTCTGCACATCTAAAATTGAAAGGAGTTGAGCCATAATGCCCAGCAAGAAAAATCTAACTAAAAGAAAATATAATCA
TAAATCATAGACTAATATCATAAAACATATACGATAAACATATGATGTAAAAATGTACTATCGTATATCGTATAGATCTAACAA
TATTGAATGGCCATTAACTCGCAATGACCGTCGGCAGTGTGATAAACCATCTAGGTCCCGTCAGTCTACTAATTGAGATAGGTT
AGATCACAGAGTACTATATGGTAAACTCTTCTAGTCCCTCTTGTCTGACTAATCGAGGTAGGGTAAATCATAATCTAAACCACG
ATAATGGATAAAAACCTTGGGGTTTTGCTATCTAAGCAACTATAACCAAAGATGATACAAAAATATTCATACATATAACATATC
AACATATCATACCATACACATAAACATTCAAAACATATATAGTCTAACCCTATTTTCCTACCAAAAAAAAAAAAAAACTAGGAT
AGAGACATGAGAACGGGATTTGGAAGACACTCCCCTAAAACCAAACAGTAAAAACCATGATTTCTAAAGATTATAGAGATTGAA
AAAGAGTAACCTAAACCATCAAAGATAGAAACCTTACCTGAAAACCCTTAAGTGTTCAAGGAACTTAAACATCTAACTATAAGG
CCAAAATAATGAGTTAGGATCTGAAGAAAAATAAAGGAACTATGGATTGGATTAAACCTGAGGATAAGAAATACCTTGGAATGA
ACTATCAATTTGATCCTACACCTCGATACCAAAATAGTCACTCTATTGTTACTTTCCAATGTTTAAGAAAAGCTTTAACCAAAA
CCCAAGTGTTTTCACTCTGAAGAGTAATTCTTTAGCAGCATGGAGGCTCTGAAGAATGCTTTGAATGAGTGAGTGAAATGCTAA
GTACTAGGTCCTATTTATAGAGTTCAAGGAGTGAAACTAACCTTACTAAAATGAATAAATAAATGAATATAATGAAATTCTTAA
ATTTTCATTGTTCAACAATTACTTCAGAAACTCGGCAAAACGTTCAAGAGCAAGTCCAAGTGTTAAGAGCTGTTTCTAGCTCGG
ATTCCACAATGTTTTCATCAAAAATCAACCCAAGGGTTCAATATATCGCCTACGCTTAGGCGATAATATCACTGCCTATGCCTC
GAGCCTTCGTGTTTCGGTTCGTGTGAAGTCGACGTGTTTTTCCGGATCTTCTGCATGTGACGATATGGCCCTATTCTGATATAT
CCGGCATCACAATATATTTTAAACAACGTTTTTGCACCACTTTCAACATATTTAAGTTTGGTTTAAACAGCTTTGACCTGAGTA
AAAGTGAATCCTAACAGCTGCTAGAAGGTTCTAAGAGTTCTAGATTCATTCATTATTAATTATTTTATCTAAAACCTTAATCTT
AAATATAACAAGCCTGTGACAAGTGTCATGTTGGTTAATTATTATATCCTAGAACATAGTTATAATTAAATAATATTTTCGATG
ACCAGAATATATTATAAATCAAACCTTATGTTAGTAATTAATATTTCTAAACTATCCTACTACTACTGTTGTTGCTTTGCTACA
CGTACTACCTACTACTACAATACTACTACTACTAATATACCTACTACTACTCCTGCTGCTACCTACTATTTCTAAACTATCTAG
TACTGACTGCTGCTACTGCTACTACCTCCTACTACTACTACTACTTGCTTTGTCGCTGCTGCTGCTGCCGCTGATTGCGCTATT
GTTAGTCTATGTTTGCTGCTACTGCTACTACTACTGCCTACTGCTACTACTACTACGTACTGCCTACTGCTACTACTACTTTGC
TGCTGTGCTACTACTGGCTACTGCTGCTACTTGCTGCTGCTACTGCGCTAGCTGCTGACTACTACTGCTACTGCTACTGCCTAC
TGCTACTGCTACTGCTACTGCTACTGCTACTCACTGCGCTGACTTGCATACTGCTGCTTACTACTATCTGCTGCACTACTACTA
CGCACTACTGCTACTAACTGCTACTACTGCTACACTCTACTACTACGCTGCTACTGCTGACTAACACTACGTCCTATACACTAT
CTGGCTGCTTACTACTGCTACTACTGCTACTATGCTACTATTCTACTACATACGCTGCTACTGCTACTACTACTACTTCTGCTA
CTACTACTGCGCTACTACTGCTACTCGCTAACTGCTGCTACTGCTACTGCTACTGCTACTCGCTACTGCTACTGCTACTTACTT
ACTACTGCTGTTACTACTGCCACTGCTGCTGTCTGCTACTAACGACTGCCCTTGCTGCTTCTGCGATGCTGCTGCGCTCGCTGC
CTGCTGTGCTAATGCTGCTATGCTACTGCTACACTGCTATGCTGCCTACTCGCTACTGCTACTACTACTACTACTTACTAGCTG
CTAAGTAAATCTGAGACGCTACAAACTATAGTGCCTGGAAGACCTAATTTGGATATATGTGTCTGATGTGCTATAATGAATTAT
GTAGTAAATGTTATTAGGCATGCATGAATTGTGTTGGATTAATATGCAGTGGGACCAATTTTCTTTAAATATGGGCGGTTTGTA
AATGCCGTTGGCATAAATTAAAATGCATGTATTATGAGTCAACCCCAGTGTTTATGGGGATATTTTTTAGAGTGTGGTCCCAAA
CGATCCCTTAGGGAATCACCGTTGAGCGGCATAGTCACAAGGGGCGAGTATCCGGCTCGGGGGTCATAGGGGTATTTGTGAACA
TAGTTTCGTTTTCAGAAAATACCGGAGTAAAGGGTGTGATTTAGTGTTCATTTGGAGTGTTGGTGGATTGAATGGGGATTTCCA
AGCCTGGAAATCCCCATTCATCATCGACACTCCAAATGCACCCCTTTTTAGGACGACTTTTAGCGAGCTCTAAAAACGTACTCA
TTGGACTTTTTAGGGCCGCAGAGCGAGTCCTAAAAGCTATGTGTTCTAAAGAGTTTTGAATTTTTTTTAACAAACACCCTTCT
TAACTTTTTAGACTCATTGCGAGTCGTTAAGAATTTTTTTAGAACAACGCGGTGCAAACCCTAAAAACTTAGTGTGTCCTAAGA
CTTTGAATTTTAAAAAAAATCACAAATTCCCTCCAATCCCTCAAATCACTAAATATATAATAAAACAGAAAAATCCTCAAATAA
TCACTCTCTCTCTCTCCTCGGTTATCTCTCTTTCCCAAGCTCCCCTCTCGTCTCTCTTAGCTTCGTCTCTGCCAAGGCCAAACG
GTCGCATTGCC
>m141121_232159_42134_c100732402550000001823141405141507_s1_p0/153246/928_9080
TCATGAGCCACCGGAAAGTTGTAAGCCCTAGCCGTGTAAGCCTTCCTATGTTTAATTAATAACGTGCATATCTGGGGGTGTTTA
GAGGGTGATACCGCCTCAAAAAGGCGAAAAAAAGTGACCTCATTAAGAAGTGACGGATTATCGAACATGGCGACATATCAGGAA
AACTGTGTGTGAGAAATTGCCCTAAAAAAGGTGTATCCAGTTATCTATTCTCAAATGTTTGAATTAAGGCACACCGTGTGTTCA

CACCTCATAGAATAAAGACGCATTTACCTAATGAGAATTCTCTTGGGGAAAGATATATCGAACTGAAGTTGAACCCAAGTGTCT
CAATACATTTTGGAGAAAATTGAGTGAAATCCTCCAATTCACCAAAAAGGGGACCCCTTTTCAGAAAGTCTTCGAGTTTCCTCT
AATATTATTGGAGCAATGGTTTTGAAAGCGATACTAAGTAGATAGGCGAGGGTCTATGAGCTCACGGAAGTCTGTAGCCCTATG
TCTTAGCCTTCTATGTTTATAATAATCGTAAAAGCATATCAGGGATGTGTTTAGAGGGTGACTAACGGTCAAAATACTGAGAGA
ATGTAATAGAATATGAGAAAAAAACTTGATGAAAATTGCCCTAAAAAAGGGTGCAGATATTTTTTCAAATGTTTGATTCAAAAG
GCACCGTTTCAAGCTCCAAATAGACGCGAGTTTACAAAGCATCGGGAAGAATCAAATCTGCGGGAAAAATATCTGAACTGAAGT
TGAACACTAAGTGTGCATATACATTTATAATGGAGAAATTGGTGAAATCTCAATTCACCAAAGGGGTACCCTTTCGAAATCTTC
GATTTCTCTAATATTAATTGGAGCAATGTTTTGAAAGCGAATACTAAGTAATAGGCCGAGGGTCATGAGCACTGGAAGTTAGCC
CCTATGTCTTAGCCTTCCTATGTTTATTAAATCTGTGCATATCTGGGGGTGTTTATAGGGGTGATACGGTCAAAATACTGAGAA
TGTGAATATGAGGAAAAAAACTTTGATGAAATGCCCTAAAAAAAAGGTGTTTGTTTTTTCAATGTTGAATTAAGGCACTCGTTT
CAACTCCATATAGATGCATTTTACCCATGAGATCCTCCTTGGGGAGAATAATATCTGATACGAGTTGAACACAAGTGGTATACA
TTTTGGAGAAATGGGAAAAATCTCCAATTCACCAAAAGGGTACCCTTTCAGAAATCTTCGATTTCTCCTAATATTATATTGGAG
CAATGTTTTTGAAAGTCGAATACTATAGTAAAGTGCTGAGGTCATGAGCACTGAAGTTGTAGCCCATATGTCTTAGCCTTCTAT
GATTTATAATAACGGTGCATATCTGGGGGTTGTTTAGAGGGTGATACGGTCAAAATACTGAGAGAATGTACGAATATGAGGAAA
AAATCTTGTGAAAATTGCCCTAAAAAATGGTGTTATAGTTTTTTTCAAAATGTTGAATTAAGTGCACCCGTATGTCAGACTCCA
ATGAGATGCATTTACCCATGAGACTCCCTTGGGGAAAAATATCGAACGAGGGCGTTGAACACAAGTGTAATATCATTTTGGAGA
AATTGGGAAATCCTCCAATTCACCAAGGGGTACCCTTTCATGAATCTTCGATTTCTCCTAAATTTAATTGAGCGAATGTTTTTG
AAAAGCGAATACTAAGGATATGCGGGGTCTATGGGCCACGGAAGTTGTAGCCCCTATTGTCTTAGGATTCTAATAGTTTATAAT
ATCGTGCATATCTGGGGTGTTTAGTGGGCAGGTGTGACACGGTCAAATACTGAGAAATCGTACAGAATATGAGGAAGGAAGAAA
CTTTAGGTGAAAAACTTGCCCACTAAAAAAAGAGAATTTTATTTCAAATGTTGAAATTAAGCACCGGTTCAACTCCAATAGATC
ATGTAACCCATGGAGTATCGGGGCTCTTGGGGAAAAATAATCAAGAGACGAGTTGAACCAAGTGCGTAATACATTTTTTTTGGA
GAAATTGTGAAATCTCCAATTCACCAAAGGGGTGACCCTTTCAGAATCTTCCGATTTTTCTAATATTAAGTGTGGAGAAATGTC
TTTGAAAGCGAATGACTAAGAATATGCGAAGGGTCCATGGCCCACGGAAGTTGATAGCCCTATGTCTTAGTGCTTCTATGTTG
TATAATATGCTGTGCATATCTGGGTGTGGTTTAGAGGGGTGATACGGTCAAAATAACTGAGAGATGTAACGAATAATGAGGAAA
AAACTTGTGAAAATTGCCCATAATAAAAGGTGTAATTTTTTTTTCAAAAATGTTGAATTAAGGCATCCCGTTTCAAACTCAA
TAGATGCATTTACCCATCGAGATCTCTCTGGGGAAAATATCGAACCTGAGTTGGAACACAAGCCTGTAATAACATTCTTGGAGA
AATTGGCTGGAAATCTCCAATTTCACACCAAAGGGGTACCCCTTTCAGAAAATCTTCGATTTCTCTAATATTAATTGGAGCAAT
GGTTTGAAAGCGAATACTAAGTAAATAGGCGAGGGTCATGAGCACGGAAGTTGTAGCCCTCATGTCTTAGCGCTTCTATGTTTA
TGAATATCGTGCATATCTGGGGTGTTTAGAGGGTGATACGGTCAAAATACTGAGAGATGTACGAATATGAGGAAAAAAACTTGT
GCGAAAATTGCCCTAATAAAAGGTGTATATTTTTTTCAAATGTTGAAGTAAGGCACCTGTTTCAAATCCCAGATAGACCGCCAT
TTACCATGAGATCTCTTGGGAAAATAATATCCGACGAGTTGAAACATAAGTGTATATACATTTGGAGAAATTGGGTGAAATCT
CCAACTTCACCAAAAGGGTACCCCTTTCAGAAATCTTCAGTTTCTCTAATATTAAATTGGAGCAATGTTTTGAAAGCCGAATAC
TAAAGAATATCGGGGTCATGGCAAAGGAAGTGTAGCCCTATGTCTTAAGGCTTCTATGTTTATAATATCGTGCATATCTGGTGG
TGTTTAGAGGCGCTGATACGGTTTAAATAATCTGAGAGATTACGATAATGAGGAATATAAACTTGTGAAAATTGCCCTAAAAAA
GGTGTATTTTTTTTTCAAAATGTTGAATTAAGGCACCGTTTTCCAACTCCCATCAAGATGCCATTTACCCATGAGATCTCTTGG
GGGAAAAAAATATCGAACGAGTTGAAGCACAGTGTGTAATACATTTTGGAGAAATTGGTGAAAATCTCCAATTCACCAAGGGGTA
ACCCCCTTTGCAGACATCTTCGATTTCTCTAAATATTTAATTGGAGCAATGTTTTGAAAAGTCGGAATAACTAGTATAGGGAGG
GTCATGAGCACGGAAGGTTGAGCCCTATGTCTTAGCCTTCTATGTTTGATAATATCGTGGCATACTCGGGGTGTTTAAGGGTGA
TACGGTCAAAATACTGAGAGATGTACGATATGAGAAAAACTTGTGAAATATTGCCCTAAAAAAGGTGTATATTTTTTCAAACAT
GTTGAATTAAGGCACCTGGTTTCAACTCCCAAATAGTGCATTTACCCATGAGATCCTTGGGGAAAATATCGAACGAGTTGAACC
ACAAGTGAATCATTTTGAGAAATTGGTGAAATCTCCAATTCACCAAAGGGGTACCCCTTCAGAAATCTTCGATTCTCTCATATT
TATATTGGAGGCAATGTTTTGAAGCGAATACTAAGGAATATGCGAGGGTCAATGGGCACGGAAGTTGTAGCCCCTATGTCATTA
GTGCTTCTATGTTTATAAATATCCGTGCATATCCTGGGGTGTTTTAGATGGGTGATACGGTCGAAAATACTGAGAGATGTACGA
ATATTGAGGAAAAAACTTGTGAAAATTGCCCTAAAAAAAGGTGTATTTTTTTCCCAAAATGTTGAATTTAAGGCACCGTTTCAA
CTCCAATTAATGGCATTTTACCCATAGATTCTCTTGGGAAATAATATATCGAACGAGTTGAAACCACAAGTGTAATGACATTTT
GAGAAATTAGGTGAAATCTCCATTCACCAAAGGGGTACCCCTTTCCTAGAAATCTTCGATTTCTCTAAAATATTAATTGGAGCA
ATGTTTTTGAAAGTCGAATACTAAAGAATATGGAGGGTCATGGGGCACTTGGAAGTTGTAGCCCTTATGTCTTAGGCTTAATAT
GTTTATAATACTCGTGCATATCCTGGGTGTTTAGAGGGTGGAGTATCGGCTCAAGAAGTACCGAGAGAGTGAACGAATATGAGG
ACAAAAACTTGTGAAAATTGCCCTAAAAAAAGGTGGTAATTGTTTTTCGAAATTTTGAATTAAGGCACCGGTCTCTACACTCCA
AGATAGATGCATTTACCCATGAGATCTCAATTTCGGGAACAAATATCGAACGATGAACACAAGTGTAATAATTTTGGAGAAATT
TGGGAAATCTCCAATTCACCAAAGGGGTACCCCTTTCCAGAATCTTGATTTCCTATAAATATAAGTTTGGAGCAAATGTTTTGA
AAGCGAATACTAAGTAATAGGCGAGGGTCATGAGCACGGAATGTTGTAAGCCTAATGTCTTATGCCTTCTATTTTATATACGTT
GCATATCTGGGTGTGTTTAGAGGTGATACGGTCCAAATACTGAGAGATATGTACAGAATATGAGAAAAAACTTGGTGAAAATTG
CCTAAAAGGTGGTAAACTCTTCTTTTCAAATGTCTGAATGTAAGGCACCGTTTCAACTCCAATAGACGCTTTACCTCATTGAGA
TCCTCTTGGAAAAATATCGAACGAGTTGAACACCAAGTGTTAATACATTTTGGTAAAATTTTTTTGGGGGGGTTGGGGGAAAAT
CTCCAATTCACCAAAGGGGTACCCTTTTCAGAAATCTTCGATTTCCCAATATTAATTGGAGCAATGTTTGAAAGCGAATATAAA
GAATATGCGAGGGTCATGGGGCCACGGAAAGTGTAGCCCTATGTCTTAGGCTTCTATGTTTATAATATCGTCATATTGGGGGG
TGTTTAGAGGGTGATACGGTCAAGAATACGAGAGATGTACAATATGAGGATAAAAACTGTGAAAAATTGCCCTAAAAAAAGGTG

TATTTTTTTTTCAAATGTTGAATTAAGGCACCGTTTCAACTCCAATAGGATGCACTCTTACCCATGAGATTCTCTTGGGGAAAA
ATTCGAACCGAGTTGAACACAGTGTAATACATTTTGGAGAAATTGGTGAAATCTCCAATGTCACCAAAAGGGGTAACCCCTTTC
AGAATCTTCGATTCTCTAATATTAATTGGAGCAATGTTTATGAAAGCGATATACTAAGTAATATGCGAGGGTCATTGAGCACGG
AAGTTGTCCCTATGTCTTAGCCTTCTATGTTTATAATATCGTGCATATCCTGGGGTGTTTAGAGGGTGATTACGGTAAAAATAC
TGAGGATGTACGAATATGAGGAAAAAAACTTTGAAAATGCCCTAAAAAAGGTGTATATTCTTTTTTCAACATGTTTGAATTAAG
GCCACCGTTTCAACTCCAATAGAGCTTTACCATGAGATCTCTTGGGGAAAAAAATATCGAACCGAGTGAACAAGTGTAATCACAT
TTTGGAGAATTGTGAATATCTCCAATTCACCAAAGGGGGTACCTTTCAGAAATCTTCGATTTCTTAATATTAAATTGGGAGCAA
TTGTTTTGAAAGCAAATACTAAGGAAATAGCGAGGGTCATGGCACGGAAGTGTAGCCTATGTTTAGCTTATGTTTATAATATGT
GCATAAATCTGGGGTGTTTTAGAGGGTGATACGGCTCAAAATACTGAGAGATGTACGAATATGAAGTGAAAAAACTTGTAAATT
GCCCTAAAAAGGTGTATTTTTTTGTTCAAATGTTGAATTTAAAGGCACCGTTTCAACTCCAATAGATGCATTTACCATGAGATC
TCTTGGGGAAAATATCGGAACGAGTTGAACACAAGTGTAATACATTTTGGAGAAATTGGTGAATATTCTCACAATTCTACTCAA
AGGGTACCCCTTTCAGAAAGTCTTCGATTTCTCTAATATTAATTGGAGCAATGTTTTGAAATGCGAATACTAAGGAATATGGCG
CAGGGTCATGGCACGGAAGTTGTAGCCCTATGTCTTAGGCTTCTATGTTTATAATGAATCGTGCATATCTGGGGTGTTTTAGAG
GGTGGATACGTCAAATTACTGAGAGATGTACCGAGATATATGAGGAAAAAACTTGTGAAAATTGCACTAAAAACAAAGGTGTAT
TTTTTTTCAAAATGTTGAATTAAGGCACCGTTTCAACGTCCAATAGATTGCATTTAGCCCATGAAGACTCTTTGGGGAAAATAT
CGAACGATTGAACACAAGTGGTACACATTTGGAGAATTGGTGAAATTCATTCACAAAGGGGTACCCTTTCATGAATCTTCGATT
TCCTCTAATATTAATTGGAGCAATGTTTTGAAAGCGAATTACTAATGGTAATATGCGAGGGTCTCATGGGCAGGAAGTTTGTAG
CCTATGTCTTAGGCTTCTAATGTTTATAATATTCGTGGCATAATCTGGTGGTGTTTAGAAGGGTGATACGGTCAAAAATACTGA
AGAGATGTAGCGAATATGAGGAAAAACTTGTGAAATCGGGCTAAAAAAAGGTGTAATTTTTTTTCCAAATGTTGAAATTAAGGC
CACCCGTTTCAACTCCAATAGATGCATTTACCCATGAGATCTCTTGGGGAAAATATCAGACGAGTTGAACACAAGTGTAATGAC
ATTTTGGAGAAATTGGTGAAATCTCCCGCAATTCCACAAAGGGTACCCCTTTCAGAAATCTTCGATTTCTCTAAATATTAATCT
GGAGCAAGTGTTTTTTGAAAGCGAATACTAAGGAATATTGCGAGGGTCAATGGGCAACGGAAAAAGTTGTAGCCCTATGTCTTA
GGCTTCTATGAATAATATCGTGCCATATCTGGGGTGTTTAGAGGGTGAAGTACGGTCCCAAGTAATACTGAGAGATGTACGAAT
ATGAGGAAAAAACTTGTGAAATGCCTAAAAAAAGAGGGAAATTTTTTTTCAAATGTTGAATAAGCCACCGTTTCAACTCAATAG
ATGCCATTTACTGCCATGAGGATCTCTTGGGAAAAAAATGATCGATACGAGTTTGAACACAAGTGTAATACATTTTTGGTAGAAA
TTGGTGAAATCTCCGATTTCACCAAACAGGCGGTACCCCCTGTTCGAGAAAATCTTCGATTTCTCTAATATTAATTGGAAGGCA
AAGTTGTTTTGAAAGCGAAATAACTAAGGAATATGCGAGGGTCATGGCAAAGGAAAGTTGTAGCCCTATTTTCTTAGTGCTTCC
TATGTTTACTAATATCGTTGCATGATCTGGGGTGTTTAGAGGGTGATATGCGGCTCAAAAATACTGAGTAGAATGTACGAATAT
GAGGAAAAAACTTGTGAAAATGCCTAAAAGAATCAAGGTGAATTTTTTTTCCAAAATGTTGAATTAAGGCAACCAGTTTCAAAC
TCCCCATAGATGCATTTTACCCATGAGACATCTTTGGGAAAATATAACTCGAACGAGTTGAACCACCAAGTGTAATTACATTTT
GGAGAAATTGGTGGGAAATCTCCAATTCACCCAAAGTGGGTACCCCTTTTCAGAAATCCTTCCGATTTCTCTAATATTAATTT
GGAGCAATTTTTGAAAGCGAATACTAAGTAATAGGCGAGGCCTCATGAGCACGGAAGTTGTAGCCCGCTATTGTTTTAGCCCTT
CTATGTTTATAATATTCGTGGGCATGTATCTGGGGTGTTTTAGAGGGTGATACGGTCAAAATACTGAGAGTATGTCGAATATGA
GGAAAAACTTGTGCCCTACAAAGTTTATTCAAATGTTGAATTACAGGGCAGCCGTTTCAACTCCAATAGATGCATTACCCATGG
ATCTCCTTGGGGAAATATCGACGGGCAAGTAGCAATACATTTTGGAGAAATTGGTGAAATCTCCATTCACCAAAGGGGACCGTT
TCAGATCTTTCGTTCCTTTAATTGGAGCAAATGTTTTGAAAGCGAATACTAAGTTAGGCCGAGCTAATCGAGCACTGGAAAGTT
GTAT
>m141121_232159_42134_c100732402550000001823141405141507_s1_p0/159115/5586_11142
ACATCTCTTCTGCCCAAAAGCAAAAAACATGGTTTATTCAAGTTAAGCAATCATGCAGAGGTTAAAAAAAAAGTATACGAATAT
TAAGGGAGCCCTACCCCCCCCGAACTGGAAGAGCCTAGGACAATTATCTTCACCGAACTGGCGCGGGTTCTAAGGTCCGGTTCT
GACCTCCGGGGCTGACTCAATTCTACCCCGTACTGTCTAACCCGGAGCATAGACACCCCAAATCCCTGAAGTGATGGACATGTG
CGTAAGTTGGTCCCCATACTCCTAAAAAAAAGATCGACCTAAAAAGCTAAGGGTGTTATCTAACTAACTACGGTACCCCTAGGC
CCGGCTCTCCTTGGTGATCCAGCACGAGCCGGATCAACCACAATGGAGTCAAGGAGTGTATTCAGGTCCGGGTCCCTCCCGTGA
CGATGGCGATCGTGCTAGGATTAGAACCTCAACCAGCGCGGGGGTCTGCAGTGGCCTATCTACATTCTTAAGCAAATAAGGGTT
ACCTTGGCCAGAAGGACCCGTGCTGGCTCCCCGGTTTGGATCATCTCCTCGTCCAATCACCCCCTGGGTCGACCTCCAAGGTCC
TTTTTCTATTCCTCACGAGGCGGAACTATCAGCCGCTCGTCGCTCCTCGTCTTCCTCTGTCGCGACCTCCTAAACACGGTGATA
GGCCTGTTGTCGCGGGCTGGGGCGGTCCCCGAGCCTCTTCAGTTTTAGAGTCTGGTTTGGGAGAGATCAATTTGCAAAGAACAA
ACCATCGTCTCGTCCGTCACGAGCACTGCGTAAAAGTCCTTTTCCCAGCCTGGGGGGCAGGCCCGCCAATGTGTCATACTGACC
CCCCGAGGGTCGCAGACTTTTCGGTCCTTGCGTAGATGGCTGCAAGATTGGCTGGAGTCAGGGTGGAATACGGGAGGAGCTCAA
AATAAAATAAAATAAATAAAACACTTAAAAGGGAGAGCTAAGGTCAGGGATTCCACTTACGAGGGCGAATTAACCCCACTCCAC
ACTGGTGCTCGCAATGCTCCGGAACCCCCCAGTTGACATGAAGAACGGTCCTTGAAAGTCATTTGGATGGCTTTGGAAGCTCGA
TGACCGCCGGCCGTATTAGGGAAAACGAGTTAAGTAAATGAACCCGTCACTCGCTCCCACTGGTCCGGGCTGGCTTTAAGGCAG
AAGAATATAGAATATCCGCAGGAGTAGGGGACCCATCCCACTCCTGCTTCTTGAATAAATCCTCAAGCCCCGCCAGCAGACGGT
AGAGTTGGGGGGGGAAAGCCCTGAAAAAAAAAAGATGGGCCAAACCCCCACATAATTCAGGAAAAATCAGCGAAGTACTGATCC
AGGGAAGCGAACACGCGCGCGCCCTGCCTTCTTGAAGCTGTTCACCGCTCCAGCGGCGCTGCGAACGATTCGTCGACGTGGCGT
GCAGTCGCTCGCCCCTCCGCAGCAGGTCGGCCAATCACCGAGGCTTCCCCAATGGAATTTTGTGGGTGAGGAAGATTTGTTGAT
CTTCGCCTGGTCGGTTAACCTTAATGAGACGATTCTCTCCGCCTCAAAGAATCGTCGGGGCCAACCTGCCCTGCTTCTCCCACG
GCCGGCCCCGAAGTTGGGGATCGGCGAGCTTGACATCACCGCCCTTTCCTTTCTCCTTGCTTGGGAGACCGAGCTTCCACGTTCT

TTTGAGGCAGATTCCTTTTGAGCCATCCTGGTCGCCTGCTGCGAGAGCAAAAGCAAATACACTTTTAGAAAACGGACCCACGCA
GAAGAAACGAAGCAACTCTATCTATTACACGAGCTGAGGTAAGCCCAGACCGTGGAGAAGTGAACCACGCGGCTTCACTGAACA
CGCGCACCCTATGCTCCAACAGGATCCCAGATTTCTACTCGGAATTCCACGCGCTGTGTCAGGGGTTCAGAGTAAAAATTTGCC
CTTGGGGCGCGAAGTTCCTCACAGGCCAGAACGTTCGCACAAAACCGCCTTCTGCACTCCCATAAGCTACCCGTTTTCGCACCC
CAAATTCCTAAAGATCCTAACCAGACAATTGTTTCTGAAGAAGCATCTTGAAACCCCATACTCTAAGCAATTTCCTACAACAAG
TGTGCCCTAAGCCTAAAGGGCTGTCACCCCTCCTACCCACGCAACCCAGAAACCTTGCATGCGGCTACAGTAATTTTTTTTACC
TCTAACTACAGGTGGCATGTTTTCAAATGAACAGGGTCAGAAAACTTAACAGTATAATGGCTGGAATGGTAAACGAGAGTGTTG
CCGTTGGTAGGGCCTCGTCGTGCAGTAGCTTCCAAAGCTTTGGAGAAACCTCCGTGCGCCCTAAAGCTTCTTGAACAAGGAAAA
TGGTGGCAGTAGAGTCGAGGGTTTCGTTCTTCTGAAAGTCAGAGAAGGAAGAAGGAGAGAGGATTTGGGAAATTTTGAAATGAA
GGGATGGCCCGTTGCGTAGGGTGGCCACCCCCTTTTTATAATAAGGGAAGGATCACGTGAAGAAGGCATCCTTGGATTGGCTAG
TGAGGATTCCGGGCCTTCCACCCTCCTATAATATGCAAACGACGGCTGGGCAAATAGCTAGGCATTAAATGCGGTTTTTTATCG
TAAAAATGTACGTCCACCAACCCCACGACGTTCCACGTATTAGACGCCTGCACAAAGAATGGAAATGTGAAAGGTGTTTGTGGG
GAAGTCTAAAAGCCCTACTATGGTCTTCTATATATGCGTCAATAACCCGAGCGCCCCCATGAGCTTGGGGGCAGGTGTACGCCT
GGATTTTCCCCACGGGCCTGATTAGCAGGACGAGCATCGGACAACATGGTTTAGTCCGAGGACTCCCCAGGTCCGGAGATCCTG
TTTCCAGATCGTACCTTCCTTAGCTTGAGGGAATCCTCAAAGCTCCGCCGAAGGCCCCCAAGACTTGAGGAAGGCATCAGAAGG
CCTGAAGGCTGAAGGTCGGTCCCAGGGAAGCAGCTCGTGGTACGAGTTTGGCCATGGATCCTCTGACCCCTTTGTAAAGTCAAC
ACACGCAAATAAACGTGCATAATGTTGACATCACGTGTTCGATTCCCCCTGACTTTCCTCGGACACGCCAAGCAGGAACGTGCG
CATTCAGACACCCACGCTGGTTGGGCCCCGTGCGGCTACATTATTCCTTACATACTGATTAGACAACACTTTGTGTCAGGTTTA
GGAATTTAATCATAAATGGTCAACAGAGTTGATATGACAAATGGTAAGGTCACGGGATGACATCCCTACCAACTTCCAGGTGCT
TCTCCCTATAAATATGGGGACCCTGGGCGAGTTGATAGGGGTGGAGAAAAAAAAATAGTAAATCTGGAGGAGCCCATTATACTTT
TGTAACCCAATACCCAGAATATATGAAATAATATTGACTAGTGGAGTAGAAGGATTTTAACCTTTGAACCACTTAAAAACGTGT
CCTTGAGTCACCTAGTTTCATTCTCTAAAGATTTCATAATCTGTGACGGTTCCTACTTTCAGTACTATTCCCTTTCTCTGTCTT
CTCTTAATTACTGTTGCCGGAAGAACCACGTCAAACAATAATAAATATCATCTTCATAATCAGCTATATTAAATCAAACCTTAT
GTTAATACTACTACTACTACTACGACTACTGCTGCTGCTACTACTACTGCTACTGCTTCTAGTGGCTTAGTGCTACGTACTACT
ACTACTACTACTACTGCTAGCTAACATGCTCGCTACTGCTAAATGCTGCTATTGCTACGCTACTGCTACTACTACTGCTACTGC
TGCTATTGATACTGCTACTCTCTACTGCTGCTGCTGCTCGCTACTGCTACTGCTACTGCTGCTACTGCTACTGCTGCTGCTGCT
GCTACTGCTACTGCTACTGCTACTGCTACTGCTACTACTACTGCTGCTCCTGCTAACCTGTTGTCCTGCTACTGCTGCTCCTGC
CTACTGCTACTGCTACTGCTACTGCTAATACTACTACATACTGACTACTGCTACTCGGCTGCTACTGCTACTGCTACTGCTACT
GATGCTACTTCTACTTCTACTGGCTACTGCTACTGCTGCTGCTACTGCTACTTCTAACTGCTACTGCTACTGCTACTGCTACTA
CTACTACTAATGCTACTGCTGCTGCTTGCTGCTGCTGCCTGATCACTTCTACTGCTACCTGCCTACTACTACTACTACACTACC
TGCTACTGCTACATGCTACTGCTACTGCTATTGCTACTGCTAACTGCTGCTGCCTGCTGCTGCTGCTGATGTTGCTACTCCTAA
CTGCTTACTACTGCTAAAAAAAAAAACTGTTACCTACTGCTACTACTACCTGCTACTCGCTGCTGACTGCTACTGCTGCTGCTG
CTGCTATTCTACTGCTACTACTACTGCTACTGCTACTGCATTCTGCTCCCTGCTTCTGCTCCTGCTCCCTGCTACTCCTGCTGC
TGCTGCTGCTACTGCTACTGCTACTGCTGCTGCTGCTGCTGCTGCTGCTGCTCTGCTGCTGCTACTGCTACTGTTCTACACCTA
CAACTACTGCCACCACCACCAACTAACCACTACTACTACGATCGATACCCCTACTCCACCAACAACCATACCACTACCATTACT
CTCTACTACTACTGCCCCTACCTGCTACGATCCACTACTATACTCCACTACTACCACCACAACCACTACCACTTACCACTCCAC
TACACCTTTTACCACTACCACTACTGCTACGCTACTGCTACTGCTACTGCTACTGCTACTGCTACTGCTACTAGCTACTGCTACTGCTAC
TGCTACTAAACTACTCACTACCGAACCACAACCATACCAACTACCCAACTCACTGCTACTGCTACGACTACCACTACTACTACT
ACCACTTACTACCACCACAACCACTACCCACTACCAACTACCACTACCTTAACCACTACCACACTACTGCTACTGCTAACTGCT
ACTGCTACTGCTACTGCTACCACCACTACACTACACACTACTGCTACTCGCTAGCTGCTACTGCTACTGTTTACTGCTACTGGT
TAATACTTGCTACTGCTACTGCTACCTGTCCTCATTGAACTGCCCTACTGCCCTACTGCCTACTGCTACTGCTACTGCTACTGC
TACCTGCTACCACAACCACTACCCACTACCACCTGACTGCTAACTGCTACTGCTACTGCTACTGGCTACTGCTAACTGCTTAAC
TGATTACGTGCTACTCGCTATCTGCTACTACTATACTACTACTTACATACCACTACTACTGCTACTACTACTACCACTACTACC
ACTGACTACCCTACTACTATACTACTACTACTACATACTACTAACTACCCACTAGCTACTACTACCACCTAACCACTACCTATA
CTAACTACTACTACTACTACCACTACACGACTACTACCTACACCACTACCACTAATCATAATACCTAACTACTACTACTACTAC
CACTACTACTACTACCTACTACTACTACTATCTGCCACTACTACTACTACTAACTATACTCACACTACCCACATACCACTACCA
CTACTACCACTA
>m141121_232159_42134_c100732402550000001823141405141507_s1_p0/56268/7078_17579
ACCCACTCCTTCCCCCGTGGAAAGGTTTTTTTTTTTTTAAAAGGGGGTTTATTATTCCCCCCCCCCCCCTTTTGTCCCCCCCC
CGGCCAAAAACAAGAAGAAACCCCCCCCAAGCTTCCCTAAAACCCGACCCCAGGAATCGAAACCCCAAACCGGAACTTTTTTTT
TTTTTCCCCCCCCCCTTAAATGGCCCCCCCAATCTTTTCCCCCCTGGAACCCCTTAGTCTTTCTTCTGAATCGGCAACAGCGGG
CGACACGCGCAATAATATATCCCCCCGCAAACCCTTTCCCTTTTAAAAAAAGCTTTTTGTTCCGCCACCCCATTTTTTTTAAAA
ATCAGCAAAAAAAAGAATCAAAACCGCTTTAACTATATTTTTTTTCAAAACACACCCCTTTAAAATAGTCCCCCCAAAAAAAAA
AACCCCCCGTGGGGGGGGCTTTTTTAGCCCGGTTTGGCGGCCCCGACCTTGCGGTTAACCCCCCATCTCTTTTTTTCTCCCCCC
AAACCCCAAAAACAAAAACAACCCCCGTGGGAAAAAGGAAGGGAAAGGAAAAAGAGAAGAGGGGGGTGGGGACCCCCAAAGTCG
CCAAAACCCCGCTTTAAAAGCACAGGTTTTTTTTTTTTTTTGACTACTTAGTGTTTTTGGAAAAAGTAAAAGCGAGTGAATCC
TTTTGGCCCCCCCTGGAAATAATGGGCCCCCCGGAGCCCCTTTAGAAAGGTTTTCTTTTATTTCGGGAAAAAATTTCCCTTCTTC
GCCTTTTTTTTCAATCCAAAACTAGGTTTTTCCCCCAGGGGGGGAAAATGGGGGCAAATAGGAATAAAAAGTTCGGTGGGGGTT

TTTTTTTATTTTTTTATTTTTCGAATTTCTTTTTTGGGGTTTCCCGTAGGAAAACCCTGGGGTTCCCCTCCCTATTTGTGCGAA
CCCAGAAAAAAAAACCATCCCCCTTTAAAACTTTTTATCCACCCGGGGAAAAGGAAAAGTGCCCCTTTTTTTTTTCCCCAAACTT
CCCCCAAAAAGGGGTTTTAAGGGGAGAAAAAGGAAAAGTTTTGGGGGGGGGTGGGGAACCCCCCGTAGGAAGTCCCCCCCCCG
AATTTGGCGGAAGGGGTTTTTTTTTTTTATTTTTTCCCCGTTATGGCCCCCCCCACCAAATTGGGGGGACCTTTTGGGGGGAGCC
TGGTCTTTACCTGGGCCCAGAAAAACCCCCCCTTTTTTTGGGGCCCTTTCAAAAAAAATCCTAGACCCCCCCCCCCTTTTCCTTTT
TTCTTTAATTTGAAGGCCCCCTTTTTATGCCCTTTTTAAGAGGTTCCCCCTTGATTGAACAGCCATTTTCAGACCTGGCCCCGT
TTTTTCGACCTGAAAGGGAAAGTTTTTACGGCCCCAAAACGGGAAACCCCGAGAAAAAGCCCACTTTTTTTTTTTTTTTCCCCA
ACCAAAATTCCCCGATGGCCTTTTTTCCACAAAACTTCCCCGTGGGAAAACAAAAGAAATTCCCCAACTTTTTGTTCCCCAAAA
AAAATGGAGGGTGGGGGCACCATATGGGGGTACCCGGCAGGAAACGATTCCCCTCCCATTCCCTTTCAGGGTTTTTTAAAAAA
GGACCAAATTCTGAAAAAGATGAGTGACTTTATTTTTTTTCTTAACCAAAAACTTAAAAAAAAAGTATAAGCCTCCCCCCTTTA
TTTCTTCCCCCCCCGATTGTTAATTTGGATTTAAACAAACGCCTCCTTTGCTTTCGGGGACCCCCAAATTTTTTTTGGCTGGGA
AGGTTAAAAGTTCAAAAATGGGCTATTTTATACCACCGGCCGGGGGTAAAAACCCGGGAAACCCCCCCTTTTGCCTTTCCCGGC
AGCCCCAAAAAGGGCCGCCCCCCCCCCCCCCTTTACCTAAAAATTTTCTAAATCACCCCCCGACCCGGCTAAAAATGGGTCCCC
CCAAAGGGGGGCCCTCCCGAGAATCAATTTTTCCAAAAGCTTCCGACTGAGGGGGGAAAGCCTCGGCAACCAATAAGCGAAATT
TTCCGGCCCTGGCAACTTTTTTGGCGTCCCCGCCTCCCACATTATCTTTTTTTTTTTTTTTTTTTCCCCAAACATACGAAGAAA
ATCGAGTCTTTTTTTTTTTTTTGGGAAAAAAATTTAAAGAAAGTTCCCGGCCAATTTTTAAAAATAACCTTTTGGGCCCCTGAAAA
GAAACCCCCATTTTTAAAAAGCCCCCTTATAAGGCCTTTTTTTTTTTTTTTTGCCCCCTTCCCCCCAAAGTTTGTGGGCTCCCCC
CCCCCCCTTTCCGCCAGAATTCCCCTTTTCCCCCCGGTGGAAAATAACCTTAAAAAAAACAAAAACAGATTGGCCCCCCCCTTT
TAAAGCTTTTTTTCAAATATTGGATAGAAAAAAACGCAACAAAAACCCGGGAAGGGGGGGGAAAGAGGGGTTAAAATAAAAAAA
AAAAAAGGGAAAAAGGTTTGGCCCCCCCCACCGTTTTCCCCCCCCGGGTAAACCCCAAACCCCCCCAAACTCCCCCATCCGGAC
CACCCCTATAATCGGTATATCTAGGAAAGCCCTTTTTTGATAAGGAAGAAAACTCGTCCCCCCCCCCGTGGGCGTTCGTTTTTT
ACGCACAACCCACTTTTCTTTACCGGAATTTCCCCCAACTTTTTTTTTTGCCCCCCCTTCGACCCGAGAAAAAAAAAAAAAAAT
TTTACCATCCCCCCCAAAGGAATGGGAAGTTTCCCCAATCCCCCCCGGGTCAGAGCCTTTAAAATATCCCCCCCCTATCCATTT
CGAAAATTTCCTCTTTTTGCCCCCCCCCGGATCCTCCTTTTTTTCCTTAATAAATTTTTTTTTTGGGGGTTTTTTTTTCCCCCC
CCCAAGAAATTTTTCTTAAATTGGGGGGGTAAAACCTAAAAAAACACCCAAAAGGGGTTTTTTTCCCCTTTGGTCTTCCCCCC
CTTACCCCCCCGAGGGTTTTTTTGGCCCGAGTTTTTCTTTTTTTTGGGGCGGAGAGCATCTGCGTTTGTTTGGCCCGTTCCTG
CTTTCACCGGGTTTAAATTTCTTTTTTGTTTTTTTCCCGATTTTTGGGACCCCTTCGCCCCAAACCGCAGACAGTTTTTTTTTT
CCCCGAACTTTTTTACCTTTTTTTTTTTTTTTGGCCAAAAAATTAGGCGGCCCCTCTTGGGGGTTTCCCCCGCCCCGCCCTTGA
AAAAAAAATTTAATACTTTTTTTTTTCTCCCCAAAAGAACCTTGGGTTTTTTTTAAACCAGAGCTTTTGGGAGTCCCAAAAACTT
AAAAAGCCCACCTGGGACGGGAAGGGGCTTTTTCACCCCTTTTAAAAGCCCTCAATTTAGCCTTAAAAACCCCCCCCTTTCCCA
GCCCAAAGCCCCCAACCAAAAAAAGGCTTTCCCGTACTCTTGCCCTTTTTTTTGTTAGACCGCAATTTTTTTGAGCCCCGAACC
CTTTTAAAAAGTTGCCGGAGTGGAAAAAATCCTATTGTTACCCCCCCCCCCTTTCCGGGGGGATTTTCAATTCAGGGAATGGATT
TTTTTTTTTTTTTCTTGGCCCGGAAGCCCGTTCCCTTCGGGTTTAAAATGAAAACCCCCAATTCAACCGGAAGATCCCCCCCC
CTTTCCCCCCCCCAAACCGAACGAAAATTTTTTTGGTTTGGGGCGGGAGGCCGGATCCCTTTTTGAGGTTAAGGGCCCCCCCCT
TTAAAATGGGGATTGGAAATTTTAACCTTCCCTAAAAGGAAAAAAAAAAAATCCCCCCCCCCCTTTGTTGCACCTCCCTTGGCAC
GAAAAGTCCGGCCCCAGGGCCCCTTGAACCCCTTTTTTTTATGGCAAAGTTTTTTCCGTGGGAGTTAATATTAAAGAAAAAATC
CTCCCGAACTGAACTTTGAGTTTTTTTTTTTTTCCGAACCCCCGCCCCCACCTTTTTTTTTTTAAAAACAAAACGTCCCCCGAT
GCTTTTTTCAAAGGCTTGTTTTTTTTTTTTGCAAAAAACCCCTTGGCCCCTTTTTTTTTAAAAGGCTTTTTTGATTTTCGGTTCCC
CCCCCATGAACCCCAAAATTTTAAAATACAGAGTTTGGGGGACCTGGATAGTTAACGGCCCCCTTTTTGCAAGGCTTTTTTTAT
ACAAGGCCCCCCGTGGAATTAAAAGGCCCCCCCACCCCCCAAAGAAACGCCCCCCCCCAACCTTTTTTCCCCCCCATCATTTTT
TGGGTTTAAAAAATATCCTTTTTGGGATTCCTGCTATCCGAGAGTTCCCCCCTCCTTTTTTTTTTTTTTTTCCTTAATTGGGTAA
AAAAACGTAAAAAAAAAAAAAGACCCTTGGCCTATTGTTTTTTTTTTTTTCCCCCCCCAAACCCCCTGAATCTCCTTTGGTCCTTT
TTTCCTTTCCCCCCTCCCGGCAAAACTAGGCCAATAAAAACCGAACAAAAAAAAAAAAAACTAAGGCCCTTTAGTCCACCCCCCC
TCAGTTGGGGAACCCGGCCAAAATGTTTAGGAAGGGAAAAAGAGGTTTTTCCCCCTCCTGCCCTTTTTTGGGGGCCCCCCCCTT
GGGGAAAAAAATAGTCAAAACCCCCAAAAGGTTGGGGTTTTTTCCCGGCCTTCAGGAATGGGGGTGGGGGGCCCCCTTTCCAAGG
TCCCCCCCCGAGGGGAAAAAAAATGCCGCCTTTTTTTTTTTTCGCACCTTCTTAAACCTTGTTCCAAAACATTCACCAAAAAAAC
TTTCCCCCCCCCTTTTTGTTTTTTTTTTTCCCCGGGTAAGGCAAGGTTTTTTTTTTTTGTAAAAAAGAAAAGGGGGGCCCAAAA
CAAACTTTGGGGGTTCGGTGGGATTTTTTTTGAAAATTTTGAATCCGGTTTTGAAGCCGCTTTTGCCAACTCCTTCCCCCCTGT
TTGGAAGGGCCCCCTTGGAACCCCCCATCTTTTTTTTTTTCCCGCAACCTGGGGAAAGGGGAAGCCCCCTCCCAAGGGCCAAGT
CGGGGACCCTTGAAATTTCTTTTTGTTCCCCCCCGGGGGTTTTTTTTTGGGCCCCATTTTCGATTTTTCCTTTCCCGGGTTTAA
AAAAAAAAATTTTTAACTTTTATGAAAAAAAAAACCAAAAAAAAGGAAAAAATTTTCCCCCCCCCGATGGCCCCCCGAACCAAAA
CCGGAAAAAAAAAGGGTGGCCAAAAAAAAAATTTATGAGGGGGCGAAAATTTGGAGCCTTCAAAAAAATTTGGGCCCAAAATAAAAC
CTCCCCTTTCCCGAGGCAGTATTGTTTTTTAAAAAAAAAAAACAATTTTACCCCCTTTTTTTCCCCCCAAGGGTCGGAAAAAACCA
TGGGTACTTTGGGGTTTTCGCAAGGTCCACCCCCCCCCCGTTTTTTTTTTGGAAAACTTTGACCCCCCAGCAAAATGGTCAATAC
CGATTGCGTAACCCACTTTTTCCATTAGGGGTCCTTTTTCTTTTTTTCTTCCCCAGATTCCTTTGCGTTCCCCCCCCCGCCGAAA
ACCCAATTTTCCTTGGGTTTTTTTACCCATAGCCCTTTTTGTTGAGTAAAGGGTAGCCCCCCCCCCCTTCCCCCCCGGCCCAAAA
AATATTCCCCCCCCCCCCCCTTTTTTTTTCAGGCCAAAATAAAATGGGCAATGACCCCTTTTTTAAAAAATAAGGCTCAAAAAAT
TAGACCCCCATTAAATCCCCCCCCCGGAGGGTTCCCCCCATGAAAGACTTGCGGTAAAAAAAAACACATGTTTCCCCTGGGTCC

CCCCGCTTTTTTCGCCCGGAATTTCATCGCCCGATCTGATTTTTATTGTATCCCCCCCTGAGTAAAACCGCGGTTCCCCCATGG
GAAGTTAAAAAAACATTGAAAGCCCTTTTTTAACAAAATTGCCTCCCCCCCCGCCCTTGTGGCAAAAATCCCCCCCCAAACCCA
GCCCTGATCCCCCCGAATTTCCCCCTTTTTGGGCCAAAAAAAATAATGAAAAAGCCAAAAAAAAAAAAAATTCCCCCTTTGGGGG
CCAAAAGGGGCTTTTTTTTAAATTTTTCAAAAAAGTCCCGGAAAGAAAGTTTTTTCCAATACAGGTTCCCCCCACCCCCCCCCAC
TTTCCCCCGGTTGGGCCCTTTGGAACATAGCAAAAAAAGACGGGGAGAAATTTTTTTGCGCAACCCCCAAATTGAAAAATTTTT
TTGGGCCTTTTTTTTTTTTTCGGCGGGAAGCGTATAAAGGCCCCCCCCCCACAAAAAAAACTTTTTTTTTTTTTTTTTTGGGGCC
AGGCCAAAAACTTTTTTTCCCTTTTTTTCCCCTTCAAGGGGGCCCCTTTTTCCGGGCTCGGGTTTTTTTTTTTTTTTTTTTTTG
TTTTTCCCCTTTTAAAAAACGCCCCTTTCCCCTGCTTTTTTTCTTTGAGAAACTTTTTGGCAAAAAAAAAAGGGGGAAAAAAA
AACCCCGCTTTTTTTTATTCCTAGGTGGGGGAAAAGCCGTGTTGCAATGAAGTGGACCCCACCTCGGGCCCCCCCCCCCCCCCC
CTTTTTGATTTCCCCCCCCTTAAAATCAAAAAAATGTCCCCTCCGTGCGACCCATGGGCCCCCCCAAAAAAATAACGTTTCCCA
GGGGTTTTTTTTTTTTTTTTTTTCCCCCCTTGCAAAAAAAGAAACGTTAGGTCCAAGCCTTCCCCCGCCCCCCTTTTATTATCA
AAACAAAGAGGCCTTTTTTCCCCCCCCTTTTTTTTTTCCCCCCCAAGCTTTTTGACCCCCGTCCCCCCCCGTGTGAATGGAAT
TATTTTTTATTTTTTTTGGTTTTTTTTTTTTATCAAGTGTTTCACAGACCTCCCCAACTTCGAGCACCTTTTTTTCCTTTTTTC
AAATTGCCCCCCCCCCCCATTTGGAAGGCCCTCCCCCGGATTCTGTTCCCTTCGCCCTAAAAACCCACGGGGGGGTCAAAAAAA
AAATGTCCCCTCCCATTTTTTTAAGGCCGAGGGCCCCTTTTTTTTTTTTTTTCCCCTTTTCATTTTTCGGAAATCCCTGAGGGTT
TTTTTTTTTTCCCCAAAATCTTTTCCAAAAAAAAGGGGAAGCCGCCCCCAAAGGGCCCCCTTTTTTTTCCCCCTTGAAGGAAAAAT
TTCCCCCTTCTTCCCGCTCCCCCTGAAAATTGGGCCCCCATTTTTTCCCCCAAGCTGCACCCCGGGGGTTTTTTTTTTATTTAG
ATTTTTTTCCCCCCCTTTTCCAAAATTGGAAAAAAAAAAATTGCCCCTGTTAAAAGTCAATAATTCCCATTTGGCAAAAGCGGAAG
GCTTTTTTTGATCTGCCCCCCCCCTTTGGATTAAAGTAAAATAGCCCCCCCCCCTTTTTGGTTTTGGAAGTTTTAGCGAAAAAA
AAACCTTTCAAAATAAATGGCGGGGGAGGTTTGGTTTTCGGATTTGGGGGAAAAAAGCCCCGATAGAGCCCCCTTTCGGAAAAA
GCTTTTTTTTATGGAGGGGGCCCCCGGGGGTAAAAAAGGCCCCCCCCCGGCCTTTTTTTTTTTCCAAAAAAAAATTGGGCCCCA
ATTTGTTAGGGGCAAAAAAAAAAGCGGAGCCCCAGTTCCAAAATTGACCCCCCCCCATTGGAACCTCCCCGAGTTAGGTTTTG
GATTACCTGTCCCCCGAGACTCACCCTTCCCCTTTCCCTAGGGGGGGTTGAATCCCCCCACCAGCTCGATCCCATCCCCCCTTA
TTTTCCGGGCGCCTTGATTCCCCCTTTTTTCCCTTCCCCCGGGAAAAAAGCCCACAAATATAGCATCAATCCGAGGAAAAAATC
CCCGCCCCTTTTTTTTAAAACCTTCCGGCCGGAAACGGTCCCCAAAAAAAAAAGACCCCCCATTCTTTTTTTTTTCTTAAGGGG
TGGGAAATTCGAAAATAGGAAAAGGGGTTTTTTTTTCAACCCAAAGAGGGCTTTTCCCCCCATTTTTTTTGTTCCCAACCCCAA
AAACACTTTTTCCTTTTCCCTTTTTACTTTTTTATTTTTTTCCCGTTTGGCCGGGGAGGCTTTTTGGGACCCCCTGCCCCCTTA
AACGAACCCCCCAAAAGGGGGGCCCGTTTTTAAAAAGAGGGGTGGGGGGGGGGGGAAATTTGGGACAAAAATGGCCCCCTTGGC
GGTCCCCCCCCTTTTTTCCTTTCCCGTGAAAAAAAGTTTTTTTTTAATCGGTTAAAAATTTTTTTTTCCCCCCCCCCTCTAACC
CGTGCCTTTTTGTTTTTTTTTGAACCCCCTAATCCCCCCCCTTTTCCTTCTTTTCAAAACAAACAAAAAAACAAAACGCGAGAGG
GGAAGGGGGGGGAAAAAAAAAAGAGGAGAAAAGGAAATTTTTTTTAAAAAATTCAAAAAAAACAGCAACGAGTTTTAAAAAAGA
GAAACTACCCCGATTGTTTAAAACTTTTCCCCCCAAACGGGAAGGGGGGAAAAAAGGAAACCCCCCGCAATAAAAGGCCCCATG
TCCAAAAATTTTCCCCTCCAAATTTAAAAACCGAATTTCCCACCCCCCCCTTTTTGGTCCCCCCCCCCCGGTAGCAAGTCCCCAA
AAGCTCCCCGCCAAAAAACCCGGAAAGGGGTAAGGGAGGAAGTGGGGTTTTTTGGTTGGGACAATTGGGGAAGCTTTTTTTTTC
CCTGTTTGAACCTCCCCTTAATTTTTCCCGGATCCCCCAACCCCCCCTTTTTTTTTTTAGAAAAAAAGGATGGGTCCCCCCCCC
TTGACCCCCCCGTCCGCCGAGTTTAAAGCCCCGGAATCCCTCCCCCGAAAAATGAGGTGGCCCCCTTATATGGCCTTTTTTTTT
TCGGGGAGAAAAAGGAAATCCAGCCAAAAAAAGCGATAACGGGGGGAAAAAATTGGAAAAATTCAGAGGTCTTGGGTGGGATT
CCCCCCCAAAAAAAAAACCCTAGGAGGAAAAGAGTCCTTCGGGACAAGTCAAAAAATCCCAAGGAACCCCTTTCTTTCGAAAGT
TTTTCATGGTCCCCACCCCCCCCCACCCCCCAAATTGGGGACCCTTTTGCCTCCGCCTTTGCCCCTACCAATGGGCCAAATTTTT
GAAAAAGGGGCCCCCGGCCCCCCCCCCCCTCCTAACCCGCCCTTTCAAAAAATTAGCCCCTTTTCCGAGGCCCTTTAAAAAAAA
AATCCCCCCGCTTTTTTTTTTTTTATACCCAAAAATTCCCCCGACCCAAAACCCTTTTCCTTTCTCTCAAGCCAAAAATTTGGA
AAAGGATTTTTTTTCCCCCACCTTTAAACTTTTCCCCCCAAACAGGTGGGTTTCCTTTATTTACCCCCTAAAACTCAAACAGGG
GGGGGCGATTTTACCCAAAAGCTTTTTTCTGCTGCCCAAAGGGGGGTGGGGAAATTGGCCCTTTTTAAAAACCCAAAGCAAAAA
ATTTTTTTTTCCCCCCCAAAATGAAAAGGGAAAATTCCCACCTAAACCACACCCCCGGGGCCCCAAGCTGAACCCAAATGGCCA
CAAATTATCAAAGAGGGCCCCGAAGGGAGGATAAATCTTTCCAAGGGAGGCCCTTGGCGGGCCGGGCTTTTTTTTTCCCTGAGA
TTTTTCTGGGAAAAAAAAACCTCCCCCCCCCAAAAAGAAATTTTTTGTTCCCCCGAAAGAGAAAAAGGCTCCGCCCTTTTTTTT
AAAATTGAACAAGGACCCAATTGCACCCCACCCCCGTGGGGGCTCTCTCTCTAGGGCCGGAAGGGGAAAAAAACCCCAGATTTC
CCCACCCCCGAAGGCCCCCCTTCCAGGGCCCCTTTTTTTTTTATTGAAAGGAAGCTTTTTCCCGAGGGAAGGTGGGAAACAAT
TGAATTTTTTAAACAAAAATCCATTTCCACACCCGGGACCCGGTCCCCCAAGCCCCCCCCCCTTGGGCGAAAAGAAGCCTCCCTT
TTTCGTTTGAATTAAAAGAGAAAAAAAAAGGGGCCCGGGAGGAGCTTTTTTTTTGAAACCTAACGATTTTCTTTTTTTCTGCCAGA
AAAAAAAAACCCTTGGAAAAAAAAAACCCCCCGTTATTTTTGGCCCCAAATGGGTATTCGGCCCCAAACCCGGAGGAAAAAAAACC
AAATTGATTTTTAGGGGATTTCAAGCCCCCCCCGAAAAAAAAGTTGGTCCAATCCATTGCACACCAAACGCCTTTTTCCCCCCC
CCCAAACCCCTAAGAAAATTTAATAAAAGCGGTCCTGCCCAAGTTTCTTTCCCCAAAAAGCCCAAAAGCAAGGGGGGGGGCCCG
GTTTTATTTTTTTATTTTTTTTTTAGGAAAAAAACACCCAGAAAAAGCCCGAAAAAGCCCCCCCTTTTTTGAAAAAAGGAAAGA
AAAAGTTGGGGCTGGGGGCCTTTTTTTTTATTTTTCTTTTTTCAAAAAAGGTGGGGGTGGGGCCTTTTTTTTTTTTTTTTTTAA
ATCCCCCGCAGAAGGGGGGGGCAAAAAAAATTTTTTTTTCCCAAAATTTTTTTGGGTTTTTTTTGCCCCCCCCCAATCCCGCT
CTGGCCCTAGGTTCTTTTTTTTTTTCCCCCAAAAGGCCCCCCCCAAAAACCAAAACAAACGGAAAAAAAAGTGGGGGCTGGGAC
C

>m141121_232159_42134_c100732402550000001823141405141507_s1_p0/57017/11747_20757
CCCATTTTTAATGATTCTCTCATGAGGTAATCTTAACCTGCACCTAAAAAACAATCTTGAACGTTCCACATTCTTTCTATCATT
CTCTACGCACAACTCACTCTGTGTTGAACATATATATATATGTATATTACTTCCTCACACATACAGCAAACCTCCATTAACCAA
CATACCCCCCTAAAAACGGGCATTCAGACAACGGCTTTCCTGTAGCCTCAAATGATCGTAGAAACTTTCACAGCATTATCTTTC
CTTATATCATAGTTTTTCACACTTCCAAAAATCGTTTGCTCAGAAACCCATATTCCGCAAAATCTTTTCCAAAAATTTTCTGAA
AGGAGGTCAAAAACTACTATTTTTTGAGACTAAGTCAGGCCCAAAAATGATTCTACATAATACTATATTCATTACCGAAAATGT
TAATATTACCCTGCTTTCAACAGAAGCGAAATCTCCCGACTTCTATAAAATTATTTTTTACGATTAATTCCATCCTACAGGGCC
TCACACACTAACGGAAAGTTTTTCAATTATAGATAATGAATTTATACAAAGAATATAGAGAGCCTATATGAAAAACTGTTGAGA
CCTAACAAGAAATAACCACGAAACCATATTGAAAAAACAAATATTTTCCCTCAATATATTAAAATGATTCGAACTGTACATATA
CTGGAATGTCTATAGCCTATATTCACTATCCAGTTAACACACAGACTAAACTATCTCACCTAGCGCTCAAAAGAACCATGAATA
TTTTTCATATGATAATGTATATTCAGAACCCTCTAAAAAATGACAGCAATCCAATCCTCAGGTATAAATTAGTGATAAGAACTA
AATATATATACACCATTATAGAAAAGACAATGAAGTAGAGCTATAAAGACTTATTCGAGAGAGCCTGGACACAGAGAACTCTCC
ATTCCTTTGCATTAATACATATTATTAAGTTTCCTATACACCAAACATAATTATCTGATGTACCATCGACACTATAATAATAG
GAAATCGTATAGTTTTTCTGAATAAGGAGGTATCCTCAATTTCGATCAGAAATTTGTTGAAAGATATGAAGTCACCTATAAATT
TGTACGCCAGAAAATCGTAATCCAAATCATTCACGATCTTCAAAGATTCAAACAATCTAAATATACCTACAGATTCTCAGATTC
AATCCATACAGAGATCCATTCAACTAATATTATATACTCATCCCCATCTAAAGCGTAACTACATATTAGGAAAATATTTATTCA
ATAATCTAAATTGCGTATTGATTAATCGTGACAAAATTAAGTTCAAAATCCAATTATTACGTGAAAAAAACTTCCCTATATTAT
AAAATATTGTGTCAAAAATACAAAGATTTATTGCATATTCTAGTAGGTAAATGTGCCCGTCCTACAAATTATATGTCAACGAAT
TTTATTAGGATTAAAAAACAAAGAAAAAAAACACACCTCAAATTAACCACCGATCTGTGCCTTCGCACACGCAACCTCACGCAT
ATGATGTCCTGTACTTACTTACTCCTGTCTCCATAACGCACCACTCTCATACTTCGCATATCGTCTATAGTCACATGGTGGCTT
ATTCAGAGGATCACTTTGCAAGATCCGGTTGATCTGCAATTTTGCGCTAATCAATAAATATCACCAGAAAACGCGTTTCACACC
AACCCTTGAAGCTCAAGCAGTCATATGTCCTGACTCAGATGATAACCGCGCCAGTATTCTGGACCGATTGTTCTGATAACCCCG
GACAGCGATGGTGAGTATAAGGGCCTACGCCTTTGGTGAAATTGGAAGATTTCACTAATTTCTCCCCGAAATGTATTTACCCCT
CAAGAGTGTTCAAACCGTTCGATATTTTTCCCCAAGCAATCTCAGTGGGGTAAATGAATCTATTGGATTTAAACCGAGCCCTAC
AGTCACATTTGAAAAAAAATTCCCCTTTTCTTATGGCATTTTCGCAGTTTCTTGCCTTATACTAGTACAATCTCGCTCAGTATAT
TGCTATATCAGACCATTTAAACGCACCTCGAGATGTGGAAATAACTTTTACACAGTTGAAGCTAAGACATACGGGCTACAACTT
TTTGCCCATGACCTCGCATATTCTTAGTTTTCACCTTCACAATCGTTGCTCGAACCCATATTAGAGACAATCGATAGAATCATT
CTGAAAGGTACCTTTGTGAATTGAGATTTCACTATCTCTCCGAAATGCTATAACACCTCAGTGTTCACTCGTTTCGATATTTTT
CCCCTAAAGAGATCTCATGGGTAAATGAATTCTATTGCGAGTTATAAAACGGAGCCCCTAAGTCCAACACATTTGAAAAAAAAT
TCCCGCTTTTTTATGGCAAATTTAGCAAGTTTTTGCCTTATATTAGTACACTCTCTTCAGTATATTACTATACACTCTACGCAC
CTCAGATGTGGAAGATATTTTACACCATTGAAGCCTAAGACATAGGGGCTACCAACTTTGTGCCCATGTACCCCCTCGCATATT
CTTTATTTCCACTTTCCAACTCGTTGCTCGAACCAGTATTAGAGAAATCGAAAGATTCTGAAGGGGAACCCCTTTGGTGATAAA
TCTGGAGATTCTCACTAATTTCTCTCCGAATGTATTACCTCCAGTTAGTACTCGTTCGAGTATTTTCCCCCAAGAGATCTCATG
GGCTAAATGAATCGTATTGATGTTAAACGGAGCCCTAAGTCAAACATTTGAGAAAAAAAGTTCCTCCTTGTTTATTGATGGCAA
TTTTTTCGCAAGTTCTTTGCCTTATAATTAGTACAGTCTCTCAGTACTATATGCTGTATTCACCTTCTATAGCACCTCGGATTT
GAAGATTATTAATAACACATTGAAGCCTAAGACATAGGGCTACAACTTTTGTGGTCATAGACCTCCGCTATTCTTTAGTTTCCA
CTTTCAAATCGTTGCTCGAACCCATCATTAAAGAAATCGATAATTTCTTGAAAGGGGAACCCCTTTGGTGATTGAGATTTCCCT
AATTCTCCGAAATGCTATTACCCTCGTGCTTCAACCTCGTTCGAACTTATTTTTCCCCAAAGAGATCTCCATGGGTAATGAATC
TATCTGGAGTTGAAACGGAGCCCTAAGTCCAACATTTGGAAAAAAAAATTCCCTTTTGATGGCAATTTTCGCAAGTTTTTGCTTA
TATTCAGTACATCTCTCAGTATAACTGACTGTATCACCTTCTAACGCACCTCCCGGAGTTGGAGATATTTAACACATTGAACGC
CTAAACTAATAGGGCTACACTTTTGTTGGCCATGGACCCTCGCTATTTTCTTTAGTCTTCACCTTCAAATCGTTGCTCGAATCC
ATAATTAGAGAAATCGAAGATTTTCCTGAAAGGGGTACCCTTTTTGGTTGATTGGAGATTTTCAACTAATTTCTCCGAAATGTA
ATTACCCTCGTGTTCAACTCGTTCGATATTTTTCCCCAAGAGATCTCATGGGTAATGAATCTATTGGAGTTGAAACGGAGCTCC
TAAGTCCAACATTTGAATAAAAATTCCCCTTTTTGGAATGCCAATATATTCGCAAGTTTTTTGCCTTATATTAAGTACATCTCT
CAGATCAACTGACTCGTAATCTACCCTCCTTACGCACCTCGGATTTGGAAAGATATTTAACACATTGAAGTCTAAAAATAGGGC
TTAAAACTTTTGTGAAAATGACCCTCGCATATCCTTTGTTTTCAACCTTCAAAATCGTTGCTCGAACACCAATATTACGAGAAT
CGAGAGATTTCTGAAAGGGGTAACCCTTTGGTGATATTGGAGATTTCACTAATTTTCCGAAATGTACTTCACCACTCAGTGATT
CAACTCGTTCGATATTTTTCCAAGAGTATCTCCATGGGTAAGAATTATTGGAGTTGAAACGGTGCCTAAGTCAACATTTGAAAA
AAAATTCCCGCTTTTTTAATGGCAGATTTTCGCAAGTTTTTTGCACTTATATTAGTACATCTCTCAGTATATTGACTATAATCA
CCTTCTACGCACCTCCAGATGTGGAGATATTTTACACATTGAAGCCTAAGACATGGGCTACAACTTTTGTGCCCATGACCCTCC
GCATATTTCTTTAAGTTTTCACCTCCTCAAATCGTTGCTCGACCCCATATTAGAGAAATCGAAGATTTCTGAAAGGGGTAACCT
CCTTTGGTGAATCTGGGAGATTTCACACATTTGCTCCGAAAGTGTATTACCGCTCAGTGTTCACTCGTTCGATTATTTTTCCCC
AGATGATCTCTGGGTAAATGAAGTCTATTGGCAGTTTAAACGGAGCCCCTAAGTCAACATTTGAAAAAAAATTCCACCTTTTTT
ATGCAATTTTTCCGAAGTTTTTGCACTTATATTAGTACTCTCTCAGTATAGTTGACTATATCCACACTTCTACCCCTAGATGTG
GAAGATATTTTACCATTGATGCCTAAGAAATAGGCTACAACTTTGTGCCCATGACCCTCGCACATATTCTTGTAGTTTCCAACT
TTCAAAGTCGTCGCTCCGAACCCATCATTTAGAGAAATCGAAGATTTCTGAATAAGGGGAACCCTTTGGTGGAAGGAGAATTTC
ACTATTTCTCCGAAATGTATTACTCCTCGTGTTCAACTCGTGATCATATTTTCCCCAGAGATCTCCATGGTCAAATGAATCTAT
TGGATTTAAAGGAGCCCCTAAGTCAACATTCTGACAAAAAATTCCCCTTCTTTTTTGATGCAATTTTCCGCAAGTTTTGCTTAT

ATTAGTACATCTCTCAGATATAAGATGTATCGACCTTCCTACGGCACTCTCGATTTGGAAGATATTTAAACCATTGAAGCCTAA
CGACATAGGGCTACAACTTTTGTGGCCATGACCGCTCGCATGTTCTTTAGTTTCCACTTTCAAATCGTTGCTCGAAACCCATAT
TACGAAGAAATCACAGATTTCTGAACAGGGAACCTTTGGTGAATTGGAGGATTTCACTAATTTCTCCGAAATGTATTACCCTCG
TGTTAACTCGTTCGATATTTTTCCACCAAGAGATCTCATGGGTAAAATGAAGTCTATTGGTAGTTGAAACGGAGCCCTTACAGT
CAACATTTTGAAAAAAAATTCCCTTTTTGATGGCAATTTTCGCCAAGTTTTTGCCTTATATCAGTAAACATCTCTTCAGTAAAT
GACGTATCCACCTTCTACGCACCCTCGGATTTGAAGATATTTAACACATTGAAGCCTAAGTAGACATAGGGCTACAACTTTCTT
GTGGCAATTGTGACCCTCGCAGATATTCTTTAGTCTTCACCCTTCCAAATCGTTGCTCGAATCCCATATTAGGAATCGAAGATT
TCTGAAAGGGGTACCCTTTTGGTTAATTGGAGATTTCCACTAATCTCTCCGAAATGTATTACCCTCGTGTCACTCCGTTCGAAT
ATTTTTCCCCAAGAGATCTCAATGGGTAATATGAATCCTATTGGAGTTCGAAACGGAGCCCTAATCATACATTTGAAAAAATTC
CCCTTTTTGATGCCCCATTTTCGCAGTTTTTGCCTTAATCTTAGTAACATCTCATCAGTATAATGACTGTATCATCTCCTACGC
CCTCGGATTTGGAAGATATCTTTAACACATTGAAGTCTAAGAAATAGGGCTAAAACTTTTGCTGACCATGACCCCTCCGACATA
TTATTTAGTTTTCCACCTTCAAATCGTTGTCGAACCCATATTAGAGGAATCGAAGATTTCTGAAAAGGGGTCACCCTTTGTGAA
TTGGAGATTTCAATAATTTCTCCGAAATGTATTACCCTCGTGTTCACTCGTCGATATATTTTTCCCCCAATGAGATCTCATGGG
TAAATGAATCTATTGAGTTGATAACGGTGCCCTAAGTCAACATTTTGAAAAAAAATGTCCCTTTTTTATGGCAATTTTCGCAAG
TTTTTGCCTTATATTAGTACATCTCTCAGTATATTGACTATATCGACCTTCCTACGCACCTCAGATGTGGAAGATATTTTACCA
CATTGAAGCTAAACATAGGCCTACAACTTTTGTGCCAATGACCCTCGCATATTCTTTATTTTCACCTTCCAACATGTTTGCTCG
AACCACCATAATTGACGAGAATCGAAGATTTTCTGAAGGGTACCCCCCTTTTGAATTGGAGATTTCACTAATTTCTCCGATGTA
TACCCTCGTGTTCAACTCGTTCGATATTTTCCCAGAGATGCTCATGGGTAAATGAATCTATATGGATGTTTAAACGAGCCCCCT
AAGTCACATTTGAAAAAGAAATTCCCTTTTTTATGGCAATTTCGCCAAGTTTTGCCCTTATGTTGAGTACAATCCCTCTCCAGG
TATAATTGACTAAATCACCTTCTACGCACCTCCAGATGTGGAAGATACTTTTTACCACCATTGAAGACCTAAAGACATAGGGCC
TACAACTTTTGGTGGCCCATTGGACCTCGCATATATCATTTAGTTTTCACCTTCAAATCCGCTCGCGACATCCATATTTAGAGA
AATCCCGAAGAGTTCTGAAAGGGGTACGCCTTTCGTGAATTGGAGATTCACTAATTTTCCCCGAAATGTATTACCGAACTCGTG
TTCACCACCCTCGTTTCGTTTTCCCCAAGAGATCTCTTGGGTAGATGAATCTGATTGGGTTGAAACGGAGCTAAAGTCAAGCAT
TGAAAAAAAATTCCCCCCCTTTTTTAGGCAATTTTCGCACGGAGTTTTTGCCTTATATTAAGTAATCTCCCAGTAATACATGGA
CTGTATCCACCTTCTAAGCGCCACGCTCGGATTTGAAGATATTTTACACCCCATTGAAGCCTAAGACATAGGCTACACCCTACT
TTTTGCCCAGTGACCCCTCCGCATATTCTTAGTTTTCCACCTTCCAACATCCGTTGCTCGAATCCATATTAAGCAGAACATCGA
CAAGAATTTCTGAAAGGGGTAGGCGCCTTTGGTGAATTGGACGATTCACCTAATTTCCGTCCCGAAAATGTATTACCCCTCCGT
GTTAACGCGTTCGATATTTTTCCCCCCAAGAGATCTCATGGGTAAAATGACTTTGGAGTTGAAACGGAAGCCCTAAGTCAACAT
TTGAAAAAAAATTCCCCTTTTTGATGGGCCAATTTTTCGGCAAGTTTTTGCCTTATATTAGTAACAATCTCTCAGCCATATAAT
CGACTGTATCGGACCTTCTAAACGCACGCGCGGATTTGGAAAGATAGTTCTAACACCATTGAGCCTAAGACACCCGGCCCTTTA
CAACTGTTTGCTGGCATGACCCCTCGGGCATATTCTTTAGTCTTCACCCCTTCCCCAAATCGTTGCTCGACATCGCATTTAAGA
AATCGAAGATTTCTGAAAGGGGTAGCCGCTTTTGGTGAATTGGGATTTCCACTAATTCGCCGAAATGTATTACCCTCGTGTTCA
ACTCCGTTCCGAATATTTTTTTCCTCCCAACCGACGATCTCATGGGTAAAATTGAACTAGTTGGAGTTTGAAACGGAGCCCTAA
AGATCAACATTTGAAAAAAAATTCCCCTTTTTGAGCCATTTCCCGGAAGTTTTTTGGCCTTATAGTTAGTAAGTAGCATCATCA
TCAGTATAATGACTGTATCACCCCTCTAAAACGCACGGCTCGATTTGAAAGATATTTAACGACATTGAACGTCTAAGAAATCAG
GGCTAAAACTTTTGTGGCCATGACCGCTCGCATATTCTTTAGTTTTCACCTCAAAAAATGTTGCTCCGAATCCATATTAGGAGA
AATCGGAAGATTTCTGAAAGGGGTACCCCCTTTTGGTGAATTGGAGATTTCCCACCTAATTTCTCCCGAAATGTATTGACCGCT
CCCGTGTTTCCAACTCCGTTTCGATATTAGTTTCCCCAAGAGATCTCATGGTAGATGAATCTATTGGACGTTGAAACGGTGCCT
AAGTCAACAATTTGAAAAAAAATTCCCGCTTTTTATGGCAATTTTCGCAGTTTCTTGCCTTATATTAGTACATCTCTCAGTAGT
AATCCGACTGTATCAACCCCCTCACTACGCGCACCCTCTGATTTGGGCACCCCGATAATTTTTACACATTTGAAGGGCCTAAGA
CATTGGGCACAACTTTTGTGCCCCCATGCCCCCCGTATTTCTTTAGTTTTCAACTTCAAATCGTTGCTCGAATCAACCATATTT
AGAGAAAATCGAAGATTTCTGAAGGGGTGACCCCGCTTTGGGTGAATTGGAGATTTCACTAATGTTCTCCCCGAAATGTATTAC
CCTCATGTGCAACCTCGTTTCGACTACTTTTTCCCCCCAAGGAGAATCTCATGGGTAATGCCATCTAAATATGGATTGAACCG
GACGGCTAAGTCACACATTTGAAACAATTCTTTTTGATGAATTCTTGCAAGTTTTTGCCTTTTCATACTTATACATCTCTCAGT
ATATTGACTGTATCTTTACCTCGGATTTGGAAGATATTTACACCATTGAATCGGCCTAAAAGACAAATAGGGCTTACGAACTTT
TTGTGCCCCCATGCCCCTCCGCATATCTTTTAGTTTTCACCTTCAAATCGTTGCTCCCCGAATCCATATTAAGAGGAAATCGTA
GCAGATTTCTGAAAGGGGTTACCGCCCTTGGTGACATTGGAGATTTCACTAATTTCTCCGAAATGTATATTACCCTCCGGTGTT
CAACTCCGTTCGATTATTTTTTCCCCCAAGAGATCTCATGGGTAAATGAATCCCTATTCGGAGTGAAAACGGTGCCGCCTAAGT
CCCGAACATTTGAAAAAAAAATTCCCCTTTTTTATGGCAATTTTTCGCAAAAGTTTTATGCCCCTTATATTAGTACATCTCCTC
AAGTATATCAGACTGTATCACCCTCTACGCACCTGATTTGCCACGAAATTTTACACCATTGAAAGCTAAGACATTGGGTCTACA
CTTTTGTGCCCATGAACCCCCC
>m141121_232159_42134_c100732402550000001823141405141507_s1_p0/59454/4370_14686
CTCCTATTTGAGCTCAATTACATACGCCCTTCTGAAAGGGGCCATCAGAGCCCACTTAGGTACGCCCTGTGACACCCCGCTGGT
TGGCCTTAGGTCAGGTGGCGTGTCGATAGCCTCCAGCACATGGGGCTCCCGACATAGCGCGCATCTTGAAGCGTGACTGGTTGT
CGCAACTATGATAGTATGTACCCCACCTTGACGCAAAATAGATCAGAGTGAAGGACATAGATCGTATACGAACTCTGAACGCTT
AATGGTGGGAAAGAGTTGCGCAAAACTCTGTATTGTTTAATTTAAATTTTTAGAGTAAAGAACAAGGTTTGAGTGAATGAAGAT
GGGAAATATTAAGGGGAAGTCATAGAATTGAATAGTAAGGATATCAGTAGAAGGATGAATTTTATTTATGTTTAAGGATTAGAT
CCTGTATATTGAATGGGTGCGTGGAATATTAGAGGGGATTTAGTGGACATCACAGCTCGGACTACATGTAGGGTTTCATTTGCA

GGATTTATTTAGGACATTACTCGTAAGAGTCATCATGATACCTGGCGCCGGCCATTGTCATAAAGAATCGGGATCAGAACATGT
CCGTACTCAAAATTCTGGCACGCACACCTGGATGAAGAGGTGTCAGCATGTGCGGTAGCCATTGTTGACAAAGCCGTCCTAATG
AATTCCACCCTTGGACTGCCAGTCCTGCCAACGGGGAGATCCCGGCCCGTTCAGGTGAGAACGATAAGGACCTCTTCTGACCAT
CGATTTGCTGGCACTGGACCAGTGTACCGGTAGGATACACGACCACGTTAGGATAAGAACTAGAGCCTTCTTGGATGCTAGCGT
GATATATCATTAAAAACCCGGCATGAGACGAGGCCCAGATAGAGACGAGAGATATGTTATCCAAACGATCCCATGGACCCACTA
TATGGAATGTTCCTAAGCTCCTATTAAGACTTGCCCACTATAAATTCAAGTGAACTCGGAATAGAATCACCATGGATAATGAGG
ATTGAAAAATTATCGATTGGCTAGTGTTTTACATAGAGCGCTTACATTATTATAGGACAATTATCTCAATGGTATGTGATTTCC
ATCCTCTCTAGTTACGTTCATTAAACTGTGTTCATTCTGAGTTTTGTCCCTATTTAAGGCGTCATCGAGTATCTACAAGTTTTT
GATTTATAACATCGTCACTTTTTAGATGATAATCTTTTAGTGTTCCTGACCTTATATGTGTCGTTGTACTTTGGAGAGTATTTA
AGGAATATCACGTCAACAGTTTGGCGACACAGTCTGTTGGGAAAGAACAAGCATCAAAGCTCAGGTTGAGCTTTCCATTCTCAC
AGTATATACTGTCCCATGATAAAGAATAACGAAGCTAAGACGTGTCACCACATGGAATGCCCTATTATGGCAAAATATCCAATA
TGATGCATGGTATTTATCCTCTCTTATGGGATGAGACAGTAGCGGCATGGAGGGCTTACCTGTATAAAGACTCCCCTCTCTTTG
CGTTTATAGCAACCTATTGTTGAGGGCAAGAACACAAGAAGGAGTCCTCTTCCCAAGGTGGAGAGGGTAAGGAAGTTCTCATTA
CATTTGACGACATGTATCACCGGCGAACGTGAGGTCATTTTGTAGCGCCATAGTGCATTTTACTTGTCACCTTTTGGGCATCAG
CAGTTAAGGCACCTAAATAAAACAGCGCTCAGTGCCACCAGATTGTACGGCGGACCAGATGATCCACATCTAACTGCATCCTGG
AGCCAAGCTAGAGTTCCTCGGGTCACTGCTCGGATCTTATAAGATGTAAGTCAGGCCGCTCAAGGACTGCCTCTACATAGATGG
ATCACTTTTTTAAATTTTTGGATGTTGGGATTGCAAGACTTTCAGATGTTAACATAAAAGCAGAACTTATCTCCGCGAATAAGC
TTGGAAATCTCCTGTATAGTACCGACCTGGGTTAAGAGGTTTTTAAATGACCTAATTGCGAGGAAAGTCTAATAGTAGCTACCC
CTTAATCTCGCAGGTACATGCGCGTAAATGGAAGGAAGCATAGTAAATAATGGGAAAATGCATCTGTGATCCTCTTATAAAGAT
GAATGGGACACCAATAGACATAAATTCGCCCTAGTAAACCCCCAAACAAGGTATCCAGTGGATGGGGGAGCCTTCTAGGGCCAA
GATCGAGTCCCTCATATAGCAATAGCGGTAGGGAATATGGGCTGATGGTCCTGTAAGATATGGAGGGCCAAGTACCCTCAATGA
AGCCCTCTGGCCTAAGGGAAAAGACCCCATGAAGAGGATAAGTGTCAGAAGTTGAATGTAAGAGAACGTAGACGCCAGTCGACG
TTTACCCCCGAGGGAACAATACAACTTGAAGGCAAAAGGCATCGCGAGCCCGAGAACCTGCATATACATAACTCCTTAATGAGC
TAAAAGAGGAAGTGGAATAGTAGCGGAGATTCCGAAAAAATTCAACGATGAGAGCCCTTCAGAGGGCTCAATTACATACGACTG
CACTTTGGGGATCCACAATCAGTCTTTGGTATATTTTCTGTTGCTGGTCTACCGATTCGGTACTATCTTGTAGAATATCTCTTA
ACTTGTTTGCTAAAACGTTAAATCTTTAGTGGATGTTACGAAGTAGTATTTGGAGGTGCTGATATTTCATACCCATTTCACAAG
CGGATGATGGCAGGCTGAGCAAGACACTATGTCCGAGCTCTGTGCTTTCTGAGAGTCCTCAGCAAGACTTCGTGACGAGGTGTT
CTACTTGAACCCTAAAATATCTTCATCGTTTTATCGATCCTTGTGTTCAAATTATATTGAATGAGATTTAGAAGAGGTTCAAAC
AACAGGGCTAAAAGAAGATGGTCCATTGCTGCTATTCATTCGTTTAGACCATCTTAAGGATGGACGTGGACTGCGCTAAAGGGG
TGTCCAAGGTAGGATATTGAGGCCTCACGATCAATTGAGAGTCATGGCATCACAATTCTTCTTGGTGAGAATTTTCTTTCGAGT
CAACTACCATTAACAAAGACCTGTTAGCATTAGACTATCTGCTGCAATGTAATATTATCCAAACATAGTTCATGGTATGCAGGC
ATGCAAGGGGTTCCATTAAGATAGTTGCCATCTTCTTTCTTGCACTATCATTCATACTGGCTTAACCAATCTATAACAACAAAA
GGCAGGCCTGGGGCCCGGGACGGGCGCGCTTGAACTCAGAACCCAGTCCCTTTTGTGTTTCCTCAACACCTAAAATCAACCTAG
ACTAACTAAAACCAGATTTAGCGGTCACACCAGCCTCGGCTATCTTACATATAAAACAACGGCTGAAAATTAAGAAAGTCTGGA
GGATCACTACAAATCCTACCAGGTTCAGAATATTAAATAGGAACCAACAATAACAGGTGAAAGGTTTTTAACCAAAGTGGAAGC
ACTTAGATCTTCGGCTAATATACAGCCTGAGATAGCGGCACGCGAGGAAAAGGGCAGAGTTGATAGGAGGAGAGAGTGATTGGC
TCAAAATAGGAGAAGTTTCGATAGATTGTGAGATCGACTTTCTTCGATGTAGCGACATAAATAGTTATCATAAACGTGTGGTAA
GATGTGGCCATTTATAGGTGATACTTCGTACGTGGATGATGATATATCGAATACGCTGTCCTTCTAGTTGTGGATTGGCGTTAA
TGGTTTTTTAGGTTAGGTTAGGTTGAGTTTTAGGGGTGTTGTTTTGGGAAACGAGATGCCCGCGTAGGCTATATCATAGAGTTA
TCGTATGAATGAGACTTACCAGGTGAAGAGGGGTTGGGGAGTGCTGTGGCATAGAGGTAGGGGGTTGTGTTCGCTGACTCATAT
GCGCTGATATGTATATGATAACGGAAATAGACTCCATCTAGAGCGATTGAGAAGGTCCCTGACCCGGAAGAGAGATGCAACTGT
CGCTCATAGAAGAGTAGAGCGCATGCGAAGAAAGAGCTCCAAATATATAGTTATGAGCGCCTGCCGAAAGAGAAAGGGGCCCTT
CCGAAAGTAGATATTCGCCTGCAAAAAGGGTCTCTCAAAGTGTGAGATGAGCGGCACACTCAGAGAATACTGGGTGTTCTCCAA
ATAGTTAGACACCTGTTCCAGAAATGGTGACTCCGAAGCATGTAGACGCCTGATTTTGTTTGCCTCCAGAGAGAGAGGGTGCTC
AAAGTAACGCGTATCATCAAATAGCTAGGAGAAATGGGGTGTGCTCAGAATGAATATAGTAGGATCGCTTATCCGACAAGTGGG
GATCTTCGCGAAATGAACCTTTGCAAATAATAGTGTACTCAGATTTGCCTTAATGATGAGATAGTCGAATCAAGGCTATCTTTG
AGTAAAGATATAATATTATTGGACGCAGCGCTCGAATATATATTATTTAAGGTATAGAGTTAACTTAAACACCAAGGGGTGGAG
TATTACATTTCCTTATAATGGTGCATAGAGACCAAGGTGCAGGCCATAAAGAGAAGATGAACCGTAGAGTCGAACCACGCGCCT
TTTTGGAAATTCGCGTTGGGTATCCATAAGGATCCTCGAGCAATAGGAACTACCAAAATATATTAGATGACCTATAAGGTTAGG
TTTAGAAAAAGAGTAAGACGTATATTTTAACAAGATAGATATTCGATATGGTTTTATGAACAGCTGAGGATAGGGAGAATCGAC
AGATATATAAAATACAGAACGCATAGAGAATAGTAGTCTTACAAGTGAGTACTGCATGCTAATAAGGCCGTCGTCCTATAGATA
GCTTAATGGGGAGGTAGGTGATAGGTGATGCCAGACAAACAATCACAACTTAATAGAATTGGCTATCTGCTGTTTAAGTGGAGA
TCCTCAAGGTGAATGAATTCTCTCTCTCACACTGATGATGGAGGATGGGAAATGTGATCAGTGCATAGCAGTAGATTTACTTGT
CCGATACAGTGGACTTTAGATTGAGTTTCCTTCTAAATTATGTTATGTGGTCCTATTGCATGAGGCTGCTCAGACGACACTAAG
TGTGCCTCTCTCCAGTCCTTATAGAGGGATGATAGGCGTTATGTGGATAGATCGTTTTCTCTGTTGGGTTAGACTCTTCTCTTT
CGGTGTTGAGGATTTTCTATCTCATGAATGTAGGATATGTATTATTTATCAATTTAAAAAATTGATCGGAATAAAAATTTCAGA
GTTTTTGATCTCGTTGGTTATGCCGAGGTCCTTTATTGTTGTAGGAAACCGCTATACGCGAGATATAGGGGGTGTGTATTGTAT
AGGATGTCTCTCTTTGGTGCAGATCCTTGAGGTGATGCACTCCTATGTACCAAAAGAGGATGTAGAATTTGAGCCTTGGGTTCT

ATTGTATATTCCGTGTTGGTGATATAAGTTTATCGCGCGCTATTATGGAATCCATTTGCGAGAAGCTGGTCCTTTTACCTAT
CATTTATGTACATAATTAGTTACTAATGTAGATTTTTTGCAAGAGCGACCACCGCGTCTCTCGCCGCGTCAGTATTGAGTAGCG
CCTATTTTACGGAAGCGTCATACTTCGAGACGGCTGTACTTTCGAGATATGAGATACATGACATACGTGTTTGGAGACCCTTTG
TCAGGAGGGCGGTCTGTAGTACTTTTGATTGAACCTTTTCTGGAGGATAGTTCTAGAGACTTTTGAGGAGACCCCTTTTTCTGT
TGGGCGTACTACTTTTGGTAGACCCTTTCCGTGCAGTGGAGCAGTCCTGGTTTGGAGGACCGCGCACAATTTTTTGGAGGCGCC
TACTTTGGAGCCCTTTTTGCAGCGCCTACTTTTGAGTTGATCATCCCCTCGGGTCGGGACTTTCCTGGCTAGATGGTCTTCGTT
CAATCTCAAAGCTTGGTCCAGCGACCCCTCTAGCAAACGCCATTCTATATGGAAATCTTCAAATACGTATTGGTAGTAGGGCAC
TGAAGGAAGGTTCGATTTCTTCCACGTAGAAGTTCTTATGGCCCTCTTCACACGTATGTACTTTTTTGTGGCTCTCTCCGAAGA
AGTCGTCCAAATTCGAAACCTTCTCCTTTTGAGCCATGCTCCCCCACAACTTGCTTCCTGGACGAACTCAGGCTGTAATAGCCA
TCTTGTGCTCCACTTTGGTTAACCTTTCACCTTTGTTGCTTCTATACTGAACCTATGGATGTAGTCCTCCAGATTTCATTCTCG
GCCTTGTTTTATGTTAGCGAGGCTGGTGGGGTTGGCATGACGTAATCAGGTTGTATGGTGGTTGCTGAAGAAATTCATCAGAGA
ATTGTTGCCATGACTCAATTGTTGATGGCTCAACCTCTTGAACCAATTGTCCGCCAACTCCTTTAAGTGTAACGATGAAGCAAT
GACACTTTGCCCTGTTTGTTGACCCCTCTTACCTCATCAAGTTATTGAAGGTGTCTAAATGATACTTTTGGGTCCAGTAAGTAC
CTCGTACGAAGTCTTGCGAGGCTCTCTGAGCAGTGGGCATCTGCTCAGCCTGAATCTCCCTTGTGAGGGTGAATCACAGTCAAA
TTCTTCGTCAATCCCATGTGATCCCAAGTGCGTGGGTGATCTTGCCTCGAGGGCTTCGTATTTTGGTGTCTAGTCCCTTCCCTC
TTATTTGCATAAGGAGTTTTGCAGGTTCTCGTGGGCGATACCTTGCCTTGGTTGCTGTCGCTCGGGGGAACCGCGGGGGGTAAC
GTCTCTTACCTCTGACCTTTCTCCATGGGCTCTTTTCTTAGGCCAAGGTGCTTTCTTTTGAGGGGACTTTGGCCTCGTTCCTTA
CGACCATCACCTCTCCGCCTTTGCTATGGGACATTTCGTTGGCCTAGGTCCCCCTGATACCTTGTTTGGGGGTTTTACTGGCGG
AAGTTTTGGTTCCCCCATCATCTATGGGGAACAGTGCTTTCCCATTTTTCCATGCTCCTATCCCCTTTACGCGCAGGAGGGGTG
TGCTTTGACTTTCCCTCGCCAATAGGTCATTTAAAACCTCTTACCATGTTCTCTATCTGGGTTTCCAGCTTTCCGAGTCCCTTT
TTATTAACTCTTGAATCTCACCTGTAGAGGCGAGTCCTGAGCTGAATACCGGAGCGTACGCCCGGGACTCTTGCTGGCGTGGCG
GTTGATGGACCTGGGTCTTGGTGGCCTGAGCGTGGTGCTACTGGAGGCCCGGTGAAGTTGAATGCCACTGGCGGCTCCAAAATG
ACCTCCGTCGGCCGAGATGGTCAGAGATGAGGCCTTCCCCTTCTCCTCCCCTGGGGGAAGAACTCCTTTCGGTTTGCCTCCCAT
GTTTAGGCCTAGAGGGGGTCCTTACTGGGTACCCTCCGCTCTACTTCCATCCCAGAGAACTCCATCATCTTGTATTTGCCATAC
GGCATTTTGAACGTCTTAGCATCTTTCTTTGCTGGGAAAGATGGATGGAAGAACATGGCTTATGCTTGTTCTTCCCCACAGACG
GCGCCAAACTTGTGACCGGTGAATCTCGTCAACGAAATTAGGTCGGAACACTTAAAGGTAATGTGACGATGTTTAAATAAAAAC
TTTAGAATGAACTCATGGACAGATGAACACAGATGAACAATGGAGGTGAAAATGTATATTGCTTAAATAGCCTCTTGCATTACA
ATGATTTTCCAACCCCCTTTCCAGGTGGTCTTAGAGTTCCTTTTTAGGTGGGCTCTAATGCCTTAGGTACTAGTGGTCCAGGGG
ACCAAGTGGTACATCGTACTGTGTAGGGAATGGCTCCAGAGGTTGTGGTCGTACACCCCGTCCCAGGAGCAGGTGTCAGGAGGA
TGTCTCCACTACTTGACCTGTATCCATGTCTGATGCGTGGGTGGCAGGCGTAATGGCCAGGAGGTAGTGGTGTCGGCTCTGACC
TAATGGCCGTAGACATTATGGACCATAATTCCAATCCTTGCATACCCACTCCTGGCGTTCCCACTACTTGTCTGTAGGGGTACG
ATATCCGTACTCTGACTCTTTAGGTTTTGTAAGTACATTCATATCATGCCTATCTTGCCCCTTGTGAGTATTCTCACCTCCAAA
GGCCATGGCCACGGGACCATGGGGCCTAGGCCATGGCGAGGCATGGAGGAGGCCATGGCCGAGGCCAGGGGCGAGGCCATGGAG
GAGGCCATGGAGTGAGGCCATGGCACCATGGAGGAGGCCATGCGCGAGGCCATGGAGGAGCCATGGTGGAGGCCATGGGGCGAG
GCCATGGAGGAGGCCATGGGGCGGCCATGGAGGAGGCCATGGAGGAGGCCATGGGGCGAGGCCAGGGGGCGAGCCCAGGGTGAG
GCCAGGGGTGAGGCCATGGCGGCCATGGAGTGAGCCATGGAGGAGGCATGGGGTCGAGGCCACGAAGTAGCCCTTAAGCGAGGA
AAAACACGGAGGAGGCCATGGGCGGGCCATTGAGGAGGCCATGGGGCCGAGGTTATGGAGGAGGCCATGGGCGAGGCCATGGAG
GAGGTCCATGGGGGCCCGAGGCCATTGGAGGAGGCCATGGGCTGAGGCCATGGAGGAGGCCTGGGGCTGAGGCCATGGAGGAGG
CATGGGCGAGGCCATGAGTGAGGCCATGGGGCGACGGCCGATGGAGTGAGTGCCATGGGGAGGCCCATGGAGGAGGGCATGGGC
GAAGGCCATGGGGTCGAGGCCACGGAGGAGGCCATGGATGAGGCCCATGGGAAGGCCATGGGCGAAGGCCCTGGAGGAGGGCCG
AGGCCATGGAGGAGGCCATGGGGCTGAGGCCATGGAGGCCATGGAGTGAAGGCATGGGCCGAGGCATGGAGGAGGCCATGGGCC
CGAGGCCATGGAGGAGGCCATGGGGCCCCGAGGCCATGGAGGAGCCATGCGAGGCCAATGGGAGGGAGGCATGGGGCCGAGGCC
CATGGGCGAGGGGGCCTCGTGAGGCTAAATGCGCCTTGGCACCCTATGCCTTTCCAACGTGGTCCACTTGGCATCGAAATCCAC
TAGAAACGAACCCAGACTCCGTTTTGTGATGGCGCGATGGCTTCCGAGTACGATGGTGGCCTTAGGGCCTTGCTGGGCCTGCAT
ACTTTCACTTGATGGATTCATGAGCATCAACATTTCTCATGAGATGATCTCCTGTATTCACAAGGCGTACAGGCTTGAGCCTAA
TAGCGTGAATAAGCGACCCTTATCCCCTATGTTTCCATCCAAGGTAAAGACGTTTTCTTTTTGGTGGACTTTTTGGCATCCACA
AGTATGCATGTAGGCCCTGATAGATTAAAAGGGATATCGTAATTTTTGGCTGTGAGGGCATAAATGTAATTAAATTTGTGATAA
ATCGTGGAGAACACATTATTATGTGGATATATTTGCAAACTTGTGGCTCAGGCGATCCTAGTGAGCGATTTGCGAAAAAAGTCA
CGGTGGGGATTATACCTTGCTCGGGGGAGCCTGGGGGTAATTTTTGGGAATTTGGGAATATATCGGAGGCTATTTGATATTGAG
GGAAATAATTGGTGAGTAGTTAGGTATCGGATGTAAGCGGTATTATTAGGGACCTCGAGGAATTAAGCGGGATTGAGAGCAATG
ACCAAAATGCCTTGGTATGTTTAAAGGCTTTAGGTTTTAATGGGAGGGAAAAATGGTCCATTTGATTTAAAAAGGGATAGGTGT
TTTGTCTATTTTTAGCCTTATGTGGGAAGATGTAAGAATGAATTAGAGTTAAAGAAAGAGCAAAAGGAAAAGAAAGAAGAAAAA
ATAGAACATCTATAATCCCCCTTTCCTTCTTCCCATTTAGTTTCCTCTCCCCTTCACCATCTCTTGAGGATTTTTGGAGCTGTA
ATTCCAGAGAAAGCCTTAGGCTGGAGCTTGGGTTAGGATCCTTCATGAAGCTAAAGGATTTAGCAGGGAAGTTTAACTACTCAA
TTGAGGTAAGGTTTAAAGGATTCTTATGAGTTTTTCTGAACTTTCTAAGAATTGTTTCTGAACTTGAGTTTTGGATGGAAGTTA
AGGTAATTAAGCTTGAAGAATTGGAGCTAAAGGCTGGAGGATACTAAGGGCAACCTAGGATTGGACTCTCCACTAAAGGTAAGG
AATTTGAGCTTGATCATCTGACTTGCTGGGTTATTTCTAGATTTTTTCTGGATGAGTTTCCTGTAATTTTGAGGGTCAGTCTTG

TTCTTTGTTTGTGTGCATGTGTAAGTCTTTATGTTGTTGGGCTATGTGGGGATGAGATTTAGAATGGTAGGCTCAATTTGTGTG
TGGTTGAGGTTTGGAGGGGTTTGCTCCAAGGAAATTCGAAGGGAGAAAATCAGAGTGCTGAAGGTTC
>m141121_232159_42134_c100732402550000001823141405141507_s1_p0/62806/5747_15077
CTACATTTGGGTTATAACCAATACTCACTTTATTTCTTCAGTAAGTGTGGATAATGTCAAATTATCTTAGCTCGTGTATTTGT
TTATATTAAATACTTTATTTATTTTAATAATACTTTTTTGAACCTATTATAATCGGGTTAACACGGTCCAGACATTTGCGATCT
TTGAGCTTTGTGTATGTCTCAAGTCATTTGAGTTATTTATTTCTTAACTTTAATGGTATATATATCAACTGATGCCCTTAATA
TCCTATGAGTGTGACCATAGGTGTATTAAATTAAGAAAGTTTGCAAAAAAAGCAACACATATTGAAAAAAAATCGATCGTTTAT
CAAAATTCGAACGATTTATTATAGAACGTTGTTAAAGAATAAAACAGCGCATGGTTCCATTATGAGACTCATTCCTCAGCTATT
GATAGTAAGGTCGTTAGATTTGATCCGCCATTCTCAATGCTCATGGTACCAATTTCCCCATTTAACTCGAATTTGTAGAAACCC
AAGGTTGAATAACTAATTCGAGTTTGGTAAGGTTCTTCCCAATTGACCGAGTCGCCAGCGGCTTGGTCGCCTTGTAGTTGGACA
CACGCCTTAACACAAGTCTCCCAATCCGAATTTCTATTCCGAACTTTTTATTGAAATATCTGTTATGCTTAGCTTCGCTATTGA
TATGCTGCATTTTTCGTTGAGCTCATCTCGCTTTCTTCAATCAAGGTCTAGACTTACTTCAAGCTGGGATTGGTTCGAGGCTTG
ATTATGCAAGCATGGCTATGAATATGTGTGGCTTATTTTACTTATAGATTGGTAACATGGCTTCTAAAATCCATATGGCCAGTA
AGAATAAATGGGGTTATTAGATCCTATTGAAAGTGTGAAGTCGTAGATTCCGAAATGCCACAATGAGCTGATTGAGGAAATCCT
CTGGCATTTTTACCTTATCTATCCTTTCCAATTTTCATATTTTTAATAGAACTCTTGAGAGATCTTATTACAGCTTTGACTTGG
CCAATATTTCTTGGGGATTGGCACAGAAGGATAGCTTTTCATTTATCCCATTTTCTTTACAAAACAACTGGTAGAAATAACTTT
TCATCTGTCGAAAAACCAATGTCGCACCCTCACCTTGTTATACAAGGAGGAATAACAATTTTTTCCTAATCCCCCTCAATGTCG
CCATGCGCATCCAAATATAAGAGAACCCCCAATATTATATATTGGTAGGCACAAAGTTTAATGTACTTAATATGAAGAGAGTAA
AAAAACCAGAAATAGTTTCTAGTAGGTTTTGTTCATGTTCCTCACCCTTAATTTAACACTCCACACGACAATTCGATCTCACTT
TTTTATTTTTATCTCTCTGAGTATCCACCTTTTATACCGGTTGATAACTGTGCTCAGTCAAAGGGTTTCACGAGACGAAGAGAT
AGAGCTCATGTGGGTGGTAAGTATACAACATATGGTTAAAGACACAATATAAGGAAGGAGAAGCCTCACGCAGACCTCCCATTC
TCTATTATATTTTTGCATTTATTCGCATATAATATACTTATATGACAGCTAGAAACGTGCGTCTCCTTTTCTGATAATGCGGCC
AAGTTTATTGTCTCTTTGTCTTTATATTTTTTCTTGCATTTTATGCTATAATGACAACACAAGTATTGGTTCTTCCACACAAAT
CCCCCTCCCATATTTTATATAGTGTGCGTTGCGTAGGGTTCCCTCTCTCTATATATAGAGCACCCAGGTGGAATGATGTACCCT
CCAGAATAAAATACACCCCCAGAATTTTTTCATATCTCGCGAATAATAACATATCCCCTTTTCTACATATTGTAGATATATAGT
GAGGAAACAAAAAAATAAAACAAATACAGTTTCTATAGACTCTTGCCCCCTCATATTCTTATGCGGGGGAAGAACAATGTTCGTG
GGTTCGAGGTAATCCGATTCTTATTTTTTGCTGGTCATTTCCGTTTGTTTTTCTCGAGTTCTCCTCCTCCAATAAGTAAATAAA
ACCAATCTTCCTATTATCGAAATTCTTCAACCTGGAGAACTGTTTTTCCGCTAGGTATCAAGTTGGGCGGACATTTCAGTTTTC
ATCATCAAGAGACCATCTCCTTCTTGGTTTGGATTGTGAGTACCCACATATGCTTGGCGGCGTGGCTTGCATTTGAATTTTTGC
TGCCGGGAACTGCTCTATCGTGTAGAACTCAAATAGTTCGAGCACGTCTGCTCCCGCTCCTATAAAATCTCACACCTGTTTTTC
GTGCTATACCCCTTTCCAAAAAAAAAGGGATTTGAGAGAGTTAACGGAAGAGACTAGATACCATACACACCTCACACACACCCGG
ATATTTGGATGCTCCCCCACATCCTAAAATAGCAAATCTCATAGTAAAATACCCCCTATTGATTGTTATCCTCCCTTAAAAGCA
TGAAGAACCAATTTATCTCTTCGCGCATAAAAACCAATCCAAAGTCTGAACTCGAGCCCTTCGTCATTCAGCCCTCATTGTTAG
ACCTCTTCTCGAAGCCACACTCTAGAAAAGGACGAGAGTGAAAAACCCCCTCTGGGATCTCCAGTTTTTGGTATGAAGTTATTG
CCCATTTTGAGGTGCCCCCCTCTCCCGCACCATCTGATTTTCTTGGTCCTTCATATGGAAAAGAAATCATCGACATAAAAGTTT
CCACAGCTCGCGGCTGGGGTATACAAAACCCAACAGCCCCAAACTGGGGGGGGCACCCCTTTTTTTGCCCCCCCCCCCAGAAAA
AAAAACCCCAAAAGAGAGGTCCCTTAAAATTTTTGTTCCCTTTGCAACCTTGGCCCCCAAGTAGCGCTTTGGTGGGGGGCGGGG
CGGGGCCCCCAAAATTTTTTTCCCCCCCCCGGGGACCCAAAAAGCAACCACCCCAAAAAAAGCTTTTTCCCCACTGTGGGTTTT
TTTTTTTTTTCCCCCCCCCTTCTCTTTCCCCCCCCTTGGCGAAAAATTTTTTTCCCCCCCCCTTTTGAAAAAAAAAAAACCCA
ACCCTTTTTTTTTTTTTTTTTTTTTCCCCCCCCCCAAAAAGAACAATCCCCCAAAAAAAAAAAACCCCATTAAAAAAAAAACCA
AAACAACAAAATCCCCAAAAGTTTCCCACCACCCCCTTTTTTAAAACCCCCCTTTTTTTCTTTCAATCTTTACAAAATTTTTTT
TTTCCCCAAAGAAACCCCCCCCCCTTTTCAAAAACCCAAAAAAAAAACCCAAAAAAATTCCAAATTTTTCCTAAACAAAAAACCAA
AAAAAACCTTTTTCCCCCCCAAATTTTTGATTTTCCCCAAACCTTATCCATAAAAACAAATTTTTCAAAAAAAAAAATCAAACA
ATTGAAGCCTTTGGGAAAAAACCTTTAAAACCAAAACCCAAAAATAAAAAAACCCCAGAAGCCAAAAAAAAAATACCCCCCCA
AAAATTGGAAAAAACCCTAGTTTTGGGGCCCTTTAAAAAAAAAAAAAAAAAAATTTTACCCTCAAAAAAATTTCCCCCAGTGTTT
GTGCGGGTGGGGGGGGCCCTTTTTTTTTTTTCCCCAAAAATGGGTGGAAAAAAAAAAACCAAACTTCCCCTCCCCAAAAACTCC
CCCAAAAAGGGCCCCCCCCCCTTACCCTTTTTTTTCCAAAAGCTTTTTAAATCCTCAAAACCCAAGAAAAAAAATCCACCCAAA
AAAAATCCAACAAAAAATGGAGATTGGGGAGGGAAAAGGAAAAAAAAAAGAAAAAAAGGTAAAACCGGTTAAAAAAAAAAAAA
AAAGCTTCAAAAAAAAACTTAACTTTGCCCCCCCTTCCCCCTTTTGTTTTTTTCTTTTTTTCTTCCCCCCCCTTTTTCCCAATT
TTCCTAACCAAAAAGGCCCCCTAAAAAAATTTCAGTTTTTAATAATCCTATCCCCTTTAAGGGGGGGAAAAAAAGACAAAAAT
TTAATTAACCCCCTAAAGGTCCCATTTTTAAAGATTTTCCCCCCTTAAAAGGCCCCTCCCCCCCCCCCCCCCCCCAAAGGGGCA
AAAAAAAATTTTGTTCCCTTTTTTTCCCCCCCCCCCAAAAAACCCTAAATCCCTTCCGTTTTAAATTCCAAAATAAATTAAAT
GCCTTTCCCCTCCAAAATTTCCCCGGATTAATTTTTTCCCCTTTTCGATTTAAATTCCCCCCCCAAAAACCCCAAAAACCAAAT
AAAAATAAAAATTTCCATAATCCCATTTACCCCGCCCTTTTTTTTAACCTCCCCCGGGTAAAAAACGCCCTCCTTTTAAAATC
AATTTCAAAAAATAAAAAACCCCGAGGAAAAAAACCCCCTTTCAAAAAAAATCCACCAAGAAGCCCCCCCCCCCCTCCCGAAAA
ATTTTTTAAAAAACCCCCCCTTTTGGTTTTTTAAAAATGAACCCCTTTAGACCCCCGCTTTAATCCAAAAAAAAAAAATTTAAT
CCCCAAAAGATTTAGGGTTCACCCCTTTCAAAAAAAAAAAATAATCCCGAAAATGGTTTTTTCCCGAAAACCAAAAACCCCAC
CAACAAAAAAATTTATAAGAATGTGGGGGGGTCCCCTTCCAAAAAAAAAACAACCATTATCCCCCAACCCAAACAAATGCCAAA

TTAACAAAAAAAAAAAATTAAAAATAAACCAATTTAATACCACTTTTTTTTAAAAACTGGGCCCCCAAATTTTTTATCCAAATC
CACAACCCCCCTGTAAAAATTTTAAAAAAATGTGGAACCCAAACCATTTGCAATGGCCCCCCCAAAATTTTTTAACAATCAAAT
TTATTAAAATTTTTTAAAAAATATAATTCCAAAACATATAACAATTGCCCCCATTTTTTTTTTAAAATTCCAAATTAATTTGAA
TAAAAATAAAAACAAAAGTTATGGCCCCTTCCCAGGCCTTTTTTTAACCTAAATTTTTCCCCCGCCCAAAAATTTTTTAAAAC
CCCCCCAATAAAAAAATCGGGAGGAAAAATTTTTTTTTTCCGGGGCCCCAAAATTTACAAACTAAAGAATCCCCCCCCCTTTTC
CCCCCCCCTTTTTTTATACCCCCAAAGAAATTTTTTTCTTGGGTCCCCCCCCTCCCCTGAAATGGGTTTTAAAAACCTTGGGAA
AACAAGCCCTTCCCCGAAAAGAATTTAAAAAAACCCTTTTGGAAAACTCACCCCCTTTTGCCCCCCAAAAAATTAATTTTTTTC
CCCTTTTTGGAAACCTTCCAAGCCCTCCCAGGTTTCCCGCCTTTTTTTCCCCTCCGACCCTTTTGCCTTGTTTTTTCCCCTTCC
AAACACCAAATACAGCCCTTTTTTAAAAAAAAAACGAAAAAGTTTTAATCCCCGGTCCTTTTAAAAAATTCCTTTGAGGGACCCC
TTTTTTTTTGGTCCCTTTTTTTTTTTTTCTGGTCCCGGAAAAAGTTAAAAATCTGAACTGGCATTCCCCCCTTCCCCCCCCCTT
CCCCCCCCCCAAAAAAAAGGGAAGAGAAAATTTTTTTTTTCCCCCCTTGCCCTTTTCCAAAAGCCCCTTCCCCCCAGATTTTTT
TTCCTTTTTTTTTTTTTCCCCCCAAAAAAATAAACTCAAAAAATAATGGGAAACCCCCCTTTTTTTTCCCCCAACCATTCAGAT
TACAAAATAACCTCCTGGAAGGAAGCTTGGAGGGGAACCAAAATGAGAAAATTTTTAACAAAATTTTATGCCCCCAAACCCGGC
CCCCCAACAACCGCCCCCAGAAAAGGGCCAAAAAAAAAGCCCAAATCTTTGGTAAGCCCCCACCCTTGAACCAAAAATCCTTTT
TTTCCAAGGAATCTCCAAAATGGGGGACCCCTACAAAAATCTTAGGAGAAACTCCCCAAAAAGCTTTTGGCCCCCCCTTTTTTT
TTCCTTTTTTTTTCCCACCCCCCAAAACTTTCCCTTTCCAAACCCCCCCTTTTTTTTTTTTTTTTCCCCCCAATGGGCGGAGA
AACCCTCTTAAAAGGAAAGAAAAACCAATAGGTTTCCCCTCCCTTTAAACCTTTTTTTGGAACTTCCCAACCGTTCCCCCCCCC
TTGCGCCTTTTTTTGGGGGGAAAAAAAAATTTTCTTTTGGCATTAAAGGCCCCTTTTTTTTTTTTTTTCTGTCTACTTTCTGAG
GAAAGGGCGAGCAAATCCCCCGAGCGCTTCCTAAATCTTTTTCCCCCCCCTTCCCCAATTGACCCCTTTTTTCAAAAACTGGTT
CCCTTTTTTTTTTCTGGAACCCTGCCCTTTCAAGAAAACCCCCCCCCTAAAAGTTTTTAGTTCTTTTTTCCCCCTTTTTTTTC
CCACCCCCTAAAATCCCCCCTCAAAAATTCCCAATGGAACTGTGGGAAAAAAAGGGGGAATTCTTTACAAAAAAAAACTTCCCC
CCCTACCAAAAAATAAAACAACAAATTCCCCGTTCACCCATAAAAGGTTTTTTGCAAAAAAACTCCCCAAAAAAAAATTGGGTTT
TAAAAAGACCTTGAATTAAAACTTGTTTTTGGTTGTTAAAAGGAAAAGAAACTTCCCTTTTTTTTTTTTTTTAAATCCAAAAAGG
GGTTAAAAAAAGAATTACCTTTTTTTTTTTACTTTTTCCCCAAAAGGTGAATCCCCACCCAAACAGTCCCCCCCCAAAAAAGCCA
AACAAAACAAAATGCTTCCCCTCCCCAAGCATGTTCCCCCTCCAAAAAAAATTCTTCTTGGAAAATTCCCGTTCCTCTTTCCCC
CAGAAATTGGCCCCCCCCCCCAATTCCCTTTTTTTTGTCCCTTTTTTTTCTGAATAGGAAAAATGTGCGATAAAAGGAATTGGG
GGGGTACTTTTTGAAAACTTCCAAAAAAAAAAATATGAGTTTTCCACCTAATAGGGGCCTTTCTGTCAAAAAACTTTTTTCCCCC
CCCCCCCCCCCAAAAAACCTTTTTGGGAAAAAAAAAAAGGTAAAAAAAGTGGGGGGGTTCCCCCCCCCCCCACGATCCCCCTTGG
AAACCAAAACCGAATCCAACCCCCTTTAAAAGGGGGTTTGGGGGGGCCACCCCTTCCCCCCCAAAATGGAAGGGGGGGGCGGCA
CGAATTCCCCTTCTTTTTTTTTTCTTCCCCAAAAAACCCAACTAAGAAGATCTTGCAAAAAATACCCTGGGTCCCCAAAACCTT
TGTTTTAAATATGTTTTTTTAGTTCCCCCCCCTCAAAAAAAAACTTGGGCAAAAAGAAAGCATGAAGCCCTTGAATTTTTTGGG
TGTAGCCGAAATTCCCACCCCTTATTTCAACAACCCCCCAAATAAAGAAAATTTTAAAATTGACTTTTGGAAACCAACAGTCCC
CCTGGGGGTTTAAAAAAGCCCCCCCCCAAACCAACACAAAAAAGACTCCAAATTTTTTGGTGAATTTGGTCTTTTCCCCGAAAA
AAAACTTTCCCCCCAAACTTCCTGGTGGAAATAATTCCCCCCCCAAGACGGGGCCTTTTTGGGGCAAATAACCCCCCCTGTCCC
CCCGGGCCCCTTCCTTTTGAAATTGCCCCTCGGCCCTTGGGGAAAACCCCCCCTTGTTTTTGACCCAATGTCCCAAAAAAAGCC
AATTTTTTTTTAAGCCCCAACAAACCAAGACTCCTTAACCCTTAAACATCCTTCCCTTTTTTTTTTCTTTCATTTCCCCCAGGC
CCTTTTAAACCCCAAAGTAAAAAAAAAAACAACGAATCCCCCCTTTCCCAAAACCCAATCCCCTGCAACAATTTTTAACCCAAT
TTTCTTTTTCGTGCGGTGGGGGGCCCCTGGAATTTTTTTTTGCCAAAGAATAAGGGGGTGGGGTTTAAAAGTTACAAAAAAAAA
AAAATGGGATTTTTTTTCCAATTTTCCCAGAAATCTTTTCCCCGCCCCTCAAAAAAAGGCCCCCCAGTGTTCCCCCATCCCCCG
CCCCCTTTTGGGTTATCCTTTTTCAAAAATTAACCCCCCTTAAAAACAATCTTTTTTTTTCCAAGAACCAACTTGGTAAAAAAT
AATTCCCCCCTGAAAAACAATTGGCTTCGTTCCCTTCCAAGCCCAGAACAAAAACCTCCATCCCTTTTCCCTTTTTCTTTGATC
CCTTTTGAATCCAGTTGTGGGGGAATTTCAAACTCCCCCAGCCTTTGACCCCTCCCTTGAATCCCCTTCCCCCCCCCTCCCAAA
TTAAAAAGCCCGTTAAGACTTTTGTAAGGGCCCGTAAAAAAATGGTTTAACCAAGCCCCAAAAAAATTTTGAGGCACCCCCCCAA
CCAAGCCAACTTCCCCTTACAAATAACCCAGCTTTTTTTTTTTTTCCCTTTGGTTCCCCCAATATCCAAATCCCTTTTGTTTTT
GCTTTAAAAAAAATTCCTCCTTTAAAGCCTATTAAGCCCTCCCCAAAAAATAAACCAAAACCTTTACAAAAAAACAAAAAAAAA
GGGAAGGCCCCCCTTACCAAAACAAAACAAACCTTAAAATATCCCCAAAATTAAAAACCTAATCTGGGATAAAAAAGTAATTTA
GGGGTAAATAAAAATCCCCAAAGATTTTAAAAAAAAATCCTGTAAATCGGGGGTTGGGGGGGTTTCCTTTTCAAAAAAAAAATTT
TTTTTGGGGGGAGCGCCAAAAAAAACGTTTTTATATTTTTTTTTAAGTGGGCCAATTTCCCCCAAAAAATTTTTTTTAAAAATT
AAAATTATTTTTTTTTTTTCTTTTTTAATTTCCTTTTTTTTTTTTTTCCCCTAAGTGTTTTTTTTTTTTTTTCACTTTAACCCCC
CTCTTTCCCAAACTTTCTTTTCCCTCTTTTTCAAGATCCCCCCTTCGCCCTTCTTTTTTTTTGTCCCCCTTTTTTTTTTTTAAAA
AAAAATCCCCCTGTTAAAGGGTTTTTTTTTTTTCCCCTTTAAAAATACCCTTCTTTTTTTTTTTTTTGGGCTTTTTTCTTTTCC
CCTTCCAAAAACAAAAAAAACTCCCAAAAAAAACCAGCAAAAGAATAAAGCCAAAGACCCAAAAAATTTTTTTTTTTGGGTTCC
TTCTTCCAATCCTTTTTTCCCCTAAGACTTTTCTTCTTTTTTTTTTAGAAATTAGGAAAAATTTTTTAAGCTTTATAGTCCCCC
CCCCCTGTAAAAGTTTTTTTTTGGAAAGCCTTTTAGGTGGTAAAGGTTAAAAAATACCCCGAGTTTTTTCCCATTTCAAAATTA
CTTGGGAAATAATCTCCCAAAAAATCTTTTTTCTAAATTAGTAATTTTTCCATTTTTTCTCCCCCCCCCCCAAAAAATAAATTT
TTCCAAAAAATCCTTTTTTTTTTTCCTTTTTCCCCTTCCCCCTTTCCCTTTTTTCTTTTCCCAATCTTTTTTCCCATCCCTTTC
TTGTAT
>m141121_232159_42134_c100732402550000001823141405141507_s1_p0/64601/5493_11268

GATTTATCAAATATATCCACCCACCCCCCCATGGCTGTACCTTTGGAACACATTGTTATAATTGGCACAATAAATCTAATCCAA
GGAATACAAAAAAATTGAGTAGATTGCTCTTTTCGCGAGCCTCAAATCGTCCTTGGTTAGTGCATAGACTCGCCGTATCAAAGG
CCCCGACTTTTGGTACAGAGAACAGACAAACATTTCCGAGGCCAGCTGTGAACCCAGCCATCAATACTGGGGTGCGCGTGCGTG
GAGCGCAAGGTCTAATTTTTTTGAACTGTTTGTCCCGCCATCTCAATGCACCCATACATCATTAAGCTATACTATGATCCATCT
CAGAATGGGGGGTCCTATATTCAACCCGACAAAAAATGATTCCTTGGCCGCCTTTCCCGCCAAATCTCCCAGGCAAGCCCTACC
AGGGCTCGGACAGCCATCTCGACGTAAGTTCACGTCATCATTTGCTCGCTGTCTAACAAGCTTAATGATTGTTGACTTCCGCGA
ACCTTTTCCCATCTCACTAATACATATACAGTAACCTATTGGCCCATGAATTGTGGGTCTCCAATTCAACCAAACAAAAAAATT
AAACCGCCCTTTCCTGCAAATCTCCACTAGCGCTGCCTAGGGCTAATTTTCAGCACTTGAAATGTGTGTTTTTCCAAGAGCCTC
CTCTACAAACGCCTCTCATAATTTCCCACAACAATAATATTTCTTTGTCATCTCCAACCAAAACAGAAACATAGCTCTGTTAAT
TTGTATTTGAACTTTTTCCCGATCATCAATAATATGCATATAGGTATTATTCTATTGAGCCCATGAATGGGGTCCAAGATTCAA
ACCAAACAAAAAAATGGCCCTTCATCCTAACAAACAATTCCCTAGCCTACCTAGGGGTTTCCGAAAGCGATGCTCGTAATTCCC
TCATATCTTCTCTCTCAACAAGCTCTAATTTTTTTTGAACTTTTCCCATCTCATAATATTCATATTGGGCTACTAATTTGACAT
GAATTGGGGTCCTTATGTCAACCAACACAAGAAAATTGACCGTTCCCTGCCAAAATCTCCTAGCAGCTGCTAGGGCTCCGAACG
ATCCTCGCTAATTTGCCACACCCCGCCCCGCCTCATCATTTCCTCTCGTAACAAGCTCGTAATTTTTTTGACCCTTTTACCATC
TCACATAATGTATTATATAATCTTGCCGTCTACTATTTTGTTATTTTCCTTTTTTTTGATATTGTTGGTGTGTTCCTTATTTTCTA
TTCTACTATTTTTATTTATATATATTTATATATATCTCTTTTCTTTTTTTTTTTTTTTTGCTTTTAATTATTTCTTGTCCTAGC
GCAAAGCCTGCCTTAGGCTCCGAAGCGATCTCAGTAATTCGACTCCATATTCTCTCTCTTACGAAGCTAAAATTTTGTTTGAAC
CTTTGTCCCCATCTCAATATGATGTCATATAGGAATCATACTAGTTTGTATATTTTCTCTAATTTTTGTATATTTTTGGGGTCC
TAATTCAACAAACCAAAAAAACTGCCCTTCTTTCCTGCCAAATTCTCCACTAGGCAGCCTATCTATGGGCCTTCGGAGCGACCT
CTCGTAATTCCACTCATATTTCTGTCTTAAACAGCTTATGTCTTTTGAACCTTTTCCCATCTCAATATATCACTATAGTATAC
CTTGCCTCTGCGAATTTGGGGTCCCAATTCAACCAAACAATAAAAAATTGGGCCTTTCCTGCCAAATCTCCTAGCGCAGCTGCC
TAGGGCTCCCAAAGCGCCTCCGTAAGTTCACTAAAATTTTCTCTCTGACAGGCTTCTTAAATTTTTTTGAACTTTTTCACACCT
CTTCATAATATCCATATAGCTATACCTATTGAGCCATGAAACTGCGGGGCGTCCTAATTCCAACCCCGACAAAAAACATTGGCT
TTCCTCCAAACTCTCCCAGCACCGCTTTACCTAGAGGCTCCGAAGCGATCCTCGTAAGTTCACGTGACCATCATTTCTGTCTTA
AAAGCTGTAATTTTTTGAACCTTTTCCCATCTATAATATCATCATAGTATACTATGGCCCATGAATTGGGGGGTCCCAATTCAA
CCAAACAAAAAATTGGCCCTTTACCTGCCAAATCTCCCTAGGCAGCACCTAGGGCCCTCCTAACACGATTCGTAATTCACGTCA
TATTTCTCTCCAAACAAGCTTCTAATTTTTTTGAACTGCTTCTTCCCATCTCCAATAATATCATATAGGTATACTATCTGACCT
ATGACACTGGGTCTCAATTCAACAAACAATAAAAATTGGCCTTTCCCGCCAAATCTCCTAGGTCCGCTGCCGTAGGGCTCCTAA
CACGGATCTCGTAATTCACCTCATATTTCTCTCAAACAAGCCTCTAATTTTTTTGCGAACTTTTTCCCTCTCAATAAGTATCAT
ATAGGTATACTATGTGACCATGAATTGGGGTACAAATTCCAAGCAACAAAGAAAATTGCCTTCCTAGACAAAATCTCCTAGGAA
AGCTAACCTAAGGGCTGTCGAAGCGATCTTCGTAATTCCATCATATTTCTCTCTAACCAAGACTCTAATTTTTTTGAACCTTT
TCCCATCTCAAATAAATATTATAATTCCGGGCCTACTATTTGACCATGCAAGTTGGGTCCTTATTCAACCAAACAAAGAAAATT
TGACAATTCCTGCCAAATCTCCTAGGCTCTGCTACGGCTCCGAAGTCGATCTCTGTAATTCAACTATCATATTCTCTCTAACAA
GCTCTAATTTTTTTGACCTTTTACTCGTCTCCAATAATAATTCATGATTGCGCTACTATTCTGACCAGTGAATTGGGGTCCTT
ATTCCCTCACAAACACAATAAAAATGAGACCTTTCCTGCCAAAGCTCTCCAGTCAGCTGCCTAGGGCGTCCGAAGCGATCTCTA
ATTCACACTCATACTTTCCTCTAACAAGCCTCTAATTTTTTTGAACCTTTTCCCATCTCAATAATTCATATACGAGTTATAATA
TTGACCAATGAATCTTGGGGTCCTAATTCAGCCAGACAAAAAAATTGGCCTTTCCTGCCAAATCTCCCTAGGCAGGCTACTTGT
AGGGCTTCGGAAGCGATCTCCGTAATTTCACTCCATATTTCCTGTCCTAAAAGCCTCTTATTCTTTTGAAGCCTTTTCCATCTC
AGATAATATCATAAGGTAACTTTTGGCCTTCCGAATTGGGGTCCCAATTCAACAAACAAAAAAAATTGCCCTTTCCTCGCCAAA
TCTCCTAGGCAGCTTGCTAGTGGCTCCGAAGCGATCTCGTAATTCACGTCATATTTCCTCTCTGACAGGCTGTTCTTAATTTTT
TTGAACTTTTTCCCATCCTCATAAATCATAATAGCTATACTCATTGACCATGAATGGGGTCTAATTCAACCCCGACAAAAAAAT
TGGCCTTTTCCCGCCAAATCTCCAGGCAGCTTACCTTAGGGCTTCATCGAAGCGATCTTCCGTAATTCAACCTCATATTTCTGT
CATTAAAAGCTGTTAATTTTTTGACACCTTTTTCCCATCTCAATAATATCAAATATAGGTATACTATTGGCATCCAGAATTGGG
GTCCCAATTCCAACCAAAACAAAAAAATTGGCCTTTCCTGCCAAATCTCCTAGGCAGCACCCCTAGGCTCCTAAACGATCCTCG
TAATTCACTCATATTTCTCTCCAACAAGCTCTAATTTTTTGAACTTTTTCCCCATCTCAAATAATATCATAAGGTATACTATT
GAACCATGAATTGGGTCCTAATTCAACCAAACGAACAAAAATTGGCCTTTCCCGCCAAATCTCCTAGGCCGCCTGCCTACGGGG
CTCCTAAACGATCTCGTAATTCACTCATATTTCTCAAACAAGCTCTAATTTTTTTGAACTTATTTCCCATCTCAATAATATCA
TATAGGTACACTATTGACCATGAATGGTCATTCAACCACACAAAAAAATTGGCCTTCTTCCCTACAAAATCTCCCTAGGCAGCT
ACCTAGGGCTTCCGAAGCCCGATCTCGTAATTCCCTCCATATCTTCTGCTGTTAAAAGCTCTTATTTTTTGAACCTTTTCCCAT
CATCAATAATATCATATAGGTGATACTTGCTGGCCTTGAATTGGGGGTCCCCAACTTTAACCAATACAAAAAAAATTGGCCTTT
CCTGCCAAATCAACCTACAGGCAGCTGCCGTAGGGCTGCCGAAACGCGATCCCCGGTATTCACTTCATATTTCTCTCCCTGACC
AGGCTCTGAATTTTTTTGGAACTTTTTCTCCCATCCTCAATAAATATCTATATAGCTTATGAGCTATTCGACCAATGAAATGGG
GGTCCTAATTCAACAAACAAAAAAAATTGGCCTTTCCCGCCAAATCTCCTAAGGCACGACCTAAGGCTCCGACGATCGTCCGTA
ATTCACTCATATTTCCCTGTCTTAAAAGCTGTATTTTTTTTAAACCCTTTTCCCATCTCAATAATATCCATATAGGTATACTAT
TGGTTCATGAATGGGTCCCAATTTCAATCCAAACAAAAAAATTGGCCTTTCCTGCCAAATCTTCCATAGGCAGCTGCCTAGGCC
CTCCCGTAACGATCTCCTCATAATTCACTCATATTTCTCTCAAACAAGCTCTAATTTTTTTTTGAACTTTTTCCCATCCTCAAT
AATATTCATATAGGTATATCTATTCGAAACCATGAATGGGGTCCTAATTCAAACCAAACCAAAAAAACTCTGGCATTCCCGCCA
AATCTCGCTAGGCGCTGCCTACCGGGCCTTCCTAAGACGAGTTCTCCGTATTCACTCATTATTTTCTCTCAAACAGCTCTAATT

GCTTTTTGAACTTTTTCCCATCTTCAATAATAGCGATCCTCAGGTGAGTACCCTATCTGACCATGAATTGACGGCGTCCCAAAA
TTCCAACCAAACAAAAAATTCCTTTCCTACAAAATCCTCCCTAGGCAGCTAACCTAGGGCTTCGAAGCGATCTCCGTAATTCCT
CATATTATCTCTCTAACAAGCTCTAATTGTTTTTGAACCTTTCCCACTTCTCAATAATATTGATGATTGGGCTACTATTGATTC
CATGCGACTATTGGGGTCCTTATTGCAACCAAACAGAAAAAAATTGACCGTTCCTGCAAGGGTTCACTAGGCAGCTGCCTAGTCC
GAAAGCGATCTCCGTAATTCACTTCCATATTTCTCTCTAACAAGCTCTAATTTTATTGACCTTTTACCATAATCAATAATATTT
ATTGCGCCTACTATTGACCATGAATTGGGTGGCCGTTATTCTACAAACATAAAAATAGACCTTTCCTGCCAAATTCTCTAGGCA
GCCTGCCTAGGGCTTCCGAAAGCGACTCGTAATTCACATCATATTTCTCTCTAACGAAAGCCTAATTTTTTTGAACCTTTTCCC
CATCTCAATAATATCATATAGGTAGTACTATTGACCATGAATTGGGGTCCTAATTCACCAAAACAAAAAATTTGGCCTTTCCTG
CCAAAATCTCCTAAGGCACTACCTATAGGGCTCGGAGCGATCCGTAATTTCACTCATAGTTCCTGTCTTAAAAGCTCTAGTTTC
TTTTGAAACCTTTATCCCATCTCAATAATATCATATACGGTATACTCTTGGCTATGATTGGGTCAATTAAACAAACAAAAATTG
GCTTTCTGCCAAATCCCTAGGCAGCTGCGTAGCTCCGAACGATCTCGTAATCTACTCATATGT
>m141121_232159_42134_c100732402550000001823141405141507_s1_p0/65248/11611_19742
ATGTGTATTTAGTTTTGCTATTGAGGCTCTGATGTGTTAGGTTAGGAAGGTGGCTAATCGTGTATGTTGTTGTGTTCTCATTAT
TTTAGTTGGTCTATATGACTTGCTTTGAGTATATTGTTGATAATTGTCGATGACTTTGACATTATTATGTGGATTATATAGGCT
GTGGAGGTGCGTGTCGAGGAATGATCTAGTGAGGCGAAGGATGTGGTCTGTATAAAAGTTAATGGCGGGATTTTATACCTGGCT
CGGGTTGAGTTAGGGGTTATAATGTGTGTTATAATATTTAGGCGATATAATGATAGCTTTGAGGTGTTGTTTTGATCTTATGAC
AATTGATGGATATATTGGTGATTGAAGGTTTGTTTTTGTGTTGTTTTTTCTATCTTGTTGGTTGGGTTTTTTTTTTGGTTGGTT
TGGTGTGTTTTTGTTATTGTTTTTTTTTTTTTTTTGTTTGTGTTGGTGTTGGAGTTTTTTTTTTTGTTTTGTGGTTTTGGGTTT
AGTTTGGGTTTGGTTGGTTGGTGGGGGGTTGGTGTGGGATTCATTTGGTTTTGGATGGTGATGTTGTTTGGTGAAGCCAAGGAA
CGTATAAGTTGACCATGGAATTGGGTCCTCAATTCAACCAAACAAAAAAATGACTTTCAGCCAAATCCCTAGGCGCTGCCTAAGG
GCACCGAAGCGATCTCCGTAATTCACTCTTATTTCTCTCTAAAAGCTCTAATCTTTTTTGAACCTTTTCCCATCTCAATAATTA
TTATATAGGGCTATTTATTAGACCATGAATTGGGGTCCTAAATTCAACAAGACACCAAAAAAATTGACCTTTCAGGCCAAATTC
TCCTAGGCAGGCTGCCTTAGAGCCTCCGAAGCGATCTCGTAATTCACCTCATATTTCTCTCCTAACAAGCTCTATATTTTTGAC
CTTTTCCCATCTCAATAATATTAATAAGGCCTATTATTGACCATGAATTGGGGTCCTAATTCAACCAAAAAAAAAAATGGAACT
TTCAGGCCAAATCTCCTAGGCAGCTTGCCTAGGGCTCCAAGCGATCTTCGTAATTCTCTCATATTTCTCTCTAACAAGCTCAAA
TTTTTTGAACCTTTTCCCATCTCAATAACTATTATATAGGCTATTATGACCATGAATTTGGGTCCTAATTCAACACATAACAAA
AAAATTGTCCGTTCAGGCCAAATTCTCCTAGGCAGCTGCCTAGGGCCTCTGAAAGCGATCTCGTAATTCTCCATATTGTCTAAT
AACAAGCCCAAATTTTTGAAACCTTTTCCCATAATCAATAATATCTATATACAGGCAAATTATTGACCCATGAATTGAGTCCTA
ATTCAACCAACAAAAAAATTGACCTTTTCAAGCCAAATCTCCTAGGCACTTGAATAAGGCTCTGAACGATCTCGTAATTCAATC
AATAATTCTCCTCTAACCAAGCCTCTAATTTTTTTTATCCTTTTCCCACTCTCAATAATATTGATAAGGCTATTATTGACCCA
TGATTGGGTCAATTCAACCACACAAAAAAATTTGACCTTCAGCCAATCTCGTAGGCAGACTGCCTAGGGATCCGAAGCGAATCT
CGTAATTCACTCATATTCTCTCCCTAGCAAGCTCTAATTTTTTTGAACCTTTTCCATCCTCCAATAAATTTAGATAGGGCTATT
TATTGACCATGAAATTGGGGTCCTAATCCACACCAAACAAAAAAATTCACCTTTCAGGCCCAAATCTCCTCGGGAGCCTGCCTA
GGCTTCCGAAAAGGCGATCTCGTAATTCACCTTTTCCCTCTAACAAGCTCTAATTTTTTGAACCTGTTTGCCATCCTCAATAAT
ATTATATAAGGCTATTATTGACCATAGAATTAGGGTCCGTAATTCAACCAAACAAAAAAAATTGACCTTTCCAGGCCAAATCTC
CTAGGCAGCTGCCTAGGCCTCCCGAAAGCGGTTTCGTAATTCACTCATATTTCTCCTCCTAACAAAGCCTAATTTTTTTAATC
TTTCCCATCTCAATAATAATTAATATAATGGGCTATTATCTGACCATGATTGGGGTCCTAATTCAACCAAACAAAAAAATTGAC
CTTTCAGGCCAAATTCTCGCTAGGCAGCTGCCTAGGGCTCCGAAGCGATCTCAGTAATTCTCTCATATTTCCTCTAAACAAGCT
CAAATTTTTTGAACCTTTTCCCATCCAACTAATATTATATAGGGAAATTATTTACCATGAATCGAGTCCATAAATTCAACCAAA
CAAAAAAATTGACCTTTCAGGCCAAATCTCCCTGGCAGCTGCCTAAGGGCTAAACGAACGCGAAATCTCGTAATCACTCCATAT
TTACTCCGTAGCAAGCTCTAATTTTTTGAACTTTTCCCATCTCATAATATTATATAGGACTATAATTGACCTGAATTGGGGTCC
TAGATTCAACCAAACAAAAAAATTGACCTTTCAGGCCAAAATCTCCTAGGCAGCTGCCCTAGGGCTCCGAAGCGATTCTCGTAA
TTCACTCATTTATCTCCCCCCCCTTTCTCTAACAACGCTCTAATGTTTTGAACCTTTTCCCATCTCAATAATATTATATAGGCT
ATTAATTGACCATGGAATTGGGTCCTAATTCAACCAAAACAAAAAATTGACATTTTCAGGCCAAATCTCCTAGCTGCTAGGGAA
GGGGGAGGGGGGCTCCTACCATCGTTACTCTTATCCATACAAGCATACTATTTTTTTTGAATCTTTTTTCCCATCTCTAATAAT
ATATTAAAGGCTATTATTGGACACATTATAAGGAGAGTATTCACTAATTCAACCAACAAATGATACCTTTCATGCAAAATTCCT
AGGCACTGCCTAAGCATCATCATAATTACTCATATCTCACTCTAACAAGCCTCTAGATATTACACTTTCATATAACTCATAATA
TTTATAGGAAACTATTGACCGATGAATTGGGGTCCTAATTCAAACAAACAAAAAAAATTGACCTTTCAGGCCAATCTCCTAAAG
GACCACAGAGGCTCCGAAGCGATCTCAGTAATTCTTCCATATTTCTCTTTATAACACTCACTTTTTTTGAACCTTTTCCCATCT
CAATTATGCTCTCTCAACCACACACAGGGGAGAAGGAGGGTTTTTGGTGGGTCAAAAAAATTGAGCTTGTAAGAAATATGAGAG
TACGAGATTTCAGGAGACTTTCGAACCTGGCAGCTGCCCTAGGAGAATGGCCTGAAAGGTCAATTATTTTTGTTTCGCGTTGAA
TTAGGACCCCAATCAATGAATAATAGCCCTATATTTATGTGAGAATGGGAGTTCAAATTGGCTTGTAAGATTGATGAATTACGA
AGATCGCTTCGGAGCCCTAGGCAAGCGCTGGATAGGGCAGAAAGGCAATTTTTATATTGATACTATTATAGGTTAGGAGAGACC
CATTTTGGAACATATTGATCAATATACCCATATGTATTGGTTGAAATTAAATTATAGGTCAATGTGAGATGGGAAAAAGATTAC
AAAAAAATTAGAGCGGTTGTGATAGCAGAGGAGAAATATTACTATACGAGACAGTCGTTTAGGGCGCGCGCAGTTAGCACGAAT
TTGCCTGAATGTATCAGTTAACACAATTAGTATATACATATAGGACCCCAATTCATCGGTCAAGCTAATATATATAGGATGAAG
GAATCAACAATTAGAGCCTTGTTAGAGAGAAGAATAAGAGTTACTCATATCGGCCTAGGCGCTGTAGGAATTTTGGCACTTAGA
TCCATTTTTGGATATAATGGATATATTGCCACCATCATGGTCACTTGCTATAAGATTTTGAGTGGGAAAGAGTTCTTAGACTTG

CTACGATATGTGATCGATGCTTCGTAGCCTAGGCACTGCCTAGGAATCCAGAGTCAATTTTTGTTATTGGTAATTGCTCGTCTG
GTAGTTCCTTAATTTTTGGTGGACAGGTTCACACACACCCATACTTGAGACGACAATGAGACGATTACGGATCGTTCGAGCATT
AGGCAGCTGCTGGAGTCCTAGAAAGATCTTTTATGTTGGATTGAATTAAGGACCCCCAATATCATAGGTCAATAATAGCCTATA
TAATATAGCGAGCATGGGAAAAGATTAAAAACAAACTTAGAGCTTGTTATGGAAGAAATATGAGTGAATTAAACGAAACCGCTT
CGAGGCCCTAGGCAGCGCCTAGGAGATTTGGCCTGAAAGGTCAATTTTTTTTGTTTGGTTGAATTGGACCCTATTCTTGTCAAT
AACTAGCCTTATATAATATATTATTGAGAATGGCAAAAGGTTAAAAAAATTAGAGTGTTAAGAGAGAAATACAGAGTGAATACG
AGATCTGCTTTCGGCCGCCCTAGGCAGCTCCGAGGGAGATTTGGCCCTGAAGGTGAATTTTTTGTTTGGTTGAATTAGTACCCC
AATTCATGGTCAATAATAGCCCTATCTAATTTTATTGAGATGGGAAAAAGGTTCAAAAAAAATTAGAGCTTTGCTAGAGATGAA
ATATGAGTGAATTACGACGATCCTTCGGAGCCCTAGGCAGCTGGCCCTACCGAGATCTGGCCTGAAAGGTCAATTTTTTTGTGT
AGGTTGAATTAGGCCACCCCAATTCAATGGTCAATAAAGCCCTCCTATAATAATTATTCGGGATGGAAAGAGGATAAAAAAAAT
TAGAGCTTGTTAGAGAGATATGATTGAATTACGAGATCGCTTCAGAAGCCTTAATTCAGCTGCCTAAGGACGATTTGGCTTGAA
AGGTCAAATTTTTTTTGTTGTTATTAGGCACTCAAATTCCATGGTCAATAAATTGCCTATAAAATATTATTTGAGATGGGAAA
AGGTTCCAAAAAATTGGCTTGTTATAGAGAGAAAAGTATGGAAAAAAAAAAGACAAATACGAGACGCGTTCAGAGCCTAGGCAG
CTGCCTAGGAGAATTGGCCTGAAAGGACAATTTTTGTTTGTTTGGATTGAATTAGGCCCAAATTCATGGTACAATAATACCCCT
ATTAAATCATTATTGAGATGTGGAAAAGGTTCAAAAATTTGAGCTTGTTAAGAGAACTAATGAAGATTACGAGCATCCGCTTCG
AGCCCTAGGCAGCTGCCTAGAGATTTGCCCTGAAAGTTCCATTTTTTTTTTTGGTTGATTAGGACCCCCAATTCATGGTCAACT
AATAGCCCTATCATGATATTATTGAGATGGGAAAAGGTTCAAAATATAGAGCTTGGTTAGAGATGAAATATTGATTACCGAGAT
CGCTTTCGGATGCTCAGGCAGCTGCCGCTAGGAGAACATGTGCCTGAAAGGTCAATTTTTTTTGGGTTTGGTTGAAATTAGGAC
CACCAATTTCATGGTCCCAATAATAGCCCTGATATATGATTATTATTAATGCGGAAAAAAAGGTTCCAAAAAAATTAGAGCTTG
TTAGAGAGAAAATGAGTGAATTACGAGAATCGCTTCGGTGCCCCTAGGCAGCAGCCTAGGAGATTTGGGCCTGATAGGTCAATT
CTTTTTGTTTGGTTTGAATTAGGACCCCAATTCATGTCAATTATAGTACTAATATAATATTATATGAGATGGGAAAAAGGTTCA
AAAAAATTAGAGCTTGTTACGACGAAATATGAGTGAATTACGAGATCGCTTCGTAGCCTATGCGCTGGCTATGGAAGATTTGGC
CTGGAAGGTCTATTTTTTTGTTTTGTTGAAATTAGGACACGAAATTCATGGTCTATATTAATTGCCTATATATTTATTTATTTA
TCGAGATGGGAAATGGTTCAAAAAATTTGAGCTGTTAGAGAGAAATAATGAGAGGCTTACGGTTCGCTTCGGAGCCCTAGGCCA
GCTGCCTAGGAGATTTTGGCCTGAAAGGTCAATTTTTTGTTTGGTTGAATTTAGGACCCCAATTCATGGTCAATATATGCCTAT
ATAATATTTTGAAGATGGGAAAAGATTCAAAAAATAGAGCTTGTTAGAGAGAAATATGAGTTAATTACGAGATCGCTTAGGAGC
CCCCGAGGCGCGCCTAGGGATTTGGCCCTGAAATGTCAATTTGTTTGTTTGGTTGATATTTTAGCAACCCCAATTCATGTCAAT
ATATAGCCCTATATAAATATTTATTTGATGATGAAATAAGGTCAAAAAAAATTAGAGCTTGTTAGACGTAGATATGAGAATTAC
GAGATCGGCTTCGGAGCCCTGGGCAGCTACCTAGGAGATTTGGCCTGAAAGGTCAATTTTTTTGTTTGGTTGAATTAGACGCAA
ATTCATGGTCAATAATAGCCCTATATATATATTATTAAGATGGGAAAAGATCAAAAAAGTTAGAGCTTGTTAAGCAGAAATATG
AGTTAAATTACGAGATCCTTAGGAGCCCACAGCCTGCCTAGGAGATTTGGCCTGAAATGTCAATTTTTTTGTTTTGTTGAATTA
GACCCCCAATTCATGTAACTAATAGCCTCATAGAATATTATTGAAGATGGGAAAAAGGATAAAAAAAATAAGAGCTTGTTAGAG
AGAATTATGGATTGAATTACGAGATCGCTTCGGGCCTTATTCAGCTGCCTATGGAGATTATGGCTTGGAAAGGTCAATTTTTTT
GTTTGGTTGAATTAGGACTCGAATTCATGGTCAATAATTGCCCTATATAATATTATTGAGATGGGAAAGGTTAAAAAAATTTGG
GCCTTGTTAATAGAGAAAATATGAGAGAATTACGAGATCTGCTTCAGAGCCCTAGGCAGCTGCCTGGGAGAATTGGCTAAAAGG
CAATTTTTTTGTTTGGTTGTAATTAGGGACCCAATTCATGGTCTAATAGCCCTATATATATTATTGAGATGGAAAAAGGTTCAA
AAAAATTACCCCCTTGTTAGAGAAGATAATATGAGAGAATTACGAGATCGCTTCGGAGCCTGGCACTGCCCTGGAGATTTGGCC
TGAAAGTTTCTCTATTTTTTTTTTTTTTGGTTGAATTAGACCCCAATCATGGTCAATAATAGCCCTATATAATCATTATTGAGAT
GGGAAAAGGTTCAAAAATATTAGAGCTTGTTAGAGAGAAATAGGAGTGAGATTAACGGACCGCTTCGGGCTCTAGGCAGATGCC
TAGGAGAATTGCCTGAAAGTCAATTTTTTTGTTTGGTTGAATTAGGACCCAATTCATGTCAACTAATAGGCCCTATATATAATA
TTATTGAAGAATGGGAAAAGGTTCAAAAAATTAGAGCTTGTTAGAGAAGAAATATGAGCTGAATTACAGATCGGCTTCGGTGCC
GCTAGGCAGCAGCCTAGGAGATTGTTGGCCTTGAAAGTCAATTTTTTGTTTGGTTGAAAGGACCCCCAATTCTATCGGTCAATA
AAGCCCTATATAATATTATTGAGATGGGAAAAGTTAAAAAAATTAGAGTTGTTAGAGAGAAGATATGAGTGATTACGTGATCGC
TCGGAGCCCTAGGGAGCTGCCTACCCGGGATTTGGTCCGAAAGGTCAATTTTTTTGTTTGGTTGAATTAGGCCCCAATTCATGG
TCAATTATAGTCATATATATATTATTGAGATGGGAAAGTTCAAAAAAATTGAATTGTTACGGAGTTGAGTGAATTACGAAGAT
GCTTCGTAGCCCTAGCAGCTGCTAGGAATTTGGCCTGAAAGGTTTCAATTTTTTTGTTTGGTTGAATTAGGACCCCAATTCATG
GTCAATTATAGTCCATATAATATATGATTGAATGGGAAGGTTCAATAAAAATTAGAAGCTTGTTACGGAGAAAATATGAGTGAA
TTACGGAATCAGCTTCGGAGCCCTAGGCAGCTCCCAAGGAGATTTGGTCTGAAGGTGAATTTTTTTGTTTGTTGAATTAGGACC
CCAATTCTATGGTAAATAATAGCCTTCATATAATATTATTTTTGAGTTGTGCACAAAGTTTCAAAAAAAATAGAGCTTGTAGAA
GAGAATAAAGTGAATTACGAGATCGCTTCGGAGCCTAGGCAGCTGCCATAGGAGAATTGGCCTGAGGTCAATTTTTTTGTTTGG
TTGAATTAGGACCCAATTCATGGTCCAATAATAGCCTATTAGATATTATTGAGATGGGAAAAGATTC
>m141121_232159_42134_c100732402550000001823141405141507_s1_p0/68845/1513_6776
CACTAATATAAGGGCAAAAACTTTCTGCTGAAAATTGCCCATAAAAAAGGGGAATTTTTTTTCAAATGTTGACTTAGGGCACCG
TTTCAACCCTCCCAATATATTCATTTACAATGAGAATCTCTTGGGGAAAAATATCGAACCGAGTTGAAACACGAGGGTAATACT
ATTTCGGAGAAATTTAGTGAAATCTCCGAATTCCACACAAAAGGGGTTCCCCTTTCCAGAATCTTCGATTTTCTCTAATATGGG
TTTCGAGCAAACGATTGAAGGTGAAAAATAAAGAATAGCGAGGGTCATGGGCCAAAAGTTGTAGCCTATGTCTTAGGCTTCAGT
GTGTATAATATCTTCACCATATCTGAGGTGCGTAGAAGGTGCATATAGTCCAAATATACTGGAGATGTACTGATATAAGGCAAA
AACCTTGCGAATATTGCCAATAGAAAAAAGGGGAATTTTTTTTCAATGGTTGACTTAGGGCCTCCGTTTCACTCCAATCAGATT

CATTTGACCCATGAGATCTCTTGGGGAAAAATATCAAACAGTTGAACACGAGGGTAATACATTTCGGAGAAATTGTGAAATCTC
CAATTCACCAAGGGGGTTCCCCTTTCAGAAATCTTCGATTTCTCTATATAGGTCGAGCAACGATTTGAAAAGGTGAAAAATAAA
GAATATGCGAGGGTCATAAGCACAAAAGTTGTAAGACCTATGTCTTTAGGCTTTCAATGTGTATAATATCGTTCCCATAATCTG
AGGTGCGTAGAAGGTGATATAGTCAATATACTGAGAGATGTACTGATATAAGGCAAAAAAACTTGCCAAAATTGCCATAAAAAA
GGGGAATTTTTTTTTCAAATTTGCTTAGGGCACCGTTTCAACTCCAATAATTTCATTTACCAATAGATCATCTTGGGGAAAAAT
ATCGAACGAGTTGAAAAACTGAGGGTAAAACACTTTCGGAGAAATTAGTGAAATCTCCAATTCACCCAAAAGGGGTACCCTTTA
GAACATCTTCGATTTTCTCTAATAGTGCGATTCGAGCAACCGATTTGAAGGTGAAAACTAAAGAATATACGGGGGTCATGGGAC
AAAAAGTTGTAGCCAATGTCGTTAGGCTTCAATGTTAAAATTTCCGTGCCAAATCAGAGGTGGCGTAGAGGGTGATATACAGTC
GATATACTTCGGAGAATGTACTAATACTATATGGCAAAAACTTGCGAAAATTGCCATAAGAAAAGGGGAATTTTTTTCAATGTT
GACTTAGGGCACCGTTTCAACTCCAATAGATTCATTTACCAATGAGATCTCTTGGGAAAATCAATCGAACGAGTTGAACACGAG
GGAATACATTTCGGAGAAATCTAGTGAAATCTCCAATTCACCAAAGGGGTTCCCCTTTCAGAAATCTTCGATTTCTCTAATATG
GGTTCGAGCAAGACGATTTGGGTGAAATAAAGAATATGGCGAGGGGTCATGGGCCAAAAAGTTGTAGCCCTAATGTCTTAGGCT
TCATGTGTATAATATCTTCCATATATGAGGTGCGTAGAAGGTGATATAGTCAATATACTCAGAATGTACTGATATAAGGCAAAA
ACTTGCGAATATTGCCATAAAAACAGGGGAATCTTTTTTTCAAATGTTGACTTAGGGCTCCGGTTTCAACTCCAATAGATTCAT
TTAACCCTGAGATCTTCTTGGGAAAAAAATATCAAAACACGAGTTGAACACGAGGGTAAAAATACATTTCGGAGAAATTTAGTGA
AATCTCCAATTCACCAAAAGGGTTGCACCCTTTCAGAAATCTTCGATTTCTCTAATATGGGTTCGAGCAACGATTTGACAGGTG
AAAAAATAAAGAATATGGCGAGGGGTCAATGGGCACTAAAAAGTCTGTACCCTATGTCTTAGGCTTTCAAATGGTATAATTCGT
TCCATATCTGAGGTGCCCGTAGAAGGTGATATGTCAATATACTGAGAGATGTACATAATATAAGGCAAAAACTGCGAAAATTGC
CCATAAAAAAAGGGGAATTTTTTTCAAAATGTTGACTTTAGGGCCACCGTTTCAACTCCAATAGATTCCCATATTACCATGAGA
ATCTCTTTGGGGAAAAATATCGAACGAGTTGAACACGAGGGTATAACATTTCGGAGAAATTAGTGAAATCTCCAATTTCACCAA
AGGGGTACCCGTCTTCAGAAATCTTCATTTCTCTAAAATGGATTCGAGCAACGATTTGAAGGTGAAAACTAAAAAGAATATACG
CGGGTCCATGGGCACAAAAGTTGTAGCCCAATGTCTTAGGCTTCAATGGTGTAAAATTTCGTGCAAATCAAGGTGCGTAGAGGG
TGACATACAGTCGATATATGAGAAGATGTACTAATATGAAGGCCAAAAAACTTGCGAAAATTGCCATAAAAAGGGGAATTTTTT
TTTCTAAATGTTGACTTAGGGCTCCGTTTCAAACTCCAATAGATTCATTTTACCACATGAGAATCATCTTGGGGAAAAATATCA
ATAACGAGTTAACACCGAGGGGTAAATACATTTTCGGAGAAAATTAGTGAAATCTCCAAATTCACCAATAGCGGGTACCCCCCT
TTCTCAGAAATCTTCGATTTCTCTAATATGGATTCGAGCAACGATTTGAAGGTGAAAACAATAAAGAATATACGGGTCATGGGC
AGCAAAAGTTGTAGCCCAATGTCTTAGGGCTTCAATGTGGTAAAATTTCGTGCAAATCAGAGGTCGCGTAAGGGTGATACACAG
TCGATTAAATGAGAGATGTATCTAATATAGAGGCAAAAACTTGGCGAAAATTGCCATAAAAAAGGGGAATTTTTTTTCCAAATG
TTGACTTAGGGCTCCGTTTCAACTCCAATAGATTCATATTACCCAATGACCATCTCTTGGGGAAAAATATCAACACGAGTTGAA
CACGAGGGTAATAAGTTTCGGAGAAAATTAGTGAAATCTCCAATTCACCCAAAGGTTCCCTTTCAGAAATCTTCGATTTTCCTA
ATATTGGGTTCGAGCAACGATTTGAAGGGTGAAAAATAAGAATATGCGAGGGTCATTGGGCACAAAGTTGTAGCCCTATGTCTT
AGGCTTCAATGTTATAATATCTTCCATATCTGAGGTGGCGTAGAAGGTGATAATAGTCAACTATACTGAGAGAGTACTGATAAT
AAGGCCAATAAACTTGCCAAAATTGCCATAAAAAAGGGGAATTTTTTTTCAAATGTTTGACTTAGGGCACCGTTTCAACTCCCA
ATAGATTTCATTTACCAATGAGATTCTTGGGGAAAAATATCGACGAGTTGAACAACGAGGGTAAATACATTTCGGAGAAATTAG
TGAAACTCCAATTCACCAAAGGGGTTTCCCCCTTTCGAAATCTTCGATTTCTCTAATATGGGTTTAGAGCAACGAATTTGAAGT
GAAAAAATAAAATATGCGAGGGTCATGGCACAAAAAGTTTGTAGCCCTATGTCGTTTAGGCTTCAATGTGTATAATATCTTCCA
TATCCTGAGGTGCTTTAGAAGGTGATAGTAGTCAATATATGAAGATGTACTGATATAAAGGCAAAAACGTTGCGAAAATTGCCA
TTAAAAAAGGGGAATGTTTTTTTCAAATGTTGAACCTTAGGTGCACCGTTTCCAACTCAATAGAATTCATTTACCAATGAGATC
TCTTGGGGAAAAATATCGAACGAAGTTGAACACGAGGGTAAATGACATTTGGAGAAATTAGTGAAATCTCCAATTCACCAAAGG
GGTACCTTTTCAGAAAAATCTTCGATTTCTCCCTAATAATGGCAGTTCGAGCAATACGATTTGAAAGGTGAAAACTAAAGAATC
ACGTACGGGGAGTATCGGGCCACAAAAGTTGTAGCCCAATGTCCTTGACAGGCTTCAATGTGTAAAATTTCGTGCAAATCAGGA
CGGTGGGTACGAGGGGTGATAAGCAGTCGATATACTCGCCTGAGAGATGTACTAATATCGACATGGTCTTAAACAGAACTTGCG
AAAATTGCCATAAAAAGGGGGAATTTTTTTCAATGTTTGACTTAGGATCCGGGCACCGTTTCAACCTCCAATAGATTCATTTA
CCATGAAAAACTCCTTGGGGAAAAATATACTCAGAAGCGAGTTGAACAGCGAGGGGTAATTCACATTTCGGCAGAAAATTAGTG
ACAATCATCCCATTCACCAAGAGCGGAGTACCCCTTTCCAGAAAATCTTCCGATTTTCTCTAATCACTGGCACTTCCGAGCAAA
GCGATTTGAAGGTGAAAACTAAAGAATAATACGGGGGTGTCATGGGGCCCCCCACGAAAAGTTGTAAGCCCGCAATGTCTTAGG
CTTCAATGTGTAAAATTTCGTGCCAAATCAGCAGGTGCGTAAAGAGGGTCGAATACAGATCGATATAACACCTGGAGAGATGTT
ACTAAATACAAGGTCACAAAACTTCGAAAATTGCCAAAAACACCAAGGGGAAATTTGTTTTTCCCAAATGTTGACTTAGGGCAC
CGTTTCCACTCCAATACGATTCATTTCCCAATGAGACCTTCTTGGGGAAGGAACAAATCGAACGAGTTGAACCACCGAGGGTGA
ATACATTTCGTGAGAAATTAGTGAAATCTCCAATTCACCAAAAGGGGTACCCCTTTCAGAGAATCTCGATTTTCTAAATACGGA
TTCGAGCACGATTTGGAGGGAAAAACTGAAAGAATATGACCGCGGGGGGCATGGGCACAAAAGTTGTAGCCCATGTCTAGGCTC
AAGTGTGTAACACTAACATTTCCGTGCAATCAGAGGTGCGTAGAGGCGGTGATACAGCTGCGAATATAGCGCTGAGAGATGTAT
CTAATATATAAGGCAAAAACTTGCGAAAATTGCCATAAAAACAGGGAATTTTTTTTCCCAAATGTTTGACTTTAGGAGACCCGT
TTCAAACTCCAAATAATTCATGTTACAATGAGAGTCTCATTGGGGGAACAAATATCGAACGAGTTGAACACGAGGGTAATCATT
TCGGAGAAATTACGTGAAATCTCCCAATTCACCGCAAAAGGGTACCCCTTTCAGAAATCTTCGTTTCTCTACGATATGGATTCC
GAGGCAACGATTTGAAAGGTGAAACTAAAAGTAATATAAGCGGGGGTCATCGGGCACCAAAAAGTTGTAAAGGCCCAATGTCTT
AGGCTTCAATGTGTAAAATTTCGTGCAACATCCAGGGCCGCGTAGAGGGTGATAC
>m141121_232159_42134_c100732402550000001823141405141507_s1_p0/68937/3910_9373

CTCCTGTCCTCCGTTCCCAAGTAAAATAAGCTACGGGTGCCTGTTGTGTATAAGACCACAAGTAGGGGGTCCTTTTTATTTTTT
AAAAAAAATGTTGAATTAAGGCCTTCCCGTTTCCGACTCCAATATTTCATTACCCATGAAGATCTCTTGGGAAAAAATATCTGA
ACGAGTTGAACACGAGGGTAATACAATTATCGGAGAAATTAAGTGAAATCTCCAATTCCAACCAAAGGGGTAACCCCTTTTCCA
GAAAATCTTCCCGATTCTCTATTATGGATTACAGCAATGTTCTGATAGTGAATATATAAAGGAATAATGCGAGGGTTCAGGGGC
ACAAAAGTTTTAGCCCTATGTCTTAGGGTTCTATGTATAAAATTTCGAGGAAATCCGAGGGGTGTATAGGGTGATACAGTCAAA
AATTCTGAGTAGAATGTACGAATATAAGTAAAAAACTTTGTGGAAATTGCCACTCGAAAAGGGGTTGTTTTTTTTTTAAATGT
TGAATTAAGGCTCCGTTTCGACTCCAATATAATCATTTAACCCATGAGGATCTCTTGGTGGAAAAATATCGAAGCGAGTTGAGA
ACGAGGGTAATACATTTCGGAGAAATTAGTGAAATCCTCCCAATTATCAACCCAAAAGGGGTACCCTTTTACAGAAATCTTCGA
TTTCTCTATTATGGATTACAGCAATGTTTTGATATGTGAAATAATAAAGAATATGCAAGGGTCATGGCCACAAAAAAATTTTTA
TCCTATGTCTTAGGGTTCTATGTATAAATTTCGTGGAAATGCCGAGGTGTGGTAATAAGGCTGATACAGTCAAAATACTGAGAG
ATGTAACGAATATAAGGAAAAACTTGTGGAAATTCCCCTCGAAAAGGGGATGTTTTTTTTTAAATGTTGAATTAAGGCTCCGTT
TTCGACTCCAATATATTCATTTACCCATGAGCATATCTTGGGGAAAAAATATCGAACGAGTTGAACACGAGGGTAAATACATTTC
GGACCCAAATTGAGTTGAAATCTCAATTCCACCAAAGGGGTACCCTTTCCAGAAATCCGTTCGATTTCTCTATTAATGGATTCA
GCAAACTGTTTTGAGTAGTGGAATAATAAGGAAATAATGCGAGGATCATGGGCAGAAAAAGTTTAGCCCTATGTCCTGTAGGG
TTCTTATGTAGTAAAATGGTTCGTGGAAATCCGAGGTGTGTATAGGCTGATACAGTCAAAATAACTGAGAGATGTACGAATATA
AGGAAAAAACTTGTGGAAATTTCCCTCGAAAGAGGGGTTTGATTTTTTTTTTAAAAATGTTGAATTAAGGCCTCCGTTTCGACT
CCAATTATATTCATTTACCCATGAGATATCTTGGGGAAAAAATATCGAACGAGTTGGAACGACGAGGGTAATACATTATCGGAGA
AATTAGTGAAATCTCCAATTCACCAAAGGGGTGAACCCCTTTCCAGAAATCTTCGATTTCTCTAATAGTAGGATTCGAGAATGT
TCTGATATGTGAATATATAAGGAATATGCCAAGGGTTCATGGGCACAAACGTTTTATCCCTATGTCTTATGAGGTTCTATGTAT
AAAATTTCGTGCCCAAATCCCGAGGTGTGTAAGTAGTGGTGATACCAGTCCAAATACTGAGAGATGTACGAATATAAGGAAAAA
ACTTGTGGAAATTTCTCATCGAAAAGGGGTTGTTTTTTTAAAGTGTTGAATTAAGGCTCCGTTTCCTGACATCCCAATATATTC
CCATTTTACTCCATGAGATTCTTGGGGAAAAATATCGAACGAGTTGAACACAGGTAATTACATTTCCGGAGAAATTAGTGAAATC
TCCCAATTCACCCAAAGGGGGTACCCCTTTTCCAGAATCTTCGATTTCTATATTATGGATTACAGAATGTTTTGATAGTGAATA
ATAAAAGAATATGCAAGGGTCATGGGCAACATAAAATTTTAGCCCTATGTCTTAGGGTTCTATGTATAAAATATCGTGGAAAAT
CGAGGTGTGGGGTATAGGCTGATACAGTCCAAAATACTGTAAGATGGTACGAATATAAGGAAAAAACTGTGGAAATTTCCCCTC
GAAAAGGGGATGTTTTTTTTTAAATGTTGAATTAAGGCTCCGTTCTCGACTCCAATATATTCATTTACCCATGGATTTGCTCTT
GGGAAACAATATCGAACGAGTTGAACACGAGGGTAAATACATTTTCGGAGAAATTAAGTAGAAATCTCCAATTCCACCAAAGGG
TACCCCTTTCAGAATCTTACGATTTCCTTATTATGGATTACAGCAATGTTTTGATAGTCCGAATAATAAGGAATACTCGAGGGT
CATGGGCACAAAAGTTTTTAGCCCTATGTCTTAGGGTTCTATGTATAAAATTTCGAAGGATAATCCGAGGGTGTATAGGGTGAT
ACAGTCAATAATTCTGAGAGATGTAAAAATATAAGTAAAAAACTTGCTGGAAATTGGCCCTCGAAAAGGGGATTGTTTTTTTTT
AAATGATTGAATTAAGGCTCCCTTTCGAATCCAATATATTCAATTTACACCATGAGAACTCATTGGGGAAAAAATATCGAACGGT
TGAAACATGAGGGTAATACATTTCCCGGAGTAAATTAGTGAAATCTCCAATTTCAACCAAAGGGGTACCCCTTTTTCAGAAATC
TTCGATTTCCCTATTATGGATTAAACAGCAATTGTTTTTTGATAGTGAATTAATAATAGAATATGCCGAGGGTCATGGGCTACA
AAAATCTTAGCCCCTATGTCTTAGGGTTCTATGTATAAATTTCCGTCCAATATCCGAGGTGTGGTATAGGGTGATACAGTCAAA
ATATCTAAGAATGTACGCAATATAAGGAAAAACTTGTTGCAAAATTTCCCTCGAAAGGGGATGTTTTTTTTAAATGTTGAATTA
GGCTCCGTTTCGACTCCCAATGATATTCATTTACCAATCGAGATCCTCCTTGGGGAAAAAATATCGAACGGTCTAAACACCGAG
GGTAATACATTGTCCGGAGAAACTTAGTGAAATCCTCCAATGTCACCAAAGGGGTACCCCTTTTCAGAAATCTTCGATTTCTTC
TAAAGTGGATTCGAGCATGTTCTGATAGTGAATAATAAGGATTGCAAGGGTCAATGGGCACAAACGTTTTATCCCTATGTCTTA
GGGTTTCTATGTATAAATTTCGTGGCAATATCCGAGGTGTGTATAGGGTGATACAGTCCAAATACCTGAGAGATAGTACGAATA
TAAGGAAAAATACTTGGTGGAAAATTTCCCTCGAAAAGGGGGTTGTTTTTTTTTTAAATGTTGAATTAAGGCTCCGTTTCGACT
CCCAATTATATTCATTTACCCATGAGATCTCTTGGGAAAAAAATATCGATACCCGAAGTTGAACACGAGGGTAATACATTTCGGA
GAAATAAAGTGAAATCTCCAATTCACCCAAAGGGGTACCCTTTTCACGAAATCCTTCGATTTCTCTATTATGGATTACCAGCCA
ATGTTCTGATAGTGAATAATAAGGAATGCGAGGGTCAACAGGGGCACAAAAGTTTTAGCCCTATGTCTTAAGGGTTCTATGTAT
AAAATTTTCGAGGAAATCCGAGGGGTGTATAGGGTGATACAGTCAAAATTCTGAGAGATGTAAGAATATAAGTAAAAAACTTGT
GGAAAATTGCCCTCGAAAAGGGGTTGTTTTTGTGTTTAAATGTTGAATTAGGCTCCGTTTCCGATCTCCAATATATTCATTTA
CACCATGAGATCATCTTGGGGAAAAAATATCGACGCAGTTGAGAACGAGGGTAATACATTTCGGAAGAAATTAGTGGAAGATCAT
CAACAATTTCCACCAAAAGGGTACCCTTTTCAAAAATCTTCGATTTCTCTATTTAATGGATTCAGCAATGTTTTTGATAGTGAA
TAATAAAGAATATGCAGGGTCATGGGCAACAAAAATTTTATCCCTAAATGTCTTAGGGTTTCTATGTTATAAAATTTCGTGGAA
ATCCGAGGTGTGTATAGGCCATGATACATCAAAAATACTGAGAATTACCGAATATAAAGGAAAAACTTGTGGAAATTTCCCTCG
AAAAGGGATGTTTTTTTTAAGATGTTGATTAAAGCCTCCGTTTTTCGACTCCAAATATATTCATTTACCCATGAGATATCTTGG
GGAAAAAATATCGAACAGAGTTGAACGACGAGGGTAATACATTTCGACAAATTAGTGAAAATTCCAATTCACCAAAGGGGTACAC
CTTTCAGAAATAATTCGATTCCCTCTAATTATGGATTCCAAAGCAATGTTTATTGATAGTGAATAATAAAGGAATTGTCGAGGA
TCATGGGCAGATAAGTTTTAGCCCTAATGTCTATAGGGGTTCTATGATAATAAAATTTCGTGGAAATCCGGTGTGTATAGGCTG
ATACACGTCAAAATACTGAGAGATGTACGAATATAAGGAAAAAACTTGTGGAAATTATCCTCGACAAAGCGGGTTGTTTATTTT
TTTAAAACATGTTGAATTAAGCGCTCCGTTTTCAGACTCCAATATAGTTCAATTTTACCCATGAGATAATCTTTTGGGGAAAAT
AATCTGAACGAGTTGAACACGGGGTAATAACTTTCGGAAGAAATTAGGGAATCTCCAATTCACAAAGGGGTACCCCCTTTTCAG
AAAATCTTCGATTCTCCTCTAATATTGGATCCGAGCAATGTTCCTGATAGTGAATATAAGGAATACGCAAGGGTCATGGCAAAC
AAACGTTGTTATCGCTAATGATCTTAGGGTTCCTATGTATAGAATTTCGTGCAAATCCGACGTGTGTATAGGGTGATACAAGTC

CCAAATACTGAGAGATGTAGAATAATAAGGAAAAACTTGTGGTAAATTTCCCCTCGAAAAAGGGGTTGTTTTTTTTAAAAAAT
GTTGAATTAAGGCTCCGTTTCGACTCCAAATATAATTCATTTATACCATAGCAGATCTCTTGGGGAAAACAATATCGAACGAGT
TGACACGGGGTAATACATTTCGGAGAAAATTAGTGAAATCATCCAATTCCACACAAAGGGTACCCTTTTCAGAAAATCTTACGA
TTTCTCATATTCATGGATTACAGCAATGTTTTGTAGTGAAAATAATAAGAATAAATGCAAGGTCCATCAGGGCACAAAAATTTT
TCAGCCCTACTGTCTTTAGGGTTCCCTTATGTATAAAATATCGTGGAAATCCGAGGTGTGTATAGGCCTCGATACAAGTTCAAA
ATATGAGAGATGTACGCAATATAGGAAAAAACTTGTGGAAATTTCCCTCGAAAGGGGATTGTTTCTTTTTCAAATGTTGAATT
AAGGGGGGGGGCGCTCCGTTTCCGACCTCCCAAATAATTCATATATACCCATGAGGATCTCTCGGGGAAAAATAATCGACGAGT
TGA
>m141121_232159_42134_c100732402550000001823141405141507_s1_p0/70785/0_6819
AAGAAAAGAGATCACTAGATAAGTTACTTAACAATTCTAGACATGTAAGAAAAAAAAAAAAAATATGTGTGCAATTATATGATAA
AAAATCGATGAAATGTGAGATGAGCAAAACACAAAATAAAATGCAATGCTTTATAATCATAAAAGGGATTGATATATAAATAAC
AATTGACCCAACAAACACCACGCGAATAATGGTCAAAGAATTAGAGATCCACACAATGAATCGATAAAAACAAAAACTTGAATA
TGATATAAAATAAAAAAAAAAAAGAACAAAAAAATAACGTATGAACAAAAGGAGATTACCCCCCCTCAGATAAGAAAGGTAGAA
TAAACAATCAAAAACCCTAAACACAAAATCGTACCAAGAAAATAAAAGTACATATAAATTTATAAGCTAAAACACAAGTTCTCA
AAGAAACCAAACCCAAAATGCGATCAGAAAAACATAGAGAATCTAAACGAGTTTACACAAGTAAACAAGGAAAAAGATACAAAA
AATGCGTATAAAAACTTGAATCATATAAAACTCCTCAAGTAAAAGAATTACAACGTAAATAATGATTCATTGACGTCCATCTCA
TTATATAGGCTCAAGAATGTGCATGCAGAATGGGCCAATGGGCCTTACATAATTTACGTAAACAAACTGAATAAAAAATAAAAA
AATAATTGATTGTCACATAAATATCTTGGCTTTTACATAGAATATATAGAGACATATATATAGATATGTGTTCCATCGAGGCAG
CATTTGGTCGTGACCATGACGACTAGTTTACTTTAAAAGTTGTTCTAACCTAGTTTTCCCTAAACTCGTTTAGAATAATACAGT
CCACATTATTGTTATCCTGGGATCATAGGTTCGATCAGTCCTTCGTAATAATGAGTTAATCTGTGCCTTCCACATGGGCCATTT
TTATGCATGGTCAAGCACATGTTGATTAATTAGTGGAAAACATTTGCCCAAGTTTATTATGATGCAACCAACATTTAAATGACT
TACCTCCGACAACAATCCTAACCTGTCAAATCAGTTGTAACTTTTATTTCCTTGAAAGGTAAATAAATCATGATTTTACGGTTT
CTAAAGGGAAAACTAATGACTCATTGCACCTCTTCAGATGCTTATAAACCAACCATTTTCAATCATGACTTTTCACCTTCCTAC
TCATTATAAAATATCCCTATATCACTTTCCTCCTTATTTTTTACCATTAACCATTTCTCTTTCCTCAAGAAATTCAAGCTTTCC
TCAAGCAAATACATACCAATCATCCGAGATGACTTCTAGATGTTTCAAGCCTTTTGAAGTGATTTCCTCAGTAATATTCTACCA
AGATAAGGTATTTTACTACATTCTTCCTATTTTTTAGCATTTTTCACGGATAAATTGCCATGATATTTCTTGTTTTTTACTTCC
ATATTGAAACATCGACTAATGCTTTGAAAAACTGAATACTTTTTGGTGAAAAATAATGTGTAATTCCGAGTCATGAAAGGTATT
TGGTAGTGTTTAGTGATACAAATAACATTAGATATATTGTACTCATGGAATCATCGAACGACTTGTATTTTGGGCATAAAATCG
AAGTAAAGAATTCGATTTTGGGGATTTTAGAGAGAGAAATACATGGGTTTTTCCCTCCCCTGTAATGAATGTGAAAAACTTTAT
CATTTTCCACTTTTTGAACGTTTTCCTCCTCTGTGACGTAGAGTTTAATCTGTTTTTCTTCCAAATATTTCTCGTCGAATAAAG
CCTTTACTTTTATCACGAGCTGTCATTAAATTATCTCTTAGTCTTACTTATTATGTGGCGTAAGAATTTCACTCCCCTTTTTAA
TTCTCAGTAATTCAAGACAAGAATGGTTTAGGTTGTGTTCTTAATGCAGTCGGGAAAGGTGATTGCATGTACATCACGTCAGTT
GAAGACTATGAACATAAGATACCCCACTCATGATCCTGAGGATTTGGCAGCAGTGGTCTTGGCATTATAGGTTATGGAGGATCA
GGAGGCCTGTTGAACCTAAGAAATTGGTCAAACGGTATGCATGTACGTTATACGTCTCTTTTGATACAAAATAAAATATAAATT
ATAATAACAGAGTACAAAGTATCTAACACAAACACAACAACAAATGTTATCGCCAATCGTCCTTCAGGTTGGTGCCCATTTGGC
TATGAAGTTTGTTAATGCTGGCTTTCATCGTAGTCCTCAGGATGAATAAAGTGATTCAAAACTGTCCTAACTCCACAGCCCATT
TGAGTAAGCCATTCCGAGGCCTCTGGCTTCTAAATAATTGTCGTAGCGGTTGATTAGTTAGGACTCGGATCGAGTGAGCTTAGA
AGTAATGGAACCAGCTTCCGAGAAGCGATTACTAAACAGCTAAGGCTAGCTTTTCAAGAAAGGGGGATACCTAGACTCCGCACC
CTGCTAGCCTTTTACTATAATAAAGTATACAGGGTGTTGTACCTGTTCTCCTGACTTTCACCAAAGTAGCACTATTTTCATGCT
CAGTGATTGCCAAATTTATGAATAACACTTCAACCGCTTTTTCTAGGACGGGAGGATTGATGAGATGCTCCTTCAATGCTTGGA
ATAGACTATATACTCACATTCTCAGTCGATTGGATATTTTGGCTGTCCATAAAGTAAGTTAAAGAAGGGGACACATTTGTCTAT
AGAACTTCGAATATGAATATGAGAGAGCAGCTCTCTGTCCAGGAGGGGTTCCGCCGAAATCACCTAAAGTGATAAGCCGATGAG
CAATCTACCACCAGCAGAACTCCTGTCATAGCACAGTCTCGCTGCCTAGGGGTATCCCCCAACCGTGACCAGATTAGTCTAATA
GTTCCTACCTGGCCTAGGCATTCTCTTTTAACCCAAATAATTACTTGGGAAAAACATGTCAAGGTCCTATGACGTGAGCTTCAT
CTTCTCAAGGGAATGATTTGTAGAATGAATATCTAAACTGAGCTTCCCGTATCCACCAAGCATATCTTCACTTGAGCATTTGCG
ATTTGAATGGAAAGGACCATAGGTCACTGTGGGAGATACCGTCATGTGCTTAGCGTCATCCTTCGTAAAAGTGAGAGTCACTTT
CATACTTCAACTTTATGAGAGACATGCTCCTTGACTGCCTGCAATGGGAGGATGTAGCGTCCCAGAAATCAGATTAGCAGTAGT
AGAAGCAGCGCGTAGTGTAGATAAGTACAGTATAGGTAGACAGTAGTAGCTAAGTAATAGTAGTAGCAGTAGTGCCGCAGCAGT
AGCAGTAGCAGACAGACAACAACGTAGTAGCAAGCAGATAAGTAAGTAGTAGATAGTAGCAGCAGTAGTAGTAGCAGCAGCAGT
AAGCAGCAATAGCAGTAGCAGTAAGCATAATAGTAGCAGTAGTAGGAGTAGTAACACTAGTAAGAAGAAGTAGTAAATATAGAG
TAGTAGCAGTATAGTAGTAGCAGTCGTAGCAGCAGTAGCAAGCTGCAGCGCTGCAGTAGTAGTAGTAGATAGTAGTAGTAGAGT
GTAGTAGATAGTAAGTAGTAGTAGTAGTAGTAGATAGTGTATAGTGTAGATGTGTAGTAGATAGTAGATAAGTAGTAGTAGTGT
GTGTAGTAGTAGTAGAATAGTAGTAGTAGCAAGCAACAGTAGCTTTGTAGTAGTAGCTTTTAGTAGTAGACTTTGTAGATAGTG
TAGTAGTAGATAGATAAGTAGCTTGTAGTAGTAGTGCTAGTAAGATAGCAAGCAGCAGCAAGTATAGTAGCAGTAGTAGTAGCT
ATAGCAGTGTGATAGCAGCAGTAGTAGAAGTAGGAGTAGCAGCAGCAGTAGTAGCAGACAGCCAGGCAAAGCAGTAGAAGTAGT
AGTAGTAGCATAAGTAGTAGACAGTAGTAGTAGCAGTAGTAGTAGCAGCAAGTAGTAGATAGTGAAGTAGTAGACAGCAAAGTG
TAGCAAAGCGCAGTGTAGGTGTTAGAGATAAGTAGGTAGTGGTGATGGTAGTGGTAGTAAGAATGAGGAGTAGTAGCAGCAGTA
CCAGTAGCAGAAGTAGCAGTAGTAGCAGTAGCATATAGCAGTAAGTAGCAGTTGCGATAGCGTAGCAGTAGAGTAGTGCTTAGC

AGTAGCAGTAGCAGCGCAGCAGGAGCCAGTAGCAGTAGCAGTAGCAGATAGCAGTAGAACAGATATTAGGATAGCAGTAGTAGA
TAGAACAGTAGCAGTAGCAATAGTAGTAGCAATAGCCAGATAACAGTAGACAGTAAGGCAGCAGTAGCAAGTAGTGTAGTAGTG
TAGTAGGTAGAAATAGTAAAGCAGGCAGCAAGGCAGTAGCAGTAGTAGAAGATAGTCATATAGCAGTGCGTAGTAGTAGTAGTA
GTAGCAGCAGCAGCAGCAGTAGTAGTAGTAGTGTAGCGATAGTAGTAGCAGTAGTAGTAGTAGTAGTAGAAGTAGTGCAGTAGT
ACAAATAGTAGCAGTAGCCAATAGTAAGCAGTAGTAGTAGTAGTATGCAGTAAAGTAGCACTAGCAGTAGTAGCAGTAGTAGTA
GTAGATAAAGTAGTAGTTAGTTAGTAGTTAGTACAGTAGTAAGCAGCAGCAGCAGTAGTTAGCAGTAGTAGCAGTTAGTAAGAT
AAGTAGTAGTAAGATACAGCAGCAGCAGCAATAGTACAGTAAGTAGCAGTAGTAGTTAGTAGCGTGTGCTGATAGCAGTAGTAG
TAGATAGTAGCACTAGTAAATTAGCAGTAGCAGTAGTAGTAGAAATAGCAGCAGTAGCAGCAACAGCAAGTAGCGCAGCCGCAG
CAGAGCAACAGTAGTAGATAGTAGTAGCGGTAGTAGTAGAAGTACGCAGTAGTAGCGATTAGTAGAATAAGTAGTAGCAGTAGT
AGAAGTAAAGCAGTAGTAGCAATTGTAAGTAAGATGTAGTAGCTGTAGTAGCAGTAGTAAGTAGTAGCAGATAGTAAGAATAGT
AGCAGTATGCAGTAGCAGTAGTATATAGAGCAGAAGTGCAGTAGTAGAAGTAGTAAGTAACATAGTAAGTAGTAAGTAGTAGTA
GATAGTAGCAAGTAGTAACAATTGTAGTAAGTAGTGAGTAACTGTAGTAGAGTAGAGTCGTATCAGTAGTAGTCGTAGACAGTA
GTAGATAGTAGTAGCAGTAGTAGTAGAATAGTAAGCAAGAGTAGTGTAAGTAACAGTAGTAGCAGTAGTAGTAGTAGTAAGTAG
CGTAGTAGCAGTAGCAGTATTATTAGCAGTAGTAAGAAGTAGTAGTAATTAGCAGTAGAGTAGAAGTTAGCAGATAGTAAGTAG
CAGTAGTAGGTAAGTGATAGTAGTAAGTAGGAGCAGCAGCACAGCAGCATGTAGCAGCAGCAGCGTAGTAGTAGGTAGTATGTA
GTAGACCAGTATTATTAGCAGATAGTAGATACGCAGTATTAGCAGCAGCAGCAGCAGTAGCAGTAGTAGTAGTAGAAGCAGAGC
AGCAGCAGTAGTAGTAAGTAGAAGCAGTAGAGTAGTAGCCAGCAGTGGTAGCAGTAGTAGTAGAAGTAGCAGTAGTAGCAATAG
TAGTGTAGTCGTAGTAGCAGTAGTAAGCCAGTAGTAGAAGAAGATAGCAGTAGTAGCAGTAGCAGTAGGAGTAGCAAGTAGCAG
TAGGCGTAGCAGTAGCAGTAGAAGTAAGAAGTAGCAAGCAGTAGCAGTAGCAGTAGCAGTAGCAAGATAGTAGTAGTAATAGTA
GTAGTAGTAGTACGTAGTAGAATAGTAGTAGTAGTATAGATAGTAGAGTAGTAGTGTAGCGTAGTAGTAGCAGTATTAAATAGT
AGCTAGCAGTAGCAGTATTATTCGCAGTAGAATAGCAAGCAGTAGTAGTAAGTAAGTAGTAGTGTAGCAGCAAGCAAGAGCAGT
AGCAGTAGTAGAGTAGAAGCAGCGCAGCAGTAGTAGCCAGTAGTAGGAGTTAGGAGTAGCAGTAGTAGTATAGCAGTAGCAGTA
GTAGCAGTGTAGAGAAGTAGCAGTAGTAGCAGTAGCAGTAGCAGTAGCAAGTAAAGTAGCAATAGCAGTAGCAGTAGCAGTGAA
GCAGATAGCAGTAGCAGTAGAGTAGCAGTAGCAGTAGCAGTAGCAGTAGCAGTAGCAGTAGCAGTACAAGCAGCAGGCAACAGC
AGCAGCAGCAGCATGCAGCAGCAGTAGCAGCAGTAGTAGCAATAGTAGTAGTAGAAGTCAGTAGAAGCACGTAGTAGCAGTAGT
ATGTAGCTAGTAGTAGAAGCAGTAGTAGCAGCATCAGTAGCAAGTAGCAGTATTAAGCGTAGTGCAGGAGTAGGAGTAGTAGCA
GTAGCAGTAGCAATAGCAATAGTAGTAGTAGTAGTGTAATAGTAGTTGTAGTAGTAGCAGATAGTAGCATGCAATGCAGTAGGC
AATCAATACAGTAGTAGCGTTGGCAGTAGTAGCAGTAGCAGCAGTAGCAGTAGTAGCAGTAGCAGCAGTAGTAGAGTTGCAGCG
TAGTAGCAGTAGCAGCAGTAGCTGTACGCAGTAGCATAGCAGCAGTAGCAGTCTTGCAGCAATAGCAGATAGTAGCAATAGTAG
CTAGTAGAATTTTTTTTAGTGGATAATTTGATTCAAATCGGGATTTTAGTTAGAAAGTCGTAGAATAGTTATGGTTTTTATA
AGTTTAACCTATGATTTAAAAAAATATTAATTTTAACATAAGGTTTGATTAAATATAGTTGGTCTAAGAATATTATTTATTATA
CCTAAGGTTTGATAGAATAAATTAAGACGTGACACTTGTCACAAGCATGTTTATTAAGGATTTAAGTATTTTTGATTAATTGT
TTAATTAAAGAAAAG
>m141121_232159_42134_c100732402550000001823141405141507_s1_p0/80462/8042_15061
TTCGTCGAGCTGAATTATATTGGATCTCTCAGGTATACGTGAGGCAGCAATTGTCGAAGTAAAATTAATAATACTGCTTTCCCG
ACGAGACAGAGAACCGAATAATTCGATATATTTTGGAGATTTCCTCAAGATATCCATGACTTCTTATTATTCAAAATTTGAGAA
ATGTTAGGTTGAGATAACCATTAATATTCATAAAATCTACAGGATATATCGATTCGTAAATCTTATTTTTCTATCTGACTCATG
CACATAGACCTAGGATCAAACGCTCTTCGATATATCAAGAATCGCTTTCAAATATCTGCGAGATTGAAATACCACTCTCTTGCA
CTAGTGTATCGCAAGGAAATACATTCGCACAAATAATCCAACCTAACGGAGCAGCACAATCAGAAAAAGCGATTCGGTATTCAT
ACAGTTTACACTAGAAAATGCACACACCACGACCATACGCTAAGAATTCCGAATTTTTCATCCTCATTGATCATTATATTCCTA
CGAGCTCGTTACTGTCAATCGGACACACAAACAATAAACATCTGAACCGAAGATAGATACGTTACTACATTTATAGGTGCGAAT
TTTGCCGATACCGGAACAATTTATAAAAAATACGTAAAGAACTCACGAATGCACTAATAATCCTTACAAGACTTATTAGACACT
GCATACCGCTATCACTTTAATCAAGAAATCTACATTGCAATAACACATCATGTCCATTGACATCTCACGCCCAAAGAAAAAAAA
AAGTCTACACCATATCCTAAACACTCAAAAAAATAATTAAAATCCCAATACCAGAAAAAAATCCGGGACATGTAGAATAAATGG
CGGTTCCGAAGGGATGAAAGTGAGATAAAATAGGGACACAGATTCCAGCACAATAAGAAGACATCATTCGATATCTATGAACCC
ACACAAAAAAACATACAAAAATATTATATGATTTCGTTATAACTATTCCATAGACCGGCACAGGTGGGGGTAAAACAGTTTACC
ACAAACATACCAACGCATTTTGAATCAGAGAGAGATTTCAGAAATGGAGATACTCATTCACCCCCTCATCATGGCGGGTCTATA
ACCAGAGCCGGATCTAGGTGTACCCTCATAAGGGATTTGCCTTTCTATTGATTCCTACAGATTGGATCAAAACAATTCTTGATG
AGGATCCTTATATAACAACTCCAGGGACTCAGTAGTTCACGGGACTAAAAGCAAGGCTTAATCTCCTTCTCTGTATCTCTCAGG
TGTTCCCTCCACTGAACCTCTCCTCTATTTTTTATGCAATAGAAAAATTGAATCTTCAGATAATTTATGCGAAGGATAGAAAAA
GAAAAATAAATGCGGTCCCAGTTTGACCATTTCATTCCTGCGGGAATGATTTGGAAGATCCAAACCCAAAATCATATCGTCTTT
GTCCTGTGTAGATGAATCAGCCCACCCCAAACATACGCAGGCGCATGGCGCTCAAAGGTGGGACGGCATGGCAGTCAAGCTGTC
TTCAACCTGAAGCCTTTCTAGCAAAATATGGTTACCCACTGTAATTCTTCTCCTCCAGAGAAACTGTATTGGCACCGACTGGCG
CTAGCAAAGACTCTTGATTGCTGCACTGCAGCCCAGTCAAAGATCCCCGAAATATAACACCCTATTGGGGTAAGCATAGAGAAC
ATGTATTGGAATGCGTTCTTTGTGTGCAATCTAGGCCGATCCGATCGCAACTTCGAAATTAAATTCAAGATCAAATAGGAAACC
CGAATACATACCTTGAGGATTCATAGAACTCACATACTGAAATATATGATCCAACATTTCTCAAATTTGAAAAAGAGTCAGAAG
AAATGGTTCGATCCCTACTCTTCATTCCTTCCTTTGTTTCGCTTTCTCGGAACACCGAGAGTACTTGCTTCCATCCATGGACGG
ATCCTAAATGCATATTAGATAACCAAAGGTCCATGGGAACAAGAATTTACAGGACATTATTGAACACTTTTCATTCTCTCTCGC

GGAGCACAAGAACAGTCTCTTGCGGCCCAGATTCCTTTTTTGGGGCCTTCTCCCCAATGTTTCCAAGTAAGTGGTTCGATCGAT
TACGTATTTAATAATATTCGATTGATTGGTCTGAGGTTTATCGACAGAAATGGAAAAGGATGTTTGGTTCTAAGTCACTTCCTT
CTCGACCCCTTCTTTTTGGGTGCAAAGTTTTGCTTTCTCTCATTTTGGTCTTAACTCACTCCTTTTTCTTCTCCTTGTGAGTTT
CGGGAATGATCCCCATTCATAGGTCTGTGAGTCCCCCACTCTCACATTGAATTTGAAAGGCCCGACGAGAGATCCTGACAGCTG
CATACATGTTTGTTGTTCGAATCAATCAGGTCTTATCAAAAGGAGATCAGTTCATTTAAAAACAATGGAAACCCTTTTTACCTC
TGGATGATCATTCTACTAAATCTCCACAAATAAATCGAAATTTCTGTGTATCGCAATGGGGAGACCAACTAATCCGACCTCGAT
TTTTGTTCCATCCGATGGCACCTAAGTTAGGTGATTGTGAAGTTGCGAGTCATGTATACCATACTAAGCTAAATACACTAAGCA
GTACTTGTGTGAGTATAAGTTCGGATGGAATCAAATTATCTTCCAAACTGATATCCACCGATCAAATCAAAGTCACTGACATTA
TGAACATCCGAGGTGAAACGCATTAATCATAACGAAGGCCATTACAATATGATAATCTCTGGTATACATCATATTTTACTATTA
AAGCAAACATAGAGACGATACATTCCTTGAATAATACTGACATCTACATCTGCTTGCATAAATGCTATAGTAACATAGAGGATT
TCCTTTGTGTATGTGGACAAAAGGCGCCGTATATCAATAATTAATATGATTTTCTACGTATGAGAGTCCTCTCCTCATACTATT
CTTGTTCATTCGCACAAAATATTTTCTTTGCGCGGCGGTAAAAAAAAATATGCTATTTTTGGAGAGAGATACTATTTCACCAAT
CGAGTTAAGGTGTCTAACATATATCACATTACCATTATCTCATTAACGATTTTCCACAAAAGCCTGGTGACTGAAAGGTATAAC
TTGTAACAAATCTTTCCATTTTCCAATTACGATCCGACTCCTCCGCGGTCTCGTATATCGTGGAGAGAACTATATTTATTCTCC
AGACCCTCTCGCCCGACAATTTCTGGATAGCTCTACACAAGGGGGACAATAGTCAATTTTGAAAGAACTTATTGTCAACCTGAT
TTCAGATATCTAATCTTTCTCCCCAGAGAGAGGAAACTTGCAATCAGTATCTCAATTTCAATTCAAACACTTGGTTTTGTTCAT
CACGCTTCCATGTTTCTGAGAAACTTATTATACCGGATGTCAGATTACACAGAGGGAAAAAAAACGGTGTGTCTTTGTCCTAAA
GAAATGTCTTGAGAAGGCAGTTATGTTATAATAGTGAACCTTATGCGAACGAGATAGGCTGCTGCTTTTTCAACTCTCTCAAAA
AATGGAATATTATTCCAAACATATGGGATGCCATGGTTCCTTACTTCGACAGGCGACTACAAATATCTAAATTTGATATTTTTA
GATATCTTTTTCAGACTATTGCCGATACAAAGTAGCAGCCCAAATTTTGTATCCATGGTTTCAATGATATTATGCATGGATTAG
ATATTAGTGATATCATCATAGGGATGGAATTCTTGAGTAAAAAATGTCTGTCTTCCCAGATGGAACTCTGATAAGTGAGAATTT
CGAGTAAAGGGGTATGTTTACATAACCTCTTCCTTCCTGTCCAAAGAAAATGATTCATCGAATAATGAGTCACCATAATGATGA
TGGCTGGAGCACATCTGATGATCGCAAATGTTCGGGTTCGGGAGATCCATCTAGGCTTCAATCCTTTTACCTTCTTCTCTGTTG
CTGGATATGCTCTCGTTCGTAAACATCTTCTTTTTTGTTTTCGAGCCTATAGTGAGTTACAGACAGAGTTCGAAAAGGGTCCAT
AATCTTTGATGATTCACTCATAGCTATCAGATTGAGTTCGAAAACATCTGGAATAGGGGTATCCTGGTCTGACGTGCAGAATTC
TTCTGGTTAAGGAGATCTCTCTTCTAGTTGCTCCGGGAACAATTTAGGCAGCCAACGTCCATAAGGAGAGAATTTATCAGGGTG
GTCTTGCTCTCTGGAGGCAAACATGCTATGGGGTGGCGGTCCCACTTATGGGGTCAAATCAATACGTTCTAAGAAGAAATTTTG
AATATCATTCTCATCCGATCATCCTCTTCCGATTTCCATAAAGTATCCCCAACACCCATGCACAATGCGAATCAGTCTTTTTCA
CGAAGTAAAGTACGAGACATCTAAGTCATACAAGTAGAGAGAGAACCTATTTTCGTTATTTGATAAGAAAAAGAAAAGCGGCAA
CAGTGATTGGATTGAATGAAGGGAAAATAGAAATCCTGGGTCTGTCGAACCCAAGTGATTGGAATTGATGATATTACAGAAAGA
GAGCTCTTAGGTTCAGTTCTCCGCCTTACACGACAGCTAAAAGGATTGATCAAATTCTATTTGGTCTGACTCATAGTTGGATCA
TTTATCAAAGAATGACTCTGGGTTATCAATATGATGAACAACCCGGGAGCAATTCTACTTACGATACTTAGTTGATCATTCATA
ATGAAAGTCTGCTAATGAATTATGAGTTCAATACATCCTGTTTAGCAGAAAGACGGATATTCCTTTCTCATTATAGCATCACTT
ATTCACAAACTCCTGTGGGGCTAATAGTTTTCCATTTCCCATCTCATGGAAAACACATTTTCGCTCGCTTAGCCCTATCCCTCT
CTAGGGTAATTTTAGTGATAGGTTCTATAGGAACTGGACGATCCTATTTGGTTCCAATACCTAGCGACAAACTCCTAACTGTTC
CCTTTCATTACAGTATTTCTGAACAAAGTTCCTGGAATAACATAGCCTAAGGGTTTTCTTAATTGATTGATAGTGACGATATTC
CGGATTGATGTGACGATATCGACGTGACTTTGTATACGGCAGGCTGAGCCTCTAACTATTGATGGAATGCGCCTAACTATATGG
GATACGTGAGTGCCGGAAATGAGACGTTTTTATAGTCACCCTTCAATTCGAATTAGCAAAAGCAATGTCCCTTGCATAATACTG
ATTCCAAAGACATTCATGGTGATCCTGGATAGTGAATGGTCGATTACCTTATCCTCCGGTCTACTCTAATATGGAACTTATGGT
CTCCAGGGATGATCGAAGAGATGTTGACCTAGAAATGAACTATCTTGTTATTGCCTTCGACTCATATTCCCAAAAAGGTGGCAT
CGCCGATCTAATAGCTCGAATAATTAAATACATGGCATTAAGATACGAAGGCTTTCTTTATTCCCACAACAACGAAAGCACTTT
TTCACTCTTTATATACTAGGGGATTGCCACTTGGAAAAGAAGAATGTTCCATACTAAATGGATTCGGGTCCATAACCATGGGTG
TCCAATGTAGAGGGATCCTGAGCATTCTCACCAATGAGGCCCTATCGATTATATTACACAAGAAGAAATCAGTGTATAGAGAGA
GCATCATAATAATGAGTATTAGTCTGCTCTTCATACGACAAACTGGGATTTGCGATCCAGGTAAGCTTCCGCGCGCTTGGACGC
TCATGGGATCCTTTTCTGGATCAGAGTAGGAAGGGCTGTATGCACAACAATGTCACTTCTAAGTAAATTGCTCCCGTAGATCTA
ATCTGCTATCTTATATGCAAGAAGAAATCATGTACACGAAGGGGATTCCCTTTTTTTTAAATTTGTACAGAATGGTACTTAGAA
CTTGGAACGAGCAATCTCCTCTCAAACAACAACGAAGCCCACCAACTAGCTGGCCGTAGAACGTACGACCGTTGCCATAACCTA
CACACACACATAAACTTCTTGCAAAAAAATACCCACCAAAACACCATAAATCTCTCTCACTACCTAATGCCAATAGAGATAAAG
AAATAACTATACTCCGTCACACCTCCTGACTTCGTTTAGGATTGTAGGTAGCATTCCATATTCTAGCGGCGTAGCTGCCCCTTG
TGATTTCTCACCTCCAGCCATGGGCACGGGACCATGGGCCTAGGCCGATGGGCGAGACCCTGGGCCCGAGGCATGGAGGAGGCC
ATGGGGCCCGAGGCCATGGAGGAGGCCAATGGGCGAGGCCATGGAGGAGGCATGGGAACGAGGACATGGAGGAGGCCTGGGCGA
GGACCAAATGGAGGAAGGCCATGGGCCGAGGCCATGGAGGAGGCCATGGCGAGGCCATGAGGCAGGCCCATGGGGCCGAGGCCA
TGGAGGAGACCATGAAGGCCCGAGGGCCAGTGGAAGGAGGCCATGGGGCCGAGGCCATGGAGGAGGCCATGGGGAGGCCATGGG
GCCGAGGCATGGAGGAGCCATGGAGTAGCCATGGGACGAGGCCCATCGGGCCAGGCCGTGAAGGAGCCATGGGGGCGAGGCCAT
GGAGGAGGGCCATGGATGAGGCCATGGGCCCGATGCATGGAGGAGGCATGGGGCGAGGCCATGGCAGGAGCTCGGGGCCGACGG
CATGGAGGAGGCATGGGCCGAGGCCATGGAGGAGGCTATGGGCGGCATGGAGGAGGCCATGGGGCGAGGCCATGGAGGAAAGG
CCAGGGCCCGAGGCCATGGGAGGAAGGCAATGCCTAGGCCAGGAGGAGGCCATGGGCCTAAGGCCCATGGAGGAGGCCATGGAG
GAGGCCATGGGGCCGAGGCCATGGAGGAGGCCATGGAAGTGAGGCCCCCTGGGCGAGGATGGGGGAGGCCATGGGGCCGAGGCC

AATGGAGGAGTCTTATGGCCGAGGCCCATGGGCCCGAGGCAATGGGTGGAGGCCATCGAGGAGGCCAATGGGGCCGAGGCCATG
GAGGGCCATGGGGCCAGGCGCATGGAGGAGGCCATGGGCGAGGCCCA
>m141121_232159_42134_c100732402550000001823141405141507_s1_p0/80860/8021_15368
CCAGTGTACATGATTCCGAACAAGCTCATCAAATCCAACTCACTCCAACTTATCTCACTTCTAAGCAGCACACAGTAAAAGGAA
CTTGAGCCAAGTGCTGTTGCTTCGCTTGGCCGAGGAACCACAATCATCTTCTTCAAAGACTTCCCTAAGCAACAGACATCATGG
GAAGTGTGATGTCGACACCCCATCGTCGACCAGCAAAACCAACTACACCCAGCCCTCCTCGCAGTGAATTTTCTGTTGTTCCTA
CTTATTTTACTTTCTCTTTTCTCTAGGAACACCGACAACCCATGCTTCGTGGTTTAAAATACACCGCCCACCTATAGTAGTTCT
CCTTTTATCTTATAATCTACATCCGACGCAGCTGAACTCGCTCCGCGGGTGTAAAGCACATTCCACTTCTAGCTGGATTATACC
AAAATCTTTTATAATTATACCAACCCATGTCTTCTGTATGACTGAATAAGCACTAACATTTATAATCTTCTGAATTGCCTCCAA
AAAATTTGACAAAAATATCCTATATGTTGCTGCGCATTGCTTTCCCTTCTATTTTTGCTCATGCGTCTTTCCTATGGTTCATAT
GCCTTATGTTACTGCCTTATACGCATTGCCCCAAAGGAATCCGACGGAGCACTAAGGTGCTTGGTTACTTCGCCATAAGACCGG
CTACCTCCGTTCTGAGACATACTACTCGTATCACCTTGCAATAGTTTAATATACAAACAACCACAGTAACCAGACCAAGATACT
TGTTATGAAACAAATACCAATCACTGCACACGAATCCCTAGCTCTGCAACATTCCTTTTCATCTTCTCGCTGAGAAAAACTGGA
ACAAGCAGAATTCCTGCCTAATACAAGTGACATAATGGTTTACCACTTATTAACTCTACCCCAAAAACTGACGACTAGCCCTTC
TCAAAAATTGTAACCTTAAAAAATGTCTTAAACTCACAAGGCACCGCACTTGACAAAAGACCAATGTTCACTGCTGAGCTACGC
TGCTGCGTACGGTGTTCTCCCATTCTCAGAATAGCGAGAATGAATTATCTAATTTACAGTCGGCAAAATTTGATACGTTTCTTG
TCCGCGTGGAAGAATTCTTCGAACTTGACTAAGGGCCTTCCCAATTCGGGTCCCAAGGACCTCCCAGCTGCCTGGTCACGAGCT
GTTCAAAAAAATCAATTGTAAGTGACTACATCACCAACAACGGAATCAACTTTTGCTCGCCATCCAGCCTAGTAAGTTAAAATAT
GAGCGACTTCTTTCTACTGTGACCCTACTGCTCGTAGTTAGCTTGTTCTCCCGACATTCTTCAAACAACGTCTAGACAAGTTTC
TCCTGCCGAACAATTGTTGTTTGTATTCTATATCTTCAGCCTCCTATGCAATGGTGATCTAACTCGCTACTAGGGGCATCGGCT
TCCTCACCATCCCACGTGCAACGGGTACAATGGAGTAATCGGCCAATCTGTTGTCTCGATGTTGTTGTTCTATATGACTCGCAG
CGCACGCCCGAGTAAATTCAGGAAATTCTTCTGCAAACTTTTTTTGGCCTTGTGTGTGGAGTATTTTCTTTTAGGTCCTAATAC
GTTTTTGTTTATTAGCTTGACTTGTCTTGTTTTCTTGCTGATGAGCACCTGAAGAGAAGCTTTTGTATTCCATGCTCATTCTCA
CCGGAAAAGCGGGCTGCGCCAGACGCGTCGCTATCAAACTGCGCTGCGTGTCGTTGGTCTGCCACTGCTTACAACGTAATCCAC
TCACGACAAAGTTATGTTTGTGACCACAAATTTTTCAATAAACAGTTCCTTCGATGTAGATGTTGGAGTTTCGGCAAACGCATT
CTCCAGTTTTCGCTTCTATTCATTTACGCTACAATTATAGACAGCTACAATTGCATAGCCTTCTTTACACCACCCTTCCTGTTC
GTAAACCATCAACATATATTCCCAAATACGAATAATGGCAGCAGACTTTCATCCCTACTTGTTGAACCATTAGGGCTCTCCGAG
GCTATCTTGAAGATCCATGCCGCCGCTATGAAACGAGTGTTACCGTATTTCCCTTTGCAATCTCCATTTCCCATGCGCTGGCCA
AAAAATACGCCCTTGCCTTCAAAACTTTTTTGGAAATCGATCTGCCCCTCAATGAGCACCCAGAACCCTGTCATGTACTTCTTG
TAGCGAGACCTGGCCTTTTCATGTGACACACCATACCGCAACTGTAGGAACCCCCCGTCGTATAGATTCCCATCACCTATATAA
ATCGAGCAAATGTCGTCTGAAAACATCTGCTTTTCCAATCAATCGGACATAGCAAATATCTCGTTGTTTCGAGGTATTGTACTA
AGTGGGATCATCCAACGTATGACGCGTGTTAATACAACTGGTTAGAAGTCTTTTTCTTGTAGTACAGCCTAAAGACGTTTGTTA
GAGTTTCAACCGGAACAAATGTTGTACAGTGTATTCGAAAGCGCTATCTTTCCCCCCTTTTGCCTATGGCTACGAACTGTTGTC
CAACGCATCTGCATTTGAGCTTTGTGGTCACAAGTACTCTGTTGTAAAGTATAACTTTACACTGAGGTGTGCGCCACAAGTCTT
TAATTTGCTAGATAGGCGACCATCCTTCATCTCGGGCCCTAATATTCTCCTAGTATGTTACTTCACCCCAATTGTAAATCACTA
ATAAATATTCAAGAGATTGACAGTTTGACGTTATATGCTAGTCGTAGTTCTCCGTGCAGGACTACTCTTGTACTATGCTCTCAT
TAATAGATAAAGTAGAAGCTCAAATTAGAGCACAAATGAATACTGGTGTCCTCATCTGAGTGATCAGAATTAAACATGCTCTGA
AGTCTCGTGTCCATTTGATGGACCATCAACCAACAAAAACTTCAATGGGTTTTAATTCTTCTGATCCTATTTGTGACTGCTTGC
TCTGTGATTGGCAGTATTGTGATAGTTCTGCTACAAAGCAGTTCAGTGCTTCCCTTCGTGTCCTGATTCGTGCTGGTAAGTAAT
GCTCAAAATTCCCCAAACTCTTCAGCCTTACTTAAGTAGTCAACCAATTGCTCCGTCAGGTTTTCTTCAACACCCCTATCTTAA
ACGGCTAGCTAGTCTATATCTCGGTTTCATCGTACTACTGAAAAAAAGGAACCAACGTCTGAGATGACAGCAAACAAACAGCTA
AGCAACTTTTTTTATGCTGTTGGATAAACCTATGATTCTGCTCCTACTAGCTTGCTCACATAATATACTCACATTTGGATCTTT
AATCATTTTCTCCAATTATCGAAAGACTCAATAGCACATACTTCGTGACTGCTAATTAATGAACAAATCCGCCTCAATTGTTTG
TAGATGACGTTCGTGCCATGATGTCTATTTGATAGCCTGGACACCCTGCTTGCATTCTCATCCATAACTCAAATTTTCCTTACT
GCTCTCAATAGATTAATTAAATGGCAAACACCACTTGTCTGTTGATTTTGAAATGAAATCACTAAGAGCAATAATTCTCCTTGT
AAACACTTTCGAACCAGTCCTTAATTCTGGTGGGTGACTTTCCTATCTTGATTCAGAGCTCTCGATTTTTTTCCCTGGTAGCCT
TATACAACGCTTGCGAGTTTAAACTGGACATCCATGAAAAATTTCCCATAACCCTAACCCAAAGGAACATTTACGAGGATTACT
TCATCCGATACTTTCTTCGACCAAGGGGGGGGGCGGGGGGGGGGGGGGGGGGGGGGAGGCGGGGGGGGGGGGGGGGGGGGGG
GGGAGGGGGGGGGAGGGGGGGGGGGGGGGGGCGGGGGGGGGGGGGGGGGGGCGGGGGGGGGGGTTGGGGGGGGGCGGGGGGC
GGGGGGGGGGGGGGGGGGGCGGGGCGGGGGGGGGGGGGGGGGGGGGGGGGGCGGGGGGGGGGCGGGGGGGGGGGGGGCGGGGGG
GTGGGGCGGGGGGGGGGGGGGGGGGGGGCGGGGGGCGGTGGGGCGGGGGCGGGGGGGGGGCGGGGGGGCGGGTGGGGGGGCGGG
GGGGGGGGGGGGGCGGGGGGGCGGGGGGGGGGGGGGGGCGGGGGGGGGGGGGGGGGGGGGGGGGGGGGGGCGGGGGGGGGGGG
GGGCGGGGGGAGGGTGGGGGGGGCGGGGGGGCGGGGGGGGGCGGGGAGCGGGGGGGGCGGGGCGGGGGGGGGGGGTTGACCCCA
GCACTCCTGGAGGTCAGCCTAACATTCCAGCTCAGTTTTCTTTGACTTTACCTAGCATGTCATCGACACTGACCTCACTTCTAT
ATTGTTGCTCGCATCTGCGGTCCCTTAAACATGTAATTACTAAGCCACTGGTAATTGCTCCAAACACATTTTTTTCCAGACAAA
ATGGTATTACCCTGTACGGCAGATACAGTCTCTGGTGCTCAGCTCCAACGCTAGTATGTTCCTCGTTTGTCGGATGGCAGTACT
CTATTTCCCTGCTGTAACCCAGAATAAGCATCCATGAAAAGGATAGTATTTGTGACCTGTGAGCATCACCACTGGCGTTCCGAT
TCCTTCTGGTACGGACCAACAATCCTTCCGGGCAGGATTCTATTGAGGCCCTTGAAATCCAACTGTCCTCCCACTGCTTACCAT

TTAGTGTCTTTGGGAACAGGATGCCGATTGTGAACCCATGACTGATAAAATGCTACCCCAGAACTGAAAGACCGAATTTCCTTA
GCCTTTTCAAGACTTCTTCTTGTTAAGCTATCAGAGCCTACCCATCCATTAAAAATCCAGCCCTTCTCCTTTGTTGTACTGTTG
GGAGTCTTTTGTCTAGCTGTGCGTCCGCATGAGCGTATGACATAAATCCCTCCAGCCAGCCATCGGATCAATTCCAACTCACCC
TCCTTGATGCAGACAGGCACAAAAACTTCTTGATCTTCTTTCTCAAAATCCACCCAACTCATTTTCTAACGTCTGTTTTCTAAG
TTTCTACACGGAACTTTCACAACTCCTTGGTCAGTTGTTCTTGTCTCTCTACACCCGGATCTCCTCCTAGATCTTCCACAGGCT
CCAATCTACACTTCCACACAAATACTCCCAAAGCAGGACCATTTCAACTTTCCTCAACCTTCGGGGCGACAACCCAATTTTCAC
CTTGTAATGCTGAAGAGTTATAAAGCATTCCCTACCCCTCCCTCTCTGGTTTCCAAAAAAGTACATACCTCACACTCCTCGAAT
CAGTTTAGATCTTCATGGGCTACATGCATACCGCAGATGTACCTACTCATCAGCTCCCCACCGCACCAATACAGTCCTTCGCAG
AGGAACTGCAGTTTTCCATCCAAGACCAATCGAAACTCACTGACACAACCGGCTATGACGCATTGTCCCATCTCTGCATGCGAG
CAGCTCCTGCCACTGGACGAGCCCACTCGAATCGATCATCGTCACGTGCAAGCCCTTCTCCCCAGTCCAACAATCCATAACTGC
GTCTGGTTGCAACAGGCCTCTCAACATCTACCGCACAACAGCTTCACTTCTCTCTACAGGATGATTTATATAGTATCGGTCCGA
CTGGAACTCCCGTTCAACTCAGCACAAACCCTTTTCACCATCATCGTTAAGCCTATTGCCGTCATCCATGATCAGGAGGTCCGT
GTGTGGGAACTGAACGTGATGTGAGTCGTACTTCACTCAATGTATAATACCAGACCCTTCTCTACTCACGAGCTTTCTCTATGA
TTGCCTCCTCAATTGTTAGCATTCGGTAATGTCTTAGCTCTGAACGTATGTCATGGCATATACTTTACCTCTTTGTGCTTAAAC
ACATGTCCGCCAAGAAATTTGTGCTCCCACAAAAAATTATAAGTCGCGGTGGCCCCGATCAGGGAAGCGCGATTAGTCGGAGGT
CCAGCTGTTGAGAGTCTATTGTCTTCCCGTTGCCAGTCGCAGCAGCCTAATTTGTTGGGGATCAACCCCGGTTCTCCGGATAAC
TCATTCCTCAATTACCTGCTGTACATATATATAAACTTGTCTCAAGTCTCATCATTAATTATTACACCTCATCTGTCTATCAAT
GCCATCGAGCTCATTGTGCCAGAGCTTCAAACTTTGCTACCTATGCCCTTTTTTGAAATGTACTTCCTGACTGGGAGCTGGTCT
ATCTGTTGAAGGTTGGTTTGAATGTGAACTTCGACAGTGCGAGCATAATGTAAGACATTCCAAATTGTGCTATAATGGCTTCC
GTGCCCTTGATGTAGCATGTAGGCGTCGCCAGGCAATACTTGCTTTCAATTATGTTACATATCCCGTGCGTTTCAATCTTGATA
TATGATGTATCCATCATGCTGAATTACTTAGATTCAAACTGAGTTCATACACACTAGATAAACATGCGTTGAATGAACTCAAAG
CCTACTATGTGGAGTGCGTCTCTTGTTAATAAAGCATCGATTTGGTAATTGTGGCATGTTGAGGGCATAAATATGGCGATTATG
TTGTGTTGTTCTAAGAACACGCAATATTCGTTGGCGAATGCTCTTGAATCTGTGGCCAAGACACTCTTTCTAAGGGAGCGGAAT
AACAAAAAGTCCAACGGGGTCAGAATACCCACAGCCTGGGTGGCCTAGGGGTATTTCTGGAATGTACCGTCGACGTGTATCAGC
TGATTACCCGGTAACAGTTGATTTAGCGATTACTTTGTATTCTGGACCTAATCGCGCTTGGCCAAATTTATAGAGGACCATTCC
GAGGATCTAGCCGCCCGAATCGGTGCACAACAGTGAGATATGCCCTCTCTGAGCATCTACTCCAGGATTTTAATCTACGGGTAG
CGCTTCTGAGTCTGTCTCTTGGAAAACTAGAAGATAACACTGGTACGCTGGCTCTACAGACTACCAGCAGAACACTTAGGAAGA
AAAAACCACAGACTCCCGCTCTCTCAATCTCTCTCTCTTAGTCATCTCCTTCTCCTCAATACCCTCCCTGAAATCGTCTCGTCT
CGGCTCTCCTCTCGTTCACTAGCTCTGAGGTTTCTGCGTCTGCAATCCTCTTGGAGATCGGCTGAGGATTTTGAAGAACTGAAC
TGAAGTGACCTGTCGCACACCCTCTCTGCGGGGGGCTGCCACATGCTTGTAACTTCCTCACTTTTGCCCTCGTTGTTCGACGTC
TGTGATCCAGTGAAATTGGATTGAACCAACAAGATTGTGTGGCGATCACAAGTGAGCTTCCGACGATTCCGTGGCTATAGATTT
CAAAAGGGTACGTCTTCAGCAACCCTAAGACCATACTTATCGACACTGTGAATGATCCACGATGAGAAAGGTGAAACACCACAT
AAAAAACCAGAGTAACTCTCTGTGGATCTGAAAAGGATG
>m141121_232159_42134_c100732402550000001823141405141507_s1_p0/90203/2412_10814
CCTCTCGCTAGCTCCTGAGAGCGCAACAGGTGCTTGCTACAATATGCAAGCTACGGCTGCCAGAAGGCGAAGAAGAATAAAATT
TCCAGCTTGAGACAACAGTAGGTTCCTAGCAGGGGAACGCTGTGCAGTACAAGCCATCGTGCTTGCCATAGTAACTGCCGATAA
CAGTAGAAAATAATACGAGAAAATTGGGAATTATCTATCATGACACTGGCTTTAAGGAACACAGACCCTCCGACTTTTGAGGGT
CAGCACAGATCCATCTCACCGGCTGAGCCGTGGATGAGTATGATGCTATCATCCTCCGAACTCTTAAGAGGGTGTGGGGCAATG
AGAGGGGTGGCTGTGCCACCAATCATCATTTTCGAGAGGATGCCTGAATCTGGTCGGGAGGTGGTATCCCAGACAGTAGGGAAT
GTTGCAATGCCCTACTTCCTTAGAGTCATTACTAGTGAGTTAAACATTCACTTCAGCCGTCGCTAATGCGAGGTTCTTTAAAAG
TTAAAAGTGTAGCTAAAATGTAATAAAACCCGGCGCGGAGTCATTGTAGAGGGGGCCGCAGTTGGTGAGCCCCGGTGAAGGACG
AGCGGGGGGCCGAGCGCGGGACGGCGTGGGCGGGCCGGGGGCGCGTGTGGGGCTGCGGCGGGCCGTTGCGAGTTCCGCGGGCGC
GGGCGGGGTGATGACATGAAGAGTTATACCATTTGGAATCCAAAATACTGTTTAGAAAATACTTTACATCTCAAAATACATTTA
AGCTCGACTTACGGCGACAAATTACCAATACATTACATCTTCCCCAAAATAACCCTGGTCTGGCACGCTAGTAGGTCGATCTTT
TACGTTTTCCCTTACTGTCAGCCGATAGATTAACCCGTGAGCCGAAAGCCAGCAAGAAAACTCACATATAACATATGCACACCA
GTCAACAATATAGCACAGCAGACAGGCAATCAGGCTAAACATATCAACGCCACGCCGTCCAACGCTATAACTGAGGCCTGGGTT
TCGCGGTCATTCAACCGAAAAGATAACTCCAGCATCCTATGAAGGTCCACCCTAATGGCCCACCTGCACATCCGCATGCTCAAT
GCAAATCCTCGGTCCCTTACCCGTCATCGGCCTTCCTACCATTGGTAAGTTCTCGGCCTTCACCGTTCCGTCATCACAAATATA
GCCATGACATAGCATAATATCACAAATATTTAAACATATACGAAACAATTCTTTTTTTTTTTAAACAATAAGGGATATGCCTT
GCAAATCAATCAATAGCGTCAACGCCTACAACCACCAACACATTAGGGCCACTCCCTGCTCTAACCAGTACAACAATGTTTCTT
ACCTTGTCGTCCTGAGCTATCTTAAACAAGCAAAAAACCCGCGATTTACGAGCCCACATTCCCCCCTAAAACCCCCCCGAAAGC
CTCCGCTGAAAAACCTTTAAAAAGTCCCCAAACCAATGCCACTTTCATAAAAATAACACCAATCCCCCCAATCCAAAAAAATTC
TAAAAAAAACCAAAAATTAAAAACCAGACTTCACAAACAAAAAAAAATATTAAATCTCCTAGCCTCCCCCAAGGAGAACCCTTT
CGGAATTCTTTACCAAAAAAAAACCCTTGGGGTTAAAAGTTAAGAAAAAAAAAAGCCTTTTTCCCCCCTTCCGAGCAAAAATT
AAAAAAACAAAAATTTCGAAGGTTTTTTCTGCCCCGAGCTTAAGAAAAAAAAATTTTTCCAAAAAAATTAAAACCCCTTACAAA
ATATTTTTTTTTTTCCCCCAATTTTTTTGAGCCTCCGGCCCAACGCCCCCCCCCACCTTTAAGGGGGCCCTTTTTTTTGGACCC
CCGCGCCCCCAAAGAGGACCCCAAGAAGGCAAAAAATATTGACCCTTCTTGTTGCCCCCTTGAAAAGGGACAAACGGGGTTTTG
CCGGCCCGCTGGGCCTTATCTTAAACCTTTTTGAGGCCCCCAAGGGGTTCCAAAAAAAAAAAAAAAACCCCCCCTTGGGTTTTT

TTTTTAGGCCCAAGAGGGGCCCCCCTTCCCCAAAAGCCACATAAAACCCCTTTAAAAGCCAAACGGGTTTTCCCCCCCACCGC
AAAGACAAAGGCTTATGGTTTGGGCCTTTCTCGCCTACCAAAACCCTCCGCCCGTTTCTTTTTTTGAAGCAATTGGGGTCCGCA
GCCAGGTGGGGCCCCCTTTCTTATAATTTTTTTTTCCCCCCCCCCTTTTGTTCTCCACCAACAATAAAAGTTTGCAAAAAACAA
AAAAAAAAAAAAATGGGTTTTTCCCCCATGGGGTTTTTTTCCTTCCCACGCAAAGTTTTGGGCCAAAAAAAAATGGGGTTTTTTT
TTCCTTGTTTTTTTCCCCTCCCCCCCACAACAGGTTGGCCAAAAACAAACTGGGGTTTTTTTTCCCCCCCCCCCTTATTCCTC
AAAAAAAAAAACGTTTAAGTGGCTAAAGATTTTTTTGGGGGTTTTTTTTTTTTTCTTCCCCCCCCCCCCCCTGGTTCTCCCCCCA
AAACAAAGAAGTTACAAAAGCAAAAAAAAATTAAAACCAAAGCCCCCCAAAAAAAAAACTAAAAAAAAACAAAAATAAGGGAAATT
TTTGAAACTTTGCAAAAAACAGCCCTTTTTTTAAAACAAATTCCCCTCAAAAACCTCTTTTTTGGCCTCACAAACAAAGAATAA
AAAAGTTTTTTTTTAAAAACTTTTCCCCACCCTTTTAAAAAATGGGGGGTTTTCCCCCACACAAAAAAAGGCCCAAAAAAAAA
AAAATTGATCCATTGTTTTTCGTTTCCCAACCACAACCAAATTAACGCTATTGAGGTTTTACGCCAAGGAATTTTACAGAGTTT
TTGGGTTTTCCAATCTTTAAATCCAAATAATTAACTAAATTGGCCGTTTTCCGAAGTTTTAAAAAAATGGGTTATTATTATATT
TTTATTTATATACAGAGGCCAAAAAGCCCCCCCCCCCTTAAACTCAAAACCTTTAAGGGGTTTTCCCCCAAAATGGAGTGCTTT
TGATTAAATAAAAACAAAACCCCTAAACTCCCCTTCCCAAACAATTTTTTATTCGAACTTAAAAGATGAGAAATGGGGAAACAA
AAAAAAAAAAAAAAATAGAAAAAATTGGGGTGGGGAAGGGGGGGGGGGGGGGGCCCCCAAAAAAAAAAAAATTTAATAATTGGCC
CCGGGAAAATTGGAATCATCGTAATTTGGGTTGAAAAATACATAGTCCCCCTTTTTAAATGGTTTAATTTGGGTAAAAAAAAGC
CGAAAAGGGGAAAAGGACTTTTATAGAACAATTTCCCGGCGACGGCCAAGTTTTTTTTTTTTTTTTTCCCCCCCGTTAAGAAAC
CCTTAATGGGGTTCCCCTTTTCCCAAGCTTTTTAAAAAAATAAATTTATAAAAAAAAAGTGAAACCCCCCCTAAAAAGTTTGGGG
AAACACGTTAAAAAAAGGTTTTTTCCAAAAAAAAAATGCTTTTTTTTTTTTTTTTGGGGCGCCTGAAAAACCACTATAAAACGAT
CTAACTTGGGTTTTGGGGGTTTTTTTTTTTATTTTTATTTTTTGACTTATAATCCCCTTTCTAAAATTCTTTTTTGTTTCTTC
CCAAAAAAGTCCCAAAAAAAAAAGATGCTTTCTTTTTTTCTTTTATGTTTAAATCAAACAAGTTTTTTTTTTTGAAAAAAACC
GAAAAAAAAACACCAGCAAAATCCAAAAACACGGTTTCTTTTTTTTTTTGGTTTAAAATTTTTCCGCAAAAATGAAAAAATTA
AAAACGAAAAAACAAAAGCCAAAAAAAAACAATTTCTTTTTCAAAAAAACCCCAAAACCTTTTTCTTTTTTTTCAAAAATTGAAA
TTTTGGGTTTTTTTTTTTCCTTTATCCCCCCCAAAAATAAATAGTTATTTTTTTTTTTTTTTTCATTGGAATTTTGCTATTGGT
TTTTAAAATCGAAAGAAATTAAGGCCCCCCCTTTTTTTTTAACTTTCCCTTTTCCCCCAAAAAAAAAAAAAAAAAAAAAAAAA
TCTTTTTTAAAATTTCTTTTTTTGGGATTGAAAGGGTTTTTTTTAATAATCTTTTTTTCTTTATCGTCAAAAATGTTTTTTAAA
TGAGGGGGGCCCGTAAATTTTTTTTTTTTTTTTTTTACAGATTTTTTTTTTTTTTGGAAACAACGAAAAAAAATGTTTTTAGGTT
TATTTTTTGGTTTTTTCCCCTAATTCCAAAAAAATTTTTGTTTTTCCCCCCTAAAAATTCAACCTTTTTATCGGAAGCCCTTTTG
AAGGTTTTTTTTTTTAAAAAGTTGGGAAAGAAAAAAAAATTTTTAAAAATTCCATTTTTTGTAAGGGTTTTTTTTTTTGTTGGTAA
ACCTTTTTTTTTGGGTTTTTTGAGAAGAATTTTAAAGAAAAATTTTTTTTTTTTTTCTTTTTTCCCCCCCAAATTCCAAAAAAAA
TTTTTTAAATAAATTTTTTATTTATTAAAACTTAATTATTGTTTGTTTATTTTCCTTTTTTTTTTTTTAGGTTACATTAAAGAT
TTTTTTGAAAACAAAAATCTTTTTGAAACTTCACTTTTAATTTACTCTTTTACTTTTTTTACCTTTTTTTTTATTTGTTTTTGA
CAAAAATTTTTAACTTTTTTTGTTGAAACCGCCGGGATTTCCCTTCGCCAACAAGGTTAATTTTTTAGGAAAAAGAAGAAAAGG
AAAGAAGAAAAAAAAAAAAGGGGAATTTTTTAAAGTTTGGCTTGGAAATGTTTAAGAAACCGTCCCAAAACCAGAATTAAAAATT
GGGAATCCCCCTTTTTTTTTTAAAAAGATTGAAAACCTTTAAAGGGTTTTTTTTTTTTTTTGAAAACCCCTTAACAAGACAAAA
CCCGTTTTTAAAGGTGGTTTTCAAAAAGGGTTTTCTAAAACTTCCCTGTTTTTTTCCCCCCTACTCCCCACCGTTTAGTCCAAA
CACTAATTATTTGAATTATAAATTTTCTGGATTACTTTTTTTTTCGGTTTTTTTTTTTTCTTAAAAACCCCAAAAAAAAAGTTGG
GGTAAAAAAAAAAAATAAACTTTTCCTTTTTTCTTTTTTCCTTGAGGTAAGTTTTTTTTTTTTTTTTTTTTTTTTTTCCCGACC
CCCCTATTTTCCAAAACTTTCAACAAAAGGGTTCCCCCTTTTTTCCCCCCAAAAATTATTTTAAAAAATAGGAGGAAAGGCAAA
AAACCCCCCCCCTTTTTTTGGAAAAAAGTTGGGTTTAAGGGAAAGGGTTTTCCCAAAATCCCCCGTTGGACCCCCCCTTTTTACA
AAAATTTTTTGTCCCAAAATAAATCCCAAAAAAACTCCCCCTTATCTGTGGGCAAATTATTTCATGAATTAATTTCCCTTAAAA
ACCTTCTCTGACCACACAGTTAAAAAACCCTCGTGGGCCCCTCTTCAACCTAAAAAAAACTTCCAAAAGGCACAAAAATCGTCA
CACTCTGCGGGAGAACTAAATGGGGCCCCCGCCAGAAACAGGCCCCCCAAAAAAAACCCCCCAGCAGCGCCCCCCCCGTTTCGGT
TTTTGGGGGGGTTCTTTCCCCTTGAACAATACCGCACTTTTTTTTTCCCCCCCTTGGCCCCCTAACCGGGTTTTGGTCCCCCCC
CCCGGAGAAAAAAAGCCCAGGGGGGGATATTTCGGAACAAAACGCTGGGGACTTGTTCAAGAAAATTTAAATTTTTTTGGAAAA
ACCGTTTTAAAAAATCTTTGCGTGCCCCGTTTTTTCCCCAAAAAAAACTTTTCCCTTCCCAGGGGGAAAGGTTCCAGAGGGCCC
CAATCCCCCCTTTGAAATTTTTTTTTTTCAAAGCCCTTTTTTTTCCCCCCGTAAAACCCACCGAGGGGGAAAAAAGCCCTTCGT
GAACAGAACGAGGGCTTTTTTTTTTTTCCCCCCCTCCCCCCCCCCCGGTGGGGGTTTGGTGGGGGCCCTTTCTTGATGTCCCC
TTTGTTCCCAGCCTTTTGGGGGGCCCCAAAAACCGAATGGGGTGCCCCAACCCCCCCCGAAGGCCCCCCCCCCCTATTGGGGAA
ATGAGAACCCCCCCCCCCCTTTTTCCGAGCTTTTTAAAAGGGAGAAAAAAAATTTCCCCCCGGTCCCCTGGAAAAATTTAGGGA
AATCTTTTTTTTTAGGTTTTTGTTTTTTTTTTTGGTGGGGCCCCGGGCCCCCTCTTTTGGGGGAACTTTAAGCCCCCCTGATTTAA
GTTTTTCCCGAGGCCCCCCTTTTTTTTTTTTTTTTCCGGCCCCCTTCCCTTTTTTTTTAAAAATTCAGCCCCCCCCCCCCCGTGG
GGGGGGACAAAAAAAACCCCAAGGGGGGGCCCCCCCGGTAACATTTTCCTGGGGGCCCCCCCCCCCCCCCCCCCCAAAAGGGC
CCTTTCCCCCTTGGCTTTTCGTGGTTTTAAAAATAAATTTGCGGGAAACGTTTCCCATACCAACAAAAAAATACGGAAAAAGAA
CCCCCAAACAGGTTAAAGGGCGGCGGGCCCCCCTTTTTTTTTCAAAGAAACTTTTTTTCCCCCCCCCCCAAAATAAAACCCCCT
TTTTTTTTTTTTTCAAAAAAACAAAAAATTCCCACCACCCTCACAATCTCTTTTTTTTTATTTTTTTTTTTCGCGGCCGTTAA
TGGGAAAAATTAACGTGGGGAACGTTCCCGGTTGGGGTGGCGGAAAAAGGGGTAAACAAATTTTTTCACCCTTTTTTTAAACG
AGAAAACCCCCCCCGCCCCAAAAAAAAAATTTAAAAAATGGGAACCCCCCCCCTTTTTTGGCCCCTTAAAAAAAAAAAACCCT
TGCCCCAAGCCCCCCCCACCCCAATTTTCGTTTCCACCCAATTTTTTTTTTTTTTTTTTCCCCGAATGGGAGGGCCCCCCCCT

TTCCCAAAAAGGCGTCTTCTTTAACCACCGTGGATCCCAAAACCCCCTTTTTGTATAAAAAAAGGCTTTGTGGTTTGGCCGCCA
ATCCCCCCTTTTACGCAAGAAACCCGGGCCAACCCCCTAAAAATCCAAAAAAAAAAAAAAAAAAAAATTTTAAAGAGAACTCCT
TCAAAAAAATATTTTTTTCTTTTTTCCCTTTTCCCCTTTTTCCCTTTTGACCCCTTTTTTTTCCCCGAAAAGAAACCGAAAAAC
CCCCCTTCAAACCCTTAAAAAAACTGGCCCCCCCTGCTTTTTTCCCCAAAAATTCCTTCCCCCCCCCATTTTTCCAACGGAAGC
CTTTTTTAAAAGCGGCCCTCCAAAAAAAAAAGGTTTTTTTTTTTTTTCCCCCCCTTTTCCCCCCAAAAAAGCCCCCTTTTTTTT
TTTTTGGGGAAAAGCAACCAAAAAAAACCTTTTACGTCCGAACCCGAAAAAAGGCCCCTTTTTTTCCCTTTTTAACCAAAACCCC
CGCAAACAAAAACTTTTCCCTTTTCGTTTTTCCCACCCAAAAATTTCCCCCCCCCAGCCCTAATAAACACTTTTTTTTGGTTAA
AAAAAAGTTTTTTTTTTTTTTCCCTGACTGTTTTTCCCCCATTTTTTTGAAAAAGCAAGAAAATTGCCCCCCCCCCAAACTAT
TTAGCCTTTTTATTTAAAGGGTAAAAAAAAAATTTTTACCCTGTAGGCCCCCCCCCCGCCAAATGCAAAAAAAAGGGGGGTTTTT
TTTTTTCCCCTTGGGTTTTTTGCCCGTTTGGAAAAAAATTTTTAAAAATTTGGGGGGGAGGGGGTGGGGGAGAAAAAAACCCCC
CCTTTTCCCTTTTTTCTTAAGAATTAGGGCCACTTTTTGTTTTTTTCTTTTTTTTTTTGTGGGAAAATTTTTCGTCCTTTTTTG
AGAAAGTAAAAAAAAAGTTGGGTTTTTTTTTTTTTTTTTTTCAAAGTTGTTTTTTTCCCCCTTTTTTTCCCCCCCCAGGAAGC
AAATTTTTTCCCCTTGAGTTAAGGGAAAAAAAAAAAACCCTTTTTAAAAAAGGAAAAAAAATTTTTTTTTTTTTTTTTTGGGT
TTGCCCCCCCAAAACCCCGGGGTTTAAAAAAAAAAGCCTTTTTTAGGGGAAATTTACAAAACGCCCCCCCCTCCCCCCAAAAAA
GCGGTTTTTTTTTTTTTTTTTGCCAATGCCTTTTCCTTGCCCCTTAAATTGGGGAAAAACTTTTCCCCCCCCCAAGAAAAAAAA
AAACCTTTTTTTTTTTTTTTTTTCTTGCTTCTTTTGAGCCCCCCCCCCCCTTGAAAAACACAAAAAGAAACGAAAAATTTTCCCC
CCGAAGGTTAATCCCTGGGGAATTCCCTGGTTGAGGGGGCCCCCCAAAAAAAAAACAAAAATAGGCCAAAAAAATGGTGGTTTCC
CAATTGAAACCCGCCGGTGGTTTTTTTTTTTTTTCCCCCCCAAACTTCCTTCCCCCCCCACGAGTTTTTTGGGCCCTTTTTAA
AA
>m141121_232159_42134_c100732402550000001823141405141507_s1_p0/92762/0_5247
CAATTCTAGTAGAGCGCGGAATACAAATAAATCAGGAGTATAGTTTGCCATTCAGAATATTTGGTGAGGTCTTCTTCAGTGGGA
TTATTATGAAAGTCTATGCTAGGGTTATAGCAGATTCGAAAATTACACAACATCATCACCACCGAGAAAGCGTTTTGGAAGAGA
GAGCAGTGGTACAGACAACAGTCATATCTGAATTTAGAGATGGTGTACAAGATATAAGGAAAAAACTTGCGAAAGATTGGCCAT
AAAAAGGAGAATCCTTTTTTTGAAATGGTTGACTTAAGGCTCCGTTTCGACTCCAATAGATTCATTTACCCACGAGATCTCTTG
GGGGAAATATCGACGATTTGAAGACGAGGGTAATACATGTCGGAGAAATTAGTGAAATCTCCAATTCACCAAGGGGTACCCCTT
TCAGAAATCTTCGATTTCTCTAATATGTCGAGCAATGTTTTGATAGTGAATACTAAAGAATAATGCGAGGGTCATGGGCACAAA
AGTGTAGGCCTATTGTCTTAGGGTTCTAATGTATAACATTTCGTGCAAACCGAGTTGTGTATAGGTGATACAGCCAAAATACTT
AGAGATTACGGAATATAAGGAAAAACTTCGCGAAAATTGGCCATAAAAAAGGGGAATTTTTTTTGAAATGTTGACTTAAGGCTC
CGTTTCGACTCCAAATAGATTCATTTACCCCACGAGATCTCTTGGGAGAAAAATATCGAACGATATGAAGATGAGGGTAATACA
TTTCGGAGAAATTAGTGAATTCCCAATTCAACCAAAGGGGTACCCCTTTCGAAATATTCGATTTCCTAATATGGATTCGAGCAA
TGTTTTGATAGTGAATACTAAGAATATGCGAGGGTCATGGGCACAAAGTTGTAGGCCTATGTCTTAGGCGTTCTATGTATAAAA
ATTCGTGCAAATCCTAGGTGTGTAGAGGGTGATACAGTCAAAATACTAGAGAATGTACGTAAATTAAGGAAAAAACTTGCGAAA
ATTTCCATAAAAAAGGGGAATTTTTTTGAAATGTTGACTTACAGGCTCCGTTTCGACCTCCAATAGATTCATTTACCCACGAGA
TCTCTTGGGGAAAAAAATATCGAACGATTTGAAGACGAGGTAATACATTTCGGAGAAATTAGTGAAATCTCCAATTCACCAAAGG
GGTACCTCCTTTTCAGAAATATTCGTTTTCTAATATGGATTCGAGCAATGTTTTGATAGTGAATACTAAAGAATATGCGAGGGC
CATGGGCACAAAATTTTGTACGCCTATGTCTTAGGGTTCTATGTATAACATTTCCCAGTGCAATCCGAGTTGTGTATAGGTTGA
TAAAGTCAAAATACTTAGAGATGTACGAATATAAGAAAAAAGTTGCGAAAATTGCATAAAAAAGGGGATTTTTTTGAAATGTTG
ACTTAAGGCTCGTTTCGACTCAATAGATTCATTTACCACACGAGATCTCTTGGGGAAAAAATATCGAACGAATTTTGAAGACGGG
GTAATACATTTCGGAGAAATTAGTGAAATCCCAAATTCACCACAGGGTACCCCCTTTCAGAAATCTTTCGATTTCTCTAATATG
GATTCGAGCAATGTTTTGATAGTGGAATACTAAAGAATATGCGAGGGCCATGGGCCCAGAAAAAGTTGTAGGCCTATGTCTTAG
GGTTCTATGTATAAAAATTCGTACAAACTCCTAGTGGTGTAGAAGGGTGATACAGTCAAAATAATTAGAGATGTAACGAATATA
AGGAAAAAACTGTCGAAAATTTCCAATAAAAAAAGGGGAATTTTTTTTGAAATGTTGAACTTAAGGCTTCCGTTTCACTCCCA
ATAGATTCATTTACCCACGAGATCTCTTGGGGAAAAATAATCGAACTATTTGAAGACGAGGGTAATACATTTCGGAGAAATTAG
GAAATCTCCAATTCACCAAGGGGTACCCCTTTCAGAAATATTCGATTTCTCTAATATGGATTCGAGCAATGTTTGATAGTGATA
TAAAGAATAGCGAGGGCCATGGGCACAAAATTTGTACGCCTATGTCTAAGGGTTCTATGTATAACATTTCGTGCAAAAATCCCC
GAGTTGGTGTATAGGTTGATAAAGTCAAAATACTTAGAGATGTAGAATATAAGGAAAACTTGCGAAATTGCCATAAAAAAGGGG
AATTTTTTTTTTGAAATGTTGACTTAAGGCTCGTTTCGACTTCAATAGATTCATTTACCACGAGATCTCTTGGGGAAAAATAT
GAACGATTTGAAGACGAGGGTAATACATTTCGGAGAAATAGTGAAATCTCCAATTCACAAAGGGGTACCCCTTTCAGAAATCTT
CGATTTCTCTAATATGGATTCGACAATGTTTTGATAGTGAATACTAAGAATATGCGAGGGTCATCGGCACAAAAGTTGTAGGC
CTATGTCTTAGGGTTCTATGGTATAAATTTCGTGCAAAGCCGAGTTGTTAATATGGTTGATACAGTCAAAATACTTAGAGGAAT
GGTAGAATATAAGGAAAAACTTGTGCGAAAATTGCCATAAAAAAGGGGAATTTTTTTTGAAATGTGCTTAAGGCATCCGTTTCG
ACTCCAATAGATTCATTTACCCAACGAGATCTCTTGGGGAAAATAATCGAACGATTTGAAGAAGAGGGTAATACATTTCGGAGA
AATTAGTGAAATCTCCAATTCACCAAAGGGGTACCCTTTTCGAAAGTCTTCGATTCTCTAATATGGATCGAGCAATGTTTTGAT
AGTGAATACTAAAGAATTATGCGAGGGTCATGGCACAAAAAGTTGGTAGGCCTATGTCTTAGGGTTCTATGTAATAACATTTGT
GCAATCGAGTTGTGTATAGTTGATAAAGTCAAAATACTCTAGAGATGCTACCGAATATACTGGAAAAAAGCTGCGAAAATTGCA
TAAACAAACGGGGAATTTTCTTGAATGTTGACTTACAGGCTCGTTTCGAAATCCAATCAGATTCATTCTACCACGAGATCTATT
CGGGAAAAACTAATCCGACACGATTCTGAAGACGAGGGTGAATCACTTTCGGAGAAATTAGTGACCAATCTCCAACTTCACCAA
AGGAGGTACCCCTTTCAGAAATCTTCGATTTCCCTCCTAAAATATGGATTCGAGCAATGTTTTATAGTGGATACTAAAGAATAT

GCGAGGGCATGGGCAAAAGTGTAGGACTATGCTTAGGTTCTATGTATAAAAATTCGCTTACAAATCCTAGGTTTGTAGAGGGGT
GATAGCAGTCCAAAATACTTAGAAGATGTCGAATATAAGGAAAAACTTGCGGAAAATTTCATAAAAATAGGGGAATTTTTTTTG
AAATGTTTGACTTTAAGGCTCCGTTCGTCTCAATAAGATTTCATTTACCCACGAGATCTCTTGGGGGAAATATCGAACGATTTG
AAGACGAGGTAATACATTTCGAGAAATCTAAGTGAACATCTCCCAATTCACCAAAAGGGTACAAAAAACTTTCAGAATCATTCG
ATTTCTCTAATATTGATTTCGAGCAAGTGTTTGATAGTCGAATACTAAAGACATGCGAGGTCTGGGCACCTAAAAGTTTAGGCG
CTATGTCTTAGGTCTATGTATTAAAAATTCGTGCAACATCCGAGTTGTGAGGGGTGACTACATTCAAAAATGGGATGACGAATA
TAAGGAAAAAAGTTGCGAGAACATCTTCATAAAAAAGGGGAATTTTTTTTGAAATGTTGACTTAAGGCTCCGTTTCTGTCTCC
AATAGAATTCATTTACCCATACGAGATCTCTTGGGAAAATATCGAACCGATTTGAAGCACGAGGTGTAATACATTTCGGAGAAA
TTACAGTGAACTCCATTACCAAAGGGTTACCCTTTTCAGAAAATCTTCCGATTCTCTAATATGGATTCGAGCAATGTTTTGATA
GTGAATACTAAAGAATATGCGAGGGTCATGGGCACAAAGTTGTAGATCCTATGTTTAGGGTTCTATGTATAAAAATTCGTGCAA
ATCCTAGTGCTGTTAGAGGGTGGATACAGTCAAAATACTGAGTGATGTACGAATATAAGGAAAAAAGTTGCGAAATTTCCAATA
AAAAAAAGGGGAATTTTTCTTGAATGTTGACTTTAAGGCTCCGTTTCGTCTCCAATAGCATTACATTACCCCACGAGATCTCT
TCGGGGGATCAAATATCGAACGGTTCCGAAGACGAGGGTAATACATTTCGGAGAAATTAGTGAAATCTCCAATTCAACCAAAGG
GGTACCTTTCAGAATCTTCGATTCTCTAATATGGATTCGAGCAAATGTTTTGATAGTGAATACTAAGGAAATGCGCAGGTCAAT
GGGCACCAAAAGTTGTAGGCCTTATGTCTTTTAGGGTTTCTATGTTACTAAATTTCGTGAAAATCGGTTGTGTACTAGGTTGAT
AAAGTCAAAATACTTAGAGATGTACGAATAGGAAAAAAGTTTGCGAAAATATGCATAAAAAGGGGAATTTATTTTGAAATGGTT
AAGGCTCCGTTTCGATCCAATATTCATTACCCACGAGATCTCTTGGGGAAAAAATATCGAACGAATTTGAAGCGAGGGTAATACA
TTTCGAGAAATTACGTGAAATCCTTCCAATTCACCAGGGTACCCCTTTTCAGAAATCTTCGATTTCCTCTAATATCCGGATTCG
AGCAACTGTTTTGACTAGTGAATTACTAAAGACATATGCGAGGGTCATGGGCACAAAAGTGCTAGGCTATGTCTTAGGGTTCTA
TGTACTAACAATTTCGTGCAAATCCGAGTTGTGCTATAGGTTATATAAATCAAAATACCTTAGAGATGTAACGAATATGAAGGA
AAAAGTTGCGAAAAAATTTCCATAAAGGGGAATTTTTTTTGAAATGTGACTTAAGGCTCCGTTTCGACTCAATAGATTCATTTAC
CCACGAGACATCTCTTGGGGAAAAAATATCGAACGATTTGAAGACAGGTAATACATTTCGGAGAAAATTATAGTGAAATGCTCCA
ATTCACCAAAGGGGGTACCCCTTTCACGAAATCTTCGATTTCTCTAATATGGATTCGGCAATGTTTTGATAGTGAATACTAAAG
AATATGGCGAGGCCATGGGCAGAAAAGTTGTAAGGCATATTTCTTTATGGGTTCTCATGTATATAACTTCGTACAAATCCTAGG
TTTGTAAGGGTGATAACCAGTCAAAATACTTAGAGATGT
>m141121_232159_42134_c100732402550000001823141405141507_s1_p0/93462/3284_14773
ACCATACAATTTCCTAAGTGAATCAGAGTGTTATCAGTGGCTTGGCGGAACCCGAGTCTTTTACTTGTACCAGGATTGGGTATG
TATACTGCCATTGCCGAATGCATCGGTATTAATCGTACTAAATAAGTCCGTTTCAATGGCAGGTTTCCATCCTATCACATTTAA
TTGGTGAAAGACCCCAGACCGCCCGTTCCTGCCATAAGTACCTCCCATATTCTGCATGTAGTGGGGTTGATGACAATGGGTTTT
GAGTCAATGATTGAAAAACTTCCCTTGTTCTCGATCTTGATTCGAGTAGAAATCTCAGCGATTACGTGTTCCTTGTGTGAACCG
GAGGAACTCCCATTCACACTGGAAATTCAGAGAACTACTTAGCTCTCGGATACCATATGATTAGGTACCGAAGAAGCAGGCCAC
GGCAAAACCACCCTTGTCAATTAAGTTGTTTTTTTTTTTTTTTTCCGAACATTTTCCCCGTCCTGGGGGAATAAAGAGAAAAAT
TTATTGCCCCCCCCAACAAGGGTAAAAAGAAATAAAGTTTTTTTCACAAAAAACCAAATTAAAATATTTACAAAGAAATTTTT
TTTCTTTTTTTTGTTTTAAGTTACTCTCTTTTTTTTAAAAAGAAGAACTAAATGTAAAAAGTGAAAATGGGGGTGTCCCAGTAA
AATCGCCTTTTTTCCAAAAAAAAAATTGGGATTCAAAGGCGTATCCCCCAAAAAAAAGATTCCAATTAAGTGCAAAGAAAAACT
TTTTTCCACGATCGTGGGGAAAAAAGGCCTTCCCCCCCGTTTCTTCTCAACTTGAACACCAAATGCTAAAATTAGACCGCGTT
TTTTTGAAAAATCGAAAGTGCCCCCCCCCCGCACCCCCGGGAAAGCCCCAACAAAAGGACCGTAAATCCTTTTTAATAAATGGC
ACATTTTCCCCCGCTTCTTTTTTTTTCCTTGCCGAATAAAAGCCTTTCCCCCAAACCATTGCCCCCCAGAAAGCGTACGGGAAA
AAATTTCCGAAAACATAACAACAAACAAACAGGGGGTTTTTTTCCTTCTTTTTAAAAAAAAATTTCATGTAAAATTTTTAAATT
CGGTATTAAATCAAGCCCAAAATTTCCCCCCCCCTGCTTTTCCCCAATTTTTTTGGGGAGATATTTTTTCCCCCTTTTCTGTCT
TTGATTTAAAGAGGGGCGGGGATTTTTTAAATTTTACCCCTAAAATTTTGGCACAAATTACCCTTTCCGGGGCCCGAGTTACCC
AATAAAACTCCCGTTTTGTAGATATAACATTAGAGCCCCCCCACAAAAACTTCTAAAGCAAAAAAAAACTATCCTTTTTGCTTT
TGAGTTGGTGTACGGGGAAGAAAAAAAAATGGGGAGAAAAAATTTTTTCCTCCCCAAAATTGAGGGCCGTTGAACCCAAACCCA
AGGAATTTTGTTATGAAAAAAGGACAGACGAGAACCCATTAAGAGGTACAACGAAAGCCCTCTCCCCTGCCTTTTTTGGAAAAA
GGGCTTCTTAAAAAGGATTAAAAAAAGGCTCTTAACCCATTGGAAAAAACAAAGCCCCCCCAATTTTGTGGAATTTTCTTTCCC
ATTCCCAAAAAGTTCCCCTTTAGAAGACACTTTTGTTTCCCCCTTATCCACATTCTAGGATGGGAGTACACAAAAATTAGCCAC
AATTTAGTCTTATTTCCATAAACCATATAAAAAAAAAATGGGGTATTTGGGGGAAAAGGGCCCTTCGTTGGTTAAAAAAAAAA
GATTGCCCCTTTAACAATCCCCTATTTGTGATTAAAAGGATGTTGGGGCGCCCCGGCCCCCCACTATTTTTTCCCAAAGCAATT
ACCGGGGGGGGGGAGTTTGCCCTTGCAAAACAATAACTGTCCCCTGAAAAAAGTAATTTTTTTCCCCCCCATAAACAAAAAAAC
CGGGCTCTTTTTTTTTTCTTTTTGTTTTTTGTTCCTTTTCGAAATTGTTTTGGGCCACTTCGCTTTTTTTAGGCAACTTTCCCT
TATGTTCCGAAAAAGCAGAAAAATGGCGCTTTTTGGTCACATTTTTTAAAAAACCACGAAATTTAACCAAAAAAAAATCTTCTGT
CCCCCCCTTTTGGAAAAAAAAAAAAGTTTTTCCCTTTTTTATTGCTATCCCCTTTTTCGGCGGCGGGCCCCCCAATTTCCACCAT
CGATTGTAGTACATGGAAAAAGTTGAAAGCGGACCTTTTTACCCGAACCCCAAGATGGAAACCCCAAAGAAACGCCCCCACCGG
GAAAATACATCCCCAACCCCCGTTTTTTGGGCCCCCAAAAGTTTAGAAAAAGGTTTCCCCCTTCACGAAAAAACACTCCCTTTC
CCTTTCCCCTTTGCCACCCCCTTTTTCAAGTTTACCATCCGGACAAAACGACCTTTTTGTAAACCCCCTTTTAAAAATTTATGA
AAAAACCGTTTTCCTTTTCCGCAAATTGGGTTTTTGGCTTCCCCCGGCCCCCGGCGATGTTCCATTTTTATCCAAGAAAGGGTT
TATCCCCACAAGCCTTTTTTCCCCTTTTTTTTGGGTAACCCCCACCAAATATATACATATACACCACCCCCTTTTGACCCTTTC
TTTATTATTCCCATACCGTAGGCCCCCATATTTTTTTTTTGGATTAAACACACCAGATAAAGGGTAATTTGGTTTCTTTTCGAT

AAGAATATTTACCACATCATATATATTATCTGAAAGAGTTTACCATAATATATATTTACCGAAACCCTTCATGTCTATCATTTT
CATTTTTTACCAAACACGGGAAGACCACACAATACTTATAATAATTTTTTTTTTGACATATAGAGGTCACACCCTTCCACAGG
AAACAAACTGGTAAAAATAGACCATAACACAACACATATATACACACCACCCTTGGGGGGAACATATGTTAGGTTCCCCCGGAC
TCTCAAAAAAACTTTTTTTTTTTATAACATATTTTTTTGGTTTCTTAAAATTTTTTACACTTTACTGCGGGGTCCCCCCACACA
TATAACACAAACAAAATCCTTTTCATTTCCTTAACATAAAACACAACTTTTAAAACAAAACGATTCGTCCTTTCCCCACATTTC
AAAGAAATTCACCCACAATAAAAAAAAGAAAACCAAACATAAACCCACCGTTGGGCCCCTTACTATTATACCCCCCTTTCTTTA
AATTTCCCCCCTTTTTCCCCCATTTCTTTCCTGCCCTTTATACACGCAAAGGCAAATCCCCTTTTTTTCTATCAAAACCTGAAA
AATCCCCCCCCCAAAATATCCCAGGAATCGCTTAATTTTGTCCCCTTCCTCCGGATAGCCCAAAAACCTGCCCCTTCCAGTTAA
AAAAAGAAAAATTTTTTTTCCCCTTCCAAGGATTTTTTTTTTCCCCGGAAAATCCAATTTTCCGGAAGCCCCGTGGAGTTTT
CAAAAGTTAAAAAAAAAAAAAAAAAATGAAGGGGGGCAAAATTGCCTTTAACTATTTCCCCCCCCCAGGAAAGAAATATTTTT
GGAATCTTTTTCCATTAATTCCGAATGAACGCCTCCGGGTAATATTCGTAAAAAAATGAAAAAAAGTAAGGTTTTTTTTCTTGT
TTTTTTTTTAAAAGCTTTCTTTCTTAATGGGCCACCCCCCCCCATAGGGGCAAACAAAAAGAAACAATTCTTCGGAATAGGAAAA
AACTTTTCCCCTTATAGGAATCCCTTTTCCCCCCTTTTTTTTTTTTTTTCTTTCTCAAAAAAGAAAAATCCGGAACCCCCCTG
GGGGAAAAGGTTTCTGTTTCCTTTTCAAATCCGGCACTTCTTTTTCCAAAAGTCAAAAAAAAAGTTTTGTTTTTTACCCACCCA
AAAAAAAAACTTTAAAACGGAGATTTTAAAAAAAAAAGGGGGGGGCTTTTTTTTTTTCCCCAAAGCGTTTTCAAAAAAAATTCTT
TTTTATAAAACCCCCCCCAAAAATTCTTAACCCTCTTTTAAAAAAAAAAACTCCCCCCAAAGTCTTCCTTAAAAAAGAAAAGAATC
CCAAAGCCAAAACCAAAAATATTATATATATATTATCACAAATTTGATTCATTTTAATATCTTTTGACGCTGTATTTATTATTT
TATATTTAATATAGATTTTTTTTTTTTTTTTTGTTTTTTTTTATTTTTATTTGGGCCTTTTTTTTTTTTTTTAAATAATATAA
TATTTTTTTCCCTGAATACTGAATTATATTATATATAATTATATATAATAATTTGAAATTAGTTAAATATCCAATTAAACACAA
AATGATTTGTGGAAGTATTATTCTTATTGATATATTAATTCCCACCACCAACCCCTACAATCACAACAATAATAATAACAAAAG
ATGACACACACACATCCCCTATATTCTAGTAAACTTTTTATTAAACAAACAATACTTTTTTTATATTCTATTTACCACCCTTTA
CCACCAAAATTTAGGGAGGAGAATTTTTTTTTATTTACTTTTAGGGTTTCATTTTTTATTTTTATCTTTATATATAATATACCC
AAATATATACAACATTTAGTCCCACCATTCTTATTTCCCACACACCCACACAGGAACAACACACATACTTATAATGTCCACGAC
CCGACTCCTATATTTAAGCCAATTAATGACCCGATATATGAAAAAAATAAAACTCGGGGAACAAACCCAATGGGGGGAACACCA
ATAATTGTGCACCTTTTTTAATATTTATAAGGAACAACAAAAATTAAAAAATTTCCCCCCAAAAAAAAAATTTCTTATATATAT
CTAATAAAACGAAAAAGGAATGGGATTAAAAATTTTTTACACAAAATACAGATAAAGAAAAATAAGGATCCATTATAATTATAA
ATTTTCCAACAGGGTTATTAAAACATATAAAAATTCGATTTAATATTATTTGTTTTTTTATTATATCCTCCATCATATTTGAC
CCCCTAAAGACCATTTTCCAACCCCCCTTTTTTTTTTGTGTTAACTTTGGGGAGTAATTTGAAAAACACAAACCCCCCTTAAAATA
AAATTTCCTGAAAGGTTTTTTTCAAAAATTGTTTTAAACCTTTTTTTTTTTTATCCCTCCCCTTTTCCCAAAAACCCCAAAAAAA
CAACCAACCCCGGAGGAAAAAAGGAGGAAAAAAAGAAAGAAGGAAAGAAAATAAAAAAGGGTTAAAAAAAAACAAATGCCCAAA
CTAAGAAAATTATTATTTGGGTTTGTTTTTGGTTTTTTTGGTTTTTTTCCCCCCCCAATAAACTTCCCCAACCCAAAGTCAAAA
AAAAAAAAAAGTGGGGGGAAAAAAAAATTGGGTCCCCCCCTAATGGTCCAATTTTTAAGGCTAAAAAAAAAAACCCAAAATAAA
ATCGAATTTTTTTTTTAATTTAGTTTTACCTTTCCGGGGGTGGAAAAAAAAATAAAAAAGAACCTTTTTATTTCTTTTTTTATG
TTTTTATGGGTTTTTTGGTAAAAAAAATGAAAATCAAAAAAAGAATTCCTTTTTTTTTCCCCCGTTTTTTTTTTTTTTTAGAAAA
AAGAAAAAAATGGAAATTTTAAAAATGTTTTTTCCCTGGTCCCCCCGCCCAAAAAATAGAAGCAAAAAATTATGTCCAAAATAGG
TTTTGGTCGGGGAGGTTTTTTTTTTTTTTTTTAATTTTCCCCCATCCGCCCAATAAATAAGGCCCCTTTTTTAAAGGGAACCCCC
CGGTTTTTTTCCCCGGTTTGACCCCCCAATAAAACCCGGTTTCCCCCTCGAGGGCCGAAAGTTTTAAAAGGGGACCTTAAACAA
AAAGATTGGAAAAAAATGAGGAAAAAAAGATTACATTTACGGAACCCCAAAGTTTTTAAAAAATTCCCCTTTTTTAAATAAAAA
AAAATTGGGAAAATTCCGAAAAAGGCCAAAAATTGTTTCCTTTTTTTTTTTACTTTAAATTTAAAGTTTTTTTAAACCTCTTTT
TAAAGGTTTAAAAGGAAGGGCGGGGAAAAAAATTTTCAATCCAAATTCCGGAAGGGGAAAAAAGTAGCTTTTTAACTCCCAAAGA
AAATTTCCCACCTTTTTCCATTTTTTCTTTTTTTTTTTATTTGGAAAATTACTTTCCCAAAATAAAGTTTTTTCCAGAATTTTT
AGCCCAATTAAAAATAAAAACAAAAAATCAAAATTAAAAGGAAAGAATGGGAAAATTTCAAATTTGAAAAATTGGGTCTTTTTT
TTTTTGCCTTTTTTTTTGGAAAAAAATCTTCCTTTTTAAGGAAACTTTGGAGGTAAAGGGGAAAAAAACAAGGTAAAGATTTGG
GGGTTTTTTATTACGAAAAAATTTGGAAAAAAAAGTTGAAAAAAAAAAAAACCCCCCCCCTTTTTTTTTTTTTTTTTATCCTTT
TTTTTTTTGGGGGGTTGGGTAACTAACTTTTTTTTTTTTGGGAGGCCCCCCCCCCGGAAATGAAAAAAAGGGAAAAAAAAACTT
TTTTTTTTTCCAACCCGTCGGGTTTTTGAAAAAAGGGATGGGGAGGGGGAATCCCCTTTAATAGGGGATCCTTTTAACTCCATT
TTTTTTTCCTTTTTTTATTGCCTAGGCCAAATTAGCGTTTTTAAAAAAAAAAAAAAAAAACCCCCCGTTAACCTTTTTCCTTACC
GATATAAAAACCGAAAGGGTTTTTCAAAAATGTCGAAAATAAAATGGAAAAAAATCCCAAAATTCCCCCCTTTGGGAAAAATTA
ATTAAATAGGGCCAATAAATCCCCCCCCCCCTTTTTTTTTTTTTTTTTTAAAAATACTTCAACGGCCCTTTTTTTTTTTTCCC
CGAAAATTGGCCAATTTTTCGAAAAATTTTTCTCCGAAGAAAATTGTTTTTTTTTTCTTTAAACCTGAAGAAGGCCAAAAAAAG
CGTGTTTTGGCTGTTAAATAATGCCCCGAGAAAAAACAATTTTAAACAGCCCCCCCCCGATTCTTTTTTGGGATTTTTTTTTTT
GCGGGGAATTTATATTTATAGATTTTTTTTTTTTTTCTTTTTTTTTCCCCGTTTTTTAGCAGAAATGGGGAAAAAAAAAAGGAAT
TTTAAGGGGGAAAGGAAGGGGCCCCCCCCCCCCCCCCACCGGGGGTTAAAGTTAAATGGGGAAAGATCGAAAAAGTTTTTTTG
GGGAAAGGAACAAGAAAAAAACGGGATTTTTGTTTTTGGGTTTTAGAACCGGCCCAAAAAAAATTAGAAAAATTAGCCCCTTTTT
TTTTAAGCCATTGTTTTTTTTTTTATTTCGAAAAAACCAAAGATATAGGAAAACCCAGAAAATGGGAAATTTGGCGGTTTTTTTT
GTTTTTTTTAAAAAAAAAATTGTTTCCCCTTAAAAATTTTTAACCCAAAAGTTTTTTTTTTTCCCACCCCTTTTTTTCCCCTCCC
CGAGTTTTTGGAAAGAACACAAAATTCCAAAAAATTTTTCCCAAAATAGTAATTTGGGCGGGGGGTAAAAAAAAACCTAATGAA
AAGTTTTTTTCCCGGGATATTATAATGTAAACCCCTCTTTTTAATAGGAACAAAAGAGTTTAAAATCCCCCCTTAATCGGAAAA

AAAACAAATTAAAAACCTTTCCCCCCTTTAATTCGGAAAATTTTTTATTTAAACAAGACCAACGTTTAAAAAAAAGACAAATT
CCAATTTTCTAACGCCCCAGGGGGGGAAAAAAAAAATGTAAAGTACAAATGTGTCAAAAGGAAGAAAATTGGTGTAACCAAAAG
AAAGCTTTGATGGGGGGGGGATAAAAAAAACCCCCCCCTTCCCCTTTTTTGTTTCTGAAATAAATGGAAAATTCCCTGCCCCCC
CCCCCCCGGGAAACCCCCAAAAACCAATGCGAAGGGGACGGGTCCCCCAAAAATAATAAATAAAAAAGGCTTTTTTACAAAGGT
TGTCCCGTTTATTTCTTCGGGAAATTTGATAAAATTTGGAAAGGGGCAAAAAAAAAAGGGGGAAAAGATTTTTTTTTTCCCGTT
GGAGAACCTCCCTACCCTTTTGTGGGCAACCGGGGTTTTTTTTGATTTAAATAAGTTGTCCCGGGGCCCCGGGTTTTTTTCCCC
CCCCTTTTTTTGTCCAAATTTTGGGTCCCCCCCCGGTCTAAAAAAAAGGTATCTTTTTTCCACCCCCCCCCTTTCCCCACCCTC
TTTTTCCCAAATATACAAATTCGGAAATGTTGGGGATTGTGGGGCCCCCGGGGCCGCGGACCAGGATTAAGCCAAAATAGAAAC
TTTTTTCCCAGAACCAAAAAACTTTTTTGTAAAAATTTTTCCCCCGGGGTTTGGTTTTAAATTAAAAGAATAAAACATAACCCA
GTTTTTTGTTGCCTTTTTTTTCGAAAACAATAAGTGGAGATTTTTTTTTGCCTTAAGAGCAAAAAACAACAATTTTCCCGAAAG
GAAAACAAAAGGAGCACGGGTTTGTAAAAAAATGGGGAAAAAAAAATACCTTTCCAGGAAAGGATTTTTTTTTTTTATGGCCAAA
AAAGGGGGCACCAATCCCCCCCCCCCCCGGGTTTAAAAATTTTTTTGGGGGCAAACTTTGGAAATTAAGGGGGGGCCCCCGGCC
CACACTCTTAACCATAGAAATTTTAAGGAAAAACACCAATAAACAAAGGGCCACCCACACAATACACACACCCAATATTGTTTT
TAGGTAGAAGGGCGCATGCTGCCGCTAAAAAAATTTTTTGTTGTTAACATCACCATAATTTGTTTTTAGTTTGTTGGTACAAG
GGAATTTTTCCCCCAAAATGTTGCAATAAAAAAAAAAATAAGAAGACCCTTTTTGGAAAAAATGGTAAGAAAACCAAAGATTTGGG
GCTTTTTTATTCAAATTGTAAACCCCCTTTTAAAATCTTTATTTTTGTTTAGGAAGGGGCCCTCCAAAAAAGCCGAAAAAAAAAA
AGAAAAAAGAAAAGGGCCTATCGTTATTTTACCCTTTAGAATGGATTTTTTTTTTTTTTTTTTCCCCTTTTTTTCATTACCAAAT
CCCTGGTTTTTTCTGGTGCCCCCCCCCTCGCCCCAGCTTTTTTATTTTATGGAAAAGAAGTTTCCCGCCCCATTTTTTCCGGTTA
AGACCAAAAAAAGAATAAACCCTTTATCAAAAAACAAAAAGATAAAAATGGGCCCCCCCCTTTTTTTAAAAATTTTTTGGGGCCC
CGCCCCCCTTTTTTTTTTTTTCCCCCCCCTTAAAAAAATTGTTCCATTTTAAAACGAGGGTGGGTAATCCGCCCCCGGAGGTTA
AAAAAAAAGTTTGGTGCCCAAAAATAGGGTTTTTAAAATACTATATCCCCCGTCTAAATCCAAAAAAGCAGAACAATTCTGTC
CAAAAACAATGAAAAAGAATTTCTGTTCGGAATAACATATAAACCGATCTAAAATTAAAGTTAAAATAGAAAAAAAGAACCAAC
CCCCCTTTTTTCGTTTTCGTGTTTTTGTTTTTTTGGGTAAAAAATAAAAAATTCCCACCCCCCTAACGCCCAACCCTTTTGCAA
TTTGGAAGGCTTTTATTCGGGCCCCAAAGAAAAAGAAATCCAAATTTTTTCTAGATAGGGTCCCCCCCTTATTCTTATGTGGGC
CTTCCCCCGAGGTGGCGGAAACCTTCCGATTTTCCCCAACCGCGCCCGGTTTTTACTTTTAAAAAACTTCAAAAAAGAGGCCCC
TTTTTCCCATGTTCCCCCGAAAAAAAAGGTTTTTTTTTTTTCACTTAAATTGAAAAATTCTTTTTTGGGGGTTTTAAACCCTA
AAAAATCAAATAAACAGGAGAATTTTTTTAATATGGGACAAAAATTCTCGTTTTAAACCTAAATAAAGTTAGAAGAAAAGTATA
AAAAAAAAGGAAAAAAATTCCCCCTTTTTTTTTTTTGGCCTAATATATTGGTTTTTTCGTTTTTTTTTCCCCCCCCCCGAAAACA
ACTACTTTTTGGGTTGTTGGTTTTCCATTAAATTTTTCTCTTTTTTTTTATCCCGCCTAAAATCGAAAAGAACCTAAAAATTAC
AAAAAGGTGGGTTTTTTTTTTGGCGGGCTTTGTCCTTGGGCCCCCCCCCACCCTTTTGTTTTTTCTTTTTTTATACAATAGCG
GTACCCCTTTTAAAATGTTCAAATAAAATTGGTTCATCCCCCCCAAGGCTTTTAAAAAAAGTACACGTTTTTTTCTTTCCTTT
TCTCCTGAATCCCCAGGTTTGGGTGAAATTCGGAAGTTTCCTTCCCGGTCTTTCTCAAAAACACAAGGGGAACCGAACCGTTAA
CAAGTTTCCCTGAAAATTCTCCCTTCTTAACATCTCTCGAACTCACGAAACATTTCGGGAGGAAACAAGGAAAAAAAAGGTCTT
TTTTTTTCCAAATTATCATTTGACTGCCACATACCCAATTTTTTTGTCCCAAACCCCCCAGAACCCTTTCGATCCCCCAGGAAA
ATATGAGGGGGCTTTTTAACCTTTTTATGGCAAAAAAAAAAAGAAAACCGGGGCCGGCCCCGGTCGTCCCCCCCCTGTTCTTTT
TGGGGGTCTTTTTTTCCAAACAAAAATTAAAAAGGTTGACAGGATAGCACTGGAACAAACTGCCCAAAGAAATTTTAAAAATCG
AACCTTTTAAGTTAGTTAGCAATTTTTAACTTAAAGGAATTTTCAAACTCTTCCCGGAAAAAAAAAAAAAATGGCCCAAAAAAAA
AATATACCCATTCCCCCCATACAAAATCCCCCCTGGGAATTCCAAAAAAAGTAAAAAAAAAAAGACCTCCTTTTCTTGGGGGGG
GGTTTCCCCCGGCCAAACACGGGCCCCAACTGGGGGCGCCCTACCAAAAATTTCTTTTTTGAATTTTTATTCCCAAAAATTAGG
AAAAATTGATGAATCAATCTCTTTCCCTTCCCAAAACAAACCAAACAAAAACCGGAGGGAAAGGGGAAAAAAAAAAGCGAAAAAA
AGAGGAGAGTCGGGAGTTTTCCATTCCCAGAATTCCCTAAAAATGAAAAAGATTAGAATTTTGTTAAGCATAAAAAGTCGTGGG
GGGGGCCTTTTTTTTTTTTTTGCCGGAAAAAAAAACCCCAGGAGGTCCCCCCTTTTTTTTTTTTTTAACTTGATATCCCCAGGGG
AAACCTGGTCTTTGGGGGATTCTGTAAAAAAAATTATTTTGCCCAATTCCCGAAAAAGGTTGGAGTGTTTTTCCCCTTTAAATT
AAATCTTACCTTTTAATAAGTCGTTTTTTTTAAATGGCACAGTTTTTTTTTTCCAATCCTTTTTTAAAAAAAAAATCCAAAACTT
TTTTTTTATTTTGTGTTCGCGAAAAACAGAACCCGAAGGACCGGCCCCCCCCGTTCTTGCCCCAAAATTAACACAAAGTTAAAA
ACCTCTCCCCCAAAATCATGTTTTCCTTGAATCTGAAGGTGGGGGTTTTTTTTATTCTTTTTGACCCAATGGGTTTTTTTCTCC
TTTTGCAAAAAGTCCAACATATTGCATTTTTGGCGGGGACAACAATTTCGCTTTTTTCCTCCGTA
>m141121_232159_42134_c100732402550000001823141405141507_s1_p0/95562/10119_21892
TATCTAGATATGGGAAGACGAAGTAGCGATGATAAATTTATTATCTCACGTTGACCACCAGAGCCCTGAACCGTTATGTCTTCT
CCTCAGAAAAGATTGTAGTTAAAGGCAGAGCGTTGGTTTAGTTGGTCATAAGTGGATCTACTACGAATTTTGTTAGATATATGA
GGCGTGTGCGCGCGCGCGGTGTATGAGTGATAAGCCAATTTAGTTGGGCGCATGAATTGCATTGAGGGAGATATGGGGCTCGGG
GTCAGATATATTTAGTTCTATTAAGATTTTTCCGTAATGAAGATTGTCTTTTGAGATCTGAGCTCGAAGTGGCATAAATGATAG
TATGTAGAGCTAGATGGTGGCGATTGCCAATATTACTTTATAGACAGAACCTCTACTTAAGAGCAGATGATTGTCAAGTTGGGG
AGGGTAAATTGGTTGAATGGTATATTACTAGCGGATTAAGGTCAGCACGAATATTGGGTTCTTGCGGATGGTGTATTAGCGGTT
TAGCTAAGGGATTATTTTCCTATATTCATGGGTTGAGATATAGGTTCGTATATATAGTCGAGACTTTGCGGTGGTTGTGTCTCA
GTGGAGGTAGATGATTGGATTCAAGACTAGGATAGATTATAGACTAAGATGGAGGCTCTCGATCACTATCACGCCGCACTCGCT
TAATCCAGGTCCAACGAAGATGTAATTAGGATCTACGGACATTTATATTGGTTGCCCGGGATGAAGAAGGATGTGGTGGACGAG
TGTGGCCAGTTTGGGACGTTGTCAACCAGGCGAGAAGCTGAGGCAATATAGCGAACCGCAGGGTTGCTTGTCAGCCTTTGGTAA

TCCCCGAGTGGAAAGTGGGAGGATATTACTATGGATTTGTGGGAAGGTTTACCCAGGACTGTAGGGCTTCATGGCTAAGTGTGG
TGATAGTGGACAGATACACCAAGTTAGCTCATTTTCTGCCAGTGATGTTCGACTTACATAGTCGGAATAGTATGTGGAGTTCTA
TGTGAGGATATAATTCATCTGTCATGGGGTTCGTAAGTTTCATATTGTAATAGGTGTTTGAAGATTATTGAGATAATGCAGGTG
TATGATGGACCCGCAGTTCGTTTTCTCTCGTAAGTCCTTGTGGGTCTCGCGCTGCCAGAATAGCCATAGGGACTCAGTTAAAGT
TTAGCGAGCTTTTAATCATCCTCAGACTGCTAGACAGTTGGCTAGAGGATGGGAATTCCGAGTGTTGGATTATTCTTTAGTTGG
GCGGTGTGTGCATTGGAGTCAAACCTGATTTGGACAAATGTTTGTCTTAACATTGTTGTCCTTGGCACCGTCGGTGGCTACAGG
AGTTTCATGTCGGTTCCATGGTGCGCGCGTTGAAGAATGTACTGTCTCTGAGTATTAGACGACTCATGTTGTTGTGGGATATAA
TTAACTCGACTGTAGAGCTGAATTGTCCGCACAGGTTGATTTATGATCCTCCCTGGAATGATCTGGTTGTCAGTAGGTTTTACG
TAGTATAGGGGAGAACAGAGGAGGCATCTATGGAGACATAAGCAATTCCGCTAGTTAGAAGGAGTTTTGTGTATGAAGTTTATA
GTAGCAGGGTTTATGGACGACCTTGGGCAGCTGTGGATCGTTGATGGGGAATCCAGTATCCGAGCTACTTCAGGTAAATTTCTT
AGGACTGATATTTTATCTTATGGTAGCTTCGTGGGATTGGTGATTTTGTGTATACGACTCTATAGACAAATCTACTTAATAGAA
GGATTCACCGGGCCTTTGGAAGGGCTTGATCTATTGGGTGCGCCCGGCAGACGATTTATTTATGTGGGTGTGCAGTTTAGGTAT
GGTAATGCCATGATCTCTTGTGGAAAAACAAAATCAAGAGAGCAACAAAAACAAGCATAGTGCTGTGGTAGTGATTCATTAGTG
AGAGCATGGCTATCATATGTGGGGTTACCAACGCTTCGCAGTAACTAGTATAAAGCAGATAGTTAGGCCCGCGTTTAGCTTTAA
AGGGGTATATTTGTAATATATAGATAAGTTTGATGGGATAAATTGTTATCCATAATATAATTGTGGACCACATTATTATGTGGT
ATTTCTCAGCCATGCGAACTTGAGGTATCCTAGGATGCCTAGTTAGCGGTAGAGTCAGACGGACCTAAGTACTTGACTGGGCCA
TCAACGGGTATTTTGGGAGTTGGATGATTATTTCAGTTATCAGGTTATGGAAATAAATAAATTGGGAGATATACTTGAGGTTAG
AAAGCTTAGGGTGGGAATACTGGGGATTTTACCATTTTTCTCTGGGGACATTTTTGAACCCCGAGCCTCGGGATTGACTAATTG
ACTAAGGATATGACTAGCAAAACCATGAATACAGTGGAAGATGTTCTAATCCGACCCCCTTTTCTCTCTTCTCTCCCTAACTTA
AGGGAAACCACAAGAAAAACTTAAGAAACTTGGTTGGAACTCCAGGAATAAATAAAGATATGGAAAGATTAACTAAAGTAGTTG
CTAAGGAATCTAAGCAGCGAACTGAGGCTCTCCAGCGGATTGAATCTATAATTTTCTGCGCGATCCAATTTCTCTTTCTGCACT
TATGCCTCAGTTTTTGGGGCTGGATTCTAAGTGTTATGGGTTCTGACTTCAAAGGTGAATTAAGGTAGAAGACTTGGGGTTTAG
TCAGCATACTTCTTGAGGATTGGTTCTTCAGTGAAGGTAAGTTTCAATTTATGGTTTAGTGTTTGAATTCCTGGGTTTCTCCAT
TGCGGTTTTTGATGTTCTTGAGCTTGTGGGTTCAAAATTATAAATGTTTGTTTTGATGAGTTTCCTAGACTAGGTTTTTGCTGG
ACTTATGTTTCCTGAAAACATGCCTAGAATGCTCCGGCTCCACTTGCTCAATTGGATTGGTGTCATTTGCATGGTTTTGGGTCA
ATTTGAGTATTCGGAGTGTAACATCTGCGTGGACTTTTCTGGTTTGAAGGGGTCGGGCCACGGCTTTGTTCTTGGTGGGTCTGC
CTTTGAAGCAGATTAGGTGCTGGCAGATAGAAGGGTGGGTCGCGACATGGCTGTGTGAACGGCCGCGACCCCATGTTTATGTTT
TGGGGTGAGGTTGGGCCTCTTAGTTCAGGGCAAGCTGCGACATTGTTGTAGCTTGACCTCAATGGCCTTCCAAGGATAATTTGC
TTTTTGGAGATTTCTTAAGGACGGTAACCTAACTTGGGTTTTCGGGATCGATTCCACTACCGGTGTTTGGGTGAATTTCGATGT
CCGAAGCTAGAACTTTAACTGACCTTTATACATTATATAAGATGGAAATCCCTTATCTTGGTTGTGACTAGGTGTAAGCTAAGG
CTGGGGAAATAGATCGTGCTCCCCAAGGGGCATCTCTACGATTTCTTCATATCAGACTAGTTCATGATGTGGAAAAATAACCAT
CAAAGGTTGTGATATGGATCTGGTTCTCTCGAGTGTTTGTTTGAATATTTGCAGTTATATTGGGACTTAAGGTGTTTCCCGTGT
TCTGTAGGTCATTCATTTGGTTTTACGATAGTCTGGGTAGGTTTTGGCTTATATGGGGTTCCGAATGATAGGATTAATATGAGA
GCGCATCTTAGAGAGAAGATGAGTTGTTTCGGTTAGGGTTATACTTAGTTTTCGACGCAACATAGGATTTGAGTGGGGGCCGTC
TTTGGAAGCTCAGCTTGGCTAGAACTCGGAGCGAGCGTATACATTAGTAATGCAGTTGATGCTCGGTTTTAAGCAGACCGAGTG
AGTTGGGAAAAGGAAAGTGGCGCACCTAAGGGCCGTCGACCCCTGATTATCCATATCATTTGTTTCATTGTTGACTGATGCATT
TATGTCATGCTGAACTAGCTTAATATTGGGTTTGTGGAGAAGTTTTCTGGTTCTTGAGGGTTGATCGTAGATATGGTCTACGGT
GTGTTTAATGATGAGGATGCTGTGTATACATGGTATTCGCATACTCTCTAAGATCTAGTTTCAGAGTCATATAGAGAGATCGAT
TTTGTGTTTGGTGCTATAGGTTATTTTATTTTTTTTTTTTTTGCGATATAGGGCCTGTGCTTCAGTGTAGAGGGTGGGGGTTAG
AGGATGCCAGACAGCTGTAGCAGCCTAGACTCATGCGTCGACCTTGAGAAATCCTAGAGGTTGGATTACATGACGTACGCCTGG
TTTTACTGTCAGCCATTTGCGATGTGTTGTAGTTTTTAGGTTGGTGACAGCACATGTGGGTGTGTTATTGCATTCCTTAATTGC
GTCGAAGAGGTCTAGTGGAGATCTCTTATGCTTCTACTGCACAGTTTTGGTGTAAACCGACTTCTTCAAATGTTGAATTTTTCC
GATTGGTTTGAGAGAGTACCGTATGTAGACTGCGTTTAAGATAGTTGACCCTATAATTTCACATATGGTTTTGATTCTTTTATG
ATCATAAAGCATGTGTGATCCTCTAGTTTCATTTGATAATGGTTTTAGTGATCACAGTTTTGGAACTCTAAATGACATAATGAC
CTTCTAGACAGTCGCGCTGCACAGATTGATGAATTACACATGTAACGGGTCTTGGCTACCTACAGGGAGTTTTACCGTCAGTTC
AGATCGGTATATACAAGCCACTCAGAGGTTGCAAGGTCCCATGACAGAAGTATGTTTTACGTGGTGTGGCGACATAATTGCTGG
TAAACAGGAATAAGAAGGCAACAGCTGATGATAGTGTATGAGGCTCATGTGTATGACAGTTCGTATTTATGCAATATTGTGTAT
GGTATCGTTGTGAGGAGGCCTTGTGGATGGTGATGGTGGTAACTTCGTTGAGAATATTAGAGCATGATTTTACTAGAAGTCTCT
ACGCTTGATGGGCATTAGAACCTCAGATATTATATAAGTTGCCCTATGATGGTTTCTCATTGTTCTGCACTATATGTCATTGTT
CGCGGTTCTTCTTGAATACTTCTATATGAGTGTATCTAAACCACGGTTTGACATGTCTCAGTCTTGTTCTTGGCCGAGGGTCGT
AAATGCGGGCATCATTAATAGATTCGGCGAAATCTGATATGGTTCAGTCGGGATGTTGAGAATGTAGCGCTCACGGCTCTACCA
CATCGATGTGTACCACCCAAGAGAGCGAGCTTCCGCCGACCTGCTCTGATATATATAGCGTCTCTAGACAGAGAAACTAGTTAG
ATAATTGCAAGACATATGTATGCCCTGCACTGGTTTCCACATGGAAGGCACTAACAGTATATTCTAAGTAATCGGGCCGATAGG
CAGGGGAGCGCGCGCGCGCAGGACGATACATGCGCTCATCAAACGAATAGCATGTTCCGGATGGAGTCGTGGGTAGAGAGGAGA
GAGTGTGATGTTCATACACAGGGATATATCTGGTCAGACATCACGTTCTGCACCATGGACTGAAGCATGAGATTAAATTGTTTT
TGGTTGCCCGGGGAAAACCGTCAGGCAAGCCTTATCGCAATATGGCATTCTCTTAGGTCAAAACTCCAGGGGAAAAAGTGTGTG
GGGAGGAATCACACTCCAAAAATTATTGGGCCGGAGAAGTTAACATACCGGCGTTTTCCGCCCTATATGTTCCTTGCTGAGGGT
GTGTACTCTCTGGGGAAGATCCGGATCAAAAAACACAAGAGGGGGGGTTGGAGGGGGGTCATAGGGCTCGCCTCTCTTTTAGTTT

TGCAACAATATATTAGTGACTTGTTCACTAAATAGACTCCCGCGTATATATAGCGCACATGGCGCGCGCTCTCCTAAATGAAAT
TTATTTTCTCCCGGCCCCAAAAAGTTTTGAGGGCCCCGGGATGGGAGAATATGAATATTATCATATATAAAACCCTGGGCCCC
CTACAAAGAGTAAGAATGAGAGTGCATAAAAAAGTCCACGATGGTTTATTTTTTTTTGGTGGGTTTATTGGTGGTAAATGGGTG
ATAATAAATGTGGATAGCAGGATAAATCGGGGTAAATATAGAGCATAACCGCGCCTACTGAGTGGCCGAAGAATATAAAAGAGT
TGGCTCGCACTTTTGTTTAGATAAACGAAATATACGAGAGCGTGCGTGCATTGGCGCGAGCGCGCCACCACTTTTTGGTCTTAT
TTAGTGATTTTGCAGAAGGGTTTTTATATGCGCGCGCGCGGGCGCGCGGCCCGCGCGCGCGCGCGCTCTAATTTTACGCTCAAT
ATATAATTTAGACAAGTCACTATCCGGCGGTAGAAATGCGTGTGTTTTCTGTTTTTGTTAAGGTTTATCAGAATCCTTTTATTT
GCGGCCTCGTTTCTCTATTTTACCAAAAAGAATAGAATTTTTCTATCGTTTGGAGAGCCTTGCGCTGCGTTTTTGCGCTTTTAT
TTTTTTTCTTTTCTATTCTCTTTCTTTTTTCATGTAATTTAAATACTTTCTGGTACCTCCCACACATCCCTCCACTAGATTTAT
TTGCTTTTTTTTTTTCCTCCAGAAATTTTTTCCTTTTTTTCGTCCGGTATTTATTAACGCACGCCCTCCGTCCAGCCCCGGGGG
AGCGCGCGCGTTACATAGGGGATCAGGTCTTTGAGGCTACGCGTATTGTTTTTCGCGCGTAGACAGAGGTTGGGGGTGTACATA
TATCTATTTTATATTATATTTATCATCCAGGATAAATTGGCGACTACATCATTTAACGGTCATTTTGGTTGCGCGGTTCCGTGT
TATTTATGAACTTTTTGGAGAAAACCATAAATCGCTGCATAGTAACATCTCACACACACCCCCCATAACACCAAAAATGAGAAT
TAGCCCAAAGACCCCGTTACCAACTTTTTGTGTTTATTTGTTATTAAAATTTGTGCCCCACCAAGGAAACCCCTTTTTTTGGCT
TTTAAACATATATCCAGTGTCGCACAGGAACCAATTTACAATGATTTTATAAAACAGAGGTATGTGGCGGAACAAAAATGTTTC
ATGTTCCCACCATAACTTAAATATACTCTTCATATTTAAGAATTATTAGATGAACGGTTAAAACATAATACAGGGGTTTTCTCT
TTGTTATAATCAAGACCGAAGAGGGTGATTATACTATTGTTTTGTTCATGTATACACAGGACTTATTTTTGATGGTGTTTTCCC
CCGATAAAGAATGAATTACGCACAAGATGCTGATTGAATTTACACCCCATTATATCGAAGCAGATTTTTGCGCACCAATTAAAA
CATACGATTATAATGTTATTATTTTTGAGGGAAAACAGTAAAGATGTGTTTTTAGCGTGAATCTACCCCCATTTTGGGGATCCC
CACAAAAGGAAATAAAAGTCTAACCATGTGGGTATCGTAGTTTTTGGGAAAAAGGTTATCGGGGTTATAATCATTAGTAATTGT
TTAATGAGTTTCTTATTATATTAGAGACACTATAGAACCCTATCATGTTTCCAAGAAGGTTAATTTTTTTACCAAAAAAAAAGA
ACCCCAACTTTTTTTTAAAAGCCCAAACTCTTTAAAGACGGCGTCGACAAAATGATAAATCTTTTTGTTTTACTAGAGAGTTAC
CTCCTCGCTCGCCATATGTTTGAGTTACTTCCCTTTTTTTTAGTATTTCTCCGCCTCATTCTCGCGCCCTCATTCACAACATAT
TGTTGCAGGCTCGTTATTCATCTAGCATGTGTTTTCATATACACCGTGTCACTCTCCCAATGTAAAAAGAAACCATTTTTTTCC
CCCACCCTCTTAGAATTTTTTTCCCCCTCCCCCACAAGGTCCCCCCCCAAGCCATGATTTTCAAGCTTTTACCCCTTTTGCCCC
CTTTTTTTTAAAACCCCCTGCAAAAAAACCAAAAAAACGTAGCCAAAAACCCCCCTTTTTAAAATTGAGGCCCAAGGGCCCAGGC
AAAAAACCAAAAAAAAAAAAAAATAATAAAAAAATTTAATTTAATAAAGCAAAAAAACCAAAAAAAAAAAAAATTTGATTGAAAAT
AAAAATTGAAAATAAACCCTCCCCAAAAAAAAAAAAATTTTTTAAAAGCCAAAAAATGGGGTTTTTCCATTACCCACCCCTAAAC
CAAAATTTTTCCCCAACAATTTTAAAAAATTCAAAAACGAAAAACAATTTCCCCCATAGGAACCCATTAAAACCCCCCAAGTAA
GAAAAAAATTCCCTTAAACCAAAAACCAATAAATTAAAAGCCCCCCCTTTTTTTTATTTTCAGCCCAAAAAAATGAAACAAACA
AAATCTTCCCAAAATCAAGTTCCAAAAACCAAATGGATTTAAAACAAAAAATCATAATGATAAAATCAAGGGTCCCCCGACCAC
CCCCCCCGCCCCCTTTTTTTTTTTTAGGGGTTTTTCCCCCCGCACTTTTTTCCCCCCCCCCCGCTTTTTTTTTTTAACCCACT
TTCCCCAAAAAACTTCCCGGTCCTTTTTTTGGGCCTTTTTTTAAACCGGAAAGGGGCCTCAAAGTGAAATAAAATTGAAAACTT
TTCTTTGTTCCCCTTTTTTTTTCCCCCCAGGCCCTTTTACCCAAGTTGGGGCCTTGGGAAAGGCCCCCCCCCCCGTGGCCAAAC
CCCCAATGGTGGCCCGCAAAAATTGTTAAAAAAAAAAAAAAAAACCCTTCCCGAAAAAACTCCCCTTAAGGCCCCCCCCCCTTT
GCGTTTTTTTTTTTATTTAATTTTTCCCCCCACAATTGGCAAAAAAAATTAACCACTCACCCAAATTCCGGGGTTTTCCCGTAA
AAATTCAAAAACTAACACAGCCAAGGGAAAAAAACCCCCCCCCCCCTTTTTTAAAAAGTCGGGCCCAAATTGTAATAATTTTTT
CCCCCCCAACCAACCGGGTCCCCAGTTTTTTTTTTTTTTTCCCCCTTTTTAACCTTTTTTCTTTAAAAAAAAATTTTTTCCCCA
AAAGAACCCCTTTTTTCTGAGTATTGAAAAAAAAAGAAAAGGAATGCCCCCCCCACCTTTTGAGGGCCAAAAACAAACGAAAAT
CCCCCCCTAAAACTTTTTTCCCAAAAGCCCCTTTTAAAGCTTTACCCCAAACTTAGTCTCCAAACAACCCCAAAAAGAATAAAG
GGGGAAAAAATTTTTCCCCCATTTAAAAAAAAAATATAATTTGGTAAATAAAAGGGTTTTTTTTTTTCCCCCCAACCAGTTTTTT
TTAAAAAAAAGTTTTTTTTTTCTAAAAGCCCCAAATTTCGGGAACCCAAAAAATCGGGAAAAAATTCCCCCCCACCCCCCCCCC
AAAAAAAAACAAAAAACGGTAAAGTTTTGGAACAATTCCCCGAATTCCCCCCCCCGAAAAAACCACCCCCCCAAAAAAAGGGTT
TTTTAAAAGGTTTTTTTTTTTTTTTACCGTCCCCCCCTTTTTAAAGGATCCCTTTTCCCAAAAAAGCAACCCCCAACAAATTAA
TCCCCCCCCTTTTTGGAGGCCCCCCCCCAAATTTGGGGGAAAAGTTCCCAAGCTACACACACCCCTGGGGTTTCCGGCAAAAAG
CTTTTTTTTTTGCCCCCCCCTTTTAAAAACCCTAGAAGGGCCCCCCAACAAAAAAAAACCCCTTCCACCCCCCCAAAAAAAAAT
TAGACATGGGGTTTCCCCCCCCGGCCGGGGCCCCCCCCCCCCTTTTTCAACAAAACCCCCCCACAAATTTTTTTGTCCCGGGCC
GACCCCCCCCCAAAAAAACCACTTTTTCTTTTTTTTTTTCCTTCCCCCCAAAGCCCCCAAACCCCTTCTTTTATTTTTTTTAAA
AAAATCTTGCCCTTTTTTCCCAAAGGGCCCCCCAAACCGACCCCCCCCAAAAACCCCAAAAAGGAAACCAAAAAGGCCCCGTTG
GGGCCCCCGGAAAACCCCCTTTTCAAACCCAGAAAAAAAAAAAAATCCCCACCCAAAAATTGTTTAAAAAAAACCAACTTTTCC
AACCTTCAACCCTTTTTTTCCCCACACAAATTTGGGAAAAAAAACCCCCCAAAAAAAACCAAATTGGGGGGCCAAAAAAAAAAT
GCAACCAAATCCCCCCCAAAAAAAAAAAATTAAATCCCAATAACCAATTCCCCCCCCCTTCTAAGCCAATGTTTTTTTTTTTTCC
CCCCCAGGAAAACCCCCCCAATATAATTTTCCAAAAGCAAAAAAAAAAACCCTAAAGTCTAGGAAAAAAACTCCAAAAAAAATCC
AAAAAACCAAACACCCAAAATTTTTTTTTTTTTTAAATTTTTCTGAAACCCCCAAAACCAAGTTCCCAAGAACAAAAAAAATC
AAAAACCCCAAAAAGCAAAGGGAAAAAAAAAAACCCCAGAAAAATTTTCAAACCCCCAACTAAAAAAAACCCAATAAAATTTGG
GAAACTTTACCCCTTCCCCAAACCCTGGAAAAGGGAAAACCCCCCCAAAAAAAATTTTCCCCTCCCCAAAAAAGAAAAAAAAG
GTTTTTTTGAATTGAACCTAACCCCCCCCAAAGTCCCTTTTTTTTCTTTTTAACCTTTTTTTTAAAATTTCCCCAAAACCCTTT
TTAAAAATTTCAAAGAAAAACCCCCATTTCAACCCCAAAAACTTTTTTTAGAAAAAAAATTTTTCCAAGCCCCCCCCCAAAAAA

AACCTTAAAAAAAAACAAAAAATAAAATCCAAAAAAATTGAATCCAAAAATTTTTAGATTAATTTTCCCCCCCAAAATGCCTTT
AACCCTTTCCCAAAAAAGTTTCCCTTTGCCCTTTAGAATTTCCTTTTAAAAAGCAAAAAAAACTTAACCCCTTTTGAGTTTTTT
TTTAAAATCCCCTTTTTTCCCAAAAAACCACTATTTTCCTTTTTTTTTCTTGTCCAATTTCCCCACTGAAAAGTTTTTTTTT
TTTCCCAACCCAAAAAAAAGGTTTTTTTTTCCTTTTTTTTTTAGGTTTTTTTTTTTTCTTTTGTGGGTTTTTTTTTTCCCCC
CCTTAAAAAAAAAGTTAAGGGAAAAAAAAAGAAAAGAGAGAAAAAAAGGGGGGTTCCCCCCCGAATTAGAACATCCCCCTTCTCC
CAACCTTTGTTTTTTTTCATGGTTTTGGGTTCTAAGGTCACTTAATTCCCCCTTTTAGTCAAAATTAAAAGTCAATCCCGTGA
AGGGGCCTTTCGGGGGTTTTTTCCCAAAACAAAAAAAAATTTGTCCCCCCGAGAAAAAAAGAAAAAAAAAATTGGGGTAAAAA
TTTTCCCCCCCCCCCCACCCCCCCCCCCAAAAGTTTTTATTCCCCCCAACCCTTCAAAAGCTTTTTTTTTTTTTCCCCTTTT
TTTAAACCCTTTTCAAAGTAATAAACCTTTTTTCCCCCAATTTTTTAATTTTAAGATTTTCCCATAACCCTGATAAACTTGAAA
AAAATCAATCCAAAATCCCCAATTTTTTTTCCCCCCCCACCCCCAAAAATAAAAACCCCCCAAACTTGTGGACCCCCCTTTTTT
GCCCCCCAAAAAGGGGTCCCCAAAAAAAAAAGTAAAATTAGGTTTTTTTTTCCCCCAACCGTTTTTTTTTTTTCCCCTGAAAAC
CCCCCCTCTCCCCCCCGCTTTAAACCTTAAAAGCCCCTTTTTTTTTTTTTTTTCCCCCTATGGGGGGAGGAAAATATTCAAAAA
AAAAACCCCTTTTTCCCCAAGTTTGGCCCGAAAAAATTGCGCCCCTGCAAAGAAAAATTAACCCCCCAAACCCCATAAATTTAT
ATGGGGTGGGTTTCCCCCCCCCCTTTCCCCAAAAAAAAACCAATTTTTAAAAATATCAAAATTATAATTCCCAAACAATTTTTT
TTTAAAATTGCCC
>m141121_232159_42134_c100732402550000001823141405141507_s1_p0/95679/6247_11460
ACCATATTGTACAGCTGTCCATAGCATATTTTTGGACACTGAAAGGTCATATTTTTTGCTTGTTGAATTATCCGCACACTCCAA
TTCCATAGTCAACTATAGGATTAGCACTCATCAATACAAATATGATAGTCTTATTCAGTAGTCCATCACTATTAAGGTTTAGAA
CATGACCTGGGTAAGACAAAGGTCACAATTTCTACCAACTCATAGAAGCCTTGGGTTATGAAGCTATGATATAACCTCATGGGA
CGCATCATCAACATTGCCACGCTTCGGCAGCCACTAGGCAGGCTCCTAGGAGATTTTGGCCTGAAAAGGTCAATTTTTTTGTTT
GGTTGAATTAGGACCCCAATTCATAGTCAAAAATAGCCCTATATAGATATTTTGAGGTTTAGAATGGAAAAATTTTTCAAAAA
TTTACAGGAATCATTTATAAGGAAATAGGTTGTAAGTGAATTTACGATCGCTTTCGGACCCTAGGCAAGCTCTTGGATATTTGG
CCTGAAAGGTCAATTTTTTGTTTGTTAATTCACCCAACCCCAATTCCTGGTCAATAATGAGGCCCTAATATAAAATATTATTGA
CATGGGGAAAAGGTTCAAAAAAATTAGAGCATTTTAAGAGAGAAATATGAGTGAATCGAAAATTCGCTTCAAGAGCCCTAGGCA
GTGCCTAGGAGATTGTGGCCTGAAAGGTCAATTTTTTTGTTTGGTTGAAATTAGACCCCAAATTAACGGTCCAATAATTGCCCT
ATATAATATTTATTAGAAGAGGGAAACCTGTCAAAAAAATTAGAGCTTGTTATGAGTGACAATATGATGAATTACGAATATCGC
TTCGGAGGCCTAGCAAAGCTGCCTAGCGAGATTTGTTCCCTGAAAGGTCAATTTTTTTGTTTGGTTGAATAGGACCCATTCATG
GTCACATAATGCCCTATATAATATTATTGAGATGGAAAATGTTTAAAAAAATTAGAGCTTGTTAGAGTGAAATTGAGTGAATTA
ACGAAATCGAATTTTTTTTCGGAGCCCTAGGCCAAGCTGCCTAGGAGATTTTGGCCGGAAAGGTCAATTTTTTTGTTTAGTTTAA
ATTAAGGACCCCAATTCATGGTCAATAAATAGCCCTATATAACTATTACTTGAGATGGAAAAGGTTCAAAAGAATTAGAGCTTG
TTAGAGAGAAATATGAGTGAATTACGAAATCCTTCGGAGCCCCTAGGAGCTGCCTAGGAGATTTGGCTTGAAAGGTCAATTTTT
TTTGTTTGGTTGAATTAGGACCCAAATTCATGTCAACTAATAGCCCTATGATAATAATTATTGAGATGGGAAAAAGGTTTAAAA
AAAGTAGAGATTGTTTAGAGAGAAATATGAGTGAATTACGAAATCGCTTCGGACCCTAGGCATCTGCCTAGGAGATTTGGCCGG
AACAGGTCAATTTTTTTCTTTGGTTAAATTAGGACCCCCAATTCATTCAATAATAGCCTATATAATATTATTGAGATGGGAAAA
GGTTCAAAAAAAATTAGAGCATTTTTAGAGAGTAAATATGAGTGAATTAACGAAATCGCTTCAGAGCCCTAGGCAGCTGCCTAG
GAGATTTGGCCTGAAAGGTCAATTTTTTTGTTTGGTTGAATTAGGACCTCCAATTCACGGTCAAATAATAGCCTATATAATATT
ATATAGAGTTGGGAAAAGAATCAAAAAAAATTAGAGCCTTGTTAAAAAGAGATAATATGAGTGAATTACGAAATCGCTTCGGGA
GCCCCTAGGCCAGCTGCCTTGGAGATTTGGCCTGAAAGGACAAATTTTTTTGTTTGGTTGAATTAGGACCCCAATTCATGGTCA
ATAATCAGCCCTATATAATATTCTTGAGATGGGAAATCATGGTTCAACAAAATTAGAGCTTGTTAAGGGAGTAAATAATGAGTG
AATAACGAAATTCGCTTCGGAGCCCCCTAAGGCAGCCTGCCTAGGAGATTGGCCTGAAGGTCCAATTTTTTTGTTTGGGTTGAA
TTAGGATCCCAAAATCATGGCTCAATCAATAGCCCTATATAATATTATTGAGATGGGAAAGGGTTCAAAAAATTAGAGCTTTGT
TAGAGAGAAATATGAGTGGACATTACGAAATCGCTTCGGAGCCCTAGGCACGCTGCTAAAGGAGATTTGGCCAGAAAGGTCAAT
TTTTTGTTTGGTTGAATTCAAGGACCCCAATTTCATGGTCCAATATAGCCTATATAATAAATTATGTGAGATGGAAAAGGTTAA
AAAAAATATGAGTTTCGTTAGAGAGGAAATATGAGTGAATTAAGAATATCTCTTTGGTGCCTTAGGCAAGCTGCATAGGAAGAT
TTGGCCTGAAAAGTCCAATTTTTTTGTTGGTTAAATTAGGACCAATTCATGGTCAATAATAGCCCCTATATAATATTATTGAGA
TGGGAAAAGGTTCGAAAAAAATTAGAGCTTGTTAGAGAGAAATATGAGCTGAATTGTGAAATCGGCTTCGGAGCCCTAGGCGGT
GCTGCCTAGGAGATTTGGCTTGAAAGGTCAATTTTTTTGTTTGGTTGACATTAGGACCCAATCATGGGTCATATAAGCCCTATA
TAATATATTGAGATGGGAAGGTTTAAAAAAAAGTAAGAGCTTGTTAAGAGAGAAATATGAGTGAATACGAAATCGCTTCGCGAG
CCCCTAGGCACATCCTGCCTATGGAGATTTGGCCGGAAAGGTCAATTTTTTTCTTGGTTAAATTAGGACCCCAATTCCATGGCT
CAAAATACGCCTATAAATATGTATTAAGATGGGAAAAGTTCAAAAATTTAGAGCATTTTAGAGAGAAATATGAGTGGAATTACG
AAATCGCTTCAGAGTCCCTAAAGGCAGCTGCCTAAGGAGATTTGGCCTGAAAGGTCAATTTTTTTTGTTGGTTGAATTAGGACG
CCCAACATGTCACGGGGTCAATAATAAGCCCCTATATATATTATAGGCGTTGGGAAAAGAATCAAAAAAATTAAGAGCATGTTT
ATAGGAATATATGAGTGAATTTATCAATTAACGAATCGCTTCGGACCCTAAGGCAGCCTGCCTAGGAGATTGGCCCTGAAAGGG
ACAATTTTTTGTTTGGTTGAATTAGGACCCCAAATGTCGATGGTCAAGAATATCCTATAATAATATGTGATTGAGACTGGGGAA
AATGTCAAAAAATTAGAGTTTGTTAGAGAGAGAAATATGAGTGAATCAACGAATCGCTGTCGGAGCCTAGGCAGCTGCCCTAGG
AGATTTGGCCTGGAAAGGGTCAATTTTTTTGGTTGGTTGAATTAGGATGCCAAATCATGTGGTGCAATAATAGCCCTATATAGA
TATTATGTGAGATGGGGAAAAGGGTTCAAAGAAAAATTAGAGCTTGTTAGAGAGAATTGAGTGAATTACGAAATCGTTTCGGAG
CCCTAGGCAGCTGCCTAGGAGATTTGGCCGAAAGGTCAATTTTTTTGTTTGGTTGGAATTAGGACCCCAAATTCAGTGGGTCCA

ACTAATATCCCTATAGTAATAGTTATATGAGAAGTGGGATAAGGTTAACAAAAATTAAGTTTGTTAGAGAGAATATGAGTGAAT
AGAAATCTCTTTGGTGCCTTAGGCAGCTGCATAGGAGATTTGGCCTGATAAAGTCAAATTTTTTGTTTGGTTAAATTAGGATCA
ATTCAGGTTCAAATAATAGCCCCTATATAATATTATTAGATGGAATTAGGTTCAAAAAATTAGAGCTTTTAAAGAGAGAAATAT
GAGTGAATTACGAAATTGCTTCCAGAGCATAGGCAGCTGCCTAAGGAGATTTGGCCCTGAAAGGTCAATTTTTTTGTTTGGTTG
AATTAGGGCCCCAATTCCAACGGTCAATAATAGCCCCTATATAAATATTATTGGAGATGCGGAAAAGGTTAAAAAAAGTTAGAG
CTTGTTAGAGAGAAATATGAGTGATGATTACGAAATCGCTTCCGGAGCCCTACGCAGGCGTGCCTAGGAGATCTTGGCCTGTAT
GGTCAAATTTTTTTTTGGTTAAATAGGACCCCAATTCATGCTCCAGATAATGAGCCCTTTCAATATAATATTATTGAGAATGGA
AAAAGGTTAAAAAAAATTAGAGTTGTTAGGGAGAGAAATATGAGTGAGATTAATGAAATCGTCTTGTGGCTGCCTTAGGCGAGC
TGCATAGGAGGACTTTGTGGCTGAAAAGTCAATTGTTTTGCTTGTGGTGTAAATTAGGACCTCAATTCATGTCCAAAATTAATA
GCCCTATATAAGTGATTATTGAGGATGGGATGAGGTTCAAGAAGAACTTAGAGCTTTTAGAGTGAAAGAGTATAGAGTGAATTA
CCGAAATCGCTTTCGGGCCTAGGCAGCTGCCTAAGGAGATTTGGCCAGAAAGGTCAATTTTTTTGTTTGGTTGAATTAGGACCC
GGCCAACTTCCATGGTCACATAATGGAGCCCTATAAGTAAGATAGTTATTGATATGCGGAAAGGACGGTTAAAAAAAATAAGAG
TTTGTTAGAGAGAATATGAGTGAATTAAGCAGAAGGTCTCCTTTGGTGCCTTAGGCAGCCTGCCATAGGAGATTTTGGCCCTGA
AGAGTCAATTTTTTTGTTTGGATTATGAAGTTAGGAACCCAGCGAATTCTGGTCAATAAGTAGCCTATATAATATTATTGAGAT
GGGAAAAGGTTCAAAAAAATTAGAGCTTTGTTAGAGACGAAATTATGAGTGAATTACGAAAATCCGCTTCGGAGCCGCTAGGGC
TGCTGCCTAGAGATTGGCTTGAGAAGGTCAAGTTTTTTTGTTTGCTTGAGATTGGACCTCCAATCTCATGGTCAATAATAGCCC
TAATATAATAATTATGAGATGGGAAAAGGTTTAAAAAAAGTAGAGTTGTTATGAGACGAAATATGTAGTGAATTACGAAATCGC
TTCGAGACCTAGGCATACTGCCCTAGGGAAATTTGGGGGAAAGAGGTCAATTTTTTTTTGGTTAAATTAGGAGGACACCATTCA
TGTCAACGATAAAGGCCTATATAATATTTTGAGATGGGAAAAGGTTCAAAAAAATTAGAGCATTTTAGAAGAGATAATATGAGT
GAATTACGAAATCGGTTCCAGAGCCCCTAGGCAGCCGTGACTACGGAGAGTGTTGGCGCTGAAAGGTCAATTTTTGTTGGTTGA
GATTA
>m141121_210323_42134_c100732402550000001823141405141506_s1_p0/4896/0_6351
AAAAAAAAAAAAAAAAAAAAGAGAGAGATAAATGTCTTAAGGGTTCTATGTATAAAATTTCGTGCAAATCCGGGTGTGGTATAG
GGTGATACAGTCAAAAATTCCTGAGGGATGTAAGAATAATAGGAAAAAACTTTGTGAAAATTGCTCTCAAAAAGGGGAATTTTT
TTTTAAATGTTGAATTAAAGGCTCCGTTTTGGACTCCAATATATTCATTTACGCCATGAGATTCTCCTTGGGGGAAAATATCCG
AACGAATTGAAAACACGAGGGTAAATACATTTCGGGAGAAAAAATTAGTGGGAAATCTCCAATTCACCAAAGGGGTAAACGCCC
CTTTCAAGATAAATCTTTGATTTCTCTAATATGGATTTCGAGCATGGTTTTTTGGGATAATGAATACTAAAGAATACAAGAGGG
TCATGGTCGCAAAAGTTGTATCCCCTATGTCTTTAGGGTTTCTAATGTATAAAAATTTCGTGCAAAATCCGGGGTGTGGTATGG
GGGGGTGGATACAAGTCAAAAATACTGCGGGATGTACGAATATTAGGAATAATTGTGAAAATTGGCCTCAAAAAAGGGGAATTT
TTTTTAAAATGTTGAATTAAGCTTCCGTTTTTCGACTCCAATATATTCCCAATTTACCCATGAGATCCTTCTTGGGGAATATAT
CGAATGAGTTGAACACGAGGGTAAAATAACCTTTCGGAGAAATTAAGTGAATCTCCAATTCCACCAAAGGGGTACCCCCTTTCA
AGAAATCTTCGATTCTCTAATATGGATTCCCGAGCCAAGGTTTTATTAAATGAATACTAAAAGAATACAAGAGGGTTCATGGTT
ACAAAAGTTGTAACCCTATGTTTATGGTTTCCTATGTATAAAATTTCCGTGCAAATCCGGGGTGTTATAGGTGAATAACAGTCA
AAATTAATGGAGGGATGTACGAATAATAGGAAAAAAATCTTGTGGAAAATTGCCCTAAAAAAAGGGGAATTTTTTTTTTAATGT
TGAATTAAGGTCCGTTTTTGACTCAAAATATATCATTTACCCCATGAGATCTTCTTGGGGGAAAAAAATATCGGAACCGAGTTGA
ACACAGGGTAAATACATTTCCGGAGAAATTAGTGAATCTCCAATTCAACCAAAAGGGGTACCCCTTTCAGAAATCTTCGATTTC
TCTAATAAATGGATTCGAGCAAGGTTTTTGATAATGGAATACTAAAAGAATACAAGAGGGTCATGGTCACAAAGTTGTAGCCCT
ATGTCTTTAGTCTATGTATAAATTTCGTAAAAATCCGGGGTGTGAATAAAAGTGATAACAGTCCAAAATCAGAGGGACCGTTAG
AATAATAGGAATAAAAAAAGTTGTGAAAATTTCCCTCCAAAAAAGGGGGATTTTTTTTTAAAATGTTAATTAAGGCTCCGTTT
GACTCCATATATTCATTTTAACCCCATGAGATCTACTTGGGAAAAAAATATCGAACGAGTTGAACACGAGGTAATACATTTCGGA
AGAAATTAGTGAAGATCTCCAATTCACCCAAAGGGTACCCCTTTCAGAAATCTTTTGATTTCTCCTAATATGGATTTGAGCAAGG
TTTTGATAATGAATACTAAAGAATACAAGAGGTCATGGTCACAAAAGTTGTAACCCTATGTCTTTTTTTAAGGGGGGGGGTTTT
CTATGATAAAATTTCGTGCCAAATCCGGGGTGTGGTATGGGGTGATACAGTCAAAATACTGCGGGATGTTAGGAATATTAGGAA
ATAAAACTTGTGAAAATTGCCCTCAAAAAGGGGAATTTTTTTAAAATGTTGAATTTAAGGCCTCCGTTTCGACTCAATATATT
CATTTACCCATGAGACTCTTGGGGAAATATATCGAATGAGTTGAACACCGAGGGTAATACATTTCAGAGAAATTAGTGAAATCT
CCAATTCACCAAAGGGGTACCACCTTTCAGAAATCTTTCGATTTCTCTAATATGGATTCGAGCAAGGTTTTATTATGAATACTA
AAGAATACAAGAGGTCATGGTCACAAAAGTTTAACCCTATGTCTTATGGTTTATGTTAAATTTCGTGCAAAAATCCGGGGTGTT
GTATAGGGTGATACAGTCAAAAATTATGAGGATTACGAATAAATAGGAAAAAATCTTGGTGAAAATTTGCCTAAAAAAGGGGAA
TTTTTTTTTAATGTTTGAATTAAGGCTCCGTTTTGACTCCAATATATTCCCGATTTACCCATTGAGATCTCTTGGGGGAAAATA
TCGAAACGGTGAACACGAGGGTTAAATACATTTCGGAGAAAAATTAGTGAAATCTCCAATTTCACCAAAGGGGTAACCCTTTCC
AGAAATCTTCGATTTCTCTAAATATGGGGATTTCAGAGCAAGGTTTGATAATGAATACTAAAAGAATACAGGGTCCATGGTCCA
CAAAAGTTTGTAGCCGCTATGGTCTTAGGGTTCCCTATGTATAAAATTTCGTACCAAATCCCGGGGTGTTGAATAAGTGATAAC
AGTCAAAATTCAGAGGAGTACGAATAATAGAAAAAGTTTGTGAAAATTTCCTCAAAAAGGGGATTTTTTTTTTTAAAATGTTGA
ATTAAGGCTCCGTTTCGACTTTCCAATATATTCAATTTACCCATGAGATCTCTTGGGGAAAAATATTCGAACGAGTTGAACACG
AGGGTAATAACTTTCGGAGAAATTAGTGAATCTCCAAATTCACCAAAGGGTACCACCTTTTCGAAATCTTCGATTTTCCTCTAA
TATGGATTCGAGCAGGGTTTTTTATTAATGAATAATAAAGAATACAAGAGGTCATGGTCACAAAAGTTGTAACCCTATGTCTTA
GGGGTTCTATGTATAAAATTCGTGCAAATCCGGGGTGTTGTAATAGGGTGTACAGTCAAAATTCTGAGGATGTCGAATAATAGG
AAAAAAAACTTGTGAAAATTGCCCCTAAAAAAAGGGGATGTTTTTTTTAAATGTTGAATTAAGCTCGCGTTTTGACTTCCAATA

TTATTCATTTACCCATGAGATCTCTTGGGGGGAAAAATATCGAACGAGTTGAAAACGAGGGTAATACATTCGGAGAATTAGTGA
AATCTCCAATTCACCAAAGGGGTACCCTTTCAGAAATCTTCGATTCCCTCTAATATGGAATCGAGCAAGGGTTTTATTAATGAA
TAATAAAGAATAACAAGAGGGTCAATGGTCACAAAAGTTGTAATCCCTATGTCTTAGGGTTCTATGTATAAAAATTTCGTAGCA
AAATCCGGGGTGTTGTTAAATAGGGTGATACCGAGTCAAAATTCTGAGAAGATGTACGAATAATAGGAAAAAAAACTTGTGAAA
ATGCCCTCAAAAAGGGGAAATTTTTTTTTAAATGTTGAATTAAGGCTCCGTTTTGACTCAATATATTCATTACCCATGAGATCT
CTTGGGGAAATATCGAACGAGTTTGAACACAGGGTAATACATTTCGAAAAATTAAGTGAAATCTCCCATTCACCAAAGGGGATA
CCCCCTTTCAGAATCTTTGATTTCTCTAATATGGATTTCGAGCAAGGTTTTGATAATGAATACTAATAGAATACAAGAGGGTCC
CATGGGTCACAAAAGTTGTAAAGCCCCCCCTATGTCTTAAGGGTTCTATGTATAAAAATTTCGTACAAATGGGGTGTGAATGAA
AGTGATACAGTCAAATTCGAAGGGACGTACGAATAATAAGGAAAAAAATAACTTGTGAATTGCCTCCAAAAAAAGGGATTTTTT
TTTTAAAATGTTTGAATTAAGGCCTCCCGTTTCGACTCCAATATTTCCATTTACCCATGAGATTCTTGGGAAATATATCGAATG
AGTTTGAAACACGAGGGTAAATACATTTCGGTAGAATTTAGTGAAATCTCCAATTCACCAAAAGGGGTACCCCTTGTCAGAAAA
TTCTTCCGTTTCTCTAATATGGATTCGACAAGGTTTTATTAATGAATACTAAAGAATACAAGAGGGTCCCATGGTCACAAAAGT
GTAAACCCTATGTCTTAGGGTTTCTATGTATAAAAATTTGTGCAAATCGGGTGTGTTGGTGATACAGTCAAAATACTGCGGGAT
GTACAAATATTAGGAATAAAACTTGTGAAAATTGCCCCCCTCAAAAGGGAATTTTTTTAAATGTTGAATTAAGGCTCCGTTT
CGACTCCAATTATTCATTTACCCATGAGATTCTTGGGAAAAAATATGAATGAGGTGAACACGGAGGGTAATACATTCGGGAAA
ATTAAGTGAAATCTCCAATTCACCAAAAGGGGTACCCCTTTCAGAAACTTCGATTTCTCTAATATGGATTCCGAGCAAGGTTTT
ATTAATGAATGACTAAAGAATACAAGAGGGTCATGGTCACAAAAGTGTAACCCTATGGTCTTCGGGTTCTATGTATAAAATTTC
GTGCAATCCGGTTGGTGTATGGGGGATACAGCAAAATTGCTGAGGGATGTACGAATAATAGGAAAAATACTTGGTGAAATTGCC
CTCAAAAAGGGAATTTTTTTTAAAATGGTTGAATTAGCTCCGTTTTGACTCCAATATATTCATTTACCCAATGAATTATCTT
GGGGGAAAATATCGAACGAGTTGAACACAGGGTAATACATTTCGGAGAATTAGTGAAATCTCCAATTCACCAAAAGGGGATACC
TTTCAGAAATCTTCGATTTTCTCTTAATATGGATTCGAGCAGGTTTTGATAATGAATCTAAGAATACAAGAGGGGTCATGGTCC
ACAAAAGTTTAGCCCTATGTTTAGGTTATGTATAAAATTCGTACAAATCCGGGTGTGAATAAAGTGATACAGTCAAAATTCAGA
GGGACGTACGAATATAGGAAAAAAAGTTGTGAAAATTTTCCTCAAAAAGGGGATTTTTTTTAAAATGTTGAATTAAGGCTCCG
TTTCGACTTTCCCAATATATTCATTTACCCATGAGATCTCTTGGGGAAATATATCGAACGAGTTGAACACGAGGGTAAATACAA
TTTCGGAGAAATTAGTGAAATCCTTACAATTCAAACCAAAGGGTACCCTTTCAGGAAAATCTTCGATTTCTCTAATATGATTCG
AGCAAGGTTTATTAATGAATACTAAAGAATAAACAAAGGTTATGGTCTCAAAAGTTGTAACCCTAATGGTTTAGGGTTCTAAT
GTATAAAATTTCGTGCAATCCGGTGTGTATGGGTGATACAGTAAATACCTGCGGAGTACGATTATTAGGAATAAATTGTTGAAA
AATTGCCCTCAAAAAGGGGAAATTTTTTTTAAATGTTGAATTAAGGCTCGTTCGACTCCAATATATTCCATTTACCCATGATCT
CTTGGGAAATATATCGATGAGTTGAACACGAGGGTAATACATTTCCGGAAGAAATTAGTGAATCCTCCAATTCACCAAAGGGGT
ACCCCTTTTTCAGAAATCTTCAGATTTCTCTAATATGGATTCGAGGCAAGGGTTTTATATTAATGAATACTAAAGAATACAAGA
GGGGTCATGGTCACAAAAGTCGGTAAACCCTATGTCCTTTCGGGTTCTATGTATAAAATTTTCGTGCAAATCCGGGGTGGTGTA
TGGGGGTGGATCAGTCAAAATCTGATGGATGTACGAATATAGGAAAAAAAAACTTGTGAAAATTGCCCTCAAAAGGGGGAAATTA
TATTTTTAAATGTTGAATTAAGGCTCCGTATTATGACTCAATATATTCATTTACCCATGAGATCCTTGGGGGAAAAATATCGAA
CGAGTTAACGAGGTAATAATTTCGGAGAAATTAAGTGAAATCTCCAATATCACAAAAGGGGTTACGCCCTTTCAGAAATGCTTC
GATTTCTCTAATAATGGATTCGAGCAAAGGTTTTGAATAATGAATACTAAGAATACAAAGAGGGTCATGGTCAAAAAAGTTGGT
TGCTATGGTCTTAGGGTTCTATGTATAAAATTTCGTAAAATCCGGGGTGTGAATAAAGTTTTGATCAGTCAAAAATTCAGAGGG
ACCGTACGAAATAATAGGAAAAAAAGTTGTGAAAATTTCCCTCCAAAAAGGGGATTTTTTTTAAAATGTGAAATTAAGGCTCG
TCTCCGACTCCAATTATTACATTTTACCCATGAGATCTCTTGGGGAAAAAAATATCGAACGAGTTGAAACCGAGGGTAATACATT
TCGGAGAAATTTAGTGAAATCTCCATTCACCAAAGGGGTACCCCTTTAGAAATCTTTGATTTCTTAAATATGGATTTGAGCAAG
GTTTTTGGATAATGAATACTAAAGAAATACAGAGGGTCCATGGTCAAAGTT
>m141121_210323_42134_c100732402550000001823141405141506_s1_p0/7734/0_10711
AAAGAGAGGAGAAGAAGATGTAGGCTAGCATTGAAGCCTCTTGTCTTTTACTTTCTGTGGACCACATCTTGCCGAATCCATTTG
GGTTTGTTGACTGCGGACAGTTTAGTCGGGTCATCAGTGTGGGGAAAAAGTTTGGTGAGCCAACCGCTTGCAAGCAACGCAGCT
TCGTGGCCAATCGGGTTATGAGCCATATCATTGTGTAGCGGGATGGGACTAAGCTTTGGTCCGAACATTCCCCAGAGACCCTAG
TACGACGTGGTTCCCCTGGTACCGCTTTTAAAACCGCTCTGAACTCTCTAGACCACATTGGGCATTATGATCCGAAGGACAGGT
AATCCAGCCGGGTATGTCGTCGCTGCTATATTGGGGCGAAGAGGTTCCATTTCATCGCCCGGATTTCATACTCGTCCTTTTTCC
TTTTTTCTGTTAGGAAGCTTTCTCCATCAGCTCCTCCATCTGAGTTAGGCGCTCGAGGTTTTGTCCCTGAGATCCTGGGTTAT
TCCGGGCTGTTCCACCAGCTCCGGACATTGTTAAAGCATATACCCTCTCAACTCGGGTGGGTTTGTACCATGTGCCCCTTGCCT
AAGTGTCTGCGATTGGTGAGACTATAAAGGGTGATCTTTGGTGACACATTTCCCTTCTTCCCATTTGGGTTTTGTAACCGCCCC
CCGGACCATTTAGGTCCGGGGATGGGTTTGTACCCCGCCCGGACACTTTGGTGTCCGGGATGGGACTAACTTGGGCCTTGCGTC
CCATTTGGGGTTTGATAACCCGCCTGGAATTTAGGTTGGGGATGGGTTTGTACCCGCCCGACCATTTAGGTCGGGAAGGGTTTG
TTACCAGCCCGGAACCCATTTGGTGTCCGGAATGGACTAAGCTTGGGCCTTGCCGTGCCCCATTTGGTTTAAACCCCCTGAACC
ATTTAGTCGGGATGGGTTTTGTACCCCGCCCGGACATGTGGTGTCCGGGATGGGACTAAAGCTTTGGCCCTTGCGTCCCATTTG
GTTTGTACCCCAACGCCCGGACATTTGGGTCGGGGATGGGGTTTGTCCCGCCCGGACCATGTTAGGTCGGGGATGGGGTTTGTA
CCCCCCGGACCATTTGGTGTTTGGGATGGACTAAGCTTGGGCACTTAACGTCATTTGGGTTTGTTACCTCCCGGACCATTTAGG
TCGGGGATGGGTTTGTACCCGCTCTGGACTTCTTGTGGTTGTCCGGTGTATGTGGACTAGGGCTTTTTGGTCCTTGCGTCCATT
TTGGGTTTGTATCCTTGCTCGGAACCTATTTGTGGTGTCCCGGTATTGGTTACTTATGTCTTGGGCCTGTGCGTTCCATTTGGT
GTTTTTTTTACCCTTGACCGGTAACCATTTGTCGGTGGAATTTGGGTTTGTACCCGCTCCGGACCATTTAGGTGGGGAACGGAT

TAGTGCCCCCCGGACCATTTGGTGTCGGATGAGACTGGTAAGCAAATGGGCCTCTGCGTCCCCATTTGGGTTTTAAACCCCCCC
GCCATTTAGGTCGGGGTGGTTGTACCACCGGAACCCAATGTGGTGTCGGAATGGATTAGGCTGGGCCTTGCGTCCCATTTGGGT
TTGGTACCCAGCCCGGACCCATTTAGGTCGGGGGATGGGTTTGAGTACCCGCTATCGTACCATTGGGGTTCAGGATGGGACTAA
GCTTGGGCCTCTGCTCCCAGTTTTGGGGTTTTAACCCCGCAGTAAACCATTTAGTCGAAATGGGTTTGTAACCCCCGGACATGT
GGTTCCGGATGGGACTAAAGCTTGGGCCTTGCATCCCCCATTTGGTTTGTACCCGCCCGGACCATTTAGGTCGGGGATGGGACT
AGAATTGGCCTTGCATCCATTTGGGTTTTGCTACCTGCCCCGGACCATTTAGGTCGGGAATGGGTTTGTACCCAACCGGAATAT
TGGTGTCCGGGATGGGAGCGAAGCTGGCCTTGCGTCCCATTTGGGTTTTAAACCCCCGGACATTTAGTCGGCGATGGGTTTGAT
ACCGGGCCATGTGGTGTCCCGGGATTGGACTATAGGCTTGGGCCTTGCTGGTTCCCATTCTGGGGTGTTTGTACCCGCTCGGAC
CAATTTAGGTCGCGATGGGTTTTACCGCACAGTGAACCAGTTTGGTGTCCGGGATGGGACTAATGCTTGGGGCCTTGCGTACCA
TTTGGGTTTGTACCCGCCTGGACCATTTAGGTCGGGGATGGGGTTTTTGTACCAAACCGGCCAGTTTAGGGTCGGGGGAAAGGG
TTTGTACCCCGCCCCGGACATTTGGTGTCCGGGATGGGAAACTAAGCTTGCCTGCGTCCCATTTGGGTTTTAAACCCCCTGAAC
CATTTAGGTTCGGAGGATGGGTTTTTGTACCCGCCCGGACCATGTGGTGTCCGGGATGGGACCTAAGCTTGGCATTGCGTCCCA
TTTGGTTGTACCCGCCGACCATTTTGGGTCGGGAATGGGTTTGTACCGCCCGTACCATTTACGGTCGGGATGGACTGAGCATTG
GGCCTTGCGTCCCATTTGGGTTTGGTAACCCGCCAGACCATTTAGGTCGGGAATGGGTTTGTAATTCCGCGCCCGAACCAATAT
GGTGTGTCCCGGATGTGACTAAGCTTGGGCCTTGCGTCCCCAATTTGGGTTTGTACCCCCCCGGACCAATTTAGGTCGGAGGAT
GGGTTTGTACCCGCCCGGACATTAGGTCGGGGAAGGGTTTGTACCCGCCCGGACATTTGGTGTCCGGATGGGACTAAGCTTGGC
CCTTGCGTCCCATTTGGGTTTTTAAACCCCCCCGGACCATTTAGGTGGATGGGTTTGTACCCGCCCGGACCATTGGTGTCCGGG
AATGGGACTAAAGCTTGGGCCTTGCCGTCCATTTGGTTTGTACCCCCGCCCTTCGGTTACCATTTAGTCGGGGAAGTGTGGTTT
GGTACCCGATTCCTGTGTGACCATTTTAGTATCTGAGGATGGGTTTGTGTACCCGCCCGGACCATTTGGTGTCCCGGGATGGG
ACTAAGCTTGGGGCCTTGCGTCCCATTTCCCAAAGTAAATTAGGAATCGTCAGGAAGAGGCCCTGAATGGAAGGTGCATCCTCA
GGTTTCCCTTGAAGGAATGGTTGACTGGGCACTGGTTCTCATTGCCTGCTGTTGTCGGTGAGAAGATCAAATAACAACATGCCC
TGTGGTCACCATTGTTGCAGAGTATTCAAAGAATCAACTCAAAGCTTTTACTCTCAATGAAAGCACCAAACCTGTTAACCGAGA
TTTTCGGTAACTATAGTCAATAAGTGGGAATTTATTGTTAATAATGAAAATAAGAAGATTGACGACGGGTTTTTACGTGGTTGG
CTTAACTTAGCCTTAGTACCACCGAGTCAATTTGTATTAGAGCTAGAGAGCTTTTACATGGAGGTTTTCTGCAAAATTTGATGC
AGGAGTAATCGCTTAAAAGCCAACCCTTGGGATCCCCCTACTGGTCACAACTGGCACTATTTATAGGACAGTCTTGTGCAATGG
CTTGGGCTGGACTAATCCTGGCCCAATATGAGATGTAATATAGTTTGACTCACTAATGATGCCAATAATACAAAGGATTTAGAA
ATATATAACATATATTAACAAAGGCCCATTGGGCCAGCCCAAACATGATCAACATTATAAGAGACTCCGACAAACAGTTGCTTC
AAGTCTGGGTGTTACGATCCAGCGAGAAACGATTCGGACCAGATCTGTCATGTAGTGGGACACTGTTTCCCCCCAGGAGACAGT
GTACCTCCATGGTAAACTTTCTTGTAAAATAGTTTGAGGAGACCACTTCTGGTTTCTCGAGGACTGTCAAGTTCCAGGGAAGAC
CAAACTTTTTCCTACACGAACTATCTTGTCCGGGATCATTAACTGGATGCCCGGACCCAATATGGAGCGGGGACCACCCTTGTC
CCGGGATCATCCTTGCCCGGGATCATTCTTGGACAGGACACCCTTGTCGGATCATCCTTGTCCGGTGATCATCTTCCGGGATCA
TTCTGGAGGGGGACACCCTTGGTCGGGATATCATTGTCCGAGATCATTATAGAACGGGGACAGACTTGGCCAGAGATCATCTTG
TCCTTAGGATCATTCTTTGATGGCCTTCGGACTTCATATGGACGGGGGGGACTATCATGACTTTTCTTCCCTGTTCATATGGGC
CCCTCCAGGGTGGGCCTGGATCTCTTTGAGGGAGAGCCGATGTACCCATCCATGTCACGCCAACTTCCGGGGGAAAACAGGGAT
AACATCCCCGAACACATAACAAACCTACTGATGAGTTCCAATAGTTCACCCTCCCTCCTCAATGTCGATATCCATATTCTATTT
GATAATGCCCAAATCAGAAATTTTCACATTCTTCTTCAAATCCTCAACTAAGCCGAAACTCCCTGTCTACCCACCCACAATTTT
CTTTAACACTCAAAGCCAGCATAAAATACTCTCTAAGATTTGCTTGGGGTTTAGAGTCTACCGTAAAATCAGAAGTATTCCCTC
CCTCAGCAGCGAGTCCCCAGTACTTCATAAATGAAGAGCTTGCTTCCCCTGTTCTTCCACCAGTTCATACCTTCTTGACTAAGA
TCTCCCGCCTCCCTCCATCGTCCCGAGAAACTTAATTGGATCCAACACTAATCAACGTCCATCCCAACTGTCTTGGTTTTCCGC
GATTCCTCTGAAGATGAAGGACCAATGTTGTAGGCCTCCTTCTCTTCCTCTTTGACATTAGAACCTCCCTTCCCATAGCCACAG
CCCCAAAGCCTGAAGAAGTAATTCAGATCAGTAGAGAGAAGTCAGAAGATTCTCTCTCTAGAAAACCTTTTTTTTGTTTTTTTT
AGCTTTAATCTCTTAAGTTTTTACTATACTTTTGTTTAAGGGTTAGCAATATACTACGCTTGACACGTTCCTATATTAAAAAAT
ATTTATAACCTTTTTTGTAAAAGCCCTATCCCTACCTTGAGCCGTTACATAGTGAGTTTTTAAAAAATAGAAATCGTGCTTTTG
ACTGACTCCTAAGTGGTTTCCTAAAACCAAAAAAGTGTGAATAAATCCAGAATTTAAGGCTGTAATATTTGAAATGTTTAGTTC
CATTGAAAACGTAAGTATCTAACCATCTGGGATCCAAAATAAGGTTTACAAAATATTTTACCAACTCCAAAATAGATTTACACC
AGGTTAACTATCCAAAAGAATGGGTTAATACAGCCATTTACAAAATGCACCCCCAACCAAAGCAGTCGGTGCAGGCCCGAACAT
GTACGCGGCACTCCACGCTCTCCCATACTCATGTGTTTGACTGACTCCCTTGCTTTTACCTGCCACATCGGAGCACCCGTGAGC
CCGAAGCCCCAGGCAAAAACCCATAATAGCACCATAACATATCAGATAATATCACTGGCATAATTCACTGATAAAGAAATCATT
AATTAAACAAGTCACGGCATACGCCCAGAGGCCGTTACCAAAAGCTTGGGGGTCTCGTCAAATTACCGTAGGATAACTCATGTA
TCCCTTGGGTCCCACCCTCGCTATAGCATCCCATGGTGCGAGCGTTACTTCCGCCCGCTGCCGTACCCGGCCTACGCCGGCTCT
CGGCCTTAGCCGTTCCATTCATTTACAATCACAACATATAACACATTCAGGAAATAACCATTCAACATAAACAATTAATTTATC
TAAGATTACACATTTCTGCACAAAACGAAGCACAAACTCTTTCGATCATCTCGTGTGAGAGTTGAGGCACGATCTCGTAGGGTC
GCCTCCCTGGGTAGTCTGGTGGACTTTGTGCCCCAAACTCCAGTCGCTAGAGCGCCGCGCCCTGCAATCACACAGTGGGCCTAT
GCCCTATTTCATGTTACGTTTTCTGTACCTGTATTGCACGCGTTCTCCTCGACTCTGACGAGCCATCACCCCCGGCGACCTAAC
CAGAATTCCCAATGGATCCTGCTGGATAGGCTTGGATGCGTCCTCTTGACCGGGATCACTTACGATCTGATCCTTCCCCTGGCA
AGCCCACGGTCCCACTAGTACGAAAGCCTCCCGAAATTCCTACGTGCGACCCAACACCAGCACAAAAGACACATTCTCAGAGAT
ACCCGAGAGCCTATACATGCCCTACAACTATCACCATTATCCCCATGGACCAATCCCGCATCATGCCGTGGCCGGGATGTCCGA
CACGGGCTGAGAGGAGATGGGTATCTGATTTGGAGAAGGGAGGTATGCCTGACGTGGGGAGGCGATCCTAATTCCGTGTCGGAT

GACAGCGAGAGAATTCTTGGTCCTTAGCAATTTCGAGTCTCTAAAAACACAGACTGCCAGCTGTGGCTCGCATACAATGCCGCC
TGGGCGCAGTGTGCCGCCCCGAGGCCGCAAGAAGGTCTTCCGTGGCGAACAGAACGCCGCTCTATAGACATCCCAACATGGTCT
GGCGCCCCGCGTGAACAATCTACATACTGGGCCTAGAGTTTTGGCCTCGGCAGGCTACTCCCTCCCGGCACAAACCACAACGGG
CCGCGGCGCGCAGAAGCCCAGGTTGCCGCCCCAGCATAAACCTGGGAAGGGCAATTGTCCATCCCGCTACGCGGCGCCCCAAGA
ACAGGGCACGCGGCGACTCGAGACGGCTGTATTTCTGTTCCCCGAATCGCGAACCCCACCCAGATTTTCGGTTTTTCTTGGCAT
TTTTAGGCTCATTCTCTCCTGAATGTATTTCCTTTCTAACTCCATCTCCAGAGAGTATTATCTACGAAGGGGTGCCACCCCCTA
GGCACAATGCACGCAGAGTACCCCACCTAGACCGAACCCAAACTTCGCAACATGGGCCCACAATTACCCTGAGCCGAGACTGTA
GGTCACTCTCATCCGAACAATCTATACCCACTCTTCCTCCATAAACACTAGACATCCAGCAGATCATAAACAACGATCTAGAGC
GTACATCGTATCAAGAGAGGCGACTAGTCGGGCTAGAATTATCCCGGATTTGCCGACCCGCGTGGTTGATTTCAAATTTCAGCA
ACAACCCAAAACTCTGTCAACCCATGCGCTCAATTTGAGCTCCCCTTATGCAAGGTGTTAAGACATATCTTCCATTCCCTCCAT
CTCACATACCTCAAAAGAAAAACAAAATAGCCCAATATTAAAAGTATGATGTTTAGATATAGAGTGACCCTCTGTGACCGGTGC
TGTGAGCTAATATGAACCAGATCCATCGACCACAGACCCTTGTGCGCGCAGCGTTATCCTCTCGGTACGTACTCTCTCCTGGTC
CCTCTGCTGTACCTTTCCAGAGATTTTCTCCATTCCCTTCAGCGATGGCACCTGGTTCTCCTCACTGCCTTCTATTTGCTTCCC
CCATCTAGCCTTGCCTTCGTTCTCCCTCTTGCTCCTCTATGCCTTTGCACTCCCGTAGAGACTTTATCCAATCAGCCAACATAA
GATCCAATTTTGTCTCTCATATTGACCGAGTTGTATTGAGCTCGGCAGCAACTAGCCGATTAAACATTACTCCCCTCCAAAACT
ATGCAAGAAAGCCTACCATAAACCTGTGTTGTATCGTCTTAGCCCAAAAGACCAAATTTACCCCTTCCTAATTATCCCTCCTTA
GCTAAACCTCAAAGGGCACTTAAGTCTTTACACTTTCATTTCAATGTCTAAACACTTTCTGATCTTAAAAACTTGTTACGCATA
CCCGTTCAAACGGTTACCCCCAGGTTGACACAATTACCAATGCTAACCCCACTCAGTTCTCAACTCAACTTATAAAATTCCCAA
ATAATCCCTAGGCCACTTCCTCCCGAGCCGGGGTAACAACCATCCCGTTGTGACTTTTAGACTAAATGACTCACTAGGACCGTC
TCGATGCTGCATCGATAAAAAAACCCACTCCACCATGGCACAATTCACGTAGTATACATATTCACGAGTTATGCCCCTATCTGG
CCAGATTACAATCATAGCTCATTATCAAGACAATCAGGTACATGCATACTAATTCACGATAGTCATGGCATCTCAAAATAATCA
ATTAGTCATATAACGTGCATTAAATGATATCATGCATAAAACTCAGATAGAAACACACACCAAAATCCATCATGCCCTCCCAGG
CACACTACTCCAGGCCCTTAAGCCCTTAATACTGATTTGGGGTCGTTACATGTTTTGGACCCAAAGCGTTTTATCTCATTATCT
TGTTTTAAACCATGATTTGTAATTTATTTTTTGGACTTATAATTTTGTAAAATAATGTCAAATAAACCCCTTAATCCGATTTTG
GTAAAATATTTTGAAGATTAAAATCACAATATAACTTACCAAATTAACAATTTAGAACAAAAGCATAGATTAATCTGTTAACAA
CTATGCTGCTTAATTCAATTTTTTCGTTATTAAAAATTAATTTTTAGGAGTCTATTTGAATTAGTTTTACAAAACTTGGAACCA
AAAAATAATTTGTTAAAATACATGATCTAATTAGATAAATGAGAACAAATATAGGGTCCAAAAAAATATAAAACCCTACAAAAA
TTCAAAATAACCTTCTTTTTTTGTAAACATATCAAAAGAAATCCTACCATTTGATTTTGGTCAACTTTTTAAAGTATACATTTT
TACCTTGTTTTTGTACAATTAATATAATTTAATAATAATTTTTTTATATATTTTTTATTTTGAACACAAATAAAAAGAGTAAAT
AATCTTATGAAAAATTCACAATGATGGATTTTTTTATATTAGTTTAAAATAACTAAATCTTTATTCGTTAATAAATAACACCA
GATAAGAAGATATTTTAATTGAAAACAAGCCATTTTACTTATGTTTATTATGATCCTAGTATAGTTTAGTATACTTTATTATTG
AAACTAAAAGTAAGATAATAATTATAATTTTTTTTAACAAGTCTCATATGAAATTTATTAATTACATAAAAAAAATAAAAGTG
CCTGTATGGAATGAACAAATTGACTAAAATCAAGTAGTGGCATGGCATGGTTTTAGTAAGATTTTAGAAATAGGGCTACCATTG
GAATTTTTCTTAAACAGTGTCCAACTTTGAAGGGCTATATTTAGACCTTAGTTTTTCCTCCCTCTCTACCCTTAGTAATAATTC
ATTTTAAATATAATGTTTGTTATAAATTTACACATACAGTTTACTAGTTATTTTAAATAATAAAATATATTTTTTCCACATACA
CTTTTCAACACATTAATTACATACAGTAATATTTATAAATATTAAATTCTGTATAATATATTTTATTAGCAAATAAGACAAGTC
AACGATATTGTAATTTATTAATTTTTTTGTGTGGTTAGTAATTTTTTTAGTTTTAACATTAAACTGTTCCTTAGTGTTGTGTTT
TAAGAAAATAAAAATTAAATTTTGTAATTTTTTTTCAAATATGGATGTATCAATTTTATTATTTTTATTTTTTAATAAAGTATG
GTCCGTTGAAGAGATTTTTTTTTTTCTTTCTTTAAATAAAGTTTTTATAACTAAATGAAAATAGAAAGGGTGTTAAAATTAAAG
GGCCTGAGGGAGTAAAAAAAAGAAAAACGTGCACTTAGTAGCAAATTTAGCTAGAACACATAAGAATAAATTATAAATATATAT
ATTTATTAAAGAATAAATGAATTTAGATGTTTTAGTCAAATGATTGAAAGCATCATTAAAATGATTTACGTCACTTTTGAAACT
ACTTTTTATCAGCAAAGATCTTTTTAAGCCGTAAAATTAGTAATTTGTCCTTAATCCAAAAAATGAATTCACCACCATGTTCGA
TCGACTTAACTGTTCAATTTTGATTAATCATACTATTTAACATCATTTAAAGTGAAGTGTGTTGATATTGTATATACATTATAT
TATATTTCTTTTAATCTAATGACGATAATCAGTGTAGGCTTGCTGTCACAATCTGGTTGTCAATAAATAAAGAACTTGCCATCA
ATTATGACTAGGCAATGATTCATTTAATTATATTCATCCTAATTAGGCTATGTATACTCTCCTATCCTAATACTAATAAAATCC
CGAATAGTACTATTTGGACATCAATTGCTTACTTATAGTCTAACTCTAGATTAGAGATATCACCATTGGCGGGTAATGTGTGAG
CGCGCATCTGGTGTCACATGATATCATTACAGTAGACAGTGAATGTGACATAAAATAGATCGAACAAAACCAAAACACCGAGAA
GGGAGACCGTCAGGTTTTTCCTTTCCTCTCTTTTTGTGTGCTCTGAGATACTGATTCCTTATGGTCTGTCCTCAGCGACCACGA
TCGATCGTACCAACATGTCCCATGTGAACAACCTGACACAATTCCTAAGCTTATAACATACGACTTCCCAACGCCAATGAAAAT
AGAGATTTCAAACCCATAAAGTGGTCAGGCTGAATAGGACACCTATTTGAATTGATGATGATCGAAAAAAATAGTTACATAAAC
TTTAACTTTGTTGGGGGGACTTTGGGGGGGGGGGAAAAATGTCTACCCAGTGGTCTAAAATCCCCATTTCTTTCTATATCGACT
AAAAACACTAAAATAGAAGGAAAGAAGAAAAAAAAGGTGATCACACCAGTCCTAAAACATTGAACATGGAGAGATCTCTAACAC
AAATTATCGACGATATTCTTCTCCAAAGTAAAAATGGTCAATG
>m141121_210323_42134_c100732402550000001823141405141506_s1_p0/14838/0_6005
AGAGAGAGATCCTAATATAATATTATTGAAATGGGAAAAGGTTCCAAAAAAATTAGAGCTTGTTAGAGAGAAATATGAGTGAAT
TACGAGATCGCTTCGGAGCGCATAGGCACTTGACTAGGAGATTTGGCAGGAAAATCCAAAATATTTTAAATTGTTTGGTTGAAT
TAGCTATCCCAATTCATGGTCAATAGGAGCCTATATACGCATATTATTGAAATGGGAAAAAAGGTTCCAAAAAAAATTTAGAGC
TTGTTAGAGAAGAAAAATATGAGTGAATTACGAGAATCGGTTCGGAGCCCTAGGCAGCTGCCCTAGGGAGATTTATGGCAGGAA

AGGCAATTTTTTTGTTTGTTGAATTAGCATCCCAATTTCATCGGTCATACGATGCCCTAATTATAATATTTATTTGGAAAATGG
GAAAAGGGTTTCAAAAAAAATTGACTTGTTAGAAAGAAAATATGAGGTGAAATTACGAGATCTGCTTCGGAGCCCTAGCAGCAG
CCTAGGAGATTTGGCATGAAAGGCTCCAATTTTTTTGTTTGGTTGAATTAGCATCCCAATTCATGGTCAATAGGAGCCCCCTAT
ATAATATTATTGAAATGTGAAAAGGTTCAAAAAAATTAGAGCTTGTTAGAGAGAAATAATGATGCAAATTACGAATCGCTTCCG
GAGCCCTAGGCAACTGCCTTAGGAGATTTGGTAGAAAGGCCAATTTTTTTGTTTTGGTTGAATTAGCACGTCCCAATTCATGGT
AATAGTAGCCCAATATAGATATTATTGAAATGGGAAAAGGTTCAAAAAAATTAGAGCTTATTTAGAAAGAAATAAGATTGTATT
TACGAGATCGGTCGGAGCCCCTAGCAGCTGCCTAGGAGATTTGGCAGGAAAGGCCAATTTTTATGTTTGGTTGAATAGCATCCC
AAAATTATGGTCAATAGTAGACCATATATAATATTATTGAAATGCGAAAAGTCAAAAAATTTAAAGAGCTTGTTAGAGAGAAAT
ATGAAGTGAATTACGAGATCTGCTTCGGGGCCCCCTAGGCAGCTGCCTAAGGAGATTTGGCAGGAAAGGCCAAATTTTTTTGTT
TGGTTGAATTAGAAATCCGCATTCAATGGTCAATAGGAGCCCCTATATAATATTAATTGAAATGGGAAAAGGTTCAAAAAAATT
AGAGATTGTTAGAGAGAACTATGAGTGAATTACGAGATCCGCTTCGGAGCCCTAGGCACTTGCCTAGGAGATTTGGCAGGAAAG
GCCAATTTTTTTGTTTGGTTGAATTAGCATCCCAATTCAAATGGGTCAATAGGAGCCCTATATAATATAATTGAAATGGGAAAA
GGTTCAAAAAAATTAGAGCTTGATATAGAGAAATAATGATGAATTAACGAGATCAGTCGGTTCGGAGCCCTAAGGCAGCTGCCT
AGGGAGATTTGGCAGGAAAGGCCAATTTTTTTGTTTGGCTTGAATTAGCATCCCAATTCATGGTCAATACGAGCCTATATAATA
TTATCTGAAATGGGAAAAGGTTCAAAAAAAATAAGAGCTTGTTAGAAAGAAATATGAGTGAATTACGAGATCGCTTCGGAGCCC
TAGGCAGCAGTCCTAGGAGATTTGGCAGGAAAGGCCAATTTTTTTGTTTGGTTGAATTAGCATCCCAATTAATGGTCAAAATAG
GAGCCCTATATAATATTATGAAATGGGAAAAGGTTCCAAAAAAATTAAGAGCTTGTTAGAGAGAAATATGAGTGAATTACGAGT
CGCTTCGGAGCCTAGGGCAACTGCCTCAGGAGATTGGTTAGGAAAGGCCAAATTTTTTTGTTTGGTTGAAATTAGCAATCCCAA
TTCATGGTCATAGTAAGCCCAATATAATATTATTGAAATGGGAAAAGGTTCAAAAAATTAGAGCTTTTTAGAAAGAAATAAGAT
TGTATTACGAGATCGGTTCGGAGCCCTCAAGGCAGCTGCCTAGAGATTGGCCAGGAAAGGCCAATTTTTTATGTTTGGTTGAAT
TAAGCATCCCAATCATGGTCAATAGTAGACTATATAATATTATTGAAATGCAAAGGTTCAAAAAATTAGAGCTTGTTGAGAGAA
TATGAGTGAATTACGAGATCGCTATCGGAGTCCTAAGGCAGCTACCTAGGAAGATTTGGCAGGAAAGGCCAATTTTTTTTGTTT
GGATTGAATTAGCATCCCAATTCATGGTATATAGGAGCCCTATATAATATTATTGAATGGGAAAAGTTCAAAAAATTAGAGCTT
GTTAGAGAGAAAATATGAGTGAATTACGAGATCGCTTCGGTAGCAGCTAGGCAGCTGCCTAGGAGATTTGGCAGGAAAGGCCCA
AATTTGTTTGGTTGAATTAGCATCCCAATTCATGGTCCCAATAAGGAAAAAGCCTATAATATATTATTGAAATGGGAAAAGTT
CCAAAAAAATTAGAGCTTGTTAGAGAGAAATATGAGTGGAATTACGGAGATCGCTTCGGAGCCCTAAGGCAGCTGCTAGGAGAT
TTGGCAAGGAAAGGCCAATTTTTTTGTTTGTTGAATTAGCACCCAATTCATGGTCAATAGGAGCCCTATAAGTACCATATATTG
AAATGGGAAAGGTTCAAAAAATTAGAGCTTATTAAGAGAGAAATAATTGAGTGAATACGAGCATCGCTTTCGGAGTCCTAGGCA
GCTGCTAGGAGATTTGGCAGGGAAAGCCAATTTTTTGTTTGGTTTGAATTAGCATCCCAATTCATGGTCATACGAGCCCTATAT
ATATATTATTGAAATGGGGAAAAAGGTTCAAAAAAAATTTTAGCAGCTTGTTAGGAAGAGAATATGATGATTACGAGATTCGCT
TCGGAGCAACCTCAGGCAGCAGCTAGGAGATTTGGGCCCAGGAAGGCCAATTTTTTTTTGTTTGGTTGAATTATGCATCCCAAT
TCAGGTCAATAGGACGCCCTATAATAATATTATTGAAATGGGAAAAGGTTCAAAAAATTTAGAGGCTTGTTAGAGAGAAATAAT
GAGTGAATTACGAGATCGCTTCGGAGCCCAGCAGCTCTGGCTATGAAGATTTGGCAGGAAAGTTTAAATTTTTTTGTTTTGGTT
GAATTAGCCATCCCAAATTCTATGGTCAAGTAGTAGCCCTATAATAATATTTATTGAAATGCGAAAAGGGCAAAAAAATTAGAG
ATTGTTAGAGAGAAATATGAGTGAATTACGAGGATCCGCTTCGGGCCCTAGGCTTGCTGCCAGGAGATTTGGCAGGAAAGGCCA
ATTTTTTTGTTTGGTTGAATTAGCATTCCCCAATTCAATGGTCAATAGTAGCCCAATATAATATTATTGAAATGGGAAAAAGGG
TTGAAAAAAATTAGACTTGTTAGAGAGAAAAAGATTTGATTTAACGAGATCGGTTCGGAGCCCTAGGCAGCTGCCCTAGGAGAT
TTGGCAGGAAAGGCCAATTTTTATGTTGGTTTAATTAGCATCCCAATTTCATGTCAATAGGAGCCCTATATATATTATAGATAA
TGGGAAAAGGTTCAAAAAAATTAGAGCTTGTTGAGAGAATAATGAGTGAATTCGAATCGTTCCGGAGGGTCCTAGGCAGCTGCC
TAGGAGATTTGCAGGAAAGGCCATTTTTTTGTTTGGTTTGAGATTACATCCCAATTCTGGTCAATAGTAGCCTATATAAATATT
ATTGAAATGGGAGAAGGTTCAAAAAAAATTAAGAGCTTGTTAGAGACAAATATGAGGAATTACGAGATCGCTTCGGAGCCCTAG
GCAGCTGCCCTAGGGATTTGGCAGGAAAGGCAATTTTTGGTTTGGTTGAATTAGCATCCCAATTCATGGTCCAACACGAGCCCT
ATATAATATTATTGAAATGGGGAAAGGTTCAAAAAAAAATTAGAGCTTGTTAGAAAGAAATATGAAGTGAATTACTGAGATCCG
CTTCGGAGCCCTAGGCAGCAGCCTAGGAGATTTGGCAGGAAGGCCAATTTTTTTGTTTGGTTGGAATTAGCATCCAATTTCATG
TCAATAGGAGCCCTATATAAATATTATTGAAATGGAAAGTTCAAAAAATAGAGCTTGTTAGAGAGAAAATGAGTGAATTACGAG
ATCGCTTCGAGCCCTAGGCAGCTGCTAGGATTTGGTAGGAAAGGCCAATTTTTTTGTTTTGGTTGAATTAGCATCCCAATTCAT
GGTCATAGTAAGCCCAATATAATATTATTTGAAATGGGAAAAAGGTCAAAAAAATTAAGAGCTTTTTAGAAAGAATAAGATTGT
ATTATACGAGATCGTGAGCCTAGCAGCTTGCCTAAGGAGATTTGGCAGGAAGGCCAATTTTATGTTTGGTTTGAAATTAGCATC
CCAATTCATGGTCATAGTAGACCTATATAAATTATTGAAATGCGAAAGGTTCAAAAAAATTAGAGCTTGTTAGAGAGAATATGA
GTGAATTACGAGATCGCTTCGGAAGTCCTAGGCAGCTGCCTAGGAGTTGGCAGGAAAGTGCCAATTTTTTTTTGTTTGGTTGAAT
TAGCATCCCAAATTCATGGTCAATAGGACCCTATATAATATCATAGAAATGGAAAAGGTTCAAAAAATTAGAGCTTGTTAGAGA
GAAATAGAGTGAATTACGAGATCGTTTCGGAGTCATAGGCAGCCTGCCTAGGAATTTGGCAGGAAAGGCCAATTTTTTTTTGTTT
GGTTGAATTAGCCATCCCATTAATGGTCAATAGTACATATAATATTATTGAAAATGGAAAGGTTCAAAAAATTAGAGCTGTTAA
GAGAATATGAGTGAATTTCGAGATCCGTTTCGGAGTCCTAGGCAGCTGCGCCTAGGAGATTGGCAGGAAAGGCCATTTTTTTGT
TTGGTTGAATTAGCTCCAATTCATGGTCAATAGTAGCTATATAAATATTATTGAAATGGGAAAAGGTCCAAAAAAAATAGAGCT
TGTTAGGAGAAATATGAGTGAATTACGAGTCGCTCGGAGCGCTAGGCAGCTGCCTAGGAGATTTGGCAAGGAAGGCAATTTTTT
TGTTTGGTTGAATTAGCATCCACAATTCATGGTCAATCTAGCCCTATATAATAATATTGAATGGGGAAAGGTTCAAAAAAAAAT
TAGAGCTTTGTTAGAAGGAAATATGATGATTACAGATGCTTCGGAGCCCTAGGCAAGCATGCCTAGGAGATTTTGGCAGGAAAG

GGCCAAGTTTTTTTTTGTTTGGTTGAATTACATCCAAATTCATGGTCAATAGGAGCCCTATATAATATTAATTGAAATGGGAAA
AAAGGTTCAAGAAAAATTAGAGCTTGGTTAGAGAGAAATATGAGTGAATTACGAGATCGCATTCGGAGCCCTAAAGGACTGCCA
GGAAGATTTGGTAAGGAAAGGCAATTTTTTTGTTTGGTTTTACATCCCAATTAAATGGGTCAATAGTAGCCAATATAAATATTA
TTTGGAAATGGAAAAGGTTCAAAAAAAATAGACTTTTTAGAAAGAAATAAGATTGTATTACGTGAGATCGGTTCGAGCCTAGGT
AAGCTCTAGGAGATTTTGGCAGTGGAAAAGCCAATTTTTATGTTTGGTTGAATAGCATTCGCCAAAATTCATGGTCAATAGTGC
CCTATATAATATTATTGAAATGGAAAAAGGTTTCAAAAAATTAGAGCTTTGTTAGAGGAAATATATGAGTGAAATTCGAGATCG
TTTCGGCAGGTCCTAGTCAATGCAGCTGCCTAGGAGAATTTTGGCAAGGAAAGGCCAATTTTTTTGTTTGGTTGAAATTAGCAT
CCCAATTCCATGGTAAAATAGTAGCCCTACCTATATAATATTATTAATGGAAAAAGTTCAAAAAAAAAAATTAGAGCTTGTTA
GAGACAAATTGTGAATTACGAGATCTGCTTCGGAGCCCTAAGCATCTGCCTGCGGATTTTGGGGGGGCAATGACAGTGCAGAA
AGGCCACAATTTTTTTTTTTTTTTGTTTGGTTGATTAGCGT
>m141121_210323_42134_c100732402550000001823141405141506_s1_p0/31095/4755_10244
ACGCCCCTCCCGGAAAACCATTCCGGGGATGCCTCATATTGAATTATAAGGGAAGAACGGACCCGCGCACATCCATCTTGAAAG
TACAACAGATGATGCAATAGCGGCGCGTCAGTGATAACGCCAAGGTTGCATGTGTCCGACTTTACCCTTTCCAAGTCCGGCGAG
GAGCCTGTGAAAGTTAGCTCAGGAATCCAATTCACCAGCCCTGGAAGGAGGCTAACATCCGCGTTCCGAAGGCAGTTTACGCTC
GCAGCCCGGGAGACCAAAGACAACGATGGAGGTTGGCTCCCTTAACCCAACATTAAGCAGCTTCCCAAGTGAGAGCCTCAAAGG
ACTTTCAGTTCCCAATAGAATGATGGAAGCTGCTGCCCAAAGACGGAAGTCGAGCGAGTGACATGAACGTGATTCCGCCCCTTGG
CAATAAGGCTTAGCCGTCGGCTCCCTGCTATGGGAGAAAAAATTTGCAAAAGGAGACGGGCCAATAATTTTTTTCTCTGGCCGA
GTTTCATGTCAAAGAGCCTCGAAGGGATGTGTGAGATATAATAAAAGGCGGGAACACAGGGGGGGGGAGGAGGGGAGGGGGGGG
GCAGACAAAGGAAGGAAGGAGGGAGGTAGGGGGGAGAGGGACCAAGAGTCCGAGAGTTGTGGACCGGAGGGTCGCGGACGGACG
TCCGGGGTATAATAATGGTTGATTACCGATGCAGGCCGGTCACCCCGGACTGATTACGATGTCGGAGTAAAGATAAAATAGTGG
AAAACCGAGCCAAAATGGTCCGGTCCCACCCCGGACGAACGATGTCGGGAGTATACTAATAATAAAGAGGGACCTGAGCCCAAA
GGCCGGGTCCCATCCTCCGGACTGACGACGTCGGTGGTACAAATAAGGGAACCGAGCCCAAGCCGGTCCCACCGGACGATGAGT
CCGGGAGTAATAAAGATAGGGAACCGAGCCCAAGCCGGTCCTACCCGGACGAACGAGTCTCCGGAGGTATAAAGATAAAAGGGA
CCGAGCCCAGGCCCGGTCCCACCCCGGACAACGATGTCCGGGAAGTATAAAATAAAGACCGAGCCCAAGGCCGGTCCCTACCCG
GAAGAACGACGTCGGGGTATAAAATAAAGCGAACCAACCAAGCCGGCCACCTCCGGCACGAACGATGTCCGGGATATAAAAATA
AAAGAGGACCGAGCCAAGGCCGGTTCCCACCCTGGACCCCGCACGACGTTCCGGCGGGTATAAATAAGGGAACCGAGCCCAAAG
GGCGGTCCAACCCCGGACGAACGATTGGGTCCCGGGAGTATAAAGTAAAGGGAACCGAAGCCAAGGACGGTCCCACCCGGCGAA
CCGACGTCCCCGGGGGTATAAATAAAGGGAACCCCGCGAGCCGCCACGGGCCGGGCTTCCACCAAGGCGGCCCCCGGGCGCCGG
ACCACGATGTCCGCGAGTATAAAGATAAAGGGAACCGACCCCAAGGCCGGTCCCAACCCAAACGAACGACGGTCCGGGAGTATA
ATAAAGAACCGAGCCAACGCCGGTCCCACCGGACGAACGATGTCGGGAGTATAAAATAAAGGGAACCGAGCACAAGGCCGTCCA
CCCCCGGACGAACGACGTCCGGGGGTATAAACTAAAGGGAACAGCCCAAGGCCGGTCCCACCCAGACCGAATGATGTCCCGGGA
GTATAAAATAAGAGGCCGAGCCAAGGCCGGTCCCCACCCCGGACGACCGACTCCGGCGGGTTATAAATAAAGGGACCGAGCCAG
AGGCCGGTCCCACCCGGACGAATGATTCCGGGAGTAAATAGGAATAAGGAACAGAGCCAAGGCCGGTCCTACCCGGACGAAACG
ATTGTCCCGGGAGTTATAACAGTACCAGGGAACCCCGAGCCCAAGTGCCGGTCACACACCCCGGACGAACGTGTCCGGGAGTAT
AAGATAAAGGGAACCCGACCACCACCCAAGCGGTCCTCACCGGAACGAACACCGATGTCCGCGAGTATAAAATAAGAGGACCGT
ATCCCCAAGGCGGTCCACCCGGCGAACCGACGTCCGGGGGTATAAATAAAGGACGAGCCCAAAGGCCGGTTCACCGGACCGAAA
CGACGGTCCGGGGTATAAATAAAGGGGAACCGAGCCCAAGGCCGGTCCACCCGGACGAACCGATGTCCGGAGTATAAAAATAAG
AGGACCGAGCCAAGGCCGGTCCCACCCCCCGGACGAACGACGTCCGGGGGTTATAAATAAAGGGAACGAGCCCCAAGCCGGTCC
CCCCGGAAGAACGAATGTCGGAGTATAAAATAAGGGAACCGAGGCCCAGAGGCCGGTTCCCACCCCGGACGAACGATGTCCGGG
TTTATAACTAGAGGACCGGAGCCCAGGGGCCGGTCCCACCCCGGACGAGAGTCCGGGGGTATAAATAACAAGGACCAAGCCCCA
AGGCCGGTCACCGGACGAATGATGTCCGGGATATAAAAAAAAGAGGACCGAGCCCAAGGCCGGTCCACCCCGGACGAACGACGT
CGGGGGGTATAAATAAGGGAACCGAGCCAAGGCGTCCCACCCCGGACGACACGAGTCCGGAGGTATAAAGATAAAGCGGGAAAC
GAGCCCAAGGCCGGTCCCACACCGGACGATACGATGTCCGGGAGTATAAAGATAAAAGGGAACCGAGCCCAAGGCCCGGTTCCC
CCACCACGGACGAACGATGTCCGGGAGTATAAGGATACAAGGGAACGAGCCCAAGGCGGTCCACCCCGGACCGACGATGTCCGG
GAGTATAAAGATAAAGACCGACCCAAGGGCCGGTCCCACCCACGGACCGAACACCGTCCGGGAGAGGTAAATAAAGGGAACGAG
CCCAAGGCCGGTCCCGAAACCCGGACGAACGATGTCCGGAGTATAACAATAACGGGGAACCGCACCCAAAGGCCGGTCCCACCC
CCGACGAACCGAGTGTCCCGGGAGTATAAAAATAAGAAGGCCGAGCCCAGCCCGGTCCACCCCCGGAGAACGACGTCCGGGGTA
TAATAAGGACCGAGCCCAAGGCCGGTCTCCACCGGACGCGACGTCCGGGGTATAAATAAAGGGAACGAGCAAAGGCCGGTCGCC
ACCCCGGACGACGATGTCCGGGAGTATAAACATAAGAGGACGGCCGAAGGCCGGTCCCACCCGGACGAAACGACGTCCGGGGGT
ATAATCAAGGGAACCGAGCCCCCAAGGCCGGTCCCTCCCCCTGGACGACCGATGTCCGGGAGGTATAAATAAGGACGAGCCCAA
GGCCGGTCCCACCCCGGACCGAAACGATGTCGGGAGTATAAAATAAGAGGACCGAGCCCAAGGCCGGTCCCACCCGGACGAACC
GACGTTCCGGGGGTATAAATAAAGGGAACCAAGCCCAAGGCCGGGTCCCACCCCCGGACGAACCGATGTTCCCGTAGTTAAAAA
TAAAAAAGAGGACCACCCAAAGGACAGGTCCCACCCGGACGAACGACGTCCGGGGGTTAATAAGGGAACCGGCCAAAGGCGTCC
CACCCCGGACGAACGACGTCCGGGGGTAAAATAAAGGGTACCGAGCCCAAGCCCGGTCCCACCACCGGACGAACGATGTCGCGG
TATAAGGATAAGGGAACCGGAGCCAAGGCCGTCTACCCCGGACGAACGACGTCCCGGGGGTATAAATAAAGGGGACCGAGCCCC
AGGCGGTCCCACCCCGGACGGAACCGATGTCCGGAGTATAAAAATAAGGGAACCGAGCCAAAGGCCGGTCCACCCGGACGAACG
ATGTCCGGGGCATAATAAGAGGACCGAGCCAAGCCGGTCCAACCCCGGATGAACGACGTCCGGGGTTAAAAAAGGGAAACGAGC
CCAAGGCCGGTTTCCACCCAAGACGAATGGAATGTCCGGGAGTATAAAAATAAGAGGGCCGAGCCCAAGGCCGGTCCACCACCG

GACGACGACGTCGGGGGTAATAAATAAAGGGAACCGAGCCAAGGCGGTCCCACCCCGGACGATATGATGTCCGGGATATAAAAG
ATAAAGGGAACCGAGCCAAGGCCCGGTCCCTACCCCGAGCGAACGATGGTCCCGGAGTATAGATAGGGACCGAGCCAAGCCGGT
CCCACCCCGGACGAACGCTGTCCGGGAGTTATAAAGATAAAGGAACCGAGCCCAAGGCCGGTCCACCCGGACGAACGATGTCCG
GGAAGTATAAAAATAAGAGACCGAACCCAAGGCCGGTCCCTCAACACGGACGAACGATCGTCGGGGGTATATTAACAAGGGGCC
AGAGCCCAAAGGCGGTCCAACCAGGACGAACGACCCGGGGGTATAAAATAAGGGGGGAGCCCAAGGCCGGTCCACCCGGACGAT
GATGTCGGGAGTATACAAAATAAAGGACCGACCCAAGGGCCGGTCCACCCCCGGACCGAACTATGTCCGGGAAGTATAAATAAG
GGAACCAGAGCCCAAGGTTCCGGTCCCACCCCGGACGAAACGATGTCCGGGATATAAAAATGAAGGGAAACCGAGCCAAGGCGG
TCCCACCCGGAACGAGCACCGTCCGGGGGGTTATAAAGTCCGTCCGACCCCGAACTACTGATCCTCGGAGCACAGAAGCAACCA
GGTCAACCCCCGGATACTCAGGACCCTAAATTCCAGGAGAAAGCCACACTCCCCGGGTTAAACAAAGCCCCAGGCCCCGAACAG
AGCAAGACTAGTCGTTGGGTTCAGTTTCCGCCGTGGAGCCCGGTGACAACACCTGTTCAATAGTTTAAGTTTCGCGACATCACT
TCTTTTACTGAATTGGTGAAAGGGTTAAGTCTGTTTAAAAACCCCCGAAAAGAATGGTAGTGGCAGTGGTGTTTGTAATTTAAA
TTGTGTTATACAATACTATGAACTATGTAACAAAATAGTTAACGGGAGGCCGGAGACAGGGCCTATACTCAGGCGGCGTCGCGG
CCGAGGTCCGCGATCCTCACCTCTTCCTTGGCTCCGGTACTCCGCACGCGTCGAGTCGGGAATCGGGGAATGATAAGGGAGGTT
CATTGTTCTTGTCCTTGGGGCACCCAGGCCCATATAAATGGCTCGGGTAAAGGGATGACGTCCACTAAGTTGTCGGCCTCCTCC
TTCTCCGATGAGGTTGGTGGTCGTGTGCCGCCCTTCTCCTGAAAGAAGGAGTCCAAAGCTCAAGAACACGAGCTTCAGGCCCGG
AAATCGTCGCTTCCTTGTCCTGGACGTGACTGTGCTTGCAGCCGTCTCCAAGAGAGTCGCCCCCTTTCGTCAGCAACTCTTCGT
CCGTCCGGACAACTAATCTTTCCACCCCGTCTTTGGCAACGGCTAACTTTCCCTCGCGCTTCCGCCTTCGGCGGCCAGCTCTTC
TTTGGAGGCGGGCCGTTTTCTTTTTGCTGGTATTGTGAGGTCTTTGACGATCCTCCCCTCTGGTTTTCTTCGGCCGCCTTCTAA
TTCCCAGCCTGGCCGTCTCCATCTATGAC
>m141121_210323_42134_c100732402550000001823141405141506_s1_p0/32238/3999_11710
CTGAGATTAGACAGGTCACCTTATATCAACTATACGGATATAATAAATAAAAAGGAATACTTCCACCTCAAACCTAAAAGATAA
AAAACATATTCAATAAATACACGCCTGTACTAAACGATATGCTTTAAGAGAGCCGAGAAGTAGTAACAACCAAAAAAATAAGAT
ACGTACACAGTAAATATAAAAAAAAAACACATACTAACAAAAACATCCTAAGTAACTAACAATCAGCCCAAGGCACCGACATCA
AAACTCAGCGGAGAATTCTAAGGGAACACGGGTGCATAATAGTAGCTAGTAATGTAAAAAAATCCGCACCCGGAAAAAATAAGT
GCAATAACTTCATTTATAGCGAGACGCGTGCCCTCCCTTATTCTCCCATCGGCAATTTCCTTAGGAACGATTCTATATTCGCTG
CTTCTGTCAGTGACCACGTATTAACACCTCAAGGAAAACGAAATAAATGAGCTTTGAAAGATTTTTCTTTAAAAAATACACAAA
CTTTTGAGCCGTAAGTGGGGTTTTTAAAGTATAGAAAAAAAAATGATATTCAGGTTGCAATCACGAGGACTTCCTAAGGAGGTCA
CCAAACATCCTATGTACTACTCTCACCAAAACAAGCAAACGCTCAACTGCGCGAGTTCTGATGGGATCGCGGTGCAATTAAGTG
CTGGTAGTATTGCACTCGAATGTCTTGAGTCCGCAATATTCATTTATAGCAGAAACGCGTGCCCAACCTTTTCTCCCTCGGATT
AACTAGGAGCTTCTTATTTACGCTGCCTTTCAGTGACCAGAAAACACCACGGTAATTAAAAACCCCTCAAGGAAACGAAATAAT
GAGCTTTATTAAGATTTTTTTCAAACCAAAAAACAACTTTTGATGCCGTAAGTGATTTTTACAAAAGAATAAAAAAAAATGGTG
TTTTCTGGGGTGCAACACGCAGGACTTCCCAGGAGGTCACCCACTACCAGTACTACTCTCGCCAAGAACGCTCAACCCTGGAGT
TCTGATTGTGTATCCGGTGCATCTACTGCTGGTTATTATTCGCATCCATAATTTCTCACATATTTCATTTATTATTATTTTATC
GCTCTTCTTCTCTTTTCTCCATTTTTTCTTTTCTTATATTTCTTATATACTGTTTAAGCAACCAAAGAGATCAATTAACTATGG
AACTCCTATTCGCTGCCTATGTCAGATGACCACCGGTATTAACCCCTCAAGGAAAACGAAATAAAATGACTTTTATAAAATTTT
TTTTCACCAAAATACAAACTAATGACCGTAAGTGGATTTTAAACGAATTAAAAAATGTATTTTCTGGGGTACACGAGACTTCCA
GGAGGTCAACCCCAATCCATAGTACTACTCTCCGCCCAACGCACGCCAACTCTGCGAGTTCTGATGGGGAAATCCGGTGCCTTA
GTGCTGGTATATATTGCACCCGGAAATGTTTGGTCCGCATATATTCATATATAGCGAAACGCTGAACCTCCCTTGTTTCTCCCT
CGGATTAAGCATAGGATGCTTATTATTCCGCTGCGTTGTTCCGTGACCACGGTATTAACCGCCTCAAGGAAGAACGAAATAAAT
GAGCTTAAAGTTTTTCTTCAACAAAAATACAAACTTTTTGAGCCGTGAATAAGTGGAATTTTTAAAGAATAGAAAAAAAAAATGG
TGTTTTCTGGGTGCAACCACGAGGACTTCCCAGGGGGATCACCCATCCCTATAGTACTGACTCTCGCCCAAGCATCCGCTCCAA
ACGCAAGTTCTGATGGCATTCTCGGATTCATTAGTGCTGGTAATGATCGCACCCAATGTATTGGTTCGAAAATATTCATTTATA
AGCGATCGCTTGCCCTCCCTTTTCTCCCTTCAGGATTAACATAGGAATCCTTATTGCAGCTGCTTGTCAGCTGACCGACTGGTA
TTTAACCCCTCAAGGAAAACGAAAATAAATGACTTTTAAAATTTTTTTCACAAAAAATACAAACTTTGAAACGTAAGTGACTT
TTTAAAGAATAAAAAAAAATGTGTTTTCTGGGTGCACACACCAGAGGACTGTATCCCAGGAGGTACCCCATTATCCTAGTACAC
TCTCCCCAGCAACGCTCAAACTGCGGAGTTCTGATGGGAATCCGGTGCATTAGTGCTGGTATGTTGCACCGCGGAATGTTTGGT
CCGCATATTCATTTAATAGCGCAACGCTTGCCCTAACCTTTTCTCCTCGGATTAACTAGGATCTCTTATTCCATGCTTGTCAGT
GACCACGAAGTATTTACAACCCTCATAGGAAACGAGAATAAATGAAGCTTTTTAAAGATTTTTTTCACCAAAAATACAAACTTT
TGAGCCCGAGTGGAGTTTTAAAGAATAGAAAAAAAAATGGTGTTTTCGGGGGAGCAAAAGGACTTACCCAGGAGGTCATCTCATC
CTAGATACTACCTCAGAGCCAAAAGACGCTTCCAAGAACTGGCGGAGTTCTGATGGGAATCCGGTGCATTAGTAGCTGGTATGA
TTGCACCCGGAAGTTTGGTCACAATATTCATAAGTATAGCCAAACCGTGCCCAGAATCACCTTTATCCCCTCAATTAACTAGTG
AGGCTTGCTTATTCGCTGCCCTTGTAGACCTACCAGGTATTACCCCTCAAGGAAACGAAATATATGAGCTTCTTAAAAAAGGAT
TTTTTCACAAAAATACAACTGTTGGCCGTAAGCTGGATTTTTTAAAAAGAATAAAAAAAAATGTTTGTCAGGGGTGCAACACGA
GGACTTCCATGAGGTCACCCATTTCCCCTAAGATACATACTCCTCGCCCAAAGCAGCGCTAACTGCGGATGTTCTCTGATGACC
GGTGCATTAGTGCCTGGTACATGATTGCTACCCGGTAATGTTTGGTCCACAAATATCCTATATAAGCAGAACGCGTGCCTCCCC
TTTTCTCCGCTCGGATTAACTAGGAGCCTTCGTTATTCGCTGCTGGTCAGCTGACACCGGTATTAACCCTCAAGGATAAACGAA
ATCAAATGACGCTTTTAAAATTTTTTCAGCAATAAATACAAACTATTTTTGAGCGTAAGTGATTTTAAAGAATAAAAAAAAATGG
ATGTGTTCAGGAGGTGCAACACCGAGAACTTCCCATGATGTCCACCCCATCCCTAAGTACTACTCTCCGCAAGCACCGCTTAAA

CTGCGAGATTCGATGGGAAATCCGGTGCATTAGTTGCTGGTATGAATCGCACCCCGGAATCTTTGGTCCGCAATATTTCATGTA
TATAGATACGTGCCCACCCGTTTATCTCCTCCGTATTAATTAGGAACATCTTATTCGCTGCTGATTTCAGTGACCACGGTACTT
AACCCTTCAAGGAAAAATACGAAAATAATGAGCTTTTAAAGATTTTTTTAAACAAAGATACAATTTTGAGCCGTAAGTGGATT
TTTTTAAAGAATAAAAAAAAATGGTGTATTTCCTGGTGCAAACCACAGGACTTCCAGGAGGTACCCATCCTAGTAACTACTTCG
CCCAAGCACGCTCAAACTGCGGAGTTCTGATGGGATCCGGTGCATTAGTGCGGTATAATCGCCCGGAACGGGGATAATGTTTGG
TCCGCCATAACATTTATAGCGATCCGTGGCATCCACCATTTTTACCTCGGATTAACTAGGAACTCCTTACTCTCTGCTTGTCAG
TGACCACTGGTATTAACCCCCAAGAAACGAAATAGAATGAGGATTTGAAAGATATTTTTTTCAAAAAATACAAACTTTTGAGCC
CGTAAGTGGATTTTTATAAAGAATAAAAAAAAATAGTTTTCAAGGGGTCAAAACGAGGACTTCCGAGGAGGTCACCATATCCTA
CAGTAACTACGTACTCGCACCAAGCAACGCTTTAACCTGCGAGTTCTGATGGGATCAGGTGCAATTAGTGCTGGTATTGATCGC
TACCCGGTGAATGTTGGTCCCGCAATATTCATTTAATAGCGAACGCGTGCCCTCCCTTTTCTTCCCTCCGGAATTAACTAGGAA
CTTCTTATTCGCTGCTTGTACGTGACCACGGTATTAATCCTCAAGGAAAACGAAATAAATGAGACCTTTTAAAGATTTTTGTTT
ATAAAAAAGTACAAACTTTATGAGCCGTTAGTGAGATTTTTAAAGAAATAATAAAATGATGTTTTCAGGGGTGCAACCCGAGAT
TCCCAAGGAGGTCACCCATAACCCTAGTTCTACTCTCGCCCAAAGAGCTTAACAGCGGAGCTTCTGATGGATCCGATGCATTAA
AGCTGGTATGGATCGCACCCGTAAGATGTCATGTTCGCAAATAATTTCATTTTATTAACAGAACGCGTGGCCTTCCCCTCTAAT
ATCCCCTCTCGTGAATTAACTAGGACATATCTTTATTCGCTGCTTGGTAGAATGAACCACGAGTATTAACCCTCAAGGAAAACA
AATAATGAGCTCCTAAAGATTTTTTTAAAAAAAAAACAAACTTTTGAGCCGTAGTGATTTTTAAGATAAAAAAAATGAGTTTGT
CAGGGGGCAACACGGAGGACTTCCGAGGAGGTCACCATTCCTAGTACTACTCTCGCCAAAAGCACTTAACTGCGGAAGTTTCTG
ATTGGACAGGTGCATTAGTGCTGGTATGATCGCGCACCGGAATGTTTGGTCCGCAATATTCATAATATAAGCCGAACGGTCGCC
CTCCTATTTCCCCTCGGATTAATACCGGAACTCTTATCGCTGCTGTCAGTGACCACGGATATAACCCCTCAAAGGAAAACGAAA
TAAATGAGCTATTTAAAGATTTTTTTAAAAAAATACAAACTTTGAGAAACGTTATGTATTTTTAAAGAATAAACAAAATGAT
GTTTTCAGGGGATGCAAAACACGAGGACTATCCCAAGGAGGTCACCCAACCTAGTCTACTCTCGCCCAAGCAAGCCTTAAAAGC
GAGTTCTGATGGGATCCAGATGCATTAGTGCTTGGTATGATCCACCCTAAATGTTTGGTCCGCAATATTAATTTTATAAGGCGA
CACCGTGCCTCCCTCTTTCTCCCTCGGGATTAACTAGGTAACTTCTTAATTTCGATGCCTTGTTCAGTGACCAGACGAGGTATT
AACCCCTTCAAGGAATAACGAAGTATAAATTGACGCCTCTTAAAGATTTTTTAAAAAACATAACAAACTTTTGAGCCTAAGTG
ATTTTTATAAAGAAATCAAAAAAAAGTAATGTTTTCAGGGGTCAAACACGAGGACTTCCGATGGAGGTCAACCCATCCTAAAAG
TACGTACCTCTCACCCCAAGCACCTTTAACTGCGAGTTCGTATGGGTCAGGTGCATTAGTGCTGGTTTATGATCCGCACCCAAT
GTTTGGTCGCAATATTCATTAATAGGCGACACGCGTCCCTCCGCCCTTTTCTCCCTCGGTAATTAACTAGGACTCTTATTCGCT
GCTTTGTCAGTGACCACGGTGTAATCAAAACCCTCAAGGAAAACAGAAATAAATGAGCCTTTTAAAGACTTTTTTACAAAATAA
AATTTTGTAGACCGTAAGTGGATTTTTAAGATATGAAAAAAAAATGGTGTATTCTGGGGTGCAACACGAAGGACTTCCAGGAGG
TCACCCCAAATCCTAAGTACTAAACTCATCGCCAACAGCGACGCTCAACTGCGGAGTTCTGATGGATCGGTGCATTAGTGCTGG
TATAATCGCCACACAGAATGTTCGGTCCGCCATAGTTCCTTTATAGCGAATCGGTGCCATCCATTTTCCTCCCTCGGATTAACT
AGGAACTCCTTACTGTCTACGCACCTGCTTGTCAGATTGACCACGGATAATATAACGCCCACAAGGAAAAACAAAATAAAATGA
GGATTCGAAAGATATATTCAAAAAATAACTAAACTTTTTGAAGCCGTAAGTGGATTTTAAAGTAAAAATAAAAAAAATGATGTT
TTTCAGGGGTGCACCACATCGAGGACTTCCGAGAGAGAACAGAAGCGTCAACCCTATCCTAACGTAACTACTCTCGCCAATGCA
GCGCTTACTGGCGAGGTTCTGATGCGGATCAGAGGGATGCATAAGGTTGCTGGTATGATGCACCCGAAAATGTTTGGTCCCGCA
ACATTCATTTATAGCGAACGCGTGCCTCCCCCTTTTTCTCCCTCGGGATTACTAGGAACTTCTTTTCCGCTTGCTGTGTCAGTA
ATGACCACCGGTATTAAACGCCCTCAAGGAAAACGAATAATGTGCTTTTAAAATGATTTTGTTATTAAAAAATTACAAACTTAT
TGAGCCGTTGTAGATTTTTAAAGAATAAAAAAAATGATGTTTCAGGGTGCAACGAGGACTTCCCAGGGGCACCCATACCTAGTA
CTATCTCACCGCCAACGCACGCTTAACAGCGATTCTGATGGATCCGATGCATTAGTGCGCCTGTGTCATAGATTGCCCCCTAAA
AATGCTTTGGTCCGCAATATTCATATTATAGGCGACGCGTGGCTCATTTATCCCTCGGGATTAACTAAGGCGAATTCTTATTCG
CTTGCTATGTAAGCCTGATCCACGTATTAACCCCTCTAAGGAAAACAAATGAGCTCCTAAGATTTTTTTCACACAAATAAATA
CAAAACTTTTGAGCGTAAGTGGATTTTTAAAAGAAATAAAAAAAATGATGTTTTCGGGGTCAACACGAGGAACTACATGAGGAA
GGTCACATCATCCTAAACGTACTCTCAGCCAAAGCACGCTTAACTGCGGAGTTCTGATGGGATCAAGAAGATGCCATTAGTGCT
GGTATATCGCACCCGAATGTTTGGTCCGCAATATTCATTTATAGCGAACGCGTTGCCCTCCTTTTTCTCCCTCGGATTTAAAAC
TAAGGAACTTCTATTCGCTGCTTGTCGTCTGAAGCCACCGGTATTAAACCCCTCAAAAGGAAAAAAATAAATGACTTTTAAAGA
TTTTTTTTAAAAACACATACAAACTTTTGAGCCGTTAGTGGATTTTTAAAGAATACAAAAAAAATGATGTTTTCAGGGATTGAC
AGACACGAGGGACTTTCCCAGAGTCACCACATAATACTACCTCATCTCCCAAAAGCACGCTTAAAGCGGGTTCTGATGGGATCC
CGATGAGATAGTGCTGGTATGTGACTCGCAACCCTATACAATTGGTCCGTGAAACGCACATATTCATTTATGCGAACGCTGTGG
CCTCCCTAATTCCTCCTCGGATTAACTAGAGAACTTATCTTATTTCGCTTTGTCTAGTGAACACGGTATAACCCTCAAGAAAAC
GAAATAAAACTCCAAAGATTTTTTTAAAAAAAAATACAAAACTTATGAGAAGCCGTATGGAATTTTTAAAGAAATAAAAAAAAT
GATGGTTTTCAGTGAGATGCAACACGAGGACTTCCGAGGAGCGTCACCCCATGCCTATGACTACTCTTCACAAGCACGCTTAAC
TGCGGAGGTTTCAGATGGGAATCAGGTGCATATAGTGCTGGTATGAAATCGCACCCCGAATGTTTGACCGCAATATTCATTTAT
AGCGAACCGCGTGCCTTCCTTTTCACTCCCTCAGGATTTAATGGAGCTTTCATCATTCGCTGCTTGTCAGCGACCAACGGTAAT
TAACCCCTCCAAGGAAAGAAAATAAATGAAATTTAAGATTTTTTTCACAAAAATACAAACTTTTGAA
>m141121_210323_42134_c100732402550000001823141405141506_s1_p0/33917/6925_12881
CCCTTCATTCCCATTGGGAATTATACCGCATTTTGCTCACGTGGGCAATACATTCCCCATCAAAAATATTTTACAAACTTGTAA
AAGGATTAACATCGAGCCATCTACATTTGTGGCCGTCACCGTTCCTTAACTGGAAGCCAACAGAGGGCGTTCTCACTAACACTG
TCAAGTGTCTTCCCACATATTTACCTCCCCGTCCCTGGAGATCCGCACACAAAGCTGAAAGCGTAAGAGCATTAACCCCTTCAC

CTCGCCCCTGAAGCTCTCAACTCATGGCTAGTCTAAACTATCGGACTTCTCACTGCCCACTGCGTCAGCCCTCACGCCAAGGCA
CAAGGTTCACTATGCGAGGCTTTTGCCCTTTTCCGCGAGCATGTGGGCGAGGCTACCCCTCATCCTGACCGCAATGCATCTGCA
AGCCACAAGGGTCTCACTATGCGAGGCTGCCCTCACCCGCGCGCCTCGCGACCCAAATGCGCGCCAATGCGTCGCACAGCAAGA
ACACCGTCACCACGCTCGTGTGCGTCTCGCGAGGTATCGACAAACTAAGGAGCCTCGTGCCACCCATCGTCTTGTGGCCCATCC
ATTCAAACCCCATCACCGCCTTGATGACTGACCCATGTTCGGCGAGCCTCGCTGAATTGCCCGGGTCGGAGTCATACATTAAGG
TGTATCACCTTAGGCACCAAGGGTCATTGCCTTGTGAGACCATCCCATAGGCCGGGTCCAATGATGTCCCAAGTCATGCCCTGG
AGGGTATCGATCGCGTTTAGGCACCATAGGTACTTGCCTTGTGAGACCCATCCCCAAGGCCTCCGTACCATGGGCAACTTAGTT
GCTTCAGGCCTGCCTTGATCCTGTTAGGTAGAGTACGGACTTAGGGTGTAGAAGTAATATGGATCGACACTTACTAAGTTAATA
TATTATTATTGAATCTAGAGAAGATATTTAAGGTAGAAGGGTAGCCACTGGGTGATGGGAGTACTATTTAATGATAGTCAAGTC
ACTGGGTATTGATGTTACATACCCTTGTGCAATAAGGGCAAAGGCATGTTTGCATCTGTTCCTAATCAAGTGTAGGGTGGGGT
ACGGTGATTTTAGTACCATGAGATAGATGGAAGCATACATGCGATCGAATCTCTGAACATAATGTAATATCCTTATGTTGACGA
CACCATAACTTTGCTAGTCATTAGGATCCATCCGTACCGACCAGTTGATTGCCACATAGCGGCCTTGAATGAATGTAGTCATGT
TGAAGTACTCAATCTTGGTCCCCTGGGACCACAATGTATGGGGAGCCCATTAGAGCTCCAAGGGTATAGGATATAGATCCGATT
TCCACCTGCTTTCTTCGTAGAAAAGGGGTTGGATTGATGGTTGTATGTACTAGGATGCTTTTTGACTAATGGATAACTCAATTT
CGATCTCCATTGCAGTTTTTACATATTTGAGGTTCCTCATACAAAGTCAGATTCAATCGGCAACGGAGGAGGAGATGGAGGAAT
AGGAAGTATGAAAGATAGATAAATAGTTAATCACTTGCAGGATTGGAGTTCGTTAATTTAGTAAGTATTTATTACATATAAGAC
CTTAGCATATCCAATCGAATTAGTTTCCAACCTCCGTTTTCTAAGAGGGGTGATGATACGTAGTTTGTATTGGTTTCATCTGGA
GCTCTAATGGCTTCCCCATACTTGTGGTCCCAGGGGACAAGATTGTACATAGGTTACATTTTCAGGGTAGTGGCACAGGTCGTG
GTTGTGTAGCAGTAAGGTTATGTGTCGGCCTGGTACATAGTCCAGAGGTATGCATGTAGTGGCAGTTCACTCTTCATGTTACAA
ATCCGTACCTCTACTACTATTGTAGGACATCAGGGATGTCCATATGAGTAATCTTGCCCTTTGTTGTATCATGGTTGTAGCGAT
CCCCGTGCTTGTACATTTATTAGTCGTCATCCCAGTGGTCCCGATACCTAACCTACATTTATCTATCTTCTTAGGTTTTTCTTT
AGTATATATTAGTAAGTGTTCGGATCATTGACTTCGTTATTATCCCGTTCCTTTCTCCAGTTGATAAGAGGGCCGTGGGAGAGA
CTGGTCTGACTGCTTACAGGCTTTTGGGGGCATTGAGTGGTTTCATCACAATGGTCATAGAACCCTATTCGGTTGCCATATAGC
GAAGTATTAGGCGACATTTGGGCATTCGAGTGTAGAGCATGGGATTGGTGATCCTTCACAAGCAAGACCTTTTGATGTCTCTAA
GTGTAGATACCATGTAGTGACCCGGGCATTCTACAGAGGCTCGGTACGATGGTCTCACAAGGCGTGATGGGGTTGATGAGGGCC
ACAAGACGATGGTGGCACGAGGCTCTTAGTTTGGTCATACCCTCGCGAGCGCACACGAGCGTGGTGAGGTGTATTTCTATGCTG
TGCGACGCATTGCGCGCATTTGGGTTCGCGAGGCGCGCGGGTGAGGGCAGCTCGCAATAGTGAGACTTGTGGCCCTTTGGCAGT
GCATGCGGGGGAGGGTAGCCTCGCATGCGGGGCAAGGGCAGCCTCGCATAGTGAGACCCTTGTGGCCTTGTGCAGGCGCATGCG
GGGGAATGGTAGCCTCGCATGCTGGGGAAGGGCAGCCTCGCATAGTGAGACCCTTGTGGGCCGTGGCAGGCGCATGCGGGGGAA
GGGTAGCCTCGCATGCGGGGGAAGGGCAAGCCTTGCATAGTGAGACCCTTGTGGCCTTGCAGGCGCACGCTGGGGAATGGTAGC
CTGCGCATGCGGGGGAAGGGCAGCCTCCGCATAGTAGACCCTTCTGGCCTGGCAGAGCGCATGCGGGGGAGGGTAGCCTCGCAT
GCGGGGGAAGGGGGCAGCCTCGCATAGTGGGACCATGCGGGGGAAGGGTAGCATCCGCATGCCGGGGGAGGCAGCCCTCGCATA
GCTGAGACTTGTGGCCTTGGCAGGCGCATGCGGGGGAAGGGTAGCCCTCGCACAGGAAGGGCAGCCTCGCATCAACGAGACCCT
TGTGGACTGGCAGGGCGCATGCGGGGAAGGGTAGCCCTCCGCATGCGGGGGGAAGGGTGCCTCCGGCTTATAGATGGATCCCTT
GGGGTTGGTAGGCGCATGCGGGGGAAGGGTAGCCCTCGCATGCGGGGGAAGGGCAGCCTCCGCATAGCGAGACCCTTGGTGGGC
CTTTGGCAGGCGCATGCGGGGGAAGGGTAGCCTCCGCATGCGGGGGAGAGAGGCAGCCTCGCATAGTGGAGACCCTTGTGCGTG
GTCGGGGGTGCATGGCGCGAGGCCTTGATGCGCCCTGCCCTTGTGACCGTATCTTGGCCTCCTAGCCTAGCAAAGGCCGATACT
TCACAGCCAAACAAAGGTGTTGCTTTCACCTTGGGAATTCAACTCTTCCCGGTTTCACAAGACTTATAGGTCTGAGCCTGATAA
TGTGACTAAGTGACAATACGCTATGTATTTTAAGACATTTATTGGGATGTTTTTTTTCTGTGATTTTTGTCCTCTCAGGGGACT
CCCATCCACAAACTTCTCAAGACTCCCACCATAAATTTGAGAATTATTTACTTAAACTCTGTGTAATTAAATATTCTCAATTTA
TTAATCTCATAATTAATTCCACTATAAATATGAGACTCACTCTTGGTGAGTTATGAAGACATTTCATTTACTGAGTACTTTATA
ATCATAAAATCTCATTATATAATCATTTCATACTAATTGACCCTCTAATAATGGTTATAATTAATCGGGAATAAAAATTACCTG
ATTTTACCATGTTGTAAGGTTTCTCGTATGTGATAGTTCCTTAAGTCACGCATCTGATGTAACTAGTGAAGGTCTAAATAATCA
TTAACTAATATGAATTTAGCTTAAATAAGCTTTCAATCCCAAAAGAAGTTTCAACCCCCCCTTCAAGAGAACACATTTCAATCT
CGTTGCGAGAAGTGCATCAGATTTCATGGGAAGAATTCTTGTGCAAAAAATAACTATGTCACCCAGCCATCATACATTAAGTAC
TTGTCTCCCAAAACCAAAGTTTCCTAAGCGCTGGAGGTCATTCCTGGCCTGAACAAGTGAATCAAAGAATCTTCATATTATGAA
CATTCAGAACAGGAGTTCATGTAGTAAATTCCAAGGATTTAAAGATTAATATTTTGCATAATGATCATTCAGTTTGATATTAGT
TATCAATAATAAATCTCTTTCAGAAAACGGTAGTTTAACTAAGTATGTTAAATAAACATATAGTTTTCTGGTCCAGTTATATAT
TACTAGAGGTACATTTCTCCTAAAGAATTGATATAAGAGCACCAATCCATAAAGTGTCATATTCACACTAGTGGAATCCGGTTG
ATCTAGGATCACATCGTAATTCGATAATACTGAGCGTGAACCGTACTTGCAGTACTTTGATTGCTTAAAGATGATCCATAGATA
TATTTACCCATGCGAACTATTCAAGTTCATTTAATCTCAACACAATCCTTCACCGTACCAATACGTGTTTGAGATCACATATAT
AAAATTAGGAATTATTTCGCTGATATTTACCTTAATATTTAAGTCACAGAAATAGAATAATATATCTGTCCATAACAAATTGAC
TACCATGATTTTGTCATAACAAAGTTCGCCAATTCATTTAATTTATTCTAAAAACAATGCTACTACATATGCTTCGGGCATCTA
TGTTCCAAATTTAGGTCATCATGTATGTATCGTTTATTTAATTATTTGTGTTTTGAAGGCGCCCATTAGACCATTATCCAATGG
GAGATATAAAATGTTGTGATGAATGGAATTTGGATGGTACAAAAAATTAAGTATGCGGGTATATTTGACACAATTTTTAGCACT
ATGCTCTAATGCAATTTTGTAAAAGTTTACTGCAAAAAATTATCTTTCATTAATGCTCATTTGAAGTATTTATTGAGAATATAC
AAAGAAAATTTAAATTTTTATTATAATTTTGAATTGTTATAGTTAGAGATAATAATAAAATAATAAGGTAAAAAGTATAACATT
TAACTGGTGATCATTAAAAATAATCCTAAATAATAAAAATGGCATAATAGTAAAATTAAAAAGTGTAGGCATTTATGCAATGCA

AAATATTCATCATGCATACTATGTGTGACAAATTTGGCAAACTTTTAGCATGTGTTCAAAGTTTAACACAACTTTTGGCCACCA
ATAGAGCAACTTCTTTTTTGTAAAATAAGCTATATTTTAGCAATGCCATCACCAATTTGACACTCTATTGGAGATGCTCTTATA
GCCCCACGCAAATCACATTTTATATGTAATATATAGTATAACATTTTTATTAAAGTTATAATATTTAAATTCAACCATATATAT
ATAAATTATACAGCGGTGCTATGGTCATATATATTACTCTTAGTTTTCTTAGCATCTTCTCTTATTATGTATATAGAAGACAGT
GGTAAGGTCCCCTTAAAAGGACCATATCATGCGATTTTTTTTCTATTTGTCGGCACATTGAAATAAGCTCTCATAACCTTAAAT
TTTTTTTATATGATGTGTATATTGCATAGTTATAGTAGTATCTCTATACAAATTTTCAAGAAATTCTTAATAATTTGAAGATGC
TGAAAAATCATAACTTGAACAGTATATTAGCACATGTGTCTGTTCGATTTTTATACGCAAACTATACAAGAGCTTGTTGAATCT
TGCGATCGATATCTAATTTCCAATATTTCTTAAAAAGATTATAGATGTTTCTTATAAATAATAATATGCCCATCATATAAACAA
AAATCAACTGAGGTAACAACTTATTCATACCCTAAAAATAGAAAAAAATATCAACCAATAGGTCCACTCTTTTGAGGGCATTTC
CAACCCATAACACAATTATATAGGTGTTTGCACAACACCAAAACTACATACTTTATGAACCATATATTTTTTCCAT
>m141121_210323_42134_c100732402550000001823141405141506_s1_p0/34176/320_8391
TGTTGATTTTATGGACTTTGTAATACATAAAGTTGTGGAAACTTCCTTAGAATTAACTCTTATACTCAGAAAGCATAAAGATTC
CGCGATTGAAACATTTATATGTTAACAAAATAAAGATACCCTAACCAAATATATAACATGAAAGGTAGACTGTTGTTGTCCACA
ATGACCTCTCGCATATTCTTTTAGTGTATTATTTCAGCTAAATCAATAACATGCTCTTAATTCATTATTAGTAAGTAAATCGAA
AGATTTAGTGGATGGTTATTAATAAGGTGAGTGAGCGGATAACCGCACAGTTTGTTGGTGAATTACGGATGAGAATTATCACGA
TGTTATAATTTGTATTACCCCTGTTGTGAAAATAGTATTTATTCTTTCCATTCTGTTGTTCAAACTCGTTCAGAATATTGATGA
TTATTTATAAAAATTTGCCACCAATGAAATCTAATCAATTGGGCCGAATTATGAATTATACTATGTATAATGGAGTCAAAACGG
AGCCTTCTTAAATGAATTACAAACATTTTCAATAAAAAAATAAAATTTCCCTGTTTTATAGATGGCAATTTTTCGCTACAATGG
TTTTATCGTTTTTCTAGTAATATTCGTACATGATCCATCATATCACGAGGAATCATTTATTGTTGACTGTTATATCGACCGGG
AACTAAACTTGATGTCCCCACTTAGATAGGGACCAATTGCTTCATAAGCCATGGTACCGGAAATCTATATTCTTATACGATTTT
TGGATACCTAGAATACAAATATTTTAACAAATGAGTTGAACCCTATGAAGACTATGAAGAGGCGTATTCAAACTTTTGGATTGC
ACATTTCATGACAACTTCATACGCATAATTTCTTTATATAGATAATTTAATTTCACTCATCAAGTAAGTTAATGTAAACTAGGA
ATAAGATTATGCTCTTAAATTCATATTAATGAGATAAGGGTACAATCGAACGAGATTTTATATTAATATAGTGTTCATACAACC
CCTTGAAAATTAATTGGAGAAGATTTACAACTAAATGATTAATATATTTTGTATGTAATCGCATGAAATATATTATTGACCCTA
AGTGTTGCAGAAACTTTATGGTTCTACGTTGATATTACATTTTACCCCAAGAAAGAATCGTATCTACCTAATGAGCGATAATAG
AAATATATTGTTTGTTTTAAATTTAGGAAAGTCGGTATAAATATCGATGATCCTATAAGTCATACATTTCAAAAAAAAAAATTCC
CCTTTTTGATGGCAATTTTTCGCGAGTGGGTTATTTTATATCCTTCGTAATTCGTACATGTACTCACTTCCGGATTATTAATAT
GATCATGGGAACACTCCTTATACTTATGCATCAGTTTTTTGGTTTAATAATATTTTATTTATTGGATATATGGAATTATTGGTA
TACGACCTCCGGAATATGGGCGAGTGGATAATATAATTATTTAAATATATATAATTGCAAACCCTGGAATGAACGATAAGGGTC
TGTAACTACATATGGTTGTGTCCACTTGACGCGTGAATGGAACTTACGCTGTAGAGATATTTCATTTATGTTATTTCATTCACG
GGATAAAATATCACTACACAAATTATAACAATTTTAGACTCTATTATATACACAATTACATTGTATAGATTTCCTGTAATGGGT
GAATTGTTATATTTTTTTCGAGAATTAGGGGTAACCACACATTAGTAGGTGAAAATATGGAGTATTATATCAACCGAATATTAT
ATTTTATCCCTTGATACAAAAGATGTAGATAATGATACACTATCATGAGTGGATTACAGTCAACTCGTTGTCTTGATTGATTAT
TATATTTATTATTTTATATATATCCCACACATAAAATCTCATGGCAAATATTAAGATATAATTAGAATTAATGGAGTCGATACA
CGAGAGCCTAATTTCTATACCATTTTCATGATATAAATATTCCAATACCCGTGTTATTTTGACATGGTCTATATTTTTCATATG
TATAATAAATTATCCTTGTATACTTTATTCTGGTATACATTCTCTCCGGTATTCCTAATTATTTATAGACTGTTACATTATAAA
ATAAATATGCATAACCATCATACAAATCACTTATTACCTCGGAGATGTGTTATCCGCAATATGAAATAGTGGTCTTACATCTAG
AGAAACTACAATCACTAAATAGAAGTTACAGGTCACAACTTATTGTGATGCTCCATTGACGGACTCAGCTTCTAAGCTATAGGT
TATTTTTTTCTCTATTATTATTTTAATTACAAACTATACAACTTAACATATTATATTCTCTATTATGTCATAGATTTATGATGA
AAATGTACAAATATGAGTTATTCCTGAAAGGGGACGTATCTCTCTTTTATTATGAGAATTTGGAGATTTTCACTAATTAATATC
TTTCCGAAAATGGTATTACCCTCGTGTTCAAGATGATCCATCCCGATTATCGTATTTATTTGATATGTATGTTTTCCCTCAAGA
GATCTATCCAAAATGCGAAATGAATGATATTGGAGTCAGTAAACAGGAGCCTTAAGTCAACCATTTAATATTCAAAAAAAAAATA
TTCCTCCTTTTTATGGATGAGCAAGTATAAATTTCACTAAGTTAGTTTCCTTGTAAGTATATAGTCGTACATTTACAAGAAGAA
ATCCATAAGAAATGTCATTTTTTCCTCACCAAAAAAGATAAAAATTTTCTCTAGTATAATCAAGAGTCTCATGTGTTGAGAATA
CTTTTGATTCCAAATTTTATTATTGTTCTCTATGTGCTCACCCACATCTTGAGATGTAGAGAACGTCCTCTTCTCTCGGTATTT
TGACTGTATCACCCTCCTTACACCTCAGATTCGCAGTGAATTTTACAAATAGAACACTATATGACATAAGGCTACAACTTTTGT
GCCCCATGACCCTCGGCAATATTCTTTAGTATTCACTATCAAACTTTTCCTAATTCATATTAGAGAAAGTCGAAGATTTCTGAA
AGGGTACCCCTTTGGTGATATTGGTAGATTTTCAACTAATTTCTCCGAAATGGTATTCCGCTCGTGTTCAACTCGTTCGCATAT
TTTTCCCCAAAGAGATCAATCGTGGAGAAATGAATATATTGGAGTCGAAACGGAGCCTTAAGTCAACCATTTCAAAAAAAAATTC
CCCTTTTTAATGGCAATTTTCGCCATGTTTTTTTCTTATATTCGTAACATCTACATCCCGGTATTTTGACTGTAACACTCCAAG
CACACTCGGATTTGTACAAAATTTTTCAAATAGAACCCTAAGACATAGGGCTACAAACTTTTGTGGCCCATGACCCCTCCGCAT
ATTCTTTGAGTATTCACTATCAAAACCATTGCTTCTAAATTCATATTAGATAAATCGAAGATTTTTTTAAAGGGGTACCCCTTT
GGTGAATTGGAGATTTCAACTAATTTTTTCCGAAATGTATTACCCTCGTGTTTTCAACTCGTTGATATTTTTCCCCAAGAGATC
TCATGGCGAAATGAATATATTGGAGTCGAAACGGAGACTTAAGTCAACATTTCAAAAAAAAATTCCCCTTTTTAATGGCAATTT
TCGCATGTTTTTTTCCTTATATTTCGTACATCTCTCGGTATTTTGACTGTAACACCTCTACCACAACCTCGGATTTGCACGGAA
TTTTTACAAATAGAACCCTAAGACGATCAGGGCTATACAACTTTTGTGCCCATGTACCCTCAGCTTATTCTTTAGTATTCACTA
CCAAAACCATTCTCTAATTCACATTAAGAGAACATCGAAGATTTCTGAAAGAGGACCCCTTTGGTGAATTGGAGAGTTTCAACT
AATTTTTCCGAAATGTATTACCCCTCCCGTGTTCAACTCGTTGAATATTCTTTCCCCAAGAGATCTCATGGCGAAATGATATAT

TGGAGTCGAAACGGAGCCTTAAGTCCAACAATTTCAAAAAAAAATTTCCTTTCTTTAATGGCAAATTTTCCGCATGTTTTTCCT
TATATTCGTTACAAATCTCTCGGTATTTTGACTGTAACACCCCTCCCTACACACCTCCGGATTCGCATGAAATTTTACAAAATA
AGAACCCTAATACATAAGCTACTAACTTCTTGTGCCCATGACCCTTCGCATATTATTATAGTCTTCACTATGAATAACATTGCT
CGAATCCCATATTAGACAAATCGAAGATTTCTGAGAAGGGGTAACCCCTTTGGTGAATCGGAGATTTCACTAATTTCTCCGAAA
TGTATTGCCCTCCCGTGTTCAACTCGCTTGATATTTTTCCCCCAAAGAGATCTCATGGGAAGATGAATATATTGGAGTGGAAAC
CGGACCCTTAAATCCAACATTTCAAAAAAAAATTCTCCTTTTGATGGCAATTTTCGCCAAGTTTTTCCTTATATTAGTACAGC
TCATAGTATTTTATCTGTATCACCCTCTACCCTACTCGGACTAGCACGAAATTTTCACATAAGAACCCTAAGAACATAGGCTAA
CAACTTTCGTGCCCATGACCCTCGCATATTATTTAGTATTCATATCAAACAACCATTGCTCGAATTGATATTAGATAAATCGAA
GATTTTGAATAGGGGTAAGCCCCTTTGGTTGAAATTGGAGATTTCACTAATTTCCCGAAAATGTATTGCCCTCGTGCGTTCAAC
TCGTTCGATATTTGTCCCCCAAGAGATCTCATGGGTAAATGGAATATATTGGGAGTGGAAACGGACCCTTAAATCAACATTATC
AAAAAAAATTCCCCTTTTTGATGGCAATTTTCGCAAGTTTTTCCTTATATAGTACATCTCTCGGTTATTTTGACTGTAACACCC
TCCCTACACACCTCGGGATTTGTACAAAATTTTACAAATAGAACCCAATAAAAGACATAGGGCTACAACTTTTGTGCCCATGTC
CCTCGCATATTCTTTAGTAATTCACTAATCAAACCATTGCTCTAAATTCAATATTAGAATAAATCGAAGATTTTTTAAAGGGGT
ACCCCTTTGGTGAATTGGAGATTTCACTAATTTTTCCCGAAATGTATTACCCCTCCGTGTTCAATCTCCGTGATATTTTTCCCA
AAGAGATCTCCATGGTCGAAATGAATATATTGGAGTCCGAAACGGTAGGCCTTAAGTCAACCATTTCAAAAAAAAAATTCCCCT
TTTTAATGGCAATTTTCCGCATGTCTTTTCCTTATATTCGTACATCTCCTCGGTATATTCGACTGATAACACCCCTCTACACGT
CGGATTTGACACGAAATTTTACAAATAGAACCCTAAGACATAGGGCAACAACTTTTTGCCATGACCTCGCTTATTCTTTAGTAT
TCTTTAAACAAAACATCTGCTCTAATTCATACATTAGAGAAATCGAAGATTTCCTGAAAGTGGGTACCCCTTTTGGTGAATTGG
AGATTTCACGTAATTTTTCCGAAATGTATTAACCCTCGTGTTCAATCGTTCGATATTTTTCCCCCAAGAAGATCTCAATGGCGA
AATTAATATATTGGAGTCCGAAACGGAGCCTTAAGTCAACATTTCAAAAAAAAATCTGCCCTCTTTTTGATGGCAATTTTCGTA
AGTTTTTTCCCTTATATTCGTACATGTCTCCTCGGGTATTTTGACTGTAATCACACTCTACAACCTCAGATTCGCATGAAATTT
TTACAAATAGAACCTAATACATAAGGCTACAACATTTCGTGCCCATGACCCTCCCATATTCCTTTAGTATTCACTATCAAAATT
GCTCCTAATTCATATTAGAATAAACGAAGATTTTTTAAAGGGGTACCCCTTTGTGAATTGGAGATTTTCACTAATTTTTCTGAA
ATGTATTACCCTCGTGGTTCAACTCGTTCCGATATTTTCCCCAATGAGATAATCATGGCGAAATGAATATATTGGAGTCGAAAC
GGACCCTTAAGTCAACATTTCCAAAAAAAAATTCCCCCCTTTTTGAGTGGCAATTTTCGCCATGTTTTTCCCTTATATTCGTAC
ATCTCCTCGGTATTTTGACTGTAAACACCCTCTACACACCTCGGATTTGTACAAAAATTTTATAAATAGAACCTAAAACATAGG
GCTAAATAACAACTTTTGTGCCCATGTACCCTGCGCATATTTTTAGTAATTCACTATCAAAAAATTGCTCTAATTTCATATTAG
ATAAAATCAAGATTTTTTTAAAGGGGTACCCCTTTGGTGAAATTGGAGATTTCACCATAATTTTTCCCGAATGTATTACCCTCG
TGTTCACTCCCATTGATATTTTTCCCCCAAGAGATCTCTATTGGCGAAATGAAATATATTGGAGTCGAAAGGAGCCTTAAGTCA
ACATTTCAAAAAAAAATTCCCGCTTTTTTGATGGCAATTTTCGCATGTTTTTTCCTTATAATTCGTACATCTCTCGGTATATTG
ACTGTAACACCCTCTACACACCTCGGATTTGTACAAAATTTTAGCCAAATAGAACCCTAAGACATAGGGGCCTACCAACTTTTG
TGCCCATGTCCCTCCGCAATATTCTTTAGTATTCACTTCAAAAACCATTGTTCTAATTCATATTAGATAAAATCGAAGATTTTG
TTAAAGGGGTACCCCTTTGGTGAATTGGAGATTTCACTAATTTTTCCCGAAATGTATTACCCTCGTTTTCAACTCGTTGATATT
GTTTCCCCCAAGAGATCTCATGGCGAATGATGAATATATTGGGAGTCGAAACGGAGCCTTAAGTCAACATTTCCCCAAAAAAAA
AATTCCCCTTTTTAATGGGAAATTTTCGCATGTTTTTTCCTTATAATTCGTACATCTCTCGGTATTGTTGACTGTAAACCCCCC
TCTACCACCCTCGGATTTGCACGAAATTTACAAATAGAACCCTAAGACATAGGGGCTACAATTTTGTGCCCATGACCTCGCTTA
TTTTTAGTATTCACTTCCAAAACCTTGCCTCTAATTCACATTAGATGAAATGCGAAGATTTCTGAAGGGGGTACCCCTTTGGTG
AATTGGAGGATTTCACTAATTTTTCGAAATGTATTACCCCTCAGTGTTCAAACTCGTTCGATATTTTTCCCCCCAAGAGATCTC
ATGTGCGAAATAATATATTGGACGTCGAAACGGAGCCTTAAGCGTCAACATTTCAAAAAAATATTCAACCCTTTTTTTAATGGC
ATTTTCCGTAAGTTTTTTCCTTATATTCGTATCTCCATCGGTATTTTGACTGTATCACCCTCTACAGACCTCGGATGTCGCATG
AATTTACAAATAGAACACTAATATCATAAGGCTACAGACTTTCTGTGCCCATCGACCCCTCGCAATATTCTTTAGTATTCACTA
TCCAAACACTTTTTCTAATTCAATTTAGGAAATCCGAAGATTTCTGAAAGGGGGTACCCCCTTTGGTGAATTGGGATTTCATAA
TTTCTCCCGAAATGTATTACCCTCGTGTTCAACTCGTTCGAATATTTTTCCCCAAGAGATCTCGTGGAGAAATGAATATATTGA
GTCGAAACGGAGCCCTTTAATCAACATTTCAAAAAAAAATTCCCCTTTTTGAATGGCAAGTTTCGCCATGTTTTTTCCTTATAT
TTCGTACATCTCTCGGTATTTTGATGTACACCTCTACACACCTCGGATTTGCACGATATATTTTCAAATAGAACCCTAAGACAT
AGGGCTACAAACTTTTGTGCCCATGACCTCCGCTTATCTCTTTATTATTCCATCTTCCAAAACATTGCTCTAATTCACATTAGA
GAAATCGAACCGATTTCTGAAAGGGGTACCCTTTGGTGATTGGAGAATTTCACTAATTTTTCCGAAATGTATACCCTCCGTGTT
CCAACTCGTTCGATAATTTTTCCCCCCAAGAGATCTCATGGCGAAATTAATATATTGGAGTCTGAAACGGAGCCTTAAAGTCAA
CATTTCAAAAAAAAATATTCCCCCTTTTTGATGGCAATTTTCGTAAAGTTTTTTCCTTATATTCGTACATCTCGTCGGTATTTT
GACGGGG
>m141121_210323_42134_c100732402550000001823141405141506_s1_p0/124662/5442_11394
ATATAACCCTACAGAAGTCTAATACCCAAAAATACAGCAGAAGATACTAACCACAGAAAACCCGCAACTATAACCGACCCACAA
CCCAGCCACAACATACAACATACCACCACACTTTCTAACATAAATACACTCACATCGGACCAGTTTTACATTTTTGTTTGAATA
ATCTGCGCGTGGCGTAGTCAGTACAATACACTTTATTTACGAAACTGATCGCTTTACCACAGAGCTCTCCCTCCTTACATAAAC
CCTAACAACCGACTAAAACCCTAAACACACTCAAACGCCTGACCAAGTAATTTTCTTCTCGCCCTAAACCACTGCACGCACACT
AAGCCAAAACCCCAACCTAAACATAAACCCTAAACCACTAAACCCCTAAACCCTACCTAAAACTCCTAAACCTCCTAAACCCCT
AAACCCTAAGCCGCTAAGAACGCTAAACCTCCTAAACCCTCAAACTAAAACCCTAAAACTAAACCCTAAACGCCCTAAACCCTG
AAAAATCTAAACCCCTAAACCCTAAACCCTGAGTCCAAACCCTGTACACAAGCAAAGAAAACCTCAAAATAGACAACCCAACAT

AGTGAGAGATCCACAATTAAGCATTTAACAAACCCAATATACCTACAATCGCTGTTCCACTGAGAATCAAAAACCATCATAACA
CACCATTACACATTTTCACCTAAGTAATCCACGCCTCTAACCCGCCAAACCGCTACAAAACTACGTTGACAACTGCACCTAAAC
CCCCGTCCTACAAAACACTCACCAAAACATCCTACATTCTACAAATACCATCCTAAATTCCAATGACTCGTACGCCCCACAAAC
CCCTACACCACATAAACCTCGACAAATCCATACCTAACAAAACCCCTAACAAACCAAAACTAACAACAATGACAAGCCTTATAA
GAGTAAGACATATCTAAAACCCACACAAACAATTCTCATCTAAACACTAAATCCCCTAAAAGCCGCGCACACAAACCCTAACCA
GTAGCGCTTCACGCTAAAGAGCCCAATGCCATCTAAATAAAACGCGTCTAGTAGACCCGTTTTCCCTTACACAATACAAGCCCT
CTTCCCAGCAAAGCCAAAGATCTAAACATACCCCCGTGTACGATCCCCTCTATAATCTGCCCCCCTAAACCTAGACACAATACC
ACGAGCCAACAAAACACCCTAGTACAGACATATGATACACCTATACCACTAAAACCGACAATTTAAACACCGTATAAAACTCCT
GAAACCCTAATACCGCTAAACTCATTCAAACCCACAACCCTACACACAGCACATTAGAACTACCCTTAACCAAAACCTCGTCAC
CTCACACAACCCATATTAGAATCGAATTCCTGAACAACACCACTAACGAACCGCCGACATCCCACTAAACAACCGTCTCCCAAA
TTCCCCGAACTAACACAACTTCACCAAAAACCTCACCAAACCAGACATCCCTAAACTCCGACTGACAAACCCTGGCGCTAAATC
CCCTTCACAAAAGACCCCTAACCCCCGATAAAGAAGCGACTCTTAATCTACCAGCATGCGCTAGTACCCTAGCAAACCCGAAAT
CAAACCTCAAAATCTAACAAGATTATCCCTGAAACCCCTATACACCCTACAAACCGTGACACAAACCCTAAAGAACCTAAACTC
CAAAACCCAGACTAAACCACCCAAACAAGCACGACAAATCAAGTCCGTCTCATTCACAACCCCAATCTAAACCCCAGAAGAGCC
CATTTCTCCACACAAACCTTCGCATGAATACCACTCTAACAACCCCAAAAGTCGACTCCTCTAATATAACACTAAAAACAACAA
CCTCCACACCAGAACAACATACACACATCACCTATAAATCCGACCCATACAAAAGACACCCATATAACATCATACACAACTTAC
CTTAAACAAACACACATACACACAATCATTGCTCCTGAGAACCATCTAGAAAGCTTCTTCAAAACTTCTCTAAGACTACAACCC
AAACGAACCTAGAAACCCCTCAATCACTAGAGACCACCTTACACACTAAGAACCCCCTAAATGCACACTGCAAAACCAACTCAA
CAATACACTACCCCCAAACCCTAAACCGCCTACAATAAATCCTCTACAACAACCCTTAAACAACCACACACAACATCAATCAAA
CCCTATACAACCCTAAATCTCTTATGACCCAAACCACACCGACACTTATGCAGTAAAAAACACCAACACAAAGGAGCCTGAACC
CTAACAACACCAAACCCTAACAACTCCATACACAAAACCCTAAACCTCTACAAAAACCTATATCCGAAATCCCCCCTATGCCAC
CAAACAAGCCTGTTTCTGACCAACATCCTAACCCCAGAAAACAACTCCTCCTAAAACCTCTGCGTAACAAAACCCTCTCACAAA
ACCTCTAGTCCTAACAACACCGCAATTAACCGTGCCTAAACACTCCCGAAACAAAAACCGGCTATAAAGCCTCTATGAAAAGAC
CGCACCTATAACACACCGTAAAAAGCCTAGGAGACATAAATCTAGAGCACCTATTGAAAACGCAGACTCCAAAACCTCAAACCT
AAACCCAAAACCAATACTCTCTTGCATTCTACATAACCCTCCTTCTAAACCCTAAATCCCTATAAAAAACCGCAAAACCCTAAA
ACCTCAAATAACCCTTTTCTCACACATCAGACCTTCTCTTACATACGCACTTAACTAAACTCCAACGTTAAAATCCCATCAGCT
ACAATAGTCCTCACTACAACAAACACAACTCCACAACCCCTAACAACAATCACACCAAACAAGAACCATGCTAGAGATACGCTT
ACCAATTATCTTAAACCCTACCCCTAAACACAACAACAACCACAAAACCAAAACCTGCTAAAACTTAAACTCACCCTAACCTCA
TAATCCGCGTACACCAACCTAAAAAAAGATCCCAGTATAACTTTACTACAACACAAGATCCTAACCAATTCCCTTACAAAAGCC
TACAATAACTCTACACAAACCCTAAAACCCGCAACCCGCAAAGCCCCGCCTTACAATAACCAAAACTATACCTCCCCTATTAAA
CCGAACTAAAGCCTCTCACTAACACACACTACTAAAAACCATCATAAACTAACAGACTCACTAAACCCCTAAACCTCAATGGGC
TACAAACAAATTACTAAAGTAGACCCCTCACTTAAAACCTTAACAAAATACTATCTATCCTCTTTATAAACCAAAACTAATATC
TCAACCTAACTACCCTAACAATCACTACAAACCTCCGATAAATACCTCAAACCCTACAACTCCTAAAAACCAATCCACTCCAAA
TAACACAACTAAACCCCTAAAATACACCCTTAAAATCCGCTCTAATATATCACCACCTAAACTCCTCCCTAACCCTTAAACGCG
CCGAAAGCCCCACACCCTTATCCCTAAACCAAGCCTCAATATACGCGTTCCTAATAATTCCAGGAGATTCTCGAAGACCCCTTA
CAAACCAGCCTAAAACTACACTCCATCAACAATGGCCTCTCAACACTAAATCAGCTCCCGCATAATAATCACTACGAGATTTCA
CATATAACACCACAAGAAGAAAACATCCTATAAAGACCTCTAAAGCGTCTTTCTCTATAAGAGCCCCTTATAACGCCTAAAAGA
AACCTATGGCCCCCAATCCTTAGTGTGTTACATAATTTAAACTCCCTCCAAAAGAAAACCTAAACACAAAGGCATAGACTAAAA
CACTAAACAAAGGCGATAACACAACCTAAAACACGCCTAGAGCCCACTGTTCAAGAGCACCCTAACACAAACCACCGTTAAACG
CTAGGGACTAAACCCCTTAAAAACGCTACTGAAAATCCGTCTGAGAGCCTCGTAACATAGAAATCCTAACTAAAACCGATTATC
AAATAACTAAACACACACTCAAAGGCCTGAACAGCCTACTAACAGAAACACCTCTTGAGAAACATCGCTTAAAATCCTCCTACA
ATCTCCTAAAACCCTTATATAACCTAGATTAAACACTCAACAAAACTTCCAACTAAACCCAACACATAAGATATCCGCATCTAC
AAAAACCACCTAATAAGTCTCGAATATAGCAACCCTCCAAAATTGCTGCCATCCCCCATAAAACAAGCTCCCGTAAAAAACACC
TTATCACTTAAACGAACGTAACCTAAACCTACACCTTAAGAGCCTCATAAAGAATCTACCTCGTGTCCTTAATAGCCGCCCCTA
AAATCCCCCAAAGGTACTAGAAATCCCTTCACTAGACGAAGACACCTAAGCACAAACCACCTTAACAAAGCCCTAAACCCACGA
AGAAAAAATATAAATAATAAAAAATAAAATATTAAATTATAATAATAATAACCTAAAACCCTAAAACCTAAAACCCTTAAACCC
TAAAAACCGACTAAAACCTATACCCTAAACCTCTAAACCACTTAAACCACTAAACCCTAAACCGACTAAAACTATAAACCCGCT
AAACCCTAAAACCCTAAACCCTAAACCACTACCCTAAACCTAACCCTAAAACCTCTGAAAACTAAAACCCTAAATCCAGCTGAA
ACCTACCTAATACCACTTAGAACCCTAAAATACCCTAACCCAAAAGTAAACCCCTAAATAAACTAAAAAAACCCTAAACCTAAA
CCTAAACATAAACCACTAAACCCTAAAAACTAACCAGTTGAGCTAAACGCCTAAAAACCCTAAAACCCTAAACCCACTAACGCT
CTATAACCACTCAATAACACTAAACCGACTAAAAAGCTAACCACAACTAAACCCAAAACCCTAATACCCCATAAACCCTAAAAC
CACCTAAACCCTAAACCTAAACATAAACCTAACCCATCTAACCACTAGAACCGACTAAAACCTCTAAACCCCTAAAAACCTAAA
ACCCTAAACGCACTAAACCACATAAACGCCTAACCCACTAAAACCTCTAAACTAAACCCTAAACCCTAAACCTAAACTAAAAAC
CTACTAAACCCCTAACCCTAAAACCCCTAAACCGACCTAAAACCACTAAACCGTCTAAAACCCTAATAAACCTCTAAACCCTAA
ACCCCTAAACCACTAAACCTAAACCTAACAAACTAAACACAAACTACATAAAACTCTAAACCTAAACCTAAAAACCACTAACTC
CACTAAAACCCCATAAACCCCTAAGAACTCTAAAACCCCAAACCGCTATAACCCCATAAACCTAACGCCTAAAACCCTAAAACC
CAAAAACCCCTAAACGCCTAACCACTAAACCTAAAAACCACTAAAACCCTAAAACCCTAAAAACCCTAAACCTAAAAAAGACCC
CTAAAACCCTAAACGCCTAAAAGACCTTCTAAAAACTATAACCCTAAACACTAAAACCCCCTAAAACCCTAAACCCTAAAACCC

TAAAACCACAAAAACCACTGAAAACCTAAACCCTATAAAAACCTAAAAACCCTTAACCCTAAAAACCCTAAACCCTAAACCCTA
AAACCCCTAAACCACTAAAACCCCTAAACCTCTAAAACCCTAAACTAAACACTAATCCTAAACCCTAAACCCACTAAACCCTAA
ATCCAAATGCCTAAAACCCCTGACTAAACCTACTTAAAACCACTAAAACCAATCTAAACCCTAGAAACTCTAAAAACCCTAAAC
ACTAAAAACTAAAACTATAAACCGCTAAACTAAACCAAACTAATAACCACTAAACCACTAAACTCTAAACCGCCTAAACCCCTA
ACCTTAACCCTATAAACCCATAAAACTAAACCCTAAAAACCCTAAACCCTAAATCCTAAACCTAAACCAACTAAAACCCTACCC
TAAACCACTAATAAAAAATAAGAATAAATAAATAATAAAAAAAAAATATAGTATATCATAAATAAAAATATA
>m141121_210323_42134_c100732402550000001823141405141506_s1_p0/128664/0_9402
AAAGAGAGAGATATACATTTCGAAGAAATTAGGGAAATCCTCCCATCAAACCAAAGGGGTACCCTTTCAGGAATCTTCGATTTC
AATAATATGGATTCGAGCAAGTGTTTGATAGTGAATAATAAAGAATTGCGAGGGTAATGGTCACCAAAGTTGTATCCCTTTATG
TCTTAGGGTTCTATGTATAAAATTTCGTGCCAGATCCGAGGGTGTGTAGAGGGTGGATACAGTCAAAATACTGAAGAGATGTAC
GAATATAAGGAAAAAACTTGCGAAAATTGCCCCTCAAAAAGGGGAATTTTTTATTTTGAAATGTTGAATTAAGGCTCCGTTTCG
ACTTCCAATATTATTCATTTACCCATGAGATCTTCTCGGGGGAAAATCGAACGAGTTGAGCACGAGGGTAATACATTTCGGAGA
AATTAGGGAAATCTCCATTCACCAAAGGGGTTACCCCTTCAGAAATCTTCGATTTCAATAATATGGATTGAGCATGTTTTGATA
GTGAATAATAAAGAATTTGCGAGGGTCATGGGTCACAAAAGTTGTAATCCCTATGTCTTAGGGTTCTATGTATAATTCGTGCAG
ATCCGAGGTGTGTAGAGGGTGATACAGTCAAAACTAAGAGATGTACTAATACAAGGAATAAACTTGCGAAAATTGCCCATCAAA
AAGGGGAATTTTTTTTGAAATGTTGAATTAAGGCTCCGTTTCGCCACTCCCAAATAATATTCATTTATCCCATGAGATGCTCGG
GGAAAAATATCGAACCGAGTTGAGCCACGAGGGTAATACATTTCGGAGAATTAGTGAATTCTCCAATTTCACCAAAGGGGTACG
CCCTTTCAGAAATTCTTCGATTTCAATAATATGGATTCGAGCAATGTTTGGATAGTGAATAATAAATGAATATACAGAGGGTCA
TGGGCACAAAAGTTGTAGCCCTATTCTTAGGTTCATGTATAAAATTTTCGTGCTGATCCGTAGGTGGTGTAGAGGGTGATACGT
CAAAAATTCTGAGAGATGTACTAATACAAGGAAAAAACTTGCGAAAATTGCCCTTAAAAACAGGGGAATTTTTTTTGAATGTTG
AATTAAGGCTCCGTTTCGACTCCAATATATTCATTTACCATGAGATCTCTCGGGGAAAATTATCAGAACCGAGTTGAGCACCGA
GGGTAATACATTTCGGAGAAATTAGGGAAATCTCCCATCTCACCAAAGGGGTACCCCTTTCAGAAATCTTCCGATTTTCAATAA
CTGGATTCCGACAAGTTTGATAGTGAATAATAAAGAATTTGCGAGGGTCAAATGGTCACAAAAGTTGTATCCTATGTCTTAGGG
TTCTATGTATAAAATTCGTGCAGATCCGAGGTGTGATAGAGGGTGATAACAGTCAAAATACTAAGAGATGGTAAACGAATATAA
GGAATAAAACTTGCGAAAATGGCCCTCAAAGGGGAATTTTTTTTGAAATGTTTGAATTAAGGCTCCGTTTGTCGACTCAATATA
TTCATTACCCCATGAAGAGTCTCGCGGGGGAAAATATCGAACGAAGGTTGAGCACAGAGGTAATACGTTTCGGAGAAATTAGGG
AAATCTCCCATTCACCAAAAGGGGTACCCCTTTCAGAATCTTCGAATTTCAATATATATGGATTCGAGCATGTTTGATAGTGAA
TATATAAAGAATTTGCGAGGGTCATGGTCACAAAAGTTGTATCCCTATGATTAGGGTCCTATGTATAAAATTTCGTGAGAATCC
GAAGGTGTGTAAGAGGGGTGATACAAGTCAAAATACTAAGAGATGTACTATACAAGGAGAACAACTGCGAAAATTGCCATCAAA
AAAAGGGGAATTTTTTTTTGAAATGTTGAATTAAGGCTCGTTTCGAACCTCAATATATTCATTTAACCATGAGATCTCTCCCGG
GGAAAATATCGAACGAGTTGAGCACGAGTAATACATTCGGAGAAATTAGTGAAATCTCCAATTCACCAAAGGGTACCCCTTTCA
AGAAATCTTCGATTTCAATAATATGGATCGACGCAATGTTTTGATAGTGAATAAAAAGAAATATACGAGGGTCATGGGGCACAA
AGTTGTAGCCCTATGTCTTAGGGTTCTAATGTATAAAATTTCGTGCTGAATCGAGGTGTGTGAGGTGATAACGCAAAAATTCCT
GGAGAGATGTATCTAATACAAGGAAAAAACTTGCGAAAATTGCACGTTAAAAAGGGGAAATTTTATTTTGAAATGTGAATTAAG
GCTCCGTTTCGACTCCAATATATTCATTTACCCATGAGATCTCTCGGGGAAAATATCGAAGAGTTGAGCAGCGACGTGGTAATA
CTTTCGAGAAATTAGGGAAATCTCCCATTCCCAAAGGGGTAACCCTTTTCAGAATCTTCGATTTCAATAACATGGATTCGAGCA
ATGTTTCTGATAGTGAATAATAAAGAAATTTGCGAGGGTCATGGTCACAAAAGTTGTACTAACCTATGTCTAGGGTTCTATGTA
TAAAATTTCGTGGCGATCCGAGGTGGTGTAGAGGGTGATACAGTCAAAATACTAAGAGATGTACAGAATATAAGGAAAACTTGG
CGAAAATTGCCCTCAAAAAGGGGAATTTTTTTTGAATGTTGAATTAAGCTCCGTTTCGACTCCAATATATTCATTTACCCATGA
GATTCTCGACGGGGGAAATATCGAAGAGTTTGAAAACACGAGGGTAATAACATTTCGGAGAAATTAGTGAAAATCTCCAATTCC
ACCAAAGGGGTACAACCTTTCAGAAATCTTCGATTTTCAATTAATAATGGATCAGCAATGTTTTGGTAGTGATAATAAAGAATA
TGCGAGGGTCATGGCAGCAAAGTTGTAGCCCTATGTCCTAGGGTTCTATGTATAAAATTTTCCGTGCAGATCCGACGTGTGTAG
AGGGGTGATACCAGTCAAAATTCTGAGAGATTAACTAATACATGGAAAAAACTTTGTGAAAATTGCTCAAAAAGGGGAATTTTT
TTGAAAATGTTGAATTAAGGCTCCGTTTGACTCCAATAAATCCTTTACCCCATGAGATCTCCTCGGGGGAAATATCGACACGAG
TTGAACACGAGGGTAATACATTTCGGAGAAATTAGTGAAATCTCCATTCACCAAAGGGGTACCCCTTTCAGAAATCTTCGATTC
AATAATATGGATTCGAGCAAATGTTTTCATAGTGATTATACTAAAGATATTGCGAAGGGATCATGGTCACAAAGTTGGTAGCCC
TATGATCTAGTGGTTCCTATGTATAATTTCGTGCAGATACGAGGTGTGTAGAGGGTGATTACAGTCAAATTCCTGAGAGATGTA
CTAATACATGAAAAAACGTTGTGAAACAATTGCCCTCAAAAAGGGGAATTTTTTTGAAATGTTGAATTAAGGCCCCGTTTTCGA
CTCCAATATATTCATTTTACCCATGAGATCTCTCGGGGGAAATATCGAACGAGTTGAACACGAGGGTAATACCATTTCTGGAGA
AATTAGTGAAATCTCCAATCCACCAAAGGGGTAACCCCTTTCAGATAATTTCGATTTCAATAATATGGATATCGAGCAATGTTT
TAATTAGTGATTACTAAAGAAATATGTCGAGGGAACATGGTCCAAAAGTTTGTAGCCCTTGGTCTAGGGTTCTATGTATAAAAT
TTTCAGTGCTGATTCCGAGGTGTGTAGAGGGTGATACAGTCAAAATTCTGAGAGATGTACTAAACAAGGAAAAAACTTGCGATA
AATTGCCCTTAAATAAGGGGATTTTTTTTGAATGTGAATTTAAGGTTCCGTTTCGACTCCAATATATTCTTTACCATTGAGATC
TCTCGGGGGAATATCGAACGAGTGAGCAGAGGGTAATACATTTCGGAGAATTAGGGAAATCTCCCCATTCACCAAAGGGGTACC
CTTTCAGAAATCTTCGATTTCAATAATAATGGAGTCGACATGTTTTGATGTGGAATAATAAAGAATTTGCGAGGGTCATGGTCA
CAAAAGTTGTACCCTAATGTTTTAGGTTCTGATGTATAAAATTTCGTCAGAATGCACGAGGTGTGTAGAGGGTGATACAGTCAA
AATACTGAGAGATGTACGAATATAAGGAAAAAACTTGCGAAATTGCCTCAAAAGGGGAATTTTTTTTTGAAATGTTGAATTAAG
GCTCCGTTTTCGACTCCAATGATATTATTTACCCATGAGATCTCTCGGGAAAATATCGAACGAGTTGAGCCACGAGGGTAAATA

CATTTCGGAGAAATTAGGGAATTCCCATTCACCTCAAAGGGGTACCCCTTTCACCAGAATCCTTCGATTTCAATAATATGGATT
CGAGCAATGTTTGATAGTGATAATAAAGAATTTGCGAGGTCATGGGTCACCAAAAGTTGTATCCTATGTCCTTATGGGTTCTTA
TGTATAAAATTTGTGCCAGACGAGGTGTGGTAGAGGGTGATACGTAAAAATACTGAAGAGATGTACGAATATAAGGAAAACTAA
GAAATTGCCTTCAAAAAGGGGAATTTTTTTTGTAATGTTGAATTAAGCTCCGTTTCGACCTTCCAATATATTCATTTACCCATG
AGATCCTCGGGGGGGAAATATGAACAGAGTTGAACACGAGGGTATGAACATTTCGGAGAAATTTATGAAATCCTCCAATTCACC
AAAGGGGTACCCTTTCGAAATCTTCGATTTCAATATATGATTCCGAGCATGTTTTCATTAGTGATTACTAAAGATTGCGAGGGA
ATGTCACAAAAGTTGTAGCCTATGTCTTAGGGTTCCATGTAATAAAATTTCGTCTGATCCGAGGTGCTGTAGAGGGTGAAATAC
AGCAAAATTCTGCGAGATGGTACATAATACAAGGAAAAACTTGCGAAATTTGCCTTAAAAAGGGGAATTTTTTTGAAATGTTGA
ATTAAGGCTCCGTTTCGACTCCCAATATATTCATTTACCATGAGATCCCCGGGGGAAAATATCGACGGAAGTTGAGCACGAGGG
TTAATACATTTCGGAGAAATTAGGGAATCTCCAATTCACCAAAGGTGGTACCCTTTCAGAAATCTTCGATTTCAATAATTATGG
GATTTCGACAATGTTTTGATAGTGAATAATAAAGAATGTGCGAGGGATCCCATGGTCACAAAAGTTGTATCCTATGTCTTAGGG
TTCTATGTATAAAATTTCGTGCAAGATCCGAGGTGGTAGAGGGTGATACAGTCAAAAACTAAGAGATGTACGAATATAAGGGAA
AAAACTTGCGAAAATTGCAATCAAAAAGGGGAATTTTTTTGAAATGTTGAATTAAGTGCTCCGTTATCGACTCCAATATTTAC
ATTTACTCCATGGAATCATCTCGGGGGAAATTATCGAACCGAGTTGAACACGAGGGTAATTACATTTCGGAAAATAGTGAAATC
TCCAATTCACCAAAAGGGGGTACCCCTTTCAGAAATCTTTTCGATTTCCAATAATATGGATTCCGAGCATGTTTCATAGTGATT
ACTAAGAATATGGAGGGACCCATGGTCCACAAAAGTTGTATCCCTATAATGTCTTAGGGTCTATGTAATTAAATTTCGTGCAGA
TCCGAGGTGTGTAAGGGTGATACAGTCAAAATTACTGAGAGATGTACGTAATATAAGAAAAACTTGCGAAAATTGCCCTCAAAA
AGGGGAATTTTTTTTTGAAATGTTGAATTAAGGCTCCGTTTTCGACTCCATATATTCATCTTACCCATGAGATTCCTCGGGGA
AAATAATCGGAACGAGTTGAGCACGAGGGTAATACATTTCGAGAAATTAGGCGAAATCTCACATTCACACAAAGGGTACCCTTT
CAGAAATCTTCGATTTCAATAATAATGGATTCGAGCAATGTTTTGATTAGTGAATAAATAATAGATTTGCTGAGGGGTCATGGT
CACAACAGTTGTATCCCTAGATGTCTTAGGGTTCTATGTATAAAATTTCCGTGCAGATCCGAGTGTGTAGAGGGTTGAATACAG
TCAAAACTGAGAGATGTACGAATATAAGGAAAAACTAACGAAAATTGCCCTCAAGAAAGGAATTTTTTTTTTTTGAAATGTTG
AATTAAGGTCCGTTTCGACTCCAATATATTCATTTACCCATGAGATCTCTCGGGGGAAATAATCGCGAGTTGAAACAGGGTAAT
ACAATTTCGGAGAAATTAGTGAAATCATCCAATATCCCAAAGGGGTACCACAATTATACAAGAAATACTTCGATTTCAATATAT
GGATTCGAGGCAATGTTTTCATAGTGATTACTAAGAATATGCCGAGGGACATGGTCACAAAAGTTGTAGCTCCTATGCTTAGGG
TTCTATGTAATATAAATCTTCTGCTGATCCGAGAGATAAGATGAAGGGTAAGTCAAATACATAAGAGGACTATACAGGAAAAAC
TTGCGAAATGCCCTTAAAAGGGAATTTTTTTTGAATAGTTGAATTAAGGCTCCGTCTTCGACTCCAATATATTTCATTTACCCA
TGAGATCTTCTGGGAGAATAGAACGAGATATGGCCAGAGGGTAATACATTTCGGAGAAATTAGGAAATCTCCATTCACCAAGGG
GTACCCCTTTCAGAAATCATATTCGATTTCAAATAATATGGAATTCGAGCAAATGTTTTGATAGTGATAATAAAGAATTTGCGA
GGTCTGGTCACAAAGTTGTATCCATATGTATTAGGGTTCCTATGTATAAATTTACGGTGCAGATCCGACGGTGTGTGAGAGGTG
AGTACAAAATTCATGAAGGAATGTTACGAATATATAGGATAAAAATTGCGAAAAATTGCCCTCAAAAAGGGGAATTTTTTTGA
AATGTTGAATTGAAGGCTCCGTTTCGACTCCAATATATTCATTTACCCATGAGATCTCTCGGGGGAAAATGAATCGAACGATAG
TGAGCACGGAGGGTAAATACATTTGGAGAAATTAGTGGACAATCTCCCAGTTCACCAAAGGGGTACCCCTTTCAGAAATCTTCG
ATTTCAATAATATGGATTCCCGAGCAATGTTTTGATAGTGGAATAATAAAGAATTTGCGAGGGTCATGGTCACAAAAGTTGTAA
TCCCTATGTTTTAGGGTTCTATGATAAAAATTTCGTGCAGATCCGAGGTGTGTAAGAGGGTGATACAGTCAAAATACTGAGAGA
AATGTACGAATATAAGAAAAACTAACGAAAATTGCCTCAAAAAGGGGAATTTTTTTGAAATGTTGAATTAAGGCTCCGTTCGA
CTCCAATATATTCTTACCCATGAGATCTCCTCCGGGGGGAAAATATCGAACGAGTTGAAAACACGAGGGTAATACATTTCGGTA
GAAAATTAGCTGAAATCTCCAATTCACCAAAGGGGTACCCATTTCAGAAATTTCGATTTCAATAATATGATCGAGCAATGTTTT
CCATAGTGATTACTAAAGAATATGCGAGGGACATGGTCACAAAAGTTGTTAGCCCTATGTCTTAGGGTTCTATGATATAAATTT
AGTCTGATCCGAGGTGTGATAGAGGGTGATACAGTCAAATCTGAGAGATTGTACTAATAAGGAAAAAACTGCGAAAATTGCCCT
TAAAGGGGAATTTTTTTTGAAATGTTGAATTAAGGCTCCGTTTCGACCTCCAATATATTCATTTACCCATGAGATCTCTCCGGT
GGGAAAAAATATCGAACTTGAGCACGAGGGTAATACATTTCGCGAGAAATTAGGAAATCTCCATTCCCAAAGGGTGTACCCATTC
AAAATCTTCGATTTCAATAAATATGGTTACGAGCAACTGTTTGATAGTGAATAAATAAAGAATTTGCGAGGGTCATGGTCACAG
AAGTTGTATCCCTATGTCTTAGGGTTCTATGATAAAATTTCTGCAGATGAGGTGTGTAGGTGAATACAGTCAAAATACTGAGAG
ATTGTACGAATATAAGGAAAAACTTCGCGAAAATTGCCCTCCAATAAAGGGGAATTTTTTTGAAATGTTGAATTAAGGCTCCGT
TTCGATCCAATAATATTCATTTACCCATGAGATCTCTGGGGGAAAATATCGAACGAGTTGAGCACGAGGTAAATAACATTTCGG
TAGAAATTAGGGAAATCTCCCCATTCACCAAAGGGTACCCCTTTCAGGAAACTTCGATTTCAATAATATGGATTGAGCCAAATG
TTTTGATAGTGAATAATAAAGAATTTGCGAGGGTCTGGTCACAAAAGTTGTATCCTATGTCTTAGGGTTCTATGTATAATAATT
TCGTGAGATCCGAGGTGTGTAGAGGGTGATAACGTAAAATACTGAGAGATGTACGAATAAAGGAAAAAACTTTGCGAAAATTTG
CCCTTCAAAAAGGGGATTTTTTTTTGAAATGTTGAATTAAGCTCCGTTTCGACTCCAATATATTCATTTACCCATGAGATCTC
GGGGGAATATCGAAACGAGTGAGCATCTCGAGGGTAATACATTCGGAGAAATTAGGGAAATCTCCCATTCACCAAAGGGGTACC
CTTTAGAATCTTCGATTTATAATATGGATTCGAGCAATGTTTGATAGTGAATTAAGAATTTGCGAGTCATGGTCACAAAAGTTG
TATCCCTATGTATTAGGGTTCTATGTAAGATTAAAAATTCGTGGCAGATCCGAGGTGTGTAGAGGGTGATACAGCAAAAAAAAA
TAAAAACTGAGAGATGTACGAATATAAGGAAAAAACTTGCGAATAATTCCCCTCAAAAGGGGAATTTTTTTGAAATGTTGATT
AAGGCTCCCGTTTCGACTCCAATATATTCATTTACCATGAGACTCTCGGGGGAAAAATATCGAACACGAGGTTTGAGCACGAGG
GATAATACATTTCGGAGAATTAGGGAAATCTCCATTCACCAAAGGGGTACCCCTTTCAGAAATCTTCGAATTTCATAATATTGG
ATCGAGCAATGTTTTTGATAGTGATAATAAGAATTTGCGCAGGGTCATGGTCACAAAGTTGTATCCCTATTGTTTAGGGTTCTA
ATGTATAAATTTTGCAGATCCGAGGTGTGTGATGTGTGTGTGGTGGTGTGTGGTGTGGTGGGGGTGTGGGTGTGGTGATGGTGT

GGGTGGTGCTGTGGTGGTGGTGTGTGTGGTGGTGTGGTGGTGGGTTGTGGTGGTGGCGTGATGTGTGGGGTGGGTGTGCTGTGT
TGTGGTGGGTGTGTGGTGTGTGGGGGTGGTGGTGGGTGGTGGGGTTGGTGGGTGTGTGGTGGATGGTGTTGTGTGGTGGTGTGT
GTGTGTGGTGGTGTGGTTGGGGCTGTGTGGGTGGGGTGTGATGGTGGTGGTGTGGTGGTGGGTGTGGGGTGGTGGGGT
>m141121_210323_42134_c100732402550000001823141405141506_s1_p0/129545/388_6868
GGGAGGTGGGATTGTTCTAGGTGTAAAAAGAAATATTTGGAATGAAAATGAAATAGTGGATTTGGGGAAATAAAAATAAAATCA
TGATAATAATATCGAATGAAGTTCCAAAAAGGATGAAATCATAGAATAATTATGATATAATTGTTGAAACGGATGAAATATGTC
AACGAAATTAGATTGGAATAACTCAAAGGTATAGTCAGGTGAACTTTATATAAGAACTGAGAACTAGACTTTAAGGAAAAAGTA
AAACACATGATAAACCACACATGCGACCTGCAAGCCTTAGTATATTTCTCAAGTAAGCGCCTGTTGCAATTACAATGAATTTTC
CAACCCCTTTCAAGTGGTCTTCCACGAGCTCTTCCCTCTTTATCAGTGGGCTCTAAAATGCTTAGGTTACAATAGTGCGTCCAG
GGGACCAATGGTACATACGTAACCTGTGTCAGCAGGGAGTGGCTCCAGAGGTTGGTCGGTCGTACACCCAGTAAGGAGTAAAGG
TGTCAAGCAGCGCCTCGTCTCCACTACTTGCTCTGTATGCCATGTCTGACTGCGTGGTGCGTAGGCGTAGTGGTGGAGCGAGGT
ACCCGTGGTGTCGGCTCTGACCTAGTGGCCGTAGATCGTTACAGACCTAACCTTCCTATCCTTGCATACCCACTCCCTGGCGTT
CCCACTAACTTGTTCTGGCTAGGGGTCATATCCGTAGTCTGACTTTCTTTAGGTTTGTCAGGTACAATCATACATTCCATTCGC
GCGTACCCTTGCCCCTTGGTGGAGTATTCCACCTCCAGGCATCGGCACGGGAACCCATAGGGCCTAGCGCCATGGGCGAGAAC
CATGGGCGAGGCCATGGAGGAAGGCCAGGGGCGAGGCATCGGAGGAGGCCATGGGCCCAGGCCATGAGGAAGGCGCCATCGGCG
TGGACAGGCCCATGGAGGAGGCACATGGAGGACGGCCCATGGCGAGGCAATGGAGGAGGCCATGGGCCGAGGCCATGGAGGAGG
CCATGGGGTCGAGGCCATCGGACGGAGGCCAATGGGCGAGGCCATCGGAGGAGGCCAGGGGGCCCGGGCCATGGGCGAGGCCAT
GGAGGAGGCCATGGCCGAAGGCATGGAGGAGGCATGGGGGCCAGAGGCCATGGAGAGGCGCCATGAGCAAGGCCATGGGGCGGA
GGCCATGGCAGGAGGCATGGGGCCGAGGCCATGGAGGAGGCCATGGACGCAGAGCATGGCGACGGCCATGGAGGAGGCCATGGC
GCGAGGCCATGGAGGAACCATGGGGCCGACGGGCCATGGAGGCAAGGCCATGGAGGGAGGCCATGGGCGAGCGCCATCGGAGAG
GCATCGGGACCGAGGCCAGTGGACGAGGCCACTGGACAGGCGATGGAGGAGGCCATGGGACCGACGGCCATGCGCGAGGCATGG
AGGAGGCCCATGGGGGCGCCTGAGCATAGAGGAGGTCATGGCGGCCGAGGCCATGAGGAGCCAATTGGCCGCAGGCCAACCATG
GAGGAGGGCATGGGCGAGGCCATGGCAGCGGAGGCCCATGGGCGACGGGCCATGAGGGGCCATGGGCGACGGGCATGGCAGGAG
GCCATGGGGCCGAGGCCATAAGGGCCAGAGGGCCCCATAGGAGGACGGCCAATGCAGGCGAGGCGCGCCACTGGGCGAGGCCAT
GGAGCAGGCCACTGGCGCGGCGCGCCAAATGGAGGATGCCCATAGGAGGAGGCCCATGAGTGAGCATGGACGCGAGGCCCATAG
GGCTGACGGCCATGGAGGAGCCATGCCAGGCGAGCGCCGTCGGGGCGACGTGGCATGGGCGAGCAGGGCCTGGTGACGGCTACT
GAGCCTTGGCAGGCCTATGCTTGCCACGTGGCGCCACCGTTTGTCAATCGACTCCACAAGAAGACGCAAGCCCCAGATCTTTCG
TGATGGCGCGATGGCTTCCCGAGACGGACGCGTGCGCCTTAGGGTTGCCGTCGGGCCTCACATACATCGTCTCTTGGATGCGGG
ATTACTGAGCAGCGTCCACATTTTCATGAGACTGATCTCCTGTATTCAACACAGGCGCTCCACAGGCCTCAAGCCAATCGCGTG
ACCTACGCGACCTTGTCCCTATGGTTCCTACTAGCGGAACCTTTAAACGACGTTTACTTTTGGTGGATTTTGGGCATCCACACT
TGCCCCCCACAGTCTATAGCGGAGGCCCTTTAGGTCGTTTTGTAGACTCTTCCTTTTCTTTTGCATTTTCTACTTCTGTGTATA
GTACGTATATTCTTTTTTTCTTTTTTTCTTCGTTTTGCTTGCAGGTCCCGTTTGGAACCACGCCAAAAAGGGGGGCTTCGATAC
CGGCTCTCTATTGGGTGCACCCTTGGCGTTGCCCCTACTCAAGGCATATATTCTGACCCTTCCGGTTCTAGTTCATCCTTTTTA
TAGTATTTCTGCCGCGCGCCTTATCTCACTGATTTGCGAGAAGCCGTCCTTCCCTCGCTCATTAACGCATAGTACTACTTGTTT
GCCAAAACGTCTTTCTTGAGAGACCCCTTTTTGCAAGCGTCCTTCTTGTGAGACACCCTCTCTTCGGAAGCGTCATACTTGTGA
GACCTTCTTTGATAGCCGATCTACACTTTGGGAACCCACTTTTTGAACGCCGTCTACCTTTGGAGACCTTTTGTGGACGTTGAT
CTACTTGGAGAAACTTGTTTGGACGGCGTCTGCCTATTGACGGTGATTGTTCCCCTTCCGGGTAGGGGACTTTCATGGCTAGAG
GGCTCTTCGTTCGACTCTCAAAACATGGTCAGGGACCCCTCCTAAAGCACCGATGCCCCCCTTCTATACCACTGGAATCTCAGA
CATACTTCTGGTGTTGAATGGCCACATGAAGGAAGGTTCGCTATTCTTCGACACTGCGAAGTTCTTATGGCCCTCTTCCACACG
TATGTACTTCTCTGTCGCTCTCTCCGAGAGAAGTCTGGCCAAATTCGAAACTCTCTCCTTGTCTGAGCACGCTCTCCCCATAAC
TTGCTTCCTGGACGGAACCTCCCGGTTAGTAATACGCATCTTCAGCTCTGCTTTCGGTTAAAATCTCCCACCTTTGTTGCCCTC
CACTACTGACTATGGAGCGTAGTCCTCTGTCAGCTTTCAGTTCTCCCAACCGCCGTGGTGTCTTTAGTTAGCGACGCGCGTTGG
CGAGTTGGCGGAACTCTCGACGGTCAGATCGCGCCGCAGTGTCGCGACCTGCACAGTCACAAGGTCCTCGCTCGAGCGGGGAAC
TTGCTGCGGTGCGCATGTCAGTTGTGTCCTGCGCTGTAACCGTTCTCTTACTGCTCATGCACTCCTTTAAGCGTACGACAAAAA
TGACACTTTGCCTGATATGTTGACCCCTCTTAACCTCATACAAGTTAAATATGAAAGGATCTTGATGATATTTTGGGTCTGTGT
ACCTCGTATGGGTCTGCGAAGGCTCTACTAGAAGCCAGTGGGAAACAAATATGCATACAGCTTTGAATCATCCTTGGTGAACAG
TAAGAGACCTACACGGTCGATTATTTCGATCTCCATAAGTGCCACACACAAAGCAAGCAGTGGATGATCTTGCCAAAGAGAGAC
TTCAGACATTATATGGATGTGATAAGAAATCCCCTCCTACTATTATTAGCAATAAGGAGTACTTGAGGAGTTCTTGGGCCTGAT
CCCTTGCCTTGGTTGTGTTCTCTTGGGGGAACCACGGGGCGGTGCGTTCTTACTTCTACCTCTTCCCATTGAGCTCTTTTCCTT
AGGCCAGGGGGTGCTCTTTTGAGGGGACTTCGGCCTCATTCTTACGACCATGCATCCTTCTCCATCTGCCTATGCTCCTGGGGG
ATCATTTTCGTCGGCCTAGTTCCCCATGGTAACTTTCCTGGGGGTTTTACCTGGTGGAAAGTTGGGTTCCCCCATCGTCTATGG
GGACATGTTCTTTCCCCTTTTCCATGCCCTCCTTCCCTTTATAGCTCTGGGACTGGGTTATGCTTGACTTCCCTTGCAAGTAGG
TCGTTTAAAACCTCCTGCATGTTCTCTATCTGTCTTCCAACCTTCTAGTCTTTTTATGCAACTCGTAAATCTCATCCTTGTACA
GAAGCGCAGGTCCTTGAGCCTGGCACTATACCGAGCGTACCCGGGACCTCCTGGCCAGTGCGTCGAGGGACCCGGGTCCCTGGT
GGTCCTGAGCGTGGGATCCTGCCGGTTAAGTGGATACACTAGTGGCTTTGAATGAGCCGTCACGTCGGACTGCGATAGCTGGGG
ACCGATGCTTCCCTTTCCTCCTCCCCCCGGGAGAAGAGGATTTCTTTCCTGGCCACACCTCCATGCTCCCGCGAGGGATTCTTT
CTCGGAGGGCCCTCCCGCTCCTACATGCTCATCTTCGGTGAACCTCCAACTCCTTGTATTTGCCGTAGGCATTTTGAACGTCTT
AGCACTCTTTCTTTGTTGGATGTGATGGAAAGAATCACTTGAGGCTTGATGGACTTGTATTCTTCCCCCCAACCCAGCCGGCGC

CAAAACTATTGACGGTGAAATCTCCGCTGAAAACAAAACCAACCGAAATTAGTATTGGAAAACTCAAGGTTAGTCAAGTGAATT
TATATACTAGAACTCGACGAATAGACTTTAAGGAAAGTAAACAACAGATACAAAATGGAGCAAGCGCTTAGTAATATTTCTAAA
AGCCTCTGCCATACAACTGAATTTTCCAACGCCCCCTTCTCAAGTGGTCTTAGGTTCTTTTTAGTGGCTCTAATGGCCCTTAGG
TACGATAGTGGTCCAGGGGACCAAAGTGGTACCTACGTTCTGTGTCAGGCGGAGTGGCTCCCAGAAGGTTTGGTCGTACACCCT
GCGTACAGGAACGCAAGTTGTCAGCGAGGATCGTCTCCCCACTACTTGTCTGTATCCATGATCTGATGCATGGGGCAGGGCGTA
GTGCGCCAGGAAGGTATGTGGTGTCGCTCTGACCTAAATCTGGTCCCGTAGACGTAGCGGAACCATAACTCCTATCCTCTCGCA
ATACCACTTCCTGGCGTTCCCACTACTATGTCTGGTAGGAGTACTATATCCGTACTCTTATTCTTTAGGTTTGTAAGGTAAGCA
TTCCCATAAAACTTCATGCGGCGTACCTTGCCCCTTTGTGAGTATTCTCACTAAAAACAAGCGCCCCATGGCACGGGGAACCAT
GGAGCGCTAGGCATCGGGCGAGGCCATGGAGGAGGCCAAATGGGCCGAGGCTAGGGGGCCCCCCCAGGCATGGAGGAGGCCCAT
GGGGCCGAGGCCATGGAGGAGGCCCATCGAGGAGACCCCATGGACACGGAGGCAATGGAGGAGACCCACTCGGAGGAGGCCATG
GGCCGAGGCCATGGAGGAGGCCATGGGCGACGGCCTCATGGAGGAGGCGCCATGGGGCCGAGGGCCATGGAGGAGGCCATGGCC
CGAGGCCCCATGGAGGAGCGCGCTGGGTCCGCCGAGCCAATGGGCGAGGGCATGGGCCGAGGCCATGGCAGAGAGGCCCATGGG
CCGAGGCAATGGAGGACCGATGGGGGTCGCAGGCCGATCGGAGAGGCCATGGGCGAAGACATGCGAGGAAGGCCATGGGGCCGA
GGCCAATGCGAGCATACCGCGCAAGGACGCGGCCATGGGCCGAGGGCATGGCGAGGCATCGGAGAGGCCATGGCGACCGGCCAT
CGGAGAGGCCATACAGAGGACGCCATGGACGGAGGGCCCTATGGAGGAAGGCCAATGGCCCGAGGCCCCATGGACCGCGAGGCC
ACCTGGAGGAGGGCATGGGCCGAGGCCATGGGAGGAGGCCCAATGGGTCCGCAGGCCGTGTTGGAGCGAGGCCCATGGAGGAGG
GGCCATGGGGCCGAGCCGATAGCACAGCGAGGACCCCATGGGCGACGGCCATGGAGCGAGCCATGGCGGGCCAATGGAGAGGCC
TGGGGCCCCAACCCGAGCCCATGGAGGAGGCATGGAGTGAGGCCAATCGGGGCCAGGGCCATGGAGGCAGGGCATGGGCGAGCG
GCCCATGGAGGAGGTCATGGCCGAGGCCATGGAGGAGGCATGGAAGGAGGCCATGGGCGCAGGCCTGGAGGAGGGCCATCGGGG
CGCAGAGGCATGGAGCGATCCATGGGCCGCGGGCGCCACACTGGAGCTGCGGATGCCGCCACGCGCAGCCCGGCATGGCCCCGA
ACGGCCATGGAGGAGGCCGATGCGGGCCAGAGCCACGAGTGGGGGGCCGAGTGCATGGGCGAGAGGCGGGCCTCGCGAGGACCC
CCTACTCAGAGCCCTTGGCACCTATGCCTTGCCCAATGTGGGGCCACTCTGGCAGATCGACTCAACGAAGACCAACCCACCGCA
ACCCACGACGTCGTGTTGTGAATGGTGCGCTGCATTCCCTGAGACGAACGGTGCCTTAGGGCCTTGCCCTCGGCGCCTCATGAT
TCTCTCTTGACT
>m141121_210323_42134_c100732402550000001823141405141506_s1_p0/130969/4281_13102
CATGAAAGGATAAAGTAAAGTAGTATTTAGTGGGGTAGCCTCAGTAAGTAGTAGTAGAAGTTGTTTTTACGTGGGTGGGGTGGG
TAAAGGTGGCGTAAGTGGTAGGTGGGGTAAAAGTGGTTTAGTGGGGTTAGTGGTAGGTGGGTAAGTGGTTAATGGTAAGTGGGT
AAAAGTAAGTTGTTTGTAGAGTAAGTCGAAAAATTGGCGGGCTGGTAGTAAATTGGGGTGGGTAGTTTAGTCGGGCAGTAAGTA
TTTTTGGGGAGGGTAAGAAAGTGGTAAGTTGATAACAGCTAGTTGGGTTGCACGTTTCTCAAGGCTTGGTTGGTGACAGTGGAA
GTGTAGTGGTAACTGGTCAAGAGGCTTTATTAACGTAACAGTTGGTAAGTTAGCTAGAAGATGACGTAGTAGTAGAAACGGTCC
CGGTAAGTCGTAGTCCGTTAGAACGTTGTAAGAGAGTAAGTAAGTCCCCGAGTCGTAAAGCTTATGGTATTCCATGACAGTGTT
GTTACGTTCAGTTGCGGCGCTAGTGGTAAGTTGGTCAGTGCGGGTAGTTTGTAGTGGGTAAGTGGCCCAGGGATCAGTGGGTTA
AGATTAAGTGGGTGGGGTGGGAAAGTTCGGGTCCAGTAAGCCAGTTCGGTACTGGTAGGGTGGTGGGGTATGGCTCCCGTAGTA
ATCCGTAGTTAAAAGCTCGCTAGTCCGGTATAGTGGCTATGGGGTAAGTCGGGGTAGCGTTGGGGTAGTGGTAAGTGGGTAAAG
TAAGTAGTAGTAGTAGTTAAGTAAGTGGGGGTTTGGGTTGTATGGTAGTCGGGGTAACCGTGGGGTAGTGGTAGCTGTTAAGTAAAA
GTTTAAGGGTAGTGGGGGGTAAAAAGTGGGAAAAAGTGCGTACGATCCCGTAAAACAGTCGTAGGTTCTGTAAGTCCGTCAAGT
GAGCTGGGTTAGTGGTTAAAAAGTGGCTAGTGGGGTAAGTAGTAGTGGGTGGCTGGGTAAGGTAAGCTTGGGTAGTGGAAAGGT
AGTAGCAACGTCGGTAATAGCCCCCCTTGTAGTAGTTAGTAGTCAGTAGTTAGCAAGTAGAGCTAAGTAGGTAGTAAAGTAGTA
GGAGGGTAGTGGGGTAGGGGTGTACTGGTAAAAAGTTAAGTAGCTGGTAAAGTAAAGGTAGTAAGTGGGCAGCCTAGCAAGTAA
GCCAGTAGCAACGTAAAGCACAGCCATAGGTAGCAGTAGCAAACAGCGTAGTAAACAAAGGCTAAGTATTTAGCAGAAGATAGT
AGTGTAGTTCCGTAGTCCGGTAAAAGTCGTAGTGGTAGAACCGGTAGTACTACAGTCAGCTTCGTCAGCTCCGTAAGAAAGTCG
GGCGTAAGTCATGAGTTTTCCTGTACGTCAGTGGGTACGTAGTAGCTGGGGGTAGTCGTAAGTGGGGGCTAGTCTTGTAAGCTG
GGGTGGGGTAGTAAAAGTTATCTCGGGGGGTGGGGGTTAAGTAGTTCATGGGCGGTAGGTTAGGCTAGTATGGGTCGCTACGCC
AAAGGCTTAGTCAGTAAGTAGGAAACGAGTTAGGTGGTGTAATCAATAGTAGTTAGTAAGTAGTTAAAAGTAGTAAGTAGTAAG
TAAAGTAAGTTAGTAATAGTAGTAGTAGTAGTAGTAGTAAGTAGTAGTAGTGTAGTAAAAGTAAGTTAGCTACGTACGTAGTAAGCG
TAAAGTTAAGAAAGTAAGTAATCACAAGCCCAGCCAGTAAGTTAGTACTAGCTACGTAATGGAATAGCTTAGTAAGTCAGCTTA
GTACTAACTAAGCTAGTAAGTAGACAAAAAGTAAGTTACACTTGTAGTAGTAGAAAATAGTAGACAGTTATTTAATAAACAAGT
AAGTAGTAGTAGTATCCTTGGCTGGGAAATAGTAGTCAAAGTAAAGGTAAAGTAAATAGTGGCTTGGTTAAGTACTGGCGCTGG
GTAAACCCAACAGTAGAAAGGCTGGGCCAGTAGTTTAGTCTGTAGTCGGGTTGCTGGGTAGTCACGCGAAAAGTGGGTAAACGC
GCCGGTAGTTCAGTAAGTGGGGGGCCAGTTACAGTGGGGGGGTTAGTTAGCAGTAAGTATTATTTTAGCTAGTATAGTAATAGT
ATACTCGGTGTTGTAGGTGTACGCTTTTTCGGTAGTTGGTTTAGTGGGTTAGAAGTTACGTGGTTGGTCGGTTAAAAAGTGGTA
AAGTAAAAGCAAATGGTAACTGGTTCAAGTGGGGTAAGTCTTGGTAGTGAAGTAAAGTCAGTAGTAAAGTCCAGTAGCTACGCT
ACGCTAGCTAAGTTTGGTAGTAGTATCAAGTCCTAGACAGCAGCTAAGTAGCTAGTCAAAGCTCATGAAGTTAAGGTAAGTCGT
AACTTAGTGGGGCTAAAAGTTAAGTACTAGTAAGTAGGAGTTAAAGTGGGTTTGTGGGTGTTAGTGGTAAGCTGTTAGTCGTCG
GTAGTGGTAAAGTAGTTTGGTAAAATATAGAGAAAATTTGGTGGTAGTCTTAATTGCGGCTGGGGTTTAAATTCCTACTTTTC
CGGGGCCAAGTAGTACTTTTGGGAGGTAAGAAGCTGGGTAGTTAGTTGTTAGTAAGAGGGTTTAGGGGGGTAGTTCGGAAAAGT
GTAAACGTGGTAATGGTATGGGGTTCATTAGTAAAGTTGTAGTTAGTAAAAAGTTAGTCGAGTAGTTAAAAGTCGTAAAAGTCG
TAAGTCCTAGAAACCGTTTGTACGTAGTAGGTCCCGTAATCGTATTAGTGGGTAAGTCCCATAAGTCGTCAGTACAACGTGGGG

GTAAGTGGGATTTGGGTTATGGGGTATTGGGTTAGTGGTAGTTGGGCCCCATGGGGTAGGTGGTAGAGGTTAGTGGGGTGGTGG
AAGGTGGGTAGTACGCCAGTGGGTTTACTGGTAGTGGGTAAAGGTTGGTCGTAGTAGTACCGTAGTTTAGCTCGTAAAGTGTAG
TAGTGGGTAGTTGGTAAAAGTGGTAGTGGTAGTTGGGTTATGTGGGGGGTAATATCAAGTACAGTAGAGTAGTAGCCTAATGGG
GATATGGGCGTAAAGTTGGTAAGCTAGGCTAGTGGGTAGTGCGGGGTAGTGGTAGGGGGTTTTTGGTAAAAGAGGCAACTTCTG
TAATTCCCCCGTCAGTCTAGGTTGCTAGTCCGTTTAGTAGTGGGTAAAAGTGGGTAGTGGGGTAAGTGATTAAAAAGTGGGTCA
AAGCCGGTAGTCTGGTAAGTGGGTGGTAAGGTCCGGTTAGTGCGGGTCTAGTAGTTTAAAGTTGGCGCTAGTAGTAGTAACCGT
GATAGTCTAAAGTAGTAGGTAGTCAAAAGTCTCCGTAAGTATCTAACGTCCCGGTAAGTGGGGTTAAATGGTCAAAACGTGCGG
TAGGTTGGGGCTGCGGGTTAGTGCGGGTATGGTAAGCGGCGCTCAGCTTGGGGTTAGTGGGGCGGGTCAGGGGGGTCAGTGGTA
GTTGGCTAAGTGGGGTAGTAAGTCTAGTCGTAGTACGTAGTCGTTAAGTACGTGGGTAGTTGGGTAGTTGGGGTAGTTTGGGTA
AGTTGGGAGTGGGTAGTGGCTGTATCGTAGTATTGTAAGGTAGTGTGGTCCGGGCGTAGTTCGGGCAGGAGGGTATCATGGTCT
AGTTGGTCCGTCGGTAAGTGGCTAGTGGTAAAGTGGGTGGGGTGTTAGTAGTTGTAAGTAAAGCTGGGTAAGTCAGTAGTACAT
CAAGTAGTAGTAAGTATAGGTAGTAGTAGTAGTAGCTGTCTAGTAACGTAGCTAAAAAGTAAGTGAGTGATCCAGCCCGTAGAA
GTATCGGTGGGTGGGCTAGCTGTCAGTCCCAGTAAGCTGGCGTAACTACAGTAGTCCTGGCTAGTAGTCCACGCTAGTAGTACG
TAGTTTTCTTGTAGAAGCGTAGTAAGGAAATTGTGGGTACAGTTATTGGTGGGGCTAGTAAGTGGCAAAGTAGTAAATTTGGTG
GCGGTAGAAACTGGTTGTAGTAAAAGTAAAAGGTTGGTTTTTTGTAACAGGTAGAAAGGGGATAGCACTGGGGTAGTGGCGGGG
AAAGTGAATAGGTGGGGGCTAATGGTAAAAGTGGGTAATGTAGTTTGTCGTAGGTAAAGAAAGTTTAAGTACACAGTATTAGCT
AGTCGTAGTCCCGTAGACCGTTTGCTTAGTAAAGTCAGTCGGTAGTCCCCGTAATAGTCCTAGTCATAGTCTGTAGTAGTGGGT
TAGTCGGTAGTTTGGGTAGTGGGTGTTAGCAAAATTATTGGTGGGTGGAAAGTTGGTAATACCACACAAGTGGGTACTTGCTAA
AGTTTTCTTGTAAAGTGGTCGTACTAAGTCCCGTAGTAGTCCGTATCCGCTTTGAGTTTAACGTGGGGTTAATTTGGGTAGTGG
GTAGCCTGCTAAGTGTAAGTAGTATTCGTACAGTATTTTAAGTCCCGTAAGTAAGCCTGGTAGTGGGTAAAGTTGTTAGGGGTA
GTGGGTTAGTGTAAGGGTAAAAGTGGTAAGTGTGTGGTAATATGGTATAACGTGAAGTAGTAAAAACAGTGGTAAAGTAGTTAA
CGGTTGTAGTTATAGTTAAGTCCGTAGGTTATGTAGTACGTGGTAAAAGTAAAGTAGTAGTGAATGGTAGTGGTAAAGTGGGTA
GTGGAGTAAACGTGGTAAAAGTGGTATGGGGTAAGCGGGTAAAAAGTGGGGGGTAAGTGGGGGGTAACGTGGTTTGTTGTAGTG
GGTACGTGGTAAGTGGGTTAGTGCGGGGTTAGTGGGTGGGTTAGGGGGTAGTCTAGTAAGTAAAAGTGGGGGGTTAGTAGTAAA
GAAAAGTTGGGGTAGTGGGTAGTGGTATGGTTAGTAGTGGGTGGTAGGAGGTGGGGGGTCAGTAGTGGGTAGTAGTGGGGTAGT
TAAAGTAAGTTGTAGTGGGAATAATGGTATCGTAAGTGGTAGTGGTAGCCGGCTAAAGTTTTTGTAGTGGTAGGGGTCGGGGGG
GTAAAAAGGAGGGGATCCACAAGTGGGTAGGGGGGTAAAAAAAGTGGTTAAAAGTGGGGTAAGTGGGTTAGTGGGCGTAGGGGG
TATGGTAAAAGTCTTGTAGTAGGGAGCCCGTAAGTAAAGTCCGTAAGTCGTCATATCTCGCTAAAAAGTTAAGTAAGTAGTAGA
AAAAGTAAGAAACGTTAGTGGGGTTAGGTGGGGGTCTACAAGTGGGTAGTTCGGTAAAGTGGGTAACGTTTGGCAAGTGTACAG
CTTGGGGTAAGTGGGGTACGTGGTCAGTTTCTTGAAAGTTGGAGAGTGGGGGGCTTAAAAGTGGGTAAAGTTTGGGTTACGTGG
GGTCGCCACGTGGTAAGTTTTGCTTTAGTGGGGCTAAGTTTGCTAGTGTTAAGTCGGGGGGTAGTCCCCGTAAGTCGTTAGTGA
GTAAGCTCGGGTAGGTCGGTAGTTAGTTAAGTAAGTTAGTAGTATAGTGGGGGGTGGTGGGTAAGTAAGTAGTAGTATAGTTAA
GTAAACTAAGTATATCTAGTAAAAGACGTAGGTTGGTAGTGGGTAGTGGGTCGTGGTGATGGTGGTTTTATGGTCGAAGGGTAA
GTGGGTAAGCTTGGTAGCTGGGGTTAAGCAAGTAGTAAATGGTACGTGGGTGGTAGTAGCAGTAGTGGGGTAGACAGTATTAAC
TCAAAAAGTACTAGTTTAAAGTAGAGTAGTAAGAAAATCACGTCAGATTAAGGTAGTAAGGTAAGTATTGTAAAGTTAGTACGA
GGGTAGTATGTAAAGTAGTGGGTAGTAGTGGTAGGTAGTGGGTTTTGTATGGGGTAAGTAGTAGTTAAGTGGGGTTCGTCAGTG
GGTTACGTAGTCAAGTAAGTAGTTAGTAGTATTAAGGCTGTAGGTAAGTAAGTTAAGTAGTAGTTAGTCAGTCGTAAGTAAAGT
AGGTAGTAGTAGTAGTTAGTTGTAAGTAGTAGTATGTAGTGAGTAGTAGTAGTAAGTTGTAAGAAATAGTGGGGAGAGTTGTGA
GTTGGTGGTGTTTTGGGTGTTTTTTAGCGGTTGGGTAGTGGGGGGGTAGTGGATATTGGTAGTAAGTACAGTAGTAGTAGTGTT
AGAAAGAATAGACAGTGTTAGTAGAGCATTAAAAGCTACTGGTAAAAGTAGGTGGTACGTTAGTAGAAGGTAGTGGGTAGTAAT
GGTAGTAAAGATAAGTAGTGGGTAGTAGTTGGGGGTAGTGGTAGTAGTAGTCAGTAAGTAGTAGTAAGTGGAAGCAGTTATGTA
AGTTAGTCGGGGTAAAAAAGTACCGACGAAAAGTAGTGCTAGAAAGTACTTTTTGTAAAAGTGGTGGTCAAAAAAGTAAGTCGT
AGAAGTAGTTTCTGTTAAGAGTAGGTGGGTGGGGTAGCAATGGTTAGTGTCAGTCAAAATGGTTTTGGTACGTAGAGGTCTAGT
GGGTAAGTTAGGAGAGTGGGTACGTGGTTAATGGTATGTGGGTTAAGTAAGTCAGTTTTCTGTAAGATTAAGAAGTAGTTGGTT
TACGTAGTCAGGTGGTAAGGTATAGTAGTAAAGCCTAGTAGTTAGTAGTGAAGTAAGTTAAGATGCCTAAAGCAAAGTCATCAA
GTAGCTGGAGTAGTAAGTCACGGTATTCATTGGTAAGTTAAGGAGTAACAAATAAGTTAAAGTTTTAAGTTGGCTGGTGTTACG
TTGGGGGTAAGTGTTGTTTTTACTGGTAAACTGGTAAGGCTCGGCGTAGTAGGTTGTAGACAAGTTAGTAGCAACATTGGGTGG
GAAGTACAATTGGTGGGGTCAGTAGTGGGGGGGCAGTAAGTAAAAATCTGGGAGGTAGAGGCTTGGGTAGTGAGTTTGTAAGTG
CAAGGCGGGTTAAGGTCGGGGTAGTTGGAACTGGGTACCAACGTGGGTAAATGGTATGGTTAGTTAAAGGTAAAGTGTGTAAAA
TAAAGTAGAATTAGTAGTTAGTAGAAAAAGCTTCGGTAGTCCGTAGTCGTAGTCCGTAAGTCGTAGTCCGTAGACGTTGTAAAA
GTAGTATCCGTAAGTCGCTATTAGTGGGTGTCCTAGTTCGTAGTAAGTGGTACGTGGTAGTGTGTAGGGGGTAGTGGCGGCGGT
AGTTGGGAAAGTGGGGTAAGTGGTAAGTCGCGGCGTAGTGGGCGTAAGATCACGTTAGTCCTAGTAGTAGTCGTAGTAAGTGGG
TAGTGGGGTAAGTGGGGTAAAAGCTGTAGGTGGGCTTTAATTCGGTTGGGTAAGCTGGTGTTAGTTTAGTAGCATGTACTGGTC
AAAGTAGTAGTAGTAAGTTAGTTACGTTCCCTTAGTTGTAAGTAGTAAACGCTGCTAGTAGTAAAGTGGGTAGTGGTAGTGTGG
GGTAGTGGGGTAGTGTAGGGGTAAAAAAGTGGTAGTGGGTAAGTTTTTGTAGTAGTCGGGTAGTGGGGTCAGTGTAGTGGGTTA
AAAGTGGTCAGGTTGGGGTACAGTATATAGTAAAGTAAAGTTAAGTGGTAATCAGCTAGTAGTTGGTCAGTGGGCTAAAGTTGG
TAGTGGTAGTGGTAGTATGTTGGTCAGTAGTGGTAAAGTAAGCTGGGTTAGTAGTGGGTAGAGTAAGTGGCGGTAGTGGGCAGG
TAGTGGGGTTAGTTTTTGTACAAGTCCGCTTGGGGAAAGGGGGGTAGGGGTAGTGGGTACGGGGTAAAGGGGGGGGTAGTGGT

CTAGTGGTACGTGGGTAGACGTGGGGGCTAGCTCGTAAGAATAAAAGCTGGTAAAGTGGTCAGTTGGGTCCGTAACGTAAGTCT
ATCGTCGTAGTCCGTTGTAAGTTTGGGTACGTGGGGGTAAAGTGGTAAATTGTTTAGGGGTAGTTGGGGGTCATTTTTTTGGAA
AATGGTAGTGGTAGTGAGTAGATGGGTAGGGTAAGTTGTAGCTGGTAAGCATTCTGGTAATTGGGTTCAAAATCGGTAGTGGTC
AGTGGTACGTGTTCTTGTAGTTGGTACCGTCGTAGTGTCGTATAGATTCTATGGGGGGTTAGGTGCCGTAGTTTTACACTAGTG
GTAGTGGTAAAGTTGGCGGCTCAGTGGTTTTGTAGTGGGTCTTAAGTGGTAGTGGGTAAGAACATTTAGTGGTGGTGGAAGTGG
TAGGCTAGCAGTGGGGGTACTGGATTGGTAGTGGCGGGTCCATAACTAGGTCGTAGTAGTCCGGGTATCGTAGTAAAGAGGGTA
GTGGGTAGTGGGGTAGTGGTAGTCGGACAGTAGGTAGTGGTAGTTGGTCAGTATGGTAGTGGTAAGTGGGTGGTGAGGGTAAGT
AGTAGTCGTAGTTTTAGCTAGTTCCGTAGTATGGGGGTAATGGTAGTGTACTTTTGGGGTTTGTGGGTAAAGCTGGTAGTGGTA
AAAGTCAGGGTGGTAGTGCAAGTAGTAGTTAGTGGGGTAAAAAGTATAAAAGTAGTAGTTAATAAGTCTAGTTTTAGTTCAAAG
TAGTAGTAAGTAGCTCGTAAGTTAGTAGTTAAAAAAAGTGGGGTAAGTGGTCAGTGGGTAAATTGGGTTACGGGGTAAGTGCG
GTATTGGGGTAGGAGCGGGGTTAGTGGGGGTAACGTGGTAAGTTCAGTAGTTGGTTACGTACTTGGTAGTTGGTAAAGTGGGGT
AGTGGGTAAGTGGGTAACGTGGTGTTAGCTTCGCAGGCTAAGTAGTGGTACGTGACCGTGGGTAGTCACGGTAGTCAAGTGGTA
ACGTGGTTAAAAGTGGGTAGTGGTACGTAGTTTGGTGGGTAAGTAAAGGTCGGTAGTAAGTGGTAGTAAAGCGTGGTGGGGTAC
AGCCTAGCTGGTACGTGGTAGTGGGTCACGTGGGGTTAGTTGGGTAAAGTTTTGTAGTGGGTAGGGGTAAGTGGGGTAAAAGGG
C
>m141121_210323_42134_c100732402550000001823141405141506_s1_p0/134133/6018_11078
ATTTCCTCAATTTTATAATGTCCACATACGGCTCATACAGATATTTTGGGTCATATTAATATAATGTTGGTTAATCTTAAAAAG
CTCATTGAAATAAATTTTGTGCGTTTTATCCTTTTGTATAGAGGTGTATTATTATTATACACAGTCAAAAAGAGTTTCCTACTA
ACAATGCAGCGAAGAAGAAGTTCCTAGTCAATCCGGAGAAAAGGGAGACACACACGCGCTCCGCTATAAATGAATTTGCGGAAC
AAACATTCCGGGTGGCCGATCATACTGAGTCACTAATGCACCGGATCCCAATCAGAAACTCCGTGCAGTTAAGCGGGGACTTGG
GCGAGAGTAAGTACTAGGATTGGGTGACCTCAAACTGGGAAGTCCTGCGTGTTGCACCCCCTGAGACATCATTTTTTTTTTTCC
CTTTTAAACCACCTACGGCTCAATAAGTTTTATTTTTTGTTTTTAAATCTTTAAAAACTCATTTATTTCGTTTTCCTTGAGGGA
TTTAATACCCGTGGATCACTAACTATAGCAGCGAATAAGAAGTTCCTTAGTCAATCCGGGGAAAAGGGAGGACACACGCGCTTC
CGCTATAAATGAAATATTGCGGACCAATACACTTCCGGGTGCGATCATACCAGCAACTAATGAGCAACCGATCCCATTCAGAAC
TCCGCTAGTTAAGTCGTGTCTTGGGCGAGAGTAGTACTTACGGATGGGTGACTCCTGGGAAGTCCTCCGTGTTGCACCCTGAAA
ACATCATTTTTTTTTCCCTTTAAAATCCACCTACGGCTCAAAAGTTGTATTTTTTGTTTTTAAATCTTTAAAAACTCATTTATT
TCGTTTTCCTGAGGGGTTAATACCGTGGGTCAACTAACAAGCAGCGAATAAGAAGTTCTAGTCCAAATCCCGGGGGTGAAAAGG
GAGGAACATCACGCCTCGCCTATAAATGAATATTTGCGGAACAAACATTCCGGGCGATCATACCAGCACTAATGCACCGGTCCC
ATCAGAACTGCCGCAGTTAAGCGTTTTTGGTGCGAGAGTAGTACTAGGATGGGTGATCTCACTGGGAATCTCGTGTTGCACCCC
TGAAAACCATCCATTTTTTTTTTTCCCTTTAATAAGATCCACCTACGCATCAATATATAGTTTTAATTTTTTGTTTTAAATATT
AAAAAACATCATTATTTCGTTTTCCTTGAGGGGAGTTACATACCGTGGTAAAACTTAACAATGCAGCGAATAAGAAGTTCCGTA
TGTAATCCGGGGGAGAAAAAGGGAAGGACACCACGCGCTCCTATAAATAATTATTGCGGAACAAACATTCCGGTGCGATCATAC
CAGCACTAATGCAAACGGATCCCATCAGAACTACCGCAGTTAGGCGTGCATTGGCGTAGAGTAGTACTAGATGGGTGAAACCTC
TCTGGGAAGTCCTCTGTGTTTGCACCCCCTGAAAACATCATTTTTTTTTTCCTTTTTAAAATCCACTATCAAAAGTTTGTAATT
TTTTGTTAATATTTTTAAAAACTCATATATTGTTTTTTGAGTTATACCGTGGTCACTAACAATCACGAATAAAGAAGTTCTAGT
ATCGGAGCGCGAGAAAAGGTAGACACACCGCTCGCTATATAATGCAAATTATTGCGGACCTAAACAATCCGGGTGCGATCTACC
AAAGCACAAATGACCGGATCCCATCAGAACTCCGCAGATTTAAGCGTGCTTGGGCGAGAGTAGTTACTAGATGGTGATCCTCCT
GGGGTCATCGTGTTGCACCCTGAATTTTTTTTTCCCTTTAAAAATCTCTATCTGAACGGCTCAAAAGGTTGTATTTTATAATAA
GAAAGAGAAATTTAAATCTTTAAAAACTCATTTATCTTCTGTTATATCTCTATATATAGAAGGGTTAATACCGTGGTACACCTA
TATCGAAGCAGCGAATAAGGAAGTTCCTAAAGTCAATCCGGGGAGAAAAGGAGACACACGCCTTCCTTATAAATGAATATTGCG
GACCAAAATTCCGGGTGCGATCATACCAGTCACTAATGCACCGATCCCATCAGAACTCCGCAGTTAAGCGTTCTTGGGCTAGTA
GTAGTACTAAGGATGGGTGACCTCCCCTGGGAGTCCTCTGTGTTGAAACCCCTGAAATAACATCATTTTTTTTTTTCCCTTTAA
AAATTCCACCTACGGCTCCAAAAAGTTGTATTCCTGTTTTGTGGTTAAATCTTTAAAAACTCATTTATTTCGTTTTCCTTGAGG
GGTTAATACCTTGGTCACAATAACAAAGGCACGAATAAGAAGTTTCTACTATTCAATCCGGGGGAGAAGATAGGGAGGACACAC
GCGCATCGCGTATAAAGTGAATTATTAGCGGACCAAAACATTCCGGGTGCGATCATACCAGCACTAATGCACCGGATCCCATCA
AGACAACTCCGCAGTTAAGACGTGCTTGGGCGATGGAGTAGTATAGGATAAGGTGATCCTCCTGGGAAGTCTCGTGTTGCACCC
CTGAAAACATCATTTGTTTTTTTTTCCCTTTAAAAATCCACTACGGCTCAAAAGTTTGTATTTTTTGTTTTATAATCTTTAAAA
ACTCCTTTATTTCGGTTTTCCTTGAGGGGTTAATACCGTGGTAACTAAACAAGCAGCGAATAAAAAGTCCAGTCAAGTCCCGGG
GGAGAAAGGGATGACACCACGCGCTCGCTATAAATAATAGTTGCGGACCAAACATTCCGGGTGCGATCATACAGCACTAATGCA
CCCGAAATTCTCCCATCAAGAAAACCGCAGTTAAAAAGCGTGCTTTGGGCGAGAGTAGTACTAGGAATGGGTGACCTCCTGGGA
AGTCCTCCGTGTTGCAACCTTAAGAAAACATCATTTTATTTAATTCCCTTTAAAAATCCACCTACGGCTCAAAAGTTTTGTATT
TTTTGTTTTAAATCTTTAAAAACTCCATTTATTTCGTTTTCCTGTGAGGGTGTAATAGTGGTTCACTAACAAGTTACAGCGAAT
AAGAAAGTTCCTAGTCAAATTCCGGGGGAAAAAGGGAGGACACAACTGCGTCGCTATTAAATGAATTTGCGACCAACATTCCGG
GTGCGATCATACCAATGCACTAATGCACCGGTCCATCAGTAACTCCGCAGTTAAGCGTGCTTAGGCGAGAGTAGTACATAGGAA
ATAGGGTGAACCATTCCTGGGAAGTCCTCTAAATGTTGCACCCCCATGAAAACATCCATTTTTTATTTTCCTTTAAAATCACCT
GAACGGCTCAAAGTTTGTATTTTTTGTTTTAATCTTAAAAACTAATTTATTTCGTTTTCCTTGAGGGGTTAATACCGTGGTCAC
TAACAAGCAGCGAATAGAATTCAATAGTTCAATCCGGGGGAGAAAAGGGAGGACATCACAGCGCTCGCTAGTAAATTGAATGAT
GTGCGGATATCAGAGACATTCCGGGTGCGGATCATACCAGCTACGTAATGCAACCGATCCCATCAGAATCCCAGTTAAGCGTGC

TTGGGCGAGAGTAGTACTAGGATTGGGTGACCTCCTGGGAAGTCCTCGTGTTGCACCCCTGAAAACATCATTTTTTTTTTTCCC
TTTATAAAAATCCACCCTATGGGCTCAAAAGTTTGTATTTTTGTTTTAAATCTTTAAACTCATTTATTTCGTTTTCCTTGGGTG
GTTAATACCGTGGTCACTAACAAACAGCGAATAAGAAAGTTCGCTAGTCAATCCGGGGATTAGAAAAAGGGAGGACACACGCG
CTCGCATATAAATGATATTGCGGTAACCAAACATTCCGGTGCGATCATACCAGCACTAATGAACTACCGGATCCCCATCAGAAC
TCCGCAGTGTAAGCGTGCTTGGCGAGAGTATTATAGGTGGTTGATCCTCCATGGGAAGCTCGTTGTTGCACCCTTGAAAAATCA
ATTTTTTTTTTCCCTTTAAATCCCCTACGCTCAAAAGTTGTATTTTTTGTTTTAAAATTCTTTTAAAAACTCATTTATTATCGT
TTTCCTTAGGGGTTAACTACCGTAATCACTAACAATGCAGCGAATAGAAGTTCTAGCCAAATCCGGGGAGAAAAGGAGGAACAC
GCGCTCCTTAAATAATATGCGGACAAAACATTCCGGGTGCGATCATACCTGCACTAATGCCCGGATCCCATCAGAACCCGCAGT
TAAGCCGGCTTGGGCGAGAGTATATAGGATGGGTGACTCCTGGGATCCTCTGTGTTGCACCCCTGAAAAACATCATTTTTTTTT
TTTTCCCCTTTAAAAATCCACCCTACGGCATCAAAAGTTTGTATTTTTTGTTTTAAATCTTTAAAAACTCATTTATTCGTTTTC
CGTTGAGGGGTTAATACCGTGGTCCACATTAACAAGCAGCGAATAAGAAGTTCCTAGTCAAATCCGGGGAGAAAAGGGAAGGAC
ACACGCGCTCCTAATAAAGTGAATGAATTTGGGAACAAACATTCCGGGTGCGATCATACAGCACTAATGACCGGGATCCCATCA
GAACTCCGCGTTAAAGCGTGCTTGGGCCACGAGAGTAGTACTAGGATTGGGTACCTCCTGGGAAGTCCTCGTGTTGCATCCCTG
AAAAACACCATTTTTTTTTTTTTCCCCTTTAAAAATCCACCTACGGCCCTCAAAAGTTTGTAATTTTTTGTTTTAATCTTTAAAA
ACTCCATTTATTTCGTTTCCTTGAGTGGGTTTAATACCGTAGGTCACTAACAAGCTAGCGAATAAGAATTCCTAGTCAATCCGA
GGGGGATGAAAAGGAGGACACACGCGCTTCGCTATAAATGAATATTGCGGAACCAAACAAATTCCGGGTGCGATCATACCAGCA
CTAATTGCATCCGGATCCCATCAGAACTCCGCAGTTGAAGTGTGCTTGGGCGAGAGTGTACTAGGATGTTCTCCCTGGGAAGTC
CTCTGTTTTGCCCCCCTGAAAACTCATTTTTTTTTTCCCTTAAAAAATCCACCTACGCTCAAAAGGTTTGTAATTTTTGTTTTAA
TCTTTAAAAAACTCATTTTA
>m141121_210323_42134_c100732402550000001823141405141506_s1_p0/134133/11124_16946
TAAATATTTTTTAAAGATATAAACAAAACAAACTTTTGAGCCGTAGGTGGATTTTAAAAGGGAATAAAAAAATGATGTTTTCAG
GGGGGTGCAACACGAGGACTTCCCAGGAGGGTCTACCATCCTAGTACTACTCTCGCCCAATGCACGCTAACTGCGGAGTTCAAA
TGATGGGATCCGGTGCATTTAGTGCTGGTATGTCGCACCCGAATGTTGTTCCGCAATATTCATTTATAGCGAGCGCGGTGTGTC
CCTCCCTTTTCTCCCGGATTGACTAAGGAACTTCTTATCGCTGCTTGTTAGTGACCAACGGTATTAACCCTCCAAGGAAAACGA
AATAAATGAGTTTTTAAAGATTTAAAAACAAATACAAACTTTTGAGCGTAGGTGGATTTTTAAAGGGTAAAAAAAAAAAATGATG
TTTTTCCAGGGTGCAACACGAGGACTTCCCAGGAGGTCACCAATCCTAGTACTACTCTCGCCAAGCACGCTTAACTTGTGCGGT
TCTGTGGGATCCGGTGCATAGTGCTGAGTATACTCGCACCCCCGGAATGTTTGTTTCCGCAATATTTTCATTTAACGAGCCGCT
GTGTGTCTCCCCTTTTTCCCCGGATTGACTAGGAACTTCTTATTCGCTGCATTGCGTTAGTGACCCACAGGTATTAACCCCATT
CCAAGGTATAACGAAATAAATGATTTTAAAGATTAAAACAAAAATACATAACTTGAGCCGTAGGTGGATTTTTAAAGGGAAAAA
AAAATGAGTTTCAAGGGGTGCATACACGAGACTCCCAGGAGGTCACCATCCTAGTAACTCTCGCCCAAGCACGCTATAACTGCG
GGTTCATGGGATCAGGTGCATTAGTGCTGGTTGATCGCACCCGAATGTTTGGTCCGCAATATTATTTATAGCGAGCGCGGTGTC
CTCCCCGTTTCTTCCCCCGGAATTGGGCTAGGAACCTTCTTATTCGCTGCTTGTTAGTGACCACGGTATTAACCCCTCCAAGGA
AAACGAAAATAAATGAGTTTTTAAAAGATTTAAAAAAAAATACCAACTTTTAGCCGTAGGTGGATTTTTAAGGGAAAAAAAAAT
GTTTTCCAGGGGTGCAAACGAGGACTTCCCAGGCAGGTCACCTCAATCCTAGTACTACTCTCGCCCAATGCACGCTTAACATGC
GGAGTTCTGTGGGACCGTGCATTGAGTGCTGGTATGATCGCACCGGAATGTTTGGTCCGCAATATTCATTATCGAGCGTGTGTG
TCCTTCCCTTTATCTCCCCCGGATTGACTAGAGAACTCTTATTCGCTGCCTTGTTAGATTGACCACGGTATTAACCCCTCAAGG
AAATATGACGAAATAAATGGTTTTAAAAATTTATAACAAAAAAGTACAAACTTTTGATGCCGTAGTGGATTTTTAAAGGAAAAA
AAATGGTTTTCAGGGGTGCCAACCTACGAAGACTTCCCAGGAGTCACCCCATCCTAGTACTACTCTCGCCAATCACGCTTAACT
GCAGGAGTTCTGTGATACCGTGCTAATGCTAGGTATGATCCGCCACCCGGAATGTTGGTTCCGCAATATTTCATTTATACGAGC
GCGTGTCCTCCCTTTTCTCCCGAGATTGACTTAGGCTTCACTATTCCTGCTTTTAGGACCGGTATACCCTCAAGGAAACGAAAT
AAATGAGTTTTTTAGATTTAAACAAAAATACAACTTTGAGCCAGTAGGTGGATTTTTAAAGGGAAAAAAAAATGATGTTTTCAG
GGTTGCAACACGGGACTCCCAGGAGGTACCCTATCCTATATACTCTCGCCCAAGCAGCTTATATCTGCGGAAGTTCTGATGGGA
ATCCGGTGCATGTAGTGCTAGGTATGATCGCACCCGAATGTTGGTCCGCAATATTCATTTATAGCGAGCGCGTGTGGTCCTCCT
TTTCTCCCCCGGATTGACTGGAAACTTCTTATTCGCGTGCTTGTTAGTGACCAACGGCATTCAACCCCTCCAAGGAAATACGAA
TAAATTGAGTTTTTAAAGATTTAACAAAAATACAAACTTGTTGAGCCGTAGGTGGATTTTTAAAGGGAAAAAAAAATGATGTTT
CAAGGGGTGCAAACACGAGGACTTCCCCAGGAGGTACCCATCCTAGTAACTACTCTTCGCCCCAATGCACGCTTAACTGGCGGA
GTTCTGATGGATCCGGTGAAATTAGCTGCTGGTATGATCGCACCCGGAATGTTTGGTCCGCAATATTCCATTCTATAGCGAGCC
GCGTGTGTCCTCCCTTTTCTCCCCGATTTGACTAGGATTATCTTCTTATTCGCTGCTTGTTAGTGACCACGGTATAACCCCTCA
AGGAAAACGAAATAAATAGTTTTTAAAGATTTAATAACCAAAAAATACAAACTTTTGAGCCCGTAGGTGATTTTAAAGGGAAAA
AAAAAATGATGTTTTCAGGGGTGCAACACGAGGACTCCCAGGAGGTCACCCATTCTAGTACGTAACTGCTCGCCCACAGGCACG
TTTAACTGCGGAGTTTCTGATGGGAATCCGGTGCATTAGCTGTATGATCGCATTCCCGGAATGTTTGGTTCTGCAATATTTTTA
TAGCGAGCGCTGTGTCCTCCTTTTCCTCCCCCCGGATTGACTAGGAACTTCTTATTCCGCTGCATTGTTAGTGACCCGGTATTA
ACCCCTCAAGGAAAACGAATAATTATTTTGTAAGATTTAAAAACAAAAATACATCTTTTGAGCCTTAGTGGATTTTAAGGGAAA
AAAAAATGATGTTTTCAGGTCAACACGAGGAACTTCCCAGGAGGTCACCCATCCTAAGTACTACTCCGCCAGAACGCATAAACT
GCGGAGTTCTGATGGGATTCCGGTGCATTAGTGCTGGTAATGATCGCAACCCGAATGTTTGGTCCGCTAAATATTTCATTTGAT
AGCGAGCGCGTGGTGGTCCTCCCTTATTCTCCCCCGATTGACTAGGACTTCTTATCGCTGCTTGTTAGTGACCACGGTAATTAA
CCCTCCCAAGGAAAACGAAATAAATGAGTTTTTAAAGATTTAAAACAAAAAATACAAACCTTTTGAGCCGTAGGTGGGATTTTT
TAAAGGGAAAAAAAAAATGATGTTTTCAGGGGGTGCAACACGGACTTTCCCAGGAGGTCCACCCATCCTGAGTACTACTCTCGCT

CCAATGCACGCTTAACTCGGAGTATCGATGGGATCCGTGCATTTGTGCTGGTATGATCGCACCCGGAATGTTTGGTCCGCAATA
TTCATTGTATAGCGATGCGCTGTGTGTCCTCCCTTTTCTCCCCCGGATTGACTAGGAACTTCTTTATTCGCCTGCTGTGTTAAG
TGTCCACGGTAATTATCCCCTCAAGGAAAACGAAATATATGAGCTTTTTAAAGATTTAAACAAAAATACAAACTTTTGACCGTA
GGTGGATTTTTAAAGGGAAAGAAAAAATGAGTTTTCAGGGGGTGCAACACGAGCTTCCCAAGGAGGTCACCCTATTCCTATGTA
CGACTCTCGCCCTAATGCTACGCTTAACTGGCGGAGTTCTGATGGGATCCGGTTGTCTATTAGTGCTGGTATGTCGCAATCGGT
ATGTTTGTTCAAAGCAATATTCATTTATAGCGAGCGCGTTTGTGTCCTTCCCCTTTTCTCCCCCGGATTGACAGGAACTTCTTA
TTCGCTGCTTGTTAGAGTGACCTCGGTATTAACCCCTCAGGAAAACGAAATAAAGAGTTTTTAAAGATTTAAAAAAAAAATATCA
AACTTTTGAAGCCGTAGGTGGATTTTTAAAGGGAAAAAAAAAATGATGTTTTCAGGGTGCAACACGAGGACTTCCCAGGAGATC
ACCATCACTAGTACTACTCTCCGCCCCCCCAAGCAAGCTTACTGCGAGTTACTGATGGGTCCGGTGCAATTAGTGCTGGTTTAT
CGCACCCGGAATTGTTGTCCGCAATATTTCATTTATAGCGAGCGCGTGTGTCCTCCCTTTTCTCCCCCGGAATTGACTAGACCT
TCTTATTCGCTGCTTGTATAGTGAACCACGGTGTTCAAACCCCTCCAAGAAAACGAAATAAATGAGTTTTTAAAGATTTTAAAA
CGAAAAAATACAACTTTATTGAGCCGTAGGTGGAATTTTAAAGGGAAAAAAAAATGATGTTTTCAGGGGTGCAACACGAGGACT
GTCCCCAGTGAGGTCACCCATCCTAGTACTACTCTCGCCCAGCACCTTAAGACTAAGCGAGTTCTGATGGGATCCGGTGCATTA
GTGCCCTGGTTGATCGCACCCGGAATGTTTATGGTCCGCAATATTCCATTTATAGCGAGCGCGTGTGTCCTCCCCTTTTCTCCC
CCCGGTTGACTAGGACTTCTTATTCGCTGTCTTGTTAGTGCCACAGGTATTAATCCTCAAGGAAAACGGGGAAATAAGAGTTTT
AAAGATTTAAAACAAAAAATACAACTTTGAGCCGTAGGTGGATTTTTAAAGGAAAAAAAAAATGATGTCTTCAGGGGTGCAACA
CAGGACTTTCCCAGGAGGTCAAACCCCATCCTATACACTCTCGCCAACCGCAACGCTTTAACTGCGGAGTTCTTGATGGGATCC
CGGTGCATTATGCTGGTAATGATTCGCACCCGGAATGAATTTTGTTCCGCAATATCATTTAATAGCGAGCGCGTGTGTCTCCCC
TTTTCTCCCCCGGATTGCTAGGAGACTTCTTATTCGCTGCTTGTTAGTGACTCCGGTATTAACCCTCCAAGGAAAACGAAATAA
ATGAGTTTTTAAAGATTTAAAAACAAAAAATACAAACTTTTTGAGCCGTAGGTGGATTTTAAAGGGATAATAAAAATGATGTT
TTCAGGGGGTGCAACCACGAGGACTTCCCCAGGAGGTCACCCAAAAATTCCTAGTACACTCTCGCCCCAAGCACGCTTAAATGC
GGAGTTCTGATGGAGATCCGGTGATTAGTGCCTGGTATGATCGCACCCGGAATGTTGGTCCGCAATATTCACTTTATAGCGAAG
CGCGTGTGTCCTCCCCTTTTCTCCCCCCCCGGAGTTGACTTAAGGAACTTCTTTTCGCTGCTTGTTAGTGGACCACGGTATTAA
CCCCTTCAAGGAAAATACGAAAAAAGTGGAGTTTTTAAAGATTTAAAACAAAAAATACAAACTTTTGAGAACGTAGGTGGATTT
TTAAATGGGAAAAAAAAAAATGATGTTTTAGGGGTGCAACACGAGGACTTTCCAGGAGGTCACCCATCCTAGTACACTCTCTGCC
CAAGCACGCTTAACTGCGGAGTTCTGTGGGATCCGGTGCATTTGTGCCCCGTGGTATGATCGCACCCGGAATGTTTGGTCCCGC
AATATTCATTTATAGCGAGCGCGTGTGTCCTCCCCTTTTCTCCCCCGGATTGACTAGAATTCTTATTCGATGCTTGTAGTGACC
ACCGGTATTAACTCCTCAAGGAACGAATATATGAGTTTTTAAAGATTTAAACAAAAAATACAAACTTTTGAGCCGTAGGTGGAT
TTTTAAAGGGAAAAAAAAATGATGTTTTCTAGGGGTGCTATATCACGAGGACTTCCCAGGAGGTCACCATTCCTAAGTACTACT
CTCGCCAAGACGCTTAATGCGGAGTTACTGATGGAGATCCGTGGCATTTAGTGCTGGTATGATCGCACCCGGAATGTTTGTTC
CGCATATTCATTTTATAGCGAGCCGCGGGTTGTGCCTCCTTTTCTCCCCCCTGGTTGACTAGGAACTTCTTATTCGCTTGCTTG
TTGTGACCACGGTAATGTAACCCTCA
>m141121_210323_42134_c100732402550000001823141405141506_s1_p0/134577/3762_13975
TCGCCGGGAGGAACTTTGGGTCCCTCCTCTTATAGGGAGTGGAATATAGATTTTATCTTGTTTTTCTGTCAATCTATGTTATGT
AATCATTCCAAGGATATTTGAGGCTCATGAGGAGATGCATTCTCTCCAAGAGATTAATCTTCCTATTGAGTCATGTCTTGGGAA
TGAGCGTGAGCGATAAGTTTTATCCTTTTTGTTCATTGGACCATGTTTCTTTTTGGGTTTCTCTCCCGTTTGACGTAGCTGATA
ACTTATTTATATTATGTTTTGTTGTTAGTATTTGTTTAAGCTCTCAATATTTTCGGCTATCGCCATTCTAAGTCCTTCTCAATT
TGGTAATAGGGCATGGGCCACGCCTATGGTTCATCCCAGTACATCTGGTGATTATTCTTCCGAGGATAGGGTATTTGAGGTGAG
TCCAGCATTCGGACCTAAATTTTCGCAGGTTCGGATCGTATACCTTCGAACCTAAGTAAACTTTTAGGTAACTCAATGGTTCTT
AAAAGGAGGTTCCGGGTCGAGGAGAGAACCAACACAAGGATGTCTTGTTGAGATAGTGCCATATCCAGTTGACTCCTGAGAAGT
GCCAGACCAGGAGGTTGCACTGACGCATACCAGGCTCTCTAGATGTGGAATTCGTATATCGTGTGTCTGTGGTGGAACATACCA
ATGAATGTTTTGAGACTTTGCTCATTTCTGGAACACTCGAAACTTTTGGGGACGATCTAGACGATAGTGTTTCAAGAATTGATT
TTGTAAGAGCGTCTCTGATAATTGTAGAGTGTCTCTTCTAGGGCTTAGGTATTTTTATAGGTGGGTTACATCTCTATTGGGCTC
CAACTTTTTCAGAGCTTCAGTGATTGTTCGTTATTGGGCGTCATCTTGCTTTTTTTCGGTTATTATTATGTTTTCATTTCTGGG
TTCTCTGACATGTAGGATTAGCTTTCCAAAAAATCCGAGAACATTCGCCTTCAGCTAGTGTTCTGTATGGTTATTTTTTCCTCC
GAGTTATGTTTCACAATTAGCGGTTACGTTCGATACACTGCTTTATTTATTTTTCTCAATCCATTTTTGTGTCCTCTTAAATTT
CTTTTTATCTAGTTTGGCATTGTTTAATTTGTAACTTTGTGTGTTTTTTTCTCAAGAAAAGTCTTGCTTAGTGTTTCTTGATGT
TCATACTAGTGTTATACTTTAGATGGGATAAGATAGGCTGTCTTTTATCCAGGAGTTTGTTCGCTAAATAGTGTTAGCGGTAAT
TCCTTTTGGTTTTTATATGAATCGAATGTTCCTGGACCTAAGGTTATATTCGCAATGTATTTTTCTAGTTTTGATTATTTATAG
GTTTGTGTTTTTTTAGGTTGATTTGTTTCTGTATACTTGTAGTTATCCTGTATTCATAAGTTTCTTTGGCAATATTTCGAGTCT
TTTTAAAAATGCCCATAGTTTTATTTATCAATTTTTCTCCTATAATTTTCTTTATAAAGTGTGTCGCAAATGTTTTTATCCTTT
TTTTGGGTTTTGGTTTTTGATTGTTTCGATTTGGTCTCTGAAAAATTGATTTGGATTTCGAAGCTACGAGTTTTGTTGAAGTTA
AATAATTTAAGATATATTCGAAGGGTCATGCGAGAGCGTAAAGTGTTGTGTTAGCCCTAAACGTCTTAGGCTTGAATGTGTAAA
TATTTGTGCAAATCCGGGTGGCGATAAAGAGGGTGATAATGCATATTGATACTGAGAGTTTTTGGTTTTTATAATTATAGGAAA
GTGAAAACGTTTTGCGATTGCTATAGTATATAGGGATTAGTTTTTTTTGTTCAAAGATTTGTTTGTATACTTTTAGTGTTGTCG
TGTCTTGTGCTGTGTAACTCCAATTAGAGTGTGCGAGTGGTTTGGTTACCTCTATTGATATTGTTTCTGTTGGGAAATAAATAT
CGGATTACGAGTGTTTGAACACGTGTATGGGTTATGAGTGGATCATTTTCGGAGAAAATTAGTATTCTATGTTTTACAGAGTGG
TACCTCTCTTCAGGGTATGTCTTTCGATTTTTTTTCTTATGGGGTTGGCGTTTGAATTCCGATTTGGGTGTATATATTAAAAAG

ATATGGCGGAGGAGTATGTGGCTTAAGATATAAGTGTGTAGTGTCCTTTATGTTGGTGATCTTTAGATCTTGAATTTGTGTTTA
TTAATAAGTTTTTTCTATGCAATATTTAGTTGCCTGAGGTGGGTGAGTGGTGAGTTATTTGTCATAGAATATCTTGAGTATTTC
TATTCTTTATATTATTAATGGCAATAACTTGCGAAATTGCCCATATAAATTAATGGGAATGTTTTTTTTTTTTTCCTATATATG
TTGAACGGGGGTTCGCGTTTCTATGGGTTGTTGTTTTTGGTTTTTGTGTGTGGTTTGGGTGTGTTTTTTTTGTGTTGTTGTGTG
TTTGTTGTCGGCGAAGTAGATGTCTTTTACCTATGATATTCTTGGGAAAAAATATCGAAACGAGTTGAACACCGAGGGTAAATAA
CAATTTCGAAGATAATTAAGTGAAATCTCCAATTCACCAAAGGGGTACCCTTTCAGAAATCTTCGATTTCCGCTAATCAATGGA
TTCGAGCCAACGATTTGAAGGTGAAACTAAAAAAGAATCGCGAGTCAAGGGGCACAGAAAGTTTTAGCCCTATGTCTTAGGCTT
CAATGTTGTAAAATTTCGTGGAAATCTAGGTGCGTAGAGGTTGATACAGTCCAAAATACTGAGTGATGTACTAATATAAGGCAA
AACGTTGCGAAAATTGCCATAAAAAAGGGAGTTTTTTTTCAAATGTTGAACTAGGGCTCCCGTTTCAAACACAATAGATTCATT
TAACCATGAGAATCTCTTGGGGAAAAATATCGAACGAGTTGAAAGACTCAGGGTAATACATTTCGTAGAAATTAGTGATATCTC
CATTCACCAAAAGGGGTACCCCTTTCCAGAAATCTTCGATTTCTCTAATATGGATATCCGAGCACCGATTTGAAGGTGAAACTA
AAGAATCCTGCGAGGGTCACGGGCCACAAAGTTGTAGCCCTATGTCTTAGGCTTAATGTTGTAAAATTTCCGTGCCAAATCCTA
GGTGCGTAGAGGTTGATACAGTCAAAATACTGAGTGATGTACTAATATAAGGCAAAAACTTGCGAAAATGCCATAAAAAAGGGG
AGTTTTTTTCAATGTTGAACTAGGGCTCCCGTTTCAACTCCCAATAGATTCATTTAACCATGAGATCTCCTTGGGGAACAATAT
CGACCCGGTTGAACACTAGGGTTAATACATTTCGAAGAAATTAGTGAAATCTCCATTCACCCAAAGGGGTACCCCTTTCAGAAA
TCTTCGATTTCTCTAATTTGGACTTCGAGCAACGATTTGAAGGTGAAAAATAAAGAATATGCGAGGGTTCATGGCACAAAAGTT
GTAGCCCTACGTCCTTCAGGCCTTGAAATGTGTAAAATTTTGTGCAAATCCGAGGTGCGTAGAGGGTGATAATGGCAAAATACT
GAGAGATGTACTAATAATAGGAAAAAACCTTGCGAAATTGGCCTATAAAAAAAGGGAAGTTTTTTTTCCAAAATGTTGCAACTA
GGGCTCTGTTTCAACCTCCAATAAGGAATTCATTTACCATGAGATTCTCCTTGGGAAAAAATATCGAACGAGTTGAACACGAGGG
TAAATACATTTCGGAGAAATTAGTGAAATCATCTAAATTCACAATAAAGAAGGTAACCCCTTTCAGAATCTTCGTATTTCTCAT
AATATGGATTCGGCAACACGATTTAAGGTGAAAAACTAAAGAATATGCGAGGGTCATGGGCACAAAAGTTGTAGCCCTAATGT
CTTAGGCTTGAATGTGTAAAATTTCATGCAAATCCGAGGTGCGTAGAGGGTGATACTTGGCAAAATACTTGAGGATGTACTAAT
ATAAGCAAAAACTTGAGAAATTGCCATAAGAAAAGGGGAGTTTTTTCAAATGTTGAACTAGGGTTCCGTTTAAACTCCCAATA
AGATTCATTTACCAAATGAGGATCTCTTGGGAAAATATCGAACGAAGTCCATAGAACACGAGGGTAATCACATTTCGAAGAAAT
TAGTGAAATCTCCCATTCACCCAAAGGGGTACCCCTTTCAGAAATCTTCATTTCTCTAATGATGGATTCGAGCAACGATTTGCA
GGAGAAAAAATAAAGAATCTGCGAGGGTCCACGGCAACCAAAGTTTGTAGCCCTTAATGTCTTAGGCTGTCAATGTGGTAAATT
CGTGGCAATCCTAGGTGGCGTAGGTTGAATACAGTCAAAATACTGAGTGATGTACTAATATAAGGCAAAAAACTTGCGAAAATT
GCCATAAAAAAGGGGAGTTTTTTTCAAATGTTGAACTAGGGCTCCGTTTTCAACTCCAATAGATTCATTACCCTTCAATGAGA
TTCTCTTGGGAAAAAAATATCAAAAAGAGTTGAACACGAGGTAATTACATTTCGGAAAAATTAGTGAAATCATCCATTCAACTAA
AGAGGTACCCCCTTATCAGAAATCTTCGATTTCTCTAATATGGATTCGGGCAACGATTTGAAGGTGAAAAACTAAAAGAATTCG
CGAGGTCATAGGGCACAAAGGAAGTCTGTAGGGCTCTTAGGTGGTCTTAGATTGTAATGGTTGTTGGGTGAGAATTTTTGCGCA
TGTCAAATTCATCGGTGCGTATGTATGGGTTTGATGATATTGCAAATGGACTGAGAGATTGTATAAATAGTGTGAAATTGGGTG
CGAATTAATTGCATAAGGGAGTGTGGTGGGTGTGTTTGCGTAATGTTGAACTAGGGGCTCCGTTTCAACTCAATAGTTTTGTGT
TGGTTTGTGTTCTATTTTTTTTTATATATGATTCGCTAGTGATTGGGTTAGTGTCTCTTTGGAGGGAAAAAATATCTGGACGAT
CTGATTTTGAACACTGTATGGTTTTTAATTTATTGATTTCTGATAGATAGTTTAGGTGAAATCTCCAATTCACCAAAGGGGTAC
CCCTTTCACCCAGAAATCTGCCGAGGTTTTTGTGCTTTAGAGGAATTTTTGGAGGTTCGTAGCAATTCGATTTTTTTGAAGGGT
GAATGAGAGAGTATTATTGGCGGACATTGCCCATTGGTCTACTATTTATGTTGTAGCGCCTATGTCTGTGGCTTGAATGTGGTG
AGAAGGTGTTGGTGCGAGAATCCGAGGTGCGTAGAGGGTGATAGTTGGCAAATTATACTGAGAGATGTAACTAATATAGGCAAA
CTTCGCGAAAAATTGCTATAAAAACAGGGAAGTTTTTTTCAAAATGTTGAACTAAGGGCCTCCGTTTCAACTCAATAGTATTC
AATTACCCATGAGATCTTCTTGGGAAAAAATATCAAACCGAGTTGAACACGAGGTAATACAGTTTCGGAAAATTAGTGAATCCA
TCCATTCCTATAAGAGGTACCCCTTTCAGGAAAATCTTCGATTTCTCTAATATGGACGTTTCGGAGGCAAGCGATGTTGAAGGT
AAAACAAAGAATGTAGTGCGTAGGGTCATTGGCACAAAAGTTGGGTAGCCCTATGTTTAGACTTGAATTGTGTAAAATTTCAAT
GCCAAATCCGAGGTGGCGTAGAGGGTGATATTATGGCAGAAAAGGGAATACGGGTGAGAGATGTACAATAAGTAAGGGAAAAAA
ACTTGGAAAATTGCGATAAAAAGAGGGGAGTTGTCTGTTTTCGGAACGATTGTTGAACTAAGGGCTCCGTTTCAACTCCAATAG
ATTCATTGTTAACATGATATCCTCCTTGGGGAAAAATAATCGAAGAGTTGAACACGAGGGTAATACATTTCGAAGATATTAGTG
AAATCTCCAAATTCACCAAAGGGGTAACCGCCTTTCAGAAATCTTCGATTTCTCTTAATATGGATTCGAGCAACGATTTGAGGT
GAAAAGACTAAAAGATTCTGCGAGGGTCAGTGGCCAGCACAGAAGGTTGTAGCCCTATGTCTTAGGCTTCATGTGTAAAATTT
CGTGGAAATCCTGATGGTGAAAGTTGATACAGTCAGAGAATATCTGAGTGATGCTACTGAATAATGGAAAATATGAGGAGGCAA
AACTTGCGAAAGTTGCCTAAAAAGGGGAGTTTTTTTTCAAATGTTGACTAGCGGCTCGTTTCCAACACCAAGTCAGACTTATC
TTAACCCATGAGATCGTCTTGGGGAAAAATTATCGAACGAGTGGAAAGACTAGGGTAAGTGCATTTGCGGGTCAGAAATATGAG
TGATCTTCACAAATTCACCAAAAGAAGGGGTTACCCCTTTCCAGAAATCCTTTCGATTTCTCCTAATATGGTACCTGTCGAGCA
ACGATTTGAAGGTGGAAAAACTAAAGGAATGCGTGCGAGGGTCCACGGCCACAAAAGTTGTAAGCCTAATGTCTTAAGGGCGTT
CAATGTTAAAATTTCGTGCCCAAATCCTAGGTGCGTAGAGGTTGACTAAGTCAAAATACTGAGTGATGTACTAATATAAAGGGC
AAAACTTGCGAAAATTGCCATAAAAGACGGGGAGTTTTTTTCAAAATCGTTGAACTAGGGCTCCGTTTCAACTCCAATGAGAT
TCGGATGGTGTAACCATTGAGATCGTCCTTGGGAAAAATATCGAACTGAGTTTGGAACGACTAGAGGTATATAAGTTTATAGAA
ATTAGTGAAATTCAGTCCAATTCACCAAAGGGGTAACCCCTTGTCAGCAAGATCTTCGATTGTCTCTAAGTATGGATTCGGAGC
AACGATTTGAAGGTGAAATAAATAAAGAATATGCGAGGGTCATTCCGCGGCACACCGTGGGGTGGTAAAGTTGTAGCCCTTGGA
CGTCTTAGGCCCTGAATGTGATAAATTTTGTGCAAATCCGAGGTGCGTAGAAGGGTGATAATGGCAAAAATAATCTGAGAGATG

TACTAATTTGAAGGAAAAAACCTTGCGAAAATTGCCTTATGAAAAATAGGGAAAGTTTTTTTTCCAAAATGTTGAACTAGGGC
CGTCATGTTTCAACTCTAATAGATTCATTTACCCATGAGATCTCTGTGGGAAAAAATATCGACGAGTTGAACCCGGAGGGTAAT
ACATTTCAGGAGAAATTAGTGAATATCATTAAATTCACTTAAAGGGTACCCCTTTCCAGGAAATCTTCGATTTCTCTAAGTATG
GATGGTACGGGCAACGATTTGAAGGTGAAAACTAAAGAATAGGTGGCGAGGGTCATGGGCGACCGAAAAGTTTGTAAGCCCCTG
AAATTCTTAGACGTTGAATTGTGTAAAAATTTCATGCAATATCGAGTGCGTGGAGAGCGGTGATGTTGCCAAAGGATAGCTGAG
GAGACGTGACTATTATGATCGCTGGGGAAGGGCAAAATACCTTGCGAAAATTGCCAATGGAAAATTAAGGGGAGTTTTTTTTC
AAATGTTGAACTGGGCTCCGTTTCAGACTCCAATAATGATTCATTTTAACCATGATAGTCTCTTGGGAAAAAATATCAACGAGTT
GAACTACGAGGGTAATACATTTCGAAGATATTAGTGAAATCTCCAATTCACCAAAGGGGTACGCCTTTCAAAGAAATCTTCGAT
TTCTCCTAATATGATTCGAGCAACGATTTGAAGGTGAAAACTCAAAGAATCTGCGAGGGTCAGGGGCACAAGAAAGTTTTAGCC
CCTATGTTTAGGCTTCAATGTGTAAAATTTCCGCTGGAAATCTACACGCCGTAGAAGGTTGATACAGTCAAAATAACTGAGTGA
TGTACTAATATAAGGCAAAAACTTGCGAAAAATTGCCATAAAAAGGGGAGTTTTTTTTCAAATGTTGAACTAGGGCTCCGTTT
CAACACCCAAATAGATTCATTTAACGCATGAGATCTTCTTGGGGGAAAAAATATCGAGACGAGATTTGAAGACTAGGGTAATAC
ATTTCGTAGAAATATTAGTGATATCTCCAATTCACCAGCGGGTACCCTTTCAGAAATCTTCGATTTCCTCTAATATGGATTCGA
GCAACGATTTGAAGGTGGAAAACTAAAAGATCTGGAGGGTCACGGGCACAAAGTTGTACCCTATGTCTTTAGAGCTTCAATGTG
TAAAATTTCGTGCAATCCTAGGTGCGTAGAGGTTGGATACAGTCAAAATACTGAGTGATGTACTCCGAATTAAGGCAAAAACTT
GCGAAAAATTGCCCATAAAAAAGGGGAGTTTTTTTTCAAATGTTGAACTAGGGCTCCGTTTCACACTCCCAAATAGATTCATT
TAACCATGAGATCTCTTGGGGAGAAATATCGAACGAGTTGAACACTAGGGTAATACATTTCGAGGAAATAGTGAAATCTCCAAT
TCACCAAAGGGTACCCCCTTTGCCCAGAAATCTTCGATGTTCTTCTAATATCGGATTCGAGCAACGATTTGAAAGGTGAAAAAT
AAAGATATGCGAGGTCATGGGCAACAAAAGTTGTAGCCCTACGTCTTTAGGGCTTGATGTGTAAAATTTTGTGCAAATCCCAGA
GGTGCGTAGAGGGTGATAATGGCAAAATATCTGGAGATGTACTTAAATATACAGGACAAAATTGCGAAAATTGCTAATAAAAAG
GGAAGATTTTTTTCCAAATGTTGAACTAGGGCTTGTTTCCCAACTCCCAAATAGATTTCATTTACCCATGAGATCCTTGGGAA
AAATATCGAACAGAGTTGAACACGAGGTAAATAACCATCGGAGAAATTAGTGAAATCATCTATTCACCTAAAGAGGTACACCCT
TTCAGAAATCTTCGATTTCTCTAATATGGATTCGGGCAACGATTTGAAGGTGAACAACTAAAGAATATGCGGAGGGTCTCATGG
GCAACAAAAGTTGTAGCCCTATGTCTTAGACTTGAATGTGTAAAATATTCATGCAAATCCGAGGTGCGTAGAGGGGTGATATGG
CAAAATACTGAGAGACGTACTAATATAAGGCAAAAACCTTGCGAAAATTGCCAATAAAAAAGGGGAGTTTTTTTCAAAGTGTG
TGAAACTAGGGCTCCCGTTTCAACTCCATAGATTCATTTAACCATGATATCTCTTGGGGAAAAATATCGAACGAGTTGAAAGCA
CGAGGGTAATACATTTCGAAGGATATTAGTGAAAACTCCAATTCACCAAAGGCGGGGTACCCTTTAAAAGAAATCTTCGAATTT
CTCTAATATGGATCGAGCAGCGATTTGAAGGTGAAAACTAAAGAATCTGCGAGGGTCAGGGGCACAGAAGTATTTAGCCCTACT
GTCTTAGCTTCAATGTGGTAAAATTTCGTGGAAATCCTAGGTGCTAGAAGGTTGATACAGTCAAAATACTGAGTGATGTAACTA
ATATAAGGCAAAAACTTGCGAAAATTGCCATAAACAAAGGGGAGTTTTTTTCAAACTGTTGAACTAGGTCCGTTTCAACACCA
TAGATTATCTTAACATGAGATCTCTTGGGAAAAAATATCCGAAACGAGTTGAAGGACTAGGGTAATACATTTCGTAGAAAATTG
TGGATATCTCCAATTCACCAAAGGGGTACCCCTTTCAGAAATCTTCGATTTTCTCTAATATGGTGGATTCGGCAACGATTTGAA
GGTGGAAAACTAAAGAATCTGCCGAGGGTCCACCGCACAAAAGTTGTAGCCCCTATGTCTTAGGCTTCAATGTGGGTAAAATTT
GTCGCAAATCCTAGTGCGTAGAGGTTGATACCAGTCCCAAAAAATACTGAGTGATGTACTAATATAAGGGCAAAAAACTTGCGA
AAATTGCCAAATAAAAAAAAGGGGGTTTTTTATTCAAATGTTGAACTAGGGCTCCGTTTCAATCATAGATTCATTTAAAACCAT
GAGATCTCTTGGGGAAAATATGCGAACGGGCTGAACCTTAGGTAATAACATTTGCGAAGAAATTAGTGAAAATCTCCATTCACC
AAAGGGGTACCTTTTCAGAAATCTTCGATTTCTCTAATATGGATTCGAGCAAACGATTTGAAGGTGAAAAATAAAGAATATCCG
AGGGTCAGTGGGCCAAAAAGTTGTAGCTCGTCTTAGGCTTGAATGTGTAAAATTTTGTGCCAATCCGAAGGTGCGTAGAGGGTG
ATAATGGCAAAATAATGAAGAGATGTACTAATAATAAAGGAAAACTTGG
>m141121_210323_42134_c100732402550000001823141405141506_s1_p0/135239/9371_18524
TGATGATGCCCTGTCCCAACACCACCCTCAACCCTGGATGTCTTTTCCCCCCCCCTATTTCCTCCCCGACACCAAAGAATGCAC
CTGACCCGCGTTTATTGCGTCACTACAGCGATCCGTCTTTAGTACGACATTACATGACCGCCTAACCATACCTATTAATAAACA
CACAACATAATAGGCGGACGGAATAGCCCCTTTGGTGCTCTCTACCATTCATATGGACAATACACACATGATCTTTGCCATATT
AACTCAAACCGAGTTCAAGGCGATGCTTGTTCCACCTCCCAAAGCATGTATGAGTGACTTCCGCGATTCTCATCCAAGACGCCG
AGCTCAAACCCATCCTTTCTCTGGGTTTGGACTCTGCAATAAAGGAGACTGCGGAACAATATAAAATGCTAAATAATATAGCTG
TCCTTCCCTAATAAGAAACGAAACCACGTGAATAATTAATACACCTAAACAATGGCATCATACATCATAATTATTTTCCAAGTT
TAACTAACATTCGCACAATCCAAACACGAGCATAGCTCAATAGTCAATCAATTTGAATTGTGCCGGTAGTACCGAGGCAAACTG
CCAACCGCAGTAGAGATAGCCGCTTCCTAAGGAACCGAGTAACTATACAGGAAACTGAATGAGCCCAAGTTGTATACACTCGTG
GAAGAGCTGTATACAACTAAAATAGATTTTGATAGGAGGATACCAATCGCTAGGATATGTCTAAAATAAAATCACCGGGAGGAG
AGGCAGAGTCCTCGTCGCGTAACGAAGTCAACTAACAGTTAATTATTGCCTTCCTGAAGATGCCCTTCACAGCGTGATAAAGGA
ATGGCGCTTCCACGCCTTGAATTATGCATGACAATTTTAAGAAACAAGTCCAAGAATCTTAACCTCGTAAAAAGTGTTTTGAGG
TCTTGATTCGCCATTGGGAGCCCGTAGGGCACATCACATACAGTGAGTGCTTTAGTCTCGATAAGGGCAATGTAGAAATCGAAT
CCCTGCGGAAGTAGCGAATGACTAATCTTCTGAGTGCAAGGGGTGTAAAGCGAAACAAATAGAAGTTCACCCTAAATACTAAGG
ACCTCGCTCGCGAACAATGAAGATATGTGTCTCGCACGGAGCCTGTAATAAATCACTCAGATGAAGGAGCTCATCGTCAGGTGA
GCGAGGAGGGTGGCGACCAAATATTGGGTCTATGCGTTACCCTATTAGGTTCGGATCAAACACCCAAAGGCATCCTTGAGACT
CAGCGATTCCTTGCATCTTCCATTGCTCTCCTCTCCGCGACTCACAACTGGCATCGCCCACTCAACTGTCCTATGTACGGATAT
GTGTAATCCAACAGGGAACGACAGCACTAATAGACCTTCCGATAAATCCGGTGGCGGAATTGAAATCTCCCGAAGCGACATAAA
ACTGCCCACTCAAAAGTGTTCCAATTGACCAGACCAACCGTATGCACGCATTGCACAGAGACAAAGGAGGGTCAGGCGGAAACT

GTATGCCGGGCGATACAAGCAACAAACCTCCGTAGGAAAAGGTAACTTCTGGACGAAAGTATGAACCGGACCGGGCGCTACAAA
CAGGGGAAATAATACGCTGTTCCCCTTTTGCGGTTGGCAGATATGCCAAGCTTTGATGCGAAGCCTCTTGAAATTGGAGCGCTG
GTTAGTCGCTTTAACAATAGGTTTCTTCCACTTCAGCCCGTGGAAATGATTGATGTCTCGCGCAGCAATCCTGTGCACTTACCA
ACCACGTCCCACTTCTTCCCATTTCCCATAACGCCTGTGAAAAAAGTAAGAGACATCCCGCTCGCAAGAACCTCAATTACAGAG
TTGTCACCGCAATCCTAGTAAATGACAACAAAATATGCGAATAATCTCATCAATAGTCATACTACGCATTCTATCAACAACGCA
GATCACACCTTTGCAAAAAATTCCCACCAATTATCACTATCGAATCGGTCTAAAAATATCGTAAATCCTGGAAAACCAAAGGAA
GAACATCTGTAGTACTCTTGCACATATAAAGAGAGCTTTTTATAGGGCTTGTGGAACCATATACATCTATAGATGCTCAGTATA
GAATGACCCTCTTCTTCAATGGCTTTATTACAGAGCAACCCTTTCCATTTACCAGTACATCCAAGCAGTGCAAAGGAATTCGAA
CTGCTGTTCTCCATGCTTTCAGGACACCCTTTCCTTCAAATGAAGGCAAACTTCTAATAACAGTCCGGCGCTCGCGTCCAAAGC
ACTGAGAGACCACCGGAAACCGACAAGTAAAAAACATATCAGACCATCGTAGAAGAAGAAGATAGACACTCTGTTTGGTTAAGG
GGCTTTGCGATCTACAATCTCATAATGCGTAGTTTTAGAGCGAGGCTATAACCATCCGTCGCCCTAATTCTATTGCCAGTGGAT
AGTTTCCATGCCGACAGAACAGAAAATGAGGTTGCGCCCTCATTCGAGGTACCACCTTCTAACAGTGGCGGGCGCGATAAGAGA
CTGTTCGCAAAGGGATAGACCATGACTCTCATTTCATACTTACGTTCTCCTCTTCTATGATCTGCTGACACGGCTCAGGCGAGC
GCAAAACTATTGTCGCTCCTCTGGCGGGAGATTTACCAAAAACTGGGGAAAATGCCAGGGGGGCACTGAACGTCTGAACAGACT
CCGACACCTAGAGTGGGGGTCGAGAAGGGCGCGATAGAGCTGAATGGAGGAAAGTCGAAGAGGTGATCCTCCAAACTCGCGAGA
TCCACCAAGTCATTCAGGTGGGCGTAAAAACTTCAAATGGCCACTTCCCGAAAAGACTGTGCCACTGTGAGGTATAATCAGGAT
ATCCTGGCATGAGTGCCACACTGATATGACAGGATTAATCCTATGTTGCGCTGTCACGCACTCACAAATAGACCATCTACAACT
CCAGTTCGCCACAAAGAGGAGGCCGTTAGACCCCGTGAGGGTAAGAAGCAGTAAAAGCTGAAGTGGGACAAGCTGATGTCATCA
GGCTTCTCAGGAGTCATTCTATCCCGTGTGGTTGGCCAACCGGTTCTGGAGAATGTTTAATTGACTTCACGGACCTGAACAAAG
CTGGCCCAAAAAGATCTATTTCCCTCTTGCCGCGAATCGATCAGATGGTAGACGCCTACTTCCAGTATGACTCTATCTTTCATG
GACCGCATACTCCGATACAACCAGATCAACGGTGCATGTCGCGGACCAGGAACACACCAGCTTCCTCACGGACAGAGGGTGTCA
TCAACTGTTACGCGGTCATAAAAAAAGCCCATATCAGGAAAAAAATAATAGAAGATAACATAGAGGAAAAGCGAAATAAAAAAA
ATACACAAAAAACAAAATAGAGACAAATGAAGAAGAAATAAAAAAATAATAAAAAGAAAAAAGTGAAAAAATCAAACAAAAAAA
CACAAACGAGAAACAAATTAAAACAAAAAAAAAAGCAAGAAACAATATAAAAATAACCAAATACGGAAAACACAGAAGAAAAAT
GGAATAATATACAGAAAGAGACAAAATACAAGAGAGAATAGAAGAAAGACAATAACACCAATAAAAAAAGAAAATAAAAAAAAA
AAAAAAAAAAAAATAAAAAACAAAGAAAAGGAGATGGAGGCCAAACCCTGAAGAATTAAAAAGGGCGTTCATAGATAATGCCTT
TCTCCCGCGAAAAGCATAAGGATGTACAAAAAAGCGTTACCGGAAGGACAGCTGCCTCTCAGCCACTGCTTCCGTATCTACAGG
CCATGACAAATCGTGTCCCATTCTTCCAATGTGTTGCGAGGAAACAAAGGGGTTGAGTGGACCCCGACATGCGAGCAGCCTTTC
CAACACCTCAGAGGAAATCTAACTCCGAGCTCCCATTCTGTCCAAACCTGTCGAAGGAGAGGCGTTTGTTCTTGGTACTTAGCT
GTGACAAACATGTCGTAAGTGCCGCTATCGTCCGGGAAGACGGCGTCTCCAGCTCCATGTGTACTTACGTGGCAAAGAGGTTAC
TGGACGCCGAGTCCAGATATTCAATGATCGAGGAAACTCTCCCTCTGCTTGCTGATTGCTTCGAGGAAGCTAAGGCCTATTTCC
AGGCGCACAACCATGCAGAGTACTCACCAACCACCCTCCTCCGACAGAGTCTTCAGAAGACCGAGTCTTCTGGCAGATTAAGTT
AAATGGCCATGGAACATGAGCCAGTTTGACATCCACTACCAGGCACGCTGGCTTTCCATAAAGTCAAGCTCTCGCGGACCTTTA
ACTGTGGAATGCTCAAAATGAGTGACCCCGGAAGATCCTGTCCCGGACTTCCCACAATGGCAGGTCTATGTAGAACGGTGCCTC
AAATGAAAAAGGAGCTGGAGCAGGGGTTATCCTAGTATCTTCCCAAAAGAGGAATCAGCTCCAGAGCGCCATTCCGTTTCGCGT
TCACAGCATCACAACAACCGAGGCAGAACCACGAAGCCATGCTGGCTGGGTTACAACGTGGCCAGGGAAGTCGGAGCCCAAAAA
ATCGAAGTATACAGCGACTCCCTACTTGGTAAACCAAAATATCGGAGAATAACCAGCCAGACGAAAGGCGAAAAAAATGGCTGC
CTAACGTGTCTCTCCTCCCGCCTAATTATGCAATATATGAACGTTCCAATGTACACTAGTAAACCAATACTACCGCTAGTGTCT
CATATAAGTTTGTCAAGCTGAGTAGGATAGAGTAGTGAATTTAGAGGGGATCGATCATAAAATTATACTAGACAACATTGCCCT
AAGCAGACCTAATTATGACATAATAACTTTCCAGACGTACCACTAGTTACCAATACTACCCATAGGTCTCATATAGTTGTCATT
CTAAGTAGATAGAGTAGTGAATTTAGAAAAGATCCCATCCCCGAAAAATTGCTACTGAGATAACATTGCACTAAGATATAGTAT
GAATTTTAGAAGGGATCTGATCACGGAAAGATGCCTACTTAGATTGACACTGCACTAAGATATATTAGTGAATTTAGAAGGGGA
TCCGATCCTGAAATTTTTTTACAAGATAAAACATTGCACTATGCAGAACCTAATTACGACATAATGGAACCGTTCCATCCTCTC
TACTTCAAACAAATCTAAGCCGCTAGGTCTCCATATAGTAGTCATTTCTGAGTTAAGATAGAGTAGGGTGAATTTGGAAGGGGA
TCCGATCCGAATAAGTACTACTAGATACAAAATTGCACTAAGATATAGAAGTGTAATTTAAGAAGGGGATCCGACTTGAAAAAT
TAATTATAGATAACATGCACTATGCAAGACTAATTAATGACTAATGAACCGTTTCATACGTACACCTAGTAAACTATACTACCG
CTGAGTCTCATAACAGTTGTCATTTTGAGTAAGATAGGTAGTGATATAGAAGGGGATCCGATCCCGAAAAATATACCACCTTAG
ATACATTCCAACTAAGAATAGTCAGTTACATTTAGAAGGAGCACCAATCCCGAAAAATTACACTAAAAAAATTGCATAAACAGC
TAATTATGCATATGAACCCGTTCCAACATAAACAAATAAAAGAAAAAACAAAATATAAAATTATATAAAAAAAAAAAAAAAAAA
AAAATAAAAATAAAATAAGACAAAAGAAACGATAAAATCCAAAAATACAATTAAAAATAAAAAATAAAAAGAGAGAGGAAGAGG
AGAGTACAAAATAATAGTTGCATTCTGAGTAAAGGAGAGGAAAGTAAGTCGGCATTTACGAGAGCGCTACTCTGAATCGTAAAA
TACTACTAAGATCAACCACATTGCACTAAGACTGATAAGTATTGATTTAGAATGGCATCCGATCCGAAAAATTATGACTAGATA
CATTGCCACTAAGCAGACCTAATTATGAACATATGACCCGTTACACACGTAACCCTAGTAACCAAATACTACCCTTAGGGTGTC
CATATATTACTCATTATGAGTAAGAAGAGGATGGGGGAATTAAAAGCGGCGATCACCGATCCGCGAGGCCAAAATACTACTAGA
TAACAATGCACTAACAAATTAGTAATGAATTTAAAGGGGATCCGATCTCGAAATAATTACTAAATAATATTGCCCTAAGCAAAC
CCAATTATGACATAATGAATCGTTCAACGTACAGTAATTAAACCAACACTACGCTAGGGTCTCATATAGTTGTCCAATATGAGT
AAGATAATGTAACGTGAATTCTCCAGAAAAGGGCTCTGACGTCCCAAAAATAGCGTCTAGCCATAACATTGCACAAAGAATATA
CAGGCGTAGTGATTTAAAGGCGGGATCCGATCCCTGAAAATTTTATTCACTGATAACATTGCACTAAGCAGACCTAATTTGACA

TAATGAGAACCGTTTCCAACGTATACTAGTAACCTATACCTACCCGCTAGACGTCTCATATAGTTGTAATTTGAGTAAGATAGT
GTAGTGAATATAGAAGGGATCCGATCTCCAAAAAATACTACTAAGAGACACATTGCAAAATAGCATAGATAGTGAATTTACATG
GGGATACATCTAAAAACTTAGAACTAGATAAAAATTGCACTAAGAGATCGAATTATGATATAATGATTGTTCAACACGATACAC
TAGTAAACAAATACTACCGCCTAGGGTCCAAATGATAGTAGTCATTCTCGAGTAACCGAATAGAGTAGGGAAATTTAGAAGCGG
GATCCGATCCTGAAAAATAGCTACTAGAACAACATTTTTACTAAGTATTTGAAGTGAATTTAGAAGGGATTCGATCCTGAAAAA
TATTACTAAGATACACCATTGTAATAAGAAGAACTAATTATGACATAATGAAACGTTTCAACGTACACCTAGTAACCAATACTA
CCCATAAGGGTCTCATATAGTTGTCATTCTAAGTAAGATAGAGTAGTTGAATTTAAGGACAGAGATCCCATCCCCGAAAAATGC
TGACTAGATACCACATTGCACTAAAGATCATCAGAGTGGAATTACGAGGGATCCGATCTCGAAAATACATACTAGATACCAGAT
TGCTCTAATCATGATACAAAGTTATTTTAGACAGCGGGATCCGATCCTTCACAAATTATTACTAGACAACATTACACTAAAGCA
GACCTAATTATGCACATAAGTGAACCATTCCAACAATCACTTAGTAACGCAATAACTACCGTTAGGGTCTCATATAGTCTTCAT
TCTGCAGACAGATAGAGTAGTGAATTTAGAAGGGGATCCGATCCCGACATAATACTACTCAGAATAACATTGCACTAAGAATAG
CAGTGAAATTAGAAGGGCCCATCCGATCTTCAAAAATTATTACTAGGATACACATTTCCACAAAGTTGACCTATATTATGACAT
AATGAAGCGTTCCACACGTACACTGGTACAACATAATACTACCGTTGAGGGTCTCATATACGCTTCTTACACCTGGGTCAAGAT
AGAGTGTTGAATTTAGAAGGGGATCCGATCTCAAAAAATACTACTAGATACATTGCTCTAAAGATATATAGAGGAATTTAGAAA
GGTAAAAAAACGATCCTGAAAATTTTATTAGATAACAAATATAGCTATATCCTAATTAATTATTAACCGTTCCAACCTACACTA
CTAAACAAACTACGCTAGGGCTCAAATAGTAGTCATTCTGAGTAGATAGGGTAGTGAATTTGGAAAGGGATGCATCCCGAAAAA
ATATACAAGATAACATTTTACTAAGATATTGTAGTGAATTTGAAGGGGCACGATCCTTAAAGGCTTATTATTAAATAACATTGC
ACTAAGCAGTCTAAATATGACCATAATAACGTGCCAAACGTAGACTAGTAACACCAATACTACCAACTACGGTCTCATATAGTT
GCTCATTCTGTGTAAGATAAGTAATGAATTTAAGAGGAGGATCTGAGCCGAAATATACAACTAGATAAATTGACTAAAATATAA
TAGTTAATTTAAAGGGGCACCAATCCCGAAAAATTAATGACTAAATATACAATGCACAAGCAGACTAATTTATGACATAATGAA
CCGTCCACCATAAACTGAGTAAACAAATACTACCGCTAGGTGTCTCTTATAGTAGTTCATTCTGAGTAAGATAGAGTAGTGAAT
TTAGAAGGGATCCGATCCCGAAAAAATACTAACTAGGATAAATTGCACTAAGAAATTGTACTGTTGAAAGGGGATCCGTATCCT
GAAAACTTACTTACTACAGATAAATTGCACTAAGCAGACCCAATTAGACGTAATGAAAGTTCCAAAAGTACACTAGAACCAATA
CTATCGTGGTATACATAATAGTTGTCTTCTGAGTAAGATAGATTAGTAAATTTAAGAAGGGGATCAGATCTCGAAAAAATACTA
CTAGAAAACATTGCTCATAAGATTAGAGTGAAATTTAGAACGGGAATCTGATCCTGAAAAATTAGTTACTAGATAACATTGCAC
TAAGCATAGACCTAAATTATGAACATAAATGAACCGTTCCAATGTAACTAGTAACCAATACTACCGCTAGGGTCTCATATGTCG
TCATTCTGAGTAAGATAGAGAAGTGACTTCAGAAGGGGAATCCGATCCTGAATAAGTACTACTGATATATTTGGCAATAAAATG
TGTGGAATTTAGAAATGCCTGTCCTATCCCGAAAAATTTATGACAAGATAACATTGCACATACACGACCCTAAATTATACATAT
GAACGGTTGCAATGTATACTAGTCCAATTCTACCGTAGGGGTCTCATATCAGATTCATCTCTGTAGTAAGAAATAGTAGTGAAA
TTTAGAAAAGGAATAAGCTCCTGAAAAAAATGCACAAGATAACATTGCACTAATAATAGAAGTGAATTTAGAAGGGGAATCCGA
TCCTGAAAAATTAAAACTAGATAAACATTGCATAACCAGACCTAATTACGACATATTGAGTCGTTCCAGACCTCACTAAACTAA
CAAATAATACCGCTAGGGTCTCATATAGTGTGTCATTCTGATAAGATAGAGTGAGTGAATTTAGAAGGGGATCCGATCACGAAT
AATACTACTAGATAAACATTGCACTAAGATATAGCAGTGAATTTAGATGGGGATCCGATCTACAAAAATTATACTAGATAA
>m141121_210323_42134_c100732402550000001823141405141506_s1_p0/143869/2630_11133
GCGTTAATGTTTATAGGTTTAATTTAGTGTGCGTCTCTCTTTGATTCTGATCCAGAACTGACTGGTCCTGAATACAGCAATAGT
CGCACATAAGAGTTATGTTGATTCGTGGATAACTTCTCCTTGATGGAAGGATCTTCGAAAAAAAAATTAATTAGAGATTTTATAA
AAAAAATCAATTTATTTCAATTTAATAAATGTGTTCAAAATTCTATTAAAAATGTGAAACCCAATTTTCCACACAAACATATGT
ATACCACACCAAATTTTTTATAAAATATATTTAATTACTACTATAAATATCGAACGACATATGTTCAACTTCCAATACAAGTTT
GAATTTGAAATTAGAATAATATTATTACTTTATTGTAAGGCGATTGTTCACTGCCAGTTAAATGCTTTTCTCATTATGCACAAT
ACACACTTATTAAATAATAGGACACCATTTCAAATTCAAACAATAATAACCAACTTTCAAGAATTTAGATATATTAAATATTTA
ATGTATATAGTTTTTTTTTTTCAAATCGTCCAAAATAATAAAGGTTAATTATTTGTATTTCTTTCAAATTTATTTATTGGGTTT
TTGATTTTGAGAAATTGTGTAGTTGAAAAATAAATAGAGTGTTCCTTTTCATTATTTTTTTTCATAAAAAAATTCCAAATCAGG
GTCATAATAATTTATTGTTGCCATTATTATTAGTTAAAATTCGATTTAGTTACATAATCAATTAAAAGCTATTTGAATAGATAT
TTGCTTTTTAGGGTAAAAATAGAACGGAGATCTATGAAGAATTTCCTTTACATAATATTAGTAGATATAACTATGACGTGAGAG
ATCTTCTCTTGAGGGACTGAAGAACATTGTGCGTGTTGATAGCGACCATCCCGATTATGGGTGTAGCGTTGCTGGTTTCGCAGA
ATACGACTGGCCCCTGTATAATGACATCTGTGTGAGTGCTTTCCCAGTCGAGTGCTTGGGTTTGTCGCTCGACCGTGGTCAAGT
TCTGGACGCTTAACTGGGAAAACTTTGTCTATGGGTGATGTATCCACGCCATCTAGTGGGAGATCAGGAAAACCTGAGGACTCC
AAGTCAAGAGCGAGCTTGGAAATTTCATTGGTAGTGTATTCTTCAAGTTATGGATCTAGCTGAGAAGATAGGATGCGCAGGGTC
GTTGTTGGAATAGTATAGTAATGGAGGTTAGCTTGACGATAGGATCAATTTATCCTATTTAAAGCCAGTTATTCTTGTAACCGT
GGTTGTATGAGCAAGATTTACGCTATTGGTACGCAGCGTAGTGTCACAAATCTTGCGAATGCGAGACTAGTGTGAACTCTTGTC
ACCACAGTGATGTGGTATTGCGCTATCAACATTTCATATCACATAATGAAATAAATTAGATAAGTACAATTTATTTTTACCTTT
CGAAGTCGTTGCCTATAAGGGATATATGATCAGAATGTTAAGGAATTGACAGTGCATAGGTTTATTTTGGTGTAGTATAATCAC
ATTGCATTTAGCATTATAGAATCGGAGGTATGATCTACTTAGTATTATCATATAAGGCTGCGGTGGGTGCAGTTATGAATAGGA
ACATAAAAAATTCTAGGTAACTTATTATTTGTGGGTAGTGCATTAGAGCGGTGCAAGATTATAGAATAATGGAATGTATGTGACC
AAACTGGATAACATAGGTATAGACAAGAGATGTGTGAAGAGTTTACCAGCGTAGTCTATTGTGCATATACGAAGATCTTCAGAG
GGATCGGTCAAATAAGTGAATGTAGGTAACGAGGTTGCAAGCTTAGTAGCTAGGTTGCCTACACCACAATTGTTGAGAGTGAGA
GAATGCCGAGGTGTGTAGTTAGTCAGCCTCTTATTAGCAAGAGGTGTTTAGGCTAAATTACCTGTTACATGATTCACAGAGGGG
TGGACAATCAGTGTTACTGGTTTAGTATTTATGTGTGTGACATAACAAACAATATGTTAGTGTAGGACCCGACTTAATTCTTAA

GACCTCCGAAGCATTAATAATCTCTAAGACTAGCTACTTATGTTCGGAAGACATGCATAAAATCAATAGAACTTTATTATGAAA
ATATAAATAACAGGATCCCATTGTTATTACATAAAAACATAAAGAAATACATAAACCTTAAATTATTTGTTCAAACACTAAATG
CGGAAAAATAAAATTACATAAATTGAAACAGATCAAAAATATAAAACTTTCATCATCGATCCTTCCTACAAGTTCATCCATTCG
AGTTCCTCCGCCAATACCATGTCAAATTGTCCATGAATCATCCCTCTTCCAAGCCTTATTTTCCTGCACCATCTAAAAAAATAAA
ACTAATGAGCCTAATGCCCACCAAGGAAAAAATATACTAAAACAGTAACACAAATCATAAGACTAGAAACATATACTATAAAACA
TATGAACGTTAAAACGTAGGTTAGATCACATTACCGTAGTAGTGATAACACCATCTAGGGTTTCTGTCTAGTAATCAAGGTAGG
TTAGATCACAAAATAGTATATGATTAAACCATCTAGGTTTTTCTGTCTACGTAATCGAAGTAGGTTTATCATAACTTAATCTAC
GATAATGACAAAAAAAAATCTTGAATTCATTTTCCTATCTAAGCAACTATATTCTCCCAAGTGACTACAACATATCATATACAT
ACAATAACAAAGCAATATAAACATAATCATAACACATATAATCCTAACCTTTTTCCTTACCAAAATCCGGGATATTGGAAGATA
AGATCGAGATGGAAACCTCCCCTAAATTCAAACAGGTAAGAATCATATGTTTCTAAAGAATGGAGATGAAAAAGAGATCTAACA
TCAACATAGAAACTTATCGAGAAACCCTTAAGTTTCAAAAACCTGACACCTAACCAAAAGCTAACAATAATTTAGGATCTGAAA
GAAAATAAAGAACTCATATTGAACTAAATCTGAGGATAAAGAATACTTGATGGACTAGACTTTGATCATACACCTCAATACCAA
AATATCCGTCTATCCTTACTGTCCAAAGTTTTAGAAAGTAGCTTAGATTTGGAAAGCTGTAATAGTAAGTATAGTAGGAGCAGC
TAGCCAGCAGCGAGCAGCAGTAGCGCAGCAGCAGTAATAGGGCAGCAGGAATAGTGCAAGGAGTAGTAGCAGAAGCAGTAGTAA
GCAGGAGTAGTAGCAGTAGTAGTAGCAGAAGTAGTAGCAGTAGTTGTCAGCAGTAGTTGTAGCAGTAAAGTATAGTAGTAGCAG
GAGCAGTAGTAAGGCAGTAGTAGTAGAAGTAGTAGTAGCAGTAGTTTGTAGCAGTGTAGTAGCAGCGAGTATTTCAGGCAGTAA
TAGCCAGTAGTTGTAGCAGTAGTAGTAGCAGTAGTAGCAAGAGCAGTCAGCAGCGAGCAAGAGCAGTAGCAAGTAGCCAATAGC
AGTAGCAGTAGCAGTAGCGTAGCAAGTTAGAAGTAGAAGTAGCAGTAGCAGTACAGTACAGTAGCAAGTAGTAGAAGCAGTAGT
AGCAACAGTACTAGCAAGGAGCAGCAGGAGCAGTAGCAGGAGTAGTAGCAGTAGTAGTAGTAGTAGTATTAGTAGCAGTAAGTAAGT
AGCAGTAGAGTAGCAATAGTAGTAGTAGTAGTAGTTACAGGACCAGTAGCAGGAGCCAGTAGCAGTAGTAGTAGCAGGGCAGCGTAG
CAAGAGTAGTAGCAGGAGCAGTAGCAGGACTATAGCAGGAGTAAGTAGCAAGAGAAGTAGCAGTAGTTAAGTACGCAGTAGTAG
CCGTAGTAGTAGTAGAAGTAGCAGGAGTATGTAGCAGGAGCAGTAGCAGGAGTAGTACGTAGTAAGTAGCAGCAGCAACAGCAG
CCAGCAGAGTAGTAGGTAGTAGTAGCAGTAGTAGTAGTAAGTAGTACAGGAGCAAGTAGCACGAGTAGTAGCCAGTAGTAGTAG
CAGCGAGCTAGCAGGAGTAGTAGCCAGCAGCAGTAGTAGTATGTAGTAGAGTAGCAGTAGTAGTAGCAGTAGTGTAGCAGTAGT
AGTTAGCGTATAACAGTAGTGCAAGGAGCCAGTAGCAAGTAGCGGTAGCAGTAGAAGTAGCAGTAGCAGCATGAGCAGTAGCAG
TGTAGCAGGAGCAGTAGCAGTAGCAGTAGCAGGAGCAGTAGCAAAAGCAGGAGCAGTAAAGAAGGACAGTAAGCAGGAGCAGCT
AGCAGTAGCAGTAGCAGTAGCAGCAGGCAGCGCGCCAGCCAGTAGCAGTAGCAGCAGCAGCAGCATCAACAGTAGCAGTAGTAC
ATACGCAGTCAGTAGTAGTAGTAGCAGTAGTAGCAAGTAGCAAGTATAGTAGTAGTATAGTAGTAGTAGTAGTAGCAGTAGCAT
AGCAGTCAGTAAGTAGTAGTTAGTAGTAGTGCAGTAGCAGTAAGCAGTAGCAGTAGTAGAAGTAGCCAGTAGTACGTAGCAGTA
TAGTAGTGCAGTATAGCAGTAGCAGTAGCCAGCAGACCAGCAGTTAGCAGCAGCAGCAGCAGTAATAGGACAGCAGGAAGT
AGTAAGCATCGAGTCAGTAGCAGAAGCAGTAGTAGCAAGGAGTAGTAGCAGTAGTAGTAGAAGAAGTAGTAGCAGTATGTTGTA
GCAGTAGTTGTAGCAGTAGTAGTAGTAGTAGTAGCAGGAGCAGTAGTAGAAGTAGTAGTAGAAGTAGTAGTAGAAGTAGTTGTG
CAGCAGTAAGTAAGGTAGCAGGAGTAGTAGCATTAGTAGTAAGCAGTAGTTGTAGCAGTAGTAGTAGCAGTAGTGCAAGAGCAG
TACAGGAGAAATAGCAGGAGCAGTAAGCAGGAGCAGTAGCAGTAGCAAAGCAGTAACAGTAGTATAGAAGTACAAGTAGCAGTA
GCGAGTAGCCAGTACAGTAGCAGTATACATGTTCTAGCAGGAGCAGCAGGAGCAGGTAGCCAGGAGCAGTAGCAGGAGTAGTTA
GCAGTTAGTAGTAGTAGTATTAAGTAAGAGTAGTAGTAGCAGTATAGTAGCCAATAGTAGTAGTAGTAGCGGAACAGTAGCAGA
GCCAGTAGCAGTAGTAGTACAGGAGCAGTAGCAAAGAGTAGTAGCAAGGGCAGTAGCCAGGACTAAGTAGTCAGGAAGTAGTAG
CAGGAGTAGTACACAGGTAGTATAGCAGTAGGCAGCAGTAAGTAGTAGTAGTAGAGCAGGAGCAGTAAGCAAGAGCCACGCCTA
TAAGTAGCAGGAAGCAGTAGCAGAGTAGTAGTAGTAGTAGAGCAACAACAGCAGCAAGCAGTAGTAGTAGTAGTAAGTAGAGTT
AGTAGTAAGTAGTAGTAGGCAGGAGCAGTAGCACGAGTAAGTAGCAGTTATAGTAGGCAGGACAGTAGCAGGAGTAGTAGGCAG
TATAGTAGTTAGTAGGCAGTATAGTAATCAGTAGTTGCTAACAGTAGTGCAGTAGTTGCAGGAAGCAGTAGCAGCTACGGTAGC
CAGTAAGCAGTAGCAGTAGCAGTTAGCAAGTAGCAATAGTAGCAGGAGCAGCTAGTAGCAGTAGTAGTAGGACGGTAGCAGAAA
GCAAGAGCTGGCAGGAGTAGTACAGGAGCAGTAGCAGTAGCAGCTTAAGCTCGCAGTATGTAACAGGAGCAGTAGCAGGAGTGT
AGCAGGGCAGTAGCAGTGTAGTAGCAGGAGCAGTAGCAAGAGTAGTAGCGGAGCAAGTAGGGCAAGAGTAGTAGGCAGTGAGCA
GTAGCAGGAGTAGTAGCAGTAGTAGTAGCAGCTAGCAGGTAGCAGGGGTGTAGGCAGGAGGCAGTAGCCCAGTAGTAAGAACAG
TGTAAGCAGCTAGAGTAGTAGTAGCAGCGGCAGTAAAAGTATAGTGCAGTAAGTTAGCCAGTAAGTAGCATAAGCAGTGCAGCA
GAAGCACAGTAGTAGCCAGCAGCCAGGCAGCAGTAAAGGAGCAAGCAGGAAGTAGTAGCAGGAGTAAGTAGCAGAAGCAGTAAT
GTGCAGAAGTAGTAGCGTATAAGAAGAGTAGTAGCAGTAGTTGTATGCAGTAGTTGTAGCAGTAAGTAGTAGTAGTAATAGCCA
GAGCAGTAAGTAGCGAGAAGTGTAGAGAGTTAGTAAGTAGCAGTAGTTGTAGCAAAGTAGTTATAGCAGGAGTAGTAGCAGTAG
TTGTAGCAGTAGTTTTGTAAGCAGTAGTGCTACAGTAGTGCAAAAGCATGTAAGCAGGAGCAGTAGCAGGACCAGTAGCGCATA
GCCAACTGCAGTAGGCAGTAAACTAGTAGAAGTAGCAGTAGGCAGTTGCAGTAGCAGTAGCAGTAACAGTAGCAGTATTAGCAA
TAGTACTAGCAGGAGCGGCAATGAGCAGTACAGCGAGCAGTAGGCAGGAGTAGTAGGCAAGTATAGTATAAGTATAGTGAAGTA
TGTAGCAGTAGTCGTAGCAATGTAGTAGTAGTACCAGGAACAGTAGCAGCGAGTAGTAGCAGATGTAGTACAAGTAGCCCAGTA
CCAAGAGTGTAGCAAGGAGCAGTAGTGCCAGGACTAAGTAGGGCAGGAGTAGTAGCAGGAGTAAGTAAGCATAGTAGTAGCAGT
AGTCGGCAGTAGTGTAGTAGTAGTAACAGAGGCAGTAGCAGGAGCAGGAGCATAGAAAAAGTAGCAGCGGCCAGTAAGCAGGGA
GTAGTAGAGTAGGGTAGGCAGGCCCAAACACAGGCAGCTAGCCAGTAGTAAGTAGTATAGCCAGCAGTAGTAGTAGTAGTAGTA
GCAGTATAGTAGTAGTATAAGCGCAGGGAGCAGTAGCACGAGTAGTCAGCAGTAGTAAGTCAGCAGGAGCCAGTAGAGGAGTAG
TAGCAGTAACGTAGTAGCTAGTAGTAGCAGTAAGTAGTACAGTATTGTAACATTAGTAGCAGCTAATTGCAGAGCAGTAGGCAG

TAGCGGTAGCAGTAGCAGTAGCGCTAGCAGTAGCAGTAGCAAAGTAGCAATAGTAGCACGGAGCAGTAGTAGGCAGTAGTAGTA
GGAGGCAAGAAGCAGAGCTGCAGGAGTCAGTGCAGTTGACAGTAGATAAGCAAGTAGCGCGAGTAGTAGCCAGGCAGCAGTAGC
AGCGAGCAAGTAGCAGCGACAGTAGCAGTAGCAGGAGTAGTAGCAGAGAGTAGCAGCGAGTATGAGCAAGATAGTTAGTAGCCA
GGACAGTAGGCCAGCGGGTAGTAGCAGCGGACCCAGTAAGCAGTTAGTAGTAGCAGTAGTAGCAGCTAGTAAGTTAGTACAGGA
AAGCAAGGTAAAAGTAGTATAGCAGTAGTAGCAGTATAGCAGGAGCAGTAGCTGGAGTAGTAGTAGTAGTAGCAGCTAGTAATC
AGGAGTAGTAGGCAGGAGTAGTAGTAGTAAGTAGAGGAGCAGCAGCAGCGCAACAGCAAGTAGTAAGATAGTAAAGAACAGTAG
TAGTAATCAGGAGTAAGTAGCAGTAAGTAGTAGCAGTAGTAAGTAAGCAGTATTTGCAGTAGTAGCAGGAGCGGAGCAGTAAAG
TAGTAGGCAGGATAGTAGTAGTAGTAGTAGTAGGAGCAGCAGCAGCAGCAGTAGTAAGTGTAGTAGCCAGTAGTAGTACAGTAGTGC
ATTTATTGTAAGTAGTAGTAGTAAGTAGCAGTAGTAGTAGCAGTAGTAGCATGGAGCAGTAGCCAAGAGTAAGTGCAGTAAGTAAGT
AGCAAGGAGCAAGTAGCAGGAGTAAGTAGGCAAGTAGTAAGCAGTAGTAGTCAGTAGTAGTAGTAAGTAAGGAAGCCCCAGGCA
GTTGTAGGGAGCCAGTAGCAGGAGCAGTAGCAGTTACTAGCAGGAGTAGTAGCCAGGAGCAGTAGCAGGAGGCAGTAAGCAAAC
GCAGTAGTAGTAGCTGGAGTAGTAGCATAGTAGCAGTAGTAATAGCAGGTAGCAGGAGCAGTAGTAGTAGTAGTAGCAGGAGCC
AGTAGCGGAGCAGTAAGCCGCGAGGCATACAAGTAGCAAGCAGTAGCCGTAGTCAGTGCCAGTAGCAGTAGCAGTAGCAGGAGG
CAGGTAGGCAGGAGCAGTAGCAGGGCAGTAGCCAGCGAGCAGGAGCAGGAGCAAGTACAGGAAGCAAAGTAGGGCAGGAGCAGG
ACAGTAGCAGGAGCAGTTTGCAAGTGCAGATACGTAGAAAAGTAGCAGTAGCAGTAGCAAGTAGCAGTTAGCAGATACAGATAG
CAGTAGTAGAAGCAGTAGTACAATAGTACTAGCAGAGCCAGCAGGAGCAGTAGGCGGAGTAGTAGCAAGTATTAGTATAGTATT
AGTAGCAGTAGGAGTAGCAAGTAGTAGTAGCAATAGTAGTAGTAGTAGTAGCAGATAGCAGTAGCATAGCAGTAGCATAAGCAA
GGTAGGCCAGAGCAGTTAGCAGGAGCAGGAGCAGGAGCAGTAGCAGGAGCAGTCATGAGAAGGACAGGAGGGCCGTAGCCAAGG
AGGCCCAGTAGCAGGAGGCAGGAAGCAGTACAGGACAGTTGCAGTACAGTAGCAGGTGAGAAGGTAAGCAGTAGCCAGTAGCAG
TAGCAGTAGCAGTAGCCAG
>m141121_210323_42134_c100732402550000001823141405141506_s1_p0/146024/0_7503
AGAGAGAATCGAGATGGACTAGGCTTTGTGCCTTGCGTCCCATTTGGTTTTGTACCCTCCTCGGACCATTTTATGTCCGGGATG
GGACTAGCATTGGGCCTTGCGTCCCATTTGGTTGTACCCTCCTAGAACATAATATGTCCGGGCTTTGGACCTAGTCTTTGTGGC
CCACCTTACATCCACTTGGGTTTCATACCCTTCGAGACCCGTTTATGTCGGGGATGGACCTGGCTTTGTTACTTGTTGTCCCA
ACTTTGTTTTGTACCCTCCCGGACCAATTTTTGTGTCCGGGATGGTTAGGAATTTTGGTCTTTGCATCCCCATTTGGTTTGTAC
TCTCTCCGAACCATTTTATGTCTGGGATGGGAATAGGCCCCATGGGGCCACTCCCATCCCATTTGTTGTACCCTTTTCGGACCA
TTTTTGTATACACGAGAGGAATTGGACTAGGCTTTATGGCCGTACGTTCCCACTTGGGTTTGTAACCTCCGTGTACCATTTATG
TCGGGGATTGGAAAAAACTGAGCTTTTGTTTCTTGCGTCCACCTTGGTTTTGTACCCTCCCGGACATTTTGTGTCCGGGATGGG
ACTAGGCTTTGGGCCTTTACGTCCCATTTTTATGATGTTCCCCACCCGGACGCAATGTGTGTCCGGGATGTTAGGCTTTGGGCC
TTTCGACCATTTGGGTGTTCCCTCCCGACCATTTTGTGTCCTGGGATAGGACTAGGCTTTGGCCATGCGTCCCATTTGGGTGTA
CCTCCCAGACAATTTTGTATATGTGATGGGGTTTAGGCCTTTGTGCTTTGCCTTCCCATTTGGGTTTGTACCTCCTCCTAGAC
CTTTTTTTGTCCGGATGGATTAGGCATTTGGCTTGTGTCCATCCTGGGTGTACCTCCTCGGACCATTTTGTGTCAGGGATGGG
ACCTAGGCTTTGTGCCTTACGTCCCCATTTGGGTGTACCTCCCAAACCATTTTGTGTTCGGGATGGGACTAGGCTTTGTGCCTT
GTGTTCACATGGTTTTGTACCCTCTGGCCTTTTTGTGGTCCGGGATGGTAGTTCATATAGGACTTTTCGTCACATCGGACACAT
TAATATACACACAGGACCATAATATACAGATGGGAATTCACCAATATGTAAGACATAACACACAACTTGGTTTGTCCGACACAA
TACATTTTGTTCCAGGAATGGGAACTAGATTCGGACCTTGCATCCCATTTGGGTTGTTCTTCAGCCAGGACCATTTTTTGTCGG
TGGCTGGCTTGTGCCTCTCACTTGGTTGGTCCTTTAACCATTTTGTGTCCAAAAAAGGGCGGCTTTGGCGCATAGGTCCATTTG
GAAACCACACTTTGTGTCCAGGATGGGACTAAGGCTTTGTACCCTGCTCCCACTTTGGGTTTGTACTCCTTCTTATCCGTTTTG
TGTCCTGGATAGGACTAGGATTTTTGGGCCAGTGTTCGCCATTTGGGTGTACCCTCTCCGAACCATTTTTTGTCCGAGATGGGA
CTAGGCTTTGTGCCTTGCGTCCATTTGGTTTTTGTAAACCCTCCTCCCCGGACCATTTTATGTCCGGGAATGGGACTAGGCATT
GGGCCCTGCGTCCCCATTTGGTTTACCCTCCTAGAACATATTATGTCCGGGCTTGGACTAGTCTTTGTGCCTTACATCCCCACT
TGGGTTTATACCCTTCCGAGACACGTTTATGTCGGGATGGGAACTGGGCTTTGTTACTTGTGTCCCACTTGTTTTGTACCCTCC
CGGACCATTTTGTTGTCGGGATGGGTCTAGGATTTGGGTCTTTGCATCCCCATTTGGTTTTGTACTCTCTCGAACCATTTTATG
TCCTGGGATGGACTAGGCATGGGCCATCCATCCCAATTTGGTTGTACCCTTTCGGACCATTTTGTATCCGGGATTGGACTAGGC
TTTGTGCCGTACGTCCCAGCTTTGGTTTTGTAACCCTCCGTGACCCATTTATGTCCGGGGATGGGAACTGAGCCTTGTTTCTTG
CGTCCCACTTGGTTTGTAACCCTCCCGGGACGCAATTTTGTGTCTGGGATCGGGTCTAAGGATTGGGCCTTTGCATCCCCCATT
AGGTATTGTACTCTCTTGGACCATTTATGTCTGGGATGGGACTAGGCATGGGCCTTCCAATAAACCCATTTGGTTTGAAGCCCC
TTTCTGGAACTTTGTGTCGCGGGATCGGATAGGCTTTGTGCCTTACGTCCCACTTTGTGTTTGTACACTCCCGTGAGCCATTTA
TGTCGGGGATGGGACTGAGCTTTGTTTCTGCGTCCCACTTGGTTTTTGTTCCTCCCCGGTCCCATTTTGTGTCCGGGATGGTCC
TAGGTTTTGGGCCTTTGCATCCCATTTGGTTTTTGTAGCTCTCTCGAACCCAATTTTAATGTCTGGGATGGGACTAGGTATTGG
GTCTTCCGTCCCATTTGGTTGTGTACGCCCCTCCCTGGAGCCTTTGTGTCCAGGATCAAGGACTAAGGCCTTTGTGCCATTACG
TCGACACTTGGGTTTGTACGCCTCCGTGACCGCATTTAATGTCGGGGATGGGACAGAGCTTTGTTGCTTGCGTCGCCACTTGGT
TTTGAGCCCTCGCCGGACCATTTTGTGTCAGGGATGGGCCATAGGCCTTTGGGCTTTGCGTCCCATTTTTATGTGTACCCCTCC
CGGACGGCGATGTGTGGTCCGGGAATGGGACTAGGCTTTGGGACTTTGCGTCCCCAGTTTAGTGTTCCTCCCTAGACCCATTTT
GTGTCCAGGATGGGAAACTAGGCTGGTGGACTGCGTCACCCATTTGGGTGTACCCCTCCCAGACGAATTTTTGGTTATCATAGT
GATGGGTAATAGGGCTTTGGCTTTCTTCCCGAATTTTGGGTTGTTACCTCCCTTAGACCTTTTTTTTTGTCCCCCCCGTGATGT
GGAACTGAGGCAATTGGCCCTTGCGTCCCCATCTAATGGAGTAACCCTCCCCTGGACCAATTTTTGTGTTCTGGGATGTGGACT
AGGCTTTGTGCCTTACGTCCATTTGGTGCTACGGCCTCCCAAAACCATTTTGTGTCCCGATGGGACTAGGCCTTTGTGCCTTGC

GTTTCCACGTCTGTTTGTAGCCCCTCCGCTGGACCATTTTGTGTCCGTGGATGGGACTAAAAATGCTTTGTGTCTTGCGTCCAA
TTTTCGGTGAACCTTCCGAGCCATTTTGTGTCGGCGGGATGGGTCCTAGGCTTTGTGGCAATTGCGTCCCACTTGGGTTTTTGT
ACCGTCCCTAAGACCAATTTTGTATCACAGGATGGGAGGCTAATGGAATTCGGCCCTTGCATCCCATTTGAGTGTTCCCCGCCC
TCCCGGACCGTTTTTTTGTCGCGGGATGGGACTAGGCTTTGTGCCCTGCTTCCCACTTAGGTTTGGTACCCCTTTAGAAGACGC
TTTTGTGTCCGGGAATAGGACTGAGTGCCTTTGCGCTATGCGTCTCATTTGGGTGTACCCCTCCTCGGCCCAGCTTTGTTCTGG
GATGGGACTACGCTTTATACCTTGGCGTCCCATTGTGGTTTTGTAAACTCTAAAGAACCAAATGTTATATTCGGGATGGACAAG
GCCATGGGCCTTGCGTCCCATTTGGTTTGACCTCCTTTTCGACCATATTTGTGTCCGGGGATTGGAGCTAGGCCTTTGAGGCCT
TAACATCCCACTTGGTCTGTCCCCTTCCCCGAGACCCAATTTATGTCGGATGGGACTGGGGGGGGCTTTGTTCCTTCTGTCCGC
AACCTTGGTTTTGTACCCCTCCCCCAGCCATTTTGTGTCGCGAAATGGGCTCACTTTGGGCTTTGCGTCCCCAATTTGGGTGTG
TAGCCCCTCCCGAACTGTGTTCGCAGGATGGGACTAGGGTTCGGATCCTTTCGTCCCATTTGGGTGTTCCCTGCCGGAACCAAT
TTTTGTCTGGCATGGGAACTCAGGCTTTGGGTCTTCCGTCCTTTGGGTGTCCCCTCCGAACCCTTTTGTGCCAGGAAAGGGACC
ATATGCTTTGTGGCCTTGGTTCCCACTTTGGTTTGCAGCGCCTCCCTGGACCCATTTTGTGTCCCGGGAAGGGAAAACCTAGGC
ATTGGGCTTTCCGTCCATGTGGGTGTACCCTCCCGGACCATTTTGTATTTGGCGGATGGGACTAGGCTTTGTGGCTTTGCGTCC
CCAAATTTGGGTTTGTACCGCTTCCCGGACCATTTTGTGTCCGGGATGGGACTGGCCTTTGTGCTTAGTGTCCGACCCTTGGGT
TGTACGTCCCCGGACAATTTATGTCGGGATGGGAAACTGGGCTTTTGTGCCTTGCGTCCCATTTGGGTGTTCCTCCTGGAACCA
TCTTTGTGTCCGTGATGAGAGCTAGGCTTTGTGGCCGTTTGTTGTCCCCCATTTGGGTTTTGTACCCTCTCGGACTCATTTTTT
TGTCGGCGGGATGGGAACTAAGGCATTGGGCCAATTCCGTCCCTTTGGTTGTACCCCTCCAAAAAGGACGCATTTTTTGTCCAG
GGATTGGACTAGGCTGCTCTGCATTTACGTGCGCGCAACTTGGGGTTTGTACCTGCTCCGGGACCATTTATAGTCGGGGATGGG
ACTAAAGCTTTGTTCTTGCGGTCACCATAATGGTTTTGTACGCCAATCCTGACGCAATTTTGGTCCGGGATGGGAAACTGGCCT
TTGGGCCTTTTGCAATCAAACACATATAAATATAATATATTTTGATTGTACCCCCTCCCCGGACAATGTGTCCAGGGTGGACAG
GCATTGGGCCGTTTTGCGTCCCATTTGGGTGTAAAAAAAAACCCCTCCCGCGGAAACATGTGTGTCAATAGGAATAGGGTCTAG
GCTAATGGTCCTTTTTCGTCCCTTTGGGAGATTTCTACACACCCGGCAACATATTTGTCTAGGTACGATAAGAGAAAACTATCC
GATCACCCAATTATATATGGTGGTCCCCTCCCCAGACCATTTTGTGTCCCCGGGATGGGACCATATGCTTTGTGCCATTTTCGT
GTTGCACCCTCCTAGACGCATTGTTGTGTCCGGGATGGGACTAAGGCATTGGCCCTTTCCTGTCTGCATGTGGGTGTACTCTCG
CCATAAACGCATTTTGTATCTGGGCGATGGGACATCAGGCTTTGTGCTTTGCGTCCCCATTTGGGTTTGTACCCCTACCCGGAC
TTTTTGTGTCCGGGATGGAACAGGCAAATTTTGTGCCTTGTGTCCCCACTTGGGTTTGTAGCCCTCCGAACCATTTATGTCGGG
GATGGAAACTAGGAAAAAAAAAATTTGTGCCTTGCGTCCCGCATTTGTTTGTTCCCGTGCCCGGACCAGTTTTGTGTTCGGGGGT
TGGGACTAGGCCATTGGGCCTTCCTTCCCATTTGGTTGTACCCCGCCGCCCCCCGAAAACCATTTTGTGTCGCGGGATTGGACT
CAGGCTTGTGCCTCAAGCGTCCCACTTTAGGTTTGTTCCCTCCCGAGAGCATTATGTCGGAGGGATGGGACTGGGAAAAAATTTG
TTTCTTGCGTCTCCCATCTTGGTTTTGTACCCCCTCCCCTGGATCCATTTTGTGTCTAGGATGGGAGCTAGGGGTTTTGGGCCC
TTTGCTTCCCATTTTTGTGTGTATCCGTCCCGTAAGCATGTGTGGTCCGGGATGGGAACTAGGCTTTGGGCCTTTGCGTCCCGT
TTGGTTGTACACCCTCCCAGCCAATTTTGTGTCCCACGATGGGAAACTAGGCTTTGTGCGCTCATTCCACTTTGGTTGGCCCTC
GCTGGACCATTTTGTGTCCGGGATGGGAAGCTGGTTTGGGCCCCTGAGTGCCCATTTGGATGTTACCCTCCCGGACCAATATTT
TTATCTGCGATGGGAACTAAAAAGGCTTTGTGCATTGCGTGGCCCATTTGAGGTTTGTTCCCACTCGGACCTTTTTTTTCCGGG
AATGGGACTGACGTAATTGGCCTGAGCCCATTTTGGTTTACCCTCCCGGACCATCTTTGTGTCCGGGAATGGCCAGGCTTTGTG
CCTTGTGTCCTACCTTGGGTTTCGTAGCGCGCTCCAAGCCATTTAATTGTCGGGGATGGGAAACTGGGCTTTGTCCCTTGCGTA
CAGCATTTAAAGGGTGTTTTGTAGCCTCCCCAAGGAACCAAAAGTTTGTGTCACGGGTAAGACTAGGCGCTTCTGGGTCTTTGC
GTCGCAAATTTGGTGTCCCCTCCGGGACATTTTGTGTCTGGGATGGGAACTAGGCTTTGGGCCCTTGTGACGACATTTTGGTGT
ACGCCTCCCCAGACCATTTTCCCTGTCCCGGTGATGGGAAAACTAGGTTTAGTTTGGCGCGTCGACTTGGGTTTTGTAGCCCTC
CCAAGACCATTTTTGTTGTGCGGGATGTGCAAAAAAAAAAAATAGGCTCTTTGGGTCTTGCGTCCCCAACTTTCGGTGAACCTTC
CCCGGACATTTTGTGTCCGGGTTGGGACAAAGGCTTTGTGCCTTAAGTCCCAAACTTGGGTTTCTACCGTCCTGCGAAGCGCAT
TTTGTAATCTGGATGGATTAGGCATTCGGCTTGCATCCGCATTTGGGTGTTCCCACACCGCAGACCATTTTTTTTCCGGGATTG
GAGCTGAGGCTTTGTGCCCCCTGCGTCCTACCTTGGGTTGGTAGCCCTTACCTAATAGACCAATTTTGTGTCCGAGATGGGAAC
TAGGCTTTGGCAATGCGTCCCCTTTTGTTGTACCCTCATCGGACCATTTTGGGTCCGCGGGATGGGCTAGGCCCTTGTTGCGTT
GCGTCCGCACCTTGGGTTGTAAACGCCTCCCGGACCCATTTGTGTCTCGGATGGGACAGGCTTTGTGCCTTGCGCTCCCCATTT
GGGGTTTGTAACCCCTCTCCCCCAGACCAAATTTTATGTCTGCGGATGGGAGCTAAGGCATTGGGATTTGCGTCGCCATTTGGT
TGTAATACGCTCCACCGCCACAACCAAATTTTGTGTCCGGATAAGGGACTAAGGCTTTGTGCCTTGCGTCCCACTTGGGTTTGT
ACCCCTCACCGCCCCAATTTTTTGTGTCCAGGATGGAACTAAGGCTTTGGCCTTGCGTCCCATTTGGGTGTCCCTCCTTGGAAC
GCAATTTTGTGTCTGGGATGGGGCTAGCTTTGTGTCTTGATTCCCCCAAAACTGAGGTTTGAACTCCCGGCAGCATTTTGTGTC
CGGACGGGGGGCTTGGCCTTTGGGCCACTTTGCGTCCAATTTGGGTGTGTACCGCCTCCAGGACCATTTTTTTGTCTGGGATGC
GGAATAGGCATTGGGCCTTTTGCGGGCCACATTTGGGTGAACCCCTCCCGGACCCATTTGTGTCCGAGATGGGACTGGCCTTT
GGGCCTTTCTTCACATTTGGCCCTTGTATCCTGCCCCAGACCTTTTGCGTCTGGGAATGGGACTAGGCTTTGGGCCCTTTGCGT
AAAAAAAAACCCATCCGTTGAAGGCGT
>m141121_210323_42134_c100732402550000001823141405141506_s1_p0/153579/772_11761
TATGTTTTTTTTATTATCTCGCTGCTGTATTCTGTGAACGGTATTGGAACTCAAGGAAAACAAATACAAGATGAGTTTTAAACG
CATTTAAAACGAAGAATAATCACAAACTTTGAGCCGCTAGGTGGGATTTTTCAAAGGGAAAAAAAAATGACTGTTTTTCAGGGG
TGCAGACACGCAGGACGGTTCCCAGGAGTCACCACTCGCTAGTAGACTATCTCGCCAAGCAGGGGGCTTCACTGCGAGTTCTGA
TGGCGGGAATCGGTGCATTAGTGCTGGTATAATCGCACCCGAATGTTTGGTCCGCAATATTCATTTATAGCGACGCGCAGTGAT

GGTCCTCCCTTCTTCTCCCAACCGGATATGACGTAGGAAACTTCTTATGTCGCTCACATGTGTAGTGACCACGGTACTCTGAAC
CCCTCAGAGGAAAACGAAATAAATGAGTTTTTAAAGATTTAAAACAAAACAATACAAAACTTTTGAGCCCGTAAGTGGACTTTT
TAAAAGGGAAAAAAAAAATGTCATTTTTTCCAGGGCGCGGTCGCACCACGAAAGGACTTCCCAGGAGGGTGCACACATCCTAGTA
TACTCTCGCCCACGCAGACCTTAAGCTGGCGGAGTTCTGATGGGAGTCCGTGCATTAGATGCTGGTGATGAGTGCGCACCCGGA
ATGTTTTGGTCGCAATATAAGCATTCTATAGGAGCGCCAGGAGTGTGTCCGTCCCTTTTCTCCCCCCGAATTGACTAGGGAACT
CTTATTCGCTGCTTGTTAGTTGAGCACGGTATTACTCAAGGAAAAACAAATAGAATGAGTTTTACAAGATTTTAGAACAAAAAA
TACACAACTTTTGGAGCCGTAGGTGGATTTTTAAAGGGAAAAAAAAAACATGATGTTTATCAAGGGCGCGCGGTCGGCAACACG
CGCATGTCCCAGGGGAGTCACCACATCCTACAGGTACTAACGTCTGCCAGAGAGGCACCGCCTTACTGCGGAGTTCGATGGGAG
TCCGTGCATTATCTGGTATGCAGTCGCACCAAGCCAATGTTGTTCCGACAACTATTGCATTTATAATGCGAGCCGCGTTGCTCC
TCGCTTATCTCATCCGCCGGATTGACTAAGGAATCTTATTCGCTGCTTCGTTAGTGACCCAGGCTATTAACCCCGTTCAAAGGA
AAACGAAATAAATGAGGTTGTTCTAAAGGGAGTTTAAAACAAAGAAATAGCAAACTTTCTGAGCCGCTCAGGTGGATTCTTTTA
AAGGGAAAAAACAAAAACCTGAAGTTCAGGGGTGCAACACGAGGATTCCCAGGAGCGTCACGCATCCTATACTACTCTCGCCCA
AAGCCACGCTAGTATCAACTGCGGAGTTCTGATGGGATCCGGTGCACGCTTAGTGCGTGGTACAAGTGATCCGCCACCCAGGAA
TGGTCTTGTCGCCCATATTCATTTATAGCAGAGCGGCCGTGTGTCTCGCCTTTTCTCACCCGATTGAGCTAGGAAGTTCTTATG
TGCTGCCTTGTGGAGACACAACGGTCCATTTACACCCTCAATGGAAAACAAACATAAAAGTGGAGTGTTTGAACAAGATTTAAA
AAAAATAAAAACCTTCATTATGCAGCCGTAGGTGGATATTTAAAAAGGGAAAAAAAAATGCATCGTTTATCAGCGGTCGCAACA
TCGAGGCACTATCCCAAGGAAGGGTCACCCAAACAACCTGAGTACACTGATTCGAGCCCAGCACGCTTACTGGCGGATGTCTGA
CTGGGATCCGGTGCCAGTTAGTGCTGGAGTATGATCGCACAGCGGCACAAACTAGTATATTATCATCCGCAGATATTCATTTAT
AGCCGAGCGCGTGTGTCTCTTTTTCTCCCCCGGATCTGACTAGGAACATTCTTATTCCGCTGCAAAAAAATTCGTTTAGTGAG
CCAACGGTATTAACACCCTCAAGCGAAACAAATAAATGAGTTTTTAAAGATTTAAAACAAAAAAATACAAACTTCTTGAACCGT
AGGTGGATTCTCTTGAAAGGGAAAAAAAAAACACGTAATGCTTCAGGGGTGCAACACGAGGACTCAGAAAAGCAGGTCCAGGAG
GCAGGACCATCGCTAGGTCACTACTCATGCGCCCAGGCACCGCTTAAAGATGCGGAGTTCTGATGGGATCTGGCGGCTGCATAG
TAGTGCTGGGTAAGATGATGCACCCGGAAGTTTGGTCCGCATATTCTTTATAGCGAGCGCGGTGGTGTCCGCGCCTTTTCTTCC
CCGGATTGACTAGCGAAACTTCTTATTCCTGCTTGTTAGGACCACGGGTGATAACCCCTCAAGGAAAACCGAAATAAAATGAGT
TTTTAAAGATTTAAAACCCCAAAAAACTACAAACATTTTGAGCCGTAGGTGGGATTTTTAAAGGGAAAAAAAAATGAATGTTTT
CAGGGGTGCAACACCGAGTGGACTTCCCAGGAGGTCACCCCATCCCCTAATAACTACTCTCGCCCAAAGCCACGCCTTAACTGC
GGAGCTTCTGATGGGATCCGGTTGCAAACGTGCCTGTATGATCGCACCCGGAATGTTTGTTCCGCAAATATTACATTTATAAGC
GAGCGCGTGTGTCCACCCATTTTTTCCACCCCCGGATTGAACTAGGAAATTTCTTATTCGCTGCTTGTTTAGTGGACCCAACCG
GAATTAACCCCTCAACGGGAGAAACGAAATAAATGAGTTTTTAAAGATTTAAACCAAAAATACAAACTTTTGAGCCGTAGGTGG
GCATATTTAAAGGGAAAAAAAAATGATGCTTTTCAGGGGTGCAACACGAGGCTTCCCCAAGCCGAGTCCCCATACTGTACTACT
CTCGCCCAGCACGCTTAACTGCGGAGTTCTGATGGGAATCCCGGTGCATTAGTGCTGTAATGCATCGCACCCGGAATAGTTTGG
TCCGCAATAATTCAATTTATAGCGAGCGCTGTGTCCTCCCCTTTTCTCCCCGAATTGGCGACTAGCGGAACTTTCTGTATTCCG
CATGCTTGTTAGTGTGACCACGGTACAGGTTAACCCCTTCCAAGGAAAACGAAATACAAGAGTTTTTAAAGATTTAAGGGAGGA
CCAAAAACTACGAAACTTTTGAGCCGTAGGTGGATTTTTACCAAGGGAAAAAAAAAAATGAAATAAGTTTTCAGGGGTGCAAAC
ACCGAGCGACTTCCCAGGAGGTCCACCCAATCTAGTACTAACTACACGCCCAAGCCAGAAGCGCGCCCTAACTGCGGAGTTCTG
ATGGGAATCCGGTGCAATCAGCAATATACAGCACCCGGAATGTTTTGGTCGCCAGCGAATATTCATTTATCAGCGAGCAAGCAG
TGTTCCTCCAATTGTTCCTCCCCCGGATTGACTAGGAACCTTCTTAATTCGCTGCTTGTTAGCTTGACCCACAGGTAGATTAAC
CCTCAAGCAAAACGAAATAAATGAGTTTTTAAAGAGTTAAAAACAAAAAATACAAACTTTTGAGCCGTAGGGTGATTTTTCAAA
AGGCCGAAAAAAAAAAAATGATGTTTATCAGGGGTGACAACACGAGGACTTCCCAGAGGTCACCCATCCTAGGTAATCAACTCTC
CGCCCAAGCAGCTAACTGCAGGAGTTCTGATGGAATCCGGTGGCATTAGTGCTGGTAATTGATTCCGCACCCGAGAATGCGTTT
GGTCCGCAATATTCATTTGTATAGCGAGCGCACGTGTGTCCTCCTTTTTCTCCCCCGGATTCTGACTAGGAACTTCTTTTCCGC
CTGCTTGTTAGTGACAAAACGGTATTACCTCAAAAAGACGAAATAAATGAGTTTATTATTATAAACAAAAAATAACTATTTAGC
CATGATGAATTTTTGGGGACAATGCATGTTTCAGGGAGTGCCCAGAACATTCACCAGGAGGATCACCCATACCTAGATACTACT
TCACACAAAAAGCCAGCATTGCTGGAGCACCAGGAGAGATTCACGGTGCAATTGTAAGCACTGGTAATAGATCGCACCCAGATT
ATTTTGTTCCGCCAATTTCATTTATACGGAGCCCGCTAGATTTTGATCTCCATATATATTTATACATACCGATTCGACCTCAGG
AACTTCTTAATTAACCAAGCTCCATTTATAACCACGGTATTAACAACACACACTCACGAAATAAGATATTTAAGGTTAAACACC
ATTTATTGAGCGTAGGTGGATTTTTTAAAGAGAAGAAAATTAGAAAATTTTTTCGGGATGGGCACCGGGCTCCCAGGGGGGGAT
TATCCCTTTCCTTCTCGCCCAAGCACAATATATATTATATCACTGCGAAAATTATACAATGAGGTCCAAATAACATATTTAGTG
CTGGTATAAAACGCAAGAAAGATTCGCAGATTCTTATAACCTATCGAGACGGAGTGGGTATTGTAACACTTTTATATATCCACC
GAGTGATGACACGTTCATTATTATACACAAGCAATAACAAGACCACAATAAAGCGCCCTCCAACGAAAACGAAATACATGCGTT
TTTAAGATTTAAAACAAATTTAACAAAAACAAACTTTTGAGCCGAGGTGATTTTATAAAAGGACGAGATCATGTTTTACAGGCG
CAACACAAGACATATTTACACCAGGAGGTACCCACGTCCTCAGTAACTACTCCTTCGCCCAAAAGCACGCTAAACTGCAAGCAA
TAATATAGATGTCCGGTAAATATACATATTATATGGATGCACCCGGAAATGTTTGGTCACAAATTTATATAAATTTACATAGCG
GAGCGCAAATAACACCTTATTTTTCATCCCGGATTGACTAAGGAAACTTCTTATTCGCTGCATTGTTAGTGACCACGGTATTGA
AACCCAAATCAAGAAAAGCAAATAAAGGTGAAGTTTTTATGAAAGATTTAAAACCAAAAAATGACAAACTTTTGAGCCGTAGT
GGCATTTTTCAAAGGGGAAAACAGAAAAAAATTAAGTGTTTTCAGGGGTGCACACGAGACTATTCCCAAGGAGAGATCACCCAT
CCTAGTACTACTCTCAGCCCAAAGACACACAAGCTTAAGCTTGCGAGAGTTCTGATGGGATCCGGTGACACTTAGTGCCTGGTA
ATGACCGCACCCGGCCAATGTTTGATCCGCAGATATTCATTTATAGCGGACGGCGCGTGAAATCCTCCCTTATATATTCTACCC

GTAGGATAATGACTAGGCAAAACTATCTTATTCGCTGCTGTGTAGTGCTAAACGGTCATTAACCCCTCAAACCACATTAATGAG
TTTTTAAAGATTTAAACAAAAAATAACAACCTGTTTGAGCGTAGGTGGCATTTGTAAGGGAAAAAAAAAATGATGTCTTATCAG
GGGTGAGCAACGACGAAGGACTTCCCAGGAGTACCCATCCTAGTACTACTCTCGCCAAGCACGCTTAACTCGCGGAGTTCTGAT
GGGATCCGGTGCAGCGCATTTAGTGCTGGTAATGATCGCAAACGGAATGTCTTGAGTCCGCACATATTCATTTAATACGACCGG
AAGTGTGGTCCTCCCCTTTTCTCGCCCCCGGATAGTGCAACTAGAGAACTTCCTTATTCCTGCTTGTTAGTGACCACGGTATTG
GAAACCCCTCAGGAAAGACGAAGATAAATGGTTTTTAAGAGATTTAAAACAAAAAATACAAAACTTTTTGAGCGTAGGCTGGAT
TTTTCAAAGGGAAAAAAAAATGATGTTTTCAGGGGTGCAACACGAGGACTTCCCAGGAGGTCACCCATCGCCTAGTACTACTCC
TCGCCCAAGACGGCTTAAGACTGCGGGAGTTCTGAATGGAACTGCCGGTGCATTAGTCCTGGTAAGTAAGATCGCAACCCCCGG
AATGTTTGGCTACCCGCCAAAATGATACAAATATATAAGCCGAGCGCGTGTGTAACTCCCTTTTCTCCCGGATTGCTAGGAGCT
TCTGTAAATGTCGCTGCTGTGGATTAAAGTGAACCGACACGGTAAGTTAACGCCTCAAGGAAAACGAAAAAATAAATGAGTTTT
TAAAGATTTAAAAACAAAAAAATACAAACTTTTGAGCCCCGCAGCGTTCGATTTAAAGGGAAAAAAAAATTATGTTTGTCGGGG
TGGCACACGAGAAGATTCCCCAGGAGTCACCCATGCTAGTACTATCTCAGCCCAAGCACGCTTAACCTGCGGAGTTCTGATGGG
ATCCGTGCAGAAATTAGTGGCGGTACCAATGATTCGCACCCCGGAATGTTGGTCCAGCAATATTCGCATTTAGGTCACACGAGC
GCGTGTGTCCTCCCTTTTCTCCCCCCAGCATTGACTACGGAACTTCATTTTTTTTTATCTTATTCCGCTGCTTGTTTAGTGAC
CACGGATATGTGAGACTCCAAGGAAAAAAAACGAAATAAATGAGTTTTTAAAGATTTAAAACAAAAAATAACAAACTCTTTGAG
CCCGTAGGTGGATTTTTAAGGGAAAACAAAAAATGATGGTTTTCAGGGGTGCACAACAGGACCTTCCCAGGAGTCACCCATCTA
TTATCTCAAAACCCAAGCACGCCTTAAAAACTGAGCCGGAAGTTCTGATGGGAAACCGGTGCATTAGTGCTGGTATGATCGCAC
CAAAAACCGGAATGTTTGGTCCGAAATAGTTCTGTTTATAAGCGAGCGCGTGGTCCGGTCCCTTTTCTCCCCGGGGATTGCTAG
CGAAAACATTCCTTATTCGCTGCTTGTTAGTGACACGCGTATTAACCCCTCAAGGAAAACGAAATAAATGATTTTTAAAGGCAT
TTAAAGAAACAAAAAATCACAACACTTTGAGCCGTAGGTGATTTTTAAAGGGAAAAAAAAATGATGTTTTCAGGGGTGCAACCA
CCCGAGGACCTTCCCAGGAGGTCAAAACCATCACTACGTACTACTCTCGGCCCAAGCCACTGCTTAACTGCGGAGGTTCATGGA
TGGGAATAACGGTGCATTAGGTGCTGTTACATCGCACCGGAATGTTAAGTCCGCAATATGTCATATTAATAAGCGGAGCGCGGT
GTTCCACTCCCTTTTCTCCCCGGATTGACTAAGAAACACTTCTTTATTCGCTGCTTGGTTAGTGACCCACGGTATTAACCCCTC
AACGGAAAACGAAATAAATGAGTTTTTAAAGATTCTAAGAAACAAAAGAATACAAACTTTTGGCCGTAGGTGGATTTTTAAAAG
GAAAAGAAAAAAAATGATATGATTTTCAGGGGTGCAAACACGAGACTTCAGGAGGGATCACCCATGAAGACTAAAAGTACGGTA
CTCTGCCAAGCACCGCTTAACTGCGGAGTGTCTGCGATGGGATCCGGTGCTTATGCCAGTCTGGTAATGCATCGCACCCGGAAT
GTTTGGTCCGCAATATTACATTTACTAGCGCGCCGCACGGTTCCTCCTTTTTCCGCCGATTGCTAGGAAACTTCTTATTCGCTG
CTAAGTGTAGGGTGAACCACGTATTAAACCCTCCAAGGAACGAAATAAAGAGTTGGTTTAAAGATTTAACAAAATACACATTTG
AGCCGTGGATATGGATTTTTAAAGGAAAAAAAAAAAATGAGGTTTTCAGGGGTGCAACAAACGAGGACATTCCAGGAGGTCAACC
CTCCTAGTATACTCTCGCCCCATCACACCGCTTAACTGCGAGGTTCTGATGGATCGGTGCATTAGGCTGCTGTATGATCGACAC
AAGGAAATGTTTGTGTCCCGCATATATGGCAATTTATAACGAGCGGTTGTGTCTACCCTTTTTCTCCCGGGATTGACGTTAGAA
CTTCTTAGTTCCCGGCTTCTGTTAGTGACCACGGTATTAACCCCTCAAAGGCAAACGAATAAATGAGTTTTTTTAAGATCCTT
AAAAAACACCAAAAATACAAACTTATTGCAGCCGTAGGAGGGTGGATTTTTACAAGAGAGGAAAAAAAAAAATGATGTTTTCGGG
GGTACAAAACACGAGGACAATTCCCAGGAGAGTCTCACCCAGTCTAGTACTACTCTCGCAAAGCACGCTTAAGACTGCGGAGGT
TCCTGATGGGTCCGGTGGCGATTAGCTGCTGGATATGATCGCACCGGATAGTTGTTGGTTCCGGCAATAAGAAATTCATATGTT
AGCAGCGCAGTTGTTCCTTTCTCCCGGATTCGACTAGAGAACTTCTCGTATTCCCTGCTTGTTTAGTGAACCCCACGTATTACA
CCCTGTCCAGAGGAAAACGAAATAGAGGGAGTTTTGAAAGCGGTTAAACAAAAATACCAAACTTTTGAGCCGTAGGGTGAAGTT
TTTAAAGGGGAAAAAAAAATGAGGTTTTTCAGGATCAACACGAGTGACTTCAGGAACACTCACCCGATCCTAGTACTACTTCGC
CCGAAGAGCACTCTTACTGCCGGAGTATCTGATGGATCGGTGCAAGGTTAGTGGGCTGGTACTGATCGCACCCGATGTTTAGTC
GCAATAATTCATTTATGAGAACAGCGCGTGTGTCTCCCTTACGTTCTCCCCCGGATTTGACCTAGGAACTCTTATTCGCTGGCT
AATGTATAGTGACCGCACAGCAGACGATATTAACCTAAGGAACAGACCACATAATGAGTTGTTTACCAAGATCTTAAAACAAAA
AGGTAAAACTTTTACACACAACATTATTTAAAGGGAAAAGAAAAGAAATGAAGTTTTTCCGGGTGCCGAACACGAGGATTCCAA
GAGGTCGACCCATGCCTAGCTGGTAAACTCCTCGCCAGGACGCTATAACTGCGGAGTTCTGAGGCTGGCGGATCGGATGCAATT
AGTGCTGCGTATGATCGCACCCGGAAATGATATTTCGTTCGCAGATATTCATTGATATACGCGAAGCCGCGAGCTGTGTTCCCT
TCCTCTTATCTCCCTGATTGACTGGAAGCTTCTTATTGCGCTGCTTGTTAGGTGACCACGGTATTAACCATGGCGAAGGGAAAA
CCGAAACAAGGGTAAATGAGCTTTTTAAAGATTTAACAAAATACAAACCTTTTTGCAGCCACAAGTAGGTGGATTTTAAAGAGG
AAAAAAAAAAATGATATTTCAAGGGTGGAACACGAGGACATTCCAGGAGGTACGACGCATCCCTGACGTCATACTCTTCGCCCA
ACAGGCGCGTTAAACTGGGAGTTCTGATGGAAATCCGGTGCAGATTAGTTGCTGTATGATGCAACGCCCGGGAATGTTTCCGTT
CCAGCATATTCATTTTATAGCGAGCGCAAGAGAAGGGTGATATGTCCTCCTATTTCTCCCCAGGATGACTAGGAACACTTCCGT
TATTCCTGCAATTGGTGTCTCAGTGACCACGTATTAATCCTCAAGAGGAAAAAAACGAAATAAAGTGCAGTTTATGTTAAAAGA
TAATAAAACAAAAAAATAACCGACATTTTTGAAGCCGTAGGTGATTTGTGAAAGGGAAAAAAAAAAAAAATAGATGTTTTAATCA
AGGGGGGTGCGAACACGAGACTTCCCAGAGTCACCGATCCTAAGTACTACTCTCGCCCAAGCACGCTTACTGCGGACGTAGACT
GATGCAGGAATCGGTGCTTTAGTGCCTGGTATGATCGCAACCCGGAATTTTGGGTCGCGAATATTAAATTTATAGAGGAGCCGC
TGTGTCCTCCTTATATTCCCCGGATGATAGACTTTGTCTATTCCGCTGCTTTGTTAGTGAAAACACGGTATTACCTCAAGGAA
AACGAAATAATAAGATAGACTTTTTAAAGATGTTGTTACGAAACAAAAAAATACAAGACTTCGTGTGAGCCGATAAAGTGGAT
TTGTGTTTAAAGGGAAAAAAAAAAGATGTCTTTATAGGGGTGCGAAACACGAGCCTTCCAGAGAGTGTACACCGATCCTAGTAC
CATATCTCGCCCAAGCAACTTAACTTGCGGAGAAGTTCTGATGGAACTGCCGGTGCATTAGCTGCTGGTATGATCGGCAACCGG
AATGCGTTTGTTCGCAATTTCATTTAAGAGCGAAGCCGAGTGTTGTCACATCCTTTCTCCCCGGAAATTGACTAGGACCTCTGT

TCGCTGTTGTTATAGGAGAGAGACTCGGGAGACCTAATGTATCGCTGAATGGTTAGAGACACGGTCAGCCGTCACGAAATATGG
AGTTTTTTAAAGATTTAAATAACAAAGATACAAACTTGGGCTGAAGCCGACATAAGTGGAAATTTTTTAAGGGAAAACAAAAAA
TAGTGATGTTATCTCAGGGGTGCAACACGAGACATTACACACACCAGGAGGTTCACATGCCTAGTACTACTCCTCGACCAAACA
CGCGTTAACATGCGAGTTTGCATGGAAATTCGGTGGCATTAGTGCTGATTACTTGATCCGCAACCCCGGAAGTGTATGGGTCCG
CAATATTATGATATATACGCGAGCGCTGTGGTGTCCTCACTTATCCATCCCCGGATTGACTTAGGAACTTCTTAATCGCTTGCT
TATGTTATGGACCACGGTATTTAAACCACATCAAGGAAAACGAAATAAAAATTGAGTTTTATTAAAGATTTAAAACAAAAAAAA
CCAAGACTTTGAGCCGTATGGATTTTTAAAGGGAAAAAAAAATAGATGTTTAATCAAAGGGGTGCACCACGAGACTTTCCGGAAG
GTCACCAGTACTAAGAAATACATGACTTTCGCCCAAGCCGCGCTTAACTAGCGGAGTTTGATGGGAATCGCCGGTGCATGAGTG
CTTATGAACGCACCCGAAAGTTTGTTCAACACGAATATTCCATGTTTAGCAGCCAGTGTGTCCCTATTTTCTCCCGGATGACTA
GGAACTTTCTTATTCGCTGCTTGTTAGATGACCGGAACCGGTATTAACCCCTCAAGGGAAAAGAAATAAGATGAGTAGTTTTAA
AGCACATTAAAACAAAAATCAAACTTGTTTGACGCCCGTAGGTTGATTTTTAAAAGGGAAAAAAAAAAA
>m141121_210323_42134_c100732402550000001823141405141506_s1_p0/60415/12404_19582
TCTACCCCACTGCAACTGGCGGTCTGGCGTGTAGCGGGTGGAGGTCTTCTGCGCCGCGATATAATATAGATGATTTCTCCGCTG
TGAGTACCTTCCCACACTTCCCCAGACATGAGAATTTTGTAGACCTCCGTTCTAGCACGTACCAAACTCGCATGAGGGCGCCGA
GGAACAATGAATTAATAAATTAGACCAAAAAGCACTGTGACTGGTCTTTGCTACAGGTATGAGCAAGCCTCTCAATAAATGGCG
GAAAGTTGGTGTTGAAATCCGGCACCTCCTAAGGCGAGCTGACGCCCTTAAATTCTGAAGCAAACTCACCCCGCTCGGATCTGC
ACACTATGTGGGGCTCCGCACGCCTAGAAAATCCTCAGCATTAGGGGGTACTCTATAAAGAACGTACAACACTCCGGTGGTGTG
CTGTGAGCCTCACGCTCCCGCACCCTATGCAGATACCGATGACTGTATAGGTCTATCTCTGGGGATCCACTTCCCTTGCTAATC
TAGAGTGGAAACTCTAATAACCAGCGTAGCTGGTGATATCATCTGTGAGTGCTCCCGTGCAACTCAGCTCCTTAGTGTGTATAG
TTTTTCACTTCACCTCTAATTCACGTCCTTCCTTAGAGAGCCTGCTATATAGTACGATCCTCCACTGCAAGGCCTTGGGTAGAC
CATATCAGGAGCGCGTTCCCTTATTCTCCTATATCGAACCAATTGGGCCTTCTGGACACACGAAAACCTTCATCTGCCTCATGG
AGGAGATGAGTGATAGTGAGATAAGGCTTTATGCACAAGCTTACCGGGGAGGAAGTCACTGGTACTGCTGGGGCGGTGTTAACA
GCATTAATCTTCCGTTATCTTTAAGAGAGACGTCTATATCGAACACAGCAAATACCATACGACCAGGGAATGAGTCACCTCTGC
CTGGTAGGACTATCCGGGGCCAACCCCAGACCATCGTCGTCCCGCAGCCTCCCGGGGGCATCCTGTTCGAGAAGAATGTCGACA
TCCGGGAGCAAGCTAGCCCATGGGACGCGGACATTGCCGACGTCTGTTGTAGGAGGACATGCGGGAGGGATATCAACCGCGCAC
GAGCTTCACGTCAAACAAGCGTAAGAAGAAACAGTGCATTTCCAGAGAGAAAGCGATTGCCTTCCGACTCGGAGCTCCCAAGGA
GAAATGGAAAGATTGCCTAGTTTGCACAAGACGTCCACTCCTAGACTAGAACGCGCCTGAGAGAGAGGAGCCTGCAATCTTCGC
CAGTGGTACCAAAGCGTAATCTGAGAGCGATGTCAATGGTAACTCCCCACTTATTTAGTCTTAACTTACGGCTCAACTGCAAAG
GTCACTGTGCGACATGGCTTGTAACCTATCTAAGACAAGTAGATGCACGGCGCGAGCCTATGAGCCGCCGGGCACACCGCGTTT
AGGGCTGATGAGAGGGGTGTGCCCCCATCTTACGGATGCCCGTCTAGTGTTTGTGGAGAGCGCGAGTTCTCAGGAGAAAAGCGG
TCCCGCACATATAATTGATATACAGACACTCGTCGCACCGTGTTGTTTCACAACGTATCGCGCAGATACGGTGCACATGAGGAG
ATCTATTAGCAATGAACCACACGAGACGAGTCTATCACCGAAACACTGTGAGTAAATCCACCCTTTTGCTTTAAGGGAATGAGT
TCCACGACCACCCGGGGCCTTTATCAACTGAAAGGGTAAATTATAGTACTCGACGGGCTACAATAAGGTATCTATTTTGGATTT
ATCTAGATCTCTTATCTGCCCGGGCTTGTCCCGTCTCGCAAGTCCATACTAATCGTAACTAGTACAGTCTTTCACTTGAGAATT
TTAGTTATTCGTATTCCCGTAGCCTTTACGCAAGAGAGGAATGTAAACGCGAACATGTCCTTAGAGCCTCAACACCCTTGAACA
GGACAAACCCCTCCCTTGCCCCAGAGGAAGGGCAGCGAGGACCGACGACTAGGAGGAGCGCGCTAAGATCAAATGGGATGCGCA
AGGCGACCGGCCCTCCGAGCAAGACCAGACAGCTGTCTGGGGTAAGCCGAGGGCGGGCCGCGACTTCAAATCTCCGGCGCTGAG
TGGGTCGCGGTGCGACTGCTGTTAACCCAAGCCCACTGGGAGAGGTCTGCGCCCAATGGGGTTCAAGTCCGACTTGACCCTGCG
AACCCAGCTCCCCTACCCCCCCTCTTTTCTCAATTCAAACCAGCCTAGAACGAGTTCATGCTGAGGAAAGATTCCCGAGGACCA
ATCACACCGTCCGCGCCATGTAAGCATCAGGACACGAGACATCTAAGCAGACTACGGCCCTGACCGAGGCCCTCCACCACCACA
CACACCCACAAGACTTGTCTCGCACAGTGTTGATCAATACGAAACTCCTGGAGAAGGGCGAGGCCAAAACCGAGCAGCACACTA
AACAACCACTTTCAACAATCCAAAACTCTCAAACCTTAAACTAATCCTTAAAATAGGATGCAAAACCAGAGAGATCAGGAGCGT
ATATGAACAAAGTTATAAGCGACCACGGTTTCCAGGTGATCCGGCCCCCTCTCTTCCTCGGGCGCGAAGGTGGAGGGCTGCTCA
GCTACCGCGGCGAGAGAGAGCAAGCTCAAGCTTAGGCCTTATACCTCTAGAGAGCACCTGAATCATCAATCTGTGCGTCCCCTG
TCCGGTCAAAGAAAATAACTCAACCGAAGAAGGAGAAAGTATCCCAGCTACGCAGGAGAGGAGAGAAGTGAGAGAGCCTAGAAG
TGACAGAAAAAGGACGCACTTCGCTTCTCTGTCCAGTCTTCTCAAGCCTTACACTTCTCCTTAAATACCCCTCCCTAGTAGTTC
TAACATACTCTCTCCATCCCGTATATGGGTCAAAAGGAGCAAGAGAATTTCTGCCTTTAGATAAACGAAGCAGCAACCCCGGCC
TTGGTTTTTCTGGTTTTTTATTTTTTCTCGGTTTTTTTTTACTGGGAGATTGGCCGAAGTGGAGGGACCCGATGCATACAAGGC
TTGAATGATCCGTCGCAGGCCCGACGTTCGCGGTAACGTCCCTGCTGAGTTCATGCGCTCCCCAAAGCCGATTGGATTCGGTAG
TCTGGCGATGTATGGGCTTCCGCCCCTAAGTTGGGCCAAGGCGTCGCCCCCATGGCTGCGGTACCTGCTCGACCAGTGTCGGGA
GTAACCATGTCCAACCTCCAGCTGAAGAAGGTATGCCACGATAGCAAAATTCCGGACGGGTCCTGTTTTCTGTTTTTCAGGTTC
TCTTCTCTCTGTTCCATCTCTTTTAGATATGTGTCTTTATCCGCGGTTAATGTTTCTTCCCTTTGCTTTTATTTCTGTTTATAT
GGCCTGGTGGCTGCCTTCTCCTTTATTATTTTCTTGTTTTTAACCCTCTTGGCCTAAATTCTAGGTCCGGCATTGCCTATCTCA
AAGGGTGCCGACTTGGTGGGCCGAGCTTGCCCCTGCAAGACTGGAGAGGCCAATCGCGGAGATGGAGGCCGGCCGTGACCAGGA
GGGAGGCCATCAGGAGAGAATCTCCAGGCAAGGAGGCCCGGCCAACCTCGCAACAACGAGTTGGCCCGCGGCCAAGAGGCAGAC
CGACCGCAACTGGAGGAAGAGCTGACTCGGACTAAGAAGGCAGCCGACGAAAAGGCGACTCTCCCTGGAGAAAGGCCCAGCGGC
ACAGTCTGTCCTGGATAAAGAGGAAAAAAGATCCGGAAGATAATAGAGAGAGAGCCGCGAGTTCCTTGGCTGGGGACTCTCTTC
TCCAGGAGAAGGCGCACTGAAGTCACTCGCTCCAGGCAGAAGAGTGGAGGCCGATAACCTGGTGGAGGTCACCTTTGACGAGGC

CCTAATTTACATGGCCTGGTGCAAAAGCAAGAACATGAAGCTCCCTGGTCTTCCCCGACCCTGACTCCAAAGCGTGCCGAGTAC
GAGGCCGCGAAGAGGGAGATGCGGATCTCCCCCGGGCCGGAATGCGCTCCCCTAGGCCTTGTCCCGCCTGCTCTTTTGTAATTT
TGCTTAGCTTCTTGTACTTAAATTTTTGACACTGTTGTTTCTTCTTCTTGCGGACAACGCTGTTTCTTCTTGTTTGGTTTGATA
AGAGATGATTTCGCTTTTCACGCATTTGTAAAGAGTTGTGTCGCGGGCCTACCCGATTGGCAGGGGTTTTCTTCCCGGCTGTAC
AGCGGCCGGGACTGCGGCCGCATCGACTAACCTTGGCCAGTTTGGGCGCCCTTCGGGACAGCTTTAATCCGGAGTGTATCTCTC
CGCTTGAATTTTAGGTCTGGTGGGTTTGGATCTGGGGCTGACCGGAGGCCTCTTTGCTTCGAAGATCAAAGTGTCCAGGTCCGG
CGCTTGCGCTCGGTTCCCTTTCTATATATACTCCGGACATACCTTACGTCGGCGGTTTGGACCGGCCTTGGGCCTCGGTCCCAC
TTCCCTTTATACCCCCGGACGTCGTTCGTGCCCGGGGGTGGGACGGCCCTGGGCTCGGTCCTCCTTCCTTATACCCCCGGATGT
CGTTCGTCGCGGCGGTGGGACCGCCTTGGGGCTCGGTTCCTCTTTATTAGTTTTTTACCTCCGGAAAAAACATCGTCGTCCGGC
GGTGGGACCAGCCTTGGGTCGGGGGTTTCCTTTATTCTTTTTTTCACCCCCCGGACATAGTTTCGTCCGAGGGGGACCGGCCCT
TACTCGGTCCTATTTTAGTTTACGCGCCGACGTCGTTCGTCGGCGGTCGGAACCGGCCCTGGGCTCGGTCCTCTTTTATTTATA
CTCGGCCGGACATCGTTCGTCCGGGTGAGGACCGGCCTTAGGCTCGTCCACCTTTATTTTACCGCCCAGGACGTCGTTCGTCCG
GTGGTGGGACCGGCCTGGGCTCGGTCCACTTTTTCCTTATGACCCCCCGGACATCGTTCGTCGCGGGGTGGGACCGGCCTTGGG
CTAAAGTCCTTTATTTTTAATACTCGGCGGACCATCGTTGCATCCGGTGGGACCGCCTGCGGCCTCGTCCACTTTTATTTTACC
CCGCCGGAGCGTCGTTTCGTCGCGGGGTGGGACCCGGCCTGGGCTCGGTCACTTTTATTGCTTATACCCCGGCCATCGTTCGTC
CGGGGTGGGACGGCCCTTGGGCCTCGGTCGGCTCCCTTATTTTTATACTCCCGGACATCGTTCGTGCCCGGGTGGACCGCCTGG
CTGCGGTCCACTTTTATTTTAACCCCCCGGACCGTCGTTCGTCCGGGGTGGGACCGCCTTAAAACTCGGTCCTAATTTTATTTA
TACTCCCGGACTCGTTCGTCCGGGTGGACCGCCTTGCTCGGTCCACTTTTATTTTACCCCCGGACGTCGTTCCGTCCCGGGGTG
GGACCGGCCTTGGGCTCGTCCGTCTTATTTTTATACTTACGCGGCACCCATCGTTCGTCCGGGGTGGGAGCGGCCCTGGGCTCG
TGACCACCTTTTATTTACGCCCCCGGACGTCGTTCATCCGGGGACAGCTTGGGCTCGGTCCTCGTTTTTTTATACTCCCGGACA
TCGTTCGTCCGGGGTGGGACCGGCTTGGGCTCGGTCCACCTTTTAATTTTACCCCCGGGACGTCGTTCGTCCGGGGTGGGAACT
GCCGGGGCTCGGTCACTTTTATTTTACACCCCGGAGCGTCGTTCGTGGGGTGCGGACCGGCTTCGGTCCACTCTTATCTTTACC
CCCGGACTCGTTCGTCCGGGGGGGACGGCCTGGGCTCGTCCACTTTTATTTTTATACCCCCGGAGCAATCGTTCGTGCCGGGGT
GGGACCGGACCTTGGGCTCGGTCCTCTTATTTTATACTCCCGGACATCGTTCGTCCGGGGGGGGGATGGACCGGCCTGAGGCC
CTCGGTCCACTTTTATTTACCCCCGGACGTCCGTTCGTCGGGGTGGGACCGGCCTTAAACTCTGTCCTAATTTTATTTATACTC
CCGGCACATCGTTCGTCGCGGGGTGGGACCGGTCCTTGGCTCGGTCGCACTTTTATTTTACCCCCGGACGTCGTTCGTTGGGGG
TGGGACCGGCTTGGCTCGGTTCCTCCTTATTTTTATAGCTCCCGGACCATTGTTCGTCGCGGCGGTGGGACCGCCCTGGCTCCG
GTCCACTTTTATTTTGACCCGCGGACCGCGTTCGTCCGGGTGGGACGGCGACCTTGCCCCAGGGCTCGGTCGCTCCTTATTTTT
ATACTCCGGACATCGTTCGTCCGGGGTGGACTGGCCTGTGGCTCCGGTCCCACTTTTAATTTACGGCCCCCGGAGCGTCCGTTC
GTCTGGCGTGGGGAAACGGCCTGGGCTCCGGTCCACTTTTATTTACCCCGGACGTCGTTTGTCGCGGGGTGGACCGCGCCTGG
GCTCCGGTCCACTTTTTTTATAGCCCCGCGGACCATCTTTCGTCGGGGTGGACCGGCCTGGGCTCGGTCCCTCTTATTTTTAT
ATGTTTTGTGCCTGGGATGAACCCCGCAACGTGGCAGACCTGGTCTGGGGTCCACTTGAGAAAAAGTAGTTAAGAAAGGAATTC
GGCCCCTGGGCACACACTTGCACATGGAAATAAAAGCCTCCCAATATGACATTTTGCAATACGTCCATTTTTCATTGTTTTGAA
AAGGGAAATAACCTGAGGCAATACAAAGGGGGGGGGGGGGGGCTTAGTGTAAACGCCCACTAACTGGGATGAGCCCTGGGGCTA
CTGGTAGTATCTTACGGAGGTGTTCTGCGTTCCAGGCTCGCGGAACCTCAGGGCCATCGAGGCGCTCAAGCGGTATGTTCCGGG
GCAGACCCTCTTGCCTGAATCTCAATAATGGTCCTTCCCAAATTCCGGGGCGCCAGAACCCCTACTCCGGGATCCTGAGTGGCA
GCGGAAGACCCTTCGGCAGGACAGATCGCGCGGTGTCCAACTTACGGCCTTGACTCTTGAGTTGAAATTGTCGAGCGATCTTCG
CCTTGGTAGCATCTTCAATGCACTTGCGACTCTTCCCGGATCTCTTCGATAAGGTTCAGAGATTCCTTGGAGTACAGGCGTGTT
GCTGGACGGGATCATACGTGTCGCGGCGATGGACGGGATGGTGTTTCAATTGGGATGACGCTTCGCAGCCCATACGCCATTGAG
TATGGGTATGCCGGTAGATGTTCGGTCGGCTCGTCCGG
>m141121_210323_42134_c100732402550000001823141405141506_s1_p0/64635/7028_16525
GGTATTCTTTTACGAAGTTGTGAACTGTATTTTCCAGGGTTTATCCGCATCCATGATAGCACATCTAAGCATAAATTGATGGGA
ATTTAGGGGGAATGCAGGCCAAGGTTAGAGGAGCAACCCTAAGTCACTAGGGATTTAACTTGCACAGCAGAATATAGTTTTCCT
ATCCCACATAGAAGACTCACTAAAGAGCATTGGGCTTATCAAGTTTTGTGCCTCCACTTAGACGCGCTTCCTGCTATAATCCCT
TTTAGTATTGTACCTTGATACAAGAAATCAAGATTTGCATCGAACGCATATTTCAGAGATCACAAATCAGAATACTTTCTGAAA
GGGGTACCCACTTTGGTGAATGTGAAGATTCTCACTAAATATTTCCCGAAAATGTAGGATGTACCGCTGCCAGGATGTTCAACT
CGTGTCGAGGAAAAGGAGATTCTATGTCATTCCACAAAGAAGATCTAACGGGTAATCAAAGTTAAGATGAATATGATTGAGGGA
GTCGAAACGAGCAGCCTTAATTCAGACATTTCAAAAATATAACTGCGCCCTTGTTAGGGCCAATTTTCAGGGCCTAAGTAGTTT
CTACACTTATATGTCATCACTAATTACTAATCTCATAAGTTTTTATTCATTAGCCGACCGTTAGGTCAGGATGAAATTTGCAGC
ACATTCTGAAAAAGTAAAAATAGCCGTAATCGCATAAAAGCACAAAAATCTCTATACATTAAAGAACCTAAAGGGAGCATTTTT
AGCGGAGAGAGCTAAAAAGCGAGTTTTTGTGCGCCAAGACCTCGGCAATAATACGGGTATAGTATTGTTTTCATCGTATCAAAA
GATTGCTCCGAATTACACCTTTACGCAATGTGTAGATAACCGACTAATATTATTTCTGATATAGGGTTAGCCTCCTTTTGATGA
ATACGCCATCAATTTCTCCGGAGATGTTTGAAACACATTTAACGAAGCTCTCCAATTAAAACATACAATTCTCATAGGTATATC
TATAAGCTCGTTTTCGAGGTATTAATTTTGTCCACACCTATAGAGAGTCTGGATATTCGGACGGGTAATATTGACATATGTAAA
TTATTTTGTATAGGAGTCGAAACCCGGAGCCTTAAAGTTCACTAAAATTTCAAAAAATAACAGATCCCCGTTTTTGATGCAAGT
TTCTATCGTAAGTTTATGTTTCTCCCTTAATTTGTGTTAATCGTTATTGACTATTACTACTTCACAACTAATTTGAATTGAGAA
CCGGTATCACCTCTCCACAACCCCTAAATAATACTTGAAGATATAGCACCTTCCCACCGATAATACGAACTAACCAAACTATTT
TATCAAAATAATGAACTCCACTAAATAGACAAGTACTAGTAGGAACACTGATGCGCGAACACTACCTAATATGCCTTTATATAT

GCAAACCTGGAGACAATCTGCTATACCACGAGACATAAAGTTCTTTACGATAATGAATAATCGACGTGATCACTACACCGGATA
TTCAAGTATAAAGTATTGAGTTGTGCATATCCAGATACTGACTCATATAGCACAAATCCTCATCATTGCCAGATAGATTACTTT
CAAGGAAACTAGTAAAATAATATCGTATATAGGAGGAGTTACACCTTGTTGGTGTAATTGGATGATTATCTATCAATTCTACAC
ACTCAGAAAAATGAGACTTGATGTTTATCTCCTGCAAAAGAGAGTTCAACCTACGTTATCGATCCAACCTATTAGAGGCTCTAA
CCCAAGACACATGAGTCCTTTCAACAATCACGGGTCAAGGAATTATTGAGATATATTAGTGCGGAGGTTGGATCACTGAATAAC
AGAATTGAGGGATGAGGCGCGTATAATATATCAACACTACTTCAAAAAATTAGAATCCTCTCCTAATAGTATATAATTGATCTC
GGAGCTACATTTTTTCGTGTAATGTGATTATTCACTCACTTATACTTATCCGCGTTATATCTGGATACTCTTTTGCTAGTTACA
ATAATGTTATGAAACCGGTAATCAAGTGAGCCCCATAAATAGTTTGAATTAACTTCCTCATCTCCTCATGGATAAGTCACTGAA
AGTTTTATATTTGCATTTATGGCTAGTATTTCCCATAACATATTTACAATACGGGCTATCCAGTTTATGGAGTATGCCATGACC
CTCCCTTAAAAATCTATTCCAATATAGTTATTCACTTTATCAACCAATCCGAAAGATTGTCGAAGTCCATAATTACTAAACTAA
GTAAAATCGAAATGATATTTCATACTGAAAATAGAGGAGGAGAGAGAGAGAGTCTAGCTCCCCACATTATATCGAATAGATTAA
GAGGATGATATCATCTTATATATATTACATCCGAAATATATATTGAGGCCCCCTCATGCCGGATGTGTTTTACAAATACTCCCC
GTTCGACTTTAAACCTTTACAACCTTCATATTAGCGTCCATCCCATTCTATATTCTGTGTGCAATCCTCCAGAGAGTCTCACAC
CAAACCGGTGCTTTCGCAGATAGTTTGATATATAGAACTCATTGAGCTAATTTCAATATTCAATTATCAAAATAAAGAAAAGCT
GGAAAAGCACACTCAACGTATCTGCTTAATTAAACAAATAAAAAAATATAATATTTATGAGGGCAATTTTCTCTGCAAAGTTAT
TCTCACCCTTTAATATATTTGCGATAACATCTCTCCAGTATTTTTTGACCGGTTAGTTTTACATAAAATTACAGTATTAAAAAT
AACCAATAAATACTAACCCTTTAATTAATTTCTCTTTTCCGGACAGTAACTTTAAACCCTTTAATTATTTTTCCACAATCATCA
AATTATGAGATACCCCACTCAGTATTAAAACCGCGTCTAAGCTATCCGAAGAAGATTGTTTATACATTAGTATTCAAGAGGAAA
CATCAAGGGCGGTAATCAATGAGGTAGTTCCTCAAGTTTGTGCCCAAAATGAACTCCCCGCATATTCTTTATATTCACTATCAA
AAGATTTGCTCCCGAGTTCTCTTTTAAAAATAATTCCATATGCCTTTGCTATTCTTATAGTAATATCCGATATAATAGAGAGAA
ACTCGGATCTTTTAATATATTTTTTCTTCGAAAGGTGTACATGGCTTAAATACCTTTGGTGAACTGTGGAAGAATAATTCCACC
ATAAAATATATTGAATCCCGAAATGATAAATTAAAGAACCGTCTTCTTGGCGAGATTTATCAATCAACCTGCTCACACCTGTTG
TACGAAAGGATGAACAATGGTAAACAACATTTTTGTCTACATACCGCAACCGTATTTATTTTTAACAACCACAAGAGGGAGATG
AAAGACGCATAGAATTATACATATTTATATAACACCACCAACTGAAGCTCGACAAAACCGCGCGCAAGCCTTATCTGTTATTCC
AAAACCATTTCATGAAAAATTGAAATAACATTCCCCGCTTTTTGATTGCATGCAATTATTCCCGCACTTTATAATGTATCCTTT
TCAAATATTATTGCGTTACCAAATTACTGCTCCCCAGATATTTTGACTGTATCAACCGCTATCAGCCACCCTCATCAGTGATTT
TACACGTATAATTGATTACCATAAGAGCATATCAGATATCCTAGCATAGAGCCATATTGGGTATACCCCCTAAAAGGTACAAGG
ATTATTGTGCCACCATATTGACCCATTCCGCATAATTATCTTTTAAGTTGACTTTATGCACTTGAAACTCAAAAGAATAAATAT
ATTTTGTCACGAATATGTCCCGATTTAAAAATTCACTCACCAGGTGATGAATAGAGTCCGACCAATTTTCTGAAGACACGAGCG
CGAGTACGAGCCCTTTGGTGGATATAGTATAGAATTAGTGCAACAAAATACTCCACCGATTAATGTATTACACTCACTCCCACC
TCGTGGTTCAAGAATCGTTCGACTAAGAGAGTTCGTTATTTGATTATTCCGCCCCCAAAGAGAAAGTGCTCGTTAAGAATTGTG
GTTTTTACTTAGCTTGGCCTGAGGGGTAGGAAGTAGACCAGAATTTATGATTGTTATATTATGAATGTAAGACTTTGTGGGAAT
GAAAGTTATGAATTATGAATGCCATAATGTGATGAATTTGGCTTGTATGAATATTGTATGATATGAATGGATGTTAGCGTGATG
AGCGGCGACACATCAAAATTGTTGCTTAAGGATAAAGAAGACGAACCCGAGTGTACTAAGGTACTATGAAGACGTAACCCCATG
GCGGCAACGCGGCCGAGGTTAATTCCAAGGACCAGAGAACATCTTATTACCTCAAAAGGGTGCATGGACACGAGTTACGGGCCA
ATGTTCAAACCAAGGAAATTGTTATAAAGTGTTATGATGTTGGTGATGTTATATTAGATGATATGTCATGATTTGTTATGATGT
TATGAATGTTTATGATATGACGTGGCCATATTTAAATGTTTATGATATTGATCCAATTATATGATGATGCTATGATGTTAAAGA
TGAGTGATCATGTGTAAATTGTGTGTCTACTTTTTGTTGTTTGTACTTCCTTAACTGGGCCTTTTAGCTTGTAAGTGCACCATT
TTTTTTTTAGTTTATAAAAATTTTGGATTTATTCCTTACTATTTGAGTAAGTGTTAAGTGCGATGAATTATTTCAATGTAAATG
AAATTGATTAGTTTTTATTGCACGGTCCCCGAAACCGGAGCCTTAATTCAACATTTCAAAAAATCTAATCCCTTTTTGTGGCAA
TTTTGCGCAAGTTCTTCTCGCTTATATTCGTACATCTTCGAGTATTTTGACTGTATCACCCCTAATACACACCTCGGATTTGCA
CGAAAGTTTGATACATAGAACCCTAAGCACATTGGGCTACGCAAGTTTGTGCCCATGCCTCGCATATTCTTTAGTCATTCAGCT
ATCAAAGATTGCTCGAATCCATATTAGAGACAATCCCGAATATTTCGAAGGGGGTAGCCCTTTGGTGATTGGAGATTTCACCTA
ATTTCTCCGAAATGTATTACCCTCGTGTTCAACTCGTTCGATAATTTTTCGCCCAAACGAGATTCTCACGGGTAACGATGAATA
TATTGGAGTCGAAACGGAGCTTAATCTCAACATTTCAAAATAAAATCACCCTTCTTTGAGGGCAAATTTTTCGCAATTTCTCCC
CCTTAATATTCGTACATCTCTCAGTATTTTGCCCGGTATCAGCCGCCCCCCCGCGCGCCCCGCCCCGCCCGAACCTAATACCAC
CACCTCGATAAGCACGAAAGTTTATACCATAGAACCCTAAAGACATTGGGCCTAGCAAGTTTTGTGCCCATGACCCTCGCAATA
TTCTTTTAGTAATTTCACTATCAAAAGATTGCTCGAGATCGCATATTAGAGAAATCGATAATTTCTGAAAGGGGTACCCCTTTG
GGAATTGGAGATTTCACTATTTCTCCGAAATGTAATTACCCTCGTGTTCAACTCCGTTCGACTATTTTTCCCCCCAAGAGATCC
ACGGGTAATGAATATATTGAGTCGAAACGGAGCCTTATTCAACATTTCAAAAAAAATAAATCCCTTTTTATGATGGCAATTGTT
CGTAAGTTTTTCCTTTTATATTCGTACATTCTCCTCCAGTATTTTGACGGTGATCACGCCATAATACACACCCTCGATAAGCAC
CGAAAGTTTAACATAGAAACCTAAGACATAGGGTACAAGTTTTTGTGCCCATGAACCCTCGCATATTCTTTAGTATTCACTTCA
AAAAGATTGCTCGAATCATATTAGAAGAATCGAATTTTTTTCTGAAAGGGGGTACCCCCTTTGGTGAATTGGAGATTTTTCACT
AAAATTTCTCCCGAAATGTATTACCTCCGTGTTTCAACTCCGTTCGATTTTTTCCCCAGAGATCTCACGGGGTAAATGAATACT
ATTGGAGTCGAAACAAAAGGAGCCTTAATTCACATTTGTCAAAAATAAATCCCTTTTTGATGGCAATTTTTCGTAGTTTTCTAC
CAATTATATTCGTACATGTCTCAGTATTTTTGCGGTATCACCCTATACACACCTCGGATAAGCACGAAAGTTTTATACATAGAA
CCCTAAAGACATTAGGGCTAAAACAAGTTTTTGTGCCCCAATCCCTCGCATATTCTTTAGTATTCACTATCCAAAGATTGCAGA
ATCCATATTAGAAATCCGAATATTTCCTGAAAGGGGTACCCCTTTGGTGAATTGGAGATTTCACTAATTTCTCCCGAAATTGAT

ACCCTCCCGTGTTCAAAACTCGTTCGAATATTTTCCCCAAGAATCTCCACGGTAATGAATATATTGGAGTCGAAAAAACGGAGC
CTTTTAATTCAAACATTTCAAAAAATAAATGCCCCTTTTTGAAGGGCAATGTTTCGCAGTTTTATCCTCCTTATAGTTGTACAT
GCTCTCAGTATTTTGACGGTATACGACCCTATACCCCGCGTCGGTATAAGCACGAAAGTTTATAACATAGAACCCTAAGACATA
GGGCTACAAGTTTTGTGCCATTGACCCTCGCATATTCTTTGTAATTCACTATCAACAAGATTGCTCGAATCCATATTAGCAGAA
ATCGAATTTTCTGAAAGGGGTACCCCTTGGTGAATTGGAGATTCAGCTAATTTCCTCCCGAAATGTATTAACCCTCGTGTTCAA
CTCCGTTTCGACCCTATTTTTTCCCAAGATATCTCACGGGTGTAATGAATATATTGGAGTCGAAACGGAGCTTAATTCAACATT
TCAAAAATTAAATCCTTTTTGATGGCAATTTTCGCAAGTTCTTTCCTTATATTCGTACATGCTCCCTCCAGTATTTTGACTGTA
TCACCCCTATACACCGTCGGATTTGCACGAAAGTTTATACATAGAACCCTAAAGCATTGGGGGCTACAGTTTTGTGCCCATGAC
CTCGCTATTCTTAGTTATTCATATCAAAAGTTGCTTCGAATCCATCATTAGAGGAAATCGAATATTTCCTGAAAGGGGACCCTT
GTGAATTGAGATTTTCACTATTTTCTCCGAAATGGTTTACCCTCGTGTTCAAATCGTTTCGATTATTTTTTCTCCAAGAGATCT
CCGACGGTTAAATGAAGTATATTGGAGTCGAAACGGAGCCTAATTAACATTTCAAGTTCAAAATAATCGCCCCCTTGTTTGATG
GCCAATTTTTCGCAAAGTTTCTTTCCTTATATTCGTACATAATCTCAGTATTTTGATGTACTCAAACCCTATACACACCTCCGG
ATTTGCACGAAAGTTTATACATTAAGAACCCTAAAGACATTGGGTCTACAAGTTTTTGTGGCCCATGACCCGTCCGCATTATTC
CTTTAGTATTCACTATCAAAAGATTGCCGAATCCCATATTAGAGGAAAATCGATATAATTTCTGAAAGGGGTACCCTTTGGTGA
TTGAGATGTTCTAATTTCTCGCGAAATGTATTACCCTCGTGTTCAACTCGTTCGATAAATTCTTTTCCCAAGAGATCTCCACGG
GTAAATGAATATATTGAGTCGAAACGGAGCCTTAATTCAACATTTCAAAAATAATCCTTTTTTGATGGCAATTTTCGTAAGTTT
TCTACTTTATTCGTACATGGCTCTCATCTCTCTCAGTTTTTGACGGTATCACCCTATACACACCTCTGATAGCAAACGAAAGTT
TATACATAGAACCCCTAGAGACATAGGGCCCTACAAGTTTTGTGCCCATGCCCTCGCATGATTCTTTTAGTATTCACTATCAAG
AAGATTGCTCGAATCCATATTAGAGAAATGAATATTTCTGAAAGGGGTAGCCCCTTTGGTGAATTGGAGATTTTCACTAATTTC
TCCGAATGTATTACCCTGTGTTCAACTCCGTTCGATTTATTTTTCCCAAGAGATCTCCACCCGGGTAAATGAATATATTTGGAG
TCGAAACGGAGCCTTAATTTTCAAACTTTCCAAAAATAAATCCCCTTTTGATGCAATTTTTGCAAGTTTTCTCCTTTATAATTC
GTCATCTCTCAGTATTTTGAGGTATCCACCTCTATAGACCACCTCGGATAAGCACGAAAGTTTTATACATAGAACCCTAAGAAA
TAGGCCTAACAATTTTGCGCGCATGACCTCGCATCATTCTTTTAGTATTCACTATCAAAAGATTGTCCGATCCATATTAGAGAA
TCGACCATATGTTCTGAAAGGGTACCCCCTTTGTGAAATTGGGAAGAATTTCACCTAATTTCTCGCGAAATGTATTACCCTCG
TGTTCAACTCCGTTCGATATTTTTCCCAAGAGATATCCACGGGTAATGAATAATTATTTGGAGTCGAAACGGAGCCTTAATTTC
AAACATGTTCAAAAATCAAATCCCCTTTTTGTGGCAAATTTTCCGCAAGTTCTTTCCTTATATTTCGTACATCTCCAGTTTTTG
AATGTATCACCCCTGATAGCACAACTCGGATTTGCACGAAAGTTTTTACATAGAACCCTAAGACATGGGGCTACAAGTTTTGTG
CCCATGACCCTCGCATATTCTTTAGTAATTCCACTATCAAAACATTGCTCGAATCCATATTAGAGAAATCGAATATTTCCTGAA
AGGGGTACCCTTTGGTGAATTGGAGATTTCACTAATTTCTCCGAAATGTATTTACGCCTCCGTGTTCAACTCGTTCGACGTTTT
TCCCCCAAGAGATCTCACGGGTAAATGAATAGTATTGAGTCGAAACGGAGCCTTAATTCAACATTTCAAAAATAAATCCCCTTT
TCTGATGGCATTTTCGCAAGTTTTCTCTTTATTCGTACATCTCTCGTATTTTGACGGTATCACCCTACTACACACTCGGATGAA
AGCACAGAAGTTTATACATAGAACCCTAAGACAATAGGGCTAGCAAGTTTTGTGGCCCATTGACCTCCGCATTATTAACTTTAG
TATTACTTCAAAAGATTGTTCGAATTCCATATTAGGAATCGAATATTTCTTGAAAGGGGTACCCTTTGGTGAATTTGGAGATTT
TCACTAATTTCTCCGAAATGTATACCTCGTGGGGGTTCAAGTCGTTCGTATTTTTTCCCAAGTATCTCACGGGTGAATGAATAT
ATTGGAGTCGAAACGGAGCCTTAATTTAACATTTCAAAATAAATCCCCCTTTTTGATTGGCAATTTCGTAAGTTTCTCACTT
ATATTCGTACATCTCTCAAGCTATTTTGACGGTAATCACTAATACACACCTCAGGATAAGCCACGAAAGTTTATACAAAACCCT
AAGACATAGGCTACAAGTTTTGTGGCCCATTGACCCTCCATAATTCTTTAGTTATTCCACTATCAAAAAAGATTGCTCGAATAA
CATATTGAGAAATCGAATATTTCTGAAAGGGGTAACCCTTTGGTGAAATTGGAGATTTCACTAATTTCTCCGAAATGTATTAGC
CCTCG
>m141121_210323_42134_c100732402550000001823141405141506_s1_p0/67577/0_7633
AAAAGAGAGAGATTCATGGACCAGTACTTGGCTTATAATCATGTGGAAATGTGGGTCATGATGGCATGGCCATTATTGTAGTG
TAGTACACGCTCCTCTGGCATAGGCGTAAGTGTTGTGCTCTGTTACGAACCTCCCACTCAAATTAAGTCGTGTTTAATTGTTTA
ACTTATGTGGATTTATGATTTATGAAAGTAATGTGAATTGCTATATATGCATGTTGGATTATTATTTAATGCCTTGTTCCTAGA
TCATTAGCTCATGGGTGCTCTATGTTCGAAGGTAAGGGCAAATGGAAGGTAGATCAAGCCATGATTTGGGAGCTTCGCAGCGAT
GCATACATCGTATCGGACCCTGCTTAACTTACCGATTGAGTTATCTGATGGACTTGACGCATCAACAAGTCTCCTCCAGTCACC
TAGGTAGACCCACTATTTTGGAACAATGATTGTAAGACATATTTTGTTTATCTAAAAGGGAATCTCTATGTAAATTTTTAAGAC
ATAGTGAAGTTGTTTACACCCTTTTTAATCAAATTTTAATGATTTTAACTTTCCTGTTTATACTTTAAATCCACACGCTTTGAG
TCGAAACCTCTTGCTTAACAAAGATAGCAATTATTATTAACGCACAAGTTGATGGTCCTGTGATAATAGGAACTCACATGGAGG
AGCTAGACAGAACAATTAGAGTGAGAAGTTTGAAGGAGTGATTAAATCCAAAAATTCTAAAAAAAAAATAAACGCCTTAATAAG
GTTAAGTCATTGACTTTGGGTCCAACCCAATAGGTTACAACCATTTATTAAAAACATTTTGGAGGCAAAAAAACATAATTTGTA
ATGATGTCAGCAAGCAGGCCTGTTGTATTCTAGATGATTCCTTGTTTGCATGCGCACTATGGTGGCAATGCTCGCTTGAGCCCT
TTCCAACTTATCCAAAGATGACCTTAAACAACTCCAAATGTTCCCAAACTTTTTGAGATTCATTATATACTAATTTTATCACTT
TAGACTTAAAGGACAATTTTTGAATGCATGCACCAAAAATTAAATTTCACATTACATTTGTGAAAAATGTTAAGTGTTTTATGC
ACCGTTGTGAAAATACACTTGACCTTATATTTAACCAATCAGTAACATTAATCCCAAGTTACCTTAATCACATGTTGCAAATAA
CCAATTTTTATTCTATGGTGGCTTCAAAAACAGCAGAAGACAAATATTTTGGTCATTTGTAATTCTGGATTGGCACGCCCATTC
AAAGGGCCTGGAACCAAATGCAAGCCAATAATTATCATGACACTCTTTCCTTGAGTGCTACAATGGGGATACATTTGCTTTTCT
AAAAGTTGCACAAGATGTAAGAGGTAAGATCTTTCCCACTTGCTTTATGTGTTGCCAATTCTAATAATGATTCTTCCTAGGATG
GTTTTTTACACAATTCAGGCAGGCTTATGGAATAAGAGAAAAACATGTGTATCTTTCTTAGATTGCACAAAGCATCATCACGAC

TACACAAAATATATACCCCAGAAATCACTCTAGATTATTGCATGTTTCATTTGTTAAGCACCTTGAAATCATCTAAAAGAACAT
GCAAGAACATACAATGGAACCATTCTTTAGGTAGCCAACAGCATACCAACAGAGGAAAATTTTGAATATTTATATGAGAGAACT
TGAAATTTTGGATAAGCGTATTTGACCTTATTTGGAAATAAAAAAATTTGGGCATCATAGATGTTTCAAGGTTCCACTGCCGAA
AAAAAATAGATATTCAACATGACATCAAACATTACTAATCATTAAACTCAGCAAATTTGGCAGCTAGAGAGCTACCAATAACAA
CACTGTCCCTGGAATGCTTGTTTGGATTTACTACAAGATTGGACCTGGAAAATAATGAGAATGAACAAAATACCTTCCCAAAAT
TGACATCCAAGCTACCAAAGAGAAACCTGACAAACATTACAAATATACTCGCTGGAGGCTTATTTGTAAGTTTATTTACTTTTT
TTATTATTTTAATTTTGTTTAACCTACTAATGTGTGATGCTCTCGTAATGTTGAGAACGTTTAATAAAGTTGTTTTTGGTTGCT
CGTGATAAGGGACATTTTTTAAATTAATATAACATACATTTATATTTTAAAAATTACAGGTACGGCATCAAATGAATATTTTTT
CAGTTGTTTAAAGAGGAAAGCCAATGTTGTTAACCTTAAAGATTGACATACCCATCAACAAAAAATTTCAAATGAATGAGATTT
TTATGCTGGTCATGACTCTTTGCAATGGTAAAGAGTGAAACCTAACATCCCTACAATACTATATCATATTACTACACAATAAAC
CATGATTAATAAACATATGAAGCACGGTATACCAATATTTACCAATGCACCTACATGCTTTTGTGAAAAGCCATCATGGTATTG
CTCCAGATGCTAGGTGAAAGCAGGGGAACCAAGAAATAAGATGGAGAGCTGTAGTAGTAGCTCTAGAATTTTAGTTTCGGTGGG
ATATTGGCCAAGCCGGGATTTAGTTGGGAAACTTGTAGAAATAGTTATGGGATTTATAAGTTTTACCTATGGTTAGAAATTTAA
TTTTAACATAAGGTTTGGATTAAATAGTTTGGTCCCCTAGAAATATTATTTATTATTCTAAGGTTTAGATAGATTAAATAAGGA
TGTGACACTTTGTCCCAAGATGATTTTATTGGATTTAAGTTTTTTGGATGGAAGGTTTATTAAAAGAAAAGATCTACAACTTAG
AACTTCCATCATTTGTTTAGGATCACATTAATACTTCAGTCAAAGTGTTTTAATCATTTCAAATTATGTTGAATCTGTGCAAAA
ACATGTTTAAAATATCACCGTATGCCGATATATCACAGCTTATACGGGTCCGATATGTCCGCCATATGAAATGGGTATTAGTTA
GTGTAACAGTAGCAGCAGTAGCAGTAGCAGCTGCAGCAGTAGCAGCAGTAGCAGTACAGTAGCAGTAGTAAGTATAGTGCAGTG
CAGTCAGGAAGTAGCAGTAGGGAGTAAGAAGTAGTAGCAAGTAGCAGTAGCAGTAGCAGTAGGTAAGCGTACAGTGCATTTAGC
GATGCAGTAGCACGTAGAGTAGCAGTAGCAGTAGCAGTAGCAGTAGCAGTAGCAGTCAGCAGTTAGCAAGCTAGCAGTACAGCT
AGCAGTAGCAGATAGCAGTCCAGTAGCAGTGCATAGCAAATTAGCAGTAGCAGTAGGCAGTAGCAAGTCGCAGAAGCCAGTCAG
CAATAGTAGTTACAGTAGCATTTATTATATCCTTAAGGGGTTTAGATAGATTAATAAGGAAATGTGAAACTTGTCAACAAGCAT
GTTCATCTGAGGATTTAAGTATTTTTTATGGATAGTTTAATTAAAATAAGATCTAGAAGCTCTAGAACGTTCCATCATACATAT
TAGGTCACGTTTACTACATCCAAATTAGTTTTAATCAATTTCAATTATGTTGAAGTGTGCAAAAATGTGGATTTAAAATATAAC
CGTCATGCGATCATCGGGCAACTATAGGGGCCGATTTATTTTGCCTATATAGATGGTAGTGGTAGTGGTTAGTGGTAGTGGTAA
TGGTAAGTGGTTAGTGGGTATGGGATAGTGGTGGTGTAGTGGTGGTGGTAGTAGTAGTGTGTAGTAAGTTGGTCAGTGGTGGTG
GTGGTGGTGTAGCTGTAGAATTTTAGTTACTTAGTAGTACTGGTCAAAGTTCGAGATTTACTTTAAACTCGTAGAATAGTTTAT
AGATTTTATAAGTTTAACATTATGGTTTTAAATATTAATTTTAACATAAAGGTTTTGATAATATAGGCTGGTACTAGAAATATA
TTTATTATATCATAAGGTTTAGATAAGTTTAAAATTAAGGATGTGACACTTGTCACATGCATGTTTTATTGAGGGGATTAAGTT
TTTATGATGGATAGTTTAATTAAAAGAATGATCTGAAGCTCTAGTACTTTCCATCTCTGTTGGATCACATTTTACTCAAGTCAA
AGTAGTGTTATTCAATTTCAAATTATTGCTAAAGTGTGCAAAACGTGTTGTAAAATATTCATCATTTGCCGATATATCGTAGCT
ATAGGGCGATATGTTTGCCTATGATTGATACAAAGAAACACATCGCTTCGCACGAACGACAAGAACCCTCGGAAACATGGTAGG
GGTGATAAATATCGCCCTACCCTACGACGATATACTCTTGCATCCAATGAATCATTTTTTTCATTTTTGTAGATTCCTGAAATA
GAAACAAACCCTCAACAACTTGACTCGTTCCTGAACTTTTTGACCGAGTTCAGGCATCTGTATGAACAGATAATTCAAATTTAT
TCAAATTTAATCATTATTTATATATTCATTTAAAGCGAGTTTAGTTCACTAAACTTGAACTTTATAAATAGGCCAACCTAAGTA
CTTAGCCATTTTTATTCATCACTCTCAAGGACATTAGAGCCTCCCAAGCTGCTAAGTTTATTCTAGAGAGAAACACTTGGGTTT
TTTGGGTTTTAAAAGCTTTTCAATCTAAGCTTTTTGAAACAACTTGGGAAGATAAGTTTTGTGTGAATTTTTTTGGGGATGT
TCGAGGTTAGATCGAGTTTTAAGATATCCAAGGTATTTTTAATTCTTCAGGTTTAGTCTATAGTTTTAATTTTTCTTCATTTTT
TTTTCAAATCCCTAACTTGCGATTAATGACTTTTTGGTTAAGTGTATCCAATTCCTTTGGAAAACTTAGGGTTCGTAGGTTTCT
TTTCCAAGTAGATCTCTATTGCTTCTTTCTTTAGAAACTTATGGTTTTCTTAATGTTATATTGGTTTTTAAGAGAGTTTTTCAA
TCCCGCTCTTGTTTCCAATATTCTGGTTTTAGTAAGGAAAATTCAGGTAGTTTTTAGTATTATGACCTAGTTATATGGTAATTT
TATGATCATGGTTATGTTTGGTATCGCCTAACATTTTTAAGGTACATATAAAAGGGTAAGTGTGTCTGGCCTAAGGGTCCAATG
ATGTATGTGTGGACCTATGTCGGTAGGCTCAGTTTGCCAAAATTTTGTGACTGGCCTAAGTATGATGCTCCGATGTATGATGGA
CCTTTGTCCGGGGGCTTAATTGCCACCCGCTATATAACACTTCTCTTTTTATTATATCTAACTGTATCACCCATTAGTTACGAT
TCAATGATATATTGACATTATGACCCCATAGTTAGTGATTATGATAATACTATGACCTATGATTTATGATCTATGACTTATGAT
TTATGCTATGAACTTAGATTAATGATTTAATGATTGTTTGAGGTTATGATAGACCTAGGGTTTATTGTTGTAACTGATTGTATA
AATAAGTTTTGGTATGACTCTTGTTGAAATTATGATTAGTTTTGATTATATATTACTATGACTAACTTTTGTTTGTATTTTTCC
TTACTTGGGCTTAGAAAGGAGCTCACCCTTATGATGTTTTTATTTCAAGAATTAAGGGATAAAAGGTCGGTGGGATGGGGACCT
AAGAGCTTTCGTGATGTTCTAGTGGGACCAAATAGTTTTGAGGAATTACAGGCCTATTTTATTATTTTTATTAAAGAAATGTTT
CTCCTTTTATTATTTTAAATTTCTCATTTTATGTAAGACAATTTTTATTTTGGAGATTTTTCTATAATCGACCAAAAGTAATAA
ACATAACTATACTTGTACCCACGGAATTTTTACTTTCGTTTCAAAAAAATTGAAAATTAAACAAAAATACTATTCCATCTCTCT
CTCAGTCTCTCTCTCTCGCGCGGCACTGGAATACTCTCTGTCAGATTACCACGGCCGGACTCCATCTACACAACATTCACTGCC
AAACAAAATGAAAAAGCAGCCCAAAACTCTATCAAGAAAATCGATGCATGATTTTGAGTAAGACGGTGGATGGTTGCCTAAAAC
AACAAAAAAGCTACATATATGAATCTTAAACCAACCAACTAACAATATAATTCAGATCTTTAAAAACAACACCACAATAACGAC
AGATCTCATCTGATAATGGATACCATCTACACCGATCCCGATTGAAAACCAGCACAACAATGGGTACTGTTTGTATTTGGGCGA
CAGGTTTACAACAAAAAGACAAAAAGTTATGTTGAAAATTGCATAGAACAATTTCAATTCTTGGGAAAATACCAAGCTCACTAT
AAGGTAGCGCAAAAAAAAACCAACAAAACCTAAAACATTAATAATCAACGCTTGCATAATCTCATATCTTTGCTTCAATTTGTA
GAAGATTCAGAATCAAGGAGAAATTGTGGAAAATAAATTGTTGTAACATTACTTCAATTAAAACATAACAATAGGTATGTATTT

CATTATAGTCTCTTCATTATTCATCCATTATGCTGAAAACTTATATTTGACTGAAGAAATGACTTGAAGAATTCAGACTATATA
TGTGTGCTCTTTAGTTGTTTTCAGTGTTTGTAATTATTGCATATAATAACTTTGCTATTTACATTTCAGCAAATGGAAATACAA
ATCTCGATTAAGAGCACAAAATGGGTAACCAAAAAGAACCTTGATTTTGACTAGTGTTGATTACAGAAATTTGCAAATAGATAC
GTTTTGCTACATTGTGTAGCGTTACCTTGCAACCCTGCAACAACAAATGCAACTCGTATCAAGTGGAGGAGTTACCACTCAAAA
TAGTGTGGTCATCTCTATCTACTTGCAGCTAAACTGAACGAGTCTTACTTCACAACATATCCATATGTGTGACCACTGTAGACA
CATATGCAACAATAAATCAATTCATTCTTACGCAATAAAGACCAACAATAACGCGAAACTGACGATGATTGAGAATGGCCACGA
ACACAGAATCACCAATAGAGCAACAAGATGAAACAAAAAATCATGAAACATTTGACTCATTGACTATCAAGTTGTACTGCAAAA
TTTGGAACAACTACATAACAACAGAATAACATGCCGCTTGACAATATAGATTCACTTGTATCCAAACCCGCCATCTCGAAATAA
ATTGTCACTCTACAAAGACCAAGGCAAACACTAAAAGCTTCTTAGTACCTTGCCAATAACAAACCACTTCCAATAAAGTGGTCA
AATCATGCTAAGAGAATACAATTATTGTTTGGACCAAACTGCATGGTCATAGGACTCCAGGAATGAACAGTGCAAAATTCATCA
TTATATATATAAATAACACAACTCTTTGAAATTAGATTCCGGGATCAACTGAACAAGCAACATAAAAATTATAACGCACTCAAA
TAAAACAAAAGTTCCTCAACTTGAAAAACATGCACTCCACTAGACTTAGAGGGACGATGATGATAATCGTATAAGAATAATATA
TGAAGATGTGAAGTAGGAACATGCCCCTCAACAAATTACGAGAATTCAAGGCATCTTACAATATACCAAGTTTACTTTGTACAT
GCTTCAAAAAAACCACCTGAACTATATTGACAGAAAGGGAATGAAATTGTTGTTCTTGCTGAATGCAATCCTTTGTGGTGGTCA
AAATCATACTAATATGTGATACTTTCTTGAAGCTGATAAGGAGAGTTGCTCCTGTTGACAGGTGTAGTATATC
>m141121_210323_42134_c100732402550000001823141405141506_s1_p0/71008/2180_12987
GAGAGAGACACATTATATCTTAATGAGTTGTATTTGGACATTTCAATCGTTCGAGATTAGCTAGTTGTATAAACTCGTGGAGAG
TTGTAAAATTGTCTCTTAAACCTCCATTAGTTGTCTTTGGATCCAATGTACCCAACACATTCTATCTAGGTTTCTGGAAAGGGG
AGGACGACAATTACACACATAGGATCTTTATGATCAAGGGCGAAATCATTCTCGTAACCCTAACCTAGAATTGATTAACTTTAG
GAACACATTATTGATTTCCAAATCACCACTTCTTACTGACTGAAATTTAGCCAGTCTTAAGCATTACCGTATTTTTGATTAGAT
AAGATAACATTGTGTACCAGACAGGTCACTTACCATTTTGCGTATGATTGGTTTCTCATCCTCGAGGTAGGCGCTAAATTACCA
ATGTGGATATTCGGAAATCAGTCGGCCCATCTACCAAAAGACGGTTCTTATTATTGTGGGTGCGCGTTGAAGAAACTTGACTGG
GCTATTTGTATCCAAATGTTCTGCCCTCTGCTTAGAGAGACATAGCTCAACTTAGGATATGGTGGTAACTATGCTACAGTTACG
AAAGTGAATGAGTGTTCACATGTCTGTGAAAAATTATTTTGACTGCACGATTTACTTTAACTGTGATTTATTGCATGCCATTAA
CCATTTACCACGAGACGAATGAATACGGTTAAGCTGATGGGAAGCTTGGCTCTTTTTCTATGATCTTGTACGAGTATGGAATTA
TTATTTTTTCAGTATGCCTCTGGCATGTGAATGAATGAGTTTCCTTAAAATATGTAGTTGTTTGCTATAGATTAGAGGTTGACT
TATGTTGTCTGTTGGTTTGCAGTGATTCATGGTGTGACTAGGAAAGTATTCTGAGTTAACCCTGTCATGATCATTGCATGACTG
TCTGTGTTGATTTCAGCAATTTAGAATTCATTATGAGCCATAATGCTATGTGTTGACTGGTGACATTATTCTTCTCTCCAACAC
ACGATAAGTACAAAGTCGGTCCGAAAAGAACTGCATATGGTTAACGAAATGTGGGGGCACATAGCATTTGAACAATTTAAGAAC
TTATAGAATGATTACCTGGATCATTCATGTGAATCACAACACAGAAAGCGAATGGAAGTACACAGAATGTTATATTGTTTTAAT
AGCTTCGTCGATGCTATATTGCAGATTCGACACCTTTTCCAACCCACCGTGTATAACCTTTTCCTGGATGGGGGTGATCATCTA
TTTATATTGGAGCTCTAATGGCTCCCTTGTACATAGTGGTCCCATCAGGACAAAATAAATACATGAATAACACTGTCAGGATAG
CATCGGTGTAGTGGTGTTTGGAAGAGGATGTCTACTCCTAAAAGTGACGTGTCAAGTCGGAGCCATTAATTCTCACATTTAAAG
TGCATCTGTTCTTTGTGGTGATCCATATCTTAAGTATAAAAGTTCTCCATCCTCGATCTTGTTCGGATACTTTGACCCAGTCCC
ATACTAATGTCGGTGCTAAGGCGCACTCCATATGACCATGTATATCTTAAGTCTAATTATTCTGATGGGACTCTTGTTAAAAGC
CACTATAATCGATCTGGCGTCCGATACTAACTATGAGCTCTGCTTCAACGTTTACACGCTTAAACTTCTCTTGGTCTGTTGGCT
CATTCCGTATCTCTCCTGATTGTCATGTCAAGATCCATTGTGAGCCTCTTCTACCTTTCCCCTCGCTTAGAAAAGACTGCATGT
CTGAGCGAAACCCTTGCGCCCCTCACTGCTATTTCATTGTGGACCACTCGGAGTGGTGCCCAGTTGGTGGCACTTACTATTCTA
GGCCAGAGAAAGGAAGTTTGCTCGTTTTCAGAACAAGCGCTGCTAGCGAATTACTGTGGATGATTGAGCAATGGTACGCTAAAT
GTGAAAAAATTACGGATCTCGATCTTGTACGACTTACCATCAATCTGAAAAAAATACTTGCACCTCTCCCGCCACATCGCGAGG
CCCTTCCGGCCACCATGGATGCCCTAGCGGTTGTTCCATTTCTGATAGTGTGGAACAGGCTACTAGTTCTCAAGCGATTACCCC
AAGGGCGCCGAGACTGTCTTTCAAGAGGCGGATGCGCGCGCGCTTAGTTTGATCTTTGCGAGGCCCTTAAGTGGTAGCTGGCTT
GGTGCGAGAGCGAAGCCCTACGAGGTCTCTTGCCATGGCCAGCGCGAGGCGGACCCAAGGCCTACAAAGGCCTCTTAGATACTA
TTAAAAGGCTATGGTGCGCTCGCGAGAAGAGCAGTCTACTGCTGAAAGGCCCTTATGTTGGTGTGCCTGATTGCCGCTGTTAGC
GAGGGCCTGGTGATGGGCCTTTAGAGGCATGTTGACGCTTGGGCTATCGAAGTGCAGCACGCCAAGAGATGGTCTCGGAGACAG
ACCACGCGCGGCAGCACGGGAGGTCGTGGCTGCGTGGTTAGCGCATAGCGATTGAGCCTGAGATTCTTGTGCATTACGCACCTT
ACACAGCACAGAAGGCGTCTTATCCCGATCTCAGTGGCGTATGAGGTCCCCTAGAAGGCGCAACAATGGAGCCGTGGTTGGAGT
CGCCTGTCCTCTGATGAAAGTATGCACGTTTACTGAGGGCCCTAGTGGTCTGTGCGTATGAACGATGGATAGACCTGAGCGAGG
GCATAACTGGGGGCCCCCGCAGCTATGTGGCTGTGTTGCTGCCAATTGGCCATGATGGGGTATTGCCTGCACGCAGAAATTCTC
GACCCCTGGCAGCCCGCGGGGCCTAGGTAGGTGCAGAGACGGCTGCTATACGGCGCGTAAAAGCAGGGACTGCGACTTAGGTGA
ATGCGAGTCTCTGCACGTTTGTGTTCCTCGCTTGTCTGCGTGAGTCAGGATTGGCAACCCGACCAGCCGATGGCGCAGACTTAC
GTCCATTTATTGCGTGCTCATAGAGCTGGCTTCGTGAGCATGGTGAATAAGAATATGCAACGAGATGGGCATGTCTATTGGAAA
AATAAGTATAGCCAATATTGTAGGGATCGAAGGTTCTATCATTACTGACCCGGGGCCTTCTTGCGCGCGGACACCACCCCTTTT
GGGATATGTTTTTCATTGTTGGTGCTTTTCGACACAAATGAGTCCAATTTTTTGCGAACATGCGTATCCCTGGACAATTCGTTA
TTATATATAGAGCTTCTAGTATAGTAGCCGAACCTGTCTAGGCACTGCACGCTTCCTGGGTCCATTCCCATCTACAATATTTAT
TTCACCACTTTCACGCCCTCTGCTGTCTCGTAAAATGTGGAGTCAAGTTTCGTTTTGCATGTAATAAATGGGAAACGCTATACA
TCCATCTCGTCAATGTCGGTGCAGGATGCTGTAATAAAGTTATTCCCTACTTTATAATATATTGTTACTGTTAATTCTTCTTTT
AACAATTAATTGTAATTAAATCTTTTGGTATGACACATCACTTGGTTGCCTTCTTCCCAGAGCGATGCGGATAGGGAGAGACTG

ATTCCTCTTTGAAAAGTCGGTTTTGTAGACTCTCTCAATATAATATCTTTCTTTCTCAATCAGATTCATTTTTCTTTTACATAT
ATATTATCAAATGACCCAGAAATTTCCGGGATTGTTTTTCCTATGCAAAAGTCGAAGCATTTTTAAGGTCCTTTATGGAGCCCA
CGTGACTATTACGAGGGGAGTTCAATCAGTGTCCCGGAGTCATCTGTGCTTAGAGTGCACCCTTGAATTTGTATTCTTACCATG
ACCAGGATATAAAATTCAGACCCCATTGGCGGATTCCTGTGTATCTTTTGCATACTTATTAGGTGCGTGCACTTTATTATTTAC
GTTCGCTGAAGCGTATGCGAATCCTTCTCGATCACATAACGCGACTTATCAAACCCCAGGTCACCTATTCCTGGAGGAAACATT
CAATGGTTCCGAGTCGCTGGTATTAGACTGATGCTCTACCTAAGAAGTCTTTCTTTTGAGATCCTTTTTTTGAAGTGTCTTTCT
TTGAGACCCTTTTTGGAAGCGTCTACCTTTGAGACCCATTTTTGCTGGCGTCTACTTTTGAGACCTTTTTGCAGGCGTCTACTT
GGAGACCCTTTTGCAGGCGTCTACTTTTGAGACCTTTTTGCGACGTCTACTTTGGAGTCCCTTTTTGCAGCGTCTACTTTTGAG
ACCCTTTTTTGCAGGCGTCTACTTTTGGAGACCCTTTTTTGCAGGCGTCTACTTTTGAGACCCTTTTTTGCAGGCGTCTACTTT
TGAGACCCTTTTTGCCAAGCGTCTACTTTTGAGAACCCTTTTTTGCAAGCGTCTACCTTTGAGACCCTTTTTGCAGGTGTCTAC
TTTGAGAACCCTTTTTGCAGGCGTCTACTTTTGAGACCCTTTTGCAAGCGTCTACTTTTGAGAAACAACCTTTTTGCAAGCGTC
TACTCTTGGAGACCCTTTTTGCCAGGCGTCAATACTTTTGAGCCCTTTTTGCCGGCGTCTACTTTGAAGGAACCCTTTTTTTTG
CAATTTTTGAGGTGATCTCCCCTCCGGTGGGACTTTCATGGCTAGATTGGTCTTTGTTCAATCTCAAACCTCGGTCAGCGACCC
CTCTAAGCGCGAACGCCCCCAGTTCGTATATGGAATCTCAAGACGTATGGGTAGTAGGGTGACTGGAGGAAAGGTTTGCCTTTC
TTCAAACATGGGAGTTCTTATTGGCACCTCTCCACGACGTATGTATTTATGTGCTCTCTCTCGAAGTCATTCCAAATTTGAACT
TCTCTTTTGAGCATTAGTTACCATAACTTGCTTTCCCTGGTCGTACTCCGGCTGTAATAGCCATCTTGAGCTCTGCTTTGGTTA
AACTTCCCCACCTTTGTTGCCTTCCATAATTGAACCTATGGATATAGTTCTTTCAGACTTTCATCCTCGCTCTTATTTTATGTT
AGCGAGGCTGGTGATTGGCACGGATGTAAAACCGAGCTGCATGGTGTTGCTGAAGAATTCAATCAGAGAAATTGCTGCCATGAT
TTGATTGTATCCTGGCCATAAAACCTCTTGACACTTGTACGCCCACTTCTTTAAGCGTAACGACAAAGCAATGGCAACTTTGCC
CTGTTGTTGACCCCTCCTTAACCTCACTCAAGTTATTGAAGGAGTCTAAGTGATAATTTTGGGCTCTGTAGTAACCCTCGGTAA
CGGAGTCATGAGAGGCCTCTGAAGCCAAAATAGCGCATTTGCTCAGCTACGATCTCCCGTTGGTGAAGGGGAATCATGGTCAAA
ATTCGTCATCCATCTTTGTGCCCCCCAAGTGCAGTGGTAATCTTGTCCCGAGGCTTTCCGCCATTTTAAGTGACCCCCCTAGTC
TAGTCCGTTCTCTCTTCTTTGCCTTTGAGGAGTCTTCGCGGTCCTCAGACTGAACCCTTGCGTTGGTTGTGCTCCTCCTGGGGA
CTGCGGGGGGCGCTTCTTTTGAGGTGACTTCTGCCTTCTTCTTGCTACCATCGTCTTTCCTTCCCCTTTGCTCCTGGGGGCATT
TCGTTGGCCTAGGTTTCCATGGGACCTCTCTGCCGGGGACTATTTTCGTGGGACGTGGAGAGTCGGGTTCCCCCATCAATCTAT
GGGGACAGTGTTTCCCCTTTTTCATGCTCTTTCTCTTTACGCTTAGGAAGGGTGTGCTCGACTTTCCTTGCACTAGATCGTTTA
GACCTCCTGCATGTTCTTATCGTGGTTTCCAGCCTTCAAAGTCTTTTTATGTAACTCTTGAATCTCATCCCTGGTAGAGTGAGT
CCTTGAGCCGGAACGTTACCGAGCGTACCCCGGGACCCTTGCTTGCCGTGCGGTCGATGGACCTGGGTCTTGTGGCCTAAAGCC
GTGGTGCCACCGGGCAGTGAAGTGGATGCACTGGCGGCTCTGAATGAACCTCCATCGGGCCGGATGGGCCGGGGAATGATGCTC
CTTCTCTCCCCTTGAGGGAAGAGGCTCCTTCCGGTCCTACATCCATGCTTGGGCGAGGGGGGCTCTTTCTTGGAAGCCCTCCGC
TCTACTTCGTCCTGGCGAACCTCATCCTCCTTGTATTTTGTCGTAGCGTTTGAACGTCTAGCATCTTTCTTTGTTGGAAGTGAA
TGGAATAACGCTGGCTGATGCTTGTTCTTCCCACAGACGGCGCCAAACTGTTTGACGGTCGAAATCTCGTCCAACGAAATTTAA
TCGGAAAACTCAAAGGTAAGTGTGGCGATGTTTGGATAAGAACTAGAAAAAATGAAGAACTTATGGAAGGATAACACAGATCAA
CAAATGGAGTAAAAAATGTAATACTTTCTTAATAGCCCTCTGCCATACAATGAGTTTTCCACCCCTTTTTCTGAGGGGTGATCA
TCTATTATTAATTGGGAGGCTCTAATGCCTCCTTTGTACATAGTGGCCCTCAGGACAAAATAGTAAATGAGTCACTGTCAGGAT
ACGCAGTACAGGTGGTAATTGGTGTCGGAAGAGTGGTGGTCTGCCTCTAGTACGTGTCAGGCGGGACTGCAAAAGTGCCTTGTC
TTGGTGCCTATTGTGCCTCCACTAACTTGTCGGGTACTGAACTCAATAAGCATGCTGAGGGAGTCTTGACCATGTAAAACGATT
CTTGTAACCTGCCACTACCCGTTTGCGTGGACGCTGTATCCTACTCATAACTCTTCTTGGTCTGATTGGACTCATTCCCGTATC
TCTCCTGACTTCATGTCCAAAATCATGTGTGAGGACCTCTTCTGCCACCTTCCTCAGACTGCATGTTCCGAGGGAACCCTTGAC
TCCTCATCTCATGTGATCATAGGGGGTAGTGGTGCCCCGTTGGTGGCACTTACCCTCAAGAAGAGAGGTAAGGCCATCTTCAAA
TGAGCCTAACCTGCGTGTGGGGATGAGGCAAAATGATGCCTACGAATAGAGATTAGATCTCATCTTTGCGAGACCCATCACTTT
GCATCCCCACATTAGCGAGGCCCTTGGTATGTGGTCAGAGGGGTGTTTTAGTGGAGGCTAATGGTCTCCGCGCGAGGCTTTCCG
AAAGGCACGAGACGCTTTGAGGGGGCTAGGGGGCTATGTCTTACGAGGCCCTAATAAGGTGCGGCTAGTGCGAGAGGCGACGCC
TCAAGGCTCTTCGGCCGTGGCCCAGTGCGCGGCGAGGCCACCTTAGGGCCTTGTCCCGTGAGCTGCTGCGCGAGGCGAGCCCGA
GGCCCTTAGCATCCGCTAGCCGCGAGACGACGCCTGGGAGTAGGTGAGGCAGGGGTCCATGGCTGCGTGGGCGCGAGGCCTGTT
GGGAGTCCTCCACGGGGCGTCACCCCGCGAGGCCCCCTAGGCGACGCGAAGGCCTGTTGGACGCATGTCGAGACGTCACCCACG
CGAGGGCCCTAGGCGCCTGAGACAGTGCTTGGGCGAGGCAGGGGCTGCAGCATGTTGTGGGGTGCGAGGCCTGTTTGGAGGTTC
CTGCACGGACTCACCCAAAGCGAGGCCCCTAGGTGGCGCGAGACAGCGCCTGGGCGAAGGCAGGGGCCGCGCTGTGTGCGTGCG
AGGCCGTGTGTGCGCCTTGCGTGAGATGGCCTCCGTGCGAGGCTAGTGGTCCATGGGCCGGGGCTTCGCGAGGCGTGGTGCATT
TGCCACCGAGATGGCATGTCTAGGAAAGTAGTCAAATTAATTTTGTAGGACCAGAAGGTTCCATCATTTGACCCGGGGCCTCTT
GCGGACTACCCTTGGGTTTGTTTTCTTTTGGCTTCCACAAAGGTCCATTTTGCGCAACGTGGCCTTATCGCTCAAGTGAAGCAT
TTAGGCCTTAGGCACTAAAGGCTCTGCCCGATTCTAAAACTTAGCTTGTGTTGCTCCGTTGGGTCAATTTCTTCATGTTCGCAA
ACCCTTACTCATCTCTATGCGGATCGTGTAATGTATGTTTTATTTATATTTTTAATGTGGATTTTTGGTAAATCACACTATGC
GTCCAAGGCGATCGAATACGACTACGGCACAGGGTCTGAGGCTAAAGATGAAAATGAATAGGTCCAGAACCCTCCTGCCCAGTC
ACCTTGAGAACTGGCAGCGATACTTGCTGATATGCCAAGCCGGTTACAAAGTCAGGAAAGAATATCTGTCTTCTTGATGACAGC
AGGCTCTGTCAGGGAGTGTTGCCCTATTAATGCCACCTGTTTGTGGCACCAAATTTGTACAGCCACCCTGAGGTTGGGAACAAG
TGGGAGCCACTATATGAACAATTCAGGAGACCACCATCCTCTAATCTTTGAGGGAGGATCGGATCACCATTGACGGTTGAGCAG
TGGATGAGTATTAAATAATACTTCCATTCTAGATTTCATAGAGGGTGGAAGGTAATTGACAGAGTGGCCTGTGCCACCATAATA

TGCTTTAGGGAGGATACTCGATTTGGTGGGAGGTGGCATCCACAGACCAGGGATTTAAAACTTTTAGAATAAATATATGAGATC
TGTTCAACCAGAATATTATAACGTTGCCGTTAGGCAGCCGAAGGTGAATGAGTTATAATATAATATGGTATTATACAAGTATGC
AATTATACTATGCCAATGAAATTTGACAGTCTTGCCGAAGTTCGCACTAAGACATTAGTCCCTACCGATGCAACCAAACAGGAC
ATATTTATCAAATAGGGCTTAATGTTATGATAGCCGAAGATGTGAGAATCACAACAGTACTTTGGGGAACAAAACATAAAAAGC
ACATGCTGTTGAGAAGGCTCTTACCACTAAAGAAGCAAAAAAAAAGATCTAGATGGAGAATGATTGGGAGACGGATGTTCACAG
GGCCGGTGCCTCATACACAGGGTCGGTAGGAAGAGAAGAGAGAACCGGAGGCCACAATAGGAAGAGGAAAGACCCGTGACAATA
TATTACAATTGTCTAATAGGGGGGGTCGGGTCTCAAGGTGGCGCAGGAGAGGCGGTTGAGCTGGGTATATTCCAGAGGTGACAG
ATGCAGAAGCGCAACAACTGGAAAAATGGAGTGTCGGTGAAAAAAAGCCTGCTACTTGTGTAGGGTGGTGGGAACACCTCAAGA
AAAACTGCCCAACGGTGAAAAGGAGGTTCAGGGAAGGTGCAAAGCTTGACTCCAGCTCGGGTGTCACCTTGACGCAGGTGATGC
TGAAGGCTAGAACCCGGTAGTAAAACATAACTTTTAGTGTTGGCTCTTCTTATACTTATTGATTGAATCATGGTGATACACATT
CATTTGTTTCATAAAAATTGATAGATTGTGTAGCTGTGAGCTTGGTATGCGGGGGGGGTTCCCAACGACTATAAACTGCTACAG
GGGATGGTAGTTTCCAAGGGGTGGGTTAGATCACTACCAGAAAATAATGGTTGGATAGTAAGAGAAAAACTGATCAATAGACTT
TATTGAGTTGGATAAAGGAAACAAATTGATGTGATTCTATGGAATGGAATTAGCTGGTCAGATATAGAGCAACTATAGATGCAG
GAAGAAGATGTAACTTTGAGCCTGAGGGAGGAAATATAAAAATGTTATCGTGGGTGTTTGAAACAGATCCACCCACAATTATAT
ACATTGAAACTAGCCAAGAAAAAACTAAATTCAGGAGGTTGTATAGGTTTTTCTAGCTAGTGTGTGGATAAAACGGTAACCAGA
GTTATAGCCAGCAAGAAAGAAAGGAGAGCAAAATTGGTATCGCGAGTTCACTTAGATGTGTTTCCTGAGGATTTACTAGGAAAC
CACAGCACGAGGAACTATCCGTTAATAAAGAGTTAGTGCCACGGTACAAAGCCATGTCGAGGGCACCAATAAGGTGGTTTCAGC
AAAACTGAAGGAATTGAAGATTAACAATTGGCAAGAGCGTCTTTGATTTGGGGTTTATTAGACTAGTTTTCTCACCATTCGGGG
CCCGCTACAGTTTTGTTTGTGAAGAAGAAGGATGGGACTCTGAGAATTCTGTTATATCTTAACAGATGAAAATTAAATAAGTTG
AATCAATAATAAATAACCTTACCAAGGATCGATGACTGTTTCGATTAGCTGCAAGGAAATCGGTATTCTCGAAGATTGATCTTC
GATCTAATTATCACCAGCTGAGGATCAAGGATGAGGAATATACCAAGACGGCCTCCGAAATGAGGTATTGGTTCATATGAGGTC
TGGGTCATGTATTGTTTCCATATGCCCATAGCATTCATGGACTTGATGAACAGGTTGTTCAAGGACGTTCCTTTGGATCAGTTT
GTGATTGTCTTAAATTGAGAACATTTTGATATACTCAGTTTAGGAGACCAGAGCATGAGCAGGCATCTTCTGGCAAGTATTTGC
ATAGACTGAGAGAGCAAATCCAGTTGTATGCCAAGTTTGAAGAAGTAACGAGTTCTGGCTTACCAGAAGTGGACATTCCATTGG
ACACCATTGTGGGTTGGAGATGGGAATTAAGGGGATTTCCAGCTATGGTAGAATGGCAGTGAGAGATTGGTGAGGCCAAGGAAC
GCCTTTAGAGTTACCGATTCTTCCTTGGATTAGCAAAATATTATCAGACGGTTGGTGGAAGGGTTCTCAAAGATTGCTTAAACC
AATATGGTGACATGAATAATTCTAAAGTTTGTCTGGTCTGACAAAGTGTTGAAGAATATCGTTCCCATGAACTGAAAGCGACGA
TTAATTAATCATGCTTCTGTGGTTTGAGTCTTCCTTTGGAAAAAGGGGGGGGAAGGGGGGGGGGGAAGTTTGTGGTATACTATT
GAACGCATAACAAGATTTGGTGGTCAGTTTGGATGCATTTTGATGCAGAATGAGA
>m141121_210323_42134_c100732402550000001823141405141506_s1_p0/75170/631_12752
CATTTTGTCGTTGCTAACTTACGTATTGCGAGCAATGAGGTCTGAAAAAGTATCCTAAAAAAGGTAAATGCAATTAGACACATA
CCCTCTGTAATCCTGTCGAAGTACAGATACACCCATGGCATATATCGTCCTCAGGAAATATAACCATTCCTCATTGTATTTCTG
AGACGATCAAGATCTGAAAAAACATAATCTTCCAGTTGTAAAAATGGTTATTTCTATCTATCACCAATCTTGCGCTCTAATCTC
GTCATTACCCATATTACCTTCTTTAGACATAAGACCCGGAAACTGACCCGATCTTAAATCCCATCTTCTACATACAGAGAATAG
AACACATTAACTATTGTCAAGAACTGGAGCGTCCGAATCAAACCCATGTTTTGCTCACCAATGTGACAAATTGAGACTACTGAA
AAGGTCATCACTCGAACAAGTACTCTTTCCGTACTTCGGAAATAAAACCAGACAATCCATTAATACATGAAAGAGGTTTGAACC
ACCACTCCAGATAAATCTGAAAACCACTATTCCGAGAAACGCACAATATAAGAACGAACACCAAGACAATTAAAACCTCAGCTA
TCCCATTAGAACAACGATCACAACGCATTAACAAGAGAAAAGGATATACCGGAAAGGAGAGACGAGAAAAAAAGAAAAGAAAGC
GAACGAACAGAGAAAAAGAATCGCACAAGAGAGAATGCAGGGAAAGTCGTCCGGATAAAACTGCAAACGATAAGTTATTATTAA
TACAGTAAGGGCGCATGATGACATAACGGGTACCAGAAACTTTCGTAGCCCAGGTGACGCTGCACACGACATAAGTAATCCGAT
AGTATTCAATAGACAAGTGAACAGTGAGGACAGACGAGAGAAGAACAGAATTTCAAAACACCTCTGCTCCCAAAATATCATATT
AGCCAAGAGAATCGTAGCATTGTCTGAAAAGAGGTACCCCCTTTGGTGAATTGCGAGATTTCACCAATTTCTCCGAAAGGTGAT
GGGGACCACAGGGGTTCAAAACTCGGTCGAGATATGTTTTCCAAGAGATCTCACTGGAAATGCATCTTGTGGGAGTTGAAACGG
AGCCTTAATTCAACATTTGAAAAAAAATTCCCCATTTTGTTTGGCAATTTCAACAAAGTTTTTGACTTATATGTCGTAAATCTC
TCAGTACTTTTCACCGTATCACCCCTCTAAAACACGTCGGATATCGCACGATATTCCTTATACATAGATGCTAAAACATAGGGC
CTACAACTTTCGCTGCCCCATGACCACCGCATATTCGCATAGTATTCCACCCTCTTCAAAAACACTGCCTCCAATTCTCATATT
AGAAAATCGAAGATTTCTGAAAAGGGGCCTACCCCTTTGCGTGATTGCGAGATTCACCAGATCTTCTCCGAAATCGCGTATTAC
AGGGCGCGGTTTCTACTCGGGTCGACGTATTTCCTTCCCAAGATCTCATGGAAATGCATCTTGTGGAGTTGAACGGAGCCCTTA
ATTAATAAATTCTGAAAAAATTCCCCTTTTTGTGGGCAACGTTTTCACAAGCTTTTTGACTTATACTTCGTAAATCTCCTCAGT
TTTTCGCCGTATCACCCTCTAGAACACATCGGATATCGCACGATATTCTTTCGACATTGAAGCGCCCAACGACATAGGACTAAA
CAATTTCTGCCATGCCGACCCCACGCATATCTCCTTAGTGCCACTTTCAAAAAACTGCTCCCAATTCGTTGTTAGAGAAATCGG
AAGAATTTCTGAAAGGGTTACCCTTGGGTGAATTGGAGATCTTCCACCAATTTCTCTCCGATAATGTATTACACGCGGGTGCAA
CTGGTCGATTATGTTTCCAAGAGATCTCATGGGAAATGCAAACTTGTGGAGTTGAAACGGAGCCTTAATTCAAAATTCGAAAAA
AATTCCCTCTTTTGTTGCAATCTTTCACAAGTTTCTTGACCTTATATTCGCGTAAATCTCTCAGTATTTTTCACCGGTATCACC
CTCGTAAACTACGTCGGATATGCACGATATTTTATACATAGAAGCCTAAGATAGGACCAGACAACTTTCGTCCGCCATGACCCC
ACGCATATTTTCTTAGTACATTTCACCTTTCAAGAAACACTGCTCCCAATTTATATTAGAGATATTGAGGATTTCTGAAAGGGG
TACCCCTTTGGTGAATTTGGAGATTTCACCAATTTCTTCGAAAGTGTATTACAACCAGGGGTTCCAACTCGGCGATATTTATTC
CACAAAGAGATCCATGGGAAATAGCTTGTGAGTTGAAACAGGAGCCCTTAATCTCAACAAGTTTGACAAAAAAATTCCCCTTTT

TGTTGAGCAATTCTTCACAAGTTTTGACCTTATATTCGTAAATCTCCTCAGTATTATTCACCGTATCACCTCTACACGTCCGAT
ATCAGATATTTATCACATAATTGCCTAAAACATAGGGCGTAAAACTTTCGTGCCCATGACCCTCGCCATACTTCCCATAGTAAT
GATCACTTTCCCAAAAACAACTTGCTCCACATTCATATTGGAGAAAATCGAAGATTTCTGAACGGGGTACCCCTTTGGTGAATT
GGAGATTTCACCAATTTCTCCGAAATGTATTGACACAGGGTTCTACCGGTCGATATTTTCCCCAAGAGATCTCATCGGGAAATG
CATCTTTGTGGAGTTGAAACGGCGGCCTTAATTCACATTTGAAAAAAAAAAATTCCCCTTTTTGATTGGCAATTTTCCAAGTTT
TTGACTTATATTCGATACTCTCCTCAGTATCTTCCCTATTATCCTCTAAACAAGTCGGATAAGGCACGATATTTTATAATAAAG
GGTATGACAATGGCTAAAACTTTCGTGCCCACGGAGCCTCGCATTCCATAGTATCGTCACTTTCAAAACACTGCCATCCAATTC
AATATGAGCAGCAAATCGAAGCATTTCTGAAAGGGGCTACCCACTTTGGTGAATTGGAGATTTCACCAATTTCTTCGAGATGTA
CTTACACAGGGGTTCAACTCGGCGATATTTGTCTCCACAAGAGATCTCATGGGAATGCAGCTTTGTGGAAGTTGAAACGGAGCC
TTTAATTCAACATTTAAAAAAAAAATTCCCCCTTTTTGTTGGCATTTTCACAAAGTTTTTGACTTATATCGTACATCCTCAGTAT
CTTCACCCCTATTACCCCTGCTAACAAGTCGCATAAGCACGATTTTTATACATGAAGCCTAAGACATAGACTACAACTTTCGTG
CCCCACTGACCAAACACGGCATATTTCCTTAGTATCTCACTGCTTCAACAACACGCTCCAATTTATATTAGAGATATTGAAGAT
TTCTGAAAGGGGTACCCCTTTGGTGAATTGGAGATTTTCACCAAGTTTCTTCGAAATGTATTAACTACAGGGGTTCAACTCGGC
GATATTTTTCGACAAGAGATCTCATGGGAAATGCAGCTGTCGTGGAGTTGAAACGGAGCCTTAATGGTCAACATTTGAAAAAAT
TCCCCTTTTTGTTGGCATTTTCACCAAGTTTTTTGCCACTTATATTTTAAAATCGTTCAGTATTCTTCACCGTATCACCCTCTA
AACACGTCCGATATGCACCAGAATATTGAGTTTATACACTACGACAGGCTATGACATAGGGCTCACAACTTCTCGTGCCCATGA
CCGCTCGCATATTCATAGTATTGCAGTTTCAAAACCCTGCTGCCAATTTCATAATTATGAGAAATCCGAAGATTTCTGAAAGGG
TACCCCTTTTGTGAATTGGACGATTTTCACCAATTGTCTCCGCACAAGGTATTGACACAGCGGGTTCAACTCCGGTCGATATTT
TTCCCCCAAGAGAACTCCAAATGGCATCAAAGAATTGAACACGGAGCCTTCAATATCACGATTTGAAAAAAAATTCGCCCATGT
TTGTTGCATTTTCACAGTTTTTGTGACATTAGTATTTCGTCAAAATCTACATCCAATATTTCACAACGTAACCACCTCCTAAAC
ACGCGGACTAATCACGATTTTTATACAATAGATGCTAAAACATAGGGCTAAACTTTCGTGCCCATGACCCTCGCATATTCCATA
AGTATTCAACTTTCAAAAACTGCTCCAAATTCATATTAGAGAAATCGAAGATTTCTGAAAGGGGTACCCCTTTGGTGAATTGGA
GATTTTCACCAAATTTCTCCGAATGTATTACACAGGGGTTCTACTCGGTCGATATTTTTCCCCAAAGATCTCATGGGAAAATGC
ATCTTGTGGAGTTAAACGGAGCCTTAATTCAACATTTGAAAAAAATTCCCCTTTTTGTGGATTTTCACAAGTTTTTGACTTATA
TTTCGTAAATCTCTAGTATTTTCAGCCGTATAACCACTCTATAACACGTCGGATATGCACGATATTTTATACATTGAAGCCTAA
AAGACATAGGACTACAAATTGTCGTGCCCATGAATCCACGATACTTCCTTAGTTAATTCACTTGATCAAAACACCTGCTGCCAA
TCGTTTTAGAGAAATCGAAGATTGTCTGAAAGGTACCTTGGGTGAATTGGAGATTTCACCGAATTTCTCGAAATGTATTACAAC
AGGGGTTCAACTCGGTCGAGCTATTTTTCCCCAAGAATCTCATGGGAACAATGCAATTGTGGATGCTTGAAACGGAGCCTTACA
ATTCAAAGTTTGAGAAAAAAATTCCTCTTGTTTGTGTTGGGCAGATTTTCACAAGTTTCTTTGCTTATACTTCGTAAAAAAATC
CTCTGCAGTATTTCACCGTATAACCCTCTAAAAATACGTCGGATAATGCCACGATATTTTATACATAGAAGCCTAAGACATAGG
ACTACAACTTTCGTGCATGACCCACGCATATTTCATAGTATTCACTTTCAAAACACTGCTCCAAATTCATATTAGATGATATTG
AGATTTCTGGAAAGGTACCCCTTTGGTGAATTGGGATTTCACACCCAATTTCTTCGAATTGTATTCTACACAGTGGTCAACTCG
GCGATGATTTTTCCACAAGAGATCTCATGGGAAACTGCAGCTTGTGGAGTTGAAACGGAGCCTTAATTCAACATTTGAAAAAAA
ATTTCCCCTTTTTGTTGGCAAAATTCTTCCACAAGTTTTGATTATATTCGTAAATCTCATCCAGTATTTTTCACCGTACTCACC
CCTCTAAAACCGTCCGACTATGCACGATAGTTTTATACAGTAGATGCGCTAAACATAGGGCCTAAAACTTTCGTGCCCATCGAC
CCTCCGCATATTCCTATAGTATTCACTTTCAAAACAACTGCTTCCAATTCATATTACGAGAAATCCGAAGATTTCTGAAAGGGG
TACCCTTTGGTGAATTGGAGAATTTCAACCATTTCTCCGAGAATGTATTACACAGGGGTATCCCTAGCTCCGGTCCCGGATATG
TTTCCCCAAGAGATCTCCATGGGAAACTGCATCTTGTGGAGTTGAACGGAGCCTTAAATTCAACATTTGAAAAAATTCCCCCTT
TTTGTTGGCAATTTTCACAAGTTTTGACTTATATTTTCGTACATCTCTCAGTATCGTACCCTATTACCCCTCTAAACCAAGTCG
GATAAGCACCGAGTATTTTATAATAGAACGGGTATCGACATAGGGCTAAACTTCCGATGCCATGAGCCTGCATATTCCATAGTA
TTCACTTTCTAAAACACTGCTCCATATTCATATTCAGAGAGAATCGAAGATTTCTGAAAGGGGTACCGCCTTTCTGGTGATATT
GGAGATTCACAATTTCTTTCGAAACTGTATACACAGGGTTCAACTCGGCGATATTTTTTTCCACAAGAGATCTCATGGGAAATG
CAGCTTGGGAGTTGAACCGGAGCCTTTAATTCAAACATTTGAGAAAAAAATTCCCCTTTTTGTTCGGCAATTTCACAGAAGTTT
TGACTTATATTCGTACATTCTCAGCTATCTTCACCCTACTTACCTCTAACAAGTCGGATAAGCACGATATTGATTATACCATAG
AAGCCTAAAGACATAGGACTACAACTTTCGTGCCCATGAACCCACGCATATTTCTTAGTATTCACTTTCAAAACACTGCTCCAA
TTTAATAATTAAGAAATCGAAGAGTTTCTGAAAGAGGTACCCTTGGGTGAATTGGAAGATTTCACCCAATTTCTCGAAATGTAT
TACACAGGGGGTTCAACCCTTCGGTCGAATATTGCTTTCCCCAAGAGATCTCATGGGAAAATGCAACCTTGTGAGAGAACGGAG
CCTTAATTCAACCATTCTGAAAAAAAATTCCCTTTTTGTTGGCAATTTTCACAAGTTTATTGGACTTATTTCGTAAATCTCTCA
GTATTTTTCACCGTATCACCCTCCCTAAAACGTCGATATCATGATATTTTATACTAGAACGCCTAAGAAATAGGACTAAAACCT
TTCGTGCCATGACCCACGCATATTTCTTAGTAATTCACTTTCAAAACACTGCTCCAATTCATCATTAGAGATACTTGAAGATTT
CTGAAAGGGGTACCTTTGGTGAATTGGAGATTTTCCCAATTTCTCCGAAGGATATTACACAGGGGTTCAACTCGGTCGATATTT
TTCCCCAAGAGATCTCATGGGAAACTGCATCTTGTGAGTTGAAACGGAGCTTTAATTCCAACATTTGGAAAAAAAATTCCCATT
TTGTTGGCAATTATACACAAGTTTTTGACTTAAATACTTCATAATTCTCCAGTTTAATTTTCACCGGTACAACCCTCCTAAACA
CGTCGGATATGCACGATAAATTTGTATAACCATAGAAGGCTAATGACATAGGGCTAAACTTTCGTGCCATGCCCTCGCATACTT
CCATAGTATTCACTTTCAAAACCCCTGCTCCCAATTCATATTCAGAAAATCGAAGATTTCTGAAAGGGTACCCCTTGGTGAATT
GGAGATTTCACCAATTTTCTCCCGAAAGGTATTACACAGGGGTTCACCTCGGTCGATATTTTTCCCCAAGAGATCTCATCGGGA
AATGCATCATTGTGGAGTTGAACACGGACGCCCTTAATTCAACATTTGAAAAAAATTCCCCAATTTTGTTGGGCAATTCTTCAC
AAGTTTTTGACTTATATTCATAATCTCTCCAGTATTTTTCACCGTACCACCCTCTAAACACGTCGGATATGCACGATTTTTATA

CATGAAGGCTATGACACTAGGGCTACGAACTTTCGTGCCATCGACCCTCTGCATATTCCATAGTATTCACTTTTCAAAACCCTG
CTCCAATTCATATTAGAGAAATGCGAAGATTTTTAAAGGGGTACCTTGGGTAATTGGAGATTACCAATTTCTCCGAAATGTATT
ACACAGGGTTCAACTCGGTCGATACTTTTTCCCCAAGAGATCTTCATGGGAATGCAACTTGTTGACGATGAAACGGACGCCTTA
ATTCAACATTTGAAAAAAAATTCCTTTCTTTGTTGGCAATTTCTCACAAGTTTTTGACATTATATTCGTAAATCTTCTCAGTAT
TTTCACCGTATCAAACCCTCTAAATACGTCGGATATGCATGATCATCTTTATACATAGAAGCATAAGACATAGGACTACAACTT
TCGTGCCCATCGACCCACGCCATATTTCTTAGTATATCACCTTTCAAGAACACTGCTCCCAATTCATATTAGAGATATTGAAGA
TTTCCTGAAAAGGGTACCTCCTTTGGTGAATTGGAGATTTCACCAATTTCTCCTCCGAAAGGTATTACACAGGGGTTCAAACTC
CGGTCGACTATTCTTCCTCCAAGAGAGTCTCATAGGGAAATGCATCTTGTGGAGTTGAAACGGAGCCTTAATTCAACATTTGAA
AAAAAATCCCATTTTGTTGGCAATTAATCACAAGTTTTTGACTTATAATTCATAAATCTCTCAGTATTTTCACCGTACCACCT
CCTAAACACGTCGGATATGCACGATATTTTATTACATAGAAGGCTATGACATAGGGGGCTACAACTTGTCGTGCCCCATGACCC
TCGTCATTTCCATCAAGTATTCACTTTCAAAACCCTGCTCCATTTCGATTATTAGGAAAACTCGAGAGATTTCTGAAAGGGGTA
CCCCTGTTCGGGTGCAATTGGAGATTTCACCATTTCTCCGAAAGGGTATTACACAGGGGTTCAACCTCGGTCGATATTTTTCCC
CCAGAGATCTCATGGAATGCATTCTTGTGGAGTTGAACGGGAGCTTAATTCAGAACATTTGAAAAAAAATGTCCCCATTTTGTT
GGCAATTTTCACAAGTTTTTGACTTATATTCGTAAATCTCTCAGTATTTTACCGTATCCCCATCTAAACACGTCGGATATGCAC
CGATATTTTTATCACCATAGATGCCTAAACATAGGGGAAGATCTAAATCTTTCGTGCCCATGCCTCTGCATATCCTCCATAAGT
ATTCACTTTCAAAACACTGCTCCAATTCATATTAGAGAAATCGGAAGATTTCTGAAGCGGGTACCCCTTTGGTGAATTGAGATT
CCACCAATTTCTCCGAGAATGTATTACACAAGGGGTTCTAACTAAGACTATTTTTTCCCCAAGAGATTCTCATGGGAAATGCAT
TCTTGTGGAGTTGAAACGGGCTTAATTCAACATTGCAAAAAAATCCCCTTTTTGTGGGCAATTTTCACAGTTTTTGACTTATAT
TCTAAATCTCCTCAGTATTTTCTCTCGCTCGTATCCCTCTCTTATTAACACCGTTCGGTATTATGCACGATATTCTTTTTAATA
TCATTGGAAGCCTAGCTCATAGGACTGACAATTCGTCCCATGGACCTCATCGCATATTCCTTAGTAATTGACTTTTCTAAAAAA
CTGCTCCCAATTCTGTTTTAGAGTAAATCGATATGATTTCTTGTAATAGGGGTACCCCTTGGTGTGAATTGGAGATTTTTCACC
AATTTCTTCCCTGAAATGTTTATTTACATAGGGGTGCAATTTACTCTGGTCGATTTATTTTTTCCCCAAGAGATCTCATTTGGT
ATATAATGCTAAATTGTGGATGTTGAAACGGAGCCTTAATTCAACAGTTTGATAAATAAAAAATTTCTTCAACCCCCCTCCTTTT
TTGTTGGCAATTTTCACATATTTTTTTGACTTATATTCTGTAAATTTCTCTAGTTTTTCACTCGTATCTACACCTCTTAATAAC
ACGTCGGATATGGTCACTGATTTTTTATATATACAATGATGCTAAAACATAGGGCTACAACTTTCGTGCCCCATTTGGACCCTC
GCATATTCTATATTATTCACTTTCTAAAAATACTTTGCCTTCCAATTCAATATTTAGTATGAAATCGTTAGATTTCTGGTAAGG
GTAACTCCTTTTGGTGAATGGAGATTTCCACCAATTTCTCCGAAATGTATTTTTTACAACAGGGGTTCTACTCCTGGTCGATTT
ATTTTTTTTTTCCCCAAGAGTCTTCATGGGAAAATGCATCTTGTGGAGTTGAAACGGAGACTATAATTCAAACATTTTGAAAAA
AAAATCCCTTTTTGTGGGAAATTTTCACAAGTTTTGACTTATATTCGTAAACATTCTCAGTATTTTCGCCGTATCACCCTCTAA
ACACGTCGGATAATGCACGAATATTCTTTATCATTGGAAGCCTAAGACATAAGGAACTACAAATTTCGTCGCCCATGACCACGC
ATATTCCTAGTTTCACTTTCAAAAAACACGCTCCAATTCGGTTTAAGAGAAATCGAAGATTTCTGAAAGGGGTACCCCTTGGGT
GAATTGGATTTCACCAATTTCTCCGAAATGTATTACACAAGGGGTTCAACTCGGTCGATATTTTTCCCCAAAGAGTCTCATGGG
AAATCATCTTGTGGAGTTTTGAAGGACGCCTAATTCAACCATATTGAAAAAATTCCCCATTTTGTTGCGCAATTTTCACAAGTT
TTTGACTTATATTCGGTTAAATCTCTCAGTATTTTCACCGTATCACCTCTAAAACGTCGGAGTATGCACGATATTTTATACATA
GATGCCCTAAAACATAGGGCTAAAATTTTCGTGCCATGATCCCTCCATTTCATATTCCATGTATTCACCTTTCAACAACACTGC
TCCATTCATATATAAGAAATCGAAGATTTCTGAAAGGGGTGACCCTTTGGTGAATTGGAGATTTCACGCAATTTCTCCGAAATG
TATTACACAGGGTTCTACCTCGGTCGATATTTTTCCCCAAGAGATCTCATGGGAAATGCTCCCTTTGGAGTTGAAACGGAGCCT
TAATTCAACATTTGAAAAAAAATTCCCATTTTTTGTGGCAATTTTCACAAGTTTTTGACATTATATTCGTAAATATCTCAGTA
TTTCGCCGATATCCACCCCCCCACCCCCCCGCCCCAACCCCCCGCCTCTACAAAACACCGTCGGATATGCACGATATTTTATAC
ATTGAAGCCTGACATAGGAACTACAAATTTCGTGCCCATGACCACGCATATTCCTTAGTAATTCACATTTCAAACACTGCTCAA
ATTCGTCATTAGAGAAATCCGAAGATTTCTGAAAGGGTACCCCCTTGGGAGTGATCATTGGAGATTTCACCATTTCCTCCGAAA
TGTAATTAGCACAGGGTGCAAACCGGTCGATATTTTCCCCCAAGCAGATGCTCCCCATGGAAATGCAACTTGTGGAGTTGAAAC
GGAGCAACTTAAATTCAACATTTGAAAAAAAATTCCCCTTTTTGTTTGCAAATTTTCACAAGTTTTTGCTTTATTTCGTCAAA
TCTATCAGTATTTTCACCGTATCACCCTCCTAAATACGTCGGATATGCAACGATACTTTTATAGCCATAGAAGCTAAGACATAG
GATACAACTTTCGTGCCCATGGACGCCACGGCATATTTCTTAGTTTACCACTTTCCAAGAACATGCTCCAATTCATATTGAGAT
ATTGAAGATTTTCTGAAGGGGTACCCCTTTGGTGAATTGGAGATTTCACCAATTTCTTCGAAAATGTATTACACAAGGGGCCTT
CAACTCGGCGATATTTCTTCCACAAGAGATCTCATGGGAAATGCAGCTTGTGGAGTTGAAAGCGGAGGCCTTAATTCACATTTA
AAAAAATTCCCTGTTTGTTGGCAATTTTCACAGTTTTTATGAATTATATTCGTACATACTCTCAGTATCTTCACCGTATACACC
CAATAAAGCTCGTGGAGATGCACGATATTTCCGCTATAGCATAGAAGCCTAAGACTAGGACTCACAACTTTTCGTAGCCATTGA
CCACGCATATTTCCATTGTATTCCCACTTTCAAAACACTGCTCCAATTCGTATTAGGATTTGAAGATTTCCTGAAAGGGGTACG
CCGCCTTTGGTGAATTGGAGATTCTTCCCACGCAATTTTCTCCGAAATGTATGTACACAGGGGGTTCAACCTCGGTCGATTATT
GTTTCGCCCAAGAGATCTCATGGGAAATGCACCTTGTGGAGCTTGAAACGGGAGCCTTAACTTCAAACATTTGAAAAAGAAATT
CCCACTTTTTGTTGGCAATTTTCACAAGTTTTGTTTGACTTTATATTCGTCAAATCATCTCAGTATCTCTTCACGTATCACCCT
CCTAAAAAACAAAAAGAAAGAAAAAACAGAAAAAAAAAAAAAAAAAAAAAAAAATACGTCGGATATCGCAGATATATTAAATACAT
AGAAGGGCCGTAAAGCACATAGGACTACAACTTTCGTGGCCATGACCCACGCCATATTTCTTAGTATTCACTTTCAAGAACACT
ACGCTCCAATGTCATGATTAGTAGATATTGAAGATTTTCTGAAAGGCGGGTACCCTCTGGGTGAGTTGGGAAGATTTTCACCAA
AAATTCTGCCCGAAATGTAATTACACAGGGGTTCACCCTCGGTCCGAGCTATTTTTTCCCCAAGAGATCTCATGGGAAATGCAT

CTTGTGGAGGTTTGAAACGGACGGCCTGTTAATCTCAACATTTGAAAAAAAATTCCCCATTTTGTTGCAATTCTTCACAAGTTT
TTGACTTATGATTCGTAAATCTCCG
>m141121_210323_42134_c100732402550000001823141405141506_s1_p0/83158/7187_16769
TCCCCTTTTTCTGGTTTTCCACCATTTAAGGAACAAACATGATTTTTATACAGCCCTTTTGACAAGAAAGTTTCCCCCAAGAAT
TAAAAAACATTTTCAAACAATCTGTTTTTTAAAACGAAAAAAAACCAAAAGCCAAAAGCCAAAGAAGAAAGTCCACTTCTTTAA
AAAGAGGCCTAAACCCCTGGGGCCAAAACGGTTTTTTTTTTTCTTTTTTTTTTTTTCTTTTTTTTTAACACACATAGGAGGGGT
TGGGGGGGGCGGCAAAAACCAAAAAACCCGGCCGGCCCCCCGTTTTCCTAAACAAATGTTTGATGAACAAAAAGTGTCCCACCA
AAAATCCTTTTAAATTTTTTTGGCCAGATTAATTATATTTCAATCCAATAAGAGCACACAGACAGCAAAAAATAAATATTTTCA
TGACAACAGGGAAAAAAGGTTTTCCCCCCCCCCTTTTTTTTTTCTTTTGGGAACCGAACAAAACAATATTACACAGACAAAAG
GAAAAAAATTCCTATAACACAAAGGCCCAATTTAACTTTGAGTTCCCCCCAATTAAAAGGGCCTCACAATATAAAGGTATACAT
ATATTTCATTTCTTATAATTTTTCTTTTTTCACTTATTTTTACTATTGTACAGACAAGAGGCCCGGAGCAACCCAGGTCCCCCA
GCCATTACAAAAAACAGACAAAACCAATCCTTTCCATTCCCTCCAAAAAACCCCCCAACCCCCAAACCAAAAACGGGGGGCGAA
GGGGGCAGGAGGACAAGAGAAGAGAATTGGTTTATTTCCTTGGTTTTAAAAAAAAAAAAGACAACTTTGGGCCTTGAAACCTTT
ATTCCCCGCCCCCCCTTTCCCCTTTTTTTCCGTTTAACCCAATATATAAGAAACCCCCTTTCATTTTGTTAAATGGAGCCCCAC
ACACCAACTTATAATTACAACATATTTATAAAATTAAAAACATATATATTTTTTGTTATTGGAACCCCATCAATATATAACATA
ATAGAAAGAGCACCCTTATTTATTATTTTTATATTATTAAAAAAAATTAGGACATTTTTTTTATTCCCTTTTTTGCCCAACCAT
CAATCCCGTACCCACCCCACCCAACACACAGGCGGGGACCCCTTTTCTCCCCCCCCCCCCCTTTGTTTCAACAAAATTCTCAAAT
ATAATAAATCCCCCCCCCATCCCATTTTGAAAAAACAAACCTCCCAATAAGGAACAAAAAAACTTGACCCCCCACCCCATTTTG
TTTCCAAAAATCAAAAAAAAAAAACAACATTTTACGGGGCCCTCCAGGTGGCCACCCCCCTTTCCTTTTTTTTACATGAGTTTT
TTTTCTTTTTTCTTTTTTTTAAAACCCCTTTTTTTGCCCCCAAAGGGTAGGCCTTTTTTTCCCCCCCCCCCTTTTTTTTTTATA
AATGAACCCCTCACCCGCTTTCTTTGGCCCTTTGCATTTTAGAATCCCCCCCCCCTTTTTAAACCCAAAAATTAATTTTTAAAT
TTTCTTTTGAACCCCTCCTTTTTTTTTTTTCCCAAGCCTTTTTAAAAATTTGTTCCCCCTTAAGGACCAAAAAGGGGGAACTTT
ATCGGGGGCGGGGAACGCGCCCCCCCCTTCCCCCCAGAAAACTTTGGGTCGAAAAAACCCCCACAGTTTTTTCTTTTTCTTTC
CCCCCCCTGGGTCCCATTTTGACGTTGGGGGGTTTTTTTTTTTTGGGAAAAAAAGCAAAGGCCCCCCCCCCAAAGAAAGAAA
AAATTGACCCCCACCCCCTGGGAGCCCCCCTTCCCGTAACGCCGACCACGTTTTGGGGCCCCTTAGTGGGAAGGCCCCCCCATC
TTACATTTGCGTTGTTTTTAAAAAAAACCGGTTTATGGGGCTTTTCCAAAAAATTGGTTTTTTTTTTTTTTTTGTTTTAACA
AGGTTTATCTTTAAGTTTTTTTTTGTTCCCCCCATTTCAAAAAAAAGGAAAAAAAAAAAATCCCAAACCGTTTTGGGGGGGGGTA
TAAAATTCCAAACCCCCAAAAACAAAAATCCCCCTTTTTGGGACACACAAAAAAGGCCTTCGTTTTTTTTTCCCGCAGGGTTTT
TTTCCACAATCTCGGGAAAAGGTTCCCGTTGTTGTTTTTGCCTTTTGTTCTTTATTTAATCCCCCCCCCCCCAAAGTCCCCCCC
CCCCCACTTGCAAGGGATTTTTCCCTTTTTGAAAAATGCCCCCTTTTTTACACCCTTTTGTTTTTCTTTTTTGGGAGAAATGAG
GTTTTTTTTGGACAATTTTTTAATTTCCCCCCCCCCCCCCCCCCCTTTTTTGGGGACAACCCCAAACAAGATTTCCCCACCC
CAAAAGGGAAGGTTTGAAGCCCCCCCCCCTTTTTTTCACCCCTTTTTTTTTTTTTTTTTTTTTATTTTTTTTGCCAAAAAAACT
TTTTCGGGGGGGTAAGCTTTTGGGTTTTTTTTCTTTTAAATTAAAATTTTGTGGGGAAAAACCTTTCCCCCCCAGCCCTCCTGC
GCCCCCCTTAGTCTCTAAGACTCGGCGGCGCTTTTTTAATACGTTTGATGGTTTTTTTTCCCCCCCGATTATCTACCCTGGGCC
TATTTTTCTTTTTTTGGCCCCGGTGGGGCCTTTTTTATTCTTTCTTTTTCTTTTTGGCCTTTTTTATTTCCAAACAATGCTTCT
GGCCCCCCCCCCCGTAAAAAAAATCGTTCTGGGCGAAAATTTTGGAAAAGGGGTTTTTTTTGTTACAGTTTTCTCTTTTTTTT
GCCCCCCCCCTGTTTTTCTTTTTTTTTTTTTAAAATTTCCAACAAAACAAAAAAAAACAATGGGAATCCTTTCCCCCCCCGAAG
GATCCAAAAGGGGTTTTTTTTTTTTTTTTGGTCCCCCCCCGTTCCTTTCTCCCCTAGGAGTTTGTAATTCTCTTCCCTTTAAGT
CTGACTTTTCCCCCCCCTTAGGCCTTCCAAAGTGGGGTTTTTTTGTATACAAAACCCTCCCAACCCTTTGAACCCCCCTTTAAC
CTTTTTTAAAAAGGGGGTTTCCCCACCCGCAGAAAGGGCACCCAAAATCCCCACACCCTATAAAACATATTATGAAAAAAACCC
CTTTTTTAAAACCAATTTTTTTCCCCCCCTGTTTTGGGATGGTGGTGGGCCCCCCCTTCCCCCCCGAATTAGCTTCTTTTTTTT
GCGCGAAAGGGGTTTGGGGGGTTTTTCCCCCCTTCCCCCAACCCCAAACTGGGCCCCCATTTTTTTTTCCCGCTTCCCAAGTT
TTTGGTTTTTTTTTTTGTGGAACCCCTTTTATGAACCAAACTGCTTGAAAAAACCTTGCTCCCCCCATACATTATTCGGGTCCT
TTTGGGAAGTACTTTTGGCCCCCAACCCTAAAATGGTCCCCCCCCCCTTGGGGGAAAGCTTTTCTCTTGCCCCTTATCCCACCC
CCCTCCCGCTGTTCCCCAGGGGGTTTTTGCAAACGCCACAGGCCCCCCTATTTGGGTTTTTTTTTCCCTGGTTTTCCTTTAGCC
CCTATAAAACCCCAAAACCTTTCCGAAAAAGGAAAAAAGGTTCCCCCCCACTCCTCTCTTTGTTGTTTCTTTGAAATAGTTTTG
CCCTTTTTAGTTTCCCCCCTCTTCCCCCCCCCCGACGCCACCAACCCGCGCCTATTAGCGCTTTTGTAAGGACCCGAAATTTGC
TAATAGGCTTAGGACCTTGATTAGTGCTGCCTGGAGGCATAAATGATTCTCAGGGTCATTATTTTATTTGAATCTCAATGTTTG
AATACTCTGTGCTAAACTTGCTTCCGATTATTTTTACTATCCCCTTCCCCTCATAGTATTTATCTTGAATTAGGCCGACGCCAT
GCGAGACACCTTTGTCTGGTGCATTCCTGGTTTAGCCAGAAGTCACACTGGTTTATCGTGGTGTTTTTGGTGCCCCTATATTAA
TTTATATTATTTGCCCTTTGGTCAGGATATATTTTTGTGAGAGATATTAAGAATTGTCTATGCCCAGAGAAATAAACCTGGGGA
CATAGGTTGTTGGATCCATTGCCTTCTGGCTCCATTCCTGGGTAAAAATTTTTTGGACGTTGTGATTGTAATACAGGCGGGGGG
AGCACTTGTGGGCCACCACTTATAGATATATATATTTAGTATTATTTTAAATATCATTGGGACCAGAGATCATGGGTGTGACACGC
CCGCGGCGCCTCAAGAATATAGACAGAAGCGGGGGCCCCCCCACCCATCCTTTATTTCCCCTCCATATCCAGCCTGTTAATATT
ATGTGCCGTATCTTCTGCGGGTTCCCCCTTCTTTCTCTTACTCGCTCACACACACACCCTTGATTTTAAGCGTCGAGAGGAGGA
AAAACTTTAGAGGAGGACTCACACACCTCCCAAGAGAGAAAATTTAGGGTATTGAGGCGACGCTTGGGGTATTTTATGTATGGT
TCCAAAGAGCCTTATGACAACAGCTTATATAGAATGTGGCCGCATTTTCCACGCGCGCGCGCGCGCAGCGGATTATTGGTTCCC
TAGAAAGCCTTTTATCCAGCAAGTGGTATAGCTTTTAATTATTATTTAATTTTTTGATTAATTTGTTAAAAATTCCCAATCCCC

CCCTCCTTGCTGAAGTAGGCCTCTCGCTTTATGAGTTTTGCGCGTGGGGGTGTGTAGGTTTAATGTTTTTTGATTCTGTTCTTT
TTGAGTTGTTTTTATGTTTGTAGTTCTTATGTAGTCTATGGATTTGATGTTGATCATTAAGTCTGTAATGTTTCGAATAAGATG
AGTGCCCGCGCTCTATATATTGCGGACATTAGTGTATATAGAGCGTGGAGACCCGCCCTCTTATATCCGCAGAAACATCAGATT
AATATTCCGGTTTTGGTGTGAGAGAGGGAGTGTGGTGGTCGTTTATCTTCGACACACAAGGTAGAGGTAAATAAATAAAATAAC
TGCAAGTAAACAAATGACACCTTCCCCAAAAAGAACACGTTTTAGGAGAGGTTTGGAGAGACGGATAGGAAGAAGATTTTCTTA
ATAAATATATTGGGCCCCGCCCCCGAAAAAAAAGGGTAGTTTAATTTTATAATATATGAGGGGGTTTTTTTCCCCCCCCCTTTC
CTCCCCACGCGCGGCCGGCGTGATAAAGCCGGCTTCTGTGTCTTTTATTAAGATCCGAGCGTATTGCTTTAGAAATTTCTAGAC
ACCGCCACATCACAGACAGCTCTCTCGGCCTCCGCTCCGCCTGTCTCTCTGGCCGGTCCTATGCGGTGGTCCCGCGGCCCCGCC
CTTTATGCTCGTGGCGAGGGGCCTCTGTTCTTCGTATGGCCTATCGCGGCTTCTCTGTGGGTTTAGTTGTTTGCACTTGCGATC
CTTAGTGCCTCTCAATTCTGAGTTTTGGTGTTTAAGTTTGTTGTTTCATGGTTTTTTCTCCTTAATCTTTAACACCCTAGTATA
ACCTTCCTGGGCTTTTGTCCCCCCCACCCCCGTTAAAACCTTCTCCACACCGCCGGCCAGATGAGGAAATGCTTATGGCGCCGA
AAACCGGTGGACACCCATTCCCTTCCGGCCCACCCGATATTGGCGGGGACCCTCCCACGCCGCCCTGCGCCCTGGAGGTAGATA
GCCCCAAGAAAAAGGGAGGGTGGGACAAGGTGGCTGGCTTAGATATATGTTATTTAGAGGGGTCCGGCCACTCCTTCACAGAAA
ATTGATGGATAGAGACCTGGATTTGTTTTATATTAGAATAAGGCCCCTCCCCGAAAAAGCCCCATGGAGAGCCTGCACCATTAT
ATGGCTGTTATGTCTTTTTTTTTCTATGGCGAGAAAGGGGGCGTTTCTTTAATGTTTTTTTATTTGCTAATGGTTTTATGCTCCT
TTATGCTTAAATTCGAAGAGTACATATTTAAAACAAAGTGACAGATACGATTTTTAGTAGTCTTTCCTTTTCCCCCCCCTCTTA
ATTGCCCCCCCCTTCACCCATGAAAAACGAAAGCCCCACCCCAAAAAAAAACTTTTTTTTTTAACCCGCCCGATTACCTTTTAT
TTTTTTTTCTTGGGGCGTTCAAACAGCTTAAAAAATTAAATGGCCAAAGGGAACAGCAGGAGAAATTTAAAATAATTAATATTA
ACCAGAAAAACACAAAGCAAGAAAAAAAAACCCTTCCCCCACAGGCCTTTCTTTTTTTTTATGTATGTCTGGGAGTTTTTTTTT
TTTTTCTCAAAAAAGAGCCCAAGTTTTTTTAAAAAAATTTGGGCCAAAAAATCCCCCAACCGAAAGTTCCAAAAATTCATAT
TAATTTTGTTTCATCCCCCACCTGTTCCAAATATCTTCCCTTTCTCCCTCAAAGGAGATATTTTCTATTATTTCCCCCCCCAAA
ATAAAATGTAATTTTTGAAAAAAAAAACTTAAAAGGTCTAAAACATTCCCCCCCTCCCAAAAGCTTACAATTGGCATGGTCTGT
CCCCCCCCCCCCCCACCATAAAAAATTTTGGAAAATTTTTCCCCCCCCCCAAAAAGACTTTTTAAAAAAAACATTTCTTCATA
AGAGAAACCCCCCCCGGTTTTGACCCAGAATAATTAACTTCCTTTCCCCATGTTTTCAAAAATTTTTTTTGGGAATTTTTTA
AACAAAACTCCAAATAATTTATTTCCCCAAATTACTTATTTTTTTGGGAAAAAAAAATTAAAAATTTACCATTTAAAATTTTAA
CAAATTAAAATATTCCCCCCCCAATTGGGAGTATTTTTTGGGGGGAAAAAGGTTTTTTTTTTTTAACATATTTTTTTTTTTT
TTTTTATAAAAAAACAAAAAATCCCCCCCTTACAAGGGAATTAAGGGGAAAAAATTTTTTTTTTTTTATTTTTTTTTTTTCCCTT
TTTTTAAACAAAAAGTTTGCCCCGCCCGTTGGGAAAGACACATTTGCGTCCCCCCCTGAAAAGAAGGTTTTTTTTTGTAAGGT
TTTTTTTTTTCATTTTTTTTTTTGGGAATGCCGCTTTTATCATTTTAACATTTTCCCCCGAGGACCAAAAGATCCCAAGAAACC
CTTTTTTTTGGGGGGGGGGGAAAGGTTTTCCTCCCAAAATCCATATATTCCTTCCCCACACCCAGTTTTCTTAGCCCTTTCCC
CCCCCCTCCACCCCCCCCTCCCCAAAATTCCTTTGTGGGGGGTTTTCCCCCCCCCCCGCCGAACAAAACCTTTTCCCCAAAAAA
TTTCTTTTCCAAGAACAAAAGAAAAAGATTTTCCCCCCCCCCAAGTGCCCCCCCCCCTTTTTTTTCCGGAAGCCCCCCCCCCTG
CAAACTTTTAAGCCCTCCCCGCAACTTATTGGTTTTAAACCCTTTTTCCCCCAATGGGACCCCCCTTTTTTTCCCGAGTGGTTTG
GGGGTTTAAAGTATTAAAAAAACCCTTCTTGGAAAACCCCAAAAAAAATTTGTTGGAAAAAAATAAAACCCTAGACCCAATGG
TGTTGTAAAATTAATGGGTTTTTTTTATTTTTCAATTGGGTTCCCAATCTAAAATTAGCAAGTTGGTACAACCCCAAAAAAAA
GGACCCCCCCTTTTTTTGTATTTTTTTTTTAAAATTTTTTTTCGGAGCAATTTTTTTACTTCCCCTTTTTTTTTTTTTCCCTT
GTATACCAAACTTTTGAGGCTAAATTTTTTTTAATTCCCCAATTCCCTTCCCGAAAAAAATTTGGGTTTTTGGTTAAACAGT
TGGGGAAACTTTTTTTTTTTTTTTGGCAAAAGAAAGGAGGGGTCCCCCCTTGAGAGATTGTCCCGGATTGTGGGACCCCGAAGG
AAAAAAATTGATTGGGTTTGGGGCCCCAAAAAAAGCCTGAACCGGGGGGGGGGTGGGGGAAAATTTGCTTGATGGGGGGGGGG
GGTTTTTTTTTTGGGGGGAAAAATTGAAAGGGGGAACCCCAAAAATACCCCCAACCCCCCCTTTGGTTAAGAAACCCTTTTTTTT
TTATTTTTTTATTTTTTTTAAATTTTAATTTTTTTGGGGAAAAAAGGGGTTTTTTTTAAATTATAGATTAAGGAAATGTTTACA
AAAAGTGTTTTGGTAAAATTAAAATTTTTAACCTTAAAACCCTTAACCCTTTTTTTTTTTTTTTTATTTATTTTTTTTTTTTTT
TTTTTTTTATATTTTAGTGGAGGAAAAAAAAATTTTTAAAAACGTTTAATTTTGTTTTCCCACTTAAAATTTCTTTAATTTTTT
TTTTTAATTTTTTTTATCTTTTCCCCTAAAAAAATTACAATTTTTAAAAATAAAGGTAGATTTTAACATTAAAAAAAAAAACAA
AAAATTTAAGTTTTAAATTTTTTATTAAAAAATATTAAATTTTTTTTTTTTTTGGGGTTTTTTTTAAAATTAATTTTTTTTTT
TTTTAAAACTAATTTTTTTTTTTTCTTTTAAAAAATTTTAAATTTTAACCCCCTTTTTTTTTTTACCCTTAACCCTTTTTTTTT
TACAAAACCCTGCGGGGTTTTGCCCCAAAAAACAAATTTTTTCGCCAAAAAAAAATTTTAAAACAATTTTTGTCTTTTTTTTT
TTGCCCGGCCGCCCAATTTTCCCCCAACCTTTTCCCCACTTTAAAATTTAAATAACTTTTTAAAACTTTTTTTTTTTTTTTGGC
CGCGGTTGCCCCCCAAATTTTTTTTGGGCCTCCCCGCCCCCGCCCCCCCCAAAAAAAGTAATTTTTTATATTTCTATTATTCCC
CCCCCCCCACCAATTTCCCTTTTGTTGGGGAAAAAAATTTTTTTTTTTCCCCCAAATCCAGCCGCCCGTCCCCCTTAAATCTT
ATTTTTTTTTTTTTTTTTTTTTTCTTGTTTTTGTAAAAAAATCTTTTCCTCCCTTCCCCAAAAAACAAACCACAAACAAAACGGG
GAGGAAGGAAAAGGAAAAAGAAAAAAAGGGAGGAAAAGAAGAATACAAACCAAAAAAAAGATATAGACGCGCCCCCTTGCAATT
GGAAAAAATTTTTTTTTTTTTTTTTTTTTTTTCCCAAAACACCCCACCCAACAGACATTTGGTTTGGGGAAAAAAAATACCTTT
TTTTTTGGCCCCCCGGGCCCCAAAAGAAAATTTTTTCTTTTTGCCCAAAAAAACCGGCCCCAACAAAAAAGTAAAAAAAAAGTTT
AGATTAGTGAATTTTTTTTAAAAGTGGGTGGAGTGGAAAAAAAAATTTTTTGCCCGCCCCCCGGACAACCTAAAAAAAGACAAT
TCTTTTTTATTTTTTGGGCCCGAAAACATTTTTTTAATTACTATTTTTTTCGTTTTTTTTATTTTGGCAAACGCCCCAGGGGGG
TTAGTTTTTTTTTTTTTTTCTTGTTCTTTAAGGTTAAGGTTTCTTTAAAAAGGTTAACATATTTTTTTTAAAAAAATTAAGCTAA
ATTCTAAACTTTAAAGCACAACATTAAAAATATTTTTTTTTCAAAATAATTTTTTTTTAAAACCTTTTTTTAATGTTTTTTAAG

GGTAGAAAATTAATTTTGTTAAAAAAAATTTTATTTTTTTATCAAATTAAACGAAAAATAAAAAAATTTCAAAAATTTTCTTTT
AAAATT
>m141121_210323_42134_c100732402550000001823141405141506_s1_p0/85317/0_6224
AAAAAAAAGAGAGAGATTCCTTTTCCTATCTAATAATATTATTAGGCTATTTTTACCATGATTGGGTCCTAATTCAACCAAACA
AAAAAATTGACCTTTCAGCCAAATCCCCTAGGAACGCCTAGGGCTCGAAGCGTCTGTAAATTCACTTCATATTTCCTTCCCTAG
AATGCCTAATTTTTTTGAACCTTTTCCCATCCCAATAATATTATAAGGCTATTATGGACCATTAGAATTGGGTCAATTTCAACC
AAGACTAAAAAATTTGACATTCAGTGCCAATCTCCTAGGCACAGCGCAGGGCTCCGAAGCGATTCCGTAATTCACTCATATTCT
CATCCCCTCTAAACCAAGCTCTAATTTTTTGAACCATTCCCATTCTCAATATATAATATTATAGGGCCTATTATTGCCCATGGA
ATTGGGTCCTAATTCAACCAAAAAAAAAAAATTGACTTTCAGGCCAAATCCCCTAGGTCAGCTCTAGGCTCTGTGAGCGATTCCG
TAATGTCCTTCATATTTCTCCGTAACATGCTCTAATTTTTTTGAACCTTTTCCCATCCTGCAATTAATATTAATAGGACTATTA
TTTACCTAATATTTGGTCTCTTCAACCAAACAAAAAAAATTGACCTTTCAGGCAAAGTCTCCTACGCAGCTGCCTAGGCTCCGAA
CGATTTCGTTATTCACATATTTCTCTCTAAGAAGCTCTTAAATTTTTGAACTTTCCCATCTCAATAATATTATATAGGCTATTA
TTTGACCATGAATTGGGGTCCTAATTCACCAAACTAAAAAATTGACCTTTTCAGGCCAAATCTCCTAGCACAGCCTAGGGCACC
AAGCGTTCCGAAAAAAATAATTCACTCATATTATCTCTCTAAAGCTCAATTTTTGTTGAACCATTTCCATTCTCAATAATATAT
ATAGGGCTATTAGTTGAACCATGAATTGGGTCTAATTCAACCAAACAAAAAAATTACCTTTCGCCAAATCTCCTAGGCAGTTGC
AAACTAGGGCCTGGAGCGATCTCGTAATTCACTCAATTTACTCCTCTAACAGAGCTCTAATTTTTTTTGACCACTTCCCATCCT
CAATAAAATAATATATAGGCTATAATTGAACCATGAATGGGTCCTAATACGACCAAACATAGAAAAATTGACTTTCAGGCCAAA
TCTCCTAGGCATACTCCTGGTGCTCCGAAGAATCTCGTAATTCACTCATATTCTCCCTAACAAGCTTATTTTTTTTGAACCATT
CCCATCGTCAAGTAATAATTAAAGCAATTATTGCCATGAATTTTTGGGGGGGGGTCCTAATTCAACCAAACAAAAAGAATTGAC
CTTTCAAGGCCAATCCCCCTAGGCAGCTGCCTAGTGCCTGAGCGAGTCGTAATTCCCTCATATTCTCCCGTAACATGCTCTAAT
TTTTTGAACCTTTTCCCATTCTCAATAATATTATAAAGGACTATTATTGTACACTATATTTGGGGTAGTCTCATGTTCAACCAA
ACAAAAAAATTGCCTTTCAGGCCAAATCTCCTAACCAGCTGCCAGGGCTCCGAAACGGATCTCGTATTCGACTCAATTTCTCTC
TAAGAACTCTAATTTTTTTGAACCTTTTCCTCTCTCAGATAATAGTATAAGGCTATATTGACCATGAATTGGGGTCCTAATTCA
ACCAAACTAAAAAATTGACCTTTCAGGCCAAATCTCTAGCAGCACTCCTAGGGCACCGAAGCGATCCGTAATTCAACTCATGAT
TCTCTCTAAACGAAGCTCTATTTTTTTGAACCATCCCATCTCAATAATATTAAAGGGCTATGATTGACCATGAATTGGGTCCTA
AATTCAACCAATACAAAAAATTGACCTTTGGAGGCCAAATCTCCTAGGCAAGCTGCCTATGGGCGTCCGGAGCGAATCTCAGTA
ATCACTTCGATTCTCTCTTAACAAACTCAATAAATTTTTTGAAACTTTTCCCATCTAAATAAAATGATATAGGGCTATCATTGA
CCATGAATTGGGTCCTAATTTCAACCAATGTAAAAAAATGACCTTCAGCGTGGCAAATCTCCTAACGCGCTGCCTAGGCTCAGA
AGCGATCAAGTAATTCACTCATATTTCTCCTTTAAGAACTCCAATTTTTTGAACCTTTCCCATCTCAATAATATTATATAGGCT
ATTATATGACCATGAATTGGGGTCCTCTAATTCAACCAGCAAAAAAATTGACCTGTTCAGGCCTAAATCTCCCTAGGCACTGCC
TAGGGTCCGAAGCGATTCGTAATTCACTCATATTTCTCTCTAACAAGCTCTAATTAGTTTTGAACCATGTCCCATCCAATAAT
ATTATATAGGGCTATTATGTGACCATGATTGGGTCCTAATCACAATAACAAAAAAATTGCCTTTCAGGCCAAATCTCCTAGGCA
GTAGCTGCCAAGGGGCTCCGAAGCGATTTCGTAATTCACTCATATTTCCTCTCTAACTCAACCTCTAAATTATTGACCATTTCC
CATCTCAATAATATTATATATGGCTATATTGACCTGATTGGGGTCCCTAATTCAACCAAGACAAAAAATTGACCTTTCAGTGCC
GAAATTCTAGCAGCTGCCTAGGGCGTGCGAAAGCGATCTCTGTTAAGTTTAACTCATATTCTCTCTAAGAGCTCAAATTTTTTT
GAACCTTTTCCCATCTCAATAATATTTATATAGGCAATATTTGACCGATTGAATTGGGGTCCTAATTCAACCAAAACAAAAATA
TTGACCTTTCAGGGCCAAAATCTTCCTTAGGCAGCTGCCTTTTGGGCTCCGCAAGCGATTCCGTAATTCACTCATATTTCTCTC
TAACAAGCTCTAATTATTTCTTGAACCATTTCCCATCTCAATAATATTATATAGGGCTATTATTGACCTGAATTCGTGGTCCTA
TTTCAACCAAACAAAAAATTGGACCTATTCAGGGCCAAATCTCCCTAGGCACGCCTATGGCTTCGGAGCGGATCTCGTAATTCA
CATCATAATTCTCCCTAACAAGCTCTAATATTTCGAAACTTTTCCCGATCTCAAAATATAATATAGGGCTACTATTGTACCCGA
TAATGGGGTCCTAATTAACCAATAAAGACTAGAAAAATTGACCTTCAGGCCAAATCGTCTCTAAGAGACTGCCCAGGGCTCGCG
AGCGATCTCGTAATCTCACTCATATATTCATCTCTAAGAACTCTTAATTTTTTGAACCTTTTCCCCATTCATATATGTTATAAT
GGGGCTTTATTGACCATGAATTTGGGGTCCTAATTCACCAAACAAAAAATTGCACCTTTCAGGCCATCTCCTTAGGGCCAGCTG
CCTAGTGCTCCGAAGCGATCCTTCAGTAACTTCACTTAATTATCTCTCTAAGAGCTTTAAATTTTTTGATCTTTTCCCATCTCA
ATAATATTATATAGGCTATTATTGACCTTGAATTGGGGTCCTAATTCACCAAACTAAAAATTGACCTTTCATGCCAAATCTCCT
AGGCAGCGCTCAGGCTCCGAAGCGAATCCGTAATTCACTCATAATTACTCCTCTAACTAGCTCTAATTTTTTGAACCACTTCCC
ATCTCATAATATTATGATAGGGCTATTATTGCCAGAATTTGGGGTCCTGACATTCAACCCAAACAAAAAATTTGACCTTTCGGC
CAAATTCCTAGGCAACTGCCTAGGGCTCCGATAGAGATCTTCGTAATTCCTCATATTTCTCCCCTAACAAGCTCTAATTTTTTT
GAACTTTTCCCATCTCAATAATATTATAGTAGGGCTAACTATTTAACATTAATTGGGTCCAAAATCAACCAAACTAAAAAATTG
GAGCCTTTGAGGCCAAATCTCCTAGGCATGCTGCCTAAGGGCTCCGAAGCGATCTCGTAATTCACTGCATATTTCTCTCTAACA
ACTCTAATTGTTGAACTTGTTCCCATCTAAGATAAAATAATATAGGGCTATATGTTCCATGAATTGGGTCCTAATTCAACAATT
ACAAAATAGTTGACCTTTCAGGCCAAATCTCCTTATGCAGCTGCCCAGGTTCTCCGAAGCGATCAAGGAATTCACTCATATTTC
TCTCTAAGAAGCCCCAATTTTTTTGAACCTTTTCCCCCATCTCAATAATATGTATATAGTGCTCATTCATTGACCATGAATTGG
GTTCCTAATTCAACCAAACAAAAAGAATGACCTTTCAGGCCAAAATTCTCCTACGCAGCTGCCCTAGGGTTTCCGAAGCGAGTT
CCGTAATTCACTGCATATTCTCTCTACAAGCTCCTATTATTTTGAACCATTTCCCCATCATCTATAATTTATATTTAGGGCTT
TACTTGACCATGAAATTTGTGGGTCCTGATTCAACCCAGAAAAAAAGATTGACCTTTGGCCAAATCTCTAGGCCGCTGCCTAGG
GCCCTCCGAGCGATCTCGTAATCCCTCATAATTCTCCTACAAGCTCGAATTATTTTCGATACTTTTCCATCATCAAATAATATA
ATATAGGGCTATTAATTTACCATGAATTGGGGTCCTAAATTCAATCAAAACAAAAAAATTGACCTTTTCAGGCCAAATCCTCCC

TAAGCAGCTTGCCCAGCGGCTCCGAAGCGATCTGCCGTTAATTCACTCCTATTTCTCTCTAAGAAGCTCTAATTTTTTGAAACC
CTTTTCCCATCTCAATAATTGATTATATGGGCTATTATGACCCTGAATTGGGGTCCTAATTCAACCAAACAAAATAAATTGACC
TTTCAGGCCAAATCCTCCTAGGCAGGCTGCCCGCAGGTTCCCGATAAGCGATTCCGTAATTCACGTCATTATTTCTCTTAAACA
AAGCTCTAATTATTTTGAACCAATTTCCCATCTCAAATAATATTATATAGGGTATTATGTGACCATGAATTGGGTCCTAATTCA
ACCAAGACAAAGAAAATTGACCCTTTCCAGCCAAAATCTCCTAGGCGCTGCCTAGGGGCTACCGACGATCCGTAAATTCACGTC
ATAATTCTCCCCTAAGAAGCTCGTAATATTTTCGAAAACCTTTCAACATCTAGAATAATATAATATGAGGGCTACTATTTACCA
TGAATTGGGGTCCTAATTCCAAACAAACAAAATAATTGACCTTTCAGGCCAACAAAATCCTATTGCACTGCCCAGTGTCTTCCG
AAGCGTCAAAGGATTTCACTATATTTCTCTCTAAGAAGCCCAATTTTTTTGAACCTTTTCCCATCTCAATAAGATATATAGGCT
ATTAATTGACCAATGAAATTGGGTTCCTAATTCAACCCAAACAAAAAAATGGACGCTTTCATGGCCAAATCGTCCTACGCAGCTT
GCCTAGGGTTCACGAAGCGATTCCGTAATTCACTCATCATTTCCTCTAACAGCTCTAATTATTTTGAACCATTTCCCCATCTCC
AATAATATTATATAGGCTATTATTGACCATGGAATTGGTCTAATTCAAACCCAGACAAATAAGATTGACCTTCAGGCAAATCTC
TAGGCCGCTGCCTAGGGCTCCGGAGCGATCTCGTAATTCCACTCATAATTCTCCCTACAAGCTCTAGATATTTTCGGAAACTTT
TCCCATCTCAAATAATATAATAGTAGGGCTACTATTTACCATGAATTGGGGTCACTAATTCAACCACAAAAATAATTGACCTTT
TCAAGGCCAAATCTCCTATGCAGCTTGCACCTCGGGCTCCGATGCGGATCTCGAATTCACTCATATTTCTCCTCTAAAGAAGCA
TCTAATTTTTTTGAACCTTTTCCCATCCTTCCAATAATATTATATAGGGGCGTATTATTGACCATGAATTGTGGGTTCCTAAT
TCAACAAAAAAAAATTAGGAACTTTCAGCCAATGATCTCTAGGCCCACTGCCTAGGCCTCCGAAGAGAGTTCGTAATTCACGGC
AATTGATTTCTCCCTAAAAAAAAGCTCTAATTTTTTTGAACTCTTTGTCCCTCCTCAATAATATTATATATGATGGGCTATACA
AATTTACCGTTGCAATTGGGTCCAAATCAACCAAACTAAAAAGTTCGACCTTTGAGCCAAAATCTCCTAGGCATGTCTGCCTAG
GGCTCCGAAGGCGATCTCGTAATTCACGTTCATATTTCCTCTCATAACAAACTCTAATTTTTTGATCTTTTCCAATCTAAATA
AAATCTAA
>m141121_210323_42134_c100732402550000001823141405141506_s1_p0/89525/4040_10426
CACATAAGCCAACTCAGTTGCTTCTAAATGTGCTCATCCCTATAGCATAATTAATGCCATCTTGCAGCATGCCATCGTAATCCC
TCAACAGTGCATAGTCCAACAGGGCCCAAGGGCCTATCAGATCTGATGCATCCATGTTTTGATGTTATTCCTCACATGCTAGCA
AAAGCAATACTGTAGCGAGGCAAAAAGGTGGAATATGGTCAACAAGTATAGATTTGAGACATAACCAATCAGATCATGACAATC
GCAACACCTGAGATCCGAGCTTGCTCTACTTGGACGCGAGCGTTGACATTCGTGGTCCAATCCCCAGACCCAATAATGACCTTG
TGGCGTCGCTCTGATACCCTAAGGTTGTAAGCCCTGACCCTTTGTCTTGTTATGAGCGGGTTCCTATAGTGAGTTTAGCTATGA
GAACGATAACGAGTGCGAGAGACTGACTCGCGTTAATCTTCGAGGGTCTTTCCAAACAGAAGTTGTGATTATAGTCAAAGAACT
TCTAAGTGCGTGAATCGCATTTTCTCTCTGAAAACTGGGCTTGACTTGTCAATTGGATTAGCTTTCAAGGAATACAACATTGCT
TGGTGTAGATCAAAATAAGGTTGTAAAACGCTAGGTTTTGCTCACTTATGAATGCATTTCACATGCTTAGATCAAGTTTATTAC
AAGCATCCTACGATTCGCATTCTACTGATCTGGCCATTTTACGTAAAAACCGCCCAGCGCCAGTGTTGTGAATCCATAGCGGAC
AGGCCTGAGAACATGGGTACGTCGTCAAGCCTTGTCACGCTCTCGTACTCATCTGGTGTTGGCTTGAGCGCAGCCTAGTTGTGC
CCTAACCCTGCAACATGCATGCACCGCGTTGAGCTCGATAGCGCGCTAGGCCCAGAGGAACGTCCATCGCTAGATACAGTTGCA
ACTACTTAGCTGGCAATGGTTCTCAGAGAAAGCTGACGTAAAGGCATTTGTTGGAATAAACGTTGAACTAGCAATAATTATAGC
ATGCGGTCGTGTCGGGTCCGCATGTCTCGGTATTGTCACACGAGTGGACCATGGCCTGTTAGATCAAGACTCCGCGGCGGTGGG
GCACGTCGCTAATTCAGAGACCACTGCGAATGGGGCCGCGCCCTATTACCCCGGCGCTGGGGGCCATGCCCTATAAACACGCAG
TGGGCTGGCCCCATGCCTAGTCTATATCCTGGGGCGGTTGGGCCCATGCGCCCTAGTAAAATCGATGCTCAGGTGGGGCCATGC
CCTGGAAAACACACAATTTGGGAGCCGTCCAAGCCCTGCAAAGTGGTGGAACTTTGGTGTACTCAACTTTGCATTTTAGAACTG
TTTGTTACACGCCTATCAACTTGGAATCCATTGAGTAGCTATGGCCGACAATCGGTTATAGATTCATCTAGTTCGGCACATTCC
AGTTTGATAGGGGTCCGTGCCCCGCAATCACAGGTGCGATGGGGTTGTGTGCCCATGCGCGCGAGAGAGCGCGAGCGTTGTTTT
AGTGTTTGCTTACCTGGTGAATTTGCCGAGCCGCGCTGGTGTGATGTCACTAGAAATGAACCTGGCGGAGCACTGTGGGTTCGC
CTAGCAGCGAGCCTTCACCAGACATAACCTATAGGGTCACGAACACCTAGCAAGAAACATCCCTCCAAATTAGCTAATCAATGT
TCAAGACCAGGTTCCGGAACCGAATCCCGCACCTCGTCCGTGTGGACTCTGAAGAACCTTAAAACAACACCCTCGGAGTCGCCC
CCCGAACACCCCCGAGCAGAAGGCCGTAGAAAATTGGCTGCAAAACACACCGCTGATTTTGTGCCTTTGGTGCCGCGGCACCAC
GTGATCTGTTGCTTGCGGCACACATCAGTTGCATGGAGGCTCGTGCCGCGGGCGATTGGCAAGAAATTCGCCTGCCGGCGGGCA
TGGCCAGCGCTTGCGCGCACCCAGAAGTTCTGGGTTTGCTTCTCTTTGCGTGTTGTTTTGCCCTGGGAGCTTGTTAACGCTCTC
AATAGTAGATCACCCGGTATAGAAATAGGATGCCCATAGAGGGACCTGGCACCTTAAGCGCGCGTGAATGACATCGAAAACCAA
CCCCCATGATGACCAACCTAACCACCCCTGAGGATGTGCAAACCTTGGGGGTCAATCCAAAAAAATCAAACATCACTCCTATAG
CCCACCACTGGGGGTTGGATGTGCATTCGCAAAATCAACATCTCAAACCAAACACCCCATAAAAAACCTGTTTGAACCTAGGCA
TTTGAGTCTTGCGCCTCAACCCTTGTAACCGAAGCAAAACAACTTTAAAGACCAGACGCATAAATTCTATACCTTCTGAAGAGT
TAATCGCCACACTTAGATGCCCTGCCTTGAATTTCCGTTCCCTAGAGAGGGGCTCACATGGTTCTCCTTCCCTGCTCTTCTTTA
CTTGTTGCGTTGTCCTAGAGTGTGTGGCCCTGTTTTGATCTCCTCTGTTCTCTCTTTTCTCTGTCTATGCTTGTAACATAAGCC
CACAAATGAAAAAGCCAAGAACCGATCCACCTAATGAAACCCCAGCTTGCAAGGCTTGATTGATTCTCCATGCGTGGTGGCCAA
AGATAGCCAAAATTACCTCGCGCCTTACTGTAAGTCCTCTGCTAAGGCTTATCAGACTGTTCAGGTACTTAAGTCATTTACGAC
TTTATTGTCCAATTCTACCACTTCGATATTCTTAATAACGTATTACTCTTCTAGGCAGTTACAGAATGATGTACCCTGGGTAAG
ACAGTGCCAATCAAGCAAACATGCCTCACGCATCAAGACTTGCAACGTTATGTTAGAGATTCTCGCGAAGTCCCCTAAGGCTCC
TAGAGCCTCGCGTGGGAGCCTGGGTACAGAATTTTGCCCGTTGGTGACTTGCCTTGTAGGTTAACTAGCTCTCTAGTGACCGGG
GTCCTGCGGCACGTGCATCGCACAGATATATAAACACACACTGCGGTACACAATTGCCCAGCAGAAGAGTAGGATTTAGTCACA
TATCATATATACCATGAGTGTTGAGTGGCCAAACACTAGGCCAAAATTGTACAATTATCGCCCTTTGCTTACACCCGTCGGCGC

CGTTACATTGGCAGTACTAATGAACGTATTCATTGGGCATCTCAAGATTAAACAAATATAAATAACCTAGTTGGCCTTAAATCA
TTAACCATTGCGTACTGTAACTCATAAAAATGCACACGCAACATGTCCCAGATGTGCCTCTGTCCTGGGCAACGCTAATTGCAA
GGGGCTGTAAAGCCTTATTATGCGAATTGTGGGGTCTTTACACCGCACCAGAGCAGTACTAATGGTACTGGGGCTGGCAGGTAC
TGGAAAGCGCCAATAACCAGTTTACCTGACGCGTAACTCGCGAACTCGTAACCATATAGAAATTGTCCGGACGGTAACCCTGCC
TAGGGGCCTCCCCGGAGGGCCGGGTGATGAGATAGATCCCCGCTGTGACTCTTTGAGGTTATGACTAGCGTCTCTAGGACCGTC
TCGGCTACGCGTTGCATCTCAGATAAATGTGCCACCTGCTCCTAGCGTGGTACTAGAATCACGGAATAAATTATTGCGACAATA
TACATTTCGCCTGCTCTTAGCGGGGCAAAAATTACAATTATGCCCTTCAGAACGACAATCAGGGGGCCCTTAGCCATTGTACCT
AATGACAGCATAGTCAGTGGCAGTCTCGAGATGAAAAAATAGTCATCCTGATATCATGCTTTTAAATTTTCATAGAGACCTTC
ATTGTGAACTCATAGAGATTGACATCCGAGTGAGATCTGCATCGTGCCTCACTGGCACGCTTAAGCAAGGTCCTGCTTAAGCTC
TGTTATGTTCGAGCTGAATTTGGTTTTTACAAGGTCTGCTAGTTTGTCCTCGAGTGGTTCACAACTAAAGTGCGTAGAGGCAAC
GTTTTCTATCCTATTGGTACCTGGATTCGTCACCGTCCTACCAGATGCGTGGTTGCTTAATAGCGTGATTCAGATACGACCAGA
TATGTGTAGAATAGTATGGCCTTTTGCCTTGTTTCTGGCTTAGTATGGGGTTGGGCTGCCTGAGGTTGCCCGTCATCTGTTGAA
GGCCTACCGTTTGTTCCTTCGATGATGTGTTGAAGAAGGGAATGGGCAATTATGTGAGTGGGTCGGACTGGATCGAGGTGAACT
TCGAAGTTGTCTCTTAACTTTTCCATCTAAGCAGTGTATTTGGTATTTTGTTCTTAGGAGGAGGAGCAGTCTCTATGTCATGGG
ACTAATTTGTGGTGGGGTTGCTCGTGATGTGGCAGACACTTGGAATTGACAATGAGCGCGCGAGAGATAATTTGTCCGGATGAA
CCCAAAGGTGCATTTTGAGGCGTGTGTTCATAGCTTCAGTCTATATACTTTCGAAGGTGTGTTGACATTTTCTTCCAGATCTCA
AGGGTGAGTTCCTTGGGCTCAATCTAAGCACTATTTTGGAAACAACATAGTGTAAGCAGTCTTGGTTTTGATCCAGGCGTTACA
CCAGTACTTGTTGTCCATCCATGCTCTCCTGTATTTGGTTAGTATAACATCCTTGTTCACTGCAACCTGGGTTTGGGTAGGTGG
CGACCTGCACTTTGTCTTTAGACCCGAAGGTCATCACTGGTTATAGGGCAATAAGCGCCTGTTTTGCTGTTGATAGAGTGGGAA
GTGGTGCTCTTTGATCATGGCTTGAGATTCAATGGTAGGTTTGGTTGTACCCTAAATAGGTGGACATCCATTGGAAGCTAAGTA
CGTCATTTGCGATTTTGTTGTCTGTTTTTTTTTTGTGTGTGTGGTGGCCTGTGTGGTGGTTGGTGGTTTGTTGGGTGTTGTGTG
TGTGGTGGGTGTGTGCGTGCCGTTGTGTGCTGTGTGGGCTGTGGGCTTGTGGGTGTGGTGGTGTGGTGGTTGGTGTGGTGTGTG
CTGCGTGTGTGTGGTGTGGGTTGTGTGTGGTGTGTGTGGTGTTGGGGGTGGGTGGGTGGCGGGGAGTGGCTGCGTGTTGTGTGT
TTGGTGAGGATGGTGGGGGGGTGGTTGTGTGTGTGTGTCGTGGTGTGTGTGGTGTGTGTGTGGTGGTGTGTCTGTGTGTGTGTG
GTGTGTGTGTGTGGGGGTTGGTGTTGTGGTGTTGTGTGTGTGTTGTGTGATGTGGGGGTTGTGTGGGCGTTGGTGGTGTGTGGG
TGTGTTGTGTGTGTGTGTGGTGTGTGTGTGTGGTGTGTGTTGATGTGGTGTGGCCTGGTGGTGTGGTGGTGGTGTGTTGTGTGT
GCTTGGGGTGTGTGTGTGTGCGTGGGTGGTGCTGTGGGTGTGGGGTGTTTGTGTGGTGTGCGGCGGGGGTGTGCGTGCGGTTGT
GTGTGTGGTGTGGCTGTGGTGCGTGTGGGTGTGGTGTGATGGTGGGTGTGGGGGGTGTGTTGCCTGGTGTGATGTGCGCTGTGT
GTGTGTGGCTGGGGGTGTGGGGGTTTGTGGTGGTGGGTCGTGTGTGCTGGTGGTGGCGGTTGTTGTGCTGTGGGATGGCTGTGG
TGTGGTGGCAATGTGGTGGTGTGCTGTGTGTGTTGTGTGTGGGTCTTGGCTGTCTGGTGGTGTTGTGGTGGTGGTGTGTGGTGT
GTGGTTTTGCGTGTGTGTGGTGTGGGTGTTGTGTGTGGTGTGGGTGTGTGTTGTGTGTTGGGTGTGTCGTGTGGTGTGTGTGTG
TGTGTGGTCGTTGTGGCTGGGGGGTGTGGTGTGTTGTGTGGTGGTGTTGTGGTGGTGGGTGTGTGGTTGGTGTGATGGGCTGTG
TGGTGGTGGGGTGCGTGTGGTGTGTGTGTGGTGTGTGGTTGTTGTGGGGCTTGTGCTGTTGGTGGGTTGTGGGGTGACTGTGGG
TGTGTGTGTGTGTGGGTTGGGTGTGTAGTTGTGTTGCTGGGTGTGTGGTGTGTGTGGCGTTGTGGGGTGGTGTGGGTGTGTGTG
TGGTGTGGGTGTGTAGTGTGAGTTGTGTGGTGTGTGTTGGTGTTTGTGTGTGGTGGTGTGTGTTGGTGTGTGGTGTGGCTGTGG
TGTTGTTGTGTTGTGGTGGGTGCGGTGTGTGTGGTGTGTTGTTGGGGTGGTGGGGTGTGTGGTGTGTGGGTGTGGTGCGTGTGG
GTGTGGTGTGGTGTGTGAGTGTGGTGGTTTGGGTGTGGGGTGATGGGGTGTGTGGGTGGTTGTGGGTGTTGTGTGGTGTGGTGG
TGTGTGTGGTTTGTTGGTTGGGTGTGGGTGGTGTGTTTGGTGTGTGTGGTGTGTGTGGGGGTGTGCTTGGTTGTGTGCTTTGGT
TGTGTGGTGTGTGTGTGGGTGTGGTTGGATGTGTGGGGTGTATGTGGTGTGTGGTGCTGGTTGTGTGTGTGGTTTGGATGTGTC
GG
>m141121_210323_42134_c100732402550000001823141405141506_s1_p0/94264/5634_11470
CATAACTCCACATTGCTTTCTATATTGGGGGCAAAGAGTCCTGGATGATTATTGATTGATATTGGTTTGAATGTAGATATATCA
ACGTACGTTTTTCGATATTGGGCTAAATGTTCTGATGATTAATTATGATCATTGGTTGATTGTCAACTTGATTACCTTTCCAAC
ATTCATGATTTGGGGCCACTTTCATTACATTTTGGCGCTGGAGAGAGTCACACCTATTTGAACTCAAAGCCATTAGCAGTCATG
CCCCAAGAGAGTCCTAGTAGGTTCCATACTACAGCTGACAATGATGTAATGATCCAATACATTGCCACATCTATAACCCATTTC
CGATCATTGTTATAGTAGTTCTATTTCATGCTGGCAACATCAGCTCACGGTGTTATGTGGTGCAGGTAAGAGGCAAAAAGAAAG
CTGGACCATCCCCTTGAGTTGGATAGCTTGAGGTGAGTGATGTGTACATATTGCCAGCTGCTCGTCCACCTGCCCAAGCCTACA
ACAACATCACATACAAGAGCATAAGCGAGCTTATTGTAAGAGGAACTAGGGATACATAAACCAAACATATAGATCATGTATACA
TTTTGAGCGCAATGTAGACAATCGTTCTATGACACATGTATAATTATAGTTTCTTATTCCTTATGTATTTATCCAGACCCTGAA
TTAATAAGTTATCCGATGATGGAGATCCCAATGTATATGGACTGTGTTTAGTACTAATTTTAGGACACATCTTACACATAAACC
ATGAAGAAATATTAGAGATGAGTCCTAAGCATCAAGACAAATGTATGATATTTAGATCCGATGAAAACCGGAAACTAAACTTCA
TAACCACTTATAGATAATATCAAAAAGACATACGATTTTAGGCCAACTTATTATAACTTCTTACGATTAGCTCTGACGGATTTT
CACGTCACACTCATTATATATGAAAGAGCAACATAGAAGCCCTGTATAGAGCGGACTCGCGACTTAGAAGAGTTTTATGAGAAG
GAAGAGAGAGAAGCGATCTAACAACCTTGGAGATAATAACAGAGCGAAAAAGCCAAAAGGAGTTTTTAACGGGTCCTGAAGAGA
CCAAATTGCCGGCATCAAAGACACTATGCAATGTATCTAACTTCTAACCGAAAGATATGCTTTCACTATATCAGGGAAACCTCA
ATAGCGGTTAATCCCCAAATGTGTTTGATCAAGTCGTAGTTGAATGTGCTTTAACTGCTTATACAAATAAGCTTTATAATAGTG
TTTCAAATCGCCCAAACCCACATGGACTTGGCATGCTATTAGTTAACATCTCCAGGCGGAGCATCCAGATTCCTGATAGACGCC
CCTTGCTCTTTCCACTCTATATTGCCTCATCAACTTCACCATTTATTAACACTACCGCGTGAAAGATACCTTTGCTTGAAACCT

TTAGAGCGAAAATAAGGTGAATAGAGATGCCTTGGTGTGAGTACTTATAAGGTCCTAATTTTACCATGCCCTGTGAAGAGTTTG
AAAGTGAATAGTTATATCTTGATTTATTATCTGTGAATGAATATATAAAATCAATAGCTGCGAGTTTGTAATGCATGGGTGGTA
TAAAATCAGATATGGTGTATATGATAGAGGCATGCAGGCAGAGGCTGAGACTCGTTCAGGATTAAATGCTGGATACTGTGAATG
ATAAAGAGTGATAGAATGTGCGTTGGGAATGTTTTTAGCACTGCAAGTGGTTTGTGAACTTGTGGTGCGGTAAGATTTATTCCG
TGGGGAAAAGCTCTTGATATGCTCACCGATGAATTAATGGGTCAATTATTGACTACAGATTCAATCAAAGCAGGTGTTATATCA
AGGAAAGATAATGAGGCCTGGTGGTGGGTTATAATGAGGCCTGGGGGTTACAGCCAGGGTATTCAAGTCTTCATCTGCTTCTAT
GAATGTTTCAGCAGATGGTTAACATGATGTTGCAACTTAAGATTGCAAACGGAATGAGGAAGAGATTAGTGTTTTGAAGCAACG
ATCGGTATCTGTTGGGGACACCTTTCCTTTTGATCTGTGCCAAGAGTGGGCACCAGTTTTGGCCTCAGCCTAGGGTCTGAGAAT
AGATGGGAAATTAAACTCTGTGGAAGACTTCAAGGAAGTATTCTTGCTCCCATTCAGCAGCTTGAGCAATGGATGGGCATGATC
AGTTCCATTCTTGATAGCATTGGACTGATCGGGTCACGATAAGGTGATATGTGCTACATGTGTACTGCGGGATGATAGCCCGGG
ACATGGTGGGAGGTGGGTATCCTGAGACACGAGACACCACTTTGATGGATGGAAAGAATTAGGCAGTGTTTATAGGAGAAGTAT
ATTGTGGATGTAAGCCAAGACGTTAAACGATGCCAACGAATCCTGAATCTTGTTGCAGGGTAGTGCATCAATGACCAAGTATGT
TACCAAATTTGATGGTTGGCCAAGTTTTCTTTGACATGGTACCTCATAGATGTGCTCCGGGAGGAAAGGTTTTATTCAGGGTTG
AAATCCCAGAATAGTTCAGTGGCATTAGAGTTGCCCCAGTGCATGAAGATCTCTACTATGCTAGGTGGTTAGGGAAGGCCTCCT
TGCTGATTGAGAGCACAGGACATCAGAATTGCGCAGCAGAGTGCCAGGGTGCACGAGCTCAAATATGGTATCTCCACTTATGTG
GACAGTAAAATAAAAGCTGGAAGACCTCGTCCAGATATGCACGCGGTGCCAAGAGGCCATCATTTGGGAGAATGCTGAGCAAGG
GCATGTTTCTCTATGTTTATGTTTGGGCATCGTAAGAGAATTGCCCAATGCGAAGGAAGGATGAGAAAAAGGGGATGGGCAGCT
CGAATCCAGCTCGATTGTGCATTCCGATGCAGCGGAATCAGGACTGAGACTGAGTTCTCAAGGTGCAGGTCCCGGTTTTCTAGC
CTAATTTTGAGTTATGCTGTTTGGCTTTGGTGCCATGTATTCCTTTGTTAGTACATATACAGAAAGGCATAAAGATGATTATGT
ATATCACATGGTTATGTTGTGATGGGAAGGCACACCGTAATCGCTAATTCATACTTTAGTTTACGCGATTTTTCAAAAATGACT
CCGATCTGGCGAGTTCTAAGCACTTTTTTAGTAAGGCAACACCGTAAAAGGTCCCCCAAAATGGAGTTTAGGGCGTTACAGCTA
GTACCAGCAGCAAGCAGTAGCAGCAGTAGTAGTCCCTGGAGAATTAGGGCGTTACCAGTAATAAGCAGTAGTACTAAGCAGTAT
AAGTCCCTGGAGTTTTAGGGCGTAGTAGTGTAGTAGTAGTAGCAGTAGTCAGCAGCAGCAGCAGCAGCAGGCAGTAGCGAG
TACCAAGTACCAGTACCATTACCAGTACAGTACCAGTACATGTGTAGTATAGTAGTAGGAGCAGTAGTAGTAGTAGCATGATCA
GCAGCAGTAGTAGCAGCAGCAGCAGTAGTAGTAGAAGAGTAAAGTAACGTAGTAGTAGTAGCAGTATAGTAGCAGCAGTAGTAA
TAAAGCAGCAGATAGCCAGCAGTAGTAAGTAGTAGCAGCCAGTAGCAGCTCAGTACTCAGCCAGGTAAGTAGTAGTAATCGTAT
AGTAGTGTAGTAGTATAGTAGTAGTAGTAGTAGTAGTAGTAGTACTAAGATAGAAGCCGAACAGCAGTACGCCAGTAGCCAGGA
GAGTAGCAGATAGGCAGCAGCAGCAGTAGTAATAGTAGTAGCCAGTATAAGTAGTAGGTTGCAGTAGTAGTAGAGCAGCAGAGT
AGGCTGCAGTAGTAGTACAGTAGTAGTAGCGCAGCAGCAGTAATAAGCAGTAGCAGCAGCAGCAGCAGTAGTAGCCAAATAGCA
GCAGCAGGCAGCACACAAGCAGCAGTAGGTACGTACAGTAGTAGCAGCAGTAGTAGTAGTGCAGTAGTAGTGCAGCACAGTAGT
AGCAGTAGGTAGTAGTAGGGCAGTAGTAACAACAATAGTAATAAGCAGGTAGTAGCAGTAGAGTATAGTAAGTACAGTAGTAGG
CAGCAGTATAGCAAGTAGTAGCAGTAGTAGTAGTAGCAGTAGTAGTAGCAGCAGTAGTAGTAGCAGCAGTAGTATGTAGCATCA
GTAGACAGGTAGGAGCAGTAGCAGTAGCAGCAGGACGCAGTAGAAAGCAGAGCATAGGGCAGCCAAGCAGGTAGCAGTACAGTA
GCAGTAGCAGTAGGTGAATAGCAGTAGTAGTAGAAGTATCAGCAGCAGCAGCGAGTAGCAATAAGTAGCAGCAGCAACAAACAA
CAGTAGCACAGCAGTAGCCAGTAGCAGTACAGGTAGTAGCATTAGTAGTAGTAGTAGTATAGTAGCAAGCCAGCAGCAGCAGCC
AACAGCAGTAGTAGCAGGTAGCAGTAGTAGCAGTAAGCAATAGCAGTAGCAGGTGTGTAGTGCAGTAGTAGTAGCAGTCAGTAG
CAGTAGCGGTAGTAGGTAGCAGCAGCAATAGTAGTAGTAGTAGTAGCAGTGGAGTAGCAGTAGAAGTAGCAGTAGCAAGTAGCA
GTAGCAAGTATGCAGTTAGCCAGGTAGCAGTAGAAGTAGCAGAGTAGTAGTACAGCAGTAGTAGTAGCAGTAGCAGTATCAGTA
GTAGCAGTAGTAGTAGCAGTAAGCAGTAGTGTAGCAGTAGTAGTACCAGTAGCAGTAGCATAGCAGTAGTCAGATAGCAGGTAG
TAGTACAAAAATAGTAGTGAGTAGTAGCATAGTGAGTAGCAGGAGCAGCAGCAGTTGGTCATTAGTAGCAGATAGTAGAGCGTC
AGTAGCAGCCAAGCATTAGCAGCAGCAGCAAGTAGAGGCGGGGGGAGTATTAGCCAGCAGTAGTAGCAGTAGTATTAGCAGTAG
TAGCATATAGTAGCAAGTATTAGCAGTAAGCGTAGTAAGGAGTAGTAGTAAGCAGTAGTAGTATAGTAACAGCAGCAGCAGCAA
GTATAGTAGTAGTAGTAGTGAGTTATCAGTAGCCAGTAGCATACAGTAGTAGCAGTAGCAAGCTGTAGTAGTGCAGTAGTAGTA
GTAGTATATTGAAAGCAGTAGTAGTAGCAGTAGTAGTAGCGCAGTAGCAGCAGCAGTAGTTGCCGCAGTAGTAGCTATAGCCAG
CATTAGTAAGCAGCAGCAGAGCAGTAGTACTAGCAGCAGTAGAGTAGTAGCAGTAGTAGTAGCAGTAGTAGTAGTAGGCCAGTT
AGTAGTAGCAGTAGTAGCAGGAGCAGCTAGCAGTAGCCAGATAGTAGCAGTAGTAGTAGCAGTAGTAGAGCAGCATTAGCAGCA
GGAGCAGTAGCAGTAGTAGCTGCAGTAGTAGCAGTAGTAGGGTAGCAGTAGTAGTGGAGTATTAGTAGTAGCAGTAGTAGTAGT
AGCCAGCAGAGTAGTTATATAGTAGTAGTAGTAGGATGTAGCAGTAGCAGTAGCAGGTAGCAGTAAGTAGCAGTAGCGACAGAA
GTTAGCAGTAGTAAGTAGTAGTAGTATTAGCAAGGTATAGTTAGCAGCAAGTAGTGCAGCAGTAGTATAAGCAGCAGTAGCTAG
CAGCAGTAGTAGTAGCAGCAGTAGTAGCAGCAGTAGTACAGCAGCAGCAGTAAAGTAGCAGCAGCAGTAGCCAGCAGCAGTAGT
AGTAGTAGGAAGCAGCAGTAGTAGTAAGCAGTAAGGGTAGGTAGCAGCAGCAGCAGCAGTAGTAGAAGCAAGTGAGTAGTAAGT
GGTGAGGGTAGTAGTAGCAGTAGCAGCAGTAACATGCAGCAGTAGCATCAGCAGTAGTATCAGTAGTAGTAGTAGTAGTAGTAG
CGTAGTAAGTATAGTAGTAGTAGTAAGTAGGTATTAAGTA
>m141121_210323_42134_c100732402550000001823141405141506_s1_p0/94332/1729_12373
TTATCGCTACCAATAAATGGAGGTGTCATTGCTTGAACTCCGTGCTCTCCAGCACTTTCCTTTTCAAGCCTGATTTACTGTGCT
CTCAACAGCAAGAGCCTTCCCTACACCTGCCACGTAGGTAGAAGATCTCATGCATTGGGACGACTCTAATGCCCTGAGCTATAC
TGGGATTCAATCTCTGAATAAACCTTTCCTTCGAGCTACATCCGTTGGTACCATAACAAAAGAAAACTTGGAAAACCCATCGAA
TTTGTTAATATACTAGTTTACTGTTGCATTTTACCTCAACTTAGGTTCAGAAACTCATTCATCTTAGCAGTCTTGGCTTGCATC

ACAGTAATATCTCTCATTGAACAGCGCCTAAATCCTTTCCCAATCCATCACAGTTGTATCTTCCATCTGGTGATACTACTTCCC
ACCCATGTCTGGGCATCCATCCCAACAATATATATGTTCGCACAGATCACCCTATCGTGACCTACCAGCCACCATACTGTCGAC
AATGGAACTGATTCCATCGCCCATCCCATACTCAGCTTCTGAATGGATCTAGGCCTCCTTCAAAGACTGGGAGGGTAATATCCT
GGAATCTTCCACAGAAAATTCCCATCTAGTTCTCAACCCTAGGCTGAGCCAAAAACTGGTGCCACTCCTGACAAACTAGGAGAG
GTGTTCCTGTCAGGTTCCGTTGTTGCTTTAAACACCTAATCTTTTCCTCTTGCTCTGCAATCTTAATTGCAAATATGTAACCAT
TGCTGGAAATTCTGAGGGGGCAGATGAAGAACTCAACCCCTGGCTGAAATTCCCAGCCCCGGTATAACCACCACCAGGCCCTCA
TTATCTGGCCTTGGATACATACCTGCTGATTAAATCTGCAGTAAATAACTTGACCCATTAAACATGATGAGCATTCAAAGAGCT
TTCCAACACGGGAACAATCTTACCACACTACAATACACAAATCCACATGTAGTGGCTAAAAACATGCCATAAGAATTCATACAT
AAATCATAATCCCTACTCTTCCAACATACTACCAATGCCTCCAACCCATCATGCAAATAATCACATTTATATAATATATATCCA
TGGAGTAGGTAATCATATAGTCAAGAGCCAGGCTTTATCAGAATCTCATGGCTCCCAAGTACAATGTGCAGGTAAGCATTTACA
TTCCTGTTTAAGCAGTTAAGCACATAACTACTGAAATAGTACCATTCCCTGAGTGAAGCTATCTTTAGTGACGATGTACATGTC
CAGCTTGTCTTCAAGGAAACGCTTAACCTTGGCTAGCTTCTGAATACAAAGTTGTAATGCCCTAAATTCCAGGGATCGTTACAG
TGTGCATTTTAACAGTGCTAAACACGTTAATCGATGTCATTTGGCATAATCGTGTAACTAAGTATGATAGGCGGTTTAGGGTTA
AAATTTTTGGTTCAGATATAACGTTTTCACTAAAACGTTACTGTATACATTGGGATCCCAAAAAAAAAATATAAGTTTAAAGGTT
AATTACAACGAAAATATTTACAACTAAGCCGACCTGAGCGAAAAAATAGGTTTAACCCTAGTTCCTCTTTCAACCCGTCGGCCG
TGGCGGTCGAGTAGCCACAATATGTACACATCGTCACCTAAGCTCTCCAACTCAAGGATGGTCAAGCTTTCCTATCTCTCTCAA
AACAACAAAAACCAACCCGGAAAAAGGAAAAAAGGAAAGGAAAAAAAAAAGAGAGAAAAAGGGATTTAGAAAGCTTTTTTTTTT
TTTTGGGAAAACCCCCCCAAAAAATCCCATTTTGGGGAGGTTTTTTTGGAGGAAGCTTTTTTTAAAGGGGGTTGAACCGATGGT
TTTTGGGGGGGGGGTTTAAAAAAACCATATGGGGTTTGGGCCCTTTTTTTTACCTTTTCCCCGACCCCGCCCCAAAAACGGCCC
CCCGAGGGGGGGTTTTTTTTTTTTGAAAGAGGGAAAACCCCCCCTAAGGGGGTTTTTTTTTTTTAAACCCTTTTTAATTTT
TTTTTTTTTTTCCCGCTTTCAGGTTCCGGCTTTTTTTAAAAAAAAAGTTTTTTGGGTAAAAAAAAAATATTTTTTTTTTTG
GGTTTTTTTTGGTAAAATTAAACCCCTTTTTAAAAAATTTTAAAATTATTTTTTTTTTTGGTTTGGGAAAATTCCCCCCAAAAA
AATTTGTAAAAAATAAAACAGTTAAAAAAAAACCCCCCGTTTTTTTTTAAAGTTGAAACGTTATAATCTTTTTTTTTTTTTT
TGAAAACCAAAAATTTTTTTTTTAAACCCACCCCCTAAAACCGCCTAAAAAAAATTCCCCATAAACTTTTTAAGTTTTTTTTTT
TACAAAAAACGATTTAAATTGGGGGGCCAAAAAATGAAACTTTTCCCCCGATTTTTAAAAAAACCCGGTTTGGGGTTTTTTTTT
AAGCAAAAAAAAAAATGTTTTTAAAAAAAAAAATGCCCAATACAAACTTTTGTAAAACCGTATTTCCCTGGAAATTTTTTTAAAA
AAAGGGGGCAAATTACCCAAACTTTGGGGGTAAAAAATCAAAAAAAAAAAGAAGCCCCTTAGCCCCCCCCCCCCCCCAAAGGGGT
TTTTTTTTTTTTTTTAAGGGGGTTTTTTTTTTTTCCCCCCCCCCTGAAAGAACCCCCCAAAGCCTTGGGGACCCCCAAATGT
TAAAAACCCTTTTCGTCCACTTAAAAGATTTAGCTTTTTTTCCCAAAAAAACCTTCCAGGGAAATGGGGGGGTTAAAAAAAAAA
CTTATTTTTTCAGTAGTTTTTTTTTTTTAATGTTGCTTTTTAACTGCTTTTAAAACAGAAAAATGGTTAAAAAAAATTGCCACC
CCCTTTTTTAAACCCCTTGCAACCCCAAAAAATTGGTAATTGGGAGAATGAAGGGAATTTTTTCCCTGATAAAGGCCCCTGGCT
TTTCTTTTTTTTGACCCTTAAATTTATGGAAAAAATTACCTTAAAAAACTTTTCCCAAATGGGGATTTTTTATATATTATTTA
ATTAAATGTGAAAAAAAAAAAAATTTTAATTTGGGCATGATTGGATAGTTTTTTTTTTTTGGGAAAGGGGGGCCAAAAAAAAAA
AAAAATGGGGTAAAAAAGGGATTTTAATGTTTTGGGGAAGAGGTTTTAAAGGGGGGGAAAATTATTTTTAATGAATTTTTAAAT
TGTTTAATGAAATTTTCCCTTTTTAATGGGGCCAAAAAAATGGTTTTTTTTTTTTTAAGCCAAATAAAACAAAGTTTTTTGATT
TTTTTTTTTGTGGAATTTTTGGTTAGTGGTTGGGGTTTAAAAAAAAAAAAAGAGATTTTGGGGTTTTCCCGTGGGGGGGAAAAA
AAAAAGCCCCCTTCCTTTTTTTGGAAAATATGCCCCCCTCCCCCAAAATTCGATGTTTTTTTTTTTAAAAAATTGGGTTTTTTC
CCCAAAGTTTTTATTGACTTGCAGATTTTTTTTTTTTAATCAAGCAGGGTTTAAATTGGTATCCCCAAAGGCCCCAGAAATTTTT
AATTGGAAGGGGCCCCTGGTGGGTGGTTTTTTTTTTTTATAAAACCGGGGGCCCTTGGGAAAAAAATTTTTTCAAAAAAGCCCA
AAAGGGGGTTTTTTTTTTTGAGTTTTTCCTTTTTCAAAAAAATCTTTGGCCCCCCCCTCAGAATTTTTTTCCCCCCAGCCAAAAA
ATTGGTTTTTTTACCAATTATTTTTTTTTTGCCCAAAATTAAGAATTTTTTTTGCAAAAGAGGCAAAAGAAGGGAAAAAAAGAA
TTTTTTAAAAGGTGTTTTTTTTTTTTAAAAAAGCCCCAAAAAAAACCCAAACGGAAACCTTGGACCCAAGGGAAAAAAAAAAAA
ACCCACCTTTTTCCCCTTTTTCCCCCTAAAAAATTGTTTTTTTTTTTGGTTCCAGGAGGTTTTGGAAACCAAAGTTTTTTTTGG
GGCTTTTCCCCAGCTAAAGGGGTTTGAGAAAAAAATTAAAGAAATTTGGGAAATTTTTCCTGTCGGAAAAGATTTTTTTTCCC
CCCCCCAGGGAAAAATTTAATTTTAAACCCCTCCCCCCAAGTCCTTTTGGAGGGAAAAAAGGGGGGCCCCCCTAGATTTCCCCC
CAATTTTTTCCCAAAGAAGCTGAAAAGGAAAAATTATGGGGGAATTTTTGGGGCCCCCCCCCCACCCATGGAAAATTCAGATTT
TTCCCCAAAAATTTTGGTTCGGACAAAAAGTAAAATGGTTTTTGGGCTTTTGGGGTAGGTTCCAAAACGATAGGGGGGTGGAAA
TCCCCCTTGTTTGCGGAACATATTAATATTTTGGTTGGGAAATTGGGAAAAAAAAAAAAAATGGGCCCCACCCCAAAGAAAACCA
ATTGGGGGTGGGGGGGGAAGTTTAGTAAAAAAAAATCCCAAGATTTGGGCCCGAGAATTTTAAAACAACCTTGGTGGGAAAAAT
GGATTGGGGGAAAAAAGGGGAAAATTTTTTTTTTTTTTTTTTTTTAGGCCAGCTTTTTTTTTGTTTTTTTTTTTTTTTTTTT
TTTCCCAATGAAAAAGAAAGGAATATTAACTGTGAATGCAGCCCCCCCAGAACCCTTGGCTTTTTAAAAGGATTTTTGAAAAAA
TGGAAAAGTTTTTTTTTTTTCACCCTGAAACCTAAAGGTTTTTTTTGGGAAAAGGGGGTTTAAATGCCAAAAAAACAGTTAAAA
AAAAAAAAAAAAAAAACTTTTAAAGTAATAAAATTAAAACAAAATTTTCCCCCGATTGGGTTTTTTTTTTTCCCCCCCCAAAAA
GGGTTTTTTTTTCCTTTTTGGAAGTTAATGGGTTTACCCAAACGGATTGGTTAGCTCGGAAGGGAAACAGGTTTTTTTTTTAAA
TTCAAGAGATTGAATCCCAAAAAGTAAATAGCCTTCCCCCAAAAGGGCCAAAAAATTTAGAGTCCCCCGTTTTCCCAATTTGCC
AAATGAGAAATTCTTCTAACCCCCCTAACGTGGGGGCCAAGGTGGGGGTAGGGGGGAAAAAGGGCTTCCCTTTGGCTGTTTTTG
GGAAAGGGAAGCCAAAAACAAAAAGGAAAATCAAGCTTTTTTTGGAAAAAAAAAAAGGGAAAAAGTGGCCCTTGGGAGGAAAA
GGGGGCCAACGGAGGGGTTTTTTTTCAAAAGCAAAAAAAAAAATTTGAAACCTCCCAAAAAAAAACCTTCCCCATTTTTTAATT

GGGGGTAGCCGGGGAGGTAAAAAATTTTTTCCCTCCTCCAAAAAAACAAACCAAACAAAAAAAAAAAACCGGAAAAAGGAGGAAA
GGGAAAAGAAGGAGAGATTTTTTACCTCCGGGCTTTAACAATAAAAATTGGAGGGGTTGGGTCAAAAATTTTTGCTTTTTTTTT
TTTTTTTTGAACTTTCCCCCCGTTTTGCCTTCCCCTCCCCCCCCAGCCCCACCCTTTTTTTTTCCCCCTTTTTTTCCAAGGCCC
TTTGGATTTTTTCCCCCCCCCCACCCCCCCTTGGGGTGGCTCTTTTTCCAAAACCCAGCAAGAGCCTTTTTTCCCCCCCCCCCT
AACCACCCCCCCTTTTTTGCCAAAACCGTAAAAGGTAGAAAGGGATTCTCCACTTGCATTTGGGGGGGAAAAAAACGGGACCTT
CCTAAAAAAATTGGCCCCTTGGGGAAGCTTTTTTTTTTTTTTTAAAATAATTAGCTGGGGGAAAATTTTCTCAAAAATTCTCTTT
TTTGAATAAAAACCCCCCCCCCATTTTTCCCCCCTTTCCCCCCCGAGGCCCCTACATCCCCCCCGTTTGGGGGTTTAACCAAAA
TAAATCCAAAAGAAAACCTTGGAAAAACCCCCAAAATCCCCCCGGAAATTTTTTTTGGGTTTTAATAAATAAATTTTTAAGTTA
AACTTTGTTTTTGCAATTTTTTTACCCTTCCAAAACCTTTTTAAGGGTTTTTTTTTTTCCCAGAAAAAACTTTCCCAATTTTTT
CCCCCCATCCTTTTTTTTTAAGCAAGTTCCTTGGCCCCCCTTTTTTGCAATCACAGTTAATATCTTTTCCCTCAATTTGAACAA
AGCTGCCTTTAAAAATCCCCCTTTTTCCCAATCCCCATCCCAAACAAAAGTTTTTGTAATTCCTTCCACCCGCCAAAAAATCTT
TGGGGAAAAAAAAAAAATACTTACCTTTTTCCCCCAAAAACCAAAAAATGTTCCCTGGGCCCCATCCCAAAAAAAAATCCCCAAA
CCAAATATAAAATAAAAATGTCCGCAACACAGAAAATTTTTTTCCACCCCCTTATCCCGTGGGGGAACCCCCCCCCCTACCCCC
AAAAGCACCAAATACCCCCCTGGGTCCCGACAATGGGAAAAAAAATTAAATCAAAAATGCCCCCCCCATCCATATCTCAAAGCC
CCTCCCTGAAAAAATGGATCCCTTTAAAGGCCCCACCTTTTTCCCTCAAAAAGAACTTGGAAAGGGGGTAAAAAAATTTAATTC
CCTTGGAAAAATCCCCCATTTCCCACAGAAAAAAATTCCCCATTTCCTTATTTTCCTCCAAACCCTAAGGGCCCTTTTTTGAAGG
CCAAAAAAAAACCTGGGGTGCCACTTCCCCTGGAAAACCAAAAACCCAAATAGGGAAAGAAAGGTTGGGGGTTTTTTCCCCCCCC
CCTTTTTGGTCCCAGGGTTTTTTTTCCCCCCCGGTTTGTTTTTTGCTTTTTTTTTTAAAAAACACCCCTAAATTCCCCTTTTTT
TTTTTTTTTTTCCTCTTTTGGGCCCCTTTTTTTTCCCTTTGAAATCCTTTTTTTAAAAATTTTTGCAAAATTTGTAAAAACCCC
ATTTTTGCTGGGAAAAATTCCCCTGAGGGGCCAGAAATTTTGAAAAAAAAAGAAAACCTTCCCCAACCCCCCCCTTGGGGCCTT
TTTTTTGAAAAAAATTTCCCCCCCCAGGCCCCCCCCCGGTTTAAAAAAATTAACCCCCAACCCACCAAAAGGGGGCCCCCTTT
TTTCCATTTTAAAAATTCTGGCCCCCCCCATTGGAAATTTAACCAAAATTTTTTTTTAACTGCCCTTTGGGATTTAAATCCTGC
CAGTCCCCCCCCCCCCCCCCCCAAAAATAACCTTTTTTGAAAACCCCCCCCATTAAAAAAACTGATTTTGAAGCAATAATTTCAA
AAAAAAAAGGAGGGCCCTTTTTCCCCCCACCCGGGAAAAAAAAAAAACAAATCTTTTAACCCCAACAAAACTAAACCAAAAATCA
AAACAAAAATCACTTGTAGTGCCCCTTTTAAAAACATTGTGCCCCATAGGAATTTCCAAAAATTAACCATTAAAAATCAATAAA
CACCCCTTTACTTCCTTTCCCCAAAAAAATACCTACCCCCCATGCCCCCTCCACACAAAAACTTTTTTCCAAAAAAATCCAAAT
GGCAAAAATAATCCACATTTTTTTTATATATATAAATAAAATCCCATGGGGGGAAAAGTAGGGTAAAAATCAATTATAGGTCCC
CAAGGAGCCCAAGGCTTTTAAAATCAAAAAGAAATCTTCAAAAATGGGCCTCCCCCAAAAGGGGTACCCAATGGGGTGGCAAGG
TTTTTTTAAAAAGGCCCCAAATTTTACCATTTTCTTGTTTTAAGCCAGTTTAAGGCAAAACCAAATTAACTTAAAACTGAAAAT
AGGTTTTTTTTACCATTCCCCCCTTTGAAAGTGGGAAAAAAAGCTTTTTTTAATCCTTTTTTTTTAAGTGACGAGTTGGGTAACA
ATTTGGGTCCCCCAAGCCTTGTTTTTTTCCTTCAGGAAAAAACCCCTTTTTTAAAAAAAACCCCCCCTTTTGGGGCCCCCCTTT
AAGGCTTCTGGGATTACAAAAAAGTTTTTTTTTTGTTTTTAATTGCCCCTAAATTTTCCCCCCAAAAAGGGGATTTCGTTTTTA
CAAAGTTTGTTTTTTGGGCCCAAAAAAAATTTTTTTTTTAAAACAGTTTGGGCTTTTTAAACCCCACCGTTTAAATTAATGAGTT
TTCCCCGAATTTTGGCCCATTAAATTTCCGTGATAACTTAAGTTTTAATGGATTTAAGCGGGTTTTTTAAAGGGGTTTTTAAAA
ATTTTTGGGGTTTTTTTTTTTTCAAATATAACGGTTTTTTTTTCACTTAAAAACGTTTTTTTTTTTTTTTTTAACTTGGTAAAT
TAAAACATTTTGGGAATCCCAAACAAAAATAATAATTTAAAGGTTTTCTAATTTAAACCAACAAAATATAATTTAAAACCCCAA
CTAAAGGGCCCCCCCCCGACCCTTTTTGAAGCCGAAAAAAAAATAAAGGGGGGTTTTAACCCCCTAGTTTTTTTTTTCCCTTT
CTTTTCCAAAACCTCCCCGGCCCCGTTTTGGGGGGGCGGTTTCCCCGAGGGTAGCCCCAACAAATATGTAAACCAACAAATCCG
TCCCAAAAAAACTTTAAAGCCCCCCTTCTTTTTTTCCCAAACTCCCCAAAGGGAAATTTGGGTCCCCCAAGGCTTTTTCCCCTA
TCCTCTCAAAAAAAACAAAACAACCCAAACGGGGAGGGGGAAGGGGAGGGAAAAAAAAGAGAAGGGAGGATTTTAAGAAAAGCC
CCCCCTTGGAAACCAAATCCCCCCCTTTTTGGAGGTTTTTTTTGGGAAAGAAGGCCTTTTTTTTAATGGTTTGGAAAACCCGGG
AAAAAATGGGGTGGGTAAAAAAAAACATTAATGTTGGGGGCCCTTACTTCCCGAACCGCCCCAACGGGCCCCCCGAGGGGTTTT
TTTGAAAAAAAGAGGGGGAAACTTTAGGGTTTTAAAAAAACCCCTTTTAAAAATTTTTTTTTTCCGCTCCAAAAGGGTTTTTT
TTTTTCCGGCTAAGTTTTTGGTTAAAAAATTATTTTTTTTTGGGTTTGGTTTAAAATTTTAACCCCCCTTTTTTTTTTAAAATT
TTTTAATAAAAAAATTTGGTTTTGGGGAAAAAATTCCCCCCCCAAATTGTAAAAAAATAAACAGTAAACGTTTTTTTAAAGTGA
AAAACGTTTATTATCTTTGAAAAACCCAAAAAAAAAAAATTTTTTTTTTTTTTAAAAAACACCCTTAAAAACCGCTTTTAAAAA
AAAAAAAAATCCAATTAAAACTTTTAGGTTTACACGATTTTTTATGGGGCCCCAAAAATGGGAACCCCCTCAAGTATTTAAAAA
ACGTGGGTTTTTTTTCTTTAAAGCCAAAACTTTTTGTTTTTTTTTTTAAAAAATTGCAAACAAAAAAACCTGTAACCGGAAATT
TTTTCCCCTTGGAAAAAATTTTAGGGCCAAAATTTAAACAAAAAACCTTGGGGTTTAAATCCAAAAAAAAAGAGAACTTTAAGC
CCAAAGGTTTTTTAAAGGGGTTTTTTTTTTTTTTCCCCCCCCTTTTTTTGAAAGAAACCCAAGCTGGGGACCCCCAAATGTAA
AAAAAACAACTCCGTTTCCCCCAAAAAACTAAAAGGATAGCTTTCCCAAACTCCCAAAGGGGGAATGGGTAACTATTTCAAAAG
TAGTTTAAAATTGTGCCTTTAAAAAACCCCTTTTGCCTTTTTTAAAAAACAAGAAAATTGTTTTAAAAATGCTTTTTTTTAACC
TTTTTGCAACCCATTGTAAAAAAAAACCCCTTTTTTTTTGGGGGAAAGGCCAAAAAAAATGGGAAAAAAGAATTCCCCCCTGATA
AAGCCCCCCCCTGGCTTTCTTTTTGGGGGGACTTTTAATGATTTTTGATTTTTACACTACCTTTCCCCCCATGGGGGGGAATTT
ATAATTAATTATTAAAATGGGGGTGGAAATTTTTTAAATTTTTTTGCCAAAAAAAAAAAAAAAAAAATGAAATGGGAGTTTTTG
GAGGCAAAAAAAATGGGTAAAAAAAGGTTAAAAAAAAAAAATGTTTTTGGGGAAGGATAAGTAGGGGGATTAAATGATTTTATGTT
TTATTGAAATTTTTTCCCCTAAAATGGGGGGCCCAAAAAAATTGGTTTTTTTTAACAAAAAACCTACAAGTGATTTTTTTTTTTT
TTGTGATTTTTGTAAAGGTGGGGTGGTAAGAAAATTTTTTTGGTTTTTTTTTTTTTCCCCCCCGTGGGGGGGAAAAAAAAGCCTT

TTCCTTTTTTTTTTTTTTTTGATAATGCCTTTCCCCCCCAAATCATTGTTTTTTTTTAATGGGTTTCCCCAAAAAAGGGGTTTT
TAAATTTGACCTTTTTTTGCAGATTTTTAATCAGCAGGTTTTTTAAATGTAATCCCCCAAAAAAGCCCCAAAGATTAATTGAAG
GGGGCCTTGGGGGGTGGTTTGGGGTTTTTTTATTTCCCGGGGGCCTTTGGGGAAAAAAAATTTTTTTCAAAAAAAAAGCCCAA
GGGGTTTTTGAAGTTTTTTTTTCCCTTTTTTCAAAATTCTGGCCCCCCTTTTTTCAAGAAAAAATTTTTTTTTCCAGCCCAAAT
GTTTTTTTTTTTAAACATATTTTTTTTTTTGGGCAAAATTTTTTAAGATTTTGCCAAAAAAAAAAAGAGGCAAAGAAAAGGAAA
AAGATTAGGGGGTTTTGGTTTTTTTTTTAAAAAAAAAAAAGCAACAACCGGGAACACCTGAACAGGGAAAAAACCCCCAACCCCT
CTTTTCCTATGGTTTTTTTTTTTTGGGGTCCCCCCAAAAGGAAAGTGGCAAAAAAAAAAA
>m141120_131642_42134_c100732402550000001823141405141505_s1_p0/60538/6179_15432
ACCCATCCTAACGTCAGACGCGAGTTACCCGACAACGCAGGGCCAACACCCGAACCAAAGCGTGGAGTTCAATATACAACCCCA
GCTCCCGGCGCGGAGAGCTGAGCCATGAGGGTCAAAACTCTGCTTTGACAACAAAATTACGTACGTCCTTGTGGGAGGATCCTT
ATTGACAGTAAAATTATCGCCTGTATTCCTCTCCGGTGTGGATCCACTTATCTCTGCTCCATGTCAAAGATTAAACTCTTATAA
AACCAACCCGACAGGAACCATTACTCATCCCACCGGGCCTCACCTTCACGATTACCGAGGACCGATCACACTTCGGCCAAATGG
GTATCACCCCTTGACGGCATTAAAAGATTAACCGCTTTGTTTAGCGGGCAGCCTTTAGTTCTTTTCAGTGCGTCAGGGATACGA
CCTTAGCGCACCTTGAAAACCAACCAAAAACACAACAAGACACCTTTCACCTCACATTCCCCTCCACCATCTCTTTTTCTCTCG
CTCAATATCCCTCTGCTCCCTCCCTTGCCAGCTTGGGAAAATTTACAGTTCTTAGAAGAAATGTGGCCTTAGGAAACATTGAAG
CGCTAGAGGCTAGGAGTTCGACCCTTGCAGGCTTCAGCTTTTCAACGGGGTCGAACAATCACTCTGACAGGTCCACCAGCTTTA
ACGCACCTGCTTACTGTGATTTTTCCTACTATTAAATTTCGGTTTTGACTAAGTCCTTAAGTGCTAGCGTTGGGCTTTTACGTT
GCGGATACCCAGATTGTGGCTGGTTTCTGGGCGTCATTAAGATCGCCTACGGATACTCTCGCTTGTCTGAGAACTTTCTAACAC
TCATTCAGTGACTTGGTTTGTGTTTAAGGTGGATTTCGGGATTTTCGCTCGGGGAAAACGCAAGAAAACATTGAATTTCATGAG
TTCCGCGGGGCTCACAACCCCGCGGAGCTGGTTCCTTGGAGCGGTCGTGGCCCGCATGACACTCAGGCCTCAGGGGGATTTGTT
GACATCCGCCCTCTATGCACAATAGCTCGCCGGTAGGTGTGGCGAGTCGCGTGTCCCACCCCACACCAGGAAATGCATAGGAGG
CCTCCTCTGCTTCTGTAAGAGGGCCCGCCCGACGCTTTAAGGGAGGGCTAAATAGGTTTTGGACAAATACGCGAGACCTTTTAC
GCTCGCGGACTTACCCTAAAGGCCTCAGGAAGAATATTACTCAATCGGTCTAGTAGCAATTTGCGGCTTCTGAAGGCTAGAAAT
TTACAGTCTGAAGCTTCTTACTCAAATAGTCCTAAGTGATTGAACTGGTTATCTGTTATTCCCGCGTCTGTGAGCTAGAATAGC
CTACTGCTAGCAGCCTTGAAATACGCGGATCCCATGCTAGGGATCGCCATACTAACTTTTTGCTCGCCGACCAACGGTGAAAAC
CTGCGCCCTTGGCCTTCGTCGACCCAAAAGGGGCGGAACCTATTCATACTCGGGATAATGACTCGTGAATTATACAATGTGACT
CACCTCATCTGAGCCATCGCTCTGTACTACTGCTGCAATCTGGATTAATGCTTTATCGCACTCTCACCATACGTCATATCCAAT
AACCTTATACCCAAAAATACATACATCTGGAAGCTTGGCCGAAGGCGCAGTCGGGGCCCGCCAGCAAGCGTACAGAACCGCAGG
TCGGGCCCTAAAAAGTCAGGGTCGACAATACGATCTTAGCAGCTCGATCCTAAGAGAGCGCGGCCTGAAGTCACATCAACACGT
ACTTCAGACATATCGATTGACTTGTCTAATTCATTGACTTGTTACTTGGTACTTCAATCCGCTGCTCTTCACATTCGAAGCAGA
TATACTTCACTGCTCAAGTGTATGATTTACTATTCATTCCGTTTTATAAACCTTTGCTGATTTATTTCTTGCCGAGCCCTTG
GCTCATGGGTTGCTGTGTGAAGTCGCAGGTAAGGGAACAGAGGAAGCTGGACAGGTCATGAGTTAACCCGAGCAGCTAAATGGG
GCTGGCGGTGTAACATTTGCAGCTACTCCGACCACCACGGGCTCGGCGGATCAACAGAGCACCTAGCACGCCAAAACAAACTCC
TAATTTTTCGGCCATCTAGTCCTCGGCCTATGTGATACAATCTGATTTAATTTCACGTCCTACTACACATGCCTTGCTAACACT
TATTTCTGGGTCCCATCGTACACGACCTCAAACTTTCTAACTGAACAGCACTATTTCTCGATCACAAATCTTTACTATAACCCT
TAATCCGCCTCAATTGGTCCTCTACTCTACATACGTTATATCACCAACATGCCGCTGATTCAGCCGTCCACCAACTATTCCTAC
CAATAACACAATGTAACCACGGTCTTGGCTATCCAGGGGATCTAGCACATCACTTGGCTATACCGACGGCGGTCCCTAGGCTTT
TAAGGGTTCTGAAGACTCGGCTCGGCCGCCATGCTACCATATCGCGCTAAAGACAAGCTCCCCGACTCGGTTTGGCTAACCTAT
ATTGACTGACTTATCCACTATCTCCACTGCTCAAATGACATCCAATAATGCCTCTACTCTGGCCCCTCCTTTACTACCACCCAC
GGCAAGCCATAGAGTCCTACCTACCTGACTAGTTCGCTGGCTCCTTCTGCCTCACCCATAATCGTGTACCATGATCGAGGAACT
GCTTAATTAGCATCGATACTGCTATGCTTAGTATATATATGACCAATAAGCCCTGTTCAGCATCTGTGATTGCATATCTTCTGC
TTGATTGTTGGCATTGTACCCTGTGGGCAGCTGACTATTTAAAATCTTACTTATCCCAATCCTGCCACTGAAGCCGCAGTTGTG
ACTACAGATCATACCTTCGGAGAGTTAGACTTTAAGGCATCAGTTCGACTAGTCGGTGCTGAGATCCAGGCCTCAGACGCCATG
ACTAAAAACCCGGTTAACGAACCCTCCGCAGTCCCTGATAACTGGCAGTAGAATATTGCTAGATTGCAAGCCAGACTTCGGAGT
CCAGAGGAGATATTCATTTATTGAGACAGCAGGTTCCTCCAGGGAACGATATGCACCCTGACTTGCCCATCATGCACAGGCACA
ACCCTACAACAGCCTGCGGATTCGAAGCCAATAGGATTGCGGAGTTCGTTGTATGAGAGAATTCGGGAAACAACCATCCTCCAA
TCTTTGAGGGACGAACCCAGCATCCACTAAGGCTAAACAATGGATGAGCCTCGACTACCCCCATTCGGATTTTATGAGAGAGTG
GTAGGATAATGCAGAGTGACTTATGCACCCCTTTAATGCTCCTCCGATATGATAGTAAAATGGTGGGCAGGTGGCATCCTAAAC
TCGAGACGTTTCTACTATGAGTTAGGATGAGTATCCAAGCGATATGTTCAACCTAGAAGACTAGAGTACAAAAATTTGGGGGGT
TCCCCGCTTAAGGGGGCCGCCGCCCAAAAAAAAAAAAAAAAAAAGCCCCGAAAGGGGGGGGGGCGGGGGGGGGGGGCTTTGCAACA
AAAAACCGTATATTTTTTTTTTTCTATCCCTCATTTTTTTACTTTTTTTTTTTATTTTTTTTCTTTGGGGGGGGGTACAGGCGC
GGGGGCCCAAAACGCTTTTCAAATTTTTTTAACACCCCACCCAAGTTTTTTTTTTCTAAACCCTTCCCCAAAAAAAGTTTTCAA
ATGGCCACAAAAAACCTGAAAAAAAAAATTTTTTATTTTTCTTTGAACAAAAAAACAAGACTTTTTATCGCCTTAAAATTCTTC
TTTTTTTGGCCCCCACCTAAAAACTTTTTAAAAAAAAAAACAGTTCGGGGGCGGGGGGGGGAGGGGCCCCCTAAAAAACAAAGA
TGTTTTAAGCCCCCCCCAAGGGGGCCCAAAAACAGGGAAAAACAAAAAAACAAAAAAACAACCAGAAATTTGCTTCTTTGGGGG
GGCGGGGGGGGTCTCCCCGACGGGGGGGAAGGCGGGGGGCCCCCCTGAAACATTGGTTCTTTTTTATGCAAATAGCCCCCCCGG
GAAAAAAAACAAAAAATGGGGGGTTTTTCTCTTTCTTTCTTTCTTCTGTTTGGCCCCGGGAAACATTTTCATTTTTTATTCAAT
CTTTAAAAATCTCCCAAAAGTTTTATACCCTGGGGGCGGGGGGCGCGCGCGCGGGGGGGGGGGGGGGGGGGGCGGGACTTTT

AAAACCTTTTTTTAACCCCTTGTTAAACTTGCCCCCCCCCACAGGAGGGGGGTTCTTTTGGCGGGAGGGGGGTTTTTTCTTTTT
TTTTTTTCTTCTTTTTTGGGGAACAACAAAAGGGACACAGAAAAAAAAGCGGAAGCGGGGCCCCCCCTCTCTTTCCAAACCAGTG
GGGCGGGGGACGGGGGGGGGGAAAAAACAAAACCAAGGCGCCCCCCCCCCAAAACCAAGAAAAAAAACCCCAAGATAATGGCGGG
GGGGAAGGGGGGTCGGGGGGGGAACGGGACAGGGTTGCCCCTTTGGCGCAAAAAAAAAAGAAAAACAAAAAAACAACCCAACAA
AATTTTTTTTATTCTTTTTTTTTTTATTTGCAAAAAGGGGGGGGCAAAAAAACAAAAAAGTGGCGCGCGGGGGGGCGGGCCCCCC
TCCAATTCTTCTTTTGGTCCTTACTCAACACAAAACAAAAAAAGGAATTTCCATTCCCCTCTTCTTGGGGGGGTTAAGAAAAAA
AACAACAACAGGGGGCGGGGGGGGGGGCGGGGCCGCGGCCCCGGGGGCAAACAGGGGCCCCCCCCCCCTAAAAGTGGGGGAGG
AAACTTCCCCCCCAAAGAAAAGAAGAAAGGAAAACACGCAAAAAAAAGACAACCCTTCTTCCCACTGCGGAACCCAACCCCCCT
CCAAAAAAAAAACACCCCCCCCCCCACCTGGTTCTTTTGGGGGGGGGGGGGGGGGTTCCGCCCCCCCTCTTGGACAATCTCAAG
GGAAAACGGGCGGGCGGGGCCCACCCCCGGGTTCTTTTCTTTTGGTGTGGTTCGGGTTTTTCCCCAGGGGGGGCCCCCCCCCG
CCCCAAAAAAACAAGTCTAAAACCAAAAAAACAAAAGAAAACACACAAACAAATGGGGGGAAGGGCAACAAAAGCGGGGCGGGGT
TTTTAAAACCCCCACCCCCAGAAAAAAAGAAAAGGGGTTTTGGCGGGTTTGGGGGGGGGCCCCCCCCCCCCCCCCCCCTAAAA
GGGGGTGTTAGGCAAAAGGCCCCCCAAAAAAACCCCCCAAATCCCCCCACCCCCCCCTTTTTTCGGGGCCGGAAAAAAATTTTGGG
GGGGTCCCCGGCTTTTTCTCTGCGGAGGGGAAAAGGGCCCCCCCATCCGCGCGGCTTTCCACTTACGTTTATCGGGGGTGGGGC
CCGGCAGGGGGGGGGGTTGGGGGTTTTCTTTGGGAACAAACTTTTTCTCCCCAAAAAGAAAAGGGAAAAAAACTGTTTTTTCCC
CGAAAAAACTTTCTTTTCTTTTTTTTTTGGACACAAAAAACAGAAAAGGAAGAACCCAAGGGGGGGGGCGGACAAAAAAAAAA
AAACAAACCCACGAAAAAACCCAAAGCCTTTCTGCTTGGACCCCTTTCCCCCCCAGCCCTCCCCCGAGTCTCTTTTTTTTTTG
GCGGGGTTTTTCTTCTGTTTTTTCCAAAAAAAAAAAACTCTTTTAAACTTCCCAGAAATTTTTAAGGAAAGGGGGGGCCCCTTT
GAAAAGGGGGGGGGGCCCTTTTAAAAGGGTCCCCCCTTTTTTCCCCCGGGGGTTTTTGGGGGGTTTATTGGGGGGGGACAAAC
CCGTCCCCAAACAAGCCCTTTTCTTTTCTTTTAAGCAAAAAAAAAAAAAATCTCGGGCCCAAAAACCTCCTCCATTACTGTATT
TTCTTTTAACAATTTTTTTTTAAAAAAAAATTTTTCCCTTAGTTTTTTTTTGGGGGGGGGTTGCCTAAACCCACCCCAAATTT
TCCATTTCCATTTTTGGGTTTTTTTTTTTTTTTTTTTTAAAAAAAAAGCAAAAAAGAGTTATTTTTTTGGGGGATTTGAAATT
AAAGAACAACATTGGGGGGGGTGGGGTTTAGGGGGCCCCCTGCCTTGGGGTGAAAAAAAAAAAAACTTTTATTAAAAAATTAAC
CCCTTTTTTCGGCCCGGGGGGGGTTATTTTTTTTTGGGGGGGAAAAAAAAAAAAAAAACCTTTTATTTTTCTTTTTTCTTTTTTT
TTAAAAAATTTGGGGGGGGCCCCCCTTTACAAGGGGGGCGCGCGGGGAAAAAAAAAGCCCCCCCCCCCCCCACCTTTTAAAAAGG
AGTGTTTTTTTTTACAAGTTTTTTCATTATCCCCCCCCCTTTTTAGGGGGCAAAAAAAAAAGGGAACACCTTTGGGGGGGGGGGGG
GGGGTTTTTTTTGAAGTATCCCCCAATTTTTACTGGCCCCCCCCCCAAAGTTTGGGACGGGGGATTGGGGGGTATTTTTTGGGAA
CCTAAATAGTCGGAGAGGGGGGGGCGGGGCGAAAAAAAAACCTTTTTATCCCAAAAAAAAAAAAAAAGTTTTTTGGGGGGGGGCGG
AAACAAAAACTTTTGGGGGAAAAAAAATTTTTGGGGGAGTTTTTTTTTTGGGGGGGGGGTTTTATGGGGAAAAAAAAAACAAAAA
AAAAAAAATTTTCTTTTTTTTATTTTTTTTTATTCTTTATGAAAAAAAAAAAAAAAAAAACCAAAAAAATGGGCAAAACCCCTTT
TTTTTTTTTTCCCGGAAAAAATTTTGGGGGGGGGGGGAAACTGGTTTTTTTTTTTTTTTTTTTTTTTTAAAAAGTTTTCAAAAA
GGGTTAAAATGGGGGGGGGGGGGGGGGGGGCCCAACCGGGAGGGGAAACCCCCCCTTTCTTTTTAAAAAAAAATTTTAAAAAACC
CCTGCCAAACAGTTTAAGAGAAACGGAAAAAAATTATTTACTTTGGGTGACTTTTTTTTTTTTTTTTTGGGGAAGGGGGGGCCC
CCCCCCCCCCTTGAGAAAAAAAGGGGGGGGGGGGGGTTCGGGAAAGGGGGGGGGGGGCCTTTTTTATCTTTTCTATTTCTTTT
TTTTTTTTGGGGGGGGGGGGGGGTTCTCTATTTTTTTTTGGGTTTTTGGGCACTTGCGTTGGGGGCAATCGGGAGGGGGGGGAA
AACCCCCCCGCCCCAACTTCCCCAAAAAAAGTTGGGAAAAAAAAAAAATTATTCCCCAACCCGGTTTTTTTTTTTGGAGGAAGC
CCCCCCCCCCCCCCTTTAAGGTTTTGGGGGGGACCCTCTTTTTTAAATTTACCAAAAAAAGGGGGGGAAAAAAAGGGGGGGTTT
TTTTCTTTTTTTTTTATTTTTTTTTTTTTTGTAATAGCGGGAATTTTCCCCCCTAGCCCCCTGGGGTTCTTTTTGGGGGGGTCT
GCGGGGGGGTTTTTTTGGGGGGGGGAAAAAAAAAATAAAAAAAACCCCAACAAGAAACCCCAAGGGGGGGGGGGGGGGGTTTTT
TTTTTCCCCAATGCCCCCCCAAAAATTTTTTTTTGGGTGGAAAAAACCCCCCCCCCAAGAAAAAAAAAAAGAGGGGGGGCGGCCC
CAAAAAAAAAAAACCCTTTTTTTTTTTTTTGGAGGGGTTTGGGGTTGGGGGGTGGGGGGGGGGCCGGAAGGGGGGGAAAAAGA
AAAAAGTTCCTTTTTTAAAAAAAAAGGAAAATTTGGGGGGGGTGAGGTTTTTTCCCCCCCCCCCTTGAGGGGGGGGCGGGGGGG
GGGGGGGCCCCCTAAAAAACCAAGGGGGAACCTTGGCCCCCACAACCGCCGGAAAAATAAGGAGGCCAAAAACCCGGGGGGGGG
AAAGAAAAAATTTTTTTTTTTGGACAAAAAAAAAAAGTTTTTTTTTCTTTTTTTTTCCAAAACTCCCCCAAAAAAATGGGGGCG
GGGTTAGTTTTTTCCTATTATTTCTGGGGGGCCAAAAAAAAAACCCCAAACAAAAGGGTTTAAACCTGGGGAACCCCCCCCTTA
AAACAAAAGTGGGGGGGTTCTTTCTTTCATGAGGACGGGGGGGCCCCCCCAAACAAAACCCCCCCCCATACACACCAAAAAGGG
AAAAAACAAACTTTTTGGGGGGGCCCCCTTTTTCTTTTTTTTTTTTTTTTTTTTTTCCCCCTTTTTGCGGCCCCAAAAAAAAAA
AAAAAGAAAGCCCCCCCCCTTTTTTTTTGAAAGGGGGAAAAAAAAGGGTTTTTTTCTTATTTTTTCTTTGAAACCAAAAAAAGG
GGGGGGGGTTTTAACAAAAAGTTTTTTTTTCTTCTTTCTATAGGGAAAAAAAACTCAAACTTGGGGGAAAAAAAATTTTTTTTTC
CTTTGGGGGGGTTTCTTTTAATATTTTTAAAATTTCCCCCCCCAAAGAAAAAAACCCCCCCCTGGGGTTTTCATTCATTTTTTT
TTTTCCCCCCCACACATGGGGGGGGGGGGGGGTTGGGGGGGGGGGGGGGGGGGGCCCCTCTTTCCTCAAAGTTTTTTTTACTTTTT
TTTTTTTTCTGATTTTTTGGTTGAAGAAAAAAGGGACCAAAAAAAAGGGGGGCAAAAAAAATGGGGGAAAACCCCCCTGAGGG
GGGGAAATTTTGTTTTTTTTTTTTAAAAAATTTTAAGGAATTTTTTTTTTTGGGGGGCCAAAAAAAAAAAAAAAAGGGGGGGGG
AGTTTTTTTTTTGGAAAAACAAAAAGGGAAATTTTGAGAAACTTCTTTCTTCCCCCTTCCCTTCCAAAACACCCAAAACCCCAA
AACGGGAAAAAGGGGGGGGGAAAAGGGGGGGGGGAAGGGAAAAAAAACAGGAAGGGAAGGAAACAGGGAAATTTTCTTTTTTTT
TTTTTCTTTTTTTTTAAATTCCCCTTGGTTTTTCCAACTTCCCCTTTTTTTTATGACCCCCAACAATCCTTTTATTTTATTAAA
AACAAAATCCCCCCCCTTCCCCCAAAGGGGGGGGGGGGGGTTTTTTTCCCCCCCCCCATTCCTTTTTCCTTTTTTTCCTTCC
CCAACCAAAACAAAAACCTGAAAAAAGCCCCCCAAAAAAAAAAAAAAAAAAAAACCCCCCCCCAAAAAAATTTTAGGGGGGATTT

CTTTTGAAACACAACAAAACCAGGGTTTTTTCCTTTGACTCTAAAAAACAAAAACACCCCCCACCCCCAGGAAAAAAAAATTT
TTTTCTTTTCCCCCCAAAAAGTTTTTTAGTTTTCCCCCCCTTATTCTTTAAAAAAAAAAACCCTGTTAAAACCTTTTCAAACC
CCCCCCCTTTTTT
>m141120_131642_42134_c100732402550000001823141405141505_s1_p0/61815/7020_14899
AGTATGATAGTTATCTTTCGCTTTATTTATAATTGTTATTACTGTATAATTATTTATGATTATTAATTTATGTCCTGTGATAGG
CATATGATATTGTATTAGCATATGATCGAAGCCCAAAGAATAATATTGTTTTAGATAGTCGCCTTCTTGCAGGTATATGACTTT
TGTTATATACAATCTTCCACTAAAGTTTATTGGAGATACTCTGGATAAATGGATGCTCTAATGACAGACGTTTCATATATGTTT
TACTTGAATAGTATAGGAAGTATCTAGGTGGTTATCTATCTATCCTATAGTCATGGTTCATGATGAGAGTATAGGGGTAAGTAT
TAAGAGAAGTTAGGTGTGGATAAGCTGTTATTTGAAGATCATTTATGAGATTATAAACACATTTAATTGGAATATTCACTTTTG
ATGAAACAATTTTGAAAAGTATGGCTATTTTCGAACAGAACGAAATTCATTGGTGTTACTAATAATACACATCAAGTAAAAGTG
GTGGTATTCTTCTTAGTTCTACTTACGTTGATGTACATGGTATTTCTCAGATATTGGGAACAATTGTTATTGATTGAGGATTTG
TCAGGTGTAAGTATATTGAGTCATGAGTTAGCTACATTCTATACGAGATATACACGCTGTTCCCAAAAGTGAAAGGATTTTTTG
CTTCTCGTCTCAAAATTTTCATCATTCTGCAAGCTTTATATGCTATCATCTCAGGTAACACTATCAGAAGTCGATTATTTGCTA
ATCTCTTCTGCATTGAAACAATGTTGGATCAGTGTACCAGTGGGGTCTAGAGTCTCACACTGAATATAACAAAGCTATATTCTC
AGTATTCTTAGAAGATTGAGAATATTCTTTCACATGGCAATCAATGACTACAATCTACGGTATGGATTGATAATGATGACTATC
CCTACATGCATAAAAAATGTCAGGGTCTTGTAGCTAGTATTATAGGTACATTAAGACTGTCTATCGCTGAGGAGGCATAGGGAAT
ACTGATCCTCATATCTTCCTATTGCCTGGAGTGTTTTAGGACACTGCTCCTTCGAAGAGATATATACCCGAAGGCGTGGCATAT
CAACCTCTTGTTGGAGTTTATCATATTAAGGCGTTCATATGCACTTATCGAATATAATTACTTGACGCACAGTTCCAAAGGTCT
TATTCTGTCTATCTCTAAGATTTTAATCCCATCAACACAGCTTGCTTCTCCAAATCTTTTCCATTTGGAATTGTTCAGGCTACC
TAGTTCTTGTAATTTTTGATAATGACAATGTAGACGTCACTCGCCAATCAGTATATGATCGATTCAACGAAGAACAAGGAATAC
TACTACGACCATTCTTTGATTTTTTTATAGCACAAGGTTGCAAATCATTTATGGGTTTTTTTCAAAACCAAACGTTTTAACTTG
TGTCATACAATTAAGTATTCAGATCTAGAAAGCTTGTTTTAGTCCATAGATGGACCTAAGAAGCTTGAGAACATTTTGGCTTCT
TGCCTTTTTATACTTTTGAACCCTTCTGGTTTGAGACGCCCATGCAGCTTATCACAATAAATTGTAGCCTTCTTATCTCGAGGA
GGTTGACACTTGCCCGGATAGCACAAGCAATTCCAGGGGTTTAACTGAATGCTAGCCTCTTATAAACTCATCTTATATTCGCTC
TCTTTCATTCTGGAGCTATCGCTCTGTATAATCTGTCAATAAACATCTGATCGCACATTCCATGAGGGGACAGGTGGAGGGCTC
TCTGGCTCCTACATTATCATTTTTGGCCTCGATAATTTGTGTCGGTCTAGTTCTGATTGACCGCCTTGGAGGTATTACTCCGAA
TTAATTTATGGATCCATGACAACGGTTTATCATGACAAATGATAACAACCACCAGAAACTGTCCACGTTCATAAACAAAACAAA
ACATACATTCCACATGTCAACAAATAATGCTCATAATCACTAACATCTCTAAGCACATTCGGATATGTCAATCAAAGGTTGGTG
CTAACAATAAATATCATGCTCCTAATCATTAGCATGATAGATAAGGAAAAACATATTATAAATAAGAAAGTAAATAGGAGTTCA
ACCCATTACCACACCTGAACATGAGTCGAGCGCTATCTTTAGCCATTGACGTACATGTTTGGTCGGTCTTTCAAGGAACACATA
AAACCTTGACACTCCCTATATCCCAAGTTTGCGATGCACTACAAAGTCCTGGACTATCACACCAATCGCTGTTTAAAATAGTAC
GTTACCTTGTTAAGCGAGTATTTATGGAACTCAAAAGTGTGAATTAAATCTAAATAAAGATTGACGTGTGAAAACTTGATAATA
GATGTAAAAAATTTCATTTAAAAAATATAAAATCATTACCTGGGACCCAAAAGTGTTTAAAATACAAGATACATTTTTAAACCA
TTCGTTCTCAAAATAAGTCCAGCTTAAGCGACAAAAATCGTACTTTTACCCTAAGTCCATCAGATTCACTCGACCCTGAGGGTG
AGTACAGCTGAACATGTACTAAAGCATTTCAAGCCATCAACACTCAATGTTGTCTACTTCCCTTTGCCCCTTGATGAATGACCG
CCTAACTTAAACCGACCTCAGACCGATTAAAACTACACATAACTACTACTTTGAATACATAACATGAGAAAATAATAGGATCTT
TATAATAAAAATCCAAAATACATCCTTTGAGGGGGGGGGTTTCATATACATAAAATACATTAGGAAAACAAAACCTTTAATTAA
CTTGTTCAAATACTAAAATGCAGAAACATAAATACAATAATTAAAAGACTTGAACAAAAACGTCTTCCTCGATCTTCCCATAAT
CCATGTCACTCAGTCCTCTCGCCAATACACATGCCAAAGCTGCTATGAATTGTCTCGCAATTCCAAGCTTTAATTTCCTGGCAA
TCATCTAAAATAAAAAGTGAATTGAGCTCTAGATGCCCAGCAAGAAAATTACTAAAGACATATCATGAATTCATAAATGTACTA
ACGCATTACAATAAGATAATAGTGATATACGAAACCATTCTAGGTCCACTCAATTCACTAATTCTAATACGTAGTAGTAGTAGT
AGAGTATGCATAGTAGCTGTAGCTGGAGTGTAGCGTAGTGGTAGGTAGTATAGTAGTAGTGGTATAGTGTGGTATCAGTAAGTA
AGTACACTAGTAGCAGTACACATCACAGTAGCAATAGCACAAGTGCAGGAGCAGTATAAGTAGAAGCAGTGAAGCAGTAGTATT
ATAGTAAGTAGTAGTAATAGAGTAGTGAGATAGTAGCTAAGTGGATAGTAGTACTAGTATAATAGTATAGATAGTATAGTAGTA
GTAGTAATGGTAGTAGTAGTAGGTAGTATATAGTATGTAAGTAGTAGTAGAGTAAGTAGTAGTAGGTAGTAGTGGTAAGTAGAC
TAAGTGGTAAAAAAGTGGTAGTGTAGTGTAGGGTAATCAGGTAGAGTATAAGTAGTGTAGTAATAGCCTGGTAGTAAGTAGCAG
CACAGTAGCAGGTAGCAGTAGCAGTAGCCAGCAGCAGCACATAGCAGTAGCAAGTAGTAGCAGTAGCAGTAGCAGTAGAGCAGC
ACACAGCTAGCAGTAGCAGTAGCAGAGCACAGCACGTAAGTCAGTATAAGTACCAGTAGTCAGCAGTATAGTAGTATAGTAGTA
GTATATGGTAGTAGTAGTGTAGTAGTAGCAGCAGCAGTAGCAGCAAGCAGTAGAGATAGCAGACACAAGTAGCAGCACCAGTAG
CAGCTACTAGCATAGCAGTACAGTAGCAGATGCAGTAGCAATAGCCAGTAGCATAGCAGTTACAAGGTAGCACAGTATAGAGTA
GTAGTGGTAGTGTATGGTTAGTGGTAGTGGTAGTGGTGTGGTAATGTAGTGGCAGTGGTAGTGGCAGTGGCAGTGGCAAGTGGC
ATGGCATGGCAGTGCAGTGGCATGGCAGGGCCAGATGGCAGTGGCATTGGCAGGGGCAATGGCCAGTGTCAGTGGCAGTGGAGT
GGCAAAGTGCAGTGGCAGTGGCAGCAGTAGCAGTTACATAGCATCCGCAGTAGACAGTAAGCAGTAGCAGTACCAGTAACAAGT
AGCAGGTAGCAGTAGCAGTAGCAGTAGCATAGCATAGCAAGTAGCATTACAGCGATAGCAGCAAAAAAAAAGCAGATACAGTAG
CAGTACAGATAAGCATAGCAGTAGCAGTAGCAGTAGCAGTAGCCAGCTAGCAGTAGCAGTTACAGTAGCAGTAGCAGTAGCAGT
AGTCAAGTGCCAGTAGCAGTAGCAGTAGCATAGCAGTAGACAGTAGCAGATAGTATAGTAGTAGTAGTAGATAGTAGTACACTA
AAACTTAGTTATAATGTTTATACTGTGGATTTTTGGTTCAAGCCGGGACTTAGCGTTGGGAAACACTCGTTAGCACACTTATAG
ATTTATAAGTTTAACCTATAGCCAAAATATAATAATAACATAAGTTTGATTAATATAGCTGATTAATAAAGATGGATTTACTTT

ATACTATAAGGGTTATATAGAACTAATAAGGATCCATCGACACTTGCAACCGTGCATGTTTATTGAGGAAATTAAAGTATTTTG
AGGAATAGTTTTTATAAGAGTAATAATTTGAAAAACCCAGATATGCCAGCACTTTAAGCATTATAGATCAGTCAAAGTTGTTA
CTCCAATTCAAATTAGGCTAAAAAAGTTCAATTACGTGTATATATATTTCAGCGTAGCCGATATATCGCAAGCCTCTAGGTGAA
TATATGCACCACCCAGGAATACAAAAAACACATAACTTCGCACGAACGTTTCGACAGCCTCAAGGAATATAAGCCAGCTATATA
TCGCCTTACCAATGGGCCGATATATTCAGCTCCAGGGCAATATTTTTGAATGGTTTGAAACCGAGCTCAGAACATTCAACCCAT
AACCTCTTGCAAGCCTCTGAGAACCTTGTTTTGACCGGTCTTAAGCTCTGCTAGAAAATAATTACCGCAATGAATTTTCACATA
AATAGTCATCATTTTATTCAGAGCTAAAGAGATATTTTATTCTTGAACCATATAATTAGGACCTAGTAACCCAGCCATTTCATT
CATTCTTCAAGCTAGAGTTCAGAGCCTTCAAGCTTCTAGGTTTATTTAAGAGTGTTAAAACACTGGGTTGGGGTTATAGAGCTT
AACCATCTTATAGCTTTTAACACTGTGGAAAGTCAAAGTTAATATGGTTGTTTTTCGATTCGAGGTGTATTCAGTTATAGTGCA
TTCAAAGAATTCCTATTCCTAGTTATTTTTGTATGTTTCATTAGTTTCTTATATTTTCTTTACTCAAATCCTAACTCATTATGG
TTCATCTTGGTTAAGGCATTTAAGTTCTTTTGAACTTGAAAGTTTCTTTTGGTAAGCTCTTCTTCTCTGCTAGTTTAGTTCATT
CCTCATTTCATCTCTTTCTTTAATAGCTCAGTCATTTCTATTGTTGGTTTTGAGATGTGTCCAAAATCTGCCTTTGCTCTCCAT
ATCCGGTATTGTAAGGAAAATAGGATAGTTTCTAATATGTATATGTTATGATTATGTATAATATAATGTTACTGATGTTAGTGT
TTTATCTAGGCTTATAGTTGCTTTAGTGACCTAGTGTTTATTCATTTTATATGTTTAATTATGAGTATACCTAACCTCAAGAAA
TTACAGAGACCTAGTGGTTATCATTAATATCTATCATGTTACTTAAGCCTACCCTAAATAAATAGACAGGGCACATAGATGGTT
ATCACATGTCAATAATGGGCCATTAATAGTGTAGTCTTATAATGGATCACGCTTTATAGTTCATGATGTATATGTTATGTTATG
TTATATGCTTTTAGTAGATTTTCCTTGCTGGGCATTTAGGCTCATTCCTTTATTTTATATTGAGAGAAAATAGTTTGCGGCAGA
AAGATTCTTGTAACTTGGATTGTGTATTGAGAAATGGATTCGGTGGATGCGTAAATGATTCGAGGACGATTTGTTTAAGTCTTT
AATTATGAGTTCTATGTATTTTCTACACTATTATTTGTAATCAATTATTTAGGATTAACATGTTTATTTTATTCTCAACCCCA
ATGAGGATCCAATAGCCATACCTGAATTTTTGATATTTTGACATTTCCTTTAATGTTGTTTAATGAAGTTATGAAGTTCATAGT
ATGTTTCTTAACGTCTAGTGCAGTATAAGCAGTTTTTAAATGGTCCAAAGTACTAAATTAGTTGGGTCTTACAATATTATGAGG
TAGGCTGTTAAACACAATATACCGTATATACTGATAAACCATCTCATCCTCTTGTACTATATCGAGTAGTACAATCTAACTATA
TCAACGACTAATAGATAAAAATATTGGGATTTGCTTATTTCTCTATCTAAGTAACTGCATTTCCCAAGCGAACTACCCATAAAA
CATTATTAACCATACATAGCTACATACACATATAGCACATACACATAATCATATACACATAAAATCCCAACCTTGACCTTGTCA
AATCTCCAACTGAGAGTATAGCACAATACTTCATCAAGACATCACAAATACAACGTTTAATAAAGTATTGAATACCCTATTCAT
CTCATCCATCAAAATATAACTTTCACTTCAGTGTCCAAACTAATATACGAAGTGCAGTCTTATTCTATCATTCTCCCTGATCTT
AGCTATAATAATCAGATCGAAGATCACTTTGGAGAATACCATCTTATTACTCAATAAATGAGCTTTTAGTTCTTACAATTCCTA
CTAAAACGTAAATACAGTACCACTAGACTGGTCTGATACCTGGCACAAACTATTCACATTCTAACTATCTATGTATAAGGTAGT
CCTAACAAACCACCCCTAGAGATAGCATCCAATAACACAACCGACTACCAATCTTCCTAAGTTTCACGCCCAACCTAAGGGTAT
TCAGCCACATACTACTTAAAGAGATTCTATGCATCCCTTCTGCACTAGTTTCTAGCCTAATACAGATGTCATAGCATAAAGGTC
CATGCAGTAAAATGGCAAGACCACAAGGGTATTTCCCAATTCTCAAAGTCAAGAGTCAACGATTTCTTCTTCCCTTGCAAACCA
TCATTGCCCCACCTCTAGGTAAAACCAATCCATATCCACGATCATTACTTGAGTCAGTCATAACAACTCTATCAAATCAATTGC
AAATGAGTCCCCTTCTCGACCATAGATAATTCGTAACCCATCAGCCCCTTTCAAAACATTCTCATCACAACATACCACTGTAAC
GACATCAATCTATGCTCGTCTAGATGCATAATAAAAAGAACAACATGGACCCAAAACCAGTCAGCCAATAAAAAACTCCAGTAA
CCTAAAAGCTTACTCCACCCACTGAGGGAACTCAACCTCATCCTCATCTCTGATTTGACTGCATCGGAGCGAAACACTCGAGGT
GAGTCAGCTTTCCACCCCATTTGCTAATCCTTCCCTTCACCTTGGCAATCCCTCTTGAGAATGCCCAGGACCATTCCTACAAAT
AGAGAAACATTCCTTTGGTCGCATTCCTCCAGAGACCGCACTCTTACACCAAGGTCATCCTGGGTAGGCTTTTCAATCCTTGTC
TTGCCAATCCAATAAATGAAGGTATTACTGCTGATCTCTGTCTCCTCCCAACCACTCTCCTTCTCCCAATATCATCTTCCTATG
CTCAACAATAAGCAAGCCAGCCTTCCTACCACCTAAGCGAGTAGAGATTATATGCATTGGCACTCTGAATCCGAAGCTATCGGG
ATTCATCCCTTGATAAACCTTTTCTTCTAAAGCTACATCCCGGCTACCATACTCAAAACAAAACTTG
>m141120_131642_42134_c100732402550000001823141405141505_s1_p0/65362/0_5039
AAGGAGAGATTAAGAGCTCTAAATATTTTTGAAACCTTTTCCCTCTCAAATTATATTATATAGGGCTATTATTGACATGAATTG
GGGACCTAATTCAACCAAACAAAAAAATTGACCCTTTCAGGGCCCAAATCTCCTAGGGAGCAGCTACGGGCTCGAAAGCGATCT
CGTAATTCACTCCATATTTCTCTCTAAAAGCTCTAATTATTTTGAACCTTTTCCCATCTCAATTATTAAATTATATAGGGGATT
AGTTGACATGAATTGGGGTCCTAATTCAACCAAAAATAAAAATTGACCTTTCAGGCCAAATCTCCTAGGCAGCTGCTAGGGGCC
TCCGAAGCGATTCCGTAATTCAACCTCAGTATTTCTCCCATAACAAGCTCTAATTTTTTTGAACTTTTCCCATCTCAATAATAT
TTACATTGGGCTTATTATTTACCATGAATTGGGACCTAATTCAACCAAACAAAAAAATTGACCTTTCAAGGCCAAATCGCCCTC
CTAGGCAGCAGCCTAGGGCTCCGAAGCGATCTCGTAATTCCACTCATATTTCTCTCTAAGAAGCTCTAATTTTTTTGAACCTTT
TCCCATCTCAAATAATATTATATAGGGCTATTTTTTACCATGAATTGGGGTCCTAATTCCAACCAAACTAAAAATTGACCTTTC
AGTGCCATATCTCCCTAGGCAGGCTGCCTAGGGCCCGAAGCGATTACCGTAATCACGTTTCATGATTTCTCTCTAAACAAGCTC
TAATTTTTTGAAACATTTCCCATCTCCAATAATATTAATATAGGGCTTATTATTGACCATGAATTGACGTCCTAGATTCAACCC
AAACAAAAAAATTGACCTTTCAGGCCAAATGTCCTAGGCAGCTGGCCTAAAGGGCATCGGATGCGATCCTCGTAATTCACTCCA
TAATTAAATCCCTAACCAATTCTAATATTTTCGAACCTTTTTCCCATATCAATAATATAATATAGGCTTTAATTTACCCAGAAT
TGGGGTCTAAAATTTCAACCAATACAAAAAAATTGAACCTTTCAGGCCAAATCTCCTAACGGCAACCTGCCTAAGGGCTCCGAA
GCAATCTCAGTAATTCACTCATATTCTTCTCTAAGAAGTCTAATTTTTTTGAACCCTTTTCCATCATCAATAATATATATATGG
CTTATTATTGAACCCCATGAATTGGGGTAATATTCAACCAACTAAAAAAATTGACCATTTCAGGCCAAATCCCCTAGGGCAGCT
GCCTAGGGCTCCGAAGCGATTCCGTAATTCACTCATATTTCTCCCATAACAAGCTCTAATTTTTTTGAACCTTTTCCCATCTCA
ATAATAATAATATAGGGGCTATTATATTACCCATGAATTGGGGAACTAATTCAAAAACCAAAACAAAAAATTGACCTTTAGCCA

AAATCTCCCTAGGCAGCTGCCTAGCTCCGAAGCATCTCGTAATCACTCATATTTCTCTCTAAAGAAGCTCTAAATTATTTTTGA
ACTTTTCCCATCTCAATAATATTAATAAGGGCTATTATTGACATGAATTGGGGTCCCTAATTCAATCCAAACTAAAAAATTGAA
CTTTCAGGCAAATCTCCTAGGCAGGCTCATAGGGCTCCGAAGCGATTCGTAATTTCACTCATAATTCTCCTCTAACAAGCTCTA
TTTTTTTTGAACCATTTCCCAATCTCAATAATATTAATAGGGCTATTAATTGACCATGAATTGGGGTCTAAATTCAACCAAAAC
AAAAAATTGACCTTTCAGCCAAATGCCTAGGCAGCTGCCTAAGGGCTCCGGAGCGATCCCTCGTAATTCACTCATTAATTATTC
CCTAACAATCGTCTAATTTTTCGAACATTTTCCCATCTCAATAATATAATATAGGCTATTATTTACCAAGATTTGGGGTCCCTA
ATTCAACCAATACAAAAAAATGGCCTTTCCAAGCCAAAATCTCCATATGGCAGCTGCGCAGGGCTCCGAACGATCCTCGTAATG
TCACTCATATTTCTCTTAAGAAGCTCTATATTTTTGAACCTTTTCCATCTCAATAATATTATATAGGCTATTATTGACCATGAA
TTGGGGTCCTAATTCAACAAACCAAAAAAATTGACCTTTCGGCAAATCTAACTAGGCAGGCTGCTAGTGCTCCGAAGCGATCTA
AAGTAATTCACTCATATTTCTCTCCGAAGAAGCTCTAAATGTTTTTTGAACCTTTCCCATCTCAATAATATTAATAAGGGCTAT
CATTGACCATGAATTGGGGTCCTAATTCAACCAAACAAAAAAAATTGACCTTTCAGGCCAATTGTCATAGCCAGCTGCCTATGG
GCTTCGAGCGATCTCGTAATTCACTCATAATTACCTAACAATCTCTAATATTTTCGAAAAAACTTTTCCCATCCTCAAATAATA
TAATATAAGGTCTATTATTTACAAAGAATTGGGGACCTAATTCAACCAAACAAAAAAATTGCCTTTCAGGCCAAAATCTCCTAT
GGCAGCTGCCTAGGGCTCCGAAGCGATCTCGTAATTCAACACATATTTTCACTTCTAAGAAAGCTCTAATATTTTTGCACCTTT
TCCCATACTCAATATATTATATAGGCAATTATTGACCCATGATTGGGGACCTAATTCCAACCAAACAAAAAAATTGGACCTTTC
AGGCCAAATCTAACTAGGCAGCAGCCCCTGGGCTCCGAAGCGATCTCGTAATTCAGCTCATATTTTCCTCCTCTAAAGAAGCTC
ATAATTATTTTGAACCTTTTCGCCAATGCCTCAAATAATATCTATATAGGGGCGATTATTGACCCTGATTGGGGTCCTAATTCA
ATCCAAACGATAAAAAATTGCCTTTCAGGCCAAAATCTCCTAGGCAGCTGCCTAGGGGCTCCGAAGGCGATTCCCGTAATTCATC
AATATTTCGTCCATAACAAGCTATAATTTTTTTGACGGCTTTTCCCCATGCTCAAATATATTATTTGGGCTATGTATTTACCAG
AATGAATTGGGGAGCCCTAATTCAACCAAATCAAAAAATTGAACCTTCTCAGGCCCAATCCCCCTAAGGGCAGGCAGCCCTACC
CCCGGCTCCCCGAAGGCGATCATCGTAATTCACTCCATATTTCTTCCTAAGAGCTCTAATTTATTTTTTGAAGGCCTTTTCCCA
TTCTCAATAATATTATAATAGGCTATTTTTTACCATGAATTGGGGGGTCCTAATTCAACCAAACTAAAAAATTGACCTTTTCAG
GAACCAACATCATCCCTAGGGGCAGCTGCCTAGGCTCCGAACCGATTACGTAATTCAACTCATATTTATCCTCCTTAACTAGCC
TCTAATTTTTTTTTGAAACCCAATTCCAATCTCAATATATTATGATACGGCTATATTACCATGAATTGGGGTCCCAATTCAAAC
GCAAACTAAAAAATTGACATTTCAGCCAATATCTCCTAGGCAGTTGCCTAGGCTCCGAAGCCGATCTCGTAATTCACTCCAAAC
CCCATATTTCTCTCTTAAGAAGCCCTAATTTTTTTAACGGCTTTTCCCGTCTTCAATAATTTATATAGGGCTATTATTGACATG
AATTGGGGTCCTAATTCAACCAACAAAAAGATTGGCCTTTCAGGCCAAATCTCCCCCCTATGCAAGTGCCAAGGGCCTCCGAAA
GCGATCTCCGTAAATTCACTCATAATTTCCTCCCTAAGAAGTCTAATTTTTTTGAACCTTTTCCCATCCTGAAGTAAGATATTA
ATATAGGGCTAATTATGTGACATGGAATTGGGGTCTAATTCAACCGAACGATAAAAAATTGACTTGCAGGCCAATTCCTTCCCT
AGGCAGGCTGCCTTACGGTCTCCCGACCAGAGGCGATTCCCGTAATCAGCTCATATTTCTCTCTTGCCATACAAGGGCTCCTAA
TTTTTTTGAAAACTTTTGCCCATCAAATCCCCCAAATAATATTATATAGGGCTATTATCTTACCATCGAATTGGGGACCTAATT
CAACCAAACAAAAAAATTGACACTTTCAGGCAAATCTCCAACTAGGCCGCCCCTAGGGCTCCGAAAGCGATTCCGTACATTCAC
TCCCCCATAATTTCTCCCTAACAAGCTCAAATTTTTTTTTTGAACCCGCCCCGCCCACCCCTCCGTTTCGCGCATCTCAAAATA
ATATTAATAGGCGCTATTATTTACCCATGAATTGGGTGCCTACACAATTCAAACCCAAACAAAAAGATTCGAGCCTTCAGGCCC
CAAATCTCCTCTAGGCAGCTCCTAGGGGCTGAAGCGATTCCGTAATTCATCTCATATTTCTCCCCTAAAAAAGAGCTCTAATTT
TTTTGAAAAAAACCTTTTCCCATCCTCAATAATAATAATATAGGGCATTATTTACCCATGAATGGGGTCCTAATTCAACCCCAA
ACAAAAAAATTGACTTCTCAGGGCCCAAATCTCCTAAGGGCAGCTGCCCTAAGGGCTCCGAAGCGATCTCGAATTCACTCATAT
TTCTCTCTAAAGAAGCCTCTAAATTTTTTGAAAACCTTTTCCCCATCCTCAATAATATTATATAAGGGCTAGCTATTGACCATG
ATTGGGGTAATAAATTCACCCAAACCTAAAAATTGACGCTTTCAGGGCCAAATCCCCATAGGCCCCCCAGCTGCCTAGGGCTCC
GAAGGCGAATCGTAATTCAACTCATATTTCTCCATAAACAGCTCTAATTTTTTTGAAGCGGCATTTCCCAATTCTCAATAATAT
TATATAGGGGATATTATTTACCATGAACATTGGGGGGAATTATTCAACCAAACAGAAAAAATTGAGCCCATTCAGGGGCCCAAA
TCTCCTAGGCAGCTGCCTAGGGCACCGATGCGATGCTCGTAAATTCACCTCATATTTCCTCTCCTAAGTAAAGCTCTAATTTTT
TTGAACCCCTTTTCCCATAATCAATAATATGTATATAGGGCTACCATTGACCCATGAATTTTGGGGGTCCTAATTCAATCCAA
>m141120_131642_42134_c100732402550000001823141405141505_s1_p0/67518/7327_12487
ACCAGACCGTAAATCAACATCAACATCTGATTCGAAATGAAGAGAGTTTAAGAGACAGAAGAAAAAAAAATTAACAGTAGCCAC
ACGGAAATGAGATTAATAGAGAAGGTTAATAGTATACTCAGTACCACAAAGAGACTGATTTCAGCTAATATGTTAAATAGATAA
TAAAGTCTTATAAATATGCTATTTACAATTAAAAAATTACATTTATCATTATTATAATCCTATAAAAGAATAAAAAATTAGCAT
TAAACTGAAAAATCAGATAGAGTGTTTGAAAGTTTACTAGAAGTGCCACACCACATCCCCTCCCTTGTGCTCCTCCTTTATATA
GATGTATAATTTATCATTGTTTTTATTCCATTTTAAATCAATAAATAATGTAATTAAATAAAATATATATACTGTATACTCGCA
ATAACGTCCGACCCCATTTGACGTGGTGGCAAGAATCTTCAATAAATATGCTACATAATCTAATTTTAAGATTTATCGATTAAC
CTTATACTTACCTAATTTGTATAAAAAGCTCTGCATGTTTTTTTACCGCCAATCCATCTCCAACAATCTCCAATATCACCCCAT
AACAACATAAACACGAAACATCCCTAAAACATGATAGAAAAATAAGAAAATGAAGTGTTTAGAATATCCCAATATCAATAATTT
AAAATAAAAACTTTAAAGATTAACAAACATTAATAATTAAAAAATGGGCCCCCAAAGTTTAAATTTAACAAACCATTAATAATT
GAATTACATACCGAGAAGGTATTGTGTTATCATCTCAACACGTAAAATCACATCCAATTTGGAGATATGAATTCGGAATTGCCC
ATTGAAAATTGACACATTGAAATAATTGTAGAGACGAAGAACGGAGAGAAAAAAATGTAGAGTTAGAGGGAAATGATGAGATTA
AATATAGAATGTTAAGAATCATATATACCATTCACAAAGATCAAGTACTTTATCTATATTTAAATAATAATACTCTTATAACAT
ACTCATTTAAATTTAAGAATTACATTATCTCATTATTATAATCTATAAAATGAAAAGAAAAATTAGCATAACAGGAGAAAATAA
TATAGAGTGGTTCTGAAGAGCTTTTAGAGTCGTCATATCATCTTCTTGGTGTATCTTTTAATTTAATATATTTAAATATTTTTT

ATTCCATTTTAAATAATAATAGTAATTAACTAAAATATAAATATATTATAACTCGCAATAACTTCCCAACCATTGAGATTGTGG
AAAAATCTCATTAATAATGCTAACAACTAGACTATCTTTAAGACTTTCATCGATTGACTTAATACTTACAATTCTATTAAAATT
TGATGTCCCTTTTTTTTCCTCAAGAACCATCTCCAAATTCGTCAATATCACCCACAGCACAACACCAATAACGGCATCCGATAG
ACATCCTAAAACATGCATGAGAACCAACCATAAGATGAAGATATAAGCAATATCAACGATCAATAATTTAAATAAAAAACTTAA
ACGTTAAAAACACCATTAATAATTGAATAAATGCCCAAGTTTAACAGATTCAACAAACATTAAATGAATTGAATAAATACCCTC
GAGAGGATTGTTTATCCATTTAACACGTAAATCAATCCCCAAATTGGAGATATGAATTGAATTCCATTGAAAATTGAAATTGAA
ATAATTTAGAGAAAAAAATTCAGCAGTGAGGAAAATACTGAGAATTCAATCAGAGAATGTTTAGAATCATATAAAAAAAAAAAA
TACGGATTACCAAGATATATATCCTATATTAAAAATAATCACTCATTATGAATATCTCCATTTAAATCTCAAAAAAATTAGCGA
TTTATTATTATTACAATTATAACAATGAATACACAAAAAATTACGATTAACAATGCAAAAATAGATATGTCGTAGCATTTCGAA
GAGTTCCTTATAGAGTCGTCACCATCATCGCATCCTCTTACGTCGCCTCTCAACTTTATATATACGATATATAGTTGTACCAAA
ATTAATAAAAAAAAAAGTAATTAAAGGACGAAAATAGATGAGTGGTTTGAAGAGTTTAAGAGTGTTACATCCACATCTACTTAG
CGTACCTCTTTTTCTTATATAACTATCAGACCCAAAACATGAAATCATAGACTGAACATAAATCGAGCTGGCGCAATCATATAT
CATAATATAAATAAATATAGTTTTGAAAAAAACTTTCATATTTGGTTAATATAATATATCTTAGAATCAAATAGTATAGTTTCT
TTGAAAAATTAATTTATATCATCGTTTTCTAAACTTATGCAATAAGAAGCCGAGTGACTATTTTTTTAAAAGAAAAATAAGCAA
AACCCTTATACAAGTGTAGTGGCTACTTTTTACAAAGTAATGTATTTTTTTCACTCTGACCAATGGTAATCATTAACCCATGTA
ACATTATTTAGCAGTAATCATTATTTGAAATTTTAATACTCAAAACCACCATGATTTTTGTTAAGCTAAATTGGATAGAGATAT
AGTCAGGTGTTATATTATTCATTAAACTTAATATAATACATAACCATAAAGATTAAACCAAGTGAAAATACAAATAAATAAATT
TATGATATCAGCAACAATTTTAACCCTAAGAGAGAGTAAACCATGCTTTGTCTTTGGTTTCTTAGACATCCTTACAAAAAACAT
ATATACGGGCTTACTTTACCACACTCAAAAGGAATAAGCGAAAATTTGATTGATACTCAATCCGGCCCGGCAGCTCACTATCAA
TTAAAAAACAATAAAGATAATAATTGCCATTAGTCAGCCAAAAATAACTTTATTAATACTTCTCTTCGTTACACCCCAAGATTT
TCGAGATAAATGAGATTATGAACCCCGAAAGTTGGCTCGTCAAGCTGTGAGCCGAAATTATGCATGGTCGTCATATGTATACCT
TTACCAGACTGCTTTGCTGTCAACTTACGCCCAACCGCCCGAAAGCCGAGGTTTGTAGCCTCCGAATATGTTTTGCACAGGCTC
AGGCAACGTCATCGAACTTCTAAATACATTCCTTTCACCTCTGTGCCTATTCGGCGCGAGATACCCCCCCAGCTTACAAGCAAG
CCGTCGCGGCAAGTACAAGCTCGATACTAAGCAAGCTTCCTTCGTCCAGCATCTACTTATCCCTCGGCGCCATCACCAACCGCG
GTGACAGTTGGTGGCGACGCCCCGTGCCATTGACTCAATAGTCTCGACGAACTCAATGCCAATCGCATGATCCTCAAAGATTTG
ATGAATCCATAGGCAATAGCAACCCATTACGCTTGCTGAACATATTTATTGTTGTGGACAGATCCCCCCAACTATTTAAGGGAT
ACACACCCTATTTCATCTAATATTCCCCATTCCCGATCTTCATGCGGACGTTAAAATCCCCGTTATGTAGGAGAAAAATGCACT
TAATAGCATACTATCCCGTTAATGATTTACAGAACCTCCCTGAACATATGTGGGAACTGAAATTCTGCAACCCTTCTCTATAAA
ATAGAGGAGGAATCACCATTGTTCAAAGGACCTTCTTTTTCCCTGACCCTTTTGGAGAGAACACTCCCCTGGTGAAACTTATTC
ACGAAAAGTTCCAGCAACAACCTCCGCCAACCGTCCTTAATAATCCATCCAGACCTCGTGGTACTAGGTACTGACAAAAAACAC
AAGTTTAAACTGTTGACACATGTAAAAACCTCCTGTTGTTTCTTCTTTATTCATTTCTCAATATTCTATTGTTTAATTCGCTC
TACTATTTAAGTTGACAAAAAACGGCGTCAAAAGCAGTTTGGTGCTTTCATTGAGAGCGTTAGACAGTACTATGCGTGAGTCTG
TTCAGTCAAAGAACCTCCCCCAAAATCAACAATGGCAGCAATGATCCCCAATGTTCCTGAGAGGACCCACGATCTTTAGATGAA
GTGCTACCCCCCAAACGGCCCTGGAAAGCACCCCATGGGTGAACTCAAACCCTGATGAAGGAGCCTGGGATTATCCCGATTCTC
AGGGACCAACCCAGGCCTAGGGACGACGAGACATGTACCTACAATCCTGAATGATATGTCCCGAATAATGGATGCCTCAAACAT
CATCAACCTGCGCGAAAGCCAGTTGCGAGGCCAAGAAAGCGCAATGAAGAAGCCTACCCAGACTAGCGGCTCAGGCCCCCACCT
TCGCCAGAGGACAAGGCCCCTTCCACCTCCGCGGGACGTTCCTGTTCCCCACCGTAGGCCCTCGAGGGCGGCCACCAAAAATGC
CTGCCACCGAAGGCCAAAGTGACCTCCGCGCTAGCAGACGCCCTGGCTCCACCCGAGAACCGGAAGTAAACCGAGCTGGAGGTT
TCTCCCCAACCGCCCAACATGCGAGGGCACCAGCGCCGAAGTAAGGAGAATAGACTGAGGCCCCTCCAGGAAGGCTCCAACCAA
AATCTCTGGTACCGCGCAGACTTATAGAACCCTGTCGGGAGAACTAAGGACCCCTTCTGTAGAGTCCAAGACCTTTTACTTAGCT
AGTTACCAATAATAGTAGTGAGTAGTAGTAGTAGTAGTAGTAGTAGTAGCAGATAGTAGTAGTAGTAGTAGTCCAGTAGTAGTAGTA
GTAGATAGTAGTAGTAGTACGTAGTAGTAAGTAATAGTAGAGTAGTAGTAGTAGTAGAGTAAGTGGGTAAGTAGTTGGTAGTAGTAGATG
GTAGTAGTGTGGTAGTGGTAGGTGGGAAGTTAGTAATGGTAGTAGTAGTGGTAGTAAAGTAGTGGTAGTGGTAGGGTAGTGCTA
GTGGTAGTGGAGTGGTAGTGGTAGTGTAGGGTTAGTGGTAGTAGTAGTAGTAGTAGTAAGTCGTAGTAGTAGTAGTAGTAGTGG
TAGTAAGTGGTAGTAGTGGTAAGTGGTGGTAGTGGTAGTCAGTGGTAGTAGTGGTAGTGGTAGTGGTAGTGGTATGGAGTGGGT
AGTAGGTAGTGGTAGTGTAATGTGGTAGTGGTAGACGTGGTGGTAGTGGTAAGTTTTGGTAGTGGTAGTGGTAGTGTATGGTGG
TGGTGGTAGTATCGGTGCGTGGTGGTATGTAGTGGTAGTGGTAGTAGTAGTAGTAAGTAGTAAGTAGTAAGTAGTGGTAGTGGT
AGTGTTGGTAGTGGCAGTGGTAGTGGTAGTGGTAGT
>m141120_131642_42134_c100732402550000001823141405141505_s1_p0/69009/3065_9373
CCAGCGAAATAGCAAGTTCTAGTTAAATCCGACGCGAGAAAAGGGAGGGCAACGCGTTACGCTATAAATGATTATTGCGGACCA
AAAACATTCGGGTGCCGAATATACCAGAACTAATGCACCGGATCCCATCAGAACTCCCGCAGTTAAGCGTGCTTGGGCGAGAGC
TAGTACTAGGGATGGGTGATCTCCTTGGGAAGTCTCGTGGTTGCACGCATGAAAACATATTTTTTTTATTCTATAAAAATCCT
TACGCGCTCAAAAGTTTGTATTTTTTTTAAAAGAAAATCTTTAAAAGCTCAAATTTATTTCGCTTTTCCTTTGGGGGGTTAAAT
ACCGTGGCCACGTCGACAAAGCAGCGAATAAGAAAGTAATCCTAGTTAATCCGAGGGAGAAAGGGAGGGCACCGCGTTCGCTAT
ACAATGAATAATGCGGAACCATACATTCCGGGTGCGATCATACGCAGCACTAAATGCCCGGATCCCATCAGCAACTCCGGCAGT
TAAGCGTGCTTGGGCGAGAGTAGTATACGGAAATGGGTGCATCCTGGAAGTCCTTGTGTTGCAGCCCTTGAAAAACATCATTTT
TTTTATTATTTAAAAAATCCATTACGGCTCAAAGTTTGTATTTTTTTAAAGAAATCTTTAAGCTCATTTCATTTCGTTTTCCTT
GGGGGGTTAATACCGTGGCCAACTACAAGAGCGAATAAGAAGTTTCCTAGTCTCATCCGAGGACGAAAAGGGAGGGCACCATAG

CGCTTCGTATAACAATGAATAATCGCGGACACAACATTCCGGGTGCGAATCTATACCAAGCACTAATAAGCACGATCCCATCAG
AAAACTCCGCAGTTATAGCGTGCTTGGGCGAGAGTAGTCTAGGATGGGAGACCTCCTGGAAGATCGATGTTGCACCCTGAAAAA
ACATCAAATAAAAAATATTATATATATAAATATAATTCTTTAAAAATCCATTCAGAGAATACAATTTGCATTTTTTATAATAAT
AAATAGATATATATAAATATATCGTTATACATATAGAGAGAGATATATACCAGTAAAGAGACACTAGAAGACAGCGAGAAAATA
TCTATATACATACACGAGAGAAAGAGGATTGTATAAGAGACAGAAATACGGTAAAAAGATACAAACCAGCAAACTAATGCAACC
GGATACCATCAGACAGATAGAGATAGCTATGAGAAGAAAGAGATAGTAGGGTAAGGGGGAGAGAATATAGATAATATAATAGAT
ATAGATACAAATATATTTATTATAATATATATATAACATATATAACGGCTCAAAAGTGTTGTATTTTTTTAAAAATATCTTTAA
AAGTCATTATATTTCCGTTTTCCTTGGGGGGTCTAATACCGTGGTCAATGCAACACGAATAAGAAGCTTCCTAGTTATATCCGA
GGGGAAAAGGGAGGGCAAAGCGTTCGCTATAAATGAATAATGCGGAACAAACATCCGGGTGCGATCATGACCAGCCTAATGCTG
ACGATCCCATAAGAACTCCGCAGTTCAAAGCGTGCTTGGGCGAGAGTAGTACTAGATGGAGACCTCCTGGGAATCTCGTGTTGC
ACCCCTGAAAACATCCAGTTTTTTTTTATTCCTTAAAAAAATCCAACCTTACTGGCTCAAATCTTTATTTTAAAAAAAAAAATC
TTTAAAGGTCATTTATTTCCGTTTTCCTTGGGGGTTAATACGTTGGCACTGACAAGCAGGAATAAGAAGTTCCTAGTTACATCC
GAGGGACGAAAAGTGAGGGCACGCGTTCGTATAATGAATAATGCGGACAAAACATTCGGGTGCGAATCATACCAGCCACTAATG
CACAGGATCATCAGAACTACCGCAGTTAAGCGTGCTTGGCGAGTAGTAAGTACGTAGGATGGGAGACCGTCCTGGGAAGTCCTC
GTGTTGCACCCCTGACAAACATAAATTTTTTTTTTATTGTTTAAAAATCCCTTACGGCTCAAAAGTTTGTATTTTTAAAAAAAA
AATTTTAAAAGGTCCATTTATTTCGTTTTCCCTTGAGGGGGTTAAAACCGTAAGGCCACTGACAAGCAGCGAATAAAGAAGTTC
CTAGTGTATATGAGGGAGAAAGGGAGGGCACGGTTCCGCTATAAAAATTGAATAATTGCGGAGACCAAACATTCCGGGTGGCGA
TCATACCAGCCTAATGCACCGGATCCCATCAGAACTCCGGCAGTTAAGCGTGCTTGGGCGACGAGTAGTACTAGGAATGGGTGA
CCTCCCTGGGAAGTTCCTCGGTCTTGCACCACCTGAAAACATAATTTTTTTTTATTCTTTAAAAATCACTTACGGCTCAAAAA
GTTTGTATTTTTTAAAAAAAATCTTTAAAAGCTCATCTTATTTCGTTTTCCTTGGGGGGTTTAATACCATCCGGCCACTGGAC
AAGCAAGCGATAAGAAGTTCCCTAGTTCAATCACGAGGGAGAAACAGGGAGGGCAGCGTTCGCTATAAATGAATACGTATCGGA
ACATTACAAATTCCGGGTGCGATCATACCAACGCAAATAATGCAACAGGATCCCATCCAGCAACTCTCCCGCAGTTAAGCGATG
CTTGGGCGAGAGTGTACTAGGCATGGGAGACTCCTGGGAATCCTCGGTGTTCCAACCCCTTCGACACACACATCATTTTTTTT
TATTTGAAAATCACTACGGCTCACAAAAGTTTGTATTTGTTTTAAAAAAAATATTTTAAAAGCTCATTCTATTTCGTTTTCCTT
GGGGGGTTAATCGTGGTCACCTGACAAGCAGCGAATAAGGTTTCCTAGTCTAAATCCGAGGGAAGAAAGGAGGGGCACGCGTTC
GCTAAATTTACAAATGAATAATGCGGAACACATTCCGGGTGCGATCATACAGCACTAATGCAACCGGATCCATCAGAACTCCGC
AGCTTAAGCCGTAGCTTTGGGCGGAAGTAGTAACTAGGATGGGAGACCTCCTGGGAAGTCCTCGTGCTTGCCACCCCCTGAAAA
CAGTCATTTTTTTTTTTTATTAAACTTAAAAATCCATTACGGCCCTCAAAAGTTTGTATTTTTAAAAAAAAATCTTTTAAAGGTC
ATTTATTTTCGTTTTTCCTTGGGGGGTTAATACAGTGCCACTGCACAAGCCAGCGAAATAAGAAGTTCCTAGTTAACACCCGAG
GGAAAAAGGGAGGGCACGCGTTGCGTATAAAATGAACTATCTGCGACCAAACATTCCGGGTGCGATCATAACCAGCACTAATGA
CGCGGATCCCCATCAAGAACTCCGCAGTTAAGCGTGCTTGGGCACGACGAGTAGTATAGGAATCGGGTCATCCCCAGATCAGTC
TCCAGTGTTGCCACCCCTGAAAACATCATTTTACTTTTTTCATTGTTTAAAAATCCACATTACGGCTCAAAAGATATTGTAT
TTTTTTTAAAAAAATCTATAAATGCTCAATTTATTCTAGTTCTTGCCCTTGGGGGGTTTAATACGTGCGCCACTGACAGCAGGA
ATAAGAAGTTCCTAGTTAATCCGAGGGAACGAAAAAAAGGGAGGCGGCACGCGTTCGCCTAATAAATTGAATATTTGCGGAACA
AACATTCCGGTTGCGATCAGTACCCAGCCACTAATGCACCCGGAATCCCATCAGAACATCCGCAGGTCCAAGCGTGCTTGGGGC
CCCGAAGAGTAGTACTAGGATGGGTGTCCTCCTGGGCAAGTCCTCGTGTTGCACCCTGAAAACGATCCATTTTTTGTATTCTTT
AAAAATCCAACTTACGGCCACAACCGGCTACAAAAGTATTGTATTAATTTTTTAAAAAATCCTTTAACAAGCATGATTTATTTC
GTTTGTCCTTGGGGGGTTAATACGTGGCGCACTGAACAGCAGCGATAAGAAGTTCCTAGTTATGTCCGAGGGAGAAAGAGGAGG
GCACGCGTTCGCTATAAATGAATAATGGCGGAACAAACATTCGCGGTGGCGATCATCCAGCAGCAATGCACCGATCCCATCAAA
TCCGCAAGTTAAGGCGTGCTTGGGCGGAGTATACTAGGACTCCGTGAACCTCCCTGGGAGTCCTCGTGTTGCCCCCCTGAAAAAC
TCATTATTTTTTTTATTCTTTTAAAAATCCCCTTACCGGCTCAAGTTTGTATTTTTTCTTAAAATAATCTTTAAAAGACTCATT
TATTTCGTTTTTCCTTGGGGGGTCATACGCGTGGGCCACTGACCAACGCCAGCGATAACCGAAAGTTATCCTAGTTAATCCGTG
GGCAGAAAAGGGGGCAGGCGTATCGCTAATAAATGAATAATTGTTGAACCCAAACACAATTCCGGGTGCGATCATTACCAGCAC
TAATGCACCCAGATTCCCATGCAGAACTCCCCGCAGTTAAGCGTGCTTAGGGGCGAGTAGTAGTACTAGGATGGGTGAGCCTCC
ATTGGGAATTGTCCATCCCGTGTTGCACCCCGCTGGAAAACATCATTGTTTTTTTATTCTTTAAAAATCCCTTACCGGCCTCAA
AAAGTTTGTATTTTTTTAAAAAAAATCTTTAAAAAGCTCATTTATCTTCGTTTTCCGCTTTGGGGGGTTAATAACCGTGGCCAC
TGAAAGCACAGCGAATACGAACGTTCCTAGTTTAATACCGAAGGGAGAAAAGGGAGGGGCAACGCGTTCGCTATAAATGATACA
TGCGGAACCAAACATTCGGGTTGCGTCATACCAGCACCTAATGCACCCGGATCCCATCAGAAGTCCCGGCAGTTAAGCGTGCTT
GGGACGAGAAGTAAGGCTACTAGGATGGGTGAACCTCCCCCAGGGAAAAGTCCCTCCGTGTTGCAGCCTTGAAAACATCATTTT
TTTTTTTATTCCTCTTAAAATCCACGTACAGCCTCCAAAAAGTTTGTAATTTTTTAAAAAAAATCTTAAAAAGCTCATTTATTCC
GTTTTCCTTGGCGGGGTTAATACCGTGCCACTAGACCAGCAGCGATAAAGAAGTTCCTAGTTAAAATCCGAGGGGAGAAAAGGG
GAGCTCGAGTTCGCTATAAACATGAATATTGCGGACAAAAACATTATCCCGGGTGCGAATCCATACCAGGCACGTATGCACCGG
ATCCCCAATCCAGAAACGTCCGCAGTAAAGCGTGGCTTGGGCGAGAGTAGTACTAGGATGGGTGACGCTCCTGGGAAGTACTCG
TGTTGCACGCCCTGAAAAACATCATTTTTTTTTATTCTTTACAAAATCCACTTACGGGCTGCAAAGTATTGTATTTTTTTGTTT
AAAAAAAAATCTTTTGAAAGCTCATTTTATTTCGTTTCCTTGTGGGTTAATAGCCGTGGCCCCACTGTACAAGCAGCAAATAA
GAAGTGCCTGTTAAATCCGAGGGAGAAAAGGGAAGGGCCAACGCGTGTCGCTAATAATGAAATAATGCGGAACAAACATTCCGT
GTGGGCGATCATTCAACACCCAAGCACCATGGCAACCGGATCGCCATCAGAACTCCGCTTCGTTAAAGCCGTGCTTGGGCGAGA
GTAGTACAGGATGGGTGGACACTCCCTGGGAAAGTAAACTCCGTGTTGCACACCCCTGAAAACATCATTTTTTTTTTTATTCTT

TAAAAAATCATACCGGCTCAAAAAGATTTGGTATTTTTTTAAAAAGAAATTTTAAAGACTCCATTTTTCGTTTTCTTGGTGGG
TTTAATACCGTGGCCCACTGACAGCAGCCCCGAATAAGAAAGTTTCCTAAGTTAGATCCGAGGGAGAAAAAGGAGGCGACGCGT
TCGCTATACAGAAACACTGCGGAAGCAAATTCGGTGCGATCATAACCAGCGCACCTAAAATACGCAGCCGGATCCACCAATCAG
AACTCGCAGTCAGCGTGCTTCGGGCGTGAGTACTACTGGATGGGTGTCCTGGGAAGTCCTCCGTGTTGTTCAGCCCCTGAAAAC
ATCATTTTATTTTTATTCTTTAAATCCCAGCTTACGCTCAAAAGTATGTATTTTTGTTAAAAAAATCTTTTAAAAGCTGATTTA
TTTCGTTTTCCTTGGGGTTAATACGCAGTGGCCACTGACAAGGCAGACGAAATAACGCAGTGTCCCTCAGTCTAATCGGGAGAA
AGGGAGGCAGCGCGTTCGGTATACAATGATATGCGGAAGCAGAATTCCGGGTGCGTCATTTAACCAAGCATAATGCACCGGATC
CATCCCAGACCACCCCACGTTCAAGCACGTGGCTTGGGCGAGAGTAGTACTAGGAATGGGTGACGCTCCGCTGTGGACGTCCTC
TTTTTGGC
>m141120_131642_42134_c100732402550000001823141405141505_s1_p0/72747/207_14016
TTCATTTTGAGACTACACTCGAAGGGAAAAATAAGGCTCCCTAGTTAAGTCAACTGCGTTGTAATAATGTTCAGCTCAGAGAGC
TAGACCCAACGAGATGTTGTTGATCACGGATTTGGTTCGCTAGGATAAGATAGGCAACATTCTTGGCTCTCAGACTAGGGGCCG
AATCGGCATGAAGAAGTACCCGAGCCTTCGTCAATACAGCAATCCCAACCGAACGAGCAAGAGATGAGGAATCCACTATCACAG
AACAGTCGCTCGATCGGCAATGAGTTGGTCCGAGCTACGTTTCACTATACCACACAGAACAGCATGAGTTAGGTGTCCGAGCTT
CCTCTTACATCGAGCCCACCTCGATCGGGCATGGAAGTGGGTCCGACTTCAACTTCTCCAAGCAAGCCCACGTCGACATGAGTA
CACAGCATGATCATAGCACTTGAACCTTCACGTTTGGCTCGTAGTGCTGTTTGCTACCATGAACGATCGGTGCTAACACTTCCC
TTTGAGGCCTAAGTGATCAGATCGTGTATATTATTGCATATCTCCTCGAACGTGGTCCGATTGACTTCCATGACATGGTCCGAT
CGGACCTTCACTGGCCTAATAAACAACACAAGTCCAGCTAATGACTTACTAACACTCGACCGGTCCGATCCCGAAGCTTGTGTG
GCACACATACAAACCCACTTAGGCATCACTGCCCGATCGGATGTACACTTTCATTGCAACCCAACATGACGACACAAACACATA
TTATCCTCACCACAACAAATGACATACCCCTTGGCCAAAACTAAGATCCAATCTTTGGCAATGCATGGGAATTCTCTTCGATAA
GTCCGAAGCTACATATTTTTGGAAGCTCAACAAGGTCGATTGGACACTTTCTTGCTTTCAATTATGTCCGATGTAGCATATTCC
TTAGATACACATTAGCACCTATTATTTTTGGACCATCATGGACCTCTCCTTGGTTGAAACAACATAAAATAGGAACTCACCAAG
CTAAGGTCCCGGAGCCAAGCATTTGGGCTCTTCCAATTATGTCTGATAGTGCATGTTGGCACACAAATGCATGATCTTCCCTGC
GAGCTTGTCGATCCGGAGAATTTCCAATCTAAGGGCTCGTCAAGCTAAGGTGCGAGCAAAGCTTTGGCTCCTTCAACTATGTCC
GATCGTCGCATGTTTCGCATCACATGCATGATCTTCCCTCGAGCTTGTTCGATTCGTAGCTTTCCAATCTAGGGACTCCTCAAC
TAAAGGTCCGAGCAAGCATGTTGGCTCCCTCCAATCACTTCCGATCGAACAACACTCTTCTTGGCATCATGTCCGATCGTGCTG
GTGTTTGACCTCGGTCCAAAGTCTAAAGTCCATGGGGGGTCATTCGGATAGACGCATCTCCCCTCGGACGAAATCCCCACTCCG
ATTCGGTATAATGCAGATTCCAAGCCACTCAAGCGTGTTTCCTCAACTCCTTGAGATTGCAGCACCTTAGGCACCAATATTGGG
CCTTTAGATCTTTGACATAGGTGAGATGTCTCCTTGATCAACATGCATCATCCCATCTCAGTATGCTTGGACATCACATTTTTT
CAAAACATTTCACCTTTAGCACTCTTAGGTACGTTCAGGGAAAAACATCAAAGCAGCATTGTTTAAACTCCCAGTTATTATTTT
GAAGATTGAGTTTCTCTCAATCAATCTTGGTGAAAACTTGACCTTCTCTTCCCGAAGACCAAATTTTTCACCATGTAACATCAT
TTTGCATGCATTTGTTGATTCACTCCAGGGAAGTTGGATCGATTATACATGCTTATAGCCGACAAAATGAGGAAGTTGTATCTG
CGTCTCCTGAGCAAGAAACTTTGCACCTGGTGAGCTTAAACTTGTATTGTGCCTTTAAGTACTTTACCTCTTCTTTACTAACTT
AAAACATTCTAAGCTCCGTCTAGACTTAAAAAGTTAATAAGTTTTTTGTGAAGAGTAAAAGTCACTCTGGGTGTGATGCCTTAG
ATTTTCGCATGAGTAATTATTTTCTAAACTTAGAAATTTCTAGACCATTTCAAAGTCCATGCTAAGTAATTACTTTTTTCCCTT
TCCAAACCAAACTCCTTTTAAAAAAAATTTGTTTTTTTTTCTAAACATTTTTTTTTTTAAAAAAATTGAAAAAAGCAATGGGAA
AAAAATTGTTATTTTTTTTTTATTGGGTCCCAAAAAACAAACGAAAATATATTTGCTGTTCCCCCCCCCCTAAAAAACCTTTTT
TTGGGGGGAAAATTTTTTTTTTTTTTTTAAAAAAAAAATTCCAAAAAAAGTTTTTTTACCTTTAAGCCCCCTTTTTTTGTTGGCC
CCCCCCCAAGCCCCAAGTTTTTTTTTTTTTTTAAGCCCCTTTTTTTAAATTGGGCCCCCTTTTTTTTTTTTTTTTTTGGGAACC
CCTTTTTTTCGAAAAATTTTTAAACCAAAGTCCCCCAGCTTTAAGAAAACCAAAAAAAAAATAATATGTAAATTGTTTTTAAAA
CCTTTTTTTTTACCCCGTTCCGGGTTGCCTCCTTATTGCCTTTTGTTTTGGGGGGGTTTTTATAGTTTTAATAATAGCCCAAAG
TTTAAATTACCCCCCCCAAAAAAATTGTTTGTGCCCCCCCCCCCCCCCAAAAAAAGTTGAAATTTTTTTTTTTTTGGAGGGTTTGA
AAAAGAATTCCCCTTTTAAAAAAAAGATCCGCCCCCACTTTTGCTAAACAACCTTTTCTTTGAAACCCCTTTGAATTCCCCTGT
TGGTTTTTTTTTTTTTCGAAAGGCAGTTTATTTTACTCCGTGGGGGGGAAAAAAACCACCCAATAGAATATTAATTGGGGGAAA
AAATACCCTCAAAAGGTTAAGTTTTTTTTTGAAAACCAACTTTGCCTTGGAAACAAAAAATATCCCCGACCAAAAGTTTTTTTT
GGGGAAGACCAAACTTTGAAAAAATTCCCCCGGAAAATAAACCTTTTTTTTTTTTAAACACAAACAATGGGGCCATTAATTTTG
GTTTTTTGTTAGCAAAAAAAAGAAGTCGAACCCCCAAAAGCATTTTGCGCGGTAAGTCCCCCCTTTTATTTTTATTTACTTTTC
CCCCCCGTACAAATTTTAAAAAAAAAATTGGGAACCCCAAAAAAAAACCAAAAATGGTCTTTTCTTTAAAAAAACAAACGAAAAGT
TTTTCCCCTTTAAAAAAAAAACAACAAAAAATTTTTTTTTTTTTTGGGTTTTTACCCCCCCAAAAAATAAAAAATGGAACCCC
CTGGTTTTTTTTTTTATGCAAAATAACCCAGTTTTTCAATACAAAAACCAAAATACCCCTTTTTTTTTAAAAAATTAACAAAGG
TTCCTAAAAAAAAAAACTTCCGAAAATTTCCAAGCTACCCACCTTCCTTTTTGAAACAAAGGCGGTAATTTTTTTAAAGAAATA
ACCCATCCTTCCTTTTTTTAGATTGCCCTTTTTTTTTTCCTGAAAAAATGGTTTGGTAACGCCAACCTTCCCCCCCAGGTTTTG
ATTTTCCCCTGCCCTTTTCCCCCTTTAGCCAACCCATTCCCCTTCCCTCCCCCAAGCATTTTACTTCCCTTCCCCCTCTTTTTG
GCTTTTTTCCCCGTTTTGCATCCCCCTTCCCAAAAACCCTCCAAAAAAAAAAGACTTAAAGGGTTTTGGGGGGGGACCCATTCC
CAAAAAACCCAATTATAGTAGTCCCCTTTTTTAAAAAACGGTGGGAAAAAAGTCCCCACAAGGGTTTCCCCCCCCCCAGGGCCC
TGGGCAAAGGGGTGGAAGAATTTTTTTTTGGTTCCCGCTTGGAAAATCCGGAGGTTTTTGGCTTTTCCCAATTTACCCCTTT
TTTCCCCCCTTTCTTTTTTTTCCCTTTTGGGGGGGGGGGTTGGGGGGGTTCCTCTTTTGCCCTTTTCCGGTAAACCCTTTTTCC
CCCTTTTTTCAAAAACCCTTTTTTTTTTTTTTGGGCCCCAACCGAACCGGAAAGGGAAAAAAAAAAAAAAGAAAAAAAAATTTT

CCTTAAAAATTCTCCGTTTTTCCCCCCCTCCTTTTGGAAAAAAGGGTGGGGGTTTTTATGTGCTAAATTTTTTCAATTTTCCC
CCCCCGTGGGTGGTCGTGAACCCCAAAAAATTAAATTTCTTTTTTTCCAAAAAAAAAAATTGGAGGAAAGCGAACAATACCTTT
TTTGGGGTTTCAAAAAATGTCCTCTTTTATATTTTTTCCCTCCCCCATCTTCCTTTTCCCCTTTTTCCCCTGAAAACATGGGTT
TTTGGGGGGGGGGGGGAAATGGCCCCTTTTTTCTTTTGGTTTAAGGGAAACCCTCCTTTTTCCCGTTTTTGGGGGCTTTTTAAG
CAAAAAAAGAAAAATTTTTTTTTTTAAATTAATCTTTCCCTTTTGCCTTTCCGAACTTGGTTTTTTTTGAGGAAAGGAGTGGTG
TAAGTTTTTTTTTTGGGGGGTTTTTAAATTTTTGGGGGAGGCCCCCCCCTCCCAATACCTTTTTTTTGGGTTTGGTTTTTTTTT
TTTTTTTTTTTTTTTTTTTTTTTTTTCAAACTTTTTGAAAACCCCCTCAAAAAAAAACCCACTTTTTTTGCCCCCCTTTTTT
TTTTTTCTTTTTTATTTTTTCCCCGTCCCAAAATGGGTTGGGGGGTTTTATAGACCCCAATTTTTTTTTTATTTTTCCCCCCAC
CCGGCCTTTGTTTTTCCCTTTTTTTTTTTTTTTCCCCACCCAATCAAACTAAAGGAAAAAAAGTAGTTCCCCAAAAAAGTTTTT
TTTTTTTTTTTTTGGTTTTTTGCCTTTGGGGTTTTTTTTTTTTTTTTTGTTTTTTTTTTTCAAAATGTGCCCAAATTTTTCCCT
CCCCCCCCCTTTCCCCTTCCCCCTTAATCGCATTTCATTCCCAAAAAAATTTTCCAAAAAAAAATAATAAATTTTTTTTTTCCCC
CCCGCCCCGGTCCAAAAAAGAAAAAAAATTTTTGGGGTATTTTTTTTTTTTTTTTGGGGAAGGTGGGGGGTTTTTGAAGGAAAT
TTTGGAAAAAAAAAAATTTTTTGGCCCCGATGGATGCCCCCCCTTTTGTTTCCCCCCCAATAAGAGGGGGAAAACCCCCCTTTTC
CCCCCCCCTATAAAATTTAAAAAAATACCCCCCAAAGCCAGATTATTTAAAGCCAAAAAAAAAAAAAAAAAAAATCCCAATCCTT
CCCCTTTTCCCGTCCCTTTCCCCCCCTAACAGCCAAGTTTTTGCCTCCCCTTTATTTTGGCCCTTTTTTTTTTCCCTCCCTTTT
CCATGAAAACCTTTTTTTATGTTAATAAATAGCCTTTCACTTTTTTTTTTTTTTTTTTAGAGACTAACCAATCCCCCCTTTTTTT
TCCCCTTTTGTTTTTTGAAAAAAAAAAATATCTGCCAAAAAGTCCCCATTTTGGGGGCAAAAATAAACCTGGTGGGGGGGAAGG
TTTTCACCCCCCCCGGCCCAAGTTGGGGCTTAAAAAATTTTTGGTCCCCCCCCCCCTTAAACCAAACTTTCCCCTTTTTCCTTT
GGAAATGCCTTTTTTTGTTTTCCCCCCAAAAAAGAAAGGGTTTTAAAAAATGGAAGGTTTTTTTTTTTTTTTTTTTTTTTTTTT
TTGGTTTTTTTTTAAAAAACCCCTCCAAATAAAAAACAAAAAAAAAAGAAACTTTCCCCCCTGGGGGGCCCAGTTGGTCCCCCC
CCCGTTACCCCCAAAGGGTTTTTGGGTGGGGAAAAAGACCCCTCTACCCAAATCTTCAGAATTTTTAATTCCCATCACTTTCC
AAAAACCCCCAAAAAGCAGTGTCCCCCCATCCCTTGGTTCCCCTCAAAAAAACTTTCCCCAAAAAACCAAATGGTTTTTCCTTT
TAATCCCCCGGTGGTTCCCCCTATTTGCACCCCCCTTTTTTTTTTTATTCCCCGGTATAGAAATGGGGGCAAAGTAAACCCGGT
GGGCTTTTTGGGGTAAACTTTTTTTGGCCTGTGGGGGTTTGGGCTTTTTGCCTCTTAAATTTTCGAAGCCCCCACCAAAAAACC
GGGCTTTGCCTTACTCCCCGGGTTTAAAGTCCCGATTTCCCCCCCCAAATTCCTAAATCTTTTTCAAAGAAGGTCCCGTTTTTT
TTTTTCCGGACAAAAAAAACCCCTTTTTTTTGGTAAACTTGCAAAAAAAAGCTTGCAACTAGCCCATTGGTCCAAAGCCCCTGG
GGGCCCCTTTTTGGGGAAAAAATTAGCCCAAAAATTGGTTTTTGGGGCACCAACCCCTATGAAGGTCCCCCCCAAAAAAGTTTT
TTTTTTTGACCCGCCTGGTCCCCTTCACTTCTTCAAAACAGACCAAAACAAAAAAACGGGATAGGGGAAAGGGAAAAAAGGAAA
AAGAAAAAGGAAGAGAATCCAAGGGGCCGGGTCAGAAAAACCTTTTTTTTTTTGGGGGGGGGACCCCTTCAATAAAATTTCAG
AGTAATTTGTTTTATTTTTGGCCCACCAAAATGCCCTTTTAAAAATTCCCAAAAGCCCCTAATGCTTTTGACAAAAATATGGCT
TGTTTAAAGGCAAGGCCCCTTGCAAAAAGTACAAGGTTTTTTTGTCCAACCGAAAACGAAACCCTTTTTCCCTTTTTTTTTG
AAGATTTTAAGATGGGAAAAAAAAGATCCGACTTACCCCGGGGGAGGTTTTAGGCCCCACCCCGTTTTTTTATTTTTTGTTGA
TCCGAATAGAAAGGCCACAGCCCAAACCACCCCAAGCAAAAGTACCAAAGCCAAAAACCGGTTAAAAACTTGCCCAATTTTTTA
TTTAAAAAAAAATAAAACCCGATAAAAAGCAAGACCCCGATTTTTTAAAGAAAAAAAACCAAAATGTTTTTTTGGGAAAAAAGG
TTTTTTTTTGGGAAAAAAAGGAACCCCAAGAATGGAAGAAAAAAAACTTGCCCTTTTGGGGTTGGGGGGGGAGTGATGAAGTTT
TATCGGATTGTTAAAAAGAGGTTTCCCCCCATTTCCCCCCCCCCCCCCAAACCCAAACCCCCTTTGAAAACACGGAACCCACCC
TTTGCCCCAGGAGGGTTGGGGGAGTTTAAAAAAATTTGGGAGGTTTTAAAACCGAAAAAACCTTTCCCATTTTTAAAAAAACCT
TTTTTTTTTTTCCCCCCTTTGGGAGCCAAAAAAATTCAAGGAAGGAAAAAGTTTTTTGGTTAGGACCCCAATTTAGTCACCTTG
CCCCGGACCCCCTTCCAAACCAAAGTTTTTTTTTAATGCCCCCAAAAAATGAAAAAACTTATTTTTGGCCCAAAAAAAGAATTT
ATCCAAACAATGAGAAAAGAAAAAATGGAGGTCCCCCCCCCCCCCTTCCCTAAAGGAGGCTTAATAAAATACCCAAAGGGGTTTT
TCAAAATGAGAAGAAGCACAAGTTTAAGAAGGCTAAATCCCTGTTTGGGTATAGGAGAACCGAAAAAGGAAAAAGAATGAATTT
TTTGCCTATAATAAATCCTGCTTTGGGGAATAATTTTTTTTAATTTGGAAGGGGGTTCCCCCTCCTAAAAATGGGGGAAAAAAA
CAAAAGCAAGATCCCCTTTCGCCCCCAATTCCCAAATATCTCAACACTTTCAAACCCAAATTTGTTTTTCCCCTTGAACCGGGG
CCCCCCACCCGGAAAAAAATAAATATTGAAAAAATTTTTTTGGAATTTTTGATGCGATAGCAGGGAAGGGGGGGGAAGAATGGC
AAAAAAAATGAAACCCAACCCCAAAAGGGGCCAAAAAAAAAAAAAACCCCAAAACCCTGACTTTTCTTTCCCTAAAAGTGGGGG
GATGGGTGGGGAAAGAAAAGGCGTGGGAAAAAAATTTAATTTGGGTTTTTCCCCCTTTAAACCAACCCCAATGAAGAACGGAAA
AAGAAAAAAAAGCAAAAGTTTTGGGAAAGGTGTTCCAAGGGTGGAAAAAAAACCCCCAAACCCAAAGGTTTTTAAAATGAGCCC
CCCCCCCCTCCCCCCAAATTTTTTAAACCCAACTTGACCAACCCAAACCCTCTTTCTTTCAAAACCCCAGTCCCGAGCCCCAAA
AAAAAAAAAAAGTAGAAAAAATTATTATTTCCTTTTTGCTTTTTAGGCCCAAAAAAAAAGAGGGAAAGGGGTTCCCCCCCCTTT
AAAAAAAAAAACAAAAGAAAAGGCCCTCCCAAAACCCCCATTCCAGGAAAAAGAGAGATTTGAGGAAAAAGGAGAGAACCCAAT
GAAAAAACCCAAGTTATTTGGTCCCCGCTTTTTTCCAAATTGGAAAAAAGATTTAAATGAGTCACAGGACAACCGATGGAATAA
TATTGCACAAAACCAAAAACCCTCAAGGAAAACCGAAGAAAAAAAAAAAAATAATAAAATATCCGGTCCCCCGGGCCCCCCCCCC
CAAGTTTTTGAAGAAGTTAAAAAAAAACCGAGCAAAAGAGAAACGAAAGAAAGGAGGGTAAAAAAGAAACACACACAAAACTTT
TCCCAGGAATTTTTATTTTCCAAAGCGACAAAAATCTCCCCACCCCCCTAAAAAAATTTTTTGCCAAGCCCCCTGGGGGACCTG
TGGGACCCCTTTTTTCACCGTAAGCAACCTAATTTATGTTGGGGGGAAAGGGTCCACCCCCTTTAAGCCTGAGGAAAGAAAAGC
AAAACCGAAGCCAAGGAGGGGAAGGAAAGGTAAAAATTAAAAAAGAGATGCCTAAGGTAAGCCAAGGAATTCAAACTTTGGAAG
TGGGGGCCCTGGTACAAACCAAATAGACAATCTTTAAGGAAAAAGAAAAATTTGTTTATTTTTAAATTTAAATACCGCCCTTT
TTTTCCCAGAGGTTGGTAAAAAGCCCCTTTTAAAATTAATATATATTATTTAATAAATAGACCCTTGGGTTTTTTTTTAATTTT

TTTTAAAAGTTTAAATAGATAATAAATATAAAGAACCCCTATATTAGAGAGAATATTTATAAATATATTAAAATGCCACTTTAT
CTATAAACCACACAAAGTTACAAAAAAATTTTTTTATCTTTATTTTTTTGAACCAAATTTTTTTTTTTTGAGGTTTTTTTTTTT
TTGTTTCTCTTTTTATTTTTAAAACAGACCCTCCCGTTGTTTAGAAACCCAAAATTTCTTTTTTTTTTTTTTTTTTTTTTTCTG
TCCCAAATCTTTTTTGTTTAAATTTTTTAAACGAGTAAAATAAAAATAGACTTACCCGGGGGCGCGCAAAAAGCGTTGGTTCCG
GGCATTCCTTGCCTTTTATTTTAAAACCCAAAAAAAAAAATAAAAATTGCCCCCCCCCCCATGTGGGGTTGGGGGGTGGTTTAGTT
TTATCCCGAAAGCCCCAAAGTTGAATATTTTCTTTTTTTTTCCCAAACCCTGGTTCCCCGATAATGGTTTTTCCCCCCCCAAGCC
CATGGTTCAACTTTACTTGGGGAAAGTAATTTTTTTCTTTTTACTTTTTTCCCCCCCCCCCCCCCCCATATATTTCCTTTTAAA
ATGTTGGTTTTTTTTTTCAAAGAAACGAGTAAGTTTTAAACCTTTTGCTTTTTCCCCGAACAGATTTTTCCCCCAAAAGTTCCC
AGGTAAAAAAAAAGCAAAGGGTTGGGGGGGAGAAACCCCTTAAGGGGAAAATCTTCAACCCCTTTCAAAAAAAATCAACTTTGGGG
GGGGGGCCAAAAACCCAAATGGGTAAAATTTACTGGTTTTAAAAAAATTTATAAAACTTAAAACACAGGAACAAATAAAATTAA
GAGCCAACGAAGGTAAAATATTAAATTTTTTGATTTTTCCCCTAGCTTTTGATCCCCTTGGTAAAAAAAAAATTTTTTTCCCGA
AGGGTCCCCCCAAAAAAAAAAAAGGCCCCCCCATTTAAAAGCTTTTAACTTTTTGTGGGCTTTTTTTTTTTTTTTTTTTTTGCC
CCAAAAAAAAAAAGCTTTAAGTTTAAAAAACCTTTGGGAAAAATTTTTTTTTGTTTTTTTAAAAAATTTTTTCCAAAGTTAAGG
AAGCCCCAAAAAAAAATAGAAAATTTAATTTTTCCGTGGTGAATCCAATAAAAACCCCCATTCCCAAAAAAAAAAAATGGCAAAAT
CCAAGTTAAAAATGTTTAAAAGAAAAACAATTTTAAAAAGGAGTTGGGGTGGGAAGAAAGTTTTAATACTTTTAGCCCCCCCCAA
AATTGGACCTTAAATTGAAATTTGGGGTTTCCCCCTTTTTTTTTTAAGAAATTTTTTTTCCTAAAGTTTTAGAAAAAAAAACTTT
AAATTTTACCTCCCCATGGCCGTAAAATCTTAAGGGCCAAAAAAAAAAATAACGACCCCCAAGAAAGTGGAAAAAATTTTTTTTT
TTTTAACTCTTATTTTTCAAAACAAAAAAAAACCCTTTTTAAAAATAAAAACCTTTTTTTTTTTTTTTTTAAGCCCCCCCCCCTT
TTTTAAGAGGAGGACTTTTGTTAAAAAAGAAAGGGTTTTTAAAAAAGGTTATAAAGTTTTTAAAGGAGAGTAAAAAAGTACTT
TAAATGCAAACCAAAACCTTTTTTACAAAAGGTTTTTTTTTTTTTTTTTTTAGCCCCTTTTTTCACCCAAGGTTTGGCAAAAA
AAAAAGTTTTTTTTTTCCCCCCCACCCCTTTGCCTCCAGGAGAGGCAGAATTTAAAACCAACCTTTTACCCATTTGGGTCCCC
CGGCTTTTTTTAAGCCCCCCAAAATGGGGTAATTCATTTCCGATTCCAACTTCCCCTGGGAGGGAAATCAAAACAAAAAAAAATG
CCAAAAAAAAAATTCCCAAAATAGGAATGTTTTTACCAAAAATTGGAGGTGGGAAAAAAAAATTTTGTCCCCCCCCCTTTTTTTG
GGAAAAAGAGGAAGTTTCCCAAAAAAAGGGGTTTTTTTTTTTTATTTTTCAAAACCCAAAAAAGATTTTTTTTTGGAATTTTTG
AGAGAAAAAATTCCAAAAAAAAATTTTTTCTTTTTCTCAAAAAAATTAATAAAATGGGAAGAAAAAGGTTTTTTTTTTCCCCCAAA
ACAAAAAGGGGGTTTTTTTGCTTTTTTTTTGGGGGATGGGTTTTTTGTCCCCTGAAACGGTACAAAAAACCTAAGAAGTGGGGGC
CTTTTAAAGGTTGAAAATTGGTATTTTTTTGGAAAAAAATGTGAATTTGGGGTCCCCCCCCAAAGCAAAAAAAAAAAATAAACCC
TTTTTTGGGGAGGAATTTGGGTTCCCCGATCCCCCCGGAATGCCCCAAAAATGTTTGATTCAAACAAGGGAAGGGCATCCCCCC
TTTTTTCAAAAAAAAAAACCCTTTTAAAAATTTGGTTCCCCCCCCCAAAAGAATCCCTTAAAGCCAAAGAGTAATTGGATTTGGCC
CCCTAAAAAGGTGCTTTGCAAATCTTTTAAAAAAGGAAAGGAGGAAAAAACAAAAACGGCTTTTTTGGAAAAGTGGTTTTTTTA
TTTTTTTTTATGGAAAAAAAAAGCTTTGCCCAAATTTATATCCCCGAAATTTCGGAAAAAAAGGGGTTGGGGGGGGGGGCATT
TTCCCGTCCGAGGGGAAGGAAATTCACGGGTCCCCCCTATTCCCATAGGAAAACACCCCCAAAAATGGGACCTTTTTTTGTTTT
AAGAACCCCCCCCCCTATTTGGGGACGAAGGGTTTCCCACCCCCAAAAAACACATGCAAAACCGGAATCCGGAAAAACATGGAA
TTGGGCCAAAAAGAAGAGGGTGTTTTTTTTTTTTTTTTATTGGGGTTCCCCGAATTCGGAAGTTGGGATTTTTTTGGAGGAAAC
CCCCAAAATGCCTTTTTTTTTGGCTTTTTTTTTCGGACCTTTTAGGCTTTTTTTTTTTTTTTTTTTTTGAAAAAGGAGTCCCCC
TAGAATATTTGGGAAAAAAAAAAAAAAGCCTTCCCCGATCGAAACCAAGCCCCTCCGAGGGGGAAAAAGATCATGGCATGTTTTG
TGGGCCGAAAACATGCCCCAACGATCCGGAACCCCCTAGTTTTTTTTGGGAAAAAGGGAGCCAAAATTGGACTTTTGGCTTTTT
TCGGAAAACCTTAAAGCTTTTTTGGACGGAGTTTCCCCCCCCCCCCCCCCCCTAGATTGGAAAAAAGCCTTCCCGAATTCCCCC
CAAGGACCCAAAAGCCCCTTTCGAAGGGAAAGAAAAATTCCCCCCCAAAATGCCCCCCCCAAAATGGTTTTTGTTTGGCCCAAA
AACATTGCCCAAACCCGAAATTTTCAGACCCCAAAGTAATTGGGAAAAAAGAAAAGCCCCAAATTTGACCACTTTTGGGCCTTC
CGGAACCCTTTTAGCCCCTTGGGGTTTATTGAAGGCCCTTTTTTTTTAATCCAAAAAAAAAATGGTTGGCTTTCCGGACCCCCCAA
GGGTTCCCCCCCCCCCCACCCCCAAAAGGGGAAGAAAAAAGGGTTTCCCAAATGCCCCCCCAAAAATGGGGTTCCCCCCCCCCC
AAAAAAGAATAAAAACTTCCAAAAGAAAGGTGGCAAAAAAATTGGCGCCCAAATTTTTTTTTTTGCCCCCCCCCCTTTTTTTT
TTATGTAAAATTTCTAAAGAAAAATACCAATGGCCCCCCAAAAAAAACCGAATCGGAAACAATAATTTGGGAAAAAAAAAAAAAA
AAGGCCCCCAGAAGGTTGGGGTCCCCCCCCCCCCAAAATCCGGGAAACCCCCCCTTTAAAAAAGGGTTTTTTTTTTTTTTTTT
TTGGGGGGAAGACCAATTTTCCCCCCAAAAAATAATGGGTTAAAAAAAGCCTCGGACTTTATTCACCCCGAAGAAGAATTTTTT
TCCAAAATTTGGCCCAAATGCCCCCAAAGAATTTTTTTTGGAACTTTTTAGGGTTTTTGTTTTTTTGGGGGCCCCCCCAAAAGG
GGTTTTTAAAAAAAATGGGCCCAAATTTGTTCCCCGATTCCCCGGACCCAATTTGAAATTGCCAAGGAAAGAGTGGGTTTCCGG
TTTTCGATCCGAAACATGGGTTTTTTTTTTGGAAAGAATGCCCCAAGGAAAATAAAAGTGGGGTAACAATCCCGATCCGGGGCAA
AAAAAAAAAATGGGGGTTTGAAAAGATTTGGGGCCCAAACGAGATTTTGGGGGGGAAAAAAAACTTTTTTAAGATTTTTTTTTT
GTTTGGGGCAAAAGGGGAGGAAAGGGGCCACCCACCAAAAAAAGCCCTTTTTTTTTTTCCCCCGAAAATTCCGGAAACCCCGTT
TTGTCCACCCCCCGAAGTGAAAAGGGAAAGGCCCCAATAGGGGGGTCCCCAAAAAAAAAATTTAGGCTTTTTGGGGAAAAAAAACC
CCTTTTTTTTTTGGGGTTTTTTTTTTCCCGAGGAAAGGCCCCCCCAAATGGGGGAAAAAAAAAAAAAAGCTCCGAAATTCCCCG
GACCCAATTTTTGGGTCAAAATGGGGGGAAAAAAGGCCTCCCTAATTCGGAACCCCCAAAAGAACGGGTTTTTTCCCCGATGGA
GAATTGCCCAAAAATAGAAAAATAACACGAATCTTGATTTTCCCCACCCATTTAAAGGGCCTCCAAAAACAAGGGGAAAGTTTT
TGAAGGGCCCCCCCCTTTCTAGGCCCCCCCCCAAAAAACCCGGATTCGTTTTTTTCCCCCCCAATTGGGCTTTTTTTTTTTTTT
TAGCCAAAACAAAAACATTCCCCACCACGAGCCCCCCCCCCCAAAAGTTGGGGGGGATAGGTTTTTTTTCCCCCAAAAGACGGT

TTCCCCGAAATCCGAAACCCCCCCCCGGTCTCACCCCCAAAAATTGCCTGAATTTTTTCGAGAACGTTTTCCCATGCCCGATTT
TTTCCGAAAACCGTGGGATTTTGGCTTTTGAGA
>m141120_131642_42134_c100732402550000001823141405141505_s1_p0/74417/5912_12449
ATAAATAATAATATTCGTTAATATTAAGTGATTGTGAAATAACATTTTGATCATGTGTCGCATTAGATCGATTAAATGATGTTG
ACAAAGCCCTTGCTTTCGTCAAAGTGACAAATAAGGAGCACGTAAAAAAATTAGTAATGGCCAAACATAAACACTATGACTCAA
TACAAAACAAAAAAATTTTACGTAGGTTCAGCAGTTAACTCTGCATAGTCGTACATCGTGTTCTGAATCTATCATATTTTTAATA
TGGCATTATAATTATTGATTTACGTGCTCTCTTTCACTAGTTGACGAAAACGCCGTCAACAGTTTGTGTTGCTCTTCAATTGAG
AAGCCTTAAGATCTCAGTCCCATGAGCTCAGTCATGAGCAAACAATCAATCACGAAAGCTCACTCAGAGGAACATTATCGAAAG
GAAAAAAAAAAAAAAAGTCCTGGGAAACAGCCCAAATTGGAAACCCAGAAATGTCGAAGAGAGGAGTGGGAATCTCCGATCTCA
GAGGAAGCCGCACATCGCCCCACCGAGAAGATACGAGGTCATACTGTACTACAATCCTGAAGCTGATTGTTATTTATATTGTGG
AACTCGAGAACCCGGATCAACTAAATAAGCTTTGGCAACAGGCAACAAACAGAATGCAGGAGTTGGCAACAGGATGCCGCACGA
GCGCGCAAGGTCCGGCATCCAGCCCCCCGCTCGCGTGAAACCAAGCACCTCCCTCCAAACGGGACATACACGTATCCTCCGCGT
ACGCACCCCCCACCGCTGGAGTAGTCCACCGAGATGCCTCAGCCACGAGCAGGCCGGCTCACTCCACGGCACCCACTTTAGGCA
CTCCGCCACTTCACCACCTCAGGTCCAAGAGGAGCTAATCGGGCTAGGACCCACCCGGTTAACCTCCACTGTGGCCAAGCAACT
GAGGGCAACACAGAATTGAACAATACGAACGCGCCCTGCCAGAGGCTCCAGAACTCAAGGCCGCCCCACAAACAAAAAAAAATA
AAACTCACACCAACAAACCAAAATTACGAAAGAGCATATACATACAAAAAAATAAACCACCCAATCTACATACACATACACCAA
CACACAACAACACAACACACCAGAAACAAACCAACAACATCAACTTATCACAACACACACACACAACAAAGCATAAAAAAAACA
TAATTATAATAAGAGCAAAAATAAAAAAAAAATAAAATATATAAGAAAAGAACAAAAAAATAAAAAAAATACAAAATATAAGAA
ATAAAAAATAAATAAAAATAACTAAAAGAAACAAAAGAAAAAAACAAAAAAAGAGAATAAAAAATAAACATAAAGAATAAACAA
AGAAATAACAAAAAGACAGAGTAAGATAATAAAATAATAACAAACCAAAAAGTAAAATATAAACTAATAAGGAAAAAAAAAAGA
AATAAATAAAAGTACATAGATAATAAAAATAAAAAGAAAAAGAGAAAAAAAATAAAAATAAAACATTAAAAATAAATAAATAAA
AAAACACAATATTACAGAACCATCATATTTCTTTATCTAAAATATCACTCTTTCCCGCTTTATGTCTTATGGTCTGTCTTTCTT
CAATAATATTTTATTATATTATGGAAGTATTTAATGGGGTTACAATAAAATATATAGTTAATATTGTATAGTCATGGTTTTTGT
TAAAGATACTAGCTATTATTTGATATAGTTGTTATAAAGTATTATTAAGATTAAAATAAGAAAAAACCATTATATTATAAAAAA
ATAAAACATATAAAAAAATAATAAAATAAAAATAAAAAATAAGATAAAATAAAAAAAAAACAAAAAAAATCATAAGTAATAAAA
AAATAAAAAAAAATTGTAGAGATGTAACGAGAGAGACAAGAGTAAGGAAAGAGAGTAGTTAAAAAAATACAGTACTAGAACAAA
TAAAAAAATAATAGCAGTAACTCAATTAACATAAACCCATGACTAACAATATTAACTAATAGACTATGGAACCATGGAATACTC
CATAAAAACAAATATAATGAAATAAAACATACCAGACAAATACAAGAGCATGTGTAATAGTCATATTTTCCTAAATATATATAT
CCACATCTAAAAGGCAATAACTCTATAACCTAGAGAAAAAAAATGAATCAAACCAGATGCAATATTTCATATATTATCATAAAA
TTATGCATAAACCACCAATATGAATCTACTTTCTCTCTAATATAGTATCAGCAGTAATATCCCACAAAAGATATCTTTCAGTAT
ATTCTAAGCCTCCAAAAGTTGCATAGTACCTGATTATCATCGACGGTATCTGATCATTCTTTTCAGTTCATAAGCCAGCTCTCC
ACCCAAATTTCGGCAAGAGCCCACCATTACTGGAAGATTTATTAATTTTTTAATAATAGAGAACGTACTAATAATACACCATCC
GGCAACAGAAAAATGTAAAGATTTAAGCAGCAATACATACTAATACTGCTAATCATTACTCAGATGGGAATAAAAACAAAGCTT
TGTTTGCTTTGTGGAGAAACTAAACCATTTATAGCTAATAAACTTGTGCATTTTTCTCTCTTTGTCCGTATTAAAGGAAACATC
CCCTGTATCATTTTCAAGTATATTGAGCTGGATAACTAGAAACCTATCAACTTAAGCTAAACAATATGAATCTACTTTCTTTAG
TTTAAATACAAGCTGTATTATAACCAAAGATAACTGTTCAAGTATACATGTTAAGCCATCAAAGTTGCAAATAGCGCCTTAATA
TGAGGTATTGACTATTAGAGTGTGTAATGCTTCAAATGGACAACATTCTTTAAAACCAGAAACATTGAACAAGTGTGTCTCATG
CAACTCATATACAAACAAAATCACTAAAATATGACACATTTTAGCTAAGAAAGAATGATTTTGATGATCATTGTTCCCTGTGA
ATAGTTCCAGTTATATACCCAGACAAGAAGACCCAATTCAAACCATAGGCCAATATGTCGACTGCAAACTAAGCTAGCGGCCAT
GTGGTCTAAAACTTGAAACAAGAAAATAATATTCCAAACTTATACTTTCTTATACCAAAAACATGGGCATGAGGAATAACTGTT
ATCGATGTTATCCCTCAACAGGGAAAAGAACTTTTCATTGTGCTTCAATCTCTATCCATTTATTGCCTTAATCCTAATAGATGT
TTTCTAAATTTCCTACATGTATATTGTAGATAACAACCGATCTTGAGTTTCACTTAGTTCTTGATCCAAAAAAAAAAACAATAA
CAAAAAGAGAGAGGGTCATATATGAGTTACATCCTACATTTATATAGTATAAAATTTAACTGTTCTACATTGATTTCAACATTC
AATCTCTATTTATTTTCCACACTCATTTCTCTCTAAAAAAATAGTCTTAAGCTTATTAATTTTATTCTCAGACCTGAAAGAATAT
AAAGATAGGTCCCCTGCTTGCACAATATAAAAATAGTCCACAAATTAAGATCCAAGTGGAAAATAAAGAGATTGGAGTCGATTG
GGCAACTGGGTATTAACAGAGCAGAGCATAAAACATAATTAATTCTAAGAAAAAACTAGCCACAAGGTACCAACACTTATAGTC
AACACGGACTGAACAGCGCAACTAGGTATTATCATGTCATGTTATTTTCAAAACTGGGGTTGTCAAATTGTCTTTCCATGGCTA
GCAAGCATTCCATATTCGCTAAAATCTCATATCCAAACACAAAATACAAACCCTATTCAAAAATTTTGTCAAGTCCCAGACAAT
CACTTTGCTAATAATTTCCAGAAAGAAAAACAACCCCAACACATTACAGGTACCAAGTCAGTAAAAACAGAAAACGAAGAAGAA
ACAGAGAATAACTTACCAGTGAAACTATGAGATTGGGTTTCTTGGCAAGGATCATGCGGTGGCGATGAGGCAGAGGTGTTACGA
TCAAAGCAACCCTTCTCCCTGTCTTGGGAGCACATCAGAACAGGGAGTGGACGTTCCATCACCAAAGTGCATCAAGACATTCTT
CTGTGATATGTTGTTGCAAAGGTTGAGTCACGAGAGATTCTTGTTTTTCCTTCTTTTAACTCGTTGCAGTTTTCTTCGCATGCC
ATTATTTCCGTTCTTCTTTCAACTCCAAATCACAAACAGTTTGCTAAGAATTGTAAAGTTGAGAAATCTGCACAAAATATTCAG
ATTCTGGCAATGTTTTTCATCATTAGACCATTGGTTGGTTTCGTAGCTTGAGTAGTTACACGAAACAAACTGTCGTGCTAACAA
AATCATGTCAATCCAAAAGAAACACCTACAAAGTTAAGACCTGAACGGCCCAAAACCCCACCAATAATTGTTTCAGTAACTTGG
GTTCCAACAATGATCTCAAATAAAACAAGAATAAAGAGTTGAAACTTGCAATTTGACTGATTAGCAAGGTTAAGTGTGGATCTA
TTCCTTCGTGTAAAGAGTTGTAATTTGCTATATATAAGCCACAACAAAAAACATCAAAAAGAAGATAATCCCAACACCAATTCA
CATTTAACTTGAAAGAAGGTTTTTTTAGTCACAAAATGAAGCTTATCATGACTTATTAGGAGAAATTTTATATCAAGTGTTTT

TGTATTCACAAATAACAATGAATTCGAAAAAGAATAAGATCGAAATGAAACCTAGACCAATTTTTGTAGTTGGAAAATGACAGC
AGAAACTATATTCTTCCTCAATGCAAGAGGAATAGGCAGAATGACTTTTTCTTGGTTCGAGCATTCGTCCCTTGTTCGTTGTCC
TCTGCTATTGGAAACTCCTTCCTATGTCTAATCCCTTTATAGCACTGAATGCCCACCATCTCTCTTCATGTCTCTCTCAACACA
ACCCCTCTGGAATATCCCAAATAAGGAATCAGAAAAAGAAGACATGTATCTTAACAAAAGATAATAATTTGTACTAAGTTTAAG
GGCTTTAAGCCAATTCTTTTCCCACAACCGTAGTTTGGGAGTTCTTCACCCAAGAAGAAGATTCTCTCTCTTTGGTCAAGTCCT
TGGAGCTGTTGATGGATGACTGCAACCCGAGTAGAGAGGGAAATTGCTGAAGATAAACAATTGAGAAAGGGAAAGTTGAGAGAA
ATGATGGAGGGTTCAGAGAGAGGTGAGGGTTTAGAGAAACATGTGACGAGGTGTTAATATTATTTTTCTAAGTTTTATTTTATT
GGGTTGAAAAAATGCTTTTACATTAATTGAGCTTTTTTTTTCCCTCCATTTTAACTTAACCAGCAATTTTTTTGGTTTCCCT
CATTGTTAACTTTATACTATTATAAATAATTTTCCCAATTAGCTAATAATTATGTATTACTATAAAGAGGTATTTGGTGTCAGT
GACTACTCACTACTAACTGCTGCAGCTGCTGCTGTGCTGCAGCTGCAGCTGCTGCTGCTGCCTGCTGCCCAGCTGCTGCTGCTG
GCTCGCTTGCTGCTCCGCTGCTGCTCCAGCTGCTGCTCCGCTGCTGCTGCTAGCTGCTGCTGCTCGCTCGCTGTCTGCTGCTAG
CTGCTGCTGCTGCTGCTGCTGCTGCTGCTGCTGCTAGCTGGCTGCTGCTGCTGCTGCTAGCTGCTCCGCTGCTCGCTGCTTGCT
GTGCCGCTGCTGCTGCTGCTGCTGCTGCCGCTGCTAGCATGCTGCTGCTGCTGCCGATGCTAGCTGCTGACTGCTTGCTGCTGC
TGCCTAGCTGCCTGCTGCTGCTGCCGCTGCTAGCTGCTGCTCGCTCGCTGCTGCTGCCGCCGCTGCCGTCGCTGCGCGCGCCTG
AATGCCGCTGCCGCCGGCTGATGCCTGCTGCTGCTGCTGCTGCCTCCAGCCTGCTGCTCCAGCTGCTGCTGCTGCTGCCTACCA
GCCTGCTGCTGCTGCTGCTCCAGCTGCTCGCTTGTCCTGGACTGCTCGCTAGCGCTGCCTGCTGCTAGTGCTTGCTGCTAGCTG
CTTGCTAGCCTGCTGCTGCGGCTAGCTGCTGCTGCTGCTGCGGCTAGCCTGGCTGCTTCGCTGCCTGCTACTGCTGCTGCTGCT
GCTAAGCTGCTGCTGCTAGCTCTGCTAGCTGCTGCTGCTAGCTGCTGCTCAGCTGGCTGCTGCCTAGCTGCTGCTAGCCGCGCT
AGCTCAGCTGCTCGCTAGCTGCTGCTCTGCCTGCTGCTGAGCTGCTGCGAGCTGCCTGCTGCTCTCTGCCCTGCTGCTGCCTGA
GCTGCTGCAGCTGCTGCTAGCTTTGCGGGAGCTGCTCTGCTGAATGCTGCTGCTGCTGCTTGCTGCATGCTGCTGCTCCGCCGC
TGCCTAGCTTCCTGCCGCCCGCCTGCTGCTCCGGCGCACCGCTGCTCGCTGCCGCGGCGCCGCTGCTGCTGCTGCTGGCTGCTG
CATGCTGCTGCTGCGCTGCTCAGCTGCTGCAGCTGCTGCTGCCTGCCGGCTAGCTTGCTGCTCTGCTGAGCTAGCCGCTGCTTG
CTGCCTTCCAGCTAGCGCTGCGCTCAGCTGCGCCGCCGCCGCTAGCCATGCTGCTGCTCGCTGCCGCGC
>m141120_131642_42134_c100732402550000001823141405141505_s1_p0/76835/9987_15611
GAGTGCAATCAGAGACTACATGGCTAATCTACAGATACATCCAGCATTCGTTTGATATTGAAATGCTAAGTCCACTCCATCAAG
GGAACAAATGCTATGCAAGGGAATATAAATTCAACATTATTCTACTTACAATATGAGACACTTTAAGATGAAAGCGGGATGAAA
CTATTGTACATGTATCTAAGTTCCAACTATACACTAATGGTTTGAATTCTCTGGCAAGGTACTTTCACAAAAAGGGAGAGGGGT
GACCAAAGAATTTGGAAGTTCACCAAAAGCCCGATCACAAGGCAAGTTGATGCCATTATCCAAGAAGCCAAGGACTCTCAACAC
TCCCATTGGAAGGACTAATTGGTTCACTCATGAACCATCCAAATTTCATGAGTGCACAAATGAAGAGGGAAGTTGAGAAGAAAA
GAAGACCATTGCATTCAATCTTCCTCTCCCGTGCCATTAGTGAAGAGTGCAATGAGGGCGGATAGCATGTGTTATGACATGGAG
GACTGGCACTCTTCACAAAGAAGTACAAAAGATTCATGAAATTCAAAAGAAGAACTTTGAGAAGAAGCCACATAGAGGAAATGA
CTCAAAGGAAAGAAAAGCAAAGATGATCCACCGATTCTTTTTGAATGCAAGAAGCCCGACCACATGGAAGATGGATGCCAATGA
GAAAGAAAATAAATACAAAGGAAGGGCAATGTTGGCGGATGGCTCAATCAGTGATGAGGAGGATCATACAATGAGGAAGAAATG
AAGTAGCTAACAATTGCATGATGGCTCTTGATGATGGGAGTAAGCATTTCTAAGCCAAAGTGAATTAGATCAAGAAAATGATGA
TGATGAACTTAGAAATACCATATGATGCAGTGCGAGTTAATGGATAATATTTTAATGATTTTGGAAAATTGCTTGCTAAAAACC
AAATTTAGAGAAAAGACCCAACATGCCTATTAAAAGAAATTGAGATTCTTGAAATTAAATGAAAAAGAACTTTAACTAAAATCT
CAATGTAAGCTACTTGCTCCTCATATAAGAAAAGAGAATTGAAAAATGCATGATAACATAAAAAAGCCTAAAAGAACTGACAAT
ATACCTTTACTAGAGGCCAAAGAAGTGCTATGAATTCAAATGCTAGGAAGCCAATCTTGTGGGTTTTGGTAAAAAAGTGGTAAT
TGGATAGTGATCCAAGTCAAGGAAACAAAGTCTTCTTTGAGAATCTTCTTGTAAAACCATCTAGTTATACCTCAATTTACATGG
CACATATTGTAACCAAGATGGTCATAACTATTTCTTATTGAATATGAAGAAATACTGCACACTAGGCAAGACTCAAATGGGTAC
CAAAGGTTTCTACAAACTAACTAAAGAATGGACCCCCAACACGCTAATGAGACGTACCCAACACAAACCAACACAAAGTAAATT
TGTTTTTGTAGGTATCCCATCAGGATAGAGCCAAAGCCTTATCGGTTCTTGGATAGTGCTTGCTCCAAAGCATATGACCACGTG
GATCCCTCAAGATGTTTACGAGCTTTAAGAGTAAGGAAAGTGGCTTGTCACTTTTGAGACATTCAAAAGGAACAAATCTGGCAT
GGCGTAGAACCTATCTGATAATGTATAACATACTACACAATTGTATCAAACAAATGTGCTCCTTGCTTATAAATCTCTAAACCA
ATTTACTTAGTATTTAGTCAACTATGCTGATAAAGCATTTAAGTTGTTGTTTTTGAATCCGTCAAAATGCTCATTCGATAAATG
CTTCAATCAATGAAGTTATTTTTGTTGGAGAAAGGAAGGATAAATGTTTATGTTAATGATGTTGAATCCTTTGATCGAAAAATA
ATGTTAACGTATGATGATAATCTTGTTGTGGCATAGGAGATTAGGACATGCTAGTATGGATTTCTATTTCAAAGTTGTTAGAAA
AGACTTGTTGTTGTTTAGCCATCTATTCCTTTGGTTAAAGATAAACTTATGCTGATGCGATGAAACAATCTGGAAAACAAATTA
CAAACAATCTTTTCATTCCAAGAAAAGAGATTACTCAACCTCTAGACCTTTGCAATTGCTTCATATTGATTTATCTTTGGTCTT
CAAGAATTGCCTAGCTAGGTGGTAAATACTAATGCATTTGTGATTGTTGATGATTTTTTCTAGATTCATGACATGGGTTCCTAT
ATTTTTTAAACTTACAAAATGATGTTTTGGGAAAATCTAAAAAAAGAATTTTGTAAAGAGTGTGAAAAAAAAGGGCGTATCTCA
ATCACCTCATTGGATGATCCAATGATTGGAGAGTTTGACAATGTTGCCTTAGAATTGTTTTGGAATGAACATGGTTTATACCAT
AACTTTTCGGCTCAAGAACTCCCACACAAAATGGAGTTGAATCGAAAGGAAGAAAAGAACAACTTCAAGAAATGGCTAGGTATG
CCATCAATGAAATCCCATTACCTATAAATACTTTTTGGGCGAGGCGATTAATCACTTCTTTGTTAATTTTGAACCGTGTTTTTC
AATTAGGCTAACATGAATAAAACCCCTTATGAGCTTCTGAAAGGGAAAAACCCAACATTGGACCTTAATTAGAGTTCTCGCTGC
TAAATGCTATATTTCTCTAAACACCAAGGACAACCTTGGAAAATTTTGATGCAAAATTCGAATGTTGAATTTTTATAGGTTACG
TTCAACCATAGTAAAGATTATAGAGATATTATAACAACAGAACATAATGTTTGTTGAAGAGGAATCTCATTCATGTTGTTATTG
ATGAGTCTAACCCTCCCTACAGCAAAGTTGTCAAGATGATGGATGTTCTATGAACCTCTCGAAGAAGCCTAAGCGCGTTAAAGC

TTGACATTGCGGAAGAAACACTCCACGCCCATCACACACTCACAACAGAGCATCACCCGTAAGCGCAACGGTAAATTGAAAGCA
AAAGTATAAACCGGATTCCTAACCCTCCCTCATGGAGTGCAATGATCAAAAGATGCCATCCCTATAACCAAAATTCTAGGTGAT
CCTTCACAAGAAGTCCACAACTCAGAAGCTCCCTTAGAAACTTGTGTAATTTCTTGCTTTTATTTCTCCAAAATTGACAAAACC
AATTCTATAATAACAAGAGCGACGTTATAGATGACATCCTTTGGTTTCTAGCATAAGACACGAACATGAAATAACCTTTTATAT
AGAAAATGCCTGTTCTGTGGAAAGTTACCGTTCCAAGACGTCACATATCCAATACGGATGATTGAACAAATGGGTCTCAATAGA
AATAAGGTCTGAAGAGACCCATGGGTCATTGCTTGCGGAAACAACCGGGCTAGACTTGAGTCGGTACCCAAAACCGGTTAACAA
TCAAGAAAAGGCAATGAAGGAGACGAACTCTCTGCCCCGTAGCAAGAACTTAGTCCATACAGATGTTATTAGCTTTTTGCATGC
CACAAGAATTTTGTCTTGTGTCAAATGGATCGTATAAACCAAAGTGCTTTCTTAATGGACGAACATTATGGAAGACGGTTTATG
TCTACTTCAACCCCCGGTTAGTCACACATGCTAAACCGCTACAACTCAAACCCTAACCACTAAACCCTAAAACCTAAACCGCTA
AACCCTAAACCTAGAACCCCTAAAGCCCCTAAACCTAACCTAAAACCTAAACCTAAACCCTAAACCTAAACCTAAACCATAAAA
CAGCTAAACACTAACCCTACCCTTAACCTAACCTAAACTAAACCTAAACTAAACCTAAACCCTAACCTAAAACCCTAAACCCTA
ACTAAACCTAAACCTAAACTGAAACCTAAACCCTATAACACTACATAAACCTAAACCCTAACCCTAAACCCCTAAAACCTAAAC
GCCTAAACTAAACCCTACTAAACCACTAAACTAAACTAAACACCTCAAGAACCTAACCTAAACCCTATAAACCTAAACCCTAAA
ACCCTAACCTAAACCCCTAAACCTAATAACTACATACCTAAACTAAAACTAAACTAAACTACCCTACAACCTATAACCTAACC
TAAACTAACTAACCTAAACTAAACCCTAAACACTAAAAACTCAATAAAAACCTAAACTAAGAACTACACCAATAATAAACCCTA
ACCTAAACAGACGCGGCTAAACCAGCTAAAACCCTGAAAAACTACAAAAAAAACCTAAACCTAAAACCCTAAACACTAAACCCC
TCAACACTAAACTAACCTACACTAAAACCGTAAAACTAAACCCTAAGCCACTCGACAACCTAAAACTAGAACTAAAACGTAAAC
GCCTAGAACCGTTGGCTAAACCGGCTAAAGCCCGCTAACAGCTAAACCCTCCAACCACCTAAACCACTACAACCTAACTAAACT
CTCTTAAACCTAAACTAAACTAAACTACCTCTAAACCCCAAAACTAACAAACCCTAAGAACCGTAAACCTAAAAACCCCTAACC
TAACATCTACACCTAACACCCTAAACCCGCTACCAAACCGCTCAGAAGAACCCGGCGCTAAACCCTAAACCAACTAATACCTTA
ACCCCTAAGACCCTAAACCTAACCGCTAAAACTCGTAAACCACTAAACCCTAACTAAACCTACAACCCTAAAACCTGAAGACTA
AATAAACTAACCTAAGACCTAAACTCTCAATACCCTAAACCCTAAACCCTAAACCCTAACCTTAAAAACTAAACGTAACCTAAA
CCTACAACCAAAACTAAAACTAAACCCAGAAAACCCTAAAAACCCTAAACCTAAAAACTAAAACTAAACTAAACCTAAACTAAA
AACCTAAACCAAACTAAAACTGTAAAACAACCCTAAACCTTAAACCCTAACCTAAACACCTAGAAAACCCCTTAAAACGCTCAA
AACCCTAAAACCCCTAAACCATAACAACTAAACCTAAAACTAAAAACGCCTAAAACCTAAACCCTAAAACACTAAAACTTAAAC
CTTAAACTAAACCCTAAACTTAAACTCGTAAACCTAAAGTCTAAAACCCTAAGACCTAAAACTAAACTAAACTAAACCTAAACT
AAAATAAACCTAAACAACTAAATATAAACTAAAACCTAAACTAAACCTAAACTAAACCCTAAACCTAAGAACCCTAAACCCTAA
ACCCTAACCCTTAAACTAAATAAACTAAACCCTAAATAAACTAAACACTAAACCTAACTACACAATAACCTAAAATACATTTAC
TACACACACTAAACTACTACAAACTACCTAATAAACCAAAATAACTCACCAAAAACAAACAAACAAACCAAAAAAATAAAACCA
AACTAAACCGTAAGCAACCTAACTAAACCCTAAACCTAAACCCTAAACCTAAACCTAACCTAGAACCACTAAATAAACCCTAAA
CACTAACCTAAACCCTAAACATAAAACCCACTAAAACCTAGACCCTAAACACTAAACCCTAGAACCTAAACACTAACCCAAAAA
CCTAAAGACCTAAACCTTAAACCCTAAACCGTAAACCCTAACCCTAACCTACTAAACCCTAACGCCTTAACTAAACCCTAAAAC
CCTTAACCCTAAACCCAAAACCGTAAACCCTTAAGAGGATTTGGCAAGAAGGTTCATTTTTTGTTTTGATCGAACAGGAC
>m141120_131642_42134_c100732402550000001823141405141505_s1_p0/77552/0_6510
GGAGAGAGAATAATGGAGATTTACTAATTTCTCCGAAATGTATTACCTCGTGTTCAACTCGTTCGATATTTTTCCCCAAGCAGA
TCTCATGGGTAAATGAATACTATTGGAGTTGAAAACGGAGCCTAAGTCAATCATTTGAAAAAAGAATTCCCCCTTTTTGATGGC
AATTTTCGCAAGTTTTTTGCCTTATATTATACATTCCTCAAGTATAATAGACTGTATCCCCCTATCCTACGCACCTCGGATTTG
GAAGATATTTTACACATTGAAGCATAAGACATAGGGCTACAACTTTTGTGGCCATGACCCCTCGCATATTCTTTAGTCTTACCT
TCAAATCGTTGCTCGAGATCCATATTAGGAAATCAGAAGATTTCTGAAAGGGGTACCCCTTCTGGTGAAATTGGAGATTTCACT
AATTTCTCCGAAATGTTATTAGCCTCGTGTTCAACTTCGTTCGATATTTTTTCCCCCAAGAGATCCTCATTGGTAAATGAATCT
ATTGGAGTTGAAACGGTGCCCTAAGTCAACATTTGATAAAAAAATTCCCCTTTTTGATGGCATTTTCGCAAGGTTTTTGCCTTA
TAATTAAGTACATCTCTCAGTATAATGACTATATCCAACCTTCTACGCCCTCAGATTTGGAAGTATATTTTACAGCATTGAAAG
CCTAAGACATAGGCTACAACTTTTGTTCCCATGACCCCCGTATCACTTCTTTAGTTTTCACCCTTCAAATCATTGCTCGAACCA
TATTAGAGAAATCGAAGATTTCTGAAAGGGGAACCCCTTTGGTGAATTGAGATTTCACAATTTCTCCCGAAATGTATTACCCTC
GTGTTCAATCTCGTTCGATATTTTTCCCAAGAGACTCATGGGTAAATGAATCTAATTGGAGTTGAAACGGAGCCCTAAGTCACT
TTGAAAAAAAATTCCCTTTTTGATCGGCATTTCGCAAGTTTTTGCCTTATTATTAGTGACATCTCTCAGTATAATGACTGTATT
CAACCTTCATATCGCACGCTCGGATTGTGGAAGATAATTTTACACATTGAAGCCTAAGACCATAGGGCTACAACTTTTGTGGCC
ATAAGACCTCGCATATTCTTTAGTTTTTCACCTTCAAAATCGTTGCTACGAATTTCCATATTACGAGAATCGAAGATTTCTGAA
AGGGGTACCACTTTGGTGAGATTAGGAGATTTCACTATTTCTCCGAAATGTATTCCCTCGTGTTAAAAAGTCGTTCGATATATT
TTCCCCAAGAGATCTCATGTGTAAATGAATCTATTGAGTTGAAACGGAAGCCATAAGTCAACATTTGAAAAAAAAATTCCCTTTT
TGCATGGCAATTTTCGCAAGTTGTTTTGCCTTATATTAGTACATCTCTCAGTATATTGACTATAATCACCGTTTTTTTTTGAAT
TTTTTTTTTTTTTTTGTTTTTTTTTTTTTTTTCTTCTACGCACCTCAGATTTGGAAGATATTTTACCATTGAAAGCTAAGACATA
GGCGCTACAACTTTTGTGCCCATGGACCCTCCGGCATATTCATTTAGTCTTTCACCTTCAAATCAATTGCTCGAAACCCATATT
AACGAGAAATCGAAGATTTCTGAAAGGGGAACCCCTTTGGTGAATTGGAGATTTCACTAATTTCTCTCGAAAATGTATTACCCT
CGTGTTTCACTCGTTCGGATATTTTTCCCAAGAGCATCTCATGGGTAAATGAATCTATTGGAGTTGAAACGGAGCCCTAAGCTC
AACATTTGGAAAAAAATAATCCCCTTTTTATGATGGCAAATTTTCCGGGCAAGTTTTTGCCTTACTATTATACATTCTCAGTAT
AAATTGACTGTATCACCTTCTAACGCAACGCTCGGATTTGGAAGAATATTTTACCTTGAAGCCTAAGACATAGGGCTACAACTT
TTGTGGCCATGACCCTCGAATATTCATTATAGTTTAATACAACCTATCACACAATCGCTAATGCATACGAAATACCAATAATTA

GAAAAAGAAATACGAAAGAAATATAAATAACTGAAGAGGAGTACAAACACCATATATAGAAGATAGAAAAATTGGAGAATTTCA
TAATAATTAATAACAATCCGAAATGTATAATACCACATAGAATGATAATACCATACACGTATCAGAATATTATATTACAACAAG
AAGAAAAATCTCTAGAGGCAGAAAAAAAATGAAAATAATATTGGAGATTGAAACAGGAAAGCACCTAAGAATCACATATTAGAA
AAAAAAATATAATATATATTAGAAATGGACAATATTTCGCAAGTTTTTTGCCTTATATATAGATAACATCTCCTCCAGTAAAAA
TAATGAATGTATCACCTTCTACGCACCTCGGATGTTGGAAGATATTTTACACATTGAAGCCTAAGACATAGGGCTAAAAAAAAA
AAAAACTTTTGTGGCCATGACCCTCGCATACTTCTTATAAAAAATAGTTTTCAACAAATATATATCAATACAGATATAGACATA
ACGATCCATATTAGAGAAATCGAAGATTTCTGAAAAGAGAAGAGATAAACCTTTGGTGAATTGGAGATATCTACTACAAATTTT
CCGATGTTTACCAAAAACATACCAGATGAATCAACTCGTCAGAAAAATATTTTTCCCAAAGAGAGTCTCATGGGTAACACTGAA
TCTATTGGAAGTCTGAAACGGAGCCCATAAGGTCAACTTTGAAAAAAAATTCCCCTTTTTGATGGCAATTTTCGCAAGTTTTTG
CCTTATATTAGTACATCTCTCAGTATAATGACTGTATCACCTTCCTAAAACCGCACCTCGGATTTGGAAGATATTTAACACCAT
TGGAAGCTAAGACATAGGGCTAACAACTTTGATGTGGCCATGGACGCCTCGCATATTCTTTAGTCTTTCACCTTCCAAATCGTT
GCTCAATCCATATTAGAGAAATCGAACGAATTTCTGAAAGAGGGTACCCCTTTTTGGTGAATTGGAGATTTCACTAATTTAAAA
ATCCGAAATGGTATTTACCTGCGTGTTCAACTCGTTCGATATTTTTCCCCCAAGAGATCTCATTGGTAAATGATCTATTGGGA
GTTGAAACGGTGCACCTAAGTCAACATTTGAAAAAAATTCCCTTTTCTGTGGCAATTTTCAGCAAGTTTTTGCCTTAACTATTA
GTACGATCTCTCCAGTATATTGACTAATATCACCTTCTACGCACCCTCAAGATTTGGAACGATATTTTACCACATTAAGCCTAA
GACATAGGGTACAACTTTTGTGCCATGACCCTCCGCATATTCTTTAGTTTTCACACCTTCAAAATGCATTTGTCGAACCCGTAT
TACGACGAATCGAAGATTTCTGAAGGGAAACCCCCTTTGGATGAATTGGGAGAATTTCACTAATGTTTCTTTCTCCCCGGGAAAT
TTGTACCTTACCCTCCGTGTTTCAAATCGTTTTCGATATTTTTCCCCAAAGAGATCTCACTGGGTAAATGAATCTATTGAGTTG
AAACGGAGGCCCCTAAGTCAACATTTGAAAAACAAATTCCCCTTTTTGATGGCAATTTTCGCAAGTTTTTGCTTATATTAGTAC
ATCTCTCGCAGTATAATAGGAACTGGGTACTCACCGTTCTACGCACCTTCGGATTTTGGAAGATATTTTTACACATTGAAGCTA
AGACCATAGGGCCTACACAACTTTTGTGGCCGATGACCACTCGCATATTCTTTAGTTTTCACCTTCAAATCGTTGCCTCGAATC
CATATCGTCCAGACCGGAGAATCGAAGATTTTCTGAAAGGCGATACCTTTGGTGAATTGGAGATTTTCACTAATTTCTCCGAAA
TGTATTACCCTCGTGTTCAACCTCGTTCGATATTTTTCCCCAGAGAGGATCTCCATGGGTAATGGAATCTATTGGAGTTGGGGG
AAAAACGGAGCCCCTAAGTCAACATTTTGAAACAAGAATTCCACCTTTTTTGATGGAAAATTTTCGCAAGTTTTTTGCCTTATA
TTAGTACACAGTCTCTCAGTATAATGACATGTATCAACTTCTAACGTCACCTCGGATTTGGAAAGCATATATTACAGCATTGAC
GCCTAAGACATAGAGGCTACCAACTTTTTGTGCCCATGACCTCGCATATTCTTTAGTTTTCACCTTCAAATCGTTGCTCGAAAA
ATCCTATTAGAGAAATCAAGATTTCTGAAAGGGGTACCCTTTGGTGATTGGGAGATTTCACTAATTTCTCCGAAATTTATTACC
CCATCATCTGTTCAACTCGTTTCGATATTTTATCCCCACAAGAGATCCTTCACATAGTAAAATGAATCTACTTGGAGTTGAAAC
GGAGCCCTAAGTCAACATTTGATAAAAATTCCCCCTGTTTTTATTGGCAATTTTCGCAAGTTTTTGCCTTATATTAGTACATCT
CTCAGATAATATCCGACTGGTATCGACCCTTACGCACCTCTGATTTGCACGAAATTTTACACATTGAAGCCTAAGAAAAAATTG
GGCTACAACTATTTGTGCACATGACCACCATATTTCTTTAGTTTCACCTTCAAATCGTTGCTCGAACCTATTAAAAATCGAAGA
TTCTGAAAGGGGTACCCTTTGGTGAATTGGAGATTTCACTAATTTCCTCCGATATGTATTACCCTCTGTGTTCAACTCGTTCGA
ATGTTTTTCCCCAAGAGATCTCATTGGTTAAATGGAATACCTATTAAAGGAGTTGAAACGGCTGCCTAAGTCAACATTTGAAAA
AAAATTCCCCCTTTTTGATGCAATTTTTCGACAAGTTTTTGCCCTTATATTACTCACATTCTCAAGTATAATGACTGTATCACT
TCTACGCACCTCGGATTTAGGAAGATATTTTACACAATTGAAAGCAAAGACATAGGGCTCAACTTTGTGGCCCATGACCTCCAG
CTTATATCTTAGTCCTCACCTTTCAAATCGTTGCTCGAATCCATATTATGAGAAATCGAAGATATTCTGAAAGGGGTTACCCCC
TTTGGTGATATTGAGAGATTTCACTAATTTCTCCGAAATTATTACCCTCAGTGTTCAATCGTTCGATCATCTACTTTTTCCCCA
AGAGATCATCCCATTGGTAAATGAATCTATTGGAGGTTGAAACGGTGAGCCCTAAGTCAACATTTGAAAAAAAAAAAATCCCCTT
TTTGAAGTGGCAATTCATTCGACAAGTTTTTGCCTGTATATAGTACATTCTCTCAGTATATTGACTATATCACCTTCATACGCA
CCTCAGGATTTGGAAGATATTTTACACATTGAAAGCCTAAGACTTGTGGCTACAACTTTGTGGCCCATGACCACCGCATATTCT
TAGTTTTCACCTTCAAATCATTGCTCGAATCCATATAGAGAAACGAAATTTCTGAAGGAGGAACAACCCCTTTTGGGAATTGGAG
ATTCCACTAATTTCTCCGATGTATTACCTTTCTCTCTGTGTTCAATTCGGTTCGAATATTTTCCCCCAAGAGATCTCATGGGTA
AATGAATCTATTGGAGTATGAAACGGAGCCCTAAGTCAGACATTTGAAAAAAAAATTCCCCTTTTTGATGGCAATTTTCGCAAG
TTTTGCCATTATATTAAGTACATGCTCTCAGTATAATGACTGAAATATCACCTTCCTACAAGCACCTCGGATTTGGAAGATATT
TTACACATTGAAGCCTAAGAATAGGCTAACTAACTTTTTGTGGGCCATGATCCCTCGCCATATTCCTTTAGTTTTTCAACCTTC
AAATTCAGTTGCTCGAATCCATATTAGAGAAATCGAAAGATTTCTGAAAGGGGTACCCCTTTGGCATGGGAATTGGAGATTTTC
ACTATTTCTCTCTCCGAAATGGTATTACCACTCGTGTTCAACTCGTTCGAATATTTTAATTCCCCAAGAGATCTCAATGGGTAA
TGCAATCCTCTATTTGGAGGTTGAAACGGAGCCCTAAGTCAACATTTGAAAAAAAAAAATTCCTTTTTGATGGCTAATTTTTCGC
AAGTTATTTGCCCTTATATTCAGTACATCTCTCAGTATAATAGAACTGATATCACCTTCTACGCACCTCGATTTGGAAGATATT
TTACACATTGAAGCCTAAGACATAGGGGCTACAACTTTTGTGGCCATGACCCCTCGCATATTCTTTAGTTTTCACCTTCAAATC
GTTGCTCCGATCCATATTAGCAGAAATCCGAAGATTTAAATGAAAGGGGTACCCGCTTTGGCTGAATTGGGAGGATTTCCACTA
ATTTCTGCCGAAATTTATTACCCCTCGTGTTCAACTCGTTCGATATTTTTTCCCCAAGAAGATCTCATAGGTAAATGAATCTAT
TGGAGTTGAACGGAGCCCTAAGTCAACATTTGACATAAAAATTCCCCTTTCTTTTATGGCAATTTTCGCTAAGTTTTTGCCTTA
TATATGTACATCTCTCGTATATCGACTGTATCACCCTCTACG
>m141120_131642_42134_c100732402550000001823141405141505_s1_p0/80751/11643_18759
GAATGAAATATAGTTAAAAGTTGTGATAATACAAGTGATTTACTGTCTTTCACATTTGAATGAGGCACGTACCGAGTACCCGTA
ACAGTAATTAGCTCCCCACTGACGAATAGACAATCCGCTTTTAATTAATGGATATGTTTGCGTGATATGCGTATATCCGTATTT
TTATACATTTGATTCTATGATCAAAGGCGGATTAATAACTTGAATATACGTATATACTGCAGTCATTCATTTCCGTCTAGATAC

ACTATACTTTAACTCCAGAAAATAAGGCGTATTGTTTTTATATTTATGATAGATTCATGCTTCATATAAAGTAGCATTGCTGAA
GCGATAAACAACATTCGTAATAATCATTAGTGTAATTTATGATTATTAACTTGTTTTGTCTACATTGACTCTTCCATGGAACAC
ATTTTTTGTTCTATTCTTATTGCGCACCATAAGCTACTGCCTAATCCGTCAACGTGCCAGTCTGATAGTCTATGGAGCTATTGT
AACTATATCCAAGACCATGAATACCAGACCGTATAACAAGTAGAGTCTATACGAGATTATTATTGAGAATTAATATGTACAGCA
TCCACCACAACACATCTTTAGTACTCTATCTGGAATCTATCAAGCTAATAGTGATTATAAATCAATCACCACCGTACATAAATA
CTAACTTCTGCTTAGACCTCAATCTCGATGTCGAGAATTGGGTTGTTTTGAAATAATGTCTCATGCTTTTCACTAATGCTAATC
ATTACTGAATTCCACAATTCGAAACTTTTAGAGCTACCTTGTGACCGTATTGATGTATCAATAAAGTCTTAATATACAGTCATT
ATCACTTTGAATGTGCATGAAAATTCTAGAAATTTTAGCAGAGAATTAATAATTCCAAAGCGAGCATATACACAATTTTCTTAA
ATACCCAATCTCAAACATACTTGAGACTTACTATTGTTATGTTTATTTATCTTTTTTTGAATCATTGAACAGTTCAACATACAA
TCATCTCATCATGTATATTACGTATAATGATATATGTGATTTTTGAGCTTTATAAGAATAAAGATCTTTTGGTTGAACAATTTA
ACAATTCAGACTATATACTTTAAGCCGGAGAAAGAAATACCATGAATGGTTCCAAATAAAGAAAAACTGTCTTTCTATCCGAAA
TTGATTGTGTCTAAAGCTTCAATCCAACCAACTAAAGAACATAAGTTCACCGATACCTGGGAAAGTTTCACATATAAATACGTT
GGGTGTGGAATAAGATTGATATTCTTAAATTGACAAGAGTATAAATTCGAGAATTCGAAACTATTGACGATTGAAATATACTGT
GTCATATTTATCATTTATGCTCCTTGATGAAATGGTGAACAAATTATACTATTTCAAAAAATAATCACACATGAATAATTTTTA
TTCATAAAACTTATTGAACTTGGTATCACTTGCTCAAATAAGTATAGAACCATCTACAAAACATAATTAATCTTAGTAATATCT
AAAGCGTAATTTAAAGTAAGATGACTTAGTTAAATTTTCTTTCATAAGTCCCAGAATCAACCATATTTCCAATACCAAATACAG
AGAAATTAATTAAAGTTGTTTATAAACCTATAAACAGATCAGAATATAATTCTAAACCTAATCTGTATATAATTTTTCTTAGAC
TAATCAGTATTCAATATCATTTCTATACCCTCAAATTTGGTTTACATAATAAACACTCTTCTTCTCTCTCAAAAGATAATATCT
GCATCTCTCGAAACAAAACATATGGAACACAAAATTATAATCAGTAGTGCATTATTTTGATAAATGAAACCTATTTCAATAAAT
ATTAAAGGAAGTATTTCCTAGCAGTAGATGTATATAATTAACCCCGGTATGTCGATCAATTGAGCAAGCTGTGCTCCACACTAA
GCGCTAAGTCATCCCTTTATGGAGCACTGCCCGCCACACATTGATTACGTTATGCAGTTTCAAATAACCATATATATTTCTTTA
ATGTCGCCGATAAGACAAAAATGGCTGATATACCTAACCTGCCAGGATCTGAGGTGTTTCAATCTCATTCCTTTCACCATTATT
TCATGGTTCTATACCAAAAGCTGGATGAAAACTTTCGTAACCCGCGCCAAGTAGGATAATCAGGAAGTCGAGCGCGTCACATTA
CAAGTTTTTGTGGTTAGATGATAGTTGCCTAAGAGAGCGATGTTAGAAATAGTATTATGATTACGTTGGGATTATAAATAAAAA
TACCAACCGCCTTGTCTTATTAGAGACCCGGATTATCTTTAATGAGTTATGTTAATTATTGTTGAATTTATTATACTCATTTAA
CTATGACGAGATTTGATAAATATTTGCGCCATTATGTCATCATGAAACAACCATCCGGAGAAATAAGCTGATTTCTTGTAGGTT
GAGTACGAGAGACATTTGGAACTGTGTCTTATACCTAAATAAAAAACCTAACTGTTGATGACGTGTCAAGAATCCTCTGACACA
CCTGGCAAGAACATTCGCCTCTCGACCAGAGATACTTTCCCGTAATGTCAAGCGTTCCAAGTTTTATACTATGACCAGCCTGGT
CGTATACTCCATTTATTTACTGTGTAAAATTCTATAACACATCTGTAATGATATCGAGAATAGTCTTCTTCATTATCACCTGAA
GATCGTTTATATTAAGGAAAGATATAATTTAATTGACTCCGGGGGGGGGGGGGGGGGGGGGGGGGGGGCGGGGGGGGCGGGGGGG
GGGGGGGGGGGGGGGGGGGGGGGGGGGGCGGGGGGGGGGCGGGGGGGGGGCGGGGGGGGGGGGGGGGGGGCGCGGGGGGGGGGG
GGGGGGGGGGGGGGGGGCGGGGGGGCGCGGGGGGGGGGGGGGGCGGGGGGGGGGGGGGGGGCGGGGGGGGGGGGGGGGGGGG
GGGGGGGGGGGGGGGGGGGGGGGGGGGGGGGAGGTAACCTCCCTTGATGCCTATAAAATACAAGAAATAGCGCAAGTGGGATCTT
TTGGAGGGGTCTCTTTCCGAATGTACAATTACCATTATCCATCATCTTGTATTCGTTTTCTCCTTCTAAAGGTCTGTAAACTCA
GAAACCCTAGTTGTTCTGATCAACACCTTTGAAGCTCAATAATCAATGAATATATCCAAGTGGACGTAGGTACCATTACAATCA
CTGGGGCCCTGAACCCACTATACACTGTTTTTGTGTTCTTTACTTTCCATTACGATTGCTTTCTCAAGCATCGTACCTAATCTG
CCTGTGTTTATCTCCACCTCTGTGCGGTTGACTAAACAAACAAGGGTCAACATCAGGTGCTTTCCATTGAGAGCTTGCACAAAG
ATCTAATGGAGCAAAAACTACCAAGAAAGACTAGACAGGCTGCCACTGCACCATCCAACCTCCTCCCCCCAAAGTGGCTGAAGA
CGAACCTCAATTTGGAGTTTGATGAGGGCAAGAGTTGGAGTATGAGACCCTGAAGGCAACACTGGGGCAGGTGTTGCAGGATGA
GTTGGCCCCAATCTGAGGGCCAATCAAGAGAATGTTGTCGAGATCATGTCGTCGTAGCAAATGGGCGAGATCAGAATGCCCAGC
GCTAGGACCTGAGCGAAGGCAGGCTGAGATGGACCATCGGCAAACGGGATGCGGCCTGCCCCTTGAAGCGGCCATCCAGCTGCG
CTAGCGAATTAGCGCTGCGCCAGCCTTCCCCAGCCAGTATCAGCCTCCAAATGGCCACCTCCAAAGGGGTCCCTAATCCCTAGT
CCCTCCACTCTAGCCAGTACAGCCCTCCAGAGGCCGGAGAAAGCCACCTATGCTCAGATGATGTCCACCCTAGGGGATCTTGAG
CAGCAGCTCCGTCTCAGGCTGGGGCCGAGGCAAATTCCCCGCTGCCCGAGGCATATAGGGCTGGGCAACAGCCCCATAGCCCTA
GACGCCCAGGGGTGAAAGCCACACACCGAGTAGCGGGAAACATCCTTCTGCAATCAGGAAGGAACTTAAAAGAGACAGCTTCT
GCAGTCAGGGGCCCCCCCCACGACATGACAATGCACGGGGACCCACCAACCCGCGCGGCCTCCCAACCTAAGGGACGCTCGGGG
AATGCAGCCCAAGGAGGCAAAGAGGGAAATATGTCGTCGCCACCATAGCCAAGACCACAGTTCAAAGATGGCCATGGCTTACAA
TGAAGCCAACTCAGGCAAGAGACAATGCCTGGTCGAGAATGAACAGACATAGAGGTGGAGGCCAGGAGCCCGCCCCACCTAGAG
ACCAGACCGACCATAGGCTCATAATCTGGGGGGCAGCCCAGGCCAAGAATAACATCTTTTAGCTCGGCTTGGAGCAAGCCAATA
GCGGCGAAGAGAGAACACCTGATTTGAGGGACGTGTCTCAACGCCGCTGAGAAAGGCACGCAGAATACATTCCCCAGGTACTAG
AGCCCCAACAATCCAGAAGCTCGTTCAGGCTCAATCGATGCCCTGAACTAGGCTGCTGCAGCAGCTCAGTCGGGGGGCAAACAT
CACACATTGAGTACGATCGGTAGGAGAGGCAACCCTTTCGTACAGATTAGATTGTTGTGGCAGACAACCAACTAGCGTAAGTTG
AAGATGCCAACAACCCTGCCCGAATTTCGACGGGTTATTGGAGACCCAGTATCTCATGTCAACAAGTTTGGGATACAAAATGGA
CATTCAGAAAGTCTTTGAGATGCTCGCTGCAGGATCTTCCCAGTGACACTATTTTGATGCGCCCAGGAGTGGGTTCTTTAAAGT
TTCCCCCCGTGAGTATTAGTTCTGGGGATGTTCGTGAAGGAGTTTTAAAAGGACAAGTTTTTATGCGGGTCGTGTGCACCCCCA
CTGGGCCAAACCAACTGTCGATGATATGCCAGAAAGAGGGGCAGCCACTGAAGGATACATTCAGCCGCTTCATGAGAGATGCCA
GCTGGAGCCAAGATAGCTGGGCCCGATGAACGCAAGATGAATGGCCCTAACTGTTGGGGTTTAGGGCGCCCATCTCCCCCTAGA
GTAGCCTCAGAAAGCATGGGGTTAAAAGTACACATGATTTTTTAGATCGAGTTGATCGGTCATCAAGCTATAGGACGCGATTTG

CATATTGAAGGAATTGCCAGAAAAGACAAGGGGCCCAGGAAGAATCTGCCAAATAGCCGCCAACGGCTCGACAGGCCCAAGGAA
ACGACAAAGGCACGGGAATAGGAATGGTAAGAATGTGGAACCGTGGGCTAGCAACGAACCCCTCACACCTCCGAGAGTAAGGCA
CCCAAGGTATCGGTATGAACCGAGATTACACCAATTACACTGCCCTTGTTTGACAAGCGAGCCGAGGTTAACCAGGCGCACAGC
TCAAGCGTAGCCCTACAAACCGACACACACCAATAAGGAAGATATCTCCAAAGGAGATTCAACAAAGTTCTGTCATTTTCATAA
CGCAACGGGAACAACGACACTAATGAGTGTAACCAGCTGATGACTGGAAATTGAGTTCTCTGATAAAGACAGGGACATTTAAGA
TAGATATGTGCGCAGCTGCGGGAAGGGTTCTCAGCGAGAGGCTTCAAGGTGGGAACGAGTCCGGTGCCTGCACCCATGCTCTGC
CGTCCTTTAACAGCGTAGCCTCCTCGTGACAGGTACCCTACTGTCACCATCTTGCGAGGCCCACATCTTGTAGGATAGATAGTG
GGCAAAGCGTAGGGAACGATATGCATTCTGAACCCTACGCCACGACCAGGGACATCGAGATGATGAGTGTTGAGGATCGAGCAC
CAAAAAAGGCTCGAACAGAGGAGGAATAATGATCACCTTCTCTACATGAACCGACACCCATACATGTGTGATGTCCCACACTGT
CGTCTGCTGGTCGTTGGACGTACAGATCTGTAACTGATGGTGAAAGTGTGGGGTGCGTATACAGGAAGTTCGTCATCATCCTAT
ACAAGTCATGCTGGACAGATGAGTTGTCGTGAAGACCTGGAGCCAGTGCAACCGAACATTTAGGTTCTTTTCTAGAGAAGGGCT
CTGCCCGGAGGGTCTATTATACTCCCAGTCACAGCAGGCCACAACATCATGCTAATCAGGAACGTTACTCACTGCTTTTATAGT
AGTTGACTTGGTCCTTCAGCGTACAACGCTGTGATAGGGCGACCAATCTGGATCCGACCTGCGAGCCGTCACTTCTATATGTGC
ACCTCACTATGAAATTCCACACAGACGCAGGGGTAGGAAGCGTGTTGGAAACCAGAGGGAATGTGAGGGAGTGCTACAACGCTC
ATCCACCAAGGTAAGAAGGTTGTATCGAGAGAAGTGCCACCCGGGAAGCGAGTGTGCAAATGGGCAACTGATTTCAAGCCTCCT
CGGGTGAATAATCTCCACCAAATAGGGCATGGCCCCACAGTGAGGACAGGGACTGTAGATCCTCCACCCTTTGGGGATTTTGAG
AAGAATGTGGGACGCCATCAAGGACCTTTAAGAGGTCCAACTTTGATACAGGAGAGTCCGACCAGAGTTGTGAAAGTCAGTAAA
AACTTATAGAGCAACAATAATACAAGCCACTGGTGGAGTTCTTAAGCGAGAACCCAGGCAGGTCTTGCCAGTCCATCAGCCATG
TCTAACATCAGACGAAAGTAGATCAGCCTCTTGAAGAGAAGTCGAGAAGCTGACGAGAATGGGTTCTCAGGTGGCATTTTACTC
ACATCGCTGATCTCTAATCCTGCGTACTATGCCCAAGCGAATTGGTCAAGTGGCGCGTCGACGTCGTTGGATTTTTAACTAGAC
CTACAATAAAGCCCTTCCCCCAAAGACTGTTTCCCACTCCCCGGGGATTCGAAGCTGTGAATGTAGGCATCGAGATCCTCTATT
CATGGATCAGTACCTCAGCGCCCAGTAATTCACCGCATCAAGTCACCAATTGATGAGGATCACACATCTCCACGCTTTCGAACT
GAACAGGGCTCTACCTAGTTTAAAGGTGAATCGCCTCTTCCGGCCTCTTGCAAAAATGTCCTGGGTGCCATTACTACGAGACTC
GTTAACCCACATTTTCAGAGCCTGATCGGCCACAAACACATGGAGGTCTACTGGTCGACC
>m141120_131642_42134_c100732402550000001823141405141505_s1_p0/87246/0_7748
AAAAAGAGAGAGATATGGGCAGCAAAAAGTTGTAGGCCTATGTCTTAGGGTTCTATGTATAACATTTCGGTGCAAATCCTAGGT
GTGTAGAAGGGTGATACAGTCAAAATACTGAGAGATGTACGAATAATAAGGAAAAACTTGCAGAAATTTGCCAAAAAAAAAAGG
GGAATTTTTTTTGAAATGTTGACTTAAAGGCTCCGTTTCACTCCATAGATTCATTTACCCACGAGAATCTCTTGGGGAAAAATA
TCGACGATTTGAAGACGAGGGTAATACATTTCGGAGAAATTAGTGAAATCTCCAATTCACCAAAGGGGGTACCCCCTTTCAGAA
ATCTTCGATTTCTCTAATAATGGTTCGAGCAAATTTTTGATAGTGAATACTAAAGAATATGCGGGGTCATGGCACTAAAGTTGT
AGGCCTATGTCTTAGGGTTCTATGTATAAAAATTCGTGCAAATCCGAGTTGTTAGAGGTGATACCAGTCAAAATAGCTGAGAGA
TGTACGAATATAAGAAACTTGCGAAAATTGCAATATAAAAGGGGAATTTTTTTGAAAATGTTGACTTAAAATTCTCCGTTTCG
ACTCCAATAGATTCATTTACCACGAATTCTTGGGAAATATCGAACGATTTGAAGATAGGGTAATACATTTTCGGAGAAATTAGT
GAAATCTCCAAATTCAAAAAAAAACCAAAAGGGGTACCCCTTTCAGAAATCTTCGATTTCTCTAATATGGATTCGAGCAATGTT
TTGATAGTGAATACTAAAGAATATGCCCGAGGGTCATGGGCACAAAAGTTGTAGACCTATTGTCTTAAGGGTTCTATGGTATAA
AAATTCGTGCAAATCCTAGGTGTGTAGAGGGTGATACAGTCAAATACGAGTGATGTACGAATATAAGGAAAAAAAGTTGCGAAA
ATTTCCATAAAAAAGGGGAATTTTTTTTGAAATGTTGACTTTAAGGCTCGTTTCGACCTACCAAATAGATTCATTTTACCCACG
AATCTCTTGGGGGAAAATATCGAACGATTTGAAGAACGAGGGTAATACATTTCGGAGAAATCTAGTGAAAATCTCCAAATTCAC
CAAAAGGGGTACCCCTTTCAGAAATCTTCGATTTCTCTTAATATGGATTCGAGGCAATGTTTTGATAGTGAATACTAAAGAATA
TGCGAGGGTCCATGGGCACAAAGTTGTAGGCCTATGTCTTAGGGTTCTATGTATAACATTTCGTGCAAATCCTAGGTGTGTAGA
GGGTGATAACAGTCAAAATACTGAGAGATGTACGAATATAGGCAAAAAACTCTGCGAAAATTGCCAATATAAAAGGGGAATTTT
TTTTGAAATGTTGACTTAAAGCTCCGTTTCGACTCCCAATAGATTATTTACACCACAGAGATCTCTTGGGGGAAAATAATCGAA
CGAATTTGAAGATGAGGGTAATACATTTCGGAGAAATTAGTGAATCTCCAAATTCACCAAAGGGTAACCCTTTCAGAAATCTTC
GAATTTCTCATAATATGACTTTCGAGCAATGTTTTGGATTAGTGAAGTACTAAAGAATATGCGAGGGTTCATATGGCACAAAGT
TGTAGGCCTATGTCTTAGGGTTCTATGTATAAAAATTCGACCAAATCCTAGGTGTGTAGAGGGTGATACAGTCAAAATACTGAG
AAGATGTTAAGCGAAATATAAGGAAAAAACTTGCCGAAAATTGCCATAAAAGGAGAATTTTTTTTGAAATGTTGACTTAAGGCT
CCGTTTCGACTCCAATGAGATTGCATTTACCCACAAGATCTCCTTGGGGGAAAAATATCGAACCCCCGATTTGAAGACAGGGTA
TATACATTTCGGAGAAATTAGTGAAATCTCAAATTCCAGCCAACGGGGTACCCCCTTTCAGAAAATCTTCGATTTCTCTAATAT
GGATTCGAGCATGTTTTGATAGTGAATACTAAAGAATAAGCGAGGGTCATGGGGCACAAAAAGTTGTAGCCTATGTCTTAGGGT
TCTAGTATAACATTTCCGTGCAAATCCTAGGTGTGTAGAGGGTGATACAGTCCAAATACTGAGAGATGTACCGAATATAAGGAA
AAACTTGCGAAAATTCCCAAAAAAAAAAAGGGGAATTTTTTGAAATGTTGACCTTAAGCCTCGCGTTTCGACTCCATGAGATCA
TTTACCCCACGAGATCTCTTGGGGAAAAAATATCGAAGCCCCGATTTGAAGAGCGAGGGTTAATAACATTTCGAGAAAAATTAGT
GAAATCTCCAATTCACGAAAAGGGGGGTACCCCTTTCAGAATCTTCGATTTCTCCTAATATGGATCGAGCCAATGTTTTGATAG
TGAATACTAAAGAATATGCGAGGGTCCCCATTGGGCCACTAAAGTTTAGGCTATGTCTTAGGGTTCTATGTATAAAAATTCTGC
CGAAATCCGAGTTGTGTAGAGGGGATACAGTCAAAATACTGAGAGATGTACGAATATAAGGAAAACAACCCTTGCGAAAATTGC
CATATAAAAGGGGAATTTTTTTTGAAATGTGACTTAATCTCCCGTTTCGACTCCCAATAGATTCATCTTACCCAGAGATCTCTT
GGGGAAAATATCCCGAACGATTTGAATGATGAGGTAATACATTTCGGAGAAATAGTGGAAATCTCCAAAATTCACCAAAGGGTA
CCCCTTTAAAATCTTCGATTTCTCCTAATATGGAGTTCGAGCCAATGTTTTGATAGTGAATACTAAAGAAAATGCCGAGGGGGG

GTCATGGGCAACAAAAGTTTGTAGACGCTATGTGCTTAGGGTTCTATGTAAATCAAAAATTCCGTGCAAAATCGCTAGTGTGTT
AGAGGTGATACAGTCAAAATACTGAGTGGATGTACGAATATAAGGGAAAAAAGTTGCGAAAATTTCCATAAAAAGGAATTTTTT
TTGAAATGTTATGAGCTTAAGGCCTCCGTTTCGACTCCAAATAGATTCCATTTTACCGCCACAGAGATCTCTTGGGGGAAAATA
TCGAACGATTTGAAGACCGAGGGTAATACATTCGGAAAAGAAAATTAGGTGAAATCTCCAATTCACCCAAAGGGGTACCCCTTTC
AAGAATCTTCGATTTTCTCCTAATATGGATTCGAGGCAAAGTGTTCTTGATAGTGAATACTAAGAATATGCGGGGTCATGGGCC
CCCACAAAAGTTGTAAGGCCTAATGTCTTAGGGTCTATGTATAAACATTTTCGTGGCAATCCTAGGTGTGTAGAGGTGATTACA
AGTCCCAAAATACTGAGAAGATGTACGAATATAAGGAAAAACTTGCGAAAATTGCCATATAAAGGAATTTTTTTGAAATTTGA
CTTAAAGCTCCCGTTTCGACTCCATAAGATCATTTACCCACCCGAGAATCTCTTGGGGGAAAATATCAGAACCCGATTTTGAAG
ATGATGGGTAATAACATTTCGAGAAATTAGTGAAATCTCCAAAATTCAACCCAAAGGGGTAAGCCCTTTTCAGAAATCTTCGAT
TTCTCCTAATATGGATTCGAGCAATGTTTGCATAGTGAATACTAAAGAATATGCGAGGGTCATGGGCAGCAAAAGCTTGTAGGC
CTATGTTTAGGGTTCTATGTATAATTCGTACAATCCTAGTGTGTAGAGGGGTGATACAAGTCAAAATACTGAGAGATGTTAGCG
AATATAAGGAAACTTGCGAAAATGCCCATAAAAAAGGAGAATTTTTTTTGAAATGTGACTTAAGGCTCCGTTTTCGACTCCAA
TCAGAATTCATCTTACGCCACAAGATCTCTTGGGGAAAATATGCGAACCGATTTGAAAAGACGAGGGGTAAATACAATTTCGGA
GAAATTAGTGAAATCTCCAATTCCACCAAAGGGTAGCCCCTTTCCCAGAAATCCTTCGATTTCTCCCTAATATGGGATTCGAGC
ATGTTTTGATAGTGAATAGCTAAAGAATATGACGAGGGTTCATGGGGCACAAAAGTTTGTAGGCGCTATGTCTTACGGGTTCTA
ATGTATAACATTTCGTGCAATCCTAGGTGTGGTAGAGGGTGATACCAGTCAAATACCTGAGAGATGTACGAATATAAGGAAAAA
CTTGCGAAAATTGCAAAAAAGAGGGGAATTTTTTTTTGAAATGTTGACTTAAGGGGCTTCCGTTTGCGACTCCCAATAGAACT
CATTTTAACCCACGAAGATTCTTGGGGAAAAAATATCGAACATTTGAAGACCGAGGGTAATACATTTCGCGAGAAATTAGTGAA
ATCTCCCAATTCACCAAAAGGGGGTACCCCTTTCAGTAAAATATTCAAGATTTCTCTAAGTATGGATTCGAGCAATGTTTGATA
GTGAATACTAAGATATGCCGAGGGTCATTGGGCCCACTAAAGTTGTAGGCCCTATGTCTAGGGTTCTATGTATAAAAATTCGTG
GGCAAATCCGAGTTGTGTAAGAGGGTGATACAGTCAAAATACTGAGAGCATGTAAACGAATATAAGGAAAAACTTGCCCGAAAA
TTTGCCATATAAAAGGGGACATTTTTTTTGAAATGTTGGCTTAAATAACCTCGCGTTCTGACCTCCAATAGATTCATTTACCCA
CGAGAATCTCCTTGGGGGAAAGATATGCGAAGCGATTTGAAGATGAGGGTAATACCATTTCCGGAGAAATTAGTGAAATCAATC
CAAATTCACCAAGGGGGGTAGCGCGCCTTTCAGAAATGCTTCGATTTCTCTAAATGGATTCGAGCAAATGTTTTGATAGTGAAT
AGCTAAAGAATATGCCGAGAGGGTCATGGGCCCCCACAAAAGTTGTAGACCATATTGTCTTAAGGGTTCTATGTATAAATAAAA
TTCGTGCAAATCCTAGGTGTGTAGAGGGTGATACAAGTCAAAATACTAGAGTGATGTACAATATAAGGAAAAAAGTTGCGAAAA
TTTCCCATAAAAAAAAGGGGAAATTTTTTTGAAATTGTTTGACTTAAGGCTCCGTTTCGACCTCCAATAAGATCATCCTTACCC
CACGAGAATCCTCTTTTGGTGGGGGGAAAAAAATATCGAACGATTTGAAGACGAGGAGTAATACATTTCGGAGAAATTAGTGAA
ATCTCCATTCCCAACGGGGTACCCCTTTCAGAAATCTTCGATTTCTGCTAATATGGATTCGAGCCAATGTTTTGATAGTGAATA
CCTAAGAATAATGCGAGGTCATGGGCCCCAGCAAAAAGTTGTAAGCCTATGTCTTAGGGTTCTATGTTAAAAAAATTCGTACAA
ATCCTAAGGTGTGAGAGGGGGTGATACAGTCAAAATACTGAGAGATGTACGAATATAAGGAAACAAAAACAAACTTGCGAAAAA
TTGCCCAATAAAAAGGAGAATTTTTTTGAAATGTTGACTTAAGGCTCCGTTTGCCCGACCTCCCAATCAGATTCATGTTACCC
CCAGCATAGATCTCTTGGGGGAAAATATCGAAGATTTGAAGACGAGGGTAATACTTTCGGCAGAAATAGTTGAAATCTCAATTC
AAACAAAGGGGGTACCCCTTTTTTTTTTCAGAAATCTCGATTTCCTCTAATATTGGATTCGAGGGGCAATGTTTTTGATAAGTG
AATACTAAAGAATATGGCGAAGGGTAATGGCCACAAAAGTTGTAGGCCTATGTCTTAGGGTTCCTAATGTTATAACATTATCGT
GCAAATCCTAGTGATGTAGAAGGGTGATACAGCTCCAACAAAAATACTGAGAGATGTAGCGAATATAAGGAAATTGCGAAAATT
GCCCCAAAAAAAGGGGAATTTTTTTGAATTGTGTTGACTTATAGGCTCCCCGTTTCGACTCCAATAGAATTCATTTACCCACC
GAGTCCTCCCCTTGGGAACAAATATCGAACGATTTGAAGACGAGGGTAATACATTATGAGAAAATTAGTGGAAATCTCCAAAAT
TCACCAAAGGGGTACCCCTTTCAGAATCTTCCGATTTCTGCCTAATATGGTTCGAGCAATGTTTTCGAATAGTGAATACCTAAA
AAGAATATGCGAGGGTCATGGGCACTAAAGTTGTAGCCTATGTCCTTAGGGTTCTATGCTATAAACAATTCGTGCAAATCCCGA
GTTTGTGTAGAGGGTGATACAGTCAAAAATACCTGAGAGATGTACGAATATAAAGGAAAAAAACTTGCGAAAATTGCCATATAA
AGGGGAATTTTTTTGAAAATGTTGACTTAAATCTCCCCGTTCGACTCCCCAATAGATTCATTTACCCCCACCCGGATCTTCCT
TGGGGGAAAATATCGAACGATTTGAAGATGAGGGTAATGACATTTCGGAAAATTAGTGAAATCTCCAATTCACCAAAGGGGTAC
GCCCTTTCAGAATCTTCGTTTCTCCTAATATGGATTCGAGCAATGTTTTGATAGTGAATACTAAAGAATATGCGAAGGGTCATG
GGCACCAAAAGTTATGTAGACCTATGTCTTAGGGTTCTATGTTAAGAAATTCGTCGCCAATCCTAGGGTGTGTAGAGGGTGAAT
AGCCAGTCAACAATACTGAGTGATGTAGCGAATATAAGGAAAAAAGTTGCGAAAATTTCCAAATAAAAAAAAGGGGAATTTTTT
TTGAAATGTTGACTTAAGGCTCCCGTTTCGACTCCAAATAGAATTCATTTACCACACGAGATCTCCCCTTGGGGGGGAAAATAT
CGAACGATTTGAAAGAGAGGGGTATACATTTCAGAATTAGTGATAATCTCCAATTCACCCAAAGGGGTAGCCCCTTTCAGAAAT
CTTCGATTTCTCCCCTAATATGGATTCGAGCCCAATGTTTTGATAAGTGAATACTAATAGAATATGCCGAGGTCCATCGGGCAG
CAAAAGTTTAGGCTATGTCTTAGGGTTCTATGTATAACATTTCGTGCAAATCCTAAGGTGTGTAGAGGGTGATACAGTGCAAAA
TACTGAGAGATGTACGTAATATGAAGGAAAAAACTTGCGAAAATTGCCATATAAAAGGGGACCCCCCATTTTTTTTGAAATGTT
GACTTAAAGCCCTCCGTTTCGACTCCAATAGATTCATTTTACCCACGAGATCATCTTGGGGGAAAATATCGAACATTTGAAGAT
GAGTAATACTTTCGGAGAAATTTAGTGAAAGTCTTCCCAATTCAACCAAAGGGGTAGCCCCTTTCAGAAATCTTCGATTTTCTC
CTAATATTGGGATTCGAGAAAATGTTTTTGATAGTGAATACATAAAGAATATGCGAGGGTCATGGGGGCACAAAAGTTGTAAGC
CTAAGGGGGGGGGGGGGGGGTGGTTCCTTCAGGGTTCTATGTATAGAAAGAAAAAATTCGTAACAAATCCTAGGTGTGTAGAG
TGGAATAAAGTCCCCAAAATACTGAAGAGATGTAAGGCCGAATATAAGGAAAAAACTTGCGAAAATTGGCCATAAAAAAGGAGA
ATTTTTTTGAAATGTTGACTTAAGGGCTCCGTTTCGGCCTCCCAATAGATTATTTACCACCAAGATCTCCTTGGGAAAATAT
CGAAGATTTGAAACGAGGGTAATCACATTTCGGAGAAAATTAGTGGAAATCTCCCAAAATTCACCAAAAGGGGGGGGTACCCCT

TTCAGAAATCCTTCCGATTTCTCTAATATGGATTCGAGCAATGTTTTCTGATAGTGAAATACTAAAAATATCGCGAGGGGTCAT
GGGCAGCAAAAGTTGTAGGC
>m141120_131642_42134_c100732402550000001823141405141505_s1_p0/87892/0_11618
AAAACCCCAGAGAGAGATTTCTAAATTCACTTACTTATTAAATCGCTCCGCTCACTCATCACCTGAGACCCTACGCGGTGGTAT
TGGTTTACCTTAAGTGTACATTGGTCGAACGATTCTCCTCATCGCCCTCCTCGCTTGAGGTATGCTTAGTGCCATGTTAGTTAG
TCAATAATTTTTCGGGATCGGATGCCATTATAATTAACTACTATAAATCTTAGTGCAATGTTATATAGGTAACTATTGGTTTCC
TACGATCGGATCCACTTAGCTAATTCACTATTGCTATCTTACTCGCATGAAATATATGACCGACCTACGCGGCCCTAGCTCTCT
CCGGTTCTACTATATGTATGTGTTGTGGAAACAATCTCAGATTATCGTCATACATTCAGAGCTATGAACTTAGTGCATTGTTAT
CGTAGTCCCCCTCACTCCTTTTCTGGCATCCGCTCCTTCTCACCATTCTCCTCTCATATCTCTCAGTGCAATTTCTCTAGTCTA
CGTAGACTACATTTGGGGATCCGAATCCCTTCTAAATTCTCTACTTTATCCTTACTCAGCATCGACAAGCTATATGGACCTAGC
GGTAGTATGTGGTTTACTAGTGTGAAGTTGGAACGATTTATTATGCATAAATTAGGACTGCTTAGGCCAATGTTATTCTACCTC
TCACTTTTTCGGGATCCGCATTCCATTTAAAATTACATATAAATAATCTTAGTATTGTTAATCTAGTAGTATTCGGATCGGATC
CCCTGTCAAAATTCACTATCTCTTCTATCAGAATGGACAACTCATATGAGACCTACGCGGTGAGTATTGGTCTTACTAGGTGTA
CGTATGGACCTGCTTCTATATGTATTATTGGCTCTGCTTAGTGCCAGGTTATCCTACGTAAATAATTTTCCGGGATCCGAATCC
TTCTTAATTCACTACTTTTATCTACTCGCAGCAACTATCATGCACGACCCCTAGCGGTGGTATTGGTTTACTAGTGTACATTGG
AACTACATTCCATTATGTCACTAATTAGGCTTGCTTAGTGCAATGCCTACCCTCCTCACGCTCACTCACCTTTTCGGGATAGGA
TGCCATTCTACAATTACCACTCTACCTGATCTTAGTGCATTGTTATAACCTACATACCATTTTTTCGGGAATCGGATCCCTTCT
AAATTCACTACTTCCATCTTACTCAGATGACAACATTGAGGCCTAGCGGTGATATTGGTTTTATAGTGTACGTTCGAATGATTA
TTATGTCATTATTAGGTCTTGCTTAGTGCAATGTGTAACCTCTAGTAAGTAATTTTTTCGGGATCAGTCCCTTCTACAAATTCA
TACTATATCTCACTCAGCATGACTACTTATATGAGACCTGCGGGTGGTATTGGTTTACTAGTGTAAGTTGGACACGATTCATTA
TGTCATACATAGTTCTGCTTAGTGCCCCCTCGCTTCCATCTAGTCATAATTTTTACGGGATCCGCATGCATTCTAAAATTTAAC
TACTATACTGTTGAGTGGCAACCCTGCTTACATCTGTAGTAATATTTTTCCCTAATCGGATCCTTCTAAATTCAATATTCTATC
TTACTCAGCCTGAAACTATATGCAGACCTAGCGGTACGTTCTGGTTTTTACTATTGTAGTTGGACACCACTCTCCTCTATGTCA
TAATTAAGGTATGCTTAGATGCATTGTTATCCTAGACCACTAATTTTTCTGGATCAGATCCTTCTAAATTCTCTAGCTATAGTC
TTAGTGCAATTTTTATCTAGAAGCGTAAATCCTTTCTGGGGATCGAACCCCTTCTAACCTTCCTACTCTATCTTATCAGCCATGA
CAACTATATGCCAGACCCTAGCGGTAGTATTGGTTTACTAGTGTCAAGCTTGGAAGATTTATTATGTCATAATTAATGGTCTGC
TATAGTCGCAAGGTTTCTCCCTCGCTAATATTCTTTGGCATTGGATGCGATTTTAAATTCCTACTATCATCCTTAGTGCCATGT
TATCTACGTAGTATTTTTCGGCAATCGCGACTCCTCCTCCAACCAATTCACCCTACTCTTTCCTTACTCAGCATGACAACTATA
TGAGACCCTAGCGGTAAGTATTGTTTTTAGCTAGATGCTACGTTGGAGATGATTCATTATGTCATTATTATGGCTGCGTTAGTG
CAAGGTTATCATAGTCAAACCTCTCTCGGCGTCGACATCCCGTTCTACAATTCCTACTATATCCCGCTAACTCAGCCATGAAAC
TATATGAGACCCTAGCCCGACTAGCTATCCCTCCGGTTTACTAGTGTCAATTGGAACTATTTATTATGGTCATAATTAGGCCCA
CTTCTGCCTAGTGCGCTGTTATCTAGTAATAATTTTTCGGGATTGGCTGCTTTTAATTCACTACTATATCTTCAGTGCAACTGT
TATCTAGTGTATTTCTTCGGATCGGAATCCCCTTGCAAACTTCACCTAGACTCTTTCTATACTCACGTGGACATATATGAGACC
TGGGTAGTATTGTTTCTACTCATGTAACGAGTTGGACCGATCCGCATCTCATTCCTGCTCATTTAGGGTTGAGGTTTATGCCT
GATGTTATCCCGGTATATTTCGTTCGGGGACTCAGACTCCTCTCTACACTCTCACTACCTATATCTAAATCAGCATGCGACTAC
TATATGAGACCCTAGCGGTGGATAGTTGGTTTACTAGTGTACAGTTGGAACGACTTCATTATGTCATGAATTACGGCTCTGCT
TAAGTGCGCTCTCTCTAGTACCTCACCTCTCTTTCGGATCACGAATGCATTTAATTAACTACTATACTCTTAGTGAAATGTTAT
CTAGTATATTTTTTCCCTAGATCGATCCCCTTGCAAATTTCACTATCTATCTTTACTCAGATGAAACTATTGGAGACCCTGCGG
TAGTGATTGGCTTTGACTATTGTTATGTTGGAAATTCATTATGTCATAATTAGGTATGCTTAGTGCATTGTTTATCTAAGTAAT
AATTTTTCTGGATCAGATCCCCATGTCTAAATCTCTACTATATCCTTAGTGCAATTTTATCTAGTGTAATTTTGGGGTGAATCC
CTTCTAATTCAGTACTCTTATCTTACTCAGCATAGAAAACTATATGAGACCTAGCGTAGTATGCTGGTTTACTAGTGTAAGTTG
GAACGAGTATTATGTATAATTAGGATGCTTAGTGCAATGTTATTTATCATGAATTTTTCGGGATCGCATTCCAGTTCCTAAATT
AAGCTACTATATCTTAGTGGATTGTTTCTAGGTAGTAATTTTTCGGAATCGGGATCCCTTCCAAAATTCACTACTCTTTCTTAC
TACGAATGGACAACTATATGGACCCTAGCGGCTAGTATCTGGTTTAGCTAGTCGTAGTTGGAATAGATCATATATGTCATTCAT
TTTGGTGCTCTGTAGTGCAAAGGTTTTTCTAGTAATAATTTTTCGGGATCGAATCCCTTCTAAAATCACTACTTTACTAACTCA
GCAGACAATATATGAGCACCTACGCGGTGGTATTTGGTTTACTAGTGTACATTGGAATATTCATTATGTCATAATATAGGGTCT
GCTTAGTGATGTTATCCTAAGTCATTAATTTTTCGGGATCCGGATAGCATTCTAAATTAACTACTATACTTAGTGCATTGTTAT
CTAATAATATTTTTCCAGGATCGGATCCCCGTTCTAAATTACTACTCCTTTACTAGAATGACACAATATGAGGCCCTAAGAAGC
GGTATGATTGGTTTTACTAGTGTACTTGGAATGATTCATTATGTATTATTTGGTCTGCTTAGTGCAAGGTTATCTAGTAAAAAT
TTCGGGATCGAATCCCCTTCTAAATTCACTACTATATCTAAACTCAGCATGAAAACTATATGAACCACTAGCGATAGTATTGGT
TTACTAGTGTACATTGGAACTTATTTAATTGTGTAATAAAAAATTAGGTCTGCCATAGTGCAATGTTATCTAGTAACTAATTTG
TTCGGGATTAGGATGCCTTTCCTCCTCAATTCACTACTATATCTTAGTGCCATGTTACCTAAAGTTGTATTTTATTTCGGAATC
CGCGCCATCCCATAAAACAATTTCACTAGTCTTTCTTTACTCAGCATGAAACTATATGGGACCTAGCGTAGTATTGGTTTACAA
TAAACGTGTCGTTGGAATGATTCATTATGTCATTATGTAGGTCTGCTTAGTGCAATGTATCTAGTAAATAATTTTTCGGGATCA
GATCCCCTTCTAATTCCTACTATATCTAATCAGCATGACTATATTGAGGACCCTAGCGGTGGTATTGGTTTTACTAGTGTACTT
GGAACGATTCTGTATGCTATAATTAGGGTCTGCTTAGTGCAATGTTTATCTAGTCAGTAATTTTTCGGGATCGGTGCGATTCTA
ATTAACTACTATAGTTTAGTGAATGTTATCTAGTATATTTTTCTAGATCGGATCCCTTCTAACTTCACTATTCTATCTTACTCA
GCTGAAACTATATGAGGAACTAGCGGGTAGTTTGGTTTCTATTAGTCATGTTTGGAACATTCATTATTGTCATAATTAGGTATA

GCTTAGTGCATTGTTATCTAGTAATAATTTTTTCTGATCAGATCACCTTTCTAAATTCTCTACTATATCTTAGTGCAATTTTAT
CTAGTAGTAATTTTGGGGATCGACATCGCCCTTCTAAATTCACTATCTATCTTACTCAGCATGACAACTATATGAGACCCTAGC
GGATAGTTTGGTTTACTAGTGTACAGTTGGAACGGATTTATTGATTCAATAATTAGGAGCTGCTAGTGCATGTGCTAGTCTAAT
TTTTCGGGATCCGGCATTCCATTTCTAAATTACATACTATATATTTAGTGCATTGTTATCTAGTAGTATTTTTCGGAATTCTGG
ATCCACTTCAAATTCCTATCTTTCTTTACTCAGAATGAAACTATATGAGACCTAGCGGTAGTATTGGTTTACTAGTGTACGTTG
GAATGATTCATTATGTATTATTCCCAGGTCTGCTTAGTGCAACTGTTATCTAGTAAGTAATTTATTCGGGATCAGATCCCTTCC
TAAATTCACTACTATATCTTAACTCGAGCAATGACTACTATATGCAGACCCTAGCGGTGGTATTAGGTTTATAGTGTTTCACAT
TGGAAAGATTCCATTATGTCATAATTAGGTCTGCTTAGTTGCAATGTTTATCTAGTCATGAACAATTTTTCGGGACTCGGATGC
CATTCTACAATTAACTAGCATATATCTTGTGCAATGTTATCTAGTATATTTTTTCCGTAGATCGGTCCCCTATCTAATTCACTA
TTCTCATCTTACTGCCAGCATGAAAACTATATGAGACCCTAGCGGTCAGATATTGGTTTACTATCTGTATGTTGGACATTTCA
TTCTGTCATAATTAGGTATGCTTAGTGCATTGTTATCTAGTAATAATTTTTCTGGATCAGATCCCCTTCTAATTCTCTACTATA
CTCTTAGTGCAATTTTTCTTAGTAGTAATTTTGGGGATACGAATCACCTTCTAAATTCACTTACTCATATCTTACTCAGCCTGA
CAACTATACTGAGATCCCTAGCGGTAGTGTTCGGTTTACTAGTGTAAGTTGGAAGATTTATTATGTCATAATTAAGGACTGCTG
TAGGTGCAATGTTATCTAGTCTAATTTTTTCGGGATCCGCATTTCCATTCTAAATTACTACTATATCTTAGTGGATTGTTATCT
GTAGATATTTTCGAATGGTCCCCTTCAAAATTCACGTATACTTTCTTACTCAAAATGACAATATATGTAGCACCCTAAGCGGTA
AGTATTGGTTTACTGAGATGCTCTAGTTGGAATGATTCATTATGTCAATGTATTTGGTCCTGCTTAGTGCAAGGTTATCTAGTA
AGGTAATTTTTCGGGATCGCAATCCACTCTCTAAATCAGTACTTTCTCTAACTCAGCAGACAACTAATATCGAGGAACTAGAGC
GGTGGTGATCTGGTTTTAGCTAAGTGTATAACATTGGAACTATTCATTATAGTTCGATAAATTAGGTCTGCTTGTGCCAATGTT
ACTCTAGTCATAATTTTTCGGGATCCGGATGATCTCCTAAACTTAACTACTATATCTAGTGGCACATGTTTCCTCTCACGCTCC
TCACCTCTCACTTTCGGAATCGATCCCTTGCTATAATTCAACTACTACATCTTACTCACATGCACTATATGAGACCTTCAGGGA
TCAGTATCTGCTTTTACTAGATGTACAAGTTCGGACGATTTATTATGCTCATAATTATGTCTGGCTATTAGTGCAATGTTATCT
AGTAATCAAATTTTTCGGTTAGGATGCATTTTAAATGCACTACTATATCTTTGAGTGCGATGTTACCTCTAGTAGTATTCTCTC
GGAATCGGTTTGCCATTCTAAATTCACTACATATCCTTAGTTGCATGTGTATTAGTAAATCATTTTTCCAGGGATCGGTCCCTC
CTAGATTCACTACCTCTATTAGACTCAGCATGAAATATAAATGAAGACCCTAGCGACTGGTAATTCTGATTACTTAGTCAACGC
TGGAACCACGGTTCAATTATGTCTATTGAGGAGCTTAGTGAAAGTTATCCTAGTCAAATAAATTTTTCGGGGATCGGTTTTCCC
ATTCTAATTCACTACTCTCCTCCTCCACCACTCAGCATGCAACTATATGAAGAGATACTAGCGGTAGTAGTTGGTTTAATAAAG
TGTACATATTGGAACACGATTCATTATTCATAATTTAGGTCTGGCTTAGTGCAATGTTATCTAGTCTTAATTTTTCGGGCATAG
GCCATGCCACTTCTCTAAATTAACTATATATCCTTAGTGCGATGTTATCTAGTAATATTCTTTTCCTAATCGATCCCTTCTAAA
TTCTCCTATTCTATCTTACTCGATGAAACTATAATGGACCCACGCGGCTAGTATTGGTTTACGTATTGTATGCTTGGAACAATT
CATCTATGTATAATTAGGTAATGCTTGTGGCATTGTTATACTAGGTAATAAGTTCCCTTTCTGTATCACGATCCCTTCCTAATT
CTCTACTATTATCTTTAGTGCAAATTTTATCATAGCTCGCTCAGCATCTCCTCTGGGGGATCGATACTTGCTAATTTCACTACT
CTATCTTATCAGCATGACACATATATAGACCTAGCGGTAGTATTGGTTTACCCTCACGCTCGCACAGTTCGCGCACGATTTACT
TATAGTCATAATTAAGGTTCTGCTTAGTGGCAATGTTATATAGCTCAGATAATTTTCGGGATCCGCATTCCATTCTATAATTAA
CTATAATATCTTAAGTGCAATTGTTATCTACGTCACGCTCATTTCTTTCGCGCTCCGGCCACTCTTCAACACTTCAATACTCTT
TCTATACTCAGATGACAACTATCAAGACGCACCTACACGCGCGTAGTCTCTGGTTTCCTCAGCTGTCAGCCTCTGGACGACTCG
ACTCGCTCATCTATGTCATTATTCTGGTCTGCTTAGTGGCAGGATGGTTCATTAAGTAAACGCCGTAAATCTTTTCGGGATCGA
ATCCCTTCTAAATTCACTACTATTATCATTAACTCAGCAAGACAACTACCTATGAGACTACCGCGGCTGGACTATCTCGCGGCT
TTCATACGCTGTCCACCTCTCGGAACTATTCATTAACTGTCATAATTAGGCTCTGCTTATGAATGGTTATCCTAAGTCCTAATT
TTTTTGGGATCGGATGGCCATTCTAAACTTAACTAGAAACTATCCTCTTAGTGCATTGTTAAATCTACATAACTATCTTTTATC
CGGGCACGGATCCCCTTCTAAAATTCACTAGCTCCTATTTACTCAGAATGACCCACACATATGAGGCCTACGCGCGACTAGTAT
TGGTTCTACACCTCAGTCGTACCGCTTGGCAACCATGATTCTCCTCTCGCGCTCTTATTCACAGCGCTCTGCTTCCAGTGCAAT
ATTCAATCTAGTAAATAATTTTCTCGCGGATCAGATCCCCCTTCTAAATTCACTACTATCACCTCTAACTCAGCATGCTACTAT
ATGAGACCTAGCGGTGGTATTCGGTTTACTAGTGTAATTGAACGATTATTATGATTCATCATTAGGTCTGCTTAGTGCAATGTT
ATCTAGTCATAATTATTTCGGGATCCGGATGCCTTCTACGAATTTAATACTATAATCTTAGTGCAAGCTTATCTAGTAAATATT
ATTCCTAGATCGGATCCCTTCTAATTCACTATTCTATCTTAATCAGCAATGAAAACAATATGAGACCCTTAGCGGTAGTATTGG
TTTACTAGTGTATGTTGACAATTCATTATGTCATAATTATAGGTATGCTTAGTGCATGTTATCTTGTAATAGATTTTTCTGGTC
AGAACCCCTTCTAAATTGTCTACTATATCTTTATGCATTTTATCTAGCAGTAATTTTTGGGATATATCCCTTCTAATTCACTAC
TCTATCTTACTCAGCATGACACCTATATGAGACCCTACAGCGGTAGTAATTGGTTATACTAGGTCAAAGATTGGAACGATTTAT
TCATGTCATAAATTGCAGCTCTGCTTAGTGCAATGTATCATAGTAATAATTTTTTCGATTGGTGCCATTTTGGGAATTCACATA
CTATAATCTTAGATTGCCATGTTATCTAGTAGTTTTTTCGGAATCGGATCCCTTCAAATTCACTATTTTTCTTACTCAGCATGA
CAACTATATGGAGCACCCTAAGGCGGTAGCATTGGTTTACTAGTGTACGTTTTTGGGAAACGATTCATTATTTCATAATTAGAT
CGATGTTAGTGCAATGTTTTTCTAGTATAAATTTTTTCGGATCGGATGCCATTCTAATGCAGATACTATTCCTTAGTGCATGTT
CTCTAGTAATATTTTGTCGGGGTTAGATCCCCTTCATCAAATTCATAGCTCCATCTTACTCAACTGCAACTTATATGGACCCTT
GCGGTAAGTAATTGCTTTATAGTGTGAAGTATTGGACGATTTTGTTATTATCGTATAATTTATGTCTTCTTAGTGCGCAATGT
TATCATAGATAATAATTTTTCGGGATTGGAATGCCATTTAAATTCACTACTATATCATTAGTCGCAGATCGGGGCGCAATGTCT
CTCCTCGTAGTATTGTTTCGGAATCCGGATGCCATTCTAAATTCACTACTATATCTTAGTCAATGTTATCTAGTAATACGGTTT
TTCAGGAGATCGGGATCCCCTCCTAGATTCACTACTCTGTCTTGCTCAGCATGACAATATCAGAAGACCCCTAGCGATGGGTCA
TTTGAATTACTAGTGGCACGCTGGACACGGTTCATTATGTCAAATAAATAATAAAAGAAAAAAGAAAAACATATAGTAGCAAGT

TATCATAAGTAAAATATTATATATACGAGAGATCAGGATCCCCTTCAAATTACATACTCTTTCTTATACAGCGCATGAACTTAT
GAGACCCTACCCCAGGGTAGTATTGTTTTACTATTGTAACGTTGAATGATTCTTATGTCTTATTGATGGTCTGCTTAGTGCAAG
GTTTCTAGTGAAGGAATATTTTCGGAATCGAATCCCCTTCTAAATTCACTACTGATGATCTAAACTCAGGCATGAAACAATCAT
TAGACCGAGCGATAGTATTGGTTTAGACTATGTGTCATTGGAACTATTCATGTTATGTCATACATTAGGTCTGCTTAGTGCAAT
GTTATCTGTCATAATTTTTCGGGATCGGATGCATTCAATCTAACTACTATATCTTAGTGATTGTTATCGTCATGAATATTTTTC
CGGGATCGGATCCCCTTCTAGAAATTCACATACTCTACTCCTTACTCCAGATGCAACTATATGAACACCTAGCGGGCTGAGTCC
TTCGGTTTACTTAGTGTCGTTGGACTCGTTCATTATGTCATCCAATTGAGGGTCTCGCTTAGTGCATATGTTAGTCTAGTCGGT
AAGTTTTTCGGATCGGATGCATTCTCAAATTAAGCTACTATATCTTAGTGCAATGTTATCTACGTAATATTCATTTCCTATGAT
CGGATCCCCTTCTAAATTCACTATTCTATCTTACTCAGCATGAAACTATTGAGCACCTAGCGGTGTATTCGGTTTACTGTGTAT
GTTGGAAACAATTCATGCTATAGTCGTAGATTGTATGCTGTAGTGGCTTGTTATCTAAGTTAATAATTTTTCTGGATCAGATCC
CCTTCTAGAATTCTCTACTATATCCTTAAGTGGCAATTTTATCTAGTAGTAATTTTGGGGATCGGAACCCCTTCTAAATATCAC
TATCCTCTATCTTAGTCTGTAAAACAGCATGAAAGACTATGATGAGACCCGTAGCGGAGTAGTATTGTATAGTGTAAGTTGGAA
CGATTTATTATTTGTCATCCGAATTAGGTCCTGCTTATGCAAGGTGTAATTTAGTAAAGTAATTTTTTAGGCATCTGGATGCAT
TGTTAAATCCACTACTATCATCTTAAGTGCCAATGTTATCTAGAGTATCTTTTCGGAATCGGATCCCCCTTCAAATTCACTACT
CATTTCTTACTCAGCAGTCGTTACACTAGTATCGCAAGACCCTAGCGGTAGTCATTGTTTTTACTATTGGTACGTGTGGCGATG
ATTCTTATGTCATTATTGTGGTCTGCTTACGTGCAAGGTTATCTAGTGAAAATTTTCGGAATCGATCCCCTTTAAAACTCACAT
ACATATAATCTAACTCAGCATGAAACTACTATGTCAAGAACCTAGCGATAGTATTGGTTTACTAGTGTACCATTGGGAACTATT
CATTATGCTCATAATTTAGTCTGGTTGAGTGCAATGTTATCTAGTCATAAATCTATTTTCGGATCGAGGGGACATTCCCTAAAT
TAACTACTATATCATTAGATGCAATTGTTATTCTAATAAATAATTATTTTTCCAAGGGATCGGGATCCTTCCTAAATTCACAAT
ATCGCTAATCTTACTCAGCATGACAACTATATCGAGACTAAGCGGTAGTATTGGTTTAACTTATGTATAGTTGGATGATTCTCA
TTATGTCAATAATATTTGGTCTGCCTTAGTGAATGAATATCTAGTAAAATAATTTTCGGATCGAATCCCTTCTAAATTCAGCTA
GAGACTATACTCTAAGACTCAGCATGACACAAATATGAGCCCTAGGGTAGTATTGGTTTACTAGTGTCCGTTGGACCGATTCAC
TATTTCATAATCTAGAATTCATGCTTGTCGCAATGTTATTAGTCATATTTTTCGGGATCGGATGCATTCTAAATGCACTACTAT
CAGTCTTAGTGCAATGTTCTCTAGGTAATATTATTTCGGGGTAGGTCCCCTTCTAATTCAATACTCCATCTACTCAACATGACT
CTATATGAAGACCTTTGCGGTAGTATTGCTTACTAGTGTAAGTTAGGAACGATTTGATTATGTCAGTATTATGTCTTCTTAGTG
CATGTTATTAGTAATGAATTTATTCG
>m141120_131642_42134_c100732402550000001823141405141505_s1_p0/88600/0_7548
ACCGAGAGACGATATTCTCAAAGATCGACCTTCGTTCTGGTTTATCATCAGTTGAGGGTCAAGGAGGGAGACATACCGAAGACT
ACTTTTCGCACCATGTAATGGGCATTATGAGTTCTTAGTCATGTCTTTTGGATGACTAATGCCCAGTTGCTTTTTATGGATTGA
TGAACAGAGTGTTCAAGGATTATCTGGATCAGTTTGTGATCGTCTTCCATCGATGATATCCTTGTTTATTCCCAGTTAGAGCAG
AGCATGAGCAACATCTGAGGTGGTACTACAGAGATTGAGGGAGCATAGACGTTTGCAAAGTTCAAGAAATGGAGTTCTGGTTAT
ATCAGGTAATCTTTTCTTAGCTAATTGTCAATAAGGAGGGGATTAAGGTAGAATCTAGCAAAAGATCGAGGCAGTCAGAGACTA
GCCCAGGCAAAGAATGCTTCTGAGGTTACGAAGTTTCCTTGGATTGACAGGTTAGTTATAGGCGTTTCGTGGAAGGGTTTCTAA
GATTTCTACTTCATTGACTGAGCTAACTACAGCAAGATCAGAGTTTATGTGGTTAGACGGGTTGTGAGAACAACTTCCAAGGAC
TAAACGCAGAGATTGATCCTACAGCTTCAGTTCTGAGTCTTCCAACAGATCAGGGAAGGTTTGTGTTTACTGCGATGCTTTCAC
CATCAAGGTTTAAGGCTATGTTTGATGCAGTAGGGAAGGTAAATTTTTGCTTATGCGTCAACGTCAGTTGAAAGGAGTAATGAA
TAGGGTATCCCACACAATGATTTGGAGTTGGCGATTATGGTCTTTGCTTTAAAAGGTATGGAGGCATTATCTTTTATGGGTAAG
AAGTGTGAGATCCATTACAGACCATAAGATCCTGAAATACTTCTTCTCCGCAGAAATATTTGAATATGTGACAAAGGCGTTGGC
TGGAGTTAGTAAAATGATTTGATTGTGAAGATTTTGTATCATCAAGTAAAAGCCCCAATGTGGCTACTGATACTTTAAGCTGAA
GGGATCCGGGACAGATTCATAGTATGCAGGCTTGATAGCCAGAGATTAGTAGATGATATGACCAGAGCTGGTATAGAAGTGTTG
TGGCCAGTTGGGCAATGTTTACGCTACTGTCTACCCTGTTAAAGAGAATCAAGGAGGGTAAATTGAATGATCCACAACTATTAA
GATCAGAGAGGGATGTTTTGGCTGGAGCATCCAGAGATTACACAGTGTCTGATTTGGGCTTGCTGAGATACAAGGATCGGATAT
GTGTTCCATTAGACACTGCTAATGAGGCGAGAGATTCTGATGAATCTCCATACTACACCTTACTCTTTGTTTGCATCCAGGACC
ATAAAGATTGTACCAGGATGTGAGATCGTGTATTGGTGGCGGGGAAAAGAGGGATGTAGTAGAGTAAGTGGCCTAAGTGCTTGA
CATGTCACACAGGTCAGGCTGGCATCATTAGGCACGACCAGGGTTATTGTAGCCTCTAGATATCCCAGAGTGGAAATGGAGGA
CATCACGATGGATTTTGTGGTAGGGTTACCCAGTGACTGTTGGTCTAGCATGATTCTATTTGGGTTATATGTGGATCGCTAATA
CAAGTCAGCTCACTTCTTGCAGTGAAGGACTACAATATTACAGTTTGATCAGTAATGCATGATAATCTATGTGAGAGAGATTGG
TGACACCTTTCATGGAGCGCCGAGGTCGATCCATGTCAAGATCGTGACTCCACTTTTACTTCTAAGTTCCTGGGGGAGTTTACA
GACAGGCCATGGAAAACAGTTGAAGTTCAGTACTGCTTTTCATCCTCAGACCAAGATGGGCAATCTGAGAGGACAATCCAAATA
TTGGAAGAGCATGCTGCGAAGCAATATGTCTAGACTTTGGTGGATCCTGGAGTAAGTATCTACCTTATGATAGAGTTTTCCATA
CACAATAGTTATCAGTTTTACATTGGGGTTGCACCTTAATGAGATGATGCATGGGTAGGAAGTCTAAATCTCCATTCATGGGAT
GAGTATAGGTGAAAGGAGATACTTGGGTCCTGAAGGCAGTTCAGAAGACCAGGTGAGGCATTGAGAAGATAATAGAGCTCGGGA
TACTTGCTTCTCAAAGTAGACAGAAAGTTATGCAGATCCCGAAGCGCGGAAACGTGGAGTCTTCCAAGTGGGGAGATACGTCTT
CCCTTAGATTCTCGCCATAGGAAAGGGGTGAAGAGATTTGGAAGAAGGTCCTAGCTGAGCCCTAGACTTTGTAGGTCTATTTGA
GATCTTGGTGAGGATTGGTCCCAGGTGGCTTAAAGGTTGTGCCTACCTCCAACATTGTCGGTCATGCATAACGTGTTTCCATGT
TTCCAGCTCTTCGAAGTATGTATCTAATGTGACTCATGTTTTAAACTATGAAGAATCCTAAGAGTTAAGGCAGATCTCCTCCTA
TGAGGAACAGCCAGTCAAATACTCTACAGAAAAGACAAGGTCCTCAGGAATAGAGACGATACCGATTTGGTTAAGGTACTTGTG
GAGGAACAGCAAGGTCGAGGAAGCGACCTGGGAGCTGGAGTGAGATAGTACGCAGTCAGCATCCCGGGAGCTGTTCAGGTAAAT

TTTGAGGACGAAATTTTTGTAAGGAGGGGATAGGTTGTAATGCCTAAAATTCCTAACTGCAGTTTAATGGTTGGATTAGTAGGC
GAGAGAGCCTAATGGATTTATTATGCCATTGAAACTATTATATGGCACTGTTTATGTGAATTATATTATTATATGGAGTGCTAA
ATGCAAATGCATGTGGGTCACATTCATCATAGGGGTCATTTTTGGTAATTTGGGCCCGTTGAGGGCGTAATTGTTATTTCCATG
AATGTTGATGAAATATTGTTGAGGCCACATCATAATGTGAGATTTATTCGAGCCCAATTCCGACATGAGACGATCTTTGAGTGC
AAATTGGCAGGTTTAATCATAATGGGATAAGTGCGGGCTCGGGGTGAGTCTCGGGTAATTTTTGATTAGAGCATTGCGGGAATA
AAAAGGGTAATGGGGATATGAATATTGGTTATTTGAGTAAATTTTGAGAATAACGGAATTGGAGGGCGTTAATTATGATTAATG
AAAGTAGGTGGAAAAGGATTGTTTTACCCTTGGGGAGCCTTTACAAAACCTTAAATTGACCATTAGGGGCAAAAAGGTCATTTC
ACCTTAAGATTTATATAAGCCATTGAAGGCTATAGAAGTTGGCTAAAACAAGCCATAAATACATTCCTCTGATACTCATTTTTC
TCTCCTTTTTCTTTTTGGATTTTTGGAGCCTTTCTTGAGGATTCAAGCTTAGGAGAAGTGGATCGTTGAGGGCTTAGGATGTGT
TCCACCATTGACAGAGGATCTAATCTGAGCTTGAAGGTAAGTTGAGCCATTGAATACTCTAGCTTTTGCTATGTTTTTATTTGG
TTTTCTGAGATTGGCTGAAGTGAATTGATGGGAGTTTTTTGGCTAGGGTTACTTTGGGTTGTGATGCCTTAGGACATGGTGTAG
ATGGTTTTTTGGTTCAATTAGGAATAAAATGAGGTTTGGAAGCTTTTGGTTCAGGTTTGAAATGGTGGAGTCGAAGGGGGGAAG
TTTTAGGGAGTTCTGGCCTAAGTGTATTGGCTGTGCGCCCACCAAGGGGGCGCAACCAGCGCTACTCAGGAGGGTTTCTGTCTG
CTTAGAGCGCTGGGTGCGCCTAGTGGGGTATAGCGCTACACGCGCTACCTTGTTCCATCAGATTCCTGTCTTCCGGGCATTTTT
AAGGGTTTGGCTCGGGGTTTCAATCCTTAAGGCTCGGGATCAAATCTACTCACCGTTTGGGTAACAATTCGGGATCCCAGGGAG
TGGAGTTTATCAAAGACCCTATTTGTCATGTTGTTGATTTTAATTATGAAGGTTAAATTGGTTATGGTTAGGTAACCGCTAAGG
AATCAAAAGTGCGATTGTTTCTAAGGTCATTCTTTACTATTTCTCGCCGAACCAGAGTAGAAAACTGCACCCCAAATATGATCA
CATGCATGGTTTATTCTTGAGGCATGCTGAATGTTTAATATGTGGACATTGATTGGCATATCGAATGCTTAGAATTTTTTCACT
TGTGCATGGCAACTGACTGAATAGTCAGAATTGGCAATGGTATACAATACTAATTGTGAAGCTGTGACTCATTAGTCCAAGTCG
GCAGTAGTACCTGAGCACTGGTCACATGGTTTGCTAAAATAAAGTCAAGAACGATCTAGCCGTGTTTAACGCAAGCCAATAAAA
GATTAGATTTAATCACATCTGCATTGAATGACCAAATGAGTATTAATTTTTCGGACCGACCTCAAGTTCGAATGAAAACTAAAA
AGCGCTTGTCTGCCCGTACAGGCTAGTCACGTTAGAGACACAAGGGCCGAGAGAGCCCCAGTGACGGTTTAAGTCACTGGCTAT
GGGTGTTGAGCCTGAAGTGACTACCTACAGTCACTCCAGTTGATTACGGAGCCAGAGCCATAAGGGGTCAAGTGACTGAATTTT
CACATGGCTATGGGTGCCGAGCGCCGTAGTGACTTGCTAATCAGATCCACTCATTATGGTTTACCAGAACCTCAATTGTAAATC
ACCATCTGATTAGAAGCTATACACATCCTTCATGGTTAATGTAAACACTAAGTGATATTCAGCTCATCTGTTTAGGTTATAAGC
TCCTGTATGATTATAATGATCCTGCATTTGATAATATTTATAATGTAGTATTGAGTTTTCTTGCTGGGCTTTGGCTTCATGGGT
GCTATGTGGTGGTAGGTAAAGGGATAAGAAAAGCTCACCGCAACCTTGAGTGAGAACTTAGGTGGCGATTGCTGTACATAGAGC
CGCTTGACCACCACGACCAAGGAAGTTGCTCAGAGGAACTAGGGGGTTTACCCTATTTTTTGCTGCTTAGGTCGCGGGTTTGTA
ATTTTAAACTGTAATGACCCATTTTTTGGGGGGGGGATGGTGGTGGCGGGTGGGGGGGGAAGGGGGTTGGTTGGGGGTGTGGGG
GGGTGGGGGTGTTGGGGGTGGGGGTGTGGGGGTGGGGTGTGGGGTGGGGTGGAGGGTGGGTGGGGGTGGAGTGGTGTGTGGTG
GGGGCTTGGGTGTGGGGTGTGGGGGGTGTTGGGGTTGTGTGGGGGGTGTGGGTGTGGGGGGTGGGGTGGTTGTGAGGGTGTTGC
TGTGTGGGGGGTGGGGGTGTGGGTTGGGTGTGTGTGGGGTGTGGGGGTGTGGGGGGGTGGGATGGGGGTGGGGTGGTGGGGTGT
GGTGTGGGTGGGTGGGGTTTGTGTGGGGGGGTGGATGTGTTGTGGGGGGGGGATTGGTTGGGGTGTGTGGGTGGGTGAGGGTGG
TGGGGATGGGGTGGTGTGGCGGGGGGGGTGATGTGTGGTGGGGTGGAGGGTGGAGATAGGTGGGGTGGGGTGTGGGGTGTGGGT
TGGGTGTGGTGTGGGGTTGGTGTTGGGTGGTGGGGGGTGGGGGGTGATGTTTGGGGGTTGGGGGGGTGGTGGCTGTGGTGGGTG
GGTAGTGAGGGTGTGGGGAGGGGGGGCTTGTGTTGGGTGGTGAGGTGGGTGGGTTGTGGGGTGGGGGTGGAAGGGGGATGGGGG
TGGGGGTTGGCGTTGGGGGTGGGGTGGTGTGTGGTGGGGTGGGTAGGGTGGGGGGTGGGGGTGTGGAGGGGTGGTGTGGTGGG
GTGGGTGTTGGTGTGTGGGGGGTGGGGTGTTAGGGGGATTGGGGTGTGGATTGGGGTGTGGTGGGGGGGGTTGGGGGGCGGTGG
TGGGATGGGGGGGGTGTGGGGTGGTGGGGTGTTGGTGGTGGGTGGTGGGGTGTGGTGGTGGGGGGTTGTTGGGGGTGCTGGTGT
GGGGGTGGGGGTGTGAGGGGGGGGTGGGGGGTGGTGGGGGTTGCGGGGGGGGTGGGGGGTGATGGGTGGGTGGGGGGTTGGTGG
GGGTGGGGTGTGGGGGGTTGGGTTGGTTGGGTGGGGGGTGTGGGGTGGTGTGGGGTGGGGGGTGTGATGGTGGGGGTGTTGATG
TGGGTGGGGTGTGTGGGTTGGCGGGGGTGTGGGGGTGGGGCGTGGGGAGGGTTGGAGGGGGTTGTGTGGGGGTTGGCGGTGGGG
GGAGGGGTGTGGTGGCGGAGGGGGTGTGGGTGGGGGTGGGGATGTGTGTGTGTGGGTGGTTGTGGTGGTGGTGGGTGTTGTTGG
GTGGTGGGGTTGGTGGGTGTGGTGGGTGTGGGGGTGGGTGGGTTGGGTGGTGGGTGGGTGGGGTGTGGGTGGGGGTGATGGGTG
TGGGGTTGGGGGTCGTGGGCGTGTGGGGAGTGGGGGTGGGGGTGTGGGCGTGGTGGTGCTGGGTTGGTGGTGTTGGGGTGGTGG
GGGTGGTGGGGGTGGGTGGGGGTGGTGGGTGGTGGGGTGGGGTGTGGGAGTGGACGTGGAGGTGGGTGGTGGTGGGGTGGATTT
TTGGGGGGGGGGGGGGATGGGGGTGTGAAGGGGGGTGGGGGGGGGGGGTTTGGGGGTGGGTGGGGTTGGGTGGGGGTTAGGGTG
GGGAGGGGAGCGGGGTGTGTGGGGGGGGTGTGGTGGGTGTGGTGGGGTGAGGGGGTTGGGGGGGGGTGTTGGTGGGGTGTGGGG
TGGGGGGTGGGGGTTGTGGGTGTGGTGGTGGGGATGGTGGGTGGATGCGGTGGGTTGGGGTGGTGGGGGGGTGTGTGGTGGGGC
GGGTGGGGGTGGGGTTGAGGGGGGTGTGTGTGGTGTGTGGTGTGGGGGTGGGGGGGTGTGGGGATTGGGTGGTGTGTGGGGTGG
GTGGGTTGGGGGTGTGGATGGGGGGGGTGGGGTGATTGTGGGGGTGGGGTGGGTGGTGGGTTGTGTGGGTGGTAGTGTGGGGTG
GGGGTGTTTGGTGGGGGGTGGGGTGGTGGGGAGGGGTGAGTGTGGTGGGGGTGGGGTGTTGATGGGGGTGGGGGGTGCGGGGTG
GTGGGGGGGGGGGGGAGGTGGTGGGGGGGGGGGGGGGTGTGGGTGTGTGGAGTGGGGTGGTGGGGGTGGTGGTGGGATGTGATG
ACGTTGGTTTGGGGTGTGACGGGGTTTGGGCTGGTGGTGGTGGGGAAAGTGAGGGGTGGGGGTGGTGGGGTGGGGGGTGTGGGG
GTGTGGGTGGGTGTGTGGGTGGGGGTTGGTTGGGTGTGGTGGGGGGTGTGGAAGTGGGGTGGGTGTGTGGTGTGTTGGGTGGGG
CTGGTGGGGTGGGGGCTGGGGGGAGCGGGGATGGTGGGGGTGGGTGGGGGTGGTGTGGGGGGCGGGTGTGTGTTGGGGGGTTG
TGTGGGGGGGTGGTGTGGGGGTGTGTTGGTTGGGTGGGGGGGGGTGGGGAGATGTGGGGTGGGCGGGGCGATGGGGTGGATTGG

GGGGAGTGGTTGGGGTGTTGGGGGGGGGTTGTGGGGGTGGTGGGGTGGAGGTGTGGGGTGGGAGGGGGGGGGGTGGGGTGGGGT
TGGGGGATGTTGGGTGGGGGGTGTTGGGGTGGTGTGGGTGTGGGTGTGGTGTTGGTGCGGGTGGGGTGGTGA
>m141120_131642_42134_c100732402550000001823141405141505_s1_p0/88681/1611_9175
GGTTGTGTGTCTTCGCGAAATCTTGGCCTCTTTAAGAGGCCCATACTTGGTTATCGCGCTGTGTATTACTAAGAATTTATCTTC
CACAGTAAGAAATGTAACCCGTGCCATGTTACATTCGTATACGTACTAAAAATAGACCCCACGTTGTGGTGCCCTCTGACTCTT
GATTTCAGAAACTGAAACAAACATAATAATATTACTTGTAGAGAAAACCAATGACTGCCAAGGTACTTACTTTTGCTCTGTCCA
TCCATTTTTCTTATACGATATTTCTACACTAAGACACCTGGATCTATTCCGTTGCGATAAAAATTCCGAGAACATTCCGAGTGG
TTCACACACCATTTCATGTATTCATGTATTTACGCTTCCAGAGATCAGAGTCCTGCGGATGGACATACAATGGGATCCCCTGC
ATATATGTAACACAATAATGCTATTGCCATCCTGCAGGTAAAAGAAAACACCCGTCAAATAAGGCCTCGGTAACACACACCAA
CGTCCGTTTACGTGCCAGGTGGGACCGGCCCTTGGCCGTCGGTTCCCTTTATTATTTATTACTCCCGGACCCCATACCGTTCGT
CCGGGGTGGGAGAGAGAGAGAGGGACCATAAAGGAGAGGAAAATATCAAAAGGGCTCGTTCCTTTATATATATAATACAGACA
CCACAGGACGTCGTTCGTAACCGAGGAAAGGTGGGCCAAAAAAAGGCCTAAGGGCCTCGTCCTCTTATTTATAATAACCATACC
CGGAACCATCGTTCCGTAACGGGTGGGAACGGCCCTTAAAAACGGAACATCCGGTCCTTTATCTTATACAACAACACAGAGAG
AGAGAACATCGTTCGTACCGAAAGACAGAGGTGGGAGACGCGGCCCCTTGGCTCGAGAAGAGATATATCCCTTTTATCTTTAT
ACATACACGGAAACAAGTACAGAGTAAATCATACCGGGGAGAAGATAGACCGGCGCTTGGGAAACTCGGTTCACTTTATTTATC
TTTTTCACATACCGGACATCATTCCGTCCGGGTGGGACGGCCCTTCAGGGCCTCGGTTCCCCTTTATTCTGTCACCCCGGACGT
CGTTCGTACCGGGTGGGACCGCCTTGGGCTCGGTCCCCTTTATTTTTATACTCCCGACATCGTCGTCCCCGGGGTGGGACCGGG
CCGTTGGGCTCGGTTCCCTTTATTTTTATACCTCCCGGACGTCGTTCGTCCGGGTGGGACCAGCTGGGCTCGTTACCTTTATCT
TATCACCTCGCGGACAACGTTCGTTCGGGGGGTGGACCGGCCTTGGGCTCGGTTCAAATTTATTTATACCCCGGACCCGTCGTT
CCGTCCGGGGTGGGACGCGGTTGGGCTCGGTTCCCACCCTTTATCTTTATAACTCCCGGAACATCCGTTCCCGTCCCGGGGTGG
GACCGGCTTGGGCCTCCGGTTTCCCTTTATTTATACCCCAGACGTCGTTCCGTCCAGGTGGGAGCGGGCCTTGGCCTCGTCCTC
CCCTTTTTTATTTTTATACTCCCGGGACATCGTTCGTCGGGGTGGACCCCGGCTTGCCTTGGTTCCCCCTTTATTTATACCCCG
GACGTCTTTCGTCCAGGGTTGGGACCGGCCCTATGGCCGGTCCTCTTATTTTTATACCCCGGAGCAATGAGTTCGTCGGGAGGT
GGGACCCGGCCTTGGCTGCCCGGTTCCCTTTAATCTTTATACTCCCGGACATCGTTCGTCCGGGGTACGGACCGGCCTTGGGCC
TCCGGTTCCCTTTATCTTTATACTCCCGGAACATCATTCGTCCGGTGGTGGACCGGCCTTGGGCCTCGGTTCCCTTTATTTGTA
CCCCCCGGACGTCCCGTTCGTCCGGGTGGGACCGGCCTTGCTCGGTCTCCTTATTTTTAATACCTTCCCCGACATCCCATTCGT
TGGTGGGAACCCGCCTTCGCTCCGGTTCCGCTTTATTTATACAACCGGACGTGTTCGTCGGGGTGGGACGCGGCCTTGGGCTCG
GTCCTTATATTTTTATACCTCCCGGACATCCCCGTTCGTCCGTGGAACGGCCATTAGGGCTCCTCGGTTCCCCCTTATCTTTAT
ATCTCCCCCGACATCGTTCGTTCCCGGGGGGTTGGGAACGGCCCCTTGGGCTGCGGTTCCTGTTTATTTATACCCCGGCGGACG
TCGTTCCGTCAAACGGGTGGCCCGACCCTTAGGGCTCGGTTCCCCTTATCTTTAGACTCCCGGACATCGTTCGTCCGGGGTGGA
AAAGGGACGTTGGGGCTCGGTTCCCTTTTTTATACCCGGACGCCGTTTCGTACCAGGTGGACGGCCTTGGGCCTGGTCCCTCTT
TTTTTTATTTTTATAACTCCCCGGGACATCGTATCGTCCGGGGGTGGGGACCGGCCTTGGGCTTGGTTCCCCTTTATTTATACC
CCGGACCGTCGTTCCGTCCGGGGTGGACGGCTTGGGCCCGGGTGGGACCGGCCTTGGGCTCGGTCCCTCTTATTTCTAATACCT
CCCGGACATCGTTTCGTCCGGGGGGGTGGGACCGGCCCTTGGGCTCCGGTTCCCTTTATCTTTATACTCCCCGGCATCGTTCGT
CCGGGGGGTGGGGACCCCGGCCTTGGCCCCTCGGTTCCCCCTTTATCTTTATAGCTCGCCGGACCATCGTCGTCCCCGGGGTAA
GAGCCAGGCCGCCTTGGCCCTCGGTTCCCTTTATCTTTATACTTCCCCGGACATCATTCCGTACCGGGGTGGGGACTGGCCCTT
GGGTCGGTTCCCTTTATTTTGTACGCACGGACCGTCGTTCGTCCGGGGTGGGGACGGCCTTGGCTCGGTCTTTATTTATACTAC
CCGGACATCATTCGTTGGGGGTGGGACGGGCGCTTTGGGCTCGGTTCCCTTTATTTATACAGGACGTCGTTGTCCGGGGTGGGA
AGGACCCGGCCCCTTAGGCTCGGTCCTCTTAAATTTTTAATACTCCCGGACATCGTTCCTCCCGGGGTGGGACGGCCTTGGGCC
TCCGGTTTCCCCTTCTATCCCTTTATACTCCCGGACATCGTTACGCGGGTGGGACCGGCCTTGGGCTCGGTTCCCGCTTTATAT
TATACCGCCCGGACGTCGTTCCGTTCAAGGGTGGACCGGCCCCTTGGCTCGGTCCTCTTTTTTATTTTTATACTCCCGGACAAT
CGTGTCGTCCCGGGGTGGGACCGCGGCCCTTGGTGGGACTCGGTCCTCTTATTTTATACTCGCCCGGACATCGTTTGTCCGGGG
TGGGGACCGGCCTTGGGCCCGTTCCCCTTTTATTTTATACTCCCGGACCATCGGTTTCGTCCGGGTGGGACCGCCTTGGGCTCC
GGTTCCCATTCCCATTTAATACCCCCAGGACGTCCGTTCGTCCGGGGTGGGCGACCGGCCTTGGGCTCGGTCCTCTTAATTTTT
AATACTCCCGGACATCGTTCGTCCGGGGTGGGACCGGCTTTGGGCTCGGTTCCTTTATTTATACCCGCCGGACGTCGTTCGTAA
CCGCGGGGATGGGACCGGCTTGGAGGGCTCGGATCTACTTAAAAATATTAAAATAACTAACCGGCATCGTTCGTCCGGAAAAGA
AAGAGCCGGCCTTGGCTCGTTCCCCTTTATTTTTATACTCCGCCGGACATCGTTCGTCCGGGGGGGGACCCAAAACCTTGGGCT
CCCCAGGTTCCCTTTAATTTTATACCCCGGACGTTCCCCGTTCGTCGGGTGGGACCGCCTTGGCCTCGGTTCCCTTTAATCTTT
TATACTCCGGACATCGTTCCGTCGGGGTGCGGAACCGGCCTTGCGGGTTCGGTTCGCCTTTAATCTTTATACCCCCGGACATCG
TTCCGTCACGGGGTGGGACCGGCCTTAAGGGCTCGGTTCCCTTCTATTTATACCCCCGGACGTCGTTCGTCGGGTGGGACTGCG
CTTGGGCTCGGTTGTCCCCTTTAATTTTCCACCCGAGTCGTTCGTCGGGGTGGGAACCCGCCCTTGGGCCTCGGTTCCCTTTAT
TTAATACCCCCGGACGTCGTTCGTCCCGGGTGGGACCCGGCTTGGTCGGTCTCTTTTTTTTTATACTGCCCGGACTCAATTCGT
CCGGGGTGGGGACGGCCCTTGGGCTTGGTTCCCTTTATTTTATACCCCCCGGAGTCGTTCGTCCGAGGTGGTGGACCGGCCCCC
TTGGTGCTCGGTCCGCTCGTTTATTTTTATACTCCCGGACCATCCGTTCGTCCGGGTGGGACCGGCCCCTTGCGGACTCGTTCC
CTTAATATTTTATATCTCCCGGACATCGTTCGTCCGCGGGGGGTGGGAAACGGCCTTGGGCTCGGTTGCCCCTTTATTTATACC
CCCCCCGGACCCGATCGTTCGTCCGGGGTGGGACCCGGCCTTGGGCTCGGTTGCCTCTTATTTTTATAAGGCAAAATCCCCGGA
ACCATCGTTCGTCGCCGGTGGGACCGGCCTTGCCTCGGTTCCCTTTATTTATACCCCCGGACGTCGTTCCGGGGTGACGACCGG
GGCTTGGCTCGGTTTCGCCCTTTATTTATAACCCCCCCGGACGTCCGTTCGTCCCGGGGTGGGACCGCCCCCTTGGGGCTCGGT

CCTCCCCTTATTTTTAAATACCCTCCCGGACATCGTTCGTACAGGGGTGGGACCGGCCCTTTGGGCTGCCGGTTCCCCTTTATT
TTTATACCCCGGACATCGTTCGTCGCGGGTGGGACGGCCGGCTTGGGTCGGTCCCTCTTATTTTTATAGCTCCGGACATTCGTT
CGTCGGGGTGGATTCTTGGGCCCTCCGGGTTCCCTTTATTTCTTTATACTCCCGGACATCCGTTCCGTCCGGGGTGGGGACCGG
GAACCTTGGGCGCCTCGGTTCCCCTTTATCTTTATACTCCCGGACATCGTTCGTACGGGGTACTAGCGGACCGCCTTGGGCTCG
GTTCGCCGTGTTATCTTTATACTCCCCCGGAACCATTCATTCCGTCCGGGTGGACGGCCCCTTGGCTCGGTTCCCTTTAATTT
GTAGCGCTCCGGACCGTCCGTTCGTCCGGCGGGGTGGGACCGGCCCTTGGGTCCGGTATCCCTTTATTTTTATCCTCCGGACAT
GTTGTCGCTGGCGGTGGGACCAGGCGGTTGGGCTCGGTTCCCCTTTTATTTTTATACTCGGAGCGTCGTTCGTCCGTGGGACCG
GCCTTAGGCCTCGGTTACCTTGTAGTCTTTATATCGCGGACTTCACCAGATCCGTTCGTCCGGGGTGGGAAGGCCGCCCTTCTG
GGCTGGTCCCGTTTATTTATAACCCCCCGGACCCGTCCGTTTCGTCGGGGGTGGGACGCTAGGCCCTTGGCTCGGTTGCCCCTT
TATCTTTATATTGCTTCCGGACCATCGTTACGTCCGGGGTGGGAAACCGGCCTTTGGGCCCTGCGGTTCCGGGCCTTTTTATTT
AATACCAGGCGCCGGACGTTCGTTCCGTCCAGGAGGTGGAGCCGGCCTTGGGGGCCTCGGTCCATCTTTTTTTAAATTTTTATG
CTCCCGGCAGCAATCCGTTCGTCCCCGGCCGGGGTGGGAACCGGGCCCTTGGGCCCCTTGGTTCCCTTTATTTATATACCTACA
GGACGCGTTCGTTCTTCAGGTGGGACCGGGCCTTGGGAGCCCCCTGCAAAGGTCCTTTCATCTTTTATACTCGGAGCCATCGTT
CGTGCTACCCGGGGTGGGAAACGCCGGCCCTTGGGCCTCGGTTCCGCTTTATCTTTATCACCTCGGCCAATCGTTCCGTCGGGG
TAGAGCCGGCCCTTGGCTCGGTTCCCCTTTATCTTATACTACCGCGGACCATCATTCGTCCGCGGGGTGGACCCGGCCTTGGGC
TCGGTTCCCTTTATATTGTTACCCCGCGGACGCGTTCCGTCCCCGGGGTGGGACGGCCGGCCTTGGGCTCGGTCCGCTCATATT
TTATATAGCCTGCCGCGGACAATCATCTCTCTGGGTGGGACCGGCCTTAGCTCGGTTTCCCTTTTATTTATACCGCCCGGACAG
TCGTTCGTCGGGTGGGACCGATTGGAGCTCGGTGCCTCTTATTTTTATACTTAAACGCCGGACAGTCGTTCGTCGGGGGTGGGA
ACCGGCCCTTTGGGCCCCTCCCCGGTTTCGGCCTTCCTAATCTTTATAGCTCCCGCTGGAGCCATCTCGTTTCGTCTCTACCGG
GGGTGAGGAGCCGCGTGGCGTTTTGGGGCTCGGTTCCTGTTATTTTATTATTTCTCCGGGTCACGTCGTTCGTTCCGGAAGGGT
GGGTTACGCCGGCCCTTATATGGGGGCTCGTTCCCTTATACTTATATCTCCGGTTTTATCATCTTTCGTTTCCGGGGTTGGACG
CTGGTCCTTGGGCCTCGGTTCCCTTTTATTTTTATTTTACCGCTCCCCGGAGCGTCGTTCGTCCAGGGTGGGACGCGGCCCCCT
TAGGGGGCCCTCGTCCTCTTTTTTTATTTTATTCTCACCCCGAGAACATCGTGTCCGTGCCGGGAGTGGGAAAGGCCCGGCTTC
CGGGCTTGGTTCCTTTATTTATACGCCGGAGCGTCGTTCTTTCCGGGATGGGAACCCCGGGCCATTGGGCCTCGGTCCTCTTAT
TTCTATAGCTCGCCGGACATACGTTTCGTCGGGGTGGGACGGGCCCCTTGGGGGCCTCGGTTCCCTTTATCTTTAATACTCCGG
ACATCCGATTCGTCCGGGGTGGGACCGGCCTTCAAAAGGAGGCTCGTTCACTTTATCTTTATACTCGCCGACATCGTTGTCCGG
ATAGCCGGCCTTGGGCCTCGTTCGGCCCTTTATCTTTATAATCCCGGAGCATCCATTCGTCGGGGTGGGACTGGCCTTGGGCCT
CGGTTCCCCCTTTATTTGTACCCCGAATTCCGTGTTCGTCCGGGGTGGGACCGGCCTTCGGGCCCTGCGTCTCCCTTATTTTTA
TACTCTCGCGCTCGCGGCACATCATTCGTCTGGCCGGTGGGACCCCCGGGCCTTAGGGCTCGGTTCCCTTTATTTATACGCGGC
CCCGGAAGTGCGTTCGTGCCGGGGTGGGACGCAGGCCTTGGGCTGCGGTCCTCTATATTTTTATAACTCCGGACATGACGTTGT
CGGTTTTTTTTGTGGACCTGGCCTTGGGCCCTGCCGGTTCCCCTTATAATCTTTAAGCTCCCGGACATCGTTCGTCCCGGGTGG
GAAAACGGCCCCCTTGGCTGCGGGATTCCCTTTATCTTATACCCCCCGGACGTCGTTCGTTCCAGGGTGGGACCGGCCTTGGGC
TCCGGTCCTCTCTTTTTTAATTTTTATAACTCCGGAATCGTTCGTCCCGGGGGTGTGGACCGGCTTGGGCCCTCGGTCGCTGCT
CCTTAATTTTTTATACCCTCCGGACATACGTTTGTCGCGGGTGGGACCGCCTTTTTGGGGCCTCCCGGTTCCTTTTAATTTTTA
ATACTGCCGCGACATCGTTCCGCTCCGGGTGAGGAGCCGGCCTTGGGCTGCGGTTCCCTTTATTTATAACCCCCGGACGTCCGT
TCGT
>m141120_131642_42134_c100732402550000001823141405141505_s1_p0/97888/0_16525
TACTAAGAGAAGGAGGCGAGACGCACATTCTATATGAGCTTCTTCTGCTGGACAATAGAGGAATACCTTGTCAATGACTGAGCG
ATCCTCCTAATTCTATGTGAGTAGAGAGAGTGACAAATCCGTTTGTTGATTCATGTCAACTTGACAATGTTGGATCCAAGAAGC
AAAAACCTGAATAGCGACATCTGAATAAACCACTAAAAATCAAAAGAAACCTTTGTTACTATTGCGAATCACACAAACACAAAT
AGAAAGAACAAAACAAAAGGGATGTTGTCGCGGTGAATCATCATCATGAAGGTGTTGTAGAATTGGACCTAACCTAAGGAGATA
AATGAATCTCTACAATCCAAATGTGTGCGATATATCAACTTTCCGTATAGGTGAATAAGATGGATTTCACTCATGAGAAAAAGT
TTAAGTGTGTGATATTGAAATTTGAACCAATCAAAATAAGCTCCTGTCCTCACTCGATTAAACCTATCATCACCCAAAAATGCT
AAACATAGGTTATCCAGACGCAAAGTAAAGAACCATCCCACCTACATAAAGGCAATCGAAAAGAACAGACCCGCCTCTCAGGAT
ACTCTGAAACAAATGCGCCTTACACAAAGCGCCAATAGGGCATAAGAGCACCTAAGAATTCTAACTAGTCACACACTCCATTGG
AAGTGTTTCTGCCGTATATGGATCACGACAAACAAGAAATTTCACCGATGAACTACCAAAATAGAATTCGAGATACGGCGAACA
ACAAAATCAAGAGAATCTGAAACATGCGTTCTACTGTCTTATGTTACGCCCAGTATCACTTTATAGGCCCATGTATGTTATTGT
GATATTTTTTGAGTGAATCCATGTATAGTCTCTCATAGTTGAATCAACTCGAACATATACAGTGATAATATATGAAGCAAGGAA
AGAACTATTAAACGATCAATCAATCCGAGAAAACTCAATATCAACTGAATTAGAAGAGTCGCTTGTGTGAATTTAAATTCACCG
AAGCATGCGAAAATAGAAAGCACATGGTCTGAGCAGATAACAGAGCACTAACCAACATCTAAACACAAAAGAGAGTCTAGAGTA
ATTCACCTCTCTATCCTTCACGATAGAGCGAGACTATTACCAGATACGTAATTTCTACAGTTGTAATTGGCGAGAAGCAGTAAT
TTACCAATAAACTCCCCTCCCAGGAACCGGAAGTTTCGTTCTAAGACAACTAGAGAGTACACTAAGAACCCCCGCCAAGGGTAA
CCCTGCTACAAACAGGGACCTATACTTGGGGAAGGGGTGCCGATTAAACTTTTTGGAGGCCATGGTAAATCCCACCCCAGGGGA
TGTTACAAGATACTATTTTCTATGTCATTGTCAGATCGATCTGAGCTATATTGGTAGATACATATGTCTCAAAAACTACTGGAA
TGCGTGAACACCCTCCGCCGGCTCACTCCTTATGAAAGCCTATTGTCAGTCCGTTGTAAAGCAACCAAAATGATCTGTTTAAAG
CATTTAGAGCACAATATTATGTGTAGTGTTCTTCAAATTAATTTATTCATATAATTAGTATGGAAAATCAGCAACCCTAGTCTA
GCCTCGATTAGCTTTATCAATAAGCGTTGATATGATTTTCTTATTTTCTGTTATAAGTTTGCATACTTAATTTTACCTAGAGGA
TAATCTCTGTTCAACAAGGACTTGTTCCCCTTCAGGACCTATCAACCCCATTCGATATAATGTTCGATTCTGGTTCTTGAGGGA

CCTATTGACCCCATAAATCCAACAAAGACTAGTCCTAGGACCTTTGTCGCCCAAACCTATTAATGTATAGATCATGCTTGAAAT
CAACATTTGTCTCTTGATAGATGTCCTGAGCGACAACCTCTCCAACACAATCAACAAACGGAGAAAACCGTCCGAGTTCTAAGG
ACCTCGAGAACATCTGGCGGCACATAAGTTTGAAGTAATTGGAGACTCCTCACATATTAGTCCGTATGTGTCGGTCAACAATGT
AGTATATACTTCTTAACATCCCATAGCGAATCACATAACTTATCGAGATTTTTAGGTTCTCTATGGTAGGACTTCTCGCCAAAT
TAATGGATCATGTTCGAAAACATATAGGTTCTTAAGGAGCCCTTATTGATCCCAACAATAAACAAACAAGCGAGAGACAGGTCC
TACGACTTGTCGCCAAATTATGGATCATGTTCGATACATTGGTTCTTGGGGAGAACTATGACCCATATGATCCCCAAACAAGAG
ATAAAGGTCCTAAAAATAATGTCGCTAAATTGATCACGTTCATCCTGGTTCTGAGGAACCCTAATCCGACCCATACAATCAAAC
AAAAGAAAAAAAAAAAAGACTGTCCGAGGAACCAGTCAACCATTATTTGATCAATGTTTTTATCCTGGTTTCTTGAGGAACCATC
GACCCCAATATAATAAATATAACAAAAAAAAAACAAGAGACCTGATTGAAAGACATCGCCATCCCATCAGTCATAATCAAATCT
GGTCCTTGAGGGACTGATTGATATTTGATCTAAATACTCGTTACAAGCTCGATTAGCCCATCTAGACTAGGCATCCAACTTAGA
CACTTCTGTCTACAGTCATATATAACGAGTACAAGAGAGATTACGTAAGAGAACGAACACTTGCTATAACAATTTGCATCTGTG
TATAGGTATCATTCTAACTGAGTATGAAACCATCACCCAACTACCAACGATGGACAACATTTGCTGCTTGCATCAATGGGTGAA
AAATGAATAAGTACTATGTGTCTAACGCTTCGAAGCAAGGCATGGTCAAGTAAGGCAAGTATGGCTGGTTATTGTATCATGAAT
CGTTATACTTTTGTAAACGATCTTTGAACTTGTATTTTTGGGATCCTATGTAAACAAATAATGTTTCTTATAATTAAAATGTGG
CTTTCGAGACAAACCAAACCATTTTAAACCCTAGTTCCTTTACAGTTTAGTAACAACGATTTTTAACTGATTGACTTGGTTAGC
CGAAGTCCTGACACTTTATAAACACACAGTGTAACGGGTCCTTGGGCTATCCCAGGACGTTACAACTTGGTATCAGAGCGTCCT
AGTTTATAGGTCCTGAAGACCTGACTGGAATATGTAAACATTCGCTGGCGGGAGAACAATCTCAACTCAGGGTTTTGGTATTTG
TTGTATGAATGACTTATGTGCCTAAATGATTTGAGTGTGATATGATTGGTTGATATCCTGATTGATGAGAAGGGAGCAATGAAG
ATACGATAGGCCTGGCCCCTTGACTATTGCATGATGTTATTGAGGCGTGCTTATTAGCCATTGCCTATGCATGTGAATGAATAT
CTGGATACAATGCTATACTTTGATTGCTTGTGAATGCATTTGGAGTTCCAGTTGATGGAATTAATGAAAGGATTATTACCCTAA
TCATCAATAGCCTAATTGGCCGAGTGTTGATTGCCAGATTGATATGGAATGTTATGCGTTCAGGAAATCCCACACGCTAGCAGC
AAGTCCTGGGCATAGAGAATGATGACCAGGGCCAGACCCTCCCGCCAGTCCCTGAGAACTAAGCAGCAGTTAATGCCCGACATG
CAGCCCGAATTCAGACCAAGATGAAACAGATTAGAATTCTGCCAGCAGGCTCCATCTGGGAAGTGTTGCCCCTGCTGTGGCCAC
CGGCAATGGTAACCTGTGGTACGCCCAGTTAACTGTTATGGAACAAGAGGGAATCGTTGTATGAGCAGTTCAAGAAAGCAACAA
CCCCGATCTTTGAAGGTGGCAGAATCCACTGAAGGCTGAGCAGTGGATGACTATGATCACTAACTATACCTTGATTTTATGAAG
AGTGGAGAGGATATGAAAGGGTGGGCTTGTGCACTTATATGCATGAGGGAGGATGCCCGAATTTGGTGGAGGTGGGCAATCACA
GACTAAGGATGTTGAGGCCCATTGACTTGGCAAGGGTTAAGGACCCTGTTTATCAAGAAGTATTATAATGTTTCCATCAGGGCA
GCGAAAGTGAAGAGTTCGTGGGGCTGGTTTAGGGCCAGTATGGACAGTTACCAGAATATGGGCCTTGGAAGTTTGATAGACTTG
CAAAGTTTGCACCAAATATTGTGCCCTACTGACGCAGCTAGGCAAGAACAGAATTTACCAGAGGGCTGAAAATGAAATATGATA
GCCCGAGTGTAAGGATTACCACAGTTCCTGGAGGGACTACTTATGCCCAGGGCTGTGAGAAGGCTTCTTACCGCTGAGGAAGCG
GAGAATAAAAGATATGGAGGGAAGAGTGGCTGTGAGAAGGGAGGGTCCGCAGGGCGGTGCCCTTCCATATTCTGGTTCTGGTAG
GGGTGAGGCCTCAGCGAATTGAAGAGAAAGACTCCTGATGCTCCGAACGCTCCTGGTCCTGATAGGAGAAGGTCCGGGGTACTC
TGGGCGGCCATCAGGGAGGCGGGTGGATTCATGGAAGGGACTTATCCAGAGTTAACGAGGTGCAAGAGACGCCATCCCCGGGCC
CGAGGTGTCGGGCTAAGGCCTGCTATGTGTGCAGGGTTGGTGGACACCTCAGGAAAGGATTGCCCGACAGTGAAGAAGGAGAGA
CAGGAAAGGTGGACTGCTTGACTCCAGCTCGAGTGTTCCCACCCTGACACGAGCGAAGGCCGAGGCTAGTCCCTCGGTAGTGAC
AGGTCAGCTTTTCTAGTGCTGGCATTCCTTTTACCTGTGTTGATTGATTCTGGTGCTAACGCATATCATTTGTATCTGAAAAGG
TTGATTGATAGACTGTGTAGACCTAGTGAGTATAGTACTTCAGGGTTTGAAACTATTTGCCTACAGAGAATTAGTAAGTATCCT
AGGAGGTGGATTAGAGCACTGCCAGTGATAGTTTGATGGTAAGGGAGTTATCTGTAGACTTGATTGAGTTTGGGTATGGAAAGA
TTTTGAATATGATTTTTAAGGTATGGACTAAGGCTGGTTAGGATATAGGAGCTACATTGACTATAGGAAAGAAAGATGGTACTT
TTTGAGCCCCAGAGGGCGAGGATCCCTTCGTCTTTGTGGGAGCGTGCATGGACCCCGCGTACCCGGATATCTGCCCATTGAGAG
CCAGAGATTTCTAGGGTGATGTATAGGCTTTTTTTTTTTTTTTTTTTGGGGGCCCTAGTGTGGGGTTGGGGATTTTAAAGCCAC
AACAGAGGTTTTTTTTTTTTTTTTTTTTTTAAAATGCAAAAGTAAAAGGGGAAAAACCCCCCCGGCAAAAGGGACCCCCAACC
AAGATTTTTTTTGGGTTTTTGAAAACCCCGAGGTTTTTTTTTTGGGGAATTTGGTGTTTTCCCCTGAGGGAAATTTTTTTTT
TTTCCCCCAAAGGGGTTTTTTGCCCCCCGCCGAGCCCCGGTAAAAAGAGGGAAGGAATTCCAGTTTTTTGGGGTTTTTTATTTG
GAGTTTTTTGGGGGCCAAAAACCCCAAGGGGAAAAAAACCCAAAAAAGAAGCCCCCCAAGGTGGTTCCAGGGCAACCCAAATAT
AAGGGATGGCCCCAAAAAAAAAAAAAAAAACCGGGCCCCCCGGGGGACAAACCTGAAAAAAGAGTTTTTTAAAAGATTAACAGC
CCCCTTGCAGGAAAAAAAAAAAAGGCCCCTTTTCCTTGGGGGATTTTTTTTTGGGGGGTTTTTTTTCCATCAGACCCTAAGCCT
TTTTTTTTTTACCTACCGCCAATGGGGGGGTTTGCCCTCCACAAGTTTTCTGTTTTTTTGGTTTTTTTAAAGAAAAAAAAAAA
AGAAAAAAGGAAAAACCCCGGGGGAAAACCCTTTTTTTATTTGAGGGGAAAAAATTGGGGGTGCCATTTGAACCCCCCCTTACC
CAAAGGGGAAAAACCCTTGGGAAAAACCCAAGTTTTTTAAACCCATTAAGAGAAATAAAGGTAACCCCCCCCCTTTTAAAAACC
CCCCCCCAAAAAAAAGGGAATTTTGAATTTTTTTGGACCCCCTTGTTTTTTTTGGGGAAATTAAGCCTTTTTTTTTTTTTTTTT
TTTTTTTTTGCCAAAGGGTTTTTTTAAAAGACCAGGTTTGTTTCCCCTTTCAAAGAAATTGGAAATTTCCCCCTTTTCGATTTT
CTTTGGTTTAAATCCAAAAAAAAAAAAAAAAAAAAACCAAAAGCTGAAGGATTCCAAACCCAAAAAGGGGGGGGAAAAAAAATTGG
AGGGGAAATATAACCTTTAATAAGAAACCCCCGGCTTTTTTTTTTTTTTTTTTTTTTTTTTTTTTTTTTTTCAACCGGCCCCGGTA
AATTTTGGGCCCCCAACCTTAAATGGAGATTTTTCTTTTGGTCCCCCAAAATGTCCCCCTTTTTTTTTTTTGGGTTTTTTGGAA
CCCCCAAAATGCCCCCCCCCCCAAGCCCCAAAAAGCCCTTTTTTTTTATGGAATTTTTCCCCCCTTTAAAAATTGAAACAGGGT
TTTTTGGTTTTCCAAAGGACCCTTCCCCCCTAGAATTTCAAAAGTTTTTTTTTTGGGTTTTTTTTTTAAAATAAAGTCCCCTC
TTTTCCAATCCGAATTTTTGGACCAATTTCTTGGTTTGGTTATTTTCCCCAAGTTTTTTCAGAGGGCCAAAAAGAGGGGCCCCA

ACCCGAAAGTTTTTTTTCCAAAAATCCTTTTTCGACCCAGGTTTTTTTTTTCCCCCCCCCCTTTTTTCCCCCCCCCCTTTGA
AGGTAAAGAAAGAAAGCAAATTCAAAAAAGTTTTTTGGTAAAACCGCCCTAAAAGTTTTTTTTTTAAGAAAAAAGGGTTTTG
TTTGAAGGTTTTTTTTTTCCCTTTTAGTTTAACCCCTATTGGAAAGGTGACAATTTCCCCCCTTTTTGGAAATACAAAATTTTT
TTTTTTTTTTTGGGGGCGCAGAAAAAATGGGAAAATTTTTTAAAAAGGGTTTTTTGGGAAAATTTTTTTTTTTTTTTTTTTT
TCCCAAAAAAAAAAAAAAAAATCTTAAAAAAAGAAAAAAAAAAAAAATAGAAGGGCCAGTTTTAAGGGAAATTTTTTTTGGGGC
CCCCCCCCCCCCCCCCCGAAGGCCCCCCCCCGCCCCCGGAATTTGGGGGCCCCCTCGAAGGGGTTGGCCCGGAGTTTTTTTTTT
TTTTTTTCTGGGGGGTTTTAAGCAAGGTTTTTTAAACTACAGGACCCCGGGGTTTTTTTTGTAGGAAGGGGTTTTTTTTT
TTTTTTCTTTTCCCAAAAAAAAGAAAAAAATAGAAAAAAAAGCCCAAAACAACCCGATTGACCAAAGAACTAAACCCAAAAAAA
GAAAAAAGAAATTTGGGGAAAAAGTTCAATCCTTGGTCCCCCCCAGGGCCCCCCCAAAAAAAAAAAAAGTGGGGGCGAAGAAC
CCAGCCTTTCCCCAAGGAAGTTGAAGCCGACCCCGGCCTTTTTTTTTATTTAAACAAAAAGGCGCCCCAAAGTGTTTTGGAAGG
GTACCTTTTTCCCCCTTCCCCCCCCCCAGGGGGAAAAGGGGAAAAAAAAAGGGTTTTTTTTTTTTGGTTGTCCCCCCTTTTAA
AAAACTTACGGATGCCATTCCCCCGAAGGGTTGGGGGGCCCCCCCCCAATTTTTTAGGCCACCCCTTGGGTGGTGTTTTTTGGA
AAAAAAAAAATTGCCCAAGAAAATTGGGAGGAAAAAAAAAAAGGGGTTTTTTTTTTTTTTTTTTTTTAAAAATAAAGCTTTATG
CCATTCCAAACGACAAAAGCCTGAAGGGGAGGTTTAATTGAACCAAGCCCCCCGATTAATCACATAAAACTTCCCATGACCCCA
CTTTTTTTTTTTTAAAGAAGTATTAGCAAAAGCCCGGTTTTTGTTTTTTTTTAAAAATTCCCCCGCACCTTGGAAATTTTTATT
TTTTAAAGCCCGGACACTTTATTCCCTTTTTGTAAAAAAACGGGGGAAAAAAAAAAGGAAGTGTGGGGAAGGGTGGGGTAAAACA
AACTTGAACCCCCCCCCCCATAAAGGAAATTTTTCCCCCCCTTTTTGAAAAAAGGTTTAAAAAAATTTTTTTTTCTTTTTTTTTA
CAAAACCAAGGAAGGAACTTTTTTTTTTTGGACAAATAATGAAAAAAGAACCCCAGAGGCCCGTTGCTTTTAAAAAAAAAGTTG
GGGTAAAAGGAATTTTTAATTGGATTTTTTGGTGGGGGGGGAAACCAAATTCCCCCCTTTTTGTTTTACCCCCCCCCATTCCCC
CCTGGGGGGGAAAAAAAGCCCAAAATGTTGGGGTGGGGCGGATTGCTTTTTTTTGGAAAGCCCGGGCCAAAGAAAGGTCACCAG
GGGTCCCCCCCCCCCCCCCCCCCCCCCCCAAAGTTTTGGGTAATAGCCCCTTTTTTCCCCCTTTGCTTTTTCAAGAATTCCTCC
CCAAAAAGGGAAAAAAAAAAAGGGTTTTTTTAGGCAATTTCTATGGAAAGAATTTTTTGGGGTTTAGAGCCAAAAAAGGGGG
GAAAAAAAATTAAAAAGAAGTTTTTGGGGTTTGGGGGGGGTTTGGGGCCCCCGGTTGGCTAAATAAATTACTTTCCTGCCCAGT
CGGCCCCTCCCCTTACCCTAAAAGAAGGGGCGAATCAACAAAAGAAGAAGGGGCCCCCCACAAAAAAACCTTTTTGGGGGGCCA
AAAAAAAAAAGAATTTGGCTTTAAAGTTTAACAGAAGGGGTTTTAAAAAGAGGGGGAGGGGAATTGGGTTCCCCTTTTTTTAAA
AAGCCAAAAAAAAGGAAAAAATAGGCTTTTTTTTTTTTTTTTAAGGAAAACTTAATTTTCAAAAAGTTTTCCCCTTGAGGTTT
TTTTTTTTTTTTGGGGGCTTTTAACCCTTTTCCCGGTTAAAATCAAGCGAAAACGGGATTTTTTTTTTGGGGTGTTTTTTTTCA
AAAAAAAGGTGATTGGAAGGGGGAATTTTTTTTCCAAAGACCCCGAAAAGAAGATTACATAAAAAAAAGATTTTTGAAGTTTCA
CCCCCCCCCCTTTTTCCCAATTTTACGGACCGCCCGAGTAAAAAACCCCTCACTTTCAAATCCCCCGGGGTTAAAACCCAACGA
AGAATTTGGGGTATCAAAGGGAAAAAAAAAAATTCCTTTAACGGACTCCCTGTTAAAAATTGGGGGGTTGGCCCCCCAAAAGGGA
TTTGAAAAAAGAAAAGGGAAAAAATTTGGTGGGGTTTTTTTTGGGGGGGAGGTAAACGGTTTTTGGCCCCTTTTAAGATGGGGT
TTTTTTTGGGAAACCCCCTTTGCCCCCCAGCCCAGGTGAAAAAAGCCCTTTTGGAGCCCCAAAAAAATTTCAGTGGGGACCAAA
GCAGGGTGTTTTTTTGGCCTTTTTTCCCCCCAAAAGCCTTTTTTTTTTTTGGGGGGGTATTTCCACTGGAATGGGGAAAAAAAA
TGGGAAGGATAAAATTTTTACTTTTTTAATGGGAAACTTTTTTTTTTTTGTTTTGGGGGGAGGGGGGTTTTTTTTTTTTGGGCC
CCAAAAAAAAAAAGGGAACCCAAGTTAAGGAAAACCCTTTTTTTTTTTTTCCAAAATTTGGCCTTTTTCCCCCCCCGGTGGGGTG
GGGTTTTGGGAAATAGTGGGGATAAAAGGAATTACCACCAAAAGGTTTTTCAAAAAAGCCCCCCCCCATTTTTTCCCCCACCCA
CTTCAATTTTTTTCCCAGTGGGGGGGAAAGGTCCCCCAAACACATTATACCCCCCTGGTTGGGATCCAAAAAAGTAATGCCCCT
TTGAAGTTTTTTTGTAAAAATGGGTGGAAGGGGGAAAAGAAAAATTTCGTAAACCGTTCCCCTCCCCCCCCCTTGGGGGGGTTT
TTTTTTTCCCCCCCTAAAAAAAAGGTCTAAAAAATTAGTTGGTCCCCCAGTTTTTCCCCGGGGATCCCCCAAAAAAATTTTCCCA
AAACTTCCCCCAACCCAAAAAGTTTTTCCCCCCCCTGGGGTGGGTTTTTGGGCCAAGAAAAGCCCCTTGGGGAAACCCTCAAGTT
TTTTTTTGGGAAAAGTTTCAAAAAAAGTACAGGCCCCCCCCTTTTTTTTTTTCCCCCCCCCAAAAAAATCCCCTTCCCCAAAAAA
GACCCCCCCCTGATGGGAACCCCCAAAAAGTTTCTTGAGAGGGGAAACCCGGATCCCCCCCCCAGGGGTTAAAAAAAAAAAATT
GGGAGGGGAAAAAAAAAAATTTAAAATTGCCTTCAGAAGCCAAAATGGTGGTTGGGGGGGGGAATTTTTTTGGATTTTTTTTTT
TTGGAAAGGTTTTTTTTTTTTTTTTTTCCCCCCCCATGGGAAGTAAAAAAAAGGTTTTAACCCTTTTTTCCCGTTGGATTTTGA
GTTTTTTTTTTTCTTCGTAAAAAATAAACAAAAATAAAGTTTTACTTTCCAAAAATTTTCAACCCCGATTTGGAAAAAGGTGCC
CCCCTTTCCCCCCCATTTAAATTGGAAGGGAATGTTTTGTTTTTTTAAATAACCCGGGGAGGAAAAAAAAAAGTGGTTAGAATT
AAAACCCCCATTTTCATTGGGGAAAATAAAAAGAAAAATGGGAGGGGTGAGAAAAGGGAGAAAAAAATAAAATTTTTTGGGGGCC
CAAGAAATTTTTTAATGGGGTTTTTTTTTTTTTTTTTCCCAGAAGAACCAAAAAAAGTGGGGAAAGGCCCCAAATAAAGAGGAA
GAGAATTTTCCCCCGGGCTTAAGGAAAATTTGCTTTTCCCCCCGCAAAATCCCCCCCAAGATATTTTAAAGGGCAAGAAAAAAG
CCCCTACGCCTGATCCCTAAAAAAAAAAAAAAAAGCCCCCGGGGTAAGGGAAAAATGGTGGGAGTTTTTTTCCAAAAATTTTGGG
GGAACCAAACGCCCCCCCTTTTTCTTTCAAAAAATAATTTTTTTTTTTTCAACCCAAAAAAATTTGCGAGGGGGTTTTTGGGGGAA
GGAGGTTATTTTTTTTTTTTTTAGAAAAAAAAAAGAAAGGGGGCAAAGCCCTTTTTTAAGCCCCCTTTAAGAAAAAAAAAATTTTTC
AAAAAAAATTTTGACCAAATATATAAACACACAAGAAAAACACACACTTTTGGGAAAAGGGGTTTTGGTCAGGTGCTTTTTTTT
TTTATATTAATATATATTTTATATATTTTTAATATATATATATATATAAACCAAGGGGTTTTTAAAGCTTTTTTTAACCGGCC
CCAATTCCCCCCCGGTTTTTTGGGGTTTCCCTTTTGGAAAGTTTTTTTCAAATGAATTTGGTTAAATGTTTTCCCAAAAAAATT
GGGTCTTTCCCCATGCCCTTTCCCCCCCCACCCCAAGAAAAGTCTTAAAATTTGTTTTCTCCCAAAAAAAAAAAGAAAAAAAAT
GTGGGACCTTTCCCCAATTGGGTTGTTTTTTGAAGGCCCAAAATGGGAAAAGAATGTTTGGAGGGTTTAACCCAAGGCCGAAAA
TTTTTGGCTTTTAAAAAATTGAAAGAGCAAAACCAAAAAAAGTTTTTTTTTCCCCCCCCCAAAATCATTGGATTTTTCCGGAAA

AAGAATAAAAGTGTTTTTTTTTGCGGGGGGAATTTAAAAAGGAACCCGATTCCCCTTTTCCTAAAGTTTAAAAAGGTCCTTTT
TTGTGGGGGGAGGAAAAAAAAAAAATAAAGTAAAAAAAGGTTCCCGAGGAGGGTGGGGAAAACCCCCCTTGGGGAAGCCCTTTTT
TTTTGGGAGGAACAAAAGCCGATTGCGGGGGAATTCAAAAAAGTAAATCCCCCCCGAAGGTTTTTGTTTTTTTTCCAAAGTAA
AAAAAAATTTTTTCCCGAAGAAAACCGAAAAATTTCCTCAAATTAGGGGAGGGGGGGGAAAAAAATAAAGTTTTTTTTTTTTTT
TTTGTAACCGACCCCCCCAAAAAAATTCCCGCCTAATAAAAGGCTTTTAAAGGATCTCTTTCCTTCAACAAAAACCCCAAACAA
AAAACGGGGAAAGGGAAAGGGAGGGAAAAAAAAAAAAGAGAGAAGGAATTCCACACTAAAAGGGGCCTTTAAAAAATTTTTTAA
GCCCCCGAAAATTTTTATTTGGGTTTTTTCCCCCCCGTTTTTTTTTAAAAAAAAACAAAACCCTATTCCCCCCTCCCCCTTAAA
TGGAAGGGAAATTTTTTCCCCCGTCCTCCCCGAAAATTTTTTACCCTTTTTTTTGAAAACCAAACCTCCGGGGAATACCTGAT
CCCCGCAAAAAATTCCGCTTGTTTTCCTCAAGCCTCCCAGGTCCCCAAAACCCCTTCCCCCTTCGACCCCTTTTTTTTTAACCT
AAAAATTTCCCCATCCCACAGACCCCCCCCATTTTTTTAAACTAGAGGGGAATCCCCCCAGGTCCTTTTTTTTTTTTTTATTTT
ATTTCCGCAAAAAAACAAACTTTTATCTTTTCCCCCGGATTTTCCCCCCAAAGAATTTTTTTTTTTTTGAACCCCTTTGGGTT
GGCTTTTTTTTTTCTTTTCCCCAATTTAAAGCCCAAAAAATTCCCCCCCCGCCTGTAAAAAAACTCCCCCCAAAATCCCTTTTC
CCCCCCAAATGCCTTTTCCAAAAACCCCCAACCAATTGGAAGGTCCCCCCCCCAACATTCCTTTGGAGGGACCCATAAACCCT
TATTCTTTTTTTTTGAAAGCAAAAAAAATGGGGGGAAGGACCCCATTTGGAAAAAAAAAAATTACAATCATGAAACCTTCCCCAA
AGAACAAACCGATGGGCGGGGGTTAAAAAAGGCCCTAACCCCCTTTTTTTGTAAAGCCCCAAAACCCATTGACCCAACCCCCCC
CTTTTTTTCCCAGGAATTTTTTTTTTTCCCCCCTTTTTTTTTTTTTGGGAAAATGGGTCCCCAAATGGAATCCTTTTACAAGGG
GGCCTTTTTTTTTTTTTTTTTTAAAAAGCCCTTTTTTGCCTTTTTCCCTTTTTTCTTAAAAACTTCCCCTCACTTTGCAAATTT
TGGTGAAAACTAAATTTGGAAAGAAAAAAGGGGCCCGGTGGGTTTTCCCCCCCCCACCCTTGGGAAAAAAAAAACCCTCCCCAA
ACATCCCCCTAAACGCTTTTAGGAAAAATCAGCCGTTAAGCTTTTTTTTCTGCCTACCTCCCCCCTGGGAAAAAAAAATGGCCGA
AGCCCCATTTTTTCAACTAAAAGCCCCCCCCGGGAATTTCCCCCACCTTTCCTTTTATGGGCCCTTCCAAGAACTTTTTTGTTT
CCCCTTTCTTTTGAACCCATTAAAAATCCTTTTTTTTTGGCCAACCCCCAAAATTATCTTTCCCCCCCCCCCCCCCCCTCTTTCC
CAAACCCCAATTTCCCCTTTTTTAAAATCCCAAAAAAAAAATTGAAAATTGGGTTTTTTTTGGAAAAATCTTTACCCCCCACTTC
CCTCCCCCCCCGTATAACCATTCCCTTTCATTAAAAGGGAGCCCCCACTTTCAAAATCGGTTTGATTTTTTTTTTGGGATTTAA
CTTTTTTATTGTTTATAACCCCGAAGAAAAAACCCCTCCAATCAAACCGGAAAAAGGTTTTAAACCTTAAAACTCCCAATGGA
ACCTTTCCAAAAAAATCCAATCAAAAAAACAACCATGGCTTCCCACCCTTGGAAGCCCCCATGATCCCCCCTTTCCCCCCCCAA
TAACCTTGGGATTCCGTCCCCCCCTTTCTTTTCCAGAACTTGTTTCCCCCAATCAAAGTTTCCCCTTTTGAGGATGAAAAAGGC
CCCTGGTTTAAACTTTTTGAAACTTTTTTCCCAACTTTTTTGAGGGGGTTCCCCCCCCCCCAGGGCCCCTTTTTCTTTGCAAAA
AACCAAACCCCCCCAGGAACCCTTTTTTGGGAAAAGTGGGAAAAAAAATTGGGGGGAATTCCCCCGATCCTTTTTTGAACCCAAC
TTTAATAAACCCCTTTTTTTTTTTAGGGGAAACCCCCAGGGGAAGGAAACCCGTTAACGAATCTTTTCCCCCCCTCCCACAAAAA
TTTACCAAACTCCCCAAAGCATACTGGATTTCCCCCCAAAACCCCAGTTTAAAATTTTTTTTAATGGTTTTTTTTTTGACCTT
TTCAAAACTTTTGGGAAAAAAAGAAAAAAAAAAAATGGGGCTTGGAACTTTTTTTTTTTGGTGGTATCCCTTATCCCCCCCAAAA
AACCTATCAACCCCAAACCACCGAGGCCCAATTGAAAAGTCCTTTAACTTGGTTCCCCTGGGCCAAAAAAACCTCCCCCCACAA
AAATTCCAACATTTATAAAATAAATCATTCCCCCATTTTTCCCCCCATTTCAAAGTGGAATAAACCCAAAGGCTGGAAAGGCAA
ACCCCTGGCCTGGTTTCCCAACTTGAATGGCCCTTTTTTTTTCCAGCCCTTTTTTTCCACTTTTGCTGGCAAAAGGTCCCCAAA
ACAAAATCCTTTAGGCCCAAAACCCCGTACTTTTCCAAAAAACCCCAAAAACATCCCACATTCCACAACCCATAACACACAAAT
AATAAAAAAACACACACCCACCTCCAGAGTCCCGCCCGTAGGATTCCCCTTTGATAAACCAATTTCCCTTTTTCGGTGGTTAAA
AAAACCCGGGAAATTTGAAAAAGGGGGTGTGAAGTTTTTTTTAAACGGGGCGGTTCCCGTTAATTGAGGAAACCTTACAAATC
TTTAAAAGTAAAATTCTTTTCCCTCAGGTCTTTGATCCCCCCCCCATTCCCCAAAAAATCCCCACCTGAAAACAAAAATTCCCC
GTTCCGCCCTTTTTTGAAAAATTTTTTTACCCCCGAAAAGTAAAAAACCCAAAAACCCCTTTTTCAAAAAAGAAAAAAACCCT
TGGAATTAGTCCCCCTTTTTTTTTAGCTTTTTTATTTCCCTTGCCCCTTTTAAAGACCCCCCAATTCCTCCTTCCCTAAACCCCT
TCATTTTTTTGTAACTTAAGCATCCCCACCTTTTGCCCCAAAAGGTTGGGCCCCCCCCCCACCACTCCTTTTTACTGAAAATTT
CCCGCTTTCTTTTTAAAAAGTTAGAGGCCGACCCCTGCCAAAGAGTAAAAATATTAGCCCCCCAACAAAGGCCCCCCAACCAAC
CAAACCCTTCTTTAATCCCTTGGGCCCCTCTAAAAACAATCCTTTTTTTTTCAATTCAAGCTAAACCTCCCCCCCCCCCCTTGG
AAAGATTCTTTTGAAACAGAGCCCTTTTTTATTTAACCAAACTGACCTGGAAAACCCCTTTTTTCCCCATTGCGGCCTCCAAAA
AGGGCCCCATTCCGCCACCAAAAAAACCCCCAAAAATTTTTTATGGCTTTTTCCCACCAAGATTTTTTTGGTAAAAAAAGGGAA
AAATTGGCCAAACAAAATTTTTCAATAAAAAAACCCCTTTTTTTTTAAAAAACCAAAACTTTTTAAAAAAAAAAGCCAAACCCCC
GGCCCTTTTCCCCTTTTTTTTTTGTTCCCATTTTCATAATTTCCAAAGGTTCCCCTTTCTGTTGTAAAAGAAAATACTTTTTTT
TTTTCAGGAATTCCCTTTTTTATGGGCCCCCCCAAAAAGTGTAAAACCACCTTCCCAAACCAAACCTTTTTTCCCCCTCCCCCC
GTAAACAAGGATTTTTTTAAAGTTGGTTCCCCCGGCTAAATATTTCAAAGGTTTGCGAATTTTTTACCCAACCCCGCTTTTTGC
CCTTAACTCCCCTAAAGTCAAATGAAAAGTTAGGGAATATTTCGCACCCTTTTTTTGTTTCATTACTCCCCCCCTTTTCAAAAA
AAGCTGGTCGTGATGCCAAAAATAACCCCTTTTTTTATAAAACCCCCTTTTTTTTCCCTCCCCAATTTCCCCCTGCAATCAAAC
CAACCCCAAAACCAAAAAAGCCCTAAAAGCCCCCAACCCTTTTTCGGATGCAAAAAAAATTCCCAGTTTTTAGTAAGAACAAAA
ACAAAAACTTTTTTTCCCCCTTTTTTCCCCCCCCCCTTGAAGGAAGACTCCCAACACTGGGCCGGCTTTGTTTTAAAATAAAAG
CCCGTCCGGCCCTTTTTTTTCAAAACCTCTTTGGGAAAGGCTTTTTTTGTCCTTCGCAACTTGGCCTGACCAAAGAAATTGAAA
CTTTTCAAAAAAAAAATTCCCCTTTTTTTTTTCTTTTTGGGTTTAAAAGTTCTTTTTGTTTTCATCCGGTTGGGCCCTGCTTA
ATCTTTTTTTTGAAGGGAAAACCCCTTTTCTAAACAAAAACCCGTCCCCTTTTTTTGTAAAAAAGTTAACCTGCTTAACCCCCA
GAAAAAAAATCCCCCGCCACCTTCCCGAAAGGCAATTCCCCGCCAGGCCCCTTTTTTCCGGCCCAAAAATTCCCCCACTAAACT
TTTTTTGGCCCTTCCTAATTCCCTTTTTTTAGAAATGGGATTTTCCCAACCCCTTAAAAAAAATCCCATTTCTTGCCGCCCAAA

AAAATGTAAAATCCAGGAAATGGTCCCCAACTTTTCCCCAGGGGTAAAACTAAAGAAAAACTTTCACAAAAAACTTTTTCTTTA
AACTTTTAGCCGGGTACAAAAAACCCTTGAATTTGCCTCTTTCCCTTTTTTTAAAAAAACC
>m141122_062923_42134_c100732202550000001823141405141562_s1_p0/102924/0_10184
AAAAGAGAGATGAACAAGGAAAGTGCCTGATCGATAAGGACAACAAGTTAGATAAATAGAGGTTGCCCTCAGTAAAATAATTGC
TAGCTACGTGCAATGGTGGGATGGCATGGCCAAGATCTTTTTCATGCAACAAATGCTACAAGGATGCAAGTTAAGGGTGCCAAT
ATAATAACACCCCTGAAGACCTAGAGCAAGAAGAGAAAAAGGACGAAGTAAGGTGAATCTGAAAAAAGGATAAAGATAAGTAAA
AATAAAAAAAAGTAGAAGAAAATGAGCTTACGTTGACTAGTTATGAAATGAGTATGTTGTTGTAGACACGCTATAAGGGTGAAT
AAAAAGTAACTCTTCCACGAACCTCCGGTTGGAGAATGGAACCCTCCCCGAGAGGTACCATGTGAGGGCCACCAACTATTAAAG
TTCATGATAAGTTAGGAGTTGATGGGTTTGTCCCTCAAGGTGAAAAAATGATAGACCCTCTTCATCGAGGGCCCACACATGATG
CCTTATCTACTAACAAAGATATATGACAACCCAAGACAACCCAACACAACCCATGAGTTTGGTGAACAACTAGAGGGCGGTATT
AAAAGTAGTTTAGTGCGATTAAAAAAAAGGATGGAGTGGCAAGGCCCCCACCAGACTAAATCACAGAATCGTTGAAATCTACAA
GCCCCTGTGTGGGTTTGGTATGCATGTCGTCGTGGGTCAGCATGGTGTAGTTGATTGTTGAGAAAAAGTTGATGGGCCTTCAGC
CATGTTGGAAATCTTAGTCCGATTCATGGTAAACTCGATGTCCACAATCTCAAAATCTTAGGGATTCTCCTTGGCATGTTTGAT
ACCTAAATAGAAACAAGATACGGTTTGTGAGCGACTGACATCATCAACCCCTAATAAGTTTCCCTATGTGTCCCTATGTAATAC
AGCCTCAGGCCTCCCCGCATACTGCTTAACTCCTTAAACGTATAGCCGTGACAGGGGAAGAAATGAAGGGAAATACCATTGAAA
GAAACTTAGGTTCAAGTGGATCACCATCCAGGTGGCGTGAACCTCGACGTTTGGGCTTAAGCGATGTAAAGGGCATGGAGGTTG
ATGGTGGAAAAATGTTCTTCTAGCAAGAAATCCAAGCTATACACTCTTGAGGGTTGATTTTAGATGAAAGGCCACGTAACTCAT
GCCACAAGGGGAAGAAACCAAACCCTTGTTGCAAGTGAAAAAATTCAAGTTCAACTCTCAAGGCCCCACAACTTATTCTAAGGT
CTGATAACGCACAACAGTGTAGGAGCATTCGCACACCGCTTCATTTGCGGAGGGTTTGTTGGTATCAGTTCCAGAGTCAGACA
ATGACAACTTGATAGGTCAACACGAGGAGGACTTTAAAGGATCATCAAACACCGTGGGCAAAGGGCCTAGACTCCGACATATAT
AAGTTGGCGATGAAAAGAACCAATATCTAAAACATGAAAGGGATGCAATTAAGGTAGATAAGGTCTATGTATACAAGCTAGCTA
ACACTCCAAAGGAAAGCTTAGAATGCGTGGAAATAGGCCTTAGATATGCATAAGAGTCTACCTTTGGAGTTACAATGACGACAT
CCAAGTAAAAACATTGTTTATACTCCCTTGAATAGTTAAGTGCGCTAAGTAGATTATTAATCTTTCCAAGTCACTAGTTACTTG
TTAGTGGTGTGTTTAGTAGATCATTAATCGTACTGAGTTCACTAGGTACTTGTTAGTACACATTTACAGATCATTACTAGTTTA
GAGTCACTAGGTTGCTAGTTAGTGTGAGATTAGTACATCTTTCTTCAATTTTTGAGTCTCTAGGTTACTTGTTAGGTGAGAATT
AGTATATCAATTATTACTCGTTTTAAGCACAAGATGACCTTGTTAATTGTGTTTAGTAGATTCATTAATCGTCTAGTCACTAGG
TTCAACTTATTAGTGTGTGTTTTGTAGATCATTACTCATTCAAACCCTAGGTGACTTGTGTAATGGGTCATTAAGTACATCTTT
AATCGAAACGACTTAGTAGGTCACTTGTTTTGTGTGAGATTGTAGATCATTAATCATTTTGAGTCCATAGGTGATTTGTTAGTG
TCAGTTTACTAGATCATTACACCGTTCCGAGTCACTAGGGTCACTAGATCACTAGTTAGTAGTGATTAGTATAATCATTACTTG
TTTCAGAAGTCACCGTCAGTTGTTAGTGTGAGATTAGTACATCATTACTTATTCTGAAATGACCAAGTAACTTTTTATTGTGTG
TTTAGTATAATCATTACTCATTGCTGTGTCACTAGGTCACTTGTAAGTGTGGTGTTTAAGTAGAATCATTACTCGTACTATGTC
ACTAATGATCAACTTCTTATAGTGAGATTAGAAGATCATTACTAGTTTAGAGTCACTGAGGTTGCTAGTTTGTGTGAGAATAGT
ACATCATTTGTACTCATTCCAATCTATATGTCGCTTGTTTGTGTACATTAGTAAGATCATTACTCTTTTGAGTAAGTAGGTGAC
GTGTTAGTGTGTGTTTAGTAATCATTACTCGTATCAAGTCCTAGGTCAATTAATAAAATACGAGATTAGCATAATCTTACCTAG
TTTTTGGTGTCACTTGGGTTTCTAGTTGGTGGTGGAGATTAGTACATCATTACTCATTTCCGACAACACTAGGTCACTTGTTAG
TGTTGAAATTAGTGGATATTACTAGTTATGAGTCACCAGGTGACCTATTTGTGTCCATAGTAGATTATTACTCCGTTCAGAAGA
TGATAGGCGACTTGTTAGTGTGACATTAGTAATTATAACTCGTTACAATGCACTAAGGTCACTTGTTTGTGTGAGATTAGTAGA
TTCTACCATTTTTAGTCATTAGGTGATTTGTTAGTGTGTGTTTAACTAGATCATTACACGTTCTGCTGAAGCACTAGGTAACTA
GATCACCTAGTTAAGTGCTGATTAGTACATCATTTCCTCTTCAGAGTCACTCAGTCAATTGGTAGTGTGTTAGATTAGTAAACA
TCTTACTTGTTCAGAGTCACTAGGTGAATTTTTTATTGTGGTGTTTAGCATATCATTTCTCATTCCGATCACTAGGTGATTTGC
TAATGTGTGTTTAGTAGAAAATTACTCAGTACTGATTCTCTAGGTCACTTATTAGTGCGAGATTAGCAGATTATTACTTGTTAG
AGTCACTAGGATGCTAGTTAGTGGTGAGATTAGTACAATCATTACTCTTAACGACTCAATAGGTCATTTCTTAGTGTGAGATTA
GTAAATCAGTTACTCTTTTAAGTCATTAGGTGACTTGTAAGTGTGTGTTTAGTGATAACTACTCGTTTTGAGGTCACTAGGTGG
TGTCAGTAGTATCCTGTGATCCGTAGGGGGGAAGACCGGGTAAAGCTGTGCAATCCCACAACCGCCTGGGGAAAGGTGAAGTGT
GATGATTCTAAGAAACTATGTAGGTATGAAGACTACATGTTGAAGAGGGCTTAAATGGATTGATGGGTACTACCATCATTGAGA
ACTCCAAAGTAATGCGTGCTTGACCTAGAGTAATCTCCAAGATGGGTGGGACCTCCTCGGAACGTATTCCAAGGAAGCGTGCGA
GTGGAGGACAAAGCACGTTGGAAGACTCGTGTTGGTTTGTAGGCCAAACGTCATTCCAGGAAGAGCCATACTGATGTGGGGCGT
TACACTAGGTTCACTAATTAATGTGAAGATTAGTAGATCAATACTGTTTCCTAGTCACTAGGTTGACTTATTAATGTATGTTTA
GTAGAACATTACTAATTCAAAGCCACTAAGTTTCACAAGTTTAGTGTGTGTTTATTAGATCATCTCTATATTGAGTTCTTAGGT
CATTGTTAGTGAGAGATTCACAGTCTATTACTTTATTTAGAGTCACTAGGTTGCTAGTTAGTGTAGATTAGGACATCATTACTC
GTTTCCGACTCACTAGATCGACTTTTTTTGTGTTAGATTAGTAGATCATTACTCGTAATGAGTCGCCTAGGTTCAAATTGTTAG
TGCAAGATTGTGTAGATCATTCTTAGTTTATATGACACTACGTTGCTAGTATAGTGTGAGATTAGTACATCATTACTTGTTTTG
AGTCTCTAGTCCATTTGTTAGGGTGAGAATTTAGTAGTCATTACTCCATTTTAAGTCACTAGGTGATATGTTATTGTGTGTTTA
GTAGATCAATACTTTGTTCCTAGTCACTAGGTCACTTGTTAGTGGTGTGTTTTAGTAGATTGATTACTCGTTTCAGAAGTCACT
AGGTGACTTGTTGGTGTGCATAGTACATCATTACTCATTACGATCACTATGTCACTTTTTAGTGGTTGAGATATGTAGATCATT
ACCCGTTTTGGAGTCCATAGGTGATTTTTTATTATGTATTTACTAGATCATTTACACGTTTTAAGTCAGTTGGTCACAAGATCA
CTGTTAGTGTGTGATTAGTACTTAATTAATTGTTCAAAGTCACATCGATCAATTGTTAGTGTGAGATTAGTACATAATTATACT
TGTTTGAGTCACTTAGTTAACTTGGTTAGTGGTGTTTAATTTTAGAACACTACTCGTTCTGAGTAACTAGGTCAAAAGTTTAGT

AATGTTTAGTAGATCATAACTCGTACGATTCACTAGGTCAGTTGTTAGTGAGAGATTCGAATATAATGTATTGATTAGAGTCAC
TAAGCTTGGGCTAGTTAGTGTTAGATTAAATACATTCATTACACATTCTGACTCCAATAGGTGACTTGTTATTGTGTTATTATT
GGAACATTACTCATTCGTGTCACTAGTTCCAAGTAGGTGTGTTTACTAGATCATTACTTTTACATAGTCGTTAGGTCTCTTGTT
ATTGCAAGAATTCAAGATCATTCTATTTTAGAGTCAGTAGGTTGCAAGTTAGCATCAGATTAGTACATCACTACTCGTTCTGAC
AGAAGATGTCTCTTGTTAATGTGTAGATAGTAGATCATTAGTCATATTGATGTCACTAGGTGACTTGTGAGTGTGTTTTCAAGT
AGATCATTTCTCTTTCAGAGTCACTTGGTCACTTGTTTAGTGTGTGTTCAGTTGATTATTACTTCGTTCCAAGTCCTAGGTGAC
TTGTTTGCGTGACATTAGTACATCATTACTTTATTATGACTCACTAGGCTCCACTTCTTAGTGTGAGATTAGTAAGAACATTAC
TCGTTTTGTGTCACAAGGGTGATTTGTTAGTGTGTGTTTACCTAGACTCATGACACGTTCCGAGTCAACTAGGTCACTCAGTCA
ATTGTTAGTGTGAGATCAGTGGCATCATAAACTTGTTCTGAGTCAGCTAGGTGGACTTTTTTATTGTGTGTTTAGTAAATTCCA
TTACTGATTCCTATGTCACAAGGTCACTTATTTGTGTGTGTTTAGTAGATCATTACTCCTACTGAGTCACTAATCATTTATTAG
TGCGTGATTTAAGGATATTTATAGTGTTAAGAGTAAATACAGTTTGGCAGTTAGAGTGAGATTGGTACATCATTACCTTGTTTT
GACTCCAATAGGTCACGTGTTTGTGTGAGATTAGTAGATCATTACTCTTTTTTGAGTGAGTATGTGACTTATTAGTCTGTATTT
AGTAGATCATTATGTTTCAAGTCACTGGAGGTTACTAGTTAGTGTGTGATTAGTATATCATTACTGCGTGCTGAGTCACTAGGT
CATTTCTAAATGTGTCCTTTAGAAGAACTTACTCGTCTATAGTAGATCATCCTCGTACTGAGTGAGTAGGTTCATTGGTAGTGA
GACATTCGCGGGATCATTAATTGTTAGAGTGGCTAAGTTGCTAGTTAGTGTCAGATTAGTACATCTATTACTCAACTTCCGACT
CACTAGGTCACTTTTTTGTGTTAGATTATTTTATCAATGTACTCGTTTTAAGTCACTGGTACTTGTTAGTGTGTGTTAAGTAGA
TCATTTACACGTTTCAAGTACTAGGGTCAATAGTTTACATGTGTGATTAGCAGATCGTACTCGTTTTGAGTCTCTAGGTTACTT
GGTATAGTGTGTTTAGTAAGAAACATTACGTCATTTTCGAGTCACTCTGTCACTTGTTAGAGTTAGGATTCCAGTACATCATTA
CTCATTCCCTGGTCACTAGTTTGACTTGTTATTGTGTGTTTAAATAGTCTCGTTCTGAGTCACTAGTCACACGTTAGTGTGTGT
TTATAGATCATCATTCGTAACTGGATTAGTAGTTCAATCATTGTGAGAGATTCGGAATATCTTACTTGATAGGGTACTAAGTTG
TTGAGTTAGTGATTAATATTAGTAGAATCATTACAGACGTTCTGATTCCACTAGGGGACTGTGTTAATGTGTGTTTATTAATAA
CATTACTTTGTTCTAAGTCACCAGGTCATAGGTTAGTGTGTGTTTAGTATATCATTACTTTACTGAGTCACTAGGTCTCTTGTT
AGGTGGAAACAATTCAAGATCATTACTATTTTAGACTCAATAGGGTTTCAAGTTAGTGGAGATTAGTAATCATTTGCTCGTTCC
GACGGACTACGGTCACTTGTTAATGTGTGATTAGTATATCATTACTTGTTTTTGAGTCACTAGTTAACTTGTTAGTGTGTGTTT
AGTAGATCATTAACCTCGTTTCCTAGTTGCGGTAGGTCCACATGTTAGTGTTTGTTTAAATAGATTTTTACTCATTAAGTTGTC
CCTAGGTGACTTGGTTGTGTGACATTCGTACATCATTACTCGTTACGATCACTAGTTCACTTGATAGTGTGAGATTTGTAGACT
CATTACTCGTTTTGTGGTGCACAAGTTGATTTGTTGTGTGTGTTTACTAGATCAATGTCAAGGCCAAGTCACTAGGTCACTCGG
TCCAACTGTTAGTGTGAGATTAGTACATCATTAATTTTTAGAATCACTAAGGTTACGTTTTTATTGTGTGTTTATGTAATGCCC
AAATCCTAATAAGGTTTAGGACTTCGATTAGAGCGGGAGGGCCATAATTGATTTAATTATGGTATTAAAAATAAATGATTAATA
TGCATGTTTATTTGTGAGTTATAATTATTAATATGATGGTGGAATGCATGCATAATGGGATCATAATTTTAATTGCAGGGCATT
TTGGTAATTTGGCCGTTGGGGCGTGATTGTGTAATTTCATGCATACGGGTGAATTATAAATTTCCCACATTATATGTGGATTA
GTTTTGAGCCATTTCGGCATGAGACCGATCATGGGAATGCAAGTTTTTTTTGGTCTAGGTCATAACGGGACTCAAGTTTCGGG
GCGGCTCGGAGTGAGTTCGGGGTAATTTGATGATTAGAACGTTGCGGGCAATTAAAGGGTAACGGAAATGCTGAATTATTGTAT
TTGAGAATATTGAGAATAATGGGAATTAGAGGGTGTTAATTATAAATTAACGGATAGGCGGGAATGACGATTTTACCTTGGGAG
TGTTTAGAAGCTTTTTTTGACCTAGGGGCAAATAGTCTTTTTCACCCCCAAGATAATATATCAAGCCTTTGGGTTTGAAGGCTG
TGGGAATTGTTAGAAAACAGAGCAAACATCAACCTCCTCTCTCTCCTCACCCGTACACCATTTCCATTCTTTCTCCCTTGTGGA
ATTTTGAGAGCCAACTTGAGGTATACAAGCTAGGAATCAAGGATTTGGGCTTAGGACTTGATCGCCATTGAAGGGGATTGAAAC
CAAGCTTGAGGTAAGGAACTCAGCCATGAAAGTCTTGGTTATACTCTGTTTTCTTTTAAGTTTCAGCTGTGAATTTCTTGGTGT
TTAGTTGGAAATTAATGGAAGTTTGGTTGGTGTCAAACTTGGGTTTATGATGAGGGTGAGTTGTAGATGATGTTTAAGTGGTTG
AATTGGTGGTGAGGTTGAGGTTTGGGGACTGGTTTGAAGGTTTTGGTTTCGAGGGATACGCAGGGGAAAGAAAATCTTACTGTT
GCTGGTTTGCGTAATGTGCCGCGGCCTGGCTATGTGTGCCGCGGGCCTGAGTGTGTTTTGGGAAGAAATTAATGCTGTTGTCTG
AGGGTGAGCGCAGCATGGCTATGGTGGAGCCGCGGCCACACAGGGCATTTTTGCCAGAAATGGGTTTTTGACTTGGGATGCAAG
CTTAAGGCCTCGGGTCGGTCATACTAACCCGGTAAGTGTGGATTGATGTCCCGGAGGCTAGATATTGGTTTGGAAACCTTTGCT
TTCAATTTTCATTGATCGAATCCTATATTTTTTTGGTTATGATAGGTGAACGCTAAAGGACTTAAAGTTTGATCCGTTCTCAAG
GTCGTTTCTTTTAATTAATTCTTGCTAAGAACCCAAGGTAAGAAGCTGCACCCCATTATGTGACCATGCATGGCTGTGATTGAT
GATGTTGGATGTTGAATGTGAACATTGGATATGCATAATGTATGCTTTGGCAATCTTGCCCATTTGCTATGATTATTATTGAGG
CATGAATGGAGTGATTAAGTGTGATTCATGTGATGCAACGAGAATCATGTGATTAGGGCGCATGCCCATGAATGATGAATATTA
GGTTTGTCAGAGCTTGAGTCAATTCTGAAGTTTGTGCATGATCATAATTATGGCTAGCAACTATTTAGTAAGCATGCTGAATGC
CCTATTATTGGATATTGTCATATGATACACATTGATAGCATTTGCTTACCTGTGCATGGTACTGTAATTAGTCAGATTTGGAAA
AGTGCTAGTATACTGTGAAGATGTGACTCCATTAGTCAGGTTTCGGCAGTGGTACGTAGGGCACTGGTCACGTTGCATCTGACT
GCATTAGTAGAACGGCCCTTCTAGCGTGTATCCACGCAAGCCAACAGAGATTAGATCTAATCGACTTTCTTGCATTGAATGACT
CAAAGAGCTTTAATGCCGACCGACCCTCAAGGTCGATGAACATTATAAGCGTTGAAGGTTAGAAGGCTTACCCAACAACACCCT
TCCATTTGAAATTTAGTGGACTTGCTTTTCAGATCACTCAGCATGGTTTACCAGAACTATTGAAGGTTTAGAGGCTTACCTAAT
AACCACCTTCCATTCTGAAATTAGTGACTCGCTTGTCAGTCCTCAGCATGGTTTATCAGCAACCTCAAGTGTTGCGACACTCAT
GCTGATTAGGATTGACTTGATAGTGCTCCAATCATAAGGCCAGGATTTTCTTAATCCTGGATACCCTAGCAATCTTATTTGAAT
TCATTTGCCATGCTGAATATGAGCTATGAATGCTAGGCATGCAGATATGAACCTGATATTGTGATATGACTGTTCATGAGCATA
TGAGTTTTTCTTGCGCGGGCTTCGGCTCACGGTGCTTTGTGGTGAGGTAAAGGCAAAAGGAAGCTGGACCGATCCTTGGTTGGA
GAGCTTAGGTGGGACGTGTACATATGCAGACTGCTCGACAATACTTGGGCGGGTTTGAAAGTGGAACTAAGGGCTGAACCCTGT

TTTGCCGCTTAGAACGGCCCTGTTGTTAAACACTTTCTTAAGTAATATAACATCTGAATTATACTTTTGGGATCCCAATGTAT
AAAGTTAAACCAGTTCTAGTTGAAACATTATATCTTAAACCAAGAGTTTTTAAATCCCTAAACCGCTATCACAACTTAGTTACA
CGTTTAATGGCAAACGACTCGCACTCACGAGTTTAGCACTGTTTGGCAATGTGCATTGTAGCGGTCGTGGAGTTGGGGTGTTTC
AGTTTAGTAAATCATTAATCATTCCATATCACTAGGTCACTTGTTTGTGATGTGTTTAGAAGATAAATTACTCGTATTAAGTCA
ACTAGGGTCACTTGTTAGTGTGAGATTAAGCAAAATCAATTACTAGTTTATAATTCACTAGGGTGCTAGATATTGTGAGATTAG
TACATCGTTACTCATGTCCTGACTCGATTGCTCACTTCTTTGTTGAGATTTAGTAGAATCAATTACTCATTTCGAAGTCAACTA
GGGTGACTTATTAATGTGTGTTTAGTAGATCATTACTCGTTTCAAGTCACTAGGTCCCTAGTTAGTGTGTGGTTAGTAGATCAT
TACTTTTGTTTGAGAGTCATAAAAAAAGCAGGCAACAAGCAAACAAAAAACAACAGAACATATATCAAAAAAAATCAATACAAA
AACAAAAAACAAAACACACA
>m141122_062923_42134_c100732202550000001823141405141562_s1_p0/105460/3717_12942
GTAATATGTCTGATATATGACAGCAATCTCATGTTTAGAGATAGTCTGGTAAGATGCGATTTACAGATTTGTTGTCGGTCAGTT
AAATTTGTATTGATTTGATTAACCTGATCTGAAAGCATAAGCCTTACAAGGAGAGTCGATGGGGGTCAGAAATAAGCATTTTGG
TAGACGGCAGCTAGCGCTTGGCTAGCGGAAACCTGGAGTACAATATGTGTCTTGTTGTTAAGGTACAGCGGGGAGTTATTACTA
CTAAAATGTTATTTAATAGTTTCACTGTTGCTTATTTGAATTTATTGATTTAGAGTTTTCTTGGCTGAGCCTTGGCTCACTTGT
GGGTGGATTACTGTGGTGCGGGTAAACCGCTAGAAAGGGGAGTTGTGATCCAAACCATGACAAGATGGGAGAGCTTGGCGTGGC
GGACCATTTCAATCTGCTCGAGCACCATGGATGAGGATTCAAGAGGGACTAGAGTCAACACTAAATTTCGCTCAGCCGGCTTTA
TGTTGTCAGCTACCATTTGGGAGTTGGAAATGACATGTAAAAATGTTTTATATTTGGGATCTCATATGTTAGAGTAAACTTTAG
ATTTGAAAGTAAACCGTTATTGATGATCAAAACTTTTAATCCCTAATCCAGTTAATTAACCCTTAGTTAATACAGCGTGTTTAT
CCAAATGACTTGGTTAACAAGTCCTAAACCCACTATTTTAAAATGACACAATGTAAAGCTTTGTTATCCAGGGCGCTACAATCA
TACATTCACATCTTCCAACCTGCAGTTGTGTAAGATGAACTTTTCTCATTGCAATAATCTTCTTAAGGTCTGGGGAGACTGTAT
CTCCTTGAAAGTGAATTGCCATGGCAGCATGGGTAAGACGATCCTTTCGGGTTGAAATTTATTCTATTGAGAAACATTCAAGCA
CTTATCAAGTGTATAGTTTTGGTGAGAGATGCCATAAGAATTCTCTGTCCTCTTCTTATCCCTTAATGATTTGGAGTACGCCAT
AGATGAATAACTCACTTCACGCTCAATATCCTTCATCTGGATTGACTGCTCAGCCAATTCCTTCACATATTGATAATTTTTTAA
CATTGTTTCCAATGAGTAATGATATCAGTGCTACATAAAAGAACTAGAAATAACCATTTATTGTGCATCTCTGCTCTTGAGTTC
GTAAACAGCGACAGCTCATCATTATTATTTGTTCAAAGCATAGGATTTTATTATCTCATTCAAACCTAAAGATTCATTAAGAAC
GAGAAGCTTGCTCATGTCCAGAAATGAACCAATTCAACTTGCAAAACTTTTTCCTTCTTGTCCAACTACCTTTAAATCCTTATG
GTTGATTTCATATTGAAGCTTCGTCAAGCTTCATTAAGAAAAGTTTGTCTTGACAGTCCATTTTTGCAAGATCCTCATAGTTGG
AGAGCGACTGCTGATTGATATGAGGAATCGGTTAATGGACTTGAGCATGGCTTACTGGACTAAAGTTCTCACACATCCACGCCT
TTCTCCTTTGTGTGTATAACCCTTTGCTCTAATTGAGCTTGTAAGTTTTGTCTTTCCATCAACACCTCGTTTTCCTTTCTTGTA
GACCCCACTTGCACCCAATGGCCCTAAATCACTAGGTGCTTCCACAAGATCCCTGCGGAATTTGCAGTACATGGACTCCATTTC
CTGTTCATGGCTTTTGAGTCATAGTTTCTTTTCAGGGATAGCCATGTGCATTTTTGAGAAGAAAATGGATCATCATCCTAGTGT
CACCAACACCATATTGTGTTTTCGAACTCCAAACATAGCGAATCCTGGGGTTCCTAGAGAACCGCCCTCCCACATGACGAGGCT
CTGATGACTGTTTGCTTCAGGAAACAATGGTACTCTTTCAATCAATTTAAATTGACTTGCGTCGGTTGGGCATGAAGACGGGAA
TTTCACCATTCAGATCTTGTGTTGATGATGATAGGAACATTGGTTGGGAGTTAATTCTTTAAACCATCTCCTCTAAAAATACTT
TGCATGCGAGATTTGAAGTTTGGACATAGTCATTATTCCAAAAGTAAGCATTCGTAAAGTAAACACTTTCTTTTTCTGAATGA
CTTATCAGAAAAGTCCAACCGAAGTTCCCTTTAGGATTGCCAAAAACATGCAACTTTTTTTCGCTTTAGATTTCCTTTCCTTTT
TCCTCAGGATGTAGGCGAAACACCCCAGATTCTATAATGGCCTAAAATGAGGTTCACGACATTCCAGCATTCCTAAATGTGTTT
GGGGATTGATTTGGATGACAAAACATTTAGAATGCCGCTAGTTTCAATTACAATGATCCCTAAGAATGAAGTTTGGCAGAGTTG
AGTAACTAAGCATCTGCATCTAAACATTTCCAATAAAGTTCTTCTTTCGGCGTTCTGCTATCCCATTTTGTTGCGGAGTACCCA
TGGCAGTAAGTGAGATAAAATTCCAAGTCTAGTACAATGCCATTTGGCCGAACTACATATCCAAGATATTCTCCACCCTATCAG
AATCGTAAGATCTTTAACGTTTTACCTAATTGGTTCTGAGCAAACGAAGGATTCCTGAAACTTTGAAAATGTTTCTCATGTTCC
TATGCCATTATGTAAAGACAATGAGTATCTAGAGTATCCGCGTCAATGAAAGTGACGAAAACTAAAAACACCCTGGCTTCACAT
TCAAAATTAGGTCCACAGACATCTGAATGTACTATTCAAAAGGTTCTTTGGCACTATCAACCATTTGCAGAGAATGGACGCCTG
TCAAGTTTGCATTCTAAGGCGTGATTCACTAGGCAGGGTAATTCACCTTAAGGTGAGTTCCCCTCAAAGGCCCGTCCTTTGTAA
GTCTTTGAATCCTACATCATAGCCAATGTGAACCATAGTCCTCAAAGTGTCAAAATACCGTCATGTTATCGTTATCAGGTCTTT
TGACGTTTATTGGTCCAAGGTTGTTAGTCTACTTTGATAATCAATTATTGAAATGCAATGGGTTTTTAGGTCATAGAAATAAAA
GTCCGTGTTTCCAAACATGCAATACACATATTGTGACCCATTGAAAGAAATATATAATTCTGAACTTGTAAAAGTCATAACAAA
TTGTTCTAATTCTATCATGGAAGCTAAATTAATATTTCTACATAAAATCCTGGGAATATAAAATTAACGAACATGTAAAAATTA
AATATTTATCGTTCCAAACTTCAGGTAATCTCTACCTCTAGCTTGGACACAACAAATGCTACCGTTCCTAACTCTAAGATTTAT
AGCCACCTGTCATCCACGTTTCTCCCACGAATTCAAAAGCTTGTAACGAATTACAAACATTGGTTAGGTAGACTAAGAATCAAA
TAATCCAAACAAATTTATCAATTCTCTAATGTGAATGCAAAATTGACTAGAGCCATAGTCAACTTGTTTCCCTCCAATTAGCCT
CAAGCAAAGCAGCCTAATATCTTGTCCAGCACAAGACCTGTTTGATGCTCATAATCTTCATCAGAGAAAAGTATGAGGCCGAGG
CAAGGTCTTAGGGCCACCGTCATCTCGGGGAAGCCCTCGCGCCATCTACAACACGAGTCTGGGTTTCGTTTCTCGTGGAGTCGA
TGCCAAAAGGCCACCCACGTTGGCAAGGCATAGGGTGGCCAAGGCTCAGTAGCCTCGCCGAGGCCTCTCCCCATCCCGCACTCC
CACGGGCCTCAATGCCCCATGCGCCTCGCCATTGGCCTTAGGCCCAGTGGCTCCTCCCATTGGCCTCCTCCATGGCCTCTCCCA
TGGCATCCTCTTTTTGGCTCGCCCATGTCCTCGCTCCGTGGCCTCCGCCCATTCGGCGTCCTCCGTGGCCCTCGGCCCATCCAT
GGCCTCCTCCATGGCCTCCTCCATGGCCTCGTGCCCAGGCCTCGTCACATCGGCCCCTCCATGGCCTCAGCCCATGGCCACATC
CATTAGGCCTGGCATGGTCGGCCTCAGGCTCGCCATTGGCCGCCGCATGGCTCAACTCCGATGGCCACCGTCCATTGGCCTCGG

CCTCCATGGCCTCCTCCATGCTCACGGCCACCAGTGGCCTCCTCCATGGCCCTCGCCCATGTCAGGGCCCCTCCATGGCCTGGC
GCATGGCCTCCTCCATGGCCTCGCCCATGGCCTCACTCCATGGCCCTCCCTCCATGGCCTCCGGCCCATGGCTCTTCATGGCTC
GCCCATTGGGCCTCCTCCATGCCAACGCCCATCGGCTCCTCCATGGCTCCCTCCATTGGCCTCACTCCATGCTCCTCCATGGCC
CGGCCCCATGGCCTCCTCCATGGCCTCGGCCCCATGGCCTCCTCCATGGCTTCGGCCCCATGGCTCCTCCATGGCCCTTCGCCC
CATGGCCTCACTCCATGGCCTCCTCCCATGGCCTCGCCACGGCCTTCCTCCAGGCCCACCCGATATGGCCTCGCCACGCCTCTC
TCCATGGCCTCGCGCAACGGCCGTCCTTCCATGGCCGTCGGCCCCCATGGCCTCCTCCATGGCCTTGCCCGTTGCCTCCTTCTA
TGGCTCGACCGCATGGCCTCCTCCATGGCCCTCGCCTATGGCCTCCGGCCCCTATCGGCCTCCTCGCATGGCCTCGCCCATGGC
TCCAACATGGCCCTCGCCCCCATGGGCCTCCTATGACGCTTCTCCATGGCCTCAGCCCATGGCCTCCTCCATGGCCTCGCCCAT
CCGGCTACTCCATGGGCCTCCTCCATGGCCCTCGCCGATTGCCTCCTCCATGCTCGCCATGGGCCTCCTCCATGGCCGTCAGGC
CGCATGGCCTCCCTCAACATGGCATCTGCCCATGGCCTCCTCCGATTGGCCTTCCTCCATGGCCTCTGGCACCATGGCCTCCTC
CGATGGCCGTCCGACCCCATGGCCTCCTCCATGGACTACGGCCCATGGCCTCCTCCATGGCCTCGCCAGATGGCCTCCTCGCAT
GGCCTCTGCCATTGGCTCCTCAATGGCCTCTACCCATGGCCTCCTCGGCCCTCGCCCCATGGCCTTCCTCCATGGCTCTGGCCA
CCATGGCCTCCTCCATGGCCTCGGCCCATGGCCTTGCTCCATGGTCCTCCGTGCCATGCCTTTAGAGGTGAGAATACTCACTAA
GGGGCAAGGTACACGCAATGAGTACCGGATACTACAAAAACCTAAAGAAGTCAGAAGTACGATGATGGTACCTACCACGACAAG
TAGTTGGGGACGCCAGGAAGTGGGTATGGATAGGATAGGCAGGTTAGGCCCGTACCGTCTTATGGCCAGGAGTCAGAGTCGACA
CTACCACTGCACTCACTACGCCTGCCACCACGCATCAGACATGGGTACAACAAGTGTGGAGACATCCTCCTGACAACTGCTCCT
GTACGGGATGTACGATCACACCTCTGAAGCCATCCCTGACATAGTACTTATGTACCTCTTGTCTCCTGGACCACCATGTACCAA
GGGCCATTAGAGCCTACTATAAAAGGACTCTAACCACCACCTAAACGGGGGTTGGAAGTTTGACTGTAGCAAAGGCTTGAGAGT
TAATGAAGAGTGATTGATTTCTACATTGATATTCTGTCATAATTCTGAGTTATTGCTTTGATTTCTATAGCTTTTTTATTGACG
ATCCTAACTTGATAATTTCTTCCAAACTCAACTTAGTTGACGAGTTCTCACCGTCAACATCTAAAATAGATGTTTCATAGGATA
AATGACGTACGCATTACCTTTGTTTTCCGATGTTAGAAACTTGGGGCAAGTTCTTGTTTCCAAATGCCCATTTTCATTGGCAGC
TCGGAACAGCATTTACCTTTACCTTTCCTTGTGTGCTTATTGTGTCTTCCCTTAAGGCCGTTCTATGCTCTCTTAGTTGCTGCA
ATCGTCTTTGCATACCCTTGGGGTTGGTGTTCATCGTGTCTGTTTCGGTGGTTTTTTGCATGCCCCTAATTATTAAATTCAATT
CTTTTGTATTTTAATCAATTTTTTTTTTCTATTAGATTTGCTAATAATAGATTGCTTAATATAAAGATAGTAATAAAAGAAGTT
TACTATTTAAAACAAAGAAAATACATGAAAATAAAACATAGTGAGCAATAGTCGATATTTTTTGACGTTGGTAATCACCATGTT
ACACTCTTTGCTCTCACATGTTTTATTTTCTGATTATTTGTTTAATACTAACTTCTATTAAATCCCGAGCATATAGCTAATCTT
ACTATGAGTGACGTAATCACAGTGGAATAACATATGATTATAATGTTAAGAAGTCAAGTTATCCTAAGATTAGTCAGTGCCACC
AGATTCTTACACTGACTTGGCCATCTATGAAAATATGATCTAGCTTACACAGTTTACAGTGTTGATGTTCTTTCCAGACCATTA
GCAAAAGTAGATAAGATAAGATGCATTGTTACATCGGATAGACCATATTGACAGCTTGATAAGATAAGGTAAAATTTACCGTTA
TTATCTATTCTAGTCTATATCATATAGTTACCATAGGTCAATTCAATCCTCAATTCTGAGTGGCTTACGCTGTAATTCTACCTA
ATTGTATTATTTTTGAGTTCTTTGACTTTGGTACCAGCTTACCCTTACGGACTAGCCCATAAGTTACGATCTTGGGAACTAGTA
GTATAATTGTAGTGGGAAGTCGGTTAATCATAGATATGAACATCTATAGCTTCTGAGAAGAAGTGAAATGGATGGTTCCTTTTC
AGTGTTGGTTTCAAGGTGTTAAATGATAAGATCATCATTTCAGATAATTAAATTAGTTTACTGAAATATCATTTACAAGGAACT
AAGTGTTTTAAGTGATAAAGATACCAAATGAAGGGGTACACAACGGTATTTTAGTCCTGATCTCATTGTGAGAACTGTCTATAG
AGGATGGAGTGAGCAATGGATGGTGAACAATGGATAATTAATAGCGAATCATAAATTGTTATAGAACGTTTATTATGAATTCAA
GAGTGAAATTCCCGATCTATAGTAGGCGTCACGAGGAATTAAATAAGTTAGTAAATTTATTTGTTAATTTAGATTAACTTATTG
GAGCTTGATTTCATAGGCCCATGGTCCCCATTGTACCTTGATAAAATCTTCATAGATGTAGTCTCAATAATTGATTTAATTATC
AAAGTTGACCTAGGTCAACGTTTTGATAGTTTTTCATAGAGTTATGTAATTTTGAAAAAGAAAAGAAAATTAATGCGCAGATTT
ATTAAATTAAGATAAAATTGGTATCTAAATTAATTATAACAACGTTTAATCAAAGTGTCAAATTATATAGTTTAATTTGATAAA
GGATTTAAATAATATTAATTAAATTAAATCAATAGAAAATAATACAGGCCCCTTGTTTTAAGTCCAATGGGCTTATAATTAATTA
AATGAGAAATTTCATGGGCCTATAACCCATGATAATTTTGACCTAGGTGCTTCAAATTGGCTATTAATTTATTGATTTTTTAAT
TAATTAAATGTGCCTAAATTGAGTCTACTAAAAGGAGTGCTTAGAGAGAAAAAAAACACAAGTTTGATAAGTCACAAGTCATGA
TTTCTTATAGTTTTTTAGATTGATCTCTAACACGAGTCATTTTTAAGCCTCCTTTTATTTTCCTCTTCTTCTCTCTATTCCTAT
CTCATGTGTTGAGAATTGCCCACACCTAGTCTAGGTGCGTTATAACGATATCTTGGAAGATTGTGAAGAAAACAGAAGATCGGT
TCAGTTTCTTGATAATAATTCTGCGCAGGAGAGGTAACAAGAGTTAGAGAATGGAATTGAAGGATTTTTTCATCCGCTGACGTA
TACTGTAAGTAATTCTATTCTTTTGTTTCTCTTCCTGAATTCAATTTTAGAACATGTTTTATGCTATTTCGTATTAATTGTTAA
TATTAGATGTACAGAAATAAATAAAGATCCTGTATAAGTATCACAAATGGCCTCAGAGCCTTTGATAATCTTTATTTTCATGCA
TGACATGTTTAAAATTGAATATCTTGATATATGTTTGAATATTGGATGGTTTTCATGTTTTATGAAGCATATTTCGATTTTAGT
GTGAATTTTTAGCAATTTTAGGTTATTTTGGTTGTGTTTTCCCATGCTATTAATGTTTTAATATAGCTTTTTTTATTTGTGTAA
ACATGTTAAAAATTAATTAGAAAAATAGTTTTGGTTGAAAAATTACTCAAAAAATTTTGGAAAAAATTTGGCCTGTCTCCCCAT
GCGTGCACAGCACGCTCCGCGCGCTGCCACCATGCTCACCCCGAACGCATGCCACGCGATGAACAATAATCGGTGAACAGTACC
GGCAGGAAAAAAAAAAAAAAAAAAAAAAAAAAAAACTATTCATCCATTTTTTTTAAAAAAAATTAAAATTGTTTGAAATTTTATTA
TTTATATCAAAAACAAAAATATTTTTTTTTTATCTTTAATTTTTTTTAAATAAGGATATTTTGTTAGTTGAGATTTAAATAAGT
TAGATATTTTCTACAAAAGATTCAAATTACAAATTAAAAATCGATTTAACAACTTAATGCTTAAATACTCTTAATATATTAAAA
TATTAGAATTATTGATATCAGATATTTGAGATATTTTTCATTTAAATTATTGGATTTTTTATTAAATCTTTATTTGAGGATATA
AATATACTTTTTATTCTATAAGTTTTTAATATTAATATTATTTGAAATTTGAATATTGTTAGTTAGATATTTTCTATGGATAAT
TGAATTTGATTACTTGTTTTGAGATATTTAGCGTTGTTAGAACTATTTTAATATTTTTTTTAATGGTTAAAAAATATTTAGTAG
ATATTTAGTAAACAACACAAGATACTTTTTGGAAAATAATTAAGATATTGATTTTAAAATTTAAAATTGCTTAAATTTTGAAAA

ATATCAATTTTTTTCAGCCATTAATCAATAAAATTTTTTTAATAAGTGAGATTTAAATTAACTTTGGTTAATTGTTGATAATC
CTATTTAAATTAAATTAATATTATTTTTTTCAATTAACTAAAACAAAGTTTGATTGTTGTAAATGAATTTAAATAACTATTGTT
AATTGTTGATAAATTTCATTTAATTGACTTATTTGTATTTTTTTTAATTAATTATTTTTTTTATAAATCTAGATATTAATTGTG
GTATTTTTGCAAATGAAATAAATTGTGGTAATTTTTTGCATAAATTCATAGAATTTCTCATAATAGTAA
>m141122_062923_42134_c100732202550000001823141405141562_s1_p0/106632/12891_18868
AACTTGATATTAAATTAACTTATCTAGGTTATGACCTGCCTGATTCCTGGAACTTAGTTCTCGTATAATTTTTATCAAAATATC
TCGGTGCTCTTTAAGAAAACAACGATCAAATTATTTTAAAATTAACTCTAAGTTTTTGACTAGGTTATAGTCGAGGCGAACTT
AGTTTGAAAACCTAGATTCGCGTTAATTTTTCCATCAAAATCTCGATGCTCTATTAAGAAAAACTTAATGATACTCGATTTACA
AATTAGTTCTAAGTTTTGAACCCTTGCTGAGCTTATTCATCACAGGAACACTAAGTATTGAAAACCTTGTTCGCGATAATGTTT
TTATCAACATCGTCGTTCTCTTTAAGAAAAACAACGATCAATTGATTCTCAAATTAAATTTCTAATTTTTATGCCTGTTAATGT
CCAGGAACCTCAGCTTTGAAAAACTTAGTTCGCGTTAATTTTTATCAATCATCTTGTGCTCCCTTTCAAGCAAACACACACATT
CATCTTGGATTTAAATTCCTTCTAAGTTTTATGACATGAGTTATGTCCTGCCACTCTAGTTTGACAACTCTAGTTCGCTCTTAA
TTCTTTATCAATAATTACACAGAATACAATGCTCTTCTAAGAAAAAAAACGATCAAACTGGATCTTAAATCTAACTTCAAAGTT
TTATGCACCTGGTTTGTCCAGCGAACATAGTTTGAAAACTATACGTTTCGCGCTCTGATTTTTAATACAATATCTCGCTGCTCT
ATTAAGAAAAGAACACCCAACGATCCAATCGATTTAAATTAAACTTCTAAGTTCGTATGACCGTCATTATATACAGGAACATAG
TTTTAATAAACTTAGCTTCCGCTTATTTTTTTATCTACATCTCCCGTGCTCACTTAAGACAAGACAACGATCAACTCTGAATTT
AAATAATTTCTAATTTTTATGACCTGGTTACTGTCCAGCGAACTTTAGTTTGAAAAAATTAGTCGCGTTAATATTTTATCAATA
ATCTCGTGCTCCTTAACGAAAAAAAAGACAATTGATTAAACTGAACTTCTGAAGCTTTTATGAACCTGGTTATGTCCAGGCGAG
AACTGTAGTTTGAAAACTTAGTGCACGTTAAACTTTTTAATCGATATCCTCGTTTTCTTTTTAAGAAAACAAATGATACAAATT
GATTTAAATAACTTCTAGGAATTATATGACCTGTTTATGTCCAGGCACTTTAGCTTCGCGTAAATTCTTTATCCAATCATCTCG
TGGCTCATTTTAGAAAAACACGACGATCCAAGTTGACTTCTAAATTCAACTTCTAAGTTTTATGACCTGGTTTTACGTGTCGAG
GAAACTTAGTTCTCTGAAACTTAGTTGGCCGTTAACCTATTTACAATATCTTGCTGCTCTTTAAGAAAAACAACGATCAAATCA
TTTAAATTAACTTCTAAGTTTTTTAGACCCTGCGCTTATATCCAGGAACTTAATTTGAAAACTTAGTTCCGTTAAATTCATTAT
CAATGTCTCGTCTTTAAGGACACACTACGATCATTTTGCTTCATTACACTCTTAAGTTTCTCTGCTGCCGTTACGCACAGGACT
TAGTTGTTCACTTCGATACTCTTTATTCATCTATACTACCCTCATAGTCTTTCAGCAAACAAACGTCACATTGATTCATTCACT
CTCTCCAGTTTTACGACTGCGTTATCAGTCCAAGGAACATAGAATTAAAACTTAGTTCGACCCCGTTAATTTTTTATCAATATC
TCGTGCATTTAAGAAAACAAACGATAAATTTCGATTTATTACTTCTAAGTTTTATGACTTGAGTTATATCCAGGAGCTTTTTTT
GAAAACTTAGTTCGCGTTAAATTTTAGCAATATCTCGTGCTCTCTAAGAAAAACATTCGATCAATTTGAATTTAATGAAATTCT
AAGTTTTATGACCCTGGTTAGGTCCCAGGAACTTAGATTTGAAACTTATTTCGGCGTTAAACCTTTCTATCAACGGTAATCATC
GCTTCTCCATTTTAAGAAAATACAATGATCAATTGATTTAAATTACATTCTAGGTTTTATGACTTGGAGTTATGTCCAGGAACT
TAGTCTCGAGACACTTAGTTCGCGTTAAATTTTTATCAATATCGTCGTGCTCTTTCAAGAAAAACAACGAATCAATTGATTTAA
TTGACTTCATAAATTTTTATGACCTTGGTTTATGTCCAGGAACATAAGTTGAAACTTAGTTCGCGTTAATTTTTATCAATATCA
TTCTGGTGCTCTTTAAGAAAACAACATCATTTGCTTAATTACTTCTAAAGTTTTAATACCTGGCTTATGTCATGGAACTAGTTT
GGCCAAACTTAATTTCCGCTTTAATGTTTTATCAATATACTCAGTGCTTTTAGAAAAACAACGATCAAGTTGAACATCTTAATG
TCTACATTCAAAGTTTTATCGACCTCGGTTATGTCCCAGCGAACCATCAGTTTTGCAAACCTTAGTCTCGCGTCTGATTTTTAT
CAATATCATAGTGCTCTTATTAAGAAAAACAGAGATCAATCGATTAAATTAACTTCTAAGTTTTATGGACACTGGGTTATATAG
CAGCCGAAACAATTAAGTACCTTTTAAAACTTAGTTCGCGTTATTGTTTATCAACATGCGCTCGCTCTTTAAGAAAGACAACGA
TCAATTGATTTAAGATTACATTTTCCTAATTATTATGACCTGGTTTTGTCCAAGGAACTCTCTAGTTTGAAAACCTTAGGTTCC
GCGTTATTATTTATCAATATCTCGCTGCTACTTAACGAAAACAAAGATCCAGTTGATTTAAATGAACTTCTAAGATTTTATGCT
GGTTATGTCCAGGACTATAGTATTGAAACTTAGTGCGCGTTAATGCTTTTACTCGATTCTCGTCCTCTCTCTTTAAGCAAAACC
CAATGATCAACTTGCATTTTTAAAATTACTCTCGTACAAGGTTTTTAATGACGCCTGGTTTATGTCCAGGAAACTTAATGTTCG
CGTTAATGTTATTTATCCAATATCTTCGTGCTCTTTAAGAAAAAACAACGATCAATTGATTTAAATTAATATCTAAGGTCTTTT
ATGACCTGGTTATGTCGAGGACTCTAGTTTGAAAACTAAGTACTCGCGTTAATTTTTATCAATAATCTCGTGCCCTTAAGAAAA
CATATGATCAACTCGATTTAATTAGCTTTCTAAGTTTACTGACCTGGTTATATCCGGAAACAATAGTTTGAAAACTGTAGTTCG
CGTTACATTTTTATCAACATCTCGTGCATTAAGAAAAACAACGATCGAATTGATTTCTTAAATTATTTTCAATTTTTGCTGGTT
TATGTCCCGGGAACATAGTTTTTAAAACTTAGTTTCGCGTTAATTTTGATCAATATCTTGTGCTCTTTAAGAAAACCCAAGATC
ATTTGACTGTTAACACTTTAACTTTAAGTTTTAATGACTGGTTATGTCCAGGGAACTTAGTTTGAAAACTTAGTGCCGCTGTAC
TATTTTATCGATATCTCGTTTCTCTTGTAAAGAAACACAGATCCATCTGATTTAAATTAAGTTCTCAGGCTTTTATAGCCTGGT
TAGGTCCAGGTTAGTTCTTCGCCGCTTAGATTTTTAATCAATGATCTCGTGCTCTTTAAGCAAAAACAGACGATCAACTTGATT
AAATTAACTTCTAAGTTTATTGACCTGGTTCAATTCGAGGGAACTTAGTTTGAAAACTAAGTTCGGTCTAATCCTTTATATCAT
ATCAGTGCTCTTTTAAGCAAAAATAATAGATCAATCCAGTTTTAAATTAGCTTCTAAGTTTTTATGACTGGGTTTTATCCGGAA
CATAGTTTTGAACTTTAGTTCGCGTTAATTTTTCATCAAACATCTCGTGATCCTTTAAGAAAACAACGCTCACGCTTGATTTAA
ATTAATCTTTCCTAATTTTTATGACCTGGTTATCGTCCCGGGAACTCCCTGGTTGTTTGAAAAACTTAGTTCGCGTTAATTCTT
TATCAATATCAATTGTTGCTCTTCTAAAGCAAAACCAACAATCATGTTGTTGATTTAATTAACTTCATAAGTTCTCTTACCTGG
TTAGGTGTCCTGCGAACTTAGTGTGAAAACTTAGTTCGCTTTAATTTATTATCAAGTATATATGCCTCTAAAGAAAAACCAACG
ATCAAATTTGATTCTAACTTAACGTTCAAAGTTTATGACCTGGTTAATGCTCCAGAGAACAATAGTTTGAAATTAGTTGCGTCT
GATTTCTTATCAATATCTCGTGCTCTTTAGAAAACAACGATCAAATCGATTTAAATTAACTTCCTAAGTTTTTATGCACCTGGT
TATACTAACAGAACATAGTTTACCTTCAGCTCGCGTTACGATCCTTTTATCACAATCTCGCTGCTCTTTAAGAACAAACAACGA

CTCAAATTGATTTACTTAATTCTCTTAATTGATTGAATGACCTGGTTTACTCGTCCGACGCCACATCACAGTTTGGAAACTTGT
TCGCGTTAATTCTTTAATCAATATAGCTTGTGCTCTTTAAGAAACACCAACCATCCTTTGATTTTCAAATTAACTTCTAAGTTT
TATGACCTGGTTATGATCCAGCGAACTTAGTTTGAAAACTTAGTGCGCGTTAATTTGTTTTATCGATATTCGCAATCGTTCTCT
TAAGAAAACAATGATCAATTGGATCTTGAAATTAACTTCTAGGTTTATAGACCTGGTTAATGTCCAGGAATACGTTCGCGTTAA
TTTTTATCAATATCTCGTGTTTTAAGAAAAACAACGATCACAAGTGGTTTAAATTAACTTCTAAAGTTTTATCGACTGGTTTAT
GTCGACGGAAACCTTAGTTTGAAACGAAGATTCGCGTTATCTTTTAATCAATATCACCAGTGCTCTTTAAGAAAAAATATCATG
ATCAATCGATTTACATTAGCTTCTCAAGATTTATGACCTGGTTATATTCCAGGAACATAGTTTGCAAACGTTAGTTCGCGTTAA
TTTTCTATCAACATCTCGGCTCTTTAAGAAAACAATCGATCACTTGATTATAAATTAATTTCTATTCTTATGACCTGGTATGTC
CGGGAACAGTTGTTTTGAAAACTTAGTTCGCGTTATTTTTCATCCAATAATCTTGTGCTCTTTAAGAAAACCAAACATATTTGA
TTAATTACCACTTCTAAGTTTTATGACCTGGTTATGTCCTGGAACTTAGTTTGAAAACTTAGTTCGCTATTAATTTTTTATCAA
ATACTCATGCTTCTTTAGAAAAACAAAGACATCAATCTGATTTAAATTAACTTCAACAGTTTATGACCTGGTTATGATCCAGGA
ACATAGTTTGAAAACTTAGTTCGCGTTGATTCTTTATCAATATCTCGTGCTCTTTTAAGAAAACAAACGATCAATCGATTTAAA
TTAACTTCTCAAGTTTTTATGACTGGTTATATAAGGAACATTAGTTTAAAACTTAGTTCCGTTAAATTTTTATCACATCTCGTG
CTCTATTAAGAAAGACAACGATCAATAGATTTAATTAAACTTCTAAGTTTTATGACCTGGTTTATATCCCAGGACACTTAGTTT
TGAAAACTTAGTTGCGTGATAGATTTTCTAGCAATATCTCCGCGCCCTCTAAGGAAAAACACACGAATCACATTGCGATTTAAA
TGAACTTCTAAGTTTTCATGACCTGTTATGGTCCGGAACTTAGTTTGAAAACTTTATCTTCGCGTTAATATTATTTAATCGAAT
ACATCGTTCTCCTTTACAGAAAAACAAACGCTAAACATTGATTATTAACATTAACTTCTAGGTTTTATGACCTGGTTATGGTCC
AAGGACTAATAGTATAAAACTTAGTTCGCGCTTAATCATTTATCAACCGTACTGTGGCTCTTTAGCAAAAACAACCGATCCAGT
CAATTTAAATGTA
>m141122_040740_42134_c100732202550000001823141405141561_s1_p0/278/0_14870
AAAAATAAGAGAGGAATTAAAGCTCTTTCGGTGTCAGACAATACAAGTTTATAGAGCCGTTAGCATTAGAAATATTTGTTTAGG
TGATTACTGGATGTCTGTAGGAGAGTTGGGCCAACTTTGGTCTAGTGTGGTGTTATTGGGTCGAGTATGCATTGCGGTAGGCAC
AAGTCTTGATTGACCTATTCAATACGATCGCGTCACGCTGCTGGTTCAGTTGCGAGGTGAGGCCCCGAAGTATTTCATGTTAAC
CTTTCTGATATTTTGGGAGATCAGACCAATATAAAGCCACCCAGGATTGAAGTTTTTAAAATGTGGCGGTTTAGATTTTGAAAC
GGGTTTCAGCTAAGACTATTCAAATAGATCGAAGCTTGTGCACTAGGGAATAGCAAATGTGAGTTTAAGATCGCGTGATAGAGG
ATGAAGGGCACTCTTGTGTGATGCTTTATGATTATTGCGTAGGGCTAGGTGTTGTGTGTCATGGTGAGTGCGGCGTAAGCCTAA
AAAGCTTGTAAATGTACCTTGCGAGTATCGTCGTGCCTACATGATTGGTAAAACCGGCGGTTATTGTGAGTGAAGGTGTCCTCG
CTGTATGCGAAAGACCTCACTATGCCGATGGGTGGCTTACTTGTTGTGACGTCGGAGCCATGCTATACATGGGGGACAAGGGCG
CCCTTGCCACGGCACTCTGCTGCCGAGCCTCCTGTCTACGCGATCGCATATCTGGCCCCTGACCCTGGCGCTTGACTGGAGGGA
TTTTTTGGGAATAATCTACATGGAAAGCATAAAGAGGATAGCGAATTTGAGGATGTTGAGGGTTGAGAGCTTGCTCTTGACCTG
AAGCATGGATTGTTGCCAATATGGAGATATCTTTATGAAGGTTTGATTGTCTTGCCCTATGAACTAGGCTCATAAGCTCCTGA
GTGATTGGGATTGAGGTGAAAATTGTGGTTGTAGGAGGGTGTGAAGGTAACGAGAGCGTTGTGATGGGAAAAGATCGTTAGCGG
GAAATATAGTAGAGATGAAAGAGCACTGAACTTGATTGTGTAGACTTGTAGTCTTGGGATCTGAGGAGAGCGAGTGGTGCACAA
GACTTGGAAGTGATTAAACTTTTGAAGAAAAATGTATATGGACGTATGCCCTGTATGTTTTGTAGGGATTGTTAGCAATGTGAA
GTCTCTAATGGCAAATCTAACCGGCACAGACTGATTACTGGATAGAAAGATGGATGGCGCGCGAGTAGAACTTTGTATTGGATT
CCTGGACCATGGCTCCGCCTTCGACACCATCCAAAAGGTCGTTACGCCGTTGCATGGATCTTGTGACCGTTTTGTTTGTTTTGA
GCATAAGGCTATTTTCAATATTTCTAGAGGAGTTATAGATCGCTGTGTAAGATATACGTTTTTTGTTGGCTATGCGGATTGTTT
TGTAAATACTGTGGCCATTGATAGAGCCCTCCGTAATTTATATGGGTGAATAGCTCCTTCATGGCTTGAAGTGAGTTTTTGGTA
TAAACTGTAGTGATACAAGTATGGAGTCTTAAGATGATATGGATGTTTGCTTCGTGGTCCTTATAATGCTTTTCATGTAGGTTT
TCTCTGTTGTTGGTTTAGTGAGATGTTTTTTCATAATCGTTGATGTTTAATCTAGCATTATCCTAGAATTTGGTACTTTAAAGT
GGAGGAAAGTTCGAAAGTTGTTTCTATAGAATTCCTATATTTAATGTAGTAACGTTGGGTTTGACTTGATTTTTTCTAAACGTC
TCATATGATTTGTGTTGCGACTGTTGTATGGTGAGATGTTTATGGCTTCATATATCTACTGAATGCTCTGTCATTCTGGAGGTG
TATTTTAAATGGTGGTTATCTTTAAGTTCCGCAAGTGGGAAAAAAATGCTGACGAACATTGTGAAGGATTAAAGGGGACTCTATG
GGGGGCAGGTCGGAAAGAATACCACCTTACATATAAAAGAAGGGTCTAATAGGATAGACTGTTTGTGAATGAGGCGTTTGTGAA
AGTCTCTTTTGAAAGAGCATCCTTCTGATCCACGTTCTCGCTAACCAACATTTCGCATGACGCTAGGCTCGCAGGGACTTGATA
GTTACGTTCATGTCAAATAAAATTCGGAGTACTAGAGACAGCTCGAGGTGGCTCGTTTATTCAGGTTCTCGGCCATCTGCCAAC
TAGTTGAGCGCGGTTGTGTTCTTCATCTCCAAACTTTCCTCCTCGAATTATCATGCGTTATTTGGAGGCCGTCCGGAGCACAGG
AGCGGGCGAGTTTTTCTTATTCTCTCCATTCTTGCGATGACCTCTTAACCTAATGTGTAATTAGCCGAACTCCGGCCGGAGACG
AGTCACTTTCTGAACGCTCAGATAGTTAGTATATTAGGAATTCCCGGTCCATGTCCGTGAGCTCTGCTGATGTAAATATATCGT
TTCTTGAGCGAGAAGGTATTACTGTTGACTGTACCCGTACAAATCAAGACTGGGACTACTATTCTTTTGAATTACGTGGCGGAA
GAGGTTATTAGCTGGAAGTAGGGCAGTTGTGTCAAGGATTAAGGTTTCGATACTAGTGGAAGTATGCTATCAGATAATGAGGTT
ACGAGAAGGGGGATCGGTTGATCATGTCTCCAGATAGAAACGATTGGGATGAACGATATTAGGACATTCATCTATATGTTCCCA
CGCCGCTCAGGGAATTACATTTAAGATGCTTCTTATGGTACATATACGCGCACCCAGGAGCCAAGGCAGGGAAATCGATTTGGA
GTGGCTCTTCTCGAAACGAATCTGAGGTCACATCAATAGAGGGGTAGGAATGCCATAAAACCAAACCAGCCTTCAGTAGCTTTC
CGCGAGGAGCTCAACAGAAGAGGGCGAATAAGAGACATACCTCTAACTGTCCCTGGGACGGCAAAGGAAAGGAAGATGTCACCC
GTCCATAGTTGAGGACTCTTTGAGAAGAGGAGGCAAGACTCGAATTTACGGAGATGAAGAGCCTGCGGAGTAAGATTGTGCATG
TGTCTGGAGGGAAAGCTTGAGGAAGAGTTCGCTATGAATTCGCTTTCCCAAAGAGATCGCCATTCGACTCCAGCACTTAAAGGG
CCTATATGACCCAGTATGAAGGAGACCGACCCAAAGTACCATCTAGACGCTTCAATGACGTTAATGAAGCTGAGGGGATAAGTC

GCGGGGTAGTGCCCTGTTTCGAGTCACGTTGAAGGGCGCTACAACTGGTTTAATGGACTTTGAAGGAGGTCCGTCAGGTCTTGG
CAACAATTTCGACGATTTCTCAGCAGATCATGCAGTGCGTGATTATACACTGCACGAACCAGCTAGCCAATGTAAACAGGGGAA
ATGAGAACATAAAAACTATATCACAGGTTCAACATGGAAGCAGCAAAGTGGGAAGCCTGAAGCAGAGAATTTAATAATGGACA
TTCCACGCCGGGTGCTTTCCGGGAAGCAGTTGTGGGATTAATATGCTCAAGGGGAAGTTCACAGATCTAGATGACTTCTACGAG
AGAGCGCAGAAGTATATTCGATTAGTAGAGATGGATTCACGAACTTAAAGGCGGGAATGGGATGAGTCCCCTGCACAACACCAA
ACAAATGAGGGTCGAATGGTGCGAAGAAAGAGGCATAAGAGGATGAGGGAATGATCACATAGAAAACCCAAGCCCGGGGAAAAA
GCGAACCCCCAATAGGCCCACCCCCCCCCCCCCCCCCAAAACACCGGCTTTTTTTTTTTTTTTTTTTACATAAACCTTTTT
TTTTTTATTTCCCACCCTTTTTAATTTAAACGGAACCCATTTTTGACGGAAAATAAACAAAGGGAAGCATAATCCCCTTTT
TTTACAAAAATTCCAAAACCAAAAAAACGAGAAAAAAAAAGCACCCCGGGGGGGGTTTTTTTCCCCCACCCCCCTTTTTCCAA
GAAAAAGGCCACCCCCCCCCCCCCCAAAAACCAAATGAGGTGGGGGAAAAAAAGGGATCGGTTTCCCCCCCCCCCACCCCCAG
AAGGGAAATTTTTTTCCCCCAATTTTTTAGGGGTAAAACCCTTTTTGCCCAAATTACCACAAAAAAGGATTTATTTGGGGTCCC
CACCACAAAAACAAAGTTAAAGAAAAATTGTAATTTCCCCCAACCAAACTTTTTTTTTGAAAAAAAAAGAAGGAAATTTTTTTT
TTTTTTTGAGGAAAAAAACTCAACACAAAATCCCGCAGGGGGGGGGCCCCCCCAAAACCCCCTAGGGTTCACCCCCTTTTTTGT
AAATTTGTCCCAGAGGGAAAAAGGGAAAGGGCTTTTTTTTTTTGGAAAAAAAACGGGGGGGAAAACCGACCCAAAAGTTGGGG
GGGTTTTTTTTTAAAATTTCTTTTAAAGAACAGCAATAGAGGGGAAGGCCCCTGTTCCCAAGAGGATTTCAAAAGGGGGGGAAA
AAAAGTTTTGGAAGAACTATTTTTTTCCGGAGGGTTTCCCAAAAGGGGCCCCCCCCCCTTTTTTTTTTTTTTTTTGGGGGGG
GGGGAGGATTTTTTTTTTTCCCCCCCCCGGAAAAAAAAAAAGGGCGAGATTCGGTTACAACAAAAATTGCCCCTTTTTTTTTTTA
AGAACGAAAGCCCCACCAAGGGCCAGGAAAGTTCCCCCACAACCCCCTGCGGGTTTTATGGAGTTTATTTTTTTTATATTTTTT
TTTCGGAAACAAACGGACCCCCCCCCCCAAAAACCCCCCCCCCAGAAGGAAGCTTTTTCAAAGGGGGAGAAAATGGAAAACC
TTTTTTTTTTTTTTTTTCCGAATTCAAAAAAAAACTTTTTTAACTTTTTGAGGAAAAAAGGGGACGCCGGCGGGGGGGGGT
TTAAACAACTTTTTCCCCCCCCCACAGAATGAATTCCCCGCTTGGTGGGGGGGTTTTTTTTTGGGAACCAGTTTTTTTTCCCCC
AGTTTTTTTTTTTTGGGGGTTTTCCCCCCCCCCAAACCGCGGGGGGGTTTTTTTTGGGGGCCAAATTCCCGTAAGTTTTTTCC
CCCTCCGCGTAAGAAACAAATTGGGGGGAAAGTTTTATTTTTTCCCCCCCCCCCCCGGGTGGGGGGAACTTTAAAACTCCCTTT
TTTTATTTCCGGCCCCCCACCCACACTGGAGGAAGGGAATTTGGGGTTTTTTTTTTTTTTTTTTTTTTTTTTTTTTTTTTTTT
TTTTTTGGGGCCAAAAATTCAAAAGGGCCAAAAACTTTTTGAAAAAAGCCCCTTTTTTTTTGGGGGGTACACCCCCCCCCATTA
CACCTTTCCCCCCTAAATAACGGGTTTTTTGGTAGGGGAAACTCGGAAATTTTTTTACCAACCACTTTTTTAAAAAGGAAAAT
GCGGAATTCGAATTTAAGCCCCAAATCCTTTTTTTTTATTTGGGGGGGGGAAGAAACCAAACCCCCACCGGACCTTTTTTTAACC
AAACGGAATTCCCCCAAACAAAAATTTCAAGCCAAAAAAAACTTTTTTTTCGTCCCCCCGTAGTTTTAAAAAAAAGAAATTTTT
TTTTTGTTGGGGCCCCCCTTTCTTTTTTTTTTGCTTTTTTTTTATTTTTCCAATGCCGGTTATTGGGGTGGGGGGGGGGAAAGA
AACCCCCCATTCCCACCCTAAGAAGAATTTTGGGAAAAGCCAGAAATTCAACATTTTTTTTTCAAATTTTTTTATTTACCAAAA
ACCTCAACCGGGTTTAAAAAAGTTTCCCCAACTTCACCCTTTTTTTGGGTGGGGGGGTTCAGCCCGAGGTTAATTGAAATAAG
GGGGAGGAAACAAAAAAGAAAAAGGGCCCCCGAGGGGGGAGGTGGTTTTTTATTTTTACAAAAAAAAATAACAAACTACCCATT
CTAGTTTCAAAAGTGAAAGGCACTTATCAAGAACCACACAAAACAAAAGTGAAAAAATGGGGTTTTTCGGGAAAAAAAATGCCCA
CCACAAAAAAATGGAGGGGGGGGGTTTTTTTAAACCCAGAACCCCCCCAAAAAAGGGGAAAACCCAAAAAGGGGGAACCCCCCAG
AAAAATTAACCGAAAAGGGGCCCACCCCAACCGAAGGGGGTAAAAGAACCCTTTAATCCACAAAATGAAGGGGAACCTTTTTTC
CCAAAAGCCTTGAAAATTTTTTTTACCAAAGTCGTTTTTGGCCCTTTTTTTAAAAAAAATTGGCCCCTTTTTAGTTTTTTTG
GTTTTTGCAAGGGGTTTTTAAAAAATGGCCCCTTTCATTTTTTTTTAAAAAAAGTAAAAGATGAGAATCCCAAAAGGAAAGGGT
TTTTTTTAACCTAGAAATAAACCCCCTTCCCCCAAAGGTAAGAAATTTTTGGGTAAAAGCCATTTTTAAAATCTTATTAAATAA
TTAATCGTTTTTGGTTTTAAATTCACTTTTGACCTTTAAACCCAAAAAAAAAAACTGAAATTGAAAAAACTTTTGTCCCTTTTTT
TTTTTCCCCACCTTTTTTTCGTTGGTGGTAAACCTTTTTTTTTTTAACTTTTTTTTTTCCACTTTTTTTTTTTTTTTTTTAAATT
GCCCCGAATTTTATTCAAAAAATTCTTTTTTAAAAGTACCCTTTTTGCCGAGAAAATTTTTTAAAAATCTTTTTCAACCGGTTT
TTTTTTTTTTTAAAAAAAACAAACTTGGGGGGGGGCCCCAAGGGGAAAAATTGAAAACAAAAAAAGTAAAACAACAAAAAAA
AAAATTTGTCTTTTTTCCTTTTTTTTTTTAAAATTATTCAACAAAGGAAAAAAAAAATTATTTAGCCTTTTTACCCCCCTTTT
TTTTTTTAAAATGAAAATAATCCCGGGAGGGGATTTAGTTTTTTTTTTTTTGTTCCAAAAAGGAATTTTGGGAAACCCCCCC
CACCCCCTGGGGAACGAAAAGCCCCGACCAACCCCCCCAAAAGGGAAAAAAAAGGGGTTGGAGGGCTGGTAAGGAGGGGAAATT
CACCCCAAATAAGTTCCTAGAAAAAAAAATTTCGCCCCCCATTTTTAAGCCCGGCTTTTTTTTAGCCCAACCCAAAAAAAGGG
GGGGTCCCCCTTTTTTTAAACAAAGGGAACACCTTTTTTTTTTTTTTTTTTTTTGGGGGAAAAAAAAAAATTAAGTAAAATTAA
AAATTGTAAAAAATTAAAAAAGGGGGGGGAACGAAAGGAAAAGGGACGACTTTTTTTTTTTTCCCCCCTTTTTTTTGGGCAAA
AAAAGTTTAACAAACAAAAAAAAAGCCCGGGCGCCGCCAAAAAAAAAAGAAAATTAAACATTAAAAGAAAACCCACCCCCAAAC
AAAACCCCCCCCAACGGGGTTTTTTTTTTTTTTTTTTTAAAGTGGGGGGGGGGGTTCCCTTTTTTTTTTACCAGTTTAAAAAA
ATAAACCAAAGGGGGGGTTTTGGGGCCACAAACCAAAAAAAAAGAAAGGGGTTATTTTATTTCCTTTTTTTTTCCCCATTTCAA
ATTTTAAAAAAAAAAAAAAAAAATAAAATTGGGGGGGTAAATCCAAACGGAACAAAAAACAGTTAAAAATAAAAAAAGAGTTTGA
TCTACCCAGAGGGCCCCCCCCCGTTTTTTTAAAAAGTTATTGAAAAACAAAAAAAAAAGAGGAAAAAAATGGGTTAACAAGGGGGG
GATTTTTTTTTTTTTTTAACATTTTAACAAAAAAAAAGCAAAAAAATACCCAATTAAAAAGGATTTAACGATGGGTTCAAAGGGG
GGACAAAAAATCCCTACGGAAAAATCATTACACCCCCCCAAACGTGGGGGAAAAATTACCCTTTTTTTCCCCCAACCTTTTCCAA
GAAAATCCCCTTCCTTTTCGCCGGGCTGGGGGGTCCCCTACAAAAAAAAGGGAGAAAATACTTAAGGTTTTTTTAACGGTGGG
GGGGGTTATTTTTTAGTCACCCTCCAACCCAAAACACAAATTTTGGCCAACGTTTTTTCCCCCCCGGATTAAATGGGGCGGATT
TCCACACCCCTTCCCCCAAACGGTTTTTTTTTTTTTTCCCAAATTAAAGCTTTCAGCTTTTACCAAGCCAACCCAAAACGAAAT

TTTTCCCCTTCCCACCCTTTTTCTTTATTTTTTTTTCCAGAGTTTTATTTTTTTTTTTTTTTTTTTTTTTGGGATTTTTGGTAGG
CCCACCTTTTTTTCCCCAAACCCCTGAAATGTTTTTTTTTTTGGGGGGCTTTTTTTTTTTTCTGAAGGAGGGGTTTTTTTTT
TTGAAAAAAAAGGGGCCCCAAATAAGCCTTTTTGGGCCCACCAACCAAAGGGGGCAAAATTCCCCCACCCCCCCTTTTCAGCAG
TACTTTTTGGGGCCACCCCAAAACCGATTAAAGGGTTTTTTTTTCCCTTCCAAAGCCCGCCCTCCCCCCCTTCAAAAGATCGGT
TGGGGTTTGGCCAAAAACCCCGTTTTTTTGGGCCGAATGAAGGAGTGGGGGGAGGGCCCCAAAAAAAAAAAAAAAAAAAAAAAG
CCAGCCGGAATTGGGGTTTTTTTTTTTTTCAAAAAAATTAAGGAGGGGCCCCTTTTTTTTGAGTGAAAAAGGAAACCCCCCGAA
AAAGAATATGAAAAAAAACAAAGCCCCCCCTTTTAGGGGGGGTCCAAGATTGATTTTAACACCAGACAAAAAAAGTTTTAAATT
ATTAAAAAAAGGGGTAAAAAAACAAATTTTTTTAAATGGGGTTGGGGCTTTTTTTTTTTTTAATTTTCCAAATAAATTAATTAAA
TCTTTCAAAAAAAAAGTTTTAACAATCGCCCGACAAAGTTTTTTTTTTTATTTTTTTTATTTTTCTTCTCTTTTAAAAAAACC
CCTTTTTTTGGCAAAAGGGGGAAGGGGGGGGGCCTCGCAATTAAATAGGGGCCAACCCCAATTCCTTTTTACAAAAAAAAAAA
GCCCCTTTTATCCCCAAAAGACCCCTTTCCGGGAAAAAAAAAAATTCCCCGGTGCCCCCCCAAAGCGGTTCCCCCCCAATTTCCC
CCCACCCCTTTCGGGAAAAGTTTTTTTTTTTTTCCCAAGTGATGGGCTTTCTATTGGGGGCTTTTTTTTCCCCAAAATAATT
TTTTGCCTTCGCCCAAGGCCCGGATGGATCCCCTTTTTTTTTTTTTTTTTGGGAAAAATTAAAGGGAAAAAACATTTTGGCT
TGGGTTTCCGGGGGGAGGCCCCGCGAGTAGCACCTTGTTCGAGGCACACGTTTTGGCAAAAAAAGAAATGGGGAATTTCGAGAA
AAAAGGACCCTGGGACCTTTTCGTCCCAAAGTAGTGGGCGAAGGAGGCAGAAGAGGGTTGCAAGGGTTTCCCCGTTTTCCTTTT
CTTGGGGGTTTTTAAAGAGAACAAAACCTTGTGGATTTTTGCCACCCCCAACCTTTGGAAAAAAAAAAATTTGCCCCCTTTCGG
GGGGTTGGGAAAAGGAAACCCCCATCAAAAAAGGGGGAAAAATTTTGGAAACAAAAAAAGAAAATTTTTTCGCTTTTCCTTAGG
CATAAAAGGGGGGGCAAAAAAGGGGCCCCAAAAAAAAAAAGGGGGCAATCAAAAAAGGGCAAAAAAAACTTTTTCGCCGCCCAA
AAGGCCGGGTTTTTTAACGGCCGAACCAATCCCGAAAAAGAGGGTAGCAAAAAAAACCGCAAAGTTAATGAAGAAAACCGTCCC
AAAAATTTTTTTCGGCTTTTTTTTTACACCCCTTTTTTTAAGCCTGCCAGCAAAAAAAACAAAGAAGTTTTACAAAAGCAAAAA
GTGAGTTAAGTTTTCCCTTTTTTTTTTTCAAGAAAACACCGCAACGGTTCCCCCCTCCTCAAACGCATCCAAATTATGGGTTTT
TCCACCCCGGGGAAGGTTCAGACCCGTTAAAAAATTTCCCCCCCCCCTATCGGCTTTTTTTTTTTTTTTGCTTTTCAAAAATT
TCATTAATTTTCCCCCTTTTTTTCAAAAAATCGTTTGGAAAACCATAATTAACCCACAGAAACTCTTTCAAAGTTTTTAACGGG
TTTTGCCGCTTGGGGGGGTTTTTTTAAATTTCAAATAAAGAGTTTTCGGGTTGACCACGGGGGGCGGGTTGGATTTTTTCCCTT
TCAAGAATTTATTTTTTCAAAGGGGGGGAAATGGGGGAGGCATAAAAGGTTAAAACTTTTTTTTTTTTTCTTGGTCCGGGGGTT
TCAGCAGCCAAAAAACCCAAAGTTTTTTTTGGGGAAATTTTGCTTTTTCCTTTTCCCGTGAAAAAGGGGCGCTTTTTTTGGCT
TTTGTTTTTTTGGCGTTTTTCCTTTTCGGCAAATAGCCCAGGTTTTTTTTTTTTTTTTTTTGTATATTTAAAAAAACCTGGGTCC
ATAGAGCTGCCGGTTTTTTTTTTTCAAATTTAAAGGGGAAAAACCAACGGACCCCTAAAAGAAAGACTTTTTGCAAAAAAAAAA
AAATTCGAAAAACGAAAGGGGAAACATTTTTTAAACCACAGTTTTTTTTTTTTTAACAAGGAAACTTATTAAAAAAAAAAAAAA
AAAAAAGAAGTTTTTCGAGAAATAAGGAAAATTTTTTTTCCCCTGGGTAAAAATAATTTTGGGGGAATTTCAAAAGTTTTTTTG
GGAAAAGGGATTTAAAAAAGCTTTTTACGTTTTTGGGGGGTGTTTTTTTTTTTGAAAAAAGTTAGAGGTAAATGGCTTGCACTT
TTTAATAAGTTTATAAGAAAGTAGTCCTTTTTGAACTTTTTTTTTTTTTCCAAAAACGCCCCCCCCCTTTTTTTTAAAAAAATTT
TTGCAAAAATTAGATTTTTTTCCTCCCAAAGGAAAAAAAAAAAAAAAGGGAAAAACTTTCCCCACTTTTTTTTAAAAGGGCCGCG
GCTTTTTTTTTTTTCCTTTTTTTTTGGAAAAAAAAATTTTTTTGGTAAAAAGAAAACCCGGGGGGTGATTTTGGTTTTTATTTT
CCTCCCTTCAAGGGGTTTTTTTGAAGAAAAGTAAATGAAAAAAAAACCTCACTTTTTGTTTTTGGAGGCCCCCTTAGAAGTTAA
CCAAACCTTTTGATTTTTGGAACAAACCACAAATACAAAAAAACCAGCTGAGCAAAATCCACCCCCAAGGGAAAGTTTGTTTTT
TTTTGGGGGGAACAATTTTTTTTTTTGTTAGTTTTGGGCGCAATTTCCCAAAAAAAAAGCGGGTAAAAAGTTCGCAAAAAGCAAA
AACTTTTTTTTTTGATTTTGAAAAGACAAGAAAGTGGGAAAGTTGGCAGAAAAGCTTTCCAACCAGATTTCCGGATTTTTTTTT
TTTATTTAGGGCGGATCGCTAAAAATTGACCCCAACGAACACCTTTTTTTTTTTTTTTTTTGCCGGGGTTTGGCCGACCGCGC
GTTTTCCTTTTTTTTTTTTGGCACTCCTTGGGTAATAAAACACAAGAACCTTTGAGGGTTTTTTTTTTTCAAGAGGTTAAAAAAA
CTTTTTAAAAATGGCCTTTTCAAGCAATTTGTTTTTTTTGCCCAAAAATTTTTTTTTTTTTCAAAAGGTAAACGGTTAGAACC
TTTTTTTCAAAAAAACTTTCATAAGAAAAAAAGGTTTGGTTTTTTCAAGACTTCTCTTCGCATTCTCCGGGTTAAAAAAAAACA
CTTTTTTTGGACAGTAAGGCAAAAAGTTTTTTTTTTTAAAAATGAAAAGCCCAAAAAAACAGGAAACTTTTTTTTCTTTTTTT
TTTAAAGAGGTTTTTTTTTTCCTTAAAAGAAAAAGAGTTCCCCCTTTTTTGCATGTATCCTACAAAAACGTTTTCTACCCGCA
AAAAGCTTTTTTTCTTTTTTTTGGCCTTTGGAAACAAACCCCTAGGGCGCAAGGAACAGGGATTGGAGAAGGGGAAAACAACGG
GCCAGCAAAAAACCAAATGGGAAAAAGGGAAAAGCCTTTTCCCGGCTTCAAAAAACAAATTTCAATTTGTCTTTTTTTAACGCG
CCTTTTTTTTTTTCAAAAGAGGAAGGATCCAACGTAATTTGGGGAGACTCGTTCAACGACCAAAAAAAAAAAAGAAAAAAAAAA
GGCCTGCAGGGGGCCCCAAGTTTTTTTTTTTCATTCTTTTAGGCAAAAGAATTTTTTCCAGAAAAAAAGGCAATTTCCCCTTTG
GAGGGGGTTGGGGTGGATTGGTAACAAAAAAAAAAAAGCCTTTTTGGGGGGAAAAAATGTAAAAATGTCTTTTTTTTTTTTTT
GGATTTTTTGTCGGCGGGAAAAAGAAGCAATCAAGGAACCCGCGTCCGGATTTTTGGGGAAAAATTCTTAGAAATCCCCTTCTT
CCTTTTTTTTTTTAAGACCAGTTCAAATTTGCCCCTTTTTTTTTTTTTTTTTAAAAAAATTTGTTCTTTTTTTTTTTTTTAAAAG
GTTTAAAGATTTTAATCTCTTTTTTTTTTTTTTTTGAGGTAAGAGCCGCCCCCGCAACACCCCGCTGCAGGTTTTTTTTAGAGGG
GTTTTTTTTTTTTTTTTTTACCATGCCCCCGGTTCGCGACCCTTTCCCCGAGTTTTTTGGAAAGGAGGAAAAACCTTTTTCAAA
ATAAAAATTTTCAACATTTTTGGGGAAAAGCCGGACTTCGGAACAAAATCGCCCTTAAGAACGCCAAAAAAAAAAAAATGGAAC
AAAAGGCTTTTTTTTTTTTTTTTTTTAAGGACCCTTTTTTTCGAGGGCGGTTTTGTAACGCAAATAAGGGGGCGATTCAAATT
ATCTTTTTTTTTACAAACAAACGAAAGTTTTTGGTTTTTTTTGAAAAAGGGTTTAAAAAAATTTTTAAGAAAAAGGGGGGCCCGG
TGTTTATGTGGTTTTTTTTTTTTTAACTAAAAGAAAAAGGCCTTTAAGTTGCGCCGAAGAAAAATTTTGCTTAAAAACTCTTTGC
AGGTTTTTTTTTTTTTTTTTTGAGTGGTCCTTGCAAACATTTTCAAATCCCTACCCCCGGTTTAAAAAAAAATTAATCAAATGCA

AAACAAAAAAAGGGTGAAGAAGAAGTTGAAAAAGGGAAAACAAAAAAGAAACAATAACCTTTGTGGGGGAAATACGAAAGCTTG
AAAAGGGGACCGCAAAGGACAAAAAGACTTTTTAAACAAAAAGGAGCCAAAGGAAAACAGCAACAAGATTTCTGTTAGGAAAAA
GCGAGGGGTTTAAATGCCAAGCGTTCCCAAAACCAGCGAAGCTTTTAAGAGAAAAAAAAAAAAAAAATTTTAAACAAAAAAAAA
AAAAAAAAAGAGAAAAAAGAAAAAAGATAAAAAGAAAAAGGAAGGGAAAACAATTTCGGGGAAAAATTTAAAAGAAAAGGGGGG
TACCCTTTTTTTTCAAAATTGCTTCTTAAATTTTGCGATAGAAACGCTTTGGAGCTTCTTTTTTTTTTTTTTTGGAAAGAGGCT
TTTTTTTTAGAGGGAATACCCAACTAAGGCTTAAAGCCCAATCTTTTTTTTTTTTTTTTTTTTTTTTTGGTGTTAAAAAAAGAGG
AAGTGGTTTTCAAGCTTTTGGGAGTTAAATTTTTTTATAGGTGGAAAGTCCCCAACAAAATTTTTGCTTTCCTTAGCCCCCTTT
TTTTTGGGGATTCCTATTTTTTTTTTTTTTTTTCCCTAATTTAAAAAATTGGTGGGGAGATTTTTCAAGAAGTTAAAAATTTTT
TGGGGTTTTAAACCTTTTTGGGAAAAAATTTTTTCCGCGATTGGAGGATTTGAATTTTTTTTTGGTTTCCCTTTTAAATTTGCA
ATCTTTTTTAAAGGGATTCTTTTTTTTTTTTTTTTTTCAATTTGGATTCACCCAAAGTTGTTTTTTTGGGGTTGGGATTTTTTT
TTTTTTTTTTTTTGTTGTTTTCTTTTTGGACACTAAAAAAGAAAAAACAACAAACCCAAAAGCTTCACGCTTTTCATTTGCTTT
TTTTCTTCAAAAAAAAATTGGCGTTTTTTTTTATTTTTTTTTTGGGAAAAAAAAATTTGCAAGGGAAAAAAAAAAAAAAATTTTCCC
CAAAAGGGTTTTTTTTTTTGGGACGGTTGGCAATTTGTTCAAAGGCCAAAAAAGAGAAAAGAGAAAGAGAAGGTTCCCTTTTAT
AAAAACCAAAACACCACTTTTTTAAAAGTTGTACTTTAATAGACTTCGGGGGGAAAAGTTTGGCGTAAAATGTGGGAAAAAAAA
TATTTTTTCCCCTTTATGCGGGTTGAATTTTTTTTTTATTTCTTATTTCCAGAGTGGGGAAGGCTTTCTAAAAAAAAAAAAATAA
CCCGAGAAACAAATCCTAAAAAAAGACGTTTTTTTACAATGTGGTAAAGGAAAGGAAGTTTTTTTTTTTTTTGGCCACCTCGCT
TTGGGTTTTTGGGAAAAAAAAAGGGCCTTTTTTTTTTTTTTTTAAGCCGCCTTTGCAAATTCAAACAATCACCCCGCTATGGCGAG
GGAAACCCCCTCCGGCTTTTTTTTTTGAAATTTTCCCCAACGTTGGGCGCACCCGTTTTTTATTTTTTTTTTCCAAATTTCAAG
GCAATCCAAGTTTCCGACGCCTTTTTGCAACTCGGGAAAAAGGAAAGGAAGTTTTCCCAAGCTTTCGCTTACCGCCCCGAGGCA
AAAACCTCGCTTTTTTTTCTAATGCGAGGGGTGGGGGGTTTTTTTTTTGTCCCCGGTGGCCATCCGTTGCCCTTTAGCCTCG
CGAAACGCTTTGCCCCAGGAGGAGGAAAGGCAGCCTTCGGTTTAATGGCGAAGGGTTTAGTTGGGCCCATTTGCCAGGCACTAC
AAATGCTTTTGCCGAGGCCCTCCCAATGTTTTACGCCAATCTGCAAAATAAGCTTTTGGCCTTAACCCCTTTGGCGCTTTTTTT
TTTGGGACTGTTTCCTTTCAGGACATGCCAATAGGTTGAAGCTTTTCCTTTTTTTGAGAAATAGGCAAAAAACTTTTGGTTTCT
CT
>m141122_040740_42134_c100732202550000001823141405141561_s1_p0/1651/6426_16298
CCGATAAGATGAAGCAGATGATTGAGTGACTCGACGAGCGACCTGAGGAGACTCACCCAAAATGCAAATAATTTGTGTAGCACA
TGCTGGATTTTGCCTTTCTAAAGTATGAGCCATTTCTCACTTACCGAGACCACGAGCATGGTACTCTCCACCCAGACTATGTGA
CGTGAACCAACTACAGGAGGATCAACATGCCAACGATGAAATGCGAAATGCAACAATTTGGGAGCTCCTTGAATCTACGAAGAG
ATCCTTGGATAGCGAAATGTACAGCAAGTCAGAAATCTCGTACTCGCCAGACACGAGGGTATACAGTGAAGGAAAGCGTTGGAA
AGTCTCAGAGGACAGCTGCTCATGCAGGAGAGCTCGGAGTGCACTATCTCACTCGTATTGGCCACGATCAACATTTGATGAATC
AGATGACGGCAGAGTCTACATCCCACAGTTCCCGGACAAGCTGCAGTGGACGGTTATAACTTCATCGTTGGTCATGCCAGTACA
ACTCCACTATGAACGGTCTTCAAGGCGTGCCCACGCCTATATGGTCTTCCTTGGATGAGTAGGTGGATCGTCAAATTCCCGAGT
CAAGGCCAGTTATAAGAAGTGACTGGAAGCTTCTGGTTTGGACAAACTGTCTAGTGGTTGATCAGTTAGAAATGGATGGGGTTG
CTGGAAATAGGGTCAAGATTTTTGAGATAGCTGGTTAGGCATACTAGCTTCCTCCATCAAGGGATAGTCTTGATTCTTCCCCCA
GTATCCTTTTACTGACGTAATACACGGGCCTCTGCATTTTTTCTTCCTCCCGTACGAGTACTGCACTTATGTGTGCTCGGTCGT
AGCAAGATACAAGTACAGAACTTCTCCCGTAATAGGTTTGACAGATAAAAGAGGTTCTGTCGTAGGGGTTTCTTGAGTTCCTG
AAAAGCCAACTCGCATTCCTCTGTCCATCCTCCTCAAACTTCTTGGCTCCACCTTCAACAGGTTAAAAAATGGAAGACAACGT
CCTGTAGATTTTGATATAAACCACTCTAGTGTCCCATCCTTCCAGTCAAACTCTGGCCTCTTTGTTGTTTTCGAGGTGAGGCAT
GTCTATCATAGGGCTGGTATCTTTATCCTGATTAGCTTCTATTCCTCGAGCCTTTAACAATGAACCCCAGGAATTGTCCTGAAG
AATATCTGAAAGAGCACTTCTGAGGGTGTAAGCTTCATGTTATATTTTTCCGGAGCACGCAAAGAATTCTTCAGGTCATCAAAT
GGTTATTGTTAAGTTGAGTTTGACTAACATCCTCGTCAACATAAAACTTCCATGTTTTACCCTATTGCTCGCAGAACATGATGT
TCACGAGCCCTGGTAAGTGGCTCTAGCATTTTTGAAGCTCGAATGCATGACGTTAGACAATATACCCTTTATCTGTTTATGATA
CGCTCGTATTTATCATGGTCTAGGGCATGCGATCGGCAAATCTGGTTATATCCAAGAATAGGCCATCCTGAATGAATTAGTCAT
GCCCTGTCGTGGCACTCCACAGAGCTGCTCAATCCGTGGTAAAGGAAAGAAGTCCTTAGGGCAGCGCCTTAATTGAGGCTAAGT
ATCAATGCAGGTTCGCCACGTCCCATTAGGCTTCGGGACCAGCGCCGATTGGCTACCCAATCAGGTAAAAGCATCCCTCAATG
AATCGTTTTGCTTTTAACCTGTCGACTTCCTCTTTTAGGGCTTTTTCCTAATCGTCACCAGTTGTCTTCACTTTTGTTTGCTTC
GCGGGAAAGACTTTTATCGAATTTACTCGTGCCACTATATTTGGACTTATTCCTATCATGTCCGAATGTGACCTATGCGAAGAA
TCCTGGTTCTTCTTAAAAGCAAATTAATTTCTATTTGGTTTCTTCGTGAAGTTTTCCCCGACCATTTACCATTTTCGACGGGG
TTGGATTCTTCGAGCTTGAACTTCTCGAGCGCTTTCCATGGTTAATCAGCTCTTTCCTCCACCTTTGCGATTCTTCATCTATCT
TTCCAGGATTGTCTCGTCTATTCCTGAAAACGGCGAGCCTTTCGCTCGCTGCTTCTTTCTCTTATGGAAATCTTAAGAAAATTC
CCCCAGCAACGGTCTCCCTTCAAACGTCCCGATGCCACTAGAGTGGAACTTTGATGGCCAAATGCCTTACAGACGAGAATGCCC
CAGCTCCTACTAGGCGGGTCTCTCGAGCAGCGACAGTTGAGGCTGATTGAAGCTCCACCTACTACAAACTTCCATCATCTTGGT
TGCTGAGACTGGGTAGTCCTCCCCACGGTTATAGATGAGTTCAAACTGCAACCCCAAATACAGGCAATGCCTCTCTTGAATAAA
TCATACAATGTCGTGCGCTAGCTTTTAGGGTCTTCGAAGTGTGAGTCCCCATCTTTCTAAGTGCCTTATAAGGAATGTTCACTG
AGCTCCCATTGATCAATGAGAACTCGGTGAACTCTCTTATTCACGATGCTGGAGAGTGGATAACCAGGGGTTCTTATGGGCGAA
ATGAAGTGGCACGCATCGTCATCAGTAAAATGTATCACGGCTGAGATATCATATCTTTTTGGCACTTTGAGCTCTAGGTTTCCG
TAGTTCATGAAGGGGACCCATCCCCAGTTTTTAGCTCTGTTCACTGCCGTATTCGCTTTTTGGGCATTCTTACCCATCCTGTGA
GATGAGGCCCGCCCCGAGATGTTATCACATCTTCTCCATCACTCGGGAGGGGCCTATCTTCTTCTCCTACTCGGGAATTACTCG

TCTGAAGTATCGCTCATAAAAAGAGATCAAGTCCTTCGTTTCATCCTTCACTGCGCTGCATTCCATTAGTAGCTGTGCCCAGTA
TCTCTGTGAATCGCAGTACTTTGCTGGAGTGCTCTCTTGGACTTATAATTTCCTCATGGGGTTCATGGACGCCTGAAAAGGGAC
CTGGATTTTCATTCCCCAGGTAATATTCATCCGACGACCGTTGAGCTCGGTGTACACTTTGTACACGGAGAAAATACCTTTCCC
CTTTCTTCTTCTTTCCCCCTTCTTCCTTGGGTTACTTCCTTCATTCTTCTTCTACTTTGAAGGGTCGTTCCGCACGGGGTTTCC
GAGGCTGATGGGTCACCAAAGGCCGAGGCAAAGTTTTAGTGTTTAATCGTTGGTAGTTTATGGATAGAGATATTATTCAGTTCG
CTTGACCTTCGCCTTTCTACAGTTGACAAATCTCTGAGCTCGTCTATCTAACCGACTCGGTTAACGGACCCCTACGACTTTCTC
TGCATATCATCCAAGAGGGGACTATCCGGCATTACCGTCAGCTTGGCGGCATGCTAGCATGACCGACTTGTCTGACATCTGAGC
TCAGGCAGACCTCCAGATTAAACCTGGTAAGGTAGGCTCTGTCAACGTTTCACTTGGCTGTTGCCGAACCGCTTGGGATCAAGG
ATCGATGCTCGGGTCCTATACGCCCGATCATGGCTTAAACTGCTTTCTTGAGAACTTTTGTGATAGCTGATCCCAAGAAGTGAT
TGAATGTCTCGTTATATTTTTCGGAACCAGTTTTTTGTTGGTCCTATAAGCGATGCTGAAAATAATATGCTCTGACTCGTAACC
CGACATTCACTTGGCTCTCATTATGGTGTTCAATTTGCTCAAATGATGTACGGGGTTCGGTATTTCCCTTTAAAGGCGGACAT
GAGGAATCCGAAACTCTTGAGGAACATGGAGTGTTTTTGGAAAAAATCATGGGAGAGCGAATGGTTCAACAAAACTCCTCTCAG
AAATTCCCGTCCTGACTCCGAAACTCGCCCTCATTCTGCAAGAGCCTAAATGCCATTTCCCTAGCTGCATCAATGTTCTTCTTC
GACTGGGTCCCGCAGGGCTCTTGCCTGAAAAATTGGCTACATTGTCACAAAATTCCTAGCTCAGCGTCTTTGCAGCGGATCTTT
GCGCCCTGTTAGGCGATGCTCTCAGGTCTGGGTTCGATGGGATTACCACTTACCCGATTCTGATTCAAAGGTTGCTCCCGCAAG
CGACGTTCCTAATGGTTTCCAGTATTTCTACGTCCTTCAATCATACATGTGACTGATTCTGCGTCCCCCCTGAGTTCGCGCACT
GCGAGGTTGTCCATGAATTGTTTCCGAGATGGATTTCTTTCATGCTGCCTCGTCTCTGTAAGTGCGACGATCAGAGACCATTCT
GGGCTTCTCGCAGCTGGGTCGAAGACGGCGCCACACACCTCTCTGTTCCCTAGTAGCCCTCCTATTCCCCTGTTTCTGTCCCTA
CCCGCCTCGCCTCTATCACCTGATTGGTTGTGCGTTCTCAGGTGGTGAGGGTTAAAATCGTATTGTGACGGAGGACCCGTATGG
GTGATGGTGGGTCTGCTAAAGCATATTCGGGCCTGGATGGTCCCGCAGTTCGCCGTGCTGCTCGGTAAACATCTGTGTGTTACA
AGGAATTTGAGGTCCGAGCCTCTGCAAGGGCTCCCATTATTCCAGTTTTCTGCTGGTACTCCTGAAGTTTGAGTTGAACCTGAG
CCTAGCCCGAGTGTTTCTTCGGGTCTTGAGGGAGCTAGGTTGCTGTTGCCGGTGGCAGAGCGTTGCGTCCGTTCTTGCTTGTGG
CAGCGTATTTTCCAAGGTCTCCACGAGGCCTAATGGGAGGAACATAACGTCCCGCAGGTGGGTCTTGGTTCTCCTACGAAGGCT
ATAGCTGAGCTGACTGCGCAATCCGCTAGCTAGACCTAACCGAACTCTTCGTCCACTGGTCGTGGGGTGGGCGGGATGCGCGAG
AGTGGTGGGGTGTCCGGTGAAGGGACTGCGGGTGGTGTTTGAGGTGCCACGGAGTGGGGGACTACCGTATCGGGAATTAAGTAG
TACATGTCCTCATCGTCCCTGGCAGGGTCGGAGGGTGCTGAAGTCTTGGAGAATCGGAGGCACTCAATCCGCGGCGTGCAGGTT
TCACCGATCGTCATCAGCTGCTTCCCAGGGCGCCGTTATAGTTTTCCTCATCAGGAATATTCTGATCATTTCGTGCCATGGCTA
ATAGGGATTGTATATGCTTAAGCTCCTCCAAATGAAAAGCACCAAACCTATTGAACGTCGTTTTCGTAACTTTTAAAATGTAAG
CGATAAACAAGGAACTAGTAACGAATAATACAGCGTAACAACAACAAGAATTTGTAGACGTGGTTCAGCAGGTGTAACTCTGGC
AGCCTAAGGGTCAGGAGTCAGATTTAATAAGACTTTGTGAATTTCAAGAAATCCTTCAGGGATGAATTCTCCCAAGAATTTCTC
CGATCAACCAAATCCTCCCCCCTCCCCCTAAGGTGTTCATGATCCTATCTATTTATACCTAACGCGTTCCAGAGTTCGTTCCCA
CATATGCCGCGGAGATACTCCTATTTATTAATGGAATAATACATATAAATTGTAACTCCTATATACCAAGGGAAACGTTCCCTG
CAAGACCATGGCATATAACAGACTAGTTAATATCCCTTTAATACGTCAGGCACAGTTAAACAATCAACTATCCATTTTGACCAT
GCCATGGATTGCCGTCTCATTCAACATGACACTTACGCCTGTCTCGAAGGTCCAGCAACAGCATCTGCCAAGCTCAGCGCTCTA
TAACGACGTACAGTTAATGAGCTCACGGCACTCCCCAAGACCAGCTTGGAGCCCCGTGTACATACCACCCGAGCTTGTCACATC
TCTAAGCTTCATACTCTATCGCCCCAAGCTCGGGTTACTTGAATCTGCAGACCACCGATCATGCCTGCATCAGCTGATGCAAGC
CTCTATTTGACTCAAAAAAGAAAACTTCGCAGGTTCGACAAAATGTCCTTCGAGAATTAGCCCATGCATTCCGCTCTCGAGCGT
TCCGAAACCCTAGAGTCACTGCATATGCGTTTCACGATATTCAAGCTCGCAATCTGCCAATCAGCATCTCTCGATCTCCACAAC
TCTCCGGTTCCCTGAAACTCTGCGGATGTCACACAGCTACTGCTCGCTACTGTACTCCACTCCTACCGCCATACGGCACGTACT
ACTGCTATTGTACTACGTACTGGCTCGCTACTCGCCTACACTCAATGACTACTACAAACTACTTCTACTTCCGCTACTATTCCT
GCATAAACTAACTTGCTGACTTCCGCTCTCTACGTTCGCTCTGCCCCTGCATGCACCCCACCGCTGCACCACCACTCACTGTGT
GCTCACCTATACTCACTGCTAATACGCCTATTTCCATACTACTACTTCGAGGCTCTGAGTGCTACTGGAGGTGCTAACTACTAC
TATTAGTAGACTGCTGCTATACTACTGCTGTACAACTAAAATGCTTGCGATAACTAAAATACCTGGTCTAAACTGCTGCTACTG
CTAAACTGGTATGAACTGCTCGCTAAAACTGCTACTGCTACTGCACTACTAAGTGCTTATAAAACTAACTCCTAAAAAAACTGC
AACTCTAACTGCTAACTACTGCTCATCCATACTGCTTCAGCTACTGCTAAAAATAACCTAGCCTTCTAACAGCTTTGCCACTAT
ACTGCTAATTGCTATCGCCTGACTACTACGCCAGTGCTACTGCTACTACTACTAACTTCCTACTAAGTTGCTACTTGCTGCTAA
ACTACTGCTACTGCTAACACTGCCACTACTAACTGCTACTCGCTTTGCTACTTCTACTGCTATTGCTACTGGCTACCACCTGCT
ACTGCCATTACTACGCTACTCCTAACTGCTGCTACTCAGCTACTATATTGTTATTTGCTGCCGCTACTAGCTGCTACAGCTAAT
GTTAATTGCTTCTAGTGCTACTGGCTGCTACTAGCACATGGCCTATTACTACTACTGCTGCTACACTGCTGACTGCAACTACTG
CCGCTACTTCTACTACTACTGTGCGGCAGCTGCTACTGCTCCCTCGCTGCTAAATCCTACACTGCTACTGCTACTACTGGGCTA
AATGCTGCTACTATACAACTGCTTACTGCTACTCCTAATGCCACCTACTGCTCAATACTGCTACTGCAGCGTAATGTGCTACTG
CTCTCTGTTGCTACCGCTCTAACTCGTGCTTCTCTAATACTAACACTGCCACTACTAACTTCATATATGCGACAGTTTACGCTA
AACTAACACTGCCACGAACTACTGCTACTGCAATCGCTACTACTTAACTCCCACCCCAACATTTACTACATGCTACCACACTTC
ATACTGCTACTGCTACTAGCTATTGCTGCTACACTGCTACTACTACTACTGGCTACTATGCTATTACTCTACTACTACCTAGGT
GCTAACAGCTGCTACTGCTAAATGCACTACTGCTATTGTTACTGTACTAACTATATTGCTACTGCTGCGACATGCTACTACTAC
TACTACCTGCTACTGCTGCTACAACTACTGCTACTGGCTTCTAGTGCTACGTTGTACTTCTAGTGCTACTGCCGCTTGCTGCTG
CTTCTGCAACTACTGCTGACTGCTACAACTGCTACTGCGTACTGCTACGCTGCCACTACTACCATACTGCTACTACTGCTAGCT
GCTGTGCTCCTACTGCTACTACTATACAGCTGCTGCTACTAACTGGCTAATACTGCTACTGCTACTACTGGCCTACTACTGCTA

CTGCTACTGCTTTAACGTACTTCCTACTAACACTGCTACTGTTACTGGCTACTGCTACTCTTGCTACACTACTGCTACTTCTAC
TACTGCTACTGCAACTACTGCCACTAATGCTTCTAATGCAGCTGCTGCTAACCGCTTACACTGCACTGGCTGCCACTGGCTAAT
ACTGACTACTGTTAACAACTGCTTACTGCTACTAATGCTACTGCCACTATTAGCTACTATTTGCTACTGGTGGCTACTACTACT
AATGCGCTACTGCCTACAAGCTACTGCTAATACCTAACACTACACTACTACTGCCACTACTACTACTATACTGCTATTACTCGC
TAACTGTACTTGTGCTACTGCTACTACTGCTACTGCTAATGCACTACTACTACTAGTGCTGCAACTGGCTGCTACTGCTACCTA
CTACTACTGCAATGCTACCACTACTAGGCTGCTGCTAGTGCTATTGATACCAACCTACTCAATGTGTTGCTATGCTACTACTGC
TATTTCTACTTTTTCTGGTTGCTACTACTGTTGAGTGCTGCTGCTTACTACTGCTATGCTACGTTCTACTACTACTGCGCTGCT
GGCTGCTACTACTACTACTACTACTAATGCTACTACAGCTACTGCCCACTATAACTACCACTACATGCCAGCTCACTGCATACT
GCTATCCGCTACAGCTACTGCTACTCCACTACTGCTACTGAAACTGCTAACTGCTACTTGCACTACTACTTGCTACTAGTGCGT
ACTGCTAATACTAACACTGCACTACTACTGCTACTTCATACTGTTATTGCGACTGCTTACTACTGCTAGCTGCTGAATAGTGCT
GCTACTGCTACTGCTACGCTACTAGTGCTACTACTGCTACTGATAACAACTACTGCAGTACTATACTGCAACTACCCCTACTGG
AAACAGATACGGATCGCTACTACTGCTACTACTGCTACTACTACTCTGCTACTACTACTAACTACTGCTGCTACTAATAATGCT
AACTTACTGCTACTGGCTACTGGCTACTGTACTACTGCTACTGCTACCTACATGCTACTACTACCACTGATGCTGTTGCTGTAC
TGCTATTGCTTGTACTACTGCGCTGCTGCTACTGCTACTTCTACTAGTGCTGCTACTGCTACTACTGCTACTGCTGCTAACGAC
TACATGCTACTGACTGATGTTGTTGCTGCTCACTGGCTACTATTGCTACTGCTACTTGACTGCTATGCTGCAACTGCTAACTTC
TACTATGCTGGCTACTGCTACTGCTGCTACTGCATACTGCTCTACTTCTAGTGGTACTACTGCAACTGCTACTGCTCACTGCTA
CTGCTGCAACTGCTGCTACTGCTGTTACCTGCTGTTAGCTGCCTACTGCCTTCGCTACCGTACTGTTACTACTAATAGCTACTT
GATACTGCTACTACTGACTACTACTTTATGCTACATCTACCTGTTCTAGTGCACTGCTGCACTGCTTGACTAATGCTACTGGCT
ATTACTACCTATTGAGGTGCTGCTGCTACTACTACTGCTGCTACTACTGCTACTGTTACTACGACTACTGCCTGGCTACTGCTA
CTACTGCTTGTTCTACAATGCACTACTATTCTATACTGCTGCTACTAATTAGCACTGCTACGTGCTATTAATGCGCTGCTGTAC
CATTAAGTGCTACTATACGCTGCTGCTACATATACTACTATATGCTACTGCTGATGTCTACCTTCTGCTACTTGCTAACCATCT
ACTGCTGGCTACAGCTACTGCTACTGCTACTACTTCTACTGCTGACTTACTATGCTTACACCTACTGCTACTGCAACTTGCTAC
TACTGGTACTTACTATGTGCTACTACTATACTGCACTTCTACCTGGCTTCTGCTGCTACCTACTATACTTACTAGCTGTCTGCT
ACCTGTACTACTACTAGCTACTACTATCGCTACTTCTACCTACTACTGCTATTGCTACTACCGGCGAGCAAAACCTTTTTTTCC
AGTTTCACTTCCCGATCCGCCCCTCCAAAAAACTTGCTTTTGTGATCGGGAATGTAGTAAAATTCTATGGCTTCTCGACTGGT
TCTCCCAACACGCACACAACTCCTGCGATCAGTTTGGGAAACTCATGGCAAAATGTCAGACCGAAGTTACGGCTCGCAAGATCG
ATGAGTATGGAGTTTTTCCAATCACAGCGTTATACTGCAAATGA
>m141122_040740_42134_c100732202550000001823141405141561_s1_p0/1974/11132_16743
TTTCATTGATCTACTTCGTCCTTATTACTCCGATATGCCTCCTTATCATGGTTCCTCGTTCTGTGCCCTTTGACTTTATAGCTT
AACGCTACCCTCACCGCTTCCTATTCTTTTTCTAGCTCCTCAAATTCACTATCCTGCCCTTTTTCTTCCTCGCTGATAATATAT
GCCTTTCGATACCAGCTTGTCCCCCAGTCCTCTAGTACGCGTCATTTCAGCTCCTTATTTGTGTTTCTCACTATAGCCTTTGGC
TCCGTAACTCTTCCCCTACATGACACTAGTGTCCTGATCTGCATTCTCTTACTATCGTACTCGCTTTGCTCGTCTCCGTGGCCT
CTCCCATGCTCTCATGCATCTTACTCATTCTGTGCTTGTTCCTATTGCCCGTCTCAACTAATTCTTGCCCTTCATACTCTCAAT
AATCTTTCCACGGCTCTGCCAACTGGTCTTATTGGTCCGCAATCTCGACTGTCCTCTCCTCTTTCCGGATGTCTGCTTGTTGTC
TTACTTTCCATACTCTCTAGTCTTCACACCAGACCCCGCTCTTCTCCTTCGGTCCTAGCCCCCGTCGTTTTAGTGCTTTCCTCT
ACTCTCTTCTTCTCGCCTTTCTTTTCACCCCTATGACTTGTCAGCTTTTAGTTGTCTCCTGTTCGACCCTAGCTGTTTTATTAC
ACTCGGGCCTCCCACAAATCCCCCCGCTGTCTCTCTAATCTCCCATGGTCGTGATGCCGCTTCGGTTGTAATTGTTGCGCCTAC
CGCGTCTCAGGCTAATAGCTGCTCGTCCCTGCGCTTCCCACTCATTGCTATTAATCCGGCCTTTCGTGATGCTGCCCCATTGCT
CTCTACAAGGCGCCTGCCGGCGGAATGCGCATACGTGGCAGTTAGATATGAGGTGTCGTAATCGGGGTCACTGGGGCCTGCAAA
TCCGCGCTCATGATGTGCCCTCCATTAATGGCTCGGAGCTCACAGATATCTGTTGTGTTGGTTCAGGCCCTTCGCAGTCTTACA
TATAAGCTCGTACCCGTGTACCGCCCTCGCTGCCGTGGTTCTCGCTGAATATTGGTTCGGATTGCGAACACTGTGGTGTTCCTC
AGTCTTTTGTAAATATCTAATTTGCGAGCCGTAGCTGGGCTCGGGCTGGCTGCTGCGAGTTTGCCCGACTTGTCCGTGTGAGAT
ACCGGTCCCGTCTGATCGCTCGCCTCTGGGCACCTTTGTGTTCGCTGCCGGCATGTATAATTTGCTCATGTGGCACTACCATGA
TTGAGGCTACCACCTCCCTCTTTGCCGATTCTCGGCCTCCCCCCCGCATTCACAATCCACCAGCGTACACAAGGTAGGACGTTT
GCCACTTCGTTTCGTTCGCGTCTCTTCTGGTATGGCTGCCCCCTCCGGTCTCGTTTTCCTTCGCGCTTGGTTATCAGGTTTGGG
CCATTCTGCTTTTTCTATGTGCTACGTAATTCGTTCTTTCTGCTCAGCTGACTGTTTGGTGCATACCGTCCGTTCTCTTATCCG
CTCTGCCTCTTCTGCTAGCTCGCCTTATGCATCAGGCCTAGTCTGTGTGCGTCTCTCTTCTCCTGGGGCTTATGAGCTTATTGC
GTTTGGGCTCGGCCTCGTTGAGTACAGTTCTTACTCGACCCTAATTCATATTCTGCTCTTCGCGGTTTTCAGACCTGCTGCGGT
CTCGGCTGTGCTCCATTCGCTTGGCGTGAGCCTCGCCCTGTAGTAAGGTCGGTTTCCCCCTCGTATTCTGTTTCGTCCCCCCCG
CGGGCCCTTGCGCTCCAGTTCACGTGGGACTTTCCGCTGGGCTTTCCCGGCTTTGTTCGGATTGGTGTCGCGCCTCGGCCTTGC
GTTGTGCTCTGGGCTCCGGCCCGCCTCGACCCGTCTCCGCTCCTGACGCTGCCCGTCGTTGCCGCCGCCCCCTTCCGGTTTCCG
TCCCCGGCGTTCTTGCCTCGGTGCCGCCGGCGTCTCTTCCCTCGTGGCCCTGTCCCCGCTCTCGCGGTCCCGGCGCCGGCCCGC
TCCGCTTCCGTCGCTCTCTCTCCTGGCCCGCCCTCCGGTCCTTGTTCCGTCTCGCTTCTCCCTTCCCCGCCTGCACGGCGGTCT
TCTGGCTCCGTCCTCTCCCTACGGCTCGCTCCCGCCTGTTTGGCCTCCGCCCGTTCCGCCCCCTTTCGCGGTCCGGTCTCTCC
ATCTCTCCCTCCCCTCACCTCCTCCCGCTCTGCTCCTGCATCCCTCGCTCCTGTTCCTGCTCCTGCTCCTGCTCCTGCTCCTGC
TCCTGCTCCTGCTCCTCGCTCCTGCTCCTGCTCCTGCTCCCTGCTCCTCCTCGTCCTCGTCCTCCCCTCTCCTCCCTCGCTCCC
CTGCTCGGATGTCTTCCTCCTCCTCGTTGCCTCCTCGTTCTCCCCTCCTTCCTCCTCGGCCCTGCCTCTTCGCCTCCTCCTTCG
CTCTCTTCTTCTCCCCTTTCTCTCGGCGCTCCCTGGCTTCCTCGTCCCTCCGTCTTTCTGCCTCGCTCTCGGTCTCGTCCTTGC

GCCGTTGCGCTGGTCCTAGCTCTCTCTCTTCCCGGCCCTGCGCGCTGTGCCCCCCCTTGTCGGCTATCTGCTCGGTCGCTGGCA
GTGGGGCTTCTCTCTCTGCCCTCTTTCCCCGCTTCTCTCTCTTCTCGTCCCTCTCGCCTCTCCTTTGGCCTCCGTTTTGCTTTC
TTCCTCCTGCTTCCCTCCGGTTTCCTACCCAGCTCTCTTTTCCGCCGCATTGCCTCCCGCTTTGCCTTCCCCTCTCGCCCTTGG
CATCCCCTCCCTCTCTGTCGCCTCTCCGCCGTGCGCCTCTCTCTCTCCCCACCCCCGCCGCGAGTGCGCGCCGCGCGCGCTCTG
CTCCTGCTTTCGCGTCGCCCGGCGCGTTCTGTGGCTCTGCCCCGTGCGCGCTGTGGACTTCCCCGTCGCGCTCCCGTCTTTCTT
CCGATCATGCTCCTCTCCAGGACGTGACGCAGTTTGGGCCGCCCGGCCCGCTTACGATACGCCCTTTCCTCGGTCCTTTTGAGA
TCTTGGGCTCCTCAGACTCTGGAGCCTCTCTGCATCTAGTCCTTTTCGCGCCACCGCTGGCCAGCTCTCGCCCCTCGCCCCCCA
CTGTGTTTCCGCCTCTCCGATGTTCGTTGTAGGATAACCGGCTCGTCAGCACGCCATGCTCACCGTCGTCAATTCGCGACGATA
GCACTGCAGAAAGACTTGAGATTACCGAGGAATGAACGCAGCTTAGCTCTCCTAGAGAAGGGATGAAGACATTAAGTCTACAGT
GTATGTCATAATAAAGTCCATAGAGGTCTCTATGGAGTCACAAACTTAGTTTGCAGCAAGCGAGAAGGTAATGCCAAGCACTGG
GAGTTGGGCATGTGAAGACATCGCGTTCGATCGCGCTCATGAGGGGTATTCCACTAGGGTATTGTCCAGTAGTACTAGTTTTTT
CCCTGTGATGGTTCGGGGTAGAGAGTAGTAGTTTCAGGGGCAGGCTTATTCAATAGGCCTCAGCTGTAATAGGGCAGTTGCAGA
TCGAAATGCTGTAGCAGATGTAGCAGTTAGCAGTAGCATGATCAGGCCCAGTAGGCAGTAGCCAGGTAGCAGTGCAGCATCCTA
AAGGAGCGCATGGATCCTTTTTCCTTAACAAATAATACTAGAGAGGCACCCCAGCGGCGACGAAGCTTGGTCTGACGAACACCC
GCTAAACTAAGTATTGCTCTGCAACTGAATCAGTTCAAGTCCTATTAATTTCTGCTGCCTTCGGTAGACGTCATTATTGTAATA
GCGTTGGAGCGTTTTAGGTATTGGCCTGATTCAGTACTCGGAATCCCAAGGTTCCTATGGGACGAGCTCAATCTCGTTCGCTGT
GAGCTAAGCAAGCCCGGCTAGCATTCCTGTCTGTACAAGTCTGAACTCCACAGCAACGCCTTGGTTCTTAACTTGCGAATGTAG
GTCGGTCCAACAGACATTTTGGCCGCGCCATCCGATTGGAGGAATGCTCAAATATATACTTCGAAGCCCGCTATATGGTCCAAA
CTTCTATGGCACCCCTCTCAATGCATCTCACGTGATCATTACGCCCTTGTAGTTGTCGGAAGTCGGAATGAATATCGTAAGGTC
ATACCTTGAACGCGGATCTCTATCCATTCCTATTCGCTCCAGCAAACACCCAAATGGTGCCGCTTTCGCATATTAGTATCGCCT
TCGCGGTTGCAAAGGTCAGGCCAATTCGTCGCGTTACAATTGAATTTGTTGCAGCCATACGCGTCACCAATCCCGATCCCTATG
ATAATTAATTCATGGTATCGGAATCGTCCATGCTATAGGTCGCTTGCTGCTCCTTGACACATATCATAGAAAGAAGCGTAAACT
CCATCCCTCTACCGTGACCACTACGCAATGATACCACGCTGGATGATTTGGCTCTAAAGCCCAGCCTCCCTAAGAGCTCACAGC
TCTCTGTAGGAATATAGTACTGAAACGCCTAGGCCATCACAAGCAACTGGTTAAAGGTTCTAACACAGCTGGATATGACATCCG
CCTAGCTAGACAGATAGATGCTAGGGCACCATCAGCAGACTCACATATATTTAAGGTTCAACAGGTAAATAAACAAGGCCCACG
CTACACTATATAGATCGTACTGCGTTTGACAAGGCTTGATGGACTTTTGGGGCCCTCTTGCTCAAGCTGATGAGGCTCGTAGTG
AAACCTACAGGGAGCGATGCGCTGTCACCTCACTGTGACCTATGGTCCTCCTCTTGTCTTCAGGCTGTCGGGCCCTCGAAATTC
TTTCATCATAGTGACTGTGGGTGCTTTCCCACTCAAACAATTGCTCAGCATTGCCCTGGCCGATTCGCTGAACTTTTCGCCCGT
ATTCGTCGCCTGACAGAGAGGACACACAATAGAGAGCCTCCAGTTGCAAACGAAGCCCCCCCTCAGATTTGTCTATCTCGCGCG
TTGGCGCGGCTCCAGCGCGCCCCCACCGCCGCCTCGCGCACCCTCACCTGTCGAGCTAAATCCAGTGGCCCGCCGGCCCTCCGC
CCGCTCCAGGCCGCCCCCCGCACCGGCCCCGCGCCACGAGTCAAATGGTCCTGGTACTCTCCTCTTCTGCTCCTCGGGCCGAAA
GCGACAACAGCGACCGACGCCCCCGCACCGGCTCCAACGGACCCGCGGCCCCCGACGCGCCCGGCACGCGCGCTCGACGCCCCT
CGGTATCACGGCTAGCAGCGTCCTCTACGATCTGATTCCTCAGCACTCTCAGGGCAGTGAGGGCCTTGTCAGCCCAGCTTGCGG
CATAGGTAGTCTCTGGATGGGCATTGTAATACTCACAGCGGCGGCGATCACTTAGCAGTTGTAATGCCCGTATAAACCTGTCGC
CTTCTGGCGCATCAGTCGGCGCACTAGCACGGGCGCAAACTTCGGCAACCTATCAATACACAGGGGTAGCTCAGTCGACAGATA
ACCTGCCCTGAGTCAGCGCGTTAACACATTTAATATCGCGCCTTCGCTGCTCTACACGACTGCTACCCTGAGTACTTCTCTTAA
AAATCACGCCTCTGATCTTCCAAGGTCATAGTTGCTAGTCACCTTCGCTGGCTCTATGCTACCGTCC
>m141122_040740_42134_c100732202550000001823141405141561_s1_p0/2483/0_6680
AAATAGAGGAGTCAAGATGTCAGTTCGATAGAGCTTATAGAAATAAAGGCCTCAAGAAGATAATTCGGGCAGGCCCTCCACAGC
TCAGAGACTGGAAGGCAAATGCTTGTTGATATGCAGATAGATTTAGTATGAGGCATGAAATAAGGATTCCTATTAGATATGGAA
CAAGTTTCGCAGGGAAGTTCCACACAGTGGGCCACCTTGCATTGGTACGGTAGTAGTATTAGCCACTGAGGTGGAAAGATTGGG
AATGCCACTTTATGAAATAGGAAAGAAATTTCACAAAAAAGAACCCTTCCAGATTTTTTTAGGGTAATGCAGATCGGTTAGGC
TGAACAGAATTGAAGTATGATTACTGCTATCTTGAATCTTATTGGGGATATTTGGTAATGAGAGAGTGGCCTGGCCACGGTATA
ATTTGTTTAGGGGATGCCAAGTTTTGTGGGAGTAATATCCCAGACAGGGAACGTGAATACCATGGATTGGGAAGAGTTCCAGAA
CATTGTTTAATGAAAAAGTATTTATATGATGCAGTTAGGGCTACAAAGGCAAATGGTTTTAATAGGCTTGCTACAGGGGAATAT
GACAGTGATAGAGTATCCATGGAGTTTTGATAGACTAGCCAATTTTGTCTGCAGATTGGTCCATCAGATGGGACATTTAGGAGT
GGAAAGGTCATTTAGGGGGTTACAATGCTATGAATAGATTGAGAAATTCGAATTACATGTGTCTGGGGTGACAAATATGCACAG
TTGTTAGAGAAATGCCTTACTGCAGAGGGCTTTGAGAACAAGACTTGGCGGCAATAAATCGACGCAGTTCTAAGAGATACAGGA
GGACGGACGTCCATTTAAATGCTTTTTGTAGGGGCGGAGGCCCTAGTGACCAGAAAAGGTAAGATCCAGATACTTTGTTAGTT
TTATGGACTCTAATAGGTATGGCAAGGGGTATACAAATCGGCGAAGGTGTCGGCAATGAGATTTGGTATACACTTTACCCAGAG
TGCACAGATGTAGAAGACGCCATCGGGAGTGTTGGCAAAGGTTTGCTTTATACAAGGTAGTGGGACAATTTTGAAGAAGTATTT
TCCAGAGATCTTAAAGATAAGGGGACCAAAGAAAGTGGACAACCTGATCAGCTCGAAGTGTTAACTTGAGCATGGCAGAGGATG
AGGCTAAGGCTTAGTCGCTTTGTTTGTGACAGGTCAGATTTCTAGTGCGGAACTTTTTGATACGTTTTAATTGATTCTAATGCT
ACACATTCCTCTTATATTTCTAGTAGTAATAATTAATAGATCTGTGTCGACTTATGTGACTATTATAACATGGGTTTGGCTTTA
TTACCCACTGGGGAGTTAGTAGTTCTGGATGGATCAGATCCTGCAGTAGTGTTGATTACATAGAGTTGTCAGTGACTTGATTTG
AATTGGTTATTGGTGATTTTGATATGATTTTGGGTATTTGGGCTTGTTAGATAAATATGGTGGCAACTATAGATCTGCAAGAAA
AAGATGGTGACCTTTGACTAAGGTGAGGATCCCTTTGTTTTTTTTGGCACTATGCATGGACCCTTGTGTTCTCTACGATTATAA
ACATTGAGAGGCAATGAACCTATTGCGAGGTGGTTGCATAGGATTCTAGCCACTGTGGTGGATACACTTGGGTCGTGCTATGTG

GGGACTGATTAAATCTTTGAGTTTCTGGACGTGTTTTCAAGTGATTTTAAGGGGTTTTTTGCGACATCAGAGAGATTAGAGCTT
TGCTATTGAACTGGCACTAGGCAAATCCTGGTCTAGGGAACCGTCATGAATAACCAGTGAGTTGAAGAGCTATAAAGGTCAATC
TACAGGAGTTGTTATACTTGGGTTTTATTAAACCAATTTTCTCTCCATGGGGTGCGCTAGTCTATATTTGTGAAGAAGAAGGAT
GGTTCTCTGAGTTTTGCATTTAATTAGCAGGGAATGAACAAGCTACCATCAAGAAAGCAAGTATCTGTTGCCAAGGATTATGAT
GAAATGTTTGATAGTTACAGGGAAGACTGTATTCTCACTTTATTAGTGAATCCTTGCGATCTGGTTACAGTCAGCTGAGGATGC
AATAGAGGAAGATATACCGAAGACAGCTTTCTCCAGATATTGGGCATTATGAGTTCCTGGTTATTCATTGTGGTTTGACTAATG
CCTCAACAAGTTTTATGGATTATGAACAAGGTGTTCAAGGATTAATTGGATCGGTTTTGTGATTTATCTTGGATTGACGATATA
TCTGGTTATTCTAGTCAGAGGCGAAATGAGTAGCATCTCAGGTTGTTTTACAGGGACTGAGAGAGCACTAGACTGTATGTTGAG
TTCTAGAAGTGTGAGTTCTGGTTACCACAGGTGACTTTCCTTGGTCACATTGTCAGTAGAATGAAATCAAGGTGGACCTAAGCT
AAGATTGAGGCATCGAAAGTATTAGCCCAGGCCAAAGAGTGATTCAGAGTCAAAAGTTTTATGGGTTTTGGCATAACAGAAGTT
TGTATGGTCAGATATAGTGTGAGAATAGTTTTCGGAAACTAAAACAGTGTTTGAATATATCTGCTCCAGGTTTATTGAAGTCTT
CCAACAGATCAAGAAAAGCTTGTGGTTTAATACGATGCTCAAAGACAGGGTTTTGGTTGTGTTCTTATATGCAGGCAGGAAGGT
GATTGCTTATTGCATCTCAAGTTGAAAGAATATGATTATTGAGTATACTCTACTCATATGATCTGGATTAACAGAAGTGATTTT
TGCACTAAAGGTTATGAGGCAATTGATAACTTATGGTGAGAAGTGTGAGTATAAACTGCTACCACAAGAGCCTGAAGTATTTCT
TCACACATAAAGATCATTAACATGAGACAAGAGATATTGGCTAACGTTGGTAAATAGGATATTACTTACAAGATGTATCATCAG
GAAATGCCAATCGTGGTGGCAGTTTTGCCTTAAGTTGGAAAGGTTTGGGACAGTTATTCAGTTCAAGCAGATATCCAGGAATTG
ATAGGGATATTGACTAGAGCAGGGATAGAATAGTGTGGGCAGTTAGCCAATATCCTTTAAGTCTACTCTTTTAGAGGGGATAAA
GAAAGTCATCAGAAGGATCTCTCAGTTGATGAGATTAAAGGGGACGTCTTAGCTGAAGTGGCTAAGACTATTACGTTTCATAAG
GATGGGTTTACTAGATATATATAGGGTTCGGATTTTTTGTTCTGATGGATGTATACTATCAGGAAGAGATTCTGGATGGAATCT
GCATACTCCCTTATTATTTTCAATCCAGGCTCGATGAGAGTATCAAGAATCTCATAAAGATTCATAATGGTGGCATGGGAATGA
AGGAAGATGTGCTGAATATGTGGCTAAGTGCTGCGTGTCAAGATAGGTCAGGTTGAACATCAGAGACAGCGGGGTAATTTTACG
GCTTTGGTATCCGGAGTGGAAATGGGAGGACTCATCATGATTTTTGTCGTTTGGGTTGCCAAGACGGTAGGTGGAGCATGATTC
ATTATGAATTATTGTGGATCAGTATACCAATCAGCTCACTTTCTAGTTATAAAGATTGACTATTTCATGTTGACCATATGCAAA
TCTTCTATGTGATGTAGAGATAGACATTCTTCATGCCATGGGCTCCGAAGTCGATGTGTCAGATCGTGACCCCAATATTTACTT
AAAGTTCTAGGGTAGTTATGCAGACGATATTTGGTACACGTTGAATTCATACCAATTACCATTCCTTAGATAGATGTGTTAAGT
CTTGAGGTATGATTTAGAACTGGTGCATGCTTTCGGAATGCGTTTTGTACTTTTGAAGGGTCCTGGGTATGTTTTTACCTTTTA
TAGAATTTTCCTACAATAAAGCGTATCAGTCGAACATTGGAGAGGCTCTCATAAGATGTTGTATGGGTAGGAAGTGTATAGAAT
TCGCCCATTTCGCTGGGACGATATGGTGAAGAAAATACTTGGGTCTTTGAAGAGTTATGAGGACCAATGAGGATATCGAAAAGA
TTAAAGGCTCGGATGCTCATCGTCGCAGTAGTACAGAAAGTTATTCAAATTTGAAACCAGGAACGTTGGAGTTCAAGTTGGAGA
ATATGTATTCCTTAGGGTTTCAATTGAAAAGGGTGAAGAGGTTAGAAGAAGGGCAAGTGAGCCTCTAGATTATGTAGGACTTTC
TTGGATTCCTGGAGAGGATATGGTCGTGTGGTCATATAGGATTGGCTCCTTACTCTGGATTTGTTGGAAGTGCAATAATGTGTT
TCATATTTCGATGCTTCAGAGATGTATAGAGATGAGACTCATGTATTGAGTTATGAATGATATGGGTTGAGACATATTTATCCT
ACGAGGATTAGCGTCCGATATTAGATAGGAAGGAACAAGGTCCTAAGGAATAAAACTATACTTTGTGTTAAGTTTATGGAGGAA
CAACAGGTCGGGATGACTGGGAATTGCAGACCCCCCCCCCCCCCCCCCCCCCCGATATGCGGTGATAGTATACAGATCTGTTTTCA
GGTATAAAAATTTGAGGATGAAATTCTGATAATGAAGGGATTATATTGTAACTCCTATTTGCTAATAAGGCGTAGCTCTTGATT
AAGTGTGCGGAGGTGCATAAATGGATATGTTATGTGCTTTTTTATGATTATATGCATGATATTACGAATTATGTGAGTTATTAT
ATGACTGAATCTATCAATATCATATTTAGGTGTTTTATGTATGCATCGTGGGCATTTCTTATTAGAAGGGTGTTTTGTAATTTT
GATCATTGATGGTATATTTGTAAATATATATATTTGTGTTTTGGTAATTTTGACTATTATTATTGTGATTATATTTTGATTTATTT
CGATGAGACGATCCAGTGAGAAGATTAGCGGAAAGTCAAAGGAGTTAAATACTGGCTCAGGATAAGCCTAAGGGTATTTTGGTA
ATTTAGTACATTATGAGATTTATCGAGTATTGGAAGTTTTTGTGATTATTTGGGATATTTGAATATTGGGAAATATGGGTGATA
ATTGATGATTAGTGGGGAATTGTGGCAATAGACCATTTTTTCCTTGATAGGGCATTTAATGAGCACATGTATTGGCCAGGGGCG
TTTTTTGGTCTTTCATGCCATGAATGGCTTAGTATTTGAAGACTTCTCTCTGATGAAAATCAAATTAACACTAAACATCTCCAT
TTCTTCCTTCTCTCTCTCTCTCTCTCTCTCTCTCCCTTTCTCTCTCCCTCTTCTCTCTCTCTTCTCTCTTCCACTTCTCTCTCTCT
CTCGTTGTTATTCCTCTTTCTCTCTTGTCTGGTTAAGTTTAATTTTAATTTTAGGGAAGGAGTCCTTGAAATGGGGGGCTTAGA
GCTTGGATAAATTGGATTAAGGGTTCAGCTTTGGGTCGTAATTACTTATTGTTAGGTAAGACTTTTTACTCTGTCTTTGGTAAT
ATTTCTGACTTGGTTTAGTGTTTTGAGTTTTAAACTCAAGGTTTGGTTTTGAGTTGTGGTGAGGTTTTGGCTTGGTTTCTTCAT
GTTATATTTGCTGGCTTTATGAGTATTGTGGATGAGTCTAAGATAATTTTGTCTTGGGTACTTGATTTGGGTGAGTTTTGTGGA
TTTGGTTCGGGAAAAGTATAATTCTGGTCCGCGAGGTTGGCGCCGTAGTACTTGAGTGTTGCCGCAATCTGAACTCGAGAGGCG
GTGGGGTTCTTCTGAACTGCTTGGCGCTGTGGCACATCTCTCTGTGCCTTTTAAGGATTGGGGCTCTCTGGTGGAGTAGTGCGT
TGTGGCTTCAGGGGCGGGTCCGCAGCTCTATTTGTGGAAAATTCAATGAGGTTTTGGTATTGCAGGGACTCAAAAATAAGGGCT
CGAGAAGTTCTTACTACCCGTTTAGCTATGAATTTTGAGGTCTGAAGGCTAGGACTCGTCCGGAGCTTTTATTTGTTTCGTTAT
TAATGTTATTTGTATTATGGTTTATGGCTAGGGTTCACCGATTGGCTCAGGTGAGAAGGGTCGTGCTCGGGGTCGTGATACATG
TTCGTTAAGGACTTGAGGAAGATAACTAACTTAAGTCTGTGGTTTAGTGGCTTAAGCCAATCAATTGAACATGGTATAACTATG
ATTTGAGATGTTTTTATTAACATGTTTGATTGTAACGAGCATATCTGGGATTAAATGATTTTATGCTTGTCTATTTTTTTTTTT
TATTGAAAGGCTTGATGATTGAGTCCTCATATTTTATTAGCTAGTTGATAGTATAGTAGTAGTAGTTAGTGAGTAGTAGTAGTT
ATAAGTAAGTAGTAGTATGTGTAGTAGTAGTTAGGAGTAGAGTAGTAGTAGTATGTAGTGTAGTAGTAGTATGTGAGTAGTAGT
AGTTAGTAGTAAGTAGTTAAGTAGTAGTAGTAGTAGTAGTAGCTAGTTAGTAGTAGTAGTATGTAGTAGTAGTAGTAGTAGTAG
TAGTAGTAGTAGTAGTAGTAGTAGTAGTAGTAGTAGTAGATAGCTAGTAGAGTAGTAGTAGTAGTAGTATGATAGTATATAGTA

GTAGTAGTAGTAGTAAGTAGTAGTAGTAAGGTAGTAAGTATAGTTAGTAGTAAGTGTTAGTATAGTATTGTTTATTCTTGTGGA
TTTTGGTTCAAGCCTGGGACATAGTTGAACACTCGTAGCAACTC
>m141122_040740_42134_c100732202550000001823141405141561_s1_p0/2483/6719_12245
GTTGCTACGAGTGTTCAACTATGTCCGGCTTGAACCAACATCCACAGTAATTAAACAATATCTATATACTACTAACTACTATAC
TACTACTACTACTACTACTTACTATACTACTTACTACTACTAATACTACTATACTACTACTACTACTACTACTACTACTAGCAC
TATACTACTACTACTACTTACTACTACTACTACTAATTTAAAAACTATACTTACTATATACTATACTACTACTACTACTCTATA
CTACTATACTACTACTATACTACTACTACTTACTACTACTACTACTCTACTACTACTATACTACTACTACTATACTACTACTAC
TATTATACTACTACTATACTACTACTACTCTACTACTACTACTATACTACTACTACTTTATCTAACTAGCTAAGTAAAAATTAT
GGACTTCTACATCATCTTCTTTTCAATACAAAAAAAATAGACAGCATTTAAATCTTTATCCAGATACTGCTCGTTACAATCAAA
CATGTTTAATAAACATCTCATCATAGTTATAACATGTTCATTGATTGGGCTTAAGCCCTAACCACAGACTAGGTGTTAGTTTTC
TTACTCAAGTCTTAAAGATACATGTATACGCCCCGAGCACGACCCTTTCTACCTGAGCAATCTGGTGAACACTTAGCATAAATA
ATACGCATTAACCATTAACTGAAACAATAAATAAAGCTTCCGGCAGAGTCTAGCTTCGGTACCTCAAAATTCTACTAACTGGGT
AGTGTAATCTTTCTTCGAGCCTTATTTTTGATCCCTGCAATGATCAATACTCATTTGGATTTTCCACAAATAGAGTTGCGACCG
CCTGAAGGGCACACGCCTTCTCACCAGAGAGCCCCATCATTAAAGGCACAGGAGATGTGCCCCAGGCAAGGCAGTTCGAAGACC
CACACGCATCTGAGTCAATGCGGGCCACCTTCCAAGTACTACGTCGCGGCGCAACTCGGGACCCAGAATTATAGCGTTTTCTCC
CGAACCAATCACCAATACTCACAAAGTTCGTACATGACAAATTGATGTCTTAAGACCTCACATATACTTTCATAGCCAGCATTT
AAAATGAAGACAAGCTCAATAACCTCACACAACTCAAAATCCAAACTTGAGTTTAAATCAAGAACTAAACCAAGTCAGAAATTT
ACCAAAGACGAGTAAAAGTCTTACTCAATTAAGTAATTACGACCAACTGAATACTTAATCCATTTCAGCTATAAGCCCATTTCA
GGACTTCCTTTCCTAAAATTATAATAAACTTAACACGAGCAAGAGAGATAAGGGGATATCAAGAGAGAGAGAGGAGAGGAGAGA
GAGAGAGAGAGATGAGAGAAAGAGAGAGAGGAGAGAGAGAGAGAGAGAGAAATGGAGAGTTGTTTTAGTGTTTAATTGTGATTT
TTTTCTTCAGAGGCTTCAAATTTAAACTAAGCATTTCATGCGGGGCATGAAATGACATAAATCGCGACCTTGCCTATAAGCATT
TGCTCTTTACTTGCCTCAAGGGAAAAATGGTCGCTTTTCACAATTCCATACCTAATCCTCATTATCACGCATATTTCCCAAATT
AATTCAATATCCCCAATATTAATCCATAATAATTCCATACTCGATAATCCTTCTAGTATATGTAATAAATTACAAATACCCTTA
GGCTTTATCCTGAGCAGGTATTTAACTGCGTTGTGACTTTTCGCTAATCTTCTCACTGGGATCGTCTCATGCTCGATAAATCAT
ATATCCACATATTACATGTGGTCAAAAAATTACAATACAAATATATACTTTACAAATATAGCCATCATGAGTCAAAATTACAAA
ACTTCTAATAAGAAATGGGCCCACAATGCATACCATAATAACTAAATGCTATGATAGTCTGTCATAATATAACTCACATGAATT
CATAATTCATGCTATATAATACATAAATTGCACATAAGTCATTTATGGGCCTCTCGCACACTAATCAATGAGACTACGCCTATT
AGGCAAGATTGGTGATGTTACATATTATCTCTATTTAACGGTAATTTCATCTCAAAATTTTACTGAACAGATTCTCGTTTGAAT
TATTTCTGATCCGCGCATATCTGACTCAATTCACAGGTTCACTTCTCGAGCTTGTTGTTGCTCATAATAATTCTTAAGCAAGGT
ATAGTTTTTATTTCCTTAAGGCATTGTCCTTCTATCTAATATGTTCTGGACTGGCTAAATCTCGTTAGATATAATATTGGTCTG
CAAATATATATGTCTTCTAATCATAGCATGAGTCTCATCTCTTACATTACTTCTGAAGCATCGATATGATAACACATTGCCTTG
CAACAAATCCAGAGGTAAGGCCACTATATGACCACCTGAACAATGTGCCTCTCCAAGGATCTCAGAGGTCCTACAAATCTAGGG
CTCAACTTGCTTCTTCTAAACCTTCTTCACTCATTTTCAATGGTGAAACCTAAGGAAGAATATTCCAACTTGGAACTCCAGTTC
TTGTGCTCGTTTCAAATTTGATAACTTTTTCTGTCTCTACTTTGCGACATGAGCACATCCGATGGCTTTTAATCTTTCGATAGC
CTCATTGGTCTCTGACTATGTCTTCAGGACCAAGTATTTTCTTTCCCCATATCTGTTCTCAGCGAATGGGCGATCTAGCACTTC
TAAGCTTCCATACAAATCTATGAGGAAGGCTCTCCATGGTCGACTGATAGTGTTATTGTAGAAAATTCTATAAAAGGTAAATAT
CATACTCAGGACCCTTTCAAAGTAGCAAAATACGCATGTCCGATAGCATGTCGTACGCAGTATCTAGATCATAGCTCTCAGCTA
CATCTATCTATAGGATGGTAAGTTGTATTTGAATTTAACTGTAGTATCCAATATCGTTCTGCACTTCCTAGAACATGGAAGTAA
ATGTTGGGCTCCCGCGATCTGAACACATAATCGATTCGGAGCCCCATCGTAAGATGTTTCTATGTCTCTCACATTAGAGTTTGC
TATAGTGGTCACTGTATAAGTCATGCTTTCTAGTAGCTATCATGTGAGTGATTTGGTATACTGATCACAATTTCATAATGTCAT
CATGCGTCACGGCTACGTCTTGGCACCAAGACAAATCCATGGTGATGTCCTCCACTTTCCAACCTCGGGATACCAAAGCTGTAA
TACGGCCCGCTGGTGCTCTGATGTCATACTATGCCTATGACACGTTCAGAGCACTTAGCACATATTTCAGTTCATCTCCTTCAT
TCCATTGCCACCATTATGAGTTTTGGATTGATAGCTTCTTCATCGAGCTCGGATGAAAATAATAAGGGGTAGTATGAGTAATTC
TAGCAGAATCTCTCTTCTGATAGTTATCATGCCCATCTTCAGTAACTAAATCCGACTTATAATCTTTAGTAACACCATCTTTGA
AATGTATAGTCTTTAGCGCACTTCAGCTAAGACGTCCGCTTTAATCTTCATCAAACTGAGGATCATTTCTGATGACTTTCTTTA
TCCCCTCTAAAAGATAGTAGCTGTATAGTGGATTATTGGCTTAACTGGTGCCCACATCTGATTCTATCGCTGCTCTAGTCTATA
TCTCTATATCAACTTCCCTGGATATGTCTGCTTTGAACTGAATAACTGTACCCAGAACGCTTTCAAACTTAAGGGAACTGCACC
ACGTTGGCTTTCGCCTTGGATGATACATATTCTTGTAGTGAATAATTCTTTTACATACGTTAGGCCACATATCTCTGTCTCATG
TTTAGTCTTTATGTGTGAAAGAAATATTCAGGCCTTGTGGTCTGTGTATATCTCACGTTCTCACCATAAAGGTATTCTGCTCCT
ATCCTTTAGTGGCAAATATATGCACTTCTGTTAATTCAGATCATGAGTAGTGGTATCTCTATTTCATATTCTTTCAACTGACGT
GATGCATATAGCATCACGCTTCTTTGCCTGGCATAAGAACAGAACTCAAAACCCTGTCTTGAGGCATCGTAGTAAACACAGCTT
TTTTTGTCTGTTGGAAGTACTCAATACTGGAGGCGTTATATAACACGTTTTAGTTTCGAAACTAATTCTCACTTAATTCTGAGC
GCATACAAACTTCTGGTTATTGGGCCAAACATAAACTTTTGATGCTCTGAAGCACTCTTTGGCTGGCTATATCTTTGATGGCCT
CAATGCTTAGCTAGGTCACCTTGATTTCATTTTTCTATCTGAGCAATGGTACAAAGGAAAGTCACCGTGTAACCAGAACCTCAC
ACTTCTTGAACTTCGCATACAGTTCTGTGCTCTCTCAGTCCCTGGTATAAACACCTGAGATGCTACTCTGTTCTGGCTCTGACT
TGGAATAATACCAGTATGATCGTGCAATCAAGAGTAATGCACAAACACCGATCGCAAGTAATCCTTGAACACCGTTGTTCATTA
GAGCCGCCCCATAAATCTTGTTGAGGCATTAGTTCAAACAAAATGAACATAACAGGAACTCATAATGCCCTATCTGGTGCGAAA
GGTCTGTCTTCGGTATATTCTTGGCCTCTTTGATCTGCCAGCTGGCTGATAACCAAGATCGAAGAGCCAATCTTTGAGAATACG

TCTTTTCTGTACTGATCAAATCATGTCATCCTATCTGGCACAGATACTTGTTCTTAGATGGTTATGCTTGTTCAGTTTCGCTGT
AATTACTGCAAATACTCAGAGAACTCATCTGTCTTCTTCACAAATAAGACTAGCGCACCCCATGGAGAGAAATTGGGTTTAATA
AAACCTCAAGTATATACACTCATGTAACTTGTACGTTAGCTCTTTCAACTCTACTGGGGTTATTCTGTATCGGTTCGCCTAGAC
ACGGATTTGTCCCGGCTGCAGCCTCACACTACGCAAGCTCTATCTCTCTGTGTGGCAAAAAACCCTTGAAAATCACTTGGAAAC
ACGTCAGTCAAGCTCAAGGATTAATCTGGTCCCACTAGCACGACCATAGTGTATTCCCACCACAGTGGCTAGGAATCTATGGCA
CCCTCAGCAATAGGTGTCTTGCTCTAAAAAAATCTGTTGATATCGTAGAAACACGGGGGTCATGCATAGTGGTGCTAAAAAAAC
AAGGGTCCTCACCTCAGTGTTCAAAGGTCACCATCTCTTTTTCTGCATCTATAGTTTGCCATATTTAGCTAACAAGCCATACGC
CAAATCAATATCGATAATATCATAACAAGTTCAAGTCATAGTGCCACTGACATACTCTTGCTATCAACCACTATTGGGCTAGTG
ATCTGATCATCTCCGAGGACTACTAATCCAGTGGGATATAATAGTCCAACCGCCACTGTATAATTAGTTCGCATGTCTACACGT
CTATTAATTATTCTACTAGAATTTAAAGAATTGTGTAGCATTAGAACATTAAAATGTATACAAAGGTTCCAGCACTAGAAATGC
TGACTGTCACAACAAAAGGGGACTGAGTTAGCCTCAACTCTTGCTTGCGTCAATGTGAACCGCCAA
>m141122_040740_42134_c100732202550000001823141405141561_s1_p0/3501/7450_14187
TCTGTGGAGTAATACGCCCGCGAGTCGCCTCAATCGAGTTTGATTGTTCAGCTGTTCGCCCTTATCATTGGATTGAATCTGTAT
GACCGCCGGCCCATCACGATCAGAGTTTCCGCTGCTTGCTCTGCGGCTGCTATTCACCCGCGTGGTGGCTCACACGGCGTGCTG
ACGGTGGTGCGACAGGTAAAGGCAAGGGCAACGTCTAATCAGCCGCGCGTAGGAGTTGAGTAGTCTCAGCCGATCGCTGGAGTC
GCTACATAGCCAGGTGGCTCAGACTGCAGCCAGAGCGGTTGAGGTCTAGCAGACGTGAACCTGAAGACCTGTTTGATTTTGCTA
AGTAGTTGAGAGAGAGGGCTAGTGAATACTCGATGTACTTTTGGAGTTTGTAAACTTGTTAACTTTGTTTTCTTTGGGATCGTC
CATGGTTTTACTACAGTTTATATACTGCAAATGAGTCTATTTTTGAGACGCAACCACTTGTATGAACCTAGGCTTTTACAACGT
TTAGTGACAATGTTTGTAAATAATGACTTGATAGGCGGAGTCTTGCGCCTTTATAGTACAACAGCATCGGTAACGGTCTTTATG
GCTATCATGGCGTTCATTTTGTGGTGCTATGTGGTGCATGTAAAAGGGAAAGAAAAAGCTCACCCCCGCTTGGGTGGCGACTCG
TCTAGGGCTGGTTGAATGGTGTTGACACACTCATTCCAGCCGGCCACGCGGCGTGCTTGCACTTACCACGGCCAAGGAGTTCTC
AGAGGAAACATAGGGGTTTTACCCTACTATTGTTTGGCGCGATTTTGCAGCTCATACGTTTATTTTATTAAATACATAAACTGC
TCTCTCAATTAAGGGCCCATTAAAAAAATATACATGTTAGTTTACACCGTCAAAATGGTCATATACAGTTTCAATAATTTCAAA
CTCGCGCTAAGCGGCAAAATAGGGAAACCCTAGTTCCTCTGAGACTCCTTGGCCGTGGTAGTCAAGCGCCCATATGTACACATC
ACCACCGTACAGCTCTCCACCTCAAGGCTGGGTGAGCATTTTCTTTCCTTTACATGCAACGACATAGCCCATGTAACGCCTGGA
AAAGCAAGACGTTACACTGTGTATTATAAAGGTGCAAGACTCGCTATTGCAAGTCATTATTTAAAAACATGTCACGTAACGTGT
AAAAGCTAGGGTTAAAAGGTTTTGGTCTCAAGCTCCATTTCATACTATTTAAACTGTAGTAACATGGATCCGCCAAAAGAAAAC
AAAGTTAGTAACAGTTTAAAACTCCAAAAGTACATGTAGTATCTACTACTAGCCATACTTAAGGCAAAACAGACAGTCTTCAGG
TTTCACGTCTTAAAGCCTATATAGAGATCAATATTAAGTTGGGGGCTGCGCACGCTATGGTATGATATACTCCATCGTCTGAGT
TTATCTCTAGAATTCAGGGCTGATTAAACTATGCCTGTGCTTCATACTGTGGAGGAGGCGGTCACGTAGCCCCGTGGCCAGGCC
CAGCAAGAACATCTTATACTATAGTCATGTAGACATAACAAAGAGAGATTCAAATCGTTATGAATGTTTTCCATCAAGATAATC
CCCATACAGATATTCAGTCATAGCAATCAGATTTAAGATACAAATCTTAAATACATATATGACCTCTAATGCACATAATATCAT
TTAGGTTCGAACGATTAGGCGCACCTTTGTTTACACACTGACTCAGGCGCTTAAAACGAGCTCAGTGCATAATAAGCTATCCTC
GGCTACCTTGCCGCAGTTCGTGTCATGATGCGCTAGTTGATTCTATCCTTGGGCATCTCATATTTTGATGACGATTTGAGAGTT
CACTAATGGTCTGTTGATACATAATACCATCTTTTCAAGCGTTTAGTAAAATAAGGGAGGCCTTAGTCCCGTCACAAATATTCA
ATAAGGTGCATTTTCTTAATTTAGTCTATATTGTTATTGAATTACGAGCACGGTCAATCGCACGAGACCTACCGCTGAGAAACT
GCAAACCCCCGAGATTAGCTCTTTGCCAAAATCGACTGGCTGGTTTCTTCACTGAAGATGACTAACATGCTCAATACCAACCCT
CAATCATACTCTCCGCCCCACCCTCCGCTAGATACTCTCTTGATTCCCCCCCTACCCCCTACCCCCGACGAGTATAAGACACCG
TACCGTCGCGTCTCCCACCCCCTCCCAGCGCGCCTCCCCAGCATCCTCAACCCCCAACACGCCAACCCCCCACGCCACTCATCA
ACTATCGATCAAACCCGCCTCTCGACTACAACAAACCCCAACCCCCACGCAACAACACGACCCCGGTCACGAACCACTCCGCGC
CCGCTCGCCCCACCCTCCCCGACACGCCCGCAGCCCACCTGCCCGCACACGCCCCCCCCCCCACAGCCACCCGCACCCGCCGCC
CCCGCCCCACCGCTCCCCCACCCCCGCCCCCCCGCCCCGCGACCGACCCCTCGGCCGTCCTGCCCTCCCCGCGCCCCCGCCCCG
CGCCCCGACCGCCCCCCCCCCCGGCCCCGCCCCCCCGCCGCGCCGCCCCCCCCGCGGACCCACACCCGCCCCCACCCCCGCGC
CCCCCCCCCCCCGCCCGCCCCCGCCCCCGCCCCCCCACCGCCGACCCCCCGCGCGCCCACCCCCGCCCGCCCACGCCCGTCC
GCCGCCCCCGCCCCCGCACCCTCCCCCCCCACTCCCGCGCCCACCGCCCCCCCCCGACTGTCGGCCCGCCCCACCCCTCCGCAT
ACCTCACCCTTCCACCCCCGCACTCCGCACCCGCTCGCCGCCGTCTCCGAGCCCCGCTCCCGGCCCCCCCCGCCCGCGCGCCCC
CCCCCCGCTCCCGCCCGCCCGAGCCCCCGCACCCACAACTCCGCCCCCCGCCCGCCCCCCCCCGCCCGCCCCCCCCCTCCTGCCC
GCCCCGCCCGCCCGCACCCGCCACCCCCGCCCGCCCCCAACCCAGCGCCGCGCCCACCCCCCGCCCGCCCCTCCGACTGCCCCC
CCGCCCTCGCCCCTCGCCCGCCCCCCCCGCCCCCCCCCCCCCCCGCCTCACGCCTCCTCCGCCCCCCCCACCCCCGCCCCCCCGA
CACACCCCCCGCCCCCCCCCGCCCCCCGCCCCCGGCCCACCCCCCCGCCCGCCACCCCACCACCCCCGCCCCCCCCCGCCGCGCG
GCCCCCCGCCCCCCCCCCCCCCTCACACCACCCGCCCCCCGCCCGCCCGCCCGCCCCCCCACCCCCCGCCGCCCCGCCCCCCCTC
CCCCCACCCCCCCCGCGCCCGCCGCCCCGTCGCTCTCGACCCCCCGCGCCCCCCCGCCCCCCCCCCCCGCCCGCGCCCCCCGCCC
GCCCCGCCTCCCCCCGCCCGCCCCAGCGCGCCCCCCCCCGCCGACCGCACCCCCCGCCCGCCGCCGGCGCCCCCCCCCCGGCCC
CGCGCCCCCGCGCCGCGCCGCCGCCCCCGCCCGCGCCCGCCCCCGCCCCCCCCCCCCCCCGCCCCCCCCCCCCTCCGCCCCCCC
CCCGCGCGCCCCCGCCGCCCGCCCCCCACCGCTCCCCGCCGCTCCCCCCCCCCCACCGACCCCCCGCCCCGCCGCGCGCCCCGC
CGCCCCCCCCCCCCCCCCCGCCCCCCCCCCGGCCACGCCGCCCCCCACCCCGCCCGCCCGCGCCCGGCCCCGCCCCCCCGGCAC
CCGCCCGCGGCCGGCCGCGCCCCGCCCCCGCCCCGCCGCGCCCCGCCCGCGCGCGCCCCGCGCCCCCGCCCCCCGCCGCCGGGC
CCCCCACGCCCGCCGCGCCGCCCGCCCGCCGCCCCGCCCTCCGCGCCGCCCCCCGGCCCGCAGCGCCCGCGCCCCCGCCGCCCC

GCCCGCCCCGCCCCCGCGCCCCCCCCCCCCCGCGCCGCGCCGCGCGCCCTCGCCCCCCCCCCCGCCCCCCCCGCCGGCCGCCGA
CCCCGCCCTCAACGCCGATCGTTCTGAGCCTCAGCTTTCTCCCTCGTACCAAAGTCTGGGTTCCATTCCCCTATATCGGTTTCG
GTTACAAATACTCTGGGAAATCCGATTCCCCAAAGCAAGGTGGTGAAACCATCTAAGCATACTAGACATTTTCCCGTGCTAAGA
ACCCTCAAACTCATGTGGCAACAAGGGCTGCGGCCCTTGGAGATTAGCCCGGCCTTATCCTTAAAACAGAAAGCTACCCACCCT
TGAACAAGAACTGCCGGCAGTTGCGTTGGGGCGCGTGCGTCTCACTTAGGCGAGCCTACTTAGGCCTGTTTCATCCTAAGGGCG
CACGCTCTTAAGAATAACATGGCCTCCCATTCGTAGCCAGAGACCATCATCTTAAAACCAAAACCTACCAAAACACCCTAAAGC
TCTACTCAACCACCCACTATTCCAACTCATGCTACACAATTTTAACTGAGAATTAGCTCAATAACATAGAGATTCACCTCCAAT
CTTGACTCCTAAGAACACAAACACCTGTTATAACAAGTCAAAATTCAAACTCAGATCATCATTCATAAGCAAGGCTTCCTTCTT
GATTGAACTCTTCCTTAGCGAAAACACGTTGATTCCAAGATTTCCCAGATCAATTCCACCTAATCACCAATATGAGTAATACCC
TCAAAGAATCCGTAAAGCTTTAGGTTGAATTTTAGGAAAACAAGATGATTATAACCTTACATTGTCTTAGATTCGTCCTAATTA
ATTCACTGAGCTACAGCTCAAGACTTACTTCTTGATGCATCTAAACTTCCAGAAATTCTTCAGCAATCCTCAATTCCCTTGAGT
GCTTTCTAGTTAGTTTCTCTTGTTTTTTCTTAAGAGTGCGATGAGAGTACCAGCTGGAGTAAGAATTTAGGTCGGTTTATATTT
TCTAAAGATGTCCTTATATCTACTTTTAACTCGTTTAAGGTAATCTCGAGGCTCGGGATGCCGGACGTCCCGAGGCAAAATAGT
GAAAATCCCCCAACATTCCCGCCTAGAACATCTACCTCGAGATATATCAATATATATTTATATTTCATGACCCGATAAGCTCAA
ATCGCTACCGATACCCAAATACCTGACTTGTTAAAGTCTCATCTTAAGCCGCTTGATTGCTTTTCCCCGCTATCTGATCTAGGA
TCTGTCTTGAGTCGTGCCCTACAACTACCCAATAATATGTGGTTCTCAATTTATCACATATATATACATTTTTTCACTATATCT
ATCCAATTATCATATAAACCATTACTAAAATATACATTCTCATTAAAATCCATTATCATTAATGCCCTCCTGGCATACTAATCG
AAGGCCCTGCATTTTAGCAATTTTGGGTCGTTACAACTATCCCCTTCTCTACTGATATCCTCTCTCCGAAATTTACTGAACACT
CGGGATACTGATCTCGCATCGATGTATCAGCTCCCAGGTCGCTATTCTCGCTTGCTATTCTCTACTCAATACTTTCCTAATGGA
ATCGTCTAGATCCGCAGAACTTTGTCTTCCGATCCAACATCTGACTGGTCTCTCCTCTATAGGCAAGTCTGCCTGAGCGCTCAG
CTCCTCTACCTAAGCACATGTGTGTATGTATCAGAAATACTTCGCACAATGAGACATGGAGTCGCAGTTATGAACTCTAGCTGT
GTTGGCAGGCAGCCTCTCAGACCACTGATCAAACCTCTCTTAGGATCCTCAAAGGGTCCAACACGGGGCTCAACTTGCTTCCCT
CAATACTCCTCACGTCTACCTTCAGGTGGTGACTCGCAGAACACCGTGGTCCTACTTGGACTCACGTTTTACGCTTAGGATCAG
CGTAGCGTTATTTCTGTCTACTCTGCGAGGACATGGTACTGCGATTTAAAGTAATACTCAAGAATCAAAAGACGAAATATACAA
ACAAGAAAAAGAAAAACAAACAAAAACACAGAAACAGAAAAAACATATATATAGAAGACAGAAAAATACCGCATCTAGCTCATT
TCTCAATGGCCTCATTAGTCTGTCAGGGGGGGGGGGGGGGGGGGGGCGACCTATCTGGCCGCAAATACTTCTTCTCAACCATCTC
AATCCAGTGCATGGGCGATCTGCACTTCTCACCATATAACATCTCGTAGGAGCACTCAATCGTGGACTGATAACTGTTGTTATA
TGAAAATCATCATTGGACGATATTTATCCACGAAAAATCGAAGTCCATCCACATGCTAAGATGTCCTCCAATACTGATCCTCTC
AGATGTATCACTCTGGAGGGTGAAGTTGTGTTACAGTTGTGGGTCCCCATCGGCTCTCATGTAGAGCTTCCAGACTTAGAGGTA
AGGATAGGTCTCAGTTGGATACAATAGATTTGACCGCCATGGCGACGCACTATCTCCCTCACATACAGACTCGCATACTGTTCA
CGGTATAAGTAACCTCACTTTGGTAGAAATGAGCTGATGGTTATCTGTCCACTATCACTACTCGAGTCTGACCCACTGTTCTGC
GTGATCCTCCACGAAATCCATCTAAATATACCTCCCACTTCATTGGGATATCCACAGCTGAAGCAACCCGCTGTCGTTGTGTGC
TTAACTCTGTTGACATGTCAAATCTAAAACATATCAACTACATCCTTCTCGATCGGGCACCAGTATGAATGGTCGTAGATCCTG
ATTATATTCGTGGTCCACGGATGAAGTGAGTATTGGGTAGTGTGAGATCATCAATATGTCACGCCTAATCCCTCATCCACGACG
AATATCCGTTCTTATAT
>m141122_040740_42134_c100732202550000001823141405141561_s1_p0/3894/7109_13939
TCTCGATACACACAACACGCTATATCTTCTATATATCTCGATGGTGGTTAGATGGTCATGTTATCCTACAGTTAGTCGCATCTG
GCGATTTGGTTGTCTTGGCAAAGCAGATGCCTATTTTTAAAATTAGTTTTTACACTCTAGCTAGTTTCTAATAATTCTTTGCAA
AACGTCTCTCATAATTTAACAGTTAGTCGAAAATTTAGATAGGTGGTCGAAAAATTTAATAGTTTGGCTCGCGAAAAATTTAGA
CGAACTGGCGTCGACCATTTTAAGACAGAGATCGATTTGCAAAAAATTAATCATTAAGTAGCCAAAGAGTGCAAACCACTTAAA
AATTGCTATTTCCCAATTCTCTATATAGTATGTATTTTTTTAATGACCATATAATAAGGTATCGCGCACATTTTGGTATAATT
TAAATTCACATAACTTTCTCAAAGGAGAGAAGAATATTCACATAAATATTAGATAATTTTATAATCTCATACATTATGTTTTTT
TTCTTAGGGATCATACATTGTTATTCATATCCAAAATAGAATTTTAAAAAAATTGAAACTGAATTTCATAATAAAAAATATAGA
TCTTCTTACAATCAGGAGAAAAGAAAGAAATAAATGGAAAAATCTATCAAAACAAAATAAGTAATAAGATTTTGTATCTTGAAA
AATAATGCACAGTTACATCTAGTGAAAAACATCAAATTAGAGATAGCTTGTAATTGGAGAACAACACTTAAAAACAGTACATAA
AATAACAACTTAAGTATAAAAGAATTCAGGAAGAACCCAAAACCAATATCACTGAAATACTCACAATACTGAATCATGCTCGAA
AAAGCTCACTACAACCATCTCACTTTGAACAGACGACCATCATCAACAGCTTTTATACGTACATTAACATATTGGTTGGTTCGG
GGTAATTTTTGTTTTTTAATTTTTGTAACACAAAATAGAAATATAATGTTATTACCTTTTATTTTTTGTTTTTTTATGCTTCTA
AAAAATAAAATTATAGTTCTCGATAACTATTTTTGGTTTTTTGTTTTTAAAACAAAAAAGTATGAAACAACGTTGTACGCTCTT
TATATTCTCTTATTGTTTTGTTAACAAATATGCCCAGGTGTTCGCTTTTGAAGAAGAAAAGAAAAAAAAAAAAAAAGTAAAAAAC
TAAAACATTCATATTTTTAAAATTTAATGAATTTAAATTAAAAAGTTAAAAATAATAATAGAGAAATAAAATAACCAAATAATT
TATGTTTAGATGTATCAATTTCTATGTAATAGAATACTAAACACTATTTTTTAAGTGTTACTTTACTTTTTCCTTCTACTCTGG
GCAACTTCGACACCAAACGCCTCTCTCACTCACAAACTAGAACAAAATCATGTTGATGTTCGATGAGAGTTTGGACTTTGAAGA
AGCTTGAGTTCGGAGATACTGCACGCTACTGATGATTCTCCAGTGATGAGTTTGGTTTCTACCATGAAACACGTTCAATGTTTG
AATTCTATTTAGTATCTCTTTTTCTCTATATGTTTGATGTAATGCATATGTTTATGAACCAGGGGAAGGACGACTCTAATTCAG
TGGCAAAGTAAAATTTTTTTGAAAGAGGTCTAATCATAAATGTTTTGAAAACAAGAGATTATCTTTAATAAACGTGCTATCAGA
CGTATGTGAGCTTTTTTGCTTTTTCTCCACATTTCCACTTGAACGGGGAGATTGTCTGAAGCTGAGTAAAAATTACACCACCGA
CAGGCACAGAAGGTATCATCTGGACAACACACATTGTTTCAGAAATTACCTAAAAATATGATTATTAATTTTTGTTAAAGATTA

ACAAAAAAAGATCAGACTTACTTCCCAGGCCTCTTGCTAAGCGCTCTCCTAATTAAAAAAATGAGGGGGGCATCCGAAAAGGGA
ATATCAGCGAAGATAAATCGTTGTAGTCAATTCAAGCCTTCGAAGTTTTTTCAGTATAAAGATCTCACCTGTACAGTTTAATAT
ACTTGTGCGTAAGTTTTTGCTCCAAAACAAGCCCAAATGGAACGTTTATCCCAGTTCCGCTCTTGGCTGATATGATGTTGAAAA
TCATCCAAACACATGAAATGATCAAGCCCAGAGCATTCTCATTGCCACCATGTCAGTTTTTACTTTCCCTCTGAGCTTAAGATT
TCCTCTGTTTCTCATTCACAAGTTATATGGCTTATCTTTTTCTCATGCTCTCCAAAACCAATCACTTCTTTACCGTAACTCTTA
AATGCTGCCTTGATCAAGCATGGTTACATCATTCTATTCACATTGGAATACGACAGTTCTCAGTGGAAGCTCACAAAAGCTTTG
AACTCTCCTTCGAACATCTCCTTTATCAGCAAGTCCAATCCAAAATATTTGAAAATCACTCTGAGTTAGCGGGGGGGGGGGGGG
GGGGGGTGGGGGGGGGGGGGGGGGGGGGGGGGGGGGGGGGGGGGGGGGGGGGGGGGGGCGGGGGGGGGGGGGGGGGGCGGGGGG
GGGGGGCGGCGGGGGGGGGGGGGGGGGGGGGGGGGGGGGGGGGGGGGGGGGGGGGGGGGGGGGGGGGGGGCGGGGGGGGCGGGG
GGGGGGGGGGGGGGGGGGGGGGGGGGGGGGGGGGGGGGGCGGGGGGGGGGGCGGGGGGGGGGGGGCGGGGGGGGGGGGGGGGGG
GGGGGGGCGGGGGGGGGGTGGGGCCTCCCAGAAACGTGTATTTTCTGCTTGCACAGCCAAAAGCTCCCTCATGACTCAAAACAC
ATCCATGTTCTGATCCTATCCAAATAGGCTGCCTGCAATGATGGCTCCAGCTATTTGACGTTGGAGTTCTTCTTCTCTTCAATA
ATTTCATGAAGCATTGCCTCTGCCTTTTCAGTCATCCTTATTACAATATCCGAGGATGAGGATATTTGGACCTCGAAAATCATA
AAACTTGATGAGATGAATCCCACTACCCTTTCACCAAAACAGCTCTTCAAATTCCCCAAGCTTCACAAGCCAGGGACTTGATGC
ATAGGGGCTCTAGTACGGTCCCTATTCGTGCTCTTGGTTCTTACACTTCTGGCCGTTTTTGAACCCCACACGCATCTCAGTCAT
CTCGTTCCTTTTTACTAGCCCCCAAGGACTGGCATACCAAGTGAAATCAGACTGGTTGTAGGCCAGACATCTTTTTATCTACCA
GTTCTTCACATTTCATAAGGGAAATCAACGATGTTATCTGGGTCACCAGCTTTTAATATAGAAATTGGGCCCAACATAGAATAA
GTAGCCCAGTCAGTCGAGATGTGGGGTTGATTCTTCATTTTTTCGAAAACTTTCTCCATGCTAATTTAGATCGGGATCTCACCG
AATGGAGTTCATCACAAACTCTATAGCTACAATATGTCAGCGTTTAACACATCTCCTTCATCTCAGACCATCACACCAGGGGTT
TTATGAGTTCACCTGAACGTAAAAATAGAGGCACATAATGATCATTGTAACTAAGAGGAGAGGAAGCAAAGCCCAGCTCTTTCA
TACTTCTTCTAATAGGTTGAGAGAGAATTTAATCAATCAGCCTTCTCTTACATAACAATTTAAAAGAGCACCGATATAGTTTTC
GTCAACATTGACTTGATCACTTAAGCTATTAAAAGTAACTCTCAGCAGAATCCAGACCACGAACTTACCAAATCGAATCTAACT
GTACAGGCATAGTCAAGATGGGTGCAAATGGGCAGATTCCCTGCTGAGTTCATGACCATTCAGAGACCTGCAATAAGTCGGAGC
AACAAAACAAACAAGATATGAAGCATAGCTGACATTTCTATGGACGAGATCCTGAAAGTTAGCGCCAAGAATCATGAAATATGT
CCCACATAGAAAAAGTTCTCAAATCATGAAAGACATAGTCACATAGTAGTACTACAAAAAAGTTTCAAGTAACTAGGAACGAAA
AATTGGTCTTGGACTGCAAATGATGTATTTTGGAGAGGCGAATTTGAGACTGATAAACATTAAGCTAGCAAAAGTTTACTATAA
AACGGCTTAATTTAAGGTCACTGATGGCATGCATACTTAAAATGTAAAATGGATTTGAAATATGGTCATGACATATGCAGCACT
GATTTGGATTAAAAGCCATTCCTGACTGCTCTATAATTTTAACCGTTGACTTTGATACATAGATGAAATAAGATTTGCAAGCTT
TGCAGATACAACAAAAACCGATTTCCTTCCTGAATATGAGAATGTTGATGCAAGTTATAATTTTTTGTGACATCCCAACAACAC
TTGTGTTAAGATTCTAATGATTGGTGAAAAAAGCGTACTGAAACTATAAACCAACAACGTGTGCTTTCTTTCCTCTAATATAAG
CTGCTGATGAATTAAACAATTTTTGTTGTGTGACATTCACAATACTTATTCTTAAGATTCTTATGGTGATGAAAGCATAAACTT
ATAAAGAAGCGACGAAAAGCAATGAATTGATCTATTTCTTTCATTGAGAAGGATGCAGATTCCAGAAATTAACCTTCTAGAGAG
AAGACTAATGTAAGTAACTAATGCATATGTTTCCAACTCAAAAATTTGGTAGAATTAATTGCAGCTTAGAGAAATAAACTTAGA
TAACCATTTTACTCCAAAAGAATTCTGAGTTCTATAGGTAAAAAGATAAAACATTTTCGTTTCTAAAGATCGAGCCTATTAAAT
AAACTAATAATCTTAAATAAACATGATTCAGAAGAGGACAACCACATGATAACTGCCAAGGGATTAAATTACATCTTAGTCTTA
CTTTTAAACAAAATTGGGAGTAGAAAAGTCCATAAGTATTATTTTTCACAGGGTCATGAAATGGCAAATTTAAAGAAATGGTAA
AACATACGCCCAAATACATATATACCAGTAAACCCCGAAAATGTTTTCCTTTTTTTTTTTCCTTTTCACTTTCTGGAGCAACAA
ACAAATCATCGAAAAGCAAATTCACTGACCTCAAGGGCCTGATTGTATCTTCGCTTGCGGCTGCGAAGCTTCGTAGGTTGAAAC
GGAGGTCATGTTCTGAGGAAGTCCTGCTTCTTCAAGACCACTGGTCAGGGAGTAGAGGAACGACACTGATGGAAAGGCAGCCCA
GGGGGCTGATTCTCGAGAATAGATTTTTCTACTTCCTCTAATGCTAGGTGGCCGCTGCAGAGACACTATTTCGGTGCAAATGAT
CTTGTAGAGATTCTGTTGACGTAAGCTTCCGTACCTTTTGATGATTGTGGAAAAATCAATCGCGCCATGGACTGAACGATTATG
TGATAAATGGCATATTGGCAGGGTTATAAGGTTTATATAAGCTCTCTGAACCTGAAGAATAATAAGAAAGAACCTCAAGTTGAC
GAAAGTAGAACATATTTTTCTTAGGGTAATTATGAACCTGGTCCCAAGCTTTAACATACTAGCTGACGTTATGTCCCAGATTTT
AAATTGTAGTTTACTAACCGTTTACTATGCATGTTCCAAATATTATTATTTTATTAAATAATCCTATTCATAATATTAAATAAG
ATTTTAGCCATAAAAAACATACTCATTTTTTCCAGTTTTAACCCTTTTTCACTAATTAATTATGATATAAATATAGTATTTTAA
TAAAATTAGGCATAACTTTAGTTATTAAGAGTAACTACAATTTATAATTTTCATTTTTTCTCACTACACACAAGTCAATTATTG
TATGTACGTTTATATAGTCAGTATGAACAGAATTCTTTATAATTTATGAGGAGATTAAGTTAGAATGTGGTGTAATAAAAAAAA
TGATAATATGAGATAAGTATCTTTATAAATGTTTTTTTTTCCGATTAACTTTTCCAAAAAAAAATAATTAAACATTTTTATTAA
AAATGATGACTAATTAATATATACGAGTGAATTAAATATTTTAGAGATTAATAGAAATAAATTTTAATATTAATAACAATATTG
AAATCAATTTTGCAAAATAATATATTTGGTTAATAATTAATGAAGGAATTAATTAAAAGGGTTCAAAAAAATAACGGTGCATAA
TTTTTGTTTTAGAAAACTTCTCTGTTGACGCGGTTCTTTCGCCGAACAGGTAATTAATAGAAGAAGAGAAAGGATTAGTGCTTG
AAAGTAAGAACCGTCACAGATATAGTGAATCTTTAAAGGTGAACTAGGTGAACTTAGAACACGATTTTAAAGTGGTAAATGGTT
AAAATCCTTCTACTCAAATAGTCAATATATTGATATATTCTGGGTGTTGGTTTACAAAGTGTATAACGTCTCTTTCTTGAGTAG
TGTTTTTCACCGGTATGAGAACCTTTTAAACTCCAGGGTCTCCCTATTTATAGGGCAGATGGCACCTGGAAGTTTGTAGGGAGG
ACCATCACCCCCGCCCCAACCCCCGTGACCTTACCCATTTGTCATATCAACTCATGGTAATATTCCATGATCTAAGTTCATAAA
TCCTGACACATAGCGTGTGGTCTAATCAGCGTGCATGGAAGTAATGGCCGCACGGCCCCAACCCAGCCGTGGTTGTCTGAATGC
GCACGTTCCTGCAGCGTGTCGAGACGCCAGGGGGGGAATCGGCAGTGATGCGTCAGAATAATTGCACGTTTATATTGCGTAGCG
TTGACTTCTCAGGAGTCAGAGCGTCCTGATCAGCTCGTACCGAGCACGTGACCCGAGCTAATCCCACTGGTGTGGGCTTCTGAT

GTCCTTGTCCAGTCTTTGGGTAACACGCGAGCTGAGGGGACGGGACTCCCCTCATGAGCTGAGGGAATGCGTCCTGGAAATAGG
ATCTTTCTGTTTAGGGCCTTTGGAAT
>m141122_040740_42134_c100732202550000001823141405141561_s1_p0/7964/4132_10435
TGACATATGAAATTGGGTGAGATACTATAAAGTTTTAAACAACATCAAGGGATGGTACATCTTGAATCCTTTTTCCAGGCCCAA
ATCTCCTAGGAGCTGCCCTAGGGCTACGAAGCGATTTCGTCAATTCACTCATATTTGCTCCTCTAACAAGCTCCTAAATTTTTT
TGAACCTTTTCCCATCTCAATAATATTATATAGGCTATTATTTGAACCATGAAATTGGGGTTTAAATTTAACAAACAAAAAAAT
GACTTTCCTGGCCAAATCTCCTAGGGCCGCAGCATACGGGTCTCCGGAAAGCGATTTCGTAAATGTCATCATATGTTCCACTCT
GAACAAGTCTAATTTTTTGGAACGTTTTCCCATCTCAATAATGCTTATAGTAAGGGGCTATTATTGACCATGAATTTGGGTCTA
ATTCAACCAAACAAAAAAATTGACCCCTTTCCAGGACATAATCATCCTAGGCAGCTTGCCTAGGGCTCCGAAAGCGATTTCGTA
ATTCACTCATATTTCTCTTTAACAAGCTCTAATTGTTTTTGAATTCTTTCCATCTCTTAAATAAATATTTATAGGGCAATTAT
TTGACGTGGAATTGGGGACCTTAATTCAACCAAACAAAAAATTGACCTTTGGAGGCCAAAATTCTCCCTAGGCAAACTGCCTTA
GGGCATTCTGAAAGCGATTTCGTAATTTCACTCAATATTTTCTCTCTAAAATGCTCTTAATTTTTTTTTGAACCTTTTCCATCT
CAATAATATTTATAGGGCTTATTATTGACCCAGGAATTTGGGGTCCCTAATTTTAACCAAAACAAAAAAATTGACCTTTCAGGC
CAAATCCTCTAAGGGCAGCTGCTAAGGCAACCGAACGATTTCGTAATTCACTCATATTTCTTCTCTTAACAAGCTCTTAATTTT
TTGAAACCTATTCCGATCTCAATCAATATTATATAGGGCTATTATTGAACATGAAATTGGGGTCCTAATTTTAACCAAACAAAA
AAAATTGGACTTTTCAGGCCCAATTCCTATGCCAGCTGGCCTAAGGCACCGAAGCGATTTCTTAACTTCACTCATATTTCTCTC
TAACAAGCTCTATTTTTTTAAACCTATTTTTTCCCATCTCAATAATATTTATAGGGCTATTATTTGACCAATGAATTGGGGTTCC
TAATAATCAACCAAACAAAAAATGTACCTTTATCAGGCCAAAAATCTCCTAAGGCAACTGCCTAGGCTCCGAAGCGATTTCGTA
ATTCACTCATTAATTTCTCTTCTAACAAGCTCTGAATTTTTTGGAACCTATTTCCCATTCTCAATACATATTTATATAGGCTAT
TATTCGACCATGAAATTGAGGTCTTAATTTAACCAAACCAAAAAATTGACTTTTCAGGCCAACATCTCCTATGCAGCTGCCTAA
GGCACCGGAAGAGATTTCTTTATTCACGTCATATTTCTCTCTAACAAAACCTAATTTTTGGAAACTTTTCCATCTCAATAATAA
TTAATAATAGCGGATATTATTTGACCATGAATGGGTCATAAATTCAAACAAACGAAAAAAATTGACTTTCCTGGCCAAATCTCC
TAGGCAGCTGCCTAGGGCCACGAAGCGGATTTTCCGTAATTCACCTCATATTTTCTCTTCCTAACAAGCTCTAATTTTTTTGAC
CTTTTTCCCATCTCTAATAATATTTATATTACGGGCTATTATTGACCATTGATTTGGGAATCTAATTTCAACAAAACAAAAAAT
TGACCTTTTCAGGCAAATCGTCCTAGCAGCTTTGCCTAGGGCTTCCGATAGCGATTTTCGTTATTCCAACACATATTTCTTCTC
TAACAAGCTCTAATTTTTTTTGAAATTTTACCATCTCAATATATTATATATGGGCTATTATTGAAATGAATTGGGGTCCTTAAA
TTCAACAACAAAATTTGACCCTTTCAGGCCAAAATCTCCTAGGCAGCTGCCGTAGGGCTCCGAAGCGATCTTCGTAATTTCACT
CATATTTCTCTCTACAATGGCTCTAATTTTTTTGGGAACCTTTTCACCATCCTTCAATAATATTATATTAGGCTATTCATTGAC
CATGATTGGGGTCCTAATTCAACCAAAAAAAAAAATTGACCTTATCATGCCAAATCTCCTAGGCAGCTGCCTAAGGTTTCCGAA
GCGATTTTCCGTAATTCACTCATGATGTTCCATCCTCTAACGAGAGCTCTAATTTTTTTTGGAACTTTTTCCCATCTCAATAAT
ATTATATAGGGCTATTAATTCCCATCGAATTGGGGAGTCCTAATCTTTATCCAAACCAAAAAATTGACCTTTCAGGCCCCAAAT
CCTCATATGCCGCTGCCTATTGGACTTTCCGAAGCGATTTCGTAATTTCACTCATATTGTCCTCTCTAACAATGCTTCTAATTT
TTTTTAAACCTTTTCCATCTCAATAAATTATATAGGCGCATTATTGGACAATGATTGGGGGTCCTAATTCAACCAAACAAAAAA
TTGACTTTTTCAGGCCAAAATTCTCCTTAAGGCAAGCTGCCTAGGGCTCCGAAGCGATTTCGTAATTCCAAATCCATAATTTCT
CTTTAACAAGCTCTTAATTTTTTTTATTTTTTCCCATTCTATAATATTAATATAAGGCTTATTATTGACCGTGAATTGGGTCCT
AATTCAACAGAACAAAAAAATTGACCTTTCAGGCCAATCCTCCCTAAAGGACAAGCTGCCTAGGGCTCTGAAGCGATTTCGATA
ATTCACTCATACTTCTCTCTAAAATGACTAATTTTTTGAAAACCTTTTCCCATCTCAATAATATTATATAGGGCGTATTATTGA
CAATGAAATTGGGTCCTAATTCAACCCAAACAAAAAAATTGGACCTTTCAGGCCAAATCTCCTTAGGCAAGCTTGCTAGGGGCA
TCCGAAGCGATTTTCGTAATTCACTCATATTTCTCTCTAACAAGCTCTAATTTTTTTTGAAACCTTTTCCCATCTCAATAATAT
TATATAGGGCTATTATTGACCATGGATTGGGGTCCTAATTTAACAAAACAAAAAAAATTGACCCTTTTCCCCGGCCAAATCTCC
TAGGCCCCGCACCTAGGGTCCCGAAGCGATTTCGTAATTCACTCATATTTCACTCTAAACAAGCTCTAATTTTTTTTTGAACAT
TTTCCCATCTCATAATATTATTATAGGGGCTAATTATTGACCAATGATTTGGGGTCCTAATTCAAACCAAAACAAAAAAAATAT
GACCCCTTTTCAGGACAAATCTCCTTAGGCAGCTGCCTAGGGCTCCGAAGCGGATTTGTAATTCAACTCATTTTCATCTTTAAC
AAGCTCTGAATTTTTTGTTGATCTTTCCCATCTCTATAAGTAATTATATAGGGCAATTATTGACCGTGAATTGGGGAACCTATA
TGCAGACCGGGAAACAAGAGAAAGATTTGACCTTTGAGGGCGCAAGAGTCGGTCCTAGGGCAGGACTTGAGTAGGGCATCTGAG
AGAGATTTTCGTATTTCACTCTATATTCTCTCTAAAATGCTCTTAATTTTTTTTGAAAACCATTTTCCGCATCTCAAATAATAT
TATTATAGGGGCTATTATTTGACCAGGGAATTGGGGGTCGCTAGATTTAACCAAACAAAAAAAATTGACCTTGTCAGGCCAAAT
TCCTATGCAGCTGCCTAGGACTCCGAAGCGATTTCGTAAATTCCTCTCATATTTCTCTTCTAACATGCCTCTAATTTTTTTTTA
AACCTTTTCCCATCTCAATAATATTATTATGTAGTGGTCTATTATTGATATGAATTGGGGTCCTAATTCAACCAAACAAAACAA
TTTGACTTTTCAGGCGCCAAACTCTCCCTAGGCCAAGGCTGCCTAGGGCTCCGAAGCGATTTTCAGTAAATTCAACTCATAATT
TCTCTTTTACAGCCTAATTTTTTTTTATTTCCTTTTCCCCCATCTTCTTAAAATATTAATATTATGCGGCGCATATTATATAGG
GCTATTATTGACCGTGATTGGGGTTCGCTTAATTCCAATCAATAACATAAAACTTGACCTTTTCAGGTGCCAATATCTCCTTTA
GGCCAGCTTGCCTAGGGAGCTCTGATCGATGTTTTCGTAATTCCGTCATACTTTTCTCTCTTAAAATGCTTCTAAATTTTTTAC
TATTTTTTTTGGAAACCCTCTCTAACCCGTTGGAATCATCCCCCCGCCCGCCTCCTCGCCCCTGGCGACCCCAATCGGGATTCG
AAAGATTAAAGTAAGTAAATACATAAAATATTATATATTAGTTTTGAAGATACTGGAAAGCAATAGGTTGAAGATTCAATATGA
ATTGTTGTTGCAATGTTGGTGGTTTGAGTGTGGATGATTTGTTGAAACCTTAACACTTTCTATATCCGCACTTCTCTTCTTTCT
TGCCATATCGACCGCTTCTTAGCCACAGCCATACGCAACATACTCCATAAAAGTAATAACCTCTATTTGCAGACAAGTACGCGG
AATATGTTGTTCTTACTCCGTCGGTTAGTTGTTCTTTTTTTTAATTTTTGTTATGATTCACCGAAGGGATCCATTCTTGATTAC

AGGTCAGTTTTTAATTTTTTTTACTATCAATTCTTATTTAATTTTTAATGATGTATAGGATATGCAATTGAAAGCTCTTTTTTT
GTTGGGACTTTTACTGAACTCCAGTTTTAACTTATTTTAGAGTGTATTTGTGTATTTATGAGCTTATTGTCTAAATTTTTGTGG
GAGTTTTCTTTTTTTATGAAACACAGACCCTTGGGTCAAAAGTATCATTGCGTAAAATGCACTTGGGCCTAAGACAGAAATCTA
TCTTTTACCAAAAGAAATGAAAGTTGTATTTTTTTCTACTCAAAATAAATATATATAATATAGGGTTAAACAGAAGCCAAACAA
AACAACCCAATAACATAACAACAACCAACTACTAAACTAAATATATAGACATTTTTGTTTTTCTGTTCTCCCTCAAGATGGGGC
AAGATGATTTGTTGAAGCATCTTGGAGACAGAGTGTGAAATTTGAGTTGATGGAAGCTTTTGTTGAAGGATGTTATGAGCTGGT
TGTTGGTGGAGACTGTAAAATTTGATGATTCATGTGGTTGATTTGTCGCGAACTAAGTGACCATCTATTCTATGTGCTTATTTT
TCTGAGACATGTTGAAGAGATATGAAGAGCTGCTTGGTGTCATACGTACGAGAAGAACTGGTTTTGTTTGAGGAAACCAGTCCT
TTTGAGTAAGGGCTAGCCATAAATTAGTGTGGCATTAGCACATGGATCTATACTCAGCTTTCCGGAGAGGGAGATGGGTGGGTT
GTTTCCTTTGACTTCCAGAGACCAAGAGTCTGTTGAAAAACACAAAACTGAAATTTGGAACGCTAAGTGTCTGAGCAGCGGCCT
ACTAGCATCACAGAAAAACTTGATGGAATGATCTAAGGGCAATGAGTGGTAGTGATAGAGGTCGCTTGCTAGTGGCACGGATAT
AGAGACCACGTCAAGGTGGGTTTGATGATAGTGTAGCTTTGGGCAGCTTGATAATGGGGTTGATCAGTGGGAAACACAAATTGG
CTCAATCTCAGATTGATGGATATGATAGATCGGGCATGTGATGGTCAGTTGATGGGTGGCCATGGCCTTCTATATGCTTTTGGG
TCCTTAAGTAGGCACCACTTCAAGCGGATAATTGAGGTTGACATCATCTGGTGAATATGTATGTTGCATCAGTGTGTCTTATCT
CTCAGGATTTGGAGTGCATATCCTCGCTATAGATACGGAAATGCCTTGTTTTCCTAGCAATTTAGGCAGGAAAAAATTTTAGTTC
CCAGATTTTTATGAACAAGATGAATAAAGTTAGAATAAGAGTGTTTTTGCAATGCTTAGAATTGCTACAATCATAATCATCAAC
ATAATTAGAAGAGCAAAAATTTGTATTGTCATGTCTTAATGAAAAATTAATTCAGTATATGAGTGTTGAAAACCAATAGGCAGT
AAGAAAGTAGTGAAGTTAGTGTTCATCCAAACATTTGTCTAGAAGCTTGCTTAAGCCATATAGACTCATGTTTACAGTTTACAA
ATGGTTTTTGAAAATTAGTCTTGCTCCCCTTAAGTGTGTATCCTTGAGGTAGAGACATGTAATCTCTTTCATCAAGATCATCAT
TAAAAAACATTATTTCATCTAATTGGTGTAATATCTCATATGGCACAATGGCCCATAAGTTTAGACGTGACATTTTGGGCACAG
GGG
>m141122_040740_42134_c100732202550000001823141405141561_s1_p0/9189/13042_19218
GGGATCGGACCCCTTCTAGATAATTCCACTACTATCGCCAAATCCTAAACTCAGCATGACGAACTATATGAGACCCTAAGCGGT
TATAATTGGTTTAGCTCAGTGTAGAAAACAAAAAGCATTGGAACATTCATTTGGTCATAATTAGGTTGCTTAGTGTAATGTTTA
TCTAGTAAGTATTTTTGCGGATGATCGGATCCCCCTTCTAAATTCACTTCTCTATCTTACTCAGGGGGCATGACAACAATATGA
GCCCTAGCGGTAGTATTGGTTTACTAGTGTAGACCGTTGGACGACGATATGCATTATGTCATATTAGGTCGCTTAGCTGCAATG
TTAGGACTAGTCATAATTTTTCCGGGATCGATCCCCCTTCCTATATTCACCTACTATGATCTACACTCAGCATGCACAAGCTAT
CATGCACAGACGCCCTAGCGGTAGTATTGGGTTTACCCTAGGTGACACTTGGAACGATTCATTGTGTCATAATTAGGTCTGCTT
AGTGTCAATGGTTATCTACGTATAGCTTTTCGAGATCGGACTCCTTTCTAAATTCACTAGCTCTAATCTTACTCGCATGACAAC
AACTCATGAGGCCCTAGCGGTAGTATGGTTTACTAGTTGGTACGTTGGAACGATTCACGTTATGTCAATTAGGTCTGCTTAGGT
GCAATGTTCCCCTCTCATCATAAATTTTTCGGCGGATCGGATGCATTCTAAATTAAGCTACTATCATCTTAGTGCAATGTTAAT
CTAGTAATATTTTTCAGGGATCGGATCCTTCCTAAATTCACTACTCTATCTTTCACCTCAGCATGAGCAAAATATGAGCGCCCT
AGGAGGTAGTATTGTTTACTAGTGTAACGTTGGAACGATTCATTATGTCATAATTAGGTCTTGCGTTAGTGGCAATGTTATCGC
TAGTCATACAATTTTTCGGGATGGATCACCTTCTATATTCGCCTACTATATCTAACTCCAGCATGACAACTCATATGAGACCCT
AGCGGTAGTTGCATTGGTTTACTAAGTGTACATTGGAACGATTTCATTATGTCATCAAATTAGGTCTGCTTAGTGCAATGTTAA
TCTGCAGTCATCAAATTCTGTCCGTGATCGGAGCATTCTAAATTAATACTATCTGTCCATAAAGTGGCAACTGATCATCCTAGT
AACATATTTTTCCGAGATCGGATCCCCCTCTAATTCACTACTCTATCCTTTACTCGCAATGACAACAATATGAGCGCCCCCTAG
ATGTAGTATTCGCGCTTTACTACGCAGTACGTTTCGGAAGATTCATGTATACTCTAATTAGCTCTGCTTAGTGCAATGTTATCA
CTAATAAATCAATTTTTGGGATCCGATGCATTAAAAATTTAACTATTATCCATGAGTGCAATGTATCTGTAATATTTTATCGAG
ATCGGATGCGCTTCTAAATTCACTTATGCTATCTTACTCAGCTGACAAACAATATGAGGCCCTTGAGGTAGTCATTGGTTTACT
AGTGTAACGTTGGCAACGATTCATTATGTCAAAGCAATTAGGTCTGCTTAGTGGCCATGTTCATCTAAGTCATTAATTTTGTTT
CGGAATCGGCATGCATTCTAAATTACTTACTATCTCCCGTAGTGCATGTTATGCTAGTAAATATTTTCGAGATCGGATCCCCTT
TCTAAATTCACTACGTCTACGTCTTACTCCCAGCATGACAGAAACAATATGAGGCCCTAGGCGGTAGTATTGGGTTTACTAGTG
TACGTTGGAACGATTCATTATTGGGGTTCCCCCAAAAAAATAAAAATTAGGTCTGGGTTAGTGCAATGTTATCTAGTCATAATT
CTTTCGAGAATCGGATGTAACTTCTAAAATTACGTACTATCTCTTAGTCAATGTTATCTAGGTCTTTTTCCGAGATCAGATCCC
TTCTAAATTCACTACTTATTCTTACTCCAGCATGACAACAATATGAGGCCTAAGCGATAGTATTGGTTTTAGTAGTGTACGTTG
GAACGCATTCATTACTGTCGATAATTAGTCTGGCTTAGTGCAATGTTATCTGCTCTAATTTTTCGGGATCGGCTCCCCTTTCTA
TATCTCACTAATATAATCTAACTCAGCATGAAACTATATGAGCGCTAGCGGTAGTAATCTGGTTTACTAGTGTACACATGCGAA
CGATTCATTATGTCATAATTAGGTTCTTGCTGTAGGTGCGCATGTTCCTCTTAGTCATAATTTTCGGGATCGGATGCATATCGT
AAATTAACTACTATATCAATGTGCAATGATATCTAGTAGTTTTTATCCGAGATCGGAATCCCCCTCTAAATTCACTACTCTATC
ATTCACTCAGCATGAAACATAATGAGGGCCTAGCTGTAGTATTGGGTTTACTAGAGTACTTGGACGATTCATTTATGTCTAATT
AGGTCTGCTTAGTGCAATGTTATCTACAATCATTACTTTTTGCGGGATCGGATGCATTCTAAATTAAGCTACTAATCTTAGTGG
GAATGTTATCTGTAGTAATATCTTTTTCGAGTCCGGATCCCCTTCTAAAGTTCACTACTCTAGTCATTAGCTTAGCATGAACAC
ATATCGAGGCCCTAGGCGTAGTATTGGTTGCTAGTGTAAGTTGGAGACGATTCATTATGGTTCATCAATTAGGCTGCTTAGTGG
AATGGTTATCTAATCATGAAATTTTTCGGAGATCGGATGCTTCCTAAATTAACTACTGATCTCTTAGTGCGAATGTTATCCTAA
GAAATATTTTTCGCCCGGGATCAGATCCCTTCTAAATTACACTAACTGCTATCTTACTCAGTCATGCAAGAAATATTAGGCACT
AGCGGTAGTACTTGGTTTACTAAGTGTACGTGAACGATTCATTATGTCATAATTAGGTCTTGTTTAGCGATGTTATCTAGTCAT
AATTTTTTCGGGTCGGATCCCCTTGCTATACTTCCCTAGCTATATCGCATAACTCAGCATGACAACTTATATGGACCTAGCGGG

TAGTATTGGCGTTTACAGTGTACATTTGGAACGTTCTTATGTCATAATTAGGTTGCTTAGTGAATGTTAGTACTTGTCATGAAT
TTTTTGCGAGATCCGATCGCTCTTTATATTTCAGTACTGTATCCTAATCCGCATGAAACTATATGAGACGCTAGCAGTAGTATT
TTTTGGGTTTTTTACTAGTGTAACATTGCGAACGATTCATTATGTCCATATATGTAAGGGTCCTGCGGCTTAGTGCCATGGTAT
CTTAGTAATATGTTTTCCGAGATCGGTCCCCTTCTAAATTCACACTCTTTCTACTCAGCATGACAACAATATGAGGGCCCTAGC
AGTAGTATTGGTTTTACTAGTGTAGATTGGAAGATTTCATTATGTCATAAATTAGGTCTGTTAGTGCATGTTATCTAGTAACTA
TTTTTCGAGATCGGATCCTTCTAAATTCACTACTGCTAATCTTACTCAGCATGAAAAATATGAGGCCCTAGCGGTAGTATTGGT
TTACTAGAGTACGTTGGAACGATTCATGTATGTCATAAATTAGGTCTGCTTAGTGCAATGTTTCATCTAGTACTATTTTCGAGA
TCGGATCCCTTCTAAATTCATCTCGATCTTACTCGCATGACAACAATATGAGGCACTAGCGTAGTATCGCTGGTTTACTAGTGT
ACGTTGGACGATTCATTATGGTCATACATTGAGGTCTGCTTAGTGGCAATGTTATCTTGTCATCAATTTTCGAGATGCGACCCT
TCTATATTCGCAGTACTGTATCGTAACTCAGAATGACAACTATACTGAAGACCCTAAGCGTAGTATTGGTTTACTAGTGTCACA
TTGGAACGATTATTATGTCATTTAAAATTTTAGGTCTGCTTAAGTGCAATGTTATATAGTAAGTATTTTCCGAGATCGGATCCC
CTTCTAATTCACTACTCTTTCTCTTACTCAGCATGAACAATATGCAGGCCCCCTAGCGGTAGTATTGGTTTACATATGTACATT
GGAGACGATTCATTATGTCATACCTTTAGGTCTTGCTTAGTGGCAATGTTATCTAGTAATAATTTTCGGATCCCGGGATCCGCT
TCTAAATTCACTAGATCTATCTTACTCAGCATGACAACAATATGAGGCTAGGCGTAGTATTGGTTTAGCTAGAGTACGTTGGAA
GAGATTCATTATGTCATGACTAGGGTCTGCGCTTTTTTTAGTGCATGTTATGCTAGTTACATTTTTTCGGACGGATGCCATTCT
AACAATTAACTACATATCTTAGGTGAATGTATTATCTAGTATATTTTCCGAGATCAGATCCTTCCTAAATTCACTACTCTAAGT
CTTATCAGCATGACAACAATATGAGGCCGCCTAGCGGTAGTATTGGTTTATAGTGTACGTTGGAACGATTCATTATATCATGAA
TGTAGTTCTGGCTTAGTGCAATGTATATCTAGTGCATATTTTTTCGGGATCGGATCCCTTCATTATATCACTACTATATCTAAG
CTCAGCATGACAACCTATATGGCAGCTAGCGGTAGTACTTGGTTTACCTAGTCGTACCTTGGCAACGATTCATTTGTGTCTAAT
TAGGCTCTGCTAGGTGTAAATGTTATCTAGTAATATTTCTTCCGAGAATCGGATCCCCTTCTCAAATTCCTACTCTATCTTACT
CAGCATGACAACAATACTGAGGCCTAGCGGCGTAGTATTGGTTTACTCGTGTGTACGTTGGAACGATTCATTATGTCAACAATT
AGGTCTGCTTAGTGCAATGTTTATCTATCATAATTTCTTCGGGATCGGATGGCAATTCTAAATTAACTACGTATGCTCTTAGTG
CACATGTTATCTAAGTAATATTTATTCCGGGATCGGATCCCCTTCTAAATTCCATACTCTATTCTTTAGCCTCAGCATGGACAA
ATGATGAGGCCCTAGCCGGTAGTGATGTGGGGTTTCCTAGTGTCACGTTGAACGATTCATTACTGCCTCCATAATTAAGTCTGG
CTCTCAGTGCAATGTTATTCTAGCTCATAATTCGGGCATCGGTCCCTTCTACTACTTGCACTAATACTGCTAACTCAGCATGAC
AACCTCTATATCGAGACGCCTAAGCGGTAGCTCATCTGGTTACTAGTGTACAATTGGAAACGATTCATTATGTCATAATATGAG
GTCTGGCTTAGTGCGAATGTATATGCTAGTCATCAATTTTTCGTGATCGGAAGGCCATTCTAAATTAACTACTATCATCATCAG
TGCAATGTTAGGTCGTAGTAAGCTATTTTTACCCGAGATGGATCCCCTTCTAAATCAGCTACTCTAATGCTTACTCCGCATGAA
AACAATATGGAGGGCCCTTGCGGTATAGTCATTGGTTTAGACTAGTGTACGTTGGAACGTTCATTATGTCAAATTAGGTCTGGC
TGTTTTGGGCAATGTTATCGCTAGTCATCAATTTTTCGGGATCGGGGATGCAACTTCTAAAATTCAACTACTTCTCGTAGTGCA
ATGTTATCTACGTAACCTTATTTTCCGAGATCGCCCGCGCTTCTAAATTCCCTACTCTACTCTTACTCGAAGAATGACAAATAT
GCAAGGCCCCTAGGCGGTAGTTATTGGTTTTCACTATGTCACGTTGGAAACGATTCATTATGTCATAATTAGGTCTGCTTAGTG
CATGTTATTTCTAGTAAATAATTTTTTACGGATCACTCACTGTCTATATTCACTAATATATCGTAACTCCAGCATGACAACTAA
TATTGGATAGCCCTACGCGGTAGTATCTGGTTTACTAGTGTACATGTGGAGAGATTTCTTATGATATCAGATCTAGGTTCTGCT
TAGTGCATGTTATCTAGTCATAATTTTCGTGATCGGAAGCCATTCTAAAGTTAAATACTATATCATAGTGCAGATGTTATCTAG
TATAGTTTTTCCGGGATCGGATCCCTTCTAATTCACTACTCTATCTTACTGCGCGCTGAACAACAATATGCAGCCGCCCTAGCG
GTAGTATTGGTTTACTAGTGTACGTTGGACACGATTCTTATGTCATAATTACGGTCTGGCTTAGTGAAATGTTATCTAGTCATA
ATTTTTTCGGGATCGGATCACTATCTAATATTCACTACTTATAA
>m141122_040740_42134_c100732202550000001823141405141561_s1_p0/10448/0_9656
AAGAGAGAATTTAGGTTTAGGGTTTAGGGTTTGATGGGGTTTAGGGTGTAGATGTAGGGATATTAGGGTTTCAGGGTTTACGTG
GTTTAGGGTTAGGGTCTTAGGTTTAGGGTATTATGGAGTTTAAAGGGTTTTATAGGTGTTTTTTATGGGTTTTAGGGTTTCAGG
GTTTTTACGGGTATTCTAGTTGGTTTATAGGGATTTAGAGGCTTTTTTGTTGGTTATTAGGGTTTAGTGGGTTTTATTGGGTTT
CATGGGTTTAGGGTGTTACTAGGGTTTTTCAGAGGTTTGAAGAGGTTCAGTGGTTATAGGGCTCTTTTAGGGTTATTAGAAGGG
TTTTTAGGGTTTTCATGGGTTTAGTTGTTTAGTGGTTTAGGGTTTCTGGGTATTAGGAGTTATTAGGGTTTAGGGTTTAGGGTT
TTCTAAGGGTTTTTGGGTTTATGGGTTTTTAGGTGTATTAGGGTTTAGGGTTTCTAGGGTTTTTAAGGGTTCTTTATGGATTTA
GGTTTAGGTGTTTACTGGTATTTAGGGTTTCGCGGTTTAGGGTTTAGGGTTTAGGGTTTAGGGTTTTAGGGTTTTCCTTAAGAG
GCTTTACTAGGGTTTCTGAGGGTTCTAAGGGTCTTTAGGAGGTTTAGGGTTTCTAAGGGTTTTTTAGAGTTTAGCGGTTTACGG
GTATTAAGGGTTTTTACAGGGTTTTAAGGGGTTTCAAGCTGAGTTTAGGGTGTTAGGGTTAATATGCGGTTCTAGTGGTTTAGG
GTTTTAGGGTTAGTAGGGCGGTGATGGGTTTAAGGGTTTGACTAGATGGTTTTTAGGCGTCGCTAGAGGTTAAAGACGTGTTTT
TAGGTGTTTAGGTGAGATATAGGGTTTTATTGGGTTGAAGGCGGGTTAGGGTCTTAAGAGAGGTTAGAGATGTTGTGATGAGGG
ATATGAAGGGGATTTATTTGCAAAACAGTTTAGTCACGAAGTTTTTATAGTGATGTACTTTTATTTAAATGATAATATACCTTA
AAGGCGATACATTATTAAGTTGTTCAGTGATGTTTTTTAAGGTTGCATTGATTTAACTTAAAATACATAAAGTGTTTTTTATC
ACAAAGTACTAGAGTATGTGTTTATGTGGATTTTAAGGAGTTGTTAAAAGGATGAAGAATAGTGAAATTACAAATAATAATCTG
TCAAATCTGGTTTTTCGTTAAATTTGGTTTACAAAGAAATAATGTATAAATTTGTATAAAAAAATCAATATACTTTATGAAAGT
AATTAGACTTAGAATAATATAAGTAAATGAAAAATACAAAATATTACAGTTGGTGGAAGTGGGTCCGCTGACCCGGGGAGAAAG
TGCTCTGCGGGCGGATTCCATAGAGAAAAAAAATTAAAGTTCAATGTGTACAATAAATATCCAAAGAATATAAAATTTAATAAA
AAGCTATAGTTATAGACAACGATTAGCCTTAGGCAGGGGGCGAGCGTTTGGAGGAGGGAGGAGGGCGGGACAGTGGGGGTGGGG
AGTGGTGAAGATGTGGGTGTCGGCGTTGTGGGTGGGGGCTTTGGGGTGGGGAGATGGGGTGTCGTCGGGGTGTGGTGGGCTGCA

GGGGGGTGGGATGGGCGTTGTCGTGGGGTGGGGTGTGATAACTTCACCTAAATAGTAGGTTGATTTTACCGACTTTTTTGAGTT
GTTGTGCTAAGGAATTTGGTGGTTTGGCGCTTAGAAGTCTTGAGTTTTGTTATTGAATTTAATGAAGTTTTTATAGTAATATGT
GAATTTATTAAGGTTTATTTTGATTTTATATATTTTTGTGTGTTTTATAGTTGTTTTGTTGTAAAATATGGTGTAGTTAAATTA
TTTAATTATTAATTGTTTAAAGTGGTTGGGTAAAAAAGATGGTTGTTTTATTGAAAGTAAATGTTAATATATATAATATGAATTAT
AAATTAATATTTTGTCAAAGAACTTACTTGGATGTTAACTTTTAGATAGTGAAAATAGTGTGATTATAATTTATTTATAATTGC
TAATGTTTTGGTGGGAAATCATCAATCCCATTTTTTGACGCGATTGAGGGAAAGGAAAAAGAAGGAAATGTGATATTTTTAACA
AAAAGAAAGCACAAAGAAGAGCCAGTTCTGATAAGTGCACGCAGTTGCTGCCAAGCTCCCAATTCGGCCCAAAGGTTTAGGTTT
AGGGTTTAGTTTAAGGGTTTCAGGGTTTAGGGTTTAGGTTTTGGCTCAGGGCCCAAGGCTTCACATCCGGGGACGCAGCAACAC
GCACACACCCTGCCGAAGGGGGGCCTCGCTTTTCAGGTGCATATATCCAATCCTTCTCAGGCGCCACACTCTGTCACTTTCCTT
TCTCGCAGCCTCCAGCGTTGACTCCGACAGTGGATAGCTCAAGCCCAAAGGCCTTTCAGCAGATCTCCGCATTCATTCCATTTA
GGTGGGTTGCTCATCAGCCGCATGCAAGCCAAGGCCGAGTCCACCTCGAAGCCTATAAAGAGCAATCTAAAGCGCCCAATTGAA
GCCAGCACAGGGATCGGCAACGCCTGACTTCTCTTCCACCAAAGCCTCAAGTCCTTTCCTTCAGGTCGCACTTCCGGCCAACTA
GCACCATCGTGCCTAGGGATGTTTAGGCCAGACGTTTAGGGTTTAGAAGCAAAAATAAGACCAAGAAGCTCACATTTTTTATTC
CCAATATTTTTTCACCAAAATATCAATTCCAATCTTTCCTATATTTCCTTAAGCCTAAAAAACATTCTAATTTATTTATTTTAC
CTTAGGACTTTTCAGCTAATCTTTTACCCAACAAAAAAATTCATCTTTCCAATACTCTTACACTTACCTCATTTACGTCTACTC
ACGTTTCCCATACACAATTAAATTCAATCGAATTTATTATTTTAAATTTGGATAAATTTTAATCTCATATTATTGCCTATAAAC
TCGATGTCTTGGTAAGACCATTTTTGGGAGGGGCTCTTGCTTCTTCAATTTCTGTTTTTTTTTGCATGGCTCTCTACGCTGCCA
GTTTTCTCTCTTGCGTTTCAACTATTTTTTCCTATCAAACCATTTTCTCTTTTGAAGAGCTGTCAAGGTATTAGTTATTGTATT
TTATTCTAGTTCAGTGAGTGTTCCTAATTCTTTTTTCAAGGTTGTATTGAAGATGATGAGGATAGGTCAAGAATGTAACTAGAT
AGGTCTGTTTATTTATTTAGTGTATGTTGATTTCGCACATTGTGTAACTTATTATGATTTCTATCAAAGTATGCATTTTCAAGT
CTATTTTGAGGTGTAAAGCATTACAAACTTAGTTTCTTCATTGATGCAAAAAGTAGTGATTAAATTCTGTTGAGTAAATGTTTT
TTCGTAAATTGTTTTCACTTATCGATTGGCTTAGGTAGGGGCATAAGTATTTACATGATTGTTGGCCTAAACATAATCTCTTGA
TTTTTTGTAGTTTGCATTAGGGTTGTAACAAATCTTAATGCTGAAATTATATCTTAATACAAGTGAAGAAAATCCTTACATTTT
GAAAGAGTAACTTGTGCTGTGAATCAGAAAATATATTTTGAAAAAAAAATAACCATAGGTGGTGATTTATTTATCAACGTATTA
TTCAATTACTGGGAACAATATACTATATAATATCTATTGAACTTGGCATTTTGTGGAGTTCCTAATTATCTTAAAGTAATCTAT
TTTACATATCTGCGCTTTGAAAACCAGTTTTATTCTATTTGATCATTAATCTTTTTATTACGTTGTCCTTTTTTTTCTAAAAAA
AGTCTCATCAAGTACTTTTGGAACTAGGGGTTAGAATGAATTAATATGCTTTGTTTGGAAATAGTTTTCTTTGATGTTACGTGG
CTAATCCTCCCATATGAGGTTGACCCCTCGTAACTTACATGAACATGTATGATCCAAAAAGCGAATCGTGCACTTGCGAGTTTT
TAAATATTCAAAACACCCTCCTTAGGGATAGCATGGGGGTAGGGTGGGGTGGGTGGGTTCAGGGTGGCACGAGCCTATTACGTC
GAAGTGGGGTGGGCCCCTCGGGGTGCGGGCTGGTGGGGGTGGTGGAGGGGGGGGTTGGAGGTGGTGTGGTGCCGTTGAAGAAGG
GAAGAAAGGGAGAGAAGGGGAAGAGAAATTGAAAATTGCAAAAAAAGGGGGGAAAATGGTCGGGCGGTAGGTTTAGCGCTGTGG
TAATACTCGTTATGATTTTGCGCGGCCGCGTTTTCGTTGCGCGGTCTAAAGATCTATCCCAGTAAAAACCCCTCAATCACGACG
ACGGTGTTTATAATAAGTATACTTTTTTTGCCGACGGATTTTGAGAACTTTTGTGGCCAACAAAACGCGCCGGCAAAGAAGTCT
GGCCACCAACCTTCTTTTAAAACGTGCATCAATTTAATATCCAACGTTGCTTTTCGCGGCAGGTTAGGTGGAAATTGCTCGCCG
GCAAAAGTTACGCGCTCGTTATAAATTGGAAATTTCCTGATATAAATTTTGCTTGGCGAGTGCGGAATAAGCGAACCAGGCAAA
AGACGTAAGTTTTTTGCCTGCCGCAGGTTTCACCGACTTGCGGGCGCGGTATGGTTTTTTTTGTCGGCGCAATTACAAATTGGC
ACAGGCCAAGGGAGTCTCATTTTTTCGTTGGAAGTGCTAGACCACCACCTCCTACCCTTTTGTGCGCCCATGGTTTACTTTCAG
TGAGCCGCTGCGGCTTTCGCGCCCCACGGGGAGCCAGTATGCGCCCTGCCGCAACGATTTTTTTTTATTTTTTATTAATTGTTA
ATATTTAATGTGTTTTTTCCAGATTTTTTTTTCCGAATAGTCGCCTCAGACTGACGCCCCGTGCCCCCCGCGAGGCGTGCCCCT
ATACCGAGCAGACGAGTTTTTTTTTTTTTTTGTTATGTTTTGTTTAATTGATTTAAAAATTATGTTTAGATATGTTAAAAACCA
ATTAATTATCGTATTTTATATTGTTAAAAGATTTTATATAATAAGGTGAGATGATGTTTTTGTAATATTGTATAAAAAAATTTTT
TTTTTGTATTTAATTGAAAAAATCAGTTTGTTTGGTAATAATGGTATTTGTGAGTAAAATAATTGTCTATTTATGTAAATGGAT
GTATATTTAATGGTGTGTATATATATATTGTTTCGTGTTTGATCTTAGAGTATTCTAGTTATATATGTGCGTTAATTTACAGGT
TTATAAAATTTGGATAGTCAGTTAATTATATTAGTGTATAATTAAATAGGAAAAATACTATTTTAGCGGCCCCGACAATTATT
AGGCAGTTCTGAACAAGTAGTCCCCCTAGATAAGATTCATATTTAGGCAGCGAGCCAAAACATTAAGTCGTCGCTGATGGTCTT
CGATTGGATAGATTTATTTTTTTAATACTACAAAATATTATTAGCGACGACATATGTCGCGACACTAATACTGCTAAAATTTCC
TTAAAAAATGTGGAATTTCCCGACCAGAAAATATTTTGGTGTTGGGCCCCCCCATTTATATCCGCCGGCAGCGCACATGAAATT
AGTCGGCCCCTAATAGTCGAGCATGTGTTAAAAAAAATTATTAGGACGCGCTATTAAGGGTGACACGATATACATCTCTTTTCC
GCATAAACTCCACATCCTCTCCCCACCTTCCCCGTTCTCCTGCCGGCAGCCTCACTCCCAATCCCGCCCCATGCCTAGCCCAGG
TGCGCCATGTCACCTTCCCCTTCTTCCCCATCTCCCCTCCTCACCTCAACCAGCCACGTCCACCGCCCCACCCCCGCCGCCAAC
GCTCGTTGCTGCCGCCGCTGCCCCTATCCCTCTGCAGCCACGGTATGATATTATATGTATATATATGTTTTTATTTTATATATT
TACTTGTTTGTTTATTTAATTTTAAATATGCGTATAAAGTATATGCATAGATATAATTTATATGTATGTTGTAATATATATATA
AATTTAGTGAATTTTTTATGTTACTTTTCTGTTTTTCTATTATTTTATATGTATTGTGTGTATTTGGTTTATAATACTATTATA
TGGATGTGTGAGACCTGGTGTGTCATGTTGTATGTATTTGATTATATATATATATACTTTGTGTAATGTTTTATGTATATATAT
AATATATGGTCGGCTGTGTTGATATTATATAGTGTGGTTTATGTGTATGTGTTATGTATTTAATTATAAGATGTGTATGTTTAA
TATATATTTGTGTAATGGTTTGAACCGTTGTTGGGTGTCGTTTTATCATATATTTCAAGGAGAAAATAAATTGGTTGTAATTTT
TGGAGCTTTTTTTTGGAGTGCTTTTTAATTGGGTATCGTGTAAATAATATTATTTATTATCTATTATTATTGTCCATTTGTATA
TTGTTAGGTCAGGAGTTTTGAATAGCTAAGTATCCATAGATAAAGTAGGTTCTGGATATACAATACTTGTGTGCGGCGGTGATT

AACGGATGTTATGAACTTGTATGGGTATTAGTGAAGTAGGTTCTGTGAAATTTTTGTGTGATGCGGAGATAAGCAGGAAGAAAC
TTAGTGTGTGCTGTTTGAAGGTTTTTTTTAACCTGTTTACATATGGTCTAGGTCCTAGTATCAAGGGGAGATGCTGCCCAATT
TTTTGTAGATTTATAGGGTCACAGATTTATGGTCTTTATAGGTCGGATAACGAGTATCTGGGGGTCGCGAGATGTCTTTATAGG
GATGGGATAAGGGTATGGGTCGATGATAGGGTCTTTTATAGGGCGGGATAGGTCAGGGGTCGAGACAGGTCTTTATAAGGTCAA
GATAGGGTCGAGATATGATACTATTAGAGGTTGGGGGTTCAGGATACGGCTTTATATAGGGTCGAGGGTCCGAGTATAAGGCCC
CCCCCCTCTTTATAGGGGTCGGGATATGGTTCATAGGGTCAGGATCAGGAGGGGTCGAGTAGGATCAGGAAAGGGTCGGGGTCG
GGATAAGGGTCGAGATAGGGGTTCAGGGGTTGGATAGGTCTTTATAGGGTCGGATATGGTTGCCATAGGGTCGGGGGTCGGGAA
AAATTACATACTAAAAACAAATTGGTCATATTTAAGTTAAAGATGCAGTGCGAGTAGCTTCATACCATGGTCGGAGATGGTGGT
GGTTAACGATCCACCAGATCCTAGCTAGCATCTCATCCAGTTGGCGGTCAAGGTATTAAATTTATTCTTTATAATTCTAATATA
CTTATTATACAACCATATAAACTGATATGATGCACTTATTTAAACAATATAGTAGCTCCACCCAAGAGATAAGGAGGTGTCTCC
GCTAGGAAAAATTTAAGCAATAGAGTAGGCGAGAAGGCGCATCCCTCCCCATAGTGGTTGACCTATCACGTACAAGTGGTAGGA
AGCAGAACATGGCGCTTTTTGTCCGTCTTTCGGGCACCCAATTCACATTAGTTGGGGGGTCCGGAAAAGGATGGGACTAAGGAT
AGTGTCATTAGTAAAGTGAAGGGTAAATTTTTTAAGAATTTACTTATAATAATTGTATATAATTCAGTATGATTAACATATTTC
TCTTGATTGTAAAGAACATACTACTCAATATGATGGTGGCAGATCACGACCACCTCATGTGGCAGTATCAATGCTACGAGTCTG
AGAGATACAGAGGATAGCGAAAACTAGCGGCACACCACTTCAAAGAAGTTCTACATAGACAAAAGATTGGACAAGTCCTATAAA
GAACCACTCAATGATTGTCAATGCGCGGATGAGTGGAAATTAAATTGTGTGACGTTTTTCAGGAGATCCAGAGTTATTGGCGTG
CTCCGGGAAGAACAAAATAAACCCGAGACGCTTTCATGAAGTACTCTACAAACAAGCATAATCATTAAGCAACCCTCATGCCAC
GAAAAATAAGTGAAATATTAACTTATTAATTGTAATGACATTCTATTATTTCTTATTAGTTGAACCTGAACCTAATTGAGAATG
AAAGGAGTATCGCTGGGACCGGCAAAAAAGAACAAGACATTTGTCAATGAGGATGGCGAACGAGATTATGTACAGTGTGAACTT
TATCATCAAATCAAATATAATATTTTCTTTGAATATAATTTTTTTTAAAAAAAACACATAAAGTGACAGTTAACTCTGAAGAT
TGAGACTGCACCACATAGTTGAATCTGGTACCGTGTTCTTCCTCGGTCAAAGTCAAATTGGAAAATACTAATCAGGTGCTTGTG
AGAAGGTGGGGCAACAGTGAGAGAGTATGACCCGTAAATGTAAAGAGAAAAGGCGTTCACCAGTCCCACTCAAAACACTCAGTC
ACAGGCACCGACTCTCAAGCGTCGACTTAAGGGAAAACGTGAAATGGTTGACAATTGTGAAGGTCATGCATAGAACGACTTCAA
TACTGTTATCACAGCTTCCACTTGAAAACCGCTCTAGGCAACCAAACGCATGGACGATGTAAGAACATAAATAGCAGACCGGTT
TTGAGAAAGGTATATAGGTCTCATCTTCTGGACTCAGACACACACATGTCTCAGCTTCAGCTCTCGACCTTCGGCCACCAGGCC
ATCCGCGAACACACTGCTACCTAACTTTGCTATCTCCCACCTTCAGTGCCCAGCCATACTAGGTATAAGCTGGTCGATATCTTC
CGTACCCTCCGGCTTATTATCCTGCATATGAGTGAGACTTCGTCCAACCCCTGCTCCTCATATGAGTAGGTCGTGCAACCTCGC
CCTAAAGATCCATATCCATACATGTATCGTGACATGGAGATCATCGTACCTTCCCACCATATCCATGGTGATGTCTGCAACACC
ACCAGGCACGCGCATGCCACCATATAGAAGGAACACAACGGATGGATGCCGAGGTTGATATGTTTTGTATATTATTGTATTAAA
ACAATACTTTTAATTAATAAATTATGATGGATATTAATTGGAGAATATTGTATTTATTTGATGTATTTTAATATTATAATTTTA
TGCATTTTAAACTTTAATATATTATTTTATGCATATTTAATATTAGTCATATTATTTTATGATATTTAATTCGATATTATAAAT
TAAAATGTATTATTTAAATATTTTATCTGTTAAATAATTTTATTAATTTTTAATTATATAAAAATATAAATATCAAAGTCACCG
CTAATAACAGTACTATTGAGGAGGACTTTTTAGTATTAGTAGTGCGAATTATGAATATGAGAATCACGATTATTAGGGGCCCGA
TATCAATTATTAGGGGAAAACAATGTCGCCGCTAATCATTGGTATGAGGAGCGATGGTCTTTTGTCACCGCTAATAAATCACCT
TTAGTGTCGAAATATTACGACGACAACGACCAGGCAGCGACATGCTCCCACCTAATGAAATAATTAGGAGTGGACTGGGATCGT
ATTAGAGTGACAATCCATCGCTGCCTAAAGACCCTTTTTTTGTTGTAGTTGCTGTCCCAAGGTATTTTGTTAGTTTAGTAAGTT
AATAAAAGTTTTAATATTAACATTAAATAAAAGTTTAAGTAACATTAAAAAAACTACATCTAAAAAATTAATTTTTTACTATAA
TTATATAGATAAAATTACGCACCCATATAATTAACAAAGTAGTTCATCACATAATGGATGATACAAAGATTGTTATATGA
>m141122_040740_42134_c100732202550000001823141405141561_s1_p0/19151/14389_20885
CTACAAGCAGAACCACCCCACCAGCAAAGCGGGCTAGCGCTACAGCGCCCCCTGACAAGCAGCTGTAGCGTGTATTAGCAGCCA
CCACCCACAAAATTGCAATTTATGCGTTTTCTTTCATTTCAACCCAATCTTAGCACAACATTATTTCCTCAAAAACACACATCA
CACTCACACTCAATACCCAAGCACCCATGAACTCAATCCAAGTAGCACCACAACTCAAAATCTACGAACAAAACCAAAAAATCA
CTCAGCAACAAGGATAAAGGTCTTAGAAAATACACACAAAGGGTAGAAATATGAACTTCGAACCCTAGATATTTTTGGCTATGC
ACTTCACCACCATTTGAATTGTTTCCAAAAAACTCATCCATTTAGCGTCAAAACACAGACTCCAAAACCAGTCCACCCTAAAAT
AGAAAATCCCAATAACTTACCTCAAATTCAAATGTGATCATTGCTAACCCTGTGCCAATCCTCAAGCCTAGCTTAGATCCTTGA
TGCTCAGTCCCCCCGTACCCTAGCCTCTCCCACTGAGCTTGACTCCAAGAAAATGAAGGAATAAGGTGGGAGAGCCCAACCGTT
CTTGGACAAAACCCTTCAGCTTTTCCTCCTTTTCCTTTCTCTCCTTCTTCTTTCTTTTCAGCCTAAGCTTTCTCTACAAAATCC
ACCTAAGTATGAAAGCCCTTTAATTCATCTCTTACAAGCCTAAAGACGAAAAATGCCCTTCTTAATAAATTCTAAACGCCATTT
ATGCCCATAAAGGGGGCATTTTAGTCATAGTTCCCAAATTCCACTAATTTTCTCAATGTGTTCCTAAAAATTCCCGCTGCTACC
CAATACCTGTACCATCAAATATACACTTCCTCGTGCATCAAGGATTAACCTCAATATCTTCCGAATATACTACATTTAAACTCC
CCAAAGGCTTAACCCGAGCGGTATAATTCCGTTTTGACTTTTCCGCTACACCCCGCTCACTCTAGATCGTCTGCGAGTCACAGA
TCACAGAATATCAACACAAGCCTTGCCCAACATGGCCAAATTCGCACATATGCTCAAAGTCAAGTCTACATGCATACTAATTCC
CATAGCTATGCATCTCAATAATCAAATATGTCATAAACGTGCATCAAAACTCATATATTCATGCATAAACTCATTAAGACAACC
ACGACAGAATTCCATTGATGCCCTCCCTGCACACTATCCAGGCCCTTAGCCTTAATACCTGAATTTGGGGTCGTTACTATCGCG
GGAACGCATTCGGACGATTTGGCGGAGGCTTTCGCAGTTTAATCCGGAAAGTACGGGATGGAAGCCTGACCCTAATAAGGTGCA
CTTTCGGCGTCGATCCGGGAAGTTCCTGGTCTTTCATAGTGGTTCCCGAAGGATCGAGAGCCAACCTGAGAAGATCCAAGGCCC
TGATCGACATGGCCTCTCCCCGGCAAGCAAAAGGACGTTTCAAAAGCCTGACGGGCAGTGGCCACCTGAGCCGTTTTGTCTCCA
AGCCACGGACAGTGCGTCCCTTTTTTCAAACGTCCTCCGGAAGCCAACGGTTCCGAATGGTCACCCGAGTGCGAAGAAGCCTTC

CAGAAGCTGAAAGAGCACTTGGCCAAGACCCCGCATCCTGGGCGAAGCCAGTCGACAGGAGAAGGGCCTTTGTACTTATACCTG
GTCATGACCGAGCATGCCATCAGCGCCGCGTGGTGCGCGATAGAAGGAAAGACACCACTCCCCAGGTATAACTACGTGAGCAAG
CGGTTACTAGACGCCCGAATCTAGTCCCGCTGATCGAAAAGCTGACTTTCTGCCTATTGACCGGTATCCTGGAACTTCGACCCT
ATCCTCCAGGCCACGCAATAAAGGTCTTAACCAACCATCCCTGCGGCAAGGTGTTGCGAAACCTGAGTCGTCGGGGAGACTCCT
TAAATGGGCATGGAGCTGAGCCAATTTGATCATATCTTACGTGCCCAGGGTGTCCATCAAGGGACAGGCCTGGCTGATTTCATA
TGTCGATGCTCCGTGCGCGATCCCTCAGAAGAGAGGCATCCCGCACCCCCCATTCGGGCGCCCGTGTGGAAATCTTCGTGGACG
GGGCCTCGAACGAGAATGGGGCCGGCGCCGAATCATGTTGGTGTCACCACAGGGCACCAGCTACAAATGCTCCTCCGTTTATGT
TCAAAGCATCGAACAACGGAGGCGGAGCGACGAAGTGTCTTAAGCCGGCCTGCGTCTGCCCGGGAGGTAGGGGCAGCGAACCTC
AAAATCTACAGCGATTCCTCAGTTAGTAGTCCCGGATAGATGTTGGGGGAGAAGACAAGGGAAACGAATGGCGGCATACGTGAA
CCCAAGGCGAGTGACGATGCTCCAGACTTCGCGGCGCATTTCCATCGTTCCAGATTCCTAGGTAGAAGAGACGTGTTCGCGAAC
ACATTAGCGAAGCTGGCAAACCGATCGCAGAGGCCGAGTTGGCCCAGGCTGGTCCCCGGCTCACCATCTCCCCGTCGCAAAGCA
TCGGGCTCCAACCGTCCATTACCGTTCGACCACTCCCAATTTCTGGATGGCGCCGGTTGATCTGACTACCCTGTCACGCGGGAG
GTTCCGAACGAGCGAGTCGCGGCCAGGAAGCCATCCCTGTACCAGGCCAAACGATACGTCATGAATGGACGGAAATCTATCGC
CGGGACTGTCTACTGCCTTACCTCAGGTGCGTGCCAGGATTGAGGCTGGGCGAACATCATGCATGAGGTCCACGGAAGGGCTTT
TGGCGGAGATTCACACAACGGGCCCAGTTTTGTCCAAACGAACGCATTCTGCGCCCACAGGACCGTAACTGTCTCTGGCCCACC
ATGGGCAAGCAGGCGGACTTGCGCATAGATACGTCGCGCAAGTGTGAACAGTGCTAGAGGTACGCGAAGATCCTCGGGCTGCCC
CAGCCCCCCCACAGAGATAAACTTCGCTCTGAACAGTCCCTGGCCCTCGTCTCGAGTGCTGGGGGCGCGGTGATCTCGTGGGAT
CCTCCTCGACGCGGGAAAGGAGGGGTGAAGTACGCCATTGGTGGCTGTAGGGACTATTTCACGAAAATGGTTGAGGCAGAAACC
CATGAATACCGATCAACATCCAAGAGGCCTTGGATTTCGATCATGACAAGCATAGTTTGTGGTGCAGCCTCCCCTACAAAACGT
GTCCGACAACGGGAAGCAATTTGACAGCCTCCACTTCACGGATTTTTGCGAAGCGCACGGTATAGTGAAGAGTTTCCTCTGCAG
TGGCCAGGCCTCAGGCGAACTGGCAGCAGAAGCCGGAAAAAAATCCTAAACAGATACACTGAAGAAAAAGCTTCGCCCTGTAAG
AGCAAGTGCCGAAGAGCTCCCCCGCGTATTGTGGGCCTACCGGACGACCGAAGGACCGTCAACCGGGCATAACCCATACTCCGA
ACTGGTGTAGTGGGTTGCGAAGTTGTCATCCCCATTGAAACGACCATTCTGTCCCTCGACACGACACGTATGATACCACCCCAG
AACCACGCTTTACTCCAGGAATACTGGATCTTATCGAGGAATTCGGGAAGAGTCCAATGGCAGTTGAAGATGTACAAGGGCAAG
AGCGCGCGGCATTTCAACCTCCAGGGTCGAGGGCCGTAAGTTCGAAATCGCGATCTAGTCCTGCGAAGGGTCTTCCCTGCCATC
AGGATCCGGGGGTAGGGTCTGGGCACAAATTGGAAGGGCCAATCAACGAGATCCAGCACGAGATCTGCCCCGGAACAGTACCGC
TTGAAGCGGCTCGATGGTTTGGCAAGTCTGCCGCGACCTCGGAACGCGGAACATCTAGTAAATAATACCAGTAGCCCAAGGGCC
TCGTCCAGTTAATCGATTTACGCCTGTAACTGTACGCTCAGGGCTAATTCCCTTTTTGACAGCAATGAAAATGGGTGGTGCAAA
ACGTCATAAACGGCGGCTACTTGTCTCCATGAAAAAGTGTGCCTCAGGGCGATTCCTTTTGAATTTCTTTCTCAAGTGACCCCA
GACCAGTCTCCGCTCCTTGCGGGGGTGTCATCCCAGGCTCCAAATGCAAAAAAAGGACCGAGCCCAAGGCCCGGTTCCCCCAAA
CCCCCGGACGAATGATGTCCAGGGGTATCAAAATTAAGAGGACTGAGCCAAGGTCGGTCCCATCCCCGGACGAAAGATGTCCGG
TGAGTATAAAAATAAGAGGACGCGAGCCCAAGGCCGTCCCACCCCGGACCGAACGACGTCCAGCGGGCTATAAAATATAAGAGA
TGACCCCAAAGGCCGGTCCCACCGCAAGGACGAAACGACGTCCCCGGGGGTATGAAAAAGGGAACCGACCCCAAGGTCGGTCCC
CACCCCGGACGAACGACGTCCGGGGGTATAAAATAAGAGGATCGAGCCAAGGCCAGTCCACCCCAGACGACGACGTCCGGCGCG
GTACGAAAAAGGGAACCCGGACCCAAGGCAGTCCACCCCAGACGAACGATGTTCAAGGGGTATAAAAATTACAGAGACGCGAAC
GCCAACGCCGGGTCCCCAACCCCGGACCGAACCGCATGTCCGGGAGTATTAGCAAGAATAAGAGGACCGCGAGCCCAAGGCCGG
TCCCACCCGGACGAACCGATGTCTGCGGAGTATAAAAATAAGAAAAGGACCGACCCCAAGGCCGGTCCCACCCCGACCGAACGA
ATGTCCGGGGGTATAAAATTAAGAGGACGAGCCCAAGGCCGTCCCACCCCGGACGAACGATGTCCGCGAGTATCGAAATGAGGA
CCAAGCCCAAGGCCGGCCCCACCCCGGACGAACAACGTCCGGCTGTATAAACAATAAGAGCCACCCGAGCCCAACCGATCCCAA
CCCCGACCGAACGATGTCCGGGGGTATAAAATTAAGAGGCGAGCCCAAGGCCGTCCCACCCCGGGCCGAACCGATGTCCGCGGA
GTATAAAAATAACGAGGACCGAGCCCAAGCCCGGTCCCACCCCCGAACGAACGATGTTCCGGAAGTATAAAATAGAGAGGATCC
GAGCCCCAAGGCCGGTCCCACCCCCGGACGAACGATGTTTCGGGAGTAAAAATAAGAGGACCGAGCCCAAGGACCGGCCCACCC
GGACGAACGATGTCTGGGGTATAAAAAATAAGAGGATCGGCCCAAGAGGCCGTCCACCCGGACGAACAACGTCCGGGGGTATGA
AAAGGGAACCGACCCCAAGGCCAGTCCAGCGCCCCGAACGAACGATGTCCCGGGGGGTATAAAATTAAGAGGACCGAGCCCAAG
GTCGGTCCATCCCCGGACGGAACGACTGTCCGGGAGTTAAACATAAGAGGACCGAGCCAAGGCCGGTCTCCACCCGGACGAACG
GATGTCCGGGGGTATAAAAATAAGAGATACCGAGCCAAACAAGCCGGTCTTCACCCCGGCACCGAACGCGTCCGGGGCGTATGA
AAAAGGAACCGACCCCAAAGGCCGTCCCACCCCGGACGAACGACCGTCCGGGGTATAAAAATAAGAGGACCGAGCCAAGGCCGT
CCCACCCCAGACAACGACGTACGGGGTATGAAAAAGGGAACCGACCCAAAGGTCGGGTCCCACCCTGGACGAACGACTGTCGCG
GGTATACAAATTGAAGAGGACGAAGCCCAAGGCCGGTCCACCCGAACGAAACGATGTAACGGGAGTATACAAAATAAGAGGGGC
CCCCCCGAGCCCAAGGCCGGTCCACCCCGGGACGAACGATGTCGGGAGTATAAAAATAAGCAGGACGACCCCCAAGGCCGGTCC
ACCCCCGGACGACAGTGTCCGGGGGTATATAATTACCAGAGGAACGAGCCCCAAGGCCGGTCCCACCCCGGACGAATGATGAAC
GGGGAGTATAAAAGATAAGAGGACCGAGCCCCAAGGCCGATCCACCCCGGACGAACGACGTCCGGGGGTATACAAAATAAGAAG
ATCGAGCCCCAAGGCCGGTCCCCATCCCAGACGAACGACGTCCCGGTGGTGTATGCAAAAGAGGGACCGACCCCAAGGCCCGGT
CCCACCCCTGGCGTACGATTCCGAGGGTATAAAATTAAGAGGACCGAGCCCAAGGCCGGCTCTCCCACCCTGGACGAAGAGTGT
CCGGAAGTATCAAAAAAAGAGACCAGAGCCCAAGGCCGGTCCCACCCCCAGACGAACGATGTCCGGGGTATAAAATTAAACGGA
TCGGCCAAGGCCGGTCCCCCAGGTGAATCAACGTCCAACCCGGACAGGTTGATCTTCGGAGTACCAAAAGCATCCGGTTGTACA
CTAGGAAAGCCAGGACCTTTAAAAAAAATGTTGAAGGAAAAATTGCCCCAAACCCTCCGGGTTAAACAAAGACACAGGCCTAA

GGACAGTGCCAAGACTTGCGTTGGGTCCAGTCCCGCCGGTTGGAACCAGGACCAAACCCCCTGCAATCCGTTAAATCGCGACAA
ACTTCTTTCTGAACGGTGAAAGGAAAAT
>m141122_040740_42134_c100732202550000001823141405141561_s1_p0/27294/11981_18410
ACATACAAATAGCTACTTATTAGCGCGACATCATGAGGTGAAGAGGTAATAGTGTGTGATTTGTTGTGTTTGATATCTGGAGAT
AACACGGGAAGAAACTTGTATCATTGTGGATTCATGCTATCATCTATCAGATTACTTGCTTATTGTGGCATATGGGTTTTATTT
TTGTTTAATATGCTACTTATGGCTATTGTAGTATAGAAATGACATCTTTGAGTTTTAATTATTTTGATCAAAAAAAACAATAAA
CAGAAGAGAGAGGTCATATAGAGTATCCTAAATTCATATATATCAAAATTTAACTGTTCTAGCTGTGATTTACAATTCAATCTC
TATTATTCTTTCAACACATCAATTTTCTCCATAAAAAATAGTCTTAGCTTATTAATTGTATTCTCAAACTGAAAGGAGAATATA
AGCATAGGTCCCCTGTTGCACATTAAAAATAGGCACAAGTTATAGATTCAAGTGGAAGTTAAAGAGATTGATCGATTGGCAACT
GGGTATTAACAGACAGAGAATAAAACTAATAATTCAAGAAACAACTAGAAAGGTAAAACTTATAGTAGGATGACAACCACTAGG
TATATCCATCGTACATGTTATTTGTTAAAAACTGGAGTTGTCATGATTTAACGGTAACAGCAGTTTCTATTCGTAAAGTTCTCA
CTATCAAGATACAAACGCCTATTCAAAAGATTTGAAGTAAGAAAAATACTTGCTAATCATAACGAAACGACAAAAGAACCCCAA
ATTAGGATAACCAAGATCGTAAAAAAGAAAGAAGAAGAAAAGAGAATTACCAGTCGAAACGATAGGAGTTGGGATTTGCTTAGG
AAAGGATCAGGGAGGAAAAGAGCGAGAGAGATCTGCCAAGAGTAGGTAGTTGAGATAAAAAACAAGCTCGCAACCTCTCTCGCA
CTTGAGCTGGCTCTTAGGGTGAGCACATCAAGAACAACGCGGAGGCGCTGTGGACTATCTCAAGATCATCCTAATGTGCATGTG
TAAGAAGGCGTTGGGATAGTGATATGTGGATCTGCACATACTTACGCTGAGTTCGCAGCACTTATTCGTTTTCTTCCTTCTAGT
CCTGCAGAAGTTGAGCGTCTTCTATGCTGATGAGGGATGTGGTGCGTGTGCTTGTCATTATACGGATGATCTCGAGCAGATCCC
TATAACAGTCGAGTGGACTAGATCCTAACGTTGTCATATAGGTTGAGAAATAATTGCACATCAATAAGTTGATCAGTTCTCGTG
TCTAGTCACCTTTGACATTCATTTCCGGCTCCGTGCTTGTTGTGAGGCTTACTCTGAGTTCTCACCGAACAATGATGAGCTAAC
ATGAGATAACGTGGATCATAATGGAAGATTAACTAAAAGGGGTGTAAGACATGACGGAAGTTCTTTTGTCTCGAGAAGTTACAA
GTCCTGCGCCTTGTTTGCGGTAATGGTGTTTTTGAATGTTCCTTCAGGATATGAAGTCAGAAGAGGAAAAAGAAAGATTAGGGG
TATCAGTTCTGACCGAATAAGAGTCGAATTGCATATGACATGATGAAGCGGACGAGGTTATCCTGGGTGAGATTTAGCTAGTCT
TTGTTGGTGAAAGAGTTGTGCGCTTTATGTATAATAATTTCATATCCGGAAGGTACAAGACCAGCTCAAATCTGGCAAACTCAT
GAGAGATAATAACACAATTAGGGATTATGGTCGGACTCTATTGAGCATATGAGGGGATCTTTAGTCACAAAATGCGATACTTTG
TTTCTTTTCTGACTTTTATATGAATCGAGTTGCAATGTAGGAGTATAAAAAAACTGATAAATGCGAGGAAAGATCATGCACGTA
TCGTAGCGATGAAGCCATAGACAATGAGTGATGTGTGAGAATGGTACAGCAGTTAATTTCGGATAATCCTCTCATGCATCAGGG
AAGAATTATTAGAGACTAAGCTGGCTCTTATATTTAGTTCGGCATTTAGGATTGGTGGACGTGATGATGAACCTCCGAGTCCGT
ATTGTGTTCGTGGCCATGTATGTGCTTTCAGTGGTGTCATTTCTCCTGTATGCGTCTAGTAATCCTTTATAGCGCTTTGTACAG
AAAAACGTCGCACCAGAAATTCGTCTGCGCTTCGTGAATCTACCCCCAACCTAGGAGCTACTATCCACTCTCCAATAATATTAA
GATCGGAACAGGCAAAGAAGGACATGTCTATCTTAACAAGAAGAGATATAATTTGTACAGTTGATACCATTTTTTCCACAAGAT
GACCAGTTGGTGGAGAGTGTTTTCACGAAGAGAGAGAATCTCTTGTGGTACAAGTCTTGGAGATTTGAATTGGGCTCGACTGGA
AACTGAGAGATCTGGATGAGAGTGAAACTTTGCTAGAGATAATAATTGAGAAGGAAAGTTGAGAGAGAGCTATGCAGTGGATGG
AGCGTTCAGAGAGAGGTGAGGGTTTAGAGAGAAAGTTTTGGCACCGGAGACACGGATATTAGTAAGTAAGCTGTTTATTAATTT
TAAGTAGGGATGATAAAGAATGTGATTTTATTAAATCGTCATTTTAAGTAGCGCTTATTCTTCCCTCATAATTATAAAACTTTT
CACTCACACTCAGCCAAATTTTTTTGGTTACTTTTTTCCATATTGATCTTGTGTTATACAGTATGGATATTAAAAGATTCCACC
TTATGTTCCAACTCGAGTAGATAAAGTTATTGTATATTATAAATGAGGTATCTAATCTCTTGCAATTCTGCTAGGTTTTCGCAT
TATCAGGATACTGTCTTTGTCACTGTCCATGCTGCTGTTGAGTCAGAGCTGTTTATTTAGCTGATGTCTGCTGGATGGGAGAGT
CCATGGAGATGCCAGTCTGCTGCTAGCGGTAACCGCGTGCATGAATAGCTTAGATGCTTGCTGTGTGTACATAATCACAGCTTC
GCTTGATTACTGTGATGCTAGCTGCTGCTAGCATAATTGCTGTTCCAGATTACAGTATGCTAGCTGCTGCTGCGGGGCTGATGA
CGCTAGCTGCTGCTGCTGCTGCTGCAGCTTGCTGCCGTCGAGCTTTGCTGCCTGATATAGAGATAGGAAGCCTTAGCTCATGTC
GTCGCTGTATGTTCCGGGTGAAGCTAGACATGGTAGCGTAGATAGACTAGAGATTTGTGACGCTGCTTAATGCACGAGCATGCA
TGCTAAGAGTGGGTCGCTGTTGAGTTGATTTTGGGGATCTTCGATTGGTTGCGTGATAGCCTGATGCGACAGTGTGCTGCTTCG
CACGGATCTGGATACACCTTGTCTCGAGGTGTGTAATGTCGAGCTAGGGCTAACGACCTGGTACGGAGCTCCCAGGCTCTCGGA
ACGCTAGGTATCGCCTGGCCCCAAAGGCTGCGCGTTACGGCACTTATCTGGGTTGTCAGAGGCGCGCTAGCTATTTAGCCTCGA
GCTACCTCACGTCAGAGTTACTAGAAGTCCTGCTTGTAGCTTTCTCAGGTGTTTGTCTGCTAGAGCCTTGTGCTTACCGATCGT
GTGAGGCTGAGCTGTTGCAATGGCCGTGCATTATTTTGGGGCTCGCACTAACACCAGCTCTGCTCTAGCTGCTGAATACAGCCT
GCATATAAGCTCTACGCTATGAATGAGATATGCTGAGCTGCCTCAAACACTAGCTCGCTGCATGCATCTCTGCTGCGCTATGCT
GCTGGTGCTTCGCGCTGCTATGTGCTGCTGCTGCGGGTATGATGTGCTAGCGGCTAGCAGCGAACCGTATGTCCGACGCATATG
ATGCTGATAGTCATGTTAGCTAGCTGCTGCTAGTTAATTGCTTATTTAGTCTGCATGAAATTATTTTTTTAGTTGCTGCTCTA
GCTCTGTAGATGCTCTATTTCCTAGCTGCTTTGGCTAGTGCGCTTGCTAGCTGCTGCTAGCTGCTGCTGCTAGCTGCTGCTATA
GCTGCTGCTGCTGCTTTTGATGAGTGCTGATGCGTGCCTTGTTGTGTTCTGCTTTGGCTAGCTGCTGATAGCTGCTTGTTGCTT
CTGCTAGCTGTCTGCTGCTTGCGCGCTCTGCTGTGCTGCTGCTGCTGCCTGCCGGCTCTAGCTGTGCCAGCCGCTGCTGTTTGT
GTGTGTGCTTGTTGCTGCGCGGCGTTTGTGGCTGCTGCTGCTGCTCGCTGTGCTGGCTGCTTGCTGCTGAGCTGCTGTAGCTGC
TGCTGCTGCCGCTGCTGGCTGAGCTGCTGCTGCTTGTTGTAGCTGTGCGTTAGCTGCTGCCGCTAGCTGATGCGCGCCGCTATG
CTGGCTGCTGCTGTTTTTGTTTGCCGCTGGTTTACTGCTGTGCTGCGTTGCATGGCTTATGATGTGTGCCTTGCTAGCTGTGCT
GCTGCTTGCTTTTGTTGTTTTTGCGCTTTTGCCGGCCGTAGCTGCTGTGCTTTTGCGCGCGCGCTAGCGGCTGCTGCTGCTGCG
CTGCGCTAGTATTGCTGTTGTAGTGTGTGTGTTGTTTTGCCGGCTGCTTGTTGTGTGTTGCCGCTGTGGTGCTTTTGCGATGCG
CTGCTGCCGGCTGCTTGCTTTGTGCTGCTACTGCCTGCCTTTGCTTGTTGGGATATGGGATAGAGAAGTTAGTGTGTGTGTGTG

TATGTGTGTGTGTGTTTTTGTATTGTATGTGTTTGTGTTTTGGATAGAGAAAGAGTGAAAAACTGAAGAATTTCAAATAATATT
CTTTTTTTATCGAAAAATCAATTTATCTTACTTCTGCTCAATAAGAAATTTGTTTTGTGTTATTCGGAGGAGGAGGTAATAGA
AGAGATTGATTCCTGTAGAATCTGACTCATTTTCTTTGGCGAGGTAGGAATAATCAAGATTGATTTTTTCGATAAAGTTATTTG
TTTTCTATTATCGTAGGTACAGGCGTTCAATCTATGTTTACTGCTACTGCATGCTACTTTCTTTTTTATATTATCCGCGGCGGC
GGCGGGCAGTACGCGGCGCACGCGCTAGCAGCTGTGCAGGTGTGTGTGTGTGTGTGTGTGTGTCTGATGTGTGTGTGTGTTGTG
TTGTGCATGTACGCAGCAGCGCAGGCAGCGTAGCGGCGGGTGCAGCGATAGCTAGCGGCGGCGGCCGAGCAGCTGTTGTGTGTC
ATGTGTCATGTAGCGGCGAGCAGCTAGCGGCAAGCGCAGCAGCAGCAAGCTAGCGGCGGCGCGCAGCAGCAGCGTCAGCGCGGC
GGGCAGCTGTAGCGCGAACTACGGCGCAGCTTAGCGCGCAGCGCGCAGCAGCTAGGTAGCAAGAAGTCTAGCAGCAGCTTAGCA
GCTGTGTGTCCAGTAGTGTGTGTGTTGTGTGTGCACGCGGCGAGCAGCGCTGCGCAGTAGCTAGCAGCGGCAGCAGCAGCAGTA
AGCAAGCCAGCGCCAGCAGCAGCAGCAGCAGCTAGCAGCGCAGCAGCAGTTGTTGTGTGTTGTGTGTGTCTGTATTAGTGTTAG
CTAAGAGCAGTCTAGCAGCAGCTTAGCAGCAGCAGCTAGCAGCGCTATGCAGAGCAGCTAGCGCAGCTAGCAGCAGCAGTAGCA
GCAGCGCAGCGCTAGCAGCAGCAGCGTAAGCTAGCGCAGCAGCAGACGCTAGCGCAGCAGCGCTAGAGCGCAGCATGCAGTGTT
GTTGTGTGTGTGTGTGTGGTGTTGTTTGCAGCAGCTGGAGCAGCAGCAGCAGCAGTGGATGCAGGTGAGCACAGCAGCCAGCAG
CAGCAGCACGCGCAGCGGCAGCAGCGGCGCGCGGATCGACGGCGCGGCGGTCAGCAGCGCGCGCAAGCACTAGAGCGGCAGCAG
CTAGCAGCGCTAGCAGCTAGCTGTGTGTAATGTTTTCGCAGCAGCAGCAAGTAGCAGCGGCACAGCGCAGTATCTGTGTCAGTG
TGTGTGCAGTGTGTGTATGTGTGTTATCTGTATGTATGTTTGTAGCAGCTGTGTGTGTGTTGGTAGCAGCAGCAGCAGCAGCGC
AGCAAGAGCAGCTGGAGCGCAGCAGCAGCAGCGCTGCAGTGCAGCAGCAGTCGCAAGCTGCAGCAGTAGTAGTAGTTGTTCTTT
TACTCTTTTATAATACATTTGTGATATTTTAATATGGTATTAGAAGTAAAATGGAGGAACATAATTGTGTGTTGTGTGTGTGTT
GTATGTTTTATATACATAAAACTTAGAAATAATTTAACCCTCGTCATTGTACTCCTCTCCTCTGATCATCATTTCTCTCCAATT
TCTTTCTCATTGTTTATCAGCATAATTTCCTCTCTCCGTCGTTGGCAGTCTTGTTTTGTGGTATGTGGTGTAGAATATGCTCAC
TACGTTGTGGGGAGAGAATTGGTAAAAGCCTAACTTAGTATATTATCTCTTGTTAAGATACTGTTCTTTTTCTGATTCTTATTG
TTGGGGAATTTTCAGGAGGTTTGTGTTGAGGATTATCATTATGAG
>m141122_040740_42134_c100732202550000001823141405141561_s1_p0/27504/10520_20105
AAATTCATTCCACCTTAAAAAGTTTGTTTTGTTTTTTCCCCCTCGTGGCCTTCTTTTCCCCCCCCCCCCACTTCCCAAAAATTT
CCCCTTTGGGGAGTAAAAATTTTTAAAAAAAATTGTCCACCCGGTCCCCCCCCCCGAATTGAACCAACAAACCGAAATGAAAAA
AATTCTTTTTAATCCAAAAAGTAGTTTCCCCCCCTTTGGAAGGACCCTTAGTTCAAAAATTTAAAAATCCATAAACAAGCCCCC
GCGGCTTTGGGGGCGCCGTTTTTAGTAAGACCAAAAAAAAGAAAACATTTAAAAAAACCCCCAAAGAAAAAACTTTTTTCCGTA
ATGTTTCCCCCCCCCATAAAAACCCGAAGTCCCCCCCCCCCCTAAAGGCCCCCCTTTTTGGTCCTTTTAGGGGGGGGAATTTTT
ATTCTTTCTCCCCCCCCTTTTTTTTACCCCCCTTTTTTGGAGCTTGAAATTGATAACCCCGACGGACCTGTTTCTAAAGAAGGG
AATTCGATCTTTTAAAAGAAAGGGAAAAATAAAAAAGTCCCCCGCCCGCCCTTCCCGGAGTTTGGAAAATTTTTTCCCAAAAAA
AAATCCAAAATTCCCCCAATTCCACCCGAGGGTTTTAGCCGGGTTTAAAAAAAAATTTTGGTCTTTTCTTTTTAATTTTTT
GCGGGTTTTACTATTTTTTTTAAAAAACTTCAGCCTTCACGGTTTAAAAAAGTCCCCCTCTTTAATTGGCCAACCCAAAACAC
TTGCCGCAATAACTTTTTTTCCCCCCCCCCGTCCCCCTTTTTCTTTCTTTCCCAACGAAAATAGTTTAAAACCCCGGGAGCACC
TTTTTCCCCCCCAAAATTGGGGGGGTTTTGGGGGGGGAAATTGGCCCTTTTGGCCCCCCTTAAAAAATGAAAACCCCCCCAAGT
TCTAGGGAGGTTCTTTTCTTTTTTTTTTGGAGTAAAAAAAAAGCTTTTTGCGTCCCTTTTTTTAAACTTTTTTTCTTTTTTTTG
AAGGTTTTTCCGGTAAAAGGTGGCCCCATCCCCCCGGGTTTTAATTTGGGTTTTGGGCCCCCCCCCCTTTTTTTTTTTTTTAGA
GAAGTAAGCCTCCCCGGGTTGCCCCGGGTTAAAACCTTTAATTTCTAAATCCCGTGAAACGCATCCCCCCCCCCCTTTTATTTTT
TTCCCTTCCTTTAAAGTTTGGGGCGGCAATCCCCTGGGGCCAAAGTCCCAAATCGGGGAAAAAAATACTTTTTTCCTTTGGGAAA
AAAAAATTTTCCCCTTTGGGGGTATTGAACCTTTCCGAACAATCTTTTCCAAACCTTTAAAGTGGGGGAATTTGTCCCCCCCCT
ATCCTTTCTCCACAATTTTTTCGTGATTGCCAATTTTTTTTTTTGGCTAAAGAATCGCCCTTTGGCCCCAAAATCCCCCCCTTTT
TTCCCCTTCCCAAATTTCATTTCCTCCCCCCTCTGAAGCCCTTTTTTTGGAGAGAATGTGACAAAAAACCCTAAAACCGGGGGG
GGGGTTTGCCGTAAGGTCTTTGGAAGCCTTTTTTGTTTTCCCAATGAAAGCCAAACAAATTTTTTTTTTTTTTTTTTTTTCCCT
TTTTTTGGCCCCCCGTTTTCCAGGAAGTCCTTCGAAAAACAACCTCTTTTTTAACCCCTTTTTTTTTTCCCTTTTTGTTTTGCC
AAAAATTTTTGGCAGTTTTCCGATCCGTTTTTTTTTTTTTTTTTTGGCGCATTGCCACCGTGCTTTTAAGCAAACCCCCAATCC
CATGCCCCCCCTTAGTAATGACGGTTCCCCCGAAAGGTCCTTTTTTTTGATTCACCCAGCTTTCCTAAATCAGGTCTTTCCCCC
CCTTTTCTAAAAGGTTTTCCTTTTTTGTCCCCCCCTCCAATCCCTTTTGATTTGGTTAACGCCCTTCGGGTTTTAAACAATT
TTTCCCCCCCCCGGTGGGGGATTGGAATAGAAAAAAAACCTACCTTTTGCCCCCAAAAAAAGGGGCCAAAACTCCCCCCGAAT
TTTTTTTTTAAACAAAAAACCTTTAAAGTTTCCCTTTAAATCTTTTTTTTTTTCCAAATTTAAAGGTTTTTTTTTTTTTTTT
TGTCTAGGTTTTAAAAAAATCCCTATTCCAAAATTAACAAAATTAATTTTTTTCTTTTTAAATTTTGGAAGGAAAAAAAATTTAG
AAAAAAAATTTTTTTTTTTTTGGGGAAAAACCTTGTTGGACCCCAAAAAAAACCCTTTTTGGGGTTTTTTTTTTTGCCTAAAGC
CCAAAATCCCGGCCCTTCCTCCCCCCCCCCTTTTTTTTGGGGTTTTTTTTTTAAAGCCCCCAAAAAAAAACCCCCGAGCCAGGG
GAAAAAACCCATTCTTTTTTTTTTTTCCCCCATCCATTCCCCCCTTTTTTTTCCTTTTTCCCTTTTGGAAAACCTTTTTTTGCC
TTGGAAAGGGGAAAAACCAAAAAATTTCTTTTCCTTTTGCCCCGGATTTGCCCCCCTCCTTTTTTGAACCCCAATTTTTTTTCG
AAAAAACCCAACCTTCCTTTGGTGGTTTTTTTTTTTTTTGGCCACGGCATTCCCCTTTTCCGGGGGGTTTTTTGGCCTTTTTC
CCCCTTTTCTTTGACAATTTGTTTTTTTTTTGCCCAAAAAAACAATGGCCGGGGTAAAAAATTTTTCCCAAAACGTAAAAAGCC
CCGAAAAAAATCTAAATTTTTCCCCCCATTTTTTTTTATTTTTTTTTTTTGGTTTCGTGCCTTTTTTTTTTTTTATTTAATTTTT
GTCCGGGGCCTTTTTTTGGACCCTTTTTGTTTTTTTTTTTTGTTCCCCCAGGGATGCCTTTTTTTTCTTCCTTCCCTTGTCCCC
CCCCGTTTTTTTACCCGTTTGTTTTTTTTTTTTTTTTTTTTTTTTTGGTTTTTTTTTGGGGGAAAACCCCCCTTGATTTTTTTTT

TTTTTGCTTGCCTTTGCCCTTAATTTTTTTTGCCTTTTTTTTAGCCCTTGAATTTTTGTTGGTTTTTTTTCCCCCGACTTTGG
CCCCTGAGGGGGTTGGCTTCCCCCTGGCCCTTGTCCCCCCCCATTGGGTTTTTTTTTTTCCCCAAAAAGCTTTTTGCTAGCTTC
CCCCCCCCTTTTTTCCCTTTTTCTTTTTGCTTTTAACCGTTTTGGCCCTGCCAACCCTTTTTTTTTTCCCCCCCCCCCACTTAA
TTCCCAAATTTTTTTTGCCCCCCGTTTCCCTCAAAAAAAAGAACCCCGTTCCCCCCCCCGGCCAAAATAATGAAAGGTGTTTTT
TTTCCCCCCCCCCGGGGTTTTTTTTTGGCTTTTTCGTTAGCCTTTTCCCCAAACCCCCCCAAATCCCTTACGGGAATCTTTTTTT
TTTTTGGTCCCCCCGGAAGTCCCCCTTTTCCTTCCAAAAACGAATTTTATTTGTTTTCCAACCCCCTTTTTTTAAAGAAATTTT
TAAAATCCGGTTTTTTTTTTTTTTTGGAAACCTAACCCTTTTCCTGCCCCGCGAAACTTTTGGCCCCCTTAAAGGGCCCGGTCC
CCCCCCAAACCCTTGACCGAGTAAATAAATTTCCGCCCCCCCACCTTTTGTTAAATTTTTTTCCCTTTTTTTTGCCCCCCTTTT
TTTTTTTTTTTTTTCAAGATTGGGGTTTGAAACCTTCCCCTTTCAACCTTCCTTTCCCGTAAGCAAAGGGTACCCGCTGAATTT
TTTTGGTAAACCCTTTACTTCCTTTTTGTGGAAGCGCCCCTTTTCCAAAACAAAAATCCGGGGTTCCCCCCCCCCACGTCAAAT
TGGTGGGGCCCCAAAGCATCCCGTGGAAAAAAATTTGCCTTTTGGACCCCAAAAAATTACCCCCACCCATAATTCCCCCCCCC
TGGCTTATTTTTTTTTTTTTTTCCCTTTTTTTTCAACCTTGGTTGGCCAACCCCAAAAGGGAATGGAGAAAACCCGCCAAGGAA
AAATAAAATTTTGCCCCCCCGAATCCACATTTTTTTTTTTAATTTTGATGGTTTTTCCCAATGTGAGGCCTTTAGGGAAAATCGG
CCCTTTTTTTTTTTTTCCCCCACAAAACCCCGTTTTTTCCCCCTTTTTTTTAAAAAAAGCCATTTTTCTTTTTTTTTTTCCCTTG
CCTTTTTTTTCAAAGGGGGTTCCCCCCCGTAGTTTCCCCCCCCTTCGAAAAGTTTCGGATTTTTCCCTTTTTCTTCCCCTCAAA
AAACAAACAAAACCAACGGGGAGGGAAGGAAAAAAAAAGGAAAAAAAAAAAAGGAGAAAAAAAGGAAGAAATCCCCCCCGAACCCCC
CCTTTTCGGAAAGGGGAAAAAAAAACCTTAACGGGAAAAACCCTTTTTGAAGGCAAAAAAAAAAAGAAAGAAAAAAATGGCCTTTT
TTAAGGAAAAAAAAAAAAAGTGGGGAGCCCGAATTCCTAAGCCCTTCACAAATGAAACACCCCTCCAAAAAAATAATGGGGTTG
GGGATTTCGCAAAAAAAAATAAATCCTGCGGTTTTTCTCCAAAAAAAAATCCTTTGGCAAAAACAAGAAAAAGGAAGCCCCAGGA
TTATGGGGGGGTTGGGGGGGGAAAAATTTTTTTGGTTTCCAAAAGCAAATCCCCCAAAACGATGCCCCCCCCTTTTTTTTTGCC
CCACCCAAATGGAAACCGTTTGGAAACCCCGCACCCGATTTTTTTTTGTGGGAGCCCTTCTTTCCCCCCCAACCAAAGAAAAGT
TAGTACCAAATTCCCAGCGGTAACTTTTTGCCTAAACGAAGAAGTTTTTTTTTGAAAAAAAAGGGGAAAGGTTTTCAACCAAAA
TTCCCCCTTTTTTTTGAAAGCAAAAAAAGGGAAATTTTTAAAAACAAGTTGGGGGGGGCGGGAAAAATTATTAACTCCCCGTCA
AAATTTTTTTGTTTTTTGGGAAACCGCTTTTTTAGCCCAAGCTTTCCCGCGGCAAAAAGAGGCTTTTCTTTTTCTAGTTTTTTT
TTTTTCCAAAACCGAATTAAATCTAAAAGGGGGTGGGAAAATAAAAACCAAGAACTTTCCCGTGGAGAAAAAGGACTTCCCCCG
ACCGAAAAGGAAAATCGAGATTGGGGGGTGGGGGGGAAGCTAGCCCAACACCCGGGAAACCAACTTTCCCAAAAAAAATAAAAA
AAAAAATTGCCCCCGACCCGTTCCCGTTTTTTTTTTGGAGACGGCAATTTTGAAAAGGTAGAAAGGGTTTTGCAAAAAAGGCC
CCCCCAAAACGTTAAGCCAAAAAAAAGAAGAAGGGCCTAGCAAAAAAAAGCCCTTTGGAAAAACCCCCAAGAACAAGCAAAAAGA
AAGCCCCAAAAACCTTCCCAAGGCCCCCCCAAAGTTTCCCCGGAAACAAACAAAAAATCAAAAAAGTCCTTAAGCAAAATAAAG
CAAAAAAGCAAGCCAATTCAGTTCCCAACAACAAAAACGGTAAAAAAAAACGGAAACAGAAGAGAAGGCAAAATCCCCTTGAAC
AAACAAAGCAAAGCAACGACAATTAAAATAAAAAAGGGCCCAAAAAAACGACCAAAAAATTAAATATGGGGGGAAATTAAGAAA
TTCCGGGCCCCCCTTAAAAAACGTGGATACCCCGCCAATGGTGCAAAAAATTTTTGTCCAAAGAAAGAAAGCAACCCCGGAGAA
ATTTGCCCGTGCCCAACAAAACCAAAAAAAGGAGTTGTTTTTTTTTTTTTTTTCCAAAATTTGGGGGGTCCCCCCAAAAGAGGG
GCAAATCGCAAGAAAAAAGATTTTTTTGGTCCTTTTTTTTCAGCAAAAAAAAAGTTCAGAAGAAAAGAGAAAATGAAAATGAAAA
AGGATGGGTTTTTTTTTTTTTTTTTTTTTCCTTGCCCTTCGGGTTTTTTTTTTTTTTTTTTTTTGGGCTTTTTTTTTTTTAAC
CCCAAAAAGGAAGAGGGCGGATTGCCCTTTAAAAGCAACCAGGTGGGTCCCCACCCAACAGGTCAAAAATTTTTCTATCCCTAA
ATTAAATTTAATGTAAAAAAAATGTGATTTAAGATAAAACTAGACAAAACCCCTTTTTTAGTGGAAAAAAAGATTTTTTAAGAA
ACTAGTTTTTTTTGTAAATCGAGTTGGCCCTTTTTTTTTTTTTTGGCAAAAGTTAAAAAAAGTTTTTTTTTTTTTTTTTTTATC
CAAAATCAAAAAACGAATTTTTGTAACGAGGCGTTTTAAAAAAAACAAAATCCCCCCAAAGATTTTTTGAAGGAACCAAGGAAA
AACTTTAGAAAAAAAAGGAAAGAACCCCCTTTGAATAGAGCTTTTTTTTGGGTTTGAATTCGAAAGAGACTTTTTCCGAACGTC
AAATACTAAGGCATTGGATTTGCTTTTTAAGCAAAAAAAAAAACGGCAAAATGGGCGCAAAAAGATCGCCTGCAAAAATTGGCAA
AGAAGGGTTTTTAGTTGGTTTTTTTTTCCCGAAAAGACTTCCCCTGACGGCCCCAAAGAAAAAAAAATGTGCTTCAAAAAAATTGG
GAAAAAAAAAAAAAAACAAAGCCTTTTTTTTCAAAAAGAAACTTTACGCAACCCCCCGTTTTTTTTTTAAGGTGTTCCCATCTC
TCCCAAAGCTTTTTTTTTTCAAAAGAGAAATGAAAAAAAAATGGAGAAAGGAATTTTTGCCCAAGCGCTTTTTTCTTTAGCCCCC
CCAAGCCCAAAATCCACGAATTGTTGGGGAGGAAGGAAGAAAAAAGCCCCATCCACTTTTTAAAGAGGTTTTTGGAAGAAAATG
TTTTTTTCCCGAGTCAAAAAAAATAACAAGAAATTTCCAAGAAAAGGTAATTTTTTTTTTCCCCCGATGACCTTTTTTTTTTTT
TTTTTTTGGCCCCAAAAGGATTTTGCGCCAACCTTAGAGAAAAAATTAGACCTTTGTCAACGAATAAAGAATTAGTAAAAAAAC
CCGGCACCCGAAGCTTTTTTTTTATCTCTTAAAAGGCAACCAAAATAACCCGGGTTGGCCAAAAAACCTTAAACCCCGGAAACT
CAAAAAAGAGTTTTTTTTAAAAAAGACGCAGCTAAACCAAAGAAACTTTTTGCCTAAGACCTTTTTGTTTTTTTTTGGGTTTTT
TTTTTTTTTTCAAAATTAGGCCCAAGCCATTTTCAAACCCATGGGGGAAAAGCCTTTTTTTTTTTTCCGGGTTTAAACCTTTTGA
AAAAATTTTTTTTTTTTCCGTGAAAAAGAAAAAGGAAAAAAAACGGAAGTTTTTTTAAAAAAAAATGGCGCAAATGTGGCAAATAGA
ACTTAACCCCCGTTGGAGCTTTGAAAAAATAAAAGTAAAACAATCTTTTAAAGAAAAACAAAATTAACCCCCCGCTTTACGTCG
GATTTTGAAAAATTTTGATTTTTTTTTTTTTTTTTTGGGAATTCCAACTCCCCCGAGGCCGCGACTTTTTTTTAATTTCTCTTT
TTTTAAGAATTTCCGATTCTAAAAACAAAAAGAAAAAAAGTTTCCCCCCCGGGTTTTTTTTTATCCATCCAAGCTTTTTCAAGG
TAAAAAAAAGGGAAGAAGAATTATCCTTAAAGACAAAAGCCTTTTAAAGGAAAACCTCGTTTTTTTTTATGGACATTTGTTTAC
GAAGTTTTTTTCCTTTGGTTTTTTTTTTAAAAAAAAATGTTTTCTTTTTTTTTTTTTTTTGGTCCTTTTTTTTTTTTACTTTAAAA
ACCGCGCCAGCCGCGTTTTTTTTTATGGATTTTTTTTTTAATGAATTAAAAGGGGTTTTTCTTTTTTTTTTTCCCCCCCAAAGG
GAACTTTAACTTTTTGGATTTAAATTTTTTCCATCGTTTTGTGTTTTTTTCCATCGACGACCATTTTTTTTTTTTTTTTAATTTT

TTTAACCTTTCCCAAGAATGAAGTTGGAAGCAACGAAGGGAAAAAAACCACTTTAAGGAAAAATTGAAATTTTTGGACAAGCGA
TTTTTGAAAGGAAGCACCAACTTCTTAACGCGAAAGTTTTTTTTTTCAAGAAAATTAACGAAAGTGAAAAATTTTGGGTTCTT
TTTCTTTTTTCAACAAAAGTGGGGCGTAATACCTCGGGAAACATCAAAAAAATTTTTATCAAAAAAAAGACGGAGTTTTGCAAA
AAAAAAAAAAAAGTTTAGAACTTCCATTCGAAAAAGGTTAAGAGGGGCCGTGGAAAAAAAAGGATTGGCCCTTAAAGGCAAAAGA
AAAAAAACGCGTCAAGAAGGTAAAAAGAAAAGCTTTTTCCTTAAGGGCCTTTTAGCCAAAGGTTTTTTTGTTTACTATAGAAAA
AAGTTTTTTTTTTGGTTAGAGGGCTTTTTTTTTTTCTTTAAAAAAAGAATAGCCACTTTCCACTCACCCCAAAACCTTGGACCC
GGAAGCAAAAACCAAAAAAAAAAGTTTAAAAATTTGGGAAATGAAAAAATTAAAGTGTGAGGGAAAAGCTTCCAGTTTTTTTT
TTTACCTTTTAAAGGATTTAAAAGCTTTTTTTTTTGCTTCCAAAACACCCCAAGTAACTTTAAGTGTCCCCCAAAAAAACACCC
CCAAAAACCGAACAAGGTTTTGTTTTTTTTTTCGTTTTGTTCCTTTTACTGTGAATGGCCATCAAAAAGTTTTTTTTAGGAAA
AAATTTGGGATTGCGTACCTGGATTGCCAAAAACGAAACAAGGTTTTTTTGAAAAATAAAGCCCTTTTAACACGCCCTCCACC
GTTTCAGGTTTTTGAAGGAAAAGTAAAAATTGGAAAAAACCAACGCTTTTAAATCCCCAAACTTCAAATGAATATTTTGGGAGT
TTTGGCAAAAAAAAAAAAAGCGGTGGTCTTTTTTTGGCCGTTTTTTAAAAAAATTTCTTTTTGGCGCGCCCATTTATTTCTTTTT
TACGGAGAACGGTGCGAAGGATTTTTTAACAAAACGGACCCAACAAAAGTTTTTTTAAAATACTTTTCTCTTAGTTTGTGAGA
AAAAAGGAGCCCTTTTTCAAACAAGTGAGGGGCAGCGGCAAAAAAAAAAGTGGTTTTTTTTTTTAGAGTTTTTTTTGTTTTA
AGTAAAGGATTTTATTAAACGACTGCCGAAAATCCCTAAAAAAATACAACCCCCCCCGGAAAGGCAAAACGTAAGTTTTTTGCC
CGATGCCCACTCAGTCGAAAAAAAGCTTAATTTTTGGGAAAAAAAAAATTTTTAGCAGCCTTTTTTTTTTTTTTTTTTTTTTAA
ATCCGGAAATTGAAAAAGCCTTCTTTACCAAAAAGCCCAGGGAAAAGAAAATTTTTTTTGCAAAAAGTCAAAAAGTTTGCCCCG
AAATTAAGAAGGTGGTTTTTTTTTTTTTTTTATAGGTCAAAAAAAGCTTTGGCTTTTTTTTTTTTTAAACTTGGTTTTCTATCC
AATCCGAAAATTCCCTTGCCTTAAGAAAAGGGAACATACCCGAAATTTTGGTTATCCCAGGGAACATTTTGACCGAACAAAAAC
AAAACTAGCAGCACAAAACATGGAAAAATTTCGGCAGTCAAACGAAAAAGGCGCCAAGAACCTAAACAGAAAATTTTTTTCCTC
AAAAAATTCAATTCCCAAAAGTCAAGGGTTTTTTAATTTTGAAAGAATTTTTTAAGGGGGAAATCCGAGTTATGCGTTTGGCC
AAAAAGAAC
>m141122_040740_42134_c100732202550000001823141405141561_s1_p0/29712/3793_14587
TACAATACGTCTTCTAAAGGCAGCGTGCCTAGGGCTCCGAGACGATCTCCGTAATTCCACTCATATTTATATCTTAACAAGATC
TAATTTTTGAACCTTCTCCATCTCAGATAGCTATCATGATCGTAGGTTATGACTATTGGGCATCGGGAATTTGGTCCTAATTCA
ACCAAAACAAAAAATTTGGCCTTTCCATTGGCCAAATCTCCTAGGCAAAACAGGTGCCTAGGATCCGAAGCGATGCCCGTAATT
CATCATAATATTTGGTCTCTAACAACCTCTAATTTTTTTGACTTTTTCCATCCCAAAATAACATATGTAGGTATACTATTGTCC
ATCGAATATATACAGGTGGTCCTAATACGACTAAACAAAAAATTGGCCTTTCCGTCCCAAATAGTCCTAGCAGTTGCCTAGGGC
TCCGAAGCGATCTCGTAATTCACTCCATATTTCTCTCTACAAGCTCGTAATTTTTTTGACTTCTTCCCATCTCAATAATAATCA
TCATAGGTATATATTGGCCATGGAATTTGGGTCCTAATTCAACCACAACAAAAAAATTGGCCTTCGCCAAAAATCTCCTAGGCA
GTGCCTCGGACTCCGAACGCGTCTCGTAAATTCACTCATCATTTGGTCTCCTAACAACCTCTAAATTTTTTGAACCTTTCCCA
TCCTCAATATTATCAATAGGTTATTATGGATGGAAATGGGGCTAAAATCAACAAAAACAAAAAAATTGGCCTTTCCTCGCCAAA
TCTCCTAGCAGCTGCCTAGGGCTCCGTAAGCGATCATCGTAATTCACTCATATGTCTCTCTAAACATGCTCTAATTTTTGAACC
TTGTTCAACATCTCAATAATATCATATAGGTGTATTATTGACCTTGCAATTGGGGGTCCTAATTCTAACAAAAACAAAAAAATT
GGACTTCCTGCCAAATCTCTTAGCACACACTGCTTAGCGTCGTAGGCGATCTCGTAGATCTCAGGTCATATTTCTCCCTAACAC
ACCCTCTAATCTTTTTGAAACCGTTCTCTTCCAATCTCAATAATAACAGTATCAGGTAACTACCTATTGTCCATGGAATTGGGG
TCCTAATACAACTAAAGAAACAAAAAATTGGCCTTTCTCCAAATCTCCCTAGGCAGCTGCTAGGGCTCCGAAGCCGATCTCTGT
AATGCAACTCATATTTGCTCTCTAACCCAGCTCTGGTAATTTTTTGAACCTTCTCCCATCTCAAGGGACATCCAAGTCATATAA
CGGTACTATACGTGGGGCCCAATGAATTTGGTCCTAATTAAGCCAACCAACAAAAAATTGCCTTTGCCTGCAAAATCTCCTAGG
CACGGTGCCTCGACTCCGAAGCGATCCGTAATTCACTCATATTTCTCTAACAACCTCCTAATTTTTTTGACCTTTCCCATCTC
CAATATTATCATATAGGGTATGATTAATTGGCCATGAATGGGTCCTCAATCACAAAAACAAAAAAATTGGCCTGTTCTGCCAAA
TCTCCTAGGCACGCTGCCTAGGGCATCCGAAGCGATCTCGTAATTCCACCTCATATTTTTTCTCTAACAACTCTAATTTTTTTG
AACCTTTTCCAATCCAATAATATCGATATTTGTATACTATTGCCATGATCTGGTTCCTAATTCATCCAAACAAAAAAAATTGG
CCTTTCTGACAAATCGTCCTAGGCCAGTGCCTGGGCCGTCCGAAGCGATCTCGTAATCCGGCCACTTCCATCGGATTGTCTCCG
GTAACAAGCTCTAATTCCCTTAGTGCGCAGCCGTTCTCACTTGGCGTCACCCTATGATCATCCTAGGGGCGCCTGGTCACCTCG
TATTTGACCTTGAATTCGGGGTCCGTGAAATTCAACCAAACCAAAAAATTCTGGACTTTCCCTGAGGCCAAATCCTCTCTAGCA
ACTGCCTAAGGGCTTCGTCAGCGATCTGCGTCCGGTACCCGTGCAGCGGTCATATTTCTCCCTAACAAGCTCTAATCTTTCTGA
ACCTTCTTCCCACTCAATAATACATAAGGTGTATTATTGACATGAATTTGGGGTAATTCAACCAAACAAAAAATGGCCTTTCCT
GCCATAATCTCTTAGGCAGCCTGCCTAGGGCTCCGAAAAGGATCTCGTAATTCACTTCATATTTCTCTCTACAGCTCTAATTTT
TTTGAACCTTCCTCCCATCTCAATAATATCATATAGGTATAATATTCCGGCATGAATTTGGTCCTAATCAACAAAAAAAAATTG
GCCTTTCCGTGCCAAATTCCTAGGCAGGTGCCTCGACTCAGAGCGATCTCGTAATTCACTATTTCGATTTGTCTCTAACAACCT
TCTAATTTTTTTGAACCTTTTCCCATCTCAATATTATCATATAGTTATTATTTGGCCATGAAATGGGGTCCTAAATCAACAAAA
CGAAAAAAATTTGGCCTTTCCCTGCCAAATAGTCCTAGGCAGCTGCCTAGGGCTCCGAGCGATCTCCGTAATCACTCATATTTCT
CGTTCTAACAAACCTCTTAATTTTTTTTGAACCTTTCCAATCTCATAAACTATCAATCAGTTGTATCCATCTATTGCCATGAAT
GGGTTCCTTAATTCTTTGCACAAACAAAAAAAGTTGGCCTTTCCTGCCAAATCTCCTACAGGCACGTGCACTAGCGCTCCGAAG
CGATCTCGTAGATCCACTCATATTTCACTCTACACAACCGCCGCGAACTCTTTGACCTTTTTCCATCTCAATAACCTAGCTATA
CGCGTGTATATCTGACCTTAGATTTGGGTCCTAATTCAACCAAACAAACAAACATTGGACTTTCCTGCCAAATCTCTTAGGCAA
CTGCCTCAGGGCTCCCGTACGCGATGCTCGTAATCCAAGTCACTCAGTTTCTCCCTTATAAACAAGCTCTAATCTGTTTTGAAC

CTTTTCCCATTCTTCCAATAATTCATGTTAGGTGTATTATGACCATGAATTGGGGTCCTAATTGCAACCAAACAAAAAAATTGG
CCTTCCTGCCAATTCTTAGGCAGCTGCATAGGGCTCCGAGCCGATCTCGTAATCACTCAATATTTCTCTCTAACAAGCTCTAAT
TTTTTCTGAACCTTCTCCCCATCATAATATCATATCAAGTAACCTAGTGTGGCCATGAATTGGGTCCTAATTCCAACAAAAAAA
AATCTGGCCTTCCTGCGCCAAAGTCTCCTAGGCAGGTGCCTAGGAGCTCCGTAGCGATCAATCGTAATTCACTAATAATTGTCT
CTAAACACTTCTAATTTTTTGAACCTTTTCCCATCTCAATAATAACATAGATAGGTAATTACTATTAGTCCATGAAATTGGGGC
CTAATAACACTACAAACCAGAAAAAAACTGGCCTTTCCTGCCGGAATTCCCTAGCTAGCTGCAAGGGCTCCCGAAGCGATCTTC
GTAATTCACTCCATATTTCTCCTCTAACAAGCTCTAATCTTTTTGAACATTTTCCTTCTCCAATATATCATATAGGTGTATAAT
TGACCATGAAGTTGGGGTCCTAATTCAACCAAACAAAAAAATTGCCTTTCTTGCCAAATCTTCCTAGGCAAGCGGCCCTAGGTC
CCGAAGCTGATCTCGATTCACTCATATTGCTCTCTAACAAGCCTCTAATTTTTTTGGAACCTATTCCCGTCTCAATAATAAATA
TAGGTATCATTATTGACCATGAATTGGGTCCTTATTGTCAACAAAACAAAAAATTGCCTTTCCTGCCAAATCTCTAAAAAGGAA
CACGAGGCGGCCCTATGGGCCCTTCCGAAGCGATCTCAGTAATTCACGTTCAATATTCTCTTACAACAACGTCTAATTTTTTTG
AACCATTTCCCGAACTCAAATAATATTCATATAAGGTATACCTTATTTGACCATGAATGGGGTCCTAAATCACCAAACAAAAAA
ATTGCCTTTCCTGCAAAATTCTCCTAGGCTGGGCAGCCTACCGGGCTCCGAAGCGATCCGTAATTCCTCATATTTCTCCTCTAA
CAAGCGCGTAATTTTTTGATACCCTTCCCTCATCTCATATAATAGTCATATATGGTATACTATTGGCCTATGAATTTGGGTCCT
AATTCAACCAACAGAAAAAAATTGGCCTTTCTGCGCAAATCTCCTAAGGCACGCGTGGCCTAGGCACCTCGTAGCGATCGTCGTA
ATTCACTAAATTTTGTCTAAACAATCTTCTAATTTTTTTGAACCTTTCCCATCCGAATACCATAAACATACTGAGGTATTAACT
ATGTCCCATCGAATTGGGGCGGCTAAGTACAACTAAACCAAAAAAAATTGCTTTCCTGCCAAAATCTCCTGGCAGCTCATCAGG
GGCGGGCTCCGAAGCGATCTCGAAATTACTCATATTTGCTCTCTACACAAGGCTTCTATCTTTTGAACATTTTCCCTTCTCATT
AATCATCATATAGTGATAATTCCGACATAATTGGCGTCCGTAATTCAACCAAACAGAATAAAATTGGCTTCCCTCCCCTGCCAA
ATCTCCTAGGCAGCGGCTACGGGCTCCGAAGCGATTCTAATTCACCTCATATTCTCTCTCTATACAAGCTCGTAATTTTTTTG
AACCTACTTCCCCGTCTCAATACATAACATATAGGTTATATTGATTCACCTCATCGGAATTGGGTCCTATTTCAACACAACAAA
AAAAATGGCCTTTCCTGCCAAATCTCCCTAGGGCAACGAGGCCTAGGCTCGAGCGATCTCAGTAATTCACTCATATCTTCTCTC
TATTACAACCTCTAATTTTTTGAAAACCATTTCCGAACTCTAATAATCATCATATAGGATATTATTGACCATGAATCCTGGGGT
CCTAATTCAACCCAAACCAAAAAATTGGCCTTTCCTGCAAAATCCCTAGGCTCGCCAGGCCCTATGCCTCCGAAAGCCGATCCT
CGTAATCACTCATATTTCTCCTCTAACAACCGCTCGTATTTTTTTTGAAAGCCTTCTCCCATGCTCAATAATATCATATCAGGT
ATACTATTGGCGCATCCGAATCTTGGGTCCTATGATCAACCCCCAAAACCAAAAAATGCCTTTCCTGCCAAAATGCTTCCTAGG
CCAGTGTGTGCCTAGGACTCAGTAGCGATCTTCCGTAATTCACTAATATTTGGTCTCTAACACTTAATTTTTTTGAATCCTTTT
CGTGTGTGATGCGGCCACCAGGTGTCCCCCGGCCTGTCTTGTCTGCGCACCCATGCTCAATAAAAAATACTAGGTATCGACTAT
TGGCCCTGAATTGGGGCCCGCTAATACAACTAAGACAAAAAAATTGGCGCTTGGATCCCCTGGGGCCAAATCCCTAGCAGCTGC
CTAAGAGCAATTTGCAGGAAAGTCCAATTTTTTGTTTGTGAAATTAGGACCCACCCTGCCAAATCTCTTAGGCAGCCCCTGCTA
CCGGGAGATTTGGCAGGAAAAGGGCCCAATTTTTTTGTTTAGTGTATTAGGCCCGCATTCATGACAATAGAACCTCATAATATG
TTATTTGTGAGATGGGAAAAGGATCACAAAAAATTAGACAGTTGTTAGAGACAAATATTAGTGAATTACGAGATCCGCTACGGA
TGTCCCTAAAAGGCACTGCCTAGGAGAGTGGCAGAAAGGCCCCCAATTTTTTTGTTTTGGATTGCAATAGGACCCAAATTCATG
GCCAATAGTAACCTATTATGATATTATTGAGATGGGAGAAGGTTCAAAAAATTAAGGAGGCTTGTTAGAGAGAAATGAGTGAAT
ACGAGATCGCCTCGACCCTAGCTGCAGCAATGAGATTTGGGCAGGAAAGGCCCAATTTTTTTTGTTTGGTGAATTAGGACCCCA
TTCATGGTGCAATAATATTACCTACTAAATGAAATCTTAGTTGATTCGGAAATGGTTCAAAAATATTAGACGTTTGTTAGAGAG
AAATATAGAAGTGAATTAGCGAGATCCCTTCGGAGCCCTAGCCGCGAATCGTTGGCTAGGAGATTTGGCGAGGAAAGGCCCAAT
TTTTTTGTTTTGTAAATACGGACCCCAATTCATGGTCAAATAATATACCTACTTAATTATAGCTTCATTGAAGACGGGAATAGG
TTTAAAAAAAATTAGAGCTTGTTAGAGAGAAAATATGAGGAATTACGAGATCGCTTCGGAGCCCTAGCCCCGCTGGCCTAGGAG
ATTGGCAAGAAAGGCCATTTTTTTGTTTGGGTTGAATTAGGACCCAATTCATGTCAAGTTATAGCACCTATACTGCATATTATG
AGAAGGAAAATGTGTCAAAAAAGATTAGAGCTTGTTAGAGAGAATAGAGTGAATACGAGATCGCTTCGGAGCCCTATGCAGCAT
GCCTAGGAGATTTGGCAGGAAAGGCCAATTTTTTGTTTAGTGTATGTAGGCCCCAATTCATGGACATATATACCTATATGTTAT
TATTGAGATTGGGAAATGAGGTTCAAAAAAATTAGAAGTTGTTAGAGACAAATATTAGTGAATTGACGAGAATCGCTACGGAGT
CCTAGGGCAACGACCTGCCTAGGAGATTTTGGCAGGAAAGCCAATTTTTTGTTTTGGTTGAAATTAGGACCAAATTCATGGCCA
AATGAGTTATCACCTATACTGATATATTTTTTTTTTTGTTTTTTTTGAGATGGGGAGAAGGTTCGAAAAAAATTGAGAGCCT
TGTTAGAGGAAATATGAAGTGAATTAAGAGATCGCCTTCGGAGCCCTAAGGCTGCAGCCCCTAAGGAGATTTGCAGGAAAGCCA
ATTTTTGTTTGGTTGAATTAGGACCCCAATCTCATGGCCAAATATGATATGCTCTACGATGATAGTATTGAGGTCGAAATGGTT
CAATAGAAACAATTAGAGCGTTGTTTGAGAGAGAAATAGTGAGTGAATTACGAATCGTCTTCGGAGCCCTAGTGCCGCGCTCCG
TTGCCTAAGGAGATTTGCAGGAAAGGCCAATCTTTTTGTTTTTGTTGAAATACCGGATCCCCAATTCATGGTCAATAATATACC
CTATATTGAATTATTATTTAGACGGGAATAGTTCAAAAAAAATAGAGGCTTGTAGAAGGAGAATTTTATGAGTGAATTATCGAG
ATCTGCTTCGGAGCCTAGGCTCGCTGCCTTAGGAAGATTTGAGCAGTAAAGGCCCAATTTTCTTTGTTGGTTGAATTAGGGAGC
CAAATTCATGGTCAATATAGCAACGCTAAAATATGATATTGATTAGAAGGGATATATGTTCAAAGATTAGTAGCTTGTTAGAGA
CGAAATATGAGTGAATTACCGAGAGTCGCTCGGAGCCCTATGCAACGCTGCCTAAGGAGATTGTGGCAGGAAAAGACAATTTTT
TTTTTAGTTGTATTAGGCCCCCAATTCATGGGACAATAGTGATACCTAGTATGATTATATATTGAGATGGTGAAAAGTGTTCAA
AAAATAAATGATTGTTAGAGCAATGATTAGTGAATTAACGAGATCCGCTAAACTGGAGTCCTAAAAAAGGCAACCTGCTCCTAG
GAGATGTTTGCAGGAAAGGCCATTTTTTGATTGTTGGAATTAGGACCCAAAGTTCATGGGCCCAAGTAGTAAACCTATATGATA
TATTAGATGGGAGAAGGTCAAAAAAAAATTAAGGCTTGTTAGAGAGAAATGATTGAGTGAATGTGACGAGATCGCTTTCGAGCC
TAGGCAGCTGCCCTAAAGAGATTTGGCCAGAGAAAGGCCCAGATTTTTTTGTTTGTTGAATTAGGAAAGCCCCATATTCATGGT

CAATAATCGACCCTACCATGATACTTATTGCAGATGGGAAAAGGTTCAAAAAGATTAAGCTTGTAGGAAGAAAAATTTGACCTG
AATTACCACGATCGCTACGCGAGCCCCTAGGCAGTTGCCTAAAGAGATTCTGGCAGGAAAGTCCAATTTTTTTGTTTGGCTTGA
ATTAGGGACCCCAAAATCTAAGTCAATAATTACACGCCTATATCGATATACTTGAAGAATTGGAAAACGGTTCAAAAAGACTAG
AGCTTGTTAGAGTCGAAATGATGAATTCAGCGATCGCTTCGGACGCTCAGGCAGTCGCCTAGGAGATTTGGCCCAAGGAAGGGC
ATTTTGTTTCGTTTTGAAGAATTAGGAACCCAATTCATGGCCAATAGTATATCAAATATGATTATTGATTGAGATTGGAAAAGG
TCAAAAAAAATTGAGGTTGTTTAGAGAGAAAGTATGGTGAATTACGGAGATCCGCTTCGGAGCCCTAGCAGGTGCTACGGAGTA
TTTGCAGGAAGGCCAATTTTCGTTTATGTTGATTTAGGACCCCCATTCATGGCCAATAGATCAGACCAGTATATAATTATTCTG
AGATGGGAAATAGGTTCAAAAAAATTAGAGGTTGTTAGAGACAAATATGAGTGAATTACGAGACCGGTTCGGCAGTCCGAGGCC
ACCTGCCTAAGGAAGATTGCAGGAAAGGCCAATCCCTTTTTTTTTGGCTTGAATTAGGACGGGCCAAATCATCGGCGCAAGGTA
AGTGAACCTATTGATATTATTAGACTCGGAGAAGGTTCAAAAAAATTTAGAGCTTGCTTAGAGAGAAATACTGAGTGAATACGA
GATCATACTTCGGAGCCCGTAGCGCAGGCTGCGCCTAAGAGCATTTGGCAGTGAAAGGCCAATTTTTGTGTTTGGTTGAAGGTA
GGACCCCATCATCGGTCAGACGTTAATTAGCAAAACTATATCGATAATTGAGAATTGGGAAAGGAGGTCCAGCGAAGATTAGAG
CTTGTTAGTGGAGAAATATGATTGGAGATTTACGAGACATCGCGCTACGGAGCCCCTAGCCATGTGTGGCCTAAGGTCGGAGAT
TTAGGAAAGCAAGTCAAATTTTTTTGTTTGGTTGAATTACCGTACGCCCAATTCAGGTCATAAAGATACACGCTATTATGATAT
TGATTTGCGAGATGGAAAAGGTGTTCAAGAAAGATTAGAGCTTGTTAAGGGAGAAATAATGAGTGAATATACGAGCGCTTCGAG
CCTAGGCACGCTGCGCTAGGAGATTTGGAGCCGAAAGGCAATTTTTTGTGATGAACTTGAGGGGCCAACCCATTCATGGCAATA
TGTATAACAACATATGATTATTGAGATTGGAAAGTTCAAAAAAATTAGAGGTTGTTAGAGAAAAAATGAGTGAATTAGCGAGAT
CGCTTCGGAGCCCTAGGCCAGCTGCCTATGGAATTGCAAGGAAAGGCAAGTTTTTTTGGGTTGTTTTGTTGATTTAGGACCCGC
ATTATCATGGCCAAGTATAATACTATTATGATAATATTTCACATGTGGATAAAAGGTTCAAAAAAATTAGAGGTATGTTAGATG
ACAAAGTATGAGTGAAAATTACGAGATCGCTTCGGAGTCCGAGGCAGACTGCCTAGGAGATTGGCAGAAAGGCCCATTTTTGGG
TTTGGTTGAATAGGACCCAATATATCATGGCGCAAGGTAGTGATACCCGTATATGATATATAGATTGGAAGGAAGGTTCAAAAA
AATTAGAGCTTGTTAGAGAGAAATAGAGTGAAGTAGGCGAGACCCCTGCTGGAGCCCTAGGCAGGCTGCCTAGGAGAATTTGGG
AGGAAAGGCCAATTTTTTGTTTAGTTTGTAGTAGGACCCCAATTCATGGACAATAGTTACTAGTTTATGTCTATATTGATCGAA
TGGAAGAAAGGTTCAAAAAATTAGAGCGGTGTTTAGGGAAAATATGACGAACAATCTACGAGATGTGCTACGGATGCCCTAGGC
AGTTCTGCCTAAGAGAGTTTGGCAGGAAAGTCCCATATTTGTTTTTGTTTGTTTGAATAGGACCCCCAATTCAAGGTCAATAAA
AGTACACCTATATGATATTATCTCGGATGGGAAATAGGTTGCAAAAAGATTAGAGGCATGTTAGACGAGAAATATGAGGTGAAT
TACGAGATCGCTTCGGAGCCCTAAAGGCAGGCTGCCTAGGGAGATTTCCAGGAAGGCCAATTTTTTTGTTTGTTGATTTAGGAC
CCCATTTCCAGGCAATAATATAACCTATATGATAATAACTTGAGATGGGAATGGTTCAAAAAAATTAGAGGATTGTAGAGACAA
TATGAGTGATATACGAGATCGTCTTTCGGAGTCCGAGGCACCTGGTCCGTAGGAGATTTGGCCAGTTGAAAGGCAATTTTTTTT
GTTTGGTTGAATTAGGACCAAATGATGGCGCAATAGTATACCGTACTATATACCCTTATGTTCGGAGAGGGGACGAGGTTCTAA
AAAAATTAGAGCTTGTAGACGAGAAATATGAGTGAGTTACGGTGATCGCTTCGGAGCCCCTAGGCAACTGCCTACGGATACCTT
GGGAGGCAACCATTTTTTTTAGATTGATTTAGGACCCCGCAATCGATGGTACCAATACGTATATCCTATATGTTACTTTATTC
GAGATGGAAAAAGGTTCAAAAAAATAGAGGTGTTAGAGACAAATATCAGGTGAGCATTACGAGATTCGGCTTCGGAAGTCACGC
CAGGGCACCTGCCTAGGAGATTTTGGGCCAGGAAAGGGGCCAATTTTTTGTGTTGGGTTATATTAGGACGCCAATAATTCCATT
GCCCAATAGTATACCTATATGATATTATTGAGATGGGACGAAGGGTTCAAAAAAGAAATTAGAGCTTGTTAGAGAGATATGAGT
GAATGTTACGCAGATCCGCTTCGGAGCCCTTAGGCAGCTGCC
>m141122_040740_42134_c100732202550000001823141405141561_s1_p0/36738/1980_13611
CCGACAGCCCTCGCCCCCTCCCCTTTTCCTCGCCTCGGATTACACTAGGACCTTCTTCATTCGCCCTGCTTGTCAGTGGCCACC
GGGCACTTCAACCCCACCACCCCCCCACCCCACCCACCGGACAAACGAAATACCCCAATGAGCTTTTAAAAGCAGTTTTTTTTT
AAAAAAAGAAACTACAACAACATTTTCGACCGTAAGTGACCACTTTAAACGAAACTCAAACAAAAAACATGAATGCTTTTCAGG
CGGTGCAACAACCTCAGGACTTCCCAGGAGGCTTCAGCCATCCCTCACGCTCCCACTCACCTCCCGCCCCCCAGGGACGCTCTA
ACCCGCCGCGAGCTTCGTCTGATTCAAGCGATCGGTGCATCTAGTCGCTACGCTCATCACCGATCCCGCCCACCCGGACACTGT
TTGTTCGCGCCACTCTATTCGATGTTATCAGGCGAACGCCGTTGCCCTCCTTTTCTCAACTCGGCATTAACCTAGGAATTCTCT
CTTATATCGGCTGCTCCCTGTCAGCCTCGGCCACGGTCATTAACCCCCCAACGGAGAAACGAAATAACTGACGCTTTTACAAGA
TTTTTTTTAAAAAATACCAAAATTTTAGCCCGTCAAGCGGATTTTCTAAAGGGATTAAAAAACAATGATCGCCCTTTTCCAGGG
TGCAAAGCACGAGGACTTCCCAGGAGGCTCACCCATACCTAAGTACCTACTCTCCGCCCAAAGCAAGCGCTTAACTCCGCCGAG
TTCTGACTGGGCATATGCCGGTGCATTCAGTGTCCGGTATGCATCGCACCCCGGAATGTTTGTCGCAATATTCATTTATCAGCG
AACGCGTGCCCATACTTTTCTCCCTCCGGATTACCACTACCGAGGCTTCTTATTCGCTGCTTGTCAACAGGCCACGGTACCCTT
CCCGAACCCCCAGGAACAACCGAACATAAACTGACGGCTCTTTTTAAAAGACTCTGTTCTTCAAAAAAACATAAAACTTTTGAG
GCCCGTCAGTCGGAATTTTTAAAGGAACTAAACCACCCAAAATGATGTTTTTCAGGGTGCAACACGAGGATCCTCCCAGGAGGC
TCAGCCCCATCCTACGGTACTACTCTCCGCCCAACCCCAACGCTCTAACAGCGAAGCTTCTTGCTGATCGGGAATCCGGTGCAT
TACTGCCTGGTATCCCGATCCGCAAAACCGCAATGTTTCGCTTCGCATTACTTCCATTTATCACGCGAACCGCCGTGCCCTTCC
CCTTTTCTCCCTCCCCGGCCGGATTAGACTAAAGGAAAACCTTCTTTTTCGGCTGGCTCTGTCAGTGACCGCACGGTAGTTCAA
CCCCGCCCCCCCAACCGGAAACGAAATAGAATGACGCTTCTTACAGATTTCTTTTTAAAAAAATACAAACTTTTAGGCCGTAAG
TGCGCATCTTCTGAAAGATTAAAAAAAAATGAATGTTTCTCTCACGGGGTGCACAAACGAGCGACTCCATCCAGCACGAGGTCC
CCCATCCTACGTACCTACTCTCTCCAAGCCGCAATCTAAGCAGCGGCAGTTCTGATGGGATCGGATGCATTAGTGCCTGGCTAA
GATCGGCACCCCGGAACTTTCTGGCTCCGCAATATTCATTTCCACTAGGCGAACGCGTGCGCCTCCCTTTTCTCCTCGCATTAA
TAGGCAACTCTCTTAACTTCGCCTGCTCTGTCAAAGCCACGGTATTCAACCCCCTCAACCGGAAAACCGAAATACATGCCGCCT

TTTAGAAGATTTTTTTAAAAAACACTGACACACACCTTTTGAAGCGTGAAGGCTCGGAGCTTTTAAAGAATCACCGAAAAAAA
GATCGATTCGTTCTTCCAGGGGTCGCCACACACCCGAGGACGTTTCCCAGGACGTCACCCCATCCTAGTACGTACTCCTCCGCC
AAGCGCACGCTTAACTGCGGAGTTCGTGATGGCGACTCCGCGTGCGATTACGTGCTAGTATCACCCGCGATCGCACCCGAATGT
TTGTTCCGCCATCCTATTCCCTTATAGCGAACCGCGTGCCCCCCTCTTTCTCAACTCCCCGCATTCAACCTAGCGAACTGTCTT
ATTCGCTGGCTGATCAGTGGCCACGGTATAACCCCCCCAACGGAAAACGAAATAACAATGAGCTTTTAAAGATTTTTTTTAAA
AAATACAGAAATTTTGAGCCGTAGAGTGGATTTTTACAAAGATTAAAAAAAAATGATGTTTCAGGGGCTGCAACACCCGAGGAC
TCTCCCAGCGCAAGCGTCACCCCATCCCCGTAGTATACTCTCAGCCGCAAGCACGCTTAACGCTGCGGAGTTCTGACTGGGATC
CGCGTCAACCTAGGTGCTGGTAATTGATCGCACCCCGGAATGTTTGTTCGCATTATTGCATTTGTAGCCCGATACGCCCCGTGC
CCTCCCCTTTTCTCCCTCGATTAAACTAGACACCTTCGTTATTCGGCTGGCTCTGTCAAGAGGCCACGGTATTAACCCCCCAAG
GAAAACGGAAATAAATGAGCTTTTAAAGATTTTTTAAAAAAATAAAACACTTTTGAGCCGTAAGTCGGATTTTTAAAGAATAA
AAAAATATGATGTTTTCAGGGGTCGCAACACGAGGACTCCCAGGAGGTCACCCCATACCTAGTACTACTCTCCGCACCAACCCA
CGCTTAACAGCGGCAGCTTCTGATGGGATGCGGTGGCATTACTCGAGCTGGTGATGATCGGCAACGGAATGTTTGTTTCCAGC
ATTATTCATTCTTATAGCCGAACGCGTGCCCCCCCCTCATTTCTCAGCTCCGCATTAACCTAGGAACTTCTTCATTCGCGGCTT
GTCAAGTGCCACGGGTATTAACACCCAACCGGAAAACCGAAATCAAAATGAGCTTTTAAAGATTTTTTTTAAAAACAATACCA
CAACTTCTTGAGCCGTCAAGTGGTTTTAACAGATTAAAAAAAAAATGATGTTTCAGGGGTGCAACACGCAGGACTTCCACGGAG
GGCTCACCCCATCCCTAGGTACTCACTCTCTCCCCAACAGCTTAACAGGGCGGCAGTTCCTGATGGATCCGGTGCATTAGTCGC
TGCGTATGATCGCACCCGGAATGCTTTGGTCCCGCGAAATATTCATTTATACGCCGACAGCGCGTGCCCTCCCCTTTTTCTCCT
CGGATTTAACTAGGAACGCCTTCTTTTCCGCTGGCTTGTCCAAGAGGCACGGTATTAACGCCCCCCAGAGGAAACCGAATACAA
TGACGCCTTTTAAAGCATTTTTTTAAAAAAATACAAACTTTTGAGGCCTTAAGTGAATTTTTTAAGTAATAAAAAAAACATGA
TGTTTTCAGGGGGGTGCAACACTAGCGACTTCCCAAGGCAGGTCTTACCTCATTCTCTAGTAACTTCTCGCCCATTGCACCGCT
TAACTCGCGGAGCTTCCTGATGGGATCCGGCTGCACTTAGTGCTGGTATGCATCGCACCCGGAATTTTGTCCGCATTATTCATT
TAATAGGCGAACGCCGTGCCGCTCCCTTTTCTCCTCCGGATTCAACTACGGAACCTTCTTATTCGCTGGCTCTGTCAGTGACCA
CCGGTATCTAACCCCCCAAGGAAACACGAAATAAATGAGCTTTTAAAGATTTTTTTAAAAATATAACCAAACTTTTGAGCGTAA
CTGGATTTTTAAAGAATAAAAAAAAATGATGTTTTCAGGGGTGCAACACGAGGACTATCCCAGGAGGTCACCCATCCATAGTAA
ACTACCTCTTCCAACCGGCCAAGCACGCTTAACCGGGGAGTTCTGATGGCGATCACGGTGCATTAGTGGCTAGTATGATCGCAC
CCGGATGTTGTTCGCCGCATTATTGCATCTTATCAGCGAACGCGTGCCCTCCCCCCTTCCTTCTCCCACTCGGAACATTTAACT
AGGACACTTCTTATATCGGCTTGGGCTTGGATCAGAACTGGACCACACAGGTATATAAACAGGAACGAAATAAATGAGCTTTTA
AGATTTATTTTTAATACAAATTTTGAGGCCGTAAGTGGATTTTTAAAGATTAAACACAAAAAATGATGTTTTCAGGGGTGCACC
GAGGACTTCCCAGGGGTACCCATCTAGTACTACTCTCGCCAAGCACGCCTTAACTGCGGATGTTCCTTGATGGGATCGTGGCAT
TAGTGCTGGTATCGACTGCGCCCAGGAAAATGTCCAGATATCAGCATTAAACTATCATTATAAGCAAGAACGAGTGGCACTCCA
TTTTCCTCCCTCCGGATTCAACTAGGCACTTCTATTCGGCTGCTGTGTCAAAGCGCCACGGTTATTAACCCCCCCAAGGAAAAC
GAATAAGAAAGAATTTTACGATTTTTTATTACACTTTGAGCACAGTAGTAGGATTATATTGTTATAATGGATTTTCAGGGTGCA
ACAACGAGGACTTCCGGGATAATCACACCTCCCTGGATACTATTCACAGCCACACAAAAACGCTTAACAAGCAGGAGTATACTG
AATGGGATCCGGTGCATGTTACTGGGCTGGTCATGGATCCGCAACCGGAAAAAAATTGTTTTTTGGGGGGTTTTTTTTTTTTCC
CCGCATTTATTCATTTATAGCCCGAACGCGTGCCTCGCCTTTTCTCCTCCGATTACCTAGGAACTTCTTATTCGCCTGTTGAAT
CAGTGACCACGGTATTAACCCCCCAAGGAACGAATAAATGAGCCTTTTAAAAACGATTTTTTAAAAAAATAACAACTTTTGGC
CGTAAGTTGGATTTTAAAGACTTCAAACAACAAATGATGTTTTCAGGGGTGCATACAGCGAGGACTTTCCAGGAGGTCACTCCA
TCTAAGTACCTACTCTCCTCCCCAAAAGCACGCTTAACAGCGGAGTTCTGATGGGCATCCGGTGCATTAGTGCTGGTATGATCG
CACCGGATGTTTGGTCCCGCATATTCTTTATCAGCCGAACCGCGTGCCCTCCTTTTTCTCCCTCCGGCATTAACTAGGAACTTC
TTATTCGGCTTGCTTGTCCAAAGGCCCACGGATTAACCCCCCCAAGGAAACGAAATAAATGAGCTTTTTAAAAGATTTTTTTAA
AAAATACAAAACTTTTGAGCCAGTAAGCTGGATTTTAAAGAATACAAAAAAATGAATGTTTTCAGGGTGCAACACGAGGACTTCC
CAGGACGTCACCCATGCCTAGTACTATCCTCGCCCAACCGCACGGCTTAACTGGCGGAGTTCTGACTGGGATCTGGTGATTAGT
GGCTGGTCATGATCGCACCCGGAATGTTTGTTCCGCATTAATTCATTTAATCAGCGAACGCGTGCCCTCCTTGGTTCTCCCATC
AGGATTACGACTAGGAACTTCTAATTCGTGCTTTCCAGTGGCACCCGGTATTACCCCCAAAGGAAACCACGAAATAAATGAGCT
TTTAACACGCATTTTTTTTAAAAAAATACACAACTTTTGACCATACAAAGTGGATTTTTAAAAGATTAAAAAAAAATGATGGTT
TTTCAGGGGTGGCAACACCAGGACTTCCGCCAGGAGGTCAGCCCATCCTAGTCTATCTCGCCAAAAACCTTAACCTGCGGAGTT
CTGATGGATCGCGGTGATTGTGCTGGTATGATCGCACCCGGAATGTTTGGTCCGCCCAATATTCATTTATAGGCGAACGCCGTG
CCTCACCCTTTTCTCCCTCCGGATTAACTAGGAACTTCTTATTCGCTGCTCTGTCCAAAGGCCAACGGTATTAACCCCCCCAAA
GGGAAAAGAAAATAAATGAGGGCTTTTAAGAATTTTTTTAAAAAAATAACAAACTTTTGCAGCCGTAAGTGGATTTTTAAAGC
AATCAAAAAAAAATGATGTTTTTCCAGGGTGCAACACGAGACCTCTCCAGGAGGTCCCCATCCTACGTACTACTACCCTCGCCC
CAAGCACCGCTTCCAACTGCAGGAGTTTCTGATGGGATCCGGTTGGGCATTAGGTGCTGGTATCGATTTAACCCGGAATGTCTT
TGCTCCGCAATAATTCATTTATCAAACGAACCGCGTGCCCTCTTTTGCTCATCGGATTAACTAGGAACTTCTTTTCGCTGCTCT
GTCAGCTGGCCACGGTACTTCCAACCCAACGGAAAACCCGCAAATAAATGACGCTTTTAACAGACTCTTTTTTTAAAAAAATAC
AAACCATTTTGACGCGTCACACGCTCGGAGTTCCTTTAACAGCATTCTCACAACAACACAATTCACGCTCAGGGAGAGGGCTGG
CACACACGAGGACCTTCCACCGGCCAGGTCAGCCCACCTCTAGTTTACTATCCAACCCAGCCCCGACGCCAACGTACTCTCGGC
CCAAGCACGCTTAACTGCGGAGTTCGTGACTGGGAATCGCGGTGCATTACGTGCTGGTATGATCGCACCCGGAATGTTGGCTGC
CGCAATATTGCATTTATAGCCGACACGGGTGCCCTCCCCTGGACTTTCCTCCCTCGATTCAACTAGGAACTTCTTATCTCGCTG
CTTGTCACACAGCGCACGGTATTGCAACCCCCCCCAACGGGAAAACGAAATCAAATCAGCTCTTTAAAGATTTTTGTTAAAAAA

AATACAAACTTTCTGAGCCGTCAGTGGATTTTTAAAGAATCAACCAAAAAAAAACTGATCGTTTTCAGGGGTGCAACACGAGGAC
TCTCCCAGGAGGTCACCCATCCTAGTACTACTCTCCGCCCAAACCCGCTTTAACAGCGGAGTTCTGCAATGGGATCCGGCTGCA
TTACTGTCGGTAGCAATCCAACCGGAATGTTCTGTTCGCGATTAATTCATTTATAGCAACGCGTGCCCCTCCCTTTTCCTCCTC
GGCATTAACTTAGGAACTTCTTAGTTCGGCTGCTTGTCAGTGACCACCGGTAATTAACCCCCCAAGGAAAACGCAAAAAGTAAA
ATGCAGCTTCTTAAAGATTTTTTTAAAAACAATACAAACTTTTTGAGCCGTAACGTGGATTTTTCAAAGATCTCCAAAAAAAA
AATGATGTTTTCAGGGGTGCAACACGAGGCACTTTCCCAGGAGAGTCACCCATCCCCTACGTCACCTACTGCCCTCCCAGCCAC
AGCTTCAACAGCGGAGGCTTCTGCATGGATCCCCGGTGCCACTCTACGTCGCTGTATCCGAAGTCGCACGCCGAATGTTTGGGG
CTCCGCAAATATTGCATATTATAGGCGAACGCGTGCCCTCCTTCATTCTCCGCCCTCGGATTAACAGGAGCTTCTTATTCGCTG
CTCTGTCCAAAGGGCCACGGTATTAACCCAGGAAAACGAAATAAATGAGGCTTTTAAAGATTTTTTAAAAAAAATACAAACTTTT
GAGCCGTAAGTCGGATTTTTAACAGACACTACACACACAAATCGCCATGTTTCCATCCCCACACAACCACACACACCCCAGTGC
CACGAGGACTTCCCAGGAAGGATCAACACATACTAGCGTCACCTAATCTCCACGCCAAGCACACGCTTAATGGCGGAGTTATAG
AAATATGGGAATCGGTGCATAATTCAGCCAAGTATGAGCGCACCCGGAATGTTGTTTCCGCATTATTCATTTATACGCCAGAAA
CGCGTGCCTCCTTTTCATACACAATCCAGGATTAACTAGGAATTCCTTATTCGCTGCTTGTCACGTGGCCACGGATAAATTCAA
ACCCCCATGAAAACGAAATAAATGAGAGCTTTTAAAGATTTTTAAAAAAATACAAACCTTTTGGCCGTACAGTCGGATCTGCT
TTTAAAGATTAAACAAAAAAAAATGATGTTTCAGGGGTGCAACGAGGACTTCCCAGGCAGGATGCAGCGCCATACCCTAGTACTA
CTCTCGCCAAAACGCACGCTTAACTGGAGTTCTTGATGGGATCCGGCTGCATTAGTGGCCTACTATGATCGCACCCGGACACCC
CCCTGTTTGTTCCGCATCTATCTTCCATTCTATAGCGAACCCAGCGCAGTGCCCAATACCCCTCTTATCTCCGCTCGATTGAAC
TAGGACTTCTTATTCAGATGCATTGTCAGTGGGCCACCGGATATTCACGTCCGCAAAGAAAACACGAATAAATGAGCTTTAAAA
GATTTTTCTTATTCTTTTAAAAAAAATCACAAACACTTTAAATGACGTAAGTCGGATACTTTTAAAGATTAAAAATAGATGTTT
CTCAGAGAGGTGGCAACACGAGAATTCCCAGAGAGGTCACCCCATCTCTAGTATATCAGCTCTCGCCAAGAGCACGCTTAAGCC
TGCGGAGTTCCTGATGGGAATCCGGTGCATTAGTGCTGCTATATATTCACCGCACACCGGAATGATTTTCGGTCCGCAATATCT
CATTTACCTAGCGACACGCGTCGCCTCCTTTTCTCCGCTCGGATTAACTAGGAACCCTCTCTTAATTCAGCTGCTTGTGGTCAG
TGGGGCCCACGGTAATTAAACAACCAGGAAACAGAAAATTCAAATGAGGCTTTCTAGATTTTTTTAAAAAATACAACTTTTGA
GGGCCGTAAGCTGAATCTTTAAAGAATACAAAAAAAATGAGTTTTCAGGGGTCCAACACTCAGGACATTCCCAGACGAGGTCAC
GCCATCCCTAAACAAGTAAAACCTCTCCCGCCGAAGCAACGGCTTAACTCGCGGAGTTCTGATAGGGATCCGGTGCATCTAGTG
GCTGGTATCCGATCGCCACCCGGAACCTGTTTGTTCCCCGCATTATTCATTTATGCAGGCGAACGCGTGCACCCTCCTTATTTT
CCCTCGGATTAAACTAGGCAACCTTCTGTATTCCTGGGCTCTGTCAGTGACCCACGGTATGTCAACCCCCCAAGGAAAAAAAAC
CGAAAAAAAAAAAATAAATGCACTTTAAGTCATCTTATTTTATACCATCAACCTTTGAGCTAACTGGATTTATACAGATACAAA
AAATGATAGTTTTCAGAGGGATGGGCACACAGGACTTCCAAGAGGCACTCCTGTACATCTCTCGCACCAACGCGCTTCCAGGA
GTTCTGATGGGTTGGATGAACCTTAGCTGTAGTATAAAAGATCAGACAGAGAATGATAGATTACAGATATATATACTATAATAT
AATAAGCGAAACGCATGACTCCTTATATACAAAAACAGAATATTAGGAACACATTTTATTACTGCATAGGACAAATAGTAACAG
TGGCCACAGAAGATATATAAAAACCCCGACACCAAGCAAAACGAAATAAATGAGACTATTTAAGATTTTCATTATAAAAAAACA
ATTTGAGGACGTAGTAGTATTTCTAGATAAAAAATGATGTTTTCAGGGTGCAACACGAGGACTTCCCGGAGATCACCCATCTAG
TACTCTCTCGCCCAGCACGCTTAAACTGCGGAAGTTCTGATGGATCCGCTGATAGTGATGGATCGATACGCACACACAGGAATG
TTTAGGTCGCAATAATTAACATTTAAAAGCGAAACAGACCTCCTCCACCTTAATTCTCCACTCGGGATTACACTAGGAATTCTT
ATTCGGCGTGCTTGTCAAAGGCCCACGTACGTTAACCCCCCAAGGAAAACGAAATAAATGAGCTTTTAAGATCTTTTTTAAAAA
AAAATACAAATTTTGAGCGGCGTAACGTGGATTTCTTAAAGAATAACAAAAAAATGATGTTTTTCCAGGGGTGGCAACACGCAG
GATTTCCCCAGGCAGGTCACCCCCATCCTAAGTACCCTAACTCTCGCCCGAACCGCAGCGCTTACATGCGGAGTTCTGATGGAT
CCGGTGCATTAAGTGGCTAGTATGAAAAAAATCCCGCACCGCGGCAATGTTGGTTCGCGCATTATCTCATTTATAGGCGAACGC
GTGCCTCCACCTTTCTCCCTCGGATTCACTAGGACTCTTATTCGGCTGCTTGGTCAGTGGCCACGGTGATTAACCCCCCAAGGA
AACGAAATAAATGACGGCTTCATTAAAGATTTTTTTAAAAATACAAACTTTTGTTTGAGCCGCTAAGCTGGAATTTTTTAAAA
GATTAAAAAAAAAAATGATGTTTTCAGGGGTGCAACACGAGGACTCTCCCACCGAGGCTCAGCCCTCCTACGTACTTACTCTCC
GGCCCAAGAACGCTTAACTGCGGAGTTCTGATGGGAATCGCGGCTGCATTAAGTGCTTGGTATGATCGCACCCCGGCAATGTTT
CGGTCCGCAATATTCATTCTTATAGCGAACGCAGTTGCCCTCCTTTTCCTCCCCTCCGGATTACATAGGAACGTTCCTTATTCG
CTGGCTTGTCATGGCACCGGTATTAACCCCCCAAGGAAAACGAAATAAATGAGCTTTTAAAGATTCTTTTTAAAAAAAATACA
AACTTCGTTGAGCCGCTAAGTGAATTTTACAGAATAAAAGGAAAATGATGGTCTTTTCAGGGGTGCAACACTAGGACTTCCCAG
CGAGGTCACCCATCCCTAGTACGTACTCTCGCCCACGCACGCTTAACGCGCGTTGAATGGGATCCGGTGCATTAGTGCTAGTAT
GGATCGCGAGCCGGAATGTTTGTTCGCATTCATTCCTTTTCATCAGCGAACGCGCTGCCCTCCCCTTTTCTCCACCTCGGATTA
CTAGGAAACATTCTTATTCGCTGGCCTGTCCAGTGGCCCACGGGTAATTACCCCCAAGGAACGAAATAAATGAGCTTTTAAAGA
TTTTTTTTAAAAAATCACAAAATTTTGAGCCGTAAAGCTGATCTCTTAAAGGATTAAAAAAAAAATGTGATCGTTTTCAGGCGG
TGCAAACAGCGAGGATCTCCCAGGCAGGTCCACCCATCCCCTACGTACTACCTCTCGCGCCCAACGCACGCTTATCTGCGGAGT
ATCTGATGGATCCGTGCATTGAGTGCTGGTAATGATCCGGCAAACCCGGAATGTTTCGGTCCCGAATAATCTCACCATTTATAG
CGAAACGGCGTGCCCTCCCTGTTTCTCCCTGCGGAGTTAACTAAGGAAGGTTCTTATTCGGCTGGCTGTCAAGCCACGGATATT
AACCCCCCAAGAAACGACAATAAATGAGCTTTAGAAAGATTCTTTTTAAAAAAAAAATACAAACTTTTTGAGTCCGTAAGTGGGA
TTTCTTAAAGAATAAAAAAATGATGTTTTCAGGGGTGGAAACACGAGGACTTCCAAGGAGGTCACCCATCTAAGTACTACTCTC
GGCCACAACGAGCGCTTAACCAGGCGGAGTTCTGATGGGCATCCCGTGCATTAAACCTGCTGGTCATGCATCGCAACCGGCAAA
ATGTTTGTTGCCGCATTATTCATTTATAGCGAAACTGCGTGCCTCCTTTCTCTCCCCTCGATTCAACTAGGACTTCTTTTCGCT
GGCTTGTCAGTGACGCAGCGGTATTAAGCCCCCCAAGAAAACGAAATAATATGCAGCTTATTAACAGATGTTTTTTTAAAAAAA

ATACAAACTTCTTGAGCCCGTAACGCTGGATTCTTTAAAAGATAATAAAAAAATGATGTTTCTCAGGGGTGCAAACCACGAGGA
CTTAACCAGGAGGTCACCCATCCCTAGTACTAACTCTCT
>m141122_040740_42134_c100732202550000001823141405141561_s1_p0/38587/0_13884
AAAAGGGGATGAGAGATGATAAAGGAGAAACGAGTATTCAATCAGGGTATGTGTTTATCCATTTGCGTGGGTGCTCTTAGATCC
GGTATTTGTATATCTCTTCAGTGCCAATATTGCTAAGGTGTTAGAACATATGTGTGGTTTGTTCACAAACTCTTGTAGATGAAA
CTCTTGCCGAGAGAATTGGTTTCTCGCTCCCAAGTGAATCGATCACGTTGGGGCTGATCGAGAGTGCCTTACTTCTAGGGGAAG
TGGAAGAGTGCGCGTCATTTTTGTTATGTGGATGCATACTCCTTGGTCAAGTGCGAAATCAGTTTATGCCACCCACTCTTGAGT
GTAGGATCACGATATCTATGCTGGATTTCAGACTGATACATGGGCTTTGAGATTTGTGATAAAGTAATGCCCTCTGCGGTTTGA
AAAATGCTGGTGCGACTTACATGCGACTAGTCAATGTCACATGTTAAAGGGGTGATCGGTACAAAACATGGAGGGTATATGTTG
ATGAAACGCTGGTTATATATTGTCCGCTCTCTCCTCCCCCGCGTCGCGCGCGGCCCTCGATAGAGGTTAGGTATGTTGAAGATG
GAACCCATAGGGGATCTTTCAATAGTGTTGTTCAACGTCTTGAAGGTAAGGTATCGAGATATGGGAGAGCGAATCCCTCATAGT
GTGCTCCTGGGTGCGAGTGTGATCGATAGGGGAAAATTTTTTGGGATTATAGAAGTTGAAACTCACGAGTGAATGGTTGGAAGG
GTTATATCGCGTAGAATGATCAAGGCTCTGGTGCGATGTGATGAAGTGAATGTGCCATAACGAATAGATTAAAGAGTGTTTTTT
AAATAGTTTAATAGGGAGAATTCGGCTGCTTGTTGCGTGATAGAGGTTTTTTGTTGATTTATCGGTCCTAAATCGAAGGGAAAG
TGCATCCCAGTTATTTTAATCTCCTTTGAGGCAATTAAGAGGAAGTGTGTGGATGTTGGAATGCGGAGGTAAGTTTTGCGTGTA
GTGCAGTGTATTTATGGGGCGTTTTCAAGGCCTTTAAATGGTTCACATTGTTCGCGAGGCCCACCGATTTCTATTTCTCTATAT
GTCGGTTGTGTCGGAGAAAAGTAATTCTGGTATTGTTTGTCATGGCGTAGCTTGGCGATCATAGAATATGACTGCGTGAGTGTG
TGTGACTAAGAGGAGGAGTGTAGAAGAGGGTTGCAAAGGTGTGCGTATAGGTGTTGTTATGTTAAGGCATAAAGTAAGATTGAG
GGGTAGCTCAGAGCTGCGGTATCTCTGCGTCGGCCGAGTGGTGAAAAATTAGTATATATGTATTGCTTGTGGATTGATGTGATT
TTAGCCTCCAGGAAAGTTGGTACTGTGCGTGTGACTGTGTGCGAAAGCTTCACGCCCTGATCGAATGTGATAGGGGTTTCCTAC
CAGGTTCCCCCTACGGCAGAAGTATCGTGCAGAAAGTGTAAAAGTTCTGTTACAGGTTATGTTGGAAGATGAGCAGTTGAACTT
GTGCGTTTTGAAATTTCTTTGACTTTACTGACTTTGAGCTATTCAGTGATAAGGGCATAGCTCTGGCCGACTTCGTGCACAGGA
AGCTTCACTGAGCTGCCTAACAGTGAACAGACAGAAAAGCATTGAACCTCAAAGCCAAACTCCCTCTTGGATAGTTTTTTACAG
ACGGTTTCTTTCTAAATGAGCACCACGATTGGAGCGGTGTGTTTTGTAACGCCTCTTGAGATATAGAGTGCATCGAAGTTTCCA
CTGTGCGTGAGTCAGGGATTCGATGCTACAGTTTTTTGTGCGTCCAACAAACGTAGAGGTAAGATGTAATGGATTGAAGCACTG
CTCGCCGGGTTATCGATTAGGTCCAGTAGTACATGTGAATTTAAGAGTCGCTTTTATATGAGGGTATGTGTGGACTCCAGTTGG
TGGGTAAATCAGATATTTATTGGGATGAATGGTTAGTTTATAGGGGCGCGATGTATGGAGAGAGAGTGCTTACGTTGCAATAGC
AAAAGAGATCTTTTAGCACATATTTGGAGAAGGGTATTCATCTGCTGTGCCAGATGTAATTACTCGGTTTGTGTATGGTTGTGG
GTTCTTGGGATGTCCAGTTAACTCTCAAGACTGTTTAGATGCCTTTTGTGGCCATTAAGGAAGAGAAAGAGCTTAAGTCGATCA
AGGGTGTGAGTGTGTGAGTAATTAACTTCTAATTTTTATGAGCGGTGGGTTAGTTTGTCCAGGAATCAATAGTGTTGGTGAAAA
GAGCTTAGTTGCGCATGTATATTGGTTTTATCAATTATTCTCGTGTGGCATCTGTGTATAGAGGAGTAACAAGATGCTATATTC
GAGATTTATGGAATTTTAATTGTCTTAATGGTGGTATTTGTATCTAGCCTAGTTATGTCCTAGTGTAACTTTAGTGTCGGAAAT
TTAGGTGTGCTATTTTTGCGTTAGATTTTTTCATTCATATATCGCTCTGTCTCGTGTAAGAATAAACCTCGTATCTTTATATAT
TGGAGTGTGTGGTATTCCTAAGTTTTCTATGGACCTGGTTTAGGTGATGTCTGAGGAATAGTCTTTAGTTTGAAGAACTTAGGT
TTCGAATATTATATGTAGGGTTGTATTGGTGTTTGGGTTGGTGGATTTTTTGGGGGTTTTGCTGGTATGGTGTTGGTTTTTTGT
TTGTTTGGTGTGGAGGGTGTTGTTGTTGTCATATCTTGTGCTCTTTGAGAGAAACAACGATCAATCAAATTAAATTAACTTCTA
AGTTTTTATGGACCTTGGTTAATATCCAGTGAACTTATTTGAAAAACTTTAGTTAGCGTTAATTTCTATAAAAACATGTCGTGC
CTTTAAGAAAGACAACGATCTATTGATTTAGTTAATTTCTAATTTTTATGACCTGGTTATGTCCAGGAACTGTAGTTTGGATAA
ATTAGTTTCGCGTTATTATTTTTATAAATATTCTGTGCTCCTTTAAGAAAAACCAACGATCAATTGATTTAAATGAACCTTCTA
AAGTTTTTAGGACCTTGGTTATGTCCAGGAACATGTTTTGTAAAACTTATTCGCAATTAATTTTTATCATATATCATGTCTCTT
TAGAAAAAAACAACGATTCAATTGATTGTAATTAACTTCCTAATGTTTATGACTGGTTAATGTCCAGGTAACATAGTTTAATAAA
CTTACTTCGCGTTAATTTTTATCAATATCTTTGTGCTCTTTAAGAAAAACATACGATTCAATCATTTAAATTAACTTCGAAGTT
TTTTCATGAAACTTGGTTTATATCCCAGGTAACTTAATTTTGAAACTTAGTTCGCGTTATATTTTTATCAATCTCGTGCTCTTT
AAGAAAACAACGATCAAATTTGATTTAAATTGACTTCTAAGTTTTAAGGACTGGTTATGTCCAGGAATCATTAGTTTGAAAACA
TAGTTTCGCTATTTAATTTTTTATCAATATATCAATGCTTCTTTAAGAAAAAAACGATCAATTGATTAAATTAACTTCTAAGTT
TTATGACCTGTGTTATGTCAGGACAAAGTTTAAAAACCTTAGTCGCGTTAATATTTTATCAATATCTCGTGCTCTTTAAGAAAA
ACAAGATCTATGATTTAAATTAACTTTCTATTGTTTTATCACCTGGTTATTCCCCAGGAGACTTAGTTCGATAAACTTAGTTCG
CGTTAATTTTTTATCAATGTCTCGTGCCTCTTTAAGAAAAACCACGGATCTAATTGATTTAAATTGACATTCAAGTTATGACCC
TGGTTATGTTCGAGGAACTTAGTTTTGAAAACTAAGTTCGCATAAATTTTTATCAATATCTTGGTGCTCTTTAAAGAAAACAAC
GATCAATCAATTTAAATTAACTTCTAAGTTTTTATGACCCTGGTTTATATCCAGGAACTTAATTTGAAAACCTAGTTCGCGTTA
TATTTTTATCAACATCTCCGTGCTCTTTTAAGAAAGTACAACGATCTATTGATTTAAATTAATTTCTAATTTTATGACCTGGTT
AATGTCCAGTGACTTAGTTTGAAACAATTAGTTCGCGTTATATTTTTATCAGAATATCTCGCTGTTCTCTTAAAAAAACAATCG
ATCAATTGATTTAAATGACTTCTAAGTTTTAGGTACCTGGTTATGTCCAGGAATCATTGTTTGAAACTTATAGTTCGCATTAAT
TTTTTATCAATATAATCATGACTCGTTTAAGAAAAACACGATCAAAGTTGATTTTAAATTAACTTCTAAGTTTTATTGACTGGT
TATGTCCAGGAAGACAATGAGTTTAAAAACTTATTATGCATTAATTTTTATCAATATATCATGCTTTTAAGAAAAACAAATACG
ATCAATTGATTTAAAATTAACCTTCTATGTTTTGAAGACCTGGTTTATGTCCAGGAACATAGTTTTGAAAACTTATTTACGCAT
TATATTTTAATCAATATATCATCGCTCCTTTAAGAAAACAAACGATCAATACTTGATTTAAATTAACTTCTAAGTTTTAATGGA
CCTGGTTAATGTCCAGGAACATAGTTTGAAAACTTAGTGTCGCATTTAATTTTTTATCAATATAGTCATGCTCTTTTAGAAAAA

CAACGATCAAATTGATTAAATTAACTTCCTAAGTTTTATGACCTGGTTATGGTCCAGGAACATATTTTAAAAACCTTTAGTTTC
GCTGTTAATTTTTATCAATATCTTGTGCTCTTTAAGAAAAACATACGATCAATCAATTTAAAATTAAACTTCCGAAGTTTTTAT
GAACTGGTTATAATCCAGGGAAATTAATTTGAAATACTAGTTCGCGTTAATGTTTTATCAATGTCTCGTGCTCTTTAAGAAAAA
CAACGATCATTGATTTAAAATGACTTCTTAAGTTTTAGGACTCTGGTTATGATCCAGTGAACAATAGTTTGAAATACATAGTTC
TGCATTAATTTTTATCAATATATCATGCTCTTTAAGAAAAACAACGATCAATTTGATTTAAATTAACTTCTAAAGTTTTATGAT
TGGTTATGTCCAGTGTAACAAAGTTTAAAAACTTAGTTCGCGTTAATTTTTATCAATATCTCGTGCTCTTTAAGAAAAACAACG
TCTATCGATTAAATTAACTTTCTATGTTTTATCACCTGGTTTATCCCAGAACTTAGTGTCGAGAAACTTAGTTCGCGTTAATAT
TTTATCATGTCTTTCGTGCTCTTTAAGAAAAACCACGATTCAATTTGATTTAATTTGACTTCTAAGTTTTATGATCATAGAGGT
TATTGTCGAGGAATCTTATGTTATTGAAAACTTAGTTCGCATATATTTTTATCAATATCTGTGGCTCTTTAAGAAAAACAACGA
TCAATCAAATTTAAATTAACTTCTAAGATTTATGACCTGGTTATATCCAGGAACTTAATTTGAAAACCTAGTTCGCGTTATAT
TTTTTATCAACACCTCATCGTGCTCTTTAAGAAAGACATACGATCTATTGATTTAAATTAATTTTAATTTTATGACCATGGTTA
TGTTCCAGAGACTTGTTGTGAAAAATTAAGTTCGCGTTAATTTTTTATCAATATCTCGTGCCTCCTTTAAGAAAAACTAACGAT
CATTTGATTTAAATGAACTTCTAAGTTTTAGGACCTTGGTTATGTCCCAGGAACATTGTTTGAAAACTTTAGTTCGCATTAATT
TTTATCATATTATTCCAATGCTCTTTAAGAAAAAAACAAAGATCAATTATTTAAATTTAACTTCTAAGTTTTTATGATCTGGTTA
ATGTCCCAGGAAAATATGTTTAAAAAACATTTAGTTCTGCGTTTAATTTTTATCAATTTCGTGCCTTTAAGAAAAACAACGATC
AATCGATTTAAATTAACCTTCTATGTTTTTAGGCCTGGTTATGTCCAGGAACATAGTTTGAATAACTTAGTTCGCATTAATTTT
TATAATATATCATGCTTCTTTAAGAAAAACAACCGATCAACTTGATTTATTAACTTCAAGTTTTATGAACTGGTTATGTACAGG
AACATAAGTTTAAAACTTTAGTTTCATGCGTTAATTTTTATCAATATCTTGTGCTCCTTTAAGAATAAACAACGATCAACTCCA
ATTCTATAATTTAACTTCTAAAGTTTTATGAAACTGGTTATATCCAGGAACTTTATTGAAAACTTAGTTTTCTGCCGTTAATTT
TTATCATGTCTCGTCTCTTTAAGAAAAACAACGATCATTGATTTAAATTTAAACTTCTAAGTTTTAGGACTGGTTTATGTAAGG
AACATAGTTTGAAAACATAGTTCGCATTAATTTTTTAATCAATATATCTATAGCTCTTTAAGAATAGAACAACGTCCATCTTTA
ATTTATATTTAAACTTCTAGTTTTATGACCTGGTTATTGTCCAGGAACATTAGTTTAAAAACTTAGTTCGCGTTTAAATTTTTA
TCAATACTTCTGTGCTCTTTAAGAAATCATCAACGAATTCAATGGATTTAAATTAACTTCTAAGTTTTTATGACCTTGGTTATA
TCTCATGGAACTTAGTTTTGAAAACTTAGTTCGCTGTTATATTTTTATCAATTATCTCCGTGCTCTTTAAGAAAAAAACAACGAT
CAATTGATTAATATGAATTCCTAATTTTTATGATTTGGTTATGTCCACTGAACTTAGTTTGAAAACTTAGTTCTGCGGTTTAAT
TTTTTATCAATCTCTCGTGCTCTTTTAAGAAAGACAAACGATCAATTGATTTAAAATTAATTTCTAATTTTATGGACCTGGTTA
TTGTCCAGGAAGTTAGTTTGAAACTTAGTTCGTCGTTAAATTTTTATCTAATATATCGTGCTCTTAAGAAAAACAAGATCCAAT
TGATTTAAATGAACTTCTTAAGTGTTATGGACTGGTTATGTCCAGGAACATAGTTTAAAACTTTAGTTTTCGCATTAAATTTTT
TATCAATATATCATCTAGCTTTTAAGTAAAAACAACGATCAATTGATTTAAATTAACTTCTAAGTTTTAGGACCTGGTTATGTC
AGGAACATAGTTGAAAACTAGTTTCACATTAATGTTTTATCAATATCATGCTCTTTAAGAAAACAACGATCAAATGATTTAAAT
TAACTTCTAAGTTTTTGATGACCTGGTTTATGTCCAGGTACATAGTTTAAAAACTTATTCGCGTTAATTTTTATCCAATATCTC
GTGTGCTCTTTAGAAAATACAACGATCAATTCAGATTTTAAATTAACTTCTAAGATTTTATGACCTGTGTTATATACCAGTGAA
CTTAGTTTTGAAATACTTAGTTCGCATTAATTCTTTTACAATATATATCATGGCACCTTTAAAGAAAAACAACGATCAATTGAT
TTAATTAACTTCTAAGTTTTATTGACCTGAGTTATGTTCCAGGAACATAGATTAAAAACGTTAGTTTCGCGTTAATTTTTTATC
AATATCTCGTGCTCTTTTATAAGAAAAACAAAACGATCCATATTGATTTATATATGATTCTAAGTTTTAGGACCTGGTTTATGT
CCAGGAACATAGTTTGAAAAACTTAGTTCTGGCCATTAATTTTGTTAAATCAATATTATCTATGCTATTTAAGAAAAAAACTAAT
CGATCAATTTGAATTTTAAATTTAACTTCTAAGTAATTATTGACCTGGTTATGTCCAGGAACATAGTTTCTAAAAACTTAGTCG
CGTTAATTTTTTCTATATCTCGTGCTCTTTATGAAAACAACGATCAAGTTGATTTAAATGACGTCTAAGTTTTTAGGACCTGGT
TTTGTCCGGGAATATATGTATTGAAACTTAGTTCTCATTTAATTTTATCAATATGATCATGCTCTTAAAGAAAAAAAAACGATCC
AATTGTATTTAAATTATTTAAGTTTTATGACCTGGTTATGTCCTAGGAAAATAGTGTTAAAAACTTAGTTCGCTGTTAATTTTT
ATCAATATCTCGTGCTCTTTAATGAAAAACGAACGGATCCAATTTGATTTAAATGAAACTTCTAAGTTTTATGGACCTGGTTAC
CTGTCCAGGAACATAAGTTTGAAAACTTAGTTCGCATTTAATTTTTAATCAATATATGGGAGGTTGAGTGGCTGGAATGGTGCT
TTTTCTTGGTAAGTTAAAACAGCATACGCGGAGTCTTAATAGTGTGTGATTGTTTAAGCATGTGCTAAGTGGGTGTTTTAGTGA
TCCGGTTTGCGTTATTTGTACTAGGAACATAGTGTGTTGAATATAAACTTAAGATTTCGCGGTTAAATATTGTTTTGAGTGTTT
TTTATTTTCTTGTGATGTGTATTTTGTTTTTTTAGTTTAATCAATGTATCTTCGGTGCTCTTAACGAAAAACTAAGCGATCAGG
GGTTCGTTTGTTGTCCTGATTGTAATTAATGCTTTCGTGTAGAGGGTTTTATGTACAGTGTGTGGTTGTATCATCTTCACAGGT
TGATGAAACTTTGTTGAAGGTAATTAGAAAATTTATGATGGTCGCATGTTGGTGTGTTTATGTTTTGGTATCGAGAATTGTATC
GATTGGCGTGGTTTGTCGCGTTATTAAGTAAAGAACAGTTACGGTTCAAGAGTTCTTTTATGCTGTTTGGATTTGGTTATGGTG
TTGTTTTTTGATTTATATTATGGGGGTAGGTGTTGATTGTTGTGTCTGATTTTAAACTAACTTAGTGCATGATATTTTGGGACG
TGTAGTTTGGGTTTAGGTTATGTCCAGTTAGTAGTGGTGTACACATTTTAGTTTTATAGTGAGGGGTGGGGAATAACTTGTGAG
TGTGCGCAGTTAATGGATGTGTGTTTTAAGTCAATGATCGGAATATGTTGTCATTGCTCTTTAAAAATAACGAAACCGATACAT
GGTGATGTGATTATTATATATTGTTTAAATCTTCTATTGTTTTTAGGACCTGGTTATGGATATGTTCCGTGAGTGTTGAGAACA
ATAAGTGTGTAGGAAAGGAGGGAGCGTGTGAGTGCGTGTGTGAATTTTTTTCGTTAATTTTCATGCTCTTATTGTAATAGAAAG
ACAACGATGCAAAGTAGTGATTTAGTATTTTAGAGAGCTTTCGTGAAGTTTTTTTAGTTGACCGTTATGTTGTTGGTATGTTGG
TGGTTGGTTTTTAGGTTAGTTTGTATAATGTCATGAGAACGAATAGTGAAAATCTTAGTTCCGTGGGCAATGGTTTTTATATAG
TATATATAGTCGAATATATGGGACTGTTTGGGATGGATTGCGGTCTTTAGATGTAATATACATTTGGGTTGTTAGATTATCAGT
TGTTAGATTTAATATTTTGTGTGCTTACTATAAGGTTGATTATGACCTTGGTATTGTCCTTAGTGGTGGGTGGAAGCGAGATAG
GGTGATTTAATATGAGATCGTTAGGTCTTAGCTGTATATATGATTGGTATAGTCATTTATGCATTGTGCATTCTTTAAGATAAA

GACAGACCGATCTAATGTTTTTGTCTGGTATGTTTGGTTTCAAGTTTTGAAATTTGTGTTTAACTTCTAGTAGGTTTTATGAAG
GCGTGAGTTTATTTTTGGGTTATTTCTATGTTGGGAACTGTTAATTTGAAAGATACTTTTAGGATCTTTAGTTTGCTGTTTGAA
TGTGCTTGGGGTGTTTTCAATATGTCATCCGGTGCTTCTGTTGGTAATGAATAACTATTACAGATCCATGTTTATGTGGAGGAA
TGTTGCGAGCTTATACGTAAGTTTTAGAGTACACTTGTATATATATGATCGCGAGGGAACGTGATTGAGATGTTGATGTAGGAT
AAGAGTCTGTGAGATGTTGTGTTAATTGAATGTTGGTATGTAGATGAGGGGTATTGTTGGTTTAGGGTATGGTGTATTTTATTA
GGAGTATATGTGTGCGAGTTGGTGTTGTGTTATGATAGTGGAGGGGTATGTAGGAGGGTATTTAATTATGGTGTTGAGGTGTTT
GGGAGAATGAGATAGTGGTATGTAAGAGATGAAAGTGATAGTTTATAAAAGATAACGTAAGGGAGGTTTAACGAATTAAAAAAA
TGAAAGATTGGAATTTGTTGGATGGGGGATGAATTGATGAAAAGATAATAAAAGAAGGGGAGGAGTAAAATTTTGTTGTTGTTA
GAATGGAATAATTAATGGAATAAAGAAAGATAAATGAATAGAGAAGTTAGATTTTGTAGTAGATATAATATGTTGATTTGGAAT
AAAGATGAATAGTATAGAATAATGGGATAAATAGTGATATGTTTATTATTTTGTAGGGAATGGATAAGAAGAGTTTTAATAGGT
ACAAAAAAATCGATTAATGGAATATTATAAAAAATGGGGAGAGAAATGGTAGAGTGAATTAGTTGGGGGGTGTGATAAGAGGAAT
GTATTGTGATACATGATTAGTAAAAAGTTGGTGAAATATATATGTTGATTTGTAGTAAAATTATGAGATATTTATTGTAGGAAA
AATAAATATGTATTATTTAGATTAAGATTTATATGATGTAATGTGGTGAGAGGTTATTTTTAGTATTTTGGTGAAGTAGTATGG
TAAAAAAAATTGGAATAATATTATTGTAGGTACGATTTAGATAAAGTAGTAAAAATAAATAGGAAAAGGGATTTTGAATTTTGT
GTTAATTAGTTAGGTGAAGAAGGGGTATATTGGGAAAATATAGATTCTGGATATTATGATAATAGAGTATTATAGTAAGAAAGA
CTTGTGGTAGGGTGTAACCTCGCTTTTACGGTGACACCAGACGCGCGTCAACCAACAGTCGCGATACACAAAGATCAATAGATA
TATTATGAATGATAATATGTATAAAATTAATTGATTTGTTATGATAGTTGGGTATATAATTGGTTGGGTAGTTAATAATAGGGG
TATAATGTATTAATAATAAAAGGTAGTATAATGATAGGTTATGTATTTTATGGACAAGAATTAATGGGAAGAAATTTTAGGGTT
GAATATTGCATGGAGATTGGATAAGGATGATTGTGATTATTAATATATAAGGATTGTATATGGGTATTTATTGATGTGAAAAGT
CATATAAATGTTAAGGTATTGAAAATACTAAGTAATTGGTTGATACTAAAAAGTTGGTAAATAGTGGGAATGTTGAATCTATTT
AATGTGTTGATGTAATATGAGATAGTGTATTGACTACACCTAATGCTCGTAATATAGAATATTGTTTAAGTTTGAAAAAATATG
AGAAGAGAATGGATTAGATCGATTATATGGGTGATAAGTATGAATAATTGATGAGTGAGTTGTGTATTGGACATGTAAATAATA
TATAGCACAGATAAATAAGGAGTATGTATATTTTATTATAGGTAATAATTTAATAATTGGTAGGAGTATTAAGGTTGGTGATAG
AAATGAGTTGAAAGTTGTAATTTTATAATAGTAATAGAATGATAAGTGATATAAAAATATATAGTTAAATTGCCATTTGAAGTA
TATATAAGAGGATATTGGTTGTATGAGAATGTATAAGAGTTGATAGGTTAGTGATTAGATTAGAGTGAGATTGGAATATGGAAG
AAAATTTTGAGAGGGATTATAAAAAGTTAGGATAGATTTTAGCAGGGGGTGAATAGATTAATATAATAGATTTTTACTGATATT
AGTATTATGGGTGTTGTATAATGTTTATAATTTTAAAAAGTAGAATGATTGGAATGTAGATGATTGAATAGATGAATAGTAATT
AATTATTGATTAAATAGGAGGGAGTTGGAAGAGGTGGTATTAGGGATTGGAGATTTATAAATTATAGTGTAATTGGTATATTGA
TGAATAAGTAAGTTAAGAAGGGAAAAATTGTTAGTAATTTTGATATGAATATAATGAGGGTATGATAATATAAGTTAAGATTTG
CAATTTGATTTGTATGGTAATTATGTATAATGATTTGATTTTATTGGGTTAGATAGATTAATGTGATTGATTTATTTTAAAAAA
ATTAAGAAAATAAAGATTTGAGAAGTTTATTATGATATTATTTATAGTATTTGAAATTATAAAAGTTTGAAAATGGATACAGAT
TATTTGATTGACAAAAATGAATAGAAGTATAATAATATTTTTGTAGATAAAGTATTGGAATGAATTATAAAAGTTGGTAGGTGG
TATGAATTATTAAAGAAAATGGAATATTATAAGTAATAATAACTTTTATATTAGGAAGTTGGTAGTTAGTAGTAAGGAGGGTGA
TATTAGTTGAATTTATAAAGAATAGTATACGATGAGAGTATATAATTTGTAGTTGAATGAATAGTTGGTATTATGGGTAGTGGG
AAGGGCATTATTTGTGAAGGTTATTGTTAAGTATTGTGAAAGTAGATGATGTTTAAAATTAAGTTTGGATTTGGGAAAGAGGGG
TTTAGATACAATATGGGGTGTAAGTATTTATATAACCGTTTGTGATTGAGAAATAGAAATTAAATAATAAGGTTGTGATAGATA
TTTAAGAAAGGAAGTATATAATTTTTTTGTGATGTGGTAGTTATTGTATAGAATTGAGGAAATGAATTATAATAAAGATGGAGA
ATTTAAATGTATATGTGAGGATGAATAGAAGTACTGGTTTTTATTAAATAAAAATGTGCATTTATTTAATTTAGTATTATTGAA
TTTGATTATAGTTAGGTAGATATAATATAGTGGAATTAGAATGTAATTAAATTATGAAGTATATAAAGTGAGGATAGTTTAGTA
AATATATTTGGAAATTGTATAATAAATATTTTATAAAGAGAGTAAACAGTAGAGTGTTTATTTTAATGTAAAATACATGTTGAA
AATAAATAGGTTATATTATTATTAGAAAAGTTTTATATGTTGTTTAACGGAATGGTATTTATTTGGAGATTGAAAATGATGAAT
TATAGATTAATTGTGATTTAGTTATAAGACTATCATATAATGAGATTATGGGTGAATTAAGATATGTAGATAGTGATATGTGAT
GTAAAGATGAGAGAGGGGTATTTAATGTAGATAAATTGTAGAGTACAATGATAATAAAAAAGGAATATGTAATTAATGAGTATA
GGATGGGGGATTAGAATTTAATGGGTATATGAAGTTATGATAGTTTAGGATTATAGTGAAAATAATTTGGGAAAGGTGTTAGGG
TAATTGAAGGTTGAGATTATATTATAGAGAATGATTTGTAGAGATTTATATGGATTAGTATTGAAAATGAATCTATAATTGAGA
ATAATGGAATTTGATGGTAAGGAAAGAGGGTAATTTGTGGGGTTAATATAGTGGATGGAAAGGTAGATAGAATTTGTAAGTGGG
AGTTTATTTAATGTTTGATTTTTGGAGAATGATATAAGATGATGGAATGGAATACATAGATATAATGGATAGGGTATTGATAA
TAGTTACGAGAATTGTTAGTATTAATTGATATTGTGGGGAATAAAATATGTCATATAGTGGATGTATAAGAGTTGAATATTAAG
TGGTTGAGATAGTATGATTAGAATTATGGATTTATGATTGGGGTTTTGGTATTGATAATATACATAAGAGTTTATGATGAGGAA
GAAAATAATATATAATAATTAATTGTTTGAATAGTTGTGTAGTGCAATATGATTATGGAGAAATTATTATTATATATTTATATG
AAAGAAGGGGGTGGTGGTTTGATATGGTATGATACTAGTACAGTTGCTAGTAAATTAGACTTGATGTGTGAGGATGGTATAATT
GGTGAGAATTGATGGTAGGGTGATATTAGGATGTTTGTAGAGGGTGATGAATTATAATTTATTTTTAAGATGTAGAATGAGGTA
TTCATTATTATTAAGTTAAAATGA
>m141122_040740_42134_c100732202550000001823141405141561_s1_p0/42794/10420_19829
GTATTGGTATGGTAGTGGTAGGTAGTGTAGTGTAGTGGAAGTGTAGTGGTAGTGGTAGTGGTAGTGTAGTGGAAGTTATCGTAT
AGTCGTAGTAGTAGGTATGTGTTTAGCGTAGTATAGGGTGAAGTTAGTAGTAGTTGAGTAGTAGTTTGGTTTGATGGTTGTATG
TGTTTAGTTACGTTGGTAATGGTTTGTTTTCGGAGTAAGTTTTATTTTTTTGTTTTTTTTGTGTTGTGTTTTGTATATTTTTTG
TTTTTTTTTTTTTTGTGGTTGTTGTTTTTTTTGTGTTGTGTTTTGGAGGTATATATATGAGAGGTTGGATGGGTGGTTTGTAGT

GTGTAAGTGTTGTGTTTTGTGGTTGGGTTGAGGTTGTATTTTTTGTGTTGTTGGGGGGTTGCTTGTTGGTTGAGTTGGAGGGTT
GGGTGTGGGATGTGTCGTGTAGTGTTGATTGGTGTGTAGAATGTAGTTTATTGCAGTTGGGGGTAGTGGGTTATGTTTGGATAT
ATGTTTAGGTTTGGATTGGTTGAGTGGGTTAGTGGGTAAGTGTTAATGGGGTGGAAGTGGGTAGTGGTGGTAGTTAGATGGGGT
TGAATGGTATGGTTGGTTCGTTTGGTGGTGTAGTAGGGTAGTGAGCGGTAGTTATGGGTAGTAGTAAGGGTGGATAGTGTTTTG
TATTTTTTGTGATATGTATAGGATGTATAGTGGTATTGTGTTGTTTATGTTTTAGTGTTGAGTTGTAGTGGTAAGTATTGTAAG
TTTGTTTTGTATATAGAAGAGTGTATTAGTAGTGTAGTTGTGAGTGGTAGTGGTATTGTTGTATTTGTGGTATTTTTGTTAGTG
TTATTAGTTTGGTGTTATTGATTATGTTAGTTGGTATGTGGTAGTTGGAGTGTGTTATAGGTAGTGGTAGTAGTGGTGTAGTAT
TTTGGTGATGTTGTGTTAGTTTGTGTAGGGTAGGTGAGTGTGGTATGGTGGTGTAGTGGGATTGTGGTGGTATGTGGTAGTGGT
GTTTGTGTATTATGTGATAGTGGTAGAAGTATTATATGAAAAACATACAGTATGGTAGTATGTAGTATGGATATGGTATAGTAG
TCTGGTGTAAGTATTAGTATGTGTGTAGTGTGATGGTATGAATGTAGTGGTGGTAGGTGTATAGTCGTAGTAGTCGTAGTAGTA
TGTGATAGTAGTAATAGTAGGAGAGGTATAGTGGTGAAGGTGGTGTAAGTTGGTTAAGTTGGTAGTGGTATGGTAGTAGTGTAG
TGATGGGTAGTTATTTGGTTATTGTGTTATTGCTGGTGTTTGACTGGTATCGTGTTAGGTTGGTAGGTGGTAGTGGGGTATGTG
TGAGTAGGGTGGTGTAGTTGGAGTTGTATGGTAGTGGTTGGTGTGTGTAGTAGTGGTGTGGAGTGGTAGTAGTCTTGGAGTAGG
TTATGGAGTATGGAGTATGTAGTTAGTATAGTAGTGAGTATAGTATTATAAGTATATGGTAATGTGAGGTTATGTGGAGTAGTA
GGTTGTAGTGGTATGGGTATAGTATGATGGTGTAGTTATTTGGTGAGTCTATGGTATTTTGTATGTTGTTGTGATAGTGGTATT
TCGTGTTATATAGTTATGTATTTTGTGTATAGAAGTAGGTTTATTTTGTATGGTTAGTGGTAGTTGGTAGTTGAGGGTGGTGGG
GTAGTGGTAGTGGTAGTGGTAATTGGTTGGTCGGTAGTGGTAGTGGTGGTTAGTTGTGTGGTGGTAGTGGATTGTGGGTTGTGG
TCCGTGTGTTGGTGGTATGTGGATTAGGTCGTAGTGGTGTAGGATAGTGAGCTATTAGGGTTTAGGGTATAGTGGTAGAGTAGT
GGTTTGTGGTAAGGTGGTAGTAGGTATTGGTAGTGGAAGAATGGTTGATGTTGGTAGTAGTGTGAAGAGTTTAGTTGGTATGAT
GTATGTGTAGTAGGTGTTATTTTTGTTGTTTGGTTGGTGGTAGTGGTAAGTTGGTGGTGTTTGGTAGTTATAGTTAGTGTTATG
TTAGTTGGTGTTAGTAGTGGTAGTGTATGTATGTGAGGCTTGTGATAGTGTATTGTTATGGTTGTGGTGGTGGTAGTGGTGGAG
TTGTTGGTGGTGGGTTAGGTGGATTTTTGTGATTGTGGTGTGGTATGGTAGTCGTTAGTAGTTTTTTTAGGGTTTTATTTTTGT
TATTTATAAGGTGAGTATGGGTTAGTGGTTAGTAGCGTAGTTTGACGGGTATTATTTGTGGTATTTGTAGTAAGTAATAAGTAG
GTTATTGTAGTGGTAGTAGGTAGTTAGGAGTATGATAGGTATGTGGTAGTGGTAGTGGTAGGATTTGGTAGTGGTGTGGTAGTG
TCGGGTAGTAGTGTGGAGTGTATGTAGTAAGTCGTTTATTAGTATATGTAGTGGTAGTTGTTAATGTAGTTAGGTAGTGAGTTA
GGTATTAGTAGTGGTGTGGTATGTTGGGTTTGTTGGGTAGTCGTTGGTAGTGGTATTTGTGTTGGTAGTTGTGTTGTTATTTGT
TAGTTGGTGGTAAGTTGGTAATTGTTGGTAGTTGTAGTGTGGTATTGTTGGGTTGTGTTATAGTTGGTTGTATGTGGGTTGTGA
GTGGTTGTAGTTGTAGTTGATTTTGGTATGGTGTGTGTGGATGGTAGTTGTTGTGGTGTGGTGGGTGGTAGTGGTAGTGGTTGA
TGTTGTATGTTGTGTAGTTTTTTAGTTGTCTCTTCTTCAATCGAACAGAAGTGTGATTCGGAGTGAGGTGAAAGAAGAGATACT
TGATCATACCACTATACCTACTACCATTAACCTACGTTTGGTTGTTTGTTTGTTTTTTGTTTTTGTTTGTGTCGATGGTTGCTT
TACCACTTACTCTACTTTTTACGACGATATATGACTCATACCTTGATGACCACTACTACACTACAACCATACTTACCGACCACT
TACAGACACTAAACCACCACTACTTACCACCACTACCACCGTACCAATACCACTACACCACTACCACTAACACTACCACTACCA
CTAACCTCTACCACTACGGACGCTACCACTACCACTACCACTACCGACTACTATTAGTCTACTACCACTGTTAGATTATCTTCT
AGTAACTTTATCTTGATTACGACTTCTACCGTACGTACTTGACCACATACTATCTACTTACCTAATGTCAGTTACCACTTGATA
CCTATCTACTCATACGTTTTTTACCATATGTACCACGTACTACTTACTTACTACTATACCAAACTACCTACTACTCATCTTGTT
TTTACTGTACTACTACTTACTACTACTACGCCATATATTACTTATAGCTTCTACTAGCCTGCTACTGTACCTGACTATAACCAC
TTGTATACTTTGATACTATTTTTCTATATATATGTTACTTACTACTACTACTTAACGACTATACTTTTTGTACTCGTCTTATAT
CTTTCTACCACCACCACCTGCTTTACCACCACTACCTGTTTGTTGATCTTGATTCGTATTTACCTTACTTTACCACGGCACTGA
CTAGTCTAACCGTCACTACTATACCTACTATCTTTGGACTAACCACTACTAACCATCGTTTACCATCTGTTACCACTATTCTTA
CTTACCACTTACCAGCTGATCTTACTACTGACTTGCGACTTAGTTCGGTGACCACGCGGGTTGGACCGTTTGTTTACTCATTAT
CCTACCGACGGGTACGGGCGGAGCGACTTATTTGGCTGTTTACCGGTACTTACCACTTACTACCTATTGTACTTATTTGTGAGA
GCTCTACTACTGTCTATCCACTTTGGGTATACCATGACTACTTACCACTCTTTTCCATCTACCGTAAGTACCCACTACCTGTAT
GATGTTTCTTTTTTGGTTTTATTCTACAGTTTTTTTGGTGTGTACTCACTTTCTATAATATAGCGGACTACTTAATCTCTGTT
TGAGATACTATACGATTGTTATTGGTCTTTATATTCTGTTTTTTGGACTACTGATCGCATACTTGATTGTATATATATACTTGG
TTTATACTTCTGGCTTACTTACCTCTTATACCTACCATACTTTTGACAATACGACCACGCACGTATGTCTTTGCTTCTTACCAC
TACCGATTTTTGATTTGATGTTTGGATCCTGTGGTTACCTCGTACTTTAGCGTTGGTTGTTTTGTCTTTGGTTGTTAGTACCTA
TACTTGTACTTCTGTACGCCTTTTTACCAGTTGTTTTTATTTGGAGTTGGGTTAGCTTGTTGGATATACGCAACTTTTTAGAGT
CTGACTTTGGAGCCTATCGGTTTGTTTTACCACGGGTTATACCGATCTACTATTGAACCTAGAGACGGTTACACTGCTACCACG
CGTACTATTTGTACTCTAGCTGGATCCTGTAACTTACTGGTTTTCGTGTAGACCTCCTTAGCTTTCTGGTGGTTGGTCTTCGCT
TACTGGTTGATGGTTCGTTTGGGTTGTTGTGTTACGACTACGAGCGAGACCACTTGTACCACTTACCGGGACTACCTTACGACT
AACCATTTATAGTATTTGTACTCATACTACGTACGCACTACTTTACTTTGACCACTACCTGAGAACTTTTTGACTCTGACGTAT
CGTATTGACTTACTATCTTAGCTAATACTGTACTACTACTTTGATCTACTTTTACTACTACTACTACTTACTTTAATCTTACTT
ACGGACACGACGTATACTATATAGCGTACTGACTTATTGACCATCTTACGTTTGGTTTTGTGGAGATTGGTTCTAGTTATATGC
TAGCCAATATAGCTTTTACTACTAGACCTTGCATCTCTGGTTTAACCACTTTACTCTTCTCGCTTTTTTTGTTGGGTTAGCCAT
TCGGAGAGTTAACGTTCACTTGTTGCTTGTATATCCGTGAGTTGTTACCACATGGTTGTGTGGTTGATTGGGGTGTTGTTTTTT
GATGTTGTTGTATTGTGTTTAGGGTGCGTTTTTTGTTTTGGGTTTTGGTTTTTGTGGGTTTGGGGTGTTTTGGGTTAGGTTGTG
GGGGGTATGGGGTTGTCTTTTTTGTTTTTTGTGGTTTCTGTTTTTATTGGATTTTGTTGTTGTTGTTATTTTTGTTTGTTTGTT
GGGATGTGGTTGGGCTTTCTTTTGTTTGGGATAATTTGTATATTTTTTTGATTTTGTTTTTTGATGGTGTTTTTTGGGTTGGTG

GGGTTGATTGTTCTTGTTGGTGGTTTGTTTTTGGTGGGTGGGGTATATTTATGTTATTATGGTTATTTGGGTTTGTTTGTTT
TGGGTTTGTTGTTTATTTCTTATAGTTTTGTGTTCATGGTGTGTGTGTGGGTGTAGTTGTTGTGCGTTGCTGTTTTGGATTTGT
TTTGCTGGGGAATTGGTGGTTGTTTTTGTTTTGTGGTGCTGTTTTTTCTGTTGGGTTTTTGGCTTGTTGGGTTTGGTGTGTGTG
TTTTGACTGACATGACCATTTAGTGGTTGACTGACTACCAGTAACCCTTTTTTACCACCTTTACCACTGGTACTTACCGTACTA
TACTTACCCTCTATTACCTATCTCTTATTCCACTATCCGACTTACTCCATTACCCTTGTTCTTCTTGTGATTGGCTACCTTTAG
CGTTACTACTACGTACTACTACACTATTTACGGGTTACTGAGTACTATTATAACTTGACTTACGGTTATATACTTTTATTTTTT
TATCGACTTTACTTACCTTTTAGTTTTTACCACCACTGATTTTATCACTTATACTACCTATTATACCTAGCTGTTACCAACTTT
TATACTATACAGTCTTACTATTACTGACTACTATATCGTGTTGGACTGCTATATAACACTGATACTTACCTATCTATACTACTA
ACTTATCTACCTATCCTTGTACTTTGTTTATCTATACTACTTTTATCTTAACTTTTTATTTACTATATACATACTACGCACTTT
TATATATATACTTGGATGATCTACAACCACCACCACTACTCAGTACGCATCTCTTTTTATGCTTGTATTGCTTGTTTTTTTTTT
TTTATACCACTTTATTACCTACTTACCATACCAGACTCACTTACATACTCACGTTACACTTACCACTTTACCACTTATCCACTT
ACCAGATACTTACCCACTACTAGCTACTACTTTACTCACTTTACACTGTTGTTTTTTGGGGTGTTTGGGTTTAATACCAGCGTA
CCTGAGACTTTATCCGTTATGTATTTCATTTACTCGTACTCATCTTAATTAGAACGTACCTAGACGTACCACGATTTTTGCTTG
TTTTACTTTATCCTTAGAATTGGATATCCGTTTACTCGTTTACTACCGACTATTGAAGGTACGGCACTACTTCTATCCGGGAAT
GACTTCCACTACTTGGTCCACTGTTACCACGTGTGACCGGAACCGGTATACCACCTGTATCCACTTGGACCTTGTGACTTCTAC
TTCCCTACTTGACTGCGCTTATCTGGGAACTTACAGGTACGTCCACATCGTATACCTGCTACTTAATCTGGGAGGCGTTTATCG
GCTTTTGGACCTTTACTTTTCCGTGAGTTTTTTTTTGGTGTGGGTGATTTTGTGTTGGGTTTGATTTTTTTTGTTTTTATACTG
TACCACCACCACTACCACCCTCTCCGCCGTCTCTGACCCCCCACTTACTTATACCACTCTACTTTAGCCCTTTTCCTATCTGAG
TCATTACCACTTACCTTACTACTCACCTTTTTTTTTATATACCGTTTTTTTTATCTTTCCATTCTTTGGTGTCCCACCCATGTT
GTGGTTTACCACATACCGACTACCTCTACGCGTGAATTTGCTTTGCCTTACTAGTTGGCTTGTCTACCTATTGGCGTTTGGTTC
CTACTACCTACTAGACTATACACGTTTATATATACCTTTCTCTTTTATTTATACCACTTATACCATCTTCTTTTATATCCATCT
TTTGTTATACCCTGTTTACATTTTTATACCTTACCTGACCACCTTGTATTTACCATACTTTTTACTCTTATTTACTTGTGGTTT
TGGGGGACTTGATTGGGGTTGACACTACGGTGACGCGGTTTGACTACTCGTTTACTGAGAGACTTTACTTATTCTACTATATTA
CTACTACTATTTGACTTTTATACTTACGTACTTTTACTATCTTAATCTTAATAGACTATAGCTATATACGACTACTTTTTTTCT
CACTTATTACTTACTACTTTCTTATCTACCTTTATACTACCTTTTTTTTGGTAACTTTACCTGGTTACTCGTTTCCTACTTGTA
CCTGTTACTATGCTACTCTTACCTAGCATTACCACTGATGCATGTGAGCTCACATTTCTTAAATTACTGACACGTTCTACTTTA
CCCACGTATTTACCACTTACTCATCTTACCACGTTTCACTTTTTTGACGATATTACTTTTTACCTATACTTATCTTTTTTGATC
TTTTACCTATATCCGTTACTCTTTTACCACTTATTCGCTTTTGGGTGACGTGACCGGGCACTGTGTGTTACCGCTCTTGACCTC
TCTCTTTGTTTTTTAGGTGTTCCCCTGACTCGCACACTGAGTAGACCACTTACTTTAGGCTGTGGGACTCTTGGATTATATCTT
GGGTAGCTTTTGCTGTGACGTCACTTTTTGTGAGCCCACTGTTATACCTTTGACTTTATGACCACTAACCGGATCTTACACTTA
CTTACTACTGATTATTTACTACTTACTACTACTTACGATACCTTACCTCTACTACTACGACTATGTTACCACTCTTTTACCACT
ACCATACCTCTACCTCAACTGACCGTACTACCACTTTTTACCACTTTTGTTGTGAGCTATACTTGACTAATTTACTTACGTTTA
CTACCCATGTACTCTATCTTACTTACTATACTAGCGACTTAGATTGCTACCAGACTCCGATCTACTTTTGCCGACTCACACTGT
TTGATTTTTGCACTCTTTGTTACCACCTACTGACCACTACGGGTTAGCTTACCTACTTTTTTTACCTACCACCGACCACTACCA
TCGTACCTTTGTTTGTGTTAGATACGACTACAACTGTCGGCTACTGTTACCACTGACCTGTTTGTCTATTTTATCAATTTACCA
CTACCTTTACTTAGCACTCCTACCATATGTTTGTGTTTTTGTGCTTTTTTTTGTGCCACTTGACCTACTTACCACTGTTTTACT
CTACCTTATTGACCAACTTACTCTTACTAGTGACCACTACTCTACTTATAACATGTGCTACTTTGGTGAGCCTACTTTGGCCTA
GACATACACCTGGACACAACTTTATTCTTGGTTTAATCCACCTATATCGACCTACTTACCGGTTTACTACGCGGACTTAGCCTA
TTGTTACTTCTACTGCAACTATTTTTTGTGTGGGTTGGTTTTGGTGGTTTACTCTACTTTTTAATCCTGTTTATCATGCCTAAC
TCACTACCGGGAACGTGTTTACCCTTTGGAGTTTTTACCACGGTTAACCTACTTTACCTTTACTACTACTTTACTACCACTATC
TTTATCGGGACCTACTTACTCACTCTCAGACGACGTCTACGTTTTACCTACTTTATCTTGTTTGTGATGTTTACGTTTGTACTC
TGTGACTGTTATCTTACTACGCGGACTTACCACGTATCCACTAACGAGCTGTTACCACGTAGCTGACCACTACCGTTACCTTAC
TCACTGTTAAACCTATGTGAGCGCGTGGATACGGGTGATCCTTATACTTGTTTTAGACTGACACCACTGTTACCGGGACGTACT
TTGATATCTGTAGATGCTGCTTTGCGTGCGAAGGGCTCACAACAAAGTGAGGTTATGGGTGGAGGGATATAAAGTGATAGGATT
GGGTTAGGTTGGAAGTGGGTAGTGGGAGTAATGGTAGTTTGTGAGGTGTTGGTAGTGGTAGTGTTGGTAGTTGGTAGGTTGGTT
AGTGTGTAGTGTTATGTTGGGTGGATTTAGGGTATGGTATGGTATGGTTGTTATAAGTTTGTGTTGAGTGTGTGGTAGTGGTAG
GGTAGTGGGTGAGTTTATTGGGGGTAGTATAAGTTGGGTGTTGATAGGGTATGTATGGGTGAGTGGGTAGTGGGTGTGTAGTTG
GGTAGGGGGGCTGTGGTTATGTGGTTATGTTGGTTGTGTGGTAGTGTTGTAGTGTGGTAGTTGGTAGTGGTTGGTAGTAAGTGG
TGGGTAGTAGTGGTAGTAGTGGTAAGTAGTGGTTGGTAAGTGTGTGGTGGGTATGGGTGGTTGGGTGAGTGTGGGTAGTGGTTG
AAGGTGTGGTGTGTGGTAGTGGTAGTGGTAATTTGGTGATTTGTGTGTTTGGGTGGTGAGGTGGTAGTGTGGTTATGTTTCGTG
T
>m141122_040740_42134_c100732202550000001823141405141561_s1_p0/43017/7738_16703
ATTAGGGAGCTATGTACTATAAATTACGTGCAATCTATCCGAGCGTAGATGTAAGGAGTGATAATGAGTCAAAATAGATATGAG
AAGGACTGTACAAATCTAGGAGAAAAAACTTGCGAGAACATGCCATCAGAAGAGGGATTATATTTAGCTGAATAATAGCTTTGA
TTTGTAAGGCTTACTGTTCTTCGATTCCCAATATGTTATTCATGATACCCATGGAGAATCTCCTTGGGAAAATATAGACGAGTT
GAGACACCGAGGGTAATATGATTCTCGATAACATATTAGTGAAATTCCAAAATTCCACCAATAAGGGGGTACCGATACCTTTCC
AGTAAATTTCGAATTTCTCTAATATGGAATTAGGAGGCATTGTTTTGATCGTCTAGAATAATTAACATTGACTAGAATATGCGA
GGGTCATGATTTGGGGAGCTCAGCCACAAAGTTGTGGCCCTATGTCTTAAGGGTACTATGTACCAGTATCTAAAAATTGGTGAA

AATCCAGAGTGTGTCACGGTCGATAAGGAGTCAAATATACATTGGGAGGAGTACTGAATATAAGCGGGAAAAAACGCTGCGAAG
AATTTGCATTAAGAAAGCGAGGATTTTTTTTTGATAATGGACTTAAGGAGATCCGTTTCGACTCCATAATCAGCTCGCATTTA
CCCACTGAAGAGCTCTTGGGTGAGGAAGTAATATAATCGGACAGCTTCACAGATCAGCCAGATTGAACACGAGAGATGGTGGCG
GTCAAAAAATGTAGGAGAATTAGAGTGAAATCTCCAATCCAGCAATATATAGGCGAGGTAGCCCCTTTCATAGATAAGTTATCT
TCGATTTCTCCTCATATTCGGATTGGAAGCATTGGTTTGATCGGAATAACGCTAAAGGAATATGCGAGGTCATGGGCACGTAAA
GTTGTCTGCCTATGTCTTAGGTGAGCTATTATCTCAGAGATAGCATCAAACGTACGTGCAATATACGAGGTGTGTCCAGGAGGG
GGGTGGATAACAGTCTATACCGAAATACACCATTAGTAAGTGATAGGTACTATAACTAAATCAGCCGAACGCAAATAGCGAAAA
TTGGGCCATCTAAATAAGGAATCTCTTTTGGAAAATGTTACTATATAGCGGCTTGTTTCCGCCTTCAATATAGTACGTCTATCA
AACATATTACCCATTGAGATCGTTCTTGGGGAGGAAAAATATCTGATGGAACGAGTTGGAGCGAGAGGGTAATAGGTAGAACAT
CACTCGGAGAAATAGCTGAAATCTCCAAATAACTTCACCAGAGAAGGGGTAGAGAAGGTACGCGCCTTTCAGAAATCTGCGATT
TCATCATAATATGGGAATTGTGAGAGCATTGTTTTGGCGTCTGTTGATATAGAATCTAAAGTAGATATTGAGGCGAGGTGGCAT
GGCGCGAAAAAGCTATAAAAACCTAATGTCTTAAGAAAGAGATACAGATGTAATTTAAATATTACGTGTCAAAATCCGATATGA
GTGTGTAGATCGGAGTGATGAGTCAAAATAAGAAGGACGACTAAAATGAACAGGATGAAAAAACTTGCGAAAATTGCCATCAAA
AAGAGGAATTTTTTTGAAATGTTTGACTTAATGGCTTCCGTTTCGATCGCCAATAATTGCATTTACCCATGAGATACTCTTTGG
GAAAATATCGAACCATTGAACACGAGGGTAATAACATTTCGGGTAGAAATTAGCTGAAATCTCCATTCACCAAAGGGGTACCCT
TTCACGAAATTCGATCTCTAGAATATCGGATTGGAGGCATTGTTTTGTCGTGAATACTTAAAGGAAATAGTGCGAGGTTCATGG
GCAAAAAGTTGTAGCTATGTCTTAGGGTATATGTAGCTAAAAGTACTGCAATCCGAGGTGTGTCACGGTGGATACCAGTCAAAA
TATTGAAGGACGTAACTAATAATAGAGTGACCAGAAAACTTGCGCAAATATGCCATCGCAAAAAGAGTGAATTTTTTTTGAAAT
GTTGATTAAGGTCTCGTTATCTGAATCCAATGTATATTATTTACCCAAAGTGAGATCTCGGGAATCTTGGGGAAAAAATATCGAA
CGAGTTGAACACGAGTGGGTAATAGAACATTTCGGAAGAAATTAAAGTGAAATTCTCCAATTCACCAAAGGTGGTATGGCGCGC
TTTCAGAAATACTGTCGATGTCTCTAATAAGAGAAATTGGAGCTAAGTGATAGATCGATAGAATACTAAAAAAATATGGCGAGG
TCATAGGGCACAAAAGATTTGATTAGACTATGTATCTATATGGTACATCATGTACGTGAAAGGATTAGGGCTGAAATCAGGTAT
ATTGTAGAGGAAGGAGTGATACAGTACAATAGAGGAATGTAATATCAATAAGGAAAAAATGCTTAGCGAAAACTGCCATCAAGG
GAATTTATTTTTTTGAATGTTGATTAAGGGATATATATCCACTTTATTATTTACCACACATGAGATCTCTTGGGGAAAAAATATC
GAACGAGTTGCAACACGTGGGTAAATACATTTCTGGGAGAAATTAGATATAAATCTCCAATTTCCACCGAAGGTGGTACACTTT
CAGCAATTCTTCGATTTCTCTAAATCGGACCCGTTGGGCATTGTTTGATCGTGAATACCTAAAGAATATGCAAGGGTCATGGCA
GCAAAAATTGTAGCCGCGATGTTCCTTAGGGTGTACTATGTATAAAATGTACGTGCAAATCGCGAGGCTGCTGTAGAGCGTGAT
AGATATACGTCAACACATATATGAAGCGAGCGTAGCCTACATATACATCCTATTGAAACAATAATGCGAAAATCTGCGATCATA
AAAAGAGGAATCCTTTTTTGAAAATGCTGAGCTTAAGGCTCCGTTCCACTGCCAATATCACTCTCATCGTTACACCATGAGAAT
CTTCTTGGGGAAAAATATCGAACGAGGTTGAACACGAGGGTAATACCATTTCGGAGAAATTATGCGAAATTCTCCAATTCACCA
AAGGAGGTACCCTTTCAGAAATCTTCGCATATTCTGCTAAATCATGGTTGGACGGCATTGTTTCTGATCGTGAATAGAGCATAG
CAGACAGTATCCGCCGAGGGTCATGGGCACCAAGTTCGTAGCCGCTAATTGTCTTGGGTACTACGTTTAGTCGACCAAAGTGTA
CGATGCAAATCCCGAGGTGTGTAGAGGGTTATAAAGAGTCAACAATATAGAAAGGACCGATTTACTAATATAGGAGGAAAAGGA
CTTGGCGAAATTGCCATCAAAGAAGAGGGATTTTTTTTTTTGAAAATGTTTGATCTAAGGCTCGCTCTCCACTCCAATTGATAT
TCATTTACCGCGCCGATGAGATTCTTGGGAAAAAATATCGATAGACGATGGTTGAACACCCGAGGTACATCTTTTCGGAGAATTA
GTGAAGTCTCCAATTCACAAAAAGGGGTACCGCGCTTTCATGAATGCTTCGAACTTCTCTAAATATGGATTGGAGCATTGTTTT
GATCGTGAGAATCAAGTATAAAGAAGTAATAGGCGAGGCGGAGGGGTCGAAAGGGTCATAGGCAAAAAGTGTAGACCCTAATGT
CTTAGGGTACATGTACTATAAAATTACGTGCCAAATCCGAGGTGTGTAGAGGGTATGAGAGTTCACAAATAGGTACGAAGGACG
TACAATGATAATACAGGAAAAAAACATATATATAGTTCTCGCGGAAAATGCGATCAAAAAGAGGATCTTTTTTGAATGTGTGAC
TTAAGCGCTCCGTTCGCTCCAAGTGATGATTTCATCTACCCATCGAGAAACATACATAATATGGAAGGAAAATCGACGAGTTGA
CACGAGCGGTTTAATATATTTTCGGAGAAATTAGTCGATAATCTCCATTCACCAAGCGGGTACGCTCCTTTCAGAAATCTTCCG
ATTTCTCCCTAATAAGATATGGAATTCGGAGCATTGTTTTAGATCGTGAATAATAAGAAGAGCATGGAAGAAAGGTCTCGGCAG
CAAAAGTATGTAGGCCACTATGTCTTACGGAAGGGTACTATGTATGAAAATTACGTGAAAATCCGAGGTGTCGTACCGAGGGTG
ATAGCGAGTCAAAATATAGGAGAGGGACGTACTAAACATCTACAGAAAAAACGTTGCGAAAATTAGCCATCCAAAAAGTAGAGG
AAATTTTTTTTGAAATGTTGACTTAAGGCGTCCGTTTCAGACTCCAATATATTCAAGTTACCCATAGAATGCTCTTTGGCAAA
ATATCGAAGACGAGTTAACACGAAACAACTACATTTCGGACAGAAATTAGTGAAACGCCCCCTCTCCAATTCACCAAAGGGGTA
CCCCCTTTCAGAAATCGTTCGATTTCTCTAATGATGGAATTGGGAGCCATTGTTTTAGATCGTGAATTACTGAGAAACGAATCA
TGCGAGGGTCTGGACCAGCAAAAAGTCTGATAAGCCTTAAATGTCTTAGGGTAGCTACATGTATTAATGAATAGTACGTGCAAA
TCCGAGGTGTGGTAGAGGGTGATACAGTCAAAATATAGAAGGACCGTACTAAGTGATCCAAAGGAAACAAACTTGCGAAAAGCC
ATCAAAAAGAGGAATTTTTTTTTGAAATGTTCGACTTAGGCTCCGTTTCGACTCCCATATAATATTACCATTTACCCATGAATT
CTCCTTGGGGGGAAATGTTTAATATCTGAACGGTTGACAGAGGGTGAATACATTGACATTCGGAGAAAATTACGTGAAATCTCC
AATTCACCCAAGGGGGTACGCCTTTCAGAAAGTCTTCGATTTCTGCTAATACTGAGGACTTGAGCATTGTTTTGGTCCTGAATA
ATAAAGAATATCCGAGAGCATCGGGCAGCAAAAGATTGTAGCCCCCAAATGTCTTAGGTATCTAATGTGTAAAATGTACGTGCA
ACATCCGACCGGTGTGTAGATGGGTGACCCCTAGAGTCAAAATTACAGAAGGACGGTATAAATATACCAAGGAAAAAACTTGCG
AAAATGACTGCCATCCAAAAAGAAAGGAATTTTTGAAATGTTGAACCCTATCCATTCTAAGGCTCCGTTTCCGAACTCCAATAT
AAATCATTTACCCATGAGATCCTCTTGGGGAAAAAATATCGAAACGAGTTGACACGAGGTAATACATTTCCGGAGAAATTAGTGA
ATCTCCCTTTCACCAAGAGGGGTACCCTTCAGAAATCTTCGATTTCTCTAATATGGATTGGAGCATCTGTTTTCGATCCGTGAA
ATAGCTCACCGGATATGCGAGGTTCCAATCGGGCCACAAAAGTTGTAGCCCTATGTCTTTAGGGTTAAACTATGTATAAAGTTA

ACGTGCAAATCCGAGGTGTGTAGAGGGTGATACAGTCAATATTAGAAGGGGAGCGTACTAATATAAAGGAAAAAACTTGCGAA
AATCGGGCCATCAAAAAGAGGATTTTTTGAAAATGTTGACTTAAGGCTCCGTGTTCGACTCCAATATATCATGTTACCCATGAG
ATGCCTCTTGGAGGAAAAATATCCGAGACGATTGAAACACGAGGGTAATGACATTTCGGAGAAATTAGTGAATCTCCAGTTCCC
AAAGGGGTAGCGCCATTTCAGAAATCTTCCGATTTCTCTAATACTGGATGTCGGAGGCATTGGTTTTGATCGTGAATACTAAGA
CATATGGCCGAAGGGTCATGGCCCCCCACAAAAGTTGTAGGGCCCTTATGTCTTATGGTACTGATGGTACGTAAACATTACGTG
AAATCCAGGTATTAAGCAGGGTGATCCACAGTCAAAATACTGAGTGATGTAAGAATACTAAGGAAAAACTTGCGAAAATTGCCC
ATCAAACAAGGGGAATTTTTTTTGAAAATGTTGACTTAAGGCCCCGTTTCCACCTCCAATATATTCATTACGCCACTGACGAAT
CCTTGGGGGGGACCAGACAGATATCGAGGACGGTTTGGAACACGTGGGTAAACATTTCGGAGAAATTAGGGAAATCTCCAATTC
ACCAAAGGGGTAGCGCGCTTTCAGAATCTCGATTTCTCTATATGGATTGGAGCATATGTTTTGATCGTGAATAAATAACCAGAA
TATGGGCCCCAGGGTCAGCAGGGGCCACACAAAAGTTGTACCGCCCTATGTCTTTAGGTACTATGTTACTACAAATTAGCGTGC
AAATCCGAGGTGTGTCAGAGGGTGATAGGCGAGTCAAACAATATAGAAGGACGTACAATATAAGCGAAACATACTTGCGAAAAT
TGCCATCAAAAAGGGGAATTTTTTTCGAATGTGTGACGTTACAGGCTCGTTTCCACTCCCAATCATACTTCCATTACACCATG
AGATGCTCCTTGGGGAAAAATATCGAACTGAGTTTGAACCACGAGGGTACATACATATTCGGAGAAATTAGTGAAATCTCAATT
CACGCAAAGGGGGTACCCCTTTCAGAAATCTTCGATTTCTCTAGATATGGATTGGAGCATTGTTTTCGATCGTCGAATAATAAA
CGCACATCAATGGCGAGGGTCATCGGGCACAAAAGTTGTAAGCCGCTATGTCTTTAGGGGTACTATGTATAATTCAGAATTACG
TGCAAATCCCGAGGGTGTGTACGAGGGTGATCAGAGTCCAAAAATATAGAAGGACGTACAATATAGCAGGAAACTTGCGAAAAT
TGCGCATCCAAGGAAAGAGGAATTTTTTTGAAATCCGTTGACTTACGGCTCCGTTTCCACTCCCAGAATCCCATCATTCCATTT
ACCCATGGAGATCCGTGCTTGGGATAAATATCGAACGATTGCAACACGAGGGTAACTCATCATTGTCGGAGAAATAAGTGAAAT
CCGTTCCAGATTCCACCAAAAGGGGTACCCTTTCAAGAAAATCTTCGATTTCTAATATCACGCGATTGGGAGGCATTGTTTTGA
TCGTGAATACATAAAGAGATATCGCGAGGGTCATGGCCAGCAAGTTGTAGCCCTATGTCTTAGGTTACTATTGTAAAGAAATTA
GTGCAAATCCGAGGTGTGTAGAGGGGTGATAGAGTCAAAATATACGAAGGACGTACCTATATATAAAGGAAAAACTTGCGAGAA
TTGCCAATCAAAAGAGGAATTTTTTTTGAAATGTTGAACTTAGGCTGCCGTTTCAGACTGCCCAGTATTCATTTACCCAATGA
GATCCTCCTTGGAGGAAAGAATAAAATCGACAGAGTTTGAAGCACGAGGGTAATCATATTCGAGAAATTAGTGAAATCGCTCCC
AATTCACCCAAAAGGGGTAGCCCTTGTACAGAAATCTTCGATTTCTCTAATGCATCGGGGACTTGGACATTGTTTTGATCGTCG
ATAATAAACGAATAGCGAGGGTGCATGGGCAACAAAAAGTTGTAAGGCCCTAGATGATCTTAGGGTACTATTACTAGAATTAGT
TGAAAACTCCGAGGTGTGTAGAGGTGACTAGAGTCAAAGAATATAGAAGCAGAGCGTACTAATGATCACCAGGAAAACGTTGGA
GAAAATTCGCCATCAAACACAAGAGGAAATTCTTTTTTTAAATGTTGACCTTAAGGACCGTTTCGACTTCCCAATCATCATTGC
ATTTACGCAATGAGATCAAGTCTTTGGGAAAAAATATAGACCGAGTTGAACACCGAGGGTACAAATACATTTCGGAGAAATTAGT
GAATCTCCCAATTCACCAAAGCGGTCACGCCCCCCCTTTCAGAAATCTTCGATTCTCCTAAATCACCTGGACTTGGAAGGCATTG
TTTTCGATGCGTGAATACTCAAAGAATATGCGAGGGTCATCGGGCCAGCACAACAGTTCGCTAGCCCTATGTCTAGGGTAGCTA
TGTAATTACACCCAGCTAGCTGCACAATCCCGAGGTGTGTAGAGGGTGACTACAGCTCAAGGGAAATATATGAAAGCGACGTGC
GACTTAAACTGACCGCCACACACGGAAAAAACTTGCGAAATTCGCATCAACGGGAAAGAGGAAATCTTTTTTGAAATGTTGACT
AGGCCCTCTTTACGACCAATACTAGTTCATTTACCATTGAGATCTCTTGGGGGGATAAAATATCGAACGGTTGAACACCGAGGG
TCAATACAGATTTCGGAGAAATTAGTGAAATCCCAATTCACCAAAGGGGTACCCTTTCAGAAATCTTCGATTTCTCTATATGGA
TTGGGAGCATTGTTGGTCCGTGATAAATACCAGAATTGCGAGGGTCATCCGGGCCCAGAAAAGTTGTAGCGCCCATATCGTCTT
AGGTACTAATGAGTCACCACCCATCTACGTGCAACATCCGAGGTGTGTAGAGGGTGATAGAGATCAAATATACGAGGACGTACT
AATATACAGGAGAAAACTTGCGAAAATGCCAAATCAAAAAAGAGGAATGTTTTTTTGAAATGTTGACTTAAGGTCCGTTTCGAA
ATCCAATATACTTCATGTTTACCATGAGATCTCTTGGGGGAAAAATATCGAACGAGTTGAACACGGGGTAACTCCGCGCACAGT
CTTTCGGGAGAATATTAGTGAAATCTCCCCATTCAACAAGGGGTAACCCCTTTCAGAAATCCTTGCGATTTCGTCGTAAATATG
GATTGGAGGCATTCGTTTTGATCGTGATACTAAAGATATGCCGAGGTTCTGGGGCACCACAAGTTGTAGCCCTATCGTCGCTTC
AGGGTACTATGTAGATAGAGTACTGCAATATCCGAGTGTGTAGAGGGTGATGGAGTCAAAATATTGAAGGACGTACTAAATATA
AGGAAAAAAACTTGCGAAAATTGCCATCAAGAAAGAGGAAATTTTTTTTAAATGTGACTTAAGCTCCGTTTCGATCCAATATTA
TTCATTTACGGCCATGAGATCTCTTGGGGAAAATATCGAACCGAGTTGAAGCACGAGGTGA
>m141122_040740_42134_c100732202550000001823141405141561_s1_p0/48977/5414_11878
GCAGCACCCCACTCGAAGGTCCTCACCTGACCAAGCCTCTTCACCCAGTTTACCAAAAGAAGAGAGTCGTCTGGATCGCTACTT
AATGGGCAGCTTGAGCTTAGGCTAATTTGAAATCAAGTAGCCAACCAAAGGACTGCTATCTAACGGTCAAGGCGCTTGGCAGAT
TTCATCGCTGAGTCGTACCCGGATCGTGAGGTCCCAGGACCACAGGACGAGTGGTTACTGGTTAAAAGAGAGAAATTCCGCCTT
GGAAACTTTTCGTTTGATGGCATCGTTGAACGAGCACCCATTTCAGCGAGGCTGGGATTATACTAGTCACCCAGAGCAAGCCAC
GCATTCATTGTGACAGCTAAGATATCCGGATTTTAATGCTTCTTAAATAACGCAACGCGGAGCTATGAGGCCTCTTAAGCAGGC
TACGACTGCTAGAGGATGTAAACGTCTTCGCCCCGGTCGTGGAGATAAATACGTGATTCCATTGGTTGGTTTGATCAAGTATGT
AGGGGCGAAGTACCAGGCAAGGGGCGCCCCCTAACGCCATCGGTGGCATACTTTAAACAAAACAAAGATTTGCTACCTAGTTCG
AGGAGTAGTACCCATCAAATTAGTTAACAACAAGGGAGCATAGAATTCAAAATGCGATGGCTCCTAGATAAGAGCTTGCTGCGC
AATAGGATGCCGAAACTCTAAATATATAATGCTCCCAGTAGAACAACTAATGGCAGACACCGAGCATATGATTTAGACCCACCC
CGCCCCCACCACCCCCCCCACCCCCGTCCCCCACCCCCACTCCCCCCACCCCCCCCCCCCGCCCTTCCCCCACCCCCCCCACTC
CCCTGCCCCCCCCCCCCTTCCCGCCCACCCCTCCACCGCTGCCCCCCCACCCCCTCCCCCCCCCCACCGCCCCCCCCTCCCACT
CCCTCCCGACCCCCCACCCCCCGCCCCCCCCCGCCATCCTCCCCCCCCCCACCCCCCCCCCACCCCCGACCCACCCCCACGCCCC
CCCTCCTCCGCCCCCGCCGACCGGCACCCCATCGCCGCCTCCTCGCTCCCCCCACCTTCCCCCGCACCCACTACCCCCCCCCCC
ACCTCCCACCCACACCCCACCACCCCTCCTCCACCCCCACCCCCACCACACCCCGCCCCCCCCGCCGGCCTCCCCCCACCCCCC

CCGCCCACCACCACCCCCCCCCCCACCCCCCCCCCCCACCCCCCACCCCATCACCCCCACTCCCGCCCACCCTCCCACCCCACC
CCCCCCCCCCTCCCCCGCCCTGTCCACCCCCTCCCCCCCCCCCCCACCACCACCCCCCCCCACCCCGCCTCCCGGCCGCCGCCC
TCCCCCCACCCACCGCTCCAACCCCCCCCCAACCCCCTCCCGCCCCTCCACCCCCACACCCCACCCACCACACACCCCCCCCCC
CACCCCGCTCCCCCACCTACACCCCACCGCCCCACCCGCCCCCCCTCCCCCCCCCCCCCCGCCCCCTCCACCCCGCCCCACCGC
CCACCACCTCACCCCCCCCCTCCCCCCCCCCCGCCACCCCCCCACCCCTCACCACCCCGCACCCCCCCGCCCCCACCCACCACC
CCACCCCCCCGCCCCCCCCCCCCCCACCTCCACCCCCCCCCCCCCCCGCCCCCCCCCCCCCCACCCCCCCACCCCCCCCCCCCCCTC
CCCCACTCGCCGCTCACTTCCGCCCCGCCCTCCACCCCACACACCACCCCAGATAGAGAGGGGGCCTTCGCCGATCACGATTAG
CCGACACTATGGATGACTCCATTCATTGCTTACCTTGAGCAAAGGGGACCTCTCCTGATTGGTCGTACAAAGCAGGGAAAGGTT
ATGAGGCAAGCGTAGAACACAATGGTGGATTGGCGATGTTATATAAGCGAGAGGATAGTCCTTACCCTACTTCAGTGCATACTC
CGCGCCAGTCAAAGACTTATTAGACAGTGCTGAGGGGTTTTGTGGAAGATCATTGCTGGGGGCGCAGAGCCTATCTAAAGATCG
TTGAGGGGCAAGGATTATTCTGGCCTACCTATGAACGAGGAACTCTATGGAATATGTCGAAAAAGTGTGACAAATGCCACATAG
ATTTTTAACAAGTACCTAGATGGCCCCAAGATCTTTTGAAGCAATAATGCAAGTCGTGGCTTTGCGGTGTGGGGGGTATTGATC
TTAGATCGGAAGTTACCAAAGGAAAGAGGAGTGCAGTTTTGCGGTGGTCGAGTAGATTATTTTACTAAATGACTGAGGCCCGAA
CCATTAGCCACGATCAGCATCGATAAGAAAGTATTAGACTTCTGTAGGTTTGAAAAACATTAATATGTGCTATGGTCTACCATA
AGAACGATAGGTGTCTGACAATGGCACCCAGTATTTGATAGCGACATATTGTACCGCATTTTTTGCCACTCGGCATGGCAGTCA
CCTAAATAGAAGTTCTCTTCGGTTGCCCATCCTCAAGCCAATGGGCAGGTTTGAAGCGGTGACAAAACTTGAAAGGAGCACTTT
AAAGAAAGGCCTGGCCAGCAAGCTAAGGGTGTTTTGGGCAGATGAGTTTACCAAGCACGGTCCTTTGGTCATATAGGCTACGCG
CTCCTCGGGCGGACGGCAACTGCCTAATATACCCCAAAATTTCTGCTTTAGCATATGGGTATGAAGCCATGTTAATCTGTGGAG
ATAGATCTCACCCTCTCCTCAGAAAGGACCGTTGTACAACCAGAAGGAGAAGCATCAGTTGTTGGGAATGTCTGGAGCTTCCTC
GAAGAGAGGATGAGAGAGGGCAAGCTCAAGAGTAGGCAGCTCACTCAGCAAAATAGTGGCACGCTACCCTGCAACTTCAGAGTG
CAGGACTCGAAATTCCAAGGTCGAGACTTTGGTCTAAGGAAGGTGTTTGTTAGAAGCCCAGCAGTTGGTGTGCTAGGACCGAAC
TGGGAAGAGGACCGTACTCAAATTGACAAGCTCTTAGCCGCCGGGCCACTTTACAATCTTCGGCTCGTTTATAATGGCAGGTGA
TCAGAAACTACTCGGAATGGGTGAGCAGCTTGAGAAAATATTTATGATCCCAAATTATCAACTTGCAGAGTAATGAACTTTGTA
TCAAGTGCTAACCTTTCGTCATTATAGACTTTTGTTTGTGAACTGGTTATTTTGCTACCCAGGTCACATTATTTTGAACAAATG
TTATGGTTTGTTTGGAAAGGCTCGTTTTGATTAGACACGTTTTTGTAATTTATTATTAAGAGTAATAAAAAGAGAGCGCACGGC
GGCAGCGTGGTCGCAGTCTTGCTAGGCAAATTTTGCATATGTGTTGATTATTTTTGCTTGGCAGTGTTTCAACTGTCATAATGA
TTTAACAAAACAGGTCTTCCTAAAAACTAAAGTGTAAATAATAGTACCCGGAAAAGTGTTCACTGTCGGCTATCGATAGATCAT
CGAAGGACCTAACGTGTAACATATATGTTTTTGCTATAGTGGTCAACTACGCTAAAATAGGTGTTCGTATACATCTTATGTTTT
CACGAGTAGTAAACTACTCAAACAAATTCATCAAAGTAACAATGCGATTCCAAAGGTTTATCAACCACCCAATCACTTGGGGGT
ACATAGGGTTATAGCTGGTAGGCATTTTCAACGACAGGCAAAAGAGCAGCGAGCAATAATAATCATTGTTTTTGGCCTGATTGT
AAAGTTTCGAAGTCTTAAATAATGCCAGTTAACGGCCTAACTTGTTTTACGCAAAGCTTAAATAACTTAGAATTTTCAAACATT
TTAAAGTTCAAAACAAGCTATTCGCTCGTAATGCTAAATATTTAATGCTCATAAAAAGTTACGAAGCATAATAAAGCAGCATGA
AGGAAGTATACTTAGTATGGAACTTATGGAATGGTAAAATTCTAAGTAGAAAACATAAATAGCTCAAAAATGTGAATTAAAGCA
TCTAAGGACCTTACTTATGTATCAATAAAACTTAAATAGTTTAAGTTGTCACTAAAGAGAGAGTAACGAGTGGATAAATGTCAA
CGAATTAGCTCACTATTCGGAGCAAGCAATATACACAAGCAAAAGCAAACTAAGTGAAGCGACGAGAGGGAGTCACATGGGCAC
GCTCCTCTAGTGGGTTGTCGACCCGCGCTGCGCGGTCGCGCGCATGCGCTGCTCCCTCCTCGAAGGATGAAGGCAGAAGCAGGC
GGGGCGAGTGCAGGCGGAGGATTCAGGCAAGCCTTCTTTTTTCAGCGGCCCTTTTTTTGCCCTTTCGCAAATCTTTGGCCAAAA
AAAAAAAAAAAGCTTTTTTTTTTCCAATTCTTGGCCTGAAGCAACCTTCCTTTTTGGAGATAAAAACATCAGCGCTTTTTTTAA
AGAAGGCTTTTTTTTTTTTTTTTTTGTTTTTTTTTTTTTTTTTTTTTTTTTCAGCTTTTTTTTCCCCCAGTTCCCCTTTTTTATT
AAAGTTAAAAAACAAGGCCAACAGTCAAGAACCTGCCCTCTTCCTCAAAGTTGAAATTTTCAAGTTCCACAGCCCTTTCCCTCT
TTTTTCTTTTTTTTTTTTTCTCTTTTTTTTTTGCTTATCCGCTTTTCCTTTTACGTGCTTTTATTCCGCCGATCTTGCGCTTAA
CCTCCCTTTTTTTCAAAATTTTTTGTCTTCGACTAACTCTTTCTTTTTTTTTAAAATCTTTCGAGGGAATCTTTTGCCTTTCTTC
CAATATTGTGAGTTTTGTCAAGGAAAAGCGTTTTTTGTTGTTGTTTTTTTTTTGGGCCTGCTTTTTCTTTGGGTGTTTTTTCTT
CCGTTCAGAAAGGTTTTTTTTTTTTTTTTTTTTTTTTTTTTGTTTTGGCAGAGAACTTTCCGTCCCCGTTTTTTTTTTTTTTTT
TTTTTTTTTCAAGCTTTTTTTACAGTTTTTTTCCTTTTTTCCTTTCCTTGCTTTTTTTTTTTGGGCCTCCCCCAGCCTTTTTGC
ATTTTTTGGCCTCCCCCCTAGTTTTGAATTTTTTCGTTTTTTACTTTGTTTTCTTTTGCAAAAACTTTGAAATTCAACCTTTTT
TTTCCTTTTTTTTTTTTTGTTTTAAAAGTCCCCCCCTTACGGCTTTTCGGCAGCCATTTTGAAAAGCCCCGTGACCAAAAAATT
AAAAAGCTTGGACAAGTGAAAAACCCCCTTTTTTGCATTTAGAAAGCCAAAGGGGGTTTTTTTTTTTAATTTGTTTAAAACGAA
AATAAAAAAAATTACTGAAATTAGACATAATTTGGTTCATTTTCAAGAAAAAAAAAAAAAGAAGAATGCTCTTCTACCTTTTTTTT
CATTCAATTGTGTAGTTTTGGAAGTTTCTCTCTTTTTTTTTTTTCTCAAGGAAACAAAAAAAAACCTTTCGGACACTGAAAAGTT
TGATTTCCTTCTTCCGATGGGTTAAAAGGGGAGTTCCCCTTGTTTTAAGGGCTATTAAATTTTTTGAAAAAAAGCAGGCTCGAT
TGCAAAATTTTCATGGGCCCAAATTATCCTCACGACAAGAAGTTTTTTCCCTTTTTTAATGCGTCCAGGTCCGCTAAAAAGAAA
CAAAGCCACCGAAAACAAGCTTTGTTTTTTTTTTCCCGCGTTTTTTTTCTGAAATACCTTACCCTTTTTGGTTTTTTGGGGAGG
TTTTTTAAAGAAGGAGAAGTTTTTAAAGAAAAAAAACCTCGCAGAAGGAGGGAAAGGGAGCAGGGGGGCCAATTTGGCCGGAGTT
TGTAAGTTTTTCCCCCCGGTGTTTTCCCTCCAGGTTTTTCTTTTTTTTGCCCTTCCCTTTTTGAACTTTTGGGCGGCGCCTACC
CCTTTTTTTTTTTTTCCCTGAATCTTTTTTTTTCCCCTTTTTTTTTGGTTGGAGGCAATAAGAAGGTCCACCTTCCCCGTCAC
CCCAAGTTTTTTTTTTTTTTCGTTTTTTTTCCTTCTTTTTGAAAGCAACCGCCAAAACTTCTTTTCCCCTCTTGAACATTTCGT
CGTTTGAAATTCTGCAAAAAGAAAAGGAAAAAAAAAACAACAAAGTAAAGTTTTTTTAGAGATTTCTTTTTTAAAAAACAATT

TACATATGAAGAACAAAGTGGCGGTTACAGGGGGTTATTTGCCTTTTTACAAAAATTTTTTAATTAACGCTCTTCTTTTAAATT
TTTAAAGCGCACAAAAGCCCTTTAATCAAAAATTATTTTTTTTCCATTTTATTTTTTTTTTTTTTACCCTTTAGACAAAAAAAAT
TTGCGTTTTCTTTTTTACTCTTTTTTTTACCCCTTGCAAAATTTTAATTAAGGGAAATATTCTTTTGGTCGAGGAACTTCAAAA
AAAATTTCCTCGTCGTCCGAGAAGGGGAAAATGAGGCTTTTTTTTTTTTTTTTTTTTTAAAAGTTTTTTTTCCCCCTTCCCCTT
CACCTTGGAATTAAAGCTTTTTTCCCCTTGTTTTCCCCAGGGCCGAGCGGAAAAAAAATTAGTAAGAAAAACCCATATAC
>m141122_040740_42134_c100732202550000001823141405141561_s1_p0/50138/1600_10232
AACTAACTGAGACCAGGCCTCTCCAAGGAGATTGTGGCCTGAAAGGTCAATTTTTTTTTGTGGTCTAAAATTAGACGCCAATTC
ATGGTCATAATAGCCTATATGATATTATTGAGATGGGAAAACGTTCAAAAAAATTAGAGCTTATTAGAGAGAAATATGACGTGA
ATTCACGAAATCGCTTCCGGAGCCCTAGGAGTTGCCTAGGAGATTTGTCCTGAAAGGCATTTTGTTGTTTGGTTGAATTAGGAC
CCCAATTCATGGTCAAAATAGCCACCTATATAAATATTATGTGAGATGCGGTAAAATGTTTAAAACAATTAGAGCTGTTAGGGA
GAAATATGAGTGATTTGCGAGATCGCTTCGGAGCCTAAGGCAGCTGCTAGGAGATTTGGACTGAAAGGTCATTTTTTTGTTTTG
GTTGAATAAGGATTCCAATTCATGGTCAATAATAGCCCTATACATAATATTAATTAGATGGGAAACAGGTTCAAAAAAAATTAG
AGCTTGTTAGAGAGAAATATGAGGGAATTAACGGATCGCTACGGGCCTAGGCAGCCAGCCTAGGAGATTTGGCCTAAAAGGTTA
TTTTTTAGTTTTGGTTTGAATTAGGACCCAAATTCCATGGTAAAAAAGTCGCTCTATATTATTAGTTTGAGATGGGAAAAGGTC
AAAAAAAATAAGAGCTTGTTAGGGAGAAATTGAGAGAATTACGAGATCGCTTTCGGAGCTTAGGCTGCTGCCTAGAGATTTGGC
CTGAAAGGTCATTTTTTTTTGTTTGGTGTTATTAGGACCCCCAATTCATGGTCATAATAGGCCCTATAATAATATTATTAAGAA
TGGGAAAAGGTTCAAAAAATTAGAACTTGTTAGAGAGACATATGAGTGAATTACAGATCGTATACGAGCCGCTAGGCAGCTTCC
AAGGAGATTTGGCTGCTGCAAGGTCCAATTTTTTTTTTTGGTTATAATTTAGGAACGCCCAATTCATGTCAATAATAGCCGCTA
TATAATATTCATTGAGATGGGGAAAACGTTCAAGAAAAATTAGAGCTTAATTAGAGAGAAATATGAGTAATTTACGAAATGCGC
TTCGGAAGACCTAGGCAGCCTGCTAGGAGATTTGTCGCTGAAAGGTCAATTTTGTTGTTTGTTGAATTAGGACCCGCAATTCAT
GGTCAAAAATAGGCCTATATAATATTATTGGAAGTGGGAAAAAATGTTTAAAAAAATTAGAGGGGGCTTGTTAGGGGAGAAAATG
AGTGAATTGCGAGAATCGCTTCGGAGGCCCTAGGCAGCTGCGCTACCAGGAAGATTTGGCCTGAAACGGTGCAATTTTTTTGTT
TGATTGAATTAGGATTCGCAATTCCATGGTCCAATAATTAGGCCCTATATAATATTATTGAGGGGAAAAGGTTCAAAAAAATTA
GAGCTTGTTAGAGAGAAATTATGAGGGAATTACGAGATCTCTAACGGAGCCGCTAGGCAAGTCAGCCTAGGAGATTTGGCCTGA
AAGGTTAATTTTTTAGTTTGGTTGAATTAGGACCCAATTCATGGTAAAAAGTCGCTCTATATGTATAGTATTGAGATGGGAAAA
GGTTCAAAAAATAAGATCTTGTTAGGGAGAAATAATGAGAGAATTACGAGATCGCTTCGGAGCCTTAGCTGCTGCCTAGGAGGA
TTTGGCCTGAAGAGTCAATTTTTTTGTTTGGTTGATATTTAGGACGCACCATTCAGCAGTCAAAATAGCCGCTATATAATATTT
TATTAAGATGGGAAACAGGTTCAAAAAATTAGAACTTGTTTAGAGAGACAAATGAGTGAATTACGAGATCAGTTTGCGGAGCCC
CTAGGCAGCTTCCTAGGAGATTTGGCCTGAAAGGTGCATTTTTTTTTTTGTTAAAGTTGGAGTGAACGCCCCAATTCATGGTCA
AATAATAGGCCTATATAATCAATTATTGAGATGGGAAAGGTTCAAAAAAATTAGAGCCTGTTAGAGAGAAATATGAGTGAATTA
CGAAATCAGCTTCCGGCAGCCCTAGGCCAGCTGCCTAGGAGATTTGGCCTGAAAGGTCCATTTTTTTGGTTTGCGTGGAATTAG
GACCCCAATTCAATAGTGCAAGTAATAGCCTATATAACATATTATTGAGATGGGAAAAGGTTCAAAAAAAATTAGAGCTTGTTA
GATTAGCAAATATGGTGAATTACGAAATCGGCTTCGGAGGCCTAGCAGCTGCTAGGAGATTTTGGACTGAAAAGGTCATTTTTT
AGTTTGGTGATTATGGACCCCAATTGCATAATCAATAATAGCGCTATATAATATTACTTGAGCTGGGTAAAGTTTCAAAAAATT
AGAGCGTGTTAAGAGAGAAAGTATGAGTGAAATTACGAAATGCGGCATCGTTGTCGCTAGCAGCTGGCCTAGGAGATTTGGCCT
GAAAAGGTCAATTTTTTTGTTTGTGGAATAGGACCCGCAATTCAATGATGCAATAATTGCCCTCTCATACATATTAATTGAGAT
GGGAAAAGGTTCAAAAAAAATAGAGCTTGTTAGAGAGAAATATGAGTGATTAGAATCGATTCGGCAGCCTAGGGGTTGCTTGCT
AGGAAGATTTGGCCTGAACGAGGTCAATTTTTTTTTTTGGTTTAATTAGTACCCGCAATTCATGGTCAAGTAATTGGCCTATAT
AAATATTATTGAGATGGGAAAAGGTTCAAAAAATTAGAGCTTGTTTAGAGAGAAATATGATGAATTAACGAAAATCGCTATTCG
GGCGGCTGGCAGCTGTCTAGGAGTTTTGGCTGAACGGTCAATTTTTTTGTTTTGGTTGAATTAGGACCCCAAGTTCATAGTCAA
TAATCAGGGCCTACTATACCAATATTATTGAGATGGGTAAAGTTTCAAAAAAAATAGAGCTTGTTAGAGAGAAATATGAGTGAA
CTTACGAAATCCAGCTTCGGAGACCTAGGGGCTACCTTAGCGAGATTTGTCTGAAAGGTCAATTTTTTTTGTTTTGGTTGAATT
AAAGGACCCCATATCATGGTCAAACAATAGCCCTATCATAAATATTATTGGATGGGAAAATGTTTAAAAATTAGAGCTTGTTAG
GGAGAACTATGAGTGAATTGGCGAGATCGGCTTGCGGAGCCCTAGGGAGCTGCCTAGGAGATTTGGCCTGCAAAGGTCAACTTT
TTTGTTTGGTTTGAATTAGGATTCCAATCATGGTCAATAATTGCCCTATATAATATTATTGAGATGGGAAACAGGTTCAAAAAA
ATATAGAAGCTTGTTAGAGAGAAATGTGAGGGAATTCACGAAGATCGCTACGGAGCCCTAGGCAGCAGGCTAGAGATTTGGCTG
AAAGTTCAAATTTTTTTAGTTTGGTTGAATTAGGACCCAATTCATGGTAAAAAATCGCTCTATATATATGTATTGCACGATGGG
AAAAGGGTTCAAAAAAATAAGAGGCTTGTTAGAGGAGAAAATAGTTGATAGAATTACGAGATCAGCTTCGGGGCCTTAGGCTGC
TGCCTAGGAGGATTTGGCCTGAAAGGATCAATTTTTTGTTTGGTGTATTAGGACCCCAATTCAATGGTCAATAATAGCCTATAT
AATATATAAGATGGAAAAGGTTCAAAAAAATTAGAGCTTGTTAGAGAGAAATATGAGGGAATTTACGAGATCGCTACGGAAACG
CCCCTAGGCAGGCTGGCCTAGGAGATTTATGGCCTGAAAGGTCAATTTTTTAGTTGGTTGAATTAGGTCCCCAATTCATGGTAA
ATATCGGCCCTATATAATATTATGAGATGCAGAAAAAGGTTCAAAAAAATAAGAGCTTGTTAGGGAGAACTCGGGGAGAATATG
AGAGAATTACGAGATCGATTCGAGCTTAGGCTGCTGCCTAAGGAGATTTGGCCTGAAAGGTCAATTTTTTTGTTTTGGTTGTAT
TAGGAACCCCAATTCATGGTCAATAATAGCGCCTCATATAATTATATTAAGATGGGAAAAGGTTCAAAAAATAGAGCTTGTTAG
AGAGACATATGAGTGATTCACGAGATCGCTTCGGAGCCTAGGCAGCTTTCTAGGTGATTTGGCTGAAGGTCATTTTTTTTTTTG
GTTAAATGAGGACGCCATGTTCATGGTCAATAATAGCAGCTAAATAATATTATTGAGTGGAAAAGGTTCAAAAATTAGAGCTT
TTAGAGGAAAATATGAGGGAATTACGAGATCTAATGGCCTACGGAGCCCTAGGCCAGCTGCCTAGGAGATTTGGCCTGAAAGGT
CAATTTTTTAGTTTGGTTGGAATTAGTCCCCAAATTTCATGGTAAATAATCGCCTATATAACATATTACTGTGAGATGGGAAAA

GGTTCAAAAAAATAAGAGCTTGTTAGGGAGAAATATGAGAGATTACGAGATCCTTTCGGAGCCTAGGCTGCTGCCTAGGGTTTG
GCCTGAAAGGTATTTTTTTGTTTGGTTGTATAGGACCCCAATTCAGGTCAATAATAGCCCTATATAATAATTATTAGATGGGAA
AAAGGTTCAAAAAATTACGAACTTGTTAGAGAGACACAATGAGTGAATATACGAAGATCTGTTTCGGAGCCAGCTAGGCAAGCT
CCTAGGAGATTTGGCCCTGAAAGGCTCAATTTTTTTTTTGGTTAAATTAGGAAGCCATCATGGTCCAATAATGCCTATATACAA
TCATTATGTGAGATGGGAAAAGGTTCGCAAAAAAATTAGAGCTTGTTAGAGAGAAATATGAGTGAATTCACGAAATCGCTATCG
GAGCCCTAGGGCAGGGCTGCCTAGGGAGATTTGGCCTGAAGGTCAATTTTTTTTGTTTGGATCGAATTAGGACCCAATTCATAG
TCATAATAGGCCCTATAGTATATATTATTGAGATGCGGAAACAGGTTGCAAAAATTAGAGCTTGTTAAGAGAGAAATATTGAGT
GAATTACGAAATCGGCTTCGGAGCGCTAGGCAGGCTTCCTAGGAGCATTTGGCTGAAAAGGTCAAATTTTTTGTTTGGTTGAAT
TAGGACCCCAATTCATAATCAACTAATAGCCGCTATATAGTATATTGAGCTGGGTAAAGTTTCAAAAAAATTAGAGCCGTGTTA
GAGAGAAATATGAGCTGAATTACGAAAGTCGCTCTGTAGGTCCTAGGCGCTGCTAGGGAGATTTGGGCCTGAAGGTCAATTTTG
TTGTTTTGGTGGACATTAGGACCCCAATTCATGATCATATTGCCCTATATAATATTATTTGAGATGGGAAAAGGTTCAAAAAAT
TAGAGCTTGTAGAGAGAAATATGAGTGAACTTACGAATCGACTTCGGCAGCCTAGGGTGGGCTGCCTAGGAGATTTGGCCTGAA
GGTCAAATTTTTTGTTGTTTAATTTAGTACCCAATTCATGGTCCAAGTAATTGGCCCCTATATAATATATATTGAGATGGGAA
AAGGTTCAAAAATTAGAGCTTGTTGCAGAGAAATTATGAGTGATATTACGAATCGCTTCGGAGCCCTAGGCAGCTGTCTAAGGA
GATTTTGGCCTGAAACGAGTCAATTTTTTTGTTTGGTTTGAATTAGGTACGCCCAATTCATAGTCAATAATAAAGCCTATATAC
ATATTATTGAGATGGGGTAAAGTTTCCCAAAAAAAATAGAGCTTGTTAAGAGAAATATGAGTGAATTTACGAAAATCGCTATCG
GAGGGCTAGGGAGACTGCTAGGAGGATTGGACGAAAGGTCAATTTTTTTTGTTTGGTTGACATACGGACGCCCAATTCATTATC
AATAATAGCCTATATAAAATTATTGAGCTGGGTAAAGTTTCAAAAAAATTAGAGCGTAGTTAGAGAGAAATATGAGATGAATTA
CGAAATCGCTCCTGTAGTCCTCAGGCAATGATGCTGCCTAGGGAGATTTGGCTGAAAGTCAATTTTTTTGTTTGGATGATTAGG
ACGCCCAATTCATGATCAATAATTGCCTATAGGTTTTTAGGAATATTCATTCTTACATCATATCATTATCCACTCATGGCCATT
AGAACCATATATAAAGATTTGGATCTAAGAGATCATATATAACCATATCATTTATTAATATCAAAAATTTGGAGAGAACGCCGT
GTGCGGGTTGGGAGTGGGGCGGAGGCACCTGTACGTGACCGGACTCCTTTGTCAATCAATATGCCGGCCCTGAAATATATCATT
GTCATCTATACTCAAACTAATAATTAAATTATTTTGAGTATGCAATATTATATATTTTCTTAAATTATTATTATAAATGTTTAT
TTGGTTATAATACTGAGGTATCTTTATAAATGGTCTTTTATATCTAATATTCTTTTTTTCTCTTTAGTTCAAGAAATGATTACA
TATCTAGGTATATCTACATGATTTTACAACTGAGATAATATACTGAATTTTTTTTTTATTGAATGGGTTCATATAATGTATACA
TATAAAATTTGATTTTCATTTCTTATCTTTAATTAATTTATTATAATTCAATTTCTGTTTAAAATTAATGATGGGCAAACCGTC
TAATTAAGACGGCGAGTCCCTCTCCCGCTCCAGGTATTATTCGGATCGGCAAGCAGGGCGAAATGTAAAGACAGCGGGGCAACC
CACCCCATTTATTGACTTTTGTTTAAATTAGTATATATAATGCTATTAGAGATGGTCTAACACTAAAAATATATTTTACTTTTT
TTGGAACCTATAACCTTGGAATAGTTGGGTGCTGGTGTGACCGGGATTGATTAATAAACTAGTTTTGAGACCAATTGACGACTG
TATTATATAACCGTGATAAAGATAGTAGTAGGATTATATTTCACTTAATCAAGTGTTGATGAAGTAACTATTTTCTTATATGAC
CTGACCTTATATTGTTGTTGTGTATTTACGAATATTTCTTACAATGGTATACTAAATTTTTAATTTGAGGCGTGTTGGAAATTT
TAATGCAAAAGCAAGGGCAATTTGAAATTGAAAAGTATCCAAAGGATACGCACTCGACTGAGTACAGTCAAGTGTCGCTCCTAA
TGAAGGTGAGACTGAGGTCTTTCGCGAACCTCGCAATTGTTCGTGTCCATGTCTCGCTCTCGACGCGATCTAACAATCTCACTC
TCTCTCACTCACCTCCAATATTAATGACGCGAAGACACGATAGCCGTTTATTGCCAATGGAGTAGGCCCGAGTCACTCCACCGA
TTCGGAAACTCCTCTCCGTATTCACCGTCGAGTATCACGCATGTTCCGCTCCTCCCCCTCATCCTCCGGGATCTCAATCAGCGC
ACTCGACTATCGGGCCAAAGCAGGCCCAGCGCCTTGCTCAGGTATGGCTCCCCAGACGGCGATGAAGACGACGACGACGAGACG
TGACCTAGGATTTCGATACGATGCCCGCCACCGCTCGCTCTCGCTGAGGACGTCACAATGGTGCTAAATCCTTTCGATTCCGCT
TCGAAGACACTCGATCTCCTTCTCCAGCGTACACATACTATACTTAAGCTACTAGAGTTTTTTTAGTTACATAAAGCGACATT
TAGTCTAATATACTCTGAGCTCGGAATGTAATGGAGCCATGAGTAATGGTCGAAATTCGGAAGATCGGTTTCCATGTTGTTAAC
TAGAATATGCTGTTTGCATTTAAAGCGGATCGTTATTGGAGTTATTCTTAATTTTGGCAAATATGAGATCGGATCTTCATAGAA
CTATGATATTTTTTTCATTATTAAGAAATGAATATAATATATACATACACTGTTTATATTGTAAGTTATATTGCTAGTAAGAAT
GGAAATAAGTAGTTTTGTAAATTTTGAAGTTTATCATGAAGCTGAGTAGGATTGCTTTTCTATCTATGAATCCGATAGTTATAA
ACTAGGGCTTGATGTCGAAAGCTTAATGGATTTGTCCTATTTAATTATTGGCCGGCCTTTGTAGAAGAAACTACATCTGACGAT
CCCACGCTCAACTGGAAGGACGATCAGTTCGTACGACATAGTGCACCATGGTCCCCCAAAAACATCTCTAAGGACTGCCGTTTC
TATGCCACCATTGGAGCTCCTGTCAACAACGTACATAGAGAATAGTAAACACAGCTAGTTCACTCTACTGATTGGCATTCCATG
AGCTGAATGGAGATATAAAATAAAAAAAGCCGATGATTGATGGCGTGGACTTCAGATTTTTTCAGAAACAGATCCTACAGCTCG
AAAGATTCTGGAGTCAGCGTGGGGCATTGTACTCGTGATGAAGTCATGTCTCCTTCGCCAACCATTTATTATTAGCATGAGACT
GCAGTTTCATCTTATTTTCTTCTGATTTGTTTATTGTCGAAGATGTAACTAACTGAAATATGCACCTTGACCTAGCAGCTTGAA
TTCTGCAGAAGAGAATGAAAATGTTCTTGACAAGGTGAGAGAGTTTATTCGTTTTTTCCCTTGATAAAATTGTTGTGTTCTACA
TATCTCTGATTAGTGATGTCTGGCACTTGTTGTACCAGCTTAGAACTGAGGAAGAAAATGCTGAGTGAAGCAGAGGCCAGGGTT
CGGGAACTTGAAAACAGGTACTAAGTTATTTTTAAATTCTAAAGAGTCATGTGTTTTGCAGTTC
>m141122_040740_42134_c100732202550000001823141405141561_s1_p0/54137/2590_9395
GATTATTAAAACGATTAAAAGACAACAAATACAAACTTGATGGGAGCCGAGGTGGATTTTTTAAAGGGAAAAAAAAATGATGTC
TTTCAGGGGTGCAACACGAGGACGTCTCCGTCCCAGGAGGTCACCCATACTCAGTACTACTGTCGCCAAGCGAGAGCGCACCGC
TTAACTGGGGTGAAGTCAGAGTTCTTAGGGTGTGGTACCGGCGGGGCGATAAGTGCTGGTATTGATCGCACCGGAATGTTGGTC
CGCAATATTCATATTTAAAGAGCGTGGCGCCCCGTGTGTCCTCCACCTTTTTTCCCCGGATAGACTTAGGACTTCTTAATTCCT
GCTTGTTAGGTACCAACGGTAAGTTTCAACCCCTCAAGGAAAACGAAATAAAATGAGCTTGTATTAAAGATACCTTTTAAAACA
ATAAAGTAAGGCGACAACTTTGAGCCGTAGGTGGATTTTTAAAGGGAAAAAAAAATGATGTTTTCCGGCGGTTGCAACACGATG

TCGATCATTCCGCCAGGAGAGCCGTAGGTCACCCACATGGCCTGAAGTACTAATGATCTGCCCAACTGCACGCTTTGACGGCTG
CGAGAGTTCTGAACTGGGCGAGAGATCCGGTGCACTTTAGTGCTGGTATGATCGCACCCGGAATGGGATTTGTTCCGCATATAT
TATCATTATTAGTAAATGCCCGAGCGCTGTGGTAGTCCCTCCCTTATTTATCCCCCGGCATTGACTAGGAAGACTTCTTATTCG
CTGCTTCGCTAAGGTGAACCAACGAGTATTAACCCCTCAAGGAAAAACGAAATAATGAGTTTTTAAAGATTTAAACAAAAATAC
AAACTTTTGCGTGGTGGAAGATTTTTAAGGGAAAAAAAAAATGATGTTTTCAGGGGTGCAAACACGAGGAACTTCCCAGGAGGTC
ACCATCCTAGTCTAACTCTCCGCCCAGACCTTAATTGCGAGTTCTGATGGGAATCCGGTGCATTAGTGCTGGTATGATCGCACC
CGAATGTTGGTCCGCAATATTTCTTGTTAATAGCGAGGTGCCGTGTGTCTCCCTTTTTCTCCCCGATTGACTAGGAACTTGTCT
TATATTCGCCTGCTTGTAGTGACCACGGTATTAATCCCAACGGAAAATGAAATAAATGGGACGGGTTTAAGACTTTTTAAAACA
AAGCGCGAGAGCATAACTTCAAGCCAGTAGGTGGGGAATTGTTTAAAGGGAAGAAAATAAAATGCATGTCTGATCAGGGGTGCA
AACGAGTCACTAGCTTTCCAGGAGGAATCACACCAATCCTAGTGTACGCCTTTACTCTCTGCCAAAGGGCGACGCTAGTACTGC
GGAGTTCTGATGGGCATCTCGGTTATATTTAATGTAGAATAATAGATCCGCACCCGGAGCTGATAGGGAAAATGGCCGCAATGT
ATTACATTTATCAGCGAGCGCAGTGTATCCTCCCTTTTCTTCCCCCGGATTTGAACTTAGAAATTATCTTAATTCGCTGCTTGT
TAGTGACCGACGGTAACTTTAATCCCTGCAGGAAAACGAAATAAGAAGAGTTTTTAAAAGATTTACAAAAAAAAATACAAACTT
TTTGAGCCGTATAGGTGGATTTTTAAAGGGGAAAAAACAACGAGTTTGTCTTGCAGGGGTGCTCACACGCAGAAGGACCACCAG
GAGGTCACTAGTACCTGGGTACTACTCTTCGTCCAACACGCTTAATCGCCAGATTCTGTGGGACATCCGTGCATTAGTGTCTTG
TATGATCGCACCCGGAAATGTTTGTTCGCGCAATATTCATTTATAGCGAGCCGCATTGTTGCCGCTCTCCCCATTACTTCTCCC
CCGTGATTGACTTAGGGATGTCGTGATTCGCTGCTTGTTTAGTTGCCCAGGACGGTGTATTAACCTCAAGGAAAACGAAATAAA
TGAGTTTTTAAAGATTTAAACAAAAATACAAACCTTTTGAGCCCCACGTCATCTTTTAAAGGGAAAAAAAAATGATGTTTTCAG
GGGTGCAACACGAGGACTTCCAGGAGTCACCCAATCCTAGTAGCTTACCCACAGCCAGAGCACGCTTTAACTGCGGAGGTTTCT
GATGGGAATCGCGTGCATTAGTGGCTGGTATGTATCGCACCCGGAATGTTTGTTCGCATATTCATTTATAGCGAGCCGCTGTGT
GTCCTCCCTTTTCATCCGCCCCCGGATTGAACTCAGAACTTCTTATTTCGCTGCTTGTTATGTGACCACGGTAAGTTAACCTAA
AAGGAAAACGAAATAAATGAGTTTTTAAAGATTTAAACAAAATACAAACTTATTAGCCGGAAGTGGATATTTTAGAAAGGGAGA
AAAAAAAAATGAATGTTTCAGGGGTGAAACACGAGGACTGCACCAGGGTCCACCCCCGCCTATGTACAACAGTCAATACTCTGC
GTCGCACAGGCACGCTATAACTCGCGGAGTCCGATGGGATCGCGTTGATTAGCGCATGGTATGAATCGCACCCGGAATGTTTGT
TCGCAATATTCATTATCGCGAGCGCCACGCTGTATGTTCATGCAATCATTTTCTCGCCCCGATTGATAGGAGACTTCTTATTCG
CTGCTTGTTAGGTGACCACGGTATTAACCCTCAAAAAACAAATGACAACATGGAGTTTTTATAAGATTATAAAACAAAAAATAC
AAACTTTGAGCGTAGGTGCGATATTTTTAAAGGAAAAAAAAATTGATGTTTTCAGGGGTCAACGGGCGGTTAGGACATCCCAGG
AGCGTCAACCGAGCTCCAGCATACCTATGTACTACTCTCGCCCAACTGTGCAGCACCTTTAAGTTATGCGAGGTCTGAGGGGAT
TCCGTTGCAATTAGTGCTGGTATGATCGCACCTGGAATGTTTTGTTCGCAATATTCATTATAGCCGAGCTCCGCCAGTAGTTCT
GTCCTCTCCTTTTTCTCCCCCGGGATTGACGTAGGATTCTTATTCGCCTGCTTGTTAGTGAACCACGGTATTAACCCCTCAAGG
AAAACGAAATGAAATGAGTTTTTAAAAGTTTAAAACAAAAAATCGAACTATCATTGGAGCGCGTAGGAGGATTTTTAAAGGGAA
AACAAAAAATGATGGTTTCAGGGTGCAACACGGGACTTCCCAGGAGGTCACCCATACCGAGTACTTACGCTCCAACATAGAGGT
TGAAAAATAATAAGAGCACGCATACCATGCGGAGTTTCTGATGGGAATCCGGTGCATTAGTGCATGGTAATGATCGGCACCCGA
ATGTTTGTTCCGCAATATTCATTTTATAGCGAGCGAGATAGTGTGTCCTCCCTTTTCTCCCGCCCACGCGGATTGACTAGAACT
TCTTAATTCGCTGCCTTGTTAAGTGACCTTGGGACGGTATTGCATGAACCCCCCAAGGGAAAAACGAAACTAATAGGATCTTTT
TAGAATTAAACAAAAAAATACATACTTTTGAGCCGTAGGGGTTTATTAATTAAAGGAAAAAAAAATTGATGTATTTACGCCAGG
GAGTGCAACACGAGGACCTCAATTCCCAGAGGTATCAACCGGCAATTACTAAGTATACTACTCGGTCGCAGAGCACGATTAACT
GTTCTCGGTGCAGTTGAGTGCTGGTAATGATCGCCCCGGAATGGAGGTGTTTTGGCTGCCGCATACTAATTCATTATAGCCGAC
TGTCGCACTAGGTTGTGTCCTCCCATTTATTTCCCCCGCAGGATTTGACCTAGGGAAATCGGCTTCTTATTCGCTGCTTGTTAG
CATGACCACGGTATTAGACCCCTCAACGGAAAACGAAATAAAATCTTTTAAGCGATTTAAAACAAAAAATAACAAAAGTTTTG
AGCCGTAGGGTGGATTTTAAAGGAAAAAAAAAAATGAATGTGCTGAGGGGAGCAACACGAGGACTTCCCAGGAAAAAGAGCATTT
CAACCATACCTAGTACTACTTCTCGCCCAAAAACAGTTACCGCTTAACTGCGGAGTTGATGGGATCCGGTGATTAGTCGCTGGT
ATGATTCGCAAGACCGGGGATAAGTTATGGTCGCAATTAATTACATTTATAGCGATGTCGCGGAAGTTGTCGCCCTCCCGTATA
TTCTCCCCGGATTTGACTAGGAACGCTTTCTCTATTACGCTGCTTGTTAGTGACCACGGTATTGGAAACACCTCGCGCGCAATG
AGAAACGAAATAAATGATTTTTAAAGTTTAAAACAAAATAAAGTAACAACTTTTGCAAGCCGTAGGGTGTATTTTAAAGGGAAA
AAAAAAATGGTATATGTCTTTCAGGGGTGCAATCACGAGGACTTCCCTATGGATGTCATCCTCATCTAGTACTACTCTCGCCAA
GCACGCTTAATCTGGCGGAGTTCTGATGGGATCCTCGGTGCATATGGATGGTCTGGATGTTCCAACGCCGCCATGTTGTGTCCG
GCAATAGTACATTGTTATAGCGACAGCTGCCGTGATGTCCCCGCTTTTTCCCTCCCCCGGAAATCTGGCCAGGAATCCTTTCTT
ATTCCGCTGCTTGTTAGTGACCACGGTATTAACCCCTCAAGGAAAACGAAATAAATGAGTTTTAAAGATTTAAAACAAAAATAC
AAACTTTTGAGCGTAAGGTGGATTTTTAAAGGGAAAAAAAAAAAAAATGATGTTCTCAGGGTGGAACACGAGGACTTTCCCAGGA
GGTCACATCATCCTGTACTACTATCGCACAAAGCACGCTTAATCGGCGGAGTTCTGAGAGTGGGGATCCGTGCATTAGTGCTGG
TTATGATCGCACCCGGAATGTTTGGTCCGCAATATTACGCTATGTCTATATAAGAGAGAAGACTGGCTGAGGTCCCCGTCGTGT
ACTATTACTGTCTTGCACGCCCTTCTGGATTGACTAGGAACTTCTTATCGCTGCTTGGTTAGATAAGTAGACCACGGTCAATTA
AAGCGCAGAGAGTTTGTCAATGTAGAGAAGACCGATCCGCGCCAAGGATACAACGAAAGAACCAAATGTAGTTTTTTAAAAGGA
TTTAAAAACAAAAAATACAAACTTTGAAAGGCGTAGTGGATTTTAAAGGGAAAAAAAAATGATGTTTTTCAGGGGTGCAACACG
AGGACTTCCAGGAGGTTCCACCCCATCTAGTACTATCCGCCCAAGCACCTTAACTGCGGAGTTTCTGATGGGATCCGGCTGCAT
TAGTGCTGGTAGTATAGCACCGGAATGTTTGTCGCGCAATATTCATTTATACGTGAGCGCAGTGTGTCCTCCCTTTTCTTCCCG
GATTGACTAGGAAACTTCTTATTCGCTTGCATTTGTTAGTGACACGGTATTAACCCCTCAAGGAAAAACGAAATAAAGAGTTTT

AAAGATTTAAAACAAAAATACAAACTTCTTGAGCCGTAGGTGGATTATTTAAAGGGAAAAAAAAAATGATGTTTTCAGGGGTGC
AACACGAGGGGACTTCCCAGGAGGTCGACCCATCCTAGTACTACTCCTCGCCCAGCCGCTAACTGCGGAGTTCTGATGGATCCG
GTGGCATTAGTGGCTGGTATTGATCGCCCGGAATGTTTTGTTCCGCAATATTCATTTATCGACGCGTGTGCCTCCCTTTTCCTC
CCCCCGGATTGACTAGGAACTTCTTATTCGCGCTGTTAGTACTGTCACGGTATTAACCCCTCAAGGAAAACGAAATTAAATGTT
TTTAAAGATTTAAACCCAAAAAATACAAACATTTTGAGCCGTAAAGGTGGATTTTTAAAGGGAAAAAAAAATGATGTTTTCAGG
GGTGCAACACGAGATTCCCAGGAGGTCACCCATCCTAGTACGACTCTCGCCAAGCACGCTTAACTGCGGAGTTCTGATGGGATA
TCGGTGCATTAGTGCTGGATGATCGCACCCGGAATGTTGGTCGCAATTCATTTATAGCGAGCGCCCAGTGTGTCTCCCTTTTCC
TCGCCCCGGATTGACTAGGAACTCTTATTCGCTGCTTGTTTAGTGACACGGTATTAATCCCCTCAAGGAAAAACGAAATAATGA
AGTTTTTAAAGATTTAAAAAAACAAAAATACAAACTTTTGAGCCCCGTAAGGTGGATTTTAAAGGAAAAAAAAAAATGATGTCT
TCAGGGGTGCAACACGGTAGGACCTTCCCAGGAGGTCTACCCAATACCTAGTATACACTCTCGCCCAAGCACGCTTAACTGCGG
AGTTCTTGATGGGATCGGTGCATTAGTGTGGTATGAATCGCACCCGGAATTGTTTGTTCCGCAATATATTTAGCGAGCGCGTGT
GTCCTCCTTTCTTCTCCCGGATTGACTAGGAAACTTCTTATTCGCGCTTGTTAGTGACCACGGTATTAACCCCTCAAGGAAAAA
CGAAAATAAATGAGTTTTTAAAGATTTAAACAAAAAATACATACTTTTGGAGCGTAGGTGGATTTTTAAAGGGAAAAAAAAAAA
TGATGGTTGTTCAGGGGTGCAACACGAGGACTTCCCAGGAGGTCACCCATCCTATGTAAGCTATCTCGCCCAAGCACGCTTAAG
CGCGGAGTTGCTGATGGGATCCGGTGCATTAGTGGCTGGGTATGATCGCAGCCCGGAATGTTGTGGTCCCAATATTCATTTATA
CGAGGCGCGTGTGTCTCCCTTTTCTCCCCCGGATTGACTGGTAACTTCTTATTCGCTGCTTGTTAGTGACCACGGTCATAACCC
CTCAGGAAAACGAAATAAATGAGTTTTAAAATTTAAAACAAAAAATACAAACTTTTGAGCGTAGTTGGATTTTTAATAGGGAAA
A
>m141122_040740_42134_c100732202550000001823141405141561_s1_p0/54277/5022_16458
GCTTTGGGGTAAAATTGCCCCCCCCGCCCACAGAAAAAACCTGGTTTTTGGTTGAAAAATCCCCAAAGAACCCCACCCGAGCCT
TTTTGGGGAAAAAAAAAAAAAAATCCCGCTTTGAAGAAAAGGCCCAAAAAAATTTGGAAAAAAAAGCCCGAAGTGTGAGATTTAGG
GGGTCCCTCTGGGATTGGGTTCATTCGCCCCTTTGCTTGCTTTATTAGTGGCTCATAAACCCCCTCTCTAGTACGGCTTATAGC
ATCTGAGCTTCCATTATATGGCCTTTCTAAGACATCTACTCCCCTTGTGTACAAAAAAAAGAAAAAAGACAGGGCCGCCCCAAA
GCCCCCCCCGCCCCCAAATATAGACCCAAAAAAGGGCCCCCCGCCTCTGCTGCCGAGGGTACTGCATTGCGTCTGCCACGATAG
TCACCATATTCGGGTTGTTCGCATAATTATGATGGGTTGGAACGAAAAAGGAATATGCCCCCCCTTTTTTTTTTTGGGGAAAAA
ACCAATGAGTTCCCCCCAATCAATATATAAAAAACAGAGAGAGTAACGTGATTGAAAGGCCCCGCCGCAATCGAGTGTCGTGTT
TTGGGGGAGGGCGGTAAAAAAATCTTCTATACATAGTCTTTGTCCCCCTCCCCCCCCCCTTTTGCCGCAGCCGGTTGAGAGGAA
AGTATTTGGATGGCAGGTCCCAAAAGTAAATCAAAAGAACAAACAGGGAGATTCCTATCCGAAGCTCCCCCGGACCCCCCGCAA
TTTGCTTTTGATTTTTATGAGTTTTTAAGCTCCGCCCCATTTTTCACAAGAGAATATGGGAATCCCCCAATCTACACACCCGG
AAAAAAACGCAATTTGCCCTACGTTACTACCCACTGGCGAGATACCTCGCCGAAAGGTTTATTTTTGGGGCGTGCACGGTTGGG
GGGCGAGAGACCCCTCCCTTCGAAATCTGAGGGCCCACCCATCCCCCTACCGGATCCATGCATCCCGCTTTCCCCCACCCCGTT
GGGCGAAACCCCGGTTAAATCAAAAAAAATACAAAATCATACTAAAGGTCCCGTATGCGTTTTGCCCCCGAGCGCAAGAAAATG
CAGTAGCCCGTAAAGAGGGGCCCCCCTGGGGTCCTTGTGTATTGTTTGCGCTTCCCATCATATTGGTGAAAAGATAGTAGGTTC
AACTTCCCCCCGGGGGGGGGAGTTTTTGGTAAAAGATAAAAAAATCCTATTGGTCGATAATCTTTTGTGACCCTTTTGTGGAA
GCTTGCTTTTTCTGTTCGAGAAACGCCCTTGGAGTCTGCGCTGGCCCAAAACCCCTCGATTCTATACTATCCCTACCACACGTC
CTCCCCCGTATTGTTCTGGCCTGTGAATCTTTCTCCATCGGTGGCGTCGTGGTGCTGTCTTCTTTCTAGGTTATGGCTAAAATA
CCCTTGACCATAAGGCCTTGTAGTGAGAAATTTCGCGAGAGTTCTTTCCCCCAAAGATGGGCCCCGGTCTAGCCGCTTGCTTGG
TACGTCGCCCTAAAAGAAATCGAGCAGAACAAACGAGAAGTTGTGTGGGGGGGCTCCCTCGAGACATAAACGTAGGGGAAGTAG
TAAGTGACTTATGTCATAACGATCTATCTTGCCTTATTTCGGAGTGGCCATTGTGTTCCGTTTCCCACATCTGCATTCCTGCCG
TTCCATTCCGGTGTCGTTGCATTTGGTTTAGGCCTTGCGGCTGGCTTCGAACAGCCGTGTTTTGCCCTCTTTCTTTTGTAAGGC
CTCTTCCGCAAATAGGCCCCGCGTGTACAAGTTTTTTTAATAACACATACAAAGAGCCACATAAGAAAGAACCCTGCCCCCCTC
AGTTGATCTATCTTACGATATAGCTTTTTTCCATGGGCGATGCGCGCCCCCCACTCCATAACCCTATTTACTTCTATCTTTTTC
TCGTCGCGGTGAAAAGTTCGGCTTAACTTTTTTCGTCGCAGTAAAGCATGGAATGTACTTGTGTAGCACCCCCACACCTTCTTC
ACGCATCCCAACAGTAGGATATAGCAGCGTCCTCGCCCGTTTTTCTTCGTTTTTTTGCCCGCAGTTAAAATTTTTGTGAAGTCA
AAGAACCTCACATACGAGGTTTCATCAGGCCCAGCTTCATGATTATCTTTGAGTTCTGAATACCCACAATCCTCTTTTTTTTTT
TTTTAGTCTCTGGGTAGAACAAAGAATCAAGGAAAGAACGGCAACTAGAAGCAGAGCTTTTAACTTCGAATATCGCCTTCAGTA
GTACCCGGGCCAAACATGTTCAACATCTTAAAAAAATGCGAAGGAGTATCGTATCCATCAAGTTCGGGAAACAAGGGCCAGTGC
TGAACTTTTCCGGATTTTTCTTCTTGCTCCCCTCCCAGCACCCTATGGGATTGTTTTCTTCTATATTTTTTTTTTTTTTGGAC
ACAAGTCAACAAAGATTATCCTGTTTCTCCTCCGAAACTTTTGTTTTCCCCCTTATCCCTTTATTATCTGCTTACTGACGCTGC
AGCAGTAGCAATGCCACATTCCGCTAACAAACCGAGAGCGAGAGAGGATTTTAACCAGGGGACATATAAGTCAGAGAAGGATCT
CCCCCCGCCCATTTGTTAGCCTCTCTCTAGTCCCCCACTATACCTCAAGCCGCTCCTTTCTCTCGCTACTTTTTTTTTTTTGGG
GAGACCAACAACTTCTATTATTTCCCCTGTCATAAAGTATCCACTCTGCTGTTGGCTGATATATGTGAGCCCTCGTATGTCACC
TACACTGAGGGGTAATACCGTCAACATGCCCTCAGTAAGAATAGTATCCCCCGGTTGAGTATGTTCCTTCTCTTTTTTTTGTTC
CGTGCCTAAAAACGAGATAGAATTGAGAACTTGACATAAAATAAAGGGAACAACCCAAGAGACTTCCCAGAAAAAAAAACCCAAA
AAGTTTCCCCCCCTACTACCATTTCCCTAATAGCAAGAAGTCACTCCCGCGCGTCGTAAGCTTTGAAGTCCCCCCCAAAGTAAA
CTTCAGTGGGAAAATGGGGCGGCGGAGAAAAAAACTGGAGCCGGGGTTTCTTTTTTTTTCCCTTGCCTTTTAAAATTTTTAAAA
ACGGGGGTAGCACTGTCGCGGGAAAGCCCCCCCCCTCCCCCAGGCCCCCGTTTTAAACCTTTTTCCCAAGAATCGCCCCACCTC
TTTTTTTTTTTTTTTTTGTGCAACCGTTTCCCATTTGGAAACCCCCGAGCGAGGCCCTTTTTTTTAAGGGCCCCAAAAAAAGG

GTTTTTTTTTTTCTGTTTTTTTTTGGGCAACAAAAAAAACCCCCCCTTGGAAAGCTAATCGGAAAAAAAAAAACGTTTGTTTGCGCC
GCCCCCTGTTTAAAGGGGTTGGGCCCCGGTTCCCCGAACTCAAAGTCCCCCAAGTTTTTTTCCCCCGGAGAAAAAATTTTTTTT
TCTTAACCAAAAAAACCCTCCCCCCCCCCCCCCCCCCGAAAAACTAAACCAATAAAAAAAAAAAAGCCCAAGGGGCCCCCCCCCC
CCTTTGTTTTTTTTGGCCCCGAAATTTTTTTTCCCGGTGGGCAAAAAAAAGGTTTTTTGTTACCGGGTTTTGTTCCAAAAGGGG
GGAAAAAGGAAATTCCGGTTCGCGTAGGGGCTTTTCCCAAACCCGTTTCCGAGGTTTCCCGCCCCCCCCCCCCCCCCCAACCAAA
CCCTTTCGGGTTCCGTGGAAAAAAGGAATGTTTTTGTTTTTTTTTTTTCGTTGAATTGGGGGGGTTTTCCCCCCCCACAAATTTC
CATTTTAAACGAAAAAAAATAATTGGGGTTCCCCGGAAGCTCTGGGACTTTAAATATCCTTTTGAAAAAATTCTTATCGCCTCC
GCCCCCCCCCCCTTTGAGGGGGACCAAAATTCCCGATTTCCCCCAAAAACTTCAAAAAGCCCCCCCTCCTTTTAAAAAGGGCGT
TTTTTTTTTTTTTTTTTTTTCCGGATTTTCCCAAAAAGGGGCCAAAATTGTCCCCCATAAAAACCAAAACGAGGGGGCCCCAA
AAGATAGGTTTAAAAAAAAAAACGAGGCCCCCCCTTTTTTTTTTTTTTTTTTTTTGTTGTTTTTTTTTTGGGCTTTTTTGGCC
CTTTTTAGGAAAAAAAATTTAAAAGCTCTTGGGTAGCGGGCCCCTTTTTAAAAAGGCGAAGCCCCTCTGGTTTTTTCCCCCCCC
CAAAATGCCCTTTTTTTTCCCAGCCCGAGTTTTTTCCCCCCCCCCCCCAGCCCCTTTTTTTCCGTGGTGGAAAATCAAAAGTT
CCTGTGTTTTTTTGGGGGGCGGAGATAACCCCAAAAGGCCCCCCCGGGGGCAGAGGGGACCACCCCCCAAGAAAGTGGCCGGG
CCCCCCCCCCTTTTAAATTCTTAGGCCAACCAAAGGGTAAAAAATTTTTTACGAGCTTTTTTGTGTTCCCGTCTTAAGCTCCCG
GTTTTTCCCCCGGGGTGGGGGGGAAATCGATTTATCGAAACAAAAAGGAAATTTCCTCATTTTCCCCGAGGAAAAACCCAAAAG
AAAAAACCCCAAAAACGCCAAAGACCGATTTTTCCCCCCCTCAAGCAAAAAAAAAACCCTTTTTTTTTTCCCCGCCAACCCCGGT
TCGGGAGAGGCGGAAACCCCCCCCTGGGGGATTTTGGGTTTCCCGCCCCAAAAAAAAAAGATAACGAAAAAGAAATTTCTTGTTT
GGGAAGGGAAAACCCAAAAAAGGTTGCTAAATTAAGGAAAAGGGAAGAAAAAACCGGAAAAAAAGGAAGGGGGGTTTTTCCGAAGG
CAATTGAAAAATATAAAAATTCCCCGCCGACCAATTCAAAAGGCCCCCCCAGACCCCCACCCCTTGGGGATATTTTGGACTAA
TTTCCCCCACCCCAAAAATTTTTTTTTGAGTAAAAAAAATCCCTTTTTTGCCGGGTCCGAAAGGGGGGAAGAAAAAAAAAAAA
GGTTTGGCCCCCGAAAGAGGGGCGTAAAATGGGGGGAAAAAAACCCCCCGCTTTCGGCCGGGGAGGTTTCCCCCCCCTTTTTTT
TTTTTTAATTCAAAAGCTTTCCCGGAGATTTTTTTTAACCTTTTTCCTTGGGTTCCCCGATGGGAAGTGTTTTTTTTTTTAAAA
TACCCCCCCGGCCGTTTGGGGTTCCCCCAACTTCCCCCCCCCTTACCCCTTTCTTTCCTCCGGTGCCGTTTCTTCTTCCCCCCC
AAAGTGGGGAGGGGCCCCCAAAAAAAAGGCGCCCCCCCCCCCAATTCCCTGGGGCAGGAAAGGGTTTTTTCCCCCCCCATTTGAAA
GGGGCATTTTCCGCATGCCCGGAGGCACCCAAACAAAAACAACTTGGGGGGGGGGCCCAACGGAGCCCTGGGGCCCCTTTTTGG
AAAAGGGGGTTTTTTTTTCCCTTCAAGGCACGAATAAAAAAAAGGGGGTTTTTTTATGTAAGTTTTGGCCCCACAAAGAACCCCC
ACAATTTTTGTTTTCACTCCCAAAAAGAAACCTTTTCACGAATCATCAAAAAAAAAAAAAAAAAAATTTTATTCTGTGGGAAAGGT
GCCAAAACAAAAGGGTGGGGGTCCAAAAGCCCCCGCCTTTTTCCGGCCCCTTGGCCCGGTGTTTCCCCGCTCGGAGCTTCCCCC
CCTTTCCCCTTTGGTCCCGAGGCCCCCCTTGAAAAAAAAAAGATGGAACAAATTTTTTTTTCCCCGGCCCCTTTTTTTTCTTTTTG
GGCCCCATTTTTTGCCTCCCTTTTTTTGGGGAAAAAATAAAAGATTTTTTTTGGGGTAAGGGCCGGCCCCCCCTTTGGCCCCCCCC
AACCCCCCTTTTTTTTTTTTTTTGAAAAAAAAAAGGGGGCAGGGGTTTTTTTTTTTTCCCCCCCCCCCCCCCCCGGTTAAACCGCCCA
CGTTTGGGGGTAAGCCCCCCCATTGATTTAACTTTCAACCCAAGTGGGCCCCCCCAGAGGGGCCCAAGAAAAAAAAACCCCGGCC
TTTGGCCGGAACCCCCCAAAAAAATAAACGGTTTCCCGAAGAAAAGGTTTTTTTTTGCTTTTAAGACTTTTCGTGGGGTTTTAAC
AAAAGGGCCCCCAAAAAAAAATTCAAAAATCTTTTTTTTTTTTGGCCCGAAATTTTTTTTTTTTTTTTGGGGGCCCCCCCCTTGCCC
TTTAAAGGAAAATAGAATTTTCCCCGGTCCCCCCCCCTTTTTCCCCCCCCCCCCGATAAATGGGGGGCAAACTCCAATTTTTTG
AAAAACGACCCCTGTTTTTTTTTTTTTTTTCCCACCGGAATTTTGTTTTTGGTGGGGAAAAAGCAACGAAATTTTTTTTTGGTGA
AAAAAAAGCCCCTTAAAATTCCCCCCTCCCCCCCGGTTGGGGGCAAATATCCCCCCCCCCTCCAGGGCGGAAACGGCCCAGGTC
GAGGCTTTGTTCCCCCCCCCAAAAAAAAAAAAAACCCAAAACCTTTTTTTTTTTTTTTGAAAAAAGGCAAGGTTTTTCCCCCCCT
TGTCCCAAAAAAGGGAAGAGGCCCCCCCTTAAGGAAATAATGTTGAGGGGCCCCCAAAAAAAAAGGGGGGGGGTTTTTTTTTTTT
TTTTTTTTTTGGCCCCAAAGAAACAAGCTTTCCCCCAAGGGTCCCCCCCCCGTTTTTGAATGGGCCCCCCCGTATGCCGGGAAAA
AACCCTTTCAACATCGAAAACCTCCCCCACCCCTTTTTAACAAAGGATTCACCCCCCCAAACCCAAACCCTTTCTTTTCTTTTC
CCCTAAAAAACCCCCAACCCTTTTTTGTTATTTGGGGAAAAGTATGGAAAAGCCCAAAAGATTTATTTTATTTTTTTCCCCTCC
CCCCCAATATAAGAAAAAGTTTTTTAAAAAAGTTTTCCCCCCCCCCCCCTGTCCCCACCGCAACCAGGGGGGGGGTATCCCCAC
CCAAGTCCCCTTCCCCGACCCCAAAAAAGGAAAAAAAAAAAAACCGAAAAACCCACCGAACTCCCTTCCCCTTTTGCCAAAAAAA
CTCCCCCCCCCCCCTTTTTTTTTTTTAAGAACCTTTTTTTTTAAATTTTTGAATGAAAAAAAGGCCGGGGGAGAAAAGGGAATTT
TTTTTTCCCCCCCCCCCCACAAAAAAAAAAGCTCCAAAGAAGCCCTTCCCCCCGCCCCATTTTTTTAAACCCCCCAAAGAAAAC
CCAAAAAAAAAAAGGATTCAAACCTCCGCTCTTGCAACAAACCAACCAATTAAAAAGATTCCAAAAAGAGCGGGATGACCCATTT
TATTTTTAACGATATCCCCCAGGAAAACAAAAATCTGGGGGGGGTCCCCCCCCCCCCCCCCCCTTGGAAAGGGGAGGTTTTTTA
TGTTTTTTTTTTTTTTTTTTATTTTTTTTTTTATTTTTATTTATTTTTATGTCCCCAACCTTTTGGGGGGCCCCCGGGAGATGATA
AAAATAGAGAATCCCCCCCAAAAAGAAAATGGGGGGGTTATTTTTTTATTTATTAAATAAGAAACCCAAAAAAACCCCCTGGGA
AAAAAAAAGGGGTACCCAAAAATACCCCAAGATTCAAACCCGGGAAAGTTTACAAATAACCAAGGATAAGGAACCCCCCCCCCT
TATAAAGAATATTATTATACACCCCGACTTTTGATAGGAGGAGACCGAGGATATCCCCCCTTTGAAAAACGCTTTTGGGAAAAA
AACGCCAAAAAAAAAAAATGTAAAAACACCCCTGGAAAAAAAAAAATACCCCTTTAAAAAATCCAGAAATTCCCCGCCCTTTTTGG
TAACAAGGGGCCTGCGAAAGGGCCCCAAAACCTTTTTTTTCTTTAATAATATATATATAAAATTACAAAGAATTCAAATGTTA
TTTTTTATCATTTCATTATATTATTATTTGGCCAAAGTTTGTGGGGGACTCAACCCCCATTTATTTTAAAAAGGTAAAAACCCA
ACCCGAAAAAAAGACCCCTTTCCCCAAAAAGATATTAACATTTGGGGGGGGCCAAATAAAAAAATGAATAAAAACCATATGGAA
TAAATATTAACCCCCCAAGGTTTTTTTTTCCCCCCCCCCTTTTTTAACGACCAAAAAAACTTTTTTAAAAAAATAAGGGTTTGT
ATTTTTTTAACGGAATTATCACTATTTTTAAAAAATTCCTAATTGGAAGAAATTTTAAAGAGGGTTTTTGATATATATATAACACA

CGAAAAACCCTTTTTTAAAAGTTTTTTCCCCCCAAAAAAAAATTAAAAAGTTTTTTTTTTTCCTTTTTTTTTTGGTTCCCCTTTA
ACCCCCGAGGGTCCCAATTAAAAAAAAAAAACTTTAGAAGTTTAAGCCCTTTTAAAAAAATTTTTTTATTTTGATCGGTCAAAAA
AGTTTTTCTTTTGTTCACTTTTTTTTTAAAAGAAAGGCCCTCCGTGGAATTACAATTGGGTAAAGGTTTAACAAAAACGAACCC
TTTAAGTTTTTTTTTTTTTTTTTTTTTTTTCCCGTTTAAAATAAAAGTTTCCCCTTTTGGGGGGTTTCCCCCCCCTTTTTAACCA
AGGGTTTTCCCCACAAAAAAAAAAAAAAACTTTTTAAAGGAAAGTTTAAACTTTTAAAAATTTTTTTTAATTTTTGATCCCCCC
CTAAAAGGTTGGTTTTCTTTTTCTTTTTTAAAAAGGAAAAGCTTCCCGTGGGAAATTTAATGGATAAGAAAGGGGGTTTTTTAA
AGCCAAACGAACCCTAAAAAGTTTTTTTTTCCTTTTAAAAAAATTTCAGAAAGTTTTTTTTTTTTTTTTTCCCCCCCCCCTTG
GTCCCCTTTTTTAAGACCCAGGTTTCCGCACCCACCCAAAGAAAAAAAAAAAAACAAAACCCCTTTAAAAGAAAAAAAGTTTAAA
AAACTTTAAAATTTTTGTTTTTTTAATTTGAATCCGTAAAAAAGAAAGTTTTTTTTTTTTTTTTTTCCCCCCCCCCTTGTTTTC
TTTAAAAAGAGGCTTTTCGGGTGGGCGAATATGGGAATTAAAGGTTTTTTTTTTTTAACAACGGGGAAAAAACTTAAAGGTTTT
TTTTTTTTATTCCCCCCTTTAAATAAAGTTTTTTTTTCCCTGTGTTTTTGTTTTTTTGAACCCGGGGTTCCCCCCCCCCCAAA
AAAAAATAAAAAACTTTTTGTAAAAAAGAAGTTTTTAAACTTAAATTTTTAATTGAAAAAAAATTTTTCGAGTTTTTTCCGCTT
TTCCCTTTTTTTTAAAAAAGAAAGGCTTTTTTCGTGATAAATGGGGATAAAGGGGGTTTTTTTAACCCAAAAACCGAAAACCC
TAAAGGTTTTTTTTTTTTCTAAATAAAGTTTTCCTTGGGGTTTTTTTTTTTTTTTTTTTAAAACCCCCGGTTTTTTTCCCCAAA
AATGGGAAAAGAAGAACTTTGTTAAAGAAGAAAAAGGGAAATTTCCCCTGTCCGTTTTTTTTTTTTTTTTTTTCTTTTCCCAAA
AAACCAACCAACACAAAAAACCCGGAGGAAGGGAGGAAAAAAGAAAGAGAGAAGGAAAGGGAAATTTCCGTTCTTAAAAAAGGTT
TTTGTTTTTTTTTAATTGGGAACCCGGGGTTTTTTTTAAAAACCCCCCCCCCCAGGGGGAAATTTTTCTTTTAAATTTTTTTTAGA
AAAACTTTTAGTTTTTTTCCCCGTTTTTGGGGGTTTTAAAAACCCTTTTAAAAAAAAAATTCCCCCCCATTTAAAAAATCAAAAA
CGGAGGCCCTTTTTTTTTTCCCCTTTTTTTTTAAAAAGGAAGAACCTAAAACGGATCAATTAAAATTTTTTAAAGTTTTTAAAAC
TTTTTTCTAAAAAGTTTTTTTTTTTTTTTTTTTTTTTGTTTAAAGGTGGACCCCGGTTTAAAAACCAAAAGGAAACCCTTTTTA
ATTTTTGTAGAAAAACTTTTTTTTAAAAAGTTTTTTCCGGGTTGGTTTAAAACCCCCCCTTGTAATCCATTAAAAAATCAAAAA
AAAAAAAAAACCGAGGCTTCCCTTTTTTTTTAAAAGAAAGGAAAAACTACCGATTCAAAAAAAATTAAAATTTTTTTAAAAGGT
TTTTCTTTTTAAAGGCGTTCCCTTTAAAAAGTTTTTTTTTTTTATTTTGATTGGGGACCTCGTTGGGTTTTTTATTAAGGGACA
CCCAAAGGAACCTGTTTTTAATTTTTTATGGAAAAAACCTTTAAAAAGTTTTTTCCGTTGTTTGTTGTTAACCCCCTTGTTTTT
TTATCCCCCCATTATCCGAAAAAACCCCCGGAAGCTTTTTCCCTTGTTTTAAGAAAGAAAAAAAAAACTAAACCGAAATCCCGAA
AAGATTTTTTAAAATGTTTGTTTTGTAAAAAAGTTTAAAAACTTTGTTCCTTAAAAGTTTTTTTTTGGGTGGGAAAAAGACGCT
TGGTTTTTTTAAGACCCCAGGGGTAAAAAAAACTTTAAAATTGTTTTTAAAAAGAAAAAAACCTTTTTTGTTTGTGTTTTCGTT
GTGAAGAACCCCTTTTTTTATCCCCATAATCAAAAAAAAAAAAGACCGGGAAAGGGCCATTTTTTTTTTCCTTTTTTTTTTTTTTT
TTTTTTAAGGAAGAACCTTTACGGATTCAAAAAAATTAAATTTTTTTTTAAAAAAGTTAACCCACTCTTTTTCCCTAAGTTTT
TTTTTTTTTTTTTTTTTTAATTGGAACCCCTTTTTTTTTCCCCCCCCGTTTTTTTTAAAGACCCCAAAGAAAGGGAAAAAAA
AAACCCCCTTTTTAAAATTTTTTTGGAAAAAAACTTTAAGGTTTTTTCCGTGTTTTTTTTTTAACCCCCCTTTTAGTCCATTAA
GAAAATTTTAAGAATCCCAAAACACCCCTAAAAGGGGGTTGTTTTTTTAAAACAGGAAAAACTTTGGTTTTTTTTTTTTTTTA
AATTTATCCCCAGGGTTAAAACCAAATTAAAAATGGGCCCCCCCCCCCCCAAACAAGGGTAAAAAACCTTGGGGAAAAGGGGTTT
TCCCCCCCCCCCCCCCCCCCTTTTCGTTTGTTTTAAAACCCCCCTTTAAAAAGGAAGGTCCCCCCCCCACAGACTTTTGGGGGC
CCAAAATAAAAAGAAAGAAAAAAACAAATTTTTTTGAAAATTTCTTTTAAAAAAAATTTAACAATAACATAGGAAAAAAGTTGG
CCCCCCTTTCCCCCAGGGCCCCCTTTTTTAAACAAAAAAAAAGGGGATCCCACTGTTTGTTTGATTTGAAAGAAGGTGCAGAAA
TTGTTGTTTTTTTTTTTCCCAAAGGGTTGGGGTGAAAGAAAAAAAATGCGGTTTTTCCCAGAAAAGTCCCCCAAAAAAGGGAGA
AAACCCGCCCCCCCCCTTTTTCCCTTTCCCAAAAGTCGGGGCCGCAAACTTTTTTTTATAAGGTTTTTTTTTTTTTTATTTTTCC
CCTCCCCCTTTTGGAAATTGGACCTTTTTTTTTTTCCGGTTTGGATTTCTGGTCAATGGGTCCCCCCCTTTTTTTTTCCCCCCC
CCAATTTTTTTT
>m141122_040740_42134_c100732202550000001823141405141561_s1_p0/121058/6761_12760
AATTCGGGATTACCGGCCAGTGCGGAAAGCGAGTACAAGCGGCAGTCGGACGAATTGAAAGCCGAAAACTCTAATCCAGGAGGA
GTTAATCCAAGGCTAAGTTTGGAAGCTGGAGTTCAGACTGCCCGCTTGCACGCGAAAAATGAACTCTTCCTCAAGCGGAAACGC
TGAGTGTATGCTCTCGAGGAGAAGCTGCGCGAGGAACAGCGTACTTCACACCGGAGCAGAAGATCCTCGCGGCATGTTGGAGTT
GGGGCACGCGGTGAAGGACGAAGAAATCTCGTCGCTTGAACTCAAGCCCCAACAAACACTGGCTGATAAGAGACGTGATGGACG
CAAAACAGGCTTCAAGCGCAAGGAGATTGACTCCTTGACGGCCGATGCAGGAGGCGTCGAAGGCCGAGCTGGAAGACTGAATGG
CTCAAGTTGTTGCTGCGGGAGGAGAAGCTGTGGCTTCCGAGGCGGTGGCGCCTGAAGGAGAAAGAAGAGGCCGACGTAGTTTGA
ACAGGACGATCAATTTATCCTACGTGGTCCTCATGCATCAACTCTGGAAGGCCAAACCCGGAGGTACTAAGCTACCTGGGGGAG
GAGGCTGCCGTCATGGAGGAGAAGATAAAGGCCGTGGGATCAAGGCTCAGATGTGTATTTCCAGATTTATGATGTCGACCGAGC
CTTGCTGAAGTCCCTGCGGCAGGTGATCCAAGGGGCGGGCCTCTGAGCCTGCCTGCGTGAGGTCTTATCCTCCAATGAACAGCC
GTGCCAGTTCCGATCGAAGCCCACCGTCATCCGATGTCTACCCGAGCGTGGACACCGACAGCTGTTGCTACGAGCCGACTTCGC
CTGCTCCAGAGGGGACGGCTGTATCGAGGGTCTGGATGCCGCAAGCTGCTTGCCACCCCAGCACCTTAGACAGGGTGCCATTTT
ATGTATTTTTCTTTTTATTTTATAGAAATTGATCTACTCAGCTGCAAGGTCTCTTTGTAATGTATTGCAGAGTCTTTTCCTTTT
TTTCTGCTTGAGTTGTCAATTTTGCCACTTAGGATAGACATTTTATACGATAGAAACTGTTGTAGGGACGCGCAGGTCTTGGTT
TCCAACGGCCAAAACTCGTGCACATTCTGACTAGGGCCCAACAGCCCGTGTCACTCTCCACGTAGTTCCTAGGTTGCGGAGGAG
TCTTGGTTCAACGGCCAGACTTCCTAAATATTTCGTGTTCGTCTTCCTAAGGCAAATAGCCGCTCCCGGGCGCGTAGCTAGTCT
ATACCGTTCGCTGGATGGCTTAAGGAATTTGTTCATCTTTCCATCGGCGATCTTCACAACGCTCGCGGGCAATGTACTTAAATG
ATTTCGCAGGATGGTGTTTGGACACAGGGGGTCTCCTTGGTAAGTGTTGGCAGACTGGAGCTAGGTCATTTGCGAGCCAAAACT

AGCTGTCGTCTAATTCGTGGACTCAGCATATGAGCTTGCGTGGCAAGTTCGTTGTGGTCGGGGAGTTGCTACACCTACAATGTG
TGATCCAGGAGCAGGGGAATCGCGGAGCGAAACTTGCTACAATCCTGGGATCAGCGAAGCTTGCGAAGCAAAGCTTTGTGGTGG
TCGAGGAGCCTCGCCACGGCTTATTTTGTGAGCGTCCCGGAGTGAAGCTGCGAAGCGAAGCTTTACAGCAGTCGCGGACAACTT
GCCTCATTCTGAGAAGATCCGGAGCTGAAGCTCTGCCGAAGCAAAGCATCCAGGGTCGCGGATCACGTCCATGTATTACTCTGT
GGGATCCGAAGCCGAAGCTTGCGAAGCAAGCTAACAGCAGTCCGGTCACGCATCTCTTGCATTACGGACCCGAGCTGGCAGCT
TGCGAAGCAAACGCTCCAGAGGTCACGGGTCACGCCATGTGTTATTCTGTGGGATCTGGAGCTGAAGAATTGCGAAGCAAAGCT
AACAGCAGTCGCGGATCACGCCCTCTCTTGCCTTAACGGCCCGGAGCTGGCTTGCGAAGCAAAGCTAGCAGCGGTGCGATATCT
GCGAGGACTCGTCAGGAAATTGGGGGTCTTTTCGGCGTAGCTCTCTGGAACGCGCCTCAACCCCCAGTCCTAGTTTATCGTGGG
CTAACACAGGAGGCAATTTTTCATGGCGCGTAATGGCGATATGCTCACGCCAATCTTTTCACATAATCACATAATGCAGCATAA
TGATTTTTCACATAAACCACAATGACATATAATTGCCACATGATAAGGCAAACAAGTTCATGGCACAAATCAAACTATGCCACA
AATCAAGTCCTAAAACAACAAGTTATAAATTTCAAACGGAAATTTAGCACATAAGCAGCGTTCTCCTTGTGTGTTTTTGTTAAG
GGCGTGTGGCTAAGTCGCCAATAGCCATGTATACCCCAAGTGAGCGTGGGGTCCAGACGTTCCGCGCGTGGATCACTTGTTAAA
ACGCAAGCTTGGAACAAAGGGGATACAAATGCAGTAAAAGCGGGAGTAAATCTCTCAGTGTTTATTTAAATTAGCCTGTTACG
GCAAGTGTTTTTACTGCTGGGAGATTATCTCCCTAATTTTACATACTTGTTACGTCCACCAAGGACTTTCGACTGTATGGTCCG
GGCCCTACTGGTAGTACGGCGGAGGTGCTCCGGTTCCAGGCGCGCGGGAACTCTGGATCCATCGAGTCCTTTAAGTGATACTCC
CATGGTCTTTTCTTTGACTTCCGTAGGGCCTTCCCAGTTGGTCCGAGCTCTACTCCGGATGCCTGCGTAGCAGGAAATACTCCT
CCGTCAGGACAAGGTCTCCAACTTCGAAGGTACGGCTCTTGACTCTCGTTTGAAGTGCGAGCTATTTTGCCTTGATACATCTTG
AGTTGGACCTGGGACTCGCCCGGAGCTCTTCGATCAATGTCCAGGGATTCCTGGAGAAGGGCGCGGTTCCGGGTAAGGGTCGTA
GTGTCCGTCTGATGAGAGGGGATCATGGTTTCGACTGGGCGATGACAGCCTCGCAACCGAAGGTCATGGAATAAGGTGTGTGGC
CCCGTTGAGCTCTCTCTGTTGTGCGATAAGGCCAAAGATATCCGCGGATAGTTCTTCGGCCAGTGAGCCTTGCATGCTTGGAGT
TTTTTTCTTAACGTGGTCTTAGGATTTTGTTTCGCTTCCACTTGCCATTGGCTTGAGGTCTGGCGACTGCGGGAGAAGCTTTTT
GATGATTCCATGCGAGCCAGAAGTCCGTAAATTGCTCACTATCAAACTGCTTACCGTTGTCGGGACACATCTTTCGTGGAAGCC
CGAACGACACACGATATTCTTGATGAATGAAAGTCCAGCTGCTTTTTTAGATGTGACCGTGTTCATAGGTTCCGCTTCAGCCCA
TTTGGTGAAGTAGTCTACCCGCGACTAGGGCAATACTTTACCCCACCTCTTCCAGTTGGGAGGGAACCTACTAGGTCAATGCCC
CGACGGCGAACCGACCAGCGGGCTGGTCATCAGGGGTGATCTCTGTAGGATGGGCTCGCGGAACTTGGCGTCACCTCTGGCATT
GCTCACAATTTCTGACATAAGCACACAATCTTTCTTCACTGGTGGGCCAGAAGTATCCTTGGTGAGGACCTTTTCGGAGAGGCT
TGGCCTGGAGGTATGGATGCGCCACAGAAGCTCCGTGAATCTCATGGAGAATGGTGCTTGATTTCGGTTCGGCAACCACTAAGG
AAAGGGTATGGACAACCCCCTGCGGTAAAAGCATTCCCATCCATAAACCACAGTATCGGGGAACTTGATATTGGAGCCTTCTGG
CGTCAGCTTTATCAGTGGGGAGCTCCCGGTGGTGAGAAATCGGGAAGGATGGGTCCTATCCGGAATGAGAATGGTCGATGATGG
CAATTTACCCACTGTGTTTTCAGTACTAGGTGCCCTAGTGCCCAATGGGGCACAGGCCATACTGCTCGATCTCCGAGTCGTTGC
AAGCTTGGCTAGTATGTCCACGAGTAAGTTTTGGGTCCCGGGGGGACTGGCGGATTTTGATAGCTCTTGAAAATGTTGCAGGAG
ACAGCGGGACCCGAGAGACACGTAGGCAGCCATTTTTTCGCCTTTTCGTCTGGTATTCTCCATGGATATTTGGTTCACACACGA
GTAGGGATCGCTGCATGCTTCAATTTTTTGGGCTCCGACTTCCCTGGCCAGTCGTAATCAGCTAGCATGGCTTCGTGTTTCTGC
TACATTGTTGGATGCTGGGAAACGGAAGCCGGAATGGTGCTCTTTGGAGCTGATGTCCTCTTGTCGTTCTGAGGCCACTAATTC
CACAATAAAGTCCCGTGAGAGCTTGACTTTTTTGGAAACACTTGCTGGCGTGGATGTCTTAACTGGCAACAGTTCCATGGCCCA
TTTTTGCGAGATCCGGAGCCAAAACTTGCGAAGCGAAGCTTTAGAGGCGGCGCAGATCTTGCTATACTTTTACTACGCGGGACC
CGTGCTGGAAGCTTGCGAAGCAAAACTTCCAGGGGTCGCGGGTCATGTCACGCGTAGATTAATTAGGGATCCGGAGCTGGTGCT
TGGAGCAAAACCTTACAGCGGTCGCGGATACATGCTTAGTCTGGGAAGTAACGGAGATGAAGCCCTTGCGAAGCAAGTAACCAG
CGGTCGCGGGAATTACCACACAATATTTCGCCATCGTTCGCCTTGCAGACCCCGGGCTGGAGCTTGCGAAGTCAAGCCTCCAGA
GTCGCAGGTCATTCATTATTTTTGGGAGATCCGGAAGCTTGAAGCTTGCGAGCAAAGCTAGCAGCGTCGCGGACCTCGCATTTG
CTCGTCATGCAGGACCCGGACTGGGCTTGCGAAGCAACTTCCAGCAATCGCAGGTCATGCATTATTCTTGGGAGATCCGGAGCT
GAAGCATTGCGAAGCAAGCTAGCAGCGGTCGCGGATCTCGCCATCTGCTCGTCATGCAGGATCCCCGGCAGCTGGAAAGCTTGC
GCAAGCAAAGCTCCAGCAGTCGCAGGTCATGCTTATTTTGGGAGGATCCGGGCTGAAGCTTGCGAAGCAAAGCTAGAGCGGTCG
CGGATCTCGCCATCAGCTCGTCATGCAGGACCCGGAGCTGGAGCTTGCGAAGCAAAGCTCCAGCGTCGCAGGTCATGCATTATT
TCTGGGTGATCCGGCTGAAGCTTGCGAGCAAAGCTAGCAGTGGTCGCAGATCTCACCATCAGCTTGTCATCGCAGGACCCGAGG
TTTGGAGCTTGCGAAGCAAAGCTCCAGCAGTCCGCAGGTCATGCATTATTTTGGAAGACCCGGAGCTGAAGCTTGCGAAGCAAA
GCTGCAGCGTCGCGGGAATCTCCGCCCATCAGCTTGTCAATGCAGGAACCCGGAGCTGGAGCTTGCGAAGCAAAGCCTCCCAGC
ATCGCAGATCATGTCATGCATGGTTCAGGCTTCTGTTACCTTGCCGGAGAGGTTAGTTTGGTTAGTTTACTCTGGTGATGCTTG
GAAGTAGGGCTTAGTTTCCTTTGAAGGCGATCAGTGAAGCAATGTGGAGAGTTTCTCGATCATTAAGTACTCTGGACTCGGCGT
CCAGGTACCTCTTGCTCACATAATACACAGGGAGCCAGAGCACGGCCGTCTTCCCAGACCGAGAGCAGCCATTCACAGCATGCC
TCCGTAGCAGCTAAGGTCAAGAACCAACGCCTCTTTTTGATAAAGTTTGGATAGAATGGGCTCCTTGAGGTTTTGAGGCTGCTC
CGCGCTCGGGCGTCCACTTCGAACCTGGTCCTCCCTTGTAACATGGTTGGAGAAAGGGGCACAAGTTTGTCATTGCGATCACGC
ATGCCCCTAGGCTACGTGTCCGTCAACTGGCGGCCGGAGCAATGCCAGGCGGATATGGGTTTCCGGTCCGGAGCTAACACGTGA
GCAATTGGTAACCGCAGGGGTCGGAGCAGGGAGCGCTGGGACTCAGCATTCCAGGAGCTTGGGGGTGACCGGTCAGGAGCGCTA
GCGGTTCCCCCTTGTCTTGGAGAGCAGGCAGCTCC
>m141122_040740_42134_c100732202550000001823141405141561_s1_p0/121058/12804_18002
GGGCTGCCTGCTTCTCCAAGACAAGGGGGAACGCTAGCGCCTCCTGGAGCGGTCACCCCCAAACGCTCCCTGAATGCAGTTTCC
CAGCGCTCCCTGCTCGACCCCTCCGGTACAACAAATAAGAGACAATTTATTGCCGTGTTAGCTCCGGACCCGGAAACCATATCC

GCCTGGCATTGCTCGCCGCCAGTTGACGGACACGTAGCCTAGGGGCATCGTCGAATGAAACTGTGCCCCTTTTCCTCCACATGT
TAACAAGGGAGGGACCAGTTCGAAGTGGACGCCGAGCCGGGAGCAGCCTTCCAAAACCTCAAGGGAGCCCATTCTATCAAACTT
ATCAAAAAGAGGCGTTGTTTCTTGTACTTAGCTGCTACGGAGCATGCTGTGAATGCTGCTCTCGTCTGGAAGACGGCCCGTCTC
TGGCTCCGCTGTGTATTATGTAGCAAAGGTACCTGGACGCCGAGTCCAAGTACTTAATGATCGGAAACTCCCACATTGCTCCAC
TGATCGCTTTCAAGGAACTAAGGCCCTACTTCCAAGCAACACCAGAGTACTAACCAACTACCTCTCAAACGCAGGTCCTAACGA
AGCCTGAACCATGCATGACATGAATCTGCGACTGCTGGAGGCTTTGCTTCGCAAGCTCCAGCTCCGGGTCTGCATACAAGCTGT
GCGAGATCCGCGACCCGCTGCTAGCTTTGCTTTCGCAACTTCAGCTCCGATCTCCAAAATAATGCATGACGTGGCGACTGCTGA
GAGCTTTGCTTCGCAAGCTCCAGCTCCGGGTCCTGCATGACAAGCTGATGGTGAGATCTGCACCACTGCTAGGCTTTGCTTTCG
CAGCTTCAGCTCCGGATCACCCAAAATAATGCATGACCTGCGACTGCTGGAGGCTTTGCTTCAGCAAGCTCCCAGCCTCCGGGT
CCCTGCAATGACGAGCTTGATGGCAGATCCGCGACCGCTGCTAGCTTTGCTTTCGCAAGCTTCAGCTCCGATCTCCCAAAATAA
TGAATGACCTGCGACTGCTGGAGGCTTTGCTTCGCAAAGCCTCCAGCTCCGGGACCTGCATGACGAGCAATGGCGAGATCCGCG
ACCGCTGCTAGCTTTGCTTTCGCAAGCTTCAGCTCCGGATCTCCCAAAATAATGCATGACCTGCGATTGCTGGAGGCTTTCGCT
TCGCAAGCTCAGCTCCGGGTCTGCATGAGAGCAATGGCGGGTCCGCGACCGCTGATAGCTTTTGCTTCGCAAGCTTCAGCTCCG
GATCTCCCAAAATAATGATGACCTGCAGACTGCTGGAGCTTTGCTTCGAAAGCTCAGCTCCGGGTCTGCAGGCGAACGATGGCG
AGATCCGAGACAGCTGTTTAGCTTTGCTTCGCAAGCTATCAGCTCCGGATCTCCCAAGACTAATGCTGATCCGCGACCGCTGTA
AGGCTTTTGCTTCGCAGCCCAGCTCGGATCCGTAAAATCACCGTGAATGACCCGCGACCCTGGAAGTTTTGCTCGCAAGCTCAG
CTACGGGTCCCGGCGTAGTAAAAGATAGCAAGATCTGCGCGCCTCTAAGCTTCCACCCCCCCCCCTCGCTTCGGCAAGTTTGGC
TCGCGGATCTCGCAAAAATGCGGCATGGAACTGTGGCCAGTTGACCATCCACCGCCAGCCAAGTGTTTCCATAAAAGTCAAGCT
CTCACGGACTTTATTTGGAATTGTGGCCTCCAAAACGAGGCAAGAGGACATCAGCTCCAGACTGCACCATCCGCTGTTCGGCGT
TCCCAGCATCCAACATGTAGCAGAACACGAAGCATGCTAGCTGGATTACGACTGGCCAGGGAAGTCGGAGCCCAAAAAATTGAA
AGCATGCAAGCGACTCCTAACTCGTGGTGAACAAATATTCATGAGTAATACCAGACGAAAGGCGAAAAAATGGCTGCCTACGTG
TCTCTCTCCCGCGGTCTCCTGCAAACTTTCAAGAGCTATCAAATACCGCCAAGGTCCCCGGCCAAAACTTACTCGTGGACATGA
CTAGCCAAGCTTGCAACAGACTCGGAGAATCGAGCAGTATGGCCTAGTGCCCATTGGGCACTTAGAGGTCACTAGTAACTGAAA
CACAGGGGTAAATGCCATCATTCGACCATATCTCATTCCTGGATAGGAACCCCATCCTCCGATTTTCTCACCACCGGGGGGAGC
CTCCCACTGATAAATGCTGACGCCAAGAAGGCTCCATATCAAGTTCCCCGATACGTGGTTATGGATGGAATGCTTTACCGCAGG
GGGTTGTCCATACCCTTCCTTAGGTGTGTTGCCGTAACGAATCAGCACCATTCTCCATGAGATTCACGGAGGCTTCTTGTGGCG
ATCATAACTCCAGGCCAGCCTCTACGCAAGGTCCTTCACCAAGGATAGTTTTTTTTTTTTTTTTTTTTATTTATTTTTTTTTTT
CTTTATCTGGCACACCATGAAGAAGTTGTGTGGCTTAATGTCAGCAAAGTGTGAGCAATGCCAGAGTACGCCAAGGTTCCGCGA
GCCGCCACCTACAAGATCACCCTGATGACCAGCCCCTGGTCGTTCGACGTTCTGGGGCATTGCCTAGTAGGTTCTCCCAACTGG
AAGAGGTGGGGTAAAGTATTGCCCTAGTCCGGCGGTAGACGTTACTTCACCAAATGGGCTGAAAGCGGAAATACTATGAACACG
GTCACATCTAAAAAAGCACTGGACTTCATCATCAAGAATATCGTGTGTCGTTTCGGCTCCACGAAAAGATTGTGTTCCGACAAC
GGGAAGCAGTTTGATAGTGAGCAATTTAACGGACTTCTGTGCTTCGCATGGAAATCATCAAAAGCTTCTCCGCAGTCGCCAGAC
TCAAGCCAATGGGCAAGTGGAAGCAGTAAACAAAATCCTAAAGACACGTTGAAAAGAAAAACTCCAAGCAATGCAAGGTCACTG
GCAGGAGAACTTCGCGAGTACTTTGCCTATCGCGACAACAGAGAAGAGCTTCAACGGGGCACACACCTTATTCCATGACCTTCG
GTTGCGAGGCTGCATCCCAGTCGAACCATGATCCCTCCCTCCAGCAGACGGACACCTACGACCCTACGCCGGAACCGGCCCCTT
CCTCCCAGGGAATCCCTGGACATGATCCGAAGAGCTCCGGGAGCAGTCCAGGTCCAACGTCAAGAAATGTATCAAGGCAAAATA
CGCTCGACACTTCCAACACCGAGAGTAAGAGCCGTACCTTCGAAGTTGGAGACCCTTGTCCTACGGGAGAGTGATTTCCTGCTA
CGCAAGGATCGCGGGAGTAAGGAAGTACGCTGCGGACCCAACTGGCGGAGAGGCCGCCTACGAAGTCGCAAGAAAAGTAAGCCA
TGGGACGTATGCACTTAAAGAGACTCAGATGGATCCAGAGTCGCCGGCGCGCCTGGAACGCCGGAGCACCTCCGCCGTTACACA
GTTAGGCCCCCCCGTCCATCCAGATTCCGAAAGTCTTTGGTGGACGTAACAAGTATGTACAAATTTAGGAGATAATCCTCCCAG
CAGTAAAACACTTGCCTAACAGAGCCTAATTAATAAAACACTGAGAAGTTTAGCTCGGCTTTTACTGCATTTGTATCCCTTTTG
CTTCGCAAGCTGGCGTTTTAACAAGTGACCACGCGGTCCGGAGACGGTCTGGACGCACGGCTCCCAATTTATACATGGGCTAAT
GCCTTAGCCAGCCGCCCCTTAAACAAAACACACAGAGAACGCTGCTTATGTGCTAAAGTTTCGCTTTGAAATTTATAACTGTTT
GTTTTAGGATTGATTTGTTTGCATAGGTTTGATTTGTTGCCATGAACTTGTTTGCCTTACATGTGCATTGATATGTTCATATGT
GGTTATGTGTCATATGTGCATTATGTGATTATGTGAAAGATTGGCGTGAGCAATAATCTGCCATTACGCGCCATGAAGAATTGG
CCTCCGTGTACGCCCAGCGATAAACAGGACTGGGGTTGAGGCGCGTTCGCAGAGAGGATACGCGCGAAAAGACGCCCAATTTGC
CTGACGAGTCCCTTGCGGCAGAGTAGTCCCGAGCCGCTGCTAGCTTTGCTTCCGGGCAAGCTCCAGCTCCCGGGTCCGTAAGGC
AAGAGATGCGCGTGATCCGCGACTGCTGTTAGCTTTGCTTCGCAGGCTTCAGCTCCAGGATCCCACAGAACTAACACATGGCGT
GACGCGTGACCTCTGGGAGGCTTTGCTTCGCAAGCTCCAGCTCCCGGGGTCCGTAAAGGGCAAGGATGGCGTGTCCGTGACTGC
TGTTAGCTTTGCTTCGCAAGCTTCGGCTTCGGATCCGCACAGAGTAATCATGGCGTGATCGGCGACCCCTGGATGCTTTGCTTC
GCAGCTTCAGCTCCGGATCTCTCAGATGAGGCAATGTCCCGCGACCTGCTGTAAAGCCTTCGGCTTTGCGCAGGCTTCAGCTCC
GGACCTCACAAAATAATGCGTGGGCGAGGCTCGCTCGAGCCACGCACAAGCTTTGCTTCGCAAGCTTCGCGATCCCAGGATTGG
TAGCAAGTTTCGCTCCGCGAGTCGCCCCTGGCTCCCCTGTCACACATTGTAAGGTGTAGCAACTTCCCGACCAAAAGCGAACCC
TTGCCACGCAAGGCTCTATGCTGGAGTCCACGAAGTTACGACGACAGCTAGGTTTTGCTTCGAAAGACGCTAGCTCTCAGTCTG
CCACATTACCAAGAGACGCGCCCTCGTTGCCAACACCATCCTGCGGGAAATCATGTCAAAGTAGCAATTGCCACGCGAGGCGTT
GTGAAGATCGGCGATGGAAAGATGGCACAATCTCCCTTAGGCCATCCCAGCGAACGGTATAGCTACGGCGTCCCGGAGCGGCTT
TTGCTTAGGAAGACCGAACAGCGATAATGAGAAGTCCTGGGCCGTTGGAACCAAGACTCCTCCGGCAACTAGGAACTACGTGGA
GAGTGACACGGGCCTGTTGGGGCCCCTAGTCAGAAGTTGCACGAGGTTTTGGCCGTTGGGACACGCAAGACCCTGGCGCGTCCT

ACAGACAGTTTCTATGCGTATAAAAGTTATCCTAAGTGCAAAATTTGACACCTCAGCAGATTACAAAAGAAAAGACTCTGCACA
TACATTAAGCAAAGAGACTTCAGCTGAGTATGATCCAATATTTCTATATAAATAAAAAGGAAAATACATAAAAT
>m141122_040740_42134_c100732202550000001823141405141561_s1_p0/124123/19302_24745
CAGTGTATAGTATAGTATCATACGCTTAGCAACAGTAACAATACATAGTACGAGGATAGGAGGGAAAACGAGTGAGAGATCTAG
GTAGTAGTAGCCGTAGAGTAGAGCAAGAAGCAAGCAGCAATAGCAGTAGCAGTAGAGGAGAGTAGTAGTAGTAGTATAGTAGCT
ATAGTAGTAGTAGTGTAGTATTCGTAACGGCGAACCAGCAACCATAGTACGCACAAAGCGCGCACTCCCCTTCATCACCCTTCT
GCACACCTCAGCTCAGTATTATTAATACGAGTAGCATGAGAACTAGCCAGCAGCAGCTAGCAGCAGTAAGAAGTAGCAGTAGCT
TACACAGCAGCAGGAACATCAGCAGCAGCAGAGGTAGCAGCTAACGGCAGCACAGTACAGACAGCTCATGCGATAGCAGTAATA
GTTGTTGTAGAGCGAGTATGCAGCCTAGCGTAGCAGGTAACGCCTAGTAAGACGATCATCGGTATCGGCAGATAACGGTATATA
ATCTACGTAGGTAGTGTAGTAGTGACGAGCACATAGCATTGTAGTCACTAGAGTAGCCGTTAACTACAAGCCCGCCGAGGAGGC
GACATACAGCATAATATACACCCGTATGGCGCAGCAGCAGTACAGCATGTAGCATCCCGTAGCAGTAGGCAGCAGAGCTTGCGC
AGTACGGCGAGCAGTAGCTAGTCACACGCTGTAGCAGTACCAGTACATAGGCAGCACAGTGCAGAGATGCAGCCGCAGTAGCAG
TAGCATTACAGCAGTAGTAGAGTAGTAGTACAGCTTAGTAGTAGTAGTAGTAGTCGTTACGCGAAGGTACTAGCTAGCGTAGTT
CATCAGTTCTTTAAGCGGCTGTAACATACCTCACAGAGACACCATTACCATAATACCCGCTCACAGTAACAGTAAAGTTAGGCT
ACGCAGGCACTCATATGCATCGCATAGGTATACTTAGCGCTCTCTGAGACACCACAGCACCCAGTCTAGCTAATGTCATAAGTT
CTCGTTAGGAACATGCCTTGCGCTCGCGAGTAGTATTGCTGCATTTAAGTCCTCGCGCCGTACAGCAGTGCATGGTCATTTAAT
GCCTTGCTAGACAGAATAGTTGTAGATGAGCATTGGAGGCAAGCGGAAATACTCAAAGAGCAGAGAGTACAGAGCTCAGCAAGT
AGCTAGCATGTAGTAGTAGATAAGTTTAGATACCTATAGAGAAGAATCGAAACTCAGTAGTAGTGAAGTAGCGTAGTAGTCAC
GCAGAATCCTCTAGGCTCTGATACGGCCGAAACAGCTATAGGTCTCGCCAGATCAAGACGGCAGGTAGAAGCGAGGAAGGTGTA
AGAGCGATATATAGAATTTTAAACGGAGCAATAGATTGACAGAATAGAGTGACAGAGCAGAGTTTTTCCCACCCATCGCAACGC
CAGACCATGATGTTGGGTTATAAGCCATTTCCTTGGGAGTTATACGAGCTATAGAGATGTAAAAATCTTCGGAATAACTTTTCA
GCGCTATGCCTATTTCTATATCGAAACCCTTATATAAGAAACGTCCATTATTCCGAACATGCGTAGCCCAGATGCGCCAACATA
CAGCGTAATAGCCAGAGTCTTGTTAAGTACTAATAGCAGACTGATGTTAATTCATTATGAGCTAGTGAAGTGACGTCTTACTAA
GTTATACTAGTACTCGATTAAGTATGCAATAGTATAGTATTTAGTTTTGTGAGTAGTAGAGTCGTGTATTTTCTAGGCCTAGGA
ACATAAACTCTTCTCCTAAATATCTAATAAACCTCTCATATCTCTGAGACACACATAATTACTTACTTGATATAACAGCTTAAT
ATAAGGACTGACGCGTAAAATTGTGCGTATGGAATATGATAGTGGAGTTAAAAGTATATAATGTTAGCAGATGGATAATAATCT
AACCTACTGCGCGCGGCGTGAGATGACGTCCCTGTTTTTTGGCTTTTGTTTTGTTTAGTCTACTATTCCCAAGTGCATACATTA
ATAAATTCATGTTTAGATGCTTATTGCAACTTAAGAAGAAACCCTCAAAATTAAAGTTTGGGGGACAGTTGTTTGCTACAACAT
ATTTCCATCATTGCTCATAACAACATTTCCACACCGACCCTTCACACTTTCGCCCGCAGCACTTGCTCCGACGGGAGGAAGCAA
CAACACCTCGGATTCGGTAATCCATACAGATAGCTTTTTCGGAATCACTCTTGTTCTGTCAATGCCACAATCGGGGCCCGACCA
GTCCAAGTCTTCTGGCAAGCAAGTAGAGGAAAGGGGGCGGCACCGAGACTCTCTCCCGCTCTAACACATACTCCCACCGCGCGC
GAAGCAAGGCCTGGATTCGCAGGCTAGGGCTTACGGCCCGTCTGCCTCAGTAGCTGGTCTCGAGAAAGAGACTTGTTCCTTAAG
ACCCCCTATATATTTAGCGCGAAGGACTAGTAAGGGGTCAAGTTACCCAACAAAAATCCGTCATGGATAAGAGATAGTAAAAGA
ACATCCAGCCTGATGTCGACGTGGCCTATATTACGTGTACTTGCAGTAGCAGTAGCAAGCAAGGCAGCAGCACCAACATGGCCC
AGTAGCAGTAGCGCCCGTCACAGACCATAGTCTGTAGCAGCTATCAGTACGAGTAGCGAGCCAGCAGTGCTAGTAGCAGTAGCA
GTTAACAACACCATCTCAAAAGCGCGTACGACACAGCACATGGTCACTACAATACAGCAGCACAGCAGAAAGTACCAAAAGCAT
AGTTAGCATGACATAAGCAGCAGCAGTATAGTATAGTAGTACCATTACGACTAAGTAGTAGTTAAGTATTATATCAGTAGCCGT
TGTTGTCAGTAGTCAGTAGTAGTGTAGCGCTAGTAGTAGCACAACAGAAGCAGCAGTAGTGGCCGTACAGTACACAGTGCAGCA
AGTAGCCTGGTAGCGTAGCAATGTAGTACAGAGTATACAGCGTAGTAGTAGTGGTAGTAGTAGTTTAACAGTTGAGTTATTGTG
TAGTGTAGTGCAGGGACTACAGATCAGGTAGCTAACCGGTCAGAGTAGTAGCAGGAGGAGCGGAAGGCACGATGAGCACAGCCT
GCGCAGTAGCTACTCTCAGAATAGTTCACTAGTATAATAGCTATTATTGTTGAGTAGGCCGGGATCGTTGATGCGTTCGTCCTA
TATTATATGCAGTAATACATTTCGTCTGTGAAGGTAGCAACAATAATACTACAGAGAACGCAGTCAGTATGGGCTCCCCCTTAG
CAGTCATAACATAGGACGTCAACCAGCTGGCAGCACTAGCGTCGGTATACAGCAGTAGACATGCGGCGCAGTACTACTTCTGAT
CTAGCGTAGCTCAGCGTAGAGTAGTCTACAGTATATAGTTGTCACAGCATTGTTCGCGAGCTCTCTCTATGTCACACACACGAG
TAGTAGTATAGCGTAGTAGTAGGCAAGCAGCCAACAGAAGCACCACACATAGCGATAAGGCAAGCTAGCAGGTAGCACGAGAGC
CTGTAGTAGTAGTAGTAGTAGTAGTAGTAGAAGAACACGTAGTAGTAGCAGTATAAGTAGCAGCAGCGCGATAGAGCGGTACAGCAC
ATAGCGACCAGCAGCAGTAGTAGCAGCATATAGTAGCAGCAAGAGAAGAGCATAAGAGCAACAGCATTGGCAGGACATATACAG
TAAGCAGTCAGCGTAGCAGCAGGACAGCATCAGCGAAGCAAAGCGTAGGAGCAATAACACAGAGCGCGCGCAGCCAGAAGCCAG
TAATTAGCAAGGCACTAGCGAGCACTCAGCAGTCAGTAGTACTACGAGTCGCTGCAGGAGGCAAATAGAAGCTAGACAGTGCGC
AGTACGTCTCACACTATATGGCACGCGTTCACAGTCTAGTACGCCATACGCGGCAACAGCAGCAGTCCGCAGAGACACACCTTC
GCGCTCGCCTCACCTGCCGCCGCTCCCTTCGCTTTTAGCACTTATCTGTCTGCGCCGCACGCTCAACAGCGTAGACAGCAGCAC
TGGCAGCCGCCACCGGTGCACGCCCGCGCCCGCTCTCAATTACGCTAGCTCGCTGCAGTCCGCGCGAATCTCTGTCGTCGCCCC
AGCAGTAGCCGCTGCATGCAGAACACGCAGCAGTAACTAGCTTCTCGCGTCACCTAAGCGCTCCTCGTGTGTAGCTCAGTCCGC
TAGTAGAATGTAGCACACAGCATAGTAGCAGAAGCAGCACTATGCACCCCCGGCGTCCGCAGCAGCAGTAGTAGGCGTCAGGCA
TATACGTAGCATGAGCAGGTAGCAGAAGAGAAGCAGCAGTAGAAAGTACAGTAGCAGTTACAGCGCAGAACATCGCAGCAGCAT
AATAGAAGCAGCAGCCGTACAGCAGCAGTACAGTACAGTATAAGTAAGCAGTTGTTGTAAGTAGCGAAGTAGCTGTAGCAGTGC
GAGCTAGTAGTGACCGTAGTAGTAGCAGTACCATACATAGCATTAGCAGTACCGTACAGCAGTAGCAGTACAGTAGCACCAGCG
CAGGCTGTAGAGTACTAGACAAGTAGGCACACAGTGCAGAGATACAAGCAGTACAGCTAGCAGATACGCAGAGTAGAAGTAGTA

GTAGGCAGCGTACGTAGTAGTAGTAGTAGTAGTAGTAGTAAGTTTGCGTAGTTGTACAGCAGAGCAACAGCAGTGGCAGTAGCAGTA
CAAGACCAGTGCGTACAGAGCCAGTAGCAGAGCAGCACAGTAGCAGGCGGCAGTGGCAGAAGCCGAGCAGCACGCTGTAGCAGT
AGAAATAGTAGATAGGCAGTACAGTAACCAGTACCTGTAGCAGTAGCCACGTACTCAGTCAGCAGTAGTAGTACAGCAACAGGT
AGCAGAGCAACAGTAGTGGCAAAAGCAGCAGCGTAGCCTTAGCCAGGCAGCAATAGTAGTAGTAACAGCGTGAGTGGTAGTAGT
AGTAGCATAGTAGTAGGTAGTAGAGCAGTAGACTAGCAGTAGCCAAGAAGTAGCAGTGTAGCATCTAGTAGTAGTAGTAGCAGC
GTCCAGTACGAGTAGTAGTAGTAGTATTCTAGTAGCCCAACAGCCCATCGTAGCAGTCGCAGCACTAGCACCAACAGAGTAGTACAC
AGTAGCAAGCAGTAGCAGGAGAGTAGCAGAAGCAGCAGAGCAGTAGCACTGGAACAGCATCCAACAGCAGTGCAGTAGCATAGT
AGCAGTAGCAGTAGCAGTAGGCAGTAGCAGTAGCAGAGTTAGTACAGCAGTAGCAGTAGTAGTAGGAGTGCGTAGTTATCCAGT
AGACTACGAGCAGTACCGTAGCACGTTAGCAGTAGCAGCAGCAGTAGCAGTAGAGCAGCATGGCAGATGCAGCTAGAACAACAG
CCTGTAGAAGTAGCAGTACAGTAGTAGCAGCATGGCAAAGCAGCAGCAGCAGCACGCTGTAGCAGTT
>m141122_040740_42134_c100732202550000001823141405141561_s1_p0/135602/5719_11934
TTCTCCTTCTAGGGTTCAACTCATTGCAGCGAATTAACCACCGACATCTAAAACCAGCCAAACACATATCTACAGCAAAAAAAC
CAAATGAAGACCTCAAATGAACCATCTACCACATCCTACTAGAGACTAGCATCAAGATCAAACAACACAATTCACACGCCAACC
AAATCCAACCTTTGATTTCTAATTTCAGAAAACTCAACCCGTAGGTCAAAACACATCCAAGCTGAAAACCTAAATCGATGCCTC
CAAAATCTTAAACCCACAACAATATGAAAACCAAGGATGTTTAAAACTCTTAACCAAATCAAAACAACGGGAATTCAACTTTGA
GACCCTCTCCCAAATCCAGCTTCAGAGCAAATCCTCTTTGATTCTTCTCCTAATTTGCGCCCAATTTTCAGCTCCCTTCCCTTC
CTCTCCTTTCCTCTTCTAGCTTTTCTTCTCTCATTTTTCACAAAACATCAATAATGGCCTAAGCTCAAACGTGTACCCCTTTTT
TCCCAGCTGAAACTTATCTTTCCCCAGCCAATGACATTTTACCCCTTCATACAAAGCCCTTTTGCTAACTAAACCTCAAGGGCA
CTTTTAGGCCCTTTCTCCTAATATTGCAATCTCTAACATCTGCTCTTCACCTACTCGTGTTACTCCCAGCAAGTAACTTAATGG
TTACCTCCAGGTTACTCAATCACCAGATAACCATTAACCGCTAAATCTCAACTTAACCATAAAATTCAAGGTACCCCTAGGCTC
CCTCACCGAGGCCAGGTATAAAATCGCGTTGCTGACTATTGGTTACACTGAGCTCTCCACGGACCGTTCGGACTGTGCATCACA
ATAATAACACACACTGCACCGGTGGTACAAATCACATCATACAATTAACACATAATATGCCCTCAGCAGGGCTAAAATTACCAT
GTATGCCCTTCTAACACAATCAGGGCCTACATCGCATACTAATCACATAGTCATGCATCTAATGCTCAATTAGTCCATATACTC
ATGCTCCTACAATCATACATCATGGCATTTTAACTCACTAGATCACACATAAATTCCATCTGCCCTCCAGGCCACTCAATCAAG
GCCCTGCAAGCGCTTAAATAGCGATCTTTGGGTCCGTTACAAAAAGGTTCTGTGGTTCTCTAGGATCCGAGCAAGATGACTGCT
CCAGAATCATGAGGTGTCTCCTCGAACCTAGCACAGATGAATGCAACAAAGGTCCTTTTGACCTACTAGGAATAAACATACAAC
GAATTCCTTATGGTTAACGTAGAACCTCATCGAACTGAGCAAGATGCAATGCTCAAAGGTTTCGGAGGTTCTCCTAGGAACCGA
GCAAGGTGAAACTGCTCCAGGTTCTGAGATTCTCCATTAACCGAGCAAGAGGTAATGATCAAAGCTTTCTGAGGGCCTCAAGAA
CCAAACAGGGGAAAAGACACCTTCTCAACGTTCTGAGGACCTCAAGGAACCAAGCAAGTGAATGGTCAAGATTTTCTGAGGTTA
TCAAGGAACCGAGCAAGATGAATGCTCCAAGATCTCAGGTTCTCCTGCAACGAGCACTGATGCCCTTGCTCAAAAGGTTTCCTG
ATGACCTCTAGGTAAACAAGCAATAAGAATGCTTTAAGGTTCTGAGGACCCCCATGGAACCGAGCAATGATGAATGCTAATTTG
TTTTCGAGGTTCTCTAGGAACCGAGCACATATAATCCTCCACGGTTTCGAGATTCTCCATGTACCGAGCAAGATGAATTCTCCA
ATTTCCAGGCTCGAAGGAACCCCAAGTACAATGATGCTCAAGGGTTCCTGATGTTTTCTAGGAAACTAGAGCAATATGCCAATC
TCCCTGGTTCGAGGTTCTCCCACAACAAACACAAGATGAATGCTTCAGAGGTCCAATGCACCTCTAGGAAAACTACAGCAAGAA
ATACGCCAGGTTTCTAGGTCCTCCATGCACCGAGCACAGCATGATGCTCCAGGTTCCGGAGGTGACTCTCGAACCAGAGCAAGA
GAAGGCTCAAGGAACCTAGGACCTTAAGTGAACGGAGCAAGATGAATGTTCAATCATGTTTGAGGGTCTCGAGAACCGAGGCAA
GAATGATATCCAGGTACCGAAGATTTTACAGGGGGAACCCGAGCAAGATGGAATGCTCAATGGTTCCGAGGACTTCTAGGGAAC
CCAAGTAATTTGAACGTTCTAGGTTCTCAGAAAACTAAGCAGATGAAATTGCTCAGCAGGTTTCTGAGGTCCTCTAGGAATGAG
GCAAGAGAATTTTCTGGTTTCGACGACCGTAATGTAATCGACAAGACTGAGAATTGAAGGAAAAGTTCTGAGGTTCTTAGGAAG
CCGAAGGCAAGATGAATGCTCCAGGAGTTCAAGGCTCACCTAAGAACTGAGTACGATGATACTGCTCAAGGTTACAATGAACCT
CTAGGAAACGAAGCAAATGCTTAAAGGTTACGCAGGAATCTCATGGAACCCGAGCAAGATGAATGCTCAAAGTGATTCTGAGGT
GTGCCATATACCGCGAGACCCAAGAAATGAAATGCTCACAGTTCTGAGATTCTCCATGATTCCGAGCAACGATGAATTCTTAAA
GGTTAGAACGGCCTCAAAGAAAGCAAAACAAGAACAGATGCTCAAAGGTTTCTGAGGACCTCGAGTAACTGAGCAAAATGAATG
CAAAAAGGTCTAAGGTTCTCTAAGCAACCGAGGAAGATGACTGCTCCAGGATCCGAGGGCTCCTTCCTGGAAGACATTGCACGA
CGAATGCAAAAAGGTTCCATTGACTTCGTAAGGAACAAAAGAAAGAAAGAAATTCTTATGGTTACGAGGACCTCGGGTGGAGAC
TGATCAAGATGAATGCTCAAAGGTTCGTCAGGTTCTCTAGGCACTGAGCATGGTGCAATGCTCCAGGTTCTCAGATTCTCCACT
GAAACTAGCAAGAGGAATCGCAAGGTTCAAGGGCCTCAAGGAACCAAAAAAGAATAATTTCTCCACGTTCCTGAGGACCTCAAG
GAACCGAAGCATAAAGAATGTCAAGGTTCTCGGTTCTCAAGGAAAGCGGGAAGAGTGGAATGCTCACAGGATCCTCAGGTGTTC
TCCAGGAACACGAGCACGAGTCCAATCGCTGCAACCCAGGTTCTGATGACCTCTAGGAACCAACGCAATAAGAATGCTCAAGGT
TTCGCAAGGACCCATGGTACCGAGCTAGATGAACTACTAATTTTCATTCCAAGGTTTTTCTAGGGACCGAGAAGAATAAAATGC
TCTAAGGGTTTCGAGATTCTCCATGTGTTCGAGCAAGAAGGATTTTCCATGGGTATCGAGGGCCATGCAAGGTAACCCAACAAG
AAGAATCCTCAACGGTTATCAGAGACTCAGGACCAACCCAAGATCGGAATGCCGAAAGGCTTCAAGTTACTCTAGGAAATGAGC
AAGATGAATGCTCCAGGTTCTGAGGTTCTCCAGGTTCTGAAACACAGATGAATGCTCAAGGTTTCGAGGAAAAATTGGAACCGA
GGAACAACCAATGCCTCAAAGCTTTTGAAGACCTCTACTCAACCGAACAAGATGAATGCGCTTAAATTGTTTCTAGGAACCTCT
AGCAACCGGAAAGATGAATTCTCCAATTTCCAAGGACACGAGGACCAAAGTAAAATGAACTGCTACGGGTTCTGATTTTTCTAG
GAACCTGAGCAAGATGAATGCTCCAGGTATCAAGGTTCTACGAAACCAACAGATGAATGCTAAGAGGTTCTGTGAGCCTCTTAG
GAACATACGCAATGGGGAAATGCGCGTACAGGTTCCTAGGACGTCAAGGCACCGAGCAGACCGATCAAGCGCTGCAAGGGATCC
AAGGCCTTTCTACGATCTCCTGAGCAAACGATGAATGCTCCAAGGTCTCCGAGGTTACTCTACGAAACAACGCAAAGTAGCAGG

CTCAAGGTTCCTGAGGACTCAAGGAACTGAGCAACGACAAGAATGCTCCAACAGGTTTTGAGTTCTTCGACGCGCGCGAGCAAG
ATGAATACTCCAGGCTACAAACTCGACGTGTTTCTACAGGAACCGAGCAAGATAAATGCTCAAAACTGGCTTGAGGACATCGTA
TGAACCAAGTAAATTGAACGCTCTACTTAGTTCTGAGGTTCCTAATAGGAACTAAGAAACCGGATGATGCTCCAAGTTTGAAGT
CTCTCCAGGGACCAAGCGATGAATGTTGAACCGGTTTTGAGAGCAAAAGGCACTTAAGCCAAAAGAAATGTTCAAGGCATGGTT
CAAGGACCCAAAAGTACTCGAGCAAATGATAATGACAAAAAGGGTTCTGCAGGTTCATCTACCGGCCAACTCGGAACGATGACG
ATGCCTCCAACGATTCACGGTTCTCAGCAACCGAGCTTCGACTGAACTGTCAAGAAGTCTATAATGTATCCGTCTAGGCACAAA
ACCTAATGCTTACAGGTTACGAGGATCTCATGGAACCGAGCAACGGATGAAGTGTTCCAAGGTTGCCTAAGGTTTCTCTAGCGA
ACCCGCAGCAACCGGTGAATCCTCGCAAGTTTTCTGAGATTAGCGCATCGAATTGAGCCAAGAGGAATGCTCAAAGGTTCTGAG
GAGCCTCAAGGTGAACCAACAAGAAGAATATCCTCCAACGTTCTAAGGACCTCAAGGAACCAAGCAAGATGAATGTCAGAAGTT
CGAGGATCTAAGAGAAACCGAGCAAGATGAATTCTCCAGGATCTCAGGTTTCCCCGAATCATAACTAGATGAAAGTGAAGAGGT
TCCCGATCGACCTCTAGGAACCTAGCATGAAAATGCTCCAGGTTCCTCAGGACCATCAAGGAACCGAGCAACGATGAATTCTCC
CATGTTCCGAGGTTCTTACAAACGAAGCAAGAAAAGGCTCAAGGATCCTAGGCCATCAGAACCGAGCAAGATGAATGCTCAATG
ATTTTGAGGTTCTTCCTAGTATCCGGAGGCAAAAAAAAGACGAATATCCAGTGTTCTGAGGTTTTACAAGAACCTACGCAGATT
GAATGCTCAAAGGGTCCGAGGAACTCTCAGCAACAAGCTAAATATGAATGCTTAAATATTCAGAGTTCTCTAAGGAACAAAGAA
GAAGAATAACTCCAGGGTTTAGAGGTTCTCAACAGAAGAGCAATACTGAATGCTCAAAGGATGTCTGAGGACATCTAGGATGAA
GAAAGAACACGAATGCTCAAGGTTTCGAGGACTAAAGGAACCGAGCAAGAGAAAACTCCATGTTCCTGAGGTTCTCTAGGAAGC
AAAGCAACGATGTAATGCTCAAAGGTTCTGAGGACCCTCCGTAGGCAATGAAGCAAGACAACATGCTCAACGGTATTTCGAGGA
CCTAAAGCTACCGAGCAAGATGAACTCGCAACAAAGTCTTTCTGAGGTTCTCGTAGACCGAGCAATGTGAATGCTCCAGGATCC
AAGGTTCTCCTAGAATCCCGGAGCACGATGAATGCTCAAAGGTTCAATGACGCCTCTGGAGAACGAATGCAAATGCTCAAGGCG
TACGAAGGACCTCATGGAACCGAGTCAGAGGATGATGCTCAAAGGTTCTGAGGTTCTCTAGAGCCCGAGCACAGATGAATGCTC
CTGGTTTCTTGAGATTCTTCATGAACGGAGCATGCATGAATGCCAACACGCTTCTCACGGCCTGTATGAACTAAAGAAGAAGAA
TGTTCAGGTTAATGAGGCCTTGAGGCAACGACAAGATGAATGCAAAAAAGGTTTCTGAGGTTTAGGAGTGCAATATGATGTCTG
CGTCGAGGTTCTCTGGAACCGAGAATGATGATGCTTAAAGGTTCCACATGACCCCTAGGAACCAAGTAATGAAGCCAATGCTCT
AAGGTTACGCTGACCTCATGGAACCGAGCAAGATGAACTTCTCAAAGGTTCTAACCAGGCTCTCTCTAAGGGAACTAGGCACCA
GATGAATGCTCCAAGTCTCTGATATTCTCCATGAACCCGGCAGAGATGAAATGGCTCAAAGGTTCTGAGGTTTCGAAAAGGACC
GAGCAGAGATGATGCTACCAACAGATCTGAGGTTTCAGGAACCAAGCACGACTAAAGTGCTGAAAGGTTCTGATGACCGCTCTA
GGAAGCGCAGGCAACCACAGAATTCGCTCAAGGCTTCCGAGGAATCCCTTGGAACTGACAGCCAAATGGTGATTGCCCCCCCG
>m141122_040740_42134_c100732202550000001823141405141561_s1_p0/139816/8213_13650
TCCTTTCAGGCAAATCATCCTAGGCACGCTGCCTAAGGGCTCCGAAGCGATTGTCGTAATTCACTCATATTTCTCTCTAACAAG
CTCTAATTTTTTTGAACTTTCCCATATCAATCAATATTATATCGGGGCTAGTTATTGACCACTGATTGGGGTCCTAATTCAACA
AACAAACAAATTGACCTTTCAGGCCAATCTCCTAGGCACGCCGCCTAGGGCTCCGAAGCGATTTCGGCGTAACTTCCCTCATAT
TTCCCTCTAACAAGTCTAATTTTCTTTGGAACCTTCCCCATATCAATAATATTATAATAGGGCATCTATGTCATGAATTACGGT
CCTAATTCAACGAAACAAAAAATTGACTTTCAGGCCAAATCTACCTAGCAGCTGCTAGGCTCCGAAGCGATTTTCGTAATTCAC
TTATATTTCTCTCTAGACAAGACCTAAATCTTTTTGAAACCATTTCCAATCTCAATAATATCTATAGTCGGGCTATATTTGTCA
TGCACTTGGGGTCCAATCTCAACCAAACAAAAAAACATTGTCCTTTCAGGCCAAATCTCCTAGGCAGATACGCTAGGGCCTCCG
AAGCGGATTTCGTTAATCCTCATATTTCCCTCTAACACTCTATTTTTTGAACTTTTCCCAATATCAAATAATAATTTATGGCTA
TTATTGTCCATGAATTGGGGGTCCTAATTCAACAAACAAACAAAATGATTTCAGGCCAGAATCCCTAGGCAGACTGCCTGGGGT
CCGAGAAGCGATTTCGTAATTCACTTATATTTCTCTCTAACAAGCTCTAATTTTTTTGACCTTATTCCCATCATCAGATAATAT
GATTACGGGCTATTATTGTCCATCGATTGGGTCCTAATTCCAACCATACAAAAAAATTGACCTCTCAGGCAAATCTCCTAGGCA
GCTAACCTAGGGCTCCGAACCGATTCGTAATTCACCAATCATTTCTCTCCACAAGCTCTAATATTCCTTGGGAACCTTTTCCCA
TATCAACTATACTTATAGGCTACTTATTGTCCAAATGAATTGGGGTCCTAATTCTACCAAAAAAATAATTGACCTGTCAGGCCA
AATCTCTAGGCAGGCTCCTGGGGCTCACAAGCGATTATCAGTATTCACTTATATTTCTCTCTACCACCCCTCTAAATTTTTTGA
CCTTTTTCCATCTCATATACTTATAGGTACGGCGCGATTAATTGACATGAAACTGCGGTCCTAAATTCAACCAACACAAAAAAA
TTGACCTTTTCAGCCAAACACTCCTAGGCAAGCTGCCTAGGGCTCCGAAGCGATTTCGTAATTCACTCATATTTCTCTCTAACT
AGCTCTAATCTTTTTGAACCATTTCAATCTCAATAATAATTATAGTCGGGCTATTATTGACCATTGAATTGGGGTCCATTCAAC
CAAAAAAAAAAAATGTACCTTCTTCAGGCCAACATCTCCAAGGCAGCTGTCCTAGTGCTCCGAAGCGATCTCGTAACTTAACTC
CATATTTCTTCTCTAACAAGCGTAATTTTTTTGTACCTTTTCCCATCATCAATTAATATAATCATAAGGGCTATTACTTGACCA
TGAATTTGGGGTCCTAATTCAACAAAAAAAAAACGTTACCTTTCAGGCCAATCTCCATAGGCAAGCCTGCCAGGGACTCCGAGC
GATTTCCGTAATCCCTCATATTTTCATCCAACAAGCTCTAATTTCTTTTGAAACCTTTTCCATATCAATAATATTCGAATGATC
TACGGGGCTAATTATTGTCCATGACTTGCCGGTCCTAATTCAAACCATAACAAAAAATTCACTTTCAGGCCAAATCTCCTAGGC
AGCTGCTAGGGCTCGAAGCGCATTTCGTAATCTCCCTCATATTTCCCTCTAACAACCTAATTTTTGAACGCTTTTCCCATATCC
CAATAATATTTAATAGGAGCTATTATTGTCCATGAATCTCACGCCCTAATCAACGAAACAAAAAATTGACCTTTCAGGCCAAAA
TCCCTAGGCAGCTGCTAGGGCCAAGCGATTCGTAATTCACTTATATTTCTCTCTAACAAGCTCTAATTTTTTGAACATTCTCCA
GTTCTCAATAATATTATAAATCGGGCTATTATTCACCATGGAATTGGGGTCCTAATTCACCAAAAAAAAAAAATTGACCTTTCA
GGCCAATCTCCAAGGCACTGCCTGAGGGCTCCGCACAGCCGATTTCGTAATTCCACCTCATCTGCTTCTCTCTACTGTTATGCT
CTAATTTTTCTTTGATCCTCTTTCCAATCCTCAATAATAGTTCATTCAGCCAGGCTATTATTGACCATGCATTGCGGTCCTAAT
TCAGCCTACAAAAAATTGGACCTTTTCAGGCCAAATCCTCCAAGGCAGCTCTGCCTAGGGCTCCGAAGCGATGTCTGGAAATTC
ACTCATATTTCTCCTCTAACAGCTCTAATTTTTTTGAACCTTTTCCCATCCTCAAATAAATAATTACAAAGGCTATTATTGACA

TGAATTGGGGTACTAATTCGAACCAAATCAAAAATTACTTTCAGGCCAACTCCTAGGCAAGCTGCCCTAAGCGACTCCGAACGA
TTTTCGTAATTCCTCATACTTTTCCCTCCAACAGCTCTAATTTTTTTGAACTTTGTCCAATATTCAATAATATTATATAGGATA
CCTTATTGCTCTCATGAATTGGGGTCCTAATTTAACCAACAAACAAAATTCGACTTTCAGGCCAACATCCTCCCTAGGCAGCTG
CCTAGTGGCTCCGAAGCGATTTGTAATTCACTCCAGATTTCTCCTCTAACAGCTCTAATTTTTTGGAACTTTTCCCATCTCCAA
TAATAATTATATCGGGCTTATTATTGTCCTGAATTGGGGTCCTAAATTCAACCAAACAAAAAAATTGTCCTATAGGCCAAATCT
CCTAGGTCAGCTGCCTAGGGCTCCAAAGCGATCATTCGCTAATCTCCTCATAATTTTCCTCTAACAAGCCTTTATTTTTTTCTG
ACTTTCTCCCATATCAATAATATTACTCATAGGGCTATTAATCTGTCCATGAATTGGGTCCTAAGTTCAACCAATACAAAAAAC
ATTGAACTTTCAGGCCAACAATCTCCTAGCAGCTGCCCTGGGGCCTCGAAGCGTTTCCGCTCATTCACTTATTTCTCTCTCTAA
CCAAGCTCTAATATTCGTTGAACCTTTTCCCATATCAATAATATTATATCACCGTACCTCTATCTCTCATGAACTCTCGGGGTT
AATTCAACCCAAACAAAAAAACCATCCTTGCACCTTCAGGCCAAATTCCTACGGCAGCGCCTACTACGGGCTCCGAACCGATTT
CGTAAATTCACTCATATTTCTCTCCAACCAAGCTCTAATATTTTTGAACCTTTTCCATATTCAATAATATTATATATGGGCTAT
TATTGTCCATGAATTGGGGGGTCCTACATTCTAAACAAAAAAATCAGGACCTTTCAGCGCCAAATCTCCTAGGCAGCTGCCTGG
GCCTCGCGAGCGATTATCGTAATCACTACATTTCTCTGCTAACAACGCCTGCTAATTTTTTGCAACGCTTTTCCCATATCAAAT
AATATATATACGGGCGGCTACTATTATTGTCATCGAATTCGGGGTCCCTATGTCAACCAAACAAAAAAATTGAGCCTTTCAGGG
CCAAATCCTCCTAGGCAGCTGCCTAGGGCTCCGAGCGTCTCGATTCACTCATAATTTCTCTCTACACACGCTCTAATTTTTTGA
ACTTTTCCTCTCATAATTATATAGGGCGATTATCTAGCATGAATTGCGGTCTAAAATTCAAACAAACAAAAAAAAATTTGAACTT
ATCAAGGCAAAACTCCTAGGCTGCTGCCTAGGGCTCCGAAGCGATTTCGCTAATTCAACTCATCATCTTCTCTCTATACGCTCT
AATCTTTCTTGAACCATTCCAATCTCAATATACCCTTGATATCGGGCTAACTTATTTGACCATGAATCTGGGGTCCTAATTCAA
CGAAAAAAAAAAAAATTGACCTTTCAGGCCAAATCTCCAAGCAGCTGCGCTAGGCAGCTCTCGAAGCGAAGTCTTCGTAACTTC
ACTCTCATTTGCTCCCTCTAACAAGAGCTAAATTTTTTTGATAACCTTCTTCCATCCTCCAATAATATCTCATCAAAGGGCCTA
TTATCTGACCATCGAATTTGGGGGTCCTAATCTCAACCAAACAAAAAATAGTTACCTTTCAGGCACAATCTCCTAGCCGCTGGC
TTAGGACTCCGAAGCGATTTCGTAATTCCAATCATTTTCATCAACAAGCTCTAATTTTTTTTCGAACCTTTTCCAATATCAATA
CATATTAGTATACGGGCTACTTATTGCTCCATGAGATTGCGTCCTAATTCACACAAACAAAAAAATTGACTTTCAGGCCAATCA
TTCCTAGGCACGCTGCCTAAGCGGCTCGAAGCCGATGTTCGTAATTTCACTCATATTTCTCTCTAACAAGCTCTAATTTTTTTT
GAACCTTTTCCCCATAGTCATAATATTATATCACGGGCTAAACATAAGCCATGAATTGGGGTCCTAATAATTCACCAAACCAAA
AAAATTGAAGCCCTTTCCAGGCCAAATCCTCTAGGGCAGCGCTAGGGCTCCGAAGCGATTTCGTAATTCCGCTCCATACATTGA
CCCTCTAACAAGCTCTAATTTTTTGAAGCCCTTTCCATATCAATAATATTATAATAGGGCTATTTATTGTCCATGAATTACGGT
CTAATCAACGAAACAAAAAAATTGACCTTTCAGGCCAAATCTCCTAGGCAGCTGCCTAGGGCCTCCGAAGCGATTTCGTAAATT
CCACTTATATTTTCTAAAATAACAAGCTCTATCCTTTTTGACCATTTCACTCTCAATAATATTACTTATCCGGGGGGCGCCGGC
ACGGTCAACGTTATCGACCGGCGGATTTCGGAAAAATTGGGGTCCCCAAGTTCAACCAAAAAAAAAAATTGACTTTCAGGCCAA
ATCTCCAAGGAAGATGCCTAGGCTCCAGAAAGCGATTTCGTAATTCACTCATGCTTTCTCTCTAACTTGCTTAATTTTTTTGAT
CCTTTTCCAATCCAATAATATTATACTAGGAGCTATTTTGACATGAATGGGGTCCTAAATTCAGCAATGAACAAAATAATCTGA
CTTTCAGCAAAAATCTCCAAGCAGCTTGCCTAGGGGCTCCGAGCGATCGGAAATGTCACTCATATTCTCTCTAACAAGCTCTAA
TTTTTTGAACGCTTTTCCCATCCTCCCAATAATATTAAAGGGATATTAGTGCCGAATCGGAATTGGGGTACCTATTCAACAAA
ACAAAAAAAATCTTACCTTCAGGCCCAACATCTCCTAGGGCAGCTGCCTAGATCCGAAGCG
>m141122_040740_42134_c100732202550000001823141405141561_s1_p0/140135/4549_10122
ATTGCTTTATTCCCATAATCTTGCCTGTCGCTTAAGATAAAGCATGTGAAACATCCTAGTACCAACCACCATAATGGTATACAT
AGCAGCGAAACAAATATAGCTAAAACCAATACAAATCTCAATCACTCTAAAAATATTATACAATTCTTGTATCATCCTCCAACA
TCTAAATAGGCACTTAAGACCTCCAAATCCAACATAACTCTAACTCCCATAATATACATCGAATAATAAACACTTGCTCCATGA
AATAACGGAATGCCTAAACTCATATAAGTGATGGACAAGCTAACAACTCATTACCCTTAATAATTATGAATCACAATTCCTTAT
ACTTATCCCTTCTAATAGAATGTAAACTATAAATGGTAATCTTAATGATATAATCGACCTGTATATCAAGAAATCTGTTCTCCT
CCTACCCCACCTGTCTAACTCGAAGTTTCTATTCTCTTGTGTGTCCCTTGTCTAAGAACTCTAGCAATACATAATGACTCATTT
GTTTATTTCTTTCGACTCATATAATTCATGGAGAAAAGAGCACAAATGAATGGTTTATCAGTAACATGTATTTTACAATAGTTA
TTGACTCTTGTTTCTCATTCCGGCCCTCTGCTCAAAAAACATTACATAGCACTCCCCCTAACATCCCATTACTGTCCCCCGACT
GAACTGCCAACAACCCACCACAGCAAGCTGGATCATAGAATAATCACGAACACTGAAAACCACCTATGAATTCAATCCAGAACA
GTGCAAGCGGAACACAACCTGGCCCCTTCTTCCAGAAAATTGACTGCACATCTAAGCCTGCTGTGATGCTACTGAGAAAGGAAT
AAAGAATCTTACGAGGACTGGGAACACCTCCCCCCAAAATACAGTCTGATCTTAGCCCAAGGCCATGATGCCCGTCATATAGCC
AGGAAATAAGTCCACACCAGACAATACTGGACGAAATCCCATTCGTGATAGTAAATTCCAAGTCTGCAGAATGCAAAGGATTTT
CCTTTTGCTGCCATTAAGGCAAAAGTAATAAGAAAATTCTCAAAGACATGAGAGGAATGAACACTTGAAACATGCTGCTCACAT
GTTGTACGCTGGGTTATCAAGTGACGAATCAAGAGCTCAACATCTGACAAAGCTCGGGGCCACCTATCCCATAAACTTGAAGGC
ATGGGATACTGGACCGCTTGGCTGAGGATTATGGTCGGTGGGTTCAGTGATGAGAACATTCCATCCCTCAAGTTCTGAGTTCAA
ATCCAAAGTAAGCTTTACACAATGCCAGAAGGCAGCAATGGGACTGTTGGGGAGATGTCTGTTATCCTGCCAAAGGAAGAGGAG
GGACATGAGAAAAGAGTGGCAGAAGCTAATCTCCATGGATGGCGACCCAACAACTCCTCACAAGAGGAAAGTTCACTTCCAACA
ACTCTAGTGACAAAAGCATCCTAATAGAAGAAAGCCCACATCCTAAGGAAATGGGCAAAGCTAGCTCATCTTCAAACTGGTCAT
AAGAACTCGAGTTTTTCAAAAGGAAGTGCAACGTCCTTTGTCAACTGCCGTTTGGCATACAAACGCTGATTGTCGACAGAAGCA
TCCAAAGCTCACCTTCAGAGAAGAAAGCGTAATTGTCTTGTGATGGTTCTTGTTTAGAGTCCAATATTATTGGATGTGCCTCAA
ACACTCTGGTGCGTTCAGATACTCAGGAGCCCTTACAATTCATGTTACTAATGTCCATTTGCAGGCAGTGACAAACCCGAAAGA
AGCCGAGTAGGTTGGAGGAGCATGTATGACCATGCGGAGACGATACTAAAGTCCCAGAGCTTTGGATTTTTTGGGGGACTCGTC

CCAAGACTTTGCGCTAAAATCTGGGCATCTTTTGGAGTTGCATGATGTTGCTTATTTCTACCCTCTATTACGAGGAATTTGATT
TCTGTATCTTATTTTGGATAGAGACTAGGTTATAGTCTTCCAATTTTGAATCTGGAAAAACTACTTTGTATGCCTGACCTCTAT
GGTTTGGTTGGAAATGGAACTTTATTGTGGAAACTTATATAAGCTAGCGGGGGGGGGGGTGGAGGGCGGGCGGGCGGGGCGGGC
GGGGGGCGGGGGGGAGGGTAGGGGGCGGGGAGTGGGGGGGGGGGGGGGGGGCGAGGGGGGGGCGCGGGGGGGGCCGGGGGGGGG
GCGGGGGGGGGGGGGGGGGGGGGGGGGGGGGGGGGGGGGGGGGGGGGGGGGGGGGGGGGGGGGCCGGGGGGGGGGGGGGGGGGGGG
GGGGGGGGGCGGGGGGGGGGGGGGGGGGCGGGGGGGCGGGGAGGGCGGGGGCGGGGGGGGGGGGGGGGCGGGGGGGGGGGAAAG
GGGGGGGGGGGGGGGGGCGGGGGGGGGGGGGGGGGGGGGGGGGGGGGGGGGGGGGGGGGGGGGGGGGGGGGGGCGACGGGGGGG
GGGGGGGGGGGGGGGGGGGCGGGGGGGGGGGGGGGCGGATTTAGCATGAGCTCTGTTCCGTGACAAAAAACTTCTACTCCGACT
CTTCTGTTCTTCCTTTCTCCTTAATGCTATTGTTGGGATCACAACGTGCAAGATAAGATTTTAACAACTGCCTTATATGCTTTT
GGAAGAGACGCTTATAAGCACCATATTCTAGAGATAGACATCGGGAGATTGGTGACGGACTGGTGCGTACCTTCAGGATCCTGT
GAGTTTACTCTGATTTCACTGCCTGTCGTGGTGATTGCTAATAAAAGGAAACGTTGACTGCCACGGTTAGCAAGAGCAAGCAAG
CACAGGGCAGCCAGATGTAATCTCGGACACTTATTCCAATACTGTATTTGTGGGCTGTTGTCTCCACCTGCTTATGGGTGGTTC
TAGATACTTTATCACCTTCATTGATGATTTTCCGGTATGGCATGTTGGCTTCATTCGTGAAAAATCTGATCTTTGGATGCCTGT
TTAAGATTTTCAAGACGAATGTTGAGCTATCAAAAGGGAAAGAAGATTAAAATTGATCAACTCTGAATCAAGAGGTGGAAGAGT
ATTTATGGACTTTAATGATGAAACTGAAAGGAACCCCGGACCTTCTCATGCCAAATACTTGCAGGAAGTGATGTTTGAATGGCA
AGATTACAATGCCCTGCCAGGGAGACACCCCAACAGAATGGGAAATTGCTGAAAGGAAGACATCGACCTCCTTCTTGACATGGC
TGCGATGTACTCGTTATATTCATTCTTAGCTTGCAGATTTTTTATGGGGGTGGAGGCATTAAAAACTCAGCTTATATCTTGAAT
CAAGTGCCCTAGCAAGTCCTGTTCCCAAAGACGCCTTATGAGCTGTATGTCAAGTAAGACAACCAGGCTTGCGTCATTTTCAAT
GTTTGGGGTTGCAAAGCAGAAGTGAGGCCTAATATACCACCAGTCTAAGAAACCTTGATGCCGAAGACACCATTAGTGGATATT
TTGTGGCTACACAATTGGATCAAAGGGTTCCAGATCTTTATTGGCTCATCTCACACCACCCGAGGGTTATAAGAATCAAGATAC
GGGCCTTTCTATCTTTTGAGGCATGATTTGGTTTCACAGTGCAGGGGTCAAGAGAACTTGTTTTTCCAGGGAAGAACGTATTTT
TTTGTCCCTCGTACCTGTCCGCTTTCCTTTTTTTGCTACTTATTGATGACCCTGTGGATTGACAGATTCCGGCGAAACTCATGA
ATGAACCTCAAAATCAAGAAACAAGGTTGAAAATTTGATATCTGGGGATCGATGAACGCATATGAAATGAAAATCACAGAGAAC
CCGCAGCTGCTATTCCTAAACTGACTACCCTTGTCTCATTTTAGGAACCATGAAGTTTGATGTGGAGACACTTTTGAGCAGTCA
CCTATTTAAGAAGCTATGCAATAGTCTCAATCTGTGTTGTGGATGGATCCAATGAAAGATGAGTTTTTGAATTCTATGTCACAA
AATGAAGTTTGGAGTTGGCTCGCACATTACCAAAAAGTTTGCAGACCATTTGATGCAAAATGGGGTGTTATAACGAATCACGTG
ACTCATGGCCAAGTGGAAAGGTAATTAGGCTAGCGTCGCTAAGGGCTATATCCAAGAGAGAAGGTATTGATTTCAAAGAACCTT
TTTTCACCTTGTTTTTATTTCCACCAAAGTTCGTTTGCGAATCATTTATGGCTAAGGTTCTGCTCCAATTTGATCTAGAAACTC
AATCAAAATTGGAAATGTGGACTGCCTTCATTGAATGGAGCATTTAATTGAAGATTGTGTTACATGACTCAACCTATTGTTTTG
AAGAAGCTCTGGCAAAGAACCACATGTTTCTTGCAAGACTCAAAAAGTTCTATCTATAGGCTTAAAACAAGCATCAAGGCATTG
GTATCTCCAAAGTCTTCGATATGATTGTCTCTGCAAATGCTCAAGGAGAAGTAGTTAGTCAATGTATTACATGAAGGTCAGTGG
GAGCAGCTATATTTTTCTAGTATTGGTTGTTATGGACAATCATGCTTGCATCTAACTAATTACTGATCTGTTTGTCTGAGACAC
AAACACTTTTTGTCTAGCCATTTCTGTATGACAGGATCTTGGTGAGGAATTTCCTTAATGCTTGGGATTCCAGAATTTCTTGTG
AGATAGGGCTAATGGTAATTTCTTTCGTTTGTCTCAGAAAGACTTACATTGATGGATCTGAGAAAGCCATTCAATATGCATACA
TGTTTCCTCCTGGGAAGGCACGATAGTGAAGGTGATAAGTTTCTCAAAGGCCCAAATGTTCCACAAAATGATAAAGAGGATGAA
AAATGAAAGTCAATTCCTTAAATGCGTCCATTGGTTTGGTAGCTTTGATGTATGCCTCAAGTATGCACACGCCATGATATTGCT
TTTGTTGTCAGCCGTGTTTGGGTAGATACTTGAGTGATCCTGGTCTTAGCCATTGGAAAAGCGGCTAAGAAAGTCATGGATATC
TATTCAGGGTAACAAAGGGATCATATGTCGACCTTATGCGCGAGCTGTGACACTCTTGGATTAGTTGGTTCAGTGACCCGATCT
ATGCAGGATCGTGGATGATAAACAAGTCACTTCAAGGCTACATTATATGATGGCTGGAGACTGTTCATGGAAAAAGTGTCAGCA
GAACATCACCAACTTTCTTTCTACGAATGGAGGCAAGAATACTGCTAGGGTGTGTACTGAGGCTACTTGCTCAGCATATGGCCT
GCGGAATTTCATTTCGCTTCTTGAATGTATGTAGACCCCTAATTTCGAGGCGTGAAATGTATTCCGTGACAAACTCCGCAGCAG
TAGCTTCTCTAAGTACACCTAAGGAGTACTTCTTCGGGCTCAAGGCATATTGATCATAAAGTACTATTTTGTTAAAGAGAAAGT
CGAGTGAGTCTCTTATTATACATATTGACGTACACGCCTACCACTAGCATGTTGGTAGACAGCACTACGACACAGCGCTACCAT
ATGTGTTTTTCCTGGAGCAATGTTGCTCGGATTTTTTTTGGGATTGTTAGGAAGCCTAGCTTGTTGAGCTCAGTGGGAGTTTTG
TTATTAATGTATAAACATGCTATATTTTG
>m141122_040740_42134_c100732202550000001823141405141561_s1_p0/142963/579_6869
GGTATGTATTTAATAGATGATTTGGAAATAGTAAAAAAAAATAGAGCTTCTTAGACTGAAAATATGAGTGGAAGTTACGAGATA
TCCAAGCATTCGGAAGATTATGTCGCTGCATTTAGGAGAAATTAGGCTTAAAGAGAGTGTCAATCTTTAGTTTTGGATTTTTAT
AAATCTAATGATTCTATAAAGTTCGATGGTCGAATTCCCCATAAAAGAACATAATTAAGGTTTGAAAAATAATTAAATATTTGG
AAAATATATAATTGAAAGGAGATGTGGAAATAAACACAAGGTATCCATTAGAAGGTTAAAGCTTGTATTAAATAAAATCGGAAA
GTTATGTGAATGTGAAGGTTAATTCAGCTTCTGAGCTCCTAAGAAGCACAGCGACTCAATGGAGATTTGGCCTGTAAACAGGTC
GTATATTTTATGATATCCGTGAATTAAAGGCCACCTCAAATGGCAAAATAACACTCGCCCATAATAATATTGAGCAATGTTCCT
GGTAGAAATGGAAAAGATTCAAAAGTCTTCAGCTATAGTGTAGAGAGAAATATGAGTGAATTACGAGATCGCTTCGAGCCCTAG
GCAGCTGCCTAGGAGATTTGGCCCTGAAAGGTCAATTTTTTTGTTTGGTTGAATTAGGACCCCAGTTCATGGTCAATAATAGCC
TAATATACGGCCCCCCGAAACTATTGTATTTGTGAAGAACAGATGCGGCACAAGCCGGATTTTCAAAAAAATTAGAGCTTATTA
GAGAGAAATTTGCAATGCGACTCTCATTCAGCGATAACGTCCCATAGAGCCACTAGGCGCATTGCGTGCCGTAGTGAGATTTTC
GCCTGTTAAGATCAAGAAATATTTTATGTTTTTGCGTATGAATTAAGGAAGCCTCAATAAATTGGCCACTTTGGTCAATACCTC
ATTGGCCATATATTGCACCCACTTATTTAGAGTAGCATGGGAACAGGCTTCAAAGAATATAGAGCTGTAGAGACGAAACGTTAT

GATGTGTAGACTATGAGAGAGATCAGTCTGTATGAGAGCCACCCGCAAGTAACGAAAGCCCAGCCTAAAAATCAAGTAGAATTT
GGCCCCCCATGAAAGGTCAAATTTATCTTAGTTTTGGTAAATTAGGACCACCCCCTAAAATTCATTGGGCTCAAAATAATAGGC
TCCCAGATAATAATAATATATACTAGTTATTTATATCATTAAAGGCGAAAAGAAGATTCAATAAAATTTAGATAAGCCTTCGTT
CAGAAGAGAAATAAAACTAGTGAAGAAATTAACGAAATAACATATTTGAATCGGAAAGACCCGCACTAGGAGCGCAACGAGTCT
CCATTATTCCTCTGCGAGATATATGTGAGCCAAGAAACAAAGGAGATCAACTTTCTTGTTTGTTGAATTCAGGACCCACACACA
TCTCAATAGGATACCAAATAATAACGCCCTACAAACGTAGCGCCTAAATATTAAATTAGGGCCGAGATGGGAGACTCGGTTCCA
ACACAAAAATTAGAGATAAGAAGGGCGGGCTCTTACCGCACGAGACACACATTGAGTGTAACCTTACAGATCCGCTTTCCGGAG
CCCCTAGGCTGCTGGCCTAGGAGATTTGGCCTGAAAGGTCAATTTTTGTGTTTGGTTGAATTAGGACCCCAGTTTCTGGTCAAT
AATAGCCGCTAGATATAAGGCTCCACTTATTTGGGGGATATATAAATAAGGGTTCATAACAAAATCTAGAGCGAGTTAGAGAG
ATAATATGAGTGAATACGTCCTAACACTCCCGCTTTCCGGAGCGCCCCTCACGCCGCTGGCCTAGGCGAGATTTGGCCCTGGAG
GGGTGCAATTGTTTTGTTTGGTTGAAATAGGAACTCTACCCAAATTTCATTTTTGGTCCAATCCAATTCAGTTGCCGCTATATT
AATATTTTAATTATATCAGATGGGAAACCAGCGTTGCAAAAGCGAACATTAGAGCCTACTTTATGGGGAGAGAAAATATGAGTG
AATAAGAGATCTGCTTTCGGAGCCACTAGGCAGCTTGCCTTACCGGAACGCACTCACCCCTGCGGCCTGAAACACGTCAACTCT
TTTTTTGCCTTTGGTCGATATTTGACGCCAGTTCAATAAAAGGAATACATGAGCCCTATAAATTATAGCATATTAAATTGAGAT
GGAAAAGGTCAAATAAATTCAGAAGATTGTTGAGAGAGAAATATGAGATCAGCATGTACGAATATTCAGGCTTCGGAGCCTAGG
CAGCTGCCTACAGGAGATTTGGCCTCTCGACAACAGATCGAGAGGTTTTTTTTGTTTGCGCCCTTGAATTAGGACCCCCCAAGA
ATTCAGCTCTCCCCGGCCACATAGAACTTTAATTGCCCAATAGACCTAATATATTATTCAAGAATGGGTAAAGGGTTCCCAGAA
CAAAGAATAGAGCTTGATATAGTGAGGAGTAATATGAGTAGATGACTACGAGAGTCGCTGCCCCTCTGAAGCCCATACGGCCCG
AGCTGGGCCTCTATGTAGATGTGGTCCTGAAAGGTCAATTTTTTTGTTTGGTTGAATTAGGACCCCAATTCATGGTAAATAATA
TTGGCCTAATAATAATTATTACTTAATGAGATGGAAAGGTTCACAAAATTAGAGATTGTTACGAGAGAAATATGAGTGAATTAC
GAAATCTGTTTCGGAGGCCCCTAGGCAGGTGCTAAGGAGATTTGGCCCCTGAAAGGCAATTTTTTTGTTGGTTGATAGGAGCCC
AAATTCAATGGTCAATATATAGCCCCATATATTCATTTTTTTTAATTTCTATGGGATAAAGATTCAATACAAAATTTGAGAGGA
GCTTCGCTCTAGAGAGAAATATGAGCTGATTACGAGATAACTTCGGAGGCCTAGGGCAGCTGGCCTTAGGATCTGGCCGAGCCA
AAGCGCTCACCCCTTTTTATTTGGTTGAATTAGGAATCCCAATTCAGGTCAATAATAGGCCCTTATATAATATTATTGAGATGG
AAATGGTCAAAAAAATTAGAGGCTAGTTTAGAGAGAAAATATGAGTGAATTACGAGATCGCTTCGGAGGCCCTATGGCCTGGCT
GCCTAGGAGATGTTGGCCTAAAAGGTCAATTTTTTTGTTTGGTTGAATAGGACCCCAGTTCATGGTCAATAATAGCCCCTATAC
TAATTTAGTTGATGATGGGAAAAGGTTCAAAAAAATTGAGCTTTCTTATAGACCCGAAATATCGAGTGAATTAGCGAGATCACT
TCAGAGAGCTAAGGCTACTGCCTAGGAATTTGCCAGGAAGTCAACTTTTTGTTTGGTTGGAATTAGGACCCCAATTCATACGGT
CAAGCTAATGGCCTCTATACCGAACCTACGCCGTAACCCAGCGGCATCCTTAAATTTAAGATGGGAAAAGGTTCAACAAAAAAC
CGTGTAGCCACGCTTCCGTTCTTACACCCACCGACGACACATACCCGTCTGCGAGCTCGAACTACCGCAGCCCCATCGCTCTCC
GCGCTGCCCCTTACACAAGGAGGCTGACTAAACGAGATTTGCCCGCTGATAAGGTCAATTTTTTTGTTTGGTTGAAAATTAGGA
ACAGGCCAAATTCATGGTAAATAATAGGCCGTATATAACTATTATTCGAGATGGAAAAGGTTTCAAAAAATGTCAGAGATTTTT
AGAAAGAAATATGAGTGACATTACGAATCGGCTTCGAAGCCTGGCAGCTGGCCTAGGAGATTTGGCCTGAAAGAGAAAAAAAAA
AAAAAAAAAAATTTTTTGTTTGGTTCATTAGGAGCCCCAATTCATGGTAAAAATAATAGCCCCTATATAATATTAATGAGATGC
CGGATAAATAGGTTCAAAAAAATTAGAAGCTTCTAGAGAGAAATAATGAGTGATTCAGAGATCAGCTTTCTCGGAGGCCCCGTG
TGCTGCATGGCCAGGAGATTCTGGCCCTGAAGGTCAATTTTTTTGTTTGGTTGAATTAGGCACCGCCCCAATTCATGGTCAATA
TCGCTTGCCCTAATACTAAATTATTGAGATGGGAAAAGGTTCAAAAAATATTAGAGATTGTTAGAGAGAAATACTAAGTTAATT
ACGAAATCGCTTCGGGCGCCTAGCAGCTCCCTAGGAGATTTGGCCTGAAAGGTCAATTTTTTTGTTTGGTTGAATTAGGCCGCC
GCAATTCATGTCAAAAATAGCCACTATATAAATATGAGATGGGAAAGGTCAAAAAAATAAGGTTGTTAGAGAGAATATGGACCC
GGCCCCCGGCCCCCATAACGAGATCCGCTCCTTCGTAGCCCTACAGGCCCGCTGCCATACCTGGAGATTCTGGCCTGATAAGGT
CAATTTTTTTCTTTGATTGAATTAGGACCCCAATTCCATGTCATAATAGACCCGAACGCTACAGAATTATTATATGAGATGGG
AAACAAAAGGTTCAAAAAAACTAGAGGCTTGTTATAGGAAAAGAGTATGTGTGAATTAGGAAACTCGCTTCGGAGCCACTAGGC
AGCTGCCGCTAACAGGGAGATTTGGCGCCCCTGAAAGGTCAATTTTTTTGTTTTGGCTTGTATTAGGACCCCAAATAATGGTCA
TAATAGCCCATCATAATAATGTATTTAGATGGGAAATAATTCAAGAATTTTTAGAGGCTTGTTAGAGAGAAAATATGAGTGAAT
ATAACGAGAGTCGCTGCTTCGGAGCCTAGGCAGAGCTGCCCTAGGAGACTTTTGGCCTGAAAGGTCAATTTTTTTGTTTGGTTG
AATTAGGACCCAGTTCATGGTCAATAATAGGCCTATATAATAATTATTGAGATGGAAAAGGTTGCAAAAAAATAGAGCTTCATG
TAGCAGCACCCCGAAATATGAGTGAATTACGAAAATCGCTTCGGAGCCCTAAAAGGCAGCTTGGACTAGGAATTTGGCCCTGAA
AGATCAATTTTTTTGTTGGTGAATTCAGGGACCCATTATGGTTCATTGCCATATAGTACCTTTTATTATTAGATGGAAACCGGG
CGTTCCAACAAGAATTAGAAGCTTGTTGGACCGCCCCAGAGAAATAGTGAGTGTGAATAACATAGCAGCAGCCCGCTTCCATGA
GCCCTACAGCAGCATGCCTAAGACGCCATTCTGGCTGAAAGTCATTTTTTGCTTTTGGTAGCTTAAGGGACGCACCCTTGGCCA
ATCCTGCGGTCACTCACTAGCCCGCCATATAATATTGCATTCTCTATATGCACAGGAAAACCGAATTTCCAAGAAATTAGAGCT
TGTTAAAGAGAGAAATATGAAGTGAAATTGACCGAGATAACTTCGGAGCGCTAGGGCCCAGCTGCCCTTCGGAGATTTGGACTA
GAAAGTCAATTAATTTTTGTTTGGTTAATTAGGACCCCTCCACAAAACATGGTCTAAGTTTCTAATAGCCCTATTACTAATAAT
TATTAGGATGGAACGGTTCAAAAAAAATTAGAGAGATAGCATTTATTGAGAGAAATATAAGATGAATTACGAGAGTGCGATAGT
TACGGAAACCCTATGGCTCGCATGGCCCTACGAGAATATTGAGACTGGATCAAGTTTTTTGTATTTGGTTGATTAGGACCCCCA
GTTCATGGTCAATATAGTATATATTCAATAATTTATTTCAACAGGATGCGGAAAAGGTTCAAAATTAGAGCTAGTTACAGAGAA
TAGAGTGAACTATAACGAATCGCTTCGGAGCCCTAAAATGGCACGCTGCCTAGGAGAATTTGCCTGAAAGGGATCAGTTTTTTT
GTTTGGATCTTGAATTAAGAGAGCGCGCGATACATGAGTCCAAAATCATTGCCCCTAACTCTACTAATTTTCTATATATTTTAC

AGATGGGAAAGGTGTTAAAGAATTAGAGCTAACTTAGAGAGAACATATTGAGTGATAATTACGAGATCTGTTCGAAGCCCTTAG
GCCAGACTGCTATAGGAGATAAATGGACCTGAAAGGTACAATTTTTTGTTTGGATGAAATATTCGGAAACGCCCAATTTTCATG
GTCAAATATGTCATTGCCCTTATATGGGGACGAAATAAAATATTTAGTATCCAGAAGAATGTGGCAGAATAGGTGCAAAGAAAG
TGTAGAGATTGTCTACGCCCAGAAGAAATATGAGTGAAGTATTAGGCGAAATCCTGCCGTCGCGGAGCCTTAGGGCAAAGCTAG
CCTAAGGAGATTTGGCCCTGAAAGATCAATTTTTTGTTTGGTTGAATTAGGACCCACAACAATTCTCATCGGTC
>m141122_040740_42134_c100732202550000001823141405141561_s1_p0/148827/11486_19412
GTCGCTGTCGAACTGCGAGTCCATTATCGGCATTACGATTTTCTTTGCAGCTCGAACCGGCAGATAATGCCTTTATGACTCGCG
AAGCCTCGACGGACTCTTCTTGGAGTGATTGCTTGCCAAAAACGGTCTGCTCAGCCCACTTTGTGGAAGTAGTCGATAGCCCCA
AGTCAGCGGACCCCGCCCTTTCCAGTAGCGGAGGCGCGCAAACGCAAGGCTCGATTCCCCAAACCCGCGAATGCACGAGGACGC
AGAATCATCTTCAGCTTCGACTGGGGGGGTGAAAAGCTCGGCGGCAACTGTGGCGAAGTCGCTGGCACTTGCTCACATTTCTTT
GACCGTAGCGAGATTGAGTCTTCTGACAGAGTGGGCCAAATATACCTTGCCTTAAGCTGTTCAGGGCCCAAGGCTTTGCCCCCC
CCACGCTGCTGGTCCCCGGGCAAAACCCTTCGTGCACTTCCTGCAGATGGTCTTTTGCTTTCGCGCTAGCAGAGCGCCACCGGC
TCAACACACACTCGTGGCAGGGGTAAAAAAAGGAACAGGCGAGTCCCCAAAAAAAAACGAGCCCGGGATTGAAAACCAAAGCAC
CCCAAACAAAATTCTTTGTTCACTTAATTCCGTGATACCCCCCTTTCGGGAAGAGCCCCCCCCTTTCGGGTTAAAAACAAGGCT
TAAAAATCCCTTTTTTTCTTGTTTTTCGTTGTTGTGCCGGTGCCCGTTTCCCCCTCAAAAGTTTACACCGCCCCCCCCTTTCCC
GGGTCAGAACTTTTCTGTTCCCCTTTTTTTTTTTTTTCCCGACTTTGCAGAAATTACCTTTTTCGGAAGGAAAAAATGGGGGGG
GGGGGCGTTTTCATCCCCGAAAAAAAAAAAAAGTTTCCCCCGGTTTCTTCCTTACAAAGTTGGGGGGGGGTTTTTTTGGATCCC
CCCCAAATCGTTCCGGTAAACCCTTTGTTCCCCCCCGCCCCCCGTTCCTTCCCTCTTTTTTTTTTCCCCCCCCCTATTAACTG
TTGCTTTGGGGTTTTTTTCCTTTTTTCCCCCAACAAGGAAAATTCCTGACCCGTTTGAAAAGACCCTGAAAGAAGACCAACCAA
AAAAAGCCCCTTCCGGTTCCCTTTTTTTCCCACCCCGGAGGGGCGTAGCGTAGCTTTTGGGGGCACAAAAAAGGCCGGTCCCCT
CTTGCGTTTTTAAAAAGGCCCCGGATTTTGGCCTCCACCTTCCCCCCCCGAAGGGTAGTTCTTTTTTGCAGTTGTTTTCCGAGT
GTTTTGTGTTGAAAAGCCCCCCGTGTTGGTTCAACAATTGCCCCCGGACAGCTCCCAAAGACCTGGTTTTGTCTCCACCCTTGG
GCCCCCAGGTTGTAGCCCCAAAGCCCAAATCCCCTTTTTTGGTTTTTTCTCCCCCCCCGTGGGCCTTTTTTGAATTTTCTCCAC
CTTAAAAGCCACCTGGGTTTTTTTGTTTTTTTTGTTTTTTTTTGTTTTTTAAGGAAAACCAACGCAGCCCCTTGGGGGAAAATT
TCCCCGGCTTTTTGAAAAAGCATTGAAACCGGAAGGGGGGCCTTGGCCCCCCCCTTTTCACACCGTGAGCGCCTTCCTTGGGCT
TTACCCCCCCCCCCCTTTTTTTTTAAAAACCTTTTTTTTTTAAGCCCCCAAGCAAAAAAGGCTTTTTCGGGTCTTAAAAATTTC
CCCCCCGCCCCCTTCTCCCCCCCCGTTTTTTTTTTTTTTGTTGGATGCCCCTTTGAGAAAAAAATTTTTTCCCGACATCTCCGA
GCCGCCGAGGGTTGGAAAAAAAATGTTTTCCCTTATGCCCCCCCCTTTTTTCCCTTTTGGGAATAAAAATCAACAAATGGGAATTT
TCCCCAATCCCCCCCGCGAAACCGCCTCCCCCGGCTTTTGGTTTTCTTTTTCCCCCTTTCCACATTAAAATTAAAATTGAGGCC
CGTTCCCCCCCCCCCCAACCGAAGGGCTTCTTTTTTCCCAATGAAAAAAACGCCTGGGGGGGCCGACGGTTGGGGGGACTCTGG
GGCTGGGGAATCTCTCCCCCAAAACAAAAAGAAAAGCCCTTTTTTTCCCTTTCTTTCTGGGAATTCCCTTTTGTGGGGCGGCCA
AATTCCCGGCCAAACGAAAAATCCGGCCCCCAGGCGGGCCCCCCCCCTTTTGGCTTTTTTTTTTCTATTTTTTTAATTCACA
CAGTTTCTGGTGGGGGGTTTTTTGGGGCGCTTAAAAAAAAATTTCTTCGGGGAACCTTTTTGAACCCCCTCTGTTTTTGGCGAG
CCCTTCAAAACCTAGCCCACCTTTTTCGACAAAAGAGCCCCGGTTTCCCCCGAGTGCGCCTTTTCCAGGGTTTTTTTTTTTGGC
AAAAAAACCCTTCTGGCCCCCCCTTTTTTTCTAACAAGGTTTTTTTTTTTTTGGATTCCGGCGTCCCAACGACCGTGGCTTAA
TTGAAGTGGGGGATTTTTTTTGGAAAACAATTAAACCCCGGCCTTTTTTCTGCAGGCCTTTTCTCTTTTTACTTTCCCCGCG
GGCAGGGCCCGCTGGGGAACTCGTTACCCAAGGCCGAACACGAATCGCCTCAAAACAAAATTTCCCTTTTTTTTCCCATTTTCA
AACGGGCTTTTTTTTTTAAAAAAAAATTCCCGGGGCACCCCTTTCCGGGCCCCCCACGAGGGGGGAATTCCTTGTTTTTCCTTTG
CTTGAATGTGTAAATAAGACCCTTGTTTGCTCCTTTTTTCTTTTTTTTCTGTTTTTCTTTGTTTTTTTTTTCTTTCTGTTCTTT
GCGGGAAACCCCCGGGTTTTCCCCCACCTTCTTTTTTATTCTCGGACCCCCAATTTAAAACGGGCAAACTAGCACAAGCAATCC
CTTTTTTTTCCCCTTTTTTGTGGAACCCAAACAGCCCCATTTTTTTTTTTTTAGGTTAAAGGGAAAAAAGGAAAGGCCTTTTTT
CTTTTTTTTCTCCTTTTTTTCCCCCCGGTTGGTTTCTTTGGGAACCCAAAAATACAGGGCCCCCAAAGCTTCGCCAAGAAAGA
AAATTTGTGCCCCCCCTTTTTTTTCCCCCAGCGGGTTTCCCCGAAGGAAAAGGGCCAAAACTTTTCCCCCCGCCACCCTTTTCC
CTCCTTGTCCAAACCTCCGAACCTTTTCCTTTTTTTTTATTTTTTCTTGTCCCCCGGAGCAGGTTTACGTTTTTTTTTTTTTG
CTAGAAAATGGGGGGGCAAAAGGCCAACCCTTTTTCTGTTTCCGGCTGGGAAAAATTCTTTTTTTTTTTAAAAAGAAAAAATTA
AAACCCCGCAAATTTCTTTCTTTCTTAAAGGGGGCGCTTTTTGTTTTCAACCCACCCACCCCCCCCCCCCTTTTTCCTCCCCTTG
TTTCCCACAAAAAGGCCTTTTAAAAAGAACCCGTCCCTTTTTTTCCCGTGGACCCTTTTTAGGCGGGTTGAGGGGGGAAATTCC
CCCAAGGGGCAAAAAAAGAATTTTTCGAAAACTTTTGGGAATTTTTCCTTTTATCGGGGGGGGTTTTGTCTTTTTTTTTTCTT
TTTTTTTTTTTGCCCCTCGTTTTCCCCCCTTTCCCCCCCCAATTTTAAGTTGGGGGTTTTTTGAACCAATGAACAAACCCCCAG
GGAAAATTTCTCTTTTTCTCGTTCCAGATCGGGCCCGGAGAAACCCCCGGAAAAAAGTTTCCCAACTGTTTCTTTTTTTTGCCG
GGGGGGTTTTTAACAGCCCCTCAAAAAATGCCCCCCCGTTAACCCCTTTCCCCCCCCCCCCTTTTTTAGGTTTTTTAAAAAAA
TTCCCCTTTTTTTTGAAAAAAAGCAAAATTCCCCCCCCCCCCTTTTAAGGTTCCCCCCCCGAACAAAAAACAATGGGTTTTTTA
TTCGCCAGCTTTTTTTCATGTTTTTTTTTTGAACCCCTTTTTGAAGCCAAGCAAAAATGGGTCCAAATCCACCTGTAAAACTTT
TTTCCCCCCCCCCAAAAACTTGGCGGTTTTCTCCCCCTTCCCCCGATCTTTGGGAGTTCTTTTGGGCAAACATTTCCCCCTTTT
TTAATTTCAACCAAAAAAGTCCTTGCTGGTTTAAAAAAAAAAAATGTTAGCCCAACCCGGCGTTTTTTCCCTTTTCTTTGTCCA
AAAAAAATTCTTTGAAAAAAACGGCCAAAAAAAAAATGAAAACCCTTTGTAAAAAAAAACCGAATTGGGGCAACCACAGTTCAC
AGTTTGGGGCTTCTCCCCCCCCCAAAATGGAAGCTTTGTTTTTTATAGTTTTGGGTGTTTTGCTTTACCCCCCTTCTGGTCAC
CCCCGCCGGGGAACTTCCCCCAAATTGGCAATCCCATTTTGACTTAATAGCCCCTGTTGAGGTTAACCCGCCATCCCCCCCAAT

ATAAAAAGGAAAAAAAAAAACAATGGGATCTCCCGGGTGCCCCCCCCCCGCCCCCCCCCCGGTGGCGTTCCAACCCACATTTTTG
GTCCGGAATCCCTTTGGGCCAAAAAAGGGGAAAAAACAGTCCCTTTTTTCTTTTTTCTTTTGGGGCCGGGGCTTTTATTTTTTT
TTTTTTTTTTTTTTAAAAAATTTTTTTTTTTTCCCCAGATTCAGAAGGAAAAAAGTCCCGATGCAGGGGTTTTCCCCTCTTG
GGCCAAAAAAAATTTCCCCCCCCCCCGTTGGGCTTTCGAACCCAAGCAAAACAAAAAAAAAAAGGAAAAAAAAAAAAATTTTTT
TTTTGGCAAACCCAAAAAAATCCGGAAACCCCTTTTGGCTTTTTTCACGAAATAAAGTTTTTCCGCAAAATTTTTCTTTTTTGAA
GCCCGGGCCCCCCCTTTTTTTAACCTTACTTTTTTTCCCTTCCCCTAGGGGTTTTTTCCCTTTTTTTCCAGCATTTTTTCGCGT
TTTTTGTTTCCCAACCAAGGTTGGGCCCTTCCCTATTTCTTGGGAACCTTTTTTTCCCCGGCCAGGAAAAACGCCCTTCCTTTT
TTTTTTGTTTAAAAATCCCAAAATTTTGGGAGGGGTGGGTTTTTGGGGCAGTTTGATTTTTCTGAAATTTTTTCTTTTGGGGAA
CCTGATTTTTTTCCCCCCCACCAAATGGGGTTTCCTTGGTGGGTTTGGAAAACATCTGCCCCAAAAAAAAAAACCACCCAAAAAT
CCCAGGTTTTCCCCCCCTTCATTCCCCTTTGCAAAGGAAAACTTTTGGATCCAGCCTTCCCCCCTTTTTCGCCCCCTTTTCTTC
CTTCCCTTGCCCAAAAAAAAAAAAAAACAGAGGGTTTTTTTTTTCCCCGCGGAGGCCCCTTTTTTTAAACCATCCCCCGGTTTG
GGGGATTTCTTTTTTGGTGGGGGGAAATCGATACTTCTTCTTACTTTTTCCCCCTTCGAGGGTTTCCTTTTTCAAATAGCCTTT
TTTTTTTTTTTTTTTCTTGGGAGCTCGATTTTTTCTTATCCCCTTTTCCCCCGCGCCCTTTATTTTCGAGGGTCCCCCCAAAAT
TTATCCCCCCCTTCTCCCACTTAGGGAAACAATATTTTTCCCCCCCCCCCCCTTTTTTCCCGGCGGTTCCCCTGAGGGTTTTTT
TTTTTTTGTTTTTTTTTTTTTTTTTTTTCCAAATTCTCAAGGAATTAGGGCAAAAAAAAAAAGGGTTTTTTTTTCCCCCCCCCTGA
AGAAAATTTTCCCCCCCACCCTTCCCTTTTTTTTTCTCCACACACCCTTTTTGGGGGGGACCGGCCCCCCCAAATTGGTTAACT
GAACGGGTTTTTTTTTTTAAAATTTTTTCCCCCCCTTTTTCCCATGGTGGAAAACTTTGCCCCCCCCCCCTGTAACAGCAAAAAA
AAAAATTTCCCCCCCCCCCTGGGCAAAAAAGCAAAAAGCTTTTTTTTTTGACATTCCGCCACCGGGGGCAACCCCTTTTTGTG
GGGGGCAAATTATCCCTTTTATTTTGGGGGGAAGAAAAGGGAAAAACAGAAATTTCACAAAGCCCCGCGAGGGGTGGTGGGATT
TTGGAGGGGGTGGGGAAAATTGGCCTTTTTTCTTTTTTTTTTTTTGTTCCCAAAAAAGCCCTTTATAAAGTGGGGGGGTAAAGG
TCCCCGACCCCCCAAATGGGTAAATTTAAATCCTTTTTTTTTCGTTCTTTTTTTGCGGGCAAAAAAAAAGGAAACCAAAGTCCC
AGGCGGGATTTTTTTTTTGGGGAAAAACCCCCCACCCGAACTTTCGAAAAGGGAAGTTTTTTTTTTTTTTTTTTGAGACTGTT
CCCCCCGAAAAGTCCCCCCCTTCTCCTTAAAAGGTGTGGGGGGAAATTTCACCCCAAAAAGCTTTTCCCGCGATCAATCCCCCT
TAATAGCCCGGTTTAAATCCCCCTCTTCCAAAGAAGGAAAAAAATCGTAAAAAAAAGCAAAATCCATTTGGAAAGGTCCAACCT
TTTTTTTTTTCCCAGCCTTTTTTCGGCGACAGATTTTTTTGTTTAAAAAACCCCAAACCTCCAATTTTCCTCCCCAGCGTCGG
GAACCCGGAAATTTTAAAAAGGAAAGGGGGGTTTTTTTTTTTTTTTTTTTTTTTACCCCAAAAAAAAAAGCTTCCCCAAATTTT
TTTTTATCCGAATTCAAAAGCAAAAACAATTTTCTTCCCCCCCCCCTAAAACCCTCCCCGAATAGCCAAAAAAGCTTTTGCCCA
ACCTTTTTTGGGCTTTTTTAACGAAACCCCAGAGGGGGTTCCGTTTTTAATGGGAGGGGAAAAACTGAACCGTGCCCTTTTTTT
TTTTCTTGGCCAAAAAAAAAAAAATTCTTTTCCCCCCCTTTTTTTTTTTTTCCCCCCAGTTTTTAAAAAAAAAAAAAATTTGAA
TTGTTTTTTGTTTTTTTTCTTTTTTTTTTTTTTTTTCTTCTTTTTTTTTTTCCCCAATTCCGCTTTGGCTTTTTTTTGCCCCTT
TTTTCTTTTTTTTGGCAAAGAATGCGCTTGTTTTTTTCCGGGGGGAGAATTGAAAAAACCCCCCCTCTTACCCTCCCAAATTA
AAATGGGAAAGGCCCCCCCCTTTTTAAAAAGTTCCCCCAAACAAATTGGGGAAACGGTAACCCTTTTCCCTTTTTTTCTTTGGG
GCAACCCTCTTTTGCCTTCGTGCCCCCCCGCCCCAAATTGAGGGGAACCCCCTTTTTTCCCCAGAAAATGGGGACAAATTGGGG
TTGGGGGGGGGAGATTCCCCTTTTTCTCCCCCCTCCTTTAATTCACCTTTTTAGAGGGAAGGGGAAAAAAAAAAACCGTTTTCCT
GAATTTTTTATCCGAAAAAGGAACCGCCCCCCGGGCCCCCTTGAATTGCCCGGGACACGTTTTCCCGGAGCCTTTGGACCCCCG
GGGGCCCTTTTTTTTTTGGGGGCGGGAACTGTCCTTTTTTCTTTGTTTTTTTTTCCCCCCCCCCCGGCCCCCCCGGGCGCTTTT
TAAAATTTTTGAAAAGGGGGGCCCCCCCCCCCCCCAAAAACAAGGGGCGCATGTTTTTATTTTCCCGGGAATTTAAAAAAAAGA
AAAAAAAAAAAAGGCTCACTTTTTTTTTTTTTTTTCCAAAAAAAAAAAGAAAATCTTTTTTCTCCCCATAAAAAAATCTTTTTTC
CCAAGGGGGGGTGAATTTTTTTTATTTTTAACAAACCAAAGTCAAAAAAAAAATCGGTTTTTTTTTTTTTTTTTTTATTTAGGAG
GTTTGAGGGGCCCCCCCCCCCCCAAAAAAAACCGTTTTTTTTTCGTTTTTTTTTTTGGGGTTTTTGCGAAAAAACAACGGGGCCC
AAAACAACAAAAAAAAAACCTTTTTGGAAGGGGGGGGAATTCCCTTTTTTTTTCGCTTTTTTTTTTTTTCCTTTTTCCTTGGGGGTG
GGCTTTTTTTTTTAATGGGGCTTTTTCCGGGGCTCTTTTTTTTTTTTCCCTTAGGGGGAAAAGGGCCCCGGAAGGGCCCCCAAG
AAAAAGGGTTTCTTTTTTTTTTTTTTTT
>m141122_040740_42134_c100732202550000001823141405141561_s1_p0/149699/4250_15193
TTTATAGTGCCAAATGAGTTATGCCGTACGGATGGTAATTTTTTACATGTATTTGGCCTCAGAAGCCATGTATCTCAACCTAAC
GCGCTCGCCCTTATCTTTGAGATTGCTTAATAGGAATCAGAACACTTCATTCCATAGGACATTGAGAGCCATTCTTTTGGATGG
AGAAAATTCTTAACACCTTGAGAATTTCGCGAGAGGAGGAATCTTGCACGTATAGAGACTATGCAAACACTGGCATATAAGATA
GATGCCATATTGGATAGGAAGACAATTCTGACAGGCACTTCACAAGAGGCTGGCCTAACTCCACTTCCTTACAAGGCCAAAGTT
AAATAACAAGAAACAAGGGCAAACGGACAAGCATACATCACATATTGGGGCAAGACCAAGAATGCAATTCTGTCAGAGGAGATA
AAATTTCAAAAGTAATTCCAGGAGCCTGGCTGTCCATCTTTGCTTTCTGTGGCGAAGAATAGACGCATTATTGCTAAGCAATTC
GTAAGGTTTGGTTCACGAGAGAGCAAAAACGAAAGTAGGAGGACCGAATGACCCATGAAATTTAATCCAAGCAGGATCATGATG
GAGTTAGGCACTTAAGTGAGGCTTCACAATGTCATTACCATGGAAGTGGAGGAAGGTGTGGCATGGTAATTTACTCGACTATGT
GCAGCCAACTCTCAATGCGTTATTCAACATACCAATCTATGCATCAGCATCTCCTAAATTCCGACCTTTTTGAATCCTCAAAGA
AGAGAGGCATGAAATCAAATGGCCAATGAGCAAACAGGTTCCAAGATTTCGCAAGGAAAAGGAACGCAAATGGATTGAATGATT
TTCTTCACAATTCGACACAAGAAAGAGGTTCTACTGTTACTCGATACTGCTTTTTGTCAGGTATATACCAACCTATTGGAAATG
ACTGTACGAGAACCCTACTTGTTTGGACGTATAGCGCTAGTCCTACTAGAGGCATCAGCACAACCAGGATCGCGTTGCTGTTAG
ATTTCGACATAGTCCATTTGATCGGATACACAAATGGTTTTTGTGGGAAGGGTTTACTTATAGTTGTGGATCAGGCTATACTGT
CTATACAACTTCTGTACTTTTGCCACACCTGGTATCACCTAGCTAAATGCACTCTTAACGTACCTATGACACACATAAAGACTT

CTTATGTCGTGTTCTACATTAATCTGCTATCACTCTATATCTCTCTAATTTACCTTTTCGTGATATCATGAGTTAAGTGAACCT
TGCACATAAGATTGGAAGTAGTGACATTTGAAGGGATCCATTCCGAACGACAATATTGAAAAACTACTAGCCAGCATTACATTT
TGTCTTATTCCTGTTGACTGAAGGCCAGTATAACTTTGATTGTTACACACTCTGATAGACACCGTGGGGATTTACCGGAATCCT
GCCAAATTATTACTATGTAGGATTATACATATATCTACATTGCACCGATGACGGACTAAACATGCGGGACTCACTAATGGCCAT
CCGAGATGACACTTGCGAATTTCCAACGTACACTAAGCTATCACCGAATACCTCACCCGGGCTAGGCCCTTTGTTTTCGATTCA
TATTGTTAACTTCCTGTTAGACATGCGTGCATACGAGTATGATAGTTTGCTACAAGTCCCCGATTTCCGGCAAAAAAATACTAC
GTAGATATACATCATGCAACTATGATACTAGTAGTTGAGTTTAAAAGGGGATCGATCCTTCTAAGAATTTTTTACTACAGATGA
AATTTCACTCGAAGCCCAGGAACTACATTTTACTGACATATCAATCTCGGTGAACCGTTCCAAGGTTCACGTACGCACTTAGCA
GTATTTATACCATAAGAGCTGCCGTCCTAGGGCCATTCACGCGACGTCATGCGGCTGCATTTGCCTGAACATAACCGATCAGGC
CGAGCTAAGTGAATCTAGATAGAGGGATTCCAACTTCCGCAAAACATAGCGTACAACATACTACATCTGCACTACAGATATAAT
AGTCGAGCGATGTCTAGATGGGGTCCCGATCCTGTACAGAAATTACCTTTCTAGATATATGCATCTACAGTTCTATATCCTACT
TGTAATTCATATGCCAACTGCTACACCGTCAACAATCGGACGCGCGCGCAGGGCTCTCATATCAGTTTTCCGTTCCTGAGTATG
AAATGATAGTGATTTAGAAGGTGAGGATCCGTTACGAAATACTACCTAGATTAAATTGCAACTAAGATATTGATCTGTTGGTGA
ATTTAGAAGGGGATCCGATCCCTGCAAAATATTACCTGTACTATGATCATGACATATGCACTAAGAAGACCATTTATGCTCATG
ATTGAAACTTTCAACGTACAACAGTAAACCAAAGTACTACCGCTAGGGTCCTCATATACGTTTTCATTCTGAGTAAGATAGATA
GTGAATTTTACAAGGGAACCAGATCCCGAAAAAATACTACTATAGCCCATATGAACCACATCTGGAACTCAAGATATAGTAGAA
TTTAGAAGGGGATCCGATCCTGAAAAAATTATCTAACTAGATAACACATTGAACTAAGGAGACCTAATTATGACATGATCACCC
GTTCCCAATTACTAGTAATACCAATACTACCCCGCTAGCGGTCTCATATAGTTGTCATCTACGAGTAAGATAGACTAGTGAATT
TGACAAGGGGATCTGATCACAGAAAAATTTCCTAACTAGATATCACCCATTGGGCACTATGATATTGGCTGTAGTGTCGAGATT
TAGCCAACAGTTGGGATCCCGACTCCTTAAAATTATTACTAGCTTAATCTGCCCACTAAAGCAGTAACCTTTAATTATGACTAT
ATATGAACCATTCCAACCGTACCTAGTATTTACCAAATATCAGGCTGCTAGACGGTCTCAATTATACCGTTGGTCATCTCTGAG
TAAGATAGCAAGTGAATTTAGAAGGGTGATCTTAAATCCCCGGTAGAAAAATAATACTAGATAATGCACCCTAGCAGGAACCTA
ATTATTATACCATAATGACTTTGCCAACTGTACACTAGTAAATGCCGAAATTACTGACAGCAATGGCGTCTTCCATATTTAGTT
TGTCATCTGAGTAACCACACTGGATGTGAATTTGAAGGGGATCCGATATCCGAAAATACTACTAGATGGACATTGCACTAAGAT
ATTGTAAGCTGAATTTAGAAGGGGATCCGAATACACTCGCAAAATTAGTTAACTTAGGGTAATAGGACATGCACTAAGCAGCAC
CTAAGAGTTATGAACATATAACTTTCATGTACACTAGTAACCAATACTACCCGCCTAAGAGTCTCATAATACGTTGCTCATTCT
GAGATGACCGATACGAGTTAGTGAATTTAGAAGGGCGATCCGATTCCGAAAAATACTTCTAACTCTTATACTATAGCACTAAGA
TATTGTAGTGAATTAGAGGCGGATCTCGATCCTGTAAAATTTATTATAGATAAATGCAACATAAGCCAGACTATTACTGAAGAA
TAAGTGGAACTTTGCCAATGTACAACTAGTAAAAACCAATCTTACAGCAAGGGTCTAATATTTATGTTGTCATTCTGAGTAAGA
ATGAGTATGAATTTAGAAGCAGGGATCCCGATTCCGAAAAACACTACCTAGAACACATTACACATAAGATACCTTGTGGTGAAT
TTAGAAGGGGATCCGATCCCCTGCAAAATTATCAATACTAGATTATGGACAATGCCTAAGAAGACCTATTATGACATATTCGAA
ACTTTCCAACGTACACTAGTAATGGACCAATGACTACCAGCCAGGGTCCGATTAATATAGTTGTCATTCTGACGTAAGATGAGT
AGTGAATTTACAAGGGCGGAACTCCGATCCCAAAAAACTACTACTAGATAACCAGGAGCGAGTTGATGGCGAGACAATGATTAA
TAGTAGTGAGTAGTTTAGAAGGGGATCCAATCTTTAAAAATTTAGGGTTACAGATAACATTCGCACTAAGCAGACTACATTAAA
TGGTCATAATGAACCGTGATCCAACATACACATCAGTATTCATTATCCAATTACGCCACCGCTAAGCTGACTCTGTCATCAATA
TAGTGGTCATCTGCAGTCAAGAAGACTAGTGAGATTATAGAAGGGGATCTGATCCCGCGACAAATACTACTAGCAGCAACATTG
CACTATGATCATGAAGTAGTGAGTTAGAGAGAGGGCACCTATCCTTAAATTATTCATAGATATTTATCACATGTGGCGAGCTAA
GCAGAACCTAATTCAGAGTGACGATTAATGAATGCGCGAGATATCCAACGTTACAAATCAGTAAGAACCTAATGCTTCCCGCTA
GGGTCTCCAAATACATTTCATTCTATAGAACGGTGAGAGCACGTTAAAATTTACAAGGATCCGTATCCGTGAATGAACATGAGC
GACCTAGATACGCACTTGCACTATGATCAATAGTAGGTCAGTTTTAGAATTGGAATCTCGACTACTCTGTAAAATTATTATCTT
ATCGAATATATTTGAGAGGCAATTGCAGCTACAAGCAGACCTAAATTATGGACATAATGTTTCAAGTTCGTTCCAGTATTTTAG
AGCGACAATAATAGACTTGAAATCCTATGTGTGAAATAAATACGTCAGCAGCGCTAGGGTCTCATATAATGGTTCATTTTGAGC
TAAGATTCATGAAAGTACGCTTGTAATTTAGACCAGGGCATCCGATTCGAAAAATACTACCATAGCTGATAAACCTAAAGACAC
CACGTTGCACTAAGATCATTGTAGTGAAATCTTAGAATGGGATCCGATCCTGAAAAATTATTACTAGATAATGCACCTATGCAA
CCTACATTATGACATAGCATGAACCTTTTGCAATGTACATATAAAAAAACCAATACTACAGCCAAGGGCTCCTCATATTCGTTG
TCATTCCTGAGCTATGAATGAGTAGTGACATTTCTAGAAGGGGATCCGATTCCGAAAAATACTACTACATACATCTGCACTAAG
ATATTGTAGGGAATTAGAAGGGGATCCAATCCTGCAAAAATTAATTAACTAGATAAACATTGCCCTAAGCAGACCCTAATTACT
GAACATATTGAACCTTTCCAACGTCACGTAGTAAACAATAATAACCTCTAGTGGTCTGATATAGTTGTTCATTCTTCTGTAGTA
AGATAAACTTATGAATTCTTACAAGGGTCGATCCCGAAAAATACACTCACTAGATAACACATTGCAATAAGATATATTACGTCG
AATTTGCAGGGGGTCCGATCACCTCGAAAAACTTATTACTAGATAACACTTTCACTTAGCCCGCACGGTTATTACATAATGAAC
CCGTTCCAGACGTACACTAGTAAAATCAGTAACTACCGCTAGGCGTCTCATATTGTTCGTCATTCTGTGTAAGATCGTGTAGTG
AATTTAGTGGGATCCACAATTCCAAAAAACACTACAAGATACATTGCACTAAGATATAGTATTGAGTTAGAAGATGATCCGATC
CTGCAAAACTTATTACTAGATACAAATTGCACTAAGCACTACTAATTATGACATATTGAACTTTCCCAACGTAACACTAGTAAC
AACACTACTACCTCTACAGGGTTGATATAGTAGGTCCTTCCGAGTAAGATAGAGTAGTGATTTACACAGTGGGATCCCGATCCC
TGGAAAATACTACTAGATTAGCATTGCAGATACAAGAAAATATTAGGGAATTTGCAAGGGGGTTCGATCCCGCAAAAATGTATT
TACTAGATAACATTGCACCTAAGCAGAGGAGATTATTTACAGTGATGCAACCCCGTTCCAAAAGTACACTAGTAAATCAATACC
AAACTGCTAGGGTCTCATATTGTTGTCCATTCTTAGTAAGACACACCTGTGTAAGTGAACTTTCAGATGGGCGGATCCAGCTAC
CCAAAAAACACATCACCAGCATACACATTGCACTAAGATATAGGTAGTCGAGTTAGGAGATGATCCCGACTCCTGCAAAATTCA

TTTCTAGATAACAATTTCACTAAGCAGCAACCTATACTATGACATACTTGAAACCCTTTCCACGAACACTAGTAAATACCAATT
CTACCTCGCTCTACTAGGCGGTCTCAATATAACATTTCAATTCTGAGTAAGATCCACGAGTACCTGAGATTTACAGCAGGATCG
ATACCAAAAAATACTACTTAAGGATCTACATTGCAAATAAGAAATACTAGTGAAATTTGCCAGGGGTCCCGATCCCCGAAAATT
ATTAACAGATACTCACATCTGCACTAAGCAAGACAGTGATTAATACACATAATGAACCTTTCCATTCAACCGTACACTAGCTAA
ATCAATACAACCGCTAGGGTTCATATTGTTGTCATTCCTGAAGTAGACGGTGTATCGAATTTAGATGGGGATCCAATCCCAAAA
ACACTACTAGATAACATTGCAGCTAAGATATAGTATGAGTTTAGAAGATGATCCGATCCCTCGCAAATTATTTACTAGATCACA
TTGCGCCTAAGCAGACCCTATTATTTATGACATAATGAACCGTTCCTACGTACACTAGTAAACCAATACTACCCGCCTAGGGTT
CATAATAAGTTTTCATTCGAGTAAGATAGAGCTAGTGATATTTACAAGGGGATCCGATCCCGAAAATACTACTAGATACACATT
GCACTAGGAAATAATGTAGTGAATTTAGAAGCGGGATCCGCATCCTTGAAAAATTATTACTCGATAACATCTCACTAGGAGACC
TAATTATGACATCAATGGAACGCGTTCCAACTACAAGTAGTACTGCCAATACTACCGCTAGGGTCTCATAATAGTTGTCTTCTG
AGTAAGGATAGACTAGTGAAATTTAGAAGGGGATCTAATCCGAAAATACTACTAAGATAGCACATCTGCACTATGATACTAGTA
GTGATGTTTAGAAGGGGATCCGATACTTACAAATTATTACAAGATAAACATTGCACTAAGCAAGACTTAATTATGAAAATATGA
CAACGTTCAAACATACACTACAGTAAACCAATACTGCCTCTAGGGTCTCCCATAATAGTTTCATTCTGAGTAACGATACGAGTA
GTGAATTTATCAAGGGAACTCCCGATCCGGAAAGTACTACATAGAGGTAACCACTTGCACTAGGATATAGTCGAGTGTATTTAG
AAGGGATCCGATCCTGAAAAATTCATTACTCGACTAACATTGCACTAAAGGAGACTAATTAACTGACAAATGAACCGTTCCAAC
GTACATAGTGTAACAATTCCATAGCTAAGGGTCCTCATATAGTCGTCCATTCTGAGTAATCACGACTAGGGATTTGAAGGGGAT
CTAATCCGAAACAATACTACTAGATAACATTGCACCTATGATCCATAGTAGTGGTTTTTAGAAGGGATCCGATCCTTAAATTTA
ATTACTAGCATAACATTGCAGCTAGCAGACCTAACTTATTGACATTCAATTGACCTTTCCAACGTACACCAGTAAACCAATACT
AACCTCTAGGTGTCTGATTAGTTGTCATTCCTGAGTAAGACAAGAGTAGTGAATTTACAAGGGGATCCGATCCCGGAAAAATCT
ACCTTGAATAACAATACAATAAGAAGTACTTTAGTGAATTGTGCCAGGGAGTCCGTCCCGAAAATTACTTATCTAGATACACAT
TGCACTAAGCGACGTGATTATTACAGTAATGAAACCTTTCCAACGTACACTATAACGATCAATACAACGCGCTAGGGTCCCTCA
TATTGTTGATTCAATTCTGAGTAAGACTGTGTCAGTGGAAATTTAGATTGGGAGATCCAATCCCAAAAAACACTACTAGCATTA
ACATTGCACTAAGATATACGTAGCTGAGTTTAGAAGATGATCCGCATCCCTGCAAAATTATTCTAGATAACATTGCATCTAAAG
CCAGACGCTAATATTATAATGACATAATGAAACCGTTCAGCACGTACACTAGTAACCTACCATATACTACCGCTAGGGTCCTCC
TATATAGTTTGTTCATCTCTGAGTATGATAGAGTAGGTGAATATCAAGGGGATCCGACTCCTGACAAAATACTGGCAAGATCA
ACAATTGCCTAGGATAATAGTATACGTGAAGTTAGAAGTCGGGATCCGATCCTGAAAATTATTACCTCCGATAACATGCACTAA
GGAGACCTAAGTACGACCATAATGAACCGTTCCAACATACACTAGCATACTAACTACCTAACCGCTAGGGTCTCATATTAGTTG
TCATTCTGAGTAAGAATAGAAACCCTTATGAATTTAGAAGCCGGGAATCTAAATTCCCGAAAAATTACTCACTAGATACGAAAT
GTGCACTATGATATAGTAGTGAGTTTAGAGGGGATCTCGAATACTTAAAATAATTATTACTAGATACCCACCATTGCCTACAGC
CAAGAACTAATTATGGACATAATGAACCGTTCAAACATCACACTACGTACAACCAATTACTGCCTCTAGGGTCTCATATATTTT
CATTCTGACGTAAAACGAGTAGTGAACTTTACAAGGGGATCGATTCCGAAAAGTTACTACTAGATAACATTGCACTAAGGCTTA
GTCAGTGAATTTAGAAGGGGATCCCGATCCGTGAAAAATTATTACTCGATAACACTTGCTAACAGGAGACCTAATTATGACATA
ATCGAAACCGTTCCCAACGTAACACTAGATGACAATTCTACTCCGTAGGTGTCTCATATAGTCGTCATTCAACTGAGTAAGGAT
AACGCTACTGACATTTAGAAGGGGATCTAAATCCCGAAAAATACTACTAGATAAACTTGCACCTATGATATAGTAGTGAGTTTA
GAAGGGGATCCGATTAACGTAAAAATTAATTAGCTAAAGATAGGCCATTGGCACAAGCAGCCAACTACCCATGTAATGACATAT
TGCAACCTTCTTCCAACTACCCCCAGATAAACCGAACACTCACCTCCTAGGTCTGATATAGTTGTTCCATTCTGAGTAAGATAG
GAGTAAGCTGAATTCTAGAAGGGGACTCAAGATTCGCGAAAATACTAGCTACATAACTTGCCACGTAAGATACTTGTAGTGAAT
TTAGAGAAGATGATCTGATCTCCTGCACAAATTATTTCTAATGATAACATTTCACTAAAGCAGTCCTGAATATATGACATACTT
GAACCTTTCCAATTGTAAGCACGAAGTAACACACGCATACAGACGCGTCTAGGGTCTGATAATAGTTGTCGATTCCTGAGTAAG
ATAGAGTTTGTGAATTTACAAGGGGATCGCGATCCCAGAATATAACTAGCTAGATAACAGCTTGCAATAAGATACCCTATCTAG
TGAATTTGCAAGGGAGTCCGATCCCCCGAAAAATTAATTACTAGATACGCACATTGCAGCTAAGGCAGACGTGATTATTACTAA
TGCAACCTTCTCCAACCGTACACCCTATTAAGCATCAATACCAAACCGGCCAGGCCGTCTCATATTGTTGTCATTCTTGAGTAA
GACGGGTGTAGTGAATTTAGAATGGGGATCCAATCCCAAAAAAACACTACTAGATAACATCTGCACTCAAGATCTATACGGCTA
GTGAGTTTAGAAGCATGATCCGATCCTGGCAAAATTATTACTAGATAACATCCTTGCACTTCAAGCAGCACTACATTATTGACA
TAATGAACTGTTCCTACCGTCATCACCTAACGTAAACCAATACTACCGCTAGGGTCTCCATATAGTCTCTCCCATTCTTGAGTA
AGATCAGATAGTGAATCTACAAAGGGGATCCGATCCCCGAAAATACTCACCTCAGATACTACATTGCACCAGGAATATAGTAGC
TGAATTTAGAAGCGGGATCCCGATAAACTGAACAAATTTATTACCTCCGATAAACATTGTTGCACCCTAAAGGAGACGCTAAAT
TAATGACATTAATTGAGACTGTTCCAACGTACAACTCAGTACCACAAACCTACTACGCGGCTAGGGCCGTCTCATATACCGTTG
TCCTTCTGAGTAAAGATAGACTAGTGATATTTAGAAGGGGATCTAATCAACCCGAAAAAATAACCTACTAAGATAAACAATTGC
ACCTAATGATACCTCAGTCAGCTGAGTTAGAAGGCAGGACCGATACTTAAAAATTAGTTAGCAAGTATAACACTTGGCATTCTC
AAGCACGACGCTAATTATGCACCATAATGAGACCGTTCATGGGCCTAACATACACTAGTACACCAATAACCGCCTCTAGGGGGC
CTCATTAGTGTTCCATTCTGAGTAGAAGATAGACGTAGTTGAATTTACAAGGGGATGCCGATTCCGAAAAGTACTACTAGATAC
GACATTGCACTAGGCTTCACCTAGTGAGTGTATTTTAGACAGGCGATCCGATCCTGAAAATTAATATACTCGATAACATATGCA
AAAGATAAGAGCGAGACCTAAGTTAACTGACATAATGAGACCGTTCCCAATCGTAACACTAGTGTCACCAAATTCTATAGCTGA
GGGTCGTCAATATAGTCGTCATTTCCCTGAGTAACGATACGACTAAGGTACAATTTAGGAAAGGCCGGAGTCTAAATCCCGAAA
AATACTGACTACGATAACATTGCACTATCGATGATAGTAGGTGAGTTGAGAAGCGGGATGCCGATTCCCTTAAAATTATTACCT
AGCATAGACATATGCACTGAAGCAGACCTAAATTATGACAATATTCGAACGCTTCTTCCAACGTACACGCGAGTAAACCCAATA

CTACCTCCTAGGGTATGATATAGTTGTCCCATTCCTGAGTAAGATAGAGTCAGCGGGAAATTTACAAGCGGGACTCCGATCGCC
GAACATCAACTACTTGATAACAA
>m141122_040740_42134_c100732202550000001823141405141561_s1_p0/160921/7999_13326
AATCTCCCATCTCCTTCAAACATCCCCGACAAGTGTGGCATCCCTGAAACCCCAAGGAGATGTCCCCGCAAACAATATTTGGAG
CAAAAGGCTCGAGCTTCTTCGTCTGAGTCGCAATGCGATCTTTTTCTTTTTCGGTAAGAGTTTTTTCATTTACTCCTTCCCATC
AAAGCTATGTCTCTCGAGGTTTGGTGCCTAGTGTCCTAGGTTAGGCTGGGGGTTGTTCAACAGCTCCCCTCCTAATCATGCCGG
CGTTTGACGGCGTTATTGTCCCTTCCACGAAGTTATTGGCTTGGTTTTTGCGGGGCATTTCCGTTATCGCACTATGGACGGACC
ATTCCTCTTTTCGAGTATAGCTAAATATCTTCGTCCTGAACTGGATACCATTCTCTGCGGTTCAGACCGATCACACAATTGGGT
TTGTGGATCATACCAAATGGGGCTCTGTGCTGAATTCAAATGATTCCTTTACGGGTCGCTTTCGAAGCCGCGCGGCTACGATGG
CTTTCGGTCCAGTAACTCTCGTTTGTGAAGCTTATCAGCTTGCGGCTGGGACGCAAGGACCTGGATTCTCCGACACATGTTCGT
GGGACGTAAGTATTGGGCAATATGGGGAGGTATTTCTCCTACCTGTCTCCATGAGCTGGAATATCACATTAAGGACGAGGTTGG
TGTTGGATGTTATACGTGATGGGGTGATGCGTTATTGGTGAGGCTATCCGTCCCCGTTTCCATCCAGACGGACTAATTAGGCCT
GCCTCTGTTCTACTCCTCAATGTCTCTAGTTCTTTGTGCCTAATGATCGCGATCCCATGGCGTAGGAGGATTTTTTGGAGGATT
TTGTCTTCGTTTAGCTATTCTAAGGCGCCCCTCCTTGTGGATTGTCATGGGGCATTCCTAGGACGCTATAAATAAGAGCAGGCT
GACTTGGGTTGCAGTCGTAACTGACCAAATGACGTTGCTGATAAGCCCCCTATGATGCGGTTGCTGGGGCAAAGCATTTGGTAA
TTTGCCGCCGCGGGTAGCAAAACTTGGAGTGGCAAATTCTAACGCGATCAACTCCCAGTTTGGACTCGATTTTCCTATGGGATT
GTATGAGACCCCCACTTTGCCTCTTTCTGACATTAACGTCCGTTGGCGAAGGGAGGGGGTAACCGGAGCCCAAAACTGCCTCAAT
CTGCTTTGTTTTCCCTAGGGCGAGATGGCTCCTTAATTGGCATGTTCTCCATCTCAACAGCCAATTAGGTAGTCTGGGTTTGGG
TTTGCGGTAATGACGTCGAAACTCCCAGTGTCGGCGCTTAATTCGGCTGGTTGTTTTCGTCGGACGTTGCTGAATCCTCAAGGC
CACATTCGTCGCCGGAACCAGCGGATCTCGGGTCTACCTGCCCCACCTATGTTGTGCCTGTCTCATTATCGTTCATTGAGGGCA
AGTGAGCTACCATGATGGGATGATCAACTGGGATTTTTGTGAAGCAGCTAATTTTGCTGCCCTCCAATGAAAGCACCGAAACTG
TTAAACCCAGTTTTTCGCCCAACAATGTATTCAAGAAATATAAGGGCAGATAGTGGCCTTAATGGGTAAACCGTAATAAAAAAT
ATCAAGAATTCACTCAACCCGAAGCACAGAAAATTTTACATATGTTTACCCAAAAAAGTTCCAGCTTGATGAGCGTGGCAAGAT
TAGTATGCACGTGAGATGCGGCCGTGCTATTTAAGTAGTTTTACGCTCTAGTCGACGGCATCCGAGTAGAGGAAGAATTCGTAT
GACAGAGGAGCATATTTCTTGGGGGCGACCAAAGCGAGGTTGGTCGCATATATTAGATATTTTTCTAGAAATATTTGATATCTG
CATTATGTAAAAAGTGTAATTCCATTATTATTCAGGCTTGTTTGTAATTAAATGGTAATCAAGGGGCCAGTCCGGCCCAGAAGG
TAGTCCTTGAGGCCCTATAAATAAGACTGAAGGGTTCTGAAGGGGGCTGCGAGAAACTAAGGAGAAAATAATTGTTCTTATCAG
AAAAAAAAAAAAAAAATTTTTTGTATCTAATGTACAATGTAGTGTCTCTAGAATTTTAAGTAGCCAGTAGCAGCTCCGCCCAGT
AGCAGTAGTAGCAGGCAGCAGTAGCAGCAGCAGCAGTAGTTAGTAGAATAGTAGCAGCAGCAGCAGCAGCAGGCAGTTAGTCAG
TAGTAGTGGTGGTAGTAGTGGTAGGCGTAGTGGTAGTAGTGGTAGTCGTAGTGGTAGTCGTAGTGGTGATAGTAGTGGTAGTAG
TAGTGTAGTAGTAATGGTAGTCGTCGTAGGTAGTCCGTAGTAGTAAGTCGTAGTAGTATCGTAGTTCCCGTAGGTTAGAGTAGT
AGTACGTAGTAGGTAGTAGTAGTAGTAGTAGTAGTAGCTAGTAGTAGTAGTAGTAGTAGTAGTAGTAGTATGTAGTCAGTAGTA
GTATACTAGTAGTAGTAGTAGTAGTAGTGGTAGTAGTGGTAGTAGTTGTAGTAGTAGTAAGTAAGTGTAGTAGTAGTAGTAGTA
GTAGTAGTAGTAGCTAGTAGTAGTAAGTAGTAGTAGTAGTAGTAGTAGTAGTAGTAGTAGTAGTATGTAGTAGGTAGTAGTAGT
AAGTAGTGTATGTAGTAGTAGTGGTTAGTAGTAGTAGTATAGTGGTAGTAGTAGTACGTAGTAGTAAGGTAGTATGTCGTCGTG
GTGGTAGTAGTAGTAGTAGTAGTAGTATCAGTAGATAGTAGTAGTAGTAGTAGTAGTGTAGTCAGTAGTAGTTAGTTACGTAGT
ATAGTTAGTAGTAGTAGTAGTAGTGGTAGTAGTAGTAGTAGTAGTGGCTAAGTAGTAGCGGTAGTTAAGTAGTAGTAGTAAGTA
GTAGTAGTAGTAGTGGTAGTAGTAGCTGGTAGTTGTAGATTTGTAGTAAGTAGTGGTAGTAGTAGTAGTGTAGTGTGGTAGTAA
GTGGTAGTAGTAGTAGTAGTAGTAAGCCTTCAGTAATAGTAGGTAGTAGTAGCCTGTAGTAGTATGTTGGTAGTGGTAGTGGTA
GGTGGTAGTGGTAGTGGGATAGTGTAGTAGTGCGTGTCGTAGTGGTAGTAGTGGTGGTAGTAGTAGTAGTGAGTGGTAGTAGTG
GTGGTGTGGTGGTCGGTGGTGGTGGTGTGTGGGTCGTAAGTGGTGTAGTTGTGGTGGTAGTAGTTGTAGTAGCAAGTAGCAGTC
AGTAGGTAGATTTGATTAATTTGTTGGTCCTAAGAAATGATTGGTTGTATTATAACCTAAGGTTTTAAGATAGAATATAATAAG
AAATGTGACACTTATCACAAGCCATCGTTTGATTAAGAAATTAAGTATGTTTGATGAATAGTTTAATTAAAAGAAAGATCCTAG
AAGGTTCTAGAAACCTTCCCCAGCAGTTATATTAGGATCTCGTTTTTACGCAGTCAAAGTTGTTTGATCAATTCAAAAATTGCT
GATCAAAGTGCAAATACATGTTTCGAATATATGCAAACGTGTATGTCCGTATACCTCGCAGCTAGTAGGCGGTTCGATTATCAG
CCTACGGGAGTTAGCGGAAAATGCGTGCCGACCTTCGCATGAAGCGAAAGCACGAAAGCTCGAGCATAGGTAGGGGGCGATATG
TCCCCTAGGGATGGTGATATCTCGGCTCCTGGGAGCGCCATTTATGAAATATTTGTAGAACCGTGCTAGAAACAACCCTTAACG
CACTTAGCACTTGGCTCTTGAACCGATTTTGACGCGAGTTCCTAGGCAATCTGTTGAACAAAAAATTGCAAATCTTTTTTATTT
ATATCCATTTATTTATTCATTCTTAAAATGGGTTAGTTCACTCCTTCAAGCTCTATAAATAAGGACCTAGTACTCAGCCATTTT
TCACCTCCATCATTCAAACGCATTGTTTAGAGTCCTCCAAGCTTGCTAAGGGTTATTATAGAGGGAAGAAAACACTTGGTTTTT
GGGTACAAAGCTTTTCGCAATCTAAGCTTTTCGTAAACACTTGGGAAAGTAAGATAAAGTGTTTATTTCAGTATCGACCCGGGT
GTAATTCGAATTGAATACAGTTTGCATCCAAAGGTATTCTTATCCTTAAGTTAGCAGTTCTTCATGGTTCTTTGTTTTCTTTTG
TTTTGCATTCGAAGATTCCTAAACTTCTTTGTTATGATTGCTTGGTTAGGGCGTTCTAAGTTATTTGAAACTAACGGATTTTAC
GGTAAGTTTCTATCTGCTATGGTTTAGTTCTCTTTTTATCTCCTTTTCTTTAGAAACTCATTGGTTTACGCTGTGATTTTAGGA
GTATTCCAAATCCTGTTCTTGTCTCCGCTATCCAGTTTTTGGTAAGGAAAGAATAGGTTAGATATATGTGTTCATATGTTTAT
GTTATGGTACTCGTTTTAATGTTATGATTGATATGTGATATGATATATTGTTGTAGTCCTTGGGCATATTGACTTACTTAGATA
CGGCAAGCGCCCAAGAATTTTTTTCAATTTTCGTGGTTTAGAATTATGATTTACCCTACCTTGATAGTAGAATAGAGGACCCTA
GATGGGTTACTTCATATGACTATAGTCGTGATCTAACTACCTTGATTAGTAGACTGAGGACCTAGATGGTTTTATCAAAACATA

CCACGTTAAGAGTTATGGCCATTAATATTGTAGTCCTAATGCATATACGTTTTTATGTCTATGTTCTTATAAGTACTATGCATT
ATGATATAGTCTTATGTTGTTATGATGTTATGACATATATGTTTTTAGGTAGATATTACATTTTTTTGGTTGGGCAATTAGGCT
CATTCGCTTTCTTTTTTTTAGATTGGTGTAGGAAACATGAGCATAGGAAGGGCAGGAAGGATTCGTGGCAGCTTGGCATGTGTG
TTTGAGGATGAGTGGATTGAATGGAATGCTAGGAGATTGAGGATGAATTGATTTTTAAGTCTTTTGATTTATGTTTTATGTATT
TCCGCCTTAGTATTTTAAACAATTGATTAAAGTTATTTTTTTTTTTTATGTTTTATGTACTAAACAATGGGTAGGGCGGGCATAA
CTTTAGCTTTGTATTCAAGGCACATATTTTGATTTTTAAAGTACAATTTATGTTATTCTTAATATATGTATCTCAATTGTAGTT
TATGTTTAGTAGTCTTTAAATGGTCTGAAGTCCTAAAAATTAATCAGGCATTACAGTAAACCCTTGTTTACGCTGATGCCTAGG
TTGCGTCCATTAGCACTTATTAAAACACTAAAGTGGAAGTAGGTCATTACCATCACTTGGGGCCCGAACCACTATACACTCCTT
GTGTATTTATCTTCTTGACTTGATTCATCTAGTTT
>m141122_040740_42134_c100732202550000001823141405141561_s1_p0/54856/12527_23542
AATTTAATTTAAAATAAAAAATTTTATCTTTATGGAACAAAATTTTTTCCTGTTGTTATTTCTGCTTTATATATTCAATTTTGG
GAGAATTTCTCTTTTTTTTTTTTTTTACTAAATTATGTGTTTTTTTTTTTAAGACATTAAAAAAATTAATATTTAAGTGTATTT
TTTTTTCTTATATTTTTTGGTTTTGTGGAATCGAATAATTAAACTTATTTAAAAAATAAAATTTTAAACATTTTCCCCTAAAAA
TTTTAAAATTTTTTTTTTTTTTTCAAAAAAACCTTCCAAAAATTATATTACAAAATTGTATAAAAATAAAACAATAACTTTTTT
TTAATTTTTTTTGTTGTAATAAAAAATAACCCATACATAAATAGGAGGTGGATAAATAATAAATAAAAAAAAAAAACCCGGGTG
AAAAAGAAAATTAAAAAAATAAAAAAAAGTTTTCCTTCCACCCCCCTTGTTTTTTTTTTACTTATGTCCAAAAAATACTGTGTG
CCTTTTCAAGAAATACAACATAATAAATTTATATAAAGAAAACCAGTTTAAAGTAAACCTTTTTTTTTTTGTTTTTGGTGTATC
CTCACCTTTCCTTTTTTTTTTTTAAAAAAAATTCCCTTAGGGGAACAAATAAAAAAAAATGGGGGGGTGGGGGGGGGGGTTTTG
GGAAAACCCAAAGAAGCCCCTCCCCAAAAAGCCACCCAAAAAAGGGGGGCGGCGCGGGGGAGGGGCGGGTGGGGGAAAAAACCC
CCTTTTAAAACCCCGGAAAAAAAAAAATCCTTTAAAACCTTTAAAAGTTTATTCCCCCAAAAGCGCCCCCTTTCCCAAAATAAGA
AATAAAAAAGAAGATGAGCATCCATTCAGCTTTACATTTTTTAATTTGTTAATTCCCCCAAAAAAAAAGAAAGAAAACAAAATT
TTCTTTGGGGGGTGGAACAAAATTTTCGTAAAAAAATTTTTAAAACCATTTCCCCCCCCCAAAATATTTTTTTTTTTAATTAAA
AAAAAAATTCTCAAAAAAAAAGGGGGGGGGGGGGGAAATTTTATTTTTTGCACCTTATAAAAAATATTTATTGAGAAAAAACTG
GAAAAAAACTACTCCCCCTTTTTTATATACTAGAGCGAAAAGAAAAAAAGATCCCCCCAAAAAATAAATATAATTTATCATAAA
ACAACAAAATAAAATACATTAAATTTTTTTTTTTAAAATACCTGAAAGATTCCCCTTTTTTATTCTTTTTTTTAATATAAATTT
TTTAAAAATATAAAAGGATAATATTATAAAGAAATAAAGAAAGAAAAATCCCAAAATTTTTTTTGGGGGGAGTGGGGGGAAAA
ATTTAAATAATTTAAATCGCCACACCCCCTCAAATTATAGAAGTAATACAAAAAAAGCCGGTTTTAAAATTTCCCCTTTTTTTA
AATTTTTAAATACCGGTTTTCCCATAAATTAAAAAATAACAATAAAGTTTTCCAATAAAAAATTTAAAAAAAAAAAAAAATTCCG
TTTTTTGATATTATATACATTTTTGTTTTTCTTTATTTTATCCTGCCCGCCTATATATTCTCCATATAACTCTTTTTTTTTTTA
TTTAGCTCCTCCTCAATAATTTAATTATGTAATAATTAACTTTTACATTTTTATGATTATTATTTTTGGGGTTTTAATAATCTT
TCCCACGCCCCCCTTAGGAAGAAATTATATAAAGATCAGCAAATAAAAAAACCAAAACCCCCCATTTTTTTTAATTGGGTGGAA
AAACCCTTTAAACAAAAAAAACCTTTTTTACAGACTTTAAAAAAAAAAAAACTCGGGGCTTACTTTTTTTTTTTTTTTGTTTTTT
AAAAAATAAATCCCCCACCAAACCACAAAAGCTTTGTGTTTTTTTTTGGCCCGCCCCCCGCGAAACCGGTTGGGAAAAAAAAAGG
GCCCTCTCATAAAAAACAAACACCCCCCCAAAAATCAAATCAATTTTATTTTTATATTTTTTTCTTTTCAAGGTTTACTTTTTA
TCCCGCGTTTTAAAGACGTTTCTAATCAATACCAACATTTTCAGGATGGGGGGGCGGGGGGGGGGGGTGTGGGGGCGGAAAAAT
AGAAGCGCGCATTTTTTTTCTAAATTTTTCCCCCCCTCACCCCCCCACCCGAACTTGTCTCTTTTTTTTATTAAGGGGTTTTAA
TTAATGGCCAACGTTTTTGGGGTTATAATAGGGCGAGGATAAACCCCCCCCAAAAAAAAAAAATTAAATATTTTTTGTCTTATTT
TAACCTAAAAAAAATTATAGGGCAAAAATAATATAAAAAAAGAGTGCGCAGGGGGTGGCGGTGGGACCCTTTTTGTGCATATGA
AGTTGGGGGTTTTAACCATCTGTGGAGGAAGAGGAGGCCCCCTGTGCGAGGGTGCGCCACAATGATTGTAAAAAACAACTTTGG
TGTGAAAATTTTTAAAAAATTACAAACAATTGGGATTAGAAAAGGCATTAAAATTGGGGCAGAAAAAAAAAATTTTTTTTTAAT
ATAAGAGCCTCCGATTTGGGGGGGTTTCTTAACTTTTTAAATTCTTCTCTCATAAAATGTTTTATTTCGCATAAAGTACATATA
TATTTTTTTTTTGGGGACAAAAATAAATTCTGTGATGTAGAAATGGTTTTTTTTAGTTTTGTGGGGAAATGTACCATGGTCCGC
CTCCCCCCCCCATACATCTCGAAAACACTAATTTTAGGGTTTTAGTATTAATAGGGTGGTTTTAAAAAATTTCAATAGAACGTT
TTCTCTCAAAATGGAGGCCCCGAAAAATGTTCTGAAAATACAAAAAGGTTGCGGCGCCCCCCCCCTCTGAATTTGATTTGTTAT
AAAATAAATACAACCGCGGGTATTCTTTGGTTGAAAGAAAATAGAAAAATGCGCGCGTGCGGTGCGCTAATAACAATTTCGTAA
AATTTTTTTTGGGGGGGCCCCCCTTTTTTCAACCCCTTTTTTGAGGGGGGGGGGGGGCCAAAAAAAAAATAAGGGAAAATTTGGG
GTTTTAAAAATAAATTTTTTTTTTTTTTTGAATTAAAAAATTTGTTTTTTGAAAGGGAAAAAAACCCCCCAAACAATTTTAAAA
AAAATTTTATTGGCTCTTGCCGGCAAATTTCCTCTCTAATATTTTTTTTTTTTTGGGGGGTTTTTTAAAAACCCCTGGTGGAAA
CCTTTGGGAAGGGGGGCCCGATTTCCTTAAGTTTTTGGGAAAGCCCCCCCCCCCCGTTTTTTCTTTTTTTAGCGGAAAAAAAGG
GGGGGGTTTTCCGGACCGGGGTTTTATTTTTTTTTCTCTTTTTTTAAAAAAAAAAATTTTTTACCCCCGGCCCCTTTTTTTTCCG
GGGGGGGGGGCCCCCCAGCTTTTGGGGGGGGGAGGTTTTTTAAAATTGGGGAAAAAAATTTTTTAAAAAAGGGAGGGGAAAATAA
AATTAAAATTTGGGGAAAAAAAAAAAGGGGGTTCCCCTTAAATTTTTTTTTTTTTCTTTGGAATGTTAATTATAATACGGAAAAAA
AAAAAAAAAGAGAAAAAGGGGGAGTGAAAAACCCAAAAATAAAAAAAATTCTATGGGGGGGTAGAGGGTGGGCGGCGGGGGGAAA
TATAAAAAAATCCCCTACACCGCCCCCAAAAATGGTATATTTTCCCCCCCAGGATAAAGCATATAAAAAGTTTCTTATAAGGCCC
GGTTTTTTTGCTAAAATAAAAAAAAGCTTTTTATGTTTTAACAATTTTAATCAAGGGGGAAAACAATTCCCCGGAAAGGGGGGG
GGGGGATTCTATATTTAAAAATGCCCGGGGGGAAACATAATAATAATATTACTTGGGGGGGGGTTTTTTTTTTTTTTTTGGGGG
GATAAAAATGAAACCTAACAACAAATGGCCCTTTGTCTTTTTAAGGAAATAGAATCGGGGGTTTTTTTTTTTAAAGAAACTTGT
GGGAGGTGTTATGCTTTTATGAGTTTTGTAAAAAAAAATTGGGGGGGAAAAAAAAAAAAAAAAATGAGGGGGGTGGCCGAATTTTT

TTGTGGGGCGGGGGTGAGGTTTGTCCCCAATTTTTTTGGATGCTCTCCCCCCTATACAATAATATATAATAGTGGCCAAAAGTG
GGGGTGCGGTGAAATAAAAAATATAAAGGAAGCAATTTTAAAAGCGTAAAATATATTTGACATTTATGTTATAAAAGGGGGTGA
GCCCTCACGCCACCCCCCCAACCTTCTCTAAGAAAAAATACACTCACATGCCTCCAGCTTCTCTTTTTTTTTCAAAAAAAGTTT
GGGGGGAAAAATATATAAAAAAAATGGAGGGAGAAAAAACAAAGGAATGTTGATTGTTATTTTTTTCTTTAATCAAGATATATA
GAACAATCGTCCCTATTTTGATGGGAGATAATTAAATAGGAAGAAAAGAAAACAAAATATAATAGGGGAAAAAAAAAGAAAAAT
GAGGATAAAAAAGGAAAGGGGGTAAAAAAAAAATTTTAAACAAGACAATTTTTTTTTTGTTTCCCTTTTTTTTTATCCCCCCCT
TTTTCCCTCCTCAAAAAAAAAAAAACGGGGGTTTTTTTGGGGGGGGGTATTTTCCCCCCCCTCCTGCCCCCCCCATCTCCTTCC
ACTTCTTTTTTTCTATATTCCCCCAAATTTCCCCCTTTAGGGGGCAAAAAAAAAAAGGTGCTTCCTCAAAAAATGGCGGGACAC
AACAAGAAAAGTCCCCCCCTTTTTTAAAAAATTTCCAAACAACAACATAAAGGAGAGTGTGGAATAGAAGCCTCCGCTCGCTTT
TTTATACGGGCCCCCACCATTTTTATAGAGATATATATAGATTAGAAAATACCCTTTTTTTTCTGTATTAAGTACTAAGAGGGG
GGCTCCACTTGGGGAGGGGGGAGGACAAAAAATTTTTTTTGGCCATCTCAAGGGCGGTTTTTCTTACTTGGGGGGGGGGGAAA
AAAGGGTTTTTTTATTATCAATTTTTTTTTTTTAAAAAAAAAAAAAAAAGCCAAGGGGGGGGGGGGGGGGTTTTTTTAATTTTC
CATCCACCTTTTGAGTGGTTTTACAGGGGGGGTTTTTTTTTTGGGGGGGAGGGTTGGAAAAAAAAAAAAAAAAAAAAACAAAT
AATTTCCAGTTTTTTTTTTTTTTTTTCCCTTTTTTTTTTTTGGAGGGGGGTTTGTTTTTCTCTTTTTGAAGAGGAGAATGTTTT
ATTTTGTGATTATTTGGGGGGTGTGTTTTCCTCCATAATTTTTGCATTAAAGTGCCCTGAACAACCGTTTTTTTTTCTTTGTTT
TTTTTTTTATATTTTTTTCCCTGTTGGGGGGGAAAAAAAAAAAAAAAAAATTTCCTAGGGGGAAAAAAAAAGAATATAAGTACTT
AAAATCCCCCGCTCCTCTCTTTAAGGCGAGAGAGGTGGGGTTTTTGCTTCTTTGGGGAGGAACGGTGAGGGTTATGACACAACG
CGAGAGAGGCTTAAAGGCGTGGGGGGGGTGCAAAAAGGTTGGGTGCATTTTAAAAAGAATTATAATATAAAGATAAAAGAAAAT
ATATAGAGAAAAAAAAATAATGTAGTATTTATTTAAAAAAACCCTAAAGAGGTGAGTGTGGGTGAGGGGGCGATATATATATTTT
TTTCTATATATATAGTATATTTTTTTCAAAACTCCCCAATTTCCCAATTCTTGACAAGAAAAAAAGGTGTGAGGGCGTAAAAAG
AAAGAAAAAAATTCTCTCTAAAGCTTATATATAAGAGGTTTCTTCGTTAATGGTTTTGAAATTTTTCCAAGTTTGAAAAAAAGT
ATTATACTTTTTTTCCCTCTTTGTTTTTTCTTGATATAAATATAATGAGTGGTCGGAATTTTTGATATTTAATCTTTTTGAACG
CTTTTCCCTAAAAAAAAGGTTATTTATTTTATTTCCCAGCCCACCCCCTCCTCTCCCTCGCCATATGGGGGGGGGGGTGGGGCGA
GGTGGCACAAAAAATTTTTTTTTGTGTGGGTAATTAGTTGGGGTAGGGGGTGCCCCTCCAAATGTGGTAATCTTGGATGTTTTT
TATGCTTATCGAGAGAGGCTTATATTTTTTATTTTTGGGGCGGGTATCCCCAAAATATATATGTTGGGGGGGTTGAGGGTTTTT
TGAGAGAGAAAAAAAAAAAAAGAGAGAGTGGTTTTGGGTAAAAGGTCCTTGTTTTATTTGATAATAAAAATTGTTTATATATTTT
CATATTATAGAGTATATTGTATATATATATTTTTGTGTTGAAGTATTTTTATTCTTGTGGGGTATAGAATAAGTAAGTTTTTTTGA
GAACCCCCGCGCGCGCTTTTTTTGGGCCCTCCCCTGGGGGGGAAAAAAAAATACTAAAATCCCGAAAAGGGATAAAAGGAATAA
GTGCCCAGGTTTTTTTTTTTTTTTCGCTTCATTTTTATGGAGCTTTATATAATATATTGAGGGTTATATATTTATTATGGGAGGG
GAGAACGTATATTAATATTTATTTATATATTATTTTTATTGAGCGCTTTTTCTTAATTGTTGTAAAAAAAGCCGTTTTATGGAG
AATTATCCAATATGAGAACCACGCCTGTTAAGAAAAAAAAAAGCCCCAAACTTTTTTAAAAGTGGCCCGCTTTGGTTAAAGTT
TGGGCCCCCTTTGAAATCCCCATAATTTCCCCCCAAAAACAAAAAAAGGGGGGTTTTTTTTTTTTTTGGGTTTTTTTTTTAAT
TTCCTTAAGGGGCGCTATAGTTTTTTTGTTCTCTTTTTTTATTTGTGGCTCCTGGGTGGCGGGGGAGGGTGAGGGGTCTTTCCG
CCGTGAGGCTGTTTGTTTTTGAAAAAGATTGGTCCCCCACCCCCCCAACCCCCGTTTTTTTTTGAAAAATGGAAAGTAGGGGT
TTTTTTTTCCCGAGGGGGGCTTCGGGAATTTGCGCTTTCCCCGAGGTGAGAGTGGCCTAGGGGGGCGGCGTTTTTTTTTTTTTT
TTTTTGGAATTGTGAGGTTTTTTTGGGGGGATTTTTTTTTTTGGTTTTATTAAACTTTTGGGTTTTGGGGGGGGGGGGGGAAC
CGTTGGGACCCTAGGGGTTTTTTTTTTTTTTTTTTAGCGGGGGTTAAAGACCGCCCCCCTAGGGGGGTGGTCTGCCCAGAACAA
ATTTGGAAAATATCTGCGCTTTTTTTTTTTTTGAGGGAATCCGAAAATATTTGGGTCAATAATAAAATTCCTATTCCGGAAAAAT
CCTACAAAGGGTAAAAAAAAAACGTGCCCAAAAAAACCCGTTTTTTTTTTGTTTAATTGAGGTTTTTTTGGGTTTTTTGTTTG
GGGGTGGGATGGGGTACCTGCTTAAAAGAAGCACTCTCTATTCTTAATTATTTTGTGCTCCTGTTTAAAATGATGTTTTCCCCC
AGAGCATAAAAAATTGTAAATTTTTATGGCCCATGGGGGGGGGTGGGTGCGGGTGAAAAAACCAATGGGGGTTTTTTTGAAAAT
TTAAAAAACCGACCCCTCCTTAAAAGCGGGGGGGTTTGGGTACACTGAAAAAAAAACAAATTAAACTTGGGCGGCCCCAAAACA
AGGGGGGGGGCCCGGGGGGCAAAACTTCAAAGAAACCCACTTGGTTTAGGCTTTTTTTTTTTTGAGGGACCAGCCCCCTTTTCT
CAATATTTTCACTATTTGAAGCTTGAAACATAAAATATTTATTTTATTATTTAAGCCGGGGGTGCCTCGCCACCACTGTTGCAC
AAAAAGTCTATCGCTTTCAGTGGGGGGAAATTTAAAAAAATTAGAGGGGTGTTTGGGGGGTCTCGGGGTGCCCCCCTCCCTTTT
ACTGGGTGCGTCTAAAACCCCTGGTTTAATTATCATCCAAAATAAATGGTGAATTTGATTTTTTTAAATTTGGGGGAAAAAAAT
AACAAAATGAAAAAAAAAATGATAATGGAACTTCTTCGGAGGTGTAACTCGCCCTCCTTTGGGGAATACTTTTTGCGGAAATAAC
AGGTGAGGTTGGATCTATATACAAAATAAAAAAAAATGGGGTTTTTTTTTATTTTATATTTGTATGAAACACAACCTGGCTTTT
TGAAGTACTTTTATGAATAAAAATTTATAAGGGCTTTAAAACCGGTTAAAGGGCGTTTTTTTTTGTTAATTCTAGAGTGTGTTC
CTTTTTGTAGCTTTTTTTTTTTGGCTTGGTTTTTTTATATATTTTTGTATTGTAATTACTTGAGATGTGTGTTAGACCTTCCTT
TGTTTGGAAACTTTGGTTGGTCTGTTTTTATATGTTTTTATCCTTTTGCCGCTGGGAGGTGACTCCGCCCATTTTTCTTTTTTG
AGCGGGGGGCCCTTCCAACGGGGGTTTTTGGGCCCTTAAAAACGTTGGGGGGGTGGGGCAAAAGGGTTAAAAAAAGGGCAAAAA
GGGGGCCCCAAAAAAGTCATATTTGGACCCATATCCCCTAAGCAGAATGGGGAGGGAAGACAGGCCTTTTTTGGGGGGGTTTTT
TTGAAAAATGTAACATAAATTCAAGCCCCTTTGAAAAAATTCCGGGCAAAAAAACCGCCAACGGGGGCTCCAAATCGCCGACGT
CATATAGAATAAAGGCTCAAAAAAGGCGCGGATTTTTTTTTTGGAAAACAATAACAGAGAGAAAAAAAGGGGGCCCTTAATAG
TGCTATATTTGGTCAATAATCATCTTTTTTGTTATGTATTATTGTTTGGAGGTCTTTAATGAGAGGTGGGGGCTTTTTTTTTT
TTTTTTAAAGTTTAACCCTTTTTTTTTTTAAAAATTTTTTTTAAATCACTGAAAAAAAAAATTTTTTTTTTTTTGGGTAAAA
AAAACTTGGGTCCTTGTTTTATAACTTTTTTTTTTTTTTTTTAAAAAAAAAACCTATACACAATTTTTGGGGGAAGAAAATTACC

CAAATTTTGGGGGTAAAAAGGGAAAAGATGTTTTTTTGGGGGAATTTTATTTAGATAAAAATAACAACAAAAAATAAATTTATT
ATTTTTCCGAAGACCCCCTACCATAACAAATTCTTTTAAAAAACCCCCTTTAGTTTCAAAAACTTTGTAAAGTAAATCCCAAA
GTATATAAAAGCAAAACTATTTTCCAAATTTTATAATATTTTAAAACTGAAAGAACCTTTTTCTCTATTTTTTGAGATTTAAAA
GCCCCTCCCCCAAGGTCCCCCAAAATGCCCCACCCCTTTTTTTATAAACACCACAATTATGGGGTTTAAACCGGGTACCCCCTT
TGGGGGAGGTTCTTGTGCAAATAAAAATTCTTAAAAAGGGGGGGGTTGTTGGTTTCTTTCTTAACCATACTTTGGAGAGGGAGG
GTTAATACCAATAATATGTGTCGCCGCTCCCTCCCTAATAGTAGGTATTATAGCAAAATATTGTTAAATAAATAATATAGATAT
ATAAAATATTAGTACTAAATTCAAATAACTATATACTTCCCTTTCTCCCCATTCTCAGTTTCTAATATAATATTATATATATAT
ATATTATAGTATCTTAACGCGCCCCCCTCCATATAATAATTAAAAATAGATTAATAAGTCCTGAAATTTTATAGAGGGGAGCGT
GGGTTCTTGGTTTCACGTAAAGCGGGATTTGTTAAAAATATATTCACACTGGGGGAACTTAAAAATAAATGCCCCGCCCCCTT
TTTTTTTTTAAATTGATTTTTTTTAAGAAAATGAAAAAAATAATGACTGTTTTTTTAGGGGATAATCCTTTGCCTTTTTTT
GGAAAAAAAACTTTTTAAAAAAATAGGGGGGGGAAAAGGCCGATGGAAAAAAAAAAAAAAAAAAAATAAACTGAAAATAAGACAG
CCCCCCCTGGGCCCCCCTTTTGAAAAAAAAAAAAAAAAAAAAACTATTTTGGAAAAAAAAAAAAAATGGAATTTTGGGGAAAAAA
TTTTTAATTTTTGAGGGGGGTTGGGGGTGCTTTCATCAGCACTTGCTAATGCCAATTTGGGTGGGGAAAATGAAAAAAAAATAT
TTTGGGGGGGGAATTTAAAATTTTTGGGAAATTTGAATTTTGGTTAAATATTTTTTGGGAATTTTGTTTTTTTTGTTTTTTGGA
AATTATTTTTTTGGAAAAAAAAAAAAAAGTCCCAAAACAAGTGGCGGGGGTTTGGGGAAAAACCTTTCAAAATGAACAAAAAAGG
AAAAAATTTAAAATGTAACTTTGTTTGGTTCCCCATCCCAACTAAAAAAAGTCCCCCCTGAACTTCTTTTTTGCCAAAGGGTTC
GTTTGAAAAATTGGCCAAAAAGAATTTGAGAAACAACCCCCCTTTTTTTGGGGAAAAATTAATTAAAAAAATTGCGTCCCCCCCC
AAGGACAAAAAAAATGCTTAAACAAAACGAAAACTAGGCCCCCGGCCAAAAAAAAAAAAACCCCCAGGTTCCCCCCTGAAAGAAA
ACTAAAAGAAAAAGATGAAATTTTGAACCCAGGGGGGGGGGCCCAAGACCCTTCCCCGGCCAATTTTTTTCCCCCCCCTGGAAA
AGTAAAAAAATTAGCAAAAAAAGCCCCCCAGCTTTTTAAAAATGGGGTAAGACATTGCAAAGCCGGCGAAAAACTTAAACAAAG
AAAAGGCCCCCCCTTTCCGGGAATTGAAAAAAAAAAATTTTTTGAATTTTCCCTACGACAAGCAAAAAATGGCCCCTCCCAAA
TCCCCAAGGGGGAAAAAGGGTTGGCCCCCCAAAAGCCCCTAGTGGGGTTGCCCAACCCTTGGGCCAGTGGGGGGGGGGTTAAAA
AAAAAAACCCGTTTTGTTACAGCCCCTTAGAAAAAGAAAGGGGTTTGGGGGATTTTTTTGGGAAATTGGGGAAAGAAATTCCTC
TTCCCTTTTCAACAAAACCAAACCAAAAACGGAAAAAGGGAGGGAGGGAAAAAAAAAAAAGGAGGCAAGAGAATTTCCCTTTTCA
CCCCATCAAAAAAAAAACAAAAACAAAAAAAAAACAAATCCCCAACCTCTTTCTAGCCCTTTTTACAACCCGGTTAAAAAAAAA
AACCCACTTTT
>m141122_040740_42134_c100732202550000001823141405141561_s1_p0/54925/0_6055
AAGAGAAAAAGAGATACAGGGTTGGAAGAGGAGTTGTCGGACACAAAAGTGCTGACGAAAGGGCGACTCTCTTGAACGGCGCGG
CACAGTCCGTCCTGGACAAATGAGAGATCCTGGATCCTGAAAGCCCGATCCCTTGAGCCTGGGCGACTCCCCTTGCTTCCAGGA
GAAGGCGGCGCACGAAAACCACCCGTCAGGAGAAGGAGGAGGTCGAAAACTTAGTGGATGTCACCCCTTTGACCGGGCCATTTA
TATGGCCTGTACAAGGACAAAGAACATGAACCTCCTTCGTCTTCCCTGATCCCGACGGCGAGCGTGCGAGTATGAGGGCCAAGG
AGGGAGGGAAGAGGCGGATCTCTGGGCGGACGGCTGTAGGCCCTTGTCCGACCTCGTTCTTCTTGTTAACGTTCTTAGCTTGTA
AATTAAATTTTAAGAAACACTGCTTCTTCCTTTGGTTTTTAAGAGATCTTTCCTTTCACGTTTAGTAAGAAGTGTCGCGACTA
ACTGATGTGCAGGGCGGTATTGTCCTGGTTCCAATGCCAGGACTGGACCCAACGATTAGCCCTCGCACGCTCGGGGCCCTTGAG
ACTTCGTTTAACCCCGGAGCATCGTTTTTCCTTAAATTTTAGGTCCTAGGCTTTGCCCTACGGGTGACCGGATGCTTTTGTACT
CCGAAGATCGACTGTCCGGTCGGTACGTCGTTCGTACCGGTGGGCCGGCCTTGAGCGTCGGTTTCCTTTTTCATACGCCCCGGA
CGCGTCGTTCGTCCGGGTTGGGACCCCGCCCTTGAGCTCGGTTTCAATTTTTCATACCCCCGGACGTCGTTCGTCCGGGGTGGG
ACCGGAATTCCTTGGGCTCGGTCCTCTTATTCATACCCCGGACGTCGTTCGTCCGGGGTGGGACCGGCCTTGGGGCTCGGTCC
TCTTATTTTTAACTCCGGAACCATCCGTTCGTCCGGGTGGGACCGCCTTGGCTCGGTCCTCTTATTTTTACTCCCGGACATCCG
TTCGTCGGGTGGGATCGGCCTTGGGCTCGGTCCCCTTATTTTTACTCCCGGACGTCGTTCGTCCGGGGTGGGACCGGCCTTGGC
TCGGTCCTCTTATTTTTACTCCCAGACATCGTTCGTCCGGGGTGGGACGGCCTTGCCTCCGGTCCTCTTATTCATACCCCCCGG
ACAATCGTTCCGTCCGGGGTGGGACCGGCTTCGTGCTCGGTCCTCTTATTCGCATACCCCCCGGACCGTCGTTCGTCCCGGGTG
GGACAGGCCTTAGGGCTCGGTCGTCTCTTATTTTTACTTCCCGGACATCGTTCGTCCGGGGTGGGACCGGCCTTTGGGCTTGTC
CTTCTTATTCATATACCCCCGGACGTCGTTCCGTCCGGGGTGGGACCGGCCCTTGGCTCGGTCCTCTTAATTCCATACCCCCGA
CTCGTTCGTCCGGGTGGGACGCTTTGGGCTCTGTCCATCTTAATTTTTACATTCCCGGACATTCGTTCCGTCCGGGGTGGGACC
GGACTTCGGGCTCGGTCCTCCTTATTCATATCCCCCGGACGTCATTCGTCCCGGGGTGGGGACCGGCCTTGGGCTCGGTCCTCC
TTATTCTTACCCCCCCCGGACATCGTTCCGTCCGGAGGTGGGAACCGCCTTGGGCTCGTACTCTTATTCATACTCCCGAACATC
GTTCGTACCCGGGGTGGGACCGGCCTTGGGCTCGTCCTCTCATTCACATACCCAAATTGTCGTTCGTCCGGGGTGGGGACCGGC
CCTGGCTCGTCCTCTTATTTTTACTCACGGACAAATCACGTTTCGTCCGGGTGGGGACCGACTTTCGGGCTCAGTTCTTATTGT
CATATTGGCGCCTGGGATGAACACCCCCCGCAAGGGAGCGGAGATTGGGTCTGGCTCACTTGAGAAAGAAATTCGAAAAGGGAT
TCGCCCCTGAGGCAACAACGTTTTCCGTCAATTTTTCATTGTTCAAAAGAATTAGCCCTGAGCGTACATGTTACGAGCGTAAAA
CATTAAAAACTGGGACGATGCCTCGGGCTAACTTGGTAGTATGTTGCCGGAGAATGCTCCGCATTCCAGGCCGCGGAACCTTCG
ACCATCGAGCTGCTTCAAGCGATACATTCCGGGCAGACTTCATGCCATGGAATCTCATTACGGCCCTTCCCAATTAGGGCTCCA
GAACCCTACTCCCGGAATCCTGAGTGGCGGGGAAAGACCCTCCCGCAAGGACCTAGATCGCCGGTTTCCGAAACTTGCGTTCTT
GACTGGAGTTTGAAATGGTCGCGCGATCTTGCCCTGGTACATCTTCAACTGCACTTGCGATTCCTCCCGGATCTCCTCGATAAG
ATACAGCCGATTCCTGGAGCAAAGGTGGTGGTTCCTGGGCAGGATCGTACGTGTTGCGTCGATGGAACGGACGGTCCGTTTGA
ATTGGGATGACGGCTTCGCATCCATAAACAATTGGAGTAGGGGTGTGCCCGGTTTGACATCCCTTGTCGGTCGTCCGGTAGGCC
CCATTAATAAACGCGTAGGGAGTTTCCCTCGGGCATTTGCTCTTACAGCCTGGAGCTTTTTTCTTCCAAAGCGTTCCCTTTTAG

GATTGTTGTTCACAGGCTTCGCCCTGCCGTTCCGCTTTTGGGGTCCTGGCGACATCCGATAAACTCTTTCACTAATACCGTGTC
CTTTCGCAAAATTCCGTGAAGTGGACAAACCTGTCCAAATTGCCTTCCCGTTGTCAGACACGATTTTTGTGGGGGGAGGCCATA
TCGACACACCTATGTGGTTGATGAACGAAATCAGGGCCTTCCTTGGAACGTGATCAATGTTCATGGCTCCGCTCGACATTTCGT
GAGTATACTAACCGCCACAATGGCATACTTTACCCCCTCCCTTTCGGTCGGGAGGGATCCCACAAAGGGTCGATCCCCATTACT
GCAACGGCAGGGACCTGTTTTCATCAAGGTTTATATATGTCGGGGGGCCCGAGGGAATATTCGCGTAAACCTCTGGCAATGGCT
CGCACTTACGGACGTAGTTTATCGAAGTCTTTCTTCATGGTGGGCCAGAAGTTATCCTTGGCGCAGATCTTCTTTTGGACAAAC
TGGGCCCCCGACTGTGGTGATCTCCGCAAAAGCCTCCATGGATTCATACATAATGGCTTCCCAATTTCAATCCGGACACGCACC
TGGAGGGTAAGGCAATAGAACAATCCCCCGCGATGATCCCGGCGCCCGGCCTGTCTCGTTCGAGGGCTCCGTCCACGAAGATTT
TCCCAAACGGGCCGCCGCGGAAGGATGCCGGGATAACTCTCCCTTCTGGGGGGATCCAGAAAATTTCCCGAACGATTAAATCGG
CCAAGGGCCTGTCCCCTTAATGGACCACCGGGGCACGTAGGATTATATCAATATTGGCTTCAACTCATCGCCCATTTTAGGAGT
CCTTTTCCCGACGACTCAGGTTTCTGTAAAACAACCGCACGCAGGCGATGGTTTGTTAAGAACTTTAATGGCGTGAGCCTGGGA
GAATAGGGTCCCGAAGCTCCGGATGCATCAATAGGCAGAAATTCAGCTTTTCGATCAAGCGGTATCTAGATTCGGCCGTCCTAG
TAACCGCATTGCTCACGTAGTATACGCGGGAGTTGTGTCCTTTCCTCTTCTCGTATACCAGCGCGGCGCCTGATTGCAAAATGC
TCGGTCACGGCAAGGTATAGGTACAAAGGTTCTCCTTCGACTGGCTTTGGCCAAGGAATGAGGGGCCCTTGGCCAAGGTTGTTC
TTTCAGCTTCTGGAAGGCTTTCTTCCCGCACATCGGGTGACACTCGAACCATTTGGCTTCCCGAAGATTGTTGAAAAAGGGGAG
CACTTGTCCGTGGCCTTGGAAACGAAACGGCTCAGGGCAGCCACTCGCCCCGTTCAGACTTTGAAACGTCATTGACGTGCTCCG
TGGAGAGGCCATGTCCGATCAGTGGCCTTGATCTTCTCTAGGGTTGGCTTTCCCGATTCCTCGGAAGCCACTATGAAGCCCAAG
GAAATTTCCGGAAGCTACCGGCGAAAGTTGGAACTTTTTAGGGTTCAGCTTCATCCCGCTACTTCCGGCAATAACCGCGAAAGC
CTCCCGCCAAGTCCTCCGAAATGGTTCCCCGCCCGACTCTTATTGACCAGCATATCGTCAAATGGTACAACTTCCATTTTCGCC
CCAGCTGGGCCCGAAACATTTTGATTGACGAGCCTCTGGTAAGTGGAATCGGCCATTTTTGAGTCCGAAAGGCATGGACTAATG
TAGCAATAAGTGCCCCTTGTCGGTTTGGAAACTGGTGTGCTCCCTGAATCCGCCACGTGCATGGAGATCTTGGTTGTAGCCCCG
AGTAGGTGTCCATGAACCTTAAGATCTCGTAACCGGCGTAAGCGTCCACCATTTGGTCCTATTCCGGGGGAGCGGGAAACAGTC
CTTCGGCACGCTTTGGTTGAGGTCCGTGAAAGTCGATTGCCAAGTTCTCCCAGGTCCCGTTGGGGGTTTTCGGCACCAAGACCG
ATGTTGGCCAGCCTGACCGGGTTATCGCTTCGCGGATTTGAAAATTGAATAGAAGATAAACCTTTCTCACCTCCAGCCTTGAGG
GCCTCGGACCTTTCCATCCCCGAAGGTCTGCGGTTTCTGGCGGACCGGGGTCGCGTTTGGGTCCAATAACTTAACGCGTTGGCA
GATGATATTGCAATCGGATCCCGGTCATAATCGGAGTGGGAAACCATGGGCAGAAAATCCCTGGTTTCCTGGCATCGGGCCGAT
AATGGCGACCCGGATCTTCGCCGGTAGGCCTCTTTCCCCCACTTGGATGGACCTGGGTCCGGGTCGGATTCACTGATGCGTACC
TCTTTCACTTCGTCATTGGCTCCAGGACACCTCGTCCCTATTCATCGGGTCCAGATTCTGGACCCACTCGCTTCGACCAGGCGG
AGGGTAGGCCCTCGACCGGCTCTCCCGAAACATTACAAAACGGGACGGGCAGAGACCTGGGTAACAACTTCCGAGCGCTTTTTT
TTGTGTCTTCCGCGGACAGTCCCTATTCCTCCGTTGCCTGCCACGGGAACTTCCTGCATAGATGGCGGATGGAGGTTATGGCGA
ACTCGACTAGGGTGGGGCCCGGCCAAAAGATGACGTTTGTAAGCGGTTTTGGCAATCCACCACAAACAAAAGGTGCAGAACTTG
ACGAGTGCTGCAACTCATCCCCAGCATCACGGCAGCTGGACCTTATCCCAATGGGGGAGGAGCGCGTCCTTCCCGTTGAAGCCG
TAGATTTTGGGGTCGGGCCAGGACGCAAATCGGCCTCCCCGTCAGGCCCAATAGCGACAAATGCTGGTTTGAACAAGCAAGTTG
ACGGAAGTATTACCGTCATCCACCAAAACACGCGGATACCAAGCTTGTTGGCAATTTGGCATCTACGACCAGCGGGGTTCGTGG
TGCGGGAAAATGGACCGTTGCGGGGCGTCGTCCTCATGAAGTGATGGGAAAGGTTCCCACCAGTAAGGGTGCTGAGCCGGGACC
TGGACAAGGGCGCAAACTTCTTGTCGTGGTCCAGCTTGCTCAGATAGCGCTTTTTGTCTTCCTTCAAGGGTCCTCCTAGGTGGG
GTCCACCGATATGGTTTGCGACGTGCCCCGTCCACCTCGAGGGGGCCGCTCCGGAGAGGTAGGGCATGCCTAGGGGCCGGGGGC
CTGCGCGCGATCGGTCCCTGAGGGGCCCTGCACTGAGGGTCCTGGGGTGCATGACCCTCCCTGGGGCAGGGTATGCACGAGTCG
GCACTGGCGCCGCGGATTGGTCCGGGGATCGCTGACACGCCCCACGGGCAATCTTACCCACTGATGGAGGTGCCCCAGCTTGAA
TGAGGTTTCAATCTCGTCTTTTGAGATGTCGACACTCATCGTGGCGTGACCCCACAAATCTTTGTGATAGCGCACCGTTTGCCT
GGTTATTCTGGGAATCCTTTTCCCCCCTTTAAATGGGGCTAAGGGCTTCCGGTATGGACTACTCTTTTCCGAGCCAGGTAGATG
TTTTTCC
>m141122_040740_42134_c100732202550000001823141405141561_s1_p0/67264/0_7713
AAAGGAGAGAGAATCAATATGTCATAAATTAGGTCTGCTTAGTGAAATGTTATCTAGTAATAATTTTGGCAGAATCGGATCCCA
TCGTCTATACTACTGCAATACTATATCTTAGTGCAATGTTATCTAGTAGTGGTTTTTTGCGATTGGATCCCCATCTAAATTCAC
TAACAACAGTCTTACATAGAATGACAACAATATGAGACCCTAGGGTAGTATTGATTTACTAGTGTACGTTGGAAACGGTTCATT
ATGTAATAATCACCGTCCGCTTAGTGCAATGTTATCTAAGTAATAATTTTTCGGATCGACCGCCTCCGCAAATTCACAAATAAT
ATCTTATTGCATTGTTTTCTAGTAGTATTTTTTCGGGATCGATCCCCTTGTAAATTCACTACTCTAATCCTTACTCAGAATGAC
AAACTGTAATAAGACCTGAAGGTATATTGTTTACTAGTGTACCGTTGGAAAAGTTCAATATGTCATAATTAGTCTGCTAGTGAA
ATGTTATCTAGTAATAATTTTGCAGGATCGGATCATCGTCTAAACTCAATACTATATCTTAGTGCAATGTTATTCTAGTAAGTG
TTTTTTGGGATTGGATCCCCATCCTAAATTCACATACAACAGTCTTACTCAGAATGAAACAATATGAGACCCTAGCAAGTTAGT
TATTTGATTTACTAGTGTAACGTTGGAACGGTTCAATTATGTAATTAAACCACGGCACCCCGTCTGCTTAGTGCAATGTTATCT
AGTAATAATTTTCGGGATTGGACCCCCTTGCAAATTCCACTAATATATATTATTGCAATGTTATCTAGTAGTATTTTTCCCGGA
TCGATCCCCTTGTAAATTCACTACTCTATCTTACTCAGATGACAACTATTCAGACCTAGAGGTAGTATTGGTTTACTAGTGTAC
GTTGGAAAGGTTCAATATTGCATAATTAGGTCTGCTTAGTGCAATGTTATCTTGTAATAATTTTGCCAGGATCGGATCCATCCT
TCTAAACTCACTACTATATCTTAGTGCAATGTTATCTAGTAGTATTTTTTGGGATTGGGACCCCATCTAAAATTCCTACACAAT
CTTACTCCCAATGACTTACAATATGAGACCCAGTGGTAGTATTGGTTTACTAGTGTACGTTGGAATCTGTTCATTATGTAATAA
TTAGGTTGCTTAGTGCAATGTTATCTAGTAATAATTTTTTGAGGAATCGATCCCCTTCTAAAACTCACATACATAATCTATAGA

GTAATGTTATCTAGTATATTTTTCAGGATCGGATCCCTTCTAAATATTCACTAGTCTATTTACTCAGAAATGACAAATATATGA
GACCATAGCCGTAGTATTGGTATACTAGTGGTTACGTTGGAAAGGGTTCAATATGTCATATTTAGGTCTGCTTATGCAATGTTA
TCTAGTAATAATTTGGCCAGATTGCTTCCCCTTCTAAAATTCACTACAGACATGCTTAGTGCAATGTTTTATCTAGTAGAATTT
TTCGGAATCGGATTCCCCATCCTAAATTCAATACACAGTCCTTACTCAGAATGACAAACAATATTGAGACCCTAGCGGTAGTAA
TTTATTTACTTAGTGTAACTTGGAAAGGTTCATTATGTATAAATCAAGTCTGCATTAGTGAAATGTTATCCTAAGTAATATTTT
TCGGATCGGACCCCCTTGCAAAATTCACTAATATATCTTTATTGCAATGTTAATCTAGAGTAATTTTTCGGGAATCGGATCCCT
TGTATAATTCACTATCCCCTTGTAAATTCACTACTCTATCCGTTACTCAGAATGACAACTATATCAGACCCCTAGAGGTAGTAT
TGGTTACTAAAGAGTACGTTGAAAGGTTCATAGTCAATAATTAGGTCTTGTTAGTGAATGTTATCTAGTAAATATATTTTGCAG
AATCGGATCATCGTCTATACTCAATACTATATCTTAGTGCAATGTTATCTAGTAGTGTTTTTTTGCGATTGGATCCCCCATCTA
AATCACTACAAAGTCTTAACTTTAGAATGACAACAATATGAGACCGCTAGCGGTAGTATGTGATTTACTAGTGTACGTTGGAAC
GGTTCATTTATGTAATAATCACGTCTGCTTAAGTGCAATGTTATCTAGTAATAATTTTTCGGGATCGGACGCCTCGCAAATTCT
ACAATATCTTATTGCATTGTTTTCTAAGTGTATTTTTTCGGGATCCGATCCCTTGTAAATTCACTACTCCTAATCTTACTCAGA
ATGACACTATACAGACCTAGAGGTACGTTATTGGTTTACTAGTGTACGTTGGAAAGGTTCAAATAGGTCATAATTAGGTCTGCT
TAGTGAAATGTTATCTAGTAATAATTTTGCAGGATCGGATCATCGTCTAAACTCAATACTATTCTAGTGCAATGTTATCTAGTA
GTGTTTTTGGGATTAGGAATTCCCCACATCAATAAATTCACATACACAGTCTTACTCAAGAATGACAACAATATGAGAACCCTA
GCAGTAGTATTGATTTACTAAGTGTACGTTGGAACCGGTTCAATTATGTAATAACCACGTCTGCTTAGTGCATGTTTATCTAGT
AAAAATTTTCGGGATCGGACCCCTTGCAAATTCACTAATATATCTTATTGGCAATGTTATCTAAGTAAGTATTTTTCGGGATCG
GATCCCTTGTAGATTCCACTAGCTCTATCTTACCAGAATGACAACTATATCAGACCCTAGAGGTAGTATTGGTTTACTTGTGTA
CGTTCGAAAGGTTCAATATTGCATAATTAGGTCCTGCTTTAGTGCAAATGTTATCTTGTAATAATTTTGCCAGGATCGGAATCA
TCTCTAAAACTCACTACTATATCTTAGTGGCAATGTTATCTAGTAGTATTTTTTGATTGGATCCCCATCCTAAATTCACTACAG
CAATCTTATTTCGAATGATACAATATGGAGACCCTAGCGGTAGTATGGTTTACTAGTGTACAGTTGGGAAACGGTTCCATTATG
TAAATAATTAGGTCTGCTTAGTGCAATGTTATCTAGTAAGTAATTTTTGAGGATCGGATCCCTTCATAAACTCACTACTATATC
ATAGTGTAATGTTATCCTAGTAATATTTTTCAGGATCGGATCCCCCTTCTAAATTCACCTAGTCTATCTTACTCAGAATGACAA
ATATATGAGACGCATAGCGGTAGTATTGTATACTAGTGTACGTTTGGAAAGGTTCAAATATGTCATATTTAGGTCTGCTTAGTG
CAATGTTATCTAGTAATAATTTGGCAAGGATTGCTTTCCCCTTCTAAATTCACTACAACATCCTTAGTGCAAGTGGTTATCTTA
GTAGAATTTTTCGGAATCGGATCCCCATCTAAATTCAATACGACAGTCTTACTTCAGAATGACAACAATATGAGACCCTGCGGT
TAGTATTTATTTACTAAGTGTACGTTGGAGGTAACGGTTCATTATGTAATAATCACGTCTTGCTTAGTGCAATGTTATCTAGTA
ATAATTTTCGGGATCGACCCCTTGCAAATTCACTAATATATCTTATTGCAATGTTATCTAGTAGTATTTTTCGGGATCGGATCC
CCTGTAAATTCACTATCCCTTGTAGAATTCACTACTCTATCTTACTGCAGAATGACAACTATATCAGACCTAGAGTAGTATTGG
TTTTACTAGTGTACGTTGGAAAGGTTCCAATATGTCATAATTTAGGTCTGCTTAGTGAAATGTTATCTAGTAAATAATTTTGAA
GAATCGGATCATCGTCTTATAACTCAATACTATTATCTTAGTGCAATGTTTATCTAAGTTAGTGGTTTTTTTTGCGATTGGAAT
CCCCATCTAAATTCACTACACAGTCTTACATTAGAATGACAACATATGAGACTTAGCGGTAGTATTGATTTACTAGTGTACGTT
GGAACGGTTCATTATGTAATAATCACGTCTGCTTAGTGCAATGTTATCTAGTAATAATTTTTTCGGGATCGGACGCCTCCGCAA
ATTCACAAAATATATCTTATTGCATTGTTTCTAGTAGTATTTTTTCGGGATCAGGATTTTCCCTCGTAAATTCACTACTCTAT
CTTTACTCCAAGAAATGACAACTGTATAAGACCCTAGAGGTAGTATTGGTTTTACTAGTGGTACGTTGAAAGGTTCAATATGTC
ATAATAGGTTCTGCTTAGTGAAATGTTATCTAGTAATAATTTTGCAGGATCGGATCATCCGTCTAAACTCAATACTATATCTTA
GTGCAATGTTATCTAAAGTAGTGTTTTTTGGATTGGATCCCCATCTAAATTCACTACACAGTCTTACTCAGAATGACACAATAG
TGAGACCCTAGAAAAGTAGTAATTGATTTATCTAGTGTACGTTGGAACGGTTCATTATGTAATAACCACGTCTGCTTAGTGCCA
AATGTTTATTCTAGTAATAATTTTTCGGGATTGGACCCCTTGGCAAATTCACTAATATATCTTATTGCCAATGTTATCTAGTAG
TTATTTTCGGATCGGATCCCCCCTTGTAAATTCACTACTCTATCTTTACTCAGAATGACAAACTATATCAAGACCCCTTAGAGG
TAGTATTGGGTTTACTAAGTGTACGTTGGGAGAGGTTTCAATATTGCATAATTAGTTCTGACTTAGTGCAATGTTTATCTTGTA
ATAATTTTTGCAGGAATCGGATCATCTCTAAACTCAACTAAATATATCCTTTAGTGCAATGTTATCTAGTAAGTATTTTTTGGA
TTGGATCCCCCATCTAAATTCACTACACATCCTTACTCCGAATGACTACAATATGAGACCACTAGTGTAGTATTGTTTACTAGT
GTACGTTGAACGGTTCATTATGTAATAATTAGGTCTGCTTAGTGCAAATGTTATCCTAGTAATAATTTTTGAGGATCGGATCCC
CTTCTAAAACTCACTACTATATCATAGAGTAATGTTATGCTAGTAATATTTTTCAGATCGGATCCCTTCTAAATTCATAGTCTA
TCTTTAACTCAGAATGACAAATATATGAGACCATAGCGGTAGTATTGGTATACTAGTGTACGTTGGAAAGGTTCATATGTCATA
TTTAGGTCTGCTTAGTGCAATGTTATCTAGTAAATAATTTGGCAGGATTGCTCCCCTTCTAAATTCACTACAAACATCTTAGTG
CAATGTTATCTAGTAGAATTTTTCGGAATCGGATCCCATCTAAATTCAATACACAGTCTTACTCAGAATGACAACAATATGAGA
ACCCTAATGCGGTAGTATTTATTTACTAGTGTACGTTGGAACGGTTCCCATTATGTAATAATCACTCTGCTTAGTGCAATGTTA
TCTAGTAATAAATTTTTCAGGGATCGGAACCCCCTTGCAAAATTCACTAATATATCTTATTTGCAATGTTATCTAGTAGTATTT
TTCGGGATCAGATCCCCTTGTAAATTCACTATCCGGCTGTAAATTCACTACTCTATCTTACTCAGAATGATACTATATCAGACC
TAAAGAGGTAGTATTGGTTACTAAGTGTAACGTTGGAAGAAGGTTCAATATGTCATAATTAGGTCTGCTTAGTGGAAATGTTAT
CATCGTAGTAATAATTATATGCAGGATCAAGAATCAATCGTCTAACTCAGATAACATTAAATATCTTAGTGCAAATGTTATCTA
GTAGAATGTATATTATTTGCGTTGGATCAACCAATCTAAATTCACATACCAAGAGAGTCTTACTCAGAATGAACACAATATGGA
CCACATAAAGCGGTAGATATTGATTTACTAGTGAGTTACGATTTGAGAACGGATATCAATAATATGTCAAAATTCAACGAATCA
TGCTTAGATGGAGCAAATGTTATATATCTAGATAATAATTATTTACGGGATCGGAACAGCCTCGCATTCACAAATAATATCATT
AATTACAATGTTTATATCAATAGATAGTAATATATTATGGAGATCGATATCCACCTTGTAAAAAAATCACAATAAGTCTAATCT
TACTCCATATGACAACTATATCAGACCACTAAGAAGGAAAGAATATTGGATTTACTAAGTGACAGTATAGGATAAGGTTTCAAT

ATGTCATAAATTAGGGTCTGCTTTAAGTGAAATGTTATCATAAGTAAATAAATTTTGCAGGATCAAGGATCATCGTTAAACTAC
AATACTATAACTTTAAGATGCATGATATATCTAAGATAGTGGTTTTTATGGATTGGATCCCATATAAATTACACTACACAGTAT
TTAAATCAGAATGACAACAATTATTGAGACAAAGCAATAGTAATATGAATTTGCTAAGTGATACGGATTGACGGTATCAATATA
TGAAATAAAATACACGATCATGACCATTATAGTGCATGATTAATCATAAGTAAATAAATATATATTCGAGGAGATCGGACACCT
TTAGAATTCACTATATAATCATTTATTGGCAATGTAAATATCTAGATTAGTATATATTTGAGGGGAGATCGAATTCACCTTGTA
AAATTCCACTACTCTATCATTACTACCAGAAATAGACAACTATAAATCAGAACACCTATAGAGAGGTATATTGGTTTAACTAGT
GTAACGTTGGAAGGAAGTTAAAATATATATATTTAGATGCATAATATATATCTTGGTAATAATTTGCAGCATCGGGATCATCTC
TAAACTCACTACTATATCTTAGTGCAAATGTTTATCGTAGTAGTATTTTTTGGGATTGGATCCACATCCCTTAAATTTCACAAA
CACAATCTTACTCCGAATGACTACAATATGAGACCCTAGCGGTAGTATTGGTTTACTAAGTGTACAGTTGGACACGGTTCTTTA
TGTAATAATTAGGTCTGCTTAGGCAAGTGTTTATCTAGTATAAATTTTTGAGGAAAAAAATTCGGATCCCTTCTAAACTCACTA
CTATATCATAGTGTAATGTTATCTAGTATAATTTTTTCAGGATCGGATCCCCTTCTAAATTTCCTAGTCTATCTTAACTCAGAA
TGACAAATATATGAGACCTAGCGGTAGTATTGGTATACTACTGTACGTTGAAAGGTTCGAATATGTCTATTTAGGTCTGCTTAG
TGCAATGATATCTAGTAATAATTTGGCAGGATTGGATCCCTTCCTAAAATTCACTACAACATCTTAGTGCAATGTTATCTAGTA
AGAATTTTTCGGAATCGGATCCCCATCGTAAATTCAATACACAGTCTTACTCAGAATGACAACAATAATGAGACCCTAGCGGTA
GTATTTATTTACTATGTAACGTTGGAAACGGTTCATTATGTAATAATCACGTCTGCTTAGTGCAATGCTATCTAGTAATAATTT
TTCGGATTCGGACCCCTTGCAAAATTCACTAATATATTTATTGCAAGTTATCTAGTAGTATTTTGTCGTGATCGATCCCTTGTA
AATTCACATCTCTATCTTACTCAGAATACAACTATATCAGACCCTAGAGGTAGTATTGGTTTACTAGTGGTAGTTGAAAGGTTC
AATGATGTCATAAATTAGGTATGCTTAGTGAAATGTTATCTAGTAATCAATTTTGCAGAATCAGGATCATCGTCTAAACTCAAA
TAGCTATATCTTAGTGCAATGTTATCTAGTAAGTGTTTTCTGCGATTGGATCCCATTCTAAATTCACTA
>m141122_040740_42134_c100732202550000001823141405141561_s1_p0/72716/0_6253
AAAAAGGAAGAGATGCTGCCGCAGGTGCCGCATACTGCTCTGCTGCAGTTGCTTCACTACTGCTAGTCAACTAAACAAATACGC
TACTACTAACTACATAATTAAGCTATATGCTACAATACTACACGACTACTGCTAACTGCTATACTGTTATTACAACTGCTACTT
CTACTACTACTTCTAACTACTATTACTTCTACTACTATTACTACTACTGCTACTTCATACTCTACTATACAATGCTATACTGCT
ACTAGTTACACTGCATCTACAATTTTTTATATTACATACAATGCTATTGCTCTGCAACTAGCTCATGATATATGCAAGCAATGA
CTGATATAACTAAAGGAAGAGATGCATAATAAGCTAACATAAAAAAAAAAAAAAAGAATACATAATAATAATGCTAATAAAGAAA
ATAATACATAAGCTAGCACTGATATACTAGCTTCATACTAGTGCTGTAGCATACTGATATACTCAGCTAAATAGAGCTATTATT
CTATAATAGCATGCTAGTGCATTGATACTGATAAATAAGATGACTACTAGCTACTATCGCATGCTGCACTAGCATACTAGCATA
CTTTGTAATGCTGCTACTAATATTAGCTACTAGCATAGCTAACTAACTATAAGCTACAGCTACGGCTAACTGCTACTAGCTACT
GCTATACATACATAATCACTAATACTACTACTACTAACTACTACTAATACACTACTACCGTAATTATTCTATATGCTATGCTAC
TGCTATTACTAACTACTACATACATGTAGTTACCTAAAACTGCTGCTGCGGTTACTCTTCACTGCTTCATGCTACACCGACTGT
TACTGCTACTGCTACTTTTACTGCAGCTACTACTAACTACATATATTCTACTGCTATTCTAACTACTGCTAGCATACAGCATAC
TCGCTAACTGATGTTCTTGCTGCTAACATACATACTACTCAGCTGGCTACTTGCTAATATACTGCTACTACTACTTGTCCTTCT
ACCTGGCAACTGCTTAACTCCGATACTATTGCTACTATATGCACACTGCTACTGCCTACTCTACTGCTATTGCAGTTGCTGCTA
CCACTACTGCTACATGCTGTCTACTGGCTATTGCTATTGCTACTACTGCTTCTACTACTTCAGTTGATCTGCTTCTAATGCTAT
GCTACATGCTGGGTGCTGCTACTGCTCCTGCTGATGCTGTTCTGCAATGGCTACAGCTGTTACTCACTACTATTGCGCTGCTGC
TACTATCACTACTACTACTACAACAACAACAATTACTACTACTACTACTACTGTACTGCTACTGCTGCTACTCTACTACTTCTA
CTGCTCTAGCTAACTACTACTACTTCTACTTCTACTACTGCTGGCTACTGCTACTACTAATGCGTACTGCCTGCTGCTACTACT
CACTATTTATATTCTACTCGTTGCTGCTACTGCTACTACTACTAATACTGCTACTACTAGTGCCACTGCTATCCCCCTACTGCT
TCTCTACTTCTACTACTCTTTTATGCTATCCTACTACTACTACTACCACCACCACTTACTACCAGCCGACCACCATTACCACTA
CTACTACTAAGTAACAAAACACTACACTACTGCTATCCCTTCTATATCTACTACTAGTGCTAACTGTTGTTTGCTACTGCTGGC
AGCTGTTACTGCTTCAGCTACTGCTACTGCTAAATTATTGCTACTACTACAATTACTGCTACTGCTCGTTATACTGTGCTACTA
CTACTACACTACTACTACTGCTAATACCTACTAACTAGTGCTTAACGCTACTGCTACTAATGTTCCTGCTGCTATCTAACTACT
ACTGCCCTGCTGAGCTTCTACTGCAGCTTGCTACTCCTACAACTAACTGCTACTGCACTATATGCTACTGTTACTGCGCTACTA
CCACAGGCACGTTCTACTTCTACCTGCTAGTGCTACTATTATTGCTACTGCTACTAATACTATTACTGGAGCTGCTGCTAACCG
CTACTGCTACCGCTACTGCTACTGCAACTGCCACTGCAGCTACTAGTACTACTACTGCTATACTACTGCAACTTCTACTGCTAA
TACTACTACTGACTACTACTACTACAGTTAACTGCTAGACTGCTTGTGCTGCTAGATACTGCATTACTACTGCTGCTACTATAC
TTCTATACTACCACCCTACCACCACTACTACCACTACAAACTAACTACTACTTACCACCAGCCACCACCACCACCACCACCATA
CCACCCTACACACCTCAATCATTACCCCTAAATGCTATTTCTACTGCTGTGCTAATGCTGTTGCTATATGCAACTGGAAACTAC
TACTTCTAACTACTAACTGCTACTACTATTTGTCTATGCTACCGCTACGGCTACTGCAACTGCTAACTCACAACTGCTACTACT
ACTACTACTAATGCTGCTACTATCTCTACTACTTCTAATATGCTACTACTACTGCTACCTACTACTACTACTGCTACTGCTGTT
GCTAGTGCTGCAGCAGGAACTGCTTCCTGCTACTGCTAAAGCTACTGTTATTGCTACTACTGCTTCTTACTACTGCCTACTACT
ACTAACTACTACTGCTGCTATTACTACTACTCTGCTACCGCTACTGCTACAGATGTTCCTAAACTGATACTACTACTACTACTG
CTGCTTCTACAGCAGCAACTAATTCTAACTATACTGTCTACTATTTGCTTTTGCTACTATCATCACCTGCTACTGCTACTACAA
CTTCTATGCTACTTCTACTACTAGTGCACTGATGTTCTGCTGCTATACTTACTACTGCTACTGCTTCTACTGCAGCTACTACTG
CTACTGCTTGCTACTGCTACTGCTTCTACTTGCAGCTAGCTACTGCTACTAATTAATGCTACATACTACAGATACTGCTGCTGC
TTACTGTTTTACTGCTTCTGGCTAGTGCTGTAGCTATCTGTTACTGCTACTGCTACTATTGCTGGCTAGTGCAATTGATATGTT
AGTGCACTGCTACTATCAGCTGCTGCTACTGCTAAACTGCTACTGTTGGCTGCTATACTATTGCTACTGCTGCTACTACTACTG
CTACCGCTACGGCTACTGCTACTGCTAACTGCTACTACTACTATCCCTATACTACTACTACTACTACTACTACTACTACTACTA

CTACCGATTAATTATTCTATAATGCTAATGCTACTGCTATTAGCTACTACTACTACTGTGAGTTGCTACTGCTCGCTGCGGTTA
CTCCTTCTGCTTCTGCTACACCATACTGTTACTGCTACTGCTACTTTTCTGCAGCTACACTACTACATATATACTTTCTACGTG
CTATTACTACTACGCTGCTACCGCTACTGCTACTGACTGTTCTGCTGCTACTATACTTACTACAGCTGCTTAACGTGCTAATAC
TACTGCTACTACTACTTGTCCTTCTAACTGCAACTGCTACTCCGAATACTATTGCTTACTATAAGACAACTGCTACTGCTACTA
CTACTGCTATGCAGTTGCTGCTACCAACTACGCTACTGTGCTACTGCTATTGGCTATTGCCTATCTACTGCTTCTACTACTTCA
ATTGATGCTTTGCTTCTACTGCTACTGCTACTGCTGGTGCTGCTACTGCTCTTGTCTGATTGCTTGCTTCTGCAACTGCTACAG
CCTGTTACTACTACTATTGCTGCTGCTGCTACTTCTATATACAAAAAAAAATACTACAACAACAACAATTACCTACTACTACTA
CAGCTGCTACTGTACTGCTGCTACTACTACTACTTCTACTGCTGCTGCTACTACTACTACTTCTACTTCTAACTACTGCTGCTG
ACTGCTACTACTAAATGCCTACTGCTGCTGCTACTCAAACTACTATTTATATACTTCTACCTGTTGCTGCAACTGCTACTACTA
CTAATACTGCTACTACTAGTGGCCACTGCTACTCCCCCTACTGCTTCTACCCTACTTCTACTACTCTTTTATGCTACTCTACTA
CTACTACTACCCCACCACTACTACAGCCACCACCCATTACCACTACTACTACTAGTAACAACACTCACTACTACTACTGCTACT
CCTTCTATATGCTACTACTGAAGTGCTACATGTTGTTGCTACAGCTGGCAGCTGTTACTGCTTCAGCTACTGCATACCTGCTAC
AATTATTGCTTAACTACTACAATTACTGCTACTGCTGCTTATACTGCTGGCTACTAACTACTACTCTACTACTACTGCTATACT
ACTACTAGTGCTACCGCTACTGCTACTATGTTCCTGCTGCTACGACTACTACTGCTGCTAGCTTCTAACTGGCAGCGCTACTCC
TACAACATACTGCCTACTGCATATATATGCATACTGTTTACTGCTACTACCACGGCCACTTCTACTTCTACTGCTAGTGCTACT
ATTATTGGCTACATGCTACTAATACTATTACTGAAGCTGCTGCTACCGCTATGCTACGCTATGCTACTGCAAGCCTGCCACTGC
AGGCTACTAGTCTACTACTGCTACTACTTACTGCAACTTCTACTGCTAAATACTACTACTATATACTACTACTACTACTAAACT
AACTATAACAGTTACTGCTACTGCGCTTGTGCTGCTGATACTGCTCTACTGCTGCTACTACTACTTCTACTACTTACCACCACT
ACACCCACTACCACCACTACTCCACTACAACTACTACTACCTACACCACACCACCACCAACACCACCATCACCTAAACACCTTA
CCTCATCATTACCCTAATGCTATTTCTACTGCTAGTGCTAATGCTGTTGCTAATTGCAACTGAAATACACTTCTACTATACTGC
TACTACTATTGTTATGCTACCGCTACGGCTACTGCATACTGCTACTAACAACGCTACTACTACTAACTACTAATGCTGCTCACT
ATCTCTACTACTTCTATATGCTACTACTACTGCTACTATACTACTACTGCTACTAGCTTGTAGTGCTGCAGCAGGTAATGCTTC
TGCTACTGCTAAAGCTACTGTTATTGCTACGTACTGCTTGCCTACTACTGCTACCTGACTACTACATCTACTACTGCTGGCTAT
TACTACTACTACGTACCGCTACTGCTACAGATGTTCTCTAACTGATACTACTACTACATAACTGCCTGCTTCTACAGCAGCTAC
TACTTCTACTACTACTGCTACTATTATGCTTTTGCTATATATGCAACTGCTACCTGGCTACTACAACTTCTACTGCTACTTTCT
ACTACTTAGTGCAACTGATGTTCCTGCTGCTACTACTACTACTGCCTAACTCTTCTACTGCACGCTACTACGCTACTATTTTGT
TTTTTCCTACTATATGCAACTGCTACTGCTACTACACTGCTACTGCTACTAACTACTACAACTGCAGCTGCTGCTACCACTACC
TGGCCTACTGCTGCTACTAGTAACCTGCTACTGCTACTAAACTTTGCTACTGCTGACTGCTACTGCTACTGCTTCTAACTACTA
CTACTACACTACTAACTACTACTATATCTACTAACTTCTATTGGCCTACTTCCTCATTTGCTACTTCTGCCTGCTGCTGCTACT
CTATCCATTGCTGGCTGCAATGCTATTGGTGGTGCTGCTTTCTACTCTACATGTTGTTCTTGCTGCTGCTTGCTGCTAGTTCTG
CTACTGCTACTGCGTGCTGACTACTACTAATGCTGTTGCTGATGCCGCTGCTGCTACTTCTACTGCTACTTGGGTAGTACTAAA
ATACTACTACTGCTACATCTGGCTTGCTACTGCTATTACTAATACCTTCTACGGTTGTGCTTCTGCACTACTAGCTATTGCTAT
GCTACTACTGTTATAGCTTGCTAATGCTACTATCTTCCTGCTGCTGGCTGCATCTGCTACTGCGACAGGTTCTGCTGCTGCAGC
TACTGCTAATCTATTGCTACTGCTGATGCTGTTTGGTGGTGCTGCTACTACTACTGCTACTGTTGCTGCTGCTGCTACTGCTAC
TGGTCGATTGTTGATGCTACTGGTGCTGTTGCTGCATACTGTTGCTACTTTCTGCTATTGCTACTGCTGTACTACTACATAATG
ATGATGCTGATGCCTGCTGCGGCAACTTCTACTGTCTGGTAGTATACTACTACTACTATTACCTAGTACTTAACTACTACTACG
TGCTACTACTACTTCTTCTACTGTACTCGCTTCTACT
>m141122_040740_42134_c100732202550000001823141405141561_s1_p0/75306/0_11890
AAAAGAGATAAGATAGTATACTAGACATGTCATAATAATTAATGTGACAATAGAATATTTTTTCAATATATATGAATAACAGAG
ACTTCCGTTTCGACTCCCCATAACTCATTAGTAAGCCCCTGAATCTCTTGGGGGCGAAATACCTCGAACGAAGTTCGAACACGA
AGGGAATGACATTTCGGAAATTAAGTGAATCATCAACCTTAACCAAACGGTACACCATATTCAGAAATCTTCAGATTTCTCTAA
TATCGGAGTTTCGAGCAGTGGTTTTGTAGTAATACTAAATATTTAATCGAGCGAGGCGTTCACAGGCACCCAACAATCGTAACA
ACACCTCAATTCTTCAGGTTCTAGTATAAAACTATTCGTGCATATCCGAAGGTTTGTATACAAAGGATTAATACAAGTCATGAG
AATGTACGTATAAGACGAAGAGAAACTATAGTACACTCAAAGAGGAAATATATTTTTATATATGTATGAATAAGCTACGTATAT
CAGACTTCACTAACTATTCATTTAACCCCTGAGATATCTGCTGGGGCAAAATATCCGAACCGAGTTGGCCAACAACGAGGGCGG
TAATACAGTTTCGGAGAAATTAGTGAATCCTCCCACTTCACCCAAGAGGGGTACGCCTTTCAGAAATATCGATGTTCTCTAATA
TGGATTCGGAGCAGTGTTTGTGATAGTGACAATACTAACTATTACGCGAGGGTCAATGGGCATAAAAGTTTGTAGCCCTATGTG
CTTCAGGTGTTCTACTGTATATTAGATTTCCCGTGCAAATCTCAGTTTTTTATAGGGTGATCACAGCAACATACTGAGAGTGTA
CCGAACATGAAGGAAAAGATTGGGAAAATCTGCCCTCAAAAAGGTGAATTTTTTTTTAAATGTTTGATTTCAGGCCTCGTTTCG
GACCAATAGTATTCATTTTAACCCTGAGATCTCTGTGGGGGGGAAATAATAGAACGAGTTGAACACCGAGGTAATCGACATTTC
GGGAGAATTAGTGAAATCTCCAATTCACCAAAGGGTACCCTTTCAGAATCGTTCGATTTCTCTAATATTGATTCGAGCAGTGTT
CGTTTGATAGTGATAACTAAATACTAGTGCGAGGGCTCCGGGCACCAAAAGTCTAACCCTATGTCTTAGGGTTCTATCCTTATA
AAGATTTTCGTGCATAATCCGAGGTTTGGCTATACGGGTGACTTAACCACGTCACAGAGATACCTGGAGACTGTACGAAATCAT
CAAGAAGAAAACTGTGGAAAATTGCCCCCCTCACAAGGGAATTTCTTTTTCAATGTTGAAGTTAAAGGCTCCGTTTCGACTCAA
TATATGCATTTTAACGCCCTGAGATCTCTTGGCGGGAAATTATCGAACGAGGTTGAACCGAGGGTAATACATTTGAGAACTTCA
TGAATTCACAATTACAAAGGGGTACTTTCAGATTCTTCGATTTTCCAACTATGGATTCGAGCAGAGTGTTTTGATAGTAATACT
TACATAATTCTGCGAGGGTCGGGGCACAAAAGTTGTAGCTCTATGTCTTAGGGTACTAGTTAGCTAATATCTCTCCGTGCCATA
TCCGAGCGTTTTGTATAGGTGAGTTACAGTCGAAAATCAGCCTGAGAGATGTACGAATCATACAGGAAAAAGAATTGTGGAATT

GCCTCAAAAAGGGGAATTTCTTTTTCAATGTGGAATTAGAGGCAACCGCGTTTCGACTCCGAATATATTATTTAAACCTGAGAG
ATCCGATTGGGGGGCGGAGAATATCGAACGGAGTTTGAACCACGAGGGTATACATTTCGGAGAAATTAGTGAAATCTCCAATTC
ACCAAAGGGGTACCCCTTTCAGAAATCTTCGATTTCCTAATAATGGACTTCGAGCCCAGCACCTGTTTTGATAGCTGGACGAAC
TAACATACTTGACTGCGAGGTCACGGGCCATAATAAAGTCGTAACCCTGAGGGATGTCTTAGGGTTCTATGTATCAAAATTTCG
TGCATATCCGAGGTTTTGTATACGGGTGAGTACAGGTCAAAATACTGAGAGAGTCTACGACCTGCACCCGGCGTCGCGCGCCGC
CGGCGGGGCGGCCCGCCCCGCCCCGGCCCGGGCGCGCGGCCCGGGCCGCGGCGCGGGCCGCCGCGCGCGCCGAGGCGCCGCGCA
ATCCGAGACCGAACTGCGGCGCGCGTGCGCGCCGCACCCCGGCGCCGAGCCCGCGACCCGGAATTGGGCCGGCCCTCCAAGACC
ACAAAGGGCCAATTCTCGTTGGTTTTACCGGACACTGTTGGAAGTCTAGCCAGCGCCTCCGTTCTCCGACTTAAACAAAACATA
AGACACCACAAAAAAAACAAATCACAGCAAAAAACAAACAACAAAGGGTTAACCTGAGATATCGTTGCGGGGGGAATATCGAC
ACCGAGTTACACACCGAGGGTCACAGTGAATTTGGTCGGGAAACCATTGTCCGAAGAATATAAAGCAGTTACGAACAAAGCGC
GAGAAAAAAATTCACAGATCAGAAGGACACAACTCACTTAAGCATTTATGAAAAAGTAACAAGATCCAATGGAGTTACGCAGAAA
GATGTTTTGATCCGTTGATACATACACAGTACTTCATAGAGGGTAATGGAATAAAAGTTGTAAGGAATAATGTATTAGGGTTAT
AGTATATATTTATGAAAGATATGAGTTTTTTATAGGGTTGGATAAAGTAAAAATAATGATAGAGATGTTAAGATATAAGGAAAG
AAAATTGGGAAAATGATAAAAAAGGTGAATTTTTTTTAGAAATGTTTGAAATTTAGGATAAGTTTTAGAATAATATATTAAATT
TAAAATGAGATATATTGGGGGGAAATGATAGAAGAGTTGAAGAAAGAGGGTAATTATAAATTTAGAGGAAATTATGAAAATATA
AATTAAAAAAAAGGGGGTAAAAATTTAAGAAATTATATTAGATTTATATAATATGGATTTAGAGAAATGTTTTGATATGATATAA
TAAAATGTTATGAGAGGGGTAAAGGAAAAACAAAGTGTAAAAATTATGTATTAGGGTTATATTTATAAAATTTAGTGAATATAA
GGTTTGTATAAGGGTGATAGAAGTAAAAATAATGAGAGATGGTAAGAATATAAGGAAAAAATTGTGAAAATTGAAATAAAAAAG
GAATTTTTTTTTAATGTTGAATTAAGGATAAGTTTAGAATAAATATATTAATTTAAAAATGAGATAATTGGGGAAATATAGAAA
GAAAGTTGAAAAAGAGGGTAATAAATTTAGGAGAAATTAGTGAAATATAAAATTTAAAAAAGGTAAAAAATTTAAGAATATATT
AGATTTACGTATAATATGGAATTAAAGAGAGAGAGTTTTGATAGTGAATATAAATATTATGAGAGGGGTAAAGGAAAAAAGTA
GTACGAAATAGTGTAGTTTAGGTTATATTTATAAAATTTTAAGTGGATATCAGAAGGTTTATATAGGGTGACTAAAGTAAAAAA
TAATGAGAGATGAAGAATATAAAGGAAAAAAATTGTGAAAATTGAATAAAAAGGGAATTTTTTAAATGTTGAATGAAGGATAAG
TTTAGATAAAATGAATATTAATTTAAAATGAGATATATTGGGGAAAATATAAGAAAAGAGTTGAAAAAGAGTTAATAAATTTA
GGAGAATTAGTGGAAATATAAATTAAAAAAAGGGGTAAATTATTTAAGCATATATATAGATTTTATATAATATAGGAGTTAAGA
AGTGTTTTGATAGTGAATAATAAATTTATGGAGGGAAAGGAAAAAAAGTAGTAGAAATATGTATTATTATATGTATAAAATTTA
GTGAATATAGAGGTTTGTATAGGAGATAAAGTAAAATAATGAAGATGTAAAGAATATAAAGAAAAAAAATTGTGAAAATTGAAAT
AAAAAAAGGGGAATTTTTTTTTAAATGTTGAATTTAAGGATAAGTTTAGAATTAAAATATAGTTTAATTTAAAATGAGATATTT
GGGGAAAATATAGAAAGAGTTAGAAAAAGGGTAATAAATTTAAGAGAAATTAGTGAAATATAAAATTAAAAAGGGTAAAATTTA
AGATATATTTAGATTTTTATAATATGGATTAAGAGTGTTTGAATAGGGAATAATAAATATATGAGAGGGATAAAGGGAAAAAAA
GTAGTAGAATATGTATTAGGGTTATATGATAAAATTTAGTGAATATAAGATGGTTTGTATAAGGGAGATAAAGTAAAAATAATG
AGGAGATGAATAAGAATATAAGGAAAAAATGTGAAAATTGAAATAAAAAAAGGGGATTTTTTTTTAATGTTGAATTAAGGGAT
AAGTTTTAGAATAAAATATAATTAATTTAAAAATGAGAATATATATGGGGGGAAATATAGAGAAGATTGAAAAGAGGGTGAATA
ATTTAGGAAGAAATTAGTGAAATATAAAATTAAAAAAAAGGCGGTAAGAAATTTTAAGATATATATAGGATTTATAGATAATAT
GGATTAGAGAAGTGTTTTTGATAGTGGAATAATAAATATTAGTGAGAGGGTAAAGGGAAAAGTAGTAGAATAATGTATTAGGTT
TAGTATTTAATAAAAATTTAGTGAATATAAGAAGGTTTGTATAAGGGTCGATAAGTAAATAATAGAGATGTAAATAATTAAGAG
AAAAAAAATTGTGAAAATTGAAATAAAAAAGGGGAAATTTTTTTAAATGTTGAAATAAGAGCTAAGTTTAGAATAAAATGATA
TTAATTTAAAAATGAGAATATTGGGGGAAAGAAATAGTTAGAAAGAGTTGAAAAAGAGGTGATAAATTTTTAGAAGAAATTTAG
TGAAATATAAAATTAAAAAGAAGGGGTAGAAATTTAAGAAATATTAAGATTTATATAATATGGATTAGAGGAAGATGGTTTTTG
TATAGGGAATAATAAACTATATGAGAGGGTGATGGAAAAAAAAGTAGTAGAAATATTGTATTAGGTTATATGTATAAGATTAGT
GAATATAAGAGGGTTTTTATTAGGGATAAAGTAAAAATAATGAGAGATGTAAGAATATAGAGGAAAAAAATTGTGAAAATTGAA
ATAAAAAAGGGGAATTTTTTTTTTAAAATGTGAATTAAGGAGATAAGTTTAAGAATAAAATATATTAATGTTAAATGAGATATA
TTGGGGGAAAATATAGAAGAGTTGAAAAAGGGGTAATAAATTTAGAGAAATTAGTGAAATAATAAAATTAAAAAAAAGGGTAAA
AATTTAAGATATATTAGATTTATATAATAAAATAAGGATTAGAGAAAGTGTATTGATAGTGAATAATATAATATTATTGAGAGG
TAAAGGGAAAAAAAGTGTAGGAATAAATGGTATTAGGTAATATGTATAATATTTAGTGAATATAAGAAGTTTGTAATAAGGGTG
ATAAAGTAAAAATAATGAGAGATGTAAGAATAATAAGGAAAAAAATTGTGAAAATTGAAATAAAAAAGGAAATTTTTTTAATG
ATAAAGTATAAGTTTAAGAATAAATATTATTAATTAAAAAATGAGAAATATTGGGGGAAAATATAGAAAAGGAGTTTAAAAAGG
GGTATAAATTTGGAAAATTAGTGAAATAATAAGAAATTAAAAAGGGTAAATTTAAGATATATTTGAGATTGTATATAATATAGG
ATTAGAGAAGTGTTTGATAGTGATAATAAATATTATGAAGGGTAAAGGAAGAAAAGTAGTAGAAATATGTAATTAGGTTATATG
TATAAATTTAAGGAATATAAGAGGTTTGTAATAGGGAGGAATGAAAGTAAAATAATGAGAGATAAGAGATATAAGAGGTAGAAA
CAAAGTTGTGATGAGAATAAAAAGAAGGGGAATTTGTTGTTTAAAGTGTTGATTGAAGGGATAAGTTTAGAATGGAAAATATAT
TGAAATTTAAAATATGAGATAATATTGGGGGAAATATGAGAAAGAGTTTGAACAAGAGGTAATAAATTTAGGAGAAATTAGTGA
ATATAAAATTAAAGAAAAAGGGGTAAAATTTAAGATATATTAGATTTATATAATATAGGAGTTAGAGAAGATGTTTATGATAAG
GATAATAAATATTATGAGAGGGTAAGAAAAAAGTTGTAGATAAATGTATTAAGGGTAATATGTATAAATTTAGTGAATATAAGA
GGTTTATATAGGTGAAATAAAGTAAAAATATGAGAGATGTAGAATATAAAAGGAAAAAAATTGTGAATAATTGAATAAAAAAGG
GGAATTTTTTTTAAAATGATTGAATAAAGTATAAGTAGAATAAAATATATTAAATTTTAAAAATGAGATAATAATTGGGGAAAA
TAATAAGAAAGAAGTTGAAAAAGAGGGTAATAAAATTTAGGAGAAATTAGTGAAATGATAAATTAAAAAAGGGTAAATTTAAGA
TATATTAGATTTATAATAATATGATAGAGAAAGTGTTTTGATAGTGAATAATAAATATTATCGGAGAGGGTAAAAGGGAAAAAA

AGTAGTAAGAATATGTATTAGGGTTATATGTATGAAAAAAATTTAGTGAAATATAAAGAGGTTATGTATAGAGGGAGATTACAA
GTAAAAAATAATGAGAGATGTAAGAATATGAAGGAAAAAAATTGTGAAAATTGAAATAAAAAAGGGGAATTTTTTTTAAATGT
TGAATTAAGATAGAGTTAGAATAAAATATATTAATTTAAAAAATGAATCTAATTGGAGGGAAAATATAGAACGAGTTGATAAAA
AGAGGGTAATATATTTAGAGAAATTAGTGAAATATAAAATTAAAAAGAGGTAAAAATTTAAAGATGATATTAGATTTATAGTAA
TGATGGGATGTCGAGAGTGTTTTGATAGGGAATTAATAAATAAGATATGAGAGGGTAAAAGGGAAGCAACAAAGTAGTAGGACT
ATGTATTAGGGTTCTATGTAAAAAAAAAATGATTAGGTGAATATAAGAAGGTTATATAGGTGATAAAGTAAAATAATGAGGAGAT
GACGAAGTATAGGAAAGAAAATTGTGAAAATTGAAATAAAAAAGGGGAATTTTTTTTGAAATGTGAATAAGGAATGAAGTTTA
GAGAAATAAATCATATTAATTTAAAAATGAGGATAGTATATGGGGAAGGGGGAAATATAGAAAGAGTTGATAAAAGAGGGTGAT
AAAATTTTAGAAGAAATTAAGTGAAATATCAAATGTAAGAAAAAGGCGGTAAAAATTTAAAAATATTAGATTTATATAATATGG
ATTGTAGAGAAGATGTTTTGATAGGTAATAATAATAAATTAAGAGAGGTAATAGGGAAAAAGTCGTAGAGATAATGTATTAAGG
GTTATATGTAGTAAAAAAATTTAGGTGCATATAGAGGGTTTATATGAGGAGTGATAAAGTAAAAATAAATGAGAAGATGTAAGA
AATATAAGGAAAAAAAAAATGTGAAATTGAATAAGGGGAATTTTTTTTAATGTTGATATTAAGGATTAGTGTTTAGATGAAAAT
GATATTAATTTAAAAATGAGATATATTGGGGGGTTTAGAAAAATATAGAAGAGTTTGAAAAAAGGTAATAGAATTAGGAGAAAT
TAGTGAAATATCAAATTTATAACAAAGGGGTAACACTTACAGAAATATTCAGATTTATATAATTATGGAATTAAGAGACAGATG
TTTTGAGTAGTGAAATAATAAATAATTATCAGCGAGGGTGAGACAGGCGAAAAAAGTAGATAGACTAATGTCTATAGGGTATAA
TGTATACAAAATAGTGGAATTATCAAGAGGTTTGTAAAGGGAGATACAGTAAAAATAATGAGTAGATGTAAGAATATAAGAGAA
AAAATTGTGAAAATGCTCCTCAATAAAGGGGAAATTTTTTTTTTTCAATGTTGAATTAAGGCTGCCGGTTTCGACATCAATATA
TTTTCATTTAACTGAGATCCCAGCCCCCCTTGGGAAAATATCCGAACGAGTTGTAACACGAGGGTCAAATACAATTCGGATAAA
TTAGCTGAAATCTCCCAATTCACCAAGGGGTACCCCGTTCAGGATTATCTTCGATTTCTCTAATATCGGATTCGAGCAGCTGTT
TTCTGATATTGAATAGCTTAATATATATCGCGAGGGTCACGCGGGCACAAAATTGTAGCTCTATGTCTTAGGGTACTGATTGTA
ATAATATTCGTGCCATATTCCGAGTTTGTATAGGGTGATAGTACAGTCCAAAAGTAATACTCAGAGATGTACGAATATCGAAGC
GAAAAAACTTGTGTGAAATTGCCCCCCTCAAATAAGGGGAATATTTTTTTTCCAAATGTTGAACTTTAAGGCCTCCGTTTCGCC
TCCCCCAAGATCATTCATCTTAACACTGAGATCCCTCTTTGGGGCGCAAATATCGAACGAGTCTGACACACGAGGGTGATTTTA
CATTGTCGAAGAAATTTGAAACCCAATTCACCAAAGGGGACCCCTTATCAGAAATCCTTCGATTTCTCCTAATATGCGATTCGA
GCAGTGTTTGATAGGGCGATACTAACGATGAATTGAATGCGCGGCATGGGCCACAAAAGCTCGTAGCCCTACTGTCTCTCAGGG
TGTCTACTAATACATTTCCGTGCAGTTATCCGACTGGTTTCTATAGGGCGTGAAATACAGTCAATAATACCCTGAGAGATGTAG
CGCACATATAAGGTAAAAAACTTGTGAAAAAATTGCCCTCAAAAGGTGAATTTTTTTTAAATGTTGAATTAAGGCACGCGTCTT
CGACTCCCGCAAATATATTCATCTTAAACCCCTGAGATCGTCTTGGGGGAAACTAATCGAACGAGTTGAACAGCGAGGGTAATA
CATTTCGGCAAGAAATTACGTGAAAGCTGCCAATGTCAGCCAAAGGGGTAAACCCCCTTCTCAGAATATTCTTGCCGATTCTCT
AAAATATCCCGGCCAGCCGGATTGCGAGGCATCGTTTCTTGATAGTGAAACTAAATAAATCTAATGCGCAGGGTCACGCGCACA
AAAAGTCGTAACCCTATGTCTTAGGGTTCCTACATGTAAACACCAAATCTTCGTGAATAATCAGGTTTGTATAAACGGGGAGTC
AACAGTCAAATACTCGAGAGATGGTAGCGAAATAGAGAAAAAACTTGTGAAAAAATTGCCCTCAAGCGAAAGGGGAATTTTTTT
TTCATGGTTCTGAATTAACACGGCTCCGTTTCGACTCCAATATACTTCCATCTTAACCCTGAGAATCGTCTTCGGGGGGCAAAT
ATCGAGCACGAAGTGAAAACGAGGGTAATACATTTCGGCAGAAATTATGAAATGCTCCAATTCAACAAAGGGTACCGCCTTTCA
GATATCTTCTCGATTTCTCTAGATATGGATTCGAGCCAGTGTTTTGATAGTGAATACGCTAAATATTTGGAATGCCGAGGGTCA
ACGGCCCACAAAAGTCGTACCCTATGTCTTAGGGTGTTCTGATTCTATAAACATTTCGTGCATACTCCGCAAGGTTTGTATACG
GGTGATACATGATCAAAGATAAGCGAGAGATGTACGAATATATAGGAGAAAAAACTTGTGAAAATTGGCCCTCAAAAAGGGGAA
TGTTTTTTCTTTAAACTGTTGAAATAAAGGCCCCGTTCTCCCTCGCTCCAATAAATTGCATTTACCCTGAAGACTCTTGGGCGC
GAAAATATGCGAGACGAGTTAACACCAGGGTGATACATTTCGAAGCGAAACTTAGCTGAAATCTCCATTCACCAAAGGGAGGCT
AACCTTTAAGAAATCTTTCGATTTCCTCTATATCACGGATCCCGAGCAGCATGTTTTTGATAGGGATGACTTAAATATGTATGC
GAGGTGCGATGGGCACAAAAGGTCGTCAGCCCCTATGTCTTCAGGGCTTCTATGTAATGAAAATCTTCGTGGCATATCCGAGTT
CTATAGGGTGATACAAGTCAAAATACGTCGAGAGATGTAACCGGAATATACAGGGAAAAAAACTTGTGGAACAATTTAGCCCTC
CACAAAAGGGGAATTTTTTTTAAATGTTGAATTAAAGGCTCCGTTCGCTCCAATATATTCATTAATCGCGCCCTGAACGGTCT
GCCTCTGGGGAAAATATCAGAACCGAGTTTGAACACGAGGTAATACCATTTCGGGAGAAAGTCAGTGAAATCTCCCAATTCACC
AAGGCGGTAGCCACCTTTCAGATATCTTACCGATTTCTCTAATATGGATTCGACGGGAAAAGTGTTCGATAGTGAGATACTACA
ATACTTGATGGCGAGGGTCAGCGGGCTACAAAAGTCGTAGCCCTATGGTCTTAGGGTTCTATGTATAAAATTTCGGCAATATCC
GAGGTTTCGTATGGGAGATACAGTCAACAATAATGAGAGATGTACGAATATAAGGCAAAAAAACTTGTGAAATTGCACATCAAA
AAGGGAATTTTTTTCCAATGTTAATTAGATACGCGCTCCGTTTTCGACCTCCAATCATAGGTTCCATTTTAACCCCTCGAGATC
CTCTTTGGGAAAAAATATCGAACGAGTTGAAGCACGAGGGTCAAATACATTTCGGAGACAATTAAGTGAAATCTGCCCAATTCCC
CAAAGGTACCTTATCAGATATCTTCGATTCCCTAATGATGGGTTCGAGGCAGTGTTTTGATAGGGAATCACTAAGATAGTTCCA
ATCGCGACGGGTCACCTCCAGGGCACAAAAGCTGTAGCTCCAAATGTCTTAGGGTATTCGTATACGAAATTTTCGTGCATCATC
CGACCGCGGTTAATTCTATACGGGGTGATAGCAGTCGAAACCATACTCGAGAGATGTACCGAATGAATAGGAGGAAAAAACTTG
TGAAAATTGCCCTCAAAAAGGGGAATTTTTTTTTTAAATGTGTGAATAATAGGCCTACCGTTTCGGACGTCCAATAATAGTGTC
ACTTTAAACCCTGAGATCTCCTGGGGGAAAAATATCGAGACGAGTTGAACACCGAGGGTGATACATCTTAGAGCAGCAGAACAA
AAAAGAAGCCGCCACCAGAAATGTTGTGAAATCCTCCAATTTCACGCCCCAAGGGGTACCCCTTTGTCAGAAATCTTCGATTTC
TCCTACTATCCCGGATTCGAGCACCTGTTTCGTGATAGGGAATACTACAATAATTATGGCCCGAGGTCATGCGGGCACAAAAGT
CGTAGCCCTATGTCTTAGGGTGCTATCTGTATCAAAATGTTCGTGCATATCCGAGGTCTGATTGATATAGCGGTGATTCAGTCG
AAAAATAGCTGACGAGAGAATGTACGAAATATAAAGGAAAAAACTTGTGGAAAATTGGCCCTCAAAAAGGGGAAATTTTCTTTT

TAAAATGTCTGAATTAAGGCTCCGTTGTCGACCTCCATTATTCATTTAACCCTGAGATCGCTCTTGGGGGAGAAAAATCGAGAC
ACGTTGACACGAGGTAAGTACATTTCGGAGAAATTAGTGAAACTCCCCAATTCCCCAAAGGCGGTACGCCCCTTCAGATATCTT
TCGATTTCTCCCTAATATGGATTTCCGAGCAGATGTCTTTTGATAGTGAGATAGCTAAGATGCTTCTCACGTGCGAGGGTGCAC
CGGGCACAGAAAGAGTCGTAGCGCCTAGTGTCTTAGGGTTCTACTGTATAAAATTCGTGGGCTCATCCGAGGGTTTATAGGAGA
TACAGTCAAATAATGAGAGAAAGATGTAACGAAAATAAGGAAAAAACTTGTGAAAATTGGGCCCTCAAAAAGGTGGAATTTTTT
TTTCGCAATGTTGAATTAAGGCCCTCGCGTTTCGGACATCCAATCA
>m141122_040740_42134_c100732202550000001823141405141561_s1_p0/82939/8045_13490
CTATCTTCCCAGCATGACAACATTTGAGGCTTGTAGCGAGTATGTGTTACTAGTGTACGTGAACATTCAGTTTATGTCATAATT
AATTGTTTGTAATATCAATTTCTTCTGGATGATCATTCTAAATCACTACTATTATCTTGTGCAACGTTTATAGTAAATTTTTCC
GGGATCACACGAGTCCCTTCTAAATTGATTTCTCTATCTTTACTCAGCATAGACAACTATATGAGACCCTAGCGGTGTATTATT
ACTAGTGTACGTTGAACGGTTCATTATTCATAATTAGTCCTGCTTAGTGCAATGTAATCTAATAATAATTTTTCGGATCAGGGT
CCCTTCTAAATTCCGCTACTCTACTTCTCGTATGAACAAGCTATATGAGTCCCTACGTAGTATTGGTTTAATTAGTGTTACGTT
GGAACTTTCCATTATGTATAATTAGGCTGCTTAATGCAATGTTATCTACTCATAATTTTTCGAGATCGGATCCCCTTGTTATTA
CTCACTTACTAAGATCTAACTCAGCATGAACAACTCAACAATGGACCCCCTTAGGGTAGTATTGGTTATAACAAGTGTAATTGG
AACGATTCATTTGTCCATAATTAGGTCCCTTTAGTGCAATGTTATCTATCATAATTTTGATCAGATCGCCATCGCCATTCAAAA
TTAAACTACTAGATCCTTAGTGCCATGTTATCTAGTAAGTATTTTTCCGCGAGATCCGATCCCCTTACTAAATTTCACTACTCT
ATCGTTACTAAGCATACAACCAATATGAGGCCCTTGGCGGATATATTGTTACAGTGTACGCTTGAGCAGCGATTCATTAAATGT
CATAAATTAACCGGTCTGCTTAGTGCAATGTAATCTAGTCATTATTTTCGGTCGGATCGCTTCTATACTTCACTACTATATCTA
ACTCAGCATGACAAACTATATAGACCCTAGCGGTATCATTGGTTTACTATTACCATTGAACGATTCAATTATGTCTATAATTAA
AGGTCTGGTTAGTGCAATTTATCTAGTCATTAATTTTCGGATCCGGAGCCATTCTAAAGTAACTACTATATCTTAGTGCAATGT
TATCTGCTAAATTTTTTTGGATCGTGTCCCCTTCTATAGTTCACTACTCTATTCTTAGTCAGCATGACACAATTTGAGGCTTAG
CGGTAGTATTGGATTTACTAGTGTGAGAGTTGGAAAGATTCATTAGTCAAGATATAGGCTGCTTAGTGCAATGTTTCTAGTCCA
TAATTCTTTCAGGATGATGCCATTCTACAATTACTACTAATCTTAGTGCATGTTAAGTCTGTACATATTTTTCCGATATCGGAT
CCCGTTCTAAATTCATACTCTATCCACACTCAGCATGACAACAAAAAGAGACCCTAGCGGTATATCTGGTTTACTAAGTGTACT
TGGAAGTGATTCATTATGTCATAATAGGTCTGCCTAGTGCAATGTGTATCTAGTCATATTTTTTCGAGATCGGATGCATTTCTA
AATTAACTACTATATCTTCGTGCAATGTTATCAGAATATTTCCGGATCGGTATCCCCTCTAAAATCACTACTCTGTCTTAGCTG
ACCAGTGACAGACAATATGATGGCCCCAGCGTAGGTATCAGGTTTACTAGTGTTACCGTTGGAATGACTCATTATTCATAATTA
TGTGCTCTGGCTTAGTGCAATTTATCTATCATACATTTTTTCGGATGATCCCTTCTGATATTCACCTATATAACTAATCAGCAT
GAACTATAAGCGAGACCCTACGGTAGTATGGTTTACCTATGTACATTGAACGATTCATTTAGCATAATTAGGTCTACATAGTGC
AATGTTCTACCATCAATTGTTCCGACGGATCCATTCTAAACTTATACTACTATATCTTAGCACATGTACTCCTTAGTAACTAAT
TTTCGGTGATCGGATCCCCTTCTAAATTCACTACTCTATCCTTATCAGTAATGACAACTATATGTCCAACCTAGCCGTAGTATG
GTTACTACAGTGTACGTGAATGATTCATTAATGTCATAATTAGGTCTTGCTAGTGCAATGTCACTAGTCATAATTTTCGGGATC
GGATCCCCTTCTAAATCACTACTTCTATCTTACTCAGTATGACAAATATATGATGACCCTAGCGTAGTATTTGTTACCTAGTGT
CACGTTCGAAACGATTCATTATGTCATTAATTACGGTCTGCCTTAGTGCAATCTTATCTAGTCATAATTTTTCAGGATCGCATG
CCATTCTCAAATTAAAAAACTATATGCTTAGTGCAATGTATCCTTGATAATATTTTTCCCGAGATTCGATCCCTTCTAAATTCA
CTACTGTATCTAACTCAGCATGAACACATAAAGAGCCCTAGCGAGTAATCCTGTTTACTTGTCACGTTGAATGATTCGATTATG
TCATATTAGGTCTACTTAGTGTAAACTGTTATCGTAGTCATAATCTTTCGAGATCGGATGCATTTCTAAATTAACTAACTATGA
TCTTAAGTGCAAGTCATCAAAGTAATATGTTTTCCCGGCGCATCGATCCCCTTCTAAATTCACTACTCTGTCTTGCACCAGCAT
GACAACAATATGAGGCCCAGCGGTAGTCACACGTTTATTGTCCTTGGCAGCTCATTATCCATCATTAGTCTGCTAGCGGGCATG
CCTCATCTAGTCAATAATTTTCGGATCGATCCCTTCTATATCACTACTATCATCTAACTCAGATGACAACTATACGAAGGACCT
AGCGCGGTAGTACTGTTTACTAGTGTCACATTGAACGATTTCATTATGTCATAATTAGGTCTGCTTAGTGCAATGTTATCTAGT
CACTATTTTTCGATCGGATGAATTCAAATCTAACTACAAATCATCATCTGTCAACTGTATCTTAGTAATACATGGCTCCCGGAT
ACGCGATCCCTTCTCTATATTCCACTACTATATCTACTCAGCATGACCCTATGATTGCAAGACCCTAGCGGTAGTATTGGCTTT
ACTATGCTACATTGGAACGATTCATTATGTCATAATTAGGTCCTGTGTAAGTGCAATGTTAGTCGTACTCATCACATTTTTCCG
GATCGGATGCCATTCTAATTGTAACTAATATATATTAAGTGCATGTCTACTAGTAATATTTTCTTGGATTGGATCCCCTTCCTA
ATTTCACGTACCCTTACTCAGCATCACAAAATTGGAGGCGAACTGTAGTACTTGCGTTTACCGAGCTACGTTGAACGATTTCAT
CTATGTCATAATTAGCTCCTCTTAGTGCCAATTTTATCTAGTCATAATTTTTCAGGATCGAATGCCATTCTAAATTAAATTACT
ATATCTTAGTGATGTTATCTAAGTACACATTTTACCGGGATCGGATCCCTTCTAAAATTGACTACTCTATCTTATCAGCAATGA
CAACTATATAGACCCTAGCGGTGGTATTGGTTTACTGGTACATTGAACTATTCATTATGTCATAATTATGTTCTGCTTAGTGCA
ATGGTTATCTAGTAATAATTTTTGTGATCAATGCCATTCTAATTAACTACTCATAGTACTAGTGCAAGTTTATCTAGTAAGCTA
CTTTCCGTGATCGGGAATTCCCCCTTCTAAATTCACTACTCTATCTTACTCAGCAAGACAACAATATGAGCCCTAGCTGTAGTA
GTGGTTTATAGAGGTACGTTGAACGATTCATTATGTCATAATTAGGTCCTGCTTAGTGCATGTTAGCTAGTCATAATTTTTCGG
ACTCCGATGCCATTCTAATAACTACTATCATATTCTCATGCAATGTTATTAGTCATAATTTTCGGAATTGATCCCCTTCCTAAA
TTTCAACTAACTCTATCTTACTCAGCATTGAACAAATAGTGAGGCCCTAACAGTATATTTGTTTAACATAGATGTCACGTTGG
AACGATCATTTGTCCATAATTAGGTCTGCTTACTGCAGAGGTATCTAGTCATAATTTTTCGGGATCGGCATTCCATTTCTAATT
AACTACTATACATTGTCAATGTTATCTAGATAATATTTTTCCGGGATCGAAATCCCCCTTCTAAATCACTACTCTATCTTACTC
AGCATGAACAATATGAGGCCTAGTTGTATATTTGGTTTAACTAACTACTTGAAAGCGATTCATTATGCATAATTCATGGTCTGT
GTATTGCAATGTTATCTACTCATAATTTTTCGGATCGGATTCATCTCTAAATTACTACTATAATTTCAGTGCAATGTTATCTAG

TAATACTTTTTCTTGGATTGATCCCTTCTAAAATTCACTACTCTATCTTTACTTCACATGACAACAATTTGAGGCCTAAGCTGT
GTATTGGTTTACTAAGTACCATTGGAACGATTCATTATTCATAACTAGGTCTGCTTAGTGCAATTTATCTATCACTAAATTTTC
GGGATCGGAAGCAATTCTAAATTAAACAACTATATCTTAGCAATTTATCTAGTTATATTTTTCCGGGATCGGATCCCCTTTCTA
ATATTCAAACTTCCTCTATCTTACTCAGCATGACAACATTTGAGGCTATACGCGCGTAGTATGTTTAGCTATTAAGTTTGGATA
CGATCTCATTAGTCATAATTAAGATCTGTTTATCAATTGTTCATGCTACTCATTTTTTTCGGGATCGATGCCATGGTAAATGCA
CTACTATATTTAGCTGCAATATAATCTATTGTACTATTTTTTCTGGAAAAATCCGCCTCTAAATTGCTAACTCTAGTCTTACTC
AGCAGACAACTATATGAGACCCTACGTGTAATTTGGTTTACTAAGTGTAACATTGGAACTATTCATTATGTCATAATTAGTCTG
CTTAGTGCAATGTTATCTAAGTCATAATCTTTTCGTATCGAAGCCATTCTAATTAACACTATATCTATTGCATGTTAATCTGGT
ATATTTTTCCCGAGATCGAGCCCTTCTAAATCACTACTTCTATCTTACTCAGCATACAACAATATGGGCCCTAGCGGTATACTG
GTTTACTAGTTACTAGAATGGAATACATTCCCTCATCAATTAGGTCTTGCTTAGTGCAATGTATCTAGTCATAATTCGTTTTCT
GATCGCAAGCCAATTCTAATTAACTTCTATACTCATAGGTGCAATGTTATCATAGCTTATAGTTTTCGGCTCGATCCCCCTTCA
TATTCCACTACTCTATCTACTCAGCATGACAACAATTTGAGGCCTATACGGTAGTATATGTTCAGCTAGTGTAGCTTTGAACGA
TTCATTATGTCATCAATTATCGAGTACTGTTAGTGCAATGCTTATCTACCTCATTTTTTCGATCGGAGCCATTCACCAATGCAC
TACTAGTATCAGTTATGCAATGTTATCCTAGAAAATTTTCTGACCCAGATCCCTTCTCAAATTGACTAC
>m141122_040740_42134_c100732202550000001823141405141561_s1_p0/85685/8400_16301
AGTGTCTAGAATAAATCTGGCCTCTCCAAATAACCAAAAGGGTAACGAAGGCCATGATTTTTCCCTAGTCAAATTTGCCTTTCG
AAGGGTCCTTACCCAAAGCTCCTAGTTTCAAGTTCATCTTTTGCTCCTCATTCTCATTACTTTTTGCTAATAAATAATCATGAA
ATGGATTATGTTCATTATGCCAAAACCGAAAGGTTACTTTGTGAGATGAATTTGGATCTTGATGCTCTACGGATGTTCTGGGGT
GATAGGTTTTATTGCCGAGAAAGATAATGCTAGTCCTTCTATCATGTGTATACTTATGGTATGTTGATCATATTGTCAATGTAT
GGCTGTATGCGGTGCCATATCACTGACGATTTCTTAATCATATGCCTTATATGTATGGCAAAGGTTGGTTTGGGGTCCATTGAT
TATTCTTCACGTTAAAGACATTTAGCGGCTCGCCTCAAATCTGCGCTTCTGGTGGTGACTTCCACTAATCCTTATGGGCTCACC
AAGCTTTGAATTTGATAGCTGACCCGGCCTCTCCCCTTTTGGATGGGGTCATCAGTCTCTGTAGACTGGTTGCGCCTGTCTAAA
TCTCGGCCTGTCCATGAAGAGAGGGTGTCCATTGGAATCCAAGTGCTATACTTGAAGTCGCCTCTCTATATACATAATTTATAT
ATGGGTTGCAAGGTGTGGTTCCTCGTATCGCTATGGCACTGCTAGAGTTTATTCCCACTACTTTTTTTTCGATTGACCAGATAA
AATAAGATGGATCGCCCAATTGTGCACTAATTTCAACATGAATCTTCCCACATATGTGGTATGTTGTCTTCAATGGTATTTTGC
TATGATATCTTTGCATGTTTTCTTAAGGAGTTTGACTCTATGGTGTCATATCAAGTATCGTCCCATTCATCTCTTATGTATGAT
CTTAGGTGGTTCCATACACTTGTTTCTGCACATAGAGGCTCGGCGATGGAAAAAAAAGAGAGGGTTCGTGATATTGGTCATGGT
TTCTCATCTACTCTTTATATACAGATGTTTTGTCTCAAAGAAAGATGTATCCTGATGGAAAATCGATTCCTGTTGAGGTTCCTC
CTTCCTTGGTCCTATAATTCAAAACCATTTGAAGGGAGAAGATCATCCTTCGAAGAAGTACAAGTCTGTGTTATTTCCCTTATT
GTCTCTACAGCACTGTTGTTGATCCAATCTGTGCTTGCTACTTGAGACACCAGAGATTCTTACTATCAAATCTGATCATGTAAT
CGGTTATTACGTCACTTGGAACCATGGAGACACTCAACAAGCAATCTTGATCAGTTGTCCGCAATATCCAATTGGTTTTTGGAT
TTTATCTTGCTTTATTTTTTGATCATTCTCGCTTTAAAATGATCCAAAAAAAAAAATTTACTTGTCTTTGTTTTTTCATTTTCAT
TCTCATTGCACTTGATGGACAAAAAGGGGGAGTAGTTCTGTATATTATTAATTTTATGTACAACTTCTGACCCTAATTTCGGGG
GAGTTGTGTGGTGTGTCTTGTTAAAATTGTTTATCCAAAAAGCTTGACATTGTGTTTTATTTTGTTAGATATGCACTTGTTTGC
AGGGGGGTTCTTAGGAACTTAATATTTTTTTTCTACAAAGTGTTTTCTACAGGTTTTTGTTCCTACAATTGCTAAATAATATAG
TTTGTCTAAAAAGTTGCCAAAGAGGGAGACTTGTAAAATCCAAAATTGGCAGACTTTTACTTTTTATATTTTTCGAATTATATG
CTAATTTCAAAATTTATATAGATGATTATAATATGGTGGATTTTGCACTTTTGGGTAAGTATGATTTTATTTTGCATGAATTAT
CCTGTAGACAAGTTTCCCCTATATATTTATGGTAAATATGATCTATAAAAGTATGTTTAATATATAATTGAGATCACAATGGTT
CAGAATTCTTGGTCTTATTGATTTAAATTGGATGTATCCTGAAAAGTCTTTGTGCCCAAAGAAAGATCAATTTCGAAATGAACT
TGGTTTGGAATTAAGATCAACGGCTTCGTCCCATTTTCTGATGCATAACAAAATGATCTTTTGTTACATCTGGATTTCTTTGCA
GTAAGCGGTAATTGAGAGTAAATTTAGAAACTCTTTGATTGATATCTGATTTTAGATAAAAAAATGAGAATTGGCTTGGCTTAGG
ATTTCTAAACTCTATAAATATTCCATATCCTAAGCAACATCTAAACTCATCTCTTCACAATTCTAAAACAGCATTTCATATTTT
GAAGAGTTCTCTTTTATGCTAGGAATAGTCCTTGTAATTCAAACTAATCTGTTTGTTTGTTTGTTTTGCTTGTGTTGTTATTGT
TCTTGATAGTAATAAAGCTTGTGAACGTAAACTACTAATCTTTGGGGAGAAGATTTCCATCAGTTCAACTTCGGAGGAGCATA
GCAGGGATTTTTGATGTTCTTGGTGTTTATCAAAATCAGATTAGTAAATTGGAGTACTTTCAAGGTTGGCCGCGCAAAGTTTAG
AGGGAGATGTAAACTCGTATATGTCATTGCTTATTGTAATTACTTGAGATCTTATATTGGATTTTATCTCTAGGCATGGCCCGT
GGACTAGATTTTTTCAAACCACGATAAAAACTTCTTGTGTCACATTACTTTCAACTTTGTTTACCTATTCTGTTCAGTTATTTT
TGATTCATTAGAAATATCTAATACATCAAGAATATCTGTTTGATCAAATAATTGGTAATAACATTCACATTAATTAATTTGTTT
TGATAAATTATGTTGGTATAATTAAAAAAATGGAAATTTTAATTAACAAGTCTTTATGTTCATTAAAAGAGCTAAGAGAATGTG
GAGTCTTCTCTTATTCCGTCATAATAAACACCTTAACCGTTTTTCCTCTTCTTCACAACGAACAAATTCGCATTTTCTCCAAAG
TGATACAACTGTGGTATGGCCTACCTAAATTGCTCTGTGTGAACTAGCTTCGATCGAGCGTGAGTGGAATTCGAGCTAATGTGG
ATTAGTCTATATTAACCCAAACACCAAATACAGTTTGCCGCATCAACCTCTAACTGAGAGTTCGTTTTCTAGTTACATCAGATT
TACGATCTCCCATTTTTTTTTTTTTTTAATAGTTTTTTTTTGGGTTTTTTTTTTTATGAAATTTCAAGAAAAAAAAAAAAAAAA
AGGCCCCCCCCTTTTTTACCCCTGTAAAGAAAAAAAAAATTTTTCAAACTCGCAAAAATAAGGGCCTTTCCTTTGCCCAAGCCCC
TTTTTTTTTCTTTTTCAACAAAATTTTTTTTTCCCAACAGGCTTGGGGGTAAAGCCCCGCCCGCGCTTTTTTTGAAGATTTTTA
AAAAACATTCCCTTTTTTTTTTTTTGCCTGCCCCTTGGGTGGGGGGTTTAAATGGTTTAAACCGCCCTTTCTTCTGGTTTAGC
AAAAGGGGGGGAAAAAAAAAAAAAAACCGGTCACAACCGGGGGTTTTTTTTGGTAAAACTTTTTTTTTTTTTTAAATTTTAGGTG
ACCCGCCTTTTTTTTTTTTTTTTTACTCCGCCTTTTTTAAAAAATTTTTTGAAAAAAAAAAAAAAAAATCCAAATTTAAGGGGGCC

ATTAACCGCCGGCCATTCCCTAAGTTTTGGGATTTAAATTTTTCATGGGGGGGGGGGGGGGACAGGGGGGGGGGGGGGGGGGTTT
TAAAAAAATCCCTTGGTTTTTTTCAAAAAAGGGAATTTTTGAAAAAAAAAAAAAAAAATTAATTTTTAAATTTTTTTTTGAAAAA
AAAACCATTAAAAAAAATTTGGGTCCCAAATTTGGGGGGGAACTATCCCCAAATAAAAAAGGTGGGTTTTTTTTATTTTAACA
AGTTACTTTATGTTTTTTTTTAAATAATTCCCAAAAAAATGGGGGGGGGGGGGTCCCCCCCATTTATAACAAAAATTTTCAAAAA
AGTTTTTTACAGTTTTCCCCTCCGCCCCCCCCCCGGGAACCCCCCCCTTAAAGGGGCGGGGGCCAAAAAAAATTAAGGGGGGGGG
GTTTTTTTTTTTTTAAAACCCCTTAGTTTTTCCCTTCCCCCCACTGAAAAAAGGAAAAAAAAAAAAAAACCACCCTTGGGGGGCC
CCAATTTGGTTTTTGGGGGTTTCCAAGCGGGGCCGCCAAAATTAAAAAAAATGGGGGTTTTTCAAAATCGGCCCAAAACCTTTA
TCATAACGGCTCCTTTCCCCCCCCCCCAACCCCCTTTCAAAAGGGGGGGCTTGGGGGGGGGGGTTTTTTTTTAAAGGGGGGGC
CCTTTTTTTTTTTTTATTCCCCCCCCCTTTTTTTTTTTAAAAAAAAAAACCCTGCCACCCCAAAACCAAAAATAGCCCCCCT
CCCCGTTTTGGGGGCGGGAAAAAAAGCCCCAAAAGGGGGCCTTTTTTTCAAAGGCCCAAGAAAAAAAAACCTTTTAAAATTTTTTT
TACTTTAGCAAAATTGGGTTATTGCCCAAAAAATAAATTAATTTTTAAATGGAAAAAAAAAAAATCTTTTTTTTCCTTGGGGGGG
GGGGGGGGGGCCCCTTTGGTTAAAAAAAAAAGTTTTCCCCCTTAATTTTCAAGATGAAAAATTTTGGAAAAAATTTTTTGTTTT
TTCTAAAAGGTTACCCCCAATTTTTCCTTAGAGGGGGGGGGGGGGTTTTTTTTTTTCCCCCTGGTTTGGCCCCCCCCTTTTTTTT
GGGGAAAAAAAAATTTGAATTCTTTTACCTTTTAAAAGTTTTTTTTCCCTTTTCCCCCCTGGGGGGGGGGGGGGGGGGGGGGTTT
TCCCCCTTTTTCCCCCGCCCTTGAAAAAAAAAAAAAAAATAAAAGAATTTTCTTTTTTTTGGGGGGGAAACTTTTTAATTAAGAA
AAAACCCATTTAAACCGAGGGGGGGGGTCCCCTTAGGACCGGGTTCCCGTTAAAAAAAAATAGAAATTTTTTGACCTAAATAAAG
AAAAAAAAAAAACAATCCCCCGGGGGGGGGGTTTTTTTCCCAATGGTGGCCCCCCTAAAAAAGGCCCCCCCCAATATGGGGGGG
TTGGGGAAAACTTTTAACAAAAGGTCACCTGGGAAAAAAAAAAAACTTTTTTTTTTTTCAAAAGCCCCTAGCTCCTGAAGGGT
AAAAAACTTTTATTAGGCCCCCCCCCATAGGACCCCCCTTACAAGGCCCCCCCCAAGGGCCGTTTTTTTTTTTTTTTTAAAAT
TTTTTTTTTCCCCTTTTTTTCCCGGGAACCCCAATTTTTTAAAGGGGGTCGGAAAGAGGCATTATAAATGGCCCCCCTCCTTT
TGGTTTTTTTTTGAAATTTTTAGAAAAAAAAAATTTCTTTTTTTATTTCCATATTTTTTGGACCCAAAGCCTTCCCCCCA
TTTGGCAAAAAAAACTTTTATTTTTTTGGCCAGGCTTTTATTTTCTTTTTTTCGAAAAAAAAAAAAAACTTTTCTTAAGTCA
AAAATTCATAATTGAAAAAAAACCCCAATGGGCCTTTTTTCCAGATTTAAAAAAAAATTATTTTCCTATCCCTTTTTTTAACTT
TTTTATAAAGGTCCAAAGAAAAAAATTCTTTTCCCCAAACGACAAAAAATTACCAAACTTTTAACAACAAAAATTTGGGGGGGGA
GCCCCCCACCTTTTTTGGGCCCCCCCCCCCCCCCCCCGGTTTTTTTCCTTTTGAACTTTAATAAGTCCAAGTTGGTCGGCCCG
GGGTTTTTTGGGGGCCCCCCCCCCCTTTTTTTTTTTTTTTTTTCCCACAGAAAATTTAAACCCAATTGCACAAAAATTTTCCC
CCAGCCCCATTATCCCCCCCCCCCCCCCCAAAAATAAAATTTTGCCCCCCCCCCCGCCCGCCGACCCAGGAAAAATTAAATTTTT
CCCCCCCCAAAATAATAGGCAAATTTTCACCAAAAAAAAAAATTGCCTATTCCCAAAAAAAAATAAATCCCAAATGGGTTATTTGT
ATTTTCCAAAAACAAAAAAATTAAAAATGGGGGGGGGGGGGTTTTTTTTGGGGGCAGTTTTTTTTTCTTTTTTTCCTTACCCCT
ATCTTTAGGACTACAAGGCCCACGCCCGGGACCGCTTTTCGAAGGCCCCCCGTAAAAAAAAAAAAAGAGTTTTTTTTTTTTTT
TTTTGAAAAAAAACGACCTTTTTTTTTTTGAGGGAAAAAAAAACGATCCCCTAAAAAAAACTTTTTTTTTTAAGGGACCCTT
TAAAAGGTTTTGGAAATCACCCTAAATTCCCCCAATTAAACCCAAAAAAAAAAAAAAAATAAATTGGGGGGAATTTTTCTTTT
TTTTTTCAAATAAATTTTTTTTTAATTTTTGGGGAAGGGGGTAATAAAAGGAAATTTTTTTTCCCCTTTGGGGGGGGAAACCCC
CCCAGTTTTTTTTTTTTTTTTTTCCCTTAAAAAACCCCCCCCCAGGGGGGGTTTTTTTTTTTCCCCCTTCGATTTTTTTTCTTT
TTTTTTCTTTTTAAAAAATGGGTTTTTTTTTTTTTTTTTTTAAAGTTAAAGAATTTTTTCCCCAAAAATTCCCCGGAGGCCCTT
TTAAAAAGGGGGGGTTTTTTTGGAAAACTTTTTTTTCAAAAATTTGGCCCCTTTAAAAACCGCAAAAAATAAAAACTAAGGGCC
CCCCCCCCTAAAGGGCCACCAAAAATTTTTAAAGGAAAAAGACAGGGTCCGGGGGGGGGGTTCCCCGGCCGACCTTGCCCCCCC
AAAGGGAGGGGGCCCCCGTGGGGGGGCGGGGCCGGCCCCTCCCAGAAACCCAAGGACCAGGGCTTCCCCCCCCTTGGTTTTTTT
TTTTTTTTTTTTTTTTTTTTTGGGTTTTTTTTTTTTTTTTGCCGCCCCGAAAGGGGGGGGGGGGTTTTTGGCCCCCCCCGGTGAA
AAAAAAAATCGCTCTTTGGGGAAGGGGGTTCTCCCCCCCCCCCCGCAAAAAGCGCGGGCTGGCCCCAAGAAACCCAAAAGAAAGC
GCCCCCTACAGTTTGGGAAGGGGGCCCCCTTTTTGTTTCCCCCCCCCTTGGGGGGGGTTTTTTTTCAACCCCTTCAAGGGTTT
TCCCCCGCGAGCAAAAACCCAAAAATGAAAAAAAACCTCCCGGTTTTTTTTTTTTTTCCCCCAAAAAAAACGATTTTTTTTTTT
GGAACCCCGGCCCCCGGAACCAAAAAAGCCTTCCCCCACCCCCCCCAAATTTGGGGGCGCCAATTTTTTTTTTTTTTTTAAAATT
TTTCCCTAAACTCAAGCCCAAAACTTTTTCAACTTAACAATCCCCACCAATCCCAAAAAAACCAAAAAACCACAACACAAAAAT
AGACACAAAACCAAATTTTCAAAAGCCAATTTAACCCAGGGCTTAGAAAACAACTTCTTTTCCCCAAAACACCAAATTTCAATA
TTTAACACCAATCTTAAGACAAAAACTCCCCCTTCCAAAAAAGGCTTTTAAAATTAAACCCCACTTTTTTAAAAAAATAACAAG
AAAAAAAGGGGGTTTTAAAAAAAAACCCCCCCCCCTTTCCCCAAAGGGGGGGTTTTTTTTTTTAACTTTTTTAAAATAAAAT
TTAACCTCAAATTAAATTTGATTAAACAAACCTTTTCCCAAAAAATTTTCCTTTTACAACTTTTCATTTTTTTCCTTAAAAACC
TTTTTTTTCAGCCCCTCAAATTTCAAAAATCATGGGCTAAATTCCTTCCTAAGCTTTTTTTAGGCCTTCAAAAAATCGGAAAA
AATCAAAGGGGTTTTTGGGGGGGGGGGGGGGGGAAAAAAAAAGAAAAGGGAAGAAAAGGGAGAAGGGTTTTTTGGGGAATTTTCC
GAGAAAGAAAGGAAAAACATTCTTGAGGAAAAAGAAAAATTCACCTTCTTTGTTATTTTTTTTTTTTTCTCCTTGATTTTTGGGG
GGGGG
>m141122_040740_42134_c100732202550000001823141405141561_s1_p0/97529/0_9071
AGAAGAGATGCGTGCTTGAGAGATATTTTTTCACTTGTACGACTATTTATTTAAGGTACTTGGAAGATTTGAATTTTTTTTTTT
TTGTGGAAAAGACAGAACCAGATTGTGGAATAAGAGTACTGAGTGAAAGGCCATAGAAACAAATGCTGAAAGTGTGCGATTTAA
CGAGCGTTATTTCGTAGCATACACTCAGGACCAGTAGTGTTAGATGCACTGGGTTGGGTCATGCAGTTGGACTGGAAATATAGA
GATGTGTGAGAGCTTTTAATGACTATCCACCGGATGACAATATTAGAGTGAGGAATGTGTTGTTAGCACGGTTTATAGGCGGGA
CTATGAAAAGTGGCGTACAAGAGATGATGAAATTTATATACGCTGGGGCTGATTATAGATTTTCATTGTTAAAGTACTAATTTC

ATGATTAATAAGATTTTTGTATTAGTTTGAGTGAATGAACAGGTGTTCAGGAATTTCGTAAGCACAACGCTCCTCAAACAGTTT
TAGTATATAGGCGCACACACAGACAGCAAGTGGTGGAGAAGTTCAAACGAATATAACCTCAACAAGTATTCAGGGTACTCCACA
CATGGGTACGTGTTAGTGGATTGATCGCATGTCTTCCGCGAGTACTTTGGGGACCGTTTTCACTACTCTGCCACATGAATCTGA
GATTCTACCACCCACACGAAACAACAGTGTTGGTTATTACACTTCGTACGCTCTCTATTATATACGACACAATTATGATATGAG
ATGAATTAGACGTTTCATTACTGGGATAATTGTTGGGAGGTAACAGGCGCATCTACTGTTACCCCATTATACCACCTCAACCCA
ACCAAGCCGATAGGTCTAGCACACATACCCATGGCCTTGACCGTCTCTCCCACCACTATAACCAAAAGCTCTTCGTGCAAGAC
AGCAAAATCCATTGTCTTCCTCATCGCTTCACGCGCACTAGCTCTTCTACACTACATTCTCTCTATATTTGGTCCTTCTCCAAG
CCACACCATAAGCCATAGACTGCTCTCTGAGAGAGGTATGCTCCCTCATTATTGACTAATTCGAACAGCGTTCGCACTCACTTT
CCACCAGTCATATGCGAATAGGAGGATCCGCATATACATAAACTATCTACGTATCCCACCCAAATTCGTAGGCCTGGCGTTTGA
TTTTGTAGGAAACTGTAAGGTGCAGTCCATGCACTGTTATTTTTTGCTGAGAGAAGGTGTCTGGTTCGACACAAAATATAGTTG
TAGACAGGAAATTTTAAGTACTTGCGAACATAATATCTTCATTTAACCGGACAGACGCCGTGATTCCACCAATACCATCCACTA
CAATCACTCCCACTATTACATCGGTGGGGGGTAGAGACATTTGAGGCTATGTGAGGATATTTGCCCGAGAAATCAGTCTCACGT
TACCTGCAGGAGCCGCAGCCGATAGCAGCGCAGCTCGCGGATCGAAGGCTATTACAGTCGTTTTTGGCTGGTTAAGCAGTCAGG
TATTGAGCAGCGGATCGGCGAATTTCGGGCGGGGGCCTTCCGTCCATGTGGATCTCAAGGTATTCTCGCTGACTTCCGTCAAGT
TAATGTGGGGGGGGTTGCCCCATTTCAGCTCCCCCAAACTCGTACCGCGTTGTTGGCGGGGTTGAAATAGCGTATTTCGGAAGC
GACGTAGTGGATGGGTACCACTCCTACGGACATTTTGTACCTTTTTCTGCCTCAGGCCAGCCCGGGATCAGCGGGGTGAGGCGC
GGGGTTTTATTACTTACTGGTTTCCAAGTACCGCTGGCGGTCGATCAGTTGCCCCAGCCACCCAGAATGACTTCAACAGTCAAG
TTCTGTTTAGTCGACATCGCGGTTACGGAGCTGCGAGCACCACTACTTCAATGCCCGTAAGTTTCTCTTCGACCTTAGCTCGTA
CTTAATGTTATAGTACAGCTCTCCTCCTGTATCCCTTTCTAATGTTAGATTAATTCCTGCAGTGCTATCTTTGTGGGATCATTG
AGAAATTCGTACCTCGGTGGCCATATACGAGACCATCTGGGCTGGGCTTTGCCCCCTCAGTGAGATATCTGGCTGTGCTCGTGA
CAGATTCGAGACGATGGTGGCCTGCAAGCTGATTTTCCAAGAATCAGATCTTTGAACTAAAGAAGGTGCTCGGGCGCTTCCCTC
CAGCCCGCGTCTTCAGGTCCCATTCATTTCTGTTGGAGGAGGTCACAGGGAATTGTAAGAAGAGTGTGAGGATCGTAGGTTTCC
CTCTCGTGAGAAATCAGAAGCGGTGCGTTGGATGGTCGCCCAACAATCGGGCGAGCGGAGGATTCTGTTCTCGGTTAGCGGGCA
GAGTCCCCTCTGGCCAAGGTAACCCTTTACTTGTTTTTAGTAGTTCTAGTATTATTAGGTGCACAGCAGACCCCGGTCTGCTGG
TTCAATCCTAGGCAGCTTCGTCATGTGCGTCACCGAGTGATCGTTACCTGAAACAACCCTTTTGCTCACGTGTCAATCCGCATT
GTGTTTGATCTATCCAATGATGCCGGTCTTGGGGTTACCTGTAGTGAGTGTATAACTACCCATGCCTTTAGTCTATCCTATCGA
GGAGTATGGACATAAACTTCGGTACATGGATTCACTTCCGGAGGGGTGTCGTAGCTCTCTGGGCTTTGGCAATATGTACGTGAT
GAATCGTATCCTGAATAGCCTCGGCCCAGAACTTCGTGCCAGTCTTGGGAAGAATCGTACTTATATTTCTTCCCTCCTCCCCCC
GCCCCCCTCTCCCCCCCCCGCCCCCCCCCCCCCCCCCCCCACCTCCCCCCCCCCCCCCCCCTCCCCCCCCCCCCCCCCCCCCCC
CCCCCCCCCCCCCCCCCGCCCCCCCCCCCCCCCCCCCACCACCCCGCCCCCCCCCCCCCCCCCCCCCCCCCCCCCCCCCCCCCC
CCCCCCCCCCCCCCCCGCCCACCCCCCACCCCCCCCCCCCATCTTCCCCCCCCCCCCCCCCCCCCCCCCCCCCCCCCTCCCCCC
ACCCTCTCCCCCCCCCCCTCCCCCCCCCCCCCCCCCCCCCCTCCCCCCCCCCCCCCCCCCCCGCCCCCCCTCTCCCCCTCGTC
CCTCTTTCGCCCCCCCCTCCCCCCCCCCCTCCCCCCCCCCCTCCTCCCCCCCCCCCCCCCCCCCCCCCCCCCCCCCCTCCCCCCT
CCCCCCCCCCCCCCCCCCCCCCCCCCCCCCCCCCCTCCACCCCTCCCCCCCCCCCCCCCCCCTCCCCCCTCGGGGCCATTTTCCC
CCCACGAACATCCTTGTTGCTACCCTCCCCACCTCCACCCACTCACAAAACTCACCCCACCACGACGCAACTTGCGGGCACATC
ATACTCCCTAAAAACATTACAAACCTGCAACCGACGCACCCGCACCAAAACCATCACGACGGGCACATTCTCGAAACACCGACG
CACACTCCACTAGACCCCCACCCTCGCACCTACCTGACAAGACATCCGTTCCAACGCGTCTCCTGCCTCGACCCCACCCGTTTA
CCTCTCCGACTCCAGCCCCCCCCCCTGGCCAACACGCTGGAACCAGTTCCCCCCGCGTACTCCCAAATTCCCCTTGACCTGTCC
ACACACCCCCCACCGCGTATCTTCCTTCGGACCCCACACAACTACGACCTGATACCTTCTTACTGTGACCCACACGCCGCACCA
GCGATCACCCTCTCCTACTCAATTTCTTCGGAAACTACTGAGCATCCCATGACCTCCTGGAACCCCCCTCAAACCTGCACGACA
CCTGCAAACTTCACTACCCTACCCCCCTCCCCCACCCCCACCTGAGCCCCCTGCCCTCACACTACTACCATCCCCTCGGTCTCT
ACTACCACACTCTTCACTATACACTTTCCTCATCCCATTCACAGCTGTAACTCTACTAATTTTACCCTTACCTTCCCCACTCTC
CGTCTTTTCTCCTCACTATGCCCTACCCCCCGCCCCCCCCCGCGTCCCGCCTACGATGATCCTCTTTAGCTGCAAAAAACCCAT
GGCGTACAACAAATCTAACCACATAATCACCATCCCCACAATCCCCTAACCCCCGTTTACGGTTCAATGCTGGCACCCGGCACC
ACACAACATGAAGAATCCCCCGAGATACCCCCCAACACAACCTCACTCCTTAAGACAACACCCTACTACCCCTCCGCACCACAA
TCCCAGCAACCCCGAAGACCGAACATCTTCAACACTCTTCTAATTACGCAACAAACCAACCGCCCAACCCGTCAAATTAATCCT
TTCTCACTACCTCATACTCCCCCCTACCTCTCTCTCGTCTACTCCTGAACCAACCTTCTCTCTAAACAACAACATCCTTCCCTT
GCTCACTCCCACTCTTTCCACTCAGGTGCTCCTCACGCCTATCCTGACCCTTCACCAGCCACAACCCCCCCCGCCCCCCCCCCA
CCTCCCCCCCCCCCCTCCCCCCCCCCCCCCCCCCCCCCCCCACTACATCTTGCACCCGAAAATCCTCTCTCTACACCATCGTACG
CCACACCTTAACACCCTTCATTGTACGAAACCTCACCACCGCCCCCAACCTGATCCACCTCACCCCCCCCCCATACCCCTATCC
CCGCTACCCAGCCCTACTAATATCCACCCCTATCCCAACCTACCCCATCTTACGATTTACATCCCTTGCCACACCCAATCACGA
CTAACATCACCCCCTTACACTATCTCCTCTTCGACACGGGCCATCCCCTAGACCCCTAAACTCATCCCGCCCACTACCCACCAA
CTCGCAACCCCCCCGCAAAAACAACTACGTATATCTAACCCCCAATCCAAGAGCTCCTCTCGCGTTCTATCACGCTCACCACCG
TAAACCTCCTACTCCCTACCTCTCCTCATTATTACACCTCGCCTCCTTTGGAGACATCCATTTTTACCTCTATCACCATACACA
TCCTCCATACCCCCAACCTCACCTCGCATCACCGCTTCTACTCACTCTCTGCCGAACCCACCCACCTCATCCCCCCCGTAACGC
TCGAGCCTTCCCGCACAAACCAACCAACTCCCCGGCACACACTCAATACCCCGCTGCGCCTCGGCACACATATCCTAACACCTT
CCACTGCACACCGTTCCGCTAGAGACCCTCTCGATCCTCTCTTAACAACCCTAACTCTCTCCGAACTCCTCGCCCCTGACTAAC
TCTTCAGAACCCTCACCGCATTGCCAAAAATCGCCACGGTCACCTTCTCCCGCTCCCTTCCCTACCACACTCCTCTTTACCACT

ATAACCTCCCTCTTCCTCTTCGCCACACCCCATGACCTCGATTTATCGATCCCTTTATCTTCTTCGATCAATCTCTTTTACCAC
ATTCTATCCTACTCCTCAAAATACTTATTAACCTACCCCCGCTCTACAGCAATACACCCACAACCATACTACCCCATCCCACCG
ACACTCAACCCTTCCTAGCCCTCCACCCACCTCGTTTACCACCTGCACTCCCACGAAACTCTCCCTAGCAATCAAACCTCCCCT
CACTGCCTACCCTACCCAATCAATCCTAACTCACCTACCCCCCCCCATCCCCCCCAAATTCCTACAAATACTCGACCCCACACA
CTCTTCACCCCTTTCCTCTATACGATTCCCTACCCCATCTACCAACTCAACTGTCTACCCCCAATATCCCAAAACCCGCCAATG
TCTTCTACTTGATCATCCCCTGGCGTCCGTCTCTCTAACGTTACTCACCCCCCACACCCCCAATCTCATCTCCTCAAATGACCT
CACTCTCTCTTATGCTTTACCTCGATCCCGTGGTCTTTCTTCTTATACAACCCCCCCTCCACTACCCCCGGCACACAAGGGACA
CCTTGGAATACCCACCACACCCTTCACCCCCCCTCATTTATCCGTCCTCCACAATCACCTATCCCCCACCTGCAACACTCTAAA
CCAACATGTCAACGCACAAAAATCAAAGTTTCCTTTCTTGTCCTGGAATTTTTCCGCGCACAAGCCTCCTTCTACACACAATCG
CATAATACCCAAATGGAACACTTCCCCCGAGCCCTTACAGTCCGCTAAAACCTCATCACCACCCCACACAACTATTCACACTCT
CCTCCCGTCCGCTCCTCACGACACAATCAAAGAAAAACTCGCGACACCTACTCTGGCCACTCCCTCCCGCCTACCCGCCACCCC
TACAACGAAAATGCACCCCCACTCCGCATTCGCTCAGCCCCCCCCCTCACCAACCTCGCACAAACACTGATAGAGCTCCTCACC
CCCAGTCAGCTCCCGCCACGCACGCACCGCACCAACTCTCAGGCCACCCCTTGCGTCATCGGGCCTCCCCTGAGTCGTCCACCC
CCCTTTTAAACCACACTCGCAAACAATCCAACCTTGTGGCCACCTCTCGACGCACAACTCGACTGCACCAACCCCCACCTGACC
GCATTCTAGTCACGTCGTCACCCGCAACTCCGAACACCTCATCCTACCCGTAAAGAACCGAACAATATCATCACCATCCCAACC
ACATCGCTATCTTATCCAAATTCACACCACTTAGACGCTGCTATCGCTCTAGCAAACACAACTAACCAACACCAAACCCTAACC
ACACCACTTCGCAATGCTTTGCTTAAAGCCGACTGATCCTGTCCTCAAAACATCTCGCAATAAACACCAATCATCCTCTTCGCG
ATACACTACCCCCCAACTTGCCATACTCTCATCTAGAATCTTCAAGTCATCCGGCACACATCACCACTTACACCACCTTCTTTT
TATATCTTATACCACCATTCAACCATTACACCAACTCACATGCACACAACCCATCAACTTATTCATCCCATGATCTACACACTA
ACCTCAACTTATAGCAATGTTTACTTTCCAATACAACAACCCATCATCCCATAGACACTTCCACCCCAAATATTCTCACTCACT
TCCAAACCTACCACCATAATACACGAATCACCCAAGTCTTTGTAGACTTGTTGAATATCAACACAGGGTTAGACGACCTACCTT
TGTATGAAACCACCAGATCCTCACCTCTTCACCGCCTCACACAATCCCACAACATCACCCCACACACATCACATCTCTACTTGT
ACCTTTCTACCGATCCTTCCAATAAACTTCGTCCCCATCCACAATCTCCAAAAATACATATTTTCTCTTCTTCTTCTTCCACAC
TTGATCTTACTGTTCTAGCCACCAACTATACCTCTTTCCCACTTTTCTCTTTTTAACTATCTGCACATCTACTTTTCTCAATCT
TACATCGTCTCCCGCCCACCCACGAGAAATGTACGCAATCTCAACACGTTACAATTACACACCCAATCACATATCTTCTTCGCT
TCGTACCGACAGACTCATTCCCAACCCAAAAGTAACACATTTACTACCCTACCTAGATCCATCAGCTGTAACTCCGGGTTACCC
CAAACGAGCGCGACTAAATTTCATCAGTCGCAAGGTCTTAGATATTGCGATCTCCCATACCCACCGACCCAACACACGACTTAC
GCTACCTACCACAAAATCGCAACACTCACAGGCACATTAACTTAATCCATCATCTATGACTCGAACGATAAAAAAACTTGCCTT
AATTCAATCTCCTACTGTGTAACTATGATTTCCCTCAACAGTTAATCAAGTGAATCTCCCAAATACTAGTGACACACACCACAC
CATCCAATTATAGTTCATACATTTTGAATAAATCTATGTTTTAGCTTAAGTAAACATACGTTACACCCGACACAAATCTCATTA
CTCATTCCTAAAATTATTTCCATCCACAATCAAAATTATCAATCGAACATTACTTACAACTTGCTGTAATAAACTCCCAATAAC
ATAACTCAAACACAAAAACAAAATAAATGAACAATTATAAAAAACAACTACTTCCCTCCCACAATTAAACTTACATTGTCCTCA
ATGAAATTATATTAAAAAGTTAACGGAAAAGAAAAATCCCCTCCAGTGAATAGTACCCACTGATTTAGACATATCTGCTTGACA
ATCATCATCCACTCTCCTGCTTCTACTCGTCTTGCATCATCGGACTGGCAATGATTCACCCAGAGCCAAGTTGTTTAGGAGCA
TCAAGCAGTCCCATATCCATGCCATGAAGATGAATGACATTCATAATGCCCGAAGCAAAAACATCCTAATATGCGCCCATTCAA
ACTCCCTGTACTTTTCATATGCTCGAATTCATTATGATGGACATGTAGACATCCTGCATGGAATTGTAGCAACCCATGTCCAAA
CTAACTCATTTCGGTCTACGCAAAGAATGGTCAGCCCATTGATGGTGGTCCTAGTGGATTGATGATCAGAGGACAAGCTTGGCT
CCAGAGGTCATTTATTTTCAAGCAACTATGTTAGCCATCAGGATCCAGGATATGCCATGGATTGCAACAATTCCATTCAAATGG
ATTCCCATTTAACACCTACTTCAATGTACTTTTGCTTTGATTAAATGGGGATGGGTAAAACGCCTTTTATAGTTCTGATGTTGG
TCAGGCGAAAGGAATAAAAAACATTACACTACCAAATCAACTTTTTTCTAATATAGCATGGTAATACGAGGAGAAAAAACTTGG
CAGTTTAATGTCAGAACTTCGTTAAAGTGCTTCGTTGGAAATAAGCTTTGCTCCAATGAAGTTATGCCAAAGATTTTGCAATTA
TACATGTGCATCTCATTTAATAGTTCGATATGCGTTGACGCATAAACCGTCAATGATAATGGATGGAAAAATTCAAAGCTTGTT
ATGAAAACTCGTAGATACAACTAGGAAAAACTTAAGAAAAGGCGAGCGCATAAACTACAATAGAGTATAATCATGCCGTATATG
CACTTTTTATCCTCTGTCTACAAATGAATTTTCCCACCCCCCTACTTGAGGGGTCGAAGCCTATTTTATAGAGGACTTTATGGT
CTCTGATACAAGGTGTCCCCAAGGACAAGTTCGACTTGACTCTGACAGAGTACTGTTGAATGGTGTAGAGGAAGAAGGGTCGT
>m141122_040740_42134_c100732202550000001823141405141561_s1_p0/99726/4250_13118
ATTTAGTGAGACACAAGAGTTCTGAAGGGGAGGAGAGAGATATATAACACCTTTGGGTGAATATGGAGAATTTTCACTAATTTC
TCCGAATTGATATTACCCTCGGTGTTCAACTCGTTCGATATTTCCCCGTCTCATGGGAAATGAATATATTGGAGTCGAAACAGA
GCCTTAATTGAAACGATTTTAAGAAAAAAATTCCTCTTTTGTGAGGGCAAGTTGGTTTCACAATTTTTTCCTTATATTCGTACG
TTCTTCTCAGTATTTTGACTGTATCACCCTATACACACCCTCGGAGTTCGTTCAGACGAAATTGTGTATAGCAAATAGAAGCCC
TAAGACATAGGTGATACACAACTTTTTTTGCCCAATGACCCTCGCATATTCTTTAGTATTGCACGTACCGAAATCGTGGCACGA
ATCCATATTGAGAGAAATCGAAGATTTCTGAAAGGGGTACCTTTGGGAGATTGGAAAGATTTGCACTAATTTCTCCCGAATTGT
ATGTGACCCTCGTGTTAAACTCCGTCGATATTATTCCCCAAGAAGATCTCATGGGTAACAGTGAATATATTGGATCGAACGGAG
GCTTAATTCAACATTTAGAAAAAAATTCCCCTTTTTAGGGCAATTTTCACAAGTTTTTCTTATATTGGGCGTACGTCTCTCAGT
ATTGGTTTGACTGTATCACCCTATACGAGTCACCGTCAGATATGCACGAAAGTTTTAATACATAGGAACCCTAAGACATAGGTG
TACAACTTTTGTGCCGATGGACCCTCTGCATATTTCTTTTAGTGATTCACGTACGAAATCAGTGAGGCACGAATCCATATTAGA
GAAAGTCGAGGAATTTTCTGGAAAGGGTAGCCGCCTGTTGGTGATTGAGATTTCCCTAATTTCTCCGAATTGTATTCCCTCATG
TTCAACTCGTTCGATATGTAGGTGGGGTCCCAAGAGAGTCTCATGGGGGGTAAAGTGAAATGAGGTAGTGGGAGTCGAGAACGA

GCCTTAATGAAAACCATTTAGAAAAAAATTCCCCTTTTGAGGGGCCAATTTTCACAAAAAGTTTTTTCCTTATAGTTCGGTACGT
CTCTGCCAGGATTTTGAATGTAATTCGACCCTATAGCGACACCTCGGATTTTGCACGAATTTTATAGCAAGTGAAGTCCCTAAG
ACATAGGTGTACAACTGTTTGTGCCCATGGACCGCTGACGCATGATCTTTATATTCACTATTGAAGAACAGGTGCTCGAAGTC
CATATTTAGGAGACATCGAAGATTTCTGAAAGGGGTACCCGTTTGTGAATTGGGAGATTTCACTAGATTTCTCGAATTGTATGA
ACCCTCGTGTTCAACTCCCGTTCGATTATTATTCCCCAAGAGATCTCATGGGTAAATGAAATATAGTTCTGGGACGGCGAAACA
GAGCCTTAATGGAAACATTGTAAAAAAGAAATTCTCTTTTTTGAGGGCAGATTGGTTCACAAGGGTGTTGTTTCGCTTATATTC
GTACGTTCTCAGTATTTGACTGTGATCACCCTATACACAACCTCAGGAGTTTTTCGACGAAATTAAGTACATAGAACCCTATAG
ACAGTAGGGATTACACTTTTGTTGCCATCGACCCTCGCAATATTTGAGTATTCACTACGAAGTCAGTGCACGAATCCATATTGA
GGCGAGAAATCGAAGATTTCGGAAAAGGGGTACCCCTTTGGTGAAGGTTGGAGAGTTTCACTAATGTTTCATCCGAAATTGTTT
ATTACCCTCCGGTGTTAAACTCGTTCGATATTATTGCCCAAGGAGATCTCATGGGTTAAATAATAGTTATGGGAGTCGAAACGG
AGGCGTTAATTCAACATTTAAGGAAAGAGATTCCCCTTTGTAGCAATTTTGCGAGCAGGTGTGGTTTTCCTAGAGTTGGGCGGA
CGTCTTCTCAGTGTATTTTCGAGCGTGCTAGTCAAACCCTATAACACACCTCAGATATGCCACGAAATTGGTTAATTACCCATA
GAACCCTAAGACATAGGTGTACAACTTTGTGCCCATGACCCTCGCATATCCTTAGTATCCACCGTAAGAAATCAGTTGCACGAA
TCCATATTGAGGGGAGAAAGGTCGAGAGATTGTCGGGAACGGGTAGTCCCTTGATGGTGATTGGAGATGGTCACTAGTTTCCCC
TCGAATTGTGATTCCCCTCCGATGTTCAAGCGTCGTTCGGAGTATAGTCCGCGCAAGAGAGCGTCATGGGGTAAATGAAGATAT
TGAGTCGAAACGGAGCCTTAAATGAAACATTTAAAAGTAAAATTCCCCTTTTCTTGAGGGCAATTTTCACAAGTTTTGTTTACC
CTTATAAGTTGCGTACGTCTCTCAGTATTTTGACTGTATCGACCCTATACACACCTCGGATTTTTCACGAAATTTTATACTTAG
AACCCCTAAGACATAGGGATACAAATTTTGGTGGCCCATTGACCCTCGCATAAAAATTTTTCCCCCCCTTTTTTTTTGTTAAGA
AATTTTTTTTTCCCCAACTTTTTTTTAACCGAAAAAAAAAATTCCAAAAGCTGCTTTCGGAATCCAATAATTTTAGAAGAAAAAA
GGAAAAACATCGAAGGAAATTTTTTCTTCTGAAAGGGGTAAAAAAACCCCCCCCCCTTTTTTGTTGAAAATTTTGGGGGGAAA
AGAATTTTCACCTAAATTTTTTTTCTCCCCGAAAAAATTTTTTTGGGGTAATTACCCCCCCCCTTTCGTGGGTTTTTTTTTTCA
AACTTTCCCGTTTTTCGATATTTTTTTTTGTTTTTTTTTTCCCCCCCCCAAGGAGGAACCCCCTCCAAAATGGCTTTTAAAATG
AGTTTATATTTTGGAGGGAGGTTCCCCCCGAACAGGAGGGGGCCCCCTTTAAAAAAAAAAATTTTTTCCCAAACCCAACATTTT
AAAAAAAAAAAAAAATTTTCCCCCTTTTTTTTTTTTTTTTTGTTTGTTAGGGCAAAGTTGTGTTTGTTTTTTTTCCAATAACAAA
GTTTGGTGTTTTGTGTGTGTTTTTTTGTTTGTTTCCCCTGTTAAGTGTTAGTTGGTTTTCCCCCCCGTAACGGTTTTCCCGGTC
CCCGCCCCTCCCCAGGTTTTTAGATTTGTTGTTTTTTTGGGAAAAAAAAACCTTTGGATTGTTTGGTTCAAAACCCCCCGCCCCC
GCCCCCTAAAAATAAGCAAAACCACCCTTCCCGGGAGATGTTGTTTTTTTGTGTTTTTGTGTGTCCGCCCCCAGAACGAAAAAT
TTGGTTTGTTTGTTAGAATTTTTACCAAGAAATAAGAAACCGCCCCTAAGAGAACCCCATAGGGGTTGGGGGGGTTAGTGCAAA
CCTTTTTGGGGGGGGGGTTGTGGCCCCCCCCCCCCAGATTTGACCCCCCCTTTCCCCGCCATTAAGTGTGTTCCCCCCGCCTTTTG
TTTTTTAGTACCCTTCCCAAACTTTTAATGAAGAAGCAAAAGATGCCCCTGCCCCGAAGGTTCCAAGATAATTTGGTAAGGGAG
ACATCCCGAAAAAGAAAAGAAGATTTTTGCCTGAAAAAGGGGGGGTGAACCCCCCCAACTTTGTTGTGGTGGGAAAGAAATGT
TGTGGGGGGAAAGAAGAGAAAAAGTTTGTTTGCCCCCAAAAAAAAAGACTTTTTAATTTTTTTTTTTTTTTCTTTCCCCCGAA
AAATTTTTTTGGGTAAAAATAAAAAAATTTTACCCCCCCCCCCTCCCGGTGTCAACTCCCGTTCCGATTTTATTTTTTTTTTTT
TTTTCCCCCCAAAAGAGAATTTCTTCCAAAAAAATTGGGGTTTTTTTTTTTTTTAAATTTTGAAAAAAATGAAATATTGGGAGT
CCCGAAAAACGGAAGGGGCCCCCCCCCCCCTCATTTTTTAAATGAAAAAACATTTTTTTTTTTAAAAAAAAAAAAAAAATTTTT
TTTTTCCCCCCTTTTTTTTTTTTTTTTTAAGGGCAAATTTTTTTTTTCCCCCAACAAGTTTTTTTTTTTTTTTTTTTTTTT
TTTGTTTTTTTTTTCCCTTTAAAATAATTTTTTCACGGTTTAAAACCGTGTCCTTTGTCCCCCGCCATTCAAAAGTAAAAAAAA
ATTTTGGGGGGAAAAAAAACCGCCTGTGGTGTGGTAAAAAAGTCACCGCGCCGTGTAAGAAAGTTTGACACAAAAAGAGCGAGCC
GGTGGTGGTGCGCAGGGAGTAGAAAAAATTGGGCCACCGCAAAAAAATTTGTTTTTTTTTTAAAATTTAACATTTTTTTAGGAAAC
CCCCCCTAAAGAAAACATAGGGGTTGGTTAACAAACCCCTTTTTGTTTTTGGGGGGGGGGTTGGGCCAAATTTGACCCCCCCCC
CCCTTTCCCCCCCCCCCGGGGCAAAAAATAAATTTCCCTTTTTTTTTTTTTTTAGTAAAAAATTTTTCCAAAACCCTATGAAAA
AAAAACCAGTGGGGCCCCCCCTTTTTTTGTCGAAAAATCCCCCAAAAAATTAAATTAAAGAGGAGCAATCCGAAAAAGAGAAAT
TTTTTTTTTTTTCCTTTTTTGAAAAGGGGTAAAAAAAACCCCCCCTTGTTTTTTTTTGGTGGGAATTTGGGGAAAGATTTTTC
CCCAAACCTTAAATTTTGTTTTATATTTTCCTTATTTCCCCCGAATTGTAAAAATTTACCCCCCTCCGTGGGTTTTTTTATCCC
AAAAAAACCCCCCTCGTTTTTCCGATTAATTTTTTTTTTTTTTTTTTATGTTTCCCCCCCAAAAGAGAGGATCCCCCCTCCCAAA
GCGCCCCTTTTTTTAAAAATGAATTATAAATCCCTTTCCTTTTTCCCTTCAACAAACAAAACACCGTGGGAAAGGGGAAAAGGG
AAAAAAAAGGGAAAAAAAAAAAAAAAAGGAAAGGAAGAGGGGAAAAAAAAAAAAATTATTCAGTTCCCAATTTTAAGCCTTTGAGAA
ATTCCTCCCTTTGGGGGGAAAAAAAAATTAAATCGAAAAACGAGTTTGAAACAACGAGGGTTTTAAATTTTTACCAAATTTCCCG
GAAAAAAAAATTTTAAGGTGGGGGGCAAATCTCCCAAATTGTTTGTTCCCCCCACCAAAAAAAGGGGGTTTTTTACCCCCCCCC
TTTTTTTTTTTTTTCCCAAGAAAAAATTCCCCTTTTTTTTTTTTTTTCCCCCGAATGGTTTCTTTTTTTCTAAAAATATGGAATTC
GAAGCAAAAACGTGGGTTTTTTTTTTTTTTCCAATAGGTTGAAAAATTAAACTAAAAGAAAAAAAAAAAATATGCCGGGAAAAGGG
GTTTTCCCACCCCCCCCCCCCCCCAAAAATGGGGGGGCAACAAAAAAGGGGTTTTTTTTTTTTTTTGGTTTTAATACACCCTTTT
AATGGGGGGTTCCTTTTTTTTTTAAAAGGGGTTTTTTCCTTATGGTAAAAGTAAAAAATTTTTTTCCCCCCCCGTGCAAATAAAA
AAATTCCCTTTTCGGAGGGTTTGTGGGGTTAAATAGGGGGGGGTGGAAAAAATACCAGTTCCCAAAAAAAAAAAAAATAAACCCT
TTTGGAAGAAAAGAACCGGTAAACGGAAAAAATATAAGAGAAAAAACCCCCCCTTTTTTTTTTTGGTTTTGGAAAAAAAAAATTG
CCCCCCCCTAAAAAAAAAGGGGAGAAAATTTTTTTTTTTTTTTTTTTTAAAATTGGGGTTTTTTTTCCCCATTAAAGGCCCCTT
TTCCCCCCCCGTTTTTTGTGTTCCCCCGAACCTCCAAAAAAATAATATTTTTTTCATTTTTAAAAACCATGGGGAGGAAAAATC
ACTTTGTTTTCTTTTTGGGGAAAAAAAAATTTATGCGAACGAGTGGTTGTGTTTTTTTTTGGGGGAAAAAACAACGAAAAGGGGGG

GGTAAATACCAATTTTCCCGGAAAAGGAAAAAATTAAAGGTGGGAAAAGAAATTCCTTTCCCCAAATTTCAAAAACCCAAAAAA
GGGGTTTAAAACCCCCCCCTTTTTTTTTTTTGTTTTTTTTTTTTTTTCCCCCCAAAAAAAAAGGAAAAAATCTTTTTTTTT
TTTTCGATGTTCTTCTTTTGTTTTTTTGTTAATAAATGGAAAATTTTTTTCGAGCAAAAACTTGGTTTTTTTTTTTTTTTTTT
TTTCCAAAAAAAAAATAGTGGGGAGGGGGGGTAACTTTAAAAGAAAAATATTGGGGGGCCCCGAAAGGGGTTCAAAAAAAGAAAA
AAAATGGGGGGGCCCCACAAAAAAAAAAGTTTTTGGGTTAAACACCTTTTATGGTTTTTCCCCCCCCCCCTTTTTTTTTTTTT
AGGGGGGGTTTTTTTTTTGTTCCCCTTAATTGGTTATAAAAAAAATTTTTTTTTTCCGGTGAAAAATTCCCGAGGTGGGGTTTT
GGTTATAGGCGGGGGTGTTTGAAAATCCTCCAGTCCCCCCAAAATACCCTTTGGAAGAGGGAAACCGTTAAAACGAAATTAATA
AAGTGGAAGAAAAAAAAAAAAAGACTGTTTTGGTGAAGAGAAGGTTTGCCCGTTAAGAAAAAAAAGAAAGAGAGGGGGAAGTGTG
TTGGTTTGTTTGGTTTTTTTAAAGAGTGTTGTTGAAATTTTTGTGTAAGGGCTTGTCCCGGTTGTGGTTTTGGTTTTTGTTTTT
TTCGGAACCCCTCCCAAAATATTTTTATTTTGCATTTGGGTTAAAGCCGAGAGTGGGGGGAAAAGGGGGGGAATTTTTTTCTTT
TTCTTCTTTTTTGGGGAAAAAAAAAGAATAAATCCGAAAAACCGAGTTTTGAAAAAAAAACCCCACGAGGGGGTTTTTAAAAAAG
TTACCCAATTTCGGAGAATTTTTAAAGTGGAAGAAAGAATCCCCCACCTCCCCCAAATTTTTCAAAAAACCCAAGGGGGTTTTT
TTTAAACCCCCCCCCTTTTTCCGAAAAAAAGAAAAAAATCTTTTTCGATTGGGTTCCCCCCTTCCGCTTTTAAAATAATTTTTTT
TTGGAATTTTTTTTCCGAAAGGCCAAACTTGAAATTTTCCGTAGTGGGGGAATTTACTAAAAAAAGAAATAAAAACTGCCGAGG
GGCTTTCCCCCCCAAAAAAAAAAAAATTTGGGCCAAAAAAACAAAAAAGTTTTTATTTTTGTTAATATACCCCTTTATTAAATTT
CTTTTTTAGGGTTTTATTCCACCCCCTAAATTGTTAAAAATAAAAAAAAAAAAAATTTTTTCGTTGGGGAAAAAAAAAAATCCGAA
AGGTTTTGGGGTGGGGGGGTTGGTCAATAGGGGGGTTGAAATAAACAAGGTCCAAAGAGAAATCATGCAAGAAAGGAACGTAAAA
AAACCGAAATTTTAGAATAAGGGAAAAAAGAAAAAAACTTGGTGGGGGTGGAAAAAAAAAAATTGGGGGGCCCCCCTTCCAAAAAA
AAGGGGGGGAAATTTTTTTTTTTTTTTTTTTTTTTTTTAAAATTTTGGTTTTTTTTTTTTTCATGTTTAAGGGGCCTTTTTCCC
GTTTGTTTTTTTTTTTTTCCGACCTTTCCCAGAAAGAAGTGTATAATTTTTTTCGACGAAATGTTTTGTTTAAAAACCCTCAAA
GGTTGAGAAAGATTTTTTCCCCGCCCCCCCCTTGTTTGGTTTTGGGGGAGAAATTAATTAAAAGAAGAAATCCGAAACCCGAG
TTTTTTTTTTTTTTGAACAACCCAATGAAAAAAGGGGGGAAAATATAAACAATTTTTCCCCGGGAAGGAAAAAAAAAATTTTTA
GTTTGGGAAATCCTTCCCCCAAAGAAAAAAATTTTCCCCCCCCACCAAAAAGGGGGGGTTTTTAAAAAAAAAACCCCCCCCTTT
TTTTGTTTTTTCCCCCCAAAAAAAGAAAAAATTCCTTTCCGATTTTCCCTCCCCCCTTTTAAAATAATTGGGGGGGATGTTCCC
CCGTTTGCCCAACCTTGAATTTTTTTTGTTTTTTCCCCCGGGTTAAAGTGGAAAATTTAAACACTTAAAGGGAAATAATTTTTG
GGCCGAGGGGGGTCCCCCCCCCCCCCCCAATTGGGCCAAAAAATAACCAAAAAAGTTTTTTTTTTTTGTGTTAAAAACACCTAA
ATGGTTCCCCCCTTTTTTTTTTAAAAGGGGGGTTGTTTCCCCCTATTTTTGGGTTAAATTAAAAAAAAATTTTCCGGGTGGGGG
GCAAAAAAAATAATCCTGAAAAAAGGTTGGTTGGTTAAATTAGGGGGGTGGAAGTTACAGTTTTTTTTTTTTCCCCAAAAAAAAA
TAAACTTGGAAGGAAGAAAACGGTTACCCCGAAAAGATATAAAGAAAAAAAAACCCCCCTTTTTTGGGTTTGGAAAAAAATTGGG
GCCCCCCCCTAAAAAAAAAAAAAGGGGGGGGGAAAAAAAAAAAAATTTTTTTTTTTTTTTTTTTGTTTTTTTTTTTTTTTA
AAAAATTTGGTTTTTTGAAAATTGTAAAGCCCCCCCCCTCCAAAAACCCCCGTTTTTTTTTTTTTTTCCGACCCCCTCCCAAAA
TATTAGAAAATTTAAAAAATTTCCCCCAAAAAAAAAAAATTTAACCCCCCAAAAATTGGAGATCCCCTCCCTTTGGGGGAAAATAA
TAAAAATCTGGAAACGGAGTTTTTTTTTTTTTTTTTAACAAAAACGAAGGGGTTAAAAATAAACCCCAAATTTTTCGGAAAAAA
ATTTTAGGTGGGAAATCTTTCCAAAATTTTTTCCCCCAACCCAAAAGGGGGGTTTTAAAAAAAAAAAAAAAAAAACCCCCACCCC
TTTTTTTTGTTTTTTTTTTTCAACCCAAAGAAAAAATCCCCCCCTTTTTTTTCGGAAATTTGTCCCTTCTTTAAAAGATTTAAA
ATGGGGGAAAAAAAATTTTCCCGGTGCCAACCTTGAAAAAATTTTTG
>m141122_040740_42134_c100732202550000001823141405141561_s1_p0/101800/5965_15235
CACACTGCTTGTCCTCGGTCCTCCCTTTGCGGGTTCCCTTGCGGGCTCGCAGGGTGCCACTTCCCTGTTGCTATAGTCCTCGCA
TTATCTGCGGGACCTTTTTTGTCTTCTTTTGCCTTCCTTTCTTCCGCCTCTTCCTTCCGAGCCACCCAGGTGACGGGGGCCGTT
TCGGGGGCCACACCGCCCCCCGGGGGGGGGGGGGGTTTTTTTTCGGCTTTGGCCTCTCACCCCGGAGCCAACGCACCCGAACCT
TGGCCCTTTTTTGGGGCAGGGGGGTTTTTTTTTTTACTCCCCCACAACGGTACCCATTCTTTGGCACAAAAGGGCCAAACGTTC
CAGGGGGAAAAAAAAAACCCGAAGGCCCCCCAAGCTTTTTTTATTTTTAAATTTCCCCTCCCACAAGCTTATCTGTGTTGTGGG
AAAAAGAGGAGGGGAGGGAGGAGAGGGTTCCCCGCCCGCCGCGACCCCCGAAAAACCCCCCCCCCTTGGCCGGGAAAGGCCCCC
CCGGAAAAAAGGGAAAAAATTGGGGAAGGCCTTTTCCCCCGGCCCAAAACGGCCTTTTGGGCCGGCCCTTTTACCCCATCCCGAA
AAGGGGGGGACCCCTTTTTCCCCCCCCCTTTTTTTGGGGGAAAAGATGAAAACACCCTTTTCCCCCGGAAAAAAAAATTAGGGTA
TTTAAAAAAAAAGGCCCTTTTTTTTTTTTTGGGGGGGGTTTTTTTTTTTTTTTAAAAGAACTGAAATTTTTTCACAACCCCC
CTTTCCAAAAAAAGCCTTTTTTTTGTTTTTTTTTTTTTTCCGGAGGGGGGTTTTTTTTTTTTTTCCCCCCTTTTTTCGGACC
CTTTTCTTTTTTTTTCCCAAGATTTTTTGGGGGGGGTTTTTTTTTTATTTTTTTTTTTATTTATTTGATCCCCCCATTTTTTTT
TTTCCCTTTTTAAAAAAGGAAAATAAGGAAATCCCCCCCAAAACCCGGTTTAAAAAAAGGGGGGGTTTTTTTACCCCCCCCCC
CTTTTTTTTTTTCGACAAAATAATTTTTTGGGGGTTGGGGGGATTAAAAAGGGGTTTTTTTTTAAAAGACCGAAAGCTTCCAT
TTGGGGGTTTTTGGGTCCCCCCCCCCCCCCTGAAAGTTTGAAAAGAAGGGGTTTTTCCCCATTTTGTGGCCCCCCCGGAAGGGGG
GTTGTCCCCCCAAAAAACCAGAGTGGAGGGGTTTTTTTTTCTCTTTTTTTTTTTTTTTTTTTTGAAACCCCCTTTTTTTTTTTTT
TTTTCTTTTTTTTTTCCTCTTTTCAAAGTAAAATGTTTCCCTTTCCCAAATTCCCTCTCCTTTTTTTCCAAAAACAAACAAAAA
ACCAACCCGGGGAAAGGGGGAAGGAAGGAAAAAGAAGAAGAAAGAAAAAATGGAATGAAAAAAATACTTGGGAGAAGGAAGAAG
GGTCCCCAAAAAACCAAAAAAATGGGGTTTTTTGACACCCCTCCCGGGCCAAAAAAAAAAAGACCCCCCCCCCTTCTTTTTT
TTTTTTCCAACCCTTTTCAAGAACAACCCATAAAATGAAGCCCCCCTATCGTATTCCCCCCCCGCCCTTAACCCCCCCCCCCCC
TTCATCGGAAAACGAGAGAGTATAATACCAGTTTTTGTTCCCGAAGGGGTAAAAAAAAAAACCCCCCTTTACCGTTGGCGTCAA
TAGGTGTTTTGTTTCCTTTAATTTTCCCCTTTAAAAGGAAAATGGGGGGAAATTCAAAAACCCCCCAATTTCCCCCCTTGAAAAG

GAAAAAAAGTTTCCCCCCCCGAAGGAAAACCCCTCCGGAAAACAGGGGGGCCCCCTTGAAGGGGGGTTTGGGGTGAAAAAAAAA
AAATCGAAGGTTCCCTAAAAAGCCCCCCCAAGCCCCCCCTTTTTTTTTTTAACCTTTAAAAAATTCCCGGGAGGGTTGGGTTT
CCCCATCCTCCCCCCCCCCCAGAGTTTTTTTTCCCGCCCTCCCGGAATGGGGTTTAAAAAGGCCGGGCCAAAAAAAAAAGC
GTGGGGGGGGCGAAAGCCCCCCCTCAATATTTTTTCCCCCCCTTTTCCGCCCCTTTTTTTTTTCACCCGGGCCAAAAAAGGG
GGTTTTTTTCCCGGGGGGAACCTTTTTTTTTTTTTTTTTTCCCTTTCCCCAAACTTTGGGGGAAAAAAAAAAGAATAAAAGC
CTTGCCCTTTCCCCCCCGTCCTTTGGGACCCCCTGCTTGGGCAAGATTTTTAAAAACCTTTGGGGGAATTAAAAACTCCCCCA
AAGGGCAAAAAAATTCCCTTTGCCCCCCCCCTTTTCCCCGGGATAATTTCCAAAGCCCCGACCCCCCCCTTTTTTTTTTTTG
GGGGGTTTTCCCCCCTTGCCCCCCTCCTTTTTTACCGGGCCTCCCCCACCGGGGCCTCGGATTGTTTTTTTCTGAAGGGGAA
AAGGGGCAAAAAAAAAAAGGCCCCAAGCCCCCCCCCCCAAAAATTTACCTTGGCCCCCCTTTTTTTCCCCCAGGGGGGGAAACC
TCAAGTTTTTAAGTTCCCACCCCCACGAGGGTTCCCCCCTTTTTTTTCCCCACCTTTTTTTTTGGGAAGGCTTCCCCCTTTTTT
TTTTTTTTTTCCCCTTTTCCCCGAGAAGGCCCCCCCCTTCGGGGTAGAGTTTTTTTTTTTTTGGGACCCCCCCTTTCCCTTTTT
TACCTATAATTTTTTTTTCCCCTTTTTTCCGTCCGCGAATGGTGGGGTTTCGGCCGGAAAATTGGCACCCCGGCCTTTGGCAAA
GAAAAATTTTTAAAAAAAGTCAAGCCCGTTTTTTAAAAAGAACCCTTCCCCGGAAGGGTTTAAAAAACCATAAACCTCTTTAA
AAAAAAAAAGCAAAAAGTCCCAATTTAAAGGGAAAAAAAAAAAGAAGTAAACTTTAACCAAGGAAACTTTTTTTGGAAAGTCG
AAGGAAACAAGGGCAAAAAAGGGCCAAACCCTTCTTCCAAAATGGGAAAAAGCCGGTCGTTCAGGAAACCAGTTTTTTATTTTC
CCCGGAGGATTTTCTTTTGGAAACCCACCCTCCCGCGGTAAATTGGCAAAAAAAAAAAAGAAGGGGCCAAAAAAAAAAAAACAAA
TTTTAAAACGTCCCCCCTTTTGGATTAATCTTTTTCTTCCAGGTTTCTTGTAAAAAAAAAAATCCAATTTTATTTTTTTCTTTTTT
TTTTTTTTTTTCTGCAAATGGCCCCCTCAAAATAGTTTTTTTTTTTTTTTGAAGACTTTTTTTTTTTTTCAAAGCCCCCCCG
TTTAAGAACAAGTACCAGCCCCCACCATTCCTTCTTTGGGGGGTAAAAGGAAGCTTTTCCAAAGTCCCGGGGGACTTTTTTTTT
TTGCAAAAAAAAAAGAAAACGTAACCCAAAAAAAAAATCCCCCCGGCCAGAAGGTTTTTTTTGTAAAAACTGGGGGGGGGAAAA
TTTTTTTTTCAAACTATTTTGGAAAGGGAAGCAAAAAAAAAATTCCTTGCTTCACGCTAAGAAATTGGCACGGAAAAATTTAAA
GCTCCCTTTTTATCAGGGGCCAAAAAAAAGTGGAGCCCCCCTTTTTCGCCAAAAAACAACCAAAAAAACCGTGTTTTTTTTTT
GGCCCAACTTTTTTTCCCAGGCGGCCCCAAAAATTCCTTCGTTGGGGCCCCTTCCTGGCTTTTGGGCCCGAACTTCACCTTAA
AGCCAAAAAAAAAAAAAAAAAAAACCGACCAGCCCGAATGGCCAGGAACAACATTTTCCAAAGCCCCGTTCGGGGATGTTTGTT
TTTCCCTTTCCCCAAAGGCCCTGCCTTTTCAAAAAAAAAGAAAGAAATATTTAGGCTCCCAAAAAACATGCGTAAAAAATTTTT
TTTCCCTTGAACAAGTTTTTTTTAGGCTTTTCTGGGTCCCCCATCCGTTTTTTCCCAAAAGAAATGGAAAAAATTTTTATTTTT
TTGATGGAACGTAAAGGGCTTAAAAGCCCCAACAAGTTTTTTTTTTTTTCCCTTTTGCCCTTTTTTTCCCCTGTTCCCCTTGGC
GCACGCGGGACCCAAAGGGGAGGCCTTTTTTTGGCTCCCCCTGGGACCAAAAGGTTTCAACCCCTTCTTACGGCCCCACGGTAA
AATCCGGCCTTTTTTTTTCCCCCTCGAAGTGGTTTTTTTTTTTTGTTTCGTCACTAAAGCCCAAAAAAAAAGGAAGAAAAAACAA
AGAGGAAAAAAACACCCAAAAAAAAAGCCCAGTTTTTCAAAAAAAAGACCCTCCTCTTTTCAGTTTAAACCCTTTAAAAAAAAA
GGTTTTTTTTTTGGTTTCAAAAAAACCCACAGGGGGAGGCACTCAGGCGTCAACACATTGGGGTTCTTTTTTGAAATGAAGGC
TCTGAGGCACGTCTACTAAGCAGCAGAAATATGGGGCCCCCCCGAGGACTGAGGCTGATGAAAAAAAAAGCAAACACACCAATG
TCAGCGTGCGCCAATGGATTGGCTAGAAAACTGGTCTGGAAGTTGTAAAACTCTGTCCCGCTCTCGGTATCGGAGCGGGAAGAC
ACCCCTTCCCCCACCGGGAAAGAAACTGCTTTTGTGAGAAGGTCCCCCTTCACCGGCGCACTCTAAAATCTATTCTAAAGTGAA
GTGGGCCTACACCCAATTTTAGCCAAAAAGTTAACAGAAAAGGACCAAACAAAAGGAGTTACGGACTTTTTTAAATCCGTTTTT
GTGAAAGCCCCCCTAAAGCCCGCTAAAAGAGAAAAGGAAAGGTTTTTTTTTTTCCCCCGAGGGAAGAACAACCGCCTTTTTAG
GAGAACGACGTTTTCAATTGCTTAAATGCCCCTAAATAAAAACAACAAAATTTCTAAAAGGCGCCGAAAAAAAAATATCAGGTGA
CAGTTATTACAAAAGGAAAGTTAAAAAAAAACAAAAACCCACCCACCCAGAAAAAAAATTCAAACAAATTAAAAAAAGCCCCCAT
GCAAAGGAATTGCCCCTTTTTTTTGAAAAAAACTTTTTTCAACAAAATCAGAACACAACGTTCCCTATCAAAAAACCCCTATTT
TCAACAAACCCATTGGCAAAACAGAGAATAAACCTTTTTTTTTTTTTTTAAAGAACGGAAAAAAAAACCCCAAAACTTTTTTTAA
AAACAGATTAATTCAAGCCAAATTAGAAAGGAAAAAAAAGCCAAAAGGCCGAAAGTTAACCTTAAAACCCCTTGAATAAATTTT
TTTTTGGACGTTTTTTTTTTTTTTTGAAAAAGGTTTTTGGAAAAAGAGTTGCTTCCGATCGTTTTGGTTTTTAACCCGCCACAAA
GTTTTTGCTTTTTTTGAGCCAAAAGGAAAAACTTGCAAAAAAGAATTTGAGCAAAAACCGGGTTTTTTTTTTAAAAGGGCCTTT
ATTTCCCTCTCCAAACCTTTTTACTTTTCAAAAAATAAACCAAGCGCCCCTTTTTTAGCAAAAAAACCGAAAAAAAAAAAGGGA
AAAAAGCCCTGGGATTTTCTTTTACCAAAATGAAAGAAGTTTAGCAAAAAAAAGGGTTTTTTGAAACCGAAGAAAAAAAAAAAG
GTAGGAAATATTGTATTTTTTGAAAAAAGCCCTTTTTTTGAAAAAGGTTGTTCGAAAAAAAATTTTTGTGAAGATTCCCCCTGA
AAAGTTTTTTTTTTTTTTTGAAGTTTTTTTTCCCCAGAGGAAAAAATGGAAAAGCGTAAAAGAGGTAAAAGAAAGGTTTCCTT
TTTTTTTTTTTTTTCCCCTTTTTTTCCAGTTTTTTTAATTAAATGGCAACAGGAACCCGAAGCCCCGTTTTTTTTTTTCGGA
TTTTTCCCCGTGAAATTTCGAGCGCGCGTTTCCCCCATGAAAATTCCTCCCTTTTCATTCCCCGCCCGTGCCCGCTTTAGAAGG
GTTTAGTGTGTCTAGTCCGACGGCTTTTTTTTTTGAGGGAAACGAACGAGCTCGTTTTCCCTTTTAAAAATTTAACTCCTTCCG
GAATGGTCGAAAAAATAAAGTAAATTTTTTATTTTTCCCCCAATCAAAATTTTTTTGTTTTTGGCCCCACCTCCGGGACCCAAA
AAAAGCCGCACCATTAAATACCAACTCCGTCACGTTTTTTTCCTTCTTTCATACAACCCAAAAGCCGTCTGGAAACGCACGTAA
GCTTTTCTCTCCAGCCCAAAGTCACCTCAAAACCGAAAACCAACGTTTGCCCTTTTTGCCGAACACTTCGAGTAACCTGAGTCT
TTTTTTGGAGGGAAACCCTTTTTTCCAGGAGCCAAAAAAAAAATAAGATGTTTTTTTGTCGCCCTTTTTCCGCCTAAGCAGAAG
AACCAAAAAACAACCCGGAGGGCCTTTGTGAAACAAGCTTCCCCGGGGATCTCCCCAAGATCCGCGACCTTTAGGGGGGGTAAA
AAAAAATTGTTTTTTTTTTTTTTTAAATCCAAAAAAAGATTCCGGAATTTGGTGTTTTTTTAGAAATTTTTGGCTGTTAAACCTT
TCGAGGGAAAGCCCGAAATTTTTTTAAAAAACTTTAAAAAAAGTTTCTCGCTCACGGAAGATAAACGTCCCTTTTAGAACTCGC
GAACGAGTGGCCCAACGGGCCTTTCCCGCCTTTTTTCAAACCTAACTTTATTGGGTCCCGGAAGATCTTTTTTTTTTCTTTTTC

GCCCTTTTTGTCCACCCGGAGAAACCCGGAAGGTTCCGTCCCAAAGACCCCTTCCCCGGACTTTCTTTGGACGAAAGGCAAAAC
TCCTTTTTTTTTTCCGAGGAAGGTTTCCCCTTCCTTTTTTTAGGCAAAGACGCCTTCCCCTAAGTTTTGTCGCCGCGGATGTT
TAAAATTTTTTTTTCCCGTTTTGCCCCCCAACAAAACGGAAAAAAAAAAAAGGGGCCGAGTTTTTTTTTTGCGACGAGACACGGG
AAGAGAACAACAATAAGTATTTTTTTTTTTTGGGGACCAATCCTCATCTGACAACAGAAAAGTTTCCCGCCCTGACAACCGCCTG
AACAAAAGGCCGAGAAAGTTTTTTTTTTGACCAACAAAACACCATTCCCCACGCCAAAACGCTTCAAAGTGCCAACCCTTTTCT
TTTTTTTTTTTTGGGCCTGCTGCCCTGTTGAACCCCGCCTGGAAAAAAAAAAACAGATTTTCCTTTCCTTCTCACCAAGCACAAC
GAGGGAAACAAAAAAAACAAGGGAGGAAAAAAACGAGAAAAAAGAAAGATCTTTGTTTCTTTTTTTTTCCCCCACAGGCCCGGT
TCAAACAAGGCGCCAAAGCCGGGCCAAAAAAAACGGCACTTTTTTTTTGAGCGTGCCTTTTTTTGGGGATTTTGTGTGGCAACT
TTGTCCCGGGCCCTTGTTTTCAAAGGCCCCCGCTGTTTCCAAAAGGGCGGGATTTTTTCCCTTTTTGTTGTCCAAGACTTTGAA
AAGGCAAATTTTCTGTCCCAAACTTTATTGTCTTTTTTTTCCTTCCCGTTGTTCCTTTTTTTCGGCAAAAACTTTCCGGCCCCC
CCCTCTTTTTTTTTTTCCGTGTTGGCGGCCGGAAAAAATACATTCCCCGGCCGAAAGGAGCAAAACTAAGGGGACAAGCTTTTTT
TTTTTCCTTCCCCTAAAAAAGAAAGGGAAACAAAAATTCCCTTCGAAAAGGAATTGGCCTTTTCGTCAAGGAAAAAGTCCCGGG
AAGGTCGACGACTTTCCGGGTCTTTTTCCGGTTTTGAAAAACAAGGGAAAAGAGAATCTTTTGCCGACAACTTAAGCCTTTAA
AGGTTTGAGGGCGGAGCCGTTTGGCCCAGCTTCGTCCACAAAACGCGAGGTCTTAGACTTTATCTTTTTCCCGTTTGAGAGAAA
CCTTTTTTTTTAAGTAAACTTTTCGGCTTTCCGCCTTTGAGGGGTTTAGCCAAATTCTAAAAAAAAAAAACACGCAAATCCGGGA
ATCCTTGGAATTTACACCATTTTTTTAACCCCCGCAAAAGGTTTCCGGCGGATATTCTTTTTGGGGAGATTTTTCCGGGAAAAG
CTTTTTTGGTTACCAGCCCCTCCGGGTGTTTTTTTTTTTTTTTTTTTTTTTTTTTTTTTTTTTTTTTGGTCTTTTTTTTTTCTTTGG
CTTTTTTAGGCGAAGGGACACAAAATTCTTTAATGCTTTTCCTTTTGGGAAAGGGTTTTTTTTTCCCCCCAAAAACTTCAAAGG
TAACTCCCGAGTGGGTTTTGGCCAAAGGCCACGTGTTTTTTTCGTCGGAAGTTTTTCTTGTTTTTGACCTGGCTTTTTGAGCCC
TTTTTTTTTTACGTTGCGTCAAAGACCGCTTGGGGTAAGAAAAAAACGTTGAAAACGGTAATTGTTTAAAAAAATGGGTGGCCGC
TTGGTTTTCCCCGGAAAGGTTTTGGGGGGCAAAACAAATGGAATTTTGGGAAAAAAAAAAATTAAAAAATTTTTTACTTTTTTTT
TTTTTAAAAAATTTTCCCATTTCCGAGGAAGGTTTCCAAATAAAGGGAAAAAAAAACCCGGCTTCCTTTTTTTTCGTTCCCCAAA
AGCCCGTCCGGATTCTTTTAGCGCCCAAACGGGGGAAATTGAGGAGAAATTTTTCATGAAACCGCTTTTTTTTTTTTTCCCGAT
TTCACGGAAATTTCGAAAACGGCTTCGTCCCCTTCTTTTTGCCCCAAAAAAAATAAAAAAAAAAAAACGAAGGAAAAAGGACTT
TTTCCTTTTTTTCCCCTTTTTTTCAAATTTTTTTTAAAACGGCTTTTTTTTTTTCATTTTTTTTTTTTTTTTTTTTTTTTTCTT
TCCTTGAAAAACCCTTTTTTTCAAAAAACTTTTTTCAGACTCACAAAATTTTTTTTTTTTTTTTTTTTTTTTTTTTTTTCGAAC
TTTCCAAAGGCTTTTTTTCAAAAATTCCCTTTTTTTTTTTTCCCCCCTCGTCCCACCCTTGCTTACTTTCTTTCCGTTTGTGGT
AAAGGGAATTCCAGGTGGCTTTTTTCCTTTTTCCGGTTTTTTGCAGGGGGTTTGGTTTTTAATTTTGAAAGGTTTTTTTGAGCC
CCTAAACCCGTTTCTCCAAATCTTTTGCCAGTTTGCCTGCCTCCAAGCACCCTTTGTGGCGGTACAAACGATCGGAGCCAAGCA
TTTCCTTTCAAAAAAAACTTTTTTTCCAAAACGTCCCAAATAAAATCAAAAAGTAAGTTACTTTTTTTTTTTTTTTTTTTTTT
TTCGCCTTTTTTTTTTGGGCTTTTTTTTTCCCCCTTTTCTTTTAATGGGCTTGAAAAAATTATTCTTGTTTAAGGTTTGGGGT
TTTTTTTTTTTTTTTTTCACCGTTCGGAC
>m141122_040740_42134_c100732202550000001823141405141561_s1_p0/103079/7060_17567
GAAGAATATAGGCCCGCCCGCCCGAGCTTTCCTTCCTCCAAGGCCCTTTTTTTTTATTTTTTTGATAGAAAACTTTCAAAGAA
AACGGTTTAATACATCGATCCCAAAAAAGAAAAAAAGGATAGGATTTTTTTCTTTTCGAAGGGTGTCCAATTTTTTTTTTTTTT
TAAGCCCTTTTTTTTTTGGGAAAAATAAAAAAAAAAAAAAAAATCTTTTAGTTTTGAACCCACCGAAAGAGAAAAGGCCGGAAC
CTTTTTTTTTGGGGTAATAAAATAAAACAAAAAATTTTTTTTTTTTTTTTTTACAAATATAATCATTTCTAGATTTTGTTTTTTT
TTTTTTTTTTTTTTTGCTTTGGTCCCCCCATTTTTGGAAAATTATTTCATAGTTGATGTTTGAAATCAAAAGCCCTCCGAGTTT
TTGTTTCCCAAAAAAATACCTTGAATATCGCTTAAATTTGGGTGAAAAAAAAACAAAATAAAGCATTCCCCCCTGCTGGTTATA
CGAAAAAATGATTCCCAAATTAAAGACCAACGCAGGACCGTCTCTTTTGGTTACCAAAGTTGTCAGTCTGTCGGGAAAAACCGC
AAGCTGAAAGCCCCCCTGGGCAAAAGTAAGTAAAAGTTTAAGGCTTTGTTTTTCGTTGCGTTTGTTTTTTCGCGGCCTTTTTTT
TTTTTTGGATAAGTGGCCCACAAAAATCGAGTTTTTTTGTTTTTTGTCGCTGACTGGTTCGCACTCGTGTGGTTCTCGTCGGAT
TGGTGCGGAGTGTCTGCGCTTGGCTGTGGTGAGATCGTTGCGGCTTTTTGGTTCGTTGTTTGGTGTCGTGCGTCGTTGTTCCGC
ATTTGCGTGTTTAGTTTGTGTTGTTTTTCGTTTTGGTGTTTCCTGGGCGCGGGCTGTCGTGTGACCTGTTGCTGTCTGTTGATT
CAAAAGTTAAGTGTTCGGTAAAGTGGAGGAACTATATTTACAAGCAGGAAATAAGTAGGATTCCTATACTAGGACCACTGCTTC
TCGCGAGACGTCTGTTTTCTAGTCTCCGCTCAAGACGCAATGAATTAAAAAGCTCACTCAGTTAATCCGTGTTTTTTTTTTTAAT
ACAGAACCACGGTTTGATTATTTGCGTAGATCAAGGCACGTATTACTTAATATATACTGTAAAGTACTCCCAAATTAGATAAGA
TATCAACATACACGACAAACATATTCAATATCAATTTATTTGATATACCCTTCATTGCCTTTGGGAACTACAGAACTTTTAAGT
GCCTTATTGATATTGAAGCTCTTAAACCTGATCATCCATGCATCTAACAATATGTGACATGTATCAGTGATCCTGCGCTTATAG
ACATGACCAATTGTGGGTTGTACCAGAGATTGTGAAGACGGCCATTGTTGTTGACAAATCACATTGTGAACATGTCACTGCTGC
GTCAGCTTTGAGTGGGTGGCGCTACTGAAGAATATTTTTTGGAGCACTCTAAACTAGCTTGAGAGCTCGAGACATGCTCCCTCT
GAAGCATGACATCGCCATGGTTGAAGAAGGTGAGAATTTAAGTCGTTGTGTTTGTTCACATACATTACTTATAGTTTTTCTAAT
ATTTACTGCTTAATTATTATTGCACGCTGTCATTAGATCATGCCAACTCTTTTGAAGGGCTGATTATGTTTTTTCTAATCACGG
AGTTCCCTATTCTAAATTCCAACTAAATTAATATTTTATGAGTTTATTTTCATTGAGTGGTGGAGAGGTGATAGTACTAAGTTC
TGATAAAAATATCACCATCGATTGTCTATACCAAGTCTCCACTTCAACCCCTTGAACTCATGACTCAACTCTTCATAATCATCA
CAATTTTTTTTTGCACTTCCCAATAATTCCACATGTCTATTAATCAGACACAGCAAATTCAATTATTTTCAGCTTAAATTATTG
AATTAACTCAGTGAGTTAAGATAACGAATAGACAATGTAAAAGGGCATGATCATGAATAATCACGAACTAAAGAAAAGCGTGAA
AAAAAGATTTTCATAATGGTCTGGATCAACTTCGTCTACTCTCTACATATTGAGCAGCGTTTTATTAATTTTAAATTCATCTGA

ATACACTTCTGTAATATTTTTTTTTCTTGTCTAATCTTAACTAAGACAAAAACCGATAAATACATGGTTTTCACAGATTTGCGT
GTTCTGGCGAGCACGCTGATATCTCGATCGGTCTGACAATATATAAATATGCGATAGATGCTCCTCGTTTAATAACATAAAAAA
CAATACAATTGTTTATAAATGTACCTTTTTAGCAGATCAACGATCAATTCATCATTTAACACACGCTCGTTCTCGCGTTATGCA
ACGCCACACGTGTCTCTCCTGCGCTTACGTCTTCCTTCCCTCATATATACTTTGGGTAATATTTCATTACGTTTCAAAAATTAA
TTTTATTTTTAAAAATTGGTACGCGAATCATTCTAGTTCTAATCATCATCAATATAATTTCTTTTTTTTTTTATATGAATGCAT
TTTACTCTTACTTCTACTTCTCTTATAATCTCACATCCACATCACTGCTTCATTAAACATTGGGGTTTCATTGTAGTCGATTGT
CAGTCAGAGCATTTTGTAGTAATATATGGGGGCTATCTTATTAATTTTTTGGTAATAATTACCGTGGCAAAATTCATATCTAAT
ACTAATCGTTTCAATTTTGTATGCACTTAACTCTTGCTTATTAAGAATTTTTGCTAGCAAACTCTCGCCATCAGGATTCTGTCT
GCTCGCTCTTGTCTAGCCTCTCCTGTTCGTCTAACTCCGACTACGCAACACACAAGCTGCTCAACTTGTTATTGCGTTTAAAAC
TAACTGAATTTCAATCAATAACGTCTTCATAATTTACCCGCATTCATCCAACCCTTTGTTTATTTAATTATATACATATGAATT
AAACCTGCTGCGAACGATCACAATGTGGTACAACAAACCTCACGTTATTTCTTGTAGCTCCACATCCTATAATACTTCTCGCTC
ACACACCATCATGGTTCAACAGTCCTGTTTACCATCAACACTAGAACTGATCCTGATATACACCTGACACTGACACCACCACAA
GTCACCTTCCCTACGTGGTGTTATCATATGCTCTTTTCAGATATTCAATAGTCACCCGCGGTTCTTTCCTCTATGCCTCAGCTT
GAGTACACGCATTCATTCCCTAGTCAAATCTTTCTCAAGTACTATATCATCTCTTACACAGTTACACCTAGACTTCGTTTCTCA
ATAGTTTTGCTGCAAGCTTGACTGTTCTTTCTCTCTTGCTGAATCGAATCTCTTAAGCACACCAAGGTTTCTTCTGCACACTCC
TTCAAAATCTGCTTGATGCATTGCATTGCTTCCTCCGATGTGCTAGACTCGGTCATGAACCACTTCCGATCAGATCGTTCCTTC
ACGTCTTCCCTCTCTGCGACACTTTTCATCTCCCAACAATCATAAGTAACCCTCTTAGTCAGAAGACATTGACACACCACCCGA
ATTTATTTCGCATAACAACTTATATCTTTGTTACAAAAGAACCCACGCTTTCAAAAGTATACGTAAACTTAAGGCGCCGAGTCG
AAGAGAATACCTTAGGGTTGATGTTGGGTCTTCGGTCATTTATCACAACCAAACCTACTTACGCAATGTCGTTACACATCTTAC
CACACTATCACCATTCTTCACTATGCAAACGCGTTTCCAAATAAACCCTCTCAATCACCAATCATGCGTCGACCACATCGCTCA
GCGCCCGCATACAATTACTGTAGTTTGTCAGAATACTTTGCTCGCAAATTTAGACTTTCGCTCATCAGCTCCAAGTCATTCATT
GTTTAACTTTGAGTAGTTACCGGCCATCTTTCGGTCAGACTTGACGCAGCTCTGTTCCAGACATCATATTCAAGCTCTCCATCT
ATAGACCCACCATCTCAGAAACTTCAAGTCTAGCTTATCCACAACTTCCGCAACGCAAATCAAATTCTGAAAGCACACCTCCGA
GAATCCACAGGCCCCTTTCTTAGACGGAATCACATTCTCTACAAAACTTCAACAAACAAAATGAAACAATCATGCAAACACCGC
TCTATGATCATCACATCAAAATATATAAATTCAATGATATTTGTCTTTTAATAATACTTGAAAATATACACATCAAATCTAAAA
ATTATTTTGTCAATCTATCTTAAATTGCAAATATGCAGATCTTCTACAATCTCCCCGCGTCCTTGGCATTTGAGGACATAAGGA
CAGGTTTATTTTTCACCTTCTATTGGTTTTAATTTTAAACACAAACACCCTGGCAAAAAGATTAGCAGCAAAAAGATTAGGTAT
TCAAATACTCCTCATGCAACAAGATCATTAAGCCATACAAACAAAATTAACAACCATGTTAACTTTTCAAAAAGTCAACACAGA
CCTCCCCCCTAAAATAGGGGTCAAGCAGTTTGTAACCATAAAAACAAAAACACCACATTTCCCCCCTTTTGTTATCAAATTGCC
CAAAATGAAACCAAAAACAAAAGAAGAAAAGACCAAGAAAACCAAAACACCAAAGTAAACATGACAGAAAAATAACAGGGTAAT
CATGTAGGCAAGGAATTCTGAACTGAACTGAGTTGCTGAAGAATAGCAGCTTTGGTTTCTCTTCCATTTACCCCAACCGACGTG
GTAAACTCATCATTCACCAAAAAACATTGTCAACACTCCAACATTATCACACTACAAACCAACCAACAGCACACAACATTGCCA
ACACTAGAGCAAAATTGGAGTTTCAAGAATTTAAAATGAAACAAGAACAAAATGCAATATATTTTACTTAGTCTCTCTTAAAAA
ACTCTCGTCCAATTTTTTTTTTATATTATTATTATAATACATGGATAAAGAAACCTAAGTTTACTTTACCAACCTCTTATCAAT
GTGTATCAACCAAACTCTTCTTGCTTTTGTCCCGTTCGATGAAAAAATCAAACTCGTGAGAAAACATAAATTAGTAGTAACAAG
TTTATCACCCCATCGTATTTTATCATAATGTTATGCAGAGAAAACAATTACCCCACGAGCCAACAATGTTAAGAAGATCAATCA
TTGTAGTGTTAGAAAGCTTTTAAACATGTTTTTGTACTATACATTTTAAAAAAAATGTTTCAAGCATTTGTGGTGAAAGTGAA
AGCAGGGGACATTGCAATTTGTCCAAAAAGCTTTGTTCTGAGAATTGTACCCAAATGAAGGTACGCTGTCCAGCACTATAACCT
CAATGTATGGCCTTTTCAATGGTAGCGGTATATTCTACATAGTCGCTCATACAGTTAGTTTTTCTAAAAAACTTACTCAGAGAT
GACTTTCACACATTGAAAGCAGATAGCCTATTCTTTCAAGAGGAGCTTGAAACATTCATTATTTTCTTTTAAAACCGGGCGCTT
GAATCATAACACATTGATGAACTCAAAATTTCTTAGGAGAAATAATTAATTTCCTGAGACTAAACATTTATAACGAGATACCAC
CTACAACAGATTACAGAAATATATTGAACATGCTTTTTTTTGAGTTTGACAGTTTCTTCTTAGAACAACAACAAGACAATTGAT
TGATTTTAAACAAGACAGATTTAGCAAGAATAACAAGCTGACAATATACACAATTAAAAGAAAAAAACTCCTAAGGACTTCCTT
AGGCGATCAAAACGAACCCGAATCAAGAACTTTTGGTAAACAATAATACTCGTTTTCAACATATTTAGACTAGAATTTTATTTC
AACAAGCTTACTCTAAGGAAGTGATGAACGAACTGTCAACTGTGTTTTATGTGTGAACGTTGAACATGATTTTTGAAAATGTTA
AATTGGCACTTGTGTTATCACAGAAAATAGTTAAAGTGTCAAGAAGAAAACCCATAATCAGTCCCACCATCTGCTTCACCACAA
ACATTGAGTGCAAACAAATTCTAGAAGGATTAGTACGTTAGCTTCAGAGCAGTAGAACAAAGATATTGAATTCTTGCCTTTTTG
ATATTCCATGGAGACAAGATTGTTGCGCCTAGGTATGAAGCATCGCTCCCACTTGTGCAATTTTAATATCCGATCTGCATTACC
TGCCAATCAGCATCACGAAAGCATCACAAGGTTAGAATTAGTTTCATTAGAATACCAAAGACATTTCCAACAGTCCCAATGTAC
AATAATGAATAATTCTTTTTAGCAACCACTACATGAGATTCCATAGGATTCCCTTGATAAGCCTAGCACAATAATAAACTATAC
GCATGATGTGCAGGACGACCTTGCGTACAGACAGTAGACAGACTACCAAAGTCATATTCTATGTAGTGTAGGGATCAACCTTTG
ACACAAATTGTTCATCCCCGTGGTTAATTTGGTTTTGTGGTCCCCATTGGAGTTTTAGATGCTTCTGGCGTTGTTTCAACGACA
ATACTTTTAACCATAATTTTTGCAACTACTTAGGGATGCAAAAGCTTATCCTCTTCGTTTGCTGTCAGTCACCCTGCAATCCTA
GAAAAAGTTGTCAATTCACCACCACCCATTCATTTCAAAATTCCATACCCTTTCATTTGTTGGAGCACCAACCACTGCACTTCA
GAGTTAGAATAGATCAAAAAATAATGCTCTCATCCCAACATATACTTTGAAAGTAATTATAGCCAGGTTTTGATGTATGTTGAT
GAAACAGAAGGTTTATCAAACCACTCCCCTATGATGAAGCCATTAGTAACCAAAAATTCGGGTTTAGCCTTTTCGGAGTACCAA
GCCTCGTGGAGTCTGTTTTTTAAGCCCATACAATGCCTTTTTTGAGCCGCTAAAACCATGATCTTTAGGAAAATTGAGGATCTT
CCAAAAACTTTACGTTGTTTCAACATATGCTGTCTTCCATTTAAGATCGCCGTTACCTTCCAAAAACAGCACGATTTTACAGTC

CGTCTAATGTACTTTGCAAACCATACTGCAAAGCACACCCAGGATCAGATAGATGCAGACTCTTATAAATTCAAGCCTAGCTAC
AGGTGGCAAAAGTTTCATCAAAATCAACAATCCTTCAAATTTGCGTGTAAACCTTCGTGCAACTAGAGTGCTTTAATTTCTTAT
TATTGTTCCCACTCATCACTTTTATTTTTGAAATCCATTAGTGCCAAATAATTAGTATTGATCTTGGTCTTTCACAAGAAGAGT
TCGCCAAACATCATTTACCTAGAAAATTGTTTCTAATCCTCTTGCAATAGCATCCTCTCCGCATGATTCATCCCGGTCCAAGAT
CTCTTTTCCACATTTTTTGGCTCAAAGAAGAGGTAAAACAATAAATTCAACACTTGACATACTCTTTCTCCTTGTAACGCATTT
ATCCGTCGAGATTGCCGGTCCAATAATCATGATTGCTGGTGATTTTTTTGCCCTTGGTGGAGGGTTCCCTTTGGATGGAATCAA
TAATGATCTATGCATGTTTTTCGCTTTGATGCTCATCTCCAATCCTGAAGAGATCTGATTATGTTTGCTCCCATTCTATGGCTA
TTGTTTTCTGGCAGTTTCCTGCTGCCTGGTGTCAGCAAACATTAGACCAAGCCAGACAGTCACTCCAGCAGGCTCCTGATTTCT
TCCTTCTGTTAAAATTCTGAAAAATATTTTGTAATCGAATCACAATTACAGACATTCAGCTGACTCCTAAAACCAGTTTGAGTT
CTCATGTTGTATTACACGACAGGCTCTACTATTAGTTGTGTAAACCAAGAAACACTCCAACATCAATCTTGGAGAGTCCAGAAC
TTACTCGTGAACTAGTTACTGGTACGCTAGTAGTAGTCAGCAGTTTAGCTAGTGTCAGGGTAGTAGTAGTATAAGTACTAGTAG
TAGTAGTAGTGGTGTAGTAGTAGTAGGTGTAAAGTGGTAGTAGCTGGTACGCTGGTATAGTAGTAGTGGTAACGTAAGCACGTG
GTAGTAGTGGTACGTTGAGTAGTTAAGTGGTAATGGTGTAGTGGTGTGGCTAGTGTGTAGTGGTATAGTGTAGTATAGAGCTAG
TGGTAGTGGTAGTTAGTAGACCTAGTAGCTAAGTAGTAGTAGCCTAGTAGTAGTGAGCTAGATTAGTAGCAGTTAGTTAGTTAG
TAGTAGTAGTAGTCTAGTATAGTAGTTTCCCAGTGTAGTGGTAGATAGTAGTAGCTTGGGTAAGTAGTGTAGTAGTCAGTTAGT
CAGTAGTAGTAGGTACTAGTAGTAGTGGTAGTATAAGTAGGAGTAAACGTAGGTAGCTAGAGTAGGTGAGCTGGTATAGAAGTA
AGTTGTAGTCAGTAGTGGTAGTAGTAGTATAGTAGTATGAGTAAGCTATAGTGGTATCGTTAGTCGTAGTAGTAGTCATCGGTA
GTTGCCCAAGTAACCCAGTTAAGTCAAGACTAGTAGTAGTAGTAGGGAGTAGGAGTAGTAAGTAGTATGTCGTAGTAGTAAGCT
AGTAGTGGTAGTGGTAGAATGGATAGTAGTAGTAGTTGGTAGGTAGTAGTGGTACGTCAGTGGTAGTGGGTAGTGGCAGTAAGC
TCGGTAAGGTGCTGGTAGCGGGACGTGGTAGTGGGTAGCTCGCGTCAGTGGAGGTGAGCTGGTAGTAGGTAGACGTAGCATGTA
GTAGTAGTGTGGTAGTCGTACGTAGCTGGTAGCCCGTATAGTGTAGTGGCTTAGTAGATAGTGGTAGTAGTAGTCGGTGGTGGT
ACTAGTCAGTAGAGCTAGTAGTGGGTAGTGGTAGTGGTAAAGTCGTAGTCTGGTGGTGGTATGGTAGTGGGGGTAGTGGTAGGT
GGTAGCTGGTAAGTGGTAGTGGTAGATAGTAGGAGTATAGTTGGTAGTGGTACCTGGTAGTTCAGTAAGGAGTCACAGTGTACT
AAGCTGGTACCGTACGTGGCTAGGAGCCTGTAGTAGTGGTAGCTTACGCCTGGTAGCTAGTGGGTCAGTAGTCGTCCAAGTAAC
GCTGGTAGCTCCTAGTGGTAGTGGTAGTAGTCAGTAGTAGTGTAAGGGCAGTAGCCCTAGTGGTAGTGGTAGTGGTAGTGGTAC
CCGTGGTACGTGGTAGTGCGGTGGTGGTACTGGTAGTGCGTGGTAGCGGTAGGGTAGTGCGGTAAAGTTGGTATGTAGTATTAA
GTCAGTGGTATGTAGTAGTAGGAGATATTAGGTAGTATAAGTAGGGAGTGGAAGTAGTGAGTGGAGCCAGTAGTTAGTAGTGGA
AGTAGTAGTAGTGAACTGTAGCAGTGGTTAGTAGTGGTAGTAGTGGTAGTTAAGTGAGTAGTAGCTGGTAGTTAGCTGGTAGTA
AGCTGGTATGTAAGTTGGTAGTAGTGGTAGTGGTAGTAGTGGTGGTAGTGTGGTAGTGGTAGGGTAGTGGTAGTAGTAGTAGAG
TATAAGTATAGTAGTAGTGGTAGTGGTAGTGGTAGTAAGTGTAGTGGTAGTAGTGGTAGGTAGTAGTAGTAGTAGTAGTAGAGC
GTAGTGTGGAAGTATGTTAGTATTAAGTAGTAGCCTGGTAGTAGTATCGGTAGTTCCAGTCAGTGTAGTCAGCTATGGTAGCTT
AGATAGTAGGGTAGTAGTAGTAGTCGATAGTAGTGCAGAGTAGTATTGGTTAGTAGCTAGTAGTGGAGTGTGGTAGTGGTGAAG
TGGTAGTCAGTGGTAGTGAAAAAAAAAAAAAAAAAAAAAAAAAACAAATAGTAGTGGTAGTGGGTATGGTAGTAGTAGTAGTGGC
TAGTGGTGGTGGTCGTAGTCAGTAGTGGTAGTGGCTGTGGTAGTGGGTAGCTGGTATGGTAGTAGTAGATGGGTAGTGGTATGG
AGTAGGTAGTCGGAGATGGTAGTAGTAGTAGCTAGTAGTCCAGTAGTAAGTCAGTGTTAGTAGATAGTGGATGGTGAGTAGTAA
ATAAAGTTGGTAAGGTGGTAGTAAGATGGTAGTTATAAGTGGTATAAGTGTCAGTAGATGGTAGTAGATAGGGTAGTGGTAGGT
GGTAGATGGTACGTGGCACGTGGTAGATGGGTAGTGGCTAGTAGTACTAGTCAGTGCTAGTAGTAGTAGTGGTTAAGCCGGTAG
TGGTAGTGGTAGTAGCATGCTGGTATAGTAGTAGGGTAGTGGTAGATGTAGTAGTAGTAGTAGCAGTGTAGTACGGGTAGCAGT
AGTTAAGTAGGCAGTAGGTAGTGAGTGTGGTAGTGTACGTAGTGGTAGGTGGTATGTGGTAAGGTGTAGTGGTTAGTGGTAGAG
GTAGTGGTATGCTGTAGTGGTAGTGGTAGTGGTAGTAGAGTGAGTGGTAGTAGTGGAGTAGTAGATAGTAGTAGTAGTAGTGGA
GCAGATGTGGTACGTAGTAGTGGTAGTAGAGTAGTAGTAGTGGTAGTAACGTAGTGGCAGTGGAGTGGCAGTGGCAGATGGCCA
GTGGCAAGTGGCAGTCGGCAGTGGCAGTGCAGTGCAGTGGTATGTAGTGGAGTAGTGGTAGAGTCTGGTAGTATAGCAGTAGCA
TAGTAGA
>m141122_040740_42134_c100732202550000001823141405141561_s1_p0/105101/9179_16425
ATACTCCAAGCTGTGCCTCTCCTATAAACTACTGGGGACCTGTGAGTTCTACAAGGGGTTGGATTCTTATTTGTGTAACAAAAA
TACCTGTAAAGAATCAGAATATAGCAAATAATATACGGCATGGTGGAGTAGAGGGTTTTAACTATTGACACCTACAAGCATAGG
TCGTCAGGTCTTAAGTGGTCATCATTAAGGACTCAATTTAATTGTGATTGGTTCATACACTCGACTAATCCCTTTCCCTTATTC
TCGTCTTATTACCTGTGCACTGGCGAAGAACTGGTTCAACAGCTCTCCTTGTGCTTTCATACTAGAGCAGCTTGTTAGTTTTCT
CTCTGGACATGGATCATACGCAAATAATCCTGCGAGGCACGGATGGGGGTGATACATTCGATCAAGACCACCGGGTATGGAGGA
GCCAGCATGATGGGAGGAGCTCATCATGCGAATCTCTGGTGAGGCAAAACCTATATCGCGAAGCTCAGCAATGGTATGGGCAAC
CCCAGTCAATAGGCAGGGATGGAAGAAGTGTCCGGACCGGCACACGAGATGTAGATATTTGCAGACACGGTTTGATCTATTAGT
ATTACACGAGTAGAGTGGAGACATGGTCTTACAGTCTGAGGTTCAGCTGGCGAAAGCTAATAAGCAGTTCAAGAGGGTGTGTGG
CACGACTTATACCCCTCTCTATACAACTGAAACTGCTAACGTGACGAATGAGGCAACGAGAGCAGAGTATCATAAGATCCTGCC
CGGAGTGCCTGTATATCCATTGCGCGCACCTTGAGGGCCTAGCTAGCGTCGGCCGCACTAACAGATCTGCTCGACCATCAAACG
TTCTAATGTTGTCACTGCCTCGGGATCGCACCGCACGAGCCAGCGGGAAACTTTTCGATAGAACTACCCGGGCGAGAACAGTAT
ATCCAGCCTTGTCGACTTCAACTCCAGGCCTCACTGCAGCCTCGAGTGCCAGGGAGGGCCGAGGGGAGTTCTAGAAGGAGATCC
GGGAGGGCTCACAGTGGGACAGCCATCAGGCTTAGCGTCTGCGCCCGCTCAGATTGCCAATGCAGGCCCGGAACGTTGGCAGGG
CCGAAAGGCCTCTACCCTATACTGTCGCCTGACGGAACCGGGGATTGCATATCCAGGTCCGCATCTATTGTCAACCGGATAAGA

TACTCCTATTTTCCTCCATCGGCCCTGTACCGACGTCATACTTCCTAGCTTATGGAGTCAGCAGGAGAAACCCCACTCGCAGGA
CCGTCCATCCCACTTCGTATAGAGCAGTTGGCGACACAGGACGCCAAGTGCCGGACCCTCTCACCAGCGCGGCACCGCCCCTCC
GAGCTTCCTCTACCCGCAGCTTCAGAGCTGCGACCCGGAAGCGTCGCACCGACGACGCTGTACTTTCCTGCGCGGCAGACCTCG
CGCCAACATGCCTAAGCCTCGGCGCAAAGCTCCTCAAGCCACCGGTCGCGGAGGCGACTCGCCGCATCCCCCCGCCCTTAACTC
CTCCATAGGGGGGCACCTACAGTGGGAGTGGGCAATCGTGCTCAACACACCACGGGCACAGGCCCTATACCCTTACGACGGATG
CACGAGGATAGCGGCACGAATGTCATCACTACCAACCTATCCTCAGAGTAGGCCCGGGGCCCCAGCGCCACAACGTGTAAACCT
GGACTCGAAGCTTGTTGACGCCAGCCCGAATAACAGGAGATCAGGCACCACAATACCATTGAGCAGCCTGGCTTCAAATGGCCA
GGAGCTGAACTGCAGCGAGACGCTCTGTCAGAAAAAAAGAAAGGGGATGATAATGATATTAGCGGAAAACCCCTGAGCTCGTTC
GCCCCTCCACGGAGGAGCATTTACGCCGCAATCAGGCATACCGATGCCGGCTTCCCGCGCAGAATTCGGAGCTTTTGTCTGTGT
GCCTGTCAACCAACTGCCAACGGAGACGGAGATTCGTCCAACATCATCTGGGTAATGTCCTCAATACCACCTGTGATGGCAACA
ACATTGGCCCGAGCTGAGGTGTTTAATGATTCCCCTCACAAACGGCTCTCTGCTGGCCTCGGCTCGACAAATGGTTAAACAAAA
GCAAGAAACAAGTCAATCAGCTCTGGAAGACCTTTCTCTTCTCCTCATTCTTCCTACCTCTTTCCTCTCCTCATCAACTTCTAC
CTAGTCTATTCTTTCGTACCTCCTACCCAGTGTCCTGGAAGGTTCTCTCGTTGTCGTTCGAAGACCGGGAAGGTTCATCGGCCT
CCGGATTCCCCGCCCCACCGCCCATCCTCCCCTCGCTCTCGCGCCCCTCCCCCCCCACCGTTCAGTCCCCCCCCCCACCCCCCC
CCTCCGCCCCACCCCCCACCACCGCCCACCCCCGCCCCTTCCCCCCACAGTGCTCACCTCGGCGCTTCTCATCCCCGCCTCGAT
CCCCCCCATTCTCACTCTCCCCCCTCCCCCCCCGCCCACCACCTCTCCCCCGACCGCCCTCGCCGCGCTCCTTCCCCTCCCCCC
CGCATCCTCCCCTCACGCCGCCTCTCACACCCCTCCCCCTTCACACTCATCTCCCTCTACCCGCCCCACTTCGCCTCACCCGCG
CCCTCATCGCCACAACCATCACACCCCCTCGTCGTCCCTACCCCTCCTCCAGCACACCTCCACTCCCCCACCCCGCCCCCCCTC
CCCCCACCCCCATCCCCCTCCCCCCACCCCACCTCCGCCCCGCCCCCCCCCACCCCCCCCCCGCCCCCCCCCCCGGCCCCGA
CCTCCCCCCCTCACCCCCTCCCCCCCCCCCCCCCCCCTCCTCCCGCCCCGTCTCACAACCCCACGCCCCCACCCCCCCCACCCC
CCCCGCCCCTCCCCCCCCCCCCCTCCCTCCCCCCACCCCGCCCCCCCTCTTCCCCCTCCCGCCCACCCCCCCCACCCCCCACCC
TCCCCCACCCCCCCCTCTCTCCCCACCCCCCCTCCCCCCCACACCACCCCCCCCCGCCCACCCCCCCTCACCCCCGCTCCTCCC
GCCCCCCCCCCCCACCCCCCCCTTTCCCCCTCCCCCCGCCCCACCCCTTCTCTCCCCCCCCCCTCACCTCCCTCAGCCCCTCCG
CCCCTCCCCTCCCGCCTCCCCCCCTCCCCGCTCCTCTCCCCCCCGCCTCCCCACTTCCTCCCCCCCCCTCCCCCCCCCCCACCC
TCCCCCTCACCGCCCGCCCCTCCCTCGCCCCCCACCCCCTCTCCTCCTCCCCCCCCACTCCCCCACCTCCACCCACTCCCTCCC
TCTCTTCCACCCCCCCCCCCCCCTCCCCCACCACCTCCGCCACACCCCCCCCCCCCACCCGCCCCCACCCCCACCCCCCACCCCC
CCCCCCCCCTCCCCCCCCCCACCACCCCCCGCCCCCCCCCTCCCCCCACCTCACACCCCCCCCTCCCTTTGACAAACGCCCCTTT
TCTATACGGAGAAGGTCTTCAGGAAGCTTCCCTAACGATCGTCTCGGGGGGGCGGGACGGAGCTCCGTGCCAGCAGTCCGGAAG
TGACAAGTGGGCTGAGACACGCGACCGAGGACCAGTTAGCGACGACTCCTCCGCTGACACAAAATCCGTCTGACTCGCCTGACA
GGTCGCCAGCTTGGCACACATTGCCGCACCGCACTTCAACAGGTGGCCCTTTTGCGCTGCAGTTCTGCCGAACGACTGGCAAAA
GACACTGCAGCTCTCCTGCGCATTTCCCATATTGCATGTACATTCACTACCTCACGTAGAATCTATTGTACGCCCAGTGCTGGC
TTACATTAATCTGTAGCCGCACTTTTATGGCTGGTGTAAGTTAATGCTCGTTGGGGGCAGGGTAAAACGCTAACCTCAAAACCT
AGCTATAGAAACCCCCTCCGATTTTAGCAGAGGAGGGGACGGCAACAAGATTTACACTAGAAGAATCAGACCGAAAAGAACTCT
CCTAGCTTCATGCTTCTTCAAGAGTACTGAGAGAAATCCTGCTGTGTGTGTGAGTTAAAGCAGATTTCTTGTATACCAGCGCGT
CACTATATTCCCGTAACTCTCTCAATATAGCGCATATTATATCCATAAACTGACGTCGTGACTAGGGCTCTATTAACGCCGGAC
CACGTAAAACTCTGCTTGTTCTTTTACATTTTAGTATTTCCACAGTTCTCGAATTTTTATTATTTATTTATAGTCTTTCCGAAA
ATGCTCGGTAAACATCTTTGGTGCTTTCATTGAGAGCGTAGGAGCAGTGTAGTCATTTTCTTTTCTGTGTACAGTGTTTGTATC
CACTCCACGCTGAAAGCTGGTGACAGCAAGAATGTCTGTTGGTCGACAAGAACGTGAGTGTCAACCCAGTGTTCAGGAGGATGT
GGTACCAACGAACCTTCGGTGTCGCCGACGTAAGGGAAGTAGTATCCTGACGACTCCGGACTACCAAGAGGACGACCCGACATC
TCACCAGGGATCGGGTCCGACACTGAAGATACGACATACGTCGAAGAAGAAGAAGATTATGCCGAGACGGCTATTACAAAGATG
GCTACCACTACGAAAAGGCCCGAACTGGTCCAAGTAGCCGAAGAGGCTAAGGGCCCAACCAAGGGCAACTTGCTGAACAGGAGC
ACGTTGCCGAAAAAATGCAACGCTGATGGAACGCTCAAGAGAAAGTTTGGAGACTATAGGGCGACTCAGTACGAGGAACGCCTC
AGTTTAGGTGGTGGCTGACGCACCACACGGATCCAGCTGCTGCTGACTCCATCCAAAGCTGCCCTAACAAGGGCAAAGAAAAA
GCTGGAGAGCTGTGCGCTCTAACCAACCCTAGCACCTCCCTAAAGGCTGAATCACAAGCCGACGCCCCCCGCGCGCAGAGGTCT
CTGGTGCTCCTAAGCGACATCCTTCATGCCCAGCGGGCACTCTTGTGGGCTAAAGGGCTCGGTGCTAAGACACCAGGCCTAGG
GGAAGGAAGCAACCGTCGAGTGATTTCGTCAACCAGGATGGTCGGGTTGGCGACACGCACTCGCGAAGACAGCTGGTCACGTGA
GCCTAAGAAGAAAATTATTAGCCAGTATGCAAAGGCCAAAGGGAGAAAAGGCATGCGGCTCTTCGCGGAATGATCTGCGAAATC
TCTCACTGACAACGCTCCGGGGGACGTACTCGGAATGTGATACGCAAAGAGGCTCGAGGAGCAGATTGGCGGCTTGGTACCAAA
CTGGTCGGAAGCAGAGGGTGGGGGGGGCCTGTCGAGCTTCTGAAGAGGAGACCGCGGGCCATGTATCCCGGAGATTGCGGAGAC
ACCACCCGGAAAACTTTAAGATGGCCTCTATTGAGTTCATATGAGGGAAGATACGGACCCGGCCATCTTTTCGTTTCATATCCT
TTGCTAAGTAAAACAAGATGATGTCAGGTAGCAGCGCTCGTCTAGTTGAGGACGCCAAGTTGCATGTGCTTATCACTCTCTACC
CTTTTTTCCAAGTCGCGGCGGAGGACTGGTGGAAGGCTAGCTTCGAGGATCGCATTACACAGCTGGAGGAGCCGTACAACTCTG
CGTTCCGGAGGCAATTCATCGCCGCCCGGGAGCGCACGACATGGAGGTTGGTCTCCTTAACACATCAAGCAGGCTGCCCAACGA
GAGGCTTCAAGGCTTTCATTCAAAGGATGACTGGAGCTGCTGCCAGACGAAGGTCAAGGCGATGACATGAAGGCTGATCGCCTC
CAATCAGGGCTTACTGTCGGCTCTCTGCATTGTGGGAGAAATGCAAAGGAGACGGCAGAAACTCTGGGCGAATTCATGTCGAGG
GCTCAAAGGAATTATCAACCTTGAGGACGCTACCAGCAGCTTTGGAGTTCGCCCCAGGCTCCGACAGGCCTCCGCGTGACTATG
CCGAAGCTTATGCTTCGCAAGCTCGACCCTGCGCCAGCCCTCACGCTTCAGGAGCCCTGTTCGGGCTCGCTAGCGTGTCATTGG
GCCCTTCCGCGTCAGCTCAGACACGAGTCAAACCCACTTCTCCGGGTTCGGAGCGGTAACCGGATGTAGTATCGCTCGCGGGTC

AGTCGCACCGCAAGCGGAAGGCTCAAAGCAAACCGCGGGCAACGTCGTCGGAAGCACACTGAAGCAACTCGCGCAGCCGGGGAG
AACCATTCGAAGAAGCCCGCGGAAGAAGCTACGAGCCAGAAGTTCCGGAGTAAACCAGCCTTGATAGATCGAGGGGAATTCTAT
CGGTCGCTGACGCTGTTTGGTCAACTACAGGTAATGCCCCACTAAATACTCGCGGTAGGAGCTAGCGGCGCCGCGAGACTCAGC
AATAGGTGTCGAATTCCACGGAGACCTGGGCACACAACCAACGAGTGGCCTTCCAAGTTAAAGGACGAGATCGAAGTCCCTGGC
AAGGGCAGGGCATCTAGGACAATGGGTGAGATATCCCATTATTCTATCCGGAGAAGTGCTCAGAATGCATACGCTTCCGGGACA
GGGGGAGTGCTAGCGGATCTCTCACTGGACGCTCGTCACCCAAGCTTCCTGGACGCAGCTCCCGGCGGCTTTCAGCTCCAACTT
CTCCGCTCCATCGGCCATGCTGGCTCGGGACCGAAAAGCCATATCGCTACTGGTCTCGCCCCCGCCAACCTGTTGACGGCACGT
GGCCACCATTTCCGGAGGCCGCCCAGCCGGGAGCACCCCAACTCCAGCAAAAGCTGAGGAATTAGAACAACGGTGGAAGTGTGG
CTTGGTCGCAATCACACGCTCAACGTCCCGGCATGATGGACTTCATTCACCTTCAAGGAGGAGGACGCTCGTCATGAACTTCCC
CAAACGATTCCGCTCCGTGTAGAAGGCCGTCAGATCGCAACAGAAGGCTGTCAGGATCCTGGTCGATACGGAAGCTCGTGATAA
CTATCCTCTTCAAGCCGCTTCCAGCGACGTCGGGCTGACCGGAGAGATCTGACGCCGCCGTTCACCTTAGGGGTTCACATGATG
CACTCTGACTCTGGCAATCCAACTCAGTCCCTCCGGGGAGACACCGATGCTTGCGCCTTCAATGCATTGCACGTTCGTGGGTAG
TCGATGTCCCCACGGCGTTATATGGCATCCTTAGGCGACCGTCTGAGTCGACTTGTGCGCATAACCTCGATACGCCATCCTGTG
CTTGAGTTTCGTGCGACAATGGGCGTATCGGAGCTCTCCGGGAGAGCTCAAGCGAAGCGCGGTAAGCTGCTATATACGTGCTCA
CTCCGATCGTTTATACGCGTCC
>m141122_040740_42134_c100732202550000001823141405141561_s1_p0/107971/251_10129
TACCTTGTATCAAATGATTTTCTACATCTCGGACAGAGAGACCTAAAAAGCCTCTGGGAGAGCCCAAGGAAGAGATTATACTCA
AGAAGTTGTCAAAGAAAGGAAAAAGCGCCCAGGAGAGGACCAAGCCGCCTGAATTTGATATGTTGGGGTCACATCCTTCAATGC
TTTTTAGATAGGCTCTAGTTATCTCTACACTAACATCAAGATGCTAAGGAGATTTGGGAGGCCTTGAAAAGAAATACAAACGAA
AGAAAAAGGTACAAAGAAATTCATTATCACTCAATATACCTTGCACCAGTTTTAAGTTCATGATACGATAACCCTGTTTCCTCA
AAATTCTTGATTTCAAGTTATTGTGAATAAATTGTCTGCACTGCCCAAGTATCTTGTATCTACCGGAAGTAATTCATTGTCGGT
GCCATGAGAGCCAAGTTACCTACTTTTTGGAGAGGCTGATAGGAGAGAGAGATTCTCCATAGGATTGGAGATTTCATTGGAGGA
AAGTTCTGCCATACCAGTTGATAGGAATGTGAGGGTGAATCTCCATTCTAGTAGGATAAAGAATGGAAGAGTAAGTCCTAAATC
AATATATGTACGGTTGCCTGCCTGTCGCGCGCGACTTATAGATACATAGGTCTCGTGAATGAGCGCAGTGTCAGGGATTCTGCA
TGGTTTGTACGGCGTATATTCAGGCGCCATGGAAGTGACTGTTCTTACCGATCGTGGGTGTGTGTGTGAAGGTAGCCATTAATT
CACAAGAATGAATTAATCTATAATCATATATAAATTTGAGCCAATAACTATTTCAGTCCCAGAATTTAACACTTAGGAAACCAA
TATTCGATCCATTAGGATTTCGGAGCCAAGTCAATAGCCTCCGGGACCTGAAATTCTACTAAACTGGGTAGTAGGACATTTTCT
GAGCTTTAGGTTTGGGTTCCCACAACCCAAAAGGACAATTTTCCACTTTTCCCCATTTGAGCCACGAACGCTCAACTAGGCAGA
GAGCCCATGCCCAAAATCCCCTGCACACGCCGCCGCAAAGCGCTCAAGCAAACACTGAGGCGCTTACCTGCGAACCTAGAAAGA
CTTGCATTTTTTCAGAAATCACAAAGTCTCCAAAAACTGTCCCAAAGAGCACTGGTTTTAACCCAATCCGACCACGATAAATAG
TCTAAACAACCAACACAGGAAAGCAATAAACAGCAACCCAAAACCTCTAAAACACTCCCAAACCCATGAATTCTCCAAATAACC
AGATTTGAGCTTCAAAACTCCATTTTCTCTCCAATCCGAAAATCAAACTTTAACTCACAGATTTTCATCTAATTTTAGTGCACA
AATAAGTTGCTTATCCTATTTTTAACATCACATCTCTTAGAATAAACGCTTAGAACTCAAGAATTTAATAAAAAGACATAACCC
CAACCTCCCTCAATAACAATGGAAGAACACAAGAAGACTCATAAAATCCATACCTCACATGAAGAATTTCATGATCCCTAGAAC
CTTGGATGATCACTTAACAACTCCTAGCTTTAGAAAATCTCCCGTTTTTTTTTTTTTTTTTTTTTTTTTTTTTTTTTTTTTTTT
TTTTTTTTTTTTTTTTTTTTTTTTTTTTTTTTTTTTTTTTTTTTTTTTTTTTTTTCCTCAAGAGATCCCACTAACGTACAAGA
ATTGCACACTTGAAAGCTTAGAGTGTGCTTAAACCATTTGAAAGAAAAAAAGGAGAGAAAGGAGCTAGTGGTATACGTCGAATG
ACCAAACCCTTTACAAAGGGTTACCTTCTTACTCTAATTAATTGCGGTTCAAATGACAATTTGCCCCCTACTCCAAACTTGATC
CTCATAATCAAACTCTATGGCATTTTAGTCATTTGCCCAAAATTCCCGCTAACCTTAAATTTCACCACAAATTTCCCAATTAAA
TTCCATTAAACCCTAAAACACTTATCAAATTAATTCCCAATTACTCAATAAAATCCCAGTAAATTACCAAAAACCTTTAGCTCA
CCCCGAAGGCCGGGTATTAATCCCTATTGGACTTTTCTGGCTATTTTGCTGCAAAAGGAACGACTCCTGTCGGATTTTAAAATA
AACCCACATATAGATGTGGTCTTAATCATAAAACATATATTACAGAATATACCTCAATGGGTAAAAATTATGAACAATGCACAT
TTAAACAGTGTTTACGCGGTTCTTCGCCAACAGTAATTAGGAAAAGAAGAAGAAAGGGATTTAGTGCTTAGGTTGAACCGAATA
GAAATGATCTTACAAATGAAATGGTGACTCAAAATACGTTTTTTAAGTGGTTTCAAAGGTTAAAATCCTTCTACTCCACTAGTC
AAGTATTATTGCTATATAGTGGGTATTTGATTACGGGTCTTTCTTACAATATCGAATCCAACCCTTATAACTCCCAGGGTCTCC
ATATTTATAGAAGAAGGCACCTGAGAGTTGGTAAGAAGGTCATCCCGTGACCTTCTTACCTATCATGTCAACTCTGTGACATTC
ATGATTAATTCCTAAAACGCTGCACATAAGTGTGGTCAAATCAATAGTAAGGGGATAATGGGCGACGGCCAAACCAGTCGTGGG
TGTCTGAATACGCAACGTCTGCTCGTGTCCGAGAAGTCAGGGGCATATCAGACACGTGATGTCTGATAATATGCACGTTTTACC
TTGCGTGATTGACTTTATGAAAAGGTCACAAAGCCTTTCACTCCAGACTCGTACAACGAGCTGGATGCTTCCCTCGACCTGTGG
TCCTCAAGGGCCTAGGCTCAGTCCTTAAGAAATCTTGGCGACCCTTAGGTTCCTGCGAGCTAAAGGAGATAGACCTCACGATGG
CAGCTCGTCTTGGGGATGTTCACGTGCAGTCCTGATTAGTCGGATCCCAGCTCGCTAATCAGCCCGTGGAAAATCAGGGCGTAC
ATCTGCCCCCCAAAGCCCCTGCTCAGTGTGCACGACGTCGTACAGGGCCAAAGATGGGGCTTTTAGGCTTCCCCAAAGAATCTT
CGTATCCCACAATCTGCGCAGGAAGACGAATACTGGAACATCGTGGTTGTGACGGTTACGTCCTTCTGAGAACCGCATTTATGG
CCTAGGCCTATTGCCCAGCCGTCGTTTCATCTTTCGAGTGGAGGGCTTGATCCCACATCAAGGGCAATCAACGTCCTCCCCACG
TGGCCCTTCATGTATATAAAAGGGGTGGCCACCTTACGCCTGGCCACCTGTTTATTTGAAATTTTTTCTAGAATTTTCTCTTCT
TCTTGCTCTCAAAAATCCCCATTTTCCTCTTTCCAGTTACCACATTCGGCGTTCCAGAAACTCCGGTGCAAGGACTCCTTCGAA
GCCTTGGATCAACTACTCCGACGAAGCCCCAACCCAAGCGGATACTTCAAGCCACTCTTCAGCAAACACTCTGTAAGTCTCTGA
CTGTGCATTTGTCCTTTGAAATTATTTTTCGCTGTAGCTTAGGTTAAAATAATTTACTGTAGCCGCATGCAAGGGTTGCTGGTT

TTTTGTGGGAATGAGGGTAAAGCTTTGTAGGTTAGGATATTTTTGGTAAAAGTAAAGAAAAAAACCCCCCTTGTTTTTTTTTTT
TTAAGGGAGCCCATTTGGGGTTTTTTACCAGGAATGCCTTTTTTTTTTTTTCTGGGGAAAAATTTTTTTAGGGGGGTTAAAA
GGAAATTTTTTTTTTTTTTTTTTTTTTGGGGATTATTTTTTAAAAGTTTCAAAAAAAATTTAAATTCCAAGTTTTTTAAAAAA
AATTTTTGGGGTGGGCAAAAAAAACGGGGGGTTTTTTTAAAAAGCTTTTTTTAAAAAGGGGGGGGAACCACGCCCCCTTTTCCA
AAGAAAAACAAAGGCGGTTTTTTTTTTTTTTTTTGCCCCTTTTTTTCTTGGCCCCTTTTATTTGGGAAACCCTTTTTTTTTTCC
CCCTTTCCCTTAAAAGGGGCCAAAAAAAAATTTCCCTTAACCTGAACCCCCCTCCCCCCTTTGAAACAAAAAACAAAAAAACTA
AATTTTCCGGAGTAAAATCGGGGATTCCCTTTTTTTTGGTTTGGGAAGCAAACAGGGCGGGCCCGTGGGGGGTTTCCAAATAA
AGAACCGCGTGGGTCCCTCCCCACTTCCTCCCCCACCGAAGCCTGGGGGCCTTTTACCCTTCCAGCCCCCCTTTTTCGTTTTT
GGGTCCCCCCCCAAAAGGAAAAAAACCCCAGAAAAAAAAATTTTTTTTTTTTTTTTTTTCCTTTTTTCCCCATTTTTTAAAG
TTTTTCCCGCTTTTTCTTTTTTCTTTAAAATTTTTAATTTCCCCTTTTTTTTTTTTTTTTTTGGTTTTCCCCCCGCTTAGGCCC
GAAACCCCACCAGAATTTTGGGACCCCCCAAAGGAAAAAAAAAGCCCCCCCCCTTTGTTTTTAAAGGCAAGCTTTCGTCCTCC
CCTCACTCCCCCCCGACAAAAAAAAAGAACAAAAAGGAAAAGGGGGGAAAAAAGGTTTTGGAATTTGGCCCCGATTTTTTCCCC
CCCCGAATCCGCCCAAAAAAAAATTTTTTTCCCGGGCCCAAAGCCCAAAAAAAAGTTTGGGAAAAAAAACCAGGGAGGCCTTTGAT
TCCGAAGGGTGGGGCTTTCCTTTTTCCCGAACGGTTTTTTTCCTTTTTTTTTTTTTTTTTTTTTTTGAGGGCGGAGGAAGG
AATTTCCCGGATTTTTTTTTTTTTTTTCCCAAAAAAAAAAAGAATCAACCTTTTGAAAAAACCCAAAGGCGAGGGGTCAACAAA
AAATAAATTTTTTTCCCCCTCCCTCAAACCCCCCCAAAATAACAAAATCGGGCCCCTTTTTGGGAAGAGGAAAAAAATTTCCCCC
CCGTGGAATTTTGCCGAACCCCCCTCCTTTTTCCCGGCAAAAAGGAAAGAGGCGAGGCCAGAGCTTTTTTCCCGGTGGGGCTGC
CCAAACTTTTTTGGGGAACGAGGGGGCCCAAAAAAAATTTTTTCCCTCGGGGCCCCCCCCTGGGAAGCAAACGGAGCCCCAAAA
AAATTTTTAAAGGCCCCCCGGGGGGCCCCTTTTTTTCCCCCCTCCCAATTTTTGGGATTTTTCAAAAAGTATATCTTTTTTTG
CTTTTTTTTGACTTTCCCCCCCCCCTTCAAATTTTTAAAAATTGTTGAAAAGGGTTTTTGGGGCCCCCCAAAATTTCCCCAGCC
CTTTTTTTCCCCCCCCCCCCCAAAAACCCTTTTCTTTTTTTTTAAAATCCGCTTTTTTTGTTTTTTCTTTGGCGGGGTTTTGAA
TGAAAAATTTTTACTTTGGTTCCCCCCCTTTTGAAAGGCCAAAAAAAAAGGAGGTTGGGGAAAGTCCCAAAAACTTTTCCCTTGG
CGGGACCCAAATTTCCCCCCTTTTTTTAATTTTTTTTCTTTCCCCTTGCCTTTCAAAAAAAGGCCCCCCCCCAAAGCCCCCCCG
GGAAGCCCAGCGGGGGGCCAACCCCCGAGGCCGACGGGTTTTGTTTTTTAAAATTTTATTTAAAACCCGGTTTTTTCCAACCAA
AACGGCCCTTTTTTTTTGGGCCGGTCCCCAAATCGGGAGGCTTTGCCAAAAAAAAAACCCCAACCCCCCCCCAAAAAATGACTT
TTTCAAAAGATTTTTCAAAAAATTTCCTTTTTTTTTAAAATGTTCGGACCCAGGGGGTTTTTTTTTTTTTTTTTTTTTTCAGA
AACTTGCCCCCGAGCCTTTCCCCAACCTTTAACTTTTTCAATTCCGGTTCCCCTCGCTAAGTGCTTTTTTTTTTTCAAAAAACA
TTTTTTTTTAAAGGCCTTTTTTTTTTTTTTTCCCGTAAGTTTTTAAAAGTACCAAAAAAATTTTTAAAGCCTCCCCTCCTTTTT
TTTTCGTAAATTTTCCCCCCTTTTTTTTTTTTTTCTTGACTTTTTAAGAAAATTAAAAATTTTTTTTCCCCTTGCAGCTTTTTAA
ATTCCCCTTTTTTCCCCCCGCCGAGGGGGATAGGAAGGAAAATTTCTTTTTTTTTTGTTTGAACCCTTCGGGGCCCCAGGTTAA
AAAAAAAAAAACGGAGAAAAAAACACTGGCCGGGGGGGCTTTTTTTTTTTTTTTTTTTTTTTTTTTTTTTTTTTTTTTTTTGCC
CCCCCAAAAAAAAAGTGAAAAAAAGGAAAAAAATTTAAAACCCCGGTGGGTTTTTTTGGGGCTTTTTTCCCATGGAACAGAA
ATTTGAAGACCGATGGGGTGGGCCCCCCCCCTTGTTTAAAGCCCCCCCTTTGGAAAAATTCTTTTTTTTTTTTCCCCCAAATC
CAAAGACCCTTCCCTGAAACCTTTTTAAAAGGGAGGGCCTTTTTTTTTTTCCCCCCAGGCCCCCCCGCCTTTCCCCCCCCGCTT
TTTTTTTTTTTTTTTTCCCCCCCCCAGTTTGGGAGGAACCACCAAAAAAGGGCAATTTATTCCAATTTTGCCCCAAAAAAAAAA
AAAAAAGAGGAAAAAGGGTCCCCCCCCCCCCGGGGGTGGGGGGGGGGGGTTGGGGGGAAAGGAAGGAAAAAGGAAGGGAAAAA
AACCCCAGAAAAAGGAAGGGAAAAAAACGAAAAGGGGTTTTCCCCCCCCAACCCTTGGTTGGAAAAAAAAAGGGAAAAAACAAAA
AGGGAAAAAAAGCCGGGGGGACGTTTTTTTTTTTTTTGGGGGGGAAAAGGTTTTTCACCCCCCCCAGAAGGGGAAAAACCCGGG
GACCGAAGGGGAAGGAGGGGAAAATTCCGGGTTTTTTTCCCCGGGAAAAAGCCAAGCCCCCCCGTTGGGGATTTTTCCCCCCC
CCCTTATTTTTTTTTCTTTTGGCCAACCCAAAAGGGTTTAAAAAAAAAAACCCCCTTTTACCATGTTTTTAAAGGAAATTTTAG
AAAATTTAAGGGCCATTTGGCCCCCGAAACCCCCCCCCCGGCTTTTAGCTTTTAAGGTTTTTTTTTTTTTTTTTTTTTTTTCAA
AAATTCCCCCCAAAAAGACAAAAAAAAATCCCCCCGTCCCAAAAACGTCCCCCACCCCGGGAAAGGTTGGATTCCCCCACCTTG
GATTAACAAACCCCCTTGCTTTTTTTCACTTTTTTTTTTTTGTTGGGTTATCCCCCGACCCAGCCCCCCCTCCGTTTTCCCCCC
TGGGATTTTAAAACCAAAAAAAGGATAAAGCCCCGCCCATTAAGGGGGGGTAAACCGTAGTCCCCGTAAAAGATAAAACCAACC
CCCTTAAGCCCCCCCTTTTTTAGGTCCCGATTCCCCCCCTAATTTCGAAGGAAAAAAAGTTTTTTAAAAAATTTGGGAAAAACC
CCAACCTTTTCCCCCGGGTTTTTTTTATTTGAACCCATTCCCCCCCAAAAACTTTATTTTTCCCGGAAAGGGGGGGTGGTCCCC
GTAAAAGCCTTCCGGGAAAACCCCTTTTTTTTTTAAAAGGCCCCAATAATTGTTATGAAGAATCCCTTAAAGCCCCCCGAAAAA
AACCCAGGTGAGGGGGGTCGGAAAAAAAAAAATCCCCCCAGAAAAAAAAACCCCCTTTTGGGGCCGCAAGCTTTACCGGGGAAA
AAAAATAATCAGAACTTTTTTTTTTAAGGAATTCCCCTTTTTTCCCCCCCCCGCCCAAAGGGAAAAAGCTCCCGGGGGGGGCCC
CCCCCCCCCGAAATGGGGGGGTTTTTTTTTGGTAAACCATTAAAAGGGTTTTTTCCTTTTTTTTTTCCCTTTCCTAAGCCCCCC
TTTTCGGGGGGGGGTTAAAAAGGAAAAATCCCCCCTTTTTTTTAAGTATAATATTTATAACTTGGAAAATTTTTTTTTTTTCTCC
CCCTTTTTTTTTCCCCCCCCTTTGTTTTTTTTTTTTGTTTTTTTTTTTGTTGAAATGGGCCCCTTTTTTTTTGTTTTAAAACTTTT
TTTTTTGGGTTACTAACCAAAAAAATAAAAATTTCTTTTTGGGTTTTTTTTGGAAACCAAAGAGGGGAAAAGGAAAATGTTTTT
CCCCCCTTTTTTCCAAAAGCCCGGGGGGGGAGTCCCTGGCGAGGCCCCCCTGGCGTTTTTTTTTTTTTCCCCCGTCCCCGGGGG
GGGGAAAAAAAAAAATTCCCCCCCAAAAACCCGGGGGGGGTTCCCCTCCTTGGCCCCCCCAAAAAAAAATGGGGAAGAAAAGCTT
TTCCCCCGGGAAAAATTCCCCCAAAGAAAAAAAAAAAAACCTTTTGCCGAGAAAACCCCCCCAACCCCAAAGGTCCTCCCCCCCC
CCGCCCAAAAAGGAGAAAAAAGGAGCAAGCCCCCCCAAAAAGGCCAAACCCCCCCGCCCCCCCCCCAGGTCCCGTGGGGGGAAA
CTGTTTCCCCCCCCCCCTTTTCCCCCCTGCTTTTGTAGGGGGGGGAAAAAGCTTTTTTTTTTGCAATTGCCCCCCCCACCCTTT

CCACCGGGCCCCCCTCCCGCCTTTTTTTTTTGGTTTTCCCCCCCCCCCCCAGGAAACAAAATAAGGGGGGGCGGGGAGCCCCCG
GGAAACCTTCCGGGGGAAAAAATTTTTTTTTTTCAAAAAAAACCTTCCCCCCGACGGTTTTGCCCCCAACATTCCCCCCACGG
TTTTTTTGGTCCCCTAGGGACCCTGGAAGGAAAGAATTTCCCCAGAAGGGCCCCCTTTTTTTTTTTTTTCCGGGGGGGAAAAA
CGGTTTTTTGGGGGGGTAAAAAAAATTGAAAAGGTCCCGGGGCCGAAAAAAACCTTAACGCCCGTCCAAAAGCACAACCATCCTT
TAAAACAAAGGTTTTCCCCCCCAACGAAAAAAGGGGAAGGAAGCTTTTTTCCAAAAGGGCCCAAAATTTTCCCCCGAAAAACAG
GAAGGCCCCCCCCCCGGGGTTTTGGGCCGTGGGGGGGTTTTTTTTTTTTTTTTTGGAGGTTCCGTTTTTCCCCCCCAAAAATGGG
CCCCCCCCCCCCCCCAATGGGGCCCCCCCCCCTAAAAAAACCCCCCGGGTTTTAAAAAAGCTTTTTAACCCCCTTCAAAACCT
TTTTTTTTTTTAGAAATGGTTTTTTTTTTTTGGGGATTATCAAATGCCTTTTTTTTGCCCCCTGTTTTTTTTTTTTTTTTTTT
TTTTTTTTTTTTTTTTTTCTTTTCCTTAAGGCAAAAAAAAATGGGGGTTGGCCCCCATTTCCTTTTTTTTTTCCAAAAATTTT
TTCCCCCGCAGAGCGGCCCAAATGTGCCCCTTTTTCCATTCGGAAAAGCCAAAAAAAAAAAAAATAGGCCCCAGGGGAAAAACC
CCCCCAAGCCCCCCCTTTTCCCCCAAAGCTTTTCAACCCCGAGGAAAAAGAAAAAAATGAGGAAAAGCCGGAAAGGCAAAAAAC
CCCACCCCAAAAAGGCCGGTTTTTTGGTGGGTTTTCCCCCAACCCCAACCAAAAAAAACCCCTCCTTCCCCCCCCCTTTTGCCC
CAGGCCCGGCTTAAAAAGGGAATTGGCCCCCCCTTGGGGCGGGGCCCCGGCCCCCCCCAGACCGGGAAGCCCCCCCCCCTTTTT
TTGAAAAAGAAAGGGCCCCCCCGGGTTTTCCCCCCCCCCCCAAAAAAAAAGGGCTTGTAAAACCAAGAAGCTTTTCGTAGAAAA
CCCCCCACCAAAAGCCGCCTCCCCGCCCGCCCTTTTCGGCGGGGGGCCCCCCCCCCCCCGGAAAACCCTTTTTTAAGACCCCC
CCCCCCCAAAAAACCTTGTGGGAAAAAAAAAGGCCCGAGGGGCCCCCCCAAGGGCCCAGGCCCCCTGGAAGGCCCCCCCCCCGGA
AAAAAAGGCCAAAAGGCCCCCAAAAAAACCTTTTTTTTTTCGGGCCAAAAACCGGGCCCCAAAATGGGGGAAAGGGACAAAAAAA
TGGTTTTTTTTTTTCCCCCCCCCCCCCCCTTTTTACCCCAAATTGCTTGGG
>m141122_014521_42134_c100732202550000001823141405141560_s1_p0/64/1631_10830
TTGGATGGAGTATAGTGCTGAGAGAATGGTCTATGAGTTATCCTTTGAAGCAGTTTATTAAGAGTAGGTTATTGCCAAAGTTGT
TTCTGGTAAAGTGGGAGATAAGATCAACTGGTTATTATAGGTAGCAAGATAGACTTGTTTGAGCAGTAGTATTGGTTAGTAAC
ATTTTATGTTTGGCTTGATTATCTTTATCGTCCTAACGCTCCCTAATGGTTTTGTATAGGTATTGTATATTTAGTAATAATAAA
TTTGTAGTAATGTTATAGTAGGGGTGTTAGTAGTAGTCATCTGTGTGCGGATCATATTTTATCGATATAAGCTTTAATAGTTAT
AGTAGTGGGAGTGATGGTTAGAAGTATTTAGCATTAGTGGTATAATTAAATATTATAGATAATGTATTTATAGTAGTTATTTTG
TTCATTTTGGGTTATTAATGGCATGCGATTGTCTATGCTTGTGTTATAGGTATATGTATTGTTAGTGTGATGTCCTTAGCTACG
TACAGTTGGTCAGTTGGTATCATGTAATTGGAGCATCGGTAGTCTCGTCCAAGCCAACATCTAAGTTGTGGTCGCTCAAGCGTA
GTGGTTACCTTGTAGTAGTAAAGTAGTCCGATATTAAGTATAGATGTCGTTAGACTATGACCTGTCCACCCCAACCTGCATAGC
CATATTACCTGGCCATTTAGGGTAGTTCGTAGGGTGTCAACATGCCTACGGGCTGCGATAAGCTATATAGTTGATATGTTGTGG
ATCAAGTAGTGTCAGCGTAGGTACGAGCAAAAAGAGTACCCTTTAGGTTTTGATATATAATAGATAATATGGTCGATTAGTGGG
ATATCGTTTTTAATGTTGAAAGATATATATATGTATTTAGTTCTTATATAAGTTAGGTTAAGTAGTGTATACGAATCATAAGTG
AGTATATGGTAAGTTTGTGTGGGTCTTATGAGTTTGGTATAGTTTGTAGATGGTGTTGTGAGGGTGGTAAGTTGTGGTTTTATA
ATTGTAATTAGAATAGGCTGTTATGGGTTAGTAGTGGGTAGTGAATAGTAGTTGGTATGAAAGTTTTATTTAGATAAGTGTAAG
TAGTGTTTCAGATATATGTCGATAATTATGTAGATTTTTTATTTGATAATGGTTAGTATATTTTATAGTTTAGTGTATGTAAGT
AAAGTTTGGTATACTCCTACATCTATGGATAATGAAGATAGTAATTAACATTGTGATAAGATGGTTTAGTGAAATATATGGAGA
ATCATAAAGATATGTAATAGTGAATAGTGGTAATGGTAGGGTAAGTAGATTTAACTAGCGTTAAATATCAGGTTATCTATAAAT
GATTTAGTAGTATAGTCAAGAACGACACCAATAACGATTAGAATATGATAATGATGATACAAGCAATTAAGGAATGGTAATTAG
GTTAGTAAGCTGTAATAAGTGGTGTGGTAGTGGTAAGTAGTGGTGTAGTGTAGTGGTAGTGGTAGTGCTAGGGGTAAAGTGGTA
GTCGGACTATGACGGTAGTCTTAAGACGGATATATCTGTCTTACATACTGAATATAGTAAGTTTGGAAAAAATCTATAGTCCCA
AGGTGTTCCAGCCAAAATAATAAGTCATAAGTATGTTTAGTACTATTAATCTTGTGAAAGGATTTGGTATGATGATTCAGTTGA
TGTTCATCTTCATGTCGGTCATGCCGACTCTATAGTTGGTTCTTCTATCATATTGTAGGAATCGCTTTAGGAATTCTGGTGGAG
ATGTCTAGATGGTTAGTAGTTGAGATTCACTTACTCTCTTAGTTTCTCGATGTTTCAGGGTGGTAGAGTGGTAGTAGTATGGGT
TTTGTTAGGCTCTTATTTGGAGGTCTGTTAGATGATATTAGTGTTATGTCTCGTTGGTTCGCGTTACATAAATGGATGGCGAAA
CTCTGGACAGATTAAGTTAGGAGGACCTTATGTCGAAATTCGGATTAAGAGATGAGATAATATTTTATTTGCGTCTTGCTCTCT
ATTCTGTAGTGCCAGTTATCTATTACCTTTTTGGTCTATAGAATTTATGCTTTTCTTTCAATATGGTACCGGTATTTTCAGTAT
TAGTTATGCGACGATTAATAAGGGTTTTATTTGACTTGTGAGCCATTAGAGTTAGTTGGTACGTATAGGATCAAAATACGATCT
TGGACGAGTTGGAATATGACGTTGTGTTATGTCTTATGACAATATAATTGGATTTTGAGAGGAGTTCTGAAGTAGCTAATAAAT
TGATAGTCGATGTGACTGGATTACGATATTGCTATGGGTATGCTTTTTCTTATAAGACAAAATGAAAGTATTGTGTAAGTTCCG
AGAGACAAAAATACGATTGGTGAACATACAGGATAGTGCTGGAAGTATCTTTGTTTGCTTATAATAGATCGAGAAATAAGTTTG
AAATCTTAAAAGAAAAATATACATAGATAGTGTGATAATAGCATAATTGCTAGATGCGGTTATTTCTTTTCTATATCTTCAATA
ATTTGAATATGTGTAAATGTGAGTAGTCAATATCACATCGTTAAGTAGTAGTGTGTGGGTAGACATTGGTGATTTGAGATGTTA
GACCAACTCACGAGTACAAAGTCAACGGTGAAATTAGATAGGGATTAGGAATATACTTGGTTACGGTTAGATAGTAAAGTAGAA
GTAGTAGTAGTTCAGTTGCTTGTAGTTTGGCTGTCCGTCTGGTAGTGGTATGTGGTGTTCGGTAAAGTAGCAAGTAGAGGAGCC
AGTAGACCTCATATCTATGCAGCTAGACTAGGAAGTGCAGGCTGCAGTCACTTAATTGTGGTAGGATGGCTCTCGGATAAGGTG
CAAGAAGTGGGTGGTTATGTGTCGGAATGTGGTAACTGCTGACAATCAAATTTTAGTTTGTGCATATCGCTTACATGCACGCTA
GGTGAATAAGGATGGTGGTCACGACATAGGGCCTTACAAAGTTCCTGAGTACATTAAGATAGTGGTAGTAAAGTGGTAGTACCA
TTGGTAGTAGTGGTAGGACGAGAAGATGTTATCAACAATACTCTTCTAATGATATGTTTTACGTTAGAGATAGTAGCTAGTGTG
TTAGTATAGTGGTAGATGTAGTGTAGTGGTAGTGAGTAGTGGTAGAGTAGTGCAGTGGAAGTATCAGTGGTAGTGGTGAGTGGT
ATTGTGGTGGTGTGGGTGGTAGCGACTCGGGCTAGTGGTACTGGTAGTGGTAGGTCGTAGTAGTCGATAGTAGTAGTCGTAGTA

GTGGTAGTATAGTGTCAGAAGTATCCGCATGTTTCCAGCCGCAGATCCGAAGAGGTGTCCGGGCTTTTTTATGTTTTTGATAT
GTTCTATCAGGTTATTTAGTGATTAGTGCGTTGGTTATAAGAATCGGATAGTAGGATAGGACAAATGTGTCCGTGTCGTCCAA
GGTCAGGAATGAGATGGAGTCGGAGTATAGGTAGATGGTCATGTGGGTATGTCGAGGTCTTACAGATAGTAACGAGTATCAATG
ATTTATGCTGAGGTAATGTTTTGGCATATGGGTGTACTTAGGCATTAAGTGCAAGTATATGGTAAGGTTAAGTTCTAGCTTGCT
TGGTGAAAAGTTTTTATAGAAACGATAATATGAGTTGTCATATTGTTATCAACGGTCTTTGACACCGATATGATCGTCACGGCA
ATCGCGGACGGTCAACTGTGCGATGCGTTGCAAGCGCTAGCCTGAACCCAGTCTGGTGAAGAAGTCCGAAGGGATTTTCCCTAG
GAAGTATGGTTGGGTAGTGGTTTAGAAGTTTGTGGATATGGCCTTATGTGGAAATGGAGGAAATAGTTGTCCTATCGCCATGTG
GGGAGGGACAGGATTAAGGCTTGGGTAAGTGGTGCGTTTATATTTAGTAGTGGATCTTTCGACTGAATTGTGTTATCGTTGTAA
GTTGTAGCTGATTAGTGATCTGGTGGATATGTTGGGTCATGCATTGTGTTATGGGTCTACCTAGGATTCTATTATAGGTGATAT
AGTAGTTTAGACGTGTTGGATGCGGTCCCGGAAAATCGTTTGTTGCTGGATTCTTGGTGTGTGCAGGGTAGTAGTGTTATCTTC
CCAGCCTGCAATGATTATTATGCATGTAAAGGGATAAAAAGGTAGTATGAGATATAAGTAGTAAGTGTGTCTTAATATAGTCTT
ATGGTGCGATGGATAATAGTATAGCCAATAATAGGTCTATTATATTGTTCTAAACCAGTTTCGTGGTGGTAGTAGTAAGGTGCA
CCTTGGCCCATAAAGGAAATTAATAGTCAATTTAGTTGAGTCAGGTCGTATAAGACAATGTAGAGCATTAGGTAGAAAAGATGG
TGGTATAGGAATCTGTGCGATTAATTGGAGTATTGCAATCGAGATTTGTCATTGTGCAGGACCAGCGCGTCTATGGATAATAGA
GGATATAAGTCGGATCTGAGTATCATAGCATTAGACTTCATGTAATATATTTTAATGGGCGTAATTATAACGTGGATTAATAGG
AGGATAGATAAGAATTGAAAATGGATCTTTATTTGCACCTGTAAAAGCCCCTAGGAATATTAGGTCCACTAGTTGGGTTAGGTT
AGCGCGTGAGGTGCATTGAATTGTATACTTCGGAGCTAGCTAGTAGTAGTTAGTGGTAATAGACGTGATGTAAGATCGCTAGCC
ACGGTAGAGTGCTTAATGTATCTAGCTAGGCCTACCAGTCTTGTTAGGCTTCGGAGGAATCAAAGAGGAAGTTATTATTGCGAT
GTTGATTAGTTAAGGCTATACGGGTTCAGCAGGTTGCGGAATGATCATCCTGGTAGGGCGTGAGATAGGACTTCGATACGACGG
CGGGCGAAGTAGTTACAAGCTTGATTGGCACCTTGCTTACACATTGTTAATATGGAATTATACAAGCCACTGCAATGTAAAGAC
TGTGCATAGTAGGTTACGTAGAGTCACGTGTCTGATGCCATGCTGGGCCCGTGTGGTGATATGGTACGTCGAGACTACGGTGTA
GTGGTATGGTAGTGTAGTAGTGGTACGTAGTCAGTGTAGTAGGGTGATAGTATGTAGTAATAGTAGTAGTAGTAGGTTAGTGAT
AGTGGGTAGTGGTGGGGTAGTGTAGCCTGGAGTCGGCAGTGGCTGAGTGGTATAGATAGTAGTGTAGCTGGTAGTGTAGTCCAG
GTAGAAGTAGTAGTAGTAGTAGTAGTATTGGTACCGCTATAAACGATAGCGTTGTTCTAGGTGTAGCAAGTTCCTGGTAGGTAGGCT
TAGCTTGGAGGTCCAGTAGGTGTGACCCAGTGTATTCACACATATAGCACCGTGATGAGGTTGCAGGACACACTTCATATGTCA
GGGAGGTTTATCGCTATCACTGCACGAGTGAATGCGGATGCACCAGACACTGACGTGATGGAGGTAAAGGCAGCGTCGCAGATA
TGACCTGCGCGTTTGCCAACACCTAGGTTCCCTCTCCCTATAGTAAATATGTGGAGACCTGGTGGAGTTGCAGGAGGGTGGGTA
AGTTCTAATTGGTTTGTGAAAGAATATCACTTAAAGGGAATATTTAAAGAGTAAAGATAGTATGTAATTAGGTTTGTTCTGGGT
GGGATCTGATAAAGTAGTGAGTGTTGTTATACCTTTGAAGCCTCACCTAAAAAAGTATTCGAGTTATAATTTTACTTTTGAGAT
CCTAATTCATCCTATTACGGTCATTCAATTAGCCACATATAATAACACTTCTCCGTTTATTCTTATAATTTTAAGATGTGCTAA
GTTGCTGAAGATGAGGATCCAGCGTAGTTCGATACTGGAGTATGTGTGTGCATGTTATTTGAGGAGATTAGTTAAGATTTGGGG
TAGTCTAGTTCGACAGTAGTATAACCTAACCCTAAATGAGGTTAGGTATTGTTCACTCCGCTCCACTGACATGGTAAGGTAGCG
AGCGTAGGTACATAAGCGTGATGGGACAGGAGCCCGACCTAGTGCTTCATGGGTAGGCCATATGCTTCGATGATCAAGAAGCGG
CTTATGAGGTTCAGCAGCGGAGTCAGGGTAGTCAAGGTATGCAGCCAATTAGGCTAAGGAATGAACTTAGGAAGTTTATAGTGT
CCGGCTCCACCGGCTCAGCTGGCTAAGTCTGAACCAAAGGTTTTACACAGGCTTAGAGATCAAAGTGGGAAATGGTTGCTCAGT
TGTGTAGGATGGGCTCAGCTGGCAACAGCAAGACCAGCGGATGTTTAGCCATAGGTGAGGATTGTTAGGCGTCTACAGCCCTCT
CATATACAAGCGGTGACACGTTAACGTTCAAAAAGATGAGCTAGGCGAGACTCATGGAAGGGGCGCGTGCGAGTGGTAAGTTGC
AAATCGGTCAGGCTTGTGCTAGTATCTGGAGTGGTCCGGTGTTTTCAGAGTAGTAGTAGTGCGTATAGTAGGTACGATAGACCC
CAACATCAAATTCTACTCCCAGCGGTGTAGGCAATGGAGTACACACCGTGGACACAGTTGTGATACGCGGACGTTGTCCTATGG
GCGCGAGACAGTGGTATCCAGTACAGCCGATTCAGAAATCCAGGACCTCAATACCGCTAGGGCTTATCTTCCGCTCGGCTCTAG
TAGGAGCGCTGTAAGGTACCACAGAGTAAGAGGTCTCGAGGAAGACTTGCGAAGAGTGGATAGTTCAGTGTGGGGAGTGGTTAG
CAGTGGAACGACAGTCCACCCTCGACCAGCGAGTTCTGCACAGTCTCGAATAGCCAGATGCCGTAGCAACGTAGTAGATAGGGA
ACGTCAGAAGGGATGGTAAGTAGGGCGGAGCGGTAGTAGTGGCTAGTGGAACCGGTAGGGAAGTGTAACTTTGGATCGCGGCCC
TCATGACTGGGAGGCGTTAGGATACGGCATTCCCGTCTAAGTCGGGTGGTAGTCGTCCTCCTTCCCAATAAGATGGATCCTGTG
CGCGCTATTCCCCAGGCTCAGATACCAGGACGTAGTGGAGTGGTAGTGGTAGTGGTAGTGGTACGGTACTCCATGCTCCATGGG
AGCATGTAGGTGAGAATTCCATCCCTGTCAGCTTGTACGGATACCTTGCCCATCGTAGGCCTGTAGGGACAGCCCTAGAGTAGA
GATATCCCGAGAGTCCTCATCCCAGCGGCTGGGGTTATGGGCTTTCTTCGAAATTGGGTAAGCGTATGGTGTAGGTGGTGAGTG
GGATAGTGGTAGTAGTGGTAGTGGTAGTAAGTGGAGATAGTGTCATAGTAGTTGTATATGGTATAGTGAGTAGTGGTAGTGTTG
TGGTAGTGGTAGTGGTAGTTGTAGTAGATTAGGTGTAGTAAGTAGATGGTAGTAGTAGTGGGTAGTAGTAGTAAGTGGATTGGT
AGTGGTATGGTGTGGTAGTGATAGTTGTAGTAGGAAGTGTGGTAAGTGGTAGTGGTAGTATAGTAGTAAGTAGTTAGTAGTGGT
AGTAGTGTAGGGTTTTTTGTAGTGGTAGTGGTAAGGAAGTACCAGTAGCGGAGAAGAGCAGAAGAAACAAGGATAGTGGTAGTG
GTAATGGTAGTAGTTGGTAGTATAGTATGGGTAAGTAGTGGTAGTAGTATGGATAGTTGGTAGGTAGTGGATGTTGTAGTACAA
GTGGTAGTAGGGTGGTAGTAAAAGTAGTGTAGTAGGGGCTAGTAAGCTGGTAGTATCGGTATAGATATGGTGGTAGTAGTGTAG
TAGTAGGCGTAGTAGTGGTGATAGGGTAGTAGTGTGGTGTAGTTTGTCAAGTAGTAGTCAGTAAGTGGTGGAGATAGTAGTAGT
AGTAGAGTGGTAGTGATAGTGGGTGTGGTAGAGGTATGGTAGTGGTGGAAGTGGTGGTAGTTATAGTGGCAGTGAAGTGGTGAG
TGTATGGGTAAGTGGGTAGGATAGAGATAGTAGTGGTACGTAGTGGTACAGTAGCTATGGTAGATGGTAGTGTGTGTAGAAGTA
GGAAGTAGTATAGTAGGAAGAGAATAAGAAGGCAGAGAAGGGAAGAGAAGGAGCAGGAGTATGTGGTTGGTAGTGTAGTAGTGG
TAGTAGTGTGGTAGTAGTGGTGTAGTGGTATATTCAGGTAGTGGTTGTGGTATTATGTGGTAGTATAGTGACGTATGGTAGTGC

ATGGTGTGGTAGGGTAGTGGTAGTGGTTTTAGTAGTAGTGTAAGTGGTAGTGTGATAGTGAAAGTAGTGCAAGTAGGTGGTAGT
GGTAGTGGTAGTGGGAGTGGTGTGGTATAGTAGTAGTGGTAGTAGTAGTAGTAGTGGTAGTAGTAATATAGTATGTAGCTAGTA
GTATTGAGTAAGTGGAGTAGTAGTGTGGTGTGTGGTGTGGATGAGTGGAGTAGTGGTAGTAGGGTAGTGTAGTGTAGTCGGTAG
TGGTAGTGGTGTGTGTAGTGGTAGTTTGTAGTGGTAGAGTAGCAGAAGTAGGAAGAAGGCAGAAGCAGAAGACGAAGCAGAAGA
AGCGCAGCAGAGCGAGAAGGCGAGAAAGCAGCAGCAGCAGAGTAGGGTAGTGGTTAGTAGTAGTATGGTAGTATGGTAGTAGTA
GTGGTAGTGATGTAGTAGTGGTAGTAGCCTGCGACGTGGTAGTGCCTGGTGAAGTCAGCTGGTAGTAACTACTCGCGGGTGAGT
AGTGGTAGTGTGGTATAGTGGATAGTAGTGGAGTGTAGTGCTAGTATGGAGTAGTTAGATCGGTAGCTAGGTGTAGATAGTGGT
AGTTAGTGTGTAGTGGTAGTCAGTGGTAGTAGTATGGGTAGTAGTGGTAGTAGGTAGTTAGTTTGTAGTAGTGGAGTAGATATA
GTAGTGCAGTATTAGTAGCGTAGTATGTAAGTATAGTGTAGTATTTAGTGTAAGTAGTGGTATAGTGGTAGTGTGTAGTAGTAT
GGTAGTAGTGGTAGTATAGTACATAGTAGCGTAGTAGAGTATGTGGTATTGTAGTGGTAGAGTGCATAGTAGTGTAGTAGTGGA
GATAGTAGTAGTGTAGAGTGTAGTGTATAGTGGTGGTAGTAGTGTCGTAGTAGTGGTGTAGTGGTAGTAGTGTAGTGATAGTGG
TATAGTGGTAGTAGAGGTAGTAGTATTTGTAGTAGTGAAGTCTGTAGTGGTAGTGATATTAGTAGTAGTAGTGGTAGTAGATAG
TGAGTTAGTAGTGTAGTAGGTGGTAGTATGTAGTAGTAGGTGT
>m141122_014521_42134_c100732202550000001823141405141560_s1_p0/2097/439_10387
ACTGTGTTCAAGGCTCGTGCTATCGGTCAATATTTCTGATATCCATGTATAGATATAAGGAAAAAACTTGCGAAAATTGCCATC
GAAAAGGGGATTTATTATAGATGTGACTTAAGGCTCCGTTTCACTCAATATAACTTCATTTACCCGTTATGAGATCTGTTGGGG
AAAAATAAATCGAACGGTTTGAACACGGAGGGTAATACATTTCGGAGAAATTAGTAAATCCTCCAATTTCACCAAAGGGGGTAA
CACCTATACAGAAACTTTCGATTTCTCAAATAATGGATTCGAGCAATGTTTGATATGCGAATACCTAAAGAATAGCGAGGGTCA
TAGGGCAAAAGTTTGTAGCCCTATGGCTTAGGGTACTAATGTGTAAAGACTTACGTGCCAATCGAGGTTTGTAAATGGCTGATA
CAGTCAAAATACTGAGAGATGTACTAATATCAAGGAAAAAACTTGCGAATGGCCATCGAAAAGGGGAATTTTTATGGAAATGTC
GATTTAATGGCTTCCGTTTCTACTCCATATATTGCATTTAACCATGGAATCTGTTGGGGAAAAAAATATCAGAACGAGTTTTAAC
ACGAGGTAAGTACAATTTCGGAAGAAAATTAGTGAAACTCCTCCAATTCACCAAAAGGGGTACACCTTTTCAGAATCTTCATTC
TGTAAATATAGGATTCGAGCAGATGTTTTATAGCGAATACTAAAGAATATACGAGGGTCAATGGCACAAAAGTGTAAGCCCTAT
GTCTTAGGTTCTATGTAATAAAAATTTCGTGCATCAGAGGTTTGATCTAGGTGATAGCAGTCCAAAATACTGAGAGATGTACTA
ATATTTAGAAAACTTTGCGAAATTGCCATCGAAAAAGGGTGGAATTTATTTGAAACGTCATTTAAGGCTCCGTTTCTAACTCCA
ATAGTATTACATTTAACCATGAGATCTGTATGGAGGAAAAATATACGACGAGTTGAACACGAGGGGTAATATACATTATCGGAG
AAATTATGAAATCTCCATTCACCAAAGGGTACCCCTTTCAGTAAATCTTCGATTTCTGTAATATGGATTCGAGCAAGTGTTTTG
ATAGCGAATACTAAAGAATATGGAGAGATCATGGGCACAAAAAGTTGTAGCCCTATGTCTTAGGGTTCTATGTATAAAGAATTA
GTGCAAATCCGAGGTATATGTAATAGGCTGATACAGTCAAAATACTGGAGAGATGTACTAATATAAGGAAAAAACTTGCGAAAA
TTGCATCCGAAAGGGGAATTTTTATTTGAAATGTTCGACTTAAGCTCCGTTTCTACTCCAATATATTCATTTTACCCATGAGAT
CTGTTGGGGAAAAAATATCGAACGACGTTAACACGAGGGTAATGACATTTCGGAGAAATTAGTGAAATCTCATTCACCAAAGGGA
TACCCCTTATCAGAAATCTTGATTTCTCTAATATGGATTCGAGCAAAAATGTTTTTGATAGCGAAATACTAAGAATAATGGCG
AAGGGTCATGGGCCACAAGTTTAGCCCTATGGCTTAGAGAGTACTATGGAAAACTTCGTGCAATCCGAGGTTTGTATAGGGCTG
ATACAGTCAAAATATGAGAGAATGTACTAATATAAGGAAAAACTTGCGAAAATGCCATCGAAAAGATTATTAATTGAAATGTCG
ATTTAAGGCTCAGTTTCTACTCCAATATTCAGTCATGTTAAATGAGAATCTGTGGAATGATCGACGATTATTCGAGGTAATACA
TATTACGGATTAGTAAAAATCTCCAATCAGGGTACCCCTGTTCAGAATCTTCGATTTCTGTAATGTGTCGGCAATGTTTGTAGC
GAAACTAAAGATATAGCGAGGGTCATGGGCCAAATTGTAGCATATGCTTAGGGTTATAGTGTATAAAATTTCGTGCCAGTCCGA
GGTTTGTCTAGGGTGATACAGTCAAACTAATGAGAGATGTAACTAATATTAAGGAAAAAAGCTTGCGAAATTGCCATCAAGGGG
AATTTTATTTGAATGTCGATTTAAGGCTCCGTTTCTACTCGCCATATATTCATTTAACCATGAGATCAGTTGGGGGAAAAAAAT
CATCGAACAGTTGAACACGAGGGATAATACATTTCGGAGAAATTAAGTGAGATCTCCAATTCACCAAAGGGGTACCCTTTCAGA
AATCTCGATTCTCTAATATGGATTCGAAGTAATGTTTTTTTGAATAGCGAATACTAAAGAATTGCTGTAGGGTCATGGGCACAA
AAGTTGTAGCCCTATGGCTTAGGGTACTATGTGTAACTTCGTGCAAATCCGAGGTTTGTATAGGCTGATACAAGTCAAATACGG
TGATGTACTTAAATATGAGGAAAAACTTGCGAAATTCATCGCAAAGGGGAAAATTTATTTTAAATTCGATTTAAGGCTCCGTTT
CTACTGCCAACTATATCATTTGTAAACATGGAGATCTGTTGAAAAAGTATCGAACGAGTTGAACACGAGGTATAACATTTTCGG
AGAAATTAGTGAAAGTCTCCAATTCACCAAAGCGGGACCCTTTCAGAAACTTTCGATTTCTCTAATATGGATTCGAGCATGTTT
TGATAGCGAATACTAAAGATATGCGAGGTCAGTGGCACAATGTAGCCCTATGGCTTAGGGTATATGTGGTAAAAACTTTCGTGC
AAATCCGAGGTTTGTATAAAGGCTGATACAAGTCAAAATACTGAGAGATGTACTAATGATAGGAAAAAACTTGCGAAAATTGCC
ATCCGAAAAGGGGAATTTTATTTGAAATGGTCGATTAAGGCTCCGTTTCTACATCAAGTATATTCAATTTTACCATGAGATCTG
TTTGGGGGAAAATAATCGAACGAGTTGAACACGAGGGTAATACATTTCGGAGAAATTAGTGAAATCTCCAATCCCAAAGGGGTA
ACACTTGTCAGAATCCTTTCGATTTCTTATATGGGATCGAGCAATGTTTGATAGCGAATCTAAAATAATGCGAGGGTCATGGGC
CACCAACGTTGTAGCCCTAATGTCTTAAGGGTCTATGTATATAAATCTTCGTGCAGTCCGAGTTTGTCTACGGGTGATACAGTC
AAAATAATGAGAGAATGTACTATAATTAGGAAAAAACTTGCGAAAATTGCCATCGAAAGGGGAATTTTATTTGAAAGCGATTTA
AGGCTCGTTTCTAACTCAATATATTCATTTAACCATGAGATCTGTTGGGGAAAAAATATCGAACGAGTTGAACACGAGGGTATAC
ATTTCGAGAAATTAGTGAAATCCCAATCCCAAAAGGGGTAAACCCTTTAAGAAATCTTCGATTTCTGAATATGGATTCGAGCAA
TGTTTGATGCGATAACTAAAGAATATGCGAGGGTCCATGGCACAAAAGTTTGTAGACCTATGTCTTAGGGTTCTTATGTATAAA
ACTTTCGTGCAAATCGAGGTTTGTATAGGCTGATACAGCAAAATACTGAGAGATGTACTAATATATAGAAAAAACTTGCGAAAT
TGCCATCGAAAAGGGGATAGTTTTTATTTGAAATGATCCGACTTAAGGCTCCGTTTCTACTCCAATATGATTTCATTTACCCAT
GAGATCATGTTGGGGAAAAAATATCGAACGAGTTGAACCGAGGTCAGTACATTTTCGGAGAAATTAGTGAATCTCCAATTCACAA

AAGGTTACCCTTTCAGAATCTTCGATTTCTCTAATAATGGATTCGAGCAATGTTTTGATAGCGAATACTTAAAGATATGCGAGG
GTCATGGGCACAAGATTGTAGCCCTATGGCTTAGGGTAACTCATGTGTATAAAATTCGTGCAAATCCGAGTTTTATACGGCTG
ATACAGTCAAGAATACTGAGAGATGTACTAATATAAGGAAAAAACTTGCGAAAATTGCCATCGAAAAAGGGAATTTATTTGAAA
TGTCGATTTAAGGCTCCGTTTCTACTCCAATATATTCATTTAACCCATGAGAATCTGTTGGGGAAAAATATAGAACGAGTTGAA
CCGAGGGTAAGTACATTTCGGAGAAATTAGTGAAATCTCCAATTCACAAAGGGGTAGCCCCTTTCAGAATCTCGATTTCTGTAA
TATGGATTTCATAGCAATGTTTTGATAGCGATACTAAAGAATACTGCGAGGGTCATGGAACAAAGTTGTAGCCCTATGTCTTAG
GGGTTTCTATGTATAAAATTTCGTGCAAGTCCGAGGTTTGGCTAGGGATGAACAGTAAAATACTGAGAGATGTACTAATATTAG
GAAAAACTTGCGCAAATTGCCATCGAAAAGGGATTTTATTGAAATGTCGATATAACGCTCGTTTCTACTCCAATATATTCATTT
AACCATGAGATCTGTTTGGGGAAAAATATCGAACGAGTTGAACACGAGGGTAATACATTTCGGAGAAATTAGTGAAATCTCCAA
TTCACCAAAGGGGTACCCTTTCAGATCGTCGATTTCTGTAATATGGATTCGAGCAATGGTTTTGATGAGCGAATACTAAAGAAT
AATGGCGGGTCATGGGCACAAAGTTGTAGCCCCTATGTCTTATAGGGTTCTATGTAAAAAACTTCGTGCACATCCGAAGGTTTG
TGTAGGGTGAATAAAGCTCAAAATACTGAGAGATGTATAAATAGGAAAAAACTTGCGAAAATTGCCATCGAAAAGGGGAATGTT
TTTTTGAATGCCGACTAAGGCTCCGGTTTCCTACTCCAATATATTCATTTACGATGGAGATATGTTGGGGAAAAATATCCGAAC
GAGTTGAACACGAGGTAATACATTTCGAGAAATGAGTGAAATTCTCCAATCACCAAAGGTACCCCTTTCAGAAATCTTCGTTTT
GTAATATGGATTTCGAGCAATGTTTGATAGCGAATACTAAGATTATGCGAGGGTCATGGGGCACAAAGTTTGTAGCCCTATGTC
TTTAGGTTCTATGTATAAATTTCGTGCAATCCGAGGTTTTGTATAGGTGATACAGTCAAAATTCTGAGAGATGTCTAATATAAG
GGAAACTTGCGAAATTGCCATCGAAAAGGGGATTTTTTTTAAGATGCGACTTAGGCTCCGTTTCTACTCAATATATTCATTTAC
GATGGATCTGTTGGGGAAAACGAACGAGTTTGAACCCGAGGGTAATACATTAGGAAGAAATGAGTGAAAAAATTCTCCCCAATT
CACCAAAGGGTACCCTTTCCAGAAATATTCGATTTCTGTAATATGGATTCGAGCAATGTTTTTGATAGCGAATACTAAAGAAAT
GCGAGGTCTGGGCAACAAAAGTTGTCCCTATGTTTAGGGTTCTATTGTATAAAATATTCGTGCAAGTCCCGAGTTTGTCTGGCG
ATACAGTCAAAATACTGAGAGAGTACTAATATTAGGAAAAACTTGCGAATAATTGCCAGTCGGAAAGGGGAATTTTATTGTGAA
ATGTCGATTTAAGGTCGCTTTCTACTCAATATAATTCATTTAACCATGAATCTGTTGGGAAAAATATGCGAACGAGTTGAACCG
AGGGTAGATCACATTTCGGCAGAAATTAGTGTAAATCTCCATTCACCAAAGGGGTACCTTTCAGAAATATCTTCGAATTTCTGA
TAATATGGAATTCGAGCAATCTGTTTGATAGCGAATACTAAAAGAATAATGCGAGGGTCATGGCACAAAGTTGTAAGCCCTAAT
GTCTTGGGTTCTATGTATAAATTTCGTGCAAGATCCGAGGTTTGTGTAGGGTATACAGTCAAATACTGAGAGATGTACCTAATA
TAAGGAAAAACATTGCGAAATTGCCATCGAAAGGGGAATTTTTTTGAAATCCGACTTAAGGCTTCGTTTCTACTCCAATAATTC
GTGTTTTATTTACGATGAGATCTGTTGGGGAAAAATATCGAACGAGTTGAACACGAGAGGTAAATACATTTCGCAGAAAATGAG
TGTAATCTCCAATTTCACAAAGGGGTACCACTTTCCAGAAATCTTCGATTTCTGTAATAATGGATCGAGAAAAATGTTTTGATA
GCGAATATAAGAGAGAGATATGCGAGGGTCAGGGCAACAAAAGTTGTAGCTAATGCTTACGGGTTCTATGTATAGAGAATTTCGTG
CAATATCCGAGGTTTGTATGGGATGATAAAGTCAAAATTCCTGAGAGATGTACTAATAAAGGGAATACTTGCGAAAATTGCATC
GAAAAGGGAATTTTTTTTAATGCCGACTTAAGGCTCCGATTTCTACTCGAATATATTCAATTTACCGATGAGATCTGTTGGGG
AAAAATATCGAACGAGTTGAACACGAGGATACATTTGAGAAATGAGTGAAATCTCAATTCACCAAAGGGTACCCCTTTTCAGAA
ATCTTCGATTTCTCTAATATGGATTGCGAGCAATGTTTTGATAGCGAATATAAAGAATATCGCGAGGGTCATGGGGCCCAGTTT
AGCACTATGTCTTATGGTTCTATGTAATAAAATTTGTGCAGTCGAGGTTATGTATGGTGATACAGTCAAATAACTGAGAGATGT
ACCAAAAAAAAATATTAAGGAGAAAAACTTGCGAAATTTGCCATCGAAAGGGGAATTTTTTATTGAAATGTCCGATTTAAGGCT
CCGTTTCTACTACCCAATATTACTTCATTTAAACATGAGAATCAATGTTGGGAAATATCGAACGAGTTGAACAGAGGGGTAATA
CATTTCGGAGAAATTAGTGAAATCTCCAATTCAAAAGGGTTACCCTTTCCGAAATCTTCGATTTCTGAATATGGATTCGAGCAA
TGTTTGTGATAGAGAATACTAAAGAATATGCGAGGGTCATGGGCACAAAAGTTGTAGCCTATGTCTTAGGGTTCTATGTATAAA
ATTTCGTGCAAATCCGAGGTTTGTGTAGAGGGTGATACATGCAAAATATGAGAGAGTACTAATATTAAGGAAAAAACTTGCGAA
AATTGCAATCGAAAAGGGGAATTTTTTTTGAAGTGCGACTTAAGCTCCGTTTCTACTACCAATCATATTACATTTACCGATGAG
ATCTGTTGGGGAAATATCGACACGAGTTGCACGAGGGTAATACTTTCGGAGAAATGAGTGAAATCTCAATTCACCAAAGGGGTA
ACCCTTTCAGAAATCTTCGATTTCTGTAATATGGATTTCGAGCAATGTTTTTGATAGCGAATACTAAGAATATGCGAGGGTCAT
GGCCAAAATTGTAGCCCTATGTCTTAGGGTTCTATGTAATAAGAAATTTCGTGCAAATCCGAGGTTTTGTATAGGGTGATACAT
CAAAATTCTGAGAGATGTACTAATATAAAGGGAAAAACTTGCGAAAATTGCCATCGAAAAGGGAATTTTTTTTGAAATGCCGCT
TAAGGTCCGTTTCTACTCAATATATTCATGTTACGATGAGATCTGTTGGGACGGAAAAATATCGAACGAGTTGAACACGAGGG
TGAATACATTTCGGAGAAATTAGTGAAATTCCCATTCACCAAAGGGGTACCCTTCAGAAATCTTCGATTTCTGTTAATATGGAT
TCGAGCAATGTTTTGATAGCGGAATACTAAAGAATATATGCGAGGGTCATGGGCACAAAAGTTGTAGCCCTATGTTTAGGGTTC
TATGTATTAAAATTTCGTGCAAATCCGAGGTTTGTGTAGGGTGATTACAGTCAAAATACTGAGAGATGTAAACGTAAATATAAG
GAAAAACTTGCGAAAATTGCCATCGAAAAGGGGATTTTTTTTGAAATGCCGACTTAACGCTCCGTTTCTTCTCCAATATATGTA
CATTTACCGATGAGACTGTTGGGAACAATATCGAACGAGGTTGAACACGAGGGTCAATACATTTCGGAGAAAATGAGTGAAATC
TCCAATTCCAACCAAAGGGTACCCTTTCAGAGAATCTTCGATTTCTGTAATATGGTATTGCGAGCAGTATGTTTTGATAGCGAA
TACTAAAGATGATGCGAGGTCATGGGCCAAAAGTTGTAGCCCTATGTCTGTAGGGTTCTATGTATAGAAATTTCGAGCAAATCC
TAGGTTTGTATAGGTGATACAGTTAAAATACTGAGAGATGTACTAATATAAGGGAAAAACTTGCGAAAATTGCCATCCGAAAAG
GGGAATTTTTTTTGAAATGCCGACTTAAGGCCTCCGTTTCTACTCGCAATATATTATTTAACATGCGATCTGTTGGGGAAAAA
TATTCGACGAGGTTGAACACGAGGGTAAACATTTCGGAGAAATTAGTGAAATCTCCAATTCACCAAAGGGGTATCCTTTCAGGA
AATCTTCGATTTCTTAATATGGATTCCGAGCAAATGTTTGATACGAATACTAAAGAATGATGCGAGGGTCATTGGGCACAAAA
TTGTCCAGCCCTGATGTCTTAGGGTTCTATGGTATAAAATTCGTGGCAAATCTGAGGTTGTATAGGCTGCATACAGTCAAAATA
TCTGAGAGATGTACTAATATAAGGAAAAAAATGAAAACTTGCGAAATTGCCATCGAAGAAGGGGAATTTTATTTTGAAATGTCG

GACTTAAGGCTCGTTTCTACCTACCAATATAATTTCATGTTACCCATGAGATCTGGTTGGGGAAAATATCGAACCGAGTTGAAC
ACGAGGGTATACATTTCGGAGAAATTAGTGAAATCTCCAATCACAAAGGGGTACCCTGTTCAGATCTTTCGATTTCTCTAGAAT
ATGGATTCGAGCAATGTTTTGATAGCGAATACTAAGAATATGCGAGGTCAGGGCACAAAAGTTTGTAGCCTATGGCTTAGGGTA
CTATGTTAAAACTTCGTGAAATCCGAGGTTTGTATAGGCTGATACAGTCAAAATACTGAGAGATGTTATAAATTATAAGGAAAA
AACTTGCGAAAATTGCCATCGAAAAGGGAATTTTATTTGAAATGGTCGATTTAAGGCTACGTTTCTACTCCAATATATTCTTAC
CATGAATTGTTGGGGAAATATCGACGAGTTGAACACGAGGGTAATACATTTCGGAGAAATTAGTGAAATCTCATTCACCAAAGG
GAAACCCCTTTCAGAAAATCTTGATTTCTGTAATATGGATATCGAGAAAGTTTTGGATCAAACGAATACTAAAGATCTATGCGA
GGGTCATGGCACAAAAGTTGTACCCTGATGTATTAGGCATTCTATGTATAAAATTTCGTGCAAAGTCAGAGGTCTTGTATAGGG
TGAAACGTCAAAATACTGAAGATTAACTAATATTAGGAAAAAACTTGCGAAATTGCCATAGAAAGGGGAAGTTTTATTTGAGAA
TGGCTCGATTTTAAGGCTCCGTTTCTACAATCATTTCATTTAAAACCCCATGAGATCTGTTGGGGAAAAATATCGAACCGAGT
TGACCACGAGGGTTAATACATTTCGGCAGAAATTAGGAGAATCTCCAATTCACCAAAGGGGTACACCTTTCAGAAATCTTGATT
TCCTGTAATATGATTCGAGAAATGGTTTTGTGAGCGATATACTAAAGAATATGGAGGGTAATGGGCACAAAGTTGTAGCCCTAT
GTCTTAAGGGTTCTATGTATTAAAATTTCGTGCAAATCCAAATCCGAGGATTTGTGGTAGGTGATTACAGATACAGTCAAAAAT
ACTGAGAGAATGTACTAATATAAGGAAAAAACTTGCGACAAATTGCCATCAGAAAAGGGGAATTATTTTTTGAAATGCCGACTT
AAAGGCCCGTTTCCTACTCCAATATATTCATTACCGATGAGACTGTTGTGTGTGGTTTGTTGTTGGTTGTTGTGTGGTATATTT
TGTAGTGTTGTTTGTTTTTTTGTTTGATGTGGTGTTGTCTTTTTTTATTTTTCCGTATTGGTTGTTATGTTTCGTTTAGTGTTG
TTTTTTTTTTGTTTTCTGTGTATTGTTTGTTGTGTT
>m141122_014521_42134_c100732202550000001823141405141560_s1_p0/6726/496_10105
CCCTATGTCGTTAGGGTTCTATGTATAAAATTTCGGTGCAAGATACGAGGTGTGATATAGAGGTGATACAGTCAAAATACTCAG
AGAGACCGTACGAATAATAAAGGAAAAAACTTGTGAAAATTGTCCCTAAAAATACAGTGGGATTTTCTTCTTTTAAAATGTTTC
ATTAAGCTCCGTTTTGACTTCCATATCATATTCATTTAGACATGAGATCTCTTTGGCAAAATATCGACGAGTTGAACACGAGGG
TATACAATTCGGAGAAATTCTGAAATCTCCAATCTCACCAAAGGGGTACCCTATCCAGAAATTATTATCGATGTCTCTAATATG
GATGAGCATGTGTATTCTAATAGGATAAACTAAATGGAATATGGTCGAGGGTTCATCGGCAAAGTTTGTACACAACCGTATGCT
CATTCAACGTTCAGTACTCTTGTACCTCTTTGGACCTCCTTCTTCGTGCATATCCGCCGGTGTTAGCTCCAAACCGGGTGAATA
CAGTCATACAATCACCTGAGAGACGCTACGATATAAGTGAATAAAACTTGAAAATGCCTCTCAAAAAGAGGATTTTTTTTTAAA
GTTTCATTAAGGCACGTTGTCGACTTCTCCAATATATACTTAATCTCCTCTTTAATAATCCTACATGAGACTCCTCGAATTGAG
GGAACCAACTACAATATCGAAACAGGAGTGGTGTAGAAACACGAGTTTCTATAATGCCATAAAGTTAACGGAGAAATTAGTGAA
ATCTCCAATTCAGAAGAAACATAGGGGGTCCCCTTTCAGACAAATCTCATTCGATGATCATGCTATATTAAATGGATTGTCGCA
ATGCAATGTGTTTCATACTGATCTATGACTATAGAACTTAAATGACGAGAGCTACTAATTCGAGGTACAATAATGTTGTATCCA
ACCATCGAAAAGTTCAAGTGGTTCTAAACTGATATAAAAACATTTTTGATTGAATAATCGAAGGTGGTGTACTTAGGGATTGAC
TAACAGATCAACAATATCTAAAGAGAGATCCCTGACGATCGAATATATATCGATAACCTAAATTTCGATGATAAAAATATTGCC
ATAAAAAAGAAGGTGGAAATTTATTTTATAAAATATATTTCTCATTATAGGACGTTCGTTATTCAGACTTCTCACATTATAATT
AAATCCTATTTTAAAAACTGATCGAGAGTCTCTTATGTGCATAAAGTACATAATATACGTAATAGAAGTTGAAACTGCAGTGGT
AATAACTATGAGGCCATAAGTCATCAATTTCAATTCAATTAAGTAAATTACGTGAAATCGTCCAAGGTTCACTCAATAGAGATG
GAGGTTAACCATTTTCAGATACTATTTCATTGCTCTACTTAATAACTGAGATTACGGAGGGCATTGTTTTCATAGTGAATACTA
AAGAATATGACGAGGTTCACTATGGGCACTTAAAAAAGACAATGTGCTAAACCTCTATAATCTTTAGGTATCTATGTAAAAAAG
ATAAGAATTATATTTTTATCTGTGCAAAATCGCGAAGGTGTGGTTTATATAGCAGGTTATAATACAGGTCAAAATACCCCGGCT
CGAGAAAAACGATAGCGACGATTCATAAACACAGGAACAAATAAACCTTGAGAAGCATGGTAAGAGTAAAGCGGGCAATTGTTT
TATATAAAGCTATTTGAATTAAGCTAGTTTCGACTAACGAATTTATATTTCATTTTAGCCAATGAGATCTTCGGGGGTCTGAGG
CCAAAAATATCGGAACGAGTTGAAAATCTGAGGTAATATATAATAATCCGGAGGCAAAATTATGAAATCTCCAAATTCACAATA
AGGGGTACCCTTTCAGAAATCTTGATGTCTCTGAATATGGGTTTCGAGCACTGTTTTCGATAGTGAATACTAAAGAATATGCAG
GGTCATGGGCACAAAAGTTGTACACTATGTTTTAGGGTTCTATGTATTAAAATTGTGAAATCCGAGTGTGTTAGGTGATATACA
GTCAATAGAATACTGAAGAGACGACGAATATAAGGAAAAACCTTGTGAAAATGTCCCTAAAAAGGGGGAATTTTTTTTTAAATT
GTTTCCATTAAGAGGCTACCGTTCGACTCAATATAATTCATTTAACCATGAGATCCTCTTGGTGATAAAATATCGAACGATCGT
TGACACGAAGGGTAATACCAATTAGGAGAAATTAGTGAAAATCTCCAATTCCCCAAAGGCGGTACCCCTTTCAACAGAATCCTT
TCGTGCTCCTAATATGGATTCGAGCAGACGTTTCCCCCTGTTATTTCAAAGTGATATCTACTAACCGATATGCGAGGTCATCGG
GCAATAAAGGCTGTACCCCTATGTCTTCAGGGTTCTACTGTCATAAAATTCGTGCAAATTCCGAGGTGATGTATAGGGTGTACC
AGTCCATGTATACTGAGCAGACGTACGCATATAAGGAAAAACTTTGCTGCAAAATTGCCCCCCTAAAAAAGGCGGAATTCTTTC
TAATGTTCTCATTAAGGCTCCAGTTATCGACTCCATATATTAATTTAGCCATGAGATCTCTTGGGCAAAAATATCGAACTGAGT
TGAACAAAGGGTACAATTCGGCCAGCTAAATTAGTGAAATCTCCAATTCACCACGAAAAGGGGTACCCCTATCAGAAATCTTCG
ATGTCCTCTATATGACGGCACCCTGTTTCATAGTGAATACTAACAGAATATGCGAGGGTATGGGCACAAAAGTTGTAACATCCC
TATAGTCTTAGGGTTCTAATGTATAAATTTCGTCGCAAAATCCGAGGTGTGTATCTACGGGTGATACAGTCAAAAACTGAGAGT
ACACGTACATATAGGAAAAAACTGTGTGAAAAATTGCCCTCAAAAAGAGGAATTTTTATTTAATTCTTAAGGTCCGTTTCGACT
ACCAATATATTATTTCATTTTCTTTACCCATGAGATCTCTTGGGGAATAATTATCGAACGAGTTGAACACGAGGGAATACAATT
CGAGACAAATAGTGAAATCTCCCAATTCACCAAAGGGGTACCACCTTTCAGAATTCTTCGATGTTCTAATAGTGGATTAGAGCA
ATTGTTTTCATGAGTGATACTAAAAGAATATGCGAGGGTCATGGGAAAAAGTTGTACACTATGTGCTTAGGGTTCTTATGTAAA
AATTCGTGCAAATTCCGAGTGTTGTATGAGGTGATAAGTCAAAATACTGGAGAGACGTAAGATATAAGGAAATAACTTGTGAAA
ATTGTGCCTAAAAAAGGGAATTTTTTTTTAAATGTTTCATTAAGGTCCGTTTTCGACTCCAATATATTCATTTAACCATGAAAT

CTCTTGTGGAAAATATCGAACGATGTTTGGAACACGAGGAAGTTAATAATTCGGTAGAAACTTAGTGAAATCTCCAATTCACCA
AAGGGGTAAACCCTTTCTATGAAATCTTCGATGTTTCTAATATGGATTCGGCACTGATTTTCATAGTGAATACTAAGAATATGC
GAGGGTCTGGCACAAAGTTGTAACCCTAAACCCTATGTCTTAGGGTTTATGTATAAATTTTCTTCGTGCAAATCCCGAGGTGTG
TCATAGGGTGATACAGTCAAAATACGGAGACCGTACGAATATAAGGACAAAAAACTTGTGACAATTGCTCAAATAGAGGAATTT
TTTTTTTTAAATGTTTCATTAAGCTCGTTTCACTCAATCATATTCTTTACATTACGCCAATGAGATCTCTTGGGGAATAATATC
TGAACGAGTGAAGCAACGAGGGTAATGACAATTCGAGAAATTAGTGAAAATCTCCATTCACCAAAGGGGATACCCCTTTCAGCA
GAAATCTTCGATGTCTCTAATTGGATTCGAGCACAGTTTTCATAGTGAATTAACTAAAGAATATGGCGAGGTCATGGGCACTAA
AGTTGTACACCTATTCTTAGGCGTTCATGTTATAAGAAATTTCTGGCAAATCCGATGGTGGTGGTATGGGTGATACATCAAAAT
ACTGAGAGAGGTACAATATAAGGAAGGGAAAACTTGTTGAAAATTGCCCATAAAAAAGGGGAATTTTTTTTTTTAATGTTTAAT
TAAGGCTCCGTTTTGAACCCAATATATCCATTTAAATCCATGGATCTCTTGTGGAAAAATATGAAGAGTTGAATCACGAGGGTA
TAAAAATTCGGAAATTATGTTGAAATCTCCAATTCACCAAAGGGGCTCTACACCTTTCAGAAATCTCGTTCCGTGTCTCTAATA
TGGATTCGAGCACACTGTCTTTCATAGTGAAACTACTCAAAGAATATGCCGCAGGGTCATGGGGCATACAAGCTTGTACACCCT
ATGTACTTTAGCGGCATTCATGTATACAAATTTACGATCGCCAACTCCGAAGCTGTCGTATAGGCGTGATCAGCTCACAAATAC
TCTCGAGAGAACGTCAACGAATATCACCACGCCGGAAAAATTGATGCAACATCGCCCTAAAAAGCAGGGGCATATATTTTCACT
TAATAATGACATTCGACATTAAGGCCTCGATTTCCTGTCGACTCAATATATTCATCTGCTACGCCCATGATGACTCTCTCTTGG
AGAAAAATATCGAACACGAGTTGACATAGGTAATAGATACATGATTTCGGAGAAATTAGTGAAATTCCAATCTCAAAAAGGGGT
ACAGCTTCTCCAGCACATCTTCGACCATTCTCATAATCATGACAGGGTTCGAAGCACATACTGTTTTTCATGAGTGAAATGACA
TAAAGAACTACTGCCGAGGTCAATGGCACAAAAGGTCTGATAACCCTATGTCCTTCAAAGGCTTCTAATGTACCAACATTTCGT
GCAAATCCGAGGTGTGTAATAGCGGTGATACGTCAAAAATACTAAGAGACGTAGGAATACTAAGAAGAAAAAACTTGTGAAATC
TGACCTAAAAAAGGCGGAAGATTTTTTTTTAAATGTTGAATTCAAAGCTCCGCTCTCGACTCAATATAATTGTATTTAGCATGA
GACATTCTTGGGGATAATATCCGAACCGAGTTGCAACACAGAGGGTAACCTAACCCATTCGGAAGAAATTTAGTACGAACATCT
ACAAAACTTCACCAAAGGCGAGTAACACATCCTCTTCAGCAAATCTATCAGATGTCTTCCTAATAACTAGGAATTTCCGAAGCA
CCTGTTTTCATAGTGAATACTAAACAGACATATGCGAGGGTACCCCCATGGGCCACCACAAAGGGTTGTACACACCACCTCATG
TCCTTAGGGTTCTATGTACTAAATTTCGTGCAAATCCGAGGTGTGATATAGGGTGAATCCCGTCAACAATCACTGAGAGACGTC
GAATATACAGGAAAAAAAAAAACAAATTGTGGAAATATCTACAAAAAAGGGGAATATCTCTTTTCATTAAAATGTTTCCATTACA
GGTCCGTTTACGACCCAACTATACTTCCCATTTAACCATGAGACATACTTGTGGAAAATATCGACACAAGATTGGAACCGAGGG
TAATAGATTCGGCAGAAATTATGAAATCCCAATATACCAAGGTACCCAACTTTCAGAAATCTTCGACCTGTCTCTAATATGGGA
TTCACAGCACTGTTTTCATAGTGAAAATACTAAAGATATGCGAGAGGCTCATGGGCATAAACCAGTTGTAACCCTACTGCTCTT
AGGCTTCATGTAATACAAATTTCCGTGCAATACGAGGTGTGTTATACCGGTGATAAGTCAAAATAACTGAGGACGTACCGAATA
TAACGGAAAAACTTGTGAAAATTGCCTCCTAAAAGGATTTTTTTTTAAATGCTTTCATTAAGAGCTCCGTTTCCGACTCCATA
TATTCATTTAAGACACCAACCATAGAGAATCATCTTGGGCAAAAATATCGAACGAGTTGACCAGAGGGTAATACAATTCGGAGA
AATTAGTGACAATCTCCAATTTAACCAAAGGGGTCACCCTTTCAAGAAATCTTGATGTCTCCTAATACCTATGGACTTCGAGCA
AATGTTATTATAGTAGAAATACTAAAGAAATATCGAGGTCACTGGGCCACAAAAGTTGTACACCGTATGTTTAGGGTTCTCTCT
GTAATAAAATTTCGTACGCAATCCAGAGATGTGTATAAGGGTGATACAGCAAAATACCTGAAGAAGGTACGAATATAGGAAACA
AACTTTTGTGAAAATTGCCTCAAAAAGAGGCATTCTTATTTTATTTTCTTAAATGCTTTCCATTAAAGCTCTGTTTCGACTCCA
AATCATATTCATTTACACCTGAAGAATCACTCTTGGGGACTAATACGAACCGACGTCTGACACCACGAGGGTAAATACATTCCG
GAGAAATTCAAGTGAAATCTCCAATTCACCAAAGGGTACCCCTTTCGCAAATATTCGACCTGGTATCTAATATGGATTCGAATG
CCTGTTTTCCATAGTGAATAATAAAAGAATATGCGCAGGGTCTCCGGGCACAAAAGTTGGACGTACACCTACATGGTACTTTAG
CCGTTCTATGTAAATCAAATTCTGTGAAAATCCAGAGGCTGTGTATCTCGGACACGTGTACCCCCCCAGCTCCGCAAAATACTG
AGAGCGTCACGATACTAATGGAAAAAACGTCATTGTGACAATTCAGCCCCTACAAAACAAGGGGCAATTTCTTTTTTTAAAATG
ATATTCATTAACGCGCTCGTATTCTGACTCCAATATTATATTACAAATTTACATCCATGAGATCCAATCTTGTGGAAAAATTAA
TCGAACGAGTTGAACACGTAGGGTATACAATTCGGCGAATTAGTGAAATCTCCAATTCCCCAAAGGGTACCCTTTAGAACATCT
TCGATGTCTCTTAATACTGGCTTCGAACAACGCACCTGTTTTCATAGTGAATAACAAGAGAACTTCGAGGGCTCACTAGGGCAT
AAAAGTATCAGTAAACTATCGTCTTAGGCGTTCATATGTCATACAAAGTTTCGTGGAAATACGACCAGGTGTGGGTAATAGGGC
TGATACAGTCACACAATACTAAGTAGATCACGTACCCGAAATATACAAGGAAAAAACATTTGGTGAAAATTGGCCTAAAAATAG
GGGAATTTTTTTTTAAATGGGTTTCATTAAGGTCCGTTTCGACTCCATCATATTACATATTAGCCATGAGATCTCTTGGGCAAA
AATATCGAACGTGTTGAACACGAGGGTAAATAAAATTCGAGCAATTAGTGAAAATCTCCAATTCAACCAAAGGGTACCCTATCA
GAAATCTTCCGATGTCATCTAATAATGGATTCGAGCACCTATGTTTCATAGTGAAATACTAAAAATAAATGCGAGGAGTTCGGG
TCATGGCACCAAAAAGTTCTAACACCATACATGTCATCTAGGGTTCTATGTACATAAAATTCCTCGTGCAAATCCGGACGTGAT
GAATCAGGGTGATCACAGTCCAAAATACTGAGAGAACGTACGAATAATTCAGGAAAAAAAACCCTTGTGAAATTGCCCTCCAAG
AAAGCAGGAATTTTTATTTTAAATGTTTCATTAAGCGCTCGTTCTGCTCCCAAATCATCATCATATTAACCCATGGAGACTCAT
TGGGGAAATAATTATCGAACGACTGTTGAACAAGACGGGTAATCAATTCCGGACCAGAAATAGTGAATTCCAATTACCAAAGGG
TACCACTTTTCAGAAAATCTTCGATGTCTCTAATATGGATTGAGCACTGTTTTCATAGTGAATACTTAAGGAATATGCAGGTCA
TGGCACAAAAGCATTGTAATCCTACTGTCTTCAGGGTTCTAATGTATAAATTTCGTGCAAATCCGAGGTGTCGTATACGGCGTG
ATACACAGACTCAAATACTGAGAGACGTACGAATATAAGGAAAAAATTGTGAAAATTGCCCTAAAAGGGGAATTTATTTTTAT
AAATGTGAATTAAGCGCTTCGACTCCAATTTATATTCTTTAGCCATAGATCCTTCTTGGGGATAATATCGGAACGAGTTCGAAC
ACTGAGGTATATACAATTCGGAGAAATTAGTGAAATCTCCAATTCACCAAAGGGGTACCCCTTTCAGAAATCTTCGATGTCTCT
AATATGGATTCGAGCAATGTTTTCATAGTGAATACTAAAAGACAATATGCGAGGGTCATGGGCACAGAAAGTTGTACACCTAAT

GTCTTATGGGTTTATGTATAAAATTTCGTGCAAATTCCGACGGTGTGTATAGGGTGATATCAGTCAAAATAATGAGAGACGTAC
GGAATATAAGGAAAACTTTTGTGAAACATTGACCCCTAAAATAAGGGGATTTTTTTTAAATGTTTCATTAAGGCTCCGTTTCG
ACTCCAATATATTCATTTACCTGAGTTATCTCTTGTGGGGAAAAATATCGAACGACGTTGAAACGAGGGTAATACAATTCGGAC
GAAATTAGTGAAATCTCCATTCACCAAAGGGGTACCCCTTTCAGAAATATTCCGATGTATCTATATGGATTCGAGCACTGTTTT
CATAGTGAATACTAAAGAATATGGCGAGGGTCTGGCCATAAAAGTGTAACCCTAATGTCTTATAGGGTTTATGTATGAAAATTA
TCGTGCAACCGAGGTTGTATAGGTGAACAGTCAAATACTGAGCGACGTTACGAATATAAGGAAAACTTGTGAAAATGCCTAAAA
AGGGGATTTTTTTATAAATGGTTTGAATTCAAAGGCTCCGTTTTCTCAGACTCCATATATTCATTTAGCATGAGATCTCTTGGG
GAAAAAAATATCGAACGAGTTGACATCGAGGGTAATATAATTCGGAGTAAATTAGTGAAATCTCCAATTCACTCAATAGGTGGTA
GCCCCTTTCAGAAATCTTCGATCTCTAATATGGGTTCGAGCCACTGTTTTCGTAGGAATACTAAGAATATGAGAGGGGTCATGG
GACTAAAAGTTGTAACCCCTATAGTCTTAGGGTTATATGTATAAAAATTTCGGCAACCGAGGTGTGTATAGGGTGATCACAGTC
AAAATATAAGAGACCGTAGGAATTAAGGAAAAAATTGTGAAATGCCCTAAAAAGAGGGGACAATTTTTTTTTAATGGGAATTAA
GGGTCCGCTTCGATCTCCAATATATTCATTTAGCCATGAGATCTCTTGGGGAATTAATATGACGAGTTGAACACGAGGGTAATA
AGATACATTCGGAGAAATTAGTGAATCTCCAAATTACCAAAAGGGGTACCCCTTTCAGAAATCTTCGATGTCTCTAATTAGGAT
TCGAGACGTGTTTCATAGTGAAGTACTAAAAGATATGCGAAGGGTCATGGGCACAAAAGTGTACACCTATGTCTTAAGGGTTTA
TTGTATAATTCGTTGCAAATCGAGGTGTGTATAGGTGTAGACAGTCAAAAATACTGAGAGACGTACGAATTAATGAAAAAAACT
TGTGAAAATTGCTCAAAAGAGGATATTATTTTTTTAATGTTTCATTAAGGCTACCGTTTCGACTCCAATGATATTCATTTACCA
TGAGAGTCTCCTTGGGGGAAATAATGATCGAAC
>m141122_014521_42134_c100732202550000001823141405141560_s1_p0/14837/12540_18269
TCGGTTGAACTGTAAGCCGTTGTTTCAGAACTGTGCTACCACTTAAACCTGGGTACAAATCTTGTCCCTGAATCTTCTCGTAGC
CCATAACACTCCCAAAGTGAGCAACTGTTTGGTCGCGAGTCTTAAAAAAAAAAAAAAAAAATGTGATCATGTTGGGCTTGGGCC
TATGGGCATTGGAAAATTAATATATGTTGTATGATATTCACATCCTTTAGTAGTTTATGGCTCCATTAGCGAGCAGATTATTGA
ATTACATCTCTTATTGGGTCAGGATGGTTTGATCCAGCCCCAAGCCACTCGGCACCAATTCCAAACTGCGCTATGAAATGAGGC
CCAGTTGATGCAAAAGTAGGGGGGGATCCCAGATTTTTCTTGTAGTATTAATCTGCTCAAACTTGCAGAAAACTCACATTTGCC
TTAAAAGCTCTCTAAGCTCTAATACAATTGATCGTGGACTAAGGCTCTGTAACCGCCCCAACCACTGTAAAACGACGAACCCTA
TTCTGCAATGCATTTTTCTTAAATATGGTTCCTTTGATGCTCTTTATCTTTTATAATTCTATTTGTTCCATAAAAAGACTCAGG
GAACATTTGGTTTGCTTCGTCATTGAGAGCTGAAGAAAGCTTTGAGTTCATCTTGAGATCTGCAACAATGGTGACACAAGGCAT
GCGGAATGTGGTGATCCTGCCTAACCAAACAACAGCCAGGCCAATGCTGTCACCATTGCCCAACATCAAAGTATTCCTTCAAGA
ACTAACCTGATGGTATACGCCTTTATTTAAGAGCCCCTCCTGGCCTGTTACAATTCCCATTTTAATGGTTTCGGTGTATGGAGG
CAAGGCACAATTGTCGCCTTCAGGGGTCCCCATCCCGATCATCGCAATCTGAGATCCGGGCAGTGATGGGCTTCGGAAGACAAC
CAAGCAAATGGAACGCAAGGCCCAATGGCCTAGTCAAATCCCGGTGGAGCATGTGCCTATTTGGGTCTTTGAGAGGGGTTAAAA
TCAAATGGACGAGGCAACAAAGACCTACAGTCCTATCCCGTTAGACATACTATTGCGTATCCGGGAGGTTTCCAAAGCCAAATG
GGACGCAAGGCACAAGCCCGTCCCATCGGCCGGACATGGAATAAGGTTCGGCAAATTTAAAACCAATGGAACGCAAGGCAACAA
AGCCCTTGTCCATCCCGAACACAAAATAGTCTGGAAGGGTTCAAACTAATGGGACGCAAGTAAAAAGCCAGTCCCATCCCGCGA
CATGAGTGGTTCCGGGAGTCTTCAAAAACCAAAAATGGGATGCAAGGCCAATGCATAGTCCCATCCCGGACCTGCTTGGTATGG
GGGGTTTAAAACCAATGGGACGCAAGGATAAAACCTAGTCCATCCCGGACTTGACATTTGGTTCTGAGAGGTTTCAAAACCAAA
TGGGCGCAAGGTACAAAGCCTTAGTCCCATCCCCGGACATAACTTACTTGGTTACTTCTAGACGAGGAGTTGAAACCAAACGGC
GCAAGGCAATGCCTAGTCCCAATCCCGGACATGGCTGGTGGTTAAGGGGTTATTGTAAAACCTATAAATGAGACGGGATGCAAG
GCAGAAAAGCTAGTCCATCCGGACATACCTAGTCTAGGGGAGAAGTTTCAAACAAAAATGGGACGCAGGCACAAAGCCCAGTTC
CATCCCAGCCATGAATGGTCCGAATATTTAAACCATGGAACGCAAGGCACAAACCCTGTCCCATCCTGAACACAAAATAGTCCG
GAGAGGGTGTTGGAAACCCAATGCGACGCAAGGCACATAAGCCCAGGTCGCTATCCCCTCATCGATGAGTGGCTTTCGTGTGAG
TTCAAAAACCAAATGGGATGCAGGCCCATGCCTAGTCCATCCCACCATTGGCGCTATGGTCTGGGGTTTACATAACCAAATTTG
AGAGACGCAAGGCACAAAGCCTAAGCCCATCCAGGACTAACAATGGTCCAAAGGTGTTTAAAACAAATGGGACGCAAGGTACAA
GCCTAGTCCACATACTGCACATAACATGGGTCCTAAGAGGTTCAACCCAAAATGGGACGCTCAAGGCCCAAATGCGCTATAGAT
CCCATCTTGGACATACTGGTCTGTGGAGGTTCAAAAAACCCAAATGGCGCAAGGCCCCAATAGCCTTTAGTCCATCCCGGACAT
GTAGTGGTCCGGGAGGTTCAAAAACCATAACAGATGGGAGATAGTGAACCGCGCGGCCATATGCTAGTCCTCCCAGGAACATAC
GCTTTGGTCTAGGGGAATTTAAAACCAAACGGGACGCAAGGAGAAAGCCTAGTCCCATCCCGGACATAACATGGTCTGGGAGGT
TTAAAACCAAATTGGTTTACGCAAGGACAAGCCAGTCCCATCCCGAAAAATGAGTGGTCCCGAGAGGTTCAAAAACCAAATGGG
ACTGGCAAGGCCAATACCTAGTCCCATCCCGGAATGCTTGGTCTAGGGGGTTTAAACAAAATGGGTTGCAAGGATAAGCGCTAG
TCCGATCCGGGACATAACATTGGTCCGGGAGGATTAAAACCAAAATAGGAACGCAAGGCACAAAGCCTAGAGTCCCATCCCGAA
TTATAACCATGATCCGGGGGTTCAAACCCAAAATGGGACGCAAGGCACAAAGCCTAGTCCCATGCCGGACATGCTTGTCTTGTG
GATTTAAACCAAATGGGACGCAAGGCAACAGAGGCCAGTTCCATCCCGGAAATAACATGGTCCGGGAGGTTTAAAACCAAATGG
GGACGCAAGGCAAAAAGCCTAGTCCCATCCCGGACATAAATGATCCGGGAAGGTTCAAACCCAAATGGAAAGCAAGGCACAAAG
CCCTGGTCCATCCTGAACATAAATGGTCTGGGAGGTTTAAAACCAATGCGGTCGCAAGCAGCAAAGCCCAGTCCATCATGGACA
AGTGGTCCGGTGAGGTTCAAAAACCAAATGGGACGAAGACAAAGCCAAGTCCCCATCCCAGACACAACATGGTACCGAGAGGTT
TGAAAACCAAATGGGGCGCAAGGCCAAAGCCTGGTCCCATTCCCGGACCATAACTGGTCTGGGAGGTTTCCAAACCCAACTGGG
ATGACAGAGGCACACAGTCCAGTCCCATCCGGACATGAATGGTCTTGGAGGTTTTAAAAGAAAATGGACGCAAGGCACAAATT
CCAGTCTCAGTCCCGACCAAAATGGTCCGGGAGGGTTCAAACCAAATGGGACGCAAGGCACAAGCCTAGTCCCATCCCGGCCAC
AAAATGGCTCAGGAGGGTTCAAAACCCAAGTGGGGCGCAAGGCACAAAGAGCCCGTCCCATCCCTGACTAAGTGGTTCGGGAGG

TTCAAAAACCAAATGGGATGCAAGACCCAATGCTAGTCTCATCTCAGCATGAGTGGTCCGGGAAGTTCAAAAACCAAAATGGGA
TGGCAAGGACCAATGCCTAGCTCCCATCCCAGACATGCTTGGTCTGGGGTTTAAAATAATGGACGCAAGGCATAAGCCTAGTC
GACCATCTCGGATTCATAAACATGGTCCGAGAGTTTCAAACCCAAATGGGACGCAAAGGCACAAAGCTAGTCCCGTCTCGAACA
TAAACATGGTCAGGAGGTTTAAACCAAAATGGGATGCAATGCACAAAGCCTGTCCCATCCCGTACATAACATTTTCTGGAGGAT
TGAAAACCAAATGGGACGCAAGGCCAAAGCCTAGTCCCATCCTGGGCATACGTGGTCCGGGAGGATTATAAAACCCAAATGGGA
CACAAAGGCACAAAGCCCAGTCCCATCCCGGACATGAATGATTCGGGAGGTCTAAACCCCAACTCCTAAATTTGCTCCCAGACC
TAGATGTGGTCTGGTCCAAATCCCTACTGCAGGTTGATATTGGTGGATTTGTCCGGAGAGTAAAGGGGTAAGTCGCTCGGCTTA
CCAATTGCAACACGGATGAAGACTTGGGGGTAAATGTGTATCCCTATTTCCCCTGGGACACGTGGATGAGACAGTGGGTACATG
GGTTCCTCAAGGGATCCAGGCCCATCCTTGCAGAGAAACTTGGAATAGGTAGGAGAGAGAAGAAAGTCTTGATTTAGCACTAAT
CGAAAATGAACCGGGACATTAATAATGCACCCGAACCAGCTCCTCTCCCGACTACCCATATCCTCTAAAGATACGAGATAAGAG
GTGGCAAGCTCACTAGTCTGGAATAGACCTTGTGGGTCAACTCGGGATTATCATCATAAGGGATCGTATGTTCGCATCCTGAGC
CCAATGAATAGGGAAAGGAGAAGTTTTGATAAGCACAATCAGATGAAAGATGAAACCAAGGCGACACTTAAGGTAAACAAACTA
GAAACCGGTAACTCAGAACATCCCTGTCCCGAACCTACCTTAATTGACAGGTATTAATCCGACCAGAACAAGAATGTCTAGATA
GCTACGAATGGGAAAGTTTGGTCTTTCCGCTGAAGCTGAAGGTCCCCGGACGAAGCTGTCTAGGGCGATGTGTGTCCCCATTAT
AAGTAACCTACAGATGGGTCTGCCTTCGGGATGTACAGCCGTTGGATTCATTAACTGTGGCCAGTTGCCACTACACTAGACAGA
TTTGTCCGGCTGAATCTTCCTTCATATTGCCCGAATCACCCACCCAGACTAGAAGAAACTGTTGGTACGCAGTTATATAATGTG
GTTCATTTTTAGGTGCTGCGCGCCCAATGGGACCTCGGTATAATATATGTTCCGATTGTATATTTCAATCCTTTGTATTCATAG
AGCTCCATTAAGTGAGCAAACTATGTTAAGATCTTCTTATATATGGTGGGCACCAGAGGAGAGAAGGTCAGGAAGTCGCAGACC
CAATTGCCATTGCACCAAAGACTTGCTACTAAACCTACATGATGGCCAAGTTCGTGCCACAGTAGGGAGAGGATCCTAGATTTT
TCTTAATAAGCAATTATCTGCATTCAATAATGCAGTTTTGTTGCAGAAAACTCCATTGTCAACAAGCTCTTTAAGCTCGTATAC
AACTGACTTGTTGGACTTAAGGCTCATTAACACCCCAACCACGTAAAACCTTGCATTGCATTTTCTAAATCCTTTCTTTTTTAA
GCCTATCTTATTATTCTTTTAATGATTCTAGTTTCTGAAAACTCGGTAAACATTTTGGTGCTTTCATTGAGAGCTGAAGAAAGT
TGAGTTGATATTCGAAACTCTACACCAGTAGAGATAGTAGATTTCCCACCAAGGCAGCAAAGCCTAGATCCCATCTCCCAGACA
TAACTTGGTCCGGGAGGTTCAACCAAATGGGAACGCAAGGCACTAAAGCTAAGATTCCGTCCTGGAACTACATGGTACTCTGGA
GGTTGTTTCGAAACTAAATGGCATCGCAGGCCAAAAAGCCCAGATACCCCTCTCCCGGGATCTATCCAGCATTACTAGGTCTGG
GAGGATTAGAAAACCAAGATGAGGACGCAAGGCCACAAAGCCTAGGTCCCACTCTCCCGAGACACACTTAAATGGTCCTGGAAG
GATTCAAACCCAAGAATGGGAACGCCAGTCCCAATTGCCTAGTTACCAGACCATTATTAATTATTTCTTTTTTTCATTATTTAG
AAGTATTTATTCCTTATGGATTTTTGAATTATCTGAGAATACTATTAAGAACAATTCAATAAGTTCGGAAAGGATAACAATGCC
AATTTATATATGATTCA
>m141122_014521_42134_c100732202550000001823141405141560_s1_p0/17374/8516_15487
ATATTGATGTTATTATTGATTATACTAATTACTGTGCATGCTATTTTAAATCCTCTATCAAATTAATAATGATGTATAGATCTT
TCTGGAATGCTGGCTTTAAAAATTTATTATTAATGAGGTTTAGATACAAATTTATCTTGGAATCTTAATTAAATCTTTTGGTCC
CTCTATATTTCATTTTTATCTAGAAATTGGACAGTTACTATTGTGGAAACTATCCAAAATTCACCTTGGATCAATCTTTGAATA
ATTTCTATTTGGGCGATGAATTAAAATCGAAGAGTTGTTTAGATACTTATCCTATGATTGTATCTTTTACATCCAATGTTAGGC
AGTGGGGGACCTGGACATGGGGCTCCTAATGAATGATTAAGCCTTAAACAATAAGATTGTCTTTATATTTTTATCAATATAAAT
TTAGTACTGTAGAGATTAATTCCTCTCTGTGGTTCCACTAAAGACTGCAGAATCGCTACTCCGCTTGATTGGCATGTAAAATGC
TCTACTACCTTGTTTGAATTCAATATAATTCTGCGTGAGTTTAGTTGTTCATACGGTTGGATATCTTAGTTAAAGCTTATTCCC
ATTTGTTATCTTACTTACTAATTTAGCTGTCGTCATCTCAATAATTGCGCCTCAATCCTCTTATAGAATCGGTCTAACAGAATG
AGATGGAGACTACAAAGACTATGCGTTTTAGCTCGCCCTCATATAGTTGTGATTTTTTTATTCGCTTTAGAACTACTAGTGTCA
CTTGGCTAGAATCGATATTATCACGTAAATCTCAATACTTTTGAATTCCTACTCAACGTAAATGCGTAAGATTTGTCTCATCAT
TTGGGTTACACTCCTTGAATCGAGTTAAGATAGTTTAATAATGCCCATCATCTTCCACCTTCTTCATCTTGATTCGAAGCTATC
GTGAGCTGCGGATCGCAATAGCTAGAGGGAGATGGCTCGTCTAGAAGTTCTAGAAATTATAGACCTAGTTGATTATCTCTTGAA
GATGCTCATAATATGTATTCTTCTTGCGTAGAAATTTTATTTAGTTTCCATGCTCTGGCAATTATATAACGATTGTTTATGACT
TTCACAAGTTTATATATCGTAGAGATCTTGCTTTTCCAAGTGGAGTCTTATGGACATTGATGTATTGCGAGATCAGCATGTAGT
GATTGATTGGTTGGAAGATTGGCTTATATATTTCTAGCGACGTTTAGAGAATGAATATTCCCTCCTCGCGCTCTTTGAAACAAG
TGCTATTTTTATTCAAAGTAGCTGAAATCCCTATGGCTATTCAATTGAACTTCATATAAGACGCGATAGACCGTTTTCTAACCT
ATCCCGCTCTTTACATCGGAATGTGGAGATACATATATGGTCACGCATTGGCTATGATGATAAGGATGATTCAAGACTATACAA
AGCGACCGGGCCCTCTTGAAGGAACTTCCCTGAGTGACGTGAATTTACCTGATCAGAGATTTTATTAGAAGTGTTAACAACTCC
AGCCAAGTTGTTCCACTCTTTACCAATGGTAAGATAGGGCCGAAAGAACCACTTGGACTTGTGCATTTCAGAATTTTTGTTGGG
CCATTGAATGTACAGACCAATGGGGAGTCCCCCCGTAGTAGCGGAGACGCCATTTTTAACCAGGCCGCGGCGGATCTAAGAAGC
AGGCAGTATGCGAGTAGAGAGAGAGAGTGAGCAGGAGCAAGGCAGAGATGTATATTATGCAGTATATAGGCCTAGTGAGCGATTTA
TAGTGAATCTATGAGAGTTTGCTAGGAGCACACAGTTGATTTTGTAGGGCTGTTATAGCAGGTATTCAGTACTCATATAGTGAT
TTTAGACATTGTTGTTTCAGTAGTAGTTAGATCTGCAATAGTGGACAGTTATAGAAGTTCAGATATGTCAGTAGCTGAATATAG
TAAGAGTTTTAGGCGAGGTTAGTAGGCAGTAGTAGTATAGCAGGCACATAGGTAGTAGCAGGCAAGTAGGCAAGAAGTAGTTAG
GGCAGTAAAGCAGGCAACGTAGAAAGTAGCCAGGAGAGATAGTATGTATTAGCAGTGTTTTAGCAGTAAGTCTTATGTGAAGTA
GGCATTATAGTATATGCTGAGTAGCAGTTAGGCTGTATTATGATAGTCAGGTGGCATAGGATAGCTGTTTAGGCAGTAAGCAGC
GGCCTAGCAGTCAGCAACAGTAAATGCTGGTTATTCAGCGTTCAGCAGTAGTAGTATAGCAGGATTATATATCGTTTCTAGGAG
GCCAGAAATTGCATCCAGATAGCAGTAGGGCAGTCTGCAGTAGTAGGAGTAGCAGTAGAAAGGCAGCATAAGCAGCAGTTAGGC

AAGTAGTTTTGGCGGGTCTTAGCAGTAGCATGGGTTTAGTTGTAGTAGCAGTTGAGCAGACCAGTACAACGTAGCTTTAGTCAG
TAGCAGTACGCAGATAAGCTAATAGAGGCAGCAACTAACAACAATGTGTGAGGCATGAGTAGAACAGTAGCAGAAGTAGGCAGT
TTAATGTGTCGTGGTAAGATGTAGGTAGCAGTAGCAGTAGCAGTTAGTAGTAGCAAGGTTGTAGCAGCTAGTAAAGTAGCAGTT
CAAGCCAGTCAGCAGTAACAGTTTAGTTAGCAGGTAGCAAGTTTAGTTGGAAGTAACAGTAGTAGTAGCGTAGAAGTAGCAGTC
AGCAGCAGCAGTAGCAACAACAGTAGCAGTAAGTAGTACGGTACTATAAGCAGTAGAAGCTAGCAGTAGCAACAGGCAGAAGCA
GCAGTAGCGAGGTTAAGGGCAGTATTAGCATTAGCAGGCAGTAGCAAGTAGTAAAGAAGTAGGGCGGTGGCTGTAGCAGAATGT
AGGTAGCAGTTCCACGGTTTTATCAGTTAGCGCGCGGTTCTAGCAGATATAGTAGACAATAGGAGTAGTAGGGCAGTAGCATAG
GAGCGCAGCAACAACAGCAAGGAGCAGGTAGCAGTAGCACCAGCAGTAGAAGTGGGTAGGCAGCAATAGCAGGAGCCAGTAGGC
AGGCAGCCAGGCAGAAGCGATTAGGCAAGATAGTCAGTTTTGAGAGCATGTTAGTAGCAGTAAGTTGCATATGTGATTATAGCA
ACAGGTGTTTTTTAGGCAGCGCAGTAGCAGTTAGGCCTCAGAAGTAACAGTTAAGCAGTTTTAGGGATTCGTTGCAGACAGCAG
TAGAACGTAGCAGTAGTCAGTTAGTATTCAGTATAGGTTTTTACCAGGCATAATAGCAGTGAGAGCAGCTGCGATAATAGCGCT
TTACAGGCAGGCAGTAGCAGTAGCAGTAGTAGTAGTAGCCCGGCAACTAGTGGTAGGCGGTAGGCAGCAGTAGAAGCAGCAGCA
GTAGCAACGTAGCGAGTAGCAGGTAGTAGTAGTAGTAGTAAGAGAAGTAGTATCAAGCGGTTAGGCAGATTGAGCAGTTCGCAG
TAGCAGCCAGGAACGTAGTAGTAGGTTTAGTTAAGTAGCAGTAGTAGTAGGAGTAGGCTGTAGTAGCTAGCAGTAGAAGGTTAG
CAGGCGAGCAGCAGCAAATAGTTAGCTTTAGGCCAGTAGCGTAGTATGTCAACAATAGTAGTTTCGGCGGCGTGCTTGAGTCAG
GCAGTAGAGTAGGCAGGTCAGCATATAGTGCAGTAGCTCTAGATTGAAGTAGTAGCAGTAGCAGGAGCCACAGTAGAGTAGTCA
GCAGCAGTAGGCGTGAAGAGCGAGCAGCAATTAGCCGAGTAGTGCAGTTAAGTTGGTGGCAGTAGTAGGAGCAGCAGCTGAGCA
TTGATTCTATGTATGCAGCAGTAGCAGTAGTAAGTAAAAGTAGTAGAAGTAGCAGTAGTTTTTTGGTATTTTTTTGTTTAGTCT
AATAGTATATGTAGTAGTAGGAGAGTGGGTGCTTGTAGTTAAGTAGTCATCAGTAGCTAGTATCATTTTTTATATAACTATTTT
ATTGGTTTATTATTTGTTTTTTATGTTTTTATTGTTACAGTAGCAGGTAGCAGTACTAGCAGGCGCTAACAGTAGCAACGTAGC
AGGCTTCGAGCAGAGGTTGGACGGCAGGTAGTGCTAGTATCAGATGTCTGGCATACCAATAGGCCAAGCAGCAGACAGATTAGG
CCAGGTCAGCAGTCGTAAGTAGAGCGGTACAGTGTAGCAGCGTCAGTGGTGTGCAACTAAGGCAGTAGAGATCAGTTAGCGGTA
GGCAGGCAGAAGTAGCAAGTAGCAAGGTACAGGTAACAGATAGCGAGCAGCGCAGCAGTAGTAGCCAGTAGCAGTAGCGTAGAA
GCAGAAAGCAGTAGCAAGTAGGCAGTAGTAGTAGCAGTAGCAGTAGCAGCAACAATAGTAGGGGCAGCAGCAACACAAGGAGCA
AATAGCAGCTAGCAGTTGCAGTAGCAGGTAGTACAGTAAGCGCGAGGAGCAGGAGGCAATAGTTAGCAGCAGGCAGTAGTAGTA
GCAGTTGCAGTAGGCAGCTTCCAATAGCAGCAGCTAGTACGGCAACAGGGCACGCAGTAGCAGCAAGCAGTAGGGCCAGTGCAG
TAGTAGTAGCAGCAGTAGCAGTCGCAGCAGGCAGTAGCAGTAGATGAGGAGCAACAAGTAGCAAGTAGGCAGTATGCAGTAACA
GAGTAGCAGCAGCTAAGTGGTAAGCAAGTAGTATAGGCAGTAGCTAACTTGTAGCAGTAGAAAGCAGGGCAGGCAGTAGCAGTA
GCGCATTAGCAGTAGGCAGCAAAGGCACAGCAAGGAGGCAATAGCAGTAGCAGAGAAGTAGTACCAGTTACAGTAGCAGGAGCA
GCAATGAGCAATATGTCAGCGCAGGCAGCAGGTACACTAGTAGTAAGGGCAGTAGTTAGTAGGCGTAGCAGTTCGCAATAGCAG
CAGCAGGCAGTAGCAGTAGCGCAGTAGTAGCAGGGCAGGGCAGTAGTTAGCAGTAGCAGTAGATGGACGCAGTAGGCAGTAGCA
GCGTAGTAGTAGCAGTAGCAGTAACAGTAGCAGCCAGCAGTATAGCAAGTGTATAGCAGTAGCAACCAGTAGCAGTAGAGCAGG
CAGTAGTAGCAAGTGAAGTAGCAAGTAGGGCGAGGAGTAGCAGTAGTAGTAGTACCAGTAGAGGCGCAAATAAGTAGCGTTGCA
GTAGCAGCAGCAAGGAGCAGTAGCGAGAAGCAGCTAGCAGTAGTAGCACGTAGAATGAGCTGCAGTAAACAACCAGCAGGCAGA
TAGGGCAAGTACCAGTAGCAGTAGGGTAGCAGTAGGCAGTAGGAGCAAGGCAGCAGTAAGCATACAGCAGCAAATAGTAGTGGC
AGCAGCAACTCAAGGAGCATAGGGTAGGCAGTAGAAGTAGTAGCAAGTAGGAGAATAGCAAGAGCAAATAGTGAAGCAGGGCAG
CAGTAGCAGTAGTAGTAGCCCGTAGGCCAGTTGCAGTAGCAGTTCCAATAGCAGCAGCAGTAGGCACGCGCAGCAGTAGAAGTG
CAGTAGCAGCAGGTGCAAGTGCAGAAGCAGTAGTGCAGTACAGCAACAACACAGCAAGCGCAGTAGCAGTAGCAATAGAAGTAG
TAGCCGTACCAGTAAAGCACAGCAGTTAGCGCAGCAACAGTAGCCAGTAGTAGTTGCAATATTAGTAGTAGGGCAGCAGCAGCA
GTAGCAGTAGCACAAAAGCAGCAGCAGCATTAGCAGTAGTAATAGAAACAGTTACATACGCCAGCAGCATTAGCAGTAGCAGCG
CAACACAGCAAGGAGCAGTACAGAAGTAGTAGACGCAGCACAGAAGCAGCAAGCGTAGCCAGTTAGCAGTCAAACAGTAGCAAG
CATAAGCACAAGTGCAGGTAGTAGTAGGAGTACTAGGAGCAGGCATAATAGCAGTATCAGTGTGTTTAGACAAGTAGAAGTAAC
AGTGTACAGCAGTAGCAAGTAGCAGCGCAGCAGCAAGTAGCAAGTAGGAGTAGTGTAGTTTTTTAAGGAGTAGTAGGAGCAGGC
AGTAGCAATGAATTAAGCAGTATCAGTAGCAGTAGCAGTAGAAGTACAGTAGTAAGCAGCAAGTAGCAAAGTAGTAGTAGCAGT
TAAATAGTAGCAGTGACAGTAGCAGCAGTATCAAGTAGAGTAGCAGTAAGCAGCAGCAAGCCATTAGTTGCAGTAGCAGTAGTA
GTAGCAGATAGTAGCAGCAGCAGTAACAGTGACATTGCAATAGCAGTAGCGAGGCAGCAAGCGCAGTAGTATGTAGCCAAGTTA
GCCAGAGCAAGTAACAGTTATCACTAGCAAAGCAGTGGCATTAGCAGCAGTAGAGTAGCCAATAGCAGTAGCAGTTCAGTTGAA
GCAGACAGTCAGCAAGTTTGGCAGTAAGCAAAGTAGCAAGAGAGCAGCAGTAGCAGTAAAGTTCAGTAGCAGTAGTAGTAAGCA
GCAAGTAGGCAATAGCCGAAGTACGCCAGTAGCGATAGTAGTAGAAGCAGAGCAATAGCAGACAGCACAGTAACAATAGCAGCA
ATGCAGTAGCAGTAGTTGTTAAGAGCAAAGCAATAAGCAGCAGCAGCAAGCACAACACACGGAGCAAGTAGCAGTTTTTCAGTA
GTTGCAGTAGTGTCACTAGCAGTAGCAGTATTAGGAGCAGCAGAGCAGTAGTAGCAGGTAGCGTAGGAAGTAGAGTAGCAAATA
GTAGTAACAGTTAGCAGCGTACGGCAACCAGTAGCAGTAGCATAGCAGCAGTAGAGCAGCTGTAGTAGTAGTACGGGAGTAGTA
GGAGCGCAGTTTGCTGCAGGTAGCAAGCAGCAGTAGCAGTTCATCAGTAGCAGTTAGCTTGGTAGCAGTAACAGTAGTAAGGCA
GCAGCCGTAGCAGTAGTGGTAGCAGTTGTGTAGTAGTAGTAAGTGAGTAGTAGAGAGCAGAAGCAGCAGTAGGCAGCAGCAGTA
GCAGTATCATTACCGTTAGCAGTAACGTAGTAGCAGTAGTAGTAACAGTAGCGAGTAGGAGTAGTATGGTATAGCAGTTGTAG
>m141122_014521_42134_c100732202550000001823141405141560_s1_p0/22204/1505_11409
GAGCAACAAATGTACGTCATATCCCGTTGACCCATGAAGCAGCAGTAAGCAACTACGCAGGGTAGTAGATGCTTGTGCAGTAGC
AGTAGCAATATTTCGTAGTCAGTCGAGCACAGTTGGAGTTCGGATTTAGGCGAGAGCAGTTAAGGTACGCTCACGGGAGGATCG

TAGGTATAGAGGAGATTATGCTTCTAAATATTACAACAAAAGTAAGCATGAGTGGTTAGGGGCATTTGCGTAGGTAGTAGTAGA
GGTGTCTTGTAACATGATGTTAGGGTGAAAGATAAGTAGATGAGTAAGGTTGTTGTATAGAGTATGTTTGTCTGTTAGGGTTAT
AGTAGAGGCGATAAGTTAGGTGATGGTATTATGTTGTGGAGTAGATTAGGTGAGTACGTTTGTAGGTGAGTAGGTTGAAAAGCA
GCCTAGGCCAGATAGTATCAGTAGCCTAGAATTAAAACTAAGCTGGTTATGAGATAGCAATGCTGATCAGGTGCTTAAGGCAAA
CATATTATCAGTCAAGACATAGATTGAGACTGTACGGGAGTTGAGCTAGGTAGACAAGTAATAGCAGAGTGTGAGTTTCGTTGG
AGATGCAGTAAGTAGGGTGAGCAATAACGATAAGTATACTGTACTAGGAATCGCAAACTCATAGTAAAGAAGATTGATGGAAGT
CGCCAGGCAGTAAGTATCGAAGCTAGCACAGCAGCGCGATAGAATGCAAGCAGCAGCAACAAACATAGTTAAGTATGTCGGTAG
ATAGCTAGAGCTAGTAGAAGTTAGCATTAGCAGTAACAGTATCAGACGAGACAGATATTTACTAGATTTAGTAGATAGTTAGTG
GTACGATAGTAGTTAAAGATCAGTAGTAGTAGTTCATATACGCTAGTAGCTTGTATGCGACAGCAGTAGCTCGATAAGCAGTAG
TAGTAGCAGACAGTAGTAGTAGCAGCTGTCAGCAGCAGTTGTAGTAGCAGTAGTAGTAGTAGTAGTAGAAAGTAGTAGTGGCAG
TAGTAGTTAGAGAGTAGTAACAGTTATTGTTAGTAGGTAGATAGCAGCACAACAGTATAAGTAGACTAGCAGCAGCATATCCTA
CTCAAAATAAAACTGATCTAACTCAGCATGAGTTGTAGTTATGTCGCAGTCAGCTAGGTATATAACAAATTAGCTAGTGTATGT
AGACAAGCTAGGCAAGATAGCAGTGATGGGTAGATACTGTGTTTATAAGGGTTATTGGCAGTAGAGTTCAAGAAGGGTAGGATA
CTTAGAACGTTGAGGCAGTAGTAGATGCTACAGAACGAATCAGGAATATGTTTAAGATTTAGGTGTATGCGATGACGCAGTAGT
CAGATAACCTTAATATGAAGTCACTAGAATGTATGAGTGAGTTCACTACAGTTGGACGACGATTAGGTAATATCGGATTAGTCA
TGTGGTTGTGTATAGCTGGGCGTAGTAGAGAGTATGTAACTCATGTCAGCTAGCTATATGTTGTTTTTAGCTAGTTAATGTAGT
ATAGTAGTATAGTTATGTCAGGTTGTTTGCAGAGGTTATTCTGTGGATAGTTATGATCTGTAGCAGCGAGTATGATAGTATGTT
ACTAGTTTTATATAGAAGCTTGTACTTAGGGGCTAGGATAGAGTTATTATAAGGTCAGAAAGTAAGTTGTAATATGCGCAGGTG
ATTTAGCTAGTGGCGAGTGGCGTGGTAGGTTTAGGTGGTTGTGGATTGAAGTTGGTTTGGCAGTTTAAACATGTATGTGTGTTA
GAAAGTAGATTGAGGAGACTGTGATTGTAGCTACTAGAATTAAGCAGTAGGCAGTTAGAGTTATTGGAGGTAGGGGAATATTGG
TTATGCGGAAATAGCGAGTTTAGTAGATAGGATTAGTTAGATAGAGTTTGTATGATTATGGTTTGGGTGTTTTCGTGTTTGTGA
TTGGATTCATGATATGGTAAGTAGATATGCGAATTACGCAGTGTGATATGATGTCTCTCGTTAGCGATATAGTCAGCTAGCGGT
AGATTCAGTTCGTGTGGGTTCATGCATATGTTTTGATGTTAGTACGCTTGTAGTACTGGATGATTTTCTACAGTTTATTTTCGC
GGTGTGTGGTTTGGTGTAGTTGGGTGGGGTTAGTGCTGTTAGTTATTGGAACTCTAGTAGACATTGTAACGTTCCTTGTGCTTT
TTTATCAGTAGTAGATTTAGTTTCAGCAGCAGTTCAGGCAATATAGAAGTATTAGCCGTAGTGAGGATAGAATGGCATTAGAGA
CGACGTTAAGTATCAATGAGACTGTTTATTAGTTAAACCTATAGTTTACGAAATGTGGGGAGTAGTTAAGATTCGAGGGGTTTT
AATGCAGTTGGGAGATAGTTATGTGTTGTTATTGTGATGGGGTGCTAATAGGTTTTTGCGTAGTATTTAAGTTGAAATTATAAT
ATTAAAGTATTATTAATTTGTGTTAGGTATAGAATGTTAGTAGATGTGTACACTTGGTGTATTTGATTTTTGGGATTGAAAGGA
TTGAATTTTTAGTACATGAGATATATTTTATATAGGGAGTATCTAGATTTCGAAGCTTAGAACTATATCAGAAAAGCCTTGGGT
GTAGGAATCTAGTGTTATGAGTATACTACTCAGAGGTCTGAATAGTATATTTTAGGCCAAACTTCAAATGGTGGTTAAGTGTGT
AATAAAGATCGTTGTGTCTTAAAATATGCCGCGGTTAATGCAGGATTAAACGTCACGGTTGTGATTCTTTGCTAAGCTTAGAGT
GATCGAGTTTCACTATGTCGATAGGAGTGGTTGGTAATATAAACAAGTTGGACAATTCGGCATATTGATATGCGAATCTACGAG
AGAGCTTCTCTCGAGATATGGTTCTTAGGGTTGAGAAGTGAGTTAGGGAGTTTTGGGATTTTGTGGATAATGTCGTTTGGTTTG
GCTCGTTCTTTATAGGTTATTTTTTTAAACTTCTTTTGTGAAAATCGCGGAATAGGAATATCCTTCTATTATGCTATGGGTTTT
GGTTAGGTTTAACTCGCATTCCAAGCATTCTTTAGATTTCCATCACGCATTGTAAATACGTTTTTTTACCTCGAGTTCTAAGGT
ATTTCGTTTTGAGCAATAGTGACAAAGTTCACATGGGATTAAAATTATTTTAATTATGTTTTAGTTTTAGTTCTTTTTAAGGG
GTTTATGTTTTTCGCAGTATCTCGTTGGACTCTAAATTATGGAACTCAGTTTACTTCTTCAGGGCCGGGATTTGTCGACAGTAC
ATATTTCGAAAGGAAAGTTGTTGGTCGATATACAGAGGCGTTCTCGCTAGGTGGTTTCTGAGTTTGATTCTGAGAGGACGTACG
TTGTAGGCGTCGGGATTTTTGGGGTCGGCTGAAGGTAGATCGTTTTCTCATGTTAATAAGCTTTTCTAACTCTTTGGAAGTAAG
AATTAGTGTCATTGGCTGAACTATTTTGTCTGGTATTTTTTGAGGTGGTGTTAGGGTCGAGGGTAATAGTTATTCAAGAGGTAT
ACTGCTTCTTAATCCTCAGGGATAGGGAGATTAGTGGGTGTGTTTATATTAGTGAATAGTTTCTTGGGTTAGTTTTTGTATGCA
TTTCTCGGGGTAGTAACCTTAGAATCCGGACGTTTGATGTGTACTAACTTCTTAAGCGTTTAATGAAGTTTAGAAAGAAGCTGA
AGATAGTAGGGTTTTGAGATAGGTTGTCTACTTACATGAAAGTAAGGTGTAAGATGCGATATTTTGTAAGGTGTGTTTTTTATG
GTAATTAATATAATTGGGATAAATTATGTTTGATTTCTAACATGTTTTTCCATTTAATAGGCGACTCTCTCGTTACCTCGGGTT
TTTATTAAATTACTATGGTCCGATTCAGATTTTTAATAAAACAGATTTACATGCGCGGTACGCTTGGGATGAGAATTGTGCCTC
TCGGTTTTCCTCGTTCTGTTCTTTGCCTAGTCTACCGTCTAGTTCTATACTTCCTTCTTTGGTTTTAGGCGTTCTGGTTCCGCG
TTCTTGGATTCTTAGTGTCTTGCTCCTCCCATAATCACCCTGGTCTTTTATGTCCGCGCACATCGTGTTGCGAGTTCACGCATC
ATGAGTGTAGTCATAATTGCTGAGCTGTTATCATAACTTGTTACATGGCTATTTGATTATTTCTTGTTTACTGCTTTTGTCTCA
CTTGGTCATGTCCATAACTCTAATCAAATGTTTTTAATCTTGTTTGTAGACTAGATTAACCTTGGGCGACGCTAGAGGTGTGGC
TGGTCCGATCCCGGGGTGTCTCGCCTTGCATCCCCCGCTTATTTATCGTTCTCGGTCTCAATGTAAAGGGTCGCTTCGCCTGCT
CAGGCCTTGACCTCCTGCTCTCGCGTCTTTGGTGGCTGTCCGGAATACGCGCCCTTCTCGCTGAGTTTTGGAGTGTTCTTCATT
GATCTTCCTCTTTTGTGCTCTCCCTTCCCTCCTTCGTCGCCCGCCGGCCTCGCTGGTTTAGTGATCTCCTGCTTGCGTACTAGG
GCTTAAGTCCTGCGTTCCTGGCCTTGCTTTAGTTCGTCCTCTGGTTTCTCTCTGGTGGTACCGTTTATATATATCACGAGTTAT
TTCACGATATTCCTGTAATACGGAATGATTTTTACTATCTGTCTTACACGTTAAGTTTTTTCATCTCCTCTCGTTTTTATCTTG
TTTTCTATGTTATCGTCGCGTTTGCCTATGCTCGGCCTTCGCTCTTCTTTGTCTTTATTCTCGCTGGTTCGCCAGGACCTAGCC
GGCTGCTGGGCTTGGGCCGCGGCGGCTATCGTTTTGTCGCTTGCCTGTTCTATATGCGATGCTGGCTATTGTGGCGGCATCGCT
GCTCTAGCGGTGTGCTGCTTTTCGCGTCTTATGCGGTATATCTGTTAAATTTATTCTGGTTTTCCGCCCTTCGTTTTGTTTGTT
CCCCTTCCCTTCCCGGGTCGTTTTATTCTTCCGTGTTTTTTCTTGTTCTAATCTTGAACTTCGCGATTGGCATCTGCTGTTCGT

TATTCTGCTCTTTAAAGCAGTGCCTAGCCTTCTGGTTGGATCTCTAATTTTCATGACTGATCGTCATTCCATAAGTTACGCTAC
CTGTTTACGTCTTAAACTCATTGCTCTCCTTCAATCACCCACAAATACTACAAAACATCGCGGAGGAACGGCGGTCGATCGACG
CGTCCGCGGGCCCGCTCTTGCATCAACTTCTTCCTCTGGCTTCCTGCGCGTGGCCTTATAAACACTCATCGTCAAGAGCAAGCT
TTAATCAACAATTTGGAACTCAAATCACACTCGCCTCACCTGCGGCTTCTCTGTGTGTTATTGAGGGTAAACTTTTGTCGCGAG
GTACCCCCCATATCAAAGGCGACATGCCCAGATTTTAATAGTTAGGGTGTCAGAATATTTAGCAGGAAACATAATTGTGGGAAT
TATGAAGATAGTTCATATTATGGGTTTTCATTAAATGGGTTTTGAGACTTAACGGAAAGCTTAACAATCTAAAATCGTTCATCT
CCTCTAGAATACATTAGCACAGGACAAATGTTACAAGATAAAAGTTACCGTCCATAATAATATACCCAATCTACACATGACCAT
ATCACTATAACGCTACAATCAGATTTGTTTAGAAATATAATTAATAAATCAATCCAGTACTAACTTTCCTGGGGCCTGTCCCCG
TCTTTTCCTAGGTGTTCTCTCTCTCGCCTATTCTTATACCCATCATTGGCGCGGCGCGCGCTCCACCTCTTAGCTAGACAATAT
CTGGCCCGGCCTATACACCTGCTAGCTCCATTATTTCCCTTTTTTTTATCGATCCTGCGCTAGTCTACTCCGCTCTCTCCACTT
TTCTTTCTTATGATTTACCAATCATCCTTGCTCATCAGTCGACCACCCTGAGATCTCGGCCTGAACTGTTCCCCATATATGACT
GCGCCACTCGCGGCACAACTTTGCTTTTAAACCTTGGCTTCTTACCCCCTTACCAAAGTTCCATGAATTTTGCTTAATTAACTG
TTTTTCTAAAGCTCAATAGGGTATTTCCTAGGATCTCCTCCTCTCTTACTCGTGTCTATCTAGTCTTTTCTACTACCTTGTGGT
TTGTTAATATTAAGACTCTCCTCCTCTCTGTCCTACACTGCTTGGTCGGCCCCTCTCTGGCTCTGACTACGCTTGATCTTCAGT
TACCTGCCTATGCAGCTCGCGTGTATTCTCCTTTCTCCCTCACATCTACCGTCTTGCTACTTCCGTCTTTGGGACCGCTTCTCT
GCATCTCGTCGCTCTCCTTAAGGTAGACGAGGGTAAAGGATAGTTGTGATTTCTGCTCTCGCTGCTAGTTTTTTCACATGGCAC
CGTCTCCGCCTCTCCATCCCTCCCCGTCTCGCATCCTGCCCTACCTTCTCCCCCTTCCTCTTCTCCTCTTCGTCTCGCTTGGCC
CACGCACGCACTGGGCTACCTGGCGCCCGCCCGAGCCTTTACACAATCACATCCTACCCCCCATGTACGCATCATGCGTTTTAT
CCCGCCTCCACGCTGAAACCCGCGATTTACCACATCAAGATAGGTAAACTTTCCGCGAAAACCTTAGTTTCAAGGAACTTAAAC
CTACAAAAGCCATAAAAAAACCAAAAAAGGTTTACGGGATCTGAAAGAAATAAAGAACTTAATGAAGCTAATCCTGGGATAAGA
ATAACCTTGATAGCTAGGACTTCGATATACACCTCAATACCGAAATATCATTATCTTTACTTCCAAGTATTTAGAAAAGCTTAG
ATTGGCAAGCTTTTAATCCAAATCCTAATGTTTTGCTCTCTAGATAAACTTAGCAACTTGGAGGGCCTGAAGAAATGCTGAATG
ATGAGTGAAATGGCTTGAGTGGCTAGGTCCTAATTAGTAGAGTCAAGGAGATGAAACTAACCCTTTTAAAAGAATAAATAAAGA
ATATAATTTATAAATTTTGAATTTTTTTTGGCTCAAAAATGACTAGAAACTCGGTAAATAAACGTTTTCGGGAGGAAATCTAAA
GTTGTTTGAGGGGCTATTATCCTATTCGGTTCAAGAAGTCTAAAATACACTAAAAGAGCGATATAGTCCCTACCTATGTCTCG
GTCTTCGTGATTTCGTTCGTGCGAAGGTCAACGTGTTTTGATATCCATAGGCAATGTATTGACTTCTAAAAGCTTGCGATATAT
CGGCATACGCTGATATTTTAACACGTTTTTAGCCCACTTCAAAGCTCATATTGAAGCTTGCTAAAAATAACTTTGACGAGTTAA
AAATGATCCTAACAGCTGGCTGGAATGTTCTACGAGATTCGAAATCTATCATTATTAATTATTCATCTAAATGCTTTAATCCTT
AATAAAAAGCATGTTACAAGTGTCACTTCTTAAACAATTCTATTCTAACCTTAATAAAATGTTAATGATTTCTAACATATAGGT
TAAACGCTATTAAATCCATAACTATTTCTAACTACTACTACTAGTAATCTACTACTTTCTAAACTATAGGTTAAACTATAAAGT
CCATAACTATTTCTATGAGTTTTACTGCTAGTACTACTTCTATTTGCTAACTGCTGCTGCTACTACTACTACTATAATAAAATC
CATAACTATTTTCTATGATTTCAACTAAATCCGCTAAAACCAACAAACACGAAACTACAATAACTACAGCTGCTGCTAATGCTA
TTGCAGTACTAACTACTGGCTGGCTGCTTACTCGCTACGAGATACTGGCTACTGCTACTACTTGACCGCAACAACACGAAAACT
AAATACTACAGCTACTACTATATAGCTACTTGATATTAGCCCGTGCTGGCTTACTGTACTTGCTGGCTGGTACAAACTGGGCTA
TACTACTGCTATGGCTACAGGGGGGGGGGGGGGGGGGAGCTACAATAGCTACCACTGCCATGCCAACTAGCTACTGCTACAACTA
GCTACTGCTACTACTTGCCTTACTACTTCTACTGCTACTTCTACTTGCTGCTGGCTGGTACTTGCTACTGCTGGCTAAACGCTG
CTACTTCTACTACGTACTGCTACTGCTACTTGCTGCTGCTCTACTACTACTACTCCTGCTACTGCTACTGCTACTGCTACTTCT
ACTATACTATTAGCTGCTACTGCTGCTGCTATACTACTGTTGGCTGGATACTACTCTGCTACTGCTACTACTACTTCTACTTCT
ACTGCCACTATACTACTGCTACTACTAGCTGTCCTGCTGCTACTGCTATTGTTACTGCTACTGGCAGCTACTACTACTGCTGCT
GCTGCTGCTACTTACTACGTGTTGCTGCTGCTACTATACTACAATGCTTTCTACTACTACTACTACTGCCACTCTACTTCTACT
ATACTACTACTGCTACTGCTACTACAACTGATGCTGCAGGCTGCTACTACTACTGCTGCTACTTACTAGCTGCTACTACTACTG
CTGCTGCTACAGGCTACTGCTACAACTACTACTACTACTACTACTATATATTATACTACTGCTAGCAGACTACTGTCTGATACT
GTTACTGCTAATGCTACTTCTACTACTTCTACTTCTACACTTATACTTTGTTGCTGCTGCTGGCTACTATGCTGCTGCTGCTGC
TCTGCTATCGTTGCTTGCTGCTACTGCTACGGTTGCTGCTGCACTACTACTGCTACTACTGCTCGTGGTATTGCTACTGGCTAG
CTCAGCTATCCTACGCTACTGCTACTGCTACTGCTACTTGTAGCTGACTGCTAGCTGCTACTGCACTGCTACTGCTACTGCTAT
ACTACTACTACTACTATATACTACTACTACTACTACTATACTACTACTTACTATACTACTAGCTAATACTACACTACTATAGCT
ACTACTACTATTCGCCATACACTGCTATGCAGACTGCTGGCTATTGCTCTTGCTAGCTGCTACTGCTACTGCTTACTGCTGTGC
TGCTATTGCTACTGCTATGCTACTGATGTTGCTACTGCTCTCGCTACTGCTACTGACTATGCTTTTGTTGCTACTGCTGCTGCT
GCTACTTGAACTACGCTGCTCGCGGCTGCTGCAGCTACTGCTACCTGCTACTCTACTGGCAACTATTACTGCTGCTGTACTACT
ACTACTACTGGCTACTGCTCTACTGGCTCTACTACGTGCTATTGCTACTGCTACTCTGCTAACTAGCTGGCTGCTATGGTACTA
AGGCTACTGCTACTACTATGCTGCTACTGGCTAATGCTGGCTACGGCTGCCTGCTGTACTGTACTTTTACTTGCTATACTGCTA
CTTCTACTGGGTACTGCTACTACTAGCTACTACTACTACTACTATTACTACTCTACTATTGGCTAATACTATTGCTACTACTGC
TGCTACTTGCTATTGCTACTTCTGCTACAGCTACTTCTACTGGTCGCTGCTTCTTCTACTGCTGTATGCTACTGCTCTTCTACT
CGGTCTTTCCCTTCTACTGCTACTTCTACTGCTACTGCACTCTGACTGCTAATACTGCTACTTGCTACTGTTTACTGTATTTTC
TACTACTGCTATCTACTATACTACAATTGTTGCTGCTACTACTACTACTGCTGCTATGCTGCTACTGCTTGTGCTAACTGCAAT
TCTACTGGGCTCTTCTACGGCTACTTCTACGGCTGCTACTACTACTAGCTACTGCTACTACTACTACCTACTACTACTAGCTAC
TATACTACTACTACTATATACTACTATTACTTATCCTGCTACTATACTACTACTACTACTCTACTACAGCTCTATATACCTACT
ACACTACTACTACTATACTAAACTACGTATACTACTACTACTACTACTATATACTAACTACTACTATACTAAGCTA

>m141122_014521_42134_c100732202550000001823141405141560_s1_p0/25879/3598_9700
TCTTATACACTAAGGATTATATATTTTTCACTTATTGTAATTTTTCCTGTACATCTCTCAGTATTTATGGACTTGTTAACACCC
TACTAAACTAGATTTGGCCTCGAAATTTTATACATAGCAACTCCTAAGAAATAGGGTACAACTTTTTGTGGCCCATGACCCTCG
CAATATCTTTAGTTTCCTTTAATAAACATGCTCGAATCCATATTAGAAGAATCGATAGATTTCTGAAAGGGGTACTCTTTGGAA
TTTGAGTATTTCAATCATTTCCGAAAATGTATTACCCTCTGTGTTCCCAACTCCGATTCCGAATATTTTTCCCAACCAGATCAT
CATGGGTAAATGAACTTATTGGCGTCGAAAAGGAGCCTTTAAGTCGACAATTTTCCAAAAAAAAAATACAATTCCTTTTCGAGTG
GCAATTTCCGCGAAGTTTTTTTTTTTTCCTTTAATATTAGTACTTCCTCAGTATTTTGACTGTATCACTATAATCATAACCTCA
GGATTTGCAGAAATATTTATACATAGACCCTAAGACTAGGTTACAACTGTTTGTGCCCATGGACTCTCGCATATTCTTTAGTAT
TCCCTTATCAAAACATTGCTTCGAATCATTAATGGAGAAATCGAAGAATTTCTGAAAGGGGTAACCTTTGGTGAATTGGGAGAT
TTCACCTCATTTCTCCGAAATGTATTACCTCGTGTTCAACTGTTGATTTTTTCCCCAACAATCTCATCGGTAATGAAATATATT
TTGGAGTAGAACGGACCCTTAAGCCGCATTTCAAAAAAAATTCCCCTTTTCGATGGCAAATTTTCGCATGTTTTTTCCTTATAA
TTAGTATCCTTCGTATTTTTAGACAGTAATCACCCCTACACACACGTCGGCTTTGCTCGAAATTTTATACATAGAACCCTAAGA
CATAGGGCTACAACTTTTGTGCCCATGACCCTCGCATATTACTTTAAAGTATTCGCTATCAAAACATTTCTCGAATCCATATTA
CAGAAATCGAAAGATTTCTGAAGGGGTACCCTTTTTGTGATTGGAGATTTCACTAATTTCTCCCGAAATGGTATTACCCTCGTG
TTCAACTCGTTTGATATTTTTCCCCAACAGATCTCATCGGTAATGAATATATTGGAAGTAAGAAACGGAGCCCTTAAGTCGGCC
ATTTCAAAAAAAAATTCCCCTTTTCGTGGAATTTTCGCAAGTTTTTTCCTTATTTAGTACATCTCTCAGTATTTTGACTGTAATC
ACCCTTAATACAAACCTAGGATTTGCTCGAAATTTTTATACATAGAACTAAGACATAGGGCTTACAACTTTTTGTGCCCATGAC
CCTCGCATATTCTTTAGTAAATTTCGCTATCAAAACATTGCCTGAATCCATATTAGAAAGAAATCGAAGGATTTCTGAAAGGGG
GTACCCCTTTGGGTGGAATTGGAGATTTCAACTCAATTTTCTCCGAAATGGTATTACCCCTCGTGTTCAACTCGTTTCGATATT
TTTCCCCCAAACAGATCTCATCGGTAAATGAATATATTGGAGGTGAAAGGCCCTTAAGTCGGCATTTCAAAAAAATTCCCTTTT
CGAGGCAATTCGCATGTGTTTTTTCACTTATATTATACATCTCTCCGTATTTTGACAAGTATACCCTAAACACAGCGTCGGCTT
TTGCTCGAAATTTATACATAGAACCCTAAGACAATAAAGGGGGCTAACAACTTTTGGTCCATGGACCCTCGCATTATTCCTTTA
GTATTCCTATCAAAATATTTCTTCGAATCCATATTAACAGAAATCGAAAGATTTCTGAAAAGGGGTACCCTTTGGTGAATTGGA
ATTTCACTTTATTTCTCCGAAAATGTATTACCTTCCGTGTTACTCGTTTGAATATTTTTCCCAACAGAAATCTCATCGGTAAA
TGGATTATATTGGAGTAGAAAGGAGCCTTATTCGGCATTTCAAAAAATTCCCTTTTCGATGGCAATTTTGCAAGTTTTTCCTT
ATATTAGTACATTCTCTCCAGTATTTGACTGTATCAACCTATCCAAACCTAAGGATTTGCCTCGAAATTTTATAACATAGAAAC
CCTAAGACATAAGGGCTACAAATTTTGGTGGTGCCATGACCTCGGCATATTTTTAAGTATTTCGCTATCAAACATTGCTCGAAT
CCATATTAGAGAAATCGAGATTTCTGAAAGGGGTTAACCCCTTTGGTGAATTGGAGATTTACTCCATTCTCCGAAATGTATTAC
TCGTGTTCAACTCGTTCGATATTTTTCCCCACAGATCTCATCGTAAATGAATATATTGGAAGTAAGAAACGGACCCTTAAGTCG
GCATTTTCAAAAAAAATTCCCCCTTTTTTCGATGGCAATTTTCGCCATGTTTTTCCTTAATAAATTAGTACATCTCTTCCGTA
TTTGACGTTATCACCCTACACAACACGTCGGGCCTATTGCCTCGAAATTTTATACATAGAACCTAATGAACATAAGGGCTACCA
ACTTTTGTGCCCATGACCCTCGCATATTTTTTTAGTATTGCATATCAAAAAATTGCTCGAATCCAATAATTACAGAAATAGAAA
GATTTCTGAAAGGGGTAAACCCCCTTTGGTGATTGGAGATTTCACTAATTTCTTCCGAAATGTATTACCTCGGTGTCAACTCGT
TTGATATTTTTTCCCAACAGATCTCATCGTACAATGAATATATTGGAGTAGAAACGGAGCCTTTTAAGTCGGCCATTTCCAAAA
AAAATTCCCCTTTTCGATGGCAATTGTTCGCAAGTTTTTTCCTTATATTAGTAAATCTCTCAGTATTTGACTGTATCCACCCTA
TACAAACCTAGGGATTTGCTCGAAATTTTATACATAGAACCCCTAAACATAGGGCTTACAATTTTGTGGCCCATGACCCTCGCA
TATTCTTTAGTATTCGCTATCAAAACATTGCTCGAATTCCATTATTTAGAGAAATAGAAGATTCCTGAAAGGGGTACCCCTTTG
GTGAATTGGAGATTTCCCACCATCAATTTCTCGAAATGTATTACCCTCGTGTTCAACTCGTTCGATATTTTTCCCCAACAGATT
CTCATCGGTAAAAATGAATATATTGGAGTAGAAACGGACCCTTAAGTCGGCATTTCAAAAAAATAATTTCCCCTTTTCGATGGC
AAATTTCGCAATGTTTTTTTTCCTTATTATTAAAGTACATCTCTTCCGTATTTTTGACAGTATCACCCTAACCACGTCGGTTTG
CCTCGAAATTTTATACATAGAACCCTAAGAACATATGGGCTTACAACCTTTTTGTGCCCATGACCCTCGCATATTCTTTAGTAT
TCCGCGTATCAAAACATTGCCTCGAATCCATATTACAGAAATCGAAGATTTCTGAAAGGGGTACCCCTTTGGGTGATTTGGGAG
ATTTCACTAATTTCTCCGAAATGTATTACCCTCGTGTTCAAACTCGTTTGATATTTTTCCCCATACAGAATCTCATCGGTAATG
AAGTATATTGGAGTAGAAACGGAGCTTAAGGTCGCAATTTCAAAAAAATCCATTTTCGATGGCATTTTCGCAGTTTTTATCATT
TATTAGTACATCTCTCAGTATTTTTGGAACGTTATCACCTTATACAAAACCTAGGATTTTGCTTCGAAATTTTTATACATAGAA
CCCTAGACATAGGGCTACAACTTTTGTGGCCCATGACCCACTCGCAAATATTCTTAGTATTCGCTATCAAAACATTGCTCGAAC
AATATTATGAAATCGAAGATTTCTTGAAAGGGGTACCCTTTGGTGAATTGGGAGATTTTCACTCATTTCCCGAAATGTATTACC
CTCCGTGTTCAACTCGTTCGTATTTTTCCCCAACAGATCTCATGGGTAAATGAATAATTGGCGTCGAAACAGGAGCTTAAAAGT
GAGCAATTTTCAAAAAATTCCCTTTTCGATTGGCAATTTTCGCAAGTTTTTTTCCTTTATATTAGTACCATCCTCAGTATTTG
ACTGTTATCACCCTATACAAACCTCGGATTTGCCCCACGAAATTGTTATAACATAGAACCCTAAGACATAAGGGCTACAACTTT
TGTGCCCATAACCCTCGGCATATTCTTTAGTATGTCCTATCAAAACATTGCTCGAATCCATATTAGAGAAATCGAAGATTTCTG
AAGGGTACCCCTTTGGTGATTGGGAGATTTTCACTCATTTTCGAAATGTATTACCCTCGTGGTTCAACTCGTTCGAGTATTTCC
CAAGAGAATCTCCATGGGTTAATGAATTATGTGGCGTCCGAACGGAGCCTTAAGTCGACATTTCAAAAAAATTCCCCTTTTCGA
TGCAATTTTCGCAAGTTTTTTCCTTATATTAGTACATCCCTCCCAGTATTTTGACTGTTATTCACCTATACAAACTCGGGATTT
GGCACGAAATTTTATAACATAGAACCCCTAAAAAAAAGACATAGGGCGTACACTTTTGTGCCCATGACCCTTCGCATATTCTTT
TAGTATTCCTATCCAAAACAGTTGCTCGAATCCATATTGGAGAAATCGAAGATTTCTGAAAGGGGTACCCCCTTTGGGTGAAAT
TGGAGATTTCACTCATTTCTCCGAAATGTATTACCTCGTGTTCAACTCGTTCGATATTTTTTCCCCAACAGATCTCATCGGTAA
AATGAATATAATGGAGTAGAAACGGACCCTTAAGTCGGCAATTTCAAAAAAAATTCCCCTTTTTCGATGGCAATTTTACCGCAT

GTTTTTTTCCTTTTATATTAGTAATCTCTCCGTTATTTGACAGTAATCACCCTACACACAGTCGGCTTTGCTCGAAATTTTATA
CATAGAACACTAAAGACATAGGGCTACAACCTTTTTGGTTGCCCATGACTCTCGCATATTTTTAGTATTCGCTATAAAAACATT
GCTCGAATCCCATAATTAGAGAAATTAGAAGATTTCTGAGGGTTACCCCTTTGGTGAATTGGAAGATTTCAACCTAATTTCCTT
CCCGAAATGGTATTACCCTCGTGTCAACTCGTTTTGATATTTTTCCCCAACATTCATCGGTAAATGAAATTATATTGGAGAGAA
ACGAGCCTTAAGTCCGGCATTGTCAAAAAAAATTCCCCTTTCGATGGCATTTTCGCAGTTTTTTCCTTATATTAGTACATCTCT
CAGTATTTTGACTGTATCACCCTATACAAACTAGTTTGCTCGAATTTTATACATAGAACTAAGACATGGGGCTACAACTTTTGT
GCCACTGACCCTCGCATATTCTTTTTAGTATTCGCTATCAATAACATTGCTCGAATCCATATTAGAAGAAATCGAAGATTTCCT
GAAAGGGGTACCCTTTGGTGAATTGGAGATTTCACTCATTTTCCGAAATGTATTACCCCTCGTGTTCAACTCGTTCGATTATTT
TTTCCCCAATCAGATCTCATCGGTAAATGAATATATTGGAGTAGAAACGGACCTTAAGTGGATTTCAAAAAATTCCCCTTTTCG
ATTGGCAATTTTCGCATGTTTTTTATATTAGTCAATCTCTCCGTATTTTGACAGATCACCTACACACCGTCGGCTTTTGCTCGA
AATTTTATACATAGCAACCTAAAGACATAGGGCCTACACACTTTGTGCCATGACCCTCCGCATATTCTTTAGTAATTTCGCTAT
CAAAAACAGATTTCTTCGAATCCCTATTACAAGAAAATCGAAGATTTTGAAAGGGGTACCCCTTTGGTGAATTGGAGATTTCAA
CTTATTTCCTCCGAAATGTATTACCCTCGGTGTTCCAACTCGTTTGATATTTTCCCCAACAGATCTCATCGGTAAATGAATAAT
ATTGGGGTAGAACGGAGCCTTAAGTCGGCATTTCAAAAAAAAAATTCCCCTTTTCGATGGCAATTTTCGCAAAGTTTTTTCCTT
ATATTAGTACATCTCCAGTATTTTGACTGTATCACCCTAATACAACCTAGGATTTTGTCGAAATTTTATACCATAGAACCTAAG
ACATAGGGCTACAATTTTGTGGCCCATGACCCTCGCATATTCTTTAGTATTCGC
>m141122_014521_42134_c100732202550000001823141405141560_s1_p0/26079/5138_10673
AGTAGTAGTAGTAGCTCAGCTGTAGTTAGTAGGTATAGAGTAGTCCCGTAGTAGTAGTGTAGTAGTAGTAGTAGTAGATACTAG
TAGTATCCGCTAGTATAGTAGTAAGTAGGAGTAGTAGAAGTAGCATACCACTAAAAGTAGAGTAGTCAGTGCAGTAGCAGGTAA
GGCGCTAGCAGTAGTTAGCAGCTAGCAGTACAGTAGCAGGTGGCAGCTAGCAGTAAGCAGTAGCAGTTAGCAGGTACCGACTCA
GGCAAGTAGGCCAGCTAGCGTAGAGTACGCAGGTAGCATAGCAGCAATAGACTAGCAGTAGCAGTAGCAGTAGCAGCTAGCTAG
GTAGGGTTATACTAGTAGTATAGCTAGTAGTCAGGTAGTCAGTAGTAGTTCAATGTAGAGATCCAGGAACGTTTACTCTGTTAG
TTAAATAGAAGTGTAGTAGTAGTGGTGATCGGTGGTGGTGGTATAGTAGTAGTAGCTATAGTGAGTAAGTAGGTAGTGCTAGTG
GTGTGGTTAGTAGTGTCGTGGTGGTGGTAGTAGATAGTAGTATAGATAGTAGTAGCTCAGTAGGTGAATAGATAGCAGTAGCAG
TAGTGTAGTGAGTAGTAGTAGCAGTAGCAGTAGCCTAGGCAGATTAAAGCGTAGCAGTAGCAGTAGCGGTAGCAGTTAGTACAG
TACGATGCATAGCAGCAGCTAGTATAGTAGCAGTAGCGGAAGTAGCAGTACAGTAGCGTGCAGTAGCAGTAGCAGCTAGCAGTA
GCAGTAGCAGTAGTAGTGAGTAGTAAGTTAGTAGTAGCTGAGCAGTAGCAGTAGTAGTAGTAGTAGTAGTAGTAATAGTAGACT
AGTAGCAGTAGTAGTAGTAGGATAGTAATATAGTAGTAGTAGTAGAGTAGTAGTAGCAGTACAGTTCCAGTAAAATAGCCATAC
AAGCTAGCAGACCTAGCAGCACAAGGCAGGCAAGTAGTAGCACTAGCAGTACAGCAAAGCAGTAGCAGTAGCGCTAGTACATAC
AGCAGTAGCGCAGTACAGCTAGTAGTAGTAGATAGCAGCCGATAGGTAGCAGTACAGTCAGCCGCTCAGCAGCTATAGGCAGTA
GTAGTAGAACGTAGTCAGAATAGTAGTAAGTAGCTAGTAGAGATAACAGTAGTAAGCAAAAAAAAAAGACCTCTCAGCAGTAGC
AGCAGTAAGCCTAAGCAAGCAATAAGAGAAAACTACAGTACAGTAGTACCTAGTGTAGTCCTTAGCTCATAAAAAAAAAAAACA
GTAACAGCAGTAGGCAGATATCAGTAAACCAATAGCAATGCAGTACGGCGTACAGCATAGGCAGTAGGCAATAGCAGCCAACCG
TAGCCGCTAGCAGCATAAAAGCCAGCAGTTCAGAGAAGAAAAATCCAAAGCATAAAAGTAGATATAGAAGGCAGCTAGCAGTAG
GCAGAATAGGCTAGCTAGCAGTAAAGTATAGGCAGTCATAGCAGTATATACGGAGTAGCAGTAGCGAAAAGCAGATCAGCCAGA
AATAGAAGTAGTAGTATGTAAGATCAGTGAAAAATAGTAGCTGTAGTAGTAGAATAGTAGAATCTCAGTAGCACTCAAGAAAAG
AAAGCTAGCGTCTAGGGAGTAGCGTAGCGATAATACAGTAGGTAGCAGTAGACAGATTGTAAAAAAAAAAGTAGTAGTACAAAA
TAGTGTAGAGTAGCAGAAAAAAAAATAGCAGTACCGCATACAGCAGCAGCGCAGCTACCAGTCGCAGAAGGGAGTAGCAGCACAC
AGTAAGGAGTGAGAACACACAAAAGAGATAGCAAAAAACAAGCCAGTAAGCGCAGTAGCCAGCAGTAGCAGTAGCAGTAGTAGC
TAGCTGTAGTAGTAATAAAATAAGCGTAGTAGTAGTAGTAGTAGTCAGTCGGGTCGTCAGTAGCTCGCAGTAGCACGTACTAGC
AGTACGCCAAGTCGATAGCAGTACCAGCTCAGCTCAGTCAGTAGACCAGTAGGCAGACTAGCAGGTAGGCAGTACGTAGCAGTA
GCTACAGTAGCAAGTAGCAGTAGTAGTATAGTCAGTAAGTAGTAGTGGCGAGTAGCGTAGTTAGTAGTAGTAAGAGACCAGTAG
CAGTAGCGTAGTAGCAGTAGTAAAGTAGCCCAGTAGCAGTAGCAGTAAGGCAGCTAGCGTAGCAGTAGCAGTAGCAGCTAGCAG
TAGCAGCTAGAGACAGTAGCATAGGCAGTACAGGTAGGGCAGTAGAGCAGTAGCAGTCAGTAGCTCGTTGGCTACGTGGTAGTC
GTAGTGGTAGTACCAAACGAGAACAGGAACCAAAACCACACGACTCACAGCCCCCCCTGCCTGGCCCCGCCGCGCGCGGGCGCC
ATGCCACAAATTGGGTCCCGTTGTTTGAAACTATCTTCCTGGGCAAAACATATCTGCATACAATGTTCTTGATGACGAAGTCAA
GAACTTTTCTTGGTCACTGATGGTTGTGAGTGGTTCCAACTTCGGCCCATATTGGTGAAGTAATGAAATCCACAACTGCGTACT
TTTACTCGCCTTTTCCTTGTGGGCAGGGATCCACACTCAAATCTATCACCATACTGCATAAAAAGCCACAGGGCTCAACCCTGC
ATCTGTTTCAGTTCCATCCTTGGAGCTGCCTGTGGAATCTTGAGAACCTTTGACCCACCTCTATCACATCTTCGTACATAATCC
TCTCGGAATCCTCGTTCATCGTATGCCCAGAAGTAGCCTTGCCTTAGAATCTTTTTTTCTGCCAAACTCTGCCCCCAGCGTGAT
CCCTGCAGAGCCTTCCATCGCACCTCTTCACGAGTTCCTTAGCCTTTGCCTGGTGTAACAACCTGAGTAGCTGGTATTGAGAAT
CCTTTCGGATACATAACACCATCGACCAATATGTCACCTAGCAGCTAGTCTTTGCAGAGTTTGACTTTGTTCCTTCTGTTGGGA
GCAATAACATTTGTCAGAACTCCAAAGTAAAGGCGCCATCCATGTATTTCCTTCATCGCGAATCTCCATCTCGACTTAGTTGCT
TGTATGCTCGGCTTACTCGTCTTTCAACTGCACATATTCAAAAGGTAGCGTCTTCTGTGCTCGCGAGTTTGGCCAAGGCATCTG
GCGTTTGAATTTTTTGATCTCGCGTGATCTGCTGGAGGGTGTACTTCGTGATGGGCTAAGCAGGTCCTTTGTTTTATTTAAGTA
GGCTACCATTTTCAAACCCGCGATTGATAATTCGTCCAAGGACCTGATTCACACCGCTGTGAATCACGCTCTGATATCACAGCA
TCTTTATGCACATATCCTTTGCCATTCTCGAATCCAGTGAGAGTGCTTCGTATTCTTTTCGTTTATTGTTTGATGAAGTGAAGT
CGAACCCTGATGGGCAGTGGAAATCGATGCCCTTCCGGCGTTATCATATCACTCCCCCGCTCCACGCGTGGTTCGTTGGAAGAA

CCATCCGTGAAATAGCTTACCACGAAGGAGTTTTGTCATGAGGCTCGGGCTGCTTCAGGCTGTTCGCACTGCTCGCTATTTGGT
GAGTTCAGCTGAAACCTCTGCAATAAGATCGGCCTAAGATGTTCTTTTTACTGCTGCTCGCGGTGAAATAGGTTTATGTCGAAC
TGCCCTAACTCGCTGCCCATTTTAACATTGCCCGCGCCTTCGGGTTTTTGGAGGAGCTTGCCGAAGGGGCTGATCAGGTTAGAA
CCGTGATAGGATGAGCTTGGAAGTAAGGGCGCAACTTTCTAGAGGCAAGGATCAAGCAATATGCTAATCTCTCGATTGGTGCGG
GATATCCAATTCTGCCCCGATCAGTCTCTTGCTGACATAGAGACTGCTTTTTGTGCACCTCTTCCTCTCGCACTTGCACCCGCG
CTGCAGCAACTTCAGTAACTTGCCAGATCAAATAAAACAAAGTTTTTCCTTCGATCAGCTTTGATAGATGGAGGAGAGCTGTGC
CATAATCGACCCTTCAGCGCCTGAAATGCTTGCTCGCAGAGTCCCTCAAATTTTGTTCCACCCTAAGTAGATTGAAGAATGGGA
TGCACTTGTCTGTTGATTTGAAATGAATCTACTTAGGGCTGCGATTCTTCCGGTTAAACTTTGTAAATCTTTTGATCCCTTTGC
TGGCGACTTCAATGTTGATCAGGGCTTTTAGTCTTGTCGGTGGTTGGCCTCGATTCCCCTTGTTTCATGAATAAACCACCAAAA
AACTTCCTGATTCGACTCCAAAGGAACACTTGAGGGATTCTACACTTTCATTTGGTACTTGTTCAACACATTAAAGCCTTCTTG
TAAATCCATACCATGCCCTTTTCTTTTTTTAGAATTACCAGCATGATCATCCACATATACCCCTCCATGTTTACTCCCAATTTG
TTATTTGAACATGTGGTTGACTTAGCCGTTGGTAAGCGGTCGCACCTGCGTTTTTCAGTGGAGGGCATTACTCTATAGGCAGTA
ACAAAGCCGCGTATGGTCCGAAAGCTGGTGCTGATCCTGCATCTGGGGGATGCATGCTAGATCTGGTTATAGCGACTACTGCAT
CGCATGAACTGCGCAGGCAATCTCGTCATGCAGTAGCAGTCGACGCAGCTTGGTCCGATCCTTGGGAGTTGGAAGGGCAGTCCT
TTCGGGCAGCGCTTTATTGAGGTCTGTAAAAAATACACACAGGTCCGCACGTTGCCGTTAGGCTTGGGGACCAGCTACCGGATTA
GGACGACGCCACCCAACGGATAACAAAGCGCACGCCTTCATGCGAAGTCCATTTCTCGCTTTGAGCCCTTTCCAGCTTCTTCTT
TTAAGGCTCGCGATCTTTCTTTATCGCACGCAAAGCTTCTTTTTTGTTGGGCACGGGTGTAAAGGTCTTGTCTATATTTCAGAC
ATGGCTGTTATGTGGGCAGGGATCTACTATCCAACCATGTCTTTATGTGCACCAGCGCGAAAACTTCCTGGTTTCTCGAGCATC
CGGCAGGAATTATCCACCCAATGGCATGCTTCGTGGTAGGCCTAAGTTTTTCCACTTTTATGACTGCCTGGTTGGGTCTATCTT
TGTCGCAGTTGACTTACTTCCAGCGTCTCGACCAGGTCCGAAAATTTTTCTTCAAATCCCAAAGAAAGGACCAATCCTCCCCGA
ATCCTCCACTTTTGGGCAACACCCTATTTTGTGACTTCACATCTATTGGGGCTTGCATATCACTTGCCACTCTGCAATCTAATC
TGGGGCAGTGGCTCCTCCGGTGGTCCGCTTTTCCGCCTTTGTGAATTGAGGCTGTTTGTTAGCACTCCCGCGCCTGGCTTCTCT
TTGGTTTCCAAAACAAGCGTCCTAGCTGCGTCTTGTGGGAACTTCATGGCTAGGTGCCACATAGAGATGACGGCGTAGACTCAA
CCCAGATATCTGCGGCTTACAATAACAGTATTGTACGCTAAAGAGGCAAAAATCGAGCGACGCGTAAAAAGATGGCGCGAGTAA
TGTCCTTAGTTGCGGCACTGTACCTGTCGTTACTGGGAGTTTGATCCATTCCGGCCTGGGGCGAGTCCTTCTCCC
>m141122_014521_42134_c100732202550000001823141405141560_s1_p0/27829/5704_12550
GTTGCAACATAAAAAAAAACATACCATATACACATATTATTCGAACAATAAAATACTAGTTGAATTGGCCATTGAATTACTTGG
GGTCCCATTAGAACTAAACAAGTCCATATGCCCATTAGATTAGTGGGGTCCTACCTATCTAAGCAGGTCATATGCCCATAATCT
ATTTGGGGTCCCGTACTAGCTTATAGCCATGGTCATATGCTCTCATAATCTATGTTGGGTTTTGTTTAGTTCATATGGGTCCAT
TATGCCCAAGCCTATATACAAACATACTCAACATATACATTTTCAATATATATCATTAAAACCATAGATTATACAGCTATAAGT
ACATATAGCATGGCATCATAAAATCCTATCCCATATTTTTCCTTATCCAAAAATAACCGAGGATATGATAAAAACTGATTTGGG
ACCTGAACACTCCTAAAAACCATTATATCAGATTAGGGTTGGAGTGACTTATTTTTAGGAAATATGTGCAATGTGGAATATTAT
GGCTTGGAATTAGGTCAGGTATTCGCGCTTTTCAATACTCATTGGAACTAAGAATAAGAATAAATATACCTGATTATATATAAT
AATGCATTGTACATATGTTCTTGTGTATGATTTCCTAGGACCAAATATTTAAGGGAGAATTAGGAGCAGGTTATTGATAGGCTG
AGTAGAATGTACCTTGGGAGACTTTGTTGAAAACCTTGAAAATTTATGTTTTATATTATGTGTTTGCGCACCCTCGCTGAAAC
AGACCTTAGAAATATGGTTCATTTTGCTTCATGTTTATGCCTCCATACCTTTTAGCCGGGGGTTGGATTAATCACTTACGTTTA
GAATAGTTCATTGTAGGAACCTTTACCTTGGAAGCACAAAATAGTCCTTAGCCGCTCAGTTCCTCTTAGCGCCGGGCACACCCT
CAGGGGTCTTAGTGCCCGTTACGCTCGAGGATTCTCTAGCCGAATCAGACACCATATCCTTGGAACCCAAAATAGTCTTCTCAG
CAACACTCCTTTAGCCGTCGGTGAGCACGCGCCAGCTACTGGCCACAGCACGCTACACTCGACACGTACTAGCCGCTTGAAACA
CACCTCAACTGCTCAAGGAGCACATTTATTTTATTTTTTTGTACTTAAAGAAATATTAATACGAATATGGACTAGAGTTTGGAT
ACTTTGAGCGTTCATCAGTGTCTCAGCTCGGATCGATCGCACCCAATAGACCACACTTGGAGACACACCCTTAAGAAGCCACTC
GTTGGAAATAGCACGCAAGCTTTCAGCTTCCAACGTTCGCTCGGTTGGTCTCAAGCCCATAAATAGAAACTCAATCTTAAGTCA
CATTGATCGCAACCCAGATCGATACTTAACACTCTCGCATACATAAGCGACATACAATTGACACAACTTTCATGCTCCTTTATA
TTTTCGCTTAAAATTTTCATCATTTCCTCAAAAATGTCTTTATCTATGGAACAAATTGATCCACTTACTCAGAGATTATTTTGT
ATTTTCACTGAGTTAGTGACAATATTAATTTTGCTTTTCATTCACTTTCTGTCTTGGTTTACTCACCTCTCCTTTATTGTTTGC
TACATGGAATTAGTTCGACTACAGGGGAGGTGGCACCATACAGACTCCTGAATTTCCTTCCAATTCCCTCTCTTCAAGATGATA
CTTTTCGGCAGTTTGGATTCCTCTTCCAAATCCCCAGCACGTAACTCCTAAGACCTGTCCTTCGTCGTCTGAAGAAAATCCTTC
GCGGTCAGACTTTATATTTCTTCACAAAGATCAGTTCCCTTAAACACAACATCAGCACCTACCTGAGATAGAGTGACCATAAGT
CTAATCAACTCCCTCAGGAACAGATCAAGCTTAACTCATAGAGCCAAACACAAAGAGGTGGTAATACGAGTACCTATGATGGAG
AAGAAATCTCTAGAGAAGCTGAGACTTCAAAGAAGCTTCATCAAAACCTATTTCGCCAAGCTCGAAAAACTATGCCAACCTAAG
AGAAATGGTAATGTCCCTTGAAATCCCTAATGTGGTTTAATGGTTGGATTAGTAGGAGTCGGATGAGGATCATAATTTTGACTT
TATTATCGCCCAATTAAACAAGTTATACGCATGTTTAATGTGAATTATATTTATAATATGTGTAATGCATGCATTGTGGGTCCA
TCATTTAATGACCAGGGGATTTTATTATTTGCCCGTTGGGGCTAATTGTATATTTTTGTATGCATGTCATGATATATTTTGTTA
GGCACATTATATAATTGGATTTTTTCGAGGATATTTCGACATGAGATGATCTTTTTGAGTTATAAATAGCGGTTGTCATAACGG
GAATTAAGTTCGAGCTTCCGGGTGAGTCTTTGGGGTGATTTTAATGATTAGAGCTGTCTTGCCTAAGGGAATTAATAGGTGTAG
CGGGAATATTGAGATGTTTGGTATATTTGAGAAATATCGAGAATATCAGGTAATTGGAGGTGTTAATTATGATTAACGAAATAG
TGGAAAGAACGTGGCTCGGGTGTTTTTTTTAACCTTGGGAGTTCTTTTTAGCAAACCTTTATTTGTACCTAAGGGGCATAAAT
TGTAATATTTTCAACCCCTAAGATATTTGATAAAGTCAGCTATTTGGCTATAGAAGCTTACCAATAAATACGAGACTCTTCTCT

TCAATTGATCATCAATTCTCCCTCCCTTCCTCCTTTTGAAGTTTTGATTCAATTTGAAGAAATAAGCTATGGAAATTATAAGGT
ATGAGCTTAGGACTTTGGTTCACGTTGATAGATGAACTTCAAAACCCAAATTGTAGGCAAGTAAATCATCCATGAATCTTTAGG
ATTTTGCTGCTATTTATTTCTTATAGTGGTTTTCGCTTATATTATTTTTGATGGTTGGATTAATTGGCCGAATTAGTGGAAGAT
TTTGAGTTTGGTTGCTTGGGTTTTGATGAGGTGTGATGTAGATGAATTTAGGGGTTGGATTGGATGTTTATGGTGATGTTTTTG
ATTTGGTTTGGAGGGTTAGTTTCAAGGCCGAAATCGCAAGGAAGGAAATACCAAGAAGAATTCTGGTCTGTTAATTGGGCACAG
CACGCCATTCCTTAGCGCAGCAGTGCTAGGCAATTGGGGCAGAGATGTTGGGCCTCTTTGCTTGAAATAGTGCCTCTAAACGCC
ATTTAATAGGGTTGCAGCATCTGTCCATTTTCCAGCAAGCCTATTTTAGGGCTCGGGATGACTTTTAAGGATCGTGGATGGTCC
TTTACCTTCCGTTTGGGTAGATTTGAGATTCCCTGAGTAGTCGGGAATTGATCTCGGAAGCGTGGTTTTGATTTTGAAACTCTT
TATGATTTTACTTTATTTAATGGATTCCATTGTTATGAATTATGGTGAACCACTTAAGGAAGCTAAAGGGCGATCATCTCAGGG
TCGCTCTTTCATTAATTCTGCTCGAACTCAGAGGTAAGAGAACTGCACCCATATGTGAACATGCATGGTTATTCATGAGGCGTT
TTGAATGTTTTAAATGTGGACATAGATTCAATATTGAATGCTTAGCAAAACTTGCTCACTTGTGTTATGGCTCTGACTGAATAG
TCCAGTTAATTGCAATGGTGTCAGTATCAACTTGTGAAGCTGTGACTCATTAGTCCAAGTTTAGGTAGTTGTACTTGGGGTACG
TTGGTCACATGTCAATTGAACTCATAAGTCAATGAAATGGCATAGCGTGTTAACTGCAAGCCAACAAAGATTAGATCTTATCGA
CATCTGCATTAAATGACTCAAAAGAGCATTAATGCCGGACTGACCTCAAGTTGATGGAATAATCTATAAGCGCTTGTCCTAGCC
AAAGGTAGTTACTTAGGGCACATTGACTACGTTAAGTCACATAGGACTGTGAGTGCCGAGCCCGAGTCACTTACCCATTCTCAA
AGTCCACTCATTTGTTAAGCTAGTTGTGACTACCCGTCAGGTTCACATCGTTATAAGCTAGTGCTTACACATTCAGTCACTCAT
ATGTTTTTTATAGAGCTCTCTTCTGTTCTTATATTTTGCAGAATATATGGGATTTAATTAATGTGGGTCCCATATTTTGCTTTT
TTGTATAGAATCTTGTTATCTAGGAACTAGCTTCGTCATTTGTTATGAGAGCTATAACCTCTTGTGATATAATGATGAAGTCAT
ACTATGATAATATTTATATCGCAATTAAAATTTTTTCTTGCATGGGCTTTGGCTCATGGGTGCTATGTGGAGTGGAATGTAAA
GGGAAGAAAGGCTCACCAGCCTTGAGTGGAGAGTGTTGAGTGGGTGATGTGTCATATGCGGAGGGTCGCTTGACCACCACATCC
AAGGAGTCTCAGAGAAAACTAGAGGGTTTTTACTCTCATTTTGTTTGCTGCTTAAGACGGCAGGTAGTACCAGATTTCCAACA
GTAATGACCATTTTGCATTATATATAACTTTAAAAATTTTTATGGGCCCATGACAGTTTATGTTTTTAAATAAAATATATCTTC
TCTTTTGATTGGTTTTCCACTTTAACCTATTAATAACACCTGAAGCACGTTTTTTTAAGACCGCGAAAGCCAACTAATTCGACT
AGTGATTGCTGTTAAATTTCATTGGTTCAAGAGTTCCAAACGTATACCGAGCTGGGTGGGTAACCAGGGCGTTACAAGAAAGGT
TTTACTCTATGGACTTTTGCCACTAGATAATCCAACTAACTGCTACACTTAAAGCTCAGGAAAAGGAAAGAAGACAAGTGCTGC
TCTTCCTGGTTTGTAGCTTAAGCCTTCTAAAGCTAAAACTCCAAAAAAATTTATAAGTCATATTAAGACTTTGGGTCTTGAAGA
GTGAATGTGATATGTCCAAAACCACACCAAAGGCCAAATATATTACCGGGATGGGCGTATTGTGTCCTGGTTCTAAGTGATCAC
ATTTATTTGCAGGAAGCTACAATCCTTTAGCATCCTTATCTGAGGTCGAATAGCAGTATTACTTTAATGTTTCTCTCCTTTACG
AGGATTGCTCCAAATGTACATGCCCTATCTGCTCTATGAGCATGTGCTCTTTACTTCACGGGCTGGAACCCACCTACTCCTCGC
GAGGTACACTATTTGTTTGAATTTTAGGACTAACCCTAGCCACAAATCACAAGTTTCTTTCCACCTTCTTTCATAGGCAAAAAG
GGGTTAAGATACCTTTCAGTGGGTATAGACCAAAAAATCGAATCCTAGGAAGTATAATATAAGGACAGGAAATACTTCCTTTTA
CGAAAGCGCAGACGATTAAAGCACAACTTGGCTTTTACATGGTCAAGCCTTTTGTCAACCTCCTCTCAACCAGAAGGATAATGC
ATGGCTCTCTGAGCATAAGATTCCGTTGCTAACATGCCACCTAGCAGGAGAAGTGTGTTGAGCAGTTGTCTCCTTTGGAGAAAT
CTCGCCGACTGGTGGATTAATATACAGGGGGGGGGGGTCGAGAGGGGGGGGGGGGGGGGGTGACATCGAGATATTGTGTGGGCA
GTAACAGCTGATGAAACAGACATAAGCGACTCGATTCAGTTAATGTTGAATAGATCGAAGTAGAGCTGACCGATCCAGTTTTCT
TCTTATACCATCCCCTCAGCCACGGCTGACCAGGTGGCCTATCATTAGAGATGCGTCTTCTATGAAAATCATCACCAACCCGCC
TTCCCCACCAAACCTGGCAAAGCAAAATCATTGAATCCTTCTAGATGAAGACAGCTCATCGTCGGGAAGATGAGGACGGAGTTG
TTGAACGAGCTCTTAACTCAGGGTTTTATCTGATAGGTAACCATATCAGATTGTGTGATGAGTTGTAGAATTTTCTTTTTCACC
TTATGTTCATTTTATTTGTTTCTCTAACCCAGTGTGCTTTACTTTTATTGCATATCCTACCATGTTCAATTGCAGTATCAACAC
TCCCTCCTGCCTCCAAAAGAGGGAAAACTGATGAAGATAGCATGCTTGAAGCTCTTCCAGAAGCAATGATTACTCGAGGACTAC
ACCACCTATGCTAGGGGAAACATCTGATCAGATCATTCATTATCCCACGCTTGACCTCCTCCATCAATTCCCTCTTCTGCTATC
CATATAGACCCTACTCGGTCAACATGTTAAGCGAGCCTCCCCCACCCCCCCCCCACCCCCCCCACCCCCCCCCCACCCCCCCAC
CCCCCCCCCCCCACCCCCCCCCCCCACCCACCCCCCCCCCCCCCCCCACACTCCCCCACCCCCCCGGCCCCCACCCACCCCCCC
ACCCCCACCACCCACCCCCCCACACCCCCCCCCCCGCCCACCCCCTCCCCCCCCACCCCCCCCCCCCCCCCCCCACCCCCCCCCC
CCCCCCCCCCCCCCCCACCCCACCCCCCCCCCCCCCACCCCCCCCCCACCCCCCCACCACCCCCCCCCCCCCCAACCCCCCCCCCC
CCACTCCCCCCCCCTCCCTTCAGCATCGTTGGTGAATATGACAAAGAGTTATTAGACATACTCTACAATGCAACGATAGCTCAG
ACCTTTGATGACTGTACCCCGACAAAGCGTCGAAGTTGTCCCTTCACAAAGGCCTTGTTCAAATTATTGGTGTATAGCATCTTT
AAAAAAATTCACTTTTTTTTGATTATTCTTATTTCTAATTTTCATGGTTATCTACCCCAAGGACTTATCCTGTTAGTTCTCGCT
CAACGCCGAGCAGTCGACCTAAAGGAGCTGATCTAAGGCTTGAATGGATCAGTTGGTGGAAGGCCAGCTAAGATTTAAACTTTG
ACTACAATCTGAAAAGATCTTCATCAAGGCTGAGCAGGCTAG
>m141122_014521_42134_c100732202550000001823141405141560_s1_p0/30423/9903_15122
GAGATTGGAAATGTTCCAAAAAAATTAGAGCTTTTAGAGAGAATATAACTGAATTATGAAATCGCTTCGGCGCCTACGGCCTGC
CTCGGACTTGGCCTGAAGGTACTTTTTTTGATTTGGTCTGAATTAGGCCCCAATTCATGGTCAATAACAGCCCTATATAATATT
ATTGAGATGGGAAAAGAATCAAAAAAATTAAGGGCTTGTTAGAGAGAATATGAGTGAATTACAGATCGCTTCGAGCCTCGGCAG
TGCCTAGAGTTGCTGAAGGTCAATGTTTTTTGTTTGTTTGAATTAGGACCCCAAGTCATGGTCAATAATACCTATAATAATAAT
TATTGAGATGGGAAGGGTTCAAAAAATTAGAGCTTGTTAGAAGAAATTCTGAGTGTATTATGAAATCGCTTCGGCGCCCTAAGC
AGCTGCCTAGGAGATTTGGCCTGAAAGGTCAATTTTTTTGTTTGGGTTGAATTAGGACCCCAATTCTGTTTCATAACAGCCCTA
TATATATTATTGAGATGGGAAAAGGTTCAAAAATATTAGAATCTTGTTAGGAGAATATGACTGAATTAACGAGATCGCTTCGGA

GTCCCTAGGCAGCTGCCTACGGAGATTTGGCCCTGAACAGGTCAATTTTTTTGTTTGGTTGAATTAGGACCCCAATTCGTGTCA
ATAAAGCCTATATATATATTGAGATGGGAAAGAATCAAAATTAGAGCTTGCTCTAGAGAGAATTATGAGTGATTTACGAAATCG
CTTCGGACATTAGGCAGCTGTGTAGTTGATGATTTGCCTGAGAGGCATTATTTTGTTTGGTTGAATTAGGACCCAATTCGTGGT
CAATAACATCCTATATATTATTTATTTGTAGATGGGGAAAAGGTCAAAAAATTAAGAGCTCTAGGAGAAATATGAAGTGCAATT
AACGAGATCTGCTCGGAGCCTAGGTCAGCCGCCTAAGAATTGGCTGAATATGTCCGTTAATTTTTTTGTATGGTTGAAATGCTC
CAAATTCATGGTATAATAGCCCTATCTATATTATTGAATGGGAAAGGTTAAAATTAGAGAATGTTTGGAGAGAAATATGAGTGA
ATTTAAAACGAAATCGCTTCGGAGTCTAGGCAGCTGCCTGGAGATTTGGCTGAGAGGTCATTTTTTTGTTTGGTTGATTAGGAC
CCTAATTTCATGGTCAATACAGCCCTATTATAATATTATTGAAGATGGGAAAGTACCAAAAAATTTGAGCTTGTTAGAAAGCAT
ATCGTGAATTACGAGATCGCTTCCGGAGACCTAGGCAGCTACTAGGAGATTTGGCTGAAAGGTCAATTTTGTTTTTGTTTGGTT
GAATTAGGACCCCAATTCATCGGTATACAGCCCTATATAATATTTTGAGATGGGAAAGTACAACAAACGTTTGACTTGTTAGAA
AGACATATGCGTGAATTACGAGATGCTTCGGAGACCTAGGCAGCTGCCTAGGAGATTTGGCCTGAAAGGTCAATTTTTTGTTTG
GTTGAATTAGGACCCCAAGATTCATAGGTCAATAATAGTCTATATATATTATTGGAGATTGGAAAATGTTTCCAAAAAAATTCAG
AGCTTTGTTAGAGAGAAATCTGAAGTGAATTATGAATCGCTTCGGCGCCTAGGCAGCATGCCTCGAACTTGGCCTGAAAGGTCA
TTTTTTTGTTTGGTTGATTAGCCAATTCATGGTCATAACAGCCCTATATAATACTTATTGGAGATGGAAAGAATCAAAAAATT
GGGCTTGTTAGAGAGAAATATGAGTGAATTACGAATCGTTATCGGAGCCCTCGGCAGCTGCCTAGGAATTTGCTGAACAGGTCA
ATTATTTTTGTTTTGTTTGAATTACGGACGCCCAAGTCATGGGTCAATAATACCCTATATAATATTATTGAGATGGGAAAGGGT
TCAAAAATTAGAGCTGTTGAGAGAAATCTGAGTGATTAGGAAATCGCTTCGGGCCCTATGGCAGACTGCCTAGGAGATTTGGCT
GAAAAGGTCAATTTTTTGTGTTTGGTTGACATTAGGACCAATTCTGTTCATAACGCCCTATATAAATTTTTGAGTATGGGAAAA
GGTTCACAAATATTAGATCCTTGTTAGGAGATTGACCTGATTACGAGATCGTTCGGAGCCCCTAGGCAGCTGCCTAGGAGATTA
TGGCCGAAAGGTAATTTTTTTGGTTTGGTTTGAATTAGGACCCAATTTGGTCAATAACAGCCCTATATAATATTATTGAGATGG
AAAGGTTCAAAAAAAATTGAGCTTCTTGGGAGAAAATATGAGCTGATTACGAGATCAGCTTCGGAGCCCTAAGGCAGCTTGCCT
ATGAGAATTTTGGCCTGAAGGTCAATTTTTTTGTTTGTTGAATTAGGCCCCAATATCATGGTCAATATCGCACTATCTATATTA
TTTCGCAGGATGGGAAGGTTCAAATAATTAACGAGCGTGTTTTAGAAGAGAATATTATTGAATTACGAGATCGCTTCGGAACCT
AGGCACGTTGCCTACGGACATTTGGCCTGAATGAGTCAATTTTTTGTTTGGGTTAATTAGGACCCAATCATGGTTCAGTAACAG
CCTATATAGATGATTAATTGGATGGAAAAGGTTCAAAATATTAGATCTATGTTTAGGGGGAGGAAATATGATGAATTACGAGAT
CGCTTCGGCCCTAAGGCAGCTGGCTAGGAGATTTTGCCTGAAAGTGCAATTTTTTTGTTTGGTTGAATTAGACCCAATTCATGG
TCAATAACAGCCCTATTATATCTTTTGAGATGGGAAAAAGACAAAAAAATTTGCAGGCTTGTTAGAAAGACATATGCGTGAATT
ACGAGACTCGCTTCGAAGACCTAGGAGCTGCCTGCGAGATTTGGCTGAAGGTCAATTTTTTTTGTTTGCGTTGAATTAGGACCC
CGAATTTCATGGTCAATAATAGCCCTATCTAATATTATTGAGATTGGAAAGTTTCAAATAATTCAGAGCTTGTTAGAGGAATAT
TATTGAATTCGTAGATCTGCTTCAGAGCCTAGGCAGAGCCTAGGAGATTTGGCCTGAAATGCGTCAATTTTTTTTGTTTTGGTT
TAGCAATTAAGGAGCCCCAATACTTGGTCAATTAGTACCTATCTATATTATTGGGATGGAAAAGGTCAAAAATATTCAGAGCC
TTGTTAGAGAGAAGTACTGAGTGAAAGTTATGAAATTCGTTTCAAGCTAGGGCAGCTGCCTAGGACGATTTGGCTGAAAGGTCA
ATTTTTTGTTTGGTTGAATTATGACCCCAATTCATGGTCAAATAATAGCGCTATCAAGTATTAACTTGACATGGGAACGGTCAA
AAAATTAGAGCGTTGTTAGCAGAGAAATATGAGTGAATTACGAAATCGCTTCGGAGCACTAGGCAGCTGGCAAGGAGATTTGGC
TGAAGGTCAATTTTTTTGTTTTGGTTGATTAGAGCAATTCAGGTCATACAGCCTATATAATATTTATTGAGATGGGAAAAGGTT
AAAAAAAATAGAGCTTCTTAGGAGAAATATGAGTGGATTACGAATGCTTCGGAGCCTAGGCAGCGCGCCTTAAGAGATTTGGCC
TGAATGGATTAATTTTTTTGTATGAGTTGAATTTGGACCCAATTCGTGGTCAATAATAGCCTATCTAATATTAATGAGATGGGA
AATAGGTTCAAAAAAATTAGCGCATGTTTTGAGAGAAATATGAGTGATACGAATCGCTTCGGCCTAGGCTGGCTGCCTTGGAGA
TTTTGGCCTGAGAGGTCAATTTTTTTTGTTTGGCTGAATTAGGACGCAATTCATTGGTCATTAACAGCCTGTATATATATTTTG
AGTGGAAAAGGTACCAAAAAATTTGAGGCTTGTTTAGAAAGACATATGCGTCGACATTAACGAGATCGCTTGAGACTAGGCCGA
GCCTTGCCTAGGGAGATTTGGCCTCGAAAGGTCAATTTTTTTGTTTGGTGACGACATTACGGACCCCAATTCATGCGGTCAATA
ATAGTCGCTATTAATATTATCTTTGAATGGGAGAATGTTTCCAAAATTCAGCAGCTTGTTAGAAGAACATATATGATTATGAAA
TCGGCTTCGGAGCCCTAGGGCAGCTGCCTAGGAGATTTGGCGCTAAAAGCGTCTTTTTATTGTTTCGGCTTGATTCAGGACCCA
TTCATGGTCCGAACAGCCTATATAATATTATTCGCGAGGGAAACAGAATCAAAAAAATTAGAGCTTGATTAGAGAGAATATGAG
TGATTAGAGATCCGCCTTCGGAGCCCTAGGCAGGCTGTACGCACCTAAGGAAATTTGGCCTGAAGGTCCAATTTTTTTGTTTGT
TTGAATTCAGGACCCAAGTCATGGTCAATAATAGTCCCTATACTACGCATATTAATAGGATGGGAAGGTTCAAATATTAGAGCT
TGTTAGAGAGAAATCTGAGTGAATTATGAATCGTTTCGGACACTAGGCGCTGCCTTAGGAGATTTGGCCTGAAAGGTCATTTTT
TTGTTTGGTTAATTAGGACAGCAATTTCAGTTCAGTAACAGCCCTATATAATATTACTGAGATGGGAAAATTAAAAATTAGAGC
TTGTTAGGGAGAAATATGAGCTGAATTACGAGATGCGCTTCGAGCCCTTAGGCAGCTGCCTAAGGAGATTTGGGCTGAAAGGTC
AATTTTTTTGTTTGGTTTAATTAGGACCCCAATTCGTGTCAATAATAGCCCTAGTATACTGTATATTGAGATGGGAAAGATCAA
AAAAATTAGAGCTTGTTAGATGAGAAAATAGTGGTGATTACGAGTGCTTCGGAGCGCCTAGGCAGCTGCCTTAGGAGATTTTGG
CCTGAAGGTCAATTTTATTGTTTGTTTGATTGGACCCCAAGTCTGGTCATTAATAGCCCTATCATAATTATTGAGAGGGAACAA
GGGTTGCAAAAAAATTAGAGCTTGTTAGAGAGAAATCTGGTGAAATGAATCGCTTCGGCTAGGCAGCTGCCTAGGAGTTTGGCC
CTGAAATGGTCAATTTTTTTGTTTGGTTTGAATTAGGACCAATTCAATGTTCAATAAGAGCCCTATATATGTGTCTTTGATATT
ATTGAGATGGGAAAGGTTCAAAAAATAGAGCTTGTTAGGAGAAATATGACTGAATACGAGATCGCTTCGGAGCCCCTAGGAAGT
GCCTACAGAGA
>m141122_014521_42134_c100732202550000001823141405141560_s1_p0/31381/6166_14514

TGAGTACTCACACACCCAGCACAAAGCCGAAACTGTCACCGAGGGAGGAAACACATTTTTTGGTAAGCATAGGAACAGTATCAT
CGCACCCGACGTCGTCAATTGCACCCACCCCCGCCCCTTTGTACCCCAATCCTTTGGGGGAGAAATAAAAAAGAACGCGTTCTT
TTTTCTTTCTCTTTAGCTTCCCCCCTAAGCCCCATCATAAATGGTCCCTCTGCTCTCTCTCGCATCCTCTAATAAGGCAAAGGC
CACCCGGGTTTTTTTTTCTTTTTTTTATTTTCAGGAAAAAAAACCCAAATATCTCTCTCTGTAGTGGCGCAGGAAATAGGCCCCC
CCCCCGTTCTTTTTGAACAGAAAGTATAATGGGTTTTTTACTTTGGCTGGAGTCCCCCTTTCATGGGGCTTTCTTTCCTCAAGC
AAAGACAAATGGGGATTATTTTGGGGCTTTTTTTTTTTTTTTTGGTTTTGATAGATCGCCCCACGCCGAAAAAGCCCCGAGGGC
GCCGCGCGCGGAGAGAGTTTTTTTGGGGGGTCCCCCCCCGCCCGCCCAACCCCCCTTTTTTTTTGGCTGGGGCTAGTTTTTTTT
CCAAAAAAACCGCCCCTTTTGGGAAAAAATAAAAGGCCCCCTTTTTTGGGCCCCCAAAAAAGAAAAAATTTTGGGCTTTTTATTT
TTTTCCCCCCCCAATTGCCCCCAAGGAAAATTTCGAAATTTCAAAAGACCCCCTTGGTGGGGATTGGCCCCAAATGGGCCCGGA
GGGCCATGGAGGGGGCCCGTTTTTTTTTTGTTTTTATTTATTTTCAAAAAAGAAAAAAGCCAAATTTGGGTTCCCCCTTCCCAA
AAATGGAATTTGTGCGTTTTTAAGAACCTTCAGGCCCTCTTTTAAAACAAAAATTCAAAAGATTTGCCCCGCGAATTGGAAAA
GAATCCGGCAAAGAAACCCCAAACAGGAAAAACCCCCCCACAATTGACCCCCCCAAACAAAGCTTTTTTTTTTTCAATGAACCC
CCCCCGGGAACTAAAATTTGTTTCTTTTTTTATTTGCTTTTTTTTTCTTTTTACCCAAGTTTTCCCCCCGATGCCCCCCATTTT
GTTTTTGGGGTCCCCCCCCTTGAAAGGGAAAAAGTTGGGGCCCCCCCCGGAAACAAGCTAAACCCCTTAAACCAAAAGGCCCCT
TAGTTTTTAAAAAAAAACCCCCCCAAGAAAGTGGGGTTGCCGCAAGACCAGATCGGGAAAAAAAAAAATAAAAAACCAATTTGGGG
AACAGTGGCCTAACGTTTTCCCGATTGGACAAAAAAAAAAAATGCCCCCCCTTTGGGCGCGTTTCCCCCCCCCCCCCCCCCCAAA
AGTTTTTTTTCCAAACAAAGCACTTCGGCCGAAACAAACCCAAAAATTTTGGGGGGTTTTTTTTTTTTTTTTTACTTTTTTTTT
TCGACCCCCTCTTTGGGAAACGAAAATTTTTTGTTTAGTTTTTTTTTTTTTGTCAACAAATAAAACTAAACCGAGAAAAAATTGA
GTGGCCCAAAATGGAAAAAGGGGGCCTTTTCCAAACCCCTCCCCCCCAGAAAAAATTGCATTTGTTTCTTTTTTTTTTTCCCGG
GTTGGTTCCCCCCGCATCAAGGGGAAAAAAAATTCTTTCCTTTCTGGGGGTTTTTTCATTAAAAAAGTCCCCCCCCCCAAAAA
TTTACCCCGAAGGGAAAAATTAAGAAAAAAGGGGGGGCCCAAAAAAAACAACCCCCGACAAGAAAATCACAAAGGGGTCCCCCC
AACTTTTTGCTTTCCCCGAGCCCTTTTGTCCTTCTTCCCCCCCCCAGGTCCCGCGGGACGGAAATTTTGCTCCCCCCCCAAGC
GCCCCCCCCCCTCACAGGGAAAGGGTCTGGGCAAAAAAAAAAAAGAGCAACCCCCCTCCAAAAAGAAACCCCGGTTTCTCTTTT
TCAATTTTTTTTATTACTCCCTTTTAAAGTTTTCCCCCAAACCCCGAATTACAAAAAGGTGTTTTTTGTACCCCTTTTTTTTTT
TCCCTTCACAAACCTCTGCCCCCCCCCCTTCCCGGGGGGAAAAAAACCAAAAAGGAAAGTTTTTTGTTTTTTTTTTTTGCGGGA
AAAAATGGGAAACCAAGAAGTAGAAAATTTTTGGCGGAAAAGAGCCCCAAAAAAAAAAAAACATTCCCCCCCCATCCGACCTGA
AGGCGGGCAACAACAATTTTCTTCCCCCCCCCTTTTTTTGGCCTTTTTTTGAAAACCTTTTTTGCCCCTTTTTTTTTTTTTTTTT
GTTGGGCTTTTTAATCCCCCCCCCCAAAAAACCCACAAAAAAGGGTTTAGGAAAAGAAACCTTTTCTTTTTTTTTTTTTTTTTTT
TTTCGCTGTTTTTTGTTTTTTTTTCCTCTACCCAATTTTGGCCGGGTTTTCAACTTTTTTTTTTAGGAGGTGCCAAAAGCCTTAAG
CCGGGGCACAAGATCCCCCCCCGCCCTTTTTAAGGGTTTGTTTGCCCGAGGGAAAAGGGAGGGAAACCCCCCCCCAAAAGTTTT
TTTTTTTTCAAGAAAAAAAACCAAAAGTCCCCTTACTACATCCCAACATTCAAGCAAGAAGGACCCTTTTTGCCTTTCCCGGAA
GCCGGGAATCCACCGCAAAGTACAAAACCCCCCAATTTTTCTGGAAATTGGAAAAAAAAAATCCCCCCCCCGTTTGGGCCGGGT
TTTTTTCTGCCCTTAAAATTTCCCAAATGGCCCTCCCCCCGGCGAAAGCCCCCCCCTTTTTTCAGGCCCCCCCCCCCCTAAAAA
CTTAACAAGTTTTCCTCCACCCTTTTTCAATTTTAACCCACCAAAGATCCCCAAAAATGGACCCCTGGAATCCAAAAGCCCCCC
TTTTTATAAGGACAGGTTTTTTTTTTTTTTATTTTGCCCAAAAAAAAAGCCTGGGGGGGCGTCCCGGGGGAGACGTTTTTTTTT
TTTGGGTTAAAAAATTTCGGGCCAAGTCCCCCCACCCCGAAACCATCGTTCTCCCCAAGAATATATTTTTTTTTTTTTTTTCGA
AGAATTTTTTTTTCCCCCCCCCCCCCCCCTACACCCACTCCCCCAACTGAAACCTTTTGCCTTAAAAAAAAAATAAAAAAAAAAAA
AGGGTCAAAAAAAAATGGGCCCCCCCTTTGGGCCCCGACTTTTTTTTGGGGGTGGGGGGCCAAAAAAAAGAGTTGCCAAACGGG
GATTTTTTTTTTTTTTTTCCCCCCCAAAAAAAAAAAGAGGGAAAAATTTTCCCCGTCAAAGAAAAAAAGGCCCTTTTTTTTAACC
CTTTCAAAAAGGGTTCCCCCCCGGCCCCCCTTTTTCCGCCCCCCCCCCAAAAATTTCCCCGTTGTGGGGGAAAGGAATCCTTTCCG
TGATTGGTTTTTTTTTTTTTCCCCCCCTAAAAAAAGATCTTAAAAAACGGAAAAAAAAACGGGCCTCCCCCGAAAGGGGCCGGAC
TTCAAAAAAAATTCTTGATATTCCACCCCCGGAGGGGGGGAACCCCCCATAATAGATTTTTTTTTTTCCCAACCTCGAACCGCCG
TTAAGGTTTTCCCCCCCCACCGGGGGTTTTTCACAAAAAGGGGGGCCGTTCCTTTAAACCAAAAAAAACCCGAGGGGCCCCCAG
AGTTTAAAAAAAAAACGAAGCTTTTTTTTTTGGTTGTTTTTTTGGGCCTTTGGCGGCCCCCCCCCTTTTGGAGGGGGGGGTTTG
GGGCCCAACGGGAAAAGTTTTTACCCAACAGGCCAAAGCTCCCGTTCCCAGTTGGGGGGGCCCTTTTTTCCCCAAAAAAAAAAA
AAGTTGACCTTTTCCCCCCCCCCCCCCCCAAAAAGCCTCCCCCGTGGTTAGAAAAAAAAAAACCAACAGGGGGGGTTTTTTCCC
CTGGGCGAAGTTTTTATTTCTCCAAAAAAGGCCGTGGGGGGGAACCCCCCTTTTTCAAAAAAAAAATTTGGCCCTTTTTTGGCC
AGCTATTTTTTTGGGGGCGGAAAAAAAAAGTAAAAAGCCCGAAAGCCCTTTGTCCCCAAAACAATTTTGTGAGCGAGGGGGGTTC
CCGATTCCCGAAATAACAAAAAGAATAAACCCTTCAAAAAGGAAAAAAAAAACAGAAACGCCAAAAAAATGCCGGGGCAATTTGG
CTCCCCTTTTGGCCCCAAAACCTTTTTTTCCCCCCAAAAACCCACCTTGCTACTTTCTCACCCGGGAAAAAAAAAGAGGACCCCC
CCAGAAGACCCCCCCCTCGGGGGTTATGGGGGGTGGCCAATAGGAAAAAAAAATTTTCCTTTTGGAAAAAAAAACCCAAGTAAATAG
AAAGGAAAAGATTTTCGGGGGCCACCCCAAAAAGAGGGGTTTTTCCCCCCCGAGATTGGAAAAAAATTTGGGAATGTCGCATAAA
AAGGCCCCCCGCCCCCCCTTTTTGGGAATGGGAACTTTTCCCCCAATTTTTTTTTTTTTTCCCCTTTTTTTTTTTTTGGGGGAGG
TTATTTCCTTTCCGGCCCGAAGGTCCACCCCCAAAGACTTTTTTTTGTTTTTTTTTTTTTTTGGCCCCCCTTGAAGGGACGTAAA
ACTGAACGCAACCGATTGAAAAAAATCTTTTTGGGGGTTATCAAGCTTCCCGAAGAAAATATTAACGGATAAAAGTTACCCGAT
GGAGGGTTTTGTTCTGTAACCCCGACCCGTGGGGCCCCACTTTTTTCCCCCCTACCTTCCTTTTTTTTTTTTTCTTTCCCCCCG
GTTGGGGGGGGCGTTTTTTTTTCCCCCCTTTTCCAAAAGTATGGGGAAAGCCGAAAGGCCCCCCAAAAAAATTCCCCACCCCTGC
CCCAAGGAAAATTTTTTTTTCAAACCGAAAAGGAATTTTTCCCCCCCCCTTTTTGCGGAGAACCCCCAACAACCCTTTGGGGGG

CGGGCCCCCAAAAAAGCCCCCCCTGGGCCCTTACACAGGTTTTTTTTTTTACCTCAAAGGCCCCAAAGGGGGTTTAATTTAA
ACTTGGGCCCCCCCCCGAGCTTTCATCCTTTACATCCCAACAAAGAACCTCCGATCATTCAAGAACAAACAAAAAAAAATAATGT
GGAAAAAAGTGGGGGCGACCAAAGTTTGGGGGGCCCACAGCCCGAATTCCGCTTTTTTGCAGGGGTATTTTGGCCCCCCCCCCG
AGCCCTTTTTTTTTTTTTTCTTCCCCCTTTTTCCCAAAGGTCCCCCGAGCCCCTTTTTTTTTTTTTTTTTTTTTTTTTTGAAA
ATGGAAATCCTTTTTTCGGCCTGTTATTTTGGGGGGGCCCCCCCATCCTCCCCGCCGTTTTTTGGTGGGGGGGGAATGTTAATG
GATTTTTAGTTAAAAAAAAAAAAAAAAAGGCCCCACCGCCGCCCCCTGCCCCTAACCCCGGAAAAAAAAGGGGCCCGGGGTTTCC
CGTTTGTAAAAAAACCCCCCGCCCTGTGGGTAAGCCCCCCCGATCCCCTACCATTTTACCCCCTTTTCCCCACTTTAAGTTGGG
GGCGAAAAGTTTTAAAAAGAAAGTTTTCCCCCGTTTTATTTTTTTGGGGCAAAACAAATAAAGAACACAATTCCGAAAAAAAAGG
GGTTACTGACCTTTTTTTTTATTTCCCCGTTGGGGTTTTTTTTTTTTTTTAAAAGCAAAATCCAAAAATCCTTTTTGCTTTTTT
TTTGGAAATTTTTCCCGGGCCACTTGCCCCAAGAAAAAAAATCCGTATCCCGACAACGAACCAAAAAAACCTCCCAGTTTTTCCC
GGACCCAAGTGGGGGGACACCTCTTTTTGTTCACCCACCCGAATTTTTTTGGTTGGGGGAAAGGCACAGGAAAATTTTGGGTTT
TGGGGGGCAAAAGGCCTTTTTTTTTTCTCCCCTTTCTTTTTTCCCCCCGTTGGCCCACTTTTAAATTTCCTTCCCCCAGAGGCC
CCGGAAAACCCGGCCCCAAGGTCCCGAGGCCTGGTCCCCCCAAAAAACAAAAGAACCCTCCCCTCCTTAAAAGGGCCGAAGCCC
CTCAAAAAGGAGAGGCTAAGAATTTTTTTGACGGTCCAAAGGGGGCCCCCTGGGCCCCCCAAAAGAAAAACAAAGCTCCCCCCC
AAAAAAGGGTCCCTGGTTTGGGCATACCGGGGAAAAGCCTCGGGCAATCGGGCTCCCCTTTTTTTTAAAACCGGGGGGTCCCCC
CCACCCCCCCGAAAAAAAAATAACTCCCTTTTTTTCCTTTTTTTTCCCCCCTAACCTTTTTTGTTTGGGAAGGTGGGGGGGAAG
CCCAAAAAAAAAAAAAGTTTTTCCCCCCCCTTGTTTTCCCCCCCCGTTGGGGAAAAGTTAAAAAAAGGTTTTTCCCCGTCCACA
AATAGAGGTTCCCCCCCCCAGCCTTTACCCGACCCCAAAAAGTGGATAATCGGAAAACCAACGCAGCCTTTTTCCCCCTTTTTT
TTAATTTGCCCAAATTCCCCCCCCCCTAGGGAACCCCCCCTAGTTCCCCCGAGAAAAAAGGGCCCCCCGAAGAAAGGAATTTT
TTTTCCCCGGCACAACCTTTCCCAGCTCGGGCCCCCATTATCAAAAAAAAAGCAAAGGAATTTACAACTTTCCCCCCCGCCTT
TTAACTTTTCCCCCAGAAACAACTTTAAAAGGGTCCCAAAAAAAACGTTTGGGCCCTTTTTTTTTTTAAGCCCAAAATTTTTT
TTTGGCCCGACCAGATGGATTCCCCCCCCCCCCTTAAGGGGGGAATAGAAGTTTTTTTTTTCTTTTTTTTTTTTATTCCCTG
GGCCCCAGGGGAAAGAAGGATTCCCCCCCAAAAAAGATTTTCGGAAAAAAGGTCCCCTTTTTTTTTTTTGGGGGGGGGACCCC
GAGCCCCCAATGGGAAAGGACCCCACAATTAAACACAAAAAAGGTCCCCATTAACCCCCGGAAAGTTCCATCAAAGAAAAGGGG
GGAAAAACCCCCTATAAAAATTCTTCCTTCTTTTCCCAACCAACCAAACAAACCGGGGAGGAAGGGAAAAGGGAAAAGAAAAAA
AAAAGAGGAGATTTTTTAAGTTTTCCCCCCCCTTTTTTCCCCTTTTTTTTGGGAAAAAAAATTTGACTTTTTTTCCGATTTGTT
GACCTGGTAATGGTCCCCCCCCCTTTTGATTTGTCCCCAGCCCTCGGGGTTTTTCCCAAAAAAGACCCTTCATCTTTTTTGGGA
AATTACTCTTCCCCCGCCTTGGCCCCCAGGAAAAAAACCTCTTCCCCCCCCTTTTAGGAACCCCATGGTTTTCCCGCCAACATT
TGCCCCCATTTTAAAAAGGGCCAAAACGTTTTTTTTTTTTTTTGAAACTTTTTTTTTTAGTTGGGTTTGAAGTAAAAGGCCCGA
GTTTGATCCTTTTTTTTTTTTTTGCTTTTTGATTAAAATTTGGGCGAGCCCCCCCATGGGAAGGTTTTTGCGAACTCCCCCCCT
TTTCCCCTTTCGGCCTTTTTTTTCCCAATTCCGGACCCCTAGGGTCCCCTAAGGGAAATGGCAAAAAAAAAAATAGGGAAAAAA
GCTTTTCCGTGGGTTTTTTCCCCGATCCCCCTTTTTGGGGGGGTTCCCCCCGTAAAGGACCCTGGGACCCCCCCCCCCCCCTTT
TTTCTTTATTTCTTTGTGGAAAAACAGAACTTTCTTTTTTTAAAAACCTTTCCCCAACCGGGGAGTGACCTTTTTTTTGCCTTT
TCAAAAAAACCCCCTTTCACCGAAAGGGGGGGTTTTTTTAGGGGGAAGAAAAAAGGAAAGGTTACTGACCCGTAAGGGGAAGTT
CCCACCCCCGATTGCCGAGGGTCCGGGTGAAATTGGCCCACCAGGGAAAAAACCCCTTTTTGGGGGGGGAAGCTTGTTTTTTTC
TTTTTTTTTTTGGCCCCAAAGCCCCCCCCCCTTTTTTTTTTTTTGCCTTTTTTTCAAAAATTCCCTAGCCCCCTCTTTTCTT
TTTTGAAAGGGGGCCTTTTCCCCGCCCTTTTAGAGGTTGCCCCCTTTTTTTTGGTTTTTTTGACAGCCCTCCCGACCTTGGCC
CCCCGTTTCCCCCGCCTTTGAAAAGGATTTAGGCCCCCCCCCCCCGGAAAGAGAAAAAGCCTTTTTTTTTTTTTTTCCAACCA
AATTCCCCCCCCCCCTGTGGCCCCCCTTTTTTTTTTTTTTTTTTTTCAAACAAAAAAATTTCCCCCCCCCCGGGTGGAAACCA
AAGGTTTTCCAACCCTTGTTTTTTCCCCCGAAACTTGGAGGGTTTTGGTCCCCCGTTTGTGTTTCCGGATTTACGGATTTTTTT
TTTTCTTTTTTTTTTTTGGCCCAAAAAGCGAATCCAACCAGATGAAAATTTTGGCTGTTTTTTTTTTTTTTTTTAACCACGGAC
GTCAAGGGTTACCCTTTTTTTTTTTTTTCCGA
>m141122_014521_42134_c100732202550000001823141405141560_s1_p0/32571/0_5007
TGGGGAAAGAGAGAAGATTGAAAGGGTACCCCTTTGGTGATTGGAGATTTCACAAATTTCTCGAATGTATTACACTCCGTGTTC
AAACTCGTTCGATATTTTTCCCCCAAGAGATCTCATGGGTAATGATATATTGGAGTCCGAAACAGAGCCTTAATTCAACATTTA
AAAAGAAATTCCCCTTTGTTGAGGGCCAATTTTCAGCCAAGTTTTTTCCTTATATTCGTACATCCCAGTATTTTGACTGTAATC
ACCCTATACAACACCTCTTATTACAGAAATTTTATACATAGAACCCTAAGACAATAGGGCTACAACTTTTGTGCCCAATAATCC
TCTACATATTATTTAAGTATTCACTATCAAACATTCCTCGAATCATATTAGTAGAAATCCGAAGATTTCTGAAAGGGGTTACCC
CTTTGGTGGAATTGGAGATTTCACTAATATCTCCGAAATGTGTTACCCTCGTGTTTCAACTCTTTCGAATTTTTCCCCAAGAGA
TCTCATGGGAAAATGAATATAATTGGAGTCGAAACAGAGCCTTAATTCACATTTAAAAAGGAAATTCCCCTTTTTGAAGGCAAT
TTTCAACAAGTTTTTTCCTTATATTCGTACATCTCTCAGTATTTTGTCTGTATCGCCTATAACACTCGGATTTGCAAGAAATTT
TATACATAGAACCCTAAGACAATAGGCTACAACTTTTTGCCCAATATCCTCCGCATATTAATTTAGTAATTTCACTATCAAGAA
CTTGCTGATTCCCATATTAGAGAAATCGAAGATTCCATGGTGGTGAATTGGGAGATTTCACTAATTTCCTCCGAAATGTATTAC
CCTCGTGTTCAACTCATTCCGATATTTTTCCCAAGAAGATCTCATGGGTAAATGAATTATATTCGAGCGAAACAGGCCTTAATC
AACATTTAAATAAGAAATTCCCCTTTTGATGGCTAATTTTCACATGTTTTTTCCTTATTACGTACATCTATCAGTATTTTGACT
GTATCTACCCTAATACACACCCTCGGATTTGCACGAAATTTTATACATAGAACCTGGTTTTTCTTAGACATAGGGGATACAACT
TTTGTGACCATGACCCTCGCATATTCTTTAGTATTCCACTATCAAAACATTGCTCGAAGTCCATATAGAGAAAATCGAAGATTT
CTGATAAGGGGTACCCCGTTTTGGTGAATTGGAGATTTCACTAATTTCTCCGAAATGTATTACCCTCTAGTGTTCCAACTCGTT

CGATAATTTTTCCCCAAGAGATCTCCATGGGTAAATGAAAAATATTGGAGTCGAAAACAGAGCCTTAATTCAACAGGGTTTAAA
AAGAAAGTTCCCCTTTTGAGGGCAATTTTCACAAAGTTTTTTCCTTATATTCGTACATGCTCTCAGTATTTTGACCTGTATCAC
CCTATAAACACCTCTTATTATTAGCACGAATCTTTATACATAAACCCTAAGACAATACGGCTACAACTTTTTGTGCCAATATCC
TCACATATATTTAGTATTCTACTAATCAAAACATTCCTCGAATCCATATTAGAGAAATCGAAGATTTCTGAAAGGGGTACCCCT
TTGGTGAATTGGAGATTTCACTAATATCTCCGAAATGGTGTTACCCACTCGTGTTTCAAATCTTTCGATATTTTTCCCCAAAGA
GATCTCATAGGGAAAATGAATATATTGGAGTCGAAACAATGAGCCTTAATTCCAACATTTAAGAAAGAAATTCCCCTTTTTGAA
GGCAATTTCACAGTTTTTTTCTTATTATTCAGTACATCTCTCAGTGATTTTGACTGACGCCCTATACACCCTCCGATTTGCAAG
ATATTTTATACATAGAACCCTAAGACATAGGGCTAACAACTTTTGTGCCCAATATCCTCCGCATATTATTTTAGTATTCACTAT
CAAAACATTGCTCGATTCCATATTAGAGAAATCGAAGATTCCATTGGTGAATTGGAGATTTCACTAATTATCTCCGAAATGTAT
TACCCCTCGTGTTCAAACCTCATTCGATATTTTTCCCCAAGAGATCCATGGGTAAATGAATATATTCGAGTCGAAACATGAGCC
TTAATTCCCAACATTTAAAAAGAAATTCCCCTTCCTTTTGAGGCAATTTTTCACATGTTTTTTTCCTTATATACTATCATTCTA
TCAGATATTTTGACTGTATCACCCTATACATCACCTCTGGTTTGCACGAAATTTTATAACATAGAACCCTTAAGACATAGGATA
GCAACTTTTGTGACCATGACCTCGCATTATTCTTTAGTATTCACTATCAAGAAATTGCTCGAGTCCATATTAGAGAAATCCGAA
GATTTCCTGAAAGGGTACCCCCCATTGGTGAATTGGAGATTTCACTAATTTCTCCGAAATGTATTACCACCTCCGTAGTTCAAC
TCGTTCGATTATTTTTCCCCCAAGAGATCTCATGGGTAAAATGAATATATGCGAGTCGACAGAGACTTATTCAACATTGATAAA
AGAAATTCCCCCTTTTTGAGGGCAATTTTCACAAGTTTTTCTTATATTCGTACATGCTCTCAAGTATTAAAACATATCACCCTA
TACACACATCTTTATTAGCACAGAAATTTTATTACAGAGAACGCCTAAGACTATAGGGCTACAACTTTTGTCCCAAATACCTCA
CATATTACTTTAGTATTCACTATCAAAACATTCCTCGAATCCCATATTAGAGAAATCGAAGATTTCTGAAAGGGGTACCCCTTT
TGGTGATTTGGAGATTTCACAAATATCTCCGAAATGTGTTTAACCTTGTGTTGAAGCTCTTTCGATATTTTTCCCAAGAGATC
TCATGGGAAAAAATGAAATATATTGGAGTCGAAACAGAGCCTTAATTCAACATTGAAAAAGAAATTCCCCTTTTTGGAGGCAAT
TTCACAAGTTTTTTTCCTTATATTCGTACATTCTCTCAGTATTTTTACTGTATCACCCTATACAGCACCTGGGGATTTGCAATG
AAATTTTATACATAGAACCCTAAGACAATAGGCTACAACTTTTGTTGCCCAATATCCTGCATTATTATTTAGTATTCACTATC
AAAACATTGCCTCGAGTCCATATTAGAGAAATTCGAAGATGTACATTGGTGAATTGGAGATTTCACTAATTTCTCCGAAAGTAT
TAACCCCCGTGTTCAACTCATTCGATATTTTTCCTCAAGAGATCTCATGGGTAAAATGAATATATTGGAATTCGAAACAGAGAC
TTAAATTCAACATTTAAAAAGAAATTCCCCTTTTTGAGGGACAATTTCCACAAGTTTTTCCTTATTGTCGTTCATTCCTCTCA
GTATTTTGACTGTATCACCCTATACACACTCTATTCCACGAAATTTTATACATTAGAACCCTAAGACTAGTGGGTTACAACGTT
CTGTGCCCAATATCCTCACATATTATTTAGTATTCACTATCACAAACATTGTCGAATCCATATTAAGAAAATAGATAGATTTCT
GAAAGGGGTACCCCTTTGGTGATAGGAGATTTAATAATATCTCCGAAATGTGTTACCTCAGTTGTTCAACTCTTTGTCGATATT
TTTCCCCCAAGAATCTCATGGGAAAATGAATATATTGGAGTCGAAACAGAGCCTTAATTCAAATTGAAAAAGAAATTCCACTTT
TTGAGGGCAATTTTCACAAAGTTTATTCTTATATTCGTGACATCTCTCAAGTATTTTGACTGTATCATCACCCAGTAACACCTT
CTTATTAGCACGAAATTTTATACATAAGACCTAGAGAATAGGGCTAACAACTTTTGTGCCCAATATCCTCACATATTATTTAGT
ATTCACTAATCAACAACATTCCTCGAATCCATATTAGAGTAAATCGAAGAATTTCTGAAAGGGGACCCCTTTGGTGAATTGGAG
ATTCACTAATATCCCGAAATGTGTTACCCTCGTGTTCAACTCTTTGATATTTTTCCCCAAGAGATACTCATGGGAAAATGAATA
TATTAGGAGTTCGATAACAGAGCCTTAATTCAACATTGAAAAAGAATTCCCATTTTTGAGGAAATTTTCAAAAGTTTTTTCCTT
ATATTTCGTACATCTCCTCAGTATTTTTACTGTAGTACCCTATAACACACCTGGGGATTTGCAAGAAATTTTATCCATAGAACC
CTAAGACATAGGGGCTACCAACTTTTGTGGCCCAATATCCTCGCAAAAATTATGTAGTATTCACTATCAAAACATGATCCAGTC
CATATTAGAGAAATCGAAGATTCCATTGGTGAATTGGAGATTTCACTAATTTCTCCCTGAAAATGTATTACCTCGTTTCAACTC
ATTAGAATATTTTTCCCCAAGAGATCTCATGGGGTAAATGAATATATTGGAGTCAAACAGAGACTAATTCAACATTTAAAAAAG
AAATTCCCTTTTTGAGGGAAATTTTTCATAAGTTTTTTCCTTATATTGTACATTCTCTCCAGTATTTTGGACTGTAATCACCCT
ATATCAGACCTCAGATTTGCACGAAATTTTATATACATAGAACCCTTAGACATTGGGATAAACAACTTTTGTGACCATGACCCT
GCGCATATTATTTAGTATTCACTATCAAAACCATTGCTCGAGTCCAATTAGACGAAATCGAAGATTTCTGAAAGGGGTACCCTT
TGGTGAATTGGAGGATTTCAATAATTTGCTCCGAAAATGTATTACCCTCTGTGTTTCAACTCGTTTCCGATATTTTTCCCCAAG
AGATCTCATGGGGTAAATGAATATATGGAGTCGAAACAGAGCCTTAATTCAACATTTAAAAAGAAATTTCCCCTTTTTGAAGGG
CAATTTCACAAGTTTTTCCTTTATATTGCGTACATCTCCTCAGTATTTTGACTGTAATCACCCTAAAATACACACCTCTTATTA
GGCACGAAAATTTTATACATAGAGACCCTAAGACGATACGGGCTACAACTT
>m141122_014521_42134_c100732202550000001823141405141560_s1_p0/36733/10088_15712
AAGACGCTCCAGTTAAGTCAAACATGTCAAACAGAAAATTCTCTTTCTATATGGCAGTCATATGGCAATTTTCGCAAGTTTTTT
CCTTATATTGTAGGCATCTCTCAGTATTTTCACTGGTACCTCACCCCTCTTACACACCCTGGGATTTTTACCGAAATTTTATAC
ATAGGAACCCTAAGACAATAGGGGCTTACACACTTTTGTTCCAATGACCCACCGCATATTCTTTAGAATTAACTATCGAAACAA
TGCTCGAATTCGTATTCAGATGAAATCGAAGATTTCTGAAAAGGGTACGCCTCTTTGGTGAATTGAGATTTTCACTAATTTCTT
CCGAAAATGTATTACCCCTCGTGTTCAACTCCGTTCGACTATTCTGCCCCAAGAGATCTCATGTTAAAGAATATATTTGCGTCG
AAACTGAGGCCATAAATCAACAGTTTAAAAAAAAAATTCCCCTTTTTTGATGGCAATTTTCGCAAGTTTTTTCCTTTATATTCGT
ACATGCTCTAAGTATTCTCCCACTGTAACCACCCCTCTACACCACCCTCCCGGATTTGTACGAAATTTTTATACATAGAACGTT
AAGACATAGGGCTACGAATTATGTTCCATGACTAGCATATTTCTTATATTCACTATCAAACAATTGCTCGAATTCCGTATTAGA
GAAATCGAAGATTTCTGAAAGGGTACCCCTTTGGGTGAATTGGAGATTTCACTAATTTCTCCCGAAATGTATTACCTTCGTGTT
CAAGGGCTAAGTTCCATATTTCTCGCCAAGAGAATCTCCATGGGTTAATGAATATATTGGAGACGAAACAGAGCCATAAGTCAA
CATTTCCAAAAAAAAATTCCCCTTTTGATGGGCAATTTTCGAGTATTTTTCCTTATATTCGTACATCCTCAGTATTGTAACTG
TATCAGCGCCTCAACACACCTCCCCCGATTTGTACAATTTTTTACATAGAACCTAAGACGTAGGAATACAACTTTTTGTTTCAA

TGACCCTAGCATATTTTTTAGTATTAGGTATCCAATACTTTGCTCGAATTCGTATTAGAGAAATCGAAGATTTCTGAAAGGGGT
ACCCCTTAATGGTTGAATTGGATATTTGCACTAATTTTCCGACAATGTATGTAGCCCCTCCGAGTTCAACTCGTTCGATATTCT
CCCCAAGGGATCTCAATGGGTAAATGAAAATATTGGAGTCGAAACGGAGCCCCATAAGTCAACATTTCAAAAAAAATTCCCCTT
TTTCATCATGCGCAATTTTCGGCAAAGTTTTTTTTCTTATATTCGTACATCTCTCAGTATTTCACTGTACCACCCTGCCTACAG
CAGGTCGGATTTGTATGAAAATTTATACAGTAGAACCCTAAGACATAGGGCTACAACTTTTGGCTTCCCATGAACCACGCATAT
CTCTTTAGAATTAACTATCGAAACATTGCTCGAATTCGTTTTAGAGAAATCGAAGATTTCTGAAAGGGGTAGCCCTTTTGGTGA
ATTGGAGATTTCACTAATTTCTCCGGCCGAAATGTATTTACCCTCCGGTGTTGCAACTCCGTTCGATATTTTCCCCAAAGAGAT
GCCCATGTTTAAAAAAATATATTGGCCGTCCGAAACGGAGGCATAAGTCAGATCATTTCAAAAAAATTCCCTTTTTGATGGCAA
TTTTCGCAAGTTTTTTCCGTATATTCGTACATCTCTAAAGTATCTCTACTGGTACCACCTCCCTAAAAACACACCTCGGATTTG
TACGAATTTTATACATAGAACGTTAAGACATAGGGCTACGAATTAATGTTCCCCATGACCCTAGGGCATCATTTCTTAGTATTA
CTCACTATCAAAACATTGCCTCGAATTCGTATTAGAGAAATCGGAAGATTTCTGAAAAGGGTACGACCCTTGGTGAAATTGGAG
ATTTCACTAATTTCTCCCGAAATGTATATTAAACCCCTCGTGTTCAACTAGTTCCATACTTTCTCCCAAGAGATCTCCGAATGG
GTTAATGAATATATTGGAGTCGAAACAGAGCCCATAAGTCAATATTTCAAAAAAAATTCCCCCTTTTGATGGCAATTTTCGAA
AGTTTTTTCCTTATAATTCGTACATCTCTCGTATTTCACTGTAACGACCCTCAACAAAGGTCGGTTTTATACGAAATTTTATCA
TAGAAACCTAAGACATAAGGGGCTACAAGTTTTTGTTCGCATGACCGACGCATATTCTTTAGGATTACTATAGAACATTGATCG
AATTCGTATTAGAGAAATCAAAGATTTCTGAAAGGGGTACGCTCGCTTTGGTGAATTGGAATTTCACTAAATTTCTCCGAAATG
TAATTACCCTGCCGTGTTCAACCTCGTTCGATATTTCTCCCCAAGGGATCTCATGGGTAAAGTGAATATATTGGATGTCGAAAG
CGGAGCCAAAGTCAACATTTCATAAAAAAAAATTCCCCTTTTATATGGCAATTTTTCGCAAGTTGTTTTCGCTTATATTCGTAGG
CATCTCTTCAGTATTTTCACTGTACCCCACCCCACCTACACAGTCGGATTTGTACGGAAAATTGTATACATAGAACCCTAAGAC
ATAGGGCTACAACTTTTGTCCATGACCCCACGCTATACTCTTTAGAATTAACTATCGACAACATTGCTCGAATTCGTATTAGAA
GAAATCGAAGATTTCCTGAAAAGGGTACCCTTTTGGTGAATTGGAGATTTCGGCTAATTTCTCCGAAATGTATTCACCCTTCGT
GTTCAACTAGTTCCATATTTCTCCCCCAAAGAGATCTCATGGGTAAATTGAATATATTGGAGTCGAAACAGAGCCCATAAGGTC
AATCATTTCAAAAAAAATTTCCCCTTTTTGATGGCAATTTTCGATAGGTTTTTCCTGTATACTTCGTACATCTCTCAGTATTTG
TAACATGTATCACACTCCCAACACACCTCGAATTTGTACGAATTTTTTAGCATAGAAGCCCTAAGACGTAGGAATGACACTTTT
GTTCCATGACCCTAGCATATTTTTTGTAGATTCAGTATCAATACATTGTCCGATTTTCGTTACCCTCCGTATTAGATGAAATGA
CAGTTTCTGAAAGGGGAGCCCTTTTGGTGAATTGGATATTTCACTAATTTCTTCCGAAATGTATTAACCTCTCCCAAAGAGTTC
CAACTCCCGTTCGATATTTCTCCCCCAAGGGAGTCTCATGGGTAAATGAAAAATATTGGAAGTCGCAAACCAGAAAGAAGCATA
AGTCAACATTCAAAAAAAATTCCCCTTTTATATGGCAATTTTCGCAGTTTTTTCTTATATTCGTAAACATCCCCTCAGTATTTT
CCACTGTACCGACCCTCGTACAGCACCCTGGGATTTACGATAAAAAAAAATTTTTAAACATAGGAACCGCTAAGACATAAGCGG
GCTACAACTTTTGTTCCCATGCCGCACGCATATTCTTTAGAATAACTATCGAAACAATGCTCGAATTCGTATTAGAGAAATCGA
AGATTGCTGAAGGGGTACCCCCTTTGGTGAATTGAGATTTCACTAATTTCTCCGAAATTATTAACCCTCGTGGTTCAAACTCGT
TCGATATTTCTCACCCAAGAGATCCTCATGTTTAAATGAATATATTTGCGCCCCCGAAACGAGCCATAAAAATCAACATTTAAA
AAAAAAAATTCCCCTTTTTGATGGCAAATTTTTCGCAAAAGTTTTTTCCTTTATATTCGGTACATCTCTAAAGTATTTCACCCT
GTACACCCTCTAAGCACACCTTCGGATTTTGTATCGAAATTTTTAACATAGAAACGTTAAGACATAGGGCTACGAATTATGTTC
CCGATGACCCTAGCATATTTCTTAGTATTCACTATCAAAACATTGCTCGAATTCGTATTAGAGTAAAATCGAAGAATTTCTGAA
AAGGGTAGCCCTTTTGGGTGAATTGGAGATTCACTAATTTCTCCGAAATGTATACCTTCCGTGTTCAACTAGTTTCCATATTTC
CTCCAAGAAGATCTCCATGGGTAAATGAATATATTGGCAGAAGCGAAACAGAGAACATAAGTCAAACATTTCAAAAAAAAAAAT
TCCCTTTTTTGATGGAATTTTCCGATAAGTTTTTTCCTTATATTCGTCATCTCTCAGTATTGTAACTGTATCACCCTCAAAACA
CCTCAGAATTTGTACGAATTTTTTTACATAGAACCCTAAGACTGTAGGAATACAACTTTTTGTTTCCATGACCTAGCATATTTT
TTTAGTATTCAGTATCAATAATTGGCTCGAATTCGTATTAGAGAAATCGAAGATTGTCTTGAAAGGGGGTACCCTTTGGTGAAG
TTGGATCATTTCAGCTAATTTCTCCGAAATGTATTACCCCTCGAGTTTCAAAGCTCCGTTCGATATTTCTCCCCAAGGGATCTC
ATGGGGTAAATGAAAAAATATTGGAGTCGATCCCAAAGTCAACATTTCAAAAAAATTCCCCTTTTATATGGCAATTTTCGCAAG
TTTTTCCTTTGAATATTCGTAAATCTTTTTTTCCAAGTATTTTTCACTGTACCACCCTCTAACAGGTCGGATTTGTATGAAAAA
TGTTATAGCATAGAACCCTAAAGACTAGGGCTACAACCTTTTGGCTCCCATGACCCACGCATATTTTTTAGAAATAAACTATCG
AAACATTGCTCGAATTCGTTTTAGAGAAATCGAAGATTTGCTGAAAGGGGTATCCCTTTGGTGAATTGGAGATTTCACTAATT
TCTCTAGGAGACTTCTACTTTTCGTTTGCAAGGGAGTCCGCCTTGACGCGAAAGCGCCTCTGAGAGGCTCGGAAATGCCTCCTT
GAAATCGACCAGAGAAACTTTTCCACGAGCTAAATAGAATGTTTCTTATATTGTCGTTAAACCCATTGCCTAAGCTTGGCCGC
GATCAGAGTCAAAGGGAATGATCAAGCATTCAGGGCTCGGGCCTGAATATCTAATGGGCAGCCCAATCATCAGGGTGTTGAAGC
ATCCCAAGTGATCAACCAGATCCTTTCCATCCCCCATCGAATTGGTAGATGGGAATCCTAAAAGCGCTGGCGGGTACATCGTTG
CTGTGATTTTTTGTTGGGGGGCCCCAAAAAGCGCTCCGAGTTCATCCCCGACGTCATATTAGTCTCTTCGCCCTTTTCTGATAG
GAAGTCTCTTCCATGTAGCTCGCTCCATCCTGACGCTACACCTTTTGAGGGTTTGGTCTTTGATCCGCTTGGTGTTTGGGCTAG
TTCCAACAGGCTTCGTCCCATGTAGCCGTTAGGTGGGTTATTGTCCCTCCAAGCTTGAGGCTCTGGTTTGGTGGGACATTCCCG
TTGTTCACGTATTTCGGTAACGGACGCATCCTCTCGGCGAGCATCCCGACTTTCATTTCTGGTCGATATCCCCGCTCTGTGAAT
TGAGGCGATCTCGAAGGTCGGTTCGTGGGGCGGCTCGAGGGCTTTGTGCCGAAGCTTAGATGATTTTCTCAGATCTCCACCCAG
AGGATCAGGCGCACTTCGGTGGCTTTCGGTTCCCAATAAACGTCCCATTAGAGAACGCTCAGAGCCTGGCTGTCGACGAG
>m141122_014521_42134_c100732202550000001823141405141560_s1_p0/37699/962_6042
ATCTTTTTTGCTTAAAACTTTAAAGCGTCGATTTTTGTAATGGGGATCATGCGTGTATCTTTCCTCTGAGGCCGGGTTATGCAT
ATAGTGTGATAGCCGTATGATCGGCATCGCCGCTCACTAACAAGCAGCGAATAAGAAGTTCCTAGTCCAATCCGGGGGACTCGA

AAGGGAAAGGTGTTTATCGTATCCGCAATATTCTCACGCCGGCCTTAATACTATCTCCGCATAAATGAATACTTTCGGCGGACC
AAAACATTCCGGGTGCGATCTTACAGCACATCAATGCAACCGGATCCCATCAGAACTGCCGCAGTTTAAGCGTGCTTCTGGGCC
GAGAGTAGTATAAGGATGGTGACCACTCCTGGGAGTCTCGTGTTGCACCCTGAAAACATCATTTTTTTTTCCCTTTAAAATCC
ACTACGGCAAAAGTTTGTATTTTTTGTTTTAAATTTTAAAAACTCATTTATTCTGCGTTTTTCCTTGAGGGGTTAATACCGTGG
TCCTAACAGCAGCGAATAAGAAGTTACTAGTCAATCCGGGGGAGAAAAGGGAGGACAACACGCCGCTCGCTACTATAAATGAAT
ATTGCGGACCATAAACATACTCTCCGGGTGCGATCATACCAGCACTAATGCACCCGATCCCATCAGAACTCCGCAGTTAAGCGT
GCTTCACAGGGGCCGAGATAGTAGTACGTAGGATGGTGACCTCCTGGGAGGTCCTCGTGTTGCACCCCTGAAAACATCATTTTT
TTTTTCCCTTTAAAAATCCACCTACGGCTCAAAAGTTTGTAATTTTTTCTGTTTAAATCTTTAAAACTCATTTATTTGTTTTC
TTGAAGGGGTTAATACGTGGTCACTAACAAGCCAGCGAATAAGAAGTTCCTTAGTCAATCCGGGGGGAGAAAAGGGAGGACAGG
ACACGCCGCTCGCACCTACAATGAATATTCGGAAGCAAACATTCCGGGTGCGATCATACCACAGACTAATGCCAGCGTTCCACG
ATCCAGAACTCCGCAGTTAAGCGTCGACTTGGGCGGAGTAGACTTAAGGATGGTGACTCAACTGCGGAAGTCTCGTGTTGCACC
CTGAAAACAATCATTTTTTTTTCCCTTTAAAATCCACCTATACGGCGTCAAAAAGTTTGTATTTTTTGTTTTAAATCCTATTAA
AAACCTCATTTAAATTTCGTTTTCCTTGAGGTTAATACCGTGGGTGGTCATAACAAAGGCAGCGAATAAGAAAGTTCCTAGTCC
AATCGGGGAGAAAAGGGACGCGACCACCCAGTGCAGTTCCGGCGGACTACCGCGTGTCGCTATACACTGAATATTGCGGAACAA
ACCATTCGCCGGGTGGATCATACCAAGCACTAATGCACCGGATCCATCCAGAACTCCGCAGTTAAGCGTGCTTGGGCGAGGAGT
AGTACTAAGGATGGGTGACCTCCCTGGGAAGTCCTCGTGTTGCACCCCCTGAAAACAGTCATTTTTTTTTCCTTTAAAAATCCC
CTACGCTCAAAAGTTTGTATTTTTTGTTTTAAATCTTTAAAAACTCATTTATTTCGTTTTCCTTGAGGGGTTAAAATACCGATG
GTCACTAACAAGCAGCGAATAAGAAGTTTCCTATCAATCCGGGGGAGAAAAGCGAGGACACACCGCAGCTCCGCTATAATGAAT
ATTGCGGAACAACACATTCCGGGTGCGATCATACCAGCACTAATGCACGACCCATCAGAATCCGCAGTTAAGCGTGCTTGGGCA
GAGTAGTACTGAGGATGGGTGACCTCCTGGAGAAGTCTTCCCGTGTTTGCACCCCTGAAAACATCAATTTTTTTTTCCCTTTA
AAAATCCACCTTACAGGCTCAAAAGTTTGTATTCTTTTTGTTTTAAATCTTTAAAACTCAATTTATTTCGTTTTCCTTGAGGGG
TTTAAATACCTGGTCACTAACGAGCAGCCGAATAAGAAGTTCCTAGTCAAATCCGGGGGAGAAAAGGGAGGACAACAACCACCC
GCTCGCTATAAATGAATATTGCGGAACAAAATTTTCCGGGTGCGACTCATACCAGCAACTAATGCACCGGATCCCATCAGAACT
CCGCAGTTAACGCGTGCTTAGGGCGAGAGTAGTACTAGGATGGGTGACTCCTGGGAAGTCCTCGCTGTTGCACCCCCTGAAAAC
ATCATTTTTTTTTTCCCTTTAAAATCCACCTAACGGCTCAAAAGTTTGTATTTTTGGTTTTAAATTCTTTAAAAACTCATTTA
TTTCGTTTTCCTTGAGGGGTTAATACCGTGGTCACCTAACAAGCAGCGAAATAAGAAGTTCCTAGTCCAATCGCGGGGAGAAAA
GGGAGGAACACCGCGCCCTCCGCCTTATAAAACCTGAATATTGCGAGAACATAACATTCCGGTGCGATCATACCAGCACTAATG
CACCGGATCCCATCAGAACTCCGCAGTTAAGCGTGCTTGGGCGAGAGTAGTACTAGGATGGGTGAGCCTCCTGGGAAGTCCTCG
TGTCTTGCACCCTGAAAACATCCATTTTTTTTTTTCCCTTAAAAAAAAATTCCACCTACGGCTCAAAAGGTTTGTATTTTTTGT
TTTAAATCTTTTAAAAACTCCATTTATTTCGTTTTCCTTGAGGGGTTAATACCGTGGTCCACATAACAAGCAGCGAATAAGAAG
GTTCCTAGTCCAATCCGGAGAAAAGGGAGACACACGCGATCGCTATAAATGAATATTGCGGACCCAAACATTCCGGGTGCGATC
ATGACCAGCACTAATGCAGCCGGATCCCATCAGAACACCCGCAGTTAAGCGGTGCTTAGGGCGAGAGTAGTACTAGGATGGGTG
ACCCTCCTGGGATCCTCCGTGTTGCAACCCCTGAAAACCATCATTTTTTTTTCCCTTTAAAAATCACACCTACGGCTCAAAAG
TATTTATTTTTGTTTTAAATCTTTAAAAACTCATTTATTTCGTTTTCCTTGAGGGTTAATATACCGTGGTCACTACAGCAGCG
AATAAGGAAGTTTCCTAAGTCAACTCCAGAAGAAAAAAGGGAGAAAACGCGCACTCGCTATAAATGAATATTGCGGAACACAAC
ATTCCGGGTGCGATCATACCAGCACTAATGCACCGGATCCATCAGAAGTCCGCAGTTAAGCGTGCTTGGGCCGGAGTAGTACTA
GGATGGGTGAACCTCCTCGGGAAGTCCTCGTGTTGCACCCTGAAGACATCATTTTTTTTTTTCCCCTTTAAACAATCCACGCT
ACGGCTCAAAGTTGTATTTTTTTGTTTTAAATCTTTAAAAACTCATTTAATTTCGTTTTCCTTGAGGGGTTAATACGTGGTCAC
TAACAAGCAGCGAATAAGAAGTTCCTAGTCAATCCGGGGAGAAAAAAGGGAAGGACAGGCACGCGCTCGCGTATAAATGAATAT
TGCGGAACAAACATTCCGGGTGGATCATACCAGCTAATGCACCGGATCCCATCAGAACTGCCGCAGTTAAGCGGTGCTTGGCGA
GAGTAGTACTAGGATGGGTGACCTCACTGGGAAGTCCTCGGTTGCACCCCTGAAGAATTATTTTTTTTTTTCCTTTAAAAATCC
ACCTACGCTCAAAAGTTTGTATTTTTGTTTTAAATCTTTTAAAAACTCCATTTATTTCGTTTTCCCTTGAGGGGTTAACAACCG
TGGTCACTACAAGCAGAGAATAAGAAAGTTCCTAGTCATAGGGGGAGAAAAGGAGGACACACGCGCTCGCCTATAAATGAATAT
TGCGGACAAACATCCGGAGTGCGATCATACCAGCACTAATGCACCGGATCCCATCGAACGTCCGCAGTTAAGCCGTGCTTGGGC
GAGAGTAGTAAGCTAGGATGGGTGGGACCTCCTGGGAAGTCCTCGTGTTGCCCTGAAAACATCATTTTTTCTTTTCCCCTTTAA
AATCCACCTACGGCTCAAAGTTTGTATTTTTGTTTTAAATCTTTAAAAACGTCATTTATTTCGTTTTCCCTATGAGGGTTAATA
CGGGTCACTAACAGCAGCGAATAAGAAGTTCCTAGTCGATCCGGCGGGAGAAAAGGGAGGACACCACGCGCTCGCTATAAATGA
ATATTGCGGAACAACATTCCGGGTGCCGAATCATACCAGCACCTATGCACCGGATCCCCATCCAGAATCCGCAGTTAAGCGTGC
CTTGGGCGAGAGTAGTAAGTACTAGGGATGGGTGACCTCCTTGGAAGTCTCGTGGTTGCCAACCCCTGACAAACAGTCATTTTT
TTTTTCCTTTAAAAATCCAACCTACAGGCTAAAAGTTTGTATTTTTTGTTTTAAATCTTTAAAAAACTTCATTTTATTCTCGTT
TTACTTGAGGGGTTAATACCGTGGTCACTAACAGACGTCGAATAAGAAGTCCTAGTCAATCCGTGGGAGAAAAGGGAGGACACA
AGCGCATCGCTATAAATTGAATATTGCGCGAACCAAACATTCCGGGTGCGATCATACCAGAAGCACTAAATGCACCGGATCCCA
TCAGAACTTCCGCAGTTAAGCGTGCTTGGGCGAGAGTAGTACTAGGATGGGTGACCCTCCTGGGAGTCTCGTGTTGCACCCCTG
AAAACATCATTTTTTTTTTCCCTTTAAAAATCACCTACGGCTCCAAAAGTTTGTGATTCTTTTGTGTTTAAAATCTTTAAACTC
ATTTATTTCGTTTTTCCTTGAGGGGTTAATACCGTGTCCTAACAAGCGCGATAAGAAGTTTCCTTAGTCCCAATCCGGGGGACG
AAAAGGGAGGACACACGCGCGTCGCTATAAATGAATATTGCGGACAACAACATATCCGGGTGCCGATCAATACCCGCACTATGC
ACCGGATCCCATCAGAACCTCCGCAGTTAAGCGTGCCTTGGGCCGAGAGTAGTACTAGGATGGGTGTACTCCTGGGAAAGTCCT

CGTGTTGACCCCTTCGAAAACATCCATTTTTTTTTTTCCCTTTAAAATCCACCTACGGCTCAAAAGTTTGTAATTTTGTTTTAA
ATCTTTAAAACCTCATTTATTTCGTTTTCCTTGAGGGGTT
>m141122_014521_42134_c100732202550000001823141405141560_s1_p0/38653/8152_16568
TTAGAAAAACATACTTACTAAGAGACCGCTCTCATCTATCTTCGTTTTCCTTGGGTTTACATTAACCAATGTCGGGGTCACTGA
CGCAACGCTCAGCGAATACAGAAGTTCCTAGTACATAACCCGAGGGGAGAAAAGGGAGGGGCCCGCGTATCGCTAATAAATGAA
TAATCGCGGAACAAACATTCCGGGCCCGCGCCGATCATACCAGCACTAATCGCACACCGGATCGCCATCCAGCAACTCCGCAGT
TAAGCCGTGCTGGGCGACCGAGTAGACTAGGATGGGCTGACTCATGGGAAGGTCCCTCAGTGATTGACACCCCTGAAAAACCAT
CATGGTTCTTTTCTTATTTCTTTAAAAATCCCTCTACGGCTCAGAAAGTTTGGTATTCTTTTTAGAAAAAAAAGTCTTTAAAGC
TCAATTTCTATTTCGTTTCCTTGGGGGGTTAATACCGTGCCACTGACAAGCCAGCGAATAAGAAGTTCCATAGTTACATCGAAG
GGAGAAAGGGAGGGCACCGCGTTCGCTCAATAAATGCATAATGCGGCACAAACATTCCGGGCTGGGCAGTCATACCAGCACTAA
TCACCGGATCACCATGCAGCAAACTACGCAGCTTAATGAACGTGCTATGGCGACGAGTCAGCTTTACATAGGATCGGGCTGACC
TGCGCTGGGAGTCTCGTGTTGCACCGCCCTGAAAACATCATCTTTTTTTATATGATCTTTAAAAATCCTACTTACGGCCCTCAA
CAAAGTTTTTTTTTAAACAAAAAATCGTTTAAAAGCCTATTTCACTTTCAGTTTCCTTGGGGGTTAAGTACCGTCGGCCCCCA
CTGACAAGAGCGAATAAGAAGCTTCCTAGTTAATCGCGAGGAGAAAAAGGGAGGGCACGCGTTCGCTATAGAAATGAATATTCG
CGGCGACCAAACCATTCCGGGTCGCCGATCTTACCAGTGCACTAATGCACCGGATCCATCCCAGCAAGCTCCGCACGTTAAGGG
TGGCTTGGGCGACGAGTAGTACAGGATGGCGTGAGCGGCTCCCTGGGAAGTCGCTCAGTGTTGGCAGCCTAGAAAACATCATTT
TTTTTTTTATTCTTTAAAAATCCCAATTAGCCTCAAAAGTCTTGTATTTTTTTTAAAAAAAAATCCTTTAAAACCTCCATTTATG
TGTGTTTTCGCTTGGGGTTAGATCCCCGTGGTCCCTGACCCACACCAGCGAAATAAGAAGTTCCTAGTTAAGTCCGAGGGAGAA
AAGGGAGGCACGCGTTCGCTATAAAAGTGAATAATGCGGCAACAAAGCTTCGCGGGTGCGATCATACGCAGCACTAATAGCACC
GCGGATCCATCAGATGACTCGCAGTAACGCGTGCTTGGGCCCGACGAGTAGTACCTAGGATGACGGTGACCTCCTGGGAAGTTC
CGTGTTGCACCCCTGAAAAACATTTCAGTTTTTTTTTTTATCTCTTAAAAATCCACTTACGGCTCCAAAAAGTTTTTTTTCTAA
AAAAAATCTTTAAAAGCCTATTTAATTTCGCTTTTCGGCTTGTGGGTTAATGACCGTGGCCACGACAAGCCCAGCGAATAAGAA
GTTCCCTAGTTAATCCCGAGGGAGAAACAGGGAAGCCACGCGTTTCGCTATAAACTGGAATAATGCGGGAACCAAACATTCCGG
TGCGATCCATACATACCAGCACTAATCGCACCCGGAATCCCATCAGAACTCCGGCAGTTAAGCGTGCTTGGCGCGACCGAGTGA
GGTACTAGGATGGGTGCACCTCCTGGGAAGCTCCTGCGTGTTGCAACCCCCTGAAAACGCATAATTTTCTTTTTTATTCCTTTA
AAAACTCCAGCTTACGCGCTCAAAACGTATTTTTTTTTAAAAGAAATAAAGTCTTTTAAAAGCTGCATTTATTTGCGTTTTCCT
TGGGGGGCCTTTAATACCAGTCGGCCACTGACCAAAGCGGGCAATCGAAATAAGAAGTTCCTAAGTTTAATCCGAGGGAGAAAA
GGGAAGGCACGCGTTGCATATAAACTGGACCATAATGCGGAATCAACATTCCGGGTGCGATCTTACCAGAGCACTAATGCACCG
GATGCCCATCAGAAGCTCCGCAGTTAAGGGTGCTGTGGGCGAGAGTAGATATAGGAAGGGTTGACCTCCTGGGAAGCATCCTCG
TGTTTGGAACCCCCTGAAAACATAATTTTTTTTTTTTATTCTTTTAAAATCCATCTAACGGGGCTCAGAAGTTTTTTTATTTAA
AAAAAAATCTTTAAAAGCTCATTTATTTCGTCTTTTCCTTGGCGGGTTAATAACCGTTGGCCACTGACCAAGCAGCGGCGAATA
AGAAGTTATCACTAGTTAATCGAAGGCGACGAAAGAGACCCCCAGCGTTCGCCTATACAATGAATATTGCGACGCAAACATTCC
CGCGGCTGCGATCTTACAGGCACTAATCAGCGGATCCCATCAGAACTCCGCATTAAGGTGGCTTGGCCGAGAGTAGGTAGCTAG
GATGGGTGACCTCCCTGGGAAGATCCTCGTGTTGCCAACCCGCCCCTGCAAAAACATCGATTTTTTTTTTATTTCTCTTAAAAT
CCAGTTCGGTCCGCTCCTGTATTTTTTTACAAAAAAATCTTTAAAAGCCTCATTTATCTCTCGTTTTCCTGGCACCCCCCCCTA
TAATCACGCGTCGGTCACTGACGAAGCAGGAATAAGAAGTTCCTAGTTAACAATCCGAGGAGAGAAAAGGGACGCGGCACGCGT
TCGCTATAAATGATACATGGCGGAACAAACATTCCGGGTGCGATCATACCAGCACTAATGCAGCCGGATCGCCAATCCCGAAAC
TCCGCAGTTACAGCGTGCTTCCGGGCGAGAGTTGCAGTACTAGGTGGGTGGACCTCCTGGGAAGATCCTCCCGTGTTGCACCCC
TGAAACATCATTTTTTTTTTTATTCCCTTTAATAATCCCTTTACGGCTCAAAAGTTTTTTATTTTTAAAAAAAAATCTTTACAAG
CCCCTATTTCACTTTCGTTTTCCCTTGTGGGGTTAATACGCGTCGCGGCCACTGACCAAGCAGCGACATAAGAAGTTCCCTAGT
TAATCCGAAGGGAGAACAGGGAGGGGCAGCCGCGTTCGCTATACATGAAGTAATGCGGAACAACATTCCGGGTGCCGATGCATC
ACCAGCAGCTAATGCACCGGATCCCATCCAGAAGACACTCCGCAAGTTTTAAGGTGCTTGGGCGAAGTAGTAGCTAGGATGGGT
GACCTTGCTGGGAAGTCGCTCGTGTTTGCACACAGCCCTGAAAAGTATAATTTTTATATTTATTCTTTAAAAATCCACTTACGA
GCTCAACAAGTTTGCTATTTTTTAAAAAAAAATCTTTAAAAGCTCATCTTATTTCGTTATTCCCTTGGGGGGTTAAATACCGGTC
CGGGGGCGGGTCACTTGAGCAGGCAGCGAATTAAGAAGTTCCTGATTAATCCGAGGGAGAAAAAGGGGAGGGCAACGCGTTCGC
TATAAATGAATAATAGCGGCAACAAACATTCCGGCCGGCCCCCAGATCATACCAGCACCTAATGCAACGCGGAATTCCCCATCA
GAACTCCGCAGTCTAAGGCGTGCTTGGGCGAGAGTAGCTCACTAGGATGCGGTGACTCAATCCGGGAACAGCCGCTCTCGTGTT
GCACGCCGCTAAACAACATCATTTTTTTTTTTATTCTTTAAAAATGCCACTTTACCGGGCTCAAAAGTTTGTATTTTTTTAAAA
AAATCTTTAAAGCGCCCCGGCGTCCATTTATTTCGTTTTCGCTCTGGGGGGTTACATAGCCAGTGGCCACTGACAAGCAGGGCG
AATACCGAATTCCTAGTTAATCCGAGGGAGAAAAGGGCGGGCCCCCACGCGTCTCGGCTATAGGAATGAATAGTTGCGGAGCCA
AAACATTCCGGGTGGCGATCATACCGCAGGGCACTAATGCACCGATCGCCATCCCGAGACTCCCGCAGTTAAGCGTGGCTTGGG
GCGAGAGTAGTAACTAGGTATGGGTGAACCTCCCTGCGGAGTCCACGTGTTGCACCGCTGAACCAACACCTACCCCCTAACATC
ATTTTTTTGTTTATTCTTTAAAAGATCCAATTACGGCTCAAAAAGTTTTGTATTTTTTTAAAAAAAATTTTAAAGCATCATTTA
TTCTCGTTTTCCTTGGGGGGTTACATACGCGTGGCCACTGACAAGGCAGCGAATAAGAGTTCCTAGTTAAATACGAGGGAGAAA
AAGGGAGGGCACGCGTCGCTATAAATGAATCATTGCGGAGCCAAACAATTCCCGGGTGCGTCAATAACCAGGCACTAATCGCAG
CCGGATCCGCATCAGACCACTCCCGCAGTTAAGCGTGCTTGGGCGCGAGTAGTACTAGGATAGGCGACGCGTCCTGGGAGTCCT
CCAGTGTTGCACCCCTGAAACGATCAATTTTTCTTTTTATTTCTTTAAAAATCGCACTAAACGCCTCAAAAGTTTTTTTTTTA
AAAAAAAAATCTTTAAAACTCATTTTATTTTCGCTTTTCGCTTGGGGGGTTAATCCGTGGCCACTGCACAATGCAACGAATAAG

AAAGTTCCCTAGTTAATGCCGAGGAGACAAGGGAAGGCACGCGTTCGGGGCTAATACAATGAATAATGCGGAAGCAAAGACATT
TCCGGGTGCGATCATACGCCCAGCACTAAATGCACCGGATCCCATCAGAATCCGCAGTTAAGCGTGCTTGGGCCGAGAGTACGC
TCACTAGGATTGGGTGACGCGTCCCTGGGATGTCCTCGTGTTTGCACCCTGAAAACATCATTTTTTTTTTATTCTTTAAAAATG
CCACCTTACGGCTTAAAAAGTTTTTTTTTTTAAAAAAAAAACTCTTTAAAGCTCTTTATTTTCATGCTTTCCTTGGGGGGTTTAA
TACCGTCCGGCCACTGACGAAGGGCCAGGCGAATAAGAAGTTCCTAGTTAAAATCCGAGGACGAGACAACGGGAAGGGCAGCGC
GTTCGCTATAAATGATCAGTATGCGACGAAACTTCCGGGTGCGATCAAAATACCAGCACTAAATGCACGCGGAGTCCCCATCCA
GAATCCGCAGTTAAGCGTTGGCTGTTGGGCGACGAGTAGTACTAGGATGGGGTGATCTCCTGGGAAGCTCGTGTTGAACCCCCG
CTGAAAACATCATTTTTTTTATTCTTTAAAATCCACTTACGTCAAAGTTTGTATTTTATTAAAAAAAATCTTTAAAAGAGCTCA
TTTATTTCGTTTTGCCTTGGGGGGGTTAAAATACCGTCGGCCCTGCGAGCAGCGAATAAAGAACGTTTTAGTTAATCCCGAGGG
AGAAAAGGGAGGGCACCGCGCGTTCGCTATAATGAATACATGGGAACAAACATTCCGGTGCGATCATACCCAGACGACTAATGC
ACTGGACTGCATTCAGAACTCGCGCAGTTAAGCGTGCTTGGGCGATGAGTAGTACTAGGCGGATGCGGTTACCCTCCCTGGGAA
GTCTCGTGTTGCACCCTGAAAACATCATTTTTTTTTATTCTTTAAAAATCGCACTTAACGGGCTCAAAAGTTGGTATTTTTTTA
AAAAAAATATTTAAAAGCTCATTTATCTTCGTTTTCCTTGGAGGGTTACCATACCGTGGCCACACCTGACAAGGCAGCGAATAC
ATGAAGTTCCTAGTTAATCCGAGGGAGAAAGGGAGGGCACGCGTTTCGCGTATACGAATGAATATTGCGGACGCAAACATTCCC
GGGTGGCGATCATACAGCCACTAATGCACGCGGATCGGCCAATCCCAGCCCAACCTCGAGCAGTTAAGCGTGCCTCGGCGCGAG
GTAGTACTAGGATCCGGGTGACCTCGTGGGCCAATCTCCCGTGTTGCAACCCGCTGGAAAACATCATATTTTTTTTTTATTCTT
TAAAAATCCAATTACGGCTAAAAGTTCTGCTATTGTTTTTAAACATCTTTAAAACCTCTTTATTTCGTTTTCCTTGGGGGTTAA
TACGCGTTGGTTCACGTGCAAGCGCGATAGAAGTTCTAGTTATACTCCGAGGGAGAAAAGGGAGGGCACCCGCGTTCGCTATAA
ATGAATAAAGTGCGCGAACAAAACTATTCCGGGTGGATCATACGCAGGCACTAATGCACCTGGATCCGATCAAGAATGCCGCAG
GTTAACGCGTGGCTTGGGCGAGGGGAGAAAAATAGTACTTAGGATGCGGTACCCTCATGGGAAGTCTTCGTGTTGGGCAGGCCC
CTGAAACATGCTCTTTTTTTTTATTCGGTTTAAGAAATCCACTTTACGGCTCAAAACGTCTCCCCTTGTATCTTTGTTTAAAAA
AAATCTTTTAAAAGCTCAGTTTATATTCGTTTTCCCTTGGGGGGTTAATACCGTGGTGCACTGAGCAGCAGCCGAATTCGAAGA
AGTTTCCTAGTTACATCCGATCGGGAGAAAAGGGAGCCACTTTTCGCTATAAATGAATAATGCGGAACAACATTCCGGCCCGCT
GATCCATCACCAGCACTAATGCACCGGATCCCATCCAGAACTCCGCCAGTTCCAGCGGTGGGCTTGGGCGAGAGTTAGTACTAG
GATGGAGTGACTCATTGGGAGTGCTCCCGTGTTCGCACCTAAAAACATCATTTTTTAATTTTTAATTCTTTAAAATCCAACTTA
CGGGCTCAAAAGTTTTGTATTTTTTAAAAAAAAATTTTAAAGCTCATTTATTTCCCGTTTTGCCGCTTCGGGGGTTAAATAGCCG
TGGGGGCCACTGACCAAGGCAGGCGAATCAAGAAGTTCCTACTTAATCCGATGGGAGAAACGGGCGGCGCACGGCGTTCGCGGT
ATAATGAAATTGCGAGACCACAACATATCCGGGTGCGATCATACCCAGACCCGCACGTAATGCAGCCGGATCCATCCAGGAACT
CCGCAGTTAGCGTGCTTGGGCGAGAGTCAGTACTAGGGATGGGTGACTCCTGGGAAGTCCACCGCTGTTGCACCCCTGAAACCA
TCATTTTTTTGTGGGTTTATTCTTTAAAAATCAATTACGGCCTCAAAAGTTTTGTAGTTTTTTAAAAAAATCTTTAAAAGCTCA
TTTATTGTCCGTTTTCCTTGGGGGGTTACCTACCGGGTGGCCACTGACAAGGCAGGGCGAATAAGAAAGTTCGCTAGTTAACTC
CCGAAGGGAGAACAGGGAGGGGCAGGCGCGTTTTTCTGGGTTATAAACCCTGAATATTCGCGACCAAACATTCGGGTGCCGATC
ATACCAGGCACTAACGCAGCCGGATCCCCATCAGCACTCCGCAGTTAAGGCGTGCTTAGGCCGAGAGTGTCACCTAGGATCGGG
GTGAATCCTGGGAAGTCCTCGTGTTGGACCCCCTAGAAAACCATAAGTTTTCTTTTTTTTCTTTAAATCCACCTAACGGCTCCA
AAAGTTTTTTTTTTAAAAAAAAATCTTTAAAAGCTCATTTTGATGTTCGTTTTCCTTGGGGGGTCTACGATAGCCCGTGGCCAA
TGACAGGCAACGATAAGAAAGTTCCTAGTTAATCCGAGGGAGAAAAGGGAAGGCACGCGTTATCGCTAACTACAACACTGACAT
ATGCGGAAACAGGACATTCCGGGTGCCGATCATGACCCAAAAGCACTAATGCACCGGGACCGTCCCGATCAGAACGTCAGCAGT
TAAGCGTGGCTTGGGGCGAGAGTCAGTACTACGGATGGGTGACCTCCTGGGATGTCCTCCCCCGTGTGTGGCACCTCGAAGAAC
ATCATTTTTTTTTTTATTCTTCTAAAAATCCAGCTTAACGGCTGCAAAGTCTTTTTTTTTAAAAAAAGTCTTTAAAAGCTGCATT
TATTTCATTTTCCTTGTTAATACGTGGCCGACTGACAGGGCAGAGCGAATAAGAAGTTCCTAGTTAGATTCCAGGGGAAAAGGG
AAGCACGGCTCTCGCTATAATGAATAAATGGCGCGAACCAAACATTCCGGGGTGCGATCATCCCCACAGCAACTAAATGCACCC
GTCCCATCCAGGCAACTCCGGTTCAAGCCGTGGGCTTGGGGAGAGTAGTACTAGGATAGGTGATCTCCTGGGGAAGTCCTCCGT
GCTTGCAGGCCCCTGAAACATCATTTTTTTTTTATTCTTGTAAACAATCCACTTACCGGCTCAAAAGTTTTGTATTTTTTTAAA
AAAAATCTTTAAAAGCTCATTTTATGTTCGTTTTCCTTGGTTAATACGTCAGGCCACTGGCGCAACGCAGCGAACTAGCGTGTT
TTAAAGTTAATCCGGGAGAAAAGGAGGGGCACGCGTCTCGGGATATACATTGAATAATGCGGAACAAACATTCGGGTGGCGGA
TCATACCAGCACCCGA
>m141122_014521_42134_c100732202550000001823141405141560_s1_p0/40005/314_8421
TTTTCGCAATAATTTTTTCGCTTTGTATGTAGAGTACATGTGCTCCTGCAGTTGTATTTTCGCGATTGGTGATCGTACCTCCTA
TGCACATCCCTCGGATTTAAACCAAATCCTTATACATAAGAACCCTAAGACACAGGGCTACACATTTTGTGCCCATGACCCTCG
CCATAATTCCTTTAGTATCTCCTATCCCCCCCAAACCTTGCTCGAATCATATTAGAGAAATCGAAGATTCCTGCAAAGCGGGTA
CCGGCCCTTTGGTGAATTGGAGATTTCACTAATTCTCACCGAAATGCATTACCACCTCGTGTTCAACTCGTTCGATATTTTTCC
CCAAGAGATCGTCAGTGGTAAATGAATATATTGGAGGTCGAAACGCAGCCTAATGTCAACATTTCAGAAAAAAATTCCACTTTTT
TCTGGCATGTTTCCGCAAGTTTTGTTTCCCTTGTAGTTAGTCACGATCTCTGTCAGTTTTTCGCGCTGTATCACCGCCTCTACA
CACCTCGATTTTAAACCCAAAATCTTATTTACCCAAAAATACGGGAAAAAACCCCTAAACACAGGGCTGAACAAATTTGTGCCA
TGAACCCTCAAGCATATTCTTTAGTATTCCCTATCAAAAGCATTGCTCGATCCATATTAGAGAAAATCGAAGATTTTCTGAAAG
GGGTACCCCCTTTGGTGAATTGGACGATTTCACTAATTTCACCGAAATGCATTACCGCTCGTGTTCAACTCGTTCGATATTTTT
CCCCAGAGATCTCCTGGGTAAAAGTATACCGGATAATTGGAGTCAAACGCAGCCTAATTCAAATTTCAGCAAAAAATCTCCCAC
TTATTTCTGGCAATTTTCGCGAGTTTTTTTCCTGTATTTAGTACATCTCTCAGTTTTCGACTGTATCGATCCTCTACAGCACCC

TCCGGATGTAAAACCACACAGATCGACTTCACTACAAGGTGAAGAACCCTACAAGACGCCCAGGCATTGACACACAAGTTTATG
TGCAACCATCTGACCCTCGCCATACTTCTTCTAAGTATTCCTACTCAAGAACATCTGCACTACGATCCCACATATTCAGTTACA
GAAATCGACACAAGAATGTTCTGCAAAGGGTACCCTTCTGGTCGAAATGCGTGGCAGACCTCACCTAATGCTTCACCGAAATCA
TGGTCCGCCGCTCAGTGTTCAAACCTGCGTTTCGAGCTGATTGGATGCGTCTCCCCACAAGAGTCCATGGCGTTAGACAGAATA
TCCCTGTCGGACGTCGAAACCGCAGACCATTGAAATGTCAACATTTCGAGAAGAAAAATTCCACTTATTTCCTTCTGGCAATTT
TCGCAAGTTTTCTTCCTCGTAGTTTGTACAGTAGCGAGGCTTTCACTACGCTCATTTTCGACTATCGTATCTCTACGCCTCTGA
CAGCCACACGGTCCGATGTGGTGGAGAACACCGCCAAATCTTATACATAGAACGCCCTAAAGAACGAGGGCTAGCAACTTTTGT
GCCCATGACCCCCTCGCGATATTCTTTAGTATTCCCTACTCAAAACATTGCTCGAATCCCATATTAGGCAGAAATCGAAGCAGT
TTCCGAAGAGGGGTACCTTTGGTGAACTTGGAGATTTCACCTATTTTCACCGAAATGCATTACCCTCGTGTTCAACTCGTTCGA
TATTTTTCCCAAGAGATCTCCTGGGAAATGTATATTGCGGAGTCGCGAACGCAGCCTTAATTCAACATTCAGAAAAAATCGTCC
ACTTTTTCTGGGCAATTTTCGCAAGTTTTTTCCCTTGTCATTAGGTACATTTTCCAGGTTTTTCCGACTGTATCACCCTCTAAG
GCACCTCCGATTTGAAAACCAAATCTTATACATCTAGAACCCTAAGACACAGGGCTCGACAAATTTTGTGCACATTTACACCGC
TCCGCATATTCTTTAAAGTATTCCCCTACAAAACATTGCTCGAATCCGATATTAGAGAAATCGAAGATTCTAAAGGGGTACCCC
TTTGGTGAATTGGAGATTTCACTAATTTCACCGAAACCCTGCATTACCCAGGCTCGTGTTCAACCTCGGTTCGACCGTAATTGT
TCTTCCCCAAGAGATCTCATGGGTACAATGAAATATAGGTTGGAGTCGAAACGCAGGCCTTCAGATATCAACATTATCAGAAAA
GAATTACACTTTTTTTCTGGCATATTTCGCAAGTTTTTCCTTGTATTATTACATCCCTCAGGTATGTTCGACTGTATCACCCT
CTACAGACACTCGGATTTCAACACCAAATCTTATACCATAGAACCTACAGACACAGGGGCTACAAATTTTGTGCACATAACCTC
GCATATTCTTTAGTATTCCCTATCAAGACATGCTGCTCGAATCCATATTACGGAGGCAATCGAAGAGTTTCCTGAAGAGGGTAC
CCTTTGGTGAATTCAGGAGATTTCACTAATTTCACCGCGACAATGCATTACCCCCCCGTGGTGTCAACCTCCGTGTCGATTTAG
AACACACAGAGATCTCATGGTAAATGAATTACGTTCGGAGTCCGAACGCACCTTAATTCGAACCATTGCTCAGCGAAAAATCCA
CTTTTTTTCTGGAATTTTCGCAAGTTTTTTCCTTGTATGTAGTACACTCCTCAGTTTTTCGACTGTCATCATCCTCTACACACT
CGATTTAAAGACACATCTTATAACATAGAACCCTAAGACACAGGGCTACAGAGATTGTTGTGCCCATGGACCCTCGCATGGATT
TTAGTATTCCCTAGTCAAAACATTCCCGTCGAACCCATCTATTAAGAAATCGAAGATTTCTGAAAGGGGTACCCCTGTTGGTGA
ATTGGGGAGATTTCAACTTTAATTTCACCGGGGAAATGCATTACCCTCGTGCTTCAACTCGTTCGATATTTTTCCCCGCCACAG
AGATCTCATGGGGTAAGCATGAATATATCTGGAGTCGAAACCGCAGCCCTTCATTCAACAATTTCAGAAAAAAATTCCACTTTTT
TCTGCAATTTTCGCAATTCGTTTTTCCTGTATTAGTGACATCCGACACGGAAGCGCCACGCGGAGTCGTCCATTTTTCGCCTGT
AGTCACCCTCTACACACCTCCGGGAGTTTAAACACAACATGCTTTACATAGAACCCTAAGACACAGGCTACAAATTTGTGTGCC
CAATGACCTCCGCATATTCTTTAGTATGTCCCTATCAAAACATTGCTCGAATCCAAATAATTAGAGAAATCGAAGATCCTTTCT
GAAAGGGGTCCCATTTGGTGAATTTGGAGAGTTTCACTAATTCACCCGAAATGCTTACCCCCCCTCGTGTTCAGACTCGTTCGA
CATATTTTTCCCAAGAGATCTCCCTGGGTAAATTAATATTGGATCGAAACGCAGCCTAATTCAACATTTCGAAAAAATTCCACT
TTTTTCTTGGCAATTTTCGCAAAGTTTTTTCCTTGTATAGTACATCTCCAGTTTTTCGACTGTATCATCCCGTACACGCACCCG
TCCGGATTTAAACAAGATCTTATACCATAGAACCCAAGACACAGGGCTACATTCTTTCTGCCCAATGAACCTGCGCATATTCTT
TAGTTTCCCTACTCAAAAACATTGCTCGAAATCCAATTAAGAGAAATCGAAGATTTCTGAAAGGGTAACCCCTTTGGTGAATTG
GAGATTTCACTAATTTCACCGAAATCGCATTACCCGTCGTGTTTTCAACTCGTTCATACCTGTTTCCCCAAAGAGATCTCAGGT
GGGTCAAATGAACTATATTCGGAGACCCCCCCCCCCGCAAACGCAGCCTTAATTCAACATTTCAGAAAAATTCACTTTTTTCTG
GCAATTTTCGCAAGTTATGTTTCTGTATTAGTACAGTCTCTCCCAGACTGTTTTCGCACTGTATATCCTTACACCACCTCGGAT
TTAAACCTAATCTTATACATAGAACCCCCTAAGACACAGGCTACAACTTTTGTGCCCATGACCCTCGCATCATTCCTTAGTATT
CTATCAAAACATTGCTCGAGATCATATTAGAGAAATCGAAGATTTCTAAAAGGGGTACCCTTTGGTGGACATTGGAAGATTTCA
GCTAATTTCTTCCGAAATTGCATTACCTCGTGGTTTCAACGTCGTTCGACCATACTTTTTCCCCAAGAGATCTCCCTGGGTAAA
TTATACTATTGGGTCGAAACGGCAGCCTTAAATTCCCGAAATTTTCCAGAAAAAATCTCCACTTTCTTTTCCTGGCAAATTTTC
GCCAAGTTGTTTTCCTTGTATTAGTACAGTCTCTTCAAGTTTTTTTCGACTGTATCATCCTCTACGACCACCTCGATTTAAACC
AAATCTTACCTACCGTACGAGAACCCACAGACACAGCGGCTACAAATTTGTTGGCCCGATGACCCCTCGCATACTTCTTTAGTA
TGTACCTATCCAAAACAGTTGCTCGAATCATCATTAGAGAAATCCGACAGATTTCTTGAAAGGGGTACCCTCTTGGTGAATTGG
CAGATTTCCACCTAATTTCACCGAAATGCCATTACCGCTCGTGTGTTCAACTCCGTTCGAGTATTTGTTCCCAAAAAGAGATCT
CTTGGGTAAAGGTGAATACTAAGTTGGAGTCGAAACGCAGCCTTGTAATTCAACATGTTCAGAAGAATTTCCACCTTTTTGTTC
TGGGCAATTTTGGCGCAAGTTATTTTCCCCTTTATTCAAAGGAGACACATCTCTCGAGTATTTTCCGACTGTATCACCCTCCGG
TACACCGCACCGATCGCATGTCCCTCAACCCAAATCATTATACAATGAGAACCCTAAGACACAGGGCTACCAACAAATTCTTGA
CTGCCATGACCCTCGGCCATATTTCTCTTAGTACTACGCCTCCCTAGCCTCAAGAACATTGCTCGAATCCATGATTAGACGGCA
AAATCGACAGATTTCTTGGAAAGGGGTACCCTGTTGGTGGAATTGGAGATTTCACTAATTTCAAACGAAATGCATTACGCCTCG
CGTGTTCAACTCGTTCGATTTCTTTCCCCAAGAGATCTTCCATGGGGTAGAATAATATATTGGAGCCGCAAAACCGCAGCCTTA
ATTCAACATTTCAGAAAAAAATTCCACTCTTGTTTCCTGCGCAATTTTCGCAAGTGTGTTTTCCTTGTATTCAGTACATCTCCCA
TTTTTCGCCTGTATCAACCGTCTACCACGCACCTCGGATTTGTAAAGCCAAGATGCTATACATAGCAACCCTACAGACAAGGGT
ACAAATTTTGTCCCATCCGACCCTGCGCAGTATGTCTTAAAAAGAAAAATTTAGTATTCCTTATCAAAACATGTGCTCGAATCC
CACTATTAGAGAAATCGAACAGATTTGAAAGGGGTACCCCTTTGTGAATTGGAGATTCACCTAATTGGTCACGAAATGCATTAC
CCTCGTGTTCAAACTCCGTTCCGACCTAGTTTTTCCCCAAGAGTCTCCTGGGTAAATTATATATTCGGAGCGTCGAACGCAGCT
TAATTCCAATTTCACGAAAAAATTCCACTTATTTGTCCTGGCAATTTGTCGCAAGTTTTTTCCTTGTATCTCAGTAACATCCTC
TCAGTTTCTTCGACTGTATCCCATCCTCTACACACCTCGGATTTCAAACCAATCTTATACAGTAGAGAACCTAGAGACAGCAGG
GCTACAAATTTTGTGCCCATGACCGATCGGCATATGTCTTTAGAGTATATCCCTACATTCATAACATTGCTCGAATCCATATTG

AGAGAAATCGAGATGTTCTGAAAGGGTCACCCCTTCTGGTGAAGTGCTGCCGCAGATTTCAAACTAATTCACGAAATGCATTAC
CCTCGTGTTCAATCTCGTTGACCTCATTTTTCCCCGAAGAGGATCGCATCATGGGTAACTGAAATACTATTGGGGAGTCGAAAC
GCAGCCTGTAATCTCAACCATCTTCAAGAACAAAATCTCCACTTTTTTCTGGCAATTTTCGCAAGTCTTCTTTCCTTGTAATTA
GTACATCTCTCCAGTTTCTTCGACTGTATCATCCTCTACACACCTCCGGATTTACCAAATCTTATACAGTAGAACCATATAGAC
ACAGGCAACTTTTTCGTGCGCATGACCCCTCGCATATTCTTTAGTATTCCCTACTCAAAACATTGCTCGACATTCCATATTAGC
AAAATCGAAGATTTCTTAAAAGGGGGTACCCCTTGTCGGTGAATTGGCAGATTTCACTAATTTCTCCGAAATGCCATTACCCTC
GTGTTCAACCTCGTCTCGATATTTTTCCCCAAGATTCTGGTAAAATTATATATCTGGAGTGAAACGGAAGCACTTTAATTCAAC
ATCTTCAAGAAAAAAATTCACTTTTTTCCCTGGCATTTTCTCGCAAGTTTCGTCTCTTGTATTTAGTGACATCTCTCAGTATTG
TGCGACTCTATCAACCCTCGTACACACTAGGATTTAAACCAAATCGTTATACATAGAACCCTTAAGACACGAGGGCTTTACAAA
TTTTGGTGCACATAACCTTAGCATATTCTTTAGTATCTCCCTATCAAAACATTGCTCCGAAGATCCATATTAGCAAGAAAATCG
AAGATCTTCTGAAAGGGGTACCCCTTTGGTGCAATTGGCAGACTGTTCACTAGAGGTTCACGGCGAAATGCATTACCTCGCTGT
TCAACTCGTTTCGATTCTTTTTTCCCCAAGAGATCTCATGGGTAACATGAATTATATTGGAGTCGAAACGCAGCCTTAATTCAA
CATTTCAGAAAAAAATTCCACTTTCTTTCTGGCAATTTTCGCAAGTGTTTTTCCTTGTATTAGTACAGTCCTCTCAGGTATTTCG
ACTGTAGTCACTCAAACCCTCTACGACACCTCGGATTTTAAACCAAATCTTATACAGTCAGAACCCTAAGACACAGTGCCTACA
AATTTCTGCGCACATAACCTCCGCATATGTCTTTAGTATTTCCCTATCAAAACATTGCTCGATATCCAATATTAGACGAAATCC
GAAGATTTCTGAAAGGGGTACCCCTTTGGTGAGATTGGAGATTTCCACTAATTTCACGCGAAATGCATTACCCCCTCGTGTTCA
AACTCCGTTCGACGTATTATTTCCCCAAGCAGATCTCATGGGTTGATATACTTGGAAGTCGGAAACGCAGCCTTTGACTTCACA
TTTCAGCAAAAAATTCCACTTTCTTCATCTGGCAGTTTTCGCAAGTTTTTTTCCTTGATTCAGTACATCTCATCGTATATTTAT
CACGACTGTATCATCCTTACACACACCTCGGACCTCTCTCAAACAAATCTGTATACATACGAACCCTAAGAGACGACAGGGCGT
GACAGAAGTGTTTCTGTGCCATGAGCGCCGTGGGCGCCTGGGCGGCATGAGTGTGTCTTTGTCTAGTAGTGCCCGTGCGCCTA
GTCGAAGCCGGGGAGCAGGACAGTTGGCCTCGAATCCCATGAATTAGCGCAACTCGACAGATTTCTGAAAGGGGTACCCTTTCG
GTGATTGGAGATTTCACTAATCTTGCACCGAAATGGCATTAACCCTCGTGTTCAACCTCGTTCGACTAATTTTCCCCAGAGAAT
CTCATGGGTAACACTGAATATAATTGGAGGTCGAAGACGCAGCCTTAATTCCAACTTTCAGCAAAAACTTCCACTTCTCTTCGG
CAATTGTTGTTCGCAAGTTTTTTCCTTCTGTATTCAGCTACATTCTCAGTTTTTCGCGCTGGGTATCCACCCCTTCTACACACC
CTCGGATTTAAACCAAATCTTATACATCACGAACCCTAAGAACAGGGCTACAAATTTCTCTGTTGCCCATGACGCTCGCATACT
TCTTTAGTAATTCCCATCCAACAACATTGCTCGAATCATATTAAGAGAAATGAACAGATTTCTGAAAGGGGTACCCTTCTGGTG
AATTGGAGAGCTTCACTAATTCCGGAAATGCCATTACCTCGTGTTCACTCGTTCGATATTCTTTCCCCAAGAGATCTCCTGGGT
AAATTATATATTGATGTCGAAACGCAGCCTTAATCAACGATTT
>m141122_014521_42134_c100732202550000001823141405141560_s1_p0/43043/8634_14649
TTATGTCAGTTCAATGATGACCAGAGTCGTGTTGTGGATCCATACCGACTCTTTTTTGTTCTGCCCATGACGACGCCTTGCAG
TAGATATTTTCTTTTAGCTAATGGTCATTTACAGGTACTTGAATAAACAATTGCTCGAATTCAATATTGGGAAATCGAAGATTT
CTGAAAGGGGTACTTTGGTTGAATTGGAGATTTCACTAATTTCTTCCGAAATGGATTACTCGTGTTCAACTCGTTCGATATTTT
TCCCCAAGAGATCTCATGGGTAAATGAATAGTCATTGGGGTCGAGACCGAGCTTAATTCAACATTTCCAAAAAAAATTCCCTTT
TTGATGCAATTTTTCGCAAGTTTTTTCCTTGTATTAGTACATCTCCTCATTATTTTTGACCGTATCACCTTCTACACATCTCGG
ATTTGCACGAAATTTATACATAGAACCCTAAGACATAGGGCTAACGACTTTTGGTGCCCATGACCCTCGAAATATTCTTAGTAT
TCACTAATCAAAACAGATGCTCGAATCAATATTGGTTAGAAATCGAAGATTTCTGAAAGGGTACCCCTTTGGTGAATTGGAGAT
TTCACTAATTTCTCCCCGAAATGGATAACCCTCGTGTTCAAACTCGTTCGATATTTTTCCCCAAGAGATCTCATGGGTAATGAA
TTATTGGGTCGAGACGGAGCCTTAATTCAATTTCAAAAAAAATTCCCTATTTTGATGGCAATTTTCGCAAGTTTTTTCCTTGTA
TTAGTACATCTCTCATTATTTTGACCGTATCCCTTCTACAATCTCGGATTTGACGAAATTTTAATACATAGAACCCTAAGACAT
AGGCTAACGACTTTTGTGCCCATGACCCTCGATATTCTTTAGTATTTCACTACAAAAACAATGCTCGAATCAATATTGGAGAAA
TCGAAGATTTCTGAAAGGGGTAACCCCTTTGGTGAATTGGAGATTCTCAACTAATTTCTCCGAAATGATTACCCCTCGTGTTCA
ACTCGTTCGAATTATTTTTCCCCAAGAGATCTCAATGGGTAATGAATATATTGGGGTCGAGACGGAGCCTTAATTAAAAATCAT
TTCAAAAAAAATTCCCCCTTTTTGAGGATTTCGCAAGTTTTTTCCTTGTATTAGTACATCTCTCATTATTTTGACCGTATCACC
TCTCTACACATCTCGGATTTGCACGAAATTTTATACTATAGAACCCGTAAGACATAGGGCTACGACTTTTGTTGCCCATGACCT
CGAATAATTCTTTAGTATTCACTATCAAAACAATGCTCGAATCAATATTGGAGAAATCGAAGATTTTCTGAAAGGGGTACCCTT
TTGGTGAATTGGAGATTTCACTAATTTCTCCGAAATGGTTACCCTCCAAAAAAAGTGGTTCAACTCGTTCGTATTTCCCCAAA
GAGATCTCCATGGGTAAATGAATATATTGGTCGAGACGAGCCTTAATTCAACATTTCAAAAAAATTCCCCTTTTGTGGCAATT
TCGCAAGTTTTTTCCTTGTATTAGTAACATCTCTCAAATTATTTTGACCGTATCACCTTCTACAACATCTCGGATGTTGCACCG
AAATTTTATACATAGAAACCTAAGACATAGGGCTACGACTTTTTTGTGGCCCATGACCCTCGAATATTCGTTTAGTATTCACTT
CAAAAGCAATGCTCGAAATCAGTAATTGGAGAAATCGAAGATTTCTGAAAGGGTACCCCTTTGGTTGAATTTGGAGATTTCACT
AATTTCTCCGAAATTTATTACCGCTCGTGTTTCCACTCCGTTTGAATATTTTTCCCCCAAGAGATCCCATGGTAAATGAATATG
ATTTGGAGTCGAAACGGAGACTTAATTCAACATTTCAAAAAAAAAAATTCCCCTTTTTGATGGCAATTTTCGCAAGTTTTTTTCC
TGTATTAGTACGATCTCTCAGTAATCTTGACTGTAATACCCTCTTCACACCTCGGATTTTGCACGAAATTTTATACATAGAACC
CTAAAAAAGACATAAGGGCTAAACGACTTTTGTGCCATGAAAACCCTCGAATATTCTTCTTTAAGTATGTCACTAATCAAAAAC
ATTGCTTCGAATCAAAATGTTGAGAAAATCGAAGATTTCTGAAAGGGGTACCTTTTGGTGAATTGGAAGATTTCACTAATTTCT
CAGAAATTTATTACCCCTCGTTCGTGTTCCACTCGTTCGATATTTTTCCCCAAGGAGATCCCATGGGATAAATGAATATATCGG
AAGTCGAAACGGAGAACTTATTCAACATTTCAAAAAAATTCCCTTTTTATGCAATTTTCGCAAAGTTTTTTCCTTTGTATTTAG
TAACATCTCTCAGGTATCTTGAGTGTAATACCCTTTTCACACCTCGGATTTCGCACGATTTTTATACATAACCCTAAGCATAAG

GGATACAACTTTTGTGCCCATTGACCCTCGAATATTCTTTAGTATTCACTATCAAAACAAATGCTCGAATCAATATTGGAGAAA
TCGAAGATTTCTGAAGGGGTACCCCTTTGGTGAATTGGAGTTTCACTGAATTTCTTCCGAAATGGATTACCCTCGTGTTCAATC
GTTCAGTATTACTTTCCCCAAGGAGATCTCCATGGGTAAATAATATATTGGGGTCGAGAGGAGCCTTAATTCAACATTTCAAAA
AAAATTCCCCTTTTGATGGCAATTTTCGCAAGTTTTTTCCTTGTATTAGTACTCTTCTCATTATTTTGACCGTGATCCACCTTC
TACACATATCCCGGATTTTGCACGAAATTTAATACAATAGAACCCTAAGACATAGGGCTACGACCTTTGTCCCATGACCCTCGA
ATATTTCTTTTAGTATTCACTATCAAACAATGCTCGAATCAATATTGGAGAAATCGAAGATTTCTGAAAGGGGTACCCCTTTTG
GTGAATTGCGAGATTTCACTAATGTTCTCCGAAATGGATTACCCTCGTGTTCAACCTCGTTCGATATTTTTTTCCCAAGAGATC
TCATGGGTAAATGAATACGTATTGGGGTCGAGAACGGTAGCCTTAATTCAACATTTCCAAAAAAAATTCCCCTTTTTTGACTGG
CAATTTTCGCAAGTTTTTCTTGTAATTAGTACTCTCTCATTATTTTGACCGTATCGACCTTCTACACATCTTCGGATTTGCAC
GAAATTTATATACATAGAACCCTAAGACATGGGTAACGACCTTTTTGTGCCATGACCCTCGAATATTCTTTAGTATTTCACTAT
CAAAACAATGCTCGAATCAATAATTGGAGAAATCGAAGATTTCCTGAAAGGGGTCACCCCTTTGGTGAATTGGAGATTTCACTA
ATTTACTCCGAAATGGATTACCCTCGTGTTCACTCGTTCCGATATTTTCCAAGAAGATCTCCATGGGTAAATGAATATAGTTGG
GGTCGAGACGGAGCCTTAATTCACGCAACAATTTGCAAAAAAAATTCCCTTTTTGATGGCAAATTTTCGAAAAAGTTTTTCGCT
TGTATTAGTAATCCTCTCAACTCCATTAATAAATTTGTGAAGACCGTTAATCACCTTCTAACACATCTCGGATTTGCACGAATG
TTTATATACAAGTGAACCTAAGACATAGGGGCCCTACGGACCCTTTTTGTTGCCCAATGACCCTGATATATTCCTTTAGTATTC
ACTATCAAAAACAATGCCCCGAATTCAGTAATGGGAGAAATCGAAGATTTCTTGAAAGGGGGTTACCCCTTTTTGGTGGAATTG
GAGATTTCATTAATTTCTCCGAAATTTTATTACCCCCTTCGTGTTCTACTCGTGATTATTTTTTCCCCCAAGAGAATCCATGAT
AAAATGAAATATAGTTGGGTAGTCGAAACCGGGAGACTTAAATTCAACCATTGTTCAAAAAAAAAATTCTTTTTGGAATTGGCAA
ATTTTCGCCAAAGTTTTTTCCTTGTATTAGTTAAAATCCCTCTCAAGTATCTGGGAAACTGTTAAATACTCCCGTTTGAAAAC
ACCCTCGGATTTGGGCACGAAATTGTTATACATAAGAACAAGACCATAGGGGGCCTACGACCACCCTTTTTGTGCCCATGACCC
TCGAAATAATTCTTTTTAGATTCACCTTATCCAGAAACATTGCTCGAATCAAAATGTGGAGAAATCGAGATTTCCCTGAAAAAG
GGTTTACCCCCTTTTGGTGGGGAAATTGGAATAGAATTTCACTTAATTTCTCCGAAAATTTATTACCCCCTCGTGGTTCCACT
CCGTTTCGACCTTTTATTCCCCAAGGAGATCCCAATGGATAAATATTATGGACGTCCAAAAACGGAGACTTAAAATTCTAACAT
TTTCAAAAAAAAAATTATTCCCCTTTTTTTATGGCAAATTTTCGAAGTTTTTTTCTTGTTATTTAGTACATCCTCTCAGTATCT
TTTGGAAAAGGGTATACCCTTTTCCACCAACCTCGGATCCTCGCAGAAATTTATTATACATAAGACCCTTAGAATGTAGGATAC
AACTTTGGTGCCCCATTGAACCCGCTCCCGATATTCATTTTAGTATTTCACCTATCAACAACAATTGCCTTCGAATCAAAATAT
TGGGAAGAGAATCGAAGGAAAAATTTTCTGAAAAAGGGGGGTCCCCTTTTGTGAAATTGGGAGGATTTCCACTAATTTTGTTCC
TTCCGAATGGGGATTATTACTTTCGTGGTTTCACTCGTTCGGACTAAAATTTTCCCCCAAGAGGATCTCCCATGGGTAAAAATG
AAGATTATATTGGGGTTCGATACGGAGGCCCCTGTTAAATTATCAACAATTGTCCAAAATCAAAAAAAAAATCCCTTTGATGGC
AATGTTTTGCAATTTTCGCAGCAAGTTTTTTCCTTGTATTTAGTACATCTCTTCCCATTAATTCTTGAACCGGTATCACCTCTA
CACAATTTGGCCGGATTGCGACGAATTTTATACATTAGAACTAAGACATAGGGCCCCTAAGACTTTGGTGCCCATGCCTCGGAA
TATCTTTAAGTTAAATCACTATCAAAACAATGCGTCGAATCATATTGGAGAAATCGAAAATTCTTTGAAGGGTTACCTTTTTGG
GTGAATTGGGAGAATAACTAATTGCTCCGAAAAATGGATTTACCCCTCGTGTTCCAACTCGTTCGATAATTTTTTCCCCAAGAG
ATCATGGGTTTTAACATGGAAATTATATTGGGGTCCCCGAAGACACGGGAGCCTTAAAAAATTCAACATTTGTCAATAAAAATT
CCTTTTTGATGGCGAAATTTTTTTTCCGCAAGTTTTCCCCCCCCTTGGTTATTTAAGTGACATCTCCTATTATTTGACCGTATT
TCAACCTTCCTTACACATCCTCCGGATTTGCAAGGAATTTTAATACATAGAAACCTAGACATAAGGGGCTTACGGAACTTTGTG
CCATGACCTCGAAATATTCTTAAGTATTCCACTATCAAAAACAATTGCCCTCGAAATCCAGTATTGGACGAAAAATCGAGATTT
CATGAAAAAAGGGGTAACCCCCCTTTTGGAATGGAGAATTTCCACTAAATTTCTTCCCGAAAATTGGAATTACCCTCGTGTTCA
CTCGTCGACCGATATTTCCCAAGAAGATTCTCTCCCATGGGTAAAATGAATTAATTGGGGTCCGAAGACCGGAGACTTAATTCA
AACAATTTCAAAAAAAATTCCCTTTTTGGATTGGCAATTTCGCAAGTTTTTTCCTTGTTATTAGTACAATTCTCTCCATTATTT
TTGAACCGTATCCAATCACCTTTAACATTCCCGGAGATTTGGCCCCAGAATTTTAATACATAGAACCCTAGAATAGCTACGACC
TTCTTTGGGTGGGGCCATTGACCCTCCGAATATTCCCTTGTATTTTTCACTGTTATTCAAAACAATGCTCGAAATCAGTATGGG
AAAATCGAAAAGATTTCTTGAAAGGGTACCTTTTTTGGTGAAAATTGGGGA
>m141122_014521_42134_c100732202550000001823141405141560_s1_p0/45837/6221_11679
CACAGGCCTCTTCCTCCATGGCCTCCCTCCGTGGCCTCTTCCTCATGGCCTCTCCGTCGGCGCCCTGATAGTTCGCCCATCGGC
CTCCCCTCCATGGCCTCGGCCCCATGCTCTCCATGGCCCTCCACAGCCCATGGCTCACTCCATGGCCTCCCTCCATGGCCACGC
CCCATGGCTCCTCCGATGGCCTCAGGCATGGCCTCTCCATGGCCTCAGCCCTCGGCTCCTCCATGGCCTCGCCCATGGCCTCAC
TCCATGGCCTCCTCCATGGCTCCGCACCCCATGGCCTCCTCCGTGGCCTCGCCCCATGGCCTCCTCCGTGGCCTTCGCTCCATG
GCCTCCTCCGTGGCCTCGCCATGGCCTCCTCCGTGGCCTCGGCCCATCGGCCTCCCTCCCTTGGTCTCGCCATGCCTCACCCCA
TGGCCTCCTCCAATGGCCTCCCCGCGCCCATGGCCCTCACTCCCATGGCCTCTCCATGGGCCTCCGCCCATGGCCTCATCCCAT
GGCTCCTCCTGGCCTCGGCCCACCATGGCCCTCCTCCATGGCCTCGCCATGGCTCACTCCAGTGGCCCTCCTCCATGCCTCGGC
CCCATCGCCTCCGCTCCATGGCCTCGCTCATGGCCTCCTCCACGCCTCCTCCATGGCCCTCGCCCATGGCCTCCTCCATGGCCT
CGCCCATGGCTCACTCCATGGCCTCCTCCATGCTCGGCCCCATGGCCTCCTCCATGGCCATGCCATGGGCTCCTCCATGGCCTC
CCATGGCCTCCTCCATGGCTCGCCATGGCCTCCTCCATGGCCTCGGCCCCCATGGGCCTCCTCCCATGGCCTCCGACCCCATGG
CCTCCTCCATTGGCCTGTCGCCCATGGCCTCGCCCCCTGGCCTCCTCCATGGCCTCGCCCAATGGGTCTCGCCCATGGCCTAAG
GCCCCATGCCTACCGCGTGCCCATGCCTGTGAGGTTGAGACAGTTCCTCACAAGGGCAAGGTACGCCGCATGAATAGTGGGAAT
GTACTACTACAACACCTAAAGAAGTCAGAGTACGGATACTAGTACCCCCTCCACCCAGACAAAGTAGTCGGGAACGCCACGGAG
GTGGGTTATTGCTAGGATAGGACGTTATGGGTCCGTACGTCTACGGCCAAGTGCGGCAGGGTCCAGCAGCCGACACCAACTACC

GTCTGGCGCACTACGCCTGCCACGGCGCTGTACCGCATCAACATGGATACAAAAGTAGTGAGACATCCCTCCTAACACCGTGCT
GCCTGTACCCGGCGGTGTACGACCCACAGCTCCCTCGGGCCACCTCCCCCCTGGACACAGGGTCGTATGTACCCAGCCCTTGTC
CCCTGGACACTATGTACACCCCCTAAGCCATTAAGCACTATGAAAACGAACTCTAAGAGCCACCTGGAAAGGGGTGGACCGCGC
GGAGAAATTCATTGTAGTGCAGAGGCTTACTTACCAGAAATATTACCATTTTTCACTCCAATTGTTCATCTGTGATTCTATCCT
TTCCATCAAGTTTAATTCCTAAGTTCTTACTCTAACTTCGCGCATTTACCTTTAAGTTTTCCGACCTACGATTTCGTCTGACGA
GATTTCACCCGTCACAGTGATATGATACCTTTCGTGGTTATGGTATATCATTCAACCAACCCATAAATAAACGAAAAAAATCAA
AATAACTTTAAATAAAAACTATTAACATAAACTAATATACATATACATATATTGAAATATTTAGGCAATAAAATAGTAAATCTT
GTTAAAGGGTGTATTCTGTAATATGGATCTTCAATATATGATAAGCTCCCATTGTTTACTACAAACATAACAAAACCATGGACC
AGCTTTACGTTACTTCTCACACCCAACCTTAGGTCATTTTGTATGCCATGCCCCAACACAAGAATTATACATTTTCTTTCACCT
TCTTTTTCCTCCCTTTCTATTTTAAAAACTATTTAAGCATTTTTACGTGCAACCGCCAAACAAGGGTTATCGTTGCAGACTTGG
AACAGGGGATTGTTTATTTATAATGCTAATTTTTTTATACTTTTTAGTCCTCTCAGACTCTTTTATCCTATTTTAGCCAGTGTG
AATTCATGATGACGATAGCGATTGGGGTGTCTAGGTTGAGGATATGCTAGAACATAACACATACTCACCACGTCTTGATCCCCC
CCTTAGCACAACCTTAGGAACTTATTACAGTTTTTCCCCCACTCTTTCTCGAAATTTTTCTTCTCACATTTTTTTCAAACATTC
GAAACCAACAGAAGGCGTTCTTGCACCCTCCCTGACATTACCTCAGTCGATCTCCCTCTCATCTCGCCTGATAAGATTCTGCTC
AGCAGTCTCACGTCTGCTGCCTTAAAATGCTGTTGAAGTAGTAGGCTGGATTCGGCTGCGTTTAAGTTGAACTCGGCTGACGCA
GGTTGAGTGAACATTTTCACTTTTGAATACGCTCCGGCTTTTCCTTCATCCCTCCTCTTCCTCTGTTTTTTCGTCAGATCTCGG
TCAACGAGACCGAGAAGCTCACCCCCTGTTCTTCCATCCTGCGGTACATTTGTCTTGCGCCTCCTCTGGTGTTTGCTGCTCTAT
TTCTCTCTTTCTGTGCCTACATTTTGTTTGTCTCGCCTCTACTTTCTAAATTTCGTCATCCTAGAACGACTTACGTGTTTCTGC
CTACTGGAGAAACTCTGTGGTGGATGCAGATCATTTGTTTCTCTTGCTTTATTCCGTTCGTCTTTAATCCTCATTCCTCTTCTG
TTCATTCTCCCATCCCGTCAAGCAACCCACACCCACTCAGAGCTGTTGCATCGTCCCACCACTCAGGCGGTCCGTGAGGTAGAG
GCAACTCTTCCTACCCCTTTCCGCACTCGTCCTCTCTATTTATATGTTGTCCTGCTGAGAAGGTAATCTCTCCTCTAATCAGCT
TCCCTCCTGTCTCCTATACTCGCGATTCCGCAGTTCAGAGCTACCAAATCGCTCCTCTCCGCATGCAACCTGCCTATTTGCCTG
GTTTTGCTTCTTACTCCTTTGCATCCTATCGCATACAACCACACCAATCGCAAACGATGAACATATGAATCATCATATGATAGT
ATTCTCTCCATAGAGAACTAATATAAAGAAAATAAGATTTGGCTGGAGTGATGTTTTACAGACTATATCCTTCTGTAGTTGCAC
TCTCTGTCGCTCGTTTTTAAAGAAACTCTGTTCTGCACTTCTTCGCAAAACTACACGTCACACTATACTGTACTCTACAGCTC
GAACCACCTCCCGCACTTTTTCCTTAAATTAAATGTGGCTTATTCACGTTACCATCCGATAGCATCCTCTCTCTCAAACCCCCA
ATCTCTGCGCCTCTTCCGTACACAGCACCGTTTGACGAAAGTCATTGTGATATTTTTTGGGTTTCACTTCAAGTTTTGTTTGTT
CTAGAAAGTCTACTGTGCAAACATGTAGTAACAACTCTTGCTGTTTACGATTCTGCTAAGCCCACTCGTTTTCATTTGCGTGGC
TCGACAATGGTAGTTTTATGAATTCATGATCCTATCTTATGATAAGTTGACCCCTACTTTATGTTGTCTGTGATAGCGCCATAC
TCAACCAAACCGGTATCTCGGCAAGAGAACGTACTCGCGGTCGCACACCCTCCTTTCCCGACACACTCACAGCGCCCAACACAC
ACCCAGCACTGAAAGCTATTAAGAGTTCACCCATGCCATCATGTCGTATCTTAGTTACATTACCTTCTCTCTACGGCCCGTAGT
CACCCTGAACTCATTCTGGCACTCGTTACAATCTACCTCTAGACCATTTTGTTACACCACCAGACAAAAAATTCATTACAAAAA
TACGTATGGAAGTAAAACTTTAGGGGTCTGCTTGTTGCGCATTCCCAGAGAAACTGTATCTCTTTAAAAGTGGAACTTCGCTGA
AATCGACAAATCTCGCAACATTTCTAGTGACTCAAGCATGTGTTCTGGAAACCTGCTGATCATGGCTCTCAATGGTCAGTAAAC
TCGTTTCTTGAGTGTTCAAAGAAAGACTTGCATCTGTGAGTCTGGGACCTTCACAACTCTCATTCGCACCAAATACCTAACAGA
AAATGAATACGTGAGTCGCCTTAGCGCCTGAAGTAAACGTCTTAGATGCTATAATTCGAGTAAACCGTCAAAATTTAATAATCA
GGGTGCATTCAATTAACATCATTAACAACATTGCATTGTCATTAAACTTTTGATTTTTTTATATAAGGTATAGTTTTGTTGTAG
GGCATGATTAGCAAATTAATGATTTAACAAATCTGTATTTGTTGAAAAATATGCGCAGTTTACTCCAAACCACACCAGCAGCTC
TCACGATTTCTAGTGTCTGAAACCGAAGTACTGTCTTACTGTCGAAACTGTCTTGCACGTCTCCGAAGTGTACCGTTGTCCGCC
TTTAACCCTATCTTGGGTGGGCGCTGTCTGTGATCAGAAATTGACAGCGTATTTTTTGCGTAACACAAGACTCTAAACAATTCC
GAATCATCTCAGTATATATACCTATTAAACTCTATTTTTGGTATAATAATCAATTGAAGTGACACAATAATCTTCATTGTTTAA
TTATTTATTGTAAATTCTCTTAATTTGATCAACTGATATTTCTTTTAGCAAACTATATTGTACACGAAATTGAAGAATTAGAAT
TATATTCCACATGAATTCATAAAGTGATTTTAATAACTTAAGTTTTCTACAATTTATCATAGAGCTACCACAAGAACAAATTTA
AAAAGCTGAGATTGACTTGTAAAAAGGACAAAAGAAAAAAAATCAAGACTATGAAGCAAAAATAAAACGGAAAGATTAGCAAGA
GTTTTTTTTTTTAAATGTAAGGAAATTATGATCGAAAACATCGAAATTTTGTTTTCAAAATCCTTGCAATTACAAAATGAAGA
TTGCTGTTTGTTGAATTAGTGTTCCAAAAACGTTGTACACCAATCCAACGTTTATGTTGTAGGGTCACAATTTTTAATTTTTGA
AAACATAAAAAATCCATTCTTGACATCCTATACACACAACTCCTCAGCCCTTAGGGTACGTAGCCTGACGCCGATCATACCACT
CACCCAGGCCAAGCGGAATTCCATACAAAATTAACCTAGTACAACACAGACTAAGGTACCCGCTGAATTAGCTGCTAATGTCAT
TCAGCCAACCCCGACCGTTTAACACACCCTGTATCTTCTTAAGCTTTTACCTAACCCAACTTCACCCAAAAAAAGAAAACAAAA
AGAACAAACTAATTAGTTTCAGCTTTTTCTTTCCTTCCTGACTATACCATCATTATTGCTTACCCACCAACTTCAAAACTTACT
CTCACACCGTCGCCAAATGACTGGTTTCTGAGCCCATATCCGGTCCAAAATAAATTAAATCAACTACCAAGATGACCCATAC
>m141122_014521_42134_c100732202550000001823141405141560_s1_p0/45994/0_5950
AAAAAAAAAAGAGAGAGAGAGATAATAGAGAGAATTCAACGTACGTTGACATACGAGCAGGGTATAATTTACATTTCTGGAGAAAT
GAGTCGAAATCTCCATAATGTTCACCAGAGGGGTACCACCTTTCAGCAAATCATCGATTTCTCTAATATGGATTCGAGGCAAAT
GTTTGATAGTGAATACTAAAGAAGTATGCGAGGGTCCATGGGCAACAAAAGTTGTAGCCCTTAATGATTAGGGTCTATGTTATA
ATTTCGTGGCAAATCGAGGTTTGTATATATGTGATACAGCAAAATAGCTGAGAAGATGTAGCTAATATAGGGAAAAAACTTGCG
AAAATTGCCCATCGAAAAGGGGCAATTTTATTTGAAATGTCGTACTTAAGGCTCCGACTTTCGACTCCATATATTCATTTACCC
ATGAGATTCTTGGGGAAAATATAGAACGAGTTGAACAACGGGGGTAACTACATTTCGGAGGAAAATTAGTGAAATCTCCATATT

CACCCAGAAAAAAAACAAAGGGGTACCTTTCGAAAATTTCGATTTCTCTGAGATATGGTTCGAGCAATGTTTTGATAGTGAATA
ACTAAAGAATATGCGAGGGTCAGGGCACAAAAGTTGTAGCCCTATGTCTTAGGTTCTATGTATAAAATTCGTGCAAATCCGAGT
TTGTATATGATACAGCAAAATACCTGAGACGATTAACTAGATATAAGGAAAAAACTTGCGAAAATTGCCAATCCGAAAAGGGGA
ATATTTTTTGAAATGTCGACTTAAGGGGCTCCGTTTCGACCGCCAATATATTCAATTTCACCTGCATGAGATCTCTATGGGGAA
CAAATATGCGAACGATGTTGAACACGAGGGTAATACATTTCGGCAGAATGAGTGAAATCTCCAATTCACCAAGGGGTACCTTTC
AGATAATCAATCCGATTTCTCCTAATAATGGATCGAGCAATGTTTTGATAGTGAATACTAAAGAATATGCCGAGGGTCATGGCA
CAAAAGTTGTAGCCCTATGTCTAGGGTTTCTATGTATAAAATTTCGTGCCAAATCCGAGGTTTGATAGGGTGATACAGCAAAAT
ACTGAGAGATGTACTAATATAAGGAAAAAACTTGCGAAAAATTGCCACTCGAAAAGGGGGAATTTTTTTTGAAATGTCGACTT
AAGGCCGTTTCGAGACGCCAATATATTCATTTACCGCATGAGATCTCTTGGGGAAAATAGTCGAACGAGTTGAAGCACGAGGG
TAATACATTTCGGAGAAATGAGTAGAAATCTCAATTCACCAAGGGGTACCCCGCTTTCAGAAATCATCGAATTTCTCTAATATG
GATTCGAGCATGTTTTTGATAGTGAATACTAAAGATAGCGAGGGTCATGGGCACAAAAGTTGTAGCCTATGTCTTCGGGTTCTA
TGTTAAAATTTCGTGGCAAATCCGAGGTTTGTATATATGTGAATACAGGCAAAATACTGAGAGATGTATAATTATTAAAGGAAA
AACTTGACGAAATTGCATCGAAAAGGGGAATTTTATTTGAAATGTCGACTTAAAGGCTCGTTTCGACTCCAATATATTCATTTA
CCATGAGATCTCTTGGGAAAAATATAAGAACGCAGTTGAAAACAGCGGGGGTAAATACATTTCGGCAGAAAATGATGAAATGCT
CCAATTCACCAAAGGGGTACCTCTTCAGAAATCTTCGATTTTTTCTCTAATATGGATTCGAGCAATATTTTGATAGTGAATACT
AAAGAATATGCGAGGGTCATGGGCAGCAAAAGTTGTAGCCGCTATGTCTGGGTTCTATGTATAAAATTTCGTGCAATCGAGGTT
TGTATAGGGTGATAACAGCAAAAATACCTGAGAAGATGTACTAATATAAGGAAAAGAAAAATTGCGAAAATTGCCATCCGAAAG
GGAATTTTTTTTGGAAATGTCGACTTAAGGCTCCGTTTCGACGCGCATATATTTCCATTTACCCATGAGATCTCTTGGGGAAAA
ATATCGAACGAGTTGAACACGAGGGTAATACATTTCGGAGAAAGAGTGAAATCTCCAATTACCAAGGGGGTACCCTTTCAGAAT
CATCGATTTCTCTAATAAAGGATTCGAAGCAAATGTTTTGATCAGTGAATACTAAAGAATAATGCGAGGGTTCATGGGGCACAA
AAGTTGTGCCTATGTCTTAGGGTTTCTATGTATAATCAATTTCGTGCAAATCCAGGTTTGTATACAGGGGTGATAATCAGCAAA
ATACTGAGAGATGTATCTATATAAGGAAAAAACTTGCGAAAATTGCCATCGAAAAGGGGATTTTTTTGAAATGCTCGACTTACA
GCTCCGTTTCGACGCCAATATATTCATTTACACCATGAGATCTGCTTGGTGGACAAAATCATCGAACGAGTTGAACACGAGGTA
ATCATTTCGGGAGAAATGAGTGAAATCTCCATTCACCCAAAGGGGTACCCCTTTCAGAAATCAATCGATTTCTTAATATGGATT
CCGAGCGCATGTTTTGATGTGAAATACTAAAGAATCATGCGAGGGTCATTGGGCCCAAAGTTGTAGGCCCTATGTCTTTAGGGT
TCTATGTAAAATTTCGTGCAAATCCGAGGTTTTGATAACGTGATACAGCAAATATACTGAGAGCTGTTACTAATATCAAGGAAA
AACTTGCGAAAATTGCCATCGAAAAGGGAATTTTTTTTGGAAGAAATGTCCGACTACTAAGGCTCCGTTTCAGACTGCCAATAT
ATTGCATACCCATTGAGATCGTTCTTGGGAAAAAATATAGAATAGGTTTGACACGGATTAATACAATTTCGGAGAAAGGAGTGAA
TCTCAATTCACCAAGGGGTACCCTTTCAGAAATCTTCGAATTTTCTCTAATATGTGATTCGAGCAAGCTGTTTTGATAGTGAAT
ACTAAGAATATGCGAGGGTCATGGGGCACAAAAAGTTTGTTAGCTAAGTCTTAGGGTTCTATGTATAAAATTTCGTGCAAATCC
CCGAGGTTGTGTATATGGCTGATACAGCAAAATACTGAGAGACGTACTAATATAGAGGAAAAAACTTGCGAAAATTGCCATCGA
AAAGGGGAATTTTTTTTGAAATGTCGACTTACAGGCTCCGTTTCTGACGCCAATATATTCATTTCGCCATGGGATCTCCTTGGG
GAAAAATATCGACGAAGTTGAATCACAGAGGGTAATAATTTCGAGAAATGCGGGTGAAATCCCATTCCACCAATGTACCACCAT
TTCAGAAATCATCGATTTCTCTAAAAAAATATGGTTCGAAGCATGTTTGTGATAGTGAATACTAAAAGAATATGCGAGGGTCAT
GGGCTAGCAAAAGTTGTAGCAGACTATGTCATCAGGGTTCTATGTATAAAATTTCGTGCAATGCGAGGTTTGTATAAGGGTGAT
TAAGCGCAAAATACTGAGAGAATGTACTAATATGCAAGGAAAAAACATGCGAAAATTGCCATCGAAAAGGGGAATTTTTTTTGA
AATGTCGACTTATAGGCTCGTTCTCGACGCCAATATATTCTATTACCCATGAGATCTCTTGGCGAAAAATATAGCAACTATTGA
ACAGGGGGTAATACATTTCGGAGAAATGAGTGAATCTCCATTCACCAAAGGGGTGCCGCTTTCAGAATCTTAGATTTCTCTATA
TGGACTTCCGAGCATGTTTTGATAGTGAATACTAAAGCATATGCGAGGGTCATGGGGCAGGCAAAGTTGTAGCCCTATGTCTTA
GGGTTCTATGTATAAAATTTCGTGCAAATCGAGTTTGTATAGGGTGATACAGCAAAATACTGAGAAGAGTAGCTAATATCAAGG
AAAAAACTTGCGAAAATTGCAATCGAAAAGGGGAATTTTTTTTGATAATGTCGACTTAAGGCTTTTTTTTTTTTTTTCCGTTTC
GACGCCAATATATTCTTTTCCCATGAGATCTCTATTTGGGGAAAAATATCGAACGAGTTGAACACCCGAGGTAATACATTTCGG
AGAAAATGAGTGAATGCCTCGCAATTTCCCACCAAGGGGTTACACCCTTTCAGAAATCATCGATTTCTTATATGGATTCAGAGC
AATGGTTTTGATAGTGAATACTAAAAGAATTATGCGAGGGTCATGGGCACAAAGTTTGTAGCCTATGTCTTAAGGGGTTTCTAT
GTATAAAATTTCGGTTGCAATCGGGTTTTGTATAGGGGTGATACAGAAAATCTGAGAGTTGTACTAAAATATAAGGAAAAAACC
TTGCGAAATTCCATCGAAAAAGGGGAAATTTTTTTTGAAATAGTCGACTTAAGGCTCCGTTTTCGACCAAATTAAATTCATTAC
CCAATGAGATCTCTTGGGGAAAATATCGAACGAGTTGAAACAACGAGGGTAAATAACATTTCGGAGAACATGAGTTGAATCTCC
AATTTCAACAAGGGGTCCCTTTCAAGGAACATCAATCGTTTCCCCCTCTAATATGGATTTCGAGCATGATTTTTGATATGGCAA
TACAAAGAAATAATGCGAGGGTCTGGCACAAAAGTTTGTAGCCCTATGTCTTAGGGTTTTATGTATAATTCGTGCAAAATCCGA
GGTTTTGTATATGTGATTACAGCAAAATCTGGGCAGATGTACTAATTAAGGAACTTGCCGAAAATTGCTATCGAAAAGGGGAAT
TTTATTTGGAAATGCTTCCGACTTAAGGGCTCGTTTCGACTCCAATAATATCTTTACCATGAAGATCTCTTGGGGAAAAATATG
AACGAGTTTGAAACACGGGGGTAATACAATTTCGCGAGAAATTAGTGAAATCTCCAATTCACAAAGGGGTTAACCCCTTTCAGA
ATCTTCGATTTCTCTAATATGGATTCGAGCAATATTTGATTAGTGAATACTAAAGAATTGCGAGGGTCCATGGGTCATGGGCAC
AAAAGTTTAGCCTACTGTCTTAGGTTCTATGTATCAATTTTGGCAATCCGAGGTTTGTATAGGGTTTTGATACAGCAAAAATAC
TGGAGATGTACTAAATATACGGAAAAAACTTGCGAAAATTGCCATGAAAGGGATTTTTTTGATGTCGACTTAAGGCTCCGTTT
TCGGACGCCAAATATATTCATTTACCCATGAGATTCTCTTTGGGAAAAATATCGAACGAGTTGAACACGGGGTAATACATTTCC
GGAGAATGAGTGAAAAATCTCCAATTCACAAAGGGGTACCCCTTTTCACGAAATCATCGATTTTCTCTATATGGATTCGAGCAA
TGTTTTTGGATAGTGCATACTAAAGAATAATATGCGACGGGTCATGGGGCAGCAAAAGTTGTAGCCCTATGTTTTAGGGTTCTA

TGTATAAAATTCGTGCAAATCCGAAGTTGTGTATAGGGTGATACAGCAAAATACTGAGGATTGTTACTAATATAGGAAAAACTT
GCGAAAATTGCCCATCGAAAAGGGGAATTTTTGAAATGTGAACTTAAAGGCTCCGTTTCGACGCCAATATATCATTTACCCATG
AGATCTTCTTTTGGGGAAAAATATCGAACGAGTTGAACACGAGGGTATACATCGGAGAAATTGAGTGAAATTCTCCATTCACCA
AAGGGGTACCCTTTCAGAAATCATCGATTTCCTATATGGATTTCGAGCAATGTTTTGTATGAATACCTAAAGATATGCGAGGGT
CACTGGCACAAAATTGTAGCTATGTTTAGGGTTCTATGTATAAATTTCGTGCAATCCGAGGTTTTATAAATGTGATTACAGCAA
AATACTGAGAGATGTACCTAATATAAGGAAAAAACTTTGCGAAAATTGCATCAGAAAGGGGAATTTTTTTTGAATGTCCGACTT
AAGGCTTCCGTTTCCGACTCCAATATATTATTCCCATGAGACTCTTCTGGGAAAATATAGAATAGTTGAC
>m141122_014521_42134_c100732202550000001823141405141560_s1_p0/50118/7907_15513
GACCACACTAATAAAATGCAACCGGATGCCCATCAGAACTCCGCAGTTAAGCGTGCTTGGGCAAGAGTTGAAGTAACATAGGTA
ATGAGTGACACCTCCCTGGGTAAAGTCGCTCTCGTAGTTGCACCCCTGAAACACTCACTTTATTTCACACTTTAAAAAATCCAC
TACGGCTACACAAAAGTTCATTGTAATATAATTTATGATTTTAAATCTTTAAACCTCCCTTATTTATTCTGATTTCCTTTGAGG
GGTTAATACGTCAAACACAGGTACAACACAACAAATCAAGCGCGAATAAGAAGTTCTAGTCAATTCCGGCAGGGAGAAAAGGGA
GTGACACAACGCCAATAATACGCTATAAAACAATGCACCTATTGCGGACCAAAACACATTCCGGGTGCGATCATAACCAGAACA
AACTAATGGCACCGGATCCCATCAGAACTCCGCAGTTAAGCGTGCTTGGGGCGAGAGTAGTACTAGGATGGGTGACCTCCTGAG
GATAGCTCCTCGTGTCTGCACCGCCCTTGAACAACATCATTTTTTTTTTCCCTTTTAATAAATCCACCTACCGGCTTCAAAAGT
TTGTATTTTTTTTGTTTTAAATCTTAAAAACTCATTTATTTCGTTTTCCTTGAGGGGTTAATCCGTGGTCTTAACAAAAGACGG
CTAACAAGCGCGAATAAAAACGCTCTCTCCGTCAGTCCGGGGACTGAAAGGGGGACACACGCGTCGCTTAAATGAATCATTGCG
GAACAATACAGCAGATCGGGTGCGACTCATCAAACCAGCACTAATGCCCGGATCCCATCAGAACTCCCGCAGTTAAGCGTGCTA
TAGCGGCGACAGCAGTAAGTGACTAGGGATATAGGTGTGAAAACCTTCACATGGGATAGATCCGTCGAAATGATCCAACCAAAC
ACATAAAGATATTATACCTAAATTTATACAATGTTGCACACCCGTTCAGAAAAACATCAATTTTGTTTTTTTTCCCTTATACAA
AATCGCACTACTAACGCCATCAAAAAGTTTGTATTTTTTTTTGTTTTAATCTTGTAAGAAGCTGCATTGGTGTAGTATGCGTCT
TATCATATGAGGGGTTACCATCACCGTGGTCACTAAAAGCAGCGAATTAATAAGTTCTTCTAGTCAAATAACCGGAGGGGAGAA
AAATAGACCCGGAGGAACAATACACGCCATCGCCGTATAAATGAATATTGCGGAAACCAAACATTCCGGGTGCGATCATAGCAG
CCTAATGCACGGTCCCATCAGAACTCCGCAGTTAAGCGTTGCCTTGGGCGAGAGTGAAGTAGCTAGGATGGGTGACCTCGCCTG
GGAAGTCCTCTGTAGTTGGCAACCGCCTGCGAAAACATCATTTTTTTTTCGCCTTTAAAAATCGCACCTACAGGCTGCAAAAGT
TTGTATTATTTGTTTTAAATCCTTTAAAAACTCATTTATTTCGTTTTACTTGAGGGGGTTAATACCGTGTCATAACAAACAGCG
AATAGAAGTTCCTAGTCAATCCGGGGGAGAAAAGGGAGGCACAGCGCGCTCGCTATAAAATGAATATTGCGACCAAAGCATTCC
CGGGTGCGATCAATACCAGCACTAATGCCACCCGATCCCATCAGAACTCCGCAGTTTAAGCCGTGCTTGGCCGAGAGTAGTAC
TAGGATGGTGACCTCACTGGGCGAAGTCCTCGTGTTGCACCCTGAAGACATCATTTTTTTTTTCCCATTAAAAATCCACCTACG
GCTCAAAAGTTGTATTTTCTTGTTTTAATCTTTTAAAAAACTCCATTTATTCTCGTTTTCCTTGAGGGATTAATACCGTGGTCA
CTAACAAGCAGCGAATAAGAATGTTCCTAGTCATCCGGGGAAAAAGGGAGGACAGCCACGGCTCGCTATAAATGACATATTGCG
GACACGGCAAAGCATTCCGGGTGCGATCATACAAGCACCCCTAATGCACCGGATCCCGCATCCAGAACTCCCGCAGTTAAGCGT
GCTTGGGCGAGAAGTAGTACTAGGTGGGTGACCTCCTGGGAAGTCCTCGTGTCTGCCCCCTGAAAACATCATTTTTTTGTTTCC
CTTTAAAATCCACCCTACGGGCCTCAAAAGTTTGTATTTTTTGTTTTAAATCTTTAAAAACCTCATTTATTTCGTTTCTTGAGG
GGTTTAAGACGGCGTGGTCACGTAACAAGCAGCGAATAAGAATTCCTAGTCAAGTCCGGGGGAAAAAGGGAGGGACACAACGCC
GCGTTCGCTAATACAATGAATATTTGCGGAACAAACATTCCGGGTGGCGATCATACGAAGCCTAATGCCCGGATCCCATCAGAA
CTCGCGCAGTTACGCGTGGCTTGGGCGAGAGTACAGTAGCTAGGATGGGTGACCTCCTGGGAAGTCCTCGTGTGCACCCCTCGA
AAACATCATTTTTTTTTTTTCCCTTTAAAAATCCACCCTTCGAGGCTCAAAAGTTTGTTATTTTTTGTTTTATACTTTAAAACTC
ATTTTATTTCGTTTTCCTTGAGGGGTTAATAACCGTGGTCCGACTAAAGCAAGGCAGGCGAATACAGAAGTTCCTAGTCAATCC
GGGGGAGAAAAGGGAGGAGCACACGCGCTCAAAGCTATAAATGAATATGCGGAACAAACGTCTGACGTTTCGTCGATTTTGCGT
GTCTTGTTTGTGTACCGTGTTTGGGATTGTGTGGCTGTCTCTTGTCATTCCGGGTGCGAATCATACCAGCATAATGCAACCGGA
TCCCATCAGAACTCCGCAGTTCAAAGGCCGTGCTTGGCGCGAGAGTAGTACCTCAGGATGGGTGACGCTCCTGGGAAGTCTCTC
GTGTTGCCCCCTGAAAACATCCATTTCTTTTTTTCCCTTTAAAAAATCCACCTACGGCTCAAAAGTTTTATTTTTTGTTTTAAA
TCTTTAAAAACTCAGTAATATTTTCCGTTTTCCTTGAGGGGTTAATACCGTGGTCAACTAACAAGACAGCGAAGTAAGAAGTTC
CGTAGTCAATCGGGGAGAAAAGGGAGGACAGCACGCGCTCAAAAAGCTAGTAAAATGAATATGCGACCAAAACATTCCGGGTGC
GATCATACCAGCCACTAATGCACCGGATCCCATCAGACTCCCGGCAGTTTAAGCGTGCTTCGGGCGAGAGTAGTACTAGGATGG
GTGACCTCCCCTGGGAATCCCTCGCCGTGTTGCACCCTGAAACTGCATTTTTTTTTTTCCCCTTAAAAAATCGCACCTACGGCT
CAAAAAGTTTGTATTATTTGTTAAATCTTTTAAAAACCTCATTATTCGTTTTCCTGAGGGGTTAATACCGTGGTCACTACAAGC
AGCGAATAAGAAGTTCCTAGTCAATGCCGGGGAGAAAGGGAGGACACCAACGCGCTCGCTATACCCATGAATGATGTTGCGGAC
GCAAACATTCCGGGTGCGATCATACCAGGCACTAATGCACGGATCCCATCAGAACTCCGCCAGTTAAGCGTGCTTGGGCGAGAG
TATACTAGGTGGGTGACTCCTGGGAAGTCCTCGTGTTGCACCCTGAAACATCATTTTTTTTTCCCTTTAAAAATGCCACCTACG
GCTCAAAAGTTTGTATTTTTGTTAAATCTTTAAAAACTCATTTATCTTCGTTTTCCTTGAGGGGTTACAACGATGGTCAGCTAA
CAATGGCAGGCGAATAATGAATCCTAGTCAAATCCGGGGGAGAAAAGGGAGGGACACCACGCCGCCTCGCTATAAATGAATACT
TGCGGACCAAAACATTCCGGGTGCGATCATACCAGGCACTTAATGCACCGGATCCCATCAGAACTCCGCAGTTAAGCGTGTTCG
CGGCGAGAGTATACTAGGATGGGTGACCTCCTGGAAGTCCTCGTGTTGCATCCCCTGAAAACATACAATTTTTTTTATTCCCTT
TAAAAATCCCCTACGTCCTCAAAAGTTTTGTATTTTTTGTTTTAAATGCTTTAAAAACTCATCTTATTTCGTTTCCCTTGAGGG
GGGTTAATAGCCGTGGTCACTACAAGCAGGCGAATAAGCAAGTTCCTAGTCAATCCGGGGGAGAAAAGGGAGGACCACACGCGC
TCGCTATAAATGAATATTGCGGAACAAACATTCCGGGTGCGATCATACCCAGCACTAATGCAGCCGGATCCCATCAGAAAATCC

GCCAGTTAAGGCGTGCTTGGGCGGAGTAGTACTAGATGGTGACTCCTGGAAGCGATCGTGTGCACCCCTGAAGCATCCATTTTT
TTCCCTTTAAAAATCCACCTAGCGGCGTCCAAAAGTTTGTATTTTTGTTTTAAAATTAAAAACTTCTTTCCGTTTTCCCTTGAG
GGATTTAATACGTCGGTCACTAACAAGCAGCGATAAGAAGTTCCTAGCAATCGGAGGAAAAGGGAACCCCGCGCTCGCTTATAA
CAATGATATTGCGACCAAACATTCGCGGGTGCGATCATACGCAGCACTAATGCACCGGATCCCCTCCAGAAAGCTCCGCAGTTA
AGCGTGCTTTGGGCGAGAAGTAGTACTAGGATTGGGTGACCTCCCCTGGAAAGTCCTCGTGTGAACCCCTGAAGACATCATTGT
TTTTTTCCTTTAAAAATCCACCTAAGCCTCAACCAAGTTTGGTATTTTTTGTTTAAACTTAAAAACTCATTTATTTGTTTCCTT
AGGGATTAATACCGTGGTCACTAACAAGCAGCGAATAAGAAGTTCCTAGTCAATCCGGGGGAGAAAAGGGGAACCCGCGCTCGC
TATAAATGAATATACGGACCAACCATTCCGGGTGCGATCATACCAGGCACTAATGCAGCCGGATCCCCAGTCAGCAACCTCCGC
GCAGTTAAGCGTGCTTGGGCGAGAGTAGTAACTAGGAGGGTGACGCCTCCTGGGAAGTCCTGTGTTGCACCCCTGAAAACTCAT
TTTTTTTTTCCCTTTAAAAATCCACCTACCGGCTCAAAAGTTGTATTTTTTGTTTTAAATCTTTAAAAACTCCATTTAATTTCG
TTTTCCTTGAGGGTTTAATACGCGTGGTCCAGCTACAAGCAGCGGAATAAGAACGTTCCCTAGTCAATCCGGGGGAGAAAAGGG
AGGGACACACGCGCTCGCCTATAAATGAAATATTGCGGCAACAAACATTCCGGGTGCGATCATACAGCACTAATGCACCCGATC
CCATCAGAAACTCCGCCGTTATAGCGTGCTGGGCGAGAGTATACTAGGATAGGGTGACCTCCTGGGAAGTCTCGTGTTGCACCC
CGAAGACATCATGTTTTTTTTTCCCTTTAAAAAATCTCACCTACTGGCTCAAAGTTTTGTATTTTTTGTTTTAAATCTTTAAAA
ACTCATTTATTCGTTTTCCTTGAGGATTAATACCGTGGTCACGAAACAAGGCAGAGAATAGGAGAAGTTCTAGTCAATCCGGGG
ATGAAAAGGGAGGAACACGCGCTCGCTAATAAATGAATATTGCGGAACAAACATTCCGGGTGCGATCATACCAGCACTAATGCA
GCCGGATCTCCATCAGACTCCGCAGTTAGCGTGGCTGGGGAGAGTAGCTACTAGATGGGGTGACCTCCTGGGAAGTCCTCGGTG
TTGGCACCCCTGGAAGACATCATTTTTTTTTCCTTTAAAAATCCTCCTACTGGCTCCAAAAGTTTGGGTATTTTTTGTTTTAAA
CTTTAAAAAACTCATTATTCGTTTCCTGTGAGGGAATTAATACCGGGTCACTAACAAGCAGCGATAAAGAATGTTCCTAGCAA
TCCGGGGGAGACCAAGGGAGGAACACCACGGCGCCGCTATAAATGAATCATTCGCGGACCAACATTCCGGGTGGCGATCAATAC
CAAAAACTAATGCCCGGATCCATCAGACACTCCGCAGTTAAAGCGTGCTTGGGCCCGAAGAGTGTACGTAGGTCGGGTGAGCCC
TCCCTGGGAAGTCGCTAGTGTCTGCAACCCCTGAAAACATCATTTTTTTTTTCCCCATTTTTTTTTTTTTTTTTTTTTCCTTT
AAAAATCACCCCTACGGCTTCAAAAGTTTGTATTTTTTTGTTTTAAATCTTTAAAAACTCATTTATTTCGTTTTCCTTGAGGGG
TTTAATACCGCTGGTCACCCTAACAAGCAGCAGCGATTAAGCAAGTTCCCTAGTCAATCCGGGGGAAGAAAAGGGAGACACCGC
GCTCCCAGCTATAAATGAATATGCGGAAGCAAACATTCGGGTGGCGATCAATACCAGCACTAATGGACCGGATCCCATCAGAAC
TCCGCAGTTAAGCGTGTTGGCGAGAGTAGTATCTAGATTGGGTGACCTCCTGGGAAGTCCTCGTGTTGCACCGCCCTGAAAACA
TCATTTTTTTTTCCCTTTAAAAATCCACCTCGGCTCAAAAGTTGTATTTTTTTGTTTTTAATCTTTAAAACTCATTTATTTCGT
TTTCCTTGAGGGGTATACCGTGGCACTACAAGCCCAGGGCAATAACGAAGTTCCCTAGTCCATCCCGGGGGAGAAAGGGAGGAC
ACACGCGCTCTGCTAATACAAATGAATAGTTGCGGACCAACCAATTCCGGGTGCCGATCATGACAGCACTAAATGCACGCGGAT
CCCCATGCAGACACTCCCGCAAGTTAAGCGTGCTTGGGCGAGAGTAGTATACTAGGATGGGGGTGACCTCCTGGGAATCCTCGT
GTTGCACCCCTGAAAACATCATTTTTTTTGCCTTTAAAAATCACCTAAGGGCCAAAAGTTTGTATTTTTTGTTTTAATCTTTAA
AAGCTCATTTATTCGTTTTCCTTGAGGGGGTTAATCCGTGAGTCACTAAAAACGCCAGCGAATAAGAAGTCCTAGTCAATCCGG
GGGAGGAAAAGGGAGGACACACGCGCTCGCTATAGAATGAATATTGCGGACCGAAACATTCGCGGGTGCGATCATACCAGCAGC
TAATGCCCGGATCCCATCCCAGAACTCCAGTTAACGCGTGCTTGGGCCGAGAGTAAGTAAGCTAGGATGGGTTGACTCCTGGGA
GTCCTCGTGTTGGGCACCGCCTGAAGACATCCATTTTTTTTTTTCCCTTTAAAAATCCTCCTAGCGGCTCCAAAAGTTTTGTAT
TTTTTGTTTTAAATCTTTAAAAAAAACTGCATTTATTTCGTTTTCCTTGAGGGATTCAATACCGTGGTCCACTAACAAGCAGCG
AATAAGAAGTTCCTAGTCAATCCGGGGAAGAAAAGGGAGGACACACGCGCCGCTATAGAAAGTGAATATTGCGGACCAAACATT
CCGGGTGCGATCATACCAGCAGCTAATGCACCGGATCCCCCGAATCAGAACTCCGCAGTTAAGCGTGCTTGGGCGAGAGTATAC
TAGGATGGTGACTCCTCGGGAAGTCTGCCCGTGTTGGCACCCCCCCCCTTGAAAAATCATTTTTTTTTCCCCTTTAAATCCACT
ACGCTCAAAAGTTTGGTATTCTTTTGTTTTAAATCTTTAAACTCATTTATTTTCGTTTTCGCCCCTTGAGGGGATTATACCCGT
GAGTCACTAACAAGCAGGCGAATAAGAAGTTGTTTAGTCAATCGGG
>m141122_014521_42134_c100732202550000001823141405141560_s1_p0/53125/12667_17964
ACCCTACGGCTCAACAGTTTGTATTCTTTAGTTTAATCTTTATAGAAACTCATTTTATTTCGTTATCCTTGAGGTTAATACCGT
GTGGTCTACTAAACAAGCAGCGAATACAGAATGTTCTAGCAATCGGGATGAAAGGAGGACACAACGCGCTCGCTATTAAAGAAT
ATTGCGAACAAACATTCCGGTGAGATCATACCAGTCACTAAGCCACCGATCCCATCAGAATCCGCAGTCTAGAGCGTGCTTGGG
CGAGAGTAGTAACTAGATGGGTGACATCCTGGGAAGTCCTCGTTGTTGCACCCCTGATAAAATCCTATTTTTTTTTTCCCTTCT
AAAAATCACCTACGCTCAAAAGTTTGTATTCGTTTTGTTTTAAATCTTTTAAAAACTCATATATTTCGTTTTCCTTGAGGGGTT
AATACCGTGGTCACTAACAAGCAGCGATAAGAAGTTCCTAGTCAGTCCGGGGACGAAAAAGGGGGGGGGCGAGAGAAACACCG
CTCGCTATAAGTGAATTATTGCGGACCAAACATTCCGGGTGGATCATACAGCACTAATGCACCGGATCCCATCAGAACTCCGCC
ATTTAGCGTGGCTTGGGCACGAGGTAGTAACTCAGGTGGTCGATCCTCCTGGGAACTCGTGTGCACCCCTGAATACATCATGTT
TTTTTTCCCTTAAAAATCCACCAATACGCTCAAAGTTTGTATCTTTGCTTTTAAATCTTTAAAAACTCATTTATTTCGTATTTC
TTGAGGTTAATACCGTGTCACTAACGAATGCAGCGAATAAGAAGTTCCTACAGTCAATCCGGTTTGGAGAAAAGGGAGGACACA
CCCTATAATGAATATTGCGGACCAAACATTCCGGGTGGATCCATAACCTAGCACTAATGCACGGATCCCATCAGAACTCCCGCA
GTTAAGCGTGCTTGGCGAGAGTAGTACTAGGATGGGTACCTCCTGGGAATCCTCGTGTTGCACCCCTGAAAACATCATTTTTTT
TTTTCCCTTTAAAAATCCATAGCTCAAAAGTTTTATTTTTTGTTTTAATCTTTAAAAACTCATTTAATTTCGGTTTATCCTTGA
GTTAATACCGTGGCACTAACAAGCAGCGAATAAGAAGTTCCTAGTCAATCCGGGGGAGAAAAGGGAGACACACTGCGCTCGCTA
TAAATGAATATTGCCGGAACAAACCATTCCGGTGGATCATACCAGCACTATGAACCGATCCATCCAGAACTCGCAGTTAAGCGT
GCTTGGGCGAGAGTAGTACTAGGATGGGTGAACCTTCCTGGAAGTCCTCGTGTTGCACCCCTGAAAACATCATTTTGTTTTTTC

CCTTAAACGTTCCACTACGCCTCAAAAGTTTGTTATTTTGTTAAATCTGAAAACTAATTTATTTCGTTTCCTTGAGGGGTTAAT
ACCCGTTTGGTCACTAACAAGGGCAGCGAATAAGATGTTCCTTAGTCAATCCGGGGGAGAAAAGGGAGGACACACCGCTCCGCT
ATAATGAATATTGCGGAACAAACATTCCGGGTGCAGATCAATAGCCAGCACTAATGCACCGATCCCATCAAACTCCGCTAGTTA
CGTGCTTGGCGAGGAAAGACTAGGATGGGTGACCTGCCGGAATTCCTCGTTGAACACCTGAAAACATTCATTTTTTTTTTCCCT
TAAAAATCCACCTACGCGTCAAAAGTTTTATTTTTGTTTTAAATCTTTAAAATCTCATATATTTTCGTTTTCGCTTGAGGGGTT
AATACCGTGGTCAACTAAAAGCAGCGAATAAGAAGTTCCTATCAATCCGGGGGAGGAAAAGGGAGGAGACACAGCAGCTCGCCT
ATAAATGAATAATTGCGACCCAAACATTCCGGGTGCGACATTACCAGCAATATAAGTTGCACCGATTCCCATCAAGAACTCCGC
AGTTTAAGCGTGCTTGGCGCAGAGTAGTATAGGATGGGTGACTCCTGAGTCCTCGTGTTCACCCCTGAAAACATCATTTTTTT
TCCCTTAAAAATCCACCTACGGCTCAAAAAGTTGTATTTGTTTGTTTTAAATCTTTAAAAACTTCATTTATTTCGTTTTCCTTG
AGGGTTAATACCGTGGCACTAACAAGCAGCGAATAAGAAGGTTCCTAGTCAATCCGGGGAGAAAAGGGAGGAACACTCGCTCAG
CTAATAAATGAATATTGGCGGACCAAACAGTTCAGGGTGCGATATACCAGCACTAAATAGAAAAAGGATCCCATCAGAACTCCG
CAAGTTAAGCAGCTTGGCGAGAGTAGTATGGATTGGGTGCCTCCTGGGAGTCCTCGTGTTGCACCCCTGAAACATCATTTTTTT
TTTCCCTTTAAATAATCCCCTACTGGCTCAAAGTTTGTAAAAAAAATTTTTTTTTTTTTTGTTTTAATCTTTAAAACCATTTC
AATTTCGTTTCCTGAGGGGTTAATTACCGTGGTCACTAACAGCAGCGAAATAAGAAAGTTCCTAGTCAAATCCGGGGGAGAAAA
GGGAGGACCACCGCCTCGCTATAAATGTAATATTGCGGAACAAACATTCCGGTGCGACCATACCAGCACATATGCACCGGATCC
CATCAGACTCGCAGTTAAGCGTGCTTGGTGCGAGAGTAGTACTAGTGGGTGACTAAAACTGGGAATCCTCGTGTTGCACCTGAA
GATATCTTTTTTTTTCCCTTTAAAAAATCTCACCTACGGCTCAAAAGTTTGTATCTTTTGTTTAAATTTAAAAACTCATTGTAG
TTCGTTTTCCCTTGAGGGTTAAAACTACCGTGGGTCACTAACAAGTCAGCGAATCAGAATTCCTAGTCAATCCGGGGAGAACAG
GGAGGATACACGCGCTCGCTATAATGAATCATTGCGGAACAAACATTCCGGGTGCGATCATGACCAGCACTAATGCACCGGATC
CCATCAAGAAACTCCGCAGGTTAAGGCGTGCTTGGCGAGAGTAGTACTAGGAGGGTTGACCATCCTGGGAAGTCCTCGTGTTGC
ACCCCTGAAAACATCATGTGTTTTTTTCCTTAAAAATCCCTACGTCTCAAAAGTTTGTATTTTTTGTTTAAACTTAAAAACTCA
TATATTATCGTTTTCCTTGAGGGGTTAATACCGTGGTCACTAACAAGCAGCGAATAAAGAAGTTCCTATTCAACCGGGGAGAAA
AGGGAGGACACACCGCTCGCATAAATGAATATTGCGCCAAACATTCCGGGTGCGATCATACCAGCAATAACACCGGATCCCTCA
GAACCGCAGTTAAGCGTGCTTGGGCAGAGTATACTAGGATGGGTACCTCCCTGGGCAAGTCCTCGTGTTGCAGCCCCAAAACAC
TTTTTTTCACTTAAAAATCCAACCTACGGCTCAAAGTTTGTATTTTTTGTTTTTAAATCTTTAAAAATCTTTATTTCGTTTTCC
TTGAGGGTTTAAACCGTGTCACTAACAAGCAGCCGCACAAGAAGATTCCTAGTCAATCCGGGGAGGAAAAGGGAGGACCACGCG
CTCGCTATAATGAAATAGGTTGCGGACCAGAACATTCCGGGTCGATCAATACCAGCACTAATGCACCGATCCCATATGAACTCG
CAGTTATACGTGCTTGGCGAGAGTGTACTAGCGATGGATGACGTCCTGGGAAGTCTCGTGTTTGCACCCTCTGAAAACATCTAT
TTTTTTTTCCCTTTAAAAATCCACCTATCGGCTCAGAAAGTTTGTATTTTTTGTTTTTAATCTTAAAACTCATTTATTTCGTTA
TTCTTGAGGGGTTATATGACCGTGGTTCGCTAACAATGCAGCCGAATAGAGAAGTTTCTACTCAATCGGGGAGAAAAGGGAGAC
ACACGCGCTCGCCATAACTGATATTGCGGAAACAAACATTTCCGGGTGCGATCAACCAGGACTAATGCCCGGCATCCCATCAGA
ACTCCGCATAGCGTGCTGTGGGCGAGAGTTCTAGGATGGGTGCCATCATGCGAGTCCTCGTGTTGCAGCCCCTGAAAACATCAA
TTTTTTTTTTTCACTTTAAATCCACCTAACGCTCAAAAGTTTGTATTCTTTTGTTTAATCTTTAAAACTCATATATTCTCGTTT
TCCTTGAGGGGTTAATACCGCTGGTCAACCTAACAAGCCAGCGAATAAAAGTTCCTAGTCACATCCGGGGGAGAAAAGGAGGAC
ACAGCGCTCGCTATAAATGAATCATTTCGGACCAAACACTCGCGGTGCGATCAACCAGCCTAATGCACCGGATCCCATCAAACC
GCAGTTAAGCGGCTTGGCGACGACTAGTACTCACGGATGCGGGTGACCTTCGCCTGGGAAGCCTCGGTTGCACCCCTGAAACAT
TCACATTTTGTTTCTAACCTTTAAAAATCCACGCTACGCTCGAAGTTTGTATTTTTTGCGTTTAAATCTTTAAAAATCATTTAT
GCCGTTTTCCTTGAGGGTTAATACCGTGCGGTCACTAACAAGCAGCGATAACGAAAGTCCTAGTCGACTCCGGGGGAGAAACAG
GGAAGGCACACGCGCTCGCTATCTAAAATCTGAATATTGCGGACCAACGATTCCCGGTGCGATCATACCAGCACTAATGCACCG
GATCGCCATCCAGAATCGCACGTACTTCAAGCGTGCTTGGCCAGTAGTAGTACTAGGATGGGTTGACTCATGGAGTCCTCGCTG
TTGCACCCCTGAAACATCACTGTTTTTTTTCCCTTTAACAAAGTCCACCTACGGCGTCAAAGTTAATTAATTTTGTTTTAAATC
GTTAAAAACTCATTTATTATTCGTTTTCCTTGAGGCGGTTTACATTAACGCCGGTAACATACACTGCCAGCGAATAGAAGTCCT
AGTCAGGATCCGGGGGAGAACAAGGGAGGGCACACGCGCTCGCTATAACTGAATATTGCGGCCATAACATTCCCGTGTGCGATC
ATACCAGCATTAATGCACCGGATCCCATCAGACACTCCGCAGTTAAGCGTGCCTTGGCGAGAGTAAGTACTAGATTGGGTGACC
TCATGGGCAAGTCTCGTGTTGCCACCCTTGAAACAATCATTTTTTTTTTCCCTTTAATAAAGTCCACCTAACGCTCAAAATTTG
TATTTTTGTTTTAAATCTTGTAAAACTCTTTAGTTTCGTTTTCCTTGAGGGTTAATAACCGTGGTCAACTAACAAAGCAGCGGA
ATAAGAGTTCTAGTCAATCCCGGGGGAGAAAAGAGGACACACGCGATCCTATAAATGAATATTGGGAACAAACAGTTCCGGGTG
CGATC
>m141122_014521_42134_c100732202550000001823141405141560_s1_p0/140079/14866_21949
GACCTCGCATATTCTTTGTTATTCACGATCAAAACAATGCTCCCAATCCATATTAAGAAATCGAAGATTTCTGAAAGGAGTACC
CCCTTTGGTGAATTGGTGATTTCAGTAATTTTCTCGAAATGTATTAACCTCGTGTTTCCAACCATCGTTTGATAATTTTCCCCA
AGAGATCTCATGGGTAAATGATATATTGAGTCGAAACGGAGCCTTAAGTCAACCTTTCAAATAAAATTCCTCTTTTTGGATGGC
AAATTTTCGCAAAGTTTTTTCCTCATATTAGTACATCCTTCTGTATTTTGTACGTATCACCCTCTAACACAGTCTCGGATATTG
CACGCAATTTTAGTACATAGAACCCTAAGATCATAGGGCTACGACTTCTGTGCCATGACCCTCGCATATTCTTTAGTAAATTCA
CGATCAAAACATGCTCCAATCATATTAGAGAAATCGAAGATTTCCTGAAAGGATACCCTTTGGTGATTTGGTGATTTCAGTAAT
TTTCTCCGAAATGTATTAACCTCGTGTTCACTCGTTTGTAATTTTCCAAGAGATCTCATGGTAAATAATATATTGGATCGAAAC
GAGCTTAAGTCAACATTTCAAATAAAATTCCTTCTTTTTGATGCAATTTCGCAAGTTTTTTCCTCATATTAGTACTCCTTCTGT
ATTTGACTGTATCACCTCCTACACACCTCCGGATTTGACGCAATTTTATACATAGAAACCCTAAGAATAGGGCTACGACTTCTG

TGCCCATGACCCCTGATATTCTTTTTATTCACCGATCAAAATATTACTCGAATCCATATTAGAGAAATCGAAGATTTCATATAG
GGGTACCCTTTGGTGAATTGAGATTTCAGTATTTCTTCGAAATGTATTAACCTCGTGTTCAACTCATTTGATAATTTTCCAAGG
ATTCTCATGGGTAACTGAATATTACTTGGAGCGAAACGGAGCCTTAAGTCACATTTCAATAAAATTCCTCTTTTTGATGGCAAT
TTTCGCAAGTTTTTTCCTCATATTTATACATCCTTCCTGTATTTTGGACTGTATCACCCTCTACACACCTCGGATTTGCACGCA
ATTTTATACATGAACCCTAAGACATAGGGCTACGACTTCTGTGCCATGACCCTCCATATTCTTTTAGTTTCACGATCAAGAACA
ATGCTCCAATCCATATTAGAGAAATCGAAGAATTTCTGAAGGAGTACCCTTTGGTGATATGGTGATTTCAGTAATTTCTTCCGA
AATGTAATTAACTCGTGTTCAACTCGTTTGATATTTTCCCCAAGAATCTCATGGGTAAATGAATATATTGGAGTGAAAGGAGCC
TTAAGTCAACATTTCAAAAATAAAATTCCTCTTTTGATGGCAATTTTTCGCAAGTTTTTTCTCATATTAGTACATTCCTTCCTG
TATTTTGACTGTATCACCTCTACACACCTCGATTTTGCACGCCCAATTTTATACATAGAACCTAAGGACAATAGGGCTTACGAC
TTCCTGTGCCCCATGACCCTCCGCATATTCTTTATAATTCACGATCAAAACAATGCTCCAATCCATATTAGAGAAATCGAAGAT
TTCCTGAAAGGAGTAACCCTTTGGTGAATTGGTGATTTTCCAGTAATTTCTTCCGAAATGTATTAACCTCGTGTTCAACTCGTT
TGATAATTTTCCAAGAGATCTCATGGGTAAATGCAATATATTGAGTCGAAGGAGCCTTAAGTCACATTTCATAATAAAAATCCT
CCTTTGATGGCATTTCCAGTTTTTTCCCATATAGTACATCCTTCTGTATTTTGACTGTATCACCCTCTACACACCTCGATTTGC
ACGAAATTTTATACCTAGAACCCTAAGAATAGGGCTACAACTTCCTGTGCCCAGTGACGCCTCGCAAAATTCTTTGTTATTCCG
ATCAAAATTATTGCTCGATCCAATATTACGAGAGAAATCGAAGATTTTCATTAAAGGGTCTTTTGGTGAATTGGAGATTTCAGT
AATTTCTCCGAAAATGGTATTAACGTCGTGTTCACTCGTTTTGATAATTTCCCCAAGAGATCTCATGGGTAGAATGATATGATT
GGAGCGAAACGGAGCTTAAGTCAACATTTCAATAAAATTCCTTTTTTGATGGCGAATTTTCGCAAGTTTTGTTCCTCATAAAAT
GTAGTAACATCCTTCTGTATTGGTTGACTGTATCACCCTCTACACACTCGGATTTGCTACGCAATTTTATACATAGAACCCTAA
GACATAGGGCTACGACTTCTGTGCCCCATGACATCGCAAATCTTTGTTTATTCCACGATCAAAATAATTGCTCGATCCTATTTA
GAGAAATAGAAGATTTCATAAAGGGGTACCCCTTTGGTGATTGGAGATTTCAGTAATTTCTCCGAAATGTATTAACGCGGTGTT
CAACTCGTTTGAATAGATTTTCCCCAGAGAGCTCATGGGTAAATGAATATATTGGAGTCCGAAACGGAGCTTAAGTCAACATTT
TCAAATAAAATTCTCTTTTTGATGGCAATTTTCGCAGTTTTTTCCTCATATTAGTACATCCTTCTGTATTTTGACTGTATCACC
TCTCACACACCCTCGGATTTGCACGCCATTTTAATAGCATAGAACCTAAGACATAGGCTACGACTTCTGTGCCCATGACCTCGC
ATAATTCTTTGTTATTCACATCAAAACAATGCCTCCAATCCCTATTAGAGAAATCGAAATTTCTGAAAGGGAGTACCCTTTGGT
GAAATTCGGGTGATTTCAAGTATTTCTACCCGAAATGTATTACTCGTGTTCAACTCGTTTGATAATTTTCCCCAAGAGATCTTC
ATGGTAAATGAATATAATTGGTAGTCGAAACGAGCCCTTTAAGCTCAACATTATCAAATAAAATTCCTCTTTTTTGATGGCAAT
TTTCGCAAGTTTTTTTTCCTCATATTTAGTACATCCCTTCTGTATTTTGACTGTATCACCCTCTACACATCCGGAATATGCACG
CAATTTTTATACATAGAACCCTAAAGAACATAGGGCCTACGACTTCTGTGCCCCATGACCTCGCATATTCTTTAGTATTCACGA
TCAAACAATGCTCAATCCCATATTAGTAGGAAATCGAAGATTCTGAAAGGATACTCCCTTTGGTGAATTGGGTGATTTCAAGTA
ATTTCTCGAAATGTATTAACCTCCGTGTTCAACTCGTTTGATATTTTCCCCCAAGAGATCTCATGGGTAATGAATATATTGGAG
TCGAACGGAGCCTTAGTCAACATTTCAAATAAAATTCCTCTTTTTGATGGCAATTTCGCAAGTTTGTTCCTCATATTAGTAAC
ATCCTTCTGTATTTTTTGACTGTAATCACCCTCCTACACACCTCCGGATTTGCACGCAATTTTATACATAAAACCTAAGCAATA
GGGCTACGACTTCTGTGCCCATGACCCTCGCATATTCTTTGTTATTTCACGATCAAAATATTACTCCAAGAATCCAATTAGAGA
ACATCGAAGATTTCATATAGGGTCCCGCTTTGGTGAATTGGAGATTTCAGCTAATTTCTTCGAAATGTATTAACCTCCGTGTTC
AACTCATTTGATAATTTCCCAAGAGATCTCATGGGTAATGAATTATCATTGGATGTCGAAACGGAGCTTTAAGTCCAACCATTT
CAAATAAAATTCCTCTTTTTGATGGCAATTTTCGCAAGTTTTTTCCCTCATATTAGTACCTCCTTCTGTATTTTGACTGTAATC
ACCCTCATACACACCTCGGACTTGCACGCCAATTTTATACATAGAACCCTAAGACAATAGGCTACGACTTCTGTGCCCATGACC
CTCGTCATATTCTTTAGTATTCACGATCAAACAATGCTCGAATCCATATTAGAGAAATCGAAGATTTCTGAAAGGAGTACCTTT
GGTGAATTGGTGATTTTCAGTAATTTTCCCCGGAAAAAAATGGGGTAAATTAACCTCGTGTTCAACTTCGTTTGATAATTTTCCA
AGAGATCTCAATGGGGTAAATGATATATTTGGAAGTCCGAAACGGAGCCTTAAGTCAACCATTTCAAATAAATTCCTCTTTTTG
ATGGCAATTTTCGCAAGTTTTTTCCTCATATTAGTATCCTTCTTGTATTTGAACTGTATCACCTCTACACACCCTCGGATTTGC
ACGCAATTTTAGTACATAGACCCCTAAGAAATAGGGCTACGACTTCTGTGCCCATGCCCTCGCAATATTCTTTGTTTATTCACG
ATCAAAATATTAAAACTTCCAATCCATATTAAAGAGAAATCGAAGTTCATATAGGGGTCCCCTTTGGTGAATTGGAGATTTCAG
TAATTTCTTCGAAATTATAACCTCGTGTTCAACTCATTTGATAATTTTCCCCAAGAGATCTCATGGGTAATGAATATATTGAGT
CGAAACGGAGCTTAAGTCAACATTTCAAATAAATTCCTCTTTTTGATGGCAATTTTCGCAGTTTTTTCCTCATATTAGTACATC
CTTGCTGTATTTTTGACGTATCACCCTCTACCACCACCTCCCGGATTTGACCAGTTTATACATAGAACCCTAAGATAGGGCTAC
GATTCTGTGCCCATGACCCGCATATTCTTTAGTTATTCACGATCAAAACAATGCTCCAATCCATATTAGAGAAATCTGAAGGAT
TTCTGAAAGGAGTACCCCTTTGGTGAATTGGTGATTTCAGTAATTTCCTCCGAAATGTATTAACCTCGTGTTCAACTCGTTTGA
TAATTTTCCCTCAAGAGATCTCATGGGTAAATGATATATTGGGAGCGAAACGGAGCCTTAAGTCAACATTTCAAATAAAATTCC
TCTTTTTGATGGCAATTTTCGCAAGTTTTTTTCCTCATATTAGTACATCCTTCTGTATTTTGACTGTATCACCCTCCTACACAC
CTCGGATTTGCACCAATTTTTATACATAGAACCCTAAGACATAGGCTACAACTTCTGTGCCAATGACCCGCAAATTCTTTGTTA
TTCACGATCAAATATTGCCTCGAATCCATATTAGAGAAATCGAGATTTCATAAAGGGGTACCCATTTGGTGAAATTGGAGATTT
CAAGTAATTTCTCGAAATGGTATTAACGTCGTGTTTCAACTCGTTTGATATTTTCCCCAAGAGATCTCATGGGGTAAGAATGAA
TAATTTAAGGAGTCGAAACGGAGCCCTTAAGTCACATTTCCAAATAAAAATTCCTCTTTTTGATGGCATTTTCGCAAGTTTTTC
CTCATATTAGTACACCCCTTCTGTATTTTGACTGTATCACCCTCTACACACCTAGGATTTGCACCAATTTTATACATACGACCC
TAAGACATAGGGCTACGACTTTGTGCCCATGACCCTCGCATATTCTTTAGTATTCATAAAAACAATGCTCCAATCCCTATTAGT
AGAAATCGAAGATTTCTGAAAGGAGTACCCCTTTTGGTGAATTGGTGATTTCAGTATTTCCGAAATGTATAACCCTCGTGGTTC
AACTCGTTTGATAATTTTTCCCCAAGAGATTCTCATGGGTAATGAATATATTGGAGTCGAACGGAGGCCTTAATCAACCATTTC

AAATAAAATTCCTCTTTTTGATGGCAATTTTCGCAGTGTTTTTCCTCATATTAGTACATCCTTCTGTATTTTTGACTGTATCAC
CCTCTACACACCTCGGATTTGCACGCATTTTATACATGAACCCTAAGACATAGGCTACGACTTCTGTGCCATGACCCTCGCAAA
TTCTTTTGTTATTCACGATCAAAATATGCTCGAATCCATATTAAGAGAAATCGTAGATTCATATAGCGGTACCCCTTTGGTGAA
TTGGAGATTTCAGTAATTCTCCCGAAATGTATTAACCTCTGTTCAACTCGTTTGATAATTTTCCCCAAGAGATCTCATGGGTAA
ATGAATATAGTTGGATCGAAACGGAGCCTTAAGTAAACATTTCCAAATAAAATTCCTCCTTTTTGATGGCAAATTTTCCCAAAG
TTTTTTCTCATATTAGTACATCCTTCTGTATTTTGACGTATCACCTCTAAACACCTCGGATTATAGCACGCAATTCTTATACAT
AGCAACCCTAAGACATGAGGGCTACGAACTTCTGTGCCATGACCCTCGCATATCTTTAGTATTCACGACAAGAACAATGCTCCA
TCCATAGTTAGAGAAATCGAGATTTCTGAAAGGAGTACCCTTTGGTGAAATTGGTGCATTCAAGTAATTTCCTCGAAATGTATT
AACCTCTGTTCAACTCGTTTGATAATTTCCCAAGAGATCTCATGGTAAATGATATATTGGAGTCCGAAACGGAGCCTTAAGCAA
CATTTCAAATAAAATGTCCTCTTTTTGATGGCAATTTTCGCAAGTTTTTTCCTCATATAGTAACATCCTTCTGTATTTTGACTG
TATCACCCTCCTACACAACCTCGGATTTGCACGGCAATTTTATACATAAACCCTAAGACATAGGGCTACGATTCTGTGCCCATG
ACCCTCCGCATATTCTTTAGTATTCGATCAAACAATGCTCCAATCCATATTAAGAAATCGAAGATTCTGAAGGAGTACCCCTTT
GGTGAATTGGTGATTTCAGTAATTTCTCCGAAATGTATAACCTCGTGTTCAACCTCGTTTGTAATTTCCCAGAGATCTCATGGG
TAAATGAATATATTGGAGTCGAAACGAGCTTAAAGTCCACATTTCAAATAAAGTCCCTTTTGTGATGCAAGTTGCAAGTTTTTT
TCCTCATATGTAGTAACATCCTTTCTGTATCTTGACTGGATCACCCTCTTAACACACCTCGGATTTGCACCCAATTTTATAATA
GAACCCTAAGACATAGGGCTACAAGCTCTGTGCCCAGACCTCGCAAATTCTTTGTTTATTCACGATCAAATATTGCTCGAATCC
ATATTCAGAAATCGAAGATTTCATAAGGGGTACCCCTTGGTGACATTGGAGATTTCAGTAAATTTTCTCCGAAATGTATTAACG
TCGTGCTCAACCTCGTTTGATTAATTT
>m141122_014521_42134_c100732202550000001823141405141560_s1_p0/143273/1561_8082
ACGCAGGGAGAGGCCGATGCATTTACTACGTTTCTCTTAAACAATGTTATAGTGCGACATGGATAAAGTGAGGTAGGGTGCATA
TTCATTCAGTTCATGGACAGGTACATAGGACCTGTTTATGGGTAGTTATATCATACCGAATCGCATAACTCTGGATATACGCAG
GTGGACCGCTATGCGGGGCTTTAGTCGATGTTCACTAATGGTTACAGTAGGCATGTTGAATGAGCAGTTAGGGGTAGAGTGCTG
TGTTAATTGTTAGGTGAGACGCTTGGTCGATTAGTTATTCAAAGTTATTCGATTTACTTACTTGATCGCAGTTCAATCTGCCTT
ATCAAGCTATTCGTGGACATATAGTCTAAGCACGTTGGCTACTCGAGTTATCAGTACCGGTTAGTTAGTTTGGTAGGGATGTAT
AGTAAGGGTAGTTAGTGGAAGTGAAGCATGAGGCAGTTGGTTACATTGAACAGGGCCCCTTCTCTCAAGGTAGGAGTAAACAGGG
AGGCAATTGTTATTTGCCAGCAGTCTCATGTAAGAATTGCAAGTATCGCCGTTAGTCCTTCCAGTTACGGCGAGGAGCGTTTAT
TGTAGATTTTGGCGGGTGGCAACAAATTAGAATGAATTGCTAATCATAGCTTATGCGTATAAAGATTCGGTATACGTAGAAATA
AAAAATCAGCAGGGGTGTCAATAAATTATAGTTATGATTCAGTTGAGTGAAGTTGTATTAAACCTGAGTAGAATATGAAGTATA
AAAGAAAATCGAGATAGTATCCACAGTTCCCGAGAACTATCGGCCTTAGACTAAGTGCTAAAAAAATGTCAATTATTATATAGG
ATATTGTTAGTATAATAACAGAGTGTATAATATCATAAAAATTAAAGGTCATGTTAATTCAGATTAGTTTATGTCATGAGACAG
TAGTCAGATAGTAGTCAAGCTAAATTATAGTTATGTATTGAGAAGCAGTAAAAGAAAGTAGAAGTAGTAGTATTAGCTAAACAG
TATAGCAATTGTTAATTTTAATTTATTTATTGTTCTAATTTTCAGGAAATACTAGTAAGATTGCATTTGGACATATATGTAATT
TAGCAAGTAGAAATAAAAGTAAGAGAATATTGTGTCATAGGTATATTGAGAAAAAGGAATTGAAAAAAACATTGGCATTTTTAG
TGGGACCCGAGCCGGCCAACAGAGAGTTTCGAGCCACAAGAGCAGTAGTACCCTATGGCCATGTTAACTGCGGCTCTCACTACT
TCTCCTCAGATACCTCCCTATCAACGTAGGTCCTAGGGGCCACACCGCTGAGGCTCACGGCTCTGCGGCTCACGACACACACCT
GTCCTGCGGGGTTAGGTACGTCATTAGGTTCGGTGTTTCTGCGGAGCAGCCACGCAGTATAGTAAGAGTACACGCGCAGGAAAA
GCTCACGCGGCCTAGCACGTACTGCTCGGACTATGCCTGCAGTGCTTTCCCTGTGTTCTTGCTTTAGAGATTCTGAAACCACGT
GGCGCAAGTATTATATCTATGAAATATCAGGCTAGTAGCGAGATTGTTAGTAGATTAGTGCCACGAACCTGAGAGTTGGTAGCA
ATAGGAGGCTGGTGTTTGAACATAGTGTAGTAATGGGTACGTAGGTGGTATAGGTGGCAGTACCAGCTCAGGACTAGTGTAAGT
AGCCCGTTTGTCAGTGTGCATGTTATGGCGAGAGCCCTGTGAGGACAGTTAGCGGGAGTCACCGAGATTTATTGGGAGCAGGTT
ATACAAGCGAGCGTACAACACATATTACTTTCTAAATTAATTGTGGATGGACTAGTAAAGAATTAAGCAGGCGAAGTGAGAGGC
GACAAGTTCAAACGTCTCGATGATCCTACGTCAGACGCACTACATTAATATTGAGTTAGTCTTTCACAAGCAGTTTCGGATTAT
GAGATTAGAGGAATAACGTTTTTCTCAACACACAAATATTCTTTAGGACTACTTGTACTTATTTAACACTAGTAGTACGCTAAC
AGCTATCAAGTAATAGGATTGGTATAGTGGTAGCAGTGGTAGTCGAGTGGGGGGTAGTGGCTGCTAGTGTACGGCACGGAAGCA
GTAGAAGTAGCAGTAGCAGTTGCAGTAGCATAGCAGTACAGTAGACAGTAGCGAGTAGCCGTAGCATAGGAGAATAATAAGTAG
TAGTAGCAGTAGTAGCACAGCCAGACAGTAGGACACGTAGTAGCAGTTGTAGAAGTAGTGTAGAGTAGTAGTAGCAACTCACGA
CCATAGCCAGCAGTAGCACAGTAGTAGCAGCAGCTAGTAGCCGTCTGAGTAGCTGTATCAGTGCTGTGTAGCGAGCACGAGCAA
TAGCAGTGCAGTAGCAGTACGTGATAGAAGTAGGAGAAGCAGTAGCAGTAGCAGTAGGATATAGTAACTGTAACCAGTGAGTAG
CAGCTAGCAGTAGCAGTACAGTAGTCAGCCCAGTATAGGATGTTGCTAGTAATAGTATGTAGTAGTAGTGGCTCGTGATTGGTA
GTAGTGCCTAAGTAGTGGTAGAGGCAGTAGCAGTAGCAGTATAAGTAGGCAGTAGGAGTAGGTGTTAGCAATAGAAGCCATACG
TATAGAGCTAGTAAGCAGATAGACAGTACCGCAGTAAAGTAAGCTCATGCAGTCACGTAGCAGTAGTACGAGCGTGTGGTAGCA
GTATAGTCAAGTAGTAGAATTAGTACAACAGTAGAGTAGAAGGAGCAGCTACTCAGCACTAGTAGCTGTAGCAGTAGAGAGCAG
TACGAGTACGTAGCAGCCTCAGGAACTAGTAGCAACAGTAGTCAGTAGCAGCAGCAATAGTAGTAGTATAGTGAGGTAGCAGTA
TTAGTAGTAGCTAGTGATGTCCGGTAGTATAACTACGAATGTAGTAGCTAGTATAGTTAATAGTATACGTGGAGTAGTAGTATG
TTGTGGTGTCTGGTAGTCAAGTAAGATAGATTTGAATCCAGTCTCAGTAGTAGTAGTGAGTAGTAGTAGTATGTATGGTCGTAC
TAGTACTTATTTGGTTGGAAGTATAGTAGTAGTACCGAGCTATGTAGTAGTAGTATGTGTCCATACGTAATACCGTAGTAGTAG
ATAGTAGTAGTAGTAGTACTTACGTAGTAGTAGTCAGTAGTAATAGTCAGTAGTATGTGTTGTTAGTAGCTCTTATAGTGGTAA
AGTAGTAGTGTAGTAGTAAGTGTCGTGGAAGTAGTAGTCATGGTGTTGTAGTAGTAGTACGCAGCAGTAGTAGTAGTAGTAGTC

TCATAGAAAGTGGGTTGGTAAGCTCAGTCAGTAGCAGCCCCAGTAGTACTATTAGTAGTAGTGGTGTTGGTACGTAGTACGTAT
AGTAGTAGCTAAGAGTATAGTAGTGGTGTAGTAGTAGTAGTAATAGTCAGTCAGTATAGTGGAAGTCAGTAGAGTGGCTGTTGA
TAGTAGTAGTAGATAACGATACGTACGCTAGTATAGTATTAGGAGTAACTGGTGGTTGGTAGTATCAGTCATAGCTATCAGTAG
TGGTAGACAGCAGTATTAGAAGCTAGTAGTATAAAAAGTTTATTAGCAGAAGTAGTAGTCGGTGTGTAGTAGTAGTCAGTAGTG
GTAGTAATGGTACGGCGCGTAGTCGGTAGTAAGTGGTACTAGCAGTAGTCAAGGTAGTAGTGGGCTGAGCAGTAGTAGTAGCTT
GGTACGTAGTGGTAGGAAGCCAGTAGGAGCAGTAGTGTAAGCTAGCAATAGTAGAAGGACTAGCAGTAGTCAGTAGCGAGAAGC
AAGTAGTACGCTAGTAGTAGTAGTAGTATTAGTAGAGTAGTATAGAATAGTATATGGTAGTCAGTGCGTAGGGTGGTGTAGTGGGAG
TAGTGGTTGTAGTGGTCAGGAGCAGGTAAAGTAGCAGTAGCAGTAGCTTAAGCAGTACAGTAGTAGTAGCATAGCAGAAAGTTA
CAAGTCAGCCGTAGCAGTAGGAAAGTACGCTAGCAGTAGTAGCAGTAGTAGGAGCAGTAAGAAGCAGTAGTAGAGCAGTTTAGA
ATGTACGATCCAGTAGAGTAGTAGTCTTTTCTTTTTATGGTAAGTAGGTTGCGATCTTATGCAGTTACAGTAGTTACTCAGCTA
TTTATTGTAGCAGTTTAGTAGTTTTTTTTTTTGAGATAGTTAGTTATGCAGTTGTATGGTAGCAGCAGTAGAGGCAGAGTAGC
ACAGTAGTCAGGAGCGCTAAGCAGCAGTCAGTACAGTAGTAGCCTACAGTAGTAGCTAGGTAGCTAAGTGGCACAGCGAGCAAT
AGCAGTAGCAGTAAGGAGTAGCAAGTCGCAGTCCTTACAGTAGGTGTAGAAGTAGCGAGAAGCCAGTAGTAGTACATAGCAAGC
AGGAGTAGCATAGCTGTATTCAGCAGACAGTAGGTAGTAGCTAGTTAGGTACGCTAGTAGTGGTAGTATAGTAGTAGTAGTACG
TAATAGAGCTAGTATAGTAGTGGTGAGTGGTAGTAGTGGTAGAGTGGTAGGAGCAGCTAGCAGTAGCAGTTCACAGTAACATAG
GGACATAGCAAAGTGTAGCGAGTAGAGCTAATAGTAGTAGTAGTAGTAGTAGTATATCAGAAGTAGAGTAAGGGAAGTAGTAGTGGC
AGTACGGTAGCAGATAAGGAGCTATCGGACAGTAGTACAGTAGAGATATTGCTAAGCAACAGTAGGAGTAGAAGCAGAGCAGAC
CAGCACCTATGCCAGAGTAGTAGCAATAGCAGTAGTAGCCAGCCAAGCAGTAGCAGAGAAGCAGTAGGAGAACGCAGTAAAGTA
GCAAGCAGTAGGAGTATAGGAGTACTAGAATAGTGTACCAGCAGCAGTAGTAGTCAGCAAGTACTAGATAGTAGTGGTGTTGGT
AGAAGTGTTAGAAGTAAGTATAGCCATATAAGCGTACTAGATAAGCTAGTATGTAGTGGTGTTGCGTAGTAGTAGTAGTAGCAG
TGGTTGTTTGTAGTAGTAGTAGTTAGCTGGTGGTTTGTCAGTAGTAGTAGTGGTGTGGTATAGATAGTAGTCAGTAAGAGTAGT
AAGAGTCAGTGAGTCATTAGAAGTTGGGTAGGAAGTGTGTAGAAGTGTACGATAGTAAACCGTAGTAGAAGTATTCATTAGGAG
TAGTAGTAGTGGTGTTGTAGAAGTACGTAGTAGTGGTAGTAATGGTAGTAGTGTACCGTAATTGTATAGCTAGAATAGTAGTTG
GTAGTGTGGTAGAGTTGTAATAGTCAGGGAGCGTAGTGGTCGTAGTAGAGCAGTAGAAGTAGCAGCTAGCAGTAGCAGTAGCTG
GTACAGAGCAGTAGCAGTAGCAGTAGAAGTAAAGTAGCCCGTAGCAGTAAGGAGAAGTAGTACGCAGTAGTACAGCCGAGCAAA
AGCAGTTGGAGCAGGTAGTAGCAGTTGTAGAAGTACGCTAGTACGTACCCAGAGGCAGAAGAAGACCAGTAGTCAGCAGTAGTA
CCAGTACGTCACGCAGCCAAATAGTAGGACCAGTAGTAAGCAGTAGAGGACGCAAGCATAGGAGCCATGCAGCAGTAGTACGCC
ATAGTATAGAACGCTTGTATCTTCTAAAGTGTAGTTGGTAAGGGAGCAGGAGCAATAGCAGTAGTGCTAGGAGTAGCACGTAGC
AGGTAGCAGTAGCAGGTAGGCTGTAGAAGTAGCGAGAAGCAGTTAGCAGTAGCAGTAGCCAGTAGCAGATAGCAGTAGAATAGC
ACTAGTAGTAGTAGTCAGTAGTTGTAGTTGTAGCGTAGTAGCTTGTAGTGAGCTAGTGTAGTAGTAAAGTAGTAGTTTGTAGTA
GCAAGTTCGTAGAATGGTAAGGAAGTAGTGCTAGTGGTAAGTAAGTGGTAGGAGCAGTATAGTGGTGTTTGGGAGTTGTAGTAG
TGTAGTAGTGATGAAGTAGTAGTAATAGTGGGTAGTGTAGTAGTGGTATAGGTGGAGACGTAGTAGTAGTAGTCAGTGTAGTGG
AAGTAGTAGTAAGTAGTGTCAGTAGTAGCCTAGTAGTAGTATATAGTAGTAGTAGTTGTTTGTAGTAGCAGTAGCAGTAGCAGA
TCGTAGTAGAGTAGTTGTTAGTATTAGTAGTAGTAGTAGGCAGCAGCAGCATAGTAGTTAGCAGAAGTAGTACAGTGGCTAGTT
TAGTAGAAGTAGTAGTAGTAATATAGTAGAGTAGCAGTAGGAGCAGTAATGGAGTAGTCAGTTGGTGCACGTAGGAAGCAGTAG
TACAGTGAGTACAGTAGTAGGAGCAGGAGTAGCCAGTAGTAGTGAAGTATATAGCCAAGAAGTAGTAGTAGTGAGATTAGAAAG
TTTAGTAGTAGTAGTGGTGTTGGTGTAGTATAGTAGTAGTATAGTGAAAGTAGTAGTAGACGGTAGTAGGCTAGTAGTAGCTAG
TATATAGTACCGTATAGTAGTATTGTGTTTTGTAGTAGTAGTAGATATTATGAGTCAGGTAGTAGTGGTTTGGTAGCGGCTGGC
AGTAGCAGTAGTAGGAAGCAGTAGAAGCAGTAGCAGTAGCAGTGAGTAGACCG
>m141122_014521_42134_c100732202550000001823141405141560_s1_p0/148605/256_9558
GGTGTGCTTTTGATGACTTCTTATCTAGACCCATTGCCTAATTAATCCCTTCCACCGATGCATTATAGAAGAAGAAGTAGAACG
TGTAGGTTTTAATTGGTTTCTGGTAGACCGGTGTTTGCATATCAGTCCATTTTGTTAAGGATTTCAGATCTTTGCAATTGCGAT
GTACGGCGGGGTACACAGGGATAAAAATTTATAACATAATATCTTAACTCCAAAAGAATAACCCGGATGACAGGAATGCACTTA
TTGTGAGCGTTAAACTAATATGTCTGCTGGTAATTAGAATATCCACAAGCTATGATCCATTGGTAACTGAGTGATAAGGTTCTG
CGTACTTGTTGGTAGAAGGTTGGGGTATGATTACATACTTACGGCTTACCATGGCTAGAGGTAAAAAGGACTCCCGTCTGGATG
GATAACGATCGTGGTATTATTCATGGACTTCCGAAGAGTCTCCGGTATGGGCTGATGCTACACAAGTGACTCATCAGCGGTTGT
TCATAATGCCTGGAACCGTCAAAGTTCAGGTATCACCTTAGGTGGCTTAGCGCAATCCTCTAAGTATTGTGATAATTGATAACT
CTATGTAGACATTATATTACTGCTAGTATTATACGTACCAAGCATTTGTCTTACCAATTGGATCTACCCCCTTCGACTATAAAC
ATCAGAATACTCATAACTAATTGGGTGTGTGCTTACTTCCTACGCCTATGTCACTCAGTAATCTCGCGCACATACCTGATGTAA
TATTTATATCATCATCTACAGGAAGGAAGATAAATTCAAGTCTTAAGATACGATAACTCCATACAATAATATAAACAACACATC
CCATGTCTCACAGTCTTGTACATCAAACATATAAAATAAACACGTGTGTGCAGTATTCAATAATATATACTAAAAAGATATTCG
GTAATCATAAAAGGAAAATCCGAAGGCTAGCTAGATATGATTATATATTAATTTAAATGATAAATAATCCTTTACATAAAGAAC
AATAATGTAAGTCCTCCGACTCCTCAAAACAGTCAAGAGAAAGGCACTGAGATCGGATGACTCTCTCTCATCCCCAACTACCCA
TCTCCTACAGCGATCAAAGAGATACTAGACACGTCGGTCAAATCTGAATATCATCTTACAACAATGCCGAATAATATCTAATAT
TCGTCAGCAAATTAATATAACCATCAAACTATGATTAATATAATGCAAGATATTACGAATTCCACACGACATATAGTCAAACGT
CAAGGTCCAATACACATCCTTAATAAATGGGCATGCAAAATAGTAATCTCCAAGTCAGATATACATTACATCTATTCAGGGTAG
TAAGTATGAGTCCAACGTGAGGATACTCTCATCTCCAATCTTCATATGTTGAATATCATGAACAAGATCACATTACAAGTCCAC
TAGGGACAAGGGCTAGGTCCGGGCGGTGAACGCAAGCTCTGGTTGAGAAAAAGGTGCTACCTACCTTTCCATTCTGTTCAAAAG

CCTATGGAAAAGCATGGTGTGATTCACTGTGTGGGATCGGTGATGCACAGTCATGTGTCGTCTGTCAGGTCAAGGAGGCAGTTC
CCACAACCAGTGGCAAGCGTTCACTCGCGTAGGGACAGTTGGGTGTGCGGGAAAAGGGGAGGGTAAACGGTGTGGTTGTAAAAA
ATAGAAACCCGTGGGAAGCCAACCCTGGACAGAGAAAGTAGAAGCATTGGTCGGAGGAATTAGAAGCAACGGGGTGGTCGCCGG
TGCGCAAAGCCAACAGCACCGAAGGCGGGGACAGGCGGAACTGAGATGTGAGCAAAACATACAACGCTGGTAGAGAGTGGTGGA
TGACGCGTGTACACCGGGTGGAGGAGTGGAATTGTTGGGTGAGACAGGGAAGAAGGACATTGAGTGGTTGACGGGGGACGGGTA
ACTGCATTGTGAGGAAAGCTCAGGAAGGCGCTGCCCAGAGGAAAGGGCCAAATGGAATTAGTGGGGAAGAGAGTGGTCGCGGTG
CGGAGAGTTTGCATGGTGATAAGCAAGATTCGAGCAAGCTTAGGAGAGTTAGGAATGGCCGCATGCCAGATGGCAGAGGTACGA
GGGCGACCTCGCGAAAGAATGAGGTTAGGAAGATGATCAAAGAGATAACGTCATGAGCATGTTGGAGTACTGCACGCCGAAGGA
AATCGTCAGCGCATTGGCCATTATCCACACTAGCCATATCCAAAAGCCCCACGAGTCCGAAGGGTGTCGGAGATGGTCGACGCG
ACCAGCAAGGGCTGGAATACAGGCTGGCCGAATAGTAGTGTTGAGTGCAGGCAACGGTAAAAGTGTGGAGCGTGGCGGAGCAGT
ACGAAGAAGTCGTATCACAAGTCACTCGTAGCGATAGGCAACCGGCAAGACGCGAGACAATCCAAGCAAGCTACCCAAGCTGTA
GAGTGGTGGCGTGGGGCACAGCGTGCTCATGTCACACGACACTCGGAAGATTAGATGGCAAAAAAGTGGAAGTAATAAGGGCAG
ACACGTGCGACGCGGCGAGCTGCAGAGATAAGGGGACCAAAACTGAAGCCTGTGAGAGAAAAAAGGCAGGAGCTCATGGTGCAT
TGTGCGTGTAGAACCAACACTCGAGAGCTAGAAGAGCGCCTCGAGTCAGTAGGTGGGTCATTGTAAGGAGTAGACGTAATTAAG
TGCGTGAACAACAGCAAGGAAGTTCACGGAAAGACCCTTGTGACTCGGAAGGAGAATGTGAGGATGATTGAGGGAGGTGATCGG
TGCAATCGAAGTGATCCAAGGAGGTGGAGAGGGCCACTAGGTAATATTGCTTCCAGACAGGAGCTTCATTAGGTAATTACCGGG
AAGGTGACTGCAGGTCCAGAGGGACGTGTAGCCGACCAAAGGGGTGGTGGAGAGTTGGGATGGAGGCGGACGGTTCAAACTCGG
GGTTGACGGAGAGAGGACCCGGGAACGGACTGGTTGTAGGAATGCGAGCAGCGCGGTAGTAGTGGAGACAAGGTGTTGTCCATC
ACATGGAGAGCATGTACCGACCCAGCCAGCTGCAGTAACACAATGGACAGGTTATGATGCTATCGATAGTGCAGCAGCGAATCC
ATGACACGTGGCCGAGACCCCCAGGCACAGAGACAGATTCAAGAACCGTGGGACGAAACGCAGATAGACGCGAGAAAACTGAGG
GGAGCAGACGGGAGGTGGGGCCGGGTTGCATGAGACAGAAGGAGGGTAGATCGAGTTGAGGTGTCGCGGTCTACACAGTTGGAG
TACCATGCAGTTCAATGAACGGTGGGAGAAGATTGCTCTGACCAAGGAACGAATTCAGGGCGTCAGGCGACCGTAACCGATGTG
GACAGAAGGCAAAAGGACTGCCGAATTAAATTAAGGAGATTGCAAAGACAGCCAGACCACACTGAGAGGGCCGACAGTGGGAAG
TGGACGCGAAGATGGAAGCGTCGAGGGAAGGCCGACATAGAGAACTGAAATTAAATCAAGCCCGCAACAAGCAAAGGGCAGCAC
TCACAACTGACAAGCAGAAGAAGACACGAGCCCTTCGAGAGGATGTTCGACCGTCCTGCCAATGTGGATGGAGAAATGTAGGAG
ACCAGTCGCAAGAAACCTTCATGATGAGATCGGGCAGGACGCCAAAAGAACAGTTTAACAGCGAAGAGTACTGGAGGTGGATCT
CCGACGACCAAACCGACGGCTCACAAAGCAGCAGGTGACATGCACATTTCACAGTCTATCGACGTGTAGAGGAAGGGGCGTCAC
ATGTCAATGGGTCGTCACAATTGACCCCCATATTGGCGTTGGTCGCACTCTCAGCTCACGAACAGTAGGGTTGTGCAGGGGCCG
TGATGATAACTGAGCTCGTGATATCCGTGTCAGGCCACCTTAGAAAAGATCAGGACTCACACTGAGCATTTGAAAGAAGTTGAC
AACGTTGTGAAACGGAGTCGAAGAAGGGACACCAGACGGATCCATTGACTCCGTGACGAGAGAACTATTCCAGTCTTCTTGTAA
CCAAGATGATGAGTTTGTGTGGTTGGATTACCCCGTCGACTACAGCAACATATGCTTCGGGAGACCTGCTGATTGGGCTGGGAC
ATATTCGTTCTATTAGCATCTGGCCATCAATTTCGAATTTACGTGGCACGCGTTGAAGGGAGACCAGCTTCGAGCATTTCCATC
GAGAGGAAAGAATCAAACAAGTGCCTAGACACTTGTGGGTGTTCATGGAGTGGATGGATTCGTCTTCGAGATAGAAGAGGTGAT
GGCCTAGGTAGAAGGAATGGGTCGATCTCGAACCACTGGGGAGGCTCGCAGAAGTAGAGGTTGCACAAAAAGGATGCCATGAAG
GTGTAAAAATGGGCAGACTTCTGAAGCCAAGATAGCATTAATTTGCTCTTGAAAGAGGAACCAGGATGCTGTTCGCATGTCGAA
CTTGGACATGGTAGATAGTCTAAATGTCTAAGCGCATGCATAACATTGTAAGACTTCCCCCAAGCAGCAAAAAGTGAAGGCTCC
CGGACGATGACAGAAAGAGGCCTTAAAGAAGAGGGTTGAGAGGTTAAAAGAATCGTTCATAGAGATGCTTTCATCCTGATTGGA
CAGCCAATCTAGTGTTGGTCCAACAAAACGGCAAAGGCGAACCTATATGACTATTCAGATTTCGAACAAGAATGCCCTAGAAAC
TGTTCCCCTTACCTCGGATAGCCAGCTCGTAGATGCAGTGCAGTCACGGCATTATGTCATTCATGGATGTTACTTGGATATACC
AATTTCCATGCAGCACCGGACCAAATACCATAAGCATTGTAACCGATAAAGATTGTATTGCTAACAACGTAATGCCTTTTGGGG
CAAAAAATGTTTTGGAGCACATACCAAAGGCCGAAATGGATGTTGGCTGAAAAAATAGGGAATAATGGAGAATTTATGTGGGAT
GATGGGCAAGGGGAGGAGCGGTGAGGTTTGCTGGTGAGAGGAAGAGGGTCGTGTGGGGGGGGCGGTCGCGGGTCGTGGGGGCGG
GGGAGGGGGGTGGGGAGAAAACAGCTGACCCGCCAGCGTGCGGTCTGCGCTGCGACGGATTGCAGGCTGCTGCTGGGCTCGCTG
CTGCTTGGCTGCTACTCTACTCTGCTACTACTACTACTATACTATATACAACTAATAACTGCTACTGGCTACCTTCTCCTACTC
CTACTACTCTTATTCGGCTACTCCTCCTAACCTATACTCCTAGCGCTACTACTCCTATACCTGCTACTGCTACTGCTACGGCTA
CTGCTACTCGTTCCTGCCTACTTCTTACTGCTACTAAGCTGCTACTACTACTGCTACTGCTACTACTGCTACGCTCCTGCTACT
ACTAGCTGTCTGAGCTGCTCCTGGCTACTACTCTGCTTACTATGGCTACTGCTACTGCTACTAACTACTACTACTGCTGCACTA
CTTGCTAGCTGCTGCTACTACTACTGGCTACTGACTACTACTGGTAGCTGCTCCTACTCCTGCTCCTGCCTGTGGCTGCCTACA
ACCTAATAACTGCTACTCACTATTCTATACTACTACTACGACTACTACCAACTGCTACTGGGCTACTACTACCTAACGTACTAC
TACTACTACTACTACTACTAACTAACTCTACTACTACTACTACTCACTAACTGTGCTGCTGCGCTGCTACCTACTACTGCTACT
CTACTACTGCTACTACACTGCTTACTGGCTACTGCTAGCTATTATTGCTGCCTGCCTGCCTGGCTACTACCTGCTGCTACTCCT
ACTGCTACTATTGCGCTGCTACTACTGGCTGCCTGACACTAACCTTGACTACTACTGCTACTCCACTACTACTGACTATCTACT
AGCAACTACTACTGCTACTGCGTACCTACTACTCACTACTGGCTGCTGCTACTACTACTGCTAACTCACTGCTACTACTACTGC
TACTACTACTACTACTCACTGGGCTACTACTACTCACTGTACCTGCTACTACTATACTAGCTCGCTAGCTCACTACTGCTGCTG
CTACTACTGCTGCTGGGCACGCTACTACCTGCTACTGCTACTACTGGGCTATAACTAACTGCTACTGCTACTACTACTGGGCTG
GCTACTATGTGCTGCTTGCTGCTCGGCTTTCTATTTTACTACTGCTCCACTACTACTACTTTACTAGCTACTATCGGGAATTTC
TTGAAATAGAATTCGCATACCATAAAAGAGTTTTCCAACAAAAATCAGATTTTAGAGATTGGGGTATGGAAAATAATGTGGATC
TAGAAATTCATTTCGGACAGCCATTGAAGCATTTTCAAAACCTGATTCTTTTTAAGAACGCAAAAATATTTGTGCCAAAATAAG

CCGGAGGACTTACGGACCGACAAGTTGATTAGGGAACGAAAAGAAAGCTACCTGATTTCGATGTACTGGGCTATGGACCAGCCA
AATGGATTCTGCAATATTACTGCTTCAACTGTCGGGCAATCATTGGTGTTATGACCCCGATATTGTGGAATCAAAAAAGCTTAA
GGGGTCTCTCTTCACCCCTTTTGAGCTTAAAGGCTTGTTGGCTTCCTTGCACGAGAGGCGATTAAGATTTTCGCAGGAAGATAT
GTCCTGGGTGTCGCGTTATGGTTGGTGTAGATCGGCATAAACGGGGCTTGAGTTTCGTTCGTAGACTTGTTATTGTTCTTTGGT
GCTGTTCTGGCGCCCCTCCCCAACGCATTCCCTTTTCTTTCTTTTTTTTGTTGGCTTCGCGCTTGGTTATTCTGGGTAACAGGT
TGAGTCGTAGCTGGCAACATCTGCCACTGTCCATGGGTTGGATAGCGGGGCCTGGCCTGATTCCTGCGATAGCAGATCGTACCT
CTTCTAGGTTTATCCCACCTCCTGGGCCTCCGGGCGCCAAGAATTCATCTGCACTTTTAAGCTCCTTTTTGTTGGATGTTCATT
GGCCACAGGTCGCCTCCCACGAGGAATCCTATCCTGCATCGGCCCATAAAGCTTTGAAGCTATCATCCAACATGCCCTCAGCTC
TTTGCTACAACATTGGTGATTCAACCAGGTTGATGTTTTACGTTGGCCAATGGGTCGCCTCTACACCGAGCTGGCCTGTGGAAA
GCTCGAAATGTTTCCTTAAATTTGGGAAGAGAATTTCTTCAGAGGGCTTATCGAATGCTTCTTGTATTGTTTAGAGACCCAGTT
GTCCTTGCCGGTCTGACCAAAAAAAAAAGTCGGCGGGGAACAAGATACACCTTAGATTGAGCCGCGACATTCGTGGGCGCATCA
TCATTGTGTTGGACCACTCCCAGAGTGCATTAGATGGATCTAATATTCCATCAACGATGTCGGCATGTTCGACATCCTACAAGC
CAATAGGATACATAGCTGTGCAATGTTTGGAGGCGAAAGGTTCAAGGCCTCTTAGATATGGAGTCGCAATCCATCTGACCCCTT
TCCCCAAATATGAGCCTCTTCCATCCTGTTCTCGCACCAGGCTAGTCGGCTCAAATGGGTCTAGGTCCCTTGGACTCTAGGTTA
GCTTGGGGGTGTTTAGCCAGGCTCCGCATAGCCTATTGTAATGGCGTTTGATGGGTTGTTGTCCTACCCCAAGTCCCAAAGCTT
TGTTAGGTGGGACTTCCCATTGTCGCGTATGATAGACAGAAGCCGCATCCTTCCATGTCGAGTATAACTTAGCATCCCTCGGTT
TCGAGCTGTGATACCTTTCTGATGAGTTCAAATGATTGCGTAGATCAGGCTGTGGATGCATACCGGCTGACTTGTGTTGAGATC
AGATGATTCCTTAGGTCACTTCCAGACCCTACCTCCATGGCTGGCTCCCCAATTTAAGTAAGCTTCCATTTACAAGCTTACAAG
CCCGCCGGTTGGAGATCGAGGACCTGGTTCTCAACCACGTGCCCATCGGGCATAAAGTATCTCCCCACCAGCGAGGGAACTGAT
TTAATCCTACTGTTCGCAACGAGCTAGGGCCCAGATCTTGTTGTGGGAGAGGTGGTTGTTAGGATGTCTAAGCCAGTGACGACA
GATGGCCTTATTGGTGAGGTGCTGTCCTTCTGTTCGCATCAGGACGAACCGTAAATTAGCCCGCCTTGGGGTTTCTGAATCTTG
GCACAGGAGGATCACTGGACATTTTGGCTTCCGTGAACACTGTCGCCAGCTCGCCTTGGTTGTAACGACCTAAATTTGCTAATA
GGCTTGGAGCGCTTGATTCGGTGCCAGGAGGGAAATAATTGTTTTTACTGCATTAATTTATGTGAATTTATTGAATTATATGGT
GAGTGGCATGTTTAGGTAAAAATAAGATATGCATCGTGGACCCGCGTTTGTTGAAGGGGATAAATTGGCAATTTAGGCGCCCCT
GAGCGGCATATATGTGATCAATTGTTTATGTGTTTGTACCCACGTGGGCGTGTGGTGTCATTAATCGTGAATGCCCGTGCCGAG
ACGGTCCTAGAGAGCTAGTTTTACTCCAAAAGTCAAACAACGGGCAATTTTATACGCTAACACGGAGGACCTAGGGTAGCCTTT
GGAATTGTTTAGTGGTTAATTGAGATTTAGCGGGTAATGGTTATTGGTGATTGAGTAACCTGGGTAACCATTGTTACCTGGCTG
TGAGTAACAGAGTTTAGTTGGAATAAAGGTAGAATTGAAATATAGTGAAGGACTAGAGTGGCCTTGATGGCTTACTTTAGGAAA
GGATTATTTGGAGGGGTAACATGGTCATTTGGCAGAAGGTTTAGAGCCCTTTTTCAGCTGGGATTATGGGGTACACGTTTAATG
GGCTTGGGGATCATATGTTGGTTTTTGATATAATTGGAGAGAAGTAAAAGCTAGAAGGAAAGCTAGGGCAATGGAGAAGAAGAG
ATGAGTGAAGGAGTTGAAATTGGAGCACTTAGGAGATGAAGGAAGCCTTAGGGTTGGATTTCTTCATTGCAAAGGTAAGGATTC
TATGTAAGTTAAGGCTTGTTTCCTGTTAATGGTTTGAGGCAATTAAGCATTGGTTTAAGTTTGAGTTTTTGGTTGTTATTTTCT
GAAGTTGAATCAAAGATGTGGATTTTGGGGATTTGATTGTTGTGTTTCGGGTTTCTTTGATGATGTTTATCGAGTTTGTTAGAT
GGTCTATGTGAAGGTTTGAATTGGTTTTGGGGCTTTGGGTTACCTGCTTGGGATGATTTGGAGTGGTGTTAGCTCGGGAAAAAA
TGCAGGGAAAAAGCCTAGAAATCTGGGTTTGCGGAAAAGGAGTGCCGGGCGACCTGGTTCTT
>m141122_014521_42134_c100732202550000001823141405141560_s1_p0/149280/292_6074
CAACGCTTAACTGCGGAGTTCTGATGGGATCCGGTGCATTGAGTGCTGGTATGATCGCACCCGGAATGTTTGGTCCCGCAATAT
TCATTATAGCGAGCGCGTGTGTCCATCCCTTTTCTCCCCCGGATTGACTAGAACTTCTTATTCGTGCTTTGTTTAGTGACCACT
GGTATAACCCCTCAAGGAAAACGAAATAAATAGTTTTTAAAGATTTAAAACAAAATACAAACTTTTGAGCGTAGGTGGATTCTT
TTAAAGGGAAAAAAAAAAAATGATGTTTTCAGGGGTGCACACGAGGACTTCCCGGAGGTCACCCATCCTATGTACTACTCTCGCC
CAAGCAGCTTAAACTGCGGAGTTCATGATGGGACTCCGGTGCATTAGTGCAATGGATGATCGCACCGGATATGTTTTGGTCCGC
AATATTCATTTATAGCGAGCGCAGTGTGTCCTCACCCTTTTCTCCCCCCGATTGATAGAACTTCTTATTCGCTGCTCTGTTAGT
GACCACGGTATTAACCCCTTCAAGGAAACGAAATATAATGAGTTGTGGTTAAAGATTTAAAACAAAAATACAAACTTGGAGCCG
TAAGGTGATTTTTAAAGGGAAAAAAAAAAATGATGTTTTCAGGGGTGCAACACCGAGGACTTCCCAGGAGTCACCTCCCAGTAC
TCTCTCGCCAAAGGGCACGCTAACGCGAGTTCTGATGGGATCCGGTGCATTAGTGCTGGGTATGATCGCACCGGAATGTTTGTT
CCGGCAATATTCATTAATAGCGAGCCCCCGCGTGTTGTCCTCCCTTTTTCCCGGATTGACTAGGAACTTCTTATTCGCCTGCTT
GTTAGTGACCACGGGTATTAACCCCTCAAGGAAAACGAAATAAATGAGTTTTAAAGAATTTAAAACAAAAAATACAAACTTTTG
ACCGTAGGTGGGATTTTTAAAGGAAAAAAAAAAATGGATGTTTTCAGGGGTGCAACACGAGACTTCCCCGGAGGTCACCCATGCC
TAGTATACTCTCGCCAAAGCACGCTTAACTGCGTGAGTTCTGATGGGATTCCGGTGCAATTAGTGCTGTATGATCGCACCCGGA
ATGTTTGGTCCGCAATATTCATTTTATAAGCGAGCGGCCGTGTGTCCTCCCTTTTCTCCCCCGGATTGACTAGGAACTTCTTAT
TCGCTGCCTTGTTAGTGACCACGGTATAACCTCAGGAAAACGAAATAATGATTTTAAAGATTTAAAACAAAAAATACAAACTTT
TGAGCCGTAGGTGGATTTTTAAAGGGAAAAAAAAAAATGATTTTTCAGGGGGCAACATCGAGGACTTCCAGGAGGTCACCCATC
CTAGTTACTACTCTCGCCCAAGCACGCTTAACTGCGGAGTTCTGATGGGATCCGGTGCATTTAAGTGCTGGTATGATCGCACAC
CGGTAATGTTTGTCCGCAATATTCATTTATAGACGAGCGCGGTGTGTCCTCCCTTCTTCTCCCCCGGATTGACTAGAACTTCTT
ATTTCGCTGCTTGTTAGTGACCACGGTATTAACCCCGTCAAGGAAAACGAAATAAATGAGTTTTTAAAGATTTAAACAAAAAAT
ACAAACTTTGAGCCGTAGGTGATTTTTAAAGGGAAAAAAAAAATGATGTTTTCAAGGGGCACACGAGGATTCCCAGGAGGTCAC
CCATCCTAAGTACTACTCTCGCCAAGCACTGCTTAACTGCGAGTTCTGATGGGATCCGGTGCATTAGTGCTGTATGTCGCACCG
GAATGTTTGGTCCGCAAATATTCATTTATAGCGAGGCGGTGTCCTCCCTTTTCTCCCCGGATTGACTAGGATTCTTATTCGCTG

CTTGTTTAGTGACACGGTATTAACCCCTCAAGGAAAACGAAATAATGATTAAAAGATTAAAACAAAAAAATACAAACTTTGAGC
CGTAGGTGGATTTTTAAGGGAAAAAAAAAAATGATGTTTTCAGGGGTGCCAACACGAGGACCTTCCCAGGAGGTCACCATCCTGT
ACTACTCTCGCCCAAGCACGCTTAACTGCCGGAGTTCTGATGGGATCGTGCATTAGTGCCTGGTATGATCGCACCCGGAATGTT
GGTCCGCAATATTCATTTATAGGCGAGCGCGTGTGTCCTCCCTTTTCTCCCCCGGATTGAACTAGGAACTTCTTATTCGCTGCT
TGTTAGTGACCACGGTATTAAACCCTCAAGGAAAACGAAAAAATTGAGTTTTAGATTAAAAAAAAAATACAAACTTTTTGAGC
CGTAGGTGATTTTTAAAGGGAAAAAAAATGATGTTTCAAGGGTGCAAGCACGAGGACTTCCCAGGAGGTCACCCATCCTAGTAC
TACTCCTCGCCCAAGGGCCGCTTAACTGGCGGAGTTCTGATGGAAATCCGGTGGCATTAAGTGCATGGTATGATCGCACCCCCG
GAAGTTTGTTCCTCGCAATATCTCCTTATAGGCGAGCGCGTTGTCCTCCCCCTTTTTCTCCCCCGGGAGCTTTGGACTAAGGAA
CTTCTTATTCGCTGCTTGTTAGTGACCGACGTATTAACCCCCAAGGAAAACGAAATAAATGAGTTTAAGATTTAAAACAAAAAA
TACAAACTTTTGGAGCCGTAGGTGGATTTTAAAAGGGAAAAAAAAAAATGATGTTTCAGGGGTGCAACACGAGGACTTCCAGTGA
GGTCAACCCCATCCAGTAGGCTACCTCGCCAACACGCTTAATCTTGTGCGGAGTTCTGTGGGATCCGGTGCATTAAGTGCTGTA
TGATCGCACCCGGAATGTTTTGGTCCGCCAATATCATTTATAGCGAGCGGCCAGTGTGTCCTCCTTTTCTCCCAACCCGGATTG
ACTAGGAACTTCTTATTCGCTGCCTTGTTAGTGGACCACGTATTAACCCTCAAGGAACGAAATAAATGAGTTTTTAAAAGATTT
AAAACCAAAAATACAAACTTTTGAGCGTAGGGATTTTAAAGGGAAAAAAAAAAATGATGTTTCAGGGGTGCAACACGAGGACTT
CCCAGGAGGTCACCCATCCTAGTACTACTCCTCGCCCAACCCGCACGCTGAACTGGCGGAGTTCTGATGGATCGGTGCATTAGT
GCTGGTATGATCGCACCCCGAAATGTTTGTTCCGCAATAATCATTATAGCGAGCGCAGTGTGTCCTCCCCTTTTCTCCCCCCCG
GAATGTAACTAAGTGAACTCCTCTTATTCGCTGCTTGTTAGTGACCACGGTATCTAACCTCAAGGAAAAACGAAATAAATAGTT
TTTAAAGATTTAAAAAAAAAATAAAACTTTTGGCCGTAGGTGGATTTTTAAAGGGAAAAAAAATGAATAGTTCAGGGGTGCAAC
ACCGAGGACTTCCCAGGAGGTCACCCATCCTAGTACTACTCTCGCCAACACGCTTAACTGCGGAGTTTGATGGATCCGGTGCAT
TAGTGCTGGTATGATCGACCCCAGGAATGTTTGGATCCGCAATATTCATTTATAGCGGAGCGCGTGTGTCCTCAAAAACCTTTT
CTCCCCGCGGATTTGACGTAGGAACTTCTTATTCGCTGCTTGTTAGTGACCCGGAGTTAACCCCCTCAAGGAAAACGAAAATAA
ATGAGTTTTTAAAGATTTAAAACAAAAAATACAAACTTTTGAGCCGTGAGGTGGATTTTTAAAAGGGAAAAAAAAAAAAATGATG
TTTTCCAGGGGTGCAAGCACGAGGACTTCCCAGGAGGTCACAGCCATCCCTTAGTACTACTCTCGCCCAAGCACGCTTAACTGG
CGGAGTTCCTGATGGGATCCCGGTGCATTAAGTGCTGGTATGATCCTGCAACCCGGAATGTTTGGTCCCGCAATATTCATTTAT
CAGCGAGCGCCGTGTGGTCCTCATCTTCCTTTTCTCCCGGATTGACTAGGAACTTCTTATTCGCTGCTTGTTAGTGACCCACGG
TATTAAACCCTCAACGGAAAACGAAATAAATGTAGTTTTAAGATTTAAACAAAAATACAAACTTTTGAGGCCGTAGGTGGATTT
TTAAAGGGAAAAAAAAAAGATGATGTTTTCAGGGGTGCCAACACGAGGACTTCAGGGAGGTCACCATCTAGTACTACTCTCGCG
CCAAGGCAGCGCTTAACTGCGGAGGTTTGAGGGATCCGGTGCATTAGTGCTGTATGATCGCACCCCGGAATGTTTGTTCGCGCA
ATATTCATTTATAGGCGAGCGCGTGTGTCGCTCCCTTTTCTCCCCCGGATTGACTAGAACTTCTATTCGGCTGCTTGTTAGTGA
CCAGCCGGTTATTAACCCCTCAAGGGAAAACGAAATAAAATGAGTTTTAAAGATTAAAACAAAAAAAAATACAAACTTTTTGAGC
CGTAAGGTTGATTTTTAAAGGGGAAAAAAAAAATGATGTTTTCCAGGGTGAAACACGAGGATTCCCAGGAGGTCACCGCATTCC
CTAGTACTACTCTCGCCCAAGCCACGCTTAACTGGCCGGAGTTCTGATGGGATCCGGTGCATTATGCTGGTAATGATCGGCACC
CCGGAATGTTTGGTCGCGCAATAATTCATTTATAGCGAGCGCTGTGTGGTCCTCCCCTTTTTCTCCGGATTGACTAGGAACTTT
CTTATTCGCTGCTTGTTAGTGACGCACGGTATTCAACCCCCTCAAGGAAAACGAAATAAATGGTTTTTTAAAGATTTAAAACAA
AAAATACAAACTTTTAGCCGTAGGGTGGATTTTTAAAAGGAAAAAAAAAATGATGTTTTTTCAGGGGTGCAACACGAGGACTTC
CCAGGAGTCACCCTGCCGCTATCTACTCTCCGCCCCAAGCCACGCTTAACTGCGAGTTCTGATGGGATCCGGTGCATTAGTGCT
GGTATGATCGGCACCGGAATGTTTGGTCCGCAATATTCATTTATAGCGAGCGCGTGTGTCTCCCTTTTCTCCCCCGGATTGACA
TAGGAACTTTCTTATTCGATGCTTGTTAAGTGAACACGGTTTAACCCCTCAAGGAAAACGAAATAAATGAGTTTTTAAAGATTT
AAAACAAAAAATACAAACTTTTGAGCCGTAGGTGGATTTTTAAAGGGGAAAAAAAAAAATGATTTTCAGGGGTGCAACACGAGG
ACTTCCGAGGAGGTCCACGCCATCCTAGTATACTCTCCGCCCAAGCACGGCTTAACTGCGGAGTTCTGATGGGAATCGGTGCAA
TTAGGTGCTGGTATGATCGCACCCCGGATGTTTGGTCCGCAATATTCATTTAGCGAGCCGCAGTGTGTCCTCCCCTTTTCTCCG
GATTGACTAAGGAACTTCTTATTCGCTGCTTGTTAGTGACCGAACGGTTTACCCCTCCAAGGAAAAACGAATAAATGAGTTTTT
AAAGATTTAAAACCAAAAAAATACAAACCTTTTGAGCCGTAGGTGGAATTTTTAAAGGGAAAAAAAAAATGATGTTTTCAGGGG
TGCAACACGAGGACTTCCCAGCGAGGTCACCCATCCCTAAGTAACTAACTCTCCCGCCCAAGCACGCTTAACTGGCGGAGTTCC
TGAATGGGATCCGGTGCATTAGTGCTGGTAAATGACGCACACCCGGAATGTTTGTTCCGCATATTCATTTATAGCGAGCGCGTG
TGTCCTCACCTTTTCTCCCGGGATTGACTAGGAACTTCTTATTCGCCTGCTTGTCTAGTGACCACGGTATAACCCTCAAGGAAA
TAACGAAAATAAATGAGTTTTAAAGATTTAAAACAAAAATACAACCTTTTGAGCGTAGGTGGATTTTTAA
>m141122_014521_42134_c100732202550000001823141405141560_s1_p0/150912/0_12573
AGAAGGAATGGCCTCAAGGAAAACGAAATAAATGAGTTTTTAAAGATTTAAAACAAATACAAACTTTTGAGCCGTAGGTGGATT
TTTAAAGGAAAAAAAAAAAAATGATGTTTCAGGGTGCAACACGAGGACTTCCCAGGAGGTCACCCATCCTAGTAACTACTCTCGC
CCAAGCACGCTTAATGCGGAGTTCTGATGGGACCGGTGCATTAGTGCTGGTATGATCGCACCCCGGAAATGTTTGTTCCCAATA
GTCCATTTATGCGAGCGCCTGTGTCTCCCTTTTCTCCCCCGATTGACTAGGAACTTCTTATTCGCTGCTTGATTAGTGACCACG
TATTAACCCCTCCAAGGAAAACACGAAATAAAATGGAGTTTTTAAAAGATTTAAAACAAAAAAATACAAACTTTTGAGCCGTAGG
TGGATTTTTAAAGGGAAAAAAAAAAATGATGTTTTCAAGGGGGGGGTGGCAACACGAGGAATTCCAGGAGGTAACCCATCCTAG
TACTACTCTCCGCCCAAGCGACGCTTAACTGCGGAGTTCTGATGGGATCCGGTGCATTTAGTGCTGGTATGATCGCACCCGCAA
TGTTTGGTCCGCAATATTCATTTATCAGCGAGCGCGTGTGTCTCCCTTTTCTCCCCCGGACTTCGGACTAGGAACTTCTTATTC
GCTGCTTGTTAGTGACCACGGTATTAACCCCTCAAGGAAAACGAAATAATGAGTTTTTAAGATTTAAACAAAAAATACCAACTT
TTTGAGCCGTAGGTGGATTTTAAGGGAAAAAAAAAAAATGATGTTTTTCAGGGTGCAACACGAGGACTCCCAGGAGGTTCACCCA

CCTAGTACTACTCTCGCCAAGCACGCTTAACTGCGGAGTTCTGATGGGAATCCGGTCATAGTGCTGGTATGATCGCACCCGGAT
TGTTTTTGTTCCGCAATATTTCATTTATAGCGAGCGCGTGTGTCCTCCCCTTTTCTCCCCCCGGATTGACCTAGGCAACTTCTT
ATTCGCTGCTTGTTTAGTGACCACGGTATTTAACCCCTCCAAGGAAAACGAAAGAAATGAGTTTAAAGATTTAAAAACAAAAAA
TACAAACTTTGAGCCGTAGGTGGATTTTAAAGGGAAAAAAAAAAAAATGATGTTTTTCAGGGGGCAACACGAGGACTTCCCCAGG
AGGTCACCCATCCTAGTAACTACTCTCGCCAAGCACGCTTAACTGGCGGAGTTCTGATGGGAATCCGGTGCATTAGTGCTGGTA
TGATCGCACCGGAATGTTTTGTTCCGCAATATCTATTATAGCGAGCGCGTGTGTCTCCCTTTTCTTCCCCCCGGATTGACTAGG
AACTTCTTATTTCGCATGCTTGTTAGTGACCACGGTAATTAACCCCTCAAGGAAAACGAAATAAATGAGTTTTTAAAGATTTAA
AACAAAAAAATACAAACTTTTTGAGCCGTAGGTGGATTTTAAAGGAAAAATAAAAAATGATGTTTTAGGGGTGCAACACGAGGAC
TTCCCAGGAGGTCACCCATCCTAGTACTACTCTCCGCCCAAGGCACGCTTACTGCGGAGTTCTGATGGGATCCGGTGCATTAGT
GCCTGGTATGATCGTCACCCCCGGAATGGTTTGGTCCGCAAATATTCATTTATAGCGAGCGCGTGTGCCTCCCTTTTCTCACCG
GAATTTGACTAGGAACTTCTTATTCGCTGCTTGTTATGACCACGGTGATTAACCCCTCCCAATGGAAAACGAAATAAATGAGTT
TTTAAAGATTTAAAACAAAAATACAAACTTTTGAGCCGTAGGTGGATTTTTAAAGGGAAAAAAAAATGATGTTTTCAGGGTTGC
CAACCACGAGGACTTCCCAGGAGGTCACCCATCCCATAGTACTACTCTCGCCCAGCACGCTTAACTGCGGAGTTCTGATGGGAT
CCGGTGCATTAGTGCTGGTATGATCGCACCCGGTAATGTTTTGGTCCGCAATATCATTTATAGCGAAGCGCGTGTGTCCTCCCC
TTTTCTCCCCCGGATTGGACTAGGAACTTTCTTATTCGCTGCTTGTTTAGTGACCACGGTAATTAACCCCTCAAGGAAACGAAA
TAAATGAGTTTTAAAGATTTAAAACAAAAAATACAAAATTTTTGAGCCGTAGGTGGATTCTTTAAAGGGAAAAAAAAAACTGAT
TTTTCAGGGGTGCAACAACGAAGACTTCCAGGAGGTCACCCCATCCTAAGTACTACTCTCGCTCCAAGCAACGCTTAACGCGGA
GTTCTGATGGATCCGGTGCAATTAGTGGCTGGTATGATCAGACCCGGAATGTTGGTCCGCAATATTCATTTATATCGAGCGCGT
GTGTCCTCCCTTTTCTCCCCCGGATTGACTAGAACTTCTATTTCGCTGCTGTTTAGTGACCACGGTATTAACCCCTCCAAAGGA
AAACGAAAATAAATGAGTTTTTATAAAGATTTAAAACAAAAAAACAAACTTTGAGCCGTAGTGGATTTTTAAAGGGAAAAAAAA
AAAATGATGTTTTCAGGGGTGCCAACCACGAGGACTTCGCCAGGAGGTCACCCATCCTAGTACTATCTCGGCCCAAGCACCGCT
TAACTGCGGAGTTCTGAATGGGATCCGTGCATTAGTGCTGGTAATGATCGCACGCCGGAATGTTTGGTCCCGCAATATTCATTT
ATAGGCGAAGCCGCGTTGTGTCCCTCCCTTTTCCTCCCCCGGTATTGATAGGAAGCTTCTTATTCGCTGCCCTTGTTAGTGACG
CGACGGTATTAACGACCCTCAAAGGAAAACGAAATAAATGAAGTTTTTAAAGATTTAAAACAAAAAAATAGCAACCACTTTATG
AGCCCGTAGGTGGATTTTTAAAGGAAAAAAAAATGAATGTTTTCAGGGGTGCAACAACAGGATTCCCAGGAGGTCACCATCCTAA
GTAACTAACTATCCCCCGCCCCAGCCGCTTAACTGCGGAGTTCTTGATGGGATCCGGTGCATTAGTGCTGTATCGATCGCACCC
CCGGAATGTTTTGGTCGCAAATATTCATTTTATAAAGGGCGAGCCGTGTGTCCTCCCTTTCTCCCCCGGATTGACTAGGAAACT
AACCCCCCCCCCCCCTCTTATTCGCTGCCTTGTTGTGACCAACGGTAAATTAACCCTCCAAGCGAAAACGAAATAATGAGTTTGT
TTAAAGATTTAAAACAAAAAATACACAACTTTTGGGCGTAGGTGGATTTTTAAAGGAAAATAAAATGATGTTTCAGGGTGCAAC
ACGGCAGGACTTTCACAGGGAGGTCACCATCCTAGTCGTACTCTCCCGCCCAAAACGCAGCGCTTAACCCTGCGGAGTTCTGAT
GGGAATCCGGTGCCATTAGTGCTGGTATGACGCACCCCCGGAAATGTTTGGTCCGCATATTCATTTATAGCGAGCGCGTTGTTC
CCTCCCTTTTCTCCCCCGCGGATGCTAGGAACTTCTTATCGCGTGCTTGTCTAGTGACCACGGTATTAACGCGCGCTCCAAAGG
AAAGACAGAAATAAATGAGTTTAAAGATTTAAAACAAAAATACAAACTTTTTGAGCCGTAGGTGGATTTTTAAGGGAAAAAAAA
ATGATGGTTTTCAGGGTGCAAACAGCCCGAAGGACTTTTTTCCCAGGAGTCACCTGCCATCCCTATACTATCTCTCGCACCCAA
GCACGCTTAACTGCGGAGTTCTATGGGACTCCCGGTGCATTATGCTGGTATGGTATCCGCACCCGGAATGTTTGGTCCGCAATA
TTCATTTATAGCGAGCGCGAGTGTGTCCTCCCCTTTCTCCCCCCGGATTGATAGGAACTTCTTATTCCCGCTGTTGTTAGTGAC
CCAACGGTATTAACCCCTCAAGGAAAACGAAATGAATGAGTTTTTAAAAGATTTAAAACAAAAATATACAAAAATTGAGGCCGT
AGGTGATTTATTTAAAGGGAAAAAAAAAAATGATGTTTTCAGGGTGCAACACGAGGTATTCCCAGGAGGTCACCCCATCGCCCTA
GGTAACTACTCTCGCCCAACCACGCTTTAATCTGGCGGAGTTCCTGATGGGATCCGGTGCCATTAGTGCTGGTAATGTATCGAC
CCGGAATGTTTTGGTCCGGTGCAATATTCATTTATAGGCGAGCGCGTGGTGTCTCCCCATTTTTCTCCCCCGGATTGACTAGGA
ACTTCTTATTCGCTGCTTGTTAGTGACCACGGTATTAACCCCTCAAGGAAAACGAAATATATGCAGTTTAAAGATTATAAAACA
AAAAATACAATACTTTTGAGCCGTAGGTGGTTTTTAAAGGGAAAAAAAAAATGATGTTTTCAAGGGGGGTGGCAACACGAGGAC
TCCCAGGAGGTCACCCCATCCCAGTACTACCTCCCCAGACGGCACGAACTTAACCTGGCGGAGTTCTGATGGGATCCGGTGCAT
TAGTGCTGGTATGTATCGCCATAACCAAGGAATGTTTGTTCCCGCAATATTTCATTTATAGCGAGCCGCCGTGTGTCCTCCCTT
TTTCTCCCCGCCGGATGTGACTAGGAACTTCTTATTCGCTGCTGTGTTAGTGACCACGGTATTAACCAGCTCAAGGAAAACCGA
ATAGAATGAAGTTTTTTAAAGATTTAAAACAAAAAATACAAACTTTGAGGCCGTAGGTGGATTTTTAAAAGGAAAAAAAAAAAT
GAATGTTTCAGGGGTGCAACACGAGGATTCCCCAGGAGGTCACCCAATCTAGTACTAACTCTCGCCCCAGCACGCTTTAACTGC
GGAGTTCTGATGGATCGCGGTGCATAGTGCTGGTAATGATCGCACCCGGAATGTTTGTTCCGCCCAATATTCATTTATAGGGCG
AGCGCCGTGGTCCTCCCTTTTCTCCCCCGGATTGACTAGGAAACTTCTTATTCGCTGGCCTTGATTAGTACCACGGTATTAACC
CCCCTCAAGGAAAACGAAAAAAATGAGTTTTTAAAGAATTTAAAAACAAAAATACAAACCTTTTGAGCCGTAGGTTGGATTTTA
AAGGGAAAAAAAAAAATGAGTTTTCCGGGGTGCAACACGAGGACTTCCCAGGAGGTACCCATCTCAGTACTACTCTCGCCCAACG
CCCCACGCTTAACTGAAGGAGTTTTCTGATGGATCACGGTGCATTAGTGCTGGTATGATGACCCCGAAATGTTTGGTCCGCAAT
ATTCATTTATAGGCGAGCGCGTGTGTCCGTCCCCCTGTTTTATCCCCCACCCGGATTGACTAGGAACCTTCTTATTCGCGGCTT
GTTAGTGACCACGTATAATCCCTCAAAAAAACGAAAAAATGAGTTTTAAGATTTAAACAAAAAATAACCAAAACTTTGAGCCGT
AGGTGATTTTTAAAGGGAAAAAAAAAAATGATGTCTTCAGGGGTGCAACAGCGAGGACTTCCCCAGGAGGTCACCCATCCTAGT
TACTACTCTCGCCCAAGCCGCTTAACTGCGGAGTTCTGATTGGGATCCCGAGTGGCATTTAGTGCTGGTATGATCGCCACCCGG
AATGTTTGTTCGCGCACATATTCCATTTATAGCGAGCGCCGTGTGGTCCTCCCTTTTTCTCCCCCGGATTGACCTTAGGAACTT
CTTATTCGCTTGCGTTGTTAGTGACCCACGGTATTCAACCCTCGAAGGAAAAAACGAAATAAAATGAGTTTTTAAAGATTAAAA

ACAAAAATACAAACTTTTGAGCGTAGGTGGATTTTTAAAGGGAAAAAAAAATGATGTTTTCAGGGGTGCAACCACCGAGGACTT
CCTCAGGAGGTCACCTCCCTAGTACTACTCCTCGCCCAAGCACCGTCTTAACGTGCGGAGCTTCTGATGGGAGTCCGTGCATTA
GTGCTGGTATGATCGCACCCGGAATGTTTGGTCCGCAAATATTCATTTATAGCGAGCGCGTGTGTCCTCCCTTTTTCTCGCCCC
CGGTTGACTAGGACTTCTTATTCGGGCTGCCTTGTTTAGTGACACTGGTATTAACCCTCAAGGAAACGAAATAAATGAGTTTTT
AAAAGATTTAAAACAAAAAATACAACTTTTGAGCCGTAGGTGGATTTTTAAAGGGAAAAAAAATGATGTTTTCAGGGGTGCAAC
CGAGGAACTTCCGCAGGAGGTCAGCCCATGCCCTAGTACTACTCTCGCCCAAGCACGGCTTAACTGCCGGAGTTCTGATGGGAA
TCGGTGGGCATTAGTGCTGGGTATGATCGCACCCCGGAATGTTTGTTCCGCAATATTCTTTATAGGCGAGCGCGTGTGTCCTCC
CCCCTTTTCTCCCCCCGGATTGACTAGGAACTTCTTATTCGGCGTGCTGTTAGTGCCTACGGTATTAACCCCCTCCAGGAAAAC
GAAATAAATGAGTTTTTAAAGATTTAAAACAAAAAAAAATAACAAACTGTTTGAGCCCGTAGGTGGAAATTTTTAAAGGGAAAA
AAAATGATGTCTTCAGGGGTGGAAAAAAAAAAAACACGAGGAGCTCCAGGAGGTCACCCATGCCTAGTAACTACTCTCGCCCCA
AGCACGCTTAACTGGCGAGTTCTGATGGGATCCGGTGCATATAGTGCTGGTATGATCGCACCCCCCGGAATGTTTTTGTTCCGC
GCAATATTCAATTTATAGCGAGCGCGTGTGTCCTCCACTTTTTCTCCCCGGATTGACTAGGAACTTCTTATTCGGCTGCTTGTA
GTGACGGGCAACGGTATTAACCCCTCAAGGAAAACGAAATAAATGAGTTTTTAAAGATTAAAACCAAAAAATACCAAACTTTTG
AGCGTAGGTGGATTTTTAAAGGGAAAAAAAAATGTGTTTTCAGGGGTGCAACACGAAGGTACTTCAGGAGGTCACCCATCCTAG
TACTACTCTCGCCCCAGCACGCTTAACTGGGAGTTCTCGATGGGATCGGTGCATTATGCTGGTATGCATCGCACCCGGAATCTG
TTTGTTGTACTCTCATCTTATAGCGACGCGCGTGTTCTCCCTTTTCTCCCCCCGGATTGATAGGATACTTCTTATTTCGCTGCT
TGTAGTGACCGACGGTATTAACCCTCAAGGAAAAACGAATAATGAGTATTTAAAAGATTTAAAACAAAAAAATACAAACTTTTG
AGCCCGTAGGTGGATTTTAAAGGGAAAAAAAAAATGATGTATTTCAGGGGTGCAACACGAGGACTTCCCAGGAGGTCACCCATC
CTAGTACTACTCTCGCCCCAGCACGCTTAAACTGCGGAGTTTCGATGGGATCCGGTGCATTAGTGCTGGTATGATCCGCACCCC
CGGAATGTTTTGGTCCGCAATATTCATTTAATAGGCGAGCGCAGTGTGTCCTCCCCCTTTTCTCCCCCCGGATTGACAGGAACT
TCTTATTCCGCTGCTTGTTAGTGACCACCGGTATTAACCCGCCTCAAGAAAACTGAAATAAATGAGTTTTTAAAGATTTTAAAA
ACCCAAAAAATACAAACTTTTGAGCCGTAGGCTGGATTTTTTAAAGGGAAAAAAAAAATGATGTTTTCAGGGGTGCAACAGCGA
GGACTTCCAGGAGGTCACCCCATCCCTAGTACTACTCTCGCCCAGCAACGCTAACTGGCCGGAGTTCTGATGGATCCGGTGCAT
TATGCTGGATGAATCGCACCCGGAATGTTTGTTCGCGCCCAATATCATTTTATAGGCGATGCCGCGTGTGTCCTCGCCTTTTCT
CCACCCGGATTTGACTAGGAACTTCTTATTCGCTGCTCATGTTAGGTGACCACGGGTATTAAACCCCTCCCCAAAGGAAAACGA
AATAAATGAGTTTTTTAAAGATTTGAAAACAAAAAATACAAACTTTTGGCCGTAGGTGGATTTTTAAAGGAAAAAAAAAAATGA
TGTTTTCAGGGGTGCAACACGAGGAACTCCCAGGGGTCACCCATATCCTCAGTACTACTCTCGCAACAAGCACCGCTTAACTGC
GGATTCTGAATGGGATCCGGTGCATTAGTGCTGGGTATGAATCGCACCCTCGGACCAATGGTTTGTTCGCAATATTCATTTATA
GCGAGCGCCGTGGTGTCCTCCCTTCATTCTTCCCGCCGATTGACTAGGAACTTCCTATTCGCTTGCCTTGTTTAGGTGACCACG
GTATTAACCGCTCGAAGGAAAACGAAATTAAATGAGTTTTTAAGATTTAAAAACAAAAAATACAAACTTTGAGCCCGTAGGTGG
ATTTTTAAAGGGAAAAAAAAATGATGTTTCAGGGGTGCAACACGAGGACTTCCCAGGAGGTCACCCATCCTAGTACTACTCCTC
GCCCAAGCACGCTTAAGCTGCGGAGTTTCTGTGGGATCGGTGGCATAGTGCTGGTAATGATCGCACCGGAATGTTTGGTCCCGC
AATATTCATTATAGCGAGCCCCGGTGTGTGCCTCCTTTTTCCCGGAATTGACTAGGAGCTTCTAATTCGCTGCTTGTTTAGTGA
CCACGGTAATTAACCCTCCAAGGAAAACGAAATAAATGACGTTTTTAAAGATTTAACGAACAAAAAATACGAAACTTTTTGAGC
CGTGGTAAGATTTTTTAACGGGGAAAAAAAAAAATGATGTCTTCAGGGGGGGGTGGCCAACACGGAGGAACTTCCCAGGAGGTC
ACGCAATCCCCCTAGTACTACTCTCGCCAAGGCACGCTTAACTGGCGGAGTTCTGATGGGACTCCGTGCATTAGTGGCTGGTAT
GTGATCGCAGCCCGGAATGTTTGGTTACGCATATTCATTTATAGCGAGGAGTGTGTCCTCCCTTTTCTCCCTGCCCCGGCATTG
ACAGGACTTCTTATTCCGCTGCTTGTTAGTGACGCACGGTATTTCAACCCGCTCCAAGGAAAACGAAATAAATGAGTTTTTTAA
AGATTTAAAAACAAAAATACAAATTTTGAGCCGTAGGCTGGCATTTTTAAAAGGGAAAGAAAAAAAAATGATTTTTCAGGGTGGC
ACACGAGGCTTCCCAAGGCAGGTCACCCCATTCCTAGTACTACTCTCGCCCAAGCAACGCTTACTAGCGGAGTTCTGATGGGGA
TCCGGTGCAAATTAGTGCCTTGGTATGATCGCACCCGGAATTTTTTGGTCCGCAATTTCATTTATAGCGAGCCGCGTGTTCCTC
CTTTTCTCCCCCCGGATTGACTTAGGACTTCTATTCCTGCTTGTTAGTGAACCACGGTATTACCTCAATGGAAAACGAATAAAT
TGGTTTTTTAAAGATTTAAACAAAAAATACAAACTTTTGAGGCCCCGTAGTGGATTTTGAAAGGGAAAAAAAAATGATGTTTCA
GGGGGTGCAACACGAGGACCTTCCCAGGAGGTCACCCATCCTAGGTACTACTCTCGCCCAAGCACGCCTTTTAACTGCGGAGTT
CTTGATGGGATCCGTGCCATTAGTGCTGGTATGATCGCACGCGCGGAATGTTTTGGTCCGCAGATATTTCACGTCATTTATGGA
GCGCGTGTTTCCTCCCTTTTCTCCCCCCCGGCATTGATAGGAATGCTTCTTATTCGCTGCTTGTTAGTGACCAACGGTATTCCA
ACCCCCTCAAGGAAAACGAAATAAATGAGTTTTTAAAGATTTAAAACAAAAAATACAAACTTTGAGCCGTAGGGTGGATTTTTA
AAGGGAAAAAAAAAATGATGTTTCAGGGGTGTCAACAGCGAGGATCTTCCCAGGAGTCACCCATCCTAGTACTACTCTCGGCCC
AAGCCACGCTTAACTGCGGAAGTTCTGATGGGAATCCGGTGGCATAGTTGCTGGTATGAATCGACCCCCGGAATGTTTGTTTCC
CGCAATTTCTCTTATAGCGAGCGCGTGTGTCCTCCCTTTTTCCCCCCGGATTTGACAAAAAAGAACGAGAACAAAAAAAAAAAG
AAAAAATAGGAAACTTCCCTTATTCCTGCTTTGTTAGTGACCACCGGATATTAACCCTGCAAGGAAAAACGAAATAAATTGAGT
TTTTAAAAGTATTTAAAACAAAAATACACAACTTTTTGAGCCGTGGTTTGGATTTTTTTTTAAAGGAAAAAAAAAAAGGGGTGA
TGTTTTCAAGGGGTGCAACACGAGGACTTCCAGGAGGTCAACCCATCCTAGTACTTACTTCCTCCGCCCAAGCACGCTTTTAAA
TTGCGGAGTTTCTGATGGGATCCGGTGCATTAGTGCTGGTATGATCGCACCCGGAATGTGGTCCCCGCTAATAATTCATTTTTA
TAGACGAGCGCCAGTTGTGTCCTCCCTTTTCTCCCCCCCGGATTGACTTAGGAACTTCTTATTCGCTTGCTTGTTGTGACCCAA
CGGTGATTAACCCAACTCAAGGAAACGAAATAATGAGTTTTTTAAAGATTTAAACAAAAAATACAAACCTTTTGAGGGCCGAGG
TGGATCTTTTAAAAGGGAAAAAAAAAAATGATGTCTTCCAGGGTGCAACACGAGAGCTTTCCCAAGGAGGTCACCCATCCCTAG
TACTTACTCTCCCGCCCCAGCCAGCGCTTAACTGCGGATGTTCTGTGGGATCCGGTGCATTAGTGATGTATGATCGCACCGGAA

TGTTTTGTTCCGCAATATTCATTTATAGCGAGCGCGTGTGTCCTCCCTTGTTCTCCGGATTGACTAGGAACTTCTTATGTCGGC
GTGCTTGTTAGGTGGACCAGGTATTAACCCCTCAAGGAAAACGAAATAAAGAGTTTTTAAAGATTTAAACAAAAAAATACAAAC
TTTTGAGCCGTAGGTGGACTTTTTAAAGGGAAAAAAAAAATGGATGTTTTTCAGGGTGGGCAACACGAGGTACTTCCAAGGAGGT
CACCCATCCCCTATGTAACTACTCTCGCACCAAAGCACGCTTAACTGGGCGGAGTTCCTGATGGGATCGGTGCATTAGTGGCTG
GTATGATCGGGGCACCCGGAATGTTTGGTCCCGCAATATTCGATATTATGCGAGCGCGCTGTGTCTCCCTTTTCTCCCGCCGGA
TGACCTAGGAACTTCTTATTCGCTGGCTTGTTTAGTGACCCACGGTATTAACCGCTCAAGGAAAACGAAAATAAATGAAGTTTT
TAAAGATTTAAAACCAAAAAATACAAACTTTTGAGCCCTTAGGTGGATTTTTAAAGTGGAAAAAAAAAAATGTGTTTCAGGGGTG
GCACACGAGACTTCCCAGGAGGTCACCCATCCTAGTACTACTCTCGCCCCAAGCACCGTTAACTGCGGAGTTCTGATGGGAATC
GGTGCATTTAGTGCTGGTATGATCGGCACCCCCCGGAATGTTTGGCCGGCAAATTTCATTTATAGCGAGCGCGTGCTGTCCCCC
TTTTCTCCCCCGGATTGACTGAGGAACTTTCTTATTCGCTGCTTGTTAGTGCCACCCGGTATTAACCCCCTCCAAGGAAAAACG
AAAATAAATGAGTTTTTTAAAAGATTTAAAACAAAAAAATAGCCAAACTTTGAGCCGTGGTGGATTTTGAAAGGAAAAAAAAA
ATGATGTTGTTTCCAGGGGTGCCAACACAGGACTTTCCCCCCAGGAGGTCCACCATCCTAGTACTAGCTACTCCGCCGCCAAGC
ACGCTTAACTGCGGAGTTCCTGATCGGGATCGCGGTGCATTAGTGCTGGTATGATCGCACCCGGAGATGTTTGTTCCGGCAATA
TTCATTTATTAGCGAGCGCGTGTTCTCCCTTTTCTCCCCCCGGATTTGACTAGGAATCTTCTTATTCCTTGCTTGTTAGTGACC
ACGGTATTAACCCCTCAAGGGAAAAGAATAAATGAGTTTTTAAAGATTTAAAACCAAAAAATACAAACTTTTGAGCCGTAGGTG
GATTTTTAAAAGGGAAAAAAAAAAATGATGTTTTCAGGGTGGCAACAACGAGGGACTTCCCAGGAAGGTCACCCATCCTAGTACA
TACTCTCCGCACAAGCAACGCTTAACTGCGAGTTCTGATGGGATCACGGTGCATAGTGCTGGTATGATCGCCACCCGGAAATGT
TTGGTCGCAATATTCATTTATAAGGCGAGCGCGTGTGTCTCCCCTTTTCTCCCCCCGGATGACTAGGAACTTTCTTTATTCGCT
TGGGCTTGTTAGTGACCAACGGTATTAATACCTCAAGGAAAAACGAAATAAAATGAGTTTTAAGGTTTAAAAAAAAATTACAA
ACTTTTGAGCCCCGTAGGACGGATTTTTAAAGGGAAAAAAAAAATGATGTCTTCAAGGGGTGCACACGAGGATTCCCAGGAGGT
CACCCATCCTAGTACTTACCTCTCGCCCAAGCACGCTTAACTGCGGAGTTCTGATGGGATCCCGGTGCATTAGGGCTGGGTATG
ATCGCACCCGGAATGTTTGGTCCCGCAATATTCATTTAGCGGCGCGTGTGTCCTCAAGCCTTTTCTCCCCCGGATTGATAAGG
AAACTCTTATTCGCTGCTTGTTAGTGACCACGGTAGTTAACCCCTCCCAAGGAAAACGAAATAAAATGAGTTTTAAAGATTTTA
AAACAAAATACAAACTTTTGAGCTTTAGGTGGATTTAAGGGAAAAAAAAATGATGTTT
>m141122_014521_42134_c100732202550000001823141405141560_s1_p0/152325/1221_12516
TCTGATAATTATGCCGGGTTCAGTATTGATCGGGGCCGTTAATACTTAGTCCTTCGCAGTCTCGAGTATGGGCTTCTAAGTGCC
CAATGGGGACTAAAGCCATACTGTTCAAAGATCTCCGGGTACCTTTCAAGCTTGGCCAGCGTGTCCGCAGTGAAGTCTGGTCCC
GGGGAGACCATGGCGGATTTATAGAGCCTTTGAAATAGCTGCATAGTCGCGGGAGAGAGACACGTAGGCAGGCCATCCTTTCTC
CTTTCCGAGCTGGTATTTCCCCGGAATATTTATTGGTAGTTTTCTTTATGGTTTACCACAAGTAGGATCCGCTTGTACACCTCG
TTTTTTGGAGCACCGACTTCTCTGGCCAGTCGTGAAACCCAGCTAAGCAATGAGGCTTTCGTGTGATCGGCCTCTGTTTGATGC
TGGGAATGCGAACGGAAATGGCGCTCTGGAAGCTGATCCTTGGGAGAATATAAGAGATGACCCCCGCTCCAGCTCCTTTTCAAT
TGAAGGCTCACAATCTAACGTGACTTCCAGGTGGGGAAGTTATCCGGGACAGGTATCTACTGGGAAAGGTCATTCATCCCAGAG
CACAGCCACAAAAAGTCTCACGCCGAGGACTTGAACTCTTTTTAATAGGAAACACAGCGTGTTTATTAGGTAGTGCGACATTCG
AACTGCTTAGTTCCATGGGGGCCCCCCCCCCAAAAAAAAAACTAATTTAAAGCAAATTATCCCTTGCCCAGAAGGGGACCTTC
CGGGCCCCTTTTTTCCTTTTTTATGCAGGAACCCCCTTTTGTGTTTCCCCCGGAAGATAAAGGGGGGGGGTTGGGGTTTTTTT
GGGGATGGGGGAGTGTTAAAAAAAACCTTTTTTTTGGGAATTTGGGATGGGGGGTGCCCCGCCCCCCCTTTTTTTTTTGAAAA
AATATGGGCCCCCCCCCCCCTCAGCCCCCCTTTTCCCCCCCGTTTGAAAAGCGATCCCAAAAGCAAAGCAGAGGGGGAGGAAAG
GTTTTTTTTTTTTTTTTTTACCCCCCCTTTTCGATCATTCCGAACAAAATAAATCCCTGTGACCCCCCTTCCGGGCCCCCCGT
TCCAATTTTAGACGGCTCCTTTTTTGCCCCCCTTTTAAAAGTTAATAATGAGAGAGAGACGCCAACAAGGAAAGCTGGAAAAAT
CTTTACGCCCCAAATGCTTGGCTTCTTTCCCTCCACCCCGGGAGACACCGAGAGGCCGGCCGGCACAAACCTTTATAAAACATG
CGTTGCTATTCAACGTTTCAAAAAACTCAGTCTCTGAGTGCTTTTTGATAATAAATAAAAGACAACGCGGCGTGACTTTCCCCC
CCTTATATCCCGGACCGCGGCGATTTTATATGGACAGAATGGGGGGGGCTCCCCCGGGGTTTTAAAAAATTTTGATTCCCCCT
TTTTTTTTTTTTTTTTTTGAGGGTTTTGTAATTGGAAAAAGGCCCCCCGCTTTATCGCCCATTTTTTTTCGGAGGGGGGGTC
CCCCCCCCCAACATTTTCCCCCGGAAAAACTTTTTCTTTGGAGGTTTCCCCCCTTCCGCAGAAAACGAAAATAAAATTTTGAAA
AAGAAGGGGGGGAATTTTAAGCCCCACAACTTTTGTTTTTTTTTTTTGGTTTCCTCCCCCCCCGGTGGCCCCCCTTTTTTTTT
TGGGATTAACGAAAAGGGCGGCCTTTTGAGAAAGCAAGCCTATCCCTTTTTCCCCCCCACGGTAACGCCATTCCCCCCCCTGGA
CCAATCCCCCTTATGCTTTTTATATCCCCACCCCCGGGGGAAGGGCACGTATCTATTTTAAATTGAAACCCCGCCCTTTTTAAA
TTTTTTTTCCCTCAAAGGGTTTTGGTCCCATTGCGCGCGCGAGCGCGTGGTACCAACCGGAGGCGGGAAAAAAAAAAGGGGATT
TGACAAAAAAAAATAAAAACCGAGGAAAAAAACCTTTTCCCCCCAAGGACCGAGAAAAAAACCCCCCCGAAATAAAGTGCAAAAAA
TTTCTTTTTTGAAAAATTTTTAAAAAAGGTCTTTATTTCTATTCCCGGTTAAACCCTTATTTTTTTCCCGGATTTCCCAAACGA
ACGAAAGCTTTTGATTTTCCCCCCCCCTTACACAAAAAGGATCCCGTTAAACCCCCTTTTTGTGTGTCACCCCCCCACCCTAGG
AAAGGATTTCCCCTTTTATTTTTGGAACCCCACTTGTTACCCAATAACAAAAAAAAAATGTATCCGTCCACCCCATTTTAACCA
CCCTTCCCCATTGTTCAAACTTCCCCCCCCATAGATTTGGGGGTCCCCCCTTTTGGAACACAAATTCCCATGGTTTTTGTTTGT
TAAACCAAAAAAGACCCCACTGGATAAAAAAAAAAGTCCGACCCCAGGCCAAAAAAAATAAAATTTTTTCCTTTTCCCCAAAAA
ACGAAAAAGGACCCCATTGATACCCAACCGTTTAACCCAAGTAAAGACCCCCCAACCCCTTTTTTTTTTTAATTTCCCGCGTGG
AGGAAAAGCTTTGGTGTTGGGTTATCCTCCCAATGAGTCCCGCCGAAAACCAAAAATGTCCATACCTTTTTTGATTCTTGGGTT
TTTTTTTTGTAATTTCCCCCCGGGTTAAGGGTAAAAACGGCATCCCAATGGAAAAGGGGGAATAAAGAAGTTTTTTTTCGTTAA
CCCCCGGAAAGTGAAGCCCCGTCCCCCCCACCCACAATCCCTTTTTGATCGAATTTCGCGGGGGAAAAGGAGGGGGGGAAAAAA

CAAAATCTTTAATTTCGAGGCAAGAGCCCCCCCCTGGAGTTTTTCTTTAAGGGGTTTTTTCCGTGAAAAGGTTTCCCCCCCAAA
GTTTGCAAAAAAACAAATTTCCCCCTTTTTTTTTCCAGGATACCCCCCCCCCAATTTCCCCCCCGGTGGTTTTATTTTTTCCCC
CCGAGGACCCCAGAAAATACTTTGGGAGTTTTTTTTTTGAGCCCAAAACCCAAAATAAAAAAAAAACCAAAAAAACGGAAAAA
TAGGTGGCCGACTCGTCCCACCCTTGGAAGTGAAAAAAATAGCCTTTAATGGGGGGACCCCAATCAAATTTTTGGTCCAACCAC
CCCAAGCTTTTTTTTGAGACACGGGGGCTTTTTTTCCCCCGGCTTGTTCCTCCGCAAACCTTGGGATTTTTTTCCCCCCCCCCC
ACCCCACGGGCCCCTGTTCCACTTCCCCTTATTTTTTTTTGGCGGAAATTTGGAGGTTTTTGCAAAAAAAAATGCATTATGGGT
CCCCTAAATTGTATTTTGGAGTTGGGCAGTGGGCCAACGGACAAAATATTTGGGGGGAAAAAAAAAAATTTTAATATTTTATCC
CCCCCCTCGTCCCAATAAATAATTCAAGCGTGGGGCGAACCATGCCCCCCCCAAAAAAAAAAAAAGGGGGAACCATTTCCCCCCT
GATTTTAAATATCCCCCCCTTTCCCCCAACCAGTTGGGGGGCCCCACCACGGATCGTTTTTTTAAAATTCCTCGAAAAACCGGC
CCCCGACCGGCATAAGGTTTTTTTTTTTTGTTACCCCCCCCCCCCCACCCCCCCCAAACCCCCCCATTGGAAATGACCTTTGGTG
GGAGTTTTATTTTTTTTTTTTCACCTTTCCCCCCCCCCCCCCGGAATTATTTTTTTGAGGGGATTACCGTCCCATTTTTTTCCAC
TATTTTTCCCCCCTTCCCAAAATACGGCCCCCTTTTTTTTTCCTATTTTTCCCTTTTCCGTTTTTCGAAATTTTCCCACCCTCC
CTTTTTTTATTTTTTGTTAGGGGTTATTTTCGATTTTATCCCGTTCCAAAGTTTAAAAAAAAAAAAATGGGGGACCCCTATTTTT
TCCCTTTTTATTTTTTTGTTGAGACCCCACGACCTCCTTAAAGGTTTTTTTCTTTCCCTTGGGCAAAATAACCCCTTTTATTTT
TATTTTTCCCCGCCCAAAAGGGGGAAAAATTATTTTGGGCCCAAAAAAAGCAAAAACGTTCTTCTCCGAGAAAACCCCGGTTCT
TGTTAAGAACCGGCTTCCCACGGGAACCCCCGGCAGGGAACGTTTTTTTTTTTTTTATAAAACAAAGCCCCTTTTTTTTTTCCG
TGCCCAACCCTTTCTTTTTGTTTGGGTCCCCCCTCCCTCCGGAGGGGGGTTTGGCCCAAAATAAAAACGGCTCCCACATTTACG
TCCCCACGAGCAAGGGGAAGACCTTTTGTTCAAGCAATTTTTAAGTTGACGTTTAATTTCGAGGGGTTGTTATGTGGGCACCCC
CACAAAAAAAATCGATTCCAAGAGGGAACCCCACCGTGAGGTCCCCCCGGTCCTTATAAGGGAATTGGCCCAAAGATTAATAAAA
ACCGGTCCCCGTGGGGGGGCCAATAAAAAAAAAAAAAAAGTCCCCGAGACCAAACACACGGTGAAAAAAATGTGGTCATTTTATT
TGCCCCTTGGATAAGGGCCCAAACAAAAGCGTTCCGCCCTTTTTTTTCCCCCCCTCCCCCCCACACCTCCCTTTTCCTGAGGGG
TTGAGGAACTTGGGGAAAGTTTTTTTTGAAAAGTTTTATTTTTATTTTTGCCCCCTAAAATATGGCATTATGGGAAAGGGATTT
ATTTTGGGGGGGGCGGTCCCCCTAAAAATTTTTTGAAACCATTTGGAAAAGCCCTTTTGGGAAAAAATTTGGGGGGCCAAAAAT
AGAGGGGCCCCCCAAAGGGTTTTCCCCCCGTCTTCCCCCCGGTCAAAAAAAAATAATCCCCCCCGATTTACACCCCCCGCAGTG
GAAAGGGGCCCGCTTTTGAAGAGGGATTTGGTTTTTCCCCCCCAAAACGGAAGCTTTTTTCCTTATTTAAATCGAATCCCCAGA
GGATCCCCCCAAGTTTGAGACCCTTTATTTTTTTCCTTTTTTTTTTTAAAAAATTGGCCAAAAAAAATACCTGGGCTTTCCCCA
CCACCGGAGTGGGAATCGAGTTTTTGTTTAGGGGGGGAAAAAGTTGCAAACATGTCCCCCGGAGCAAAAAAAAAATCCCCCTGG
TCCCCCAACTTTATTTACTATGGTTGGAGGGGTTTGGAAATGGGAAAGAATCTTTCCCATTTCATATTCGGAGGGGAAACCCCT
GAAAAAGCCCCCAGTAGTTTGGGGGGGACAATTTGGGGACCCACTCCCCCAAGGGCCGCAAAACAAATCCCCTTATCCATCGGC
CCGTGGCCCCCCTTTTCCCATTAACTCCCTATTTCCCAAGGGGTTTAAACCCCTTATTTTTTTTTTCCCTTGGGAGTTATTTTT
TTGCGGGTGGAGGGCCCCTTTTTTTTTTTATTTTCCCCCCCCCCCTGGCAAAGGGGTGGGGGGTTTTTTTTCCCCCCCCCCTCC
CGGAAATGGGGTGCCCAGAACCCGTTGTTCCCCCCCGTCAAAAAAACGGTGGGGCCACCCCGGGAAAGGCAAGAAAGGGCCCGT
GGGCCCGGATTTTATGGGTTTTTTTTTTTTTTGCCCGTCCCCTTTTGGGAGCCCCAAAACCAAGGGCAAAAAAATTGGGCCCCG
GCCGAAGCAAGGTGGGGAAAGGAAAGGAAATGGCCTGGGGGAAAAACTTGAGGAAAAACCCCCCCGGGGAAGCTTTGGGGGGTT
TTTGACCGGCCCCCTTTCCCCCAAAGGAAGCGGCTTTAAGCGGCCCCACCCTTCTTTTTTTTTTTTTTTGTTTTCCCCCCGGGG
GGGGGGAAATTTTTTTATGCCAAAGCTTTTCCGCCCGTGAAACCTGTAACCCCCCCTTTTTTGGGGTTTTTTCCCCCCCCCGCC
GGGAAAAATAGAAATTTCATTGGGTTTTTAATTTCTTTTTTTCCAAAAACCCCCCCCACTGGGACCGAGGGGTTGTTTTTCCCC
CCCCCCCCGGCTTCTTTTTGCCCCAAGGGGAAAAAAAACCGCCCCTCCCCGAGTCCCCTCCAAATCCCTGTTTTTTTTTTCCCC
CAAAGCTTTGAAGGGCAAAAAAAAATACTCCAAATTTTCCGTCGGGATTGGGGTGTGCCCCCAAAAAACGTCCTCCCCCCCGTGG
GAAACCTCTCCCCAAAAACACCCCTTTTTTTTTCCTTTTTTTTTTTTTTTTATTTGGGAAAGTTCCTTCCTCCGCGGACCGGCCCT
TTTTCCTTCCCGTTGAAAAAAAAAAAAAAGAAAACCCCCCACTTTTTTTTTCCGGGGGGCCCTTTTTTTTCCTGTAAAAAAGGTT
TTTTTTTTTTTTTTTTTGACCAAATATTTACAAGGTCCGCCCAAATAGAAACCCAATTTTCCCCCTACCCTTTTTTTTCCGCGAG
TTTTTTTCCACCCCCCCCCCCCTAAATTTTCAAAAAAACTGGGTTAATTATTTCCCGGTGGGAAACCTTGGGGGCTTTCCAATA
GTCCCCCCCCCCCTTTTTTTTTTTTCCCCCCGGGTTTTCTTTGAAAGATGGGTTCCCCTCCCCGGTTGGAAAGTTTTCCCCTT
TGTTTCGCTTTTTTTTTCGTTTTTTTTTTTTGGCCCTCCCGCCGAATTGTTTTGGGCCCTCCAAAAAACCTTTCCCTCCGGTTT
TTTTCCCGGGGGCCCAAAATCCCACCCGCCCTTTTTGCCCGGCCCTTTTTTTTTGCCGGCAATCCCTTTAAGGGAAAGCTGGGA
AATACAATGACGTTTACCATTCCCCGGTTTTTTTTTTGTTAAAAAACCTGCCTTCCCCCCCCAAAAATTAAAACCCCAAGGAAG
AAATGGGGGGTTTTTTTTTTGACCCCCCGGCGTCCCCCCCTTTTTTTTGATGCCCCCCAAGACCCCCCGCCCCGGAAGGGCCAC
AATACCCAAAAAAAAAAAACACCTCCCCGGAAGTTGGGAAAATCCCGGGCCCCCTTTTTTTCCCCCCCCTTGGGAAAGTGCAGTT
GGGGAGGGCCCCCTGGGTTGCGGGGAGCGCTTTTGCCCCCCGAAAAAGCAAAAAAGCCCCTCCGGCCCCCAGTTTCCCCCCCCC
AAACGGAAAAGGGGTTTGGAAAGGGGGCCCCCCCGGGAGGGAAACTCCCCCCCCCCAAAAAAAGGCCCACCCCCCCCTTGCCCCC
GGTAAAAAGGGCATCCCTCAATGGGATTTTTTGAAATATGAAATTTTTTTCGCCCCCCCCCCCCCCCCCCCTTTTTGAGGCTTTT
TTTTTTATTTGACATGAAAAAAAAAAAAATTTTCCAGGACAAGGGGGAGGGTTTTTCCCCCTTTTTTATTTTGCCCCCAGTTCCT
TTTTTTTTTTTTTTTTTTTCCTTTTTGGCCATTTTTTTCCCCCCCCCCAATTAGGCCCCCCAAAAGGGGGAAAGCCCCCCCCCG
ACAAGATTAAAAAAGGCCCCCCCCGATTTGTTTCGAAAAAGGGGGCGGATCAGCCCCCCCCATTTTTCCAAAAATTTCATCGCT
TTTGACTCTTTTTTTGTTTTTTTTTTTTTTTTTTTGGCAGCCAGCTTCCCCCCCCCAAAAAATCCCCCCAAAAAAATTTTTTT
TCCCCCCCCTTCCCTGAATGGAAAAAGCAAAAAAAAAAAATTTGGAAAAAAAGGTTTTTTTCCTTCAGTGGGGTTTTTTGGCC
CCCCCCCCCCTTGGCCCTTTTGATTGTTTTTGGGTTAAAGGAAACCCCAGAAACCCCTCCAAAAATGTCCCCGGTGTCACCCCC

CGGCCGAGGCCCCGGCAAAAAAATAAAAAACTTGCCCTTCCTTTAAACCGGCCGGATGCTTCCTTTCTCAAACAAAACCAAAAA
AAAAACGGAAGGAAGGGAGGGGGGGAAAAAAAAGGAAAAGAGAGAATCAACCCCCGGCCGTTTTTTTTAGGGAAGGGCCAAGT
TTTTTTTTATGTCTTATTTTTTGCATTTTGTTTTTGGCCCGCCCCCCCGCGACCCCACGGACAAAAAAATGAGGAGGGGTCTTT
GGGTTTCCCCTTTTTTAAACCCCCAATCCAACCAAAAAAAAACCCAAAAAACCCAACCCTGAAGGAAAAAAAAACCCTCAAAAAAA
AATTGCTTTTTTCATTTCCCCAAAAGAGAAATGAAAAAAAATTGGGAAGCTGCTTGCCCCAAAAAATAAAAAAACCCCCAAGGG
GGTCAGCGATGACCATGAAGCCCTTTTGAAATTCGCCCTTTTTCCCAAAAAAGTTCGGTGCCCCCCCCCTTTAAAAAAAACTGT
TCCCCGGGGCTTTCCCCCCCCGCTTTTTAATGGGGGGGGGGGAAAATGCCCCAAAAAAGGAAACGGGCAAAGAAGCAAGTAAAA
AATATATGAACCTGTTTTTTCCCGGAATTTCCATGGTCAAAAAAAGCCCCCTCCAAAGGGAAAATTTGCAAATTCCCAAACCCT
TGAGGGGATTTTGCCCCCTTTTTACCCAAGGCCCCCAAAGCCCCCCTTTTTTTGGATGAAGGTTCCCCCCTCCGGCCCCTTTTC
CCAAGAAACCCCCCCCTTTTTCCCGGTGGAACTTTGCCCCGGCCCCCCGAGCCCCTTTTTTTGGCTTTTTCCCCCCGCAAAAAG
CTTTCCCCCGGCCCCCCACCCAGCCCCCTCCAACCGCCCTTTTTTTCCCCAAAGGAAGGCCCCCCCGAAATTTCCCCCCAAACT
TTTTCCGGAAAAGTGTTGTATGGGGCCCCCCTTTTTTCCGCCGTCCCCCCCTGGCTTTTTCCCCCCCCAGACCCCCGCGGGTTT
CAAACCCCCCAAATTTTTTTTTTCCTCCCTGTGGGGTTTTAAAAAAAAATGGAAAAGCAAGTAAAAACCAAACCGGAATGTA
GGTAAAAATCGCCTTTTCCCCCCTTAAAGCCCAAATGGGGGGCCCCCGGCAGCCGCAAAAAAAGGCCCGGAAATTGCCCCCCGG
AACGAAGTTTTTTGAGCCACAAAATCGCGGAAGCAAAAAAACGAAAAAAGCGAAAGAAACTCCCAAAACCGGGGAAAAAAAGAA
AAACCAATTTCAAAGAACCCCCGAAAAAAGGACCTTTAATGAAAAAAAAAAAAAGCCCAAAAAGTTTTCAACCGGAAATAATTAC
CAAGTTTTTTTTGAAAAAAAATTAGAAACCCCTCGCCGGAGAGAATTGTTTCTTTTAATTGGGCCCCCGGACCTGTAATTAATG
GTCCCCAAACTAAAACCAAGGAAAGCCCCCCCCCGGAAAGTTTGTTTTCCTCCAAAACGGGAGGGAAGGCGATCCCCCCCGCCG
AAGAAACTTTTCCAAAATTAAAAAAAGAGGGTGTAGGAAAGTTTCCCAAAAAACGGAAGAACCGTTGGGGCCAACAACCCAAAA
CCGATTTTTGAAAAAAAAGAGTGGCCCCTTTTCAAAGCTGAAGGATTTGAGAAATTTTTTTTTTTCGAGTTTTCCACTGGCAA
AAAAGAAAAGCGGGAAAAAAACCCAACCTTTTTTTCCGGTCCCCCCAAAGTGGTGAAAGAAAAATACCCATAATCCTTATCCCGG
GACAGGGGGGTTTAAAGTTTTTTTTTTCAACGGAGCTTGCAATTATTTTTCCACGGGAACAAGAGGGGGGCCCCGTCCTTAA
AGCGCTCCTTTGGGAGGGCCCGGTCAAAACCCCCCAAAAAAGCCTTCCCCCGGGTCCCCCTCAAAAAAGTTTCCCCAAGCAACT
TTTTTGTTCCCTCCTTCCAAACCTTTTTTTGCCTCCCCCGGCGCCCATTTTGCCCCCCTTTTGTTTTTTTTTTGGCTCAAAGG
ACAGGGCCAACCCCCAAATAAATCCGCCCCGGCCTTTTTTGCCTCCGCCCAAACCCGTTTTTGGAACGGCACGTTTCCGCCGCA
ACCATTGTTTCCGGAGGAAAACACCCACCTTTTTGCCCCGGAAAGCAAAAAACCCGCAACTCCCCAAAAAGAAAGGGTACTTTG
AGGGAGTTAGAAAGGCCAAAAACCCGAGGAAAGAAAATTTGTTGCGCCTTGGTCCAATTCACCTTGCTTCCAGCCGTCCCCGAA
AATTGATGGATTTTCTTCCCAATTCCAAACCTTTTTTTCAAACAGTGGGACCGAATTGCTTTTCGGAACAAATTGGTGCAACCT
TTTTTCCCCCACAAAACCCGAATTCCCCCCCTTCGTGGTGGAAAAGCAAATAAAGGCCCCAGATTTGCCAAAATAAAGAAGGTT
TTCCCTTTCAAAACCGGAATCCTGGAATTCGATAACGGAAGGCTTTTCCCGTTGAAACCATCTCCTTTTCAAAAAAAAAAGCGGC
CTTTCCCCAAAAAAACTCGAAAATCCGGTATGTGAAAAAACCCCCGAGACGGACCCCCCCTGGCCCCATTGCCCCCCCAATTCC
AGCCCTTGTCAAAGGATTTTTCAATGGGGGAACGCCAACTTTCATGGCATTTAGGCCCCCAAAAAAAAAATTTTTTCAAACTTG
TTTTCCCCAAGTCCAACCCCTCAAAAAAAAAGAGGAGAAAGCGACGCCTTGTGCTTTTTCAAAGCATAAGCAAACAAAATTTCG
TTGGGTTGTCCCGACTGCCAAAAAAACGGCGCGTAAATAATTTGCCAATCTTTTTTAGGCCGACCGGTCCCCCTGAAATCGGAT
TTTTTTTTTCTTGTGGGCCATAAAAACTCTCGATACGTCAAACTTTTAAAATGCTTTGAAAATGTTTTTTTTTTTCCCTGCGA
ACGTGGGCCCCCCGGTAATTGGCCCCAAAAAAACCCCTCCGAAGGGAAAAAAAAAGAAAACCCAAAAGAAAGGTGCCAATACCG
GAGCCCTGCTTTAAATAAACGTCCCTTCCGTTCCGAAGCCGTCTAACAAAACGGTCCCCGAAAGAGAAAAAAAACGTTTTTTT
GGCTTTTTTGGCCATTTTTTCCTTTTTTTTTGGCGGAAAAAGGTTAATTTGCAGAAAAACTAAGGGGTTATTTTTTTTTTTGC
TTTTTCAAAGAAGGCAAAAAAAAAAAAAAATACCTTTTTGTTTTTTTTTTGACGAACTCGACCCCAAAAGAGTGGGGAAAATC
GAGAAAAAAAAAGAAAAAAATTAAAAGAAAAGCCCGATTTTTGGAGCGAAAAGTTCCCCCGAAAGAGGTAAATTCCCTCAAATT
TCCCGGGAAAAAGAAACCCCAAACCCCAAAGGTCAAAGAATTGTTCCAAGGTGGGGAAAAAAACCTTGCCCGTTTCGGCCCCCC
CAAGGGTTTTTCGAGATAAGATCGTTGCAAAACTTGTTGAAAGGAAAATAATCAAGGATTGTCCCCTGGCAATGGTGCCCCCAC
GCTGATAAATGACCGGGGAAAAATTTAATCCCCAAATAATTTGGCAAGGTTGCCAACGGCAAAAAACTTTTCAACAAAATTAGA
AAACCCAAAAAAGCCCTTGCCAACTTTCCCCCACAAATT
>m141122_014521_42134_c100732202550000001823141405141560_s1_p0/155405/0_5677
AAGAAGAATAGGCCATGGAGGAGGCCATGGGCGAGGCCCATGTAGGAAGGCCATGGGGCCGAGGCCATGGAGGAGGCCCATGGG
GCCGTGGCCATGGAGGAGGCATGGAGTGAGGTCTATGGAGGAGGCCATGGGCCGAGGCCATGGAGGAGGCCATGGGGCCGAGGC
CATGGAGGAAGGCCATGGGCAAGGCCATGAGGAGGCCCATGGGTGAAGCCATGGAGGAGGCCATGGCGAGGCCATGGAGGAGGC
CCTGGAGGAGGGCATGGGGCCGAGGGCATGGAGGAAGGCCATGGGCGAGGCATGGAGGAGGCCATGGGCGAGGCCCATGGAGGA
AGCCATGGGCCCGAGGCCATGGAGGAGGCCATGGGCGAGGCAATGGGGCGAGGCCATGGAGGAGGCCATGGGCGAGGCATGGGG
CGAGGCTGTGGGCCGATTGCATGGGTGAGAAGGGGCCTCGCGAGGGCTACTGAGCCTTGGCACCTATGCCTTGCCAAACGTGGG
CCACCTTGGCATCGACTCCATGAGAAACGAACCTGACTCATGGTTGTGGAATGGCGCGATGCTTTCCCCGAGATGACGGTGGCC
TTTAGGGCCTTGCCTCGCCTCATACCTTTCTCTTGATGGATTATGAGCATCAATGATTTCTCATGAGATGATTCCTGTATTCAA
CAAGGCCTACAGGCTTGAGACCAATAGCGTGAATAAGCGACCCTTATCCCCCTATGTTCCAACCCAGGGACTAAAGAGATTCTT
TTTGGTGGATTTTTTGCATCCAAACCAGCATTAACAATGCCTACTCCACTTGACTGCCTCGATGAATAACTTTTAAATCGGTCT
GCAGTCAATGACTCAATCCCATCAGAGTGAATCCCCATCCGATACCACTCCCTTGCAGGGCCCAGACAATACAATGGCCACATA
CAATAAGCATATCAAAAGACAATGTATTAATGCTCTTGCCTTAATTACAAAGTAGATAAGGCAATTTAGATAAAGAACAAGACA
AATATTTATAATACCAACAGACCCTGAGTCGACCTTGTCTCTGGCAAACGAACGTACATGTTCGGTCCAATCCTCACAGGAACC

ATAAACCTTGGCATGCTCTAATACCGAGTAAGTAGTAAACACCACCTACTACCCTAGAGTCGTTAACCAGTGATTTTAAAATCT
TCATTAGCCCCACTCAATAATTGAGTATAGACCCAAAAAGTGTGGTTAGTTAAAATGAAGACTCGTTAAATAAAATTTTGGAAC
AAATGGGTTGCAAATTCATTTTAAATCATGAAAGTGTTTACATTAGGATCCCAAAATACTTTTTAGAAACATATTTAAACTCAA
AAGTTACTTTTATAGTCAACACTAAGCATACCAATATCCAAACATTTATAAACATGCTCCTCAAAATACTCCAGTCGTGGCGGC
CGGTTTTTAAAAAAGGCCAAAAAACCCCCAAAAAAAAATGGGGGGGGTACGCAGCACCCAGCCCTCCCAACTCATGGTTGATCAA
ACCTTTCCTTGCCCTTACCTATGCCATAGAGCAACGTGTGAGCCAAGGCCCAACCAAGAAAACTTCACAGCAACAATAGAATAA
TTTATTTCAACAAATACATACTTAATCAAGAAACAAACAATTAACAGTTTCCTATTAGAATACACAATAGCTGGCCTACGCGAT
CAAGCGTGCATTACTAGGCACCAAGTTCACGGGTCTTAACTGAGTTGGTGTGTAGAGCACCCTTAAGAGTCTCCGCCCTAGGCG
ACCTGCATGCAATGCTTAACACAGATCCTCGACCTTCTGGTCGTTCATAACCTCTGCGCTCCTGGCCCTTTCCCACTCTCTGAT
CTTTGCCATCGGTCACATATCACATGTATGACATAATGCTATAAATACATACAACACTAAACATCAACATCAGCTCTATGGGCC
ACAGACAGTTCTCTTTAACTTGTGTCCCAAGCTGACCACCCATATGTGATCCTGAGCACGATCCCTAACCCCGAGCCCGAACGA
TTAAAACCTAGTCAACGCAGTAATGATAATAACGCATCATGGATGTAAATCAAGTTAAAGTCTTCAGAATAATAATACCCTATG
TCTCTGGGACCCATCGAATCTCTAAACTAAGGTAAGTAGAATCCATACCCGAGCTTAAGATATGAGTTTCTGAGCTTAAACCCT
ATTTTGGTCATTCCCCCAAATTTTAGTCATGCCTACCCCTAAGGCTATGGCCTGCAATACAAGTCAGAGAGAGCTGGGCTCTAG
CCAAGGGGCTTGGGTCGTAGCATGCATCAGCCTGTGCATCGGCACCTGGGGCAAAAATCAGATGCTCCCAAGCCTCCTAGGTTA
ATACGGGTCGCAGGGCCTGAAGAACAGGGTCGATGGCTTCGGACCTGGGAACCCAGTTTTTTTCCAGCTTTTCAGATTTCGAAC
TCCTAAAATTCAGTCTAAACATACTCCATAAGGCCAAATTAAGTCCATCAACGACTCTACTCACACTTTAGGCAGTACTAACAC
CTTAGAAAGCTAGCTAAATCACCTATTCAGAAACAAATTAAACTTATGCCTTAGAATCTTATCTAAGCTTCAATTCTCAACAAA
ATTTACCAAAAGAAACATTGAAAATAACCTTCCCTGTTCTGTGTGATTTTGACCCTGAGCTAATTTCCAACACTTGCCTATCTT
GTTTTCCCCCAGTCCCCAGCCTTCAATTCTCAGCAATCCTCGAGAATTCTACCAATGCTCCAAGCACACCAAAGAGATGGACAG
ATCCAAGTGGAAAGGAGAGGAAGAGATCCCAATCTGGAAAGAGAGTGTAAAGACCCAACAATTCCGTTATTTAAGGCTTAAAGG
GCCTTGATTAGCCGTGCCAGGAGGGGCACGATGGGATTTTATGTGTGATTTTATGAGTTAAATGCTTGGTTATGAATTTAAAGC
ATGATTATTTGAACTAATTTTTAATCTGAGATGCATGACTATGTATAATAGTATGCCATGTAGGCCCGGATCGTGTTAGACAAG
GGGGCATAATTGGTAATTTTGGCCATGGTTGGGCATAAACTTGCTTTGGATGTGTGATATTGTTGAGACCACACTGTGAGGTGG
GATGTATCTGTGATTTGTGACTCGAGCCGATCCCAGTGGAGCCAGGCCTAGCGGTAAGTCTTGACAGGAATTTTATACCCGGCT
TCGGGGCAGCCTGGGGTATGATCAGGAATTCAGAGAATAGATTGAGATTATTTTGATGATGGGGGATACTTAATTTGGTAGTTT
ATCAAGAGTGTTGGAAAGCACGGGAAAATATTGGAGACACTTGAAGATTAGCGGGAATTGGGTAAAATGACTAAAATGGCTATT
TGGACAAAATGGATTTAGATTTAATAGGAAGGGCATTTGGTCATTTGCTTTTAGGGATTATTTATGAGGCTTTTACCACTTAGT
GGGATTTATAGAGAGTGTTGGCTGTGAGAGAAAGAAAGAAAAGAAAGAAAAAGGAAAGAAAGAAAAGAGGGAAAACAGAGGGGT
TTTCAAAGGTCCCGGTTTGTACATTTTTGCAGCCAATTCCGCTCCATTTTTTCTTGGAGCAAAGCTTAGTGAGAGCAATGGTAG
GACTGAGCATCAAGGATCTTGGGTTTAGACTGAGATTTGGCAAAGACACCATCAACTTTTGAGTAAGTTCTTGGAGATTTCCTG
TTTTAGGTTTTGATGTTTGAGCTGTGTTTCTGAGCTGAGTTGATGGAATTTTAGGGAATTTCTGGGAAGCTTTGATGATGAAAG
CTTAAGGGAAAAAAAAGGGGGCTAGGGTTGAATCAAGGTCGAAATTTGCATTAATGGTATGATTTCTAAGACTCTATCTTTATG
GTTTGCATAAATTTCTAGTTTTTAGGGTTGTTTCATGGGTAGATTTTGAGTTTTGGGTTGCTTGGAGCTTTGGGATTGGGTCGC
TTAGGATGCTGGGATGGAGTGTGAGTGTTTATAAGCTTGATTTTGGGCTTGGAAAAGGTTTGGACAAGTTTTGGGTTGAATTGG
TGAAGGAAATCACAGAAATGTCGATTTTGGGTTTTGGGCTGCAACTAGCGCTACAGGCTAGTCAGTGGTGCTGTAGCGCTAGCC
CCTGTCTGGGGGTGCATTCTGCTTGTAGCGCTACAGTGCCCTCTTTAGAGCGCTGTATGCGCTGCACTGTTCCAGAGGTGTAAT
TTTTTGGGTTTTATGAGCGATTTTGACCTAAGGGTTCGGGGCTCGATTCCCGCCACTTTGTTTTGGGGATTTAGGACTTCCCGG
GGGCTTGAGATTGGTCCCGAGGCTAGGTATTCGGACTTGGGGGTTCGGTGTTGACTTTGACTATGTTTGTGCCTAGGTGTGCGC
TAAGGCTCGAGTGGGATCGTGCTCAAGGAGTTAGGTGATCTTAACTATTGAAATGGAAAGGTAAGAAAACTAATAACACCCGTA
GGATGGGGCATGGGCCCCATTAGTGTGATATGCGGGGAGCGGCCCTAATTGCATGCTGCATGATGAGATGATTGCTGGGCACGG
CCCTATATTCATTACATGTTAGGGTGCAGATTTAAATAATTGGTATTTTGTTTGAATGTTGCTTTGATTTATGCTATATATGGA
TTATAATGAAATGACGGCTATGGCCGAGGGGCCGGCAAGGCCGAGAACGGCCAGCGGAGACGGAAGTACACTTAGCACATGGGA
TGCTTCATAGTCAGGGCTGGGCCGATGGATACAATGGGATATCCTTACGGTGAGGACCGAGACCCCAGGCTTCGGTAAGGTTCT
GGGCGGCATGGCCCGTGTTTGCTTAGTCTAATGGTTGACTTGTCTATCTTGTGGACCTATCTGATGTGATTAAACTGTTGTTTG
CATATGTTATTGTAACGCCCTACTACCTTAGAGCCGTTACCAAGTGAAGTTTAAAACTAAAATTTGTGCCATATAACTGACTCA
AAGTGGTTTCTAAAACCAAAGTGTGTTTAAGCTAGAAATATTGAAAGGCTGTATTACTTTTGAAATGTTTAAATTTATTGAAAA
CGTTAGAATATAAAACACTGGGATCCCCAAAAAAGGTTTACAAAACATTTAACAACTCAAATAGACTTACAACAAGTTTGACTA
TCAAAAGAATAAGGTAATAAGCCATTTACAAAAGAACTCCCAACCAAAAGCAGTCGGACGAGGCTGAACATGGTACGCGCCGCC
CCCACGCTCTTCTATACTCATGGTTCGGTTGACTGACTCCCCCTTGCTTCTACCTGCCACATGCAGCACCCGTGAGCCCGAAGC
CAGCAAGAAACTCCATGCAGAATATAACATAATGCAGTTCAATACAAATATATATATCATGGCAGATCCATCCACATATAAACA
ATTCAACCCTTAGTCTAAGCAAAATCAACCTTAGACTAGGCAAACACGGCCATGCCGCCCCCCGAAGCCCGCCGAGCCTGGGTC
CTCGGTCCACACCGCGGGTCCCTACCCTCACCATAAGGCCATCCCGGGTTGCTAGGTGGTATTTCCGGCCCGCTGCCGTACCGG
CCCACGCCGTTCTCGGCCATGCCGTTCAATTCACCTATAATCCTATATAGCATACATCAAACAACACCTCAAACGACTAACAGT
TCATTTAAATCGGCACCCTAACGCATGCAACATAAATAGGGTCGTGGCC
>m141122_014521_42134_c100732202550000001823141405141560_s1_p0/155425/0_5398
AAGAGAGATCCATGAGCAATATTGGGAAAATTATCGAACGGAGTTGAACACGAGGGTAACATTTCGGAGAAATTAGTGAAAATC
TCCCAATTCACCAAAGGGGTAACCTTTCAGCAAATCTTGATTTCTCTAATATGAATTTACGCAATGTTTTGATAGTGAATTCCT

AAGGGAACATGCACGGGTCAGGAACAAAAAGTCTAGCCCCTGATTGTCTCAGGGTTCTACTGATAAAATTTTGTCGCACACCAG
AGGTGTGAGAGGGCGATATCCTGTTCAAAAATACTTAGAATTACCAATATAATGAAAAAACTTGCGAAAATTGCCATCAAAAAC
GGGAATTTTTTTTGAATGTTGACTTAAGTCTCCCCGTCTCGACTCAATATATTCATTCATGAGATCATCCTTTGGGCGGGCGAA
ATATCAACGCGTTGAACACGAGGTAATCAACATTCTCGAGAATAGTGAGAATCCTCCCATTCACCCAAAGGTATCCCTTCTTCA
GAAATCTTCGCATTTCTCTAATATGCAGATTTGAGCCAATGTTTTGATAGTGAATACTAGAACTATGCGAGGTCAAGGTAACAA
AAGCTTCTAGCTATGTCTTAGGAGTTCTCATGTAAAAATTTCGTGCAATCCCGAGGTGTGTAGAGGCGATACAGTCAAAATACT
TGAATTCACAATCATAATGAAAAAATTCGAAAATTGCCATCAAAAACGGGAATTTTTTTTGACAATGCATGATTAAGTATCCG
TCTGGACTCCACATATTTTTTACATGACGATATCTCTGGGGAACACGAAGATTACAGGCGGGTACTCCTTTCACGGCACTTAGC
GAACATTCACATTCACAAGGTATTCTAGGATCTTCATTTCTCTACACATCTGATTCAGCATGTTTTCGATCGGCAACACTACAG
GAACTGCAGGCGGTATTGGAAATAGTACTCAGCCTTGTTTAGGGTTCTAATGTATAAAATTTCGTGCAAATCCGAGCTGTGTAG
CAGGGTGATACTGTCAAAATACTGAGAGATGTACGAAAATAACGGAAAACACTTTCGAAAATTGGCCATCAAAAACGAATTTTT
TTGAATGTTGACTTAAGTCTCCGTTTCGACTCCAATATAGTTTTTCCCCATGAGATATCTTGGGGAAAATATCGAACGATTGAA
ACACGAGGTAATCACTTTCGGCGAACATTAGTTGAAATCTCCAATTCACCAACAGGGGTACCCTTTCAGAAATCTTCGATTTCT
CTAATATGAAATTAGAGCATTTATTCTTGATAGTAATACTAAGAAATATCGAGGTAAAGGGGAACAAAAGTGTGTAGCCCTATT
CTTAGGGTTCTAGTGTATAAAATTTCGTGCTATCCGAGTCTGTGTAGAGGGCATACATTCAAAAGTACTTTAGATATGTACATA
TACAGGAAAAAACTGCGAAAATTGCCATCAATTAGGGGAATTTTTAATGAAATGGTGGACTTAGAGGCTCCGTTTCGACTCCAA
TATATCCTATTTCGCCCATGAGATATATTCGGGGAAAATATCGAACGTACGTTGACACCGAGGGAATACATTTCGGAGAAATTA
GTGAAATCTCCAATTCACCAAGGGGGTACCCTTTCAGAAATCTTCGATTTCTCTAATAGAATTGGCAAGTGTTTTGATTACGTG
ACATACTAAGGAACATGCGAGGGTCATGGGACAAAAGTTTCTAGCCCTAGTGTCTTAGGGTTTTCTATGTATAAAATTCGTGCC
AATCCGAGGTGTGTAGAGGGCGATAAACCAGTCAAAATACTTAGTACGATGTTCACCAATATATGAAAAAACTTGCGAAAATTG
CCATCAAAAAAACGGGATATGTTTTTTTGAAATGAAATGCTTAAGTCTCCGTCTCGACTCCAATATAGTTGCTCCCCATTGAGA
TATCTTGGGGAAAATATCGAACAGTTCCACACGACGGGTAATTAAATTTCGAGAAATTAAGTGAAATCCTCCAATTCACCAAAT
GGGGAGAAGAAGTACCCCTTTCAGAAATCTTCGATTTTCCTCTAATTGAATTTGAGCAAAGTGTTTTGATAGTGAAATACTAAG
GACATGCGAGGGTCATTGGAACAATAGTACTAGCCCTATGGTCCTTAGGGTTTATGTAAAAATGTGCCAAATCCGAGCTTGCTA
ATGAGGTGTAACTGATCAAATACTGAGAATGTAGTACGAAAATAAGGAAAACAAATTTCGATAAATTGCCATCAAAACGGGAAT
TTTTTTTGAAATGTTGACTTAAGTCTCCGTTTCGACTCCAATATATTCATTTCCCCATGAGATATTGGGGGAGAAATATCGAAC
GAGTTGAAACGAGGGAATACATTCGCGAAATTATGAAATCTCCAATTCACCAAAGGGGGCTTACCCCTTTCAGAAATCTTCGAT
TTCTCTAATATGAATTAGAGCATTAATTTTGATAGTGAATACTAAGAAATATGCGATGGTCCATGGGAACAAAAGGTGTACCCT
ATGTCTTAGGGTTCTATGTATAAAATGTCGTGCGTAATCGAGTTTGTTGTAGGGGCGATACATTCAAAAATACTCTAGATATGT
ACGAAGTATAAGGAACAAAACTTGCCGAAATATTGCCATCAAATAGGGGAATTTTAATGAAATGCTGACTTACAGGCTCCGTTT
CCGACTCAATATATTCATTTACACCATCTGAGATAAGATATTCGGGAAATTCGAACGAGTTTGACAACGAGGCGTAATACCATT
TCGAGAAATTCTAGTAAATCTCCAATTCACCAAAGGGGGGGACCCCCCTTTAGTAAATCTTCGATTTCTCTAAGTCAGTGAATT
ATGAGCAATGTTTTTTGATAGTGAATCCGCCTCAAGGAACATCAGCGAGGGTCATGGGAACAAGAGTTCTACGGCCTATGTCTT
AGGGTTCTTATGTATACCAAATTTTTGGTGCAAATCCGCCAGGTGCTGTAGAGGGCGAGCTATTGTCAAAATACCCCTATGAGA
TGCTACCAATATAATGAAAAAACCTTGCGAAAATTGCCATCAAAAACGGGAATTTTTTTGAAATGTTGACTTAAGTCTCCGTG
CTCAGGACTCCCATATATCATTCCCCATGAGATATCTTGGGGAAAAATATCAACACGATTGAACACGAGTGGTAATAATTTCGC
AGAAATTAGTGAAATCTCCAATTCACCAAAGGGGTACACCTTTCAGAAAATCTTCCGATTTCTCTTAACCTCCCGGATTGTGAG
CAATGGTTTTGACTAGTGAACCATACTAACGGAACCATGCGAAGGGTCATGGGAACAAAAGTTCTTAGCCTATGTCTTAGGGTT
CTATGTATGAAAGTTTCGTGCAAATCCGGGTGTGTAGCAGGCGCGATACAGTCAACAATACTTAGAGATGTACCAATATAAATC
AATAAAACTTGCGAAAATGCCATCAAAGAACGGGAATTTTTGTTTTGAAATGATGACCTTGAAGTCTCCTTCGACTCCAATATA
CATCGTCCCCATGAGATATCTTGGGGAAAATTAACCGAACGAGTTCCACACGAGGGGCTGATACCATTTCGGAGAAATCTAAGT
GAAACTCCAATTCACCAAAGGGTACTTCAAGGAGAGGGCGTTCGAATTTCTCTAATAGTGGAAGTTTGAGAATGTTTTGGATAG
CTGAATACTAAGGAAGCATAGCGAGGTCATTGGAACAATAGTATAGCCCTACTGTGCTTAGGGTTTATGTTAAAATTTTCGGCA
ATTCGGAGCTGTGTCAGAGGGGTGATACTGTCAATAATACTGAGAGAGTGTTACGCAAAATAAGGAAAACACTTTTCGAAAATT
GCCATCAAAAACGGGAATTTTTTGAAATGCTTGACATTAAGTCTCCGTTTCGACTCCCCAATATATTCATTATCCCATGGAAT
ATCTTGGGGAAATATCGAACGAGTTGAACACGAGGGTAATACATTCTTGGCGAAATTAGTGAAATCATCCAATTCACCAAGGGG
TACCCCCTTTCAAAATCTTCCCCGATTTCTCTAATATGGAATAGGAGCATTATTGTGATAGCTTGAATACTAAGAAAATATGCG
ATTGTCAGGGTACCAAACAGTTTGCTAGCCCTAGTGTCTTAGGGTTCTATGTACGTACAAATTTCGTGCTCAATCCGAGTTGTG
TAGAGGGCGATACATTCAACAATAACTTTAGATATGTACAATTATAAGGAAAAACACTTGCGAAATGGCCATCAACATAGCGAG
GACATTTTTAATGAAAATGTGGACTTAAGGCACTCCGTTTCGACTCCAATATATTCATTTCCCATGAGATTCATTTCGGGGAAA
TATCGAACGATTGAACACGGCGCGCCTACTTTCGGAGAAATTAGTGAATCTACAATTCACGCAAGGGGTACCCTTTTCAGAATC
TTCGATTTTCACATTTCCAATTGACAAACAAAACAAAAACACATCTCCTGAGCATGTTCTCTGACGCTGCGAATACAAGGAACA
TGCGAGGGCAGTGGGAAACAAAAGTTCTACAGCCCCTATGTCTTAGGCGGTTTCCTATGTATAAAATTTGTAAATCGCGAGGTG
TGTAGAGGGGCCGAGTACTTGTACAAAATACTTAGAGATGGTACAATATAATGAAAAAACTTACGACAAATTGCCATCAAAAAC
GAGAATTTTTTTGAATATGTTGAAGCTTAATTCTCCGTCTCGACTCGCCAAAATATATTCATTACCCATGAGATAGTCCTTGG
GGAAAATATCAAAGAGTGAAACACCGAGGGTAATCACATTTCGGAGAAGATTAGTGAAATCTCCAAATTCCACCAAAGGGGTAC
CCCTTGTGCAGAAATCTTCGATTTCTCTTAAATATGAATTTGAGCAATGTTTTGATAGTAAATTACTAAGGAACATGAGAGGGT
CATGGGAAGCCAAAAGTTCTAGCCCTCATGCTCTTAGGTTCTATGTATAATTTCGTACAAAATCCGAGGTGTGTAGAGGGCGTA

CAGTCAAAGAATATCTTAAGATGTACCAATATAATGAAAAACTGCGAAAATTGCCAATCAACCAAACGGAATTATTTTTTTAA
TAATGTGACTAAAGTCTCCGTTCGACTCCAATATATTCATTTTCCCATGAGATAGACGCTCCGTGGGGAAAATATCGAACGAA
GTGAACACGAGGGGTAATACATTGTCGGAGAAATTAGTGAATGACTCCAATCCTCAGCCACACACAAAGGGGTAGCCACCTTTC
AGAAATCCTTCCGATTTCTCTATATATGAATTAGAGCAACTGATTTGATATGGAATGCTAAGAAACATGCGAGGGTCTATGGGG
AACAAAAAGTTCTAGCCCTATG
>m141122_014521_42134_c100732202550000001823141405141560_s1_p0/157696/8154_15740
ATTTGGTGGTTGAAGTTTTTCCTCTTTTTATTTGAGTCGGGAAACTTTTTTTGTACTTCAGGAACCTTTCATGAGTTCACGCCT
GCGCGTGATTGTGAACAAGTTGAAATTATCTTAAGATTGTATCTTGCCTGAAGGCTTTCCAATTGGTGCAATCACGTGGAAATC
CACCAACTTGGAGGAGATAGGAAAATAATACCCATAAAGAATGGAGGTTTACACTGCCTGGCTAGGAAGAGAGTCCAAGATTAA
TACATGAGAGATTGTATTCAGAGGATCTGAGAATAAGGACCGACATTTCATTGGCGCTCGAGAAATTTGTATGTCGACGGAGAT
GGAAATTCTATCCAGATAGGTTGCGAGAGTTGCGTGTTCACTACGAAGGCGACAGCCCGTGCCGAGGGCGAACACTAACATAAT
GGAAGTGGAGCAGTGGGATTGTTGTTATCGTTCTCTTACAGGATCACATCGAATGCCGACAATTGGTGTCAAGAGGGTCACACG
TGTGGACAGGTGGCGAGTTGCTTCTCTGATGTTTGCTGCGAAGATTCCCTGGACGTGCAATATGCTATGCGTGGGAGGAGGGGG
GTAGGGGATCCTTGAGGAAGGTTCTCTCACTTGGACATTGAGGTCAAGTATGCATAGAAACATACAACGAATTTTTGAGATCCT
ACATGATACGATTTCAGTTGTTGTTCATTCCGGGCATGCTTGTTGGGTATAATATTTGTTAGTTTCTTGCAATGAAGAGCGTGT
GGTACTCTTGTAATGTCGTATCTATTTGTGCGAAACTTAATCTAAGAGGTATTTATCCTATCTTGATTAAATTGTCTTTTACTT
TGAGCTAATGATGACACTTTGGTGAGAATGAGATGTCTCTAACAACGATTGTTTCTTCTGCTTTTGTTCTTTTCGGCGTTCTTC
TTTTCAGTACTTTTGCCGTCAAAATGGAGACTGGGTCTAGAGAACGAGCGGAGTAGTATTGGTTTAGGTGTAGTATCCACACAC
TTGAGGTGTGTTGAATAAGATGTTGAGGTCGTGGTTCTCTTGTTCTCACCAGAAGATTATAAAAATTAAATTTGTGGAAAGATT
TTTTATTTGGTGAAATTAAAATAAAAGAGAAATAAATAAAGTTGCTATGCTGTACCTAATACAGTGCTCAATTGATTGGTAGTA
TACAGTAAGGGTACAATGAAAGTTATAAGAGCATCTGGGAGGTGTTTGGTGTCGCGCGTTTAGAGTAGGTTGAGTATGTGGCTT
ATGAGATCGTTGAATGTAGAGGGAAAGTTACTTCATGACGTCATTATAGATCTCATAGAGTATAGATTTTATATTTCTGTGGAG
TGATAATTATACTAAAAAATATAATAAATTGTACGCATGTTCAGGATAGGAGAGAGAGTGATAACTGATATAGAGTGAAATCAT
TAGTATATTCTATTAGGAGTATAATAGGTAATAAGTTTATCACAAGTATACAGGTGCGATTAGTTTCTTATATTTCAACCATC
ACTAGAGACTGCATGTCACTCGAGGAGAAGGTAAAGGCTTGGATCTATTTGTGATTTGATTTAATCTCACTAAAGAAAGGCAAT
AAATAATATTTACATAGCATTAGTTCGTGTGTTATTCTTTAGGGTGTGTCAATTCTATTTCTTTATGCTACTGTAAAATCAGAT
AAATATATGTGTGACAAAGTTTACTCATTTGCGTGGGTTGAGGCAATTTGAGTACAAAAGTCAGCATATACGAAACTGATGAAA
TCCTCACGACGTACAGTGAGTACATAGATCTTATAAAATAGCTTCGAATCTTGGTTCAGAGAGATATGCGTAACTTTGTCTTGT
ATTATTATATTTTGTGCTTACATGAATACGTACAAAAAACATCTTGCACTTCAATAGAATACATGATTCAAATCCGACATGCTT
TACTGAGGAGCTAGTTCGTGTATCCACCTACCTACTACTTCTTATATTTTAGGTCTGCCATACATCTTAAAGCGAGTACATAGA
CTTTCATTGACAAAATAAAAAATTCTTAAGAGGCCGGTCCTGGGGGGCTTTGCGCCCACTCTCTCCTTCGCTGTTTGGTTAAAAT
ATGATAAACATATAAAATTGTTATCATTGAATGATAGGATAGGTATGAGTTATTCTCTATATAGAATTTAAATATTTCTAATGT
GGGTACTTCTAAGTTAATAAATAATATAGAATAGTGAATGGATTCCTTTGAGAAAAAACAAACCCGAGATAATGATTACATTTG
TTACACTCCTTGTAATTCACGAATTATGTTAACATTGAACTTATTATTTCTGAAATATGGACGAGGTGTAAGTTAAATGAATCT
TCTTGTCTGCTTGGGGTGCTCCCGACATAATTGGGGTGATTGTGCTGAAAAAGAATTGGAAATTCTGTTTTCTTGAATGTGATA
ATTATTTGTAGTAATAACTTAAAAAAATTGTCATTATGTACTTTACTTAGATTAATTATTAAATTATAATTAGTATTTCTGTTG
GATATATAACTAATGTTGTCTAATCTTATATATTAGACGGTTGTCTCATTTGAGTGGTCGGCATTTTCACTGTTGTAACATCAT
CACAATCTAATTGTATTAAATGTATCTGTGGCTTATATTCTTTTTTGAATGAAATTAACTCTCGTTAAAAACATCTGAAAGGAA
TAAAGGTAGAATAATATATCTGCAGTATAAGCTCTCTAATGATTGCTACATAAATAAATTTGTTCAATATATGTACACTGGTTT
GACGCTGTATTACACATCAGTTTTCTAAAACGTCACATATGCTTATACAAGATAAATTAAAAATTATTCATCGATGACAAAATT
AATTTACACTTTTATAAATTATGTATATAGGAATTGTAATTCACATAATAAAAAGGACTATGGAAAAAATTATACTTAGGTTTT
GGAGTTTGGTACCTTTTTTTGTATTTACATCGAGGTAACTTATAATACATTACTAGTACCTACTGCTTTTATTACTGAGCAGTT
TAGAATTATGTTGATTCATACAATACAAAAGATGTGTGTAATATTTATAAGTACTTAACTATTATTTCTTATTAAAATAATTTC
GGCGAGTTGTAGCTTTTAAGATGGATCTGTTATGAAGGTGAAAAGGATGAATAAACTGTACTGTATCATAGGATGGATATATAT
TTGAGTGTTTGTAAATAAGGACAATCTTAACCTGCTCGTACTTTTGAAAATAGGCGGTAAATCTGGGACATATCAAAAGAATAT
TAAATACTCATAAAGGTATTTCTGTGTTAATCCGATATTCGCATCGGTTATAAACATTAATGTCGAATCACAATATATAATAAG
GTAACATCGCGCAAGGTTGTGATCTGTTAGGAGAATTATATTATACTTCTTGCATTCTGATATACAATTGGTGCTCAAATGGGA
AAAAAAAGCACATTAAAATATATTATGTACATCAAAAATAAAATAGAAAATTATAATCTACATTCCTTGTTGATTAAAGAAATA
TTAAAAATAAATTGGATACAAAAACGATCTCATAACTAGATATTGAACAACAAGAGACATAATAGTAAGAATTTCAATTTATTA
TACATAAATATATATCAAAAATAACGTATCAAAAATAACATATGTAAACTATCCTGCAATATGTACATGACACAGATTAAACAA
CATCACAATACATATAATAATCGTAAAAGAAGCAGAACAAATATGCAAGTCTCGTGATAAGAATTTATTACAATACAAAATAAG
TACTACACAACTACAAAGAACCAAGCTTGTGATGTTCAGATGTTCGGATTATAAAAATTGAAGAATTTGATTCTCAAAAAAATA
TTGTAATTATCAACGATTGTGTGGGCATACAATAGTTCATATAAGAATACATTACGTGGCCAACTACTATATCAGCAACATCAG
TCGTTTATATGGAAATTTATTAAAGAGATCCTCACGCACTTTAATCTCGCGGTTCTGTTTAATCTTTTTTGTACAGAAGAGAGT
TTGTATTCGATTGGCATTGACAAAAATGCTGTGTCCTTCAAATAGCACGTATACCTTCTCACGGTAGCACTCTAACGTTGTAAG
CTATCGAGTTCTTGCTGTTAAACGAAGAAAACTCATATTCTTCATGTGCAATATTTAAAATTGAGAAAAAAATAAAAGCGATTG
CGTTGTGCTCTCATACAATCCTTCTACGTCTTCTCTAACGCGCTTGCGTTTATCCGTGTCACTGACTGCTAGAAGTTGGTATGC
ATCTGGGAGTTAGGTTTTATGTAGGCCTTAGGATCAACACCGTCTTACAACTGATATATGAAATACGCACAATAAATACTGTCA

GGATCATGTAAAGGGAAGAGAGAGAAACTGGCGCAAACCGACTCTGCTGATTCAGGCGATCGTTGGGTAGAGCGGACAACTGGA
GATATGAAGAAGAAGATATGGGACATACTACAGAGATATACGCATCGATGTAGAGATATTTTAAATGAAAACAGAGAGATGCAC
ATCTTTTTTTAACATGAACACTCTCAGCTCATAAAAATAACGTTAAATATCATATAATTCTAAAATAGAGGGCCTGCATTTCTT
GCTATAAATAACATAAAGAAATATACATGCGTGTACGCTTTTACATCACATCTGCTACTCACAACACAGAAATGATTTAGGACT
GCTACATGATTGGTTGGAACACAATACTTATAATAGTGTGATATATATTGTGAACACTTATAATATATCCTCTAGTGTTAGCTT
GGGTCTTTTTATTTTTTTTCTTAATTTCTTCTTTCTTCACTTCCTTTTTCACTTTTCGAATGTTTAAATAAAATTTGCTCAATT
TGAATTGCATCGTGACATATAATAAATTATTGTTAATAATATTTGGATTTTTTAAAATTATATATAGAGTATTCTTCGCTTTAA
ATATATATTCTCATTCATTTGCTTCATATAATATCGTGTATCAAAACATTTTTCTATATATATTCTTTCACTCTCTTAATACTA
CAAAATATCTTTCACTATTTTTTCTTGCTCGTACACATTAATAATCACCCTCACTCACTTGCTATATTCCATACTTTTATTTAT
GGACTTCTCCGCTGTTCGTCCCGGTGTGGGGAAGGCCTGGCAGTTCATTGCATAGAGTACCGTACACACAGTTATCTATAATAT
ATTATTATAATGTCAGGTTGTCATTTAAAGCTTCAGCACGACAGGTATCTAATATAGTAAATATAATTTTCGTATGAGCTGATA
TTACGATTCGTAGACTAGAAATCGACTTGCGCAAAACTGCTGAATGGATAAGTAAAACACTGCTATATTTCACTTCGCTTGAAA
CACATATGAATTTTTACTTTCGAATATATAATGAGTTGCTACAGCCGGTAAAAGTTAACATCGGTATAGAAGTTACAAGCAGTA
CTATTCTTTCGAATAGCGAAAATGTACAACTGTGATTGAGCGAATGTAATAAGAGTGCTACACGTTATCTATATATTTTCTAAA
CCCCATTCTTGGCGTAATTTAATTTAAAAGCTTAAGTACTCCACGATAGAATGATATAAGACAGGTTCTTTGTTAAAGTAGTCA
GTCTAGTTTAGCACGGTGAGTAATAGTAGGCGAGAAGTAGAGAAGAAGCTATGAAGCATAGTGAGGTAGAAGTTCACTCACGTT
ATGGCGATTTTATGCAAGTACCACGGTTAGGTACGTAGTAGTGGTTCTAAGTACTCAGTAGTTGGTCACGTCATTATCTACGAG
TAGTCAGTATTTAGTAGTAGGCTGGTAGCTCTGTACTAAGTATAGTGGTACGTACTCAAGTATGTCACGTTCAGCACGCCAGTA
TGCTCATTTAGTCTATAGTAGCTCAGTACCTAGTTCACAGTAGTTCACGAGACGATGTAGTAGTAAGTATATTAGGAGGCGTAT
AGCACAGTATGGTGGTGTAGTAAGCTGATCGCGCTGGCGTATGCTCAGTAGTATTGTTAGCTGTGATAATAGTAGGCATCGTTA
GTCGTTGGGCCGAGTAGTTTCTTACCTCGTAGTTCTCAGTGTACGCTTTTTCATTATGGTGGTAGTTACACGTACCGGTTGATG
TAGTATGTATGCTTAGCTAGTTCAGGTACCGTACGATAGTCTTAGTAGTAGTATCTAGTTTTACTATGCTCACGTCAAGTATCT
ATCTTATCAGTATGCGTTCAGTGGAGTAGTAGTACGCTCAGTCGCTTAGCTAAGTTCGCTTCATTCTCACTGTAGGTTAGTAGT
AGTATAGTAGTGTAGTAGTAGTATAGTAGTAGTAGTAGTAGTGTAGTAGTAGTAGTAGTAGTAAGTAGTAGTAGCGAATAGTAG
TAGTAGTCAGAAAGTAGCAGATAGTAGGAAGTAGTAGTAGAAGTAGTAAGTAGTAGTTCAGTAAGTAGTAGTAGTAAGTATAGT
AGTAGTAGTAAGTAGTAGTAGTAGTAAGTAGAGTAGTAGAAGTGGTAGTAAGTGGTAGAGTGGTAGTAGTGGTAGTAGTGTAGT
AGTGGTAGAAGTGGTAGTAGTGGTAGTAGTGGGTAGTAGTATGTAGTAGTATAGTAGTAGTTAGTAGTAGTTAGGTAGTAGTAG
TGGTGGTGGTGGTAGTAGTGTAGTGGTGTAGTGGGTGGTAGTAACTAGTAGTAGTAGTAGTAGTAAGCTAAGGTACGTAGTAGA
TAAGTAATAGTAGTAGTACAGAACGAAGAAGAAGAAAAAGTAGTAGTAATAAGTAGTAGTGTAATACGTAGGAGTAGTAGATGT
AGTAAGTAGTAGTGGTATAGTAGTAGTAGTGGTAGTAATAGTAGTAGTGTAAGTAGTAGTAAGTAGAAGGTATAGAAGTAGTGA
AGTAGTAGAAGTAGTAGAAAGATAGTAGAAGTAGTACGAAGTAGTAGTAGTAGTGTAGATTAGTATACGTAAGTATAGTAGTAG
TAGTATTAGTAGTAGTATGTAGTAGTATAGGTAGTAGTAGTAGTAGATAAGTAGTAGTAGTAAGTAGTGAAGTGGTAGTTAAGT
GGTAGTAGTTGGTATGGTATAAGTGGTAGTAGTGGTAGTAGTGGTAGAAGTGAGTAGGGTAGTAGTGGTAGTAGTAGTAGTTAG
TAGTGTTAGTAGTAGTTAGTAGTTAGTAGTAGATAGTAAGTAGTAGATGTGGATGGTAGCTCAGTGCGGTCGTGGTGGGTAGGT
GGGATGGTATTAAATTAATAGTGTAAAGGATCAGTATAAGAGTCAGTAAAGTAGATGTAGTAGTAGTAGTAGTAAGTAAGAAGA
AGAATGAACAAATAGTAGTAATAGTAGATGTGAGTATTTTAGTTTTAGTGGTATAAATGGTCAATAGACACAGGATTTAGTTAG
TAAATTTGCCAAGGCCAGTGAATATTTTATGAATTTTTATAAGTTTTAAAACTATGTTATAGAAATATTAAATTTTAAATAACA
AGTTTGATTAATTATATGAACGGTACCTAGAAATATTATTATATTATTACCTAAAGGTTTAGATAGACATAATTAAGAACATGA
ACACTTGGTCACATGTATGTTTATTG
>m141122_014521_42134_c100732202550000001823141405141560_s1_p0/159514/2078_9962
AGGAATTGCTACGGAGTGTTAAAGCAAGAGAGTTCTCTGGGTTGGTCCGGATCGCATCTGTTCCCGTGCGACTAGTAGGTTCAA
TGGGCGAAATCAGTCACACATGGGGTTGAGCTCAACTGGTCCTGGATGGTCCCTCTCATCCCAGTACCTAACGATTGGGGATTC
CACCCGTCGGACCCCCCTTTGCCTGGCGGATGGGTTGCCCGACCAGGCAAACTTCAGTCCAGCCGCCCCCCTCATCCAGTCATT
GATGAATCGCCCACACTTTATCTTAGGGGGTTGTCCCTGCCATATATCTCGACGCTGCAGTATCAAGGACAGAATAAGTGTACA
CCTCAACATGCGATGAATGCACGAAGGTCTTTATGCGGAGATCACACAGCGAATCAGCTTGCATCCCAGAAACTCCTAAGGCGA
AATTTTTAGGCCAACGATCGAAAAGGTTGTCGTTGATTACGTTCGCCGAAGTGTGACGACAGTGCCCAAGGCCCCACGAAGATC
CTGGTGGCCCTCCGAGAGAAATACAACCCTGACTGCACCAATCCTGGCTTGTGGCTGGTAGGTATCCGGGGAATCAGATTTAGT
AGGATCGCTCCAACTAGGAGGGTGGCGTACCATACGCCATTGTGTGCTCGCACTATTACACGAGTGGGTCGAGCGAAAACATGG
ACAGCCATCATCCAAAGGAGGCCCTGCGGACTTCTCATCCAATCAAATTGTGTGCGATACGAACCGTCCCTCGGCAAAATTGTG
TCTGAGAAGGAAGCAATTTGATAGCCGTCAACTTCACTGACTTCTGTGAAAGACATGTCATCCATCAAAAGCTTCTCTGTAGTT
TCAGGCCTCAAGAAAATGGGTAAATAGAGGCGTGACAAGATTTTGAAGGGACAGTTAACGAGAACAAGCTACAAGCCATAAGAG
TAAGTGGCAGAAGAGCTTGTCCCGCGTCTATGCGGCCTCACCGAAACCACAGAAAGACGTCGACCGGTCACATCACTCCCTATT
GCGGTGGTGATACGTATGACATGAAAGCGGCCCTTCCCAGTAAAAATGGTTGCTCCGTCTCACAGACGGACACGATATGTCCCA
TCCAGAACCGACGCTTACGCACCCAGAACAGGCAGCAGGGTCGCCAGAGGATGCGCGCTTCGACATTTTTTCACTCGCGTATCT
CTCGAAGAGAGTCGTGGGACGGATCCAAGGGTGCAGGCTCGTAGATGTCTAGGGAAAGATCACTTGACACTTTAACCTCTACAG
CTTACAGAGTCGGCGAGATTTGACACCCAGCATACTAGTCCCTGTAGAAGAGGTCTTCCCTGCGATTCGAAAGATGCAGGAGTG
GGGGTTCCTAGGACCCAAGCGTCAGAGGAAGGACCCTATGAGTCCAAATGAAATATGTCTGGGAACCGTATTGGTTAAGATGGC
ACTAGGATGGAACGGAGGTCCGCAAGGGTCTGGAACGGCAGAACACCTCCGCCATATATGCTCGTAGTCGTGGCGGGTGCTGAT

TCTGTTTGTCCTTTCCTAATCTCTATTTTGCTTCAATGTACCACACGCTAGGGGCGATTTCGCTGTTTAATAAAACATGACTGT
ATACAAAATGACTCTATAAGAACATCGCTTGTGAGAGAAAGAAGAAAGTTCACGTTCGTCCCCTTCAATTTTTTTTTTTTACAC
AACAATGACTAAGGCTACATTCTTCAATCACTAGAGGGTGTGGCCAGACATACACGGTCTGGGGTTCAAATAGGATGCCATCTC
AGGCTTATTCTTCTAGTGTCCGACTCGGTATAGACCTGGGGGTACATAACCCAACGCCAATAAATAAGGGCGCAAGGCTCAAGC
CAGCTCCTACACTTCGGACATACCGTCCGGGGGGTTTCATCACCAATAGGGCACAAGGGCTCAACAGCTAGTCTAACTGGACAT
ACATGGTCCAAGGGTTTAAACGATAGCGACACAAGGCTCAAAGGCCAACTCCTAGCCTACCGCTCCCTAAAAATTGGTTGACCT
ATACGTGGTCCTCAAATACAGCCATCCTATTCATTCCGTCTTCCTACTTATAATAGCCAGACCATTGAACGTGAGTTTTACTAG
GTTTAACGTTGTTTAAGTTATCTTATAAACGTCCAGGGCCCCGTGGAACGCTTGAACCGCCAGGTTTGCATAAGATACCTCAAC
TAATCCCTCCTAAGCTGGGCCTCTAGACATTGGACCACCTAAACTAGCGCTCAGAGACAATACACGATCCCGACGCCCCCTCGA
ATCCGTGGTATTGAATCTTTAAAGCGCCAGGACCGTCTGCACACTGACCTCGCGTGGCTCTCGCGACTGCGTTACATTTCATGA
CGTCCCGACGCCCGCCCCCCCGCCCGCGCCCGGCCCCCACCCCCCACCCCCCCCCCCCCCCCACCCCCGCCCCCCCCCCCCC
ACCCCACCACCACCCCCCCCCCCCCCCACCCACCCCCCCCACCCCACCCCACCCCCCCCCCCCCCCCCCCCCCCCGCCCCCCAC
CGCCCCCCCCCCCACCACCCACCCCCCCCCCCCACCCACACGCACCCCCCACCCCCCCCCACCACCACCCCCCCCCCCCCCCCA
CCACACCCGACACCCCCCACCCCACACCCACCCCCCACCACCACACCACCCACGCACCACACCCCACCCCCCCCCCACCACCCC
CCAACACCAGCAACAACCCAAACCCACACAACCACCCCACCCCCCCCCCCCACCCCCCCCCCCCACCACCCCCCACCCCCCCCA
CCCCCACACACCACCCCACCCCACCCCCCACACACCCCCACCCCACACCGCCAACCCCACCCGCCCCCCCCCCCCCAATACTCTA
AAAAATCTATACGTCCAGCTGTTGGACACCCCTAATGACAGAGTTCAATAAACGGTTGAGTTAACATTGGTTAGTCCAATAGAC
CGGGCGTCAGACCGAAAGTGGGTCTCCAGATAAGGATTTAAATACTTCTATACATATTTCTCTAGTGGTCCCGACCATTGGACT
GGACCCAATGTTTCACTTGGACTTCGTGAGGTAGGTAAACTTCAGTGAAATTTAGAACAAAAACAAATCACGAATATAATTAGA
AACATTGTGTGACAAAGTCAACGTGATCCGAATACGGATTAAAATTTCCTTCGACGCATCCACGTCCATCCAAACTATGAATAC
CTTACAAAGTGAAAGGAGCCCTAACAAAATACAAAGCCCAGGCCTAGGACTTCCGTTATGATATTTGCTTCAGAATTAGCCTCT
TGAAGCTGGATCCAGATAAGAGGAGGAGGTTCATGTTCTTGTCTGGAGCCAGCCATGTCACACGGCTCATCAACAAGCTGGCTC
CTACGACAAGGCATCAAACCCGCTCCTCTCGCCTTTTGCAAGCCTCTTCGCTCCATCTCATCCCTCTACGTGTGAAGTGTGCCC
TCCAAAGCTGAATTTCAGCGACTCACGGTCCTTGCTACCCTCTCACTGCCTCCTGGGAAGTTGGGCCATCAGCTGCTCAGAAGG
GCCTTGATCGACAGCCTCCCGAGCCACAATCATGATGCGCTCATCTAACTCATAGGTTGTCCACCAACTTCTTTTGTTTGACTG
TCCTTTCTGGACAAGCTTCCTCAAGCTGAGCTGACGTTCAGGTAGCCATCCTCACGCACTCCAGGCCCTCAATTGCGAGCAGAG
CTCATTGGCAAGGGCTTCCATAGCTAGCCCTTCCCCACTGATCTAACGCATCCTCCTAGCTCATCTGAGTTAATCTCCAGCTTC
ACTACGCCATTCTTGCCCCCAGATTCCGCGTCTCCTATGGGCCAAGCAGGGGGGATCTGGGAACAAACCGAAACGTTAGAAAAT
GTACAATCAATAATGATTGGTCCAAATAATTCAGAGGGGACCATGCGTGCTCACCCACGGTGGTTTGATGAAGAACTGGTTGGG
TTACTGAAAAGGCCCATCTTGGCTGGTGCTATCACTTGAGCTGAAGAGTGGACCCTGGTAGCCCACCACGCCGCAACGAAGCCT
GGCCAACAGTTCTGTAGCCAGGCGAAGCCATATCCTGGCCCCAACTTTTTTTTGGAGCTCTTAGCATACAAGTTGGTCCTGATC
TGTTGGGGAGTATTGAATGGCCTTCCAGACAGAGTTGGTGATCCACCTTCTGCTAGGTGATCAGGGCCCGGACCACCTCATCCT
CTATTTCCCGTTGGTCATCCCAAGCCGTGACACATCTTCATCTCTAGTTCCGTAAGGATCAAGTCCTTCTCGGGGAACGGCTGG
TTGACGGAGAAGTATGGGCGCTCCCGCAAGCTGGTGCGTGATGATCGCGCCTCTCCCAGAGGAGTGCTCTCAACCAGAAGCAAG
ATCCCTTGGTCTGGCCTCTTGGCCGCTTTGAGGACTCGGGGATGACGAGTCCGCTGCCTCTTTCTTCGCCTTCCAGCTTTAGAG
GGCCTCATGTTCCAGTTAATACTTTGGAGGAGCAGATTGGGACAGGCTTTTGTCGATAGGTGCCGTCGGCAATGGAGGTTGCTG
CTGACTCTCCTCGGGATCCCTTTGCGAATACTGTTTGTGAGCACCCCCGAGGATCGCCTCTATGACCTTTCTTCTTGGTCGGCC
TTGAGACCCTCTTCGCCGAAGCTTGGCTACGGCTGACCTCCGACCTCCGATCATGTTCCTCCATTTTGCCAAAGGGTTGGACTC
CTCTCCGGATCCACTGAGATAGATAGAATCAGGAACTAATCAGTCAAAATCATGAATAGGGGATACGAAGTCAAAAAATCAGGG
AAATACTAGTCTAAAGTAGCCTCCCAGGAGCCCGAACCCTTATCAAAGAACGGGCATGACTTAGACTCTAGTGTGAAAGCTGAA
TTCCAATCCCCCATGTCATCAGGTCTAACAGATCCACGCAATGGCGGCCAAAGGAAGCCATGCGTCAAAAATAGTCTTCGACGA
ATACATCGGTGATACTAATTGCCTTATTCCTAGCGTAGCCCTCAAAGGGCAAGGGACATAGTAAAAATAGAGGGATTTTTACTT
GCCTTGATGATAGCTCTGGGAGACACAGTGTTCGGCTGCCTCCCTCAAGAAGGGTGCTGCCAGGTCTTCAGCACACTTCGCCCC
TCTCCGCTGACGGCCTGCTCTCATTTGCCTTCTTCACATCTGCCTTGGTCTGGCCTCCACAACCTCGCATGTGGATAGGTCTAG
CTCCTCCTCAGGGGATGGTCCCTCTCAACATTGCAGCCCTCAGAATCTCAGGGGGATTCTCATCGATGCTAGTTACAGAGGCAA
GCTCCCAACCAGACGAAGGGTTGTCGTGGTCAGAGCCCCTACGATCCAGTTCCTCATGCTACATCGGTAGCTCAGTATTTCTTG
AGCCGATCAGGAGTGGTGTTGGTGAGTTGAGTCTGAGGGCTACGGTACCTGTTTCTAGGACATGATGGTCAGCAAACAAAACTA
AGGAGACGTACAGTGATAAGGCAGAAAGAGTGACTTACCACGATTGGATGGTCCGCAACAATGTGGGGTTCTTCTCGCAGGGGA
CCTAAATGTCATACAACCAGTAATGTTGACATCTAGGGTGCTTCCAAGTGTTGACAGTAGCGAATGGATACCCATGGGATTTCG
TCTGCTAAACGACATCTCGCGTCTTGTCCTTGAATTCAGCTGCGAATCCAGGCTATAAACAATATAAACCTCAAAGAGGTAGGC
GTTGGGATCCTTCGATGCGTAAAATATACGCCATCCCGCTTCAGCCAGCTTGTACACTTAGGGCAGAGCTGGAATGATCTGACT
CCTATGATTTAAGGACATGGTGAAGTAATCATTGCGCGGGAGCTCTCGCGTGATTGTGGCCGTATGCTGCGGTCCAAATCGCCC
ATCGCCGATATGGGCCTTCTCCTCCTTCCGGTTAACTAGTTGGTAAATCCCTGGAGTCGACTCCACTCAGGTGTTCTCCCAGGG
TAATCTAAATTCGGATAGTTTGCAAAACTCATTCTTCTCCCCGTCCACTCCCTCCCATGCATACCTTGCCAGTGGCGCAATCTG
TGCCTATCCAGAATACCCCGCAATGACGAAGTCTTCCGGATGAAGATACGGCGCGACTCATGATCAGCCCCAGGACGCTAGAGT
GACAAGAAAATGAGAACCAACGCAGTTAGGCACCAAAACCGAAGAAATTTTTTGGTTAAGGTTGCCGGGTTCTCCTAAAACTGC
TTGGAGAATATCCCCTCCAGACCCGTCCAGGAACTCAATACAGACCCCCAAGTCTTCAGTTGGGAATAAAGATTTTCTACTCAC
CCAGACGCCTAAGAGGCGCCTGCACTTTATTGGGACAACCCGACTAATAGCAAGTTATTATTACGAAAATCTATGGTCTCATAC

TTACCGCGTCTTATTTCATGCACCAGCCTACACGGCTGGTCCCACCGCCTAAATATTTCACGTCGCCAGAGGAATAACAGTGAC
GACAGAAAAAAACTAAAAGGAACAACAGATGAGAAATGACAAAAACTCCTCTTGATGGGATATTCAAACTTGAAATTCGTAGAG
TCTCAGGTGACAGCGTCGGCAAACAACTAGTCTTGAATCTGAGAAGATGATGTAGAGGCCAGTTCTCTCCGTGGTTGAATCAGG
ACACAGCTATCGTTCTATTATGACTATATGGAATGTGTAAAGCTTTGAACGCGTTTGGTCGGGTATTTATGAGGGAGAAATCCC
TCATTTGATCAACGTCCCACAGTGGGGATCCCCAAGAGTATCCCTCAACGGTTCGGATGGCTGCAGAGGCTTAATTACGCTAGT
CGAACATAATCATGGACTTCATCACGCACGATTGCACCTGCTACATTCCATGGACGGTCCATGTCGACAACTCTCAAGGTTATC
CCATACTAACGGTGTCCAAATGATGTAGAGCAGCATTAAATACCGCCACTCGAGTACCCCAATACGATCCTCCTCCCCCCCTTC
CTCCTCCTACAACGGAGACGGTCTTGAGACGATTGTGCTAACACCCATAGGAACTAGCATTTGGATATCAAGAAGATTCTTCTG
CCAGTCCTTTAGAGGCGGCGAGTAGGCATACAACGACCATCAACTTGGAGGCACATGTCTAAGACGTTGAGAATTTTGAAAGAC
CGAGATTTGTCCAAATGGCCCAAAGTAAGAAATCGACTTCTGGTAAACAACTGGATATTTGCAATTGTCCGGCGCGGGAGCAAC
GGCAGCTCTCAACCATGCAACCACATGGATGAAGCCTTGGGGGCGCGGACGTAAATGTCTCCTCGTTCTCTCCACCGCAACATA
TGCTCCGGGGGGACCATCGTGTCAGAGGGCCCTCTTAAAGAGGTCCAACGCATCCTTGATAGCGCAATCATCACCTCCGCGAAC
ACAACAACCTTCTGATGGCACCGCGAGCTATCGACAAGAGCCGGGACTAAGCTGCAGGCCTTCGACTCATGCTCCATCCCTTCC
GCGGATCCTCGACCATATCCTGCTATCCCGTCCGGGACGGCCAATGAGCGTGCAGACTAACTAGCTGAGGGAGAGTAGACTATT
ATTGGGTGTCTCATAGGAGATATTGTCAGGCTGTCACCAATGTTCGATCTTGGCGATATGAAACCCAAGTCTTGGTGCACGACA
CCACCGCGCCCTTGGGAGTAAACCGGCCGACATATTATGAACCAGCCGACAGTACACCTGCTAGACGGGGCAACGCGCTGACCT
TCCTTGTGTTGCATGTCCGCTGGGAAAATTGAAAGTTGGTCTCAGCTCACTCACTTTGTAGAGCGGGTAGCCACGGAATGCACA
CGTTCTGCTAGGAAATGAATCTGTGCACTACTCTGCAGAGTCCTGTCTCCAGGCATCTCCTCCTTAAAGCCC
>m141122_014521_42134_c100732202550000001823141405141560_s1_p0/160154/0_7654
GAAGAGAGATCCCTATAAGATTAAATAGATGGAAATAGGTTCAAAAAATTAGAGCTGTTAGAGATATATTGAGTGATTTTATGA
AATATCTTTCGAGCCCTACAGCTGCTCTAGGAGATTGGCCTGAAAGGTCAATTTTTGTTTTGGCTGAATAGGACCCCAATTCAT
GGTCAATAACAGCAACTGATAATATTTGAGATGGGAAAAGTTCAAAAAAATTAAGAGCTTTTACCAGAAGTAAATATGAGTGAA
TTACGAAATCTGCTTCGAGCCCTAGCAAGCTGCCTATGAGATTGGCTGAAAGGTCAATCTTTTTGATTTTGGTGAATTAGGACC
CCAATTCATGGTCAAAACAGCCCTATATAATTATTATTAGAGATGGAAAGAGGATCAAAAAATATTAAAGCCTTGTTAGAGAGA
AATAGGAGGGAATTACGGAGATCGCTTCGGAGCCCTAGGCAGCGCCTAAAGGAATTTGCTGAAAGTCAATTTTTTTGTTGGTGA
AATTAGGACCCCAAATTCATGGTCAATAACAAGCCCTATATAATGTACTTATTGAGAATGGGGAAAAAGGTTCAAAAAAAATTA
GAGCTTTTTAGAGAGAAACATATGGAGTGAATTTACGAAATCTCTTTCACGGAGCCCTAGGCAGCTGCTAGGAGATGTTGCCTG
AAAGGTCAATTTTTTGTTGGTTGAATAGGACCCCAATTCATGTCAATAACAGCCCTATAAGTAGATATTGTATTGAGATGGAAA
TGGATCAATAAAATTAGAGCTTGTTATGAGAGAAATATTGAAAGTGAGATATGAAAATAATCTTCCGGGCCCTAGGCAGCTGCC
TAAGGAGATTTGGCCTGAAAAGGTCAATTTTTTTTGGTGAATTAGGAATCCCAGATTCCATGGTCAATAACAGCCCCTATATAA
TATTTTGGATGGGAAAAGGTTCAAAAAATAGAGCTTTTAGAGAGAAATATGAGTGAATTACTAAATCCCGCTTCGGAGCCTAAG
CAAGCGGCCTAGGAGATTTGGCCCGAAAGGTCAATTTTTGTTGTTGTGGTTGAATTAGGACCCCAAATTCATGGTCCGATAACA
GTCCCCTTATAGATATTATTGAAGATGGGAAAAGGATCAAAAAATATTAAAGCTTGTTAGAGAGAAATATGATGGAATTTACGA
GATTCGCTTCGGAGCCCCTAGGCATTGCGGCCCTAGGATGATTTGGCCTGAAAGGTCAATTTTTTTGTTGGATTGAATTAGACC
TCCAATGTTCCATGGTCAAAACAGCCCTATATAATATTATTGAGATGGGAAAGGTTCAAAAAAATTAGAGCTTTTTAGAGAGAA
ATATGATGAATTACGAAATGCTCTTCGGAGCCCTAGGCAAGCTGCCTATGGAGATTTGGCCTGAAAGGTCAATTTTTTTGTTTG
GGTTGAGATTAGGACCCAAATTCATGTCAATAACAGCCCTATATAATATTTTGAGATGGGAAATGGATCAAAAATTAGAGCAGT
TAAGATGAGAAATATGAGTGAATTACGAGATGCTTCGGAGCCCTAGTCAGCTGCCTTGGAGATTTGCATGAAAGGGTCAATTTT
TTTGTTTGGCGGAATTAAGGACCCAATTCCATGTCAATGTAATTAGCCATATATAATATTATTGAGATGGGAAAATCTTCAAAA
ACATATTAGAGCTTGGTTAGGGAGAAAATATTGACTGAATTACGAGATCGCTACGGATGCCCTAGGCAGTCTGCCTTAGGAGAT
TCTGGGCCTGAAAGGTCAATTTTTTTTGTTTGGTTGACTTTAGGACCCCAATTCTATGGTCAATATACAGCCCTATATAATATT
ATTAGATGGGAAAGGTCAAAAAAATTAAAGCTTGTGTAGAGAAGAAATATGAGTGAATTAACGAGATTGCTTCGGAGCCCTAGG
CAGCTGCCTAGGAGATTGGCCTGAAAGGTCAATTTTTTTTTTTTGTTTGGTTGAATTAGGAACCCAATTCATGGTCAATAACAG
CCCTATATAATATTATTGAGATGGGAAAGAGGATCAAAAAAATTAAAGCTTTGTTAGAGAGAAATTATGAAGTGAATTACGAGA
TCGCTTCGGAGCCTAAGGCCATGCTGCCTAGGATGATTTGGCCTGAAAGGTCAAATTTTGTTGTTTTGGTTGAATTAGGACCTC
CAATTCATGCAATAACAGCCCTAATATTAGATATTATTGAGATGGGAAATGATCAAAAAAATTAGAAGCTTGTTAGAGAGAAAT
TTGAGTGAATTTACAGATCGGCTACGGAGCCCTAGGCTGCTGCCTAGGACGATTTGGCCTGAAAGGCAATTTTTTTTGTTTGGT
TGGAATAGGACCCCAATTCATGGTCAAAACTTAACAGCCCTATATAATATTATTGAATGGGAAAAAGGACAAAAAAATTAATGC
TTGTGTAGAGAGAAATATGAGGGAATTACGAGATCGCTTCGGAGCCCTAGCAGCTGCACTACGGAGATTTGGCCTGAAAGGTTC
AATTTTTTTGTTTGGGTGGAATAGACCAAATTCATGGTCAAGTTAACAGCCCTATTAATTTATTGAGATGGGAGAATGGACAAT
TAAAAATTAGAGGCATGTTAGAGAGAAATATGAGTGAATTAACGAGATCTGGCTTCGGAGCCCTAGGCAGCTGCCTTGGAGATT
GCCTGAAAGGTCAATTTTTTTTGTTTGGTTTGAATTAGGCACCCCAATTCATGGTCAATAATAGCCCCTATATAATATTATGAG
ATGGGATAAAGGTTCAAAAATTAGAGCTTGTTAAGAGAGAAAATAGAGTGAATTTACGTAAATCGCTTTTGGAGCCCTAGGCAG
CTGCCTAGGAGATTTGGCCTGAAAGGTCCAATATTTTTGTTTGTTGAATGTAGGACCCCAATTCAAGGTCCCAACAACAGCCCC
TATATAATAATTATTGAGAATGGGAAAAGGTTCAAAAAAATTAGATGCTTTTAGAGAGAAATATGATGAATTACGAAAATCGCT
GTCGGATGCCTAAGGCACTGCCTAAGAGATTTGGCCATGAAAGGTAAAAAGTTTTTGTTTGGTTGAATTAGGTATCACCAATTC
ATGGTCATAACAGCCTATATAAATATTATTGAATTTGAAATGGTTCAAAAAAATAGAGTTGTAGAGAGAAATATTGAGTGAATT
ACGAAATCCGCTCCGGAGCTCCTAGGCAGCGGCCTAGGAGATTTGGACTGAAAGGTCAATTTTTTTGTTTGGTGAATTAGGCCC

CAATTGCGATGGCAATAACAGCCCTATATATATATTATTGAGATGGTAAAAGGATCAAAAAATTAGAGCTGTTAGAGAGAAATA
TGGAGTATATTACGAGAACGCTTCGAGCCATAGGCAGGAGAATTGCCTATAGGTCAATTTTTTTGTTGGTTGAATTAAGGACCC
CATTCATGGTCAATAAATAGCCCTAATAAATTATTTGAATGGGATAAGTTTCAAAAAATTAGAAGCTTGTTAGAGGAAAAATAT
GAGTGAATTACGAAATCGCTTGACCGGAGCCCTAGGCAGGCGGCCTAGGAAATTTGGCCTGAAAGGTCATTTTTTATGTTTGGT
TTGAATTAGGTCCCCAATTCATGGTCATTAACGAGCCTATTAATATTATTGAGATGGGAAAATGTTCAAAAAAATTAGAGCTTG
TTAGAGATAGTAATATTGAGTGAAATATGAAATCGCTTCGGCCGCTAGGCAGCTGCCTAGGAGAATTTGGCCAGAAAGGCAAGT
TTTTGTTTGGTTGAATTAGGACCCCCAATCATATTCAAATAACAGCCCTATATAATATTAGTTGAGATGGGAAACAGGTCAAAA
AATAGAGCTTGTTACGTGGAGAAATATGACGAATTACGAGATCACCTTCGAGCCCTAGGCAGCTGCCTAGAGATTTGGCCTGAA
AGGTCAATGTTTTTGTTTGGTTGAATCTAGGACCTCCAATTCCATGGTCCAATAAACAGCCCTATATAATATTATTGAGATGGG
AAATGGAGTCAAGAAAAATAGAGCTGTTAGAGAGAATAAGAGTGAATTACGAGATCGCTTTGGAGCCCTAGGCAGCTGCCTAGG
AGATTTGCTCTGAAAGGTCAATTTTTTTGTTTGGCTGAATTGGACCCCAATCTATGGTCCAATAATAGCCCTATATTAATATTA
TTGAGACTGGGATAAAATCTTCAAAAAAATAGAGCTTGTTAGGGAGAAATATGACTGAATTACGAGATCGCTACGGACCTTAGG
CAGCGCGCAAGGAGTATTCGCCTGAAAGGTCAATTTTTTTGTTTATGGTTGACTTAGGACCCCAATTCCATGGTCAATAACAGG
CCTATATAATTATTGAGATGAAAAGGATCAAAAAATTAAAGCTTGTTAGAGGATATAGTGAATTACGAATGCTTCGAGCCCGAG
CAGCTGCCTAGGAGAATTTGCTGAAAAAGTCCAATTTTTTGTTGGTTGAATTAAGGACCGCCAATTCATGGTCAATAACAGCCC
TATATATTATATTATTGAGATGGGATAAGATCAAAAAATTAAAGCTGTTAGAGAGAAATATGAGTGAATTACGAGAATCGCTTC
GGACCCTAGGCAGCTGCCTAGGAAATTTGGCCTGAAAGCAATTTTTTTGTTGGTGAATTAGGACCCAATTCATCCAATAACAGC
CCTATATACGATATTATTGAGATGGGAAATTGGATCAAAAAAATAGAGCTTGTTTAGAGAGAAATTTGAGTGGATATACGGATC
GCTACCCGAGCCCTAGCTGCTGCCTAAGGAGATTTGGCCGAAAGGTCAATTTTTTTTTGGTTTGAATTAGGACCCCAATCATG
GCAAAACAGCCGCTATATGAATAATTATGTGAGATGGGATAAAAGGATCAGAAAAAAGTAAAGCTGTTAAGGAAATATGAGAAT
TCGAGATTCGCTTCGGCCTAGGCAGCTTGCCTAGGTAATTGCCTGAAAGGTCAATTTTTTGTTTGGTGAATTAGGGACCCCAAT
TCATGTCAATAACAGCCCTATATTAATATATTGAGATGGAAATGGATCAAAAAAATTAGGAGCTATGTTGAGAGAAATATGAGT
GAATTACAATCGGCTTCCGGATCCCTAGGCAGCTGCCTTGGAGATTGCCTGAAAGGTCAATTTTTTGTTTTGTGAATTAGGACC
CCCAATTCATCTGGTCAAATAGCCCTTATATATAAATATGAGATGGGAAAAGTTTCAATAAAAAGAGCTTTGTTAGAGAGAAA
TAATGGAGTGAATTACGAAATCGCTTGGAAGCCCTAAGGCAGCTGCCTAGGAGAGTTGGAACCTGAAAGGTCAATTTTTTTTGT
TGGTTGAATGGACCCCAATTCAAAGTCAACAACAGCGCCTATATAAATATTATTGAGATGGGAAAAGTTCAAAAAAATTAGAGC
CTTTTAGAGAGAATATGAAGTGAATTACGAAATCTGCTTCGAGCCCGTAGGCAGCTGCCTAGGAGATTGCCTGAAAGGTAAATT
TTTGTTGTGAATTTAGACACCAATGCATGGTCAATAAAAGCCCTATATAATGATTCTATTGAGATTGTAAATGGTTCAATAAAA
ATAGAGTTGTTAGGAGAAATATAACGTGAATTTACGAAATCGCTCCGGAGCCCTAGCAGCGGCCTAGGAGATTTGGACTGAAAG
GTCAATTTTTGTTTGGTTGAATTATGACCCCAATTCATGGTCAAAACAGCCTATATACAATTATTGAGATGGAAATGGATCAAA
AAAATTAGAAGCTTGTTAGAGAGAAATATGAGTGAATTACGAGAACGCAATCGAGCATAGGCAAGGAGATTGGCCGTGATAGGT
CAATTTTTTTGTTTGTGAATTAGGACCCCAATTCATGGCATATAAATACCCTATATTAATATTATTGAGAGGAAAGGTTCAAAT
AAAATAGAGCTTGTTAAGAATTAATATGAGTGAATTACAAATCGCTGCCGGAGCCCTAGGCACGGCCTATGGGAATTGGCCTGA
AAGGTCAATTTTTTTTGTTTGGTTGAATTAGGTCCCCAATCCATGTCCATTTAACAGCCCTATAGTAAATATTTATTGAGATGG
GAAAATGTGTCAAAAAAATTAGAGCTTGTTGAGATATCGAATATGAAGTGAATTATGAAATCGCGTCGGAGCCTAGGCAGCTTG
CCTAGAGGATTTGCCAGAAAGGTCAATTTTTTTTGTTGGTGAATAGGACCCCAATTCATATTCAATAACAGCCTATATAATATA
TTGAGATGGGAAAAGGTTCAAAAAAATAGAGCTTGTTAGGGAGAAAATGACTGAATTGACGAATCTGACTCTCGGAGCCCTAAG
CAGCTGCCTTAAGAGATTTTGGCCTGAAAGGTCATATTTTTGTTGGTTGAATTAGACCTCAACAATTTCATTGGCAATAACAGC
CCTATAGGTATATATTATTGAGATGGGAAAAGGATCAAGAAAATTAGAGCTTTTAGAGAGAAAAAGAATTAAAATTACGAAATC
GCTTCGGAGCTAGGCAACTGCCTATGGAGATTGGCCTGAAAGGTCAATTTTTTTGTTTGGTTGAATTAGGACCCCAATTCCATG
GTCAATAACAGCCCTATATAATATTATATTGAGATGGGAAAAGTTCGAAGAAAAATTAGAGCTTGTAGAGATATATCATGAGTG
AATTTATGTAAATAGTCTTCGAGCCCTAGCAGCTTGCCTACGGAGATTTTGGCCTGATAAGGCATATTTTGTTTGGTTGAATTA
GGACCCCAATTCATGTCAATAACAGCCATGTATAATATTTATTGAAGCATGGAAAAGTCAAAAAATAGAGCTTTTACAGAGAAT
ATGAAGTGAATTACAATCGGCTTCGAGCCCTAGGCAAGCTGCCAGAGTATTGGCCTGAAAGGTCAATTTTTTTTTGGTTGAAAG
TTAGGACCCCCCAATTCATGTCAATAACAGCCCTATTATAGATATTATTGAATGGGAAAGGATCAAAAAAATTAAACTTGTTAG
AGTAAATAATGAGAATTACAAGATCCCGCTTTCGGAGCCCTAGGCAGTGCGGCCTGAGAGATGTGGCCGAAAGGCAATTTTTTT
TGTTTGGTTGAATTAGGACCCCAAATTCATGGTCATAACCAGCCCTAATATAATATTATTGAAATGGGAAAAGGTTCAAAAAAA
TAGAGCTTTTAGAAACGAGAAATATGAGTGAATTACGAAATCTTCTTCGGTAGCCCTAGGTCAGCTGCCTAGGAGATTTTGCC
TGAAAGGTCAATTTTTTTGTTGTTAATTAGGACCTCCAAATTTCATGGTCAATAACAGCCCTATATTAATATTATTGAGATTGG
AAATGGATCAAAAAAATTAGAGCTTGTTAGAGAAAATATGAGTGAATTATGAAATATCTTCGAGCCTAGCAGCTGCCCTAGGAG
ATTGGCTGAAAGGTCAATTTTTTTGTTTGGATTTATTAGGACCCAAATTCATGGTCCAATAACACCCTATAGTAATAATTTAGA
ATGAAATAGTTCAAAAAAATAGAAGCTTTTAGAGAGAAAATGAGTGAATTACGAATCGCTTCGGAGCCCTAAGCACGCGCCTAG
GAGATTGCCG
>m141122_014521_42134_c100732202550000001823141405141560_s1_p0/160975/2695_12773
AAATAAAAAATTCATGTAAAAATAAAGTACCTTGTAATAAAATAATAAACCTATAGTCAAAATCGAGAATATATTAGGGCATTT
TATGAGGTTAGCAAATTTACAATAACTTTTACCCTAAAATTATTGCCTCACCACTATATCCTATTCCATGAATGGACCATGTGA
CTTCACTTTTAATCGATCATTAAATAAGGCCCGTTAAAGTGCCGCAGGACATTAGTCAAATTCTGTGACATTAACTGAAATGTT
ATGAGTTTTGTGTTTAAACCTTAATAGATGAAAAGTTATAGGAATCATACAACATTTAATTTAATTCGTCTTGGGCCAATACTC

TATTTTAACCTTGCATAAATATAAAGCCTTAACAGCCACCAAAAAAATAGCACGAGTATACCATAAACAATCATTATTCCCGCC
ATAGTTTGCGGTGTCCATTCCCATAATTGATGAATCGAACTTCAAATGGAGATGACAAAATTTAATTGAGTTTAATCCCTCCTC
CAATTCCAGTTATTCTCGATATTCTAAAGATACCAATAATTCATATCCTTATTACTTTAATTCTCCGGTTATATTTTTTAAATA
TTCTATTATCCGATATATCTTCAAGAAGTAATCACTACCAGTTGTTTTGATAATTTTAGAGGTTATACAATCTACCCGACACAC
GCCACTTCCAAACCCCCCCCGCCCCCCCGCGGCCCCTACAAACCTGGCCACATCTCCCTTCTAAATAGCCCTCCAAATCCCTAA
TATAATTAGTTTGATCTACACCTAGACCCAAGGATGCTGGGAGGGTTCATAATGTTTATTATGTATTTAATGATGTATATGCAT
TTTATGAATTATATATAATACGGACATAAATGTACAATGGGTTTATTTTAATTATGAGGGCATTTTGTATTGGGACCCGTTTAG
GCGCGTAACCACTACCACTGTACCTACTTTTACTTCAACTTACCGAGAAAAATAACTTGTACGTTACTACCTACACATACTGAA
CACCACTTACTCGACATACTACCCCGCTAAACCTGACGCTTCAAAAGCCACATAAGCATACTAACCACTACCACTTTAAACAAC
TTACCACTGGTTATACCTTCACTACTTACCACTTATTTCAACATACTAGCTACTACCACTACGACGTACTACCACTACCTCTAC
CCCTCATACTACTACGCATGGAAAAAACCACTACTATTAACACTACACAACTAGCCCTCAAACCCGGCCAAATTACCAAAATGC
CCTCTCACACTACCACAATATACCACAATACACATAACACTACACCCACTATACCACTGTCTAACCCCATACATCTATTCACCT
GACATACATTATAACCACTATATAACCTACGTCACACCCATAATACCACATGTACCTACTACTTAATATACCCTTTTAAATCTT
ACAACTCATACACACTAACTCACACCATACAATTACCACATGTACACACCCCTCTCAGACCCACTATTACACTCAAACGTGACG
TACTCATATACCACATACAAAACACTATCTCTAATCAGTATACCTTGGGCATTACAACCGAGGATGACACTTGACGTCACAGCA
CTACTTTACTACCAAAGGGGGGGCGAGCGGGAGCGGAGAGCGCGCGGGGTGACCACGTGACGCGGAGCGGGAGCGGACGAGCGC
GGGCGGTTAACAAGATCTACTTAACTTACTACTACTACTAAACACTACTTACCCCCTTTACCAAAACTTATTCACCCACTTTAA
CAAAAACCACTCAATGTACTCACTTACACAACTAACAACCTACCACTTCCACACCTAACCTTGACTACACTTACGACTTAACTA
CCTACTACTCACCGACTCATAAACGACTTACATTATTACATAACTTGGGAATTACAAACTACACTCTACCCAGTAACTTAAAAA
ATTACGACTCTACTACTGTCATCACCTACCTACTTTACTAATTACTCACTTTCACACATATAATGAACGCCGAACCACCACGTA
CCACACTACACACCTACACCATACGTATTACCACTAACACTAATTCACACACCACAATACACCACCACTACACGATACCACCTA
CTACTCACCACACTACTTTTACTTATCTTATAATAAGAAACATACCTTAAACTTAACACAACTTACCACTTCACCCAAGACAAG
AAATAATAACCATCTGACTTACTCCTACATAAACCATAATCTTACATTCCACACTTCATCCTACATTAATAGACCATTACTATC
TTAACTACCACAACACAAACTACTAAACCATTACAAATACCACTATACCACTACACAACTATTAAATTGTACCACTTTCCATCC
ACTACACACCTTCTATCACAATGATCATCACAATCACACCACTACACTACCACTACCACTACCACTACCACTACCACTACCACA
CCACTACCACTACACACCACTACCAACTACCACTACTACCCTACTACCACTACCTACACTATACCACTACCACTACTGTACCAC
TATACCACTACCAACCACTCCACCAACTACTACCAGACTACTACCCTACTACCACTACTACACTATCAAATACTACTACTACAC
TACATACTTATCTATACTAAGACTTAAGAGCACTCACCACTCATCCAGACCATTACACACACACACTACTACTCCATACTGTTG
GTAAACAAACTCACCTAGCATAAACAACATAAATACGCCCATCACACACATACTTATCCAGTTTTCCCTTAGGTTTAGTTATTT
TTTACCTACCCAACTTAATTTTCTCTTTAATCCCAAAATATAGTTTCATAGTTTTTTCATTTTATTTTCCTTGTTTTCCCGATC
AGAGCGCTTTTAGCGCTGTCGGTCCAAGACCCGAAGTCTTATCTTGAATCTCTACCTAAATTCACAACTACCACTACCAAATCT
AATTAACCATTGGACTATGCACCACATCATTGACGACATTAACTACTTTACCATCAACCAAAATTCACACAACCGATACTACAC
TTTACTAAAATAATAACTCTCAACTCGAGCGGCAAAAAAACTCCGATCACGCGACGTCGTTTTACATAAGACGTATCAACTATG
ACACACTCGCAGGTACCCGGTTACAGAAGCGCTGGGAATAAATGTACCAACTTTCCTCATCTCACGCATCTGTCCACCTAGCCT
TAGCGCGGGACATGTTCAGCGCGGGCCATACGTATACCACTACCTACCTACTCTCTAACCTTGCCTACTAAACTCAGGCGTTTA
CCAAGACACTTCACTCGTAGCGATCGCCTGGAGGCAATACAGACTGATTTACATACTGAATACTACATTACATACTACTATATC
GAATACTACTTTACTTACAATAATGAAACTACTACTATCTACTTAACTATGCATGTACTGACTACGGAGCGTACTTAAACTACT
ACATTAACCTGCCCAATACGTAGCCACTCCACTCGTACTGTCATGAGACCACGTAGACCACGGGGGGTAGCGCGGAAAATTGGT
AAACTTACCTTACGCCCGTTACGCGGCAATATAACCAGCGTGATAATATATCTTAACTACTCTGACCTTTGACAACTACGTGGA
ATGCCGGTACGTATACATACTTATGTGAGACAAACCTACGTACTATACCACACGTGTAACGACTACTAACCACTACCACTGCCA
ACTGCCTACTGCCCTCCCACTACCACTACCACTCCTTTAGAAGCGCACCTTACGCATAAACTCAAAATAGTACACCAACGACTT
ATTACCTTACATCGGCTCGGGAAGAGCTAGGCTATTTATCGATGCGGTATCTTATCTTAACTTTACCATCTCGAGCGACTGTTA
ACCACTTTTACCTAACTGGTTACCACACTGGGTAACTCTGTTGACTATACCGTAGACCAACTTGCTACTGACTAAGTAACAAAT
TCAGATAGCCAGAAAACTATATCGTGACTTATATGCGTTAACCGCTAACACGTACTGCAAGGCATACCAAGTTGCCACTGAGGA
CTTAGTTATACCAGCGATAGGGAATTTTAGTGGAGGGAACACGCTGGTCTTTTGCGCACGAGGACTTAGATGAGACATCTCTGG
ATTGATACGCGGACACCACACTCGAGACTACTACACGATACTTTATGCGAAGTACTGGTGAATATAAGTTTGGAGGGCTAGAGA
AATCAAGAGGGAAAGGAAAACTAAAGAACTAGTAGAAGTAGCAAAAGTGGAACCACGCGAGGAGGCGCAACACGTGACCACCAC
GCACCTTACGAGACTTCTACTATCTAGCGTACTACTGACCACTACTACTACCACATGACGACTACCACCACGACAAGCTGGACA
GATGCATCCGGACGTGTGTGAATCCAAACTCAGGTACAACATGAGCCATCACTCAGAGGTTAAGAATTCTACCTGCCTTTTTAG
CGCGTATACAATAGTTACTTTGATACCTTAGATAACTACATATCAATACTAGCGTAAGCGAAATATACTTGAAAACAGAGTCTA
TCCCATAGCGCTACGACTATAACCACTGGATCTTACTGCTATGAGCTATATCAGCTAACTCCTCACATGTAGAGCTTTTGATTC
CATCATACATGACTAGGTTGGAGAGAAAGCATCAACATCGTAACTTTCACTCTGACTTTAACTGTAAGCCCACATGTATCTGAA
ACCAACTACGTTTACTACTTGAAAATTCTCAGTACACACTACTTATGCGATCTTAACTACTACCTTCAGACGAACCAGTCCTCG
GTACTCTTGACACACATCTTCTTCCTCAAAGTACTCACTTGGTTTAGTATACAATCAGTGCAATACTCCACAGCTGTGGACTGT
TTGGTGTGAATTGTGTGTTGTGAACTTGTTGGGGTGGTTTATAGGTTGGGTAGATGGTTTATTTGGGGTTTTTGAATTGAGCGC
GATTTGGGGCAGTACGCACTCACCTTGCTTTAGGTACACACTCCTTACGGCAACTGGACTCGGACATCTACTTTAGCCACTTCG
ACGGTATCACCACTAACCGGGAAAACACGCCAACCTGGGAATCTACAAACCCACATACATTACCCATCCTACCTACTCGGGCTC
CCTGCACCACCACTACTACCACTACCAACTACTACTACCACTACTACTACCACTACACACTATGACACAAACTACTACCGAACT

ACACTACTACCAGCTATGTACTACACTACTACTACTACTAACACTACCACTACACCATACCACCCACCCGGGGCCTGTTTTGGG
TGCCTGCGGGCTACACCACCACTAACCACTCTACCACTACTACCACATACTACCACCTGACACTACCACTACCACTACCACTAC
CACTACCACTACCACTACCAACTACTACTACTACCTACTTCCTACTATACTACTACTAACTACTACTACTCCGTCGCCGCGCGC
GCGCCACCACACACCACCCCACTACTACTACTACTACACTACTCGTATAAAAGAATTTCTCCTCCGAAATTGTACTTACCAAAC
ATCATCGGGTACCGCTGCCTCATTTATTCTCTCCAATTCCCATGTGGCCCTCTTTTCCGTGCTTTTGCTCGCCCAAGGGGATTT
TAACCATAGGGGACACTTTTAGGACCTTAAATCCTTAGTTCCCCGTTCTAGACCCATACTGGTCATTCTTAATAAACCATGAAG
GGATCACTTTTGGACCTTAACTCCTTAGTTCTCCGTTCTAGAACCCATATGGTCATGTCTTCATAATAAAACTTATTTTATACG
TAGAGCCTCATAATCGAGCACATGGGATGGATCTGATATTATCTCAACATCGATACATGAAAAACGTTATGTGCGTTCAGCTAA
TGATTGGCGGTAAAGTCAAGTCGAATGCTAACATGCCCTACTTTGATCCTAATATCTCAAAAGGACCTATAACCCCGGCTTAAC
TTCCTTTCTTCCAAAATGCAATAAGCTCTCTTCATCGGAGAAACCTTTGAAAAGACTTGATCACTTACTGAAAACTCATCATTT
TGCCTCTTAACGTCAGATAGCTTCTTCTGAGGTGAACTTCAGTGCAATCATTCGTTGTCTTACTTTTTCTACTGCCTCAGTTGC
TTCTCTTACAGCGCTTAGGTCCTAAATAAATCGACTTTCACACAACTCGTCCAATGAAGTGGTGATCGGCAATTCTGGTCTATA
AGTGTCTCATAAGGACCACCATGCCCTATCGCAGCGCTTTGTTTTAACTATTGTTGTAAGAGAATTTCTATTAATGGAAAGATA
CTTGCTATATGATCTGAGAAGTCCTAAAGCACAGCCTCAGATATATCATCTAATATGTAATGGTTACATTTCCTAACATGACCA
TCGTTAGAATGAAAATGTCATACGTAAGTTTAATGTAGTGCCCCATTGCACCCTGTAACCTGCGACAAAGACTTGTGATGTGAT
ACTGGATCCCCTATTAGCAGAACTATCGTCTTGTCGGAACTCAGTGTATCTCACTATCTCTGCCACAGTAAAGCTCTGCATACT
GCTCAGACATTATATACTATCTTACTGGCAAGAAGTGAGCAGATTTTGTGATATATCTACTAATCCACCCAAACAGAGTCAGTG
CGGCTTTGTGTCCTTGGTAAGTCCTGTCACAAAGTCCATAGCTATATCTTCCCATTTCCATTCGGAATCTCTATGGTTTGCAAA
ACTTCCAGCGGGTCTTTTTATGTGTCAGCCTTACTTGTTACATGTAAAGGCATTTGCTACAATTTCTGCTATGTCTTTCTTCAT
TCCCAGGTCCACCAGTACATTGTCTTGACATCGTTGGTACATCTTGGTCGATCCCGGGTGAAATGAAATATGGAGTAGATGTGT
GCTTCTCTCATAATACTTTCTCTTAATTCACCACTTTCGGGCCACACATACTCGGTTTCTATATCGTACATGCCTTCTTTCTTG
TTACGGAGAAATCAGCATTTTCTGAGCTTTATTATTGCTGCCATTCATCTTAAGAAGAAACTCGTCCTCGTTGTTGTTTCTCCT
TTTATTTCCTCCATCAGCGAGTTGACTTGATTGTGCAGGTCAGCTAGTCTGTCCCAGTTATTTAACTCAATTCTGGAACCTCCT
TAATTATCATCTTGTAGTGTTGAAGCTAATCTCCTTGTTAAGTGTTGACAAACTCCGTAACTCCGCCTACTAGTGTGTCTGCCA
CGAACATTAGCCTTTCCGCGGGATGATACAAGAACTCACAATCTAGTCTTTTACTAACTCTAGTCACCCTTCCGTTCATCATAT
TTGAGCTCTCTTCTACGTTAAAGAAATACTTCAGACATTTTATGGTCTGTATAGATTTCGCACTTTTCTCCGTACGATAGTGCC
TCCAAATCTTTAGAGTAAAAACCACCGCTGCCCAATTCCTAAGTCTGGGTCAGATACCGTTGCTCGTATCTCCCTTTAGCTATC
GCGTCATATCCCCTTAGCTATCAAATCGAGGCATATGCACCACTCTTTAATCATCAAAAATGCATCCAAGTCTCTGCTTCAAAC
GCGTCGCAATATACTAACAAACTTGCCCTTATCATGTGGGACGCATAGAACTTAGGGCTGATTATGAGTTTGTCTTTTAACTTT
GGGAACTTTCTTCACATTTCTCCGTCCCATTCAAATTTTTGGTTGCTTCCATGTCCCAAATTTGTTAAGGGTGTAAGCATATTC
TGGAGAATCCCTTAACAAATCGTCTATTAGTATCCCTGCCCATGCCCAGCAAATACCTTTCACCTCCGTGGCACTCTTGGGTTT
GGGCCAGTCCTTCACGTGTTTCAACTTTACGGATCTACTTCCACCGCCATTTTTTAGACACACGTGTCCTAGGAATGCCACCTT
CTTCTACCCAGAATTCACATTTCTTTAACTTGGCATATAGTTGGATGACTTTTGAGTCTCCCAACGTCAGTCGCAGGTGCTCTT
CAGCTCGTCTTTCGTCCTTGAGTAATCAATATGTCATCTATAAACACCACAACGAACTTATCCATGTAGTCCTTAAATACTCTG
TTTCATAATATCCATGACAAGTTGCTGGTGGCGTCCGTTAAGCCAAAAGACATCACCAGAAACTCTAGTGTCCATATCTAGTTC
GACAAGTTCGTCTTTGGGATAAGTCTTCTTCTCTGTCTCTTAGCTGAGTGATACCACTGGCCCAAGTCTATTTTGAGAATACCG
TAGATCCTTATAGTGTGATTAAAAAGTCGATCTATTTCGAGGTATAGGTATTTTTTCTTGATCATCACTTTATTATAATTCTCG
ATAGTCAAAGCACATCCTCATACTTCCCGTCCTCGCTTTTTTTACAAACAACACTGGGCTCCCCAGGTGAATAACTTGGTTAGA
TGAACCCCTTGTCTAAAAGTCCTGCTCAGTTGCATTCTTTTAATTCCTTTATCTCGGTAGCCTTCTATATGAGCTTTCTGAGATT
GGCCCATTCTCAGGGTAGCCAATTCTATTTCAACCGATTTCCCTACTCAGAGGTACTCCCGTAGATATTCTAAAACATTCGGAA
TTCAACATACAATATGTACATCCTTTGGTTCAAGCTGCATCTCTCGTCTTTGTCCCACCACCACTACTAGAAAATGCTTGGAAA
ACCGCTATTGATTAAACTGTTGATGCTTTTAGAGTTGAGACTATCGGAATATGCGAGTGCGGCGAAACTGATCTCCTTTTGAAG
GTAGAAACTGCTCCCCGCCTTTTGCTTAACCTAGTCCATGCTTAAAACGTGAGCACCATAACATCCTTAAGGTCTAACTCATCA
AATCTACTGGTATTCCTTCCGTCGATTCCTAACTATAGCGCATACCACCCCTCTACTAATATGCATCTTTCCCCGGATGGTAAA
AACGTCAGTAGACAAACTTGATCATCTAAAAGGCCTTGCATCCTGTATCTTAAGCCTAATCATCATATTCCATAGAGACAAAGG
GAATGTGTAGCTCCTGAACTCAAACTAAAAACTATTGCATAGTTTTTAACAAACAGAGAATAGGACCTGACCGACCTAACCAGC
TTACCACTACTCAGTCTCGGTTTTCCCTCCAGTCAAATTGGAAGCAACAATCTCTGGATGGCACAAGCTTTTTGTCATATTTCT
TTTGGTCCTTCAACTTTGGGAATCCTTTTTATGTGTCCTTCTTCTCCACAATAGAAACAACCTTTCGTTTTATATCGGCATCCC
GTGATGCTCCTCTTGCAGATAGGGCACCTGGGGATATTCCACCATAAGGGCTTCTTATTCCCATTTCCTTCTGATGAGTCTTAT
TCTCTTGATGGTCCTTTTGAATTTTGACCCACCGTCATTTCACATTTGGTTCGTCTGATCATTGTTTTTCCTTTGGAATCCTCT
AACTGTGAACTTCCTTCCAGATTTCTTTCCATTCAGCCTGCTCAGCTTCCAAGTCCCCTCTCAATTGTTCTGCATACGGGTTAT
CCCCGGATCAACTATTACGTAATGGGCTTTCAATAGGTTTTCAGTTCCTTCAATGAATCTTTGAATTCTTAGCGTCTCAGTGGT
ACATGTTTCTGCGAACTAGCCATCCCATTCAAAACTTGGCATACTCCTATCACAGTCGATTCATTGATAAGCTCCGATGAATTC
GTCCACCCTTGTGCTGAATCGCAGGCACTGTAATACTTCTCATTGAACACTTTGGATAAACTCGTCCATGTCATAATGTTCACA
TCTCTAGTTTTTTCACTCTATCCCACCAGATGCGGGCGTCTTTCTTTTAGCAGCTTGAAGCGCAAGGACTCCGATCTTCGTTTC
CTGCCGCATGTGATTCTAGGTATAAACTCCTCGATCGAAGCATTCTTCGACTTCATTTGGTTCAGATCTGCCTTCAAAGTTCGG
TGGTTGCTGCTTCATTTAACATTCGTACACGCCCGTCATACAGGTTCTGCCATAACTGTATAGTTGCTGCCTAGAGGAATGATT
TCTGTGCCACATTGTTGTTGTTGCCCTGAGTTTATTGGAACTCTTCTTCCCTTCTCCTTAACTCTGCCCTAAGGCGGGCATT

>m141122_014521_42134_c100732202550000001823141405141560_s1_p0/161024/0_7076
AGGAGAGATCAACTTTTGTTCATGGCCCCTCGCATATTGCTTAGTATCTCACAATCAAAACATTGCTTGAATTGATATTCAGAG
AATCCAAAGATGTCTGAACGGGGTACCCATTTGGTGAATTAGTTATTTCACTAATTTCTCCGAAATGTATTACCCTCCCGTGTT
CCACTCGTCTCGATATTTTCCCGCCAAAGAGATGCTCATGGGTAAATGAATATATTGGAGACGAAACGGGCCTTGAAAGTGAAC
ATTTCAATAAAAATTCCCCTTTTTAATGGAAATTTTTGCAAGTTTTTTTCCCTATAGTCGTACAGTCTCCCAGTATTGTTGACT
GTATCAACCCCTCTACAAACCTCGGATTTGCACGAAATTTTATACATGGAACCCTAAGACATAGGGATAGCAAACTTTTGATTC
CATGTCCCTTCCCGCATATGAGTGAATTACGAGATCGCTTCCGGAGCCCTAGGCAGCCTGCCTAGGAGATTATGCCTGAAAGTC
ATTTTCTTGTTTGGTTGAATTACGAACCCCCCAATCATGGTCAATAATAGGCCCTATATAATATAATTAATATGGAAAGGTTCA
AAAAAATTAGAGCTTTCTAGAGAGAAATATGAAGTGAATTACGAGATCGCTTTGGAGCCCAAGGCAGCGCCTTGAGATTTGGCC
TGAAAGGTCAATTTTTTGTTTGGTTGAATTAGGACCCGGCCAATTCATGTCAATAATAGCCCTATATAATATTATTGAGATGGG
AAGAAGGTTCAAAAAAATTAGAGCTTGAAAGAGAGAAATATGAGTGAATTACGCGATCCGCCTCGGAGGGCCCCTAGGCAGCTG
CTAGGAGATTTGGGCCCTGAAAGGTCATTTTGTGATTGGTTGATTAGGACCCCAATTCATGGTCATAATAGCCCTATAAAATAT
TACTTTGAGATGGGAAAAAGATTAAAAAAAAATTAAAGAGGCTTGTTAGAGAGAAATATGAGTGAACTTACGAGATCGCTTCGG
CAGCCCTAGGCCGGCTGCCTAGGAGAAATTTGGCCTGAAAGGTCAATTTTTTGTTTGGTTGAATTAGAACCAATTCATGGTCT
ATAAATACCCTATATAATTATTACTTGAGTTGGAAAAGGTTAAAAAAAATTAGGGCTTGTAAGAGAAGAAAATATGAGTGAATT
ACGAAAATCGCTTTCGGAGGGCCCTAGAGCAGCCTGGCCTAGGAGATTAGGCCTGGAAAGGTCAATTTTTTTTGTTTGGCCTGA
ATTAGGACCCCAATTCATGGTCAACAATAGCCTTAAGTAGTAATATTATTAGATGGGAAAAAGTTTCAAAAAAATTAGAGCTTG
TTACAGAGAAATATGAGTGACAATTACGAGATCGCTTCCGGAGCCCTAGGGGCAGTTGCCTAGGAGATTTGGCCTGAAAAGGTC
AATTTTTTGTTTGGTTGAATTAGGACCCAATTCATGGTTCCAATAATACCCCTATAACAATATTTTCAGATGGGAAAAGCTTC
AAAAAAAATTAGAGCTTGTTAAGAGAGAAATATGAGTGATATTACGAGATCGCTTCGGAGCCTAGGCCAGGCAGGGCCTTGGAG
AGTCTGGCCCTGAAAGGTGAGATTTTTTTTTGGTTGAATTTAGGATCCAATTCATGGTCAATAATAGCCCTATATAATATTGA
TGGATGGGAGAAGTTCAAAAAATATTCAGAGCTTGTTAGGGAGAAAATATGAGTGAATTAGCGAAATCGCTTCGGAGCTAGGCA
GCCTGGGCCTTGGAGATTTGGCCCTGAAAGGTCAATTTTTTTGTTTGGTTGAATTGGAACCCCAATTCAATGTTCAATAATAGG
CCCTACATAATACTTATTTAGATGGGAAAAAGGTTCAAAAAAATTAGAGCTTGTAAGAGAGAAATATGAGTGAATTACGCGATC
TGCCTCGGAGCCCCTAGGCAGCAGCCTAACGGAGATTTGTCCTGAAAGGTCAATTTTGTTTGATTGGTTGAATAGGACCCAATT
CATGGTCAAATAATAGCCTATAAAATATTATTGAGATGGAAAACTTCAACAGAAATTAGAGCTTGGTAGGAGAAATTATGAGTG
AATTACGAAATCGCGCTTCCCGGAAAAAGCCCTAGGGCTGGCGTAGAGATTTGGCCTGAAAAGGTCATTTTTGTTTTGGTTTGA
ATTAGGAACGCAAATCCATGGTCAATAATAGCCTATATAATATTATAGAGATGGGAAAAGGTTCCAAAAAGAATTAGAGCTTGT
CTAGAGAGAAATATGAGTGAATTAGCGAAATGCCGCTTTCGGATGCCGTAAGGCCAGCCATGCCTAGGAGAATTTGGCCTGAAA
GGTCAATTTTTTTGTTTGGTTGAATTAGGAAAGCGCAATTCATGGTCAATAATAGCCCTATATAATATTTTGAAATGGGAAAAG
ATTAAAAAAAAATTATAGCTTGTAAAAGAGAAATATGAGTGATTACGAGAATCGGCTTCGGACCTAAGGCAGCCGCTTAGGAGAA
TTTTGGCCTGAAAGTGCAATTTTTTTGTTTGACTGAATTAGGACCCCAAATTCATGGTCAAATAAAGGCCCGTATATAATATTA
TTGCGAAGGGAAAAGGTTCAAAAAAATTAGAGCTTGTTTAGAAGAAGAAATAGTGATTGAATTACGAATCGCTTCGGAGCCCGC
TAAGGCAGCATTGCGTAGTAAGATTTGGGCCCCTGAAAGGTCATTTTTTTGTTTGGTTTGAATTAGGACACCATTCATGGTAAA
AAATAGCCCTATACTAATATTTTTGAGATGAACAAAAGATTATAAAAGATTGAGCTTGTTAGAGAGAAATATGAGTGAATTCAC
GAGATCGGCTTCGGAGCCTAGGCAAGCTGGCCTAAGGAGATTTGGCCCTGAAAGGTAATTTTTTTGTTTGGCCTAAATTAGGAA
AGCCCCCAATTCCATGGTGCAACAATAGCCCCTATATAATAATTATTGATGCATGGGAAAAGGTCAAAAAAATTAGAGCTTGTT
ACGAGAGAAATATGGAGTGAATTACGAGATCGCTTGGAGCCCCTAGGCAGTTGGCCTAGGAGATTTGGCCTGAAAGGTCCATTT
TTTTTGTTTGTTGAATTAGGACCCAATTCATGGTCAATAAGTAGCCCTATATATAAATATTATATCCGATGGGAAAAACTCAAA
AAATTAGAAGGCTTGTTAGAGAGAAATATGAGTTGAATTACGAGATAGCTTTCGAGCAACCTAGGCTGCTGCATAGGAGATTAA
GGCCCTGAAAGGTCAATTTTTTTGTTTGTTTAAATAAAGACGCCAATTCATGGTCCAATAATATCCCCATATAATATTTTGAT
ATGGGAAAAGGTTCAAAAAAATTAGAGCATGTTAGAGAGAAATATGAGTGAATTACGAGTTCGCTTCGGAGCCCTAGGCAGCTG
CCTAGGAGATTAGGCCCTGAAAGGTCAATTTTTTTTGTTTGGTTGAATGAGGAAACCAAATTCATGGTCAATAATAGCCTATAT
AAATTAATTAGATGGTAAAAAGGTTCCAAAAAAATTAGAGCTTTGTTAGCGAAAAATATGGAGTGAATTCGAGATCGCTTCGGT
GGCCTCTAGGGCAGTTGGCCTAGGAGATTTGGCCCTGAAAGGTCAATTTTTTTGTTTGGTTTGAATTAGGACCCAATTCAAAAA
ATTGGTTCCCAAAATAATAGCCCTTATATATCATATTATTCGAGATGGGAAAATTTTTATATAAAAAATAAGCAGATTGTGTTA
GAGTGAAATATGAGTGAATTACGAGAATCCGCTTCGGAGCCCTAAGGCAGCGGCCTTGAGATCTGGCTGAAAGGTGAAATTTTT
TTGTTTGGTTAATTAAAGGAACACAATTCATGGTCAATAATAGCCCTATATAATATTATTGAGATGGGAAAAAGTTTAAAAAAA
AATTTAAGTTGTTACAGAGAAATATGAGTGAATTACGAGATCCGCTTCCGGAGCCTAGGAGCCTGGCCTAGGAGATTTTGCCTG
AAAGGTGCCAATTTTTTTCTGTTTGGCTGAATTAGGACGCCAATTCATGGTCAATAAACCACCACCCTACCCCCGAAGCCGCACC
ACCACCCACACACCCACAACACATACAGATACATCATACTTGCGATGGGACCACAACGTGTTCAACACACACTTAGAGCTTGTT
AGAAAGAAATATGGAGTGCAATTCGGAATCAGCTTCCGGAGTCCTAAGGGAGCTGGCCTAAGGAGATTTGGCCTGATAGGTCAT
TATGTTTGTTTGTTGAATTAGGACCCCAATTCAAGGTCAATAATTGCTATAATAATATATTATTGAGATGGAAAAGCTGCAAAA
AATTTGAGCTTGTTAGCATGAAATAGTAGTGAATTACGAAATCGCTTCGGAGCCTAGACAGGTGCCTAGGAGATAAGGCCCCTG
AAAGGTAAATTTTTTTGTTTGGTTGAAATTAGGACAAATTCATAGGTCTAATAGCCCTATATAATATTATTGAGATGGTAAAAG
GTTCAAAAAATTTGAGAGCTTGTTAGAGAGAAATTATGACGTGAATTACGAGATCGCTTCCGGAGCCCTAGGCAGGCTGCCTAAC
GAGAATTTGCCTGAAAGGTTAATTTTTTTGTTTGGTTGAATAGGACCCCAAATTCTCGAGTCATAACAGGCCTATATAAATATT
ATTGAGATGGGAATGTTTAAAACATAAGAGACTTGATTAGGAGTGAGTGTTACGACATCATATTGGAGCCACTAGGCGCAGCTT

GGAAGATCTGGCATGAAGGTGAATTTTTTTAATATGATGAATTAGAGATCCCAATTCATAGTCAATAATAGCCCTAATATTAA
TTATTGAGATGGGAAAAGGTTCAAAAATATTATGAGCTTGGTTAGGGGAAAAATATGAGTAGATTACGAAATCAGCTTCGGAGC
CCTAGGCAGCTGCTGGAGATTTTGGCTGAAAGGTCATTTATTTTTGTTTGGGATTGAATTAGGACCGAATTTCATGGTCAAATA
ATAGCCCTATATATATTATTGAGATGGGAAAGGTTCAAAAATAGAGACATATCTTGAGGTATGGGTGTTATAGTCGTTCGGATC
CCTAAGGCAGCGCCTAGAGGAGATCTGGCCTGAAGCGAATTTTTTTTTTGTTGAATTAGGACCCTATTACCGTGTCAATAATAG
CCCTATATAATTATTTGATGGGAGTTAAAAATATTAGCTTGTTAGAAAAATATGAGTGCATTACGAATCGCATCGGAAGCCTAG
CAAGCTGCATAGGAGATTTGGCCTGAAAGGTGGAATTTTTTCTGTTTTGGTGAATTGACCCATTCTGGTCATATCGGCCTTGTA
AATAATTTTTTTTATGAGATGGGAAAGGTTCAAATGAGCTTGTTAGAGAGAATATGAGTGAATTACGAAAATCGTTCGGAGCC
TAAGGCAGCTTCCTGGACTCATGGACCTGAGGTCAATTTTTTTGCTTTGGTTGATATGTTCCAATACATGGTGCAATAATAGCC
CCTAATATAATATTATTGAGATGGAAAGGTCAAAAAATTTACACTTCCTTAGAGAGATTGAGTGAAATTCATATCGCTCGGAGC
AACTAAGGCCAGCTGCCTAGGTGATTTGGCCCGGAAAAGGTCAATTTTTTTGTTTGGTTGAATTAGGTCCCCAAGTCATGGTCA
ATAATAACCCTATATAATATTATTAGATATAGGGAAAAGGTTCAAAAAATTAGAGCTTCTTAGAGAGAATGATGAATTCGATAT
CGTTTCGGAGCCCTAGCAGCTGACGTAGGAGATTTGTTCGGAAAGGTCAATTTTTTTGTTTGGTTGAATTATGACCCCAATTCA
TAGGCAAATAATAGCCCTATAGCTATATTATTGAGATGGGAAAAGGTTCAAAAAATTAGAGCTTGTTAGGAGAAATATGGTGAA
TTACGAATCACTCCCGATCTAGCAGCTGCTAGGAGATTTGGCCTGAAGGTCAATTTTTATGTTTGGTTGAATTGGACCCGCAAT
TCATGGTCAAATAATAGCCCTAATATATATTATTGATTGGGAAAAGTTCAAATAGGCTTGTTGAGAGAATATGGGTGTTCAATG
TTTCGGAAGCGCTGGCAGCTGACTAGGAGATATGGACACTGAAAGTGAAATTTTTTTGTTTGGTTGTTAGGACCCCATTCTGGT
CATCATAGTATATGAATATTAATAGGATGGGCAAAGGTTCAATTAGAGCTTGTTAGATGAAATTGAGTGAATTACGAATCAGCT
TCAGGATCCCCTAGGACTGCCTAGGAGTTTGTTTCGGAAGGTCAATTTTTTTGTTGGTTGAATTAGTCCACAATTCATGGTCTA
TGCTATAATATATTTATGTATGGGAGGTCAAAATAGGCTTGTTGAAGAATTGTGATACGAATTAGCTCCGGAGCCCTAGCAAGA
CATGCCTAGGAGATTTGGCACTGAAAGTCTTTTTTGTTTGGTAGAATTAGGACCGCAATTCATGGTCAAGTAATGCCCCTATAA
ACTATTATTGATATGGGAAAAGCGTTCAAAAAAATAGAGCTTGTTAGAGAGAAATATGAGGGAATTACCGAAATCGTTTCGGAG
CCCTAAGCAGCTGCTAAGGGATTTGGCTGAAAGGTCATTTTTTGTTTGGTTGATTAGGAACCGCTATCATGAGTCAATATAGCC
CTATATACATTTATTGTTTGGGAATGTTCAAATTGAGCTTGTTGAGAGAAATATGAGTGAATTACGAAATCGGGCTTCGGAGCT
CTAAGCAGCTGCCTAGGAGATTTGGCCCTGAAAGGTCAATTTTTTTGTTTTGGTTGAATTAGAATTCCGATACATGGTCATACA
TAGCCTATATAATATTATTCGAGAATGGGTACAAAGGGTCCAAAAGAATTTAGAGCTTCTTAGAGAGAAATATGAGTGAATTAC
ATATCGCTTCGGAGAACCTAGGCAGCTGCCTAGGAGATTTTGGACCGGAAAGGTCAATTTTTTTGTTTGGTTGAATTAGGTCGC
CCAAAGTCATGGTCCCAATA
>m141122_014521_42134_c100732202550000001823141405141560_s1_p0/161832/0_8540
AAGGGAGATGAAGTTTATTGTCGATCAATTTGCATCAGTTAGTTTTATCTTAAAGAGCACGCAGCATATTGAAATACTCACTTC
CCAGTCGCGCTAAGCTGTGGCTTCCCCTGGTGGATTGGATATACGGACTCGAGTCGCTTAGAGACTGTGAGAAGTTTTAATTTA
AATACAGATTGAGATACGTCCCGCTATGAGCTGTTTCTTCTATCGAGCAGTATATTCGATACCACATTGACACGCTGAATTGAA
ACTATGTTCCTGGGACATAGTCATGTCTTAGGAACCATAGAGAGCGTTTATATTTAAAGGAATACGAGATTGATCGTTATGTTT
TCTTAAAGAGCCAGTTTAGAGGTTGATTGACTCAATCGATCTAATTGAATGCTGGACTATTATGTCTGAACTATTGTTACCTGG
ACGTACACCAGGAGTCTATCAAAACGCTTAGGAGAGTTAAGCTTTAATATATTGATCGTTGTTTTTCTTATAAAAGAGCGCGAA
CACGCTTGATAAGGGAAATTAACGAAGAACAAAATTTTCAAATTAGTTCCTGGATATAACCAGGTCCATAAAACCTTAGAAGTT
AATTTAAATTGAGTTGATCGTTGTTTTTCTGGTAAAGTGCACAGGGAGAGTAGGATGGGATTTAAACAATTAAAGGCAGTCTAC
ATGAAGTGGTTGGATCAAACTAGAAGTTCCTCGACATAGAGAGGTCATTGGAAAGAATCGTAATTGTTGAAGTTAATTGTACAA
TCATACTTGAGTACGTTCATGAATTATTGCGTGATAAAGGAGCACGAGATAAATTTGATATAAAATTCATGGCGAACTAAGTTT
CGAACCTAGTTGCCGGGACATAACAAATCATAAATCCTAGAAAGGAATTATTTTAATCATCTTCGTCTGCTTGGGTTTTTGGTC
TGTAAAGAGAACGAGATATTGGAAAAGGTTAACGCGAACTAGTTTTCAACTAACGTTCCTGGACATAACCGAGGTCATAAAACT
TAGAAGTTCATTACTCAATTGATCGTTGTTTTTCCTTGTAACAGAGCAGCGTGAGATACTGGGAAGTTGAGATTGTAGAAAAAA
TTGGAAGGCGAACTAAGTTCCAAAACGTAAAAGTTATCGCTAGGTATATTAACCAGAGTCATCGAAATTAGTAGTGAATTAGGT
TTATTGAGGATAAATGAGGTTGATCGTTTGTTATTTCTTAAAGAGCAGCCCTGAGATATTTGGATAAAATATTATCGCGAACAA
GTTTTTAAACTATGCTTCCTGGACATAACCAGGGTCATAAACGTAGAAGTTAAGTTTAAATCAATTGATCGGTTTTTCTAAAGA
GCATGATGATATTGATAAAATTAATCGCGAATAAGTTTTTCCAAACTAAGTTTCTGGACATAAACCCAGGTCATAAACTTAGA
GTTAATTTAAATAATTGATCGTTGTTTTTCTTAAAGAGGAGAGACATTGATTAAAAATTAAGCGACAAAATTTTCAAAATTAA
GTTCCTGGATATAACACAGGTCATAAAACTATAGAAGTTAAATTATATAATTGATTGATCGTCTGTTTTTCTTAAAGTGCACAA
GATATTGATCAAAAATTAATGCGAACTACCTAAGTTTCCAACATAAGTTCCTCGACCATAACCAGGTCATAAACTTAGAAGTTA
ATTTAAATCAATTGATCGTCTGTTTTTCTTAAAGCGCGAGATATTGATAAAAATTACGCGAACTAAAGTTCCGGGACATAACCC
AGGTCATAAAACCTAGAAGGTTAATTAAATCAATTGATCATACTGTTTTTTCATAAAGAGAACGAGATAATTGATTAAAAGTTA
ACGCGAGCTATGTTTTCAACTAGTCCCTGGACATAACCAGGTCATGAAAACTTAGAAGTTAATTCAGAATCATTGATCGTTGTT
TTTCTTAAGGAGCCCGAGAAATTGCGATAAACAACTAAGCGAACTAAGTCTTCAAACATAATGTGTTCTGGACATAACCAGGTC
AAAAAATTAGAAATTAATTTAAATCCAATTGATCGTTGGTCTTTCTTAAGAGAGCACGATATGTTGAAAAAATTAACGAGAACT
AAGTTTTAAACTATGTTCCTGATATAACCAGGTCATCAAAACTTAGAAGTTAATTTAAAAAAATCATTGACTGTTGTGTGTTCG
TAAAGAGCACGAGATATTGATACAAAATCAACGCGAACTAACGTTTTCAAACTATGTCCTGGAAAAAACCAGGATCATAAAACT
TGAACGTATTAATTCAATTGCGTCCGTTGTTTTTCTAAAGAGCATGAGTATATTGATAAAAATATTAATCGGAACAAAATAAGT
TCTCACACTAGTTCCCTGGACATAACCAGGTCATAAATCTGTAGAATCCAATTTAAATCAAATGATCGTTTGGTTTTTCTTAAA

GAGCCCGAAGACATTGATGAAAAATTACGCGAACTAACAGGTTTTCAATTAAGTTCTGGATATAACCAGAGTCATAAAACTTAG
AAGTTAAATTTAAATTGATTGATCGTTTGTTTTTCTTTAAAGAGCACAAGATGTTGATAAAAAATTAACGCCACTAAGTTTTC
AAACTAAGTTCCTGGACATATACCATGTCATAAAACTTAGAAGTTCGATTTAAATCAATAGATCGTTGTTTTTCTTAAAGGACA
ACGAGATAATTGGATATAAAAAATAAAAATTAACGGCGAACTAAGTTTTCAAGCTAAGTTCCTAGGACATAACAGGTCATAAAA
TTAGAAATTATATTTAAAATCTAATTGTCTCGTTGTCTTTTCTTAAAGAGCACGAATGTGGATAAAATTCACGCGACTAAGTTT
TAAAACTATGTTGCCTGGATAGTACCAGGTCAATAAAACTTAAGAAGTTAATTTAAATTGAATTGATCGTATTGTTTTTACTTA
AAGAGCACAAGATATTTGATAAAAATTACTGCGAACCTAAATTTTCAAACTGAAGTTCCTGGACATAACCTGGTCAATAAAACT
TAGAAAGTCATTTAAATCAATTGATCGTTGTTTTTCTTAAGGAGCACGAGATAATTGATAAAATTACCGCGAACTAAGTTTTC
AAACTAATCCTGGACGTACCAAGATCATAAATTAGAATAATTAAATCAATTGATCGTTGTCTTTCTTAAAGAGCACTGAGATGT
TTTAAAAATTAAACGCAACTAGTTTTAAAATATGTTCTTGGATAGAACCAGGTCATAAAACTTAGGAAGTTGTAATTTAATCGA
TTGATCGTTGTGTTTCTTAAAGAGCACTGAGATATTGATAAAAATCAAGCGCGAACTAAGTTTTCAACTACTGTTCTGGACAAT
AACCATGTCATTAAACTTAGAATTCAATTTAAATCAAGTTGATCGTTGTTTTTCTTAAGGTTAGCACGAGATATTGAATAAAAA
ATTAACTGCGACTAAGTTTAAACTAAGTTCCTGGATGTAACCAGGTATAAAAATCTAAGAAATTAATTTAAATCAATTGATCGT
TGTCTTTTCTTAAAGAGACGAGATGTTGTAAAAATTAACACGAATAAGTTTTAAAACTATGTTCTTGGATATAACCAGGTCATA
AAATTAGAAGTTAATTTATAATCGATTGATCGTTGTTTTTCTTAAATTGCATGAGAATATTGACCTAAATCGACGCGAACTAGT
TTTCAACTATGTTCCTGAGATAAACCAGGTCATAAAACTTTGAAGTTCAATTTAATATCAATTGACGTGTTTTTCCTTTAAGAG
CATGTAGTAGTTGTATAAATTAATGCGAACTAAGTGTTCAAACTAAGTTCCTGACATAACCAGGTCAGTAAAATTAGAGAAAGT
TAATTTAAATCGAATTGATCGTTGTGTTTATCTTAAGAGCACGAGATCTTGAATAAAAATTACGCAGCACTAAGTTTTAATACT
ATGTTCCTGGATATACACCAGGTCATAAAACCTTGAAGTTAAATTTAAATCGAATTATACAGTTGTTTTTCTTAAAGAGCTAGC
GAAGATAATTGATAAAAAAATCCACGCGAACTAGTTTTCAAACTATGTTTCCTGGAAAATAACCAGGTCAATCAAAAACATTTG
AAGTTATTTAACATTAAAATTGATCGTTGTTTTCTTAAAGAGCATGAGGTATATTGACAACAATTAATGCGAACTAAGTTTTCA
AATAGTTCCTTGACGATAAACCAGGTCTATGAAAATTAGAAATTATTAAATCAATTAATCGTTTGTTTTCGTAAAGAGCACGAG
ATGTTTGATAAAAATTAACGCGAACTAAGTTTTAAAAGCTATGTTCTCGGATAAAACCAGGTCATAAAACACTTAGAAGTTAAT
TTAAATTTAGATTGATCGTTGTATTTTCTTAAAGAGCACGAGATATTGTAAAAATCACGCGAACTAAAGGTTTTCAAATATGTT
CCTGGAAATTACCGGTAAATGAAAACTTTGAAGTTAATTTAATTCAATTGATCGTTGTTTTTCTTAAAGAGCATGATATATTCG
ATAAAAATTAATGCGAACTAAGTTTTTCAGAAATAAGTTCCTGGACATTGAACAGGTCCATAAATTAAGAAATTAATTTAAATC
AATTGATCGTTGGTTTTTCTTAAAGAGCACGAGATGTTGATTAAAAATTAACACCGCGACGAACTAAGTTTTAAAACTATGTTC
CTGGATATAGACCATGGTCCATAAATTAGAAGTTATATTTAAATTGATTGATCGTTGTTTTCTTAAAGAGAAAGATATTCTGAT
AAAAATTATACGCAGCACTAAGTTTTCAAACCTATGTTCCTGGAAATAACCGAAGGTCATGAAATATTTGAAGTTAATTTAATA
TCATTGATCGTTGTTTTTCTTAAAAGAGCATGACATGATTGATAAATAATTAATGCGAACTAAGTTCTCAAACTAAGTTCCTGG
ACATAACAGGTCCATAAAACTTTAGAGATCAATTTTAAATCAAATGATCGTTGGTTTTCTTAAGAGCACGAGACATTGATAAAA
ATTACGCGAACTAAGTTTTTCAAATTAAGTTTCCCTGGATATAACCAGGTCATAAAACTTAGAAGTTTAATTTAATTGATTGAT
CGTATGTTTTCCCTTAAAGAGACAAGAATATTGATAAAAATTAACGCGCACAGTAAGTTTTCAAACTTAAGTTCCTGGGACCAT
AACGCAGGGTCAATAAACTTAGAAGTTCATTTAAATCATTGATCGTTGTTTTTCTTAACGAGCACGAGATATTTGATAAAAATT
AACGCGACTAAGTTTCAAACTAAGTTCAATGGACAAAACAGGTCTAAAATTAGAATTAATTTAGAATCATCTTGATTCGTTGAT
CTTTCTTAAAGAGCACGAGAGTTGATAAAATTAACGCAGAATAAAGTTTAAACTTGTTCCCTGGATAAATAACCAGGTCATAAA
CTTAGAACGTTAATTAATTTGATTGATTCGTTGTTTTTCTTAAAGAGCACAAGATAATTGATAAAAATTAACGCGAACTAAATT
TTCAAACTAAGTTTCCGGGACGATAACTGGATCATAAAACTTAAAAGTTATTTAATCAATTGATCGTTTGTTTTTCTTAAGGAG
CACGAGTATGATAAAAATTAACGCGACTAAGTTTTTCAAACTAAGTCTTCTGGACGTAACCAGGTCATAAAATTAGAAAATTAA
TTTAAATCAATTGATCGTTGTCTTTCTAAAGGCACGAGATGTTGATTAAAGAATTAACGCGACTAAGTTTTAAAACTATGTTCT
TGGTAAAACCAGGTGTCATAAACTTACGAAGTTAATTTAAATCGATTGATCGTTTGTGTTTCTTAAGAGCACGAGATATTGATT
AAAATCCACGCGAACTTATTTTCAAACTATGGTTCCTTGACATAACCAGGTCATAAACTTAGAAGTTCAATTTTAAATCAATTG
ACTCGTTGTTTTTCTTGAAGGAGCACTGAGATATTGATGAAAAATTAACGTAACTAAGTTTCAAACTAAGTTCCTGGACGTGA
ACCAGGTCAATTAAATTAGATAATTAATTTAAAATCATTGATCGTTGTTCTTCTTAAAGAAGTCAACGAGTGTGATAAAAATTA
ACACGAACTAAGTTTAAAAACTATGTTCTTGGATACTAACAGGTCATAAACTTATGAAGTTAATTTTAATCGATTGATCGTTGT
TTTTCTTTAAAGTGGTACGAGATAATTGATAAAAATCAACGCGAACAAGTTTTCAAACAATGTTCCTGGAGATAACCAGGTATA
AAACTTTGATTAATTATTCAATTGATCGTTGTTTTCTTAATAGAGCAATGATACTAATTGATAAAAATTAATGGGAACTAAGTT
TTCAAACTAAGTGTCCCTGGACATAATCCAGGTCAATAAAATTAGAAATTAAACTTAAATGCAATTGACGTGTTTTTCTTAAAG
AGCACGAGATGTTGATAAAAATTAACGCGAACTAAGTTTTAATCTATGTTCTGGAATAACGTCAGGTCATAAAAACTTAGAAGT
TATTTAAATCGATTCGAGTCGTTGTTTTTTCTTAAAGAGCACGAGATATGATCAAAATCAACGCGAACCTAAGTTTCAAACTAT
GGTTCCTGGAAATAACACAGGTCTAAAACTTTGAAGTTAATTTAATTCAATTGATCGTTTTTTTCTTAAAGAGGCATGATATAT
TGATAAAAATTAATGCGAACTAAGTTTCTAAACCCTATAGTTTCCTGGACAATCACCAGGTCATAAATTAGAAATTAATTTAA
TCAATTGATTCGTTGTTTTTCTTAAAGAGCACGAGATGTTGATAAAAATTAAACGCGAACTAAAGTTTAAAACTAGTTCCTGTG
ATTATAACCAGGTCATAAAACTTAGAAGTTAATTAAATTGATTGATTCGTTGTTTTTCTTAAAGAGCCACCAAGACTAATTGAT
AACAAAGTTAAGCTGCGCACCTTAAGTTTTCAAACCTATGGTTCCTGAAATAACCAGGTCGATTAAAACTTGAATTAATTTAAT
TCAATTGATCGGTTGTTTTTTCCTAAGGCATGATATATTGATAAAAATTAATGCGAACTAGTTTTTTCAAACTAAGTTCCTGGA
CATAACCAGGTCAATAAAAATTAGAAATTAATTTAAATCAATTGATCGTGTCTTTTCTTAAGAGCACGAGATGGTTGATAAAAA
TTAACGGAACTGAAGTTTTAAACTAATGTTGCCTGGACATAACCAGGTCATAAAACTTAGAAGTTAATTTAATCGTTTGATCGT

TGTTTTCTTAAAGTAGCACAGAGATATTGATAAATCAAGCGATACTAATTATTCAAACTAGTGTCCTGGAAATTTACCAGGTCA
ATAAACTTTGAAGTTAATTTAATTCAATTGATCGTTGATTTTTCTTAAAGAGCATGATTATGTGATGAAAATGTAATGCGAAAC
TAGTTTTCAGAACTAAGTTCTCTGGACATAACCAGGTCATAAATTAGAAATTATTTAAATCAATTGATCTTGTTTTTCTTAAAG
AGCACGATGATATTATAAAAATTACGGCGACTAAGTTTAAAACTATGTTCCTCGGATATACACAGGTCATTCAAAAACTTAAAG
TTAATTTAAATTGATTGATCGTTGTTCTTTCTTAAAAGAGCAAAGATATTGATAAAGAAATTAACGCGCACTTACAGTTTTCAA
CTAAATGTTACTGAAATAACAGGTTTCATAAAACTTTTGAAGTTTAATTTAATCCAATTGATCGTTGTTTTTCTTAATAGAGCA
TGGATATATTGATAAAAATTAATGCGAACAAAGCCCAACTAAGTTCATCAAACTAAGTTCCTGGACATAACAGCGTCATGAAAA
CTTTAGAAGTCAATTATAAATCAAGATGACGTTGTTTTCTTAAAGAGCCCGAGACATTGATAAAAATTAAACAGCCGACTAGTT
TCAAATTAAGTTCTGGATATAACCAGTCATAAACTTAGAAGTTATATAAATGATTGGAGTCGTTGTTTTTCCTTAAAGAGCACA
AGATATTGATAAAAATTAACGGCGCACGTAAGTTTTCAAAGCTAAAGTTCCTCCCGGACATAACCAGGTCATAAAACTTAGAAG
TTCATTTAAATCAATGATCGTTGTTTTTTCCTTAACGAGCAAGCGAGAATTATTGTATAAAAATTAACGCCGAACTAAGTTTTC
AAACCTAAGTTCCTGGCTGACAAAACCCAGCGGTCAAAAATTAGAATTGAATTAAACCGCTATCATTGATCGTTGTCTTTCTTA
AAGAGCACCAGATGTTAGAATAAAATTAACGCGAACTAAGTATTTAAACTATGTTCCTGGATATAACCAGCGTCATAAAACTTA
AAGTTAATTTGAAATTGATTGATCGTTGTTTCTTAAAGTAGCACAAGATATTGATAAAATTAAGCGAACTAAATTTTCAAACTA
AGTCCTGGACATTAACCTGGTCATAAAGACTATAGAAAAGTCAATATTAAACAACA
>m141122_014521_42134_c100732202550000001823141405141560_s1_p0/57038/5979_11842
ATGAGCGATGAATGGTTACACATACGTACCTATATACAATGGACTAATAATAAACTATCGCTGATGATAAATGCTGCTGGTGCG
CCAATCAATAAAGGGGAAAATCAGCTGATTTTATTAGTTGAAATGTTGTATTAAGGCTCGTTTTGAAACTCACAATATATTACC
ATTTTCCCAATGAGATCTCTTGGTGGGAATATCGAAGCAGTTGAACCACGAGGGTAATACCATTTTCTGAGAGTATTAGGTGAA
ATCTCCAATTCAACAAAAAGGGGTACCGAACTTCAGAAATCTTCGGATTTCTCGTAATCATGGATTTCGAGCATGTTTTGATAG
TGAATCACTAAAGAATATGCGAGGGTCATGGCAGAAAACTTGGTAGCCCTATGTCTTAGGGTTCTATGTATAAACATTTCGTGC
ATATCCGAGGTGCGTATAGGGTGATTACAGTGAAAATACTGATGAGATGTACGAATATAAGGAAAAACTATGAGAAAATTGCCT
TGAAAAAGGGAATTTTTTTTGAAAATGTTGTATTTAAGGCTCCGTTTTTGACTCCAATATATTCATTTTCCCCATGAGATCTCT
AGGAGAAAATATCGACACGAGTTGAAACACGAGGGTAAGAATACATTCGGAGAAATTAGTGAAATTCCATTCCACCAAAAGGGG
TACCACATTTCAGAAATCTTCGATTTCTCTAATATGCGATTCGAGCAATGTTTGATTAGTGAATACTAAAGAATAGTGCGAGGG
TCATGGGCAGAAAACTTGTAGCCCTATGTCTTAGGGTTCTATGGTATAAAATTTCGTGCATGATCCGAGGTGCGTTATAGGGTG
ATACAGTCATAAATATGAGGAGATGTACGAATATGGAAAAAACTTGTGAAGAATTGCATCAAAAGGGGAATTTTTTTTTAAAAT
GTTTGAATTAAGGCTCCGTTTTGACTCCAATATATCTTCATTTCCCAATGGAAGATCTCTTGGGGGAAAGATATCGAACGAGTT
GAACACGAGGGTAATAGCATTTCGGAGAAATTAATGAATCTCCAATTCAAACCATAGGGGTACCCTTTCAGAAAAGCTTTCGAT
TTTCTCTATATTTGGATTCGAGCAGAGTTTTGATAGTGAATAACTAAAGAATATGCGAGGTCATGGGCAAAAAAAAAAAAAGAA
AACTTGTAGCCCCAGTGTCTTTGGTGTTCTATGTATTAAATTTCGTGCATATCCGAGGTGCGTATTAGGGTGATAACATCAAAA
TAACTGAGAGCTGTACGAATATAAGGAAAAAACTTGCGAAATTGCCTTGAAAAAAAGGGGAAATTTTTTTTGAAATGTTGTATT
AAAGGCTCCGTTTTACTCCAATATATTCATTTTCCCATGAGATCCTCTAGGGGAGAGAAATATCGAACGAGTTGAACACAGAGG
GTAATACATTTCGGAGAAATTAAGTGAATCTCCATTCACGCAAAAGGGGTACACTTTCAGAAATCTTTCGATTTCTCTAATATG
GATTCGAGCAATGTTTTGAAGTGAATATAAAGAATGATTGCGAAGGGTCTCTATGGGCAGAAACTTGTAGCACCTATGTCTTAG
GGTTTCTATGTATAGTATAATTTCGTGCATATCCGAGTGCGTATAGGGTGATACAGTCAAATACTGAGAGATGTACGAATATAA
GGAAGAACTTGCGAAAATTTCCCTGAAAAAGGGGAATTTTTTTTGAAATGTTGTATTAGGGCTCCCGTTTTGACTCCATTATAT
TCATTTTCCCATGAGATCTCTAGGGAGAAAAATATTATCGAACGAGTTGAACACAGAGGGTAATACATTTCGGAGAAATTAGTG
GGAATCTCCAATTCACCTAAGGGGTAAACCACTTTCAGAAATCTTCGATTCTCTAATATGGAAAGTTCGGCAATTTTCTGATAG
TGAATACTAAAGAATATGCGAGGTCATGGGCAGAAAACTCTGTAGCCCTATGTCTTAGGGTTTCTATGTATAAATTTTTGTGGC
ATCATCCCGAGGTTCGTATAGGGTGATATTCAGTCCAAAATACTGAGAGATGTACCGAATATGAAGGCAAAACTTGCGAAAATT
GCCATCAAAAAGGGGACTGTTTTTTTGAAATGTTGAATTAAGGCTCCGTTTTGACCTCCAATCGATCATTACATTTACCAAAAA
TGAAATCTCTTGGGTGAAAAATATCGAACCGAGTCTGAACACGAGGGTGAATGCATTTTCGGCAGAAATTAGTGAAATCTCAAT
TCACCATACGGGGTACCCCTTTCAGAAATAATCCGATTTCTCTAATATAGATTCGAGCATGTTTTGATCGTAATACTAAAGAAT
AATGCGAGGGTCCATGGGCAAGAAAACTTTGCCCGGACTGTCTTAGGTTATAATGTATAGACAATCTTCGTGCATATCCACGGT
GCGTAATATTGGGTGATACAGTCAAACTCACTGAGAGACTGTACGAATATAAGGAAAAAACTACGAAAATTGCCTTTGAAAAAG
GGGGAATTTTTTTTGAAATGATGTGTATTAAGGTCCGTTTTGACTCCAATATATTCATTTTCCCATGAGAAATACCTCTAGGT
GAGAAAAATATCAAATCGAACGAGTTGACAGAGGTAACTACATTTCGGAGAAATTATGAAATCTCCAATTCCCCCCCCCACCAA
GGGGTACCACTTTCAAGAAATCTCGATTTCTCTATATGGTCGGAGCAAGATGTTTTGATAGTGATATACAACAGAATATGCCGA
GGGTCCATGGGCAGAAAACTTGTAGCACTATGTCTTAGGGTTCTATGTTAAATTTCGTGCATATCCGAGGTGCGTATAGGGTGA
TTCAGTCATAATACTGAGAGATGTACGAATTATGAAGTTGAAAAAACTTGTGAAAATTGCCATCAAAAAGGGGAATTTTTTTTG
AAATTTGATTAAGGCTCCCGTTTTGACTCCAATATATTCATTTTCCCATGCGATCTCTTGGGGGAAAATATCGAACGAGTTGAA
CCGAGGGTAATACATTTCTGAGAGATTAGTGAAATCTCCAATTCAACAAAGGGGTAACACTTTCAGAAATCTTCGGATTTCTCT
AATATGGATCGAGCAATGTTTTCATAGTGAATACTAAAAGAATATGCGAGGCTAATGGGCCAGAAAAGCTTGTAGCCCTATGTC
TTAGGGTTCTATGTATAAAGATTTCGTGCATATCCGAGGTGCGTATAGGGTGATACAGTCAAAATGCCTGAGAGATGTACGAAT
ATAAGCGAAAACTTGCGAAAATTGCCATCAAAAAAGGGAATTTTTTTTGAAATGGTTGAATTAAAGGCTCCGTTTTGACTCCAA
TATATTCAATTTACCCATGAGATCTCTTGGGGGAAATATTCGAACGATTTGAACCGAGGGTATTAACATTTCGGCAGAAATATA
TTGGATCTCCAAATTCAACCAAAGGGGACCTCCAGAAATCTTCGATTTCTCTAATACTGATTTCGAGCAATGTTTTCATAGTGA

ATACTAGAATATGCGAGATCATGGGAAAGAAAACTTGTACCCCTATGTTTAGGGTTCCTATGTTTAAAATTCTCGTGCGATATC
CGAGGTGGTATAGGGTGATACAGTCAAATTCTGAGAAGATGTACGAAATATAAGGAAAAACTTGCGAAAATTGCCATCAAAAAA
GGGTATTTTTTTGAAATGTTGAAATTAGCCCTCCGTTTTGACTCCAATATATTCATTTACGCCATGGATCTCTTGGGGGAAAAT
ATCGAACAAATTTGGAACACGAGGGATTACATTTCGAGAAATTAGTGAAATCTCCAAATATACACCAAAGGGGTACCCCTTCCA
GAAATCTTCGATTTATCCTAATATGGATTCGAGCATGTTTGATAGTGAATACTAAAGAATATGCGAGCGTCATGGGCAGAAAAA
CTTGTAGGCCCTATGTCTAGGGTTTCTATGTATATAAAATTTCGTGCAATATCCGAGGTGCGTATAGGGTGGATACATCAAAAT
ACTGAGAGATGTACCGAAAAATATAAGGAAAAACTTACGAAAAATTGCTCCTTGAAAAGGGAATTGTTTTTGAAAATGTTGTAT
TAAGTGCTCCGTTTTGACATCCAAATATATTATTTTCCCAATGAGATCTCCTAGGGAGAAAATAATCGACGAGTTTGAAACACG
AGGGTAATCATTTCGGAGAAATTAGTGAAATCTACCAATTCACGCAAAGGGTACCACTTTCCAGAAAATCTTCGAATTTCTCTA
ATATGGATTCGGCATGTTTTGATAGTGAATTACTAAAGAATAATGCGAGTTCATGGCAAAAACTTGTAGCCCTATGTCTTAGGG
TTCTATGTATACAAATTTTGTGCATATCCGAGGGTGCGTATAGGGTGATACAGTCAAAATACTGAGAGAGTGTTACAATATAAA
GGAAAAAACTTGCGAAAATTGCATCCAAAAAGGGGAATTATTTTTGGAAATGTTGAATTACAGGGCTCCTTTTGACTCCAATA
ATATTCATTTACCCAGTGAGATCTTCTTGGGGGAAAATATCGAACGAGTTGAACACCCGAGGGGTAATGCATTTCGGCAGAAAT
TAGTGGAAATCTCCAATTCACCATAGGGGTACCCCCTTTCAGAATAATCAGAATTTCTCTAATATAGATTCGAGCCAATGATTT
GTGATATGTGAAACTAAGAAATTGCGAGGGTCAATGGGCAGAAAACTTGTAGCCCTATGTCTTAGGGTTCTAATGTATAAAATT
TCGTGCATATCCGAGGTGCGTATAGGGTGATACAGTCAAATACTGAGAGAGTACGAATATAAGGAAAAAACTTGCGAAGAATTG
CCTTGAAAAAGGGGATATTTTTTTGAAATGGTGTATTAAGGCTCCGTTTTGACTCCCAATCGATATTCATTTTCCCATGAGAT
CTCTAGGGAGAAAATATGAACGGAGTTGAACACGAGGGTAAATACTATTTCGGAGAAATTAGTGAAATCTCCAATTCACCAAAA
GGGGTACCACTTTCAGCAAATCTCGATTTCTCTAATATGGATTCGAGCAACGTGTTTTCGATAGTGAATACTAAAGAATATGCG
AGTTCATGGGCAATGACAAACTTGTAGCCCTAATGTCCTTAGGGTTCTATGTATAAAATTTTCGTGCATATCCGAGGTGCGGTA
ATAGGGTGATACGCAGTCAAAATACTGAGAGATGCTACGATATAAGGAAAAACTTGCGAAAAATTGGCCATCTAAAAAGGGGAA
TTTTTTTTGGAATGTTGAATTTAAAGGCTCCGTTTTGATCCAATATATTCCAAAATTTTTACCTCCATGAATCTCTTGGGGGAA
AATATCGAACGAGTTGAACACGAGGGTACAGTGCAATTTCGGGAAATTAGTGAAATCTCCAATCACCATAAGGGGGTACCCCTT
TCAGAAATATGATCGATTTCTCTAATAAGATTGCGGAGCAATGTTTTGATAGTGTATACTAAAGAATTGCGAGGGTCATGGGGC
AGAAACTTGTAGCCGCTATGCGTCTGTAGGGTTCTAATGTACATAAATTTCGTGCATATCCGAGGTGCGTATAGGGGATACAAG
TCAAAATACTGAGAGATGGGTACGCATATAAGGAAAAAACTTGCGAAAAATTGCGGAACACAAAGGGGAGATTTTTTTGAAATG
TTGAATAAGGCTCCGTTTTGACTCATTATATTCATTTTCCCATGAGATCTCCTTGGGGGAAAATATCGAACGACGTTTGAACAC
GAGGGTGAATACATTTCTGAGAAGATTAGTGAAACCTCCAATTCAACCAAAAGGGGTACCACTTTTC
>m141122_014521_42134_c100732202550000001823141405141560_s1_p0/57114/0_8228
AAAAAAGAGAGAGATTCACTCGCCTAGTCTCATGCCATAATTAAGTTTTCAGAGTGTTTCACACTTTCGATTAATCGCTTAAGT
CGTTGGAAGTCAAAGGCAAGTACCACATAACTTTCTGAGGAGATAAGTTTCCTAATCTCCTTATACTCTTGTTAGGTTTTGTAG
GCAGGGTGAATCACGATTCTGGGCAAGTGTCCATGTCTGATACTCTTTACGATATACAAAATCGTTCGGACATGCATGGATCTT
ATTCATACTTCAAGCCTATGAAATTTAAAGTTTTCTTAAGCTTACATACGTAGACTCAGGAAAGCAATTATCCTGACGGCAAAA
TCTTCTTTAAAAGTAGCTAAAAATTGACTAAAACATTTATCGCTAACTTCAATTTTCTGATTTTAATGTTGGATAAAATTTTCC
GATCGTTGGTAATTTTTTGTTTCAGAAAACCATTGAATAATGGTTTGTTGCAATCTATATGGTTATACTTTGGAACAACAACGA
GTTTCGAGCCTGAGAATTTATCATGTAAGGATGATTTCAAGAATGGACTTAGAAATATAATAAAGACTGGCTTATTTTTCTAAG
TTACAACCCACTCTAATGATAAAGAAGGCGTTATATAATTTTCATAAGAAATCATAATTAATAGGTCCGAGTTATGTTTGCATA
GATATTATGTTTTTGCCTTAGAGCCTATTAGGGTAATTCCTAACATGTTTTTCTCAACTATTAGAACTCTGCTGAGATGTCACA
CCCCCTCCCCCCCCTCCTGAAGGTCTGCTCGCAACAATGCTAATGTCTCCAACGCTGCTCTGAGAGGCAATTGAACCCAATCCA
GTTTGCAGAAGGGGAGTGCGTTCTACTGCCAACTGAAATGCGTTGCCACCGGTCGCCAGGTTGATAGCACTGCAGAAATTGCTA
GACTAACGAAGCAAGTTGAGGAATTACTGGGCAGCAACAACGACAAGAGGCTCAGGGCTTCAGACCTCAGCCTCTGTCGGCAAA
CCACCAAACCAGCAGGGGCAAATGGCTCCAGCACCCCGCAACAAGTTGCTGCATATGGGGATGGCACAATGGAGAACTATGCGC
CAACCCAGCTCAGCCATGGAGCTAGTTTATAGCGGCGGTTTCGTAAGCAACCACGCTTCGGAAGTTTGAAAGGGACACAGACCC
CTTCAAGGCAGAAGAATGGCTCAGAAATGTGGAGCCAATCCTGACACACAATAGAATCTTGGCAAATGCGAATCGCATATCCCT
GCATTTCGTCTTTGCTCCAAAAGAAAGATGCTAGAATAATGTGGGAACTTAGTTCAGGCAGACTCACGATGTTGCCACCATGAC
TTGGACTGATTTTGTGCGTGGCTGTTCCACAAAACACTACTAAATTCGGCAGTCAAATCATTTCAAGAGTTCGAGGAGTTCGCT
GGCATGAAGCGAGGGAGTTTATTAGTGGCAGAATATGCTCGACAGTTCAACCGATTTAGCTAAGTTTTGCACAAAAATGTTCCA
ACTAACTTTTCTAAGGGTGACCAAGTTCCTTAGAAGACTTTTACCAAAGATTGAGATAGGGGGTTAAGCTAGCAAACCAGGAAA
TACTACAAAAAAAAAAATTATTGCCGATGTTCTAGAAACGGCAATGAGAAGTAGAAGAGGATTCAAGGCAAATGTTAGTAAAGA
AGAAGCGCAGTAAAGCCTGAGCCTAAGACAGGAGAGTCAACCGTAGACTAGTTTGGAACAACAACCACCACATACAAGCAGCAA
TCAAGTCCAACACAGCAATAACGGATCAGAAAAGAAGGCATCGCTGAACAATAAGCCAGAGCAAGTCCGATAGGCAATAAAAGG
GCACAGACCGAATAATGGAGGTAATGGCTGGGTTATGTAGAATTACCCACAATGTGCTAGAGTGCAAGAAGAAACATCCTGGTG
AATGCGCGGGCAAACACCAGGGATGCTACAACTGTGGATCAAGAAGACACCGGAAGAGAGACTGTACCCAGCTCAAGCCAGAGA
GGGAAAAAGGAAGCAAGATGGTTTCCTGCCAGGGTATTTTGTCTTAACCAAGGAGAAACTAATGCTAGCCTAAAGTGGTCAGCA
GGCCAGGTTTCTATCCTCAATAATATATGTTCTGTAAAATTATTTTATTCGGGAGCCCACTCATTCGTATATCTCATTAGGAAT
GATAGAAATCTAGGAAACCTTGTGAAAGATTTAGACTAAGGTTTGTAACAGAGTTGCCGTTCCGGGCAAAGTAGTTCTATCATC
ATGGATACACGAGGCATACCAATCAAAATTGAGGAACGTAGAACTAAGAAGGAGACCTGATAAGAACCTGGAGATCAAGACTCG
ACGTGATACTAAGCATGGACTAGTTAGCAGGCATGGTGCAACCATCGATACAAACACAAGAAAGTGACGGTTCGAGAACACCTG

ACAGGACAGAGCTAATGCTTCATCGGGCAAGTTTCAGCCTAGCACACTGCTAATATCATCCTCTCAAAGCTCAAAGGATGATAG
AAAAGGATGTCAAAGCATTCTTAGCCAGCATCTACGGATGTGGTATAAGGAAACAACTACTTAAGGTGAAGACGTCCGCCATTT
GTAAAAGAATTCCAGAAGTATTTCCAGATGACTTTCCGGGTTGCCGCCAACTCAGGAAATTGACTTCACAATCGAATTAAGTAC
CAGAGGCACGCAAGCCTATCTCCAAGGCACATACCAAATGGCACCCACCGAACTCTAAGGAGTTAAAGAACTGTTGATGGTAAA
GAACTCGTCAAGCCGGATTGACTGGTTAGAAACTATCAATCTCTATACTATAAGAAGCTATGAATTAGATAGAAAGATCTAAAA
CACCAGGAGAAAACTCAGGCAAGCATGAGAACCAGAGCATATATTTCATAAGTCTCATTTCTACATTCAGTTTTCCAACCCTTA
CCTGAGGGGTTGGAGCCCTATTTATAGGGAGGTCTCTATTGGCCCAGGATACAAACAAGTCCAGACACTGGGACCTGTACATAG
GTACTCTGATAAAGTAGTTGGCCCAGGGTGTAGTGGTGTCTACCCCTGATGGTGTCAGAGTCGTGGCCCAGGACAAAGTACTGT
CGGGATTCTGGCGTAATGGTCGGGGTGTCCAAGTGGTAACCACTACTCTTCGTACAGGTCCGTACTGCCACTACTCCTCAGTAG
CGCAGATCATAGTCCGTGCCTCTGTTCTCTCCACAACCGTACAAACCCCGTGTCAGCGCACGTGTTCCGTACTCGGTTGTTCAC
TCATCTAATGTACCGTTCAACCCTGCTCCAGTGTTCGTTTGGCCATAATAAGCAAGGTTCTTCCCAAGTGAATCTCGTGCCTAT
GCCAAGGAGGCTTCAACCCATGCATGTCCTGAGAAGTCAAGCACCAGGGGCCAGGCCGCTTAAATGCGGGTCACATGGTACACG
AGACTCGGAGTATGGACGCGTGGCAGGCACACCTCGCTATAGCGAGGGTACCTCGCGTAGGAGGTTGGCCCTTTTCCTCAGGCG
TAGGCATTGGCTCAGGACACGGGCGTCATAACAAGGCAGCACACCTCGGCATAGCGAGGCGCCTTGCCTTCCCTCGGGTGCAGC
GTCGCAAGGCTAGGGGCATGGATGCCACACGAAGCCTCACCCTCGCATAACGAGGGTGCCTCGTGTAGCGAGGCTGAACCTTCT
TCTTCCCGAGTGTAGACGAGGCTTGATGCTGCTGGGAATGGGGCGGCACGCGATGGCCAGCTGGGACCCTCGCATAGCGAGGGT
GAAGTTTGGATGGGCAAAGTGGCCGAAGTCCCTGCGCCTAGTGACCGGGTAGAACATCTCTTACCCAATCTCCGTCTCCACCAT
CATCGTGATGCCGCTTAGCTCAGGATGTCTCCGTAGCATGGAGCTTCGACTCGTGAATAGAATTCACAAGGCAAAAAATTCCAC
ATTTACACTTCGATAGGCTCTATTGGCCCTGGATACAAACAAGTCCAGACCACTGGGACGTACATAGGTATCTTAAAGTAGTGG
CCGCAGGGTGTAGTGGTGTCTACCTGATGGTGTCCAGAGTCGTGGCCCAGGACAAAGTACTGTCGGCTCTGGCGTATGGTCGGG
GTGTCCAAGTGGTACCACTACTCTTCGTACAGGTCCGTAACTGCCATATCCTCAGACGCAGATCATAGGGTCGTGCCTCTGTTC
TCTCCACAAACCGTAACACCCGCGTTGTCACGCGCACGTGTTCCCGTACTCGGTTGTCATCATCAATTACCGTTCAATGCTCAC
ATGTTCGTTTGGCATATAGGCAATGGTTCTCCCAAGTGATCTTCGTGCCTATGCCAAGGAGGCTTCAACCATGCATGTCCTGAG
AAGTCAAGGCGCCAGGGCCAGGGCCGGCATTATGCGGGTCACATGGACACGAGACTCGTAGTATGGACGCCGTGGCAAGGCCAC
ACCCTCCCGGCATAGCGAGGGTAACGTCGCGTAGGAGGTTGCCTTTTTCCCTCAGCGTAGGCATGGCTCAGGACACGGGCGTCC
ATAACAAGGCAGCACCCTCGGCATAGCGAGGCTGCCTTGCCTTCCCCTCGGGTGCGAGCGTCCGCAAGGCTAGGGGCATGGATG
CCACCCACGAAGCCTCCACCCTCGCATAACGAGGGTCGCCTCGTGTAGCAAGGCTGACCTTCTTCTCCCCGAGTGTAGAACGAG
GCTTGATGCCTGCTGGAATGGGGCGGCAGCGGATGGCCAGCTGGGGGACCCTCGCATAGAGAGGGTGATAAGTTTGGATGGGCA
AGTGGCCGAAGTCCCTCGCCTAGTGGAGGTGACTCTCTTACCCCGCAACTCCCCTCAACCATCATGATGATGCCCTTTCAGGAT
GTCTCGTAGCATAGAGGCTTCACTGCGTGAATAGATTCACAAGGAAAAATTCCACATTTACAAAGACCTCCAGCTACAAGAACT
CCTAGACTTGGGTTTCATCAAAACAAGCCAATTCACCATGGGGAGCACTAATGCTTATTTGTGAAGAAAAAGGACGGAAGCATG
CAGATGTGGCATAAGATACCGCGAGCTGAATAAGGTAACGATTAAGAATAAGTACCGCTACCTTGGATTGACGCTTGTTTGATC
AACTCTAAGGATGCGCCGTGTTTTTCAAAGATTGATTTACTGTCCAGGTATCTATCAGCCTCAAGGTACAGGGAGAAGATTTCC
CTAAGAACGATCTTCTTAGAACTCGTTATGGCATTACAAGTTCTTAGTTAATGTCTTTTGGTCTTACCAACACTCCAGCCCGCA
GTTTATTGATTTATGAATAGGGTCTTTAAGGACTACTTGGATAAATTCATCAATAAGTATTCCATCGAGACATCTGATATACTC
GTAAGAACGAAGTCGAGCACGAGGAACATTTGAGGTTGATCTTATTATGATAAAGAACATAAACTTTTACGCCAAGTTTAAGAA
GTGCGAGTTTTGTCTTTCGCCAGTAGCATTCCTTGGGCACATTGTATTCAAGGATCAAGAAGTTGGAGTAGACCCATCCAAGGT
AGGGCTGATGAAGGATTGGCCAAGAACAAAGAGCGCGTCAGATGTAAGAAGCTTTCTGGGATTAGTAGGTTATTTTATTGGAGG
TTTGTAGAGGGCTTATCTAAGATAGCAGCTCCACTCACAAACTTGACCCGGAAGGCAGGCAAAGGTTCAACTGGACCGATAAGT
GTGAAGAGAGCTTCAAGTTGCTTAAGGATACAGCTATGCTCAGCACCGTAATCTCTGTGTACAACACCAACCGAAAAATTTATA
GTCTACTGCGATGCGTAAAGCAAGGGTTGGGTTGCATGCTGATGCAGAATGACAAGTGATAGCCTACGCCTCAAGGCAATGAAG
TGAGTACGAACAACGGCTATCAAACTCATGATATGGAGTTGGCAGCGTGGTTTTGCGTTAAAATCTGGCGCCATTATCTTTAAT
GGAGAACGGTGTGCCAGGTTTATACAGACCCCACAAAAGTTTAAAGTACTTCTTCACTCAGAAGGAGCTCAACATGAGGCTAGC
GTAGGTGGTTAGAGTTAGTAAAGGATTATGACTGGCGATTCCTACACCACCGCTAAAAGGCAAACGTAGTTGTGATGCGAGGCT
AAGCAGGAAGAAGTTATGGAAGTAATAGCAGCCTAGCTGAATAAGAAAAAGCGCTATCAGGCAGGAGCGTGATTAGTGCGGAAT
AGAAGTAGTCATCGGTAAGCTGGCTAATTTCAGTCTATCTCAATCAAATCTGTTAGAAGATATAAGGATGAGCAAGGGCATGAT
GACACCATTAGAAGTAGCATATTGATCGCAGTTTAAGAAGGCAGGCCACATATTTCTTTCAAATATCAGGATGGGGTTATTAGA
TATAAGGAATCGGGGATTGCGTGCCAAAAAGATAAAGGGATTGAAAGGTGAGATTTTTACGAAGAAGCACCCAGTACCCCATAT
TGCAGTTCACCCAGGGTCGACGCAAGATGAATCACGATCTCAAGGCAATGTATTTGAGTGGCCAGGAGTTGAAGAAGGATATTA
GTGGAATTTGTTGTCTAAATGTCTAGTATGCGAGCAAGTAAAAGAGAACATCAGCGGCCTGCAGGCTTATTGCAACCTCTTAGG
CTAGCGAATGGAAATGGGACGAATAATGCTATTTATTTCGTGACGAGTTTTCCACAAAAGAAAAAAGTAAGGAGCATGATTCAG
CTTGGGTAGTAATCATTAGACTACCAAGTCAGCTCATTTCCTGCCTGTTATGGACTTTCATACACAGCGAATCAATACGCAGGC
ATCTATGTCCAAGAAATAGTACAGGTTTGCATGGATTTCCGCAGACAATAGTGTTCGATACCGAGGATCGGGTGTTTACATCGA
GAATTTTGGGGAAGTTTTAGCAGCAAGCCATAGGGATACTCAAGTTAAGTCTTAGTAACATCTTTTCATCCTGCAGCCACAGAC
GGCCAAGTTCGGCGTTACCATAAAAAACAAATTTTAGAGAGTGTTCGGCACTTGTGTACATTGATTATCGGAGGATCGTGGGAA
GTAAGATATTGCGCTGATAGAGTTCTCGCTACAAGCAATACTACCAGTCAATGATTCGGGATGGCACCATATTGAGTTTCTTTA
ATTGGGAAGAAGGTGTCGATCACAGTTACACTAGGACGAGGTAAGGGACAAAGGCAGCTTCTTGTTCCCGAACCTGTTAGAAAT
GCTCAGGAAGTAAGTAGCATTATTAGAAAGCGTATGCTCACTACACCCCCCCCTCAAGCCGTCAGAATAGTTAGTGCGGATGCC

AAGCGGTAGGTGTGGAATTCAAAAGTCGGAGATCAAAGTCTTCCCTAAAAGATATCTCCTATGAAAGGTGTGAAATGGTATGGG
AAGAAGGGCAAGCTTAGTCCCCATTTTCTAGTCCTTTTGGATATGGACAAGGTGGGAGCAGTTGCATATAAATTAGCCCTACTG
CTAGCTCTAGGCAGCATAGTCACAATGTGTTCCACATCTCAATGCTATGAAGTAATGTGTCAGCCATCTCACGAGTCTTCAAGT
ACGGATAACAGATAGCACTCCAGAAGACTTGAGTTATGATAGGACGACCGGTTAGCATCCTAGATAGAGGGATGAAGCAGTTAG
AATTCCAAGTAGTATTCTAATAGTCAAAGTCTTATGGAGTAATAGTTCCGAATGGGAGGAAAGTGGGAGTTGGGGGGGCAATGC
AAGCCCCAGTATCCCGAAATTTTTTGGGACGAAATTCTTTTTATTAGGGAGAATTGTAGTGTGGTATGGTGTGGTAGTGGTAAG
TGGTATGTGGGTAGTGGTGGTAGTGGTGGTTAGTAGTGGTAGTGGTAGTTGTAAGTGGTAGTGGTAGTAGTGGTAGTGGTAGTT
TGTAGTGGTAGATGGTAAGTGGTGGTGGTGGTGGTGGTGTGGTGGTGGTGGTGGTGGTGTGGTGGTGTGGATGGTGGTGGTGGT
GCGGTGGTGGTGGTGGTGGTGGTTGGTGGTTGGTGGTGGTGTGGTGGTGGTGGTGGTAGGTGGTGGTGGTGGTGTTGGTGGTGG
TGTTGGTGGTGGTGGTGTGGTGGTTAGTGGTAGTGGTATGGTAGTGGTAGTGGTAGTGGTAGTGGTAGTGTAGTGTAGTGGTAT
AGTGGTAGTGGTAGTCGGTGGTGTAGTGGGTAGTGGTAGTGGTGGTAGTCAGAGGGGGGGGGGGGGGGGGGGGCGGTGGTA
>m141122_014521_42134_c100732202550000001823141405141560_s1_p0/64706/316_13955
CAGGACTCGAATCGAATACGCCTATTTTTATAGGTTAATGTCATGATAATATGGTTTCTTAGACGTTCGGTGGCACTTTTCGGG
GAATGTGCGCGGAAACCCCTATTTGTTTATTTTTCTAAATACATTCAAGATAAAGTATCCGCTCATGAGACAGATAAACCCTG
ATAATGCTTCAATATAGTTGGAAAAAGGAAGAGTATGAGTATTCAACATTATCGTGTCGCCCTTATTCCCTTTTTTTGCGGCAT
TTTGCTTCCCTTGTTTTTGCTCACCCAGCAACGCTGTGAAAGTAAAAGATGCTGAAGATCAGTTGGGTGCACGAGTGGGTTTA
CATCGAACTGGATCTCAACAGCGGTAAGAACTTGAGAGTTTTCGCCCCCCGAAGAAACCGTTTTCCAATGATGAGCACTTTTAA
AGTTCTGCTATGTGCGGCGGTATTATCCCGTGTTGACGGAGTCCGGGCAAGAGCCAATCGGTCCGCCGCATACACTACTTCTCA
GAATGACTTGGTTGAGTACTCACCAGTCACAGAAAAGCATCTTACGGATGGCATGCACAGTAAGAGAATTATGCGTGCTGCCAT
AACCATGAGTGATAAACACTGCGGCCAACTTACTTCCTGACAACGAGTCGGAGGACCGAAGGAGCTAACCTGCTTTTTTGCACA
ACATGGGGATCATGTAACTCGCCTTGATCGTTTGGGAACCGGAGCTGAAATAAGCCCATACCAAACGACGAGCGTGACACCACG
ATGCCTGCAGCAATGGCAACAACCGTTGAGCCAACTAATTAACTGGCGAACTACTTACTCTAGCTTTCCCCGGCAACAATTAAT
AAGACTGGGAATGGAGGCGGATAAAGTTGGCAGGACCATTCTGCGCTCCGGCCCTTCCGGCTGGCTGGTTATTGCTGATAAACT
GGAGCCGTGAGCGTGGGTCTCGCGGTATCATTGCAGCACTGGGCAGATGGTAAGCCCTCCGTATCGTAGTTTATCTACAACCGA
CGGGGAGTCAGGCAACTAAGGACTGAACGAAATAGACAGATCGCTGAGATAGGTTGCCTCACTGATTTAAGCATTGGTAACTGT
CAGACCAAGTTTACTCCTATATACTTAGATTGATTTAAAACTTCATTTTTAATTTAAAAGGATCTAACGGTGAAGATCCTTTTT
GATAATCCTCATGACGCAAAATCCTTTAACGTGAGTTTCTCGTTCACTGAGCGTCAGACCCTTGCCCCGTAGGAAAAGAATCAA
GGATCTTCTTGAAGATCCTTTTTTTCTGCGCGTAATTGCTGCTTGCAAACAAAAAAACCACCCGCTAACCAAGCGGTGGTTTGT
TTGCCGGATCAAGGAGCACCAACTCTTTTTCCGAGGTAACTGGGCTTCAGCAGAGCGCAGATCCAAATACTGTCCTTCTAGTGT
AGCCCCGTAGTTAGGCCCCACCACTTCAAGAACATCTGTAGCACCGCCTACATACCTCGCTCTGCCTAATCCTGTTACCAGTGG
GCTGCCTGCCAGTGGCGATAAGTCGTGTCTTACCGGGTTGGACTCAAGACGATAGTTACCGGATAAGGCGCAGCGGTCGGGCTG
AACGGGGGGTTTCGTGGCCACAGCCCAGCTTGGAGCGAACGACCTACACCGAACTGAGATACCTACAAGCGTGAGCGTGATGAG
AAAGCGCCACGCTTCCGAAGGGAGAAAGGCGGACAGGTACAACGGTAAAGCGGCAGGGTCGGAACAGGAGAGCCGCACGAAGGG
AGCTTCCAGGGGGAAACGCCTGGTATCTTTATAGTCCTGTCGGGTTTCGCCACCTCTGACTTGAGGCGTCGATTTTTGTGATGC
TCGTCAGGGGGGCGGAGCCTATGGAAAAACGCCAGCAACGCGGCCTTTTTACCGGTTCCTGGCCTTTTGCTGGCCTTTGCTCAC
AATGTTCTTTCCCTGCGGTTATCCCCTGATTCTGTGGATAACCGTATTTACCCGCCTTTGAAGTGAGCTGATACCGCTCGCCGC
AGCCGAACTGACCGAGCGCAGCGAGTCAGTGAGCGAGGAAGCGGAAGGAGCGCCTGATGCGGTATTTTCTCCTTACGATCTGTG
CGGTATTTCACCACCCGCATATTGGGTGCACTCTCAGTAGCAATCTGCTCTGAATGCCGCATAGTTTAAGCAAGTTATACACTC
CGCTATCGCTTACGTGACTGGGTCATGGCTGCGCCCCGACACCCGCCAACAACCCGCTGGACCCGCGCCTCCTGACGGGCTTG
TCTGCTCCCGCATCCGTTACCAGACAAAGCTGTGACCGTCTCCGGGAGCTGCATGTGTCAGAGGTTTTCACCGTCATCCACGAA
ACGCGCGAGGCAGCTGCGGTAAAAGCTCATCAGCGTGGTCGTGAAGCCGATTCACAGATGTCTGCCTGTTCATCCGCGTCCAGC
TCGTTGAGTTTTCAGAAGCGTTAAATGTCTGGCTTCTTGATAAAGGCGGGCCATGTTAAGGGCGGTTTTTTTCCTGTTTGGTCA
CTGATGCCTCCGTGTAAGGGGGATTTCTGTTCATGGGGGTAATGATACCTGATGAAACGAGAGAGGAATGCTCCACGATACCGG
GTTACTGATGATGAACATGCCCGGTTACGGAACGTTGTGAGGGTAAAACAACTGGCGGTATGGATGCGGCGGGACCCAGAGAAA
AATCCACTCAGGGATCAATGCCACGCGCTTCGTTAATACAGATGTAGGTGTTCCACAGGGTAGCACAGCAGCATCCTGCGATGC
AGATCCGGAAATAATGGTGCAGGGCGCTGACTTCCGCGTTTTCCAGACTTTACGAAACCACGGAAACCGAAGACCATTCAATGT
TTTGCTCCAGGTTCGCAGACGTTTTTGCAGCAGCAGTCCTTCAGTTCGTCGCGTATCGGTGATTCAGTTCTGCTAACCAGTAAG
GCAACCCCGCCAGCCTACCGGGTCCTCCAACGACCAGGAGCACGAGTCATGCGCACCCGTGGCCAGGACCCAACGCTGCCCGAG
AATGCGCGCGTGCGGGCTGCTGAGAATGGCCGGACGCGAATGGATATGTTCTCCAAGGTTGGTTTTGCGCATTCACAGTTCTCC
GCAAGAATTTGAATTGGCTCCAATTCTTGGAGTGGTGAAATCGTTAGCGAGGTGCACGCCGGCTTCATATCAGGTCGAGGTGGC
CCGGCTCATGCACCGCGACGCACAACGCGGGGAGGCAGACAAGGAAATAGGGCGGCGCCTAACAATCCATGCCAAACCCGTTCC
ATGTGCTCGCCGAGGCGCCATAAATCGCCGTGACGATTCAGCGGTCCAATGAGTCGAAGTTAGGCTGGTAAGAAGCCGCGAGCG
ATCCTTGAAGCTGTCCCTGATGGTCGTCATCTACCTGCCTGGACAGCATGGCCTGCAACGCGGGGCATCCCGATGCCGCCGGAA
GCCGAGAGGAATCATAAGGGGAAGGCCATCCCAGCTCGCGTCGCGAACCGCCAGCAAAGACGTAGCCCAGCGCGTCGGCCGCCA
TGCCGCGTAATGGCCTGCTTCTCGCCGAAACGTTTGGTGTGGCGGGACCCATGTGACGAAGGCTTGAGCGAGGGCGTCAAGAT
TCCGAATACCGCAAGCGACAGGCGAATCATCGTCCGCGCTCCCCAGCGAAAGCGGTCCTCCGCCGAATAATGACCCAGAGCGCT
GCCGGCACCTGTCCTACGAGTTGCATGATAAAGAAGACAGTCATGAAGTGCGGCGACGATAGTCATGCCCGCGCCCAACCGGGA
AGAGCTGACTGGGTTGAAGGCTTCTCAAGGGCCATCGTCGACGCCTCTCCCTTATGCCGACTCCTGCATTAGGAAAGCAGCCCA

GTAGGTGGTTCGAGCCGTTGAGCACCGCCGCCGCAAGGAATGGTGCATGCAAGGAGATGGCCGGACCCCAAACAAGTTCCCCCC
CCCCCGGCCAAAACGGGGGCCCCCCCCTTTGGCCCAACCCAAAATAAAAAACCCAACGCCCCCCGGGAAAAAAAACAAAAGCGG
GGCTCAATGGAAAGCCCCCGGGGGAAAAGTGGCGAGCCCCCCCGAAAAAATTCTTTTTTTTTTCCCCCCCAAAATTCGGGTTG
GGGGGGGGGGGAAAATTTTTGTTCGGCCGATATAATTAAAGGCCGGCCAAAAAAAAGCAAAACCCCGCCAAAACCCCCCCTTGTT
GGGCGCCGGTTTTGGGGAAAAATTGGGGCGGGGCCCCCCCCAACCACCGGAAATTGCCGGGGTTCCCCCCCCGGGGCGTAAAAA
GAAAAGGAAATTCCCCAAAACAAAAGGAACGGGGGGTTTTTTGGGGGGGGGGTTGGGGGATCCGGCCCATGAAAATTCGCCCGG
GGTAGTTCCCGGAAAATAAGGTGGGGGGCCCCCCCCCTTTTTTTTTTTCCCCAAAGGTAAAAAAAGCCCGGGAAAATAAGGCGG
AGGGCCAAAAAAGGAAAACTTGGGGCCGGGGCGGGGCCCAAACGGGGTTTTTCCCCCCGGGGAACCCCCCCCCCAAAGTGCCT
CCGGAGGAAAACCGGGGTTGGGCGCATTAAAAGAAAAAAATTGGCAAAATCACGGCAAAATATAGCCCGCTAAGCCCCCAAAGC
CCAAACGGGCCCAATAAAGTTGGGGAACTGGGCGGGGGATTGCCCCCCCCCTGGTTTTTCCGGAAAAAAATTGGGACCCCCCCC
CCGGGATAATCCCCCTTTTTTTTTCTTTCCTTCAAAACAACAACCCCAAACCCGGAAAAAGGGAAGGGGAGGGAAAAAAAAAGA
AAGGAGAAAGGGGATTTTTTATCGTTCCCCATTTCCCGACAAAGCCAAAAAAAATCGCCCAGGTCCCCCCCCCCCAAAAAAACT
TATGGGCCCCCGTGGGGGCCCCCCTTGCTTTTTTTTTAAAGCCCCGCCTAAAAAAAAAAAATTGCGTTTTTTTTTGGAATGCC
CAAATTTTTTTTTTTCCCCTATGCGCAAAAAAAAACCCCGGTTTTTTTTTTCCACTTCGGGAAAGGCAAAAAAAAAAAAAAAC
TTGGGTTCCCCCCCCGAAAAACCGGGGGCTTTTTTTGGGGCCCCCGCCCCCCGGCCCCCAAAGGGTCCCTGCTTTTCCGGCTT
TTTCGCCCCTTTTTTTTAACTTGGAAAGGCCCCCAACCTAAAATCCGGACCTACGCGGGGATTCATGGGCGGAACCCCAACCAA
ACCCCCCCGTCCCCCCCCCCTTTGTGGGGGAATTTTTTTCCCCATCCCCCCTAACGGGGGGGCCCCGGGAAACCGCAATCCCCCG
GTTGGGGCCCGGGCCCCCAAAAAAAAAAAAAAAAAATCCCGGGCCCCCGGCCCCCCCAACAAAAGGTTTTTGGGGGGCGGTTGA
CCTGGGGCCCCCCGCCCCCTTTTTTTAATATTCGCCGACAATCAACCCGATTGGGGGGAAAAAAAGAATTCGGGGCTTTTTTTT
TTTTTTTCCGCCCAAACTTTTCGGGCCCCCCCTTTTCATGAAGGGCCGCCATTTTTTGTTTTTTTTTTTTTTCCGGCGTGGGGG
GGGTTTTTTTATGGTGGGCCAAAGGGGCCCCCCCCGGGTGGGGGGCCGGGGGGGACCTTTTTTTTTTTTTGGGGGGTTTTTTG
GGGCCCGGGGGCCAAAAAATCCCTCCATTTGGCCCATTTGCCCCAACCCCAAAATTCCCCCTTTTTTTGGGCGGCGGCCGGGG
GGTTTTGGGCTCCCAAACGGCCCCCCCCCTTTTCACCCCCTACTAAAAAACTTTGGGGGGGCCTGCTTTTTCCCTTAAAATTGC
AAAAGGGAAAGGGTCCCCCGCAAAAAAAAAAAAAAAAAATAAAAGGGGAAGGGAAGCCGGTGACCGGACCCCGATTTTTTGCCC
CCCCCCCTTTGAAAAAGAAGCCTTTTTTTCAAAAACCCCCCAAAAAAAAGTTCAGCCCTTTTTTTTCCCCCCTTTTTTTTTT
CCCGGGTTTTTTGGGGGCCCGGGCCGGGGGGGCCCCAAAAATGGGGAACCTTTAATCGGTCCCCCCCGCCCGGAACTTTATTTG
ACCTGTTTCCTTCCCCCCCCCTTTTATCCCCATTGCAAAAACCTTCCCGTTAAAAGGGAAAAACAAAGGTGGGCCCGGGCCCCA
AAAGCGCCCCTTTTTCTGGGGTCCCCCCCCAAAAATTTTTTCCGGGGGGCCCGGAGGGAACCCCGGCTTTTTTTTTTTTTTTCC
CACCGGCCCTTGGAGGGGGCGGCGGAACCGATTGATTTTCCGGCCCCCCTTGGTCCCCGGGGGCCCTTTTTTGCCGGGGGGGGG
TTAATTCCCGGGAAAATCTTTTTTGGCCCCCAAAAAAAAAAACCGCCCCCCCCCCTTTTTTCCGCCCTCAAAGGCCTTCCCGTC
CCACTGGTCCCCCCCCCGCCCCCCACCAAAACGTTTTTTTTTCCGGGGCCCCGAGAATAAAAGCAAGGCCCCCCCCCATTTTTT
TTTTTTTATCGCCCCCCCGGGGGCCAAAAAATGGGGCCCCCCGGGCCCCGGGGGGACCCGGCGGGCCTGGGGCCCCCTTTAACC
GTTTTTCCCCCTTTTTGGGCCCTGGCCGGTTTCCGCCCGAACCCCCGCCCCGGGAAGGGGGGCCCCTTTTTTTTTTTGGAAAAA
TTTGGCCCCCCCTTCCCCCCCCCCCCAAATTTTAAAATTGAACAATTTTTTTTTCCTTCCTCGCCCCCTGTTTCCCCCCCGGCG
GGCAAAATCCGGGGAAAATGCCCCGCCGGTTTTTTTTTGGGCCAAGGCCCAAAATGGGCCCCTGGTTCCAGGCAAAGGGTTTAA
AGATGAAGAACCGACCCCCCCAAAATTTCAAGGGGGGGAAACAGCCTTTTCCCCAAGGAATTCTCGCCTTTTTTTCCGGGCGGGC
TTTTTTTTTTTTTCCCTTTTTTTTTTAAACCCAAGCCCTTAACTTTTTTTTTTCAGGGAATCATTTTGGGAAACCGCTTTTTGG
ATTTTCGTCAAACGGGGGCCCCCCGGGAATTTTTTTTTTTATGCCCGGCTTTCGGCCGAAAAAAAGCACAATTGGAAAAAAACGG
GGGTTTTTTTGGCAAAAAAATGGGATTGGGGTAAGGCCCGGGGCCGGGGGCACCCCCCCCCCTAAAATTAACCCTTTTTGGTTCC
CTTTTTTGGCCCTTTTTTTTCCGCCCCGTTTTTTGCCCGGTCCCCCGGCCCCGGTTTGGGCAAATTGGGAGCCCACCGGCCCCC
CAAACCCCCCCTTCGGAACCCCCCCTGAAAAAATGGAAGCCCCGGCCCCGGCAAACCCCCTTCGCCCTAACGGAATTTTCCCAA
AAACCCCCAAACTTTTCCAAAGAATTGGAAAGGGCCAACCCCCCCAAAAAAAAATCAAAATTTTTTCCCTTTTTGCCGGAGAAA
ACCTGTTGGGGGGAAAATGCCAGGGCAAACCCAAACCCCCCCCCCCCCTTTTGGGCAAAAGAACCATTTATCCCCCAAAAAATC
CGGCGGTTCCCCCCCCCGCCCCAAAAATCCCCCCTTTCCCAGCAAAAAGCGCCCCCCACGCGGGGGGGGGCGCAATCCTTTTTC
GGGGGGGCAAAAAAGCCCCGTTTTTGGGGTTTTCCCCCCCCCCTTTTGGCCCCCCCCAACGGGGTGGGCCGGGGGGCAAAAAA
ATGATTCCGTGGGGGGCTCCCTTGTCCCGTTTTGAGGAACCCCCCGGGCCCCCCCTTTTAGGGGGGCTTTTTTTTGGCGGGGT
TTTTTGGGCCCCTTACTTTTTGTTTAGCAAGAATTGAAATCCCAAAAAAACCGAAATGTACGCGAGCGGAAAAAAAACCGGTTT
TGGAAAAAAAAAGCCCGGAAACTTTTTTTGCTTTTTGGCTTTTGGCAAATAAAAACCGTCCCCCCCATTTGCGAAAACCCTGAA
GCCAAAACACCCAACCAAATTTGAAAAAAATTTTGGGGGTTTCCCCCCCCTTCGGGGTTTTTTTTTCCCCCCGTGTTTTTTTT
TTTTTTCCCCGTAAAGGGTTCCCCTTTTGGAAAAACCGCCGGGGAAAAGTCCCCCCCCAGCGCCCCCCCCCCTGCCCCAAAAAA
AAAAAAAAACCCAATTTAAAAATTTGGTTTCCGGAAAAAAATCCATGCATCCCGCCCAAAAAAAAAAAGGAATGCCCTTTTGCCA
ACTGGGCTTTTTTTTTTTTAACCCCCCCTGGTTTTTGGGTGAAACCAAACCCTAACCAATTCCTGGTATTAACGAAAAAAAAGC
CGCTTGGGCAATTGACCCCCCCTTGGAGTTGAAAGTTTTTTTTTTCCTCTTTTGTCCCGCCCCCCCGCCAAATCACCCCCCCAA
ATTTTACGGCCCCCCCCAGTTTTTTTGGGTTTTAAACCCCCTCCAAACAACCGGTTTTTTTTTCCCAGTAAAAAAAACCCCC
GGGGCCCCAATTGGGTTTTTTTTCATCAATCCCCAACCAACAAAAAGTTAAAAAAAAAACCCCCGTAAAAAATTCCCCCCCGGT
GGGGAAAGCATTCCTTCTCCTTCCGGGTTTTTTTTTCCATCGGTTTTAAATCCAAAAAAAAAAATTTACCCCCCCCCCAAAATG
AAAAACAGAAAAAAATCCCCCCCCCCCTTTTTTACAAAACGGAAGGGGGGGCAAAATCCCCAAAGGTGGGAAAAACCCCCAA
ACCAAGGAAAAAAAAAAAAACCGCCCCCCCTTTAAACCATTGGCCGCCTTTTTTTTTTTTTTTTTTTTTTTTTAAATCAAGAA

AAAAAGCCCCCAGAAAAACCCAAAATTTTTAAAAAACACGGGGCTTTTTTTTTTTTTTTTCTGGAAAGGAAAAAAAAACCTTTTT
CAAACGGAGCCTGGGAACCCGGGCCGGATGGGAAAAACCCAAGGCAAAAAGACCCATTCCCCTTTTGTTGGGGGGGGGAATCGC
TTTTCACAAAACCCCGGACCCCCCCAACGGCGAAATGGAGCCCCCCCCCCTTTTAAACCGCCAAAAGGGCCCTTTTTTTGCCCC
CCCCCTTTCCCCCGGCCGGGGGGCGGGTTTTTTTTCGGGTGGATGACGGTGAAACTCCTTTTGGAAACCCAACAATGCCCCAAG
CTTTTTTTTTTTCCCCCCCCGGAAAAGGGACGGTCCAAAAACAGGCTTTTTTTTTTTGGTTATTTTTTTTTTTGTAAAAAGCCC
GGAAAATGCCACCCCCCGGGGGAAAAAGCAGACAAGCCCCCCCCGTCCCCCCCAAAGGGCCGGCGGTTCCCCCCCAGCGGGTTT
GGGTTTTTGGGCGGGGGTTTTGGGGGGTTCCCGGGGGCCGCAAAAAAGCCCAAATTGGGGGGGACCCCAAAGTTTCCCAAAGTA
AAAGCGGGATAGCCGGAGTTGGGGTTAATAAAACTTTGGGGGCCTTTTTTTTTTTTTAAAACTTAATTTTGCCCGGGCCCAAAA
ATCAAAAAAAAGAAAAGCAAAAGAAAATTTATTTTTTGTTTTTTAAAAAAAAAAAACTTGAAGAGGTTGGGCCAACCATTAAAAA
TTGCCCGGGGGTTTTTTTGTGGGAAATTAAACCGGCAACAAAAACCAAGAATGCCCGGGGGGGGGTAAGGAAAAGAAAAAAAA
AATTACCCCCGGCCCCAATCCCCAAAAAGGCCCCCGGCCCCTTCCTTTTTTTTTCCGCTTTTTTCCCTCGGCCCCTCCACTTTT
TTTGACCTTTTCCCCCCCTTTGCGCCTTCGGGTTCCCGTTTTTTTTTTTTCCGGCCCCCCCTTTTTTTTTTTGCCGGGGCCCCG
AGCGTTAAAAAAAAAAAAATCAAAAGCATTTTTTTCCAAAAAACTTTTTCCAAAAAAAAAAAGGCCCGGGGGTAAAAAATACGGGG
GTTTTTTTTTTTTAAATTTCCCACAAAAGGAAAATTCAGGGGGGGGGAATTTTTTAAAACGGCAAGGAAAAGAAAAACCCAATT
TGGTTTTTTTTTGGAAGGCAAAAAGGCCAAGCCCAAAAAAAAAAAGGCCCCCAAAAAAAGGAAAAAAAAAAACCCCGGTTAAAAA
AAAAAAAAAAAGGCCCCCGGCGTTTTTTTTTGCCCCTTTTTGGCCGGTTTCTTTTTTTTTTTTTTTTTTTTTTCCAAAAAAAAT
AGGCTTTTTTTTTTTTTTTTCCCGCCCCCCTTTGGGGAACCCGAAAAGCATCAAAACAAAAAAAAAATCCCGACGCCCCCCTTCC
AAGGTTTTTCCCCCCAAAAAAAGAGGTTTTTTTTGGCCAACCGAAAAAACCCACCGGGGGAAAAACAAAAGGAACCTTTTAAAT
TTAAAAGATTAACCAGGCCGGTTTTTTTTTTCCCAAAAACCCCCCTGGAAAAAGCTTTCCCCCCCTCGGTGCGCCCCTTCCTCC
TTGTTTTTTTTTTTTTTTTCCCCGGACCCCCCTGCCCCCGCTTTTTTTTTACCCCGGGAATTTTTACCCTGGTTTTTCCCACCC
CGCCCTTTTTTCCTCCCCCCCTTTTTTTCGGGGGGAAAAAAGCGGTTTGGGGCGGCTTTTTCCCCCCCCCTTTCCAATTAAAAG
CCTTTTTTTCCAAAAAAACGCCTTTTTTGTAGGTTATTCCTTTTTTCAAAAAAAGTTTTCGGTTTTTTTTTTTGGTAGGGGGGT
TCCGGTTTTTTTTTCCCCCCCGCTTTTTTTCCCCCCCCAAGGCTTTGGGCCCCTTTGGGTGGGTTTTGGGCCAACCGAAAAACC
CCCCCGGGGTTTCAAAGCCCGAACCCCGCTTTGGCCGGGGGGCCCACCCTTTTTTTTTTTAAATCCCCCCGGTAAACCCTTAAA
AAAAAAATCGTCCCCCCTTTTTTTTGAGTCCAAAACCCCCCCGGGGTAAAGAACCCCCAAAAAAAAAAAACGGAACCCTTTATCGC
CAAAAAATTTGGCAAAAAAAAAAAAGGGCAAAGCCCCCAACCTTTGGGGTTAAAACAAAAGGAAAGTTTTTTTTAAGCAAGAGAG
GGGGGAAGGGTTTAATGTAAAGGCCCCGGGGTGGGGGGGGCTTTTTTAACCAAAAAAGAAAGGTTTTCACCCCTTTTTTTTTTT
TTGTTGAAAAGGTGGGGGGGTGGGGGGCCCCCCTTTTTTTAAAACTTAACGGGGGCCCCCTTTTTTTTTTTTTTAAAAAAAACA
AAAAAAACCTAAAGAAAAGGGGACAGGTTTTAAATTTTTTTTTTTGGGGTAAAAAAAAATCCTTTTTTTTTTTGGGGCGGGCCA
CTTTTTTTTTTTTCTGAGCTTTTTGAAAAGCCCCAAAAAAAAAAAAAGTTTTTTTTTTTAACCTTCCCCCGAAAAAAGGAGTTTG
GGGTAAAGCTTTTCTTTTTTTTGGATTTCCGGCCAAAAAACCCCAAACCACTCGGCTTTTGGTTAAAGCGGGGGGGTGGGGTTT
TTTTTTTTCTTTTTTTTTTTTTTTTTTTTTTTTTTTTTGTTTTTTTTTTTGGCCCAAAAGCAGCAGGGAAAATTTTACCCCGCCC
CCGGCCAGAAAAAAAAAAAAGGGGAATTCTCCCAAAAGAAGAAATCCTTTGGGGATCTTTTTTTTTTTCCCTACGGGGTTTCTTG
ACCCGGCCTTCCACAAAAAAAAAAAAGGTGGAAACGGAAAAAATAAAAAAAAAACCCTCCAAACGGTTTTAAAAAGGGATTTTTTT
TTTTTTGGGTCCAATTGGGAGATTTTTGATCAAAAAAGGGAAAAAAAATTTTTTTTCCTTTATCCCAAAAACCCCTTTAGATTT
TCCTTTTTTTTTTAAAAAAAAAAAAATTAAAAAAAAAATTGGAAGTTTTTTTTTTTTTTTTAAAATCAAAATCCCTTTAAAAAGTAA
AAAAAATATTTTTATTTGAAAAAAAAAGGTAAAAACTTTTTGGTCCCTGACCAAAAAAGGGGTTTTAACCCCAAAAATGGGGCC
CCCCCTTTTTAAACAAAAAAAAAGTTGGGGGGGAAGGGCCCCCCCCCACCTTTAAATCTTCCAAAAAAAAAAAAAAAAAAAAGCGG
GGATTTTCTTTGTCTTATTTTTTTTTTTTTTTCCGTTTTCAAAAAATCCCCCAAATAAAGGTCTTTTTTTTTTGCCCCCTGACTCCC
CCCCGTCCCCCCCGTGGGGTAGAATAAAAAAAAAACTTTAAAAAAAAACGGGGAAAAAAATTTTTAACGGGAGGGGCCCCCTTACCC
CAATTTTTCTTGGCCCCCCAGGTGCTTTTTTTGGGCCAAAATGAAAAATAACCCGGGAAGGGGAACCCCCCCACGGGGGCTCCC
AAAAAAACCGCCCCCCCCCCCCTTTTTTTTTTTTTCCAGAATTTTTTTTTTTTTTTTAAAATCCAGCAAAAAAAAAAAAAAAATAA
AAAAAAACCAAAAAAAAGCCAAGCCCCGGAAAAAAAAGGGGCCCCCGAGCCGGCCAAAAAAAAAAAGAAAAAAGTGGGGGTCCCCCC
CTGGCCCAAAAAAACCTTTTATTCCCGCCTCCCAATTTCCCCAGTCCTAAAATTTTAAAAATTTTTTTGGTTTTTTTTTTGCCC
CGGGAAAAGCCTTAAGGAGTTAAAGTTAAAAAGTTCCGGCCCCAAAGTTTTTTTTTAATAAGTTTTTGGCCGCAACCGTTTGTT
TTTTTTTTTTTTTGCCCCAATTTTTTTTTGGGCCCCCCCCCTTTTTGCAGGCCCAAAATTCGTGGTTTGGGGTCAAACGCTTTTTT
TCAGTCCCCGGTTTTTTTTTTTTTTTTTTTTTGGGGGTTAAAATGGGGCCCTTTTTTCCAAAATTTCCAAGCCCCCTTCCCCCGGT
TTTTTTTTTCCCCAAAAAAAAACGGAAATCCAAGGCGGAAAATTTTTAAACATTGAATCCCCCCCCAAAAAAAAAATGGTTTTTTG
GGGTTTTTTTGGCCAAAAAAAAAAAAAAAAAGGCCGGGGGGGGTTTTTTTTTTTTTTTAAGCATTTTTTTTTTCCTTTTTTTTTTCCG
GGTTTTTTCCCCCCTTCCGAATTTTTCGTTTTTTTTTTTGGTTCCCCCAAAAAAGAAGTAAGTTTTTTTTGGGCCCCGCCAAGTT
GGTTTTAAATCCAAACTTCCCAAAAAATTGGTTTTTATGGGGCAAAAAAGCCCAAACCTGCAAAATTTAATTTTCCCCTTCTTTT
TTAAAAAAAAAACTGTTCAAAATGCAAATTTTCCGTTTTAAAAAGGATTGCTTTTTTTTTTTTTTTTTTTTTTTTTTTTTTTTTTCC
CTTTTTTTGTTGGGACCCTTGGTGAGTTTTTTAAAAAAACTCAAACCAAAAGGTCAATTTCTTTGAAAAAGAAAAAATTTTATG
TGTTTTAAAGAAAATTTGCGGCCCCGGGAACGGAAAAAGTTTTTTTTTGCTTTTTTTTTTTTTTTCCCTTTTGCACCGGCCGGTT
CAAAACAACGGGGATAAGATAAACCCGAGCGCAACAAAAAAAAAAAAAAAATAAGCCAAGAAGCTTTTTTTTTTTTTTTTAAAAAA
GTTGCTCATTCATTTTTGGGAAAAAAAACGTTTTTTTTTCCTTTTTTTTTTTTTCGGGGGCGGAAAAAAAAACTTCTCCAAAGAATT
CATTTTTTTTTACCGCTTTTGTTTTTGAGAAATTCCAAAAAAGTTCGATTTGTTAAACCCCAAACTGCGTGCACCCAACTGAAA

TTTTCTTTCAGCCAAATTCTTTTTTTTTTTTTTTTTTACTTTTTTTTTCACCAGCCGTTTTTCTTGGGTGGGAAGCAAAAAAAA
CCACGGAAAAAGGCAAAAATTTGCCGCAAAG
>m141122_014521_42134_c100732202550000001823141405141560_s1_p0/64948/10593_18158
AGCTAATGAGTCAATGATAATATATCATACCATTGGGGGTCATAGCACATTTCGATAAGCGGAGACCATCCAAGACTATTAGGC
AGTTCTTACGTATGGAAACTGGAAGCATTCATCCTATTCATAACCTTAAGGAGAGCGGAGGGCGACAAGTATCACCAGATAGTA
GCTAAATACGGCCTCGTGGTTATGTGGAGGGACATGTATGAGCTTCAGGACCTCGGTTATTCATCATGTCATGTCATGTGAAAA
ATCCGCCGTAGGATCGACAGAAGTGTGCTTTCATCCAAACAGGGTAAACATTATGGACTACCCAGGTAATTATCTATGAGCAAT
GTCAGAGCATATGCTGTCCCTAAAGGTTATTAGTCCAGAGATCGTCTAGGAGGAATAAGCTCACTAAGCATCCAAGGGCCAAAT
CGGATATCGGTGTGTTCTGTGTGTAGGGGAGCAGCGCATCTCAGTGGCTTTTCTTCAATGCGCAGCAGACAATGAAATGTGTTA
ACAACAAACACGTCAACCAGACTTACTCACATAGGCAATCTATAGACGTTGGCGTACTCGCGCTATATGAAGTTTTAGCTACCC
ACATTTTCTTAAAGACAGTATCAGGCGAGGAAATACTATACACTTCCTACACAGACCATTGCGGTATATATATTAAATTGGCCG
CCGTTACTCGGCTCCACTATTGGAACTCCAGAGAGCGCACACTTGCCAAACGTGAGCCCTACCGCCAGTAGTGAGAACTCTTCA
TGATATATTTTTGACGTACGTGGACAGTCATACGCTGTCCTCTCTAATGTATACTCATCAGCAAGTGTCGACACTGCATAGCTG
ATAAATGCAGAGGGCGTAAGCTCTACCCTAGCAAACTCAATCATTCTATTTAGGCATGGAACCCTTGGCCCTTGCCAAGTTATC
GTAGCACGTAAGGGTGGGTCTCATCCTAAGAATGAAACTCGGTAGCGTGGGTACGGCGTACATACCACATTCGGGGCAATCCCT
GCGTGAGGGCATAACCCTAAGCGTGGTGGTTCTCGAAACATCCACCCCCCGCGTCAGTTGGCAGTCCAAGCGCTAACGCCAGAC
GGTCTCGGCGCGATTTTCTACTCTTAGCAATTGAAGTAAGCATCATTGGCCGCCCATGTCGCAGTCTTGTGAACGACGTGCGAG
CATCCTTTCTCTTCGCCTCCAACTCCCTGTATGCGTATGGTCTTCATCATTGTCAGCGCAACCGATAGAACGAGCACAATTAGA
CCCGCACCTTCCAGCGACCAGAACCTAGACGAGATGGAGCTACCTTACACTAGTAGAACAACGAGGAACGTACAGGGCGGTCTC
AACAACACGTAAGTGCTTGACTATCAGTCGCAGACCTCAGTCTCAGAACCTCTTCCCCTGGTGGCATGCGCCATGCAAAGGCAC
GAGGGTCTCACAACCTGGATGCGGTTTTCAAACACCCATTATTACCCACCGCATTGTCAGACATAGTCTGTGGTCGTATCACAT
GCTGTTTTCCGACGACCCAATACGGATCATCCCGCATTGGTTTGCCTCTTTAATTGACGACGAAGATAGAGGCCAATACGAGGT
GGTTTCTCCTACTTAATTGGTCATTAGGTAAGCACCTTGCCGCAGACGTGGACATTAGTCGCGAGAAAGTGCTACCCAGACAAT
CCAAGATTCGGGCAAGTTGCTCCTCAAAGTTTGGGGCGTATGGTTCCCGAATGGCAAGCATGGAAAACAGGGGTAACGAAGGGG
TAGTTTCTTCAACTAAAATGAAAGAGGGTACTGACCATGTTGCTCCGAACCTGGCCAAGACCGCTCGCCGGGTATACGACTGGC
GATGCTCTCATTTTCTTTCTACTTCACTTTCGCTGATACGACCCATCTTGAGGACTCAATTTCACAACCATTAGGAGACACTGG
CGAAGAGCCATGTCAAATCCGGAGCTGTGCAGGGGGTCTTCACTTTAGTGCGATGGACCTCCTGGCCCCATACTCGCCTAGCAC
CCTTTGTTATGGAACACACATCCACCCCCTAACTTCAGAGTGGGAGCGGCACACCCAGTACAGGCCTACCCCAACTTAATTAAC
TTCCTTGTAGGGTCTAACAATAACCACATGGTACAGGGTACCCTTGTTCCCCCGCATGCGTGCTTACCCTTCCCTCCGCATTGC
GCCTGCCAAGGGGGTGCCACGAGGGGGTTCACTATGCGAGGGTACGCTTCCCCGTATGTGGAGGTCTAGAGGTATGCGTAGGTG
TGTAGATGCCCGCCCGTACGTTCAGATAACCCTTCCCCCATGCGCCTGCCAAGGCCACGCAGGGTCCTCATTAAGCGAGGAACC
TTCCCCAGCATGCATGGCTACCATTTCCCCCGCATGCGCCTGCCAAGGCCACGAGGGTCGACTATGCGAGGGGTACCCTTGCCC
CCGCATGTGAGGTTACCCTCCCCCCGCATGCGCTCCCAACCCACGAAGGGTCTCATTACTGCGAGGTGCCCTTTCCCCCACATG
CGCCTGCCAAGGCCACGAGGGTCTCACGTATGAGTACCTTCTTCCGCATCGCGAGGCTACCTTCCCCGCTGCGCACTCGCGAAG
GCGACGAGGCGTCTCACCTATGCAGGGTACCCTTCCCCGCATGCCGAGCTACCGCTCTTCACCGCATGCGCCTGCCAAGGCACA
AGGGTCTCCACTATGCGAAGGGGTACCCCATCCCGCATGCGTGGCTACCTTCCCCGCATGCGCTGCAAGGCACGAGGTCCTCAC
TAATGCGAGGTACCTTCCCCCGCATGGGAGGACTACCCTTCCCCCGCACGCGCCTGCAAAAGGCCACGAGGGTTCTCATTATGC
GAGGGTCCCTTCCCCGCATGCTATGGCTACCTTCCCGCATGCGCTGCCACGGCACGGGGTCTCACTATGCGAGGGTACCCTTCC
TCGCATGTGTTTACCTTCCCCGCATGCGCCTGCCAAAAGCCACCGAGGGTCTCATTATGTCGAGGGTATCTTCCCCCCGCATGC
GTGGCTACCCTTCCCCGCATAGGCCTAACCAAGGCCACGAGGGTCTCACTATGCGAGGGTACCCTTCCCAGCATGTGAGGCTAC
CCTCCCCCGCATGCCCTGCAAGGCCACCAGGGGTCTCACTTACTGCGAGGGTAACCCTTCCCCCTGCATGGTGGCTACGCTTCC
TCCGCATGCGCCTGCCAAGCCACGAGGGTCTCACTATGTTAGGTACCCTTTCCCGAATGTGAGGTACCTTCCCCCGAATGCGCC
TGCCAGGGCCACGAGGGTCTTCATTTGCGAGGGTACCCTTCGCAATCGTGGCTAACCTTCCCGCATGCGCCTGCCAAGGCCACG
AGGGGTCTCACTATCGCGAGGGTACCTTTCCCCGCATGCGGGCTAGACCCTCCGCCGCAATGCGCCTGCCACAGGCCACGAGGT
TAACATGCGAGGGTATTCCCCGCATGTGAGGCTACTTTTCCCCCGCATGCGCCTGCCAGGGCTCACGAGGGTCTCCTTCATTAT
GCGAGGTACCTTCCCCCGATCGCGAGCTACCCTTCACCCGCATGACGCCTGCCAAGGCCCAGGGCCCTCACTATGCGAGGGTAC
ACTTCCCGCCCGCAGTGCCAGGCCTAACCTTCGCCGCATGGTGCTTGCGAAGGCACACGGGTCTCACTCATGCCGAGGCTACCC
CTTCCCCCCGCCAATGCCCCAAAAGGGGCTACCTTAACCCACTATGCGCCGCCCAGGCCACGAAGGGTCTCACTATGCGAGGCT
AACCTCACCCGCGCGGCTCGCGGACCAGCAGATGCGCGCCAATGCTCGCACGAGCAAGACACCCTTACCACACGCTCGTGCTGT
GTCTCGCGAGGGTAAGGGACCACGCTAAGGACCTCATGCCACCAGACCGTCTTGTGGCCTCATCAACCCCATCACGCCTTGTGA
CCCATTCCGAGCTCGTGCCAAGTCACTGCCTGACTACTCCGCTGAGGCACCAAGGGGTCTTGCCTAGTGAGACCCATCCCCCAA
CCTCGTACATTGCCTATTCCCAGGCCTCCTACCTGGAGGTAAGGACGGGATAAAAGAAGTAGGATGACAACTTACTAAAATATA
ACAGAAACCTAGAAATATGATTTAGTAGAAGGGGAACCCTAGGATGAGTAAATGTACAAGCACGGGATGTACAACCTGAAAAGG
CAAAGGCATGACCTGACATCTGTGTCACAAGTAAGTAAGACGATTCGTACAGAGATGGAAGCATACATGCATACCTCTTGACAT
GTACCAGGCGACACCATACTTCTCTCCAACCACGACCTGTGCCACTACCCTGGCAATGTACCTCTATGTAATCTTGTCCCTCGG
ACCACAATGTTGGGGGAGCCATTAGGCTTCCAGTATAAATAGATCATCACCGCTAGAAAAGGGGTGGAAAACTGATTGCCATGC
TAAGGCTATTAAAGTAATATACAATTTGCACTCCATTACGAGTTCATACATATTTACTCTTCAAGTTTCCTCCCATCTTCTTCT
GAGGAGCTTTGGCAATATATACTTAGTTTTTCTAATCTTATTATCGTTGACGAGATTCATGTCAACAGTTTGGCACTTCTTGGG

ACGTAGCGACCGAAGCTACTGTCTACCATTTACTTCGGCAAGGAGAAATGCCAAAGACGTTCAATCAGCTGACTCAGGGAGCGA
CGAGAGGATGGGTCCACCCTAAACAGATCCAGCGAAGGCCTCCATGTTAGGATCCATCTCCGCCCGAGACGTGGAGCCCCCAGG
ACCTGAGGTTCTTGAGATGAAGGGAGGCCTCGTCACCAGGCGGCCATCCGTCCGGTGATAATCCTTCCAAGGACAAACACCGCA
CCACAAGGATAGCCAACGAATTCCCTCCTCCCAAACCACCGCAGTATTGAACTCCGGCCGCAGGATCCAGGCCCCGAAATCGAT
GGTAGACATGACAAAAACCCGGCTCCATTCCGTGACCGAGTTCTAAATCCGTCGTTTCTATGAAGAGGTGATTCGTCAGCTGCA
CCGTAAAAACCAAGACTGGAGACCACCCGAGGAGAACAGTGCAGGAGGGTGCTGAATGGCCGGTTGCAAGGTAAGTCGAACGTG
ACCTTGCCCCAAGAGGGAAGAGAAGGTCAGGAAACCACTGTCCCGGTAAAAGGGGGAGACAGAACACTCCACGGCAGCTACAAC
ACCCCAGAGGGAGAAGCCCACATGAGCGATGCCCGGCCCCATCGAGCCACCTCAGGGGCCAAGGCAGACGGACACAAGTGTTT
GGCTCTCACCAACCGACATCGAGGTCACCTCAAAAGAGGACGCACTCAGGTCTACGAGAAAGGCTCAACAAGGGAGGGGCCAAG
ACAAGAACTACACCTCATACTGTCCCCCGAGACGAAAATGACAGGAGAACTGCTCAACCCTCCATCGGCGTCGAGCGCACACCT
CGAAGGAGCATGGACCGTAACAGTCCTCAGAATACAGAGACGAGACATGCCGCGAGCCCAGATTTCGCTCGCACCGAGCCGCAA
TCCATGCAAAGACCTGAGCTTCGATCATCGAATCGCCCTCACTAAAGAGATCCAAGCCATCCGAACTCCCAGGCCAACTTCAAG
GAGCCACAGATGACCTATGAGGAGGACACGACCCCAAGTACCCACTTCTGACGCCATTCAAGATGATTGAGTGAGGGCTCAGAG
GGATCAGAGCGGGGCTACCCGATGCCCACTCATTGTCGTGGTTCACATCGAAAGGGCTGCGATGACAAGTGGTTCAACAAGACT
TTGCCCAGCCACATCAGGTATTCGGCCGCAGCAGGTTTTCGGATGAGTTTCATCAAACAGCCATCACGTCGCTACGTGATTAGC
AGATCAGAGCGCCACCAGCTCGCCAATGTGACAGAACAAGGAGAAACAGAGTCCTAAAGGGTTACCATTGCCCCCGAGTTTAAC
AGGAGCGGCAGCGGCCAAGTCGGGCTTGACCCGCAAGGGAAATTAAAATGGCCATCACACCGAGTGCGTCCGGAAGCCAAATTG
TGGAGCCAAACATGGGCCGAATGAGGGCGAAGTCATGACTAGGATGACTTATGCAGAGGCGAAGTATACCTTCGGCATGGAGGT
GGCCCCCATGCACAGACTTAAAGGCAGCGAAACCAGCGAGTCCCATGGAACCAGCTCCGCCAATCGAGCCGCGGGTCGCAATAG
AAGCACACCAGCTGACAGAGAGAGCCCATCAACGACTGCGCATGTAGCACAGAAAACGCCGAACTTCCTTACCAGTAACCCCTA
CGATGAACGTCTAAAGACGTCAATATAGAACGGGAGCAGCTGCTAAAGTGGTCGCCGTTGCGACCGCAGTTTACAACCACAGTC
GGACGCCCACGGCAGCCCATATAAGGCATCACTAAGCTCCTGAATAGGCACAGCGCACGTCACCCCTCTAACAATTGCCAAAAA
GGCCCAAATAACGCCTATGAAAAGGGGTCCACGAGACGTTCGCAAAAAAAGGTCCTCCAGAGTGACGCTTGCAAAAAAGGGTTC
CAACGTACGACGTTTGCAAACAGTCTCAAAGTCAGACGACTTGGCAAAAAGGGTCTCAACCATAGACGTTCGCAAAAGGTCTCA
GCAGTAGCACGCCTTTGCAAAAGGCGTTTCCAATGTAGACGCTCGACCAAACGGATCTCGCGAGTAGACCCTGCAAAAACGGTC
TCCAACGTCAGAGCGTTTGCCAAGGCACTCTCACGTAGACCGTTTGCAAACCAAGTCCAGGCCAAGTACACGCTTGCAAAAAGG
GTCCAAAGAAAGGTGCCTCTGCAAAAAAAGGTCAACGAAGACGCTGCAAAGGATCTCAAAGATATCTGCTAAAAAAGTACTATG
TAAACGACGAGTAGGACGCTTCTCGCGATAGATACAGTAAGCGGCACGCAAAAAATAGGATAAAAGGATACGCTAGAACCATAA
AGGGTCAACAATATCCTATAGACAACCAACCACGGTGGCAGAACCAAAGAGAGACCCCTATTGAACCCCCATCTGCGAGGTCGC
GTGGTGTCCCGAGGACCCCCGAGCATAAAAAAAAACAAAAAAATATGATAAAAAATAAAAAGGAAGAGAAAATCTACAAGAACG
CCTAAAGGCCTCCATATAGCTCCTCAAGTGTGGATGCCAAAAATCCACCAAGAAATAAAATCCCTAGTAAATGGTTAAATACGA
AGGCTATAAGGTCACTTAGTCACATTATACCGGGCCTCAGACCTGAAGCTCTTGTGAAACCGGAGGTGAATATTATACCCAAAG
GGAAGGCATCACCATTTGTCAGACTGTGAAGTATGGCCTTCGAGAGGCCACACCGCTAACACGTGTCACAAGGGGGCCAGGCGC
ATCCAAAGGGCCTCGCGACAGCATGCACCCCCCGACCACCGCACAAAGGGATCTCGCTATGCGAGGCTAACCCCCTTTCCCCGC
GCGCCTGCCAAGGCCACGAGGAGTCTACTATGCGTAGGGTACCCTTCCCCCCCCAATGTGAGTTACCTTCCCGTGCTGAGCCAT
GTGGC
>m141122_014521_42134_c100732202550000001823141405141560_s1_p0/66819/9172_14823
TATTGAAATGGGAAAAAGTCAAAAAAAAATTAGAGCTTGTTAGGATGTATATATGAGTGAATTACGAGGATAGCTTCGAGGCTA
GGCAGCTTGCATAAGATTTGGCAGGAAAGGGCAATTTTTTGTTTGGTTTGAATTCAGCAATCCCAATTCAATGGCAATGTAGCC
TAATAATTATTATTGGAAATGGAAAGGTTCAAAAACAAATGAGCTTGTTAGAGAGAATATGAGTGAATTACGTAAGATCGCTTA
GGAGGGCCCTTAGGCGCTGCCTTAGGAGATTGTGAGACAGGAAAGGCCAGTCTCTTTGTTTGGTTGAAATTAGCAATCCCGAAT
TCATGGTTCATAGGAGCCTAATACAGTAAATATTATTGGAAATGGGAGAGCGTTGTAAAAAAATAGAAGCTTGTTAGAGAGAAA
ATATGAGTTGAATTACGCGATCGCTTCGGAGCCCTAGGCACGCAATGCTAGAGATTCTGGTGGGCCAGGATGAAAGGCCATTTT
TTTGTTGGTTGAATAGCATCCAATTCATGTCCAAAAGGGGAGCCCATGAGTAATAGTTATAGATGAAAATGGAAAGAGTGAGTA
AAAGAATTAGAGCTTGTTAGAGAGAAATGTAATTGAGTGATTACTAGATCGCTAAGTGAGAGCCTAGAGGGCAGCTCCTACATA
GGAGTTGGCAGGAAAGGCCAATTTTTTGTGTTTGGTTTGAGATTAGCATCCCATCAATGGTCACAATACGAGCACCCTATATAG
ATTATATTGAATGGGAAAAAGGTTCAAAAAAAAATTATGAGCTTGGTTAGAGGAGAAATATAGTGAATTACGAGATCGCTTTCG
GAGACCTAGGTCCAAGGCGCTTGTGAGATTGTGGCAAGGAAAGGGCCAATTCTATTTCTGTAGTTGGTTTGCTAATCAGCATCC
CAATTCAAATGTCCAATAGTGGGGCCCCTGATATCAATAATTACATCCTGAAATGGAAAAGGTTAAAAAAATTAAGAGCTTGTT
AGAGAGAAATATGAGTGGAAGATACGAGAGTCGGCAATCAGGCAACACCTAAGGCGCTTGCCTAGGCAGATTTGGCAGGGAAAG
GCCAATTTGTTTGTGCTTTGGTTGAATATACATCCCGAATATCATGGCGTCAATAGGCCTATCATACCCTTCATACTGATTGAA
AGTGGGAAAGGTTCAAAAAATTAGAGCTTGTTAGAGAGAAAGGTAATGAGTGATTACAGATCGCTTCGGAGCCCTATGGGCATG
CCTGCCCTAGGCAGATTATGGCTAGAAGTCCCAATTTGTTTTGTTGGTTGTCATTGGATGCATCCCAATTCCTTGGATGGCTTC
AATCAATCCGTACCTAATAAAGGTAATATGGAATACTTCATTGAAATGGCGAAAAGGGGACAAACAAAACTTAGAGCTTGTTCA
GGAGAAAATAGTGAGTGGAATTACGAGAATCGCTTCGGAGCCCTAGGCCAGCTGCTAAGGAGATTTGGCAAGAAAGGCAATTTT
TCTTGTTTGGTTGGAATTACATCCCAATCATGGGCAAACTCAGTAGCCCTAATATATTCAGGCGGTTGTTATTGAAATGGGGTA
AAAGGGTTCAACAAGAAGATTAGAGTGTTAAAAGAGAAATTAAATGACATTACGAGAGTCGGTTCGGAGCCCCTAGGCGAGCAT
GCTAGCGAATTTTGGCAGGAAAGGCCAATTTTTATGTTTGGTTGAATTAGCATCCCATTCATGTCCCCCAATGTAGCCAATATA

ATAAGAGGTTATTTGAGAAAATAGGGAGAAGGGTTCAAAAAAATTGGAGTAGCCTTGATATAGAGACGAAATCCAAGTTGAAAT
CGAGATTTCGGTCGGCAGCCGCTAAAGGCAGCGTGCCTAGTTGAATTGGCACGGAAAGGCAAGTATTTTAACTCGCTTATTGGT
TGCGTGGAAGGTTAGCATCCCAATTGGTCAGGGTCAAAAGAGCTTAATAAATCTGTCGGAATAAATATGATTTAATAAGAGAAG
TGGTGGAAAAAGGGCTTCTAAAAAATACCTTGAGCTTGTTTTTGGTCAGAGGAAAAATATGAGTGCCATACGAACTCGCTTCTG
GACGCCCTTAGGGCCAGAAGCATGCCTATGAAAGGAGATTGATGCAGGAAATGGCCATCTTTTAGATTTGTCTTGTTGAATTAG
CGATCCCAATGTCGATGGTCACATAGGACCTATATTACATTACTTTATTGAATGGGAAAAGGTTAAAAAAATTAGAGCCTTGTT
AGAGAGAAACCTATGAGTGCTTACGAGATCGCTCGGGCCACTAGGCCGCCTGCTGTGAGTGTTTGGCAGGAACGGCAAATTTTT
TTTGTTTGGTTGAATTAGCATTCCCAACATCTCATGGTCCAATAACCCATATAGAATGATTATTGAAAATGGAAAAGGTATCA
AAAAAATTCAGAGCTTGTTCAGACGAGAAATATGATTGAACTTACGAGATCGCTTCGGAGCCCTAGGCAGGCCCTAGGAGATTT
GGCAGGAAAGGCCAATTTATATTTGTTTGGGTTAAATTAGCATCCCATTTCATGAGTCAATACGAGACCTATATGTAATTTAT
TGAAATGGAAAAGTTCAAAAAAATTGAGCTTTTAAGAGAGAAAATATGAGTGGCAATACGAGATCGCTTCGGAGGGCCTAGGCG
CTGCCTAGGAGATTTGGCAGGAAGAGGCCAATTTCTTTTTGTTTGTGTTGAATTAGCATCCCAATTCCATGGTCAATAGTACC
TATATATATTATTGAAATGGGAAAAGGGTTCCAAAAAAGAATTAGAGCTGTTAGAGAAATATGCAGTGAATCTACGAGATCAGC
TTCGGGAGGCTAGGCACTAGCCTAGGAGATTTGGAGGAAAGGCCAATTTTTTTGTTTGGGTGAATTAAGCACTCCCAATTCAT
GGTCAATAGGAGCCCTTATATAATCAAATTATTGAAATGGGAATAAGGGTCTTTTAAAAAAATTAGAGCTTCGTTAGAGAGAAT
ATGAGCTGACAATTACGAGATCGCTTCGGAGCCCTAGGCCAGCTGCCTAGGAGATTGGCATGAAAAGGCGCATTTGCGTTTTTG
TGTTGGTGATAGCATCCCCAATTCATGGTCGAGGAGGGAGGCCGCTAAGTATAGTGATTGAGGGAAATGGAAAAGGATTCCAA
AAAAATTAGAGCTTGTTTAGAAGAGAAAATATGAGTGAATTACGAGATCGCTTCGGAGCCCTAAGGCAGCTGCCTAGGGAGATT
TGGCAGGAAAGGCCAATTTTTTGTTTGTTGAATTAGCAATCCCAATTCAGTGGTCGTAGGAGCACCTATAGTAATATTCATCTG
AAGATGGGAGAAAGGTTCAAAAAATTAGAGCTTGTTAGGAGAAATATGAGTGGAATTTACGAGATCGGCTTCGGAGCCCTAGGC
AGCGCTAGGAGGATTTGCTGAAAGGGCAATTTTTGTGTTGAATTAAGCATCCCAATTCATGGTCAATACGGCCCTAGTATAATA
GTATAGAAGATGTGAGAAAAGGTTCAAAAAAATTAGAGCTTGGTTAGAGAAAATATGAGTGAATACGAGATCCCTTCGGAGCCC
TAGGCAGCAGCCTTAGGAGATTTGGCAGGAAGGCCAATTTTTTGTTTGGTTGAATTAGCATCCCAATCATGGTCAACAAAGTAG
CCCTATAAATTTATGAAATGGGAATAAGGTCAAAAAAATTTAAGAGCTTTGTTAGAGAGAAATATAGTGGACTATTACGAGATC
GCATTCCGGAGCCTCTAGGCAGCTGCCTAGAGATTTGGACAAAGGAAAGTTCCCATTCTTTTGTTTGTTTGATTACGCATCCCA
TTCATGGTCAATAGTAGCCATATACTATTATTATGAAATGCGAAAAGGTTCAAAATTAGAGCTTCGTTAGAGAGAAATATGGAG
TGAATACGAGACTCGCTTCGGAGCCTAGGCTAGCTGCCTAAGGAGATTTGACAGGAAAGCCCAAAATTTTTTTGTTGGTTGAAA
TTAGCATCCCAATTCAATTGGTTCATAGATCACCAACCTATAATTTATTGAAATGGGAGAACGGTTCAAAAAAATTTAGAGCTT
GTTAGAGAGAAATAGATTTGAATTACGAGAATCGGATTCGGAGCCCTAGCAGCTGCCTAGGAGATTTGGCAAGGAAAAAGGGCC
CATTTATGTTTTGGTTGAATTAGCTATCCCATTCAAATGGTCATAGTAGCCCATAAATATTAATTGAAATGGGAAAGGGTCAAA
AAAAAGAGCTTGTAGAGAGATAATAAGGATTGAATTTTCGAGGATCGGTTCGGAGCCCTAAAGGGCCCCAGGCTTGCTGGAGAT
TTGGCAAAGGCAGGCCAATTTTTTGTTTGGTTGAATTAGCTCCCAATTCAGATGGTCAAATAGACCCTATATAATATTTATGAA
TGGGAAAGGTTCAAAAAATGAGCTTGTAAGAGAGAAATATGGTGGAAATTACGAGATACTTCGGAGCCATAGGCAGCTGCCTAG
GAGATTTGCGGAAAGGGCCATTTTTTTGTTACGGTTGATTAAGCAATTAGCAATTCCATGCGGTCAATAGGACCCTATACAATA
TTATTGAATGGGAAAAAGTTCAAAAATTAGAGCTTGTTAGAGGATGAAATTATATTGAGGAATTACGAGATCGCTTCGGAAGCC
CCTAGCAGCTGCCCTAGGAGATTTGGCAGGAAAGGCCAATTGTGTTTGTGTTGTGTGTGAGATTAAGCATCCAATCATGGTCCC
AAGTAGGACCCGATATAGATATAGGTGAAAGGGGAAAGGTCAAAAAAATTAAGAGCTTGTTTAGAGAGAAATATGAGTGAATTA
CGAGATCGCTTCGGAGCCCTAAGGCAAGCGCCTACGGGATTTTGGCAGGGAAAGGCCATTTTTTGTTTGGTAATTAGCATCCTA
ATTCATTGGTCAATGTAGCCCCATATAAATTGAAAGGGAAAAGGTTCAAAAAAATAAGCTTTGTTAGAGAGATATAAATTGGAA
ATACGAGATCCGGTCGGCCTATCAGCTGCCTAGAATTTGGCAGGAAAGGCCAATTTTTATGTGCTGAAATCTAGTCTTGCGAAA
TGGAAGTTGTCAGGTTTTAAAGATACGCGAGAGTCAATATAAGAAGATGACAGATAATGTTCATTTGAGTTTGGGTGAAGATAT
ATGATCTGATCTACTTGCATACGGGAATGTGGCTGTGAACGTACGTAGTGGTTGTGTCGACTACTTAAATGGGCAAGCTGAGCC
ACTCGACAAATGTTGCAGCGTCATGACATTATTAATCTTTTCTTCAGTCATTAACATCAAAAGAACAAAAATAGACAGATAAAA
GAGAGAGAAAGAAAATACATTAAGAGAAAAAAGGTAACTAGAAGAAAGAAAGACTTGAAGAAAAAAAACTAAAGGGAAAGCATG
CAAGAAAAGTATGTAGAAACAAGTCATGATAAATCATGGAGAAGAAAATATACACACATTAACAGAGAAGAAAACAGGTTCATT
ACGATTATTTCCTTGGCATCTTGGAGTATCAAGCTGTTTGTTAAGTTGTCGGATGCTCTCACGCAGATCTAGGTAGACAGGATT
CTACTCAGCAGTTATCATGGAAT
>m141122_014521_42134_c100732202550000001823141405141560_s1_p0/71429/0_6553
CACACACACCCGGGCTGTTCGCCGCCGTCGGCGCGTGCCGCGCTCGCGCACCAGACGCAGCGGCGCCGCTTTCGGCCGATCGTG
CGCTTTCGGAAAAGCGGATTGCACGCGGCGCGCGCGCCCCTTTTCCCACTTCGTTTCCTTCTGCGCTTAGATACGTGTCCACTG
ACAACGCACGCGAAAAAAACCTAGGTTAATTCCCGAGGGGAGAAAAGGGAGGGCACGCGTTCGCATAGAACGCAAGTAGTTGCG
ACCAAACATCCGGTCGATCATACCAGCATAATGCCCACGGATCCCATCAGACACTCCGCAGGTAAGCGCTTCGGGCGAGAGTAG
CTACTAGGATGGTGAACCCTCCTGGGAAGTCCTCGTGTTGCACCCCTGAAAACATCATTCTTTTTATTCTTAAAAATCCACTTA
CGGGCCAAAAGTTTGTATTTTTTTAAAAATAATCTTGATAAGCTCATTTATTTCGTTTTCTTGGGGGGTTCATACCGCGCCGAC
TGACAAAGGCCAGCGAATAAGAATTCTAGTTAATCCGAGGAGAAAAGGGAGGCAACGGCGTTTCGCTATAAATGCAATAATGCG
GAACAAACATTCCGGTGCGATCATACCAACACTAAATGCACCCGGATCCCTCAGAAACTGCCTGCAGTTAAGCGTGCTGGGCGA
AAGTAGTACTGGATGGGTGACCTCAACGGAAGTCCTCGTCGTTGCATCCCCTGAAAACATCATTTTTTTTATTCTTTCAAAAT
CCACTTACGCTCAAAAGTTTGTAGTTTTTTAAAAAAAATCTTAAAAGCTCATGTATTCGTTTTCCTTGGGGGGTTAATACCGGG

CCCTGACAATGTGTGTGAGCAGCGAATAAGAATTCCCTATGTAATCCGAGGGGAAAAAGGGAGGGCACGGCGTCGCATACAATG
AATAATGCGGAACAAACCATTCCGGTGCGATCACGACCCGAGCACTAATGCACCGGATCTCCATCAAACTCCGCAGTTAATCGC
GCTGCAGGCGAGATAGAGCAGGATGGGACTCCTGGGAAGCCCGTGTTGCACCCTGAGAAACATCATTTTTTTTATTCTTAAAAC
CATCCACTTACGGACTCAAAAGTTTGTATTTTTAAAAAAAATCTTTATAAGCTCATTTATTTCTTTTCCTTGGGGGTAATACCG
GTGCGCCACTGACAAGGCAGTCGAATAAGAAGTTCCTAGTTAGATCCGAGGAGAAAAGGGAGGGCAACCGTCGCAACAATGAAT
AAATCGGAAACAAGACAATTCCGGGTGCGAATCATACCACGCACTAATGCACCGATCCATCGAGAGACTCTGCAGTTAGCGTGC
TGGTCGACGTATCACTAGGATGATACCTCCAGGGAAGTCCATCGTGCCACCCCTGAAAACATCATTTTTTTTATCATTTCAAAA
ATCCACTTACGGCTCAAAAGTTTGTATTTTTTTTAAAAAAGTCTTAAAGCTCATTTATTTCGTTTTTTGGGGTAATACCGTGGC
AAAAACCTGACACAACAGCAGAAATAAGAAGTCCTAGTTAACCGAGGGAAGAAAAGGGAGGGCACGCGTTCGCTATAAGAATGA
ATATTGTGATGGGACCAAACATTCCGGTGCGATCATACCGAGCACTAATGCACCGGCATCCCACTCAGAACTCCGCAGTTAAGC
GCGCTTGGGCAAGAGTAGTAAAATAAAAAAAGTAAAAAATAGTGAAGAGATGGGTGACTCCTGGAAGTCCTCGGTGTGCAGCCC
CCTGAAAACATCATTTTTTTTATTCTTTAAAAATCCACTTAACGGCGAACAAGTTGGTATTTTTTAAAAAAAAAATCTTAAACG
GCTCCACAATTATTTCCGTTTCCTTGGGGGGTGAAACCGTTGGTACTGACAAGCAGGCGAATAAGAAGTCCATAGTTAATTCCG
AGGAGAAAGGGAGGGCACGCGTTCCTATAAATTAATAATGCGGAACAAAAATCCGGTGGCGATATACCCAGCACTAGCACCGGA
TCCCATCAGAACTGCCGCAGTTAACGTGCTTGGGCGGAAGTAGTACTATGAGGGTGAACCACCCCTGGAAGTCCTCGTGTTGCA
CCCCTGAAAACATCATTTTTGTTTATTCTTTAAAAAATCCACTTAACGGCTCAAAAGTTTGTATCATTTTAAAAAAAATCTTTAA
AAGCTGCATTTAATTTCGTTTTCCTTGGGGGGGTTATATACGTGGCCACGACAAGCAGCGTAAAATGGTTCCTAGGTTAACCCG
AGGGAGAAAAAAAAAGGGAGGGCACGCGTTCGCCTATATAATGAATAATTGCGGACAAACATTCCGGTGCGATCATACCAGCAC
CTATTGGCCCACCGGATCCCACAGAACTCCGCAGTTAAGGCGTGCTTGGGCGAGAGTAGACTGAGGATGGGTGATCCTCCCTGG
AAGTCCTCGTGTTGCACCCCTGAAAACACAATTTTTTTTTTACTTCTTTAAAAATCCACTTACGGCCTCAAGAAATTTGATTTT
TTAAAAAAAATTTAAAAGCTCATTTATTTCCTTTTTCCTTGGGGTTAAACCGTGGCCACTGACAAGCCAGGCCGAGATAAGAAA
GTTTTCCTAGTTTAAGTTCGAGGGAGAAAAGGGAGGGCACGCGTTCGCTATAAATGAAGAATGCGGACAAACATTCCGGGTGCG
ATCATACCAGCACTAAATGCACGGATCCCCATCAGAACTCCGGCAGTTAAGCGGTTGCTTGGCCCGAGAGTAGTACTAAGATGG
GTGACCTCCTGGGTAAGCTCCTCGTGTTGCATCTGGAAGACATCATTTATTTTTTTATTCTTTAAAAATCCACTACGGGCTCAA
AAGTTTGTATGTTTTTTAAAAAAATTCTTTAAAAGACTCATTTATGTTCGTTTTCCTTGGGGGTTAATACCGGGCCACTGAAAA
GCAGGCGAATAAGAAAGTTCCTAGTTAATCCGAGGAGATAAGGGAGGGCACGCGTGTCGCTATAACGAATAATGCAACAAATTC
GGGTGGACATACCAGCACTAATGCACCGGATCCATCAGAAACTCCCGCAGTTGTAAGCGTCTTGGGCGAGAGTAGTACAGGATG
GGTGACTCCTGGGAAGTTCCCGTGTTGCACCCCTGAAAACAGTCATTTTTTTAGTTCTTTAAAAAATCCCACTACGGCTCAAAA
AGTTGTATTTTTTTTTTAAAAATAAATCTTTAAAAGCTCAATTTATTCGTTTTCCTTGGGGGGTTAACAACGGTGGCCACTGAC
AAGCAGCGAAAAAGAATGTTCCTAGCGAATCCGAGGGAGAAAAGGGAGGCACGCGGTCGCATAAAGTGAATGATGCGGAACAAA
CATTACCGGGTGCGATCATACAGCACTATTGACCGATCCCATCAGAACTCCGCAGTTAAGCGTGCTTGGCAGAGTAAGTTACTA
AAGGATGGGGACCTCCTGGAGCTCTCGTGTTCACCCCTGAAAACATCATTTTTTTTATTCTTTAAAAATCCACTACGCATCAAA
AGTTGTATTTTTAAAAAAAGCTTTAAAACTCATATTATTTCGTTTCCTTGGGGGTTAATACCGGTGGCCACTGACAAGGCAGCG
AATAAGAGTTCCTAGTTTAATCCGAGGGAAAGGGGAGGGCACGCCGTTCTATAAATGAATATTGCGACCAGCATTCCGGGAGCG
AGTCATACAAGCACTAATGCACCCGATCCCATCAGAACTCCGCAGTTAGCCCGCTTGGGAGTGTAGTACTAGGATGGGTGACCT
CCTGGGAAGGATCCCTCCGTGTTGCACCCATGAAAACATCAATTTTTTTTATTCTTTAAAAACCACTTACGGCGAAAAGTTTGT
ATTTTTTAAAAAAAATCTTAAAAGCTCATTATTCGTTTCTTGGGGGTTAATACAGTGCCACTGACAAGCAGCGATAAGATTCCA
TAGTTAATCCGAGGGAGAAACAGGGATGGCAATGCGTTCGCTAAATAGAGTGAATAATGCGGAACAAACATTCCGGGCTGCGAT
CATACCAGCACTAATGCACCGATCCCATCAGAACTCCCGCCAGTTAAAGCGTGCTTGGGTCGAGAGTATACTAGATGGGGACCT
CCTGGGAATCCTCGTGTTGCACCCCTGAAAACATCATTTTTTTATTCTTTAAAAATCCACATTACTGGTCAATAAGGTTTGTAT
TTTTTTAAAAAGAATCTTAAAAGCCATTTATTTCGTTTTTCCTTGGGGGGTTAGCTACCGGGCCACTGACAAACAGCGAATAAA
TTTTAGTAATCCGAGGAGAAAGGAGGGCACGCGTTCCCTTAATGATTTGCGGAACCAAACATTCCGGGTGCGATCATACCAGGA
CTAAGTGCACCGACACCATCAGAACTCCGCAGTATAGCGCGTTGGCGAGAGTAGTACTAGGATGGTGACCTCCCTGGGAAGATC
CCCGTGTGCGCACCCTGACAAACATCATTTTTTTTATTCTTAAAAATCCACTACGAGCTCAAACAGTTTGTACCTTTTTTAAAA
AGAAACTTTATAAGCTCATTTATTTCGTTTTCCTTGGGGGTTAATACCGTGGCCACTGACAGCAGCAATAAGAAGTCCTAGTTA
AATCCGAGGAGAGAAAGGGAGGGAACAGCGTTCGCTAAAATGAATAATGACGCAACAAAACATTCCGGGTGCGATGCAGTACAG
CACAATGCACGCGATCCCATCAGAACTCTGCAGTTAAGCGTGCTGGCCGAGGAGATAGTACTAGGACGGGTGACCTCCAGGGAA
GTCCTCGCTGTTGCGAGCCCCTGAAAATCATCATTTTTTTTTGATTCTTCAAAATCCACTTACGGCTCAAAAGTTTGGATTATT
TTTAAAAAAAACTTTATAATCTCATTTATTTTTCGTTCCCTTGGGCGGGTTAATACCGTGGCACTGAGCAATACAGCGAATAAG
AATTCCTAGTTATTCCGAGGAGAAAAGGGAGGGCACTGCGTTCGCTATAAATGAATTATGCGGACCAAGACATTCCGGGTGCGA
ATCATGACAGCACTAAATGCACCGGATCCCATCAGAACCTGCCGCAGTTAATGCGCGCCTTGGCGAGAGTAGTACTAGGATGGG
TGACATCCGGGAAGTCCTCGTGTTGACAGCCCCTAAAACATAATTTGTTTTTTTATTCTATTAAAAATCCATTACGGCTCAAAG
TTTGATTTTTTAAAAACATCTTTAAGAAGCTCATTACTTCGTTTTCCTGGGGGGTTAGCACCGTGCCACTGACAAGCAGCGAAA
AAGTTCCTATAACCGAGGGAGAAAGAGGGAGGGCAACGCGTTCGCGTATAAAATGAATAATGCCGGAACCAAACATTCCGGGTG
CGATCATACCCAGCAACTAATGCAACCGGATCCCATCAGAAACTACCGCAGTACAGCGTGCTGGGCGAGAGGTAGTACTGAGAT
GCGGTACCTCCTGGGAAGTCCTCGTGTGCACCCCATGAAAACTTCATTTTTTTTTTATATCTTTAAAAATCACTTACGGCTCAA
AGTTGTATTTTTTAAAAAAAATTCTGAAAACGTCATTTATTTCGTTTTCCTTGGGGGTTAGACACCGTTGGCCACTGACAAAG
TAGGCAATAAGAAGTTCCTATTACAGTCCAGGACGAAAAGGGAGGGCCACGCGTTCGCTATAGATGAATAATGCGGAACAAACA

TTTCCGGTGCGATCTGCACCAGCACTAAGCACGGATCCCATCAGAACTTCCGCAGAATAACGCCGTGCTTGGGCGATAGTAGTT
AGCAGTGATGGGTGACCTCAAAATCTGGGAAGATCCTCCGTGTTGCACCCCTGAAAAACATAATTTTTTTTAATTCTTTAAAT
AATCCTCACTTACGGCCAAATTTGTATTTTTTTTAAAAAAATCTTAAAGCGTCATTTATTCGTTTTCGCTTGGGGTTGATACCG
TGGCCACTGACACGCAGTACGAATAAGAGTTCCTAGTTAATCCGAGGGAGAAAAGGGAGGGCACGCGTTCGCTTATAAATGAAT
ATTGCGGACCAAACTATCCGGTGGCGATCAATACATAGACACTATGCACCGATCCCATCAGAACTCCGGCAGTTAGAGCGCGCT
TGGGCGAGAGGTATACTAGATGGGACCTCCTGGAGTCCTCGTGTTGCACCTCCCTGAAAACATCATTTTTTTATTATTCTTTAA
AATCCACTACCCGAAAGTTTGTTTTTTAAAAAAAATCTTTAAAAGACCATTTATTGTCGTTTTTCCTTGGGGGGTTAATGACCG
TGGCCACGACAGTGACAGCGAAGTAAGAATTCCTAGTGTAATCAGAGGGAGAAAAAGGGAGGGCACGCGTTCGCCTATAAAAAT
GATAATGCGGAAACAAACATTCCGGGTAGGCGATCATTACCAGCACTAAGGCAGCCGGATCCATCAGAACCCGCAGTGTAAGCC
AAGGGGTGCTTGGGCAGAAGTAGTACTAGGATGGGTGAACCCTCCTGGGAAGTCTCGTGTTGCACCCCTGAAAACATCATTTTT
T
>m141122_014521_42134_c100732202550000001823141405141560_s1_p0/71689/7020_17581
CACTTTGTCCCTCCTTCGGTCCGATGTGGGTGACGCGTCTACACTATGACATCTGCCACGCACCTGTCCATTTGATTGGAGGCC
CTTAGCAGTGATAGACAAATCAAAGTTGACTTAGCTCAATGCATTTCATTAGTCTCCTGAAGTCCTGGCTTGATGGTAAGACTT
GTCTCAAAGGACTGTCTGTTAATGACATCAATTGTATTGTGCATGGGAAGCTAAGTGCTTCATTCCAACTTCCTTGACGCCGAC
TACTAGCCATAGGGCCAAGTTTTTCGATCTTCTGGATACTGGCTTCGCCGTCTACCAGGCGCTTTGCTGACATAGTAATACGGT
TGTTGTTTCTTCTTTCTCTCCCTTAACAAGGGCTGCGCTGATGGCATGCTCAGACCACTGCCAAAGTGATAGGTACAGCTTCTT
CGCCCTTCTCTGGCTTTCGCTAACGAGGGGCGGCTTTCCCCCAGGTGCTCTTTCAACCTGACAAACGCTTCTCACATCCTTTCT
TTCCCAGCGAACTTCTTTGCCCCCTTTAACGGTATGTCAAAGAATGGGAGGCACTTGTCGGTGGACCTTTGAGATGAACCTATT
TAAGGCCGCCACCCTTCCTGTTAGTATGCACCTTCCCTTCTTATTCTTCGGCGGCTTCATTTCTATGGTGCTTTATGCTTGTCT
AGAGTTGGCCATCGATTCTCCTAGAATTGGACCATGAAACCCAAGAATTTTCTTGAGGAGACCCCGAAGGTGCACTTGGCGGTT
CAACTTCATCCGGTATTTTTCTAGCGTGTCAACATCTCACCAAGGTCGATTGTTGGAGCTCTGCGCATACCTTGGTCTTCACTA
GCATGTCATCCACATACACTTTCCATATTCCTTCCTATCTGATTCGGCGAACATTTTATTCACACAGCCTTTGATAGGTGGCTC
CCGCGTTTTTGAGCCCGAAGGGCATTCAACCTTATAAACGTAGAGGCCCTTTGTCTTGTAATGAATGACGTGTGTTCTTCATTC
TGCAGGGTCATTGGGATTGCTTGTACCCCGAGTAGGCATCCATAAAAACTCAGTAACTCATGCCCCGCTGTTGCATCCACTAGC
TGATCTATCTGGGGGAGTGGGAAGCTGTCCTTAGGATCAAGCTTTATTCAAATTTCGGAAGTCAACGGCAAGTTCTCCACTTCC
CGTTCGTTTCGGCACAGTCTTGGGTTCGAGTCCACAACAGGGTAGAAGGGACTCACGGATGAAACCGTGGCCTTCAACTTTCAA
CCGTCCTTCCTTTAACGCTGCGTACTTGGTTTGGATCTACCGCCCGCTTGTTTCTGCCCTTGATGGGCAAACTTGCTCCCCGGG
GAGATGTTCAAGTGGTGACATTACGCTGGGGTCTATGCCCACCCATGTCCTCTGATGAACTCCCATGCAAACACGTCTAGATTT
TCCTTCAAAAATCTTATCAATTCCTTCTTCCACGTCACCTTGTAGCTTGCCCTTATCTTTCCAATTTTCTTAGGGTATCTTCCA
TCAAAACCGTAGCCTCATCCTACCTCTTCTACTGGTTTGCTCTTTGGCCTCATCCACGGATCCGAGGGTCAAGCTCGCTGCGGA
CCCGTTGGCATGGCTCCTTGCCTCATGCCAACCCACCATTGGCCTATTGGCTCTCGCTGTGCTTTGCAGGCGCGCGGAGTGAGG
TGTTGGTAACACGTTCCCTCGGCTTCTTTCTGGTTTCTCCTTGTCCATACTTGCGACCCCCAACTGGGGTCGGGAACCTGACAA
AACCAAAGGGTTGAAATATATTGGAAGTTTTTATGGCCTTTTTTTTTCCAATTCTTTTCTCAAGGGGAGGGCCTTCCCCCCCAA
ATAAAAACCGGGGGCCCCGGTTTTTTTTTTGAGGAAAAAAGCTGATGGGGCCACAATTCCCCCTTTACCAAAAACGACCAAAGG
TTTAAGACCAAATGACTTTTTTTTTTTTGGGATATAGTTCCCCTTTTTTTTTTGCCAAGGGGTTTTTTTTTTTTTGCCCCCCC
CTTTTTCCCCCCCCCCCCCCAAAAATTGGTAGAAAAAATAGTGTCCCCATAATTTTTTTTTCCCCCGATTTTCCATTCCCCCCC
AAAAGGGGGGGGGTTTTGCCCAAAAAAAAAACTTGAAATTCCCCTGGTTTAATAATCCGTAAATTTAGGGGAAAAAAAAAAAC
GATTTTGGGCCAGGGGTTTTTTTTTTTTTTTTTTTTTGTTTTTTTTTTTTTTTTTTTTCCAAAGGTTTTGGGGCCGGGCATA
CCCCCCCCCCCAATTTTCCCCCCAAAAAAAAAAATCTTTTTTTTTTTTTTTTTCCCCAAAAGGCCAAAAGGGGGCCGGTTTAAA
AAAAACAAAGGAAAAAAATTAATAATCCCACTGGAAAGCCCCCCTTCCCCCAGTTTTTGGTCCCCCCCCAAAAACCAAAAGACC
TTTCCCCCGATTTGCAAAAAAACAAACCGCCAAAAAAATTTAATTTTGGGCCCCCTAGCTTTGAGGACTTTTGTCCCAAAAAAAA
AAAAGCCCCACCAAAAGCGGATTCCCCCCAATTGGGTTTGGGGGGAAAGTGTTTAACCCCCCCTTCCCCGTGCCCATTCCCTTT
TTTTTTTTCCCCTTCAGTTGGGAAAAAACGGTTTTTTTTTTTTTTTTTACGAAGTCCCCGTTTTTTTTTTTCCCCCCCCTTTT
TTGGGGGGGGGGAAAAAAAACTCATTTTCCGGGGGGGGGGGTCCCCGTTTTGTTGGTTTCCCTAAACTCCCCCCCATGAAACC
CGGCCTTTGGGGGAGGGCGGAACCTTTTATTTTCCCCCCCGTCCCCCCGGGCCTTCTTTTTCCTTTTTTTTTTTTTTTGCCCAT
AATTTTTTTAATCCCTTTTTTTTTTTTCGTTCCCTTTTTTCCCCCCCTTAGAAATTTTCTTCCCCCCCCCCCCACCCCCTAAAT
TTCCCTGGGGGACCCCCTTTCCCCCAAAAAAAAAAAAAAAATTGGGTGCCCCGGACCCCCCTTGTTTTTTCCCCCCCCCCTTTG
TAAAAAAATTTTTTTTTCCTTTAAGGGGCCCCCTTTTGCCTCTCCCTGTGGGCCTTTTTTTTGGGGGGTTTTGCCCGGCGGGG
GGGGCCTTTCGCCCTTTTTTTTTTTTTTTTTTTCCCCTTTCCCCCCTGGGCCGGTTTTTTGCCCCCCCTCTCCTTATTTCCCTT
TTTTTCTTTTGGCAATTAGCGGGCCCCCCCAAAAGTTTGCCCCCCCGGGCCCGGGGAAAATTAAAAGCGCCTCCTCCAAAATTT
TTTTGCCCCCCTTTTCCCCCCCCCCCCCATGTTCGAACAAGCGGTGGGGCATAGTAAACACTCCTTCCACACCCCTCCGCTAAA
AGTGGTTAATACTAGCGTCTTCCCCAATGGGTGTCATCCCCCCTATTTGTTGGGGCGGTAGGCTCATAAATGGGACAGTGCATC
CCAAAAGCAAGAATACTACAACACAGTTCTTTAACCCCCCCCTTGGGGGTTCCCCCCACTCCCCTTAGAACCCGGATTTCACCC
CTTTTTCCCCTCATTTGGGTGGGGGGGGTTTTTACCTTTTTTTGTTTTCCTTTTCTCTATTCGGCAAAAAACTTCAAGTACGG
GTTTTTTCCGGCTACGTATTCGTTAGTTTTGGGGGGTGGGCAGCAACCGTCTGATAAAAAAGAATAGGTGATAATAAAAAAGTT
CGGGAGTTTTTACACCCCCCATGGAGAGAGGATGGCCCTCTCCCCTCGAGTGGGAAGCGGGAGTAGTTGAGGGGTACAACATTT
CTAAAGTACCTACAGAGCGAGACATTAGGACACCATTTGTTTTGGGGGGGCCTAAATTGAATCCCCAACCCCCCACCCCCCTCC

CGGGGGCTTTGGTAATTTTTTTTTCTTCCTAATCTTTTTTCCCTCCTTTAGCTAATGTTTCCTAGGCTTCGGATCTTCCACCC
CTCATAAAAGATCTTTTTCTTCCATTTGGTACACTTTCCCCGTACACACCCCAACCTCGGTATCACGTGCCCATCCCAATTACC
GCCGTGCTTCTTTTTTTTTTCCGGTTAAGCACAGCCCAAATCCCATAAATGTGCTCCCCCCCGACCCCCTTTCCCAATTTGTGC
GCTTGGCGTTCGCGGCCCGCGCGTGCCCCCACCAAGGGACCCTTCGCCCCCCGCAAACTATGGGACTTCCGTAAGCCGACCTAG
TTCTAGTTCGAAATAAAGACAAGGCTTCTTTCTTCGGCCCAGGTTGGGCCCACCCCCCATTCCTTTATATAACGATGTGTACAA
CGGAATATAACAATTACCCTTACCTTAGAATGCTCAATCTGTCTATCCCCTAAGTATATTATTCTTTTTAACAGAAGGGGTTCC
CCCCCCCCCATCCCTAAGAACTTTCCCATTCTTTTGGATCTGGGCAACTTTGCCCTTATCCATTCTTTTAAAGCCCAACTCATT
AAAATCCCAAAATTTTGACCCATACGGTACTACTGCTTTTTCCCCCACCCGAGAAAAAGCGGAACCCCGGGGCCGGTTGGAGAG
CGAATATTGCGGTGGCCCATTTATATTTTTTCTATAAAAAAATCCTCCTGCGGGGCCCCACCCCCCCAACAACGGCCCCCTTCCA
ACCCCATTTTTGGGCTGGCTTTTTTTTTTTCTTTTTCCCCCCCCCATGTTTTTGGAAAACCCTGTTTCGAGCAGGGGGCGAAAAG
TGTTAAATTAGTTCCGGTTTGTCGTGTTTTAAGGCCCCCCTCTCAAAAATTTGTTTTACCAGCACTCCTTGATCTTTCTCTATC
TCATTTTAGGCCCCTTTTTGATTACGCGCACCTACTTTGGGTATATGCCCTCCCCACCCCCCCCACCCGCACATACCCTATACT
ATTATATAGGGCCAAAAAATTTTGTCAAAACTAAGATAATTCACCGACCACACCAACTAGAGGGTACCGCGAAACTGAGGGATG
CATTTTTTGGCCTTTTTTGGAGGAAAATCATTCCCGTGCCCACCCCCCTTGAAAAAAAAAATTTGGTTTATGGCCCCAAAGACC
CCTTTTTTTTGATGGGGAACCCCCCCGGGGGGTTCCCCGAAAAAAGTATCCCCCCGCCTCTTAAAAACCCCATTTTTTTTTTTT
GGGGTTAAAAATTGCGAAGGCCCCCCCCCCTTTTTTCAACGTGGGGACCCCCCCCCTGGGGGCGGGAATAAACAGTTGGGGGGAC
AAATCCTTTAAGCACCCCGCTACTTAATTTCCCCCCTTTTCTCCCAAAAAAAAGGCTTTTCCCCCAAATTTAAAGTCCCCCCAT
TATGGAAATGCCCCCCGGGTTTTTCCCCCCTAGATGCGTGTAAAAAACTTAGGGTTTTTCCCCCCCCCGGGTTTTGCCCCCCTT
TTTTCCCCCCCCTTTCCAAAAATAAAACCGGGGGTTTCCCCAAATAAAATGAAAAAAAAGGCTCCCCCCCCCCTTTTTTTGGGA
AAAGTTTTTTGGCTTTTTTTTCTGGGAATCCCGAATGGCCCAACCCCCTTTTGTTGGAAAAATTCTCTTTTTTTTTTGGGTGGG
GGAAAGGCGGGAATATTTTTTTACCCCGTAGTCCCCGAACTTTTCCCTTTTTTCCCCTTTCAAAAGGCCCCCCCCTTTTTTTTT
TTTTTGCCCCCCAGATAAACGCCCGGTTTTTGGACCAAAAAAATCCTTTAAAAAAAAAAAAAAAAAACTCCTTTCCCTTTCCCA
CCCCCCGCCCCCAGGGCCTCTTTTAAAAAATTTCTTTTCCCGTTATCAGAAGGGGTTCCCCCCCCCCTGCCTTTTTTTTTTT
CTTCCTTTCCCAGGGGAGGTCCCCTTTAAACCATGGGGAAAAAAAACCGGGGGGGTTTTTTTTTTGTTGACCAACCTTTTCCC
TTTTTTTCCCTTTTTTGCCCCCGTCCCCCCCGGGGAAAAAAAAAAAAAAACAAAGTAGAAAGGGGGGGGTAAAAAAAGTTTTTTT
TTTTCCCCCCTCCCTTTTTTTAAATTTTCCCGCCCTCTTTTTCCTTTTTTGTTTTTAGCCTCCCCCCTCTCCCTTTTTTTTAA
AAGGTTCTTGGGGAAGGCCCCGGGTCCCTTTTCTTTTGAAAGGTGGAACCCCCCCCTCGGGAATTCCCGGTTGGTGGGAGAGCC
CCCACCCGTTTGGTTTTTTTTCCCCTTCGCCTTTGGCCCCTTTTTTTCCCCTGGGGGGGGGTTGGGCCTTTTGACTCGTCCTTG
AAGGAAAATGCTTTCCCCTTTGTTTGGATTTTTTTTTTTTCCCCCTGGGGGGGGTTTGGTTTTTTATGGGGTGGGGGAAAGTTT
TGGTCCCCCCTTGGGTCCCCCCCTTTTTCCCCACCGTTTCCCCCGGTCTTAACTTTGGGACCAAAGTGGGGGGGCGTTTTTTTT
TTTTCCCTTGAACCCTTTTCCCTTTTTTTTTTCCTTTTTATTTCCTCTTGGGCCCCAAAAAGGTCCAAAAAAACAAATTTTTGA
ACTTTTTTTTTTGCCCCCCTTTTTTTTGCCCAACCAGAGGCCCCCAAATTTTCCCAAAGCAAACCTTTTCCCCCCCCCACCTGC
CATGGGGTTTTTTTCCTCCCCCCCAGGGTAAAGTTCCTCCCAGTTTCCCCCTTTTTTTGAAATTTTTTTTTACGAATGGTAAGT
TGTTTTTCCCAAACCCGAAAAATCTTTAACCCCTTTTCCTTTTTTTTCGGTAAGAAAAACGAGATTTTTTAAAGGACTCGAAAG
CCTTTTAATTCAAAAAAAAAAAAACAACGGAAAATGGAAACCCCCGGGGGGGGTTGGGTTTTTAAATCGCTGGCCTTGGTTTGG
CCGTTTTTTTTTGAGGGGCCCGGGATTTTTCCCTTTTTGCCCGGGGGGGCCCCCCGGGAAGTTTTTCCCGGGGGTAAAAAACTA
ATTTGTTGGGGGGTTTTTGGAAGGAAGGGGAAAACCCCCCCTCGTTTTTTTGGGGCCCCCCCCGTTTTTTTCCACCGTTGGG
GGTGGAGGGGGCCAGGTAAAGTTTTTTTGTGCCTTTTTGGGGAGGATTTCCCCTCCAAAAAACCGGGCGGATTTGGGCCCCGG
GAGGGAACCGAGGCCCTTTCCCCCCCCCCTTCCTTCCCCCATTTCTTCAAAACCAAAAAAAAAAAAAAAACATGAAACCTCAAAA
GGGGTTCCCCTTTTGGGGGGGGTCTTTAAAACCGGGTCCCCTTTTCGGGGGTTTTTTTTTGGGGAAGGGTGGAATTCCCCCTTC
CCCAAAAAAAAAATTTGGGGGAAAAGGGGCCCCTCCCCCAAGCTTTTTTTTTGGGAAACCGTTCCCGGTAAAAAAGGTTTTTTT
TTGGGGAAACCCCCTCCCCCCAAAAAAAAAAAAAAAACCTTTCCTTTTTATTTCGGAGGGGATCTTTCCCCTTTTCCCCAGGGTC
CGGCTTTTTTTCGAATAACGTCCCCCCTCCCCCCGGGCCAAAATTTTTTTTCCCCCTTCCCCCTTTTGGTCCGGAAGTAATGGC
AAAAAAAAAAAACAAAAAAAAAAGAAAAACCAAGTTAAAAAAAGCTTTTTTTTTTTTGGTGCCTTTTAACGTTCCCCCCCAAA
AATACAAAATTTAAACGGCCCCCCCCCCGCCAAAAAAAAACCCTTTGGTTTTGTGAACGGGGGTGAAGAACCTTCCCCGTCCCC
CCAACGAAGTTTTTTTTTTTTAAAACCGTTTGGAAAAAAAAAAAAAAAATTTTTTATCGAAAGGTTTTTAAAGGAAATTTTTTGG
TTCCCCCCCCCCAAAATAAAGAAAAAAAGCCTTTTTTTATTAAGAAATCAAAAAAGCCAAAATAAAACCTTCCCAAAGGAAAAA
AGCTAAATCTTTTTTTCAAAAGAAATAACCCAAAATGGGGGGGGGAAAATTTCAAGTTTTTTTTTTTTTTTTGGGTCCCCATTC
ACACCTTTTAATCCCCCTCTTTGCCTACAGCAAAAAAAAAAAAAATTTTTTTCCCCCAAACCCCCCCCTTTTTTTTTTTCCCAGG
GTGGTCCCTTTTCCCAAAAAATTTTTTCACCCTTTTTTTTTTTTTTTTTATAGTAAGGCTTTCCTAATGGGCCCCCCCCTTTAAA
AAAAAAGGTTAAAACATAAAAACGGGTTTTGGGGGGGTTTTCCCAAAGGGGGACCCCCAAAAAAAAGGTTGGTACAAATTCCCCG
TACTTTTGGGTTTTTGGGGTTTTCCAAGGGGAGTGGGCTTTTTCCCCAAAAAGAGGGTTTTATTCCCCCGGGGGTGGGAACCCG
TTTCAAATTCCGTTCCCGTAACATGGGGCCAAAAAAGGTTTTTTTTTGGGGGTCCCCCCCCAGGAAAAAAAAACGTTCCCCCCC
ACCAAAACAAAATTTACTTTGTTGCCTTCCCCTTTGTCCCACCTGATTACGTGGGCAAAAAAAAAAGCAAAAAAGGGGGCATAA
AAGGGTGGGGCCGGCCAGGAGGTTTTTTTTTGGTTGGGGGGTTTTCCCCCCCCGGGCCTTTTTTTTTTTCCTTGTCCCCCTAA
TTGGCCCCCCCCCCGTAGACCGCAAACGGGGAAAACCCATGGGAAAAAAATTCCCCCCCTTAACCTCCAGCAAATCCCCTCAAAAC
CTTGGCCCCCTTTGGCCCGGGGTTCCGTTAAAAACCCAGTTAAAAACTTTTTTGGTTTTTCCCCGGTAACCCAAATAATTGTCC
CCCCCCCACCCTTCCCCCAATTTCCGACCCCCCGTTACTTAAAAAGATTTCTCCTTTTAAGCCCTAGGGGGGGAAAAAAAAACC

CTTTTCAAAAAAAAAAAAAAAGGTTTGTGAAAAAAAAGGGGGGGGAAAAAAAAAAAAAAAATTCGTCCGGCCAACCCGCCCAAAAA
AAACTGGCCGTTGGGGCGGGGGGTTTTTTTTTTTTTTTTTTTTTTTTTGAAGGTAAGGGGGGGTTTCCCCCCCCCCCCCCT
TGGAAAAACCCCAAAATACGCCCCGTTGGTTCCCAAAAAGTCCAAAATTGGGGTTTTGGTGGGCCCCCCCTTCCCCCCCCTCC
CCTTTTTTGAGAAAAGGGAAATGGTGGGGGGCCTTTGATTTTTTTTGGGGGAAAACCCCCTTCCTTTCGGGGGGGACGGTGGGC
CCCGCCGGTGTGGGGGGGAACCCCTTTTTTTTTTTGGGCCGTTTGGGGCCCCCCCCTTTTTTTCCGCCCGCAATTTTGGGGGGC
GCCCAAAAAAAATTAAGGAAAACCCGGGCCCCCCCCTTCGCAGCCCCCTTCCGTTACACTTTCCGGGCGAAAATTTGGACCAAAA
AAACCCAAATAGGGGTGGCCTTTTCGCAGCCCCCTCCTGTGGGGGGCCTTTCGCCGGCCAACGGGGGGGGGGGGAAAAAAAACC
CCCCCAAAAACTTTAATTTCCCCCATGGCCCCTCGCCGGGCCCCCCTTTTTTTTGGTTTGGCTCGCGGGGCCAATGGGGCGCT
TAAATAGGTTGGCCTCCCGAGCAAAAACTGGGAACAACCTATTTTTTCCCAAAAAAAAAAAATTTTGGTTCTTCCGCGGCCCCC
CTTCCCGTTGGGCTCGGCGGCAAAAAAAAAATGGGCCGCCCTTTTTAAAAAAAATAAAGGTTTGGGGCCCCCTCGCAAGCCCCCG
TTTGCTTTTTCCCGTCCCGAAAATGGGAAACCCACCAAATTTTCCAAAATGGCCTTTCGCGGCCCTATCCCGTTGGCCTTTTCA
AACGCAATGGGAAACACATCAAGGTTGGGCCTTCGCAAGCCCCTTTTTTTTCCCGTGGCTGTCCCTGCGAAATGAAAACAAAAC
CAAAAAATGCCAATTGGCCCCTTTGTCGCGGCCCCTTTTTTTTTTGTGGCTTTTTTTGCGCACGGGACACTATTTCATGGGCC
TTTTTTTCTGGCGGCCCCCTTTTTCGTTTGGCCCCCCTCGGCGCATTGGGGGCGCTATAAAGGTTTTGGCCTTTTTCGCAGCC
CCCGTGTTGGGCCCATTCGCGAAAATGTTTGGGAAAAAAAACCAACCAAAAAAATTGCAATTTGCCCCCCTGTTTTTTTCCCGCG
GCCCCCCTTTTGTGGCCTCAGCGCACGGGAAACACTTAAAAAATTTTTCAAAAAATGGCCCCTTTTTTTTTCCGCCGGCCCCTT
TTCCGGTGGCCCCCTTCGCGCAAATTGGGGCGCCAATTCGTTGGCCTTCCGCGGGGCCCCCCAAATTTTCGTGCCTTTTTCGCC
GCCAAATGGGGGCCGCTTAAATAGGTTTGGGCCTCCGCAAGCCCCTCCCCCGTGGCCCTCTGAAGAATTTGGGAACACCAATTC
AAAAAAAAATGGCTTTTCGCGGACCCCCCTTTTTTCCCGTTTGCCCTTCCGCCCGAAAATTGCGGAACACCAATAGGTGGGCCC
CTTCGAAAAGCCCCCCTTCCGTGGGGGCCCTTTTTTTTCGCGAGAAACGGGGTGCCTTCGCGGGGACCGGTTTTGGGCCCTCGC
GAAAAAAAAATTTTTTGGGAACGGGCCCCTTCCCCGCCCCCAAAATATGGGTGGCTTTTTTTTTTTCCGTGGAAATGGGGGGG
GGGGGGGGCTTTTCCCCGCCCCAAAAAATTGTGAAAACAAAGCCTTTTTGCCCTCTTTTTC
>m141122_014521_42134_c100732202550000001823141405141560_s1_p0/72645/0_7409
AAGAGAGGAAGATTACACGACAGAGACTGACGTTGATACCGTACTACTGCTATACTGCCACTAACTTACACACGTCACACCACT
GCAACGTACATGTATTTTAATCCATTCACATCATCTGTATTACGATACATTATACATTTCTCCACAGATCTCTCATGATCAATA
CCCGTTACCTTCCTATTATAACAGGTTGCTCAAAAGGAATCCCCGCGTTTCCATCAAATATTCTGCTTTTGCCCGGATACTCGG
GTTCAATACCCATGGATTCCGCTGTTTGCTTACTGGCTTCATATAATTCAGAAAGACTAGTTCGCGAGCCAATCTTTTGATCTC
ATACATGCCTTCATCTCTATCTCAAAAGGTGCTAGCTTTCTCGATTAACAGGCTACTATCTACACATAGCGCTCCCCGCTAACA
CACCTGAGCGAGCCATTCAAACATTCTTCCTACAACTTGCTACACATACTACACTCGTTGAACGTGTTTACACTCCCTAATGGT
ACTTGAAGACCATAATCAACCAAGGTCTTCATCTAGAGCAGACTACATAAGGCATAACTCTTGCTCAGGCGTAAGCATATTTAC
TGAACACGACACATTACCACCTTACTATCTGTCCACTCTCACTTCCCAGCTACCTTTTATCACACCACACCCTCAACCATTGAT
TTCACGTTTTTGTGAATATACACGAATCGTTTCTGGATATAACTAGAACCCCTTTTTTCTGATCCATCTCCACACTTAGTCTAA
CTACACTCCGAACCACTACTACTACTCTACCTATCACTAACCACTTACGTGATCTATACACGTTACTACTTTAGACAAGCTCAT
AGCTCCATCTTGACATGTAGACATATCCTTGAAACTGAACTCAAGACACTAGGCTCGAACTCAAACTTCCTTTATAGACACGCG
AGCATACAGCTTGAATCTCTCCACCCTACCTAGAGCGCTCAACTATTGCGATGGAGACGTATATAATTACTTCACACTACTTAC
ATCATGATACAAGTCAAGCGCCTCGCTCCGACCACCATAGACCGCCTAACTACATCACAACATGTCCACTGCTACTGCTTTAAT
GTTGCCTAACATTTTCGGGTAGTAAACTATGAGAGCGTCTACTACATGAACTGCCGGCGCTATTAGACCACCTCATTATAGACT
AGTGATTCTTTCAGGCCCGTGGCTTGTCACATGAGTTTGAATTCATATTTCCCGTATATGAAAAGATAGTATAAATTATCCCCA
TATACCAAAAACGCTCATGTAATGAGTACTGCATCCTCAAAATTGTACCCCTCCCATGCATATATAGCTAGCTAATACATTTTT
TCCCCAAGATAGTTCGCCGCCAACTGTCCGCAGCACCGTTGCTAAAATCTGCCCATTTTTAATACATTATCGCGCAACTTTGGG
GGTTTTTGATGCATACAATATTTTTGTTGGAACGTTGATATATAACTAATGGAAATGCCTTAAAGTATCTCCATTGCCCCGATA
AAATGATCTTGTCAGTATCGGTATAAATGATCTTTTCCCTCATGTTCGGCATAGCGGGAACTCCGTGACTAGACCCACCTGGTG
ATTCCAACCCCCAGTTCCAACAATGCACTTTTTTCCGATCGAGAAGGAGGAACTGCTTGACCGATTGCATATTAGAACTCATTA
AAGCACGATTCGCATCATTATGCCTCGATAAAAGGAATGAGGAAGCTCCAATAGAAAAAATATTGGAAGGGAAATATTTCGTAA
GATGAATACCTGTTTCCCATTCAAAGTCAGGTAATCTTGACGGTATCGAGCCGAACAACCTGTTTCTTCCTTGAACTACCTCGA
TTCAGTGACCAAAAGAATTTCCTGCCGCTACCCATATGTATTCATTCTTCGACTTGGTGATATATTAAACATCCGTACTTTTTT
TGATCTCCTCAGAGATTTCATAAAACGGACTTTCTAGAGATCCCCAATGACTCAATCCTCGCAATGAATTGATAAGGATCCAAT
AAGTCCAACATTGATTCCTTCGACGTATCAATAGGACAAATGCGTCCAGTAGTGACTAGGGTGAATATCTCGTATCCGAAAAAC
TAGCAAGTTCGTCCCTGTCAATCCCTCCAGGGCACAGATAACTCCGATTTCCCCATGAACAATTTGTGTCAATGGATTTGTTCG
ATCCAAAACTTGAGATAATGGGTGTAATCCGAAAAAAAATTCATAAGTAAGTTGTTAATGTAGTTGAGTTACCAAATTCTGAGGG
TCGGTATCTAATTTTATGCCTAAATTGGGCCCCCACTCATAGTTCCTCTAACATATTTTCTAACGAACCAGGGCCAATCCGAAT
TGATACTTGTAAAAGATCCGCTACAGAAACGAATACGTTTTTATTTTCAAATGATCATATCGTCAAGTATACCCATTTCCGAAT
TTCATTCCAATCATAATGATCCGCAGCCTGCCCAATATATCTCGTGGTAACAAAAATGTATTGTTCTGAGGTATATCAAGATTC
ATCTACGGTTCATATTTCCGTTCGACCAATTCTTCCAATTCACATCTTTGTTAAAGAATTTCTTTTGTAATTCCTTACATAAGG
ATTCCGAAAATACCGGATCTCCGCCTACACAAGCAAATTGTTGATAAAACTCCAAAATGGCCATTTTCCCTTTTGACTTAATTT
TTTTTTTTCTCCTTATCCATAACCTCAGAAAGACAACCCAAGAAAAACGCTAACAACAACATTCTCATACGAATACTTCCTCTA
TAGATTTACCCCAATCTCACGCACCGATGACAGAATCTACAAATAACACCGTACATACTACTTTAACCTAGTTTCCACCTACAC
TCACACCCAGCGCCCATATCCCTTTACTACTCTTCTACTCAATCCTCTAATTCTACTAACCTTCTCCTCCCCCACAATCTGAAT

ACTCTTACTACATTGAGCCACCTGAGACTAACATTACCTAGACTCCCTACTACCGCGAAACTCGCTCTTAATACTGAGTCCAAA
TTCTGTACCGGTAATTAGCATGACATACTTACCCAGGAGCTTCTACTGCTACTAGATCTGTCTATCTGAAGATCCCAATTCTGC
TACGTACATAGCGTTATGTCCATTTATACTACTACGAAAAAGTTATCCCAGACGTAGAACTAATCATTAGACGTAACGTACATG
GTGCCTTACAACATAAAAACTGTCATAACTATGGCTTGTACTTTTACTACTGCATATCCCCTACATCTTGGCTACTTGTCTCCA
CATATTAATCCGGCAGAAGAACACTAATTCATATTAATTCAGCGTAACACCGAAATGTGAGTGACCTCTCATATCATACATGCA
TCCTTTCTTTATCAAAACTTGGATATCAGCTCTCAACTTTAGACTCACTTTCCACAAATAATTGGAATTCAATTTTCTTTGATC
CGTATTCTTCCATTTTTGGAAACTTAAAAGTTCTTCTGTAAGCCCTGATCAATGACCACTATCACACAAACCCTTCAAACATTG
TATCTCGAAACTTATAAATACCCGGGTAACCACCTGTAGACATTTACACCATTTCCCTCCCCACAACATCTACTTTTAGACTCT
TTTATTTCCGTACTTCTACTTATCATTAACTAAGACAAATCCCACATACACATATAACTTCCATATCGTCATCAATAAAAACAC
ACCAAACTCCGAACTCATAGCTACTCCTGACCTGGAATTTAATTCTGTTTATTACTGAATCACACCTGAAATTTTCACTACACT
CCATATAATTATAAATAACTAAGCATGGAAGCAATACGTATAGAGACACCGACGCAGGAATAAAGACTAATAGATACTACTTAC
TCATTCACCAAAGACAGCGTAATAGAGCAACAGAAGCATCAACATACTGGAACACGACGAGAGGTGATAAATTCCACTTAGACT
AGGGAGTTTTGGTATAGAATAGCAATCAAACAAAAAGTGATTCAATTTCTACCATGTATTAATGATATTCCGTATTCCAAATTC
CAATTGAATAACCAGAATGTATTCAGGATTTGATCTGTTCAAGTGAGATAAAGACATAATAATCAGAAACAACATAGAATTTCC
TTTTTTTAGCACTTAACGTTTCCTAGCAAATTACACAAAAGATATCTCTAACACGAGAAGAGACCTAAGACTCGTTTCACAAAC
TGCTACTTTAGAGATCTGAAACAACTAGATACGAACAGATGACTTGAACTACTACTTACTACAATTAAGCTACGTTACATGGCT
ACTTCAACTAAAGCAGAGAGCTAACCTACTGCAACAGCTATTCTACATACTAATGCTACTACTACTAACTACTAACTACCTAGA
CTATCTATACACTTTACAGCTTACTATCTTCGGAACGCACCTGCCAGGCTTTATCAAAACTTACTTCACGGTTTATTACTTAAC
AATACCACTACTAGACATTTCAAACATTGAGACTACAACCAAAACATATACTTTGAGCACGAGACAACTACTGCTATTACTGCG
AAACATACTACCTTATTAAGAACCACTACTACCATATAGTCAGATGCATACTAGAACACCTGCTTTCAGAGCAATATCTAGCTC
AACTGCTACCTGCTGCTTTACCTGTTTGTACATGTCTATATACTTATGTTATGGGGTTTGCATCAATCTATTACGCCTAAATCT
TGCTGTTATATTCAGAGTTGACATTACTTTTACTTGCTTAACTGACAGAAAAAGAGACTTCATGTGCTATCACCTACTATACTC
ACTATGATTATCTGAGCTTTATTCTTCATAACACCCACATTTACCATTAGGGAAACACAATTCAACTATGAAGATCCCAAATTC
ACAGAATCGTTCACATCTTACTACACGAATCGACCACAACAATCAACACTACAGACTTTACCAATACAGTCAACTAAACTCGGA
CCACAACTACTTGACTCCTAGCAAACTTTGCTTTGATCGTACCGAGAGCGATAACACCAAGACTTTGATACTTTTCAATAAAAC
TCTGCATATATAAGAAAGTACTTTTAACGCAACCTAATCTCGACTTACATAGACTTACTTAAGAACTTAACATCAACAATACAA
TCACTACTACCTCGACAGGAGATCTGAACTCAGACACACACATCAAATAACGTGAAAAGCATATCTCTTTAACGGGCAATAGGG
ATTGGACTAAACGTAAAACGCATGAACTCAAAGCTATCTAGCTACAAGTACAAAACAATAGCATACAAGCATAATTACTTGGCT
CTAATTGCTCTTATATTACTTAGACATTCACCTACCACTATCGGCGGTCATCTCGAGAGCGCGAGTGGGTACGAGGCGGGGACA
CTTGCAATCCTCACTTTCTCCCACAGTGAACACTCCTGAGCGAGCCTACCACTGCCTTGAGACAACAATAACGCAATCACACAA
CTACTCTCATCTGTCTCTAGCTTTACATCACTAACAATCGTCAACATTAGACTTCCCAGCAGAAGCAAAGCGCAGACACGACGA
CTCTTTGAGACTTACCTTCGATCGACGTATCCTGACAGATTGATCATCTATCTCAACACAGTACGAATACATGCTGACTACACA
ACACCTCCTTTCCTAAGGGTTAACCGGAAGGAAACACTTAGACGTCGTTAGCGAAAAACAAAACACACGTAAATACTACTCTGG
ATATGATAACCACTGCCTTATCCCCACTCTATACAAATCTGGAAGTGTCGACAGACGAACGCCCGGACTATCTGAATTACGCAT
TGGAACTATACTACCTAGCCGGACACCGAGTTACCTAATTTACGCTAGAAGAACCGAAAAGACAGCCTGAAGATACACAACTAA
TACTACTACTATACTACTGCTACTGCTGCTACTACTGCTACTGCTACTGCTACGCTCTGCTACTACTACTGCTACTGCTACTGT
ACTACTACACTACTACTACTACTACTAATACACTACTACCACCACTAACTACCACTACCACTACTATACGACTACTACTACCAC
TACACCAACGTACTACCACCTATACCTACCACCACTACTACCACTACACCACTACTGCTACTGCTACTGCTACTGATACTACTA
CTACTACTACTACTACTACTACTACCACTACTACTACTACTCTATATACTACTACTACCACTACTACCCCACTACTACCCACTA
CTATACTACTACTTCTACTACTACTACTACCGACCCTTACTACTACTACTACTACTTCTACTACTACTACTACTACACTACTAC
TACTACTACTACTACACTATACTACTACTACTACTACTACTACTACTACTACTACTACTATACCACTACCACTACCACTACCTACTA
CATACACTACTACCACTATAACCACTATACTACTACTACTACCACCATACCGAACACTACCACCACTACTACTACCACTACTAC
TACCACTACTTACCACCACTACTACTACACTACTACTACACCACTACTACTACCACCATACTATTACTACTAATACTACTCTAC
TACTACTACACCAACTATACTCTACTCACTACTACTACTACTATACTACTACTACCACTACCTACCACTACTACTCACTACCAC
TACATACTACCACTAACCACTACCACTACACTACACTACCACTACTACTACTACTACTAACCACTCTCTACTACTCCACTACTA
CTACCACCTACTACTACCACCTAACTACCATTAACTACTACTACTACCACCACTACCTACTACTACTACTGAACCACCACTACT
ACCTACTACACTACCAACCACTACTCTATAGCTACCACACTTCTACCACCACTACTAACCACTACTACCACCACTACTACTACA
CCACTGCCACACTATACCACCACTACTACTACCACCACTACTACTACTACTATTACTACTACTAACCTACTACGGCTACTGCTA
CTGCTACTGCTACTGCTACTGCTCTGCTAACTGTACTGCTACTGCGACTGCTACTGCGACTGCAGACTGCACTGCGACTGCTAC
TGCACTGCTACTGCGAGCTGCTACATGCGACTGCGAGCTGCTACTGCGACTGCTACTGCTACTTTTTTTTTGCCTACTGCTACT
GCTTATCTGCTACTGCTACTGCTACTGCTACTGCTTGCTACTGCTACTACGTCTACTACTACCACCACTACTACTACCACACTA
CTACTAACCACCACTACTACTACTACACTACTACAACTACTACTACCACTACTACTATACCACTACTACTACCACTACTCTACA
CTACTACTACCCTTAACTACTACTAATAACTACCACACTTACTACTACCACTACTACTACACTACTATAACCACTAACTACCCA
CTACTATGACCACTACT
>m141122_014521_42134_c100732202550000001823141405141560_s1_p0/74776/3223_12964
CCTAATAATTCAATTCCCGGTAATTCAACCAAATTACCAAAATACCCCTAGGCTTCACCCCGAGCCAGGGTATGTAATCACTGC
AATTTGTGACTACTTCACTAACTTGCCTCCAAAGAACCGACTCGTGCCGAATATCTCAAATATATGCCACCATAATGTTGGGAA
ACTTTATGCATGAATCTTTTATTTATTTTCCCATGTAGATCTACCATCCATCCTAAACAAGTTCATAGAGTTGATTGAAGATGT

AGCGATGGGATCCTATGTAACATTGTTCTTCGCTTATAGATTGAAGTTTTTCAGAGACGAAATAATGATTGTATAGTGAGGATT
AAGGCTTTACTATTTTTAATATAAATGTAGTCTAGTCGAAGATATAATAATTGCAGTCATTTCCTTTTGATCTGTTAGTTTGTC
TCTTAATCTCGGTAATGTACTATTCGGTCTATATCATCTTGATCGCAGAGTATTACAAAAAACCTGAACCGATCTTCAATTTTC
TTCACAATCTTACAAAGTATTCTGAAATAACTGTTGATCCAAAAAGAGGGTGTTTTAATAATTAAACTCGAGTGCACGAAGTCG
TTCGGAATATAATGGTTCATGTAAAGTACGAGGTCGAACCCATGGGAGGTTGACTAACATCAAAAGAAACTATTTCAAAAACAA
AGCCAATAATATTCTAACCTAGATTCCCAAATATTTGAAGATTTGTTTATAGAAACAAAAAATAAAAGACAAGTAATAAAAATA
TTTAAAGATTAAATAGAAAATTGTTTTCAAATGATATACTAAAAAATAAAGATTATTAAGAATGTTAGAACATCCCACAAAATG
CAAGTTCAATAGTATTTATAAGTATATTGATTCCCAAGTTAAATATAGTTGAAATAAATCACACTATATATTTTCGAAAATATA
TTTTCTATTCAAGCACAAGTTACTCTTTCAAAAGGTAGGATTTTTCTTCCACTTATATAAAGATATAATTTCTAAGCATTAGTT
GTGTTACAATCTAAAATGAAACTACAAAAAATCAAAGAGATTCATGTTTAGGGCAAAATATGATACTTATCCCGCTCTACAAGA
ATTTAGATGTGAACAATTTAGATGAAAAAACAATTTAAGTCAAAGAAATATCAAAATTTTTTTTGCATAATGAAGAACTAAGTG
TAATTGCTTTAACACTCAATAATAGATGAAAAAAGCATATCATTGATAGAAAAATCCATAAACAATGTTACACAAATGGGAAAT
CCAACATAGCAATAAAAAAATACTATCTAGTTACAATTTTGCTTCATCATCATAATTAATAAGCCTTTGAAAAAGATTAGAATC
TCATTAACTAGTATTCTTCTGTCATTCATGGAATAGTGCATACTGATGTATATTGCCTCTTCATATAGACATAAGATTGAATAA
TGGTAGAGCGTTAAGTGTAGATATATAGTGATTCGCAAGAAGAGAAAAATGTGTAGAAGAGGTTGAAAAATATACTGGAGAATG
CTCTTTGAAGGAAAACACGTCGGTCCCCATAATGGTCTTACTAAGACTTCTATATATTATATACTTCGTCGAGCATTTATTGGA
TATGGTTGTGTGATTTAGTTATATAATTTAATTATCTTACACATATAGAGTTATATTAGGTTGATTTAGATTTATATTTGTGTT
CCGGGTACAGGTGCTCCTGGGAGAGAGGAGAGGAAAAATAGAGTAAACACCTTTTCAGTTTAGGCCTGTGTGTAGATGTCCGGT
TATTTTGGAATGATGAAAATTATGTTTACCCTTGGTTGTGGTTGTCTGACAGGTTGTAAGAATTTAAGTGAATTATATCGAAGG
GTAAAACATTAGAATTATATTACGCGCCAATTTGTTCATCTACTTCTATTTGTAGTGTATTAGTGACACAAATATTGGGAATGG
TGTCCACGGCCGCATGCATTGATATTTTAGGTCAGATATCGATAATAGCGTTTGTGTATGACTAAAAATGTTCATCTTTTCGAT
GACCTTTTTGTGGCTGTGGCAGGTGCAAATTGGGTCAGGCATTTGGGCGCTTGCGGACTGGCTGGCGTTTGCGTGGCAAGGTGC
AATGATTGCGGTACAAGAGATATTGTGAGATATGTTCAAGTGTCTTTTGATGGGTGTGGTCACTCGGCTTGGCTTTTCGGAGAC
CGAAGGTTATAGGCTCTGGAGGGATGTCAATGCACATTTTTGTGCGAGATTCTGAGTTTGATGGCTGCTGTCCTGTGAGGGTTA
TTCCATTTATTTGTCTTACCTTATCTGTCGCATGAGTTATGGTCATTTATATATCCGGCCATGTAGTTGGGAGATTTGGTGGAG
TCAACGTGGGAAGGCAGGTGGGAGAGAAGTCCGGCACTATACTCGACGTTCAGGGTCTCTTGGCTGGGCTTTTCGCGCACTTTT
TGGGATCACATTGGTGGAGATATGTCTCAATTGCTGGGCTCACAAATTGCGTTGATTTTTTCCATTCCAGTATTTACTCTATCT
CTTATATTACTTGCATTATAGTTTCGCTTTCTTCCTGTTCACTCTATTCGAAAAAAGATCGTCATGCACCGATTAAATATTATA
TTCTGTAGGATCCATCCACTAGAGTTTTTACAATTCATTGTATACACTTAATAAGCTATACATGCTATGGTTGTGCAGGAATTT
TAGTTTTGACATACACTGTTTGATAGAATTGTTAAATACTTTCTGGAATTCTCTAAATTTATCAATATCTTATATGATATATGG
ATCAAGAGCGATAAATTTCGCACTTATTTTACTATCTTACTACGATATATAAGGCTCGCGCTTTAGTTCTTGAATTATTCTCAC
ATATATTATATCACTTGTACGCGACTTCATTATCTACAGGTGTTAGTTTTATTTCCCGAACCATAAAATATCTTAATAAAAATA
ATATAAAAATCTGAAATTAATAATAAGACTAATAATGTAAAATTACTTAAAACTTAATAAATCAATTGAAGAAGCTCAAGAACT
AAGCAACAATTTAAACACATAAAAAAAGTGGGTAAAATAACTCCTCGTGTAGTATTGTTGTAGGGTGAAGTCTCAATCAATATA
TAGAGACGCTATGTACAATTTTATAATAATGATGATGTTCAATTAGTTGATTTATGTGAGCTGGTTGATAGTGTATCGCAGATA
TAAATTATTTTTCTATCTAATTACTTTAGTTTTGTCAGACTAAGGTATTAAAGTTTAACTAGACTGAGATTACAAAAGAAATAG
TGCTTGAGTTTTTATATATAGTTAGCAGGCATATGGATAAGACCCTTATACAAATTTTGCAAGTGTCACTTTTTTGCTTGTTTT
TAGGCCAGGTTAGATGTCAGGCCTTTTGTGCACTTCACCGTTCGCTTCCGTTTTATCCGCCACCACGTCATTGAACTAAAATTT
TGGGGATAACATAATATTAATGAATAAAATAGTCAGTACATATTAAACAGTAAGGAATATTTAATCAATGATTTGCTTAGTACG
TTCCTGTATGCCCCTCAGATATATATGTCTATGAGTAAGCACCTGGTGTCTGATGTGAGTAATTCTTAGTGATTCACACCTGGA
GCTTGACATGTTGGTTATAGTCCTGAGTTGTGTATGCAATGGGTCGGATTGGTACATCTTGGACCTTTAGACATCGTTGCTTTT
GATAGATTGCATTAGGGTTTTTATATTGACTAGATTATAATGCCAGTGGCGATGTTAATTAATTCTTTATAACACTTACGTTTG
CCATGAAGATTTAAATTCAACATCTTCAAAAGATTCCAATCATTTGTAGCTCGGTCTAAATCCTGAGTAATCAATGAACTCATT
GTTCCATTGTTCTATTTGAATCTTTTGATTCATTTGTTAACGGTTTCTTAATATAATGAGAGCTTAATGACTTTTGTTTTAGAG
ATTTCAATATTCTTGAATGGCCGGAAACATGAGTTTTACAATAATGAAATCCATGCCTTTGCCTATTCCGGGGAACTATTGTAA
TAGGTAGTCTTTCTCGCTTTAATGGATTTTATTATATTTTCTTGGGAATAATAGTTAGATAGTGTTATTTTGGGCATGGTTCGC
TCTAAGTTCTAGAGTTTATTTCTTAGTTCGGTTTTAGTATTATCGGATTATACCGATTAATTATTCACTCTGGTTGGATAAAGA
GCATATAAAGAACCGGTATTCATTAGAGGTTACAAGAGCGTTTATCACATAGATTCTTAGTAGTATATAACTATAATAGGACCT
CTTGGCATTTTTTGGCTCAGCTGCTATTGCTCTAAATTAGTCTCACTTGTCCTCGCTAAAAATAGATGAAAAACTTCATATATT
TGATTATAGTCCATGGACTGGGAAGAGGTTACGTCTTTAACTATACTAAAGTTTATAAATTCTAAGAGTGCAATTCCTCATATT
TATAGTGGAGTAATAATGGCAATTTAATAAATAAGATTAATGTTTAATTAAAGAGTTTACATTAATGTAGTGGTGAGTTTATTG
GGCTTGGTATTATAAGTTCATGGTCCACAAACAGGTGTCTCTCCCCAAGTCGCCCTTCTGTACTACACGGTCAATGGTAAATAT
GTCAGCAAGCAAGATTAATAGAGAAAAGGAACTAAATTCCAAATGATAATTTAACTTTTTCCCCGGACTGTCAAGCTGATTGCT
GTTAGTAAGTTTCCGACACTGTGCTCTATCGGATGGGTATTTTTAACTTCTCATTGGACTGGTTGTGTCCCAGGAATATTAAAA
TTTGAAAACTTAGTTCCGCAGTTAGTTTTATCCGACAAACTCGTGCTCTTTAGGGAAACACTAGTTTATCCAATTTAACTAACT
TCTATGTGTTGTAGCGACCTGGTTTTGGTCAGCATATATTAAAACCTAGTTTGCGTTAGTTTTATCGACACCGTCGTGCTCTTT
AGGAAAACCTACTTATCAATTTAACCAACTTCTATGTTTTAGGTCTGGTTGGTGTCCAGTATAATAATATTAAAAACTAGTTTC
GGCGTTAGTTTTTATCGACACCTCGTGCTCTTTAGGAAAACGACCGGTCAATCAATTTAATAAACTTCTAAGTTTGAGGACTGG

TTGTGTCCAGGTATAGTTATTTAAAAATTTAGTCGCGTTAGTTTTAATTTGAACTCACGCTGTTGTGCTCTTTAGGGAAACATT
GGTTAAATCAATTAAACAAACTTCTATTTTTTAGGCCCTGGTTGTGTCCAGGATATGTAATTGTAAAAACTTAGTTTCGCTTAG
TTGTATCGAAGGCAATTGCGCTCTTTAGGAAAAACCACTGGTCTGATCAATTTAACTTAACTTCTAAGTTTAGTGACTTGTTGT
GGCCCAGGATATTAATATTGAATTTGGTTCGCGTTAGTTGTATCAAACCGTGGTAGCGCTTTAGGAATGACAAGCTGTTAATCC
AATATAATCATAATCATAGTATAGGAGACGTGGTTGTGTCGCAGAAAATTAAATTTAAAACTTTGTTGGCGTTTGTTTTATGGA
CACCTCGTCCCCGTTTAGGAAAGAACACTGGTTAATCAATTTAACAAACTTGCTATGTTTTTAGGACCCTAGTTGTGTCAAGGT
ATTAATTTAAAAACGTAGTTCGTGTTGTTTTTTTCGAACCATTGTGCTATTTAGGGAAAACATTGGTTAATTATTTAACAACTT
CTAGAGTTTTAGGACCCTGGTGATGTCCAGGATATTAATTTTAAAACTTAGTTCGGCGTTAGGTTTTATTGACCCATTGTGCTC
CTTTAGGAAAAAACATTGGTTAATCGATTTAACTAACTTTCTAAGTCTTTATGGAACTTGTTGTGCCAGGATATTAATTTGAAA
TCTTTGTTTCGCGTTAGTTTTATCGACAAGTCGTGGCGCTTTTAGGAAAACATTGGGTTTAATCACTTATTTTTTAACTGAACT
TCTACGTTTTAGGACCTGGTTGTGTACAGAGATATTAATTAAACTTAGTCGCTGCGTATAGTGTTTCATTGACACATCGTGTCT
TTAGGAAAAAAATTGGTAAATCAATTTAACAAACTTCAATTTTAGGACCTGGTTGTGTCGCAGGATATTAACTTTAAAAACTTTG
TTGCGTTAGTTTACGACACCAAGTTCGTGATAATTAGGGAAAAACACTGTGTAATCAATTTAAAAATTCTAAGTTTAGGGAGC
TGTTGTAGTCCATGATTTAATTTAAAAACTTAGTTCGGGCGTTTGTTCAATCGACCCCTCGTGCTCTTTAGGCAAAACACTGGT
TATTCATTTAATTACCTTCAAAGTATTAGGACGCTGGTTTGTGTCTAGAAATTAATTTAAAAAACTTAGTTCACGTTGTTTTAT
GACACCGGTCGTGCTCTTTAGGAAAGAACACTTGCCATAATCAATTAACAACTTCTAAGTTTTAAGGAACTGGTTTTTGTCGCG
TTATAGTTCATTTAAGACTTAGTTGCGCGTTGTTTATTATCGACAACCCTCCGTGCACTTTAGGAAAAGACACGAGTTTAATCA
TTAACTAACTTCTAGTTTTATGGCGAAGGTTGTATCCAGGATATTAAATTTAAAAAATTAGTTCGGCTTTAGTTTTATCGAGCA
ACGACCGTCCTCTTTAGGAAAAAATCTGGTTATAATTTAACTAACTTGTCAAAAGTATTAAGATTGGTTGTGCCTAGGAGAAAT
TATTTAAAACTTAGTTTGCGTTAGTTTTATCAACACGCTCTTTCCCTATAGGAAAACACTGGTTAATCTTTTTTAACTAAGCTT
CTAAGTATTATTAGGTCCTGGTTGTGTCCAGGATATTGAATTTAAAAAACTTAGTTCGCGTTAAGGTTTTTCGACGCGCGATTC
GTGCTGTTGAGGAAAAACATTGGTTACATCAATTACAAATACCTTCTAAGTTCGGACCTGGTTGTGTCCGGATCATAATTTAAA
ACTTTAGTTCGGCGTTAGTTTATCGACACCATCGGTGCTCTTTAGGAAAAACATGGTCCCAATCAATTTTATTAACTTCTAAGT
TTGAGGAGCCTGGTTGTGTCCAGTATAAGATTAATTTAAAAAATTAGTTCGCGTTAGTTTTATGATCCTTGTGCCCTTAGGAAA
ACATTGGTTATTCAATTTAACTAACTTCTATGAATTTATGACCAGGTTGTGTCCAGGCATATTAATTTGAAAACTTTAGTTCGC
ATTAAGTTTTATCGACCCTCGTGCTCTTTAGAAAAAACACTGGTTAATAATTTAACTAATCAAAAAACTATGTTTTAGGACCTG
GTTGTGATCTAGGATAATTAATTAAAGACTTCTTTTGCGTTAGTTTTATTGACACCGTATCGGGCCCTTTAGGAAATACATTGC
TAATCAATTTATCTACTTCTAAGTTTTTAGGACTGGTTGTGTACAGGATTTAATTTCAAAAACTTAGTCTGCGTTAGTTTTTTG
ATTGACACCTGGTGCTCTTTTTTTTGGAAAAAATTGGTTAATCAATGAACAACTTGTAAGTTATAGGACGTGGTGTGTCCCAGA
GATTTAATTTAAAAACTTAGTCGCGTTAGTAAGTATCGACGCTCGTGCTCTAGTAGGAAAAACACATGGGTAATCAATTTAAAA
AACTTTAACATTAGCGACCTAGTTGTGTCCAGGATATTAATTTTGAAAGCTTAGTTCGTTAGTTTTATCGCACTCGGTGCTCCT
TTAGGAAAAACACTCAGGTTTATCAATTTAACTAACATTCTAATTTTTTTAGGCCCTGGTTGTGTCCAGGATATTTATTTAAAA
CTTAGTTGCGCATTAGATTGTATCACACGTTGTGCTCTTTAGGAAAACACTGGTTAATCAATTTAACTAACTTCTAAGTCTTAT
GAACTTGTTGTGCCCAGGATATTAATTGAAATCTTGGTTCGCGTTAGTTGTTCGAACACCCTGGTGCGCTTTAGGAAGAGCACT
GGTTAATCATATAATATAACATATAGTATTAGGCCTGGTTGTGTCCAGAAATTAATTTAAGAAACTTTGTTTGGTAGTTTTATG
GACAGCCCTCGTCCGCCTTTAGGAAAACATGGTTAATCAATTTACAACTTCTATGTTTTAGGACTAGTTGTGTCATAGGATATT
AATTTAAAACGTAGTTCGTGTTAGTATTTTTTCGACCCGTTGGTGCTATTTTAAGGAAAGACATTGGTTAATCATTAACAAACT
TCTAATTTTTTAGGCCTGGTTGTGTTCCGGATATGAAGTTAAAAACTTAGTTTCCGCATTAGTTGTATCGACCCTTGTGCTCTT
CTAGGAAAAACACCGGTTAAGTAATTTAACTAACATTCTAAGTCTTATGAACCTTGTTGTGCCAGGATATTCAAATTTGAATCT
TGGTTCGCGTTAGTTGTACATCACACTGGGTGCGCTTTAAGGGAAGAACACTGTTTTATCAATATAATTAACATCAATGGTATT
AGGCACCTGCGTTGTGTCTAGAAAATTAATTTAAAACCTTGTTGGCGTTAGTTTTTTATGGAACACCGTCGTCCCGTTTAGGAA
AAACACTGGTTAATCAATTTAACAAACGTTCTATTGTTTTAGGAGCCTAGTTGTGTCAAGGATATTAATTTAAACATGTAGTTC
GTGTTTAGTTTTTTTTTCGACACCTTGTGCTATTCTTAGAAAATTGGTTGATCTATTTACAAACTTCTAAGTTTTAGGCCTGGT
GGTGTCGGATTTAATTTAAAAACTTAGTTCCGGCGTTAGTTTTATTGACACTTGTGCTCTTTAGGAAACAAACACTTGGTTTAT
CATTTAACTAACTTCTAAGTCTTATGATTGTTGTCCCCAGGATATTAATTTGAATCTTGGGTTCGCGTTAGTTTTATGCGAAGT
CGTGCGCTTTGGAAAACAATTGGATGTAATCAATTAAGACTAACTTCTTAAGCCTTTTAGGACCTGGTTTGTGTACAGGATATT
AATTTAAAAACTTTGTCGCCGTAGTTTTTTGACCAGAGTCGTGCTCTTTAGGAAAAGCATTGGTAATCAATTTAACACTTCAAA
GTTTTAGGACTGGTTGTGTCGCAGGATTTAATTAAAAACTTTGTTGCGTTAGTATTATCGACACCGGTAACTCGTGGCTAATTA
GGAAAACACTGGTTTACATCAATTTAAAACTCTAAGTTTTGGAACCTGGGTTTGTGTCCAGGATATTATTTAAAACTTAGTTGC
GGCGTTTGTTCAATCGACACCAAATCGTGCTCTATTATGTTTTTTGTGTTCAACATCACGATGGTGTTTATAATGTATGGATAA
TTTAAATGAAACTCTTTTTATTATAATTTATACGTAATATTGGATTCTATCAACTGACATTTCATGTGATGTTTAGCTTTATGT
ATAAGTCCTTTGGTCACCGTCCTGTCTCATCGCATTGGCATTTCTCAACTCTGCTTCTCTTCCCTCATCATGCCCTTTAAGCCC
TTTTCATTCTCATCAGCCTCCTCTTCAAACCAAAATAAAAACTCCCTATTTTTCTTTCTTTCATTCTTTCTTTTTCTCTTCTTT
TTCCTTCGGAAACAAGACATCAGAAAACAAAACCATGGTTGTTTTTCTGTCTAGCCCAGCATTAGACCATTTCTTCCCATATC
AAAAACATATAAGCTCCAGCCACTTACACTTCATGTTCCGTGTGGTCGACCCTTGAAACATTTATTTGATTGTTTGCTTGA
>m141122_014521_42134_c100732202550000001823141405141560_s1_p0/75081/7588_14482
CCGAGCGCCCCTGAAAAGACACAATAGTTGACTTGAAGACATATAAGGGTGCCTCCATCATGGTAGATGTTGTTTTAAATCTCA
AGAGAATGCATCCTAACCAAAGAATTGGATTTTTAGAGTCTGCATATGAATGGTGCGACATGCGTCAGAAAGCAACACACAAGA

CATATCTACGTTAGATAGGGAGAAGAAACTAAGCTGGCTGAGGCTTAAGGGCCTTAGGATATAGGATAAGTGGCGGTGACACAG
GAATCATAAATGTCAGGTTTATGTGTGATTTAAAATGGGTTTGTACGAGAATGCATTGATAAAGAGATGAGTTTAAGATGGATC
TGCATATAATACACCGCGGTCAAACTATTTACCCACTAGCTGTAGGGATACTATAAAAATCAATAGGAGGGACACAATTATCGA
GCAGTCACAGGAACTACAAAGTGGAATGGACGGATTGAGGACGTTGAAAAAACCTAATGCCAGCAATTGAGAGGTATAGAATGC
ACGGTTAACGTGTTTCTATACTCGGAGGAACACACAAGAGCCAAGTGTCACGTATCTGTTCGAGCCACGGATGCTGAATACCTG
CGTTGCTCACCTGGCGGAAGAAGAGAGGAGCAAAGAATAGACTGCTCCCTGAAATTCTCTTTGGCCTGGCGTGGAGGGGCGGAC
ACAATGAATGCTAAACCTGCGACCAGGACCCATCAAGATCGCTCCTGCCTCGCGGTTAGGATTTTTTCTTTTATTCTTTTAAGT
ACCCAACCTATGAGATCTGATGTATGAGACAATGATGATATTTTTTTAGTACTGTACGGGCATACTTTTCTTTGGCGGAAGCG
AGCGGGAGGGGGTGTAACAGGGGGTTAAGACATTAATTAATTTGCGTTTGTTATATATCTATATGTTCGCATGAAGGAATTAGC
GAGTACTTATGGTGTTGATTTAACAATAATACACAAATTTTGAAAAAATACTCTAAGTCCCTAGCATGTTTCACTTATTTTGCT
CATGTGTTTACATACCTTTCGTATGCCTTGCTTTACTAGACTGCCATTTATATGCATACCATCAGTGATTGGGAGCTTTAGGTC
CTTAATCACGTTTTGGCCCCCCTTGGACCCCCCCAAAACCTCCGTTTCGAAATTACTACTCGAGCAAATAATTAAAATGATATA
TACATACAAAACACACACGTATATGAGCAAAAACTTGTAATATCAATACAATAATTAGCAAGAATGACTAGCTGGCTGCATAAG
TCCCTTATATTCTCAGTAATAAATGGAAAAAACTGTCCTACGAATGATCAATATAGACTTACACTTATAAGCGATTAGTCATAT
AAAATGACCAACCCTTTTCATAACTTGTAGAAAGTAAAATTTAATACAATGCCAATTCTTTAGAAGAATTGGTTTAGTTGATAG
TACTTGCGCAGGTGTTTCCCATTCCAATAGTCATGGATGAGATTTCCATTTAATGCTGGGCAAGTTTAGTAGGTGCCTGGGATG
GAGGACTTCTTCAATCTGGTATGGTCCTTCCCAAATAACGTCCGAGTACTCCAACAGCCTGGTCGCGGGGTATTTAGGAAAACT
CTTCAAAGTACTAAATCTCTGAGTTGAACTTTCTTTCAAGTACGGTTAGAGTTGAAATATCGGCGATCCTTTTTTTGCTGGATA
CGCAGCTACTCGAAGTGGTTTTTCACGTCTTCTTCATTCGTCAAGTCCAAGGGATCCAATCAAAGTTGGCGTATTAGTAAGGCT
ATGATCATACGTTATCCTGCGATGCGAGGGTGGATCTAACTCAACAGGCAACATATGCCTCATACCCTTATAGCAAAAGAAGAA
TTGGAGTATGCCTGTCCCTTCTGTTCGTGGGAAGTGTGTACGACCAAGATGGATGTTCAGGCCTTGCCTGGCCATGCTCCCTTT
AACTTCTTCCAGTCTGTTTCTTTCAAGGTATCCTGTCTACATTTATTGACTGCCTCGATTGTAACATTTTCTTGGGGATGAGTG
ACTGATAGAAAAGCTTTTGATAATCCCATGTCGTTCGCTAAAAGTCCGTGGAATAATGCACTATCAAGGGACATGTGTGTCGTT
ATCTATAGCGATTTCCTTGGCAATCCATTACGCCAAAGCGATGTTTTTTGACCACAAAAATCCGCACCTTCTTGGTCGTTCTAT
GGTTGCGAGTGGCTCTGGGCTCTCTTTTTCAGCCCATTTAGTGAAGTAATGACGGTGAGCACAGCATATTTTGAGACCGCCCTT
TCGTGTTGGGGTAAAGATTCCGATTAAAAGTTTATCCCTAAGTGAGTAGTAGTAGTAGGAGGGCAGCAGCAGCAGTGGTATTGG
TAGTGGTTAGTGTGTTGTGGTAGGTTGGTAGTGGGTAGTAAGTCGTTGGTAGGGTAGTGTAGGTGGTAGTGTGTGGTAGAGGTT
GGTAAGTGGTAGTGGTAGTTGGTAGTGTAGTGGTAGTGGTAGTGGTAGTGTAGATGGTGGTAGGTGTGGTAGTGGTAGTGGTAT
GTTGGTAGTGGTAGTGGTGAAGTGGTGTGGTAGTGGTAAGTGGTAGTGGTAGTGGTAGTTGGTATGGTAGTGTAGTGGTAGTGG
TAGTGGTAGTGGGTAGTGAAAGTGGTAGTGGTAGTGGTAGTGGTGGTAGTGGTGGTTAGTGTGGTAGTGGTAGTGGTAGTGGTA
GTGGTATGGTAGTGGTAGTGGTAGTGGTCGTGGTGGTACTGTAGTGGTAGTGGTGGGTAGTGGTAGTGGTAGTTGGTAGTGGTC
GTGGTCGTGTAGTGGTAGTAGTATAGTGTAGTAGGTAGTGTATAAGTTAGTGGTAGTGGTGGTGGGTTGTCTGGTGGTTGTGGT
GGTCGTAGTTAGCAGGTAGCATGTAGCAGTAGCAGTAGAGTAGCAGTAGCAGTAGCAGTAGCAGTACAAAGCAAGTACAGTAGC
AGGTAAGCAGTAGCAGTAGTGGGTAGATTAAGTTTCCCCACGACTGCAAACGAACACTGACGTTTGCATCTGTTTCAGTTCATT
AGGTGGCTTGCTTGCGGGAACTTTTTGGAGAATCTCTGGCACTTGGTCAATCCGTTACGAATTACTTCAAGTCTTCATTCATTG
CTGGCCAGAAATACCCTTGCCGTTCGGATCTTTTTCGACAAACCTTTTCCCCAGCATGGTCCCCGCAAAAGCCTTGTGTACTTC
TTTCATCAATTCTGTTGACCTTTTCTTTCAAAACACATTCTGAGGAGTGGCATTGAGTAATCCCCTTCGGTAGCCATAGATTTT
CATTGACTAGGAATGTAATCCGTAGCAGCCTGTCGTTGAAGGGTCCTAGCTTGTTTTTGTTGTTGGTAAACGCCTTGCGTGAGA
TACTCTACGTATGGTTCCATCCATGTATTCTGCCACTTGGGATCACCAAGTGGTTCTCCTCAGCTTGGATGCTTGGTACTAAAT
AGTCGTTCAACTGGCACTATGGTTCAGAGTATGCAGCATCTTTTGCACTTGCTAGCTGGCCAAAGCATCAGCGTTTAATTATGG
TCGCTGAGGTACATTGCTGAAGGGTGTACTTGTCAACTATGCTGAATAGATCTTTGTTTTGTTTAGAATAGGCAACCTCTTTAA
ATCCCCGGGCTTGGTACTCTCCCATGACTTGATTTTCTACCAGCTTGAGAGTCACAGTATGTCAAGTATCTTTGCATTTCATGT
CCCTGGCTAATCGTAACCCAGCGAGCAATGCTTCATACTCAGCCTCGTTGTTGGAAAGCAGGAAATCGAAACTGATTGGTCAGT
GGAAATCGATTCCCTTCAGGTGTAATCAAGATCATCCCCGCACCTTGCATTGATGCTCATTTAGAAGTAGCGTCTGTAACAACT
TCCATTGACAGGGTTGGTTTGAGAATTCAGGCTCATCAGGCTCTTCCGGTCTTGCTCACTATCTGTAAGTTTCTGTGAACTCTG
CAGATGAAGTTGCCAGGGCTTGTCCTTTTATCCTGCTCTTGGTAAGTATAGAGATATTGGAATGCCCAAGTTCGACTGCCCATT
TCAACAATCTTCCACAGCTCTGCTTTTGCAGGCACTTGCCGTAGACGCTGGTGTCAAAACTATTGATTGGGTGGGCAATTGAAA
GTAAGGTGCAACTTCCTTTGGCAAATTAAGCAATAGGCTAACTTTTCAATTGGTAGATATACGTAGCTCCGCTCCAATTAGCTC
TTTCTTACATAATAAACATAATCCTTATGCAAACCCTTCTTCTTCCCTTACTAAGACAGCACTAAGCCGCATAGTCTGTAATTG
CCATAGTATAAAACAAGGTTTCTTCAGTCGACCGGCTTTGATAGAACGGGTGGTTGCGCCATGTTGATGTCTTCAATGCTTGGA
ATTCCTGCTGTGCACTCTCCATCCACTCAAATTTCTTGTTGCCTCTAAGTAGATTATAAAAATGGACGGCACTGTCCATTGATT
TTTGAATAGTCTACTGAGAGCAAGCAATTCTTCTGTAAAACTTTGTGTTTTTTTTATTGATCAATCAAAGATATTATACGCAGG
GAACAACATCAACATTGTTAATTTAATCTCTATGAAGATTTCTAGGTTTGTTGATGCTAATAATTTCATCAAGAGGAATGCAAG
GCCATCTCGGAGGCCTTCTCGCGAGGCCACGTTCCGCCGAGGCCCTCAGCATGGGCCCGTGCCCATTCCGCGAGGCGAGTTCCG
CGAGGCATCGTGTGGCGAGTCCCACCGTTCCGCGGAGCCATTGCGTCTCGCAGGGGTCATCTCGCGAGGCTTCTCGCGAGGCAC
GTCGCGAGCCCTTTTCAAAAGCATGGGGCCGCGCCTCCATTTCCGGCGAGCCGAGTTCCGCGAGGCATCGTGTGCGAAGGTCCC
ACCGTTCTGCGGACCCCATTCGTCTCGCAGGGTCATCTCCGCGAGGCCTTCCCCGCGATGGCCATTCCGCGAGCCACTGTCCGC
GAGGCATTCGCGAGGCCCGTTTCCCGCGAGGTCCCTCTGCATGGCCGCGCCTCCCATTCGCTGAGCGCGTTCCGCGAGGCCATC

CGCCTACGTCTTCGCGAGCCCGTTGCGCGTCTACACCCCACCGATCCATCTCACGAGGCCTATCTCGCGAGGCCATCCCGCCTA
CGTCTCTGCGAGGCCCGTGCGCCTACTCCCGCGAGGCCATCTCGCGAGGCCTATCGTCTCGCGGGGCACCAGCACGCCAATGCG
CGAGGCATGCGAGGCCCTTGCGAGGCACTCCGAGAGGCCCGATAGGCGTCATCTAACACAGGTGATTCCGTTGCGACCGTCGCA
ACCACACCCTGGGTCGCAGCCACACCAGTCAGGGGGTCACTGTTAAGGGGAGACCACGCCGAGGCGAAGCCCGCGTGGAGGCTT
GCGGGGGAGACAACCTAAGCCTCGCATACTTAGACAGTCTGGGCGGGCCAGGTGTAATCACCGTGTTAGTGAGTGAATTTCAGC
GGGCATTCAAGACCTCGTGGCGAGGGACCCGAAAACGCTTCTCATTTCGTACCTGACGATTATATGCATCAAGTTCGCCCTTCC
TCGTACTCCTGAGATACAATGCTTAGAGCTCCAACACGGTCGAAGGGGACATATTTGTATGCCAAGTACTATGGTACCGGATAC
CAGGGCAGTGAGGTTGCTGGGATAAAGTCATGGTCCGTATCTACGGGCCATGGGACAGGCCGACCCATGACCGTCCTCGCGCAC
TAATGCCTGTCACCGCGTATCGACGGAGGCAGCAATGTGGTGGAACGTTTTCCTGACACCTGCTTCATGTAGCGAGATGTACTC
CAGCCCGACTCTGAAACCACTCCCCTGCCAGTACGAATGTACCAGCTTGGTCCTGGACCGACTTATGTACTAAGGCCATTAGAG
CCTACTATCAAAAGGATTCTTAAGACACCTGAACAGGGGGGTTGGGAAAATTGGCGGTAGCAGAGCTTAAGGTGAATGACAAAC
TGAATTCTCCATTGTTATTCCTGATATAATTGATTGAGTTAATTGCTTTGACCAAAATAGCTTTTTATTGACAATCCTAACTT
GATAATTTTCGCAACTTAATTCGTTGACGAGTTGCTCACCGTCAACATAGGTCTACTTCTTCACTCATATATATAGTATATATG
AATCAAAAGATAATGAATAGAAAAATTAACCTCAGGTCCTTCCTTACTTGCTATCTTTTTGTATGGCAAAAATCCTTGAGATCT
CACACCAAAGATCTTCAAAAATTAATTCTTAGGCACATGAAGAAAATAGTGTGGGCTCGCTATACAAAATAATAGGGGCAAAAT
ATATTTATCAGATGTTTCTCCAACCACATGAGTATGCTGATAAAATTTGGACCTAGGCTTTTGGTAAGAACAGTGACTTTTGTT
TGTATCACTGATCTCTTTCTCTGAGAGAGAGTTCAAGGGATATTTTTCTATCCTGAGAAAGTTAACGGTTCTGAAAAACTAATA
TCCTATAATTAATATACCAATATATAAAATTAAAAATATAGTATTTTAAACAATTTGAAAATAACTAATGAGTATCAATTAATA
ATATTGAATCATTATTATGAATATCTCATTATTTATTTAGTATTGTAAATAACTAATAATTTGTGGAAGCCAATAAAACTGGCT
TCAGAATCTCTTATGGTGTAGAGCAGCAGTGATTGTTGCACTGTGCTACACGTGTAGCCTAATTTCCAGTGGATGGCTTGTGAT
TTTTCCCAAATTTTATAATTATTTAAATCAAAAATCCCAAAATATAATTAATTCAAAATTCAATTTAATATTTCTGTTAAATGC
TAAATAATTTAATTAATTAGCACATATATACAATAATTATGTTTGATGCATAGAAAAATATTTTCTTAATAAAATAAGTCATTT
TTCCCC
>m141122_014521_42134_c100732202550000001823141405141560_s1_p0/75709/5853_15140
GTGCAATCTCGCGGGTGTCACCTTTGGCCTGAAAGGTCAATTTTTAGTCTAGGGTTATGAATGAATAAGCCTCCCAAATCCTCA
TCCGGCGCCTAACATAAATAGCCCATACCTCATACATGGTGACCAATAAATTCTGAAAAGGATGGGGAGATGGTGGGTCGAGAA
CCCAATAAGAGCTTCCCCTACCCCAGCAAATACCTGCTAGGGGACATTCAACGATTTGTTCCGAGTCCTAGTGCCTGCCCCCGC
CTGAGGGAGATTGGTTTCGCCGAAAGGTCAACCTTCCTCCTTTTTGTTTGGTTGTAGTTAAGGTGAGCCCATTTCATGGGTCAG
AGGTAGGATCAGCATATATATATGCAGGGGTGAGGCCGCTAATCGGAGGATGCCGCCTATTGGATTACCGCCAAGCATATCCCA
CAGCGACTTTGAATGGTATGATGTTCTGTGTTCTTGCCTTACTAGAGGACAGAGCACTTTCCTTAGAGTGGGAGCTGATGTACG
AGTCACCTGCTTCGGGTCGGCCCCTCAGGTCGGAGTAAAAGTGCCTCCTCTATGCGAAGATCGCACACCCTGAAGGGAGCCAAT
CTTGGTGCATTTTTGGTTTAATTTACGGTGGACAGCCAGTAATCATGGTCAAATATACACAAGTTCGTGAGCCCCTATAAGAAG
GTTGGACCCTGCATCCCGCCCGCGGATATTGAAGAGGGCAACTACCATAGGCGAATGTTCATCAAAAAAATTTGAGCTATATTT
AGGATATCTGCAAAGCAACAAGACAATAATGAACTAGAACTCTAAACTGTAACCGGGATCGGTAGCCTACGAGCACGTCTGCTA
GGAGCACTTGTGGGTCGCAAGGTCAGAAAGCTTTGTGTTAGTATCCGTTAGCTTGGTTATGTTAGGACTCCCAATTCATGTGTT
GAATGGTATTAATGGGACCTAGATTAATCTACCTAAACGAATGGTCTACTTTACCTCAGCGGTGCCGTCGCCCTGGGTGAAAAA
GTGCATGCGCCGCAATCTGGTAGATTTCACCTACACATATCAATAAAGAAAGAAACCCTAACGCTTTCATAGGGAGAGAATTGT
GAAGAGTGGACCAATTAATCGAGTTCTGTTACATCTCGCGGATCGCCTCTACGAGCTACTGTCACATCTTCCAGTGGCCGGTAG
GGGTGGGAGGCGTCTAGGTGTGTGGTCGCACTGTTGGTCCGTGTTCCATCGAGGCTACGGACCCAATTCCATGGTCCAAATCTG
AGGTATTAAGAGGAGCAAACCATTCCATGCGCTCTATATGTCTCCTTAGGTCCCTTAGCATTAAGTCCGGTGCTCCAGCTTTTA
TGGTGCAGAGTACGCAATACTACGCATTACCACCTTATGCAGTGGGATCGAACTCGCTTGGACAGCCATGAGTGGGCCGGTTTT
AACGCAAAGATCCTCGCGCCGCGCTCGAGACACGCAGATTAGAAATAACCCACCACGCTGCTGGCAAAGCACCAAGGTAGGATC
CTATGGGCAAAGATCTGGTAACTAGGCTGCGACATCCCCCATCGCTCGATACGTTTTTGGTTATAGAATAAAGGTTGCGGCGAA
AACTCATATTCTCATGTCGAAGATCCTGAGCCCTAGAGTACGTTAAATGGCTCCATTGTATATATATGTGGCCCACCGTCGCGA
ACGATGGGGCACGACAAGAGAGAAAAAATGATACACCCCACGTGTGTAGAGTCTGTGCTAGAGTATGTATCACGTATGCGCAGA
GAAGTCTGTTTGTTGTAGACGATAGGACTCGGTTGTCGGAGGCTGATGGCCGCGATGCCTACACCACGCAGCCGAATGGAGAGG
GCTCATCCCACATACCAATCGATTAATTCCTGATATAAGCTTTAACTTAGCCGCTCGCGAACTTAGGACTCACATCTCGGTCAT
GCCACTAGAAAGTTACCATGCTTCAGTCTAAGGTTGGAATGGGAGCGTGAACCTGCTTCCCTGGGAAGAGGACGTTCGAAGTCA
TAGCTGATGGAGATGCAGAGGAGAAAAAACAGAGGGTTCAAAAAAAGCAGCAATAACAAAACCAGATAGCAGACAGAGCCATCC
AGACTTACAATCCCCTAAAAAGCTTCGATTATACGTGAGCGATACGGCAGGTGCTGTCCAAGAGACGTATAGGCCTCTCGAATA
GGGACAGCATGTTCACGCTGTCTCTGGTATGGGATTACCAGGGAGTCCCAAAAGGGGTACCACAAGAGCCTAATCTATTGACAG
GGTCAAATACACAACTCACCCGTATGGAAATGCGTTACACCAACAGTCATTCTCTCTCGCGAATGCCCAGCCTGATCACCTTTA
TATACGTGTAACCCGATGGGAAATAAGGGACTCATCAAAGCACTATACGAGAGATAGAGAGAACCGTGAAGTATAGGGTGAACC
CAGAAGAACATTCCTCACCGACCTAAGAACTCGCGCCCTAGAAGCTTGCGCATAACCACGACCGAGATCATCTATGGCTGAAAA
TATGAATCTATATTATTATGTTGTTATTGGCACGCTCTGAGAAGACAGGGCCCACTGAAACAACTATGCATAGTTAATAAATCC
GAGGCAGCGCTAAGTAATAATACGGCAGAAGAAGACAAAGCAAAGCAATGAATAACTACAGATATCCGCGGTAGTGACGATATT
GGAGAAAAGAGGTGGCTGTCAAACCAGAGATATCGAGCATGCCGTCTGTTGAAGAGAAGACATCCACCTTGAGAAAATGTAGAA
CATGAGGACCTCACCGCATAAGACGTCCTCAGGTCGGTACACAGCCGGAACACGCCCTGCCGTATCAGGTGAGAGATGGACGTG

CGCAGAGTACCCAAGGTGGTAGTATGTGGGTTGAATGATAAAGACCACGCGGCAACTGGCATCGGAGGTGGGATAGAATAGCTG
ATGAAGGTGGCGCTGACTATCGGCTGGAGGAGCCACACTCAGTCATTTAATAAGCGATGATCCCTTGGTCGGACGAAACAGAGG
AGATAATGCACATATGATAGGGACGATCACAGGGTATTGAGAGGTGTAGATTATTAACACGATAGAAACAGCGAGTAACTCAGA
CCTAGGCTGCTGCTTAGAGAGAAATTGCCCCCTGAAAAATGTACGAGCAGGTACTATTCTGTTCTGGGTTGAATCCGGGATTAC
GCACCGAATCTCCTACCGCCACCATTGCGAACTAACCGTCAATAATCCATCCTGCAAGAAAGTCCATCCAGTCGGGTGACTATA
TCTATTTCGAGATGGAAAAGTGTTTCACACAAAATAAGCTTGTTTAGATGAGACATATGAAGTGAAATACGATATCTGTAACAT
CGAGCCTAGGCAGTGTTCCTAGTGAGATTGGCCTGAAGGCCAATTTTTTTTTTTTGGTTAAATTGATAGGTACGCCTCAAATTC
CATCGGAATACAATAATACCTAACTTATTAATTAATTGAGTAGTCGGAATAAGGCTCAAGGAAAATTGGGAGCTTTGTGTAGGA
CGAGAAATAATCGTATGACTAGAACACTCAGCTTCGGTAGCCCGTTTAAAGGCAGCTGCGCTCACATGGAGATTTGGCCTGAAA
AGGTCAATTTTCTAGTTTGACTTTGTATAGGACTCCAATTCAATGGTAAAAAAGATCCCCATATATTAATATATACCATTGAAG
ATGGCAACAAGGCCAGACAACAAGATTAGTAGCTTGTTAGAGAGACATTATAGAGAAATTAAACGAGATCGGGCTTCGGAGCCC
CTTAGGCACGCTTCCTAGTGTGACTCCTGGTCTGAAAACGGCTCAATTTTTTTTTTTTGGTTAAATTAGGTCGCAATTTCACTG
CGGGTCAAAGGTGCCTAAATAGCCCTAAATATATATATTGAAAAAATGGGAAAAGGTTCAAAATTAGATGCTTGTTAGAGAGAA
TAGAGTGAATCACGAAATATTGTAACGCGAGCCCTAGGCAGCGTGCTTAATTTCGAAGCGAGCATTTGGCCCTCGAGCAGTTGG
TGTTTTTTCCGTATTGGCTTAAGATTAATACGCCAATTTTGTACCCTTCAATGGTCAATAATAGCCCTTCATACTCAGGCTGAC
TTCGATTCATTATTGACGACCTGGAATAAGGTCTTTCAACAAAAATTACGAAGCATTGCTTCCTCTGAGAGAAATATGATGAAT
TTTACGAATCGTCGGAGCCCTTTGGCAGCCTGCCTTTCGGGACTTAGGCCCTGAAGAGGCTCTATTGTGGCCTTTCTGTTCTGA
GATTGATTAGGACCCATGTCCATAGTCCACACCTAATAGCCCTGTATAGTATTATAACCTTATTTCGAGATGACAAAGGTCTCA
AAAAATAGAGCTTGTTAGAGAGACAATAATGAGTGAATTTCGAGATCGCTCGGAGCCCTAGGCAAGCTTTCTAGGAGATTGGCT
GAAAGGCATTTTTTTTGTTTGGAATTAGTGCCCCAAAATTCATATCCAATAATATGGCCACCGCTATACAATTCCTATGTTATG
TGATTGGGAAATAAAGGTATGCAAAAAAATTAAGTTTGTTTGAGAGAATATTGACGCACTTATCACGAACATCGTTTCGCCCTA
GGCAGCACCTCAGGAGATTGTCTGCCTGAAAAGGTCAATTGGGTTTTTTTGTGTTATGCAATCCTAGGCACGCCAATTCATTGG
TACTTAGTAGCCCGAATAAAAGGATTCATTATCCGTCAGAATTGAATTAGACGTGGGGATAGGTTAAAAAATTAGAGCTTGTTT
AGAAGATCATATGGTTGATATGTCCAGAGATCCGTCATTCGAGCCTCAGGGCAGCTGCCTAGAGATTTGGCCTGAAGGTCAATT
AGTGTTTTGGTTTGTTAAATTAAGCGACCTACGACCAATTCATGGTCGAGGTAATGAAGCCCTCACTGTAAGTCACTTGTTATC
TTGAAGTTGGGAAGGATTCAAAAATTAGAGCCTTGTTGGTAGAAGAGACCAATGAGTGAATTCTGGGATCGTTAGGCGGATCCC
ACTCCGACAGCCATGGCACACCGTAGGAGAGTTATGCCTAAGAGTCACATTTTTTAGGTTTGGAGCTGAATTAGGACCAAATCT
AAGGTAAAATTATGAGGGCCACCCTATAATTTGGGGCACCATTTATATAGCATGGGGAAATGTTTAAAAAAATAGAGCTTTTTA
GGGAGAAATATGGGGATACGAGTCGCTTCGGAGCCCTAGGCTAGCTGCCTAGAGATTGGCTGAAAGGTCTATTTTTTGTTTGGG
ATTGTATAGGAACCCCAACCTTCATGGTCATGATGCTTACGCACTTTATTGGACATCGAGGGAAGAAGGTTTCTAGCTTGTTAG
GGAGCAATATGGGGAATTCGGATCGCTACGGATGCCTAGGACAGCCGCCTAGGGATTTTGCCCTAAGGTCAATTTTTTTGTTTT
GGTTGATTAGGTACCCAAATTCATGTAAATAAAACCTATATAATATGTATTGAGATGGGAAAAAGGTTCAAAAAATTAGAGCTT
TAGAGAGACATTGGCAGGATTCAACGAGATCGCTTCGGACCCTAAGGCAGCTTCCTAGGAAGATTTGGCCTGAAGGTCAATTTT
TCTTGTTAAATTAGACGCCATTCATGTCAATAATAGCCCTAATAAAATATTATTGAGATGGGAAAAGGTTCAAAAAATTAGAGC
TTGTAGAGAGACATATGAGTGAATAGAGACGCTTTCGGAGCCCTAGGCAGCTGCCTAGAGATTTTGGCTGAAAAGGTCGAATTT
TTAGTTTTGGTTGAATAGGACCCCAATTCATGGTATAATAATAGCCTAATATATATTATTGCGATGGAAAGGGTTCAAAAAATA
CGAGCTTTTTTAAGGGAGAATATGGGGGAATTAGAGATCGCTTTCGAGCGTGGCTGCTGCTAGGAGATTTTGGCCTGAAAAGTT
CTATTTTTGTTGGTTGTATTAAGGACCCAATTCCATGTCAATAAGCCAATTTATCTATTGAGATGGAAAGGTTCCAAAAAATTT
AGAGCTTGTTAGAGGACATATGCAGTGAATTATCGATTCTTCGGAGCCCTAGGCAGCTTCCTAGAGTTTGGCCTGAAGGTCAAA
TTTTTTTTTTGGTTAAATTAACAGAAGCCAATTCAGCAACGTAAAGGCCTATATAATATTAATGGAATGGGAAAGGTTCAAAAA
AATTAGAGCTTGTTAGAGAGAAAATATGAGTGATTTACGAAATCGTTTCGGGCCCTAGGCAGCCTGCCTAGGAGTATTGGCCTG
AAAGGTCATTTTTTTTTAGTTTGGTTGAATTAGGACCCAATTCCATGGCAAATAATAACCCTATATATAATTATTGAGAATGGG
AAAAGGTTCAAAAATCTAGAGCTGTTAGAGAGACTTATGAGTGAGATTAACGCAGATCGTTCGGAGCCTAGGCAGCTTCCTGAA
TTGCCTGAAAGGCAATTTTTTTTTGGTTAATTAGAGCAATTCATGTCCATTAACCATATATTAATTATTGAGATGGGAAAAAGG
TTCAAAAATTAAGAGCTTGTTGAGAGAATATTGAGTGAATTACGATCGCTTCGGAGCCCCTAGGCAGCTGCCTAGGAGATTTTG
GCATGAAAGGTCAATTTTAGTTGGTTGAATTAGGACCCCAAATTCATGGTCAATAATAACCTATATACATATTATTGAGATGGG
AAAAGGTTCAAAAAATTAGAGCTTGTTAGAGAGACTTTATGGAGTTGATTTACGAGAATCCGCTTCGGAGCCCTGGCAGCTTCC
TAGGACGATTTGGCCTGAAAGGTCAATTTTTTTTTGGTTAAATTAGGACGCCAGATCATGGTCAAATAAAGCCTATATATATTA
TGGAGATGGGAAAAGGTTCAAAAAAATTGGCTTGTTACAGAAGAAATATGAGTGATTACGAAATCCGTTTTCGGAGCCTAGGCC
AGCTGCCTAGGAGATTTGCGGCTGAAAGGGTTCAATTTTTTAGTTTGGTTGAATTAGGACCAATTATGGTAAAATATAGCCCCT
ATATAATATTATGCGATGGGAAATTGGTTCAAAAAAAGAGCTTTGTTAGGGAGAAATATGAAGTGATTACGAGATCGCTTCGGA
GCTAGGCTCTGCCTATTGGAGTATTTGGCCTGAAAGGTATTTATTTTTGTTTGGTTGTATTAGACCCAAATTCATGGTCAATTA
ATACAAATATATATAAGTTATTGAAGATGGGAAAGGTTCAAAAAATTAGACCTGTTTAGAGATACAATGAGTTGAATTCGATCG
CTCGGCCCTAGGCAGTTCCTAGGTAGATTTGGCCTTGAAGGCAATTTTTTTGTTCAAATTAGGACGCCAATGCATGGTCAATAA
TGCCCTATACTAATATATTGGAGATGGGAAAGGTTCAAAAAAATGAGTTGTTAGAGAAAATATGAGTGAATTTACGAAATCGTT
TTCGGAGGCTAAGGCAGCTGCCTAGGAGAATTTGGCCTGAAAGGTCAATTTCTAGTTTGCTTGTTTAGGACCCCAATTCATGGA
TAATACCCCATATAATATTATTGAGATGGGAAAAGGTTCAAAATAGAGCTTTGTTAGAGAGACATATGGTGAAAAAATTACGGA
TCGCGTTCGGAGTGCCCCTAGGCAGCTTCCTAGGAGATTGGCTGAAAGGTCAATTTTTTTTTTTTTTGGTTAAATTACGAGCCC

AAACTTCATGGTCAATAAGCTATAACTAATTCTATTGAGATGGGAAAGGTTCAAAAAAATTAAGTGTTAGGAGAAATATGAGTG
ATTACGAAATCGTTTTCGGAGCCTAGGCATGCTGCCTAGGAAGATTTGGCCTGAAAGGTCATTTTTTTTTGGTTAAATTATAC
GCCAATTCATGGTCAAAATAGTCCTATTAATATTATTTGAATGGGAAAAGGTTCAAAAAATTAAGAGCAGGTAGAGAAAATGAA
AGTGAATTACGAAATGCTCGGCAGCCCTTGGCCAGCTGCCTAGGAGATTTGGCCTGAAAGGTCATTTCTTTTTGTTTGGTTGAA
TTAGGACCCCAATTCCATGTCAATATAGCCCTATAATAATATTATTGAATGGGAAAAGGTTCAAAAAAATTAGAGCTTGTTTAG
AGAGACAAATACTGAGTGAAATTACGAAGATCGCTTCCTCGGACCTAGCAACTTTTTAGGAATTTGGGCCTGAAAAGGTTCAAT
TGTTTGGTTAATTAGCCAATTCATAGTCCAATAATAGCTAATACAATATTATTTAGGATGGGAAAAGGTCAAAAAAATTTAGAG
CTTGTTAAAGAGAATAATGGTGAATTATACGAAAATCGTTCGAGCTACGGCCAGCTGCCTAGTGAAGCATTTGCCTGAAGGCAA
TTTTTTAGTTTGGTTGAATTAGGAAACCCCAATTCATGGTAAATACGATAGCCTATATAATATTATTGCGATGGGAAATGGTTC
AAAATAAATAGAGCTTGTTAGGAGAAATGAGTGAATTCGAGATCGTTGGAAGCCCCTAGGCTGCTGCCTAGGAGATTTGGCTGA
AGAGTCAAATTTTTTTGTTTGGTTGTATTAGGACCCACATTCAATGGTCAATAATAGCCTATATAATAATTATGGATGGGCAAA
AGGTTCAAAAAAATTAGAGGCTTGTTAGAGATAATATGAGTGAATTACGGACGTTCGGGAGCCCTAGGCATTTCCTAGGAGATT
TGGCCTGAAGGCAAATTTTTTTTTGGTTAAATTGGCGCAATTCATGGTCATATGAGCCCTATATAATATTATGAGAGGGAAAGG
CAAAAAATTAGAGCTGTTAGAGGAAATATGGTGATTACGAATCGTTTCGGAGCCTAGGCAGCTGCTAGGAGATTGGCCTGAAAG
TCAATTTTCTAGTTTGCTTTGTATTAGGCCCAATCATGGTAAATAATAACCCCATATAATATTAATTTGGAGTGGGAAGGTTCA
AAAAAATAGAGTTGTTAGAGAGACATATGAAGTGAATTACGAGATCCCTTTTCGGAGCACTAGGCCAGCTTTCCCTAAAGGGAA
TTTTGGCCTGAAAGGTCCATTTTTTTTGGTTAAATTAGGACGCAATTCATGGTCAAAATAGCCCTATATAACATTATTGAGATG
GGAAAGGTTTCAAAAAAATTAGAGCTTGTTAGGAAGAAAATATGGAGTGATTACGAAATCGTTTCGGAGCCCTAGGAGCTGCCT
AGGGCATTTGGCCTGAAAGGTCATTTTTTTTGTTAAATTAATACGCA
>m141122_014521_42134_c100732202550000001823141405141560_s1_p0/76256/0_10852
ACAAAATACACGAGAGAGAATAACACAAAAAAGGCGAATTAACACTCAGGACTCCCACAAACCTCAATATAGATATTATATCCT
GCCTTAGTAACTAGGGCTATGTTTACTTTTAACTGTTGAAGTTTATGCCATCCAGCTTTTAAATATAATCTTTTGGGGATCCCC
TTGTATTTGATCTGGTTATAAATCTTATAAATAATCTTTTGAACCAAAATGTCTTTAAACCTAGAAATACTTAAACCTAATGAC
ACTTTTTAAAATTAATGACTTGACTAGAAAGTCTTGCACTTTATAAGTACAACAGTGTACAGTCTTGGCCTATCCAGGGCGTCT
ACAGTCAATGTTCTTAATTATTCAATCTAAACTCTTAGGTTATAATAAATAATCCATTTCTAGGACCATCTTATATTAATCAAC
CATGTGGTTAAAATTAATATTTCTAGACTGCCAGTTAAAGACCTTATAAATCCATTAACTATTTCAAAAGAGTTTCCAACTAAA
TCCCGGCTTGAACCTATAATCACGAAAACTAAAAATCTACAGCTACTACTACTATCTAAGCTAAGTAAAATTCTGAGTGCATAC
ACTACACATGTAATCTTAATCCTAAGGTTGCCTAATAAACTCTTATTTATGTCATCAGATCCCTTTATTTGTGGCATTAAGGTT
TGTCAGAATATCAGGCTAAGAACAATTGTTTTGGGGACTCAAATGATGTATAATTGATGGGGACATAGTTTTAATGATTGGAAT
TTTAGCTTCTTTATAGATTGAATAAATGGTTCCTTATGGTTGACTTTTTGGAATTGAAAAGGTTGAACGCCTCAAGTCGAAACT
TGTTGTGACACAACCTTCTCTAGTCAAGTCAACGGTAACTCTTAGGAACAAGATAATAACTAAAAGGGTGGAAACGGTAATTTT
ATTCCCATTTTAATTATGACCAATTAAATAGAGGATTAACGTTGGTATGTATGGATTATATCAATGGAAAACTTTATTCTTTAA
TAAAGTATCTGTAAAGATTCTATAAATTATCAAGGCAGTTAATCCAATAATTTATAGTGGGAAGTAATCATTGAGGATTTATAA
TATATTATTTTTAATCAAAGTGCTTTGAATTAATAATCTACTATTTTATTGGAGCTTGATATTATAGGTCCATAGGTTCTCCAG
GGTTGCCTCTATCAACATCATATAATTTAAGAGTTGATATAAAGGTAAAAATGTAATTTGGAAATTGAGAGAATTTTATTCTTC
TAGTGGAAGTATGATTATAATGATAAGTTTGAAGGTTTGAACATTAAATTATTAATTTTTGAAGATATATTTTTATTTTGGATT
ATTATTTTCAAAATTCATATATAAAAATAAATAATTAAATTTTGAAATTAATTAATTTATTTGAGTGGTAGAAAATAAAATTGT
CAAGTCCAAAATAGTTTTTTTGATTTAATTGATTTTAAAAGATGGGTGATAATGATGATCATCAATTTTGATTTTGAAATTACA
TTTAAAAATTTTTAAACATGGTTGTCCCATTTTTCCTTTAAAAGAAAATACCTTATTTTTTTTTGGGAAGATTGATTTTAGGTT
AATAAATAAAATTAAAGAAATACAATATCCCTGAAAAATGAAAATAAACAGTTCACCGCATGGGACATAGTCCAAGGACTGTGT
ACCATGTGTGGGACTACTAACACTGATATTGTATGATATAGAATAAATAGAAATATACAAGACATAGACATTAAAAAAAATCAA
TTTAGAATAGTTCCTCATAATTTTTTTCCAGACAAAAAAGTTTATCATCTTCTTTCTTTTTTCTAACATTCTCGACCATAATCC
AAGCTTTCTCATGTGTTGAGAAATACTTATGAATTCTTAAAATAACAAACTCATGTTCTCTATGTGCCCACACACATCTGAGGT
AGAGAACACTCTGGAAAGATCCCGTGATTTGAGTGCTCAAATGCATTTGGATAGGAAGAATCCCGATAAATTAATTTGAAGACT
CAAGGACAACCTTTGGATAGGCTTTCAAGGAGGTAATTTTTCATGTTTTTGAATTTTTATGGTGAATTGTATGTTATATATATA
TATTTGTATATTTATATATATGTACATGTTAAATAATATTTGTATATTAATGTTTTTTGTCTAGATATTTTTTGTTCCATATAA
ATTAATTTATATGAGTTGGATGAAATTTGGGTATTCTTGTAATCAATGGGAACACGGCCTATTCTCCTGCGTACTATGAATGTT
TATCAAAAAAAATGTCGTTTTGACAATTAGACTAGAAAAGTTGGACAAGGCTAAGTGTTCTGGATCATTCATGTACATCTCTTTC
TCTATCCTTAAGTTTTTCTTCCTTCAAAAGGGTTCTTGTAAGACAAACACATAGGCCTAGAAACACAACTCCCTTCATTTTTTT
TTTGTGTTGAGGATTCAAGGTTAAAGTAGAGCTTTATGATGGAATGGGCTGCAAGCAAAAGGGATACTTTGACTAGAGATATTT
TTCCGAATTCCAAATCACTATTGGTATAAATTCCCAACTTCTTCTTCTTGTATAGCTTTTGTGTTTCTTGAGAGGAACTTGGGG
TTGGGTTTATTTTCCCAAATAAGGATGAATTGCTAAGTTGTTTTGGATGGGTGTAGATTGTAGTGAAAATGTATCCTTGGTTTT
GGATTTTATTTTATTGAGAAATAGAATTTGAATTAAGGTTGTGTTAATTTTTTGGATATTGAGGAATTGGAGAATAGTGACCTA
AAATTTTGGAATTTTTTTTGGTTTGGGAACTGAATTTGGGTGTTTAGTTGTTGGTATTTTATGATTTATGAGGTGTTGTTTTGT
CTGAATGGCCGAATGTTGTGAATGGAATTTCTGCAAGGTTCGGGGAAAGGATTGTACTCGAGGTCCGTTTCCCGTTGGAACACA
GGAACTCGAGGTAGATAAACTGCAATATGCACATAAAGCGGATTTGTTTGGCTATGTATGTAAGCATTGATATATCAAGTCCGA
TGATTGATACGATCTATTTTTAGCAAATCGTGTGTAACGGTGGGCCGGTCAGGAATCGTGCGCAACATGGTCTTTCAAGGGCCC
GGAGGTTAAGTTAAGGGGTGCAGTTCCCAATTTGGCGTGGCATGGACTTGTAAAGCTTTTGATTGTCGGATGCCACACTTGGCT

CGGTAGGATGATTCCTATGAGAAATTGCTCCGAATCATATTAGAGGAAAATCGAAGATTTCTGAAAGGGGTTAACCCCTTTGGT
TGGAATTGGAGATTCACATAATTTCTCGAAATGTATTCCCTTCCGTGTTTCAACTCTTTCGATATTTTTTTCCACAAGAGATCT
CACGGGTAAATGAATGTTATTGGAAGTGGAAACCGACCCTTAAATCAAACCATTTCCAAAAAAAATTCCCCTTTTTTTTGATGG
CAATTTGCAAGTTTTTTTTTCTTATATTGTACATCTCACCAGTAATTTTGAACTGTATCACCGTCTTACGCCATCTCGGATAAG
CACGAAATTTTACACCATAGGACCCTAAGAATAGGAGATACATTTTTGTTTCCTGAACCTTGCATATTATTTAGTATTACTTAT
GAACAACATTGCTCGAATCGATATTAGAGGAAAATCGAAGATTTTTTCTGAAAGGGGTACCCAACTTTGGTGAATTGGAGATTT
TTCGACTAATTTTCTCAACGAAATGTATTGCCCTCGTGTTTCAAACTCGTTTGATATTTTTTCCCCAAGAAAGATCTCCATGGG
GTAAATTGAATTATTGGAGTGGAAACGGACCTTAAATCAAACATTTCAAAAAAAAGTTCCCTTTTTGATGGCAATTTTCGGCCA
GTTTTTTTCCCCTTAATATTAGTAATCTCACAGTATTTTGATGTTCACCGTTCCTTACCCATCTCGGACTAGCACGGGGAAAAA
AAAAATTTACACATAGGAGCCCTAAGACATAGGGGATAACACTTTTGTGCCCATTGACCCCTTGCAATATTATTTAGTAATTCA
CTATGAAAAAACATTGCTCGAAATCGATTTAGAGAAATCGAAGAATTTCTGAAAGGGGTACCCTTTGGGGTGAATTGGAGAATT
TCAACTAATTTCTCCACCGAAATGTATTGCCCTCGTGTTAACTCGTTTTGAATTATTTTTCCCCAAGAAGATTCTCATGGGTTA
ATGAATATATTGGAGTGGAAACGGACACCCTTTAAATATCAACATTTCAAAAAAGTTCCCCTTTTTTGATGGAATTTTCGCAAG
TTTTTTCCCTTATATTAGTACATCTCCACAGTAATTTTTTGACTGTATCACCCTCTACCAACTCGGACTAGCAACGAAATTTTA
CAACATAGAACCTAAGCCAATTGGGCTACAAACTTTTGTGCGCCATGACTTGCCATATTATTTAGTATTCACTATGAATAACAT
TGGCTCGAATCGATATTAGAGAAATGAGAATTTCTGAAAGGGGTACCCCTTTGGTGAATTGGAGTTTCACTAATTTCGAAATGA
TTATTGCCCTCGTGTTCCAACTGTTGATATTTTTCCCCAAGAGATCTCATGGGTAAGATGAATATATTCGGAGTGGAAACGGAA
CCTTAAATCAACATTTCAAAAAAGTTCCCTTTTTTATGGCGAATTTTCGCAAGTTTTCCTTATATTAGTACACTCACAGTATTT
GACTGTATCTACCGTCTACCCATCTCGGACATACACGAAAATTACACATAGGACCCTAAGACATAAGGGCTACCTAGCAACTTT
TTGTGCCATGACCTTTAGCATAATTATTTAGTTATTCACTTATGAGAACATTGCTCGAATCGATAATTAGAAAATCGAAGATT
TCTGAAAAGGGGTACCCCCTTTTTGGTGAATTGGAGATTTAACTAATTTCCCCGAAATGTTTGCCCTCGTGTTACTCTTTCGAT
ATTTTTCCCCAAGAGATCTCACGGGTAAATGAATGTATTGGAAGTGGAACGGACCCTTAAATCACCATTTTCAAAAAAAATTCC
CCTTTTTTTTGATGGCAAAATTTTTCGCGAAGTTTTTTTCTTTATATTAGTATCTCACAGTTATTGACCGTTACCGTCCTAACC
CATCTCGACTAGTCAACGAAATTTTTACAACATAGGTACCCTAAGACAAGGGATAACAACTTTTGTGCCCATGACCCCTGCATA
TTAATTTAGTCTTCACTATGAAAAAACATTGCCTTAGAATCGATATTAGAGAAATACGAAGATTTCTGAAAGGGGTACCCCTTT
GGTGAATTGGAGATTTCAACTAATTTCTCCGAAATGTATTGCCCTTCGTGTTCAACAATCGTTTGATATTTTCCCCAAGAGATC
TCAATGGGTAAATGAATATATTGGAGTGGAACGGACCCTTTAAATCAACATTTCAAAAAAAGTTCCCCTTTTTGATGGCAATTT
TCGCAAGGTTTTTTCCCTTATATTAGTACATCTCCAACAGTATTTTTTGACTGTATCAACCCTCCTACCATCGGACTAGTACAC
GAAATTTTTAACAACAATAGAACCCTAAGACATTAGGCCTACAACTTTGTGCCCCATGACCCTTGCTATTTATTAGTATTCCAC
TATGAAAACATTGCTTCGAATTCGATATTAGAGAAATCGAAGATTTCTTGAAAGGGGTACCCCTTTTGGTGAAATTGGAAGATT
TCACTAATTTTCTCCGAAATGTAATGCCCCTCGTGTTCAACTCGTTTGATATTTTTCCCCAGAAGAATCTTCATTGGGTAAATG
AATATATTGGAGTGGATAACGGACCTTAAATCAAACATTTCAAAAAAAAGTTCCCCTTTTTTTGGCAATTTTCGCAGTTTTTTT
CCTTTATATTAGTACAATCTCCCCAGCAGTATTTTGACTGTATCACACCGTCTACCCAATCTCCGGATACCGAAATATTACACA
TGGGGAAAAAAACCCTAAGACATAGGGCTACAAACTTTTGTGCACATGACCTTTGCCATATTAAATTTTTAGTAATTCACTATGA
GAACATTGCTCCGAATTCGATATTAGAAGAAATCGAAGATTTTCTGAAAAGGGGTACTCCTTTTGTCGAATTGGAAGATTTCAC
TAATTTCCTCCGAAATGTATTGCCCTCGTGTTCACCTCCTCTTTCGATAATTTTCCCAAGAGATCTTCACGGGTAAATGAATGT
AATTGGGAGTGGAACGGACCGCTTTAAATTCAACATTTCAGAAAAAATATTCCCCTTTTGATGGCAATTTTCGAAGTTTTTTCC
TTATTTATTAGTAAATCTCAGCAGTATTTTTTGGACTGTATCTACCGTCTACCCTCTCGGACATAGCACGGAAATTTTACACAT
AGACCCTTAGACATAGGGATACAACTTTTGTGCCCATTGCCCTTTGCATATTATTTTTATATTCACTATGAAAACATTTGCTCG
AATCGATATTGAGAGAAATCGAGGAGGAGATTCTGAAAGGGGTACCCCTTTGGTGACATTGGAGATTTCACTAATTTCTTCCGA
AATGTAATTTGCCCTCGTGTTCCAACCTCGTTTTGATATTTTTTCCCCAGAGATCTCATGGGTAAAATGAATATATTGGAGTGG
AAACGACTTTAAATCAACATTTCAAAAAAAGTCCCCTTTTTTGATGGCAATTTCGCCCAGGTTTTTTTCCTTATATTAGTACAT
CTCACAGTATTTTGACTGTATCACCGTCTACCCATCTAAGGACTAGCACGAAATTTGTACACATAGGAACCTAAGACATAGGGC
GATACAACTTTTTGTGCCATGACCTTTGCATATTAAATTTAGTATTCACTATGAAAACATTGCTCGAATGATATTCAGAGAAAT
CGAAAAGATTTTCTGAAAGGGGTTACCCCTTTTGGTGATTGGAAGGTTTTCACTAATTTTCCCCGAAATGTATTGCCTCGTAGT
TCCAACTCGTTTGGAATTTTTTCCCCAAGAGAATCTCAAATGGGTAAAATGAATATACTTTGGGTGGAAAACGGACCCTTAAAT
CATACATTTCAAAAAAAAGTTCCCTTTTTGATGGCAATTTTCGCAAGTTTTTCTTTTAATATTAAGTACATCTCACAGTATTTT
GACTTGTATCACCCTCCTACCCAACTGGACTAAGCAAGAAATTTTACACATAAACCCCTAAGACATAAGGGCTACACTTTTGTG
CCACCATGACCCCCCTTGCAATATTATTTAGTTATTCACTATGAAACAAAATTTTGCTCGAATGATATTAGAAGAAATCCGAGA
TTTCTGAAAGGGGTCCCCTTTGGTGAATTGGGATTTCACTAATTTCCTTCCGAAAATGTATTGCCTCCGTGTTTTCAACCTCGT
TTGATATTTTCCCAAGAGATCTCAATGGGTAAAATGAATATATTGGAGTGGAAACGGACCCCTTAAATCAACATTTTCAAAAAA
GTTCCCCTTTTTTATGGCATTTTCGCAAGTTTTTTCCTTATATTAGTACATTCTCACAGTATTTGACTGTTCACCGTCTACCCA
TCTCGGACTAAGCAACGAATATTACACCATGGCCCTAAGACATAGGGCTACAATTTTGTTGGCCATGACCTATGCATATTATTA
GTATTGCACTATGAAGAAATTGCTCGAATCGATATTAGAGAAATCGCGAAGTTTCTGAAAGGGGTACCCTTTGGTTGAATTGGG
AGATTTTCACTAATTTCCTCGAAATGTATGCCCTCAGTGTTTCAACTCCTTTCGATTTTTTCCCCCCAAGAGATCTCACCGGTA
AATGAATGTATTGGAAGTGGAAAACAGGACCCCTTAAATCAAGCATTTTCATTCAAAAAAAATTCCCCTTTTTTTGAATGGCAA
TTTTCGCAAAGTTTTTTCCTTTATATTAGTACATCTCACAGTATTTTGACTGTATCACCGTCTACCCATCTCGGGACTAGCACG
AAATTTACACATAGGCCTAGACATAAGGGGATAGCACTTTTGTGCCCATGACCCTTTGCATATTAATTTAGTATTTCACTAATT

GAAACATTGCTCGAATCGATATTAGAGAATCGAAGATTTCTGAAAGGGGTAACCCTTTGGGGTGGAATTGGAGATTTCACTAAT
TTCTCCGAAAAGTTATTGCCCTCGTGTTTCCACTCGTTTGATATTTTTCCCAAGAGATCTCATGGGTAAATTGAAATATATTGG
AGTGGGAAACGACCCCTTAAATCAACATTATTAAAAAAAGTTCCCCTTTTTATGGCATTTTTCCGCAGTTTTTTCCTTAATAT
TAGTACATCTCACAGTATTTGAACCTTGTATCACCCCTTTACACAACTCGGACTAGCACGAAATTTTTACACATAGAACCCTAA
GACATAGGGCTACAACTTTTTTGTGCCCATGACCCTTGCATATTAATTTAGTATTCATATGAATAACATTGGCTCGACATGCGA
TATTGACGAAATCGAAAGATTCTGAAAGGGGTACCACCTTTGTGAATTGGAGATTTCACTAATTTTCCCGAAATGTATTGCCCT
CGTGTTCAACTCGTTTGATATTTTTCCCCAAGAGATCTCATGGGTAAATGAAATACTAATTGGAGGTGGAAACGGACCCTTTAA
ATCAACATTTCAAAAAAGTTCCCCTTTTTTTATGGCAAATTTTCGCAAGTTTTATTCCTTATATTAGTACATTCAAAGTATTTT
GACTGTATCATCACCGTCTACCCATCTCGACTATGCACGGAAATTATTACAGCATAGGCCCTAAGAACATAGGGCTAAACAAAC
TTTTGTGCCATGCCCTTGCATATTATTTAGTATTCACTTAATGAGAAAATCTCGAATCGATATTAGAGAAATCGAGATTTTTGA
AAGGGGTACCCTTTGGTGGAATTGGAGATTTCAAACTAATTTTCTCCGAAAATGTATTGCCCTTCGTGTTCAAACTCTTTCGAT
ATTTTTCCCCAAGAGATTCTCATAACTCACCGGGTAATGAATGTTTATTGGGAGTGGAACGGACCCTTAAATCAACATTTCAAA
AAAAAAATTCCCCTTTTGATGGCAATTTTTCGCAAAGTTTTTTCCTTATATTAGTACATCTCAACAGTATTTTTGACTGTATCA
CCGTCTTACCCATATTCTCGGACTAGCAACGAAATTTTACAACAATTAGGACGCCTAAAGACATAGGGGATACAGACTTTTTGT
GCCACCATGACCCTTGCATATTATTTAGGTATTCCATAATGAAACATTGCTCGAATCGATATAGAGAATCCGAAGATTTTTCTG
AAAGGGTACCCTTTTGGGTGAAATTGGAGTTTTTCACTAATATTTCTCCGAAATGTATTGCCCGTCGTGTTCACTCGTTTGATA
TTTTTTCCCCCCCAAGAGATTGTCATGGGTTAAATGAATATATTGGAGTGGAAACGGACCCTTAAATCAACATTTCAAAAAAAA
GTTCCCCTTTTGATGGCCAATTTTTCGGCCAAGTTTTTTTCCTTATAGTGTAAATCTTCAAGCAGTATTTTTGACTGTAAATTC
ACCGTCAATTAACCCATCTCGGACTTACACGAAATTTTACACCATAGGAACCTAAGACGATAGGATACAACTTTTTTGCCCATC
GACCCTTTGCAATATTATTTAGTATTCACATATGAACAACATCATGCTCGAATCAGTATTAGAAGAAATCCGAAGAATTTCTGA
AAGGGTAGCTTTTGGTGAAATTGGAGATTTCAGCTAATTTTCCGAAAATGTATTGCCGTCGTGTTCAACTCGTTGTTTGAGTAT
TTTTTCCCCAAGAGATTCTCATGGGTAAATGAATATATTGGGTGGAAACGGACCCTTAAATCAACTTTTCAAAAAGTTCCGCCT
TTTGTGGCAATTTTTCGCAAGTTTTTTCCTTATATTATACATCTTCCACCAGTATTTTTGACTGTATACACCCTCTTACCCAAC
TCGGAACTACGCACGAATTTTAACACAATAGAAACCCTAAGACATAGGGCTTTACAACCTTTTGTGCCCATGACCCTTGCATAT
TATTTAGTATTCACTATGAAAAACATTGGCTCGAATCGATATTAGAGAAATCGACAAGATTTCTGAAAGGGGTTAACCCCTTT
TGGGTGAAATTTGGAAGATTTTCACTAATTTTCTCCGAAAATGTATTGCCCTCGTGTTCAACTCCGGTTTGATATTTTTTTCCC
CAAGAGATCTCATGGTAAAAAATGAATATATTGGAAGTGGAAACGGCCCTTAAATCAACCATTTTCAAAAAAAGTTCCTTTTTT
GAATGGCAAATTTTCGCAAGTTTTTTTCCTTATATTAGTACATCTCACAGTATTCTTGACTTGTATCACCCGCTAAACCCATAT
CGGACTAGCACGAAATTTTACACATAGGACCCTACAGACATAAGGGGATACAACTTTTGTGCCCATGACCCTTGCAAATATTAT
TTAGTATTCACTTATTGAAAAAGCATTGCTACGAATCGATATTAGAGAAATCGAAGATTTCTGAAAGGGGTACCCCTTTGTTGA
ATTGGGAGTTTTCACTAATTTCTTCCCGAAATGTATTGCCCTCGTGTTCAACTCGTTTGATATTTTTTCCAAGAGATCCTCTAT
GGCAAATGAAATATATTGGAGTTGGAAAACGGACCCGTTTAAAATCAACATTTCAAAATAAAGTTCCCCTTTTTGATGGCAATT
TTCGCAAGTTTTTTCCCTTATATTTAGTACATCTCCAAACAGTATTTTAACTGTATACCCTCTACCCAGACTCGGACTAGCAGA
TTTACAACATAGAACC
>m141122_014521_42134_c100732202550000001823141405141560_s1_p0/76666/0_6625
AAGAGAGAAGATTTAGGCTCCGAAGCGATCAATCGAATCACTCATATTTCTCTCTAAACGCTCTACAATTTTTTGAACTTTTA
CCATCTTAATAATATTATAATAGGCGATATTATTGACAATGAATTGGGTCTAATTCAACCCCAAAAAAAAATTGACATTTCAGG
CCCAAATCTTCACACTCCAAGGCAGCCCCTAGGGTCCGAAGCGATCTCATGTAATTGCCACTCAATGTTCTCTCTAACAAGCTC
TAAATTTGTTTTGATCCTTTTCGCCACTCTCAAATATATTATATAACGGGCTGTTATTGACCAGTGAATTGGGGTTCCTAATTA
CAACCAAACAAAAAAAATTGAACCTTCAGACCAAATCAATCCTAGGCAGCTGCCTAAGGGCTCTGCAGCGATTTGTAATCTCACC
TCTATTTGCTCTCTACAAACTTAATTTTTTTTAACTTTTTACCATTCTCAAAATTAATAAATCATGTGCGCTGTTCATTGACCA
ATGAAAATTAGAGGGGTACTAATTAACCAAACTACAAAAAAAAATTGACTTTCAAGGCCAAACTCCTAGGCAGCTGCACATAGGC
TCTCGAGCGATTTCGTAATTCAACTCATATTTCTCATCCTAACACAATCTAATTTTTTGAACCTTTCCCTACTCAATTAATTTA
TTAGGTATAGGGCCTATTATTAACCATGAATTGGGGCCTAATTCAACAAACAAAAAAATTGACCTTTCAGGCCAATCTCCTAGG
CAGCTGCCCTATGGTCCTGAAAGCGATCTCGTAATTCAAAAAACAAAAAAAATTGTCCTTTCAGCCAAGATCTCCTAAAAAGGT
CCTGCTCAAGGCTCGAGCGCCATCTCTAATTTCACTCATATTTCTCTCTAACAAGCTCTAATTTTTTTTATCCTTTTCCCATCT
CAATAATATTATATACTTGCTGTATATTGAATTGCATTGGGGTCTTAATTCAACCAAAAAAATTGACCCTTCAGGTCAAAATTC
TAAGGTTGCTGCCTAGGGCTCCGAAGCGATTTCTATTACACTGATATTTACTCTTACAGCCTCTGAATTCTTTTTTCTGAACC
ATTTTCCCATCTCAATTAATATTAATATAAGGGCTATTATTTAACATGAATTGGGTCCTAATTCCAATCAAACAAAAAATGATT
GACCTTCAACAGAAGCAAATTCGATAACGAAACAAGTAGGCAACTAGGCCAAGACTGCCTAGGGCTCTGAGCGATCTCGTAAAT
TCAACCAACGAAATAAAATTGACCATTTCAGGCACAATCTCCCTAGGCAGCTGCTAGGGCTCCGAGGCGATCGTGTAATTGCAC
TCATATTTCTCTCTAACAAGCTCTAACTTTTTTTTTATCCTTTTCACAGTCTCAATAATATTATATACGGGCTGTGTATTGAAC
TACTGCATTGGGGATCTTTAATTACAACCAAACAAAAAAATTGTACCTTCAGGCCAATATCTCCTAGGCTCTGCGCTAAGGGATC
CGACAGCGATTTCATAATTCCAACTCATATTTCTCCTCTAATACAAGCATCTAATTTATTTATGGAACCTTTTCCCATCTCAAT
AATATTTATAGGTATAATGATCCATGAATTAGGGTCCTAATTACAAGTCACACAATGACAAATTGCCTTTCAGGCCACAATCTC
CTAGGTAGCTGCCTAGGGCACCGAAGCGAATCTCAGTAAATTCATCATATTTCTCTCTAAAAGCTCTAAATATTTTTGTATCCT
TTTCCCATCTCATAATGCATGATATCATACGGAGCTGTTATCCTGACATGAATTTGGGGTCCATAGAATTCAAACAGACAAAGA
ATTTGACTTTCAGGACCAATCATCCAAGCAGCCTGCATAGCGGCTCCGACAGAATATTTCCAGTAAATTCACTCTATATTCTCA

TAACTAACTCTAATCTTATTTTGAAACCTTTTCCCATCTCCAATTATATTCATAATAGGGCACTGTTTATGACAAATGAATTCG
GGGGCATCCTAATTCAATACAACAAAAAAATTGACCTATTCAGGCCAAATCGTCCTAGGCAGGTGCCTAGGGCTCCGAAGCGAT
CCTCGTAATTCACTCATATTTTATTCTAACAGTTTCTAATTTTTTTGACTGTTTCATCTCAATTAATTATCATTACAAGGCTGT
TCATTTGACAAGCATTGGGGTCCTGAATTCGAACAAAACAAAAAAATTGACCTTATAGAGCCAAATCTCCTAGGCAGCTGCCTA
TCGGAACTCCGAAGCGATTTCGTATTCACCATCATGATTTCTCTCATAAAAAAGCTCTAATTTTTTATGATCTTTTCCATCTCA
AATAATATTATACATATGGTCTGTTATTGACCATCGAATTGAGGGTCCATATATTCAGGACAAACTAAAAAAATTGACCTTTCA
GCCAAATCGTCCTTAGCAGCTGCCTAGGGTCTGAACGAATCTCGTAATTCAACCACACAAAAAATATTGACCTGTGTCAGGCCA
ACACTCCTAGGCAGCTGCTAGGGCTCTCGAAGCGATTTCGTAATTCAGTAATATTTCTCTTCTAACAATGCTCTAATTTCTTTA
TTATCCTTTTCCATCCTCTAATAATATTTATATAAGGGCTGTTATTGAATATGAAGTAAGGGTCTTAATTAACCAACAACACAA
ATAATTGACCTTTCAGGCAAATTCCTCAGGCTGCTGCTAAGGGACCGCAAGCGATTTTATCGTCAATTCACGTCATTTTCTCTC
TAAAAAGCCTCTAAGGTTTTTTTGCAACCTTTTCCCATCTCATCAAAATTATAATAGGGCTTAGTTATCTGACCATTGAATTTG
GGGTCCTAATTCAACAAGACAAAAAATATGACCTTTCAGGCCAAATCTCCTAGCAGCTGCCTAGGCTCCGAAGCGATCATCGTA
ATTCACTTCATATTTCTCTCTAACAAGCTCTAATTTTTTTTGTCTTTTCATCTTCAAATATAATTATATACAGGCTGTTAATAG
ACCATGAATTGGGGTCCTAAATTCATATCAAACAAAAAAATTGAACTTTCAGCTGGCAATCTCAAGCGCCTGCTAGGGTCCGAA
GATATTTCATAATTCACATCATATATATCTCCTAACAAGCTCTAATTTTTGAACCTTTTCCATCTCAAATTATATTATATATAG
GGCTTGTTAATGAACCATGAATTGGGGGTCCTAATTCAACCAACAAAAAAATTGACCTTTCCAGCCAAATCTCCTAGGCAAGGT
GGCTAGGGCTTCCGAAGCGATTCTCGTATTACACATCATTATTTCCTTTCTAACAAGTTCAATTATTTTTGAACCTTTTCCATA
CTCAATAATCCTATACATATGGGCTTGTGTAATTGACACATGAAATTGGGGTCCTAAGTTCAACTCAACAAAAAATTGCTTTCA
GGCCAAACTTCCCTAGGCCAGAACTCCCCAGGGCATCGAAGCGATCTCCGTAGATTCAACTCATATTTCTCTCTATACAAGCTC
TAATTTTTTTGATCTTTTCCGATGCTCAATAATATTTAATATAAGGGCTGTTATATGACCATTAGTTGGGGTCTCCTAATCCAA
TCAAACAAAAAAATTGACATTATCAGCACAAATGTCCAGGCACACTGCTAGGGCTCATGCACGATTCTCAGATAATTCCATCAT
ATTTCTCTCTAAAACTCTAATTTTTTGATCTTTTCACCATCTCAATAAAACTTATATACGCGCTTGATTATTTGACCGATGATA
TGGGGTCCTAATTCACCACAAACAAAAAATTGACCTTTCAGGCCAAAGATCTCCTAGGCATGCTGTCCTAGGGCTCCGACAGCG
ATTTCGTAATCTCACGTACATATTTTCCTCTAAACAAACGATCTAATTTTTATTGACTTATTCCCATCTTAATAATAGTTCATA
TATAAGGGCTGATTATGTTGACATGAAAATTGGGGTTAATTCAACAAAAAAAAAATTGACCTTTCAGGTCCAAGATCTCCAAGG
CAGCTCCCTAGGAGCTCTCGAAGCCCGATCTCCGATATTCACAGTCATATGTTTCTCTAACAAGCTCTATTTTTTTGATCCTTT
TCCCATAATCTCCAATAATAATACATACTAGGAGCTCGTTATATCTGACCATCGAATTGGGGTTCCTAATTCAAACCAAAAAAA
AAATTGCCTTTCAGACCAATCTCCCTAGGCAACTGCTCAGTGGCTCTGCCAGCGATTTTCGTAATTCACTCATATTTCTCTCTA
AAAACTTATTTTATTTTGAATCATTTTCCATCTCACATAATATTACGTATACGCTGTATTGACGCATACGAAACTTGGGTCTCA
ATTCAACCAAAACAAAAAAAAATTGAGCCTTATCAGGCCAAAATCTCCTAGGCAGACTGCTAAGGGCTACCGAGCGATTTTCGT
AATTCTCATATTTCTCTCTAACCAAGATCTAAATTTTTTTGAACCTTTTCCCATCTCAATAATATTGTTAGGGTATTATTAACA
TAGAATTGGGTCCTAATTCAACAACAAAAAATCTGACCTTTCACAGGCCAAACTCCTAGGCAGCTGCCGTAGGGCTCTGAAGCG
ATCCTCGTAAATTTCAACCAACAAAAAATTGTCCTTTCAGGCCAATATTCCTAAGGCAGCTGCCTAGGGCTCTCCGAAGCGATC
TCGTAATTCATCGTCTATTTGCTCTCTAACAAGCTCTAATTTTTATTATCTTTTCCCAATCTAATAAGACTGTAATATAATTGC
TGTATATTGAATTTGAATTGGGGTCTTTAATTCAACCAAACAAAAAAAATTGACCTTTCAGGTCAAATCTCTAGGCTGCTGCCT
AGGGCTCCGAAGCGATTTCAATACATTCACTTGATATTATATCTCTAACACAAGCTCTAAATTTTTTTGAACTTTTCCATCTCA
TAATATTATGATAATGGCGTATTATTAAAATTAGAATTGGGGGGTCCTAATTCAATCAAAAAAAAAAATTGACCTTTCAGAGCA
AATCTCCTAGGCAGCTGCCTAGGCTCTGAAGCGATCTTCGTAATCAACCCACAAAAAATTGACCTTTCAGGCCAAATCTCCTAG
GCAGCTGCCTATGGCTCCGAGCATCGTCGTAATTACATCATATTTCCTCTAACAGCTCTAATTTTTTTATCTTTTCCCACTCAA
ATAATATTATAGGGCTAGTTATTGAATATGATTGGGGTCATTAATTCCAACCAACAAAAAAAATTGACCTATTCAGGCAAATCT
CCTAGGCCTCTGCCTAGGGAATCCGAAGCGATTTCATAATTCACTCATATTTGCTCTCTAAAAAAGCTCTAATTTTTTTGAGCC
TTTACCATCTCAATAATTATTATAGGGCTATAATGACCATGATGGGTCCTAAATTCAACCAAACAAAAAATCTGACCTTTCAGG
CCAAATCTCTAGGCAGCTGCCTAGGGCACCGAAGCGATTCTCGGTAATATTCACTCATATTTCTCCTAACAAAGCTCTAAATTT
TTTTGATCTTTTCCCATCTCAATAATATTATATAGGGCTGTTATCTGACCTTGAATGGGGGTCCTATATTCAACGCAAACAAAG
AATTGACCTTTCGAGGCCAAATCTCCAAGGCAGCTGCCTAGGGCTCCGAAGATATTTCATAATTCACCTCATATATTGAATCCT
AACAGCTCTAATTTTTTTGAACTTTTACATCTCAATTATATTATATAGGCTGTTATATACATGAATTGGGGTCCTAATTCAACC
AAACAAAAAAAATTGACCTTTCAGGCCAAATCTCCTAGGCAGGTGCCTAGGGCTTAGAGCGGAATCTCGTAATTTTCACCTCTAT
TTCTTCTAACGATTCTAATTTTTGATTTTCCCCATCTCATATAATTATAGGTGTTATTGACAAATTGGGGTCATAATTATAAAA
AAACTTGCCTTTCAGCAGCCAAATCTCCTAGGCCGCTGCCTAGGCTCGAAGCGATTTCGTAATTCCCTCATATTTTCTCTAAAA
GTCTAATTTTTTTGATCCTTTTCCTCTCATAATATTATATGTCTTGTTTTGACATTGATTGACTATTCAACCAAACAAAAAATT
GACTTTCAGGCAATGCTAAGGAGCTGCTAGGGCTTGAGGATCTCTAAATTCAACAAAAAATTGACTTTCAGGCAATCTCTAGGG
ATGCTGCCTGGGCTCGAAGCGTTTGTAATTCCCCATATCTCCTCTAAACAGTTAATTTTTTTATCTTCCATCCTCAATAATATT
AATGGGCTGTTAGTGAATTATGATTGGGGTCTTAATTCAAAAATACAAAATGACCTTTCAGGCAATCTCCTAGGGGCGCTAGGA
ATCGAGACGATTTCGTAATTCACTCATATTTCTCTCTAAAAATGATCTAATTTTTTTGAACTTTTCCATCTCAATTAATTCATT
ATACGGCTATATGCATCGACTTGGGGTCTAATTCAACCAAAAAAAATGACTTTCAGGGCCAAATCTCCTTAGGCAGCCTGCTAG
GGCTCCGAAGCGATCTCAGTAATTTCATCTACTTTTATCCATCTAACAGCTCTAATTTATTTGATCTTTTCCA
>m141122_014521_42134_c100732202550000001823141405141560_s1_p0/79931/3239_9088

ATCATCGTAGTCTGTGAGGTGAGCATTCTCGAGCTTTGATCTTCTCAATAGCCTCAATTAAGTTTCTCTAAACCATCTCAGTCC
TAAGTACTTCCTCTCCCTTCCATATCGTTCGCAATTGAATGGGTGATTCTACAAATTTCCTGCCATAACCACATCTCGTATGGT
GGCTTCTCCCAATATGTTGATACTTGATAGATTTTTATTGTAATGAAAGCTCAATCAATGGAAGATACTGTTACTCAAGATCGC
CTCAAAATCTACATACACATGGCCCTAAGGGCAATGTCCTCCCTAGTATACGAATCGTGCCTCCTTAGACTGTTCCCACCTCAG
TTTAAGGGTGCTCGGGTTATTTACTGAAAAGTGTGAGATGTTTATCGAAAGTTCGCTTCGGTTGTTTGGGCAGTAAACCCCTTGA
AGCTTAAATTTGATTTGTCCCCTTTTATAGATAAAATATATAGAGATAGAACCCTTCCCAAAAATTAGAGTAAAATCGAGAGAG
TCCCTTGGTTTATGTAACTTTTTATATAGGAATAAGAGATTATATAAGAATAGAGAGATTCGATATAGAGGTGGTCTTTTAATA
TTTGGAGGGGGTAAATACCAGTGGTCAAACTAAACAAGCAGCGAAAAGAAGTCTAGGTCAATCCGGGGGAGAAAGGGGGAGGAC
ACACCGTCCGCTATAATGGCAATTTGCGGAAACCAAACATTCGGGTGCGTCATACCATCACTAAATGCAAACGGATCATCAGAA
ACTCCGCAGTTAAGCGTGCTTTGGGCGAGAGTGTAAACTAGGATGTGACCTCTGAGAAGAAAATCGTGTAATGAACCCTGAAGA
AATAATAAAATATTAAAAAATAATAATAAAATAATACACACATATTAAAATATAAAATTTGTATTATATACATATATTTTAAA
ATCTTTAAAACATCCATTTAATTAAAATACAGGTAGAATAATAATTAGAGAGGAGAAAATAATACGTGAAGGTCCAACTAAAAG
CAGCGAATAAAGAGTTCTAGATAAAAAAAAATCAGAAAAAAAAGGGGACGAAAGGGAGACAACGCGCCGCTATAAATATAGTTG
CGGACAAACATTCCGGGTGGCGCATCTACCAGCATCTAATGCACGGTCCCATCAGACTCCGCAATTAGCGTGCTTGGGCAGAAG
TAGTACTAGGATGGGTGACTCCATGGGAATCCCTCGTGTTTGCCACCCTGAAACATCATTTTTTTTTTCCCTTTAAAATCCCAC
CGTACGGCAAAATTGGTATTTTTTGTTTTAAATCTTAAAAACTCAAAATTAATTTCGTTTTCCTTGAGGGTTAATACCGTGGTT
CAACTAACAAGCAGCGAATAAGAAGTCCTAGTCCCAATCCGGGGACAAAAGGAGGAACACACGCGCTCGCTATAAATGAATTTG
GCGGACGCAGGAAACATTTTCGGTGCGATCAATACCAGCATATGCACGGATCCCAATCAGAACTCCGCAGTTAAGCGTGCATGG
GCGAGATAGTACCTAGGATGGGTGACCTCCTGGGAAGTCTCGTGTCACCCTGAAGACTCATTCTTTTTTTCCCTTTAAAAATCC
ACCTACGGCTTCCAAAGTTGTATTTTTGTTTTAAATCTTTAAAACTCATTTATTTCGTTTCCTTGAGGGGTTAATACCGTGTCA
CTAACAACAAGCGAATTAAGAAGTTCCCTAGTCAATCCGGGGGAAGAAAAAGGGATGGAACACTGGCGTCGCTATAATGAACTA
TTGCGGACCAACATTCCGGGTGCGACATCAGTACCAGAACTAATGCACCGGTCCCATCAGAACTCCGCAGTCAAGCGTGCTTGG
CGAGAGTTAACTAGGATGGGTGATCTCCTGGGAAGTCTCGTGGGTTGCACCCCTGAAAAACATCATTTTTTTTTCCTGCTTAAA
AATCCACCTACGCTCAAAAGTTTTCTATTTTTTTGTTTTAAATCTTTAAAAGACTCCATTATTTCGTTTTCCTTGGAGGGGTTA
ATACGTGGTCACTAACAAGCCGAATAAGAAGTTTTAGTCAATCGGGGGAGAAAGGGAGGGACACACGCTGCTCGGTATAAATGA
ATGATTGCGGACCAAAAGTTACGGGTGCGATCAGTACCAAGCACTAATGGCACCGATCCCATCAGAACTCCGCAGTAACGTGCT
TTGGGCCGAGAGTAAGTCACTAAGGCATGGTGACCTCCCTTGGGAAGTCCTCGTGTTGCACCCCTTGAAAACATCATTTTTTT
TTCCCTTTAACAATCCACCTACGGCTAAAAAGTTTGTATTTTTGTTGCTTTAATCTAAAACTCAATTTTATTCGTTTTCCTTGA
GGTTAATACGTGGTCACTAAACAAGCAGCGAATAAGAAGTTCCCTAGTCAATCCGGGGGAGAAGGAGGACACACCGCGCAATTG
ATCGGCTATCTAAAAATGAATATTGCGGAACCCAAACATTCCGGGTGCGATATACCAGCACCTACTGCCAGCCGGATCCCATCA
GAACTCCCTGCAGTGTTAAGCGTGCTTGGGCGAGAGTAGTACTAGGATGGGTGACCTCCTGGGAAGTCCTCGTGTTGCTACACC
CTGAAGACATCATTTTTTTTTTTCCCTTTAAAATCCACCTACGGCAAATAAGTTTGAAGGTTTGTCTTGTTTTAATCTTTAAAA
CTCATTTATTTCGTTTTCCTTGAGGGTTAATCCGTGGGTCACTACAGCAGCGAATAAGAAGTTCCTAGTCAATCCGGGGGAGAA
GAGGGAGGACAACACCGCTCCGCTATAAATGAATATTGCGGACAAAAGTTCCGGGTGCGATCATGACCGACTAATGAACGGATC
CATGCAAGAACTTCCGCAGTTAAGCGTCTTGGGCGAGAGTAGTACTAGGATGGGTGACCTCCTTGGGAAAGTTCCTCTGTGGTG
CCCCCCTGAAAACATATTTTTTTTTTCTTCCCTTTAAAAATCCACTACGGCTCACAAGAGGTCGTTGTTATTTTTGTTTTTAAT
TTTAAAAATCCATTTATTTCGTTTCCTTGAGGGGTTATACGTGGTCACTAACAAGCAGAGCGAATAAGAAGTCCTAGTCAATCC
GGGGAGAAAAAGGGAGGAACACGCGCCTCGCTATAAATGAATTGCGGACCCAAAATCCGGGGTGCGATCATACCAGCACTAATG
CACAGATCCATCAGAACTTCCGAGTTAAGGCGTGCTCTGGGCGAGAGTAAGTACTAGGATGGTGACCTCCTGGGAATCCTCGTG
TTGCACCCCTGAAACATCATTTTTTTTTTTTTTCCTTTAAAAATCCACCTACGGCTCAAAAGTTTGTATTTGTTTGGTTTTAAAT
CTTAAAACTCTTTTGATGTTCGTATTTCCTTGAGGGTTAAGATACCGTGGTCACTAACAAGCAGCGATAAGAACGTTCCTAGTC
AATACCGGGAGAAAGGGAGGACACACGCGCTCGCTTAATTGAATTATTGGCGGACCAAACATTCCGGGTGGCGAACATACCAGC
ACAATGCAAACCGGATCCCAATCAGAACTCCCGCAGTTAAGCGGCTTTGGGCGAGAAGTAGTATAGATGGTGACTCTGGGAAAG
GTCCTCGTGATTGCCCCTGAGACATCATTTTTTTTTTTCCTTTAAAAATCACCTACGGCTCAAAAGTTTGTATTTTTTGTTTTT
AATCTTTAAAAACTCATTTATTTCGTTTTCCTTGAGGGGGTTAGATACCGTGGTCACTGAACAAGCAGCGAATAAGAAGTTCCT
AGTCAATCCGGGGGAGAAAAGGGAGACGACACGCTGATCGCTATAAATGAAATTGCGGAACAAACATTCCGGGTGCGATCATCC
AGCCACTAAGCCCGGATCCCATCAGAACTCCGCAGTTAAGGTGCTTGGGCGAGAGTAGTACTAGGATGGGTGACTCTGGGAGTC
CCTACGTGTTGCACCCTGAAGACCATCATTTTTTTTTTTCCCTTTAAAAATCCACCTAACGGACGCTCCAAAAGTTTGTATTTT
TTGTTTAAACTTTAAAAACTCATTATGTTGTTTTCCTGAGGGGTTAATAACCGTGGCACTAAAAGCAGCGAATAAGAAGTTCCT
AGTCAATCCGGGGGAGAAAAGGGAGGACACCGCGCCCGCTATAAATGATTTGCGGACCAAACATTTCGGGTGCGATATACCAGC
ATAGCACCGGAAATTCCCATCAGAACTCCGCAGTTAAGCGTGGCTTGGGCGGAGTAGTAATAAGGATTGGTTGACCTCCTGGGA
AGTCCTCGTGTTGCCCCCCTGAAAACATCATTTTTTTTTCCCTTTAAAAATCCACCTACGGCTAAAAGTTGTATTTTTTGTTTT
TAAATCTTTAAAAAACACTCATTTATTTCGTTTTCCTTGAGGGGTTAATACCGTGGTCACTAACAAGCAGCGAATAAGAAGTTC
CTAGTAATCCGGGGAGAAAGGAGGACCACAGAGGATCGTGCGTATAAATGAATATTGCGGAAACAAACATTTCGGGTGCGTCAT
ACCGACTAAGCACCGATCCAATCAGAACTCCGCAGTTAAGCGTGCTTGGGCGGAGAGTAGTAACTAGGATGGGTGACCTCCTGG
AAGTCTCGTGTTGGCACCCTGAACACAACATTTTTTTTTTTTCCCTTTTAAAATCAACCTACAGGCCTTCAAAAGTTTGTATTT
TTTTGTTTTAAAATCTTTAAAACTTCATTTTGTTTTATCCTTGGGGTTAATACCGTGGTCAACTAACAAAGCAGCGAATAGAA
GTTCATAGTCAATCCGGGGGGAGATAAGGGAGGACACCCGCTCGCCTATAATGAAATATTGCGGACCAAATATTGCGGCCAAAA

TTCCGGGTGCGGATCATACAGCACTAATCCACCGGATCCCATCAGAACTCGCAGTTAAGCGTGTTGGGCGAGAGTGTCTAGGAT
GGGTGACCTCCCTGGGAAAGTCCTGCATCGTGTTGCACCCCTGAAACATCCATTTTTTTTTCCTTTAAAAATCCACTACGGCTC
AAAAGTTTGTATTTTTGTTTTAAATCTTAAAAACTCATTTATTTGTTCGTTTTCCTTAGAGGTTAATACGTGTCCCCACTTAAC
AAGCAGCGAAATAAGAAGTTCCTAGTCATCCGCGGGGAGAAAGGGAGGACACCGCGCTCGCTATAAAATGAATATTGCGGAACA
AAATTCCCGGGTGCGATCATCACCAGCACTAATGCACCGGATCCCATCAGAATCCGCAGTATAAGCGTCTTGGGCGACGAGTAT
ACTAGGTGGGTTACCCCTTGGGAGTCTCTGTGTTGCCACCCTGAAGACAATCATTTTTTTTCCCTTAAAAATCCACCTACGGAG
GTCAAAGGTTTGTATTTTTTTGTTTTAAATCTTTAAAAATCATTTATTTCGTTTTCCTTGAGGAAATTAATAACCGTGGTCAAT
ACAAGCAGCGAATAAAGTTCCTAGTAATCCGGGGAGAAAAGGGAGGCACACTGCGCTCGCCATATATTGAATATTGCGGACCCA
AATTCCGGGTGCGATCATACCAGCACTTAATGCACCGGATCCATCAAGATCCGCAGTTAAGCGTGCTTGGGCGAGAGTAGTACT
AGGTGGGTGACTCCTGGGAAAAGGATCTCGTGTTTGCACCCTGAAACATCATTTTTTTTTCCCTTAAATCCACTACGGCTCAAA
AGTTTGTATTTTTTGTTTAATTTTAAAAATCATTTATTTTAAATTTCGTTTTCCTTGAGGGGTTATACGTGGTCAACTAACAAG
CGAGAATAAGAAAGGTTCTGAGTCATCCGGGGAGAAAAGGAGAACACGCGCTC
>m141122_014521_42134_c100732202550000001823141405141560_s1_p0/85098/460_5914
GCGGGGAGTATCCAGTGGGAGTGATTGAGGTGGCGCGGTAGAGAAGAAGTCGCGTAATCGGGGTTGTTTCTGTTTCGTTGAATT
AGGAGAGCGGCGGGTGTCCAATTCATGGTCAGGTAGGTATCGTCCCTATACTGATGCATTATGTGAGCATGGAACACCGGTACA
AGAAATTAAGCTTTTTTAGAGCGACCGAGAATAGAATTCGAGTGAAAATTACGATATCGCTTTCGGGATCCCTAGGCACGGCTG
CCTACAGGAGATTTGCAGAGAGAAGCGGCCAAGTTTTTTCTGTTTGGTGTGTGAAATTACGCCACCCAATTCATCCGGTAAAGT
ACGCTAGCCCGAATAGAAATGATTATGTGAGGATTTGGGAATGATTTTAAAAAGAAGTTAGAGCTTGTTAGAGAGCAAACTATG
AGTGATTACGAGATCGTTATCGAAGCCTACGGCAGCTGCCTACGAGCATTTGGCAGGAACGGCCATTTTTTGTTTGGTTGAGAA
TAAGGACCCCCAATTATGGCCATAAGTAATACCTATATGATTTATTGAGATGGAAAAGGTTCAAAAAAAATTAGAGCCTTGTTA
GAAGAAATATGAGTGGAATACGATTATCGTTCGGAGCCCCTAGGCTGGCAGCCCTAAGGAGATTTGGCAGGAAGGCAAATTTTT
TTGTTTGCTCGAATTTAGGACCCCAATTCATGGTCAATAGTATACCTATACTGATATTATTGAGATGGGAAAAGGTTCAAAAAA
ATTAGAGCTTGTGGAGAGAAAATATGAGTGAATTACGAGAACCTTCGAAGCAGTAGGCGCAGCTCGCCTAAGAGATTTGGCAGG
AAGTTCAAGTTTTTTTTCTTTTGGTTGTATAAGACCCGAATTCATGTAATAGAGTAGCCCAGATGAGTAATCATTATTGATATG
GGAAAGGTTGAAAAGAATTAGAGCTTGTAGTGAGAAATATGAGTGGAATTTAAGAGATACGTCTTCGGAGGCCTAGGCACTGCC
TAGGAGATGAGTGCGAGGAAAGCGCCAATTTCTTCTGTTTGGATGCAATTAGGACCCAATTCCAAGGTCAATAGTATACCGTAT
ATGAACTTTATTGAGATGGGAAAAAGCATCAAAAATATTAGAGCTTGTTAGAAGAAATGATGAGGTGAAATTTTTTTACGAGAT
CACTTCGTAGCCCTAGGCAGCGTGCCTAGAGGGACGATTGGGCAAGCGAAAGGCCAATTTTTTTGTTTGTTTGAATTAGGACCC
AATTCATGGTCAATAGTATACTAGAGATATGATCATATTGAGATGGGAAAAGGTGGAAAACAATTAGAGCTTGTACTGGAGGAA
ATATGATTGAATTACGAGATCGCTGTTGGAGCCCTAGGCTGCTGCCTAGGTGATGGTTGGCTGAAAGGTCAATTTTCATTTTGT
TTGATGTTGAATAAGGACCTGAATTCATGGTCAATAGTCAGGCCCAATGATACCATAGTTATTGATATGGAAAAGGATGAAAAA
AATTAGAAGCTTGTTAAGTGAAAATATGAGAGAATACGATATCGTGGTCGGAGCCCCTAGGCACAGCTGCCTAGGAGATTGGTG
CGCAGAATGGCCAATTTTTTTTGTTTGGTTTGAATTAGGTTAGGACCCAAATCTCAACTTCAATAGTATACCGATATGAATATT
GAGATGGAAAAGTTCACAAAGCAATTAGAACTTTTGTAGAGAGAATATGAATGAATTACGAGAGTCACCGCTTGCGTAGCTTCT
AGGCAGCTGCCTAGGAGATTTGGCAGGGAAGGGCGAATTTTGTTTGTTTGGGTTTGAATTAGGACCCCAATTCGTGGTCAATAG
TACCCAATATACATATTCATTGAATTGAGATGGGAAAGGTTCAATAAAATTAGAGCTTGTGTAGAGTAGCAAGTTATGAGTGAG
ATTACGAGATCCGCGTCGAACTCAGGCAGCTGCCCCTAGGAGACGTTGGCAGGAAAGCCAATTTTTTGTTTGGTTGAATTAGGA
CCAAAATTCATGGTCAAGTAGTCAGCCCAATATAATGCTTATTGAGAGGGGAAAAGTTTCAAAAAAATTAGCAGCTTGTTAGAC
CGAGAAAATATGAGTGAATTGAACCCGAGACCTCGGTTTGGAGCCCTAGGCGCGGCCTAGGAGATTTCGGCAAGGCAAGGCCCA
ATTTTCTTTGTTTGTTTGAGATTAGAGTCACCGAATTCCACTGGCCAATAGTTGACCTATACTGATATCTATTGAGCATGGGAA
AAGGTTCAAAAAAAATTCAGCAGCTTGTTAGGAGAGAATAGAGGTGAAATTAGGGATCCACTTCGGAGCCTTAGGCGCTGCCTA
GGGAGATTAGGCAAGAAGGCCAATTTTTTCTGTTTGGTTTGAATAAGGACCCCAAATTCATGGTCAGGTAGTAGGCCCAATAGT
AATATTACCTCTCAGATTGGAAAAGTTTCAAAAATACTTAGAGCTTGTTAGTGAGAATATGAGTGAAATTACGAGATCGGTTTC
GGAGCCCTAGGCAGCTGGCCTAGGGAATGTTGGCAGGAAAAGCCAATTTTGTTGTTTGGTTTGAATTAGGACCCCAATTCATGG
CCATAGTAATATATATGATATTATTGAGATGGGAAAAGGTTCAAAAAAATTAGAGCTTGTTCGAGAGGAAATATGAGTGAGATT
ACCGAGATCGCATCGGAGCCTAGGCTACTGCCTAGGAGATTTGGAGCGTAAAGGCCAATTTTTTTTGTTTAGTTGAATGTCTGG
ACCCCAATTAACTGTCCAATAGTTTCCTATATGATATTAATTGGAGATGGAGGAAAAGGTTCAAAGAAAAATTAGAGCTTGTT
AGTGGAGAAAATGATGAGAGAAATTACGAGAACGCTTCGGAAGCCCTAGGCAGCTGCCTAAGAGATTTGGCAGGTAAGGCAATT
TTTTGCTTTGGTTGAATTAGGACCCCCAATTCATTGGTCAAGTAGTATCCCAATATAGATATTATTGAGATGGGAAAAGTTTCA
AAAAATTTGAGCTCGTTGCAGAGACATATGATTGAATTACGAGATTCGTTTTTGGGAGCCCTAGGCAGCGGCCTGGAGGATCGG
CAGGAAAGGCCAATTTTTTTTGTTTGTTTGAATTAGATACCAATTCATGGCCAATACGTATACCTAGTATGAGTATTAAGTTCT
GAGATGGGAAAAGGTTCTAAAAATTAGAGCTTGTAGATAGAAATATTGAGTGAGATTGACGGATCGCTTCGGAGCCTAGGCAGC
CTGCTTAGGAAGATTTTGGAAGGAAAGGCCAAATTTTTTTGTTGGTTGAATTAGGAACCCCAATTCATGGTCAATAGTTGCCCA
CATATAATCTTATTGAGAGGGAAAAGTGTTCAAAAAAAATTGAAGCTAGTTAGCGAGAAATATAGTGAATTACGAATCCGTTTC
GGATCCCTAGGCAGCTGCCGTAGGAGATTCGGCAGGAAGGCCAATTTTTTGTATGTTTGAATTAAGATTCCACCCAATTCATG
GCCAATAGTATCAGCCTATATGATCCATTGATTGAGATGGGAAAAGTGTTCTAAAAAATTAGAGCTTGTTAGAGAAGAAATATG
AGTGAGATTACGAGATCACTGATCGGAGCCTTAGGCAGCTGCCTAGGAGAATTAGCAAGAAAGGCCAATTTTTTTGTTTGGTTG
AATAAGGCCCCAAATTCATGGTCATTAGTAGCCAATATAATATTATTGAGATGGGAAAAGTTAAAATATTAGCAGATTGTTAGT

GAGAATAATGAGTGAATTACGAGATCGTTTTCGGGCCCTAGGCAGCTGCCTAGGAAAATTTGGCAGGAAAGGCCAATTTTTTTG
GTTTGGTTGATTAAGGAACCCAATTTCATGGCCACTAGTAGTACCTATATGAGTATTTTGTGAGATGGGAAAAGGTTCAAAAAA
ATTAGAGCTTGTTCGATGAGGAATATGAGTAATTACGCAGATCGCATCGGAGCCCTAGGCTACCTGCCTAGGAGATTTGGCAGT
GAACAGGCCAATTTTTTTGTTTAGTTGAATTTGGACCAATTAATTCCAATAGTATTTCCTATATGATATTATTGGAGCATGGGA
AAAGGTTCAAAAAAATTAGAGCTTGTTAAGTGAGAAATATGAGAGAGTTACGAGAACGGCTTCGGAGCCCTAAGGCAGCTGCCT
AAGAGATTTGGCAGGGTAAGGCCAATTTTTTTTGTTTGGTTGAATTGGACCCCAATTCATGGGTCAATAGTATCCCAATATAAA
TATTATTGAGAGATGGGACAAAGTTATCAAAACAAATTAGAGCTCGTTTGAGAGACATATGAGTGAATTTACGAGATCTGTTGT
TGGAGCCCTAGGCAAGCGCCTAGGGAGATCGGCAGGAAGGCCAATTTTTTTGTTTAGTTGAATTAGATCCCAATTCATGGCCAA
TATCATACCTATTAGTGATATTATTGAGATGCGGAAAAGCCTCGTTCGTACGGAACGATTAGAAGCTGTTTTAACCTGAAGTAT
GACGTTGATTAGAGATTCGTCCGTCCGGAGCCCTCAAGAGGCAGAGTGCCTCTAGGAGACTTTGGCAAGGGGAAAGTGAGCCAA
GTTTTTTGTTTGGTTAGATTAGGACGCCAATGTCATGGTCCAAGTAGTGTGCCCAATATATCTTGATTGAGGACGGGGAACAGG
TTTCAGAAACAATTAAAGTAGTTAGAGAGAAATATGGAAGTGAATTACGAGATCAGTTTCGGAGCCTAGCACTGCGCTTAGAGA
TCGGCAGGAAAGGCCAAATTTTGTTTGTATGTGTTGGAATTACGATCACCCAAGTTCATGGCCAATAGTATCACATCATATGCA
ATATTGATTTTTTGAGATGGGAAATGTTCTAAAATTAGCAAGCGTTTTGTTGAAGAGAAAGATGGAGTGAATGTAACAGATTCG
AATTCGGAGCCTGTAGCAGCTGCCCTAGGAGATTAGGCAGAAGGGCCAATTCTTTTTTGTTTGGGTTTGTAATAAGGACCCAAT
GTCATGGTCAATATAGGCCCCAGAATATAAGTATGTACTTGAGAATGGGAAAAAGTTTTCGAAAAAAATTAGACTTTGTTACTG
GACGAATACTGTTCGATGAATTTACGCAGATCGTTTCGGATGCCCTGAGGCAGCTTTTGCTAAGGAAATTTGGGCAGGGAAGGC
CCAAATTTTTTGTTTGGTGAATTAGGACGCGCCAATTCAACTGGCCAATAGTTATCGACTATAGAAATGAAGTTAATTGAGATG
GGAATAGGTTCAAAAATTAGGAAGCTTTGTTAGTGGAGAAATCATAAAGAAGAATTTACGAGAAAGCTTCGGAGCCCCTGGCAA
GCTGCCTAAGAGATTGTTAGGTCTAAAGGCAATTTTTTTGTTTTTGAATAGACCCCAGATTCTGGGTTTCAATAGTAA
>m141122_014521_42134_c100732202550000001823141405141560_s1_p0/87595/3355_8536
TTTTATTGTGTCGTTTCTGGTGGAATTAGGTACCCCACAATTTATTAGGATCTACCAACGGATTGGCCAGGGTACATGCCCTAG
CCTAACTCCTGACAATAAAATGACTGCTTCTGAACGATGGGAAAGAGGCTATATTCACAAAAATTATAGAGCATGCTTGTTAGA
GAGAAATACTGAGGGAAGTTAACGGAAATCGCCTCGGAGCCCTACAGGCAGCTTGCCTTGTGACAATGTGGCCTGAAGGTCTCA
ATTTTTTTGCTCTTGGTTAATCATTAAGGGACACTCGCCAATTCATAGTCTCCAATAGCCCTATCATAACTGTTAGTCTCGGAG
ATGGGAATAATGTTTCAAGAATAAAATTCAAGACAGCTTGGGTAGAGAGGAAATATGAAGTGATTATCGAAACCTCGCTTCGGA
GACCTTGGCTGCAGCCTAGGGAGATTTGGCCTGAAAGGTCAATTTTTTTGTTTGGCTTGAATTTAGGACCCCGATTCCATGGTA
AACTAATATGCCTATAGTAATATTACTTGGACCGATGGGAAAAATGTTCAAAAAAATAAGAGCTTGTTTAGGCGAGAAACTATG
AGCTGACATTGCGAGTCCCCCGCTTCGGAAGCCCCTAGGCTCGATGCCTAGGAGCATTTGCCTGAAAGGTCAAGTTTTTGTTTC
TGGCCTCTGTATTCCACGCGAGCCCCAATTCATGGTCAACTAAAATAAGGCCCTATATAGATAGTTATTGAGACTCGGGAAAAA
CCGGTTCAAAAAAGTTAGAGCTTGTTAGACGAAATATGAGTGAATTACGAGATCGGCTTCGGAGCCCTAGGATGCTTGCCTAGG
AGATTTGGCTGAAAAGGTGTCAATTTTTTTTTTTTGGTTAAATTAGGAGACGGCCAATTCATGGTCAACTAATTGCCCTAAATA
ATATGCTGAGTTGTGAGATGTGGAAAAGGTTCAAAAAAATTCAGAGCGCCTTGTTAGAGAGACAATACTGAGGGAATTACGAGC
ATCCGCTACGGAGCCCTATGGGCAGTGGCTGGCTACGGAAGCATTTGCCTGAAAGGTCACATTTTTTAGTTCGTTGTTGAAATT
AGGACCCCCAATTTCATGGTAAACGTAATCGCCTATATAAAATTATTGTGAGATGGCGAAAAGGTTCCAAAAAAATAGAGCGGC
TTTTTAAGGAGAAATATGAGGTGAACCTGCGAGATCGCTTCGGAGCCTAGGCCAGCTGGCCTAAGGAGATTTGGCCTGAAAGGT
CAATTTTTTGTGTTTGGTGTAATTAGGACCGTACAATTCATGGTCATAATGGGCCTATATAATATATATTCTACAGATGGGAAA
AAAGGGATTCAAAAACAATTAGATACGTTGTTAGAGAGACACTATGAAGGTGAATTACTAGATCCTGTTTCGGAAGGCCCTAGG
CTGGCTATCCTAGGTAGATTATGGCCCATGAAAAGGGTTCAGATGTTTTTTTTTTCCTGGTTAAATTAGGAGCGCCAATTCATG
GTCAAATCAATAGCCCCTATTATAAATATTAACTTGAGATGGGAACAAGGTTCAAAATAAATTCAGAGCTTGCTCTAGCAGAGA
AATATGCAGTGAATTAGAAATCGTTCGGCCGCCCTTACGGCAGCTGCTACGGAGATTTGGCCTGAAAGGACACATTTTTTTGTT
GGTTGAATTAGGACCCCAATTCATAGTCCAATACTAGCCCGCGTATATAATATTATTGAGAATGGGTAAAGTTTCGAAAAAAAT
TTAGAGCTTGTTAAAGATGAAATATGAGTGAATTAGGGGGGGGGGGGGGGGGGGCGAAAAATCTCCCCCGCTTGCGGAAACCCTA
GGCAGCTGGGTAGGAGATTTTGGCCTGAAAGGTCAATTTTTTTGTTTGGTGGAACTTAGGACCCAATTTCATGCGGTCAATACT
TGCTCTATACCTACATTATTATCTCGAGATGGGAAAAGGTTCAAAAAAAATTAGAGGCTGGATTAGATGAGAAATATGAGGGAA
TTAACGGAAATGCTTCGGAGCCCCTATGGCAGCTGCGCTAGGAGATTCTGTCCTGAAGGTCAATTTTTTTGTTTGGTTTAAATT
CGGGACCCCAATTCATAGTCAGTAAGTATGCCCTATATATAGTATTGTTTGAGGATGGGAAAGAGGTTCAACAAAAATTAGAGC
TTGATTAGAGAGAAATAATGAAGTGAATTAGAAATCGGCTTCGCGAGACCTTGCTGCAGCCTAGGAGGATTTGGCCCTGAAGGC
TCAATTTTTTTGTTTGGTTGAATTTAGGACCCGCCAATTAATGGTCAATAATAGCCCTAATATAATATTATTGAGATGGGAAAA
TGTGCTTTTTTTAAACGAGATAGAGCTTTGTTAGGGATGACAATAATGAGTGATATTTGCGAGAATCGGCTTCGGAGGCCCGTA
GGTCAAAGCTGCTAGCGACGTACCTTTTGGCCTGAAAGGTCAATTCATTTTTCTGTTTGGGTTGTATTAGGACCGCTCCAATTC
ATGGGTCAATACATAGCCTATTATACTATATTGTATGAGATGGAAAGGTTCAAAAAAAATTAGAGCCTTTGTTAAGGAGAGAAT
ATGAGTGAATTACGGAAATCAGCTTCGGGCCCTAGGCAGCCTGCCTAACGGATTTGGACTGAAAGGTCAATTTTTTTGTTTGGT
TGAAGATTAGACCGCCCCATATCTCATGATCAAGGTAATAGCCCTATAATATATATTATTGATGATGGGGTAAAGTTTTCCAAA
AAAAATTAGAGCTTGCCTTAGTAGAGAAATATGATGAATTACGAAATCGGTTTCGGAGCCCTAGGCACTGCCCTAGGAGATTTG
GCCTGAAAGGTCAATTTTTTTTGTTGGTGGAATTAGGACCCCAATTCATGATCAATAATATGGCCTATAGTAATATTTAATTGA
GATGGGAAAGGTTGCAAAAAAATTAGAGCTTGTTAGAGAGAAACTATTGTAGTGAACTTACGAATCGATTCGGCAGCCCCTAGG
CATGCTGCTACGGAGATTTGGCGCCTGAAAGGTTCAATTATTTTTGTTTGGTTTAATTATGTAAAACCCCGAATTCATGGTCAA

CTAATCTGGCCTATATATTATACGTTATATTGAGATTGGGAAAAGGTTCATAAAAATTGAGAGCTTGTTTAGAGAGAATATGAG
AGTTGATTAAACGAATCCGGCTTGCGGAGACCCTTGGCTGCAGCTAAGGGAGATTTGGCCTGAAAGGTCAATTCTTTTTGTTTG
GTGAAATTAGGACCCAATTAATGGTTCAAGTAATAGCCCTATATAAATATTATGTTCGAGCATGGAAATCCGATGTGTAACAAA
AATTAGAGCTTGTTAGGGAGAACATATGAGTGAATTGCCGAGATCCTGCTTCGTAGCCTAGGCACCTGCTAAGCCGGAGGGATT
TGCGCTGAAAGGTCAATTTTTTTGTTTGGTTGTATTAGGACCCCAATTCATGGTCCCAAACCGTAATAGCCCTATTAGATTATT
ATGTGGAGAATGCGGAAAAGGTTCAAAATAAATTAGAGCCTTTAGTTAGAGAGAAATATCAGTGAATTACGAAAAATCGCTTCG
GAGCCCTAAGGCAGCTGCCTACGAGTATTGTGGTACTGAAATGGTCAAGTTTTTTTGTTGTTTGGTGAATTAGGACCCAATTCA
TGGATCAATAATACCCTATAAATATTATGTGAGATGGGTAAAGTAGGTTTCAAAAAAATTAGAGCTTGTTAGAGAGAAATATGA
GTGAATTACGAAATCGCTTCGGGTAGCCCCTAGCAGGCTGGGCCTAGGAGAATTTGGCCTGAAAGGTCATTTTTTTGTTTGGTG
GAAATGAGGACGCCGCAATTCAATATGCAATAATTTCCCTATATATTGATGATTAATTGACGATGGGAAAAGGTTCAAAAAAAT
TATAGAGCTTGTTAGAGAGAGAACTAATGAGTGAACTTACGAAAATCGATTCGAGCCCTAGGCAGCTGCCCTAGGAGATTTGCC
TGAAAGGTCAATTTTTTTTGTCTTGGTTTAATTAGTACCCCCAATTCATGTCATAATTGGCCTCATAATACCCCATATTATTGA
GATGGGAGAAAAGGTTTCAAAAAAATTAGAGCTTGTTAGAGGAGAAATAATGAGTGAATTACGAAATCGGGCTTTCGGAGCCCT
AGAGCCAGCTGCCTAGGGAGTTTGGCCCCTCGCAAAGGTCAGATTTTCTTTGTTTGGGTTGACATTGGACCCCACATTCATGAG
TCAAGTAATAGCCCATAATAAATAATCTATTGAGATGGGTAAGAGTTTCACAAAAAATAGAGGATTGTTAGAGAGATAGAATAT
GCAGTGAAATTATACGAAATCTGCTTCGAGCCCTAACCGGCAGCCTGCTAGGGAATTTGTCGCTGGAATGGTCATTCGTTTTTG
TTTGGTTGAATTAGGACCCCAATTCATGGTCAAAAAATAGGCGCTATATTACTATTTCTATCTGTTCGAGATGGGAAAATGTTT
AAAAAATTAAAGCTTGTTAGGGGACAAATATGAAAACAAAAAAGTCTAGAACATTGCGAGAGTCGCTGGTCGGTGCCCTAGAGC
AGGCTGCCCTTAGGAGATTTGCTCTGAAAGGTCAATTTTTTTTTTGTTTGGTTGAAATTAGGAATTGCCAACTTCATGGTAATA
ATAGCCCTATATAATATTTTGAGATGGGAAAACGGTTCAAAACCAAGATTAAGAGGCTTGTTAGAGAGAAATATTGGAGGGAAT
TACGAGATCGCTACGGAGGCCCTAGGCAGCTGCTAGGGAGATTGGCCTGAAAGGTCAAGGTTTTAGTGGTTGGTAGGAGTTGA
GGACCACCAATTCAGGTAAGATAATGCGCCCTATATAGACGGAATTACTATGTGAGCATGGGAAAAGGTTCAACAAAAAATCTA
AGAGCTCTGTTAGGGAGAAATATGAGTGAATTGCGAGATCGCGTTCGGAGGCCTTAGGCGGCTGCCTAGGGAGATTTGGCCTGA
AAGGTCATGTTTTTGTTTTGGTTACATGAGATTCCAATTCTGGGGTCAATAATAGGCCCTATATAATAATTGATTGAGATGGGG
AAAATCCCTTTAAAAAAGAATTAGCAGCTTGTTAGGAAAAAGTATGAGCGTGATTTG
>m141122_014521_42134_c100732202550000001823141405141560_s1_p0/98769/6368_14448
TCTCTTTCTTGGGCTGGCGTTCCCCTACCTCTCTGCGCTTGTTACGCGAGCCCATATTGTCGAGATTACGAGACTCAGCTTCGG
AGCCTAGGCGCGCGATATTTTGCTTGCCCGGATCATTTATTTTTTATGATATCGGAAGTTGAATTTATTGGTACCTTTCAATTT
TTTTGTTTTCATCATTTTTTTTTTTTTATTATGTCCCTATATTTATATTTTTTTTTTTTTTTTTTTTTATTTTTATTTTTTTATT
TTGATATTATGGGTTATATATATTTTATTTAGGTTCAATAGACATTAATGTGAGAGCATTTGTTTAGAGTTGAGGAACAATTTA
TATTTGAGATGACTTGTGTACGAGGAATGTGGTTCCGGAGCCTTATGGCATGTTGGAGAGCTGTAAAGGGAGGAGGGTATTGGT
CTACTGAATTGTAGAAGATCATATTTTTTTTGTTTGCGTTGGTATTTCATGGAACTCCTCTCAATTTCTATTGGTTAGAATAAT
TTAGGATATATTGAGTATTTATTGTTAAGTGGTGGGGAAGGTTGAGATAAAATATTGAGAGCTATTTGTTATGGATTTAGACTA
ATATATGGATGTGATTACGAGATCTCCTTCGGAGCCTAGGCAGCTGCCTAGGAGTTTGGCTGAAAAGGCCAATTTTTTTGTTTG
GTTGAATTAGGACCCAATTCATGGTCAACTGATAGGCGCTATACGAGGGGTTAGAATTGTATTATAGACATATATGAGAATATG
GGAAACGGTTTTAAAGAATAATTAGAGCTTTGTATACGTGAAATATTGAGTGAATAGTAAGATATCGGTGTGGTGTTCTATGAT
TTGAGCTTGAGTGTGTTTGTTTTATTTTTATTTGGGATGATTTTTGTTTTTTAAGTTTTTGTCGGGAGCCCCATGCCTAATTTA
TGGGGTTATTATGGCCTTAGAAAGAGTTGCAATATGTTGTTTTTTGTTTTGGTTTAGTGATTTAATTCAGGGATATCTCCCAAA
TGTGAATGGTTCATTTGTGAATATTAGCTATATAATATTGAAGGTTGGTTTATGTTAGATTTAGTAATTTATGTGGGATATTAG
GTTTTATGATTCAGAAATTTTAGGATCATTAGTAGAGATGAAATATGTAGAGAGGAATTTAGAGATCGCTAAATGGTGGATTCG
GTAGTCCTATAGATATATGTGGGAATTCGTTACCTTTTTGAACATTATAGGACCTGAAATGAGTCAACTGTGGTTTTGTGGTAT
TTGGATTTGAAGATTAGTTGTTCCGGCCTAATTGTGCTATGGGTTTAATATTATATTAGGCCTTATTAGTATTGATGTTTTATG
AGATATTTATTTTGGGATCATTTAGGGAAAAGGATTCAAAAAATATATATAGCCTTGTTAGAGGGCAGAATAAAATGTATGTGA
GTGACAATTAGACGAGGAGGCTTTGTTCGGAGTCTCCGGCTAGGCAGCTTGCCACCTAGTGGGGATATTGCCTGATATGGTCAT
ATTATTGTTTAGTTGTGTGAATTTTGAATATTTAGGATACCCCAGATTCATGGTGCTAGAGAGTTTGTTAGTTGAGGTTTTATT
ACTTAGTTGCCTTGATAATGAATTATGTATACTTTATTGAGATGGGATGTATAAGGTTTCAATATATAACATTAGAAAGCTTTT
TTTTTATGGAGGAGAAGTACTACAGTTGTGATGGAATATACTCATCGCTGTCGGATGCGACGTGTGCATGCTCCCTAGGAGGTT
TTGATCCGGTATCGCATTTATAATTTGTTTGTTTTGAATTAGGACCAAATTCATGGTCAATAATAGCCCTATATATACATGTGG
GTTTTATTTTAGTGAATGATGGGATTGAATGGATTTAAATCAGTTAGAGTTGTTTGTTAGAAGAATATATGATTGAATTACGAG
ATCGCTTCGGATCCCTAGTTCAGCTGGCCTATATGGGAGATTTGGCCTGAAATCATTTTTTATTTTGTTTGGTTGGGATTCTGG
AGCAGCGCGAATTCATTGGTCACAGATGAATTTAATTAGTGCCTATATAATTTTAAGTTTAATATTTTGAGGCTATTGGGTTTG
GGGTAGCTGGAAAATGGTTCAAAGAACAGGCAAAATGTGGCTTGGTAGATGAGTAAATAATGGTATGAATTTTACGAGATCGGC
TTTGGATACGCGGAGTATCCCATTAGCGGTCTAGGATTTGGCCGCTGAAGAAGGTGCTGAATTTTTTTTGTATGGGGGTGGGGG
ATAGCTGGTGAGCGACGGAGCCAATTCATATGTCAATAATATGGGGGTGTATAATGATTGTAGTATTTATTTGATTTTGGATAA
TTGAGATGTTGGGAATAGTGGTATGATTCATAAGAGAAAATAGTTTGGGAGCTTGTTTAGATGAGATAATTTTATTGGGTTTGT
ACGTGGTGAATTACAGATCGCTTCGGACCTAGCAAGCTGCCTAAGGAGTATTTGGCCTGAAGGTCAATTTTTTTGATTTGGTTG
AATTAGGACCCCAATTCAATGGTCAATTAATAGTCCTATATAATCTTATTGAGGATGGGAAAAGGTTCAATAATTAGAGCTTGT
TACGGAGAAATATGAGTGAATTACGAGATTGCTTCGTAGCCCTAGGCTGGCTGCTAGGAGATTTGGATTGAAAGGTCAATTTTT

TGTTTGGGTTGAATTAGGACTCGAATTCAAGTCATAATGATGCCTATATATAATTACTTGAGATGGAAAGGTCAAAAAAATTTG
AGCTTGTTAGAGAGAATGATGAAAGAGAATTGACGAGATCCTTCGGAGCCTAGGCGCTGCCTATGGAGATTGGCCTGAAAGGTC
AATTTTTTGTTTTGGTTGATTAGGACGCCAATTCATGGTCATAATAGACTATATAATATTTTGAGATGGGAAAGGTTCAAAAAA
ATTAGAGCTGTTGTTGAGAGAGAGATATGATGAATTACGAGATCGCTCCGGAGCCCCTTGGCAACTGCCTAGGAGATTTGCATG
AAAGGACATTTTTTGTTTGTGGTGGAATTCGACTCCAATGTCATGGTCAATAATAGCCCTAATATAATATTATTGAGATGGGAG
AAATATTCAAAAAAATAGGGATTGTTAGAGAGAAATTGAGTTAATTACGAGATCGCTTTAGGAGCCCTAGGCAGTGCCTAGGAG
ATTGGCTGAAATGTCAATTTTTTTTTGTTTGGATTGGATTAGGACCCCAATTCATGGTCAATAATAGCCCTATACTAATATTAT
TGACGATGGGAAAAGATTCAAAAAAGTTAAGAAGCTTGTTAGAGAGAAATATGAGAGAATTAGCGAGATCCCTTAGGAGCCCGT
AGGCAGCTGCCTAGGAGATTTGGCATGAAGTGTCAATTTTTTTGTTTGGATTGAATTAGGACCCCAATTCATGGTCAATAATAG
CCTAATAATATTATTGGAGATGGGAAAAGGTTAAAAAAATAGAGCTTGTTAGAGAAAGATATGGAGTGACATTACGAGATCGGC
TTCGGGAGCTCTAGCAGCGCCTAGGGATTTGGCCTGAAAGGTGAAAATTTTTTGTTTGGTTGAATAAGGACCCAAATTCATAGG
TTCAATTATAGTCCTTAGTATGATATTACTATTATTGAGATGGGATAAGTTCAAAAAATTAGAGCTTTTACGGAGAAATATGAG
TGAATACGAGATAAGCTTCGTAGCCCTAGGCAGCTGCAGGAGATTTTGGCCTGAAGGTCAATTTTTTTGTTTGGTTGAATTAGG
ACACAAATTCATGGTCATAATTTGCCCTATAATATTATTGAGATGGGAAATGGTTCAAAAAATTGAGCTTGGTAGAGAGAAATA
CTTAAGAAAAATTACGAGATCGCTTCGGAGCCCTAGGCAGGCTGCCTAGGAGATTTTTGGCAGAAAGGTCAATTTTTTATGTTT
GGGTTTGAATTAGGACCCCAATTCATGGTCAATAATAGCCCTATATAATATTCATTGAGATGGGAAAAGATTCAAAAAATCAGA
GCTTGTTAGAGAGAAATATGAGTGAATACGAGATCGCTTCGGAGCCTAGGCAGGCTGCCTAGAATTTGGCCTGAAGGTCAATTT
TTTTGGTTTGGTTGCATTAGGACCCGGAATTCATGGTCAATAATATCCCTATATAATCATTATTGAGATGGGAAAAGGTTCAAA
AAATTAGAGATTGTTAGAGAGAAATATGATGAATTACGAGATCTGCTTCGGAGCCCTAGGCAGCTGCCTAGGAGATTTGGCCTG
CAAGGTCATTTTTTTGTTTGGTTGAATTAGGACACAAATTCATGGGGTCAAATAATTGCGCTAATATAATATTAGAGATGGAAA
TTGGTTCAAAAAAATTTGAGCTTGGTAGAGGAAAGATGAGAGAAAATTACGAGATCGGTTCGGAGCCCTAGGAGCTGCCTAGGA
GATTGCCTGAAAGGTCATTTTTTTGTTTGTGAATTAGGACCCCAATTCATGAGGTCCAATAATAGCCCCTATATTATATTATTG
AGATGGGATAAAGATAAAAAAAATTAGAAGCTTGTTAGAGAGAAATATGAGTGAATCTACGAGATCGCTTCGGAGCCCTAGGGG
TAGCTGCTAGGGGATTGGCTGTAAAGGTCAATTTTTTTGTTTGGCTTGAATTAGACCCCAATTCCTGGTCAATAATAGCCTTAT
ACAAATAATTAAAAAATTTTTTTTTATATGGGAAAAGATTCAAAAAAATTAGAGCTTGTATAGAGAGATATATGAGGGAATTA
CGAGATCGCTATCGGAGCCCTTAGGGAGCGTGCCTTGGACATTTGGCATGAAGGTCAATTTGTTTGTTTGGGTTGAATTTAGGA
CCCCAATCATCGGTATACATTGCCCTATATAATATTATTGAGATGGGAAAAGGTTCAAAAAAATTAGAGCTGTTAGAGAGAAAT
ATGAGTGATACGAGAATCGATTCGGAGCCCTTAGGGACAGCTGCCTAGGAGATTTTTGGCTGAAAGGTCAAATTTTTTAGTTTG
GTTGAATTAGGACCCCCAATTCCATGGACAATAATGCCTAGTATAAATATTATTGAATATGGGAAAAGGTTCAAAAAAATTGAT
CTTGTTAGGGAGTAAATATGAGTGAATTACGAGATCGCTTAGGGCGCCCCTTAGGCCTGCAGCCTAGGGAGATTAACCTGAAAG
GTCATTTTTTGTTTGGTTAAATTAGACCCCAATTCCATGGTCAAGTACATAGCCTTATAAATAAATTGAGAATTGAAAAGGTTC
AAAAAATTAGAATCTGTTAAGAGAGAGATATGAGTGAATTACGAGATCGCTTCGGAGCCCTAGGGCAGGCTGCCTAGGAGATTT
GGCCTGAAAGTCAATTTTTTTTTTGGTTGAATTAGGACCCCAAATTCATGGATCAATTATAGTCCTATATAATATTTTGATTGG
GAAAAAGGTTCAAAATAATTAGAGCTTGTTACGGAGAATAGTGAGTGAAATTACGGGATTGCTTCGTAGCCCTAGGCTGTTGCC
TAAGGAGATTTGGGATTGAAGGTCAATTTTTTTTGTTTCGGTTGAATAGGAACTCCGAATTCATGGTCAATAATTGCCCTAATA
TAATATTATTGAGATGGAAAAGGTTCAAAAAAATTTGAGCTTGTTAGAGAGAATATGCGAGAATCGTACGAGATCGCTTTCGGA
GCCCTTAGGCGCCTAGGAGATTTGGCCTAAAGGTCAATTTTTTGTTTGGTTGAAATTAGACGCCAAATTCATGTAATAATAGCC
CTATATATATTATTGAGATGGGAAAAGGTTTCAAAAAATTAGAGCTTGTTAGAGAGAGATAGTGAGTGAATGACGAGATCCTCC
GGAGCCCTTGCAGCTGCCTACGCGAGATTTGGCATGAAAATGGATCAAATTTATTTTGTATTGGTGGAATTAGGACCCCAATTC
ATGTCAAAATAGCTATATATATGTATTGAGATGGAATATCAAAATTAGAGATTGTTAGAGAAGAAAATGAGTTAAGTTACGAGA
TCGCTTAGGAGCCCTAAGGCAGCGCCTAGGAGATTGTGGCCTGAAATGGTCAATTTTTGTTTGGTTGGATAGGACCAATTCATG
GTCAAATAATAAGCCTATATATATTATTGAGATGGGAAAAGATTCAAAAAAATTAGAGCTTGTTAAGAGAGAAAATATGGTGAA
TTACGAGATCCCTTTAGGACCCTAGGCAAGCTGCCTAGGAGATTTGCTGAAATGTCAATTTTTTGTTTGGTTGAAATTAGGAAC
CCAAATTCCATGGTCAATAATAGCCCTATAACTTTATTGGAGTGGGGAAAAGGTTAAAAAAAATTTGAGCTTGTTAGAGAGAAT
AATGATGAGAATTACGAGATCAGCTTTGGGCCCTAAGGCAGCTGCTAGAGGAGATTTGCCTGAAAGGTGAATTTTTTTGTTTGG
TTGAATTAGGACCCCACAATCATGGTCAATTATCAGTCTATAAGAATATTATCTGAGATGGGATAAGGTTAAAAAATTGAGCTT
GTTACGGAGAAATATGAGTGATTCGAGGATCGCTTCGTAAGCCCTAGGCAGCTGCCTAGGAGATTTGGCCTGAAAGGTCAATTT
TTTTGGTTTGTTGAATTAGGACACAAATTCATAGGTCAATAATTGCCCATAGTAATATTACTTGAGATGGGCAATGGTTAAAAA
ATTTGGTTGTAAGATGAGAATATGAGTAGAATTACGAGATCGTCGGAGTCCCTAGGCAATGCCTAGGAAGTATTTGGCCAGAAA
GGTCAATTTTTTTTGTTCTGGTTGGAATTGGAACCCAATTCATCAGGTCAATTTATAGTCCTATATAATATTATTGAGATGGAA
AAGTTCAAAATATTTAGACTTTACGGGAATATGAGTGAATTACGAGATTGCTTCGTAGCCCTAGGCTGACTGCCTAGAGATTTG
ATTGAAAGGTCAATTTTTTTTGTTTGGTTGAATTAGATCGAATTCATGGTCCATAATTGCCCTATATAATATTATAAGATGGGA
AAAGGTTCAAAAAATTGAGTTGTATAGAGAGATATAGAGGAAATTACGAGTCCTTCGGAGCTAGGGAAGCTGCCTTAGGACATT
TCCGAAAGGTCAATTTGTTTTGATTTGTGAATTAGGACCAATTCATGGTAAAGTAATTGCCCTCATATAATATTTATTTGAGTA
GATGGGAAAAGGTTCAAAAAATTAAGAGCTATGTTAGAGAGAAATATGAGTGAATTAGAGATCGCTTCGGAGCCCCTAGGCAGC
TGCCTAGGAAGATTTGCCTGAAAGGTCAAATTTTTAGTTTTGGTTTGAATTAGGACCCCATTCATCGGACAATAATAGCCCTGT
ATAATATTATTGAGGATGGGAAAAGGGTTCAAAAAATTAGATATTGTATAGGGAGGAAATATGAGATGATTACGAGATCTGCTT
AGGCGCCTAGGCTGCAGCCTATGGAGATTTAAACCTGAAAGGTCAATTTTATTGTTTGTTAAATTAGACCCAATTCATGGTCAA

TAATAGCATATATAATATTATTGAGACTGGAAAAAGGTTCAAAAAAATTAGAGCTTGTTAGAGAGAAATATGAGTGATTACGAG
ATCGCTTCGGAGCCCTAGGCAGCTGCCGTAGGGAGATTTGGCCTGAAATGTCAATTTTTTGTTTGGTCTGAATTAGGACCATTC
ATGGTCAATAATAGCCCTAATATATATATAATTGAGATGAAAAAGGTTCAAAAAAATTAGTCTTGTTAGAGAGAGAATATGAGT
GATTACGAGATCAGCTTCGGAGCCCTAGGCAGCTGCCTAGGAGATTTGCTGAAAGTCAATTTTTTTGTTTGGTTGAATTAGGAA
CCCAATCATGGTCAATTATAGTCCTATATTCATTATTGATATGGGAAAGGTTCCAAAATAATTAGAGCTTGTACGGGAGAAATA
ATGAGTGATTACGAGATTGCTTCGGTAGCCCTAGGCTGATGCCTAGGAGATTTGGAATTGAAAGGTCAATTTTTTGTTTGGTTG
AATTTAGGAACCGAAT
>m141120_105518_42134_c100732402550000001823141405141504_s1_p0/134/0_8933
CCCCACCCCAAAAACAAAATGAGAAGGAAGAGATCTCCCATGACCCTCACGCATATTCATTTAGTATATCACTATCCAAACATT
GGCTCTAATTCCATATTAGAGAAATCGAAGATATCTGAAAGCGGCGCTACCCCCCTTTGGTGACATTTGGCAGATTTTCACGTA
ATTTCTCCGAAATGTATGTACCCTCGCTGTTCAACTCGTTCGATATTTCCCCCGGAATCAATCTCAATGGGGAAAACTGAATAT
ATTGGAGGTCGATACGGAGACTTAAGTCAAAAAATTATCAAAAAAAATTCCCGTAACGTTTTATGGCAATTTCGAAAGGTGTTC
TTTCCTTAATTCGTACATTAGCTCTCAGTACTTCTTGACAGTATCGCCCTCTACCACATCTCCGGATCTTGGCACGAAACTTTA
TACACTAGGAACCCTCGAGACATACGGGCTACAACTTTTAGTTCCCATGACGCCTCGCATGATTCCTTAGGTTATTGCATATCA
AAAATATGCTCTAATTACATATTAAGAGAAATCGAAGATTTCTGAAAGGGGTACCCCTTTGGTGAATTGGGAGATTTCACTAAT
TTCTCCCGAAATGTATTACCATCGTCGTCAACTCGTTCGTATTTTCCCGAATCATATCTGCATGGGGAAATGAATATCATTGGA
CGTCGAAACGGAGACCTTAAGTCCAACATTTCAAAAAAAAAATTCGCCTTTTTATGGCAATTTTCGAAAGTGTTTTTCCTTAATA
TTTCGTACATGCTCTCAGATTGACAGTATCGCCCTCGGACACACTCTTCGGATTTGCCACCAAAATTTCTATATCATAGATCCC
TGAGAATGACGGGTTAACAACTTTGTTCCCATGACCCTGCATATTCCTAGCTCTTCACTATCAATGAACATTTCTCTAATTCAA
TATTAGAGAAACGACGATTTCTGAACAGGGTCACCTTTGGTGACATTGGAGGATTTCACTAATTTCTCCGAAATGCTCATAACC
TCGTGTTCCAACTCGTTGATATTTCCCGCCGCAACTATATCTCATCGGGGAAAATGAATATATTGGAGTCGAAACAGGAGCTTA
AGTCCAACATTTTTCAAAAAAAGTTCCCGTTCTTTTTATGGCAATTTTCCGAAAAGTGTTTCCTTATATTCTAATCTCCTAACA
GTAATTTTGACTGTATCCCGTCCATACACAGCTCCGCTATTGCACGAAATAACTTATACATAGAACCCTAAGACGATAAGGCTT
AAACACTTTCTGTTCCCATGACCCTCGCACGCTATCCCTTAGTATTCACTATCAACAACATTGCCTATATTCATATCTAGAGAA
AATCGAAGATATCTGAAAGGGTACCCTTTGGCTCGATTGGCAGATTTCACTAATGTTTCGAATGATTACCCCTCGTGGTTCAAC
TGTTCGATATCTTTCCCCGAATATATCTCATGGAAATGCATATATTGGAGGTCGATAACGGAGAACCTTAAGTCAACATTCAAA
AAAAATTCCCGTTTTTTATGGCAATTTCGAAAAGTGTTTTCCTTATATTCGTACATCTCCTCAGTATTTTGACTGTATCCGTCA
ATACAACTTCGGAATTTGCACGAAATTTTAATACATAGAACACCTAAGACAATAGGGCTACAATTTTATGTTCCCAATGACCCT
CGCATATTCCTTAGTATTCACCGTATCAAGAACATTGCCTAATTCATATGAGAGAAAATTCGAAGATTGTCTGAGGGGTAGCCC
TTTGGGGGAATTGGAGAATTTCACTAATTGTCTCTGAAATGTATTACCTCGTGTTTCACTCGTTCGAATTTCCCCGAATATATC
TTCATGGGAAAAAAGTGAATATATTGGAGATCGAAACGAGACTTTAAGTAACATTTCCAAAAAAAATTCCCGCTTTTTATGGC
AAGTTTTTCGAAAGTGTTTTTTCCCTTATATTCGTACATCTCCAGATATTTTGACAGTATCACCCTCTACAAACAATCTCGGAA
TTTGGAGACGAAATTTTGTACCATAGAACCCTGAGAGAAAAATAGCTACACAACTTTTGTTCCTTGAACCGTCCCGCATATTCT
ATAGTATTCACTTTCAAAACCATTGCTCTAAGTTCATATTAGAGACCAATCGAAGAGTATTCTGAAAGGGGTAACCCTTGGTGA
ATGGAGATTTCACTAAATTTCTCCGAAATTATTACCCTCCGTGTTCAACCTCCGTTCGATATTTTTCCCTGAATATATCTCATG
GGGAATGAATATATTAGGAGTCGAAAACGGAGACTTAAGCACACTTCCTAAAAAAAAATTTCCCGTTTTTTTATGGCAATTTATT
TCGAAAGTGTTTTCCTTATAATTCAAGTACATACTCTCAGTATTTTGATTGGTATCCCGTCCAAATACACACCCTCGGATTTGC
CACGAAAATTTCATATACATAGAACCTAAGACATAGGGGCTACAACTTTTGTTCCATAGACCCTCGCAGTATTCCTTAGTATTC
ACTATCAAAAACATTGCCTCTAATTCATATTAGAGAAATCGAGACTTTCTGAAAGGGGTACCCTTAGGGTGAAATTGAGATTAT
CACTAATTTCCCCGAAATGTATTACCCTCGTGTTTCAACTCGTTCGATATTTTCCCCGGAATATATCTCATGGTGAGTTGAGTC
GAAACGGAGACTTAAAGTCAACCATTTCAAAAAAAAATCCCGTTTTTTATGGCAATTTTCGAAAGTGTTTTTCCTTATATATCG
GCACATCTCTCAGCTATTTTTGACAGTATCGCCTCCGTACACATCTCGGATTTGGACGAAATTTTATACACTAGGAACCCTGAA
GACATAGGGCTACAACTTTTGTTCCCATGACCCTCGCATATTCCTTTAGTATTCACTAAATCAAAACATTGCTCTAATTCAATA
TTAAGAGAATCGAAGATTGTTCTGAAAGGGTAACCCTTTGTGAATTGGAGATTTCACTAATTTCTCGAAATGTATTACCATCGT
GTTCACCCTCGTTCCGATTTATTCCCCGAATATATCTCATGGGGAAATGAATAATATTGGAGTCCGAAACGGAGGACTTAAGTC
CAACATTTTCAAAAAAAATTCCCGTTTTTTATGCAATTTCGAAAGTGTTTTCCTTATATTCGTACATCTCTCAGTTATTGTTGA
GAGTAATCGCCCTCTACACATCCTCGGATGTTGCACAAAATTTTTATACATAGAACCTGAGAATCAGGGCTACAACTTTTGTCC
CATGAGCGCTCCGCATATTCCATTAGATCATCACTATCAAAAAACATTGCTCTAATTCATATTAGCAGAAATCCGAAGATTTCT
GAAAGGGGTACCCTTTGTGAATTGGAGATTTATCACTAATTTCTCCCGAAATGATATTAACCATCGTGCTTCAAACTCGTTCGA
GTATTTTCCCAGAATGAATATCAGGGAAATGCATTACTTGGAGTCCCGCAAACGGAGAATTAAGTCAACATTTCAAAAAAAATTC
CCGTTTTTATGGCAATTTTAAAAGTGTTTTAAACTTATACTCGTACATCTCTCAGACTATTTTGACGAGTATCGCCCTTCTACA
CATATCGGATTTGGTACGAAATTTAATACATAGAACCCTGAGACATACGGGCCTAACAACTTGTTGTTCCCCATGAACCCTCTC
GCAAATTCCTTAGTATTCCACTTTCAAAACATTGCCTATTCCTATTAAGAGAAATCGAAGATTTCTGAAAAGGGTACACCTTTG
GTGAATTGGAGAATTTCACTAATTTCTCCGAAATGTAATTGACCCTCCGGCTGTTCAACTCTTTCGAGTATTTTCCCCAGAATA
TATCTCATGGGGAAAATGATATATTACCAGGAGTCGACAACGGAGACTTAAGTCAACATTTCAAAAAAAAATTCCCGTTTTTTAT
GGCAAGTTTCTGACACAAGTGTTTCCTTATATTCCGTACATCTCTCAGTATTTTGATTGTACTCGTCCGAATACACACTCCGGA
TTTTGCACGAAATATTTATACATAGAACCCTAAGACATAGGCGTACAACTTTTGTTCCCATGACCCTCGCATCTTCTTAGTATT
CACTCATCAAACATTGCTCGTAATTCATATTTAGAGGAAAAAATCGAAGATTTCTGAAAGGGGGTACCCCCTTAGGTCGAATTG

GAGATTTCACCTAATTTCCCGAAATTGTTATACTCGTGTTCAAACTCGTTCGATAATTTTTTTTTTTTTCCCGAATATAATCTT
CATGGGGGACAAATGAAATATTGGGAGTCGAACGCGAAGACTGTGTGAGAGTCACATTTCAAAAAAAATTCCCGTCTTTTTATG
GCAATTTTCGAAAGTGTTTTTCCTTGATATTAAGCAACATCTCTCAGTATCTTGACAGTATCGCCTCTACACATCTCGCGATTG
GCGAAATTTTTATACATAGAACCCTGAGACATAGGCTACAACTTTTATGTTCCCATGACCTCGCATATTTCCTAATAGTATTCA
CTATCAACACATTGCTCTAAAAATTCATCATTAGAGAAATCGAAGATTTCCTGAAAGGGGTAACCTTTGGTTGAATTGACCCGA
TTTCACTAAGTTTCTCCGAAATGTATTACCATCGTGTTCACCCTCGTTCGAATATTTTCCCCGAATATATCTTCATGGGAAATG
ACTAATATTGGAGTCCGAAACGGAGACTTAAAGTCAAACATTTCAAAAAAAATTCCCGTTTTTTATGGCAATTTTCGGAAGTGT
TTCTCCTTATATTCGTTACATCTCCTCCAGTATTTTGACAGTATCGCCGCCCAATCCTACACATCTCGGATTTTGCACAAAATT
TTATAGCATAGAGACCCTGAGACATAGGGCTGACAACCTTTTGTTCCCATGACCCTAGATATTCCTTAGTATTCACTATTCAAA
ACATTGCTCTAATTCAATATTAGAGAAATCGAAGATTTCTTGAAAGGGGTACCCCTTTGGTGAATTGGAAGATTTCACTAATTC
TCCGAACTGTATTCCCATCCGTGTTCACTCGTTCCGATATTTACCCCCCCCCTCCCAGAAATATCTCATGGGGAAATGGAATAT
ATGGAGTCCGAAACGAGACTTAAGTCAACATTTCGAAAAAAAAATTCCCCGTTTTTTGGCAATTTTCAAAGTGTTTTCTTATA
TTTCGTACATCTCTCAGTAATTTTGACGGTATCCGCCCTCTAACACATCTCGGATTTGGACGAAATTTAAATACATAGAACCCT
GAGACATAGGGCTACAACTTCTTGTTCAACATGACCCTCGCAATATTCCTTAGTTTCACTTTCAAACAATTGCTCCTAATTCCA
TATTAGAGAAATCGAAGATTTCCTGAAAAGGGTACCCCTTTGGTGAATTGGAGGATCTTCACTAATTTTCTCCCGAAATGTATT
ACCGCTCCGTGTTCAATCGTTCGATATTCTCCCCGAATATATCTCATGGGAAATGAATATATTGGGTCGAACGGAGACTTAGTC
AGACATTTCCAAAAAAAAATTCCCCGCTTTTTTATGGCATTTTTCGAAAGTGTTTTCTTATATTGTAATCTCTCAGTATTTTGA
CAGTTAATCGGACCTCATAAAATCATCGATTTGCTACGAAATTATTATACATAGAACCTAAGACAGTAGGGCTAGCAACTTTTG
TTTCCATGAGCCCTCGCATATTCCTTAGTATTCACTATCAACAACCATTGCTCTATTCATATTAGAGAAAAAATCGAGAATTTC
TGAAAGGGGTAAACCCTCTTGGTGACATTGGAGGATTTCACTAATTTCAACGAATGTTTACCCTCGTGTTCGAACTCGTTCCGA
TATTTTCCCAAGATATCTCATGGGGAAATAACTATACCTTGGGAGTCGAAACAGAGAACTTAAGTCAACATTTCAAAAAAATTC
CCATTTTGATGCAATTTTCGAAAGTGTTTTTCCTTATATTGTCATCTCTAAGTATTTGAATGTATCGTGCAATACACACCTCGG
ATTTGCACTAAACGTCTTTAATACATAGAAACCCTAAGACATAGGGCTACAACTTGTTCCCATGACCGTCTTCGCGATTAATTT
CCTTGACATATTCACTATCAAAACATTGCTCTAATTCATATTAGAGAAAGTCGAAGATTTCTGAAAGGGGTTACCGCCTTTGCG
TGATTGTGAAGAATTTCATCTTAATTCTCCCGAATGTAATTACCCTCGTGTTCAACTCGTTTTCGATATTTTCCCCCAACAGAT
ATCTCATGGGGAAATGAATATATTGGAGTCCGAAATAGAGCTTAAGTCAACATTTCAAAAAAATTCCATTTTTTTGATGGCAAT
TTTTCGAAAGTGTTCTTCCTTATATTCGTACCATCTCTAAGTATTTTGAATGTATCTGTCACAATACACACCTCGGATTTGCGA
CTAAATTTTATACATTAGAAACCCTAAGACATAGGGCTACAACTTTTTGTTCCCCATGACCCTCGCATATTCTTATATTCACGT
AATCCAAAACATGTGCTTCTAATTCATAATTAGAGAAATCGAAGATTTCTGAAAGGGGTTCCCCTTTGGTGAATGGAGATTCAC
TAATTTCTCGAAATGTATTACCTCGTGTTCAACTCGTTCGATATTTTTCCGTTCCGATATATCCTCGTGGGGAACTGAATATAT
CTGAGAGTCGAAATCGGAGACTTTAAGTCAACATTTCAAAAAAAAATCCCCGTTTTTATGGCAATTTTCGAAAGTGTTTTCCT
TATATTCGTACTCTCTCCAGTATTTGACAGTATCGCCTCTACACCATCTCGGATTTGGAGCCGAAATTTTATACATCGAACACT
GAGACATAGGGCTACAACTTTTTGTTCCCATGACCACCTCGCATATTCCTTAGTATTCCACTATCATAACATTGCCTCTAATTC
ATATTAGAGAAATCGAAGATTTCTGAAAGGGTAACACGCCTTTTTGGTGAATTGGGAGATTTCACTAATTTCCTCCGAAATGTA
ATTACCATCGTGTTTCAAAAAACTCGTTCGATATTTTCCCCGAATGATATCTTCATGGGGAAAATGAATTATATTTGGAGTCG
AAAACGGAGACTTAACGTCAACATTTCAAAAAAAAAATTCACGTTTTTATGGCAATTTTTCGAAGGGTTTTCCTTATATTCGT
ACATCTCTCCAGTATTTTGAAGTATCGCCGCTTCTACACCATCTCGGATTTTGCACAAAATTTTTATACATAGAACCTGAGACA
TTAGGGCTACAACTTTTGGTTCCCATGACCCTCGCATATTCCTTAGTATTCACTATCCAAAATATTGCTCTTAATTCATATAAG
AGAAAAATCGAAGATTTCTGAACAGGGTACCCATTGCTGGTGAATTGGAGATTTCACTAATTTCCCGAAAATGTATACCCATCG
TGTTCAACTCGTTCGATATTTTCCCAGAATATATCTCATGGGGAAATGAAATATATTTGAGTCGAAACGGACTTAAGTCAACA
TTAAAAAAAATTCCCGTTTTTTTATGGCAATTTTCGAAAAGTGTCTTTCTTATAATTCGTACATCTCTCAGTATTTTGAAAAGT
ATCGCCTCTACACAATCTCGGATTTGGACAAAATTTTAATACATGAAAACCCTGAGACATAGGGCTCAACTTTGTTCCCATGAC
CCTCCCGCATTATTCCCTTTAGTATTCGACTTCAAAAACATTGCTCTAATTCATATTGAAAATCGAAGATCTTCTGAAAGGGGT
TACCCCTTTGGTGAATTGGAGATTTCACCTAATTTCTCCGAAATGTATTTACCATCGTGTTCAACTCGTTCGATATTTCCAGAA
ATATATTCATGGGCAATGACATATCACTTGGAGCTGGAGTCGAAACGGAGATTAAGTCCAACATTTCAAAAAAATTCCCGTTTT
TTTATGGCAATTTTCGAAAGATGTTTTCCTATATTCGTAACATCTCTACAGTATTTTGGATTGTATCGTCCAAATACACACCTC
GGATTTGCGAATTTTAATACATAGAACCTAAGGACATAGGCGTAGACAACTTTTGTTCCCATGACCCTCGCTATTCCTTAGTAT
TCCACTAATCAAGAACAATTGCCTCGTAGATTACATATTAGAGAAAATCGAAGATTTCTGAAAGGGTACCCTTTGGTGAATTGG
AGAATTTCACCGTATTTCTCTCGAAAGTATTACCCTCGTGTTTCAACAATCTCAAGTTCGATATTTTTGCGCCCGAAATATATC
TCATGGAGGGAAATGAATATATTGGAGTCGAAACGGAACTTAAGTCAAAATTTCAAAAAAATTCCCCCGTTTTTTTAATTGGCA
AGTTTCCGAAAGTGTTTTCCCTTATAATTACGTACATCTCTCAGTATTTTGACAGTATCGCCCTCTACAAATCATCTCCGGAAT
TTTGGACGAAATTTTATACATAAGAACCTAAGACATAGGCTACAATTTGAGTTCATGACCCGTCGCGATATTCCTTAGTATTCA
ATGTCACCTATAAAAACAAGTTGTTCCTACATTCATATTAGCCGAATCGAAGAGCATTTCTGAACAGGGGACCCTTTGGTGATG
GGGACTTTCCAAATAATTTCTTTTCCTGAAGATGTATTACCGCTCCGTGTTCAACTCCATTGATATTTCCCCCCGAAGATATCT
CATGGGGAAATGAATATATTGGAGTCGAACAGACGAGACTTAAGTAACATTTCAAAAAAAAAATTCCATTTTTTTTGATGGCAA
TTTTTTCGAAAGTGTTTTCCTTATATTCTTACATCTCTAGTATTTTGATGTATCGTCCAATACACAGCCTCGGAAATTTTGCAC
GTAAATTATTATACATAGAACCCTACAGA
>m141120_105518_42134_c100732402550000001823141405141504_s1_p0/477/355_7463

CGATTAACTCCCTTCACTACTGCTATTACCACCGCGCCCTCGTACTATCTCTCTCGTCTACCGCCCCCCCCCCCTTCCTCTCTCC
TAATAATGACCAACACACTCTTCTGCGCCGAGAGTCGCCTCCTCGCCCCATCACGCTCTTCGACTCAAACCTCCATACAGCGCT
TTCTCTTCAACTTTCAATTTGACAACAACAAAATCCCACTCCCGCCTCCAAGCGAACTTCGCACGTTTTGTTCTCCCCCGAACC
ATCTCATGAATTACGATTAACGCTTTGAGTGTAGTGTTCGTAAGCAACCAGAAATTGGGGACAGCAAGATAACTCTCTTTTATT
GAACGCTATTTGGTTCTTGCTGCACGACTTTCATCAATTCTAATTCTATTTTGTTTTCATATCCCTGGAAATTCCTCACTCTCT
CCCCCGAGTTCTCACCCTCGCCAGGGACCCTCGTCTTTAACGAACAGGTCAACTGCGATCTCCTTTCCAATCTCTCTCTTTTAA
GCTCCTCGCAATTTGACAATCATATAAAGCACAATTCCTATTCTATCATACTAGCCTATTTGTGCAAGTATTTCTACGCTCCCG
CAGCAGACTGCTTTACATCTGTACAGACATCTGTGTCAAAGTCTACTATAACCTGGTAGTATACCTTATTGTCTCGTATACTAG
CATTTAGCGCGAGTGATCCACAAACGACGATAAACTATCTCTTTTGCTTACATCTATGCTGATGAGCCGAAACAAAAGCTATTA
TTTTCTGTTGATTAATAGCTATCGAGTAAAGCTCGTCTGATGCAGCCCTCGAAAGCTTGATGCAAAGAAACGATTTGTCTGTTC
CACGTCTTTCGAGCTATACTATCTCGATGTTTATTCTAGTATTGACCTAAATGAAACTTTATGATGAATAACTAATTGGGATTT
TTTCTTTCCCAGTTATATGTCCTTGACATTTTCTAGTCTATTCACTAAGAAAACGGATTCTTGCCACATGTATACCCAAATCAA
AAATGTCCATGCTTTTTTGCTACTATAACCTTCCACCACCGATTTTATTTTTTTTTCTAGACTGTCTCAACCCTAGAAATCAA
GAATGTATTGATCACTATAACCAAATTAGTAACATACACAAAGCTGAGTAACATACCTAATCAAATATACGGATATTACGGTGG
GCTTCAATCGGTTCTTACTGGCTATCTCTTAAGACGGTAAGGTACCTCTCGTAGATCAGCCGAGCTCTTCCCTCATTACTCAAT
GTTATTGCTTTAACCGTTGTACCGTTTTCACCATCTCTTTGGCTCCTAACCCACTACATTACATCCAGTCAACCCTCAGGTCTT
TTTCACACCGCGCACCCTCACTCTCAAAGTTACCCCGGTGATCCTAACTTAATAGTTCCATGAGAGATTAGTCTCGAGGTACGC
TGAATGTCTCAGTCGTTCTCTTTGCAACGAGCTCAAGAGTAACGGCGCACGTACCCTATACTTCTCGGCTTTTTTACTAGGACT
TCTTCCTGCTTCATGTACGATACCATTCAATTGCTCCACATCCCTGGGGATTCTTTCTCATCTTAAACTGACACCTGGCACCTG
GATTGCTTTGTTACCCAACTCAACAGCGAACCATAATTAATTCGATAACCACAACCACAACGACGATACCCTGATAAGCTTTTT
TAGATTATTTCGATATTCTTCATTTTTTCAATCTGATAGTGAATGGTGTTTTCCAGATTCTTCCCCTATGTCACTCTTACTGCA
TCACTGACGACGGACTCATTGTTCCTTTTTTTTTTTTCTCTTGGCCTACTCCATGTTCTGAAAGCAGTCCGCACATACCCCTAT
ACATGTTCCTCGTTCGCTGAGGACACCCCCGAAGAGCGGGGGGATTTCGTGGACATTTTCCTGCATCTTGCTGTTCTTCGTCTT
TCGTAATAGAGTTGTTGCAATAATGTGGGATGTTGAATATCATCATACATACGATGCGGCTTCGAGTTTAGCACGCTCCCTACA
CCCGGATAAATGATTCAATCGAATCACTCTTCCTTTTACTTCAATACGATTCCATCAATACATATATTACTCGTTCTTATGTTA
TTATAGTTCAAACCTGGTACAACCGAAGAGAACATAATCAAATCTGCATTTTTGCGGGAAATTCCTGTATCTTTTTCATGTCCA
CCGCCTACAGACTCGAACAAGGTCCATGAGCTGTTGGCGCTCTCCTGTTGCTCGCACAGATACAACACATCCTCCTTTCATGTC
TACTTCGAACACCATAAACCATTTGCCCGCTTCTTGTCATCCAACCGCTTCTCGTTGAAGGTTACCTCGCGCAGCTTCCGTACA
GTTCTTCCGCTGCAGGATGAGATTCCCGTCGGTGCCTATAAAAAGACTAGCGCGTTGTGGACTCAATTCGATGACATATAACAA
TAATCAATAAATTCTTTATCTCGGCATATGAACAGGCGAGAAAGATATTAATAGCGAAAAAAGCCTAGAATTGAACAACCGTCA
CACAGGCCATCTTCTGTATATATCCTGCGCACTACCGGCTCTATAATCGCGGAAATTCCTTTTCCCTTTTTTTACCTTACATAG
AAGAATTATACAATAAATTCCGAATGATAGGCGTCTATCCTATTCACATAGGTCAAATGATCATATCATCGCTACTTCTGAGCT
ATAAGCCAAAAATACTATGAATGGTTCGTTGGCTTTATCATAATACAGATTCTCGACTGTTACTTCTAGAAATCCCAAAGCTTA
TTTTTAGATCTCTGAAAAAAAATATAAACTTAATCTACAAGGTAAAACTAGCGAACCTTCTACTTTCGTAACAAGCTTGTGGCA
CCGCTGACTGGGTCTCCTGATTATACTCCGCTAACACAATTGGATACTTAATCTCTAGTTACTCTTTCGATACAATAATGTACA
CAATTCTTGCAAACCAAAAACATTCTACTATCGAAGTCGAAATGCATGCTATTTTCTTAATCATATTTCTTCTACATCGCGAAG
GCATAGTCGTTTTTTTCTCTCAGGGATCAATAAAAAATAAAACTCTTGCGGCCAAGCGTGAGGAGACACTGATAGACGTTAGGG
CGGGGGGGGGGGTGGGGGGGGGGGCGGGCGGGGGGCGGGGGGGGGGGCGGGGGGGAGGGGGCGGGCGGGGCGGGGGGGCGCGGG
GGGGGGCCGGGGCCGCGGGGCGCAGGGGGGCGGGGGGGGGCGGGGCGGGGCGGGGGGGGGGCGGGGGGGGGCGGGGGGGGCGG
GGGGGGGGGGGGGGGGGGGGGGGGGGGGGGCGGGGGGGGGGCAGGGGGGGGGGGGGGGGGGGGGGGGGGGGGGGGGAGGGGGGGC
GGGGGGGGGGGGGGGGCGGGGGGGGGGGGGTGGGGGGGGGGCGGGCGGGGGGGGGGGGGGGGGCGGGGGGGGGGGCGGGGGGGC
GGGGGGCGGGGAGGGGGGGGGGGGGGGGCGGGGGCGGGCGCCGGCGCGGCGGGGGGGAGGGGGGGGGGGGGGGCGCGGGGGGGGG
CGGGGGGGGGGGGTCCGTATTTCTCCTCCGAACGACGAACTAAACCAGATCCATTGACAGCGGCTAATCCCTCATGCTTATCTG
TGTTGTACGTCTGGTTGCACAACTTGCATAGTAATCACTCCTAATAGCTACATCAGGATTAACCATCCGCCGGAGGCGCTAATA
TATAGGAACAAATACAGATCTTGTATCACCTATCCTCTATACTGAGATAACCTATACCATAAGACCAATAAACGTTGATTGTGA
GAGTAATCCGCTATCAACCTCGTTATGGCTACAAGAAAGTAACTGTTCTCGTAAAGTCCGGCTTGCTTGGGAGTACATCTGTCA
TCCTTTGGACACCGTACTGCATCCCTTTTGATGCATCGACGGTTGCACAACAAATGGCGAAAAAAAAGATCCATGTGTAGGATT
ATAGTTTTTTTTTTCGTTGTTTCACTCGCGGGCGCTGCTTCGTACTGGTCAATAACACAGAGGCATCCTTCTGGTGCTTGCATT
TCTGTAAAAAAGGATGAGTTTTGGTCTGTTTAATGTTTCTCCCTATTTATTCTGATATCACCTCTCTTTTTTCTGATATAATAG
AAAATACGAACAAAACATTCACTACAAACTTATACACAGATTATCGAACTATCTTCTTCATTGTTGTAGATTGTTTTTAGCATC
GGATCAATCACGATGTACATTTTCTGTGTGTCCTGAATGGTCTTTCTTGAATTGCTTTTAGGTTTATGCCTCTACCGTCGAGCT
CACAAGATCTGCGGAGAGATTTGGATTTGGCTATATACGACAAATGGACAACATACTATCGGTCTTTCTGCTACGACTTCCTTT
TTTTTTTTACACTTTATGTCACTCTCGCTACCCTAAACTCACAACACCTTAAATATGTCATTCGAAAGTCCACGCTCTATCCTC
GACTCAGCTTACGCACCACTTCCAAAGCCAATCCGAGTAGTAAGTAATCACAGATCCATGCACCCCCAATATGATCTATCCCTA
AGTTACAATATCCTACAGCTATCTACTTCACCCATCTTCTTTCGTTTCTAAACGGCTAGCCTGGAGTTACTCTATTTTACTTGG
TCGACCGCAAGCCACCATAAACTTCAACCCCCCCGCCCTTCGCTAATACCTTCAACTTCCTCCTGTATACCCCCCCCCGGTGGA
CGCCAAAAATCAGCATCACCTAAGTGGGCCCTTTTTCTCCCCCTCGACTCCTTCGCATCGCACAAGGGTGTTTCCCTGCTACAC
TCCACTCACTCGCTCTCTCCCTTCGGGCGAAAGGGAGGCCCTCACCCCTCTCGATCGGGAAGACAGACACGCGGGGGACTACTA

CATCCACCTGCCCACCGCATATATCTCTGACAGTCGACTATCGCCTCAACCCGCACGATGCACTGCTTCGGTTCCAGGACTTTC
GAAAGGTTCTTTTGGAAGCACCAGATAGGCATTAACGTGAACGAACATTTACGCCTAGTCCCACTATATCAGAAATCACTTTGA
GGTGAAAGGCGCGAAATCGGCGCCTGTAACATTCGGCTCGGCCCTCAGACAATCCAATCCATCCCCTGTCCACTCTTAGTGCTC
TTTTATCCTCAGCATCAAGACCTTCCGAACTAACCCCCGCCACGGCTCCGCTCCTCCGTGTTCCATTCAGTCCCGCCGCCCACA
CCGCGCAAACTTGGCTGACGCACAACTCGGTCCCGTCCCTGAACTGCATCGCAACACACATAACAGACGGCCCATGTTACTCCC
CAGCATCACCGAATACAGGCCAATCAAGTCCGACCCCCCCATCCGCGTCGCGTTCTTGGTATCCTCTCGGCGCGCTGCCTCCGA
ATAACTCCTGGGCGTTCTTGCTTCCTAACCCACGAGCAAACCACATTGTTCCATTCGCTCTCCCCTGCCTCAACCTCAAACAAC
AATTCTTCCTAACACCGCACCCTTCCCGACCGAAAGAGCTCAAACCATCCCGCACTACATTCTCCCCCGCCAGATACAGCTCAC
ACTACAAAAAGTCACAGGTCATAGTCGAGAGTTTTTCTCGCACCAAGTCGGCACTACCGTTAAACTACCCCCAGTCCCGTCCTA
CCCCTTTTCTCCACTTGATATCACAGCAGGCGCTCATAACCTTTCTACGCGGGCTCTTGCCTTCCGGTCGTCGCGTGTTTCATC
TCCCACGGCTCGGATTGATGATGCCTCAGGCCGTTTTGATTTCCATGTCATCCCCCGGGGCACCCCGCCGACGATGCAGCCACT
CAGCACCCTCGTAGCGCTCGTGCCCCGGCGCCTGAGAAAGCACACCAGACACACTATGCGCCTGGACACAAAATACACATACAA
CAACAATATAAAGCAACAGAACCAAACCAATACCACATCTTAAATACAAACAGACTCGTCTGACCCTGTTGCTCGAAATAGAAA
TACAAAATAGACACCAAAAGAGCAGACGCGGACGTACCCCCAAGCTAATCCTGGCCAATCCCACTCTACAATATTCATACGACC
GACGCCGTAGAGGGCACCCCTCCCCGAATTAGTCGGGCGGTGCCCGATCTGGTGTGATGCGCCCCCGAGGGCGGCGAACTTACC
ACGACGGAACCCCGGCGTCACCCGCACACGCCGCGGCCCGCAGGCATGCGCTCGGCACGTCCCCCCCTCCGGCGGCCACAGCCC
GTGCCCGTCGAGGCTGCCTGACACCCATCACCTGCCTGTCTCTCTGCTTGCTGCTTGTGTCGTAGCAGCGCCTATCTGTGGAGA
TCCCACGCTCTCGGGTGTCATCTGCGACCGATCCTACAGCAGATACTTTTTCCTGACCTCGCACCGTACAGCCGAAAACCCTCC
TGTCTGGATTTCGCCCGAACGATTCTGCTTCGGAATGTACATACCTTTATCTTCCCCTCAGAGGGTGGCTTTGCTTTCGGATTT
TGGCGCATTTCCTGTAACAGGAATTGTATGGTCCTTGGAATTGGGTATCCAGATCCTTACCTGGCAACTAACCACCCACGGAAG
GGTACACCAAGAGTGCTGTAAGACTCCCAGCATGCGGGCACCGCGTCGGAAAGCGTTCTTTGATCCTTTTGCATTCGGGGAGGC
AATAGCTTCTCAGCCCGATCATATTCAGTGCAGCGGGACGAACGCCTTGCGGCCATCATGGACGGGTTATTCGGTCTAGCTGTC
CGCTCCGCCCAACGTCACCTATATCATCACGGATCCCCTCGTATGGGAACTTATTCGACTTCGCACGTCCTATGTCCACGTCAG
CTCCCTACCGGCTGCGGTCTTTTTTGGCAGCTTTGTAGTAGCTGCTGGAAGTATGTGGTATACGGGTCTCGGCACTACCAACCC
GCATTGACTCTGATCTTGGCGCGCCCGCAGACGTCGTTATCCATCGCGGGACTCTCTACAGGATAACCGTTCTTCACACAAGCA
AGTAATATCGCCGCCTCCGAAGAGCGCCGCCTAGTGGGCTGGGGGCTAACAGCCGAAAATGCAAGCTTTACTCGTTGAGAGCTT
CGCTCTGAAAATCCAGAACCATTCACGCCCTTTTATGATGTAATCGGCAATA
>m141120_105518_42134_c100732402550000001823141405141504_s1_p0/1316/0_7108
ACACAAGAAGAGATAATAAATGACATGGTAAGGTCGGTTCAACACAACCGACCTAGCAATGTCACATGTGAAGTCCTTTTCCAC
ACAACCGACTACATTGTCTTCTAAAAAATATTAAAGAGATACACTAAATTATGTTATAAATTTTTTATTTTAAATTATCACTAT
TAATAAATTAAAAAATAAATTTTTTTTTTATAAAAATGAAATAGAATGTATTTAATTATAAAAATTTATTAATTTATTTCATA
TTTCAAAAATTATAATATATGTAGTATCTAATAATTATATACTTTTTATACTTATTTCATACCCAAAAAATAAATTTATATAA
GTAATTGTTTAAAACTAATTAATATATTTTAAATTTTCAATAATATAACAAATTAGAGTATATTTAATAATGTATTTTTTTTAT
ATGACATGGTCAGGGTCGGTTCTACACAACCGACCTACAATGTCCATGAGAAAGATCATATTTTATTTCCCACACAACGACCTA
CCATGTCCTCTAAAAATTATTAAAGAGATACACTAAATTATATTATAAATTTTTTATTTCAAATTATCACTATTATTAAATTA
AAATATTTTCTTGTTTATAAAAATGAATAATTCATTTAATTACATAATAATTTATTAATTTATTCATATTTCAAAAATTTATA
AATAATATGTATATTAATAATTATATATCTTTTTATACTTATTTCATACCAAATAAATTGTTACTATATATATATGTTTAAATA
ATTAATATCATTTTAAACTTTTCAATAAATATAACTAAACAAATTATGATATATTTAATAAATGTTATTTTTATGATGACATGG
TAGGTTCGGTTCAACACAAGCCGACCTACCATTGTAACATGTGAAGTCCTTTCCCACACAACCGACTACATGTCTCCTAAAAAA
TTATTAAAGAGATCACACTTAAAATTTATCTTATAAATTTTTTTTATTTTAATTATCTCAATTATTAAATTAAAATATTTTTTC
TTGTTTATAAAAATGAAATAAATCATTCATTAATAAATAATTTATTCTTATTCATTTTTCAAAAAAATTATAATATATTGTAGT
ATCTAATAGATTATATATTTTTTTATACTTATTTCATACCCAATAAAATTTTATAATATGTATTGTTTAAATAAATTAATATAT
TTTATTATTTTCAATAATATAGAACAAATTAGAATATATTTAATAATGTATTTTTATAATGACATCGGTAGGTTCGGTTCAACA
CAACCGAACCTAACCATGTCACATGTGAACGTCTTTCCCACACAACACGACCTACCATGTCTTCTAAAAAATTATTAAACGAGA
TACACTAAATTATGTTATAATTTTTTTATTAAATTATCACTATTAATAAATTAAATATTTTCCTTTTTTATAAAAATGAAATAA
ATGTATTTTAATTATAATAATTTTATTAATATTCATATTTCAAAAATTATAATATATGTAGTATATAATTATATATTTATACTT
ATTTAATACACAAATAAATTTTATATAAGTATTGTTTAAATAAATTAATATATTTTTTAAATTTTCAATAATATAAACAAAAT
TAGAGTATAATTTAAATAATGTATTTTTATAATGACATGGTAGGTCGGTTCTACACAACGACCTACCATGTCACATGAGAAGTC
ATTTCAAACCAACAAGACCTACCATGTCATCATCAAATATTATTAAAGAGATACACTAAATTATGTATAAATTTTTTTATTTTA
AATTATCACTATTATAAATTAAAATAATTTCTTGTTTATAAAAATGAAATAAGTTTATTTAATTAAAAAAATAATTTATTAATTT
ATTCATAATTTCAAAAATTATAATATATGTAGTATTAAAATTAATATATATATTATACTTTATTTCTACCCAAAAAAACTTTTA
TATATGTATTGTTTAATAAATTAATATATTTTAAATTTTCAATAATATAAACAAATTAGAATATATTTAATAATGGTATTCTTT
ATAATGACAATTGGTAGGTCGGTTCAACTCAAACGAGACCTACCATGGTCACATTGAGAAGTCATCTTCCACAAAACCGACCCT
ACATGTCCTCTAAAAAATTATTAAAGAGATACACTAAATTATATTATAAATTTTTTTTTATTTTAAATTATCACTATTAATAA
ATATTAAAATATTTTCTTTTTTTATAAAATGAAATAAATTTATTTAATTATAAATAATTTATTAATTTTTCATATTTAAATTAT
AATATTATGTAGTATTAATAATTATATATTTTTAACTTATTTCCATACCCAAATAAATTTTATATAAGTTTGTTTAAATAAATA
AATATATTTTAATTTTCAATAATATAACATATTAGAGTATATTTAATAATGTATTTTTATAATAATGGGTAGGCTCGGTTCTAC
ACAACAGTCCTAACCATGTCCATGAGAAGTCATTTTCCACACCAACCGACCTACAATGTCCTCTAAAAAATTATTATAGAATAG

ACAAATTATGTTATAATTTTTTTATTTAAATTATCACAATTATTAAATTAAAAATTTTCTTGTTATAAAAATGAAATAAATTCA
TTTAATTATTAAAAATAATTTTAATTCATTTATTCATTTTTCAAAAATTATAATATATGTGTATTAATAATTAATATATTTTTA
TACTTATTTCATACCCAAATAAATTTTATATAGTATTGTTTAAATAAATTATATATTTTAAATTTTTCAATAATATAAACAAAG
TAGAAATTTATTTAATAATGTATTTTTCTAATGACATGGGTAGGTTCGTTCAACACAACCGACCTAACCATGTCACATGTGAAG
TCCTTTCACACTCACCGCCTAACCATCGTCTTCTAAAAAATATTAAAGAGATTACACTAAATTATGTTATAAATTTTTTTATTT
TAAATTCATCATATTATTAAATTAAAATATTTTCTTGTTATAAATGAATAAATTCATTTAATTATAAATAATTTTTTTTTTTTT
TTTAAAAATTTATTCATATTTCAAAAATTATAATATATGTAGTATTATAATTATTATTTTTTATACTTATTTCATACCAAATAA
ATTTTATATATATATTGTTTAAATAATTAATATATTTTAAATTTTCAATAATATAAACAAATTTGAGTATATTTAAATAATTGT
AATTTTTATGATGACATGGTAGGTCGGTTCAACACAACCGACCTACCATGTCACATGTGATAGTCCTTTCCCACACAACCGACC
TACATGCTCTAAAAATTATTAAAGAAGATACACTAATATTATGTTATAAATTTTTTTATTTAAATTATCTCAATTATTAAATTA
AAATATTTTCTTGTTTATAAAAATGAAATAAATCATTTCATTATAAATAATTTATTCATTATATCATTTTTCAAAAATTATAAT
ATATGTTAGTATTAATAATTATATATTTTTTATACTTTATTTCATACCCCAAATAAATTTTATTATAGTTTGTTTAAATAATTA
ATAATTTTATATTATCAATATATAAACAAATTAGAATATATTTAAGTAATGTATTTTTATAATGACATGCGTAGGTCGTTCAAC
ACATACCGGACCTACCATGTCAACATGTGAAGGTCCTTTCCCACAAACCGACCTACCATGTCTTCCTAAAAAAATTTTAAAGAG
ATACACTAATTATGTTAAAAATTTTTTTTTTAAATTATCAACTATTAATAAATTAAATATTTTCTTTTTTATAAAAAAATGAA
AATAAATGTATTTAATTAATAATATTTATTATTTTTCATATTTCAAAATTATAATATATGTATATTAATAATTATATATTTTTT
TATACTTATTTAATACCAAAATAAATTTTTATATAAGTATTTTTAATAAATAAATATATTTTTAAATTTTCAAATTAATATAAA
CAAATTAAGAGTATATTGTAATAAATGTATTGTTTATAATGACATGGTAGAGTCGGTTCCTACACAACCGACCTACCAAATGCG
TCCACATGAGAAGTCCATTTCCCACACAACGACCTACCATGTCCTCTTAAAAAATATTAAAGATATACACTAATTATATTATAA
ATTTTTTTTATTCAAATTATCAACTATTATTAAATTAAAATATTTTCTTGGTTTATAAAAATGAAATAAATTCATTTAATTAT
AATAATATTATTAATTTATTTCATATTTCAAAAATTTAATATTAAGTAGTATTAATAATTATAATATTCTTTTATACTTATTTC
ATACCCAAATAAATTTTTATTATATATTGTTTAAATAAATTAATATATTTTAAATTTTCAATAATATACAACAAATTTGAGTAT
ATTGATTTATAATGTTATTTTTATGATGACATGGTAAGGTCGGTTCAACAAACCGACCGTACCATGTCACATGTGAAGTCTTTC
CCACACAAGACCGGACCACCATGTCCTCTAAAAAAATTATTAAAGAGATACACTAAATTATGTTATAAATTTTTTTATTTTAAA
TTATCTCAATATTAATTAAAATATTTTCTTTGTTATAAAAATGAAAAGAAATTCATTTCATTATAAATATTGTATTCATTTATT
CATTTTTCAAAATTATAATGATATGTATATAATAATTATATATTTTTTATACTTATTTCATAACCAACATAAATTTTATATATG
TATTTTTAAATAAATTAATATATTTTATATTTTCAATAATATAAACAATATTAGAAATATATTTAAACAATGTATTTATTTTAT
AATGACATGGTAGGTCGGTTCAACACAACCGACCTACAATGTCCATGGTGAAGTCCTTTCCAGCACCATACCGATCCTTAAGGC
CAATGTCTTCTAAAAAATTAAATAAAAGAGATACACTAAGATTAGTGTTATTAATTTTTTATTTTAATTCATCACCTATTAAAT
AAATTAAAATATTTTCTTTTTTATAAAAATGAAATCATGTATTTAATTATAACAATAATTTATTAAATATTTCAAAAATTTAAG
TAATATATGTATATTAATAACTAAGTATACCTTTTTTTATACTTATTTCATACCCCAAAAATAAATTTTTATATAAGTTTGTTT
AAATAAATTAATATATTTTTTAATTTTCAATAATATATACAAAAATTTAGAGTATATTTAATAATGTAATTTTTATAATTGACA
TGGTAGGTTGGTTTCTACAGCAACCGTACCTACCAAATGTCACATGAGAAGTCATATTCCCAAACAAACGCGACGTACGCCATG
GTCCTCCGAATAAAATAATTATTAAAGAGATACACTAAATATGTTTATAATTTTTTTTATTTTAATTATCACTATTATTAAATT
AAAATATTTCTTGTTATAAAATAAATCAAGTTTATTTATTAAAATATAATTTATTAATTTATTCATATTTTCCAAAAATTATAA
TATGATGTAGGTATTTAATAAGTTACTATATATTTTATAGCTTATTATCCATACCAAATAATTTTTATATTGTATATGTTTAAA
TAAATTAGATATTAATTTAAATTTTCAATAAGATACAACATTAGAAATATATTTAAGTAATGTATTTTTATAATGACATGTTAG
GGTCGGGTTCAACTCCAACCGACCTGACCCATAGTCACAGAGAAGTCATTTTCGCCACACAACCGAGCCCGCTGACATGGTCCT
CTAAAAAATTAATTAAAGAGATACACTAAATTATATTATAAAATTTTTTATTTTAACAATTATCACTATTATAAATTAAAATTT
TTACTTTTTTAAAAAATGAATAATTTATTAATTTAAATAATTTATTAATTTATTCATTTTATTTAAAAAAATATAATATATGTA
TATTAATAATATACCTATTTTTATACTTATTTCATACCCCAATCACAATTTTAATATATGTAATTGTTTAAATAGAATTAATAT
AATTTTAAATTTTCATAATATATAAGCAAATTGAAGAATATTATTTAATAGATGTATTTTTATAATGACATGGGTAGAGTCGGT
TCCAAGCACAACTGACCTAGCCATGTCACATGTGAAGTCCTTTCCCACACAACCGAGCTACCCGATGTCTTCCTAAAAAAGATT
ATTAAAAGAGATAGGCACTAAATTATGTTAACAAATTTTTTTTATTTTAAATTATCACTATTATTAAATTAAAATATTTTCTTG
TTTATAAAAATGAAAAAAATTCATTTAATTATAAATAATTTATTAATTATTTATCATATTTCAAAATTATAATATATGTAGTAG
TTAATCAATTATATATTTTTATACTTATTTCATACCAAATAAATTTTATATTATGATTGTTAAATAAATTAATATATTTTAAAT
TTTTCAATATATAACAATTTGAGTATATTTAATAACTGTATTTTTATAATGACAATGGTAGGTCCGGTTCAAGACAACCGATAC
CATGTCAGCATGTGAAGTCTTTCCCACCACAACCGACCTACCATCGTCCTCTAAAATATTTTTAAAGAGATTACACTAAATTAT
GTTAATAAATTTTTTCTTTTAAAATTATCAGCTTTATTTAAATTAAAAATATTTTTCTTGTTTATAAAAATGAAATAAATTTAT
TAATTATAAATATTTATTAATTTTATTCATATTAAAATTATAATATATGTAGTAATTAATAATTTATATATTTTTTATACCCTT
ATTTATACCCCAAAAATGAAAATTTTATATATGTAATTGTTTAAGAATAAATTATATATTTTAAAATTTTAATAATATAAAAAC
ATTAGGTATATTTAATCAATCGTATTTTTATATATGACATGGTAGTGGTTTACCCAGCAATCGACATACCATGCCAGCATGAGC
AAGTCTTTTTCCCAGCACAACCGACGCTACCCATCGTCCTCCTAAAATATTATTAAAGAGATACACTAAAATTATGTTATACAT
TTTTTTATTTTAAATTATCACTAATTATTAAATTAAAATATTTGCTTGTTTATAAAATGAAATAGACTTTATTTAAATTATAA
ATGAATTTAATTAAATTTATTCTATTTCAAGAATTTAATAAATATATGATAG
>m141120_105518_42134_c100732402550000001823141405141504_s1_p0/2045/3797_13360
CTCCAAAAATCCAAAGGAAAAGAGAAGGAAAGATGTATCAAAGGGGAGGATGCTTATGCTCTATATTTGCAACTCTACAGCTTC
ATGCTATAAATATACATTAAGGTGAAAAGGACCATATTTTCCCCTAAGGTTCAAATAAAGTTTTCTAAAGGCTCCCAAGGGTAA

AACTGTCATTTTCCTGCCTATTCGTTAATCATAAATAACACCTACCCCAATTCCTGTTATATCCCAAAATAATCTCAAATACCA
ATAATTCATACATGTTAGCCCTTTAAATTTCCCGCAACGCTCACCAAATTACCCCAAGACTCCCACCCGAGCCCAAATTAATCC
GTTATGACCAAAGACGCTAAACTTGCACTCAAAGATTGTCTCATGCTGAATGGCTCCAAAATCCAACAATTATATATTGGACTT
TATCAATAATTCACCGAACATGTTCATGAAGATATACAATTACGCCCTCCAATGCCCAAATTACCAAAATGACCTATAATAAAA
TGATGGACCCATATGCATGCATTTAACTCATATATAAATAAATTCACATAAATATGCAATAACATTTAATGGAATAATAAAATC
AAATTATGGCCTCTCAAGCTACTAATCAACCACTAAACCACATTAGGCATTTTAGGGCATTACAATTAATGCTTTGACACTTGG
AACAATTTTTGGGAGTGTTGAAATTTGGAAATGTTGGGGAAATTTCTGGGTTCGCAGGGTCGCACTGCGAACACTGGTTCTGAA
TGCAGCTGCCCGCTCGAACTTCCCAGGAGGTTTGGCGTTCCAATGAATCCACCTCAGGTGCCATGACCTTGGGGTAGGGCGCGT
CGAGCGGCGCATGTCTAACGAGAAATACGATAGTATGGGCTTTTTGACTTGTACAGGACCACGACTCCTAAATGTAGGGGCGCT
GCAGCTTAATTAGGGCGTCGCGACGCGAACAAGGGTTTGGCCAATCTAGGGTTTTAAGCTCGGGAACCCAAACTTAAGGGCTTG
AAAGGATCCTACACCTGGTTTAAGTAGGATTCGAGGTCCGGAAGCTAGAATATTAGATTCAAATATCTTAATGTATTTTAAGGG
CTTAATGGACGTTATCATTATTGCGTGTGACTAGGTTTTACGTCAAAAGCTCGGTTAGGGGATCGTGGCTCGGGATCCCGCAAT
AATGTCAGCTTGGAAACACAGTAAGAAAATGTTTGTGCCCGGTAGAGAGCATGGTGTGGCCCATATTTTTTGGGGTTTTTTTTT
TTTTTTTTTATTGGTTGGGTTTCAAGGACCCAAATTGGGAAAACCCCCCCTTAAAAAAAAAATTTTTAAAAATTTTTTTTTT
TTTTTGTTTTAAATGTAAAAAAAAAATAATGTGGGTGGGGGAAAGTACCTTTGTAAAAAAAAATGCCCCTTTTTTATGCCCCCTTT
TTAACGGTGGGGGGGTTAAAATGGACTAAAATTGAAAAATTTGAAACGGCCCCAAAGGGGGGCTTTTTTTGAAAGAAACCCCAG
CCGGAAAGGCTTTGGGAGAATGGGGGCCAAAAAAAAAAAAAAAAAAAAAAAAGGGCCCGGTTAAAAATTTGGTGGGGGGGAAAAGC
AACGCTGAGTTTTTGCAGGCCCCCCTTTTTTTGCCTAAAGGGGGTTGGGGGGGGAGGACCCCCCCCTTTTTATTTTTTAAAGGG
TTGGGGGGTTTTGTAAAATTCAACCCACCTTTCCGGTTTGAAAAAAAAAAAAATGGACCGTGGAAAACCCCCCAGTGTCCACCCT
TTTTTAGTTAAAGCCACCTGGGTTTTTTTTTGGGGGTTTTAAAAAAACAGGCCCCCCCGCTTTTAATAATTGTTTTTTTTTTAC
CCTTTGCCCCTTTTTTTTTATTTGGTCCCCACCCCACCCCCCACACCCCCCCCCCTTTTTAAAAAAGCTTTTTTTTTGGTGGTT
AAATTTAATCCAAAGAATTTTTTGAAAAAAAAAATTTTTTTATAATTTTTAAATGTTTTTAATGCGTTTTTTTTTGAGGTTTTTT
CCCTTTGTTGGGGGCCCCTTCCCCAGCCCTTTTCCAAAATGAAAGGGTTATTTTTTTTTTTTCCCTTTCTTATTGGGTGGGGGG
TAAGGTAAGAAAGCAAAAAAAAAAAAAAGGGAAAAAAAAAAAAAAAAAAGGTCCACCAAAACCCAAAAACCCAAAAATGAAGTTTT
GGGGAAGAGCCCCCCGTGGGGAAGGGGGGCCGGCCCGGGTGGGGTAAAAAAAAACAATTGTTTTTTTCCCCCAAGCCCCTTTAA
ACCCTGGCCCTTTTTTTTTTTTCCCCCCCCCCAACCGAACAAAAAAGGGTTTTTTTTTTTTTTTTTTTTTTTTTTTTTTTTGG
GTTTTTTTTTGTGGGGAATTAATGTTTGTAACCCAATTTGAAAAAAAATTTTTTTTTTGGGGTCCAAAAAACCTTTTTTTTTTT
TAGGTCCCCAGACTTTCCCCTATCTTTACAATTTTTTATTTTTAAGGCTTTTTTTTTTTTTTTATTTGTTAAAAAAAAAAAAAA
AAATACAAATTTTTTTTTTTTTTTTTATTTTTACCGAAATAAGTTAAAAATTTTTTGGGAAAAAAAATTCCCCCCAAAATGTGGGG
GGGTTTAAAAAAAAAACTTTTAATGAAATTTTTTTAAAAAAAAAAATTGGGAATGGGTTTTCCCCCCCCCCCCCCCCCAAAAAA
TAATTTTTTTTTTTTAAATAACTAAATCTGTTTTTTTGGTTCCCCCCAAAAATTGAAAAGGGTCCCCCCCCCCCCCCTTCTTTT
TATTAAAACAAAATTTTTTTAAATCCAACAAAAACTTTTTTAACCTAAAAAACCCTTTTTTTTCGATTTAGCCGAGCTTTAAA
TTGGCAAAACAAAATTTTTTATTAAAATCACATTTGGGGTTTAAAAAAAAAACCGACCCCTTTTTCCTAAAGGAGGTAAGGTTT
AAAAAAGGGCCAAAAAATTTATAACAAAACCATTTTTGGCGGTTTAAATCAGAGCCGTTTGTCCCTCCCCAAAGGGTTTTTTTT
TTTTTTTAAAAAAAAAAAATGGGGTTCCTGAAAGATTCCAAAAAAAAACCGAAAAAACCCCAATTGTTTTTTAAAAAACCGCCCCT
CCCCGTTTATTGTCCAAAAAATGAAACAAAAAAAGCCACTTCGACCCTCCTAAAAAGGGTTTGGGGGGTTCGGTAAAAATTTTT
AAATTGAAATTTTTAAAAATGTGGGCCCCCTTTTTTTTTAAACCTTTTCCCTTTTTTTTTAAAATTTTTAAAGTTTTTTGCCCC
ATTTAAATATTATAATTTTTGCCCCTGAGTGGGGTTTTATTTTTTATAAATAAAAACCTTTTTTGGAAATTGAAAAATTGGGTG
GGGGGTTTTTTTTTTTTTTTTTTTATAATATGTTAAAAATAAAAATTTGAAAATTTATTTGCCCCAGGGGGGGGGCAAACGACCC
CCCCCCCCCCCTTTTTTTTGAATTGGGGGTTTGGGGTAAAAAATGCCCCCCTTTTTTTTAATTGGTTTGTTGGGGGGGTTGTTT
GGGGGGGGAAAAAAAAAAATTGTTTGGGTTTTTTTTTTTTTTAAATTCTTGTTTTGGGTTTTTTTTTTTTTTGTTATTGAAACCTAT
ATGATTTTTTGGGGGGGATTGGGAACCTGGGAAAAAAAGGTTGGGGGGTTTTTTTTTTTTTTTTTTTTTTTTTTTTTTTTGGATT
TTTTTTGGTTTTTGTTTTTTTTTATCCCAATGGCCCCTTGAAACTTTGGTTGCCGGGCCGTCTCCCATTTGATTTTTTTTTTTG
CCAAAGGGCATTATTTTTGGAAGTTTTATGCCCTTTTGGGGGCCCGATTTTCCCGATTTAAGACCCCCCCTTCCGCCGGTAAAA
TTTAGGGCCAAAAGATTGAAAAAAAAAATGAGCGGGTTTAAAAAAAGGGGAAAAAACCTCCCCCCCCCAAAAACCTACCCCCA
ACAGAAAATTTTTTGGGGGGGTTTAATAAGTTTTTGGGTTTTTTTTTTTTTTAAATCGAAAAATGGGGGGAAAAAAAAGGCCC
CAAAGACCCCCCTTTTTTTTTTCACGAAAAAAAAACCCAAGAATAGGATTGAGCTTTTTTTTTCGAGGAAAAAAAATTGAAAGG
GGCCCAAAAAAACAAAAGCCCCCCCCCCTTTTTTTTCCCCAGGGTTTAAATTTTGGGGGGTTTTTTTAAAACAGATTTTTCCC
CCCCCCCCAAAAAAATCGGTATTTTTTTTTTTGTGGGAGGAGGGGCCAAAAAACCAAGTGCCCCCCAAGCCAGGCCCTTTTTTTT
TTTTTTTTTTTTTTTAATTTTTGGTTGAGAATTTGGGAATGAAAATTAGGTTTTTTTTGGGGAGCCCCCCCTTTTTTTATTTATG
AAAAAAAAGAAAATTTTTTCAGGAAAGGCAAATCCAACCCCTCCCCAAAAACCCCTTTTTTTCTGAGGGCAGTCCCTTTAGAAC
CCCCCACTTTTTTTTTGCCGGAACAAGGGTTTGCCCAAAAGCCGGAAAAAAATTGAAAAAATTTTCCAATTTTTTTTCCCCCTT
TTCCCAAATCCCTGGATTTATTTTTTTTTTTTTTTTTTTCAATGAAGGGGGTTGGGGAAGGGAAAAAAATGAGGAAACGAGGGG
GGGTGGGCCCCCCCTTTTTTTTAAAAAAAACCCCACTAGGTTTTTTTTTTTTAACCCAATGTTCCAAATTAAAGGGAATTGCCC
CCCCCGCCGTCCAAGGAGGGAATTGTTACTTTTTTTTTGGGTGGGTTCCCCACCCCCCCCCCCCCCAAAAGAAAAACGAGGGGG
GGGGAATGGGTGGGGGGGGCAAGTTAAATGGAAAAACCTTGGAAAAAAGAATTTTTTAAGGGGGGGGGGGAGACCATCTTTTT
TTTTCCAACGAGAAAAAAAAAAAAAATTTTACTAAAAAAAAAAAAAAAAACCAAAATTGGTTTTTTTTTCCCCAGGTTCCCCCTTT
TTGAAGGGGTTTGCAAAAAAAGGTTTTTTTGGGGGGGACCCCCGAGTTTTTTTTCTTTTTTTTTCCCCAAAACCCTTGGACACTT

CCAAAAAAAAGGCTGGGTTTTTTTTAAAAACGGATTTTTTTTTTATAAAGAAATTAAGTAGCACTAAAAAGTTGTTTTTTTTG
GACTTGGTTTTGGGGCGGGAAAGGGGTTTTTTTTTTTTCGGCAAACCCACTGGCCCTTTTTTTTTTTGGGGTTGGGGGGGG
GGCCCCCCTACTAAAAAAAAAAATTTATTGGGGCAAAAAAAACATTAAGGAGGGGAAAAAAAAATCCAAGCTTTTTTTTTTT
TGGGTGGGTTTGGGGGGAAAAAAAATTTTTAAAAACCCGTTAAAAAAAAAAAAATGGAATTAAAAAGCCCCCGTTGGGAAATTC
CCCCCCCCCCAAAAAAGAAATTTAAACGTTTGGGAATCCTTTTGAGGGACCTAAAAACCAAAAAAACCTAAGCTTTTCAAAAAAA
GTTAGTTTGGGAAAGAAAAAGGTCCCCCTTAATCAGCTGAAGGGGCCCCGAAAGGGGATCCCAAGATTTTATGGGGGAGAGAGA
GGGCCAAAACCTGCCCTTAGGGGCACGATTTGGGTTTTTCCCCCCCCCGGAGGGACCCCCGGTGCCTTCAAAATTTTTTTTTAC
TGGTTTTCCTTTTTTCATTTTTCCGGGGGGAAAAAAAAAAGTGGCCCCCCCCCCCCCTTTAAAAAAAATTGGGAGCCAAAAGAAA
AAAAGAAAAAGGCCCCCCCCAATATTTCCCCTTTGGTTTTTTCCTTCCCTGGGTTTTTAAAAAAAAAAAAAAAAAAAAATAACA
GAATAAGGAAAGGGGGGCCACCGAAGGGGGTTTTGGGGGCCCCCCCCCACCCCCCTTTTTTTTTTTTTTTTTAAAAGTGGGGGG
GCCCCCCGTTTTTTTTTTTAGGGGCCCCCCCCCCCCCCGAGGGGGGAGGGGGACAACAACCTGGGGAGGAAGTTTTTTTTTCCC
CCCCCCAAAAAAGTTGGGGGGTTTTCCCCCACCCCCTATCCGATTGCCCCAAGAAACCGAACCGACAAAAAAATCAAGGGGAA
AAGAAGGTGGGGGGTTAAGGAAGGTTTTTTTTTTTTTTAAGATGCCCCCCCTTTGGGCCTTTCACCAACCTTTTTGTTGGGGGA
AGGTTTATTTTTTTTTGGGGGGGTTTTTTTTTTTTTAAGTTTCCCCCCAATCTTTAAAGGAAAAAAAAAGGGGAACCTTACCCC
ACAAGCCCCCAGAAAAAAAAGGAAGGAAAAACCAAAAAGAAAGAACGACAAAGCCCCTTCAAAAAACATCCAAAAGCCCTGAAAA
AGGTTTTTTTAATTTCCCCCACCCCTTTTTTGGACCCCCCCCCCCCCCAGAAGGGTTGGGAGGCTTTTTTTTTTTGGAGGGGCCT
TTTTTAAGTCCCTTTTTTAGTTTTTTTTTTTTTTTATTGGAAAAAAAAAACCCAAATGTCCCAAAAAAGAAATTCCCTTTTTTTAA
AGTGGGCCAAAAAAGTTTTTTCCCCCCTTTTTTTCCCCCCCAAATTTAAAATCTCTCCTTTCCAACCCAAACAAAAACAAAAAC
CCCGGGAAAGGGGAAAGGAAAAGGGGGAAAAAAGAAAAGAGGGGGAAGATTTTTTTAAAATTTGGGAAGAAAAAAAAAAAACTG
GCCACCCTAGGAAAATTTCCCTTTTGAAAACATTGTTTTCCAAATTTTAAAACTAAGGGACTTTTAAGCCCCCCCCCTTTCCCAA
AAAAAGCCCCTTTTCAAAACTTTCCCCTTTTGGGTTTCCAAGGTGAAAATTACTCCCCCCCCCCCCCAAGGCCTTTTGGAAGTG
GAGGCCCCCTTTGGTTATCCGTTTTCCTTTTTTTTTTTCCCCTTTTTTTTTTTTTTTGGGTTTTTTTTTTTTCCTTTTTTTCCT
CTTTTTTTCTTTGGCTTTTTTTTTTTTTTTTTTGTTGAGGGGTTTTTAAGTCCCCCTTTCCTTAAGATTTTGAAAAAAAACTTT
AAACCCCAAACCCTCCCCCCAAAACAAGTGAAGCAAGGCTTTTTTTTCCCCTTTTTTTTTAAAACCCCTTTCCCTTACCCCACT
CCCTCCCCACCTTGAATGTCCCCCCCGTCGTCCCTTGGGCATTCGAAGGAAAACACTTTTTTTTTTTTTTTTTTTTTTTTGGG
AAAACTTTCCCCCCCTCCCCCAGTTTGGTCCTTTTTTTTTTTTCCCCCCCCTTCCCGCTTAAAAACGGGGCCCCAAAATAAAAA
AAAGCCAAAAAACCTTTTATCGTTTTGGCCCCCCCCCCCCCTCCCCCCTAAAATTTTCTTGTTTAAAAACCCAAGAAAGAGGAA
AAAAAAGGAACCAAAAAAAAAAAAAGGGAATTATGGGGGGGGGGGCCCCCCTTTTCCCCTTCCCTTTTTTTTTCCCCTCAATTAG
GGCCCCCCCAACTTTTTCCCCCCCCCGAAAAATAGGAAACCCCCCCAGTAAAAAAAAAAAAAAAATGAAAAAGGCCCCACCGGGTC
CCCGCTCCCCGTCCCTGCCCGACTCCTGATCCCCCCCCGAAAATTGTGTGCGGGTGGCCCCCCCCCTGAAAGTCGCGAAGAAGGT
GTGGGGGCCCCCGCTCCCGTCCTTGTTGTTTTTCGCCCCCCCCTCCCCGAAAATTATTGCTTGTTTTTGAATTGTCCTGTTTCT
TTTTCTGGGCTCCCCCGCCCTCCGTCCCTTAGCCTTTGTGTTGAAATGGGATAGACCTTGTTGTCCCCCCTGGTCCCTCATTGG
ACTACCTTGTGTTTTGGGAATGCTTGTGTATAATGTTGTTTGGTGTTATTGAATGATGTCCCCCCCTCCCCCCCTTTTGGTCCA
GATTGTTTCCCGAAAGAACGTTAATTCGGGGTTTGATTGTTTTGGAAAACAAGTCAGACGGGGGCCCCCCCCTTTTTTTGTTTT
TTGTGGGGGTGAAGGTAAGAGTCCCCAATGGTGTTGGTGTGTGGAGTTGTGAAGAACGCCCCCGGTTTTGTTTAAAAATGAAGT
TCCCCCCTCCCGCGCTATTATAGATTTACACCTCTCATATAAACCTGGAAGTTGTGTCCCCCTCTAATTTTGTGGCCCCGAATT
GTTGATTAAAGTGTGATAGGCCCCCCAGAAAAAACCAGTTAAAAAGGCCCCCCCCACAAAGTGGTGCGGAATACCCCCCTTGTT
CGCCCCAGAACCGCAAAAAAAAAAAAAAGTTTCCCCCCCGCCCAAAAAAATACTTTTTGTTGGTGGGCCCCAAAAATGTATTCCCC
CCCCCTTTTTGTTAAATTGTTAGACCCGCGTTTAAAAAAGAGACGCGGTAAAGCACGCCCTTTGTTTCCCCCTTTTGTGAAGGG
GTTTCAGGAGGGTTTTGTTGTGTTCAATTTTTTTGTGGAAATAATAAATACCCCTCCTGTCTCCACCGCGTTTTGTTTGGTTTT
TTTTGTGCCCCTCATAACCCTTTGTTTCAAAGAAGTACCCCCCCCCCCCTTTGTTTTTGGAAAACGCATTGTAGGTGTAGATAG
AGTAATTTGTGGTGTCCACTCCCGTTGTTGTTTGTGAGAATAAAGAAAAGAAGATGGTTCCGCCTGTAAAATTTCCCCCCCCCT
CCCTGTTGTGTTCCCTCTTCCCTGAGAAGAGGTCAGAGAGTGAAATTTGTGGCCCCATACGAATATAAAGATAGTTTTTCCCCC
CCGTTGCCCCCGCCGTTCCTCCCTCCCCGCCCCCTGCCCTTGTGGTGAAACCGCCAATGTCCAAAGATAACTAAAAAAAAATCG
CCTTCCGCCCCCTTGACGCGTGGCCGAATAATTGCGGTCACTTGTTTTTGTAAAAATGGAATAAAACGAAATGGGTTTTAAAAT
GGTCCTTAAGGTGGGAGTGTGTGAAAAGGGGCCAGAAAAGACTTGGTATTTCTGTCACCCAGTGTTTTTCCCCCAAATTGTCCG
CCCCCCCCCTGTTTGCCCCTCCCGCCCGAAAAAAAAACCCCTGCCCTCTCAGGGAAGAGTAGTATGAAAATCCGCTGCCAGGAG
AAGATTGAAGAAAGGGTAAATAAAAAAGAAATTTGAAATTTGTGTTTGTTTTTTTTGTTTCCCCCATGAAAAAAAATCCGCCTT
TTGGCAAAAGAAAGTAAGATAAGGACTGTGGGGTCCACTCCCCGGCCCCAAGTGGGGGTGGTTTTCCCCCCGCTTGTTAGAGAA
ACGGTGTATGCTCGCCGTCGTCCCCCCTTTAAAAATAAGGGGTTGTGTATGGTTGAGGGAGAGAAGAGGTGGGGGGGGGGAAAA
AGTGGGGCCCGCGTGCCCCCCCCCTTTTGAGATCCCTTTTTTTTTATTTTGTTTTTTCCCAATATAAAAAAGGCCCCCCCGCCCA
CCCCCCTCCCGAAAAAGACCCATAAAATTTTTTCCTCCCATCCAAAACAATAAGGCCCCTGGGGGGCCCCCCCCCGCCCCCACC
CTTTTTGGCACAACCTGGGGTGGGGGGGCCCCCCCAAAGAAACAACCCCACCCGATGGAAAATTTTTTCCCCTGGTTTGTAAAA
CCACCCAAAAAAATTACCCCCCCTGGAGGGGGGGGGAAGGGGGGCTTTGATTTTTTTTTGGGGGGTTTTT
>m141120_105518_42134_c100732402550000001823141405141504_s1_p0/11980/2707_9505
TTTGGGGGTCTTTTTTTTCTTCCTTTTTTTTTTTTTTTTTGGGTTTTTTCCCCCCCCCCCCAAAAAGAAACCTTCCCCCCTTT
TTTTTTAAAAAAAATGCGCAAAGATGGAGCCCCCTGAAAAAAAAAAATTAAAGGGGGCCCCCTTTTTAAAGCTTTTCCCCCCAA
GAATTTTGCGAATTTTTTTAAGTTTAAAGGCCCAAAAAAAAACCCATTCCCAAAAAAAAAAAAAACTTTTATTTTTTTTTTTTT

TTTTGATTAAGGTGGGGGGGTAAATTGGGGGGTGGGGGGGGAATTTTTTTTTAATGGAAAAAAAACCTAATAAAAGCCCTTTT
TTTTTTTTTTTTGGCTTTTTTTACCGTTTAAATAAAGAACCTTAGGAAGCCAAACCTTGGGGGGGGGCCCCCTTCCCCCACAA
TGTTTTTTTTTTCCCCCCTTTTTTTTTTCCCCCGGTTTTAAAAAACTAAAAGGTGGGGGCCCCGGGACAAAATTGATTTTGGCC
CCCGGCGGTGGGTATCGGGTTTCCCCAAAACCGAACCCAAAATTAGAGGGGGTTAAAGAACCGGGATTTCCTTCCCTTTCCCAC
CGGTTTTTTTTTTTTTTTTCCAACTAGAAAAAAAAAAAAAATTTGGGGGTTTGGGGGATTCGGGGCCCCCCAGAAAATGTTTTTA
TTTTTTCCTTTTTTTTTTTTTTTTAAAAAGTTCTTTTTTTTTTTTTTGGGAAACCCCCCACCTTTTTCCCACAATTCCAAAAAAA
AAAAAGCCCCCGACAAAATTTTCCCGGGAAACCCTTTTTTTGACCCCCCTTTTCCCCCCCCCCCCAAAGGAAAAAGATAAACC
CGTTTTTGAAAAGGGGCCAATGGGGGATTGCCCAAAAAAAAAAAAAAACCTTTTTGGTCCCCAAAAAAAAAAATTTGGCCTTTT
TTGGAAGGGACAAAAAGCCCGGACTTAATCCCCCGCCCCTTGGGTCCCCCCAGGGCTCTGAAAAACATCCATTTTGTGGGGCCC
TTTTTTTTTCCCCCCCCGGTTTTGGCCCCCGAGGGCCCCCCATTGGGGTCCCCCCACAAAAAAAAATTTCCAGTTGGCCTTTTT
TTTGATTTTTTTTTTTTTTTTTTTTCCTTTGGAAAATTTTTTTGGGGGTCCCTCTTAAATTTTCTTCTTGGGTTAAAAGCCTTT
TTTTTTTTCTAAAACCTTTAAATGAAAAAATACCTTAAGAAAAAACCTTTCAAAAAAAAACGGAAATTGCCCCCCACCAACCCA
AAAAAAGGGTTGGCCCCATTTTTTTTTTTTTATTTGTTTTTGGGGAATTTCAAACTTATCCCCCCCATTAACCCTTTTCCCGA
AAAAAATGAATTCCCCCCGTTGAAAGGGCCCTTTCCCCCGTGCCCCAAATTCCCAACATTTCACCAAAATTGGTGGGCCTTCCC
TGGGAGGGGGGGGGGTTCCCCTTTTTTGGACTTTGGGGAACCAAAACAAAAAACAATAATTCAAAATCGATGGGGTAGGCCCCC
CTCCCCCCCCCCCAATTGTTTCCCAACCCCCCCCCCTTTTTTCCTTTAAAAAAGCCCCCCTGGGGGGCCTTCCGGAAAACCATT
TTCCCTTTTTATTGGGACCAGCCGCTTGATTAATGTGGCCCTCCAAAGCCGGTTTTTTTTTTTTTTTTTGAGTCCCCCACCCCC
CAAAAAAAAGGGGCACCAAAAAAAATTTGGGGAAAAAAACGACGGTTTTAAGGAAGTTTTACAACCCCCCCCCTTTTTTTGGTTC
CAGGTTTTTTTTGGGGAAAAAAAAAAAAAAAAAAAAAAAAAAGCTTTTGCGGTTTAAATTGGGTTTTTTCCCCCCCCCCCTAACA
GTCCCCCCATTTACAATTTTGCAATAGAAGAAAAAAAAATCCTGGTTTTTTTGGTTTTAAAGCCCCCCCCCCCCCCTGGGGAAAA
TTAGGCCCCCCCCCAAATCCCCAAATAAAAGGCTCCCCCAAAGAATTTTCCCCCCGGTAGCCAAAGGATTTTGGTTTTTTGGGC
AAAAAAAATCCAACCCCAAAATCCTTTTTTTTTTGAATTTCGATTTCCCCCGGAGGGGGAGCGGGAAGCAAAATCCCTTTTTTT
TTTTGTTTTTTTTTTTTAGGACCAAGCCCCCTTTTTTTTTTATTTGGAAAGGAATGTTTTTCCGTGAAAAAAAAAGGGTCCCCCC
CCCCGATTGCAAAACAAAAATGGGTTCCCCCTTGCCCCCAAAAAAAGTTCCCCGTTTTTTCCATTTCCCCCCCTTTAAAGGCCA
AACCCGAACAAACCAAGAATTTTTGGCCCCCTAGCCCCCCCCCCAACACGGGGGGAAAAAATTAAAAAATAAAGCTTTTTTTTT
TGGTCGGATTAAAGTTTTTTTGATTTATTTCCCCGGGAGTAAAAAATAACTTTTTTTCCAAACTTTTTTCCAAACTTTTGTTTA
AAGAGCCTTCCCCGCCTTTTTCATTTTTTTCCCCACCCCCCTTTTTTTTGGGGGGGTCCCCTTCCGTTTTTTTTTTTTTTTTTT
TGGGTCCGGAAAAAACCTTTTCTGGGGGGTTTTTTTTCCCCCCCCCCGTGTTTCCCTTTTGCGGTTTCAAATTATTTTTTTTTT
TAAGGCGTTTGGGGGGCCAAAAAAAAATAAATTGAAAATATGGTGGTTTCCCCAAATTCCACCCCCCCCTGGGGTGCGGAGTGT
GAATCCCCACCCGGAAAAAAATGAGGGAAACCAGGGGCCCAAAGGAAAAAACAATCCCTGGTTTTTCCTTTCCTTTTTTTCCCCC
CCCCCAACCCAAGGCGATGAAGTTGGCAAAAAGCCCCCCGGACCCCTCTTTTGTTTTAATACCTTTTTTTTTTTAGCTTTTTTT
TTCCCTTCCCCCGACCTTTTTTTTTTGAAAAAAATTATACCCTTTTTTTGGGGTCCCCCGGGGGGGTTTTTCCCCCGGGTTTG
TCCCCGCCTTTTGAATGCAACACCCCCTTCCAAAAAATTTTTTTTTTTTTTTCCCCTCCAATGGGGCCCCCCCCTTTTTTTTT
TTTTCCCAAAGTATAAAAAAAAAATGCGATATCCTCCCCCCCCCTATCCCCCGTTGGGGGTCCCCCAAGCCCTTTTTTTCCCCG
TTTTCTTTTCCTTTTTTTTTTCCCCAAAAACCCCAAAATCCCCTTTTGTTAAAAAAATTGGCCCCCCGGGGGGAAAAGGAAAGG
CCAAGAACCTGGAAAATCGAGGGTTTTTTTTTTTTTTCCCCCCTCCTTCCAAGGGGAAGGTTTTTTTTTTTCTTTTTCGCGGA
ACCCCCCATTGTTAAAAAAAAATGGGTTTCCCCCACCCCTTGGGCAAAAAAGATAACGTGGGGGGTAACGCAAATTTTTTTTCG
GGCCCCGTTTTTTCCCTTTTCCCAATTTTTGATTTTCCCCGGGGCCTTTTGTACCCTACCCCCCCCCTTTTTTCCCCCCCAACAA
CGTTTTGTTCCCGCAAAAGGGGGAAAAACCCTTTTTTTTTTTCCCCAATGCAAGAGGGGGGTGGGGGGGGGGGCCGGAATTGGAG
GCCAAAAAAATTAAGCCCCCCCCGGAAAGGTCCGAACCCCAGTTTGGGTTGGGGGGGCCCCCCGACTTGAAAAGTTTTTTTTTGG
GAAAAATTGTTTTTAAAAATACCCGGCCGGTGGGGGGGGGGAAAATTTTTCTTCTTCCTTCCCCTCCCAAAAAAACAAAACAAA
ACAAAACGGAAGGGGAAGGGGAAAAAGAAAAGGGGGGAAAAAGAGAGAGGATTTCCCCCCACCCCGCCCCCGGGGTTTATTTAA
ACAAAATTCCAACCCCACCCTTTTTTTTTTTTCCAAAAAAAAAAAAAAATAAGTCCGGCCCCCCCAACCAAAACTGGGTCCGAC
CCTTTTTTTTTTTTTTTCCCCGGGGGCTTTAAATGGCCCCACCCCCCCCTTTTCACCCCCCATTTCCCGCCCCAAACCTTCT
TTTTTCTTCCCCCCCCCCCTTGCCAATGAAAAGTTTTCCCCCCCCTTTGGCGGACCCAAAAAAACGGAGGGGTTTAAAAGGGCC
CCGGGGGGAGAAATTTTCAAAAAGAAAGAACGCCCCGAAAAAAAAATGGGTTTTTAACCCCGAAGACCGTTTAATCCCCCCCTT
TTGCCAGACCCCCCCCCCAAAATTTTTTTACAATCTGGTTCCCCCCCCCCCCCAGCCGAAAGAACCCCTCCCTTTTTTTTGGAA
AGGGAGGGAAAAAAACCCTCGATCCCCCAGGTTTATTTTTTCTTGGCCCTTTTTTCCTTTCCCCCCCCGCCCCCAATTACCCCA
GAAAATTTTTTTTGTTTTTTTTTTCTTTTTTTTTTGGGGAAAGGAAAAAAGAAAAAAACCCCCGAGCCCCATGGGACCAAAACC
GGATAAGGGGGGGAGGAATTTTTCGCCATAAAACTTTTGGCAAGGCCCCCCCCCATGGGAAAAAAAAGGAAAAATTTTTTGAAA
GGTGGTGGGGCGATTCAAAGCCCCGGACCACCCGAACCCCCCGACCCGAAAAAGGGGGGTTTTTTAATTTCCAAAGTCCACCAC
GGGAAAAGAAGCCTTTTTAAGTACCAGAGGTTTTCCCCCCCCGGGGCCCCCCTTTTGCCCCCAAAAATCACCCAAATCCCCCGC
CTTGGGTGGGGTGAAAGAGAACCAGGGGGGGGATTTGTCCCCCCCCCTGGGGGACCCCCTTTGGGGGTCACCACCTCCCCAAAAA
ATTCCGGGATTCTTAAGTTTGAAAAAAAAAAAAAAACCCCCAAAAGGAAATTTTGAAAACCCCCACAAATATCAAATTTAAAAAA
ATTGCCACAGCCAAAAACGGCCCCCCCATTTTAAAAAAAATTATTTGGAACCCAAAGGGAAAACCCCAAAAAAAAACGACCCCCC
AGTCCCACCGACAAAAAAACCGGGGAGAAAAAAAACCCCAAGGGGTGGGTCTTTTTTTTTTTGGGGGAAAAGCAAAGCGGAAA
AGCCCCCTTCCTTTTTTTTTTAAAAAAAAAGTTTTGGGAAAAAAGTTTTAATTTGGAAAAAAAGTTATAAAACCTTCCGATCA
ACTGTTTTTTAAATCCCCCCGACAAAAAAGCCCTTTTTTTTTTTTTTTTTTAATTATCCCCGTGTGGCTTTTTTTTAAGCCC

AATCTTTTTTTGGTGGTTTTTTGGTTTGCCCCCTTTTTAAAGATAAAAAAAAAACAGGGAAACCCTTATGGGAGGAAAACAAAA
AAAATGCAATCGACTTTTTTTCAAAAGGATTTTCCTCAAAATTTAAAGTCCCCTTTTTTTTGTTCCCCCTTAAAGGATGTTTG
GCCCTTTTCCCCCGCCCTTTTTTCCCCCCCTTCGGATTTTTTCGAATTTTTTTTTTTTCCAAAAAGATGGTTGGAAAATTTTTG
CAAAACCATCCCGGCCTTTTACGAAATTTCTTTTTTGAGCTTTTTTTTTTTATTGGAAATTTTGGCCTTTTTTTTTTTATTTTT
TAAATCTTCAGGCTTACAACCAGAATCCCTTCTTTTAATGCCATTTGTTAAATGGACTTTTTAGAAAAACCAAATAAACCCCAA
GCCTTTTTTTTTTTACCAAAAAAAAAACCTTTTTTGACCCCCAAGGGGGGGGTTTGGTAAAACTTTTTTTTCTTTTTTTAAAAC
ACGTTCGTCAAATGGCCCCCCTTTTTTTTTTTTGGGGGACCCTCCCAAAAAAACCGCTGGAAAGCCCCCACAATGAAATCAAG
CGGCGTTGTTTTTTTCCCCCAATAGAAAAAAAAATTTGTTTTTTTTTCCCGAAGGGGGCCCCCCAAAAGCCTTAGGAAAAAAAA
AGGGAAAAAAACCAATGGAGGGCCCCTACCCCCATCGAATGAAAATAATTTTTTTTTGGTCAAAGTTTTCCCCAAGACCTCCAG
AAGCAAAACAAATTGTTGAATTTGAAAATTTTTTGGCCCGAAGCCTTCAACGAAATCAAAAATTTTTTATATTATCGAAGGTT
TTTTAATGGGGTAATTTTGGAAGTTTTTGAAAATCCCAAAAAAAAAAAATTTTTGAAACCCCCTTTTTTTTTTTTTTTTTTTGG
TTTTTGGGTGGCTCCCCCGTTGAAAGTTTTTTTTTCCCTTAAGGAAAATTTTTTTTCCCAATAGTTTTAAAAAAAGCCCTTAA
CCCAAGAGAAAAATAGAGAACCAAGAATTCCAGAAAAAAAAATAAAATCCCAATAGCAACTTGATTTGACAAAAAAAAATGCCCC
CTCGCCCACGGAAAGCCAAAAAAAAAAAAACCCAAAAAATGATTGTTTTTTTTTTCAAGAACAGCCCCTGGAACAGGGGCCGGA
TTTTAGTCGCCCGTTTTATTTTTTGTTTCCTCAAAAAGGCCCATTATCGGAAACAAAAGTGGCCAATCCCCCTAATTCTTTTTC
AATACGTCCCCTTTTTTTGGGGAAGGTTTTTCCCAAAAGGCTTTCCCAGAAACATTGGTTTTTCGGCCTTTTTGAAACATGTGA
AAAAAAAAAAGTTGGGCTTTTTTTTCCAAAAAGAACCCTTAAAAAAAGAAAAAAAAATCTGGCCCGATTTTCCAACCAAAAATT
CCCTAGTAGAAAAACCGGTGGAGGGAAAGAAATCCGTTTCTAAGCCTTTCTTTTTTAAATTTTGGTTTTACCCGTGACCCGAAA
AACAAACGCCAAAAATCCAAAAATGGTCCAGCAAAATAGTACGAAGTAAAAAAAACAATTTTTGGGGGAAACAAGGCCCGCTTT
TTCCTAAGTTCCCTAAATTACGGTAAGCCCTAAAAAAAAAAGGCTTAAAAATTTTTAGGTTTCAAAAAAAAAAATTAA
>m141120_105518_42134_c100732402550000001823141405141504_s1_p0/15326/2038_14450
TCCTACGTACACCAAAGAGTGTCCCATTAAATGGAATCGATAACAAGGATTAGATAATATCCCTCTAAACATCTTGAATTGTTC
AACCCAATAAATATTTCAGCATATAACTATTAAGCTCAAGATACGAATTCAGGCAAATCTTCCGAGATATCTGTCGTCACGACT
CGTTTACACGTTTGATTTTGTTTTTTCTCTTTTGCTATGCTCAGAACAAATACATTGACGATGTCGCCACCATTCGAACTCACC
CTTTCTGAGCTCGAACTAGTCTAGAAGGCAAGAGATGGTTCAGGATATATCCAAGTGTCGGCAACCATGCTGTTTTGCGAGCTC
ATAGCATACTTCGAGGCCTGGCACATCCTCGAACCTTGAGGTTTTGTTTACATAAGAACGTTCGGCTGAAGATCATGTGACCGA
CGCGTTCTATTTCGAGCTTTCACCTAACGAGCTACGAGAATTCCGGGATCATCAAACTTCTCATGGTTGAAATCTGGGGTGTAA
CATACCCTAGAACATGATTCCAGGGCCCATGCCAATTATATAAATGTTGGTTTGCACTATATGTGGTATTTTGCCATTGGGTTT
CATGCATATAAGTGCCCAAGTAAAATGTTTGTTATGCATATAATGGAATTTTTCCAAGCATAAATTATTCATAGATGGAATGTT
AATTTGGCCATTATAAAATGTAAGTTTAAATTCTATCTCTTGTGATTCCACCTTGAAGGCCCCATTTGCTTGCCATGGACTATA
GTGGGTCTAAAATCAATAATAACAATTAATAACAAAGACAGGTTTTAAATTCTGTCTTTGGACCCTTTGTATCAGAATATAGCA
TTTGTAGTGGGAAGGACATACTGGACCCCCAGCCCCATCCTCCGCATACAGGACCACAATTGTTAGACGGCAACATGTGTTAGC
CTGAGTTGAGCTGTAATTGGTATACGGGATGTTGCATGGGATATAGAGTGGCAGTTAGGAGGACGCTAAGATAGGATTACGAAC
GAGATTACATTCTAAAGTTAATTGAATGTTGTTTCAATTACGACTTTAGTAATTAGATAGGGAAATTAAACGTTCTTGGTTAAA
AGTTGCTAATCTTACATGTTTTGGAGAGAACCAGGTCAGTATTCGAAAAATAATAAAACATACTATCTTTTTTAATTTTATAAA
TGGACGCTTATTTAAGATTTTATTCGATTCGACACTGTTGGTTTAAACATAGTAATAGCTTAATGCGTCCGATGCCGTTTGACT
TCGTCCAATAGCTTAATAGTCACGTGTAGTTACGTTTTGACAAGGTGTCAGATTTGAAAGATAGATAATTATAGGTCAAAATTC
TACTCAGATCCACCCCGTACGATGACTACTAGAACTATATCGTATGATTATGCGAAACTGTGGCGTCTAGCCTCCATAAAATAA
GACGATTTGTTTCGTATATTGATCGAATAGTTTGTTAATAGCTGTGAGTCCATTATTAAATGATTTACAACTCTATTTAAACTA
GTGGCTATTTTTCACTTTCTCGCCCAACCGGGAAAACACAGGATATTATAGATAGTTAAAATCCTAGAAATAGTGATATGATTG
TTTTTGTATGTTTTCCTCATATCTATATACATATTTTTTGGTATATGTTGTGCTAATTTCTGAAAATTCTGTGTGAATAGAAGT
TATTGACAAACATGTGATTGATTTCTAATTTTATTAATTGATAATTTGCTATTTTAAGTTAAGTAGTGAGTAGTGCTCTGTGTC
CTACCTCCATCCTTCTTAACCTTTCGACTGGAGAATTCACTGGAGAAAACTCTCCTTAAATTGAGCAGAAATGCAACATAGAAG
TATAGTGATGACAACTCTGAGTTTTCATGATTGAGGGAATCCCCAGAATGAGCATTGTGTCCGACGTTCGTGATGTCACCCGTC
AAGTGCTCGTGATGACACACCTCCCCTCACAAAAAGAATGCCGGAGCAGCAGCGCTATTCAGAGGTCTCACTACAATATACGAC
GTGATATGTACTTAAACCGAGCCCAGTCTCGTGAGGGGCGGTAGCAATGGCACTGGTATGCTACAGCGCTCATCTGGAAGTACA
TACATCCAACCTTCGAGATATAGTTCGCTATAGACCATCTTCAATGGTACACAGGACCGACCGTCCAGATCCCCTACGGTGAAC
TGACTCTCGAATTACTAATGTTGGCGGAGGCTATTCTGTAGATATGATACCGGAAGTAACGGATGAACTAAACACTGGAAGAAA
AATCAGCTTACTGTTGGCACTTCAGGGTCGTGCTGCGCTCCGGGAACTGATTGCTTACCTAGGGACATTGAATCCATCAGATGA
GGTGTTTCCCCGTTGAGGGACTCCTTGAAAGAGCTAAGGATACTCGAGAAGATCGAAGCCAGTGAGTCAGGCATAGGTACAAGG
ACCAACATACACAACTCCCCAGAAGAAAGCCTTAACGACAAAGAGGCTCGCATATAACGAGTGCACAACCCAAATGCACAGATT
GCCTAAGGGAAGAACAAAGAAAAACAAGACATAGACAGGGCGTATAAGGATAAGTGTGAGTAGTGCGATCCCAACCAAATAGAA
GGCGCCCATTGGAAACGAAGGATCGTCCTCATAGATATCTCAAGAGACCACGGGACACATGATAGAGAATACACCTGAAGGGTA
TTTTATCAAATCATCCGGGGTGAAGCCAGCGGTTCGATGGCACTTTACGAGTTGGTGCCGCATGGCTTAAGAATTCCATGAGGT
TCTTTAGAAGATTAGACACTTTGGCAGATGCACCCCAACTCATGCGCACAAGTTCCAGTTGAAGGGGTTTAGCCTTTGTAATCG
TATACCCTCGCGCTCGCGCGCGCTACGGCGTTGGTGGCGAAGAGGCATGGACCACGCTGGCTCGAGCTCACCTCCACCCTGAGT
CACACGCGTTGTGCTGGACGCTCGCCCTCCAGGCGCTCCGCTGATCGAACAACTAACCTTTCTCGCTCCACACACCTGCACCCG
CGCTCCCGCGCACACGCTCTCGCACCCTTATTGTCAGGCACACGATAAGGAGAGATATACACACACCTCACCTCAGAGCAGCTC

GGGCACTGGTGTGGGGGTCTGAATGGCATGGAATGAGCAATTTTATATTTCACGTGCTAGGTGTCCCACTCTCACCCGCGCGCG
CGCGCCTTCGCGCCCTCTCTTCCGCTCGAACCTCGCCTCTCCGGCGATCCGCGCGCGCAGTACCCTGCGCCCTCCCGCTCGCTC
CCGCTCTTTGTAACTCTTCGTTGTACGGGTAGAAGCCCGCGAACGAGAGGATACGGACGACAGCCGCGCGGCGGAATCCATCCC
TGGTGATCCCAGGGCACCAGCTACAAAAAAGCCCTCGCGCTTCCAGTTCCAAAGCATCGAAGCAACGAGGAGGAGCATGAAGAG
AACTTGTCATACCGCCTGCCGTCTGGCAAGAAGAGAGATGGGAGAAAACTCGAAGTATAGCAGCGACCTCTCAGCTGGTAGTCA
AGACCCAGAGTTTAGGGGAGTATCAGACCAAGGGAGCAACGAACTGGCGGCATAAGTGTAACCCAAATGAAGGGAGAATTTGCT
CCAAGACGTTCGCGGCAACATTCTATCCGCCAGATCCCCCGGGAGAAGAATGTGTTCGCGGACAACACTAGCGAAGCTGGCCAA
CAGACGGGAGGCCGAGTTGGCCAGGGCTGGTCCCCATCAAACCGTCTTCCCGTCCCGAGCGTCGGGGTTTTCCACCGTTTCACG
CCGTCGACCCACTCCCAATTCTTGGATGGGGCCCCGCTGCTCCGCTACCTGTCCGCCGGGAGCGTTCCCAAACGAGCGGCCAGC
AGCGGCAGGAAGCTCCGTACCCAGGCCACACCGGTTACGTCCTAATGGACGGAAAACTTTATCGCCGGGACCTGTCTATGCCTT
CTCGGTGCGTGTCCTGGGACTGAGCTGGGCGCATTATGCATGAGGTCCACGAGAAGGGTTTTGCGGGGATCACACCAGCGGGCC
CAGCTGTCCAAGAAGTTCTCGCCAAGGATACTTCTGGCCCACCAATGAAGGAAAGGACCCTGCATAGACTACGTCCCAAGTGGC
GAAACAGTGCCAGAGGTACGCGAGATCCCTCGGCCCTCCCCAAACACAGAGAATAACTTTGATGAACAGCCCTGGCCCTTTGCC
GTATGGTTTTTTTGGGAGTCGATCTTGTGGGATCCTCCCGACCGGGAAAGAGGAGTGAAGTAATGCCATTGTTGGCGGTAGAAC
CTATTTCGACCAAGTGGTTGAAGGTGCGGAGCCTAGTGAACAACGATCCGTCCAAGAAGGCCTTAGATTTCGTAATCAGACAAC
ATAGTGTGTCGGTATGGGCCTCCCTACAGATCGTGTCCGACAAACGGGAAGCATTCGAGCAGCCTCCACGTTCACGGATTTTTT
TTTTGCGGAGCCACGGCATAGGTGAAGATTTTCCTCCACGTGGAGGCCCAGGCGAACGGGACAGGCAGAAGCCGTGAACAAAGT
CCTGAAGGGGACACTGAAGAAGAGAAGCTTCAGGCCCTGTAAGGAGCAAGTGGCCCGAAGAGCTCCCCCGAGTGTTGTGGGCCT
ACCGGACGAACCGAACGGACATCAACCGGCATAACAGACCTTACTCAAGGAGTATGGATTGCGAAGGCCCGTCATCCCAATTGA
AACAGACCATCCGTCCCATTCGTCGCGAACAAACGTAATGATCCCGCCCAGAAACCACGCCTCTACTCCCAGGAATCACTGGAC
CTGATCGAGGAGGAATCGGAAGAGTCGCAAGTGCAGTTGAAGATGTACCAGGGCAAGATTCGCATCGGAGCATTTTCCAAACTC
AAGAGTCCAAAGAGCCGTAAGTTTGAAACCGGCGATCTGGTCCTGCGAGGGTCTTCCCTGCCCAACTCAAGGACCCCGGAGTAG
GGGTTCTGGGCCCGAATTGGGAAAGGCCGTATGAGATTCAGCAAGAGGTATGCCCGTGGAACCAATACCGGCTTGAGCGGCTCG
TGGCTCCGAGGTCCGCCGAGCCTGGAACGCAAAACACCTCCATAAACTGCAGCTACAATAGCCCCAGAGCTCATCCCACTTTAA
GTGTTTACACTACATGTATACGCCATCCAGGGATAATTTCCCTGTTCTAAACAATGAAATGACGTATGCAAAATGTCATAAAGG
GCGTTTTGTTTATTTCCATGCGAATGTGTGCCTCAAAGCGAATTCGTTTTTAAACTACTTTCTCAAGTGACCCCAGACCAGTCT
CTGCTCAACTTGCGAGGGGTGTCAATACCCCAAGGCACAAAAATATAAATAAAAAGAGGGACCCGACAGGCCGGTCCCCACCCC
GGACGACACGATGTCCGCGGATATAAATACTAAGTGGACGGCCCAGGCGGTCCACCCGGACGGAAACGCGATGTCCGGGGATAG
TAGAATATAAGCGACCGAGCCCCAGGCCGGTCCACCCCGGACGAAATGATGTCTGGGGGTATAAATATAAGTGGACCGAGCCCA
AGCCAGTCCCACCCAAGGATCGAACGTTGTCCGGGAGTATAAATAAAAGCGGAACCCGAGCCCAAGGCCGGTCCCACCCCCGGA
CGGACGTTTTCCGGGAGTATAAATAGAAAGGACACGAGCAAGGCCGGTCCACCCCGGACGAACGATGTCCGGAGTATAAAATA
AAAGAGGACCGAGCCCGAGGCCGGTCCCAACACCCGGACGAACGATGTCCGGGGGTATAAACCATAAGTGGACCGAGCCCCAAG
GCCGGTCCCACCACCTGACGAATGATGTCGGGGGTATAAATATAAGTGGAACTGAGAGCCCAAAGGCCGGTCCACCTCAAAGGA
CGAACGTTGTCCGGGAGTATAAATAAAAGAGGACCGAGCCCCAAGGCCGGTCCCACCCCGGACGAACGTGTCCGGGAGTATATA
AAAGAGGATCGAGCCAGGCCGGTCCCACCCCGGACGAACCGATGTCCGGGGGTAATAAATATATAAGTTGGACCGAGACCAGGC
TGGTCCCACCCCGGACGAATGATGTCCGGGGTATAAATATAGAGCGGACCGAGCCCAGGCGGTCCCACCGCCCCGGACGAAGAC
GATGTCTGGGGGATAAAATAAAGCGGACCCGAGCCCAGGCCGGTCCCACCCGGACGAATGATGTCAGGGGTTATAAATAATAAG
TGGACCGAAAGCCCAAGGCCGGTCCACCCCGGAACGAACGTTGTCCGGGAGTAATATCAAATAAAAGCAGACCGAGCCCAGGCC
GGTCCACCTGCCGGACAGAACGTTCGCTCCGGAGTATAAATAAAAGCGGAACCCGAGCCCCAAGCCGTCCACCCCGCCGGACGA
ACGTTGTTCCGGGAGTATAAATAAAGCGGACCGGAGCCCCAGGCCGGTCCCCACCCCGGACAGAGCAACGATTCCTGGGAGTAA
TAAATAAGAGGAACCGAGCCAGGCCGGTCCCACCGCCGGACGGAACGATGTCCGAGGGTTATAAATATAAGTGGACCGGCCCAG
GCCCGGTCCCACCCCAGACGAACGATGTCCGGGTATAAATATAAGTGGACCGAGCCCAAGGCCGGTCCCCAACCCGGACGACCA
CGTTGTCCCGGGAGTGATACAATATAAAGTGGGACCGACCAGGCCCGGTCCACCCCGGGACGAACGTTGTCCTGGGAGTATAAA
TAAAAGAGGAACCGAGCCCAAGGCCGTCCCCACCCAAAACCGGACGACGTTGTCCGGGGGTATAAATATAAGGGACCGAGCCCA
AGGACGGTTCCCCACCCTTGGGCGAACGTTTCCGTGGGAAGTAATAAATAAACAAGAGGACGAGCCCAGGCCGGTCCCGACCCC
GGACGAGAACGATGTCCGGGAAAGTATATAAAAGAGGACCGAGCCCCAGGCCGGTCCCACCCCCGGACCGAACGATGTCGGGGG
TAAATGAAATATAAAGTGGGACCGAGCCAGAGGCCGGTCCCCACCCCTGGACGACGAGTGTCCGGGGTACAAATATAAGTGGAC
CGAGCCCAAGGCCGGTCCCACCCCGGACGAGAAGACGTTGTCCGGGAGTATAAATAATAAGTGGAACCGAGCCCAAAGGCCGGT
CCCCACCCCGGACGAAGACGTTGTCCGGGGTCATAAATAAAAGGAAGGACCGAGCCCAAGGCCGTCCCACCCGGACCGGAGCGA
CGTCCGAGGAGTATAATAACAGGGAACCAAGCCAGAGGCCGGTCCACCCGCACGAGCGCCGCGTCGCGGGGGTATAAAATCAGT
CCCGCCCCAGACCCGGACAGTTTTGGATCATTCGGGCACATAAGCATCCGGACTTCAGCCCCCGGGAATAAACCGGGACCCTCG
TAAAATTCAAGGAAAAATATGACTCGGGTTTAACAAAGTCCCAGGGCCGAACAGGCCAAAACTAGCTCCGATGGTTCCCAGTCC
CTGGCCGTTGGAAAGACCGGGACAAAAACCCCATGGGGCAATCGTGGTGTAAGGTCGGCGACAACCAACTTCTTTACCGAATGG
GTGAAAAGGAAAATATATTTAATAGATACCCCAAAAGCAGGAGCAGTGTTCAAGAAAGTTAAAGTTACAAGGCATAAGCCAAA
ATTACACGAGAGAGGAACAGGCCGGAGACAGAGGATAGAGCGCGTCGCCCGGAGGTCCGCCATCCTCCTCTCCTTGGCCTCGT
ATTCGGCACGGCTTTCGAGTCAGGATCAGGGAAGACCAAAGGAGGTTCATGTTTTTTGTCTTTGCAACCAAGAGGCCAAATATA
AAATGGCCCTCGTCAAAAGCTGACATCCACCATGTTGTCGGCCTCCACCACCTTCTCCTGACGAGTGGACTTGTGCCGCCCGCC
TCCTCCTGAAGAAAGAGAGTCCGCCCAGCTCAAGGACTCGGGGCTGGCCCAGGACCCGATTACTCCTCCTTATCCAGTGATGAG

GACTGCGCCGCGGCCCGTCTCCAGGAGAGTCCCGCCTTTTCGTCAAGGCTACCTTTTTTGGTCCGGAACAGCCTCTTCCTCCAA
TTCCCGTCCGCGCTCTGCATTCGCGCGGGCCAACCTCTTGTTTGGAGGATAGGCCGTCCTCCTTGCCTGAGATTCTCCTTGATG
GCTTTCCCTCGTGGGTTTCACGGCCGCCTCCATCTCACAGCTTGGCCGTTCTCCCATCTTCATGGGCAAGCTCGCCACACAAGG
TCGGCACTCCTCTTTGAAAGATAGCAATCGACCCTAGAACAAACAAAGGTTAGAAAAAGGAGAAGTCTCCCACCAATTGATGAC
TAGGAGCGTGAGTGGAGGAAAACTTTCACCCGCGTAGCCCTGGTGGGCGGCAGAGCCTGAGAAATGAACAGCCCGTTCCGGCGT
TTGGCATCGTGGCATACTCTTCAGCTGGAGGTTGGAACATGGTACTCCCCGAACCTTGGTCGAGGCAGGTCCGCAGCACTGGGG
CAACGTCCTGGCCCAACTTGGGGCGGAGCCCATCTCGGCACTAGGCCGATCCACGGATCGGCTGGGGAGCGCTGACTCGGCAGG
CATTCCGAGAAACTCGACCCTTTGACGACGATCTCGTCCAAAAGGGCCTTGGTAAGCATCGTCCCTCCAAGCTTCGCCGACCTT
CCCTCCAGTCCGGGTTGACCAGACGGGACTTCAGGCTCGTCCCGTAGAAGGCAGACTCCCCCCTGCCTCGAGAGGTTTGCGACG
GAGGGGAAGAGCCAGAGCGGCGGGGATGCTTTTTCGCGGTTAGCCTGCTCCTTCCCCGTGAGACTCCTTCAGCTGTTCTCTGAC
GGCCGTCGTCCGAGGCTTCTTCCCGGGATGCTCCCATCCCGGGCATCACCTACTGGGCCTCCTCTGTTCCCTGAGGCATCCGGC
TACGCGGCCTGTCGCCGTCAATTTCCAGGTTGATGAGTACGGGGGCCTGGTCCGCGGGCAGTCTGCGCCCCGCCTCTACGGCCC
CGGAGCGGGACACGGCCCAAGTGACTCGCCGGGGGCGGGCGCGGCCCGGGATTTGGGACCTCCTGGCCGCCCTTTTTCCCTTGA
GGATTCCTCTCATATTCGCTAGCCGGGGTAGACATGCGGAATCCTCTGCAAAATACAGGGGAAAAAAAGGGTCCCAAGTGCGAA
TTAAGAGCGGAAAGCAACAACAGAAATAAGAAAAGGGTAAATGAGCCGGAACAAAACCCTCGAAGTCTAACGCCCCGGGCGAGA
CTCAATTCCTTAAAGCAAAAAGAGTTGTAATCCGCTCTCCTACCGGTCGGTCCTGGGTTAGAAACCCCCCCGTACTGGAGGAAC
CTGGAACAAGGAACATTAGGGTTTCTGACCCTACAGGGACGGGAAGATGAGGGCCTCTCCCCCCTTCAGCCCCGAAGACGCGGGG
CCCGGCGGCTTAGCTGTCCCTAGCTACAGTCCCAACCTGAGGAGCGTAAGTTAAGACTAAGGGGAGTTTACTCGCCCAGATACG
CTAGCCCGGGAGTTTCCCAACGTTCGGCAGGGCATCTTCAAGACAGCTCTTTTCCAGCTCCCTCCGACGGTGGCCCCTGTCTTC
GCCGGGAGCTTTGTCTTCTTCGTTACGCTCAAGCCGCGGTGACCCGCGCTGTCTCACCAACCGTAGCGATGTTGGTCCCAAGGG
CTAGAGCTGCTCCGAACGTCAACATTTTTCTCGAACGGGATTGCCCCGGAGGCTTGGGGACAACAATGGTCCCTGGGTAAAGCC
CGAGTAGCCCCACCAGCCGGAGATTTTTCGTCCGTTGACTAGCCCCCCAACGTCCAGCTCCTTTCCCGCGGTCAGCTTGGCGTA
AAAGCTCCTCCGTCTCCCAGGGAAGAAGTGGCGGTGAGGGGGTTTTGGTGAAGGGACATGCAACCCGGGAGGAGATGATGAGAA
GGATGAAAGTCGAAAGAGTGAGTAAAATAGAGGGTCTGGGAACTACTTACCTCACTCGCTTGGAAAGTCCCAGCAGCAGCGTGG
GATTTCTCGGTCAACGGGAACCCCGGATGTCATAAAACCAATAGTGCCTTGACATCCGGGGGTGCTTCAGGTTGCTGGTAGGG
CCGGGTAAGCCCTCACGGCTGAGCAGCCCTATAGAAGCCGTCAGGCTTTGTCTTGGTGACGTTTTACCTGCGGAACCAGTTTTG
GTAGAAGTACAAAGACTTCCCGCGGGGGTGGGCTCCGCGATCCCTCTTTCGACGGCGCAGAAGACCTTCCACCTGGCAAGCAGG
ACGGTAAAGGCCTGGGCAAGTAATTGGGAATGGTGGCGACCCACATACGTTGAGGAACCCGCAACGAGACAGTTATTTATGAAG
CGGGAGCGGTAGCTTCGCGCGATGTGGCCGGCGCTTCCAGGCCCCGCGAATCCATTACGGACGTGTGCGCCCGCTCCTCTATCC
TGGCCATGACGGTTGTGGAACAGGCCCGCAGAGTCGATTCGATGCCAGAACGCCTCTCCAGGTAGCAGCCATCATCCGGTAGTT
TCCGGAACAGATTGGCGTCGAGTCCATCTCCCCACCCCCGTGTTGCCCTTTTGGCGTAACTGGAGCCGGGAAACATCGACTACG
GAGCCCTGTTGAACGTCTTCATCCTGAGTGCAATTGTGACGCCCGTCGGTGCCATGATTGCGTCTGGGGACAAGGAAAGGAAAA
CCAAGCAGTCAGTACCTAAACCAAAGGAGTCGGTCGAGTAGCGGGCGAAGATCTCCATAAAGGACTGCTTGAGTCCCCGTCGAG
ATCAGGTCCCCGGACCTCCGGGAAAGCCCCGGACATCCTGAATCGGAGAGGAAAGCTACCTAAGGTCTGCCCTTGTCCAGGGAG
CATTTGGAAACGGTGCAACCCAAACCAGCCGCCTTCCCCTCAAGCAAAGCTCTTAAAGAGGGCACTGAGCCTAGGCACAATGCG
TCTAAAAGGCCTAAAATTCACAAAAGTGGGAATGAGAAGTAAAAAATACCTTACTTGTGTGCAGTCGGACCGTTGAGGGCTGGC
GGTTGTCCGATCGTAAGGATTGGCAGGAACCTTCGTTCTTGAAGTGGGCGCAGCCGGTGGCAGCAGTTCGTCAGCGGGCGCAAG
TCGAGAAGCGCCGTCAGGTTGCCAGAGCCAAAGGCTGAAGCCCTTTGGGAACAGGCGGTGCGTGAACTAGCAAACGGCGGATGT
ATTTTGTCGCGAGGTCGGAACATGCAAGGCTTTCTAGCGAGCGTTCCTGGTTTTCCTTTTTTCTTAGACTGGTTTCGGAAATGG
AAAAGTGCCGAAGTGTTGTTCTTCTGGAGTTTTTAAATTCAGCCCCAAAATCCTGGCCCCCCCGATCCAAACGGTCAGCGCGCA
TCTTCAATCAACGACGATTCGAGGATCGCGCAGGGACCATTTATGACCTTTTTTTTGGTCGGGATCTCAGCACCTCCAATCTAA
CGGTCAAGAAGGTGGCGGATGCCGAAAAGGACGCCCATCCTACGGTCGCCCTGTTTTCGAAGGAAATTGATGATGAATCTCCCG
TGGCGGAGTGGGAAATCGTGACGTGCGCTGACCCACCTAACCTAGGGCCTAAGCAGACCTTGGGAGCCTAGGCGAATCTTCGAA
GCCCTTGGCAGCTTACCTGAAAAGCCACATATGCCACTGTTCTTGAAGCGGGGCCAAGGGGTAAACGCGTCCCGGAGCACAGCG
AGTGGTGGGCCCGGTCCCTTTTCACGACCTTGCCCGGTGACCTCCTGGCCCTGACAGGAAAATTGGGGAAGGAAACGCTCGGCA
GGAACCGCGAGGGTGGTGCAAACCCCTCCCCCCGGGCTAGCCCGGGCGAAGGCTTTGGGGGTAAAATTGTTATCCCCCAATTTC
CTGTCCAGGGTTTGGGCCCCCCTTACTCGGTGGCCCACGTCCCTGGGACAAATGCGGCTGCCTTGGGGCCTTTTGCCCAGGGAA
TGCCAAACTGGGCCTGTGGGGAACGGGTTCCCGGGACCATCAACTCCGGTTCATCTTCAAGAGTGGAACGGTCCCGGCCGTATC
CCGAAGGGCCCGGAATCGTCGCCGGCCGTCGCGTCCCCTTGTCCCGGAGCCCGTTGGATGGTCCTTGGCTATTCGAAGTAAACG
TAAGTGAGCCAAAAGAAGGTAAACGACCCCGGACCGTTTTCTTCCCCCAGGCGGCCAGGACACTGAAATAAACAGACTAAAGTG
TGCCTCATCTACGCAGCGTAGCCTTTATAATAGGTCCCCTGGTCCAAACTATAGATTTTACGATCAACTGCGGTCGTCTGAAGT
TCGGAAATAAAAACTTACATCTTAACAAGGGATGTATTTGTATTAAATCTTGAGGTCATTCCTAGTAGAAATTTAATTTCCATT
TCCCATGGGTTGCAATTAGAACGGATTTGGTTATGAACTTTCAACAAGTTCTAATATAAGTCTA
>m141120_105518_42134_c100732402550000001823141405141504_s1_p0/17236/3783_13605
ATTTAAGTAAGTAGAAATATTTTTCTTGCACTAAAACCATATTATTGTATAATTAATGTGTAATTAATTATATTGAGAATTAAT
CAATATTTCTAATTTAAAAGAACATTAATTTTGAATTATTTATTTTTTGGGGATTTTTCTGGTACTTTAAATAATGAGAAAATT
GGGAAAAATCACAATGCCCTCCCTGGCATGAGTTGGGCGTGTAACACAGTGCACAGTCACTGTGCTACACGAATAAGAAGATCT
AGAAGAATCCCTTGGTTAAACAATCTTGTTATTTAAATATTTAATAAATAATCGAGATATTATTAATATGATTAACAATATTAT

TTATTTAAACAAAAATTTGGATAATGATAACTTATTTAAATTGTTTAAATAACTAAATATTATTTATTTTAATATCACATTGG
ATATATTAAATATAGGAGATCAGTTTTTTCAGAATTGATACTTTCTTTTCAGAGATAGAAGAAATATATCGTGAAAATCTCCTG
AGAGGAAATAGAACAGTGCTACAAGCAGTAGGTCAAATTGTTCTTCAAAATTTAGGTCGCAAACGTTATCAGCATCTCATGTGT
TGGGAACATTTGATAAATAATTTTGCTAATTGTTTGTATAACGAGCCCACACTCGTTCTTCTGTGTTGTTGAGAACATTTTGGA
AGATCCTGGTGTGATCTCAAGGATTTTTGCTTACAAAAAGATTGCAGCAAGGAAGGACTTGAGGTAATTTTCTATTCCATCTCT
TTGATTCAATATATATGATGTATGTGAGAGAAGTAGATCTAGAAAATCTTGTGGGGTTAAATTAGCAAATTTTTGATTGTTGTT
CTGTTGATGGTGAGAACTCGCAACTAAAGTTAAGTTAGAAAAATTGCAAAGTAAAGATTATCAAAAATAAGCTTTAAAAATCTA
AGTTATAAGACTCCAATAACAATTGAGAAGAATGAATGTGAAATCAGTTTGTATTCTCTATGGTGCACTGCTACAGTAATTTTT
CCTTCGCCAATGTGGAATTGGGTTCATTATACTCAGTGGGCTCTAATGGCCCTAGATACCAGCTATGGTCCCAGGGGAACCAGG
TGGTACATAAGTACACTGTCAGAGAGAGTTGGTTTCAGAGGGTGGTGTTGTTTGTATAAGTACATGGCCAGGTACCATGAGGAT
GCCTCCACTACTCAATCGTACAACCTGTCGGAAGGAGTGGCGGCAGGTGTATGGTGTCGACTCCCTGACATTTGGTTGGAGCA
TGCAGCCAGGCCCTCTCTCCCTAGGGGCTCCACTACTTGTCTTGCATGAGTGCCATGGTCCAGGCGTACGGGAGTTCCGTAAGA
GTCAAAGGGTGCACAGGCTAGGTCGCGCGTGGTCAAACCGATCTTCTCGAATGCAGATCTTTGAATAGGAGATTCACTGAACTC
CCATGTATCCACCAGGATCCTGAACCACCATCTAATTGGACACCCAACCTCTTGATCACCAAAAGGTTTCATGGTGTAGGAAGT
TGACTCCTCCGAGCATTGTCCTCAGAGAATGTGATGATTTGGAATCAGTCAACCATGGCATTTGGGCTGGGCAGTTGAGTTAAG
ACCAACACCTCATCATCGTGATTTAAGGCTCACGCCGTATTTATTCTATGAGACTTTGGAAGTGCCCCCCCAAGTGGGGGTCCC
TCTCAAAATGTTCCCTAACTCTAACCATTCACCGGAGGGGGTCAGTTTTTCTATTGGTTGACGACTGAGCTATCGGCCACTAGG
ATTCAGCGATATGGAGCAAAGTAGGGTTGCGTAGGTATTCAATACTATGAATCGATGGTTTGCGAGGCAAGCTCCAAGCATATT
GGTGGAGGTGTCCCAACTTGATGAGGTTTTCGATCTTCGTCCTTTGATGCTGGTGTCACTCATTGGTGTGGTGCCCTATGTCGT
TATGAAAACGACAAAAATTCGGGATCTCACCTTTCCCTGTCCTTTCGGATGGGATGTGCTTTCGGTAGTGAACATGCTGCTTCG
TGGCCCAAGATATTCTCTTGGGAGTCTACCAGAGTAGCTCTTGTTCACATACTTCTCGCCAGAGGTAGCTCCTTGTTTAGCCTG
TGCGATTCCATTTCCCTTGGCTTTTGGCCCTTTGCCCCTTGGCCCTTTGCCCTTGCCCCAGCATCTACTACCGTGCGGGGGTTT
TTGAACCCCGGTGCAACAGAATGGGAATGGAGCAAGCAACTCCGGCATCCAATGCAAGCTAAAAAGCATTGTATCCAGTGGGAG
CTGCCTCTGTATCCCGTCGGAGCCAACGCTGAACCAGTGGCGACACGAACTGAGAACCTGTGGGTTGGGAACACTCAGCCTGGG
ACTAGACTCCCCCCAAACCTGGTTGGGACAGAGCGTAAGGCAGATATTGGATCTCTTCCTCCAAAATGCTCCAATCTTGGCCAT
GCCCTCCCAACTGAATATACTCTTGTGCTCGATTGGAATGAATTCTTTGAGCCATCAGTATCCTCTCGTTGGAGGTCATCCCAC
ACGAAGAACGCCCATGGATGCCAGCCTGCCAAGGCCATCAATAGCTAGCCATCGTCAACCCATCTTGGTTCTCCGCAGCTTCCT
CCTTGAACATTTGATATATGCCTTGAGGGTCTCGAATAGGCCCTTGCTTTATGTTGCCAATGCGTTGACCCCGAGATTTTTTCA
ACCTTTTTTTTCTTGAGTTCCCCCTAAAAAAATAAAAAATTTTGGGTCCCTTTTTTTTTTTTTTTTTTGGAAGGGAAAAGGGGA
CGATTTTTAGGGGTTGGGTTTGGGGCCCAAACCCCCGATATTTTTTTTTGAAAACTTGGGGATCTTCCCCTTAAAAAAATGGA
TGCCCCCCCCTTTTTCCTTTTTTTTTTTTTTTGGGGGGGGGAACCCCCACCTTTCAAAAATTTTTTCCGCTTCGTCCCCCCGTT
AAAAACCAGTTTTATTGGGAAAAAAAAAAAAAAAAAAAAATGAACATTTTAAGCCGTCCCCCCCCCCCCCCTTTTTGAAGGAACGC
CGGTGGGGGGGGCCAAAAAAAACCCGACAAAATCAAAAAACCCCCCTGTTTTTTTTTTGAAACCCCCCCCCTTAGAAAAGGGGT
GGTCCCCCCCCCCCCGGGTGGGGGTTTCCCCAAAAAAAGCCCCCCCCCCCCGCCAATTTCATAAGGGCCGGGAAAAAAAAAAGGC
CCCCCCCCGACCAATAATCCTTTGAAAAAAGCCCCCCTGGGGCCAAAGCCCAAAAGGGGGGCAAACCCCCACCTTTTCCAACAA
AATTGGGGTTGGGGGAAGCCAACCCATTTTGGGGCTTTCCCCCCCTTTTTCCTTTTTTTTCCCCGTCCCCTCCTTTGAAGTCCC
CCTTGGTTTTTTCGTTTGGCCCCCCCCCCTTTTTTTTTTTTTTTTGGTTTTGGGGGGTCCTTTTTTGGGGGCCCCATTTTTCCTT
TTTGTAAAAGCCCATTAAATCTTTTTTTTTTTTTTCCCAAGACTTCACCTTTTTTTAAACTGTTTTCCCCCCCCTTGCGCCATGG
AAAAGCCGGGGTCACCAAAATTTTTTTTGGTTTTTTTTTTTTTTTTTTTTTTGAACCAACTCTTTTTTTGGGCAAAATCGGTTT
TTTTTTTTTTTTTTTTTTTTTTTTTTTTTCCCAAATTCCTTCTTTTTTTTCCTTTTTTTGGGGCATTTTTTTTGGGGGAAAGAAA
AATTTTTGTCCCCCAAAAAAATAAGGTTTTCCCCCCCCCTGAAACGGACCCCCCTTTTTTGGGTTTGGCCAAAAGGCCTTCCCG
CCAACCCCCCGGGTTTTTTTTTAGTTTTCCACCCCACCCTTAAAATTTTTTCCCCACCCACCCCCTTAAAAAACGAAGCCCCC
AAATAAAGGGGGGTTTTTTTTTTTGGTTCCCCCCCCCCCTCGTAAGGGGTTTCCCTTTTGGCCCTTTTGACCCTTTCGGAACG
CGTCCCCGGGTTTTAATTCCCCCTTTTTGGGGGAAAACCCGAGCCAAACCCAAAAAACTTCCCAAAAAAAAGTTTTTTTCCACC
CCCCAAAAACTTTTGGAACCCCCTCAAAAACCCATTTCAACCTCCCCATTTAATTTTTGGGGGGGAAAAACCGGAAAGATTTTT
TATACCCTGAGGGGTTTCCGGCCGGGCCTTTTTCCCCCCCCCCCCCCCCCGCTTTGGGACAAAGAAGGAAAAAAAAAAAAAACT
TTGGGGGGGCCCCTCATTTTGCCCCCCCTTTTATATAGGTGGGGGGGGCAAAATCTCCCCTCTCAACCCAACCAACAACGGGAA
GGGGGAGGGAAGGGAAAAAAAAAAGGAGGAAGAGGGAAAATTTTGCCCCAAAAAAACTTTTAAAATTTAATAGAAGGAGGGCAA
AAAAAAATGGAAAGGGGCCCCCCAGTTTTTTTTTTTTTCCCCTTTTCTGTCCCCCCCCCAAAAAAGCCGGGAAGCCCCCGCGG
GGAAAACCCCTCCCCCAAAAAAGGTAAAAATTCCCCCCTTTCGGGGTCCCCCCAAAAAATAAAAAATGAAGGAAAAAATTGTTT
TGAAAAAGTTTCCCCCCCCCAAAAGTTGGGCGAAAACCTGAAAAGGTTTTTGGCCCCCTTTTTTTTTTTTTTTTTTTCCGGGTC
CCCAAAAAGGGATTAAAAACGGAAAACCCCCCGCCGTTTTTTTTTTTTTCCCCCCCCCGGAGGGGTTTTTCCCCCCCAAAAAAA
GCCAAAAAGAACCCTTTTACGAGAACCAAAACCCCTTAAATTTTTGGATTTTTCCCCCCCCCGAAGGTTTTTTTTTTTTTTTG
GGAAAAAAATTAAAGAACCTTTTTAAAAAACGGGGTTGCCGAGGCCCCCCCCCCCCCCCCCTTTGGCCCCAAAAAAAAAAAAACA
AAAGGGGTTTTTTCCCCCCCCCCGGTTCCCCAGAACCCCTTTTAATGGGGGGGGGGGGAAAAAAAACCAAAATCCCTTTTTTTTC
CCCAAAAAAATGGCCCCCAAAGAAAAAGAAGAATTTTTTGGAAACCGATGCCAAAAAAAAAAAAAGAGGTGTTCCAAAAAAAAAA
AAAAAAAAAAAACCCGAAATTTTTGTGGGGGACCCCGCTTTCCCAAATGCAAGAAGGTTTTTTTTTTTTTTTTAGGTGGAGTC
CCCCCCCTTTTGGAAAAAAAGAAAATTAAATGGCTTTAAAGAATGGGCCCAGACCCCAAACCAAAAGGGCCACGAACTTTTTTT

TTTTTTTTCAAGAACCTTTCAAGAGGGGGACCCCCCCCCCCCCCCCCGGAAAGAAAAGGGGGGGGGAACCCCCCCAAAATGGT
TTTTGCCCCTTTTTTTTTTCCCCCCCCAACATTGGTGGGGGAAGGCCCCCCCCCCTTTTTTTTTTTTTTTTGGCCCCCTTTTTG
CAAAAGGGCCTTTTTTTCCCCAAAGAAAAATTTTTTGTTTTTTTTTTCCGGCCCCATACCCCCCCCGCCCCTTTTTAAAATG
GAAATTTTTGGGGGCCCCGGGCCCCTTTTTTTTTTTTTTTTTTTTTTTTGGGAAAAAAAACCCCCACCCCGAACCCAAAACCCC
CACTTTTTCCCCCCCTTTTTTAAAAAGGTTTTTTCAAACAAGGGGGTTTTTGTTTTTTTTTTGGGGGAAAAAAAAAAAATTTTT
TTTGGGTCCCCGGTTTGGCCCCAAAAAAACCCGCGGGTCCTTTTTTTTTTCCCCCAAAGGAACCGCTTTTTAAAATGGTTCCCC
CATTTTTTTTTTTTTTTTTTTTTTTTTTTTCCCCAAAATTTTAACTTTGTTTAAAAAAACGGGAAAACCCGGAAGGGCCGAAAA
AAATGAAGGGGGTTTTTTGGTTTCCAAGAAAAAAGCAAAAATCATTTTTTAGGGAATTCCCATTTCAAATCCCGTGGCCCCAA
AAAAAAAAAAAACCCCAAAAACCTTTTATCCGTCCCCCCTTTTTTTTTTCCCCCCTTTTTCCCCAAAAAGACCCCCAAATTTT
TTTATAGGAAAAAAAACTCCAGGAAAAGGGTGGGGGAATCTTTTTTCGGGTTCCCCCCCAACCGCAAAAATTGGCCCCCCAAAA
AACAAAAATAAAAAGCCAAGGGGGGGCCCCCCCCCCCCTTTTTTTTTTCCGAGGGGAACCCCCCTTCCAAAAAAAAGGGGCCA
AAAAATAATATTCAAAAAAAAATGCTTTTTTTCCGCAAACCCCAAAAGGGAAGGGGGAAAGCCCTTGCCGAAGGAACCAAAGGG
AAAAATGGGGGTTTTTTTTTGAACCCCGGATTGGGCTTTTAGCTTTTTTTTTTTTTTTTTTTTTTTTTTTTTTTTTTTTTATTT
GAAAATGGCCTTTTTGTTTTCCAGGCCCCCTGGCCCAAAAAAATCCCAAATGGGGGGGGGTTTTTTTCCCTTTTTCCCCGTATT
TTTTTTTGTGGGGAAAAAAAAAAGCTGGAAAAAAAAAACCCCCTCCCCCAAAACGGAAAGGAAGGGGAAAAAAATTAAACTTTTGGG
ATTGGCTTCCCAAAAAAAAAAAGAAATTTCCCAAAAATTTATCGAGCCAAAAACAAAAAGAAGTATAAATCCCCAAAAAAAAGTT
TTTTTTTTTTTTTGGGGAAAGGGGGGAAAAAATTTGCCCCCCCCCCCCAAAGAAATTTTTTTTTTTGGAAGGCAAATTTTGGGGAG
GGGAAAGAGAAAAAATTTTCCCAAATTTATTGCCTTCTTTAAAAAAAAAAAACGCTTTTCCCCCTTTTGTCCCCCCCCAAACCCA
GGTTTTTTTTTTTTTTTTTGGGGGGGGAAAAGGTTTCCCCTTTAGTCCCACCCCAGCTTTTTTTTGGAGTTGTCCCCCCCCAAA
ACCCCAAACAAAGGGGTTTTTTTTTTTTTTCAAGTTGGGGGGGGGTTCCCCCGTTGGTTTTTTTCCCCCCCCCCGGCCAAAAC
TGGTTTTTTTTGTATTTCGTTTTTTTTTTTTCAAAGGCGTGGGCCCCCCCCTTGCCCCCGAGACGGGAAATTTTTACAAAAGAG
CAAAAAAAGCCTTTTCCCCCCCAACTTGGAATTTAAACAATGGGCCCCCCCCCCCAAATTTTTTTTGTTTTTTTTTTTTTTTT
TTTTTTTTTTTTTTTTTTTAAAGCCTGTTGAACACATTGGAAAAATTTTGCCCGGGAAAAGGTTTTTTGCCCTGTTTTTTTTTT
TTTTTTTTTTTGCTTCCAAAAAAATTTTCCCCCCGAAAAATTTTCCCCCCCCCCCCTTGGTTTTTTGGCCCAAAAACGGCTTCC
CCCCAAACCCCGCACGGTAAAAAAAGTTAAAAAAAAAAAACGGAAAAATTTTTTTTGGGCCGCCCTTTTGGGGGGTCCAAAGG
GGCCAAAGGGGGCCCCAAAAAAGGGCAAAAAAGGGCCAAAAAAGCCCCCCCCCCAAAGGGGGGGAAAATTTGGAAAAAAAATTTT
CGCAAAAACAAAAAGGCTTTTTTTAAACCAAGGAAAGGCTTTACCCCTTCCCCTTGGGCGGGAGAAAAAAAAAAAGTAAAAAAAT
GTTTTTGGAACAAAGGGAAGCCCCTTTACTTTTTTTTTTTTGGGGGGGTAAAAAACGGACTTCCCCCCAAAAAAAAAGAGACAAAA
TTTATTTCCTTTTTTTTTTTTTTTGGGGTGGGGCCCACCCGAAAGGCAGCAAAAAATGTTTTTTCACTTAAAAAAAAACCCCTGGG
GAAAGGCCCCACAAAAAAAAATTTTCCCCCCCAAAAAAAAAATTTTTTCCCCCCGAAAAGGGACCAAAGGGGGAAAAAAAAAAAA
AAGGGTTGGGGAGAATCCCCCCCGACAAAAATGGGTTTTTTTTTTTGGTCCCGTTTATTTTTTTTTCAAAATAACGACCCAA
TAGGGGGGCCCCAAACCCAACCCACCCCAAATTTGAAAGGTGGACACCCAAAAAAAAAAAGCTTTTTTCAAGAAAAACCCGAGAA
ATTCGAAAACCCCACCTCAAAAAAAAAAAAAAAAATTTTTTCAAAAAGGTTTTTTTGGGACAAAACCCTTCCCAACCCAATGATT
GGCCCCCCTTTTTTGGAAGCGGAAAGGGGCCCCCCTTTACGGCAAAAAAAAAACCATCCCGATTTCCCCAAATAGTAGGAAAATTT
AAACCTTAACCGCAAACCCTTAAACCTTGCTTTCCCCATTAATCCGGCTTTTTGAGCACCCTAAAAAGGTTTTTGCCCCCCCGA
AAATAAAGCATCCCCCAAGTCCCCTCCCAAAAAAAAAAAAAAAAAAAACCAAATTAGAAAACTTTTTGACCCCCCTTCCGGTGG
AAAATGTGGTTTAAAAAAAAAAGAGGTTTTAGGGGGGGAAACAAAAAATTTTTTTTTTTTTTTTTTTTTGAGAGGGACCCCC
AACCTTTTTTTGGGGGGGGCAAAAAAACTTTTTTTTTTTCCCCCCCCCCAAAAAAAAGAGTCCTTCCAAAAATAGAATTTAA
AAAATTAAGAACCCGCCCCCCCCGTGGGAAAAAAAGCCCCTTTTTTAAAAAAAATTTCAAAAAACGATTTTGATTGAAAGGTTTT
TGTTTTTGTTTTTTTTTTTGGGGTTTTTCCCCCCCTTTAAACCTCCCCAAACCCTGCCACCAAGCCCCAAAAAAAAAATTGCCCC
AATGGTTTTGGGAACCCCCCTTTTTTTTTTTTTTTGACCACAAATTTTTTTTTTCATTTCAACCAAAATTCTTTCCCCCCTTAT
GAAGGAACCCAAAAAAATGCCCCTTTCCCCCCGGAAGGGAAGTTCCCCCCAAAAAAACACCTTTTTTTTTTTTTCCCCCCCTAC
ACCCATGGACCCTTTTTTTTTTTTTTGGGTGATCAAAAGAAGGTTTTTTTTTTTTGGGTTGGTCCCCCCCCCAATAAAAGAT
GGGTTGGGGTTTAAAGGAATCCACCTTGGGTGGAATTAATGGGAAAAAAAAAGTTTTCAAAGTGGGGGGGGAAAAAAATTCTCCAC
CCCCTTTATTTCAAAAGAAAAATTCTTTTTTTGCAAATTTCCCCGAGAAGAAAAAATCGGTTTTGTTTTTTTTTTTTTGAAACCC
CCCACGGCGGCCCCGGAACCCCTAGCCCCTTTGGGGTGGGGGGGCCAAAAACCCTTTTTCGGAACCTCCCCTTTTTTTTTTTT
TAAACGGAACCCCCCGTAAAAACCGCCCCTGAAAACCCCCCAAATGGGGCAAAAAACCTTTCCAAAAAAAATGCCCCAAAAGAA
CAAGGTAAAAAAAGTTTTTTTTTTGGAAGCCCCCTTTTAGGGGAAAAGAAAGGAGGGCCCCCCTGAGGCCCCCCCTGGCATTT
TGGCTTTTTTTTTTCCCCCCCCCCCAAAAACCCAAAAAATAGTCCAAAAAGAGTTTGTCCCCGACCAACCCACTTAAAAACCCAA
AAAAAAAACCCCTTTGGCCCCCCGCAACCTTCCCCCTTTCCCTGACAAAAAGTTTTTTTTTGGTACCGATTTTTGAGGGGGGTT
TTAAAGTTGGGGAGGGGCCCAAAAAAAAAAAAAAAATCCCTTCCAAAATGGTTAAAAAAAACCCCCCCTTGGCCCAATGATACTT
TTTTTTCAATAAACCCAAAACAACCCAAAAACCCAAAACCATTCCCTGAAAAAAACCAAAAAACCCTTTTTTTTCCCCCTCCCT
GACCAGTGTTAACTTTTTAAAAAAGATGTTAACCACCGTTGGGGGGTCCCCCCCCTTTTGGACCCAATTAAAGTGGGGGGTAAA
AAAAATTCTTAGGGGGGGGCCCCAAATTAAGAAAGCCCCCCCCCCAACTATATAAATTTGGAAAAAAAAAACCCAATTTTTTTC
CCCCCCACCCCCATGGGGGAGGGGGGGTTTTTTTTTTTTTTTTTTGGAAAAAAAAATTTACTTTTTGTAAAAAGGC
>m141120_105518_42134_c100732402550000001823141405141504_s1_p0/18582/353_10095
AAAAAAAATCCGCCACACCTGGGCCGTCAGATCTGAGACGAGCGAGGATCGCCAACCTGACCAGGAACCTCCACTAAATGTCTT
CTGCGCCGAGGCTCCTGACGCGTCTGGATGAAGACGATCATCCCGTTGGACGTGGCCAAACCACTTCTCACATACACCCCCACG

AAAACCAACCATTTGGACTTTTACCTCTTCTAACAGCGTATAGAACCGATAACCACTACTCGAGCCTTGCATGTTCCGACATCG
CCAAACGACATAGCATCGCCACCTGCTCCACTTTCTGCATCCGGCGCTGTTCCCAGAACCCCAATTTCCGTCTTCCACCTGAAG
ACGTAACTTGGGTTTGCCCTGCCGACCGAGGCTACTCCATCGGCTGCATCCATTTCAAGAACAGGTTCTGCCATCCTTACATCA
GACGCCATCGTCTTCTACAATCGACACCGACACAGTAAGATCTTTTTAACTCTCTTGGTAACAAAATATTGTGAACTTATGTTC
TTTTTGATGCGATGCGTTTTAGAACTGCGTGATATTCAGATATCCTGTTATGGGGAGTAACGGCTGTCCTGGGTATCGTTCATC
GGGTCTGACTATTGTAATGCCATTTTCTGGATTTGCTTCGAAAATAGAGGGCGGCCTTTACAGATCTACAACATACATCCCGGA
TCCAGATTCTTCTTTAGTCTTGGTTACACCCAAAGGGGAACACAAGCCTCGATGTGGCCCGGTCTGAACTGATCGCTACCAACT
ACTATATGGGACTCCGTCTTGACCGACTTTCTGGGTTTAGGTAACTGCACTGACTTGATCATCTCTTTATCATATACTGATCAG
AATCGTAATTAATCGGGCAACGGGCAAAGTTACGCTCCATTCCGAAGAAAGAAGATCAATACGGCCCCAACTCACCGTTCTCGA
TCCTAGACGCCTAACAAAGCGGGCAACAGGCTCGGGAAATGACTGACGACCACTCGTTCTCGGAATACCGGATTGCCTGCTTCT
GAGCAAGCGTCTGGGCATCGAATCAACGTCGGGCTCTTTTTCACAACGGGCTTGGCAGGCTCAGAGGTTAGCGGTGCGCGGATA
CGCCAGTGGAAGGATATCTAGCCTGGAGTACTGGACCCAATTATCTAGCGAAAACGCGGAGCTAACGCTCCCAGCTTCAAGCTG
ACATTTTATCAGTGCGTTCCTGACGTACATGGGAATACGCAACGTTACCAAGCTTTTTGCCACCGAATCCGTCACCGTCTACTG
CTGGGTGGAGGTGTTTGGGTGCGCGAAGGAGAATCTGCGGAGTCCCACCCCATGCGGAAGTCCTTGTACTTCTACCAGCTGGAG
CTGCGGTTAACGTCAAAGGACAAACCCTGGACGGCTTTTACCAGGCTGAAGCCCGCGGGCAGCTATCCGCTCTACCAGCTCCTA
GATGGCCACCCCCCCGGACGTCATGCAAATTATTGGTTTATGACATCCGGTTCCTCGTAGACAAGAACCCACACTGTTGCTGGA
CTTTCACGAGTGGGTAAGTATTTCCCTAGGACCTTTCTCTTTTATTCGTCTTTTCTACTCGTCTCTTATCCAATGGCTTCCGGT
GGCAGGCTTCGCTTAAAACCCCCTTACCGAGTTCATACCTGGACAAGAGGAGGTTTTATGCGAAGTTGACCGCAGAGATCTGGA
TGTAGGGGGCCTACGTGCACGAACGATAACCTCCGCTGGTTGGGATGCTCGCGGCCACCCAGACCATTGTTATCCCGAATCTTC
GGGGCATCCCTGGTGAGAAGAAACGTCGACGCTGGAGTCAGTGGCCCTCGACCACATTGCACTGTGGTAAGGGCGGCGTGGGCG
GACGCGCCACGAAGAAAGAAAGAACCAGCCTCCGGCGAGGATGGCCTCCTCCAGAAGAGCTGGAAGGACTCTTTCGAAGATGCC
ATACCAAACGTCGGGAAACTCCCGGGGCTAAAGCGTATCGGGGCGAGGTAAACTCCCCTTTAATCTTAACTTTATGCCTTACCC
GAATCGGGGAACTTAGCTAGGGACAGGCTACGTACAACACGAGCCCCGCGATTTGGGGGCGATGGGGAGATGAAGGCCCTTCAG
TTCCCGTCCTGTAGGCTCAGAGTCTCATGTTTCTTACTCCGGGTTCCTCCCAGTAAACGGGGCGCTTCTTGCAACGGCTAACAC
CGACGAACATAGGGGAATCAGGTCCATACATACATATTTCTATTAAGGGAATTAATCTAAGCCCGGGGCCTAGAATGAGGGTTC
GTCCGGGCCATTTCTAACTTTGATTTGTGTTTTCATCTGTTTTGGAATTTACCCGCCCTAAGCGACTCCTTTTTTTTGTACTTT
TGGCAGAGGACCAAACCGACATGTCAATACCCGCCAGTGAATGAGGACAGCTCATCAAGGATAGGCGCCCAAGAAGGCGAACAG
GAAAGAAACCGAGGAGACGGGCCGGAGCCCACCATTCACCGAGGAACTGTCTGAGGACGGAGCTCCGTCCAGGTATGAAGGAGC
TCTGCCGTGTCCCTCTTCCAAGGGCAGTGGAGCCTGCATAGCAAAATTCGACCTCCCGACCCGCCCCCCGTGATTGATTTGGAG
CGGGCGGCGCTGCCAGCCGGAGTTCCGGGAATGAGCAGCCGGATGCTGACGCTGGAGAAGGAAACGCCGGGAATGAGGCACCGA
AGATGCAAGCCGGCTCGACAGTATGAAGATCACATGGGAGATGTACGCCGAACCCGCAGAAGGAAAAATCCCCTCACCTCCCAG
TTCTTCCCCTACAGCCCACTCTTCTGAAGGAGGGGGAGTTAGCCCTGTCGGATGAGCAGTCCCGGCTGATCAACCAGACTTGGG
AAAAGGCCGGTTGGCTAAAAGAGATACACTTACAAGGCCCTGAACGATTCCGTCGCCAGTTTGAAGTTCTCAAGATCGTCCTGC
CGAGCTTCAGCGCTCCCACAAGCCGATCGTGGACCGGCCTATCACCGAAGCAGGGCCTTCCCGCCCTACAACTCGGCCAGGAAC
CCCCCCTGGCGGCTGACCTGGCTGGAGCCAGGTCCAGGACACCATGTCCAACTTCAGACTCAGAGGTACGCCCGATAGCCAACC
CAGGGCGTACTGTTTTCCATACTCTTCAGGCTCTCTTCGCCACCAAGGCACCGCATAACTTTTTCACTTCTCTTACACTCCTTT
ACCTTACGCAACACTTGTTGATGAATGGTTTTGTTTTCTAACTTTTCTTTGCTCCAGAGTTGATCTGTTTCCCATATGAAAGTG
CCGATCTGGTGGCCGAAGCTTGCCATACAACGAATGGAGATGGCTCAAGCTGAATTAAGATGGCGGCCGCCGATCAGAGGGAGG
GACCGCCAAGAGAACCCTTAGGGCCGGACGAGACGGCCCCGCCTCCAGGAGGAGGTGGCCGAGTGCAGGCAGAGAGCCGACGGA
GCTCGGCCCGGCCAAGGTCGGTTGGAAGAGGAGTGTCATCCGGAGAATAAAGGAGATCGATGAGCGGGCAAACGCTCTTGGAGA
CGGCCGTGGCTCAGTTCGCCCTGGAATAAGTGAGGAGGATCCAGGCCTTAGAAAACCCGAGTTTGATGAGCTGGGCGATTCTCT
TCCTCCAGGAAGAAGGGCGGCCATGAAACGACCCGTCGAGAAAAGAGGAGGTGAAAGGCCCTACTAGATGTCACCTTATGACGA
GGCCATCATACATGGCCTGAGTGCAATGGATAAGTAGCATGAATCTCCTTGTCTTCCCCTATCCTAAGTCAAAGCGGGCCGAGT
ACGAGGCCAAGGAGAGGGAGGAATGCAGAACCTCCGGGCGGACTGCGTTGTAGGCCTAAACACGACTTGTAGTTTTTTTTTTAT
TTGTGTTATTTGGACTTGATACTTAAATTAAAACACTGCTACCTTCTCTGGTTTTTATGCAAGGCTTCCTTCCCATTGTTCAGA
TAGAAATTTTCATTTTTGTTGCCGCGACTAACCGATTGCAGGGGTTTAGTTCCGGGTTCCAACGGCCTGGACTCAAAAGACCAA
AAAAGCTTACTATTCAAAGTTTCCTAAATCTGGCACCCTAAATTCCAGGGCCCTCGGGGCTTGGTTAAACCGGAGTTTTTGTTC
TCCTTTTTATCAGCCTTTAGACCTGGCTTAGCCCCGGGGTAAACGCGGACGCTTATTTAAATACCCCCGGACAATCGTTGGTCG
AGGTGGGACCCGCCTTTAGGTCGGTAAACTCTTTTACACCCGGACATCTTTCCGTCCCGAGCTGGACCCTGCTTGGGCAACGGT
CTTCGTTTTATACCTCCGAACATCATTCGTCGGATTGGACGGCTTGAGCCGGTCCTTTTTTTATACCCCGGACATCCGGTTCGT
CCGGGGTGGACTGCCTTTAGGTCCGGTCCTACTTTATATACCCCAAGACATCGTTCCGTTCGGGGCTGGACTACCTTGGCACG
GTCCTTTTTTATAGCCACCCGACATCGTTCGTCCGGGCATGGACTGGCCCTTGGGCGCGGTCCTCTTTTTATACCACCGACATC
GTCGTCGAAGTTGGACGGCCTTTAGGCACGGTCTATTTTATACCCCCGACATCGTCTCGTCCGAGACTAGGACCAGCCTTGGGC
AACCGGTCCCTCATTGTTTATACCTGACACCATTCGTACCCGGGTCTGGACCGGCAATTTAGGTCGGTCTCTTCTTGTACGCAC
CGCATCATTACGTTTCGCGACTCGGACGGCCTGTAGCACGGTCCCTTTTTTATACCCCCAGACATCGTTCGTCCGGGTGAGGAG
CGCACCTTTAGGTCGGTCTCGTTTTTTATACCCCCGGACTCGTTCGTCGGGACTTGACCGCTTTGCACGGTCCTTTTTTATACC
CCCGGACATCGTTCGTCGGGGTGGACCGCCTTTTGGTTCGGTCCTCATTTATACCCGGACAATCGTTCGTTCGGGACTGGACC
GGACCTTAGGCACGTCCCCTTTTTTTTAAACCCCGGACATCGTTCGATCGTGGTTAGGACCGGCCTTGGCACAGTCCTTTTTTA

TACCCCGACATCGTTCGTCCGACTCATGACCGGCCTTTTGGCACGGTCCTTTTTTTACCCCGGAGCATCGTTGTCCGGGGCGGA
CCGGCCTTTAAGTCGGTCCTCTTTTATACCTGGACATCGTCGTCCGGCTAGAACCTGCCTTGGGCAGGTCTTTTTTATACTCCC
CGGACCAACGTTCGTCCGGAGCTGCACCCGGCCTTGGGCACAGGTCCTTTCTTTATATCCTGGACATCGTTCGTTCGGGCTGGA
AGGCCATTGGGCCGGTCCTATCGTTAATACCGGACATCGTTCGTGTGGGGCTGGACCGGTCTTTAGGTCGGTCCTCTTTTGTAC
CCCCCGGACATCATTTCTCCGGGACTAGGACCGCCTTTAGCAGCGGTCCTTTTTTATACCCCTGGACATCGTTCGTACCGGGAC
TGGACCGACATGGGCAACGGTCCCTTTTATACCACTCGTACTCGTGATGGTCCAGGTGGGAACTGCCTTTAGGATCGGTCCTCT
TTTACACCCCCGGACATCGTTCGTGCGGGCTGGACCTGCTTGGGCCGGTCCTTTTTTTATACTCCGAACATCGTTCGTCCGGTG
AACCTGTACCGGCCTTGGCACGTCCCTTTTTTTTTATACCCCCAAGACATCGTAGTCGTCTCGGGGCTGGAGCGGCCCTAGGGC
CTGGATCCTTTTTTATACCCCCGACATCGTCTCGTCTGGGGTTGACCCGGCTTTGGGACACGGTCCTTTTTTATACCATCCGGA
AATCATTCGTACGGGATTGGACCGGCCCAAATCGGGCACGGTCCTTCCGTTATAACCCCGGACACATTCGTCCGGATGCAGACA
GGACCTGGACCGCCTTGGTCAGTCCTCTTTTTGTACCCGCATCATTCGTCCGGGACTGGAACAGCTTATTAGCAGCGGATCTTA
TTTGTACCCCCCGGACATCGTACGTCCGGACGGACCGGCCTTTAGCAAGGTCCTTTTTTTAATACACGGACATTCGTTCCGTCC
GAGTTGGGACTGCTTTAAGGTCGGTCCTCTTTTGTACACCCGGACATCGTATCGTCTGGGACTTGAACCGGCTTTCTAGCACGT
CCTTTTTTTATATCCTATGCCGGACATAGTTCTGTCCGGGTGGATACTCGCCTTTAGGTCGGTTCCTCTTTTGATACCACTCCT
CTCGGACATCGTTTCGTTTCGGGGCGGGGACTGGACGGGCACCTTGGGGCCAACGGTCCCCTTTTTTTTATATACCACCCGGAC
ATCGTTAGCCGGGGTGGGATCTGCCTTTTATGTTCAGTCCTCTTTGTATACCCCGATGACATCTTGTTCGTATTCCGGGCTGGA
CCTGGCCTTGGCAAGGTCTGATTTTTATACCCGCGGACCATTCGTTCGTCCGTGGCTGGTATTGGCTTGGGTCCAGTCCTTTTT
TATACCCCCGCATTCGTTCGTTCGGCTGGAACAGTTATTTACCCCTCGGACATTCGTTTCGTTCCGGGGAATGGATCGACCTTG
TGGCAGCGTCCTTTTTTTATACCCCAAGACATCATCTCGTCATGGGCTGGACCGGCCCTTTAGGTCGGTCTCTTTTTATACGTC
GGACATCGTTTTCCGTCCAGGGCTGGACACCTGCCTCTTACACTACGATCCTTTTTTATACCCCCGAACATCGTTCGTTCCGGG
GCTGGTGGCCTTTGCACGGTCCTTTTTTTAATACCCCCGGACGATCGTTCGTCCGGGGCAGGGACCGGTCCTTGAGCACGGTCC
TCTTTTATACCACTGGACAGTCGTTCGTCCGAGACTTGGGAACCGGCCTTGGGCACGGTTTTCCTTCTTTTAATACCCCGGACA
CCATTCGTCCGGGCTGGACCGACATTTAGGTCGATCTTCTTTAATAACCCGAACACCCATTCATCCCGGGCTGGACCGACCTTT
TAGGTCGATCCTCTTTTGTACCCCGGCCATAGTATCGATTCGGGAATGGACCCGGCAATTTAGCAACGGTCTTTATTTTATACC
CAGCACATCGTTCGTCTGTGGTGGGACCGACTTTAGGTCGGATCCTCTTTTATACCACCGGAAAATTGTTCGTCCAGAGGTGGG
AATCCAACCTTTAGGTTCGGATCCTACTTTATACCCACCGGACATCGTTCGTCATTGGGACTGACCTTGCCTGGCACGGTCCCC
TTCTTTTTATTCCCCGGACATCGTCGTCCGAGGCTGGAGCTGTCTTAGGGCCAACGGTCCTTACTTTTATACCCCCCGAAATCG
TATCTCCCGGGCTGGACCGGCCTTGGGCACGGTCTTTTTTATACCCCAGACACTCGTATCGTCCGAGGCTGGAACCGGCCTAGG
CACAACGGTCCTTTTTATATCACCTAGAACATCGTTTGTCCGGGTCTGGACCGCCTTTTAGGTCGGTCCTCTTTTAATACCTGG
ACATCGTTCATCGGGAACCTGGACCTGCCTTGGGCACGCATTTTTTATACTCACAGACATCGTTTGTCCGGACGCTGGACCGCT
TGGGCACGGTCCTTTTTTAATCCCCGGACATCGTTCGTCCAAGTCTGGAAACAGGCACTTGGGCACGGTCCTATTTTTATACCC
CCGGAAATCGTTCGTACCTACGGCTGGCCGGCTTGGGCCACATTTTCGTTATACCCCCGGCACCATTCGTCCGGGGCTGGACGG
CCTATAGGTCGGCTCCTCTTTTTTACCCCCCGGACATCATTCCGTCTGGGACTGGACCAGGCCCTTTAGACACGGTCCTTTTTT
TTTATACCTCCGGCCTCGTTTTCGTACGGATGACTAGGACCGGCCTTGGCACGGTCCCCCTTTTTATTTATACCCACGGAAAAT
CGTTCGTCCGGGTTGGGACTGCCTTTTCGGATCGGTCTACTTTATATAACCCGGATAAGCGTTCGTCCGGCTTGACAGGCCTTG
GGCATGGTCTTTTTTTAATACCCCGAATCGTTCGTCCGGGTTGGCGGCTTTGGGCACGGTCCTTTTTTATACCTCCAAAAATGT
TCGTCCGGGATGGACCAGGGCATATGGGCACGGTCCTTCGTTATACCCCCGGACACCATTCGTACGGGGCCTGGACCAGGGCCC
TTATTTTATAGGTCGGTCCTCATTTTGTACCCCCGGACAGTCATTCGTGCCGAGACTGGACCGGCCTTTAGCACGTCCTATTTT
TGTACCATACGGACATACGTTCGTTCGGGATTGGAACCGGCTTTAGCAAGTTTCCTTTTTTTATACCTCCGAACATCGTTCATCC
GGAGTAGGACCGCCTTTAGTCGTCCCTCTTTGTAACCAACGGCATAGTTCGTCTAGGACTGAACCGGCCTTTAGCACGGTCCTT
TTTTATAACCCCGGACATCGTTCGTCCGGGTGGGACCGCCTTTAGGTCGGTCATTTTTTGTACGCACCTGGACATCGTTCGTCG
GGACTGGGACCGCCTTGGCACAAGTCCCCCTTTTTTTATACCTTCGGACATCGTTCGTCCGGGTGGGAACTGCTTTAGGTCGGT
CCTCTTTTATTCCTCGAAATCGTTCGTCCGGGGCTGGAACCTGCTTTGGGCACGGTTCCTTTTTTTATACCCTCGAACATTGTT
CGTCTGGGCCGGATTGGCTTGGGCACGGTCCTTTTTTTATAGCCCCCGACATCGTTCGTCCGTGGCTTGGAATGGCCTTGGGGC
ACGGTCCTTTTTTATACCCTGCGGGAACAGTCATTCGTCCGGGCTGGACCGCCTCTTTCAGGTCGTCATCTTTTGTACGCGTCG
GAACATCGTTCGTCCGGGTGGACCTGCCTCCGCACAGATCCTTTTTTATACCCCCCGAAAATCGTTCGTCCTTGGCTTGGACCC
GGCCCTTGGGCACGATCTTTTAAGTTATACCCCGACGACCATTTGTCTCGGGGCTGGACCGGCCTTTAGGTAGTCCTCTTTTGT
ACCCCCGGACATCATTCGTCTGGGACTGGACCGGCATTTGCATGGTCCTTTTATACCCGGCCATCGTTCGTCGGGACCTGGA
CCGGCCTTAGGGCACGGTCCCCTTTTTTTTATACCAAACGAAATCGTCTCGTCCCCGGTTGGGAACCTGCTTTTGGCGGTAACT
CTTTTATACACCCGGACATGTTTCTTCCGGGCTGGACAGGCTTGGGCATGGGTCCTTTTTTTATACCCCCGGACATCGTTCGTT
CCAGGGGTTGGAACGGCCTTGGGCACGGTCCTTTTTTATACCTCTGAAAATCCGTTCGTCGGGATTGACCGGCATCGGGCACGG
TCTTCGTTATACCCCCGGCACAATTCGAGGGGCTGGACCGGGCCTTTAGGTCGATCCTTTTTTGACTCCGACATCATTCGTCCG
AGACTGGACCGGCCTTCTAGCACGGTCTCTTTTTTTTGTACCCACGACATCCGTCGTCCGGGATTGCGAACGGCCTTTAGCGAG
TTCCTTTTTTATAACCTCCGGACATCGTTCATCCGGGTGGGACCGCCTTTAGGTCAAAGGTCCCTTCTTTTGTACCCCGGACAT
CGTAACGTCTTAAGGACTGAACCGACATATAGCACGGTCCTTTTTTATACCTCCGGCATCGTTCGTAAACCGGGTGGACCGCC
TTTAGGTCGGTCCTCTTTTGTACCCTGTCAGTCGTTCGTGCTGGGACTGGACAGGCTTCGGGGCAAGTCCCCTTTTTTTTAT
>m141120_105518_42134_c100732402550000001823141405141504_s1_p0/19522/2523_9426

CATATACAAATAATACATAACTTTATGAGGCCGTAGTTGGATTTTTAAAGGGAAAAAAAAAAAATGATGTTTTCAAGGGGTTGCA
AACACGACGGACCTTCCCAGGAGGTCAACACCAATCCTAGTACTACTCTCGCCCAAAAGGCACCGCGCTTCAAACTGCGGAGTT
CATGATGGGATCCGATGCTATTAGTGCTGTATGATCGGACCCGGAATGTTTGGTCCGCAATATTCATTTATAGCGGCGCGTGTG
TCCTTCCATTCCTCTTTATATGTCACTTGCTTGTTCGCCCTCTTTGCATGTGGCACTTATGAAGCGTGCTCTTCTAATCTCACT
GTTTGTTCCATGATGGACTACCTTCACGGGAGTAAAAACTTACAAACATCCACTCTAAAGAGAGAGCCGAGCGGAATAATGCAA
ACTCGACTGATAGTATTTTAACAAAAGATTTAAAAAAAAAATACAAACTCTTTTGAGCCCGTAGGTGATTTTAAAGGGAAAAAAA
AAAATTGGATGTCCTTTCAGGGGTGCCAACACGAGGACTTCCAGGAGGTCACCCATCCTAGTGTAATTCACAACAATAAATCTT
CTCCTCAGCATAACATTTTAGAAGAGCACGCTGCTTAAGCTTGTCGGCAGCAAATTCTATGGGAGGAGCCTATCTCATTAAAAG
CCCGGCTTGCATTATAGTGGCTGGTATGTATCGCACGGAATGTTTGGGGTCCGCAATATCATTTATTAGCGAGCGCTGTGTCCT
CCCTTTGTTCTCCCCCGATTGACTGGAGAGCTCTTTATTTCGCTGCTTGTTAGTGACCAACGGTATTCATCCCTCCAAGGAAAA
CGAAATAAATTGAGTTTTTAAAGATTTAAAACAAAAAATACAAACTTTTGAGCGTAGTGGATTTCTAAGGGAAAAAAAAAATGA
TGTCATTCAGGGGTGAACACGAGGATTCCCAGGAGGTCACCCACCTAGTACTACTCCTCCGCCAAGCACGCTTAACTGCGGAGT
TCTGATGGGAGCCGGTGCGGTGGTAGTGAATGGTATGGATGGTGCGCTACGCCGGGTGAAAGGATTGTCCGCATGGGAGTGAGT
GTTAGTGAGCGAGCCGGGGGTGGTGGTCGCTCCCTGGGTTCGGGTGACCGGCGGCCGCGAGTTGACTGAGGAACTTCTTATTCC
ACGCGCTTGTTAGTGACCACGGGTATTAAACCCTCAAGGAAAACGAAAATAAATGAGTTTTTAAAGATTTAAAACAAAAAATAC
AAACTTTGAGCCGTAGGTTGGATTTTTAATAGGGAAAAAAAAAATATGTTTCCTCAGGGGAGCAACACGAGGACTTCCCAGGAG
TCACCCCATCCTAGTGAACGGACGCGGGTCGACCGAGCACGGCTTGGAACTGCGGGTTAATGAGTGGGAGTCTGCTGCGGGTCG
GGTGCAGTTAAGTGCGTGGTATGATCGCACCCGGAAATGTTTGGTCCCGCGGAGAGGTATTCAGGTTAGAAGCGGAGGCGGGGA
GTGTTGGTCCCTCGCCGTTTTCTCCCCCGATTGACTAGGAAACATTCTTATTCGCTGCGTTTTAGTGCACCTTACCGGTATTAA
ACCCTCAAGGAAAACGAAATAAATGAGTAATTTAACAGAAATTTAAAACAAAAAATACAAACTTTTGAGCCGTAGGTGGATTTT
TTAAAGGGAAAAAAAAATGATTTTTCAGGGGTGAACACGTAGACTTCCCAGGAGGTCACCCATCCTATGTACTACTCTAACAGC
CAGCACAGCTTAACTGCGGAGTTCCTGATGGGATCTACCGTGCATTAGTGCTGGTATGATCGCACCCGGAATTGTTTGGTCCCG
CAATATTCATTTATAGGCCAGAGCGCGTGTGTCCTCCTTTTCTCCCTCGGATTGACGTAGGAACTTCTTATTCGCTGCTTGTTG
TTGACCCAGGTAATTAACCCATCAAGAGAAAACGAAATAAATGAGTTTTTAAAGATTTTAAACAAAACAATACAAACTTTTGAG
CCGTAGGTTGGATTTTTAAAGGGAAAAAAAAAATGATGGTTTTCAGGGGTGCAACACGAGGACTTCACACAGGATGGCTCACCC
ATCCTAGTACTACTCTCGCCCCAAGGCAGCTTAACTGCGGAGTTCTGATGGGAATCCGGTGCATTAGTGCTTGGTAGTAAACGG
CACCCCGGAATGTTTGTTCCGCAAATATTCATGTTATAGCGAGCGCTGTGTGTCCTCCCTTTTCTCCCCCCCGGATTGGACTAG
GAACTTCTTATTCGCTGCTTGTTAGTGACACGGAATTGCAACCCCTCCAAGGAAAACAAATAAATGAAGTTTTTAAAGATTTTA
AAACAAAAAATACATAAACTTTTGAGCCGTAGGTGGATTTTCAAAGGGAAAGAAAAAAATGATGTTTTCAGGGGTGCAACACGA
GGACCTTCCCAGGAAGGTCACCTCATCCTAGTACTTACTCTCGCCCCAAGCAACGCTTAACTGCGGAGTTACTGCCCATGGGAT
CCGGTGCATTGTGCATGGTATGATCGCACCCGGAATGTTTGGTCCGCAATATTCCATTTATAGCGAGCGCGTGTTCTCTTTTCA
ATCCCCCGGATTGACTAGGATACTTCTTATTCGCTTGCGGTATGTTAGTGCCACGGTATTAAAACCACTCAAGGACAAACGAAA
TAAATGAGTTTTTAAAGAATTTAAAACAAAAAATACAAATTTTGAGCCCGTAGGTGGATTTTTAAGGGAAAAAAAAAAAATGATG
TTTTTCCAGGGGTGGCAACACGAGGACTCTCCAGGAGGTCACCAATCCTCAGTACTACTTCGCCCAAGCAACGCTTAACTGGCG
GAGTTCTGATGGGATCCCGGTGCATTAGTGCTGGTAATGACATCGCACCCGAATGTTTGGTCGCAAATATTCATTTATAGCGAG
CGCATTGTGGTCTTCCCTTTCTCCCCCGATTTGACTAAGGAACTTCTTATTCGCTGCTTGTTAGTGAACCACCGGTATTAACCC
TCAAGGAAAACGAAATAAATGAGTTTTTAAAGTTAAAAAAAAAATACAAACTTTGAGCCGTAGGTGGATTTTTAAGGGAAAAAA
AAATGAATGTTTTCAGGGGTTGCAACACGGAGGACTTCCCAGGAGGTCACCCATCCTAGTACTACCTCGTCGCCCAAGCACGCT
TAACTGCGGAGTTCTGATGGGATCCGGTGCATTAGTGCTGGTTGATCCGCACCCGAATGTTTCTTCCGAAATACTTCATTCTAT
AGCGAGCGCGTGTGTCCTCCCTTTTCTCCCCGGATTGACTAGGACTTCTTATTCCGCTGGCTTGTTAGTGACCACGGTATTTAC
CCCTCAAGGAAAACGAAATAAATGAGTTTTTAAAGATTTAAGAACAAAAAATACAAACTTTTACCCGTAGGTGGATTTTTAAGG
GAAAAAAAAAAAAGTGATGTTTTTCCAGGGGTGCAACACGAGGACTTCGCCCAAGGAAGGTCACCCATCCTAAGTACTACTCTC
GCCCGAAGCACGCTTAACTGGGCGTGAGTTCTGGATGGGATCGACGGTGCATTAGTGCTGGTATGATCGCACACCGGTAATGTT
TGGTCGCGCAATATTCATTTATATGCGAGCGCGTGTGTCCTTCCCCTTTTCTCCCCCCGGATTGACTAGGACTTCTATTCAGCT
GCTTGTTAGTGCCCACGGTATTAACCCTCAAGGAAACGAATAAAAGAGTTTTTAAAAGATTTAAAACAAAAAATAACAACTTTT
GAGACGTAGGTGATTTTTAAAGGGAAAAAAAATGATGGTTTTCAGGGGCGATGGCAACACCGAGGACTTCTCCAGGAGGTTCAC
CGCATCCTAGTACTACTCTCGCCAAGCACGCTTAACTGCGGAGTTCTGATGGGACACGTGCATTAGTAGCTGGTATGCATTCCA
CCCGGAAAGGGGGGGGGTGGGGGGGTGGGGTGTCCAAATGTTTGGTCCGCAATATTCCATTTATGAGCGGAGCCCGCATGGTGT
CCTCCCGTTTTCCTCCCCCAGGACTATGACTAGGAAACTTCTTATGTCAGCTGCTTGTTAGTGAACCCAGTAAGGGATTAACCC
CTCAAGGAAAACGAAATAAATGAGTTTTTAAAGATTTAAAACAAAAAATACAAACCTTTTTAGCCCGTAGGTGCGCATTTTTA
AGGGAAAAAAAAAAAATTGATGTTTTCAGGGGTGCAACACGAGGACTTTCCAGGAAGGTCACCCATCCTAGTAACTAATCTCTCG
CCCAATTAATATGCAACGCTTAACTGCGGAGTTCTGATGGATCCGTGCATTGTGCTGGTATGATCGCACCCGGAGATGTTTGTT
CCGAGCAATTATTCATTATAGCGAGCGCTGTTGTGTCTCCTTTTCTCCCCCCGGATTGACTAGGAACTTCTTATTCGCTGCCTT
GTTAGTGACCAACGTATTAACCCCCCTCCAAGGAAACAACGAAATAAAATGAGTTTTTAAGATTTAAAACAAAAAATATACAA
CTTTTGAGCCGTAGGTGGATTTTTTAAGGGAAAAAAAAAATGAGATGTTTTCAGGGGTGCAACACGAGGACTTCCCAGGAGGTT
CACCCAATCGCTAGTACTACTCTCCGCCCAAGCAACGCTTAAACTGGCGGAGTTTCTGATGGGATCCGGTGGCATGTAGTGCTG
TATGATCGCACCCGGAATGTTTGGTCCGCAATATTCATTTATAGCGAGCGCCGGTGTCCTCCCTTTTCTCCCCAGGATTGACCT
CGGAACTTCTGTTTATTCCGCTTTGTATAAAGTCGACAGACGGTATTCCAACACCCACCCTCCATAGGAAAACGAAATAAATGA

AGTTTAAAGATTTAAAACAAAAATACAAACTTTTGAGCCGTAGGGTTGGATTTTTTTAAAGGGAAAAAATAAATGATGTTTTTC
AGGGGTGCAACACGAGACTTCCAGGAGGTCACAATCCTAGTAACCTCACATTCGGCACCAAGCAACTGCTTTAATGCGGGAGTT
TCTGATGGGAGTCCGGTGCTTAGTGGCGGTATGTCGCACCCCGGAATGTTTTGGTCGCAATATTCATTTAATAGCGAGGAGCGC
GTGTTGTCCTCCCTTCTTCTCCCCGATTGACTAGGAACTTCTTATTCGCGGCTGTTTAGTGAACCACGGTATTAACCCCTCAAG
GAAAACGAAATAATGAGTTTTTTTAAAGATGTTAAAACCAAAAATACAAACTTTTGAGCCGTAGGTGGATTTTTAAAGGCCGAA
ACCAAAAAAAAAAATGATGTTTTCAGGGGTGCAACACGAGGACTTCCCAGGAGGTCACCCATCCCTAGTAACTACTCTCAGCCCC
AGAAATGCACGCTTAACTGCGGAGTTCTGATGGGATCGGTGCATGTAGTGTGGTATGATCCACCCGGATGTTTGGTCGCAAATA
TTCATTTTAGCGAGCCGTGTGTCCTCCCTTTTCGTCCCCCGGATTGACCTAGGAACTTACATATTATTACGCTTGCTTGTTAGT
GACCACGGTATTAACCCTCAAGGAACCAACGAAATATTTTTTGAGTTTAAAGATTAAAACGAAAAAATACAACTTTTGAGCCGT
AGGTGGATTTTTAAAGGGAAAAAAAAATATGATGATTTCAGGGGTGCAACACGAGGGACTTCCCAGGAGTTTCACCCATCCTAG
GTACTACCTCCCTCCCCCCCAAGCACGCTGTAACTGCGGAGTTTCTGATGGGATCCGGTGGCATTAGTGCGTTGGTAATCGAGA
TCGCCACCCCGAGGAATGTTTGTTCCGGCATATTTCATTTAAATATGCTGAGCCGCTTGATAAGTGTGTCCTCCCCTTTCTTCG
CCCTCGGATTTGAACTAGGTAACACTATCTTATTCAGGCTGAGAGCTTGTATTAGTGACCAAGTGACGGAATTAACCCCTCAAG
AAAACGAAATACAAGAGTTTGTTAAAGATTTAAAAGCAAAAAATACAAACTTTTGAGCCGTAGGTGGATGTTTAAAGAGGAAAA
AAAAAAAAAGGAATGTTGTTTTCAAGGTGCAACCACGCAGGACTGCTCCCAGGAGGTCACCACCTAAGTACACTCCTCAGCCCC
AAGCACCGCTTAACATGCGGAGTTCCTGATGGATCCGTGCAGTTAGGTGCTGGTATGATCGCACCCGGTAATGTTTGTTCCCGC
AAAATATTCATTTATGAAGCGAGCCGGCGTGTGTCCTCCCTTTTCTCCCCCCGGATTGACTAGGAAACTCTTATTCGCTGCTTG
TTATGTGACCAGCCCGGTAATTCACCCCTCAAGGAAAAAGAAATACATGAGTTTAAAGATTGTAAAACCAAAAAATACAAACTT
TTTAGAGCCGCAATAATGATCAAGTATATTACTAAGCAGTTAAACACACATTCTAATAGGATTGAATATAAGAAGAAATAATGC
AAATAAGAACATCATTAATTAAATTAAAAGTAGAAAATTTCCAAGTCACCTTACATTAAAACCCTACGATAAGAAGAATGTAAG
TTTCATGGGAAAAGAATAGAAAAGAAAGGGTAGAGAGATAGGAGAAATAAGAAGAAAGTCAAGAAGAAGATAGAGAGATGAGAA
TCGCCCATACCTAAAAATGGTGGGTCCTAGAAGCATTTCTTTTTATTGAGGGTAGATAAGATTTGCAAAAAGGTTTTCATAAGT
GTTTTCATTTGTTAACTTTGATATGCCGTCAAAATATCCTTTATTTAAAATTTCAATTCAGTAACTGCTATAGCCGTTGTGTAA
TTTGTTACGGTCCTTCAAATATCCCAGTAAACATCCTAAGTATTTTGACCTTTTTTTTTAGACCCAGATTTCGTAGGTCTTCTG
AATAATCTTTACACC
>m141120_105518_42134_c100732402550000001823141405141504_s1_p0/20675/169_10368
CCACAACCGACTCATGTCATCGGGGATAGATGCGAAAACTCTATCTCACCGCCCACAGGCGTTTTTTCCACTGCCTTACCTCAG
GTGTGGTGTCCGGGACACGCGCCGCGAGCCTGGAAGCACCATTCAAGTCCCGGCACTCGAACGTGCCACGAACGGCTTTCTGAG
GAGATAAGACACTGCGCGCGGCACAAACCAGGTCCTACTCCAGAGAAGATCTGCGCCACAGGCATCACTTCTGCGCGCCGCGCC
CCATCGCCCCGAGACCACGAGCTTGTAATCCGATTACGTCCGCCAAGCTGCACACACGGTGCCAGCGAGACTGAGCGCGAACGC
GTCCTGCCGGGCACCCACCCGCAGGACAATACAACCTTGAATGACTAGCCCCTGCCCTCCTCGCGATGAGGGGGGCGGATCGAC
TCTGGTGGGCGAGACGTCCCAACCGGGAAGGGAGGGGTGAAGTATGCCATCGTGGCCGTGGACTCACACTACACCAAGTGAAGG
TTAAGGCGAGCCCATGAACCACAATCACTTCCAAGAAGGAGCATTAGGATTTCCTCATCAACAACAGTAGTGTGTCGGTAGCGC
CTTCCCTACAAAAGTCGCGTATCTCATACACGGGAAGCTGTTCGACCAGTGACCACTCCCATCACGGATCGTTTCCTGCGAGAG
ACATGGAGTCACCGTCAAGAGCTCTCTCCCGCAGCTCGCGCCACGGCCCCAGCATACAGGGTAGGGGACAGAAGCCGTGAACAG
AATCCTAAAAGGAGGGCCGGCCCAGAACCAACAAAAGCTCCAGGCTCCTGCCAGCAGCAACCAGTTGGCCAGCAGGAAGCATCC
GCCCCCGCCCCCGCGTATTGTTGGCCTTACCCGAGACGACCAGAGGAGGAACATCAACCTGCACACGTCCTTATCCACAGTCTA
CCTGGACCACCGTCAGTCACCATCGAAACGACCATCCCCCCACGTCTCACCGCGCACACACGACACGTATCGACCCCCTGCCCA
CGCAACGCGCGCGCCTTGCACCTCCAATAGCATCCACTAGAACTCTCATCGCGCAGGAGGCATCTTGGGGAGTCGCAGTGCTAA
AACTGAAGACAGTACACAGGAAGAGATCCGCGCGACCCGGGCACCTGATTCACGAACGAAAGTGCAAGACCGCAAGTTCGGAAA
TCGACGACTTACCCGTCTTGCGAAGAGTCTTCCTGCTACTGTGCAAGATCCGGAGTGGGCGGTCCTGCCCAGAATTGGGAAAGG
GGTCGTATAAATCCAAGCATGAAATATGCCCCAGGGACAATCAAACCACTTAAAGCGACTCGACCTGGCTTCGGAAAGTCTCGC
GAGCCTAGAACGCGGAACATCTTCTCGTAAATAGTTATCAGGCCTAGTGGGAGAGCGCTGTTCCAATATTAGTATTTTCGTTGT
AAGCAATGTACGACCTCAAGGGCGACCCTTCTTTCCCGAACTAACTCAACAAACCTGCGTATGACAAAACACTCATAAGAAACT
GTTGTAAAACAATGATAATCTTTCTCCAGTGACCCATACCAAGTCTCTGCTCAACTTGCCGGGGGTATCCTGGTATAAGCAAAT
ACCCAGAGGGTGCAAGCTCAGGCTAGTCCCGGCTCCGAACCAACGAGGTCCGGGTGAAAACAAACCCGTGGTCCCAGCACCGGA
CCGAGTGCCGACTGTCTGGGGGCTATAAAATTGACAGATGACCCGAGACGCTGCATGCTAGTCCCACCCCTGAACCGAGCGTCT
GTCCCAGAGGCTATAATTAAAGAGACTGGCTAAAGGCCGGTCCCGCCATAGCGACGAAGTTGTCCGGGGGTATAAACTAAAGGG
ACGCAAGCCCAAGGCCTGTCCCACCCCGGACGACGCCGTTGCCCTGGGGCTATAATTAAAGAGGTCGAGCTAAGGCGTCCCGTC
CGGACGCAACGTTGTCCGGGGTAATAATTAAGGGACCAACAAACGCCAAAGCCTGTCCCACCCTGGACGATGTTGTCCGGGGGT
AAGAAAATTAAAGGATACGAGCCTAAGGCCGATGCCCGCCCAGAGACGAATGGTTGTCGGGGGTCCTAATTCCCACAACGACAA
GCCCCGACGGCTGCGTCACCCATGGACCCCAGCGATGTCCGGGGGATACGTAAATTAAAGAGAACTCGAGCCTAAGGCCGGTCC
ACCCCCGGAGCAACGTTTTCCGGGGGTATAAGTTACCCAGGACCCAAAGCAAAAGGCTGGTCCCGACCCAGACGAGCGATGTCG
GTGAGCATACAATTAAAGAGGAGCGAGCTCAGGTCGGTCCTGCCTAGACTAACGTTGTCCTGGGGGGGTATCAATTAAAGGGTA
CCAAGCCCAACGGCTGGTGCCAACCCGGACGAGCGACGTCCCGGCGGTATAAATCAAAAGGGACCCAAGCCAAGGCTGATCCTA
CCCTCGGACCGAGCGAGTCTGTCCGAGGCACTAAGTCTAAAAGACGACGCCCAAGGCCGTCCCACTCCGGATCGAGCCGAGGTT
GGGAGCGAGAAGCATGCCTGGTATGCCCAAGGACAAATCGTCGGCGTACCGAACCTGTAAAGCGGGAAAGCAAGATATCAAAAC
AATAAAACTCAGCAACGTTGGTCCTGGTCGTTGGAGCGGATAAGACACTTGGCGGTTCAGGTCTGAAAGCTAGTCAGTGATTGC

GTCGTACGTTTGAGGCCGTGAATCCGGGACCCAAACACTCAGAATCCCTCCAATCTGGTACCCGTACTAGTAAGTAGTATACGT
AGGTACTAGCTAGCCTAAGGTAGGCGTAGTCGTCAGCTGGTTCAGCTGGTAGTGGTATCGGAGCGTGGTCAGTGTAGGTGGGTA
GCTTGGTACTGTATCGAGCTAAGTGGTACGTCGTAGTCAGCTACGTGACGTAAGCGCCTCAGTACTCCTAGCTAGCTGGTAGTA
ATGGTAGTAGTGGAGTTAGTGTAGCATCGCGTAGTCAGTACCCCGTGCATAGTGGTAGTGGTACGTAGTTCAGTGAGCTCAGTA
ATAGTGGTAGTGTAGTGGTCAGTGTAGTGGTAAGCTGGTACGTGGTAGTGCTTCAGCTGGTACGTGTTAAAGTGGTATCGTACC
CGTAGTAGTATAGTAGTAGTCAGTACGATAGTAGCTAGTAGTACGCTAGTAGTTAGCTAGTAGTGTGGTAGTAGTAGTAGTTAG
TAGTAGTACAGTTAGTGATAGTAGTGAGGTAGTGAAATGCGTTGGGTAGTGGTGGTAACGGTACGTGCTAGTAGTGGTAGTGCG
TAGCTGGTAGTGGTAGTGGGTAGATGGCTAATCGGTAGTGGTAGTAGTAGTAAGTAGTATAGTAGTGTCAGTACGTAGTGTAGT
TCGTAGTAGTAGTGTAACGTGTAGTCGGTAGTGGTAGTGTAGTAGTCAGATGGTAGTGTAGTGGTAGTGGTAGTAGTGGTATGT
ACGTCTGGCTAGTGGTAGTGGTAGTGGTAGTAGTGGTGTTGGTAGTGGTGGTAGTGCGTAGTGGGTAGTTGGTAGTGGTAGTCG
TAGTGGTAGTAGTCAGTAGTATAGATAGTAAGTAAGTAGCTGGTAGTCCGTAGTAGTAGTGGTACGTGTGTACGTGGTAGTGGT
AGTGGTAGTGGTAGTCAGTAGTGGTAGTGGTAGTGGCTAGTAAGTGGTAAGTGGTATGGTAAGTGGTGCAGTGGTAGTGTAGCT
GGTAGTGGTAGTGGTAGGTGGTAGTATGGTAGTCAGTAGTAGTGAGTGAGTAGTAGTAGTAGGCTAGCTCCGCTAACGTACTGG
TATCGCTATAGATAGTAGTAGTGTAGTGGTAGTGTAAGTCGTAGTCTGCTACTACCGTAGTAGTACAGTGGTAAAAGTGGGGGG
CGTTTAGCGGGGTGTATAGTAGTAGCTGTGGTACGTCCCGTAGTAGTCTGTGGTAGTGGTAGTTGGTAGTGGTACGTGTAGAGG
TGGTGATGGTAAGTGGTGGTAGCTGGTAGCCTGTAGTAATGGTATGGGTAGTGGTAGTAGTAGATAGTAGTAGTAAAGTAGTTA
GTAGTAGTACGTAGTGGTAGTCGTATACAGTAGTGGTAGTCGGTAGTGGTAGTGGTAAGTGCTAGTATAGCTGTAGTGGGGTAA
GTGGTAGTGGTAGTGGTAGTGTGGTAAGTGGTAGTGGGTAGTAGTGTGGTGGTAGATGGGTAGTGTAGTGTAGGTGGTAGTATC
GGGTATGGTAGTGGTATGGTAGTTAGTAGTAGTAGTGTAGTGGTAGTGTAGTGGTAGTGTAGTGGCTACAGTAAGTGTAGTGGT
AGTGGGCTAGTGGTAGTAGATAGTAGTAGTACGTAGTAGTAGCTATAAGTAGCTAGTAGAGTAGTAGTAGTAAGTAGGTAGTAG
TAGTAGTAATAGTCGTAGTTGTCGTAGTATGTCGTGTAGTAGTTGGTAGTCGTAGTCAGTAGATGTAGTGGGTATGGTAGTCAG
TAGTGCTAGTAGTAAGTGGTAGTAGTGGTAGTAGTAGTGGTAGTGCGTAGTGGGTACGTCTTCGTCGAGCGTAGTAGTAGTCGT
CGTACGTAAGCTGCGTAGTAGTGCGCTAGTAGTGGGTAGTGGTAGTGTAGTAGTGGTAGTAGTGGTAGTGGTAGTGGTAGTGGT
AAGTAGTTAGTGAGTAGTAGTAGGTAAGTAGTAATAGTGACGTAGTAGTAGTAGTAGCTGTAGGTGGTAAGTGGTAGTGGTAGC
TAGTAGTAGTAGTCGTAGTAGTAGTAGTAGTAGCTGTAGTCGGTACGTGGGTAGTGTGACTGGTACCGTGCTAGTGGTAGTCGT
ACCAGTGGGTATGGACGTAGGGTAGTGGTATAGGTAGTAAGCTAGTAGTCAGTAGTATAAGTGGTGGTAGTAGTATAGTAGTAG
TAGAGTATAATGTGTGTAAGTGGTGGTTGGTAAGTGGTAGATGTATGTAAGTGTAGTGGGTAGTTAGTGGTAGTGGTAGTAGTA
TGGCAGTAGTAAGTAGTTAGTGGTATGGTGGGTATGGTAGCTGGTAGTTGGTGTGGCTAAGTGGTAGTAGTAGTGGCTAGTGGT
AGTGGTAAGTAAGTAGGTGGTAGTGCTAGTGTAGTAGCTAGTGGGTGTCGGTGAGTAGTAGTCACGTGGTACATGTTAGTGGTA
GTGGTAGTCGTCCGTCGTAGTCAGTAGGTCTAGTCGTCGTAGTAGCTGGTCTAGTAGTAGTGGTACGTAGTGTGTGTAAGTGGT
TATGGTCAGTGGTAAGTGTCAGTCGTCGTAGTGTAGTGGTAGTGGAGTGGCAGTGGTAGTGGTAGTGGTAGTAAGATAGTAGTA
AAGTATAGTAGTAGTGGTAGTAGAAAAATATATAAGTGGTGGTAGTGGTGTATATGCTGGTAGTGGTGGTAAGTGTAGCTAAGT
GGATATGATAGTAGTGCGTAGTGGTACAGTAGTGGTGGTAGTCAAGTGCGTAGTAGTGGTAGTAGTAATAATGTGGTGTTGTGG
TGGTGGTGGTGGGTGCGTGGTGTGGTCAGTGGTAAGTGGTAGTAGTAAGTAGTTAGTCGTAGTCCGTAGTGTAGTCCGCTAGTA
TAGCTAGTAGTCAGTACCTCAGTATAGTAGTAAAGTGTAGTGCCCGTAGTGGTAGTCGTAGTGGAGTGGTAGTATGGTAGTAGT
AGTACTATAGTAGTGGTAGTGGTAGTCCGTAGTGGCTAGTCGTAGTCGTACGTAGTAGTGTAGTGGTAAGTGGTACCGTGTGCT
GGTAGGGTAGTGGTTAGTGGGTAGTGGATATGTAGTATGGTTCCATTACTATGGGATTTGGTTCAAGCGGCGACTTTAGTTGGA
ACTCCTAGCAATAATTACTGATTTTATAAGTTTAACCTATAGTTTAAGAATATTAATTACTAAATACAGGTTTTGAATTGAATA
TTGCGTTAGTTAATTGATACATTTATTAAACCTAAGGTTTAGATAGAGTCAAGTAGGATATGCACACTTGTCCATGAGCATGGC
TTATTCCTAAGGTTATGATTTTTAAGAATTATTATTTTAATGAAAAATAAGAAGAAATATAAAGACTTAGTCTTCACCAAAATC
TGATGGGAGCATGATTATTTGTCACATTTTATTTACTTAATTTGTGGATTATGATTAAATATGGATTTTTCGTATCTTTTAGGG
TACTGTAACTTCAGACCTGTCTTTTTGACCAGTTATGTTAGAAGTGGTGGCGAAAAATAGTATTTCTAGAAACTTTGTAGATAA
TTTAATTAGCTTGTCTACAAGGAATAAGGAGAGTCAAATCAGAGTTCTAAAGCTCCAAATATGTTGATCTTACTGAGGGGAGTC
TTAGAGTTACCGAGTTTGGAGTTAGAAGTTTAGATTCTGATTTGTTTTTTATTATTTTAAAAATCAAATCTTTGAGTCCTTAAT
CTCTTCGTGAAAAATGTTGACAATTCCTAAGCCCATTGGCTGAAAGATACTTCAATAATTTTCAATTAAATTTATTATTTTGTA
ATTCAAAGCCAACAAAGAAGATTTTATTCCTAAACTCGATATACCATAGGCACCGTTAGCACAAAAGCCTTTTCAATTCATTCT
TCAAGCATTTGATACAGAGCCTTCATGTCTGGTGAGACTATAGAGTGACAAGCACTTGGATTCGAGGTCTACTAAGCGTTTAAT
CATTATAGCTGTAATAAAAACTTGGAAGTAAGGTTATAGTGCTATTCTGGTTTCGAGTATAGTTATCGACTCATAGAACATTCA
AGTGTTATATGCCTGAATTCTAGTTCCATTTTCTGACTCTGTTCATTATACCGTTCTTCTCACTCAAATGTCTAACTCAGTCTT
TTCTATTCTAGGGCATCTAGAGTTCTCTTCGAACTTAATATTTCATTGAGATAACTAAACTGTTTCGATGGTTGTACGTTTATT
CTTCATCCATCTTCTTTTCTTTAGTATATCTCCACCTCTATAGTTTCGATTTTTAGGATGTTCCCAAAATCCCCCGACTCTTTT
TCTCTATCATCCCGGTATATGGTAAGGAACAATGGTAGATTTTAGTATGTTATATATATGTGATAGGTTTTCTTATGTTATGTT
GTGTTATGAAATCGTATATCATCTTGTGTATGTTTTTGTAACTTGGGCATATCCAACCTAATACCTACAGCCCAATAAATTTGT
ATGGCATATGACTTGCTTAAGCTCAGCAAGCCCCCAAAAATCTAATGGGCATATGATTGTTTAATTTATGGAACCCCAAGTAAT
AATGGCCATTATAGTAGTTGTGACATATGTTTGATAATGATACTGTTCTTTCTAATTATATGCTACGTTATAAGTTTTATGTAA
TCATGAGTTATGTGTTTAGGTTTCCTTGCTGGGCATTAGGCTCATTCCTTTATCTCTTTTTTTTAGTGTGATGCAGGAAACTAG
ATACCGGAGCGGAGGATCTCTTGGAATCTTGGCATGTGTTTGAGGATGAACGAACTGAGCTGACTGCGTGTCGATCGAGGATCA
GAGTATGTTATATTTACGTCCTTTATTAGATTATGTTTCTTTCCGCCAATTTAGTTTTTTTAAACAATTGAATTAAAGTTTATG

TTTTTCTTTTAAACAATGGGTTAACATGCCATATTTTCTAGTTATTATGTATTTTGATACAATCATTCTTGAGATTCAATAAGT
TTATATTATTTCTTATGTAGTCTTTCCCAGAAATAGTCAAGTTATGTAGTAGTAGATCTACATGGTCCCAAAGGTCATAGAAAT
AGTTGGGTCAATTACACAATCGCGCAGGCAAATTAAAGTGAAATTTCTACTCAGGGAATAAAAATATTATAATATAAAAAACCG
GGAGAATGCATATGTGTTTGTGGACGCGTACGCGTCTGAAAAAGGTAAAAAAATTACAAAGAGAAAAGAGGTAGAGGAGACAAA
AGGAGATCCGGGCCTAGGCTTCACTCCCCCAGAGTTCCACCGTCCTCCTCTCCTTGGCCTCGAATTCGGCCCGCTTCGATTGCC
GGTTCAGGAAGACCAGGAGGTTCATACTCTTGTCCTTTGCACCAAGACCATGTAAATGGCCTACTCGAAGGTGACGCTTTCAGC
ATGCGCGTCCCGTTGCCTCCTCCTTCTCCCGCGGCGCGGATGCTGTCGTGCACCGCTTCTCATCCGAGGAAGAGCACATCGCCG
CAGTTCGGGCAAGACCCGGCGCCTTCCAAGGTCTGGATCTCCTGCCTTATCCCGGAGCATGACTGGAGCCGCGGCGGCCTCTAG
AGTGTTGCCATCTCATCAGCCGTCGTTTGTCGCCTTTCTGGACAGAAGCCTCTTCAGTCTGGCTTGGACGCGGTCAAACTCCTC
ACGATCCGCACCGGGACATGGGCCATCTCCCAGTCAAGGGCCCCTTTGGCGGCCTCCCTTATGACTTCACGGCCGCCCTCAGCT
CCAACTTTGGCTACTCATTCCATCTCATGGCCAGCATCGGCCACTAAGATTCGTCACTACCGATGGACACATAGATCTAACCCT
GGAGCAAAGAAAGTTTAGGAGAAACATCATAATTAACAAGTAAGAAGGGGAAAGAAAAAGACGAAGTGAAATCACTGCGGTGGC
CGTGGTGCCAGGAGCGCCTGGGAGATGAACAGTACATCATCGGGTTTGGCTATCGTGCGTACCACTTCTGCTGTGAGGTTGGAC
ATTGTGCTTCCTCCACCTGGTCAAACAGGTTCCGCCACCAGGGGGGCCGGTGTCCTGCCAGAGCTTGGGACAGAAGCTCGTCTC
GGCGGCTGGACGATCCAACAGGATCGATCTGAGAGGCGATGAATGTCGCTGGATAACTCTTCGCAAATTCTACCTGACGGCGAT
CGCAGGGCCCTGTCAAGTTTCGTCCCGTCTACCTCCGACCATTTTTCCCAGGTCTGTTGATCACCGGACTGCTCATCCTGCACA
AGCGGAACTCCCACATCCCTCAGGAAGATGAGGTCGGACATAATGGGGAGACGAAAGGGCCTTCACGGCGAGCCCGACTCTCGC
CGTGCGAACTCGTCGCGACGCCACGCCCGCCATCATCCCGGGCCCTTCCCGGGTTCTGGATCCTTGGTGGCCAGCATCAATGCC
CCCTTTTTCCATAGCCTTGGATGCGCTGCCCCGCCTCAAAAAAGTCTATCACCGGTTTGGCAGCGGCCGGAGACTGCAGCGCCG
ACTCGAAGCGCTTGAACTGGCGGACGAGTAAGGGCTCCCCCATGCGGGACGAAGTTTCATCTCGACCTTCCTTGTTGATGTCGG
GCTCCGGCCCGCACCCCTGTTTGCTTTCCTAAGCAGCTTTCCTGGCCGCACCTCGTCCTTTGAGGAGCTACGTCATATTTCCAG
CGGGAAGTAGACATACCAGAGTCTCCTGTAAAAGCAACAGAACACACGGGACATTAGTATGGGTAAACAAAAAAAAGGCAAAAA
TAAACATCAACATCAGAACTATAATGAACCCGGCGCGACGAACCCTTGTCTATCGCCCGGGCATGCGGACTTTAAACATCCCTA
AAGCGGAAGGAATGACTCGGTCCCCCATGTCCGTCCAGGTTCGTGAAATTCCATACTGAAGGAACACGGATAAAAACAACCTAA
GAACTCCAAGCCTAACCTTGGAACGGGAGCGAGGGTCTAAGTCTCCCCGTTAGCCCGAACGCGGGACTCGGAGTACTGACTATC
CCTAGCTGGTCCCCGACATATGGAGGGAGCATAAAGCAAGATTGAAAGGGAGTTAACCCTCGCCCCGATAGCTAGCCCGGGAGT
CCCGTGTTCGTAGGGCTACTCGAAGCGATCCTTCCAGCTCTCCTGAAAGTAGGCCACCTCCGTCTGGGCCTGGTCCTTCTTTCA
CCTGGGCCGCCGTCAGCTCACACGCACCTTCTCCACTTCTGCAAAATGTGGTCCAGGGCCAGTTGGTTCACCTGGATGGCGACG
ATCTTCTCGCACTGAGTGCCCGAAGGGTTTGGGGAACAATGGTTTGGGTGGCCGCGAGCAGTCCCAACCAGTCGGGCAGGTTGA
TCCCCGTGTGACATAGACCTTTTTCCCCCCAAACATCCAGGTCGTCCGTGCTCAGCCCGCAATAGACACTTCCTCCGTTCCATA
GATGAACGGGGTGAGGGAGTTTGAGCGAAAGGTCCTGCACCGGAAGAAGATAATAAGAAAAGAAGAAAAAGGGAAAAATAAAAC
GAAAAAGGGACAAGGTCCGGAGCACTTACCACGTC
>m141120_105518_42134_c100732402550000001823141405141504_s1_p0/21150/3347_13310
TCTAGGCAGCTGCTAGGAAGATTTGGCTGAAAGGTCATTTTTGTTTGCTTGAATTAGGACCCAAAATTCATGTCAATAATACCC
TATATATTTATTAAGATGGGAAAGGTATCAAAAATTGGTTTGTTAGGAAGAATATGAGGGAATTACGAAATGATTTGGAGACCT
AGGCAGGCTGCAAGGAGATTAGGCTGAAAGGTCATTTTATTATATTTTGGGTGAATTAGGAATACCAATTCATGATCAATAAAT
TGCCCATTTTAATATTAGAGATGGAAAGTTTCTTGGCTTTTTGAATATGAGTGAATTACGAATCGCTTCGGAGCTTAGGCAGCT
GCCTAGGAGATTTGGCTTGAAAGGTCAATTTTTTTGTTTTGGTTGAATTAGTACCCCAATTCATGGGTCAATAATAGCCCTATA
TAATATTATTGAGATTGGAAAAGGTTTCAGAAAAAAATTAAGAGCTTTTTGGAGAGAAATAATGACGTGAATTACTAGATCGCTT
GAGCTTTAAGGCGCTGCTGGAGATTTTGGGCCTGAGGTTTTTTTTGTTATGTTGAATTAGTACCCCAATTCAAGGTCATAATAG
CCCTGTATAATATTTTGAGAGGAAGTTTCAATTTAGAGCTTGATTAGAGAGAAATTGAGTGAATTAACGAAATCGCTTCGCTGC
CTAGGCAGCTGCCTAGGATATTTGCCCTGAAGGGTCAATTTTTTTGTTTGGTTGGAATTAAGTAAACCAAAATTCATGGTCAAT
AATGCTAATATAAATTTTTGTGGGGTTCTATTAGGCATATTGGTTAGAGGAATTGAGTGGTTACGAATCGCTTTGGGAGCCCTA
GGCAGCTGCTGGAGATTTGCCTGAAAGGTCATATCCTTTGTTTGTTTGGTTGAATTGTACCAAAATTCCATGGTCCAATATAGC
TATATTATATTGAGATGGGAAGGTTCAGAAAAATACAGAGCTTTGTATTGAGGAAATATGATGAATTACTAGATCGCTTCGGAG
CTTCTTTAGGCCAGCGCCTACGGAGATTTGGCCTGAAAGGTCAATTTTTTTGTTTGGTTGAATTAGTAACCCCGAATTCAAGGT
CAATAATAAGCCTGTATACAATATTATTGAGATGGGAAAAAGTTACAAAAAGTATTAGAGCTTGTTAGAGATAATATGAGTGAA
TTAGAAATCGCTTCGGTGCCCTAGGACCAGCTGCTAGGATAAATCTTGGCTGAAAAAGAGTCAATTTTTTGTTTGTTGAATTAG
TAACGAATTCATGGTCAATAATAGCCCTATTATAATATTAGAATTGAGATGGGCAAAGTTTTCAAAAATATTAGAGCTTGTTAG
AGAGAAAATATGAGTGAACCTTACGAATCGCTATCGGAGCCCTAGGAAGTATGCCTAGGACGATTTTCCTGGCCTGAAAGGCAT
TTTTTAGTTTGTTGAATTAGGACCCCATTCATGGTCAATATAGCCCTATAATAAATAATATTGAGATGGGAAACGCGCAAAAAT
TTAAAGCTTCAGTTAGAGAGAAATATGAGAAGGATTAAACGAAGATGCTACGGAGCCTTAGGCAGCTGCCTACAGTGACGATTT
GGCCTGAAAGGTTCAACTTTTTTTGTTTGGTTGAATTAGGACCCCAATTCATGGTCAATAATAGCCCTATATAATTATTATTGT
AGGAGGAAAATGTTCAAAAAAAGATTAGAGCTTGTTGGAGGGAAAACTATGAAGTGAATTAACGAAAAATCGCTCTCGGAGCCT
AGGCTCCTGCCTAGGAGATTATGGCCTGCAGGTCCATTTTTTGTTTGGGTGGAAAATTAGGACCCCAATTCATGATCAATACCT
TGCCCTATAATAATAAATTATAATAGAGATGGGAAAAGGTTTCAACCACAAAAATTAAGCTTGTTAGAGAGAAATATGAGTTGA
ATTACGAAATCGCTTCGGAGCCCGTAGGCAGCTACCTAGGAGATTTGGCCTGAAGGTTCAATCTTTTTATGTTTGGTGAATTAG
GACCCCAATTCATGGTCAATAATTACCATATAATAATAATTATTCGAGATGGGAAAATGTTCAAAAAATTAGAGCTTGTTAGGG

AGAAATTATGAGTGAATTGCGAGATCGCTCTCGGAGCCCTAGGCAGCTCCTATTTAGGAAGATTTGGCCTAGAAAGGTCAATTT
TTTTGTATTGTGAATTAGTAAACTATTCATGGCAATAAAATAGCCTAATATATGAGGTTATTGAGATGGGAAAAGGTTCAAAAA
AATTAGAGCTTAGTTAGAGAGAATATGAGGGATTACGAGATCGGCTACGGAGCCCTAGGAAGTTGCCTAAGGAGATTTGGCCTG
AAAAGGCCAATTTTTTTAGTTGGTTGAATTAGGACACCCAATTCATGGTCAATAATAGCCCTATATAATATTTATAGAGATGGG
AAAAGGTTCAAAAAATGGTGGAGAGCTTGGGTTAGAGAGAAATATGAGGGAATTACGAGATCGCTAACGGATTCCCTAGGCAGC
TGCCTAGGAGATTTTTGGCCTGAAAGGTCACATTTTTTTGTTTTGTTGAATTAGACCCAAATTCATGCGGTCATAATAGCCCTA
ATATAGATAATTATATGAGATGGGAAAGAGGTTTCAAAACAAAATAATAGAGCCTTGTTTAGAGAAGCAAAGTCATGACTGGAA
TTACGAAATCGCTTATGGAGACCGTAGGCAGCTGCCAAGGAAGATTAGGCCTCGAAAGGTCAATTTTTTGATTGTGGAATTAGG
ATCCCCAATTCATGATCAATACTTGCCTATATAATATTAATATGGATGGGAAAGGAAAAGTTCAAAAAAATTAGAGCTTGTTTA
GAGAGGAAATATGAGTGAAATTACGAAAATCGCTTCGGAGCTTAGGCAGCTGCCTAGGAGATTTGGCCTGAAAAAGGTCAAATT
TTTTTTGATTGGTTGAATTAGGACGCCCAAATTCATCAGGCAATAATAAGCCCTATATAAAATATTATTGAGATATGAAAATTT
CAAAAAAATTAGATCTTGTTTTGGAGAATTATGAGTGAATTGCGAGAATCGCTTCGGAGCCCTCAGGCAGCTCTTATAGGAGAT
TTGGCCTGAAAGGTCAATTTTTTTGTCTTGGTTGACATTAGTAAAACTAATTCCATGGTCATAATAGCCCTATATAATATTAT
TGAGATGGGAAAAGGTTCCAAAAAAATTAGAGCTGTCCTAGAGAGAAATATGAGGGAGTTTAACGAGATCGCATACGGAGCCCT
AAGGAAGCTTCCTAAGGAGATTTGGGCTGAAAGGCCAATTTTTTTAAGTTTAGGTGGAATTACGGACCCCCCAATTCATGATCA
ATACTTGCCCTATAATAATATTATTGAGATGGGAAAAGGTTCAAAAAATTAGAAGCTTGTTAGAGAGAATATGAGTGAATTACG
AAATCGCTCGGAGCCTAGGCAGCTGCCTACAGGAGAAAATTTGCCTGAAAGGTCAACTATTTTTTGTTTGTTGGAATTGGACCC
CACATTCATAAGTCAATACAATAAGCGTATTAATATTCATTGAGATGGGAATAAGTTTCAAAAAACAGCTGCTTACACCCTCCC
ACCTCTCACGAAATCGCTTTGGAGCCCTTAGGCATGCTTGCAAGGAGATTAGGCCTGAAAGGAAATTTTTTGTTTGGTGGAATT
AGGATCCCAATTCATGATCAATACTGATGCCCTATATAATATTATTTGAGATGGGAAAAGTTTCAAAAAATTAGAGCTTTGTTA
GGAGAATATATGAGTGAATTACGAAAATCGCTTCGGAGCCATAGGAAGCTGACCTAGGAGATTTGGCTGAAAGGTCATTTTTTT
GTTTGGTTGATTTTAGGACGCACAATTACAATAGTCAATAATGAGCCCATATATAACAATTTCATGAGATGGGAAAAGTTTTCC
AAAAAAATTAGAGCTTGTTAGAGAGAAAATATGAGTGAAATAATAACGAAATCGCTTCCGGAGCCCTAGGAAAGCTGCCTAGGT
AGATTTGGCCTGAAGGTCAATTTTTTTGTTTGGTTGGATTTAGGACCCCAATTCTAGTCAGTAATAGGCCCTATATATCCATAA
TTATCTGAGATGGGGAAAGGTTCAAAAAAAAATTAGAGCTTGGTTAGAGAGAAATATGAGTGAATTACGAAATCGCTGTCGGAG
CCTTTAAGGCTAGCTGCCTAGAAGAAAGATTTGGCCTGAAAGGTTCAATTTTTTGTTTTTTGTTTGAATACTACCGCATTCATG
GTCAATAAAGCCCTATATAATATTTATTTAGATGGGAAAAGGTTCAAAAAAATTAGAGCTTGTTAGAGAGAAATATGAGGGAAT
TAGAATGCTACGGAGCCCATAGGCACCGTTGCCTAGGAGAATTTGGCGCGGGAAGGTCAATTTTTTGTTGGTTGAAAATTAGGA
AGCCAATTCATGGTCAATAATAGCCCATATATAATATTATTTAAGATGGGAAAAATGTTTAAAAAAGATTGAGAGCTTGTTAGG
GAGAATATGAGTGAATTAACGAAATCGAATACGGAGCCCTAGCAGCTGCCTAGGGGGAGCATGTTTGGCCTGAAAGGTCAATTT
TTTTGTTTTGGTGGAATTTAGGCCCAAAAATTCATAGATCACAATACTTGCCCTATAAATAATATTTATATGGAGATGAAAAAG
GTTCAAAAAATTAGAGCTTGTTAGAGAAGGGGAAAAAATTTAATGAGTGAGATACGAAATCGGCTTCGGGAGCCCTAGGCAAGC
GTGCCTAGGATATTTGGGCCTGAAAGGTAAATTTTTTTTGTTTGGTAGAATTAGGACCCCAATTCATGGTCAAGTTTATAGCCC
TATATATATATATATTGATAGGGAAATAGGTTTCAAAAGAATTAGAGCTTGTTAGGGAAGGAAAATGAGTGAATTACGAATCGC
TATCGGAGCCCTAAGGAAGCTGGCCTAGGAGATCTTCGGCCTGAAGTCAATTTTTTTGTTTGGTTGATTTAGGACCAATTCATA
GTCAATAAATAGCCCTATAAATAAATATTATTGAGCACTGGGAAAAGTTTTCAAAAAAAAATTAGAGCTTGTTAGAGAGAAATAT
GAGTGAATTACGAAATCGCTTCGGAGCCCTAGGCAGTACCCTGAGGAAGAGTTTGCCATGAAAAGGTTTTTTTTTGTTTTTTG
GTGGAATCTAGACCCCAATTCAGATGGTCAAGCTATAGCCCTATAATAATGATTATTGAGATGGAAAATGTTCAAAAAAATTCA
GACGCTTGTTAGGGAGCAATTATGAGTGATTGCGGAATCGCTGTTCCGGAGCCCTAGGCAGTCTGTTTCAAGGAGATTGGCCTG
AAGGTCCAATTTTTTGGTTTGGATTGAATTAGTAAAAACAATTCATGGTCATAATAGCCCCTATATAATAAATTATTGGAGATG
GGAAAAAGGTTCAAAAAAATTAGAGCTTGTTAGAGAGAAATATGAGGGAATTACGAGAATCGCAGGACGCCCTAGAAGCTGCCT
AGGAGATTTGGCGCTGAAGGCCAATTTTTTAGTTTGTTGAATTAGGACCCCCAATTCATGGTCAATAATAGCCCTATATAATAT
TATTGAGATGGGAAAAGGTCAATAAAATTAAAGCTTGTTTAGAGAAAATATGGAGGGAATTACGAGATCGCTACGGAGCCCTAG
GCAGCTGCCTAGGAGATTTGGCCTGAAAGGTCAATTTTTTTGTTTGGTTCAATTAGTACCCCAATGTCGTGGTCATATAGCCCT
ATACAATATTATTGAGATGGGAAAAGGGGTTCAGAAAAAATTAGAGCTTGTTATATAGAAATATGAGTGAGTTACTAGATCGCT
TCGGAGCCTAGGCAGCCGGCCTAGGAGATTTGGCCTGAAAGGTCAATTTTTGTTTGGTTGAATTTAGGCCCCGAATTCATGGTC
CAATAATAGCCCTATAAAAAAAAAATATTATATTGAGGATGGGAAGTTCAAAAAAATAGAGCTTGTAAGAAAGAGAATATGAGT
GAATTACAAATCGCTTTCGAGCCTAGGCAGCTACCTAGGAGTTTGTGGCTGAAAGGTCATTTTTTTGTTTGGCTGAATTAGGAG
ACCCCAATTCCATGGTAAGGTAATAGCCCTATTAATATTATTGAGATGGGAAATGTCAAAAAAATTAGAGCTTGTTAGGGAGAA
TTACTGAGTGAATTGGGAATCGCTTCGGAGCAAACCTAGGCAAGCTCTCTGATTTTGGAGATTTGGCCTGAAAGTCATTTTTTT
GGTTTGGGTTGAAATTAGTAACCAATTCATGGTCAATAAGTAGCCCTATATAATTTATTGAGATGGGAAAAGGTTCAAAAAATT
AGAGCTGTTAGATAGAAATATGAGTGAATTACGAAATCGCTGTCGGAGCCCTAGGCAGCTGCCTAGGAGATTTGGCCTGAAAGG
TCAATTTTTTTGTTTGGTTGAATTAGGAACCCAATTCATCAGTCATAATAGCCGTATATAATATTATTGAGATGGGAAAAGGTT
CAAAAAAATTAGATCTTGTTTAGAGGAGAAATATGAGTGAATTACGAAATCGCTTTGGAGGCCGCTAGGAGGCTGCTAGGAGAT
AGGCCTGGAAAGTCCATTGTGTTATGTTTGGTTGAATTAGGTCCCCAATTTCATCGTCAATATCATAGCCATATATAATATTAT
TGAGATTGGGAAAAAGATGTTCAAAAAAGATTAGAAGCTTGTTTAGGTGAGATATGACGTGAATTACGAAAATCGCTTCGAAG
CCCTAGGCAGCTGAACCAGGAGATTTGGCCTGAAAGGTAATATTATTTGTTTGGTGGAATTTAAGGACCCCAATTCATTGATCA
ATACTTGCCCTAAATAATATTATTGAGATGGAAAAAGGTTCAAAAAAATTAGAGCTTGTTTAGAGAGAAATATGAGTGATTACG

AATCGCTTCGGAGCGTGCCTAGGAGATTTGGCTGAAAGGTCAATTTTTTTTGTTTGGTGAATTAGGACCCAATTCATGGTCAAT
AATAGCCCTATATAATATTATTGAGATGGGAAATGTTCAAAAAAATTAGACTTTGTTAGGGAGAATTATGAGTGAATTGCGAGA
TCCCGCTTCGGAGCCCTAGGCAGCCTAATTAGGAGATTGGCCCCTGAAAGGTCAATTTTTTTGTTTGGTTGAATTAGTAAAACC
AATCATGGTCAATAATAGCCCTATATAATTATTATTGAATGGGAAAGGTTCAAAAAAATTAGAGCTTGTTAGAGAAGAAATATG
AGGGAATTACGAGATCGCTACGGAGCCCCTAGGCAGCTGCCTAGGAGAATTTGCCTGAAAGGTCATTTTTTTTTTGTTTGGTTC
AATTAGTACCCAATTCGTGGCAATATAGCCCTATATCAATATTAGGTTGAGATGGAAAAGGTTCAGAAAAATTAAGCCGTTGTT
ATATAGAAAATATGAGGAGTTACCTAGATCGCTTCGGAGCCTAGGCAGCGCCTAGGAGATTGGCCCTGAAAAGGTCAATTTTTT
TTGTTTGGTTGAATTAAGGAACGGCCAATTCCATGGTCAATAATGACCCAAATTAAAAAAATTTATTGAGATGGGAAAAGGTTC
AAAAAGCAAGAGGTTAAGGAACCAGGAGCTTAAAAAGCAACTCCGCAGAAGCCCCCCATGCTTATCAAACATATTACCGAAGAG
AAGTAGCCTATACTTATATCGTTGGCACACTACCGAACACGCAATAGTGCAGTGCTCGTGCACGGAAAGAGAAAAGTACAGAGG
CAAAAAAAACGCATAATTAACACTCAGTAAAGATTAGCCTGGGGGGCAGAGTCGAGATACCCCTTGATGGGAAAAGCTAGCTGC
TCAGTCTTAATTCCTTCCATATCATAACGTTCGACCCTCACTTTCAGGCCGCAGCCACATCCCATGTATTAACCGATCAACCAC
TGAGGCAAAGTCTTGTCTAAAGCTAGAAGCTTCAGCGTCGACTTCCTTAATGGGCCGGTTGAATCTTCCCGGGCAATTCGAGAT
CACTCTACCCACCCCCGAAGGATGGACCATTAGGGCAGCAGGCCTTGGCAGAGCTTTATAGTAGAATGTAGCTGGCATACCAAA
CGATGAAATGATAAGCCCCGGCCCCAACCGAAGATGTGGACCCCAGACTTTAACGTCGATGGGTCATCTAATGAGAATGGCTCG
AGGGTAAAGAGGGTCATTTTGATTACTCCCCCGCAGGAAGAAGATTTCATTAATGCCTTAAGATTCGACTTCAACTGCATTGAA
TAACGAGGCAAATACGAGGCTTTACCTCGCGAGAACTTCCGTATAGACCCAACAGAGCTGAAAGCTAAGCAATAGCATTGCTAC
AGCGGACTTCCCAGCTTTGTGGTTAACCAAATTTTGGAGAATACCAGGCTCCGTGGCACGAAAATGGTGAGTTACTTAGAAAAG
GCAAAATTCCCCGCTTGGAACATATTTGCGGTTTTATGCGATTGAACAGGTCCCCCAGAAGCAAAACTCAAATGCAGATGCCTT
AGCTTGGCCTCTGTTACTTCCCACCGAGAATGATGAGCTAAATGTTGTAGCCAATAGAACATCCCTCTGCGGCGCCTAGCATTA
ACGAGCTGGAGGCAGGAAACGTGTTGTATGTAATTGATTCCGAGCCCTACCCTGGATAAACCCCGATGTGCGAGATATCTTCCC
CCGGAGACCCACAGGAGTCCTTCCGCCAAAAGAATCGAAAGCCCAGCTCCCGAAAGTTAATGTATCAACTCCCGTTACCCCATT
TTGGATGGAGAAGCTGTAATACCAAGGGGATATTGCCAATGCCATTACTTCCGGTGCGTGACCTCACCGCGAAGCAAGAGGTAT
CATTGAAGAAATTCATTGAAAGGGTTCCTGTGGAGGCCATAAAAGCTGGGGCATAGCTGTCTAAGAAACATAATAACGCCAAGG
TGTATTTCTGGCCCCTACATGCGAAATCCAGATTCTTTCGAAAAATCCCAGTAAAAGAAGTGCTGATAAGGTGGCGCCAGCAGT
TCACGCAGCCGATTCCTCGAGCTCCACCAATCCGAGCTGAGCCCCATCGATGACTTCGCGCCATGGCCAATGTTTTGGTATGGG
GATCAACGCTGCATAGGCTCATCTCCCCCCGCAACCCTGGAAAAGGCGGATGAAGTATGGTTGTAGTTGCGCGTAGATTACTTC
ACCCAAAAGTGGACAGCAAAGCTGAACGCGATTAGCAGACAATAACTTCCAAGGAAAAGTACTTGAGCGTTCGTGGTAAAAGCA
TTTAACTGCGATATGGGGATGCCGAGGAAAAAAATCTGTATCCGACAAACCGGAACCCCAGTTGCGGATACGCAACTTGTTCAC
CGCAAACTTTTGGCCGAGAAAAATGGACATAATAAGAGTTTTTTCGTCCAGTAAGCGTCATCCCGCCAGAGCGAATGGCCAGAT
CAAAAAACTGTAAAAAAAACTCCCTGCCAAAGAGTTCGCTAAAAGAAAAAGTTGGAGGAAGCAAAAAGGACTGATGGCCTGCGA
AGAATTGCTCCAAAGTCCTATGGGGAAATATAGGACTATACGCTCGAACATCAACGGGGCCCATAACCGCCGCGTTCTCCTCTG
GGGCATACCGGTTGTAGAGGCTATGTTGCCTATTGAAGGTCGAAATTCCTACAATTCGAACCCCACCCGCTTCATGACGCGAAA
GGCCTGCGAACCCCAACAATCAGCTCAAGAAGAAACGCTTACACCCCTGTCGAAGAAGAGAAAAATGAAGCCCAGCCTGAAGGA
ATGCCGCATACCAAACAGCGAGCCCTACCCAGGTACTTCCAACAGAGGGTTCAAGATCGATAAGTTCCGGTGTGGGAGATTTGT
GCTAGGGCCGTGTAATTTTTGGCAAACACGAGATCCAGGCAGGCTGGGCTG
>m141120_105518_42134_c100732402550000001823141405141504_s1_p0/32707/14230_21100
AAAAAGCTTGATTCTATATTGAAAGGCTTATTGGTAAAGGTAGTGAAAAGTCATTTTAAAAGTGCTCAAAATATAGATTAAGCA
CCATTATTTATTTCGTACTTCATAATAATTGTTTTCATAAACATTACATGTTTCAGCAAGAACATAACATTTATACTTTTACTT
TATACTAATCATACAACATATAAAATCTACGTCATAATATATGCCTAAAGGGTCAGTCAAATGCGTAGGTAGGAATCAATAATC
TATATATACAGTTATAGTCAAAAGTACATAGTTATAAAAAGTTTATATTGAGGGCGAGTTAGTATATAACTAATGAATTCCGCG
CTAAGCAGCGTATGACATAGGTACAGGTCATAATCCGATGTTTATAGAATCAGTTAAGTCTTCAATTAACAATGTACAGATATA
GGAAAATAAATAGATTAAGATGTACCCTTCATAAACAATGTGGTCATATTAAAGTGTTCCCTCAACATAAGTCTGTTCATAATA
AAGATTTGGAACAATATTGAAGTGCCTACAGTTATTTTTTTTTTTTTTTTTGGTACATCAAACTTAGGTCAGTCATACAAGTTT
TGAGCACTGAGCCTTTACCTTGGAACATAAGTACTGTCTATTATCATTCATTCGCGGAAAAACACCTTTTAGGTCCAGTCCACA
CTTTCTCATATAGCTATCTCCAACCACTTAGTACAATCTTTGAAAAACCTTATTATCAATCGACAAACCCACATAAACCAATCC
CAACTTTCCAAATATTTATCTTAATTTTTAGCGAAGATAATATCCTATTACAACACGTGATGTTAATTGGTGAGTGTGTGTTCT
ATTATTTTGAGATCAATGATACATTCTACAGCCTAACTCTTCCCGTAAGAACTTATAATTTTCTCCTGTTTATCTCTTGTGCAA
TTGGAGTGAGTGTATGGCTAGTATATTATCATGCAAATCACTTAATTTCATCTTTTGATGTTTTTTCTTCAATAATCTAATTAA
TTTTTGCTTCTTCTATTGCTAATAAATATAATCCAACATTATTCAAAACATCCCAATATTACTCCAAACACCATTTACCCCAGC
AACTCACTTTTACATATGCTACATTGGATATATCAATAGGAAAGAAAAAAAAATAAACAAAGGCCCTTCAAATAAGTCTAGCAC
ATAAAATAATATATATAAACAAAAAAGAAATACACAATTAGGTACTCGAATTGGAGTAAGTCTTAGGATTTTAATAAAATCAAG
GCTGGCTTGAGGCTTCAACATTCCAAAAAGAAATTGCCTAATCATCTTCCTAGCATATAATCATCTCCCCAGATTATCATCTCA
AAATAAGCAAGCAAACAATCGTTCTAGGATATTTAGTACATTTTCATAATTTTTATTTTCAACATTCAACAAGCATAAAAAACT
TTTTTAGTTAACACAAAATTATATTAATAATCATGATCAAAGCTCTTAAATTTTTACGTAGACTCAAAGTATGTTCCCAAAGAT
GGAAAGAACTATTCAAACCGCAAGCACATAGGAGGAATTTTTTCATAAGCTTCATTCCATTCAATTTATACAAGGCATAATTCA
TTCAACTCATATTATCCATTGCATAATCAATATTTTGTCTTAGTTGTTCAACTAACACTCCTTAAACAACCTAAGACAAACACC
ACTAAAAAATAAAAAAAAAAAAACTTAAAACAAACAATTAAGTTCCTTCCCCCAAATGAATATCTTAAAAAACCCCCTTATACA

ACATTTGTCCCTCAGATGTTAGAGTAATAAAATAGCTTAAGGAAAAGAAACTTACCTGTACCTCAGACTACTTAAAAATGATAT
CGAATCAAGAAATGTGAATTTGAAAAATTGGAAAAAGAAAATTCTCAAGTTACCCATAATTCATTATGCCATTGTTTAGGATTG
CGTCGATATACGCGTTGAAGTATAAAATGTAGGAAATCTCAATATTTTAATGGAAATTAAAATCAATAAAAATAAAAAACATAA
TAAACTAAAATTCACAAATTATGTTCCCATAATCAAAAACCTTAAAATAAAACTATAATAAACAAAATGCCAAATAAAAGATTG
GAATTGCACTTCCCAAATGCGCCATCCGTTAACCGTCATTTAGAATCCAGACACTTGCTTTATTTTTGTTCATATGTGCAACTT
TTGGACAATGTCCTCCTGCCTCCCAACATCCCTTGAGAGCCATAGTATTATGAGTTACGATACTTCGTTATTCTGGATGATTAG
AAATTGTGGAACTTTTTTTAACCCTGCTCCATAGTAAAAATTCGTAAATATATGATGCCTTTTACCTAAAGACCTTTTTAAACA
TGCACGTCCGGAGTTTATTCGAAGTCCAGTTTTTAACCACAGATCTTTTCCTTAGAGACTGTTCTCACAAACTTGTTCTCTCCT
TGAGTTTTGTAACCACTTCAAATCTAACACCAAAGTTGGAAGTTTCTTGTGCATGCAAACAACGTTAAAATTATTTACCTCGCT
CTTTTTTGCACTCCGCAAGCCTTCGTTAACCTCACGCCTTTTGTTAACATTGATTAATGCCCTACCCAGATAGCCAGAATGGAA
CTTCTACAGTAATTTATTGAATATTTTTTTTCATCTTCCTCCATATTCAAAGATGATAAATTTTTTGTTCTATTTTAAGTCCGT
AGGAAAGATGAATTTATCCACTTTTCCACCAAATACATCCCCTCATCACCCCACGAGGATGTTTGACTGATCCAAACTGCCTTC
TGCGTATAAAGGAAGGACAATGGTTGAATAGGCCGAAGCTTATGGGCTCGCCTAAATTCCAACTTCTGACAAAGACTATTGAGT
AGGGAGACCCACTAAAGATTTTCACATTGGCCACATTAAAATCAACATAAAGCCTAATGCCGTTTTAATATCGATCCATATATA
GAATCATTAGAGAATAATTATAGCAATACATACCAGGCCGATCTTTTAAGCCTTGGGAGGTGTTTCCTTTATCCCTCTTGAATT
AGTACTCTGGCGACTTGCAAGTTACCTCGATACAAGTGCCTTACTCTCTCTTTTATAAAGTGCGCGTGGCCTATACATATTTAG
TTATAGGGCCTTTTATTTGGTTTCAATTTTACACACATTCTATTTTCAATATGAAACCTTGAAATCACATGAGACTTTAGGCAC
TTTGTATGCGACCCAGTGCATGTCGACCAAAGGAAATGTTTGTGTGAAAGCTCAAAAACGACCTATATAAAATTTAGAAAATTG
TTTGTCTAGTTTGGAGGGGGGTGTGCCTCTTCTTTTGAAGCCCCTCTCTGATGTAATGAAGTGTAAACCAGGTATATTATAAGT
GTGATGCTCATATATTCCTCTAATATATACATGGACTCCTGTTAGTATGCGTCGAATTTATTATTTTCAGGTTAGACTTTCCAT
TCGTTTCAATTTTACCCTTTTTTTCATTGACCGCAATTTTGTTAACTGATAACCATAGAGCTTCTTTATTTGGGCCCTATCCAA
CTTCTTACCTTTACTCCTCAAAAGAATAATTTGGCATTCCGTATTGTTTTTTTTTGGTTTACGTCAGTGGAGAAGCTTGGTAAA
AGTTCCATTCGGAGTGCTCAATTAAGACATTAACTTTGCCAGTTGACCCAGTTTTTGTGTCTCCCAAACTTCTCTTATTTAATT
GACACAAACCAGTTGGTTTTCAGTCATAAGAGTATGGAATTCAAACATGTCTGATTTGTGCTTCTGATAGGATTCTACTATGAG
GATGGCGAGTGGTGGGATTGTTACACTGCATTAGCTTGGCGAGGATCATAATAAGGTCCGAAGGATTTAGGCCATAACTAAATT
GATTGATGGGGTGGAGTATTGGTGCGGGAATGTGTATTCTTGTGGGTCATATTGCGGTGTGTTGCTTTCCTTGATCTTCACTCC
TTTTCCCAAAGGTAACAAAAAGTTATAGTGTGATTTCTGCTAAACCGGTTTTCTTGAAGTTGGCAAGAACATGGGAGAAAATAC
ATGGATATATTGCTAGCCTTTGGCTATTAATCCTAATTGACTTGAAACTTGTTTCCAATAAGGCAAAATGATTTAAATCCATTG
CCAGGAGACAGGCTGGTTAGGTGGATGACGCCATTCAACCACCATTTCATTCCTACACATACTTTGCTAATTGAATGGGCTATT
TGGAGATGAATGAGGTGTAGTTTCTGTTGGTAGGTGGGTGTTTCATCCAAAAGACGCCACTCCGGAGCTGTAAGCATTGAGATT
TGGCATCTAATTCCAAATCATAGGCTTCCCAGCACATTCTTTGTATTTTCGCCTCCTTCCAAGTAGCCACTGATAATTAGTTCA
TGGCCATCTCTTCCTAAAGTAGATCATAAGCTTTCGTTGGCACTCTTTCTCATAAATACCCCCTCCCTGCTGCCTGGCATCTAG
ATAGTGCAAGTGTAACCGCCTAAAAACCCCCATTGTAAAAATTAATGGACTAGCATCCCCACTTCTTTAATTCCCATGATGTTG
ACACTATACTCTGACAATTCTAATACAATTCTCTTTCCCATGCATCCTACAATGAACTCCTTCTCTCCTCTCCCATATGAACCA
GAAATTATTAATCTCCCCTCGTCAACCGTGCAGTCTTGGGATGGAGGAAGAAATTTGGCCAAGGTACATTTGAGTGAGCTCCTC
CCATCTGTAAAGTCTGAGTGTTATTTTGTCTCCAAGAAGATCCTCTCCCTCTTTATATCATATGCTATTGTTATAGTGGTAGTA
GTGCATTGAACAAATCAAGATAGGTAGGTAATGGGATAAGGTGGGTAGTGATAGGTGTGATGTGAAGTAATTTGTGTGAGCGGT
GCGCTCTATTGGTAGTAAGGTAGTAGCCGCCGAGAGTAGTGAGAGTGATGAGAGGTATTTGAGGGAGATAAGATAGTATGGAGG
AGTGAAATGTGAATGTGAGTACCGTGGTAAAGTAGGCAGTAGCAGTAGTAGCTAGCGTAGCAGTAGCAGTAGTAGGTAAGCAGT
AGCAGATGGCAGTGGCAGTGGCAGATAGCAGACTAGCAGTAGCAGCTAGCAGTAGGAGTAGTAGTAGGCAGTACAGTAGTAAGG
AGTAAAGGAGTAGTAGTAGTAGTAGCAGTAGTAGATCAGTAGTAGCAGTAGTAGCAGTTTCAGTAGCTAGAAAGTAGTAGTAGTAAG
TAGCAGATAGCAAGTAGTAGCAGTAGTAGCAAGTAGTAGTAGAAGTAGTTAGTTAGTAGTAGTAGTGAGTAGCCAGTAGCAAGT
AGCAGTAGCCAGATAAGACAGTAAGCAGATAAGCAGTAGCAAGTAAAAAGCAGTAGCAGATAACAGATAATAGAAAGTCCAGTA
GCAGTAGCCATAAGTGAGTAGCAGTAGGCAGTAGTAGCCAGAGTAGCAGGTAGCAAGGAGTTAGATAGAGTAGCAGGAGTAGGA
GTAAGCAGCTAGTCAGATAGTAGTAGTAGTAGTAAGTATCTAGTAAGTAGTAAGCAGATAATAGTAGCAGGAAGTCGTAGCAAG
GAGTAGGAGTAGGAGTAGCAGTAGGAGTAGCAAGTAAGGGTAGTAGTGCAGTAGCAGTACAGTTAGCAGTAAGCTGTAGCAAGT
TAGAAATAGCAAGCTTAGTCGTAGTAGCAGATAGTCGTAAAGTGGGTAGTAAGGTAAGTAGTGGTAGTAGTGGTAAGTGCCGTA
GTGGAAGTTAGTAGTAGTGTTTAGTGGTAGTCGAATAGTGATAGTAGTAATAGTAGTAGTAAGTAAGTAGTAGTAAGTGGTAA
GTAGTAAGTTAGTGTAGGGTATGCAGTGGTAGCGCAGCAGTAGCAGCGTAGTAGCGTAGCCAAGTAAGCAGAAGCAGTAGTTAG
TTAGCAGTTAGCTAAGTAGTAGTCAGTGGTAGTGGTAGTGGTAGTGGTAGTGGAGTGGTAAGTTGGTAGTTGTAGTAGTATGGT
AGTGTAGTTTGGTAGTGTAGTAGTATGTAGTAGTAGTAGTATAGTAGTAGTAGTAGTAGTAAGCGGTAAGTAGTAGGTAGTAGT
AGTAGTGGTAGTGAAGTAGTAGTGTAGTAGTAGTAGTGGTAGTGGTAGTAGTGGTAGTAGTCGTAAGTGGATAAGTAGTCGTAA
GTGGTAGTTAGTGGTAGTAGTGGTAGTGGTAGTAGTGGTAGCTGGTAGAAGATGGTAATGCAGTAGCAGTAAGCAAAATTAGTA
GTAGCAGTGGTAGTGTAGTGGTAGTAGTGGTAGTGGTAGTGGTAGTCGGTAGTTGGTAGCAGTAGTAAGTAGTGGTAAGTGGTA
GTGGTAGTAAGTAGGTAGTGGTAAAGTGGTAGTAGTACGTAGTTGGTAGTGGGTAGAAGTGGTAGTTGGTAGAAGTAGTAGTGT
AATGTGGTAAGTAGTGGTGTAGGGTAGGGTAGTAAGTGGTAGTGGTAAGTAAGTTGGTAGGTAGTGGTATGTGGTAGTGGTAAG
TAATGGTAGTAGTGTAGTGGTAGTGGTAGTAGTGGTAGTAGTCGTAGTGGTAAGTAGTGGTAGATGGTAGTAGTGGTCAGCAAG
TAGCCAGTACGCAGTCAAGCAGTAGTCGTGTAGTAGTGTAAGTAGTAGTAAGTGGTAGTATAGTAGTGTAGTAAGGTGTAGTAG

TAGGAGTAGCGTAGCAGATAAGTAGTAGCAGTGGTAGAAGCAGTAGCAGTAGGCAGTGCCAGCAGTCGGCAGTAGTAGTAAGCA
GTAGTAAACAGTAGTAGCGTAGCCAGCAGTAAGTAGCATAGTCAGCAGTCAGTAAAGTGGTAGTGGTTGGGTGTGGTGTTGAAG
AGGTGGTGGTGGTAGATGGTCAGTCGGTATAGTGGTAGTGGGCTAGTGGTAAGTGGTAGTCGGTGG
>m141120_105518_42134_c100732402550000001823141405141504_s1_p0/33129/2743_8330
GGCCTGTTAGCAGGGTGGGCAAAGTCTATGCAGCGTTCCCTGGAAAAAACCTGTTGTCTTAGAAAGGAAAGAATCCTTCCTGCC
TTTGTCTTTGGAAGTCTAGCTTTGGCATATTAGCAAGCATCTGCTGCCGTTCCCAGAGACTGTAGAGGGTTGTTGGCCCTTGGT
CTCATCTCTAAGCCTCGATCCTTATGTGCCGACTGGTCGAGCCTGATTCACCTTGGAGGGCATAACTCTTACAAGGTTTATCCG
TAAGCCAACGACTCTGCACGATCCTAGCAAGTGATAACAGGTTTTTCTGAAAATCCACCACCACGGTCTAAAGCCAATACTCG
AGCAATCAATAATGACAATTAAATCCAACGGCGTTTTAAATATTATTCACATAAATTTAGAAGAATGCTAATAAGTACGCCCGT
CATCAATATAACATAGCAGTCAAAGGGCCAGGCCTATCAGATAATATCTCTCAGGCTTCTAGTAAACAAGCAGATAAGGTATGT
TTTACATTGTCATTAAAACAGTTAATTCATATAATCACATATAGGTTACCAAACCTGAGTCGTGCTTTGTCTTCAGTGGAAAAG
TGTACATGCCAACTAGTCTTCAGGTAACCATTTAAACCTAAACCACTCTGATACTGAATTTGTGGTAATGCCCTATGAATAAGA
CAAGATGCAATGGACCCACCTATTTCTAAGAACCTTGGACATTAATACTAACTAGACCATAGCTACCATTTTTTTTTGAAAAC
ATACATAAGAAATATCATAACTTTATTGAAAAACCTCCAAAATTTTAATATTGCTACCAATGCCCGGCCCGCGAGGCTCGACGG
CCATCGCGCCGTGGCCCCCACGCCTGAAGGTAGTAGGTAGGAGTTTAGTAGTAGTCAGTAGTGGTAGGTATGTAGTAGTAGTAG
CAGCTGCAGGGAGTAAGCAGTAGGGAGTACGGTGGTTAGTAGAAAGTAGTAGTAGACAGTAGCAGTAGCAGTAGCAGCTAGCAG
TAGCAGGTAGCAGTACGAGTAGCAGTTTGCAGTAGCAGTAAGTAAAGGCAGCAGTAGCATAGGAGTACAGTGCAGCTAGCAGAT
AGTAGTAGTAGTAGTAGTAGTAGTAGTTGGTAGTAGTAGTAGTAAGTAGCAGGCAGTTTGCAGTAGCAGTAGCAAGTAGCAGTA
GCAGCCCACCACCGCAGCAAGTAGCAGTAGCATTGTAGTAGGGAGTAGCCAGTAGCAGCTGTAGCAGCAGTAGTAGTAAGCAAG
GAGCAAGAGCACAGCAGCAAGCAGTAGTAGGAGTGTAGCGTAGTAAGCAGCAGTAGTAGTAAGTAGCAGTAGCGGTAGCAGTAG
CAGGACAGAGCAGCAACAGCAGTAGCGTCAGTAGTAGCAGTAGCCGCAGTAGTAGTAGTAGTATGAGTAGTAGTAGTAGTAGTA
GTAGTAGTTGGAGTTTGGAGTAGCAGCAGCCACAGGAGTAGGTGTAGTAGTAGCAGTAGCAGTAGCAGTAGGCAGTAGTAAGTA
AGTAGTAGTAGTCGTTTAGTATTAGTTTGGTAGTTTGTGGTTAGCGTGGAATCGTTGGTTTTAAGTCGGGACTTAGCTTGGAAA
CCTCATAGCAGAAACTTGTGGATTTTATTAAGTTTAACCTATAGTTTGAAGAGATATTGAATTATACAATACAGGTTTGATGTA
GTATATGCTGGATCAAGAAAATATCATTTATTATAAACCTATTGTTTAGCTATAGTGCTAAATAAGAGTGTGACACTTGTCATA
ATCATGATTATTAAGGGAGATTAAGTAATCGTTTTGATGAATTGTTTTAATAAAGAAAGATTAGAAGCTCTACGAACTTGCCAG
GAGTTGTTAGGATCGTATTAGACTGCAGTCAAGTTGGTTTCTCCAATTAAAATTATGCTAAAAAAGTGCAAATATGTGTTTAAT
ATATCAACGTATGCCGGATATAGTCGCAGCATAGGGGCGATATATCGCCTAAAAAGACAAGGTTCATCGACTTTGCATGAACGA
TCGAACGGGCCTCGGGAATAGTGGGGGGAAGGGATATATTGGCCTTAAGGGGCGATAATAGTTGGCTCCATGTTTGTATTTTTG
AAAGTTTGGTTTTTCAATTTTAAAAAATAGCCTTAACCACTTTAAACCTGCCTTTGAACGATTTTGACCGATGTTCTGGGCAGT
CTGTTGAACGAAAATTCCAAATCCTTTTCATTTTAATATTCATTTATTTTTTCAAATTAAAGGGGTTAGTTTTACTGCTTGAAC
TCAATAAAGTTAGACCTAGTGCTCAACCATTTTCTCATTCTTTAAGCATTTGATCAGAGCCTCCCAAGGTAACTAGATGTTACT
AATAGAGAGATAACACTTGGCTTTGGGGATAAAGCTTTATCATTCTAAATCTTTCTAAACACTTGGAAGTGAGAATAGTAGTGT
GATTTCGATATTGAAGGTTTAGATCAATCCATAAGATCCAACCAAGGTATTCTTATTCTAAATATTATTCTTTTATGGTTCTTC
TAATTTTTCTGTTTATTCAGCAATCCTAACTTTTGTTTATGAATTATTGATTAGGTAATTTAAGTTCTGGAGAACTTAAGGTTT
CTTGCTTGGTAACGTTTCTTCTATGATGGTTTAGTGGTTCTTTCATCTCTTTATTCTTAGAGAATACATCATCATTTTACTGTT
GGTCTTAAAGGAGTGTTCTACATCTCGTTCTTGGTTCCAAAGTATCTCGTTGTTTGGTAAGGAAAATAGGATAAATTTTATGTG
CTTATTATGTTATGGATATGTATAATGTTTATGTTATTATATGTATAATGTATAGTATGTCGCCTTATAGTCCTTAGGCATATG
ACTTGATTTAACAACAAGCCCAAGAAATATGTTGTTCAGTCACTTGGGCATATGACTTGCTGTAGATAAGCAAGCCCTGAGAAT
TTATGATTGGGCATATGACTTGTTAACCGATAGCAAGCCGCCATAATTTATGAAGTGGGCGTATGACTTGCTTAGATAAGGAGC
CAAGAAGTTATATTGGGGGCATATGACTTGGCTGTAGATATGCCAAGCCCCACAAATTTTATGGGCCATATGACTTGCTTAGGC
AACAAGCCCAAGAATCTATGATGGGCATTATTATTATAGTCCTAATATGGATATATGTTTTTATATAGTCAACGTTTTTATAGT
ACATGATTATGATCAATAGTCCTTTATGCCTTATGAAGATAGTGATGTTGTTGTTAGGTAGATTTTCCTTACTGGGCAGAGCTC
ACTCCTTTATTTTTAGAGTCTGATGCAGAAATGAAATTATGGAAGGCCAGAAGGATTCTTTGGTAAGCTTGGTTGGTGTTTGAA
GAATGAATGGATTCAATGGACTGGCGTGTCGAATCGACGGAACGACGTTATTAATATTTCTAGTCCTTAATTATGTTTTCTTGA
ATGTAATTGCCGCATTAGTATTTAAAGTTTAAGTGTTTTATATTTTGTAAACAATGGGTATCCCATACCTATCACGTTTTATTT
TATACTTTTATGTATTCCGGTAGCAATATAAATTTTGGGTTTTCGAATAAAGGTTAAATGATTATTTCTTATGTATGTTTTCTA
AAAAAAAATGGTAGCTATGTCTAGTATATTATGGTCCAAGTCTTTAGAAAAATGTTTGGGTCATCTGCATCTTGGCTATCTAGG
GCATTACAATTAGATCAGGAGTGTTTAGGGTTTTAAGGTTCCTGAGAGACTAGTTGGCCCATGCGTACACTTGCCACTGAACTT
ACAAGCACGACTCAGGGTTTGGTAACTCTTATATGTGATTATATGATTAACTGTTTAATGAAATGAGAATACCTTATCTGCCTG
CTTTACTGGAAGCCTGAGATGATATTGATAGGGCCTGGCCCTTGACTGCTATGTGTATATGATGAGGCGTACTTTATTAGCATT
TCTAATGTATGTGAATAATTATTAAAAAGCCTTGTTTGGATTGTAAATCTGATAATTGTTTGTCGAGTATTGGCTTTAGACCCG
TGGGGTGATTTCAAAAAACCTGTTTTATCATTGCTAGATCTGCAGCAGTCGTTGACTACGGATAAACTTGTAGAAGTTATGCTC
CAAGGTGATCAGCTACGACCAGTCGGCACTCAAGATCGATGGGCTAGAGATGATGACCAAGGCCGAAAACCCATCTAACCAGTC
TTCTGGGAAACTGCAGCAGATGGCTTTGCTATATGCAAGCTAGACGTTCAAAGACAAGAGGAAGGGATTCTTCGTTCTAAGACA
ACAGGTTTTTCCAGGGAACGCTGCACTAGACTTGCCACCCCTGCTAAGGCACCAGTAGTACCAGCAACCTGAAGATTAGGAATA
GGTGAGAGTCGTTTTGTACGAAGGTTAGGAAGGCAGCATGCCCTCCAAATCTTTGAGGAGACGCAAATCCCAAATTAGGCTGAA
ACAGTGGATGAGCTTGATCACCCCGCGGGATCCATCTTTGGACCTTCATGAGGGTGGCAGGTAATGATAGGCGTGGTTGTGCCA

CTTACAGTGTTTTGAGTATGATTCCCACATTCGTGGGAGGTGCATCAGACTCGAGATGTTTCTAGACCTAGAGTTGGATGAGTT
CAGAGATTTGTTCGCCATAATACTACAGCAGTTTGTCGGTCAGGGCGGTGAAGGTCGGAACGAGTTCACCAGGTCTGGTACACA
GGTAACATGACCAAGTTTAATTGAGTTGCCTAAAAATTGAACATCACTAGCAAATTTTGCACCTGATCTAGTGCCTACAGATGC
CACCAGGCAAAGAGATGTTTGTTCTGTGGCTGAATGCTATGATAAGACCGAGGAATGTGCAATTCACATTAGTCACCGTGGCAG
ACTTAATGTAATGCCTAAAGGTTGGTTGAGAAGCCCTCCGAACAACTGGAGGAGGTAGGAGAACGAAGATATGGAGGAAGAGCG
CTGCAAGACGGATGCTTCGGATGGAAGGTGCCTGCACTTATAGATCTGGTAGGGCTGAGGCCCCCAGTGATCAGCAGAAGGTGG
AAGACCCACTCTGACACCTCGACCATTGATGTTCCTACATAGAGGAGGGCCAAGGTGTCCCACGGGTGGCCCGCAAGGCAGAGG
TGAGACATGAGAAGTTACCCGTCCAGAGGTGGTGCTTAGATGTAGATCACCATCCGCGGTGGGTCCGAAAAGCTTATTATTACT
TGTGCGGAGTGATTGGGCACCTCAAAGAAGGACTTAGCCGCGAACTTTTGAAGAAAGAAGCAAGCAAGGAAGGAAGATAGCTTG
ACTCCACGTCGAGTGTGTGCCACCTTGACGTACAGGCAGAGGCCTGAGGCTAGTGCCTTGTTGTGACAGGTCATGCTTTTTCTA
GCACTAGCACTTCCATATTCTTGTATTGATTTGACTTTGATGCTACACATTCATTTGTTTTCTAGCAAAGCGTGATTGATAGAT
TGTGTAGGCCTTATGAGTATTATTACTGCGGGGTTGAACTATACTGCCTTACAAAAGGAACTGGTAGTTTCTAGGAGATGGAGT
TTAGATCCACATGCAAGATGATGGGGGTGGAGCACAACCAGAG
>m141120_105518_42134_c100732402550000001823141405141504_s1_p0/38850/0_7007
ACAGGAGAGAGAGAACTATGGTTCAAAAAATATTAGAGCTTGTTAGAGAGAAATAATGAGCGAATTACGAGATCGCTTCGGAGCGC
TAAAGGCAAGCCTGACTAGGAATTCTGGCACGAAAGGCCAATTTTTCTTGTTTGCTGTTTTAGGACCCGCAATTCATGGCTAAT
TAGCATTACTATCAATGATATGTATGGAGATGGGAAATGGTTCCAAAAAAATTAGTAGCTTGTTAGGAGAGAAAATACTGAGTG
AATTCCCGAGCTCGCTGCTCGAGCTCTAGCGCCCAGCTGCCTAACGGAGATTGGCAGGATAGGCAAATTTTTTTTGTTTGCTTG
AATTAGAATCCAATTTCATTTGTCATAAAGTAGCCTATATTATCACGTTATTGAGCATGAGGAAAAGCGTTCAAAAAATTAGAG
CCTTGTTAAGCAGAAATATGAGCGAATTACGCAGATCGCTTCGGAAGCCCTAGGCAGCTGCCTAGTGACGAATTTGGCCAGGCA
AAGGCCACATTTTGGTTTGGTTGAATTAAGACCCTAAGTTCATAGGTCAACAGTATACCTATATGAAATCATAAATGGACGATG
AATGGGTCAAAAAATTAAGAAGCTTGTTAGAGAGAAATTATGAGTGAATTCGAGATCGCTTCCGGAGCCCTAGGCAACGCTGCC
CACGCAGACTTTGGCACGAACAGGCCTAATTTTTTTGTTTGGTTGAATTAAGGGACCCTGCAATCATCATGGTCAAAAGTATAC
CTATACTGATATTGATGGAGACGGGAAATGGTTTCAAAAAAATTAGAGCTTGTTAGAGAGAAAGATGAAGTGAATTACGAGATC
GCTTCGGAGCCCTATTGCATGCTGCCCTACGAGATTGGCAGGATAGGCCAATTTTTTGGCTTGGTGAATTAGGATCCCAATTT
ATTGTCAAAAGGTAGCCCTAATGTTAATCCACGTACTCTGAGCATGGGAAAAGGATACAAAAATAATAGAGGCTTGTTAAGAGA
GAATATGAGCGAATTACGAGATCGCTTCGAGCCTTAGGCAACTGCCTTATGGAGATTTGGCAGCGCAAGGCCCAATTTTTTTGT
TTGGTTAAATAGGACCCAAAATTCATGGTCAAACATAGTATACCTATCATGATATTAATGGAGAATGGAAACATGGGTTCAAAC
AAAAATTAGAAGCTTGCTTAGTAGAGAAATACTGAGTGAAATTACGAGATCGCCTTCGGCAGCCTAAGGACTGCTGCCTAGAGA
TTTCGGGCAAGGATAGGCAATTTTATGCCTTTGGCTTGAATTAGGACCAATTTATTGTCAGAAGTAGCCTATATAGATAGTATG
TGAGATGGGAAAAGGTTCAAAAAAATTGGAGCATCAAGTTGAGAAGAGAAATATGAGTGAATTCGAGATTCGCTTTCGGAGCCT
AGGACACAGCCTGGCAATATTTGGACACGAAAGGTCAATTTTTTGTTTGGTTGAATTAGGACCCCAATTCAATGGTATATAGTA
CCCAACATACACTACTTAATTGAAATGGGGAAAGGTTCAAAAAAAATTAGAGCTTTGTTATAGACGAAATATGAGTGAACTCCG
AGATCGCCTCGGAGTCCCTAGGCCCTGCTGCATAGGACGATGTTGGCCAGGAAAGAACCCAAATTTTTTTGTTTGGTTGAAATT
AGGACCCCCAATGTCATGGTCAATAAGTATACCCTACATCATGATATTATCGGAGACTGCGGAAATGTTTCAAAAATTAGCGCT
TTGTTAGAGAGTAAATACTGAGTGACTTACCGAGATCGCTTCGGAGCCCCTAGGGTCAGCTGCCAACGCGATATTTCTGGTAGG
AAAGGACAATTTTTAGTTTTGGGTTGAATTAGGACCCAATTCATGGTCAATAAGTATTCCTATATGATATTATGGAGATGGGAA
AAATGGTTTCAAAAAATAGAGCCTCGTTAGAGAGAAATATATGAATTACGAGATCGCTTCGGAGCCCTAGGATGCTTGCCTAGG
AGATTCTGGCAGGAAAAGGCCAATTTTTAGTTTGGCTGGAATTAGCATTCCCAATTTCATGTTCAATAGTAGTCCCCCTATATA
ATATTATTGAAATGGGAAAAGTTTCAAAAAAATTGAGCTTGTTAAAGAGAAAGATGAGTGAAATACGAGATCGCATCGGCAGCC
CTACCCGGCAGCTGCCTAGGAGAATTTGCAACAAGGCCAAATTTTTTTTGTTTGGTTAAATTAAGGACTCAAATTCATGCGTCA
TAGTAGCCTATAGAATCATTATTGAAATTGGGAATAAGCGTTCAAAAAAAATTAAGCTTGTTAGCAGGAGAAATATACGTCAAT
TACCTAGATCGCTCGAGCCGCTAGGCAGCTGCCTACGGAGATTTGGCAGGAAAGGCAATTTTTTTTGTTTGGCTGAATCCTAGG
ACCCAATCTTCATGGTCATAGTAGCCTTATAGAAATATTCGTTGAAACGGGAAAAAGGTTCAAAAAAATTAGCAGCTTGTTAGA
ACCGAAATATGAAGTAATTACGCAGCATCTGCTGCATCGGAGCCCTAGGCAGCTGGCCTAGGAGATTGGGCAGGATAGGCCATT
TTTGTTTGTTCCTTGAATTAGAATCCCAATTTATTGATCAAATAGTAGCCCTATATTAATAGTATTGAGATGCGGAAAGAGGTT
CAAAAAAATTAGAAGCTTGTTAGAGAAAATATGACGGAATTACCGAATCGCTTCGGAGCCCTAGAGCTGCCTAAGGGAGATTTG
GACAGGAAAGGCCATTTGTGTTTGGTTGAATTAGACCCCAATTCATGGTCACAGTATACTATATGCATATACATGGAGATGGAA
ATGGGGTCAAAAAAATTAGAGCTTGTTAGAGAGAAATATGGATGGACATTACGCAATCGCTTCGGAGCCCCTACGGCACGCTGC
CACGAGATTGGCACAAAGGCTCAATTTTTTTGTTTGGTTGAAATTAGGAACCTGCAATTCATGGTCAAAAGTATACCTATATGT
ATATTATTATCGGAGATGGGAAAACCTGGTTCAAAAAAAATAAATAGAGCTTGTTAGAGAGAATATGAGTGAATTAAACGAGAT
CCTTCGGAGCCTCTAAGGCACTGCTACGAGATTCGGGCCAGCATAGGCCATTTGTTTTTGCTTGGTTGAATTCAGCGATCCCAA
TATGTGTCAAAAGTACCCCTATGTAATAGTATATGAGAATGGGAAAGGATAAAAAAAGTAGAGCTTGTTAGAGAGAAAATATGT
AGCGAATTATCGAATCTGCTTCGGAGCCTCTAGGCAACTGCCTACCGGAGACTTTGCAGGAAAAGGCCAATTTTTTTGTTTGGT
TTGGATAGGACCCCAATTCATGGTCCAATAGTATACCTATATGATATTAGAGATGGAAAAGGCTTCAAAAAATAGAGCTTGTTA
GAGAGAATATGAGTGACATTCACGAGATCGCTTCGGAGCCGCTCAGGCCTGCTGCCTAGGAGATTGGCAGGATAGGCCAAATT
TTTATGTTTGTTGAATTAGGATCCCAATTTATTGTCAAAAGTACCCCTATATACATAGTATTAGAGATGGAAAATGGTTCAAA
AAAAATGGAGCATGGTGGAAGAGAGATATATGAGTGAATTACGAGATCGCTTTCGAGCCCTAGGCAGAGCCTGGGATATTTTGC

ACGAAAGGTCCAACTTTTTTTGTTTGGTTGAAAAGTTTATGACCCCAATTCATGGTATATGACTGTAGCCTACATAATATTATG
AAAATGGGGAAAGGGTTCAAAAAAATTAGAGCTTGTTATAGGAAATCAATGAGTTGAATTCGAGATTCTGCTTCGGAGCCCTGG
CTCTGCCTAGGAGATTCTGGCAGGGAAGACCAATTTTTTTGTTTCTGGTCTGAATTAGGACCCCAATTCATGGGTCAATAGTTA
TACCTATATGATATTATGTGAGATGGAAATGTTCAAAAAAATAAGAGCGCTTGTTAGAGAGAAATATCTGAAGTGAATTACGAG
ATCTGCTTCGGACCCTAGGCACGCTGCCAAGGTGAGATTTGGTAGGAAAGACAAATTTTAGTTTGTGGAAGTTAGGAACCCAAT
TCCCATCGGTCAATTTATATTCCTATATCAGATATTATGGAGATGGAAACCTGGTTCAAAAAAATTAGAGCTCGTTAGAGAGAA
TATGAGTGAATTACGAGACGCTTCGAGCCCTATGGCAGCCTGACTACGGAGATTTTGGCAGGAAGGTCCAATTTTTTAGTTTGG
TGGAATTAGCATCCAATCATGTTCAATAGTAGCCCTAATAATATTATTTGAAAGGGAAAAGTTTCAAAAAAAATTAGAGCTTG
TTAGAGAAGAAATTGAGTGAAATACGAGATCGCATTCGAGCCCCTTAGCAGCTGCCTAAAGCGAGATTTGGCACAAAGGCAAAT
TTTTTTTGTTTGGTTAGAATTAGGACTCAAATTCATGGTCAATAGTAGCCCTAATAGAAATATTATTGAAATGGAAAAGGTTC
AAAAAAATTAGAGCTTGTTAGAGAAAATATGAGTCAATTACTAGATCGCTGTCGGAGCCTAGGCAGCCTTGCCTACGGAGATTT
GCAGGAAAGGCGCAATTTGTTTTTGTTTTGGTTGAATTAGGGACCCCAATTTCATGGTCAATAGTACCTTATAGAATATGTGTT
AGGAAACGGAAAAGGTTTCAAAAAAATTAGAGGCTTGTTAGAGAGTAAATATGAGTGAATTACGAGACCGCATCGTAGCCCTAG
GTAGCCTGCTTAGGAGATTTGGCAGCAAAAGGCCAAATTTTTTTTGTTATGGTGAATTAGGACTCAAATTCTGAGTAAAATAGA
GCCTATAAGAATATTTATTAAAGGGAAAAGGTTTCAAAAAAAATTAGGCTTGTTAGAAAAAATATGAGTGAACTTACGATATCG
GCTTCCGGAAGCCCTAGGCGCCTAGAGTATTTGCAGGAAAGGCCATTTTTTTTTGTTTGGTTTTATTCTTAGATCCCTATTCAT
GTTCAATAGTACCCTATATAATACTTTATGTGAAATGGGAAAAAGGTTCAAAAAAATTAGAGCTTGTTAGAGAAAAAATATGGT
GAATTACGAGATCTCTTCGGAAGCCCTAGGCAGCTTGCCTATGGAGATTTGGCTGGAAAATGGCAAAATTTTTTTGTTGGTTGA
AATTAGATCCATTCTGTTCATACTAGTGACCCTCATATAATAATAATTGAAATGCGGAAAAGGTTCAAAAAAATTAGCAGCAAT
GTTAAAGAGAAAATATGAGCGTAATTACCGAGATCGCTGTGCGGACCCCTAGCGCAGCTGCCTAGGAGATTTGGCAAAAAAGGC
CATTTTTTAGTTTGGTTGAATTAGCATCCAATTCCATGTTCAACCCCTATAGCCCTATATAGATATTATAATATGAATGGGAAA
AGGGTTCACAAAAATTAGAGCTTTTTAGAGAGAAATATGAGTGTAATTACAATCGCCTTCGGAGCCCTAAGGAGCTGCCTAGGA
ATTTTGCTGGATCAAGGCCATTTTTGTTTTGGTCGAAATTAGGATCCCAATCATGTTCAATAGTAGCCCTATATATATATTAGT
TGAAATTGGAAAAGATTACAAAAAAATTTAGAGCTTATTAGAGAAAATATGAGCTGAATTACGAGATACGCATCCGGAGCCCCT
AGGCAGTGGCCTAGGAAGATTTGCAGCAAAGCAATTTTTTTTTGTTTGGTTGAATTAGGACTCAATTCAATGGTCAATATAGCC
CTCATACAATATTATAGAAATGGGAAAAGGTTCAAAAAAAATAACCTTGTTAGAGAGAAATATGAGTCAAATTACGAGATCCCC
CTGCTTCGGAGCCCTGGGCAGCATGACTACCGGCGATTTGGCAGGAAAAGGCCAATTTTTTTTGTTTGGTTATTAGCTTCCACC
AATTCATGTTCAACTAGTAGCCCTATATAATATTATTGAAATTGGAAAGTTCAAAAAATTAGAGATCTGTTAGAGAGAATATGA
TGAATACGAGATCGGGCTTCGAGAGCCCTAGGGCAGCTGCCTATGGATATTGGCAGGAAAGCCTAATTTTTAGTCTGTTGTATT
AGAATCCCAATTCATGTTCAATAGTACCCTATATATATATTATTGAATGGAAAAAGTCCAAAAAATTAAGCTTGTTAGAGAGAA
ATATTAGTGAAATACGAGATCGCATCGACCCCTAGGACAGCTGCCTAGCGAGATTGGCAGCGAAGGCCAATTTTTTTGTTTGGT
TGAATTAGACTCAAATTCATGTCACGTAGTAGCCCTATAAAAGAAATCTTATTGAAATGGGAAACATGCTTCAAAAAAATAAGC
TGTTAGAGAGAAATAATGAGTGAATTACGAGATCGCTTCGGAGCCCTATGCAGCTGCCTAGGAGATCTGGCAGGAAAGGCCAAA
TTTTTTGTTTGGTTTATTTAGCATCCCAATTCATGTTCAAATAGTACCCTAATACAATTTATTCTGGAAATGGGAAACAGTTCA
AAAAAATTAGAGCTTTTAGAGAGAAATTAGTGAATTACCGCAATTCGCTTCGGAGCCTAGGCAGCTGCCTAGGCAGAATTTGGC
TCTGGGAAAGGCCAATTTTTTTATTGTTGGTTTATTAGCATCCTATTCTGTTCAATAGTAGCCCCTATATATAATTATGAAATG
GGAAAAGGTTCAAAAAAATTAGGCTTGTAGAGAGAAATATGAGTGAATACGAAGATCGCTTCGGACCCCTAGGCCCTCTTGCCT
TGAAAGAAATTTTGGGCATGAAAGCCAATTTTTTTGTTTGGTGAAGTAGGACCCCCAATTCATGTGAATAGTATACCTATATCA
TATAGAGATAAGGAAATGGTTTAAAAATATTAGAGCTTGTTAGAGTTAAATATGAGCGAATTAGCGAGACGCTCTCGCAGCCCT
AGCAGCCTGCCTATGGAGATTTGGCAGAGAAAGGC
>m141120_105518_42134_c100732402550000001823141405141504_s1_p0/44568/12016_17411
CCTGGATGAGGTTAAGGTAATTTCGTAGAGACACGAGTAATGGTCGAAAGGAATGACCTGGGTTAACTGGAATCGAGTGTGTGC
GGAAGTGTATATTGCTAGGACGAGTTACAGATAAGAATTTCGAGGAATGAAAGTTTCTTGTGAGGGGAGGTAGGTTTGTAACGA
CCCAAATTTTCCTAAAGTAGGTTTAGGACCTTGATGTAGGAGGCCAGGAGGGCCATAATTGGAAGGTTGATGTTGCTATTTAGG
ATTATATGAAGTGATTGATGTGGGTCGATAAGTACTATATGATGGAGTAAGAGGCATGCATGGGGGATAGTATAGCTTCTGTTG
TGGAGTATTTATTGGTAATTTGGCTCGGTTGGGGAGCGTAATTGCATATTTTGTTGGTATAGTTGGGTAGGCTAAGTGTTGGAC
CACCAATATTGATGTGAGTATGTTTGAGCTTGTGACTCGAGGCGGATCCTAACAAGCGGTTTAAGCGGGGAAGGGTCAGAGCAG
AGAAATTTATACCTAAGCTCGGGGTGAGCCTAGGGATTGTAGGTCTGAGAATTTGGGAGAATATATTGGAAATTATTTGATAAT
AGGAAAATAGTGTGGTGAGGTGATTAGATGATGGGTGTGGGGAGGCTCAATATTACCACGCCCTTAAAGATCGAAGAACGCGTG
CTAGAGGTCCCATAAAGGGCCTAGAGTGAGTGGTTAGAGCCGACCTGCGTGTCGCTGTTGGACCGCACCCAGGGCGCGAGCCGT
TGGTCACATCTGCCTCGTCCACCGCGAGGCCAACGCCCCGAGGCCCACTACGAGGTCGTGGTCCGCAGCCGCCTCGTCCACGCG
GAGGCCGACGCCCGAGGCCCAGGTCCCGAGGCCGTGTCCTGCCACCTTGTCCGATGCGAGCCACCCCAGGCCCAGCCGCGAGGG
CCGTGTCCTACCACCTCACCCATGCAGGCCATGCGTGCCGTCCGGCCGTGTGAGGCCCTCGTCATGGCCCAGGCTGTGAGGCCA
TGTGCCACCTCTCGGGATCAGCATCGTCAGTGAAGACCTCTCCCAGAGCTACGCGTGTGGAGACAACCTCGCCCCGATGGATCT
CGTCCCAAGGCCCGCGTGTATAAGTGACAGTGGTCGAGGTAGGACCAAAAGAGACAAAAGAGAGTACGGAACAAAGGTAGCCTC
AAAGGGGTACGTACATGCCGTTGGCTAGGGGATCGAGGGGACGGGCCTGACACCTGTGCCGACAAAGTATGGCGGGTTGGGGAT
GGTACGAAAGTGGGCTACACATCACCACGTCTCTGACACCCGTTACAAGCGAGTGGTGGCAGTCCATGAACTGGGTACGAAGAA
GCTGAGTGGCTGCCATGGATTCTGGACATGTAGCCAGAAGCCGAAACCACTTCTCCTGTTGTGTACTCAGTCGTACGATTGTTG

GTACCCTTGGACCACCTATGTAACCAAGGCCCAATTAGAGCCCCACTATATAGATGAACGCCCAATTCCCACGATGGATGGGGG
TTGAAAATTGACTGTAGCGAAGTGCATCTAATGAAATGAAAAGTTGAATGTTCACATTGTTCATGTAACAATTTTCTTAGAGTT
GATACAATAAGATATCCGTTAGAGTTTTTCTTTGCATAGATCTTAGCTCTGTGATTTGTTCCAACTTAACGATTAGTTGACGGA
GCTTCTACCATGGCGCAACAGTTTTGGCGCCGTCTGGGGAACTTAAAGTTCAACTGTGGTTTCCTACATGGTGTCTTCCGCAAG
AACGAAATGCCAGAGACGTGTCGAAGGAGGTGACTTAGGGAGAGCCACGAGAGGGGGGTGGCCAGGTTCCAGACGCTTGAGACG
GGGGGTCGAGCTGGAAGGCTCCATGAAGGATCCCCCTCCACCGAAGAGAGAGGTGAAGGGGCCCCAAGGGACCCGTGAGGGGTC
TCACGAGGGTAGGGAGTAAAGAGGCCTCGGTCACGTGGGGTGTTCCCCGCCTATAGAGAGTCCTTCAAGGGCACCGTCGGGAAA
ATTCCAACGAGTTTCCACCTCCCTAAACCACAGAGCAACTACCGAACTCCGGCGGCAGGACCCGGGCCCTTCTACACATAGGCA
GCGATAAGCCCCCCGGGTCCCATTCCAGTGCTCGAGTTCGAAATTCGATTCTATGAGAAGAATATGTGGAGCTCGCATCGTAAG
AACCAAAAGGTCGGAGACCACCAATCGGAAGAACATGCAAGAGGTGTTGAATGGCTATTGAAGAGTAGTCAAGCGTGACCCTGC
CCCAAGACGGACAGTGAGAAAGGTCGGGAAGGGGGCTAGAGACCACCGTACTAGTAGTGATGGGTAGACCCGACAACTCTACAG
TAACCCCAACGAGCGAAGCACGCGAACATCCTAAACTACCGAAGTAGGCCCCAGAAACCGGAGCCCAAGGACCAACGCTGCCCC
TCGCAAGAGGACGAGGTCACCTCTAAAGAGCACCCTCGGTTTACGGGAGGTTAGTTCCAATAAAAAAGAGGGCGGTAATAAGTG
GACCACGCGCCCTATTGGAGCCTCGAGAGGCAAGAAGAAAGGCAGATCCTTAACCGTCCGCCTTTGAGGCGACTCTCTAAGCCA
AGAGCAGCAGGCGCTGAGACACAGAAAATGAGCAGCCTGCGAAGCCAAGATCGCTCCGCTCTGGCGGATCAGCGCGTTTGAGGA
AGAGATTCGACCAATGAGTCCACCCTTCATGAGGGAGATTTAGGCCATTCGGACTCATCTGCCAACTTTAAGGAGCCAATATGA
TTACCACTATGAAGAAGCACGGTCAAAATATTATCTATGTATACATTTAACGACCTGATGAAGGTGAGGGGAAATTGGCAGCGG
GGCCGAGATGTCATTGCTTCGCTGTCAACATTAAAGAAGGACTGCATCAAATGGTTCAAAGAGGACTTAGACCGGGGTCGCATC
CAATCTTGGCAGCAACTTTCTAATGAATTCATTCCAGCAGCACCACGACGTGCGAGATTACACGATGCCAGGCCACCAGCCTCC
CAATGTGAAGCAAGGAGAAAATGCGAGTCTGAAGGGCTTATATCCACCGGTTCAATATGGATGGCAGATAAGGTAGGGAGCCTG
ACCACCGAGAGCTGAAATATGGCCCTTCATAAGCGGAGTACGCCCTAGGGAGCAAGTTATAGGGAATAACATGCCAACGAGAGG
TCACAGACGTTGGTGATTTCTACGAGCGGACAAAAGTACATTAAGCGTGGGTGATGGTCCACGAACTCTCAAAGCGGAAAAGGA
AACATCGCCCCCACAAACCTTCAGTCTGGGAAACCTAAAGCAGTGCAAAGAAGAAAGGGGGTTTATGAGGGAACGAGGGACGAT
CGACCCCGGACCAGCACCAACGCTCTGATGAGCCACATACAGAGGCCACTACAACTTTTTACACAGACCTCACCCACGCGAGAG
AGGATATTTTTGTTCGATGAAAACCAGGTCCCTTTCAAAAGGCCCCCGCAATCAAAAATAGATCGGTCTAAAAGAGACCAATAG
CAAGTATTTTTTAGCCAAATGTGTGGACGGGTGATAATCTCGTCAACTGAAATTAGGTCGGAAACTCAAAGGCTCATCGCGGCA
AATCTCTAGATGGAAGACACTTAGAATAACTCTTCTGAAAAGGTAAACCACAGATGCAAGATGGAGTGAAAAAAATTGTATTTT
TTAATGGGCCTCTGCATACAATCGAATTTCCCACATCCTTTTCAGGTGGGTTTTTAGAGTTCATTTTCATTAGTAGGCTCTAAA
TGGCTTAGTGGTACAGTAGTGGTCCCAGGGGACCAAGTTGTCCATACGTACTGTGTCAAGGGGAGTGGCCTCCAGAGGCCTGTG
TTCGTTACACACCGTACAGGAGCAGGGTTGTTCAGCGAGGCATGTTCCACTACTTGTAATATATCCATGTCCTATTGCGTGGGT
GGCAGTGCGTAGTGGCGCGGCAGGTAGTGGTGTCGGCTCTGACCTCATGGTCGTAGACGTTACGGACCCATAACTCTACTCCTT
GGCACTCAACCCACTTCCCTGGCATTCCCTTACTTATCTTTGTTGGTAGGGGTAACTATTAATCCCGTACTCTCGTGACTTCAT
GTAGTTCGTAGGATACCATTCAGTATTCAATCGCGTGTACACCCTTTGCCCCCCTTGAGGCGGCGGGGTTGCCTGGGGGGGGCG
TGGGTGAGTATTCTCACTCCCAAGGCCATGGGCAAGGCCATGGAGGAGGCCATGGGCCGAGGCCATGGAGGCAGGCCATGGCGA
GCCATGGAGGAGGCCATGGGGGGCGAGGCCCATGGGGAGCGCATGGGAGGAGCGCTTGGGGGCGCGGCCATGGAGGAGGCCCAC
GGGGGCCGGGGCATGGGAGGAAGGCCATGGGCGAGGCCAATCGGAGGAGGCCATGGGGGAGGCCATGGAGGAGGCCAGGAGGAG
GCCATGGCGAGTGCGGCCATGGCGGCCGAGCCTATGGAGGAGGCCAGGGGCGAGGCCCATGGAGGAGGCCATGGGAGTGACGG
CCATGGGCGCGCATGGAGGAGGCCATGCGGCCCGAGCAACCATAGAGGCAGGCCATGTGGGCCGCAGGCCATGGAGGCAGGCAC
CATGGGGAAGAGGCCAATCGGGCGAGGTCCATGAAGGAGGCGCAATTGGGAGGAGGCCATGGCGAGGCCATGGAGGAGCATGGG
GGCCATGGAGGAGGCCATGGGGTGAGGCCACGGCAGGAAGCTGGCCATGGAGGAGGCCATTGGCCGAGGCCTGGAGGAGGCCAT
GGCGAGGCCATGGGGAGGCCATGGGGGAGGGCCTAGAGAGAACCGCGGCCATGGCAGGAGGCCATGAGCTGAGGGCCCCATGGA
GGAGGCCATGGGCGAAGACCATCGAGGAGAGGCATGGGGGCTGAGGCGCATGGGAACGGAGGCCCATGGCTTTTCAAGGCCCTG
GAGGACGGCCAACCGGGGCACAAGGCAATCGTGGAGCCATGGGGCGGACGGCCATGGCGAGAGGCCATGGGTGAGGCCATGGAG
GAGGCACATCGGCGCGAGCCACGGAGGAGGGAAACATGGCAGGAGGCCATGGGGCGAGGCCACGAGGGGCCATGGAGGCAGGCC
ATGGGGCGAGGCCCATGGAGGAGCCATGGGCGGCCAATGGGGGAGGGCCATGGAGGAGGCCCATGGGCGAGGCCAATGGAGGGG
CCATTGGTCCGAGGCCATGGGAGGAGGCCCATGGAGAGAGGCCATGGGCGACGCCATCGTGAGGAGGCCATGGAGGAGGCCATG
GCGCCGAAGGCCATGGAGGAGGCCATGGAGTCGATGGCCATGGAGGAGGCAATGGAGAGAGGCCTGGGGCCGAGGCCCATGGCA
GGAGGCGCATGGGGAGCCG
>m141120_105518_42134_c100732402550000001823141405141504_s1_p0/49708/10418_16638
GCTTTAGTTCTCTCTACGGAAGCGTATGTTAATTCACTCATATTTTTTCTTCTCTAATCAGTCTCATTTATTATATGAACCCTT
TTCCCATCTTAATAATATTATATATGGGTGGTGGGTTATAAGAGGGCTATTTATAATGACATTGGGGAAGGGGATATGATGGTG
TGGGTCGCGTAGGAGTATAGTCATAAGCCTGAAACAAAAGTATGGAACTTTCGCCGGAGATAATGTGGCATTTAGGGTGCATCG
TCTTTAGGGCTCGGAGATAAGACGGGATTCTCCGGTAAGTAATCTACTCATTATTTTCTCTCTAACAGCTACTAATTTTTTTTT
GATCCTTTTCCCATCCCAATAATATTTATAGGGGCTGTTATTTGTGATATGAATTGGGGTCCTTAATTCAACCAAACATAATAG
AATTGACCTTTCAGTGCCAGATATCTCCTAGGCCGCTGCCTTAGGGGTGTTCTCGAAGCGAGTTCATAATATTTTCCTCTTGAT
ATTATCTTCTCTAAGAGCTGATTTTTTTGAACCTTTTCCCCAGATAGTCTCAATAATTAATTTATATATGGGCTCTTATTATTG
GATCATTGAATTGGGGGTCCGTATTTCAATTCCAAACAAAAAAATTGACCTTTCGGCCAAATCTCCTAGGCAGCTGCTTAGGTC
TTCCGAAGTCGATCTCGTAATTCACTCATATTTCTCTCTAACAGCTCTAAATTTTTTGAACTTTTCCCCATCTTAATAATATTA

TAATTTGGGCTATTATGTTGAGCAATGATTGGAGTCCTAATGTCAACCCAATACAATATAAAAATTTGACCTTTCAGGCCAATA
TCTCCTGTGAAGGCATGCTCGCTTAGATAGATGGGCTGCGCGGAAGCGATCTCGTAATTCACTCATATTTCTCTCTACAAGCTC
TAATTTTTTTGATCCCTTATTTCCCATCCTCAATAATATTAATATACTGGCTGTTCATTGACCATGAATTGGGTTCCTAATGTC
TACCAAACTATGAGAAATTGACTTTCAGACCAAATCTCCTAAGGCATGCGTCCTATTGGGGGCATCCGCCAGCGATTTCGTAAT
TTCACTCCATATTTCTCCTCTAAAATCTCTAATTTTTTTGAACGTTGTTCCCATCTCAATAATATTATAATAGACCGCCTGTTA
TTGACAACATGAATGGTCCCTAATTCAACCCAACAAAAAATTGACCTGTAGGCCAATATCCTTCCTAGGCATGAGTGTCTGCCT
AAGGGTCCGAAGCGAATTTCGAAATTCACTCAATTATCTCTCTAACAAGGTCTAGAATTTTTTTGAATATATCCTTTTTACCT
ATTCATCAAGATAATATTGTTAGGTGCTATTATTGACCATGTTGGAAGTCCAATTCACCAAATCAAAAAAATTGACCTTTGCCA
GGCCAAATCGTCCTAGGCAGCAGCCTAGACGGCGTCTGAAGCGAGTCTCGTAATTCAACCAAACAAAATAAATATGTACTCGAG
CTTTTCAGGCCCCAAAATTCATCCTAGGCAGCTGCCTAGGGCATCCGAAGCGTCGGAGAATTCCTCGTAATTCCTCATATTTCT
CTCCTAACAAGCCTAATTTTTTACCTTTTCCCATCTCAATAAATATTCATATGCGGGGCTGTGTGTATAGGAATGTGATGATGG
AGGTGGTGCGTTGTAATTCAACTCGAGTACAGATAGAATTGACCTATGCAGAGGTGAGAGAAGTCTCTAGGCTGCTGCCTATAG
TGGGCGCTCCGAAGCGATTTCATAAATTCACTGATATTTATCTCTAACAAGCTCTAAAGTTTTTGTACCTTTTCTAGTCTGCAA
TAATAAATGTATATACGGGGCTGAATTATGGACCATGGGTAATTTGGGTGTTCCTTAATTCTAATAAACAAAGTTGACTTGTCA
GGCCAAAATCTTGTTAGGCAGCTGCCTAGGGGCTCTGAAGCGTATCTGGTAAATTCCACCAAACAAATAAATTTGGCTTTCAAG
GCCATATATTCCCATAGGCAGTGCCTATGGGGCTCCGAAGCGATCTCTTGTCATTCCGTTTCTCGCATAATATTTCGTCTTACA
AGTCTCTTAATTTTTTTATCTTTTTTCCCCATGATCTATTATATTTATTATATTTTGTGTGTTTATGTATTATTATGATATTTG
GGGTTTCTTTTAATTCAACAAACAAATAAATATTGACCTTTTCAGGCCAAAATTCTAGGTTGCTGCTCTAGGGCTCCGAAGCGA
TTTCATATATTCTCTGATATTTATCTCCTAACAAGCTCCTAATTTTATTTTTGATCCTTTTCCCATTCTCAATAATATTTATT
TTTATGTGGTATTTTGGTTTTTATTTGATCATTTTTGAATTGGGCGTCCTAATTTCATTCAATTACTAATTTTGTAGTCTTTTT
TTTCTAGTTGTCATATCTACGGCAGTTGCTTTTAGGTTCGATGTCGTGATCTTTTGTCGTATTCACGTTGCTCGATATTTCTGC
TCTTAGACAGCTCTTTTTTTTTGCGGTTTTTTCTATCAGTTAATTGAAGTTTCATTTTTTTATATTTTAGGCTATTATTGACCA
TGATCATTGTGGGGTCTAATTTCTATCCAACACAAAAAAAAATTTGACCTTTCAGGGGCCAATCTCCTAGCAGCGCCTATAGAG
GGCTTCCGAAGCGTTCTTCTCGTGTAATTCACTCCATGATTTCTCTCTATAGAACAATGGCTCTCTAATTTTTTTATCTTTGT
TTCTCATGTCATAATGAGTTTTTGGATTTATAGGGCTGTTTTATTTTGGATATGATATGAAATTGGGGTCTTGATTCAACCAAA
TACAATATAAATTGGACTCTTTCATGTATTGTTCTAATATCTCTCCTAGAGTACTGTCTGGGCTATGGGGCTTCCGAGCGATTT
CAGTTAGGTTTCCCTTGATATTTATCTGCTACAATGGCTGCTAAGTTTTTTTGATGAGCGCCTGTTCCTGATCTCAATAATAA
TTATTATATATGGGCAATTATTAACCATTGAATGTGGGGTCCTATAGATTTCAATTTGATTCAGGACAAGATAAAATTGACTTC
GTGTGGTCCAAGTGCCAAATCTCCGGTAGGATGCTGCCTATATATATATTGTGTGCTATATGGCTCTGATTCTCTCTCAGTGTG
CGACCAACAAATTGACCTTCAGGACTCTCCTGGGCTGTAGGGCTTCCGAAGCGATCTCGGTGTATTTCACTCTTATATTTCTCT
CTAACAAGCTCTAATTTTTTTATCCTTGTTTCCATCTCAATAATATTAACATAGGGCTGGTTATTGAAATAATGAATTGAGTG
GTCTTAAATTCACCCAAACAAAAAAAATTGAACGCTTTCAGGGATTATCGGTCCTAGGCTTTTGTTTAGTCTGCCTAAGGGAGT
CTCGAAAGCAGTATTTATTCCGTAATTCACTCATATTTTCTCTCTAAAAAGTTTCTATTTTTTTTTTGTGAAACCTTTTGTCTC
GCATCTCTCAATAATATTATGATTTTAATTGGGCTATTTTGACCATGAATTGGGGTCCTAATTCAACCAAACCAAAAAATATTG
ACCTTTCAGGCCAAATCTCCTAGGCAGCTGCCTAGGGCTCCGAGCGATCTCGTAATTCACTCATATTTCTCTCTAAAACAAGCT
CAATTTTTTTGAATCCTTTTCCCTCTCATATATATATATGGGCTGTTATTGACCATGAATTGGGTCCTAATTCAACCAAACAAA
AAAATTGAACTTATCAGGCCATCCTCCAAGCAGCTGCCTAAGGGCTCCGAAAGATATTTCATAATTCCACTCATATTTCTCTAA
AGCTCTTTTTTGACTTTTCCTCTCTTATTTGATTAGGGCTGTTAATGGACCATAGAATTGGGGTCCTATTCCAACAAATTTGAC
TTCAGGCAAAATCTCATAGGCAGCTGCCTGGCTCCGAAGCGATTTCGTAATTACTCATATTTCCTCTACAAAAGCTCATAAATT
TTTTGGATCCTTTTCCATCCTATATTATAATAGGTCTGTTTATTTTTATTGGGAAAAAAACATGAATTGGGTCCTAATCAACAA
ACAAAAAAATTGACCTTTCAGGCCATCTCCTAGGCAGCTGCCTAGGGCTCTTGACGATCTCCGTTCCAACAAAATTGCTTTCAG
CGGCAAATCTCTAGGCACTGCCTAGGGCTCGCGAGCGATCTCGTAATATCACCTCATAATTCTCTCTAACAAGCTCTAATTTTT
TTTAATCCTTTTCCCAATCTCATTTTATATAAGGGCTGTTATTGAAATGAATATGGGGTCTTAATTCACCAAACAAAAAAACTT
GCACTTCTAGGCAGATCCTCCTGGCTGCTGCCTAAGGGATCCGAAGCGATTTCGTGATTCCTCAATAATTTCTCTCATAAAAAG
CTCCTAATTTTTTTGAAACTTTTTCCCATCCTCAATAACTATTATATAAGGCTATTATTGGACCAATGAATTGGGGTCCTAAAT
TCAACCAAACAAAAAAATTGACCTTTCAGCGCCAAATCTCCTAGGACAGGCTGCCTAGGGCTCCGAAGCGATCTCGTAATTCAC
TCATATTTCTCTCTACAAGCTCTAATTTTTATTGATCCCTTTTCCCCATCTCAATAATATTATATAGGCGCCTGTTAATTGACC
AATGAATGGGTTAATTCAAATCAAACAAAAAAATTTTGAACTTTCAGGCCAAATTCCAAGGCAGCTGCCTAGGGCTCCGAAGAT
ATTTCATATTCACTCATATATATCTCTAACAAGCCTCCTAATTGTTTTGAAACCTTTTCCCATCTCAATATTTTATAGGCTATT
ATTAACCATGAATTGGGGGTCCTAATTCATCAAACAAAAAAAAATTGACCTTTCAGGCAAATAATCCTAGGCAGCTGCTAGGGCT
CTGAGCGATTCGTAATTCAACCAAAAAAAAAAAATTGACCCTTTCAGGCCAAAATCTCCTAAGGCAGCTTGCCTAGGGCTCGAAG
CGATCGTCGTGAATTCACTCATATTTCCTCTCTAAGAAGCTCTTAATTTTGTTTTATCCTTTTCCCCAAATCCTCCTATATATT
ATATAGGGCTGTTAATTGAATATGAATTGGGGTCTTAATTCAACAAACAACAAAAATTGACCTTTCCAGGCCAAATCTCCTAGG
CTGCTGCCTAGGGATCCGAAAGCGATTTCGTAATTCACGTCTATTTCTTCTAAAAAGCCTAATTTTTTTGAACCTTTTTCCCAT
TCAATAAATATTATATAGGGCTATTATTGACCATGAAATGGTCCTAATTCAACCAAAACAAAAAAATTGACCTTTCAGGCCAAA
TCTCCTAGGCACGCTGCCTAGGGCCTCCGAAGCGTCTCGTAATTCACTCAATATTTCTCTCTAAACAAGATCTAATTTTTTTTG
ATCCTTTTCCATCTCCAATAATATTATATATGGATGTTATTGACATGAATTGGGGTCCTAATTCAACCAAACAAAAAATTGACA
TTAGGCTCTCCGGCGCTGCCTAGGGCTCCGAAGATATTTCATAATTACTCATAATATATCTCTAACAAGCTCTAATTATTTTTG

CCTTTTCTCTCAATTATATCATTAGGGCCTGTTAATGACCATGAATTGGGAGTCCTAATTCCCACATTGCCTTTCAGGCCAAAA
TCTCCTAGGCAGGTGCCTAGGGCTCCGAAGCGATCCCGTAGATTCACCTCATATTTTATCAACAAGTTCTAATTTTTTTGAAC
CTTTTCCCATCTCCAATAATATTATATAGGGCTGTTATTGACCAGAATTGAGGGTCCTAATTCAAACAAAGAACAACCAAGACA
AAAAAATTGAACCTTTCAGGCCAAATCTCCTAGGCAGCCTGCCTAGAGGCTCCGAAGCGATTTCGTATTACATCATAATTTTCT
CCATAAAAAGCTCTAATTTTTTTGATCCTTTTCCCATCTCAATAATATTGTAAGTACGGTCTGTTATTGACCATGATTGGGGTC
CTAATTCAACAAAACAAAAAAAAATTGACCGCTTTCACAGGCCAAATCTCCTAGAGAAGATGCTAGGGCTCTGAAGCATCTCGTA
ATTCAACCAACACAAAAAAAAAATTGACCTTTCAGGCCAAATCTCCTAGGCCAGTCTCAGGGCTCGAAGCGATTCTCGAATTCCC
TCTA
>m141120_105518_42134_c100732402550000001823141405141504_s1_p0/116729/698_7195
ATTGATATTGATCTCAAGGAGGAGTTATGGCGCGCTCCGTCCATATCTGACATGAATTGGGGTCCTAACTACCAAAAAAAAATT
TACCTTTCAAGGCCAAATCTCCTAGGCACTGCCTAGGTCGACAGTGATTTCGTAAATTCATCATATTTCTCTCTACAAGCTCTA
ATTTTCTTTGAACCCTTTTCCATCACAATAATATATAGTAGGGCTATTCATTGACCATTTGAATTGGGGTCCTAATTCAACCAA
ACAAAGAAATTGACCTTTCAGGCAAAATCCTCCCTAGGCAGCTGCCTAGGACTCCGAAGAGATTTTTTCGTAATGTCACTCAA
ATATTTCTCTCTTACAAGCTCTAAATTCTTTTGAAACCTCTCCTATCTTAATACATATTATGATATGGGCTTAATTATTTACC
ATGAATGTGGGGTCCTAATTCAACAAAGAAAAAAAAAATTGACCTTCCAGGACAAATCTCCTAGGCAGTTGGCCAGGGCTCCGAA
GCGATTTCGTAATCCACTCATATTTCTCTCTTAGCAATCCTCTAAAGTTTTTTAAACCTCTTCCTATCAGTCAATAATAATTG
ATATAGGACTACTATGGACCATGAATTGGGGTCCTAATTTCAGGACCAAACAGAAAAAAATTGACCGTCTCTGGCCAAGATCTC
CTAAGGCAGCTGCCCCTAGGGCTCCGAAGCGATCTCGTAATTCACTACTTTTCTCTTCTAACAAAGCTCTAATTTTTTTGACT
TTTCCCATCTCAATAATATTTATATAGGGCTATTATTGAACCATGAGATTGGGGTCCCTAAAATTCCAACCAAACAAAAAAAGA
TACTTTCAGGCCAAATCTCCTAAGGCAGTCTGACTAGGCTCCGAAAGTGAATTTCGTAATTCATCATATTTCCTCTCTAACAAC
TCTATTTTTTTTGCATCCTATTCCTATCTCAATAAATTAATATATTAGGGCGGTATTATTGACCCATGAATTGGGGTCCTAATT
TCAACCAAACAAAAGAAATTGCGACCTTCCAGGCCAAATCTCCTAGGCAGCGAGCCTAGGGCTCGAAACGAGGTTTCGTTAAGG
TTCGTCATATTTCTCTCTGTACAAGCCTAATTTTTTTGAACCTTTTCCCATCTCAATAATATTAGTTATAAGGGCTATTATTGA
CCATGAATACTGGGGTCCTAATTCAACCAAACAAAAAAAATTGAACTCTCAGGCCAAATACTCCTAGGCAGCTGCCCTAGGGCT
CCGAAGCGATTTTCGTAATCTCACGGTACTATTTCCTCTCTAACAAGCCTCTAATTTTTTTGAACCTTTCCCATCTCAATAAT
ATGTATAGTACAAAACGGGCTAATTATTGACCATGAATCTGGGGTCCCTAATTCAACCAAACAAAAAAATTTACCCTTTCAGGC
CAAATTCTCCCTAGGCAGCTGCTAGGGCTCCGAGTGATTCTCGTAATTCCACTCATATTCTCTCTACAAGCTCATTAATTTTTT
TGATCCTATTCCCATCTCCAAATAATATTATATAGGGCCTATTATTGACATGAATTGGGGTCCTAATTCAACCAAACAAAAAAA
TTGGACCTTCCAGGCCAAATCTCCCTAAAGGCCAGCAGCCTACGGGCTCCCGAAACGATTTCGTAATTCGCTCATATTTCTCTC
TTACAAGCTCTAATTTTTTTGAACCCTTTTTCCCATCTCAATAATATTATATAGGGATATTAATTTGACAATGAATTGGGGTCC
TAATTCCAACCAAACAAAAAAAATTGGGACCTCTCCTGGCCAAATCTCCTAGGCCAGCTGACTAGGCTCCGTGGGAGCCCGCAT
ATTCCGTAATTTAATAATCCTCATATTCTTCTTCTCATAACAGTCGCGTCTAATTTTTTTCGAACCTTTTCCCATCTCCAATA
AATAGTTATATTAGGTGAATTATTGACATGAATTGGGGTTAATTCAACCAAACAAAAAAGATTTAAATCTTTCAGAGCCAAAAA
TCTCATAGGATGCTGCCTCTATGGGAGCTCCGAAGTGATTTTCGTTAAAATATCACTACCATAATTTATATCTCTCTAACAACC
TCCCTAATTTTTTTGAAAAACTTTCCCATCACAATAAAGAATTACCCAAATCATATACTGGGCTATGTATTGACCATTAGAAT
TGATGGTCCTAATTCAACCCAAACAAAGAAATTAGACCCTTTCCAGTTTAATTCAAAATCTCTTAGGCCAGCTGGCCTAGGAC
TCGAAAGAGATTTCGTAATTCAGCCTCATTATTTCCTCTCTTAGCAAGTCTCTAATTTTTTTGAACGCTCTTCCTATCTTTATA
ACATAAATATAAATATATGGGAGCCCCTATTAATTTAACGCAATGCAAGATGGGGTCTAAACTATCAACCAAAAAGAAAAATAA
TTGACTTCCAGGACAAAATCTCACAATAGGTATGTTATGCTAGGGCTCCCGAAGCGATTTCGTAATTCACGTCATATTTCTCTC
TTACAATCTCTAATTTTTTTAAACCTCCTTCCCTATTCCAATAATATATATATTTAAGGGTAATTATATGACATCCAGATATTG
GGGTACCTAAATTAAAACCTAAATCACAAAAAAAAATTGACCTTTCCAGCCAATCTTCCTAGGCCAAGCAGCCTAGGGCTTCCGC
GATTTGGTAATTCGTCTCAATCTTCTTCTTTACAAGCATCATAATTTTTTTTGAAACCTTTCCCATTCTCAATCTTATAATATT
ATTTAAATGTCATAGCTATTATTGAGGCATTCGAATCTGGGGGTCCAATTCAACCAAACAATAAAAATTTACCTTTCAGGCCAA
ATCTCCGCTAAGGCAGCCTGCCTAGGGTCCGAAGTGATTTTCGTAATTAACTCCATAATTTCTCTCTAACAAGCTTCTGAGATT
TTTTTGAATCCTATTCCTAATCTCCAATAATATTATTATTGGGCGAGGTTCTATTATTGACCATGAATTGGGGTCCCTATATTC
AAGCACAAACAAAAAACATTGACCTTCCCAGCCAAATCCTGCCTAAGGCAAAGCAGCCTAAATTGGGCTTCCGCGAAACGATTT
TCGTAATTCGCTCCATAATTTCTCTCCTTACAAGACTCTAATTTTTTTGAACCTTTTCTCATCCTCAAATAAATATTATCCATA
GGGCCTATTATTGAAACCATTGCGAAATTTTGGGGTCCTAAATTCAAGCGCAAAAGCAAAAAGAATATGAGCGCTCTCTGGCCC
AAATCCTCCTAAGGGCCAAGCAGCCTAAGGGCTCCCCGTAGGCGATTTCGTAATTCACTCCTATTTTCTCTCGTAAAACAAAGC
TCTAATTTTTTTTGAACCCCCTTTTACCACAATCTCCAAATAATAATTTATAGTTTGGGCTATTATTGACATGGGAAAAAAAAA
AAAAAAAAAAAAATTTGGGGTCCTAATTCTACCACCAAACAAAAAATTTACCTTTTCAGGCCAAAAATGCTCCTAGGCAGCTGCC
CTAAGCGGCTCCGAATGATTTCCGTAAATTCAGCTCCATATTTCTCTCTAACAACTCCTAATTTTTTTGAACCTTTTCGCGAAA
TTCAAAATATATTATTTAATAGTACAGGGCTATTATGTGACCATTAGATACCGAATTGGGTCTATTCAAACCCAAAGCAATACG
AAATTGAGCCCTTTCAGCGCGCAAATATCCTAGGCAGCTGCCCTAGGACTCCGAGAGATTTAGTAATTCACCTCCATATTTTCT
CTCCTTAACAAAGCTCTAATTTTTTTGAACAATCCCTTCCTATCCTTAATAATATTATACTGGGCTTATTATTTGTTATCCATA
ATTGGGGTCCTAAATTTTTCAAACCAAAAAAAAAAAAATTGACCTTCCCAGGCCCAAATTCTCTTGGCAGTTGCCATAGGGCTC
CGAAGGCGATTTCCGTAATTCAACTCATATTTCTCTCCTAACCAAGCTCTAATATTTTTGATCCCTATGGCCCTAATCTCAATA
ATATTATATAGGGCTAATTATGGACCCATGAATTGGGTCCTAATTCAACCAAGCACAAACAAAATTGACCCTCTCCTGGCCAAA

TCCTGCCTAAGGCGCTGCCTAGGGCTCCGAAGCCCGAATTTTGTAAATTCACCATACTATTTCTGCTCTAAACAAGCTCTAAAA
TTTTTTGAAACCCTTTCCCCCATCTCATAATATTACTAATAGGGCCCTATTATTGAACCATGAATTGGGGTCCGACTACATTAA
CAAGACAAAAAAATTTATTTCCAGGCAAATCTTCCCTAGGCAGCTGCCTAGGGGCACTCCGCGAAGTGATCTCGTAATTCACTT
CATAATCTTCTCTTCTAACAACGCTGCTAATTTTTATCCTGATCCAATTCTATCGCCAATTAATATTATATAGGGCTATTATGT
TCAGACGCATGAATTGGTGGTCCTAATTCAAACAAACCAAAAAAATTGACCTTCCAGGCCCAAATCTCCTAGGCAGTGTTGTAT
GACACAGCCTAGGAGCAATCCGAGAAACTGATTTCAGAAAAAGTATTTTCAGAGCTTCATATTATTCTTCTCTTACAAGACTCT
AATATTTTTTTGAAAAAACTTTTCCCATCTCAATATATTTATCCATGTAGGGCTAATTATATGCCCCATGAACTTGAGGTTCCT
AAATTCAACCCCAAAACAAAAAAATTGCCTCTCCAGGCCAACATCGTCCTAGGGCAAAGGCTTGCCTAGGGCTCTCGAATGCCG
ATCTTCGTAATTCACCTACTATTTCTGCTCATATTTCAAGGCTCTAATGTTTTTTGGAACGCTTTTCCATCCTCCCAAGTACCC
CATTATATAGGGCCTATTATATGACCATCGAATTGGGGTCTGCTAATTCAACGCAAACAAAAAAACTTTACCTTTCCAGGGCCC
CCGAAATCCTCCTAGGCAGCTGCCTAGGGTGCTACACGAAGTGATTCGTAATATATTCCACTCCAATTAATTTTCAATACATAC
ATAACAATATGCTCTTCAATTTTTCTTTGAACCTTTCCCATCTCAATATATGATGTTGTGTTATTTAGATGACAAATACAATTG
AATACAATTGTAGAGATGACCTAACTTTTATTGAGAATAGAATTGGAGAGTCCTAATTCAACCATAACAAAAAAATTTTATAAG
CCTTTACAGGTCCAAATCAATCCTCTAGGCCAGGCATGACCTATGTAAGAGCTCCGGAATGTGATCTTCGTAATTCACTCACTT
TAATTTCCCTCTACAAGCTCCCTAATTTTATTTTGAAGCTTTTTTCCCATCCATCCTAATAAATAAATTATTTATATACAAAAT
AGGGCTATTATTCGCCCTGTATTGACTTTTTATTTGGATTTTAATTAATTGTTTTATTATTGTTTTTTATTCTCCTCTGCGTTA
CATTATTATCTAATGCAATTAAAACTAATTACACATTTCAAAAAAAAAAAATTATATATAAACATTCATTGAATGTATCAGAAA
ATCAAGTCCTATAATACCTTTAACAAATTAAGCATTCATGATTAATAACTTCTTTAACTTTTTCATCTTCGGTCAATGTAATAA
AATCCAAAAGTAAATTTTCGGAAAGATGGTATATAATAAGATAGTTGTATTCCTTCTCGACAAACTTTATACGCACGTTGGTTA
TAGACCTGGTTCTATTATCGTGCATCGCAATTGGAATGGTTCGGGGTCATAATTCAACCAAACACAAAAAGAACTTGAGCCCTT
TATTACAAGGCCCAAATCTCCTAGGCCCAGTGCTGGCTAAGGACCTCCGAAGAGATTTCGTAATTCACTCATATTTCCTCTCTT
TTAGCAAGCTCGTAATTTTTTTGAAGCCATCTTCTACTCTTCAATACATATTATATTGTTGGGCTAATTCTATTAATCCATTGT
TTTCTGACATTGGGGTCCTAATTCACACGCAACAAAAAAAAAATTGACCTTCCAGGCCCAATCTAAAAGCTTGGGGGCAGTTTG
CCTTAGGGCTGCGCGAAAGCGATTTCGTAATTCACTCAATATTACTGCTCTCCCTTACCATCCCTCTAATTTTTTTAAACCTC
CTTCCTATCTCAATAATATTAATAGGGCTAGTTATGCACGGACCATCCCGAATTGGGGTCCTAATCAACGCAAACAAAAAAATT
GACCCTCTCTGGCCAAATCTCCTAGGCGAGCTGCCTAAGGGGCTCCGAAGCGATTTTTGTAATTCACTACTATGTTCTCGTCTA
ACAAGCTCCTAATTTTTTTGAACTTTTCCCATCTTCAATAAATATTATATAAGGGCTATTATCGTGACCATGAATTTGGGGTCG
GGCTAATTCAACGCAAACAAAAAAATTTTAACCCTTTCAGGCCCCAAATCTTCCTAGGCAAGCGTGCCCTACCGGGCTTCCGAA
GTGATTTCGTAATTCCACTCCATATTTCT
>m141120_105518_42134_c100732402550000001823141405141504_s1_p0/120405/7766_13109
TTCATTTGAGGACGACAATTTATAAATTAGAAGCCGTAAAGACATAGGGCTACAACTTTTGTCGAAACCATGACCCTCTCATAT
TAATTCGTAGTATTCACTAACAAACACTTGCCTCGAATCCATATTAGGAGAAATCGAGATTTCTGAAAGGGGTACCCATTTTTG
GTGAAATTGGAGGATTTCACTAATTTCTCCGAAATGTACCCTCTCACCCTCGTGTTCAACTCGTTCCGATATTCTTTTTCCCCA
AGAGATCTCATGGGTAACAATGAATATATTTGGAGTCGAAACGGAGCCTGTAATCAACATTTGAAAAAAAAATTCCACTTTTTG
AGGGCATTTTCCACAAGTGTCTATTTTCTATGATTCGTACATCTCTCAGTATTTTGACTGTATCACCCTAATAGCACCCTCATT
TGGGACGAAATTTTATACATATTAACCCTAAGACATGGGCTACAAACTTTTGAGGCCCATGACCCTCGCATATTCTTCTTTAGT
ATTCACCTATCAAAACATATGCTCGAGTCATATTAGAGAAATCGACAGAGTTTCTGCAGAAGGAGGTACCCCCTTTGGCTGAAT
TGGAGATTTCACTAATTTCTCCGAAATGTATTACCTCCGGTTCAACTCGTTCGATATTTTTCCGCAAGAGATCTCCATGGGTAA
ATGAATATACTTGGAGTCGAAACCGGAGCCTTACATTTCCAACACTTTAAAAAAAAATTCCCTTTTTTGAGGGCAATTTTCACA
AGTTTTTTCTTATATTTCGTACAATCTCTCAGTATTGTGACCTGTATCACAATAACACACGTCGGATTTGGACCGAAGATTTTA
ATACATATAACCCTAAGACATCGCCCGCTAACAACTTTTGAGGCCATGACCCTCGCATATTCATTTAGTATTCACTTTCAAAAC
ATAGCCTCAAGAATCCATATTAGAGAAATCGAAGATTTCTGAAAGGGGTACCCTTTGGTGAATTGGAGATTTCACTAATTCTCC
GAAGTAATTACCCCTCGTGTTCAACTCGTTCGATATTTTTACCGCAAGAGATCTCATGGGTAAATGATATATTTGGAGTCCGAA
ACCGGAGCCCTTAATTCAACATTTAAAAAAAAATATCCCTTTTTGGAGGGCAATTTTCAACAAGTTTTTTCCTTATATTCGTAC
ATCTCTCAGTATTTTTGACTGTATCACCTTATATCTATCCTATCTCTTCGGATTTGGACGAAATTTATACATCATCATACCCTA
AGACTAGGGCGTACAACTTTTGAGGAGCCATGACCCTCGCTATTCTTCTAGTATATCACCTATCAAAACATTCCCGAGTCCATA
TATTAGAGAAATCGAAGATTTCCTGAAAGGGGTACCCCTTTGGTGAATTGGAGATTTCACTAATTTCTCCGAAATGTATTACCC
CTCGTGTCCAACTCGTTCGATTTTTTCCGCAAGAGATTCAATGGGTAATGAAATAGTAGTTGGAGGTCGAAACGGAGCCGTATT
CAACATTTAAAAAAAATTCCCTTTTTGGGGCAATTTTCCACAGTTTTTTCCTTATAATTCGTACATCTCTCAGTATTGGTGACT
GTATCACCCTATACACAACTCGATTTGGACGAAATTTTAATACATATAACGCCTAAGACATAGGGCTACAACTTTTGAGGCCAT
GACCCTCGAATATTCGTTTAGTATTACTTTCAAAGACATTGCTCGAAATCCACATATTAGAGAAATCGAAGATTTCTGAAAGGG
GTAACCCTTTGTGATTTGGAGATTTCACTAATTTCCGAAATGATTACCCTCCGTGTTCAACTCGTTCGATATTTTCCACCAAGA
GATCTCATGGGTAAATGGAAATATATTGGAGTCGAAACGGAGCTTGAATTCAACATTTAAAAAAAAATTCCCCTTTTTGAGGGC
AATTTTCACAAGTTTGTTCCTTATAGTTCGTTAATGCCTCATCGAGGTATTTTGACTGTATCACCTATACAACCTCGGATTTG
GAACGAAATTCTATATAAATACGGAACCGTAAGACATCGCGCTACAACTTTTTGAAAGGCCCATGACCCTCCATCATATTACTT
TAAGTATTCAACACTAATCCAAAAACTGCTCGAGATCCATGATTAGAAGAAATGGAGCATTTCCTGAAAGGGTACCCTTGTGGT
GAATTGGAGATTTCACTAATTTCTCCGAAATGTAATTGGCCTCGTGGTTCAACCCTCGTTCGAACGTATTTTTCCGCAAGAGAT
CTCATGGGTAAATGAATATACTTGGAAGTCGAAACGGAGCCTTAAATTCAACATTTAAAAAAAAATTCCCTTTTTTGAGGGCAT

TTTGCACAAGTTTTTTTCTTATATTAGTACATCTCTCAGTGATTTTTTGACTGTATCACCCTATACACCACCTCGGATTTGGA
CGAAATTTTATAAATAGAACCGTATAGACGATAGGGCTACACTTTTGAGGCCATGACCCTCTCATAATTATTTAGTATTCACTA
TTCAAAACATTGCTCGAATCCATATTAGAGAAAGTCGAAGATTTCTGAAAGGGGTACCCCCTTTTGGTGATTGGAGATTCACTA
ATTTATCCGGAAATGTATTAAAACCCCTCCGTGTTCAACTCCCGTTCGATATTTTTCCGCAGAGATCTCATGGGTAAATGAATA
TATTGGAGTCGAAAACGGAGCCTTATGTCATACATTTAAAAAGAAAAATTCCCCTTTTTGAGGGCAATTTTCACAAGTTTTTTC
CTTATATTCGTACATCGCCTCTCCCCAGTATTTTGACTGTATCACCCTATAACACAACCTCGATTTGGACGAAATTTTATAAAT
AGAACCGTACAGACATAAGGCTACAACTTTGTGAGGCCATGACCCTCTCATATTATTTAATATTCACTATCAAAACATTGCTCC
CGAATCCATATTAGAGAAATCGAAAGATTTCCGAAGGGCGTATCCCCTTTGGTGAATTGGAGCATTCTTTCCACTAATTTATCC
GAAATGTAATTACCAATCGTGTTCAACTCGTTCGATATTTTTCCGCAAGAGATCTCATGGGTAAAATGAATATATTGACCGTCC
GAAACGGAGCCTTAATCAACAATTTAAAAAAAAATTCCCCTTTTTGAGGGCAATTTTCAACAAGTTTTTTTCTTTTTCGTACAT
CTCGTCCAGTATTTTGACTGTATTCAACCTAAATACACACTCGGAGTTTTGGACGAAATTTTTAAAATAGAACCGTAAAAGACA
TAAGGGCTAGCAACTTTTGTGACCATGACTCTCCTCATATTATGTTTACGTATTCACTATCAAAACCATTGCTCGAATCCATAT
TAGAGAAATTCGAAGATTTTCTGAAGAGGGGTACGCCCTTTGGGTGAATTGGGAGGATTTCACTAGAATTTCTCCGAAATGTAT
TACCCCTCGTTGTTCAACTCGTTCGATATTGTTTCCCCAAGAGATCTCATGGGTAAATGAATATATTGGAGTCGAAACGGAGCC
TTAATTCAACATTGTTAAAAAATTCCCCTTTTTGCAGGCAATTTTCACAAGTTTTTTCTAAATATTCGTACTTCCCTCAGTATT
TTGACTGTATCACCCTAATACACAAACCCGGATTTGCACGAAATTTTATACATAGATCCCCTAAGACATAGGCTACAACCTTTT
TTGTGACCATGACCCTCTTGTATTTCTTTAGTATTCATTATCAAAAACCATGCTCGAATCCATATTAGAGAAAGTCGAAGCTTT
CCTGAAAGGGGTCCCCTTTGGGTTGAAAAAGTGGAGAATTTCCTAATTTTCTCCGAAATGTATTTACCTCGTGTTCCACTCCGT
TCGATATTTTTCGCAAGAGACCTCCAATGGGTAAATGAATTATTGGAGTCGAAACGGAGCTTAATGTCAACATTTAAAAAAAAA
ATTCCACTTTTTGAGGCAAAGTTTTTCACAGTTTTTCCTTATATTCCGTACATCTCTCAGTGATTTTGACTGCATACCACTATA
CACACCTTCGATTTGGGACGAGAATTTTAGTACATATAAACCCTAAGACATAGGGCATAGAACTTTTGAGGCCCAATGACCCTC
GCATATTCTTTAGTATTCACGTATCAAAAACATTGCTCGGTCCCATATTAAGCAGAAATCGAAGATTTTCTGAAAGGGGTACCC
CTTTGGTGAAATTGGAGATTTCACTAATTCTCCGAAAATGTATTACCACTCGTGTCCAAGCTCGTTCGAATATTTTTCCGCAAG
AGATCTCATGGGTAAAAGAATAAAATTGGAAGTCAAAACGGAGCCCTTAATTCAGACATTTAAAAAAAAAATTCCCCCTTTTTG
AAGGGCAATTTTCCAAGTTTTTCTTGATATTCGTCATCTCTCAGTATTTTGACTGTATCACCCTTAACACACCTCGGATTTGA
CGAAATTTTATAAATAGGAACCGTAAGACATAGGGGGCTACAACTTTTTGAGGCCATGACCCTCTCCATAAAATTAAAATTTAG
GTATTCACTATCAAAACAATTGCTCGAATTCCATATTAGAGAAAATCGAAGATTCTGAAGGGTACCCTTTGGTGAATTAGAGAT
TCACTAATTTATCCGAAATGTATACCCTCGTGTTCAATCCGGGTTATCGATATTTTTCCGCAAGAGATCTCATGGGTAAGATTG
AATATTATTAGGAGTCGAAAACGGACCTTAATTCAACATTTAAAAAAAAATTCCCCTTTTTGAGGGCATTTTCAAGTTTTTTCC
TTATATTCGTACAATCCTCTCAGTATTTTTGGACTGTATCACCCTATAACACACCCTCGGATTTGGACGAAATTTTATAAAAT
AGGAACCGGTAAGACATAAGGGCTACCTACTTTTGTGACCGATGACCCTCTCATTAATTATTTAGTTATTCACTATCAAAACAT
TGCTCGAATCCATATGTAGAAGAAAATCGAAAGATTTCTGAAAAGGGTAACCCTTTGGTGAATTGGAGATTCACTTAATTTATC
CAAATGTATTACCCTCGTGTTCAACTCGTTCGATATTTTTCCGCAAGAGATCTCATGGGTAGATAATATATTGGAGTCAAACGG
AGCCTTATTTCACATTTAAAAAAAAATTCCCCCTTTTTTGGAGGGCAATTTCCACAAAGTATTTTTTTCTTATATTCGTACAT
CTCTCCAGTATTTTGACTGTATCACCCTAATACACACCTCGGATTTGGACCGAAATTTTATAATAGAACCGTAAGACATAGGGC
ACAAACTTTTGAGGCACATGACCCTCCTCATATTATTTAGTATAGTCACTA
>m141120_105518_42134_c100732402550000001823141405141504_s1_p0/122511/6279_11971
AAACTGTAGTTCGGGCATTTATTCTTTAATTGATTTTCTCGTGCTTTTTAAGAAAACAATCGACTTATGAATTTTGATCAATGC
TCTAATTTTTAGGACCTGGTTTCATCCCCCAGGACATGATTTTGAAAACACTTAGTTCGCATTAGATTTTTTTTATTAATATCT
CCCCGTGCTTTTAAAGAAAGACAAAACTGATCAATTTAAATCAACAGGAAAATTTCGATGTTTGAAAAACTTAGTTCGCGTTAA
TTCTTTATTGATATCTCCCCCGTGCTTTTTTGAGACAAAAACATCGATTAACTCAATTTGAATCAAACTATAATAACAACACCA
TAAGGTTATTTCCGATACAGTTGATTTTGAAACACTTAAGTTCGCATTAAGTCCTTTATTGATATCTCGTGCTTTTTAAGAAAA
AACATCGATTATCAATTTGAATCAACGTTCTAAGTCAACTGGTTGCATCCCAGGACATTGATTTTTGAGACACACTTGAGTTCG
CGTAGATTCTTTATTGATATCCTCGTGCTTTTTAAGAAAAACATCGATTAATCAATTTGAATCAACTTCTTATGTTTAGGTCCT
GGTTCTTTCCCAAGATATTGATTTTCAAAACATAGTTCACGTAATTCTTTATTGATATCCGCCGTGGCCTTTTAAAGCAAAAAC
TCAATTAATCAATTTGAATCAACATCTTAGAGTTTAGGACCAAAAACGTTTATTTCCAAGATAAAAATTGATTATTGAAAAACT
TATTCGCGTTAATGTCTTTATTGATATCTCGTGCTTTTTAAGAAATAACATCGATTAATTAATTTGAATCAACTTCTAAGTTAG
GACCTGGTTAATTTCACATAGATTTGAAAAGCTTAGTTCACTACTCTTTATTAATATCTCCATTTTTTAAGAAAAACATCAAAA
ATGCAATTGAAACAACTTCAAAGTTTTAGTACCGTGGTCATTTCCAGGATATTGATTTGAAAGAACTTAGTTCGCGTTAATCTT
TATTGATATCTCCAGTGCTGTTAAGAAAACACAATCGATAAATCGAATTTGAATCAACTCTCTTAAAAAGTTTTCAGGACCTGG
TTGCATCCAGGACATTGATTTTGAGAAACTTAGTTCGGCGTTAATTCTTTATTGATTATCTCATAGGCTTTTTTAAGAAAAAAT
CGATTAACAATTTGAATCAACTTTTAAGTTTAGGACGCCTGGTTGCATCCCCAGGACATTGGATTGTTGACAAAAACTTTAGTC
TCGCATTTTCTATCTCTTTTACAAAAATTGATATCTCGTGCTTTTTAAGAAAAACATCGATTAAAGTGAATTTGGATGCAACTT
CTAATTCTAGACCCTGCGCTTTCCATCCAGGACATCTTGATCTTTGAAAAACTTAGTTGCGGCATTAACTTTTTTCACTGAATA
TATCGCTGCTTCTACCACATCCAGATCAATTTAATCAATTCTTAAGTTTAGGACCTAGTTATTTTTCCAGGAAATATTGCATTT
TGAAAAACTTAGTTTCGCTTAATTCTTTATTGAAACACAAGAACTTTTTGAGAAAAACATTCGATTAAGTCAATTTGAATCAAC
TTCCTAAGTTTAGGAGCTGGTTATTTCCAGGATAGTTGATTTGAAAAGCCTTTGTTCCACGTTATTCTTATTGACTATCTCATG
TTTTTTTTCCAAGAAAAACACAATTAATGCAATTTGAATCAACTTCTAAGTTTATACGCTGGTTATTGCCAGGATATTGATTTG

GAAAAACTTAGTTCGCGTAAATTCTTTATTGAATCTCGTGCTTTTAAAGAAAACATACGATTAATCCAATTGAAATCAACTTCT
AGTTATGGACTGGTTGCATCCAGGACATTGATTTTGGAAAGCCTAGTTCGCCCTGTTAATTGCTTTATAGATATCTCGTGCTTT
TTAAGAAAAACCATCGATTAATCAATTGAAATCAACCTTTTAAGTTTTAAGGACCTGGTTGGCCTACAGGACATTGATTTTGAA
AAACCCTTAGTTCGCATTATTCTTTAATTGATATATCGTGCTTTTTTAAGAAAAAACATCCGATTAGTCCCAATTTGAATCAAC
CCTTCCTAATTTTAGTCTGGTTTTCATCCCAGGACCATTGGTTTTGAAAAACTTTAGTTCGCATTAATTTTTTTTATTAATCAT
ACTCGTGGCCTTTTTAAGAAAGAGCATCGACTGATCAATTAAATCAACTTCTAAGTTTAGGACCTAATTAATTTTCCAGAAAAT
ATAATGAAAAAAGTTATTTCGCCGTTAATTCTTATTGATATCTCTGCTTTTAAGAAAAACATCCGATTGATCCACATTTGAAAC
ACAAACATATCTAAGATTAGGACTGGTTTTTCCAGATATTCAAAATATGAAACAGTTCGCATTAATATCTTTATCATCATCCCT
CGTGCTTTTTAAGGAAAAAACATCCGATTAATCATTTAAACAACAACATAGACCTGGAAATTGCATCCAAGGAACATTGATTTT
GAGAAACTTAGTTCGCAAACACTTTATTGATATCTCCCCGTGCTTTTTAAGAAAACCATCGAAATTAATCAAATTTGAAATTCC
AACTTCCTAAGTTTAGGCCTGGTGCATCCAGGACATTGATTTGACGAAATCTTAGTTCGCGTTAATTCTTTATCAGATATATCC
GTGGCTTTTTAAGCAAAAAACCATCGATTAATCAATTTGAACATCAACTTTTTAAGTTTAGGACCTGGTTTGCCTACCGAAGCA
TTGATCTTTGAAAACTAGTCTCATCTCTTTCCTTTATTTGTTTCCTCGTGCCGTTTTTAAGGAAAAACATGCGACTTTATCATT
TGAATCCAACTTCTAATTTTAGGAACCTGGTTTCATCCAAGGACATTGGTTTTGAAAAACCTTAGTTCGGCATCTAATGCTTTT
TATTTCCTATCTAGTGGCTTTTTCAGAAAGACATCGACTGAGCAAGTTCAAATCAACTTCCAAGCCTCTAGGACTAGTTATTTC
CCAGGATATTGGATTTGAAAAGAACGTTATTCCCCACGCATTGCATATCTCGTGCTTTTAAGAACAACCCACCGCGATTGATCA
CATTTGAATGCAACTCAACAGATTCAGGACCCCGCTTGGTTCTATGTATCCAGCGCACCACCGAGTTTGCGAAAAACCTAGCTC
CGCATTAATCACTCTTATTCATATCTCGTCCGGTTTAAGACACAGAACATCCGATTAATCAATTTGAATCAACTTCCTAAGTTT
AGGACCTGGTTGCCTCCAGGACAGTTGATTCGAGCACAACTAGTTCGCGTTCACGATTCACGTTGAATATCCTCGTGCTTTTAC
AAAACATCCGACCTAACCGTCACTTTGAATCAACTTCAAGTTTAGGACCTGGTTTCTTTCCCCAAGATATTGATTTTGAAAACT
TAGTTCCGTTAAATTCTTTATTGATATCTGCGTGCTTTTTAAGAAAAACATCGAATTAATTAATTTGGATCACCTTCTATGTTT
TGGACCTGGTTATTTCCCAGGATATTGATTTAAAGCCTTAAGTTCAGCCGCTATATTCTTTATTGATATCTCATGTTTTTCCAA
GACAAACCCATCATTCAATAAATCAATCAACCCTTTCTAAGTTTTAGCAGCCTGGTTACTTTCCAGGATTACCCTTGATTTTGA
AAAACTTAGTTCGCGCGTTAATTACTTTATTCGATATCTCGCTGCTTTTAACGAAAAAACATCCGATTAATCCAATTTGAATCA
ACGTTCTAAGTTTTTGCCTGGCTCATCCGCGGAAATCTGCCACCTTGACAAACTTACTCTTTCCCGGTTTATTTTATTCATATC
TCGTGCGGCTTTTACGCAAAAAGCATTCCCGCATTAATCAATTGAATCAACTTCTACAGGTTTAGGACCTGGTTTGCATCCAGG
ACAATGATTTTCGAGGAAACTAAGTTCTTTAATTCCCTTGCGTTGATATCTCTGCTTTTTAAGAAAAAAACCATCGATTAATCAA
TTCTGAATCAACCTTTTAAAGTTTCAGGACCCTGGTTGCATCCAGGACATTGGATTTTTTAAAAACTTAGTTGCGGGCCCATTT
ATTCCTTTATGATGTATCCGGGGGGGGGGGGGCGGGGGCGGTCCGTGGCTTTTTAAGAAAAACACCCGATTAATCAATTAGCATG
CCAACCCTCTAATCTCTGTAGGGACCTGGCTTCTCATCCAGGACAGTGATTTTGGACAAAACCCTTAGGTTCGGCACTTACCAT
TTTCCTGTATTAAATAGTCTCCTGCCTTCTTTAAGAATCAGACATCGACCTGATGCAATTTAAATTCAAGCTTCTAATGTTGAG
AACCTAGTTATTTCCAGGATATTGATTTTGAAAAACTTTATTCTCGCGTTACATTCTTTATTCGATATTCGTGTTTTTTAAGAA
AGACGATCGACTGATCAATTTAAAGTCAAACTTCTACAGTTTGGAGCCTAGTTATTTCCAGGATATTGATTTGCTGCAAAAACA
TTTAGTTCTGCGTTCAATTCTTTATTGATATCCTCGTGCTTTTTAAGACCAACCATCGATTAATCAATTTTGAATCAAACTTCT
AAGTCTTAGGACCCTGGTCCATGTTCCAGGATATTGGTTTGAAGCTTAGTTCACGTTAATTCTTTATTTGATATCTCATGTTTT
TTAAGAAACACATCAATTAACTCAATTTGAAATCCAACTTCTAAGTATACAGGACCTGGTTACTTTCCAGGATATTCGATTTTG
AAAACTTAGTTCCACGGTTAATTCTTTATTGATATATGTTTGTTTCAAGAAAACATCCCCAAGTTAATCACACATTTGCAATCA
ACCACCTTTCTAAAGTTTTACGTACTGGTTCCATCGTGTCCTAGGATAACACGTTGATTTTGAAAAACTTAGTTCGCGTTATTA
TTTATTGGATATCTCGTGCTTTTAAGAAAACATCGATTAATCATTTGACATCAACTTTCTAAGCTTTGGCGACCGGTTTGCAAT
CCAGGGACATTTGATTTTGCAGAAACTTAGTTCGCGTTAATTCTTTAATTGATATCTCGTGCTTTTTAAGAAAACATGATTAAT
CAATCTTGAATCAACCTTTCTAAAGTTTAGGACTGGTTCTGCACATCCGCCAGGGACAATGATCTTTGAGAAACTTAGTGTTCG
GTTACATCTCTTTGTTCATATCTCCGTGCTTTTTAAAGAAAACATCCGCTTAACTCCAATTTGAATCAACTTTTAAAGTTTTAG
GACCCTGGTTTGCAATCCAGGACATTGATTTTGAAAAAACTTAGTTCGCATTTATTCTTTATTGCGACTATGCTCCGTGCTTTT
CTAAGAACACCATGCGATAATCAATTTGAATCAAACTTCTAATTTTAGACGCTGGTTTCATCCAGGACATTGATCTTTCGAAAA
AACTAGTTCGCATTAATTTTTTTTAATAATATCTCCGTGCGCTTTTTTAAGAAAGACATCGACTGATCCATTTAGAATCAATTC
TAAGTTAGTACCGCTAAGTTATTTCCAGGAATATTGATTTTGAAAAAACCTTATTTTCGCGTTAATTTCTTTAATTGATTAACG
CGTGTTTTTTAAGAAAAGAGCCCATCGACTGATCAATTTAAATCAACTTCTAAGTTTACGGACC
>m141120_105518_42134_c100732402550000001823141405141504_s1_p0/122511/12020_17126
GTCGCTAAAACTTACGAAAGTTGATTTAAATTGATGCAGTCGATGTCTTTCTTAAAAAACCACCGAGATATCAATAAAGAATTA
ACGCGAAATAAAGTTTTTCAAAATCAATATCCTGGAAATACTTAGGTCGCTAAAACTTAGGAAGTTGATTTAATTGATCAGTCG
AGTCTTTCTTAAAAAAAGCAGCTGAGATATTAATAAAAAAATTAATGCGAACTAAGTTTTCCCAAAATCAATGTCCTGGATGAAA
CAGGTCCTAAAATTAGAAAGTTGATTCAAATTGTTAATCGATGTGTTTCTTAAAAAAAGCACGAGATACGTCAACCTAAAGAATA
AATGGCGAAACAAGTTTTTCAAAATCAATGTCCTGGATGCCAACCCAGGTCCTATAACTTAAAAAGTTGATTCAAATTGATTAA
TTCGAATGTTTTTCTTAAAAAGGAACGAGATACCGAATGAACAAAGATTAAGCACTGAACTACAAGTTTCTCAGAAATCCAATG
TGTCCTGGATTGCAGACCAAGAAGTCCCTAAACTTTAGAGTTGATTCAAATTGATTAATCGATGTTTTTCTAAAAAGCAGCGAG
AATATCAATAAAGATTAACGCGAACTAAGTTTCTCAAAAATCAATGTCCTGGATGCAACCAGGTGCCAAAACTGTAGAAGTTGA
ATTCAAATTGATTAATCGATGTTCTTTCTTAAAAGCACGAGATATCAAGTAAATACCCCATTAAGCGCGAACTAAGTTTTTTCA
AAATCAATATCTAAAAGGAATAACCAGGTACTAACTTAGAAGTTGATTCAATTCTGATTAATTGATGGTTTTTCCTTGACAAAA

GCATGCAGATAATCAATTAAAGAAATTACGTGAAGCTAAGCTTTCAAAATCAATATCCTGGATAACCAAGGTCCTAAACTTACG
AAGTCTGATTCAATTAGAAAAAACAATTAATTGATGTTTTTTTAAAAAACATGAGATTATCAACCCTAAAGAATTCAAACGTG
AACTAAGCTTTCAAAACCAATATCCTGGGAAATGACCCAGGTCTAAAACTAGAAGTTGATTCAAATTGATTATCGATGTTTTTC
CTTAAAAAAGCCAACGCAGATATCAATCAAGACATAACGAGAACTAAGTTTTTCAAAATCAATATCTGAAATAACTGGTCCAAG
GTCCCTAAAAACTTAGAAGTTGATTTAAAATTGATCAGTCGATGTCTTTTCTTAAAAAATAAACTAACCGAGATATAATAAAAG
GAATTAACCCGCGAAATAAGTTTTTCAAAATCAGATATCCTGGAAATAACCTAGGTCCCTAAACTTTAGAAAGTTGATTTAAAT
TGATCAGCGATGTGTTTTTCACACAAAAAGCACGAGAATATTATTAACAAAAAATTAATGAACTAAAGTTTTTCAAAAATTCAA
CTTGTCTGGATGAAACCAGGTCCTAAAATTAGAAAGTTTGATTCAAATTGTTTAATCCGGTGTTTTTCTTAAAAGCCCACGAGA
TAGTCCAATAAGCAATAAAAGCCGAATAAGTTTTGTTAAAAATCAAAGTGTCCCCTTGGATGCCAACGCAGGTCCTTACACCAA
ACTCGTAAAAGTTTGATTCAAATTGATTACTCCGATGTTTCTTCTTAAAAAGCCAACGAGATACTCGAACAAAGAATTAAGCAG
CGAAGCTAAGTTTCTCAAAATCATTTGTCCTGGATGCCAACCAGGTCCTAAACTTAGAAGTTGTTCAAATTGATTAATCGATTG
CTTCTCTTCAAAAACGCACGGATGGGCAACTCAATAAAGAATTAACGGCGAACTAAGTTTCTCAAAATCAATGTCCTGGATTGG
CAACCAGGTCCAAAACTTAGAAAGCTTGATCAAATTGATTAATCGATGTTTTTTCCTGTTTTTTAAAAGCACCAAGAGATATC
GATAAAAATAATTAACCGCGAACCTAAGTTTTTTCAAAAAATCAATATCCTGGAAATAACCAGTGTCGAAACCTTAAAGTTGATC
AATTTGATTAATTGATGTTTTTCTTGAAAAACATGAAGATAATGTCAATAAAGAATCTAAAGTGAACTAAGCTTTTCAAAATCA
ATATCACTGGAAAATAACCAGGTCCAACCACCGCATAAAGAAGTTGATTCCCAAATTAATTAATCGGAATGGTTTTTCTAAAGG
CCACGCAGATATCAATAAAAGAAATTAACGGCGCAACAAGTTTTTCAAAAAATCAATATCTTGGAAAGAAACCCAGGTCCTAAA
ACTTAAGAAAGTTGATCAAATTGATTAATCGATGTTTTTCTAAAAAGCACGAATACTCACCTTCGAAATAAAGATTAACGCGAA
CTAAGTTTCTCCAAAATCAATGTCCTGGATGGCAAACCAGAGTCCGTAAACTTAAGAAGTTGATTCAAACTTTGATTAATCGAT
GGTTTTTCTTATAAAAAGCACGAGATATCCACACTAAATGAATTAATGCCGAACTAAGTTTTTTTCAAATGCAATATCCTGGA
AATAACCCAGGTCCTAATCTTTGAAGTTGATTTCCAATTTGATCATCGATGTTTTTCTTAAAAAGCAGAGATACTCAATAAAGA
ATTAACGCGAAATACTTTTTCAAAAATCAATAATCCTGGAAATAACTTAGGTCCTAAACTTTAGAAAGTTTGATTTAAATTGAT
CAGTCCGATGTCTTTCTTAAAAAAAAAGCACGAGATAACTTAATAAAAAAATTAATGCGAACAAGTTTTCAAAACCTATGTCCCT
GGATGAAGCCAGGTCCTAAAATGAAAGATTGATTCAAATTGATTAAATCGATGTTTTTCTTAAAAAAGCACGAGATATCCCAAA
TAAAGAATAAATGCGAACTAAGTTTTCCAAAAAAATCAATGTCCTGTAGGCCCAACCAGGTCCTAAAACTTAAAAGTTGATTCA
AATTGATTTCAAATCGATGTTTCCTTAAAAAGGCACGAGAATTATCTAATAAAGAATTAACGCGAACCTAAGTTTCTCAAAATC
AATGTCCTGGATGCAAGCCATGGTCCTACCAACTTAGAAGTTGATTCAAATGATTTAATCGATGTTTTTCCTTAAAAACGCACC
GAAAGAAATATCAATAAAGAAATTAACCGAAACTAAAAAAGTTTCTCCAAAATCAATGTCCTGCGATGCAGACCAGGTCCTAAA
AACTAGAAGTTGATTCAAAATTGATTAATCGATGTTTTTCTTAAAGAAAATTAAAGCACGAGATATGAAATAAAGAAGTTAATG
CGAACTAAGTTTTCAAATCAATATCCTGGAAATAACCAGGTCTCTAACCATCCTAGAAAGTTGATCAAATTTGATCATCGATGT
TTGTTCTTAAAGGCCACGAGATTCAATAAAAATTAACGCAAATAACTTTTCAAATCAACGTATCCTGGAAATACATTAGGTCTA
AACTGTAACCGAAGCTTGATTTAAATTGATCAGGTCCGATGTCTTTCTTAAAAAGCACCGAGATATTAATAAAAAAATTAAATG
CGTACAAGTTTTCCAAACCAATGTCCTGGAATGAAACCCCAGGACCGTAAAATTAAGAAAGTTGATTCAAAATTGATTAATCGA
TGTTTTTCTTAAAAAGCACAGAGATATCAAATAAAGAATAAATGCGAAAAACTAAAGTTTTTTCAAAAAAATCAAATGTCCTGT
AGGACAACCAGGTCCTAATTAAAAAGTTGATTCAAAATTGATTAATCGATGTTTTTCTCCCAAAAAAAGCCACGAGATATCTCA
TTAAAGAATAACGCGAACATAAAAGTTTCCTCCCCAAAAATCCAATGTCCTGCGGATGCAACCAGGTCCTACTTAGAATTGATT
CAAATTGATAATCGATGTTTTTCTTAAAAGCACGAGATAATCTAAAGAATTTACGCGAACTAATTTTACTCACTATCTAATAAC
CAGGGTCTACTTAGAGATTGTTCATGATTAATTGGATTTTTTCTGGAAAACATGAGATATCAAATAAAGAATAACGCTGAACAA
GCTTTTCAAAATCACCATAGAATACCAGGGTCCTAGAACTTAGAAGTTGATTGCAAAATTGATTAATCGATGTTTTTCTCAAAA
AGCCGAGAATATCAATAAAAGAATTAACGCGACTAAGTTTTTCCAAAAATGCAATATCCTGGAATAACTAGGTCCTAAAACTTA
GAAGTTGATTTGAAATTGATCAGCTCCATGTATTTCTTAAAAAGCAAGCCGAGAATATTAATAAAACAATAATTAATGCGAACC
TAAGTTTTTCAAAAAAAATCAAATGTCTGGATGAAACCAGGTCCTAAAGATTAGAAGTTGATCAAATTCATCTAATCGATGTTT
TCCTTAAAAAAGGGCAGAGATAAATCAATAAAGGAAATAAAATGCGAACCTAAGTTTTTCAAACAATCAATGTCCTGGGATGCA
CCAGAGGTTCACTAAACTTAAAAGTTTGAATTCAATGATTAATCCGATGTTTTTCTTAAAAAGCCATGAGATATCAATAAAGCA
AAGTTAACGCGAACCTAAGTTTCTCAAAAAAATCAATGTCCGCTGGATCGCAACCCAGGTCCTAATACTTAAGAAAGTTGATTC
AAATTGATTTAATTCCGATGTTTTTCTTAACAGCAGCGAAGATATCAATAAAGACATTACGGCGAACTAGAGTTTTTCAAAAAT
CAATTCCTGGAAATGACCAGCCGTACTAAACTTGAAGTGTTTCAAATTGATTAATTGAAATGTTTTTCCTAAAAAACATGAGAT
ATTAATAAAGAATTGAACGTGAACGTAAAGCTTTTGCAAAAATCAATATCTTGGAATAAAACAGGTCCTAAAATTAGAAAGTTG
AATTCAAATTAATTAATCGATGTTTTTCTTAAAAAGCACGAGAATATCAATAAAAGAATTAACGCGAACTAAAGTTTTCAAAAT
CAAATATCTTGGAAATAGACGAGGTCCTAAAAACTTAGATGTTGATTCCAAATGATTAAAAATTGATGTTTTTCTTAAAAAGAA
CAGATATCAAAAAAGAATAACGTGACTAAGTTTTGAAAAAATCAATATGCTTTGGAAAGAACCAGG
>m141120_105518_42134_c100732402550000001823141405141504_s1_p0/124279/4079_14068
CAAGGAGTCATAGGGCATAAAGTTATAATGCCCTAATGTCTTAGGGTTTCTATTTGTTTGATAAATTTATTTTTCGTTGCGTAG
TCCGAGTTGGTTAGTATGGGTGATATCATCAAAATATTGATGAGATTACTATTGTGTTTAATTATAAAATAATAGGAAAAAACT
TGGCGAAGAATTGCCAATAAAAAAGGGAACTTTTTTTGAAATGATGTGTTAATTTAAGGTGTCGTTTCCAACGTCCAATATATT
CATTTACCTCCTGAGAATCTCTTGGGAAAAAATATCAGAACGATTGAACACTGATAAGGCAATACATTTGCGAGAAATTAGTGAA
AGTCTACAATGTCCAAGGGGTACCCCATTTTCAGATAAGTCTTCGATTTTCATTTCCTATAGGGTTCGATTGCGATAAGGGGTC
GTTTGTTATGTCATAGTGATATCTAAATAGTTTAATGAGTGCAATGGTCATGGGCTCACACAAATAGTTTGGAGTCCATATGTT

CATTAGGGTTCTTATGTGTAATTTTTCGTTGCTAGTCGAGGTGTTTGTTATGGTTTAGGGTTGATTCACAAGTTCATTAACATA
TGGAAGATGTACGTAATATTGTTTGGTAAAAAGCTTGCGAAAATTTGCCATTCAAAAAGGGGTAATTTTTTTGTTATATGTTT
CGTATTATGGTTCTTTCGTTATCCACTCCAGAATATACGTTTACATTAGCCCATCGATGATCTTTTTCTGGTGGGAGATAAAGT
ATCATATACGAGTTTGTAACTACTGATGTGAATTCTTATACTTTTCGGAGTTATAATTTAATGAAAATCTCTCATTCACCCAAA
TGAGTACCCCTTCAGAAAATCTCGATTTCACTAATATACGATCTTCGACAAAAGTATTGTTGTTTCCATAGTGAAATAATAAAT
AATAATTTGCAAGGTCAATCGTGGTGCTCACAAAAGTTGTAGCCCTATGTCTTAGGGTTTATGTGTATAAAATTTCGTGCTAGT
CCGAGTTAGGGGGGCTGGGAGTGATACAGTCAAATAATGTGAGGATGTACTAATATAAAAGGAAAAAACTTGCGAAATTGCCAT
ATAAAGGGAACTATTTTTTGAAATGTTGATTTAAAGGTCCGTTTCCAATCCAATATATTCATATTACCCATGAGAATCTCTTGG
GGAATAAATATCAAACGAGTTTGAACCCGAGGGGCAATACATTTTCGGAAGAAATTAGTGAAATCTCCAATTTCACCAAAGGGG
ATAACCCCCCTTTCAGATCTTCGATTTCTCTAATAATCGTTCGAGCAAATGTTTCATAGTGAATACTAAATAATATAGGCAAGG
GTCATGGCACATAACAGGTTGTAGCCCTATGTCATTAGGGTTCTATGTGATAAAATTTCCTGTGCTAGTCGAGATTGGGTAGAA
GGGTGAAACAGTACAAGACAATACTGTGGAGATTGTACAATAATACAGGAAAAAAACTTGCGAAAAATTGCCATCAAAAAGGGG
AACTTTATTTTTTTGAAATGTATGACTTTAAGGGATCCGTTTCCTCCTCCAAATATATTATATTCCCATGTTACCCATAGAGA
TCTCTTGGGGGTGAGATAAAATATTCAAACAGAGTTGAACACGAGAGGAAATACATTTTCGGAGAAATTAGTGAAATCTCCCGC
ATATATCACCAAAGGGGTACCTCTTTCGCAGAAAATTCTTCCGATTGTTCTCTAAATATGCGATTCCGAGCAAAGGGATGTTTT
GTTCAGTATCGAATGTTGAAAATTAAAAATGATATATGTGCAAGGTGTACCATGAGGCACAAAAGTTGTAGCCTATGTCGTTAG
GGTTCTAGTGTAAAAATTGTTCGTGCTAGTCCGAGTTGGGTATGAAGGGTGATACGATCAAAATATACTGTGAGATGTTTATAA
TATTAGGAAATTAATCTTGCGATAATTATCAATAAAAAAATATGGTTAAGAAGGGGGAACTTTATTTGAAAATGTTTGATTTAG
GGTCCGTTCCACTTGCCAATATTTGCAAATTCAATGTGTACCCACTGAGATCGGGTCTTGGGAATAAGATGACGAACTCAGTTG
ACACGTAGGGCAAATACATTTTCTGGCAGAAATTTAGTGAAATTTCCAATTTCACCAAAAGGGTACCCCTTTTCAGTAAATCTT
CGATTTCTCTAATATCGATTATCGACAATGTTTATGGTTTGCTTAGTGTATACTGGAGGTACGGGAGAAGATATATGTGAAGGT
CATGGGCACAAAAGTTGTAGCCCTATGTCTTAGGGTTTCTAGTGTGTAAAATTTCGTGTGCTAAGTCCGAGTTGGGTAGTAGGT
GATACGTCAAAATACTGTGAAGATGTAACTAATATAAGTGAAAAAACTTGCGAAAATCTGCCATATCAATAAAGGGGAACTTTT
TTGTGAATAATGTTGATGTATAGGGTCGTTTCCAACTCCCAAATAATATTCATTTTACCCATAAGAGATCTCTTGGGGAAATAA
CAAGTAATCTAAACGAGTATTAACACGAGGGCCATATACATTTCTGGAGAAATATTTAGTGAATCTCCAATTCACCAGAAGGGG
TAACCCTTTCAGAAATCTTATCGATTTCTACTAATATCGATATCGAGCAATGTTTTCTTAGTGAATACTACAATATATATGAAG
GGTCATGGCCAAAAGTTGTAGCCATATGGTCATTAGGGTTCTAAGTGGTAATAAATTTCGTGCTATTCGAGTTGGGTTAGCAGG
GTAGATACAGTCCAAAATAACTGTGAAGATGCTAAACGCAATCACTTAGGAGTCTTGAAATAAAGGCTGTGCGAAAATTTCCAT
ATAAAAAAGGGAATTTTTTTTGAATTGTTGTTATTTAGGGTCGCTATTTCCACTCCAATTAGTATCTTTCATTATACCCTATTG
AGATCTATTCTTTAGGGGATAACATATCTATCATACACGAGTATGAACATACTTGAGGGCAATACATTTTCGGTAAGGATAAAA
TTACGTTAGAAATCTCCATTCACTCAAAGGGCTCACTCTATCAGAGAATTCTTACGATTCCTCATATTAAGATCGAGAAACTGT
TTTCTTATAGTGAATACTAAAATAATAGTGGAACGGGTCATGGAGAAAAGTTGTAGCCTATGTCTTATAGGGAGTTCCTATGAA
TATAATAATAATTTCGTGCTAGTCCGAGTTGGGTAGCGAGGTGAATACAGTCAAAATTACTGAGAGATGTACTAATATAAAGGA
AAAACCTTGCGAAAAAAAAAAAGGAGACTTTTTGAGAATGTTGATAAAGAGGTGTTTCCACCTCCAATATATATCATCTTACAC
ACATGGAGATCTCTGGGAAAAATATCAAACAGCTTGAACTTAACCGAGGAGCAATACATTATCGGAGAAATTAGTTAATAACAT
TCACCAAAGGGGTCCTCCTTCAGAAATCCACGATTTCTCTAATATAAAAAAAATAATAAATACTAATAAATATACTGGAGAGCG
GGAGTCCATTGGGAACAAAAGTTTTAGCCCTATGTCTTTCAGGGGTTCTATGTTCAAAAATTTCGTTGATAGTCAAGAAGTTGT
AGATGGGTGAAATACAGTCAAAATACTGTATGAGAAGTAGCTATATATAAAAGAAAAATTTACGAACATTTATGAAGAAAGGAT
AATATTACATAATAACCAAAAGAAATTGCCTCAAATACATAAAAAGCAAAGGGGAATTTTTTTGTAAACTGTTGAATTTAGAGG
TTCCGAGTATTTCCACTCCCAATATATTTCAAAAATAAGATCATCTTGGGGAAAAATATCAACGAGTTGAACACGAGGGACCAA
TACATATTTCGGAGAAATTAGTGAAAATCTCCAAATTCACAAAGGAGGTAGCCCTTTCAGAAATGCTTCGATTATTCTCTAATA
TACGATTCGAGCAATGTTTTCATAGTGATACTATAAATATATGAACAAAGGAATCATGGAGCACAAAAGTATAGCCCTATGGTC
ATGTAGGGTTCTATGTGTAAAAAATTTTCGTGCTAGGTCCGAGTTGGGTAGAGGGTGATAGTAACAGTCAAAAATACATGAAGA
ATAAATAAATATAAAGGAAAAATACTGACTTGTCGAAAATTTGCCATCCAAAAAGGGGTGTGAAACTTTTATTGAAATAACTTG
ATTTAAGGGTTCCGTTTCCACTCCAATATATTCCAATTTAAAAAGTATATATAATATAAAATATTGGGGAAAAATTCAAATCGA
GTATTAAACACGAGGCAATACATTTCAGGAGAAAATTAGTTGAAATCTCCAATTCACCAAGGGGTCTTAAATCAGAAAATATAT
ACGAGAATTTATCATAAAATATAAACAAAGATGTCCGATGCTAAATGATTGTTTCTAGAATAATATAAATATATATGCAAGGGT
CTGGCACAAAATTGTAGCCCAGACTATGTCCTTAGGGTTCTATGTGTAAAATTATCGTGCTAGTCCGAGTTGGGGTAGACGGGT
GATACAGATCAAAATACTTTGTTGAAAAGATGTACTAATGATACAGGAAAAAAACTTGGCGAAAATTACCCTCAAAAGGAACTT
TTCTTGAAAGTGTTGATATAACAAGGTCCGTTATCCACTCCTCAATATATTTCATTACCCATGAGATCTCAAATTGGGGAAAAA
TATCAAACGAGTTTGAACATGAGGGCAATACCATCTCGGAGAAATTAGTGGAAATCTCTCAAATTCCACACAAAAGGTACCCCT
TTTTTCAGAAATACTTCGATTTCTCTAAATATACGATTCGAGCCAATGTTATCATAGTGAAATACTAATAAATCACTTACCGAA
GGGTCACTGGGCACGGATAAAGTTGGTAGCCCTATGTCTTAGGAGTTCTATTGATGTAACAATTTCGTGCTATACCGAGCTCTG
GGTAGAGGGTGATACAGTCTAAAATACTGTGAGATGTACTAATATTAAAGGAAAAAATTTGCCGAAAATAATCAAAAGAAGTGT
GAAAATACTGATGGTCTCTTGTAAATAGTTGAATTTAAGGAGTCCGTTTAACACTCCAACTATATTCATTTACCCATTCGAGAA
TTATCCTCTTGGGGAATAAATATCAACGAGTTGAACACGAGGGCAATTACATTTGGATAAATTTGTTGAAAAAATCTCCAATAA
TCTACCCAAAGGGGACCCCCTTTCGAAAATTCTTCATTCTTCTAAATATATAGATTCGAGCAATTGTTTTATTTTTGCATGAGT
TGATTTATATCTATATAAGGTATATTATATAAATACCTATCGATCATAGGCAGGAAAGTTGACGCCTATGCATTGGGTTCTAAG

TGTTGCTACAAAATTTCGGCGATAAGTTCCTGAGGTGTGTAAGAAGGCGTGATACAGCTCAAATATACTGTAGATGTACTAAAA
TGAGAAAAATCTTGTCGTATATATTGCCTCATATTAAGGAAAGGATTTTTTGATAATGTATATTGATTTATTATGGGTCGCGTA
GTATCCACTCCAATTATATTTTGGCTCATTTATTCATGAGAAGTCTCTTGGAAAAAATAAATTATGAAGTAATAATAAATTAAA
AAAAAATAATAATCAAAACGTATGTGTAATCACGAGGGCATATTACATTCTTTGAGAAATTGAGTGAAATTCATCCAATTCACA
CAAAGGGTACCCCTTTCAGAACTCTTCGATTTCTCTCTTAATTGATCGATTTGAGGCAAGGATTGTTTTTCACAGTGAATAATA
AAATAAATAATCAAGGTCATAGGGCACAAAAGTTGTAGCCCTATGTCTTAAGGGTTCTATAGTGTAAAATTTCGTTGCTAAGTC
CCAGAGTTGGTAGGGGTTGATACAGTCAAAATAACTGTGAATGTACTAAATATAAGGGAAATAAAACTTGCGAAAATTGCCATC
AAAAAGGGGAACTTATTTTTTTGAATATGTTGAATTTAAGGGTCCGTTTCCCACACTCCAATATCATTCAAATTTACCCAGTGG
ATCTCTTGGGGAAAAAATATCAACGATTTGAATCACGAGGGGCAATTACAGTTATCGGCAGAATTAGTGAATTCTTCCAATTCAC
CAAAGGGAGTATAACCCCTTTCAGAAATTTCGATTTCTCTAATATAGATTCGAGCAGTTGTATTTCATAGTGAATAATAATATA
GATTATGCTACGGGTCATATGGGCACAAAAGTTGTAGCCCCTAATGTCTTAGGGTTCTAATTGTGTAATATTCGTGCTAGTCCG
AGTTGGTAGAGGGTTGAGAATACAGTCAAAATACTGTGAGATGTACTGATTTCAATTAATAAGTGAAAACTTGCGAAAATTGCC
AATAAAAATAGGGGAGAAACTTTTTTTTGAAATGTTGATTTTAAAGGTCCCCGTTTCCCCCAATTATTTCATTTACCCATGAGT
TATCTTTGGGGAAAAATATCAAACGAGTTTGAAAACACGAGGCTGATAGACCTTTCGGAGAAATTTTAGTGAAATCTCTCAATC
AACCAAAAAGGTGGATACGCCCTTTACAGAATCTTCGATTTCTCTAATATCGATTCGAGCAATTGTTTTCTAAGGTGAATAATA
AATATCAATAAAATATGCAAGGGAGTCATGGCGTCGAAAAAGTTTAGCCCTCATACTCTTATGGTTTTATGTGATAAAATTTTG
TGAAACCGAGGTGTGTGTGAGGGTGATACAGTCATATTAATCACTCGGAGAGATGTACTAATATTAAAGGAAAAAACTTATGCG
AAAAAATTGCCATAGCAAGAAAGGCGGGAGAACTTTATTTTTTTGAAATGTTGATGTTAACAGGGTCCGTATTCCACTCCAATA
TATTCAATTTACCATGAGATCCTCTTGGGTGAAAATATCAAACGATTGAAACACGAGGGCAATACATTTCCGGAAGAAATTAGT
GAAATTCACTCCAATTCAACACAAAGGGGTACCCCTTTCAGAAATCTTCTCGATTCTCTAAATATAGATTCGGCAAATGATTTT
TCATAGTGAATATCTAAATATAATTATGCAAGGGTACAATGGCCAAAAAGTTGTAAGCCTATATGACTTAGGGTTCATGATGTA
AAATTTCGTGCTAGTCCTAGTTATGGGTAGCAGGGTGATACAGTCAATAATAAGCTGTGAGATGTACAATATATAAAGCGAAAA
AAACTTGCGAAAAATTGGCCATATAAACGGGGAACATTTTTTATGAATGTTGATTTAAGGGTCCATTTCCACACTCCAATATGA
CTTCATTTACCCATAGATCATCTTGGGAACAATATCAACACGAGTTGAACACGAGGGCAATACATTTTCGGAGAAATTAAGTGA
AATCTCCAAATAAACCAAGGGGTAACACCCTTTCAGAAATCTTCGATTACTCTATATAATTACGATTCGAGCATGTTTTCATAC
GTGTGAAATCAATAATAATCATGCAAGGGACATGCGGCACAAAAAGTTGATAGCCCTATGTCTTTCAGGGTTCTAATGTGTAAA
ATTTCGTGCTAGTCCGAGTTTGGGTTAGAGGGTGATAACAGTCAATAATAAACTGATGAGATGGTACTAATCATATAGGAAAAA
AACTTCCGAAAATTGCCATAAAAAGGGGAAACTTTTTTTTGAAATTTGAATTTAAGGGTCCGTTTCCACTCCAATATACGTTTC
ATTTACCCATTGAGAATCTCTTGAAAAATAATCAAACGAGTTGAATACACGAGGGCAATACATTTCTCGGAGAAATTAGTGAAT
CTCCAATTCAACAAAGGGGTACCCATTTCCAGAAATCATTCAGGATATCTCTAATAATCGAATTCGAGCAATGTTTATCATAGT
CGAATAATAAATAAGATGATGCAAGGGTCATGCGCACCAAAAGTTGTAGCCCTAATATCATTATGTTTGAATGTGTAAAAATT
TTGTGCGAAGCGAAGTGTGTGGTGGTGTACCAGTCATAATACTAGAGAGAATAGTACTAATATCCCAAATAGAAAAAACTTGCG
AAAATTATCAAAAAGGGGAATTTTTTTTGAGAAATGAATTGAATTAAGAGGTCCGTTTCCAACTCAATGTTATATTCATTTACC
ATGGGAAAAAAAAAAAAGATCTCTTTGGGAGAAAAATATCAAACGAGTTGATACACGAGGGCAACTACATTCTTCGGAGAAATT
AAGGGAAAATCTCCATTCACCAAAGGGTAACCCTTTCAGAAATCATCGATTTTCTCTAATATCGATTCGAGCAACTGTTTTTCA
TAGTGAATACTACGAAAAATATATAAATGCAAGCGGTCATAATGCAATGCAAAGTTGTAGACCTATGTCTAGGAGTTCATATGT
GTAAAATTTTCGTGCTAGTCCGAGGTTGGGTAGAGGAGATACAGTCAATAAATACTCCGTGAACTGGATACTAATATAAAAGGA
AAAAACTTGCGAAAATATCCATCAAAAAGGTTGGAACTTTTTTAAAATGTTGATTTAAGGGTACCGTTTTCCACTCCAATATAT
TCATTTACCCATGAGGATCTACTGTGGGGAAAAATATCAACGAGTTGAACACGAGGGCAATACATTTCGGGAAAATTAAGTGAA
ATTCCAAACTCACCAAGGTACCCTTATTCAGAAATACTTCGATTTCTCTAAAATACGATTCGAGAGCAAATGTTATTCATACTG
ATATAATAAAAATAATCATGCAAGGGTCATGGGCAAAAAGTTGTAGCCCTATGTCGTTAGGGTTCTATGTGTAAAATTTCGTGC
TAGTCCGAGTTGGGTAGACAGTGATACAGTCAAATTAACTGTGAGATGTACTAATATAAGGAAAATAAAAACTTTGCGAAATTG
CCATGAAAAAGGGGATACTTTTATTTGAAATGTTGATTTAAGGGTCGCGTTTCCACTCCAATATTATACATTTTCAATTACCCA
TAGAGGATCTCTTGGGGAAAAATATCAAAAGATAAACACGAGGGGCAATACATTTCGCAGAAATTAGTGAAATCTCCTATTATT
CACCAAAGGGAGTACTTTAAGAATCTTTCATTATTCTATATATCGATTCGAGCAAAGATGTTTTCATAGTGAACTAATAAATAA
TATGCAAGGGTATTGACGCACAAGAAGTTATGTAGCCCTTATATCTTATGGTTTTATGTGTGAAAATTTTGTGCGAAGCCCGAG
GTGCTAATGTGGTGTGAGACGTCATATATACACTGAGAGAATGTACTAATATAAAGGAAAAAAACTTGCGAAATAATTTTGACA
ATCAAAAGGGGGAATATATTTTTTGAAACCAACAGCACCACTGCTAAAATATAATAAAACCTATAAAAACAAATTAAAAAAAGA
AACAAGAAAAACAGCTTAACCAATATAATAATGATAAGCACCGAAAAAACGTAAAAAAAAAAAAAAAAAAAAAAAACAAAAAAA
TAAAAAAAAACAAAAATAAAAAAAATAAGAATCATATGAGCAAAACAAACTAAAACATAAAAACACAAAAAAACACAACACAAC
CAAAACACAAAAAAAATAAAAAAAGAGATAATAAAAAAAAAAAAATCAATAACAAAAATAAAACTCATAAAAAAACTAAACCAT
AAATAACTACATATAAAATAATTTTAAAATAATAATGATAACAACAAAATATTAAAAAAAAATAAACAAGATAAACAATAAATA
TACCAAAAAAGTATAAAATAAAAATAAAGCAAAAGTAACGAAAAATAAATACAGCAATAAAAAAATAAATGATAACAGAAAAAA
ACAAATAAATAGATAATAAAAAAATAATAATATAAGACGACAAATGAGCTAACTTAACCTAACTAAAAGAATTTTCTATAAAAT
AATACACGAAGCATAAATTATCATAAAAATATAATATACAGATAAATAAATAATAAAAAAAAATAATAAAGTAAAAG
>m141120_105518_42134_c100732402550000001823141405141504_s1_p0/126400/13190_20067
GCCTAGCCAATGTTTAAGGGGGGAAGGAACCGAGAAGACACCCACAAACGGTGCGCCTATGACAAAGACGTGGGCCAACACCAC
CCAACGAGTGTTCGGCCAAAGCTAAAGACGAGATTGAAAAACCTCATTCACAGCCTGGGCACCTCAGCCAGTTGGGTAAGATGC

CCCGTGGGTGTCCCGGGCATGTCAGCAAGCCCACGGGCAATCCACGGCACCTGTGACAAAAAGACTGTTCGTGCATTAAACCAA
AAACCCCAGGGAGGGTAATGCGGCACAGTAAGGACCTCCCTAGCGACAAGCCCCTCAGGGGACCATTGCCGCGCAGGCTCCCGC
TCTGGATGCCCCTATCCTTCCGGGGAAAGCCAATACCCCCATCGAAAAAGTGACAACATGATACGGATACCTGGTGAACGACCA
TTCCAATCTAAGCCGTAATGTTACCCCTACCAAATAGGAGGACCTTTTATAATTCGAAATCCTGTAAAACTAAGAAAAAGACAC
AATTAAATACAAATTAACATGAAAGGAAAAAAAAACAAGCGACTATCTGAGTTGAAGCGGCACCAATTACCAAGAAGGTTTGCG
CCCATTCCACCGCCAAGCCCCGCTCAAGCGCACCTAAAAATCTAAGAAATACACACACACACACCACGTTATATCACAATATTA
TGAATTAAAAACCAGTTGATGGAGGGACGACGCCTCCGGAAACACGTCCTATAATTTTATTACCGCCGCACAACGACGCCTGGA
TCATAAGATGCTCAAATTGCCAACAAGTATTCGTAAATAACAAACGCATATGATTTTGGTGGATGACGGAAGCTCACATATATC
AACTTGTTGTTTCAAACCACACCTTTTATTATAGAAGTCATTGGCCTGACGGAAGGGGATCTAGCAATAATCCTGCCCAAAACT
AAAATCATTACGAGCTTCTCAACGGAGAAATATACATTCTCCCTCATTTGAGAGAATAGGTCCAGCTGCCGGAATTGACGCTGG
GAGAGAGGGAGAGTGAGATAGACTTCGAGCAGCACTCGTTCCAAAAAGGTTTCCTCAACCTTATATCGCGAAAAAATGAGGAAG
CGATGGAATTAACCCAAACCGCTCTACAAGCGATACATCTGTGTCAATGGCGCAGACCCAGCACGTATGAGGTCGAGTATCAAA
GAAAGAGAGAGAGCCTATACACCTCTATCCGCCATAAAACCCATTTGCATGAAGTTCCCGTGCAGACAACAGAAGGAAATTAGG
AGGATCTGAATCCGGGGAGACAAAAAAGCGCTCAGAAAAATAGTAATTACCTAGATATCTATCGCCCAAATACCCTGAATAAAA
AATAATATAGGTCCGGAGGAGACCGTTAGAGCACGCCTACACATATATATCCAGGCTGAGCAAAATGTAGTCCAGGAAGAAGAT
GATCTGGACCCGCCGAATAGAGACGATCGCGTCCTAGAGCCAATGGACAAAAATAGAAGAGGTATGCATCTAGCGAACCAAGAC
CCGACCAGAGGTCCATCCAAATTAAGTGGGAGAAGAGCTTGCGGCGAACATTCGGAATACCGAAAGCCGCCATTAAAAAATCGC
ATCCCGAGTCAGGAAAACCAGGATCTTCTGGCCTGGTCCCATAACTCTACGATATGACCAGGATCGACCGCAATATCATACCTG
CCACGCATTTTAAGTATTGAACACCCAAACAACGCAGACATCAGGTCCGCACCAGACAGTTCGCAGATCTTAAATGAGGGAAAA
ACGGAAAGGGTCCCGAGGCCCTTAATTTTTTTCAAGCTAGAGGTAGAGAAGTTGTCTTCCATCACTTCAGTACGCCAAAGAGG
ACTGTGTACCCCCGTGTGGGTTGGCCAACCCACGTCTATAGAAATGTAATCGAAACCGTAACGGTGAACCAATCCGGAACAATG
CATATATAGAGACTTTCACAAATCTATCAACAAAGCGTGCACAAAGTGAACTGTTTCCCGCTCTTCCGAATCGCGGATCCAAAA
TCTGGAAAGGATGCTACGTCGGACTACGATAGCGTCTTAAGTTTCAACACGCCCACGCTTACTCAGGCTACAACCAGTCTCCAT
GCCCAAGTGGCGGAAATACAGAACATAAACCAAGACTAAAATCCCAGACCAATAGCAAGCGGATTATTGCTAATAAATAAGACA
TGCCTTGTCAGAGACTCAAAATGCCAGAGCAACATAACCAAAGGAAACTCGCTCTAATTCGAAATATGAATAATATTCGGGCCC
AGCTAGGGTGAAACATGGAGGTGTTACATCGAGATATGCTGGTCAAGTCCAAGACGTCCGGGACGATGTCGGACGATTTGGCGA
GGCTTTCGCAGTTTCCGGAAGTAACGGGATGAAGCTGAACCCTAAGAAGTGCACCTTCGGCGTGGCTTTCCGGGAAGTTCCTGG
GCTTCATAAGTGAGCTTCCCGAGGAATCGGAGGAGCAACCCCAGAGAGAGAGAGCGAGCGAGCGAGTCCGATATGGACCTCACC
CGAATACACAAGGACATCCAAAGCCTTGACGGGCCGAGTCTGGCACCCCTGAGCCGTTGGATCGTTTCTAGAGCGCGACGGACA
AGTGCGTCCTGTTTCTTTCAACGTCCTTACGGGAAGCCAGCGGTTTGAGTGGTCACCCGAATGCCGAAGAGGCCTTCCAAAATA
GGCTGAAGAGATACCTAAGCCAGAGCCCCCATCCTGGCGAAGCCAATCGAAGGAGAGCCTTTGTATCTACTACCTGGCCATGAC
CAGAGCCACGCTATCAGCACCGCGTTTGTGCGAGAAGAGGAAAAGACAGAACTCCCGGGTAATACTAACGTGAGCAAGCAGTTC
TCGCAAAAAACGAGTCCCAGGTAACGTAAGTTCCGTTTGATCGAAAGACGTGACATTCTGCCTGTTTGATGGCATCCCGGAAAG
CTTCGACCTTAAACTAAAAAGATCCCAGGCTCATGCCGATAAAAAAAAAAAGTCTTAACAACATCCCCCCTGCGACAGGAAGTT
GCCCAGAACCTGGTCGGGAGAACATCCTGAAGTGGGTCAATGGCGAACTGAGCCACATTTTGTGATATTATCTTTTATGTGCCC
CGGGTGTCTCAAGTGCCATAGGGCAGGCCCTGAGGCCGATTTCATCGTCTCCGAATGTTCCGGGATCTCCCACGAAAGAGAGTA
ATCCCGCAGCACCAACGACGCCCGTTTGGAAAATCTTCGTGGATGGAGCCTCGAACCGAGAATGGGGCCGGTGCGGGTCATCTT
GGTGTCACCGCAGGGCCAGCATATCAAAATGCCCTCCATTTCCCAATTCAAAGCATCGAACAACGAGGCGGAGCATGGAAGCTG
TCCTAGCCGGCCTGCGCTGTGGCCCGGGAGGTAGGGGCAGCAAACCTCAAAAATTTAAAGCGATTCTCAGCTAAGTATCAAAGA
AAAAAATCTCGAAGGGTTCAGACCAAGGGAGAAAAGAATGGGGCAATACGTGACTCAAAAAGAGCGAGGGGAATGTATGCTCCA
GAAGTTACACAGAGAGCGCGGCGAGTATCTATCCGCGCAGATTACGACTCTCCAAAGCAGAATGTCTTTTCGCGTGAATTACGT
TAAGAAATAGCCTGGCAACCGACGCCAGAAGGCCTTTGAAGCTAGGCTGGTTTTTGGTTAGTTCCCTGCAACCATGCTTTCCTT
TGTCCAAGCAGCGGCCGCGCCCCGCGCGTCCACTACCGTTTTGGTGCGCGTTTCTGATTCACTTATTAAGTTGGATGCCATGTT
AATCCGGTAGGCGCATCCTGGATGGGACCGCTGATGAGAAAATATCCCTGTCCACTTCCTGGTCGGGAATGCTCCACAAAAGTG
ACCTGGGACGCAGCCCCAGGAAACTTTGTACCTAAGCCGTCATGGGTAAATCGTCATTGATGGACGGAAAGAACTCTACCATGC
TCGCAAAGTGGCTGTCTATGAAACTTACCAGATCCAGGTTTCGCGGGTGTCCGAAAACTGAGCTGAGTGCCATAATGCACGAGG
TGGCACAGAGGAGATTTGCGGAGATCGACACGCAGGAGAGAGGAGTGCCCCAGTCTGTCCAAGTAAAGATCTCTGCGCCAAGGG
GTACTTCTAGCCCACCATGAAGAAAGATTGCATAGGGCTACGTCCGCAAGTGCGCAGTCGGCCAGAGGTATGCGAAGATCCGTC
GGGCCCAACAAAAAATAAAAAACTTGATGAATAGCCCCCATGGCCCTTCGCAGTGTGGGGGGATCGATCGTACCGTTGGATCCT
CCCGATGAGGAATGAGGAGGGGGTAAAGTAAGTGCCAGTTGTGGATGTGGACCTATTAACACGCAAAATGGGTTTGAGCTAGAA
CCCGTGAACACGATTTTTACTTCCAAAAGGGCTTGGAATTTCGTCAGTCAGACATGCATTTAAAGTAAATGTCGGTACGAGGCC
TCCCCTACAAAATCGTGTCAGACAACGGGAAGCAGTTTTGACCAGCCTCGCACTTCACGGATTTTTGCGAAAAGACCACGATAT
GTGAAGAGCTTCTCGGCGGTGGCCAGACCTCCAGGCGAACGGGCGGCAGAAAGCCGTGAACAAAATACCTAAGAAGGGCAACTG
CAAGAAATAGTCATCTAGCCTGTAAGAGCAAATGGCCTGATGGAGCTCCCCCGCGTACTTGGGCCTACCGCGGAATGGAGCCGA
AAGGACGTAACCGGCACACCCTAAAACTCTGAAATTGGATTAATGGGATGCGAAAGCCTGTCATACCAATCGAAACGATATCGC
CGTCCCATCGTAGCGTACACGTGAGGTGAGGATTCACCACCCGAATCCAACCAGTTTTGCCTTGACTCAGGGATCCGTTTGATC
TTGCAGGAGGGAGATCCGAAGAGGTCGCAAGTTGCAGTGAAATATGTATCCAGGGGCAAGATCGGCGGCATGTTCAAGACTCCA
GGGTTTATAGGCCGTAAAGTTTGAATACCGAACGATCTAGTCACTGCGGAGGGTTCTTCCTCTATTTGCCAACTCAGGATCCGG

GAGTTAGGTGGTTCTGGGCCCCTTAAATTGGTGAAAGGGCCGTATGAATCCCAGCACGAGGTCTGCCTCGGAACGTACCCTTGA
AGTTGACTAGATGGTTCGAAGTTTTTCCGCGAGCCTGGAACGAAGGAGCATCTCCATAAAATACTACCCAGTGAGTCCTAGGAC
GTCGTCCCAGTTTAAATGTTAAAGTTGTTAATATGTACGCCTCAAAAGGGCTAATTCCCTTTTAACAGATGAAAAATGACCGGT
GTGCAAAACGTATAGAGGGGTATTGTCACCATGAAAGAATGCGTGTTCGAAGGGCGAACATTGCCTGTTTTCACTGAAGTGACC
GACAGAACCCCGTCTCCCTCACTGTGCGGGGGGGGTGTCATCCGTAAAGGACAAGCAAAATTAAGAGGACCGTGCCCAAGAGTC
GGTCCACCCTGGACGAAGACGTCCGGGGGTGTAAAATTAAGAGGAAACCGACCCAGGCCGGTCCCACCCCGGACGAACGGACGT
CCGGGGGTATAACAAAGGACCGAACCAAGGCCGGTCCCACCCTGGACGAACGACGTCCGGGGGTAATACAACAAAGGACCGGCC
AAGGCCGGTCCCACCCTGGACGAACGACGTCGCGGGGTAATAAAATTAAGAGGACCGACCCAGGGCCGGTCCCACCCCCGGAGA
ACGATGTCCGGGGGTTAAACAAAGGACCAAGCCAAGCCCGGTCCCACCCTGGACGAAACGAAAGTCCGGGGTATAAAATTAAGA
AAAGGAAGAAGGGGTCCACCACCCGGACGAACGACGTTCCGGGGTATAAAAAGAGGACCGGAAGCGCAAGGTCGGTCCCGGCAC
CCGGCGGACGTCCGAGGTATAAATTAAGAGGACCGGACGCCGGAGACGGCCGCGTCCACCCTGGACGAACGGACGTCCGGGGGT
GTAAAAATTAAGAGGACCGAACTAGGGCCAGTCCCACCTGGACGAACGACGTCCGGGGTATAACAAAGGACCGAGCCGGCCGGT
CCGCCCCGAGACGAACAAATGTCACGGGGGTATAAAATTAAGAACGGACCGACCCAGGGACGGTCCACCCCGGACGAATGACGT
CCGGGGGTAATAACAAAAAGGAACCGAAACCAAGGGCCAGTCCCGCCCCGGACGACGACGTCTGTGTATAAAAGTTAAGAGGCC
GAGCCAAGGCCGGTCCCACCCCTGGACGAACGACGTCCGGGGGTAGTAACAAAGGACCGGGAAGCCAAGGCCGGTCCACCTTGG
AACGAACCGACGTCCGCGGCAGGTAGAAAATTAAGCAGGAAACGACCCAGGCCGTCCCAGGGGGCCGGAGACGAGACGTCCGGG
GGTAGTGAACAAAGGAGCCGAGCAGGTACGGTCGCCGCTCCGCGACGAACGATGTCCGGGGTAGCTGAAAATTAGAGAGGATCG
CGCCCTAAGGCTGGTCCCACAACCTGGGACGAACCGACGTCCAGGGGTATAACAAAGGACGAGCCCAAGGCCGGTCCACCCTGG
ACGGAACGACGTCCGGGGGTATAAAATTAAGAGGAACCGACCCCAAGGGCCGGTCCCACCCCCCGGACGAACGACGTCCGGGGG
TATAAAAAGGACGCGAGCCAAGGACCGATCCCGCCCCGGACGAACGACGTCCGGGGGTATAAAATTAAGCAGGACCGTGCCCAA
GGCCGGTCCCACCCTGGATGAACGAATGTCTGGGGGTATAACGAAGAGGACCGAGCCCAAGGCCAAGCCCACCCTGGACGAAC
GACGTCGCGGGGGATACAAAGGACCGAGCCAAGGCCGGTCCCCACCCCGGACCGAAACGACATCCAGGGGTAT
>m141120_105518_42134_c100732402550000001823141405141504_s1_p0/127222/1681_12839
TGTCGGCTATAGGGTTCTTATGTGTAAAATTTCGTGCTAGTCTGAGTTTTGGGTAGAGGGTGATATCATTTACATACATACCTG
TGAGATGTACTAATAATTATGATAGTTTATAGTTGGAAAAAACGTTGCGAAATATTTGCCATTTCAAAAGGGGAATTTTTTTT
TGAATATGTTGATTTTAAGGGTTCACGTTTCCACACTCCATATATATTCTCATTTGCCCTATGAATGATCTCTTTATGGCGGAG
AGAGAATAATATCAGATTACGATGTTGACACCACCGAGGTGGCAACAATACATTTTCGGAGATTTAAGCTGAAATTCCAATATA
CAATTCAACAACAGGGCGCGTACCCTCTTATTGGCAGATAATACGAATTCGAATTTCTCTAATATACGAATTCCTGAGCAACTT
GTTAATTAATAGGATAAGAGTAATACTAAACTATACATTATCTGTCAAAAGGGTCATTTTGCGGGGGCATCCAAGGTTGTATCC
CTATGTCTAGGGTCCTCACACTGCTGTAACAATTTCCGGTGCTAGTCTGTGAGATGAGGTTTAGTACGCGTGATATCAGTCTAA
AATACTTGTGAGATGTTATTATTATTAAAAGCAAATATAACTTGCCCGACACTATCCTGCCATTTCATAGGATAGGGGAACTTT
TTTTGAAAATATTTGATTTTAATGGTCCGTTTCCTACTCACCAATATAATATACCATTAATATGGTCCCAATGAGATCCATCAG
TCTTGTTGGGGAAAAAATATCAATCACGAGTTGAATACACGCAGGTGCATACGTACATTATCGTTAGAAATTAGTGAAATCATTC
CATATTCACCTAAAGGGGGTATCGCCCCCTTTTCAGAAATTCTTCGATTTCTCTTCTAATATTCGTTTCGACGCATAATGTTTT
CATGTTAGTGATACTAAAATACAGTTATATGCAAGGGTCACTTGGGCACAAAAGTGTGTATGTGTCACTTTCTATGTCTTACAG
GGTTCTATATAGTGTTGAGATATATAATTTCGTTGCCTTAAGTCCGAGTTGGGTAGCACAAGGGATCGCATTAACAGTCAATAA
TACTGTGAGATGTACTACATATACGATGGAAAAAACCTTTTTGTCGTACAAAATGCCATTCGTAAAAAAATGGAATAAGAGGGA
ACTACTTTTTTGAAAAGTTCTTGAATTTTAAACGGGTTCCGTTTCCACTTCCAACTATATCTGCATTTCCCATGCGATCCCTCT
GTGCGGAAAAAATGATCAATTATCGATTTTGATACACGAGGGCAACCCCCTACATTTCAGGAAGAATCTTGTATTGGAGCAAGAT
CTATCCAATCACCTAAAGGGGTACCCCCATTTTCAGAAATCTTCGATTTTTCTAAATATGATTCGAGCATTTTCTTGTTTTATA
AATATAATGTAGAATACTAAATTATATAATTGTTGCAAGGTCATTGGGCTATCGAGAAAGTTAATGATATCCTGATGTTCTTTC
AGGTGTCCTATTGATTGTTTAAAATAATATCGGTGTAGTTCGTAGAGTGGGTAACGAACGGTGATACAGTCAATAATATACTGT
GAGATTACTAATATAGTTTCAGGAATAAAACTTGCGAAAATTGCCTATAATAAAAAAGGGAATTTTTTTGAAACTGTTGATTAA
GGTCCGTTTCACTCAATATATTTTATTTTACCATGGATTCTTTCTTGGGGAACAATATCGAAGCGAGTTTGAACACGAGGCATA
TCTTTTGGAGTACAATTATGATAAAGTTCCAATTCACCAAAGGGGTATTTTCAGAATCTTTCGATTTTCTCTAATATGCGGAAT
TTCGATGCAATATGTTTTCCATAGTGAATCTACTCTAAATATATATTAGCATGAGACCCATAATATGTGGCACCCAAAAGTTGT
AAGTATGTCTTAGGGTCTATGTGTAAAATTTCCTGCTATGTCGAGATGGGTTAAAAGGTGATATTGATCATGTCAATTAATACT
TGTGAGTAATGTTACTAATGTATATAAGGTAAAAAAATTGCGAAAATTGCCATCAATAAAGGGGATTGTATTACCTGTCTTGAA
ATGTTGAATTTAATGGGTATCCGTTTTACACTTCTCAATATATAATTTATTTTTACTCCGTGATATTCTCCTATGGGGGTATAA
AATGTTTATGAAAAAGAGAAATTGAACACCGAGGGCAATACATTTCGGAGAAATTTGAGCGTTGAACATTTCTCAATTCACCAA
GGGGTACTCTTTTCAGAAATCTTCGATTTCCTCTAATTAATCAAGGATTCGATTGCAATGTTTATAAGAAAGTATCATAAATAC
ATATTGCATAGTCGATAAGAGGGGACATACAAAAAAGTTGTTACCTATATGTCTTTAGGAGGTTACACATGTGTTCAAAATTTA
CGTGCATATTCCGAAAATGGGATAGAAACGGTTGAGATACAAGATACAAAAAGTACCGTTGAGATGGTATTAACTTATATAAAG
AAAAATACTTGCGAAAATTGCCATCTAAAAGAGGAACTTTTGTGTGTATTAATTGAGAAATGTTGAATTTAAGAGGTCGTTTCC
ATCAATATAATTCACTTTTACAATATTAGAAGAATTCACCTTGGGAAAAATATCAAACACGAGTTGAATCTACAGAACGGAAAA
GTTTTGGAGAAATTAGTGAATTAATTCACCCAATAGGGTACGCCTTTCAAGTAAAAATCTTCGATTTGCTCTAATATCGATTCG
ACAATGTTTTCATAGTGAATAATAAATAATATTGCAAGGTATGGGCGCACACAAGTTGTAGCCTATGCTTAGGTTCTATGTGTA
AAAATTTCGTGCTAGTCCGAGTTGGGTTGAGGGTGATACAGTCAAAACTACTGTGAATGGTATAATATAAGGAAAAAACTTCGA

CAACATTGCATCAAAAAGGGGAATTTTTTTTTTGATTTGATTTAAGGGTCGTTTCCACCTCCAATATATTCATTTTACCATAGA
TCTCTTGAGAAGAATATCAAACGAGTTGAAACGAGGGCAATTACATTTCGGAAATCTAGTACATCTACATCCACAAGGGGTACC
CCTTTCAGAAATCTCCCTCGATTTTCTATATCGATTCGAGCAATGCTTTTAGTGCGAAACTACAATAATATGCAAGGCATGGGG
CACCAAAGTTGCTACTCTATTCTTAGGGTCTATGTGTAAATTTTCCGGCTAGTCGAGAATGGGTAGACGGTGATACAGTCAAAA
ATATGTGAATGTACTAAATATAAGGAAAACTTTGCGACAATTGCATACAAAGAAAAGGCGGGAACTTTATTTTGTAATGTTTGA
TTATAAGGGTCCGTTTCCACTCCAATAATTCATTTTTACCCATGAGTATCTTTCTTGGAAATATATCATAACGAGTTGAACAAC
GAGGGCAAAATCATTTCGGTAGAATTTCAGTGAAATCTCCAATTCAACCAAAGGCGTACCCCTTTCAGAAATCTTCGATTTTCC
TAATATCGATTCGAGCAATGTTTCATAGTGAATATGATAATAATGATGCATAGGGTCTATGGCGCAAAAGTTGTTAAGCCTAT
GTTCTTAGGTTCTATGTGTAAAATTTTCGCTAGTCCCGAGTTGGGTAGAGGGTGATATCAGTCCAAAATACTGTGAGATGTACT
TTAATATTAAATAAAAAACTTGCGAATAATTTGCCCACTCAACCGAAAAGAGGGAACTTTTTTAAAATGTTGAGTTAAGGTCC
GTGTATCCTACTCCAATATATTCATTTACCATGTAGATCCTCTTGGGGAAAAATATCAAACGAAGTTGAAACAACTGAGGGCAA
ACTACATTTCGGTTAGAATATTAGTGAATCTCCATTCACCTAAAAGGGGCCCTTCAGTAAATCATTCGATTTCTCTAATATTCG
ATTCGAGCAATGTTCTCCATATGAAGTACTAAATAATTATGTTGCCAAGGGTCATTGGGCAAAAAAGTTTAGCCTATGTCTTAG
GTTCTATGGTAATAATATTTTCGTGCTAGTCGCGAGTTGGGAGAGGTGAATATCCAGTTAAAATACTGTTGGAAGATGTACTTA
ATATTAAGTGAAATAAACTTGCGAAAATTGCATCAAAAAGGAACTTTTTTTGAAATGTTGATTTAAGGGTCCGTTTCACTCCAA
TATATTTTCAAATTCCATGAGACCACTCTTGGGGAAAAATATCGAACGAGTTGACCACGAGGGGCTAATAATTTTGGAGAAAAT
TAGTGAAATCTCCCAATTCACCAAAGGGGTACCCCGTTTCAGAAATCTTCAGATTCTCTAATATCGATTCGAGCATGTTTTCAT
AGGTGAATAGCTAAAATATTATATGCAAGGCGGTCATGGGCACAAAAGTTTAGCCTATGTCGTAGGGGTCCTATTGTGTAATAT
TCGTGCTAGTCGAGTATGGGTAGACCGGTGAATCACAGTCAAAATATACATGTGAGATGTACTAATATAAATGGAAAAATCTTG
CGAAAAATGGCATAAAATAGGGAACTTTTTTTGAAAATGTTGATTTAAAGGTCGTTTCCACCTCAATATAATTCATTTACCCAT
GAGATTCATCCTGGGAAAATATCAAATCGGTTGAACACGAGGGCAATACATTTCGATAGAAATAGTGGAAATTCTCCAATTCCA
CTTCAAAGGGGTACCCCTTTCAGAAAACTTCGATATCATCTAATATTCGATTCGAGCAAGTGTTCTCATAGTGAATTACTAAAT
ATATATGCAATGGGTCATGGGCACAAAAGTTGTAGCCCCTATGTCTTAAGGGTTTCATATGTTAAAATTTCGTGCCTAGTCCCG
GAGTTGGTAGAGGGTTGATATCAGTTAAAAGTACTGTGAGATGTAACTAATTAATAAGGAAAAAACTTGCGAAACATTGCCTAT
CAAGAAAATGGGGATACTTTTTTTTGAAATGTTGATTTATAGGGATCGTTTCCACTCCAATATATTCTTATTTGCCCATGAGAT
CTATCCTTTGGGCGGAATAATATCAAACGAGTGAACACGAGGGTAATACATTTTCGGAGAAATTAAGTGAAATCTCCAATTCAC
CAAAGGGTACCCCTTTCAGAAATCCTTTCCGATTTCTCTTACAATATCATTCGAGCAATGTTTTCAATAGTTGAATACTATAAC
TAATATGCAAGGGTCATTGGGCACAAAAGTTGTATCCTATGTCCTTAGGTCCTATGATGTAAAATTTCGTGCTAGTCCCGAGAT
GGGTAGACGTGTTGATTACAGTCCAAAATACTGTGAGATTTAAAACCTAATATAAGGATAAAAACTTGCGATAAATTGCCATCA
AAAAGGGGAATTTTTTTGAAATGTATTTAAGGGTCCGTTATTCCTACTCCAATATATTCATTTACCCATGAGACTTTCTTGGGA
AGATAATTAATCGAACGGAGTTGAAGCACGAGGCATAAACATATATCGGAGAATTAGTGAGTAATCTCCCAATTCATCACCAAA
GGGGTACCCCCTTCCCCAGAATCTTCATTTTCTCTAATATCGATTCAGACAATGTGCATAGTGAGATACTAAATAAATAATGCA
GGGTCAATGGCAAGCAAAAGTTGTAAGCCCTATGTCTTAGGGTACCTATGTGTTAAAATTTCGTGCTAAGTCGAGTTGGGTAGA
TGGTGATTACAGTTTAAAATAACTGTGAGATGTCTTAATATAAAAAGATGAAAAAACCTTGCCGAAAATTGCATCAAAAAAGGG
GGAACTTTATTTTGAAAATGATTGATTTAAGGGTCCGTTTCCACTCCAATAATATTTCATTTTTTTTTGCCTCCATGAGATCTT
CTTGGGGAAAAATAATTCAAACCGAGTTTGGAAACACGAGGGGCATATACTTTCGGAGAAATTAGTGAAATCTCCAATTCACCA
ATAGGGGTACTCTCCTTTCAGTAAATCTTTCGATTCTCTAATATCGTATTCGAGGCAATGTTTTCATAGTGAATTACTAAATAA
AATTGCAAGGGTCATGGCAAAAATTGTAGCCCTATTGTCTAGGGTTCTATGTGTAAAATTTCGTCTAGTGATGTTGGCGCGGTA
GAGGTGATACAGTTCAAAATACTGTGAGATGTAAACTAATAATATACGAAAACTTGCGAAATTGCCATCAAAAAGGGGAAAACT
TTTTTGAAAAATGTTGATTTAAGGGTTCCGTTCCACTCCAATATTATTTCATTTACTCCATGAGTATCTCTTGGGGAAAAAAATA
TCAAATCGAGTTGATACACTGAGGGGCAATACATTTCAAGGGAGAAATTAGTGAGATATCTCCAATTCACCAAAGGGGTACCCT
TTTTCAGAAATCTTCGATTGTTCTCTATATATCGATTGAGCAATGTTTTCAATAGTGAAATTAATAAATAATATTGCAAGGGTC
ACTTGGGCGCAAAAGTTGTAGCCCCTTATGTCTTAGGCGTTCTTATGTTATAAATTTCCGTGCTATGTCCGGTTGGGTTAGAGG
GTGATATCAGTCAAGAATATCTGGTGAAGAATTGTACTTAAATATTAGATATAATAACTTGCCGAAAGAATTGCCCATCAAAAA
GGGGAACTTTTTTTTGAAATGTTGATTTTATTAGGGTCCGTTTCCACCTCCTAATATAGTTTCATTTATCCCAAGAGATCTTTG
GGGAAAAAATATCGAAAACGAGTTTGTACACCGAGGGCCATTACATTATTGGAGAAATTAGTGAGATCTCCAATTCACCTAAAGG
GGTATCCCTTTTCAGAAATCTTCGATTTCTCTAATTATTCGATTTCGAGCAAGTTCTCTATAGTGAAATACTAAATATATAATG
CAAGGGTCATGGGCACAAAAGTTGTAGCCCTGATGTCTTAGGGTTCTATGTGTAAAATTTCGTGCTAGTCCGAGTTGGGTAAGG
GTGATACCAGTTAAAATACTTGAGATTACTAATATGAAGGAAAAACTTGCGAAAATTGCCATCAAAAAGGGAACTTTTTTTGAA
ATGATTGAATTTAAGGGTCCGTTTCACTCCAATATATTCATTTACCCATGAGAATCTCTTGGGGAAAAATATCGATAACGAGTT
AACACTGAGGGCAATACTATTTGAGAAATCTAGTGAAAATCTCCATCACAAAGGGGTAACCCTTTCAGAAAATCTTCGATTTTC
TCTAATATACGATTCGAGCAATGTTTTTCATAGTGAATACAAAATAAGTGCAGGGTCATGGGCACAAAAGTTGTAGCCTTATGT
TTAGGGTCTATGTGTAATATTTCGTGCTAGTCCGAGTGGGTCATATACGGTGATACAGTCAAAATACTGTGAGATGATACTAAA
TATAAAGGAAAAATACTTGCGACAAATTGCCATAAAAAAAAGTGGGAACTTTTTGAAATGTGATTAAGGTCCGTTTCCACTCCA
ATATATCATGTATACCCATGAGGATCTCTTGGGAAAAATGTATCTAAACATGTTTGATTACACCAGGGCAATACATTTCGGCAG
TAAATTAAGTGAAATCTCCTCAATTTCCACCAAAGGGGTACCCCTTTTCAGAAATCTTCCGGATTTCTCTAAATATCGATTCGA
GCAAATGTTCTCATAGGAATACTAAATATATATTGCCAAGGGTCATGGCACAAGAAGTTGGTAGCCTATGTCTTTAGGGTTCTA
GTGTGTAAAATTTCGTGCATAGTCCCGAGTTGGTAGAGGGTGTATACCAGTATATAAATACTCGTGAGATGTAAACTAATATAA

GGAAAAAAACTTGCGAAAATTGCCATCAAAAAGGGGAACTTTTTTTGAAATTGTGATTTACAGGGTCCGTTTCCACTCCAAAT
ATAGTTTCATTTGCCATGAGATCGTCGTTGGGGAAAAATATCAAACACGAGTGAGACACGAGGGCAATACATTTCGGAGAAATT
AGTGAAATCTCTACAATTCACCAAAGGGGTACCCCTTTCCCAGAAATCTTCGATTTCCTCTAAATCGATTCGAGCAATGTTTTC
ATAGTGATATATCTTAAATAATATAGCAAGGGTCATGGGCAACAAAGTTGTGCCCCTATGTCCTTTAGGGTTCTAGTGTAAATT
CGTTGCTAGTCCGAGTTGGGTAGAGGGTGTACGTCAATAATACTGTGATGATGATACTAATATAAGGAAAAACTTGCCGAAAAT
TGCCCACAAAACAAGGGGAACTTTTTTTGTAAATGATTTGGATTTACAGGGTCCGTTTCCACTCCAATATATTCATTTCCCCAT
GAGAATCTCTTTGGTGGGAATAAATATCAAACGAGTTTGAACACGAGGGCAATACATTTCGGAGAAATTAAATTGAAATCCTCC
AATTCACCAAAGGGTAGCCCTTTCAGAAAATCTTCGATTCTCTAATTCGATTCCGATGCAACTGTTTTCATAGTTGAAATACTA
ATATCAATATGGCCAAGGGTCATTGGGCAACAAAAAGTTTGTACTCCTATGTCTTGGGTCCCTAATGTGTAAAATTTCGTGCTA
TGTCCCCGAAGATGGGTAGACGGTGATTAACAGTCAAAATAACTGTGAGATGTACTAAATATAAGTAAAAACTTGCGAAAATTG
CCGACAAAAAAGGGGATTTTTTTTGAAATGTTGATTTAAGGGTCCCGTTTTCCACTCCATATATAATTCATTCCCATGAGATCT
CTTGGGAAAAAAGTATCGAACTGAGTTGAACACGAGGGCAATACATATTCGGAGAATATGAAATCGTCCAATTCACCCATAAGG
GGTACCTTTCAGAAATCTTCCGATTTCTTCTAAATATCGATTCGAGCAGCTGTTCTCATAAGTGAATACTAATATAGATATGCA
GGGTCATTGGGCACAAAGTTCCGTAGCCTAATGTCTTAGGGTAACTATGTGTAAAATTTCGTGCTAGATCCCGAGTTGGGTAGA
TAGGTGATAAGTTAAATACTGTGAGATGTACATAATATAAGGAAAAAACTTGCGAAAAATTGCCATAAAAAGGGGAACTATTTT
TTGAAATGTTGATTTAAGGGTCCGTTTCCACTCAATATATGTTTCAATTGTTGCCCATGACGAATTCTCTTGAGGATAAAATAC
AACGAGTTGAATCACTGAGGGCCAATACATTTCGAGAGAAATTAGTGAAAATCTCCAATTCACCAAAGGGGTACCCCTTTCAGA
AATCTTCGATGATTTCTAATATCGATTCGAGCAAGTGATTTTCATAGTGATATACTAAATAATAAAAAAAAAATGCCCAAAAGG
GGCCCTGGGCCACAAAAGTTGTAGCCCTATGTCTTAGTGTTCTATGTGTAAATATTTCGTGCTAGTTCCGAGTTTGGGTAAGAA
GGGTGATACAGTCAAAATACTGTGAGAATGTACTAATATTAAGGAAAAACTTGCGAAAATTGCCATCCAAAGAAGGGGAAACTA
TTTTTTGAAATGTGATTTAAGGGTCCGTTTCCACCTCCAATATATTCATTTACCGAATGAGATCTCTTGGGGAAAATATCAAAC
GAGTTGAACACAAAGAGGGCAATACATTTCGGGAATTAGTGAAATCTCCAATTCACCAAAGGGGTACCCCCTTTCGAAATCTTC
GATTTCTCTAATGTCGATTCGAGCAATAGTTTTTCATAGTGAATAACAATAATATGCAAGGTCTGGGCGCCAAAAGTTGTACCC
TATGTCCTTAGGGTTTCATGATGTAAAATTTCGTGATAGTCGAGTTGTGGTATGTAGGTGATATCAGGTCAAAATACTGTGGAT
TCGTACTAAATATTAAATATAAAGAACTTTCTGAAAAGTTGCCATCAAAATATGGGGAATACTTTTGAAATGTTGATTTAAGGG
CTCCGTTTCCACTCCAAATATCAATTTACCCATGAGTATCTCTTGGGAAAAAATATCAAACGAGTTGAACACAGAGGGCCAATAC
ATTTTGGAGAAATTAGTTGAAATCTTCAATTCACCAAAAGGTACCCCTTTTCAGAATTTCTTCGATTTCTCTATATATTTCGAT
TCGAGCCAACTGTTTTCATAAGTGAATTATAAATAATATGGTCAGGTCATTGGGCACAAAGTTGTAGCCCTATGTCTTTAGGGC
TCATATGTGAAAATTTTCCGTGCTAGATATCCGAGAATGGGTAAACGGATGATTACAGTTCAATCAATTACTGTGAGTATGTAC
TAATAATGAAGGAAAAACTTGCAGAAAAATTCCATAAAAAGGGACTTATTTTTTGAAATGTTTGATTTTAAGAGGTCGTTTCCA
ACTCCAATATATTCATTTACCCAGAGATCTCTTTGGGGATGAAATATTCGAAAAACGAAGTTGAACACAGAGGCCAATACATTC
TTATGGCGAGAAATAGTGAAATCTCCCAAATTCACCAAAGGGGTACCCCTTTCAGAATCTTCGATTTCTAATATCGATTCGACC
AATTGTTTTTTCATAGTGAATACTAATATAATATGCAAGGGTCGATGGGGCACAAAAGTATGTAGCCCTATGTCTTTAGGGTCC
TATGTGTAAAATTCGTCTAGTCCGAGTATGGTAAACGGATGATACAGTCAAAATACAATGTGAGATGTACTAATTATATAGTGA
AGAAATATTGCCGAAAATGCCATCAAAAAGGGGAATTTTTTTTGAATATGTTGATTTAAAGGTCCGTTTCCACTCCAATATAT
TCATTTACCCGTGAGATATCTCTTGGGACAATATGAAAAGAGTTGAAAGCACCACGAGGGCCAGAATACCATTTATCGGAGGAA
TATGTAGTGAAATCCCATTCACTCAAAGGGCCCCATTTCAGAAATCTTCGATTTCTCCTAATATACTGACTTTCGAGCAAGATG
TTTTTCATAGTGAATAACTAAGATAATTATGCCAAGGGTCCATGGGCACAAAAGTTGTACCCCTAGTGATCTTAAGGGTCCTAT
GTTGTAAGAATTTCGTGCTATTCCGAGATGGGTGACGGTGATATCAGTCAAAATATCTGTGAATTACTATTATAAGGAAAAAAC
TTGCGAAAATTGACAATCAAAAAGGGGGAACTTTTGTTTGAAATGTGATTTAGGGGTCCGTTTCCACTCC
>m141120_105518_42134_c100732402550000001823141405141504_s1_p0/128356/1510_11380
TTGAGCGAAATACAAAGAGCATACCTTACGAGGGTACACATGCGTACTTACCCACACCCTGTGAACCCTATGTTCTAAGACTGG
TCTCCGTCATAAAATATCTGTGAATAGTCCCTGGTATTTGCCCGAAGGTTGTGTGGATATGTTCACCAATACATTGTCCATGCT
CGATAGAGTGTACGACATGAGTAGGAGAAAAGCAGAACTTTGAAAAATTGCCTAACACATTTACGTGGGTAATTTACTTTGTTT
CAATGTTGAAGTTTAAGGCACCGCTATCAACTCCAATAGATGTCATTTAACCACCAATGAAAGTCCATTGGAGAAAAAAAATAT
ACAGAACTACACGATATTGAAACAAAACACACGTAAAAAATAACAGATTCGGAAAAATTGATGAAATCTCCAATTCACCAGGTA
AACCCTTAATCGATCTTCGATTTCTCTAATATGAATTCGAAAAAATTTTTAAAATGAAATTCTATAGAATAAATAAGAGTCATG
GCATGGAAGTTGTAGCCCTATGTCTTAGCTTTTATGTATAAATGATCGTCCATCTAATCTGAGGTGTTTTAAGGTGATATGTTC
ACAATACTAAGAGCATGTTACAATATAAGTGCAAAAACTTGTGAAATGCCAACCAAAAGGGGAATTTTTTTTTCACAATGTTAT
AATTAAAGCCACCGCTTTCAACTCCCAATAGGATGCATTTACCCATGAGATCTCTTGGAGAAAAAATATCAATGAGTTGAACACC
AGTGTAATCACATTTCAGAAATTGCGAGCGAAATCTCCAATTCACCAAAAGGGGTACCCCTTTCAGAAATCTTCCATTTCTCTA
TATGAATGCTTCGCACCGATAGCCTTGAAAGTTGGAATCATCTAACGATATACTGCGTAGGAATGTGCCATCGGCATAAACCTG
AATAACCCATATGTGCTTAAGACCCATTCTCAGTATACAAGATATCGATGCGATACCTCTGAGGCTGGTTTAGGGTTGATAGGA
TCACAGGGTATGCACTTAAACGATCAGATGTACGAGAGAAATAAAGGGCTCGTCCACAGAGACATATGACAATACCGCAATAAT
GTGTCAACAACACCAAGGAGATTTTCACAATTTCAATATGTTTCTCTAATTATAGAGCACCCGTTCTCTCACTCCAATAGATTC
GCATTTACCCCATGAGTTCTCTAATGGGGAAACATATATATCCGATCCCATGAAGTTTGAACACATGTGTAGATACATCTTTCA
AGAGAAAACCTCTGGTCACAATACTTCCATCATCACAACACGGGTCTTACCCTTTCCAGCAAATCTCTCGCTTCTCTACATCTT
AGGATTGAAAACTGCTACTTTGAACAAAGCGAGATATACATAACTAAAATCATGCGAGGGTCCATGCGCATCGGCAGTTGTAGC

CTCACGTCGTCTTAGGCTTCCTACATGTAGTTCAATATCCGCCTGCATATCTGGACCGTGTCTTAAGAGGCGTCGATTCACCTG
TTCACCAATACTAAGAAAATGTACTAATATGAAGGCAAAACTTGTGAAATTCCCACACTAAAAAGGGGATTTTTCTTTCAAT
ATTGTTGTATCGCCAAGCAGCCGGTTTCCTATACTCCATACGTAGATTTGCATTTACCCCATTTGTCAATCACATGGCACAGAA
GAATAATATCAGAACTGGTGATACAAACCCAAGTCTCACCAGATATCACTTTCGGAGAATTGGTGAAATCTCCAATTCACAAGG
GTACCCTTTTCAGAAATCTTCCATTTCTCTAAATATGAGATTGGAGGCAATTGTTTTGAAAGCGAGATACTAACGGAATTAACT
GCGGGTGGGCATCAATGCACGAAACTTGTAAAGACCCTAATGATACATACTAGCGACTTGCTCAGTATAAACATATCCGTCGGC
CATCATACATCCGAGTCGTGTTTTTAGAGTTTACTAGGCATTTCGCACACTCACCTGAGAGAATCGCGCTACGATAATAAAAAT
AGCAACACCACTTGTGAATGCACAAGCAAGCGGCGAATTTTTTCTTCAAGATGAGTCAGAGCGCCCACCGTTTCTCACAGTGTC
ACAACCTAGGGTCACCATGATTGCAGCTTTTACCCAATCTGCCATGATATCTCTTGTGGAATAATATCGAACCGTTGAACACCA
GTTAACATACGCATTCAGCAGAAATTGGTGAAATCCTCTAATTCACAAAGGGGCGTCACCCCTTTCAGAAATCTTCGATGCGCC
TGACATGCCAACCTAATATGGAACAATATTCTATGAATTGCAAGACTATGGGCAGCATCCTCTTGAGGGTGCATTGGTGCATGA
AGTTGTAGCCCTATGGTCTTAGGCTTCTATGTATACAAAATCGTGCATATATGAGGTTATCTTGAGGAGTTAGACATCGTATCA
ACAGAAATAACTGTACTGAGAGGTTGTTATTACTTGAATATTAAGGCAAAAACTTTGCGAAAATTGTTGCCCAACACAGAGAGG
GCGAATTTGTTTTTTCAAAAAATGTTGAATTAAGCCACCGCTCTCAGACTCCAAATATGATGACTCATTCACTCCATGAGATCT
CTTGGGGAAAAATATCGACTAATTGAACAAACATGTAATACAGTTCAGAGAATTGGTGAAATATCAACTTACACACATATATAG
GGGTAACCTCTTCCAATACATTATCGATATTCTACTAATGAATTAAAAACTCTGAATCAATGTTACTTGGAAAATGCAACTTAC
GAAAGATCAATTAGTCCGAAGGAGATAGGAAATACCCGGAAGTTGTACGCTCTAATATACTTAGCTATTACTGATTCAAAGCAA
ATATCCCGCTCCCATTATCTCAGGTGTCTCTCAGAGGATCTATGAACTACAGCACACACTAACATAAAGAAGCTCAAATAAATA
ATATACACGGCAAAAACCTTGTGAAAATTTACCATCCCAACCAAAAAGGGGATTTTATATTTTCAAAACATGTTAAAACTTAAG
GCACACTTTCAACTCTCCAATGGAGCATTTGTATTCTTAAAATAATATTCCAGTCTTGAAGATAATATTTTGTAAAAACGACGC
TCTACCAACAACTAGAAGTAATCACCCATTTCGAGAGAACTCCGGTTGAAATCTCCAAAATTCCCAAAAGGGGGTACCATCCTA
TTCAGAATCGTTCGATTTCTCTAATATCGCATATTAGGAGCAATAGATTTATTGAATAGTAAAAAACTAAGCGCACTATGCGAG
AGCGTCAATGGGCCCGAAACTTGTAGCCTATGTCTTAGACTTCTCTGTACTAAAATATCGTGCATATCCTGAGGTGTTTAGAGG
ATTAGAGACTCAGTGAATACATCAATAACCTGATCAAAGAAATTGTATGCGCAATAATAAAGAGCAACTAAACTTGCTTGATAA
TATGCCTCACCAGGGATATAAGTGTTGTTTCAAATGTGTGAATGAGTACCGTCATACAACATTCAAGAGATGCCTTTTACCGCA
ATAAAAGGATCTCTCCTGGGTGAAAAAATATCGCATCGAGGTTATACCCTATTCCACAATAGAATATTATCCATACGATGTCGG
CCCCATATACTTTAGATGAGAACTTCTACATCACCATAAGCGCTTCAGCTCCTTTCAGATAATTCTTGTATTTCTCCTAATTCT
CAGAAAACCTTGTGTGTAGCAATGTTTGATAAAAGTCCGCAACTCACTCAAACAGGGCAAATACAAACTCCAGGGATAGCCCAT
GAGGCGCACGAAACACCTGTAGCCCTATGTCTTAGACTTCTCTGTAAATACCCAAATCCGTTGAATTACATCTGAGATTTTTTT
AGCTAGTTTTATATTCCACAATACTCACACAAGAGATTCTCACCGATATAGAAAGCAAAACATGTCGAAAATGTCCATACATAA
GGAGGGACATTTAATTTACATATTCAATAGTTAAAATATCACAAGGCAGCGCTTCAAAACATTCAATAAGTGTATGCATCTTAC
CCTATTGACGATTCTCTTCGAATGCAAAATAATCAGCCCATGAGTTGCAACAACAGTGTAATACAGTTTTCGTAAGAAATTGGT
AAATCTCCAATGTCCTTCATTTAAACCAAAGGGGTAACCCCTTTCGGAAATCTTCGATTTCCTTAATAATGGGTGAATTTTTGA
TAACTATATGTAGTGTTAAAATCGCAATTCTAAGGAATATTCGCAAGGGATCATAAGAACACCAACCGGTGTTTATCCCTGATT
GTCTTTAGGCTTTCTTATGTACCTCCTTAAAGAGATTATTCGTCCATTTGAACATTCTGAGATTTATCAAGAGGAGCGTGCTCA
TCCCTTTGCTTCACATATATCTTATACCCGATGAACTGCACTTATATTATCTTACTTAAGGAGGCCAATAAACTTGTGAAAATT
GCCCACAACAAATGCTTTTTTATATCAAATTTTAAATTAAGGTCACCTGTCTTCCAAACTCCAAATAAGGATGCCATCTTAAAC
GCATGAGACTCATCTTTGAGAAACATATCGATAAATGATTTAACACCAGTTGTAAAATACATAATATCGAAGTAAATTAGCGAA
AATGCTGCCAATCTCTCCAATGCTGGTACCCTTTCAGTATAATCCCTTCCATTTCTCTCAATATGAAGTACAATATAGGATAAA
TTTGTTTTTGAAAGCGACATCTAAGCATATTAAAAACCTGCGAAAACAGGCCAGTTTTTACCCCGCCTTTCCATTCCTTAAATT
CCCATTTTTCTAACTAAAAACAAACCTCAAGGGCATCCCTAGACCACTTCTCCACATCCCTCTAACAAATATAAAATTTTACCT
CACCGTCTTCTCACAGAGCGTACTTATCCCTCTGCGGTCTTCCCTACACACATCTTGGGTTTCAACTTCCAACAACCCATACAA
CCATACAATTCTCCACTACTCGCCTCTACCACCACTGTCACACTCTACGGTTCCCTCTCTTCACCGACGATACCTTAAAAAATA
CTAAGGCTTCCTCCAAGCCCCAGGTATTTTATCTGTTGTTTAACACTTTTAACTAATAGCTCCCTAGGGCCACCGTAACCCTCG
GAATGTGTGGCATCCCCCGCCTCGCAGTCTCACCTCCACGCAAAAAAAACCCCGCGCGATCCAATTAATATCATAAATTCTCCA
TATATATTTAAAATTTTAATGCCCCTCAGGGGCTTAAAATAACCTAAGTTTATCGCCCTACGCAACCAACTTAACTCCCGGGGC
ACATCGCCACTCATTCGTATCTAAACCTAAACATGCATTCTAATCCACATAATCTTATTAAATTCACATCAGATTTCCACAACC
ATGCGAGCAGGGTCCTGGCAGCCGCACATTGTAGCTTTTCCATTGTTCACCCAGACTTCTCAGGCTATCAAAATCCACGTTCGG
GACTATCTAGGTGTTTAGAGCGGATTTGACGCCTTCAAGGCGGCGTCAAATAACAGAGATTTACCATATACCACCGCACGCGCG
CGCAACAACACTTGCTGAATAATTGCCAAACAAAAAGCGGGGAATTTTATTTCAACATCGTTTTCACTTAACGGGCAGCAGCCG
CTCTTCTACTCCAATAAGACTGCACTTTACACCACCTCGAGCTTCCTCTTGGGGCGAAAAATTACTCGAATGGAGTTTGCAACC
ACCCAAGTGTACATCAGCATTTCAGAGAAATTGGTGGAATCTCCCAATTCACCCAAAGGGGACTACCCTTTCTTTCCAATCGTT
CGATATCTTAATACGACCGACAACCGCCTTCGAGACAAGTTTTGAAGCGCAATACTAACACCAAACTATGCGAGGGCATGGCAC
TGGGGCACAAACACAATCACCATAAGAAATATATACTCGTGCAATCAAGGATACAATCATAATAACCACAAGTACCATAAGAGA
TGAACTATTACAGGCAAAAACTTACAACATAGCCAACAACCACCCCATATATTTATTCATTTGTAATAGGCACGTTTCAACTAA
GAGATGCAAATATCACCAATGAAAAACATATAAAACAAAACAAAATATCGAAGGAGTTGAACCATTACATAAACCACATAACAA
ACAATCTCCAATTCACCAAAGGAGGTACCCATTATAACAGATCTTCCATTTCTCTAATATCTGAATTGGAGCTAATGTTGTGAA
AGCCGAATACTAAGAATAATGCGAGCGTTCACTGGGCCCGAAACTTGTAGCCCTATGTCTAAGACTTCTCAGTATAAATATCCG

GTGCAATATCTGAGGGTGTTTAGAAGTTGATATGCATCTCAATACTGAGAATGTACATATACAGGCAAAAAAACTTTGTGAAAT
TGCCAACAAAAAGGGGATTTTTTTTTTATAATGTTGAATGAAGTTCACCGTTTCAAACTCCAAGAGATGCATTTACCCATAAG
ATTCTTGGGGAAAATATTCGAACGAGTTAAACAACACAAGTGTAACACATTGTCGGAGAAAATTGGTGAAATCTCCAATTTCAC
AAAGGTACCCTTTCAGAAATCTTCAATTTCTCTAATGACATTCGGAGCAATGTTTTGAAAGCGAATACTAAGGAATATTTCGAA
CGGGTCATGGGCACGAAACATGTAGCCCTATAGTCTTAACTTCTCTGTATAAAATATCGTGAATATCTGAGGTTTGTAGAGTTT
ATATGTTCAGCAATACTCGCAGAGATGTAACAAAATATCAAGGCAAAACCTGTGGAAAATTGCCCCAACACAAAGGGGAAAATT
TTTTTTTCAAATGTTGATTAGGCAACCGCTTCAACTCCAATAGATGCATTTACCCATGAGATCTCTGGGAAAAACATAGTCGAA
CTACGCTTGGAACAACAGTGTACTACGTCAGCAGAAATTGGTGAAATCTCCAATTCACAAAAGGGGTACCCCTTTCGGAAATCT
TTCGATTCTCTATAGTGAATTGAAACAATGTTTTGAAAATGAATTCGAAGGTAATACTGCGAGGTCATGGCATGGAAGTTGTAG
CCCTTATGTCTTAGGCTTCTATGTACTAAAAATAATCGCTCCATATCGTGAGGTGTTCAGAGGTGATATGTTCACAAATACTAA
GAGATGTACTAATATGAAATGGCACAAAACTTGTGAAAATTGCCAACAAAAAGGGGATTCTTTTTCTTCCAAATTGTTAAATTA
AAGGCACCGTTTTCAACTCCAATAGCATGCATTCTACCCATGAGATCTTCTTGAGAAAAATATCGAATGAGTTGAACACCAGTG
TAATACATTTCGGAGAAATTGGCGAATCTCCAATTCACAAAGGTAACCCCTTCTCAGAAATCTTTCCATTTCTCTAATATGCAA
TTGGAGCAATGTTTTGAAAAGCGAATACCTAAAGGAATAATGCGAGGTCATGGGCACGAAAACTTGTACCCTATGTCTAGAGAC
TTCTCATATAAAATATCGGTGCATATCTGAGGTGTGTTAAGAGTTTGATATTGATCTCACATACTGAGAGACGTACCGAAGTAT
ACGAGGCAACAAAAACTTCGTGAAAATTTGCCAACAAAAAGGGGATTTTTTTTTCAAATGTTGAATGAAGATCACCGTTCTCA
ACTCGAAGAGATGCATTTACACCATGAAGATCATCCTTGGGGAAAGATATCGAAACGAGTATTTGAAACCAACAAGTGTATACA
TTTCGTAGAAATTGTGAAATCTCCAAATTAACCAAAGGGCTTACCTTTCAGAAATCTTCATTTCCTAATTATGAATTGGAGCAA
TGTTTTTGAAAAGCGAATCTAAGGAATATTCCGAGGTCATGGGACAACGAAACATGTAGCCCCAGTTGTCTAAGACTTCTCTGT
ATAAAATATCGTTGATATCATGAAGGTTTTTAGAGTTTGATATGTTCACAATACCTGGAGATGTAACGAATGATAAGGCAAAA
ACTTGTGAAAATGCCACACAAAGGAATTTTCTTTTCAAAATGTTGAAATTAAGGCCACCGCTTCACTCAATAGATGCAATTTAC
CCATGAGATCCTCTTTGGGAAAAATATCGAGACTAGTTGAACAACAAGCTGTAATGACATTCAGAAGATTGGTGAAATCCTCCA
AATTCACCAAGGGGTACCCTTTTCGGAAATCTTCGATTTTCTCTATAATGATTGAACAACATGTTTTGAAGATGAATTCTAAGG
GATATTTGCGAGGGTCCATGGGCACCAGGAAGTTGTAGCCTATGTCTTAGGCTTTCTATGTATAGAAATACTCGTCCATAATCT
GAGGGGTGTTGAGGGTGATATGTTCACCAATACTAAGACGATGTAACTAATATAAGGCAAAACTTGTGAAAATTGGCACAACAA
AAAGGGGATTTTTTTTTCAATGTTAAAATTAACGGCACAAAAACGTTTCAACTCCAATAGATGCATTACCCATGAGATCTCTGA
GAAAATATCGAAGATTGAACACCAGTGTATACCATTTCGGAGAAATTGGCGAAATCTCCAATTCACCAAAGGGGGTCCCCTTTC
AGAAATCTTTCCATTTCTTCTAATATCGAATTTGGGAGCAATGTTTTTAGAAAGCGAGATACTAAGGAATATGCGAGGGTCATG
GGGCACCCGAAACCTTGTAGCCCTAATGTCTAGAGACTTCTCAGTATAAAATATCGTGCAATCTGGGGTTTAGAGGTTGTATAG
GATCACAATACTGAAGAGATGTAAACGAATATAAGGCAAAACTTGGTGGAAATTGCCAACAAAAGGGGAATTTTATTTTCAAAT
GTTTTAATTAAGCGCCACCGTTTCTACTTCCAATAGATGCATTTACCCCATGAGTTCCTCTTGGGTAAAATATCGAACGAGTTA
CAACACCAGTGTAATACTTTCCGGGAAATTGGTGAAATCTCCAATTCACCAAGGGGTACCCCTTTCAGAAATCTTCGATTTCCT
ATTATGAATTGGAGCCAATGTTTGAAAGTTAATACTAGGAATATGCGAGGGTCATGGGCAAAACTTGTAGCCCATATGTCTTAA
GACTTCCTCTGTATATAAATATCGTGCATATCGAGGTGGTTTAGTGGTTGATATGATGCTCAATACTGAGGACGATGTACGAAT
ATAAGGCAAAAACATTGATGAAAATTGCCACAAAAACGGGGATTTTTTTTTCTCAAATGTTCGTATGAAGCACCGTTTCACTCC
ACCAGAGACCCTGCATTTACCCTGCGAGATCTCGATGGAGAAAAATATCGAATGAGTTTGAACACCAAGTGTCAAGTACATTTC
AAAAAGCGAAATTGGTGACAATCTCCAATTCACGAAGGGGTACCCCTTTTCAGAAATCTCATATTTCTCGTCAAATATCGAATT
GGACAATGTTTTGAAAGGAATACTAAGAATAGTTCGAGGGTAATGGGCGCGCACGAAACATGTGCCTATAGTCTTAGACTTCTC
ATGTATCAGAAATATCTGAATATCTGAGGTTTTTAGAGTTTGATATGTTCACAATACTGAGAGATGACTACGAATAATAAAGGC
CAAAACTTGTGAAAATTGCCAATCACAGGGGGATTTTTTTCAAATTTGAATAAGAGCACCGCTTCAACTCCAATAGATGGCACT
TTACCCATGAGCATCTCTTGGGAAAATTATCGAGACTAGTTGAACAACAGTGTAAAACAGTTCGAGAAATTGGTGAAATCTCCA
ATTCACCAAAGGGGGTATACCCGCTTTCCAGGAATCTTTCGATTCTCTAATATGAATTGAAACAATGTTCTTGAAAATGAATTA
TAAGGCACATATGCAGGGTCCATGGCATGGAGTTTAGCCCTC
>m141120_105518_42134_c100732402550000001823141405141504_s1_p0/135525/0_11533
AAGAGAGAGATTTAGGACCCAATTTATGGTCAATAGTATACCTTTAATATGAAAAATAAATTTTTATTGGAGGAAATGGAAAA
AAAAAAAGGGGTTCCAAAAAAAAAAAAAATTTAAAAGAGGCTTGGTTTTTAAAAAAAGGGAGGAAGGAAAAAAAATAAAATTTG
GAAAAAGAAAAAAAGAAAAATTTTTACGAAAAAGGATTTCCCCGCTTTTCCGGAAGAGCCCCCCCCCTAGCGCAACGCCTTTTT
GCCCCCTTTAGGAAGGATTTTCTTTTTTGCAAGGGATTAGGCCCCCAAAATTTTTCTTTTTGGTTTGGTTTTTAATTAGGCAAC
AAACAAATTCCCATGGGTCAAAATAGTATACTATATGGATATTATTGAGGCATGGAAAAGGTTCAAAAATATTAGAGATTGTTA
GAGAGACAATATGAAAGTGATTACGAGATCGCTTCGAGCCCTAGGGCAGCTGCCTAGGAGATTTGGGCTTTGGGTAGGGCAATT
TTTTTTGTTTGGTTGAAATTAAGGATCAATTCATGGTCAATTAGTATACCTATATGAATATTATGAGATGGGGAACACCTTCAA
AAAAAATAGAGCTTGTTAGAGTGAAAATATGAGTGACATTACGAGATCGCTTCGGAACCCTAGGCAGCTGCCTAGGAGAAATTT
TGGCAGGAAAGGCCAATTGTTTTTGTTTTGGTTGAATTAGGACCCAAGATAAGGCCAATAGTATACCTCATATGAATAATTATT
GAGATGGGGAAAAGGATTAAAAAAATTAGAGCTTGTTAGAGAGAAATATGAGTGAATTAACGAGATCCCGCTTTTCGGAGCCCT
AGGCAGCTGCCCCTAGGAGATTTTGGCAGGAAAAAGGCCAATTTTATTTTGTTTGGTTGAAATTAGGCCCAAATTCATGGTCAA
TAGTACTATATGATAATTTATTGAGAATGGGGAAAAATTCCAAAAAAAATTAGAGCCTTGTTAGAGCAGAAGAAGGACGTATGA
TGAATTCCGAGAATCGCTGTCGGCAACCCTAGGCACGCTGCCTAGGAGATTTTGGGTAGGAAAGCAATTTTTCTTTGTTTGGTT
GAATTAGGACCCAGATTCAATGGTCAATAGTAGCCCAATATAGTAGTTTTGAGAATGGAATAGTTCAAAAAACTAAGAGCTTTG

TTAGGAGAAATGTGTGAATTACGAGATCGTTTCGGAGCCCTAGGCAGCTTTGCCTTAGGAGATTTGGCAATGGGAAAGGCCAAT
TTTTTTGTTTGGTTGAAAATTAGGACCCAATTTTCATGGGTCAATAGTATAAATATATTGATATTATTAAGATGGGAAAAATTC
CAAAAAAATTAGAGCTTGTTAAGAGAGAAATTGAAGTGCCATTACAAAGATCGCTTCGAGGCCCTAGGGCAGCCTTAGAGATTC
TGGGAGGCAGGCCAATTTTTTTTTTGGTTTAATTTAGGCCACAATTCCATGGTCAATAGTATACCAATATGATATTATTGAGAA
TGGGAAAAGGGTTCAAAAAAATTAGATCTTATGTTTAGAGTAATATGAGTGAATTCGAGATCGCTCATCGGAGCCCTAGGGCGC
TGCCTAGGAGATTTGGCAAGGAAGTCCAATTTTTTTTGTGTTGATTTGAATTAGGAACCCCAAATTATGGCCAATGTAAATACC
TATATGATTATTATGGGGATTGCCAAAAAGGTTGTCAAAAAAATTAGAGCTTGTTAGAAGAAATATGAGTTGAATTACGAGATC
GCTAAACGTAGCCCATAGGCAGCTGCCTAGGAGATTTTTGGCAAAGGAAGCCCAATTTTTTTGGTTTGGTTGAATTAGGCCCCA
ACTTCATGGCAAAATTGTATACCAATATGATATTGTTGCAGATGGGAAAAGGTTCAAAATATTAGAGCTTGTTTTAGAGAGAAA
TATGAGGTGAATTACCAATCGCTTCAGAGCCCTTAGGCCAGCTTACCATAGGAGATTTGGCAAAGGAAAGGCCAAAATTTTTTT
TGTTTGGTTGGAATTAGGACCCTAATTCCCCATGGTCAAAACTAGTAGCCCAAATATAGTATTTATTGGAGTTGGGAAAAGGTT
CAAAAAAGAAGAGCTTGTTTTAGAGAGAAAATTGTGTGATTTACGAGAATTCGTTTCCGAGACCTAGGCAGCAGCCTAGGAGAA
TTATGGCAAGAAAAGGCCAATTTGTTTTTGTTTTTGTTGAAAGATTAGGACCCAATTCAATGGCCAAATAGTATACTATATGAT
ATTATTGAGATGGGGAATAAGGATCCAAAAAAAATTAGAAGTTGTTTTAAGAAGAGAAAATATGAGTGAATTACGAGATCGCTT
CGGAGCCTAGGTAGCTGCCCTTGGAGATTTGGCAGGAAGGCCATTTTTTTTGTTTGGTTGAATTAGGAACCCAATTCCATGGCC
AAATGTAATAAACCTATCAATGAATATTATTGCGCTGGACGGTTTCAAAAAATTAGAGCTTGATTAGTTAAGAGAAATATAAGT
GCATTATGAGAGATCGCTTCGGAGCTTAGGCAAGCTGCGTAGGAGATTTTGGCAGAAAAAAGGCCAATTTTTTTTGTTTGGGTT
GCTCGGCCCAATTCATGGAACAATAATATACCTATATGATATTATTGATGGTTCAAGGATCACAAAAAAAGTTAGAGCTTGTTT
AGAGAGAAAATATGAGTGAAATACGAGATCGCTTTGCCTAAAGGCAGCCCTGCCTAGGAGCATTGGCAGGATAGGCAAATTTTT
TTTGTTTGGACTTGAAATTAGGACCGCCAATCAGGTCAATAGTATACCTAATATGATTATTATCGGGATGGGAAAAGGTTCAAA
AAAATTAGAGGCTTTGTTAGAGAGAAATTTTGAGTGAAGTTACGAGATCCGCATCGGAAAAAGCCCTGGCAGCTGCCTTCAGGG
AAAAAGATGTCTGCAAGGAATTAGGCCAATTTTTTTGGTTTGGTTTAATTTAGGACCCCAATTCATGGCTCAATAGTATACCTA
TATGATATTATTGAGAATGGGAAAATGGTTCAAAAAATTAGAGGTCTCTGTTAGAGAGAAAATTGAGTAAATTTACGAGATCCG
CTTTCCGGAGCCCTAGGGAGCTGCCTAGGAGATTTGGCCAGGAAGGCCAATTTTTTGTTTGGGTTTGAATTAGGACCCCAATTC
AATGGTCAAAAATAGTATAAATATATGCATATTAATTAAAGATGGGAAAAAATCCAAAAAATTAGAGCTTGTTTTAAGAGAGA
AAATATGGCTGCATTAACAAGATCGTTTAGGAGCCCTAGGCAGCCTAAGGAGCCATCTTGGATAAGAATAGGCCAATTTTTTTT
GTTTGGTTTAATTGGGACCCCAATTCATGGTCAATAGTATACCTCATATGATGTTATTCAGATGGGAAAGGTTCAAAAATAGAA
TCTTGTTAGAGAGAAAATATGAGTGAAATTACGAGATCGCTTCGGAGCCCCTAGGGAGGCCTGCTCAGGAGGATTTGGCAGCGG
AAAGGCCAATTTTTTTTTTTTGTTTGGTTGAATTAGGACCCCAATTCATGGTCAATAGTAATATATATATATCGAAATAAGTTCATT
AAGCATGGGAAAAAATTTCCAAAAAAAATTAGAGCTTGTTAGGAGAAATAGTGAGTGCAATTACGAGTTCGTTTCGGAGCCCTAG
GCAAGCCTTAGGAGATTTGGCAGGATAGGCCAATTTTCTTTGTTGGTTTGAATTAGGACCCAAATTATGGCCAATAGTAATACT
TTAAGATTTAATGGAGAAATGCGAAAAGGTTCAAAAAAAAATTCAGAGCTTGTTAGAGAGAAAATATGAAGTGGAATTACGAGA
TCGTTTCGGAGCGCTAGGCAGCTTGCCTAGGAGATTTGGCAGAAAAGGCCAATTTTTTGTTTGGTTGAATTGGTTCCCAATTCT
CATGGTCAATAGTCCAATTAGTATCTATTTAAGAGGGGAAAGGTTCAAAAAAAGTAAAGAGCTTGTTTAGAGTGAAATATGAGT
GAATTAACGAGATGTTTTTGGGCCCTAAGGCAGCTGCCCTAGGAAGATTTGGACAGGAAAGGCCATTTTTATTTGTTTGGTTTT
TGAATTGGGACCCCAATTCAATGGTTCAATAGTTTGCCCAATATATAGTATTAATTGAGAGGAATAGGTTCAAAAAAATACAGT
GCTTGTTAGAGAGAAATATGTGTGGAAATTTACTGAGTTTCCTTCGGAGCTAGGCAGCCTTGCCTGGAGATTTTGGCAAGAAAG
GCCAAATTTTTTTTTTGTTTGGTTGAATTTGGAACTCTAATTCGATGGTCAATTAGTAGCCCCAATATAGGTATTTTTGAGAGG
GGAAAAGGATTCAAAAAAGTAAGAGCTTTGGGTTAGAGCGAATATGAGTTGATTTGAGAGTGCGGTTTTCGGGAGCCCCTAGGC
TGCTGCCTAGGGAGATTTGGCAGGAAAGGCCAATTTGTTTGTTTGGTTGAATTAGGACCCCAATTCATGGTCAATAAGTATACC
TTTATATTGAAAATATTATGAGAGAGATGGGAAAAAGTTCGAAAAAAAAATTTTAGAGCTTGTTAGAAGGAAATATGAGGGAAA
TTACGAGATCGTTTCGGAAGCCCTAGGGTAGCTTGCCTTGGAGATTTTTGGAAAGGAAAGGAACAATTTTTTTTGTTTAGGTTG
AATTAGGACCCCAATTCAATGGCCAATTAGTATACCTATATGATATTAATTGAATGGAATAGGTTCCAAAAAAATTAGAGGCTT
TGTTTGCAGAGAAATATGAGTGAAATACGAGATCGCTTCGGAGCCCTAGGAAGCTTGCCTAGGAGATTTGGCAGGAATTTAGGC
CAAATTTTTTTGTTTGGTTTTGAATTAGGAGCCCAAATTTTCCATGGTCGATAGTAATACTATATGATATTATCTGAGATTGGT
AAAGGTTCAAAAAAAAATTAGAGCTTGTTAGAGAGAAATATGAGCATGAATATACGAGATCGGCTTCGGCAGCCCTAGGCCAGA
CTTTAAGGAAGATTTCTTGCAGGATCAGGCAATTTTTGTTTGGTTTAATTAGGACGCAACAATTCTGGTCATAGTATACCTATA
ATGACTTATTATTGAGATGGGAAAAGGTTTCAAAAAATATTAGAGCTTGGTATTAGAGAGAAATATGAGTGGAATTACGAGATT
CGCTTTCGGAGCCCTAGGCAGCTGCCCTAGGAGATTTGGCTGAGATAGGCCATTTTTTTGTTTGGTTTGAATTAGGACCTTCCA
ATTCATGGTCAATAGTATACATATGATAAGTTATTGATATGGGAAAAGATTCAAAATAAGAAGCTTTGTTAGAGAAGAAAATAT
TTGAGTGAAATTACCGAGTTTCGCTTGCGGAGCCCTAGGCCAGCTGCATAGGAAAGAATTTGGCAGGAAGCCAATTTTTTTTTG
TTTTGGATAGAATTAGGACCCCTAAATCAGGGCCATCGTATCACCTATATGTATTAATTGTTGAATGGGAAAATGTTTCATAAA
AATTTAGAGCTTGTTAAGAGAGAAATATGAGTGATTTTACGAGTTGCTTCGGAGCCCTAGGCGAGCTTGCGCTAACGGAGATAT
GCAGGAAAGGCCATTTTTTTTGTTTGGTTGAATTAGGACCCCAAATTCATTTGTCCAATAGTAGCATAATATATATATTTTTCGAG
AATGGGAAAAGGTTAAAATATATGAGCACATGGTTAAGTAGAGAAATATGAGTGAATTACGAGAATCGTTTCGGAGCCTAGACA
AGCAGCTTCCTAAGGAGGATTTGGCAGGAAAGGCCAATTTTTTTGTTTGGTTGCAATTATTAGGACCCAATTCATGGCAAATTA
GTATACCAATATGATATTGTTGAGATGGAAAAGGTTTTCAAAAAATTTAGAGCTTGTTTAGAGAGAAATATGAAGTTGAATTT
ACGAGAATCGCTTTCGGAGCCCAGGCAGCGTGCCTAGGAGATTTGGCAGGAAAGGCCATTTTTTTGTTTGGTTGAAATTTAAGA

CCACAACATATCAATGTGAATAGTGCCAATATAAGATAACTTATTGAGAATGGGAAAAAGTTCCAAAAAATTAGGCTTGTTAAG
AGAGAAATAATGGTGAATACGAGATCGTTTTCGGACCATAGGTAAGCTGCCTTGGGAAGATTTGGCCAGGAAAGCCTATTTTTT
TTTTGTTTTTGTTGAAGTTAGGACCAAATTCAAATGGCCAATAAGATACCTATATGATTATTAATTTAGAAGGGAAAAGGTTTC
AAAAAAAATTAGAGCTTGTTTAGAGAAGAAATATGAGTGAATTACCGAGATCGCTTTGGAAGCCTAGGTAGCCTGCCTTTGGAG
ATTTGGCAGGAAAGGCCAATTTTTTTTGTTTTGGATTGAACTTTAGGCCCCTTACCATTCCAATGGCCAATAGTGATAGCCCTA
TATGATTATTATTTAGATGAGGAAAAGGTTCAAAAAAAATTAGAGATTGTTTTAGGAGAAATATAGGAATTAGCGGATCGCTTT
CGGAGCCTAAAGGCAGCTGCCAAGGAGATTTTGGCAGGATTAGGCCCCAATTTTTTTTTGTTTGGTTGAATTAAGGGCCCCCAA
TTCATTATCCATAGTATACTATATGATACTATGTTGCAGATGGGAAAAAGGTTTCAAAAAATTAGAAGCTTGTTAAGAAGAGAA
ATATGAGTGGAATTACGATTTCGCTTCGAGCCCTAGGCAACTGCCTAGGAGATTTTTGGCAGAAGGCCAATTTTTTTTTGTTTG
GTTGATTTTTAGGACCTTCAAATTCATGGTCCAATAGTTATAGCCCATATGATATTATTTGAGATGGAAAAGGTTCAAAAAAA
TAAGAAGCTTGGGTTAGAGAAGAAAAATGAGTGAATTTTCGAGTTTCAGCTTCTGAAGCCCGTAGGCCAGCTGCCTAGGAAGAT
TTGGCAGGAAAGGACCATTTTTTTGTTTTGGTTGAAATTAGGACCCCAAATCAGGGCCCAATAGTAATACCTATAATGAATTAT
ATGAGATGTAAAAGGGATTCAAAAAAATTAGAGCTTTGTTTTAAAGAGAAATATGAGGTGAATTCACGAGTTCGCTCTTTCGGA
AGCCCTAGGCTGCTGCCTAGGAGATTTGGCAGGAAAGGCCAATTTTTTTTTGTTTTGGGTTGAATTTAAGGAACCCACAATTTC
ATGGTCAACTTAGTACCCAAATAATAATATTATTGAGATGGGAAAGGTTCAAAAAAATAAGAGCTTGTTTGATGAGAGAATACA
TGAGTGAATTACGAGTTCGCCTACGGAGCCCCTAGGCAGCTGCCTTAGGAGAATTGGCCAGGAAAGGCCTATTTTTTTTGTTTG
GTTTGAAAAATTAAAGGAACCGCAATCTTGGCTAAATAGTAATACCTATAACGAATATTATTGAGAATGGGAAAAAGGTTTCAA
AAATTAGAAGCTTGTTTTAGGAGAGAATAATAAGTGCATTACGGATGGCTTTCCGGAGCCCTAGGCAGCTGCCTGGAGATTTGG
CAAGGAAGAGGCAATTTTTTTTGTTGTTGGTTTGATTTAGGACCCAATTCATGGACATAGTAATACTATATGATATTATGTGAG
ATTGGTAAAGGAATCAAAAAAAATTAAGAAGCTTGTTAGAGAAAATATGAGTGAATAGCGAGATGCTTCGGCAGACCTAGGCAG
CTGCATAGGAGATTTGGCAGGAATAGCGCCAATTTTTTTGTTTGGGTCTGATTAGGACCCAATTCATGGTCAAATGTATCACTA
TATGATATTAATTTTATATGGCAGAAAACGTTTTCTAAAAAATTAGAGCCTTGTTCAGAGAGAAATATTGAGTGATTTACGAGA
TCGTGTTCGGAGCCCTAGGTTCTGCTTATCTTCGGAGATTGGCAGAAGGCCAATTTTTTTTGTTTTTGGTTGAATTAGGACACC
CATTCCATGGCCATAGTATAACGCGTATAATGATCAATTATTTTAGATGGGAAAAAAGGGTTCAAAAAAATTAGAGCTTGTTTA
GGAAGAAATGAGTGAATTAACGAGATCGCTTCGGAGCCTGGGCGCTTGCCTGAGGAGTATTTGGGAGGATAGGCCAATTTTTGT
TTTTGTTGAATTACGGCGCCCAATTCAATGGTCAATTAGTAATACCATATAATGATTTATTGAGAATGGAAAGGTTCAAATAAA
TTAGAAGCTGTTGAGAGAAATATGAAGTGTTAACCCCGAGTTCGCTTTCGTAGCCCTAGGCCGCTGCTAGGAGATTTGGCAGGA
AATGCCCCAATTTTTTTTTTGTTTGGTTATGAATTAGGACCCGCAAATCATGGGCTTTAAATGTATACTATATGATATTATTGA
GATGGGAAGGTTCAAAAAAATAAGAAGCTTGGTTAGAGAGAAATTAATAAGTGAATTTAGAGATCGCTTTTTTTCAGAGCCCTA
GGCAGCTGCCTAGGAGATTTTTGGCAGGAAAGGCCAATTTTTTTTTTGTTGGTGTATTCAGGACCGCCCAATTCATGGACAGGT
AGTATGAACCTATATGATAATTAATTGAGATGGTAAAGGATCAAAAAAATTAGAGATTGTTTAAGCAGAGAATATGAGTGATTA
ACGAATCGCTTCGGAGCCCTAGGAGCAGCTTGCGTAGGGATTTGGCAAGGATAGGCCAATTTTTTTTTTTTGGTTGAATTAGGA
CCAACAAATTCAATGGTCAATAGTATACCTATATTGAATAGGTTCATTGAGATGGTAAAAAGGTTCAAAAAAATAAGAGCTTGT
TAGAAAAGAATATGAGTGATTGACGAGATTCGCTTCGGAGCCCTAGGCAGCTTGCCTAGGGAGATTTGGCAGATAGGAATTTCT
TTTGTTTGGGAGATTAATTAGGACCCCAAATTCATGGTCAACTACTATACCTATACGTATTATATGAGATGGGAAAGGTTTCAA
AAATTAGAGCTTGTTTAGAGAGAATATGAGTAGAATACGAGATCGCTTCGGAGCCTAGGCAGCTTGCCAAGGGAGATGGCTGGA
TAGGCAATTCTTTTTTTGTTTGTTTTGAATTAGGACCTCAATTTCATGGTCAAGTAGTATACCCATAATGATATTAATTTTGAG
AATGGGAAGAGGTTCAAAAAATTGAGCTTGTTAGAGAGAAATATGAGTGATTAAGAGTTTTTGCTCGGAGCCCTAGGCCAGCTT
GCTAGGAGAATTTGGCTGGATGGGCCAATTTTTTTTGTTGTGGGTAGAATTAGGAACCACAATTCATGGTCAATGTATCTAATA
TGAATATTATTAGATGGGAAAGGTTCAAAAAATTAGAGCTTGTTAGAGAGACAATTTATGAGTTGAATTACGAGTTTCGTTTGG
CGCCTAGGCAGCTGGCCTAGGAGATTTGGCAGGAAAAGGCCAATTTTTTTGTTTTGGTTTGATTAGGACGCCCAAATCAGGGCC
AATAGTATACTATATTGATATTATTGAGATGGAAAAGGTTCAAAAAAATTAGAGCTTGTAAAAGAGAAATATGAAGTGAATTTA
CGAGATTCGCTTTCGGAGCCCTAGGCAACGTGCGTAAGGAGATTTTGGCGGGAGCCAATTTTTTTTGTCTGGGTTGAATTAGAC
CACCAAATTTCATCGGTGCAATAGTAGACCAAATAATATTATTGAGATGGCGAAATGGTTCAAAAAAATAAGAGCTTGTTAGAG
AGAAATATGAGTGAATTTCGATTCGGCTTGGAGCCCTAAGGCAAGGCTTGCTAGGGATTTGGGCAGGAAAAGGCCAAATTTTTT
TTGTTTTGGTTGAATTCAGGACCCCAAATCCAGGGCCAATAGTATACCTTATTGGATATTTGGATGGGAAAAAAAGGTTCAAAA
AAATAAGAGCTTGTTTACGAGAGAAATATAAGTGAATTTAGACGAGAATCGCATCGAGCCCTAGGGGCGCTTGCCCTAGGAGAT
TTGGCAGGAAGGCAATTTTTTTTTGTTTGGTTTAATTAGGACGCCACCAAATCATGGCTAATAGTATACCAATATAATGAATTT
ATTGAGATGGAAATGGTTCAAAAAATTAAGAGCTTGTTTAGAGAGAAATATAAGTTTGAATTGCCGAGATCTCTTCAGAGCCCC
TAGGCAGCTGCCTAGGAGATTTGGCAAGGATAGGCCAATTCATTTGGTTTGTTTGAATTTAAGCGACCCCAATTCATGGTGCAA
TAGTATCGCTTATACGATATTAATTGAGGATGGAAAAAGGTTCAAAAATAGAGCTTTGTTTAGAGAGATAATAATGAGTTGATT
AACGCAGATCCTATTCGAAGCCCTAGGCCAGCTTGCCTAGGAGATTTTGGTTTGATTAGGCGCAATTTTTTTGTAATTTTTGAA
TTTGGACTCCAATTCATGGTTCAAATAGTATACCATAATGAAAATATTAATTAAGATGGGAAAAGGTTCAAAAAAATTAGAGCT
TGTTAAGAGAGAAAAATATGAGTTGAATTACGAAGTTCGCTTCGGAGCCCTAGGCACTGCCCTTAGGGATTTGGCTGGATAGGC
CAATTTTTTTTGTTTTTGGTAGAAATTGACCACAATTCATGGTTCAAGTAGTATACCATATATGATATTATTGAGATGGGAAAG
GTTCAAAAAAATTAAAGAGCTTTGTTAGAGAGAAATATGAGTGAATTACGAGTTCGCTTTGGAGCCCTAGGCAGCTTGCCTAGG
AGATTTGCAAGGAAAAGGCAATTTTTTGTTTGGTTGAATTAGACCCAAAATCAGGGCCATAGTACCTATATGAACATATTATTG
GATGGGAAAAAGGTTCAAAACAATTAGAGCTTTGTTTAAAGAGAAATGATGAAGTGAATTTACGTTGTTCGCTTCCCGGAGCCC

CCTTACGCAGGCTTGCCTAGGAAGATTCTTGGCAGGAAAAGGCCAATTTTTTTGTTTGGTTGAATTAGGACCCCAAATTCATGG
TCAACAGCTAGACCAATAAAATATTATTGAGATGGGAAACTGGTTCAAAAAAATTAGAGCTTGTTAGAGAGAAAATTGAGTTGA
AATTAGAGTTCGCATTCGGAGCCGCTTAGGCAGCTGCTAGGAGATTTGGCGGAAAGGCCAATTTTTTTGTATTTGGTTGAATTT
AGGACCCCAAATCAGGGCCCAATAAGTATACCTTATAATGATATTATTGAGATGGGGAAAAAGGTTCAAAAAAATAAAGACTTG
TTTAGAGAGAAAATTAAGTTGAATTACGAGATCGGGCTTCGGAGCCTAGGCAGCGCTGCCTAGAGAGATTTGGCTTGGAATTGG
CCATTTTTTTTGTTTTGTTTGAATTAAGGAACCTCAATTGCTTGGTTCAATAGTATAACCTTTAATGACTATTTGAAGAATGGG
AAAAGGGTCAAAAATTTAGAGATTGTAAATAGAGAGAAATATAACGTGAATTACGAGTTCGCCTTCGAGCCTAGGCAGCTGCTA
GGAGATTTGCGCTGGATAGGCCCAA
>m141120_105518_42134_c100732402550000001823141405141504_s1_p0/142397/8758_20595
TTCAATTGGAAATTTTTTTCAAGGTAAAACGTTTTTATCAAAAATTAAAAGGCAAAAAACAAAAAAAGTCCCAAATTTGATAAG
AGTTCTAGTTGCCTCTTTTTCTCACTCTTTGAGGGTGTATTGGGGCCCCCTTTTCCCCTTTTTTCCCCAGGAAAAAAAAAAATCA
AAAGTCTTTTTGAACCCCAAAGTTCCCGTGGGGACATTTTGGGTCTTTTGTTCCCCCCCCAAAGTGGGGAAAAAGGCCCCATTG
CCCCCGAGGGCTCCCCCCCTTTTTGCCCCCTTCCCGAAGAACCCGAGAGGAATTTTGTCACTTTTCCCAAGGATTTTGAGGCCG
CGCATCCCCCCACCCAAAAATCCCTTTTTCCCGAACCCACAAAAATCTTCCAAGGTCCCCATGGATGTCCCCCCCCGAAACCCG
AGTGAGGCCCCCAACGCCCTTGGGAAAAGGCCCGAAGGGAAATTGTTTTTCTCCGTCTTATGTATGGGAAGTGTTTTTTTTTCC
AAATGGTCCCCAAAAAAATTGGAGGCTTTTTTTTTTCTTTTTTCCCCCCCCCCTTTTCACTTTTCCCGCCCCATCTCCCCCCCC
TCCAAAAGGGGTAAAACAGGGAAATTTTTTTTTTTCCCCCTTGAATTCCCCTTCCCCCAAGAACCTGGTTAAAAAAAGTCAAAT
TTTGCCTTTTTTTTTAAGGTTTGTCCCCCAAAGTCCCAACCTACGGGTTTTTTTTTGGGCCACCAAAGAAAACACGGGGGAAAA
AATTCAAACACAAACTTCTTTCTGTGTTGGGATACAAATACTTTTCTTTTTTTTTTTTTGGGGGGTGAAGCCCTCGATTTAGTT
GGGGTGTTCCCCCCCCCCGAACGGTTTTTTCGTAACCCTTCGAACAATTTCGACCAAAATTTTTTTTTCCACAAACCAAACT
ATTTGCCCTTTTTTTTGGGAAAAAACAAAAAACCGAAGGGCAAACCCGTTTTTGCCGCCCCGGCAAAAAAAAATTCCCCCTGATT
ACTGTTTATCCCCAAAAAAAAAGCAGGCCCCAAATGGTTTCCCCCTTTCCCCAATAAAAGCCCCCCTCTAAATGAAACAAACTT
TTTTTTTCTTTTTGCACAAAAAATTGGGGCCCCCAAATACAAAATTCCCGCCCCTTTTTTGTTTTTTTTTGGGGTTCTTTCTCC
CCCGTTTTACAAGACAAAAAACAGTCCCTCCCCATCTTTTATAAACTAAAAGGTCCACAAGCCTTTTTTTTTTTTTTTTTTTT
ATTTTTGGTATTTAATCTCTTTCTTTCTTCAAAACCAACAAAAAAACAAAACGGGAAAAGGAAAGGAAAAGGGGAATAAAAGAA
GAAGGGAGAAAAAAAAAATTTTAAAAATTAACAAAAAAAAAGCCTGGGAAAAAAAAAAACCCTTATATAAAAGGAACCCTAACGA
CCCCCCCGGCCGGATTATCTGTTTTTTAAAAAAAATGGGCGGGGCCCCCATTGGCCCCCCACCAAAGAAAAAAAAGTTTTGTTT
TTTTTTCCCCCAAATTTTTAAAGCACCCTTTTTTTTAAATGAAGGGAAAACCAAAATTTGCCCATTCCCCTTTTTTGACAGTTT
TTTTTAATTTTTCAAAGGGAATTTTGCCCCGGCCGCAACGTTTTTTTTTGGGCTTTTCGGTGTCCCCCAAGGGGCCCAGTTGGG
GGTTTTTTTGTCGGCGAAAAAAAGTGCCCGAATTTGGAGGTCTCCCCACCCCCGGAGGGGGGTGTTATACGAAAAATTTTTTTC
GACCCCCCCAAACCAAAGGCATGGTTACACCTTTTTTATCCCGAGTGGCGCGAGCCATATACAACCAAAAAAAGGTCTGTCAAG
GGGTGAAATTCCTTCCCCCCCCTTTTTCTTTCCTGGTGAGGGGCCCCCCCAAAATACATATATATCACACACACACACACTCC
GGTAAGTGCGGAACTCCTCCTGGAACAAACCTAAAAAATGCCCAAAAAAAAAAATATTTTTTTGTATGAACTTTGTTTTTTTCTT
TCTTACAAGGCTCAGAAAGGGGGAAAAAATTTTTTTTTTCCAAAAGAAGATCCTGGTAAACCCTGAAAGAACAGACAGGGAGAG
GGGCGAGTTGGGAAAGAAATAACACCCCCCCTCCAAAAAAAATGCGAAACAAATGAACCCCCCTTTTCCAAAATAACCCCTGCA
ATAAGGAAATATCTCAAATCCCCTTTTTGCCCCCTCTCTTTCCCATATATAGAGAGGGCGTTTGAGAGGCTCTTCCCCCCACCA
CACACGGCCCGACCAAAAAAAAATTTCCCAAATATATAATATTAGTTTCTTCTTTTATTATGAAGAATAAAAAAGAAGCACATTT
TTTTTGCAGAGAGGAAATTGGGTTTTCCCCCCGAAGGGGGGAATGGGTGTGCCGAGGTGGCCCTCCCCCGAGCCTTTGGGGAGG
AATAGAAATACGAATACAAAACCAAAAAATTTCCCCTCCCCGGTTCCCCCTTCCCTTTGCGTGGCGAGCGGCGAGCGCCCCCAA
AAGACCCACCTCTACTTTCCGGGGCCCCCCCCAAATGGGGCCCTATTTATCAAAACTTTGGAAGAAACATCCAACCCTGACTTT
TTATTGGTTTTTGGGAGTTCCCCCAGAACTTTTGGTATTTATGAACCTTCAATGTGTATTCTTTTCCCCCCTCCCCCCACACTC
TTGTTGGCGGAAGGAAAGGCCACCACCACGCGATAACTGTGTCTTCCCCCCAAGTGATAAACCTGTTTAGTCTGTTTTTTCCTT
CCCAAATGACCCTTTTTTTGGGGTTTTTCTTTTTTGCTTTTAAATTTTCTATTTATACCATTCCTTTAAAAAATACTTTTCCAA
ATATGGTTCTCTTTCTCTCCAATGGTTTGGGTTTAATTTCGTATATAGAAAAACAAACCGAAAAAAACCCAAAAAAACAAAAAA
AATTTTTATTTTATTTTCCTTTCTTCCTTCGCACCACAAGTTTTCCCCCCCTTTTTTTATGTTTCCCGCAGCTCCGATCCTGCT
GTAAAAATAAGTAAAAAAGAAAGAAAACCCCCTTTTATATCGTAACGATTTGAAAGGAAAAAAAGGCGAAATATGTTATTTTTT
GAATAATAGGTTTTTTTTTTTCCTTTCCGGAGCGGGGGGGGGGGGGGGAGCGGGGAGGAAAAAAATTGTTTTTTTTTTTTTTT
TTTTTTTTGGGGCTTTTTTTTTTTTTTCCCTTTTTTGAAAAACCCCGGTTTCAACCTTTTCCTTGGAAAAAATTTTTTTTTAA
AAACCGCCAAACCGTAGGTTTTGACCCCCAATTTCCCCTTTTTTTTTATTTGGCCTGGGGCCCCTCTCCCGCGAGTCCCAATCA
AAAAAATTGCAAAAATTAATTTCTGAAATAAATAGAATGGTGGGATTGTTTCTCGAGAGAATAATTTCGCCCCAACACCAAACA
CGGCCCCCAAAAAACCAACAAACATTCCTATATCCAAAAAATGGTCCGATAACCCCCCCCCAATAATAGTGCCCCCGGTTCCCC
TTCCCAACCATCTCAGCTTTTTTTTTATTGTGGGGGGGGCCCCCCCCAAACAACCATTCACCCCCTCTTTTCAGTTTTACCAAA
AAATCTTTTTTAAAAAATTGATCCCAAACAAATTCCCTTTTTTTGACGGACTTTGGGGGGTAGGAGGAATTTTTTTTTTTTTT
GCTTTCTCATAATTTGTCGCCTCACTCTGGCACCCCGAAGAGGGAGTTTTTCACTTGGTTTCTTATATTTTTTTTTTTTATTAT
TTTTTTTTGGTTGTCTGTTTGAGTTTTCTAAAGTAAAAAAAAGAAAAATGCCGCCCCTTTTTCTTATTTGAATTGCGCAAGAC
GGAAAAAACTTATTATGCCCCCTTTTTTTCCCCAGATGGGCAAAAACCTTCAATCAACATTCTTTTTGCTTCTTCGAAAAATTA
AACAAAAAAAAAAAAAAAGCTTGTTTTTTCTCGCCCCCAAAAATTAATCCCCCCTTCCCTTTTTTTTCCCTCCAAAAAAGCTTT
TTTTCCCCAGTCCTTATTTCAAAACTCCCCCCGTTCCCCTGATCCATTCCCCCTTTTTTTTTTTTCCCCCATTAAAACAAAAAT

TTTATTATTTGGGAACTCCCGGTTTTTTGATGTGGGGGTATTGATAATGGAAAGAAAAAAAAAATCCTTTGAGCTTTTTTTCTTT
CCCCTTAACAAACTTTCTTTAAAAAAATCACAGGACAACAGACCAAAAAGAGGCCAAAATAATTACACTTTTATTTTTTATAGC
TCCCCCGTTCGATATATATTGACTACTGTATTCCGCCTATATGTATGTTTCCTTTTTCTCTATCCAGCAAAAACCTATTGTCTA
CCTTTGGCATCTCACATCCACCCAAAAAAGAGCTCATTGTTTTTGCCTCTCCGAGGTAAAAAAAAAAAAGTTAAAAAAATGATTT
TTTTGTGTTTAACCCCTGGGTTTTTTTTTTTTTTTTTTATTTCTCTTTTTTTTTTGTGGGGGGGGGTTTGCCCCCCCCCCCAAAAA
TCCGAAAGTTTTTTTTTTTTTTTTTTTTTTTTAAAAATTCTTTTTGGATGTAGGTTTTTCTTTAAGATTGGCCCCCCAGAAAAAAAA
AAAAATAATTTTTTTTTAGGAACCCAAAAAAATGGCCTTTTTTTTTTTTTATTTTTGTGGTTCTTTAAAAAAAAAAAAAAATAAAA
AAAATTTTTAAAAAGGGGGGGGGTTTTTTTGGGGGGCCCTTGTTTTTGACCCGCCGCTTTTTTTTTAAAAAATACGAAGTTCCC
TCCCTTTTTTTTTTTTTTTTTTTTTTTTTTTTAGGCACAATTTTTTGGGTTGTTTTTTTTTTTTTTTTTTAAAGAAAACCCTGGGG
TTCCCCCCCCCCAAAAATTGGCCCTCAACAAAAACACAACACACACACCCTATTAATATTAGGAGTCCATAAAAGAAAAATTTT
TTTTTTATGGGCCCTTCTTTATCTTCTTTAAGCACAACAAAAATCTTTATCGGACCTCTGGAGACGATAGATTAAAATATACCT
GGTGGCCGCTTTTGGAAAAAAAAATTCCCCCCCCAAGAAATATAGTTTTCCCCTCCCCTCCGGACGGTGTTTTTTCAAAATAATT
GATCCCCCCTCTCTAGGTGGGTTGGGATGTGTTTTTTTCATGTGACGTTCTTCTTTGTTTCCCCTTTTTTTTTCCCCGGGTGGG
GGGGAACCTTTAAATTTCCCCCATTTGTCTTTCTTTTTTATTTTTGGGCCCCCCCCAAGGAACACCCCACCCCCACCCGTTCCC
TTTTTTTTTTTTAGAATTTTTCCCTTTCAAACCTCCTTTTCCTCCCCCCCTTTTCCAAAAGGCAAAAAATTTTTTTTTTTGTGT
AATCTTTTTTATAGCGGGATTTATTTGATCCGCCCCCCCTTTTCCCTCACTCTATTGTTTTTTTTGTGAAAAAAAAATGTTTCCCC
CCAAAAAATTACTTTTCTTCTCTTTTCTTTGTTGTTTATCGCGGGGTGCCACAAGGTAAGCCTGTTATGTTTATTTTTTTTATA
AATAAAAAACCCCTTAACACTCCTTTTTGATTTGTTGTGTTTGTTTTATTGTTTTTTTAGTTTTTTGGTTATGTTTTTTATTCCA
AACAAAGTGTCCACGAAGGGAAAAAAAAGCCCCCGTTTTTTATATTTTGTTTATTTTTTTTTTTTTATCCCATTGAAGAAAAAAA
AACAACCTTTTTTTAAAACAAACAAACAAAAAGGGGGGGGGTTTTTTTTTTTTTTTTTTTTGCTCCCTTACCAAAAAAAAACCTG
TTTTTTGAGCTTTTCGAAACGCCCGAAAAAAATTTTTCCCCCCTTTTAAACCCCCCAACAAATATACTCTGGAGGCTTTTTATT
AGCGGGAAAGGAGAATTTTTTTCTGTCAAATTAAAAAGGAAAAAAACATTGTTCTTATGAGGAAAAGGGAAAAAAAGTCCCCCG
GAGGTGCGACGTTCACCTTTTTTTTTTTTTAAGAGGCAAGCCCAAAAAAGTTTACTCTTTCCCCACCCCGCCAAAGCCCCCCTC
CGACGTGGCTCCTATTGTGGTTTAAGCAAAGAAAGGGAATTGTGCCTCGCGACCTTTTTGCCTTTATTGAGACCCGAGCCTTGA
CCAAATACAGGTAGAGAAAACCCCCCCTCCACCCCAATTTGCTTTTTCTTTGAATGGGGGGGTTTTTTTTGGGTCCCAAAAAT
TCCTGCGAGAAAGGCTAAAAAGGGGTAAAGATCCAAATATAAGGACAAATGGGAAGGGGGGGGGTTCCCCCCCCCCCCCCAAAA
AAAAGAATGCCCCCCCTCCAAATGGGGAAAAGATAACATTTTGTGAGAACAATTTCGGTGGGATATATATAAAAAAAAAAGAATA
ATACATGAGGGGCTAAAGAAAAAAATATATATTTTTCGAAGGTGTCGGGTCAAGGGGGGGCGCCAACTTCCCCCCCTTTTTAAAA
CGAGCCCCTTTTTTTCCCCCCCCGAAAAAACCCCAACTTTCAAAAATGTGGGGCTTTTTTATTTTTGGAGAAAGATTTTTTTTTA
CCCCTTACACACTCCCAACAAAAACCCTCTAAAAAAAAACGGAGGTGGGGAAAAAACCAAACCTTTGGGGTGGGGGAAGTAAAAAG
CATTTGCGCCCCCTGGGTTAAAAGGGATAGGCCCCGCTTTGTTCCCCTTGTTATTTCCCGGGGAGGGAACGAAAGGAAAAAAG
GGGGCTGGGGGCCAAGTGAGAAGAGAGAAAAAGCAAAATCCCCCCATGTGTTAAGCCCGTTTTTTCCCCGGGCCCCTTTTTGG
GGGGGGTGGGGCCTCTTTTCTTTTCAAAAAAAATCTCTAAACAAGGCCCCCCTTTGGTGGCAAAACCCCCCCTGTCTAAAAAGA
GATAGAAACAAGCCAAATGTGTATATCAAATATTTTGTTTTTAAAAAGGTAAACTGTAAAACTAAAAAAAAAAATTGTATTCTC
GCGATGTTTTTCTCACCCTCCTCCAAGCATATTTTCCCTGCCGTTTGTTGTGTTGTTGGGGCCAAACATTTAAGCCCCCCCCCG
CCACCCCAAATTTCCAAGCTGTCATCCCCCCGAAAATTGGGGGTGCCGCTATATAGAAAAAATAATTTTATCATTCCTCCTACG
GGTTTTGGGGGGCCTTTTGGGTCCCCCCCCCCCAAGGCCCCAAAAAAAAAAAAAAATTTTAAAAATATTCTCCCCCCTTTTTTCCC
TTTCAAAACCAAACAAAAACCACCGCGAAAAAGGGGAAGGGGGGGAAAAAAGGAAAAATAAAGAAGGGAAGAGAAAAATATTTA
AAAAAAAATGCCTTTTTTTTTTTTTTTGAAACCAGCAAAAAAAAAAAAAAAAACCCCGACCATTTTTTTTAATTTCCCAATTTTT
CCGGGGGGAAAAAACCCAACTTTGGAAAAAATTGGGGCACCGGCCTTACCTATGCCCCCCAACGAAAAAACCACTTTTCTGGAG
AAAAAAAGGGGAAAACCTTCCGAAACCCATAGGTTAAAACCTAAAATGTAAAAAAAATGGGGTAGTCGCGCGTATGCCTCCAAA
AGCACAGCGCCCCGCGCTTCTATTGTAAGGAAGGATTTTTGGAAAAAAAGGGAAAGCCCAAAACTTTCCCCCCAAGGCCCCCCC
CGAAAAAGCCCCCCCTTTTACAATGGGAAAAAAATTCCCCTTCCCCCCTTCCAAAAAAAAATGCAAAAACCCCTCCTCCGGTCC
TTTTCCCCCTCCCACCCCCGAAAAGACCAAAAGCCCCGGCACTAAAAAAAAAACTAAAAGCCCAAAAAACATTTCTCGGCAAAC
CAAGGGTTTGTGGTGTTTTTTCCCCCCCCACTCCCCCCACGGGGAGTAAGTGGGGAAAAACCATAGTTCCGAGGAAGTTTTCC
CCTTTCTATTCCCCCCCTTCCCCAAAAAAGCCAACCAATCGGGAGAAGTTGGGTTCGGGGAAAAGCCCTTCGAGTGTTTTTTTG
TAAGGGAGGTTGGAAAATAGATATTGGTGTGCCCCCCTTTCCCCCAAAAAAACCCCTTTTTTCGAAAATTCCCTTTAACCCACC
CCCGGAATTTGGTAAAAAAAAAGGGGGTTTTTTTTTTTTTTCCCCCCCCCCAATTTTGGGGGGCATCCCTGAACCCCTCCAAA
AAAATTTTTGGCCCCTAAAACTTTTTTCCCCCCCCTTTCCCCGCAGGCGAGTAACATCGCCCTCCCCCCCCAAAAAACTTTTGT
CCAAAGAGGGGGGTTTTAATCTATTTCCCCCCCCCCGCCCCTGTCCCCAAATCGTTCCCCCAAAAGGCCCAAAAAAAAAGGGGAA
TCTACCCCCCCCCCTCCTCTTGTTGAACCACAAAATAACCGCCTGCGATGGGGGCCCCCTTTGGGAATTTAACCTGGCTAAAAG
CCCCCCCCCAAGCTCCTTTTCACATTATTTCCCCTTTTTTTTTCTTATTCTCAAAGATGTTTGTTTGTTGAGTTTTATTCAATTTC
TCTCTCCATTATCCCATTTTCCCTCAAAAGGCACCCAAAAAAATTTTTTTTTGTATGTGGGTAAAAAAAAGAAATTTTTTTTTA
TTTTTTTCTCGGCGTATCTCGAAAGGGTGGGGAACAGAGTAAACCCTTTTTGTGTGTCTGTCTTGGAGTTTTTATATTCTTGTA
AAAAGTTTTGGGGTTAGAAAAGGCCCCCCTTTCCCGGGGCCCATTTTTTTATTTTTTTTTTTTTTGGGGAAAAACAACAAAATGC
GCGCGTACATATATTAAAAGAAGAGGTGTTTTTTTTTTTTTTTTTTTTTTTTTAGCTTATTTCCCCCAATTTAATGGGCCACCCG
AAAGAAAAAAAAAAAAAAGATGCGGGGAAATTCCCAAAAAAATGGAAAAAGAAAATCCCCCCCCTAAAAAGAAAAAAAACAAGAG
ACCCTTAGCTTATTTTTGAGGGAAGAAAAAAGGAGGGTGGGGAAGAAAATTTTCCTTAAAAGGGACCGGGTTTGGGGTTGTCCC

CACCTTTTTTTTTTTGGGGGGCCAAAAAAAAGAAATAAGTTCCACAACCCCCCCCGGAGAAAAAAAAAACCAAACCCCACACAAG
AAAATTTAATAGAAAAACCACCCCGCGGGAAGATCCTTTGGGAATATAAAAAAAGATTTTGTTTTGGTTTATTCCCCAGACAT
TCCCAAAAAGAAAATGTGTCGGCCGAAAGGGTCCCCCCTAAAAACACAAAAACTGGCAAACAAAATTCTGGGGCCTAAGGGTTT
TGGCGTGTTTTTGCGTGGGTGGGGAATAGCACGCCATTTTTTTTGTTTTTTTTGGGGGGGGGAAGAACCCCACTTTTTTTTT
TTTTTTCCTAAAAAAACAAAACCAAAAAAAAATTGGTTTGGCCTTTTTAAAAGACCTCCGTATTAAAAAAATTATACCAGGGGTT
TTTTTCAAAACAAAAAAAAAAAGGACCCCAAAAAACCCCCCTTAAAAAATACCCCCAAGGAAACCCAAAAAAAAAAAACAATTGTT
TGTTGTGGTCCCCTTTCTTGTTCCTTAATTTAAAAATTTAAATTGTCCCCCCCCACCTCCTAAACCCCTCTATATTCCAATAAA
GTTAATACTACCATCCGAAAATTTTGGGCCAAATAAAAACACAAAAAAAGAGAACCCCCAAAAGTAAAAAAGAAAAAAAAATAC
TTTTTTTGGGTTTGAGACAAAAAAGAGTCGTGTCGAGAATCTTTGGGTTTTAGTTTTATAATAAGGTATTGGGCCAAAGTTTTA
GTGTTTGGTGCTTCAAAAAATCCGAGGCCTTTTTTTTCTGATTTGTTGCTTCGAAGAACGCCTAAGAGAAAGAAATATGGGCCC
TCTAGGTATTTTTTTTTGTATTATTTTGGGCGTTCGTTTTTTTCCCCGCCCTTTTTAAGGAAAGTAAAGAAAAAGATTTTCCTT
TTTTCCCAAAACAACCCCCGGTTCCCCAAAGAAAAAGTTTGGGGTAAAAAAAAAAAAAGAAAAGGGGTGAAAGAAAAAAAAAAT
CAAGGGGACGAAGTTTGGGAAAAAAAAAAAGGGAACTGGGGAAAAAGGCCCCTTTTTTTTTTTTTTGGGAAAAAGGAAAAGAAAG
AAATTTTTTTAAATTTTTTTTTTTTTTTTTTTTTTTTTGGGAAAAGCTTTTTTTTTTTTTTTTTTTTTTTTTTTTTGGAAAA
AATTTTCCCAAAAATGGGGTTTTGGGGGGAAGTGCAAAAAATTTTTTCCCTTGAGAGGCCCCCCCAAAAGTTTTTTTCCCCCCT
TTTTTCCAAGGTTTTTTTTTTTTTTTTTTTTTTTTTAAATACCCTCCCGCCCAAACAGAAACCCCTGGGGGGGGGTGGAAAGCC
CCAAAAATCTTCCAAAAAAAAAAAAAAAAAAAAACCCCCAAAGCCCCCGTTCCAAAAGCAAAAGTTTTTTTTTTGTGGAAAAAAAA
AAAAATTTCCCCCAAAAATGTTTAAAAAGAAATTTTGGAAAAAAAGGGATGTTCTTTTGTGGGGCCAAACAAAAAAAGCCCCGAG
GGAAAAGGGACCCCCGCTTTTTGGGGGGTTTAAAAACCGAAAAAAATTTTAAGGATTTGGGGGTTTTTTTTTTTTGGGGCCCCG
TGGGGGGGGAAAAATTTTTTGGGGGGGGGACCCCCCTTCATTTTTTAATTTTTTTTTTTGCCAAAAAAAAAAAAAATTTTTTGGA
AAATTTTGGGATGGGCCCCCAGGCAAAAAATAAAAAGGGGGGGGAAAGGTTCCCCCCCCCCCCCAAAACCTCCGTTTGGGGGGG
GGCGTTAAAAAAAAACCACCCTTTTTCCCCCCCACCCCCCCAGAAAAAAAAGGTGGGGGGGGGGGAAAAACGTCCAAAAAAAAGA
AAGCCCCCAAAACCATCCCTTTTCCCCCCTTTCCCCCCCCCCCCCCCGAAAGAACCCCCCTTTTTTTTTTTTCCCCCCAAAAAT
TTTGGGGCCCTCCCCCCTTCCCAAAAGGTTTTTTCCCCCCCCCTTTAAAAGGTCCCCCCCACCTCCCTTTAAAACCCCTTAAAA
TTTTTTCCCAAAGGAACGAGTTTTGGGGAAGGGACTTTTTTTTGGGGGGAAGGGGGGAAAAAAAAATGGGTTGTTTTTGTTTC
TTTTTATTATACGGTGATTTTTTTTTTTTTTTCCCAACCAACAAATTTTTGAATAAACAAAACAATGGAAAAAAAATTTTAGGGA
AAAAAAAAAAAAAAAAGGTAATTTTTAAAAGCAACAAAGGTTTTTCCAAAAAAATGAAGGTTTTTCCCCCCCTTCTTTGAAGTTGG
GGGTGGGGCCTTTTTTTTTTTCCTTTTTTTTTTCCCAAAGGAAAAAAAATTTCAAAAAAGGTCCCTCTTTTTTGAACCAAAAGGC
CTGGAAAAAAAAATGTTGGTCCTTTCCCCAAAGTGGAAAAAAGCCCAAAAAAATAAAAAAAAAATTGCCCCCCCACCCCGAAGGGG
GGGGGTCCTTTGCCCTTTTTTTCCCCCCCGAAAGGAACGAGGGAAAATTGTCCCCCCCCCTCCCCCCAAAAAAAAAAAGCGTTT
TTTTTGGGAAAAAAAGGCCCCCAACACAAAAAAAAAAAAATCCCCTTTTTCCCGAAAAAAACCCCAATTTTCCCCTCCAAGTCCA
CAAAAATGGATTTTTTTTTGGGTCCCCACCCCCCCGCCCGAGTGGAGGGGCCCAACCCCCCCCCCGCCCTTTTTTTTGGAGGGG
CAAAAAAAAAGGATGTTAAAAATTTTCCCCCCCTTTTAAAGGGTTTTTGGGGGGGGGAAAGGTCCACCCCCCCCCCATTGGGTT
TGTCCCCCCCCCACCCCCCCAAAATGAAGGGCCTTTTTTTCCTTTTTTCCCCCCCCCAACATTCCCAGCCCCATCTT
>m141120_105518_42134_c100732402550000001823141405141504_s1_p0/145404/0_10211
AGAAGAGAGATGACAGAGACTGGAAGATATAATACTAAGCACATATCTTCATATTCCCCAATGATTCAGACTTTTAGGTGTAGG
ACTAGAAAATACATCTATTTAAGTAAGACAAAGAAATGTGTGTGAAATCTCAAATAGTACCAATTGCTATCACAATACTTTTTT
CCCATAAGCTAGCTTTTCAAAGTAATATATAAAAAATAAGGAATAGGAAATTGAAAGGGGGGTGTTTTACTCTTTTTTTTTTTT
AAAATTAAAAAAAAAAACAAGAAGAAAGAAGAAATTTGTCTTCATAGAGTCAAGTCTTCACAAAAATATAAAAATAAAAAATGA
GAGTAATTCCTCGTAAATAAAAAAAAAACAAAAAAAAAAAGCAAAAGTAGTTTGCCATAATTTTCAGATGTAAAACCAATAACA
AAAATAGATATGGAGAGGTAATAATAGAAATAAATAAGCTAGTTGCAAGAATCTATTTATCTCTGCAACATCCGGGCAACAAAG
CCAAATGGTTCAAATCAGGCCATACTTCCTTTGTATAGCAATACTCCAACTAACAAACTGAGCAAGAGCAACAAGACACTAGCT
CAAGCATATTCATTAAAACTTAACACCACCAGCAAATAGACCATACCTTTTCAAATAATCAAAAAATTATTTTCATCAATTGAT
TAAATTTAAATGCCACAGCAGAAGATTTGAATGAATACAGACATTCCCCAATGAGAAGACATGTTTATGGTTGTAACTAAAATA
TACATCATTTAGTAATGGAAAGGAGATGGTGTTTAATCTCAATAGCAATTTTGGGCTATCGAAATTACTTTTCCATAAGTTAGC
TTTCAAAGATATATAAAATAGGAAAGAAATTGAAGGGGGTTTTCCCTCTTTTTTTTTTTAATAAAAAGAAAAAGAGAAGAAAGG
AAAAGAAATTTGTTTCATAAGTCATCTCACGAAATATGAAAAGGAAAATGGCGTAAATTCTCATAAATAGGAAAAGAAAAGCCA
GAGTAGTGTGCCATTAATATTCAGTTGTAACAATAACAAAAATTAGAGTATGGAGAAGGTAATATAGAGAATAATAAGCCTAGT
ATGCAAGAATCTATTTATTCTGCAACATCGCCGGGCAACAAAAGCCAATGGTTCAATGCAAGCCTACTTCCTTTTAAATAGCAA
TACTCAACTAAACAACTAGAGCCAACAAACCTCGATCAAGCATAATTCATTAAACTTAAGCACCACCAGCAAGATAGACCGTAC
TTTCAAAGCATCAAAAAATTATTTTCTCAAATTGACTTAAATTGAAATGCGCCACAGCAGAAGTATTTTTGAATTGAATACCAA
CACAATTCCCATGAGAGACATGTTAGGTGTAGACATTAGAAAATATCTCTTTATATATGCAAAGAATGTGTTTAATCTCAACAT
AGTCAAATTGCTTTATCAAATTACTTTTCCATTAACTTCAGCTTTTCAAAGAATATATAAATATAAGGAATAGAAATTGAAGGG
GGTTTTACTCCTTTTTTTTTGAAAAATAAAAAAAAACAAGAAGAAAAGAAGGAAATTTGTCTTTCATACAGTGCATCTTCACAA
ATATGAAAATAAAATGGGAGTTAATTCTCCGGTAAATAGTAAAAAAAAACAAAAAAAAAAGCAAAGTATTGCCCATAAATTATT
CAGTTGTAACCATAACAAAAAATTAGAGTGATGGAAGGGTATATAGAAAATAAATAAGCTAGTTGCCAAGAGATCTATTTATTC
TGCAACATACGGGCAACAAAGCCAATGGTTTCATCAAGCTAACTTCCTTTTATAGCAATACTCCAACTAACAACTAGCAAGCAA
CAAACATAGCTCAAGCATAATTCATTAAACTTAAGCACCACCAGCAAAATTAGACCTACTTTCAAATAATCAAAAATTATTTTC

TTCAATGATTAAATAAATGCCACAGCAGAAGATTGAAAATACCACACAATTCCCAATGAGAGTACATGGTTAGGTGTAGACTAG
AAAATACATCATTTAGTAACAAGAGAATGTGTTTATCTCCAATAGTCAAGTTTGCTTATCAAATAAACTTTTCCATAAGCTTAG
CTTTCAAAGAATATATAAAAATAAGGAGAAGAATAAAGGGGGGTTTTTCCTCTTTTTTTTTTTAAATTAAAAGAAAACAAGAAG
AAAGAAGAAAATTTGTCCTTCATAAGTCATCGTCACAAATATGAAAAAGAAAATGTAAATTCTCATTAAATAGAAAAAAAAAAA
AAGCAAATAGTGTGCCATAATTTTCAGTTGTAACAATTAACGAAAAATTAAGATATTGGAAGGGTATTATAGAAAATAAATAAG
CTAGTTTGCAAGAATCTAATTTATTCTGCATACATCCGGGCAACAAAAGCCAATGGTTCAATCAAGCCTACTTCCTTTTATAGC
AGATACTCAACTATACAACTAGCAAAGCAACAAACACTCGATCAAGCATAATTTGCATTAAACCTTAAGCACCACCAGGCAAAT
TTAGACCTACTTTTCAAAGCATCAAAAATTATTTTCTTCAATTGATTAAACTTAAATGCCACCCCAGCAGAAAGATTTGAATGA
AATACCACACCAATTCCCAATGGAGGACATGTTAGGTGTAGACTAGAAAAATACAATCATTTATTAAGCAAAGAATGTGTTTTA
AATCTCAATAGTGCAATTGGCTATCAAAATTACTTTTCCATAAGCTTACTTTCAAAGAATAATAGAAATAAGGAATGAAATGAA
GGAGGGGTTTTTACTCTTTTTTTTGAAAATAAAAAAAAAACAAAGAAGAAAGAAGAAATTTGTTTCATAAGTCATCTTCACAA
ATATGAAAAATAAAATGGAGTAATTCTCGTAAATTAGAAAAAAAAACAAAAAAAAAAGCAAGTAGTGTGGCCATAAGATTTTCA
GGTTGTAACCAATAACAAAATTAGATTATGGAAGGTATATTTAAGAAAATAAATAAGCTAGTTGCAAGAATCCTATTTTATTCT
GCAACATCCCGGCAACAAAAGCCAATGTTCAATCAGCCTACTTCCTTTTAATAGCATACATTCAACTAACAACCTAGCAAGCAA
CAAAACTACTAAGCTCAAGCATAATTCCAATTAAACTTAAGCACACTGAATACTATAGCAAATAGAACCTACTTTCAAAGAATC
AAAATTATTTTCTGCAAGATTAAATTAAATGCCACAAGCAAGAATGATTTGAATGAATACCAACACAATTCAATGAGAGACATG
TTAGGGTGTTAGCTAGAAAGGATATACATCATTTAGTAAAGCAATAGATGTGTTTAATCTCAAAGTCCATATGCTATCAAATGA
CTTTTTCATAAGCTTTAGCCTTTCAAAGAATATATGAACACAATAAGGAGAAGAAACTTAAAGGGGGTGTTTCTCTTTTTTTTT
TAAATAAAAATGAAAACAAGAGAAAGAAGAAATTTTGTCTTCATAAGTCATCGTCACAAATAATGAAAAAGAAATGGAGTAATT
CTCATAAATAGAAAAAAAAAAAAAAAAGCAAATTAGTTGCCATAATTTTCAGTTGTAACAATAACAAAAAATTAGATATGGAAGG
TATATAGAAAAATAAATAAGCTAGTTGCAAGAATCTATTTTATTCTTCAACATCCGGCAACAAATCCAATGGTTCATCAAGCCT
ACTTCTTTATAGCAATACTCAACTTAACACCAGCAAGCAACAAACAATAGGCTTCAAGCATAATTGATTAAACTTAAGGCACCA
CCAGCAAATAGATCTACTTTCAAAGCATCAAAAATTATTTCCCCCTCCAATACTCCAATGATTAAATTAAATGCACAGCAGAAA
ATTTGATGAATACCAGACAATTCCCAAATAGAGAAACATGTTTTTAGGTGGTAGACTAGAAAAATAAATCATTTAGTAAGCAAT
AGAGTGTGTTTAATTCTCAAATAGTCAATTTGGCTTATCAAGATTATACTTTTCGATATGCTAGCTTTTCAAAGAATATATAGA
ATAAGGAAAAGAAAATTGATAGGGGGTTTTCCTCCTTTTTTGTTTTAATAACAAATAAAATAAGAACGAAAGAAAAAAATTTGT
CTTCATAAGTCATCTTCAACAAATAATGAACAAGGAAAATGGAGTAGGATTCTCGATAAATAGGAAGAAGAAAAGCAGAGTAGT
GCCATAATTTTCAGTTGTAACAATAACAAAAATAGATAGGTGGATGAGGGTAATATAGAGAATTAAATATGAGCTAGTTGCAGA
ATCTATTTATTCTGCAACATCCGGGCAACAAAAAGCCATGGTTCAAATCAAGCCTACTTCCTTTTATGCAATACTCACTAAACA
ACGCAAGCAATGAAACAAACACTAGCTCAAGCCTAATTCATTTAAACTTAAGCATCTCACCGCAAATAGATCTACTTTCAAAGC
ATAAAAAATTGATTTTCTCAATGATTATAAATTAAATGCCCACAGCAGAAGATTTGAATGAAAACAAGACAAATTCCATGAGAG
ACATGTTTTGGTTGTAACAGTCAATTGGATATCAAATTACTTTTCCATAATTTAGCTTTCACAAGAATATATAAAAAGGAAGAA
AATTAAAAGGGGGATTTTCCTCTTTTTTTTTAAATAAAAGAAAACAAGAAGAAAGTAGAAATTTGTCCTTCATAAGTCATCGTC
CACAAATATGAAAAAGAAATGGAGTAATTCTTCTAAATGAGATGAAAGAAAAAAAAAAAAAGCAAATTTGTTGGCCATAATTTTC
AGTTGTAACAATAACAAGAGAGATTAGCTCATGGAACGGGTATATAGAAAATAAATAAGCTTAGTTGCAAAGAACTATTTATTC
TGGCAACCATCCGGGCAACAAAATCCAATGGTTTCAAATCAAGCCTCTTCCTTTTATAGCAAGTACTCAACGTAACAAGCCAAC
AAGCAACCAAACACTAGCTCAAGGCTAATTGATTAAACTTAAGCACCACCAGTCAAATAGATCTACTTTCAAAGCATCAAAAAT
TATTTTTCTCCATGAATTTAAATTAAATGCCAACAGCAGAAATTTGAATGATAACCAGCAACAATTCCATGAGAGACATGGTAG
ATGTAGACTAAGAAATACCAATCATATTAGTAAGCAAGAAGAGTAGTGTTTAATCTCAATATCCAATTGGCTTTCAAAATTAAC
TTTTCCATAACTTAACTTTCAAATAATATATAAAATAAGGAAAAAAGAAATTGATGGGGGTTTTTCTCTTCTTTTTTTTATTTTA
ATAAAAAAGAAATAAGAAGAAAGAAGAAATTATGTCTTCATGAAGTCATCTTCACCAAAATATGGAAAAGGAAATATGGAGTAA
ATTGGTACATAAATAGGAAAAGAAAGCAGAGTAGTTGCCATAATATTTTCAGTTTAACAATAACAAAAATTAGATATGGAAGGG
TAAATAAGAGAATAGAAATAAGATAGTTGCAAGAATCTAGTTTATTTCCTACATGCACATCCGGGCAACAAAAGATCAATTGGT
TCAATCAAGCCTACTCTTCCTTTTATTAGCAAATACTCAACTAACAACCACCCAAAGCGATCAAACACTAGCTCAAGCGAATAA
TTCATTAAGACTTAAGCCCACCAGCAATAGATCTACTTTCCAAGCATCCACAAATTTATTTTCTCAATGATTAAATTAAAATGC
CACAGCCAGTAAAGATTTAATGAATACAGACAATTCCCATCGAGAGAACATGTTAGTGTGTAAGACTTAGAAAAATACATCATT
TAGTGAAAGTAAGAGTGTGTTTAATCTCAATAGTCAGATTGGCTATCAAGAAAATTACTTTTCCATAAGCTTAGCTTTCAACAG
AATATATAAGGAATAAGGAAAAGAAATTGAAAGGGGGGTTTTTCTGGGTCGTTTTGTTTTTGTTTAATAAAAAAAAAAGAGAAG
AAAGAAGAAAGATTTGTGCTTCATAAGTCATCTTCAACAAATAGGGTTGAAGGAAGGAGAAGAGTGGAGGGTAATATCTCATAT
AGGGTAAGGAAAAGAAAAGCAGAAGTAGGTGGTGCCCAGAAGTTGTTCAGTTGGTACAATAACAAGTAAATTAGATATGGACAG
GGTATATAGATGAATAAATAAGCAGTTGCAGAATCTATTTATTTCTGAACATCCGGCAACAAAGCCAATGGTCAATCAAGCTAC
TTCCTTTTATTGCAATACTCAACTACAACCGCAAAGAAACAAACACTAGCTCCAAGCCTAAATTCATTAACTTAAGCACCTACC
AGCAAATAGACTACTTTCAAAGCAATAAAAAATTATTTTCTCCATGAAATTAATTAAATGACCACAGCAGAAGATTTGAAATGA
AAACAAGACAATTACCAATGAGAGACATGTTTGGTGTAAACAGTAAATTGGAATATCAAATTAACTTTTTCCGATAAGTTTAGC
TTTCAAAGAGATATATTAAAATAAGGAAAGAAAGATTAAAGGGGGATTTTTCCTCTTTTTTTTTTAATAAAAAGAAACAAGAAG
ATAAGTAGAAATTTGTCTTCCATAAGTCACGTCACAAATATGAAAAAGAAAATGGAGTAATTTCATAATAAGAAAAAAAAAAAA
AGCAAATTTGTTGCCATATTTTCAGTTGTAAACAATAACAAAATTAGCTTATGAAGGGGTATTATAAGAAAAATAAATAAGCTA
GTTGGCAAGAATCTATTTATTCTGCAACATCCGGGCAACAAAAATCCAATGGTTCAATCGAAGCCTACTTCCTTTTATAGCAAT

ACTCTAACTAACACCAACAAGCAACGAAACACTAGCTAAGCATGGAATTGATTAAACCTTTAACATCCGACCAGCAAATAGATC
TAGCTTCAAAGCATCAAAATTATTTTTCTCCATGAATTAAGATTAAATGCCACAGCATGAAAATTGATGAAATACCAGAACAAT
TCCCAATGAGAGAACTTTAGATGTCAGATAAAAAAACATCAATTGTAAGTATAAAAGCAAAGAGTGTTGTTTAATCTCAATAGT
CCAATTGGCCACATCAAATTACTTTTCCATAAGCTTAAACAGTTTTTTTTTATTTTTTTTATTTTTTTTTTTTTTTTGTTTTTT
TTTTTTGTTTTTTTTTGTTTTTTTTCTTTTCATTGTTTTGTTTTTTTTTTTTTTTTTTTTTTTTTTTTTTTTTTTTTTTTTTTGTT
TTTTTTTTTTTTATTATTTTTTTCATTTTTCAAATAATATATAGAATAAAGGAAAAGAATTGAAGGGGGGTTTTCCTTTTTTTT
TTTAATTTAAAAAATAAGAGTAAAGAAGAATTTGTCCTTCATAAGTCCATCTTCACAAATATGAAAAGGAAAATGGATAATTGA
TCATAAAATAGGATAAAGAAAAAGCAGAGTAGTCGCCATAATTTTCAGTTGTAAACAATAACAAAATTAGATATTGGAACGATA
TATAGGAAAAGTTAATAATAAAGCTAGTTTTGCAAAATCTCATTTATTCTAGCAACATCGGCACAAAAGCCAATGGTTCAATCA
AGGCCTACTTCCTTTTTATAGCAATACGTCAACTATCAATCCAGCAAGCAACAAACCACTAGCTCAAGCGTAATTCATGTAAAC
TTAAGCACCACCAGCAAATTAGATCTACTTTCAAAGTCTATCAAAAATTATTTTCTCAAGTGATTAAATTAAATGCACAGGCAG
AAAATTTATGATACCAGAAAAATGTCCAAAATGAGAGCATGATATGGTGTAATGACTAGAAAGATACATCATTTAAGTAAGGCA
AGAGTGTGTTTAATCTCAATAGTCAATTGGCTAGTCAAATTACTTTTCCATAGCTTAGCTTTTCGAGAATAGTATAAAATAAGA
ATAGAAATTGAAGGGGGGGGTTTACTCTTTTTTTGAAATAAAAAAAAAACAAGCAAGAAATGAAGAAATTTGTCTCCATAAGG
TCATCTTCACGAAATATGAAAAATAAAATGGAAGTAAGTTCCTCGTAAATAAAAAAAAATACAAAAAAAAAAAAGCAAGAGATT
GCCATGATTTTCAGTGTAACAATAACAAAAATTAGATATGAAGGGTATATAAAAATAAATAAGCTGAGTTGCAAGAATCTATTT
ATTCTGCAACAATCCGGGGGCAACAAAAGCCAATGGTTTCAATCAAGCCCCTACGTGTCCTTTTATTGCAATAGCTCAACCTGA
ATCAACTAGCAAGCAACAAACACTAGCTAAGCATAATTCATTAAACTGTAAAGCACGACCAAAGCAAATAGACCTACTTTCAAG
ACTCAAAAATTATTTCTCCAATGATATTAAATTAAAATGCCAACCAGCAAGAAATTTGAAGTGAATACCACACAATTCCAAAAT
GAGAGACAGTTAGGTGTAAGAATAGAAAATACATCATTTTAGTAAGCAATAGAATGGTTTAACTCAATAGTCAATTGGCTATCC
AAATATTACTTTTCCCATAAGCTTAGCTTTTGCAAAGAATAATAAAATAGAGGAAAAGAATTGAAGGGGGGTTCTTCCCTCTAT
TTTTTTTTTAATAAAAAATAAGAAGAAAGGAAGAAATTTGTCTTCATAAGTCATCTCGCAAATATGAAAGGAAATGGAGTAAAT
TCTCAATCAATAGGAAAAAGAAAAGCAGAGTAGTTGCCATAATTTTCTGTTGTAACAATACAAAATTAGATATGGAAGGGTTAT
AGAGAATAAATAAGCTAGTTGCTAAGAATCTATTTATCTGCAACATCCAGGCAACAAAAAGCCAATGGTTCAATTAAGCTACTT
CCCTTTTATAGCAATACTCCAACTATCCAAACCCAAGCAAGCAACAAACACTAGCTTCAAGCGTAATTCATTAAACTTAAGCAA
GCCACCAGCAAATAGATCTACTTTCAAAGCATCAAAAAAATTTTTCCCCAATGATTAAATTAAATGGACCACAGCAGAAATTGA
ATGAATCCAGACAATTCCCAATAATGATAGAACTATGTTATGTGTAACTAGAAAATACAGTCATTTAGTATAGCAAAGAATGTG
TTATAATCTCAATTAGTCAATTGGCTTATCAAATTACTTTTCGCATAAGCTTAGGCTTTCAAAGAATATATAAATAAGGGAATA
GAAATTGAAGGGGGGTCTCTTACTCTTTTTTTGAAAATAAAAAAAAAAACCAAGAAGAAAGAAGAATTTGTCTTCATAAGTCAT
CTTTCACAAATATGAAAAATAAAATGGAGAAAATTCTCGTAAAAGAAAAAAATACAAAAAAAAAAAGCAAAGTATTGTTTGCCC
ATATTATTCAGTTGGTAACAATGAACAAAAAGTTTTTAGATATGGAAGGGTTAGTAGAAAGATAAATAAGCTAGTTGCAAGAA
TTATTTATTTCTGCAAGCATCGGGCAACAAAAGCCCAATGGTTCAATCAACTACTTCCTTTATTGCATACTCAACTAAACAACT
ACAAGCAAAAACACTAGCTCAAGCATAATTCCGTTAAAACTTAAGCACCACCAGCAATAGACCTACTTTCCAAACATCAAGAGA
ATTATTGTTCTCAAGTGATTAAAGATTAAATGCCACAGCAGAAGATTTGAATGAATACCACACAATTCCCAATGAGAGAACATG
TTTTGTGTAGGACTAGAAAATACATCATTTTAGTAAGGCAAAAGAATGTGTTTAATCTCAATAGTCAATTGGCTATCAAATTAA
CTTTTCCATAATGCTTAGCTTTCAAAGAATATATATAATAAGGAAAGAGAAATTGAAGGGGGGTGTTTCCTCTTTTTTTTTTAA
TTAAAAAAAAAATAGAGAAAAAAAGAAAGAAGAAATTTTGTCTTATAAGTCATCTTCGCAAATATGAAAAGGATAAATGGAGTA
ATTCTCATAAATAGGAAAAGAAAGCAGAGTAGTTGCCATAATTTTCTGTGTAACAATACACAAAAATTAAGATATGGAGGAAGG
GTAATATAGAGAATAAATAAGCTTAGTTGCGCAAGAAACGATTTATCTGCCAACAATCCGGGCAACAAAGAGCCAAATGGTTTC
CAATTAAAGCCTACTTCCTTTTAATAGCAATACTCAACTAACAACCAGCAAGCAACAAACACTAGCTCAAGCAAATTCATTAAA
CTTAAGCACCACCAGCAAATAGATCTACTTTCAAAGCAATCAAAAAT
>m141120_105518_42134_c100732402550000001823141405141504_s1_p0/150771/0_7118
CAACGAGTCATACCCTAGATAGTAAGGATCGACTAATGAGTCACACTCGTATAGGAGAGCTAATGAGTCTCACTCTGTATAATC
GGTGACTAATGAGTCACACTTATGGGTTATTTGTTTGGTTGCAATTTGTATATGATCCATATGTTCTCTCCTTGGCGTTATTTT
TTTATAATCACTCGCTTTTCCTTTCTACAAGAAAAGAAAAACCGATCACAATAAGATTTAAATTAATTTCTAATTTTGAATGGA
CCCCCTGAGTTTGTTGGTTCCAAGGAACTTAGTTTTTTTTTGAAGACTTAAGTTCGCGTTTAAATTTTTTATCAATTTCTCGTG
CTCCTTAAAGAAAACAACGATCACTTGATTTAAATTAACGTTCTAAGTTTTCAGAACCTGGTTATGTCCAGGAAACTTAGTTTG
AAAACTTAGTTCGCGTTAACTTTTATCAAATATCTCCGTTCTCTTTAAGAAAAACAATGATCAATTGATTTTACACATTATTCT
AGGTTTTATGACCTGGTTATGTCGGAACTTAGTTCGAAAACCTTAGTTGCGCGTTTAATTTTTATCAATATCTCGTGCTCCTTT
AAGAAAAACAACCGATCAAATTGATTTAAATTAACTTCTAAGTTTACCTGTTGTTTTTGCTTGTAGGATCTTTTATGTGAAATT
ATCTTATGTGGTCTATTAATTTATCAATAATCTGTCTTCTATTAGTAATTAATACAACTAATTCATTTTTTCTTAAGTTATTAT
ATCTTTCTATTAGTTTTTATTGGTTATATCCAGGAACTTGAATTTGAAAACTTAGGTGTCGCGTTAATGTTTTATCAAGGTGTC
TGCGTGCTCTTTAAGGAAAAGACAACGATCCAATTTGATTTAAATTAACTTCAAAGTTTTATGACGGTGGTTGATGGTCCAGAA
GCTTAGTTGAAAACTTAGTTCGCATTAATGTTTTATCAAGTATATCTGCTTTTAAGAAAAACAGACCGATCAAATGATGTTATG
ATTTAATTCTAAGTTTGAACGGACCTGGTTATGTCCAGGATACATGAGTTTTGAAAACTTACGTTCGCATTAAGTTGTTTAATC
ATATGATCATCGCTCTTTAAGAAAAACAACGATCAATTGATTTAGAATTAATCTTCTAAGTTTTATGACCTGGTTATGTCCAGA
ACATAGTGTTAAAAACGTAGTTCGAAGTTAATTTTTATCAATACTCGTGCCTCTTTAAGAAAACAACGATACAATCGATTTAAA
TTAACTTCTAAGTTTTATGACCTGGTTGATCATCCAGGCATGCTTAGTTTGAAAACTTAGTTCGCGTTAATTTTTTTCAATAAT

CTCGCTGCTCTTTAAGAAAACAACATCAATTGATGTACAATGAACTTCTAAGTTTTATGGACCGTGGTTATGTCCCAGGATCTT
AGTTTTGAAAACTTTAGTTCTGCGTTAACTTTTATCAATATCTTCGTTCCTTTAAGAAAAAACATGAAATCAATTGATTTAAAT
TAACTTTCTAGGATATATGACCTGGTTACTGTCCCGGAACTTAAGTTCGAAAACTTAGTTCGCGTTTAATATTTTATCAATATC
TCGTGCTCTTTAAGAAAAAACAACGATCAATTGATTTAAATTAATGTCTAAGTTTTATGACCTGGTTATGTCGAGGAACTTATT
TTGAAAAACTTATTCGCGTTATTTTAATCAATATCTTGTGGCTCTTTTTAAGCAAAAACACGATCAGTCAATTTTAAATTAACT
TCTTAAGTTTTAGAACTGGTTATATCAGGAACTTCATTTGAAAACTTAGTTCGCGTTAATTTTTTATCAATGTTACGTGCTCTT
TTAAGAAAAACAAAAGATCAATTGATTTAAATTAAACTTCTAAGTTTTAGGACCTGGTTAATGTCCAGGAACATATTTGAGAAA
CTTAGTTGGCATTAATTTTTATCAATATATCCATGCTCTTTAAGAAAAACAACGATCAAATGATTTAAATTAACTTCTAAGTTT
TATGAACTGGTTATGTCCAGGAAACATTAGTTTTAAAAAATGTAGTTCCGTAATTTTTATCAATATCTCAGTGCTCTTTAAGAA
AAAATGATCAATTGATTTTAAAATTAACTTAAAAGTGTTTAATGACCTGGTTTTAAATGTCCAGGAACATAGTTTGAAAACTTA
GTTCGCGTTGATTTTTATCAATATCTCTGTGCTTTAAGAAAAACAACGATCAATCGATTTAAATTAACTTCTAAGTTTTTATGA
CTGGTTATATCCAGGAACATTGTTTTAAAACTTAGTTCGCGTTAATTTTTTCAACATCCTCGTGCTCTTTAGAAAGACCACGA
TCAATTGATTTAAATTATTTTCTAATTTTATTGACCTGGTTATGTCCAGAACCTTAGTTTGAAAACATTAGTTCAGCGTTAATT
TTTATCAATATCTCGTGCTCCTAAGGAAAAACAAACGATCAATTGATTTAAATGAACTTCTAAGTTTTAATGACCTGGTTATGC
TCCAGGAACTTAGTTTGCAAAACTTAGTGCGCGTTAATTATTTATCAATATCTTCGCTGCTCCCTTTAAGAAAAACACGATCAA
AATCAATTTATTAACTTCTAGTTTTATGACCTGGTTAATCCAGGAACTAATTTGAAAACTTAGTTCGGCGTTAATTTTTATCTA
ATGTCTCGTGCTCCTTTAAAAGAGAACCACAGCGATTCATTTTGATTAATTAATTTAAGTTTTATGACCTGGTTATGTCCAGGA
ACTTAGTTTGAAAACTTAGTTCGCATTAATTTTGTATCTATAAATATCATGCTCTTTAAGAAAAAACAACGATCAATTGAATTA
AATTAACTTCCAGTTTATGACCTGTTATGTTCCAGGAACATAAAGTTTGAAAACTTAGTTTTTCGCGTTGATTTTTAAATTAAT
ATCTTCGTGCTCTTTAAGAAAACCAACGATCATGATTAAATTAACTTCTATATATTTTTATGACCGTGGAATATCCAGGAGCAG
TAGTTTTAAAACTTAGTGCGCGTTATTTTTATCAACATCTCGTGCTCTTTAGAAAGAAAATCGATCAATTGATTTAAATTGAAT
TCTAATTTTAATGAGCCTGGTTTTGTCCCAAGGAACTTAGTTTGAAGAGCCTTAGTGTCGCGTTAATTTTTATCATATCTTTCT
CGTGGCTCACTTAAGAAAAACAAGCGATCAAGTTGTATTAAATTTGAACTTATAAAGTTTTTTATGAGCCTGGTTATGTCGCAG
GAAACTTAGTTTGAAAACTTAAGTTCCGCGGTTATTTTATCAATATCTCCGTTCTCTTGTAAAGAAAAAACAGATGATCAATTT
GACTTTAATATTAACTTCTAGGTTTTATGACGGGGTTATGTTCGCCGAACTTAAGTTCGAAAAACTTTAGTTCGCGTTATCTTT
TTATCCATATCTGCGTGCTCTTTAAGCCCAAAAACAACGATCCAATTGTTTAAATTCACCTTCCTAGAAGTTTTATGCCTGGTT
ATGTCGAGGAACTTAGTTTGAAAACCTTAGTTCGGCATTAATTTTTATCAATTCATGTGCTCTTTAAGAAAAAATCAACGATGC
ATTCAATTTAAATTAACTTTCTAAGTTTTATGACCTGCGTTATATCCAGGAACTTAATTTGAAACTTAGTTCCGGGCGTTAATT
TTTATCAATGTCTCGTGCTTCTTTAAGAAAAAACAACGATCGAACTTGATTTAATTAACTTCTAAGTTTTTTATGACCCCTGGT
TATGTTCCAGGAAACCATAGTTTGAAACTTAAGTTCCGCATTAATCCTTTTGATCAATATGATCAATGCTCCCTTTAGAAAAAC
GACGATCAATTGATTGAAATTAAGCTTCTAAAGTTTGAGAGACCCTGGTTATGTCCAGGAAGCATAGTTTGAAACTTAGTTCGC
ATTATTTTTATCAATATATCATAAGCTCTTTAAGTAAAACAACGATCAAATTGATTCTAAATTAACATTCTAAGTTGATGACTG
GTTAATGTCCGACGGAACATGAGTTTAAAAAAACTTGTTCCAATTAATTTTTATCAATATCTCGTGCTCTTTAAGAAAAACAAC
CGATCCAATCACTGAAATTTATAATTTAACTTCCTAGTAGTTTTATAGAACCTGTTATTATCCAAGCGAAAGCTTAGTTTGAAA
AACTTAGTTCGGCGTTAATTTTTAATCATATCTTCACGTGCTCTTTACGGAAAAACACGATCAAATTGATTTCAAATGAGACTA
TTAAGTTTATGACCGTGTGGTTACGCTGTCCAGGAAACTAGTTTGAAAACTTAGTTCGGTTAACTTTTAATCAATATGTCGAGC
CTCTTTAAGAAAAAACAACGTCAATTTGATATTTAAATTAACTTCATAAAGTTTTATGACCCTGCGGTTATGTCGAGGAAACTT
AGTTTGAAAACTTTAAAGTTCGCATTAATTTTTATCAATATCTTAGCGGGCTCTTTAAAGAAAAACAACGATCAACTCAATTTA
AATTAAGCTTCTAAGTTTTTATGACCTGGTTAGTGATGCCCAGGACTTAATTTGAAAAGCTAGTTAGCGTTAACTTTTTATCAA
ATGTCCTCGTGCTCTTTAAGAAAACACGATCAAAATTGATTTTAAATTAATTCCTAAGTTTTATGACCGTGTGTTAGTCCAGGA
AGCATCAGTTTGAAAACTTAGATTTCGCATTAATTTTTATGCAATATAATCCATGGCTCCCTTAATTAAGAAAAATACAACGTC
AATGTAGATTTAATTACCTTCTAAAGTTTGAGGACCGTGGTTATGAGTCCGGAACCATAGTCTTGAAAACTTTAAGTTCCGCAT
TAATTATTTCATCAATATATCCATGCTCTTTAAGAAAAACAACGATCAAATTGATTTAAATTAACCTTCTAAGTTTTTGAGCCT
GGTTACTCGTCACAGCGAACATACGTCAATAAAACTAGTTCGCAATTAATTATGTTTATCAATATCATCCAGTGCTCAATATAC
GGAGAAAAACAAGCGATCAAGTCGACTTGTAAAGTTAAACTTCTAAGTTTTATGACCTGGTTGATATCAGGAAAAGCTTAGTCT
GTTGAAACCTTAGTTAGCGTTAATTTTTATCAAGTATCTCGTGGCTCCTTTAAGAAACAAGCCAACGATCAGATTGATTTAAAA
TGAACTTCTAGTTTATATGACGCCTGGTTTATGTTCCGGAACGTAGTTTGAAAACTTAGTCCGCGTTAAGCTTTTATCAATATC
TCGTTCTCGTTTAAGAAAAAAACATGATCAATTGATTTTAACTTTATTAACTTCTAAGGAATTTATGACCGTGGTGGGTTATGT
CGAGCGGAACTGTGTTCGAAAACCTTAGTTTTCGGCGTTAATTTCTATCAATACCTCCGTGCACTCCGTGCTCCTTTAAAGAA
AAACAACGATCAATTGATTTTAAATTCAAAGCTTCTAAAGTTTTATGACCCGCGTTAGTGCTCCGAGGGAACTTATTTTGAAAC
TTAGTTCGCGCCGTTAATTTTTATCCCAATATCTTGTGGGGCTCTTTAAGAAAAAAAGCGTCAAACTCATTTAAATTAACTTCC
TAATGTTTCATGAACTGGTTTATATCCAGCGAAACCTTAATTTGAAAACTGTAGTTCGCAGTTAATGTTTTATCAATTGTCTCG
TCGCTCCTTAAGAAAAACACACCGATCAATCTGATTTAAATTCAACCACTTCTAAAAAAGTTTTATGACCCTGGTTAAGTCAGA
CATAGTTTTGTTTGCGTTAATTTTTATCCAATGTTCGTGCTGCTTTAGAAAACAACGATCCATTAGATTTAACATAACGTTCTA
AGTTTTATGACTAAGGTATGATCAGGGTGAACGATAGGTTGAAAACTTAGTTCGCATTAATTTTTATAACTATCATAGCTCTTA
AAACATCAAAATTGATAAATTAACTTCTAAGTTTGATGACCTGGTTATGTCAGGACATTAGTCTGAAAACTTAGTTCGTTAATA
TATTATCATTTCAATAAGCTCTTTAAAGCAAAACACATCCAACATTTAAATTAACTCCTAAGTTTGTTATGACGCTGGTAGATG
TCCAGGAACATAAGTTGTAAAACTTAGTCGGCATCTAATATTTATCACTATTCGTGCTCTAATCACAAAAACACGATCATCGGA

TTTCAATTAACTTCAAAGTTTTATGACCTGGTTATATCCAGGAATTAGTTTGAGAAAACTTAGTTCGCGTTATATTTTAATCAA
TTATCTCGGTGGGCTGCTTTAAAAAACAACGATCAATTGATTTAATGAATTCTAAAAGTTATATGAGCTGGTTAGTCCAGGAA
TGCTTAGTTTGAAAAATTAAGTTCGGCGTTAAGCTTTTATCAATATTCTTCTGCATTTAAGAAAAAACAATGATCATTGTTTAA
ATTAACTTCTAAGGATTTTGACGCTGGTTTATGTCCCGAACTTAGTTCTGAAAAAGCTTAGTTCGCGTTAATTTTATCATATCC
GACGTGGCTGCTTTAGAAAACAACGATCAACATTGAATTAAATTACTTCTAAAGTTTTATGACCTGGTTATGTCGGCAGGAAAA
CATAAGTTTCATAAACTTAGTGGCATTAATTTTATCAATATTCGGCTCTTCTAAAGAAACAACGATCAATTCGAATTAAATTAA
ACTTCTAAGTTTTTTAATGACACTGGTTATATCCAGGAACTTAGTTTGAAAACTTAGTTACGCGCTTAATTTTTAATCAATATC
CGTGCTCTTTAAGAAAAACAGCGATCAAGTTGATTTAATAATGAACTTCTAAGTTTTATGACGCTGGTTTTGTCCAGGAAACTT
AGTTTTGAAAACTTAAGTTCGCGTTAACTTTTATCCAATCTCGTCCTTTAAGAACAATGATCATTGATTAAATAACTTCTGAGG
ATTTATGACTGGTTATGTCCGGCGGAAGCTTGTCTCGAAATTAGTTGGCGGGTTATTCTACTCAATATACCTCGTGGCTCTTAA
GAAAACACGATCATTTGATTTAATAATTACTTCTTAAGTTTTATACCTGGTTATCGTTCGCA
>m141120_105518_42134_c100732402550000001823141405141504_s1_p0/152466/6069_12455
GGAGAATTTGGCCTGAAAAGGTCCCCCAATTTTTTTGTTTTGGTTGAAATTAGGAACAACAATTCAATGGTCCAATAATAGCCC
AAAAATAGTAATATTATTGAGATGGAAAGGTTCCAAAAAAAATTAGAGCTTGTTAGAGAGAAATAATGAGAGAATTACCCGAAA
GATGGCTTCCGGAGCCCCCTAAGGCCAAGCTGCCTAGGAGATTTGGCTGAAAAGGTCAATTTTTTTGTTTGGTTGAATTAGGAC
CCCCAATTCCATAGGTTAACCTAAATAACCCCGCATATAATATTAATAGAGATGGGAAAGGTTCAAAAAAATTAGAGCTTGTTA
GAGAGAAATATGAGTGGAATTACGAGATCAAGCTTCGGAGCCCTAGGCGGCTGGCCTAGGAGATTTGGCTGAAAGGTCAATTTT
TTTGTTTGGTTGAATATAAGGACCGCCCGAATTCCATGGTAAAAATAATAGCCAATATTTATATTATTTACAATGGGAAAAGTT
TGCAAAAAAAATTAGAGCTTGTTTAGGGAGAAATAGAGTGAATTACCGAGATCCGGCCTTCGGAGCATAGGTAGCTGCCTACGGA
GATTTGGCCCGAAAGGAACATTTTTTTTTTGTTTGGTTGAATTAGGACCCCCCAATTACATTGGTCAATAATAGCCCCCATATA
ATAATTATTTGAGATGGTAAAACAGGTTCAAAAAAATTAGAAGCCTTGTTAGAGAGTAAAATGAGTGAATTAGCGAAATGCGCT
TGCAGAGCCCTTAGGGCAAGCTAACCATACGGCCAGATTTGGCCTGAAAGGTCAATTTTTTAGATTTGGTTGAATAGGCACACC
GCAATTCATGGTCACATAATAAGCCCCCATATAATATTTATTGAGGAATGGGAAAAAGGCTTCAATAAAAATTAAGAGCTTGTT
AGAAGAAATTATGAGTAGAATTACGAAGATCGCTTTCCGGAGCCCCTAGGGCAGGCTGCAGGAGATGTACGGCAGAAAGGTTCA
ATTTTTTTAGTTTGGTTGAATTAGGACCCCAAATTCCATGGTAAATAATAGCCTATTATAATATTGATATTAGATGGGAAAATA
GGTTCAAAAAAATTAAGAGGCTTGTTAGAGAAGAAAATAAGAGTGAATAAACAGAGATCGCTTCGGAGGCACCTAGGGCAAGCT
GGACGTAGGAAGATTATTGGGCCTGAAAGGTCAATTTTTTTGTTTGGTTGAATTTAGGACCCCAATTCCATGGTCAATAAATAG
TCCCTTTACTAATCATTATTTAGATTGGGAAAGGTTCCAAAAAAAATTAGAGCCCTTGTTTAGGGAGAAATATGAGTGAATTCT
ACAGAGATCGGCTTCCGGAGCCCCTAGCCTAGGCAGACCAAGGAGAATTTGGCCAGAAAGGTCCAATTTTTTGTTTGGTTGAAT
TAGGGAACCCAATTCATGGTCAAATTAATAGCCCTATATAATATTCCCACCCCTGACGATGGGAAAAAGGTTCAAAAAAATTTAG
AGCCTTTATTAGACGAAGACACTATGACGTGAATTACGAGATCTCTACCGGACGCCCGTAAGGCAGCGGCCCTAGGGAGATTTG
GCCTGAAAAGGTCAACTTTTTTTGTTTTGGTTGAATTAGGACGCCCCAATTCGTGGTCAATAATAGGCCCAATATAATCATTAT
AGAGATGGAAAGGTTCAAAATTAGCATCTATGTTAGAATAGAAAATGAGTGATTACGAGATCGCTTCGGAGGCCCTAAGGGCAG
CCTGCCCTAGGAGATTTTTGGCCCTGAAAGGTCATTTTGTTTTGTTTGAATTAGGACCCCAAATTCATGGTCAATAATAGCCCC
CCATATACATATTATTGTAGAGATGGGAGAAACGGGTTCAACAAAAAATTAGAGCTTGTTAGAGAGAAATATGAGAGAATTACG
AGATGAGCTTCGGAGCCCTAGGCCAGTTGCCTAGGAAGATTTGGCCTGATAGGTCAATATTTGTTTGGTTGAATTAGGGACCCC
AATGTCATGGTTCAATAATAGCCCCATATAAATTATTGAGATGGGAAAGGTTCCAAAAAAATTAGAGCTTGTTAGAGAGAAATA
ATGAGTGAATTACAGAAGATCGCCTTCGGAGCCCGGCCTAGCCGCTGCCCCTAGGGAGATTTGGGCCTGAAAGGTCAATTTTTT
TGTTTGGTTGAATAGGGACCCGAATTCAATGGTAAATAATAGCCCAAGTATAATATTATTTAGCCATGGGAAAAAGTTTCAAAA
TTAGAGGCCTTGTTTAGGGACGAATATGAGTGAATTACGAGAATCCGGGCTTCGGAGGCCTAGGTAACGCTGCCTAGGAGATTT
GGCCCTGACAAGGACAATTTTTTTGTTTGGTTGAATTAAGACCCAAGTTCATGGTCCAATAACTAGGCCCATATAACCCATTAT
TGAGATGGGAAAGAGGTTCAAAAAAATTAGAGCTGGTTAGAGACGAAATATGAGTGAATAACGAGATCGCTTTCGGAGCGGCCC
TAACGGGCAGGCTGCCATAGGAGATTTGGCCCTGACAAAGGTCAATTTTCTGTTTTGGTCTGAAATTGAGGACTCCCAATTCAT
GGTCATAATAGCCCTTTATAATATTATTTAGATGGAAAAAGAACGTTCAAAAAAATTAGACTTTTTAGACGAGACATATGAGTG
AATTACGATGATCTCCTAACGCGGAGCCCCAAGGCAGCCTGGCTAGGAGATGTGGGCCTGAAAGGTCAATTTTTTAGTTTGGTT
GAATTAGGACGCCCCCAATTCGTGGTCAATAATCAGCCCCCATATAATATTATACAGAGAATGGGAAAAGGTTCAATAAAATCT
AGAGGCTTGTTAGATTCGAAATAATGAGTGAATTACGAGATCGCTTCGGAGCGCCTAGGCAGCCCTGCCCTAGGAGATTTGCCT
GAAAGGTCAATTTTTTGTTTGGTTGAATTAGGAACCGCGCTAATATCATGGTCCCAAGTAATAGCCCATATAATATTATCTGAG
ATGGGAAAGGTTCAAAAAAATTCAGAGCTTGTTAAAGGAGAAATATTTGTAGAGAATTCACGAGATGGCTTCCCGGAGCCCTAC
AGCCTGCCTAGGAGATTTGGCCTGAAGGTCAATTTTTTTGTTTGGTTGAATTAGGAACTGCCCAATTCATGGTTTTAATAATAA
CCCCATATAATAATTATAGAGAATGGAAAAGCGTTCAAAAAAAATAGAGCTTGTTAGAGAGAATATGAGTGATTAGAGATCGC
TTCGGAGCCCTAGGCGCTGCCTAGGAAGATTTGGCCTGAAAGGTCAATTTTTTTGTTTGGTTGAAATTAGGACCCGAATTCACT
GGTAAATAATACTAGCATATTTAATCATTATCTTAGATGGGAAAAGTTTTCAAAAAATTAGAGCTTGTTAGGGAGAAGTATGAG
TGAATTAGCGAGATCGCTTCGGAGCCTAGGTAGGCTGGCGCCTAGGAGATTTGGCACTGAAAGGACAAATTTTTTTGTTTGGTT
GAATTAGGACGCCCCAATACATACAAAATAAGCCCCTAAAAATATTATTGAGATGGTAAAACAAGGTTCAAAAAAAATTAGAGC
TGTTAGGAAGTAACAATGAGTGAATTTACGAATCCGGCTTCAGCAGCCCTAAAGGCAGCTCCACATAGCGAGACTTTGGCTCAT
GAAGGTCAATTTTTTTAGTTTGGTTTGAATTAGGCAACCGCCAAATTCATGGTCAATAAATAGCCCCCAAATATAATATTATTT
GACCGATGGGAAAAGGTTCAAAAAAATTAGAGCTATGTTATAGAGAAACAATCCGATGAATTACGATATCGCTTCGGAGCCCTA

GGCGACCTGCCCAAAGCGAGATTCGACACAGAAAAAGGTCAATTTTTTTAGTTGGTTGAATTGGACCCCCCCAATTCATGGCT
AATAATAGCCTATATAATATTCATGTAGACTGGGAATAAAGGATTTCAAAAAAAATTAGAGCTTGTTTTAGGAGAAAATATGAG
TGAACATACCGACCGATCGCTTCGGGAGCCCTAGGCCAGCTGCCTAGGAGATTTAGGGCCTGAAAAGGTCAATTTTTTTGTTGG
TTGAATTAGCGACCCCCAGTTCTCATGTCCAATAATCAGGCGCCTTTATAATATTTTTTAGATGGGAAAGGTTCAAAATTAGAG
CTTGTTAGGGAGAAATGCTGAGTGAATAATACGAGATCGGCTTTCCGGAGGCCCATAGCTAGCAGGCCAAGGAGATTTGGCGCA
GCAAAGGTCAATTTTTTTGTTTGGTTGCAATGGAACCCAAATTCAATGTCAATAATAGCCCCTATATACCGCGATCATATTGAG
ATGGGCAAAAGGTTAAAAAAAATTAGAAGGCGTTAGAGACGATATGAGGGTGATTACGAGATCTCTACGGAGCCGCTACAGGCA
AGCTGCTAGGAGATTTGGCGCTGAAAGGTCCAATTTTTTTGTTTGGTTGAATCTCAGGACCCCACAATATCGTGGTCAATAATA
GCCCCATATAATACTTATAGAGATGGGGAAGGTTCAAAAAAATTAGCATTTTGTTAGACTAGAACTATGAGTGAATTACGAGAT
TCGTTCCGGAGCCCCTAGGCAGCTGCCTAGGAGAATTTTGCGCGCGAAAGTCAAGTTTTTTTGTTTGGTTAGAAAATTAAGGAC
CGCCAATATACATGGTCAATAAACTAGCCCCATTATAATCATTATCTGAAGATGGGACCAAAGGTTCAATAAAATTAGAGCTTG
TTAACGACAAAAGGGAAAGAATATGAGAGAATTACGAGATGGCTTTCCGAGCCCCTAGGCAGCTGCCTAGGGAATTTGAGCCTG
AAAAGGATATCAATTTTTTTGTTTGGTTGAATTAGGACCCAAAATTCATGGGTCAAACTCAATACCCCATACATAAATTAATTT
TTTTTTTGCAGATGGGAAAAGGTTCAAAAATTAGCAGCTTGTTTAGGAGAGAACATATGAGTGAATTACCCGGAGGATCGCTAT
GCGAGAGCGCCCTAAGGCCCAGCTGCCTAAGCGACAGATTTGGCCCCCGCAGAAAAGGTCAATTTTTTTGTTGGTTGAATTAGG
ACCCCGAATTCATGGTACAATTAATAGCCCAATATAAATATTATTTACATGCGAAAAGTTTCAAAAAAATTAGAGCTTGTTAGG
GAGAAATATGCAAGTGAATACGAGATCCGCTTCGGAGCCCTAGGTAGCTGCCTAGGAGAGTTTGGCCCCAGAAGGACAATTTTA
TTTTTGTTGGTTGAATAGGCCCATACATGGTAATAATGAGCCCGTATAATATTTAGAGATGGAAAAGGTTCAAAAAAATTAGAG
GCTTGTTAGAGAGTAAAATGAGTGAATTACGAAATCGCATTCGGGAGCCTAACGGCAGCCTACATAGGCAGACTTTGGCCTGAA
AGGTCAATTTTTTATTTGGCTTGAATAGGAACCCCAATTATGGTCAACTAATGAGCGCTATACCCATAAATAAAGTTTAATATT
TACGATGGAAAAATGTTTCAAAGGAAAATTAGAAAGCTTGTTAGGAGAGGCGGGAACAATACATGAGTGAATTACAGAGAGGAT
GAGGCTTCAGGAGCACTAGGGCAGCACACTTAGGAAATGCGGCCTGAAAAGGGAGCCCAGATTTTGATTAGTTTGGGTTGAATA
AGAGCACGGCCCAATTCATGGTAAGATAATAGCCCTATATAACATTTAGTTTAACATGGAAAAGGTTCAAAAAAAATTAGAGCT
GTTAGAGAGTAAACAATGAGTAGATTACCGAACAATAGTCAGAATCATAATCGGCAAGACACACATAAACAAGGCAAGCAATAC
ACAGTAGCGAGATTTTTGGCGCTGAAAGGTCAAAGACTTTTTTTGTTTGGTCCTGAAATAGGACCCCACCAAATTCATGGGTCA
TAATAGCCCAATACAAGTAAATACTATATTTTAAGCAGATGGGACAAAAAGGTTTCAAAAATATTAGAGCATGCTGCCGTTAGA
GAGACAAATAATGCACCTGACAATTACGAGATCCGCTCATCGGAGCCCTAGGCAGCCTGCCTAGGAGATTCGGCCCAGCAAAGG
TCAAATCTTTTTAGATTGGTTGAATTAGGAACCCCCAATTTTCATCAAGGTAAAGAAATAGAATGTCCCCTATACATAATCATT
ATTTAAGATAGAGGAAAAAAGTTCAAAAAAATTGTGCTTGTTGAGAGAGAAATAATGATTGACATCAAGCGAGATCCGCTTCGG
AGCCCTTAAGGAAGGCTGCCTAGAGAGACTTTGGCCTGAAAGGTCAATTTTTTTGTTCTGGTTGAATTAGGACCCCAATTCAAT
GG
>m141120_105518_42134_c100732402550000001823141405141504_s1_p0/153673/6347_13678
GGCTAGCGCTACCAGCTGCCCACTGACTAGCGCTAGTCATGCGCCAGCAAACCAAAAAATCAATTTCTGTGATTTCTTTCAACC
AATCTTAACCCAAACTTCCCCAAATCCTTTACAAACTCAAACACACTTATGAAACACTCACACACACATATACACCTAAGAACA
TTTATTTTCAATTACTCAAAATCTACCCATCATCAACCCTAAACCTCAGAAATTCGCCAAAACAACAGAGTTACAGGACTTAGA
AAATCATACACTTTGCTGCAAATTTCACTTGATCAACCCTAGCTAAGCTTGCTCCTCTCTCCAATGCTTGCCCAGAAATTCCCC
TTAAGATTTCCCATCAATCCAAGCTTAGATACATAGCCCAAACCAGTCATTAACCCCAAAACTTGAAAAACTCACTAAAACTTA
CCTCAACATTGATAGTGTTCCTTGCTAATCCTACACACATATACCTTAAAGAAATCCTTTATGCTCAGCATATCTTAGCTCACA
TTTGAGCTTATGTCAGAAATAGAGGATGTGCAAGGATCACAACGATCCTTTAGAAAAACCTGCGTAGTATTCTCTCCTTTCTTT
CTTTCCTTCTTTTCATTCTGTTCTCTCCACGCACATCTCTTTACATATAGCCTTCCATAACAACATACAAAAAGCCAAATGACC
AAAATTCCCTCCCACTAATTACAAACCCTTTTAATCCAAGTAAGCTGGCCATTTTAGTCCATTTTAAATTTCCGCTAATCCTCA
GTGTCCTCTTATTTTTCCGTTGGCTTCCAAACACCTGAAACACAAAATAAGTATACACCAAAAATAAATCTCACAACATAACAC
ATAAAATTCCTGCTCATACCCCAGGCTCGCCTGAGCGGGTATAAATTCCCCGTCATGACTTTCCGCTAGCCTGCTCACTGGGAA
TTGCCTCGAGCCACCAATCCAACAGATACATCCACCATCAAATGTGCTCTCAACCAATCAATCACAATATCAATACAGCTATGC
AACATGGCAAATTATAATTACGCCTTCTAAACACGAAGCCGGGGCCTACATGCATACTAATATACATAGTCATGCATCTTCAGA
TAAGCAAATAAGTCCAAATAACATGCTTTAAATCATAACCATGTATTTAATTCATCAAAGTCACACAGAAATCCCATTCATGCC
CTCCCTGGCAACGCTAATCAAGGCCCTTAAGCCTTATTAGCGCAAATTTGGGTGTTACATACAGTCACGCTCATGTGCGTACGA
CGTTTCGACCTTAAGCTGATAATTACAACGCGCATGGTGGGATCACCATTCGGCTTCGTATAATCCGTAAATGCGATTAAGAAT
TTTGAGGGATTGTGTAAGATTGTTAAGAAAGCATGATTACGACCATATCATTCGTACTCATTATGGTATCATTATTAACTGTTT
TTATTGGTCTTTTATAATTTAATGTTATCGCCAAGCCACTAAACCGCCCGGCATAGGTCAGATCCTTTGTTGGCGTTAAGCGAC
CCGCCCCGCTTTAAATCAATAGCTTGCCTCGGATAACCCCTTTGCTGAGAATACACCAACACTATTGGATACCACTAATGATCA
TATGTTGGATGCTGACCAATTACCCCATCAATCTTCTCTTTAACTAAAAGTGACGATCCGCTTCCCCGTGTTTTAGATTCTGTC
ATCATGTTTCGAGGGTTGCTGTGCAATACTGCATCCGGTAAGATTGATTATCCACACGTAAACGGTGTGAATGAGAGCTTTATA
TTCCAACTCTTCTAGTTCTCCTAAACAGGTGTCTGATCCATATCGCTTCGCATAATTCATGGGCCATGCTCTATACTCTGCTTC
TGCAATGCTTTCTGGCAACCACTGTTTGCTTTTTACCTTCGCCATGTTACGCAATTGCCCCATACAATTGTACAATAACCAGAT
GTAGATTTCCTAAATCATCACTGACCCAGCCCCAGTCTTGCATCTGTGAATACTTCAACTTTTTCTCTATCGGTCTTTTATGAA
AGAAAAGACTTTTTCCTGGTGGTTTGCTTTAGTACATCAGAATTCTATAATACTGCATTAAGATGTCCTTGACCGATGGATCAA
TGCATGTATTTGACCTGACCACCACTTACTACAAGGGAAAATATCTAGTCCTGGTATGAAGAGAGGTAGATAAGAGTCCGTTAA

CTATTTGCTCAGTACTTTCATTTGTCAACTGGGTTTCCCTTCAAACATTTCCTCCTTGTATCCGATCGAATTGAATTGGAGTTT
TGCTAGCGTTTACTGCCTAGCATTCCTGTCTCATTCAGAGTTCAAGAGTTGTATTTTTCTTTGAGAACATAAAATTCTCTTTTT
GCTTCTAGCAAAAATTCCATTCCCAGAAAATACCTGAGTCACCGAGATCCTTGGAACCTTTAAAATCCCTCTTGCCAACCATTT
CTTTAATCCGAATCATCTTCTTCAGTATGTCTGCCAGTGACAATGATATCATCAACCATATACAATAAGAACTGCACCAATCTT
CCTCTTTCTGAGTGTTTGATGAAGAGAGTGTGGGTCAGTATGACCCTTGCTATGGTATCCAAAAGTCCTTAATAACTTTCAGAA
TCAGATTGAACCATGCTCGAGGAGATTGTTTTAGACCCGTAGAATGATTTGTTTAGTTTGCAACTTTGGTTGTATTGGGAGATT
CTTCAAAAACTCGGAAGGCCTGACTCATGTACACTTTTCCTCCAGCTCACCCATATAAGAAATGCATTTTTTACATCAAGTTTG
AATCGCAATTCCCAATTCTAAGTTGATTGCAAGCGAGAAGCAATACTCCTGATGGTTATTGAGTTTGGGAAATTGGAGCGAATG
TTTCAGTGTAGTCAATTTGTAGGCTTTTGAGTAAAAACCCTTGCAACTGACCGAGCTTTGTACCTCTTCAATAGATTCCATCTT
GCATTTATTTGACTGTGAACACCACCTTGCACCCTAGTATCACCTGTTCTTGTCTGCGTGGTAATCCACTAATTGCCAAGTACC
ATTATGCTCAAGTGCTTTCATCTCTTCGAAGAAAACCCCTTGATGTGCTTTCCATTGAGGGATACCATGGTGCTTGCATTTGGT
ATTTGATGGACTTCAGAAACGGCGTATTCGTTTTAAAAATTTATACTCGCAACGCGCACGAATCGTAATTATGAATATAGTGGT
TCGTGTGACAGAATCGACACCCAAGGAGTTGTCTAAAATCAAAAAGAACAACTATTTTAAATCAGAAAGTAATAAATTCTAACC
TTAGTCCAAAAGATTGATGAGTTTTTTCAGTGTTTGAACAATAAATAAAAAGATTGAAAAATAAAGTTATTTAAAGAGAATAAA
AAAATAAACCAATAATAATTTGAAATAAGTGGTAAAGAAGATTATTAACGCATACTAGAATCCACAAAATTAAAGTTTACATAA
TATTTATTTAGCATATTGATTCCCAAGTTTTTAGTGATGTTAAAATAATTCAAACTATCAATTTTCCAAATAGATTTATAAATT
TTAAGCACAAAATTACTTCTAAAAAGATAAGGATTTTTCTTCACTTTCAAATTATAATCTTCAATCAGTATTTAATCGTGATAC
CCAACCTAATGAAAAACAAAAAATCAAATAAATGTATTTATAAAGAAAAAACATAATATTTTTGTTCTAAGCAATGGAATGTGT
ACAATTATGATACATTTACACAAAGAATATCTATGTTTTCTTGCAAAAATGAAGAACAAAGTGCATATTATCTACAATCCAAAA
TATGAGATATTAAAGATGAAAACAGTATCACGAAGAAGAAAAATCCATAACTTTGTTGCATTACAAGGGAAATCAATACAACAT
AAATACTTATTACGTTAACAAGCTGCTTATCAATGATCTTAATAATCTTATGAACAAAGATTTAGAAGCACATAACTAGAATAG
AAATACAAAATAAACAAAATACATACTTACATAAAATGCTCTTTGAAAACCCAAAATGGAAGAGAAAAGGGTAGAAAGAAGATC
GGTAGACCAAAAATTAAGCACCCCAAAATGGTCTTGAATTCCTATTTTATTTGCCAAAATGAATAGCATTAAAATATAACTTAA
ATTAATTAAAATTGATTACATTAATTAAATAAAATAAATTTGATATGTAGAGGTAAATTATAGGGTATAATGATGATGTTGTGG
TTAAATGTGTCAAAAAAGGGTAACAAAGTTGGCATTTTGGAATAGGAGAACAAATGGTACCACTTAGGCAATTTATGGGCTCAA
GAAGGGGAAATGGCAGTCATTGTGGTGGCTGGTGGGAAGCTTGGTCTAGCATGGGCCTTGGAGTGATACGTAAGGCCACATGCG
GCAGCTGAAGGCTGGAAACTTTGGGCACGCTGAAGGCTTGGCTGAGATGGAGCAGCTTGGAGTGGGGGTGGTTGGGCGGTGGAG
CAGGCCCAGGCGGCTGGGAGGCATGCATTCAGGTGGCAGTTTGGCGATGGGCTTCTGGTTTTGGGCCGAGTTAGCCTGGTGGCA
TTGGCTGGGCAAAGGCGTCGGCTGGGCTTGGTGAGCAGGAGGTTAACAGCTGGCGAAGAGAGGAGTGGGAGCTCGGGCCAGGCG
GCTGCTGAAGGCTCGGGTGGGCTTGGGATTTGAATGGTGGGGCTTGGCTTGCTGAAAGAGGAATGGGGCCGAGTGGAGGCAAAG
GAGGCTAGCTGGGCCATGGGCTGGGGCAAAAGGCTTCATTTGTTATCAAAAATTGCCAATTTTTTTTCTTTCTTTTCCTTACTT
TTCAAAAAGAAAAGAAAAAATACAGCATACCTCCTACAAAAATAAACGTACATTAAATCAAAATCAAATATTTTCATTATAAAAT
AATTCATACTAATTTTTGAAAATATTCATTAACATTAATCTTAATTTACATTTAGTTTCAATAAACACACATTTTTTTTACTTC
CTAACTAAACACCAATAAATTTAAATAATTAAACTACAACAAAATAACTATAAAAAACACAAAAAAATATTAAATCATGATAAA
CCAATAAATTCAAATTACTTAAGAATTAATAATTAATTAAATAATCAAGAATACAAAAATTAGCATAATAAATATGATAAAATA
ATCTAATGATTGTACGAGTTATCAATTATTCCTGGGAATCACAACATTGATAGACTAGTGGTAAAAGCTTTAAATGGAGATGAT
AATTTGGGCATAACAAGATGAATTTTGAATTAGGCTGTTGGGTTGCAAGATTCTACTTCCCTTTTTTTAAAGCAATAGGAGTAT
CTGAGTCTGATTGGAGTGTTTGAAGAGTAGAGTACCTGAATCTTTTAACTGAGGTGGTTCTATCAGTGACCGGCTCTTGACTGT
GATGAGATTCGTCATTAGTTTTATTTCTTTCTGCTTCTGTATGATACCCGAATTTCTTTCTGCATGTTTTTGATTTTGAGAGCA
TGAGAAGATTGGAAAGAGTTGAAGATTTTCTGGTTCGGGAATGATTTCATTCACTGGAGGAAAAGACATGTCTAGGGTAAATTC
TACAACCACGACACTAGAAGTTAATTGCTCCTATGAATAGCTCCCCCTAAAGTGGAAGTGGGAGTGAAGTGATGGAGTATTTTC
AAAAAAGGTTAGCTCGTTGGGAAGAATGTAGATTTTCTTGGTGAGGGATAGTTTGCAACGATAGTATAAGCGTCTTCCAGAATT
TTACTTAGCTTGGATTTAGTAGTAGTAGTAGTAGTAGTTAGTAGTAGTGAGTAGTAGTAGTAGTAGTAGTAGTAGTAGTAAGTAGTA
GTAGTAGTAGTAAGTTATCAGTAGTAGTAGTAAGTAGATAGTAGTAGTAGCTAGTGTAGTAGTAGTAGTAGTAGTAGTAAGTAG
TAGTAGTAGTAGTAGTAGTAGTAGCTAGTAGTAGTAGTAGTAGTAAGTAGATAGTAGTAGTAGTAGTAGGTCAGTAGCTGTAGA
ATTTTAGGTTTTTCGTGGATATTGGTTCAAGTCGGGATTTAGTTAGAAACTCGTAGAAATAGTTATGGATTTTATAAGTTTAAC
TATGGGTTTTAGAAATATTATTTTAACATAAAGGTTTTGATTAATATAGTTGGTCTAAGAATATTTATTTATTTCTAACCTAAG
GTTTAGATAGAATAATTTAAGAGGCGTGACACTTGTCACAGGCATATTTCAGTATAAGGATTTTAAGTATTTTGATGAATAGTT
TAATTAAAAGAAGATTGAAAGCTTCTAGAACCCTCCATTTATACTGTTAGGATCACGGTTTTTAACTTCGATAGTAGTTTTTTA
ATCAATTTCAAATTATCTGAAATGTGCAAAAACGCTGTTTTAAAATATCAGGTGTACATAGGTTATAATCGCAGCTATAGGGGG
CGATATATCGCCTTGATCTGATACGGAAAAACACGTCAACTTCGCACGAACGAGAACAATGGACTCGTTTATATGGTGGGCGAT
ATATCGCTACCTAGGCGATATATCGCTCTGACTTTTTTCTTCCAACACACGGGAGAGGAGGGGAGAATTCTGTCTTGGGTCGAT
TATCGCTAGGTGGCGATAATAATCGCCTATTTCGGGGATCCATGGTTCGTTCTGCGAAGTTGGACGTGTTTATCCGTATCATCA
TAGGCGATATATCCGCCTATAGCTGGATATAACGCATTACCTGATATTTTAAACCACGTTTTTTGCCATTTCATGATAATTTTG
AAATTGATTAAAAAACTACTTTGACTGAGTAAAACGTGATGCCTAACAGATAATGGAGGTTCTAGAGCTTTCAGATCATATTCT
TTTAATTAAACTATTCATCAAATACTTAAATCCTTAATCAAATATGCTGTGAAAGTGGTCACGCTCTTAATTATTCTAATCTAA
ACTTAGGTTAGAATAAATATATTCTTAGGACCAACTATATTATCAAACTTATGTTAAAATTAATATTTCTAAACATACGGTTAA
ACTTATAAATCCATAACTATATTCTACGAGTTTCTAACTAAATCCGACTTGAACCAATATCACAAACTAAAATTCTTTACAGCT

ACTACTACGTACTACTACTATAACTACATATACTACTACTACTACTAATACTACTACTACTACTACTAACTACTACTACTACTA
CTACTATACTACTACTACTACTACTACTACTATAACTACTACTATACTATACTACTACTACTACTACTAATCTAATATACTACT
ACTACTATACTATACTTACTCTACTACTACTAACTAATATCAGCTAAGTAAAGAATTCTGAGACGCCATACTATCGTTGCAACT
ATCCTCACCAAGAAATCTACATC
>m141120_105518_42134_c100732402550000001823141405141504_s1_p0/154800/5076_15221
AATTACCGTGAAAAATTTTTTGAACTTATTATTCGATGCCCAAATATGCAGCAGCAACCCCTTAAGTCTCCAAGTAATATTTGT
ACATGTTTTTTGGATAAAAACTTTAAAACAATATACTAACTTAATCATATGCTAAAATCTTTTATTAATACTAAATCAAAAAAA
AAAAAAAAAAAAATAAATAATTATATTATTAAAATTATAATTATTGTTATAATTCATAGAAATCGTACCTTAAAAGTAATTAAA
CCTTAAATTAATCATTTTAAATTCTACGCAATCCAAATTTATAGACTATAAACTAAATCAAACTGAACCTTATAACGTAACTTA
AAAGTACAAACACAGGAAGAGGAATAAAGACCCAGTAGCCTGTGTTCCAGTAACTATAGAAAAGAGTAACCATCATCCTATTTT
AATTTATTAGAGCCCTGGACATGGGGCTGTAAGAAGTAAATTGAATGGGATTGGCTTGTCAATATGGTTAGAGGCAGCCATAAT
CTACAATCACCAATGAGAGTTTTTGAATAGTAAGAAAGCAACACGAATAACTGGATTGGGACTGGTGTCGAGGACCTGGTTCAC
TTAGTGGGAATTTGGTGTTTTAAAAGCTGATGGATTTTGTGAACCTGCTTTGTCTGTGAATGGGAACTGGTTCTTGGTTTGGGA
GCAGTATGTGCACGTCCTTCTAATGGAGTGCGAGGGCGCGTGTGGTCCAATATGCTGGTCTTTCCACTGGAAAGATGCCAACAT
GTCTCCATAGTAAAAATACCAGGTCTGCTGCATTGATACGACATGGGACGCTCTTCCAAGATGCGTGTTCGCATGAGAATGCAG
CCACAACATTAGGAATGGCGGAGCAACCAAGGGCATGAAATGGTCGACTTTTGCAACAGGGCCCATCATGACACCACTTCGACT
TCTTCTCTACACACCTCCTGCAAAGGTCAGCACAAAAGGGAAGTGGAGACTTCACAATCCTACCCCTAGAACCTCATCGCAAAC
TCCCACAGTTGCAAACATGAAGGAAAAATCAAACAACCCGTTTGGCTCGATCAGCATTTTTTTTATAAAAAAGAGGCATTTTTG
CATTTCTAGTCAAGGTACATACACTACATTAATCTATTGCCACATATTATAAGATAGGCATTTAAAATTGTGTTACCCGAGAGA
TTCTGTTGGGCCGCTGGTCCTAGAGTTTTTGGACCTTGAGTCAAACACTTGAGAAGAGTTTTCAAGGTCAGCAATCGGCCCTCT
TTTGACATTGTCCCAGACCTCACTAGCAGTGGAGAGGGATAAGTAATGTTTTCCCCAATATCTAACCTCCATGAAAGCCAAGGC
CATGATTAGAGAGTTTTTCAGAGTTCCATTGTGCGAAAGAAGGATCATCAGTATTCTAGAGCCAAGAAGTGTCACCCATTCAGA
TAGCCAAGGCGAACCTGCTACCACCGAAGATACAAAGTCAGCCTAATCGGGAGCCATTGTAAGAAAATTTTTGACCAATTCAGT
TTTGGTGAATGGTGTTTGGAGAGAGGACTGATTTTCCAAAGGACAGATATGGAAGGCATCATGTGTGTTTTCTGGGCAGAAGAG
ATGACAGAAATTTTCATCCCAGAGATGAATAGAGTTTTTGTGTTGGCAGCCATCGGACGATTTTTGGGGGGCTGGAAGAAAGAA
TAAGATGCAGGAAACTTAGTCAGAGTTTCCGCTTCTAATACCAATAATAGAGATAGTGATAGGAATCTGATGTATTATTCTTGA
TAGAAAGAAGCTTAATAGGCTAAGATTACCAAACAATTGTACAATAGGCTTAGCTGTACTAAAACTTCTTTAAGTCATTTAATT
CTCGTAAAAATTTGGTATAATACATTATATTCAGGAAATAATAAACTAGGTTTTGCTTAAGGAATAATACCAATTAATACCCAA
ATATACTGATTAAATATAAAATATTCTACAGGCTCAATTTTTACAGAAAATGGTAACGGTTACTTTATCTGCAAATCTAAAAAG
AACAATCAAATTTGAATTTTTAAAAAAAATGATAATAAATCAAAGCAATCAATAAAAACTAAAAAAAAAACTATTGAAGAAAAG
CAAGAAGAACACTTCCATGTTGGAGAAGGAATAAGAATAAGATATATTTTTAAGTAACATCAAAAGTAATAATTATTCAAAAAA
CTCATAAATATACACATATAATGTATGGAAAGGTCACCATTACCTAATAACATATTAAAGATAGAAAAAAATATAAGAAACTAC
ATTTGCAATAATACGATTAATCGGGGCTTACCTAAAAAAATTTTTAGAGCCAATGTTAAAGCCGATTTTTTTTTTTTTTAAAGA
TTGAAGGGGCAGAGAAACAAGAATTATGAACGTTGCCAAAGACCAAAAAGAAGAAGAAGAAGATTGAAATAGCCGGTCCGGAGG
CTTTAGGAGGTTGTTGGATTTTGGTTGGCTCTTCATTTGAAATTTTAGATTGCTAGGCTATTCCACTCTTCGTTTTTTTATTTG
GTCTGCTAAGTGAATTCATCCTTGGTTTGCTTATCAATTTTTTATTTTGAAAATGTGTTCTTTCCGCATTTTTTTGTAGTTCCG
CTGGTTTTTGTTCCATAATTTATTTAATTTTTTTTTTAATTTTTCGCAATAAAAAAAAGTATAGAACATGTAAATTAATAAAAG
AAAAATATTTCTAAAATTTTCCTATAATACCAAAACAACAAATAGTGGAGGCCGGCCAGTTGAAAGATTAGGAAGGAGGCCCCA
ATTCAAGTTATGAAACGAAAAATTCCATACAAATACCGCGGAATGCTCTCTAGAAATGGCCATTAAATCAAATCCTTTTTTGCC
GCCAATTTAATCAACCTCAAATTTGAGAACAATATTCAAATCTCCCCAATCTCTCGCTTATTTTTTCTTTTTTTCTTTCTCCAC
TGCCCAATGCATCCGACTTCTCCCTTCCCCAGCGCGCATCTATTCCTCCATTGTACTCTTCGGCAAGTTGAAACATCTGGTAAA
AGATTTTATTTTATTTCCTTTTCGCCTTCTCCTTGATACTATATTCCAGTTTTGAAGTTTGGATTCTTGATGGCCTAGAAACTA
TGTTTGTTCGTTTCTTTTTGTTCCTTGCTGCTCTTTCCACCCCAAACATTTTCATTGTGCTCTTCCAGCTCATAATGATTCTAG
ATTAAGCGATTTCTCCTTTCTAATTTGTTTTGGGTCCTTGCTTGTCTTCGGGTTTCTAGAGACAAAATATTGAATCTTTTTGAG
AAAATTTGTAATAACAATATACTCCACTGCTGGCCGAAGGTGGATTTATCAGATTATTTTGAATAAACATAAAAAAGGATTTTT
CCTGAGGTTGTGATTTGGGCCAAGGCAGAAAATTGCATAATAATGGCAGTCAAAATCGGTACCGTTTTTCCAAAAACCTCCCTC
CTTTTTCTGGCTTCCCGATTGAATCCGCCTAACCCCACCTCCCAGTTTCTGAAGAAGATAAGCGAATTAGAGCAGTCTGGGGGA
GGAAGAAAGAACACCAGTTTATTCAACAATATAACGTCGAAGAGATCCGCGACCGAAACAGGTCTTTGCCTTTGTATTTGTATA
AATGGTTGTATATAAAATGAAAGATGTCGAAAGTGAATTCAGAATTGTGGGTTTGTATTTTTACTTTTTTGGTGAGTTCTGTAA
GTCCGAACATAGTTGTCTTCATCACTGAATTTTATTTTTCCTTCCTGTTTTGATGAAGTGGGGAAAGAGAAAATGGCAAAGATG
GCCAATGGCTAAACAAACTTTTTCGGAGCCAAGGGAGAAACAATGGGGAAAAACAAGAATGAGAAGCTATGCCGCAGTACGCAT
TTAGATTTGGGGTCAGTCTGATTTTTAACCCTTCCATAGTTGTTCCCATATGTCTCATAAGGATTTTTCATAAGAAACTAATAG
CGCCCATCGAATTCAATTTTAAGGTAGAAATATTTGATCAAGTCATTTTGAAATGGGCCTTTGTGTTATCATTGCTTAGTTCGA
AATTAAAAATTGGACCAAATTGCCGATAAGTTTCTGGCAGAGTGCAGCTGGACTATCGTCCGTTCTCTTTAAGACGTCTTCGGC
GGTTCCTGCCCGCGCAGAGTGTGCACGACAGTCTATATTAACCACGCCCGTCCTCAGGATAAACAATCACGTGTATACGATACC
TCTTCTGCACTGAAAGGCTTTCGTCTCGTAAACACCCTCAAAAGTCAGAGAAAAATTTGATTATTTTAGAAAAAGATCGTGTTT
TGCTGTAAAACAGATGTATTCTTCTGTTATTCTAATATGACTTTAATGTAGAGAAGTCGACTCCAGCAATAAAACAGTAAGAAC
AACGTTTTATTCCAATCATAGTCGTGGGAGGAATTGTCGCGTGGTCCATCAATGGCAGTTTAACAACGGATCACATTTCCGCGG

GCTCTTGCTTAATAAATCCGTTTTAAAATTAATTAATAAAATAAATAATATTTTATTTTATTAAATAATTTTTTTTCCAACCA
AAAAATAAAGTACCACACCCCACGCGCCTGCCCAGGCTCAGGTCTATCGACGGAGCGCGCACGCCGTGTGGTGGTCAACGGTGT
GAGTCGCTCACCCCATTCCGTCAAACAAAAATGGGTAACCTTTCAGCCTCCATCATGGCACTTGCAATTTTCAATATACAGCT
CTTGGAGGAGTGTTCCAAATCCATATGGGAGCCTTTTCCCATATTACACCAACAAAATACTTTTTTTCAATTTTTAAGCATTCA
CTTTTTAAACTTTAGCCGACAAAAAAGTTCCCAACATCCCCCCCACTTGAAATTTTAAAACATATTTTTTCGAGCAATATCAAA
ATCTATACAGATTGGTGGCATAAACAATGTATCTTTCGATTTTCTTAGGAAAAACAGCTGGTTAAATCCCAATTTAACTAACTT
CTAAGTTTTTAGGGAGCCTGGTTGTGCCCAGATATTAATTTTAAAAACTTAGTTCGTAATTAGTCTTTATCCGACCACCCCTGT
TTCGTGTTTTTTAAGGAAAAACACTGGTTAACGTCAATTTAACTAACTTCCTACAGTTTTAGGACGGCTGGTTGTGTCGCAGGA
TATTAATTTAAACCCTTAGTACGCGCTAGTTTTCCTCGCCACGCGCGGTTTCTTGCTTGTTTAAGAAAACACTGGTTATCAATT
TAACTAACTATCTAAGTGTTTAGGACCCACTGGTTGTGTCCAGGATAGTCAAGTGTTAAAAACTTAGTTGCGTATTACCGTTTT
TTATCGACCTCCTCCGTGCTTTTTGGAAAAAGCAGCTAATTAATCAATTTAACTAACTTCTAAGGTTTTAAGACCTGGTTGTGT
CCAGGGATAACTCCATTTAAAAACTTAGTTCCGCGTTAGTTTTATCGCGACCACACCTCGTGCTTTTTAAGAAAACACCTGGTT
AATCTATTTAACTAAATTCTAAGTTTAGGTCCTAGATGTGTTCCAGGCCCCTTATTAATTTAAATACTTAGTTCGGCGTTAGTT
TATCGACACCGTCGTGCCTTTGTTTTAAGAAAAACACCTGGTTAATCAATTTTAAACTAACTTCTAAGTTTTAAGACCTGGTTT
GTGTCCAAGATACTCAAATTGTAAAAACTTAGTTTCGCCATTAGTTTATCGACAACTTCGTGCCTTTTAAGGAAAACAGCTGGT
TAAATCTAAATTTTAACCTAACTTCTAAAGTTTTTAGGACTGGTTGGTGTCCCAGGATATCAATTTTAAAAACTTATTCGCGTT
AGTTTTATCGCACACCTCAGTGCTTTTTTATGAAACACTGTTAATCCAATTTAACTAACCTTCTAAGTTTTAGGAGCCTATTGT
GTGCCAGGATATCAATTTAAAAACTTAGTTTGGCATTAGTTTTATCAACAGCTTCGTGGCTTTTTAGGAGAAAAACACCTAGTT
TATCAAATTTAACTGTAAATTCCTACAGTTTTTAGGACGTATTTGTGTCGCAAGGATACTCAATTTACAAAAATTAAGCTCACG
TTAGTTTTATCGACAACTCGTGTTTTTTTAGGAAAAACACTGGTTAATCAAATTTAACTTAATTCCTAAGTTTTTAAGACCTGG
TTGTGTCAGGATATCAATTTAAAAACTTAGTTCGGGAGGGGGGTTTATCGACCACGTCGTGTTTTTTTAGGAAAAAAACTAGTT
ATATCAATTTAACTAACCTTCTAAGTTTTGGACCTGGTTGTGTCCAGGATACCTTAATTTAAAAACTTAGTTCGCGTTAGTTTT
ATCCGAAACTCATGTTTCATAGGAAACACTGGTTAATCAAATTTAACTAACTTTCTAACGTTTTAGGAGGGCGGCTGGTTGTGC
CCCAGGATAATTAATTTAAAAACTGTAGTTTCGCATTAATTTTATCGACACCGGGTCCGTGTTTTTTTAGGAAAACACTGGTTA
ATCCAATCTAACTAACCTTCCTAAGTTTTAGGACCTGGTTGTGTCCAGGATATTAACTATAAAAACTTGTAGCGGCGTTAGTTT
TATCGAGCCACCCTCTTGCGTTTTTAGAAAAACACTGGTTTAATCAATTTAACTAACTTCTAAGTTTTAGGACCTGGTTGTGTC
CAGGATATCAATGTTTAAAAAGCTTAGTTCGTATTTAGTTTTATCTAAGCTCCCTCGTGCTTTTTAGGAAACCCACACCTATTA
ATCAATTTAATCTAACTTTCTAAGTTTTTAGGACCCGTGGTTTGTGTCCAGGATATCATTTAAAAACTTAGTTTCGCGTTAGTT
TTACTCGACCCCTCCGTGCTTGTTTAAAGAAAAAAAACACTGGTTAATGCTATTTAACTAACTTCCTAAGTTTTTAGGGCCCTG
GTTGTGTCCAGGAATACTCAATTTAAAACCCTTAGTTTTTGCAATTAGTTTTATCAGCAAGCTCGTGCTTTTTAGGAAAAACAC
TAGTTACATCAATTTAAATAACTTCTATGTTTTAGGACCCTGGTTTGTGGTCCAGGATATCATTTCAAAAACTTAGTTCGAGTT
AGTTTTATGCGACACCGCCGTCGTGTTTTTTTAGGGCGAAAACCACTGGTTAATCAGTTTTAAGCTAATTTCTCTAGCAGTTTT
AGGTCCTTCGATCTGTCCAGGCATTAATTTAAAAACTTAGTTCTTGTTAAGTTTTTTATCCGAAAAACCTGAAGTGCTTTTTTA
AGAAAAAAGCACTGGTTAAGATCAATTTATACTAACGTTCTCTTTTTAGGAACCTGGTTGTGCCCAAGATATCAATTTAAAAAC
ATAGTTTGGCATTAGTTTTATGGACCCGCCTCGTGTTTTTTTCGGAAGACAGCTAGTTATATCCAATTTAACTAACTTCTAAGT
TTTAGGACGCTGGTTTGTGTCCATGATATCCAATTTAAAAACACTTAGTTCGTGTTAGTTTTATCCGACCGCCTTCGTGTTTAG
GAAAAACACTAGTTTAACATCAATTAAACTAACTTCCTTACAGTTTTAGGACCTGGTTGTGTCCACGGATTATTAATTAAAAAA
CTTAGTTCGCGTTAGTTTTTATCGACACCGCACCTGTGTTTAAGAAAACACTGGTTTAAATACAATTTTAACTAACAGCTTTCC
TAAGTTTAGGTCCCTAGATGTGTCCAGGCCTATTAATTTAAATACTTAGTTTCGCGTTAGTTTTATCGACACCCGTCCGTGCTT
TTTAAGAGAAACACTGGTTAGATCATTTAACTAACTTCTAAGTTTTTAAAGACCTGGTTGTGTCCAAGATAATCAATTTAAAAA
CTTAGTTTGCATTAGTTTTTTAATCCGAGCAACTCCGTGCTTTTAAGGAAACAACACTGGTTAATCTATTTAAACGTAAACTTC
CCTAAGTTTTAGGACCTGGTTTGTGTCAGGTTATCAATTTAAAAACTTAGTTCGGCGTTAGTTTTATCGACCACCCATCCGTGG
GCTTTTATGAAAAACACTGGTTAATCCATTTAACTAACTTCTAAGTTTAGACCCTGGTTGTGTCCAGGATATCCAATTTAAAAA
CCTTAGTTTGCATTAGTTTTATCAACAGCTGTGCTTTTTAGGAAAAACAAATAGTTATCAATTTAACTAAATTCTAAGTTTTAG
GAGCGCTATTTGGTGTGCCAGGATATCAATTTAAAAATTAACTGCCACGTTAGTTTTACGACCAACTCGTGTTTTTTTAGGAAA
AACACTGGTTAATCAATTTAAACTAAACTTCTAAGTTTTTAAGACCTGGCTTGTGTGCCAGGATATCCAAATTTAAAAACTTAG
TTCCAGTGTTTAGTTTTTATGCGAGCACCACGCTCCGTGGTTTTTTAGGAAAACAACTAGTTATCAATTTAACTAACTTCTAA
GTTTTTGTACCTGGTTGTGTCCAGGATATTAATTAAAAACTTAGTTCGCGTTAGTTTTATCGACACCCTTCAATGTTTCTTAGG
AAAAACACTGGTTACTATGCAATTTAACGTAACTTCTAAGCTTTTAGGACCTGGTTGTGCCAGGATATTAATTTTAAAAACTTA
GTTCGCATTAGTTTTATCCGACCCACCTCTGTGTTTTTTAGGAAAAACACTGGTTAATCAATTTTAACCTAACTTCTAAGTTTT
AGGAACCTGTTGTGTCCCAGGATATTAATTTAAAAACTTAGTAACGCGTTAGTTTTATCGACGACCTCCCCTTGCTTTTTAAGA
AAACACTGTTAATGCAAATTTAACTAACCTTCTAATTTTAGGAACCTGGTTGTGTCCAGGATATCAAACTTTAAAAACTTAGTT
TCGTATTAAGTTTTATCAACGGTCCTCGTGGCTTTCCTAGGAAAAAACACTAAAATTAATACATTTACTAAACTTCTAAGTTTT
AGGACCTGTGTGTCCAGGATATCATTTAAAAACTTAGTTCGCGTTAGTTATTCCCGACACCGGTCGTGCTTTTAAGAAAAACAA
GCCTGGTTAAATCTATTTAACTAACTTCTAAGTCTTTAGGACCTTGGTTGTCCAGGATATCAATTTAAAAACTTAGTTTGGCAT
TAGTTTTAATCAACAGCTCGTGCTTTTTAGGAAAAAACACTAGTTAATCAATTTTAACTAACTTCTATGTTTTAGGAACCTGGT
TGTGTCCAGGATATCAAGTTCAAAAACTTTAGTTCTGGCGTTGTTTTAATCGACACCCTCGTGTTTTTTTAGGGAAACACTGTT
AATCAGTTTAACTAATTTTCTACGTTTTAGGTCCTTGATCTGTCCAGGCTATTTTTACAGTTTTTGTTAGTTTTATCGAAAACC

GTCGTGCTTTTAAGAAACAAACACTGGTTAATCAATTATACTTCTGTTTTACGGACTGGTTTGAAGATATATTTAAAACCAATA
GTTTGCATTAAGTTTATCCGATCGTGTTTTTTCGGAAAGACACTAGTAAATCAATTTAACTAACTTCCTAGTTTTAGGAACGCT
GGTTGTGTCCAATCGATATCAATTTAAAACCTTAGTTCGTGTTAGTTTTATCGAGCCGCGCTCGTGTTTTTAGGAAAAACACTA
GTTTAATCAATTAAACTATTCAAGTTTTAGACGCTGGTTGTGTCCCAGGATATCATTTAAAAATTACGTTCGGTAGTTTTATCG
ACATCGTGCTTTTTATGAAAACACTGGTTAATCAATTTAAACTAACTTCCTAAGTTTTAGGACTGGTTGTGTAGTTAATTTAAA
ACTTATTTGCAATTAGTTTTAATCAAACAGGCTCGTGCTTTTTAGGAAAAACACTAGTTTATCAATTTAACAGCTAAATTCTAA
GTTTTAGGACCTATTTGTGTCTAGGATATCATTAAATTAATCACGTTAAGTTTTTATCGACACTGCTCGTTTTTTTAGGAAAA
ACACTGGTTTCAATCAATTTTAACTAGAACTTCTAAGTTTTTAAGACCTGGTTGTGTCCAGGATTCAATTTAAAACTTACGTTG
TGTTGTCTTTATCGATGTGTTTGGCAAAACACTAGTTAATTCAATTTAACTAACCTTCTAAGTTTTTTTGGAACTGGTGTGTCC
CAGGATAGTTAATTTAAAAACTTAGTTGGTTAGTTTTATAGACTCCCTCATGTTTCCTTAGGAAA
>m141120_105518_42134_c100732402550000001823141405141504_s1_p0/161652/0_6058
ACATAGAAGGATGTCTATAGCATGGAGGAGTGGCTCTAAGGACTTTTCTATTCCCAATGTGGGGTTCTATGATGAAGAATATGA
TAATACTCGGAGTTACGTTCAGCCCCTAATACTAATATTATTGAGATTGGAAACAGGTTCAAAAAAATTTAGAGCTTTTAAGCG
TGAGAAATATGAGTGAATTACGAGCATCGCTCCCCGGAGCCTAGGCAAAGCTGCCCTAGAGATTGGCTTGGGAAAGGTTTCAAT
ATTTTAGTTTGGGTTTGAATTAGGACCCCAATTCGCATGGTGCCAATAATTCCAGCCCCTAATATAAATATTTATGCTAGATGG
GAAAAAAGGCTTCAAAAAAATTAGAGCTTGCTTAGAGGAATATGTGTGAAGTTACGAGATCCGCTATCGGAGCCCCCTAAAGCA
GCTGCACCTAGGAGATTTTTGGCGCTGAAAGGTAATTTATTTTGTTTGGTTTGAATGAGGACCCTAATTCATGGTAAATAACTA
GTCGCTATATAATATTAATGAGAATGGGAAAAGGTTCAAAAAAATTAGAGCTTGTTTAGGCGAGAAATATGAGTGAATTTACGA
GATCGCCTCCGGAGCCCTAAAAACCGGCAGCTGCCTAGTGAGATTCTTGGCTTGAAACAGTTCTCAATTTTTTGTTTGGTTTGA
ATTAGGGACCCCCCAATGTCATGGTCACTAATGCGACGCCCTATATAATCACCTTATCTGAGCAATGGGACAAGTGTCTTCCAA
AAACAAATTAGAGCTTGTTAGAGACGAAATATGAGCTGAATGCTACGAGATCGCCCATTCGGAGCAACGCCGCACAACCGCTCA
ACAAGAGAAGCCACAAACCCAACCAACAACGAAGCCCACAGAAACGTCACGGCCAGCTCCTTATGAGAAATTTGGCCCTGAAAG
GTCAATTTTTTAGTTCTTTGATTGATATTAGGACCACCCAATTCATTGTCCCCAATCATAGCCCTATAAAATTTATTGAAGAAT
GGGAAAATAGGTTTCCAAAAAAAAATTAGAGCTTTCTTAGAGAGAATATGAGTGAATTAGCGGAATCGCTCCCGTGAGCCCCTAG
GCAACTGCCTAATGGAGACAGTTTGGCCTGAAAGGTCCAATTTTTTTGTTTGGTTGAATTATGACCGCACATTAATGGTGCAA
TGATAGCCCCTATATAAATTTTATTTTAGATGGGAAAAAGAGGTTTCAAAAAATTAGATCGTTGTTTTAGAGAGAAATATTGAG
TGAAGTTACGGAAGATCGCTTCGGAGCCCTAGGCAACTGCCGCTAGGAGATTTGGGCTGAAAAGGTCAATGAATTTTTTGTTGT
GGTTGAATGTAGGACCAATTCATGATAAATACATAGCCCTATATAATAATTATTGAGATGGGAAAAGGTTCAAAAAATTAGAGC
TTTTAGGGAGAAAATATGAAGTGAATTACGAGATCGCTGTCCGGAGCCCTAGGCACGCTGCCTACGAGATTTGGCTTGAAAAGT
TCAATATTTTAGTTTTGGTTGATTAGGAACCCAATTTCATGGTCAATAATAGCCTATATAATATTATTGAAGATGGGAACAGGT
TCAAAAAAAATTAGAGCTTCTTAGAGAGAAAATATGTGTGAATTACGAGATGCTTGCGGAAGCCGCTAAGCAGGCTGCGCTAGG
AGATTTTTTGGCCTGAAAGGTCAAAATTTAATTTGTTTGGTTGAATGAGGACCTAATTCATGGTAAATAAATAGTCCTATATAA
TATTTAAGTGAGATGGGAAAAAGGTTCAAAAAATTAGAGCCTGTTTAGGGAGACAATAATGAAAGTGAATTACGAAGATCGCTA
CGGAGCACCTAGGCCAGCGCTGCCTAGGAGATTTGGCTTTGAAAGTTCATATTTTTTTGTTTGGTGAGATTAGGACCCCAAATT
ATATGGTCAATTAATAAAGCCCTATATAATCATTTATTGAGATGGGAAATGTTTTCAAAATAAAATCAGAGCTTGTTAGAGAGA
TATATGAGTGAATTACGAAGATCGTTCGGAAGCCGCTAAGGCAGACTGGCGCTAGGAGATTTGGCCTGAAAGGTCAATTTTTTA
GTTTTGGTTGAATATAGGACCCCCAATTCATTGTGCAATCATAGCGTATGAAAATATTATTGAGATGGGAAAAGGGTTCAAAAA
AAATTTAGAGCCTTCTTAGAGAGCAAATGATGAGTGAATTACGGAATCGCCTTCGGAGCCCTAGGCCAGGCAAGCCTAGTTAAT
ATTGCTTGGCTGAAAGGGTCCAGAATTTTTTAAGTTGGGTTTGGAATAAGGACCCCAATTCATAGTCAATAATAGCCCTAATAT
AATATTATTGGAGAGGGGAAAAAGGATCAAAAAAATTAGAGGCTTGTTAGGGAGAAATATGAGTGAATTACCGGAATCGCTCCG
GAGCGCCTAGGCAGCAGCCTAGGGATTTTGGCCTGAAAGGTAATTTTTTTTTGTTTGGCTTGAATTATGGAGCCCAATTACATG
GTCAATGATAGCCGCTATATAATTTTATTTAGATGGAAAAAGGTTCAAAAAAAATTTAGAGCTTCTTAGAGAGAAATATGAGTG
AATAGCGAGATCCGCTTTCCGGAGCCTAGGCCCAGACCTGCCTAAAAAAGGAGGATTTGGGGCTGAAAGGTCAATTTTTTTTGT
TTGGTTGAATTAGGACGCCCAATTTCATGGTAAAAGTAATACCCTATATAATATTATTTGAGAATGGGAAAAAAGGTTCAAAAA
AATTAGAAGCTTGTTAGGGGAGAAATATGAGTGAATTACGAACATCGCTCCGGAGCCATAGGATGCTGCAGGGGAGATTTGCTT
GAAAGTTCAATTTTTTTGGTTTGGTTGAATTAGGACCATTCAATGTTCATAATACGCCCTAATATAGAATATTTATTGAAGATG
GGTAAATGGTTCAAAAAAATTAGAAGCTTGTTTAGAGAGAAATATGAGTGAATTACGAGATCCGCTTCGGAGCCCTAGGCATGC
TGCCTAGGAGATTTGGTCCTGAAAGGTCAATTTTTTTGTTTGGTTGAATTAGACACCATTCATGGTCAATATTAGCCCTATGTA
CATATTATTTGAGATGGGAAAAATGGATCAAAAAATTAGAGTTTTTAGAGAGATATGAGTGAATTACGAAGATGCGCTTTCGGA
GCCCCTCAAGCAGGCTGCCTAGGAAGATTTGGTCCTGAAAAGGTCCATTTATTTGTTTGGTTGAATGAGGACCGCTAATTCATG
GTAAATAAGATAGTCCTATATAATATTAATTGAGAATTGAAAGAGGTTTTCGAAAAATTAGAGCTTGTTATGGAGAAATATGAG
TGAATTACGAGATCGCTAGGACCTAGGCAGCTGGCCTACGAGATTTGGCTTGAAAGTTCAATATTTTTGTTTGGTTGGAATTAG
GACGCATCAATTCATGGTCAATAATAGCCCTATTAATAATATTATTAAGATGGGAAATGGTCAAAAAAAATTAGAGCTTGTTAG
AGTGAAATATGAGCTGAATTACGCAGATCAGCTTTCGGAGCCCTAGGAGCTGGCCTAGGGAGATTTGGCCTGAAAGGTCAATTT
TTTTGTTTGGTGAATTAGGACCCCCGAATTCCAATGGTTGCAATAATAGCGCTATATAATATTATTGAGATGGGAAATGGATCA
AACAAAAATTAGAGTTTCTTAGAGAGAAATATGAGTGAGATTGACGAGATCGGGTTCGGAGCCCTAAGCAGGCTGCTAAAGGAG
AATTGGCCTGAAAAGGTCCAATTTATTTGTTTTGGTTGAATGAGGAGCGCCTAATTCATGGTAAAATAATAGTCGCTATAAAAT
ATTAATTATGATGGGCAAAAAAAGGTTCAAAAAAATTTAGATGCCTTGTTAGGGACGAAATAAATGAGTGGAATTACGAGAATC

GCTGCGCGGAGGCCCAAAAGCAGGCTGGCTAGGAGATTTGTCTTGAAAAGTTCAATTTTTTTTGTTTTGGTTGGAATTAGGGAC
CCAAATTGAATGGTCAATATAGCCGCTATATAATATTATGAGATGGGAAAATGGTTTCAAAAATTTAGAGCTTGTTAGAGAGAA
ATAGTGATGAATTTAAACGAGATCGCTTCGGAGCCTCAGGCAGGCTGCCTAGGAGATTTGGCCTGAAAGTCAATTTTTTTGTTT
GGTTGAATTAGGACCGCCCAATTCATGGTCAATAATAGCCCTATATATATATTATATGAGATGGGAAATGGATCAAAAAAAATA
TTAGAGCTTCCATAAGAGAGAAATAATGAGTGAATTACGAGATGCGCCTTCGGAGCACTAAGGCAGGCTCCTTAGGAGATTTGG
CCTGAAGGTCCAATGTTTTTTTTTGTTTGGTTTGAAATGAGGAACCCCAAATCATGGTAAATAATGAGTCTATATAATATTATT
GAGAAGCGGAAAAGGTTCAAAAAAATTATATTCATGTTAAGGGAGAAATATGAGCTGAATTACGGAATCGCTCCGGAGCCCTAG
GCAAGCTTGAAACTAGGAGATTTGGCCTGAAAGGTACAATTTTTTTGTTTTGGTTTGAATTATGGACCCAATTTCATGGTCAAT
GATAGCCTATATAATTTTATTTAGATGGAGAAAAGGTTCAAAAAAAATTAGAGGTTGTTAGAGAGAAATATGAGTGATTACGAG
ATCGCTTCGGCGCCCTATGCAGCTGCCTATGGAGGATTTGGCCTGAAAGGTCAATTTTTTAGTTTGGTTAAATTAGGAAGCCCA
ACATTCATGTCCAATAATTGCCCTAATAATAATATTATTGAGATGGGAAAAGGGTTCCAAAAAAATTAGAAGGCTTGTTAGGAA
GAAAGATATGAGTGAATTACGGAAATCGCTTCCCGGAGCCCCTAGGCCAGCTGCCTAGGAGAATTTGGCCTGAAAGGTCAAATT
TTTTAGATATGGTTTGATTAGGAACACCCAATTCATTGGTCAATAATAGCCCTATATATATTATTGAGATAGGAAAATGGTTCA
AAAAAATTAGAGTTTGTTAGAGAGATAATATGAGTGAGATTACGAATGCGCTTCGGAGCCTAGGACCAGCTACTTAGGAGATTT
GGCTTGAAAGGTCAATTTTTAGTTTGGTTGAAAATTAGGAGCCCCAAAATTCATTGTCAATCAATAGCCCTAATATAATATTAT
TGAGATGGGAAAGGTTCAAAAAAAATTAGAAGCTTCTTAGAGAGAAATATGAGTGAATTACCGGAATCGCTTTCGGAGCCCTAG
GGCAGCTTGCCTAGGATATTTGGCCTGAAAGGATCAATTTTTTAAGTTGGGTTGAATACAGGAACCCCCAAATTCATGGTCAAT
AATAGCCCTATATAAATATTATTGAAGATGGAAAAGGATCCAAAAAAAATTAGAGGCTTGGTTTAGGGAGAAATATGAGTGCAA
TTAACGGAATCGCTTCGGAGCCCTAAAGGCAGCGGCCTAAGGAGATTTTGGCTGAAAAGGTGAATTTTTAGTTTTGGGTTGAAT
TGAGGACCCCAATTCATGGTCAGATAATAGCCCCTATATTAAATTAGTTATTGGAGATGGGAAATTGTTCAAAAAAATTTGAGC
TTGTTAGAGAGAAATATGAGTGGGAGATTTACGAAATCGCTTGGAGCCCTAGGCCAGCTGCCTAGGAGATTTCGCCTGAAAGGT
CGAATTTTTTAGTTTTGGTTTTGGAAATTAGACCCCCAATTCATGGTAAATAATAAGCCTATTAATATTTAATAGAGATGGGAA
AGAAGGTTCAAAAAAATTAGAGCTTTGTTAGGGAGAAATAATTGAGATGAATATACGAGATCCGCTACCCGGAGCGCATAAGGC
AGCTGCCTACCCGAGATTTTGGCTTGAAAGTTCAAGTATTATTAAGTGTTGGTTGAAATTAGGAGCCAAAATTCATGGTCAATA
ATAGGCCTATATAATATTATTGAGATGGGAAAAGGTTCAAAAAAAATTAGAGCTTTGCTTAGAGAGAAATAAATGAGTGAAATT
AACGAGATCC
>m141120_105518_42134_c100732402550000001823141405141504_s1_p0/65907/13313_19331
ACAACCCAATTCAACAAACCCAAATTTGAGACTAAGTGGCATGGAAGAGCAAGATGGTAGAAAATCATCGTTAAGAACAATTGC
TTAGAACCTAGGTGCAGTCTCTCATGCTTACTTTATATTGCGAGTGTGAATATAGGAAAGCATTGTAGATTCAATATCAAATAA
TAAGACGATGCTTATATTACACATGTATCATGCAAATAAGTGGGAGCTGCGCAACGAGGAATTGACAGTATATATAATGATCAA
GGACATCTCACACCTTATACAAGCATCTTAACATAGGCTTTTCCGTCTACATATTGTAATTTTTATCAAATATAAGTATATTTC
AGCACTAGTTACACACCTCTGTTTCGAAAAACAAAAATATTGAGCATAAACAAGGTACGATTGTTGATATGTTTGGAGTTAACG
AGAACAACACTGTGGTTTCACAGGAAGGTAGGGTGTTTTGGCAGCTGTGAAAGGTGGTGAGATTTTGTTGTTGCCATAGATGCA
ACATAGTGTAGACCAGTGCGCAGGGAACAGAGAGGAAGGAGAAAGCGGTAGAGATTGGAGTGTGAATGGATGGGAGACAAACAA
GCGAACACGTGGAAAGAACAAGAAAGACTAGGATGAGGAAAGAGAACACTGTGGCCCAAAAAGAGCACTCAACCATCTATTGTT
AGAGCAACTCCAAATATTAAGAGAGACTAAAATCTGGAATAAGAGAACTGAGAACTAACGGTAGCTCCTGCGATCAGGTAGAGA
TATTCAAATTTCAGGAATGGCGCCCTAGAACACAACCAATCAACGAAGGGGGCGAAGATCACACCCTAGTCTTCCTGCATCCTC
TTTCGCGGGAGCAGTGGCCGCGCGCGACGACCCTCGTCACGACGCTTTCCGCATAAGAGAAGAGAAAGGCTGAGTATGCGAATA
CCGGTTGCACACATTCATCAAGAGAGGACAAGATGTGTCCGCAATGTGAAGCCAGAACTCAGCCGATTTGAGCAAGAGCACATA
GATAGATAAAACATAATCCTATTTGAACGGAAATGGTTGTCGAACATCAAGAACATAAAGCTGAAAGAGTAAAAGAAAATAATA
AATGTTATGCTAGTTTAAATCCAATAAATTGATCATATATCTAGTTGAGAAGTGCCACCCCATCTCCACTAAGGTCAATTCTAA
AAAGGTTGACAATCGGCCACAGTTTGGTAGAAAGGCTGTTCGGTGCATGGTTTGGCGTCACGTACACGGCAGGAAAACAATCTA
TCTTGATCTTCTCTCTTTTTCCAGAACCCCCAGGCAGTATAACCCAAGGCGTCCGCCGCGTCGAGAATCGTGGTAACGCCACAG
TATTTGAGGAAGTGTGGATGTTAAGGTTGGTGCTAAAAGTAGATGTAGATAAGACGAGTGGTTTATTATTGGGAAAGTAAATTA
GAAAGTCGCATTGTAGTCTTTAGTAAGTGAGGTTGGTTTGTTGATTAGTTATTGAATAATTATTATAATATGTATAAAATTTTT
ATAATTAATTAATGAGAGTGAATGATCTTGTCATGATATGAAAGGGTTAAGAACATAGATAATGAGTTAAGGGAATTATGGCAA
CATGGTTTAAAGGATTAAGTGGTTGCTATACGGTTAACTAACATTTTTAAATGCATTTAAAGAGTGCTGATCTTGTATGCAGCT
ACGAGAGGTTAAGATCATAGATAATGAATACGGAAATTATGGTACGAGTCGCCAGAACTATTACTTAAACTAGTTAGGTAGAAT
ACGTAGAATAGGTAGTAGGTAGTAGAGTAGTAGCAGTACAATCAGTACAGTCCAGCAGCGTAGTAGCAGTCTAGTAGTAGCAAG
CAGTGTCAGCCAGCAGTAAGCAGGCCAGTAGCTACGAGTATAGCAAGTAGTAGTAGCAGTAGTAGTAGTAGTAGTACGCAGATA
TAGCAGTAGTAGCTAGTAGTATGTTATTTTTAGCAGCAGTACAGCAGCAGAGTAGCAGCAGTAGTGAGCAGCAGTAGCAGCAAG
CAGTGTAGACAGTACAGTAGTAGTAGTAAGTAGACAGCAGTAGCAGCCCAGTAGCAGAGCAGTAGCAAGCAGTACAGGCAGCAG
TAGGCAGCCAGCAGTAGTGCAGCAAGCAGTCAGTAGTACCGCAGGTAGTAGTAGCTACAGTAGCCAGTAGGCAGTTAGCCAGTA
GTGCGCCCAGTGCAGTAGTAGTACAGTATTAGTAGCAGCAGTAAGTAGCAGTAGCAGTAGCAGTGTAGCAGTAGCAGTAGAAAG
TAGCAGTAGTAAGCAGTAGCAGTAGCAGCAGTAGTAAAGCAGGTAAGCAGTAGTAGCAGTAAGCAGCAAGTAGTAGTGTGGTCG
TGGTGGTGGTCGTGGTGGTGTCGTAGTAAGTAGTAAGACAGTAGCAGTAGCTAGTACAGATATGCAGTAACAGTAGCAGTAGCA
GTAGGCAGTTTGCAGTAGTCAGTTAGTAGTAGTAGTAGTAGTAGTAGTAGTAGTAGTAAGCACGGTAGCAGCGCAGGAGCCAGT
AGCAGTAGCAGTAGCAGGGGAGGAGTAGTAGCAGTAGCAGAAAGGAGCAATAGCAGTAGCAGCATAGCAGTAAGCAGATAGCAA

GTAGCAGTAGCAGGTAAGGAGCAGTAGCCAGATAGCAATAGTAGTAGTAGCAGTATAGTAGTAGCAGTAGAGTACTAGTAGTAG
TAGTAGTAGTAGCAGTAGCACCGCAGTAGCAGTAACAGTAGTGCAGTAGACAGTAGCAAGTAGCTTATACAGTAGTAGCAGTAG
CAGTAGCAGTAGCCAGTAGCAGGTAAGCAGCAGTAAAAGCAGTACCTTCGTAAGGCAAGTAGCAGTACAGTAGCAGTAGTAGCA
GTAGCAGTAGCAGTAGCGTAGCAGTAGCAGTAGCAGTAGTAGAATAGCAGTAGTAGTAGGCAGTGTAGGCAATAGTAGCAGTAG
TTGTAGTAGTAGTAGTAGTAGTAGTAGTAGAGTAAAGTAGTAGCAGTCAGCCCAGGAGGCAGTAGCAGGAGCCAGGTAGCAGTAGCA
GGAGCAGTAGCAGTAGCAAAGCATAAGTAGCAGTAGCAGTAGCATAGTAGCAGTAGCTAGCAGTAGCAGCCTAGTAGTAGTAGT
AGTAGTAGTAAGTGAGTAGTATTAAGGGTAGATAGCGGTAGTAGGTAGTTTAGTAGTAGTAGTATTAGGGGCAGTAAGCAGCAG
GAGCAGATAGCAGTAGCAGTAAGCAGATAGCAGTAGGAGTAGTGGTAGCCCACATAGCAGTATGCAGTAGCAGGATAGTATCAG
TAGCAGTAGTAGTAGTAGTAGTAGTAGTAGTAGCAGGTAGTAGTAGTAGTAGGGGAGTAGTAGCAGAACAGTAGCCCAGGCTAGCAG
TACAGTAAGGAGTAGGTAAGAAGTAGCCAGGTAGAAGCAGTAGAATAGCAGTAGCAGGAGTAGCAGTAGTAGCGTAGGCAGCAG
TAGCAGCAGTGGATCGTGGTGGTGTCGTGGTGGTGGTCGGTAGTAGTAGTAGTAGTAGTAAGTAGAATAGCTCAGTGCAGCAGC
AACAAGACAGAAGGGGACAGCCATTAGCAGTACCGAGGGCCATAGGCCGGAGTAAGCAGAAGAGACAAGTGTTCAGCAAGAAAC
AGCTACGGTCGAGAGGCGACATGGGAAACTGGAATCAGATTATGCAGCAGAGATGAGCGAGAATGATCAATATTCATAGCAGTA
GCCCGCAGTTATGTCAGCAGCAGTAGTTAAATAGTTAGCTCGAGGTACAGACACATAGCTCTCATCAGAGGTAGCGAGCAGGTA
GCAGCAGTGGATTGCTGGTGTGGTCGTGGTGGCTGTTAGCATGTATGACAAGGTACCAGTAGCAGGTCGAAGTAAGATTAGCGG
TACGACGAGAAGCAAGCAGCAGCAGCAGTAGCAGCCAGCAGCAACACAGTATGCAGCAGCTAGCAGCAGATAGCAGGTAATAAG
CAGCTAACAGACAGCAGTGCAGTAGGAGTAGTACAGTAGCAGTAGCAGAGTAGCAGACAGTAGTAGTAAGTAGCAGTAGTAGCA
GTAGCAGCAGGTAGCAGCAGTAGCAGCAAGTGGTGGTGGTGGTGGCGTGGTGTGGTCTAGTAGTAGTAGTAGTTAGTTAGA
GTAGAATAGTAGTAGTAGTAGTAGTAGTGATAGTAGTAGTAGTAGTAGTAGCATAACCAGTAGCAGTAGCAGTAGCAGTAGCACAGT
AAGCAGTAGCAGTGACAGCAGTGAGTAGCAGTAGTGAGCAGTAGCAGTAGCAGTAGCAGCAGTAGCAGTGCAGCAGTAAAGTGC
AGTAGCAGCAGTGTCGTGGTGGTGGTCGTGTGGTGGTAGCAGTGCAGTAGCAGCAGCAGCAACCAGCAGAGAAGCAGCTGGGCA
GGGAGCATGAGCAGCAGCAGCAGTTATAGTATGGTTTTATTACTCTGGTTTTTGGTTCAAGCCCTGGGAACTTAGGTTGGTAAG
TAGTAGTAGTAGTAGTAGTAGTGTAAGTTATAAGTATGTTTATTACTGTGGTTTTTGGTTCAGCCGGGACCTTATTTTTGAACA
CTCGACAACACCTGATAGATGTTATTTAGGTAGTTTAAGAATATATAATTATAACATAAAGGAATTTTGAGTAATATAGGGTGA
TTAATAATGATGTCATTTACTTAATACTCGATAAGGTTTAGATAGAACTAATACAGGTCACTGCACACTTCGTCGGTGCATGTT
TATTAAAGGGGTTAAGTATTTTGATGAATATGTTTTAATTAACAAGAAAGATCTGAAAGCTCTAGATACCTTCCAGCAACTTTA
AGAATCGTATTATGACTCAGTCAAAGATGTTTATCCAATCAAATTAAGTGTGATAAAGTGCAAGTTAATGTCTAATATTTCAAT
GTTGCGTGATAGTAGGTTCGCAACACTACATAGGGGCCCGATAATCGCCACACAAGAGATACGAAAAATGCGTCAACTTCGCAC
GATCAAGAACCACGAGCACTTCAGGAACATGGCCAGATGAATATCGGCCCTAGGACGGCATTTTCAAACATAATAGTAGGATAC
GAGCGTTGATCATCCTTAACCTTGAACGTTTTTGACCGAGTCTTAAGACTTGGTGTGAACAAATATTCAGAATATTGGTTCAAT
TAATAGCAGCTCATTATTTTATTCAAGCTAAAAGAAGATACTTTCACACCTTGGAAACTCTATAAAATAGGATCTAGTAACCAG
CACATTTCTCTCATTCTCAAAGCTGTTGTATCCAGGCCTCAAGCCTGCTAGGGTTATATAGAGTGAATACACTTGGGTTTGGGA
TCAAAGCTTTATCACCTTAAGCTTTAAGAAAACACTTGGGAAGTGAGATCATAGAGTGTGTTTTCGATATCGAGGTGTAGATCG
GTCCTTCCTGAGTACATCCTAAAGGTTATTCTTATTCTTATTCTTAAGTTCATTTCCGTGTAAGTTCTTTAGTATTTCTGTTAT
TTTCTTTTCTCAAATCCTAACTCGCTGTTTTCGATTCTGGGTTAGGTATTTAAGTTCTTTGAACTTAACGCGCCGTTTTTTTCG
GGTAAGCTTCTTCTTGGTGGTTTAGTCTCTTCTTCTTATCAATCTCTTATCTTTAGAAATACTCACCATTCTTATTCGTTGCGT
CCGCTTTAGGAGTGTTCCAAACTCCGTCCTTGTTCTAAAAATAGCGGGGTGTATGGTAAGGCAAATAGGAATACGATTTTATGC
TGCTTTATGTTTTTATATGCTATGTTTTGAGTATGATTGGATGTATGATAATGGTTTTTAGTTCTTGCAGTATGATGTGCTGTT
ATAGATACACAAGCATACTACACGGTGGTTCTAGATAACACCATCATACATTTGTTTAGATAACAAGTCCAGTAATATGATTCC
CAATGGGCATTATGATTTGTTTAGATAACGAGCCATAAATCTATATGCTTGGTTTAGATAACTAAGCGCGCCGTAAATTTTATA
GGCATATGGTGCTAGATTAGCAAGCCCAAGAGTATGATGGCCATTGTAATACTT
>m141120_105518_42134_c100732402550000001823141405141504_s1_p0/67652/4167_10877
AAAAACAACGAATCCAACTTGATTTTTACAATTAACATTCTTAAAGTTTTATGACGCTGGTTATGTCCAGGAACTTAGTTTTTA
AAATACTTAGTAGGGGCATTAATTTTATCCCACTAATATCAACGCCTCTATAAGAAAAACAACGATCAACTTGATTTAACATTA
ACTTCTACAAGTTTTATGAACCTGGAATATGTCAGAGGAAGCAATAGTTTAAAACTTAGTTCGCGTTAATTATTTATCACTATC
ATCGATGGGCTCTTTAGAAAAACCAAAGATCAGAGCGATTTAAATTACACTTCTAAGTTTAATCGACTGCTTAATATCCAGGAA
CTTAGTTTGAAAAGCTTAGTTCGTGTTTAAATTTTTACAATATCATCGTGCTCATTTAAGAAAAACAAGATCAATTTGCTTTTG
CTTTTAAGTTTATCGCACTGGTCATCGCGAACCTTAATTTGAAAACTTAGTTTCGCGTTGAACTTTTAATCAATATTCCGTTTC
TCTTTAAGAAAAACAATGATCCTTATTGACTTCTCACACTTCACTGCTACACGTTACTGCACGTGGTATGTCCAGGAGACCTTA
GTATCGAAAACTAGTTCGGCGTTAATATTTTATAATATCATTCGTGCTCCTTTCAGCACACCACACGACTCAGATCGATTTAGA
ATAACTTCTAAGTTTTTATGTGTTATACTCGCGAACTATTGAAAACTAGGTTCGGCGTTAATCTCTCTCTATCACACCGCTGCT
CGCTCTTTACGACAAACGATCAAATTTGTTTATTACTTCTAAGTTTACTGAACAGGTTATGTCGAGGAACATAGTTTAAAACTT
AGTTCAGCGTTACATTTTTTTTCACCATATCGTGCTCTTCTAAGAAAACAAACAACGATCAACTCGATTTAAAATTAACTTCTA
AAGTTTCTATGACCTGGTTATAAATCAGGACTTAGTTTGAAAACTCACGCTCTCCGCCGCTTTTCTTTCATATTCTCGCTGTGT
TCTAGAAAACAACGATCACCGTGATTTAATTGAAATTCTAAGTTTTATCCACCTTGGTTAATGTCCAGCGAGACTTAATTTGAA
AACTTAGTTCGCGTTAACTTTTATCAATATCAGCGTTCCCGTTTTTAAGAAAAACAATGATCAAGTTGATTTAAATTAACTTCC
CTAGGTTTTATGACGTGGGGTTATTGTCCAGGAACTTAGTTCGAAAACTTAGTGTCGCACGTTAATTTTTATGCAATACTCTCG
TGCTCTTTAAGAAAAAACAACCGCATCAACATCTCGTTGATTTAAATTAACTTCTAAGCTTATGCAAACTGGTTATTGTCCAGG

AACATACGTTTATAAAACTTAGTGTCCGCGTATAATTTTATCAATTCTCGTGCTCTTTAAGAAAAACAACGATAAAATTGATTT
AAATTAAACTTCAAAGTTTTATGACCTGGTTTATGTCCAGCGAACATAGTTTGAAACTTAGTTCGCGCAGTTTGATTTTTGAAA
TCAATATCTCGTGCTTTTTAAGAAAAACAACGATCAATCGATTTAAATTCAACTTCTAAGCTTTTCATGACCTGGTTATAGTCC
GCAGGAAAAAAACAATAGTTATTGAAAACCTTAGTTCGCGTTAATTTTTTATGCATATCGTCGTGCTCTTTAAGAAAAGCAACTG
ATCAAATTGATTTAAATTGACTTCTAACGTTTTATGACGCTGGTTATGTCGGGGATACTATAGTGATGAAACAGCTTTAGTTCG
GCGTTAATTTTTATCAAATATCTGTCAAGCCCTTTCAAGAAAAAAAAGATCAATTCAAATTTAAACTTCAATCTAAGTTTTATG
ACCTGGTTAATGCCCAGGAGACTTAATTTGAAAACTTCTGTCAGCGTTGATTTTTTCAATATCTCGTGGCTTTTTAGAAAACAA
ACGATCACTCCAAACGAGTCGATTTAAATTAACTTCTAAGTTTTATGCGACTGCGTTATGATCCAGCGAACATAGACTTCTAAA
ACTTAGTTCAGCGGTTAATTTTCTATGCAAACATCTCGTGGCTCTTTAAGAAAAACAACGATGAAATTGTTTAAATTTAACGTC
TCTATGTTTTAGGACCTGGTTATCGTCCGGGAACATAGTATTGATAAAACCTTAGTTGCAGGCATTAATCATTTATCAATATAT
CCCATGCACTTTAAGAAAACAACGATCAATCTGATTTAGAATTAACTCTAAGCCTTTATGACTGGCTTATGCTCCAGGAACATA
AGCTTTAAAACTTAGTCTGCGTTAAGTTTTTATCAATATCCATCAGTGGCTCTTTTAAGAAAAACAAACAATCAATTGATTTCA
ATTAACTTCAAGTTTTATGACGATGGTTTAATGTCCAGCGAACATAGTTTGAAAATTAGTTCGGGCGTTGATTTTTATCAATAT
CCTCGTGCCTTGGTTTAAGAAAAACAACGATGCATTCTCTCCGAATTTAAATTAACTTCTAAGTTTTATGACTGGGTTATAATC
CAGAACATAGTTTTAAAACTTAGTTCGGGCGTTAATTTTATCAATATCCCCGTGCTCCTTAAGAAAAAGCAACGATCAATTGAT
TTAAATTGAACTTCCCTAAGTTTTAATGAGCGGCTGGTTATGATCTGGTTCGGAACTTAGTTCGCCGTTAATTTTTTATCAATA
TCTTGTGGCTCTTTAAGAAAACATCCGATCAAAACTTTCAATTTAATATTAACCTTCTATGTTTTATGACATGGTTAAATATCC
AGGAACCTTATATTTGAAACTTAGTTCGGGCGTTAATTGTTTATCATGTCTCGTGGCTCCTTTAAGAAAAACAAGGGGGGGGGG
GGGGAATCAATTGATTGTAAATTAACTTCTAAGTTTTATGACCAGATGTTATGTTCCGAGGACTTAGTTTGAAACCTTAGTTC
GGCGTTAATTTTTTGTAAATATCCTTGTCTCTTTAAAGAAAAAACAACATCAAATTCAATTAAATTAACTTCTAAGTTTTTTAT
AGACCTGAGTTCATATCAAAGGAACTTAATTTTGAAAAACTTAGTTCGGCGTTAATTTATCAATGTCTCGTGGGCTCTTTAAGA
AAAACATCGGATCAATTGATTTATTAACCTTCTAAGTTTATGACTGGGTTATAGTCCAGGAATTAGTTTTAAAACTTAGTTCGC
ATTAATTTTTATCAATAATCATGCTCTATAAGAAAAACAACGATCAATTGATTTAAATGAACTTCCTAAGTTTTTACTGACCTG
GATATGTCCAGGAACATCAGTTTAAAAAAACCTTCCCGTTCGCGTTAATTTTTATCCAATATCTCGTGCTCTTTAGAAAACGAA
CGATCATACAGATTTAAATTAACTTGCTAAGTTTTATGACCTGGTTATATCCCCAGGAACTTAGTTTGAAAACTTATGTTTCGT
GTTATATTTTTATCAATATCATCGTGCTCTTTAAAGAAAACAACGATCAACTTGATTTTAATTGAACTTTTAAGTTTATGAACC
TGTTATGTACAGGAACTTATTTGAAAAACTTAGTTCGCGTTAACTTTTTCAATTATGCTCGTTCTCTTTAAGAAAACATGATCT
AAAAATAATATAAATATAATAATGACGAATAGAACTTAGTAATAGAAACAACAAAGTTAATATAATACAAATATACAAAAAAAA
CAAAAAAAAAGTGCAAATATATATAAGACAAATCGATTTAATTAACTTTCTAAGTTATGGAACGCTTCGGTTATATCCAGGAAC
TTTAGTTTGAAACTTGTTCTGGGTTACATCGATTATGTATCAAATGTCTCGCTCTTAAGAAAAACAGCACGATCAACCGCTCGG
TTTAAATTACGACTTCTACAGTTTTATGAACAGCGTTACTGTCGATGGAACATCAGTTTCCAGCAAAACTAGTATCCGCGTTAA
TTTTTATCAATACTCGTGAGAGAGACTCTTAAGAAAACGATAATGACGATTTAAACTTAAGAATAAGCCTGGTTATCATCCAGG
AACGTTAGTTTGAAACACTTAGTTCCGCCGTTTCAATTTTTTACTCAACGTATCTCCGTGCTCTTTAAGAAAAACGAACGATGC
CAATTGATTTAATTGAACTTCTAAGTTTTTATGCCACTTGGTTATGCGTCCAGGAACTTAATTTGAAACCTTAAGTTCGCGCTT
AAACCTTTTATCAATATCTCGTTCCGTTTTAGAAAAACAATGATCAACCTTGATTTAAATTCAACTTCTAGGTTTTATGACGCT
GTTCATGTCCAGGAGAACTTTAGTTCGAAATCTTAGTTCGGGCGTTAATTTTTATAAATAATGCTCCGTGCTCTTTAAGAAAAA
CAACGATCAAATTGAGTTTAAGATTAACTTCTAAGCTTTAATGAACTGGTTATATCCAGAAGCATAGTTTAAAAACTTAGTTGC
GTTAATGTTTTATCGAACCTATCTCGATGCTCTTAAGAAAAACAACGATCAATTTGATTTCAAATTAACTTCAAAGTTTTATGA
GCCTGTTATGTCCAGGAACATAGTTTGAAAACTTTAGTTCCGCGTTGATCATTTTATCATATCTTCGTGCTTTATAAGAAAAAC
AACGATGCATCGATTTAAATTAACCTATCTAAGTTTTATGACCTGGTTATCATCCAAAGAACATAGTTTTTAAAAACCTTAGTT
CCGCGTTAATTTTTATCAATATCTCGTGCTCTTTAGAAAAACAACGATCAATTGATTTAAATGACCTTCTCAAGTTTTAATGAC
CTGGTTTATGTCGGGGATTTTTAGTATGAAAACTTAGTTCCGCGTTAATTTTTATCCATATCCTTGTGCACTTTAAGAAAACCA
ACCGATCAATCAAATTTAATTAACCTTCTAAGTTTTTATGAACGCTGGTTAATCCACGACTTAATTTGAAAAACTTTGTTCACG
TTGATTTTTATCGAATATCCTCGTGGCCTTTTTAAGAAAACACGATCCAAATCGATTTAAATTAACTTCTTAAGTTTTATGACG
CTGGTATATCCCAGGAAACATAGTTTTAAACTTAGTTCGCCGTTAATTTTTATCAAAACATGCTCGTGCTCTTTAAGAAAAAAC
AACGGATCCAATTGATTTAAATTAACTGTCTACTGTTTTTAGAAAGCTGGTTATGTCCAGAGAAAGCATAGTTTGAAACCTTAG
TTCGCATTAATTTTATCAATATAAGTCATGGCACTTCACAGAAAAACAACGATCAACCTGATTTGAACATTAACTTCTAAGCTT
TATGGAACTGGTTATGTCGCAGCCGAACATAGTTAAGAAAGATTTAGTTCGGGCGTTAATTTTATCAAATATGCTCGTGCCTCC
TTTAAAGAAAACCAACAAATCAACTTGACTCTAACACTCACTTTCAACGCCTTTCACTGATGCGCTTATGTCCAGGAAAAATAT
TTCGAACAACTTAGTTCGCGTCTGATTTTATCATATCCTCTGGCTCTTCTTCAACGCACGCATCAATCGATTTATTACTTCTAG
TTTCATGAAGACCTGCGTTATAGCCTAGGAACATGAGTTTGAAAACTTAGTGCGCCGTTATTTTTATCAATGTCTCGTGCTCTT
TAACCAAAACCAACGATCAGTTGATTTAAATTAACTTCTAAGTTTTATGACCTGACGTTAACTGTCCAGGAACTGTAGTTTGAA
AAACTTAGTTCGCATTAATTACTTTATCAATATATCATCGCTGCTTTAAAGAAAAACACGAAAACGACGATCAATGAATTTTAA
TTAACTTCTAAGCTTTTATGAAGCTGGTTATGCCAGGCACATAGTTTAAAAACTTAGTTCCCGCGTAATTTTTATCAATTGTCT
CGCGTGTTTTAAGAAAAACATAAACGATCAAAACATTGATTTTAAATTAGACCTTCTTCTAAGTTTCTAGGAACGCGAACTGGT
CTTTGTCAAGAACAACAACCTCAAGATTTGATAACGGCATTCTATTCACTGAAACTTAGTTTCGCATTAATTTTTATCCAAACC
TATAATTGTCAATGCTCGCTTTAAGAAAAAATACAAACGATCAAAAAGTCAGCATTGATTTAAATCTAACTTCTAAGTTTTTAT
GAACTGGTTATGTCGCAGGAACATAGTTTAAAAACTTAGTTTTCGCGTTAATGTTCTTATCAAATATCTCGTGCTTCCTTGTAA

GAAAAACACACGATCAAATTGATTTAGAATTACACTTCAAAGTTTTATGACCTGGTTACTGTCCGGACCATAGTGTTGAAAAAC
TTAGTTCACGTTGATTTTATTCAATACTCTCCCACGTGCTATTATAGAACACAACGATCAACATCGATTTAAATTAACTCTAAT
TTATGACCTGGTATTCAGGACATAGTTTTATTAAACTTAGTTCGACAGAATATAATTTTTATAAACAACCGCAGCTTCCCAGGA
GCATAATCCTCGTGCTGCTTTTAAGAATTAACAACAAAGTGACCACGATCAATCTTGATTTAAATTGACTTATAAGTTTTAGGC
ACCTGGTTATCGTCAGGAACATAGTTTTGAAAACTTAAGTTCGCATCTATTTTTTAATCCAATATATCATCGCTTTAAGAAAGA
AACAACGAATCAATTGATTTAACAATTTAAAAACCCTGCTCTAAGTTTTAATGAACTGGTTACATGTCCAACGG
>m141120_105518_42134_c100732402550000001823141405141504_s1_p0/68203/7759_15486
ACAATTGGTATTAGACGTGATATTTTACGAGATCTGATTTCGATGCCTATAGTTTCCGCAGAGCCAATACACAAGTAATATGGA
GAATTTCTGGACAGAAAGGTCCAACATATATCATTTGATTTGGTTGGGAATAGAAATTCAGGACACATCAATTAAATACATGTC
CATATTCTTGGTCAATTAAATACTACCTAACATAAAGTCAATTTTGAGTTATATATTATGAGATGGGAAAATGTTCAAAAAGAT
TAGAGCATTGTTAGAGAGAAATATGAGTGAATTACAGAGATAGCTATCGGAAGCACTAGGCAAGCTGCCTAGAAGATTTGGGGC
AGGAAACCAATTTTTTTGATAATTGGAATTAATTAGGAACCCCAATATTCACCATAGGGCCAATAGTATTGGAAAACAAATATG
AATATTATGAGAGGGATAAGGTTCAAAAAATTAGAGGTTGTTAGAGACAGAATAATGAGGAATGATAACTTAACGATGATCGGT
TCGGAGTCCTAGGCATAGACTGCCTAGGAGATTTTGGACAGGAAACGCCAATTATTATTGTTATTGGTTGAATTAGGACCCCAA
AATTCATTGGTCAAAATAATATACCTATAATAGAAATATTATTTTGAACATGGGAAAATGGTATCTAAAAAAATTAGAGGTTGT
TTAAGAGAACAAAATATGAGGTGGAATTACGAATCGAGCTTCGGAGACCTAGGACAGCTGGCCTCAAAAGGAGAAATTATGGAC
AGTGAACGCCAATTTTTTTATTTTGTTTGGTATAGAATAGGACCCAAATTCATGGCAATTAATATAACCTATATAGAATTTTGA
AATGGGAATAGGTTTCGCAAAAAATTTAAGAGCTTCGTTAGAGGGAAATAATGAAGTGAATTACGAGGATCGCAGCTTCGGGCC
CTAGGCCGCTTAGCCAATGGAATTTGGCAGGAAAGAGCCATTAATTTTTTGTTTTGTTGAAATTAGGACCCCAAATTAATGGTC
AAATAATACTGACTAGTATGATAATTATTGAGATGGGAAACGGTTAAAAAAGATTAGAAGGCTTAGTTAGAGAGAAATATGAGT
GAAATTAACGAGATCGCTTCGGAGCCTAGGCAGCTGCCTAGGATGATTTGGTCAGGAAAGGCCAAATTTTTTATTGTATGGATG
AATTAGGAAACCCAAATTCCACTGGCCAATAGTATACAAATACATGATTTAATATGAGATTGGAAAAGGTTCAAAAAATTTAGA
GGATAGTTAGAGAGAAACTAATGCAGTGAATTACGAGAAATTGCTTCGGAGCCCTAAGGGCTGCATGCCAGAGGAGATTTGGAA
GGAAAAGGCCAATTTTTTTGATTTGTTGATTTAGGACCCCAATTCATGGCAATAATATCACCTAAATATATATTTAGAGGAATG
GTTTAAAAAAAAAAAAAAAAAAAAATAAAAAATACAAAAAAAAAATTAGAGCTAGTTAGAGAGCAAATATGAGTGAAATTAAC
AGAGATCGCTTATCGGTGGCCACTAAGGACAGCTGCCAGAGAGGAGATTATGGCAGGAAATAGCAATTTATTTTGGTTTAAGGT
GAAATATAGGACCCCAAATTCATTGGTATCAAATAGATATAACCTTATAATATATTATTAGGAGAATGGGGAAAAGGAGTTTAA
AAAAATTAAACAGGTTGTTAGCAGAACAATATGAGTATGATTATACGAGATCGATTCGGAGCCCTTAGGCACCTGCCTAGGAGA
TTTAGGAAGGAAAGGCTTATTTTTTTGTTTGGGTTTAAAATTAGGACCCAAATTACATGTCCAGATTAATATAATCTATATGAA
TTATTTGAAGATGGGAGAAGGTTCAATAAAAAAATATAGATCTTTTAGAGAGAATATGAGTGACTATAACGAGATCATAGCGTC
GTGAGCCTTAGGCAGCTGACTAGGAGATTTGGCAGGAAAAGGCCCAATTTTTGTTCGGTTAATTAGGGCCCCAATCCATGGTC
AATAATATAACCTATATGAATTATAGAGATGGGGAGAATGGTTACAAAAAAATTAGAAGCTGGTTAGAGAGACAATAATGAGTG
AATTACGAGAATCAAGCTTCGGAGCACTAGGGCAAAGCTGCCTAGGAGATTTGGAGCAGGAAAGGCCAATTTTTTTTGAGTTTG
GATGGAAATAAGAGACAAAATTCATATGGCCAAATAGTGATCACAAATATGAAATTATTGAGATTATGGAAAAGGGTTCTATAA
AAAAATTAGAGGTTGTTAGAGACTATAAGATGAATGGAAGATGTAATTAACGAGATCAGCTTCGGAGATCCTAAGGCAAGCTGC
CTAGGAGATTTGGACAGGAAACGCCCAATTTTTTATTTTTGTTTTGGTTGAATTAGAGACCAACAATATATTATCAGTGGTCAA
AATATATCCTATATGATATTTATTGAAATGAGGAAATGGTATCCAAAAAAATTAGAACTTGTTAGAGAGAAATATGAGATGAAT
TACAGAGATCGCTAATCGGAGCCCCTAAGCCGCTGCCAATACGGAGATTTGGCAGGAAAGCCAATTTTTTGATTAGGTATTAAA
TATTAGACCCCAAATTCATGGATCAAAATAATAACACCTATATGGAATATTTCATGAAGATAGGGAAATGGTTCAAAAAAGATT
AGAGCTTTTAGAGGACGAAATATGAGTGAAACTACGAGATCCGCTACGGAAGCCCACTAGGACAGCTGCCTAGGAGATTTCAGG
AAAAAGGCCAATTTTTTTTATGTTTGGATTAATTAGGACCCAATCATGGCGCAAAAAATAGTATACAAAATAATGATATTATTG
AGGATGGGAAAAGAGTTTCAAAAAATTAGAAAGGTCTGTTAGAGACAAATATGAAGTGATAATTACAGATCAGGTTTATGGATC
CTAGGCAGCTGCCTATAGCGAGAATTTGGCAGGAACCCAATCTTTATTTGATTTGGTTGAATTAGATCCAGCCAAAATTCATGG
TCAATAATAATACCTATATGATATTATTCGAAATGGAAAATGGTCAAAAAATTAGAAGAGGTTGTTAGAAGACAAATATATGAA
TTACGGACGATCAGCTATCGGAGTCTATGGCAGCTATGCCTAAGAGAGATTTTGGCAGTGAAACGCCAAATTATTTTTTGTAAT
TAGGTGAATCTAGGACCCCAATTCATGTCAAAAAGATATACCTATATGATAAGTTATTAAGAAATGGGAAATGGGTTCCACTAA
AAAATTAGAGCTTGTTAAGAGGAAATTATGAGTGAATACGAAGATCAGCTTCGGAGTGCCGCTTAGGCCGCTGCCTAAAAGGAA
AAATTTTGGCAGGAAAGGCCAAATTTTTTGTATATATAATGTTGAATATAGGACCCCAATTAATTATGGTCAATAATACTCTAA
ATGGATTAATTATTGAGATGGGAAAAATGGTTCAAAAAAGATTAGAGGTTGTTAGAAGAGAAAAAATTTAGAGTGATTACGAGAT
CGCTTCGAGAGCGCGCTAGGGCAGACTGCTAGGAGTTTGGCCAGGAGAAAGGCCAATTTTATTTGCATTGGATTGAATTAGGAA
CAGCAATTCATGCGCAAATAGTCAATATAACAAAATATGATATTATTGTTAGAGTTGAAAAGGTCAAAAAAATTAGAGGTTGTA
TAGAGAGAAATAGATAGAGTGAAAATACGAGATTGGCTATATATCGGAGCCCTAGGCTGCTTGCTAGGAGAAATGGCAGGAAAG
GCCAATTTTTTGATTTGTTGATTTAGGACCCCCAAATTCTTGGCATCAGAATAAACCTAAATATGAAAGATATTGAGATGGGA
AATGATTCAAAAAAATTAAGAGCTATGTTAGAGAGAAAATATGTATAGTGAATTACGAGATCGCTTTCGGAGCCCTAGGAGGCA
CCAGTTGCACTAGAGAGATTATTGGCAAGGAAAAGGCCAATTTTTTTTGTTTGGTTTGAATTAGGACCACCAGAAAATTCATGG
GTCAACAAATAATATACTATATGAATAAATTAATTGACAATGGGAAGAATGTTCAAAAATTAGAGCATTTGTTTAGAGAGAAAA
TATGAATGATGATTAACAGAGAATCGCTATCGGAGACACTAAAAGGCCGCTGCTAGGAAAGATTATGAGGCAGGAAAGACCAAT
TTTTTATATAGTTTTGTTTGATTAAGGACCACAAAATTAGTGGTCAATAAATAACTTACCCATAATAAATGAATTATTATTTGA

GAGATGGTAAATGTGTATTTCAAAAAGATTGAGAGAGATTGTAGAGAGAAATATGAAGTGAATTATCGAAGCATCAGCTTTCAC
GGAGCTTCTAGCAGCTGCCTGAAAGGAGAATTGGCCTGGAAAGAGCCAAATTTTTTTGAGTTGGATAAGAATTTAGGAACAGCC
AAATTATAGGCCAAATAGTATAAAATTATGATATTGCATTAAGGATGGGAGAAAAGGTTCAAAAAAAATTAGAGTATTGTTAGA
GAGAAATATGAGTGAAATTAGCGAGATCCGCTATCGGAGCCTAAAAGGAAAGGTGCCTAGGAGATTTGGGCAAGAAAGGACAGA
TTTTGATTATTTTGTTGATTAGGACCACCAAATATCTTATGGCCAATAATATAACATATATGATAATTATTGAGAATGGGGGAA
AAAGGTATCAAAAAAATTAAGGATTGTTAGAGAGCAAATAATAAGAACGTGGAATTAGCGAGATTCAAAGCATATTCGGAGTCC
TAAAGGAAGCTTGCCCTACGGAGATTTGGCAGGCAAACGCCAATTTTTTTGTTTGCTTGAATTAAGGACCCCCAATTCTGGAGT
CAATAATATACTAGATACAATAATCATGGATAAATTATTGAAATGGGAAATGGTTCCAAAAAATTAGAGCTTTGTTGAAGAAAT
AATAAGTGAATATCGAGATCGCTTCCGAGTATCCTAGCAGGTTTGGCTAAGGAGATTGTGGGCAGGAAAGCACCCAAAAAATTT
TTTTTTGATTTGGTTGAATTGGACGCCGCAATTCATGGTCACAGATAATATAACCCCGTATATGCCATCATTTTCAGGAACCGA
CTGGGACACAAACCTGGTTCGCAAAAAATCTACGGCAACGACTTGTATATAGCAGAAGCAAATAATGAGTGAAATTACCCGAGA
TCGCCTTCGGAGCGCCCGACTTAGGCCCTGCCTAGGAAGATTTGGCAGCGCAAAGCCAATTTCTTTTGCTCCTCGGCACGCCCA
AAATTACCATGGGTCAATAATGAGCTCTATATGCCATACTTATTCGCAGAATGGGAAAAGGTTCAAAAATACTTTAGAGCTTGT
TAGAGAGAAAATATGAGCACCGCCCAGGCAGCTGCGACGGAGATTTGCGCAGGAACGCGCGCCAATTCGTTTTGGTTTGGATG
CAATTAGGAACGCCCCCACATTCATGGCCAAGGTAGCTATAGCGCAAATATCGATATTAGTATCTTGATGAGATTGGCAGCAGA
AGGTTAAACAAAAAATTAGAGAGTTAGTTAAGACGAGAAAATATGGAGTGAATTACGAGATTCCGCTTCGGAGCTCCTAGGCTG
CTGGCCCTTTGCACTAGGAGATTTGGCAGGAAAAGGCCCAAATTTTTTTGATTGCTGTTGATATTACTCGGACCCCAATTCATC
CGGCCAACCTAAGTATCCTATATGACCAATACTTCGAGATGGGACACAATGGTTCCAAAAAAAATTAGCAGCTCTGTTGAGAGA
GAGAGTACTAAGCTGATTACGAGATCCGCTTCGGAGCCTAGGCAGCTGCCTAACGGAGCATTTGGCCAGGAAAAGGTCCAATTT
TTTTGTTTGGTTGAATTAGAACGCACCGAATTCATGGTCAAGAATAATATACCTACTATGATATTGATTCGAAATGGAAATTTG
GATTCCAAAAAATTAGAAATTGTTAGAGAGAAATATGAGTGATTACGAGATCCCGCTTCGGGAGCCCTAGGCCGGCTGCCCGTA
GGAGACTTTGGCAGGAAAGGCCAATTTTTTTGATTGTCTGACTTAGGACCCCAATTCAATGGTCCATAATACACCTATATGATA
TTTATCGAGATGGGAAAATGGTTCAAAAAGGATTGAGAGCTTGTTAGAGAGAAATATGAGCTGATGATTAACGAGATCAAGCTT
CGGAGGCCACTACCAGCTGCCTTAGGAGATTTGCAGGAAAGGCCATTTTTTTTGTTTGGATTGAATAGGAAAGCCCAAAGTTCA
TGGCCAATAGTATACAATATGATATTATTGAGATGGGACCCAAAGGTTCAACAAAAATTAGAGGTTGTTAGGGAGACAAATATG
CAGTGAATTACGCAGATCCGGATTCGCGAGTCTAAGGCAGCTGCCTAGGAGATTTGGCAAGGAGAACGCCAATTTTTTTTGTTT
TGGTTGAATTTAGGACGCCCCCAATTCATGGTCAATAAATATAACCCTATCATGATGAGTTATTGAAATGGGAAAATGTTCCAA
AAATTAAGAGGGTTGTTAGAGACAAATATGAGTGAATTAGCGGATCGCTTTCCGGAGTCCTTAGGGGCAAGGCTCCCTAGGAGA
TATTGGCAGAACACCGCCAATTTTTTTGTTTGGTTGAATTGGACCAATTTCATGGTCAATATAATGACGCTATATGATATTTAT
TGAATGGAATGGTTCCAAAAAATTAGAGCTTGTTAGAGGGAAATAGTGAGTGAATTAACGAGATCGCTCGGAGCCCTAGGCCCG
CGCTGCCCAGCGAGATTTGGCAGGAAAGGCCGAATTTTTTGTTTTGTGATTGGACCGCAATTAACTGGTCAAACTAAATCCTAT
ATGATATTATTGAAGATGGGAAATGGTTCCAAAAAGATTAGAGCTTGTTAGAGAGAAATATGAGTGGAATTAACGAGATCCGCT
TCGGAGCCCCCAGGCAGCCCTGCCTAGGGATTTGGCAGGAAAGGCCAAATTCTTTTTGTTTGGATGAATTAGGACCCCCCCCAA
ATTCATAGGCCCAATAGTATTAACAAATACTGATATTGATTGACGATTCGAAAAGGTTCAAAAAATTAGAGGTTGCTTAGAGAG
AATATCGAGATGAATTACGAGATTGCTTCTCGGAGCCCTAGGCCTGCCTGCCTAGCGGAGATTTGGCCAGGAAAAGGCGCCAAT
TTTTTTGATTTTGTTGATTTAGGACGCCCAATGTCATGGCCCAATAATATACCCTACTATGGATAAAGTTCTTAGAGGGAATGG
TTCAAAAAAAATTAAAGAGCTAGTTTAGAGAGAAATATGAGTGAAATTACGAGCTCAGCTTCTGCGGTGCCCTAGGCAGCTGGC
CCGAGGAGATTTGGGGCAGGGGAAATGCCATTTTTTGTTAGGTGAATTACGGACCCCCAAATTCATGGACAATAAGTATACTAT
ATGATATTTTGGAGATGGGAAAAGGTTTTTAAAAAACATTACAGGTTGTTTAAGACCCCATATGAGGTGATTTACGAGATCGCT
TCGGAGCCCTAGCCCACCTGCCTTCTAGGAAGATTTGCAGGAAGGCGCTATTTTTTTTTGGTTGGTTTTAATTAGGCGTAACGG
CGCAAATTCATGTCCAACGTACATATATCCTTATATGATATTTTTGAGATGGGAGAGAGGTTCAAAAAAAATTAGAGCTTTTT
>m141120_105518_42134_c100732402550000001823141405141504_s1_p0/69383/4241_15149
CCCCCACGGGAAATCGGAGAACCAAAATTTGTTTTGGGGGGGAAAAAAAAAACCCCCGCCCCCCCTTGCGCGCGGTGTGTGTA
AAGAGAATCCCTTTTTGGAAAAAGTTTTTTGTCAAAGTGGGAACGCCCCTGAAACTTCAAAAAAAAAAAGGCTTTTTTAAACA
AACCCCCAAGAATTTTCCTTCCCCGAAAAATGGGTCCTATATCTGTGTTCTCTCCTCAAAACGAATCCCCCCCAGGAAAAAAAA
AAAAGCCAAAAAAAAAACAAAAAACCAGTTTTTTTTCCCCCCCCAAACTTTTGACAAAGGGGGCCCAAAATCCCTTTTTTAACC
CCCCTTTGCTAAAAATGGGTTCCCCGGGTCCCCCATGCCCCCCCCCTTTTTTTTTTTCCCCCGGGAACCTCAAGAAAATTTTAT
GCCGGGAAGGCAAAAACCCTTTTAAAACCCCGAAGGGGCCCCCCCTTTTTTTTCCCGTCCCAAAATTTCGAAAATGTTCTTTTA
GGGGCCCCCCAGCTTGGGGGCCGGAAACAAAAAAAAAATGGGAAAAAAAGGTTAAAAAAAATAAAAAACAATCCGACGATATTGG
CCTTGGTTTCCCCCAAAACAGTCCCCCCCCAAAGAAAAAACGTCCGGGCCGGGGAAACCAAATTTCCCCCCCCCCCGGAAGCAT
TTTTTTTTTGGTTGGGGGAGGCTTTTTCGGGTTTTTTTCTGGGTTTGGGAAAAAAATGAAAAGCTTTTTTGAAACTAAAAAACGA
AAAGTGACTTTTTTTTTACGGCGTCCGAAAAAATCCGGGGAAAAAAAAAAAAAGTTTTTTTTTTCCCTTGGGCCTTTTTTATGATGA
GTTTTTTTCCGCCACGGGACAATTGGGACGGGCGGGGGCACGCAGAATCCAAAAGGGCTGAATTCCAAACAAAATGGGCTCCCC
TTTTTTCCCCCGGAAAAAAAGCCCAAAAAAACAAGGGGGGAATTGGGTTTTTTGTTTTTTTCCCCCAAAAAGCCCTGAAACCCC
CGGGGGGCCCCGGAAGGTGGGGGGGCCTTGCCCCTAAAAAGCCCCGCTTTTACTTTTTTTTTTTGGGGTCTTTTTCCCAAGGCC
CCACAAACGGAAAAAAAACCAAGGTGAGAGGGGCGTCCCCCCCCCCTTTTTTTTTTTTTTTTTCCCCAAAACGTCCCCCCCCCC
CTTTCCCCCGGGGAAAAAAAGGCAGCTTTTTTTTTATTTTTTTTTTTGGGTTTTTCCCGAATTGGTTTCACCCCCCCCGAAGGG
GTGGGGGGGCGAAGAAAGCGCCCCCTCTCAGAAGACACAATAAGAAAAAAAGAGCCAAAAAAAAAAAACTTTTTTCTGGCCCAA

GGCCAAATTCCCCCTGGCCGGGAAAAAAGCAAGTAATAATATTCAGAGGAAGAAAAAGCACTATTATAAGATATTGGTATTAAC
TTTTTTATACCTTTTTGGCCCGTACCGGCCCCACAAGAACATTTACAAAAAAAAGCGCCCGCCGTTTTTTTTTTTGGTGGGG
GGCGCGAAAAGAAAAGGGGGGAAAGAAAAAAGGGAAACCCTTAAAACCCCCTTTTCCCCCCCGTTGGGTATATATATAATTATA
TATACCCCTTTAACGTTTGGAAAGCAAAGCCGATATTTATTTTACTAGAAGACACAGCCGAAAAATATGATATAATATACACCA
AAAACCTAAAAATTTACGGAAAACTTTTTTTTTTGACCTTTATATCCTTTTGTTTCCTGCCTATGGACGGCAATCCCCCCGGAA
AAAGCTTTTTTTTTTTTTTTCGACTTAAACCCATTTTTTTTCCCCAGGGGGCCAACGAAATTAAAAAGGTTTCCACTTTTACAAA
ACCAACCCCCTTTTGCGGCCAAGGGTTTTGGGGGGGGTTTTTTTATTTTTTTTTTTATTTAAAAATATAGAGACCAAGAAAAC
CCTTGAAAGGGCCCCGTCGGGGGAAAGGGAAAACCTTTTCCCCCTTGGAAAAAATGGGGGCCCCCCAAATGGAGGGCCTTTTTG
AGGCCCCCAAAAAATTTTTTTTTTTTTTTTTTTTGAAATTTATTAAAATGCGGGGGTTTTTAAAAAAAACCGGTTTTGGGGCCC
CCCCGGGGGGTGGGCCCCATTTTTTAAAGGGGGGGGAACAAAGGGCCCCCCTTCGGGGCCCCGAACTTTTTTTCATCGGTTTCC
CGGAAAATGGTTTTTTTCCCCCGGGGGGTTTCCCAAACCCCCCAAAAGAAAAAAAGAAAAGAGGGTGGCCCCCCCCGCCGGCCC
CCCGCCCACTTTTTCCTCCCCCCGTTTTTTTTTTTTTGGGAAAAAAAGTCTTTTTTTTTTTTTTTCCCCCCCCCGTTGGGGGA
CCCCGGAGGCCCCAAAATCGAAAAACGAGAAAAAACCGGGGGGGGCCCCCGGGCGCCCCCGGGGAAAAAAATCAAATCCACTGG
TAAAAATTCCCCCCCAACCCCCAAGGGGGCCCAATAAAACCCCAAGCTTTAACCAAAAAAAAACCCGCCCCCCTCCCCGTTTTT
TTTCCCAAAAAGTTTTTCAAAGCGTTTTTGAAAACCCAAAACCGAAGGCCAAAAAAAGAAAAAAAGGCCAAAATGAGGGCCAC
CCCCCCCCCGGTTCCCAATAGCCCCCCCGGCCCCTTTTTTTTTTTTTTGCAAAGTCTGGGCCCCCCCCCGAAAGAAGTTTAAA
AGGGGGCGGCCCGGAAAAACCCTTTTTCGAAATAATTACAAAGCAAGGATTTTTCTTCCCCCCCCCCAAAAAAAAGCCTTGGTA
GGTACCAGAAACCCCAAGAGTTTTTTTTTTTTTTTTTTCAACCGGGGGGGAAAAGTTTTAAAATATCCAAAAGAACCCAAAAA
GGGGGGGAAAAAAAGGAAAAAAGAAAAAAAAAAGCGGAAAAATGGCGGTCCCCATATCGTTTAACCCCAAAAGGTCCGATGGGGA
GAAAAATTTGGCTTTTTTTTTCCCCAAAAAAAGCCGGGTTTTCCCCGGCCGGGGCCCGGCAAAAAAAAAAATTTCTCCCCCCCA
AATCCCGCCCAAGATTAAACCCCCCGGGAAGGAAAAAAAAGAAAACCCGCTCCTTTTTTGGGGCGGACAAACATTTTTTTTTAA
AAAAGGCCCAAAAACAAAGCTTTAAAGCAACCTTTGAAAAAAAAAACCGCCAAAAAAAAAAAGAAGGCCGGAGTTTGTTGGCCC
CCCCCCGGGCCCCCTTGGGTTTCCCCCCGTTTTCAAAAAAAACCAATTCCCCCCTTTCCCCCCCCCCGGTCCCAAAAAAAAAA
GGCACAATAAGGGAAAACCCTCCCCAGACCGTTCCCCATTTTTGCCCCCCGTTTTCCCGGAAAATGTTCAACTCCCAAAATTTT
TACAAAAAAAAAAATGGATTTGGGGGGCCCCCGCCTGATCCCCCGCCTACCCTCTGTCCCCCCACAGCCGAGGGGGGAAGGGTT
TTTTTTTTGTTTTTTGTTTCTTTTTTTCCCGAAAACCGGACGCCGAAGGCCCCCGGGGGCCCAAGGGAAAAAGGCCTCCCCCCC
CCTTTTTGTTTTTTTACAGGCCCACCCCCTAACCCGGGAATACCCGTTTACAAATGAAAAAAAAAAAAAAATTTGGGGACCCGGG
GAGAAAACCTTTTAATAGCCCCACGGGGGGGAAAAAACCCATTTTTGTTCCCCACCTTTTTAAATCTTGGCCTTTTTTTGAAAA
AAAACCCTCCGGTTTGGGCGTTTGTTTTCCAAGAGGAACCCTTTTTCGAGGCCCCCCCTTGGGGTTTTTGGCCCCAAATCAATG
CCCAATTTTTTGGGGAAAAAGGGGTTTTTTCCCCCCCAAAACCCGAAAAAGGCCTTTTTTTTTTTTTTTTTTTTTTTTTTTGG
CCGGGAGAAATCACAAAACCAGCCGGGAACCCCCAAGTTTTTTTTTTTTTTTTTTTGATCGTCATCCAACAGTCCATCGGCCC
GCAGCAGGAAGAGCGAGAGGGCGGTGAGGCGCTGCGAGCCGAAGAAAAAAAGGGGGGAATTTAAACCTTTTTTTCCTTGGCCC
GCCCCCAACAAAAGGTGAGAGAGAAAGGGAATACCTTTGGGCAATAGACCCCTTTTTTACCGTGCGCGCGCCATCCGAAACGAA
AGCCCCCGCAGAGAGAGGAGAAGGCGCGCGAGAGATCCGCCGCGCGGTGCCGCGCGGCGCGCGCGCGCGCCGGCGCCCAAATTT
CTATATATTAGTATAAAAATATGGATTATACCCCTTGATGGGGAACCCAAGTCCCCCTTGGCCCCCCAATTTTTTTTTTTTTT
CTGCAAAAGTGTTTGGGGGGACTTTCGAATTTTTCCCTTTGTTGGGGGAATTTCCCCTTTCCCCCCCAAACCCCAAAAAAAGGG
AAAGAAAGGGGAAAAAGGGGGGGGGAAGAAAGGGTTAAAATGGCCAAATTTTTGGTGGGGCGGGTTAAGAAAAACCCCCCCTTA
AAATTTCCACGAAATGGGGGTTTTTGGGGAGGGCGGAAGCCCCCCCTTTAAAAAAAAAAAAAAAAATGAAAAACCAACGAATCA
AACCGTCCCAGGGAAGGCCCCCTTTAAAAAATATTTTCCCCGGTCCCCCCAGAGTTAAAAACAAAAAACCATAGTTTTGGTTGT
CGGTTCAATGGCCCTTTTTTTCCCCCCCTACAGGAAGTGAGAGGCGGGTTCCCCCCCCTTTTGAACCAAAACGGGAAAAAAAAC
AAAATTTTTTTTTGACAGCCTTTTTCCCCACTTTTTCACGGGAAATTTTTTTTTTTTTTCTTTTTGCGAGAAGGGGGCCCAAAA
AAACGGTTTAAAAAAAAAATAAAGTTTTGGGGGGGGGGAAGGAGGGTTTTTTTCTTTTCCGCGGTGGGGGCCCCAAAAAAAGG
CCCCCCAGGGGGGGGTGGGGGAAACGGAAACAGGCCCCCCCCGAAAGGCCCCCGTTTTGGAAAAAAAAAAAACAAAAAATCCCT
TAAAAAAAGGAACCCCAAAACTTTGAAAGAAAAAAAAAGCCCTTTTTTTTTTTTCCCAAGGCCCCCCCCCTGTAAGGAAAGCCA
AAGGGGTGGTCCGAAAAAAGGGCACCCTTTTTTTTCCCTTCCCGCGGGTTGTTTTTTTTTTTTTTTTTGGGGTGGGGGCCCCCC
CCCCCCCTTTTACCGGGACAATCAAAACCCCGGCCCACAAAACCCCCCCAATTTTTTAACCCCCTCCCCAAATTTGGACCCCCC
GTACCGGAATTGCGAAGGCCCCCCGTTGTCCCCCAAAATCCCCAAAAATTGGAAAACACCGAACCAAAATTCCGGGTCCCCCCC
ATTGCTTTTAAGTTCGCGACAAGCGTAAAAAAATTTGAAATCCCCAAACCCCCCCCCCCAAAAAGAAAAACCAATGCCCCCCCT
TTAAAAACTCCCCCCCCCAAAGGGAAATCTCTTGGACCCTTAAAAAAAAATTTGGAGGAAAAAAAAATCCCCGGGAAAGGAAGGGG
TTTCCCCGCAAAAAAAGGGTGGGGGCCCCAAAAGTTTTTTTTAAAGGAAAATTTGGTAAACCAGGGGCCAAAAAGGAAAAAAA
ATTCCGCTCCCGCAAAAACCTTTTTTTTTTTCACTTCCCCCCCCAAAAAAAAAGAAGGTCAAAGGAGGGGCCCCCCGTAAAAAG
TTTTTTTTCCCGAAAACCCGGCCGATTCCCCTTGGGTTCCTGCGGAAGGGGTTTCCCCTTTTTTTTTTCCCCCTTTGCCACCTT
CCAGGATCCCGGGGGGTTAGGGGGGAAAAAAAATTTTTTTTTCCCCCCTGGGCCCCCCCCAATTTTGGGGAATAAAGGGCCCC
CCGTATGAAAGAAAAAATTTTTTTTCCAGCCAAAAGGAGAAAATCCCTTGCCCCCCCCCCCCGGAAAAAAAACTATTTAAACC
CCGCTTGTTTTTTTGGGAAAAAGCCCCCCCGGCTTTTTTTTTTTTTTTTCCCAACTGATGGGGTTTCCCCCCCCCGAGGGTT
CCCCCCGCGAAGGCCCTGGGAAAAAACGCTTGGAAAACAGAAAACCCAAAACCTTCCCCCCGTAAATAACCCTACCCCCAAAA
AAAGTTTAAAAGCCTAAGGGGGGCCTTCATCCCCCGAGGTTTTTTTTTTTTTAAATGGGGTTTTACGCCTGTTAACCCATTTAA
AAAACGCCCCTTTCAAAAAGGGGCCCCCCCCCTTTTTTAAAAAAAAATTTTTCCCCCCTTTTTTTTTTTTTTTTTTTTTAGAAA

ACCAAAAATGGGAAAAAAAAAAAAATTGACCGTTGAGGGGGGTGCAAAAACCGTCAAATAAAAAAAGGGGCTTTTAATTTTTTT
TTTTTTTCCCCCAATTGGAAAAAATTTGGTTGGGGGTTTTGCCCCTCAAAAAGGGCCTTTTTTAAAAAAAAAATTTCTTTTTA
TCCCTTTTTCAAGTTTGGGAACACCCCCCAACAAAGGAACCCCAAGGTCCCTTTCCCCTGCCTTTCCAAAAACTTTGCCCCCGC
CCGGGGGGGGGGGGGGGGTTGGGGGGGGTCAAAAAATCCCCCAGGGCCCCCCGGCGAACAACCAGGCAAAAATTTTAAAAAAAA
AAGGGAAAACCGAGCCCCCAAAGGGGCCCCTGGCCCCCCACAAACCCCCGGGAACCGAAAAAAAAAACGAACTTTTTTTTTTT
GTTCCCGGGGAAAGTTTTTAAAAAATGTTAAAAATAGAAAGAAAGGGAAAAAAGCGTTGGCCCAAAAAGGGGCCCCGGTATTTT
TTTTGTTTATTTTTTCCCCCCCCCACCCCAACCCCCCCCGGACCCGAAAAAAACGAACCCCCGTTCCGGGGGGGTAAAAAAAT
TAAATTACAAAACCCAAAGGGAAAAAAAGAACCCCCGAAAGCCCCAAAGGCCCGGGGGGTTCCCACCCCAAAAACCCACCAGGA
AACGAAAAAAAACCCCCGGATGTCCCCCCCGGAAAGTTAATAAAAAAAAAAATACAAAAAAAGAAAAGGGGGACCGTGGCCCCC
AAAGGGCCCCGGTTTTCTTTTCCCCCCCAAACCCCCCCGGGGGAAACCCGAAACCGAAAAGCCGTCCCCCACCCGGGGGTTTAA
AAAAAAAAAAAAAAATTTAAAAAAATAACCAAAGGGGGGAACGCCCCCGAACGCCCCGCGGAGAGCGGCGCCCCCCCCGGTTCCC
CCAAAAACCCCCGGAAAAAAACGAAAACGGATGGGTTCCCGAGAAAAAAAAGGTAAAAAAATAAAAAAATAAGGAGGACCCCCG
AGGCCCCAAAGCGGCGGCCCCCCGGGTTTTTTTTTCCCCAAAAAACCCCCAAAAGAACGAAAACCCGACGTCCCGGGGGGGGT
TAACCAAAATTTTAAAAAAAGGGGCAGAAAAAACCCGAAAGGGCCCCCAAAGGCCCGGCCCTAAAAACCACCCCCCCGGAAAGA
ACGATAAAAGAATGGTTTCCCCCGCCCCCCCCCCGGGAACAGAAGTTAAATAAAAAAAAAAAATTTAAGAAAAAGGGGAAAAAAC
CGGGTGGGCCCAAGGCCGCCCGGTTTTTTATCCCCCCAAAAACACCCCCAACCCCCGACCGAACCGAACGTCCCCCGGGGGGGG
GGTATAAATAAACAAGGGGAAAAAAACCGAAGGGGGCCCAAAGGGGCCCCCGGATCCCCCCCAAAACCCCCCCCGGAAACGAAA
AAGAAAAGGATGATTCTCTCTTCCCCCCCGGGAAAAGTTACCAAAAAAAAATAAAAAAAAATTTAAGAGGAACCCCGTGCCCCC
GAAAAAAGGGCCCCGGGTTTTTTTCCAACGCCCAACCCCCGGGAACGAAAAAGAAAAACCTTTTTACCGTTCCCCCGGGGGG
GTAATAAAATTTAACAAAAGGGAAGACCGAGCCCCAAGGGCCCGGCCCAACCCCGGCCGGAAAAAACCGATTCCCCGGGAGGGAA
AAGGGTTATAAAAATAAGAGGGACCCCCGGCGTTGGGCCCAAGGGCCCCCCCAAACGGTTTGTTTTTTTATTTTTCCCCCCCC
CCCAACCCCCCCGGACCGAACCGAACCCCGTTCCCCCCCGGGGGTTTTAAAATAAAAAATAAAAACAAGGGAAAACCGAGCGG
AGGCAAAAAAAGGCGCGGGGCCAGCCCAAGAACACCCCCCCGCCAAAGAAACCCGAACCCGATGGGGGGTTCCCGGGGGAAAAA
AGTTCTTTTCTTTTTAACAAACACATTTAAACAACAATCAAGTCAAGAGGGCAACCGTGGCCCAAAAAACAGGGGGCCCGGGTT
CTCTTTCCAGACCCCACACCCGCGGGAACCCGGAAAACGCACGACCCCGGGGGGTTTTCTTTTAAAAACAAAAACTTAACATAA
TTAAAAAGGGGGAAAAAACCCGAAAAGCCCCAGGAAGGCCCCCCCGGTCCCCCCCCCAAAACCCCCCGGAAAACACAAAACAA
AAACGAACCGGTCCCCCCCGGGGGGAAAAAAAAATTTTAAAGAGGGAGGTAGAAAGGGGAACAAAAAAACCGGAAGTCCCCAAA
GGCCGGGTTTCCCCCCCCCCCACCCCCGGAACCCAAAAAACGCAAACGTTTTTTCCCCGGGGGGAGAAATTAAAATTAAAAAAA
GGGGCCAAAAAACCCGAAGCCCCCCCAAAAGGCGGCCCCGTTTTCCACCCCGGGAAACGGAAACCCGGAAAACGTCCCCCCCGG
GCGGGGGGTCTGTTTTAAATAAAAATAAAAGGGGTAAAAACCGAGGCCCACAAAAAAAAACAAAAAAGGGCCCCAACAAGTCAC
CCAACCCCCCGGAACGAAACGAAACGTTTTTTCCCCGGGGGTAATTTAATTAAAAAAAAAAAGCAAATTTAAAGAAAAAGGGGG
GGCAACCGAGGCCCCAAAAAAAGGGGCCCCAGGGGTTCCCACAGCCCAAAACCCCCGGAAGCGAAGTGGCTGGTTTTTTCCCCC
CCCCTGGGGCAAAAAAAAAAGTTTTAAAAAAATAAAAGGTTCCCCTGCTTCCGACCCCGGAAAACCCCGGTTTTTGGGGCCCT
TTTTTTTCTTTTTCGAATTCCTCCGGGCCCCCAAAAACGGAAAAAAAAAAGCAGATTAGGCTTTTTTCCCCCCCAAAAAAAAAA
GCCCCCCGGACAAACAAAACCCCTTCGCCATAAAAAATTTCTCCCCCCAAAGGGAAAAGGCAAACAAAAAAAAAGAACCTCCGC
CGGGTTTTTTCTTTTTAAGGTTTTTTTTTAAAAAACCACAAAGTTCCCCCCCCAACGGGGGCCCCCCCCGAAAAAAAACAAAAA
AACAACCCAGAGGGCAAAAAAAAAGACCTTAGTTTTCCCGTTCTGGGGGTTCCAAAGTCCCCCGGCCGTTGGGGAAAAAAAGAA
ACCCGGGCGGGGGGGGAAAAACAAAAAAACAACCCCCCCTTTTTGCAATCAGTTACGGCTCCCCCCCCGCGACATCTCAAAAAA
AAAACTTTTTTTCCTTTACTTGAATTGGGGTTTGGAAAAGGAGAAAATCTCATTCTTTTTTTTAACACAATAAAACCCCAAAAC
AAACGCGGAAAAAAAAAAAAAGAAAGCCACAAAGCTGTTTCTTTTTTTTTTTTGCCAAAAACAAAAATTTTAAATTAAACAAGC
TTTTCAAAAAAAACTTAAGAAATTAAACAAAAAAAAAAAAAAGAAAAAAACCGAGGGCGAGAAAACACCCCAAAAGGGGGCCCCC
CTTTAAAAACAGCGCCCGGTCCCCGGCCCCGGGTGAAGGGGTTCAACCCCCCCCCGCAAATCCTCCCCTCTTTTTTTTCCCCCTT
TGGCCCCCTCAGTTTACTCGGCCCAAACGCTTCCCCCCCGACGTCGCGGAATTTCCGGGGGAACACAAAGAAAACTTTTAAGGA
AAAAAAGGTGTTTTTTGTTCAATGGTTCTTATACTTTCTGGTCCCTTTTTTTGGCAAAACAAACCCGAAGGGCCCAATTAAATT
AAAAAAAAAAATGGCCCCTCGGTTTTTAAAAAGGGTTCTGCAAAAAACCATCGCCACCTAAGTTTTTTTGTCCCCGCCGCGCG
TTTTTCCCCCCCTCCCCTTTCCTGTTCCCCCGGAACGGGTGGGGGGGGGTTGGGGGGGTCCCCCCGTGGTGCCCCGCCCCCCCT
TCCCCTTGTTCCTTGAAAGAAAGGGGGGAAAGGGGGTTCCCACAAAACCCCCCCCAAAAAGCCTGCTTTTTCCCAAAAGCCAAC
AAACGGGGGCCTTTTTTCTCCAACCCGGCCCTTTATTTTCTTTTTCCGGAATCTTCCCTTTTCCCCTTCCCCTCTTTTGTCCCC
AGGAAAAAAAAAAACCGGAACCTTTTTTTTCTTTGTTTGGGCCCCGCACCGCGTCATTTCCCAAGACGAACGTTTTTTCCGCCCC
CCTTTTTTTTTTCCGTCCAGCAAAACCCCGTTTTTTTTTCGTTCCGTCCCGGGGACCCCAAACCCTTTTTCCCCCTTTTTTTCC
TTCCAAACCGCCCCCCGGTCCCTTTGGGGCCCCCCCAGAACGGCTTTCTTTTTTTTACCAAAAGATTTCTTCCCCTCCGCCCGC
TCCGCCCTTTTTTTTTTTGTCTTTAACCGGCCGCGGGCCCCCAAAGCCTTCACCCCTATTCCCCTTGGGCCAGGCCCGGGCGCC
TTGCGTTCCCCCTTCCCCCCCACCCCCCTTGCCCCTTTGAGATTTTTTTTTTTCTTTTCCCTTTTGACGGGTCCCCTTTTATTT
TTCCCCTTTCCCTTTTGGGGTTTGTAAACGGCCCGCCTTTTTTTTTCTAAAAAAATTTCTTTTCTTTTTCCCCCAAGCTTTTT
GCCGTTCCCTCCCCCCAAAATTTCCTTCCTTTTTTGGGAAGGGCAAAAGCTCGGCCAAAATTCAAAAAAAATCCGGCCAACTTC
CCTTTCTTTGGGAGATTTTTAAACAAGGGCCCCAAACCTTTTTGGGGAAAGCCCCAAAAAACGAAAAAAAAA
>m141120_105518_42134_c100732402550000001823141405141504_s1_p0/71108/0_5898

AAAAAAAAGAGAGAGATTTTAGCGAGCGGGGCCCGGTGTGGTCCCTCCCCTTTTTCCTCCCCCGGATTGAACTTAGGAACTTCT
TATTCGCTGCTTGGTAGTTGACCACGGTATTAACCCCCTCAAGGAAAACCGAAATAAATGAGTTTTTAAAGATTTAAAACAAA
AAATACAAACTTTTGAGCCGTAAGGTGGATTGTTTAAAGGGAAAAAAAAAATGATTGTTGTTTCAAGGGGTGCAACACGAGGAC
CTTCAACCAGGAGGTTCACCCATGCCTAGTACTACTCTCCGCCCAATGCACCGCTTAACTGCGGAGTTCTGATAGGGATCCGGT
GCAGTTAGTGCTGTATGATCGCACCCGGAATGTTTGGTCCGCAGATATTTCATTTAAGTAGCTGGAGCGCGGTGGTTCCTCCCC
TTTTCTCCCCGGATTGACTAGGAACTTCTTATTCGCTTGCTTGTTTAGTGACCACGGTTATTAACCGCTCAAGGAATAACGAAA
TTAAAATGAGTTTTTAAAGATTTAAAACAAAAATTACAAACTTTTGAGCCGTAGGGTGGATTGTTTAAAGGGAAAAAAAAAATG
ATGTTTTTCAGGGGTGCAACACGAGGACATTCCCAGGAGGTCACCTCCATCCTAGTACTACTCCCGCCCAAGCAGCGCGTAACT
GCGGAGTTCTGATGGGTATCCCGGTGCAATTAGTGCTGGTAAATGCATCGCACGCCGGAATGTTTGGTCACGCATATATTCATT
TATAAGCGAGCGCGTGTGTCCTCCCTTCTTCTCCCCCGGATTGACTAGGAACGTTCTTATTCGCTGCAATTGTTAGGACGCAAC
GGTATTTAAACCCCCTCAAGGAAAACGAAAGTAAATGGAGGTTTTTAAAGAGTTTAAAACAAAAATACAAACTTTTGAGCCGTA
GTGGATTTTTAAAAGGGAAAAAAAAATGATGTTTTCAGGGGGTGCATACCACGAGGAACATTCCCAGGAGGTCAACCCATCCTA
AGTACTAACTCTCGCCCCCAAGCACGCTTAACGCGGAGTTCTGAGTGGGATCCGGTCATTAGTGCTGGTATAGATGCGCAAACC
GGAATGTTTGTTGCCCATATTGATTTAAGGCGAGCGCGTTGTGTCCTTCACCTTCTTCCTTCCCCCGGATTGACTAGGAATTCT
TATTCGCTGCTTTGTTTAGTGACCACCGGTATTTAACCCCTCAAAGGAAAACCGAAATAAATGAGTTTTTAAAGATTTAAGAA
CAAAAATACAAACTTTTGAGCCCGTAGGGTGGATTTTTAAGGAAAAAAAAAAATGATGTTTCAGGGGTGCAACACGAGGACTTCC
CAGGAAGGTCACCCATCCTAGTATACCCTCTCGCCCCAAGCACGCTTAATCTGCGAGTTCTGATGGGATCCGGTGCTATTAGTG
CTGGTAGATGAATCGCAACCCGAATGTTTGTCCGCAATATTCATTATAGGCGAGCGCAGGTGTCCCTCACCTTTTCTCCCCCGG
TATGTGACTAGGAACTTCTTATTCGCGTGCTGTGTTAGTGACTCACGGTAATTAACCCCTCAAAGGAAAACGAAATAAATGAGT
TTTTTAAGGATTTAAAACAAAAAATACAAACTTTTGGCGTAGGTGGATTTTTAAAGGGAAAAAAAAATGATGTTTTCAGGTGCA
AGCACGAGGACTTCCCAGGGGTCACCCCATCCTAGTACTACTCTCGCAAAACAAGCACGCTTAACTGCGAGTTCTGATGGGAAT
CCGGTGCATTAGTGCTGGTATGATCGCAACACGCTGGAATGTTTGGTCCCCGCAATATTTCATTTATAGCGAGCGCGTGTGTCC
CTCCCTTTTCTCCGCCCCGGATTGACTAGGAACTTCCTGTTATTTCGCTGGCCTGTTAGTGACCCCACGTATGTAAACCCTCCA
AAGAAAACGAAAATAAATGAGTTTTTAAAAGATTTAGAAACAAAAAATACAAACTTTTGTAAGCCGTAGGTGGGATTTTTAAA
GGGAAAAAAAAAATGGATGTTTTCAGGGGTGCAACACGAGGACTTCCCAGGAGGTCACCCATTCCTAGTACTAACTCTCGCCCCA
AGCACGCTTAACTGCGGAGTTCTGATGGGATCCGGTGCATTAGTGCTGGATATGATCGCACCCGGAATGTTGGTCCCGCAATTT
CATGTTAATACGCGAGCGCGTGGTCCTCCCTTTTCGTTCCCCCCGGATTGACTAGGAACTTCTTTATTCGCTGCTGTGTTAGTG
ACCAACGGTATTAACCCCTCAAGGAAAACGAAATAAATGAGTTTTTAAAAGATTTAAAACAAAAAATACAAACTTTTGAGCGTA
GGTGGATTTTTAAAGGGAAAAAAAAAATGATGTTGTCTCAGGGGTGCACACGAGACTCCCAGGAGGTCACCGCATTCCTACGTA
CTACTCTCGCACCAGCACGATTAACTGCGGAGTTCTGATGGGAATCCGGTGCATTAGTGCTGTGATGATCGCACGGCCGGAATT
GGTTTTGTTCCGCAATAGTTCATTTATAACGAGCGCCTGTGTGTCCTCCCACCTTTTCTCCCCCTGGATTGACTTAGGAACTTC
TATTCGCTGCTTGATTGTGACCACGGTATTACCCCCCTCAAGGAAAACGAATAAAGAGTTTTTAAAAGAGTTTAAAACAAAAAA
TAAAACTGTTGAGCCCGTAGGTGGATTTTTAAAGGAAAAAAAAATGATGTTTCAGGGGTGCATACACGAGGACTTCCCAGGAGG
TCACCCATCCTAGGTACACTCTCGCACCAAGCCACACGCTTAATCTGTACGGAGTTATGATGGGATCCGGATGTCATTAAGTGC
TTGGTATGATCGCACCCGAATGTTTGGTCCGGCAATGATTCATGTATAGCAGCGCGTGTGTCCTCCCTTTTCTCCCCCCGGATT
GACCTAGGAACTTCTTATTCGCTGCTTGTTAGTGACCACGTGATTAAACCTCAAGGAAAACGAAATAAATGAGTTTTTAAAGAT
TTAAAAAAAAATACAAAACCTTTTGAGCCGTAGGTTGGATTTTTAATAGGAAAAAAAAAATTGATGTTTTTCAGGGGTGCAACA
CGAGGACGTTTCCAGGAGGTCAACCATTCTAGTACTATACTCTCGCCCAAGCACGCTTAACTGCGAGTTCTGATGGGATCCGTG
CAGTTAGTGCTGTTATGATCGCACCCGGACATGTTTTGTGGTCGCAATATTCATTTATAGCGAGCGCGTGTGCCCTCGCCTTTT
CTCCCCCCGGATTGACTAGGAACTTCTTATTCGCTGCTTGGTTAGTGACCAACGGTAATGTAACCCTCAAGGAAACGAAATGAA
TGAGTTTTAAAAGATTTATAAACAAAAAATACAAACTTTTGAGCCGTAGGTTGATTTTTAAAGGGAAAAAAAAATGATGTTTTC
AGGGTGCAACACGAGGACTGTCCAGGAGGTCACCGACATCCCTACTTGTACTACTCTCGCCCAAGCACGCTTAACTGGGCGGAG
TTCTGATGGGATCCGGTGCATTATGCCATGGATATGATCGCAGCCCTGGAATGTTTGTTCCGCAATATTCATTTATAGCGAGCC
GTTTCCTCCCTTTTCTCCCCCGATTGGACTAGGAACTCTTATTCGCTGCTTGTTAGTGACCACGGTAATTGAACCCTCAAGGAA
AACAAATAAATGAGTTTTTAAAAGATTTAAACAAAAATACGAAACTTTTGAGGCCGTAGTGGAATTTTAAAGGGAAAAAAAAAT
GATGTTTCAGGGGTGCAACAAGAGGACTTCCAGGAGGTCACCCATCCTAGTACTGACTCTCAAGCCCAAGCCACGCTTAACTGC
GGAGTTCTGATGGGACCGAGTGGCATTAGTGTGGTATGATCGCACCCGGAATGTTTTGTTTCCGCAATATTCATCTTATGAGCG
ACGCGACTGTTGTCCCTCCCTTTTCTCCCCGGAATTGACTAGAACTTCTTATTCGCTGATTGTTAGTGACCACGGTATTAACCC
CTCAAGGAAAACGAAATAAAAGAAGTTTTTAAAGATTAAAAAAAAAAAATAACAAACTTTTGAGCCGTAGGTGATTTTTAAAG
GGAAAAAAAAAAATGATTTTCAGGGGTGCAACACGAGACTTCCCCAGGAGGTCCCCATCCTAGTACACTCTCGCCCAAAGCACG
CTTAACCTGCGGAGTTCTGATGGGATCCGGTGCCAGTTATGCTGGTATGGATCGCACACGTGAATGTTTGTCCGCAATATTCAT
TTATATAGCGAGCGCGTGTGTCCTCCCTTTTCTCCCCCCGGATTGACTAGGAACTTCCCTTATTCCTGCTTGTTAGTGACCGAC
GGTTATTAACCCCTCAAGGAAAACGAAATAAATGATTTTTAAAGATTTAAAACAAAAAATACAAACGTTTCTGAGCCGTAGGTG
ATTTTTTAAAGGGAAAAAAAAGAATGATGTTTTCAGAGGGTGCCATAACACGAGGAACTTCAGGAGGTCACACCATCCTAGACT
ACTCTCGGCCAAGCAACGCTTAAACTGCGAGTTCTGATGGATCCGGTGCATTAGTGCTGTATGGATCGCACCCGGAATGTTTGT
TCGCAACTATTTCATTTATAGCGCAGCGCGGTGTGTCCTCACCTTGTTTCTCCCCCGGATTGACTAGGAACTTATTATTCGGCT
GCTTGTTAGTGACCACGGTATTAACTCCTCAAGGAAGATACGAAATAAATGAATTTTTAAAGATTTAGAAACCAAAAAAGTACA
AACTTTTGAGCCGTAGGTGGAATTTTTTAAAGGGAAAAAAAAAAATGATGTTTTCAGGGTGCAACCACGAGGACTCCCAGGAGGT

CACGCCATCCTGAAGTACGTATCTCGCCCAAAGGCACGCTTAACTGCGGGATTCTGATGGGTCTCCGGTGCATTAGTGGCTGGT
ATGATCGCACCCAGGAAATGTTTGCCCGCAATATTCCGATGTTATAGCGAGCGCGTGTGTCTAACCTTTTCTCCCCCCGGTTGA
CTAGGATCTTCTTATTCGCTGCTTGTTAGTGACCACGGTATTACCCCTAAGGAAAACGAAATAAATGAAGTTTTAAAGATTAAA
ACAAAAAAAAATACAAAAAAACCTTTGAGCCGTAGGTGGATTTTTTAAAGGGAAAAAAAAAAAAAAATGAATGTTTCAGGGGTGCAA
ACACGAGGACTCTCCAGGAGGTCACCGCAAGATCCTAGTGACTACTCTCGCACAACGCACGCGTAACTGCAGGAGTTCTGAGGG
ATCCCGGTGCATTAGTGCTGTATGATCGCACCCCGGAATGTTTGGTTCCGCAAATATTCATCTTATACGAGCGCGTGTGTCCTC
CCCTTTTCTCCCCGGATTGACTAGGAACTTCTTTATTCGCTGCTTGTTAGTGACCACGTATTAACCCCTCAAAAAAGGAAAACG
AAATAAATGAGTTTTTAAAGATTTAAAACAAAAATACAAACTATGTCTTTTTTTTTGAGCGTAAGCTGGAGTTTTTAAAGGAAA
AAAAAATGATGTTTTCAAGGGTGCAACACGATGGACTTCCCAGGAGGTCACCCATCCAGTACTACTCTCAGCTCCAAAGCACGC
TTACACTGCGGAGTTCTGATGGGATCCGGTGCATGTAGTGCTGTATGATCGCACCCGGAATGTTTTGTCCCCGCAATAATTCAT
TTTATACGAGCGGCGTGTGTCCTCATTTTTCCCGGATTGACTACGAACTTCTTATTCGCTGCTTGTTGTAGTGACCACGGTTTA
ACGCCCTCCAAGGAAAACGAATAAATGAGTTTTTTAAGATTTAAAACAAAAAATACAAAAATTTTGAGCCGTAGTGGATTTTT
TAAAGGGAAAAAAAAATGA
>m141120_105518_42134_c100732402550000001823141405141504_s1_p0/72674/8494_15164
TGCTCCTTACACACCATGCTTTTTGTATCTTCATGCCGGCTTCCCGGATTTAGCGCCAATGCCGAGCACCGCGATATTCTTTCC
CAGTGTCTGATAAAGTAAGGAACATTACTGCATTAATCAGCACATGATAGTTTCTCCCTATCTTTTTTGTTCGATGAATATCTA
AAATGTTTATAGGTTACCTTATAGAGTACGTCGCTCATTGCCTAGGTCACATGGTTACTCACTAATTAACATGAATTATAAATC
TGGTATTGTAGATGTGTGTGATTTTGTATTTGGGCGTTATATGTAGTTGTGGAAGTAGACTATATTGAAATTTAATGTACTGAT
ACATGAATGATGTTCAAAGTACTCAGTTATACATTGTATGGTACCTATTTGATTTGGAGTATTGGTAGTGTCTGACACATACAA
CATATATTTGTTCGTTAAGAGCTGACCTTAGTAGCCATGCAATAGAGTAATCTACATAAAGTACTCAGGCGTTTCGTCTATCCG
TCAGTGGTTGAGCATGATAGTACTAGGCATCCTTTTAATATTTGTGTGACAATATTTTTGGGCAGATCATTCATTTCTGGTGTT
TAAGTGCTCGCTGATAATGAAGAGGAGTTACAGTTCTTGCGGTAGGGTATAATAATTAGATTGTGGAAATAGAATCATGGATTG
AATATGTGATATATTATGTGATAGATATTTGGGATAAAGTAACAAAATTCATGTGTAGACCGAGATATTTCGAGCATGAATATA
AATGAGTAATTAGTGATAATTCGATTTAAGAGACGACCCATGGTATTCTCATGATTGGTTAAATTGGGTCCGTCTGGATTTCGA
AGTAGTGAAAAGGACATTTACTGATCGGCTTATTTCTCGGCCATGAAAATACCGCTCTACGCCATATTATGCAAGGAGGTACTG
AATGCACCCTGCTGATAGGCTTAGGCCCTTTAAAGATGTACTCACCGGCATATCAGGACACGGGTTTCCTTATATGATGGGGTT
AATAGAGAACTTGGGTATTTCCTTTTGTCCTTACACAGGACTACAAAAATTACATTCTGCGTAAAAACGTGAATTGGTACGAAA
TTATAATTCGGAATGATAACAATATATTAAATCAGATTGCGAGGCAATAAAATATGTTAGTAAATATAAAAAAAAATATAAAGA
AAGAATATTAGTTGAATAATTAATAATTAAGAAGTAGTCTATAATTATTTGGATAAAACTATTTAATGATTGTATCAGAAATAT
AAATTGTAATAATTAATATTAAAACAAACTAAAAATTCTGACCGATAATTTAATAAATTAGACCCATTGTAAGTATACATAAGT
TATGCGCCGCGCCGCCCTCGCGCCCGCCTTGCTCCTGTCTGCTCGCGCCTCCGCCGGCTACAGCTCGGCTCCATCTCCCTAGTC
CTTTGGTGCCTTTCGCGCTTTTGGGCTTGGTGGTGCCGCCCCCGCTTGGGCGGCCCGCCTTGCGGTTGTGCCGCTCGCCGTCTC
GCTGCTGCCTCGTCGCCTTCTCGTGCGGCCGGTTCCTCGGTTCTCTCTGTTTCCTTCTCTCTCCTTGCCGTTTTCCTGCGCGTT
GCCCTTTCGTTTGCCTTTTTGCGTGTTGCTCCGAGGTCGCCTCTTTTCCTGGGGGTTGTTGTGCTGTCGGCTGGTTTGGTCCGG
CTTCCCGGCTTCCTGTCCTGGTTTGCCCTCCGTTTGGGTGTGCTTTGTGGTCCCAGGCTCGGGCACCCTGTCGCCCCTGGTGGG
CTTTCTCGGTTTTCCTGCTCGCGCCCCGCCCTGTTCCCGGCTCTGCCCGCTGCCTCTGCGGCCTTGGGGCTCCGCCCGTTTCGC
CACGGTGTCGCGGCGCCCGCGTGGGTGTGTCGCCGCCTGTGTCCTTCCGCCGGGCGCCCTCTTCTCTCTGCCTTGCGTGCGCCC
CTTGCCCTTCGGCTGGGCGTCTCCCCTGGCCTCCTGCCTTTGCGGGTTTTCCGCCGGCTCCCTGTTCCGGCTGCGGGCCGGCTT
ATCCTCTTCGTTTGCTCGCCTTCGCGGTCTTGCGGCTCCTCTCTCTGCCGTTCTTCTTTCGTACTCGGGTTGCTTTTTCTTCTG
GCTGTGTTGTGCCACGTTTCCGCCCCGCTTTGGGTTCGGGGCTCGTGCTCCGTCTCGCGGTCGTCGCCCCTTCGGCCGGTCGCC
ACTTGTGCCCGTCGCCGGCCGGCCCTGGCCCCTCTTGTTTGTGTTTCCCGGGCCTGGCCCTCTTCTTTGGTGTTCCTCCTGTGT
GCCTCTTTCTGCTGTTTTGGTTTGTCCTCTTCTGCCGCCTGCGGCTGCGCCCCGCGCCGGCCGCTCCCTGTTCGGCCGCTGCGT
GTCGGTTGCCGGGGCGCGCCCTCCGGTGTCGTCCCCCCCTGTCACGTCCTCGGCTTGTCTGTCGTCTGTGGTCGTCGTCGTCGT
GGTGCTTCCGGGTGTGGTGGTGGTCGTCGTCGTCGTGGTGTGGGTCGTGGTGGGGTCGTGTCTGGCCGTCGCGGCCGTCGTCGT
GGTCGTCGTTGGTCTGGTCGTGGTTAGTGGTCGTTGGTCGTGGTACCGTGGTCGTGGTCGTGGGTCCTGGTCTGGTCCGGTCGT
CGTCGTCGTCGTGTCGTCTCGTCGTCCGTCGTCTGGCAAACCCACAAACAAGTGGTCGTCGTCGTCGCGTCGTCGTCGTCGTCG
TAGTCGTCCTGGTAGTTGGTCGTGGTCGTGGTCGTGGTCGTGGTCGTGGGTGTGTCGTGGTCGTGGTCGTGTTCGTGGTCGCGT
TCGTGGTCGTCGGGTCGTGTGTGGTTGTGGTGGTGGGTCCTGGTGGTGGGGTGGGTCGTCCGGCCGTCCGCCGGTCGTGGTCGT
GGTCGTGGTCGTCGTTTTGTCGTGGTCGTGGTCGTCGTAGTGGTGTGTGTCGTTGTCGTCGTTGCTGTCCGTCGAGAGGTCGTC
GTCGCGGGTCGTCGTAGTCGTGGTCCGTCGTCGTGGTCGTCAGTGGTCTTGTAGTGGTAGTGGTCCGTGGTCCGCAGTGGTCGT
TCGTCCTGTCGTGGTCGTAGTCGTCGTGGTCGTCGTCGTAGTCGTTGGTCTGTGCGCTGGAGTGGTGTCGTCGGTGGTACTCGG
AGTAGTCTAGTCGTGTCGTGGTCGTGGCTGGTAGTGTCGTGGTAGGTCGTACCGTAGTACGTGGGTGTGGTAGTGGTAGTGGTA
CGTGGAGTGGTAGTGGTATGGTAGTATAGCAGTAGCAGTAGCAGTAGCAAGTAGCAGTAGCAAGTAGTAGTAGTAGTAGTCGTA
GTAGTCGTAGTGGTGGTAGTGGTGGTAGTGGTCGTAGTAGTGGTAGTGGTAGTGGTTAGTGTAGTAAGTAGTGGTGGTAGTGGT
AGTGGTGGTGGTGGTGGGTGGTGTGGTAAGTAGTTCACGTATTGGTTCCAAGCCAGGATTTAGTTGGAAACTCATTGAATAGTT
ATGATTTTATATTGTTTAACCTATAGTATAGAAATATTAATTTTAACATAAAAGTTTGATTTATAAAACGTCCTGTCGGTTTGC
GGGTACCTTTCTAAACGATCGGGTCCGGGATTCCGAGTTAAGAATATTATTTTAAATAATATATATTTGTTTGGAATTTATTTC
CATTGAGTTATTGCTAGCCAAATTATGAATTTTTTGATTAAAAGTCGAGATAAGACTTTCGGTCTTTGGACCGACACAAAAAAC

CTTGAATCCCCGGTGTAAAAAATCTCGGAAAATAAAATGAAAAAACTAATGGCAAATATATTTTGGGCAAAAATCACATTCATA
TAATTTAAAAAATTTTTGTTCAAAATATAATAAACCTTAAGAAATGGAAAATTTTTAGGAACATTTTGTGTTAAATACTTAAAA
TTTATCGAAATGAGGGAATTTTGATTCCCAATAAATAAGACCTTGGGTAATTTTATTGTGCGATTAATTAAATTAAATGTGAGA
GATATTTAATTTAATTATTATATGTTACAAGTTTTTTTTTTAAAACTTAATAAAGTGAAAAAGGTATAGAAAGTCAAATTGGGG
TTTTTCTTCTTATGATACATATTTATTAACATGTAACGCCCTGGGTAACCAAAGACCGATACACTGTGTATTTGAAATAGACAA
GACATGCTAATACAAAGTTATTTACAAACAAAAATGTGATCTAACGACCAAAACGGTTTAGTGTTTTAAAAGATTTTGGTTATA
GGAACGAATAAGTTTTCCATTGAAACAAAATATTTGTATACTGGGATCCCAAATCAAGGTTTAAATAACGTAATTGTAGAATTT
TCAAAAGCAAATAAATAACCAAGCATTCTAATGGAAAAATACACATTCTAGGTTCGTGTCCCTGTACAATCCCTCGACCGTGCA
GCCGAGCAGCTGCAAGACACCTTCGACCCCAGAGCTCTCTTACTCATGTCACAATCATCTTATCCCTAAGCCCTTTTCTGCACT
ACGTAGCACCTGGAGTCGAGGCCCTGCAAGAAAACATAACATAACCAGTATATTAACAGCTAAGCAATTCCAGAAGCAATAACA
AATAATTTACAAGGCTTTAATCAATTACCCAGCAAGCCATATCATACATTCAGTGCAGCACTAACCGGTCAATAACCAATGAAT
CTAGTTTAAGATGACTCATCACATCATACACAAAATAACAATAGAAATACATGTCAAGCACAACTAGGTTTAATGCCCGTAGGC
TGCCCCTCTATTTAATCCACTATCTAATGGCTCACTTAGGCCAAGCCGCAGTGGTTTACCCACTAACTCGGCCGGAGCTTAACC
GAGCCTCGTGATTATTAAGCTGTCCTCAGCTTTACCAGTGGCCGAGCCAGCGCGCCTGGCCAGGTAACTCCCGGCGGCTCTGTA
GGCCACTCCATTTACTATTTACATGGCATAATACCATCCTGTATAGAAGCATACAAACAGGGAAACATTTTAGTCCCATTGATA
AATCCACAACGGTGTCAAGTTTTCTACCTTTAATTCCAAGAGGGGGGGGGGGGGGGGGGGGGGGGGGGGGCTTAATTAGGGAAAGAC
GACCCCCGAACACGATCCTAGCCCCGAGCCCGAAGCGTATACCTAAGTCACACCATGATAAAGGATTTCATCAATAATTATATA
GGGTTTTTCCCAGATAAAAGTTCTAGCTCACGGACCTCGAAATTCCACCAAACATGGTAGTGGAATCGATCCCGAGCATCTAGG
TTAAGTTCCCATGCCTTAAAACCCAAAAACAAAAGTATCCTTAAGGGTCAAGCTCAGCCTCCCATGCCGCGCTGTGACCCAAAA
CAGAGGCTCAAAAACTCTCCCCGAAAATGGACACGAGCGCGCGGCCCTACATAGCCCCATGTCGCTACACACCCTCACCTTCTC
CAGCACCATCAGCTTCGTAAGGGTCGCCGGCTCACCCAGAACTATGTCGCAGCCCGACCCCAATTTGAACTTAGACAAATCCAC
CAATTTATAATTTTTCCTCTAACTTATCCTACGTAGTCAGTTAATACTGAGACTCGGGTACCTGAAAAATGTCCCCAAGGGCAA
AATGGTAAAATTCCCCGATATTCCACCCTAGGCTTCCTAACCTCATCAAATATATCCTGCAATTATATTTGTTTCCATTAAACC
AATAAACCCCACAAAATGACTATCCTAGATATCATAGAACCCCATGAACTCACCCGAGTCCGAGTATCGGGTCCCGTTGTGAAC
TTCCCGCTTCTTGGCTCCCCTAGGATTGCCTCGCGCAACGAAAAGCTGCGAACACGATATCATATATCTAACATAATAATGTGG
TCTAAACAGTTTATCACAAATTAATCACGTTTAATGCCCTAACGGGCCAAAATTATAATAAATGCCCTTACTAGCCAAACAGGG
CTGCATCTTATCCAATACTCCCATAAAACATGCATTCCTCACAACCATTTGTAACGACCCAAACTGTTTATAAACTTTTGACCA
CTTTTTAAAAAAAAAAAAACTACTAAAACTAATCTTAGAAACTACATACGAAATAGCTTATAACTTTTTTATAACTGTAAGAGA
AGGTTGAAAAACCATTAAAAACTCATAAGTAGGGAAAATATTGGAATCCCATTGTTTTGAAAATAAAATATAACATGATTAACT
CAAAATTTGTTAGCAACTCAGATGCGGAAAAATACATATAAGCAAAATTTAAAAAGACTAGAAAACACTTCATCCTCAAAATCG
TTTGCGCAGTTCCACGCGATTACATTCTGCCTCATACACATCTCCAAGCTGCAAGACCTTTTTGCCGCCATACATTTTACCTGC
CACAAATATCAAACATAAAAGGAATGAGCCCAAATGCCCAGTAAGGAAAATCTACTACAAGTCATGAAACATATATCATTACAC
AATAAACTATAAAACTAATAAAATAACTGTAAACTATAATCATTGACTATAAAATACTACACTATTACAATGGCCATCATACTT
CTTGGAAGCTTGCTAGCTAAGCAAATCATATGCCCAAAAATTTACGGGACCTAGTTATTCTAAACAAGTCAAATATAAATTTAT
GGGGCTCGTATCAACAATCATAATGCCCAAGGAACTACTATTGGGACTTGTTATCTAAACAAGTTAATATTGTTATCTAAACAA
ATTAGTGATTATTGTTATGTATTCAATTCATATT
>m141120_105518_42134_c100732402550000001823141405141504_s1_p0/75980/409_14437
GCGGGAGGAGAATGTTTTGTACTATCTTTTCGAGCTACCCAAACAGCACACGAGACTCTTGCACACACTCACGAACCCACTCCT
GACTTCAGAAGGCTTTTTTTCTGGACAGAGTGTGTCTGACACCGTTCGTCTCAGGCGGATTCTCAAGGTATTCTCGATACTTTT
TCAATTTTGGAGCTCGGAACTTTTAGCCCCTTAAATCCAGTATTTAGTTGAAGTGTATCTTGCTTCCTCTAGCTAAACTTCTAG
CGTCCCACCCTGACGAGAACAATGAAGGGGCACAAGAGAGACCCTTGCATCCGGCTCCCCCTTAGGCAGGACCAGATTCTCTCA
TTTCACACATGAGGACAAGCTACGAGCTTGTGGGTGTTGCGACCAGGGCCAATTACTCCTGACGTCAGTCCAGTAAAAAATATG
AACGCTGGAGGAAGTGCCCACTGCCCACAGCCATCCCTTCCCTCCGAGGCACGAGAAAGGAGGGGCACCCTCCCAGATTCGCTC
GGAGAAGTCCGCGGTCGGGGGAACGAAGGCCAAGACGAACACCTCGAGTCGGACTCAGATGGAGGTAAAACCCTTCCTACTTCG
CGGAAATGTGTCCACTTTACTTAAACACAGGCCAATAGAACTAGTCTCCGTGCCCACCAGGAATACGTACCGACTTCTACACTA
GTTGGAGTTGCGGTAGACGACTGTGTCCATAGGTTTGATAGTGGGTCGGAAATACGATACCTATGTACACCACAGGACGAGGTG
TGGAATGGGATAGCGGTGCAGGGAACGGACAATGACGTATAGGGACCTCTCGACGGTTATCCCCTACCTTACTAAGGGAAGGC
ACTTCCCCCCCGCCTCCTTCTGAAGACCGGGGGGAATTTCATGGTCCCCAAGCCTCGAGCCTCGGGGGAGAGTTCCAGTTAGGT
TTTTTTCCTTCAACTGGTTTCCAATCTTTTTTCAAAGTTATTATTACATGTTAACCACTGTCATTAGTGCAGGTGGCCATGAAT
CCCGACCTCGACGCCGTAGCTTTTTAAGGCGATGGGGGAAAAGCTGGCGCCCCAAAAAGAGTAACGCCCGAGGGATACCAAAGA
GATCCGACGACGTGCTAAGGTACCTAAACCATCGCGAGCGATCCACGACCCAGACACTGGCACAACCTTCCAAGAAAAGCACTG
CGCAGATTGATGGGTCTCAGCCCGACTCGGCGCATCCAACCTCGCTCGCTGCTTCCCACCGAAACCCGGTTAACAGTTGTTCGG
CCCGAAGAAAACCTTTCTAGTCAACGGGTTCAGCTGCCCACGCCTCGACCACAACTTGAGGATATGTGCTTGATGGTGCCTTGG
ACAGCCAGGGGCGGTTTACCTCAGCTCGGACGTCTTGGGGCTCTCGGGTAGTCGCAGCAGTTTTTCTGGCTTCGGCGCCGACCA
TGGGAAATCTGTGCGCAAGGCTCTTCGGACTGCAACACCTCTTTATAGAGAAGAGTAATCGAACTCACCGCCGCGGTAGGCCTT
CTCAACTCCTATTTTAAGTCATTTCTATGCTCAATTCGCAATTCTGATTTTGTTTGTTTTCAGCCCTTGTTGTCCTTCAGCACA
GCTCAAACTATAAACCGACCAACGAGGTGCAAACGATCTTGTCTTCTGGCTCAGAAAGTCGAAAGGAATTCTCCCCGAGCTTAA
GATGTTGATGGAGCTCAAAACTCGAAGTCTGAACTCTGAGAAAACTGAAGACCCGGTCTCGAAGAGCTGGAAAAGTCAAATGCT

GAGCTCGAGAAGGCCAATGCCAAGCTCGAAGAGGAGAACGCTGCCACCCATCGATCATCGGAGAGCGAGAAGGTCGCCCTCCCT
GAATGAGTCTGAAGCGAGCAGAAGGCAGAAGCGACTGACCAGGCCAGTGTACGAGCTTTTGGACCCGACAATTCCGACCTTGAC
ACCAGCTTTCCTGGTCCTTCACCGAAGAACACATATGTTGACGCGGTGGAAATGCCCGTCTTTTGGCAAGTGTCAGCTGCTCGA
GAGCGGCCTACGCAGGATAGCCCATGGTTCAGTTCCGGTCCTGGACGGGCTCTGGTGATACTGATGCTGAGAAGGCCCAACGGA
TACCTCCTCTTCCTTAAATATCCGATCTCGTAGAAATCAGCCTATCGGGACAGAACCATCAGTTATTGCGGGGCTGCGCATCCC
GCTTCACTCTTCTCGTAACTATTTAAATTTGGTTGCCACGGGGCTGACACAATTTCTTCTAACTAATTCCTTTTTCATGATGGC
TTTACATTCCTTGGCTGACGAAATATTTTGCACATTTTTTATAATTTGATGAGCTACGTTACTGTTTTATTTAACATTCATACA
AACATAGTTTTGGCATTTAGGCATCGACAATTTGACTGCATTCATGCAAATGAGCCTTTATTCGATTATCCGCTTCCAACCTTC
GTTCATTCGTACGTCAAGGTCAGGACATTTAGCTCATTAACCATGCAACTGAAAAGTGTACTCTATCTTGGCATGTAATCATCG
AACTGACTTTGCGTTATAATCGTTTTTTTTTTTTTTTTTTTCTCTTAAGTTCAGCCTTTCCTTCATCCTCAGTAATTCTTGGTC
CGCAGGTTCTAGAGTTCTCGAACTACTTTTTCTCAAGATATTCCAGCTCGACTCTTGACTTTCTCGCCAATAGGTGTAGCTCCC
AGTTTATCGTCGATCGATAACCTTGGCTGGTTTGTTCCAAACCTGTTGTGTTCCTGCACATCTGCGTTAACTCCAATATTTAGG
GTTCTTTGATAATTTGGTTTTGTTCGAACTACTCTACAAAGCCCTCTGCCGTATTGGTCATGTCCAAGATACCCTTCACTCGCT
TTTTGATACACCTTCCGGTTTATCGTCCGAACTATCAACAACGAGTCGCCGTACCTGGTTATCACTCCAAATACTTAATGTTTT
CTGATAATCTTTAGTGGGAGAACTAATCTAACACGAGCTCGCTCCGGGCATTTACAGTCCGAACTATGCTTACAGCTCCGCGTA
ATTCTGGTTATAACTCCAAATAACTTTATGTTTCTCATTTATTTTTTTAAATTTTCTTTTAAGCTGGTGGGTATGTATACCACAA
CGTGCAATGGCCCCTTAATATCTGAGCTGAGTGTGACCAGTCACGTTACTTAAATTAAACGAGAGATTGCAAAGATAAAATAAA
TTACATTGAAAAAAAGTAGACCTTTTCATTTGATGAAATTCAAAACATAAACTAAATAAATAAGAAAAGTATAGGTACGCGGAC
ACTGCCTTTACCATATTGAATACGAATATGGTCCTCACAGCGCTGTGTCGCCATTCCCACGTGCTCGAAGGGTATCGCGCTCCC
CATCTAATCTCGCAAGTTTTATACCCACCAGGTCGGAATGACTGATTCTATCTGGTATGGTCCTCCAGCTTCGGCCCGAGCCAC
CCAAGCTGTTGGGCTCTCGGGTTTGCCAAGAATACTCGTCTCAAAAATCAAGGATCTCCCACTCTGAACTGTTCGACTCTCCGA
ACCTCTTATTGAACATAGCGGTTCGTGGTTCGTTGCTGGCTAAAGGCAGCATTTCTTAGCTGAGCTTCTTTTCCTCTTTCTCTT
TCTTCGTAAGTCTAGCGGTTTCCTCGACGCTGAGAGTATGCATTCGGAACTTCTGGATCGCGTAAATTGGTTCGGATCGTCCGG
AATTTCGAAATCGAGGGCCCAACATGCGCCCTCGCAGCCCGTACGCTAGAGAGAACGGAGCTACTGTCCTGTCGATGTCCCGAG
CAGTAGGTTCTATATCCCCAAAGGACTTCGGGCGGAGTTCCCTCGGGCCATCAATCCCTTCTGCCTCCTGCCCAACTTTTTTTT
AAGACTTGTTGTGACGATTTTTGTTAACGGCCCTCCGACCTGACCATTCGCCTGAGGGTGAGCCACTGACGAAAAGCTCTTTAT
TGATACGGTTCCTGTGTTACAAAAGTTGTGAATAAAGTAGATCGACCAATGGGGCTTCCGCTTATCAGATACCAATCTTCTCTT
GTACTCCCATATCGACATACGATGATCTTTACCACGAAATCAAGGATATTTTTCGAAGTTATGCGGTCACCAATGTTCAGCATG
CCGTCCATTTTGTGGAAGTAATCCAACGGCGACCACAGCAATTTTAACTCCGCCTTTGGCCAGTTGGGAGTTGATCCTATGAGG
TCGATGCCCATACAGCGAAAGGCCATGGGGGATGTCATTCAACTGGTCAGGTTCGGAACGGTGGAGGCTGGCAGGGGTATCGTG
GCGAACTCCTGCGCATTTGTCCGCCACTTTTTCACGTACATCGAAGGATCCCGTTTTAAATCGGTTGGCCAGAAATATCCAAAT
GGCGTACTAAATTTTCTTGGATAGCGCTATGCCCACGGTATGGTGCTCGTACGACCTTCATAGAATTTCTTCAATGATCTTCTT
AGCCTCGGGAGGAGTTTACACACTCTAAGCACGCGGCATGGAGTACCTATTACTGTTACATTCAATCCAGAATGGTGTCAAGAG
GAACGTTTCGAGTACATTCAACGCTTCGAGCTTGATTTCGATCTCTCGGAGAAATCCCCTAATTTTCCGATAGTATCGACTATC
GACTCATCAGGTTTTGGTTCTGTTTCAATCATACACACATCTTCCTCGTCTGCTCAGTAATGCTGGCGTGCTGATTAGGTGGTT
CCTAAGCGGGTATAAACAATTAAGCTCTTCATTTTGCCGCAGTGGCGAGCCGGAGCTAAGGCATCTGCATTTGAGTTTTCGTTC
TCGGGAACCTGCTCGATTGCCATTAAAATCGCAAATACTACAACTGCGGATTTGCCCTTCTCAGATACAGCTGCCATTTTGTGC
CAACGCAGCGTGGTAATTCTCCCAAGATTTGATTACCACCAGCTGGGAGTCGCTGTAGCACTGGATGTAGCTTTGGCCTTCGAG
CTCCTTTTGCCTATTCGTAGTCGATGAGTAGAGCCAATCATACTCAGCTTCATTATAGATGGCCTTGAAGCCAAATCTTAAAGC
AGCAGTGAGGATCTTCTCCCTGCAGGAGTGCCTCATACTCCAGCACTTCATTATTACGATGCCTTGAAGCCAAAAATCTTAAAG
CAGAGTGAAGATTTGCTCAACCTGCAGGAGTCGATCAGAATGAACTCCTGCCCCCGCTCCCCATTTTCATGTTGACGAGCCGTC
GACCGTAGAGTTTCCACAGTCGTGGCTGTGGTTTTACTCGTCGTCCGGCTATACCAGTGCACTGCACTATACAAGTCTGCCAAG
GCTTGTGCCTTAACATGGTCCGTGTCTCGATGGTAGGTGATCTCTGAACTGTCCCGAGCTCAACCAGCCCATTTAACGGAGTCG
ACCAGACGCCTCTGGTTCTACACAAGACTTGCCTTAGTGGTTGATCTGTTAGTAACATGGATAGGATGTGCCTGAAAGTAAGGC
GGAGCTTGCGAGATGAGTGGATTAGACTGAGCGGCGAGCTTCTCCCCAATCAGTGGATATCTGAACCTGCCCCCAGTAACTCTT
CTTAGCTGATATAGTAGACGGGTTTCTTGTACTCTCTCCCTTCCTTCCTCGAACGAGCACCGCGCTTATCGCATGCGTTTCGGT
CAGTTGAGAGGTATAGGAACCTAGTACTTCTCCTCTCAGGCTTCGATACGGATGGGCGGTTCGCGGCTAACGGTGCTTTTCTGA
GCTCCTGAAAGGCTAGCTCGCCCCGACTTCGTCCTGTCCATTCGACTTCTTACTTCCTTTCAATAGTTGAAGAAATGGCGAGAC
CGCGGTCCGTTGACTCGAGATGAACCTTGCTTCCAGAGCAGCCAATCTGCAGTCAAGCTTTGAACATCCTTTATGCTTCCGAAG
GCGAAGGCATATCCGATCAGCCGGCCTGTATTTTGTCGGCGATTACCTCAGATCCCACGAGAGTTGACAATGAAACCCAGAATT
TTCCTGAAAGACACCCCAAAAGTGCACTTCTGAGGATTCAACTCTCATGTTTGTACTTTCCTGCAGCATGCCCAAAAGCATCTT
CGAGGTCATCAACATGGTTTCTTGTTAAGTTGAGACTTTACAAGCATGTCATCAAACATAAACTTGTCCATGTTGTTCCCTTAT
TTTCTCTGAGAAATCAATGTTTACGAGCCGCTGGACCGTGCTCCGGCATTCTATGAGTCCGAATGGCATTGACACATTGTAGGC
AGTAAGCCCTTTTAGTCTGTGATGAGCTCGTAATGCTCCCTTGTTTATTTTCGGGGAGCGTGCGCCATGGGAATCTGGTTATCA
GTCCAGAATAGGCATCCATGAACGACATCCAGGCCATGCCCCGCCGTGGCAATCCCACGAGCTGGTCAATTCTTGGCAGCGGAA
AACAGTCTTTCGGGCAGGCCTTGTTGAGGTCCGAGTATCAATGCAGGTTTCCCACCGTCCCATTGGGTTCCGGGACCCAAAACC
TGGATTTGGCTACCCAGTCCAGGGTAAAAAGCGTCCCTGATCAAAATTGTTTGCTTTTAGCCTGTCAACTTCCTCTTTAGTGCT
TCTCTTCTATCCTCCGTCCGCCTGTCCTTCGCTTTTGTTGCTTCGGGGGAAAGCTTTTGTCTATAATTCAGGTGGTGGCTCGCA

ACGCATTTGGGCTTATCCCCACCATGTCTGAGCTGTGACCAGCGAAGACATCCCTGGTTTTTCTTGGGAAGGCAAATTAATTGC
TATTTTGCTATCCGTCTTGGAGGTGTTTTCCGACCTTCGTACCTTCTTCGAGGGTAATCAGCCATCCCGTCGAGCTGAACTTCT
TCGCAGCTCTTCCAATGGTTCGCAGTCACTTCTCAACTCGATCCTTGATCCGATTTCCTCGTCAATTTCTAAAAACCGTCCCAA
TCTTAATTTTTGGTAAAACCGATTAAACACGAAGTTGCCTTTTTTTTTTTTTTTGAAAAAGCCCCCGGCCCCCCTTTCGTTTTTT
GTTTTTTTTCCCTTTTTTTTTTTTTTTTCCACCTTTTCAAAAAAAAAAAGAGAAAAATACTTTGTTTACACCACTCATTTTTTTT
CTTTTTCTTTCTTTTTTCTTTTTTCACCCTGAGCCCCCCAGAAAATTTTTTTTTGATGTCCTTTTCACGCCCTTTATTTCCCAA
ATGGTTTTTACGACCCCCCCCGCAGCCCTTCCGGAGGGTTGGGGGGAAAAAAAAAAAACTTTTTTTTTAAAAAAGGGCCCTTAAA
GGGTTTGGGCCCCCCCTTTTTTTTTTTTTTTTTTATTTTAAAAAAACCAGAATTTGGAAAAGACTTTGCCCCCCCCCAAGCCC
CCCCCCCCGACCCAGGGGCCCCGGGGGGGTTTCCCCCACTTCCCGGAGCAAAAAAAAAAAGTTAAAAACCAATTGTAAAAGGCC
AAAGAATTGGGGGTAAACCCCCTTCCCCCCCCTTTAAACTTTACCACCCGAACCTTTTTTTCCCAAATCCCCAAAAAAAAAAAA
ATCTTTTTTTGGTCCCCCCCCACCCGAGACTAGGAATTTTTAGTCCCCTTTTCCCCCCAAAGGGGGTTATCCCCCCCCCAACCA
AGGGGAAAAAAAAAGTTTTTTTCGGAATGGGGACCCCAAATAAAAAAAAAACCGGGCGGGTTTTTTTTCCCCCCCCCCTTTTT
TTCCCCCCTCCCAAGAAAAAAAGCCCGGGTTTAACAAAAAAGGGTGGGGGGGTTTTTTCTTTTTTTTTTGGCCCACATAAAGGG
GCCCCCCCTTCCCCAAAGGTTTCGCCGAAGGGGGAAGAAAAGGTCCCCCCAAAATTTTTTTCCACCTCCTTAAGTTGGGGGTT
TTTTTTTTTTTTTTTTGCTTTTTTAAAAAAAAAAATTAAAAAGAAAATGTCTTTTTTTTTTTTTTTAAACTCTTGAAGCCCTTTT
TTCCCCCCCATTTGGTCTATGAAAAGAAAAAAAAAACCCTTTTTTTTTTTGGGGGGCGGCAACTTCTTTTTTTTTTGGATTTTTT
TTTTTTGGCAACAAAAGGCCCCCCCTTTTTTTTTAAACCAGGGTTTGCGGGGAATGAACCCCCCCCTTTAAGGGGTGGGGGAAATT
TTTCTTTTCCGTGGGATGAGGGGGAAAACCCTTGGAAAACCCCAAAAAAAAAAATGGGGAAAAAAAGGGCAAAAAAAAAAAAAAA
TCTTTTTTCCCCCTTTCGGGGTGGGGAAAAGGTTTCTTTTTTTATAAGGCTTTTTTGAAATTTTTTTTTTTTTTTTTTTTTTT
TGGAAAAAAAACCCCTTTTTTTTTTTTGGGGCTTTTTTTTTTTTTTTTTTTTTGGGTTGGGGGCCCCTTTTTTTTGGGGTTTTT
TTTTTTTCCCGGGGTTTTTTCTTCAATAAGGGGAGACCCCCGTCAACCCCCCCCGTTTCTTTTTCCAAAAACACCCCAATTTTTT
CCATTTTTCCAACGGTTTGTAAAAAAATTCCCGCTTTTTTTTTTTATCATTGGAAGCAATTTTTTTTTCCTTTTTTTTTCCAAC
CCCCTTAAACCCCCCCTTCCCCCTGGCGAAGATGAACAAAAGACCCCCCCTCCCCCCAACCCGAAGAAAAAAATGGGTTTTATT
ACATCCAAAAAATCCTCCAAAAAAATTCCATTTCGGTTTTGGGGGGGCCCCCCTTGTCAAAAAAAAAAATCCTTTCTTTTTTCC
CCCCCCGAGAAAACCCATGGGGAAGTTTTTTTTTTTTTTTTATTTTATTTTTCCCCCTTGCTTGCCCCCCCCCGGCAAAACCCG
GAAAAGGGGCCGGGCACGGGCCCTATTTCCCTTTCCTGTTGGCTTTTTTTTTTTGGCAGGGTTGGGGGGGTTCTTTTTTTGTC
CCCCCACCCCCCCCAGTAAAAAAAAAAAATTCCCTTGGTTTTTTTTTTTTTTTTTTTTTTTTTTTTTTTTTTTTTTTTTTTTTT
TGTGGAAAACAAATTTAAAAAACTTGCCCCCCCCCCTGGAACAAAATTTAACCCCCCTCCCCCTTCCGGAGAAAAAAAAACATCA
AAAACCCTTCCCCCGAATTTTTTTCCTCGTTTTCCCCTTTCCCCAAAAAGCCTTGTAACCCCGGCAAACTTCCCCCAATCAGTT
TTTTGGTTGGGGTTGGTCCCCCCGGGTTAATTTCCACCCCTTCCCCCTAATTTGAAAAAAAAAAAAACCCCGGCCAAATACTTA
AAACCCCTGGGAATTTCCCCCTTTTTCTTTGGACCTTTTTGGATTTTCCCCTTTCCATTTGGGGGGTTTCCCCCCCCCCCCAC
CCCGGAACGCCCCTGGAAAAAGGGGGGGGAAAACCCCTTTATTTTTTGGGGGTTTTTTTTTTTTTTTTTTTTTTTTCATTAAG
CCCAAAGGGGGTTAATTTAAAAAACAAAAAAAAAAAAATTGTTTTTTTTTTTTCCCGAGACCTTTCCCGTTTTTGAGCTTTTTTT
CCGGTGGTGGGGCAGACTCTTTTTAAATATAACGGAAGAAAAAGTTACCTTTTTCTTTTTTTTTTTTTTTCCCTTTCCCCTTTTT
TTTCCTTTCTTTTTTCCCTTTTTTTCCCTTCCAATCCCAAAGGCCCCATTTTTTTTTTCCGGGGGTCTTAAATTTTTCTTTTCT
TTTTTCCTCCCGCTTCTTTTTTTTTTTTTTTTTTTTCCCCCCCTTTTTTCCTTGGAGGGGAAATCTTTTTTTTTTTTTTCCCT
TCCCCCCGAGGGTTTAAAAAAAAAAGAAAAGACCCCCCTTTGTGGGGGAAGCTTTTGCTTTTGGGTCCCCCAAAGCCCCGAGG
GTTTTTTTTTTTTTATTTTCTTTTTTGGAGGGCCGGGAAAGGTTTTTAAAAGTTGTTTTTCTTTTTTTGTTTTTAAATTTCCG
TTTTTTTGGTAACAAAAAAAAAAAAAAAAGTTTTTTTTTTTTTTTTCCAGGGTCCCTGCGAGGGTATCCCCACCCGCCTATTTTG
AGCCGTCTGGAACCCTCCCGCAAAAACCTCCCTTCCCCTTACAATTTTTTTGGGAAAACCAAATCTCCTGCGGCCCCGTCTTTG
GTTTTTTTTTTTTTTTTTTAAAAAAACCCCCTTTTCGGGTTTTTTTAATCGACACACCTCAAAAAAAAAACCCGGTTTTTTTTT
ATTTTTTGTTTTTTTATTTTCCCCCCCCCCCCCTTGCCCACCAAAATTTGGGGTTTCCCCCCCCCCAAAATCCCACCCAAAGGG
GGGGCCAAAAACTCCCCCGGGGTTTTCAAAAAAAAAAAAAAAAACAACCAAGCTCCGGAATAAAAAAGCCCCCAAATTTTTTAA
AGGTTTCTTTTTTTTGCCCCCCCAACCCCCCTTTTTTTTTTTGTTCCAAAATCCCAAAAACCCAATTTTCCCCTTTCGAGGCCT
TTTCCGGGGCCGGAACCCCAACCTCAAAAAAAGAAAAAAATTTAAAAAAAAAATCTTTATTTTGGTCCCCCAAGAATAGCCCCT
TTTTTTTTTTTTTTTTCCCCAAAAAAGTGTTTTTTCTTTCTTTTTTTCCCCCAAAAAACCCCCGGGCTTTGTTGGTCCCCCCCG
GGAAAAAAAAACGTTTGGGGGGGGAGGGTTCCCCAAAGTGGAAATTTTTGCCCCCTTCCCCTGGGGTTTTTCCCCACCCCACTTT
TTTGGAAAACTTTCCCCCAAAAAAAATCATTCTAGCCCCCCGGGAAACCCCCCCTGCCCCTTTTGTTTTCCCCTTTTTTTTTTT
TTGGAAAAGTCCCCCCATAAGGAAACCAACCTTGAATCCCCCCCCAAAAAATTGAGGGTTTTATCCGAGTGGAGAGAATTTCCC
CCCGTTTTTTCTTTTTTTTGTTTTACTTTTCCCCTCCGAGGCCCCCCCCCCAAAAACTTCTTTTGGCAGGTTCCCGGGGACCCC
AGATTGAAGTGGTTGTTTTTTTTTTTTGGGAAAAACCAAACCATGGCCCAAAACCCCCATTTTGGAGCCCTCCGGGTAAACCCCC
CCAACGTTTTTTTTTAAACTTAAACAGCCTTTTTTTTTTTTTTTTTTTCCCTTTTTTTTTTCCCCAATTTTAAATAAAAAAGTGTTT
TTTTTTTTGGGAAAAAAAAATGGTTTAAAAAACTTCAAAGGGTGGGCCCTACATCTGGGGTCGGGTTTTTTTTTTTTTTTTTTT
TTATTTTTTTTTTTTCTTTTTTGTCCCCCCTCAAAAAAAAAAAACGCTTGGGGGGGGGAAACCCACCCCGTGGGGGGAAAAGGG
GGGGAAAAAATTCCCCCCCCCCGGACCCCCACTATTTTTTTTTTTTTTTGGGGAAAAAACTCTTTCGAGGTGGGGGTTTTTAAAA
AGGAAAAAAAATTTATTTTTTTGGGGGGGGGAACAAAGCCCCCAAGACCGGCTTTTCGGAAAAGCCTTTCTCCCCCTTTCAAAA
TCAGAGTCCCCCCTTCCCCAATTCTTATCCCGGGCAACCCCTGTTTTTTTTTTTTTTTTTTCCCCCCATTCTCCCGGTTCCCC
CAAATTTTTTTTTTTTTAAAAAAAGCCTAGAAAGGGGGCTTATTATTTTTTTTTTTTTTTTCTTTTTATTCCGAGCCCTGAATTTT

CCAATCCCCTCCCCTTTTTTCCCTTTTTTTTTTTCTTTTTGGAAAAACCCTTGGGTTTTCCCATTTAAAGGGGCCCCGGTGGCC
CCCCCTGCGGAAATTTTTGCCCCCCCCAAAAAGTCCCCATATTGGAAATTTTGTGGGGGCAAATTGGATTAGGCCCCCCTCAC
CGCGTTCCCCCTCCCCCCCACGTGGAGGGGGGGAAAATTCCCCCCTTTTTTCCCTTTTTTACGACCCCCCCCAAATTTTTTAA
GGGTGGGATTTTTTTTTCCCTTTTTTTAGGAGTTTTCCGGAATTTTTTTTTTTTTGGGTCCCTTTTTTTGAAATTTTCCCCCCC
CCCCCTTTTCCCACCCCCACCCTCTGCCTTTCCCCTGAATTTCCCAAAGATGAAAAAATTTTTTTTCTTTTTTCCCGCCAGGG
TTTTCCGGGGAACCGGGGTTTTTTCCCTTTTTCGTTTTGGCCCCCCCCGGCTTTTTTTTTTGTCCCAAAAGCTAAAACTTTTT
TACACAACACTTTTTCTTTCGGTGCCGGGTTTTTTTTTTTTAAACTTTGGACGGAACCTTGGTTGTTTTTCCCCCCCTTCCCA
GAAAACCCCCTTTTCCGTTTCCCCCGAAAAAAAAAAAAACCTTTTGGGGTTGGGGAAAACTTTTTTCTAAAAAAAATTTTTGA
AATTTCCGGTCTTCATCGTTTTTTTTTTCCCCCCCCCCAACCCCGAAGGAAACTGGGGGTTTTTTTTTTTTTTATTTTCTCCTT
TTTTTTCCCCAAAAAAAATGGTCGGCCCCTTTTGGGGGTCCCTTCCGCCCAGTTGGGGCGAGACCCCCACGGGCAAACCCCGAT
CTTTTTTTTTTTCCCTTCCTTCAACCAAACCAAAAACCAAAACGGAGGGGGAAAAGGAAAAAAGGGGAAAAAGAGGAGGAAAAG
GAATTCGTCCCCGGTTTCCCCTTTTTTTTTCGCACTGCGGAGAACCCCCAAAAGGCCGAACAAATGGGAAAAAGAAAAAACCAA
AATCCCCCCCTTCCCGAAAAAAAAACCGACCGATTTCACAGACACCTAAAAACGGTTTTTTTTCCCAAACGCCAGTTGGAACGG
GAGTCTGGAGAAAAACCCCCCAGGTTTCCACCCCGTTCCAGTTAAAAAACCCGCGACCCCCGAAGGGGGGGTTTCGATCTAAAA
AATTTTTAACTTGGGAAAAAAAAGGAAAAAAAAAGGCCCCGAGAAAAAAAGACCCCCCGTTTTCACCCCGACCCTGGCCGGGAA
AAATTCAATCCCCCCCCCTTTGAAATTCCAAGAAAGCCCAAAGGGGGGGGGGAAAAAAGGGGGGATTTTCCCAAAGAACCAAAA
AAGTCCCCCGGACCCCTAGAAGAAATTCAAACCCCCTAAAAAAATGCGGCGGCATTTTAAGCGACGATAGTTCCCCCTTTCACA
AACCGGGAGCGCCGCAGGCCCCTTAAAAAAAGCCCCCCAAAAAATCAAACAATTCAAAAATTTTTGAAACTTGGGCAACATTTT
TGTTTTCCCCCCCAAAGGCCAACCCCCCGCCCCCCCTTTTAAGGACCCCCCAAAAGCTCCCCCAAAAACGACAAGAAGGGGAA
AAATTTGAATCAGCTCGAAAAAAAAAAGCCTTTTAAGGGCTTTTTTGTTTTTAAAAGACAACCTGGAAAAAAAACCCGCAGG
GAAACCCCCAAGTCCCCCCACCCCCGATTAGTGAAAAAAGGGGGAAACCTCCCTTTTTTGAAATCGGGAAAGGGGAAAAAAAAA
AGCCCTCCGGGACAGGGCCCGTTTTGCTCCCAAAAAAAATATTTTTTTATTTTTTTTTCCCCCCCTTTAAAACCCACCCTTCTC
AAAAGTTTCTTTTTTTCCCCCCAAACGGGGTTTTTGTTTCTTTTTTTTTCTTTTTTTTTTTTTTATTTTTTTTTTTCCCCCCC
GGATCCCCCCTTTTTTTTCCCCCAAATCCCCAAGGCACCCCCCCCCCGGGTCTTTTTTTTTTTTCTTTTCTTTTTTTGTTTCTG
AGGGGAAAAAAAAAAAACCCCGCGACCCCAAATTGGTAAAAAAAAGCCCACCACCAATTTGAAGTTAAAACAAAAAAGTTCCCC
CAACCAACCCTTAAAAATTTATTTGCAAAAAATAAGAAAAAAAGCCCCCTTTTAAAGGTAAAAACGTGGGGTTTTTTTTTTACCG
AGGCCTTTTCCAAAAGGTGGGCCCAAAAAAAAAAAAATTTGGGTTTTTTTTTTGGGTTTTTTTTTTTTTTTTCCAAAAACAATCC
CCCAATTTGGGGGCCCCGAAAACCTGCCAAAAAAGGAAGTTTTTTTGGCCTTCGGAGGAAGTTTAAAAAAAAAAAAAAAAACA
AAGAAGGGAAACCCACCACTTTATTTTTTCGATAACCCCCCTCCAATGGGGGATCCAGTTCTTTTTATTTTTTTTTTGTTCCCC
CTAGGGGCCACAAAAACCCATTTTCCCCCAAGAAAAAGGCCCAAGTTTCTTTCTCCCGGGTTTTGTTTTTTTTTTTTTTTTAAA
>m141120_105518_42134_c100732402550000001823141405141504_s1_p0/76513/1992_7721
GCTGAATTATCCTACACACACCTCCCAAATCGGGAGTTGACGCTGCCCCAGGGCCATACTTATAGCAAGCAAGCATTGGGGTCA
GTGGAAGCTCGTGCAAAATGCCCATACTATATAGCGACCTCGCGCACCCGTGCGCACACAGGTTCGCGCTAACCATAGCATTTA
TATTAGCTGCCTTCGCACGTTCGGATTCTCTGCGGGGCCATCGCAGCCTTCCGCTGCTGCATCCATACACCTCGACAGCACCAT
ATATACGCGCACTACAGGGCCTATATCTGCAGCCCCCGTCGCGCCATGCGCCTCGTGCGCCCATGCCGCCTCGCGCAGTGACCA
TATGAATAATTTGGATTGTAACGAAATGGGTTCATTATCATATGATTAAAAAAAAAGAACATATAATTTATTTTATGCAGTAAA
TGTTGGTAGTATGGAGGTGCGCCTCGCCGCCCACGCGCCTCGCGCCCGCCGCATCCAGGGGCCTCGCGCCCTGCGCGTACCAGG
GGCCTCGGCCCCACGCGCGCGCCAGGGGCCTCGCGCCCCGCACGCACCAAGGGCCTCGCGCCCCCGCGCGCACGCAGGGGTCTC
GCGCCGGCGCATGCGCCCCCGCGCGCACAGGGGCCTCGCGCCCCCGCCATGCGCCTCGCGCGCACCCACGCGCATCGCAAGGCC
TCGCCGCCCAACGCCGCGCCCATACGCGCCTCGCGCCCCGCGCGCACGCAGGGGCCTCAGCGCCCGCGCGCACAGGGGCTCGCA
ATGACGTATGGCCTTGGCCATGGCCCTCTCCATGGCCTCCGACCAATGCCTCGCTCATTGCCTTGCCCATGGCTCCGCCCATGC
TCGCCATAGGCAGTGCCCATGGCTCTCCCATCGCCTCGCCCATGGCCTCTCCCCATGGCCTCAAGCCCATGGCCTCTCATGGCC
TCAGACCATAGTAGGCTCGTCCATGGCTATTCGCCCATGGCCTCGCCCATGGCCTGCCCGATGGCCCCTTGAGTGAGAATACTC
ACAAAGGCAAGGTACATGCATGAATATGGAGATTGCCTACATAACCTAAAGAAGTCAGAGTACGGATATAGTCAGCCTTACCAG
ACAAGTAAGTGGGAATGCCACGGCAGCTGCGCTTATGCAAGAATAGGAAGCTAATGGTCCTAACGTACCTACGCCCACATCGGT
CAGCAGCTGCACCAACTAACTTCCCTGCGCCACTAACCGCTGCCACCAGCATTAGACATGGACCGACAAGTAGTGGGACGACAT
CCTCCCTACACCTGCTTCTGTAACGGGGTGTAACGACCGAACCTCCTGAAGCCACTCCTGACCACAGTAACGTATGTCACTTCC
TGTCCCTGGACCACTATGTATCTTAAGGCCCATTAGAGCCTACTATAAACAGCCGAACCCTAAGACCACCTGAAAAGGGTTGGA
AAATTCATTGTAAAGCGAGAGGCTATTGAGCAAATATACCTAGGATTTGCCTCCATCATTTTATCTGTGTCTACTTTTCTTAAA
AGTGTATCTCTTAGTCTTACAAAACCTCACTAGACTTACCTTTGAGTTTATTCCGATCTAATTTTCGTTGACGAGATTTCACCG
TCCAACTAGTTTGGCGCCGTCTGTGGGAAGAAACAGCATCAAGCCAGTGTTCTTCCCTTCATTTCCAAGCAAAGAAAGATGCTA
AGACGTTCAAAAACGCCTACGGCAAATATCAAAAGGTGGACGGTTCACCGAGACGAAGTAGAGCGAGGCCTTCCAAGAAAGACC
CCCCCTCCTGCCCGAGCATGGAGATGGACCAGAAGAACCCTCTTCAACCCCGGAGAGGAAGGGGAAGCCATCGTCCTCCAGCGG
CTCAACCCAGCCCGACGGAGTCATTCAGAGCCCGCTAGTGTATCCACTTAACCCGCCCGTTTGAACCCCACGGCTCAGGCCACC
GGGACCCCAGTCCCTCGACGCACTGGCCAGGCCAAGGGTCCCGGGTATACCAACGCCTCCGGTAAGTTTCCAGCTCAGGGACGC
GCTTCCTACAAGGATGAGATTTCAACGAGTTGCATAAAAAGACTAGGAGGATGGAAACCATGATGGAGGAACCATGCAGAAGGT
TTTTTAAACAACTACGGCAAGAAGTCAAGCATCCGCCCCTCACCAAAGCATAAGGGAAGGAGCATGAAAAAGGGAAAACACTG
TCCCCCATAGACGGTGGGGAACCACTTTCCGCCAGTGAAACCCCCAGTGCAAAGTACCATGGGGAAATAGGCCAAACGAAGT

GTCCGCCAGGAGCAATAGGCAGAGGAGAGATGAGCGGTCATAAGAAGATGAAGTCCCCCCAAAGGGTCACCCCCCTGGCCTAAG
AAAAGGTCCATGAGGAGAGTCCAGAAGTGAGAGACCGGCACCCCCCGCGGTTCCACCGAGGACACAACGAAGCGAGGAGTCAAG
CCCGAGAACCGCAAAATTCCCTTATACGAAAAGAGGAGGGGACTAGACACCAAAATGCCGATGCCTCAAGGCAAGATCACCACT
GTACTCTGGGAGGGCACAATGGATGGTGGAGAATTCGACCGTGATTCACCCTTCACAAGGGATGATTCCAGGCCTGAACAAATG
CCCCACTGGCTTCAGAGGAGCCTCGCATGACTCCTAATGAAGGGTAACCTACAGACCAAAATATCACTGTAGACTCCCCTTCAA
TAACTTCATGAGGTTAAGGGGGCAACAGCAAATGAAAAATGTCATTGCTTCCGTCCGTTACTCTTAAAAGGAGTGGCGTACAAA
TGGTTCAGGAGGTTAAGCCAGGAACACATTCAAGTCATGGCAACAATTCTCCTGATGAATTTCTTTCTTCAGCAACACCATGCA
CGTGTGGACTCTACGTCATGACAACCTACCAGCCTCCGCTAACATCAAACAGGCCGAGAATGAAGTTTGAAGGACTACATCCAT
AGATTCGGTATAGAAGCAACCAAAGGTGGGATGTTTAACCAAAGCAGAGGCTCAAAGTGGCTATTACAGCCGGAGTTCGTCCAG
GAAGCAAGTTATGAGTAACATGCTCCAAAAGAGAAGTTTCGGATTTGGGACGACCTTCTTCGAGAGAGCAAGAAGTACATACGT
GTGGAAGAGGGCCATAAGAACTTCCATGTCCGAAGAGAGCGAACCTTCCTCCCAGTGGCCATACTACCAGTATCCGTCTGGGAA
TTCTATAGACAGGGGGGCATCGTGCTAGAGGGGTCTGACCAAATTAGAGATCAAGACCGACAGACCATCTAGCCATGAAATCTC
CACCAAAAGGGGTCTACAAAGTAGGACCAACCAACCAAAAGGGGTCTACAACGTAGAACACCTCCCAAAAGGGTCTCCCCAAAG
TAGGAGCCTCAAAAAGGGTCTCCCAAGTAGGAACGCTATCTCAAAAGGGTCTCAAAGTAGACGCATAATAAACCGAAAAGGGTC
TCAAAGAAAACGCTTGCAAAAAGGGGTCCTCAAAGAAGACGCTTGCAAAATAGTAACTATGCCAAGTGACGAGAAGGACGCTTC
TTCGCAAGATATAATGAAGCGCGCGCCGAAGATATCATAAAGGATAACTAGAACCAAGGGGTCAATAATATCCTTATAGGTGCA
ACTAAAACGCATCAACAAGAGAGGCCCCTACCGAACCCCCCTTTCGCGTGGGTTCCAAAGGACCTCGAACATGATTAATATATCA
AAAAAAAAAAGCAAATAAGAGAATAATAAAAAGGAACGGAAGAGTCTACAAGAACGCCTAACAGGCCTCCCTATAGACTAGGGG
GGGTAAGTGTGATGCCGAAAAATCCCACCAAATAAGAAAAACACACAAAGAAGTTTCTAGTCCTTAAAGAAGTAAATAGCTAAG
GGCCACAGGGGCGCCAAGTAAGCAATGAAGGCGAGGTAATTATAAGGCCATCGAAGTGTTAGACACTCGCAGAGAACCTAGGCC
TCTGAGGTCATTCCAGGCCTCCCAAGCGTGCAAATTACCTTTGTTGACGGTTTGTCAAGCACACATGGCCCTTATCCGTGCCCC
TCCGAGGAGTTTAAGGCCCCAGCCTCCGAACAACCACTCCAGGCATCACCCCGCGCAATGCCAACCACCTCCCAAAATGCGCCC
TAGCCTGCCCCGAGGTGTCCCACAAACATAAGTACTCACGCCAAGAAGACGTTTCGCACCCTAGGATGTATCACCCATGTATCC
ATCTAAGCATGCGCCTCGCGCCCCGCGCGCACCCAGGGGCCTCGCGCCCCGCGCGCATCAGGGCCTCGCGCCCCCGCGCGCAGA
CAAGGGGCCCTACCGCGCTCCCGCGCGCCATGCGCCTCCGCCCCGCCGCGCACAGGCCTCGCGCCCCGCGCGCCCATCCGGCTC
GCGCCCGTGCGCACAGGGGTATCTCGCGCAAAACGGCATGCGCCTCCGCGCATCCGCCGCGCCACCAGGGGCCTCGCGCCCCGC
GCGCCATTGGCGCCTACAAGCCGCCCACGCGCAACCAGGGGCCTCCCGTGCCCCCGCGCCCATGCCCTCGCGCACGCACCATTC
GCGCCTCAAGCGCGCCCGATGCGCCTCGCGCGCCCAGACTTCCCAGCGATCGCGCCGCCCATCCGCGCTCGCGCGCCAAGCGCC
CTCGCGCCGCGAGCACCATGGCCTCCGCGCCACCGCGCATCAGGGGCCTCGCGCCACGCGCACCAGGGGCCTCGCGCCCGCGCG
CCCATGCGCCTCGCGCCCGCGCGCACCAAGGGCCTTCGCGCCGGCGCGCCCATCGCCTCGCGCCCCGTGCAGCACCAGGGTCTC
GGCCCAGCGCATCGCGCCTCGTCCGCGCGCACCAGGGGCCTCAGCGCGCGCGCCCCATCAAGCGCCTCGCGCCCCCACGCGCAC
CAGGGCTCGCGCCCCGCGCGCCCATCCTCGTGCGCCCATGCGCCTCCGCGCGCCCATGCGCCTCGCGCGCCCGACCGCGCGCCT
GCGCCCTCGCGCGCATCCAGGGGCCTCCAGCGCTGCCGCGTACCAGGGGCCCTCGCGCCCCGCGCGCGGCCAGGGCTCGCGCCC
GCACGCACCAAGGGGCCTCTCGCGCCCCGCGCGCACCAGGGGTTCGCGCCCGCGCATGCGCCCGCGCGCACCAGGGGCCTCGCG
CCCCGCGCCCATCGCCTCGCGCCCACGCGCACCAGGGGACTCAGCGCCCCGCGCGCCCATCGCGCCTCGCGCGCCCGCGCGCAC
CAGGGGCTCGCGCCCCGCGCGCACAGCAGGGGCTGCAATGAGGTACTGGCCTTGCCCAATAGGCCTCTCCCATGGCCTCGACCA
TCGCTCGTCCATGCTTTGCCATGGCCCGCCCATGGCCTCGCCCATGGCATATACCATGGCCTCCCCATGCCTCGCCCCATGGCC
TCTCCCATGGCATCCGGCCATGGCCTCTCCCATGGCCTCTCAGACCATAGGCCTCGTCCATCGGCTTCGCCCATTGGCCTTCGC
CCATCGGCCTCGCCCATGGCCTTGGAGTGAGAATACTCACAAAGGGCAAGGTACATGCATGAATAATGTGAATTTTTGCCTACA
AACCTAAAGAAGTCAGAGATAACGGATATAAGTACCCTTACCAGACAAGTAGTGGGAATGCCAGGAGTGGTTAATGCAGAATAG
GAGCTATGTCCGTACCGTCTCGGCCAATTGGGTCAGGCGACACAACTACCTGGTCTGCCCACTTTACGCCTGCCACCGACGCAT
TAGACATGGGTACGGCC
>m141120_105518_42134_c100732402550000001823141405141504_s1_p0/78451/2754_9707
CTTGGGATCGGATCCAGCGGCGATTCTGCTACTATATCGTCAGTGCAATGTCTGTATTCTAAGTAGTGATCTATTTGGGGTAGT
CAGGTCCCTTCTAAATTCATTGTTCTCGAGTCGGAGCTTATTTTTACTGTCTACTGACAGACTGACCTCCATGACGCACACGTA
GGCGGTAGTCAGCTGGTGGTTTAATACTTGTGCAAGCGTTTGGATCATGAGTTCATTATGTCCCATAGAATTATGGTTCTGCAT
TAGTGCAATTATCGTAGTAATAAGTCATAGTTTCTGTCAGCGCATCTGTTCCCTTCTAAATTCTCTAGACTTTAATATTAGTGA
CGTTGATGTGCTAAGACTTGGTTACTCGTTTATGATTTTTGGGGATCGGAATCCCTTTAGAATCATCATCTACTCTATCATTTA
ACTGCATGCAGTGCCATAGCCACGGGATCATTTGTGAGCTAAGCTGGTCAGGTGATGATGGTGTTGATGCCTAGATGTATCGTT
GGTCACGGTTCATATTATTGTCAATAATTGTTTCTGCTAGTGCAATGTGGTTATTCTAGAGTATTCTTTAACTTTGTCCCGGAT
CGGTATGCCAATGCGAATTCACTACTATTGGAGGTTTTCTTAGAGTGCAATGTGTGTATTCTAAGTAGTTATTTTCGTATCTGG
ATCCCTTGCTCTAAATTTCCACGATAGGTTGCTTATCGATTAATCTTCGACATGACCATATCTATTTATGTCCAGTTCCCTAGC
TAGGTATATTGTTTTACTAGCTGTAGAGTCGTTGGAACGGTTCATTATGTAATAAGGAAGGGGGTCTGCTTAGTGCCACATGTT
AAGTCAGTAATAATTTTTCCGGGATCGATCCTTTCCTAAATTCTCTACTATATCTTAGTGGGCAATGTTATCTAGAAGGTTTTT
CGAGATCGGATCCCTTCTAAAGTTCACTACTCTATCGTTTCTCCGCATGACAACTATAAGAAGACCCTTGCGGTAGTATTGGTT
TACTAGTGTTATCGTTGGAAGGTTCCATTATGTCATATATTAGGTCCTGTTTAGTGCAATGTATATCTAGTATAGTTTTTCGGG
ATCGGATCCCATTCTAAATTCTCTACTAATATGTTAGAGCAAAAACGTTTCTAAGTAGTATTTTTCGGGATGCGGTATCCCCTT
TCGTAAATTCACTACTCTATCTACTCAACATGACAACTATATGAGACCCTAGCGGTAGTATTGGTTTGACTTAGTGCACGTTGG

AACCGATTTTTATGTCGTAATTAGTTCTGCTTAGTGCAATGTATCTAGTAATATATTTCGGGATCGATCCCCTTTCAATTCTCT
ACTATTCTTAGGGAAATGTATCCTTGGTAGTAATTGGATCGGATCCCCTTCTAAACTCATTACTCTATCTGTACGTCAGCATGA
CAACTATATGAGACCCTAGCGGTAGTATTGGGTTTACTATTGTACGTTGGAAAGGTCATTTGTCATTATTAGTCTGCTTAGTGC
AATGTTAATTCTAGTAATAATTTTCGGGATCGGATCCCTTCTAAATTCTCTACTATGATCTCAGTGCAATGTTTCTAGTAGTAT
TTTTGGGATCGGATCCCTTCCTAACTCATTACTCTATCTTTACTCAGCATGACAACTATATGAAGACCCCTAGCGGTAGTATTT
GGTTTACTTTGTACGTTGACGGTTCTTATGTCAGAATTAGGTCTGCTATAGTGCAGTGTTATCCTAGTAATAATTTTACGGGAT
CGGTTCCCTCCTAAATTCTCTATATATCTTAGTGCTATGTTATCTAGTAGTAATTTTTGGGGATCGGATCCTTCTAAATTCACT
ACTCTATCTTACACAGCATGACAAACAATATGAGACTGCTAGGCGGTAGTAATATGGTACTTAGTGTAGGTTGGCAAGGTTCAT
TTATGTCATAAATAGTTCTGCTTAGTATGCAATTGATTATCTAGTAAATAATTTTCCGGGATCGGATGGCAATCTAAATTTCCA
AAAACCCTACTATATCTAGTGCAATGTTAATCTAGTGTATTTTTTTCGGTATCGGATCCATTCTAAATTCACTACTCTATCTTA
CTCAGCATGACAACTATATCAGACCCTAGCGGTAGGTATTGTTTACTAGTGTACGTTGGAACCGGTCATTATGTCATAATTAGT
TCTGCTTAGTGCAATGTTACTAGTAATAATTTTTCGGATCTGGATCCCCTTCTAAATTCTCTACTAATATCTTAGTGAATTTTC
ATTGAGTATTTTCGAGATCGGTCCTTCTAAAATTCTCTACCTATCTTTCTCCGCATGACAACTATAATGAGACCCTTGCGGTAG
TAACTTGGTTTTACTAGTGGTAATGTTGGGTTTTGTCATAATTCAGGTCTGCTTACGTGCAATGTTAATCTAGAATAGTTTTTC
GGATCGGATCCCCTTCTAAATTTCTCTACTATTATGTTAGAAGCACCGATTTTCTAGTAGTATTTTTCAGGGATCGAGATCCCC
TTTCTAAATCACTACTCTATCTTCACTCACATGACAATTATATGCAGACCTTGCGTAGTATTGGTTACTAACCTGTCACGTTGG
AAACGACTTTATTTGTCGTTATTATTCTGCTTGTCGCAATTTATCTAGTAATAATTTTTCGGGGTTCGGATCCCTTTCCATAAT
TCTCTACTATTTCTTAGGAATGTTATCTATGTAGTATTTTTGGGGAATCAGGATCCCTTTAAACTCATTACTCTATTTCTCTGA
CAACTATATGAGACCCTAGCGGTAGTTTTGGTTTACAGTGTACGTTGGAACGGTTCCATTATGTCATAATTACGGTCTTCTTAG
TGCAATGTTATCTACTAATAATTTTTCGGGATCCGGCATCCCCTTCACAATAAATTCTCATACTAATATCCTTAGTGCAATGGT
TATCTAGTAAGTAATACTTTTGGGAACGGATCCCCTTATGAATTCATTACTCTATCTTACTTAGCATGACAACTATATGAGACC
CTAAGCGGTATATTGGTTTACTAGTGTACGTTGAACGGTTCATTATATGTCATAATTAGTTTCTGGCTTAGTGCAAATGTTTAC
CTAGTAAATAATTTTTCGGGATCGTATCCCCTTCTAAATTCTCTAAATATACTCAATTAGTGGCAATGTTATCTAGTAGTATTT
TTGGGACGGATCCCCTTCTAAACTCATTACTCTATCTTACTCAGCATGACAACTATATGAGACCCTAGCGTAGTATTGGTTTAC
TAGTGTAACGTTGGAACGGTTTTTGTCATTATTAGGTCTGCTTAGTGCAATGTTATCAAGTAAATAATTTTTCGGGATCGGATC
CCCTTCTAAATTCACTACTATATCTCAGTGCAATGTTATCTAGTATTAATTTTGGGGATCGATAAACTTCTAAACTCATTACTA
TATCTTACTCGCATGACTACTATATGAGACCCTTGCCGGTAGTATTGGTTTAATATTGTAACGTTGGAAACGGTTCCACTATGT
CATACATTAGGTCTGCTTAGTGGCAATGTTAATCTTAGTAAAATTTTTCGAGATTAGGTAATCCCCTCTAAAGTCTCTACTATA
TCATTTAGTGCCAATGATTTCTAGTAAGTATTTTTGGGGATCGGATCCCCTTTCTAAATTCACAACTCTATCTTACTCAGCATG
CAACTATATGAACTCTAGCGTAGATTGGTCAACTAGTGTACGTTGGGCCACGGTTCATTAATGTCATAATTACGTTCTGCTTAG
TGCAATGTCTATCTAGTAATAATGTTGTCCCGGATCGGATGCCAATCTAAAGTTCCTACCTATATCTTAGTGCAAGTGTTATCT
AGTAGTATTTTTCGGTATCGGATCCTTCTAAAGTTCAATACTACTCTATCTTATCTCGCAATGACAACTATATCAGACCTAAGC
GGATAGTATATGGTTTACTAGTGTACGTTGGAACGGTACATTAATGTCGATCATTATTCTGCTTAGTGCAATGTTATCTAGTAT
ATTTTCGGGTCGGTCCCCTTTTAAATCCTTCTAATATCTTAGCTGCAATGTTATCTAGTAGTATTTTCGAGAATCGGATCCCTT
TCTAAATTCACTACCTATAATCTTTTCCGCATGACACCTATATGAAACCCTTTGATGATAGTAATTCGGTTTCGAGTGTAAGTT
GGAACGGTTCAATTTTTGTCATATTTAGGTCTGCTTAGGGCAATGTTATCTAGTAATAGTTTTCAGGATCCAGATCCTCTTCTA
AATGCACTACTATTACTGTTAAGTCAATGTTAATCTAGTAGTATTTTTGGGGATCGGATCCCTTCTAAACTCATTACTCTTCTT
ACTCGGCATGACAACTATAGAGACCCTAAGCGGTAGTATACGCTTTAACTAGTGTACGTTGGAACGGTTCATTATGCCCATAAT
TAGTTCTGCTTAGTGCAATGTTATCCTAGTAAATAATTTTTCGGGATCGGATCCCCCTATCTAATTCTATACCTATATCTTAGT
GCAATGGTTATCTAGTAAGTATTTTTGGGATCGGATCCCCTTCTAATCACTACTCTATCTTACTCAGCAATGACAACTATATGA
GACCCCTAGTACGTAGTAATTGGTTTACTAGTGTACGTTGGAAACGGTTCATTATGTCATAATTAGGTCCGCTTAGTGCAATGT
TTATCTAGTAAATAATTTTTCGGGCTCAGATCCCTATTCTTAAATTCATTATCTCTATCTTACTCAGCATGACAAACTATATGC
AGACCCTAAGCGGTAAGTATTCGGGTTTCTAGTGGTACGTTTGGACACGGTTCATTATGTCATAATTAGGTCTGCATTAGAGCA
AGTGTTATGACATAGTAATAATTTTTCGGGATCGGATCCCTTCTAAATTCTCTAACTATACTTAGTGCAATGTTATGTAGTAGT
ATTTTTGGGGATCGGGTCCCTTCTAAATTCACTACTCTATCTTACTCCTTGCATGACCAACTATATGAACTCTAGCGGTAGTAT
ATGGTTACTAGGTGTACGTATGCACGGGTTCAATATGTCATAATTAGTTCTGCTTAGTGCAGTGTTATCTAATATAATTTTCAA
GATCGGATGCCATTCTAAATTCCCAACATATGTTAGTCAATGTTATTTAGTAGTATTTTTGGATCGGATCCCCTTCTAAAATT
CACTACTCTATCTTACACTCCAGCAGTGACAACTATATGAAGACCCTGCGTGTATTGGTTTAACTAGTGTACGTTGAAACGGTT
CATTATGTCATAATTAAAGGTCTGCTTAGTGCAAACTGTTATCTATAATAATTTTTCCGGGATCGGATCCCCTCTAATTCTCTA
CTATTCTTAGTGCAATGTTATCTAGTACTATTTTTGGGGATGACCCTTCTAAACTCATTACTCTATCTTACTCCGCAATGAAAC
TATATGAGACCCAGCGGTAGTAATTGGTTTACTAGTGGTACATTGGAACGGTTAATTATGTCATAAATTAGGCTCTGCTTAGTG
CAATGTTAATCTAGTAAATAAATATTTGGGATAGGATACCCTTTCTAATTCTCTACTATATCATTAGTGCAATGTTTTTTCTGT
AGTATTTTTGGGATCGGATCCCGCTTCTAAATTCACGTACTCTTCTTACTTCTGCATGACAACTATATGAGACTCTAGCGGTAG
TAATTGGTTACTAGTGATACGTATGCAACGGTTCATTATGTCATATTAGTTCTGTAGTGCAAATGTTATCTAATAATGAATTTT
CAAGAATCGGATGCCAATTCTAAATTCACTACTATATCTTAGTGGGGCAAGTTTAATTAGTAGTATTTTTGGGGATCGTCCCTT
CTAAATTCACTACTCTATCTACTCCAGCAATGACAACTATATGAGACCCTAGCGGATAGTTTGGTTTAACTAGTGTAACGTTGG
AACGTTCATTATGTCTAATTTGGTCTGCTTGAGTGCATGTTATCTAAGTAATAATTTTTTCGTGATCGATCCCTTCTAAATCTC
CTACTATAAATCTTAGTCAATTATCTAGTAGTATTATTTCGAGATCGATCCCCCTCTAACATTTCACTACCTCTATCTTACCTC

AGCATGACCAACTATATGAGACCTAGCGTAGTATTTGTTGTCGTAGTGTAATGTTGGAACGGTTCAATATGTATAATTAGGTTG
CTTAGTGCAATGTTTTTTTATAGTAATAATTTTTCGGGATCGGATCCCTTTCTAAATTCTCTACCTATATCTTAGTGCAATGTT
ATCTAGTAGTATTTTTGGGGATCGGATCCCTTCTAAACTCATTACTCTATCTTACTCGCATGACACTAATATGAACCTAGCGGT
AGTATTGGATACTAGTGTACATTGGAACGGTTAATTATGATCATAATTAGAGGTCGCTTATTGCAATGTTAATCCTAGTAATAA
TTTTCGGGATGGCATCCCCTTCTAAATTTCTCTACTATATCTTAAAGTCAATGTTATCTTTAGTATTTTTGGGATCGATCCACC
TTCTAAATTCACGTACTCTATCAGTACTCTGCATGTAAATATATGGAGACTCTAGCGGTTATTGGTTACAAGTGTACGTATGCA
CGGTTCATTAATGTCATAATTAGTTTCTGCTATGCAATGTTTATCATAATAATAAATTTTTTTTCAAGATCGAATGCCATTCTA
AATTCACTACTATATCCTAGTGCAATGTTATTTAGTAGTATTTTTGGGATCGATCCCTTCTAATTCACTACTCTATCTTCATCA
GCATGACATTATATGAGTCCCTAGCGGTAGTATTGGTTTACAGTGTACGTTGGAACGGTTCTTATGTCATAATTAGGTCTGCTT
AGTCATGTTATCTAGTAATAATTTATTCGGGGATCGGATCCCCTTTTACTAAAATTCTCTACTAC
>m141120_105518_42134_c100732402550000001823141405141504_s1_p0/78610/0_5598
AGAAGAAGAATAGATCTGATACATAGAGAATTATGTTGGTCTAGCGGCACTGGCAGATTGGGATTTTTTAAAAAAAAAAATGGGT
TTTTTAAAAAAAAGAAAAAAAAAATTTTTTGGGGGGGGAGGAAAAAAGAAAAGAAAAAAAGAAAAAAAAATAAAGAAAAAAAAATAA
AAATAAAAGAGAAAGAAAAAGAAAGAAGAGAAATTTTTTTTTTTTTTTTTTTTGAAATTTAAAAAAAAAATTCCCCCCCAAAA
ATTTTTGAAAAATTTTTTTAAGGAAAGCTAATATAAGACATGTAATAAATAATTCTTGTAAAATTTTTAGAAATAAACTAGGC
CTTACGGTGTAAGAGAAATGAATTTTCAACTTACCGGGATAAAAATCTGGTTGTAAAAACTTCTGGCCAGCAGGCCAACTGCCG
GATTTGCGCTTTACAAATTTTGAATGAGTTTGCCACCGGCCATAAAATTAAATGAAATTTTGAGAGAATTAGTTAAGAGTATGT
AGTAAGGACCGTATGTATGAATGGTTAGAGAAAATGTTATCCGGACCGGTGTAAGATGAAACAATTTTTTTTTTAACTTTTCCC
TGAAAGAACTGAAAGGTAGTATTTTCAAACCCTTAACAAGAAAAAAATGTTTTGGGAGGTGTAGTTCTTTTAACAAAAGACTATT
TCTGCACGATGCTTTCGCTCTAGTTCCCTTACTTATGACAAAGAACGACGTGAAAATAATAGTTTAAGGGTTTTTTTCCTAAAC
TCAACTTAGATAAAGGTTTTAAAAAGAAGATTTTTACCCCTTAAGATTAAATAGTAGGAAGTAATTAGTGTTTGTAAAGTGAGA
AGAAATAATATATTTTGTAATGAATATAGCGTAATTGGATTTTGGAGAACTTAACCGATCCCAAATCAGCTTGAAGGTTAATTT
AAAATTTGATTTTTGCTATTTTAAGTTTAATGGGGTCCTAGGGTTATGGATAAAAATGGTAGAGAATGGGAAAGTTGGGCTTTT
GTGTGAGAATCTTTAAGAGAACGGGAGGAAACATATCCATATAGTTATTTGTAAAGGATCGTAAAAAGTAGTTATTTTGTAAAT
TATTGAAAACGGCAGAACTTTCCGTGAGAGGACAAACAAGTTGGTAATTTTTCTAAGGATGAGGAGTTGAGTAGAACTCCTTTT
TCCCAAAAAAACCCAAAAATTACGAGTTGTCCAATAAGAAGAGTGTCTCTAAGTTAAAAAATAAATATGTAAGAGAGTGCATGGG
TAGCATGGTAGAGCGTGTTGGTGGTGAGGCTGTTGATTTTCTCATGATAACTAAGGTAAAGGACTTGAAATGAAATCCGGGTCT
TATATACTGTATTAAGGTGTATAAGAGGCCTATAGTATAGGAGTCTCTAAGAAGGAACGTTATTTTGCATGTAGCTATCCAATA
GAATATTTAGCTGATAAGAGTATGCCATATGCATTTAAATCCGAGTACATTGTAGTTGAATTAGCGTTAGGGTGAATGTGTGAG
GAGTGGTATAATAGAATCGTGAAGGGAGAAAACAAATAGCGGGTGTTTTAAAAATCCAGCTGGGAGCTAAGGTCTCTATGTAAG
GTAAGCCTTGTCTCTTTCTGTGGCTACAACATTAATATGAATTAGTGTGGCCTTGTAGTAGTAGAGTAAACACTAGTTTGTTGT
TTGGTCATTAAGACTATGGTGTTGCTTTAATGCCCTTACTTACTCTTGGCAATTGCTTTGGCATTCCTAATGGCTAAATGGTAA
GGTAGTTCAAGGTTTGGAGCAGAACCGCTAGACGTAAAATAGTTGCCGAATGTGGGAGCCAAGACATGGAAAGTATATAGTCTG
GTTGGAGCAGAAGCATGGTATAGTATATATGTCCAGTTGAAGCCCAGGAAACAATGGTAATTAGTAAGTCCAGGTTGGAGCTAG
GGCAATGGTAAGATATGGTATGGGGTTTGAAGCGAGGACATGGTAAGAAAATATAGTCCGAGTTGGAAGCACAGGACAATGGTA
TATAGTCCGGGTTGGAGCCAGGAACATAAATGCTAGAACCGGGGTTAGCCCGAATCCTTTGCTGTTGATTAATTATATTAGTTT
ATGAATATGTGGTTTTATATATCTCGATGTTCTAAGGATTTTTTTGTGAGTGCAGGTTAGCTCATGATATTTGGTTGCAAATGT
TGTTTGCTAGTAACAATCAAAGTATTTGATTCTGAATTTGGCTAATTTGTTTGAGAATTGTATGATAAGGGTTGGGATTTATCC
AATTAACCTAATGATTCGGTAGTGATAACTACCTTTGGTGCAACTCATTTACTGGGTTTAGTAAGTTTGTGTCAAGGCTATTTC
TGTACAAAGCATTTCACTTACCGTAGTTGTTTTCCATGTTGTATGTAGAATGCAAAAGCAAGAGTGAAACAGTGAACGTCCCAG
AAAATATGCTTGTGGATATGTGTACATTGTGGCCCTGACGTGGGGAGGTTTTTATGGAACCCAATTTTGAATAAAAACACTGC
GAAGGTTAATATTTCTTTTTTATGACGATTTTGTTTGTTATACTCTGTGAAAGAGTAGTTGTATGTTCCTTTTTACTAAACAAA
AAGTTGTGCGCACAAGTCTGTTAAACATTTTTTTATATGGATTTTTTTCGATTTTACAATGAGTGTCAAAGTGAAAAAGTTTTA
AATTTCCGCAATAAATTTGGTCTTGTATGTTTGAAGGTCATTACATAGTGGTATCAGAGCTCAATTTGAGCCGATCCTGTAGAA
CAAACGACACAATAATATCATTCTGTAAGGATAGACTGGGCCACTCACTGTATAGTAAATACTTTTCATATTCGTTGTTTTCTT
TTGCTTTCTTTATTTCCCTCGGAGTTTGTAAATTGGTAGGTTAGTTGCTGATATATTATCTTGAGATAATTGGTGACCCTGGCC
CGATAGAGGATCCTATCAACGATGATCCTTTTACGCCTGTGATTGATTAACTATAGATTCTTTGACAACGAGAATCTCGACACT
TGGTCGAGTATTGAATCAACGACATTGACGTGTCAGTTTGGGGCAATGGGTCGAAATTACAGTGACAGAAGTCGTTGATAGGGC
TTAAGCACACTCCGAGCGACGGAGAGTGCGACATGCCAGCAACTTGTCTGGAGAGGCTCGGCATAGATATGGTTTTTCATCGCA
TTTCAGTAATGTGGAGATCGACCTAGGGCTTTGGGCCATGGATTTTGCGCTATCTCCATGGTTGATAGCGATAACTTGAGGCAA
GGCGGTAGAGCCAGAGGAGGATCCCGAGTAGCAATGGCGAGTATGTTCCGATGTAGAGCTGGGAGTGTGTTGATGGATAGGATA
TATTTAGGTGTTTGCTTTGAAGTCTTGGTTTGATTTACTGTAGTGGAAACGACTCGGAAACCTTACTAAGGTTAAGTAGAGATT
CTTGTATAATGGTAGCAGGCGTATGTAGAAAACCAACGCGTGTTCGGGTTCTCTTTTGTATTTACTGGTTCTGCTACCTAAAAC
TTTATAGCATACATGATCATTGTTTTGCTAGTCAATGATCCCGAATGATTGATTAGCTGCTTTGAGGAAGTGTTGTTAATTATT
GTTTCTTTTCCTTTCTCTTTTTGGCCGTTGGTGTTAGTAGAAATTTACTAGTACAGCTCCAATGGTTTTAACATTTCGTATATA
AATTTGCGATGGTGCCGTAATTTAACCGTGATGCCAGAGGAAAGGGAGGTCGAGGGCTGTTAACCCATCAGACCCAGCCGAGAG
AGCAGTGGTGTTGACCAAGAGGCAGGGTCGTTTTGGCCGAGGTAGAAAAAGGGGTCGATGGCGAGGGGAAGAGGTCAAGGACAA
AAGGGTAGCAGCCAGAACTCAAAATCTGGAAACTCGTGATTGAGGATTTGGAGAAGGATTGTGGATGAGCATGTGAAATGCGTA

AAACAGTCAGTATGGGATGAGGCATTGATGCAGAGATTCCTGGAAGCGGCCAACACCCAACCCTCACACCCATGGTAAGGAGCT
TCCCAAACAACAGGAATCCCTGCGCAAGAAGAACCGCACTGGTTTTGGATGCAAAATCCGCTAGCGGGGATGCCCCTAGACCTG
AACAGCCTATTTATCAATCTCTGAAACAGTTTAGCAGACAAAACCCACATGGTGTTTTGAAGGGACATTAACCCCTAATGGGAT
GGCGGAAGAATGGGTCAGCATGATCGGAGAGAATCATTTGACTGTTGTGGCGGCCTACTGAGAAGGGAAAAAGTAAATATGGTG
CGGTGTATGGATGTGTGAGGAAGGACACCCCAAACGATCCTGAGTGGTGCATATTGATAGGAATGGGTGTGATGTTGCACAGAA
TGGAATGGGCGGAAATCCTACCCTGTTTCAAGATGGCAAGTACTACAGCCCAGACCATGATAGATCTGAAGGTGGTTGAGTTTC
AACCCAACCTAGTCCAAGGGTTATATAGCGCACAGGAGTGACGTTGCCAGCAAGTTATTGACCACTCGGTCAGATTTGTGCTCT
GGATTCTTTGGGTACACACTGAAGCCAAATGTGGGTATGCGGAATTGTTCATGAAGTGGGTGGGGTGGGTGGGTGTGGGTTGGA
GGGGGTGTGGTGTGCTGTTGTGGGTGGTGGTGTGTGGTGTGTGTTGAGTGGTGGTGTGTGGTGTGTGTGGTGGTGGTGGTGGTT
GTGGTGTGTGGGTGTGTGTGTGTGGGGTGTTGGGGTGGTGTTGTGTGATGTGGGTGTGGTGTGGGTGGGGTGGGGAGTGTGGT
GTGTGGTGTGGTTGTGTGGTGTTGTGTGTGTGTTGGTGGTGTGTGGGTTTGTGTGGGTGGTGGGGTTGGTGGTGTGTGTGTTGT
GTTGGTGGGTTGTGTGTGTGGTGGGGTGGTGTGGTGGTCGGTTTGTGTGTGGTGGTGTGTGTGGTGTGTGGTGTGTGGGGGTGT
GGGTGGTGTGTGGTGTGTGGTGTGTGTGTGGGTGGGTGTGTGGTGTGGTGTGTGGGGTGTGTGGTGTTGTGTGTGGTGTTTGTG
TGGGTGTGTTTAGTGGTGGTGTGATGTTTGTGGGTGTTGGTTGGTTTGTGTGTGGTGTGTGTGGTGATGATTTGTTGTGTGTGT
GTGATGTGGTTGTGTGTGTGTAGTTGGTGGTGAGGATGTGGGTGTTTGAGTGATGGTTTGGTGTGTGGTGTTGTTGTGTTGGTA
AGTGGGTGGTGGGTTGGGTGATGGGGTGTGTGTGGTGTTGTGTAGGTGTGGTGTGTTGAGTTGGGTGTGTGTGTGTGTGGGAGT
TTGGTTTGTGTGTGGAGTGTTTGTTTGAGTAGTGGTGGTTTGGTGGTTTTGGTGTGTGTGTGATTGTGTTGTGGGTGTTTGGTT
GAGGTGTGTTTGGTGGTGGGGTGTTGTGTTGTGGAGTGGGTGGTGTGGGTGTGTGAGTGGGGTGTAGTGGTGTGTGGTTGTGTG
TGGGATAGAGGTTGTTGGTGGGTGGTGTGGTGGGGAGGGTGTGGATTTGTGGTGTGTGGTGTGGTGTTGTGTGTGTGTTTGGTT
TGTTGGGGTGTTTTGGTGGTTGTGTGTGGTGTGGGTGTTGGGGGGTGGGGTGGT
>m141120_105518_42134_c100732402550000001823141405141504_s1_p0/87211/875_6819
GATCCCTTCAAATTCTCAACTCATATTTAGTGCAATGTTATCTAGGAGTAGTTTTTGGGGATCAGTCCTTCTAAATTTCATTAC
TCTATCTTACTTCAAGCATGACAATCTATAAGCAGACCCTAGCCGGAAGATATTGGTTTAACTAGTGTACGTGGAACGGTTCAT
TATGTCATAATTAGGTCATGCTTAGCGCAATGTTACTTATAGTAAATAATTTTTCGGATCGGATCCCTTCTAAAATTTCTCAC
TATATCTTAGATTGCAATGTTATTAGTAGTATTATTTGGGGATCAGATCTTCTAAATTCATTATCTATCCTTTCAGCTTGACAA
CTATAAGAGACTAAGCGAAATATTGGTTTATAGTGTAATCGTTGGAACGGTTCATATATGTCATTATTTGGTCTGACTTTGCGC
AATGTTATAGTAGTATAATTTTTCGGGATCGATCCCCTTCTAAATTCTTTTACCTATATCTTAGTGCCAATGTTGTCTAGTACG
TAATTTTTGGGATCGGAATCCCTTACTAAATTAATTACTCTGTCTTACTCTGCATGACAACTATATGAGACCTAGCGGTAGTAT
TGTTTCTAGTGACGTTGGAACGGGTTAACTTATGTCATAATTAGGTCTGCTTGTGGCAATGTATGTAGTAATAATTTTTTTGGG
ATCAGATTCCCTTCTAAATTCTCTGACTATTTCTTAGTGCACGTTATCCTAGCTGTATTTTTTGGGGATCGGTCCCCTTCCTAA
ATTCACTACTCTTTCTTACTCAGACATGACAACTATATCAGACCTTAGCGGTAGTATTGGTTTACAGTGTACGTTGGACGATTT
AATTTATGTCATATATTAGTATGCTTTAGTGCACATGATTATCTAGCAATAATTTTTCGGGATCTGGATTGCCATTTTAAATTC
ACTACTATATTTTAAGTGCAATGTTATCTAGTAGTTATATTTGGGAGATCGGATCCCCTTCTAATTCACTACTGCCTTTCTTAC
TCCAGCATGACAACTATATGAACCCTAGCAGTAGTATTTGGTTTTACTAGTGTACGTTGGAAACGGTTCATTATGTCATAATTA
GTGTCCGCCTTAGTGCAATGTTTATCTATAACAATTTTTCGGGATCGGATACCCTTCTAAAATTCACTACTATTATATCATTAG
TGCAATGTTATTAGTAGTATTTTTCATAGATCGATCCCCTTCTAAATTCACTACTCCTATCTTACTCAGCATGACTACTAATGT
CAGACCCTAGCGGTATAGTATTGGTTTAGCTAGTGTACGTTGGAACGGTTCATTATCTTCATAATTAGTTCTGGCTTATGTGCA
ATGGTTTATCTAGTCTAATTTTTTCGGGATCGAATCCCTTCTAAATTCCTCTACTATATCTTAGTGCAATGTTATCTAGTAATT
TTTCGGGAAATCGGCATACCCTTTCTAAAAATCACTACTATATCTAGCTGCAATGTTATCTAGTAGTCATTTTTCGAGATCAGG
ATCCCCTTTCTAAATTCAACTACTCTACTCTCTACTACCGCATGACCCACCTATATGAACCCTTAGCGGTAGTATTGGTTTACT
AGTGTACGTTGGCGGTTCATTATGTCATTTATTGGTCTGCCTATGTGCAATGTTATCTAGTAAATAATTTTTCGGGATCGTATC
CCTTCTAAATTCTCTACTATATCTTAGGCAACGTTATCTATTAGTAATTTCGGGATCGGATCCCCCTTCAAAATAAAGTCACTT
ATCTATCCTTACTCAAAAAACATGTACAAATATATGAGACCCTAGCAGGGTAGTATTGGGTTTAACCAAGTGTACGGTTGGAAC
GGTTTATTATGTCATAATTTAGGTCTGGCCTAGTGCAATGTTATCTAGCAATAAATTATTTCGGATCGGATGCCATCTTCTAAA
ATTTACTAACTATATCTTAGTGCAATGTTATCTAGTAGTATTTTTGGGGATAGGATCCCCAATTTCTATAATTCCACTACTACT
ATCATTACTCCAGCTGAACAAACTATTATGAGATAAAGCGGTAGTATTGGTTTACTAGTGTAAGGTAGACGGTTCATTATGTCA
TAATTACGTTCTGTTAGTGCAATGTTATCTAGTAAGATTTTTCGGATCGAATCCCTTCAAAATATTTCTACTTACTATATCTTG
TCTGTTATCTGTAAGTATTTTTGGGGTCGGATCCCTTCTAAATTCACTATCCAATTTTAACTCCCCAAGCCATGACAACTATAT
GAGCCTAGAGGTATATGTGTTACTGTGAACTTTGCAACGGTTTCACTATGTCTAATTTGGTCTGCTTAGTGCATGTTATCAGTA
ATTAATTTTCGGGATCGTTCCCTTCTATTCTCACTATATTAGTGCAATGTTAATCTAGTGTTTTTTGGGGATCAGATCCCCTTC
TAATATTTATTCTCCTATCTTACTCAGCATGACAACTATCAAGAAGACCAGGCGGAAGTATTGGTTTACTAGTGGTACGTTGGA
ACGGTTCATTATGTCCATAATTAGTGTCTGCTTAGCGCAATGTTATGTAGTAAATAATTTTTCGGGATTCGGATCCTCTTCTAA
ATTCTTCCTACTATATCTTAGTGCCAATGTTGTCTAGTAGAATTTTTGGGGATCGGATCCCTTCCTAAATTAATTTATACTGGT
CTTAGCCTCTGCATGACAACTATATGAAGACCTACGGTAGTATTGGTTATCCTAAAGTGTAGCGTTGGAACACGGTTCAATTAA
TGCATAATTAGGTCTGTTAGTGGCCAATGTATATCTAGTAATATTTTCGGGATCAAGATCCCCTTACTAAATTCTCTACTATAT
CTTAGTGCAATGTTTATATAGTAGATATTTTTGGGGACCGTATCCCTTCTATTCACTAACCTCTGTTCTTACTCAGCATGAAAA
CTAATATGAGACCCTAAGCAGGTAGTATTTGGTTCTAGAGTACGTGGAAACGGTCCTTAGTCATAATAGTTCTGCGGTATGCAT
GTTCTTGTTCTTTTTCGGGTCAGATGCCATTCTCAATTCACATAATATCCTTAGTGCCAATGTTATCCAGTAGCACAGTATTTT

TGGGATCGCGATCTTACCTAAAATTCACTACTCTGTCTTACTCAACAGAATGACAACTGGACCCTCCTGGCTAGTATTTGGTTT
TACTAGTGTCGTTGACGGTTAATTTTCATTTTAAGGTCTGTTAGATGCATGGTTATATGTAGTAATAATTTTTTGGATCAGATC
CTTCTTCTCTCCTATTTTGTAGGCAACGTTTATCAGCTGTATTTTGGGGATGCGGATCGCCCTTCTAATTCACTAACTCTTTCT
TACCTCAGCATGACAACTATAGTCCAGACCCTTAGCCGTAGTATTGGTTTCCAGTGTCTTGGAACGTTTATGTCCCATGGTTCG
GCTTGCTGTTATCTAGCAATAATTTTTCGGGATCGATGCCCATTTCTAAATCACTAACTTATTTAGTGCAATGTATCTAGTAA
GTTTTTTGGAGATCGGATCCCCCTTCTAAATACTACTCTTTCTTACTCGCTGACACTTGCAGCCTGCAGTATATTGGGTTTTAC
TATGTCGTTGGACACGGTTCATTTGGTCTTTGGTCGCTTTAGTGCAATCGTTATAGTAACAATTTTTTCGGGATCGGATACCCT
TCCTAAATTCCCACTACTAATATCTTAGTGCAATGTTATCTACGTAGTATTTTTCTAGATCGGATACCCTTCTAAATTCACTAC
TCTATCTTACTCAGCATGACACTATATCAGACCCTGAGAGGTGTATTGTTTACTAGTGGTCGTTGGAACGGTCATTAGTTCATA
AGTAGTTCTTGAGCTTAGTGCAATGTTATCTAGTAATTATTTTTCGGGATGAATCCCTTCCCTTCTCTACTATATCTTAAGTGC
AACTACTTTAAACAATTTTCGGGATCCGGATACCTTCAAATTCCACTACTATATCTTAGTGCTGTTTCTAGTAGTTTTCGAGAT
CGGATCCTTCTAATTAACTACTCTATCTTAACTACGCATGACCACTATATGAGCCTTGCGGTAAGTATGGTTACCTAAGTGTTA
ACGTTGAACGGTTCATTATGTCATTATTAGGTCTGCTTTGTGCCTGTTATCTAGTAATAATTTTTTCGGGATTCGTTCCCCTTC
TAGAATTCTCTAACTATATCTTAAAGTGGCAACGTTATCGTATTAGTATTTTTCGGGATCTGATCCCCTTCTAAGTCACTACCT
CTATCTTACCCAACATGACAAATATATCAGACCCTAGCGGTAGTATTGGTTTACCAGCCAGTGTACGTTGGACACGATTTATTA
TGTCATAATTAGGTTGCCCTAGTGCATGCTTATTTTAGCAATAATTTTTCGGGATCGGATGCCATTCTATAATTCACTAATATA
TCTAGTGCAGATGGGTATCTAGTAGGTATTTTTGGGATCGGATCCCCTTTCCTAAATTCACTACTCTATCTTAACCCAATGACA
ACTATATGTAGACCTCTCAGCGGTAGTAATTCGTCTTTTTACTAGTGTGAGGTTAGAAGGTTCATTATGTCATAATTACGTCTG
CTTAGGTGCAATGTTATCTAAGTAAATAATTTTTCGGGATCGAATCCGCCCTTCAAAAATTTCCTACTAAAATATCTTAGTGCA
ATGTTATCTACGAGCATTTTTGGGAACGGATCCCTTCCTAAATTGCAATACTAATCTTACTCAGCCATGACAAACTATATGAGT
ACCACCTAGCGGTATTATTGGGTCTTTACTAGTGTACGTTGCAACCGGTTCATTATGTCATAATTAGGCGTCTGCATAGCGCAA
TGTTATCTAGTAATTATTTTCGATCGGATCCTCCTTCTACCCCAATTTTTACTATAATCTTAGTGCAATGATTGTCCAAGTAGT
ATTTTATGGGGATCGGATGCCCTTCCTATTAATTACTCTGTCTTACTCCCAGGCATGACAACTATATCATTACCCCTAGCGGTA
GCTATTGGTTTACCAGTGTAACGTTGAACGATTTATTAATGTCAAAATAATTAGGTCTGCTAGTGCAATGTTATCTAGTAACTA
ATTTTTTCGGGAATCAGGATGCCCCATTCTAAATTCACTACTATATCTTAGTTCAATGTTATCTAGTAGTAATTTTGGGGATCG
ATCCCCTTCTCAATTCACTATCTATCTTACCTCAGCATGACAACTAATATGAAGAACCTAAGCAGTAGTAAATTGGTTTACTAG
TGTACGTTGGAACGGTTCATTATGCATAATTAGGTCCGCCTTAGTGCCAATGTTATCTAGTAATAAATTTTTTTCCGGGGATCC
GGGATCCATTCCTAAATTCTCGTACTATTGTTAGAGCAAACATTTTCATAGTATATTTCTTCGGGATCGATCCCATTCTTAAAT
TTCACTATTCCCATCTACCTCAAGCCATGACAACTCATATGAGACCCTAGCGCGTAGTATTGGTTTACTAGTGTAACGTTGGAA
CAGTTCCATTAATGTCATAATTAGTTCTGGGCTTAGTGGCAATGTTATATAGTATAATGTTTGGATCGGATCCCTTCTAAATTC
TATCCTATATTTAGTGGCAATGTTAATCCTAGTAGTATTTTTGGGGGATCGGATCCTTTATAAATTTCATTACACCTATCTTA
CTCGCATGAAAACTATATGAGACCCTAGCGGTAGTGATTGGTTTACTAGTGTACGCTTGGAACGGTTCATTATGTCATAATTAG
TTCTGCTTATGCAATGTTAACCTAGATAATTTTTCGATATCGGATCCCCCTTCTAAATTCTCCACTATTTCTAGTGCAATGTTA
TCTTAGTAGTATGTTTTGGGATCGGATCCCTTCTAAACTCCATTACTCTAATCTTTACTAAGCA
>m141120_105518_42134_c100732402550000001823141405141504_s1_p0/99387/7352_14436
AGTATAGACGTATGCATCCGATTCTGTAAAATTATTTCTAATAGATTCAATATCATATATTCAAACTACGCACAGACCTACAAA
TTCTCATAGCCTACTCATAATGGTCGATCTTCTAACGTAACTTAGGTATTCTTAACAATCCATTACCTTTCTACCTCTCTAAGT
CCTTACTGAAATATTACGTGCCGTTTCATTTCTGTGAGTTATAGTATCTTCACAAGGATTGTAGTTGAAATATTACATGAGAGG
AAATCTCGATTATCCTGACATAAATACTACTAGCAATAGCACTATTCCAAATATGCAAAAGATATATTTTAAACATTGGGTGAA
GTCGTTTGCACTGGGAGAATGATCCCGAAAAATTATTATAGATAACATGTATCGCACTAAGCAGATACGTTGATATTTACATAA
TGAACCGTTCCATAACCGTAAACACAAGTATAGAATACCAATAGGAAATCATGAAACCTACCGCTAGGGTCTCATATTGTTGTC
ATTATTGAAGTAGACTGCTAGAGTTTGAATTTAGAAGGAGTTTTCAGTTAGTTAACTTTTACCATACTCGATTTCCGCGAATAC
AATTTCTAACAACAAGATAACACATCTGCACTAAGATTGTTCGTACAGGGTGGAGAATATCGGTAGAACCCGGTGAGTCCAAAA
CCGTCTTGCCAAAAGATTACTTGAAAACACTCCAGCCGAAGTATACATCTATTTGCACTACAAGATAACTGAACTTCAATAAGA
AACCTCTAAATTATGACATACTGTAACAGCCTGGTAGTCCAAACACGTACTACTATTCATTACCTAAAAATGACTTACACAGCC
GCAGGGCTCTCATCATATTTGTCCATTTCTGAGTACACAAGATAGCACTGTGATTTATTTATGAACGCGGGATCCGACCCCTGA
AAAAATATTACTAGATAACAATCCTCTTTTCCAACTATGATCTATTCCCAGAATAAAGCAGGTGAGTTTTAGTCAAGGGGATCA
CGATCCTCAAAAATTATGTACTAGATAACATTGAGCTAGCAGACCTTAATTATTACATATGAAAGCCGTTCCAGACGTACACTA
GTAAACCCAATACTCACCGCTAGGGTCTCATATTGTAGTCATTCGGAGTAAGATCTCGTGTATGAATTTAGCATGAGGGATCCA
ATCCAAACAATCACTTACTCAGAGATAAATTGCGCACTACACAGATATAGTAGAGTTTAGAACGATGATCCGATCCTGCAAAAT
TATTACGAAGATACATTTTGCGCACCTAAGCACGACCTACACATTATGCAATATTGAACCTTTCCAAACGTACACAGTAAACCA
ATACTACCTCTAGGGTCTGATATAGTTTTCAATTCTGAGTAACGATCAGAGTCAAGTGATTACAAGGGGATTCCGATCCTGAAA
CAATACTACTAGATAACATTGCAATAAGATATACTTAGTCGAATTTGCAAAGGGGGTCCGGATCCCGAAAAATTATTAGCTAGA
TAAACATTGCACTAGACAGACGTGGTTATTACCATAATGACCGCTTCCAACGTACCATAGTAAATCCAATACTACTGCTAGGTG
TCCTCATATTGTTGTCCATTCTGAGTAAGACTTGTGTAGTGAATTTCAGAATGGCGGATCCAATCCCAAAAAACACTACTAGAT
ATACATTGCCACTAAGAAAATATAGTATTGAGTTTACGACGATGATCCGATCCCTGCAGAAATTATTCACTAGATAAACATTCT
CACTAAGCACGACCTAATTATGACATATTCGGAAACCTTTCCGAAGCGGCTAACTAGGTAAGACCAATACTAGCCTCTAGGCGT
CTGAATACAGTTTCCATTCTGTAGTAAGAATAGAGTAGTGAATTTACAAGGGGATCCGATCCCCGAAAAATACTAAAATAGAAA

ACAATGCAAATAAATTTATTTGCTTGAATTTGCGAGGCGGTCCGAATTCCCGAAAAGTCCTTACTAGATAACATTGCAGCTATG
AGCAGACGTGATTATTAACATACCATGAACCGGTCCACGTCACACCTAGTAAATTCAATACTACCGCTACAGGCGTCTCATATT
GTTGTCTTCCTAAGTAACGACTGTGTACGTGAATTTAGAATGGGGGATCCCAATCGCACAAAAACACTTAACTAGATACCATTG
CACTAAGACACTTACAGCTATCTGAGTATAGACGATGATCGATTTCTGTAAAATTACTTCAACTAGCAATACATTTCACTAGCA
CGAACTAGAGTTACCTGACCATATTGAACCTTTCAAACGTAACTGAGTAACACCAATAACTACCTCTAGGGTCTGATATACGTC
TGCTCATTCCTGAGTAGATAGGAGTAGGAATTTACAAGGGGATCAACGTCCGAAAGAATACTAACTAGATAACATTCACATAAG
ATACTATTGTGGAATTTTGCATGGGGGTCCGATGCCCCGAAAAATTATTACCTAGATAAATCTGCACTAAAGCAGACGCTGATG
TTATTGACATATGACACCGTTCAGACCGAGTACACAAGTAAAGATAACATACTAACGCTAGGGTCTCATAATGTTGTCATTCAT
AAGACTGTGTCATTCTGAATTTAGTGGATCCGATCCGAAAAATTCCTACTAGATAATTGCTAAGATGTTGTAGTGAATTACGAA
GGGATATCTGCAAATTCATTACTAACTAACATTGCACAAGCAAGCTAAATATGACATTTTTATTGAACCTTTCAACGACACTAG
TATAAATACTACGCTAGGGGTCTCATATATATGTCATTCTGAAGTAAGATAGACTCAGTGAATTTAGAGGGGAATCGATCCTGA
AAATATTACTAGATAACATTACACTATGATTATAGTAGTGATTTAGAAGGGATCGTCCTCAACAATTATTACTAGATAACATTG
ACTAAGCAGACCTAATTATTAATAATCGAACGTGTCCAAAGTACACTAGTAAACCAATACTACCGTAGGGTTCACTTTGTAGTC
ATTCGGAGTAAGATGTGTAGTGAATTTAGATGGGATCCATCAAAAAATATACTAGATAACATTGCACTAAACATATGTAGTGAG
TTTAGAAAATGATGATCTGCAATTATTACAAGATAAAACATTGCACTAAGCAGGCCTAATTCTGCCATATTGACCTTTCAACGT
ACACTATAAACAATACTACTCTAGGGTTTGATATAGTGTCATTCTGAGTAAGATAGAGTAAGTGAACTTTACAAGGGGTCCGAT
TGAAAATACTACTAGATACATTGCCAATAAGATATAGTACGCTGAAAAACTTTGGCCAAGGGGGTCGATCACCGAAACAAATTA
TTACTAAGATAACTTTGGCACTAAGCAGACGTGGTTATTCACATAAATGAACGTTCCACACGTACACTAGTAGATCCAATACTA
CTGCTAGGGTCCTCATAACATTGTTGTCCATTCTGAGTAAGAACTGTTGTAGTGAATTTTAGATGGGATCCAATCCAAAAAAACA
CTACACTAGATAACATTGCACTAAGAATATAGTATTGAGTTAGACGATGATCCGATCGCAAAATTATCTACTAGATACATTTCA
CTAAGCAAACGACCTAAATTATGACATATTGGAAACTTTCACACACGTACACATAATAAACAATACTACCTTAGGGTCTGATAC
AGTTTGTCATTCTAGTAAGATAGGTAGTGAATTTACAAGGGATCCGATGAAAATACTACTAGAAAACAATGCAATAAATATTTT
TGGATTTGCGAGGCGGTCCGCTCGAAAATAATTATAATAACATTGCACTAAGCAGACGTGATTATTCAACATAATGAACCGTTC
AACGTACATAGTAAAATATAATACGCTAGGGGTCCATATTTTCTTCGTTTTCAAGTAAGACTGTTGACGTGAATTGTGGGGATC
CAATGCAAAAAACACTACTAGATCAACATTGCACTAGATATAGTATTGGCAGTATAACATGATCCGATTCTGTAAAATTATTAC
TAGATATTTCACTAAGCCAGACTAATTATGAATTTGAACTTTCAACGTCACACAATATTAAACATACTAACTCATAGGGTCTGA
ACCTATCAGTTTCATTCTGAGTAAGTAGAGTAGTGATTTACAAGGAATCCGATCCCGAAAAATACTACTAGATAACATTACAAT
AAGTATTATTTGAATTTGCATGGGGTCCGATCCGAAATATTCATAGATAAAATTGCACTACAGCAGACGTGATTATTATAATGA
AGTTCAAAGTACACAATAATAAATATACGTAGATTCTCATTTGTTGTATTCTGAGCTTAGCAGTGTGTATTCGATTTTAGATGG
GGATCCGATTCGAATTCTACAGATAACATCTGCAACTAAGATGTTTTTAGTGAATTAGAAGGGATCCATATCCTGCCAAATTAT
TACTAGATAACATTCCACTAAGCGACCTAAAATATGAATATTGAACCTTTCCAACGTACACCTAGCCTACAATACTACCCGCTA
GGGTCCCTCATATATTTGTCATTCTGAGTAAGATAGACTAGTGAATTTAGAAGGGGAGGCCGATCCTGAAAAAATATTACTAGA
TAACATTACCTATGATATAGTAGTGAGTTTTAGAGGGGAATCCGAACCTCCTCAAAAATTATTATAGATAGCACATTGCACTAA
GCAGCACCTAATTATTACATAATGCAACCGTTCCAACGTACACCTTAGTAAGACCAATACTACCGCTAGGGTCTCCATATTGTA
GTCATTCGAGTGAGATTGGTAAGTGAATTTAGATGGGATCCAATCCCAAAAAAAATACTACTAGATACATCTGCAGCTAAGATA
TAGTAGTGAGTTTAGAAGATGATCCGATCCTCGCAAAATTATTACAAGAATAGACATTGCCACTAAGCATGACCCTAGATATGC
AAATATTGCCTTTCCCAACGTACACTAGTAAACCAATACTACATCTAGGGTCTGATATAGTTTGTCTTCTGAGTAAAGATAGAG
TAGTGATTTACAAGGGGATCCGATCCCGAAAAATACTACTAGATAACATTGCAGTAAGATATATTTAGTGAATTTGCAAGGGGG
TCCGATCCACGAAAAATTATTACTAGATAACAATTGCATCTAAGCAGACGTGATTATTACATAAGAACCGTTCAACGTACCTAG
TAAATCAATACCTATGCCTAGGGTAAATCATATTGTTGGCATTCTGAGCTAAGACTGGTGGTAGTGAATTTGATGGGGATCCTA
TCCCCAAAAACACTAACTAGATACACCATTGCACCTAATATAGTATTGAGTTTAGACGATGAATCCGATCCTGCAAATTATTAC
TTAGATAACATTTCACCTAAGCAGACCTAATTATGACATATTGCACCTTCAACGTACACTAGTAAAACCAATACTACCTCTAGG
GTCTGATACAGTTGTCATTCTGGTAGAGATAGAGTAGTGCGAATTTTACAACGGGAAAATCCAATCCCGAAAAATAATACTAGA
AACATTGCAATAAGATATTTTTGTGAAATTTGCGAGGCGGTCCCGATCCCGAAAAATTATTACATAATAAACATTGCACTAAGC
AGACGCTGATTATTACATAATGAAAACCCGTTTTTATTCTCCACGTACACTAGTAATGCAATACCACCGCTAGGGTCTCATAT
TGTTGTCATTCTGAGTAAGACTGTGTAGTGAATTTAGATGGGATCCAAAAAAATCGCAAAAAACACTAACTAGAATAACAAGTC
TGCACTAAGATATAGTATTTTTTTTGGAGGTATAGCGATGATGCCGATTCTGTACAAATTATTAACTAGATAACAAAAAAATTT
TTTTTTTCTCTACGCAGACCTAAATTATGAATATTGAACCTTTCCAACGTACATAGTAAACCAATACTACCTCTAGGGTCTGAA
TTATAGTTGTCATCTGAGTAAGCATAGAGTTAGCTGAAATTTCAAGGGATCCGATCCCCGCAAATACTACTAGATAACATTACA
ATAAGATATATTATAGTGAATTGATGGGGGTCCGATTCCCGAAAATTATTACCTAGAATAACATTGCACTAAGCAGACGTGATT
ATCTTCAACCAATTTAAATTGAAACCGTTCCAACGTAACAACAAGGTAAATAAAATAACTATCGCTACAGGGTCTCATATGATG
TTGTCATTCTGAGTAAGACTGTGTATCTTGAATTTAGAGTGGGTCCCGATTCCCGAAAAAATTCTACAAAATACTTGCACCAAG
ATGTTGTTAGTGAAATTTAGAAGCGGATCCAATCCTGCCAAATTATTACTAGATAACATTGCACAAGTAGACCTAAAGTATGCA
CATATTGAACCTTTCCAACCGTACACTAGTATACCCAATAATAACCGCTAGGGTCTCATATATTTGTCATTCTGAGTAAGATAG
ACTAGTGAATTTAGAAGGGGATCCGATCCGAAAATTATTACTAGATAACATTACACTATGATATAAGTAGTGAGTTTTAGAAGC
GGATCCGATCCTCAAAATTATTACTAGATAACCATTGCACTAAGCAGACCTAATTATTACATAATAACCGTCTCCACGTACACT
AGTAATACCAAATACTACCGCTAGGGTCTCCATATTGTAGTCCATTTCGAAGTAAGCATGTGTAGTGACATTTAGATGGGATCC
AATCCAAATAAATACTACTAGATAGCACATCTGCCGCAACGCTAAATATACGTATGACGTTTAGAAGATGATCGATCCTGCAAA

ATTAATATACAAGATAAACATTGCATAAGCAGCCCTAATTATGCAATATTGAACCTTTCCAACGTATCACTAGTAAAGCCAATA
CTAAGCCTCCTAGGGTACTGATATATTGTCATTCCTGAGTAAGATAGAGTAGTGAATTTACAAGGGATCCGATCCGAAAATACT
ACTAGATACATTGCAATAAGATATAGTACTGAATTGCAAGGGGTCCGATCCCGAAAAATTATTACGTAGATAACATTGCACTAA
GCAGCACGTGGTTATTTACATAATGAAC
>m141120_105518_42134_c100732402550000001823141405141504_s1_p0/99387/14485_20717
TCATTATGTAATCAACCACGTCATGCTTAGTGCAATGTTATCTAAGTAATATTTTTCGGGTCAGGACCCCCTTCTGCAAATTCA
CCTACTAATCTTATTGCAATGTTTATCTAGTAAGTAAGTTGATTCTCGGATCCGGCATCCCCTTGTAAATTCACTAACTCTATC
TTACTCAGATGACAACTATATCAGACCCTAGAGGTAGTATTGGTTTACGAGTGGTACCGTTTGGAAGTTCAAAATTGCACTAAT
TAGGTCCTGCGTTAGTGCAATGTTACTTTGGTAACTAATTTTGCAGGATCGGATCATCTTCTAAACTCAGCTAACTGATATTTA
GTGCAATGTTATCTAGTAGTATTTTTTGGAGGTTGGATCCCATAAAATAAATGTCACTACACCAATCTGTACCCGAATGACCTA
CAATAGTGAGACCCTAGCGTAGTATTGGTTGTACTAGTGTACGTTGGAACGGTATCATTATGTAATAATTAGGTCTGGCTTAGT
GCAAGTTATCCTTCAGTAATAATTTTTGAGGATCGTATCTTCTAAACTCACTACTATATCCATAGTTAATGTTATCTAGTAATG
AATTTCAGGATCGGATCCCCTTCCTAAAATTCACTAAGTTCTATCTTGACTCAGCAATGAAAATATATGAGCCACCCTAGCCGG
TAGTATTGGTATACTAGTGGTCACGTTGGAAAGGTTCAATATGTCATAATTTAGGTCTCTTAGTGCAATGTTATCTAGTAATAA
TTCTGCAGATTGGATCCCCTTCTAAATTCACTACAACATCTTCAGTGCAATGTTATCTTGTAGAATTTTCGAATGGATCCCCAT
CTACAATCAATACACAGTCTTACGTCAGAAATGACAACAATATGAGACTGCGCTAGCGATAGTATTTATTTACTTGTGTACGTG
GAACGGTTCATTATGTAATAATCACGTCTGCTTAGATGCAATGTTATCTAAGTAATAATTTTTTCGAATCGACCCCATGCAAAT
CTCACTACAAACCTATATCCTATTGTAATGTTATCTAGTAGTATTTTTCGGGATCGGACTCCCGTTGTAATTCACTACTCTATC
TTCTCAGAGACAACTAATATCAGCACCCTAGAGGTAGTATGGTTTACTAGTGTACGTTTTTTGGGAAAAAAAAAAAAAAGGTTCA
ATATGTCATAATTAGGTCTGCGTAGAGAAATGTTATCTAGTAATCAATTTTACAGATATCGAATCATCGTCTATACTAAATACT
ATATCTTAGTGCAATGTTATCTAGTAGTGTTTTTTTGCCGATTGGATCCCATCTAAATTCACTACACAGTCTTACTCAGAATGA
AACAATATGAGACCCTAGCCGGTAGTATTGATTTACTAGTGTACGTTGGAAGCGGTTTCATTTGACATACATCACCGTCTGCTA
GTGCAACATCGTTATCTAGTATAATTTTTCGGATCGGACCCGACCTCGCAACTTTCCCACAAATATCATCTTATTGCATGTTTC
TAGTAGTATTTTTCCCGGATTGCGGATCCGTTGTAAATTCAAATACTCTATCTTACTCACGATGAACACTGTGATCCAGACCCT
AAGGTAGTATTGGTTTACTAGTGTACTTGGAAGGTGCATATGTCATAATTAGGTCTGCTTAGCGCGTGAATGTTATCTAGTAAT
AATTTTGCAGGATCCGGATCATAAGAGTCTAAACTCCATACTATAATCTTACGTGCAATGTTATCTAGTAAAAGTGTTTTTTGG
GATAGGATCCCCATCTAAATTCACTCACACAGTCTTACTCAGAACTGACAACAGATATGAGACCCCTAGCAGTAGTAATTGACT
TTACTAGTGTCACGCCCTTTGGACGGTTCATTATCGTAATAATCACGTCTCGGCTTACGTGGGCAGTGTTTATCCTAGTAAGTA
TTCTTTCGGGATCGGTACCCCCTTGCAAATTCACTAATATCATCTTACTGCAATGTTATCTAAGTAGTATTTTTTCGGGATCGG
ATCCCCTTGTAATTCACTACCTATCTTACTCAGAATGACCAACTATATCCAAGACCCTAGAGGTAGTATTGGTTTTACTAGTGT
ACGTTGGAAAAGGTTCCAAATATTGCATAATTAGGTCTGCTTAGTGCAATGTTATCTTGTAATAATTTTGCAGGGATCGGATCA
TCTTCTAAACTCAACCTACCTATATCTTAGTGCAATGTTATCTAGTAGTATTTTTGGGTATTGGATCCCATGCTAAATTCACTA
CACAATCTAAAACTCCGAATGACTACAATATGAGACCCTAGCGTAGTATTGGTTTACTAGTGTACGTTGGAACGGTTTCATTAT
GTACCTATAATTAGGTCTGCTTAGTGCAATGTTAATCTAGTAATAATTTTTTGAGGATCGGATCCCCTTCTGAAACTCCCTACT
ATATCATAGTCGTATGTTATCTAGTAATATTTTTTCAGGATCGGCTCCCTTCTAAATTCACTAGTCTATCTTACTCAGAATGAC
AAATAATGAGACCCTAAGCGGTAGTCATTGGTAATGCTAAGTGTTACGTTGGAAAGGTTCAATATGTCATATTTAGGTCTGCTT
AGTGGCAATGTTATCTGTAATAATTTGGCAGGATTGGATCCCCTTCATAGATTCATACACATCTTAGTGCAATGTTATCTAGTA
GACATGTTTTCGAATCGGATCCCCATGCTAAATTTCAATACCAGTCTTACTCAGAATGACAACAATATGAGAACCTAGCGGTAG
TATTTATTTACTTGTGGTACTTGGATACGGTTCTTATGTAATAATCACGTCTGCTCTTTAGTGCAATGTTATCTAGTACATAAT
TTTTCGGATCGGACCCCCCATGCAAATTCACTAATATCTCTTATGGTGAATGTTATCTAGTAGTATTTTTCGGGATCGGATCTCC
TTGTAAATTCACTACTCTATCTACTCAGAATGACAACTGATATCAGGAACGCTAAGAGGTATATTGGGTTTACTAGTGGTACGT
TGGAAAGGTCAATATGTCATGAATTAGGTCTCTGGCTTAGGAAACGGTTATCATAGTAATAGATTTGTACAGAATCCGGATCAT
CGTCTATACTGCCAATAATATATCTTACGTGAAAATGTCTATTAGTAGTGTTTTTTGCGATTGATCCCCATATAAATTCCACCT
ACACAGTCTTACTTCAGGAATGACAACAGATATGAGACCCCTAGCGTAGTATTTGATTTACTAGGTACGTTGGAACGGTGTCA
TTAAATGTAATAATCACTCTGCTTATGCAAATGTTAGTCTGTAATAATTTTTCGGGATCCGACCGCCTCGCAAGATTCACAAAT
ATACGTCCTTATTGCATTGGTTTTCTAGTAATTTTTTCGGGATCGGATCCCCTTGGTAAATGTCAAACCCTATCGTTACTCAGA
ATGACAACTGTAGTCAGCCGACCCTAGAGGTGTATCTGGTTTGACTTATAGTGTACGTTGGAAAGCGTTCAATATGATCATACA
TAAGGTCTGCTCAGTGAAAGTCTATCTAGTAATAATTGTTGCAGGAATCGGATCATCGTCTAAAACTCAATACGTATATCTTAT
GATAGCAATGTTATCTAGTGTGTTTTTTGGATTGGATCCCCATCCCTAAATTCACTACACAGTCTTACTCAGAAATGACAACAA
TATGATACCCTAAGCAGTAGTTAATTGATTACTAGTGTACGTTGGAACGGTTCATTATGTGGAATACACCAAAGTCTGCTTAGT
GCATACTGGTTATCTAAGTATAATTTTTCGGGATCGGACCCCCCTTTGCAAATTCACTACTATATCTTATTGCCAAGTTATCTAG
TAGTATTTTTCGGGACATCGGATCCCCCTTGTAAATTCCACTACTCTATCCTTACTCAGAATGACACTATATCAGACCCTAGAG
TAGTATTGGTTTACTAGTGTACGTTCGAAGGTTCAATTTGCATAATTAGGTCTGGCTTAGTGCATGTTATCGTGTGTAATATTT
TGCAGGATCCGATCATCTTCTAAACTCACTACTATATTTAGTGGCAATGTTATCTAGCTATGTTATTTTTGGGATTAGATCCCC
CGATCTAAAATTTCACTAACATCTTACTCCGAATGCTACAATATGAGACCTAGCGGTAGTATTCGGTTACTAGTTTACGTTGGA
ACGTTGATTATGTAATATGTAACGGTCCTGCTTAGTGCAGATGTTATCTAGTAATAATTTTTGAGATCCGGATCCCTTCTAAAC
TCACCTTACTATAGTCATAGTGTAAATGTTATCTAGTAATATTTTTCAGGATCGATCCGCCCTTCTAATTCACCTAGTCTATCT
ACTCCCAGAATGACAAATATATGAGACCTAGCGTAGTAACTTGTATTAGTGTACTTGGAAAGGTTCAATATGTATATTTAGGTC

TGCTTAGTGCAATGTTATTTAGTAATACATTTGGGCAGGATGTTGATCCCCTTCTAAATTCACTACAACATCTTAGTGCAATGC
TACGTCTAGTAAGAATTTTTGCGGAATCGGATCCTCCATCTAAATTTCAATACACAGTATTAACTCGAAGACAACATTATGAGA
CCCTAAGCGGTGAGTATTTTATTTACTTGTGGTACGGTTGGAACGGTTCATTATGTAAATAAGGTCAACGTCTGCTTAGTGCAA
TGTTATCTAGTAAGTAATTTTTCGGGATCTGGACCCCCCATGCAATATTCACTAATATTCTTATTGTACAGTGTTATCTAGTAG
TATTTTCGGGATCGGATCCCCTTGTAAATTCACTACTCCTATCTTACTCAGAATGAAACTATATCAGAACCTAGTAGGTCAGGT
ATTGGGTTTACGGTAGTGTACGTTGGAAAGGTTCAATATGTCATAATTAGGTCTTGCATAGTGCAAATGTATATCTAGAATAAA
TTTTAACAGATATCGGATCATCGTCTATACTCAAATACTATATCGTTAGTGCAAATGTTATCTATAGTGTTTTTTGCGATTGGA
TCCCCATCTAAATCACTACACAGTTTACTTAGAATGACAACATATGAACCCTAGCGGTAGTATTGATTTACATAGTGTACGTTG
GAACGGTTCTATATGTAGATAATCACCGTCTGCTTAGATGCAATGTATCTAGTAATAATTTTTCGGGATCGGAGCCCTCGAAAT
TCACAAAATCATTCATTATTGCATTGTTTCTAGTAGTATTTTTCGGGATCGGATCCCGCTTGTAAAATTCCACTACTCTATCTT
ACTCAAGATGACAACTGTATCCAGACCTAGAGGTAGTTATGGTTTACTAGTGTACGTTGGAAACGGTTCAATATGCATAATTAG
GTCTGCTTAGTGAAATGTTATCTAGTGAAGTAATTTTGCCAGGATGCGGATCATCGTCTAAACTCCAATCACTCGAGTAGTCTT
AGTGCAATGTTATTCTAGTAGTGGTTTTTTGGGATTAGGAATCCCATCTATAATTCACTACACAGTCTTTACTCCCCCAGAAAG
TGACAACAATATGAGGCCCTAGCAGTAGTATTGATTTACTAAGTGTACGTTGAACGGTTCATTTATGGGAATAACCACGTCTGC
ATTAGTGCAATGTTATCTAGTAATAATTTTTCGGGATCGACCCCCTTGCAATTCACTAATAGTATCTATTGCAATGTGGTATCT
AAGCTAGTATTGTGTTCAGGACGGATCCCCTGTAATTTCCCCCCCCCACCCCAAACCCTTAAAAAACTTTTCCTAAAAAAATCT
TACACTCCAGAATGACAGAAACTATATTCAGACCCCCCCCCTAGAGGTAGTAATTGGGTTTTACAGTGTACGTTGGAAAGGTTC
CAATATTGCATAATTAGGTCTGCTTAAGCTGCAATTGTTATCTCCTGTATCAATTTTCAAGGATCGATCATCTTCTAACACTCA
CTACTATATCTTAGTGCAATGTTATCTAGTAGTACTTTTTTGGGATTGGACCCCATCTAAATTCCTAACACCAATCTTCACTC
CGAATGACGACAATTATGAGACCCTAGCGTAGTAGATTGTTTACTAGTGTTACGTTTGGAACGGTTTCATTATGTAATAGATTA
GGTCTGCTTAGTGCAATGTATCTAGTAATAATCAGTTGAGGATCGGATCCCCCTTCTAAACTCACTACTATATCCATAGGGTTG
TAATGTTATCTACGTA
>m141120_105518_42134_c100732402550000001823141405141504_s1_p0/101868/161_14634
TTGCCTGGGATTTTGGCCTGAAAAGGTCTAAATTTCTCTCTTCTCTGGTAACATTAGGACCCCTAATTCTTGTCAATAATAGCC
CTAATATTACATCTACTCTCACATTTTGAGACACTCGCAAAAAGGGGTTACTAAAACAATTTAATGAGGGCTTTGTGTAGAGAG
AGGTTAAATATGAGTGATATTAACGAAATCGCTTTTGGAGACCTTGGTAAGCTGCCTAGTGAGCATTTGGCCTGAAAGGTTCTA
ATTTTTTTGTATTGAGTTGAACTTAAGCACACCCCAATTCAAGGTATTCTAATTAAGCCTAATCATCACGATATCTTATGGGTG
GCCCTGGGAAGGTTCATACCAGAAAAATTCAGAGCTTAGTATAGAGAGAAATAATGCAATGAACTTCACTGAAATCGCGTACTC
TGGAGCCTCAGGCCACTTACAATTATGATAATTTACTCACGGCAGCTGCCTAGCCTATTGGCCTGATACAACTTTTTTTGTTTT
GGTTGAATTAGAACCCCCAATTCATGGTCAATAATAGCTATATTATACTAATACTTCTATCACCGAAAATTATTATCATGTAAG
AGATGGGACCCAAAGATTCAAAAAAAATAGATCTTTGTTATGAGAGAAAATATGAGTGAATTGCGGAAATTCGCGCTTCAGCGC
GCAGGCAGCCGCCTAGGAGATTTGGCCTGAATAAATGTCATATCTATTTTTGTATTTGTCTCAGAATTAAGGGCACCCCCAAAT
TCATGGTACAACTAATTGCCCTTATATTCACACCACATATATTAATAACATTTGAGTTTGGGAAATAGGTTCAAAAAAAAAACG
TTTAGAGCTGTGTCGAGGAGATTTAATATGAAGCTTGAATTATCGAAATTCGTTATCGTTAGTGCCCTAGGTCAGCTGAGGCCT
ATAAGGGGATTATGGTCCTGTAAAGGTCTTCAACTTTTTTTTTTGGATAAGAATTATGGGCCCCCTATTCTTGGTCCCAATAAT
AACGGCCCTATATAATGAGTTACTATAAGAGATTGGGAAAATCACGGTTCTAAAAAAGTTAGGAGCTTGTTAGGGTAGAGAAAT
ATTGGAGTGAATTACGAAATCACGCTATCGAGGCAGACCGAAGGCAACGCCGCCTATGGAGAGAGATTCGGATCCTTGAAAGGT
CAATTTTTTTTTGCGTTTGGTCCATATCCACATCCCCCCAACTTTCCAATCGACACAACTAATAAGCCCTATATTAAGTATTTA
ACAGGTTGAGATGGATAACCTATTAGGTCATATTTATATTACTTTTAGCAAGCTTGATTTTAAATGAGATGAAACATAATAGAA
GCGAATTACGAAAATCGCTTCGGAGCCCCTACGGCCCAGCTGCCTAAGGAGGATTGGCCCTGAAAGGACAATTTTTTTGTTGGT
TGAATTAGAACCCCAATTCATGGTCATTAATAGCCCTATATAATATTATTGAGATGGGAAAAGAAATTCAAAAAATTAGAGCCT
TGTTAGAGAGAAATATGAGTGATTACGAAAATCGATTCCGGAGCCCTGTTAGCACTAGCCATACAAGGAGAGGAGATTCCTGAC
CCGAGCTCAACATTTTTTGGTTTGGCTTGCGATTATTAGGAACCGCCAATATCAATGGTCAATATTATTAGCGGCCTTATATCA
CTAATAGCCTTCAAAAAATGAGCGGCAGATGGGTTCTATAAATCAAAAAATAAGAGCCTTTGTTCCTAGAGAGAAATATGAGTG
AGATTAATAAATAGATCAGCAATCGGAACCCCTACACAGGATGTTGTATATTCATGTCATAAGGAGATTATGGCCTCAAAGGCT
TCAATTTTTCTTGTATTATGAGTTTGAATTAGGAGTCCCCACAAACATGGGCTCAAAAACCATAAAAAAACCTAATATACGGAA
ATATTATGAGAAATGGGAAAAGGTCAAAAAGATTACGAGCGCTATGTTAGAAGACGCGAAATATGAGTGAGGCCACACGAATTC
GCGAGCTGGATCCCGCGAGCCCTAGGGACCCAGGCTGCCTATTCGTATTTGGTAGCTTTGGCCAAAAGGCAGCATTAATTCTGT
TTGGCTTGCGAATGTAAGGAAAAACACCAAGCAATTCATGGTCAATAATAGCCCCCTATATAATATTATTGAGAGGAAAAAGAT
TCAAAAAAAATAGATCTTGTATAGAGAGAAATAATATGAGTGAATTGTGCGAAATCGCTATCCAGCCCCAGCGCCCACCTTAAA
TGAATTCACACACCCACCCTCTAACCTTTACATATTCTAGAAATATATACTCTAAGAAGGCGACAGCCCGCCCTAGGAGATATA
ATAGCCCTCTGAAAGGTCAATTTTTTTTGCGTTATGGGTTCTGCATATTAAGACTGGCCCCACATTTCCATTGGTCACTCATCA
ATTAATATTGGCCCTTACAATATTAGTTTCACCAAGATTCATATTGAGTTGGGAAACGAAGAAGTCAATAAAATCTAGAGCTTG
TCCCTATAAATCCGAGAATTAACAGACCAACCGTTTCGTAGCCTCAGGCCCAACGCCTAGCGGGATTTGAGCCTATGATAGGAG
ATCTACATTTATTTTTTTGCGCGCTCACTAGAAGTAGCCGCCGCCTATCTTTGTGCCCAATAATAGCCCCTTAATATACTATA
ATATTGAGATGGGGATAACGGTTCAATAAATTAAGAGCTTGTTAGAGAGAAAAATAAGAGTGAATTAAGGCGAAAATCGCATTC
CCCGGCGACCTACCGGCAGCCCGCCAAGGGGAATACGGGCCTGAAATAACCCACAGTTAGTAAATCTCTAGAGGGAGAGGAAGG
CAATACCAGAGTTAGGCGCTTCACATTTTTGAGTTATCTGGTTGAATTAGGTTCCGAATTCAGTGGGTCATATATATACAATTT

ATTGAGATGGAAAAAGGTTCAAAAAGTGGAGAGTTGTTACGAGAGATATGATGTGAAGCAATTCTACCGGCAAATCTGTATTGT
CCGGAGACCACTAAGGCGACGCAGCTGCCTAGGAGAATTTTGGCCGCCTGAAAGGACCAATTTTTTTGTTTGGTTGAAATTAAG
AACCGCCAAATTACATGGGTCAAGCCTAATAGTCCCCTATTACACCCCTATATATATAGTTGAGATGAGGAACAGATCGATTCA
ACATAATAATTAAGTACGCATTTCGTTAGAGAAGAAATATGAGTGGAATCTAGCGAAATCCGCATTCGGAGCCCTAGGTACCAG
CTGGGCCTAGGCTGGGATCAGGAGGGCTTTGCCTTCGAGCAGGTTCTATATTTCCTTCTTGTTGACTTGAATCTAAGGACGTCA
ATTTCCATGGTCATAATCCACGCCCTGACTATATATTAATCTGGAGATGGGGCGAATCAGATTCAAAAAAATTTAGAGCTTGTT
AGAGAGAAATATGAGTGAAAAATTTTTTTAAAAAAAACCAAAAATCGAATCGGAGGAGCTCTAGGAGCCCTGCCTAGGAGATTT
GGCCTCAAAGGTCAATTTTTTTGTCAGTCTTGATCTCAGGTCCCCCCCCAATTCATGGGTCAATAATAACCCTATACTATATTT
AGTTGAGAATGGGAGAAAGGTTCAAAAAAACGTTAGAGCTTGTTAGTAGAGAAAGGTATGAGTGAACTAACTGAAACGCTGCAT
CCGGAGCCCTACGAGGGCGAGAAAGCCGACAGCCGCCTGTTAGGGACAGATTGTATGAGCACCTGAAAAGGATCACATTTTTTT
TGTTATAGACTTGATATTAGTAACCGCACAAATATCATGGGTCAAAATTAATAAGGCCGCTGATATTAATAATAAACTTTACGA
GAGGAGATGGAAAGAGATTCAAAAAAAAATCAGATATCCATTTGTTAAGAAGAAGAAACCCTAATGATGTAAGGACATCAGCGA
AATGTATCCTGCATTCCAGCGCCCTAAACCCCACATTGCCCCTACAAGGGAGCCCATTACTCGGCTGACAAAAGGTCAATTTTT
TGATATATGGTTATCTAGTTATAGTTGGTAGGCTTCGCAATTTCATGGTCAATTCATCTGCTCCCTGACCTTAAAATACGAACT
AAATGAAATAATTCCAATAAATTGGTTCTACCCACTGAGACAATGGGCAAAAGAGACTTCAATATTCATAACCTTCCGAAGCTT
TCTAATTTCAACATGAAAAATAAGGACGACGCGAGAACGTTAACAAAATCGCATCGGAGCCCACACTAGGGCAAGCAGCTGGGC
CTAGGAAATTGGCGCTCTAAGTGGGGATCAACTCCATTTTATGCGTTTGGTTGCAATTAGAACCCCAAATTACTATTGGTTTAA
ACCATAATCCCTACCCTATATATAATATATATTCCGAAGATCAGGAAAAAATCTAAAAAAAATTAGAGCTTTGTTAGAGAGAAA
TATGAGTGAATTAGCGAAATCAGCTTCGGAAGCCCTAGGCAGCTGCCCCATTTTTGTAGAGATTTGGCCTGAAAAGGTCCAATT
TTTTGTTTGGTTGATTTAGGAACCACCAATTCATGGTCAACACCCTTCAATTCCCCACCCCACAAAATTCGAAATAGGAATTAC
AATATTATTAAGTATGGGAAAAGGTATTCAACAAAAAAAATTAGATCTTGTTAGAAAGAGAAATTTGAGTGAATTACGAAATCG
CTTCAGGATGCCGCTAGGCTATACGCAATGGCCTTGAAAGTGTCTATTTTCTTATTCTTTCAGAAATAAAACACATCGACGAAT
TCATGGTCAATAATAGGCACTATAATAGTAATATTATTGAAGAACTGGGAATTCAAAGAATCAAAAAAATTAACTCTAGATCCT
TGTTAGCGTAAATAAGTGAATATTCAGGATAAATCGCTTCAAGCGCCTAGCATTATAGCAGGCCGCCTAGTGCGTATTTATGGC
CTGAAAGGTCAAATTTTTTGTTTGGATTTGTATTATTAACCCAGGATTATCAACTTGCACAAATTAAATTTGACCTATAATAAT
CAGAAATTACATTATTTGATGTTAGGAGGAAATGGTTCCAAAAAAATTTAGGACTTGTTTAGAGAGAAATCATGCAGTGAATTA
CGAAATCCGTTTGCGTAGCCCTAGGCTCAGCGCACTGCCTAACTGGGATTTGGCCCCTGAAAAGGATCCCTTATTTCTTTTTTT
GGTAGACCCACTTAGGCCCCTACTCTCATCACCTAATCAGACCCTAATAGTCATTCCGCAATTTCTAAAACTCTACCGTTATAT
TATTGAGATTGGGAAATACAGGGTTCTAACAAATCAGAGGCTTGTTAGGAGAGAAATATGATGGATTATTATTCTGTTATATCT
TGTCTTTCGTGCGACCCTATGGCAGGCGCCTAGGGATTCGTTCTCTCTGTATAGTTGTCTATTGTTGGTGAATTTGTGTCTCTA
ATTCATTGTCTAATATTATTATTGGCTCTATATAACAATTTGTTAGTATTGGGTATATTATTGTTCTCTATATATCACACGTTC
AGCATGTCCTTAGTTTAGAGAGAAATATGAGGTGATCACGCATCGCTTCGTGAGCCCTAGGCCAGCTGGCCTTAATGGAGCATT
TGGCCTGAAAGGTCAAGTTTTTTTGTTTGGTGAATTAGACGCCTAATAATGGTCATATATAGTCTCTATATTATAAATATAGA
TGGGAAAAGATCTATAAAAATATGAGCTTGTAGGTATGTAATAGTGTGAAATTACGAAATCGTTCGGAGCCCTAAGGGCAAGCT
CCGGTGGATGTCTGAGGGTCTATTGTTTGGTTTGAATTAGGACGCCAATTCTATGTCAAAATGCCTATATTATATATTATTATA
TTGAGAATGGGCAAAGATTCAAAAAAATTAGAGCTTGTATTGTTAGTATGTATAAATATTATGTATATCTTCAAACTTTATCTA
TTTATATCGCATCGGGCCCTAGGCAGGCTGCCTAGGAGATTTGGCTCATATATGTTGTTCATATTTGTTGTGTGTAATATGTAT
ACTCTCTATATCTATGTGTATATATATATATTGGCCTATAAATATTATTGAGTGGGAAGATAGTATCTAATAAATTATTAAGCT
TTGTTAGAGAGAAATACTGAGTGATTATATTCTGATACTTCTTGCGTTCGTATGCTAGGTCAGCTGCTAGGAATTTGGCCTGAA
AGGTCATAACATTTTTTTGTTTTGTTTGAATTGGACAGCTAATGTCAAGGTCAATAATAGCCGTATATATATATTTGAGTATGA
GTAATATAGGTCAATTAATAATTAGTATGCTTGTTATGTAGAAGAATTATTATGTTATTGAATAACGAAATCTGCATCGGAGCC
CGCTAGGCATGCTTGCCTAGTGTATGATGGGCCTGTAAAGTCAATTGTTTGGTGAAAGAACCTCAATCATGGTCATATATAGCC
CTATATTATACTGTATATTATATGTATGGGTATATATAGTAAATATATAATTAGATCGTTTAGATGAGAATATGAGTGAATTGC
GAAATCGCTTCAGCAGCCCGTAGGGTCAGCCCGCCCTAAGGAATTTGTGCCTGAAAGGTCCAATATTTTTTTTGTTGGTTGAAT
TAGGGCCCCAATTCATGGTCAATATTGCCTATATAATATCATTGAGTTGGGAAGAAGGGTTCATTAAAAATTAGTATCGCTTGT
TGAGAGTAAATATGTCTAGGGGAATTCAACACTGCTCTTCGCTTCGAGCCCCTAGGCAGCTGCCTACCCGGGGTTCGGCAAAGG
TCTATTTTTTTTCTGGTGATTTATGCCTTCATTTTGTCCAATTTATTAGCCATATTATTATAAAAATATTCTGACTGATGGCGG
AAACAGATTCTGACAAAATTTAGACGCTTGATAGAAGAGCACACCACTGAGTGATTACGAAATCGGGTACGGCCCGCCCTACGT
CTCGGGTCATGTCCGCCCTAAGGGGTTTTCGGCCCTGAAAGGTCCAATTTCTTTGTTTGGTTGAATTAGGTTCCCCAATTCATG
GTCAATAATAGCCCATATATAATATTATTGAGAGGGAAAAGGTTGCAAAAAGTTAGAGCTTGTTAGACAGAAAATATGAAGTTA
GAATTTCGGAAAATCGCTTCCGGAGGCCCTAGGCAAAGCTGCCGTAGGGAGAAATTTTCCCATGTGTTCCTGAAGGTCAAACTT
TGTTTGTTGAATTAGAAGCCCCAATTCATGGTCCAATATAGCCTAATTTCCACATCAATATATCTACTTTTCCGAGAGATGCGC
GCTATAAGATTCAACACAAATTAGAATTGTTTTAGAGAAGAATACGCGTATTTGTCCCACAAATCAGGAATTCGCGCACCCTAA
GGCAGGCTGGCGCTAAGGAGATGGTTCCTGGAGACGAGATTCCAATTTACATTGTCTTGGAATTGAATTAGGGACGCCAGAATT
TCATTAATTTCCAATAAAGTAGCCCCTATATACCGGAATGAATAATATCGAGATGGGCCGATAAGAATTCCAAAAGAGAAAAAA
TTAGAGCGTTGTTGAAGAGAAATAAGAGGGGAAATTAACAAATGCGCATCGGAGCCCTAGGGTTCAGCTGCCTAGGAAGATTTG
AGCCTCCCAAGGTCAATTTTTTTTTGTTATTTATGGTTGAATTAGTCCCAAGGTTCATGGTCAAACCACTCCACCCCTTAATAT
TACCTCCTGAATATTATGTTACTTGAGATGGGAAAACAGGTTCACACACATAAGTTAGCTCGTTAGAGAGAAATATGAGTGACT

ACTTACGAAATCGCTTCGCGCAGCCCTAAGGCAGGCTGCCTAACGGAGGATTTGCCGGAAAGGTCATAAATTTTTTGTTTTGGT
TGCTAACATGAAACAACATTTCATGCAATAGGCGAATATATTTAATATATTATTGAGAATGGGAAAACTTAGATTGCAAAAATA
AAATTTAAAGCTTTTTAGAGAGAAATATGAGTGATTACGAAATCGGCTTTGGAGCCCCTTAGTAGACTAGCCTAATTCCGGAAG
ATTTTGGGCTGAAAGGTCAAATTTCTTTGTTTTGGATTGAATTAGAAACCCAACTCTCAAAGGTCAATAATAGCCCTATATCAA
TCATTATTGGAGATGGGAAAAGGTTCAAAAAAATTAGAGGCTTGTTTAAGAGAGAAATATGAGTGAATTACGAAAATCCCGCAA
TCGGAAAAAAAAAGCCCTAGGGCAGCTGCCTACGGAGATTTGGCGCTGAAAGGTCAACTTTTTTTGTTTGTTTGAATTAGAACGC
CCAATTCATGGTCCAATAATAGCCTATATAATATTAATGAGATGGGAAAAGATTTAAAAAAAAAAAAAATAGATCTTGTTAGAG
AAGAAATAATGAGTGAATTGCGAAATCCGCTTCACGCGCGCTACGCGCAGCCGCCTAGCGAGATTTGGCCCTGAAAGGTCAATT
TTTTTTGTTTGGTTGAATTAGGGGGCCCCCAGATTCCATGTCAATAATTGCTATATATATTTATTGAGTTGGGAAGAAGGGTTC
ATAAAAAATTAGAGCCTTGTTAGAGAGAAATATGAGTGAATGTAACGAAAATCCGCTTCGTAGCCTAGGCAAGCTGCCTAAGGG
ATTTTAGGCCATCAAAGGTGCTATTTTTTTTTTGGTAGAATTAGGACCCCTACTTCTTGTCCAATAAATAGCCCTATATAACGA
TATTATTGAGATGGGGAAAAGGTTCTAAACAAAATTAGAGCTTTGTTAAGAGAGAATATGAGTGAATTTAGCGAAATCGCTTCG
GCGGCCTAGGCCAAGCCCCGCCTAGGGGATTCGGCCCCTGAACAGGTCCAGTTTTTTTTGTTTGGTTGAATCAGGTGCCCGGC
AAATTCATGTTCAAGTAATAGCCCCCTATATCATAATTATTAGAGATGGGAAAAGGTTCAAAAAAGTTGAGGCTTGTTAGAGAG
AAAATATGATGAAAATTACGAAATCGCTCTCGGAGCCCATAAGGCAGCTGCCTAGGAGATTTGGCCTGAAAGGTCAACTTTTTT
GTTTGTTGAATTAGAACCCCAAATTCAATGGTCAATAATAGCCCTATAATAATATTAATATGAGATGGGAAACGAAGATTCCAA
AAAAATGTAGAGCTTGTAGAGAGAATATGAGTGACATTACGAATCGATTCCGGAGCCCCTAGGCCCCAGCTGCCTAGGGGATTT
GGCCTGAGGGTCAGATTTTTTTGTTTGGTTGAATTAGGACGCCATTCATTTTTCAATAATAGCCCTATAAATATTAATCTGAGA
TGGGCCAGAGAGATTCGAAAAAACAATTAGGAGCGCAACATGTTAGAGAGAATATGAGTGAAATTACAAAATCGCATCGGAGCC
GGAAAGGACCAGCTGCCTAGGAGATTTTGGCCTCCAAAAGGTTCAATTTTTTGTTTGGTTCCCCATTAGGTCCGCCCAATCTCA
TGGTCAATAATAACCCTATATAATATGTTTGATGAAGAGGGAAACAGTTCAAAAAAGTTAGAGCTTTGTTGAGAGAAATATGAG
TGAATTACGAAATCGCTTCGAGCGCTAAGCAAGGCCTGCCTAGGAGATTTTGGACTGAAAGGTCAATTTTTTTTGTTTGGTTGA
TTAGACCCCAATTCATGGTCAAATAATCAGCCCTATAATAATATTATTGAGAATGGGAAAAGATTCAAAAAAAATTAGAGCTTG
TTAGAGAGAAATATAGTGAAATGAAAATCCGCTTTGGAGCCGCTTGGTAGACTACCGTAGGAAGATTTGGCCTGAAAAGTCAAC
TTTGCTTTTGTTTGGTTGAATTAGGACGCCCCAATTCAAGGTCAAGTAATAGCCCTATAGTAACATATTAATTGAGAATGGGAA
AAATGTTCAACAAAAATTAGAGCTTGTTAGAGAGAAATATGAGTGAATACGAAATCGCATCGGAGCCCTAGGCAGCTGCCTAGG
AGATTTGGCCTGAAAAGGTCAATTTTTTTGTTTGGTTGATTAAAACCCAATTCATGGTTAATAATGAGCCGCTATAATAAATAT
TATTGAGATGGGAAAAGATTAAAAAAAATAGATCTTCCTTAGAGAGAAATATGATTGAATTGGCGAAATCGCTTCAGCGCCCTA
CAAGGCAGCCGCCTAAGGAGATTTGGCCTGAAAGGTCAAATTTTTTTTGGTTTGGTTGAATTAGGCCCCAATTCCATGGTCAAT
AATTGCCCTAATATAATATTATTGAGTGGGAGAAAGGTTCAAAAAAAAATTAGACGCTTGTTAGACGAAGAAATATGAGTGAAT
TAGGAAAATCCGCTTCCGTAGCCCTAAGGCAGTTGCCATAAAGGGGAATTTTGGCCGCCTGAAAGGTCATACTTTTTTTTTTT
TTTGGTAGGAATTAGGAACGCCCCTAAATTCTTGTCAATAATAGCGCCTATTACCTAAAATTATTGAGATGGAAAAAGGTTTCT
AAAAAATTAGAGGCTTGTTAGAGAGAAAGTAACGCAGTGAATTACGAATCGCGTTCGGGCGCCTAAGGCAGCCGCCTAGGGGAA
TTCGAGCGATGAAAGGTCAATTTTTTTGTTTGCGTTGAATTAGGTCCCGCAATTCATGGTCCAATAATGCTATATACCGATGAT
TATTGAGAATGGGATAAAGGTCAAAAAAGTTAGAGCTTGTTTAGAGAGAAATATGAGTGAATTAACAGAAATCGCTTCGGGAGG
CATAGGGCAGCTGCCTAGGAGATTTGGAAACTGAAAGTCAATTTTTTTTGTTTGGTTTGAATTTAGAACCCCCAAATTTCATGG
TCAAGTGAAAAATTACCTATACCTAATATTATTGAGATGGGAAAGAGATTCAAAAAAATTAGAGCTTGTTAGAGAGAATATGAG
TGGATTACGAATCGCTTCGGAGCCCTAGGCAGACCCTAAGGATTCGGCCCTGAGAGGACAATTTTTGTTTGGTTGATTAAGACC
GCAATTCATGGTCAATAATACCTATATATATTACTTAGATGGCGAAGATTCAAAAAAAATTAGAGCTTGTTAGAGAGAAAATAT
GAGTGAATACACAAATCGCATCGGAGCCCCTAAGGCAGGCTGCCTAGGACAGATTTGGCCTCAAAGGTCCAATTTTTTTCTGTT
TTGGGCTTGAATTAGGTCCCAATTCATCGGTCAATAATAACCCTATATAAATATTATTGAGATGGGAAAAGTTCAAAAAGTTAG
AGCTTGTTAGAGAGAAATATGAGTGAATTAGCGAAATCGCTTCGGAGCCGTAGGTCAGCTGCCTAGGAGATTTTGGCCTGAAAG
GTCAATTTTTTTGTTTGGTTTGAAATTAGAAACCCGCAATTCATGGTCCAATATAGCCCTATACAGATATTTATTGAGATGGGA
AGAGATTAAAAAAAACCTATAAGATCTTGTTAGAGAGAAAATGAAGTGAATTGCGAAATCGCTTTCAAGCGCCTAGGCAGCCGC
TAGGAGATTTGGCCTGAAAGGTCAACTTTTTTTGTTTGGTTGAATTAGGGCCCGACTTTCCTGGTCAATAATTGCCCCTAGTAG
TAATATTATTGAGTTGGGAGAAGTTCATAAAAATTAGAGCTGTTTAGAGACGAAATATGAGTGAATTACGAAATCGCTTGCCGT
AAGCCCTAGGCAGCTGCCTAGGGGAAATTTGGCCCCTGAAAAGGTCCATTTTTTTTTGGTGAATTAGGACCCCCTTAATTCTTG
GTACATATAGCCCTAATATAAATTATTGAGATGGGAAAAGGTGGTCTAAAAAATAGAGCTTGTTGAGAGAAATATAGTGGAATT
AGCGATCCAGCTTCGCGCCCCTACGGCTCCTAAGGGGATTCCGGCCTGAAAGGTCCAATTTTATTTGTTTTGGTTGGAATTAGT
CCCCAATTCATGGTCAATAATAGCCCTATATAATATTATTGAGATGGGAAAGGTTCAAAAAATTAGAGCTGTTAGAGGATATAG
TGACATACGAAAAATGTTCGGAGCCATAGGCAGCTGCCTAGGGATTTGGCTGAAAGGTCAACTTTTTGTTTGGTTTGATTAGAA
CCCATCTGTCCTATAGCTATTAATTTTTGAGATGGGAAAAAGATTCAAAATTAGAGCTATGTTAGGAAATATGAGTAGAAATTA
CGAATCGATTCGGAGTAGGCAGGCTGCCTGGGCGGATTTTGGTGAGAGTCAATTTTTTGTTTTTTGAATTAGGACCCAATTCAT
GGTATCAATAGCTATATAATATTATTGAAGAAGGGCGAAAGATTCAACAACATTAAGTTGTTAGCAGCAGAAATATGATGAATT
ATAAATCGCATCGGCAGGAACTAAGCGCAGCATGACTACGGAGATTCTGGCTCAAAAGGTCAATTTTTCTTGTTTTGGCTTGAA
TTAGGTCCAATTCATGAGTCAATAATAAAGAATATACTAAATATTATAATGAGTGGGAAAAAGGTTAAAAAAGTTAGAGCTTTG
TTTAGAGAGAAAATATGAGTGAAATTACGAAATCGCTTCGACCTGGCAGCTGCTAGGAGATTTGGCCCTGAAAGGTCATTTTTT
TGTTTGGTTGAATTAGAACGCCCAATTCAATGGTCAACACTAATAGCCCTATATAATATTAATTATCTGAAGAAATGGAAAGA

TTCAAGAAAAATTAGAGCTTGTTTAGAGAGAAATATGAGGTGAATTTACGAAAATCGCTTTGGAAGCCCCTTGGTAGCCCTGCC
TAAGGAGCTTTGGCCTGAAAGGTCAATTTCTTTTGTTTGGCCTTCTTGCGAATTAGGCGAAGCGCCCCAATTCAAGGCGACAAT
AATAGCCCTATATACATATTACTTTTCGAGATGGGAAAAGGTTCAAAAAAAATAGAGCTTGTTACACGAGAAATAATGAGCTGA
ATTACGAACATCGCATCGGAGGCGCCTAGGGCGCTTGCCTAAGGAAGATTTGGCCTGAAAGATAATTTTTTGTTTTTGTTAATT
AAAGAACCCCAATGTCATGGTCAATAATAAGCCCTATATACATATTTATTGAGATGGGAAAAGATTAAAATAGATCTGTTAGAG
AAGAAATATAATTGAATTGGCGAAATCGCTTCAAGCCCCCCTAGGCAGCCGCCTGGAGATTTGGCCTGAAAGGTCAATTTTTTT
GTTTGGTTGAATTAGGCCCCCCAATTCATGGTCAATAATGCCCTATATGAATATTATTGATTGGGAGAAGGTCAATAAAAATTA
GAGGCTTGTTAGAGAGAAATATGAGTGAATTACGAAATCGCATCTCGTAGCCCTCAGGCAAGTTGCCTAGGGGATTTGGCCTGA
AAGGCTTATTATTTTTTGGTAAGAAATTAGGACGCCCTATTCTTGTCAATAATGCCGCTATATACATATTAATGTAGAGATGGG
AAAAGGTTCTAAAAAAATTTAGAGCTTGTTAGTGAGAATATGAAGTGAATTACGAAATCGCTTCGGCGCCCCTATGGGCAGCCC
GCCTAGCGAAGATCGCTATGAAGGTTCCAATTTTTTTGTTTCGGTTGATAGGTCCCCCAATTCATGGTCCCAATAATTAGCCCA
TAACATAATATGTATTGAGATGGGAAAAGGTTCAAAAAAGTTAGAGCTTGTTACGGAGAAATATGAGTGAATTGACGAAAAATC
CTCGGAGTCTCTAAAGGCAGCTGCCTAGGACGATTTGCTGAAAGGTCAATTTTCTTGTTTCCCTTGATTAGAACCCCCAATTCA
TGGAGTACCTAATAGGCCTATATTTAAATAATTATTCCGAGATGGGAGAAAGGTTCCTAAAAAAATTAGCAGCTTGTTAGAGAA
GAAATAATGAAGTGAATTACCCGAAACTGCTATCGCGCCCCCAAAAAACCCAATAGGCACGCGCCGCCCTAGGGGATTCCGGCC
CTGAAAGGTCAAAATGTTTTTTGTTTGGTTGAATTATGGATCCCCCAATTCATGGGTCCAAAATAATAGCCCGTAATATAATAA
TATTCGCGAGATGGGAAAAGAGTTCAAAAAAGTTAGAGCTTGTAGAGAGAAATATGAGTGAATTACGAATCGCTTCGGACCGCC
CTAGCAGGCTGCCTAAGGACGATTTGGGCCCTGAAGGTCAATTTTTTTGTTTTTGGTTTGAATTAGAACCCAAATTAAATGTCA
ATACATAGCCCTATATAATATTATTGAGATGGGAAACCAGATTCCAAAAAAATTAGAAGCTTGTTAGAGAAGAAATATGAGTGA
ATTACGAAATCGGCTTCGAGCCCTAAAGGCCAGATGCCTAGGGGATTCGGCCCTGAGAAGGAACAATATTTTGTTTGGTATTAG
AATTTAGGACGGCCAATTCATGGTCCAAATAATAGCCCTATATACATACTTGATTGAAGAATGGGCGAAAGATTCAAAAAAGTT
AGAGTTGTTTAGGAGAAATATGAGT
>m141120_105518_42134_c100732402550000001823141405141504_s1_p0/102314/927_10458
ACTAAAATAGAGGGTCCTTACAAGTATTGTGCCACACTAGAACACAATCTTCGTATTTGCTGCACCATATATGGATTTTCATCT
TATATCGCGATTGAGAGCCTTAACCTGTTTTAGATTAGTAGAATCCTACAAGAGTATAATATCTGCGAGGGTGTACATTTACA
TTATACGGCACAATTCATAAAATAAATATTAACACATCAATAAACAAAGTTTGTCACGCCTTTATATCATTGACAATGCCAAAA
GGTATCTATATAACTATATGAGTGACAACCCTTAGAGACCTCTCGTGCAAATCCCCGAGGGTAGTGTAGTATGGGTTTAGATAC
AGTCCAAAAAAAACAAATAAGTGAGAGATGTACGAGATATAACGAAGGGAAAAAAGTGCGAGACAAGTTGCAATAAAAAAAACT
CGCAATTTTCTCTTTTCCGAAAAAACAATGTTGACCCAGCTTCAAGGCTCCGTTTCGACTCAAAAATGAGATTTAATTTGACCG
CACGACCGATTCCCCTCTTGCGGGAAAAAATTATCCGAACCGAAGTTGAACAAAAAGATGAGCGGTACTAACCCAGTGTGTCGC
AGCTCCAGATTAGTGAACATCTCCCAATTCACCAAAGGGTACCCCTTTCAGAACAATCTCATCGATTCATCTCTAATATCCGCG
ATTCGAGCACCATGTCCTTCTGATAGCTGAATCAACAAGAAAACTATCGAGGTTCATCCGCGGGCACAAACAAAGTCTGTAGGC
CTTATGTCCTTAGGGTTCTATGTACAACACATACTATTGTGCAACTCCACCGAGGTGTGTAGAGTGCTGATACCAGTCAAAATA
GTGAGAAGCACCGACACCATGTAGAATACTACCGCAGGAAAAAACAAGATTGCGCGCAACAAATTGCCTTAAAAACCCAAGGGG
AATTTTTGTTGCCTTGCGCAATGTTGACTTCACGCTCCGTTCTTCGACTATCATAGAATTGAAATTTACCACGAATCCAACCGT
CTTTGAGGGAAAAAATATCGAACGAGTGGAAGATGAGGGTAATACATTTCGGAAGAAATTAGTGAAATCTCCAATTCACAAAGGG
GTACCCTTTTTCAGAAATCTTCCGATTTCCCTCTAATACTGGCATTTCGAGCAACTGTTTTGATAGTCGAAGTCCTCAAAAGAA
CTATGCGAGGCGGTCCATGGGGCAAAAAGTTGTAGGCCGTATGTCTTAGGGTTCTATGTCTAAAAACTTCGATGCCAAATCCGA
GGTCGTGTATGAGGGTGATACAGTCAAAGTAGTGAGAGATGACTACGAAGATCACGGGAACAAAAATGTTGCGAAAATTGCAAT
ATAAAAATGGGAATTTTCTTTTGAAATGCTTTGACTTAAGGCTTCTTTCGAACTCCAATCAGATTAATTTTCCCACGGAGATCT
CTTTGCGGGAAAAAATATCCGAACGAGTTGAGCAGAATGCAGCGGTAATACATTTCGGAGTTAACATTAGTGAATCTCAATTCAC
CAAAAGGGGTACCCCTATTTCAGAAATCTTCGATCTTCTCCTAATATGGAGTTCGAGCAATGTTTTGATAGTGAATCCTAAAGA
CATACTGGCGAGGTTCATTGGGCACAAAGTTGTAGGCCTATGTCCTTAGGGTTCTATGTAAAATACTTTGTGCAAATCCGAGGT
GTCGTAGAGAGTGTGATACAGTCCAAAATACGTGAGAGATGTACGAATATAAGGAAAAGTTGCGAAAATTGCCCTTAAAATAAG
GGGAATTTTTATTTGAAATGTTGACTTTAAGGCTCGTTTTCGACTCAATAAGATTAATTTTCCACGAGATCTCTTGGAAATATA
TCGAACGCAGCTTGAAGACGAGGTATAACAAGTTTCGGCAGAAAGTTAAGTGAAATCTCCAATCTCAAAAGGGGTCACCGTTTC
AAAGATCTTCGCATTTCTCTTATGGATTCGAGCCAAACTGTTTTGATAAGTGATCCTAAAGAATTAATTGCCAGGGGTAATCGG
GGCACACCAGACCAAAGTTGAGCCTATGTCTTAGGGCTTCTCTGTCATAAAAGTTCCGTGCAATCCGGAGAGTGTGTAGAGGGT
ACTCACAAGTCAAATACTCGAGAGATGTACCCGGAATATAGGGAAAACAATTTGCGCAAATGCCATTAAAAAACTGGGAATTTT
TTTTGAAGTTGAACTAAGGCTCCGCGTTTGCGACTCCAATGAGATTAAATTTACCCCACGAGATCCTCTTGCGGGAAAAAATATC
GAAAGAGTTGAAGTGAAGGGTAATACCTTTGAGAAAATTAGTGAAATCTCACAATTCACCAAAGGGGTACCCTTTCAGAGAATC
TTTCGATTCTCTAATATGGCTTTCGAGCAATCTGTTTTTGACTAGTGAATACTAAAGAATATACGGGAGGGTACATGGGCCACA
AAAAGTTGTAGCCTATGTTTAGGGTTCTAGTATTTAAAACTTCGTGCCAATCCGAGGGTGTGTAGAGGGATATACAGTCGAAGA
TAGAGTGAGAAGATGTACGAATATTAAGGAAAAAAGTTGCGAAACATTGCCTTAAAACAGGGGAATCTTTTTGAAATGTTGACT
TAAGGCTCCGTTCTCCGATCTTCAATAGATTAATTTACCCACGAGATCTCTTGGGAAAAAATATCGAACAGAGTTGAAGACGAGG
GTAATGACCATTTCGGAGAATTAGTGAAATCTCCCCAATTCCAAAGGGGTACCCCTTTCAGAAATCTTCGATTTCTCTAATCAT
TGGGATTCGAGCAACATGTTTTCAGTAGTGATCCTAAAGAAAGTCATGCGAGGGTCAAATGGGCAACAAAAGTCTGTAAGGACT
ATGTCTTAAGGGTCTATGTATAACACTAGAACCTTCCGTCAAATCCGAGGTGTGGTAGAGGGTGATGACAGTTTAAATAGTGAG

AGATGGCTACGAATATAAGGAAAAAAGTTGCGAAAATTGCCATCAAAGAAAAGGGTAACTTTTTTTGAAATGTGACTTAAGGC
TCCGTTTCAGACCTCCATAGATTAATTTACCCACGAGATTCTTGGGGAAAAAATATCGAACGAGTTGAAGACCGAGGGTAATAAC
ATTTCGGAGAAATTAGTCGACAATCTCCAAAAAAAATTCAACCAAAGGGGGTACCTTTTCAGCAAATCTTCCGATTGCTTCTAA
TATGGGCATTCGAGCAATGTTTTGATACGTGAATCCTAAAGATATGCAGGGATCATCGGCCACAAAAGTTGTAGCCTATGTTAG
GGTTCTATGTATAAAAACTTCGTGCAAATCCGAGGTGTGTAGAGAGTGATACAGTCAAAACTGTGAGAGATGATTACGAATATA
CGGGAAAAAAGTTGCGAAAATTTGCATAAAAAATGGAGATTTTTTTTGAAATGTTGGATTAAGGTCCGTTTCGACTCCAATAGA
TTAATTATACCCCACGAGATCTCTTTGGGGAAAAAAATATCGAAACGACGTTGAAGATGAGGTTAATACATTTCGGAGAATTAGT
GAAATCTCCAATTCTCCAAAGGTAACCCTTTCCGAAATCTTCGATTGTCTCTAAGAATATCGGTTTCGAGCAATGTGTGATAGT
GAAACCTAAAGAATATACGAGGGTCATGGGCACAACAATCTGTAGGCTATGTCTTAGGTTCTATGTATGAAAACTTCGTGCAAT
CGAGGTGGTGTAGAGGGTCGAGAGCAGTCGAAATAGTGAGAGAATGTACGGAATATAAGCGAAAAAAGTTGCGAAAATTGCCTT
TAAAAAAGGGGAATTTTTTGTTTGAAATGTTGACTTTAAGGCTCCGTTTCGACTCAATAGATTAAATTTACCCACTGAGATCTC
AATTGGGGAAAAAATATCGAACGGGTTGAAGATGAGGGTAATACAGTTCATCGAGAAATTAGTGAAATCTCCAATTCACGCAAAG
GGTACCCCATTTTCAGAAATCTTCGATTTCTCTAATATCGGGTTCGAGCAACTGTTTTGATAGTGAACCTAAAGAATATGCGAG
GCGTCATGCGGGACAAAAGTTGTAGGCCTATGTCAATCAGGGTTCTATGTATAGAAAGTTCGTCGAATCCGAGGTGTGTATGAG
GGTGATACAAGTCAAAACAACTGAGAGATGTACGAATATAGGGAAAAAAAAAAGTTCGAAAATTGCCCCATAAAAAATGGATTT
TTTTTTGAAAATGTTGACTTACAGGCTCGTTTCGACGTCCGAAATCAGATTAAGTTTAACCACGAGATTCTTGCGCGGGAAAAA
TATCGAACGGGTTGAACAGATGAGGGTAATACATTTCGGAGAATTAGTTGAAAATCCCAAATTCACCAAAAAGGGGTACGACCT
TCTCCAGAATCTTCGATTTCTCTAATAGGGCTTTCGAGCAATGTTTTGATTAGTGACTCCTAAAGAATATGACGAGGGAGCATG
GCACAAAAGTTTAGGCCTATGTCTTAGGGAGTTTTGTAACGTCTCGTGCAACTCCGAGTTGTAGAGGTGTACATGAATATGAGT
GGTTGAAGTTGCGAAATTGCCTTAAAAAAGGGAATTTTTTTTGAAGTGTGCTGACTTGAAGGGCTCCGTTTCGACTCCAATAG
ATTAATTACCACGAGACTCTCTTGGGGAAAAATATCGAACAAGAGTTGAACGATGAGGGTAATTACATTCGGAGAATAAGTGAA
ATCTTCCAAATCTCACGCAAAGGGGTACCGCCTTTCAGAAATCTTTCGATTCTCTCTATATGGATGCGAGCAATGTTTTGATAG
TGAATCCTTATAAGAATACTGCGAGGTCATCGGGCACAAAAGTTGTAGGACCTATGTCTTAGCGGTTCGTTATGGTAGAATAAA
AAGTCTCGTGCAATCCGAGGTGTGTAGAGGTGATACAGTCAACAATAGTGCAGAGATGTACCGATATAGGACAAAGATTGCGAA
AACTTGCCATAAAAAAACCTCGGAATTTTTTTGAAACTGTTCGACTTAAGGCTCCGTTTCGATCCAATAGACTTAAAGTTTACC
ACGGAGATCTCTCTGGGGAACTAAATATGCGAGAACGAGTTTGAAGATGAGCGCGTAATAGCCATTTCGGAGAATTAAGTGAAA
TCTCTCAATTCACCAAGGGGTACCCCTTTCAGCAAAATCTTCGATTTCTCATAATAATGGTTTCGACAATGTTTTGATACGTGA
AGTCCTAAAGAATATACGAGGGCTCATGGCACAAAAAGTTGTAGGCCTATGTCTTTAAGGGCGTTCTATGGTCCCTAGAAACTC
TCGTGCCAATCCGAGGTGGTGTAAGAGGTGATCACAGCTCGAAATAGTGAGAGATGTACGAATATAAGGAAAAAAGTTGCGAAA
TTGCCTTAAAAAAGGGAATTTTTTTTGAATTTCACTTAAGGCTCCGTTTCGATTCCACTAGATTAACTTTAACCCACACGAGAT
CTCTTTGGGCAAAAATATCGCAACGAGTGAAGACGAGGGTAAGTACATTTCGGCAAAATTAGTGAAATCTCCAATTCAACCCAA
AGGGTACCTCTTTCAGCAAAATCTTCGATTTCTCCTAATTGGATTCGAGCAGGTGTTTTTCGATAGTGAATCCTAAAGAATATC
GAGGTCCATGGGCCCAAGTTGTAGGCCTATGTCCTTAGGGTTCTATGTCAATAACCTCTCGTGCCAATCCGAGTGCTGGTAGAG
GGTGATAACAGTTAAAATAGTGAGAGATGTAGCGAATTATAAGGAAAAAAGTTGCGAAAGATTGCCATTAAAAACAGGGTAATG
TTTTTTGAATGTTGCACTTACAGGCCTCCGTTTCGACTCATCAGATTACAAATGTTCACCCACGAGATCTCTTGGGAAAACATA
TCGAACGAGTTGAAGAACGAGGGTAATAACATTTCGCGAGAAAATTAGTGAACATCTCCCATTCACCACAAAAGGGGTAAACCC
TTTTTCCAGAAATCTTCGATTTCTCTAATATGCCCTTCGAGCAACTGTTTTGATAGTGACAACCCCCCCACCCCCCCTAAGAAT
ACATGCGAAGGTCCCATCGCACAAAAGTTGTAGGCCTATGTCTTAGGGTTCTATGCCTAACCTTATAAACTTCGCTGCAAATCC
GAGCTGTGTAGAGGATGGATACAGTCAAACATACTGTCGAGAGACTGCTAACCGAATTATAGGGAAAACCAAGTTGCGAAATTG
CCATAAAAATGGGAATTTTTTTTTGAGAACTGCTTGCACTTACACGAGGCTCGTTTCCGACAAACCCGCCCCTCCCAGGCCGGA
CACCCGAAGACGCGCCAGACTCAGATAGATTAATTTAACCCACGAGATCTCTTGGGGCGAGAGAAAAATATCCGGAACAGAGTT
GAAGATGAGGGGTACATACATTTCGGAGAAATTCAGTGAAATCTCCAATTCCCAAAGGGGTACCCATTGTCAGAAATCCTCATT
TCTCTACATATGGTTTCGAGCAATGTTTTGATAGTGAAGTCCTAAAGACAATTACGAGGGTCATGGGCACAAAAGTTGTAAGGC
CTAGTCTAGGGTTCCTATGTATAAAACTTCGTGCCCAATCGAGGTGTGTAGAGGGTGATACAGTCGAGAATAGTGAGAGCATGT
ACCGAATATAAGGAAAAAGAGTTGCGAAATACTGCTTAAAAAAGGAATTTTTTTTGAAATGCTTGACTTACAGGCTTCCGTTTC
GCTCCAATACGATTAACTTTTACCCACAAGATCCTCTCTGGGGAAAAATATCGAAACGGCGTGACAGAGAGGTAATCACATTTC
GGAAATTAGTGAATCCCCAAATCACGCAAGGGGTACCCCCTTTCAGAAATTCTTTCCCAGATATTCTCTAATATTGGGTTCGAG
CAATGTTTTGATAGTGAACTCCTAAACAAGAATCAGTGCGAGGGCTTCATGGGCACAAAAGTTGTAGCGCCTAATTCATAGGGT
TCTATGTATAAAGTTCGTCGCGAATCCGAGTGTGTAAAGGGTGATATCACGTCAAAATACTGAGAGTGTACGAATATTAAAGGG
AAAAAAGTTGCGGAAAGATTGCTCATAAAAAAAATGGGGATTTTTTTTTGAAATGTTGACTTAAGGCTCCGTTTCGACTCCAAAT
AGATTAATTTTAACCCAACGACGATCGTCTTGGGAAAAAATTCGAACGGAGTTGAGATGCAGCGGTAATACATTTCGGAGAATGT
ATGAAATCCCCAGATTCACCAAAGGGTCCCTTTTCAGAAATCCTTCGATTTCTCCTAATATCGGTTTCGAGACAACTGTTTTGA
TAGTGAATCTAAAGAATACACATACGAGGGTCATGGTGGCACTAAAAGTTGGTAGCCTACTGTCTTAGGGCGTTCTATGTATAC
CCAAACTTCGTGCCAATCCGAGGGTGTGTAGAGGGGTGATACCAGTCGAAAATCACGTGGAGAGTACCGACAGTACACATAGAC
GAAACACAACGTTTTGCGAAAATTGACTTAAAAAAGGGGAATTTTTTTGAATGTTGACTTACAGGCTCCGTTCTCGACTCAAT
AAGATTAATTTAACCCACGAGCATCTCTTGGGGGAAAGTATCCGAACCGATGTTGAAGACGAGGGTGAATACATTCTCGGCCAG
AAGCCATTAGCTGAAATCTCCAATTCACCAAGGGTACCTTTTTCAGAAATCTTCGATCTTCTCTAATATGGTATATCGAGCAAC
GTGTTTTGATACGTGAATCCCTAAAGAATAATGCCAGGGTAGGGGCACAAAAAGTCTTGTACAGCCTATGTCTTAGGGTTCTAT

GTATACACAAACTTCGTGCAAATCCCGAGGTGGTTGTACCGAGGTGATAAAAGTCAAAATAGTGAGAGATGTCACGAATATAAG
GAAAAAAGTTGCGAAAATTGCCATAAAAAGGTAATTTTTTTGAAATGTTGAACTTAAGGCTCCGCTTTCCGATCAACCAGGAA
TTCCAATTTTCCCACGAGTCGTTCTTTGGCGGACACAAAATATCGAACAAAGCCGGAGTTGAAGACGAGGGTAATACATTCGAG
AAATTAGTGAAATCTCACATTCACCCAAAAAGGGGGTACCCTTTCAGAAATCTTCGATTTCCTAATATCGGATTCGAGCAAACT
GTTTTGATACGTGAATCCTAAAGAATATGCGACGGTTCCATGGAGCACAAAAGTTCTAGGCCTCCATGTCTTACCGGGTTCTAT
GCATAAAAAAACTTTGTGCAAATCCGAGGTGCTGTAGGAGTGGTGATACAGTCAAGAATAGTCGAGAGCTGTACGAATATAAGAC
ACGAAAAAAGTTCGGCCGAAAATTGCATTAAAACAAGGGAAAATTTCTTTTTGTAATGTTTGACTTAAGGCTCCGTTTCGACTC
CAATAGATTGAATTTATACCCACCGAGATCTCTTGGGACAGGCATAATCGAACGAGTTGAAGAAAATGAGGCCGTAATACATTT
CGGAGAAACTTAGTGAAATCTCCACATTCACCATACAGGGGTACCCTCTTCTTCAGAATCTTCGATTTTCTCGTAATTATCGAT
TCGAGCAATGTTTGTTATAGTGAATCCTAAACAGCATATACGAGGGTACATAGGGGGCACAAAAGTTGTAGGCCTATGTCTTCG
AGGCGTCTCTATGCTATAAAACTGTCGTGCCAAATCCGAGGTGGTGTACGACGGGGTTGATACGACGTCGAATAGTGAGAGATG
TAACGAATATACGACAGAAAAAGTTGCGAAAAATTGCCTTAAAAAAAGGGGAAATTTTTTTTGAAATTGTTGACTTAGCAGGCT
CCGTTGGACTCCAATAGATTAAATTTACCCACGAGATCATCTTCGGGGAAAAAATATCGACACGAGTTGAAGACGAGGGTAAAAT
ACATTTCGGAGAAATTACAGTCGAAATCTCCAAATTCACCAAAGGGGTACCCCTTTCAGTAAATACTTCTTTCTCTAATATGGA
TTCGAGCAATTGTTTTGAATAGTGAATCTAAGATAGACAGGGTCAATCGGGCACGACAAAGTTTGTAGGCTAATGTCTCTAGGG
TTCTACCTGTATAAAACTTGCCGTGCCAATCGAGGGTGTGTAGAGGGTGGATTACAGTCAAAATAGTGAGATGGGTACGAATAT
AAGGAAAAGTTGCGAAATTGCCAATATAAAAAGGGTAATTTTTTTTTGAAATGTTGACTTTAAGGGCTCCGTTTCGCACCTCCA
ATGAGATGAATTTTACCCACGAGATTCTTTTTGGGGGGGAAAAATAATCAACGAGTTTGCAAAGACGAGGGTAATACAATTCGG
AAATTAGTGGAAATCTCCAATTCCACCAAAGGGGTACCCTTTCAGAGAATCTTTCGCATTCCTGCTAATATGGATTCGAAGCAA
TCCCGTGACTGCAATGCTTTTTGATAGTGAGATCCTAAAGAGATATCGAGGAAGTCAATGGCAGCAAGTTCTAGGCCTACTGTC
TGTAGGGTTCTACTGTATAAAACTTCGGCAAACGAATCC
>m141120_105518_42134_c100732402550000001823141405141504_s1_p0/102894/6164_11701
GTCTAATCAATTTAACTAACTTCTAGTTTAGGACCTGGTTGTGTACAGGATAATAATTATCAACATCCCTAGTTCGTGTTAGTA
TTTTTCGACTCCTCGTGCTCACTTAGAAACAAACATTGGTTATCATTTTTACTAACTTCTAAGCTAGCTAGGACCTAGGTTTGT
GTCCAGGATATTTAAATTTAAAAACTTAGTTCGCGTTAGAATTACCCCGACACCTCGTGCCTCTTTAGGAAAAACCACTGGTTT
AATCAGATGTTAACTAACTTCTAAGTTTTAGGACCTAGTTGTGTCAGGATATTTAATTTAAAACTTAGTTCGCGTTAGTATTAT
CGCACCCCTCGTGCTCTTTTAGGCAAAAACACTGGTTAACTAAATTTAACAAACTTCTAAGTATTAGGACCTGGTTGTTGTCCG
GGATATTAATTTAAAGACTTTGTTTCAGGTTTAAGTTTTTTATGACACCTCGTGCACTTTAGACAAAACGATTGTTATATCAAT
TTTACCTAACTTCTAAGTATTAGGACCTGGCTTTGTCAAGGATATTAATTTAAAAACTAGTTTGCGTTAGTTTTATCGACACCT
CGTGCACTTAAGGAAAAACACGGGCTTAATCAATTTAACTAACTTCTATGTTTTAGGAGTCTGGTATTTACTCCAGGATATTAA
TTTAAAAATTAATTCGCGTTAGCCTATCGACAACCCGTGCTCTTTAGGAAAAACATGGTTAAGTCAATGTAACTAACTTCTAAG
TTTTAGGACCTAGTTGGTGTCAAGGATTTTAATTTAACACTTAGTTCGCGTAGTTTTACTGAAACCTTGTGCCTCCTTAGGAAA
AACCAATGTTAATCAATTTAACTAACTTCTTAAGTTTTACGACCTGGTTGTGTCCAGGATATTAATTTGAAACTTAGTTCGGCG
TAGTAATCTATAGACACCTCGTGCTCTTTAAGGAAAAACCACTGGTTAATCAATTTAACTAATTCTAAGTTTAGGACCCTGGTT
GTGTCCAGAATATTTATTCTAAAAACTTAGTTCGCATTACTTTTTCTATCGACACCTCGTGCTCTTTAAGAAATACACTGCGGT
AAATCAATAAATTTAACAAACTTCTAAGTTTTAGGACCTGTCGACTGGTCCAGGATATTACTATAAAAAACTTAGTACGCTTAG
TTTTTATCGACACTTGTGCTCTTTAGGAAAACACATAGTTAATCAATTTTAACTAAATTCTAAGTTTTAGGACCTAGTTGTGTC
CAGGATATTTAATTTAAAAACTTGTTCGCGTTAGTATTTATTCGACACCTATCGTGCTCTTTAGGAAAACAACTGGTTTAATAA
ATTAAAACAAACTTCAAAGTTTTAGGACCTGGTTGTGTCCAGGCATACTAATTTAAAATTTGTTCAGGTTAGGCTTTTCATGA
CACCCTCGTGCACTTAGAAAAACACATTGGTTAATCAATTTTACTAACTTCTAAAGTATAGCCTGGTTTCTTGTTCCCAGGATA
TTAATTCTAGAACTACTTACGTTTGCCGTTAGTTTTTCGACACTCGTGCACTTAGGAACAAACACGGGTTAATCAATTTTAACT
AAACTTCTAAGGTTTTAGGGCCTGGTTTTATCCAAGATAAATTAAAAAAATTAGTTCGCGTTAGTCTATCCGACAACCCGATGC
GCCTCTTTTAGGAAAAACACTGGTTATCATTTAACTCACTTCTAAGTTTTACCGGACCTATAGTTGTGTCAAGGTTTTAATTTA
TAAACTTAGTTTCGCTTTAGATTTACTGAACCTTGTGCTCCCCTCCCAGGAAAAAACAATGGATAATTATTTAACTACTTCTC
ATGTTGTTATGACCCTGGTTTGTGTCCAGGATGATTAATTTGAAAAACTTAAGTTCTCCCGTTAGTATTATCGCAACCTCGCTG
CACTTAGGTAAAAACACTGGTTAATCAAACCTTTAACCTAACTTCTAAGTTTTCAGGACCTGTTTGTCTCCAGGATATTGTATT
TAATAAACTTATTTCGCGATACTTTTATCGACCACTCGTGCTCTTTAGAAATACCACCTGTTAATTCATTTAACAAACTTCTAA
GTTTTAGGACCTGGTTGTGTCCAAGGATATTAATTTAAAAACTTAGTACGCGTTTATTTTATCGACACATGTGCTCATTTAGGA
GAAAACACTAGTTAATCAATTTAACTAATTTGAATGTTTTTAGGACCATGGTTGGGCGTCCAAGTTATAATTAATTTATAATAC
TTATTTACCGTTAGTTTTCCTATCGCCACCTCGGTGCTCTTTAGGAAAAACACTGGTTAATCCAATATTAATTTCAAAGTATTA
GGACGATTTGAGTCCACGAAATTAATTTAAAAAATTAGTTTGCGTTAATTTCATCGACACTCGTGCCCCTTCTAGGAAAAACACA
GGTTATCAATTTAAATAACTTCTATTTTTTATGACCTGGTTGTGTCCAGTACTATTATTTAATAACCTTAGTTTCGCGTTAGTT
CTATCCGCACCTCGTGCATCTTTAGGAAAAACACTGGTTAGATCAATTTAGTAACTTCAAGTATAAGGTCCTGAGTTGTGTCCA
TGAAATTAATTTAAAAACTTAGTTTGGCGTTAGTGTTATCGACCACTCGTAAACCCGTTTGGAAAAACACTGGTTAATCTATTT
ACACTAACTTCTAAGTTTTAGGACCTAGTTGTGTCCAGGATATTATTAAAACTTAGTAACGTCTTAGTTTTATTTCGACACTCC
ATGCACTTTAGGTAAAAAAAAAAAAAAAAAAAAAAAAAAAAAAAAAAAAAAAAAAAGAACCACTTGTAAATCGAAGTTTAACTAACTTTT
TGTTTTAAGGTCCTAGTTGTGTACCAGGATAATAATGGTAAATACTAATAGCTTATGTATAGTTTTTTTCCAAAAACTCGTGCT
CTTTAGGAAAAACACTGGTTAAGATCAATTAAACTAACCTGTCTAAAGTTTAGACCTGTTTGTCCAGGAATTAATTTAAAAACT

TAGATCGTGTTAGTTTTATCGACACCCGCCCTCTTGCAGGAAAAAATCTCGGTTAATCCCAATTTAGCTAACTTCAAAAGTATT
AGGACCTGGTTGTGTCCAGGATATTAATTTAAAAAGCTTCGTTTGCGTTAGTTTTATTGGACACCTCCGGGCCTTTAGGAAAAA
CATGGTTAATCAATCTTAACTAACTTCTAAGTTTTAGGACACCTGGTTGTGTAACAGATAATAAATTTAAAAACATAGTTCGTT
TAGTATTTTTTCGACTCCTCGGTGCTCATTAGGAACAACACTGGTTTAATAATTTCTACTAACTTCTAAGTATTAGGACCTAGT
TTGTTGTCCAGGATATTAATATTAAAAACATAGTTCGGGTTAGTTTTTTTTCGACAACTCTTTCTCTTTAGGAAAAACATTGCT
TAATCAATTTTACTAACTCTTAGTTTTTAGGACGCTAGTTGTGTCCAGGATATTTATTTGAATTTAGTTTCGCCGTTAGTTTTA
TCGACACCTCGTGCTCTTTAGGAACACACTGGTTAAATCAATTTTCTAAACTTCTATGTTTTAGGACATGGTTGTGTCCCAGTA
TATTAATTTTAAAAACTTAGTTCCGTGTTAGTTTTATCGACACATCCGTGCTCTTTAGGAAACATTGTTATATCAATCTAATAA
ACTTCTATGTTTTAGGAACGGGTTTGTGTCTGGAAATTAATTTAAAAACTTGTTCGCGTTAGTTCTATCGACACCTAGTGCTCT
TAGAAAACACATGTTATCAATTTAAATTAACTTCAAATTTTTTAGAACCTGGTTTGTTCCATAGAAATTAATTTAAAAACTTAG
TTTTGCGTTATTTTATCGACCACAATCGTACCTTTAGGAAAAACACTGGTTAATCTATTCTAACAAACTTCTAAGTTTAGCGAC
CTAGTTCGTGTCGCAGGATATTAACTTTAAAAACTACAAAAAAAAAAAAAAAAAAAAAAAAAAAAATAAAACAAAAAAAAAAAAAAA
AACAAAAAAAAAAAAAAAAAAAAAAAAAAAAAAAAACAACAAAAAAAAAAAAAGAAAAACAAAAAAAAAAAAAAAAAAAAAAACAAA
AAAAGTACGTCCTTAGTTTTTTTGACACCTCATGCTCTTATTAGGTAACGAACACGTTGTCCTACACTCAGTTTAACTACACTT
TTATGTTTTAGTCCTAGTTGTGTCAAGGATAATAATCGTAACATACTTTAGTTTAGTGTTAGCTTTTTTCTTCGAAACCTCGTG
CCTCTTAAGGAAAACACTGTTAATCAATTAAACTAACTTCTATGTTTAGGACCATTTATTGTCCTGGATATTAATGTTGAACAC
TAGTCGCGCTTAGATTTAATCGACACTCGTGCTCTTTTAGGAAAACACTGGTTAAATCAATTTTTACTAACTTCTATGTTTTTA
TGACCTGTTTGTGTCGGATATTAATTTGAAAACTTAGTTCCCGTTAGTTTTTATCGACACCTCGTGCTCTTAGGAAAAACACT
GGTTAACCATTATAATAACTTCTACGCTTTGAAGGACCTGGTCGTGTCAGTAATATTGAATTTAAAAACTTATTCGCGTTTAGC
TTTTATTGACCCCCTTGTGCTCTTTAGGAAAAAAACTAGTTTAATCACTTTAACTAAATTCCCTAAGTTTTAGGACCTAGTTTG
TGTCCAGGATATTAATTTAAAAACTTAGTTAATGCGTTTTAATTTTTATCGTCACCGTCGTGGCTCTTTAGGACAAAACACTGG
TTAATCAATTTTGAAACTAACTCTAAGTTTTAGGACCTGGTTGTGTCCAAGATATTTATTTCAAAAACTTAGTTTCCCGTTATT
TTTATCGACCTCGTGCTCTTTTATGAAATATACACCTGGTTAGATCAATTATAACTAACTTCTAAGTTTTTAGGACCAGGTTGT
GTCCAAATATTAATTTTAAAACATAGTTCGCTGTTGTTTTTTTCGACACCTCTTGCTCTTTAGGAAAAACATTGGTACAAGTCA
ATTTTACTAACTTCTAAGTTTTAGACCTCGTCGTGTTCCAGGATAATTAATTGAAAAACTAGTTCGCCGTTAGTAATTATCGAC
CACCTCGTGCTCTTTAAGGCAAAAACACTGGTTAATCAATTTAAACTAAAAAATTCATAACGTTTAGGACCTAGTTGTGTCCCA
GGATAATTAATTTCCAAAACTTAGTTCGCGTTAGTATTATCGACACCAATCGTGCTCTTTAGGAAAATGCCTGGTTAATAAATT
TAACAAACTTTTTAAGTTTAGGACCTGGTTGTGTCCAGGATATTTAATTTAAAACCTTTGTCAGGTTTGTATTGTTATGACACT
CGTGCACTTTGGAAACCACACTGGTTAACATCAATTTAACTCACTTCTAAGTTTTAGGACCTAAGTTGTGTCAAGGTATTTTAA
TATTAAAACTTAGTTCCGTTAGTATTTACTGAAACATTGTGCTCCTTAGAGAAAAACAACTGGTTAATCAATCTTAA
>m141120_105518_42134_c100732402550000001823141405141504_s1_p0/108707/5257_11338
ATAATAAAACCAATATATACATGCTAGGGTCTCAATATAAGAAATGTGTCATGCTGAGTAAGATAGAGTAGTGAATTTAGAAAG
GGATCACGATCCCCAAAAATACTACTAATATACATTGCACTAAAATATAGTAGTGAATTTAGAATGGCATCCGATCCCGAAAAA
TTATTGCTATGATATACAATTGCACTAAGCAAGCACCTAATTATGACAAAATAAAACGTTCCAGACGTACACAATGGTAACCAA
TACTACCGCTAAGTTCTGTATAGTTTGTCATGCTGAGTAAGAAAGAGTAGTGAAATTTACAAGGGGATCCGTCCCCCAAAAATA
CACTAGATAACGTTTGCACTAAGAAATAGTAGAGAATTTAGAAGGGAATCTGATCCCGAAAATTAATTACTACATAACATTGCA
CTAAGCAGACCTAATTATGACATAATTAACCGTTCCAACGTACACTAGTAAACCAATACTACCTCTAGGGTCTCCATATATGTT
GGTCATTCTGATTAAGGAACATGAGTAGTGAATTTAGAAGGGGATCCGAATCCCAAAAATACTAATAGATAACATTGCACTAAG
ATATACTTAGTGAATTTAGAATGGCAATCCCATCCGAAGAATTCATTGATAGAATAACATTGGCAGCTAAGCAGACCTACATTA
TGACATAATAACAAACTTCGTTCCAAACGTACACTGGTAACACCAATATACCGCTAAGGGTCTCATATAGTCTGTCAATGCTGA
GTAAGATAGAGTATATGAGTTTAGAAGGGGATCCCGATCCCCAAAAAAATACTAAACTAGATAGACATTGACTGAGATATAGT
AGAGAATTTAGAAGGGGATCCGATCCCGAAAAATTATTACTAGATAACATTGCACTAAGCCAGACCTAATAAATGACATAATGA
ACGTTCAACTACACCTGGTAAACCAATACTACCGCTAGTGTCTGATATAGTTGTCATGCCTGAGTTAAGATAGGAGTAGTGTAT
TTGGAAGGGGTCCGATCCCCCAACAATAAACTAGATTAACGTTGCAAACTAAATATATAGGAGAATTTAGCAAGGGATCCGATC
CCGAAAAATTATTACTAGATAACACTTGCACTGAGCAGACTAATATGACATAATGAACCGTTCCAACGTACACCTAGTAAACCA
ATACTACCGCAAGGGTCCGTCATATAGTTGTCATGCGGAAGTAAGATAAGAGTAGGGAACTTAAAAAGGGATCCGATCTTGAAA
ATACTACTATGAGTAACATTGAAACTAAAGATATAGTAGTGGAATTTAGAAGGGGCCTCCGATCCCGAAAAATTGTTACTAGAT
AACATGCACCTAAGATATAGTAGAGAACTTAGAAGGGATTCGATCCCCGAAAAAATATTAGCTAGATAAGACATTGCATCCTTA
AGCAGAACTAAGTTATGACATAATGAACCGTTCCAAAACGTACAACTAGTAAACATACTACCGCTAGGGCTATATAGCTTGTCA
TGCTGAGTAAGATAGAGTAAGTGAATTTTGAAGGGGATCCGATCCCCAAAAATACTACTAGATAATCCATTGCAGCTAAGATAT
AGTCGTGAATTTAGAATGCATTCTAATCACCGAAAAAATTATTACTAGATAACATCATGCACTAAGCAGACTAATTAATGATAT
AATAAATCGTATCCAACGTACACTGGTAAAAGCAATACTACCGCTAGGGTCATGATAATAGTTGTTATTGTCTGAGTAATAAGT
AGTGATTTATAAGGGGATTCCGATCCCGAAAAATATTACAAGAAAGCTTGCATACAGATGATTGTGAGAATTTATAAGGGGATC
CGATCCCGAAAAATTCAAATTACTAGATTAACATTGCACTAAAGCAGACCTAATTAATGACCATTTTTTTTTTAAAATGAACCG
TTCAACGTACCACTAGTAAAAACCAATACTACCGCTAGGTCTTCTATATAGTTGTCATGCTGAGTAAGATAGAGCCGTGAATTT
AGAAGGGATCCGATCCCGAAAAATATACTAGATAACATTGACTAAGATATAGTACAGTGAATTTAGAATGGCATCCGATCCCGA
AAAATTTTATTGCTAGATAAACATTGCACTAACGCACAAGCTAAATTAATGCACATACAATAATATCGTTCCAAGTACACTGGT
AAAACCAAATACTACCGCTAGGGTCTGTATAGTTTGGTCATGTTGAGTAAGTATGAGAGTAGTGGCTTTAGAAGGGGATCGATC

CGAAAAAATACTACTAATAGACGTTTGCACTAAGATATAGTCTAAAGAGAATTTAGAAGGGTCCCGATCCCGAAAAAATTATTAC
TAGATATACCATTGCACTAAGCAGACCTAATTATGACTATATGAACCGTTCAAACGACACCTAGTAAACCAAGTACTACCGCGT
AGGGTTCTCATATAGTGTCATGAACTGGTAAGCATAGAGCTTGTGAATTTAGAAGGCGGAATCCGATCCCAAAAAAAATACTACT
AGATAAACATTGCACTAAGATTATATAGTGATTTAAGAATGGCATTCCGATCCTAGAAAAATATAATTACTAGATAACATTGCA
CTAAGCAGAAACTAATTAATGACATAATGAATCGTTCCAAACGTAACACAAGTAACCAATACCTACCGCTAAGCGTCTCTATAT
AGTTCAATGCTGAGTAAGATAGGTAGTGATTTTAGAAGGGATCGATCCCCAAAAATACTACAAGCATAACATTGCACTAAGATA
TAGTTAGAGAATTTAGAACAGGGGATCCGATCCCGAAAAATTATTACTATATACACATCTGGCACTAAGCCAGACCTAATTCAT
GCCATTAACATCGTTCAAACGTACCACTAGTACACCAATAACTACCGCTAGGGTCTCATATAATTGTCACTGGCTGAGTAAGAT
AGAGTAGTGATTTAAGAAGGGAATCCGATCCCCAAAAAATACTACTAATAACATTGCCAAAGATATCAGTAATGGAATTGAGAA
GGCTCCGATCCCGAAAATTATTGCTTAGATAACAACATTGCACTAAGCAGACCTAAATTATGACATAATCATTATCATTCAACG
GTAGCACTGGTAACACCAATACTACCGCTAGGGTCTGATATAGTTGTCATGTTGAGTAAGATACGAGAAGTTGACTTTAGAAGG
GATCCGATCCCGAAAAATACTACTAGATAACGTTGCACTAAGATATAGTAGCAACATTTAGAAGGGGATCCGATCCGAAAAATT
ATTACTAGATAACATTGCAGCCAAAGCAGACCCTAGATAATGACTAATAAACCGTTCCAACCGTACACTAGCTAAAACCAAACT
[truncated: 547,074 more chars]
